# Dynamic Changes of Urine Proteome in Rat Models Inoculated with Two Different Hepatoma Cell Lines

**DOI:** 10.1155/2021/8895330

**Published:** 2021-01-07

**Authors:** Yameng Zhang, Yufei Gao, Jing Wei, Youhe Gao

**Affiliations:** ^1^Gene Engineering Drug and Biotechnology Beijing Key Laboratory, Department of Biochemistry and Molecular Biology, College of Life Sciences, Beijing Normal University, Beijing 100875, China; ^2^College of Information Science and Technology, Beijing Normal University, Beijing 100875, China

## Abstract

Urine can accumulate systemic changes with no mechanism to be stable, which may reflect early changes associated with physiological or pathophysiological processes. To explore the potential value of the urine proteome, two rat models were established by intrahepatic injection of two different hepatoma cell lines, CBRH-7919 and RH-35. Urine samples were collected and analyzed. Compared with controls, the two models exhibited different numbers and types of differentially expressed urinary proteins despite having similar histological results. The results were compared with the urine proteome of a Walker 256 (W-256) liver tumor model. The differentially expressed urinary protein patterns in the three models were different. These findings demonstrate that changes in the urine proteomes of the two models can be detected at early stages and that the patterns of differentially expressed urinary proteins can differ even when the histological results are similar. Urinary proteins have potential utility for distinguishing among different tumor cells grown in the same organ.

## 1. Introduction

Urine, an ideal biomarker resource, can accumulate systemic changes and sensitively reflect changes associated with physiological or pathophysiological processes at early stages [1]. Due to the lack of control by a homeostatic mechanism, urine can tolerate more and higher-magnitude changes than other body fluids, including blood and cerebrospinal fluid [1]. It can also be obtained easily and noninvasively in large quantities [2]. However, urine is affected by various factors, including sex, age, diet, exercise, and lifestyle [3]. It is difficult to sort out disease factors due to the changes in clinical urine samples caused by other factors. The use of animal models can help avoid the effects of such factors in related research [4]. Specifically, animal models can minimize the influences of confounding factors and can be used to monitor whole processes from disease onset, which will be helpful for discovery of early biomarker candidates and future clinical validation. Animal models such as myocarditis [5], Alzheimer's [6], liver fibrosis [7], glioma [8], pulmonary fibrosis [9], intracerebral W-256 [10], and chronic pancreatitis models [11] have been widely used to research different diseases. The related studies have indicated that animal models are effective tools for identification of early urinary protein biomarkers, which can help elucidate the starting point of a disease and the dynamic changes in the urine proteome that occur throughout disease development.

Recent studies have shown that the urine proteome has great potential not only for discovering early biomarker candidates but also for distinguishing some subtle differences, such as the differences between the same types of tumor cells grown in different organs [12]. Different animal models have been established by injection of W-256 carcinosarcoma cells into different organs, including the W-256 subcutaneous model [2], the W-256 intracerebral tumor model [10], the W-256 liver tumor model [12], and the W-256 lung metastasis model [13]. For these four models, changes in the urinary proteome can be identified at early stages, even before histological examination or magnetic resonance imaging (MRI) reveals changes. Previous research has indicated that urinary proteins can be used to differentiate among tumor cells of the same type grown in different organs [12]. In addition, the urine proteome has been reported to reflect pathophysiological changes with extremely high sensitivity [14]. In one study, changes in the urine proteome could be sensitively detected even when only approximately ten tumor cells were injected subcutaneously in rats, and changes in biological pathways have been reported to be associated with tumors. These findings led us to investigate the sensitivity of urinary proteins. We hypothesized that urinary proteins have the potential value for distinguishing among different types of tumor cells grown in the same organ.

In this study, we established two rat models by intrahepatic injection of two different hepatoma cell lines, CBRH-7919 and RH-35, at the same dose. Urine samples were collected and analyzed by liquid chromatography-tandem mass spectrometry (LC-MS/MS) on days 0, 5, 7, 14, and 28. Dynamic changes in the urinary proteome were analyzed and compared with those of the W-256 liver tumor model [12]. This study aimed to discover dynamic changes in urinary proteins during the growth of two hepatoma cell lines in the liver and to investigate the usefulness of the urine proteome for differentiating among different tumor cell types grown in the same organ.

## 2. Materials and Methods

### 2.1. Animal Models

All experiments in this study were approved by the Institutional Animal Care, Use and Welfare Committee of the Institute of Basic Medical Sciences, Peking Union Medical College (Animal Welfare Assurance Number: ACUC-A02-2014-007), and all methods were performed in accordance with the relevant guidelines and regulations. Two different tumor-bearing animal models were established in this study. Fifty male Wistar rats (180 ± 20 g) were purchased from Beijing Vital River Laboratory Animal Technology Co., Ltd. The animal license was SCXK (Beijing) 2016-0006. All Wistar rats were randomly divided into different groups: the control group (*n* = 10), the CBRH-7919 experimental group (*n* = 20), and the RH-35 experimental group (*n* = 20). Food and water were withheld from all rats for 12 h before urine samples were collected for the modeling experiments.

The two rat hepatoma cell lines, CBRH-7919 and RH-35 (China Infrastructure of Cell Line Resource), were all cultured in DMEM (Gibco) supplemented with 10% fetal bovine serum (Gibco), 100 IU/mL penicillin G, and 100 *μ*g/mL streptomycin. The cells were maintained in a humidified atmosphere with 5% CO_2_ and 95% air. Before each implantation procedure, the viability of cells was tested via trypan blue staining (confirming >90% cell viability for each tumor implantation procedure). The cell suspension concentration was 1 × 10^8^ cells/mL. In the CBRH-7919 model, all rats were anesthetized with sodium pentobarbital first; then, the rat abdominal cavity was opened, and the left medial lobe of the liver was exposed. CBRH-7919 hepatoma cell suspension (0.1 mL) was visually injected under the hepatic capsule into this lobe. An equal volume of PBS was also injected into the same location in each of the control rats. After the injection, the wound was sutured. In the RH-35 model, all procedures were the same as those described above.

### 2.2. Histological Analysis

The livers of twenty-four experimental rats and two control rats were randomly harvested at 5, 7, 14, 21, 28, and 35 days after injection, and the rest of the rats were sacrificed at day 42. The livers were used for histopathology. All the samples were fixed in formalin, embedded in paraffin, sectioned, and evaluated with hematoxylin and eosin (H&E) staining.

### 2.3. Urine Sample Preparation

Urine samples were collected from all rats before sacrifice, including the experimental rats who were randomly selected for histopathology at different time points. During urine collection, food and water were withheld from all rats. The rats were placed in metabolic cages alone overnight to collect urine. The urine samples were temporarily stored at −80°C for later use. Before LC-MS/MS analysis, the urine samples were thawed and centrifuged at 12,000 ×g for 30 min to remove impurities. The supernatants were transferred to new centrifuge tubes and mixed with three volumes of precooled ethanol. The mixtures were precipitated at −20°C for 12 h. Then, the samples were centrifuged for 30 min, the supernatant was discarded, the precipitates were dried, and lysis buffer (8 mol/L urea, 2 mol/L thiourea, 50 mmol/L Tris, and 25 mmol/L dithiothreitol (DTT)) was added until the precipitates dissolved. The samples were again centrifuged at 12,000 ×g for 30 min, and the supernatant was retained. The protein concentration was measured by Bradford assay. All proteins were digested by the FASP method [15]. One hundred micrograms of each sample were added to a 10 kDa filter device (Pall, Port Washington, NY, USA), and prepared urea buffer (UA; 8 mol/L urea and 0.1 mol/L Tris-HCl, pH 8.5) and 25 mmol/L NH_4_HCO_3_ were used to wash the protein several times in sequence. Then, 20 mmol/L DTT (Sigma) was added to the protein samples, and the samples were incubated at 37°C for 1 h. Next, 50 mmol/L iodoacetamide (IAA, Sigma) was added to the samples, which were incubated for 40 min in the dark. After centrifuging the samples at 14,000 ×g for 40 min, UA and NH_4_HCO_3_ were added again to wash the protein several times, trypsin (enzyme-to-protein ratio of 1 : 50) was added to the protein, and the samples were digested at 37°C overnight. The collected peptide mixtures were desalted with Oasis HLB cartridges (Waters, Milford, MA) and dried by vacuum evaporation. Then, all peptides were diluted to 0.5 *μ*g/*μ*L with 0.1% formic acid (FA). The concentrations were determined by BCA assay.

A total of 76 samples were chosen for LC-MS/MS analysis: 8 urine samples from control rats; 32 samples from CBRH-7919 experimental rats on days 5, 7, 14, and 28; and 36 samples from RH-35 experimental rats on days 5, 7, 14, and 28. In the CBRH-7919 model, a pooled sample of equal amounts of polypeptides from the 40 individuals (8 control samples and 32 CBRH-7919 model samples) was used for library generation, whereas the 40 individual samples were used to analyze the urinary proteins. The 44 samples for the RH-35 model were also treated according to the method mentioned above. The two pooled samples of polypeptides were separated with a Pierce High pH Reversed-Phase Peptide Separation Kit (catalog number: 84868, Thermo Fisher Scientific, USA) according to the manufacturer's instructions. Ten effluents of each model mixture were obtained after separation and evaporation to dryness in vacuo, labeled, and stored at −80°C.

### 2.4. LC-MS/MS Analysis

Both models were treated before LC-MS/MS. A calibration kit (iRT kit, Ki3002, Biognosys AG, Switzerland), which contained a mixture of nonnaturally occurring synthetic peptides, was used in this study. iRT was added to each sample before measurement at a ratio of 1 : 10. For data-dependent acquisition (DDA), ten isolated samples from each model obtained by the above method were added to 25 *μ*L of 0.1% FA and centrifuged at 14,000 ×g and 4°C for 30 min. Twenty microliters of each sample was mixed with 2 *μ*L of the iRT polypeptide. Then, 2 *μ*L of each peptide sample was loaded on the trap column and separated on a reverse-phase C18 analytical column with an EASY-nLC 1200 HPLC system (Thermo Fisher Scientific, USA) at 0.25 *μ*L/min (the column flow rate) for 90 min. A Thermo Orbitrap Fusion Lumos Tribrid Mass Spectrometer (Thermo Fisher Scientific, USA) was used for the analysis. The MS data were acquired using the following parameters: a spray voltage of 2.4 kV, an ion transfer tube temperature of 320°C, a first-level full scan range of 350–1,550 m/z with a resolution of 60,000, a secondary scan range of 200–2,000 m/z with a resolution of 30,000, a cycle time of 3 s, and 30% HCD collision energy.

For data-independent acquisition (DIA), to prepare a mixed sample for quality control for each model, 3 *μ*L of each peptide sample was taken, and the iRT polypeptide was added at a ratio of 1 : 10 (20 *μ*L of each sample was mixed with 2 *μ*L of the iRT polypeptide). The 40 samples from the CBRH-7919 model and the 44 samples from the RH-35 model were processed by DIA individually to assess proteome differences. The variable window parameter was set to 36 isolation windows, and the maximum injection time was 50 ms for the full scan and DIA scans. The MS1 parameter settings were as follows: a resolution of 60,000, a range of 350–1400 m/z, and an automatic gain control (AGC) of 1e6. The MS2 parameters included a resolution of 30,000.

### 2.5. Data Analysis

For each model, 10 raw files from DDA were searched using Proteome Discoverer software (PD, version 2.1, Matrix Science, UK) with the SwissProt_2017_02 database (taxonomy: *Rattus*; containing 7992 sequences). The search conditions included trypsin digestion, two missed cleavage sites, cysteine as the fixed modification, methionine oxidation as the variable modification, 10 ppm as the peptide mass tolerance, and 0.02 Da as the fragment mass tolerance. The applied false discovery rate (FDR) cutoff was 0.01 at the protein level. Then, the PD results and 10 DDA raw files were imported into Spectronaut *X* software (Biognosys, Switzerland) to generate the spectral library used for DIA data analysis. All the single DIA raw files were also analyzed using Spectronaut *X* software. The *k-*nearest neighbor (K-NN) method was used to fill the missing values of protein abundance.

### 2.6. Statistical Analysis

Comparisons between two groups were conducted using two-sided unpaired *t*-tests. The selection criteria for differentially expressed urinary proteins were a fold change ≥2 or ≤0.5 and a *P* value <0.05. All results are expressed as the mean ± standard deviation. The results for the W-256 liver tumor model were used in this study for comparisons [12]. All the differentially expressed urinary proteins in these three models and their biological processes were compared.

### 2.7. Bioinformatics Analysis

For functional annotation, all differentially expressed proteins were analyzed with the Database for Annotation, Visualization and Integrated Discovery (DAVID) 6.8 (https://david.ncifcrf.gov/). The proteins were described according to three categories: biological process, cellular component, and molecular function. For ingenuity pathway analysis (IPA), the UniProt accession numbers of differentially expressed proteins were uploaded to IPA software (Qiagen, USA). The proteins were mapped to available canonical pathways and ranked by *P* values.

## 3. Results

### 3.1. Bodyweight (BW) and Histological Features over Time

**Figure 1:**
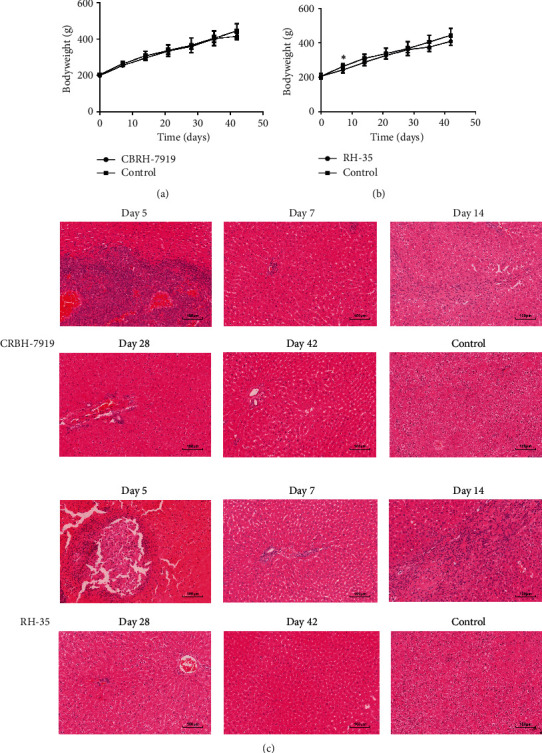
BW and histopathological characterization of the two models. (a) Change in BW in the CBRH-7919 model (CBRH-7919 group: *n* = 7; control group: *n* = 8). (b) Change in BW in the RH-35 model (RH-35 group: *n* = 8; control group: *n* = 8). ^∗^*P* value < 0 : 05. (c) H&E staining of a control rat and two model rats.

All rats in the two models except for one rat from the CBRH-7919 model that died after anesthesia lived until they were sacrificed on day 42. The BW and daily behavior changes in the two models were observed and recorded after modeling. Compared with controls, the two models exhibited no significant differences in daily behaviors. On day 42, 7 rats in the CBRH-7919 experimental group, 8 rats in the RH-35 experimental group, and 8 rats in the control group were sacrificed. Statistical analysis was performed on the BW data of all the rats. On day 0, the mean BWs of the CBRH-7919, RH-35, and control rats were 199.4 g (±5.8 g), 206.3 g (±16.0 g), and 205.6 g (±8.3 g), respectively; on day 7, the BWs of the RH-35, CBRH-7919, and control rats were 255.3 g (±10.2 g), 243.1 g (±16.9 g), and 266.8 g (±18.4 g), respectively; and on day 42, the BWs of the CBRH-7919, RH-35, and control rats were 415.6 g (±16.6 g), 400.1 g (±20.4 g), and 447.1 g (±41.8 g), respectively. In the CBRH-7919 model, there were no significant differences in BW during the 42 days (Figure 1(a)). In the RH-35 model, there was a significant difference in BW between the tumor-bearing and control rats on day 7 (Figure 1(b)).

Two rats from each experimental group (CBRH-7919 and RH-35) were randomly selected for histological examinations on days 5, 7, 14, and 28. Two control rats were also randomly selected for histological examination on day 5. The remaining rats in the two models were sacrificed on day 42, and their livers were used for histological examination. H&E staining showed that the histological results of the two models were similar when the two different hepatoma cell lines grew in the liver over the whole experimental period (Figure 1(c)). On day 5, white lesions were clearly seen on the liver. H&E staining revealed the presence of numerous hepatoma cells and necrosis of some liver cells in the two models. On days 7, 14, and 28, the presence of some inflammatory cells was observed in the two models. Some necrotic and apoptotic hepatocytes were also stained partially with hematoxylin. Moreover, no obvious hepatoma cells were found. On day 42, no hepatoma cells appeared in the livers from the two models.

### 3.2. Changes in the Urine Proteomes of the Two Groups

Seventy-six urine samples from eight CBRH-7919 rats, nine RH-35 rats, and eight control rats at four time points (on days 5, 7, 14, and 28) were selected for analysis. Urine samples from before day 28 were selected for investigation of the early changes in the urine proteome caused by injection of the two different types of hepatoma cells because the histological features of the liver did not change substantially thereafter.

**Figure 2:**
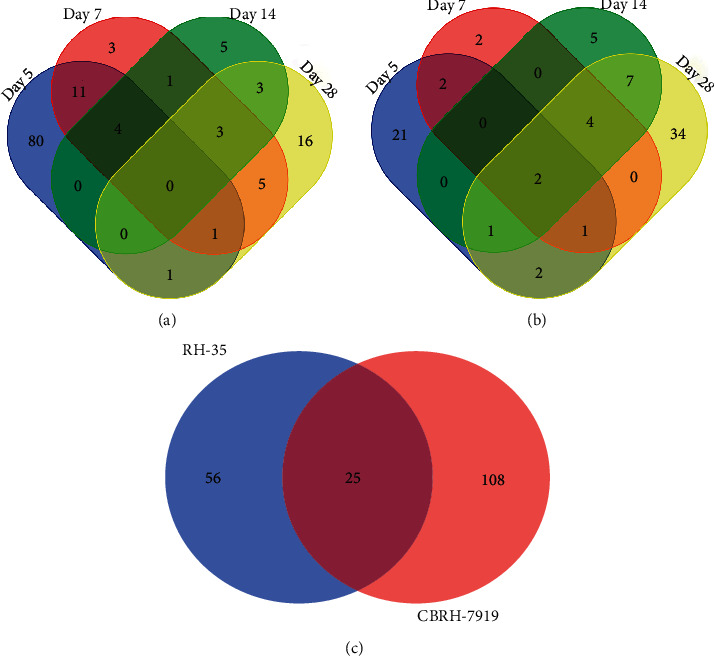
Venn diagram of differentially expressed proteins at different time points in the two models. (a) Differentially expressed proteins in the CBRH-7919 model rats at four different time points (days 5, 7, 14, and 28). (b) Differentially expressed proteins in the RH-35 model rats at four different time points (days 5, 7, 14, and 28). (c) Comparison of differentially expressed proteins between the two models.

In the CBRH-7919 model, a total of 973 urinary proteins were identified (Supplementary Table 1). The screening criteria were a fold change ≥2 or ≤0.5 and a *P* value < 0.05. Differentially expressed urinary proteins were selected that satisfied the following criteria: the peak area for the rats (*n* ≥ 5) in the upregulation group was greater than that for the rats in the downregulation group, or the peak area for the rats (*n* ≥ 5) in the downregulation group was less than that for the rats in the upregulation group. With changes in the numbers of rats (e.g., *n* ≥ 6, 7, and 8), the numbers of differentially expressed urinary proteins varied (Table 1). A total of 133 differentially expressed proteins had human orthologs (Table 2). Moreover, 97, 28, 16, and 29 differentially expressed proteins with human orthologs were altered on days 5, 7, 14, and 28, respectively. On day 5, 64 upregulated differentially expressed proteins and 33 downregulated differentially expressed proteins were identified; on day 7, 7 upregulated and 21 downregulated differentially expressed proteins were identified; on day 14, 3 upregulated and 13 downregulated differentially expressed proteins were identified; and on day 28, 5 upregulated and 24 downregulated differentially expressed proteins were identified. The Venn diagram in Figure 2(a) shows the overlapping differentially expressed proteins at different time points.

In the RH-35 model, the screening conditions were similar to those in the CBRH-7919 model. The numbers of differentially expressed urinary proteins were different for different numbers of rats (e.g., *n* ≥ 5, 6, 7, 8, and 9) (Table 1). A total of 903 urinary proteins were identified (Supplementary Table 2). Eighty-one differentially expressed proteins with human orthologs were identified (Table 3). In addition, 29, 11, 19, and 51 differentially expressed proteins with human orthologs were altered on days 5, 7, 14, and 28, respectively. On day 5, 6 upregulated and 23 downregulated differentially expressed proteins were identified; on day 7, 1 upregulated and 10 downregulated differentially expressed proteins were identified; on day 14, 2 upregulated and 17 downregulated differentially expressed proteins were identified; and on day 28, 11 upregulated and 40 downregulated differentially expressed proteins were identified. The overlapping differentially expressed proteins are shown in a Venn diagram in Figure 2(b).

When the differentially expressed proteins of the two models were compared, 25 common proteins were identified. In addition, 108 and 56 unique proteins were identified, respectively (Figure 2(c)). Although the histological results for the two models were similar, the numbers and categories of the identified urinary proteins were mostly different. The results indicate that the patterns of differentially expressed urinary proteins are different when different tumor cells grow in the liver.

### 3.3. Random Allocation Statistical Analysis

To further confirm that the differentially expressed proteins were influenced by hepatoma cells, we randomly allocated the data for 16 samples (8 experimental and 8 control samples) at each time point in the CBRH-7919 model and the data for 17 samples (9 experimental and 8 control samples) at each time point in the RH-35 model. The criteria used to screen differentially expressed urinary proteins were the same as mentioned above: a fold change ≥2 or ≤0.5 and a *P* value <0.05. At each time point, 16 samples of the CBRH-7919 model were randomly divided into 2 groups (*n* = 8 in each group). A total of 6,435 random allocations at each time point were performed, and the details are shown in Supplementary Tables 3 and 4. At each time point, 17 samples of the RH-35 model were randomly divided into 2 groups (*n* = 8 in group 1, *n* = 9 in group 2). A total of 12,155 random allocations at each time point were performed, and the details are shown in Supplementary Tables 5 and 6. In each iteration, the data for group 1 were set as the control data, and the data for group 2 were used as the experimental data.  Table 4 shows the results of the random allocation statistical analysis. It should be noted that the random allocation results included the actual grouping results. The results show that there were false-positives in the actual grouping. The highest false-positive rate was 0.177. However, when tumor cells grew actively in the liver, the number of differentially expressed urinary proteins was large, and the false-positive rate was low. For example, on day 5, the false-positive rates of the two models were 0.049 and 0.087, respectively. Therefore, most of the changes in differentially expressed urinary proteins were caused not by random allocation but by the tumor cells.

### 3.4. Functional Analysis of Differentially Expressed Proteins

By using the DAVID and the IPA database, functional analysis was performed for the identified differentially expressed proteins from the CBRH-7919 and RH-35 models. The proteins were categorized on the basis of their related biological processes, cell components, molecular functions, and canonical pathways.

**Figure 3:**
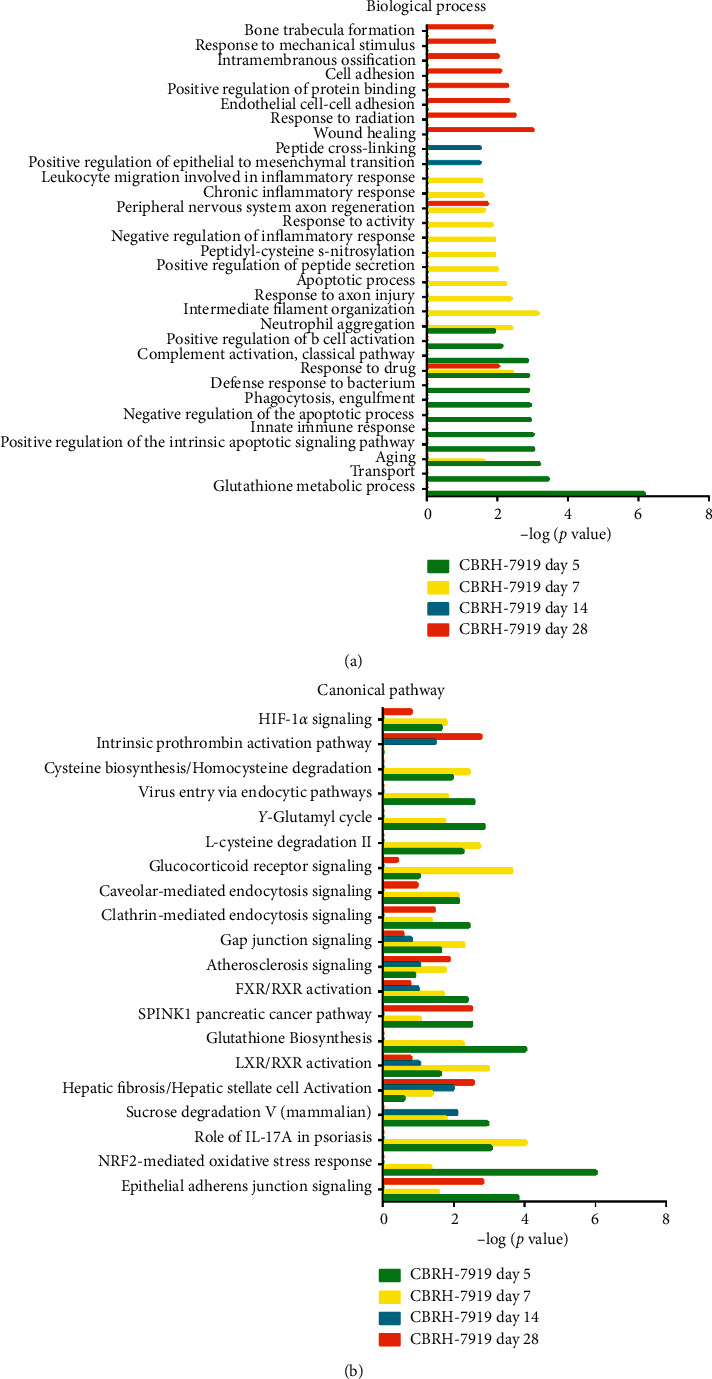
Functional analysis of differentially expressed proteins at days 5, 7, 14, and 28 in the CBRH-7919 model. (a) Biological processes for the CBRH-7919 model. (b) Canonical pathways for the CBRH-7919 model.

In the CBRH-7919 model, a variety of biological processes were associated with the differentially expressed proteins on day 5. Specifically, the glutathione metabolic process, transport, aging, positive regulation of the intrinsic apoptotic signaling pathway, innate immune response, negative regulation of the apoptotic process, phagocytosis and engulfment, complement activation and classical pathway, defense response to bacterium, response to drug, complement activation, classical pathway, positive regulation of B cell activation, and neutrophil aggregation terms were associated with significantly changed proteins. On day 7, the differentially expressed proteins were associated mainly with the following biological process terms: intermediate filament organization, neutrophil aggregation, response to axon injury, apoptotic process, positive regulation of peptide secretion, peptidyl-cysteine S-nitrosylation, negative regulation of inflammatory response, response to activity, peripheral nervous system axon regeneration, chronic inflammatory response, and leukocyte migration involved in the inflammatory response. On day 14, the positive regulation of epithelial-to-mesenchymal transition and peptide cross-linking terms were associated with the responses to the tumor cells. On day 28, the biological process terms included the wound healing, response to radiation, endothelial cell-cell adhesion, positive regulation of protein binding, cell adhesion, intramembranous ossification, response to mechanical stimulus, and bone trabecula formation terms (Figure 3(a)). In the cellular component category, most of the differentially expressed proteins were associated with the extracellular exosome, blood microparticle, apical plasma membrane, extracellular space, brush border membrane, focal adhesion, cytosol, cytoplasm, lysosome, extracellular region, and extracellular matrix terms, which represent the main sources of urinary protein (Supplementary Figure 1(a)). In the molecular function category, the differentially expressed proteins were associated mainly with metallodipeptidase activity, antigen binding, protein homodimerization activity, transporter activity, dipeptidase activity, glutathione transferase activity, protein binding, carboxypeptidase activity, immunoglobulin receptor binding, cytoskeletal protein binding, structural molecule activity, carbohydrate binding, and Toll-like receptor 4 binding (Supplementary Figure 1(b)).

IPA software was used to analyze the canonical pathways associated with the differentially expressed proteins. Liver fibrosis/hepatic stellate cell activation, LXR/RXR activation, FXR/RXR activation, atherosclerosis signaling, NRF2-mediated oxidation stress response, HIF1*α* signaling, and gap junction signaling were enriched (Figure 3(b)). Several signaling pathways have been reported to play important roles in tumor development. First, tumor cell apoptosis and proliferation are promoted in hypoxic environments, and hypoxia-inducible factor 1*α* (HIF1*α*) is a significant hypoxia-inducing factor that is related to the movement and adhesion of liver cancer tumor cells in such environments [16]. Second, NF-E2-related factor-2 (NRF2) is an important transcription factor that can regulate oxidative stress and the expression of a series of detoxification genes and antioxidant defense genes in the liver [17]. Third, liver fibrosis/hepatic stellate cell activation is a prominent pathological feature of liver fibrosis [18]. Finally, the liver *X* receptor/retinoid AX receptor (LXR/RXR) activation pathway is related to the regulation of cholesterol transport, glucose metabolism, and the inflammatory response, and increasing evidence has shown the involvement of LXRs in various malignancies [19].

**Figure 4:**
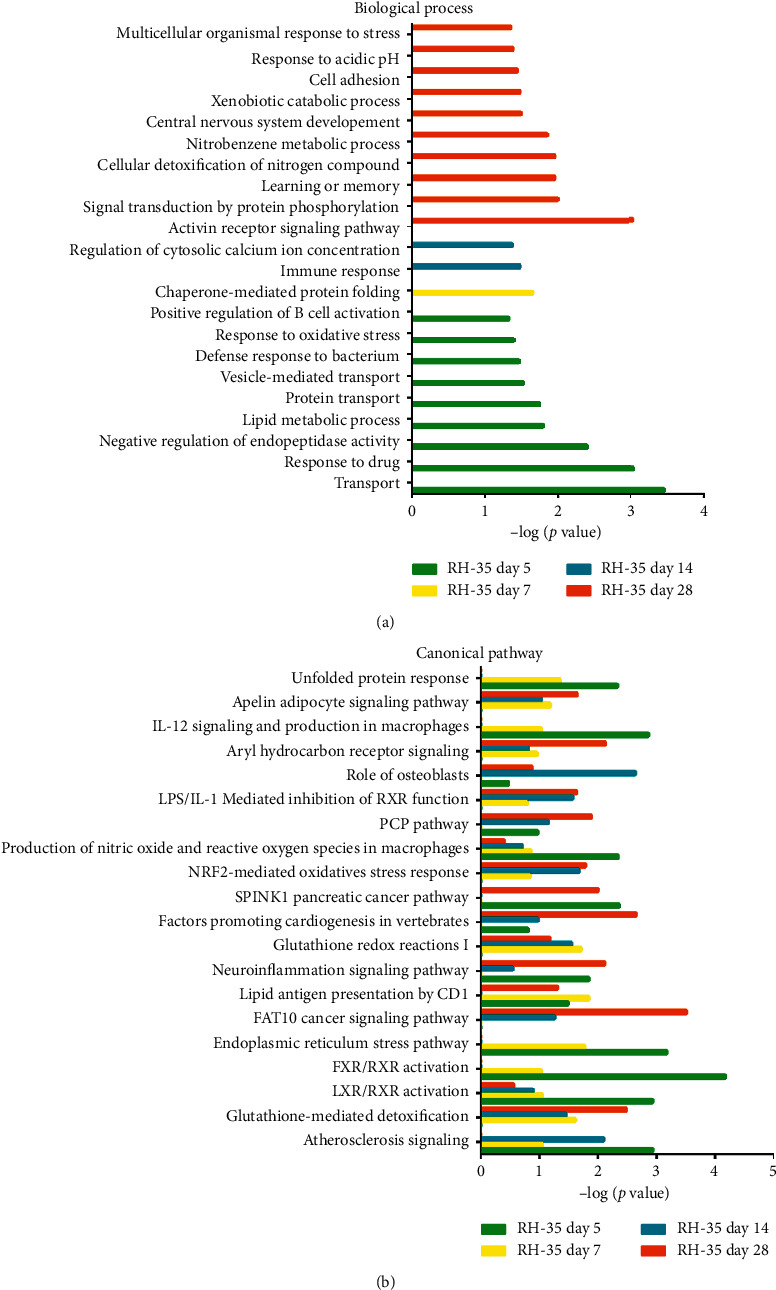
Functional analysis of differentially expressed proteins at days 5, 7, 14, and 28 in the RH-35 model. (a) Biological processes for the RH-35 model. (b) Canonical pathways for the RH-35 model.

In the RH-35 model, the differentially expressed proteins were associated mainly with the transport, response to drug, negative regulation of endopeptidase activity, lipid metabolic process, protein transport, vesicle-mediated transport, defense response to bacterium, response to oxidative stress, and positive regulation of B cell activation terms in the biological process category on day 5. On day 7, chaperone-mediated protein folding was independently enriched. On day 14, the immune response and regulation of cytosolic calcium ion concentration terms were associated with the differentially expressed proteins. On day 28, the activin receptor signaling pathway, signal transduction by protein phosphorylation, learning or memory, cellular detoxification of nitrogen compound, nitrobenzene metabolic process, central nervous system development, xenobiotic catabolic process, cell adhesion, response to acidic pH, and multicellular organismal response to stress terms were associated with the responses to the tumor cells (Figure 4(a)). For the cellular component category, the differentially expressed proteins were derived mainly from exosomes, the extracellular region, and the extracellular space (Supplementary Figure 1(c)). For the molecular function category, on day 5, protein binding, antigen binding, metalloendopeptidase inhibitor activity, misfolded protein binding, transporter activity, immunoglobulin receptor binding, and GTP binding were overrepresented. On days 7 and 14, the differentially expressed proteins were associated with glycoprotein binding, metal ion binding, and transmembrane receptor protein serine/threonine kinase activity. On day 28, the growth factor activity, receptor signaling protein serine/threonine kinase activity, activin receptor activity, type I calcium ion binding, glycoprotein binding, activin binding, protein homodimerization activity, and transmembrane receptor protein serine/threonine kinase activity terms were enriched (Supplementary Figure 1(d)).

IPA software was also employed to analyze the canonical pathways in which the differentially expressed proteins of the RH-35 model were involved. The atherosclerosis signaling, glutathione-mediated detoxification, LXR/RXR activation, FXR/RXR activation, FAT10 cancer signaling pathway, NRF2-mediated oxidative stress response, production of nitric oxide and reactive oxygen species in macrophages, and IL-12 signaling and production in macrophages pathway terms were enriched (Figure 4(b)). In addition to the pathways mentioned for the CBRH-7919 model, some other signaling pathways have also been reported to play important roles in tumor development. For example, high expression of FAT10 has been found to be positively correlated with proliferation and poor prognosis in liver cancer [19]. In addition, M2-polarized macrophages have the ability to generate nitric oxide (NO), which has different effects on different types of tumors because it can promote either the growth or death of tumor cells (depending on the cell source) [20]. Furthermore, IL-12 can induce the production of other cytokines, thereby exerting its biological functions and regulating the occurrence and development of inflammation and tumors [21]. Clinical studies have shown that IL-12 can kill tumor cells by activating and expanding natural killer (NK) cells in peripheral blood [22].

The above results indicate that when two different types of hepatoma cells are injected into the liver, there are differences in the biological processes and the main signaling pathways of the responses.

### 3.5. Comparison of the Urinary Proteomes of the Three Models

**Figure 5:**
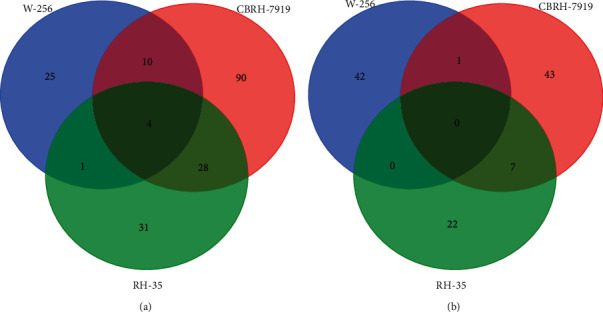
Comparison of differentially expressed proteins in the three different models. (a) Overlapping differentially expressed proteins on day 5. (b) Overlapping differentially expressed proteins on day 7.

To investigate the differences in urinary proteins exhibited with the growth of different tumor cells in the same location of the liver, the differentially expressed urinary proteins of the CBRH-7919, RH-35, and W-256 models were compared [12]. Briefly, the differentially expressed proteins at a common time point (on days 5 and 7) were selected from each model for comparison, and the criteria used to screen the differentially expressed urinary proteins were a fold change value ≥ 2 or ≤0.5 and a *P* value <0.05. The comparison results for the differentially expressed proteins are shown in Figure 5. On day 5, four common urinary proteins were identified, including aminoacylase-1A (ACY1A), vomeromodulin (VOME), probasin (PBAS), and complement C4 (CO4). The numbers of unique proteins in the CBRH-7919, RH-35, and W-256 models were 25, 90, and 31, respectively (Figure 5(a)). On day 7, there were no common proteins among the three models, and 42, 43, and 22 unique proteins were identified in the CBRH-7919, RH-35, and W-256 models, respectively (Figure 5(b)). After injection of tumor cells, the histological results for the W-256 model were different from those for the CBRH-7919 and RH-35 models. The tumors grew more aggressively in the W-256 model than in the other models, which may be the main reason why its differentially expressed urinary protein pattern was different from the patterns of the other models.

**Figure 6:**
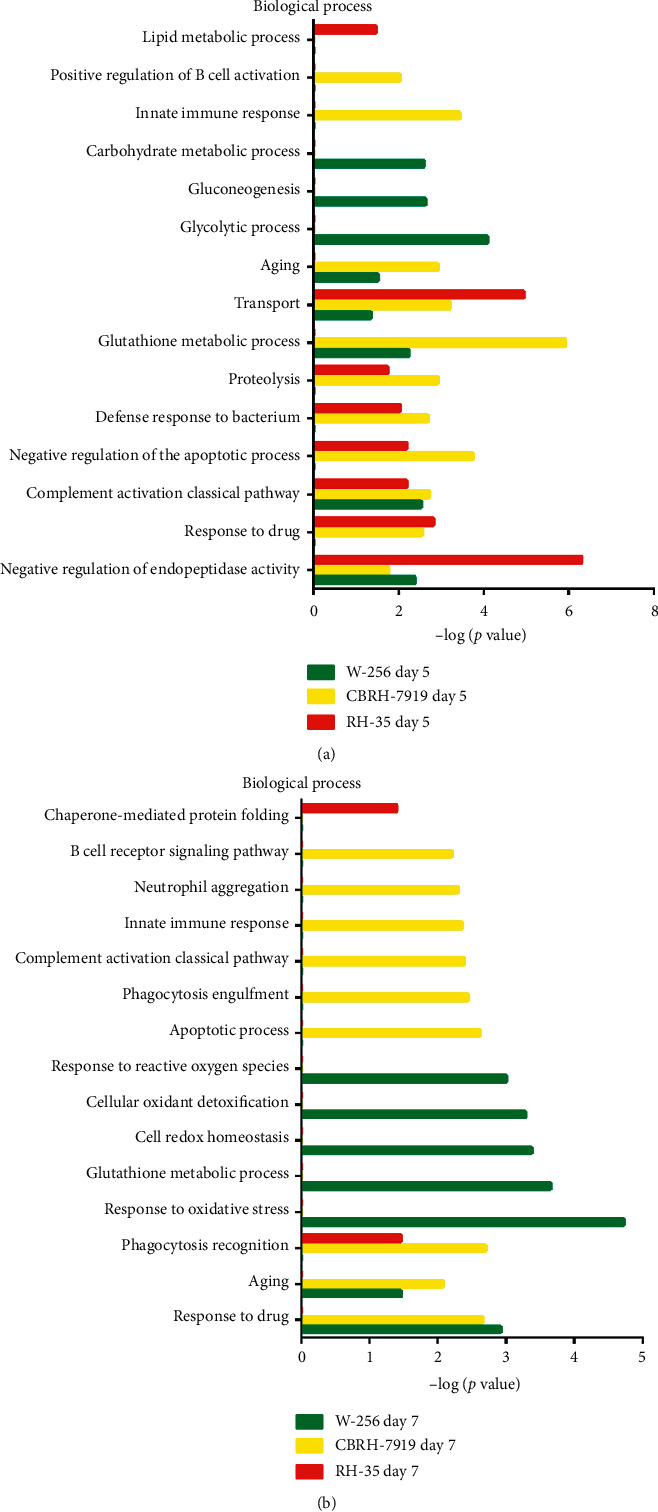
Functional analysis of differentially expressed proteins in the three different models. (a) Biological processes on day 5. (b) Biological processes on day 7.

The DAVID was applied to screen the differentially expressed proteins by using the above criteria. On day 5, the common biological processes of the three models were the negative regulation of endopeptidase activity, complement activation classical pathway, and transport processes (Figure 6(a)). The unique biological process terms for the W-256 model included the glycolytic, gluconeogenesis, and carbohydrate metabolism terms. The unique biological process terms for the CBRH-7919 model were the innate immune responses and positive regulation of B cell activation terms. The lipid metabolism process was enriched only for the RH-35 model. On day 7, there were no common biological processes among the three models (Figure 6(b)). The unique biological process terms for the W-256 model included the responses to oxidative stress, glutathione metabolic process, cellular redox homeostasis, cellular oxidant detoxification, and response to reactive oxygen species terms. The unique biological process terms for the CBRH-7919 model were the apoptotic process, phagocytosis engulfment, complement activation classical pathway, innate immune response, neutrophil aggregation, and B cell receptor signaling pathway terms. The unique biological process of the RH-35 model was chaperone-mediated protein folding. According to the above results, the W-256 model tumors grew aggressively over time. Tumor cells rapidly generate ATP and growth substrates by using aerobic glycolysis, thereby consuming large amounts of glucose [23]. The unique biological process reflects mainly tumor growth [2]. Based on the histological results, it can be inferred that the hepatoma cells of the CBRH-7919 and RH-35 models may have been recognized by the immune system and gradually cleared after the injection. Therefore, the immune response term was enriched in the biological process category for the CBRH-7919 model. The RH-35 model exhibited fewer enriched biological processes than the other models because of (1) its small number of differentially expressed proteins and (2) the diverse responses to the different hepatoma cells after the injection.

In summary, it can be concluded that the early changes in urinary proteins are different when different tumor cells are injected into the same location of the liver.

### 3.6. Differentially Expressed Protein Analysis

In both models (the CBRH-7919 model and the RH-35 model), a total of 25 proteins were identified, 8 of which have been reported to be associated with liver cancer; these proteins are here described. (1) Carboxypeptidase Z (CBPZ). The specific constitutive expression of CBPZ in the liver protects against acute liver injury by combining the mechanisms that inhibit apoptosis in hepatocytes and promote cell cycle progression and proliferation [24]. (2) Apolipoprotein D (APOD). APOD may be a new tumor suppressor gene of hepatocellular carcinoma (HCC), and its expression status may be an available biomarker for predicting patient outcomes [25]. (3) Aquaporin-1 (AQP1). AQP1 may be a highly selective marker of differentiated bile duct cells that can help to diagnose liver tumors [26]. (4) Clusterin (CLUS). Abnormal expression of CLUS in the tumor tissues or serum of patients with primary liver cancer is considered a useful biomarker for diagnosis and monitoring [27]. (5) Filamin-C (FLNC). FLNC is a potential marker of the progression of HCC that can be assessed by using quantitative proteomics analysis [28]. (6) Galectin-1 (LEG1). LEG1 plays a role in the migration and invasion of HCC cells. It was initially identified for its role in the pathogenesis of HCC and has since become a potential molecular therapeutic target [29]. (7) Thrombospondin-4 (THBS4). THBS4 may play a role in the development of HCC and may become an independent prognostic biomarker and/or therapeutic target for patients with HCC [30]. (8) Collagen triple helix repeat-containing protein 1 (CTHRC1). Overexpression of CTHRC1 in solid tumors leads to enhanced tumor cell migration and invasion and is associated with decreased overall survival and disease-free survival. CTHRC1 expression is enriched in patients with hepatitis B virus infection and is highly correlated with the progression of HCC [31]. In the current study, in addition to changes in urinary proteins that have been reported as biomarkers of HCC, there were some changes in common urinary proteins that may have been related to the growth of animals during this period (28 days).

Twenty-four of the unique proteins in the CBRH-7919 model have been reported to be associated with liver cancer (Table 2), of which 17 could be clearly identified on day 5, including CO4, macrophage migration inhibitory factor (MIF), alpha-crystallin B chain (CRYAB), dimethylargininase-1 (DDAH1), chloride intracellular channel protein 1 (CLIC1), annexin A6 (ANXA6), brain acid soluble protein 1 (BASP1), monoglyceride lipase (MGLL), beta-ureidopropionase (BUP1), LIM and SH3 domain protein 1 (LASP1), ATP-binding cassette subfamily G member 2 (ABCG2), fructose-1,6-bisphosphatase 1 (F16P1), cathepsin D (CATD), matrilysin (MMP7), deleted in malignant brain tumors 1 protein (DMBT1), angiopoietin-related protein 4 (ANGL4), and protein S100-A9 (S100A9). Among them, CO4, CRYAB, CLIC1, BASP1, BUP1, LASP1, ABCG2, F16P1, and ANGL4 have been reported as biomarkers for the diagnosis or prognosis of liver cancer [32–40]. In addition, the combination of S100A9 and other biomarkers in urine has been reported to be useful for the early diagnosis of HCC [41]. The expression of MIF is significantly positively correlated with HCC; therefore, MIF plays an important role in the progression of HCC [42]. DDAH1 is important for the regulation of angiogenesis in human HCC, and its expression is increased in patients with liver cancer [43]. It has been reported that the specificity of ANXA6 is decreased in the context of human HCC, indicating its function in the development of liver cancer [44]. MGLL has been identified as a unique tumor suppressor for HCC [45]. CATD has proteolytic activity, and its expression is a prerequisite for cancer invasion. Its expression is also valuable for predicting the prognosis of HCC [46]. It has been reported that suppression of MMP-7 expression can inhibit the invasion and metastasis of HCC [47]. DMBT1 may play an important role in the proliferation of hepatic progenitor cells (HPCs) in HPV-related liver disease, and decreases in DMBT1 may increase the risk of malignant transformation of HPCs [48].

Fourteen of the unique biomarkers of the RH-35 model have been reported to be related to liver cancer (Table 3), of which 5 were clearly identified on day 5 and are here described. (1) Alpha-1-acid glycoprotein (A1AG). Notably, glycosylation of A1AG gradually changes with the progression of cirrhosis to cancer [49]. (2) Guanine deaminase (GUAD). Due to its favorable specificity, combining assessment of GUAD with assessment of other enzymes (such as AST and ALT) in liver function tests may effectively predict liver disease with a lower false-positive rate than other methods [50]. (3) Transaldolase (TALDO). TALDO can be used as a novel serum biomarker for HCC metastasis [51]. (4) Latexin (LXN). LXN has the potential to suppress tumor cells in HCC [52]. (5) Thioredoxin-dependent peroxide reductase (PRDX3). PRDX3 can be used as an early and sensitive biomarker for the early detection of HCC [53].

On day 5 after injection of hepatoma cells, large numbers of hepatoma cells were observed in the livers, and a variety of urinary proteins related to HCC were identified at the corresponding time point. These findings indicate that urinary proteins can sensitively respond to changes in hepatoma cells at an early stage.

## 4. Discussion

Urine is an early and sensitive source of biomarkers. It has been reported to be useful for early diagnosis in a variety of animal models. Urinary proteins have the potential to be used to distinguish among the same types of cells growing in different organs [12, 54]. In this study, two different hepatoma cell lines were injected into the livers of animal models. To reduce the impact of individual differences, during the screening process, proteins were only considered differentially expressed if they were upregulated or downregulated in more than half of the total number of animals. Although the changes in histological features were similar after the injection, the changes in urinary proteins were quite different. These findings indicate that urinary protein patterns can still be used to sensitively differentiate among tumor cells even when histological findings are similar.

In the random allocation statistical analysis, it was found that under the same screening criteria, random grouping could also generate some differentially expressed urinary proteins. As the growth of tumor cells decreased, the number of differentially expressed urinary proteins decreased and the false-positive rate increased. However, the maximum false-positive rate was 0.177, indicating that most of the identified proteins were related to the physiological response to tumor cells rather than the result of random allocation. The existence of false-positive proteins is undeniable. The number of differentially expressed urinary proteins was increased, and the false-positive rate was decreased, when tumor cells grew actively in the liver. The false-positive rate can be reduced by increasing the stringency of the screening criteria, but the false-negative rate will increase at the same time. How to select appropriate screening criteria may still be a challenge for future studies.

Compared with the previously reported W-256 model, the two models had different urinary protein patterns and associated biological processes. Even when they are grown in the same organ, tumor cells can lead to different changes in the body, which indicates that urinary proteins can be used to recognize such changes at the early stage regardless of how the tumor cells grow. Urinary protein changes may also predict the trends of subsequent tumor-related changes in the body. Different types of tumor cells may be able to be classified by their different urinary protein patterns. This study demonstrates that urinary proteins have the potential to be used to distinguish among different types of hepatoma cells. This possibility needs to be further validated in clinical samples in future studies.

Interestingly, the urinary proteins identified at each time point were different even after the same numbers of CBRH-7919 and RH-35 cells were injected into the liver. The largest number of urinary proteins was identified in the CBRH-7919 model on day 5, while the numbers of proteins decreased at the following three time points. The numbers of urinary proteins identified for the RH-35 model were decreased at the first three time points and increased on day 28. At the same time point, the types and quantities of identified proteins in the two cell lines were quite different, which may have been related to the different categories of hepatoma cells. On day 5, the unique biological processes in the CBRH-7919 model included the innate immune response, complement activation, classical pathways, and neutrophil aggregation, which indicate that the immune system quickly responded to CBRH-7919 cells with numerous protein changes after the injection. Positive regulation of B cell activation was a common biological process in the CBRH-7919 and RH-35 models on day 5. However, no other immune-related biological processes were identified in the RH-35 model, indicating that RH-35 hepatoma cells did not immediately cause a strong immune response and that relatively few changes in urinary proteins occurred. On day 7, the unique biological process terms of the CBRH-7919 model associated with the inflammatory response included the chronic inflammatory response and leukocyte migration terms. At the following time points, no biological processes were related to these immune and inflammatory reactions, and relatively few differentially expressed proteins were identified. The immune response biological process term was enriched in the RH-35 model on day 14. More proteins were identified on day 28 than at the other three time points, which may have been due to the late appearance of the immune response caused by RH-35 hepatoma cells.

## 5. Conclusion

In this study, changes in urinary proteins caused by the injection of two different hepatoma cell types were identified at an early stage. Even when similar histological results were obtained, the patterns of the urinary proteins could be used to sensitively distinguish the tumor cells. Some of the differentially expressed proteins have been reported to be associated with HCC. Urinary proteins have the potential to differentiate among different tumor cells grown in the same organ, which may provide clues for clinical diagnoses in the future.

## Figures and Tables

**Table 1:** The number of differentially proteins under different screening criteria in the two models.

Model	Time	Fold change (FC ≥ 2.0 or ≤0.5)	*N* ≥ 5	*N* ≥ 6	*N* ≥ 7	*N* ≥ 8	*N* = 9
CBRH-7919	Day 5	132	115	86	57	19	—
	Day 7	52	41	27	21	8	—
	Day 14	23	18	11	8	5	—
	Day 28	36	30	24	18	10	—

RH-35	Day 5	64	35	30	22	18	4
	Day 7	29	17	12	10	9	6
	Day 14	36	24	20	16	8	4
	Day 28	69	53	47	42	27	10

*Note*. *N* indicates the number of rats; — indicates no data reach the criteria.

**Table 2:** Differential urinary proteins in the CBRH-7919 model.

Accession	Protein names	Trend	Fold changes				Reported to hepatocellular carcinoma	Reported to other cancers
			D5	D7	D14	D28		
Q9Y694	Solute carrier family 22 member 7	↑	3.41	—	—	—		Colorectal cancer
P53004	Biliverdin reductase A	↑	3.34	2.29	—	—		Breast cancer
O76082	Solute carrier family 22 member 5	↑	3.33	3.17	2.17	—		Breast cancer
P48507	Glutamate–cysteine ligase regulatory subunit	↑	3.13	2.09	—	—		
O00338	Sulfotransferase 1C2	↑	3.08	—	—	—		
P11234	Ras-related protein Ral-B	↑	2.83	2.32	—	—		Nonsmall cell lung cancer
Q01523	Neutrophil antibiotic peptide NP-4	↑	2.80	—	—	—		
B1AK53	Espin	↑	2.77	—	—	—		
Q96KP4	Cytosolic nonspecific dipeptidase	↑	2.76	—	—	—		
P0C0L4	Complement C4	↑	2.69	—	—	—	Serum	
P32929	Cystathionine gamma-lyase	↑	2.64	2.04	—	—		Melanoma
P14174	Macrophage migration inhibitory factor	↑	2.62	—	—	—	Serum	Gastrointestinal tract (GIT) malignancy
P21399	Cytoplasmic aconitate hydratase	↑	2.57	—	—	—		
Q86YJ6	Threonine synthase-like 2	↑	2.50	—	—	—		
P02511	Alpha-crystallin B chain	↑	2.49	—	—	—	Tissue	Colorectal cancer
O94760	Dimethylargininase-1	↑	2.48	—	—	—	Tissue	Prostate cancer
Q96S37	Solute carrier family 22 member 12	↑	2.48	—	—	—		
Q8N5Z0	2-Aminoadipate transaminase	↑	2.47	—	—	—		
Q13113	PDZK1-interacting protein 1	↑	2.46	—	—	—		
O95154	Aflatoxin B1 aldehyde reductase member 3	↑	2.45	—	—	—		
O00299	Chloride intracellular channel protein 1	↑	2.44	—	—	—	Tissue	Gastric, colon, lung, and glioblastoma cancers
P13866	Sodium/glucose cotransporter 1	↑	2.42	—	—	—		Pancreatic cancer
P36543	V-type proton ATPase subunit E 1	↑	2.40	—	—	—		Pancreatic cancer
Q9Y696	Chloride intracellular channel protein 4	↑	2.39	—	—	—		
Q96FL8	Multidrug and toxin extrusion protein 1	↑	2.38	2.10	—	—		
O43708	Maleylacetoacetate isomerase	↑	2.36	—	—	—		
P35579	Myosin-9	↑	2.35	—	—	—		Colorectal cancer
P26038	Moesin	↑	2.34	—	—	—		Breast cancer
P12955	Xaa-Pro dipeptidase	↑	2.33	—	—	—		
Q15599	Na(+)/H(+) exchange regulatory cofactor NHE-RF2	↑	2.27	—	—	—		
P08133	Annexin A6	↑	2.27	—	—	—	Tissue	Melanoma, cervical cancer, epithelial carcinoma, breast cancer, and gastric cancer
P80723	Brain acid soluble protein 1	↑	2.26	—	—	—	Tissue	Pancreatic cancer and cervical cancer
P48506	Gamma-glutamylcysteine synthetase	↑	2.26	—	—	—		
Q9UHI7	Solute carrier family 23 member 1	↑	2.25	—	—	—		
Q03154	Aminoacylase-1A	↑	2.25	—	—	—		
Q96KN2	Beta-Ala-His dipeptidase	↑	2.24	—	—	—		Cancer cachexia
O15400	Syntaxin-7	↑	2.23	—	—	—		
Q99685	Monoglyceride lipase	↑	2.23	—	—	—	Tissue	Gastrointestinal stromal tumor
Q14894	Ketimine reductase mu-crystallin	↑	2.23	—	—	—		
Q6ZQN7	Solute carrier family 21 member 20	↑	2.23	—	—	—		
O00161	Synaptosomal-associated protein 23	↑	2.22	—	—	—		
P49189	4-Trimethylaminobutyraldehyde dehydrogenase	↑	2.21	—	—	—		
Q9Y6I3	Epsin-1	↑	2.20	—	—	2.18		Prostate cancer
Q9UBR1	Beta-ureidopropionase	↑	2.17	—	—	—	Serum	
Q14847	LIM and SH3 domain protein1	↑	2.16	—	—	—	Tissue	Gastric cancer
O43704	Sulfotransferase family cytosolic 1B member 1	↑	2.15	—	—	—		
Q9UNQ0	ATP-binding cassette subfamily G member 2	↑	2.15	—	—	—	Tissue	Right-sided colon cancer
P30041	Peroxiredoxin-6	↑	2.14	—	—	—		
O14745	Na(+)/H(+) exchange regulatory cofactor NHE-RF1	↑	2.13	—	—	—		
P68032	Actin, alpha cardiac muscle 1	↑	2.12	—	—	—		Glioblastoma
P27449	V-type proton ATPase 16 kDa proteolipid subunit	↑	2.11	—	—	—		Prostate cancer
Q5T2W1	Na(+)/H(+) exchange regulatory cofactor NHE-RF3	↑	2.09	—	—	—		
Q16348	Solute carrier family 15 member 2	↑	2.09	—	—	—		
Q9H0W9	Ester hydrolase C11orf54 homolog	↑	2.08	—	—	—		Renal cell carcinoma
P60709	Actin, cytoplasmic 1	↑	2.08	—	—	—	Tissue	
P09467	Fructose-1,6-bisphosphatase 1	↑	2.08	—	—	—	Tissue	
P46721	Solute carrier organic anion transporter family member 1A1	↑	2.07	—	—	—		
P05062	Fructose-bisphosphate aldolase B	↑	2.07	—	—	—		
P29972	Aquaporin-1	↑	2.06	—	—	—	Tissue	Bladder, brain, breast, cervix, colon, lung, nasopharynx, and prostate cancers
Q08257	Quinone oxidoreductase	↑	2.03	—	—	—		
O75083	WD repeat-containing protein 1	↑	2.03	—	—	—		Ovarian cancer
Q9H8S9	MOB kinase activator 1A	↑	2.02	—	—	—		
Q14019	Coactosin-like protein	↑	2.02	—	—	—		Small cell lung cancer
Q8TF66	Leucine-rich repeat-containing protein 15	↑	2.02	—	—	—		Solid tumors (e.g., breast, head and neck, lung, and pancreatic)
P05090	Apolipoprotein D	↑	—	3.38	—	2.43	Tissue	Breast cancer and prostate cancer
Q12792	Twinfilin-1	↑	—	—	2.48	—		Lung adenocarcinoma
P55259	Glycoprotein 80	↑	—	—	2.47	4.00		
P06870	Prostatic glandular kallikrein-6	↑	—	—	—	3.37		
P02741	C-reactive protein	↑	—	—	—	2.16	Serum	Gastric cancer
O00115	Deoxyribonuclease-2-alpha	↓	0.50	—	—	—		
P37173	TGF-beta receptor type-2	↓	0.49	—	—	—		
Q9BRK5	45 kDa calcium-binding protein	↓	0.49	—	—	—		
O94985	Calsyntenin-1	↓	0.49	—	—	—		Lung adenocarcinoma
P10909	Clusterin	↓	0.48	—	—	—	Serum	Lung adenocarcinoma
P07339	Cathepsin D	↓	0.47	—	—	—	Tissue	
Q6P4A8	Phospholipase B-like 1	↓	0.46	—	—	—		
P34096	Ribonuclease 4	↓	0.44	—	—	—		
P04233	MHC class II-associated invariant chain	↓	0.42	—	—	—		
Q8NFL0	Beta-1,3-*N*-acetylglucosa-minyltransferase 7	↓	0.42	—	—	—		Breast cancer
P30740	Leukocyte elastase inhibitor A	↓	0.41	—	—	—		
Q8TD33	Secretoglobin family 1C member 1	↓	0.40	0.31	—	0.42		
Q16674	Melanoma-derived growth regulatory protein	↓	0.40	—	—	—		
P15309	Prostatic acid phosphatase	↓	0.40	—	—	—		
P01834	Ig kappa chain C region, A allele	↓	0.40	0.46	0.49	—		
P01859	Ig gamma-2A chain C region	↓	0.38	—	—	—		
Q14315	Filamin-C	↓	0.38	0.49	—	—	Tissue	Prostate cancer
P16444	Dipeptidase 1	↓	0.38	—	—	—		Colorectal cancer
P09237	Matrilysin	↓	0.38	—	—	—	Tissue	
P27797	Calreticulin	↓	0.37	0.42	—	—		
Q13217	DnaJ homolog subfamily C member 3	↓	0.36	—	—	—		
Q9UGM3	Deleted in malignant brain tumors 1 protein	↓	0.36	—	—	—	Tissue	
Q9NZU0	Leucine-rich repeat transmembrane protein FLRT3	↓	0.35	0.47	—	—		
Q08188	Protein-glutamine gamma-glutamyltransferase E	↓	0.34	—	—	—		
Q3LXA3	Triokinase/FMN cyclase	↓	0.33	0.18	0.13	—		
Q9BY76	Angiopoietin-related protein 4	↓	0.29	—	—	—	Serum	Colorectal cancer
P08118	Beta-microseminoprotein	↓	0.23	0.16	0.23	—		Prostate cancer
P15086	Carboxypeptidase B	↓	0.21	—	—	—		
Q99895	Chymotrypsin-C	↓	0.19	—	—	—		
P34913	Bifunctional epoxide hydrolase 2	↓	0.18	0.32	—	—		
P05109	Protein S100-A8	↓	0.16	0.29	—	—		Breast cancer
P06702	Protein S100-A9	↓	0.14	0.23	—	—	Urine	Esophageal adenocarcinoma
Q9UPX8	SH3 and multiple ankyrin repeat domains protein 2	↓	0.09	—	—	—		
P36896	Activin receptor type-1B	↓	—	0.49	—	0.42		
P08253	72 kDa type IV collagenase	↓	—	0.48	—	0.50		Rectal cancer, laryngeal cancer
P02760	Protein AMBP	↓	—	0.48	0.47	—		
Q6UY14	ADAMTS-like protein 4	↓	—	0.47	—	—		
Q8N8N7	Prostaglandin reductase 2	↓	—	0.45	—	—		
Q9Y678	Coatomer subunit gamma-1	↓	—	0.45	—	0.30		
Q13145	BMP and activin membrane-bound inhibitor homolog	↓	—	0.43	0.50	0.29	Tissue	Melanoma, colorectal cancer
Q66K79	Carboxypeptidase Z	↓	—	0.43	—	0.22	Tissue	
P48745	CCN family member 3	↓	—	0.42	0.41	0.44	Tissue	Breast cancer, gastric cancer
P09382	Galectin-1	↓	—	0.40	—	—	Tissue	Gastric cancer, colorectal cancer, and pancreatic ductal adenocarcinoma
Q03403	Trefoil factor 2	↓	—	0.33	0.39	0.34	Tissue	Colorectal cancer
P0CE71	Oncomodulin	↓	—	—	0.47	—		
Q9UBC9	Cornifin-A	↓	—	—	0.45	—		
O95388	WNT1-inducible-signaling pathway protein 1	↓	—	—	0.43	—		Oral squamous cell carcinoma
P49221	Protein-glutamine gamma-glutamyltransferase 4	↓	—	—	0.35	—		Prostate cancer
P02452	Collagen alpha-1	↓	—	—	0.22	0.16		Colorectal cancer
P57740	Nuclear pore complex protein Nup107	↓	—	—	0.14	0.35		
P35443	Thrombospondin-4	↓	—	—	—	0.49	Tissue	Gastric cancer
P49747	Cartilage oligomeric matrix protein	↓	—	—	—	0.48	Tissue	Breast cancer and prostate cancer
O15197	Ephrin type-B receptor 6	↓	—	—	—	0.48		Colorectal cancer
P35613	Basigin	↓	—	—	—	0.47		
P16112	Aggrecan core protein	↓	—	—	—	0.47		
Q04721	Neurogenic locus notch homolog protein 2	↓	—	—	—	0.46		Laryngeal squamous cell carcinoma
Q96CG8	Collagen triple helix repeat-containing protein 1	↓	—	—	—	0.43		
Q14112	Nidogen-2	↓	—	—	—	0.43	Serum	Ovarian cancer
Q12805	EGF-containing fibulin-like extracellular matrix protein 1	↓	—	—	—	0.42		Meningioma
Q96IU4	Protein ABHD14B	↓	—	—	—	0.42		
Q9NP85	Podocin	↓	—	—	—	0.41		
O60462	Neuropilin-2	↓	—	—	—	0.39	Tissue	Nonsmall cell lung cancer, squamous cell carcinoma, and melanoma
O95336	6-Phosphogluconolactonase	↓	—	—	—	0.39		Breast cancer, nonsmall cell lung cancer, and ovarian cancer
P11684	Uteroglobin	↓	—	—	—	0.21		Prostate cancer

*Note*. — indicates no data reach the criteria compared with control. The results of two parts (reported to be related to hepatocellular carcinoma and other diseases) are annotated from previous studies.

**Table 3:** Differential urinary proteins in the RH-35 model.

Accession	Protein names	Trend	Fold change				Reported to hepatocellular carcinoma	Reported to other cancers
			D5	D7	D14	D28		
Q03154	Aminoacylase-1A	↑	2.38	—	—	—		
Q66K79	Carboxypeptidase Z	↑	2.33	—	—	0.49	Tissue	
P02763	Alpha-1-acid glycoprotein	↑	2.18	—	—	—	Serum	Bladder cancer and lung cancer
P05090	Apolipoprotein D	↑	2.11	—	—	—	Tissue	Prostate cancer and breast cancer
Q9UP38	Frizzled-1	↑	2.03	—	—	—		Follicular thyroid carcinoma
P29972	Aquaporin-1	↑	2.03	—	—	—	Tissue	Bladder cancer, brain cancer, and breast cancer
P09488	Glutathione S-transferase Mu 2	↑	—	2.02	2.17	3.58		Nonsmall cell lung cancer
Q9H3Z4	DnaJ homolog subfamily C member 5	↑	—	—	2.05	3.24		
P06870	Prostatic glandular kallikrein-6	↑	—	—	—	5.44		
P31639	Sodium/glucose cotransporter 2	↑	—	—	—	3.10		
P08754	Guanine nucleotide-binding protein G	↑	—	—	—	2.87		
Q15833	Syntaxin-binding protein 2	↑	—	—	—	2.74		
O94832	Unconventional myosin-Id	↑	—	—	—	2.35		
O95968	Prostatic steroid-binding protein C2	↑	—	—	—	2.23		
Q9H2G2	STE20-like serine/threonine-protein kinase	↑	—	—	—	2.02	Tissue	Glioma
O14773	Tripeptidyl peptidase 1	↑	—	—	—	2.02	Tissue	
P02144	Myoglobin	↑	—	—	—	2.01		Lung adenocarcinoma and breast cancer
Q9H6B4	CXADR-like membrane protein	↓	0.49	—	—	—		
Q9Y2T3	Guanine deaminase	↓	0.45	—	—	—	Serum	Gastric cancer
Q92932	Receptor-type tyrosine-protein phosphatase N2	↓	0.45	0.39	—	0.48		GRN-associated frontotemporal dementia
P15309	Prostatic acid phosphatase	↓	0.44	—	—	—		
P34096	Ribonuclease 4	↓	0.44	—	—	—		
Q99757	Thioredoxin	↓	0.43	—	—	—		
P61204	ADP-ribosylation factor 3	↓	0.43	—	—	—		
Q9UGM5	Fetuin-B	↓	0.42	—	—	—		
P07384	Calpain-1 catalytic subunit	↓	0.42	—	—	—		
P05937	Calbindin	↓	0.41	—	0.40	0.37		Lung cancer
P01859	Ig gamma-2A chain C region	↓	0.39	—	—	—		
Q71U36	Tubulin alpha-1A chain	↓	0.39	—	—	—		Renal cell carcinoma
P37837	Transaldolase	↓	0.39	—	—	—	Serum	Ovarian cancer
P08962	CD63 antigen	↓	0.39	—	—	—		Melanoma
P10909	Clusterin	↓	0.39	0.48	—	—	Tissue	Lung adenocarcinoma
P11021	Endoplasmic reticulum chaperone BiP	↓	0.37	—	—	—		Gastric cancer
Q9BS40	Latexin	↓	0.35	—	—	—	Tissue	Gastric cancer and prostate cancer
Q14315	Filamin-C	↓	0.34	—	—	—	Tissue	Prostate cancer
P57740	Nuclear pore complex protein Nup107	↓	0.27	0.09	0.21	0.17		
P0CE71	Oncomodulin	↓	0.25	0.29	0.33	0.36		
P30048	Thioredoxin-dependent peroxide reductase	↓	0.23	—	—	—	Serum	Endometrial cancer and prostate cancer
P27797	Calreticulin	↓	0.22	0.31	—	—		
P08118	Beta-microseminoprotein	↓	0.15	—	—	0.21		Prostate cancer
Q6P587	Acylpyruvase FAHD1	↓	—	0.49	—	—		
P31150	Rab GDP dissociation inhibitor alpha	↓	—	0.48	—	—		Nonsmall cell lung cancer
Q5KU26	Collectin-12	↓	—	0.46	0.45	0.47		
Q6ZVN8	Hemojuvelin	↓	—	0.46	0.49	0.49	Tissue	
Q96DG6	Carboxymethylenebutenolidase homolog	↓	—	0.45	0.38	0.39		
Q9HCB6	Spondin-1	↓	—	—	0.50	0.48		Ovarian cancer
Q12805	Fibulin-3	↓	—	—	0.49	0.46		Meningioma
P47755	F-actin-capping protein subunit alpha-2	↓	—	—	0.49	0.43		
P20333	Tumor necrosis factor receptor 2	↓	—	—	0.48	0.50	Tissue	Cholangiocarcinoma, colorectal cancer, and myeloma
Q9NWV4	CXXC motif containing zinc-binding protein	↓	—	—	0.48	—		
Q14332	Frizzled-2	↓	—	—	0.43	—	Tissue	Prostate cancer
O95716	GTP-binding protein Rab-3D	↓	—	—	0.41	—		
P04003	C4b-binding protein alpha chain	↓	—	—	0.39	0.48	Serum	Pancreatic cancer and epithelial ovarian carcinoma
Q03403	Trefoil factor 2	↓	—	—	0.36	0.27		Colorectal cancer and cholangiocarcinoma
Q6P1J6	Phospholipase B1	↓	—	—	0.19	—		
P02452	Collagen alpha-1	↓	—	—	0.19	—		Colorectal cancer
P07108	Acyl-CoA-binding protein	↓	—	—	—	0.50		Lung cancer
Q96RD6	Pannexin-2	↓	—	—	—	0.50		
Q9Y5Q5	Atrial natriuretic peptide-converting enzyme	↓	—	—	—	0.49		Small cell lung cancer
P13591	Neural cell adhesion molecule 1	↓	—	—	—	0.49	Tissue	Lung cancer
P07602	Prosaposin	↓	—	—	—	0.48		Gallbladder cancer and breast cancer
P51884	Lumican	↓	—	—	—	0.48		
Q04771	Activin receptor type-1	↓	—	—	—	0.48		
P13667	Protein disulfide isomerase A4	↓	—	—	—	0.48	Serum	
Q9H461	Frizzled-8	↓	—	—	—	0.48		Prostate cancer and renal cell carcinoma
P09382	Galectin-1	↓	—	—	—	0.47	Tissue	Colorectal cancer and gastric cancer
P51687	Sulfite oxidase	↓	—	—	—	0.46	Serum	Prostate cancer
P35443	Thrombospondin-4	↓	—	—	—	0.44	Tissue	Gastric cancer
P36896	Activin receptor type-1B	↓	—	—	—	0.43		
Q96CG8	Collagen triple helix repeat-containing protein 1	↓	—	—	—	0.43	Tissue	Osteosarcoma and ovarian cancer
Q8TER0	Sushi, nidogen, and EGF-like domain-containing protein 1	↓	—	—	—	0.43		Papillary thyroid carcinoma
Q9Y678	Coatomer subunit gamma-1	↓	—	—	—	0.42		
O60667	Fas apoptotic inhibitory molecule 3	↓	—	—	—	0.41		Chronic lymphocyte leukemia
P16112	Aggrecan core protein	↓	—	—	—	0.40		
Q6UY11	Protein delta homolog 2	↓	—	—	—	0.39		Melanoma
P04155	Trefoil factor 1	↓	—	—	—	0.36		Prostate cancer
O95336	6-Phosphogluconolactonase	↓	—	—	—	0.32		Breast cancer, nonsmall cell lung cancer, and ovarian cancer
P36957	Dihydrolipoyllysine-residue succinyltransferase component of 2-oxoglutarate dehydrogenase complex, mitochondrial	↓	—	—	—	0.30		
Q9BQ08	Resistin-like gamma	↓	—	—	—	0.29		
P58062	Serine protease inhibitor Kazal-type 7	↓	—	—	—	0.21		Esophageal cancer
Q9UBC9	Cornifin-A	↓	—	—	—	0.18		

*Note*. — indicates no data reach the criteria compared with the control. The results of two parts (reported to be related to hepatocellular carcinoma and other diseases) are annotated from previous studies.

**Table 4:** Random allocation in two models.

Model	The numbers of differential urinary proteins	Day 5	Day 7	Day 14	Day 28
CBRH-7919	Actual grouping	132	52	23	36
	The average of random grouping	6.45	5.07	4.06	4.69
	The false-positive rate (the average of random grouping/actual grouping)	0.049	0.098	0.177	0.130

RH-35	Actual grouping	64	29	36	69
	The average of random grouping	5.59	4.44	4.22	5.10
	The ratio (the average of random grouping/actual grouping)	0.087	0.153	0.117	0.074

## Data Availability

The data used to support the findings of this study are available from the corresponding author upon request.

## Supplementary Materials

Supplementary Materials Supplementary Figure 1. Functional analysis of differentially expressed proteins at days 5, 7, 14, and 28 in two models. (a) Cell component for the CBRH-7919 model. (b) Molecular function for the CBRH-7919 model. (c) Cell component for the RH-35 model. (d) Molecular function for the RH-35 model. Supplementary Table 1. All urinary proteins identified in the CBRH-7919 model. Supplementary Table 2. All urinary proteins identified in the RH-35 model. Supplementary Table 3. The details of 6435 random allocations in the CBRH-7919 model. Supplementary Table 4. The occurrence of the protein in 6435 random allocations in the CBRH-7919 model. Supplementary Table 5. The details of 12155 random allocations in the RH-35 model. Supplementary Table 6. The occurrence of the protein in 12155 random allocations in the RH-35 model.
