# Supplementary material for: Dynamic Changes of Urine Proteome in Rat Models Inoculated with Two Different Hepatoma Cell Lines
Source: J Oncol. 2021 Jan 7;2021:8895330. doi: 10.1155/2021/8895330 (PMC7810548; doi:10.1155/2021/8895330)
Supplement: Supplementary Materials — Supplementary Figure 1. Functional analysis of differentially expressed proteins at days 5, 7, 14, and 28 in two models. (a) Cell component for the CBRH-7919 model. (b) Molecular function for the CBRH-7919 model. (c) Cell component for the RH-35 model. (d) Molecular function for the RH-35 model. Supplementary Table 1. All urinary proteins identified in the CBRH-7919 model. Supplementary Table 2. All urinary proteins identified in the RH-35 model. Supplementary Table 3. The details of 6435 random allocations in the CBRH-7919 model. Supplementary Table 4. The occurrence of the protein in 6435 random allocations in the CBRH-7919 model. Supplementary Table 5. The details of 12155 random allocations in the RH-35 model. Supplementary Table 6. The occurrence of the protein in 12155 random allocations in the RH-35 model. [file 8895330.f1.zip › 8895330.f1/Supplementary Table 1.pdf]

Title: Dynamic changes of urine proteome in rat models inoculated with two different hepatoma cell lines. Full author: Yameng Zhang, Yufei Gao, Jing Wei & Youhe Gao*.

Table S1 All urinary proteins were identified in CBRH-7919 model.

Protein ID	1<br>CON-rat1	2<br>CON-rat2	3<br>CON-rat3	4<br>CON-rat4	5<br>CON-rat5	6<br>CON-rat6	7<br>CON-rat7	8<br>CON-rat8	9<br>D5-rat1	10<br>D5-rat2	11<br>D5-rat3	12<br>D5-rat4	13<br>D5-rat5	14<br>D5-rat6	15<br>D5-rat7	16<br>D5-rat8
A0JPJ7	2964.621	3659.552	5869.095	4650.362	3120.421	2723.599	3585.343	4386.939	3761.597	2181.831	5380.314	3198.702	2166.383	3525.171	2414.443	3760.716
A2RUW1	7093.979	8175.456	10496.82	7318.543	3536.441	6880.646	8101.218	10758.55	21906.32	9020.619	36303.8	16513.68	9600.726	9912.034	9443.653	15279.47
A4KWA5;A4KWA6	6368.625	5753.219	6154.302	9242.325	10012.46	9175.779	5582.427	6551.714	10638.54	8133.956	6005.537	5191.39	7565.572	9087.402	7147.057	9140.103
B0BNA5	78073.74	69025.75	56987.84	55640.7	105946.6	70993.93	65543.72	83059.46	180141.4	133975.3	213407.8	145187.8	141158.3	151197.4	129451.2	87351.98
B0BND0	21178.4	28367.38	28627.2	24475.73	29212.05	18674.85	22791.62	29048.03	50558.27	21934.59	32362.51	29873.55	26369.38	31869.91	29127	37322.87
B0BNE5	41713.88	40672.64	25788.65	34237.91	68228.14	42262.48	34846.03	45824.05	49349.89	32375.34	97410.92	61170.32	56261.9	77356.52	49005.84	35232.35
B0BNN3	47182.57	30822.72	40755.96	75922.8	9666.452	9536.402	32572.26	35264.51	7941.877	10611.28	20619.15	7112.283	38511.46	34868.95	13144.93	27082.09
B0LT89	11268.87	3041.778	3017.893	5099.187	5796.908	6799.42	4223.267	8531.273	32973.49	9673.813	19833.84	10905.22	13540.71	8653.619	8132.586	6763.917
B1H234	44589	35315.21	15579.1	52406.43	29224.68	90746.63	29942.72	12586.84	12655.6	10066.48	21460.42	7529.69	17064.44	15146.62	12098.33	13405.34
B2RYW9	3290.897	5438.727	14455.1	8518.077	7620.694	7109.127	4894.804	6036.168	16636.04	9950.7	9767.934	13563.33	10490.05	11639.45	2831.989	3489.34
B5DEN9	8814.863	4645.824	8305.754	11544.3	8043.484	7044.059	7608.218	5910.881	14315.98	13358.9	10528.48	9722.336	8096.372	5610.007	3756.495	6893.036
B5DFC9	452260	774483.4	468714.1	563410.9	668093.6	958093.3	598780	819367.4	605715.4	821986.5	325503.5	574591.5	625076.9	506497.9	382342.2	472877.5
D3Z8L7	24975.06	19946.44	10718.06	34642.28	15355.79	24047.05	25293.88	23300.7	29978.65	21144.92	38262.27	17372.82	21459.77	31931.06	21122.1	19478.04
D3ZHA0	48224.84	59728.97	46751.52	90731.93	51091.65	49719.93	72763.3	88110.75	25559.13	21884.47	14914.24	22860.91	26061.21	33725.49	17057.87	31383.89
D3ZTD8	16736.49	26151.42	21472.59	27550.22	22667.62	17539.52	24086.21	20354.49	50356.79	25270.12	17397.99	18825.68	23552.59	28137.03	26348.9	12953.85
D3ZTE0	86859.09	142026.7	147198.4	221199.2	130993.8	144995.3	166958.7	183249.6	177836.1	180503.1	141822.9	85267.68	123155.9	116488.7	167477	185259.4
D3ZTV3	17916.63	13582.36	12870.67	15514.25	15530.22	11273.74	21617.62	18435.07	10426.72	20185.9	12161.29	11060.11	9391.606	11384.3	10729.8	17247.13
D3ZTX0	7247.252	28772.42	8026.898	8279.075	16320.59	23065.54	11267.17	9167.845	13875.45	15376.83	4755.367	8154.854	9281.967	4316.985	7350.737	7823.297
D3ZUC6	277648	491903.7	54330.86	260631.9	42150.28	252328.2	107208.9	79873.08	402414.4	249497.6	205611.1	165212.1	195107.6	59017.19	168117.1	461959.4
D3ZUK3	32411.98	70145.43	44105.5	50723.33	42744.2	40340.48	59758.69	51489.43	16660.47	50156.15	30592.06	27157.53	26113.86	31752.25	41448.52	38810.21
D3ZW55	5149.304	7634.2	5717.624	6899.54	4225.671	8811.155	6242.303	8464.86	5384.54	4969.938	3503.046	7090.962	7287.974	4157.664	4296.322	5564.522
D4A1J4	18078.52	16884.32	22363.45	23572.04	35726.38	14587.6	16601.09	18192.54	26935.08	26303.21	47647.29	30923.57	37776.05	41986.64	34791.98	30448.77
D4A1R8	50824.81	72951.88	50119.79	53481.91	56723.16	44982.36	49855.86	51797.06	122924.9	48505	63603.06	92200.76	64080.11	59555.46	67987.93	37420.04
D4A5U3	12281.59	26517.73	15150.76	25076.96	10935.58	12438.9	19417.06	46658.42	9646.978	5414.751	7178.457	4982.992	9988.503	8533.885	3065.045	7795.702
D4AE59	4558.505	4576.222	3146.757	3545.75	3660.509	4860.633	3980.867	2332.662	4800.035	3645.855	8003.456	6329.06	3590.696	4256.587	3432.037	4878.299
E9PT87;P20689	8245.892	5572.765	9652.221	5903.691	5156.484	7203.293	5445.682	9789.456	21390.41	10041.47	16028.5	9500.338	12965.11	11614.24	10281.57	5150.742
F1LM93	22842.81	20179.63	13865.24	24424.92	17725.46	27365.47	26261.97	19895.91	19634.34	16001.2	56230.94	25152.43	23038.85	13759.06	17178.93	23929.36
F1M3L7	21620.87	17051.92	9661.711	8180.292	35337.9	9587.537	4981.521	15187.51	12273.32	7542.05	14739.99	14659.15	12564.94	11669.03	10360.68	9760.049
G3V686	290375.6	194803.3	105185.7	350147.8	68790.07	117228.8	121432.9	161468.5	140956	355103.2	133216.7	137636.1	136065.3	107139.3	146070.8	156564.8
G3V7W1	136790.6	171374.6	172329.2	183289.6	201251.5	119960.2	168146.6	178069.8	207141.6	129268.2	196814.3	154390.2	116191.7	108589.1	194780.1	134476.1
H1UBN0;Q5BJS7	87477.83	86796.36	104085	69727.26	103280.9	99118.91	62314.2	71066.42	148747.5	65235.12	149252.9	134927.1	106364.8	91141.45	96246.12	82441.04
iRT-Kit_WR_fusion	314310.8	219963.1	76710.41	47478.96	83609.05	125164.9	265528.2	310262.4	114410.8	56747.88	101973.7	153493.3	72172.77	53891.78	93717.84	87978.55
O08557	22382.25	10820.95	25519.07	24151.14	18841.77	18800.34	9852.977	36877.13	77213.26	34674.74	45902.23	60805.32	55780.95	54147.85	36008.62	50904.01
O08628	113868.5	153561.5	98282.7	67930.91	98879.98	169191	138911.6	126401.5	117281.7	156409.2	62513.73	85777.94	74394.78	83715.46	104235	131433.6
O08651	8111.181	6883.398	11190.18	8502.909	10383.5	12658.01	8487.616	8587.119	7413.055	8212.823	20036.76	15437.37	7986.864	14485.41	9143.103	6083.496
O08815	14260.35	5889.616	3505.464	11418.74	5084.695	8610.202	9651.856	9859.953	9315.535	6252.914	32254.78	13379.59	9475.342	6051.189	9779.232	13541.12
O08839	1942.485	6448.475	3715.946	4053.86	5342.862	6290.107	4392.148	2707.011	6268.832	6112.383	3904.284	5542.93	5200.146	3973.906	4261.654	4959.004
O09175	3325.211	6024.681	6000.085	6797.917	9341.714	8647.943	6745.165	7711.937	9728.804	9259.661	17441.8	7967.54	6280.862	9367.184	6068.764	5979.688
O35077	3601.948	6991.7	2116.656	4077.52	2817.17	4886.502	2991.724	1581.269	10368.17	2628.905	7255.621	10664.31	4476.163	4578.863	3565.985	1351.131
O35112	76746.63	100250	84150.78	97536.98	125854.8	147341.3	101608	91102.81	83490.12	89088.7	52262.84	83401.49	63000.9	90244.91	65593.21	74423.55
O35142	35594.11	51945.11	39941.19	47105.62	54838.07	35143.39	56761.81	55872.33	20957.1	34844.98	24943.27	25543.84	23751.68	24572.2	26132.17	43162.28
O35217	474965.2	635319.5	492452.1	641442.7	409175.6	453141.6	497149.2	450181.9	443954	499498.7	539023.4	422270.9	572054.4	371643.3	418337.5	456222.7
O35244	152605.1	106969.6	149533.8	146547.8	100880.3	154354.2	116700.9	138644.4	340594.7	207722.4	339722	262765.8	311600.8	317908.9	251315.1	249198.9
O35264	21982.8	11027.84	13846.62	21400.03	18442.36	21797.7	12937.8	32557.05	34356.25	25949.15	30618.92	25710.12	35472.6	36287.71	27551.7	18041.7

O35276	13201.7	20088.93	7323.63	21271.14	17865.17	16886.1	13147.4	13812.55	15649.34	22110.87	13343.25	14424.86	15778.27	15839.1	13876.34	14051.52
O35331	16238.48	13892.69	20036.73	14520.5	18948.48	17211.57	14258.24	12586.62	20969.66	23119.54	26308.96	24506.21	24900.87	23796.94	16782.71	22834.79
O35509	102098	74060.81	60944.32	92294.84	79304.98	83671.87	66273.54	73233.51	148286.1	48236.21	342757.9	108696.2	119181.1	93589.54	81052.87	82279.7
O35547	43964.2	14400.21	9183.27	24283.96	14107.75	25852.61	19860.12	21482.89	4394.158	1211.177	87474.78	16123.36	12306.57	6145.291	4387.396	24732.93
O35568	838045.4	1126460	1204937	1482628	1461789	2246605	1377105	1443124	1461343	1525160	767373.5	1168860	1175163	1134645	871903.9	1010635
O35760	34579.34	36327.87	53134.52	64032.93	21253.4	37597.78	56332.07	72623.09	31139.48	94564.89	23497.12	38986.43	45774.36	46420.6	33194.91	39130.78
O35763	211369.2	216994.6	129046.3	251343	308904.9	267713.9	171002	151738.3	480334.8	220080.6	852153.1	494532.8	524478.5	617574	400389.4	405693.4
O35952	47327.48	47363.41	45687.64	45643.39	61139.55	28962.3	27987.43	23639.52	66441.12	38316.01	91538.7	52716.63	61515.37	60978.25	46179.08	63833.26
O35956	10501.02	14439.48	19074.86	6108.15	17829.1	9646.932	7339.626	10875.88	22517.38	12178.4	25089.28	20148.75	4472.235	17806.99	15965.01	11751.59
O54715	167400.2	162588	76984.88	72493.25	105267.9	136152.3	117791.9	145290.2	160334.4	127753.2	61595.04	115290.1	91588.15	66745.29	55295.36	60119.17
O54728	1546470	30737.06	3452.724	8093268	4662.995	18070.9	11445.46	2611471	12461.98	10000.39	1934124	13317.95	2623100	4108686	10558.71	2830185
O54800;Q5DWV2	392386.2	478836.5	367176.5	153631.3	96029.99	447831	182608.3	148685	239755.9	395330.8	123699.8	227933.1	357390.5	361761.3	152031.6	281851.1
O54858	63041.32	110710.1	13662.63	79522.91	92228.05	158255.6	87016.55	282207.9	110001.6	106004.3	64470.23	75943	80892.27	80750.65	88182.55	64118.1
O54861	23378.14	21916.95	96367.41	47940.32	102808.1	51725.65	106187.8	47140.59	152406.3	96342.88	43365.73	83640.77	52577.49	68155.34	71143.09	70758.24
O54975	14763.94	10307.4	20176.93	15805.34	16259.16	12208.27	10583.57	17640.46	29012.58	14493.33	29499.4	23304.21	20134.61	26668.2	17472.56	12079.62
O55004	333482.4	564227.6	277077	1023559	189966.9	469845.5	414426.5	589124.5	135787.7	383156.9	119236.9	150871.6	262719.7	142611.3	198230.2	296708.8
O55006	579527.3	873939.6	558659.7	738207.1	726542.8	666684	618326.3	949109.3	1337025	1454274	729659.7	1454083	796046.1	809590.3	995011.3	635207.3
O55096	14187.57	15930.3	14363.92	8767.832	10208.78	16289.59	14086.83	15765.96	14181.6	16833.72	17326.51	16872.86	17033.63	16058.27	12293.76	17337.81
O55145	9238.83	18049.36	11565.3	30585.76	9613.256	22997.63	8225.387	7927.396	18389.57	14736.89	14391.45	17044.83	13066.67	9332.273	12606.68	5270.691
O70215	13165.72	21762.48	18158.84	19941.12	23456.64	22228.94	20115.54	25099.22	26967.66	29945.15	14784.91	18769.61	13282.67	10724.96	18244.01	15856.95
O70244	144362.3	183948.4	212371.8	237905.1	296875.9	152253	240017.1	203375.2	133606.2	161326.3	176920	177078.9	172773.3	175213.1	174145.2	186067.7
O70257	3577.351	3772.753	3138.135	1806.325	4511.451	2248.116	2329.898	506.8445	9508.106	2955.656	8284.4	7927.531	3790.902	6613.026	5738.055	4067.754
O70352	29895.8	34010.41	26212.79	33765.94	27604.93	30225.74	30674.48	34548.15	32396.33	21094.66	61284.43	44696.93	45005.23	22507.7	23612.34	34428.04
O70377	8878.562	8499.397	3144.473	11253.97	16763.36	11906.05	9662.743	5815.531	25199.33	7614.571	43603.2	23052.44	20005.17	19899.43	13966.97	14847.58
O70417	726470.9	326482	2445750	1903038	257046.5	1246295	1599236	2470080	1028943	1066372	510782.4	1040309	189591.6	1543743	259208.9	529713.1
O70489	392419	449295.6	402812.8	361187.3	456560.9	384960.8	463068.3	397948.4	337805	417482.5	327670.4	399755.8	337775.7	334568.2	383316.4	433317.5
O70513	706572.4	733108.8	640625.2	1038093	969815.4	792271.1	1185553	828129.1	1026798	901280.8	539823.3	925891.3	1458903	720154.5	793709.8	763398.6
O70535	171782.8	213533.9	207030.7	196319.3	245597.1	166573.4	198073.3	193583	203076.4	246245.3	112491.4	180636.5	172845	162237.2	168891	156181.1
O70540	12258.9	15093.5	20709.71	13687.02	14504.15	29861.18	7919.516	18585.77	19436.49	22584.14	15009.68	10293.9	15419.74	7195.796	12614.21	9231.341
O70594	23985	17964.56	13361.6	25549.51	22087.56	20554.18	14213.52	17682.3	69156.16	23330.71	100501.4	94458.09	57781.14	73939.77	48470.42	49754.34
O88204	18178.37	27552.71	28475.86	23299.86	22055.28	34950.27	19845.07	24909.99	31288.96	30486.78	18957.58	26482.46	20469.1	20712.67	24967	22859.75
O88267	18081.74	17959.54	29991.3	8671.573	13344.49	15064.11	19196.54	14039.26	37678.13	14983.95	54469.88	23097.5	34902.46	51962.13	23664.98	20436.1
O88339;Q4V882	2564.241	1416.406	1567.346	2745.288	2813.804	2104.472	2400.669	1913.722	5350.658	1496.547	8125.587	7263.616	4542.685	4610.155	2397.315	4741.37
O88600	5956.096	6718.449	4440.219	7130.717	6236.824	5134.75	6252.144	3851.051	291201.7	10104.44	18519.43	12872.49	13265.41	17904.48	8848.262	7940.597
O88766	260129.1	408011.3	196692.6	574460.3	242513.8	364971.9	225922.3	318049.9	178479.5	401435.3	272886.7	268533.4	290786.1	231776.3	202740.4	210234.1
O88767	247487.9	181022.1	129767.9	158594	118601	257351.8	172224	141186.6	118494.1	151194.8	197326.7	145429.5	141127.5	152191.8	128957	137089.1
O88775	14082.62	23302.13	22421.73	20637.56	24250.76	25649.57	18473.88	12963.52	21346.97	22053.52	15656.26	20576.53	20126.93	16528.04	18715.21	19904.97
O88797	4021.545	2298.658	5135.237	3262.938	4167.036	2124.024	826.3646	6544.43	4861.998	2320.572	13876.92	3596.569	1353.006	2324.303	2076.376	4364.925
O88917	31121.32	41083.27	62837.05	21194.63	34275.28	27676.26	33581.43	6802.843	95909.91	42722.03	16789.2	53766.19	43219.35	52104.73	52222.16	36352.3
O88989	428616.7	332056.4	381135.4	258987.1	512404.8	450238.1	396849	501083.7	511329.2	450862.2	852001.8	665929.7	558955.3	633725.9	360782	327313.7
O89117	102356.2	103020.3	72734.7	10714.89	37958.5	125622.6	56832.18	111567.8	127208.4	121409.9	146600.7	59003.41	112538.1	119660.1	7037.138	143123
P00502	329680.4	432326.6	342238	321722.9	561263	423005.8	313412.4	330966.1	667679.4	362685	1072091	643856.8	703737.2	785512.1	496769.1	468183.4
P00507	4697.74	6295.315	8681.891	6192.504	6259.701	6109.308	18332.91	6052.355	5498.761	12125.58	4352.046	10720.6	4437.786	9322.289	6568.369	9131.591
P00689	15917559	24833654	25014754	15105843	22239254	13244222	20283910	17464414	21955660	27514396	17816412	17974592	19677128	18718806	17312914	15561839
P00714	57244.08	93041.03	99946.19	491084	81584.92	261967.3	259005.2	162317.3	118227	162924.9	306751.8	145797.6	263696.4	69627.91	83661.21	286180.6
P00731	9536.903	29433.01	9416.697	7455.204	11088.28	7334.162	8509.153	11435.47	178864	5158.002	5550.708	5605.079	6617.294	5497.206	6374.376	5050.044
P00758	752212.1	8005153	7988823	6945413	9935169	6362597	6638053	6871663	5657593	5463190	3735830	5864784	4871928	5102725	6012706	876892.4
P00762	1153784	1491709	9027107	8035095	849146.1	816427.8	1377755	7616463	448636.4	901183.3	4402054	3527245	4203583	2725946	2647870	8815534
P00774	5663.003	41112.31	29933	31628.47	10073.65	10004.19	12524.91	25999.87	15230.6	4403.088	11523.82	9706.696	9492.911	1187.546	7365.431	8225.191

P00786	274288.9	262854.1	298772.1	368324.6	136325.9	244680.4	271871.8	221373.9	203252	264175.7	132169.9	185624.7	200310.9	184265.3	236054.1	254890.6
P00787	1079279	1279438	1015213	1778105	771682.1	958625.9	927515.4	1387545	505828.3	771395	709016.5	516173.4	697492.4	591653.1	592645.3	806361.6
P00884	335254	259001.1	365540	307078.4	392539.6	373962.9	307789.4	293929.5	949258.1	514991.3	839023.7	677176.1	621238.8	806467.9	489402.3	553944.7
P01015	1010999	1400526	467188	1968025	660059.8	679228.4	655834.7	1218362	369807.1	605511	546728.9	493106	833631.6	637785.8	721526.1	1219275
P01026	368395.4	243513.4	316315.4	298490.3	184893.1	312682.8	457581.1	287278.6	328544	330606.1	148297.6	231422.8	191686.9	302592.5	581820.4	476067.5
P01039	44889.35	683108.4	44046.32	96308	227963	302149	106642.8	18497.63	3568.651	17906.34	134719.4	16129.35	23807.21	29147.71	21156.63	49895.52
P01041	118571.6	162003.2	100019.5	168175.6	47762.57	134342.1	82099.08	45091.62	204282	220563.3	57626.54	163083.4	96537.3	171013.9	69522.28	117651.1
P01048	1451887	2226795	2755746	3465699	1422138	1791952	1760127	1815746	3104883	3368741	2519530	2734542	2107884	2528333	1981457	2350242
P01681	4521165	342879.8	3214822	2451824	1781376	297931.5	799220.6	2647360	1674849	488725.4	517145.7	365545.1	162649	452664.8	1123249	253885.4
P01830	228804.3	256105.1	235054.4	250536.4	131652.2	164091.6	194970.4	185311.1	129391.3	199811.6	132567.5	127746.7	157348	170112.8	148590.9	169773.9
P01835	240537	53405.49	15264826	20807488	13130864	61158.5	62184.6	21432868	17375856	13294916	17092172	10518664	71946.8	7531820	8883400	9864880
P01836	31372496	40681180	36940708	35923552	32896732	34746664	40744684	37144504	15437518	25003220	525711.3	15123225	17605606	10493264	11719807	19268634
P01946	510245	10419.95	285446.9	8566.391	61845.49	188981.9	10186.85	13566.38	7966.267	30590.18	11803.99	5567.516	11109.66	28070.48	184647.5	12842.41
P02091	375522.5	2656.641	251154.5	5089.108	26853.61	140330.4	13236.51	23217.63	13306.36	32650.13	11735.26	7222.685	7773.986	14634.76	197298.2	15141.53
P02454	137295.2	306474.6	75306.25	216777	174210.9	1055905	186204.3	460786	205635.2	297686.4	66107.06	96880.84	79574.17	45210.42	51622.45	72564.46
P02625	223628.8	274795.5	612043.9	343913.8	153309.3	291150.9	240818.2	649204.4	59492.41	339995.3	282608.7	349860.6	295266.3	220553.9	191766.9	674510.4
P02631	39789.8	85795.92	25306.59	53979.41	24481.6	100178.8	66064.38	93553.11	19780.04	55709.32	14496.56	31631.91	41574.81	14701.52	12615.93	25446.07
P02650	2476017	2427414	1332769	2290532	3078401	1810551	2522010	2402296	2983466	3296272	1640234	2042692	2255445	2722523	1815709	2481972
P02651	219649.3	104798.4	148446	107146.3	109110.3	137720.6	141975.2	152505.8	99185.98	138367.6	43028.19	62554.26	79271.7	93353.72	114018.9	150879.1
P02680	15516.32	20429.41	15768.88	20190.03	3453.635	12451.79	23036.47	20697.52	10642.53	23586.61	8772.914	11742.2	13756.71	17890.83	14675.3	12870.35
P02696	10765.41	6385.831	15888.58	10643.7	7871.475	6238.836	7911.426	5082.166	10483.28	6538.377	6244.549	5777.488	6186.47	9210.964	6230.974	6280.876
P02761	1.04E+09	1.13E+09	6.55E+08	1.58E+09	5.59E+08	6.05E+08	1.02E+09	5.34E+08	90276864	3.4E+08	4.51E+08	1.97E+08	6.05E+08	6.19E+08	4.28E+08	8.74E+08
P02764	4776683	5334554	4147064	6474199	2547571	3930331	2483201	3952927	6628034	9427169	7213125	8114967	8582181	7360724	6093078	4757822
P02767	1850545	1494942	1430032	1841914	1411066	1588200	1892146	1637253	1438278	1256504	730411.5	938698.3	996027.1	1693556	1582513	1490729
P02770	37906696	29407012	44543632	20924428	21188202	18980014	43426764	24287298	23117050	20736688	7298988	14261236	14957868	27102160	28142342	28367710
P02780	43283976	25689228	22468876	73834672	29543678	50269764	67702936	70995360	9015505	8864812	73150632	19497886	13549271	8906730	6886310	43253628
P02781	29816660	14419548	9496852	33066184	11735669	23484706	29470758	30768968	1848406	3218916	26554888	8025886	9075513	6102920	3110920	24784478
P02782	18294970	6310083	4791524	13772715	5580238	10520581	14089984	19026422	1807451	1856558	18579874	4245075	6483600	3394836	2010708	16431866
P02783	44307.59	8793.584	81753.81	23441.23	5001.551	53334.43	12358.79	12702.2	2646.398	5949.293	13486.03	5375.812	2310.334	15047.89	11889.75	37776.96
P02793;Q7TP54	11427.95	9738.619	10737.46	7247.268	16782.38	11967.24	10813.87	15328.97	5522.718	10243.17	11168.63	6703.471	8025.839	9479.041	43241.61	3090.68
P03994	7298.53	5884.325	11368.96	3062.672	10389.76	9184.494	7156.208	4302.225	14804.2	21720.4	9398.218	9331.994	11733.39	8551.622	9766.14	12931.94
P04041	25938	24315.99	21375.26	64785.42	34715.33	28405.17	40758.27	42325.16	30826.79	21849.76	34871.13	19154.01	23711.58	29921.44	26531.91	19198.55
P04073	116939.8	216064	161891	150557.2	125170.1	144235.4	142144.8	174273.8	111270.7	163401.2	73152.59	104572.9	177926	127784.5	88322.49	58509.26
P04218	52698.34	79284.06	56642.17	72320.95	82544.34	59290.15	46200.72	88089.55	88208.79	157775.3	40483.71	120352.9	95712.77	84108.69	126305.5	96724.93
P04276	1350038	732519.8	1623264	1178288	762765.3	813757.6	1780600	1114190	972206.4	1316597	438116.9	726557.3	779043.9	872679.4	1733233	1665315
P04355	50843.9	38650.31	27512.56	34866.15	44380.91	53829.28	23601.13	21689.28	25087.53	49741.5	39122.26	36085.11	38234.25	48567.13	42908.86	30787.45
P04636	22932	31463.38	39066.69	33381.95	35631.38	37650.22	37648.91	66457.66	19653.26	40185.81	17791.83	33423.75	24566.93	31986.91	14040.91	17604.21
P04639	90484.33	101216.8	119182.3	80394.2	69584.62	84739.73	110214.5	75702.23	72424.74	123456.2	26927.06	55689.66	54026.22	97915.09	86574.34	92159.24
P04642	168520	114469	174184.5	118735.7	76421.7	128460.5	151287	116478.8	121286.7	117179.5	66270.68	170345.8	134571	173789.3	124797.2	68324.09
P04762	6835.334	5486.944	14550.23	4362.769	6704.337	7005.945	8490.317	5498.875	6736.613	9179.663	13419.47	13810.71	5679.575	5152.424	4148.774	9904.438
P04764	454271.5	350564.8	395489.3	376895.8	516327.8	474478	513526.3	539820.3	543832.7	604789.7	530043	575789.9	504310.5	830974.1	527949.7	418735.8
P04785	98764.07	84495.73	71731.03	97558.24	99749.19	101627.1	96425.65	112811.7	47901.16	72182.56	82747.8	58444.92	72657.17	57217.64	61996.2	60186.07
P04797	331661.6	283040.6	207032.4	239283.3	259487	361793.9	249968.7	405083.8	578693.1	357832.5	794114.4	546958.1	539701.9	512360.8	417874.9	373326.8
P04897	72447.98	37777.18	27730.44	76225.55	58385.88	84120.55	49799.31	77140.16	95865.93	37114.3	169316.1	83927.93	88401.91	68004.26	59365.28	61551.84
P04903	20227.52	17145.65	14361.8	24276.33	23640.96	18417.76	18153.96	20633.82	32359.01	22293.98	52975.22	26248.73	37595.31	34066.24	21271.95	27013.62
P04904	690861.8	649199.7	716335	610728.3	751895.7	693860.2	555518.4	647392.1	1857891	1050569	1756963	1671522	1502093	1908214	1214010	1129200
P04905	43041.71	21961.22	13126.93	43655.62	25533.15	31543.13	36479.29	56378.35	18645.57	20661.61	113806.1	24412	15436.92	18283.08	12199.57	37186.76
P04906	51547.2	32438.17	37029.05	56414.77	66794.71	36426.82	37816.28	54233.13	90193.82	29558.85	102983.2	77305.38	114263.5	80885.68	50110.26	63520.5
P04916	46089.91	42134.83	30955.12	67756.26	25622.71	27395.16	47418.89	62032.24	24791.65	18832.23	11928.03	13876.62	30333.29	26310.72	40209.3	28854.35

P04937	6490167	6705706	8111581	8858278	10012564	9523141	7702872	7763942	9669818	8590389	7218001	6723682	9221299	11460643	6496128	8238205
P05065	82391.16	54359.17	45618.64	45734.19	35597.02	80532.63	81485.68	69956.14	53417.06	46698.78	94809.3	57164.74	53768.58	66772.74	43373.15	67705.89
P05197	31145.34	27258.54	25875.13	30386.2	23224.58	23534.51	32014.54	27481.17	41784.77	31466.27	67258.13	53302.04	43717.34	47034.04	32639.75	31337.39
P05369	15063.39	13079.44	12543.49	13044.76	6630.313	14218.65	13553.22	10599.72	5547.777	3622.717	46650.7	11531.92	13654.96	3929.808	5018.061	16032.55
P05371	811986.1	611916.6	312549.3	475120.7	278840.7	585571.5	373826.4	772017.6	214992.5	338292.6	280756.8	243876.3	294497.4	165479.4	217120.9	279985
P05539	32357.33	12023.95	14950	5396.631	19432.42	81513.48	27670.97	8274.945	24742.74	26119.27	22100.61	24426.21	26889.99	33256.13	25850.89	17518.95
P05544	38397716	59073708	40745652	35600116	54750136	26845284	50332456	48216732	9605594	35775884	24454784	27777408	29315800	38404664	28679630	30237710
P05545	2844374	4460140	5198211	2262112	3602507	2320250	3788048	4861104	5245906	5396715	2023170	3566625	3494539	3474791	3206994	5388572
P05712	11166.19	11257.95	8892.997	10508.36	12937.72	17338.25	7810.957	16225.96	16003.9	5142.048	22276.08	12587.94	11671.86	11508.52	9992.641	10650.63
P05964	153336.1	234160.3	114848.2	129597.9	61746.14	146153.8	169071.7	127101.4	44467.4	111322.5	286157.1	88284.09	95467.11	135298	85855.33	201257.4
P06214	13735.8	12251.15	17578.52	11260.31	17036.65	12091.9	10259.11	13473.03	26163.97	12354.88	23774.8	18147.74	20829.8	19209.14	14370.65	13238.41
P06399	11396.13	19554.64	7114.586	14916.27	10685.14	16338.95	15941.5	20416.02	6505.718	16558.17	5207.527	10213.82	5289.789	12964.84	8371.685	13549.85
P06685	40373.46	39512.05	32075.11	45845.09	33259.91	37584.82	38876.04	38957.06	34050.65	37827.56	49796.39	57501.5	33372.26	37372.45	43055.34	28272.67
P06760	762039.1	854591.9	291610.8	1508822	6014131	558709.9	4486735	5431289	1096889	236554.8	1244928	186855.3	220409.7	490196.3	121974.7	96143.22
P06761	152339.2	89329.04	83889.55	229748	113414.8	296968.1	417900.8	298607.6	52902.34	66131.96	161837.8	71603.34	79361.94	67333.74	49104.62	98351.31
P06866	3251309	4046398	1158261	3042665	957487.1	1900490	1319514	1480449	1765930	5081949	2327181	2384859	3503407	2429195	2289539	2761473
P06911	502933.7	218697.9	147156.4	341767.3	71234.02	481938	374189	819828.4	44416.08	22901.89	269901.2	200173.5	84800.2	128612.5	16293.12	81030.46
P07150	50004.07	67172.52	45468.96	98873.18	68392.72	83627.66	169715.6	219138.6	63520.8	48490.05	134470.3	47082.68	31189.74	60458.3	45314.04	48283.87
P07151	2198024	2532522	2052356	3547179	1309445	2774416	2190964	3746743	818798.5	2235197	1050090	1099345	1583596	1147374	1490914	6861406
P07154	241685	243405.7	257974	257706.9	217075.7	232209.3	270806.3	193038.3	160367.7	183101.8	163772.6	170827.1	193097.6	193255.9	155935.3	181552
P07171	679258.1	857771.1	496865.6	1722031	1006817	1457967	1858518	1325891	493684.5	854812.3	511068.2	626178.6	1093193	733286	634281.9	362851.5
P07174	21876.68	29650.17	97928.54	12398.14	37080.67	37856.83	55787.51	46614.37	51919.62	75652.28	29057.88	61165.54	51150.17	60882.59	47194.47	54767.39
P07314	2493113	2770579	2270072	2814251	2864006	2740064	1997597	2694689	7011824	2214312	5459877	5367493	5357570	5137860	4053774	4181730
P07335	9559.578	10337.92	12006.16	8740.351	11564.69	5664.338	12437.27	11459.19	13054.06	19705.6	10037.15	8115.262	3218.627	11041.89	7539.445	8916.197
P07340	61463.95	69697.5	38061.61	61998.27	53611.28	83406.11	242196.7	94319.95	39701.27	65801.3	54710.5	56371.42	43407.83	47371.39	52091.96	38792.49
P07379	12420.5	5736.761	26301.51	8492.093	19704.76	8749.633	10562.09	13719.83	14159.04	16553.93	16845.19	15685.99	21370.41	33231.29	12941.51	10138.19
P07483	124257	98553.9	66979.38	145899.5	149952.6	145685.9	122405.5	144621.3	109844.5	160322	66357.96	101806.7	99183.48	118506	106695.8	42194.51
P07522	28173678	31800770	25845552	29711428	38936652	32429902	27815218	27841156	19390030	26232102	23948356	25854948	23124160	18933912	12084216	19724706
P07632	5007607	5791240	8069196	8331117	6286375	5288325	5636095	10573693	2578349	3481748	3541946	2652450	3156557	4271144	4555416	3821759
P07647	16232454	8062896	4311253	17457222	5603915	14640544	12630295	9608512	1487932	2195149	12455841	3325185	4125045	2558164	1345867	15399079
P07861	1652736	828400.1	554866.9	1469804	1060177	1101193	896565.6	1613154	1886588	644340.5	3943972	1520016	1364442	1425373	1309227	1295948
P07897	548471.6	698414.8	699507.9	738374.1	899473.4	843865.5	768395.8	724529.8	893929.3	926171.3	490954.4	751092.5	857432.4	965189.2	829228.7	688459.9
P07943	47620.3	41186.17	26141.57	27323.41	41577.71	27445.92	29229.33	51570.04	59356.34	45541.49	128860.8	19995.22	31439.24	37386.9	78586.75	31993.87
P08010	32701.95	14818.67	23143.28	21413.28	23414.83	22288.23	27971.44	46564.67	10984.93	16961.28	77990.16	18436.99	14825.96	13753.56	13578.77	29747.21
P08289	204273.3	207683.9	162825.2	265936.2	177709	236297.5	166234.3	226916.1	188392.2	120354	336259.4	165349.9	196667.2	219726.5	185352.5	189699.9
P08290	21232.33	39037.73	13229.54	24639.5	23357.12	32288.58	19454.21	19980.81	10577.65	19987.45	14242.73	8482.384	22200.11	28388.41	24471.09	13848.25
P08460	353043.4	497864.8	362643.5	428245	525122.7	596852.8	400891.6	506045.8	540100.9	672653.3	356747.2	517269.4	387070.5	362825.5	272777.8	389900.9
P08494	32225.63	11863.6	7166.693	22960.35	17740.76	32196.17	16985.93	20012.3	22301.11	5573.737	20303.46	17960.13	16556.89	11745.37	10419.66	19233.82
P08592	199063.7	269463.2	275508.7	223327.1	221877.1	238587.9	170126.9	214919.7	300726.9	333651.9	115391.3	256504	177762	135404.3	188669.6	224413.6
P08644;P20171;Q0	46997.21	28207.95	39706.45	44736.57	27161.51	57364.39	48060.29	66140.27	44132.37	27031.69	141533.4	50681.62	43448.77	40714.23	40064.65	36821.75
P08649	959702.5	1726548	1091754	1508117	453981.1	4034112	631552.6	2152613	3619076	9277057	2271455	4837017	4080932	3049628	3474863	3115375
P08650	80073.87	80098.72	63445.98	57600.9	60655.23	53812.13	73993.2	65172.12	44777.13	46724.97	46981.73	41772.02	61499.28	42429.87	34255.96	50452.42
P08721	6039798	2215849	3037185	2925774	3280813	3780574	4745465	1383190	1138405	1650461	1775490	869023.8	1078372	709056.8	3103431	1500426
P08723	13453379	4550732	3425770	10309830	806137.7	9408406	13063826	15707275	1273133	1315073	20230854	1599333	1693245	872044.3	1405452	9576301
P08753	30697.45	23491.82	27040.98	27612.11	25026.88	35555.23	19823.22	69297.01	57773.62	23060	98972.56	33474.02	40488.3	22905.36	36551.29	35483.06
P08932	1507240	1779192	2051408	2246080	1180431	1435769	1696516	1358925	2678299	3806209	2419667	2423831	2427129	3155626	2897104	2494783
P08934	28803.46	20885.14	28333.06	30479.21	20606.26	22013.48	35023.79	18350.12	21763.69	25146.21	8133.646	15171.18	16485.04	14028.08	37289.22	35848.28
P08937	479845.3	371758.2	1362541	399573.4	782615.8	711403	582781.5	1554192	302773.8	350443	318598.7	223649.3	227427.5	527123.8	71949.37	117391.4
P09006	519171.5	327577.7	352292.7	467898.4	158421.9	212273.3	506999.1	404388.2	231394.5	416261.4	271502.5	323891.3	352656.7	269790	469814.9	514519.6

P09034	99262.04	68346.71	102112.8	49199.89	95645.73	64991.96	58847.61	97955.06	214774.3	140719.8	194219.5	141797.6	144234.3	136550.5	146855.3	92931.5
P09456	17269.73	12385.15	3714.292	12585.62	10120.71	15199.72	20747.38	23316.97	7822.765	3828.557	43441.43	5317.634	9470.12	6278.451	3713.837	18565.8
P09527	13768.11	10300.2	29833.59	11340.5	13737.92	18046.92	9672.364	30654.52	39057.26	8472.423	42337.09	19264.84	16376.25	14781.83	14638.24	11404.09
P09606	68386.29	97654.64	38359.46	94201.68	100687.7	88694.62	51461.18	83169.34	188579.8	104951.6	248687.8	173069.6	183849.6	146894.9	90235.23	92968.88
P09656	16041.78	10455	43314.13	12861.94	4900.129	24680.49	6515.002	6783.784	14562.91	8553.936	15130.93	12345.09	3953.821	16333.67	20120.59	913713.2
P0C0A9	32060.06	12999.31	9780.76	51227.45	8728.021	33369.75	45484.52	41298.07	4913.622	10600.03	86372.09	9145.97	9697.144	14515.91	12699.51	36108.45
P0C0K7	14731.05	27222.57	13235.07	28974.81	12631.4	27111.12	16018.38	19787.6	6930.726	20780.58	9511.949	12203.01	12384.88	13504.02	11236.57	17155.36
P0CG51;P62982;P6	696862.1	624343.6	227322.3	1064309	546786.1	528408.8	498802.1	736667.6	687229.1	304895.8	1323677	638519.1	669492.6	480988.2	322446	591219.7
P0DMW0;P0DMW1	61741.38	51874.96	36935.15	17390.47	22584.59	38346.15	42867.22	28925.31	125691.4	5945.185	70302.22	11823.58	59541.97	78723.46	47563.19	19315.77
P0DP29;P0DP30;P0	142506	143480.6	59150.19	112191.8	158943.2	169725.2	139290.6	66333.34	128979	137162.9	183116.5	131063.6	102260.6	102797.3	60087.35	77924.3
P10111	169941.4	176402.5	129207.3	171556.9	184290.1	150071.9	145637.7	167782.2	216652.5	139570.5	386577.8	229082	208022.7	217916.4	122504.2	222489.1
P10247	136083.8	237646.8	176309.7	126194	279912.6	213873.7	101465.8	226666.4	74402.97	103429.8	80709.33	81725.34	92302.46	49126.7	100014.9	51897.07
P10252	1194445	1738542	1478397	1522408	1892130	1307871	1370853	1397766	1087760	1535015	806505.4	1339997	984977.9	1053004	996381.9	751665.8
P10536	7334.337	7833.686	7036.965	9849.048	6058.896	12110.14	6259.698	8966.346	24538.83	5306.159	24530.23	14807.63	14369.21	13898.74	9773.008	14527.54
P10719	19397.73	23805.29	49642.27	16706.04	20407.03	17300.2	16065.51	47687.93	34623.75	35286.24	43420.16	45952.68	26194.93	26726.04	13189.62	18702.49
P10758	615688.6	228741.5	329731.8	948911.8	141979	172429.5	381578.8	209947.5	211715	374129	110715.7	420305.2	190112.1	66295.48	426123.9	357442.5
P10760	35535.26	38741.62	23279.45	37256.68	33096.2	39837.8	33436.27	36709.2	68375.19	32476.65	103216.2	80764.47	76520.38	79553.41	54680.25	52428.53
P10824	18694.98	22361.23	20510.59	19545.4	12137.74	26936.76	22773.23	29283.73	27974.18	13288.82	54246.72	36994.32	30916.21	34841.3	30448.35	25119.37
P10959	1648081	2280854	1913554	1873725	1381609	1285750	1821027	2163354	1082252	1456909	490884.9	663503.1	1125684	1626465	1171444	1548944
P10960	5321687	6478264	8328513	6295902	9183846	7973352	7531028	8063606	7278626	7423814	3336881	4785865	4414654	5069977	7298835	5774797
P11030	50438.57	42392.07	40526.41	75481.35	54014.8	46837.71	62015.95	52398.78	50226.95	58616.33	58431.92	48756.9	25946.81	24298.83	33504.57	49902.14
P11232	829090.2	1006616	545092.4	1130388	758950.1	1091682	712537.2	1217268	552972.1	601246.3	695738.4	616953.3	776526.5	732923.6	499744.2	536219.2
P11348	80583.84	63758.01	54954.38	90140.18	88476.86	62352.66	72976.52	59217.21	154802.8	99758.33	157163.2	113268.6	110821.6	127409.1	90792.2	82938.66
P11442	7798.829	7725.147	12788.63	15173.59	11230.66	8112.102	9351.276	11168.44	13260.88	13383.86	36721.83	23417.12	14224.94	22207.82	9291.512	13033.99
P11598	72609.14	44997.12	31853.88	139301.4	36434.36	149644.6	215468.1	61825.17	23060.95	36493.74	105180.4	35454.96	44172	36547.5	25672.64	55075.65
P11762	12258.82	12607.83	10697.06	15630.78	5235.069	17161.87	7824.685	6866.349	2114.961	5532.396	6061.321	10158.82	4583.237	6234.382	6178.69	3686.677
P11883	10499.64	22357.96	22555.91	9411.195	11762.67	6202.024	23831.02	31055.2	16197.18	10307.93	33353.65	33099.15	8967.958	36225.87	12696.93	9185.18
P11980	36124.38	46688	36613.07	40790.56	32645.5	52098.07	33414.9	52498.45	43637.66	49837.63	63306.89	59112.94	52793.3	57083	42029.21	40848.65
P12020	808303.7	841510.7	270530.8	703910.2	185979.8	1511338	787265	2574464	78598.65	122389.2	1014382	755504	278286.8	391564.9	130281.4	328826.9
P12346	10433577	6630541	11187211	6010606	4676522	6931144	11817196	8250420	9487045	10244692	2778463	5019594	5666281	10514982	14689199	11002464
P12368	25348.4	16951.51	9897.974	19136.63	13586.33	23998.15	14702.91	14632.43	23919.35	7571.275	72034.21	18113.45	27333.21	25037.5	13114.84	22172.07
P13221	52630.95	57264.93	60248.26	52474.94	67212.34	51023.73	58822.24	74990.49	69754.44	76239.49	56103.47	71609.91	67677.5	95260.94	63919.59	45164.73
P13265	75562.7	105814.3	37479.89	100772.6	107955.2	51531.15	69587.87	27900.76	54435.16	95076.23	63420.39	101253	67898.56	94773.15	113100.7	94112.23
P13432	992373.8	52008.48	33324.31	325832.5	355610.3	568377.3	243795.3	1674410	94127.05	78161.08	9834.808	60355.6	63254.56	82206.2	151847.6	111215.7
P13596	77487.03	70084.43	108153.2	98618.96	68920.88	97038.1	88871.74	154415.6	129341.4	138926.9	57268.44	93733.57	69856.06	55252.17	74436.3	58532.39
P13635	537437.6	649659.9	688119.2	427279	506237.9	388089.6	714737.7	417540.7	904608.9	765501.4	964725.4	593789.4	608727.7	1031521	875407.1	1008246
P13676	6905.299	10147.81	13879.13	8551.468	9953.397	7327.059	15323.82	18601.14	15459.58	4026.483	8016.176	7432.75	10726.33	7263.884	4063.299	8260.429
P13852	104992.4	251365.6	168944.9	260607.5	184711.4	287993.5	232242.1	287388.2	162508.5	241022.3	145003.1	99589.37	145463.1	82124.41	139294.1	195735
P14046	1173792	1775396	1712665	1130233	907290.4	1098232	2000366	977340.8	2632008	2131012	842151.1	1116753	1507056	3119235	2486030	2492234
P14173	883.213	314.6875	12924.81	8260.317	7127.444	7714.948	6261.028	11452.32	7207.546	9234.522	7842.048	9775.917	8689.044	15253.67	7464.754	8528.389
P14408	6400.274	7424.597	12287.9	8100.979	9375.904	8444.508	7853.053	9503.126	16263.86	9097.6	11687.28	12839.98	14229.21	15575.57	7337.732	8899.199
P14480	27752.21	39986.71	21706.99	35430.72	16148.22	35308.22	71030.75	60202.74	23394.28	56491.96	17594.58	28300.96	26392.88	34214.34	30075.24	26058.27
P14562	116057.7	98939.06	141647.5	165117.5	196908.9	95032.41	186061.1	335229.3	126091.7	128638.5	151014.4	107552.1	92580.24	105681.8	211102.8	197292.8
P14630	653085.4	681483.4	1247705	758872.3	845902.7	761227.6	708203.9	777950.9	476892.3	456979.7	460533.3	399573.8	465323.1	531400.6	509626.4	417254.7
P14668	24679.82	41633.87	64179.66	85965.01	71587.78	29434.15	87234.34	66022.63	95254.58	24733.96	147627.8	65224.3	45011.81	30517.01	72597.98	35055.41
P14669	152719.3	327057.2	196447.4	280207.7	461138.4	276887.3	299070.1	233207.3	400637.3	230912.3	186339.7	244531.8	445440.8	159548.1	236774.2	194248.3
P14740	1252438	1170674	1298674	1467904	1708862	1900391	1070872	1900060	3573697	1622352	2559997	2301253	2001163	2615298	2570145	1597932
P14841	3313221	5289279	3590869	4542200	4618165	4491556	4244557	5194166	2538167	5322763	3146618	2638018	2801769	3155485	2376340	3231210
P14925	134662.2	120088.7	98418.6	141316.9	124793.6	158362.1	118845.3	129383.2	58525.14	92999.14	69626.34	108840.6	113638.9	115085.4	105881.3	86926.51

P14942	45159.49	43546.59	31308.77	35894.69	56784.84	31624.55	30190.3	53488.12	111589.9	36546.27	75715.52	59043.25	51198.92	68176.46	44282.13	39186.07
P15083	3131190	2571899	2297531	2998153	2136238	3009840	4731373	5862630	1817251	2482739	3434745	2563199	2504798	2413824	1991739	2502050
P15087	7200.545	9595.519	8554.615	8188.408	8062.35	5816.416	6397.319	7651.034	9591.678	10316.01	9080.622	7565.79	10622.02	5634.475	5889.6	8344.049
P15390	199996.4	229040.5	245495.5	294718.9	230315.3	245500.1	264714.5	142332	134926.8	208765.7	152991.8	204174.7	192425.4	173503.4	211636.4	226004.7
P15399	45268848	16031368	7674598	13923194	2781524	18614548	3480781	27314234	1439226	10856434	6395794	1594319	4298318	4979099	6985139	9617074
P15473	88833.56	136320.9	87485.93	131922	129318.9	153961.6	87262.4	78486.59	121722.1	190242.1	77058.28	112505.3	104983	108537.3	137619.6	97475.05
P15684	2532870	2588687	1863789	2523265	2952536	3143319	2204046	2772303	5249896	2189624	3956771	4585912	4219105	4146520	3199018	3108299
P15800	8814.884	11291.7	8331.5	8450.953	9015.984	13689.09	11231.51	71976.38	11758.46	8988.319	7264.062	5183.448	6616.041	4466.819	5438.761	9147.729
P15943	68735.16	88897.07	61673.88	112202.6	80597.95	114444	94299.56	90364.45	72870.63	120547.3	44577.8	84582.35	88061.95	73943	88041.1	79899.52
P15978	999416.3	989418.5	542338.1	995842.3	1069994	1197047	1184187	396143.6	1200435	246794.5	508297.8	472622.5	718329.6	578464.9	665263.9	745393.3
P15999	8725.081	6212.787	24303.97	8601.602	10717.65	9358.882	6599.77	7498.983	10379.62	26596.37	17942.56	13494.35	15070.1	12150.82	5289.991	11803.88
P16086	8145.422	6655.739	4630.514	6916.241	5498.67	6957.654	75731.63	11812.3	9638.537	6661.979	8715.91	8620.86	7364.591	7257.74	5693.959	21313.27
P16228	42523.46	60623.88	31577.86	56236.7	31395.96	48509.18	60501.54	63315.11	36161.09	23985.63	21441.61	28253.37	36858.94	29203.67	24366.5	20357.25
P16290	64529.64	34902.01	34919.26	24435.7	17337.87	52558	47506.46	49828.86	57844.07	46097.41	44196.56	62429.83	48568.77	40657.28	77811.9	22332.79
P16310	42699.2	60742.47	100952.5	55094	68548.38	51313.6	54719.44	56866.8	89218.44	77538.08	29625.92	50036.53	46039.04	45013.81	39204.15	59153.56
P16391	477165.1	633972.5	790232.1	611729.8	626379.1	569616.6	640279.2	371793.3	835332.8	305991.5	425836.6	250555.2	833218.3	553176.6	573734.3	620329.4
P16446	7688.755	7509.968	6889.609	3896.628	10067.37	7544.788	6780.081	7775.618	13221.68	8554.959	17097.03	11822.46	13880.78	11088.91	11514.57	10025.67
P16573	1598977	2019402	1055335	3447788	3424678	3260485	1503020	5862140	2838431	1842544	1327414	1962342	1491273	1902396	2005656	2369698
P16617	28929.15	34737.84	37912.01	27205.51	33531.57	42824.93	28641.22	30964.87	41425.88	44646.82	49269.18	49405.88	52158.62	57115.08	31426.37	32668.59
P16636	17772.17	37437.45	11705.28	19608.14	46061.36	102147.9	18257.21	16974.46	268863.7	61166.38	21717.49	66436.55	34529.07	54010.41	32868.07	18741.57
P17046	50488.05	51377.43	104665.6	45133.15	97294.11	86139.34	65126.84	184454	116485.3	115365.2	52133.61	77289.86	91243.52	70328.3	143972.9	89658.81
P17164	597679.9	874238.1	837356.3	700970.8	654846.6	444207.6	502331.3	650867.5	676397	552551.9	401331.9	695511.8	544127.4	714129.6	635415.9	686866.6
P17475	7199641	6398165	4665910	5556928	2880936	4004945	6891013	5567379	6292039	6859089	2298924	3562262	4434772	7850305	6540985	7042562
P17559	46131.54	210180.9	78528.42	124350.1	87844.72	536273.4	51801.7	152202.1	164708.5	152560.1	26820.19	63677.98	77226.13	55428.22	40123.31	35910.93
P17988	3798.248	3752.873	6113.284	3062.041	4281.396	3379.991	3335.475	10184.34	14101.62	7018.095	7393.951	10413.76	16035.41	10612.28	1952.563	3373.555
P18292	3845749	5564064	13621469	5145133	4092251	5554874	5782226	4454204	6510780	8146235	3612355	6195886	5331292	5823286	5603300	5187393
P18297	5322.632	4743.66	3894.447	4896.822	8448.445	5946.458	3229.54	31617	13352.73	4313.449	16929.19	9882.839	9636.541	9690.897	8580.402	5047.317
P18298	8881.92	5923.836	5043.644	9522.792	8686.229	9022.683	9687.945	14205	15547.81	6372.342	22234.59	13030.4	14646.61	11872.09	8763.378	8610.172
P18418	145663.8	69596.05	28402.52	184107.5	83474.91	119958.7	81995.01	110263.2	38896.11	55101.47	39495.95	29398.24	36808.24	28961.74	27757.61	46819.14
P18421	8610.395	6795.735	5348.838	5682.061	6831.626	10054.08	10056.95	14025.87	10400.1	8107.581	12176.83	3902.886	4815.132	3184.262	4037.861	4781.808
P18427	16539.64	34128.31	16263.91	21904.16	13022.26	30813.16	24688.34	22614.62	8334.139	18953.29	11592.11	8265.842	10835.6	12774.16	11010.51	19421.31
P18757	35963.92	44600.96	38117.38	47951.59	57243.39	42315.69	28321.15	41176.85	143710.9	50716.56	116806.8	125088.8	133472.7	118371.8	107814.3	90795.38
P19112	39294.13	46378.14	58345.95	39789.52	59737.37	37400.69	31409.63	36289.83	131677.7	55825.26	119472	114859.7	79314.17	97681.84	64344.3	60394.57
P19132	18106.31	18215.71	9158.504	7988.114	23722.6	10264.3	12185.63	25570.35	11317.54	9606.321	2792.773	10589.37	7020.303	7914.862	27899.66	4071.894
P19218	50161.74	191644.4	160740.7	134729.9	45998.31	59122.66	119515.6	66961.77	253348.4	198272.2	68283.44	114463.6	151029.3	35235.02	163858	165013.3
P19223	18042.91	28907.7	9935.458	50860.02	10207.31	11778.78	15060.17	14512.61	4043.248	5851.246	2727.739	1097.438	6859.123	4681.775	2135.965	5736.046
P19468	280763.3	311584.5	255706	353143	543626.1	440091.4	204135.3	401099.8	1495236	285500.3	1298861	848630.9	645140.3	828925.6	510207.6	392878.8
P19629	103092	40049.3	21481.56	24787.14	8702.332	80491.02	47549.43	57828.52	7403.505	27725.22	11079.02	16003.83	5992.674	3851.099	29546.49	20796.52
P19804	173984.8	100843.4	120973.6	104154.5	192128.6	139160.5	167888.3	200469.8	185294.4	174241.4	205729.7	162721.5	153415.2	226780.1	151109.3	130514.4
P19814	585.7106	1245.879	3220.135	2745.288	837.8235	1721.405	1870.386	2170.544	3504.286	2420.591	7748.832	1022.462	2566.193	2928.229	2248.686	4015.897
P19939	214188.8	248786.5	104066.2	261920	216259	117762.1	155114.7	106200.2	324357.7	184754.6	301025.3	213134.9	142253.8	116345.2	66264.91	198566.4
P20059	10258569	11614993	11164358	16486486	8110312	11130343	10107279	15884482	10983231	13527524	8241101	9663293	11237236	12705905	11020061	12693801
P20611	1667443	2388295	2226405	2092859	3012585	2256913	2426164	2058999	1956626	2589567	1649877	2378273	2377433	2449471	2577757	2424657
P20646	10288.83	7999.762	8827.133	24103.99	8381.194	4400.288	26762.24	16427.84	8674.63	5673.844	4605.561	2197.609	3503.288	4909.007	6847.799	6431.417
P20673	11025.84	5434.69	12549.08	3990.726	6846.841	7344.095	5678.616	6324.654	22551.94	14266.61	11743.87	17406.33	13699.81	14949.06	12717.73	9685.828
P20759	66041.09	245968.3	166825.9	286168.4	485228.6	144129.6	160878.9	169594.7	282578	166954.1	114164.8	113965.6	128040.1	230672.1	152953.9	201841.1
P20760	527449.9	617741.3	448259.2	1538188	1210398	658850.2	525249.7	1569424	538750	374210.5	188863.3	211735.1	255033.1	423671.3	237560.6	485655.1
P20761	123961.2	292327.4	317705.5	728309.2	151565.8	253101.8	310348.6	422029.2	64190.74	92680.72	56712.43	50443.24	89267.88	104187.7	93277.04	149076.2
P20762	730792.8	2505572	768001.8	2074482	1089887	1002102	1166563	2289905	406592.5	966175.8	398708.2	406255.7	343011.2	803924.8	820063.7	927862.6

P20766	18791.96	112123.2	61602.13	18045.69	19934.05	35645.43	86929.52	38314.52	32329.41	144823.2	31145.77	25106.95	21923.58	8378.222	28637.36	34542.3
P20767	14755905	13531451	12977212	40559360	14343109	16281158	13173159	27486456	22903512	27982044	19674668	25244332	8988231	14677165	19983714	13685501
P20786	225398.4	220413.3	204943.2	175933.7	224852	197043.3	211371.2	206748.3	162641.7	351720.7	122366.4	184101.8	164564.3	93626.44	176362.1	132406.3
P20961	28835.31	33041.29	7773.934	27647.79	32925.16	22157.87	39395.14	23058.54	39544.5	37246.43	20615.5	36963.74	28112.44	31392.05	39295.22	38677.2
P21581	53335.61	62290.99	78297.02	74697.76	73310.52	67456.64	62815.5	79476.42	55226.04	57126.25	32347.01	65755.2	59469.12	73249.44	47944.24	40859.59
P21588	6007.263	6374.271	10275.89	2926.067	3385.111	3361.059	9432.22	17244.66	14137.52	14947.89	2062.445	22832.07	14437.72	14556.57	13654.46	189339.1
P21670	11071.3	10582.23	4869.292	9182.447	9796.516	11713	13212.51	20431.89	5075.835	10033.26	8501.442	3416.014	8228.999	13178.81	5514.747	6411.018
P21674	1639.214	8925.461	7995.831	7438.26	8987.258	4917.552	2602.888	2432.014	2045.465	3934.989	4272.961	1632.738	8619.436	3186.606	10607.29	2231.47
P21704	25180212	29998370	28656648	23303782	25891496	21207456	24483670	26441934	13283524	25226556	12984202	25992828	16124590	13068026	19379932	12362729
P21708;P27704;P63	4773.709	4662.88	2023.23	3260.702	5173.992	5234.239	4568.993	3432.49	10573.4	5005.46	7141.423	5938.323	6944.617	4039.177	4973.364	2784.742
P22006	199956.1	81191.48	103378.3	264561.9	32210.9	464961.6	139112.7	82543.21	180323.8	48040.55	96342.83	44273.74	61881.67	65333.7	61019.98	713517.2
P22057	4294373	3820218	4747513	4603079	1945250	5340232	3216355	4348578	1351012	2455707	2485292	2086643	2977213	2105723	2667294	2509969
P22273	15950.65	185253.1	172875.8	1970634	396816.5	1255662	1392529	2255907	59102.89	141269.3	1898634	195678.9	239254.9	56015.94	13116.48	255203.3
P22282	27314188	9267078	9925492	66206936	17135424	45272340	49175296	89012448	4805341	7377161	47678740	12732267	13502011	5140367	4582339	17308206
P22283	50834872	11873985	12605979	44566912	15131644	57181004	68849568	41248688	3790389	7800001	62132196	13522218	17301506	6389613	5756600	29756452
P22734	142505.7	94488.4	58610.52	62552.94	85944.68	83796.22	103078.6	44476.26	170871.8	163794.2	198546.3	129969.3	87374.43	207241.9	133699.9	168656.7
P22985	6031.385	5786.802	12616.16	7832.673	8317.35	5846.315	6188.568	11214.6	4739.691	6227.906	7665.034	4472.994	6819.198	7982.163	6042.085	4111.499
P23377	164116	170634.6	116056.9	144235.4	147983.5	146849.9	188844.7	166690.3	106090.5	131585.1	92397.49	114584.1	138590.5	156020.8	76879.03	147075.4
P23593	15744.94	16480.44	14173	9801.637	20996.5	14476.11	23610.76	12861	5253.201	27368.44	55583.08	46907.66	20613.52	16086.86	16973.84	9975.234
P23680	2337685	2628423	2514054	2070862	3040112	2971775	2473508	2274328	4083892	2619347	1825107	2559585	2267683	2275827	3212309	2085143
P23739	37990.19	15736.88	16381.16	24209.91	68114.07	11980.84	16032.15	27220.53	18352.57	24944.33	45499.21	44091.2	65787.25	49669.72	45604.69	31995.9
P23764	305782.4	355885.1	419390.9	362765	295709.3	176861.6	288553.3	296845.6	191123.4	322487.7	177720.8	181695.2	169790.1	181116.4	384127.6	266759.7
P23785	730579.4	735064.2	497829.5	425561.4	1177159	621495.1	461836.1	588965.4	979260.7	802905.5	367611.1	688000.5	537372.2	546984.1	561417.3	311037.7
P23928	2887.542	9142.174	12227.36	11677.05	6494.64	6101.347	6889.265	11082.24	20670.63	9438.51	29430	25458.46	23949.65	20488.41	19093.68	16768.38
P24090	46655300	62100636	46700452	81878464	59095704	69491040	61599268	47578160	72748992	89508632	34359900	52740164	57442936	55832772	52455316	64163216
P24268	1042849	892315.4	870719.8	1542183	1269480	930987.5	2092299	1530920	555643.8	785843.4	618926.6	632902.9	426946.4	636676.7	538117	588720.9
P24368	76893.48	44999.14	36384.92	65655.1	20644.23	60200.82	81936.66	88012.03	27076.11	38785.21	128881.5	33491.71	55232.06	19330.47	28416.27	89687.4
P24594	107049.5	128890.8	109176.7	112021.6	105527.9	156166.3	99919.95	132495.4	80792.8	150534.2	79470.96	85796.88	114311.5	103366.7	192010.3	134380.7
P25031	65364.56	64057.49	46386.88	60576.6	120333.8	146807.4	51272.78	152437.2	50231.85	143793.1	20317.74	48023.55	39459.94	34090.25	25798.74	53684.23
P25093	81488.6	88497.31	87090.48	105657.3	126896	67580.42	62184.8	113441.3	206328.6	89592.02	248355.1	176894.1	149553.8	177228.5	147327	127818.7
P25113	95075.73	54528.29	66720.84	53251.36	83599.59	73046.77	77367.32	94804.5	125513.8	92809.23	176821.8	103057.8	102920.8	109111.5	99568.55	83726.16
P25236	19764.05	16395.31	21889.35	23042.64	16361.11	13101.1	38201.73	24883.42	19023.37	23938.78	11801.25	13198.18	14825.54	10821.66	22911.16	22394.89
P25809	9717.846	61716.62	11098.71	12988.88	15614.93	12063.23	19717.22	40899.55	5861.107	14936.18	6873.568	6682.195	19002.99	12810.39	5594.704	5003.113
P26051	1708503	2158399	3225120	1890852	2673205	2408032	1914658	2794485	2694424	2832297	1663991	2267726	2434694	2648634	1927432	1781231
P26342	5862.907	5558.777	12748.67	9882.906	12534.67	10670.73	9646.813	25659.24	9847.371	12371.49	951.1843	11597.63	8737.615	8542.418	10817.71	9372.189
P26453	44545.76	75465.83	102301.9	65583.82	81480.88	66410.65	80518.85	91726.26	72462.75	83014.71	54550.18	63569.02	55500.39	57421.44	54794.48	43561.45
P26644	1252062	1092458	1442684	1867698	1049747	1130738	1497750	1492117	1717914	1662434	685265.8	895318.6	1219481	1100598	1440611	1624563
P26772	10044.01	14337.83	32713.89	22559.06	17314.5	22678.52	21477.03	25315.77	15831.54	12633.74	9754.271	15294.05	6283.055	9455.674	5201.699	11048.29
P27139	207636.5	113621.8	133229.5	149711.7	188901.6	226478.1	245911.1	271633.2	186334.9	236423.5	423989.5	211001.6	288378.5	308864.3	190384	166411.8
P27274	4420909	5267736	4125321	6134550	3671971	5060431	4361261	8300742	3534594	4130892	2693587	3408113	4706353	3291549	3549138	3990129
P27590	34550032	22322208	29072612	41981740	39653744	14364630	29610396	56684064	24414592	23637356	37061688	24500652	14989292	13892220	11321710	8772180
P27605	19533.39	12389.36	8119.572	10282.9	14416.48	12154.95	11682.49	10485.55	14101.05	14094.45	18422.03	18345.17	10395.61	19655.18	20383.03	10414.11
P27653	9507.258	12169.22	7326.652	6215.798	9424.394	15386.48	10529.23	9478.194	27852.75	16712.87	19594.87	16448.09	13008.27	26924.3	10536.07	12501.53
P27867	84435.55	54689.98	50563.43	46917.76	100930.7	71505.61	75489.96	67486.9	88705.36	78042.5	118417.5	108930.8	92866.85	144214.9	93725.35	93269.87
P28037	10819.89	10622.63	10665.06	10708.19	21153.19	14707.1	10230.29	14709.36	29014.08	12922.35	21872.23	17472.57	13811.01	34618.27	13741.94	4567.188
P28075	3690.834	4848.581	3558.678	4272.838	2902.282	7428.467	3656.978	3995.052	3315.402	977.04	1894.63	4097.89	3464.135	2985.625	3417.332	3814.7
P28480	4754.156	2803.929	2465.118	3545.75	2749.07	4586.495	3656.978	2691.861	7374.585	1789.851	5689.514	5809.16	4534.678	4728.076	3284.629	2941.957
P28494	57688.64	66837.52	110971.7	47898.34	61776.91	32728.2	44502.3	57415.07	62020.36	68957.91	28886.21	50028.36	48137.44	32759.55	68793.78	41243.35
P28570	10216.78	6415.769	6578.154	3820.971	10950.54	7875.305	6441.991	7235.869	37707.8	6630.926	11687.14	15177.31	8476.51	14788.72	13495.93	5380.829

P28648	140398.7	129812.6	179437.3	160819.4	182812.3	82266.27	124313.4	176511.7	88754.7	87162.83	137615.5	120059.4	93723.74	44450.88	107727.3	48533.59
P28826	901913.4	896317.9	1085088	1205535	998120.1	1265255	636704.9	838413.1	3129248	823235.5	1678213	1586147	1730422	1548626	1372376	1103550
P29288	33015.76	26938.4	24018.89	39520.98	29662.21	23499.58	32580.41	18518.3	18195.3	27890.13	19267.57	19956.96	17909.52	20437.03	20008.06	15176.38
P29315	17625.78	39520.73	261078	141516.4	20876.68	17621.74	12724.86	19772	37688.17	28895.77	54408.34	29873.38	31032.13	31736.07	77247.05	197970
P29534	86079	122173.1	75643.9	107946.6	87337.18	108316	103416.1	128503.6	74646.59	117284.6	62794.22	77082.08	72978.16	82390.87	77036.23	78860.13
P29598	1153747	1216040	1065772	1315430	1527070	1330816	1295114	1284313	1070167	1638768	851948.2	1294377	1398245	1253549	1451585	1473374
P29975	380486.1	410074.4	142923	542031	680292.4	423917.3	221030.3	516772.4	1187761	384105.8	1129680	1149616	849547	935734.1	704149.9	502312.4
P30120	108729.7	58926.77	33235.88	83850.94	36457.52	73899.09	96538.19	209012.7	23520.88	28673.8	157948	49862.23	80544.56	38866.77	22750.21	169868.4
P30121	46921.63	76039.72	55051.8	44442.86	85493.13	61464.34	55072.05	52063.41	49614.7	71093.88	35193.74	47142.88	43828.6	56365.95	53059.22	41648.34
P30152	1617824	2032800	1725913	1616017	1301006	3336149	1950378	2254719	2438509	2544927	2459537	2508898	1947356	1636819	1335230	959694.4
P30713	28436.33	38547.82	18140.67	40303.99	29119.3	30335.6	32889.49	57216.45	94376.84	34772.73	89274.53	56299.57	56490.66	42546.04	59876.34	38211.85
P30836	14878.56	23596.78	10661.89	28096.36	16915.58	6606.657	15507.33	13765.63	15515.94	20559.12	7486.072	16112.5	19327.27	14469.72	18429.42	18875.82
P30904	591825.1	503124.1	427552.4	412510	610269	575286.3	394728.1	269198	1968782	523102.1	1325128	959782.6	1174606	1488493	988466.1	1469662
P30919	47222.96	43122.4	22702.93	29171.16	25612.1	20951.3	173214.8	50318.83	53977.29	42951.94	19298.57	45871.84	24262.92	470565.4	47743.65	376538.6
P31044	1772565	927831.4	531493.3	585604.4	676445.6	1775727	1402447	1280315	277648	712326.4	645356.8	564106	543115.6	512357.4	468933.3	401186.5
P31211	871380.4	1192546	1753035	712044.9	1930398	854201.1	1267265	664828.7	1968420	2137602	356981.6	1103656	1325066	2123542	1283535	1332289
P31430	18368.3	24844.21	8920.807	20289.38	12330.94	20679.62	14562.36	18341.18	5530.03	6970.297	14147.08	8780.157	5249.369	5477.816	4122.82	2446.111
P31977	1868877	1257213	704110	1533760	1661482	1575053	1392474	1616449	2263319	1170874	4856646	2664177	2639316	2982757	1682602	2216939
P32038	879222.1	1336767	680441.4	1391677	966583.3	1170328	1020734	1234689	1215804	1213372	696073.8	808011.4	1016970	716346.6	649070.1	833791.3
P32755	35881.83	24229.62	37982.17	20356.88	49973.41	24775.34	28600.45	20733.76	52363.32	38608.06	37073.64	44697.1	33831.15	48536.73	36126.98	22057.91
P33436	9044.677	9698.015	9775.782	9210.922	6303.309	10487.32	4925.354	4945.374	4937.368	11599.51	7548.006	8502.476	4310.616	5751.733	1581.207	12483.88
P34058	32612.04	17405.89	23583.18	28352.33	27234.67	32633.51	25811.29	29857.9	75303.98	24806.57	82371.52	32551.84	53063.29	43184.48	45805.39	34499.48
P34080	48701.84	43999.15	61475.49	89225.75	24187.45	87288	43721.32	42056.15	73938.51	105093.3	121715.9	66420.13	36626.06	62052.12	72258.22	41765.48
P34158	840440.4	126881.2	268254.6	185423.3	538131.3	956002.9	624263.9	11558715	14006.76	656232.6	159423.7	84163.95	347717.1	186797.6	578954.6	6014.494
P34900	86029.71	132978.8	75250.04	92603.88	91621.64	83256.38	100193	105739.8	76423.23	99230.25	86182.02	83668.09	11148.8	13320.52	112428.5	84241.16
P34901	93980.85	146872.4	133547.2	89869.48	119551.8	26478.21	64822.2	110399	89382.75	116896.7	41209.2	140661.2	20456.87	48259.62	144439.2	34737.48
P35053	21076.14	17058.91	20433.82	29147.47	27901.15	19241.71	28709.23	15467.58	4061.704	4975.145	26201.7	27057.35	15234.8	10529.15	16334.66	12156.86
P35213	144137	100379.3	57227.3	120047.7	101423.8	144208.1	139359.4	109229.1	137766.3	68978.9	304475.6	135561.8	97791.25	122521.6	88830.8	108369.5
P35280	12104.25	78093.69	2754797	5218.782	3136.38	29154.74	3031529	2323566	3228134	6541.214	1275846	5325.877	3241.761	25994.53	1897521	2984.129
P35444	66920.28	80619.88	47733.94	53106.74	66586.52	84263.72	53976.38	49140.08	53393.52	91422.9	44509.61	59496.9	66578.9	68266.35	61255.5	61080
P35446	6839.095	6072.222	2836.184	5659.489	5391.818	8615.803	7760.753	6061.237	4580.79	7822.059	7571.825	7300.986	4611.907	5929.764	3936.296	4370.726
P35704	32863.1	32917.9	38873.38	51133.39	43711.94	38174.84	47107.57	51036.63	37376.93	33704.25	114777.8	49670.51	35266.95	51244.73	30417.48	29435.41
P35745	2062.662	13213.58	26521.29	15847.53	6807.916	7589.998	3913.439	3029.533	3797.299	5599.733	11187.19	5657.652	9529.739	9544.168	4194.405	10348.38
P35859	40675.73	41326.95	47438.17	39185.47	35916.19	42881.34	37027.15	52910.86	74725.16	34399.64	21523.53	32506.28	26333.06	48577.15	51496.98	19731.13
P35952	40674.55	25358.4	12198.83	27459.67	26090.36	42516.77	21824.98	64161.24	12077.4	22427.92	18237.18	14552.42	18986.82	14183.13	15474.07	14750.97
P36373	46160412	36496512	18755636	63002112	40881436	55311236	52331600	74543752	33490086	31118752	44926864	35746156	29242304	23771838	30720190	40002060
P36374	3590852	825372.4	330791.6	4747786	1223785	2302472	2845830	3226157	362274.2	383006	7986766	1773488	920068.3	439950.3	872239.5	2051596
P36375	68259.59	87571.13	52694.02	63045.15	159863.3	72568.98	42603.73	118185.9	44976.29	47497.6	21971.47	51952.78	45935.63	58337.21	49491.64	40739.88
P36376	740348.8	1982893	2229708	1006105	1446078	845712.3	322830.8	2390605	1919953	647238.9	697875.4	1555110	447432.3	526135.7	2009476	333092.2
P36860	2274.356	10958.71	24877.24	10932.97	2147.534	2702.295	10829.19	22699.97	26692.15	14935.39	35814.6	26995.68	21001.44	51595.93	24730.71	45868.31
P36953	1203270	1504801	1463970	922929.7	634333.1	576634.4	1178895	643536.1	1457420	1625423	942622.1	1121425	1204605	1630888	1345397	1613636
P36970	48141.43	18481.01	26907.55	38988.91	19702.23	25231.05	27648.65	38455.64	89978.67	31995.66	96581.05	46180.23	53290.25	51431.1	69356.47	28111.13
P36972	24235.8	23764.23	20091.6	6630.254	15586.69	13373.27	22838.66	30849.91	38636.86	24183.16	34440.47	25673.07	17086.38	33862.16	26578.54	13514.92
P37996	12696.99	14650.61	13505.72	13270.61	14806.72	19415.69	9151.429	8813.253	33946.37	17241.11	41980.61	27068.91	19074.93	11052.91	12764.67	21115.48
P38438	102239.8	178483.7	140254.9	204530.5	159266.4	189372	150606.7	362056.3	85030.14	137387.3	53333.35	98390.47	74714.34	95005.16	93695.05	95119.73
P38444	4871.71	9602.226	7987.35	7333.817	8143.296	8443.779	5745.648	4244.042	8288.865	10006.27	4822.673	7224.871	8327.542	5494.071	6737.14	6584.212
P38652	12076.46	9806.316	11035.11	9171.152	12305.95	9841.021	14649.74	8672.513	29894.87	14072.63	23835.74	24906.97	22823.16	26631.9	16207.03	11673.31
P38659	42171.93	89631.36	53894.01	83546.16	52247.95	72439.27	69752.73	54710.87	64266.68	63076.83	71477.95	53544.77	56045.5	50523.16	51451.71	52441.72
P38918	83827.05	76704.37	64446.12	129465.2	121491.7	73121.34	71877.86	89065.63	265396.5	107759.2	337872.2	213502	245956.6	256719.6	145128.1	168166.3

P39069	32201.05	22401.85	127535.4	18930.51	16182.19	35861.69	17141.34	14014.78	21826.1	35459.6	26557.99	16488.31	28615.12	30581.31	17116.02	16363.28
P40241	99864.59	50893.86	42687.79	94414.38	44680.14	65068.75	85018.97	94284.83	22171.12	23145.33	135615.3	45664.1	41443.92	19251.27	25124.55	45958.77
P41498	37096.38	31884.99	34662.7	36370.46	34774.27	54205.61	32198.58	48081.51	69890.09	32057.76	89858.6	57098.7	69144.7	68284.2	54848.4	52048.27
P41562	99905.76	83022.61	97346.55	61625.65	142809.8	85713.46	100785.7	81171.41	139301.8	182343.4	170435.2	178714.5	180448.8	258706.6	199282	96070.66
P41740	9578.208	13465.3	16889.04	8585.544	13676.76	2578.827	6618.264	9777.302	17101.57	10433.11	11906.87	12456.28	6722.533	17998.19	9146.83	8232.688
P42123	345145.2	230398.3	324159.9	227003.8	364100.7	298953.6	306059.1	329217.3	493628	357796.6	290196.4	436687.4	381910.7	524006.2	383577.3	157807.9
P42854	6491893	9627841	7888272	11007495	6736645	14116303	5209432	6386550	8514738	16993210	3308484	10200136	10993575	7804610	4361314	13357078
P43303	335079.2	464709	409277.8	550764.2	541673.2	394674.7	602700.8	570800.8	429545.3	412911.9	254454.3	355867.3	270934.1	347643.6	318807.7	306541.6
P43427	30040.02	28258.35	26134.15	38632.93	26235.25	34205.4	20249.47	39203.84	81039.01	36249.76	57194.48	69950.45	80703.52	61560.51	45134.69	35155.29
P45479	54449.38	62001.03	91822.32	62605.75	63476	45744.59	64860.59	50439.01	36963.42	67638.98	40582.64	43201.91	47127.8	37502.44	30356.79	52156.11
P45592	19420.02	17971.43	11040.79	15614.65	18500.58	19754.55	11832.94	14467.48	37541.78	18738.36	63385.83	30025.22	31363.89	17515.46	17005.77	28009.54
P46413	140960.6	137088.8	89784.56	130077.8	160457.5	206630.5	123162.4	158464.2	287811.3	100780.9	547631.3	217525.9	278283.2	245955	189774.8	146082.8
P46462	67126.51	39711.75	30705.31	80291.38	36954.36	95549.41	88940.28	116183.7	24286.79	20221.34	175644.7	42394.98	43690.84	37429.88	22581.21	61185.2
P46720	112753.8	111209.2	38219.3	128602.2	154137.9	141252	75877.88	99284.3	325949.8	122056.4	296208.1	250013.8	223979.2	243060.6	194278.2	129670.8
P46844	7625.535	10768.31	4522.167	10040.51	17134.76	11666.85	7480.315	8888.46	48881.23	14469.01	62349.38	30493.38	27760.9	33020.33	29179.31	15032.49
P46953	46071.81	56017.56	38790.29	51321.41	72204.65	38789.11	64352.38	46378.24	74185.01	36371.33	127120.4	64938.73	82644.63	74224.52	66014.53	68479.1
P47727	37608.64	27740.87	7993.056	26671.17	8393.09	27116.73	36505.6	67757.88	16074.15	35387.14	303728.1	60493.75	29459.72	41883.39	29619.9	64982.86
P47820	114179.3	114426.6	136185.6	106139.1	151862.5	138104.6	142535	110988.3	116668.9	83239.27	94785.54	111249.4	88109.75	89059.16	87057.53	87934.39
P47853	281761.1	332176.6	263085.7	173009	408023	191847.8	303833.1	191452	261011.3	305921	247450	247426.6	338106.6	304754.9	183343.3	257828.2
P47967	1031159	1809954	1321.711	1784601	11290.69	2314452	4777.388	2046917	3931970	1924490	814928.8	618800	890169.6	3994915	3411034	2647175
P48032	4225.181	6586.233	6606.223	2982.983	5493.601	5111.41	2157.548	4176.529	7401.494	9308.636	2390.973	6497.466	2547.03	3790.068	7985.908	5984.287
P48037	25239.57	19418.39	16186.61	22590.88	23209.61	14677.18	22064.43	42627.84	78457.96	32306.59	48259.73	64928.71	63536.84	37649.43	59290.44	37054.98
P48199	1361876	1174285	1637973	1000849	608877.1	795451.7	1156525	1038265	1924052	1432450	898866.4	931899.3	1158792	1363462	1744864	1206925
P48284	14829.08	20603.82	15053.09	17627.74	11753.89	15731.49	18384.44	15467.35	11085.68	11775.59	16108.38	9552.783	13504.64	7924.962	13780.52	9888.417
P48500	110542.5	98168.21	117855.6	105983	126143	128927.9	162981.5	223316.3	125863.3	158338.6	162001.1	144123.1	128701.5	223695	151446.9	83810.14
P48508	113748.6	137939.5	114304.5	157884.5	161654.2	162269	114317.9	145504.9	572429.4	197791.8	722202.8	435321.5	459144.8	446641.6	321369.9	311493.7
P49002	4294.443	4599.471	5210.724	7702.545	7557.956	6215.005	3598.611	7567.717	7334.487	7977.505	8339.083	2726.773	3986.39	6554.622	4822.298	3789.353
P49134	14804.42	85857.4	31303.31	20552.89	18739.94	28227.51	15320.14	9080.391	13920.04	17801.97	14821.28	26550.92	35777.73	37642.3	23860.36	22461.72
P49744	14338.3	11266.81	7266.813	16849.78	9919.633	13121.05	12109.03	9550.403	10047.35	11527.77	7871.109	9369.716	13753.98	13441.69	12629.06	17892
P50115	14187.37	51377.51	36619.28	35265	57615.39	76515.75	136002.8	79248.84	9782.252	9932.17	15949.1	5860.642	10569.78	6546.194	8617.467	12307.24
P50116	21310.26	95067.42	22404.46	60909.47	144951.2	131884.8	212142.7	49179.98	22375.81	22349.42	20608.98	12185.78	3831.834	10108.3	5707.234	4145.992
P50123	1223430	1112722	1086470	1164498	2101081	859426.5	905499.5	1535532	1453844	811348.6	1070519	1534961	929463.3	1097166	1648836	1121182
P50137	16542.61	19430.77	23997.77	16348.92	26182.65	21195.8	33219.67	29117.68	43206.85	29274.86	31295.55	40918.83	23680.99	39085.47	20597.91	19836.61
P50280	22829.28	28993.95	10586.84	23509.8	8601.291	30594.96	20992.1	47463.16	4535.977	5373.712	22630.14	4350.346	2864.47	8386.029	11347.16	13281.9
P50398	43768.04	41942.96	27680.72	45839.18	42995.78	34417.76	36939.68	33306.88	43428.38	36250.78	75290.64	47657.82	52761.5	61715.15	41056.59	38994.56
P50399	60802.1	43723.99	37380.36	32963.36	59245.23	37979.2	44788.42	64015.56	94343.73	27774.52	91861.34	60579.07	42372.02	65965.46	45975.93	50806.55
P50430	342754.9	393720.4	393314.3	409383.6	362338.9	384107.8	296421.6	292553.3	234280.3	359985.1	261881.1	274307.6	255773.4	356896.2	370117.3	409328.3
P50503	8929.769	6452.952	6389.413	9865.293	17905.04	13751.77	7952.485	7535.398	15003.32	7502.892	27576.42	11767.27	13147.93	13277.94	9813.501	11658.18
P50609	18145.55	23815.85	16938.25	24584.14	17686.5	24165.29	18010.5	24470.16	14243.14	22741.97	19204.9	14904.68	24218.99	18358.75	32204.17	14491.46
P51635	615183.8	341155.6	505199.5	351405.5	495458.5	342857.9	496030.1	567415.9	1037581	824959	917033.9	817850.1	675501.5	1186584	534357.7	527855.9
P51647	16839.35	19054.24	30479.09	21223	21163.12	24604.24	25552.96	25708.05	22491.09	24624.15	23533.43	26577.35	21596.85	28679.3	30544.9	12301.22
P51740	16519.93	13591.63	6104.298	19510.73	7029.33	163496.2	16127.05	19090.33	12509.03	8821.024	12310.2	6517.471	14349.67	10293.64	7547.117	6883.272
P51792;P51794;P51	4636.492	8982.425	5733.07	6297.671	16347.05	6477.247	4184.522	10220.31	11525.26	3400.997	12891.9	10159.36	5251.271	8586.506	5811.798	5630.925
P51886	37830.53	60293.5	27652.53	39410.47	55365.58	41265.55	40494.66	32132.61	52498.3	79288.91	31663.94	54045.76	39197.26	51815.82	51502.76	33672.22
P51907	37646.18	26247.55	44279.88	32270.78	54685.88	37730.51	23089.81	30755.17	94148.86	19718.61	124733.9	91973.27	50755.46	79662.99	43495.71	49736.37
P52590	6040092	5710925	2444312	3188662	8901668	7821860	1971415	1824090	1022650	146551.3	701476.9	476881.6	1175107	3653831	216541.4	2150571
P52759	565875.5	452681.8	390788.7	679925	551444.8	510984.9	465265.8	797467.3	990607.5	686856.8	1199796	890720.1	701344.5	705082	639423.8	708729.6
P52796	39859.51	45225.17	30818.44	54206.18	39965.08	42949.18	43629.19	29818.79	11653.89	24485.67	22453.42	19554.49	22771.05	31872.14	29472.19	32955.83
P52847	13102.84	11835.67	11193.63	11818.63	12422.5	2552.397	11682.6	15709.19	33668.74	17494.88	25552.19	27937.84	25736.42	22366.59	26977.69	14422.31

P53369	75558.26	99321.97	151081.1	86541.94	104860.8	103025.6	87237.41	72411.3	84471.96	98205.71	70482.91	85671.16	74322.16	87365.82	75999.12	66493.86
P53790	59869.47	50233.64	29330.78	64065.68	75255.55	54439.19	40127.27	37401.25	181777.1	40152.6	203082.7	136485.8	139416	139780.3	89162.43	62812.13
P53792	25610.83	18145.38	23894.02	10778.24	26579.91	18792.71	7888.743	5955.185	28376.51	17371.76	13218.34	35332.23	20454.02	22261.88	19839.5	19363.99
P53812	11533.11	8968.102	7243.561	13874.62	10716.51	12732.67	7331.255	6882.987	16215.01	11835.99	20468.69	12982.78	10218.47	18610.08	10464.98	6595.484
P53813	119908	95368.56	56400.99	136517.8	97466.05	123568	88016.45	77476.95	35173.57	68741.55	99697.23	66805.37	97398.46	73692.74	60231.29	69616.06
P54311	59061.23	51220.56	30159.5	45698.37	36316.62	79179.78	42344.67	88341.44	69944.78	31907.91	140079.8	60241.18	65779.94	43914.21	36403.47	66085.77
P54313	239491.9	144301.1	103354.4	174931.6	190884.3	193466	162869	233962.8	245326.3	108639.1	483444.2	279083.5	236179.4	190772.6	215303.9	229350.7
P54921	3160.813	6434.007	6539.378	2619.853	6806.219	2763.888	9135.195	2840.587	24793.19	1613.669	43067.61	15227.25	19599.35	3011.005	2375.27	14940.43
P55018	8023.58	15336.35	17490.31	24636.51	17867.98	17289.14	21613.57	12269.61	23176.36	24354.83	17925.69	14180.34	19807.14	22798.12	15233.53	10270.28
P55053	15123.72	23727.36	17154.04	13696.6	12234.39	13341.46	16326.72	13785.55	13779.02	13915.52	22399.57	14395.54	14961.18	13122.28	11222.59	17416.21
P55091	25356.19	79408.07	10563.95	35578.62	15264.24	44441.27	31930.53	31773.35	8887.547	12975.94	9865.27	5311.429	6954.288	3128.455	2280.699	1638.589
P55146	58270.36	60684.12	76216.37	86495.79	54507.81	52831.67	50152.57	77191.29	69867.97	68737.02	38757.75	66253.33	68774.04	45594.34	69904.88	53866.55
P55159	19454.5	21848.63	28419.35	18390.29	12276.48	21810.95	8523.785	13445.72	20478.29	21609.76	4637.361	9994.738	15599.15	19164.74	15625.73	31430.29
P55260	33441.3	42719.54	28710.64	59537.04	38445.07	36752.27	45638.73	83115.42	54333.31	34591.43	126293.3	66683.8	59521.07	52267.52	52293.83	50360.79
P55281	8889.339	13674.63	10462.34	9760.33	14690.82	18743.71	9374.571	9057.028	19885.36	10665.57	8409.44	14753.52	8833.657	10988.09	6030.863	10913.79
P55314	5599.949	11147.9	6038.257	4061.147	657630.3	336940.4	8510.264	2873.647	4980.314	6795.516	263363.3	3788.018	3282.273	5373.849	7796.335	8401.27
P57097	48274.21	62413.23	58395.45	54119.76	66152.84	49267.14	47685.29	46884.21	50761.41	50861.03	42463.39	41080.23	52627.9	65655.25	44837.04	30532.18
P57113	2095.518	5542.324	4009.999	5710.014	5638.108	1715.934	649.1592	4273.851	18037.37	2412.57	12518.26	8922.033	8119.069	7125.079	7824.975	4987.58
P59647	39142.57	35259.21	43064.88	36955.65	40053.64	52792.47	46153.13	45157.69	65861.6	77612.51	42518.53	46426.36	64568.69	16733.02	33886.18	48848.54
P60711;P63259	1361207	1394741	681882.3	1523924	1524137	1234619	1139639	1395172	3378018	1312344	4356966	2746226	2856009	2543997	2285033	1851425
P60901	6955.236	7120.992	6602.671	6243.799	5586.099	4910.7	6558.307	7890.931	10127.41	3307.97	6780.92	8334.093	10056.12	7296.498	2733.642	3952.36
P60905	21547.18	7483.346	4969.347	18365.71	9022.697	13369.93	10580.9	20854.08	10987.07	5484.976	45651.07	10093.18	10965.67	7170.399	7842.456	13744.01
P61107	14040.07	13789.21	15057.15	11593	8472.633	16860.64	8949.666	14116.42	6556.51	11525.64	15378.2	8378.729	10979.09	12523.66	7361.818	15178.23
P61206;P84079	34256.87	27857.49	23778.26	38983.21	36104.27	52383.93	20915.61	33314.49	53923.61	10056.92	98083.88	40479.7	27078.46	27047.35	18904.45	19173.29
P61459	104941.4	93692.72	87499.11	17105.84	133224.5	75079.19	80783.8	78105.34	114575	99174.26	82442.16	88154.59	70097.89	119295.2	75110.6	56362.57
P61589	43129.47	23428.74	25904.22	36346.96	37338.32	47225.6	34630.81	68976.66	39983.1	25353.42	151003.1	57735.94	36515.85	40946.37	36814.98	53784.39
P61943	16000.34	14453.46	12952.42	16903.01	14463.24	18366.2	16182.75	21214.98	25719.46	23910.64	12756.16	24546.25	15711.64	16918.36	11821.82	14902.07
P61972	249279.5	333023.4	340972.8	387615.2	357093.6	304689.9	177872	448189.5	688932.8	447985.6	207187.2	457689.2	309662.7	406480.5	390881.8	390406.7
P61983	29919.34	35600.18	10948.32	35263.26	34756.1	49839.78	38195	55076.07	52254.87	26327.78	48798.24	43225.74	37415.58	50308.86	34957.67	23815.07
P62260	74958.84	67582.38	52340.13	84680.48	73549.83	56271.52	75009.17	85044.77	80325.71	74161.33	202582.4	87367.35	78019.27	85986.14	68553.48	69544.39
P62630	201664.3	201665.8	156131.5	201147.6	255205.8	267993.5	203299.6	265128.4	607066.3	211476.1	668492.2	447950.8	395930.7	436793.8	320832.6	272792.8
P62749	29996.35	26057.97	18620.01	39575.65	43289.53	36448.95	24828.57	46816.22	99510.73	32448.96	72433.73	38717.59	40180.7	49360.98	43822.65	33800.57
P62775	6089.899	11091.37	12971.36	9840.344	9982.859	8710.743	7801.752	11226.35	28513.02	9316.951	6109.452	12500.06	12994.88	12532.09	12592.95	14689.63
P62804	17866.02	119722	29777.81	39600.66	38282.86	36694.3	109817.6	94353.36	47270.92	70134.77	125762.6	105268.8	42280.15	82827.34	17949.15	106189.7
P62815	20230.24	16592.61	36820.56	14352.8	19460.88	18568.94	17900.69	24889.18	39795.58	34659.06	32508.81	44280.44	37925.93	42142.15	26787.84	29308.38
P62828;Q8K586	13593.24	13179.32	14121.54	11548.03	14627.76	13030.48	14136.12	9820.609	19006.27	13637.93	25601.73	17498.72	17636.58	16787.61	9905.699	14531.39
P62836	36945.92	32979.23	27702.83	54591.51	32596.62	45637.72	40634.39	69065.5	44882.46	31905.51	181275.8	39884.48	46924.53	43861.21	30814.13	56964.29
P62898	17960	41662.86	65805.28	62829.54	46440.91	67048.89	87761.1	81149.15	39631.54	44800.13	28536.48	47080.26	34473.65	46547.77	31093.24	37389.31
P62959	60105.24	45585.46	50180.91	37690.59	127685	52859.11	46796.95	79277.74	80373.25	30965.83	127182.7	54544.64	51081.91	49683.33	46132.16	59704.52
P62963	73647.96	52049.25	47217.11	54039.95	37539.09	43497.59	61905.23	67235.19	106212.6	58210.36	120723.2	74165.36	88676.81	92615.76	65474.34	85792.01
P63018	350380.6	267480.2	261700.3	299461.2	330208.8	294502.4	348377.3	391319.7	424890.8	286797.7	772385.6	455197.3	446929.3	473833.8	321138.8	370288.6
P63029	31833.34	42436.61	30573.03	7945.103	19746.8	36910.31	16340.16	25633.94	14161.6	17527.25	44190.55	12941.68	16647.27	18965.15	8353.563	10770.55
P63081	17616.53	19836.24	15367.47	20074.06	19085.57	2049.845	11549.9	17352.63	31963.96	22378.92	66433.34	41604.41	21296.04	19539.5	33009.36	22657.24
P63095	115097.7	83558.72	69728.22	130308.6	119036.1	144153.2	83750.26	238436.3	271930.9	61400.8	354395.9	180266.6	114370.7	117840.4	98047.77	100735.7
P63102	175900.3	134371.8	128570.8	116623.7	148865.8	124379.9	130169.5	163155.2	119255.4	109929.4	323327.1	200061.7	180742.4	198817.7	144369.4	149994.7
P63322	8767.484	6956.545	3250.379	9130.752	5755.774	7698.818	10198.2	7275.406	9934.985	3535.001	26578.14	11585.81	7283.595	6255.421	4613.426	11890.59
P68035;P68136	2075501	2106299	1308116	2063729	2721587	1883873	1976881	2040810	5264873	2131122	8624289	4361131	3847117	4194291	3111997	2794971
P68255	23679.48	16926.75	23644.37	24411.83	15402.21	17260.08	15905.29	13131.41	40457.17	21355.95	26415.34	43234.27	28283.99	28875.24	21677.86	16177.99
P68370	70267.19	45843.88	19377.57	32438.08	33941.34	67808.7	47238.01	62007.1	43652.21	32443.6	49630.07	47058.27	34815.09	40134.8	20665.15	29140.53

P68511	2693.06	5613.602	8315.558	7708.573	4780.663	5435.749	6017.315	4658.555	6939.611	7545.819	14737.16	4749.496	6333.852	7705.821	4634.78	4303.978
P69897	54819.43	35789.98	21007.39	27017.35	22769.67	58211.59	47098.27	49410.33	40905.88	27472.46	44956.9	34457.37	26530.04	22139.23	18631.97	21704.55
P70470	21845.06	18545.23	15287.07	27231.93	15337.31	25259.41	23093.21	30200.9	15791.41	15904.27	34911.31	24888.87	16393.06	39060.29	19320.33	19583.86
P70490	117456.6	100985.5	97003.2	100843.3	78966.19	102968.1	129883.4	136182.7	99664.94	98009.63	81583.93	106296.6	82842.66	53907.27	90197.3	51363.54
P70502	128466.7	133358.1	20937.28	184102.8	108725.3	86429.49	87975.19	69717.4	124217.2	78532.43	97225.22	133441.5	124995.4	117364.1	77353.4	75266.22
P70545	33362.63	26221.08	36659.6	29753.97	28827.37	31527.96	26942.58	20561.56	13148.68	16519.41	42903.49	51679.76	80458.13	74787.95	84300.41	38890.29
P70549	1631.996	22407.67	6910.908	12783.79	18023.75	3065.455	7678.419	7122.958	12300.08	9557.574	20544.65	6683.016	289.5305	8474.301	4549.247	11817.91
P70619	34117.63	15853.7	39380.3	19719.17	12241.75	26967.03	23982.6	55592.82	53903.98	50581.46	41321.75	60221.29	52096.27	73487.06	47448.79	52371.53
P70709	32190.91	53051.66	59765.72	53121.64	40573.2	39326.89	84771.8	51795.86	40915.11	26414.11	65856.84	67148.44	15551.05	15065.74	13859.98	18276.26
P70712	1484.779	4536.971	3558.678	4530.984	3772.071	1821.299	3043.549	1636.497	2126.316	4005.847	3857.935	2849.889	2349.187	3517.929	1848.426	4143.419
P80020	87335.89	153728.7	57548.1	607701.9	36043.79	80472.61	33743.6	51840.25	22460.1	28942.39	25129.52	10742.32	34842.35	20731.28	22408.31	70900.81
P80067	567179.3	916665.6	830697.1	804324.2	786326.5	825613.8	660966.9	789176.4	670446.2	612407.4	298019.7	512068.1	390551.2	748864.3	721511.9	525351.9
P80201	58654.2	159463.8	113614.2	75211.16	67884.38	96992.73	50103.19	133204.9	123573	143980.7	41116.29	108173.4	87530.6	55866.29	115853.9	70198.38
P80202	234732	350641.3	305610.2	148686.3	188665.7	329062.1	196268.7	463603.3	380015.7	298732	146697.9	257315.2	200311.3	120356.4	212517.7	311252.5
P80204	47058.4	69073.75	73506.66	53079.08	35750.25	67977.13	36286.06	63269.38	57527.89	96183.69	35433.25	67191.8	57247.88	67648.7	66486.66	61299.37
P80254	105020.3	73209.97	116094.6	119953	105433.1	79921.2	55902.64	147616	144278.9	64286.93	88730.44	168753.5	98548.96	142702.5	109928.8	105490
P80299	493939	887702.3	224455.4	752332.4	146962	444706.4	686763.6	5039.603	72725.96	187979.6	8987.923	84476.83	254838.5	14212.45	20015.34	4107.433
P81556	13811.99	11193.6	9479.148	12704.9	7765.229	15225.49	18817.28	7795.833	9009.027	2450.949	53544.57	7460.432	7363.952	7403.366	8239.798	13461.4
P81827	1.08E+08	1.27E+08	1.33E+08	1.26E+08	95881192	1.43E+08	1.61E+08	1.53E+08	1.86E+08	1.7E+08	85260312	1.74E+08	1.19E+08	62254400	1.63E+08	1.81E+08
P81828	1.44E+08	1.52E+08	1.26E+08	1.31E+08	1.53E+08	1.02E+08	1.19E+08	1.36E+08	95644728	1.18E+08	96724688	1.48E+08	1.09E+08	91808440	1.02E+08	1.63E+08
P82252	26281.81	24809.28	24385.85	25213.16	30738.12	34274.78	23734.28	27799.9	58501.86	17502.19	61517.47	52180.24	41056.93	46666.86	35084.13	24695.05
P82450	163126.3	236489.8	197705.5	261156.5	240714	124206	164083.8	268208.4	191595.8	318277.2	120826.7	308195.6	222753.6	273448.4	282518.2	274463
P82471	5594.516	16302.23	9066.799	83825.35	14692.49	10042.31	146656.5	58021.79	19655.71	5331.688	22663.15	13600.86	15719.79	10568.34	7336.154	9721.623
P82995	39420.83	46840.03	73837.67	36167.32	37148.96	53945.97	35039.01	40720.78	33266.05	27479.44	89633.36	38903.74	28929.86	30108.17	136867.9	41627.56
P83121	67528832	71422264	80330096	62695536	60804640	69429056	56496572	62730440	5698826	57879444	38817124	45690584	42482828	39050300	48996976	66976496
P84039	21352.29	41152.46	27392.43	29881.52	20596.76	37912.29	37968.55	68038.46	38480.41	54149.86	18551.01	34365.92	35506.13	29152.81	44752.57	57843.36
P85968	16645.7	16967.01	13457.66	16659.22	17187.65	15958.16	14446.64	18175.57	27508.32	18554.41	45023.8	29890.64	27625.45	37267.95	22282.96	21188.5
P85971	961244.2	1393672	2103569	1079802	763416.9	1264577	843322.1	1273811	1675354	1030957	628031.9	1126689	736055.6	914590.7	791705.7	885685.6
P85973	25980.5	30362.69	17654.03	39059.99	36271.57	34165.79	39390.67	35814.2	34355.27	28278.76	60281.34	39199.84	33767.94	51680.29	29881.4	28170.49
P97523	19255.12	15717.3	10112.5	20349.32	12745.38	19555.88	21151.54	34771.71	17423.88	12394.36	68846.87	16477.49	13360.36	12633.47	12327.32	24626.32
P97532	16711.49	9171.118	23885.4	17080.88	8005.416	25038.61	12494.75	15140.62	4623.57	15922.76	10559.72	8799.141	11489.62	11016.69	6895.701	12130.87
P97546	753976.8	1147450	986102.9	1096135	1345264	1049369	933376.8	1160306	888486.6	1188381	541856.4	946005.8	890746.1	731954.8	863547.6	838402
P97553	84814.23	64147.64	71456.39	20955.48	53816.05	45278.42	55428.27	74897.05	80748.02	70020.98	44354.98	65395.23	45503.55	39016.2	47555.29	36329.04
P97574	119655.2	152701.9	84238.45	193542.4	89790.33	113495.8	174205.8	83863.75	89972	67588.64	59943.77	68197.5	74910.2	70768.36	88900.47	57602.58
P97580	6317562	3521654	2089922	2306476	336668.8	2145402	475837.8	4355145	56520.07	1320933	184219.8	44554.73	619291.7	1221181	449319.6	1089808
P97584	7922.899	10636.85	7834.111	8714.035	7453.425	8304.484	15043.9	19677.01	18195.55	13316.08	43074.55	13931.22	10341.98	17770.12	11363.66	11043.58
P97603	67600.76	82908.7	70664.17	108289.7	59596.9	93082.08	80511.34	95735.71	92549.3	90015.34	75620.27	69705.17	65591.3	77526.98	74029.91	68393.81
P97605	70247.25	104449.5	101416.8	18984.82	99116.7	107821.6	83238.38	102285.8	41921.84	117318.1	32451.53	18368.27	62717.39	44388.18	72993.77	88848.45
P97608	3918.186	7865.31	6530.202	7075.42	8074.612	9151.911	3418.832	4066.27	10130.5	5250.222	12718.48	10188.79	15773.15	7467.365	6876.842	1809.288
P97615	6481.814	13245.92	11918.86	6134.068	2764.582	6226.903	5829.081	6947.05	7730.119	6546.941	3241.919	7164.438	4100.271	5665.742	3174.79	5947.225
P97675	123822.4	188858.2	108311.4	128108.9	100484.4	97798.69	104144.5	168359.7	107205.2	80900.76	199013.3	123576.2	153556.3	111455.4	92191.49	111772
P97697	25433.63	29463.94	12300.49	46038.43	42996.74	19067.23	33373.95	35588.88	34688.81	14649.21	27895.83	16036.34	30904.6	14630.37	10351.64	19327.03
P97710	38769.11	40880.77	20891.56	54115.42	61123.38	46476.5	33767.82	43233.36	29615.42	36614.91	35913.39	30563.74	29456.7	36436.12	38335.24	18596.29
P97829	69040.21	217992.9	72567.92	93999.09	120033.4	113434.9	99424.74	68274.48	47444.41	94773.79	61284.2	56356.05	65999.39	66216.32	62118.93	59071.77
P97840	130381.1	390538.2	35034.77	34636.76	44711.89	356877.7	22847.58	332149.5	519676.8	238997.8	181515.3	91332.52	181351.1	529913.3	343696.7	284904.9
P98089	19166.14	158723.7	13752.58	79239.82	15037.69	50681.55	43623.25	74460.34	2263.645	3142.149	3697.92	16221.95	7416.181	4146.482	6195.788	2437.678
P98158	1080423	1304589	1197544	1348882	1436792	1178783	1336734	1230503	1075545	1510482	947649.9	1730698	1412063	1357064	1336007	1339396
P98166	13251.49	11009.85	14107.76	12492.08	5825.262	15061.28	10550.35	20709.5	9463.482	9019.424	4783.257	9474.04	7375.764	3601.529	6573.498	7988.222
Q00238	259464	284484.1	262348.5	276335.7	344478.6	346269.1	256263	239718.6	287826.2	299556.8	170378.8	243201.8	247239	229725.2	244230.4	206501.7

Q00495	41214.73	64128.82	81617.34	43111.63	44947.17	61982.84	40274.53	96383.84	66572.53	116172.8	35537.82	97934.13	94882.91	98835.27	93671.2	62961.26
Q00657	205750.9	200273	171884.5	296999.5	583192.8	183981.2	144426.5	169359.3	247964.9	222263.6	119700.6	276073.3	415261.9	235529.9	184250.2	114382.5
Q00715	52210.59	226306.2	59430.63	61753.51	67139.91	74712.87	112223.5	109017.3	20057.88	62168.12	166053.7	91447.07	38058.13	60266.7	23769.16	114810.1
Q00918	11313.87	10503.85	6275.547	13064.21	9515.629	16825.88	6867.098	8826.435	8478.078	12598.69	103283.3	7567.413	7749.607	7878.562	7614.715	9959.939
Q01177	2409367	3387514	2475801	4327182	2268317	2540378	2975477	2041057	3284154	3656347	1154798	2720339	3346738	2691850	2040974	4369523
Q01205	336390.5	607276.6	389601.7	1021819	515579.6	482802.3	531104.6	917869.9	854422.4	741398.9	671730.3	715003.6	474081.7	346018.5	723331.5	824249.6
Q01460	1257549	1607373	1226025	1410278	1663235	1118148	1001927	1248672	1128327	1199907	742995.1	994728.7	1059094	1180116	942682.8	844528.2
Q01984	8880.681	7297.33	8040.617	14842.13	8997.039	8952.565	11561.19	10505.98	17100.21	5155.478	23788.09	20901.91	14565.58	17211.63	11604.8	10821.42
Q02445	20095.49	23455.63	18398.13	14753.96	14687.78	15312.59	18029.29	16065.96	13072.02	18642.5	21645.08	14123.89	14733.71	14465.63	9021.756	12219.6
Q02765	83583.44	126915.2	117697.8	112853.7	42931.77	62471.61	77174.49	117532.6	78566.4	124291.6	38606.78	79404.46	71503.61	63745.28	71112.8	93259.62
Q02974	80893.24	60838.46	150575	79902.86	91288.34	70602.32	76148.57	88533.93	147069	74454.39	201265.3	102402.1	172093.2	180828.6	113704.8	128355.1
Q03191	416320.4	259877.3	61528.78	240769	474053.8	155183.7	196313.7	80144.09	873800.8	182700.5	283593.2	552380.6	173522.9	133837.9	78298.52	169917.9
Q03248	8899.327	11203.84	10204.5	7841.914	12747.13	7271.789	5887.112	10632.77	45027.86	8743.458	27789.21	17120.15	20568.54	19001.55	13365.94	10146.78
Q03336	166197.3	210379.3	159966.5	190205.4	313538.5	241325.6	181875.7	192800.8	244322.9	129277.4	466888.6	298957.8	353063.6	413445.9	209498.5	226461.3
Q03626	244605.3	427957.1	361937.2	294584.1	329764.9	322019.2	468596.2	303704.1	491400.6	393076.1	191278.6	246521.3	310374.4	584313.3	395160.1	448550.4
Q04589	44547.62	80656.22	33029.67	82229.35	51345.1	31368.35	53527.56	71901.39	43446.86	87811.29	28104.16	74243.95	114301.3	52703.99	51914.5	91580.73
Q04807	2166780	2631433	1716558	3879797	1945049	2149545	2722324	2379786	3528687	1803159	1753542	3771329	2356576	1136174	2241089	2480311
Q05030	21892.22	38106.8	50647.13	30965.05	48842.87	30212.62	36061.22	29516.15	32566.81	36842.91	19114.49	34350.83	26635.51	29319.75	29883.1	17115.11
Q05175	177230.4	160773.3	89528.44	211099.2	206402.6	174360.7	92318.71	68756.81	385375.2	113743.9	678949.5	352051.6	368360.5	327812.4	197185.7	244903.2
Q05511	16636.06	6819	21093.22	9305.461	13625.84	5353.254	10014.13	14168.13	14103.82	10126.56	11042.45	9370.994	8048.286	11543.84	14448.61	9376.162
Q05695	97532.16	137444.4	136020.1	182556.9	115149.7	156921.2	189511.5	188113	80135.63	130911.8	101366.8	83607.51	83853.9	124378.4	87613.53	68696.06
Q05702	8338.973	8925.715	13119.43	11331.7	7097.762	14598.27	4995.476	14668.92	5274.367	4708.094	3674.97	5113.15	3386.023	2982.079	3829.904	4585.588
Q05820	944916.9	862623.4	662656.9	788882.7	419513.8	1194954	1117272	1060675	581020.3	1046820	844322.7	519968.9	695457.1	608718.3	530067.2	834357.2
Q05982	21752.04	19568.32	11218.16	13371.36	20344.22	16727.21	21366.77	9557.462	31271.89	24167.57	27667.83	24454.85	15968.5	11210.35	20411.76	10329.93
Q06000	36280.14	26931.8	38712.23	30044.03	24354.54	36660.59	8559.963	20248.58	30309.33	58008.44	16887.14	11328.63	24715.09	30349.07	4324.486	15772.76
Q06496	58263.24	76134.98	45032.86	79848.69	133994.7	95294.41	84328.18	49647.38	155226.4	110717.9	117074.7	191588.3	121594.3	202172	133697.9	113293.8
Q06880	298412.9	359349.8	270988.8	291912.3	304285.8	393780	259258.3	246235	263004.5	300248.3	140753.2	239575.1	118962.4	133556.8	179888.3	105795.6
Q07116	16484.76	20774.31	36768.22	24482.68	23830.31	23633.05	16284.73	15398.48	21767.44	26906.56	14636.46	16857.53	17739.8	19968.2	25571.79	19107.55
Q07523	12523.28	11482.97	38294.89	9377.604	13331.07	17768.58	13274.85	6709.237	15234.88	14052.4	14204.18	29748.86	17844.6	14698.34	14895.24	13142.98
Q07936	19868.11	30860.14	18669.68	35898.62	31204.15	33504.88	47939.11	82069.59	41449.85	22666.02	57676.23	29341.61	33663.28	41767.33	39789.08	31718.63
Q08163	4792.246	6856.774	4803.204	5806.761	8069.071	13904.23	10370.6	7645.375	9521.719	6524.888	14003.95	7186.819	5261.003	5093.049	3598.593	5862.88
Q08406	37263.52	52913.47	47139.64	58664.54	54873.9	40981.44	47188.03	73540.76	49292.93	69932.03	30876.34	46419.82	23285.19	33008.61	37283.41	38103.86
Q08415	20795.28	21635.17	16040.23	22050.92	18508.25	11322.13	20275.89	18535.46	30503.05	22264.02	22848.94	33876.52	25698.71	26556.8	24562.82	19407.88
Q08420	130933.4	158303.3	196261.7	200255.9	97309.28	162251.9	132639.2	247785	191834.4	202220.5	121324.4	149062.5	153564.1	194312.8	170033.8	127000.6
Q08463	51567.25	194187.4	139979.3	128933.9	175692.8	222982.7	82288.64	268028.7	425432.6	273430.6	168357.9	291723.2	200124.7	269973	254696.6	95319.62
Q08464	32318.78	67916.59	74911.37	60079.82	65604.48	65873.48	52440.42	55023.08	135063.6	67163.16	25122.14	63796.41	50566.87	62179.85	42452.08	24442.3
Q08849	12088.42	9044.318	11787.23	15925.87	13972.24	14639.12	20641.45	13204.48	17487.5	8729.877	32997.85	14982.1	18392.33	14768.11	17016.07	17034.54
Q09030	1111167	795916.4	1017187	534844.1	735350.5	1109107	443488.1	618137.2	2098750	456500	272705.6	530324.9	539212.2	587910	195925.8	634283.7
Q09326	15328.63	51495.73	71836.94	54682.55	55662.11	40710.24	47904.66	32065.81	59511.81	157009	18116.18	59145.16	53237.23	56857.11	61915.9	149636.8
Q0PMD2	629961.4	1013229	733957.5	816587.4	939429.6	986931.1	671084.9	850216.9	863281.4	658189.4	584511.6	669584.4	645328.8	488794.7	573183.2	511525.2
Q10743	18660.71	19866.9	22463.55	41909.41	18316.66	22869.29	6471.679	31444.81	17559.94	18999.28	28056.08	7687.059	23368.18	35583.76	29513.85	7815.269
Q10758	26276.61	24270.17	13779.2	28069.16	14736.97	13066.65	8468.138	32399.48	5841.699	9013.666	27758.11	6188.471	8639.666	7301.014	14898.08	10806.03
Q1WIM1	87703.09	127178.4	128554.7	113107.5	111221.4	127543.4	103063.4	131740.3	90375.68	137815.1	93315.67	137665	111374.5	110844.2	116285.2	89858.85
Q1WIM3	15492.24	17397.04	26271.76	31089.87	7085.137	19023.56	18757.79	11770.9	9723.824	14305.55	5701.94	8810.479	12033.97	10379.31	10731.99	20215.95
Q30KJ2	1285841	554172.8	218873.4	488350.4	49951.3	341551	78341.88	935027.4	24718.26	219498	12797.65	7808.834	76108.02	95949.16	85330.46	256728.2
Q32KJ6	85679.57	119148.2	146848.8	125696.8	155376	76087.45	106934.8	146640.8	96175.45	104551	48974.2	85103.02	61444.36	86717.96	102554.3	94589.94
Q32PY2	72519.25	86026.23	47388.77	83194.96	31498.04	42849.07	64250.4	56464.19	36275.74	24596.33	60896.02	51154.39	35179.29	25760.78	39472.12	36898.46
Q3KRC4	185439.2	213835.1	156858.5	225001	297330.8	204651.7	214907.2	204901.4	236860.5	209967.1	251330.1	289066.8	296317.5	222835.3	239347.6	206898.2
Q3KRD8	38756.27	28219.92	26574.3	34158.45	22865.19	46081.26	30426.36	31841.52	18952.58	42637.1	24899.39	27355.29	40001.75	35226.5	16395.14	19699.17

Q3MIE4	95410.75	74490.14	26070.81	49654.69	93981.63	77938.77	54804.2	76310.87	145955.8	42396.63	225745.7	139050.8	129979.9	111003.5	81796.5	103264.2
Q3MIF4	5008.555	6611.204	4517.658	6400.198	5028.724	6739.732	5503.527	3877.833	8528.549	5527.738	7366.263	5948.818	7354.116	4298.549	7525.959	4126.695
Q3T1J1	16499.07	13237.24	7974.61	20706.42	13643.84	17219.95	10782.22	26498.99	12128.46	5698.385	32050.51	10768.49	10182.52	10777	7491.927	8604.737
Q3T1J9	33904.78	27441.39	18045.2	34851.09	40372.02	22935.88	24594.65	25996.08	67524.61	21363.31	90857.58	68978.49	54383.85	70890.23	48853.96	37973.05
Q3T1K5	4262.302	4096.931	3246.358	3260.702	5807.185	4881.279	3607.507	3147.319	9030.559	4052.585	9295.834	4688.059	7815.511	6370.669	3716.707	4517.348
Q3ZAV1	21409.23	21026.14	21555.3	24209.32	31815.33	23434.43	11850.08	6738.732	64699.13	17869.72	68589.59	57599.62	52809.39	65301.76	48647.46	26196.8
Q497B0	33565.88	23443.8	39629.59	31394.5	32408.67	34664.37	28208.68	43720.08	72419.84	39515.94	57651.28	40520.68	39221.57	52075.34	28145.2	24318.13
Q498D9	7084.405	3638.448	6322.647	6996.541	6609.159	6026.771	14199.16	6411.773	17864.73	7061.567	24255.96	12129.65	10828.24	19693.68	6143.353	15654.81
Q498R7	22666.55	29052.71	41017.68	24657.3	10936.02	30449.77	14668.81	14197.04	28218.01	14736.61	8654.13	13380.63	13059.73	11899.09	10618.11	20698.71
Q498S8	72581.08	123247.3	115309.1	138759.4	122175.5	143721.7	134473.4	132451.5	60688.18	127021.8	61199.5	98018.06	96190.36	84723.33	87672.84	79329.1
Q499T2	361835.3	506885.3	521650.7	457002.6	443891.4	433103.1	485008.4	460939.6	341186.3	466591.7	273482.7	349717.8	304237.3	339371.3	385882.4	394566.2
Q4AEF8	19799.16	23397.46	18843.93	25612.15	20325.29	26000.1	19715.44	13218.91	14465.01	5804.394	9536.074	13711.15	11679.73	11376.66	13818.73	12519.36
Q4FZU2	1477918	360117	896617.8	991593.1	1008799	310546.5	509443.8	989290.2	138608.1	271819.3	1792270	249497.8	275450.1	295894.6	998689.2	383546.5
Q4FZU4	12200.42	16887.58	7485.554	8483.925	9147.371	17907.54	11449.33	9017.348	8486.691	10170.66	4071.923	7177.512	4227.075	6006.031	6841.214	4188.243
Q4FZU6	9939.621	12608.82	10480.39	9756.981	12829.82	9649.229	18915.91	30358.49	10310.92	9455.55	1345.626	7122.879	8645.714	7238.953	9163.088	4037.672
Q4FZV0	306536.3	256374.1	288478.9	393571.8	465255.8	270446.3	356351.1	477132.1	157213.3	144867.3	217339	169708.3	189292.4	200026.9	199885	238748.1
Q4G063	13153.12	11450.98	10131.17	18692.39	12566.58	18538.25	32897.21	21176	5471.007	7850.85	16529.64	5882.668	7741.845	6870.994	8457.691	9383.951
Q4G075	29801.55	42149.66	24447.1	63801.68	43891.59	33668.68	115688.6	115868.3	14185.44	17965.87	41255.51	17814.64	24794.14	29353.62	21093.96	25016.79
Q4KLZ6	5620344	8116036	4558106	11471350	5160965	4634893	67526.38	53036.38	101036.8	45694.43	4581325	150153.9	139311.9	3885673	76966.73	3933497
Q4KM73	23153.61	14139.12	9847.122	35341.25	28968.17	23517.81	16785.32	24132.71	35659.61	24890.21	40846.04	28019.5	29861.36	26639.46	16819.41	20235.83
Q4QQT4	2468.635	2876.185	3558.678	1657.178	2624.45	1832.023	1529.144	1560.745	4525.709	1384.262	13817.03	4022.606	3435.469	3355.544	1977.797	2075.726
Q4QQV8	66475.27	81528.94	63983.88	96778.94	70897.99	83693.48	71093.09	51093.21	66399.91	75244.51	70869.09	74525.99	83813.58	65803.47	60089.87	98376.34
Q4QQW8	117347.3	153752.2	148563.7	182493.2	144600	145864.5	136845.3	106441.3	90481.1	157789.9	129070.2	127271.2	137375.1	148246.1	154151.4	199060.5
Q4TU93	6511.987	9345.091	9217.818	8549.332	10876.22	8800.616	4386.383	1980.379	5086.453	8455.384	5439.105	4699.048	9803.15	9804.156	6944.609	4428.702
Q4V885	599581.4	686000.8	631792.8	824142.3	284359.3	624467.1	556471.1	631350.3	250488	472258.8	289520.8	283032.3	519309.3	446345.3	412420.8	455604.5
Q4V8I1	30073.64	18960.19	15203.22	27643.46	45397.88	16073.32	34290.47	34253.01	26736.54	29437.05	15251.9	38393.11	31913.05	20701.34	30416.96	18765.29
Q4V8K5	6949.86	8612.087	5646.924	9243.804	7766.391	7335.416	7230.883	11038.43	13308.95	5621.182	12757.09	6050.102	6412.449	6428.199	8807.45	4959.459
Q561R9	2008.424	3618.733	3558.678	2745.288	3139.325	2256.276	1887.32	1897.988	3722.062	3558.038	7865.406	5123.282	4229.191	3192.333	2597.761	4774.77
Q562C9	195558.3	187485.8	313366.3	238006.1	141186.2	223044.8	159986.5	206686.7	113544.1	140346.6	173949.9	138927.8	138228.3	140749.5	105662.9	129701.8
Q568Z6	119816.6	153291.5	170164	151688	161289	110454	119790.5	120881.6	170835.3	151908.9	160346.7	163400	152558.1	136872	146298	117431.6
Q56A20	10378.21	11478.21	7582.522	18698.54	12899.39	25525.42	10617.09	13746.77	7852.018	7345.417	13540.56	9988.864	7603.364	9109.105	9097.181	6574.775
Q5BJP3	9375.824	10571.66	5743.244	13556.27	7756.957	12792.2	10100.48	15792.01	8110.902	7497.3	28781.81	9426.832	7163.96	3696.462	12956.99	10376.2
Q5BJY9	8538.703	21365.79	16638.35	17976.23	8296.583	10524.23	9968.372	24604.1	6575.861	14715.54	10549.47	6537.578	9134.619	7045.172	2969.317	10796.96
Q5BK81	32474.65	27570.81	44062.76	33763.19	20162.11	36080.38	20967.36	26422.01	21769.37	24934.18	22807.97	33028.55	25464.31	38786.51	23319.97	27445.25
Q5EGZ1	23345.21	55279.35	11453.37	18476.27	19832.56	30329.6	18108.49	25186.98	18267.75	15938.84	16197.32	19642.02	19382.95	22823.2	15415.97	17418.05
Q5FVF9	275699.5	327035.9	343969.6	395557.1	158501.6	237771.5	296430.5	218967.8	250267.9	335504.3	219943.6	276869.8	355269	311897.4	241302.4	269544.8
Q5FVH2	230417.9	223496.4	327062.1	289724.7	460357.2	285871.6	346842	447312.4	157531.7	226713.9	220359.9	232044.8	254690.5	261701.8	298821.3	284563.5
Q5FVI6	434570.5	16564.8	3670.163	9795.414	3479.97	46961.46	522407.8	15644.33	5895.357	5434.695	9837.519	10036.71	5301.435	8818.039	5584.941	6911.723
Q5FVR0	1081165	1320104	1487551	1183889	1297232	1493468	1036208	1394601	2607757	2084222	831852.6	1839648	1318151	1648613	1923944	1058050
Q5FVR3	31608.78	42763.02	41456.4	44667.89	29295.92	36952.46	43374.19	35785.54	22860.29	31506.08	32199.18	26707.8	23408.85	26423.68	19050.49	27133.59
Q5GRG2	205686.1	138161.2	24104.39	95491.7	21791.74	192709.4	109094.5	171878.3	13491.1	11950.27	162677.2	61362.23	23981.96	62296.63	15923.05	27061.96
Q5HZW5	165340.1	221244.2	231874	216383.4	230196.4	193781.2	206486.2	287113.5	139682.1	251000.2	128819.5	182976.2	146782.5	171484	183575.3	151142.3
Q5HZW7	50958.96	48019.98	54595.64	55444.74	35288.36	40536.45	46251.55	41609.76	26764.3	30079.27	39723.22	43745.06	51668.9	24603.73	36878.21	25100.4
Q5I0D1	12860.42	8532.304	13695.75	13164.56	17344.93	8459.629	16618.83	10896.83	3396.837	9690.15	26740.86	19025.28	12181.44	4166.189	2673.292	2127.725
Q5I0D5	106599.5	119703	303748.7	222207.8	120384.2	131362.8	100632.3	136273.9	164496.1	162649.2	137155.9	179468.5	138617.2	186197.3	150515.2	133486.6
Q5I0D7	44109	27279.09	14936.73	26683.9	23877.5	27776.17	42594.94	60838.68	133317	76853.7	96955.34	67590.2	66191.48	82521.77	67581.93	34335.72
Q5I0E9	57134.88	49402.4	43794.85	57350.88	67006.09	63778.64	25841.54	65155.24	203002.9	47800.23	151216.3	190299.6	129307.4	128682.3	87749.88	83832.23
Q5I0J9	6785.788	8069.367	4257.061	1645.998	11758.3	10491.99	2026.823	4296.796	8681.717	3414.066	7571.664	5045.069	2672.802	7339.072	2896.292	4179.352
Q5I0M2	7190.006	5623.81	7706.955	9249.176	12295.81	13009	7621.963	10547.25	13512.05	8190.21	25342.47	12220.99	16717.75	14574.73	15413.25	16241.64

Q5M7T9	22264.54	19650.26	19059.62	12610.89	30720.42	16413.48	9038.119	13191.45	80168.88	23136.96	50489.83	46925.88	44059.28	50395.05	33663.62	28300.88
Q5M819	24038.57	19848.18	16433.85	20396.15	33576.07	20104.91	23138.09	19778.34	45727.23	39605.47	30757.28	36158.53	20340.84	32409.58	40250.35	15841.87
Q5M843	16450.49	22419.18	17116.17	18544.16	16588.15	16634.19	20786.99	18158.1	20966.75	20611.75	8966.518	15341.44	16365.97	12883.25	14045.17	12179.76
Q5M871	102713.2	172957	91132.01	136351.3	131201	192236.4	146769.8	131208.5	178069.7	143768.4	58421.35	119168.9	99814.04	125729.3	90993.39	84520.11
Q5M872	7889.373	6809.528	17910.62	4983.468	11402.14	6344.155	4439.711	15808	14704.86	9618.021	3834.075	10696.35	12154.45	12166.68	13544.44	5732.346
Q5M876	224046.3	167332.4	235988.2	207094.4	214379.8	133926.7	168403.3	153176.5	337105.2	119235.1	381006.7	195005.5	244437	252877.4	194241.4	149710.2
Q5M8C6	61112.4	27028.28	48048.83	94316.34	40882.72	77139.98	99479.91	133814	16118.27	20428.55	92859.1	33056.56	27176.11	24568.51	11004.24	59391.02
Q5PPH0	20385.24	39542.11	35412.83	24204.49	11936.83	22853.29	13983.91	16048.02	11490.24	17505.88	17147.71	19301.51	20167.75	22253.36	14827.04	17298.89
Q5PQL7	2315.696	7353.756	7014.459	6452.848	5393.058	1208.931	4788.25	7103.789	3295.557	3157.53	5550.508	7121.091	1717.504	1436.22	2728.38	3864.764
Q5QE79	15653.44	22468.1	37016.01	33309.88	23315.38	37343.68	63867.61	32017.55	24685.63	13887.29	10452.57	8845.287	15076.66	28085.07	3267.572	4687.233
Q5RJL6	28557.15	35935.5	39646.15	24181.6	26128.33	27513.22	29717.61	23152.64	29867.9	33041.82	19887.11	26203.48	21039.72	25571.23	29882.88	16741.65
Q5RJR2	79927.41	89848.55	102275.7	51970.95	38589.42	70702.3	111434	49475.55	112049.3	131328.7	107128.2	157189	141027.9	134834.6	155583.7	139501.3
Q5RKH6	2553.758	4182.512	4895.789	2745.288	3534.943	2823.697	3214.075	1554.524	2861.106	3254.041	2486.418	2115.636	2463.902	2955.008	1157.472	2467.818
Q5RKI0	18284.7	17791.37	15422.69	11166.87	20966.84	13978.2	17642.86	19367.6	36907.41	20577.18	54410.89	36528.8	35054.36	38771.93	27863.82	23413.83
Q5RKI1	9025.865	6531.284	52229.77	5830.463	5117.508	7952.266	57988.25	10423.78	10576.68	62003.95	7324.773	9037.434	21462.91	10744.28	49968.03	4958.793
Q5RKI7	225354.9	368104.4	296592.3	172355.5	305646.9	437144.6	176572.7	227012.2	816265.2	185948.5	430938.2	387384.7	354805.8	367702.3	306913	291907.9
Q5RLM2	30277.54	21684.65	29294.94	37207.52	13672.06	55480.59	18971.79	37695.18	245015.3	45562.96	133853.4	51239.42	126770.2	42180.82	105779.5	82642.44
Q5U1Y4	1073575	619521.7	794748.2	575132.5	1132155	654997.9	789395.4	925509.4	1993547	1576571	728902.5	1615436	1342666	2476132	980880.7	927139.4
Q5U2P2	33467.89	35388.02	50461.95	35519.67	34748.77	40031.05	48126.96	51238.8	41961.25	84089.6	29559.41	98559.12	27606.06	64717.07	29762.57	40550.6
Q5U2Q3	101037.8	92492.71	122285.9	86797.24	144448.7	96430.8	117558.8	109230.9	361570.2	159615	337145.1	204925	193939.3	272821.1	156850.9	124052.1
Q5U2V4	13689.41	19586.7	22391	14825.5	15165.63	15255.96	25907.31	25329.4	12877.66	5951.383	11257.85	6493.745	7119.446	7902.663	9091.211	9459.338
Q5U300	12581.02	7935.59	9290.955	5312.695	10233.12	13127.38	9376.902	12896.01	11792.1	6452.218	22759.03	8805.347	10507.38	13600.86	4984.614	10715.18
Q5U316	4274.226	4393.741	9964.371	10394.46	3236.859	5485.76	7764.431	89322.62	8766.863	3676.169	54090.21	7828.765	33216.59	5371.819	5236.834	6581.546
Q5U367	77422.21	71570.22	48495.9	62481.49	60238.7	56343.11	41913.83	56343.57	43527.99	71299.11	41671.7	41606.77	62090.1	69247.48	42321.34	32768.63
Q5XFX0	14244.46	19109.76	10020.45	12361.99	12678.06	13789.63	14257.55	21902.69	13688.09	13588.42	33931.08	12780.95	8112.043	13476.77	11697.27	12432.4
Q5XI43	4474302	6878136	4877548	5389951	5705155	6231071	4564653	5060097	6079682	6770031	3603912	5162481	4602921	5840595	4024661	3870431
Q5XI73	88747.41	75273.93	51630.11	90010.15	124074.2	99342.98	85907.58	155162.5	75625.93	58339.61	197178.1	116848.5	146417.3	167250.8	116853	96103.3
Q5XI89	2648.821	6963.464	5919.037	4505.445	7665.489	4668.951	5312.408	3838.035	5260.844	5773.891	9087.82	5175.344	4308.125	4903.121	5497.127	4058.177
Q5XID1	2218.636	2923.136	3220.135	3066.414	2624.45	1618.846	1667.491	1554.524	5077.393	1450.281	17937.78	3626.152	2341.686	2235.982	1413.169	4074.302
Q5XIE8	22411.12	27982.02	26096.61	26925.8	27477.94	24005.5	16117.88	22523.95	16956.01	27596.18	20277.13	23157.28	17167.29	14051.1	22723.78	20419.15
Q5XIL0	31816.66	48595.53	36474.27	40932.14	70752.55	48561.15	35116.26	41084.11	24759.11	30056.55	20058.39	23776.51	18552.31	19009.04	37334.68	18791.09
Q5ZQU0	6539.781	11484.02	10315.28	8414.722	7649.614	6930.401	4564.109	6429.933	11415.76	18157.52	7652.5	16509.25	15982.94	13919.79	11098.5	12377.02
Q62632	165234.3	172766.6	140539.3	168959.1	288972.3	197040.6	158188	207826.2	252041.8	277391.2	131810.3	242791.3	194830.1	167248.3	236144	176084.7
Q62635	77579.72	411146.7	25336	145208.1	21034.61	92212.52	108683.4	243303.8	15033.35	6796.67	36055	30777.76	27479.19	76596.45	10760.61	16123.3
Q62638	89848.84	69680.3	67277.26	95717.14	86301.21	100480.7	93636.29	104606.9	63145.39	84947.28	77527.45	61012.19	53858.24	56391.69	58413.44	56158.29
Q62687	59011.8	55729.41	28267.97	60434.09	79351.66	70565.53	30172.16	40179	145927.7	38133.82	135031.3	122228.8	87513.19	102680.8	85339.6	67707.88
Q62714	13704.83	18745.32	15698.59	71193.56	11363.69	12256.87	12661.61	20895.94	80695.1	61419.63	131740.7	86403.23	31528.78	29152.06	26037.05	47704.58
Q62718	6540.316	10877.91	12755.39	13306.29	11484.17	10960.94	7816.925	13407.76	6041.304	9650.139	6966.41	6931.606	6998.609	9170.408	10314.55	8472.879
Q62740	252953.7	454007.4	277800.8	506916.8	390857.2	469090	339623.9	513684.4	550426.5	697041.5	231909.8	377879.1	431595.9	298107.3	392121.6	439573.5
Q62745	56578.95	73237.17	104144.7	62740.55	58938.19	67665.18	52871.41	85001.53	82584.57	84932.07	72785.84	116956.1	41224.46	35118.7	42629.17	40814.14
Q62753	16436.83	9509.574	8121.653	13982.42	10457.03	15762.22	12068.6	10636.67	23725.44	7483.051	66416.22	22417.1	18527.39	16747.08	12642.44	21451.43
Q62761;Q62762;Q6	24386.38	12542.26	11994.09	15149.7	13819.41	11073.84	14703.72	735.2054	43010.88	14535.87	37245.9	28602.9	18174.76	16240.71	18102.54	4925.057
Q62786	100676.8	111862	126418.4	119291.4	138054.3	136607	118763.2	111906	131955.8	166066.7	92420.09	133615	117302.1	149980.8	155319.7	121313.6
Q62795	25113.27	52063.59	19594.72	29465.75	37962.52	37904.71	31558.75	26843.82	65833.82	48183.08	51227.96	64296.84	48666.9	57215.68	47063.48	58681.56
Q62812	3047.639	3935.126	3381.993	7432.219	5663.906	3412.953	295.6559	5558.669	9690.723	10056.07	10065.85	13097.75	10940.08	7395.263	8390.566	7256.388
Q62867	2523264	3248495	2427390	3021740	2436712	2191697	3443679	4668997	2382403	3129589	2219977	2650448	3615055	4540628	3062648	4137111
Q62894	11558.29	12450.83	13176.93	9701.383	4021.095	18448.53	30205.04	12081.74	9209.298	9927.395	10732.16	4741.695	8920.18	7164.635	11428.03	9611.115
Q62902	28984.93	18097.34	39263.99	52712.13	17443.03	34276.54	38886.09	45791.07	19010.8	6690.757	37470.92	7018.768	27527.49	8418.133	9752.221	31894.7
Q62930	180363.7	189489.9	146883	295581.8	85537.74	92859.8	184081.3	172942.9	147786.2	270868.5	111205.5	225257.3	161510.6	169779.3	243419.9	231930

Q62946	181282.1	142987.8	172579.7	223913.3	139740.9	167033.5	287220.9	271293	94565.13	67500.7	100752	97303.05	90219.85	77991.94	48541.03	62169.05
Q62975	650.3278	5710.856	3600.819	5631.506	6040.995	4796.124	4214.993	8539.254	7163.29	7509.819	7134.705	4500.252	6219.417	5349.113	10109.87	8278.49
Q62997	13507.67	26845.29	13022.68	25417.97	21092.3	15068.51	12179.96	13939.28	19768.49	22476.93	4048.668	21703.19	14487.39	11635.61	22736.82	28158.44
Q63041	6437343	6729582	7557998	6676238	10641240	7383068	8374010	6849592	5527218	8288757	5562191	7840935	5985147	7858975	7988021	6279047
Q63060	5613.568	8252.788	7638.247	6243.853	10703.18	9877.514	5776.486	14184.8	20024.97	5520.079	16690.88	11355.47	15914.3	9899.457	8617.909	7005.036
Q63072	16851.42	34436.95	28972.13	27568.44	28026.78	20597.85	26175.7	29344.42	30671.01	17432.07	19616.25	16764.38	16939.12	23270.2	19404.2	14464.13
Q63081	10790.55	11171.86	7252.424	12355.54	9239.746	16479.54	24626.88	9903.992	8498.856	9999.32	23953.74	11652.1	8719.832	8164.111	7372.505	9096.18
Q63083	736706.3	768225.1	405002	325977.1	316644.7	413997.1	291747.4	533765.3	341805.1	425955.7	397548.7	344233.1	489040.7	279175.7	233830.7	249482.7
Q63135	17852.08	40716.24	28651.47	23079.03	24231.89	21016.85	23194.8	22729.75	47991.95	23995.56	20737.13	24517.99	19476.85	3801.267	21101.72	19833.24
Q63149	15211.85	22309.91	29533.47	21825.8	15468.66	20076.01	15625.88	14738.04	21063.1	6040.475	17543.23	4933.182	13429.67	19990.76	14316.45	11347.17
Q63150	7919.298	8108.763	7045.541	6505.704	3172.311	3423.578	7815.004	9301.363	11438.36	13834.69	13360.87	11150.05	16702.76	14008.9	10159.9	9881.803
Q63199	2846.072	6795.545	5149.192	1917.195	4102.898	4590.013	5950.028	3373.249	2460.26	4922.943	3812.593	4598.811	1801.653	2172.67	2622.806	2119.259
Q63228	6435.984	7215.049	13613.95	8522.011	9651.268	8239.436	6774.324	6353.907	12193.47	6871.657	7578.983	6970.694	6613.034	10113.14	7622.523	6258.498
Q63257	3104702	4116213	4672133	3761888	3397356	3792118	3723647	4444041	5103829	4274069	2804681	3297773	3128629	3449850	3285040	3167061
Q63270	32637.49	30329.93	22912.69	23916.12	27748.96	26244.8	21574.06	27568.95	119412.5	33918.81	88572.73	61209.82	73572.29	86430.93	41606.76	43052.5
Q63317	970804.5	1398047	2394096	875393.9	739262.2	727286.7	862574.9	774920.9	418939.8	508885.7	391788.2	405591.7	406276.7	466944.2	475136.7	598770.3
Q63355	47550.39	34048.86	31459.63	36074.03	41147.99	67818.09	25610.42	37166.91	124933.4	21391.81	122159.4	98612.13	64163.06	65676.79	50333.65	51278.44
Q63357	33444.73	15654.97	11777.38	23885.23	14750.99	24417.64	22795.97	31671.57	35697.84	10071.6	83541.27	28829.7	24564.62	21764.14	21004.49	29823.12
Q63416	209251.1	187503.9	149453.9	249916.9	161867.1	181989	180825.3	187975.2	165172.7	267309.7	110415.5	164310.5	200570.1	174999.1	274265.9	221227.8
Q63424	102952.9	103907.2	53277.82	96213.24	180922.2	158851.9	48974.7	99331.23	405260.4	47238.87	293208.1	280448.6	191246.5	260744.3	178276.3	107821.7
Q63467	387203.5	514680.1	238846.7	426843.4	341067.3	488915.1	331731.5	266007.4	690626.3	393183.5	111037.6	364122.1	300336.6	196479.8	130002.2	238832.3
Q63474	3908.712	31505.47	94869.95	29003.43	46951.5	21965.97	28720.5	18955.86	36024.95	18263.75	45864.89	42564.73	41634.54	41013.66	20063.41	65941.05
Q63475	30909.04	30030.84	36813.43	50340.76	16805.29	19618.01	16895.94	26622.94	23612.39	17745.8	11592.2	13070.33	32167.93	25262.16	18141.04	27352.38
Q63493	42928.72	9993.768	16429.1	29554.72	13419.86	27337.81	35699.21	40045.94	8651.08	5692.979	67307.37	10669.83	9607.376	5195.715	7484.006	8867.928
Q63514	67744.88	70799.12	131619.5	79593.55	32525.86	56409.36	51536.5	72374.27	71236.65	61165.38	69992.94	80288.48	83376.8	104220.4	81390.2	75858.43
Q63515	66179.75	92140.09	138731	106937.3	75217.79	140865.2	108610.9	130259.6	179966.4	150473.3	80708.57	110059	97120.63	113123.9	104953.8	144363.3
Q63530	100671.3	92101.43	150549.8	62083.06	149014.8	95840.55	111660.3	122139.1	212744.6	111004.3	205021.3	154490.6	174737.8	223811.6	198409.7	123162
Q63532	189957.4	246720.1	126375.1	206275.8	116257.6	434052.7	117308.6	231920.6	234940.9	231888.1	211782.6	204405.7	67965.52	118519.5	102976.2	190510.5
Q63556	200257.8	260007.2	141196	155307.2	184306.7	92379.72	230484.4	121475.7	116449.5	384189.2	135246.6	179829.3	136964.5	230588.3	271640.5	284953
Q63598	10206.74	15673.53	21587.83	29407.68	67312.01	20189.32	12296.49	14666.74	53481.29	15265.12	86029.09	39180.13	48062.89	61189.35	31783.2	35910.3
Q63617	30870.25	10532.56	11534.52	57936.4	9073.61	68261.7	114815.7	43962.29	21603.11	6143.473	71070.76	11299.22	20053.25	4232.998	3847.084	17390.29
Q63618	22854.91	7808.073	24708.88	12489.3	29728.13	26873.28	20161.33	17923.77	66116.88	12644.24	97698.73	65533.61	38595.95	71039.56	45327.68	53167.64
Q63621	215677.5	276001.2	356004.8	250388.7	205274.6	170969.4	329439.3	420514.5	375800.3	416466.7	186425.8	306514.9	334067.7	357331.7	289272	408552.2
Q63678	94553.32	90824.1	122401.1	87195.84	48343.8	81397.02	74591.48	66732.71	89226.99	93643.86	18829.33	47315.85	61175.87	64206.56	84971.69	103361.5
Q63691	193563.5	220554.3	240588.2	263088.5	231884.5	210275.7	232274.3	200112	236085.8	278748.4	242882.4	250104.1	281750.4	158988.9	116552	237502.8
Q63716	208687.5	245425.8	206746.9	209507.6	281439.4	222207	197323	385957.5	424001.3	236577.8	517037	410750.6	400057.3	384997.6	322904.5	406123.2
Q63751	369372.7	234087.3	849481.5	368887.6	330499.1	403748.2	439705.6	425393.6	278501.9	180568.4	237380	192827.9	97612.13	523362.5	37230.74	57901.24
Q63772	130471.5	133927.5	114933.5	139888.3	129720	106293.3	70018.75	100171.2	112464.4	164467.5	90242.23	155807.1	116506.9	108905.6	128227.8	91908.5
Q63797	11740.6	11566.18	9005.605	10755.37	9700.907	7971.953	6881.669	8469.337	10639.62	8843.851	3402.163	7743.451	8996.314	9025.43	7938.53	8004.277
Q64057	20821.53	19032.07	27303.63	16941.92	21474.61	20247.06	15421.07	16829.92	47338.06	23749.21	59400.41	31946	28866.29	32680.09	18198.44	15298.36
Q64093	19542.72	23502.57	11281.42	30630.53	50764.98	52941.37	5964.932	20222.31	77706.61	10085.36	72498.66	60663.72	45576.16	66472.06	35243.25	24723.71
Q64119	94565	164800.1	62634.54	27113.06	188254.8	109751	94677.77	130879.7	76451.93	104661.1	168042.8	131870.7	114152.6	119024	108663.4	116299.7
Q641X3	92789.23	128286.8	170214.5	116053.7	128711.7	86668.64	179965.6	113020.8	98648.19	123690.8	92188.5	117527.7	93011.16	101107.7	111807	109284
Q641Z6	62226.65	70990.41	41871.36	67723.77	86986.2	109757.7	48358.85	74325.31	118325.4	41510.42	175154.1	133018.5	93378.9	104270.5	58682.83	94359.32
Q641Z7	66571.97	55156.02	78486.9	79810.08	51197.63	60436.13	72213.41	46689.46	27605.47	45662.22	32062.69	40154.05	27106.2	38729.11	53610.78	44257.05
Q641Z8	25736.68	42046.14	35201.81	27326.32	31565.58	22157.04	31597.65	29643.97	53256.59	36358.91	37373.57	36844.33	42300.05	28460.75	34829.34	31039.86
Q64230	3757346	3476369	4534024	3593383	4108832	3910617	3821601	3277705	4161046	3273456	2584120	3407020	3172940	3801143	2809357	3098865
Q64240	19329536	32052724	25219366	24810810	22870120	40898220	19253214	34295968	24191158	33424646	7529539	13798047	21191510	18210516	13885459	19415770
Q64268	24008.33	11470.54	15673.52	16984.39	6398.73	10740.4	24904.26	13533.67	13839.83	22161.38	6402.683	13009.45	11742.91	13341.84	36514.69	25528.01

Q642A7	674202.7	1125758	851174.9	623927	1216380	713931.3	730126.9	1072521	805279.4	1164304	790527.9	1201805	975630.1	1002163	1332533	839915.5
Q64319	1921748	2279178	1552142	1988680	3715216	3690563	1427306	2127591	6450519	1538283	5131000	4685590	4368983	3859396	3846147	2840499
Q64335	17462.33	21458.5	6033.522	17647.92	33260.59	47096.5	33133.75	22370.05	18789.01	47563.95	9168.477	16147.17	13029.92	21962.35	32598.53	34117.3
Q64361	57639.37	65390.01	49592.25	118630.1	30284.57	62596.95	37342.58	66856.18	56175.82	69404.82	25709.65	55041	60142.64	54034.64	50257.65	62669.64
Q64537	18630.25	15900.32	11179.9	24335.36	20562.91	26371.32	33947.94	21212.08	10161.29	16881.29	38414.02	17315.39	18104.57	22570.08	12350	12247.52
Q64573	168809	156865.8	132593.8	169976.8	110633.2	102636	149281.2	128287.6	169517.7	116093.8	50187	58122.18	147263.7	141243.6	115424.8	107518
Q64602	191568	223344.2	181597.3	254153.4	251220.3	215577.3	125109.5	221149.3	776928.5	248809.9	686899.6	494413.4	629625.3	466171.7	466647.3	344002.4
Q64604	94011.63	102364.3	94952.49	94867.93	79482.09	101949.1	85178.14	119713.2	89454.16	98961.35	108338.7	95151.57	89578.46	106209.8	89360.17	98179.69
Q64605	16650.03	23808.68	25335.2	26388.27	22717.56	17428.36	15503.7	12528.65	20716.1	15567.54	13513.66	12480.47	19708.66	21646.51	17678.56	16867.26
Q64611	5458.088	9601.515	7718.654	5225.961	8879.675	7572.658	5488.5	11116.24	10920	8798.485	10642.89	7796.934	9200.092	10214.12	8054.479	7007.751
Q64640	14412.85	11651.63	10097.14	9738.205	12754.39	12918.29	10358.03	7876.894	31746.54	11850.79	27784.34	15511.34	18385.9	20518.41	15954.73	20604.2
Q66H12	124826	162807.9	115471	100618.4	147464.2	126227.5	126638.6	114620.3	124121.4	138723.9	117168.5	117675.4	141178.3	143512.1	160143.9	127746.3
Q66H69	25222.39	24834.61	10028.53	13565.43	11077.61	8448.045	21612.76	35542.53	1511.879	10232.73	10981.27	11213.82	13509.69	4486.965	5543.205	5967.688
Q66H94	5931.099	10014.6	11659.71	9528.537	6875.785	12135.22	7407.817	6226.109	6656.842	12652.48	5740.528	3958.147	7578.012	9924.492	5423.532	8221.955
Q66HG3	7091.977	11000.4	10843.04	10946.89	15693.12	6194.354	8540.928	11837.27	25014.05	10329.59	44426.07	15024.63	19425.83	21704.8	26329.47	21948.22
Q66HG4	44960.16	37800.19	61234.83	68184.47	65809.45	42762.24	45820.11	52079.94	65351.57	65265.78	64763.34	70991.71	82879.41	96448.91	56762.2	63399.54
Q675A5	45001.96	48402.75	45071.63	77170.47	65077.41	54461.24	53878.34	41793.15	24448.01	41511.5	31015.91	49044.95	41990.59	37499.18	29650.28	34034.39
Q68FP1	4689033	6301154	4646626	11466562	5407202	6827554	6338597	10262763	3679669	5922405	4100012	2884498	5969506	5295289	4724932	6407498
Q68FQ0	4438.804	3772.753	2933.486	4291.882	3202.557	2554.484	2381.772	3216.914	5686.507	3283.511	5071.104	3846.782	5273.901	4503.112	3109.25	2841.269
Q68FQ2	51812.58	71914.8	54536.41	100218.2	73643.17	69003.52	51136.82	47927.24	50979.96	60369.13	52457.68	42899.55	36805.27	35623.86	39464.94	37780.28
Q68FS4	13541.55	13539.28	16780.87	12047.91	19166.73	11751.33	15335.2	7013.248	14453.16	13072.99	25659.21	20418.08	19572.79	23403.47	15890.53	12848.26
Q68FT5	19899.17	21967.91	22001.11	21886.19	24667.62	13646.92	14476.98	25860.47	68040.21	19387.56	57707.02	43405.38	40427.37	45238.63	31656.63	21255.83
Q68FX1	8374.633	4420.43	4225.065	6839.058	3382.695	5472.368	4084.903	4639.902	7262.178	3149.229	6579.222	3261.622	3767.798	4622.73	3263.398	3681.054
Q68G31	763516.9	1335546	22908.89	28072.11	31683.81	731538.2	28843.92	624746.5	36704.38	425413.2	362646.1	26973.79	504838.8	242925.4	232990.4	40329.7
Q6AXR4	124950.9	180864.3	199598.8	157087.3	203846.1	116657.1	326449.6	164623.1	111739	139290	156185.7	143944.6	115663.6	125068.8	142847.6	189352.8
Q6AXS4	172818.4	118238.2	118157	71721.98	70941.1	96364.64	66509.52	92199.85	61408.83	85608.44	69860.85	87574.32	78742.27	55582.21	48345.01	47932.31
Q6AY33	15911.43	11158.36	10551.21	10678.42	8553.919	12351.37	12285.95	14748.01	5384.008	9977.029	7329.796	5516.756	1897.118	4564.496	5579.979	14052.9
Q6AY41	26873.7	33907.14	20494.23	47525.05	47241.64	37436.84	20512.57	11115.01	97456.36	20845.1	89305.13	53924	52934.59	66956.41	45009.76	30389.27
Q6AY61	2619.315	817.228	1464.741	7056.253	959.5469	4088.97	1032.011	7664.715	4022.245	3199.883	6092.192	680.6194	3471.843	3143.269	2848.71	3952.095
Q6AYC4	6590.398	22006.35	12717.29	12168.62	10275.04	17294.18	8293.503	11518.54	15406.85	9845.125	12843.87	11439.83	8499.856	11500.64	9807.288	9792.604
Q6AYD4	537902.7	782654.3	711408.7	525420.5	609605.8	763473.8	593480	692489.4	765510.3	677791.4	372788.6	631104.8	599194.1	690973.2	554945.1	633521.7
Q6AYE5	19939.27	43959.86	19742.13	21353.26	69390.05	36565.72	34372.37	55709.37	40109.96	60178.13	22677.48	19506.77	24596.36	16013.29	28411.43	33505.32
Q6AYH6	8141.819	15208.6	10032.74	6489.248	10154.11	12323.03	11864.62	6487.142	15976.31	13485.6	5883.718	7290.859	11801.83	7839.596	7370.614	10629.8
Q6AYP5	81281.52	102946.2	126592.7	171777.9	116173.9	100992.6	96567.98	114722.8	124507.9	116107.9	68095.63	101508.4	74413.39	80508.86	85776.7	95677.02
Q6AYQ8	105.4462	2336.177	3867.115	2497.734	7825.085	9106.313	2274.307	6691.904	9139.852	6454.557	6345.559	10273.39	4584.066	8011.532	5921.222	3290.276
Q6AYR5	12877.95	7500.229	3705.856	9787.142	10323.36	11795.72	10558.68	10147.61	16435.79	8699.579	16659.17	18893.04	12348.55	15172.84	3744.822	11810.49
Q6AYR6	25653.62	20500.75	25976.87	30419.58	16073.26	23663.87	17606.45	13651.46	20142.27	27172.03	25450.75	23243.91	23973.74	22481.23	14215.71	20673.08
Q6AYR8	6747.097	10721.23	24770.41	5838.312	13176.76	6637.578	8140.874	17488.45	8858.351	8825.699	9954.244	11349.87	10777.26	22154.56	10377.81	14219.25
Q6AYR9	33297.78	12739.14	7812.789	25341.1	6933.689	21062.7	22180.61	37545.98	14682.89	4094.49	95353.96	8108.787	14950.93	11898.43	11756.4	8418.873
Q6AYS4	22871.17	19254.01	22576.59	15750.26	33591.13	20677.13	25880.45	23736.58	23301.85	16260.05	14510.88	20870.85	14025.51	14465.24	13608.17	20243.77
Q6AYS7	221839.2	239522.5	184759.8	197684.6	304861.9	199681.8	138528.6	186428.1	562932.3	323032.3	700743.8	480290.7	434994.7	491131.5	359788.3	408969.5
Q6AYT0	107712.4	104277.5	131302.7	104612.1	171179.3	141319.8	114410.1	119650.6	324428.3	165634.8	294138.4	237053	283079.9	318937.7	171408	228440.6
Q6AYT8	71509.16	95015.63	95140.55	52800.21	71642.73	105021.4	83236.39	94674.8	33448.95	53037.72	60872.55	55252.13	55206.1	69762.3	38871.59	93954.39
Q6B345	20599.34	27554.24	28727.15	61035.46	35904.51	28124.55	38545.16	42115.56	24455.82	34500.52	90267.25	9248.529	13695.2	23406.12	17525.95	18395.86
Q6BEA2	23482.86	20025.22	15299.65	17737.38	15413.97	24755.01	8564.229	29804.71	13007.14	14032.17	13038.01	25759.57	5489.7	9616.496	13948.16	11033.25
Q6DGG1	719743.4	777250.3	3196203	1422706	892279.6	555372.4	634368.3	1221937	443674.8	974829.8	680129.8	1066770	825139.9	949937.8	859545.6	765640.6
Q6GMN2	21536.57	14473.63	5423.311	22533.62	20654.99	20916.35	14426.46	22349.81	11670.04	6235.796	38641.25	11556.76	17080.16	13490.71	8122.166	18424.07
Q6I7R3	2608.078	6178.201	6858.979	6535.508	4405.085	5560.903	3100.486	9951.967	11187.23	7531.726	8331.595	10882.33	7763.173	6202.157	7768.956	4539.554
Q6IE52	253436.1	347841.4	262560.9	170189.5	209561.8	234099.5	360673.1	268145.3	364514.4	284444.5	127000.3	188384.1	206309.6	325860.5	226411.2	312353.3

Q6IE64	20259.71	16329.19	22313.14	17158.56	20663.27	25041.56	16647.7	21897.75	19767.74	22799.04	12655.47	10434.23	18200.67	16356.29	9757.764	7919.046
Q6IFU7	10290.8	6149.854	10828.9	5866.386	8738.699	4817.742	3046.629	23254.84	1501.515	4303.954	11811.8	3190.139	4323.24	4381.106	7877.921	4153.686
Q6IFU8	716685.8	556792.1	406745.8	608104.1	549025.3	185239.8	321643	781932.5	168355.5	189853.2	1482575	190759.9	188964.5	237859.2	547776.2	280134.3
Q6IFW6	676002.5	178185	598866	405306.8	377572.7	115034.3	225833.6	489078.3	85143.82	140290.4	1040904	139472.1	115090.4	128091.6	600903.4	175437.6
Q6IG02	176730.4	51058.11	212651.4	181900.9	116890	49371.34	54722.98	124938.3	24618.74	46467.5	384847.6	54079.41	60580.8	52744.93	195811.1	55319.32
Q6IG05	55585.21	20490.25	19557.25	5936.555	23574.96	13382.34	10430.06	38808.25	5765.098	6104.166	15152.66	14140.81	6664.355	7377.229	15466.74	8610.215
Q6IMF3	679594.8	217608.9	346373.5	348042.5	492343.5	126829.5	226173.8	573665.1	77091.58	117022.6	1073053	109461.3	99529.2	118943.3	488855.4	145121.8
Q6IRE4	17874.08	21278.34	22577.43	20410.31	26466.04	16378.94	19409.38	23621.46	18668.21	19864.48	34732.46	20702.37	16186.83	14687.41	16395.21	16279.67
Q6IRK9	2630517	3677933	3262946	2966763	3347960	4078411	2497718	2421400	3841603	4152581	2628549	3740508	2519764	4046210	4516253	3252399
Q6IUU3	1048962	901128.5	899136.7	905676.1	617402.1	946516.8	850783.3	1170953	819338.1	599566.8	743601.6	692583.3	667745.5	633122.1	665569.1	3423448
Q6MG61	261665.9	303762	191071	263169.5	312266	312853.7	186918.7	221442.5	992454.8	243387.8	942585.1	600529.2	619774	710724.1	438916.9	459285.3
Q6MG71	628981.5	491189.8	540545.9	487859.8	398593.9	523669.4	504003.9	696631.7	612883.9	354394.4	1360176	583535.3	476817.2	433499.6	457040.6	543890.6
Q6MGD0	10777.96	13576.01	14147.76	9387.745	13453.12	5447.445	8937.349	11946.65	20638.71	17393.38	15108.09	25031.55	17414.88	14512.97	10861.17	12304.49
Q6NX65	6370.442	5875.978	4876.665	5978.616	6696.959	5079.937	3876.697	9659.367	6513.503	8078.022	11606.62	11572.63	10746.36	7318.366	5882.127	4898.684
Q6NYB7	101295.1	95194.35	73725.33	105870.9	81003.77	93139.13	70547.91	110779.6	123439.5	61593.89	231999.3	110409.3	108827.1	90560.64	75412.48	99522.7
Q6P6Q2	299388.9	80801.12	137453.6	202008.5	216665.2	55718.56	88719.81	221564.2	43073.61	53043.14	431160.6	55091.9	56038.44	61905.93	210427.6	70189.13
Q6P6R2	5278.008	4677.126	7811.116	8556.902	5114.201	8238.885	14060.08	20291.72	9316	7364.309	7670.54	2600.365	3271.072	3989.653	4345.306	2469.407
Q6P6S4	9109.814	7789.929	8013.559	6183.471	3593.449	10694.74	6037.289	22533.03	10710.05	5337.534	14090.45	3897.78	3224.236	2016.746	6890.931	3843.854
Q6P6S9	25820.66	25773.24	31232.61	29417.35	17869.36	20740.96	24992.25	21907.73	10887.34	21230.8	12010.42	12032.6	28255.61	23477.09	19644.14	24769.4
Q6P6T1	7863.362	9447.945	10291.63	9597.733	13299.68	18030.9	11041.9	7514.524	10151.92	19052.78	21143.4	15306.04	11600.05	23288.17	10409.14	14783.3
Q6P6T4	5027.579	4694.85	6665.863	7219.168	5732.329	4959.682	4153.984	9190.883	22638.77	7227.62	8980.357	7779.322	8977.691	14913.85	10419.24	8668.729
Q6P6V0	56051.97	37545	25269.11	35132.49	48748.08	42431.43	50707.02	35258.73	62948.12	45406.93	80323.19	57469.58	74790.72	74481	48682.76	32484.71
Q6P734	117809.5	170702.5	126669.6	139007	93387.8	95384.48	108196.9	137105.5	127267.4	159003	66990.34	112947.8	147363.8	101009.3	136746.8	154546
Q6P767	484083.9	542402.1	886381.5	645293.4	1093565	399719.7	422329.7	552576.3	724923.3	601699.5	631391.8	667255.9	649740.8	445734.9	981575.5	495816.2
Q6P777	6400.618	9218.939	7066.214	10659.18	11819.63	8902.909	10538.95	10314.97	8587.017	19069.85	9789.406	19803.28	11197.01	18342.42	15005	16292.27
Q6P7A9	725125.2	686548.4	694771.3	788587.4	671900.8	555211.1	635159.4	597206.4	655688.2	747146.8	577302.6	615535.9	590668.7	750944.3	930272.1	756472.4
Q6P7Q4	38589.11	17544.77	56769.64	31796.7	23552.4	29995.66	33299.31	31281.08	32781.54	35170.11	46120.14	48028.91	42908.67	55761.09	40971.68	26829.49
Q6P7S1	90771.57	130273.2	162605.8	117387.4	220359.1	81395.12	104493.5	93057.55	192047.4	131133.5	130934.2	153118.1	107951.7	141388.3	169107.8	113735.1
Q6P9T8	83332.22	69496.34	35508.63	44233.03	24959.29	75692.02	50095.2	75205.02	34198.27	45013.16	45718.76	65963.14	31259.73	33649.91	32183.71	36319.2
Q6PCU2	31369.58	17219.63	43507.14	19890.83	13469.77	31024.46	23693.42	14854.07	66005.66	47841.09	84960.3	78187.7	48614.98	49487.11	37434.86	55758.49
Q6PEC4	14106.19	17067.29	14172.85	22719.43	24832.76	24621.57	18462.04	30634.15	52568.62	16460.71	41919.41	19033.79	21382.1	30048.59	42963.6	15410.79
Q6Q0N0	15175.5	9421.276	13111.53	9126.685	5966.626	10701.8	16662.63	14560.91	5364.537	5256.317	8549.857	4963.072	5412.323	3143.168	6460.857	6876.819
Q6Q0N1	47948.1	50799.36	42798.75	42443.49	88273.28	79801.8	50232.68	48086.56	181405.9	79754.02	323161.5	151961	131687.6	164956.8	99669.3	111633.4
Q6Q7Y5	271371.7	105512.2	161795.9	312518.3	101460.2	176355.5	188214.1	312336.5	394861.8	75011.03	296758.3	235327.8	122583.6	136162.6	74388.2	118240
Q6RUV5	92409.74	47866.42	61125.92	129625.1	84831.5	140726.2	98277.54	155418.7	106824.1	44015.8	315779.9	83524.98	99519.8	85892.38	65288.83	126421.4
Q6RY07	24207.66	58101.84	14206.36	33552.8	22947.57	33290.02	25325.04	29598.53	19839.31	35715.7	12742.25	18113.89	22495.34	15261.13	16682.37	15419.86
Q6TMA8	26617.2	49007.5	23494.39	88908.2	41860.84	27767.76	53976.68	48648.87	8988.991	11032.44	9847.581	9983.791	13702.45	14676.38	13426.77	23934.59
Q6TUD4	18440868	28278152	20858956	17253314	24579376	32248396	16812942	38324664	44941428	28366654	13999607	31215060	24599572	27858214	25141636	22177178
Q6X936	12042.26	21226.63	30649.45	21677.72	23021.5	21834.06	16488.43	14592.69	17238.79	21625.84	13372.4	17022.18	18735.95	15927.67	12566.79	16477.58
Q6XQN1	12352.06	13291.16	9119.197	8074.263	16163.35	10109.83	7092.467	14316.29	19432.66	7699.678	15995.93	14904.74	10749.32	11519.34	15809.84	8530.839
Q711G3	53774.36	51289.67	66233.95	70213.29	88775.13	35228.26	66687.77	55155.19	103523.6	91329.04	171061.3	127750.6	116690.9	143248.7	81447.2	94994.56
Q71MB6	14568.01	13270.24	11264.04	16291.88	20607.92	18973.8	13243.41	29265.73	56176.68	15881.78	53815.24	40725.23	52547.79	35694.48	35490.39	15605.41
Q76HN1	73163.28	69131.91	73832.2	84289.98	50425.28	53136.5	57127.86	82205.42	64126.91	113454.3	55771.77	62473.54	74226.96	64986.92	112656.1	74157.12
Q78P75	32502.41	16678.03	8083.596	13072.03	8560.836	33271.24	24843.05	20566.11	13064.89	8401.854	15504.02	10847.42	12689.37	9497.874	9215.181	5760.99
Q793F9	42492.25	82298.63	47037.38	161796.5	25705.77	107414.3	50652.85	66177.55	72351.8	63503.19	94128.28	74184.4	66923.48	59537.34	62943.81	50949
Q794F9	7190.102	8244.545	23942.17	17839.25	19268.06	18821.04	13199.53	9038.046	23379.06	21377.59	15257.82	20273.81	19828.92	15877.18	22350.15	14261.46
Q7M0E3	40543.28	36627.86	34974.64	43606.66	27230.05	38594.43	31233	53910.98	102209.9	42334.7	100967.2	89639.48	77652.34	73592.34	43168.8	62611.87
Q7TP52	31102.79	40764.11	15491.04	36942.55	45850.44	25989.86	30783.32	23480.7	93281.1	39666.51	54551.02	46186.58	41623.59	59092.88	42856.88	24246.11
Q7TPB4	42749.17	75166.73	54229.77	51688.98	66932.79	76385.75	70946.92	68262.84	104086.7	78238.64	40006.22	64851.5	73844.53	76555.48	57014.2	56388.72

Q7TQ94	33517.18	34026.43	24126.76	34609.23	40233.07	28227.16	27952.04	27585.74	67692.63	36787.83	86866.7	62394.05	67703.71	76213.32	50641.85	46443.76
Q80W57	73786.73	100069.2	49180.7	107887	148428.3	131423.9	57016.99	93893.23	334624.6	65681.48	310771	219714.2	199444	227369.1	148223.2	129927.8
Q80WD0	45811.21	80187.02	62835.55	64794.9	66125.01	70878.48	61634.95	73797.22	74522.3	84382.13	49503.18	64268.34	48246.73	50274.68	65072.63	56942.78
Q80WD1	73161.7	123997.3	113467.5	87037.41	118573.7	106693.1	73874.93	118852.3	126669.5	139889.9	61508.14	117747.1	103324.1	140328	112582.2	80698.17
Q80WF4	28982.73	49223.29	25282.13	27569.45	45242.38	29348.14	36207.28	36488.23	38170.75	34219.22	21182.38	45552.07	36613.79	50122.02	40577.97	27889.35
Q80WL1	20425.68	58901.19	21612.75	68636.83	16063.28	50370.72	50390.37	9687.123	24359.18	38821.49	22988.08	30481.31	9009.336	45717.89	22159.34	23247.27
Q80WY6	20114.96	25316.79	35846.25	38151.79	26825.72	41223.4	38471.3	24468.27	16563.93	55346.52	15392.33	19119.63	27844.43	13248.75	37882.98	34537.41
Q80YN4	10876.61	15731.46	12090.09	17666.68	16327.27	13119.68	5530.785	11807.47	25217.13	15593.11	8087.005	13858.81	6849.095	13238.35	13439.37	14235.84
Q810F4	55942.67	69511.26	51281.27	71762.98	39878.36	55831.71	91553.5	55134.92	38959.68	71179.46	24853.2	56537.05	39541.1	42842.4	44212.55	30293.81
Q811A3	13266.55	10378.04	10890.37	9894.156	8234.611	4853.208	9238.578	5871.055	8277.281	8636.833	6009.478	4782.627	7746.783	7852.262	7802.045	8159.581
Q811M5	5624.336	9390.504	6733.234	11614.58	10361.64	36612.81	5635.603	5737.716	20516.45	23577.74	4888.808	6136.937	5448.346	5129.578	13213.08	10643.12
Q811X6	73334.44	54466.77	77620.27	50221.75	96660.68	51240.2	64914.75	73759.83	165324.9	95708.22	116778.1	135422.9	98940.65	125469.4	100045.1	78655.4
Q812E4	9688.908	3944.206	1960.554	5667.225	2255.838	5166.739	9075.742	12032.56	9042.283	854.9191	20467.84	3158.531	2955.378	5819.293	942.5548	6979.181
Q812E9	127347	133669.3	75130.5	133051.1	151601.8	166078	83295.75	133975.7	211514.8	73887.52	192234.4	190048.6	251826.7	216314.5	154121.7	184421.2
Q8CFN2	32594.27	23388.72	27352.79	32605.07	28256.96	46607.79	38700.66	87355.35	26689.69	15834.99	143434.5	35955.29	24948.41	33121.82	16170.83	46871.03
Q8CG08	78104.35	101778.4	117685.9	113116.6	124330	101706.1	81051.55	106964.9	154039	171850.3	85483.32	120628.8	156666.9	198323.5	166294.5	120452.3
Q8CG45	51577.48	42516.59	28245.26	40712.04	69800.73	48083.98	37317.87	43488.54	54355.99	43215.61	123684.4	75534.56	78788.75	85488.86	57958.93	53482.02
Q8CGS4	19927.7	13219.17	11419.4	19293.04	8861.479	11544.85	17745.27	8063.704	24260.16	20792.01	39784.08	27246.37	19592.84	31674.8	15358.33	35875.4
Q8CGU6	9880.534	4823.996	9748.976	10592.21	11938.17	23889.29	9903.272	9516.53	9151.454	2887.647	12198.63	6729.345	5116.818	5544.674	9986.785	7036.007
Q8CHN3	2492906	2859150	1510637	1939151	2935265	2789057	1730708	1961193	2010305	2758183	1660138	2351682	2115764	2208451	1881715	1832846
Q8CHN8	14957.59	27268.15	17800.25	22271.87	7712.702	23798.19	16645.19	17195.78	20135.99	21611.51	13722.2	17316.62	18736.74	14190.03	16153.34	15158.2
Q8CIZ5	33167.87	94486.68	44750.86	80907.69	34068.92	38806.8	48573.01	117784.8	19483.75	23218.39	22123.16	23231.03	31674.8	23834.98	20746.09	14598.79
Q8CJ52	17776.34	6920.233	4552.353	13327.43	5903.495	12382.81	16664.03	10213.12	6275.064	2337.86	18684.7	3094.823	4136.276	6006.905	35087.81	9514.982
Q8CJD3	9296.99	14508.51	5182.398	6300.583	14180.17	8931.173	9148.667	13493.04	18331.71	6008.455	5157.634	38040.47	9129.816	10839.76	8628.143	10645.44
Q8JZQ0	129200.8	142473.6	128464.1	170162.5	149655.7	200200.9	125032.8	156050.3	106753.6	148906	113002.3	120026.3	151086	141527	101369.5	111677
Q8K1G0	5823.641	6857.119	11353.69	10734.8	3217.253	5799.1	5308.231	1813.362	8104.4	7227.945	2222.737	3003.371	3352.042	4115.954	4937.649	4896.212
Q8K3P7	6996.168	6906.551	8499.978	7678.421	7967.496	6850.5	6856.937	9342.468	18342.81	7196.331	14842	21743.3	17328.07	17175.76	8999.245	16534.09
Q8K3V3	17293.35	28297.05	36717.34	18304.25	27814.57	29988.72	29874.67	31103.13	39203.15	31682.03	27646.29	34178	22771.89	26497.74	28728.39	25455.34
Q8K4G9	13055.52	21340.44	20362.8	15083.07	21959.38	17988.79	12273.71	11854.32	17145.56	16015.6	18238.34	26535.15	23539.97	10293.55	16346.87	14216.91
Q8K4Y7	9328.145	14029.46	12233.74	8835.063	12102.25	11053.01	11537.46	17781.68	8361.325	11549.85	10264.21	12738.57	11524.82	6926.847	8056.188	9433.869
Q8N7M5	15090.51	12813.92	13615.59	19673.88	19911.85	15524.31	13593.25	14839.77	10162.92	10169.7	18471.86	5613.471	15025.91	9411.373	6970.848	11876.81
Q8R431	17908.71	30263.27	16309.99	16402.3	22750.46	28744.46	12716.47	14444.23	75138.19	14844.64	64622.59	50406.02	43255.43	52669.04	30375.21	24576.36
Q8R491	46186.64	75738.62	57800.11	56148.89	38226.34	64940.23	31185.47	76527.59	51261.98	55391.48	92317.53	70615.39	45050.16	34275.95	33350.85	64739.48
Q8R4C0	16198.26	11408.88	10776.16	15521.7	5334.776	14180.8	12711.92	16004.4	3622.354	7222.068	12741.24	5811.792	9507.615	9512.871	7772.571	7000.148
Q8R4E1	33909.67	41960.16	26432.58	36383.6	32505.26	41058.66	31591.42	45038.26	47694.5	43908.29	39158.19	40739.23	35238.57	18216.04	16214.32	29210.5
Q8R5M3	48044.11	43514.77	60605.95	45248.38	31530.95	53470.64	22875.17	68715.41	98191.39	143642.8	62887.69	114604.4	79565.45	107708.7	98416.41	49890.38
Q8R5M5	28335.02	17172.57	21957.22	13703.38	26322.9	9965.9	9601.949	11996.35	15559.09	16201.04	16959.79	14489.67	14128.03	22911.07	20016.56	12685.82
Q8VBX1	13401.88	10500.69	5934.323	8703.305	3027.826	9646.339	6512.653	10790.31	11855.25	6975.948	26267.82	9321.437	9183.06	10735.69	11983	6398.068
Q8VD89	62255.89	175382.7	133394.8	56701.77	106857.1	47293.12	138123.8	116576.2	113546.5	185916.8	106594.6	147341	188142.8	148165.6	100488.4	177897.1
Q8VI04	23278.1	19930.8	11340.53	17303.64	24832.54	15139.69	10100.05	29063.06	30189.81	12544.44	57029.43	32170.06	37277.32	34723.83	27739.07	23016.87
Q8VIF7	109070.5	97795.52	85122.26	96995.16	157495.1	88165.98	101601.2	110132.5	175014	113286.1	158864.4	124742.6	152064.7	180364.7	131654.1	98949.82
Q91XN4	135467.2	155024.4	150752.8	90997.91	126416.6	192896.7	92441.59	107183.7	97146.33	110434.4	63767.87	108152.8	76721.11	57753.18	76973.92	95471.41
Q91XT9	17602.43	22298.59	22637.77	17039.21	21464.9	20065.04	20646.56	19347.76	11738.56	9143.68	13447.56	11942.4	8594.23	12916.69	13223.75	8336.566
Q91Y81	5500.886	5345.66	9664.935	5506.233	4301.779	4774.919	4095.327	2810.854	13636.92	10862.85	10774.77	10072.13	9540.382	8949.531	6785.551	6568.29
Q91ZS3	17943.03	21805.27	7549.988	8389.996	10244.05	13063.65	10276.19	14827.15	6704.158	9359.662	5998.111	8496.646	6600.738	6006.096	1896.308	5726.159
Q920A6	955418.4	1078793	995027.1	1058399	980555.9	1220244	765318.8	1110769	677797.4	933685.4	696329.5	876635.9	585645.4	548401.2	1148009	806853.7
Q920G2	9930.617	2796.4	9041.855	11709.49	3618.79	7836.055	7713.238	7516.723	14778.85	13517.21	16552.6	15212.25	17755.59	26705.51	15679.69	16150.23
Q920J4	23118.65	17538.56	15198.11	26084	20779.77	27660.51	18644.88	28350.39	24419.68	17198.54	23430.98	7564.231	14129.03	12414.35	16439.27	9725.416
Q920P0	21855.09	14038.49	23976.39	19421	18350.27	10573.83	15437.66	16115.36	40674.03	29677.23	33400.34	34309.39	27368.12	38210.6	21901.91	19626.33

Q923M1	14152.08	12911.13	13836.37	11063.05	22937.67	16155.36	12891.39	31700.59	29836.96	7288.897	34254.37	32764.65	17859.56	35592.17	15111.4	18241.59
Q923S2	71049.89	74498.09	39669.89	65578.29	92753.84	72392.49	40761.31	46113.65	190577.8	52524.16	239609	173665.3	213427.3	128662.5	135252.2	104525.3
Q923V8	8758.258	8292.441	17289.7	9060.559	3247.949	6647.497	12448.27	24189.83	7006.903	10341	3191.856	11602.26	8612.396	5560.2	5535.499	8254.23
Q924B5	14596.68	9163.35	6562.501	10549.52	8023.906	13641.15	9676.105	10684.36	4871.556	8444.544	19207.27	9480.471	7958.781	5989.268	5671.791	5632.976
Q99041	1561490	400629.9	892341	4331648	1489221	4242733	4097409	3691872	211351.7	1438026	6875437	472504.8	2319323	2509942	1133380	2165982
Q99068	8947.154	9834.113	11871.6	9628.402	15876.62	13871.63	8102.169	9691.085	11758.48	15168.33	6754.436	10015.38	9447.054	10497.34	7914.478	8705.079
Q99376	4745.258	7120.443	5100.815	2644.293	3925.326	2838.15	1821.269	4410.213	3846.232	5979.918	2560.64	3640.339	6659.578	2083.29	7348.743	1845.537
Q99J86	148568.7	192673.9	243404.1	129066.3	221032	147345.4	164654.3	215008.8	170356.9	175779.9	203522.5	144625.1	113079.6	100097.3	112268.8	132352.3
Q99M75	30401.21	44209.92	29316.02	34793.31	43336.25	16255.99	34297.12	28953.24	20402.06	33636.27	24096.03	20250.25	12762.27	17285.11	17778.45	19807.8
Q99MA2	322598.5	318231.3	2378613	388815.5	284573.2	304288.8	238172.1	333774.1	702359.3	292422.8	982226.7	573860.4	706072.4	617292.4	534515.9	401170.6
Q99MF4	52971.56	55635.65	38052.54	46392.29	41150.9	63417.39	63666.73	69503.45	43330.07	51262.02	24790.47	45745.86	17819.31	43059.53	18718.3	48296.88
Q99MH3	1050262	2802666	1315431	50503.91	724888.2	1401386	331329.2	907364	1885239	1114777	1050500	1070005	468426.6	301213.5	1439315	72480.86
Q99MZ8	11866.02	19039.92	9621.491	15168.82	15813.39	18106.8	11243.99	17589.44	37130.07	20502.27	57568.98	32411.47	33073.69	29214.52	20545.96	25845.92
Q99PD4	3146.441	4064.709	4740.805	5398.821	3618.865	3503.877	2954.916	5165.078	5941.767	3485.493	8592.987	3508.11	5089.345	4212.016	2859.688	4079.06
Q99PP0	10566.38	24085.7	20660.06	21463.9	14352.84	30559.31	15995.71	17228.06	19704.1	31188.7	9672.195	11934.26	10097.73	12337.04	14261.03	13060.49
Q99PS8	558049.7	822806.6	538146.9	552836.1	1041361	582657.2	760771	712102.4	665000.2	755477	400842.6	598850.1	561687.9	664503.9	732238.3	522335.3
Q99PW3	137702.1	196869.2	261574.9	137715.5	174026.5	160181.1	149194.1	304342.5	144477.7	168742.4	61806.1	92089.26	102899.1	117513.5	210082.8	136165.2
Q99PW7	10289.39	9587.336	9991.923	11072.43	9451.748	12956.39	11772.04	6552.984	5460.528	8576.979	6509.774	9153.536	5666.326	9087.38	7675.484	5825.753
Q9EPB1	5241431	7513651	6020243	6315764	6725388	4454389	5044317	5267229	4571187	6514046	8374120	6753912	4926680	5442559	7298172	6070115
Q9EPF2	43673.21	64585.25	59473.46	58203.67	70257.13	60764.25	45663.28	44102.05	61245.63	76304.22	27433.84	54098.61	46977.11	48671.04	58116.94	43554.51
Q9EQS0	2436.59	2435.752	15472.52	7478.342	6308.134	3865.877	10037.26	21929.79	8443.494	13722.29	9713.949	14448.16	5478.26	11875.33	12934.92	6128.583
Q9EQT1	11439.7	10191.68	8290.964	9502.009	11474.65	11614.5	10085.6	10851.51	10998.48	17515.35	15515.68	7293.162	6362.575	9610.161	14190.79	7659.11
Q9EQV6	136879.3	128644.2	123002.6	173540.7	113374	133475.1	126618.4	136659.5	95706.36	143393.7	132163.9	111424.5	136489.5	116625.4	131161.6	151091.3
Q9EQV9	99062.2	88990.09	84822.78	98260.27	28440.86	64845.51	75587.73	48883.23	46609.23	75803.68	32165.49	42643.8	55411.07	81951.7	99505.85	90357.98
Q9EQX9	14303.13	17373.86	10793.58	13170.9	18817.03	16521.14	16703	14147.78	32722.73	20555.27	46158.89	19646.99	17782.4	22734.25	14516.43	15169.38
Q9ES87	78983.87	110293.6	96885.26	213099.3	124383.1	87568.41	13762.99	70912.3	101028.8	139090.2	48206.47	159580.9	135245.1	115419	109264.9	97933.15
Q9ESG3	31918.77	44003.43	35812.69	51631.56	60109.95	47890.31	24477.31	47564.55	101175.7	38589.88	75219.11	69733.01	83165.09	86628.88	46313.92	68607.73
Q9ESS6	194574.3	225226.1	148818.2	224047.8	233965.1	225563.6	166375.1	204971.4	192579.7	185359.7	94205.58	163329.7	162692.6	170292.6	141549.3	129571.2
Q9ESW0	17462.45	23337.13	21888.02	25551.27	14291.92	20144.43	16407.71	19181.84	10806.54	12754.1	13211.04	14580.54	9863.412	21958.62	12992.18	13275.45
Q9ET32	21665.13	9275.021	16808.27	13786.73	8050.466	21712.21	9447.758	16750.31	11647.96	4960.233	20967.15	14024.74	12244.44	12966.24	5124.013	20609.16
Q9JHB9	665208.2	239530.1	122288.7	306865.9	142657.5	328500.9	446734.9	431910.2	68724.06	72088.92	470538.2	140562.1	63929.69	83639.9	55334.65	407482.9
Q9JHW1	23800.63	21901.38	18037.05	27799.46	20406.53	21089.69	26853.55	30153.61	14423.88	18120.93	13607.02	13989.47	21144.79	13228.83	12331.93	15417.28
Q9JHY1	1102272	1184459	1212532	1023247	904899.9	1092048	1164232	1243701	1246236	1536512	732535.5	1004194	829634.3	907530.8	1061723	695790.8
Q9JI85	523896.6	249607.9	128217.6	306368.4	85359.61	470394.3	226415.3	434397.8	41199.09	60515.22	297023.1	122068.7	174709.6	53948.03	28615.88	110043.8
Q9JI92	209088.5	180417.6	138043.5	219893.5	192793.8	178486.9	230281	282039.1	58896.36	134965.7	180318.8	129888.8	146914.4	135553.3	141497.2	148571.4
Q9JIK1	716415.4	627897.4	635719.9	483206.6	602894.4	441408.5	965163.6	663870.2	791860.2	870700.9	366469.8	865029.6	725822.9	699006.4	584680.4	508980.6
Q9JJ19	696689.3	588942.9	523674	682294.4	752748.4	816039	426565.7	585153.3	1771220	583644.4	2301338	1323045	1242978	1525162	1010842	1029237
Q9JJ22	14918.68	12155.33	8271.395	7993.317	8772.274	13088.87	9376.887	5979.235	17200.2	10291.82	12597.83	7958.135	8466.744	6582.733	8249.509	8577.691
Q9JJ40	490162.3	487337.8	428667.6	455356.7	747850.3	624737	309930.3	536182.4	2126121	407624.7	1358955	930213.6	1018565	1216246	938703.1	549066.3
Q9JJ50	15298.01	2229.491	20246.78	27414.44	1344.888	11433.3	18593.8	22537.12	18242.11	21151.66	27040.61	28589.09	25418.27	33978.19	28216.58	26840.17
Q9JJS8	10887.96	11912.54	10293.15	19832.74	11353.46	8759.301	3206.914	15941.67	11702	16879.7	3323.145	7886.452	10033.85	11659.52	17016.78	3983.116
Q9JLJ3	40575.28	41244.6	38767.88	42354.02	55307.48	37515.48	37987.43	41205.08	116134.2	47741.51	135444.2	94839.34	105406.5	110099	68462.75	61809.31
Q9JLS4	34495.47	80351.32	80425.87	85416.48	63219.62	67609.63	54664.16	103285	87913.04	95651.66	44175.45	85143.56	83715.02	117175.7	82544	74387.64
Q9JLZ1	5806.538	6889.602	12676.48	10336.99	10172.91	14518.86	7404.479	7704.177	7413.879	6619.753	8594.185	6486.67	7017.185	18653.48	8169.089	6676.935
Q9QUL6	1471.905	4536.971	3558.678	3515.921	1317.558	4123.969	3043.549	2665.402	3146.301	3718.357	4705.862	2726.587	3229.403	1918.199	1905.421	6396.246
Q9QW07	23730.79	3340.824	7166.561	12124.35	4012.592	23777.26	17946.74	22518.34	9079.203	5475.777	27517.68	12709.35	1247.617	7431.29	12215.48	16607.11
Q9QW30	25058.22	36295.89	19807.48	35882.87	32686.34	49324.93	37968.57	34053.09	21135.55	32974.59	19156.3	18050.19	20921.34	19598.58	26189.37	30117.99
Q9QWJ9	15332.9	20840	13636.61	15803.28	20365.53	17431.96	15845.29	14854.73	9470.543	14157.57	10515.2	18555.49	15876.39	13838.52	16161.64	16203.66
Q9QWN8	23714.51	25471.43	17319.13	25095.95	17791.32	24649.19	15623.98	118212.8	14825.09	5171.688	16598.93	2762.076	30742.43	27996.06	6172.115	7527.593

Q9QX74	977431.4	508793.6	724766.1	1245412	1595986	1030202	2932904	4051348	233008.3	165279.7	266993.5	49497.43	81402.7	111367.7	185840	117971.9
Q9QX79	1636906	898083.6	1581173	1093300	994004.8	1239638	1503747	785493.2	900072.9	981170.7	286090	455398.8	479247.2	636544.7	1039919	924656.3
Q9QXN4	11928.32	10620.26	2795.936	12196.18	15091.19	9994.11	12159.87	11992.56	11166.06	5242.447	24676.39	7060.561	8119.425	14566.26	9437.947	11657.99
Q9QXQ0	8433.485	11780.17	14966.91	11088.33	15002.73	10990.62	14587.49	26226.62	30527.29	13351.53	30116.87	21981.77	21995.71	21884.2	12122.28	9187.591
Q9QY17	28116.51	21403.58	11603.33	21493.74	23821.17	26987.43	19899.39	17576.18	33052.26	13455.41	74220.57	27176.66	33579.43	31359.04	22124.91	32740.9
Q9QYP1	584547.6	313199.8	107033.1	1687032	17643.65	168780.2	266928.8	35055.55	4378.992	437986.2	17101.01	102566	107788.1	290123.6	321038	451346.2
Q9QYP2	6839.26	11874.62	8398.73	10835.56	7628.673	7636.054	6073.367	7774.735	1993.207	17772.46	5575.463	18232.72	14825.73	11888.96	12134.51	14152.74
Q9QYU4	23038.53	22199.19	21731.67	28232.73	31212.9	22320.53	22477.15	31366.42	82646.98	28456.66	78432.62	44505.48	57321.16	77729.11	43932.38	38436.95
Q9QZ76	21399.55	17892.83	41990.89	19442.64	8084.307	19802.21	15035.19	36368.32	12136.33	26271.03	17990.43	35170.21	29126.66	25858.5	17543.18	28862.78
Q9QZA2	170163.9	185213.7	151583.5	167901.3	188425.8	134259.5	164722.3	170409.2	173004.8	145283.6	237406.4	220173.9	215356.5	211188.1	168689.7	160646.4
Q9QZA6	12544.71	8998.95	6060.023	13453.57	8773.57	11257.29	10600.9	7963.944	4272.917	5653.74	24655.38	6391.975	8995.061	6306.842	5221.116	10261.16
Q9QZH0	124743.1	75800.39	84645.27	141347	88655.55	109348.6	140125.5	114331.3	106906.5	123143.7	51893.17	88944.95	57512.55	127012.6	87955.27	128779.2
Q9QZK8	33708.8	22548.83	18424.65	39919.34	18734.78	33938.87	30506.59	42141.45	10586.53	14084.94	23659.13	13498.76	14546.66	12819.23	11328.4	18253.05
Q9QZK9	29389.63	16634.94	35029.19	28190.11	16948.68	49279.88	42257.16	61667.41	9922.857	12258.94	95910.64	25665.71	4685.003	31474.33	39113.76	207807.6
Q9QZQ5	79113.99	180948.8	96176.05	128924.6	115843.1	353522.9	89519.89	133558.6	125198.9	225892.5	70597.66	117088.8	109576.5	93736.91	74565.91	92414.66
Q9R044	11333.11	16487.08	12460.89	11258.35	17089.63	8881.189	8999.775	6411.245	11123.09	8014.277	8667.24	9776.299	7129.249	6559.749	9227.441	7532.363
Q9R063	139188.5	97304.55	105023.5	82939.02	67996.33	101032	106992.8	120313.9	148254.4	104751.2	207904.8	99786.95	105345.3	101414	75987.41	120111.2
Q9R066	90399.29	113689.5	90521.78	75400.55	90822.77	110128.5	89822.13	126055.5	77252.55	119521.9	47451.46	77226.38	61953.85	67710.39	65911.55	75529.1
Q9R0D6	128460.4	171903.1	160825.4	137216.1	154173.6	139253.3	142415.2	164873	211170.6	134910.4	94739.41	136671.4	119233.4	95944.2	100744.2	133380.5
Q9R0J8	47381.81	100855.4	84662.35	72070.21	59360.37	70021.32	52891.88	45473.43	44145.95	53095.25	63168.6	41553.74	43955.12	45671	55769.62	79585.23
Q9R0T3	32629.3	27859.1	25014.47	91819.52	32552.36	51157.81	54305.57	37337.3	14649.73	18034.84	28654.09	17923.88	16752.37	9624.116	5528.889	17531.77
Q9R0T4	7046158	11925518	9084906	7251290	10787157	10649002	9399055	11347194	3114002	11355200	4419639	8347045	6774438	9006068	4515842	6275580
Q9R168	1573.723	903.2755	5821.417	2895.599	3911.102	3653.973	521.8255	12148.93	438.8409	3161.786	1968.817	7088.812	2672.713	2035.155	4128.205	1781.424
Q9R1T3	118557.4	155932.3	116614.1	150977.6	95551.64	121116.6	133984.6	108044.2	60664.98	117306.8	70092.13	67636.24	87780.66	93832.98	73109.81	86067.45
Q9R1T5	19019.95	18508.88	26313.99	24006.05	43189.18	17619.12	15859.43	15742.6	81222.81	25947.92	50695.18	46364.45	43492.88	49507.21	29609.8	26030.08
Q9WTQ2	333753	313335.3	260549.2	284730.9	330731.7	249626	253910.8	192638.2	168967.7	181155	296954.7	360084.8	248530.3	196327.4	256165.6	165697.4
Q9WTT6	6449.038	7025.339	5593.703	7687.497	8522.07	6307.022	31245.44	29201.67	5261.457	4439.603	10294.13	4146.802	4717.744	4455.731	5311.718	3944.116
Q9WTW7	126692.9	118094.5	81808.21	168205.9	296364	179476.4	72540.03	131274	758673.7	127210	338591.1	320329.9	340984.1	394492.3	203423	160323.9
Q9WU49	2476.233	2291.172	3246.358	3551.998	2910.753	3191.573	2224.242	2579.96	3524.615	1724.784	15098.42	4188.343	4420.291	3064.036	2144.469	4277.79
Q9WU74	4257.849	7085.559	3743.003	7975.885	2626.821	8172.231	4155.218	4704.879	5254.084	6713.748	4337.59	3716.471	3215.596	3174.905	4409.814	4363.612
Q9WUC4	194731.9	560000.8	441584.5	511326.2	311738	316597.1	141461.1	312693.8	559346.9	496888.3	261459	496151.4	273058.4	335017.1	263618.6	251219.6
Q9WUK5	198310.4	281865.6	222229.4	293542	237813.2	284601.1	311775.7	463520	287755.2	322425.7	224071	340135.2	312005.2	347749.1	391785.2	281105.6
Q9WUW3	79019.3	56596.02	74671.27	79959.34	34116.7	63507.68	105571.4	67795.27	62191.51	84066.24	35959.02	37754.38	52721.36	60519.52	133558.5	120704.6
Q9WUW8	17982.08	11321.53	7772.278	27799.67	12670.82	41232.61	15184.15	28914.51	96410.79	43118.61	91173.62	79814	40255.25	37216.66	72000.97	41410.12
Q9WUW9	62330.06	63766.95	57597.36	61665.27	83348.38	88919.46	53024.1	61889	234040.3	73695.62	279812.7	157349.4	117346.8	108051	150375.8	101136.4
Q9WVH8	23978.52	63679.5	22610.17	34632.37	22749.76	63492.99	42843.45	20878.97	45647.48	51248.4	14968.68	16301.22	27347.93	20965.25	21659.07	26545.85
Q9WVK7	7145.32	18485.54	23403.11	13052.5	8671.267	10645.97	9816.744	13070.28	18441.9	13354.63	15432.39	25625.6	8572.129	19742.52	11930.35	8560.881
Q9Z0J6	34459.38	29773.53	94471.85	7282.885	12464.15	18072.2	15820.67	41767.93	39108.16	55172.21	36716.98	63862.56	42288.67	74437.33	52904.16	23147.08
Q9Z0J8	4977.063	9253.384	9344.724	9455.254	6119.99	10028.31	6676.177	6746.923	2755.38	7375.471	3483.062	5819.157	4432.082	5649.547	6325.754	3841.074
Q9Z0T0	11152.46	12499.32	8665.079	13118.13	14305.08	9897.161	12562.53	16715.79	29928.65	10911.75	40627.04	19633.3	24001.03	18100.56	11608.61	11769.83
Q9Z0V6	53139.29	51456.69	30474.13	62912.22	55963.23	35860.72	85588.64	160213.7	43776.26	36450.15	26526.93	22087.25	19863.99	15709.79	55640.64	26954.47
Q9Z0W7	93761.16	85045.59	62072.21	94381.6	135396.4	110726.6	60679.89	79741.52	282065.7	99543.57	365226.6	224299.6	203475.3	272030.4	155918.8	122783.1
Q9Z1F2	3656.027	7795.289	28855.7	5504.741	20626.22	17419.57	2111.945	6314.532	17028.07	8647.033	21207.28	17483.34	16528.1	16002.39	15579.81	47221.13
Q9Z1Y3	840022.2	930166.3	882677.6	577613.6	1365608	999967.9	1064746	426250.3	437241.6	976963.6	385668.2	593520.4	645710.5	863332.3	657430.4	575982.1
Q9Z2L0	12105.55	29521.65	23028.41	11902.7	27445.71	18225.27	7897.843	9014.681	39899.74	33302.21	79802.71	17046.06	10021.91	11575.82	19202.44	21152.58
Q9Z2Y9	107028.1	64054.23	83582.43	78342.38	51243.29	66432.98	82967.92	49553.87	73690.33	104741.6	77887.58	93143.55	93411.59	83101.64	90237.71	73851.75
Q9Z339	111558.9	106635.5	100676.3	130105.2	49833.05	92926.72	90791.81	121749.5	117615.9	130426.2	173855.1	135280.8	153326.8	142803.8	92720.06	112463

17	18	19	20	21	22	23	24	25	26	27	28	29	30	31	32	33
D7-rat1	D7-rat2	D7-rat3	D7-rat4	D7-rat5	D7-rat6	D7-rat7	D7-rat8	D14-rat1	D14-rat2	D14-rat3	D14-rat4	D14-rat5	D14-rat6	D14-rat7	D14-rat8	D28-rat1
1919.766	2643.188	4824.7	2330.602	1282.103	2131.455	1439.455	1154.202	1697.099	2496.585	4426.349	8057.217	2305.404	1310.707	2002.03	3117.718	2396.435
8287.629	8419.541	10744.44	15708.75	7058.671	8668.59	5745.223	9397.341	7485.921	5840.699	9610.224	15940.97	10915.17	7731.211	8106.85	15392.95	10516.84
8755.765	8465.674	7600.805	6477.061	5700	5672.431	4779.194	6393.557	6907.784	6438.863	6765.586	2227.715	6676.911	4618.235	5842.749	4182.896	9417.421
97940.58	88638.01	107183.7	137283.6	85499.74	72491.2	86230.43	78663.72	82032.55	55890.99	58577.7	87996.2	94153.66	84638.56	104986.9	91085.44	79398.83
24685.57	22300.41	23887.91	49907.57	25932.55	27193.93	30089.01	36820.43	27726.15	29252.32	28532.24	19238.02	23236.84	29948.04	29805.46	35190.22	26264.23
41961.08	38718.74	66945.84	48352.19	54235.96	35456.63	32133.48	33220.57	44120.12	24816.88	45135.31	49340.18	47463.45	45621.16	43299.92	43069.03	26568.73
26697.71	13948.2	52296.24	25732.82	46949.14	32330.12	21060.71	29535.34	22042.84	26487.16	36816.95	16045.38	55889.96	41388.21	40337.28	57548.26	72172.61
10362.39	8100.998	15536.83	27111.54	9502.433	5812.13	4202.554	5684.448	3614.468	5777.59	13772.77	12523.42	3977.529	7586.813	5925.294	10546.4	5463.271
16515.95	19090.6	18871.48	15947.69	25391.88	16212.7	13807.2	19083.26	21609.07	25667.94	24374.67	25617	75779.91	31825.6	35871.54	24667.66	52560.88
5476.513	4951.538	3994.026	5974.649	11971.24	3444.67	4012.946	5306.601	6920.229	4794.619	10138.44	10564.17	9679.765	3044.994	2068.097	4077.135	4853.165
9910.273	2457.88	5887.265	5482.493	7624.169	2976.305	6666.014	3263.091	1195.154	3665.153	4928.077	6687.019	4073.171	5460.232	3229.717	6686.428	8264.326
447499.9	478297.6	348805.8	326699	549668	215426.8	272273.1	313636.8	486964.2	437326.3	332443.9	421310.6	433820	311076.2	303815.9	352566.2	273679.3
22366.43	22022.79	22979.17	24919.01	21263.2	30565.42	26464.54	19453.3	21225.79	12920.56	39367.76	24346.98	31397.67	21030.62	17227.01	26482.97	26122.46
24329.82	28358.12	30268.28	21801.65	33317.48	31635.23	42738.46	35779.03	48866.79	35466.18	52162.12	31617.68	48841.05	29504.61	43574.23	51863.25	73370.64
27568.03	25614	23038.13	18111.82	21398.22	16917.39	28865.28	20856.88	22561.92	20757.34	27462.44	20319.6	26253.98	20002.3	30105.46	19712.6	22665.91
175512.7	137302.6	86667.52	95754.59	85086.94	117746.6	199410.2	149529.1	109968.1	104635.2	189987.3	61137.32	121719.8	70614.05	122606.5	80745.48	174910
25241.27	10955.61	11395.82	15511.03	15915.36	10325.88	10393.64	19588.06	27329.09	17434.28	8080.561	17723.81	6574.783	17127.06	19642.4	18789	13897.33
10565.5	9629.414	8031.232	6763.482	10827.57	6583.961	7428.118	8304.375	7887.954	13682.71	9548.096	7877.572	8319.696	13051.54	4801.008	12221.71	22915.45
297613.4	206312.7	182409.8	79658.99	79618.77	340678.5	212583.1	328538.5	1077791	400245	286082.3	121447.8	295767.1	577421.6	365939.4	514639.1	608612.8
39546.38	37090.96	42785.26	38575.68	17091.36	26971.75	54692.72	31866.15	32840.79	39259.93	45296.55	49614.27	22940	44754.62	52449.77	40817.03	60423.82
5866.951	5330.805	3295.934	5347.402	2513.449	4676.148	4512.813	5319.919	6888.675	5436.76	4183.168	4149.291	3572.503	6257.284	7295.368	2593.134	5264.752
19933.63	22049.77	24619.01	33621.24	29383.26	20743.76	17551.87	22887.84	22559.43	15106.24	22707.14	20704.5	26882.44	23077.07	19960.46	19313.01	15001.11
47155.09	36082.09	33098.99	52832.07	66939.41	31380.72	50756.95	41472.34	53590.92	47047.1	100668.9	36021.66	81911.59	56042.79	68314.03	53507.44	52133.54
18020.21	5944.984	11098.3	51302.75	14043.91	4647.355	3795.62	4595.787	11722.17	14431.72	114731.1	7654.981	14606.35	16881.11	8845.487	14554.02	10335.98
2993.97	3459.255	2987.207	4979.671	3912.036	1792.017	1934.727	3111.389	3050.643	3377.349	7681.155	3769.119	3590.135	2585.092	2621.446	3765.736	2787.91
4398.737	7528.436	12730.44	12521.66	10503.23	21165.48	6448.492	7523.606	7560.494	7186.451	11206.99	14378.07	11892.74	6682.921	6656.07	9853.536	5538.727
20112.83	23047.29	35683.52	53490.65	23572	12981.6	12674.33	28902.11	13384.8	17478.22	27832.7	54702.15	35318.91	13778.65	18998.34	30259.73	20079.43
10054.81	11688.59	12091.2	22877.68	19622.41	14624.56	9474.373	10281.18	12227	11244.99	14097.82	24365.74	14759.65	19219.47	15786.72	17527.91	10866.14
235683.5	234964.5	143998.2	143863.2	51032.57	125711.2	84204.8	100582.8	155417.7	135047.6	78801.19	78579.15	47839.74	106824.3	97114.48	95886.59	161548.3
199642.7	175297.3	152114.3	160464.8	210095.9	147060.1	207302.5	132342.5	196322	156721.9	185149.7	137462.7	162569.2	207335	205594.3	170345.8	146682.5
122863.2	66965.81	95688.65	124999.1	75253.09	88214.08	52915.27	43501.91	70741.21	54954	79119.22	74131.27	72948.18	94853.5	124200.5	98575.84	73223.02
54192.71	30471.73	120218.8	86791.96	129948.5	72118.66	76958.06	53167.03	173580.2	214735.4	265453.3	252503.1	127594.1	149498.7	234117.8	101721.4	156112.8
29624.03	36477.52	52602.35	56062.45	38081.73	38193.7	26776.82	23519.76	33410.75	15380.71	25325.06	22272.94	37087.6	26471.35	30016.94	34150.09	31534.46
102986	125232.3	55239.27	72847.09	83784.81	70778.81	101185.7	113284.1	116958.4	114733.3	103809.3	60439.52	72868.31	68467.99	109395	132176.3	133595.9
8775.507	8998.105	11035.99	9059.575	10164.95	6957.301	6788.654	4595.002	8635.829	8820.146	12180.92	9374.545	10388.22	11249.15	10786.33	9976.979	10024.07
7722.653	10274.47	13784.73	25083.98	12687.98	4336.637	2725.568	8754.836	8279.646	7105.442	18716.05	17645.77	13844.76	3830.112	8835.99	14968.04	12652.89
4521.627	3116.662	2029.85	4875.786	1853.57	3536.025	2347.868	3070.552	2794.208	2250.108	5342.721	1294.924	3974.682	1461.174	1939.195	2415.584	2428.574
5766.79	7760.931	6713.822	8362.336	10714.46	8020.389	7098.365	8940.479	4750.06	6212.258	9341.737	8976.774	9896.589	4528.169	5488.068	17702.04	6872.026
7136.015	3905.991	3022.993	12675.87	3432.15	4341.03	3265.036	6423.169	1937.437	2075.147	44594.91	4957.636	4872.03	3616.361	3263.172	3713.909	6148.359
55432.91	81953.25	63220.53	47083.47	42521.25	53986.01	51478.28	91927.38	80948.63	91944.96	78449.74	94498.56	88407.67	88137.52	96389.77	84789.09	76445.34
32299.81	38016.05	36863.43	27968.16	53865.33	22483.51	30305.75	24616.14	36424.06	40189.97	30461.82	26012.25	35023.04	25851.03	31068.87	26348.11	25515.7
410828.8	541229.1	603850.2	550938.3	643675	407346.2	499484.6	505293.3	444046.3	816457.3	452991.6	778004.3	781714.3	528855.3	519212.2	622670.8	433578.6
181961.5	199201.9	240293.1	283342.1	167575.4	218651.7	172820.9	178885.4	192836.5	153128.9	199550	223147.8	193313.3	206589.3	233651.9	213343.3	200653
20304.34	39334.65	45441.41	43500.14	33180.83	24654.37	23727.27	18869.16	19485.74	26018.72	22453.16	52398.85	33642.07	33298.75	26905.35	29651.9	18242.1

9262.364	13816.5	8068.045	7422.848	11133.37	8385.983	6632.263	9421.795	8983.301	7951.102	8781.854	3948.693	7782.984	9187.688	7119.425	11810.88	6822.379
15457.5	19127.12	18676.09	30207.61	16768.11	21442.12	20898.87	17255.25	14520.6	11115.44	12697.54	14628.81	17054.49	21168.21	23264.75	23947.07	21580.22
85362.36	81556.52	128814	248304.2	97086.62	78073.02	68190.16	109255.7	85272.57	61968.45	122912.5	149402.4	89310.93	97344.72	80322.51	117450.5	76476.06
15309.17	10693.97	34071.73	58363.93	30370.9	10997.77	12033.91	27215.67	16644.92	15549.18	42337.57	46305.54	43151.48	12128.74	17097.03	43401.7	18372.31
1127794	1312195	798049.5	764523.3	756221.7	1002805	717257.1	914286.6	949239.1	1082617	265712.7	845806.6	842876	853180.3	577562.5	740383	316913.8
24655.76	58501.84	40698.71	36958.12	54268.91	14632.45	37675.69	30378.36	36332.5	39426.54	18965.61	35510.86	50574.49	45862	33107.06	53364.55	60416.64
343165	263645	477330.9	750160.4	405503.9	393536.7	261197.2	330615.9	269317.3	167893.2	315731.5	309113.3	350962.3	357705.3	234037	426850	278269.2
39883.54	46768.76	41161.43	86685.43	48679.47	63177.92	41547.49	52356.51	72819.98	58343.52	89281.05	51717.07	66143.35	50710.25	79497.78	61467	53058.9
10453.6	10809.32	8739.587	10068.95	8591.134	6322.863	11397.32	6040.521	8779.755	13059.05	11819.54	13018.58	7274.867	15766.17	15642.57	7334.552	7355.805
77372.58	153334.2	74276.07	140703	76722.94	50242.39	56337.93	74587.29	104167.3	114668.3	75533.59	106179.7	80301.01	50602.2	64414.89	121961.8	104513.4
101231.3	14501.83	3659789	129981.7	15370.36	5042.089	69797.8	4010822	5759.158	3530235	384283.4	2627512	15550.55	16717.06	756979.8	4824732	2771095
151838.3	249423.5	118429.5	139971.8	117485.3	143494.5	114880.7	126923.8	208536.1	194564.1	89949.34	78837.46	128115	168046.1	121076.1	193304.8	337395.8
30634.21	43492.38	31697.4	17480.56	31752.63	83974.59	75821.39	62167.89	74540.03	57813.27	46067.74	45029.25	15610.95	63746.26	76022.29	61094.23	63220.16
120816.2	104454.6	56837.32	98692.73	59465.9	66192.05	67942.13	82879.5	88264.39	93746.98	50385.7	95505.94	52741.54	71864.3	91429.61	73405.1	70243.37
14016.85	13143.68	16700	20225.52	17775.26	14805.62	15140.34	12298.23	15203.9	12614.94	19956.3	15778.88	16884.46	15738.05	11376.3	15363.74	10484.3
177411.5	366420.2	298273.3	311098.9	302136.9	193372.7	223166.8	279390.7	266133.1	357167.5	206002.8	206126	257165.8	201056.5	273713.7	484375.6	802681.4
989378.9	1313989	1196503	620952.6	518797	868775.1	1084245	622063.4	1061877	989544.4	423776.9	817234.6	577105.8	632829.8	1122819	511585.5	1050080
15848.42	14053.94	15804	15294.31	15017.18	13062.75	14616.44	14896.32	19057.05	14817.66	16852.65	12515.02	16135.25	10824.53	13976.62	15944.59	9449.415
5981.471	9020.777	6601.734	17713.06	16171.73	9317.127	6690.521	7560.564	8386.02	7295.258	6335.182	8086.938	17407.36	16273.07	8504.996	27075.31	11709.77
28338.28	26727.03	12343.88	11283.05	15569.21	14556.08	21783.8	20194.42	28436.73	37917.2	19170.11	25462.13	20223.02	23796.64	27860.29	26717.85	56602.16
238755.5	191554.9	223419.1	197068.1	146297.1	187602.3	244779.3	210087.3	249793.8	225700.3	240551.3	184526.9	161562.9	184207.8	265621.9	170873	119939.6
5623.656	2864.231	7222.912	7662.932	3664.003	3528.69	3582.892	3299.579	3934.884	2612.245	5340.38	2141.984	1188.469	1732.75	2305.913	3423.9	3089.549
26874.34	31753.87	54745.05	67519.04	36006.28	28515.71	21537.48	43887.44	38819.95	34787.07	27537.73	48963.74	32104.81	26943.32	28940.39	42098.37	25184.14
9323.787	9760.951	11074.16	27338.01	15734.23	11108.08	8200.979	12925.23	11406.06	6441.427	16148.54	12693.46	10329.15	9296.007	12559.69	13490.29	13407.9
1288072	685550	608246.6	1130203	122623.5	846728	216984	507422.6	1278137	559261	970268.5	834743.9	214536.1	1526647	206844.3	533300.8	1067246
417122.4	461931.8	457707	416746.8	317959	339454	364160.9	511124.4	464425	568097.4	438799.8	494442.3	358674	470451.9	631132.1	520907.1	529069.9
861312.3	1167612	687032.4	687583.8	1539603	528202.3	580279.9	968281.5	938059.6	2027009	659198.5	777602.1	2039074	657651.8	843619.8	916765.1	1227840
211188.8	227135.3	173792.2	181270.8	182283	151099.9	194359.2	199453.3	207642.1	222229	152059.9	198896.2	222357.8	176054.9	234880.3	213726.3	213387.4
16584.15	15175.8	9926.932	12706.74	17456.6	9763.389	11557.12	10525.46	9859.101	12774.61	9076.725	12339.45	13375.06	15557.22	10878.95	8320.883	16523.78
29981.09	41600.27	63503.61	118934.7	58443.13	74944.98	38879.34	66304.86	39949.59	34040.21	47127.76	43218.1	18007.54	58060.49	33748.07	62689.99	21775.7
29098.96	20774.78	15459.38	15657.78	14137.7	20711.38	17725.68	21092.7	12004.46	20275.47	17692.62	16235.47	12105.82	14366.95	17137.09	19412.05	29031.55
23118.96	16997.86	19997.5	36808.84	23788.38	26491.04	25908.46	22073.75	26952.47	20976.88	23613.54	38108.31	29835.89	22574.8	21267.6	24356.56	20930.86
2910.915	2273.763	5565.902	5852.026	3761.092	3170.044	3112.848	3749.316	1915.448	3430.197	2309.853	1968.531	4919.831	4251.786	3868.397	4777.796	3974.775
8932.46	8307.156	10587.29	16427.72	9601.382	8703.298	8243.824	5382.445	8355.586	7790.243	11157.31	8964.595	5579.112	5858.72	7428.465	11564.61	5706.798
205543.3	279070.4	390971.5	174756.5	319563	183116.8	182128.8	209010.8	265863.9	296424.6	292455.3	224221.3	305025.8	233656.9	176455.3	259677.6	193828.3
114090.9	147777	157438.3	172533.2	128880.9	103113.5	105519.9	138205.3	194641.5	131546.4	165333.1	160109.4	149843.6	192944.4	241870.3	203638.4	236024
21387.08	20027.39	16533.34	16443.96	18251.79	14838.06	19345.97	20329.05	19842.37	20385.84	15319.4	17460.72	15259.55	13381.47	25044.07	18864.99	22365.08
2048.185	2045.595	3726.942	4050.862	3164.382	3329.014	1248.932	1892.862	2954.682	2134.97	859.481	1202.662	2926.647	7023.051	1146.834	3046.906	4125.543
55983.9	57131.66	51515.83	26024.55	13591.56	46930.79	46761.17	37865.95	55177.12	42005.59	47067.34	39533.38	27012.78	33921.2	40621.89	31380.78	37707.73
404196.5	500597.9	420267.2	494536.8	545690.4	378579.6	264457.9	291660.8	355176.8	366786.9	356227.8	384184.6	445367.2	436065.6	346823	364918.8	295328.2
240255.3	6882.668	150870.3	163529.6	22139.91	157877.1	72675.77	67692.48	13631.54	85542.58	25562.21	81176.17	2326.245	100138.1	101398.9	119322.2	197372.4
388225.8	372598.1	590475.3	653583.3	520327.9	451884.1	411622.5	437359.4	387715.4	280694.9	442023	449584.4	513515.1	474703.6	314781	405440.5	220866.7
9637.828	9719.293	5496.287	26903.55	13041.03	2301.897	5403.778	5850.48	3651.422	3336.791	18040.71	13788.54	7951.008	6074.705	5590.878	3305.434	7160.601
21513678	19215620	18108928	18894842	16317583	14331335	16378173	14035172	17316070	19539790	16068776	15628922	18491154	16954984	9087015	9416069	6697781
52570.34	52062.01	153604.3	121772.7	160642.5	48446.35	35464.4	134320.8	41144.12	34421.39	209140.9	33570.35	188275	149331.1	131464.3	638852.4	179651.1
4810.147	6088.48	6293.012	9887.645	9162.69	5506.176	5404.312	4594.513	5451.01	5136.329	27184.19	4982.089	8513.866	9615.492	6981.132	3562.353	12081.04
7248776	5695043	7119243	5414422	1088430	5095457	6889322	4980817	5628496	5710791	1761232	3380776	720336.6	6049220	8580335	1431461	6670208
2558569	2369597	2034140	5348000	5771061	3130629	3257491	4554504	7900789	4558763	10066341	2269810	4289042	6146466	5622772	12280705	6794211
123800.4	32376.36	5503.056	220234.6	21685.86	5838.207	12465.07	4966.24	3879.489	1852.066	585431.3	7252.349	4674.96	8132.784	7884.353	8014.768	5666.559

188330.8	241237.8	204773.4	207777	157906	210415.3	258523.4	264367.8	244506.6	307616.5	197722	236539.5	172536	239387.2	341194.9	332828.5	305567.2
700016.3	775550.3	1132807	778003	867813.6	606598.5	742618.5	686814.3	808756.7	958462.6	1069766	611251.6	998028.7	946764.1	988142	986930.2	1396671
493559.6	512833.3	560791.5	795997.4	553038.2	471779.7	258195.8	445301.8	538592.8	413471.3	401027.9	370016.1	414072.3	477383.1	368754.5	516422.2	344014.4
446282.3	1004679	1114297	785496.9	1108691	537670.3	1097782	1361380	851071.7	1423417	1248175	1022455	1572312	763956.7	828555.6	1572355	977376.6
287960.5	329505.1	253160.5	181873.5	220583.1	646431.1	2611977	424234.3	224302.7	264774.5	238910.1	226981.1	220760.5	217023.1	375830	196312.3	147946.5
348386.4	37807.49	20451.92	10943.01	57495.86	18601.55	9288.477	375157.5	53247.11	78718.61	459026.5	7626.842	10579.12	97257.9	104696.7	90609.82	495084.1
153083.5	124409.3	55002.95	130585.1	131154.7	49351.38	62466.47	77416.95	139934	158941.1	99176.77	102431.1	145487.3	54125.79	70979.7	122411.1	141051.9
1705109	2274227	2664483	2493738	1242074	1696524	2633604	1787790	1325948	1210686	1283972	961791.6	1300629	1823173	1151895	1629565	2151901
839311.9	408381.1	945721.7	187513.6	255107.2	491446.8	664750.8	362981.8	2786295	260014.4	770264.7	412646.8	263963.9	628226.9	966079.1	2663305	4925177
154978.4	199040.1	218642.5	154434.8	172820.9	178672.2	160708.7	165214.7	231939.4	234373	164423.7	174996.8	229676.3	219977.9	231765.3	212104.7	325382
17406142	10703178	19030824	6228824	43743.63	8621022	8640034	7296651	18821022	8663022	15956442	9024039	35188.43	6560158	8469511	7006785	27898000
13562264	19333706	13651976	27875716	17865962	12148783	11263275	18941548	19851380	12966996	21133992	10876383	15421612	26746306	14700331	21525610	18671680
132588	9241.803	68884.91	8719.424	60944.35	427705.4	38499.43	26189.88	536597.1	249363	140825.9	30455	127182.8	2623385	1144082	20511.29	13083.01
141901.6	7586.146	75314.98	5205.108	41575.57	501933.7	38323.89	23197.34	428675.9	190640.8	94835.69	21228.63	113636.6	2801891	1173239	15043.75	18692.38
135045.3	143557.8	49723.36	138958.9	91605.66	43237.94	48136.34	38095.94	102909.5	64775.2	51561.88	99742.67	23255.63	33616.32	51462.25	149593.1	102781.3
146781	309867.7	97705.6	104266.7	365734.4	174841.4	97303.38	255041.9	396599.3	170550	204819.5	165298.3	136297.8	774221.9	362095.6	417933.5	379303.2
30537.2	35722.95	25522.94	49227.58	85961.52	16425.23	18011.73	17250.94	46858.79	21103.88	24058.53	38650.07	23876.19	26933.89	12377.35	35481.95	51911.2
1931479	1832804	1448855	1312416	1192984	1922009	2411219	1395630	1985719	2256046	1186005	1261127	1416682	1838615	1736167	1815080	1700100
79665.65	154202.2	53569.35	58791.42	87885.36	77948.39	208058.6	146545.1	142804.6	140776.6	46228.64	80045.37	79486.38	69471.6	147039	116535.8	174168
11209.83	22075.37	19961.49	11070.69	12623.05	103017.4	528832.3	12417.39	9431.059	15202.27	15167.98	12901.99	13197.57	17045.56	22422.22	14196.53	11053.79
5377.261	6465.923	6453.038	6130.941	5310.223	5398.825	4857.335	6919.482	6869.725	7149.477	6109.415	2602.165	4910.583	13050.01	6160.224	13034.03	8661.587
2.14E+08	3.93E+08	2.43E+08	5.12E+08	6.34E+08	5.37E+08	5.22E+08	6.92E+08	7.14E+08	6.97E+08	9.78E+08	3.7E+08	8.98E+08	6.57E+08	9.27E+08	1.25E+09	1.51E+09
3200942	3948922	5356792	3684127	3936417	3207841	3031816	2194572	2234772	2363821	1078531	2233069	3297741	3864156	2976009	2826739	5287891
918424.9	1088824	769076.1	615704.6	1151793	1146642	1949178	1332169	1553908	1188117	1029521	752578.6	1107136	1451120	2431807	1556073	2185077
23632314	20030050	9718478	10258899	20735914	24762192	43740276	29205414	21911626	22682998	12242249	19293742	19550316	38803264	37049952	21735158	29025606
11357430	20538112	32789620	1.27E+08	29998402	17180750	4869543	17262438	21711074	31205840	43061508	71734592	51807432	33702992	26024368	91719840	56875012
18136634	13020377	21926260	38172572	27974782	7189034	2328587	27359492	21704152	19770186	31561198	46599176	23984808	11632574	11803642	31419398	29278810
9852235	5395926	10284511	12170486	20374788	5330686	1669832	17945228	7973550	13141739	16780034	20726424	10288528	6054368	7219019	8729329	13401751
5179.239	3840.061	8897.97	10330.63	12280.99	40045.19	70395.66	4755.372	1119.455	12879.98	12885.38	892.5995	15249.02	9419.576	7745.29	7889.106	1255.072
10113.15	12701.4	4004.617	9638.357	8554.364	10539.01	46092.88	1214.925	33301.16	22328.51	27952.28	11051.19	12301.79	16444.82	142837.8	3720.588	30767.36
6713.344	6671.677	3860.485	9788.563	5666.583	5011.706	4641.207	4358.027	7474.986	6340.621	9641.451	7917.322	5616.976	7684.123	5306.821	7570.124	7212.818
29650.69	30313.38	14656.33	39164.17	31796.77	17622.71	27070.83	19976.9	30686.85	34189.11	29726.92	26237.77	33101.28	31011.29	67810.25	36751.86	52868.21
142109.2	118305.2	79957.77	88300.81	196352	110056.8	47928.72	72408.66	121602.6	117262.2	109843.8	108658.1	201941.1	133449.8	80920.23	83243.66	87926.22
102206	115808.1	104911.8	92358.72	45164.24	73199.2	91723.8	69599.06	98866.68	98783.25	74466.61	90421.43	70451.58	37303.64	79086	61897.2	112742.5
1243123	1187115	603498.9	734369.1	747659.3	994954.6	2386683	1454497	1018585	1229257	402446.9	985838.3	849405.3	565609.2	1543975	1086520	1089892
33521.76	78206.32	25562.49	88576.81	13093.75	53214.8	92921.52	34739.96	43373.15	37413.57	36192.44	25230.98	25631.62	23913.36	58878.54	37255.56	51485.04
44735.62	33209.55	38022.33	39923.06	73094.24	24465.03	25836.5	17873.46	29178.28	22936.27	63359.14	54417.57	48401.02	17549.46	13975.87	11352.13	6092.384
60916.04	69351.37	39373.38	38708.26	60478.63	172024.8	548209.8	72015.9	65399.09	110165.6	45250.3	43161.91	51300.58	100590.8	83577.81	83128.91	141657.1
98056.73	194828.6	128631.4	165319	228754.4	145552.5	145400.1	87599.51	150233.7	169729	177578.8	243325.5	204997.4	173566.4	204601.1	148654.9	95582.82
6506.394	3998.413	2082.058	4093.171	19315.02	3115.107	3952.65	6845.967	6621.033	5246.755	12422.12	5956.813	10645	9835.207	6796.923	4405.028	6448.033
361284.6	510545.7	472389.3	547444.2	465650.6	477461.9	553785.3	388996.5	418203.9	349358.7	437321	567298.7	449924.5	522858.4	723545.4	528071.5	431164.3
81261.83	83997.47	96255.99	98958.37	76464.81	53394.3	53337.22	71764.34	62810.41	77736.87	102977.1	116334.6	75000.02	69006.94	81784.02	97387.88	82094.75
394877.7	545789	487026.7	584708.4	381328.5	447252	363094.6	387961.2	378070.7	374256.3	607780.6	507385.9	375721.8	413561.4	505208.3	461131.9	430104.9
63503.88	41727.55	100576.2	125501.2	79803.52	44751.32	44471.77	66179.16	54667.48	50004.78	89192.88	111681.7	103814.6	57018.39	54528.86	81575.77	43643.8
21909.47	19558.1	36225.66	42563.87	21870.49	28640.87	21783.67	25808.06	21567.8	16154.88	39380.14	19075.03	26987.31	22872.09	19621.34	24328.6	18037.93
790464.3	876527.2	1070611	1402318	1238251	1195334	812242.2	1013419	853587.1	716664.3	919136.9	1031578	1219648	978421.3	799511.7	978056.6	568048.1
28939.63	25562.54	53838.38	68515.56	47528.48	13061.61	9320.327	24543.23	24188.04	22468.19	47221.53	73434.45	39059.05	19716.92	14842.7	55656.46	20121.23
69004.66	36009.11	70035.16	92816.02	88851.85	55787.77	41195.37	48126.49	77038.55	34399.19	90458.05	34889.39	71855.55	38546.96	33470.91	42613.63	49173.83
30823.13	23893.82	18723.93	24685.11	30128.64	35392.24	54274.04	29704.73	36093.39	25137.71	16356.18	24860.21	49271.77	25263.59	57904.45	30866.03	58409.21

7187839	7344261	6108439	4890942	6016590	7141039	6942556	6233429	6288145	5973300	5763096	5833266	5843608	8523959	6837586	4766042	6374512
47798.32	80324.67	67259.66	102347.5	56179.27	39819.5	35915.31	57486.91	80103.05	50378.54	81444.44	68107.74	70125.84	42960.82	57242.27	60937.9	70078.95
26639.2	34571.64	48030.27	48010.95	41313.82	33885.72	25618.91	32752.04	22993.36	31843.92	46822.15	60896.54	42062.14	26192.61	29353.96	41475.38	26081.5
7288.469	6010.898	26050.36	22289.72	22979.66	5116.691	6326.276	9684.211	8723.077	7935.344	17108.06	15971.1	22414.8	8741.555	7194.337	23155.84	12602.68
246379.5	339252.3	330132.1	286438.3	294262.1	220489.2	274113.2	271317.1	374914	485075.8	321058.3	343343.2	435844.4	256923	372165	374281.3	441464
45140	15673.88	18442.29	13928.78	37479.95	16548.94	6522.787	15424.57	27577.94	21024.18	30540.27	32321.39	44224.59	18066.35	23683.93	17842	28134.06
45269236	33822004	16605225	22211070	29338690	26944912	28204380	27894126	42493864	35172808	34887744	25658344	31142152	43042796	37123560	32693228	44450436
4148126	2629729	2464071	2297978	3457316	3786105	3690853	3488799	4088822	4719886	3410330	3010777	3174998	5760032	3300077	3303550	4840191
11943.85	9860.035	13108.86	19629.45	12180.99	9945.214	8820.77	8926.917	10316.48	8717.963	18065.26	20488.63	10946.61	11463.89	8669.253	14782.7	9177.008
108709.4	87973.76	93007.09	342314.3	117308.1	67818.41	63094.51	55796.72	89979.67	125143.2	570418.5	209379	103383.3	67698.27	125290.7	172082.8	88510.63
9401.768	12878.59	14798.7	21249.58	20046.8	13482.12	14948.67	13229.71	11250.66	11218.27	16362.21	11855.96	16813.95	18033.88	10820.92	16206.19	9033.269
9356.243	22250.96	7968.597	6630.538	11773.79	90393.3	500232	10993.89	14446.53	14445.18	15676.8	13895.25	10692.85	11987.16	18737.52	12751.23	17914.71
34469.78	48225.68	41526.36	34882.62	27101.79	26090.82	36678.46	32386.37	37950.01	57523.38	44719.42	60498.47	27912.59	38733.66	51387.59	29792.76	23819.68
2822871	1424165	2680988	2857182	5974074	391750.8	392975.5	108827.1	1806764	2532021	2119432	2023034	7165446	242120.8	863166.9	180127.1	2313664
107301.9	123839	174083.5	209252.9	158384.1	61685.78	48671.79	89819.52	100879.6	132870.3	175908.7	205412.4	153906.8	61280.82	83589.3	99532.36	131736.5
964329.2	2961479	3273450	1661061	2407627	1433108	2591257	1867403	1074329	2087972	1391385	1498385	2107856	1025857	1163376	2359482	2928568
250556.6	128458.6	62489.52	646539.6	45424.05	74591.38	38781.29	76857.39	296453.4	102746	169004.8	233212.9	149062.1	120762.6	183668.2	86030.97	180191.6
93817.47	93141.71	132323.5	81570.7	80734.24	45125.59	51596	26106.94	58845.37	168335.3	109970.6	131793.6	45095.3	49325.36	48447.48	56479.09	91425.45
1037340	1770820	1446641	538811.2	1171651	1041211	1184403	2156072	1698218	2155470	1416475	1030799	1899430	1541838	1393024	2579966	3357925
164274.1	190270.8	212883	148952.4	181929.1	139348.6	135330.5	152945.4	214428.4	235540.2	156503.9	203420.1	202904.4	188019	226683.1	206445.6	277803.4
739156.9	1524565	1387817	1248880	947435.9	738861.2	829305.2	721550.8	420589.9	453120.3	477032.6	1063231	682480.9	820039.8	744111.1	643813	850753.3
62160.63	59492.45	35866.44	54991.87	26970.36	53469.64	51056	17143.5	35166.19	30673.35	25647.92	34333.18	41386.33	29808.72	64576.75	28286.64	49245.68
3017688	2733260	5485974	4412752	3539598	3948942	3404185	3785798	3405045	2259175	3820489	3021718	3190399	3297018	3128234	4203844	4102365
7344.788	15390.36	13131.94	17927.54	17733.1	2271.215	332.3965	7372.14	8046.485	11690.16	14546.86	11576.89	2549.667	7988.227	7696.2	8288.986	5853.781
52542.96	68195.08	56142.38	49567.04	46722.11	45266.29	66309.68	60314.31	61804.4	101444.5	72145.08	63993.59	53341.29	53139.09	101777.7	59439.25	84444.43
14463.71	22475.75	23303.87	20760.35	23581.44	29994.96	23653.13	13941.32	20443.65	12185.98	18874.36	14927.06	32063.08	24201.22	16364.89	14896.15	7576.05
76907.45	131634.5	112353.2	101117.5	102760.1	80353.26	142540.5	83739.13	90056.25	106315.5	76877.7	100783.6	109140.7	127065.9	192748.2	99824.61	133727.4
19318434	21580806	25405442	19834158	26800006	17462206	24341034	22109584	19840730	26360780	26461736	29071136	26192710	22782050	25483984	24037022	15971605
4619571	4772955	3249366	3953580	4325319	4064867	6604564	5145500	5082546	4875787	4281931	4132310	4103439	6899632	11075898	6217549	10663459
8254330	7210212	10051431	14952568	15170648	3343682	914420.4	10625981	9891702	8291753	9314768	19749002	12433622	4856983	4295457	18293032	13126962
1329561	774202.3	1849521	2220550	1748837	1279288	893525.1	1326030	1377582	850614.2	2029358	2427035	1955181	1121670	919086.3	1732648	1305099
702324.8	627957.4	644678.7	482207.4	538582.7	676198.3	637890.3	613848.4	598899.8	545286.8	459719.8	532054.6	508617.4	523705.1	619801.4	423572.4	554767.4
37793.3	35525.05	82379.45	50620.72	17963.66	21337.69	37767.75	55149	38250.77	30939.37	60345.18	84255.63	52863.21	26667.17	30308.01	68090.88	66337.69
35365.15	30573.75	51774.82	42594.97	55372.48	16604.44	12998.98	27060.13	26427.94	30296.99	46154.42	70661.92	53730.48	14507.73	14972.2	34613.4	21509.61
140049.9	169329.6	288175.7	283755.3	146988.9	192699.8	172729.6	200343.7	114642.8	194904.1	140714.5	206663.2	158756.7	290881.5	247929	339915.6	239284
14045.9	20707.97	6845.656	10690.8	9971.97	30946.92	13692.37	22447.07	31468.78	32962.5	18501.57	6710.208	2226.418	28759.7	31063.23	26529.61	29220.99
377272.8	573430.7	394734	279906.8	407224.6	305764.9	264167	398815.5	325160.5	514138.6	377687.5	554060.5	394895.4	379816.6	255083.1	380441.8	266093.9
13408.47	26462.82	15712.89	5506.229	14551.63	20875.53	20926.81	18493.99	9490.936	29773.32	22261.98	24821.23	19688.26	33017.46	34085.13	32734.38	47566.29
263493	237988	127530.5	194762.5	145612.4	194497.2	246032.1	204456.8	233070	128836.4	174406.9	171766.4	176009	146445.5	197089.7	162513.6	229177.7
33447.47	26493.67	67083.3	83488.36	53005.82	28630.62	25332.75	30378.57	38029.18	36568.69	70309.41	88404.91	65137.88	50801.71	48862.51	66338.78	65336.24
2577493	4675174	1766343	1303909	783529.3	1622259	1232596	1219393	1657140	1715522	741835.7	1441077	626625.3	560170.4	435084.6	800796.4	1818005
64544.83	62714.55	65188.35	53969.8	78095.09	54650.26	76544.26	69257.63	63784.26	63250.73	77956.65	109939.9	103474.3	79736	56042.66	68206.86	49497.84
3553759	2183436	5092240	1985780	472827.3	2585894	2494305	4073314	3520606	3788491	2467454	2592997	641568.8	6057866	4791810	4871555	9762981
5981658	4454506	11721853	16898224	8944192	3733713	1150759	13325119	7346581	6571585	9236338	21870616	11183651	4977767	6269344	17911124	20818824
43763.19	13378.8	50022.29	73475.52	43996.34	25541.51	34690.16	27945.67	23210.5	30694.44	41814.61	48335.48	21256.86	23402.13	20905.23	54301.93	24520.75
1758930	2085095	2929576	1446439	1637740	1979247	2020539	1795708	1352088	1524569	1079794	690214.3	1173295	1485849	1117699	947347.2	1659207
18595.07	23997.8	12712.51	12719.06	19222.75	23568.47	89356.15	34829.39	18229.82	23464.77	7998.089	20340.19	18566.72	17502.98	42037.23	23466.72	36073.85
304139.8	482922.8	676470.7	406141.8	784012.8	418448.5	269186.6	241150.7	555422.6	610877.4	746266	992363.8	835048.3	777482.4	672949.4	461332.3	794531.8
411632.6	319276.6	300875.6	367312.5	315518.3	306601.2	699561.4	400585.4	312438.9	432501.4	317491.2	238395.7	323192.3	284655.7	482644	335412.9	326938.3

87674.58	118613.3	121416.5	103662.2	108279.2	119418.2	110884.2	90768.22	91551.05	121558.2	72288.68	135008	98578.2	141759.3	181154.6	111239	58722.86
14277.77	5353.637	15563.16	32681.51	17768.48	5440.051	3354.477	18901.61	19363.96	12810.19	16861.69	33087.13	17920.69	9476.517	6596.009	15908.97	16177.65
20233.79	15253.8	24474.37	35081.58	19165.06	14239.78	12937.64	15046.74	13586.62	22876.08	34306.4	33499.66	17206.46	16848.13	12910.54	18984.85	10036.81
66959.15	117338.6	123968.2	135833.8	111172.7	103718.2	64753.95	81245.16	64551.62	61470.09	102430.7	86613.74	94915.2	102299.5	59032.66	108903	56622.77
4050.609	3278.336	2510.504	11362.66	13210.32	10771.87	25479	6928.713	6861.719	8364.955	12363.12	10196.49	12148.97	7676.507	2288.772	7110.4	8700.725
7052.341	18151.95	38514.07	33246.97	29053.05	13882.09	14111.18	21261.87	46961.07	17744.47	46577.82	57233.77	34403.48	10386.67	12394.7	37743.56	29660.39
15486.6	16926.54	16082.23	11114.62	11921.96	11521.11	11114.75	8571.295	12591.26	16227.15	7380.337	10229.73	11338.65	8389.771	9907.543	9972.422	10813.36
326717.6	393874.3	571111.3	1104566	720727.4	424677.7	327070	622346.5	498984.8	458878.7	778070.9	399592.9	352632	779673	627916.6	1042803	497370.8
39966.95	38408.29	51166.75	29526.58	52065.43	21501.81	52514.27	50293.13	37224.5	43740.09	80109.88	92450.16	41739.19	11365.25	35054.89	18059.7	23634.91
70461.76	60897.89	95828.01	166339.7	81877.81	47938.96	38507.73	52262.41	77571.44	41138.59	68033.05	66352.77	48636.34	83008.17	56106.09	119443.3	93608.26
152978	163375.9	208175.2	392479.9	224780.5	122782.8	103596.8	140925.4	164725.6	113986.3	177450	183090.9	185975.3	149199.8	165914.5	241253.6	268439.9
155017.3	107221	92315.02	59905.12	123540.5	86524.06	87329.63	146089.2	68673.88	142235.6	81056.18	125213.1	105743.2	83157.11	158047.9	86491.56	259473.4
1062335	996827.8	840767.6	672890.3	872598.4	888382.2	871918.1	722571.2	926196.5	834726.9	778372.2	667225.1	646501.6	1408666	1161008	932831.4	1244754
10664.89	6404.042	17930.85	20730.13	17293.45	10817.88	6974.056	12121.64	10439.55	8706.299	16576.64	12384.61	8585.939	9808.725	10019.67	15179.66	13805.3
40644.12	31185.34	34160.1	31080.97	62379.47	18650.29	22394.42	24345.72	17532.16	23313.97	34515.15	32764.34	39286.78	21919.23	13001.64	15359.78	9939.969
72391.87	144521.9	454108.7	701372.4	107633.3	78838.62	632986.2	138125.8	362196.4	293412.8	474350.1	313739.6	479850.2	121491.3	314409.4	287057.3	164333.4
40606.89	45960.98	62470.54	81690.5	72786.69	61866.88	38791.24	47557.93	39307.38	33609.34	37515.29	61906.45	74468.99	53795.12	38822.1	45316.78	32181.62
23227.94	16299.03	48583.21	36266.12	30328.21	29274.4	19517.63	30204.02	26205.25	36274.88	38869.5	46833.57	29427.62	28615.49	35758.22	40793.88	49842.96
1169698	1367959	742551.7	967622.9	1541570	1414808	1489804	1964072	1219563	1466564	824764.5	1005230	1143471	2380093	1240355	2265570	1574009
4842157	5154353	2971253	3107591	2223509	4402771	7759394	4297326	6373075	5463164	4440781	3988405	1590295	4758843	9044161	5203686	7549675
25548.14	30322.48	25390.91	25611	33059.24	29611.27	48074.96	30463.48	55244.41	31548.67	41528.41	33920.85	26268.3	73225.09	76103.55	47091.48	48449.2
595981.9	525028.9	572300.6	1059873	673563.7	387619.2	307642.4	587813.1	478672.8	578652.1	679361.4	723108	648656	710311.6	519744.4	1090504	1030101
66008.45	76171.33	118625.7	89370.88	84253.2	86399.63	99999.21	72981.62	74907.51	66118.88	56413.16	81206.37	101961.1	104712.9	91570	94338.02	44900.73
7553.418	13066.32	13265.04	15313.95	19866.17	9174.035	10260.6	13787.59	3710.368	6821.649	17816.61	23191.92	15113.32	8780.075	6304.492	10970.37	9959.709
29071.32	45901.33	51704.35	88378.52	62271.94	20446.71	25798.14	33281.94	27389.66	45377.56	55812.48	73265.72	44127.32	21338.69	34938.88	51104.4	50198.14
3537.267	4423.762	5975.268	5199.867	3914.917	4724.299	3453.839	4515.881	6625.951	3036.625	8827.707	13432.42	5920.236	7098.034	5994.803	5856.974	10647.59
7371.953	6344.16	12775.19	7790.083	17153.28	12128.77	7388.278	8610.645	13478.97	18251.52	30756.56	18892.95	14630.01	16439.28	9491.018	15245.25	13355.34
43363.24	57802.76	52364.76	84397.05	49214.46	43432.96	36348.34	41851.41	18624.13	38872.2	99390.6	65546.41	47852.45	41335	36982.39	51815.02	27818.78
400859.2	556324.2	240669.7	1548657	142668.6	423310.3	126766.6	275551	587412.7	269923.4	302189.6	573557.8	507595.4	365102.3	717402.1	296123.9	479233.8
8044337	9440090	4463053	4482136	7276964	10095747	18412184	10518333	8421080	10096420	4507778	5516878	6904210	9491391	12777509	6428228	10202987
14334.69	12916.28	37474.12	43222.99	25750.03	22102.43	8161.183	19651.18	15370.29	8817.399	21906.16	27680.05	18821.13	19407.59	13753.92	19557.39	19834.68
50936.2	65677.59	73998.3	97715.61	59548.64	54856.25	60241.42	50584.84	55184.54	57561.25	60171.29	74435.86	68240.66	55195.27	56126.76	48549.05	35454.81
95682.95	140120.2	103777	76832.1	55193.41	43099.11	25782.27	67499.7	72333.02	103050.9	60601.46	96649.04	69630.19	80593.83	70987.34	47004.2	52881.56
541601.8	455227.8	147234.1	277657.6	86318.49	120321.9	48149.99	165946.3	571369.7	450725.6	166746	144341.7	343999.3	9834.672	220578.4	552605.4	527780.1
64401.49	70369.6	62402.69	75634.67	84826.55	41943.64	45480.97	37647.39	57853.1	44703.64	35746.98	66387.75	51123.93	87793.63	41334.3	49528.06	65577.24
597900.4	634338.2	532769.4	695017.8	334114.5	735679.2	1164372	695171.9	544987	438319.8	498069.9	427801.8	460136.6	833945.6	637971.3	577455	555353.8
26989.63	7235.277	12390.75	38656.06	11274.19	4081.262	5195.557	6143.369	6417.045	10880.65	82935.58	9422.807	10529.19	6365.537	4907.948	8506.407	10746.47
141099.6	140860.5	130173	89603.39	103667.1	126658.2	103403.7	78426.47	132717.2	153626.1	65670.65	116158.3	136531.2	116180.3	107530.8	63352.83	198534.3
2340759	1920305	1294378	1282438	1295080	3862904	8391770	2338878	2763547	1688841	1487691	1074367	1606966	3942298	2345154	1923268	2547591
9079.921	13526.66	5319.042	7767.355	6554.622	12756.31	6046.646	6024.504	16084.96	7369.635	8996.002	7332.604	8421.66	20972.65	8465.957	7203.886	8622.841
8846.196	11373.49	12012.66	12399.19	14687.08	9834.754	6546.512	8770.916	11031.02	5560.791	11695.51	9037.756	11479.06	5756.733	5129.74	7012.126	6935.525
27652.6	60817.28	36006.78	24463.18	41359.8	375826.1	1561781	33418.21	36771.15	44219.5	43651.33	39230.99	32077.52	39496.55	58149.56	27328	32919.74
162949.8	203636.7	133066.4	152517	118651.6	69937.43	185751.7	133590.5	215005	232168.3	135294.2	149699.4	92593.91	125990.7	473765.6	131437	242616.6
501733.3	459485.4	385949.1	267170	440796.7	414874.3	408152.5	574096.5	432039.1	488890.7	335430.4	505220.2	269276	725316.1	615495.9	722655.2	789615.8
90163.57	108229	100009.3	102715.6	88611.19	71489.38	80822.72	75882.16	44693.32	66083.75	56211.68	108102.9	50086.97	101578.8	92998.31	96757.52	52079.6
232658	121974.9	110029.4	228162	217686.1	132747.4	169987.3	153739.8	241604.1	184655.8	197254.8	115671.1	126616.5	136030.6	201385.6	352319.9	232612.1
2418954	1680564	1940368	2264465	1531908	2511119	1948383	1854421	2401427	1699670	1957764	1707464	1294661	1672679	2357684	1541082	1694896
3124388	3237577	2548967	2995859	2932742	2409918	2524847	2613718	3594782	3814556	2715653	2816602	3469563	2590657	2972882	2962168	4476928
114733.6	98852.94	119407.6	102076.2	96710.7	89895.98	89039.75	94534.99	115021.2	95454.95	94851.59	124972.8	126334.3	111645	99849.66	102139.5	109728.8

37735.11	30120.95	58588.38	52956.1	47086.02	34456.7	33631.32	33186.34	34982.49	22224.5	37960.29	39582.31	43518.36	40214.82	25793.35	35071.11	26075.49
2931639	2538695	5092293	4053377	2873926	1909272	1839423	2772023	2670712	2683219	3938427	4418203	3295212	1962597	2531698	3092888	3085241
11304.4	8315.137	9765.505	2647.684	8624.825	8205.824	10066.48	7455.085	6139.457	5023.046	7488.783	4797.373	23642.93	6364.58	4761.931	2260.566	7149.806
193531.9	174783.6	169844.6	196530.9	195668.2	152193.2	179277.4	232751.5	256236.3	313858.2	391091.3	209910.8	192550	384375.4	312484.3	389693.9	320865.6
3501120	4702394	8051501	7522967	2084756	12224963	2073169	4118209	6643700	14719541	20355664	9721659	3986011	8590980	4334867	8242205	7215070
116223.5	83412.1	69690.07	64792	48429.05	73245.72	100305.1	102372.7	113883.2	63504.7	75912.59	70339.73	51943.62	97421.22	112669	92968.88	135502.8
3126815	2703891	3104033	4176425	3444643	3308315	2807967	3198056	3156942	2452028	2809461	2756171	2575382	4153572	3177509	3645807	2255192
11343.52	8744.805	11575.42	11832.58	4834.886	6896.893	7151.678	5400.572	7849.946	6063.101	8849.433	27469.6	9034.338	5377.571	6870.52	8815.446	5691.501
76661.4	90640.4	68648.7	84016.63	76325.52	63529.89	61704.41	63887.96	54669.45	83378.41	60465.06	88750.9	70212.16	55475.07	87675.92	71088.43	110195.5
518176.1	152264.8	638743.8	578088.9	828521.4	357293.7	479020.7	805815.7	660664.7	349495.3	656035	306014.3	1140276	724771.9	1021134	1086701	1202622
12179.26	10896.56	13721.66	12642.19	29816.52	7950.126	10983.7	8725.593	12944.5	13365.67	16883.74	22904.27	20403.99	8996.42	8214.112	8418.785	12153.13
11355.94	7911.401	10044.83	16695.57	10573.59	6182.056	5451.569	4671.063	6730.746	2114.988	14020.46	9374.85	4937.74	9393.972	2208.327	8483.76	4740.765
19023.76	21381.76	17230.85	34360.98	56024.04	22917.7	20382.34	34298.81	33367.88	31849.01	50618.19	33103.97	50482.78	56031.85	38495.93	51953.18	38225.01
33119.05	33918.29	41441.3	45423.3	59491.1	37246.09	38462.18	49772.95	64761.42	29829.35	52446.42	75423.8	49985.59	45886.46	54263.22	42075.14	63218.13
65824.69	52434.88	54931.85	43033.23	33688.81	28117.1	42423.55	38052.86	55189.7	50486.68	49686	36178.93	37405.63	48229.27	60054.56	48755.54	65223.51
569708.1	237795.3	553184.3	820437.8	572856.5	426099.5	513698.1	354216.3	530451.3	145208.7	362947.7	99578.58	742837.6	369051.4	444903.8	503412	591140.4
8685.195	8981.358	12376.57	14153.56	8635.024	8785.514	8679.192	6584.958	7553.384	5818.037	6530.06	8993.63	7055.779	6224.538	6174.777	8192.646	5858.489
1798883	1822035	1947697	1384831	2566996	2486847	1962486	1985024	2647280	3065280	2329902	2443669	1430741	2680027	3173756	2095943	3821753
33185.64	48498.19	44802.45	50319.81	42425.68	34204.35	32177.72	29101.75	33741.56	35721.31	33297.13	41754.91	36665.3	45628.85	43511.02	36978.21	34409.74
16744.31	35494.56	4244.373	12217.11	6842.314	17522.2	30131.96	7687.43	13473.91	7513.602	15299.04	13118.09	12435.25	13870.85	12833.85	19487.08	35445.3
146220.1	124510.8	120392.7	92749.46	59691.38	40105.91	132787.5	154195.7	146338.2	148886.1	217327.9	125636.7	69739.66	64794.17	134211.8	114761.8	194929.3
518107.4	507474.7	510298.9	482785.6	379660.7	387351.8	427773.1	520848.7	615056.4	444042.3	528201.8	480414.2	271137.6	401569.5	451220.7	466762.4	227642
3784738	5636702	3196798	3626671	4541733	5873593	9417107	6301838	6319503	6916249	3680359	3966295	4301068	7718440	6666133	5829236	6583399
104395.7	54127.23	7802.15	206274.8	35215.58	24995.18	89406.25	18235.46	95362.97	113612.3	198481.6	52298.24	34891.28	40998.27	43733.16	34948.23	69353.38
5489.392	7887.527	2401.143	8097.332	6746.818	6380.614	4411.417	3458.633	4326.3	4170.322	15274.68	5361.722	6287.341	3945.302	3597.987	4931.71	5569.865
4513511	4557788	4229387	2635598	4425184	4686539	4578809	3601641	5006744	3970138	3160002	2822985	2635542	4042434	4625212	3414361	4725120
7461.301	8651.912	9973.631	9413.746	5864.425	10762.36	7160.525	6484.806	6932.502	3197.798	8121.626	5358.367	5471.746	5643.737	1871.379	5131.182	6523.07
11196.45	5500.821	10724.75	23291.63	10853.35	4720.677	5451.099	8303.928	7030.978	5185.585	34951.41	13026.58	10380.88	10048.05	4172.244	11079.64	11134.11
50901.41	58846.86	47211.39	50902.32	45247.05	17419.05	29632.25	47997.73	60282.84	125030.3	71535.42	82310.61	48839.73	30031.32	60495.11	117432.3	123721
8226.48	6981.674	9943.779	11374.1	17674.42	5166.293	4816.628	7506.268	5644.935	9241.099	14222.98	11713.8	10518.28	6680.698	5262.832	6034.894	7323.057
13890.28	19744.84	10128.22	7392.829	12703.99	12455.39	12655.68	15797.27	12425.38	12722.83	20564.23	13216.52	11440.39	14975.57	15245.85	15662.61	17660.41
80101.64	59517	128930.4	138425.3	46147.27	81850.49	78936.87	72168.61	74663.3	37811.15	91621.8	55383.54	64283.47	52244.2	34445.08	64015.16	37696.77
59603.24	46170.95	44413.89	87971.34	64912.14	72716.99	64864.22	50938.43	51134.49	42566.45	49186.35	38431.61	63305.09	65573.14	55517.77	59895.95	30732.53
11904.39	10098.59	2807.006	16545.59	13042.25	6199.805	28935.54	5017.292	25828.21	21883.06	166601.4	16703.72	15307.86	20218.85	108520.3	8773.98	40252.69
264645.5	328648.8	83765.23	99299.98	164315.9	50957.95	151793.3	206420.8	402188.4	444887.9	82230.4	169975	281756.6	52926.29	317676.3	293565.6	771208.9
40424.34	8134.292	6299.807	65253.11	17326.74	5293.813	5792.249	4943.17	9798.518	8995.122	78187.56	4624.045	18767.13	11761.82	10471.16	7010.741	17688.98
442326	342357.2	672989.4	709113.7	661315.3	598302.9	482513.4	418147.8	391026.4	314336.9	597898.3	563714.1	550694.8	618889.9	396747.8	580596.9	369793.2
1883.281	6996.248	9299.537	44570.21	28448.62	11497.36	20314.29	750.5003	68982.44	30060.57	51134.26	50033.16	26820.72	25568.3	35442.46	35020	35737.34
123125.2	121956.8	143710.1	210835.1	148154.6	118286.9	166441.9	98870.11	134106.4	116609.8	211585	190229.2	145938.2	128518.4	154970.6	144295.8	117262.4
1299.098	1270.243	10675.87	3475.692	1520.832	2586.147	604.9907	2969.631	3369.455	3198.078	2127.083	2675.862	3550.48	2644.799	3015.619	3016.925	4879.687
189917.7	111543.8	98132.29	56618.24	191064.4	60790.71	36215.43	174833.4	273040.1	167234	174966.5	134207.2	176864.5	160671	120637.9	158328.5	252123.8
11511833	14383037	11597223	9757007	11607215	12924050	12536122	14401358	10604974	12189893	10644204	8193951	11909207	13527264	12804750	12127825	18549268
2045222	2000744	2219096	1355125	1008373	1757776	2184009	1941670	2358401	2042951	2650912	1896078	1395258	1872896	2393201	1626296	1346972
26889.84	10648.81	22395.66	15238.98	11830.15	8028.911	4918.125	3093.428	19279.38	28261.51	19622.2	32965.75	24945.77	6823.53	8746.286	3799.017	15278.51
9178.972	9502.104	12011.14	10312.54	9588.657	6173.521	7731.27	7051.169	7366.702	6219.533	8354.767	7623.177	9434.83	8633.037	9097.744	8162.265	2751.1
292364.4	192258	160371.9	195339.7	175347.6	314707.9	402283.6	264263.7	349109.9	190210.8	218061.9	244074.9	194424.5	482843.2	298500.7	339834	658030.4
491982.2	369751.8	421565.9	327619.9	252641.9	514655.8	628930.2	511920.9	729918	679469	616318.1	498915.1	412287.1	728535.9	490100.3	751038.9	1567031
104012.1	114828.6	126579.9	83332.42	140563.3	160730.8	337010	178310.9	178233.9	264099.2	202210.7	122341.4	197857.1	184264.4	122302.4	196919.7	333221.4
445855.1	1275039	590192.9	495986.4	472368	798091.6	1159620	663597.7	548869.8	1969635	972350.8	715063.8	531392.3	906893.3	534778.3	575053.3	342631.3

22929.82	75667.58	40015.95	14739.17	14161.27	8836.815	28203.92	34394.99	21917.29	31690.71	26852.11	6869.003	11922.64	17238.31	92733.62	20084.36	20151.75
32139354	20818706	22240896	16132457	8786784	11947939	16589824	13276238	22486824	16351744	18041218	18848206	6341496	13941418	21529880	11593708	28616004
105038.2	189695	241963.3	83056.41	155221.3	188234.3	194726.3	146289.9	177173.1	275628	34039.75	329917	210341.6	193472.6	268077.6	214939.4	304866.7
26080.41	33527.99	27323.36	22030.94	14436.49	26199.1	29669.61	27872.16	26460.86	39719.3	30620.88	28343.84	17472.02	24632.57	35642.63	33152.54	31305.88
60131.86	45793.66	48973.52	49122.16	44748.6	34353.92	43396.64	33231.09	27901.22	25768.33	39298.95	33088.53	38215.64	51977.3	48446.22	42446.05	61174.5
5325.794	10129.88	3625.587	13462.09	95647.55	3600.952	5263.245	2333.128	14818.65	2072.135	4058.395	10142.24	10656.75	5289.152	26557.2	19458.03	8502.686
19222.15	3976.232	10751.68	14290.99	18362.46	6939.466	5679.906	10059.11	13969.83	10866.06	23867.86	17329.52	20679.41	9718.845	9192.483	7871.148	10419.06
2147.792	6050.759	2330.052	7537.952	4296.162	9819.908	2034.856	11759.2	15452.48	9527.412	13195.24	1754.227	1547.578	10745.74	9143.127	12427.68	2296.587
21397284	22674154	19175502	21931510	16050217	15461909	23096200	14483908	27937420	26476908	25645432	34264764	21982652	15475344	21018250	15237175	17301108
5487.062	4406.576	6704.616	8107.721	2248.012	3808.92	2513.972	3465.813	3723.418	2561.029	11576.52	7137.931	4429.183	4509.364	4093.155	2603.391	4721.513
59394.94	184851.5	138304.1	320217.6	33805.42	466700.1	149763.6	560850.2	17272.65	178675.9	202014.8	323329.2	16023.27	213785.7	739964.8	681394.5	446711.5
2283764	3192325	4110956	2661183	3205546	2684185	3453418	3707780	4013185	4296109	3106472	3119934	4487959	4982275	5152025	2990051	4578930
189764.6	282500.8	227600.7	1384179	624952	59314.06	28468.88	143558.5	173176	342495.9	738559.5	596063.6	463565.6	13886.8	11164.38	15756.07	257906.8
15006361	16436339	36804988	35315692	25453998	5763702	3858716	16163690	14011459	16713524	47317464	37916008	20130252	9707652	10674128	28147796	24690060
19371438	22063016	43299212	55848532	48519100	9905405	4823003	21902024	21561144	22985500	53511520	63509508	43015904	10081169	17817778	32974358	31873594
119912.9	128519.9	131900.7	98592.59	43811.73	89734.42	90182.09	122444.9	128977.3	99790.84	169557	92622.53	70008.33	159146.7	136257.2	172140.5	89309.28
7941.968	3749.482	5490.278	5504.863	5940.169	5908.301	8618.229	5785.878	6902.17	4022.8	10640.78	4748.143	5482.835	6819.204	6499.198	6157.705	6408.166
114957.1	122259.2	125407.7	115085.9	140085	79591.09	58709.96	122030	126119	137106.5	107493.1	114237.3	124771.6	101123.4	84357.05	144323.4	128386.1
43727.94	49731.02	89176.18	65112.4	71431.91	18701.13	50979.14	43919.97	11801.03	28889.67	26492.9	34651	16008.75	18227.09	14238.95	22076	31380.06
2493701	1611501	2116838	1289927	1342316	2147744	2751065	1729710	2772661	2201830	2047009	1853917	1469811	2500545	3057866	2021785	3768541
19289.26	6474.584	20122.13	61287.04	10912.7	12367.17	80163.88	23064.23	25105.54	14829.64	45105.97	17905.43	6735.949	20796.76	57776.22	25590.38	22048.54
351655.9	426406.1	331305	294982.2	207730.9	228780.6	649147.1	277112.8	390581.7	337232.8	326665.3	271224.3	183308.6	270770.1	441844.7	326542.6	488885.4
386418	493475.5	188609.4	356631.8	142526.8	316480.6	812884.9	382492.4	497170.6	479099	482709.8	378257.2	106183.3	600352.1	690921.4	642931.8	905078.4
10916.61	8961.125	15753.14	9179.537	12338.21	12014.07	7836.737	9145.264	10193.33	5404.79	8382.965	8050.263	4278.287	10613.71	8872.373	12355.44	11210.3
56568204	49732964	43573256	33995700	56731460	46691620	45170652	47295968	43209020	59989324	33848404	33545278	44796928	35070236	42983076	45523892	50617764
1029706	807616.5	1068765	704321.4	1056308	562118.8	591783.5	607979.4	943997.9	1375972	1255416	1688690	1189746	778345.8	738610.6	666277.6	856689
64930.45	47150.5	105904	116921.4	117331.2	24264.46	14998.57	55882.26	69278.36	63949.84	64589.09	137840.4	76201.59	23945.17	22784.56	104731.2	128306.4
91846.07	125869	72411.28	68243.92	49575.4	88909.5	148484.6	79960.08	137962.3	132645	108406.7	79528.67	43179.91	104089.5	157206.6	169646.8	182546.3
21864.74	12039	20363.04	410266.7	19633.02	16596.05	50306.93	5894.157	30147.61	26539.55	1364953	29941.1	65218.89	24139.47	14041.32	27953.29	92950.23
112368.2	99997.63	157879.4	154909	121586.1	132148.2	113950.9	113090.7	142758.4	76611.19	127480.3	85429.39	147818.8	135700.8	83796.52	125314.3	49675.5
64340.19	60777.24	78373.76	77323.8	115502.8	77664.71	105283.4	71774.3	63322.4	79352.52	82311	103357.3	90055.04	110540.5	102630.9	85049.41	83078.25
18954.49	16521.31	13585.79	16744.98	17139.41	28492.82	56202.84	32184.61	10276.68	31020.12	21330.95	14820.65	18608.25	16698.17	27785.77	18892.27	27894.82
32354.87	34335.61	20013.94	57088.7	38053	16196.5	7230.6	6119.212	10454.77	19304.91	60912.73	21358.44	24390.31	9445.765	7865.555	12972.3	19767.24
2562347	2405354	1797219	2235557	1441631	2478535	2716221	1880382	1401928	2182528	1304988	2111980	1709688	2257497	3187760	1602652	2307104
23608.9	16151.88	20325.41	16226.16	14298.43	9587.277	12700.83	15406.96	23419.78	26853.62	42823.07	28512.34	17664.45	6561.325	10596.88	5766.576	9898.817
98282.9	40668.72	44372.16	58756.94	35231.31	68046.34	95645.95	78293.21	49717.45	51071.71	44431.73	25374.1	36553.96	50000.13	50444.13	33956.29	42783.71
1251089	1222610	1411052	1044804	944500.2	858793.1	1790721	1212979	1028851	928990.6	806119.3	908990.9	1045327	898512.1	1196220	1218298	1294978
27961.95	22885.36	10383.81	21929.43	59296.77	8121.867	10837.15	11188.21	19023.77	23938.71	40728.92	16954.19	19992.31	8403.179	13564.85	6904.982	29835.73
156750.8	184260.1	226296	208859.8	179801.6	212383.3	172684.3	114463.5	178122.9	154106	113967.2	209857.5	221377.3	233651.4	258689.3	164240	123539.7
3447404	3375938	3549101	3543864	3581154	2878697	2759692	2865937	4536134	3196050	3490358	3199621	3602883	3637273	3363915	4327060	7289915
19193060	22144942	15950885	12757103	10692134	50947512	20961492	13615413	25278256	21463540	17316436	15287115	22910848	37976800	21283764	14622122	40954584
11974.12	11931.87	21253.18	11196.34	15707.97	13142.81	12908.28	12458.37	12669.82	9064.879	13427.06	16671.54	15668.48	12924.03	8453.811	12557.41	7745.768
9568.356	9680.821	17440.94	12281.27	15168.14	12959.29	17430.5	11084.38	14056.13	9218.679	14512.03	10795.34	11649.01	14632.75	10742.04	17542.54	10596.65
59444.78	60296.27	76852.02	83510.31	85945.54	59849.32	65654.29	82082.02	64028.43	49632.13	75856.96	86137.59	88747.66	60093.94	69292.48	93568.71	44618.75
13318.97	18915.08	17238.36	18407.49	19930.97	24364.2	15133.1	14697.37	7818.292	5070.819	30166.78	15607.16	10857.77	15185.43	10915.42	13075.84	7161.746
4520.519	3849.815	2374.15	4710.05	4533.744	2825.158	2736.913	4062.716	3043.904	2371.198	2682.505	5496.621	3974.682	2167.026	1668.557	2188.942	1914.767
3627.662	4488.704	4513.178	3279.427	3263.486	3808.498	3288.581	3192.874	5134.021	3036.371	4424.979	4279.522	4594.517	3254.131	2839.47	2371.359	3265.261
37602.76	55224.02	71917.51	2459955	42624.24	48570.4	76008.02	41928.2	44196.79	63565.24	48208.83	100410.1	82946.18	35219.38	46456.03	43993.07	38567.59
5592.868	5597.264	12718.18	8778.961	3851.447	7679.771	4959.827	4318.865	6416.922	8150.539	12282.07	8098.891	6033.381	8850.447	5029.767	8318.422	6153.13

131981.3	62481.3	54747.51	140923	60243.01	46190.68	114207.9	51446.38	139442.8	99731.79	152096.1	49818.94	67994.68	149557.7	228944.4	147322.2	227226.4
1481424	722482.3	1081969	1630702	1141006	1109338	968117.4	943360.4	848648.7	523452	1692785	778945.6	1268161	959788.2	1017886	955054.2	1056185
27163.11	25676.7	23972.09	20008.27	15158.74	21029.71	23285.39	26696.94	30484.21	31998.45	26729.09	40695.96	25010.15	28286.94	30541.93	24599.96	33006.91
23119.31	22520.85	26647.85	25086.54	131604	18988.55	16432.23	13346	414831.4	25574.56	23312.75	20875.69	14331.84	12443.74	180229.7	13619.09	17339.45
86600.73	85458.55	69914.91	58625.44	64298.2	66697.71	60163.08	84394.48	101970.9	93222.49	63879.35	93211.82	83348.95	79558.34	80155.05	99553.23	134217.1
1207521	1394557	1332629	897488.4	1170373	1311153	1474289	1313605	1055172	1492844	1110813	1321667	1216492	922468.3	1336736	1286756	1096973
575923.3	521229	772980.6	934215.6	684061.1	757155.9	401973.1	619353.4	647891.6	451633.6	725797.6	577757.1	399486.4	692615.8	463410.7	764838.7	415405.4
101884.4	66329.8	119232.8	214118.5	162939.2	52677.67	16037.82	142436.2	78999.59	78621.32	67986.68	143723.3	162336.5	43975.97	56298.74	194163.6	108019.6
42162.81	55275.59	58873.75	47411.81	36449.89	50785.02	53281.63	47279.06	59000.63	66119.91	56126.71	64396.34	42085.77	51772.08	64887.94	45437.27	75247.58
1771096	1592253	2484300	1347286	1190595	1159233	266010.9	934783.6	1263712	1456817	1134173	2063259	1427456	1387525	904425.7	1094125	972526.6
31920.71	43997.54	39420.44	50732.17	63872.51	42155.57	42305.4	38023.29	33626.59	33609.13	36690.78	57312.15	53542.85	43951.61	35740.5	37847.2	38733.41
16810.01	21607.57	20836.15	10476.34	18392.21	17269.32	15709.7	18248.34	18465.52	22388.7	10922.44	13314.67	19589.81	15434.64	21006.74	18274.88	40986.06
492713.8	606357.7	935676.6	1107510	578541.5	712735.9	534657	593410.8	752727.9	432108	593528	813019.5	659666.1	614137.1	737486.3	1021981	864685.7
257110.5	243267.6	132824.6	359786.4	46583.19	66673.84	125691.7	31826.59	114734.9	44618.63	56507.86	28451.44	50889.97	17899.94	52396.3	608009	60288.03
534784.7	691820.3	695239.5	867611.7	450468.9	379111.5	449658	372198.3	1190055	496976.7	881695.4	794891.8	690463.7	695969	1367173	729172	1457944
1868612	1618824	1011871	901433.6	1089548	2204898	1471018	1410396	1761971	2171618	620466.9	1201452	1339465	1864149	972706	1112769	1548669
222164.6	15290.35	13578.86	523669.8	33166.54	39418.86	11716.92	5401.805	3532.842	3529.505	502395.4	7579.021	5377.281	12599.82	1682.483	8539.59	15613.17
1666456	1538514	2906348	3214512	2180169	1715396	1134184	1666074	1559393	1130817	2220819	2504066	1974704	1612170	1385343	2429360	1723019
939096.5	826904.3	593101.4	518924.5	800645.4	608000.3	541607.1	555135.4	643719.3	700080.4	707968	539434.5	879085.9	757368.2	602566.9	669093.7	1239289
28553.84	26493.29	30339.43	25024.32	28902.16	21127.25	34533.98	17803.29	33725.98	26645.83	26740.22	35013.92	42763.54	34445.9	39276.56	31098.64	13484.67
6753.1	5749.373	2873.518	4673.279	2783.76	723.6812	3875.26	3344.985	4574.627	3434.84	4834.403	1163.933	3697.891	5321.874	5449.05	4052.325	4133.443
25898.48	40883	57189.61	53170.64	43318.8	44746.12	35188.24	45400.54	32440.36	29853.04	79173.02	34285.83	37558.76	51139.38	54161.8	50549.07	36157.47
41204.95	114560.3	34629.8	68034.92	36858.08	65479.28	102843.7	45123.91	47876.57	56107.72	42508.27	77460.74	48467.3	56093.16	73244.42	53572.88	51193.07
404800.8	139826	1112702	307076	710047.7	48043.97	63668.91	91514.9	16092685	96175.55	166790.7	513438.4	640277.6	10539.73	64840.29	33697.58	771121.9
85256.95	16249.07	27840.15	76321.23	54795.18	62763.5	67863.29	52222.26	89238.62	136280.2	76182.88	81204.11	38658.32	110908	42651.36	77545.87	85313.43
59882.02	38817.86	36612.59	70859.44	41534.97	69687.92	90417.21	67144.48	95858.46	99337.09	100701.6	39825.3	41226.96	118061.8	71820.25	66220.42	107792.3
22361.46	10527.3	22110.09	17425.36	33965.38	4541.274	2657.179	15204.46	23032.09	26326.4	22130.96	36437.3	30870.34	15573.25	17226.69	7981.759	15819.38
115170.6	104354.9	137895.8	229969.5	156307.2	106812.9	99314.17	127869.7	116469.9	100385.2	135166	247954.9	207694.4	123097.2	107934.8	158652.2	106847.2
102232.7	2767.675	6318.532	36166.86	27557.91	2491894	10823.23	34314.12	2729.747	2233.989	4037.34	35655.67	41287.52	3468221	16729.1	2327576	73318.28
48063.82	39993.93	29564.66	50558.31	38890.43	44355.49	50910.82	45341.05	42587.7	26171.6	42259.1	34948.95	29926.85	36756.81	42550.69	35356.69	46808.13
5542.88	4609.219	2277.176	3684.524	3105.94	3638.869	5477.002	2806.54	2955.567	4756.185	7779.341	3165.295	3172.539	4169.629	4241.69	5910.218	3875.253
29971.41	32374.12	49470.82	43155.96	43410.41	34412.21	28405.17	30181.46	29865.39	33960.72	48298.25	59724.54	40462.16	81035.4	46062.79	33523.71	29819.58
6631.217	9678.815	12361.25	6848.33	7167.616	8691.114	7477.717	9232.736	10799.15	8464.429	12522.04	6067.021	9394.557	3794.46	5980.646	10543.47	15211.36
53030.02	46211.06	27076.48	28444.13	29963.94	50626.67	73309.91	28709.11	45515	34693.75	21052.97	38438.27	32741.75	43018.24	33176.88	36528.1	53825.82
13464.07	16452.49	20046.19	19403.12	26045.16	12494.51	13412.29	16565.43	23026.57	22927.6	17919.31	13111.28	10927.31	24195.58	21031.37	12802.77	42000.5
49025984	36613368	46610220	61016344	35423800	20622230	35770808	38649248	43121452	39888308	48142576	63011380	45916056	25373472	49488628	49034644	53450600
2685998	1200930	5206815	8152895	2559043	775432.7	523046.3	2323648	3278075	2913614	5732459	13622624	4269919	1873301	4622359	4741820	10391154
66645.44	51656.84	39360.34	134548.8	67621.84	5749.416	47375.91	45544.01	61006.11	68827.71	94012.31	41242.29	8030.08	63044.54	66463.78	69634.05	78992.38
1390261	564961.7	642112.8	1841260	426409.7	671487	2169871	421960.8	1072641	339979.3	737192.3	1164128	395445.3	539049	1673900	397971.3	334609.2
17204.69	27860.27	42232.16	41638.64	2786.193	32949.28	25093.78	13078.23	21854.79	8660.704	18626.96	24217.79	3600.532	535.3962	702.6844	2445.147	1769.055
1164288	1259371	1585927	1071669	1019129	1389972	2087562	1524342	1579636	1398290	1282635	1390916	1241603	1637045	1283313	1523789	1400244
50892.11	30049.98	41576.13	77842.73	33526.94	56234.27	31556.52	24090.55	27108.85	23485.21	34722.01	92692.89	44859.6	28677.4	35584.4	47827.48	40349.53
12209.37	22998.95	45720.78	18152.79	23783.62	24576.57	24360.45	14265.74	13851.79	17020.81	21664.14	26134.04	16333.75	14154.79	15919.97	19887.9	18045.25
19507.37	21857.95	29440.81	22901.23	18200.4	20833.19	17357.83	10934.53	17170.23	12639.65	11437.31	18728.51	14385.55	20016.95	13190.72	23798.12	22144.75
97863.05	112047.8	82130.3	62659.44	108516.8	112911.9	95111.7	77654.59	104370.2	123894	91077	105479.4	106451.1	127160.3	103847.2	118354.3	159596.2
7753.737	8474.169	8052.558	9139.668	5851.761	7510.691	4168.384	6518.676	5518.015	4410.782	5638.912	5015.553	7316.011	4744.078	5461.984	6257.379	8064.211
15874.94	15742.64	23170.96	26772.25	16718.43	13726.46	13154.07	14453.92	16096.66	12342.91	17173.97	13394.81	15481.62	8395.012	8983.309	13655.34	6835.596
51183.68	54232.19	63441.82	56484.42	39899.41	39650.13	48793.48	46052.39	39503.66	53212.39	45451.11	34992.84	40640.63	61103.66	49430.76	39205.53	53838.31
123709.3	117720.5	248288.2	319236.8	181689.1	156200	101075.8	151743.7	103542	71588.16	175352.7	150536.4	171571.7	149308.2	70332.79	179971.3	66755.58

21274.04	46620.09	53521.13	29754.2	21686.41	22866.69	21480.6	18287.99	14072.7	33038.76	33244.5	19508.57	28259.37	38619.1	35154.29	28213.69	20197.38
51482.74	41260.33	82488.2	106535.4	115280.9	25005.12	22772.95	60706.28	55732.37	50608.54	97827.98	108646.5	99062.55	54017.34	39914.9	98654.88	59624.1
69455.59	67681.94	82730.8	46258.23	37395.48	71714.66	52600.34	47341.05	62875.79	41212.8	50020.4	44644.92	54649.53	63691.03	84330.06	60269.6	81445.84
128260.3	152165.8	104223.8	212274.3	139796.1	119920.6	137273.6	94740.8	136108.8	122610.2	153719.3	149645.2	133992.5	109091.6	152993.5	114322.1	58618.55
10132.42	8721.148	11624.7	9798.587	11069.39	7484.438	7670.795	8223.701	6032.092	12768.83	9312.438	6720.378	4545.349	8045.588	11501.42	7532.334	6789.27
325480.7	432346.8	382987.5	337278.8	414264.8	301038.3	402674.6	251263.8	287653.8	370696.1	306699.8	459685.3	370929.6	400651.8	461731.4	270799	183913.6
735171.2	1194287	5269417	4610351	1818652	3106722	5760669	739014.1	3737237	3829559	3253655	3394298	7631531	2531197	1714102	4281187	5397968
454318.5	431904.1	400723.5	317077.6	212225.6	337154.2	431463.8	399810.7	567021	457353.6	396145.6	397367.5	310761.5	375562.8	447262.2	422945.3	690708.9
54804.78	62118.57	54091.98	72008.25	42504.55	76761	51825.54	41922.07	61374.25	35850.28	46150.43	30247.14	32241.92	48725.19	42701.19	45279.95	46256.95
61039.11	64035.39	62428.21	37399.55	43578.34	41811.04	31750.46	51006.35	67980.28	60808.29	47931.21	54777.35	50109.72	57863.24	48209.98	52142.78	60856.23
26452.16	26184.86	23476.83	27594.62	24212.34	27443.38	15970.86	23598.37	19852.33	18342.01	10524.27	12844.38	16206.06	30572.71	19839.17	28609.03	31566.72
135898.9	105833.8	247218	224748.9	233145.8	159936	140852.4	130545.4	102063.2	74085.43	165837.5	187468.4	226209.4	175645.4	96404.84	155886.4	148037.3
46404.26	35303.43	72675.73	138722.6	62809.44	29821.37	15860.26	47444.83	50231.98	36247.02	91625.02	102561.5	65338.19	33648.86	42160.3	70059.31	56419.79
123792	176312.4	325176	159348.4	180718.1	316016.6	76286.8	138795.9	124026.5	80536.58	120844.9	183362.4	89781.13	266184.5	55567.5	138239.9	68879.63
20411.33	17683.07	26084.91	42297.65	17089.19	24135.27	15430.11	16016.54	18716.26	14678.25	21033.78	25225.17	18138.54	15127.74	13750.18	19458.08	13763.91
68112.75	40036.32	55869.46	109677.9	70151.78	60393.93	49233.05	62549.32	25003.95	38615.18	65130	45928.04	73912.13	78747.42	61909.4	79826.61	57284.09
45160.46	39436.03	38829.78	118707.5	38132.09	17836.81	3698.454	32562.73	29863.35	21095.68	44248.05	69807.8	54313.38	13484.83	18005.34	33432.47	40864.44
140803	86216.36	89318.25	171863.4	102505.1	65208.14	79884.55	106866.4	120742	100659.8	77297.92	122384.6	108592.2	92349.83	78714.62	121279	73034.4
321513.5	446847.4	394129.8	198932	418901.8	134976.6	107478.5	267866.1	329018	446915.3	252461.3	276330.3	335628.1	243131	208452.2	285898.3	384376.6
3186092	1719319	1318610	337119.7	800624.9	2886850	3574771	3165657	4054538	1930150	1305788	432253.9	1020913	2633607	3724797	2668890	1905667
2501.363	7288.292	4806.906	5697.657	2422.296	8380.672	10578.26	5995.51	3682.6	6681.226	7426.662	7278.139	1657.776	6476.958	12110.63	11280.35	5179.834
39489.21	53191.74	43631.4	46208.5	33264.49	51681.02	48471.3	36029.43	29036.19	29265.61	24518.96	35360.75	35155.98	34103.16	36722	37413.02	16875.74
1672259	1389876	1025098	779292.3	1139968	2177752	2472279	1692860	1732944	1681167	716128.4	1679200	1482456	2300286	1855553	1610826	2093635
13794.08	14126.8	10993.05	21422.43	14031.11	18796.94	9663.541	16241.11	17664.89	24803.77	217674.2	16132.69	329755.5	17581.31	241055.3	16783.07	24345.1
137105.3	180017.3	171928.6	203832.7	129858.6	110923.9	112777.6	95328.96	122835	125720.1	138319.2	204537.7	173792.1	136416.8	217838.4	148923.7	169680.1
222508.3	220743.6	339722.6	440732.6	333896.4	282990.6	234695.5	241501.7	230530.2	162803.3	256070	299918.4	285862	232554.7	217260.9	247563.2	132964.1
4139.225	11590.45	4850.13	6596.583	5780.565	9026.892	9902.433	8540.083	4614.02	10385.3	5347.644	3820.848	6753.514	4775.24	6172.697	4919.449	8350.571
39047.24	26737.97	66609.91	62893.7	39327.71	30915.86	33515.52	28486.6	43698.99	29535.87	96706.28	45388.74	35778.2	38117.21	40338.29	57356.54	82817.21
12218.35	5560.963	8625.671	12102.93	8259.788	3971.534	7874.674	10135.54	3711.073	4156.745	11440.9	3844.371	6040.863	15374.19	7485.187	7132.749	3819.05
32420.1	8062.11	30893.74	10840.53	19352.84	12048.2	20332.76	7416.937	36060.03	27580.95	61744.48	22199.98	18445.84	11305.56	18799.08	11413.88	52753.88
43958.5	12719.91	20692.63	29059.11	33272.05	6535.336	15347.97	7619.563	26674.22	35361.26	189778.1	25992.13	15028.6	9186.474	16068.01	14716.6	89257.69
1160983	911882.3	815512.3	1356293	909134.6	1179297	1955514	1267771	1459950	1134875	1030983	1114021	1010350	1086367	2400913	873059.8	1193714
26676.02	27804.59	22337.91	33011.33	33626.54	20462.58	26587	10857.11	20592.74	11754.72	57528.81	26723.42	33391.97	19045.2	9499.765	20022.48	78629.28
6409.122	14258.2	14744.21	25521.63	7963.73	9865.185	6093.852	8079.075	12776.49	24740.43	30291.51	20916.56	11136.79	11530.34	8803.684	26058.78	9435.716
32499.93	36144.52	57242.82	72088.79	47366.38	37943.5	24701.5	37826.89	42351.32	36586.25	55847.99	46598.27	50669.69	42137.41	38223.07	42846.75	32856.79
41107.25	47564.3	61400.93	68579.75	73320.33	54989.98	47667.68	58570.94	53465.06	47687.4	59320.49	71193.2	70258.38	54300.23	50264.79	58141.17	38244.01
242241.4	338140.8	391242.4	287230.4	228825	267630.4	333402.6	425184.3	344865.7	437127.2	408994.1	359101.5	248440	418529.1	511022	455605.9	446244.3
8567.629	8415.226	12662.59	14399.36	10364.37	9925.02	7208.393	13432.19	9342.175	9661.248	13055.97	12799.42	6633.593	13917.01	13606.21	17226.68	16883.52
11433.61	14649.52	18383.7	10234.37	11020.95	15130.05	19918.91	9790.918	11889.24	7654.105	17462.93	11448.45	19318	17689.66	15284.29	11463.86	21506.53
592304.1	621652.3	631480.3	670031.7	789193.6	447216.6	483538.2	422199.1	692499.6	621805.7	527814.3	738902.6	785354.9	557039.1	636444.3	732083	418182.4
21544.83	29575.02	21067.38	30674.18	28948.16	22174.14	24175.42	19831.28	15337.51	13098.1	10626.76	30518.84	27144.14	14480.55	12766.81	14580.95	13002.87
4624.355	57336.57	18525.4	1503406	147980.4	4284.459	1863.847	7531.76	15528.85	5811.855	74123.34	21646.63	45856.38	9776.161	7644.349	6346.937	16275.26
6180.177	3523.941	9306.525	15036.14	6048.146	7918.811	7633.162	5440.915	4208.359	3267.273	8779.224	8735.526	7782.55	9911.684	3959.018	5992.036	7457.234
43082.04	56204.45	22574.91	85006.27	26936.99	39667.32	64325.02	38162.65	33597.8	39877.33	28749.49	31379.41	13053.68	50780.68	37621.5	45174.87	53564.98
43417.34	46911.13	74489.53	80079.29	50214.38	65512.06	42495.8	49516.55	50675.89	24551.18	85875.55	34006.98	30613.03	51058.19	32795.32	60486.23	65153.29
1309237	799238.2	3178987	804335.8	917106.1	518309.2	610934.6	342359.8	213973.5	504160.3	533356.4	853758.8	1363251	546421.1	498527.4	782468.7	2371530
534343	543485.8	597560	723688.7	727032.4	612156.7	499510.4	485590.9	729020.1	647765.4	767639.2	694984.4	794648.7	865601.2	875683.4	879387.3	865654.3
23759.69	28795.05	30428.62	20926.41	33670.77	20043.95	21693.61	23564.29	24650.87	30610.58	27962.52	24798.72	49156.77	37817.55	34623.48	26518.8	41295.01
8739.268	6596.272	17831.44	22632.18	13122.09	10390.75	9895.581	9954.539	12052.06	5761.939	16477.65	19460.75	10172.77	23460.78	12716.95	17384.74	11108.76

98537.5	92319.97	98731.03	59919.5	75647.13	78859.82	77361.45	66559.77	93759.73	82537.21	63656.79	62710.65	53576.96	79239	86654.82	77220.8	77818.06
81997.45	79409.63	141285.9	154897	113088.6	108152.5	66752.84	71092.58	81866.38	54258.64	102147.4	65001.96	48803.58	96346.16	59788.09	107325.8	56583.05
24545.44	24620.85	19523.5	19242.21	7252.727	16057	30307.72	18757.35	22263.09	18626.27	30551.14	18224.13	7201.754	25592.39	25569.35	21163.34	8853.552
8823.869	7655.977	16940.78	20684.38	11132.53	10516.33	7527.383	6414.73	9274.282	6797.735	17563.78	11304.92	7672.417	8660.386	7462.646	10411.96	7368.35
93234.77	105902	130508.6	120301.7	82737.13	73794.51	64490.43	97537.59	88213.46	122748.8	89943.58	198180.2	119899.6	87087.16	78630.59	102066.4	76277.34
45284.84	37124.95	78669.05	112277	56237.55	46591.29	33412.51	67013.72	54621.88	64753.58	62290.86	94058.33	68239.89	42684.86	44519.18	77697.9	66877.04
217175.1	172987.3	367687.2	496679.7	228811.3	179538.7	155461.3	211987.1	232219.5	164442.1	237313.8	345768.6	321063.7	153021.2	182144	259580.1	157255.8
12914.54	2648.486	23607.29	5349.167	4653.669	2749.853	2271.201	13600.1	2337.307	2907.867	3922.646	24431.38	18381.78	3294.526	2273.804	5472.565	9259.074
20504.19	58841.96	39799.57	13609.4	15364.35	16045.41	13396.89	19824.63	18782.46	38307	24745.3	11538.56	16064.15	17880.86	16504.64	12073.94	18090.96
13861.26	13444.1	16046.77	30079.08	10477.25	7067.428	7441.758	8568.456	19439.63	17696.65	26465.35	13807.15	12050.52	12232.5	9102.225	11251.85	34616.11
150539.2	32772.75	9459.354	195521.6	23641.03	9655.218	23539.51	3404.336	7256.87	14137.45	775490.9	9098.121	5862.88	17093.54	7648.029	4502.054	48317.27
67341.86	72810.89	68370.7	57415.27	59754.2	65827.98	69504.56	66254.02	87710.36	75368.66	51722.84	49515.46	61928.11	71959.04	91208.51	76356.16	89333.08
21575.02	24820.05	13873.81	12343.89	7583.525	34894.94	81707.28	22352.01	22065.88	15222.55	15569.71	15640.8	14414.06	30016.23	21392.54	18222.93	14420.45
65198.65	54872.23	68562.61	82094.97	50983.31	45783.84	29168.88	44476.61	50637.63	44544.58	48477.29	60395.17	49055.23	41836.78	55884.92	51495.13	47100.64
12565.61	8115.613	8289.099	44157.2	6887.645	3363.413	5454.813	7912.356	8876.44	8401.387	23167.98	15927.73	8293.346	7040.196	4548.058	7190.422	10893.55
3439.563	6008.186	2282.907	2604.449	4909.525	9061.543	28266.86	9484.519	3014.144	5249.76	3379.791	3000.725	3201.817	3106.656	8697.709	4986.878	2319.138
66459.9	43758.11	64497.43	52187.51	42872.93	37502.46	41207.52	42150.26	61352.85	45149.2	51284.47	34930.4	52885.7	53754.14	64830.97	40707.44	70781.56
4134.043	5811.472	8105.848	8946.639	6927.157	7854.544	6198.347	6375.397	5637.341	3200.207	15868.92	5833.482	6420.145	6383.146	3287.892	4900.524	6614.203
39830.43	55923	34712.62	43351.47	21547.5	47095.55	28767.15	37298.87	54714.98	46705.02	31757.96	24338.79	21703.23	50518.18	20533.84	52543.16	35615.66
1644295	1534045	2146420	2473114	2160080	1727906	1388638	1649510	1476270	1084785	1559725	1479489	1866731	1491144	1062508	1338222	872323.1
6369.082	7827.454	2530.049	8333.34	7397.593	5685.038	2731.967	4953.164	7312.475	2189.479	5400.503	11370.47	7100.162	7021.259	4690.693	4894.751	3897.137
15249.33	10527.45	24138.94	30574.76	14855.59	7586.083	4776.85	25034.24	10563.92	11750.51	23609.97	23606.28	18225.65	15634.4	11778.75	18547.53	18027.66
12696.05	16018.34	9898.303	24351.66	14289.75	14844.84	13080.4	11184.31	13911.01	16142.73	16099.75	14735	7100.67	13853.94	13529.03	12593.89	16514.72
28122.75	32192.42	36080.59	102696.9	43189.59	23424.94	17415.61	23014.32	20480.3	18518.1	17764.79	36064.7	21668.51	28673.08	28380.47	61337.41	25774.13
57333.06	59391.24	88784.05	25666.39	74652.75	48196.49	71776.56	57478.03	72811.97	78957.71	118313.7	62480.89	101122.7	101131.1	161903.1	84513.4	113312.8
34040.06	26645.44	36425.62	114397	41321.93	22473.22	19727.78	25164.21	52653.08	25235.69	61615.06	74704.88	49650.09	38250.25	28765.86	65483.78	32617.51
22466.52	12336.8	19611.49	13702.72	15009.45	10735.86	14811.36	16837	14802.84	18412.23	16675.05	18423.57	14930.83	11121.69	16149.61	17432.85	10887.35
262016.7	343240.3	220500.6	204864.5	175933.7	448417.5	566154.8	230969.1	249737.4	414287.6	253341.6	291145.3	141728.7	351733.6	412760.1	329142.3	373898.4
33348.16	20895.54	61390.18	62895.01	26044.58	32566.11	27334.06	31178.59	31302.42	33410.21	53133.44	38182.34	43861.18	42556.8	49448.05	37631.13	25809.74
69309.41	70309.73	103396.6	182536.9	82914.63	52978.91	44049.86	65942.29	70506.34	46683.89	86129.91	94530.23	66696.33	72194.73	48145.06	100759.7	45037.33
256148.6	281129.4	394604.1	441866.2	278991.1	270321.8	233904.5	228913.2	214679.5	169920.8	314939	386588.6	275524.1	291058.2	227722.7	342179.8	219852.3
47275.57	20889.39	47313.19	59672.45	30420.8	23704.45	18955.77	21073.98	35862.42	18398.98	44916.48	22171.12	24068.56	75505.93	29388.27	51862.64	50967.89
13146.83	11603.08	7398.703	14335.95	1690.872	7646.542	12698.12	5133.375	6716.524	20455.42	25232.66	8092.337	5958.159	11770.7	14227.33	12420.6	7031.751
59150.57	62599.24	69072.27	68817.3	125395.5	27080.02	22837.72	13666.29	30134.2	26665.98	39583.04	47537.21	63100.28	42298.33	11994.33	10175.21	10371.7
22865.52	20876.37	28741.75	27599.28	23150.52	22724.81	24669.35	19363.87	18809.76	20294	25756.16	26771.54	35615.28	20801.37	20050.21	20301.08	14017.27
12988.45	10900.64	10299.67	18703.58	19799.23	12006.55	13482.79	8660.764	15214.74	12863.88	14617.56	11246.04	14302.13	21572.56	10439.17	20091.07	10731.07
36135.81	28055.81	38375.66	76221.75	51391.82	39034.74	26660.87	30289.81	48042.17	35007.94	69018.22	63590.52	47556.82	51926.92	45128.11	60336.4	34907.17
82349.55	54699.6	82926.87	52820.05	92435.95	44387.93	30756.18	33672.99	69147.23	86917.02	72027.62	95657.76	112601.2	33163.4	64519.69	41592.94	116425
51783.98	51005.8	40808.96	68860.62	50690.08	50055.78	40573.67	49250.02	51205.17	35039.3	84580.86	60557.29	56714.34	54770.69	70109.16	75884.73	150409.1
53885.33	62576.35	87814.74	101151.6	77763.47	66411.91	42578.97	61166.15	64663.83	41708.8	33152.83	93701.09	79011.27	62965.03	59381.58	77238.27	52597.29
375862.6	305656.1	516067.8	458925.5	439694	322223.8	299438.9	371564	342383.3	296191.1	507589.6	590684.8	476118.9	376379.7	402575.1	404774.9	352502.9
73157.09	16950.31	17524.61	91436.04	30373.98	22937.93	26150.37	17182.82	9938.988	23496.12	249446.3	27982.86	12954.87	43371.51	34996.58	22057.2	91576.02
16030.45	24460.39	27691.18	10164.93	14443.75	18269.76	43370.79	11840.68	10438.88	14680.85	4004.424	11940.16	18696.06	35692.85	43882.51	25902.11	31999.72
105822.8	84218.39	179998.6	188107.6	161950.4	104665.1	71877.32	113910.9	111711.7	82237.05	160996.7	172050	145739	150583	102679.3	151176.2	98927.63
127869	122845.6	223822.5	262944.1	142712	111603.6	120508.5	117074.1	161000.8	102241	213526.5	236480.6	156622.4	102422.1	131755.7	166757.7	138268.4
4431.5	3764.52	6380.173	15347.29	6791.226	4962.525	6582.024	8550.697	11047.46	5965.36	10688.66	11815.84	5192.162	6006.594	5773.669	15458.36	9294.905
2264586	2394903	3302547	4955828	3403302	3502015	2221554	2200260	2326748	1478876	3209255	2157380	2896328	2855211	1724154	2558565	1316390
23108.66	24909.06	35248.02	48004.64	28499.46	27671.17	13600.74	20107.45	26957.85	20260	17814.49	28588.26	24457.85	21273.3	16185.61	20601.6	15983.99
32664.07	45857.46	35286.3	63721.42	32044.95	30336.68	24759.1	29858	62358.97	32409.03	41796.8	60603.97	49130.17	31074.82	31620.19	41138.5	28279.57

3165.106	4472.947	7739.066	15248.35	3997.91	3090.385	2131.192	6561.518	5407.712	2493.192	8747.601	7458.276	12370.24	5148.055	2799.215	6140.071	4700.657
20363.2	29665.28	23189.58	48209.27	25025.51	18420.74	14670.48	20413.89	34923.34	32122.38	28918.8	41261.05	33456.64	25573.67	32850.98	37572	24814.2
17145.86	18827.85	26458.47	25446.69	21386.19	26714.42	15274.45	21007.51	14058.34	21463.87	26171.94	24955.34	21156.48	36808.48	20152.26	24471.93	15831.65
82440.48	64434.51	132725.3	101416	46579.46	59419.2	60140.57	58568.18	98751.85	60092.41	62125.13	79618.66	80709.82	67182.76	76229.04	77845.74	90854.21
55571.09	120432.8	75259.51	150016.2	64408	101241.5	81881.69	112501.7	50596.05	115467.7	102555.8	68984.61	87819.14	116026.4	65900.59	90165.39	132929.8
36539.4	38706.74	52449.88	99871.33	44012.33	58963.84	30029.86	30726.34	37846.7	27508.67	34579.98	45232.62	39539.43	44296.31	34148.98	29611.11	30963.45
2866.098	5588.161	564.146	8823.712	12499.53	4353.021	4257.167	1541.848	7136.536	1681.919	9299.565	6421.884	975.613	31938.37	5028.684	12143.86	5807.747
39920.93	55696.97	50239.33	68508.16	45019.33	35614.07	33752.02	24711.42	31458.83	23198.03	41059.75	49889.02	45441.34	49644.11	28886.63	36412.25	29057.46
33810.65	35571.4	31040.48	25473.18	51563.52	59606.5	47600.07	25750.14	19872.17	23900.38	36078.31	50167.41	45311.93	91424.19	75907.38	34026.56	47712.86
3009.172	1717.876	2748.898	4041.343	3273.713	3198.645	1605.411	2160.138	1471.514	1429.53	3884.724	3799.393	3871.951	2531.001	2051.163	2847.688	2740.242
24233.18	75802.13	85695.06	32871.82	54328.25	19240.7	16908.46	85855.26	35649.91	60022.98	142531	18699.77	102779.4	47416.18	76457.45	90147.24	166242.5
770466.2	527158.4	727200.9	404711.4	353746.9	625015.9	687679.1	701040.4	795003.8	773273.6	751068.2	725543.8	495818.3	887620.8	1187383	628184	815290.8
109509.7	56126.11	81065.33	82641.05	70440.98	59269.91	121221	82665.75	112816.8	107396.6	59842.78	77864.95	51837.31	104096.1	103816.6	37463.86	150027.4
92240.77	159586	96028.34	107858.9	97542.03	184873.5	215034.2	135835.5	187718.3	163095	112566.7	104395	44522.03	168650.6	168329.1	186349.9	205346.3
55272.87	85763.06	44376.39	47852.11	27515.52	40296.1	26757.3	43855.34	45265.86	46562.55	22710.33	48142.46	37591.1	38540.56	50942.5	33367.55	53511.62
59698.8	63644.52	80181.77	116270.7	84481.23	81815.86	86604.15	77914.1	45967.61	52069.47	121641.3	194278.6	124386.3	85935.92	160410.2	96199.07	88618.76
79557.88	111655.1	203511	193470	145373	236721.8	172329	20115.24	18045.2	11745.32	615350.9	300040	556108.9	271224.9	861205.8	685368.8	755126.1
14354.26	7410.602	15272.07	27055.51	13031.57	6839.229	6057.429	17941.66	10752.77	8004.006	12423.8	26733.75	16846.44	8050.773	3587.015	20744.71	15531.34
97855376	1.14E+08	81058936	66564364	47115016	94529824	1.27E+08	92154552	3.04E+08	1.37E+08	1.97E+08	92941984	55181752	1.17E+08	1.39E+08	1.35E+08	1.99E+08
80264296	85092656	1E+08	99155960	61515440	58173500	83347712	1.22E+08	1.15E+08	77811136	83235128	1.02E+08	85123792	1.42E+08	95473744	1.24E+08	1.57E+08
24915.78	32877	51716.13	48785.39	53721.59	48018.41	37196.29	36178.47	42445.73	27200.86	35432.12	42613.57	27738.06	37023.9	22396.73	37026.64	25986.42
102122.6	162139.2	151226.9	200372.1	140581.1	226295.4	218381.8	152093.4	137937.2	194485.6	176986.4	118190.7	148963	163954	257526	241374.4	149515.2
8670.928	7707.081	15118.13	19490.35	9730.465	6477.344	5317.923	16874.06	11710.76	9442.302	12799.08	19351.84	20888.69	15326.18	84816.7	21533.74	171489.8
33759.05	29993.39	60143.28	52804.79	43301.04	33688.5	35426.76	42260.12	28700.75	32458.35	54418.86	46570.54	33127.35	35290.29	38639.69	41829.41	29023.02
36088336	34771772	24280902	27353928	34548972	50636568	48462108	51497668	68096392	14707770	63010744	26821552	18942368	46035268	65356040	61030272	88518032
47039.05	59038.96	52559.71	62901.84	36681.68	23095.97	53295.62	64517.97	58578.48	79204.35	42631.59	56664.06	46596.53	20766.32	98663.72	68866.78	60409.62
19710.1	21255.55	22685.15	30432.7	26018.99	22656.09	19085.73	22360.33	19725.99	17131.02	25297.29	28153.98	21762.95	24044.72	14586.57	19480.27	14271.99
851914.6	1099215	826435	457484.9	444351.9	974671.2	1032590	610048.7	827290.1	1034853	764540.6	596477.9	373946.4	1090738	834928.1	795406.4	596211.3
29139.07	27917.08	36210.13	64011.19	39270.78	30383.09	28015.35	25988.52	17944.03	17976.39	51972.68	39126.79	45816.42	24931.84	21974.57	31984.82	16665.17
12732.44	14892.1	18917.75	32321.54	16221.93	9155.383	10861.49	16058.31	13667.48	20958.95	46621.64	63204.71	21630.8	14336.04	28482.84	19428.56	22314.15
12197.15	15485.46	12838.79	9393.186	11047.25	9564.574	3466.556	10294.81	13005.04	18173.36	15120.46	11229.93	11015.63	11631.75	10451.37	15756.81	11848.79
1142533	958144.4	969397.3	943194.4	627024.1	942516.4	923720.1	809341.9	961906.9	911410.5	660705.3	543539.3	660920.4	962236.6	1074792	814333.5	1029263
40808.59	45205.95	90870.44	27897.6	68710.4	72951.05	74989.3	64079.28	66969.35	62081.8	61832.68	80595.5	64637.63	62701.41	88779.9	59177.92	103116.4
111680	79269.05	119015.9	90774.38	66644.64	94125.67	169050.6	100586.3	129113.8	55315.11	66815.48	103804.9	102232.9	79449.75	135005.8	75702.27	138409.7
546129.9	542919.9	619873.9	52069.57	73939.73	1609517	82632.36	8726.075	561926.9	1186717	1869799	186723.7	261398.8	811076.9	69097.94	100640.9	337608.6
18921.59	10719.54	14438.94	16334.14	11668.52	6952.511	7157.651	5934.443	9275.404	12825.39	34777.3	24535.7	15139.78	9641.648	8573.334	9005.188	8674.825
89854.17	62976.44	57145.7	80311.76	93363.97	71503.19	82822.66	77656.8	66882.17	88799.23	79052.34	122344.1	75844.6	86014.99	91723.86	85625.09	88866.17
76843.6	64091.22	64556	60455.3	65355.57	62970.12	60461.09	85645.7	101236.4	63361.27	88620.45	60617.5	34606.35	69533.95	87911.35	86258.37	107227.6
7483.18	6112.481	11131.93	12648.05	4741.558	6381.576	4018.797	5056.429	3683.311	4812.558	8944.77	7206.729	11950.69	6721.096	2527.008	6418.973	6561.358
4552.692	7592.847	5022.224	4340.337	3111.949	2089.163	2440.867	3813.712	2941.36	4629.192	1930.769	2081.961	2205.943	7068.508	4571.654	7628.599	5096.413
265426.8	101969.5	140039.6	519437.8	154175.2	101653.9	68619.86	118856.5	98541.23	116672	571237.6	127340.5	126975.6	141753.9	107531.1	127581.3	131215.1
29828.12	12316	15597.56	39310.23	18881.18	8398.379	13148.88	14663.8	19107.5	16562.46	20513.55	16727.22	22846.69	21289.07	20934.64	39093.89	31505.1
33861.69	31659.26	39334.57	27504.11	28596.99	21793.32	26087.11	27453.43	30316.87	32514.93	32207.62	33431.13	36829.03	49776.22	32450.99	30016.01	47026.6
69313.91	99744.96	60186.23	109703.5	90942.34	110060.7	71491.7	64146.93	63593.51	114812.4	179886.2	100764.3	196420.3	89811.82	70161.14	128090.6	304707.9
317613.9	129871.4	172431.7	91291.91	156696.9	236461.6	292320.7	323277.1	457426.3	247511.5	231148.5	72660.1	184765.1	270672.9	432129.8	318154.8	307941.5
439418.1	32183.76	13313.24	692006.5	93242.38	38702.16	16220.59	4407.433	31009.03	2742.884	1306832	9841.818	3946.752	19205.56	6981.728	16789.32	12246.85
1428365	1314950	1369707	1239868	1048592	1114846	1326728	1326162	1411729	1483282	1375908	1273774	1245815	1429485	1554553	1384310	824684.4
9815.525	7617.615	12726.13	9324.042	8082.16	2005.543	6464.442	6295.124	10667.36	6486.342	25413.71	5822.294	8945.052	5801.479	6532.229	21086.02	10815.05
274553	257243.9	257463.7	176791	212883	203699.2	218578.3	234749.3	305361.1	302614.5	169993.8	220853.4	279544.4	234204.3	275860.3	232147.7	395281.3

60559.87	61705.52	64948.94	67608.41	66015.64	55154.85	41102.97	59212.83	55832.8	54233.13	39788.62	71042	54232.58	90257.97	69498.39	52131.21	76536.33
190076	184873.8	126343.3	104313.6	327446.6	142489.3	137117.8	119810.5	181275.1	155342.3	129771.5	189056.1	326603.7	149448.6	138995.7	114815.6	131381.5
58597.31	83635.28	59921.64	64379.52	217563.7	25626.18	23464.7	31120.22	46892.93	46477.74	98287.84	85463.48	112278.8	25510.36	30125.62	21516.49	78917.96
11126.05	10613.76	7536.015	8806.607	11185.76	46659.48	6359.219	101575	8668.512	7453.737	29093.96	5704.931	14634.79	12288.33	10093.83	9345.813	12865.19
1920431	2768091	1970241	1754291	2730123	1221843	3016817	2332653	2942111	3145962	2288832	1554926	2752469	2731673	2670008	3303111	3769118
457336.1	386965.1	361503	596200.4	259688	190740.9	360652.8	209538.3	523400.8	287999.1	593097.8	274730.1	147947.2	1046888	349044.7	874627.1	760572.5
1047426	993188.1	936648.6	864151.1	912610	847499.9	860745.2	975795.4	1083266	1079173	1101283	710925.3	791096.9	1405722	1348412	1228439	1187713
12403.71	9176	13767.72	35917.47	8983.438	16228.92	26506.78	11481.11	20082.27	3548.785	19805.82	7345.07	6855.569	6062.071	7409.791	19438.97	10607.29
11940.49	14508.84	14930.67	15910.57	14919.83	3054.86	15921.9	9994.391	17792.24	12449.62	19003.74	14457.35	9990.661	15011.75	11582.92	14248.37	20533.89
77805.66	93650.02	80872.04	102257	66682.1	89254.13	76500.84	68029.73	96727.58	116464.1	97726.6	67011.51	94598.84	81283.23	80869.17	93897.58	194102.2
86873.59	92131.2	135366	155288.7	143572.5	105707.9	79524.53	104355.7	101919.3	91983.46	104190.3	124205.3	150796.7	94777.41	87062.16	102513.9	72095.59
80106.82	161807.3	32674.49	187861.7	105696.4	148284.8	160546.9	93747.33	153470	73703.89	501663.4	69124.63	66500.91	97113.39	110480.8	165410	317299.7
7026.645	7949.454	10668.67	5347.195	18200.14	8694.196	8236.529	7724.732	7852.191	7858.977	9594.272	8973.151	14663.48	9901.01	7793.922	11558.46	9510.355
135832	132223.9	228615.9	231634.8	308914.9	289865.6	137892	260910.5	138630.1	106318.3	191870.3	269314.4	303974.8	321764.5	164746.2	264821	138514.8
509727.4	411784.8	299670.1	304390.3	301802.3	633605.4	1279623	465862.6	505792.5	390219.6	370467.8	280059.7	414352.4	773793.6	475171.9	479061.5	494577.3
65970.22	65669.33	64430.72	45242.41	55133.29	49274.31	56155.94	75490.36	74031.37	72633.31	22179.48	54172.89	75276.64	15683.04	69720.42	66779.11	103590.1
3121858	2488828	1673278	3223001	1666034	1725033	1713913	2470403	3570341	3298806	1184062	4434238	2912151	2536407	3344886	3882432	4312139
17595.13	32567.74	34710.52	25711.57	19875.92	24103.54	26398.03	12620.29	28059.85	26938.09	28525.12	29190.99	18322.98	36822.72	32886.48	23638.08	32368.3
85642.34	149177.5	140444.1	408643.9	137698.6	323638.9	176921.7	179512.9	176108.2	101511.4	130248.9	125800.5	86966.69	280763.1	175983.2	288625.8	237946.6
12130.85	11248.69	9499.521	11860.05	5174.162	8509.055	16321.46	7204.311	20507.56	8826.612	10436.62	2653.399	2960.867	17039.01	7293.744	7286.037	9627.667
148953.6	130883.5	107279.4	208367.4	87587.53	88742.2	125621.8	74899.48	100942.5	111444.5	575078.9	90219.02	108909.7	101862	116211.4	68367.86	149660.9
3315.897	3550.21	2487.364	4273.721	3640.149	1699.193	3884.856	3150.899	5043.91	5997.19	3226.601	2108.572	4390.928	4802.338	9030.372	5342.373	6805.908
628067.4	557644.8	461675.1	819023.9	1002216	537744.6	426442.1	459797.6	477658.9	639337.8	698778.5	850545.4	751321.8	430500.2	395670.5	826531.3	916750.7
6210.567	6860.538	12566.46	16258.34	18563.82	7659.739	10810.57	9329.165	6072.849	10790.18	15701.5	11579.45	12454.43	16604.41	22688.02	20512.03	15606.61
41005.67	62450.54	30002.51	22706.05	9830.25	25512.56	6734.721	16341.24	52255.11	54680.57	19941.15	17913.72	3883.217	22471.19	6114.881	27759.17	49135.79
116002	124839	129269.4	147372.9	81593.29	104968.1	109186.3	108063.2	106962.8	125837.1	159783.3	136472	56660.43	104316.5	93318.64	110746.4	70233.59
245334.8	187763.5	175092	110491.5	155582.7	175061.1	219330.1	154128.3	251876.4	256958.5	183508.8	152385.5	99625.89	316809.7	281949.3	215166.8	413374.1
22684.28	19250.35	11794.87	23893.44	8904.129	17329.14	14555.04	10391.57	30677.91	18072.98	26558.65	15591.33	15188.26	14213.68	25183.84	14005.29	20604.87
14665.67	9586.928	10015.13	12891.82	58473.73	10150.58	15101.58	11126.07	10319.42	4953.888	18029.96	9946.001	24093.6	9035.534	9999.86	10850.54	7604.488
46440.18	46625.24	40993.1	43630.01	45066.15	52457.26	30696.88	23974.61	31176.63	73219.47	45142.57	36504.88	25734.15	42667.76	30241.61	30464.54	34616.5
9228.141	7133.248	7703.909	11188.43	7116.585	3299.714	2839.335	4200.284	5394.959	4116.134	18611.63	3275.66	5992.655	5574.885	4140.714	5713.782	6763.616
68099.69	64634.27	43521.5	59236.3	46323.46	46884.1	49344.45	42306.67	50346.4	65778.29	37323.47	50000.53	46618.04	53857.17	65519.74	47417.97	42889.51
19755.2	22086.37	28073.38	26264.44	26071.68	21352.36	27551.26	19446.14	16321.37	14850.59	20753.54	25777.45	31961.67	25200.28	28513.29	16514.9	9673.456
145717.3	177562.8	172808.9	166423.7	121445.6	116798.8	160055.5	124144.8	148126.6	128488.5	95857.67	192770.9	171035.5	150331.4	191095.6	187395.9	248873.3
265299.5	182783.8	222998	133781.4	50777.16	163098.3	169949.6	131619	144731.5	140128.6	56374.58	224193.5	142143.1	169145.7	197542.2	140669.8	130075.9
58531.9	50844.62	59531.11	31903.71	36709.61	55588.21	54742.66	32621.19	42264.7	45662.72	34540.23	54489.64	39047.88	38009.7	47949.02	37404.62	68687.95
13650.38	12777.42	15321.82	19114.26	14812.41	14892.67	10230.04	14239.84	13448.4	11384.73	10268.05	17306.63	15213.18	12291.29	15365.3	20082.13	12396.6
254599.2	192520.8	77209.34	122159.3	270029	389928.5	495302.3	276663.9	622751.3	118313.4	93113.88	333238.9	89593.38	427194.8	460954.3	338681.2	666278.6
53440.73	62125.1	154584.1	51090.28	49898.5	113923.6	52090.34	31133.89	53555.98	36362.7	46386.04	29935.1	56473.11	25885.44	41609.81	23410.44	34475.5
857884.6	833047.2	830916.1	653512.1	654323.1	804981.3	572654.5	680987.3	765432	686398.6	702475.4	594785.3	615418.1	854161	633740.1	676554.9	481106.8
5582.176	5967.013	11171.15	15031.16	20829.9	14469.39	449.5216	20509.58	4045.988	30817.03	22182.34	13594.13	30241.07	21036.52	24934.66	20285.75	14735.12
16176.13	13910.86	8028.567	40907.95	17958.66	5260.721	10304.73	8876.066	6873.335	7464.717	67474.74	10586.86	10801.81	17913.52	9309.252	7836.707	11253.26
123537.6	119396.1	101257.3	77102.74	108232.3	104498.5	120546.9	107805.3	86951.59	113298.7	92733.72	116792.8	113385.3	125015.4	152254	103954.6	133234.8
10161.87	17168.57	13083.08	8047.834	8540.021	15398.46	8220.59	11185.06	14186.92	13780.88	11341.41	15865.49	15451	17659.14	8922.986	21078.18	32950.74
76971.81	106615.3	77022.73	12174.14	9054.451	251242.2	18905.77	9046.075	117916.4	261987.1	339531.1	42521.72	33027.99	159045.9	13850.95	12605.28	85760.91
101699	86657.73	57993.9	59108.9	42083.52	66441.66	94656.23	79854.97	110077.9	98952.23	93731.75	68072.14	33380.2	71397.22	102856.6	66644.87	67946.48
32302.56	27345.39	66181.52	48496.59	47667.36	12166.5	38191.61	34779	42784.9	31524.27	72158.52	51292.86	39942.05	68967.48	51879.13	44756.63	41251.02
223717.7	277668.5	254678	298621.8	264569.1	233689.5	239525.2	257400.4	263630.3	232923.3	300258.1	202377.9	191079	291590.7	239835.9	272037.6	148322.1
28674.97	24775.01	24394.14	16180.2	18251.19	23820.8	36007.03	20452.58	29317.26	22083.64	37961.59	18992.82	38009.16	35128.57	23422.72	34093.02	16803.04

81107.8	52855.74	126109.1	126277.7	124454	89609.96	59745.71	92562.66	92710.94	57852.7	105976.2	149726.3	150900.7	62998.84	65987.53	98961.52	73341.06
2826.513	5401.111	6533.089	4514.66	4454.345	5333.572	5186.345	3832.311	2701.522	4154.481	4242.484	5655.549	5282.346	5223.786	5644.449	4523.829	8476.191
8474.559	8043.816	12764.53	17386.63	11927.14	14265.17	8359.488	11288.96	5180.89	12782.62	17964.32	20714.12	13636.68	14002.2	17171.03	18025.18	38229.84
33411.45	36402.33	56029.5	59124.42	38156.5	45604.88	32023.28	34377.11	32308.94	15271.74	34968.28	48748.11	49963.3	36236.33	21604.14	43301.54	18372.8
4106.349	3369.224	5781.147	8046.298	4059.954	4641.539	3557.557	5996.173	3842.859	2894.489	3714.375	3511.231	6971.834	6078.869	3827.932	4117.729	3645.664
36192.02	23943.58	55407.18	70824.87	44896.39	36286.26	24019.81	23354.54	25694.36	16194.19	34381.96	23797.29	11323.24	42573.85	27450.13	25514.29	17627.61
30076.1	27911.25	38671.96	35638.47	43053.8	33996.3	21645.57	23252.74	28464.16	20208.84	54317.09	20255.83	37871.62	43548.77	19883.58	27838.07	22247.47
7785.427	8070.339	8100.19	13828.21	9923.773	6498.956	7040.9	12483.11	10208.92	5101.419	13729.62	14969.39	5645.233	15345.71	6568.661	9082.553	11803.88
21876.3	30726.38	31831.26	19989.07	17937.57	20330.19	16731.33	24378.2	31305.15	26075.95	18789.54	13981.35	18527.76	18278.05	19283.69	30492.22	34367.33
99892.55	85171.02	88979.97	66759.2	67602.77	81037.7	63320.04	85962.95	101943.2	86084.02	61935.67	77623.27	86070.42	74328.02	93074.09	79321.05	104193.8
377621.7	440420.7	289507.8	434924.8	316307.9	291644.1	330856.8	414971	363025.1	536524.3	314162.8	497943.5	349952.3	410603.5	515935.3	539150.2	585807.4
12508.93	11276.15	11797.61	13284.08	12502.95	2376.6	6218.812	4907.801	22431.86	13222.01	10637.43	13950.87	15804.56	7754.653	5310.496	6826.531	8546.269
542518.7	111323.6	219710.8	366038.2	493344.4	150924.3	236236.6	521932	557088.6	289456	526084.3	203252.4	489877.9	1672953	470065.7	439770.3	561263.1
4627.407	5910.14	7625.129	3294.872	5148.245	7275.734	6260.063	3271.818	12208.36	4807.799	6872.152	7691.009	5803.259	9931.586	6296.011	6563.545	22272.11
10489.42	13630.66	7540.235	6924.143	12440.98	8807.294	8273.455	6433.39	10527.67	17776.22	81357.13	7729.252	15526.37	6262.363	3218.847	2879.885	8924.026
252408.5	209886.2	310458.7	220467.5	222178.6	180698.2	267425.4	221038.4	256091.1	296149.3	255960.8	329714.3	257193	184149.6	356767.3	253722	204381.6
11712.62	9420.404	13142.09	21967.06	9545.02	8064.765	9126.329	9423.506	9036.911	6891.96	12407.25	15009.79	8325.943	10661.58	10495.77	16612.81	16690.12
30251.77	19105.8	25859.03	108854.1	14396.33	26696.65	25509.28	12390.49	41915.52	32388.82	147266.8	38943.2	61660.74	25803	23432.8	19744.86	36339.85
78989.46	2067099	115685.6	140414.1	128035.5	79974.97	56083.2	4607276	4418780	75902.91	82405.22	88039.23	100816.9	79411.87	73071.23	127300.8	65639.58
22586.33	23803.23	29537.45	34793.28	21267.21	17112.04	14361.6	20273.66	18011.88	17970.33	19817.82	20830.34	21320.7	22481.61	25509.24	29566.34	25393.43
1753.94	2130.747	2978.859	2177.858	3098.208	2107.725	1783.41	2433.95	2148.773	2491.87	3458.384	3734.709	2152.48	2680.142	1090.234	1511.231	1479.742
45562.25	54136.63	61007.94	66571.55	52362.8	66172.67	72707.32	50250.95	72079.5	52002.31	70898.27	55016.91	57143.82	81057.58	67785.54	61793.91	134885.2
124318.9	165124.8	157020.5	150031.5	123709.9	113820.9	130515.8	162958.2	148556.2	206880.9	181608.9	176771	120567	162459.5	198351.6	177820.1	178907.3
8858.602	7322.253	11913.79	7242.917	4145.486	6434.743	7023.443	2644.761	6041.079	9394.941	4771.607	8282.434	7783.825	5201.196	8288.782	6276.154	1590.369
326616.6	309696.5	404958.1	271111	339720.6	316039.4	234902.3	236084.8	423893.9	306456.5	214358.3	223109.6	331061.3	333578.1	302432.4	348701.3	847562.6
40915.61	31652.17	9472.034	20420.62	26076.09	19863	33330.14	31597.32	26356.06	40571.98	20353.39	31424.58	20156.04	15638.42	28777.69	33093.96	32351.22
6387.161	4526.789	4738.077	9109.473	6023.178	5627.823	4954.148	9003.479	13918.18	7953.975	928.6613	8813.254	7745.928	8409.072	5490.167	62206.34	6319.351
1699.28	2398.9	1898.628	5837.958	4567.574	4483.332	2887.656	3976.834	2575.094	1836.37	2943.406	1469.75	3536.995	2256.207	1787.612	3411.329	2684.968
153547	133722.2	154609.5	158126.6	137042.8	119771.8	97125.59	149610.4	136662.3	130513.8	142672.5	142073.5	120440.3	132620.6	118705.8	173474.1	167853.8
138492.4	132660.8	129675.3	132816.2	125941.1	135024.5	128851.8	123404	175529.5	126225.5	145406.3	146089.5	151696.4	117752.7	86138.04	119142.7	89578.44
12816.08	8680.531	7330.218	5868.707	8073.147	7250.448	6449.517	8794.757	9571.206	16010.55	9251.714	7291.06	10254.13	12179.79	10809.77	8958.842	27607.47
6387.806	6772.338	7201.202	14016.29	14312.69	5478.834	6813.837	7706.846	7504.259	9974.243	9616.434	16488.79	8238.903	5080.24	5051.995	12013.42	15411.91
9758.774	16622.78	11245.19	35662.41	12614.16	11623.46	18132.33	6276.854	11710.36	19671.62	44465.39	11008.06	8679.503	5720.252	11272.25	5152.39	6683.452
14510.34	17717.79	12321.35	13020.76	14778.26	10102.54	16198.01	10464.29	26524.52	9743.662	36057.14	21207.94	18183.62	19656.06	28793.71	27765.99	5887.231
17155.94	21064.34	18425.04	17654.79	22996.42	14598.42	13780.67	18136.92	17508.33	25190.45	20361.25	10123.61	23455.75	20876.01	9029.928	24045.78	11820.39
244692	385252.3	297562.4	287645.9	378744	331066.8	212786.8	309145.6	391068.1	567547.1	296146.1	515937.2	392216.7	491887	319643.8	468091.1	256492.1
307008.1	295440.3	254836.8	214318.2	242692.7	249540.5	317059.8	364256.4	374303.1	348661.3	310330.2	235729.3	250667.7	301074.7	404569.3	271338.8	194734
4538.748	3869.354	7387.918	6818.354	5349.351	148794.9	3457.29	9647.597	3809.539	17945.52	16293.86	5457.966	20413.45	19113.59	10824.77	8418.938	9595.992
1471611	1114918	768148.2	1167186	962543.9	778236.3	960090.9	814506.4	1296939	1471089	878242.6	1188612	793154.3	1090943	1464180	1095102	2124028
31079.47	33767.84	33064.8	28189.17	21459	27065.95	21395.8	21987.79	30031.77	31664.66	16523.65	23230.88	20756.17	38060.83	23766.69	26158.97	25943.75
81925.37	56364.59	32443.82	217545.2	25453.33	24664.35	5829.276	29601.82	123175.2	37509.39	87980.8	127468.6	77210.89	47995.08	114338.3	49497.38	62059.43
282530	194402.8	166076.1	161775.5	126252.9	155812.5	150927.5	145949.8	172254	191348.6	99134.34	275492.9	121470.7	136865.1	164081.8	209507.4	291562.4
43071.19	36310.91	62364.73	56920.4	35599.74	24341	24370.4	19494.04	36264.92	41806.27	102308.1	35274.77	35617.37	37640.84	47860.72	31969.94	39155.65
2439.487	7356.744	15398.99	25019.92	11468.61	1884.719	1711.058	1829.228	2550.152	7351.581	15561.13	9248.274	10304.59	3265.348	2510.577	4058.464	2288.042
158892.8	144098.3	160605.4	142838.2	127672.9	129465.7	118809.7	110736.5	116044.4	160872.5	91002.24	122362.4	86665.51	109251.4	120565.6	101999.1	88886.78
43715.46	71489.6	56891.84	493757.8	109080.9	63797.2	64113.61	37847.78	49115.29	59437.68	38307.03	96800.26	61219.48	75170.94	66706.74	52167.25	28826.9
101431.7	73410.8	125228.7	234157.3	87894.01	111213.3	84211.87	83377.02	95633.26	45874.02	96088.06	60949.07	37377.58	70607.02	54496.54	77093.56	53905.22
4759.77	2715.559	5185.35	8302.549	6336.095	4444.808	1824.234	4137.029	1688.647	2414.156	5158.647	3739.477	3960.246	7299.522	2554.06	5840.168	4527.619
10225.32	11832.85	12545.23	11749.27	10812.83	14050.36	16697.67	8770.027	9331.856	7373.433	12602.76	11199.6	14815.95	10314.28	5874.869	7635.468	9244.578

24940.75	27643.41	35588.38	40348.94	35193.49	35342.71	24486.66	30116.63	26508.03	20529.42	25531.85	27084.88	31931.41	24250.93	24586.63	32736.03	13831.1
19819.11	33025.47	31467.41	26601.38	18301.53	20488.47	31711.3	13676.71	28863.87	32154.68	28658.61	31902.01	39962.05	35324.43	41339.1	28785.09	16482.42
16700.02	15695.08	13994.29	12317.19	14058.02	9486.558	16664.71	10165.97	20030.24	25275	15426.25	13239.08	17311.17	12851.29	9788.592	9937.716	12360.89
112799.3	143279.9	110113.8	72909.05	45633.69	96932.57	79184.87	74937.85	111278	106246.9	62220.09	101877.8	85125.46	116600.5	112658.9	110966.4	202893.5
16225.52	10402.92	8161.959	14273.53	11474.54	13319.8	16894.55	5661.981	11644.33	7401.679	11486.37	10512.45	14505.22	11965.9	17279.95	6178.112	15039.16
181124.9	123466.8	194540.3	229234.1	279449.3	165461.2	145094.9	137554.5	196128.6	120293.3	207656.3	194810.3	232904.8	232679.8	180203.6	189031.2	186978.8
47803.75	34987.69	51336.3	104906.7	75310.08	18994.87	15600.92	59635.16	48660.76	44733.25	59906.92	100688.3	59183.45	24744.24	29745.61	61056.43	31198.24
11873.92	15030.43	25485.87	8761.82	14025.58	16039.81	11220.39	11543.18	22266.86	15027.1	16629.46	13181.64	16746.72	15895.16	16985.56	20327.82	19597.15
6977.04	3465.305	3435.616	2174.048	2631.468	2358.01	19840.42	7729.906	1827.587	4859.427	13512.69	4416.989	10459.51	4347.277	10439.79	3635.277	10212.04
60694.18	21025.41	49201.63	12789.63	49785.83	21464.54	6911.293	12887.34	48061.03	14331.68	56050.09	31792.25	21258.93	28687.46	8321.229	17543.5	6727.131
19048.82	24294.26	20867.73	10335.83	17521.88	19888.7	28215.92	24092.89	24831.28	31878.34	24715.35	34349.09	26179.09	14841.94	35431.25	21678.88	19016.96
136061.8	135468.8	148782	133872.5	91453.96	104783.7	131564.3	170647.4	195479.4	123773.9	432712.3	267737.3	95625.27	77112.12	87044.54	193925.2	155317.3
2589.503	3717.631	4215.37	5654.036	997.502	2295.132	2328.005	1174.724	2542.515	2033.74	3212.539	1690.515	931.5272	2022.4	1110.038	1861.633	2288.461
22948.02	24814.26	33025.31	30918.5	29190.52	24327.21	19831.14	20890.14	23356.73	17646.27	29928.95	31309.18	36649.74	23726.44	20823.41	23253.83	9400.639
5760.019	6866.43	7623.665	10181.63	27669.91	5579.022	6881.032	5581.195	5898.743	4323.902	13827.23	4491.255	8734.97	7388.296	3374.621	6737.933	54266.37
313475.8	180788	351658.1	394749.6	288798.1	315437.4	239765.2	329681.9	375254.6	156620.2	348952.7	234993.3	124503.5	331789.4	245329.1	343228.6	265715.2
61273.2	47581.98	80240.68	59882.51	78026.96	41234.3	47650	31922.43	102622.2	23482.51	51745.96	30705.37	77383.06	70197.6	46739.38	75700.7	77650.41
1110877	1176952	1253858	1080533	1076561	811455.9	848029.2	735178.4	1083183	1150086	985380.2	663311.4	1414582	813547.1	1185566	1167837	639840.3
38403.4	25580.34	46903.85	29640.07	45036.88	33915.88	38855.6	23821.45	24450.5	25433.05	29320.6	46568.28	41546.95	29658.41	24041.95	34857.57	44425.67
128114.5	95121.49	150251.8	153204.6	142405.9	182896.5	148982.3	138937	144971.3	104193.4	184467.7	179573	156655.3	176007.3	168401.3	174751.5	165041.4
27415.65	13812.57	16275.6	34215.76	11977.18	6314.472	8832.617	6963.193	11810.66	22570.89	63169.28	19252.5	19744.48	9414.152	15146.45	7993.81	15650.72
7763.273	11904.55	10407.02	15968.93	9310.152	8799.594	5169.685	8653.46	7721.797	7259.576	15652.54	12573.5	10784.28	10213.73	12619.16	16234.66	4780.173
5483.725	4199.72	6777.917	15441.75	4118.704	26723.36	3707.251	6193.071	5907.025	11362.64	9234.594	8041.866	4648.809	6598.469	4753.312	5684.514	3351.377
27592.4	67695.91	64133.37	50193.18	66718.19	48695.7	34346.48	32415.55	32695.37	75931.16	57728.77	47343.78	75334.44	47393.47	31382.96	32484.73	20772.99
13436.11	8912.32	9957.502	11060.17	15247.5	9267.42	11661.1	13443.82	18008.89	15259.31	12123.29	22070.07	11352.4	15453.57	17433.75	14016.63	16383.56
5689815	5142662	3552711	4117095	3826335	4450345	4117116	3748849	4717175	4742864	3389346	4743078	3787560	4389744	4815037	4159066	5979349
85749.45	84107.91	135378.4	132340.6	115046.5	94233.91	79834.05	104091.7	85288.17	62107.69	102923.2	109379.4	125109.3	100292	113114.9	108631.3	109910.5
3990.367	9174.123	7451.498	7684.01	2998.5	3999.251	5040.259	4671.833	7774.657	7608.742	1212.479	6488.463	4353.143	3100.499	4233.284	4230.293	7988.796
1044.679	1870.336	1487.73	1808.047	2037.437	1848.859	1836.449	1342.728	2911.013	2491.87	3413.902	2898.154	1612.734	2365.832	1156.847	2656.849	3525.785
17993.63	25222.33	20614.62	35238.36	15289.58	14461.05	24564.56	22197.71	21567.21	31938.98	18243.05	28271.88	11895.94	25246.61	24046.87	35278.68	21824.19
22114.36	34633.27	29278.72	17336.07	23192.83	24304.19	34878.16	28570.15	24330.74	29799.92	16785.83	34165.95	23797.82	34793.59	63485.78	19074.42	38758.96
6365.524	11056.01	10010.48	8171.504	5229.835	7972.592	11617.18	5796.33	8669.515	9027.223	9120.107	8741.791	5346.552	4515.658	6002.855	8629.705	5988.246
177399.2	256507	184625.6	150637.8	132026	147089.9	221340.6	142153.7	162568.4	196084.7	135431.9	201050.2	181620	163399.7	252618.7	165051.4	193416.3
1240087	124009.4	189957.1	2195744	179422.8	68130.42	51736.83	15355.04	43653.02	11800.52	2908156	16918.38	19916.9	34954.2	26094	8506.407	60801.73
64205.11	84966.16	76591.9	104104.9	76528.48	68896.44	74111.45	64034.13	60073.31	98556.01	96985.55	94905.2	66030.78	73885.36	81163.57	82285.63	75019.55
79203.95	64321.04	97835.99	137018.6	81652.48	104126.7	60213.75	72104.96	70590.27	36461.36	101130.1	35206.69	33703.03	84679.02	42438.18	84378.97	50991.01
171336	79841.13	63417.46	64109.53	27097.23	19214.4	23019.72	33357.29	35945.17	23116.21	24609.94	47003.03	35627.2	33974.08	17092.23	19607.32	11656.69
6901.708	6964.491	7742.45	6239.632	6384.852	8309.234	7544.609	7112.436	9878.905	10748.36	4330.716	6135.481	7366.796	8058.614	8974.422	5970.802	7490.314
469515.8	353077.2	190008.6	281367.1	403024.5	306204.4	299059.8	304399.7	357117.1	307080.5	159116.2	285357.4	430886.2	179400.7	298430.2	225531.2	470081
57774.48	52214.97	74028.79	48427.19	66458.59	38234.59	44245.68	40939.38	47023.28	52738.61	67378.99	60011.16	37764.7	60616.27	50694.89	40489.2	47002.06
15404.03	12552.85	16273.35	38812.61	18488.55	17531.31	12657.96	16026.92	18582.33	11907.06	25552.03	29700.54	22579.38	14832.4	9553.103	20687.44	11296.81
11815.17	2526.023	30922.24	7626.725	14612.91	8337.725	10949.16	14629.23	11225.07	7169.624	22492.45	33460.67	14100.12	4172.649	7218.903	23399.76	9183.748
148452.4	123672	116578.7	89921.61	107107	121594.8	120776.2	101816.3	109262.4	117379	104298.8	120675.8	106796.7	105085.3	113706.1	88923.18	93042.43
34340.81	29064.07	48149.93	59633.07	37570.47	38889.9	39320.73	32350.11	28599.38	20355.22	51095.7	5161.188	1427.511	32044.67	28261.97	33828.01	20539.48
7009.002	6777.248	6553.726	9275.178	7290.209	4656.67	6568.269	4307.516	4422.696	5287.15	10534.68	3795.411	4276.335	4820.54	3726.327	3222.998	5810.042
2565003	3782394	3577740	3350149	3124849	3925336	2411054	3564444	2881186	3406529	4059138	2586536	2545510	5005475	3698018	4564051	3571519
8809.667	10707.71	6210.651	13212.33	6649.907	10616.29	30771.43	9196.563	8479.474	10976.54	18326.54	6743.78	5330.664	5446.244	14026.55	4503.901	13404.81
15136.98	15335.33	38697.06	30010	24945.61	18984.58	11103.21	27850.55	11607.55	19265	39306.88	62425.54	27665.23	13106.48	18296.78	44362.3	40471.89
104956.2	221566.3	164493.2	124193.8	112491	240729.7	315134.2	252901.6	146106.9	197332.1	88433.95	100102.7	159020	146175.2	231094.6	207709.9	173593.5

156880	68089.97	105707	186918.8	125433.7	42327.06	46132.05	59698.11	137209.4	97265.23	119435.6	159949.9	142255	84291.95	70719.69	97449.73	123359.9
6553.164	5451.499	4464.656	8778.275	3691.548	6178.413	13752.92	7309.913	1626.362	5755.463	16506.7	2403.699	7446.112	3494.801	6452.319	3232.861	5426.925
15049.72	21945.86	20389.74	14711.52	10389.02	16912.84	18111.49	17731.78	17962.65	19576.1	32576.13	15417.48	11024.76	16073.5	20880.85	21680.37	35222.92
6340701	7397153	8291699	7073392	5060897	6147356	8788722	6387694	7171351	7625825	7972107	8317408	6511294	8206685	8459361	8630184	5620052
10078.03	4219.465	9488.19	12681.52	13846.71	5999.586	3425.631	9024.399	8677.525	7172.631	19760.63	5352.635	10799.61	7272.479	5087.866	11893.64	8096.905
59560.94	18616.89	27514.2	55905.96	26687.5	20813.05	16028.04	29729.97	18914.5	17031.94	159139.2	19677.54	17070.11	19405.72	17027.35	15269.62	47958.12
8018.415	12418.15	15311.38	24730.96	9680.678	9066.456	8481.691	7399.083	9216.114	6758.812	19723.51	25315.21	10416.51	9200.186	5151.501	11481.9	9790.485
307755.3	337042.8	423704.9	284662.2	337026.1	275510.9	273782.7	328272.7	272592	413750.5	393574.1	327443	387987.8	324732.5	275307.3	342919.2	426492.7
13574.29	22540.41	16302.46	14115.8	21017.52	17862.24	25320.86	16763.76	23615.72	28470.63	26865.08	27812.19	23229.82	25932.34	29745.87	22666.21	35255.5
16856.55	17741.35	14238.46	15473.8	13196.12	14674.78	10609.32	16110.52	31474.33	13937.9	22312.03	13043.25	14608.83	16244.78	8287.752	13314.72	8264.107
8804.829	7437.263	10824.88	17641.33	8891.457	6175.211	7403.801	7613.716	6379.474	5386.295	13359.45	14284.65	11690.37	14865.5	6201.441	20416.94	4938.68
4315.684	3434.33	3751.882	3219.017	2135.415	1779.532	3879.952	3884.617	2710.451	3433.466	5290.541	5166.777	5214.429	3454.53	1878.08	3819.47	5323.222
7444.214	2562.049	4240.416	8053.861	4551.664	6211.588	8242.332	7387.43	4973.717	3331.937	8561.954	5754.774	6061.985	3486.071	7553.202	6454.637	7321.539
3552577	3586646	3457698	2613733	2328064	3118473	3587134	2762324	3930987	3000192	2381586	2807805	2447700	3058038	3362284	2759139	3800075
42102.73	34022.97	59336.78	55651.26	46839.59	46821	28799.24	37317.35	42514.91	32904.66	41194.41	51089.61	44957.44	43836.82	29632.21	51938.85	28710.6
540020.8	759381.1	568128.3	512682.4	438545.3	562481.3	526074.2	494266.5	700864.3	716937.9	523617.3	549116.1	503972.5	586834.6	731654.9	559917.6	1029413
35445.24	44220.63	68245.89	87726.41	45705.34	64403.18	37689.48	49864.99	45332.17	28081.53	42036.26	95643.86	47342.72	59174.68	38394.07	76132.99	32100.99
22103.56	18125.1	39592.92	59655.69	21741.37	19591.25	11756.19	30450.49	27171.49	20628.55	35952.44	51047.34	29658.02	16142.27	17171.55	49954.98	24708.34
130512.6	178121.9	158161.6	108999.9	115644.4	129326	343149.6	185517.8	143245.3	150576.9	152626.3	91234.85	123630.4	119156.3	147164.8	123009.2	137085.7
157263.1	96754.71	232318.3	260621.7	132847.9	194749.1	115839.4	123750.7	128339.6	52243.02	176906.8	80822.38	46642.4	193574	99471.73	183500.1	88196.34
506254.9	363032.3	117193.2	786048.1	179465.8	168544	506798.9	291034.2	402805.2	172590.3	264154.2	138029.2	117358.4	362394.6	301417.9	146101.9	264808.5
38508.04	75740.85	45078.84	69228.8	2249.731	74616.02	41282.77	23027.92	43488.12	22135.62	30825.84	19113.07	4578.366	22685.24	4437.591	21837.37	23534.24
11396.89	23648.67	30836.16	49420.28	14762.56	18911.69	17473.64	20518.61	21962.08	27590.59	33447.25	15135.48	7451.481	25999.79	27947.8	48640.16	37605.92
16411.29	10448.16	24939.96	26057.25	31320.84	6834.568	9966.432	6163.519	10421.43	9389.417	19510.93	35899.33	36372.72	7312.463	19965.54	20526.6	32333.29
102294.1	52440.18	90944.7	105863.3	26226.53	40615.61	57167.42	46223.53	51292.23	52290.1	45904.89	40848.11	47267.11	67775.69	50054.76	58232.92	71226.91
108280.4	104724	112064.1	78715.65	61522.92	72810.38	79953.71	80249.57	85286.22	65874.79	67893.14	57315.54	58843.38	61631.78	71696.7	68834.66	121143.8
136841	115456.1	123351	182062.5	148637.7	129145.2	146722.2	107625.5	135052.3	124438.5	138742.8	181178.1	172727.5	134912.6	143398.1	144415.5	73482.18
63813.7	87704.88	64594.93	41844.56	120698	162627.3	304001.7	81234.63	73788.55	74209.22	56721.89	99852.8	28731.62	82907.99	247570.5	93345.56	220819.2
103662.1	132327.7	47181.26	56093.61	118104.4	142416	248764.1	105356.5	211095.4	111501.1	87383.2	60659.98	234858.7	199046.4	155620.8	125864.2	154485.4
28536.39	34168.98	67437.54	57346.23	44025.5	52303.98	30841.33	30625.36	16364.49	12854.33	24650.57	13817.61	40589.88	31154.64	14860.66	32399.83	20323.13
9160.633	13727.03	57120.59	81380.29	54031.5	9126.075	2820.494	15487.67	9835.935	15445.04	30019.89	67106.59	37030.01	8556.242	13265.05	22344.92	18556.17
33776.59	22432.94	30778.3	66657.48	33026.18	24452.12	41472.84	43846.2	30415.3	22257.48	37785.64	24051.85	30529.55	38281.52	36113.49	35175	50667.22
402052.2	359687.3	362834.6	379359.1	190671.6	245683	264276.4	314552.6	396149.2	344793.2	232118.3	324788.2	315277.6	291891	266415.1	339220.8	516516.4
65070.77	35702.97	49812.03	45320.54	65341.35	53015.47	101228.9	97540.33	93267.19	79738.24	57052.41	61341.99	90709.13	58486.02	106558.9	67539.18	107392.7
231175.8	208395.3	326024.4	221319.9	203525.4	133305.1	102464.3	210427.5	226940.2	195564.2	187777.2	279270.2	216900.9	122085.5	122774.9	230003.4	237989.1
312128.1	257296.2	367009.2	416917.3	380645.4	256247.7	254811.3	214363.8	254387.3	219715.4	294984.4	289917.8	340875.7	239395.3	279101.6	302797.3	289722.9
336103	468406.2	884560.9	273427.8	705905.4	333721.8	135443.3	177561.2	486478.3	518102.1	946578.1	1060468	506858.9	664622.4	372264.4	391486.2	229155.1
68447.73	132167.4	107112	74906.3	100133	96546.03	93360.46	81947.14	93872.41	142503.6	93575.39	106587.5	82944.17	149743.5	135297.8	149747.4	98416.81
7190.53	8304.8	10300.69	9409.989	10328.02	6608.109	6687.784	9030.519	5691.587	15874.28	15013.28	9398.396	12673.88	16657.82	13245.14	17120.47	12214.11
19299.43	18103.41	17430.46	19491.77	29342.01	21612.64	20307.61	17561.28	15024.9	21862.27	25389.45	31787.28	22571.88	36774.78	27819.35	22612.38	15489.6
33003.38	31738.54	51880.17	49654.75	39585.94	53589.6	31360.09	32170.63	29615.55	19377.94	33416.1	22502.88	7332.375	37714.49	23723.44	31543.38	14276.1
92784.72	143675.4	102346	85390.06	77446.43	96551.41	147854.5	139211.2	77658.67	22573.2	115394.1	109688.2	85762.52	102360.3	115353.3	79883.49	84144.21
89511.02	107831	98940.42	99920.02	66654.86	86123.53	119128.6	98116.22	109705.2	112257.2	112491	108130.1	75150	90357.25	103712.4	74186.89	79774.88
56738.12	47275.07	87196.85	125534.8	73005.17	80271.92	46544.23	100353	69618.95	46822	79720.23	81318.33	55137.98	98430.38	51927.02	134784.8	55205.34
58484.64	70465.41	56662.15	50333.77	32364.76	45152.2	66811.11	64208.84	69099.6	64302.85	62886.18	58060.74	41021.03	70268.6	70967.02	52305.24	50929.8
25535.45	23778.07	12791.85	31496	30807.47	24200.94	27205.84	21873.67	46175.34	27833.47	25566.93	16680.04	30489.14	38501.25	27314.92	33326.46	31180.13
3036504	2564091	3435244	2236344	1985118	2746049	2640530	2746952	3001315	2242210	3315498	2345022	2528447	3261995	2647459	2845075	2464074
18903488	19534654	11094683	10273504	13211355	10710139	9812378	10567656	15992900	19073472	8388698	11774733	12379257	8044027	11313415	15489757	28706218
17208.05	24836.87	10303.04	10647.32	12961.43	20152.96	74292.36	30394.09	15568.39	25466.96	5848.975	16406.76	14484.02	8735.732	28776.26	12077.85	13834.04

843317.3	1007746	836964.5	931568.7	841626.6	776278.8	1230601	743576.4	749834.4	1048709	1206311	1119292	879621.3	921865.6	1527057	855257.1	608721.4
3001128	1925793	3357940	4130810	2770370	3204835	2785862	2724120	3351768	1444950	3048787	2043015	1778754	3972249	2774041	3547639	2250908
36462.12	25074.19	12544.37	18302.09	14051.82	20057.01	17802.86	5863.304	26140.94	17894.62	22542.21	19492.99	14874.89	15473.23	16787.89	17122.51	27189.88
48515.46	63591.63	45166.63	69515.24	58096.29	41731.22	27450.98	39230.66	37921.04	44402.86	62266.49	30757.07	44477.69	64276.01	27474.16	34853.69	38177.7
20875.43	19787.54	30376.99	31632.58	23759.51	13382.41	14940.12	12932.26	14169.77	22718.83	37376.29	28255.04	21703.16	15604.46	18526.72	16254.42	13807.17
129259.3	159433.2	141697.7	119457.3	113834.9	189073.5	191133.4	191331.3	129570.6	149046.7	68168.99	132391.7	179866	202478.8	169620.1	193097.5	130126.5
299448.8	329292	352224.8	539173.2	543208.6	443314	340778.7	287270.1	310032.3	218679.4	250263.1	312225.1	331997.8	346080.4	228160.8	324460	198206.8
96856.9	108329.4	108458.1	116932.7	82297.34	81891.63	96223.44	91213.88	120817.3	113095.7	88798.06	113135	109655	93400.89	116384.3	96561.59	80674.64
24914.47	18351.1	24961.37	21204.61	6683.969	13442.62	8246.574	12968.82	13485.66	7805.533	14780.82	11140.31	11548.63	12915.27	13200.1	17732.23	8733.973
4106.211	10597.86	9213.734	6623.632	7949.715	6924.418	6455.468	6151.656	12106.9	7680.42	5543.585	13958.26	7242.001	8600.153	5360.854	4464.958	1227.688
11363.76	11805.66	12520.3	19244.95	12816.95	14101.2	9231.021	10151.81	13858.53	15406.54	15245.91	12641.22	21953.35	19078.9	16710.46	19116.55	12949.49
130642.5	126153.2	129583.6	95867.72	98517.52	72916.89	78036.72	100927.2	116257.4	127476.9	171121.9	112287.8	81504.19	92217.74	84108.19	87743.54	76214.89
13954.69	11747.09	35074.5	7438.412	16925.05	3590.021	4441.02	8098.98	8893.346	11238.03	28101.19	9944.265	11611.66	8623.479	6551.81	8155.226	7216.054
7533.58	8784.396	8044.11	7509.311	5253.378	4711.759	5992.286	5274.438	8133.964	6885.558	4986.888	4565.023	6593.219	2766.279	6803.397	5188.497	6510.176
12765.87	13805.95	21357.61	42122.77	10085.23	11498.81	17349.61	15532.7	12662.27	10883.31	27976.74	12602.68	9925.99	17205.28	12729.96	16396.39	10958.1
56896.45	57177.2	59617.39	59265.3	58430.26	64605.93	54623.93	41452.45	50065.21	38425.96	51685.53	44034.61	74445.35	66908.73	58792.22	65134.94	25136.09
43451.02	47361.71	41316.07	50907.95	44312.9	22192.7	41692.18	61082.81	48772.11	74607.39	50674.86	50255.04	37698.06	58059.55	51831.59	77043.62	39189.4
4255761	5014788	5237989	4286480	5492849	4687974	4330501	5138126	5153172	6856046	5720240	4833275	6977483	5624818	4894843	6025152	7952544
2653.518	4055.627	5133.889	6095.876	1274.145	2152.448	2549.23	2329.684	2204.224	1665.94	4008.38	4085.787	4035.116	2958.165	2380.573	2561.843	2658.309
42659.93	33572.61	41741.45	41782.06	38940.62	35775.41	30451.96	39696.33	46038.86	42756.48	30286.34	25746.7	29373.68	56615.33	38810.09	68483.3	48347.37
14677.95	11351.09	18716.42	17571.86	17345.6	14735.1	18387.76	14548.7	18459.06	11492.2	14634.99	20494.62	22228.41	24652.96	16724.49	16813.56	9826.602
22134.63	19687.46	39225.56	35609.7	27586.58	25962.3	21942.75	25474.46	43173.85	22196.99	28466.96	21780.53	26929.5	21388.3	13231.99	19185.75	5969.926
2519.668	3766.095	5077.134	1549.491	3315.143	2938.675	1924.518	2678.717	3043.956	1800.148	4267.993	8153.198	2611.51	2685.942	1621.366	2829.158	5604.934
184186.5	356064.1	816751.2	600263.4	477769.8	35824.66	56484.88	588340.6	642606.1	556006.6	380514	533556.7	549631.4	854433.9	22498.86	1048800	1172042
107447.6	142831.7	140537.3	121033	115970.8	118717.6	124821.1	133451.7	191099.2	160393.3	195711.3	155582.9	113510.3	119474.3	140578.5	113763.8	95893.47
69987.67	122098	97035.45	55366.59	81227.72	52333.7	55116.26	82355.45	105708.7	144574.1	57054.45	105628.1	80015.59	68681.05	56638.04	67734.69	51854.77
7797.045	12832.55	5234.997	18171.16	4955.903	7475.343	9883.352	5757.453	11082.22	8516.946	8674.23	481.6935	4578.698	5282.3	7914.912	6281.599	9322.852
27976	37922.01	62563.59	62261.73	42569.9	48429.71	40249.96	47131.95	29523.94	34828.74	54023.86	42370.22	35521.66	50072.99	30966.37	51446.52	28246.33
962.8876	6969.37	4802.83	4974.887	1499.725	15411.86	7397.824	707.8312	3878.283	6070.768	2184.466	1263.28	1272.704	18106.62	4625.17	1176.64	4285.784
5913.309	5457.022	7300.264	4635.5	20987.9	10685.21	4430.868	6545.316	7336.374	9173.27	10179.44	6764.91	6288.489	6983.578	6057.335	9064.696	9922.81
566468.1	741009.6	581872.1	593854.4	605113.9	527380.3	538173.7	449044.8	646801.8	682233.4	492641.1	504326	564870.3	482982.2	650592.2	515142.1	692545.8
35202.4	27700.44	25617.44	22366.24	37023.04	28692.32	27417.42	34021.71	28370.74	41842.25	39557.09	33837.61	37980.03	37084.25	51292.3	26639.11	35145.17
10498.96	10425.48	9857.689	9766.426	8836.096	4494.491	5573.479	6513.705	9885.35	8433.9	11211.65	2759.895	6903.747	4076.731	5636.331	5691.061	10191.61
111949.1	114472.2	69010.22	97241.77	63110.8	70936.71	78335.74	95148.48	98423.78	67823.33	37000.95	68716.98	54019.68	84770.22	99628.3	84062.98	96675.66
15296.08	5682.059	6363.205	37445.08	12749.37	5814.563	3575.704	5075.369	5485.738	3590.473	46703.9	8017.806	9081.808	3992.52	5233.232	7021.299	2525.286
17472.56	13230.21	11977.62	14866.54	8546.58	15609.9	22135.94	17344.76	11021.38	9869.124	12261.82	6248.505	6939.284	9332.807	12595.56	7115.95	11437.3
12808.52	21549.2	19070.67	21985.52	20941.96	16368.1	11215.2	19191.09	16065.01	17412.64	12662.03	18949.43	15244.74	18406.21	12117.39	28961.87	12332.09
7660.145	8550.943	8846.508	9461.188	7856.539	9341.117	4757.844	6882.325	4090.954	7412.697	9377.967	8383.886	4388.44	12604.61	11321.68	3540.588	6803.91
15024.08	5804.364	8884.134	21611.92	32079.04	12636.68	12703.2	23920.05	18183.74	17425.49	51852	74472.86	27904.37	13500.29	10677.51	40370.48	20015.83
31241.59	17711.26	16246.67	27247.29	22725.88	10824.39	14165.71	29275	20817.34	32014.72	18540.85	26215.14	14021.87	16356.02	12248.54	22807.41	17109.45
325825.8	277241.3	341217.3	423385.5	293347.8	345073	291585.4	312766.9	314769.7	204977.5	199957.8	245741.4	325220.7	298235.7	262396.6	340792.6	295971.5
180295.5	156134.4	221304.2	233977.2	212180.7	225545.4	141421.7	171512.3	159985.8	110548.6	157886.2	135173.6	154068.7	171132.3	110455	158585	120394
41292.32	38163.16	41348.43	70168.33	24146.88	67290.16	72481.45	56166.52	40904.57	68210.13	44362.06	59923.68	65140.41	93264.81	68733.86	41849.58	66219.56
33747.95	31109.79	37833.58	22496.59	51463.47	18277.61	23678.74	20132.79	17588.72	37970.5	67302.66	29493.21	35695.7	28730.88	34515.42	22561.98	24527.57
16742.84	16763.02	14725.87	10251.11	14325.94	11435.77	13314.98	15955.61	22827.27	13920.5	14227.24	33592.01	15992.79	13339.96	10793.16	8835.516	5891.445
871095.2	949632.5	736811.1	1097415	498674.3	863812.1	700082.8	574597.1	856144.1	670656.5	829162.3	599454.5	531293	698425.9	345806	753652.3	441250.3
11410.21	9693.242	20356.69	29971.04	20803.44	10132.84	7408.534	20148.95	18872.31	12873.16	23061.19	34818.65	26263.69	12791.67	13768.99	22560.51	17760.6
6707.7	7178.416	10487.22	12641.5	4414.761	6002.888	7494.456	5785.766	4810.648	7040.222	11890.74	6944.396	9708.723	5898.797	4058.53	5755.513	6488.822
270970.2	198456	98504.7	194891	212789.8	316661.2	674554.3	272815.1	323908	187555.5	252326.4	112052.8	191088.5	427365.3	272832.9	320802.8	275207.8

30430.69	25317.47	11208.43	16909.04	36729.71	18547.46	17806.66	14587.29	26218.86	33712.94	23749.53	22227.96	40622.02	19303.34	17095.94	16340.36	17717.84
4429.429	3872.851	1517.852	4662.22	4195.392	3513.375	3392.66	3619.558	4113.028	2292.408	6122.304	3846.987	5560.079	21467.59	2123.325	3500.011	8097.227
281658.3	134796.2	178239.7	304012.4	351802.1	113644.6	184414.7	307519.8	322151.1	199209.5	442841.1	139033.9	377415.5	1142327	265046.2	176859.5	313478.6
186143.6	63257.08	92770.19	198535.6	274144.6	98661.58	127728.2	266179.6	284693.8	95891.35	285537.7	77498.34	271615.3	760204.5	179956.4	106918	175719.9
62386.35	22754.67	32935.95	97040.76	72746.78	26357.64	42793.43	63028.46	74163.64	31045.74	115324.1	28176.66	132609.7	269259.4	57238.83	30236.46	51422.78
8288.706	6711.502	7672.442	10564.51	8671.927	4822.313	8704.267	6506.47	10633.91	6105.325	14551.99	5403.706	6094.83	77307.48	10878.09	6151.242	14175.16
188112.2	45795.39	133715.8	178276.5	206942.8	70266.2	124870.2	197759.6	201624.8	96362.8	246299.9	111458.9	266388.8	953331.8	206296.7	162927	219630.2
16816.83	13674.98	23988.56	26759.56	20970.19	20699.74	16494.64	15919.07	21206.65	20847.17	24740.77	22402.64	19002.94	24207.59	19898.19	17842.29	14053.21
2614174	3191593	3044269	2720688	2016874	2578270	2936042	2539339	2694309	2756636	3593853	3480001	2034689	2939455	4417774	3470205	2592971
675720.6	585632.3	587794.6	1001797	739848.9	455817.7	475627.8	568652.4	600551.8	672851.3	673064.3	958466.1	649796.3	523858	566196.1	562529.5	369831.8
364437.4	293446.6	574518.8	625165.6	481816.7	435492.4	264519	378293.1	313732.2	205333.7	408331.4	336762.1	387716.9	332937.2	254246	423045.4	256332.7
503506.6	356251.8	712835.6	904096.7	620001.1	374701.1	326754.6	484889.7	665441.1	469525	957732.6	953166.7	557111.6	529271.9	516991.3	729515.9	526962.1
9140.878	11240.2	5311.597	15897.78	7048.904	9443.674	9251.693	8867.656	15008.08	11219.14	14743.12	6086.987	12238.14	13081.35	9936.577	11507.02	8639.771
6423.745	4799.445	10842.3	13311.22	6483.373	5977.666	4040.197	7857.407	4791.875	4831.443	9810.08	7275.288	10089.3	8927.984	5289.439	7949.867	5926.229
97739.51	89850.19	115187	173703.9	94371.93	82660.94	67769.8	111955.7	114569.9	77275.08	128602.8	121803.5	87207.67	100762	81704.77	142195.3	99530.28
88092.41	29330.79	44431.67	102493.1	96133.88	35735.9	50307.22	97077.36	76260.02	38819.27	117734.1	54732.33	94630.46	333741.2	66528.65	65201.01	92321.84
30879.9	16985.57	26947.82	24590.46	32343.87	5455.727	7782.92	3274.985	8424.977	25515.07	14495.05	50947.02	29903.12	2627.009	6860.603	2531.174	14442.54
5936.153	6603.829	9692.586	11037.21	10545.81	7477.316	5006.261	6326.938	4129.564	5675.729	10199.79	18718.77	5843.067	8183.54	4855.18	8312.177	6850.355
18309.18	24533.96	26713.59	25773.1	25738.25	20317.43	16069.71	30018.01	23010.53	25242.22	9000.553	20438.74	23906.54	20251.14	13380.97	30575.68	16595.19
9267.214	8791.678	4238.703	15541.08	6910.181	14036.18	12078.01	10539.74	11521.74	11613.5	13023.77	3844.484	11103.02	1989.551	9130.752	11239.18	12174.98
3502.011	5166.815	8619.406	10126.42	5788.332	5207.85	3430.796	4152.915	6461.412	4178.741	9830.154	6067.556	5092.124	7297.938	4035.573	6456.827	6286.354
48713.33	55081.99	69714.16	88145.79	65422.14	42214.44	39251.85	40540.56	45131.8	37435.01	39487	57988.6	50721.64	43994.21	50526	48400.19	30565.21
106228.3	139213.8	119192.1	113919.1	156544.3	115150.9	206706.8	144483	141535.3	150630.4	96960.55	103110.6	205606.9	73272.12	100902.2	125197.8	173559
687109.9	661988	593504.9	529231.8	394448.3	343320.3	879084.9	524910.3	768477.4	843399.9	503669.8	671270.6	182004	587141.1	1421737	608916.8	282735.9
14443.72	9565.276	20829.62	22216.65	12913.13	14474.19	6720.862	7946.894	4624.423	7726.122	15562.21	14274.77	27218.65	6775.954	4155.52	9965.184	9321.007
734795.1	571853.1	824199.3	465901.5	396608.3	443632.4	790150.3	709720.2	807334.3	763320.3	914468.2	646757.7	517304.8	551615.4	1048817	597201.2	649449.2
34462.04	27686.49	45309.61	69290.62	40353.85	31151.76	26754.16	29245.69	33562.67	27159.13	36743.38	61954.6	53597.58	41497.31	54293.35	53597.67	40865.66
108631.6	110548.8	144433.6	92957.98	80076.22	122282.5	138740.7	115795.7	136013.5	108399.8	95981.41	99878.15	75216.36	139866.6	165643.6	125744.4	108813.2
31460.47	34960.79	42754.98	60230.67	23067.16	37837.59	22029	16564.41	52435.33	39152.62	46487.75	41523.14	29176.84	36755.59	32670.12	42954.98	49004.91
31640.7	47314.66	34586.33	46584.46	40182.09	38510.13	28295.2	40573.98	43070.82	41394.8	37306.35	45578.07	37120.95	43759.55	34577.35	55162.58	60815.99
32480.05	18816.67	27047.73	30469.84	24377.76	20354.99	17255.67	28266.16	20929.04	19141.12	25446.76	34288.33	25770.79	20257.6	25401.2	27137.36	35606.51
15347.33	8544.485	9088.602	9230.125	9486.58	4852.185	4579.217	6374.566	10703.26	15509.76	90973.21	11956.72	7706.106	5673.502	6489.666	10023.49	5765.006
82341.84	73149.05	126648.3	163120.3	77474.45	82310.35	64929.65	61112.33	84092.02	43244.97	102194.2	66116.78	60765.55	91136.82	53556.96	80946.15	43356.07
82728.93	66843.21	213449.5	158865.7	194663.1	89200.47	87725.96	120097.2	246869.3	181634.4	191788.4	213473.8	177856.8	190969.2	239353.5	179595	224665.7
110101.5	62533.41	162840	247663	62981.26	56329.79	54290.51	93535.89	76650.73	60472.61	147626.8	260324.3	63838.95	94639.97	76218.63	146411.9	75773.37
11948.09	30928.27	19629.47	38765.91	19711.1	11798.96	16212.74	14393.71	24733.95	28577.54	32042.36	20345.67	17631.89	18247.85	27917.21	27727.82	29005.68
27018.87	14444.34	43416.59	41299.5	17872.95	14074.73	18871.54	32282.95	30017.21	14800.36	54090.98	13846.11	38136.15	17909.23	30058.93	61172.07	53848.42
15605520	25509762	13115842	15904034	11631898	23969624	36743256	20730138	23868306	19676640	15603040	14241171	8661581	20101022	23223258	21845404	29222454
18620.33	18054.34	14385.25	13799.55	13015.3	16225.65	9770.355	19504	18062.11	15184.94	12372.92	11887.44	15238.56	18261.34	15496.35	14877.54	15596.9
6990.078	9396.474	10714.53	11756.09	10297.35	7492.906	10021.83	3509.687	8416.221	7109.125	13667.03	10717.6	12352.16	14209.22	8122.719	10913.13	6418.094
79118.44	84286.49	108756.7	115519.1	81618.86	75606.09	69759.35	57776.92	75852.38	49386.73	81575.49	68088.44	66864.99	78583.39	48931.63	101247.3	29751.29
23252.68	18074.46	34026.71	28274.28	25481.33	33110.2	21141.9	23753.88	31180.58	15003.42	10475.61	18338.65	16481.91	30427.48	24381.46	25385.41	28390.58
63078.13	59020.15	54961.82	50370.75	39176.98	54614.07	82346.47	47439.38	70345.16	73665.72	49207.56	50601.56	68666.38	90290.37	87679.87	55875	49067.14
11716.68	10166.57	8015.903	19502.74	15519.13	9861.124	8881.047	10735.5	23731.53	18140.8	18183.08	16136.39	11215.5	11923.68	20892.43	27609.34	30987.87
62614.99	45014.26	40022.49	53673.99	44198.48	52381.18	51540.04	51661.57	80207.66	152020.7	65444.79	63116.27	47101.85	79126.66	66195.13	85551.72	52867.2
23450.08	20082.14	25575.01	19759.77	14821.02	10562.69	16819.11	14231.04	16473.14	18666.39	16728.71	25165.78	17240.26	11873.62	15057.65	12493.46	15304.77
48584.91	39034.96	54947.48	82287.48	45094.55	41173.1	25309.54	47412.79	38148.41	22908.57	56416.27	51849.48	50252.54	37125.69	27180.94	48118.4	47574.28
37668.28	34597.55	38358.96	34060.89	38103.36	32699.75	31435.48	25657.75	33310.77	29805.14	40269.55	37280	38980.93	47264.55	36875.26	32179.18	16432.36
70920.72	60876.78	55773.98	52902.48	50332.2	57620.84	53859.94	51298.61	53782.63	56479.34	41249.35	58243.39	54820.84	48639.8	54942.03	46964.61	72661.84

43589.3	35084.65	46992.67	52734.08	40607.94	48027.17	36100.89	32936.41	35462.66	21018.15	43658.54	35677.73	40740.02	46565.68	25973.98	31171.92	22826.84
125093.2	117094.9	198288.8	225628.5	156837.1	141214.9	86400.18	118104.9	127548	77134.01	115476.7	96465.69	67081.73	144620.7	96239.25	136316.6	88531.99
59160.79	69339.67	56400.2	52467.01	52892.66	52261.16	64329.23	52907.79	67777.21	84563.91	63953.08	67870.84	55887.22	51774.67	65997.98	55483.86	36638.16
90369.14	110237.2	143799.1	87690.38	56706.55	106852	94358.66	70050.54	100984.2	84289.07	77591.41	100172.8	68532.56	94879.96	77945.13	70885.46	85039.27
33484.25	39097.7	32270.61	23604.49	32519.88	26192.97	31318.66	28513.41	41629.09	39577.62	33152.64	32753.69	39238.41	30815.99	26568.55	24385.19	15388.62
24274.19	53035.53	32688.57	50256.05	23842.77	33380.32	21082.89	5876.032	12217.4	39891.9	29995.46	26187.4	30051.12	64595.57	10181.96	10132.7	16870.27
28068.32	35518.97	56493.65	21992.38	7489.006	21706.59	12190.08	15127.53	43510.81	10865.99	17193.51	32711.26	23241.51	21143.73	19875.75	16198.65	26285.04
16829.96	12005.86	12274.42	9000.402	7040.583	12040.2	15406.96	13583.61	14318.41	12162.82	13518.1	13291.86	4396.763	12318.42	15716.77	12800.12	10994.27
43898.47	57099.05	51466.72	65385.2	26814.27	46480.73	44203.89	36624.26	54157.82	63158.17	47760.51	35824.75	33585.74	49063.25	33348.71	65619.09	51940.59
6783.285	9852.053	5099.964	2636.785	7650.712	9147.272	6239.224	13522.17	9548.935	12168.29	8716.738	8159.613	8214.131	9765.521	8905.273	11600.53	12019.42
4141.045	10281.22	19603.31	4284.369	8049.341	31922.83	30243.27	15491.47	11518.03	36710.16	44571.99	5085.111	37300.27	58729.93	4950.012	12061.27	15622.73
75911.18	72445.67	96001.05	94676.2	80133.93	78545.41	71823.19	73360.58	81107.72	64939.34	74166.3	70208.47	110662.6	85640.37	79415.04	81486.4	44327.92
3303.514	2689.649	6271.838	17450.22	6925.771	1752.837	4535.767	6519.698	5327.166	3540.318	12076.08	20563.87	12195.06	2600.416	2004.583	9047.214	5999.738
173650.6	120024.6	213866.3	262788.3	150220.7	205369.6	144571.3	207211	185446.7	98130.74	170264	127559.8	108489.6	174250.7	120465.6	246230	117504.5
28630.17	18513.41	32568.86	53452.39	38644.75	24290.15	24317.29	36202.33	37704.47	46339.77	66771.51	121491.9	71024.41	35105.47	22887.58	63018.7	51634.58
128903.1	123862.9	115048.9	101093.3	92194.47	132845.1	129136.2	132158.2	111336	98178.19	80596.82	70108.98	81629.43	82714.77	77964.28	73059.13	53968.95
53028.09	47424.01	47510.43	67552.08	60658.89	49485.64	37971.39	46288.56	55888.74	39189.14	61144.64	52433.79	68103.94	64220.57	44774.41	66185.52	32365.19
25046.41	15401.05	27182.25	53817.07	20235.24	18713.39	16758.29	17692.99	18548.48	16775.26	28071.46	36638.32	85147.46	5906.887	11897.65	25789.87	27436.81
11282.42	5458.324	9595.904	18776.26	6804.622	4879.118	8334.722	15374.91	6113.07	6340.026	17941.12	16081.76	8486.188	6922.623	17879.92	8267.761	11240.11
2055702	2313763	1613292	1619024	1730005	1897141	1727718	1739411	2041161	2292948	1855796	1762478	1209238	1990840	1996796	2014287	3125991
14844.78	17006.82	11588.43	12427.45	16181.64	11420.86	10736.18	10671.11	20402.56	17992.2	13440.85	17679.42	16572.87	13738.39	13412.66	13206.9	8432.183
204833.3	73377.81	131036.4	1214533	135358.5	15281.45	27596.99	21291.99	21373.05	16442.02	865474.7	60542.18	35824.98	23707.59	21960.54	25445.21	40882.26
4839.085	4801.063	8771.017	11241.21	11525.66	2171.52	6717.186	9144.151	7028.553	12317.18	11388.25	18813.28	11338.16	4234.287	6772.634	13192.34	10331.06
10297.83	1047.541	29905.2	11146.36	6082.646	4928.778	13039.75	4376.083	2229.822	8998.022	64604.41	2325.638	3020.365	21176.89	4213.33	4068.635	35594.8
105317.1	132560.4	117432.8	94600.38	137321.2	95845.63	89181.5	104539.6	128211.3	103307.4	85391.55	112376.4	121475.1	95193.23	91895.13	107037.2	127882.9
8452.11	7333.611	6380.031	7190.776	3439.395	4405.538	2072.291	3616.821	2912.648	3389.677	4241.47	6068.795	5366.297	6480.673	4091.781	6047.6	15831.3
9272.669	8481.648	19098.8	21504.86	10049.02	10496.24	6330.104	8126.667	14374.13	5842.879	12764.09	9809.189	9910.635	8277.669	6833.212	6494.334	10009.42
22728.59	25459.21	31441.17	11333.03	34487.17	21036.21	29627.83	25455.41	24286	29864.43	31328.44	38562.31	32468.27	28147.84	34451.52	23541.34	42769.55
20716.47	24386.83	25562.65	5991.96	13932.17	5706.902	16846.83	13244.62	17985.04	16593.91	9101.027	13825.94	12749.93	8859.616	14163.06	4815.323	2719.884
9673.573	10434.29	8840.885	9123.014	11085.48	7827.31	7619.289	10857.96	9387.384	15180.57	15611.46	13104.51	18247.33	12369.98	6984.381	16650.69	13068.55
11097.82	7388.132	11607.51	8829.587	9157.06	7662.195	7012.217	13572.93	12524.37	9890.526	14578.73	5880.583	8886.86	14923.41	8606.714	10471.26	10961.65
28992.41	21521.46	40938.61	63275.93	30265.42	39964.91	19389.64	29990.74	32263.08	17408.52	28164.07	32031.53	24615.27	27213.81	17200.54	34071.87	29926.58
36209.47	54998.46	51717.7	63416.01	49242.17	30969.36	31175.95	40949.05	35817.5	46202.57	43371.17	80248.02	43890.09	30339.56	30953.75	54590.2	43641.7
14179.51	6549.74	9172.245	31051.47	16697.22	8483.656	7755.477	12400.28	13651.95	18003.24	32305.43	27862.53	23316.04	12737.81	10780.41	13205.85	12577.46
28260.1	36677.09	33649.82	46920.45	41743.48	16807.44	17788.21	34373.46	32860.43	37453.5	18394.92	36909.92	32874.16	25155.66	25570.87	52942.79	29879.37
78117.77	92000.59	66245.5	62512.5	54646.03	65025.82	55283.45	40269.81	58493.43	75816.24	54132.05	92864.28	43859.99	51417.31	36163.17	33956.3	31479.2
10302.21	11227.84	11034.76	16121.15	13555.59	15075.4	19085.62	18285.58	17194.45	17345.17	15967.07	13050.02	23545.03	11228.33	30788.88	18753.62	12635.19
5888.14	7300.822	6330.61	11216.96	1886.782	6673.298	6967.969	12533.32	8397.963	3535.672	7711.414	5938.212	7072.98	4910.216	10119.51	7568.044	5591.455
67175.28	82281.83	48837.65	102383.3	145426.6	87145.44	111208	98541.63	183713.5	108234.2	82254.12	65148.64	347082.2	31176.3	113501.8	53922.99	128359.9
20399.56	17307.98	23024.12	40478.33	23062.8	24279.61	17474.73	17829.05	17528.05	9119.512	11509.33	24940.1	19143.9	20066.54	16015.76	17722.07	25064.57
82353.27	95092.85	103660.6	577819.6	157847.4	96990.38	95053.02	97315.02	89819.57	73295.52	116953.8	94854.28	108011.5	137201.5	141941.3	138378.2	58608.62
50191.67	80142.28	5240.805	46570.38	53463.97	74421.4	67674.84	69830.61	68042.12	68108.1	73066.36	53466.38	51042.45	66871.41	62985.05	80446.02	74832.88
15210.35	16448.89	8873.092	14828	13710.4	12938.25	17070.91	13137.94	14406.85	11707.73	36055.06	14889.75	10061.41	14410.88	13789.06	9425.063	12011.11
6588.375	1421.128	3272.51	8700.712	6523.537	5700.035	5427.006	4888.71	4290.956	6166.047	11111.8	12341.76	9115.577	6794.558	5780.454	5035.5	4813.039
1565.105	10299.26	1936.309	9047.871	8133.324	4344.349	3482.19	4511.014	8728.893	7267.002	9669.624	4453.203	6678.335	6955.622	6534.174	4566.9	9664.583
803250.2	740077.8	708621.8	738052.3	596910.8	586982.9	921600.8	961973.1	1088599	898983.8	1050105	757082.6	484640.6	847400.4	1176744	770287.9	769135.6
9343.196	7993.372	12847.41	9467.594	9961.592	12526.73	9256.793	6240.593	11061.04	2909.737	9511.72	7325.034	8150.611	11621.46	5379.226	8671.376	16079.46
24867.13	13738.69	38413.88	22907.16	16141.91	24729.99	12990.11	23495.13	23044.31	18467.33	28772.54	26718.58	21393.67	22827.05	24030.69	26181.41	23150.87
22314.68	27279.54	25270.57	24585.96	33265.38	19588.22	16833.49	16079.34	24749.62	32394.67	18471.54	23572.07	44057.23	38182.14	31949.47	28093.5	17571.1

10401.41	7773.703	21439.73	29720.9	19974.82	16287.74	9272.96	13556.23	18108.46	12064.16	17792.92	13803.21	18116.32	18749.25	14213	18378.54	18623.13
79344.45	70672.49	133014.2	246529.1	109623.1	75432.18	51792.44	80741.61	77500.81	39220.4	85276.93	57008.94	56025.37	98452.01	44428.78	84492.32	58850.72
8025.834	13176.97	9205.69	9859.083	14657.77	8360.115	10406.38	7845.915	11975.95	11049.71	4766.945	13987.51	2350.721	6373.107	14630.2	12863.62	8751.403
9009.578	9090.869	5640.262	13996.16	6750.507	5573.989	7650.783	7434.315	9473.247	10773.58	7590.861	11525.64	11454.76	5914.392	12850.15	9911.821	13897.64
254327.2	600438.1	1469819	1112706	1140516	2491679	396642.3	218362.2	50173.63	1026505	1560755	1428458	632965.5	1363699	306405.4	966877	53084.31
8835.272	11129.26	9664.988	12245.39	10106.62	9150.055	7950.495	9729.982	7639.029	13791.46	13277.94	10563.01	8357.399	8088.106	9920.199	9152.612	10592.34
6360.444	8760.188	5565.698	5477.292	6392.53	3910.142	8606.642	4870.77	3040.576	6479.932	2753.009	12196.58	6635.296	3337.554	5972.323	4575.317	8963.351
162816.9	101792.9	163661.3	183299.3	92374.88	112475	171433.6	137264.6	174754.2	137362.4	168999.7	127513.2	140524.2	162122	167067.2	99955.51	137269.9
35021.95	24508.2	22398.34	38118.89	19138	23284.55	38338.82	15262.71	25683.5	34140.21	32298.5	24076.02	17674.5	30851.6	27034.12	27250.94	46806.54
547642.3	359848	523348.5	794765	553826.7	557639.3	452936.4	458473.4	468230.2	365614.7	733388.4	430200.7	445350.7	538133.3	515581	486377.6	396885.1
38530.72	35948.9	27146.91	41646.34	68999.59	17533.89	35659.83	13970.21	24934.98	26464.26	35539.67	28345.06	39870.19	28506.88	26774.84	31273.28	62064.33
286405.3	745557.4	27539.39	513907.8	58580.64	938734.9	2042554	32060.66	432565.9	1403011	126468.3	365081.2	8602.667	920029.6	652220.4	801406.4	1857686
25438.65	16490.28	20327.25	33815.86	19714.5	25295.59	18669.03	24059.43	15943.42	11440.8	18524.71	18399.36	14566.01	14444.95	17647.7	36768.95	28763
2467.764	3236.382	1593.62	6000.569	3676.32	4385.096	2518.074	3251.014	2677.967	1912.582	4346.905	4680.748	4258.188	2965.78	2313.659	2843.462	2875.035
23213.78	15926.45	6438.651	7038.718	6544.871	7518.368	9367.507	7509.812	11667.9	11330.34	4396.337	10946.18	6087.24	5625.063	9198.95	7485.942	17691.21
658094.9	547999.3	377876.6	537713	501184.4	154980.1	538636.4	783361	580055.3	427098	476945.4	541922.9	636832.9	544757.1	813645.1	541545.4	815139.5
172544.8	197830.6	92226.98	80134.84	78953.33	98991.46	195701.5	156511.1	215516.4	236138	111640	142752.5	102317	134667.1	296128.3	122654.3	130785.9
6589.426	7588.236	7761.751	6073.971	6399.714	5292.463	5045.102	3818.021	5532.907	4919.946	5512.383	8046.932	7462.153	7286.586	3769.581	7963.848	9034.134
4468746	4751530	5026334	3252083	3437684	3740755	5418899	4762548	6017909	5909843	6142806	5756010	4073906	5418370	6054042	4217343	3128350
49093.61	68996.84	43647.02	43726.13	16642.92	37033.14	45619.39	31473.88	38094.74	44883.85	27213.72	47968.81	27586.54	46117.85	43022.61	32640.68	40173.73
11215.66	4804.986	9774.795	12989.81	14472.37	2267.101	3188.204	10204.95	6871.688	5059.671	14459.54	11848.85	13393.08	5936.019	8304.042	8732.924	5326.688
2603.184	9166.91	6916.732	5539.222	10506.46	9619.403	13601.17	7812.738	8220.651	11419.55	9072.771	12762.59	6503.27	9678.447	7553.292	13051.29	14672.87
141940.9	178410.4	168959.4	167994.5	132978.5	125287.3	145214.2	206010.4	164153	236909.6	146039.5	217549.5	143091.7	141987.3	158801.5	175643.8	122385.9
50527.42	82110.54	77933.53	44976.03	64164.89	94369.5	118966.5	82761.81	70779.43	85065.23	60225.56	43985.27	88628.38	76621.07	97270.66	95240.76	128430.6
16795.82	15419.66	26316.07	39534.57	19653.02	14712.65	12636.2	15955.39	14649.08	9857.889	17675.5	24457.14	19608.6	21721.2	15589.55	28133.38	10370.95
90795.65	90943.2	87049.02	136038.7	86596.7	92315.45	92480.98	74436.65	120098.4	85293.43	72746.94	45983.31	72901.6	161674.6	83353.72	134986.8	44678.59
51287.88	47542.43	55204.5	66194.8	64417.37	70713.54	54157.07	55922.96	50984.5	27724.86	57144.29	24574.12	35843.45	54496.38	38245.03	58070.21	54985.91
150295	167421.7	175754.4	157984.5	142409.1	181770	150797.3	122663.6	192647.1	165987.6	137868.3	141802.4	125262.4	164681.6	164913.1	154637	183557.7
16314.58	13866.64	17866.7	29954.22	18188.03	4527.138	11337.28	16732.87	16628.89	21843.14	45993.05	12635.91	13748.85	17726.57	16428.32	15431.07	13564.25
17686.69	9801.086	14319.21	37278.04	14060.1	13137.2	8676.367	15027.85	19785.77	6968.012	20665.81	31278.79	14557.79	11653.94	11706.24	18030.13	17158.21
184023.4	175737.9	131632.6	451733.7	228164.6	121170.8	29980.5	284197	431892.5	316646.4	523586.7	311563.9	185624.2	277855.8	238490.9	702218.9	459354.2
15108.03	17664.82	25644.22	24626.8	20638.28	8197.155	12845.74	15043.11	20284.32	22603.8	26221.08	21139.18	26577.38	16785.88	17463.94	17772.45	20974.33
896411.6	1037951	772225.1	723859.5	671016.9	781460.5	796929.1	651228.4	811123.2	1026425	667088.8	983143.9	835611.4	1240845	1214948	993691.3	1244142
137270.3	122152	279139.5	330324.3	263392.6	53421.66	33785.7	125305.3	107737.8	164098	219062.4	447024.3	199810.8	60044.55	91597.69	227426.3	170039.2
168372.2	168800.6	245323.1	234148.4	204805.4	128817.4	171223.7	174989	171962.6	194067.6	203683.8	246441.6	211649.3	205241.4	211646.1	205159.1	166052
1136964	783696.8	593424.8	1219130	834883.9	744631.8	769804.4	465209.1	842239.9	851942.4	1009484	782075.6	722128.9	761214.4	729328.3	468552.4	1014311
842522.4	658080.6	1313659	1363140	760109.9	1094113	824382.9	970732.4	1005164	453107.2	1009887	788439.5	776990.2	919966.6	778668.6	962447.5	1253179
11034.94	12746.42	11033.92	10673.39	16815.87	6553.503	8379.02	12745.42	13607.52	16513.98	14843.92	23658.29	16138.79	10961.72	11423.95	17241.71	12445
875262.3	602418.5	1054675	1208901	735909.1	952340.7	730862.6	701163.4	737508.1	319170.3	622559.5	491341.5	417672.1	784733.9	569602.5	701393.5	670110.9
33349.64	15455.79	31044.86	35985.19	18042.3	50678.5	10936.82	20030.81	28619.48	11953.48	1792.428	36827.09	18670.49	29081.79	38388	20931.95	31469.83
21752.53	12880.86	8817.849	4678.281	9555.957	8177.695	10498.15	3625.716	7141.776	18709.27	25918.11	6623.165	13107.02	7373.876	8588.992	11025.53	20623.41
74315.94	61782.45	78813.65	106562.8	71580.31	73465.38	45993.07	56378.5	70188.1	36036.88	83926.89	64264.47	59629.49	59666.63	37721.26	62140.45	31047.72
73554.3	77747.13	76510.44	57951.49	60997.77	75764.44	60028.25	58238.79	59573.55	41436.64	67405.55	45311.16	44296.1	48186.63	62610.82	44748.53	63461.15
9676.018	6460.72	40721.58	8813.604	4665.179	11666.15	7503.787	9553.427	4151.615	7671.812	16160.8	7612.816	9459.304	6202.114	7527.039	9924.048	6794.242
2639.588	669.6315	3824.378	3165.1	2632.584	2000.219	1448.727	2602.353	2205.715	2009.267	3863.775	2612.708	2492.353	1354.586	1388.035	3298.29	975.9822
3149.279	10638.57	13550.98	26656.44	9675.264	6830.372	5660.897	13910.02	13353.05	10201.38	9437.368	45419.04	13134.43	6406.178	1590.405	10092.47	14346.34
20605.72	26601.96	11290.53	24602.86	13678.82	20079.04	25495.84	16478.5	23581.44	15168.35	22435.45	20661.03	12004.97	11784.05	20226.97	18610.73	31618.63
16635.6	17875.11	12784.76	8517.232	16224.76	9763.821	18026.67	14966.03	17363.11	19584.64	21699.21	14624.84	15703.99	15659.1	17894.19	9497.591	12501.59
74583.02	12963.5	16520.23	239943.7	32889.82	13604.54	14056.83	10399.29	27383.06	116512.4	214255.2	226358.6	9178.567	14074.6	12755.81	198951.5	101860.7

3977343	1688686	2033260	1048149	2637999	547509.5	629202.6	238236.9	1135454	2742364	1280124	1205811	2901883	408788.6	1274007	186653.8	3182191
670469.4	1024508	524907.3	489230.9	489956.1	803105.1	1953512	790999.6	473249.1	783827.3	379677.7	543035.8	501918.3	718296.6	1702269	717057.5	869663.6
9213.451	7202.741	11635.89	13399.08	3075.909	10379.34	6741.309	5980.86	9433.49	3545.482	9252.378	11954.54	2419.556	8318.733	9041.466	7053.149	6715.989
17732.95	14729.38	17416.98	24414.61	24936.9	13175.5	14673.46	9228.293	9896.863	14244.18	25750.81	17628.72	25393.06	9754.879	10158.47	9926.303	8544.042
28822.72	20159.7	43334.28	55493.28	32326.31	28417.24	17932.87	34159.91	20203.08	18954.14	39947.13	42208.09	39022.63	23905.61	22932.4	40593.16	37523.49
242723.4	222850	29197.39	962843.3	69683.25	33857.08	504639.4	555755.4	647362.2	228757.7	667791.4	18139.99	26120.36	485135	909267.3	338415.1	459661.4
9748.604	12987.96	11000.33	8669.145	6664.471	6446.983	7560.417	8453.616	8295.315	11747.01	7908.401	10544.97	8483.396	8992.911	8367.19	7205.695	5200.269
27076.2	30442.54	47097.13	34500.9	38771.7	44901.15	30461.04	35747.84	30884.33	32458.06	44921.92	36161.29	40147.8	33049.09	29515.89	36177.87	21412.57
20677.22	53686.07	26196.06	26426.11	36466.04	24320.67	15661.48	36112.5	33528.23	57260.24	28483.24	35776.5	42862.23	34757.46	27790.57	41414.77	26486.16
181423.5	184576.7	208244.2	275242.8	202359.6	173036.1	165995.9	168034.5	203105.3	199548.8	226582.7	238727.6	192103	171720.8	159732.1	191252.7	100376.7
11055.68	7803.176	14846.58	23389.24	12942.94	5066.733	4921.979	12335.37	11817.64	9395.55	14477.57	13669.62	13244.78	9409.735	10660.57	15734.89	8620.07
175203.4	142054.8	253672.4	93799.77	93156.23	80523.2	107721.6	80122.72	103657.8	138044.3	31461.29	207799.4	176106.3	79783.95	103522.9	99950.12	121691.6
20626.86	17640.76	28808.73	32656.6	22197.03	9700.707	5196.109	19044.44	20317.61	27365.35	41360.55	39988.84	30565.52	22285.88	19591.43	31894.81	44282.95
28017.81	3457.36	42563.86	34817.04	12608.89	37627.76	16926.51	29677.01	11621.88	8217.251	38509.77	31479.26	7246.625	26246.15	49401	40617.36	8913.447
82821.11	85158.09	57974.54	67362.31	71671.63	44710.39	35757.13	43790.66	72479.59	53624.31	78546.53	70164.12	43746.96	36849.95	52047.47	78915.03	114656.7
11600.39	8516.901	11242.77	7652.601	10889.71	7518.44	6782.804	8035.285	8255.201	14215.51	6422.134	13721.46	7437.766	5528.33	7791.146	6702.423	6812.418
90572.82	122027	157701.6	172798.3	120838.2	75956.51	63409.54	100606.2	94818.12	91684.5	125016.8	207299.2	127518.4	106195.7	102194.1	162545.4	168289.3
85364.09	95011.51	53438.9	86097.06	60923.19	54459	57890.04	55305.27	84487.2	89581.29	55394.77	80460.97	70994.39	65844.71	74265.48	64781.57	91826.16
115465.9	117251.6	108119.6	106313.1	116725.2	90598.03	99529.2	86941.21	116896.9	130218.9	129908.7	138571.5	121705.2	92987.97	99644.95	112676.7	102434.5
37462.23	53216.61	69904.05	35146.36	45799.65	42469.87	50404.83	66268.21	46572.68	57091.63	40378.54	50515.94	37018.74	61782.3	72682.43	82027.63	109565.4
25603.95	21462.62	37733.05	50548.25	34759.38	2973.582	9104.535	19950.23	23510.96	31689.23	37525.84	57990.05	27439.23	27192	41170.8	42304.02	37174.51
6291417	7289769	7391199	7401286	4997562	5291021	4208282	4009580	6094689	4860969	6257794	6885865	4193079	6724667	5110455	4790939	7259840
748.3622	10338.03	3994.736	9556.576	929.9695	742.5103	3916.273	3185.818	2286.438	2500.312	4196.643	3238.954	3416.199	747.3382	7439.877	549.9781	862.3909
109893	98117.31	75312.4	91741.47	104667.9	83690.7	77086.28	88366.62	128300.5	126060.6	93232.27	99286.38	155104.1	98904.06	94918.81	115411.3	149656.2
26991.39	22252.36	35015.59	34383.58	32272.79	28481.59	24457.13	24530.6	26285.66	13443.94	21896.23	23179.54	21740.42	22628.23	15440.17	18965.46	8032.033
261944.5	229161.9	339301.7	261506.4	242943.6	192777.8	221068.1	223724.7	272269.5	282761.2	293933.5	348613.6	220227.6	310030.3	275984.3	251911.4	214658.2
18498.55	10110.66	17167.15	26191.11	15339.12	5742.208	6617.703	3381.698	11923.11	21384.3	15965.51	15923.97	13350.81	4248.752	5657.515	2302.773	4143.463
273225.1	193770.8	220941.8	428636.4	268425.5	331807.9	216435.1	258009	251734.8	151772.4	232690.5	186407.9	131533.9	265724.8	136237.2	225055.4	180634.8
3472.716	2557.749	4542.576	3339.175	4597.861	4390.742	3395.279	4187.017	4068.471	2502.439	2339.641	3402.899	3780.547	5347.561	2181.739	5851.628	7608.546
7098.797	11021.7	5207.907	4621.53	3638.102	8146.408	8962.835	2863.161	6448.586	8137.431	4710.605	5600.376	4258.133	6885.219	3881.187	6390.105	9661.036
411069.7	591845.8	123936.8	257063	150222.9	371381.6	392304.7	295609.1	207349.3	284002.7	230888.4	142400.6	80877.44	479872.6	411211.3	335592.4	401734.3
197154.4	190634	198585.2	148728.4	183692.9	227420.4	214642.3	186593	180789.8	157882	225747.9	186354.4	146301.8	201774.7	207375	253175.6	305482.5
61487.36	85508.09	57753.26	35900.56	53903.69	85565.96	226351.5	91910.86	45754.09	73371.79	25584.71	52733.8	44698.57	56757.18	93425.01	59257.06	66634.23
38417.92	30786.52	54612.41	28913.02	31334.54	21980.85	60218.57	30924.76	44051.98	29744.61	59884.91	35587.43	28208.12	23306.82	54322.91	35437.22	22242.71
92293.59	76514.24	186948.5	103193.2	91598.04	69393.81	95525.41	92070.7	95375.38	47057.7	83900.2	71013.68	67709.22	62799.83	75291.8	112085.9	52983.21
32507.47	35810.26	17278.23	18464.08	26894.95	14200.97	17481.01	21160.9	29559.93	30172.48	7090.733	15528.62	19514.86	12261.94	25200.87	21098.65	36406.07
59343.1	9492.872	21255.28	13835.12	45368.57	10351.11	7600.068	8898.447	6230.146	10690.66	47266.9	29479.55	16081.16	8920.615	9144.866	8596.316	8419.146
13184.94	31741.81	51142.83	6986.382	37615.15	21321.66	63533.04	38454.36	80410.64	37023.53	28567.08	38217.68	60142.03	36956.34	28383.6	34255.83	88226.64
4513.176	6073.35	4910.946	3175.37	4791.552	4106.87	5767.594	3702.762	5798.289	5601.64	3554.149	4655.784	3822.554	4508.503	5059.85	5173.731	9345.437
14224.52	11155.98	14640.92	25171.33	17424.27	13285.88	5953.215	12872.53	10083.54	7091.99	13649.86	10581.93	11304.68	19682.64	7634.542	19321.35	16241.13
78590.2	57764.77	44009.44	31962.52	69184.08	30527.9	82095.2	19466.69	89293.42	74118.38	55483	51335.77	36359.79	30003.59	107159.3	25423.92	38807.38
126586.6	121117.6	245402.1	272651.1	170295.5	193970.7	127478.3	149228.5	100400.1	69052.4	142019	124064.7	158806.9	131430.5	77098.45	146408.1	100171
10854.22	10373.27	22303.47	22946.6	10837.24	13171.15	19243.63	12859.6	11038.25	6291.158	11323.31	8259.152	60108.05	9999.958	11258.15	15269.38	44094.78
648277.2	589823.6	655806.9	307006.5	449160.2	558836.1	579577.8	359971	634483.3	573957.1	530685.7	700950.3	549017.1	760794.6	648376.6	512241.7	957172.3
3478.67	11745.22	6567.399	5016.457	2324.58	11472.05	15747.92	4173.24	14852.42	22248.82	7813.805	1938.382	3145.781	19963.42	28769.14	12952.85	18641.56
76959.79	86285.08	70719.98	89675.49	80162.16	65705.05	69454.64	74594.52	86602.16	107859.4	94688.68	77614.64	84448.58	91650.75	109775.5	102732.7	81278.84
77393.3	136487.9	169461.3	81348.23	135359.9	96276.48	63309.88	88516.39	120530.7	133570.9	113390.8	129914.7	129773.6	121901.7	72819.71	130571.3	100811.7

34	35	36	37	38	39	40
D28-rat2	D28-rat3	D28-rat4	D28-rat5	D28-rat6	D28-rat7	D28-rat8
4513.814	2268.739	1999.651	5203.31	3319.014	2899.722	1766.46
7709.329	10908.64	17321.83	26776.28	5563.078	8067.92	9596.89
6865.884	4946.885	5365.436	4926.121	4366.361	5434.863	3577.776
68520.23	85339.8	83247.87	45042.65	58804.84	47113.05	50614.88
14579.3	26066.6	18995.68	20151.82	21924.27	20256.29	34396.05
25030.61	48973.38	38367.77	47432.14	32207.46	16749.2	28769.61
59656.6	58602.53	20336.38	122296.2	71893.12	29690.26	36776.14
4396.022	7724.032	8638.05	11694.94	5960.982	2914.353	6649.74
46775	72798.83	56070.7	65383.04	50819.12	114789.3	95823.74
7375.258	9729.805	4882.466	8747.127	5751.689	2751.599	2579.066
6075.995	7479.191	2506.032	6651.236	5756.491	4583.923	4377.549
305628.8	212196.4	293747	341140.8	270761.3	220183.1	373878.2
19782.53	23767.95	34659.39	23129.96	8098.87	35086.64	16477.58
62655.41	57568.23	45969.2	56486.84	96091.41	46305.83	87935.75
22266.93	25930.65	18676.49	15873.33	17488.09	16504.97	14560.24
102870	62485.86	52607.06	45137.41	62016.38	326078.6	90700.35
17919.56	16280.74	9966.472	13004.57	16934.06	5758.66	9330.566
7499.353	6982.611	8726.644	12868.02	3788.159	9733.746	5439.693
575096.8	399002.8	329818.4	128659.7	167678.8	135626.9	126843.1
42600.52	31416.31	34803.9	17592.93	20674.52	22005.02	33131.28
6233.729	2882.905	8127.062	2807.49	2548.794	3274.211	4674.485
7004.375	24131.73	16932.94	19533.71	13196.89	12617.81	14707.46
53331.39	78118.64	37539.83	48010.37	60256.46	46338.35	45696.64
19642.84	8820.412	7334.609	14835.07	32445.65	45996.45	23273.13
2768.999	3056.724	4413.007	5432.273	3773.533	3533.547	4005.543
6782.868	7908.554	8089.885	19058.16	5380.907	5721.912	9509.529
9950.468	119398.5	22777.19	54660.46	17303.64	24072.98	28507.59
4978.928	20352.75	17239.48	25024.03	7291.157	6297.357	8218.962
182359.3	84997.59	153675.6	33466.38	80207.8	95747.09	47444.96
121834.3	194397.8	137408.4	178992.2	123684.5	115261.7	123591.6
59605.71	134496.1	59019.88	116536.4	33768.84	40908.13	54430.48
180976	113258.7	137043.7	73085.02	152822.3	58827.9	104067.8
36694.76	46851.56	40196.22	24556.77	23634.64	14607.17	23246.14
141728.4	75070.55	79266.99	79929.69	45670.25	96240.21	153969.4
7892.359	6161.799	9462.513	14823.19	6587.575	7835.904	9676.298
4443.492	6599.659	14612.32	18936.03	6307.698	4526.609	16502.09
3258.903	1422.87	3564.828	5631.606	3081.172	3229.576	2128.848
6573.706	8259.569	9733.231	8973.255	7176.769	7690.315	6065.909
4979.779	4086.705	2238.434	4616.107	2420.69	4090.199	3843.646
100628.4	74515.04	98745.01	66217.84	85991.05	57463.18	97858.73
22988.98	31731.23	17426.85	32957.94	27641.11	24221.5	29253.88
579934.4	692158	537930.4	812238.6	465094.8	422842.4	501358.1
159604.3	223633.5	204830.7	215482	146359.2	100093	150433.8
14822.25	17434.87	25891.91	44254.98	19728.31	17296.97	18284.36

9125.238	5000.12	5344.788	4405.727	8569.839	4382.591	5041.351
18788.2	16323.3	20563.63	18191.96	14279.5	10410.54	8778.943
59692.3	117753.6	117985.9	160971.3	56950.12	58433.9	84190.75
7742.44	18939.14	27963.33	104002.8	9151.372	23731.16	34569.8
769737.4	545759.8	379687.6	615626.8	729981.8	519629.6	822783.9
53921.88	30024.81	38085.2	35348.5	26094.9	26544.02	26876.78
211678.6	397743.8	327789.8	301720.8	166648.7	141476.4	215230.7
69599.37	67703.75	63665.44	35689.12	40759.26	30375.46	40521.79
7212.872	13395.78	8122.583	5881.953	8218.219	7017.598	5843.216
164103.2	95415.18	113503.1	87666.86	70857.27	91974.37	98296.44
21858.55	32217.68	3669675	3192.467	47155.37	30243.67	37732.14
227853.6	119735.9	103665.8	110919.1	173738.3	97174.64	100413.9
36391.61	22856.58	13815	6588.342	11850.02	13173.65	23050.19
72545.6	64874.49	78027.43	44872.67	86875.49	73187.09	80734.74
12184.2	15932.34	12392.01	18321.67	10902.86	9841.618	10579.96
1052419	305708.2	434624.9	427113.2	263534.3	224246.9	282396.8
1031755	742313.1	1133461	391878.7	587728.1	658388.6	465138.2
13400.32	13969.09	18475.78	13808.01	7865.215	10838.63	11848.22
21957.5	7771.424	14342.35	11820.96	8140.618	4595.331	7973.171
38120.99	16551.3	40736.46	14860.66	9180.516	30628.06	26901.5
145485.9	272590.9	178015.4	121479.6	269994.2	210866.6	250864.7
3591.678	2873.291	2799.223	1445.497	2762.481	3440.205	3256.477
26022.07	31876.31	38576.67	33551.05	22765.55	19308.03	22483.06
7064.053	11806.48	12873.46	11707.9	8192.969	4964.271	8731.9
800006.1	1142565	928804.4	91580.69	2089026	271025.4	776267.3
562578.1	580415.8	687296.3	400198.5	474808.9	433365.7	561665
3505891	748184.1	1175661	1763061	861244.5	772546.3	1130142
305788.3	168844.5	245661.1	175654.8	205627.8	189386.2	194212.3
15637.44	9803.967	13076.96	9319.199	14413.93	9541.763	12006.49
14154.63	48306.58	39394.85	35034.35	16103.19	13861.07	32745.95
26068.44	12491.96	15620.85	12730.2	16560.05	10428.94	15119.31
12340.97	23610.69	14197.49	18729.07	16976.37	18415.54	17583.43
4334.867	3880.29	6105.705	8101.035	3081.172	4420.524	4283.087
6127.475	6790.505	10612.47	15450.4	7141.814	7239.403	9504.773
277359.3	262153	279636.1	318714.8	265108.6	301459.3	216875.3
229701.9	132047.6	247916.6	233263.4	171106.9	124086.9	187080.1
23960.75	14182.59	16799.91	13007.46	17549.16	12700.08	16161.57
4111.574	5461.199	3715.727	1503.946	3655.71	2679.885	2498.05
26251.01	37581.79	24700.94	20902.76	17138.57	55966.92	36121.5
407342	436936.3	456468.5	469697.3	337996.5	209763.4	294363.1
141077.6	112112.4	76350.36	66068.67	49868.17	1802.047	32863.25
196655	410136.5	317388.6	428151.3	280831.5	217043.6	314103.4
7475.764	6522.71	3106.762	13506.63	7889.967	6330.27	1800.401
6422948	15277523	6915419	16348626	18876616	16041500	15455536
203488.2	286597.1	157697.2	263316.3	282418.7	90323.7	405447.6
7929.706	5062.062	5449.522	5850.301	6482.73	5994.062	17499.81
1139460	5751568	5449385	3423737	4731079	6418407	1049631
10709252	4360767	4335114	2640004	5257243	3228220	3628544
12786.33	106161.9	1905.683	6159.65	26413.76	30639.36	56920.43

381311.3	214265.5	325496.8	208691.8	285586.4	276979.9	301476.4
1444437	1213768	962964.1	1161022	1047514	783902.5	767061
417841.9	350468.4	362924.2	578841.3	245819.6	153836.2	266584.4
1388258	1490757	908762.6	2652348	1144670	1481439	1459927
126666.7	236730.3	178527	208022.7	613447.8	10266431	276133
112277.1	49011.37	29020.03	12248.02	326072	191023.5	541064.3
147623.8	74788.99	105822.6	82250.92	133056.8	122129.7	106534.2
1497993	1157249	2323545	1046284	3115210	3507287	1185752
446633.5	1397799	2946324	190854.8	617205.8	1035320	2313181
290348.3	266510.7	192961.2	165144.2	131897	81494.66	149245.8
11865808	24727484	15262961	21865.88	9356492	12274072	9749100
26393818	18247084	26972166	13322742	29001574	16592774	15756711
574667.6	3574.94	32742.83	9160.368	349206.1	29801.34	14056.47
459522.1	8162.17	27434.9	14072.26	309461.1	16884.25	8750.742
55810.67	38583.8	67972.41	33231.82	14823.13	16057.07	76047.41
840618.1	160267.5	373731.9	189877.9	423670	124296.2	148423.6
40982.89	17913.58	17962.15	31273.36	27334.46	26007.09	38932.38
2436933	1830540	1213745	1113916	1480306	2247202	1940063
182566.5	75890.12	81890.53	55224.58	67482.28	407658.1	73707.25
16250.42	21639.07	15839.7	22024.49	29302.65	1641016	17260.12
9353.96	5435.089	5663.836	5186.083	4615.315	8251.5	1928.628
1.65E+09	1.32E+09	6.63E+08	9.91E+08	9.99E+08	4.64E+08	7.98E+08
4360649	3457356	7727952	3343178	5215623	4529248	2298196
2057717	1344096	808995.4	977044.4	2836498	1860748	997996.8
21829028	17533432	13199262	23331592	1.17E+08	1.16E+08	26332690
68193048	33881260	51741972	95964496	43914044	44224936	71472872
23008960	11151267	30387252	48278288	20631300	16484948	24815464
11082164	8960722	15740985	38952932	7488531	11808341	20648656
7260.138	8156.112	297204.1	13471.41	3317.569	15029.3	24243.81
25966.04	3464.478	6243.632	14344.07	15643.89	29442	2964.996
11156.1	7004.569	5610.711	4379.565	5416.264	6632.523	6605.743
60969.39	28555.28	37559.52	41701.28	46082.32	39218.02	20603.86
98412.73	85703.2	74310.14	202620.5	171788.5	76439.39	81027.34
88481.56	63864.48	67757.33	29337.76	47127.83	44039.07	51499.71
1023125	586989.4	609797.8	699661.1	861003.5	4681588	838073.1
74387.08	29990.1	49353.66	11914.63	28575.77	42712.61	18853.95
10232.96	26448.04	33159.2	46438.36	34262.64	25064.64	23629.18
92393.86	58496.86	38546.58	43209.55	233219.5	1256942	55267.64
115187.6	120535	155309.7	252321	149051.8	157599.5	131227
13483.18	5937.254	9439.027	8708.194	7615.062	6077.909	5804.408
480895.6	415245.3	489456.6	416400.5	369227.1	261551.8	309340.9
91577.05	85139.16	93556.72	82469.05	70229.52	65898.73	68062.52
455995.6	411424.2	515255.2	437352.9	237428.1	294509.2	316950.8
30694.97	73426.33	61584.11	126853.8	48262.75	39799.46	75888.27
14421.35	31104.81	24986.82	19311.71	14838	18077.38	21184.32
578775.6	935731.6	767559.9	973204.9	692302.4	404895.4	787848.3
28975.24	28921.6	39759.77	76689.38	34791.13	30557.47	47489.56
43147.41	63218.54	36551.38	58062.29	39340.48	34512.54	46388.91
29146.24	20441.99	15831.09	41156.77	31812.05	98864.54	29678.25

4795209	7318804	5097071	4641213	6025052	4724993	6174354
78500.7	55907.3	124836.9	172549.1	57358.04	63028.83	67301.63
25323.58	31088.45	36291.05	73649.85	12034.9	22586.02	27789.77
6054.257	12062.11	22909.63	31219.34	9899.914	21033.56	24849.43
798498.8	338079.9	573050.9	364767.4	364005.7	533789.3	531148.3
6376.288	19062.95	16170.97	15500.48	16630.7	16245.49	19637.16
44228248	27610584	23731982	24689368	62980776	30607418	34537960
3720024	2949535	3032408	3714741	6835949	3912408	3824603
9957.673	11484.48	12130.89	13872.04	9586.883	10533.42	7474.485
164721.2	82676.85	123961.9	211891.2	167666.1	142702.3	259579.1
3454.75	11052.45	9951.907	18048.67	13741.45	10517.06	9663.358
25384.13	19144.49	18807.29	13231.22	27620.96	2430817	16012.54
20270.22	27623.28	44231.34	31530.98	31859.61	25363.73	36383.55
4825823	1354369	2068734	3883194	1620318	1368061	448913.8
150458	110342.3	151510.8	199224.1	103788.4	102142.3	107187.6
3903781	2507479	3877188	2974112	2627032	5195687	1826107
138509.9	67513	463382.8	483779.9	337904.9	298647.1	409922.8
194595.5	77638.5	83589.23	70857.94	96073.3	78381.46	56949.27
5856597	2077384	2859867	1690923	1068028	998799.1	2103656
362208.5	219426.9	248760.4	188251.2	220711.8	183629.5	224813.6
479497.3	631394.7	1628221	1138949	720716.6	635329.3	580519.1
35776.09	32151.85	24895.11	31142.7	83011.44	204942.4	15231.73
2837963	5108885	3975332	3109812	2090261	1768011	2962619
12549.1	7024.978	20241.18	7659.441	9817.38	13453.95	5886.684
98384.21	52532.88	73066.78	47652.47	58880.36	46825.48	59196.39
9837.697	18364.95	9319.649	28783.97	19550.81	9849.271	10032.67
134678.4	84978.09	113901.6	97969.72	88134.73	64827.91	73747.11
19356376	30449068	19020256	20582294	22879050	19767416	28482110
7765800	5374690	4970954	4369105	7375498	7835975	5978450
14638220	6477748	13497392	27048208	10128326	7074030	15319075
713057.7	1599760	1618093	4106026	893921.3	1123581	1727638
374846.9	387919.3	366272.6	242473.3	316458.3	263657.6	299113
34905.37	26787.7	70798.91	23916.69	19765.61	31028.99	49986.31
29275.86	28851.71	41443.84	78525.22	18750.61	32297.4	40454.36
135587.1	258066.4	233431.3	143605.2	114898.9	64147.95	152074
24739.04	16482.63	13327.34	7014.673	7964.567	15171.25	19194.2
341795.2	272405.3	435162.1	290062.3	312516.6	219368.2	373615.7
51010.61	17672.33	42078.86	8230.293	8914.964	15658.56	15967.09
163580	120367.7	200886.5	87954.19	164737.6	159931.2	170391.4
37445.59	68554.99	72316.88	78061.36	34565.04	41402.38	57643.45
2022646	533045	2352911	487469.3	362230.5	738359.8	427075.2
68050.05	42465.09	55790.86	104852.2	71214.56	210977.8	97763.27
4396780	3412824	4440531	1089837	1393659	574548.9	486515.5
9503722	7070288	18129926	24639240	6729150	4579445	7484688
17220.98	29295.21	29546.43	73502.47	24856.77	32253.21	30385.06
1514934	1146725	2155904	1198714	2325056	5233907	1204963
30537.4	16490.42	19603.25	13543.04	26365.38	355719.8	20227.52
771929.1	845840.9	492709.3	507763.2	1289916	1234213	664113.4
399172.1	322828.5	391198.7	447780.9	734538.3	2198958	418232.8

69933.2	100598.7	77266.33	78872.21	63821.67	47249.14	55094.48
15260.99	6647.576	26170.94	40285.91	13920.45	9764.111	20185.51
6648.376	15561.32	18278.89	21914.28	13461.95	10858.33	13589.56
51499.24	129024.2	112632.2	82527.12	46858.83	33195.41	59680.43
2337.985	8674.482	40180.36	17253.93	8777.357	5930.527	4476.752
24391.3	14863	24428.38	95969.86	11023.16	19326.58	18380.51
11485.21	8460.362	11048.38	12184.82	6279.928	8110.439	8581.316
589231.4	796401.2	618160.8	741279	559359.3	346931.3	529802.1
19339.06	53559.5	54890.49	78453.8	37593.73	55966.48	62263.89
71351.41	62662.11	90760.92	50861.96	121230.5	59968.78	91714.56
268276.1	183653.2	299870.9	227135.5	115154.4	92321.03	139558.8
158756	160734.6	115007.5	78456.63	131328.4	120102.8	173614.3
1271246	1203987	1191196	691651.9	781869.8	591641.1	731280.6
8490.736	15212.45	14718.74	14922.35	6549.428	5071.522	10497.3
16380.21	22125.75	27614.62	35889.56	29228.85	31228.42	15314.73
294402.8	336595.7	186407.6	113772.6	350575.3	383034.3	496302.9
23011.57	51765.48	45405.69	57532.68	32805.82	30545.16	40539.72
26251.69	31138.88	35812.48	38588.25	25401.64	26147.83	25363.65
1349326	817527.6	645423.6	1430762	4968996	2786575	2077503
5032604	3404112	4016197	2076211	2944427	4808696	5424078
57499.45	19401.82	26964.14	27877.24	16958.96	15223.59	18155.38
741016.6	435148.3	651387.5	543090.2	808070.3	399979.5	511983.8
47364.46	80082.72	63503.06	83587.06	65072.06	39205.24	51186.63
9008.741	12790.15	19847.76	16584	14075.56	19145.81	10450.29
58841.47	24967.14	59051.03	79028.09	30147.16	28803.08	32815.44
7021.551	10676.92	16861.49	5328.478	6640.031	7058.935	4851.13
15165.01	19599.49	5016.083	13754.08	63644.17	32428.33	19793.18
39004.21	42406.09	62042.32	56828.78	39142.97	45339.52	40243.2
645598.1	167496.3	919381.2	975182.6	826536.9	593811.9	878457.8
9099527	4517316	5981317	11750952	23725700	51362064	6712251
9846.343	35324.32	28863.97	37076.93	7506.306	6595.624	16922.1
34373.61	55979.61	51929.84	79551.91	52153.49	43418.17	46121.08
86643.3	68026.81	74056.05	48937.61	80080.49	32856.18	40920.7
907147.3	324234.8	588091.8	805401.2	31896.24	390222.1	851888.4
45661.33	66633.92	61678.42	75224.73	16670.21	44789.04	52164.23
372622.9	493834.2	497708.7	523783.8	1477112	3206671	458457.2
10139.34	8329.581	7545.931	7431.975	13456.86	12965.52	8494.19
147469.2	110433.4	119042.5	86684.53	136697.8	83322.68	94198.09
1250335	1709402	658246.6	1705126	6548590	19212532	2163615
8175.498	6510.363	6582.172	835.4737	1518.242	8647.381	4996.405
6450.013	7436.484	7836.895	12243.73	6410.025	5832.239	7141.347
54684.89	57707.37	43103.48	52698.7	76304.08	7792869	44400.47
200304.3	152432	128277.6	121062.5	118867.8	248570.6	129631.4
548767.7	577836.3	566565.7	363597.7	266178.5	699062.7	645699.3
93713.49	112682.5	101541	55563.18	49767.99	56169.95	69418.84
140644	144016	159634.1	382033.2	210131.8	219242	84665.94
791017.9	2368537	1384380	1196037	1224531	1486754	1454570
5399244	3411862	4315827	2601458	2582813	3796220	3308486
109248.9	123786.5	109588.4	109616.9	129892.7	97809.31	114316.6

15228.15	36605.19	26190.16	40385.34	24699.78	21615.57	24452.99
3745975	2981052	3172882	3568509	3302927	3081358	3695050
10869.57	6608.878	6465.578	16573.35	5641.055	6529.69	5160.338
339384.4	182758	162031.7	181975.9	239404.4	194254.1	240308.4
51602684	10867675	6305694	7361889	15085428	12592834	28730464
134119.6	60573.82	95622.8	28700.96	75611.47	104664.5	80939.84
2010091	3227302	2924243	2094300	2617636	1650108	2580264
3776.643	5816.464	4723.11	15271.85	9449.879	7501.089	8044.963
107004.1	62300.35	96244.85	57893.87	61433.02	44676.34	62827.71
408287.6	982100.1	584662.4	851975.6	680008.8	654982.9	884136.1
13237.19	7225.306	14610.8	11750.31	12070.7	9460.953	10665.88
7489.252	6072.848	4503.759	19727.06	10394.9	6303.546	6464.448
47194.05	37405.41	30782.03	48623.99	88164.62	37359.72	39229.34
75442.85	42630.63	106000.3	78407.56	62851.32	50481.95	77943.51
76448.91	48090.04	40424.82	28741.89	43450.71	40804.63	43396.07
229663.7	456333	152593.1	438544.7	373087.2	360134.4	295279.8
7340.78	9210.131	9339.378	12716.28	5527.381	5654.333	8347.959
4617629	3147156	3137103	1218444	3144690	3050892	2731891
53010.54	25449.02	40778.39	40047.8	29554.53	22712.67	27697
13915.37	7865.731	17872.11	12109.55	8285.879	11842.95	10151.63
84800.73	124686.1	97444.44	81160.86	76367.77	69500.88	44462.54
194873	341584.9	381156.7	513424	550342.8	463270.6	648870.1
6042472	3437842	3611820	6347864	21024152	15609170	6211731
40931.93	32230.88	25831.68	23037.61	33811.89	11871.05	29578.2
5049.304	2828.274	2070.603	4012.029	4821.887	5287.322	4186.264
4033939	2957092	3681732	1800333	3048489	3322837	2851957
4307.387	7114.487	4786.235	5246.362	7723.833	17874.39	5395.592
8496.89	11501.33	8887.119	13140.72	3870.389	5715.735	9642.2
213279.7	59906.07	100104.4	65364.79	60440.14	60493.95	74876.8
11946.17	7058.202	12767.89	19348.04	9532.051	16121.56	12711.87
26987.75	13855.67	13528.81	6922.68	13487.85	10731.76	22805.61
23202.29	77733.03	44696.35	58830.82	46572.37	42827.67	48700.48
20975.51	57555.86	38975.89	41542.59	39115.61	24090.02	36856.63
30616.84	7189.916	12697.57	4220.988	48027.98	61534.86	16735.27
962144.3	100013.2	165853.8	225010	92824.91	522441.6	476185.7
15006.54	17596.64	3307.864	29954.18	30927.17	19698.41	24557.23
220144.2	637137.6	466606.4	392835.2	305793.9	265680.3	356822.9
42715.27	22998.13	103547.8	129683.6	63049.33	74724.27	118327.6
156419.9	103729.8	145364.2	215746.1	91311.83	107637	96732.85
6446.441	1169.982	2210.937	2287.864	757.4189	2473.261	2250.641
274013.3	108986.9	139550.8	111902.7	112670	43129.73	69872.96
15896220	11806301	12640447	9310410	13644198	18197616	11049192
1710500	1656317	1486203	1011919	2273865	1751537	2656340
28591.07	8619.636	13388.04	10647.82	10038.21	12513.4	9167.73
5832.274	5410.312	6387.375	10035.18	8060.299	5383.865	7645.854
256113.8	264586.2	353655.7	236013.7	929116.1	3237916	359258.7
1001848	798059	1274975	456764.9	1376311	6231981	897691.9
374269.1	375949.5	216683.3	381774.5	857641.2	6094393	199341.9
1328388	550822	378392.2	371180.7	556913.3	1772416	432868.9

50179.85	23406.98	23049.79	4416.343	22518.65	39700.89	25166.37
14667450	19177262	39553076	3912108	14758203	28500514	11975488
223822.2	159278.9	244602.6	147541.3	118699.6	139650.6	140514.3
45490.88	31145.87	32264.68	14062.76	29905.45	32759.46	46011.07
35038.33	40438.46	43870.45	28002.37	48229.69	31107.06	44655.23
10681.68	12900.47	12535.95	11271.03	10308.88	90378.38	12147.67
15716.99	9802.397	16365.23	17080.46	13221.93	16583.86	11708.51
4202.042	10643.54	9614.551	1870.959	8085.375	1110.434	2063.805
14887541	25974340	25526268	22137992	19197918	19980874	24816028
3591.678	4702.257	4182.097	5918.802	3997.763	3252.345	3939.334
2652738	515164.2	1780770	104588.6	120008.3	578374.2	294127
5703173	5329252	4165096	3645107	2846802	2550403	2990020
26186.46	76897.23	19603.52	893945.2	219516.3	298473	317691.4
36876400	24130844	26628400	46764224	15271671	25589534	19756236
44672704	24869796	52194968	71620568	22020192	35922676	29316656
77116.73	141109.7	79286.16	46977.13	54060.87	42573.65	80674.73
6635.408	4546.658	6526.488	1779.321	4581.579	17852.17	4653.264
147857.9	128172.9	109971.6	133997.7	202888.7	143048	157853.6
58417	26899.67	55218.38	30249.11	53499.82	22349.46	32814.46
2094474	1906759	2004555	1094445	1812029	1793999	1582278
20581.6	45613.97	51952.26	13654.51	10359.54	42158.79	15317.8
642820.1	455410.8	407845.1	375433	377072	953056.1	273355.2
862840.6	599190.1	727234.5	180691.5	381193.8	788485	617166.5
8431.555	9933.251	13335.59	4232.498	8259.315	2161.459	7787.223
41456912	26646524	26423792	27342584	23351036	28852794	32817200
787175.3	876505.8	899390.9	1180040	1148711	1338228	1213119
117189.2	52764.73	79327.1	228686.6	42070.98	59032.45	116787.4
291149.5	110429.2	122439.6	54446.25	103930.2	126283.7	146321.3
36394.59	23158.9	19351.63	14574.18	103252.4	66067.41	173127.3
38087.68	139403.9	88130.63	102128.6	83471.95	67052.7	94625.92
85345.57	78347.38	87812.62	67848.71	51657.41	52333.91	46862.05
20986.27	12055.21	14870.52	9425.766	40705.62	105809	22812.13
58873.12	37399.91	16667.17	23610.63	26428.15	22523.05	8326.432
2007802	1621950	2153144	1196552	1452375	1642503	1685286
11985.66	8102.293	9691.586	13425.38	9603.315	6691.933	8168.017
46305.52	36148.45	32696.48	24067.47	52195.19	29638.34	24498.82
1252192	800301.8	747286.9	874496.1	784867.9	3004825	870170.3
63051.96	13345.88	33268.38	14810.63	12052.56	11325.73	7480.244
225014.3	191367	198331.3	212665.6	174697.7	116391.9	128383.2
5364059	4088532	3365203	3582234	3472169	2513584	3512226
59423200	44280032	34803824	12479468	48549256	20296876	23025054
10582.35	16302.1	21705.36	15932.87	11757.19	8159.101	5515.221
7007.789	12779.65	16186.61	10368.33	9618.547	8130.041	11224.28
36415.17	62346.35	65421.58	150247.7	64436.96	55131.14	119113.2
11510.8	8461.461	10483.56	15040.78	12000.62	10014.47	10543.39
3364.931	4391.875	5655.655	6499.901	3081.172	5130.751	6128.009
2425.067	3477.165	4980.649	5888.041	3445.862	3435.842	4663.663
40719.63	63191.34	60707.86	82406.52	48420.07	66478.49	65866.92
9488.366	17160.95	8030.204	5481.081	8345.532	1221.033	5884.536

140615.5	92225.69	93836.5	67183.5	172680	119006.9	76572.91
664762.8	1771904	1091714	847360.4	856098.1	659877.7	955028.8
31107.59	39144.26	36707.64	24935.77	35305.96	52563.13	34229.83
1180375	39663.11	453544.5	108256	365700.8	14533.05	29697.74
113984.4	69614.07	78765.64	66301.22	76716.95	54478.05	77683.16
2352476	1357659	1419877	1261039	1122364	1227417	1428301
333552	749010.5	505935	377773.3	327466.9	280825.1	380261.2
92104.01	52610.25	96509.2	247987.1	71584.52	54653.2	159812.2
80156.03	71798.48	59936.64	35040.95	48745.69	43769.18	71136.82
1279069	1184973	1874047	1422111	984888.4	1162607	1304190
22707.72	42877.48	32482.39	67552.64	44736.15	45281.88	37713.84
42400.84	19764.12	24963.54	18059.29	15828.21	25265.53	17511.57
760095.4	944284.6	803731.4	623951.9	296226.8	205819.8	393784
56529.85	38521.87	197212.2	45507.32	51611.94	33397.7	54160.76
1395984	623628.6	2198539	2102841	1422885	1197658	2064729
1178779	707130.9	844687.3	1001750	2387693	1323630	1230623
9842.437	33721.93	3967.001	4822.208	137604.5	26310.08	49834.54
1296256	2229688	2186451	2478759	1082505	1252857	1657811
1263693	783219.1	749118	633130.8	925868.7	646796.4	806000.2
17737.19	23784.55	16240.02	26801.55	16388.27	9035.576	13796.62
6302.515	2482.527	2606.903	5350.796	3544.058	3092.228	4404.037
20174.39	41912.68	47916.25	50852.39	19482.46	30924.85	23454.29
103395.6	67821.82	89165.38	42615.14	61995.76	41165.07	65810.84
509546.4	559204.7	9275.829	156411.2	786907.7	115164	423259.6
83725.49	67969.74	72507.77	68784.39	83314.03	63227.8	73982.82
101951.6	77190.35	75793.57	56502.66	70647.86	63023.69	72125.08
19885.29	18534.95	7466.317	24763.65	15480.88	5764.076	4757.51
100822.2	147893.1	184486.8	254245.3	90122.84	118024.9	166226.8
1773.285	23898.66	8312.471	26717.41	4997399	59750.58	21660.6
42228.7	24614.45	36661.55	22590.77	19881.8	20192.03	30194.18
7044.574	3457.17	3873.218	2546.928	4723.771	2747.036	5777.667
29675.33	282612.7	53023.22	50853.84	46330.22	34563.02	33497.26
6671.42	4416.582	5754.574	3355.342	4724.19	4150.994	6009.471
50603.09	30117.54	33218.71	40113.37	54007.33	135043.4	27113.16
28342.55	24629.31	19256.91	22065.63	15345.24	17381.88	12625.46
49252792	39274728	48808176	56733080	35630088	35214560	53282448
6672196	4928193	9409955	14493033	5221181	6750094	6423190
66198.8	29148.73	34935.66	54427.23	98914.7	103414.5	54223.64
329713.8	643243.6	317714	181976.1	542927.1	1697918	634926.7
10896.21	18106.01	1017.63	6253.444	1550.192	5494.746	2039.863
1293890	1209786	1068751	1202383	2347982	2443865	1395867
20067	59356.65	44423.93	37368.99	40890.39	37941.23	42808.18
18119.43	27247.89	12361.64	24241.96	22129.08	14712	8318.521
18937.4	16849.88	23072.87	13468.58	10288.08	13267.85	14271.95
187696.8	88670.61	91707.92	85252.17	129887.2	110445.3	119460.7
7347.421	4761.998	7557.915	5205.052	6030.896	5347.798	6905.42
6535.667	12515.23	11488.62	15774.48	9868.14	7705.523	5912.533
50294.25	40193.41	61121.79	27713.66	42811.75	48879.88	67184.44
57461.36	191259.5	92994.36	156116.8	80419.43	62707.15	111290.2

61186.62	32346.1	26947.53	28418.74	16778.04	9683.174	17915.17
44609.52	72350.3	76788.13	183861.3	52194.06	61105.87	107837.2
56797.25	68355.69	62766.73	32860.78	29850.7	21768.14	37659
89856.92	89804.44	101703.3	117253.3	78610.93	85826.09	82064.25
9078.584	8017.243	7870.561	6026.628	15598.28	6615.319	3841.527
220396.2	243608.1	223279.9	331598.4	245868.4	226287.1	169501
4385834	3174430	3865866	615494.4	1771053	2021380	3839005
572474.3	415993.6	461482.4	215114.6	506271.5	373264.5	409309.7
23647.5	55195.9	48999.82	44619.2	36152.57	28272.3	38113.93
80141.57	52197.21	59392.17	62726.23	91354.83	42042.18	60922.4
29875.6	25899.13	34944.07	21235.88	13752.26	8232.159	14178.09
77693.09	264716.1	187212.9	166025.3	105070.2	111969.3	129444.4
50667.38	38677.27	84247.11	157142.6	31316.91	53879.59	53467.13
57072.45	267502.1	155011	118061.5	66712.19	41690.01	98524.3
6462.284	21117.04	16768.97	13819.52	10308.85	15212.93	10206.4
94568.09	79335.47	59248.13	55519.37	57084.88	32511.7	39418.61
39724.16	21549.49	56678.28	68643.15	31917.24	24783.86	46734.33
82543.96	93607.41	91313.86	121354.4	81671.16	62400.04	122981
654315.9	279531.6	297935.7	284489.8	293845.1	93709	191526.2
1747019	1807622	497549.8	1084029	2611410	4019946	3461336
10087.43	5059.736	11015.02	5131.103	8005.773	15412.9	11978.53
22181.54	24309.63	31523.3	33097.19	17450.74	23039.41	24544.38
1207451	1325650	1361959	1738392	3000802	5809840	2384311
21340.08	17271.68	10966.98	9742.909	12390.68	11268.15	17069.31
226828.2	130161.9	216283.6	121213.5	113607.4	61811.77	101624.9
113290.1	317639.7	238673.9	185079.5	96000.73	113628	175122.5
7552.374	7130.443	10389.96	3001.482	5161.622	8513.532	3910.621
10346.31	72918.97	52968.9	5168.534	38725.63	38376.04	17844.73
3732.706	7429.921	5173.794	4349.23	7442.358	6263.6	7608.101
28983.78	15372.93	38765.35	15108.71	79122.3	41111.69	27065.99
66102.46	11724.39	41660.21	9522.08	154898.1	40346.97	60845.96
838300	1130782	1197616	730088.8	861376.9	1720062	1074464
4706.667	12639.92	17603.82	26875.6	22292.72	17813.58	20853.68
34311.36	27224.98	19877.9	16627.7	13785.69	10554.58	19754.06
18181.48	37284.33	40151.57	59689.68	33406.69	26807.85	39450.01
25032.49	64133.34	59309.83	78356.48	34880.88	42633.55	57730.41
434280.8	442357	331099.3	303328.7	295610.7	270484.4	408441.4
4995.246	13432.27	15491.4	14583.95	2825.542	6460.359	10683.39
16768.06	8199.399	4638.888	8919.001	16092.54	12324.37	11108.56
411209.4	470187.9	405793.4	911088.5	401786.4	316705.8	365600.3
20297.07	10850.12	25070.41	45106.6	16622.63	19719.6	21439.5
10400.89	41426.57	10207.05	10480.49	258918.6	133628.1	150289.6
7308.569	7488.059	6174.034	4688.862	6750.477	6197.318	6026.686
38300.66	22323.01	26897.91	17006.33	22993.45	40949.57	24668.69
50650.14	56471.77	33307.05	26552.43	24697.09	19427.58	31252.6
1560270	1253910	2654628	1465362	922837.9	478520.8	2484641
881084.2	874080.7	632079.6	653761.1	692116.3	511383.7	552106.5
48800.54	45133.36	37450.05	31299.83	30700.59	26892.94	37728.66
7423.147	21109.67	13616.44	12580.81	10097.04	9955.377	13768.59

70513.47	70166.36	55476.71	53346.95	67199.94	49192.26	57164.98
40005.14	115437.8	63036.55	58184.3	45818.21	36046.53	63051.26
7453.382	16557.14	19021.53	11223.51	8579.193	4648.034	15491.53
7446.89	11183.34	13056.84	14057.87	7797.179	6561.451	9167.451
88448.65	90155.17	107117.3	155183.3	81497.42	123376.3	99980.71
49446.71	67208.2	79570.75	115934.4	49022.48	55182.77	56662.2
101379.6	211508.4	217241.6	350630.7	123848.1	120675.3	211311.4
9459.955	4679.873	6178.73	21634.6	6282.791	1745.859	1883.884
33986.65	25529.43	12190.4	19788.2	9457.008	8113.343	16485.04
37094.12	19760.08	13058.1	11823.91	15062.12	14061.86	8178.91
28470.05	47883.24	4886.211	7152.674	264593.2	281026.3	284490.7
99037.39	64012.8	75291.7	68838.9	76431.62	46001.94	62220.88
13746.65	12226.36	11461.95	14929.78	32699.47	218918.4	11605.13
76891.95	49807.5	64368.31	39225.73	26331.61	42201.15	34283.45
6610.656	11186.35	6814.266	5569.445	8741.2	12796.26	12004.81
4934.021	4125.59	5674.254	75709.16	9953.886	141400	6275.744
59621.77	54341.37	46987.65	27945.91	56427.06	34964.65	42929.42
5802.376	8521.073	4744.64	9276.856	3366.802	5407.265	4185.221
38266.9	44033.71	34727.24	24122.02	36620.85	13047.73	25165.27
713450.9	1754113	1298232	1620411	1223517	868193.8	1099656
6898.28	7154.509	9364.9	9191.842	12327.39	12918.21	4667.115
10529.81	36404.27	26642.94	38740.8	10667.07	12648.51	29307.4
9272.32	17004.97	12803.45	14243.23	8678.76	10209.62	10520.2
14130.08	24722.26	27535.78	30693.33	19548.65	3006.039	19235.95
127142.9	67604.9	66081.03	48306.43	79286.33	89878.06	52657.45
43750.1	36871.91	59384.28	83893.84	24009.35	31906.69	48875.52
19770.47	19671.34	19758.79	15112.93	14663.03	14210.03	20293.47
249743.6	333412.9	267673.8	125548.2	268068	316049.8	295633.2
46129.37	23491.15	48476.35	51040.14	23464.44	15858.9	25422.27
54295.38	57857.33	77954.32	90447.12	54426.4	41598.71	61393.12
173982.2	294151.8	289629.8	416927.1	171562.8	170689.5	203752.7
15703.44	39721.87	19372.81	39392.12	18382.82	26626.45	28388.22
10550.18	9262.952	5091.834	11494.25	9446.619	7838.159	6578.232
106375.9	20796.18	59789.09	96722.19	70906.86	39306.26	64383.8
9832.11	21162.22	17121.48	29362.72	27028.93	13558.99	11826.25
11840.56	13111.91	16081.26	13086.28	12557.23	12547.82	13344.79
38326.64	49868.98	46967.77	56098.05	46130.91	40106.83	51514.8
194255.3	58642.17	104560.9	93310.88	63573.2	54832.84	101453.3
81617.82	68251.9	88505.91	87571.68	89528.47	60988.33	45829.81
52508.41	71984.28	90796.6	111317.2	42096.99	46854.62	59706.43
434241.9	491763.1	522868.6	592716.9	271162.2	326608.4	398212.7
42523.36	45865.03	38634.96	23216.76	92342.08	11065.6	56996.06
27284.63	34220.34	27209.36	8450.335	15234.06	21489.74	13870.42
54371.02	143010.3	122580	232784.1	81281.59	101098.9	137856.1
133485.4	153356.1	221435.6	229330.5	118758.1	124900	163680.8
10660.83	7924.164	11154.35	15963.99	7400.43	7246.938	9271.212
1063022	2574816	1937560	2559056	1712177	1504113	1904656
14449.68	24168.22	19722.36	27944.54	19893.22	19173.73	14793.04
33926.62	25099.77	60068.87	87329.2	37966.79	35856.5	41172.27

3519.803	3602.372	5690.405	6628.623	9179.235	5059.777	3717.798
43380.13	17421.86	59534	58767.75	25831.32	30686.2	31136.3
16350.54	20849	30595.96	26764.01	23985.49	20804.02	19493.22
103505.2	55845.92	115935.9	54799.7	92982.45	80498.82	67833.88
96123.53	131460.8	59956.18	84057.21	337725.9	181197.3	87569.09
30713.17	33243.68	38681.36	48082.11	27895.78	11908.76	30884.18
8845.424	4581.464	841.3276	167.1668	8092.828	5399.535	3980.718
28485.05	31514.43	35483.16	60869.63	29896.17	42932.32	25042.11
24388.93	37268.07	55299.64	19242.8	51425.24	90299.27	71772.3
3787.246	1232.925	1352.358	1388.836	2762.481	2633.7	2297.854
359261	156172.6	47938.43	152073.5	55914.1	35088.52	67785.5
725064.2	642472.6	631186.8	374550.3	976014.1	764167.6	827473.1
113171.6	81739.93	84858.13	50545.59	73903.52	39958.18	50345.43
146296.8	97576.56	106864.3	55906.38	65857.11	110592.6	147004.5
60569.31	40505.15	55155.1	27569.77	38717.86	38193.39	34048.8
107612.7	104119.3	159337.5	106997.2	105044.3	76590.64	81788.98
1179448	280397.8	322408.4	5808.892	708964.9	233726.4	202136.5
10325.3	4951.357	16181.74	22462.38	13067.25	14200.43	17880.68
1.62E+08	1.2E+08	1.03E+08	47740952	39622184	72673328	78631808
1.47E+08	1.78E+08	1.6E+08	65764068	90589144	85188384	1.07E+08
14416.42	53973.41	35232.98	34023.29	28540.57	17922.48	32558.37
219688.9	171274.9	125417.2	123743	190432.7	160010	203599.3
325118.2	87726.14	100015.1	34482.12	118298.5	96082.65	12439.13
24084.97	266379.9	52283.11	50553.46	23116.95	62026.27	307786.4
62872976	58579272	45230912	32044680	30384310	42628080	44657556
64044.53	58321.68	60620.3	45636.12	36237.72	67135.65	90336.48
11179.49	20223.84	19549.97	27885.45	18601.09	14939.34	20116.23
365292	700123	437836.1	360466.2	378815.1	485332.6	453327.8
12448.22	29075.18	31009.61	47411.42	29411.46	25362.56	28809.68
13549.78	17070.59	16098.59	33944.38	13014.31	16288.39	24780.26
8712.596	12928.16	14255.18	15373.55	7124.417	8296.004	10819.65
1044456	749595.2	683003.1	537089.1	852372.3	595830	646651.9
113776.3	74930.47	94131.33	63485.11	56316.95	55087.46	74293.74
87668.73	133952.1	131311	79023.27	108340.6	138732.1	116043.7
6167681	1172660	103375.4	85267.21	2279748	1384101	3322911
7807.653	14102.28	6431.444	11949.54	28586.32	23229.64	15489.96
83771.87	72617.9	86261.44	77057.8	82267.83	73044.59	77769.61
121297.7	77243.77	103080.5	48848.67	74091.97	46143.37	71259.28
7166.734	5221.731	6047.234	3788.22	5339.449	6046.68	4398.333
6772.189	2230.718	8378.063	2327.199	2198.187	3467.521	3454.514
102167.4	230614.5	105328	144563.7	129113.5	110310.3	166065.7
25348.87	15172.1	27102.52	18875.89	16031.12	15154.32	15000.21
39170.55	34856.59	36764.6	15840.9	39982.66	28313.43	29996.65
359709.9	128011.7	72272.46	76548.16	173448.5	95547.56	115012.8
323348.3	222865.3	51261.34	159324	389609.6	525823.6	466064.3
16434.79	185547.7	4092.378	9436.135	299068.4	173476.5	192457.6
883199.3	1075330	1062725	876677.6	1334939	924077.4	1444167
8784.399	6845.905	8709.477	5409.017	10859.56	11348.25	8589.36
370252.1	244712.3	309052.2	171309.5	211274.6	165759	235674.4

40853.71	52839.92	49456.75	28296.51	42384.43	25279.59	37718.51
95552.38	133123	137664.3	283443	120770.3	90867.56	111083.1
243868.7	72206.49	177915.3	112193.6	97318.73	69529.77	67689.35
15436.98	9400.863	14632.69	18306.95	15383.91	13527.98	10530.35
4176392	1864585	1881141	2262266	2953616	3196489	2234561
560545.6	540340.6	288013.4	158904.9	363708.8	363716.7	441534.1
1042421	942969.4	832268.4	735060.7	1246960	797035.3	1049138
7819.931	12759.5	7866.347	22369.2	8039.298	7122.024	9159.275
28523.66	21927.38	19738.21	9647.397	15709.11	16527.59	23466.38
203621.6	122798.8	128629.5	82903.95	114665.1	69746.83	90295.02
58847.7	116079.1	76914.1	124683	59524.15	57997.68	83713.47
127890.3	217123.9	299301.8	72498.78	345269.7	180993.1	203495.4
9919.486	12401.61	10716.32	9785.542	5363.779	3234.358	5464.375
99781.88	313628	235037.2	158915.5	148264.9	117398.6	247854.4
268982.4	265350.1	143647.3	283272	983098.5	3566798	463100
104822	53866.21	99153.16	34537.96	56490.12	54479.76	71900.33
1783725	3236883	3789305	1611875	2298058	1371357	2399163
43209.6	33146.64	30848.03	16414.82	35562.32	20706.75	19268.25
132258.1	264042.3	243299	59759.27	126100	92409.8	84754.03
14847.58	9325.509	7442.669	6270.725	8335.657	2608.835	5050.441
145581.6	308177.3	96083.79	65938.33	318212.3	246159.7	428501.9
6891.74	1521.795	4233.52	4598.579	3192.373	3373.453	3962.431
846600	518584.3	555348.6	845853.8	486688.2	514273.3	610103
16265.93	13627.55	20486.02	18290.74	20358.4	10464.25	13283.59
72357.67	38741.84	13908.18	13571.13	35444.28	22625.69	40787.23
70923.62	109609.7	94263.39	69559.26	62533.72	59285.79	83690.24
339691.7	266038.1	347717.9	145973.7	188356.9	269293.2	203439.9
17922.73	13781.11	17252.3	16555.09	11931.59	6035.52	13387.66
8902.847	8883.83	11581.57	18742.67	7952.554	9937.594	7893.063
90370.41	37602.37	42990.61	22490.33	38179.1	48587.47	37125.46
9496.491	7215.989	5191.724	8287.261	5479.949	5362.189	6880.417
50535.27	39764.24	42089.07	38297.9	42670.79	30434.94	44367.62
19470.31	16849.83	19540.66	28336.69	10706.73	21307.46	23615.34
187472.8	144966	169074.8	112783.5	131272.6	143452.6	180861.2
151274.2	159526.9	226199.2	101833.3	59242.8	144148.2	111248.5
49447.72	56762.49	33860.32	27532.3	38611.1	26467.93	36924.68
16114.3	11689.24	15350.71	15265.49	17291.2	23516.21	14123.83
214266.6	261230.3	407415.1	145564.1	152394.8	160059.5	168956.8
33046.79	53210.53	26801.69	51673.76	24290.93	23640.31	38488.33
442752.9	614610.6	431825.6	415516.9	632820.1	368911.1	610818.6
20620.69	26920.35	23921.46	21599.81	14293.73	17013.23	21632.92
20698.98	13255.01	13999.73	15861.15	27717.15	30340.18	19423.35
124652.1	124288.6	129238.3	74462.04	95199.84	83027.87	91245.09
35497.86	18556.55	16613.73	19771.72	16049.68	12732.91	15205.61
1635919	206810.5	15235.11	19612.78	287903.4	180574.1	321993.5
54977.36	58336.63	58237.86	41223.05	76904.47	93530.1	98484.48
37174.19	37892.07	41338.3	53968.48	49209.6	29403.1	42478.27
174652	302820.3	201619.2	244002.9	160578.5	118020.5	218825.9
11107	35192.88	29425.92	11442.89	29625.37	32820.78	36360.02

38007.73	106555.3	103700.3	197942.4	60422.55	80433.7	100740
5963.674	5429.98	4062.886	5802.865	4472.343	3581.18	4984.882
32304.08	17641.1	16773.58	14807.62	11885.46	17383.1	9809.644
8830.335	42041.34	24450.03	36281.45	20501.11	14159.42	27129.47
3591.678	4644.589	3739.333	6101.929	4242.711	3462.974	4456.221
13298.32	53872.11	26292	25974.47	10993.62	13286.33	20223.52
26076.54	46818.56	27670.31	34735.84	26282.38	19370.21	21599.84
10719.26	7571.938	8293.269	7020.818	6962.086	6472.616	10895.85
48363.04	27218.91	29318.4	19931.43	16672.04	13376.92	16093.97
89099.81	60152.63	89991.97	56079.43	73180.57	64416.09	75794.05
613282.8	379740.9	441702.6	284760.3	414639.2	355639.9	453098.2
3738.646	8629.396	4530.879	7026.602	4055.038	7356.12	5540.105
761597.4	232300.2	232050.3	823835.2	1006819	485854.7	625745.1
11763.41	9636.132	10640.94	4987.69	6989.691	5336.203	6514.543
12704.65	6567.382	4734.363	9488.176	11637.69	24756.08	20352.6
243877.1	315603.3	267085.1	301744.9	300579.4	327395.4	419627.9
12755.49	9389.418	13840.05	5381.936	11054.85	8190.54	6881.822
38599.93	30480.41	30898.54	24674.65	63114.52	43928.19	27550.53
9043246	109089.7	4938580	6647162	8393533	36250.84	5486305
25895.61	20540.38	29082.61	21904.91	16377.49	16148.82	15548.65
3798.401	2372.2	1728.497	2537.001	2762.481	1679.21	1494.146
85319.98	121231.8	88760.81	54296.09	82058.27	63042.25	90168.21
168611.9	155991.1	159213.1	172111.5	188689.8	177708.1	172031.7
6075.877	7526.564	3522.159	4752.834	5571.622	3447.796	3078.898
515897.8	353971.4	306828.7	277705.7	240622.4	133485.6	208120.5
23076.21	34874.35	40545.89	15830.78	32334.22	30264.14	26991.33
7108.154	12979.28	11843.1	8403.013	12619.17	5858.108	7688.486
3364.931	4125.434	4602.929	3578.798	3118.013	2999.849	2799.835
156119.2	146738	156310.8	171922.7	103227.2	67680.87	135657.9
87470.39	118178.6	92224.48	133061.4	126907.1	110257.3	138046.8
10255.76	7093.771	11705.19	10670.91	9469.304	7499.115	11378.45
16459.21	5301.896	13171.38	20207.17	6517.347	6093.017	13379.93
11665.81	10560.94	15213.45	6477.436	18107.34	46540.1	15772.21
19909.81	21971.33	17474.61	18882.8	13891.74	16418.24	14941.74
10367.84	47168.45	9175.142	14728.4	28062.4	29212.89	28936.81
244118.8	341551.9	343366.5	424806.6	467314.3	330421.9	406565.7
182005.4	258390.1	207960.8	255016.3	325831.5	337900.9	458111.3
11303.73	5890.699	4741.216	3185.612	14008.18	19867.05	12334.98
1467353	1077894	1217838	506383.2	726571	461754.7	829245.4
25907.17	24224.08	29161.82	19583.98	25291.35	18011.54	24018.26
34225.89	27005.75	140358.1	218853.1	142389.1	95923.6	200516.8
206712.1	135241	191267.3	110908.5	141847.3	139682.1	204682
44583.49	61022.47	36134.84	25996.16	36469.87	23069.12	35070.88
7681.958	10456.67	14467.97	13337.59	3858.138	1627.943	1407.528
99587.09	110730.4	94331.7	69023.49	101712.6	68501.36	76081.48
18490.26	38987.22	36298.25	65416.17	132766.9	89398.99	91927.98
28235.45	97631.78	78283.71	59125.99	42497.45	41674.96	52922.84
4381.963	8289.282	4317.684	6525.283	8925.709	2340.541	5446.62
7170.511	14465.23	12300.16	11529.95	7161.297	6504.041	8652.68

15762.2	29307.03	21694.01	26259.03	15928.76	9706.135	15695.4
26324.66	20952.81	27265.65	21561.89	19406.08	19738.7	16079.44
18044.48	15383.06	13058.65	14020.65	11225.41	10889.92	15695.76
158024.6	117077.1	131989.7	42598.23	88698.21	60852.2	67250.84
10680.35	9633.459	8358.643	11259.14	11202.19	12609.83	7357.536
84114.61	259241	155633.4	292190.5	189231.6	154183.1	226886.6
35963.15	25801.94	63968.6	82201.22	47684.65	58516.66	52339.11
11100.17	37786.01	10046.55	11572.96	9173.876	13556.29	11109.19
6007.698	4339.327	4474.114	9972.49	13121.92	4614.251	8660.342
12356.9	24058.3	11896.85	30161.24	136939.4	57384.15	37783.33
29901.82	26156.8	31383.13	18879.21	21657.27	17524.62	20430.9
175554.5	120694.2	137131.1	103710.8	95292.81	101885.7	37804.29
4036.821	5133.094	4189.809	2555.855	2477.949	1350.579	2060.875
9184.68	21482.56	20804.96	28844.72	17915.66	11594.31	18964.51
9118.16	5080.471	4550.068	9147.037	8316.555	91400.45	7865.587
138750.4	270514.8	497228.4	147166.9	130306.6	107957.3	226629.9
27508.53	56587.2	25732.02	40459.06	17478.4	43719.23	25676
639893.4	728927.6	627207.6	1213586	737302.9	486093.9	590354.6
37357.64	5125.035	31589.18	40237.75	49623.15	62394.97	29099.84
100450.4	200264.1	164761	128969	123192.1	76943.36	85068.96
14285.06	15700.24	17589.83	14600.29	31562.66	35627.82	23017.04
10011.71	7662.153	8697.873	23572.2	3952.21	12503.52	15447.82
10845.33	3562.171	5366.935	50330.46	9914.046	8013.183	3777.081
31716.25	48860.55	27061.96	81104.49	58178.93	30653.82	35853.43
27991.17	23810.84	19165.78	16980.28	16070.24	8268.253	9286.032
4057145	3728133	4007881	2385018	3201294	2415542	2857263
87121.66	129025.2	119538.3	105833	83842.24	77384.49	89986.43
5170.554	5700.94	4902.304	4432.07	4486.923	4306.702	4713.285
2015.186	2539.596	1965.998	1206.03	2762.481	2699.426	2538.302
34097.47	26661.92	34678.77	18654.55	11674.51	17845.45	20594.72
40164.98	38501.31	34450.66	31635.57	25028.24	39174.84	34951.45
6494.432	6639.204	5422.384	6458.639	7294.354	5835.801	5423.958
197814.4	165044.3	207229.2	93929.59	95653.02	135775	122809.8
39217.6	318687.7	43366.23	50565.68	590080.8	350880.4	506367.7
86324.56	83718.02	81708.23	88094.51	74338.91	86892.06	79633.22
41081.77	108066.5	63751.92	51516.88	33441.36	29583.61	46051.32
16568.62	32938.21	33916.33	8984.608	12651.93	32386.7	4637.606
6354.583	4304.343	5318.162	3825.3	5407.442	7662.848	5517.712
325008	132594.1	208436.3	210639.8	164181.6	204851	215326.3
33632.82	69487.22	53732.05	50687.64	45788.42	45907.54	52688.05
6354.796	15127.92	17182.61	35037.59	5963.548	9653.368	15897.33
10546.85	20580.45	16242.77	32768.41	12157.91	9409.184	15460.12
79917.67	77831.22	79505.3	68501.83	50853.99	28532.51	84919.02
16971.7	36404.77	21531.3	17885.97	24976.12	15583.61	34512.13
5145.381	4228.831	5518.459	6400.398	2548.687	18571.13	4104.326
5532962	3562509	4146974	2913454	4321243	2576323	5538775
16242.56	4890.772	3922.988	2029.006	6211.715	78893.05	5629.533
50451.22	18699.61	29744.42	53828.06	65484.77	30426.67	24273.08
213118.2	138438.6	172155.8	157351.6	255427.2	884728.8	171437.9

176318.6	131661	136864.7	148748.4	127423	91432.97	133747.4
3739.287	4452.069	4633.262	6273.533	10462.57	72486.56	5421.882
30991.12	23234.67	28010.89	15307.16	16638.24	4838.819	14967.61
7536607	7642279	6652781	5358242	7609508	14663563	7276766
8156.238	8283.615	8122.376	11551.93	6510.095	5458.135	6908.184
18495.25	38620.78	17875.61	11556.79	22232.98	16534.88	23899.96
10125.44	7446.283	11244.3	15760.73	10098.96	7979.09	8690.233
604638.1	594929.3	413925.1	309379.6	255812.6	235602.8	310362.7
33002.09	27513.21	38560.63	26732.66	19056	19861.55	20646.93
5166.687	19628.73	6232.526	2671.607	10513.34	10355.16	9569.405
5701.615	9019.822	6008.475	11014.59	9938.326	5835.771	10146.61
3906.873	1730.682	3352.46	2913.393	3441.908	3239.551	3349.571
8873.285	6474.582	12838.32	4643.256	4969.971	8224.423	6752.961
2861131	2762594	2527546	1738459	2432755	2375410	2810356
14018.3	38943.63	24581.31	41253.8	26345.25	15571.06	29805.99
1115683	719728.5	561392.6	528241.9	547607.9	406682.5	626780.6
18487.44	55694.28	65304.37	73697.44	26769.65	26065.8	53333.82
11028.19	20845.41	27343.14	53415.31	17441.37	20630.26	32854.15
151174.6	129641.6	94450.02	88364.23	111523.7	946147.3	76587.93
33760.25	209079.6	123809.7	87830.06	77464.5	61469.3	100262.4
232774.7	102104.6	216267	91549.11	213212.9	210755.5	198715.1
24872.7	37026.28	2435.299	3837.177	4180.021	20107.19	19151.81
23895.22	12350.19	8925.387	6838.929	13264.66	7144.354	12682.43
8838.361	16500.18	21601.07	23138.13	14409.02	12068.34	13683.96
57004.13	46970	56018.18	33219.9	47520.18	220457	32298.17
95887.87	52499.47	102756.1	35883.45	61029.12	93284.77	72374.43
102732.4	144673.8	103718.3	161694.8	130250.5	141388.4	139938
219618.7	77663.57	104263.2	56929.64	154917.3	230090.3	115800.4
116707.3	96936.53	60994.68	48765.61	265368.2	360847.3	100738
10630.02	38180.8	21726.46	20822.56	23913.57	25500.59	20612.39
14266.79	16975.61	28392.13	45842.09	10861.42	11250.9	9313.648
19280.28	47120.41	50230.65	23793.81	17558.62	7598.889	25935.86
355597.2	236525.7	240456.7	274530	342296.8	324904	270572.1
101129	63366.39	41914.77	75763.13	138318.2	142626.2	78619.47
221454.5	176712.8	194601	334777.9	159804.8	102446.4	221431.2
290714.8	274994.8	312385.8	273507.3	150178.7	120488.1	159352.4
428616.9	747311	412193.3	538495.3	1375048	1400378	667482.4
145220.8	130986	121229.9	84736.81	88467.72	78226.08	84582.82
24321.12	8074.463	14842.76	11907.25	16635.14	32912.24	17025.68
14610.04	22339.31	17973.58	25797.28	17201.3	9891.405	12636.96
9844.873	41133.02	18118.52	24863.7	8605.952	2988.791	21430.2
31803.24	86590.3	124706.6	50917.74	96679.74	142782.5	114946.9
57529.92	99796.73	85810.05	105284.7	114052.6	150079.6	124128.8
29737.15	73900.29	79350.1	78543.06	57095.64	32041.47	100118
60235.25	58570.93	53748.45	51624.27	67332.3	52795.72	52997.13
18313.09	22353.84	10982.59	26590.36	22432.92	20173.41	19777.91
2370672	3337244	2290370	2038589	2900286	1800263	2733030
24626720	10911236	11819714	11565230	11276504	11678677	14985918
13619.31	7322.87	12310.46	13204.41	17097.66	245483.6	17403.44

1076420	1115224	1040104	774072.6	855633.3	982931.6	853774.8
744974.3	2952299	2601727	1408753	1544589	1146105	2060520
21545.99	18421.79	18726.11	12380.64	20962.12	15053.39	26196.65
32386.95	34349.12	35682.14	28821.48	24306.92	37306.38	30579.41
14819.75	16881.45	19284.04	23316.35	21039.66	20586.94	24409.16
108415.2	105823.7	81693.97	164354.3	276607.1	342942.7	243662.6
135791.2	291367.8	241894.4	310044.2	172849.3	168494.6	212653.6
71796.09	111187.9	94986.13	106975.7	70949.78	78892.89	91833.28
11877.96	8859.08	9426.768	8963.916	15076.38	10400	12368.15
6591.225	4759.868	5510.583	8977.467	6709.291	7579.418	6148.871
9448.948	16759.83	16688.45	16735.69	8179.453	12862.67	18597.29
48942.84	117374.8	73966.63	127551	109977.6	120185.6	136081.3
14152.69	11834.64	12436.07	14910.23	428.9696	8542.48	11794.85
7872.549	5011.715	3864.833	5197.25	5407.442	4710.859	4918.792
7562.78	26136.48	11910.56	6889.84	8987.348	12074.58	13702.99
30403.85	52563.3	34983.79	52033.03	46257.9	30815.29	34599.46
54450.71	48304.73	48901.38	44476.85	68638.07	47734.86	68921.45
8146973	6092933	5283304	6714814	6467767	5385764	6704653
3364.931	3441.568	5328.395	10355.68	2350.874	3367.379	4175.095
49487.26	35599.06	29552.6	16167.43	49159.6	20541.74	28906.7
21694.08	12088.93	14562.5	14071.23	15708.82	10771.62	12491.24
11539.25	29116.03	15622.27	23539.01	14743.59	12772.13	11539.32
3986.188	2996.644	4925.55	12489.35	4307.222	4549.795	5543.129
21000.51	859596.2	515887.3	937939	1043532	721566.8	667702.6
97315.81	138658.9	113818.3	137012.4	154906.7	170050.4	199867.9
60108.52	100797.1	88474.55	93651.39	92320.01	112771.6	92369.73
7273.808	4827.896	7747.055	9383.795	7245.113	18952.5	4981.438
18267.52	56662.09	19241.52	25878.2	20529.25	7303.544	28815.01
2419.265	8804.147	10155.6	1723.055	2925.835	4093.595	3539.314
12359.08	4871.116	9799.053	3357.715	7244.571	4555.43	6175.356
668563.9	599388.2	615126.9	450490.4	566127.8	425531.7	496376.2
53136.2	33790.42	41490.16	26621.12	21589.67	24600.1	47071.49
12667.39	9333.245	12796.06	5470.119	6069.623	6923.295	3769.255
85122.34	62679.44	81004	43770.34	71579.2	39068.99	61834.34
6088.877	13720.33	11676.06	4839.46	9874.919	3106.421	7748.129
7764.28	15197.35	9913.063	10109.88	8116.53	6583.914	13126.85
14174.33	15155	14934.14	16310.6	12831.5	9412.07	15697.86
7473.143	6142.367	6811.554	8927.745	4687.629	2812.645	6564.079
17259.79	17043.01	32957.15	71791.8	13876.16	22333.1	24386.12
15487.7	14864.4	19128.49	28784.54	24044.33	19752.74	37701.32
140783.6	348158.7	261801.1	270651.5	172569.6	150767.6	190585.8
92773.72	144415.5	139887.4	160107.5	94124.56	87532.38	111138.4
91954.45	55784.98	54860.7	36108.48	42281.14	45897.92	57867.7
44561.11	43010.65	36341.23	30064.36	41864.16	30580.52	26407.51
11327.99	11891.17	18143.97	15296.71	11817.38	23835.57	12918.65
343771.4	719422.7	504993.8	644522.6	587622.3	276179.9	418643.6
11402.3	15231.85	22917.81	33102.14	11237.44	13353.25	21496.82
6446.435	6231.781	6280.802	8484.939	5098.396	19854.43	4833.667
266796.3	137205.7	104085.7	159878.3	806090.8	1893791	290907.8

22915.38	20782.66	18790.7	44161.67	18165.45	68168.66	15562.36
6982.476	3699.519	4795.683	2755.316	8239.68	6539.023	6276.679
527018.8	230047.2	171575.5	404884.7	695498.1	241632.3	233947.4
303565.3	85943.81	99631.07	355130.6	354484.8	228622	206331.7
98651.09	59122.63	37313.83	145159.1	117238.3	80416.98	71687
15925.08	6500.294	7176.577	12600.34	24602.08	1873.14	6004.855
322885.6	149273.3	126759.8	307208.8	331538	139833.2	239165.6
30667.24	25209.68	14839.61	28452.18	6225.096	17353.26	19142.17
1903602	3218169	2761333	2866984	3103763	3769510	4330044
556348.1	608737	749439.4	822919.2	681164.3	595008	770059
209308.9	535887.9	350270.7	381671.5	214542.8	205237.5	281797.1
298771.4	685065.9	639269.8	960479.4	405461.4	361688	549966.6
9596.246	12383.1	20853.78	9206.991	12395.48	7211.476	9612.496
5890.887	7283.674	5188.591	7173.793	4901.977	6062.061	5209.957
84793.42	110466.2	112304.3	140181.2	67699.73	47975.32	84684.63
113206.5	46513.61	39296.71	130205.5	193420.4	89754.88	98686.96
17693.11	9787.443	23043.24	23675.95	4040.707	12942.17	6100.79
4279.559	9725.015	20130.5	12325.93	6785.096	9503.768	12402.73
13560.89	22831.36	12140.57	30694.15	26688.36	11525.16	20914.46
16946.06	12440.04	16222.04	12071.22	16208.13	21825.6	13348.03
7217.65	6627.002	4016.768	4253.625	3298.032	5218.061	3467.572
30136.46	40816.74	50627.02	72822.14	59296.04	43212.23	57752.44
252419.2	123574.3	133096.7	211037.6	115053.7	529135.3	143243.3
835203.9	711854.9	666448.7	543584.2	622426.5	577441.1	643159.9
7688.284	5408.449	9443.362	22449.22	3275.988	3739.841	12345.09
525228.9	693779.6	506233.2	410162.8	488361.8	599981.3	707212.3
40590.46	35824.26	50701.66	53828.96	35627.91	37193.03	42008.28
104593.8	97526.02	88779.84	50970.6	148810.1	122955.3	121633.9
64489.64	44565.82	94560.73	64548.07	57023.6	40942.34	44509.5
55746.43	67029.27	52285.11	34262.77	9021.697	19474.1	22957.15
19885.07	28401.2	24167.6	22263.05	20346.6	17311.46	18076.52
12780.52	9062.559	4781.204	8704.691	14641.44	12022.95	15101.97
39360.83	96867.35	67633.1	49201.38	34279.17	29807.49	37878.29
124665.5	170344.2	153062.7	198628.5	181468	128940.8	168539.3
65004.75	109416.4	139648.7	157693.8	96389.38	103618.4	121707.8
15729.38	15100.79	18350.23	16867.67	30161.62	13789.79	29366.72
59626.9	56157.61	24161.02	24613.25	40412.3	63794.44	51027.42
18882396	14375275	18824900	7551768	12380870	11155192	18228066
17633.06	10485.08	13370.02	8689.238	13407.39	9385.035	9756.587
7358.722	10440.99	8916.14	8218.812	10069.5	6884.292	7590.115
34497.95	67889.55	49285.61	81459.36	47425.29	32465.06	35434.93
11909.82	38217.48	15256.09	18557.18	11135.33	17555.21	21336.38
67474.09	52910.02	51765.59	54493.76	51428.2	62691.05	64335.48
28019.7	12314.49	35641.19	30656.33	20528.99	24528.42	42637.23
80287.92	90102.93	59173.26	51921.73	67064.1	63703.59	56602.7
20999.78	14483.45	7229.663	19030.88	18386.36	17120.14	17192.8
44549.65	68691.51	61673.56	60671.97	39119.36	27753.22	42604.29
14791.57	34273.54	19349.67	28711.8	54456.58	12045.23	18475.07
62025.3	54646.91	56821.88	31392.07	40907.54	40255.41	42169.18

10228.99	51153.53	33944.93	39415.24	27189.81	21526.94	26987.87
46515.57	165362.8	96909.93	89600.3	60536.48	44909.88	67309.13
39856.18	52844.27	53010.12	55896.11	56125.49	53497.42	63695.43
79379.94	98343.46	71521.56	73878.14	73693.53	64630.82	64398.27
26996.37	24315.32	27630.62	30014.28	33630.45	24332.01	32986.95
91806.42	29186.18	37411.32	25079.46	27316.28	21904.81	25380.49
37577.05	19834.91	21658.73	14557	14087.01	16349.59	12808.21
10763.04	9514.936	12568.81	7067.712	5502.342	8390.293	9518.484
66914.59	44611.08	42537.21	23553.23	73513.83	21321.96	48192.9
8693.286	8399.193	6866.854	7959.131	8990.286	6841.086	11175.55
5201.08	64087.34	5625.165	2472.969	8067.173	92206.19	4407.416
36318.86	63554.57	45836.4	72676.02	58637.88	37106.28	43919.99
5593.782	4122.633	10647.17	29688.25	4241.641	10293.76	9579.133
76925.53	180365.1	128318.2	89179.48	97009.98	53146.55	118062.5
46177.22	60573.67	41155.96	77260.62	55243.55	62837.3	64746.37
53426.27	48826.03	42133.32	39813.63	40172.11	33410.66	45912.45
25766.65	65679.85	51128.05	54126.76	35617.18	24029.52	32913.16
21184.36	21026.32	28952.12	35441.77	21334.36	25616.88	19116.86
12594.65	8509.484	8427.173	11985.38	6100.823	7553.385	9365.525
3011252	1993248	2176577	1175986	1923396	1625398	2023976
15467.24	10623.53	12799.76	12721.69	14002.93	16263.74	10072.96
56219.18	45116.83	47006.39	34232.75	279545.8	196263.8	465711.5
10855.15	8718.822	9887.403	22144.38	6240.69	1749.126	11026.96
22959.94	17175.43	6597.558	4909.849	366392.1	354812.3	185909.2
153340.2	73062.33	106869.8	89569.8	82305.91	68896.79	95143.11
7143.103	5064.741	4473.923	4264.255	4111.635	4135.146	4255.642
7044.53	16243.75	12379.91	12170.32	8481.08	4395.303	11681.09
43729.61	45692.64	35518.97	31062.87	30315.88	29811.27	26374.4
12913.85	5091.234	11206.05	5139.94	3848.028	11130.7	2600.212
17723.03	12274.22	16139.74	9761.563	19963.33	11272.6	13101.52
9637.887	10410.61	6588.7	10600.67	8647.018	6189.411	10489.87
8297.332	46738.19	33602.2	26093.25	20086.84	14304.34	24875.61
51846.58	24092.28	42652.32	89203.64	23674.91	36370.7	44893.08
11506.25	12504.81	19874.04	28717.7	16279.26	12205.35	28314.95
43429.83	21319.48	32561.77	32594.41	26744.5	16530.22	19955.06
23977.17	36866.66	40966.39	46552	33997.66	32433.25	29617.18
13826.49	16711.88	7827.202	16666.18	13636.12	10346.17	9017.9
8022.105	7110.54	4696.52	12636.88	8802.495	4524.969	8364.988
155105.5	58658.65	73190.13	147506.3	85288.3	216566	304636.6
14995.63	27001.86	34484.98	19406.46	13917.72	15159.01	15473.21
40424.36	127566.3	67875.14	94253.63	160881	91419.02	117206
61296.11	10056.03	60875.94	17049.74	24620.21	5134.651	51323.77
18627.45	19462.73	20513.94	17855.67	26060.12	23331.12	24410.39
5668.356	11382.58	7056.639	10426.61	3384.265	6255.669	5926.651
17037.61	5564.039	12422.28	5052.049	8651.428	4641.461	6219.736
734076.3	894151.5	788733.3	637279.3	1059240	1106560	1047500
7108.845	18560.61	16947.73	6911.382	6301.38	4039.852	8184.578
22502.41	25503.47	21054.56	26865.08	19272.28	22605.36	20355.85
23798.69	18376.25	14945.48	31485.67	23484.95	10899.06	15331.15

8093.354	19702.25	14056.72	8992.779	11205.61	9630.154	6026.69
47077.74	119898.8	85268.05	74256.45	35959.05	23655.32	59491.19
21953.95	15437.21	5947.005	38761.62	5850.718	14159.9	16778.47
16877.07	7465.869	10532.73	23731.95	18661.61	12161.65	13742.54
950009	2659875	792874.6	3058773	1759897	420354.3	2625474
19917.31	10813.99	15507.92	6137.082	13271.63	8888.662	12301.44
5858.053	3501.838	3381.778	11291.12	4116.906	8701.506	3485.855
129326.8	136373.8	132993	81616.67	137992.4	170451.7	145170.4
40181.84	28167.08	19877.93	21913.4	44329.22	22775.3	27897.6
274485.7	741375.9	429299.8	368445.1	350765.9	292989.4	327939.2
24374.36	27940.69	26556.6	34908.55	64713.63	33443.74	42706.75
1164870	374090.2	1065017	180062.6	13728.77	943928.4	186504.1
18842.61	24646.14	25571.27	14938.94	5489.353	15741.51	11122.24
3935.915	2146.229	3588.235	4791.921	2717.578	2811.263	3282.443
11372.68	4767.937	11254.86	3742.084	4411.911	4871.228	5088.31
572152	384670.9	401094.4	263811.9	499547.6	701548.9	709563.1
165184.5	101060.1	113357.7	70372.02	170511.3	257198.7	159474.3
11549.79	5851.462	9210.762	7489.797	8519.539	8385.3	12701.69
3735622	5420750	3940566	2690017	5241593	4952898	5392371
50504.47	34735.91	42734.3	27121.76	34809.37	30956.99	36976.48
7736.173	2211.157	4206.81	9430.714	22351.57	9470.735	2205.298
20307.2	16420.73	13774.1	11469.08	9087.268	8157.136	9956.666
138362.8	173381.3	190771	222219.2	178013.1	189235.3	188366.8
98191.42	75787.16	52018.48	110765.2	125623.8	305964	65811.28
8390.979	19053.77	22196.72	25905.39	14097.05	10966.61	16673.33
66397.29	64431.77	31393.39	61219.5	81498.77	71476.41	78452.96
38138.97	69062.13	45814.19	30998.6	28019.54	25578.16	32446.45
177859.4	142944.9	158595.4	102789.9	136294	112724.8	132978
11559.76	8250.478	12590.84	13284.83	11096.68	13118.2	14815.37
12149.83	12173.76	18374.8	34658.43	18911.38	9627.126	23096.73
515779.1	155667.8	317216.1	371379.1	234566.5	106872.9	285674.9
29727.17	24627.83	24209.2	33483.83	17523.72	19742.89	23309.41
1211097	982469.8	1061475	674126.5	997035.4	767914.8	858637.5
298655.1	192016.2	405778.7	418922.3	111400.4	85597.67	153316.7
205515.7	205404.6	197202.1	280570.3	108914.3	152460.5	170657.9
833242.3	575282.1	852960.6	579250	1032356	712858.6	612368.1
770004.1	1438864	1155420	656137.9	440128.9	377548.8	660470
13417.53	12605.08	10639.36	19002.49	15097.46	16232.38	15612.9
396445.6	926257.4	667183.1	442140	370605.9	294511.9	389252
19409.61	57866.5	20079.94	15080.69	32362.3	39183.45	25413.26
17451.23	7906.583	4454.928	12133.34	20976.54	25447.35	19013.66
21262.44	61862.4	41678.18	68745	33086.19	21120.96	42165.11
50404.61	52132.91	33005.92	30020.75	79860.91	35610.34	55560.62
11584.91	7741.795	9430.806	9468.563	7809.5	11575.66	8204.623
3787.246	1726.508	2502.872	3543.595	2762.481	3165.198	3073.327
11833.73	8317.22	8436.043	30688.67	3746.026	5189.169	24028.71
28156.93	12863.29	15946.59	4303.289	9281.563	11601.54	10109.95
12810	10723.94	14228.14	11669.69	13315.95	10724.51	12797.78
30155.36	26490.65	18929.1	76349.82	23579.98	52804.08	46208.96

4568054	1821595	2006626	1007212	499500.2	1399484	318532.4
1117830	540001.9	568267.4	472520.7	919200.4	3034641	579847.7
8517.881	3597.021	10028.7	4020.65	7297.914	6317.834	8455.12
13685.32	17205.21	10712.88	13721.68	15269.92	11236.56	20425.38
26799.47	33460.73	40316.54	44751.6	20714.23	27853.78	31206.14
296275	178053.3	109144.6	24016.92	396953.7	161849.6	453782
8769.291	7294.226	6406.301	2306.956	8699.934	6524.9	9794.985
12879.26	41634.19	22461.55	26033.96	20004.12	22470.38	23155
69327.68	33892.44	28535.72	22634.88	35925.08	21782.83	40755.21
89878.76	186886.8	145363.8	223340.3	134222.4	130987.9	160900.9
6593.468	12764.72	13699.39	23361.99	8105.426	6958.033	14455.21
126713.5	86503.84	111573.8	110583.3	114186.1	139855.8	125544.3
48013.98	23624.2	35570.39	48738.39	18242.73	27031.21	25333.78
42216.8	27102.34	37400.61	41855.45	29384.5	58573.89	39256.14
71321.34	49137	96415.48	47142.24	41214.41	46965.02	51823.21
8860.681	5677.918	8565.011	6328.234	5103.869	7208.245	9040.087
136470	110572.5	192278.2	198025.1	80567.93	67529.25	112291.1
94690.49	61054.42	62793.44	56572.31	65839.9	48226.89	71132.7
122065.8	126358	125111.1	127360.3	122930.9	113594.1	137841.1
77132.16	106614.9	82990.55	47703.36	68825.59	83724.05	94804
44494.61	31863.76	40030.41	51474.15	52040.46	20804.46	34023.14
6126586	5757756	5369736	4414109	6738365	3701870	3696380
616.2892	1264.384	2423.877	2099.487	1400.337	8837.063	3140.496
151945.9	91031.43	85038.55	101206.4	110291.8	72005.16	88401.22
13835.18	25294.36	18789.8	24963.1	15541.31	12387.17	16089.14
205553.5	447077	389125.8	195741.1	214110.6	199132	287745.6
10337.16	7993.738	5045.109	6591.469	8227.633	10116.64	4248.43
85374.9	181649	165288.6	162787.1	105118.2	78030.7	144502.3
5336.309	6629.213	7085.318	4873.551	2762.481	2876.821	3166.228
6516.466	4660.333	9042.138	3051.763	5685.292	6584.013	3346.426
625254.6	313757.9	229633.8	38031.88	291064.1	242757.5	119491.9
220848.8	111634.2	185123.2	89934.7	199313	169039.6	187567.9
63624.38	41352.85	64362.73	35087.16	56903.49	825926.5	57169.92
16992.68	42061.53	26889.63	26785.35	23326.86	57716	19470.56
29337.58	104095.8	62061.25	64972.21	38344.61	44005.29	52202.84
38821.58	10243.15	18160.92	25395.33	24961.88	27976.16	21760.06
9401.432	21505.99	13023.49	20533.89	16913.61	11967.59	8423.524
87062.85	52654.61	70544.78	47834.2	47585.59	42166.65	67634.95
8035.074	3518.787	7602.215	4713.269	4237.388	3281.175	5514.153
4606.83	17270.59	19632.16	15269.89	7793.317	8850.014	12010.37
103587.6	45426.62	26806.04	37042.89	38970.75	161052	29144.32
77832.02	203283.7	129920.5	121593.1	74990.11	76725.99	109327.4
8114.436	16891.25	10924.59	45534.36	77728.63	7023.248	68200.84
633682.6	673761.5	495503.2	429710.9	863925.9	355380.4	437749.9
17644.48	13797.25	7834.762	2914.999	10608.25	11873.74	6031.31
79601.44	88554.81	82789.99	80038.34	71429.79	72258.43	98462.79
142925.3	153060.2	112388.5	170551.7	94450.75	61208.14	82865.87
