# Supplementary material for: Dynamic Changes of Urine Proteome in Rat Models Inoculated with Two Different Hepatoma Cell Lines
Source: J Oncol. 2021 Jan 7;2021:8895330. doi: 10.1155/2021/8895330 (PMC7810548; doi:10.1155/2021/8895330)
Supplement: Supplementary Materials — Supplementary Figure 1. Functional analysis of differentially expressed proteins at days 5, 7, 14, and 28 in two models. (a) Cell component for the CBRH-7919 model. (b) Molecular function for the CBRH-7919 model. (c) Cell component for the RH-35 model. (d) Molecular function for the RH-35 model. Supplementary Table 1. All urinary proteins identified in the CBRH-7919 model. Supplementary Table 2. All urinary proteins identified in the RH-35 model. Supplementary Table 3. The details of 6435 random allocations in the CBRH-7919 model. Supplementary Table 4. The occurrence of the protein in 6435 random allocations in the CBRH-7919 model. Supplementary Table 5. The details of 12155 random allocations in the RH-35 model. Supplementary Table 6. The occurrence of the protein in 12155 random allocations in the RH-35 model. [file 8895330.f1.zip › 8895330.f1/Supplementary Table 2.pdf]

Title: Dynamic changes of urine proteome in rat models inoculated with two different hepatoma cell lines. Full author: Yameng Zhang, Yufei Gao, Jing Wei & Youhe Gao*.
Table S2 All urinary proteins were identified in RH-35 model.

Protein ID	1	2	3	4	5	6	7	8	9	10	11	12	13	14	15	16
	CON-RAT1	CON-RAT2	CON-RAT3	CON-RAT4	CON-RAT5	CON-RAT6	CON-RAT7	CON-RAT8	D5-RAT1	D5-RAT2	D5-RAT3	D5-RAT4	D5-RAT5	D5-RAT6	D5-RAT7	D5-RAT8
A2RUV9	19759.47	23196.6	121018.3	27273.58	3471.622	10574.48	42519.95	12749.93	16957.71	22374.99	2030.106	59970.19	34762.6	78475.52	45446.24	2134.288
A4KWA5;A	8003.971	15954.47	11579.6	11950.99	18339.97	14902.49	14760.52	12669.5	14290.54	14795.25	13137.04	17512.4	22343.92	25631.18	19283.43	12787.38
B0BNA5	82218.27	86841.09	73358.23	67294.95	114631.4	78830.42	75158.77	85012.68	106061.6	164717.3	139061.4	80626.16	123346.6	91431.52	129987.2	121318.3
B0BND0	35782.93	43558.06	70446.34	34698.08	81112.37	35202.73	37751.84	31120.47	37794.13	34860.86	44996.86	60184.18	35108.2	28840.71	25402.22	41058.36
B0BNE5	77734.44	54256.74	78625.64	44304.48	142344.7	55732.69	76521.42	65961.74	114714.8	118375.6	132108.9	83875.26	66782.26	64128.1	88346.77	89354.95
B0BNN3	52635.48	35268.57	82767.03	165454.5	12738.76	59348.73	3272.119	49127.65	5108.656	14090.92	18327.22	47758.46	5289.173	30254.15	80707.84	3564.313
B0LT89	12170.37	6224.09	4809.591	6266.775	9378.964	5756.564	10560.34	7071.839	7493.734	11164.45	12243.47	7096.247	4609.789	4573.117	6983.643	8530.447
B1H234	43582.54	74461.66	82110.98	43637.38	137297.7	72845.54	45593.53	48135.78	43359.78	40843.62	32802.91	39792.05	24253.52	54177.08	48245.56	50627.38
B2RYG6	8598.708	3925.858	11149.07	12850.35	19697.79	9270.36	9837.142	9375.033	10831.45	18468.11	15195.45	9623.774	6155.83	3974.12	11951.23	6998.159
B4F795	21525.28	8297.336	17372.83	12520.98	9663.919	1589.718	13336.94	15231.95	11217.88	4983.792	11990.27	2644.388	7230.171	8655.23	11808.03	25846.29
B5DEN9	16625.61	16475.42	13031.46	25310.53	23438.23	11976.04	14187.04	14191.28	12537.48	12792.18	15223.71	10108.89	14473.87	11956.76	7964.048	13223.86
B5DFC9	355590.5	668056.2	495623.7	493664	239075.1	599266.9	960197.6	449625.7	551546.2	677890	504946.3	510793.6	661076.2	610013	633599.2	803911.9
D3Z8L7	16148.87	16754.27	12945.43	18049.98	13700.6	12439.66	14362.32	14528.21	29746.25	19744.02	31780.27	13170.27	7824.842	16049.54	9472.729	19897.85
D3ZHA0	40015.35	55427.29	39264.83	82803.67	31698.55	45400.15	43253.56	87153.47	23962.28	35709.91	10403.01	26567.85	10936.34	15561.94	19747.21	11948.53
D3ZTD8	21476.84	24949.8	20011.5	23617.57	35895.97	14252.6	12842.21	16237.1	16481.87	13708.04	22737.29	18753.24	16301.88	24533.83	21822.28	16035.29
D3ZTE0	191828	289668.7	255800.9	469402.3	201959.2	282981.7	265262.5	334167.1	374962.9	168543	261039.7	438610.5	403120.3	434136.5	354302.5	306131.6
D3ZTV3	10035.86	10757.13	25267.16	25727.77	34859.77	22129.94	20949.07	17766.76	14439.17	17620.98	16057.75	15874.91	21665.87	22324.95	26100.42	29748.49
D3ZTX0	9191.115	19709.65	14142.96	23748.61	19562.63	18449.51	17067.43	23423.73	12597.55	17195.61	11518.31	19489.73	13519.12	11787.81	14801.52	8056.403
D3ZUC6	536069.1	740563	91044.08	482811.8	81058.71	115123.8	400542.3	167146.1	313557.4	147352.7	374679.5	623418.3	312205.3	698102	410402.7	451158.5
D3ZUK3	61917.8	134516.9	90232.87	105698.2	20699.66	74897.36	109490.6	103302.4	61921.95	16975.06	60118.29	78628.77	61742.18	83925.67	78216.43	92691.79
D4A1J4	20404.75	24871.37	23618.14	25190.5	44840.15	21667.54	17133.55	22149.75	38113.79	50694.16	49158.65	22992.13	35918.58	36870.96	33843.23	42651.78
D4A1R8	28181.47	31179.77	23916.75	25411	33476.67	21356.15	18445.86	25486.43	30492.27	27777.74	41357.65	30690.5	23945.93	19093.67	24274.93	33703.6
D4A5U3	19478.7	28374.95	17877.33	29582.31	16625.01	27392.46	14009.63	26769.67	14898.08	23022.73	10318.14	25651.68	14678.12	3334.073	2469.834	16016.87
E9PT87;P2	7720.304	8531.716	10800.34	8369.758	12508.24	10844.07	10131.84	8073.918	9737.037	13380.5	15752.09	7708.666	8241.856	6985.874	7955.313	8466.112
F1LM93	74390.24	11039.89	19161.89	31698.17	41596.68	45576.3	37576.03	44235.42	16997.47	38842.94	25331.54	63438.21	14939.88	13386.74	15751.12	16392.73
F1M3L7	4971.543	6996.323	10947.46	21730.07	15475.82	13607.17	12189.08	7474.663	9401.078	14544.42	10482.88	11596.45	8022.269	11786.52	5769.777	7779.26
G3V686	219115.1	658213	203426	396573.5	185765.1	309196.9	233968.1	79561.16	135571.9	95200.81	205677.1	232547.9	231760.8	195998	96721.31	321289.7
G3V7W1	165561.4	218674.7	230014.5	238166.5	280160.5	189403.9	154361.5	218857.3	195779.1	187862.6	330956.9	195526.4	189129.6	265115	230322	231339.3
H1UBN0;Q	37881.57	125854.7	86181.76	116168.2	100279	128597.5	47277.01	48134.03	197103.7	96796.56	188780.4	131648.8	92392.96	96636.88	114744.3	146230.2
O08557	16079.59	21888.06	23704.22	19090.07	47748.11	14657.94	24673.57	5576.705	49685.54	52053.01	54514.72	20111.05	27618.37	20904.2	27610.69	48878.65
O08628	86432.27	110113.4	68023.95	95701.02	75681.52	94708.55	146920.3	121977.8	179960.2	158677.6	111824	133493.5	73227.61	225491.8	127265.1	117759
O08815	20380.05	5166.871	11774.44	14843.87	7656.572	18152.76	11101.33	7677.527	8841.946	21801.23	17096.7	19679.03	6954.443	7662.435	2295.584	4840.472
O09175	16520.77	24164.35	18870.63	20121.04	14355.73	31591.78	17486.01	30046.73	16576.14	22698.27	35428.5	20041.41	14743.09	25243.01	23865.28	11610.26
O35077	51028.71	19442.84	24091.08	23664.32	17334.47	16991.48	35580.15	19524.79	26044.09	45063.18	43211.83	20502.78	15212.25	10500.77	44889.38	25122.12
O35112	113113.4	148939.1	146912.9	123269.6	227508.1	116005	167147.3	161170.4	103277.5	107288.8	83530.68	80357.8	114904.7	131790.1	111004.4	127356.2
O35142	33368.29	79140.41	49526.09	104095.5	60684.44	109890.7	49695.94	117022.9	60094.68	54320.11	49611.37	59186.11	47945.27	64412.85	68589.52	33251.64
O35217	468569.2	544078.3	483262.5	537871.3	383406.7	394804.2	385706.1	450628.2	464171.9	514043.1	812530	553397.4	447893.7	572325.8	643557.9	480955
O35244	299163.1	197279.4	219359.1	249626	269440.4	186217.7	250170.8	211685	324812.1	289835.9	371558.1	270928.9	241040.3	229800.5	270887.4	255657.2
O35264	33008.39	14844.86	29599.21	20535.83	27242.16	26020.88	24275.5	33116.28	39869.82	27509.47	36378.38	20426.86	41365.69	30884.95	48612.72	38233.79
O35331	22809.59	40195.63	20929.97	24796.35	27851	20040.76	24415.28	15207.51	25261.85	37088.44	65483.2	19688.17	19266.54	22014.33	25180.37	29059.8
O35509;P6	123059.5	114193.5	67196.88	132153.4	112791.5	110903.5	115966.3	61428.32	101028.9	108233.9	146352.2	106812.6	81547	26274.81	45812.63	125414.5
O35547	43287.28	15725.22	12891.63	17615.64	20626.09	37192.23	7920.547	26616.43	8272.101	17818.13	34876.05	39463.59	11222.94	13594.28	12457.34	13298.03
O35568	634001.7	1192155	802467.4	1323031	1182789	1169301	1587219	684732	404499.3	1025841	779187.8	981480.4	1348590	961436.4	625521.4	1204328
O35760	17234.3	23304.2	28957.92	43025.71	36630.15	48864.74	23678.63	23089.87	48880.86	30256.76	21576.9	45324.54	15897.74	33362.04	34394.64	14018.37
O35763	316178.5	284390.3	189296.5	338867.7	452540.9	214112.5	294827	155383.7	363917.3	479384.7	652335.1	278669	309624.1	174777.7	251431.8	378078.1

O35783	12668.55	8495.176	9046.728	6857.267	3297.921	5590.973	10606.97	4313.938	5066.834	7680.615	5866.969	9914.582	4241.067	3925.547	1825.334	3293.859
O35952	49750.82	43941.06	50753.72	26431.37	65047.8	17270.31	47700.68	33927.22	60137.86	46214.96	59903.15	46530.7	48873.91	37939.39	47810.8	56507.79
O35956	7441.617	12934.88	15306.27	8712.472	14854.55	9210.346	10259.74	6906.373	8808.804	24063.35	22393.05	7602.475	7890.676	13215.06	12381.96	17393.58
O54715	250014	281205.3	105606.1	133210.3	94864.59	145439.1	121430.7	166395.5	79151.52	82210.22	169105.7	99836.69	56554.53	53446.61	63010.2	76068.54
O54728	10513353	74262.72	11446194	5075667	8041462	24143.14	3853602	2704878	7132811	2544877	3619506	125419.4	3357641	3554551	8535455	4509536
O54800;Q5	313662.5	416576.2	371515.5	297052.6	95393.41	242790.5	482595.6	235200.8	331112.4	393911.5	253401.6	260201.1	245276.2	224017.9	75123.14	257061.4
O54858	129884	159498.7	284426.4	182833.8	107663.6	247829.2	295833.4	255782.1	547982.2	356349	358795.8	428355.1	549470.4	619546.2	727256.7	491719.6
O54861	31245	27625.34	124539.9	76397.33	125126.1	55805.74	38126.87	106281.6	72694.05	65593.4	25317.65	65211.02	127722.9	134771	87497.39	96975.81
O54975	15787.13	10200.48	15085.3	13962.62	10921.01	10368.73	12454.95	15488.71	18116.73	28279.28	24329.6	12730.91	12119.1	8820.794	12101.92	15035.29
O55004	506429.9	718955.1	324717.4	1216139	281425.2	596218.5	673452.3	509052.1	270366.9	219092.8	380685.9	406828	139166.3	142498.2	291600.6	166766.2
O55006	323975.7	475917.2	411096.1	390269.8	360850.2	228072.5	356321.9	370483.6	415232.3	546523.9	978515.4	899546.1	1192318	1286260	710266.8	1116952
O55096	19342.87	8696.669	6482.036	11727.51	14895.93	14069.98	13149.4	10877.69	25551.92	34624.14	25252.37	20137.89	25082.99	13418.55	17419.37	21304.84
O55145	15612.15	12935.09	57301.67	20509.89	8329.045	19628.3	21764.74	28040.03	25470.05	59298	20535.81	423355	17001.74	26786.64	20501.13	13543.22
O70244	239371	285714	302830	387070.4	357257.9	274158.9	247112.3	452895.1	359317.6	300541.3	320921.9	369577.1	346244.2	538275.3	355699.9	521538.2
O70352	65327.1	89648.36	58446.89	70437.46	64942.79	97377	79748.72	154138	52204.92	48165.09	61568.14	80596.86	44017.25	56264.98	36006.75	58841.88
O70377	18074.17	19280.59	12469.87	11761.03	9866.043	9847.276	6082.909	7959.933	30654.21	13884.73	26556.44	18455.09	18196.24	13152.79	18551.03	21177.27
O70417	1546703	485809.6	5018792	2757697	400601.2	2401733	1738544	2609203	2024772	1375264	1172131	1739080	1466396	1099835	1833793	2150488
O70489	343186.2	485742.8	432845.5	426783	445699.1	404043.6	425990.9	437659.3	348387.9	317728.1	436760.6	324163.3	331914.4	510217.7	405787.4	349967.7
O70513	568794.1	897173.9	906475.6	1326430	1126076	840285.8	726132	1255435	622096.4	720322.1	775726.4	813050.8	847825.1	1426718	1274490	908257.8
O70535	212784.2	244283.8	240978.2	154307.6	264011.4	165267.8	196957.4	234519.6	170070.5	162065.1	152840.9	136560.1	169661.6	219090.7	170579.4	234747.5
O70540	47326.09	32741.85	65155.52	32765.02	30486.1	29059.49	56599.33	2062.429	38799.05	35836.14	40835.05	71473.16	32685.41	32101.09	33730.47	37560.17
O70594	42852.98	91074.66	14327.98	77637.92	37018.26	37276.05	46030.06	18291.44	54810.73	73454.55	65822.1	38584.75	35775.46	17594.72	52334.08	49015.66
O88204	39646.56	101437.3	25048.63	54437.1	23922.06	54314.47	43775.01	56434.26	46213.07	46725.88	48455.43	53659.68	65468	71965.98	58322.2	80926.87
O88267	10956.87	4074.419	18145.48	14125.87	9217.36	9739.915	6533.811	13947.85	43920.77	46852.82	45132.96	24932.52	30573.53	32237.27	49160.22	33455.31
O88600	7413.108	10712.55	14230.88	11418.65	10524.91	19985.3	15685.15	7894.872	7980.868	8530.498	9292.707	12967.29	5866.784	6794.071	2403.53	6374.632
O88766	243118.5	304859.3	244214.3	516451.3	206646.6	215198.3	306397.2	163227.2	179813.8	269284.5	340001.3	245931.2	186864.3	224282.6	235138.2	250799.7
O88767	236428.4	125517.5	129487.3	163152	95410.73	97910.06	211037.1	141912.3	119295.9	165274.3	154158.6	104511	107233.2	100810.8	122612	99389.86
O88797	5789.746	6377.762	4950.873	7236.786	7757.784	4374.915	6632.623	4183.156	9823.811	7554.334	18332.66	1879.089	4285.695	6796.986	5928.133	5655.794
O88917	35058.23	85566.6	85783.03	81932.75	183533.8	90181.41	105373.9	78553.44	84850.41	82473.66	86006.73	76218.34	102150.3	113911.3	133445.2	130970.9
O88989	371539.8	262670.2	305620.4	202616.3	414709.8	323740.2	388078.8	366044.1	481343.6	984898.3	777754.6	263531.3	449069.7	334146.3	455078.3	383425.2
O89049	26791.09	17507.68	18427.57	24411.3	30631.09	17006.21	20246.52	24021.86	20906.36	29348.91	33125.4	14678.73	16156.73	14378.97	20783.01	23153.68
O89117	289855.5	209454.2	99189.25	283779.7	113676.3	70638.02	117506.8	88969.91	114921.9	78630.98	136453.8	162659.6	108797.6	195019.2	141141.1	155761.4
P00502	431195.4	533154.6	462804.9	395396.6	642431.4	353536.4	515981.1	350286.9	693173.1	945954.1	774810	438472.8	411282.8	338646.4	430320.9	679200.5
P00507	4236.025	5699.067	4401.92	6380.291	2236.304	6693.64	789.1814	6310.223	3952.081	6578.254	5464.77	8029.494	5625.206	5231.608	3855.761	8098.419
P00689	27026836	39776524	25401078	28908728	31139394	25287134	18753898	28000298	24046930	19014000	20605524	22555988	30481344	28616550	26971166	26846476
P00714	60920.34	81464.38	105515	855044.4	133087.2	159582.3	394008	456797.1	200177.6	471493.1	408253.2	193746.7	26157.96	148630.6	85513.2	129382.3
P00731	24662.82	17982.9	20058.58	11446	17550.43	7848.917	15299.03	19832.41	8294.808	26165.89	16878.72	19463.51	6391.207	6965.992	20056.29	9269.652
P00758	13291510	21708284	20750368	18707378	10792490	16489252	19570210	18903786	14257280	11487132	15433826	14294633	13089333	19322604	17928068	16002964
P00762	2507868	5725375	12470178	14591307	1777095	14007383	1966717	5845576	8141231	11994610	13897041	12117495	4746253	6492354	5834182	6004867
P00774	2107.438	29339.38	27313.55	22428.15	11946.28	21915.58	12045.12	12250.82	16121.89	19775.24	14724.3	212306.3	17190.39	24100.39	17641.36	1694.042
P00786	349983.9	364185.2	449334.8	524868.1	253116.5	228605.4	394428.1	427762.5	271637.4	345099.3	450054.2	399570.4	319796.9	475009.1	462861.8	299833.5
P00787	2933578	3180848	2410618	3930150	2526699	2366528	2523507	2512775	1429591	1620267	1929208	2916661	1132372	1766351	2125148	1129735
P00884	498526.6	379070.8	526291.8	317751.9	671364.3	424753.3	437319.1	378526.5	569313.4	957043.4	678416.1	366271.6	499931.2	392078.5	445008.1	549337.5
P01015	1263793	1861755	622365.9	2681415	467741.3	1251960	998758.9	1231681	642622.3	940761.6	1386482	1492993	564736.8	513007.2	1518032	354121.5
P01026	472885.9	410332.3	421423.1	483684.8	294934.3	283407.1	458462.5	583327.3	219889.7	300327.8	456244.3	324035	231093	260060.1	351062.8	243115.3
P01041	167588	248640.8	173346.9	171805.5	50224.98	103589.5	364612.1	126822.5	60780.38	87312.48	69151.81	135547	44356.4	74303.1	110754.6	75162.95
P01048	1802432	2913515	2390182	4666721	1233637	2022974	2136576	2385410	2520854	3403059	2325371	4423887	2066402	3833001	3390628	4464829
P01681	5099428	660233.1	4462352	3854984	3130094	3306720	491200.1	1180277	502773.5	540244.6	1271744	1591522	488292.9	713340	173623.4	544419.3
P01830	432537.6	488362.1	420982.4	539411.5	236734.7	279268.2	310748.2	245318.1	186354.8	150114	193258	232925.3	227933.4	237023.1	302593.4	144798.5

P01835	29124	39180.47	25853842	29961946	24675922	39939720	67027.91	104498.7	28177574	21947.7	16676923	36936388	43298.53	24227224	140553.5	237579.4
P01836	9677561	16669750	13364846	18350630	188169.5	1001926	10296823	1044017	1041522	917068.9	6253268	8952432	5662954	1093345	5761904	5592980
P01946	477111.2	231077.3	343658.2	22481.26	71651.15	17833.78	250328.5	30258.29	10483.36	16048.32	33580.77	26797.84	39466.09	24609.78	2888.483	20029.27
P02091	1023742	30592.21	679729.5	16137.32	69860.71	30182.59	445101.5	15918.1	10151.53	34083.13	31382.52	47149.81	40063.16	8496.959	16095.6	22958.85
P02454	278130.8	326987.4	159270.3	250090.8	212900.4	208614	1481466	225366.2	90479.32	230585.1	203661.7	141936.8	113037.4	81316.12	107687.4	51807.88
P02625	240566	265174.6	803481.5	729977.5	162193.8	532520.3	259089.4	236112.1	302646.4	139933	445488.1	419734.4	316406.1	284802.4	673678.9	103088.7
P02631	90426.58	185943.2	69071.93	89934.59	53585.27	219510.5	185017	75676.2	35019.98	8954.168	45757.16	24519.83	11321.54	47968.29	57892.64	19598.58
P02650	4112451	4459813	3422523	4171191	5679170	2821794	3499706	5068796	5088578	3804672	4781715	4321409	5772209	6530258	4736926	6308584
P02651	690150.8	296024.4	501042.7	273797.6	299538.4	316860.1	431479.8	443051	393660.8	187552.8	297752.6	312848.5	262355.3	270633.7	314663	323951.1
P02680	12458.82	28227.61	15787.27	19306.04	7649.709	30541.59	16529.73	25402.96	16241.46	19203.11	19003.73	23505.96	17075.85	21067.22	15591.33	21958.28
P02696	15385.79	38775.74	37221.16	28176.4	11725.56	31892.12	22919.24	19909.93	20221.52	33656.29	16186.35	24702.17	15464.97	11717.4	12041.75	13251.73
P02761	9.78E+08	1.55E+09	9.26E+08	2.35E+09	3.51E+08	5.92E+08	7.03E+08	1.44E+09	4.98E+08	6.96E+08	6.67E+08	4.16E+08	1.95E+08	4.41E+08	8.95E+08	1.03E+08
P02764	6142145	5437805	7663979	6570563	3564438	3219703	4543042	2862947	8034612	9276863	9764209	12501134	6909063	11771210	14414401	13584332
P02767	2760023	2061450	2311359	2513638	1648794	1702515	2069395	2780947	1210073	2026248	1781176	1397576	1057984	1160803	1400068	1220768
P02770	1.07E+08	72003072	1.08E+08	64565668	22026062	56549152	63290256	82184416	40468984	41225624	37840200	36526064	35305232	30635110	32385164	27724308
P02780	55487004	38656080	22567888	94993736	35778008	81917376	59019004	95111080	31625394	24746460	48878664	76931520	52239956	32861936	1825231	4350554
P02781	25086520	10004956	11251461	23054306	14048735	27165898	19427440	31688442	8702154	12825217	22709020	30151052	17058054	10218433	1432897	1648971
P02782	20924404	12832733	7348560	13959537	4791692	38866012	10946492	43217028	8874284	6191124	13057770	30560856	18058054	12374326	1338493	1362379
P02793;Q7	9167.545	7295.353	9864.903	11941.7	14894.82	14095.21	2066.624	14278.15	6272.101	2983.649	6840.689	4531.442	3359.941	13006.14	4385.026	3401.746
P02803	2637.874	9926.743	41028.17	9410.211	15123.64	24682.76	18901.77	6151.427	3560.992	12093.04	5557.721	3132.837	3532.087	21899.72	34030.65	18238.1
P03994	15502.96	30829.07	21100.73	19284.2	23855.33	24747.61	23197.49	20198.11	26590	29122.72	12931.13	26758.29	35191.08	32274.11	33897.73	46247.66
P04041	39903.88	19079.35	36673.24	34716.86	71446.87	39064.84	33639.21	50256.86	21722.95	30117.73	45919.46	20605.38	14183.67	16008.95	13559.59	12595.1
P04073	81260.02	179645.1	178791.4	312895.3	100050.3	170875.1	150535.3	115707.1	99831.35	133483.6	53509.09	175767.5	69507.97	172020	89089.02	211011.3
P04176	5677.231	8692.936	10752.43	10732.32	6863.974	6744.14	5358.877	6334.287	6015.982	11658.76	7393.04	5709.049	5809.836	2143.925	5625.05	4123.283
P04182	15824.42	8138.863	52216.72	11862.59	6517.893	8571.419	7756.702	9527.689	6663.251	8175.698	8049.452	7148.885	8069.621	9600.137	8171.236	5907.231
P04218	9796.565	111424.3	46660.49	154004.8	72101.43	66566.74	79472.75	47180.64	34019.57	170058.3	35070.57	67688.69	59275.42	46204.8	52948.62	46739.44
P04276	2108648	1327776	2036148	1945966	1025131	1621070	1241055	2855706	1358754	978249.1	1164016	1317964	1195553	1394180	1235944	858886.8
P04355	51010.76	58422.57	23836.84	29519.27	31637.72	54211.01	41326.3	29472.59	34733.25	8566.139	12626.88	47616.04	48318.15	48776.11	29937.29	40118.5
P04636	41613.26	47265.72	79935.33	63948.52	53892.27	84434.86	51155.59	79291.02	18539.11	38368.45	36138.74	65975.13	29563.29	56669.7	36634.81	20325.45
P04639	113876.4	173551.8	112697	103557.4	88571.3	103998.2	103810.8	143185.1	69147.52	76022.8	78107.49	77725.3	63847.73	97162.75	81613.25	130561
P04642	144466	151685.3	126222.8	167597.5	178728.3	161650.6	174896.3	168571.7	142431.3	328707	213325.5	139033	113202.8	129441.1	169484.2	156448.8
P04762	12883.03	13724.45	38826.81	15635.06	16986.78	10289.92	8243.613	20715.83	11537.41	14544.08	15888.02	14325.52	5874.219	11322.99	4997.395	7888.713
P04764	771241.3	430791.8	682546.8	640366.4	839137.4	714306.1	768127.9	909216.8	746000.3	1066043	826714	516100.8	702131.4	686824.8	898619.3	722655.1
P04785	109068.9	160342	85010.97	167586.5	139602	144188.4	128007.6	124706.1	77937.06	67937.2	104459.9	94429.11	85435.19	93189.7	83330.37	80443.96
P04797	686035.5	580304.1	469695.2	450615.4	449034.1	505876.1	731370.1	469915.1	945653.6	911003	1028777	714864.7	547521.4	564580.9	733376.8	606627.9
P04897	207659.5	146932.6	97101.14	217555.2	199955	246113	272845.9	197779.6	222411.8	239141.7	346659.1	289985.9	148305.1	169100.7	164939.8	188633
P04903	17597.28	28569.83	12286.57	18034.29	23492.7	26806.93	31167.87	14205.63	38029.31	24269.16	33523.16	21112.96	20848.77	15137.25	22071.77	33716.64
P04904	1202866	1141620	1090705	1061547	942616.9	1043795	851814.6	888247.4	1729408	1973993	1363428	874392.9	1157864	853313.3	1218031	1366674
P04905	47158.48	14679.83	14573.72	40766.97	16243.13	51344.17	25908.07	30305.09	16136.06	23373.42	45701.21	52775.9	26884.86	27803.42	5419.492	7759.617
P04906	46583.72	52813.77	71855.18	56355.95	78183.89	28557.44	52164.48	35169.77	38457.64	46738.93	66732.34	39234.29	32496.03	40882.22	61560.52	40561.75
P04916	295594.3	175368.1	157168.9	224065.9	191958.7	191079.9	182458.9	400962.7	112619.3	128263.2	101937.2	156481.9	71778.52	98999.33	149566.1	124641.2
P04937	10150328	14198394	13984261	16904362	23101294	13660484	17078210	16051756	16314510	12844973	13363685	14311723	16384811	18854624	18834240	15117705
P05065	124392.5	77103.09	98712.12	57844.54	120772.7	79548.67	146230.7	123562.1	113233.1	178495.6	126226.5	84372.58	105990.1	85181.99	121715.9	87688.22
P05197	37948.94	27409.01	28932.22	28031.94	41967.64	35406.89	33818.54	31591.18	29168.03	34830.69	76555.03	37073.63	25088.72	23581.85	21634.15	25891.46
P05369	25397.66	11667.94	7674.947	10649.54	16508.33	10568.95	11153.03	12409.7	10074.34	13785.37	71398.82	13965.56	7258.022	5521.1	14580.93	17917.91
P05371	1552353	792291.2	557684.7	586115.3	490202.6	693088.7	998529	442458.3	285377.2	258364.3	443435	287929.7	212074.1	311732.8	280721.2	324961.7
P05539	39537.93	16403.3	23127.87	79844.23	42159.61	33442.38	45974.05	9239.486	30085.85	7025.284	38742.39	18505.24	58825.47	20160.21	27341.44	6069.639
P05544	34425740	1.02E+08	60660180	64853400	74802512	57255424	34706000	77011776	49471376	54134684	41207568	32722956	46688556	54137372	37999564	41951148
P05545	28253224	42217840	38812456	34582896	39795084	25693024	14257737	31072266	20686540	20979314	18731640	17328990	20894236	24179440	16883328	18796232

P05712	15177.9	13257.25	5130.99	11229.43	22810.35	18353.37	25412.13	7808.348	14715	15951.17	25744.31	13471.02	9733.192	9291.509	6513.953	17493.62
P05942	25865.18	66345.91	16024.28	22402.65	20897.04	42079.49	24663.54	20500.96	35605.53	37216.68	41009.63	31899.38	22512.91	36047.02	29514.75	26493.29
P05964	555461.5	780560.7	350132.6	497277.6	179719.5	312051	441823.9	258621.6	172053	435615.3	509922.3	425624.2	153072.3	179863.1	127196.8	118415.7
P06399	26911.45	29414.14	12504.12	20023.99	23008.38	24357.6	33711.62	28783.79	20087.95	37430.61	33923.71	25642.36	30139.16	29533.86	21686.74	46071.63
P06685	50790.64	53040.74	46593.61	50497.49	51132.17	36395	34638.26	40494.8	37932.39	59964.39	52204.11	35621	19455.26	46299.07	46469.38	39119.92
P06757	24848.81	17471.93	16631.71	23241.99	15119	18424.72	19219.66	11134.72	16199.06	56082.45	33905.66	54602.71	10958.4	7667.047	10420.85	20862.13
P06760	733368.3	889555.5	326174.7	2069502	6218203	4829474	490190.7	5220790	79079.18	1170440	757078.1	983156.9	252774.8	216087.1	115105	116733.5
P06761	99403.27	56802.6	45875.7	159679.5	71427.51	163816.5	170623.1	259470.3	35422.32	37304.81	40294.06	85956.29	81021.3	54542.56	27935.59	18619.08
P06866	5209719	5580444	1853816	4105614	880730.1	1442559	2426539	1985221	2606144	4188544	3628882	9776140	2125983	2385145	5163133	4612144
P06911	579472.6	266609.2	166792.3	417263.7	80258.64	705367.8	473614.3	422997.7	18792.76	150776.8	49709.23	118793.9	761336.9	430316	2164.313	18973.3
P07150	43294.73	41953.83	38466.26	75962.77	49916.55	112790.5	65022.43	159313.4	23151.61	38690.25	101166	29487.89	26595.6	30577.05	26685.45	37581.58
P07151	11639615	9375837	11521533	10987968	3383988	12761795	10233710	8415956	7277302	4602364	5549070	14779028	5934729	6563081	11792446	5141899
P07154	149751.3	195034	205610.2	209642.4	147961.3	138960	183820.9	193934.9	180941.2	180367.4	113664.4	175226.1	144688.1	170712	143184	150068.4
P07171	804394.2	904683.6	579239.8	1792367	1527430	1175379	1722054	2335911	413630.6	491887.9	1029595	241983.9	429945.9	890489.1	923616.4	350116.9
P07314	5333162	5975792	4310544	5696335	6706320	4701562	6074835	4101992	10006451	7682880	9981522	4911070	5800158	5492867	7479811	8931961
P07340	139533.3	138464.5	117906.9	115921.1	126121.6	144948	136026.4	129050.1	94742.21	94274.95	120860.2	106513.3	103140.4	147015.5	106800	127826.7
P07379	25531.46	19047.35	21141.38	30624.45	39285.63	45041.03	24507.74	35707.8	25272.98	59406.42	43951.24	12643.39	27051.22	14025.68	21328.45	30332.07
P07483	220597.1	188907.2	250955.4	329455.1	206045.5	236203.9	192089.8	245482.7	179694.9	254093	165256.8	114128.8	146852.7	195742.4	227277.4	174430.6
P07522	49094736	64683956	41576908	55571180	77717656	38938908	50259176	47447712	48331120	52316916	42137856	46973836	53482772	68345152	46264748	53390284
P07632	9418382	10359422	16663800	15941276	7467506	17545092	9469346	8022608	5732204	6728701	4878018	6358577	5542048	5446957	7409545	5982170
P07647	16584894	11728224	5350398	22938728	7418852	23538566	12879336	17657060	4641001	4948608	8557326	13820979	10217665	4512479	550042.1	647216.5
P07861	2343438	1134707	774316.4	1935901	1594991	1746427	1686720	1265869	1880091	1718449	1939713	2172727	1224448	1144533	1074943	1238160
P07897	595047.4	601009.5	693966.2	793746.6	976094.7	615075.1	835862.8	647204.4	647956.4	572920	559357.1	570021.3	859759.1	993474.2	829553.5	972083.6
P07943	61161.41	61821.57	22668.57	97704.24	55345.18	67232.6	38313.89	68721.38	103189.1	50808.49	85174.36	71183.33	63079.39	87324.34	66532.72	81768.16
P08010	36066.67	19806.93	8346.359	25462.63	34000	53500.59	26889.66	55957.73	27381.7	68602.37	53682.04	69076.83	24581.75	33769.72	44590.27	14083.9
P08289	205718.7	173985.2	180626	214661.7	204745.8	148114.9	229308.4	165561.3	209886.3	149526.3	463593.8	117069.5	95350.65	99738.74	111924.5	113446.9
P08290	34746.51	57401.09	31170.56	31921.04	32533.65	48904.18	49444.82	23752.98	13545.32	15932.53	29945.89	25033.32	20903.21	57458.74	36237.92	27904.68
P08460	339602.3	424752.4	472635.7	388236.2	547215.3	398255.4	564116.1	525894.8	538194.9	674997.6	764208.1	469259.8	783858.6	757163.3	725071.9	639784
P08494	39847.21	19135.39	23731.91	14924.78	14997.24	21486.23	18957.3	19385.16	5616.605	15841.81	17250.87	11687.92	13916.57	16478.83	18076.74	18531.06
P08592	446815.6	695080.4	594245.6	681580.3	630593.7	610315.1	583235.2	741146.1	478359.5	492402.8	365960.8	506649.2	359554.2	353508.4	540060.9	556392.1
P08644	55300.55	38684.74	39319.66	67111.71	9583.903	107326.2	30787.74	69251.43	53990.42	69393.55	15628.01	65932.38	39563.56	42009.02	45970.14	39175.93
P08649	1201620	1441496	1393051	1535806	417679.1	1549150	3687692	741036.1	2704060	1123914	1360468	4653033	4915676	3380153	3346281	3248281
P08650	141794.5	147189.7	156775.7	147135.2	125697.1	68233.18	116888	133741.4	148426.6	154298.3	167286.6	138714.8	180724.1	163600.7	175208.9	160340.8
P08689	2965.51	13012.48	1880.235	16771.59	10814.31	8441.584	7596.958	11304.6	7648.952	5016.767	7887.608	7579.671	22869.03	6803.11	8915.068	11834.01
P08721	8393994	3337454	3956844	4827164	3116057	1835742	4006939	4035005	1175738	4935357	1105694	3257503	567613.6	2970096	2247918	7603520
P08723	20748070	5983865	5064982	13384018	1334537	20894230	11950869	15582900	3239588	5274867	6182452	16619078	5586874	5334991	608444.3	640951.4
P08753	19771.09	19583.76	17527.83	63028.23	6870.912	30228.85	44414.25	18587.89	39711.95	41901.57	78558.05	53377.66	11802.56	25628.63	30161.6	32115.58
P08932	2193110	2673176	2546216	3787418	1395350	1770455	2260952	2232974	3219112	4236353	2824708	5596268	3594223	4745862	3687974	4866000
P08934	28914.35	17239.85	32672.63	33568.68	9965.225	24703.41	19608.47	38908.17	20439.97	18816.97	20884.33	20518.17	29207.33	25073.66	17756.15	12260.09
P08937	660478.4	526851.7	1944825	649631.9	1189990	1034535	960591.1	760164.8	145643.6	282467.9	351249.1	525206	376394.1	213779.9	226544	144590.9
P09006	869018.3	541533.3	527145.8	637744.4	232050.9	458702.4	333428.9	754348.2	460390.6	528362	557787.6	742409.9	408299.2	464721.7	684802.6	530894
P09034	131038.1	124919.7	140689.7	98966.56	169083.6	151547	117868.7	99550.47	218654.9	160612	251172.9	126431.3	162865.2	144710.3	212586.1	176174.3
P09456	20521.34	10738.75	6544.942	15010.98	6967.426	20312.4	26997.34	12539.08	12850.95	10687.98	13103.51	16141.71	14170.51	9189.352	2029.456	4116.718
P09527	32218.4	19716.93	34752.51	27025.46	22338.41	24784.2	46234.67	21143.4	30375.99	62211.37	54810.51	28764.43	22417.43	14860.65	9318.276	17597.15
P09605;P2	7152.517	14180.69	11332.03	17466.33	4694.644	23696.66	20177.21	6351.419	10755.44	2590.98	8899.327	10793.13	5091.311	2976.144	3717.911	9082.658
P09606	84721.77	100656.1	45954.33	170315.6	110791.3	71935.09	86675.88	53684.58	104219.4	80491.98	143250.3	56116.91	111269.8	77867.7	90072.92	90035.55
P0C0A9	79109.7	40541.95	16309.16	137941.8	38506.7	130924	55498.19	104583.7	49490.39	58668.2	97178.11	153423.4	60143.03	47787.04	8503.063	14007.13
P0C0K7	10982.79	19923.06	9710.347	13790.79	13944.21	18231.71	26656.08	13016.02	11870.41	14273.12	11408.65	13395.59	11221.05	12794.32	14946.2	10499.01
P0CG51;P(	1620418	1219591	826275	1768555	1033469	1288891	1401867	1320438	1068584	1295684	1217978	1460863	720228.1	802649.4	898659.6	907606.3

P0DMW0;I	22273.39	21538.31	31919.04	24986.36	33520.45	26172.85	44768.86	38866.29	30001.87	33235.73	38112.38	41211.08	26374.01	41679.55	21851.71	29505.91
P0DP29;PC	277738.2	242495.2	143801.2	203700.8	280469.9	214751.6	339294.4	311054.5	163828.3	194043.2	237889.7	115112.9	161098.7	141362.8	88054.13	124819.2
P10111	184994.3	171133.5	108820.9	191255	174969.9	129597.6	152884.8	158760.5	139617.3	168624	197703.2	128018.9	93466.05	74505.8	73548.29	83720.53
P10247	194247	202426.3	199143.8	126408.3	63544.4	192965.5	44557.4	156231.5	141609.1	128916	117976.2	154136.7	231543.1	210108.1	209979.5	179273.3
P10252	1092741	2236259	1474629	1647659	1094079	1589776	1629699	1481705	1481594	1038471	933824.5	1179449	1358143	1462294	1372760	1733699
P10536	7304.439	11079.47	10096.99	8657.921	7326.242	9939.934	12143.24	5766.894	21856.89	24884.33	50670.94	18561.98	15611.36	10670.47	10859.87	20111.64
P10715	92451.62	26686.54	20176.09	12076.74	20031.62	21029.67	76832.41	63178.31	20412.93	4691.187	33654.78	42942.09	4467.449	2203.626	17172.74	18997.52
P10719	26383.59	36729.07	49388.68	34251.94	34806.2	44677.78	27312.46	42438.34	17708.26	39887.96	64147.61	29012.02	33236.03	45772.65	25285.94	6663.245
P10758	770528.2	292734.3	491526.5	1095392	276053.2	262055.3	253362.9	576011.6	807949.8	607563.8	557053.7	1309187	721268.8	692995.1	941021.9	101624.5
P10760	52315.71	56103.78	54726.65	40703.56	49980.25	52349.72	49366.19	28082.15	69265.84	50534.66	76387.47	41910.11	48765.8	54105.56	63115.87	73724.47
P10959	2643016	3585512	3526762	3125147	2017043	1842861	1836787	3091977	1104752	1943883	1410164	1054248	1712999	1278793	1547136	1105716
P10960	5115500	7914533	8843627	6414152	9225224	10636343	8840829	9155366	5990423	5242646	5155260	4867788	9307806	15366065	7041872	8763402
P11030	96722.99	54426.12	65300.58	65958.23	86311.94	80013.82	51719.44	54741.45	100251.2	137782.6	86938.12	93045.72	65600.88	113957.1	107989.9	93817.38
P11232	799124.4	1177598	742360.4	1468079	556993.9	1320546	1115911	938075.6	616207.4	922644.8	944477.3	669341.8	532929.8	538722.8	519018.9	415109.7
P11348	83947.95	89146.2	81945.1	125904.9	149507.2	121929.9	72803.55	80359.94	124791.2	141134.8	125135	73049.3	88452.78	86055.42	114163.2	119203.3
P11442	4511.4	9624.853	11179.2	9493.665	8005.667	5364.706	7241.416	6178.608	8101.209	11292.28	15361.77	5681.7	7338.044	6976.184	6787.164	9031.133
P11598	41153.3	29427.64	18030.79	56201.85	4919.222	60149.97	89870.02	116851.3	19820.46	26758.84	28410.73	39007.4	43188.34	35275.88	26790	15782.42
P11762	11470.35	21960.46	20369.21	17502.8	18709.95	11560.36	33265.16	10884.17	9013.265	9739.986	8049.054	15013.4	7715.152	14290.17	14785.98	3906.16
P11883	5804.301	33897.65	35485.17	17329.08	18234.62	38408.58	17387.23	20950.68	14484.04	19485.13	25034.4	17700.8	11575.08	12328.61	2010.689	12229.62
P11980	43006.09	68856.26	47112.47	65891.77	38718.79	44783.11	52231.84	46986.84	53828.59	61472.21	96882.5	64133.33	40784.63	65253.51	54493.14	54728.83
P12020	1217074	923416.4	312474.3	868568.3	228623.7	2135782	1914867	888320.6	49374.11	1059412	122003	272409.6	1230455	1128924	37674.66	58534.81
P12346	16986736	10648107	17657112	9376882	8024298	9740147	11626362	16137366	7177070	10315179	8383710	7847454	6951424	7855057	8852608	7048708
P12368	38915.83	15888.39	15883.02	10435.93	18189.75	20607.87	23306.83	9893.232	21651.01	24027.89	62542.08	35129.32	15931.91	11229.61	14134.16	27939.93
P12785	2256.439	1959.182	5806.881	5770.31	1802.759	1722.179	1769.491	844.3473	3423.823	3880.968	16257.49	1074.04	2924.111	1927.701	1511.001	2133.207
P13221	88479.23	81746.77	85825.59	61465.64	130048	68090.36	59771.24	91294.34	105834.6	158531.1	94081.17	60368.38	75099.11	84401.53	117424.3	87979.02
P13265	75582.77	70952.62	64328.63	79995.62	84964.04	17077.26	36593.34	62748.5	16382.5	30158.78	41097.44	49559.97	56719.68	50561.18	34421.09	33562.55
P13432	1011349	153614.1	114064.6	322703.3	183214.2	1047901	435540.6	418470.5	199617.7	18377.07	187728.4	1660698	131240.5	156910	87232.03	115560.6
P13596	110810.8	156108.9	170957.3	156514.1	93514.64	128468.6	170827.9	14649.99	160812.3	97115.16	141092.3	95835.17	165387.7	168135	192189	164806.4
P13635	801305.8	802813.8	901592.1	752658.5	490431.9	642941.6	563976.8	853469.9	488036.1	587562.1	581077.5	600414.8	587312.3	572963.7	637634.2	512357.5
P13852	83795.48	111750.1	114178.7	280207.2	96597.35	104958.3	171459.9	79054.9	83390.27	57846.66	99574.67	73378.04	116789.3	103845.3	108252.9	95750.95
P14046	1453302	2592194	2565435	1559971	1214855	1900188	1377607	3487950	2056263	2305762	1568448	1030763	1667825	1676787	2142422	2055373
P14173	5715.248	15036.64	9607.602	8562.819	5742.44	8627.128	3240.393	4159.41	10551.88	21216.27	8006.434	5641.232	5827.536	5043.497	3808.302	5466.64
P14408	18917.98	17605.09	6700.781	55298.87	26638.75	8451.828	19441.27	7206.573	8670.776	11476.17	25536.75	12634.4	12854.03	10010.93	17440.45	21297.75
P14480	26111.61	61222.74	16515.79	54391.59	8179.346	43532.47	32914.66	13553.1	16825.6	30787.46	17377.98	36190.77	32581.76	25424.7	9447.506	47850.47
P14562	170416.1	117401.7	76107.91	168919.1	290359.6	215467.1	166107.6	245948.8	97931.08	125945.7	176049.1	90439.29	125794.7	233214.3	96413.92	105574.7
P14630	728347.3	617239.8	1851458	693108.4	1187434	662134.8	1046721	900005.8	861519.6	739262.4	747923.1	942067.9	1291932	1591657	1552691	1558615
P14668	28911.2	33608.06	55159.59	124676.3	101046.9	94019.59	26209.86	108747.1	36077.44	25387.16	102169.5	25979.63	10889.91	33976.29	34940.46	65680.86
P14669	34341.78	35815.85	22617.19	54403.07	31138.36	70624.73	40393.45	107924.2	13551.92	20542.37	117291.6	17005.56	12083.61	14501	16859.82	2464.1
P14740	1546820	2130879	1759187	3027177	2470637	1943989	2574227	1633843	3420167	2737787	2792222	2526245	2600531	3222745	3651581	3710571
P14841	9211234	13686527	9772049	12829807	12517460	10585513	11760517	10617841	6979768	6678591	7337498	7963355	7027660	8108238	7523050	6464805
P14925	198427.8	147815.5	227439.4	180290.5	204753.3	143392.4	141449.1	174252.9	110279.4	103051	128382.7	149026	124322.6	151782.8	147369.6	140333.3
P14942	89445.27	67569.96	83791.63	61160.99	135883.4	48029.21	43556.37	52925.37	141570	163550.6	120451.4	74343.34	91593.45	75395.16	117420.3	122022.8
P15083	5190122	2881894	3624862	4807259	4585853	7071715	4543290	7949768	2625929	2755533	3645315	4764668	3577803	3627864	2406921	1745466
P15087	10814.35	12514.27	10481.43	27498.9	2021.529	9689.956	8462.458	12826.17	11865.11	11311.05	8124.217	8200.238	11321.1	11789.46	13223.48	14417.98
P15399	58310136	18863514	10114836	16576696	3039642	34141080	21204404	4313771	3533840	8381482	16480074	5777281	2954581	6776227	1232697	1646074
P15473	151061.2	124087.5	60172.82	98761.76	123775.7	81533.75	141023.9	126464.6	150856.7	72885.51	117985.4	160058	117836.3	114341.3	99207.3	121526
P15684	3551616	3834305	2764391	4019504	3703954	3359882	5275172	3478342	4032915	4022464	5230961	4117463	3259374	3002939	3044068	4474864
P15800	6346.112	8217.502	3064.972	9658.524	8001.691	9541.516	7162.991	8630.513	4692.311	4932.156	7575.167	9100.559	6405.424	5243.624	3992.967	3648.952
P15943	25283.52	40335.95	31269.11	51201.93	36843.68	47369.56	37353.7	39141.33	25958.04	25921.82	29181.98	41724.04	37418.04	37634.63	34264.27	34204.8

P15978	1089081	1174054	954347.8	1381931	660448.4	341696.3	1227437	1350972	754952.2	865120.9	881295.5	1235772	311163.1	287853.2	978183.2	1009303
P15999	44891.64	30686.82	20827.28	8584.965	2432.999	28406.24	18457.83	56764.72	14112.06	28590.63	16894.99	26481.92	55209.41	29063.06	61741.98	14737.58
P16086	20214.34	28766.93	42764.63	15044.15	3605.028	38958.25	64245.2	15139.37	2795.367	3555.005	9246.829	1806.625	6723.388	9795.271	2369.34	4323.937
P16228	58447.94	79687.23	57001.01	104500.8	48563.55	66507.55	79781.93	100901	43154.66	52276.73	35734.62	69572.91	28136.96	36620.57	38045.57	22925.96
P16296	14923.27	24100.64	19414.15	18593.01	14467.98	12683.92	24517.57	25832.91	12913.29	16676.05	10803.3	9485.949	9357.491	8360.64	10729.11	9459.683
P16310	29149.37	52630.68	64376.08	45114.78	51323.71	42419.69	43584.81	37323.33	25477.59	31490.15	40359.21	35197.71	59318.98	44256.26	33324.8	47405.65
P16391	776956.3	925785.9	818778.4	860836.5	1112247	315114	1084731	1494169	467407.9	1141965	946712.6	892413.6	166882.1	196339.5	203119.2	601091.7
P16573	2964964	2295139	9964660	4788885	9461499	5198500	8310607	9747273	6834555	12588412	9881937	4921536	8117883	11298510	9138708	5075012
P16617	38039.97	31525.41	62094.55	26022.94	38938.75	27801.11	48154.31	40394.26	65293.99	52558.72	67332.34	44718.44	36036.48	41099.19	49870.34	41265.03
P16636	28945.75	41961.46	7999.323	20375	61437.31	20616.47	129364.7	21594.62	44744.6	28497.47	32905.17	20075.06	43120.39	84296.28	105147.2	47404.73
P17046	273851.7	53542.54	250284.2	26521.15	269532.7	129234	217088.5	35439.46	39093.41	44970.38	197968.4	200928.8	153696.3	67652.2	73560.99	226951.8
P17164	280041.7	495586.2	403048.3	391271.3	366115.3	360186.2	305743	312824.3	284356.7	348752.4	327120.5	436029.9	369765	664031.1	409807.9	1127148
P17475	12506106	10196152	11464025	8385092	3737992	6431226	5327787	9565979	3950666	5255916	4777062	5104728	4632378	4452780	5538054	3816816
P17559	42812.06	274742.4	87164.43	160639.2	129078.5	175845.3	878050.8	56373.6	27631.23	129119.6	62483.58	168860	55937.47	40778.94	61517.86	50635.71
P18292	4430161	7148439	10650456	6238562	5411281	5734609	5927609	8287560	5663017	14199290	6335177	6025224	5927945	6197947	8915691	12464038
P18297	5852.814	13178.07	7868.468	12465.97	12493.09	10986.83	13469.8	9013.617	13555.09	8855.127	18899.22	9279.284	9993.028	8793.616	14311.96	13576.49
P18418	228530.1	94190.7	56999.61	266804.7	80314.29	110807.1	183808.6	115654.2	27807.71	60373.68	32216	40709.02	25898.88	34950.77	23779.08	17486.81
P18427	10356.08	39713.23	7973.339	28280.54	5511.157	29145.57	22413.71	24359.72	20264.72	7534.305	18696.81	34162.64	33120.52	17176.52	24493.94	32551.5
P18484	13229.27	20060.17	19950.17	14172.11	12276.57	8505.044	9016.458	20099.03	11960.02	10837.52	17988.55	16408.74	12460.84	13482.16	16741.54	18699.27
P18757	66495.88	67296.59	60200.38	70387.52	82765.96	50032.05	66748.36	42508.5	125829.9	92075.4	179688.1	74563.5	67654.31	70214.99	91130.69	135322.6
P19112	75463	91149.73	117877.8	89525.82	115230.4	78544.58	72049.79	66410.42	141216.6	191800.4	187095.4	59075.92	68429.45	84659.65	101219.9	133428.1
P19132	7024.496	7114.869	8329.18	7063.329	9367.877	7097.77	4326.374	10270.83	3934.667	4697.804	3649.061	6139.65	2510.524	6635.374	3706.7	2434.231
P19218	41232.34	132908.6	119922	126866.3	45406.74	40072.73	41940.3	100123.9	67257.98	65788.03	244857.4	36682.35	35119.2	172917.3	82696.74	56139.77
P19223	20741.5	34766.05	16155.72	45838.43	6749.634	13882.39	24003.6	25703.17	13753.52	11668.06	8172.882	30446.3	3605.594	3496.366	4464.77	3473.463
P19468	432358.9	516640.4	392738.1	542240.6	866926.1	424020.5	673428.9	412130.8	947514.6	914326.9	1539073	523771.1	580382.1	478171.9	726628.9	971584.4
P19629	204502.8	60203.4	33162.56	12220.5	23065.01	101822.4	173578.7	120046.9	16950.57	8664.848	34422.61	45255.63	15415.14	65818.63	37958.83	17246.2
P19804	287293.7	144828.7	186836.7	140796.5	208109.8	242101.1	199034.1	182527	219368.8	335157.4	183081.5	128801.5	197106.5	164786.3	178712	152875.2
P19814	44051.84	37269.52	24212.12	30221.89	48746.63	18178.29	37072.41	36524.84	9005.4	19181.96	25031	18683.53	17628.59	23938.37	19227.61	17067.48
P19939	246607.3	309667.7	300797.2	405477.2	411681.8	140326.8	128880.5	201407.3	322808.9	190670.6	316792.1	413175.5	408117.7	602234.3	391876.8	442457.8
P20059	22936820	25648398	22291050	41860568	3542152	24636776	18290156	26004100	18989676	16633185	18938708	22502444	15850968	17950716	23123360	15840240
P20171	5357.66	8393.375	12087.83	9147.599	6167.025	11149.07	12958.49	9498.55	19517.74	15996.58	24570.81	17898.22	12750.63	12436.09	9468.216	9684.521
P20611	2237803	3025856	2677507	2542038	3780333	1561941	2509584	2418350	1830853	1672668	1816667	1686520	2481987	2929853	2291022	3560478
P20646	10847.67	13958.71	11313.92	28607.47	29478.22	25975.11	17683.87	16375.3	11339.46	10317.85	10316.43	9912.073	2000.898	3077.645	7307.011	8717.118
P20673	15671.17	16443.15	17379.66	13950.48	8456.787	6331.523	8938.592	15753.8	12938.32	26984.52	19574.48	6555.391	14457.95	10742.54	13167.21	8876.628
P20759	169718	536723.3	542833.3	621558.4	965114.8	429794.1	375602.7	287062.8	308283.2	470469.3	343292.4	415490.8	336206.3	416671.2	441829.9	252187.4
P20760	473201.1	1009313	584771.3	2465311	1438062	1490411	934970	725316.8	265630.3	407107.2	319068.4	356207.8	383334.5	413090.2	641916.8	327818.5
P20761	145258.3	345718.6	421338.4	948631	188824	577169.8	366208.5	332558.8	52952.96	60604.67	64828.93	113249.6	68303.17	96498.05	146532.8	44780.19
P20762	1187096	3816268	1261874	2736043	1691749	2452958	1785162	1795911	561509.9	411260.5	952672.1	774927.3	479234.9	1151438	745531.6	183978.3
P20766	10233.82	137373.9	77064.29	43050.17	17008.02	29238.45	42560.78	98285.56	59423.05	30915.33	42070.9	24125.21	129279.3	38407.12	28429.05	24655.54
P20767	20453248	25241364	18297700	56690736	15448808	37149848	20281784	19544512	29937242	24265378	27734794	42232320	23807450	49603708	17391244	45101508
P20786	194394.2	208388.8	275711.8	268087	257829.6	285106.9	242624.7	245230.8	268704.4	174841.8	191230.7	222911.8	345900.1	325557.2	352189.3	175581.6
P20961	26386.09	37965.28	20333.43	24966.38	31970.31	20845.3	12111.1	45409.05	30172.38	23763.98	27863.18	29380.7	30483.5	40371.6	37705.71	30150.18
P21581	7044.135	12651.02	4070.263	8546.887	10901.04	12262.91	22796.05	18578.52	7139.546	8502.559	8318.396	9481.703	12516.81	9912.436	6581.848	10028.7
P21674	5389.892	5561.121	3245.687	7734.456	7621.983	5851.47	3378.62	7670.605	6345.668	4023.025	5991.979	5141.25	3944.594	4080.346	6453.848	8377.394
P21704	48344440	51419316	57218172	39926200	26401744	39644364	30155828	44100724	34583336	34396272	28794426	35066588	32648770	38780300	48217696	26524218
P21743	173269.9	106741	78147.1	67539.34	43878.19	60414.33	71725	110949.9	87085.97	66942.41	51028.19	54796.14	49241.63	100871.6	74834.6	106915.8
P22006	230919.4	63542.8	99461.55	246935.1	36113.08	130384.3	602061.8	157971.1	102297.4	148588.4	244233.8	105587.1	98831.7	147036.8	715515.6	43625.2
P22057	4573264	4396938	5856811	5484556	2169663	3738856	4807240	3633132	3022384	2982548	3799376	4452582	2655282	3021741	2957106	1883258
P22273	16542.81	15618.22	7808.375	18743.71	18830.65	15428.44	24451.39	26394.18	15490.11	17819.39	18526.87	22224.32	14725.21	11820.01	20881.74	22015.35

P22282	61099736	24605238	21244044	79387376	25715366	98475888	83345440	84426568	18389916	15584187	29080660	42730156	43922248	23681766	2697991	2467990
P22283	54539048	18120648	17812272	61316620	23254028	73971480	75933520	83358440	17247060	20372090	31412536	41446796	36599080	20232508	2073634	1949803
P22734	161018	122391.1	159931.2	159142.6	149557.4	85042.87	136791.3	105325.6	180615.1	305953.6	144515.1	149713	164723.8	101816.7	85511.9	118380.9
P22985	7088.889	6900.498	10954.26	10641.7	11142.48	5703.853	6291	6936.999	5769.403	13665.48	10982.97	5734.5	6669.216	6565.287	6246.46	8215.363
P23377	178647.7	178877.1	142867.3	141057.7	152071.8	187370.3	172201.4	168315.3	131034.1	133170.6	152596.5	218532.7	124111.9	117094.1	80641.1	90168.45
P23593	5252.213	15217.15	6781.513	9371.92	5827.083	6449.549	24121.93	16112.41	11177.06	22756.1	34237.16	22705.74	25303.83	48190.09	21543.65	14239.46
P23680	3290062	3736313	4240770	2651066	5586596	2637467	4103111	4100330	3954719	3037673	3031197	4088404	2713224	5197325	4800296	3876619
P23739	23197.04	20229.07	24280.54	15492.95	59869.52	28019.19	14316.43	19769.74	24580.84	28064.7	38745.16	34553.46	5456.673	23188.57	22033.01	20541.78
P23764	348242.6	353417.9	394373.9	333292.3	342631.5	305260.3	347451.5	261967.1	253487.2	252815.8	265609.8	294237.8	178591.8	253041.3	241912.6	195598.9
P23785	682128.7	790637.9	418071.5	264982.8	1033402	557138.6	600808.1	369086.5	687605.8	513575.2	525067.6	631138.8	791036.9	1374256	1371321	1229185
P23928	7826.428	13001.03	13335.83	10091.79	7501.16	12029.96	9111.645	6930.52	26689.7	16843.47	39281.77	13302.34	13476.19	18107.41	23643.16	11531.31
P24090	25888452	33745828	31433334	36552600	41164656	32830378	38501816	63365776	50460940	75006440	37718816	37196312	39930432	35571392	60514524	58628788
P24268	1496320	1394742	1859933	2398781	2049454	2204173	1326819	2670912	874020.3	1203802	1057146	1088176	1152089	2073179	1396321	1022900
P24368	209601.7	203163.7	81089.51	294205.3	89967.78	266710.5	229769.2	294562.8	117275.5	150062.4	233261.6	293772	165411	146308.2	45637.79	65785.44
P24388	63220.35	225321.5	166969.1	232874.8	98962.54	189099.3	31151.03	160058.9	200981.6	182051.5	127580.1	140731.3	261406.2	296673.2	323017.8	192477.3
P24594	200266.1	297081	161148.5	250139.5	115787.2	112069	279775.7	257103.2	232115.6	383162.6	254040.2	160672.6	193176	195699.9	175073.5	196037.2
P25031	130973.3	242276	152065.9	164224	168677.2	187500.1	220497.3	34166.38	140104.5	76419.45	268326	380574.6	217195.2	306160.3	241899.5	85787.21
P25093	112836.5	77333.73	104355.9	97151.22	166552.3	75522.47	87929.95	81248.09	203414	165549.3	208762.4	110828.4	134262.9	120006.6	150712.3	192372.3
P25113	69996.21	88768.69	111458.6	63817.68	101120.9	105828.5	115811.4	109884.7	180857.7	295522.7	234234.6	111187.6	152280.9	153065.2	162225.5	129903.3
P25236	20582.45	47461.26	7483.792	56342.05	816.722	33616.26	9984.134	11604.97	8100.137	6422.294	7134.82	19223.99	23740.98	22724.31	9060.153	8315.93
P25304	21112.1	19480.33	16761.26	43558.09	20567.36	19308.16	18469.87	36304.31	27673.31	6091.383	21869.3	28106.16	33141.88	35232.68	12866.4	25490.62
P26051	2935347	4076117	3724202	2724164	4263821	3161570	3258159	3193749	2890211	2134388	2355684	2270771	3316131	3909089	3499895	2986423
P26342	13781.3	11775.23	12134.54	21002.91	19735.9	10876.06	16335.41	9895.091	9918.377	7302.055	11037.7	9901.76	5156.454	11354.41	8710.807	11694.43
P26453	44567.31	69837.8	47376.86	74717.14	69385.41	46411.57	59381.32	53980.03	46718.47	54197.95	67088.67	37830.5	53298.28	56350.04	54590.04	72572.11
P26644	2118316	1897511	1715274	3406251	1956877	1661848	1600783	2459645	1252894	1262950	1654341	1320050	1318148	1406344	1605137	1310799
P26772	12654.77	20024.97	41048.98	29400.31	18079.04	25588.29	32219.78	28262.3	13155.55	21116.38	20720.55	20410.96	10960.63	22420.7	32614.99	9751.053
P27139	200749.5	192343.6	181781.7	232975.5	248021.4	273970.2	194602.8	272246.8	395343.4	326637	322281.4	357782.3	284761	274093	362111.7	278477.1
P27213	8402.771	7126.385	5419.64	43113.06	4212.746	9009.269	7477.943	10544.94	8376.267	9920.614	8727.86	16322.12	27426.99	16246.67	14400.71	21679.45
P27274	8095926	6301165	8068799	8141571	5864066	6184950	9827252	6889811	4821451	3746232	5551537	4817463	4091975	5502035	6244387	5276553
P27590	59688024	48963504	46821548	82501664	36435812	68055424	22549616	39754564	29088726	20220378	33972496	25078144	29541540	27496686	34992460	26835988
P27605	10600.33	6644.17	10090.89	7398.418	7180.314	8778.073	6870.233	12112.83	25665.35	13835.9	9040.183	10550.4	10967.06	7429.042	11632.21	10776.75
P27791	7365.046	6419.634	5190.02	6857.267	4037.406	5860.24	4318.921	3788.121	1582.327	5499.754	2594.76	4569.599	4293.193	4253.768	3451.262	3669.788
P27867	108915.9	59803.29	99491.08	58170.13	107422.6	73336.97	86247.07	95136.53	129174.9	181894.1	118005.1	72468.12	102690.7	65213.86	100317	96909.48
P28037	15352.27	24961.32	24973.17	19615.85	42713.06	17005.72	21937.43	15419.95	25983.84	30537.82	43778.66	16269.95	20296.18	16453	22091.39	28741.26
P28494	38838.75	36698.44	45475.61	38085.64	66111.95	34663.08	33132	32252.23	34608.07	39881.37	46349.75	41390.26	52061.7	52593.31	54971.84	43277.68
P28648	179990.6	221442	141123.9	199020.5	281279.6	131426.4	145118.5	116980.9	65228.9	123004.7	113292.1	64281.65	57851.45	50807.18	40893.55	43231.83
P28826	1691917	1582222	1810022	2412588	2112520	1377416	2584659	1088692	1942791	1511575	2465048	2500661	1761002	1504931	1861236	3019702
P29288	48714.56	60750.68	63873.84	68620.66	58151.73	35669.84	23497.78	46000.57	24959.8	31646.12	23648.55	32511.47	18360.88	47981.76	24918.13	28765.97
P29534	138425.2	191465.6	114348	169374	116825.4	145969.4	162715	182092.2	110653	105677.4	109178.5	143559.5	99033.22	101713.1	118084.1	118752.6
P29598	1584985	1811612	1855247	2252398	2464844	1701664	1969920	2279100	2186546	3248148	2499432	2348443	2455140	3359775	2773951	2842581
P29975	332920.1	348621.2	328381.9	487859.2	806008.4	341699.5	481281.3	361592.5	858381.7	1410383	1468699	556493.6	470200	572844.8	876645.8	1052272
P30120	174421	91410.14	42977.03	141740.5	70350.59	237409.3	118030.6	157319.5	52771.34	57442.9	108874.1	302883.3	100986.8	73235.35	9548.254	20046.9
P30121	90878.63	115862.9	89960.91	70930.82	103194.1	65313.58	77395.76	67014.68	70828.45	63779.81	45860.74	65981.05	89321.39	91665.27	54110.41	55473.77
P30152	1631165	3112104	1935820	2531771	1503999	2008747	3080611	1567922	1827697	2075554	2169701	2868589	1881595	3854173	3282881	3167144
P30713	66310.6	67781.73	53882.3	70033.23	64264.3	62887.01	43072.16	56565.73	70813.68	88109.2	101273.9	61365.73	76737.01	58469.48	79824.69	64981.96
P30836	26775.09	43413.34	22051.57	47820.16	23406.51	20902.73	44648.18	31276.77	27284.57	15186.37	26021.6	45286.4	21122.29	11062.36	30700.06	11361.45
P30904	580146.9	433641.2	444776.7	170109.1	673993.1	344878.3	395103	209288.2	406754.6	361870.8	435556.2	266333.9	194651.3	218379.5	347447.8	282996.5
P30919	6111.598	36618.81	62354.15	30945.68	51322.47	367478.3	40845.23	27069.69	14196.19	24301.13	27879.34	66209.94	81030.38	83137.12	67279.54	78232.86
P31044	2506607	1200065	692820.4	1037610	681250.8	1345586	2537013	2372037	624213	859203	662735.4	480890.3	644953	630608.6	572902.4	545557

P31211	1117307	2183334	2412921	1367278	2795895	1437331	1289511	1368734	1545437	1811219	1773576	752303.7	1672858	1603659	1223972	1481314
P31430	29320.14	41118.03	26562.46	36095.79	18835.96	14467.28	22097.51	26702.04	10309.96	14911.18	12644.32	85291.7	14973.9	51564.87	11100.59	14104.38
P31977	2658365	1558396	1076113	2145557	2140212	1605749	1855022	1534554	1851046	2161479	3315632	2141617	1899630	1294402	1357682	2171752
P32038	1295331	2371963	1204160	2926373	1084440	1762794	2264089	1907698	1183144	1573115	1742650	906736.3	1379112	1267876	1192384	2115707
P32755	61171.48	31003.93	70119.35	24467.71	101015.1	30467.91	41619.29	38707.13	36721.4	94786.13	42848.88	31690.41	33784.48	34392.11	46495.11	62811.8
P34058	20498.16	21139.51	10558.92	14308.32	14281.16	12000.69	14550.68	21808.95	14190.3	19212.28	25524.39	6193.471	13841.98	11898.33	16559.23	17336.73
P34080	28679.31	47859.71	49119.95	88983.82	48108.52	100348.8	82932.92	51090.02	30505.93	38227.38	67308.14	34336.84	32109.46	87689.2	35150.98	33608.28
P34901	175544	239267.1	277491.1	213399.9	178341.8	63314.48	56092.49	18604.08	52219.31	64594.49	62608.78	90660.53	26387.56	82698.65	47340.81	53816.08
P35053	25658.19	36384.37	29356.45	45993.23	43980.8	19383.43	14322.32	46469.37	12599.12	17347.62	25862.53	47021.79	21097.84	19797.82	26674.18	5690.396
P35213	20723.13	18064.18	15334.98	13049.39	13990.49	18297.32	15116.96	17546.72	16251.67	19511.68	23482.64	24214.88	15164.25	11180.09	8942.238	15369.09
P35280	100543.4	420935.8	1483923	383072.5	9202618	71296.08	99260.39	107239.4	4193441	70429.15	137362.1	2894679	6037825	1709439	41864.33	4766102
P35281	69384.46	18912.06	21155.81	35783.14	19982.23	37919.18	21108.95	53569.94	17573.68	36927.75	26842.01	33287.33	17531.93	11889.38	25006.5	20293.72
P35434	30280.2	17669.29	37790.25	18863.42	14956.47	26885.27	14183.8	17872.87	13027.02	7995.112	19370.74	13670.17	11710.64	26765.69	15396.02	3355.522
P35444	41472.53	88700	46117.16	86999.86	45284.28	26163.33	87369.57	51221.7	73001.82	57486.04	55489.84	88766.05	92418.13	103636	98581.2	73175.09
P35446	20125.19	10381.82	12842.86	13662.59	12360.18	11141.86	16100.13	8048.263	13598.36	15763.64	12162.85	14309.82	14957.49	14122.16	19203.25	7503.489
P35467	1333.963	1164.891	5500.506	2299.673	1708.786	1764.227	1387.378	4538.121	3820.376	3253.489	6662.776	2505.316	2924.111	1458.656	1208.266	1580.872
P35704	42921.39	66671.8	36237.49	64375.15	59925.62	130078.5	73508.73	117373.4	79737.98	102155.5	177194.6	109134.8	68357.52	72020.93	75081.65	52411.3
P35859	44869.83	33393.85	38778.14	34432.03	34986.75	30085.45	20477.65	30929.54	20194.31	15137.62	22357.4	17107.63	16685.41	13635.99	15801	23354.22
P35952	62168.01	35700.34	81045.26	73024.38	94961.95	47924.41	100794.8	40982.23	53683.82	79165.89	76255.23	50475.37	49263.67	61838.05	89242.87	84828.42
P36373	95157528	79467632	45292164	1.06E+08	73206696	89821240	70005592	93608960	50503828	46952084	70762888	84232200	63732380	60547080	45136012	47275140
P36374	6714255	1224224	490983.9	5728554	1677874	2703333	3750625	3322773	2424982	2315878	3721590	6962460	4306320	1560000	214637.7	140492.1
P36376	787660.7	4084337	1661032	2243895	2496577	1680366	1682218	754985.1	1046838	1430618	898808.6	715518.3	1020655	1491614	1349957	950666
P36953	2172063	1946712	2381743	1260496	1084958	889124.3	922799.7	1305099	814532.4	921628.4	1091543	1390358	1200560	1277650	1323380	948626.5
P36970	28457.58	13737.4	8443.805	8808.729	42546.56	80161.23	31327.03	8689.066	55345.89	47089.96	71749.22	34868.63	32003.03	21608.25	49814.93	66550.39
P38438	78760.87	101467.2	261662.6	253745	129929.9	203804.3	136876.4	273461.4	174458.2	183026.5	174820.3	188689.7	178056.5	270486.4	238474.9	234082.1
P38444	4059.074	13847.95	10212.48	17007.65	13228.4	10319.86	8387.44	6899.671	6017.858	12926.41	4307.787	4186.479	8937.644	9786.762	9371.067	7671.594
P38652	19897.61	23600.88	15006.6	14602.6	19080.41	17228.04	19265.64	15228.48	22298.8	26689.47	25808.96	14748.79	17612.18	10740.69	15949.36	18931.22
P38659	70423.37	79445.17	69437.41	84018.11	97813.43	82501.05	87136.13	66270.4	58500.25	50918.04	65606.36	68602.59	79241.3	98899.45	79223.24	71521.69
P38918	197549.2	198366.8	144253.3	356793.8	171364.8	219515.3	174361.2	179222.3	453907.3	352564.1	583224.4	292537.3	384212.8	279629.1	393888	423957.7
P39069	30478.29	18226.97	33215.75	17598.91	14086.56	17424.39	35219.7	17344.06	33559.8	34185.1	24110.93	19021.81	24257.06	25812.39	28614.98	34874.24
P40241	134137.2	59047.14	61380.36	92901.67	44147.03	111641.8	60050.51	77607.7	35994.05	43754.04	63521.98	127097.9	47502.83	53155.64	29239.08	44600.09
P41498	36551.15	41730.96	22606.92	43396.18	39766.09	39209.22	41093.66	36266.54	44081.58	35478.93	46993.49	37766.21	35298.48	28323.14	39475.64	34764.3
P41562	144272.6	126555.3	139044.1	95854.37	199594.4	123084.5	105189.4	136318.7	179103.9	262084.4	199851.2	135967.4	196243.2	178572.8	158856.4	217546
P42123	438361.3	284768	331436.9	286742.8	422157.4	333227.2	284124.1	272340.7	348644.1	837285.9	384354.6	246946.8	337161.4	246923	384862.7	344550.2
P42854	16577253	23317742	19066640	24313612	9456349	10724493	27542412	11558305	25675094	19547254	34179692	32061872	25661314	20634914	38511388	9162181
P43303	246300.3	459647.4	278582.3	503865.5	373319.5	337867.7	344294.9	574581.5	216610.3	322425	240766.3	225228.1	245264.1	362458.3	306106.8	293292.2
P43427	58451.62	34148.48	32814.33	75895.29	46214.71	41461.88	64305.2	33419.63	50850.98	75561.3	100750.1	52881.73	41673.7	46566.77	52045.77	75363.97
P45479	89862.22	74683.44	119990.6	83501.25	91378.31	54198.18	69710.02	92220.96	40130.68	43946.11	28880.08	50110.44	55432.18	76036.01	39912.85	44138
P45592	30389.68	23845.54	22918.61	21932.33	20207.63	28233.22	25360.07	10785.38	31115.04	22396.87	49854.84	37106.27	30044.22	18786.12	19681.03	34193.42
P46413	191586.3	238252.4	192736.5	181115.3	340723.3	162820.7	232381.8	182444.1	484053.1	315867.2	608112.4	144548.4	205503.2	206033.3	244978.9	271320.3
P46462	117407.2	51747.46	47135.73	107768.8	77329.2	151639.5	132418.6	122664.4	55821.78	54842.14	120754.3	112375.5	82667.46	73855.36	26354.33	26624.19
P46720	115779.5	154655.5	79469.59	196847.4	253939.3	97951.98	217991.1	90826.38	185948	239234.5	290611.6	91248.73	95939.2	88926.94	197127.9	194764
P46844	13545.63	16355.89	15149.11	9955.854	22161.92	23487.97	15579.11	12724.21	33205.79	17922.79	75343.85	26937.77	24984.5	31434.73	29340.59	32969.01
P46953	104145.1	123011.1	85684.68	143133.9	138781.5	107775.6	78526.63	68440.61	229598.3	151286.1	238454.3	115971.3	183770.4	127719.1	154380	201318.8
P47727	56875.09	29021.64	10749.14	31307.69	13963.53	82418.67	38440.47	53156.29	16063.92	52354.87	115660.8	54446.78	56143.18	73519.69	6789.557	6981.916
P47820	122842.2	129857.1	147014.5	98770.89	133911.9	105497.2	141759.1	153238.3	42304.37	64183.48	75445.67	68859.42	80639.41	105011.5	86546.75	85890.43
P47853	384933	475805.4	410613	242833.9	653351.4	244107.4	293216.9	405537.2	230441.5	241944.2	504620	302459.5	249269.9	326007.2	215335.4	437771.7
P47967	1929349	2427118	1397621	1979886	32843.5	2132716	3152978	1263749	1583839	3283715	1783764	3022284	1734115	418484.2	414614.1	2903089
P48032	10780.15	14934.75	13837.58	23093.26	23954.39	12019.63	14954.17	9645.983	13481.37	9420.088	8905.385	9277.36	10371.75	15846.87	10237.63	9314.858

P48037	21802.5	17086.35	14109.19	25394.33	26897.92	42526.57	15495.29	33947.93	30167.32	28523.97	34265.24	47418.37	23297.75	32635.39	18729.18	55701.44
P48199	1831281	1622181	2841666	1625499	843828.8	1397184	1065410	1570353	1761133	1211033	1433467	1481772	3179904	2811174	2675857	1934981
P48284	28516.56	31212.11	24569.3	26657.15	24654.11	24368.74	27019.46	24446.22	22863.8	21888.04	25376.49	28413.76	26833.32	25146.59	26340.32	32996.65
P48500	203586.2	172972.4	277844	201391.6	261840.3	313541.4	231002.9	310380.5	294187.8	313308.6	279411.5	178610.2	211325.3	190389.3	299666.4	252550.7
P48508	167710.2	184398	163310.5	174330.5	345727.1	196915.2	204537.2	156407.3	416865.8	327426.4	589804.1	242342.2	261933.3	211996.6	266328.3	346444.4
P49002	37836.4	39275.69	33234.22	36118.54	35032.56	31888.39	22295.72	34577.25	17961.13	14407.57	20121.26	35725.61	27904.88	38309.52	11873.56	11702.44
P49134	41320.86	61107.23	44160.69	38072.17	37644.39	35792.28	40568.41	37313	22538.9	23395.31	26202.65	21412.79	22418.17	28837.27	32071.38	40263.42
P49744	8652.523	8641.966	16333.75	15637.36	22088.89	10325.17	11175.83	9967.594	6090.97	17079.14	8536.922	24313.07	15170.95	7682.549	7162.32	10023.25
P50115	8343.233	53247.2	41711.45	31641.51	59532.63	54823.4	76002.76	140904.6	15860.89	5629.344	28558.45	26687.96	4614.298	5538.532	4093.592	3793.746
P50116	24969.66	112965.3	70428.23	64644.57	172533.4	73278.45	140033.4	238706.1	23686.8	8970.117	51096.45	40933.75	4794.82	19557.05	3780.884	778802.9
P50123	1697824	1143839	1675749	1195410	3376386	1239013	1076095	1048888	1027703	1055679	1128635	1019386	1064443	2160241	1364345	1664669
P50137	12136.19	15330.45	15914.18	193789	24718.81	15779.26	16619.32	20826.86	15197.35	31246.94	24532.65	7945.773	9336.791	10665.22	14486.15	10085.13
P50280	25926.38	37926.8	11581.56	36168.13	13350.08	59471.66	21880.39	29938.71	7638.614	12750.59	18991.68	19205.24	16313.56	15203.55	13131.57	7031.299
P50398	62487.64	48435.58	36078.1	42688.48	79425.92	40017.14	51844.21	46884.83	33044.48	37462.58	39982.66	33162.78	25944.76	27239.71	22606.37	32877.29
P50399	152016.8	105542	94113.04	96866.05	173477.2	131523.2	88596.44	130807.5	137285.1	159196	159897.1	119304.5	96531.03	89931.17	101915.2	125471.3
P50430	449279.4	572159.5	604129.1	562761.4	784623.6	379019.9	473033.9	444280.3	294609.4	409239.9	425521.9	512580.5	382981.7	472639.7	672515.5	419322.1
P50503	26447.82	17884.75	11754.6	12920.99	13660.36	15602.81	26830.74	13464.92	18137.7	23749.58	24155.07	18472.47	10283.98	11873.15	11796.22	18540.7
P50609	21170.7	50543.68	44420.38	29727.09	45284.87	25787.15	49792.75	27612.3	35358.27	28779.45	50288.35	48151.84	46769.36	55200.91	61554.93	38256.22
P51635	936921.3	449108.8	886867.7	409871.8	946144.1	638272.9	425103.6	605117.1	1161869	1797645	969738	650912.9	937603	532794.5	860459.4	836919.2
P51647	19571.54	31861.61	38622.85	20660.04	29480.74	40849.11	31231.03	36465.94	21279.65	38501.94	27512.04	20960.38	29402.31	37469.32	27523.19	25341.45
P51886	50450.64	105184.3	33670.41	72178.16	54368.62	44944.53	61799.84	47785.82	53245.14	46020.5	30024.33	43794.58	34020.1	37329.73	34958.75	33476.96
P51907	113169.8	164921.7	16594.1	264331.3	120528.7	20110.25	138726.9	20013.64	136038.6	185735.3	347308.3	221801.9	16480.71	125787.2	255942.4	163444.5
P52303	4700.319	4288.724	3458.517	6140.985	3861.768	3294.417	2417.136	4110.017	1628.808	3343.115	4862.346	2466.331	2954.598	2064.877	3914.405	1262.964
P52590	8231301	7723512	3688049	3913818	10784968	2092918	9593842	2347956	3922640	2576697	713432.8	727126.9	162535.3	331130	851642.3	32326.96
P52759	1065041	720717	1213228	1141761	587262.3	960879.1	681199	852370.1	2476478	2154118	1602335	1048560	1519338	1177344	1859226	1809671
P52796	42040.83	86460.08	57890.28	74652.12	57528.5	54396.04	53589.54	53302.49	35826.87	45448.32	50440.8	52188.56	51536.4	55647.88	51620.59	40666.2
P52847	23144.01	19696.37	17854.84	18158.22	35234.83	14712.91	16127.02	16370.57	36360.21	27087.84	43558.65	17940.18	22568.43	7393.914	14520.57	38126.94
P53369	122318.7	144883.8	181346.1	98884.39	118478.1	107376.2	155877.2	119037.4	83501.11	84261.97	88463.59	95191.13	104119.1	124874.7	93412.8	105325.9
P53790	52615.55	48168.27	37218.93	50845.18	68679.63	38058.64	49536.09	26549.48	76659.66	106907	118305.9	62915.05	23460.79	39430.05	59824.38	113640.6
P53792	9484.445	17333.34	14366.02	40720.91	6774.046	8568.754	8617.743	16345.26	7658.846	43497.15	31615.23	25142.03	8689.246	12841.78	13403.07	23337.98
P53813	146507	125423	73451.77	89882.41	134519	92114.18	131349.8	123747.9	88461.25	100890.9	114819.8	122014.8	126264.5	116253.3	104353.8	103402.2
P54311	78889.23	52632.34	41070.24	68112.35	14039.75	83453.66	114244.4	102886.7	64165.58	49363.17	93325.97	83091.02	54625	51351.14	46702.32	46268.87
P54313	131533	99353.84	84709.73	100466.7	152241.8	133904.3	123553.4	106529	136275.9	128171.4	182101.4	137077.9	128049.5	98483.99	108363.8	135432.9
P54921	9221.44	2632.664	9297.619	7665.38	8398.226	8428.117	7828.243	9167.069	11536.41	8689.612	17543.11	10222.78	7174.966	4551.905	9093.709	5654.68
P55016	10170.37	25836.48	43507.58	42081.02	63681.25	30403.64	40712.55	38571.79	46486.34	35569.07	53431.66	20928.19	28614.79	46303.09	14672.3	60703.53
P55018	20871.83	14095.43	8488.233	18249.65	41046.98	16446.7	13878.25	16078.57	7830.209	36862.78	11746.54	8505.662	8506.599	13055.73	12885.54	13644.67
P55053	24592.21	22816.73	21252.59	22907.23	17110.64	25956.63	47256.4	10367.41	16552	9731.547	29721.78	18370.98	15269.15	13983.83	7815.093	18978.79
P55091	41128.5	99262.09	25108.67	50877.12	24739.51	32201.15	70961.61	55378.32	6740.143	8010	10819.32	66873.34	20093.28	11487.18	6951.347	2533.569
P55146	131695.6	114804.8	103110.5	112247.1	174633.5	75920.36	112507	125467.9	73159.55	59030.09	70853.03	94278.16	85578.86	126504.7	75964.73	93291.83
P55159	27951.67	26281.77	36333.07	16579.35	15239.57	12744.74	16661.02	13179.87	10513.29	14669.35	17160.53	9006.053	16359.38	5222.488	10242.81	18712.98
P55260	29921.91	40285	27901.67	59362.77	48177.55	58319.34	30720.44	42111.84	49654.83	48107.79	86177.11	44787.85	38973.19	35899.13	39034.89	50075.17
P55281	15528.73	17367.27	12486.1	10948.06	12253.49	10180.46	18655.9	12173.46	8823.777	11981.11	8104.271	39956.06	11796.82	15516.63	14623.42	13905.2
P55314	1419883	302786.8	648356.9	187708.8	273574.6	237449.7	79897.44	11994.8	3431.635	120890.1	865271.1	11162.99	3944.625	5521.899	8055.146	198109.6
P57097	84165.8	83556.31	101395.4	81815.61	101765.4	61962.66	69634.78	88467.03	84822.32	58587.2	77041.25	71534.44	87829.1	116616.4	84988.95	108053.8
P57113	5256.667	3926.97	5783.652	7688.268	3222.885	5626.49	4016.33	5308.202	12346.55	6865.386	9234.28	6553.303	6422.419	3960.288	6314.577	5667.684
P58195	16901.14	18586.08	14068.15	24114.65	19989.59	11151.56	17869.68	13849.82	6733.251	16252.08	16702.23	12752.24	11679.36	20472.48	13918.27	20836.81
P60571	360474.2	337849.6	453601.7	557721.6	405491.3	255980.3	312079.9	378652.1	138797.2	369211.8	338803.3	199588.6	286058.7	303808.1	164762.6	417319.5
P60711;P6	1483982	1583041	1432338	1539796	2944925	1411589	2147291	1355200	2120760	2566204	3015846	1909801	1037409	1588163	1288179	2286206
P60905	12677.73	17413.73	3567.15	17831.99	13513.33	37103.57	23454.99	29129.8	13551.73	21723.86	22867.33	34728.16	13202.9	10475.77	6423.872	9974.31

P61107	14035.69	22816.58	27143.83	12342.83	19634.31	16035.23	16914.61	11711.86	12757.62	10539.82	15332.5	16879.54	13194.81	12455.37	11231.27	13829.39
P61206;P8	76984.14	57199.78	39987.44	117917.5	109813.6	91098.55	152402	37950.48	30661.79	60216.01	82313.59	33262.58	22838.01	22215.69	12652.29	51601.05
P61459	116794.9	91637.14	105528.5	85551.16	97588.12	84130.36	91303.16	113104	117741.1	212037.2	126982	79263.31	131705.1	122150	117394.5	163058
P61589	94963.92	68602.49	76614.84	75426.66	122663.1	132260	87647.77	84638.02	151329.7	103788.9	155367.9	118473.5	97213.48	110278.6	112085.8	65738.74
P61943	33296.99	38160.07	27225.67	26184.8	47621.77	35927.53	30934.01	40777.86	28601.59	30700.18	40016.23	31759.95	19181.82	25237.29	31436.5	35260.43
P61972	330995.6	504447.1	452260.2	425390.5	450593.6	398925.3	392303.6	304828.1	612680.4	479665.6	380423.9	596210.2	664958.3	1269564	701645.3	812156.2
P61983	36227.9	68016.74	40787.02	66584.98	81624.86	68916.35	28044.95	81652.25	41525.31	50788.02	62429.13	47342.84	37492.18	33844.36	32091.66	38236.58
P62260	119719.1	68851.24	66137.05	111116.3	117685.8	110855	105735.3	106663.5	87318.07	110916.9	109196.9	88430.77	71334.43	62496.05	48232.2	67593.59
P62630	239561.2	209044.7	141623.2	213254.3	187643.8	254386.1	261078	160647.3	209896.4	234030.6	266427.2	181568	130759.5	139857.8	140134.3	167684.9
P62804	30949.11	202360.8	39645.35	38350.7	51524.21	74364.95	35776.36	97593.48	29559.69	79561.52	115738.6	110651.2	23618.43	73547.09	41481.13	14553.7
P62815	33347.03	26136.74	26439.34	7156.816	34256.45	10501.79	23751.78	27322.28	56930.67	98974.67	70625	33059.52	35108.38	38553.6	39825.43	55582.86
P62836	61637.52	36165.29	48753.81	73385.03	44474.87	82880.8	83550.22	63515.93	109140.9	110751.4	116383.7	81618.43	56465.04	58736.78	67697.27	57916.63
P62898	91341.79	86922.37	96554.8	119618.1	135242.2	159687.7	129545.8	208652.9	54961.13	87283.86	61401.74	69162.47	51714.54	65514.92	57239.45	41769.23
P62959	65872.88	49157.71	50456.67	89303.66	57225.98	49111.1	71072.58	37566.03	84653.08	93771.7	100680.3	53117.25	49679.37	33938.84	55378.16	53166.27
P62963	90216.58	81533.43	54883.75	70509.25	68841.28	67441.38	72067.67	66211.3	81298.35	75111.79	101423.2	54644.2	56743.82	46682.88	38096.02	51428
P63018	401040.1	388550.4	327283.3	454966.9	410938.8	497035.6	417072.1	496395.6	408906.9	498747.1	484609.3	368302.8	356827.8	321925.5	310308.8	349209.3
P63029	12417.9	10534.23	16587.91	14450.87	20299.03	17107.03	9913.306	6602.659	25089.54	31538.79	48670.35	12807.6	13583.25	22242.46	24347.9	12138.13
P63036	2483.047	11420.29	7759.083	4563.91	4075.591	2728.048	2663.578	3011.758	1115.557	3498.567	5080.365	3784.421	3158.935	4274.58	5419.604	3266.43
P63095	35874.78	19233.57	13278.42	34481.33	26568.2	42740.98	56624.79	44309.91	48274.7	59985.8	69908.44	31538.76	26770.51	28719.79	25739.31	48476.41
P63102	210925.5	163853.5	122949.1	163147.9	185273.9	165094.5	172112.6	179373.7	184038.5	133678.9	342692	182848.3	172019.7	133718.4	118834.1	133862
P63322	47248.92	18646.58	22027.1	30545.26	49987.8	63215.42	58149.88	51995.92	66866.27	55221.9	69182.72	59147.47	35734.51	42330.96	37164.43	31301.67
P63331	24012.25	15858.59	15652.31	21456.63	27949.32	48625.31	23965.15	25920.15	30968.48	29801.91	31236.06	23733.87	14460.43	19616.01	24363.52	25858.14
P68035;P6	3761561	3356862	2504389	3489959	4051129	2750372	3115040	2923252	3685032	4415114	6318573	3551801	3782360	2643623	2744089	3981760
P68255	324772.5	244588.3	204651.9	306933.4	226927.6	244036.6	224761.3	239611.2	255602.1	300522.6	476365.9	282244.8	255792	229523.5	182934.8	183936.3
P68370	259542.9	149467.3	56632.1	80914.74	85118.42	140976.5	170246.8	147202.7	46251.92	70852.09	77300.23	55927.88	55882.35	53837.82	43329.14	42841.74
P68511	6558.682	10184.4	9600.801	17464.24	10695.28	18319.86	6152.182	6193.812	21378.07	8778.304	10132	10746.4	10895.61	11226.79	8002.977	6548.13
P69897	117493.4	67910.09	31590.2	56311.16	34676.15	97412.7	100391.6	97905.81	48004.61	58364.82	65316.82	41881.11	47400.09	50581.3	43823.99	38822.84
P70470	50290.91	50961.85	48373.71	62838.1	55262.44	43725	72516.09	37805.97	48925.88	59254.18	77571.51	45680.65	40414.18	38620.81	27850.38	24764.78
P70490	93768.51	95231.2	88972.26	113478.6	59995.27	149166.1	83323.33	120813.6	68845.86	92850.75	92251.66	71519.24	76835	93224.34	91194.85	77335.73
P70502	182237.1	244835.2	91665.87	271311.7	164755.9	168410.9	272874.8	72149.65	139579.1	191951.5	259960.7	137393	106243.6	140796.6	113246.7	184467.7
P70545	50749.23	8793.444	51013.59	53370.11	55156.41	15995.81	36934.3	32408.45	77429.68	81650.5	97942.02	54561.18	38334.64	75831.32	84223.85	84483.02
P70619	82193.98	53277.88	66939.79	77610.2	27485.46	70510.25	53342.45	76024.77	123861.7	125573.5	137837.5	120774.4	113855.1	76397.31	69278.52	69653.02
P70709	143084	200264.8	57281.8	141731.2	59054.88	166819.1	69126.22	66927.11	78397.31	140709.8	70154.84	189901.8	184145.9	81370.14	98524.18	82380.01
P80020	114942.5	219788.6	54660.85	825359.3	45404.98	80835.77	58605.27	23532.95	18705.3	27183.94	39001.14	116751.1	7967.181	31213.05	46870.18	16218.86
P80067	267017.2	257730.2	275022.9	303379.6	360841.8	217481.7	278958.4	281184.7	174633.9	332064.6	210827.2	132606.5	259295.8	289068.4	255438.9	336220.8
P80201	108255.2	178312.2	271709.8	115092.1	302070.8	137937.6	298304	111881	78236.45	89618.3	169531.8	168120.3	246982.2	234066.3	91070.45	205421.7
P80202	345104.8	501017.2	468793.2	363168.9	369684.3	430173.9	479614	330057	406031.8	250577.6	365961.7	356929.5	455862.3	815619.3	499732.8	603385.2
P80204	76788.02	95738.91	30299.64	77746.3	65846.89	36734.02	99553.38	66092.86	89098.3	34888.42	38104.05	83450.77	67528.8	127837.4	44416.53	46522.74
P80254	90398.69	80011.69	140654.1	116924	140185.9	75281.13	89824.89	164381.5	242358.3	439196.6	176544.2	98925.99	122995.3	127972.5	224493.1	132784.4
P81827	1.19E+08	1.88E+08	1.67E+08	1.67E+08	1.3E+08	1.56E+08	2.1E+08	2.49E+08	1.66E+08	1.12E+08	2.96E+08	2.13E+08	2.31E+08	2.93E+08	3.08E+08	2.33E+08
P81828	1.1E+08	1.55E+08	1.04E+08	1.78E+08	71199648	1.49E+08	84113864	1.01E+08	90976768	1.01E+08	82923328	1.35E+08	1.19E+08	1.35E+08	12636776	1.08E+08
P82450	314878.4	461293.9	417758.2	413262.8	730914.6	380506.3	243216.3	389454.7	447847.3	551035.8	329438.9	357521.9	518088	605517	453280.7	616512.5
P82471	5467.695	13083.74	11140.14	17118.8	20352.29	23321.01	24569.13	27722.84	7473.613	23059.98	13552.66	9908.858	4893.18	9034.799	7575.991	11764.87
P82995	44700.92	84712.45	16697.96	63574.55	45123.58	17583.02	166865.2	67243.23	88410.86	75849.98	92125.19	58291.09	21054.59	90437.12	51561.26	76389.34
P83121	85134240	93949352	94749560	13135651	81216952	5697866	15285030	77360424	7468095	58381784	5096446	86713936	66064252	13865606	86450480	79096592
P83748	33766.16	74319.59	15881.12	47777.48	71022.71	77763.41	89112.69	22373.12	15945.03	17761.85	4652.434	40860.48	20805.34	16962.35	27132.77	22128.37
P84039	29358.49	73720.29	41681.53	81756.09	29508.09	142751.8	64597.1	59482.94	63554.66	42567.73	46763.96	43916.84	65555.7	59523.27	98816.86	34906.17
P85968	46153.34	45014.97	33910.43	37819.14	55815.42	52296.83	37339.99	35602.78	47366.69	59370.05	59746.87	33404.21	33308.76	32782.32	36734.68	43086.2
P85971	1261619	1577132	2555987	1084897	581937.8	859247	1325541	812886.6	1196075	586372.8	989149.4	1036430	634121	1491811	1174775	1509838

P85973	33375.76	36356.05	25189.06	65224.45	35872.27	29492.48	33081.46	40154.8	37858.61	32695.32	41353.03	36597.73	27586.91	29171.62	41011.07	39735.8
P97532	20567.16	17825.37	20087.21	18021.29	10148.83	11690.32	34243.57	12254.3	18417.18	18058.04	16685.45	16589.16	7357.386	7332.264	14023.16	6364.938
P97546	1139913	1222952	1315669	1111930	1330704	982655	1397496	1145300	851710.9	676533.9	621275.5	688776.3	888898.7	1140915	854613.9	1102333
P97553	94915.41	223845.9	147947.6	38869.35	47575.48	106332.8	78111.48	98014.73	93026.74	97757.78	95095.89	75434.72	101310	138230.4	151837.6	102139.8
P97554	24404.38	18489.42	15545.49	12651.19	17912.78	14006.3	22140.47	21107.27	18144.34	11080.08	10924.9	50316.81	21754.92	4975.155	12977.24	17363.44
P97571	14692.61	14286.51	12323.98	15705.38	15044.38	12421.22	29535.08	16878.63	6845.375	12782.04	3942.915	5608.462	5694.121	5677.103	7170.65	5806.893
P97574	228959.7	245563	156620.5	308899.5	111573.6	125896.1	175542.5	300142.5	112497.7	128996.6	109516.5	88120.91	79120.45	320860.5	139514.6	277977.3
P97580	9524796	3116383	1587455	2311527	425388.1	4013923	2681182	397537.7	65367.32	569015.4	1196075	103995.3	14952.2	367895.4	30239.3	114870.5
P97584	9442.501	8284.055	10858.39	7171.899	9059.47	22054.86	3148.844	17208.47	10487.18	13822.65	19714.01	19132.55	14355.08	14730.37	13003.64	17750.7
P97603	109759.4	142204.1	134080.5	217144	90810.87	128456.6	108593.8	138119.3	115330	127701.8	111704.3	109946.9	133105.9	136588.9	119969.7	155155.6
P97605	23933.79	166153.7	121526.3	102708.6	172074.2	88548.82	122749.9	82187.78	95677.36	52370.37	47912.37	52426.74	64706.77	88392.7	81314.02	61665.88
P97608	14706.26	9328.374	10205.07	17441.84	14316.29	5820.447	10606.6	16465.66	14607.3	19115.48	20051.67	12455.2	8313.063	5987.429	13396.19	13946.25
P97615	4619.033	6420.732	11658.82	4371.955	4815.302	5112.812	5916.227	4364.42	1289.44	4834.772	2239.939	2112.61	1823.702	3198.93	3003.565	1790.809
P97675	123710.8	166131	132727.5	112730.4	99216.13	109111.7	109691.7	119892.7	121908.3	95903.59	244481.6	348468.8	94829.01	94064.54	126967.4	107838.5
P97697	56620.41	30245.04	35723.13	39045.76	67933.45	40353.33	48895.87	33698.14	23176	39370.84	27533.58	32812.48	25491.04	21901.11	25171.95	17617.16
P97710	121583.1	97260.96	144760.5	110967.2	123832.3	93205.57	66806.2	116055.2	100216.6	92357.55	113159.6	111524.9	103057.3	134111.1	112994.1	125863.3
P97829	129515.3	115489.6	179224.7	148074.8	153693.4	118586	162039.9	122999.4	127499.6	135199.5	120318.9	74536.45	74941.27	88215.67	101458.4	150774.1
P97840	603873.3	834773.1	19298.76	25414.59	28197.29	839663.6	1029039	22502.54	577787.6	1206861	20698.51	995094.7	402529.8	99366.9	157658.8	690566.4
P98089	25812.2	304382.8	18148.78	123993.2	4158.865	88728.22	42323.4	47498.28	15315.04	9738.723	12204.16	629092.2	3815.235	24100.39	17641.36	15361.85
P98106	15965.97	23039.57	15180.35	17245.46	13864.95	11979	10602.14	20422.18	14935.08	18076.61	22317.46	44726.45	18076.63	22156.53	17519.46	23089.97
P98158	1611813	1916589	1765899	2417625	2815123	1782731	1746160	2028438	1902744	2779565	2150480	1579662	2281642	3104119	2403086	2385006
Q00238	235297.3	308267.8	289870	284383.7	287963.3	272409	315531	242311.3	197635.2	209319.2	226265.2	198940.3	228237.6	244024.1	208491.9	267725.3
Q00495	75600.2	164684.6	131983.6	119670.2	107173.3	116805.3	133419.2	111891.1	197991.7	156516.5	187852.1	111692.7	160267.8	150509.2	126051.4	188701.4
Q00657	215126.9	239904.8	217107.1	372640.2	536090.4	156555.9	220805.7	189546.9	264085.4	191273	203115.3	190186.3	210751.4	251613.1	260416.6	309472.8
Q00715	127028.7	556216.7	123105.7	135937.5	85876.65	198627.9	115476.7	248898.9	82923.32	170458.3	301859	273207.1	90029.75	194704.1	147544.6	43197.92
Q01177	4608945	8856030	5414276	10149108	5969117	7074413	5415275	7324924	7637117	9340906	7235909	6181873	5576239	5929842	9940382	7697722
Q01205	655702.1	2235084	934738.3	1443058	1406364	1024414	1242618	1383939	957457.3	1428293	1252866	963855.9	1118089	1539902	1240852	1092255
Q01460	1212798	1451779	2177850	802524.3	1298552	1102104	1258675	1179247	1315139	1028305	917313.9	1186668	1548496	1701017	1413857	1827945
Q02253	17439.81	20114.9	94026.89	12315.84	20397.26	20400.79	15066.19	16104.18	25980.15	24269.2	31204.76	23237.62	15964.08	18692.01	11760.36	7217.058
Q02765	97461.24	142677.6	133181.8	111504.8	66333.69	108664.9	110800.8	181601.7	58442.68	109265.2	127098.8	164117.3	92670.7	102971	107273	94630.59
Q02974	109164.4	92135.29	96019.61	114847.7	168989.5	91017.09	105741.5	79159.42	163290.3	169192.4	180392.2	83489.55	117555	88388.16	147529.1	168299.9
Q03191	1891565	2384632	682429.4	982792.5	2273816	498544.4	1402361	1348294	1568580	1082594	1570866	1417971	1371884	4639638	2225735	1189664
Q03336	209738.9	259882.3	204273.7	296137.1	549596.1	178308.3	294969.8	207958.5	494827.8	230860.1	488375.8	177519.8	314531.3	249485.1	310002.6	271500.7
Q03626	203403.1	574211.4	320580.2	377926.8	401947.5	352681.3	301293.7	453006.2	329487.3	254068.6	353021.2	283007.4	491293	436883.4	465622.5	378490.5
Q04589	51675.51	26449.92	42886.65	50538.79	22712.06	45981.77	39293.14	67812.38	41599.89	47284.09	45923.17	68530.27	62397.03	56764.65	57127.5	54958.31
Q04807	4896423	5039971	3265486	7930318	3006524	3570421	3544365	6786912	3354995	1661368	2479524	5867043	3055792	4257792	4565402	2805860
Q05030	22550.56	39949.06	40877.34	42532.1	59618.46	34505.29	30798.19	33440.43	31734.69	27336.02	36896.84	36772.87	29225.63	31234.77	25905.79	40178.08
Q05175	65229.7	103617	61698.56	140777.6	115532.8	60181.05	101255.1	100999.8	118703.6	17630.03	197641.2	33438.18	24319.94	69575.72	96459.68	111604
Q05511	16150.75	23262.63	16472.97	30062.13	30170.28	18391.44	19342.57	10933.05	18363.87	13436.69	17302.66	10544.44	16217.72	16344.84	16284.44	12151.62
Q05695	155829.3	168580.3	128366.4	284030	180933.1	187004.1	267425.9	301406.9	99064.45	193993.1	96639.28	113638	111247.9	139035.4	106390.8	116347.8
Q05820	692970.3	760440.8	669439.8	795384.9	198803.6	891417.5	860434.7	928709.1	587852.3	449132.2	783606.6	1097972	924000.4	803434.9	1017232	837596.9
Q06000	67918.11	56957.79	74874.42	55747.86	7938.721	58397.06	70017.48	19335.03	55457.34	60275.84	40726.18	43682.69	58339.27	69150.72	59531.77	47347.58
Q06496	57179.82	57496.93	50568.38	67486.98	182952.7	51021.23	92587.78	70841.04	76656.15	223756.6	150459.3	73590.72	78911.46	114898	146449.8	128850.3
Q06880	317921.4	437843.9	315232.8	316318.6	223266.3	247690.1	531835.8	385951.6	333987.3	269797.5	269145.2	253529	270760.3	478168.1	381067.9	293902.4
Q07116	20177.85	35950.17	57052.65	44753.06	51680.21	44337.48	51638.47	42090.53	35174.58	25427.58	32781.71	35162.36	40415.71	55740.73	42969.87	47101.65
Q07523	7927.252	14482.05	65381.34	3779.78	15344.9	10726.02	13887.22	10971.25	16216.33	16339.7	75507.59	12898.55	10672.45	15281.55	14012.86	7621.891
Q07936	22136.79	40487.59	17131.93	47144.01	42547.09	82612.32	44961.59	47763.9	12853.59	24165.33	48687.93	31158.01	14072.08	22380.29	19946.45	35373.25
Q08406	29309.13	56032.82	43978.6	60610.22	40114.99	53771.45	27674.57	68784.93	49050.2	36547.55	42526.78	31883.17	53302.21	41317.3	37476.37	44034.53
Q08415	22187.88	17751.72	26498.47	14915.86	21529.85	25549.16	20280.71	30447.35	38832.27	58808.75	31779.49	22794.26	28554.7	22482.62	29990.5	32085.51

Q08420	246276	202704.5	223248.9	276542.5	149239.8	187758.9	231819.1	166319.5	226969.1	192572.7	225850.3	161390.7	167254	181571	291072.6	229437.7
Q08463	81113.73	246657.6	193200.9	247975.8	230974.2	202789.7	359399.8	225293	432683	273490	419883.4	317176.1	443914	742146.1	471603.2	712627.6
Q08464	54815.07	82390.2	83858.38	87168.61	83199.94	35137.86	89824.3	67402.19	37512.09	40217.16	37394.96	34763.07	39489.58	60160.53	49569.4	84115.48
Q08849	15575.76	27430.85	10806.18	52060.39	43462.55	77501.66	24648.81	12883.77	37213.7	39294.2	43849.82	51627.14	21589.43	7140.929	22755.01	29606.7
Q09030	698111.6	865478.4	745954.7	562053.4	608140	224226.7	1413235	458903.9	37142.43	445227.6	528012.9	448550.5	1282231	659526.2	1220463	613447.8
Q09326	19821.56	30502.47	31398.28	22101.15	21895.2	21177.62	27538.12	18821.32	23094.14	34747.7	32826.37	18828.18	26942.51	24342.44	26003.63	19944.68
Q0PMD2	845578.1	1358756	1071761	1125949	1293966	995393.1	1175945	805983	705148.6	947051.6	712094.9	688408.8	862211.3	669475.4	865743.3	985996.6
Q10758	128277.7	55555.73	53203.59	73887.69	72481.85	79474.83	24826	34546.9	15935.93	76655.43	30320.27	140166.9	15773.37	39234.42	11801.25	33746.16
Q1WIM1	89113.91	117464.1	148733.8	120249.7	87147.11	96866.49	130085.1	105713.8	116655.9	109666.1	134515.7	128825.9	167201.8	146525.8	150943.5	166761.4
Q1WIM3	25221.94	29922.25	18797.2	30506.47	23795.35	28071.01	25796.09	31093.55	16487.63	20699.76	15394.89	20221.44	13626.84	13731.19	13732.19	13569.29
Q30KJ2	2154560	797909.3	367316.4	629917.1	57108.42	1182692	685666.4	112084.3	26249.87	75941.16	237086.8	13864.75	10272.98	90658.63	7716.135	25469.41
Q32KJ6	119838.1	237668.3	214880	225974.3	210434.9	192217.3	97893.37	179232.1	97684.85	114392.9	95395.41	158042.6	155442.2	214959	153287.6	203867.9
Q32PY2	77336.94	73229.25	57338.07	69371.94	57630.06	61747.11	70608.45	71885.63	30205.53	43919.1	59242.78	63090.91	39166.45	38572.19	32289.2	36185.77
Q3KRC4	264447.3	378660.4	203036.8	345968.6	408617.8	256391	233583.5	263018.3	271607.7	422643.5	335642.3	262593.3	202065.7	270317.9	292175.6	320802.9
Q3MIE4	139094.4	138054	65150.77	61399.44	174635.2	95412	139687.7	70832.14	132910.6	160677.2	243396.9	156157.5	117768.8	78384.08	74015.92	104799.8
Q3T1J1	20180	15124.19	10910.95	25331.52	13428.96	19319.59	30926.36	18049.86	27963.92	14667.96	21793.09	30111.99	13647.66	11780.67	24850.04	16804.97
Q3T1J9	12653.61	11283.96	10855.34	13851.31	23473.43	13077.01	11745.71	10945.44	20406.06	20006.35	34262.46	13782.79	12381.94	9390.243	13315.58	21782.28
Q3T1K5	39206.72	31178.18	34079.21	49395.23	36170.71	55298.98	34874.72	27791.38	29542.27	31803.55	46823.24	11493.73	24700.85	26658.85	31968.65	39830.63
Q3ZAV1	32722.66	33464.25	17248.1	52398.16	48154.64	26930.6	34884.13	19953.08	44440.92	77645.5	61773.54	33785.23	23619.88	26151.12	32530.46	60391.41
Q497B0	64905.72	48842.83	66721.59	52585.3	79147.23	41423.66	58794.2	34936.41	91689.67	96136.26	79485.43	50934.88	57932.46	65781	47036.41	68259.52
Q498D9	3656.323	13738.04	7432.31	6721.108	4305.414	3956.956	6010.119	6424.023	13422.14	5325.005	20236.94	5148.821	4706.737	4743.714	6688.042	12690.47
Q498R7	29808.5	59305.3	45035.29	39544.2	24585.33	29855.43	41107.54	17576.31	22858.31	11293.96	25752.62	41284.23	15680.41	11311.83	14690.87	9895.09
Q498S8	86662.86	129262.8	124106.1	178085.4	191028.2	158229.2	190064.3	160204.8	96945.94	75819.78	93169.85	122965	126306.5	136270.7	122997.8	118306.8
Q499T2	519887.3	611448.9	755597.3	556943.3	642638.9	648912.6	666798.2	532240.6	562059.5	492519.6	464229	551625.3	695245.6	801716	747596.1	640769.1
Q4AEF8	41739.63	12838.49	46981.3	61655.66	62480.9	46682.68	43021.68	47051.73	29263.05	41842.19	41595.55	42373.17	40525.34	34072.01	22363.35	41019.43
Q4FZU2	613276.5	206563.3	1409795	690894.5	571120.8	596990.3	146879.7	189072.5	124304.5	313219.8	425024.8	662971.3	109263.2	155109.5	216331	200734.2
Q4FZU6	677.8254	13023.32	6533.434	1927.022	1302.235	17753.63	1465.059	3121.954	4239.447	8362.537	5766.931	3755.233	4953.783	3184.58	7308.804	3033.892
Q4FZV0	240270.4	254019.5	248072.5	425126	380561.8	395531.3	232503.2	292405.2	80938.61	169415.5	184462.6	201030.1	140434.3	208102.1	144560	142568.9
Q4G075	74200.8	72537.23	64299.26	146922.8	75827.74	135557.1	90211.4	187167.4	16005.25	53676.39	57083.99	102599.3	36382.38	32954.77	44732.22	41763.81
Q4KLZ6	97683.47	60814.78	60082.74	52469.97	86978.11	65203.79	103678.2	40684.7	101646	141851.1	174812.4	64254.5	59247.88	54016.91	87659.31	109755.7
Q4KM73	69950.66	45397.12	53034.1	43267.85	69024.45	59240.86	39652.98	26910.97	97865.12	56369.5	62970.91	50572.66	36420.33	31138.16	73618.81	70866.05
Q4QQV8	102804.9	100993	175661.8	148399.8	188870.8	102444.9	98346.57	112667.1	99304.7	132555.2	114842.2	90697.73	102060.6	170680.1	101873.8	127241.3
Q4QQW8	414497.8	563061.1	661876.6	647951.8	636186.1	440384.3	532276.8	523616.5	445133.1	589030.8	631770.8	492536.5	431461.3	723104.2	676890.9	519618
Q4TU93	8264.588	9527.603	11533.27	5106.676	12596.76	6018.992	9859.328	6799.451	7928.292	8202.271	9479.679	6745.252	7285.692	12276.03	11542.89	7925.82
Q4V885	501942.2	743999.6	528462	634619.3	457611.4	396403.6	740341	441689.5	365010.8	282936.8	337819.7	427127.3	284398.9	328423.9	338751.1	289401.4
Q4V8I1	26091.16	37702.61	23791.1	36789.48	34966.22	31608.32	57551.82	29709.55	28212.13	10472.14	15436.13	63616.24	38148.22	23775.94	18504.38	37430.42
Q4V8K5	27961.04	40670.98	53701.79	35495.75	37658.04	33087.66	26595.76	25626.87	26153.06	27577.03	17636.73	20621.12	22091.49	27649.75	20995.4	25691.73
Q561R9	15605.24	10708.2	2322.983	47035.74	1943.149	2215.29	14414.08	17055.45	22020.26	23258.35	29185.59	11964.58	15507.32	9368.983	24939.64	13807.01
Q562C9	197043.1	185609.1	379771.9	349215	146095.3	238656.1	331118.8	242422.5	171219.2	138973	173154.8	216852.4	192459.4	205519.4	177192	171070.2
Q566E6	8357.263	15534.35	22232.3	12966.34	8373.082	7483.219	11677.93	13046.98	8863.312	6222.958	4077.938	11542.25	13549.59	43901.09	10364.77	20233.71
Q568Z6	135085.2	248036.7	201570.4	232572.8	189556.7	149675.8	141512.3	146994	181572.9	210191.3	164633	188199.9	205601.4	305417.1	178710.6	227819.3
Q5BJP3	23308.43	23304.21	23478.7	41238.87	17775.03	25981.26	34563.25	36732.19	25492.64	19990.19	50166.46	48851.77	30452.81	29474.57	35789.86	11848.06
Q5BJY9	9845.248	8874.163	7407.796	13732.43	7892.159	10443.89	10170.66	11112.79	1529.068	13036.22	11058.56	2547.364	2666.926	7027.073	4673.141	4598.86
Q5BK81	60062.06	73039.98	100664.6	81094.8	52036.43	57693.11	59114.2	34432.79	46763.18	61726.63	69653.61	63994.43	51547.62	72316.64	51723.53	39967.7
Q5EGZ1	19405.82	37360.47	16935.77	16162.67	10278.42	13830.01	14416.24	17464.43	21017.08	12708.82	13581.34	13814.66	14111.39	25385.69	16236.95	18173.02
Q5FVF9	254935	318877.9	251477.2	373051.4	280866.5	249475.3	210506.6	290278.3	300554.7	265358.4	247117.7	226749.5	309902.7	317474.3	293989.7	274095.2
Q5FVH2	167194.9	211314	278766.4	255328.4	430586.7	249651.9	213191.4	268976.3	154309.8	176798.4	211910.9	181608.7	201903.3	329847.4	187921.9	252423.2
Q5FVI6	17084.77	4133.2	14685.04	50469.35	9610.492	22137.64	33850.19	8442.642	12387.88	35787.07	25277.3	11929.29	7363.675	5788.285	14529.1	18524.22
Q5FVR0	1915265	1882712	2909272	1775778	3302294	1562495	2574345	2079977	1807712	1234487	1275639	1619377	2061738	2463423	2286718	2853211

Q5FVR3	45955.25	48182.31	58855.14	52033.1	37049.16	47202.8	45669.91	43848.41	38386.67	48121.41	30644.34	34126.17	40014.01	40806.68	38226.14	37668.69
Q5GRG2	291885.3	170505.5	41126.22	155522	39069.69	237696	246723.8	181392.9	9559.979	93570.92	47922.69	46025.84	254578.5	149130.5	83673.04	10791.21
Q5HZV9	12193.85	22362.62	15370.5	27978.32	10465.97	75690.09	8469.82	5476.427	21133.9	8162.177	10320.39	17812.81	4405.63	29431.26	21668.7	18948.39
Q5HZW5	292084.4	482798.6	360817.5	604952.9	470591.8	379199.9	386603.8	353714.8	282711.2	298212.9	265754.8	329864.5	376027.3	485389.4	365159.4	412429.2
Q5I0D1	35072.38	12718.52	23854.93	20368.92	32883.71	20161.35	12756.77	22238.76	8543.447	7945.964	38210.18	12375.04	3530.009	15135.17	23145.64	13067.76
Q5I0D5	153180.1	190267.3	380638.3	202353.7	166050.4	160139.3	190956.1	115327.5	166951.7	147545.7	137827	153719.7	228518.9	237008.5	217880.8	205956.7
Q5I0D7	88809.38	54824.89	95363.69	59743.69	96611.23	89387.41	52142.13	88262.99	112983	141119.3	112210.7	67542.77	95988.88	82981.54	136401.8	113666.5
Q5I0E9	100880.4	121566.2	74725.32	155040.8	151152.3	101740.4	136702.2	81465.08	151928.2	279367.6	298111.3	130892.1	69242.72	85760.88	157757.1	213292.4
Q5M7T9	37452.22	39613.32	48703.01	36632.59	48420.94	34563.92	47546.52	31300.92	62856.65	82326.2	70028.45	38981.17	43517.68	26414.73	41093.56	61903.1
Q5M819	36481.09	20356.52	11411.36	19947.38	7921.583	31086.11	19582.33	7934.407	30922.03	58344.77	36100.95	26980.9	21757.31	24916.94	40101.29	32665.27
Q5M871	150675.3	212484.3	207398.6	182399.9	223955.5	155266.6	228847.5	193496	125915.7	92641.33	206902.9	126287.4	136243.4	176954.9	71391.45	162066
Q5M872	8300.955	12108.56	10205.45	7421.987	7689.553	9113.961	12366.5	6660.55	17910.02	13184.03	13262.59	6870.381	13508.9	13404.49	22865.71	10490.85
Q5M876	196150.5	137964	229877.7	168228.9	357003	118116.1	205227.1	199845.6	334244.7	271053.7	341195.1	213234.4	244424.5	191102.7	306275.4	301367
Q5M8C6	156212.4	91717.95	87507.92	125078.7	113441.8	125828.8	33258.4	146925.8	49535.24	44132.25	50814.91	118357.4	53790.6	38269.13	22006.87	17065.87
Q5QE79	32308.98	48384.8	42104.23	38211.48	4429.05	29349.44	12232.34	66270.45	67740.15	3908.364	14705.07	11850.62	13087.97	10917.93	154363.2	19902.76
Q5RJL6	23885.36	38461.97	24176.33	26960.49	22646.55	22728.89	20024.75	20546.56	19062.44	22276.18	20405.77	27193.81	21576.46	27232.75	17915.13	22046.37
Q5RJP0	119874.4	108722.1	68437.11	108195.2	145911.7	99018.75	133641.3	69120.39	270340.1	133956.6	288634.3	133522.1	139212.9	114041.5	151871.7	175795
Q5RKH6	23841.04	18405.35	14022.3	19646.92	19792.63	16790.46	34744.04	14124.41	23385.71	8930.917	11114.34	28988.63	19702.56	18692.06	13722.11	20753.28
Q5RKI0	26429.66	24186	25747.81	18341.11	27842.95	25917.7	24908.48	21994.72	31842.47	39187.46	43415.93	26936.55	29269.67	29175.64	30619.37	28058.41
Q5RKI7	236388	236167.5	267144.3	378742.7	392951.5	177100.3	475940.8	317158.5	260112.3	354352.3	430349.9	208170.2	88678.17	94839.37	270988	304926.6
Q5RLM2	54473.48	33207.82	58423.37	58135.3	26492.19	30244	48317.05	35966.78	107783.2	59273.93	74073	102636.4	27414.74	48120.16	78510.22	103046.8
Q5U2Q3	139722.9	113300.9	133038.6	127897.5	146442.1	127615.4	156082.8	114252.7	272415.7	241029.8	208042.3	114276.8	178320.7	131822.1	199080.6	224021.3
Q5U2V4	14981.53	33399.72	13684.91	25746.04	18560.16	19251.46	13278	48285.2	9281.441	16649.71	28487.75	24225.09	5566.236	26617.6	16666.82	26390.37
Q5U300	21547.96	8826.786	12876.86	8865.896	11785.96	9794.932	13661.93	4867.964	11051.34	12737.9	15988.73	15113.52	12301.13	5663.677	4285.091	21564.38
Q5U367	51134.47	36178.83	36145.94	26677.75	60363.09	21699.38	43656.19	46329.06	21355.24	27916.96	37619.51	45814.63	53280.49	27940.12	28731.08	43890.07
Q5XFX0	4679.04	16617.57	11785.64	22223.76	16260.74	15573.68	19168.35	10057.8	25903.6	33099.98	24745.04	10826.51	13807.69	25192.51	11088.36	7693.894
Q5XI20	2350.569	43629.66	4845.365	2709.001	2884.224	2895.045	3679.011	3386.467	11237.95	6453.331	16347.27	2583.553	4722.371	6664.87	4941.772	5028.307
Q5XI22	2796.73	4288.724	4983.132	4380.406	943.8269	3113.733	2572.261	4245.696	1885.009	2095.977	5399.208	2408.712	2939.595	500.6129	2195.836	1050.145
Q5XI32	17589.91	18361.98	27537.69	16126.2	29551.93	21070.93	23482.69	15676.27	21575.02	19506.51	17854.25	19203.61	16071.13	16718.9	12718.24	15486.17
Q5XI43	6296457	9994286	8331730	7751279	6981886	7296389	9128444	6604185	7199899	6273073	4540163	6630783	8259601	8393884	7469826	7850695
Q5XI73	98501.9	74764.66	51090.64	29080.74	98036.3	88817.57	41951.7	149754.3	90831.72	115713.4	227278.6	77750.65	59195.62	58098.86	85886.43	97452.03
Q5XI89	22083.18	19959.71	15664.54	10228.46	14732.25	12874.16	17757.11	5417.311	10928.16	33183.14	11751.21	12063.99	10458.94	12487.99	13143.51	17897.16
Q5XIE8	34266.05	44076.02	32364.1	41814.05	37099.04	33080.84	35500.04	42110.36	35868.45	21965.24	37974.42	43682.73	29084.52	33486.91	30280.77	21989.71
Q5Y4N8	22147.85	31637.01	17928.41	23214.6	20164.53	15341.28	16533.58	12025.35	7420.912	20591.48	6787.825	7812.379	18303.72	17699.32	9795.354	12864.06
Q5ZQU0	11002.02	16623.14	8000.06	14943.58	13533.63	6700.027	11314.86	9988.874	7085.045	12065.13	15095.85	8270.433	9225.69	9321.482	12343.88	11628.82
Q62632	172200.3	210075.4	269010.8	185608.7	356304.5	247998.4	240932.4	208381.8	294769.9	192951.1	383286.6	236039.9	301083.7	476623.5	436602.4	459165.1
Q62635	58622.51	241900.8	19197.79	91224.16	28802.52	215279.3	63227.65	147896.9	7393.156	31425.85	41737.26	322423.8	109515.7	52839.6	29778.03	10733.32
Q62636	30270.38	29894.68	20809.64	34033.64	22381.02	11305.17	23603.54	13593.94	23379.03	31324.62	65117.13	36985.84	20140.69	11623.1	17280.05	7833.357
Q62638	128178.3	94027.52	98881.68	119112.6	96909.29	130176.1	124106	102154.6	52405.77	73871.09	83575.05	90076.16	93979.05	109062.1	39034.7	74416.34
Q62687	43228.9	77313.24	31204.99	87397.56	100848.1	50337.78	92466.23	35662.82	76747.6	125047.2	104830.4	75776.13	25171.98	26354.49	58346.62	123327.9
Q62740	201244.8	532477.6	350460.1	760092.8	317606.1	599115.3	484653.9	293311.9	558153.9	539148.8	522798.4	342461.7	612298.8	611354	687743.4	964966
Q62745	115229.6	99706.16	110298	178678.5	90754.58	71793.67	59746.5	99759.56	64029.89	92798.73	98580.15	83971.56	54470.01	97393.31	84036.19	92440.2
Q62753	25941.67	13104.85	14888.27	16269.98	28334.7	15337.35	19775.27	20484.29	29326.51	27663.35	30812.4	31431.01	20604.68	20838.37	14212.81	18827.06
Q62786	89661.09	192026.9	134509.6	225256.5	113333	177996.6	121877.7	177653.4	186383.1	199271.3	125195.7	122032.2	193503.1	209708.1	200143.4	179063.1
Q62795	61809.8	63220.28	36573.15	67714.85	83481.14	51845.43	86538.18	45964.62	71880.84	110720.7	116607.4	74008.13	37947.29	34223.22	73631.71	108526.8
Q62812	5794.693	15873.15	4940.516	10468.99	8647.323	7826.221	5590.793	6838.262	15087.69	14567.45	16566.16	7019.492	5377.96	7185.155	9482.716	8043.314
Q62867	2529321	6203728	4284009	5297041	3633542	5373524	4366487	6587994	2378247	4788433	4347852	2389253	3929477	4455311	5382483	3240942
Q62894	14416.91	6620.253	14833.39	9644.083	7956.416	3471.985	5457.045	11693.71	5286.702	2591.795	3423.289	5981.231	5178.737	2486.344	7951.066	6163.228
Q62902	63545.02	31439.72	27603.34	53697.75	18755.77	102917.4	101978.5	85524.61	29841.15	18375.86	51996.44	71287.77	104636.9	48320.01	6038.265	29116.83

Q62930	234755.5	176092.8	163390.3	183326.3	99646.76	145882.7	186326.7	256250.3	166887.1	224746.8	262168.7	284198.7	157351.7	185708	273940.1	233023.1
Q62946	170484.9	170876.4	261955	252345.7	142747.1	259710.8	153177	276544.8	75266.36	124936.1	150374.6	161676.3	136243.5	131212.3	95558.05	96185.1
Q62975	13820.2	14362.39	33767.49	5086.888	14258.62	10056.57	21673.72	11479.17	21804.9	7645.491	12400.46	9322.634	10450.3	17274.02	18228.84	14991.19
Q62997	23978.26	38563.77	24265.75	46089.31	29471.4	16158.07	18563.05	18318.66	11782.2	65419.8	3954.784	9005.551	22362.04	17460.18	20505.08	133574.7
Q63041	12305592	12834413	13021689	12653896	19435032	8597733	13274399	12279335	12037120	13450579	11140309	9148360	14133939	15924172	16284043	13445249
Q63072	16389.84	33975.73	26176.32	20889.46	30390.77	21526.62	16688.44	30309.46	18838.79	17813.33	25710.08	49296.37	13870.28	23356.67	18104.06	25520.82
Q63083	1517934	1401109	634422.6	560727.3	502530.4	586592.3	746729.2	476857.1	827610.6	909739.8	2063457	797062.4	698610.5	725907.8	682120.8	747831.5
Q63150	5100.509	17396.72	21777.45	17204.79	22289.25	13762.43	18134.7	10972.15	31898.31	20532.47	18867.11	11761.55	18316.73	13127.67	7441.979	13916.01
Q63203	23371.54	35605.67	26821.96	29882.2	10929.15	25395.64	28523.04	24296.17	38596.14	24765.2	17231.92	25997.42	30394.39	85767.49	45703.45	40762.93
Q63257	5879115	8164118	9422862	7656773	5580665	5537279	7274765	7378966	6665650	5961096	7775080	6137079	7746988	10106391	10081228	10901453
Q63270	62531.66	56911.46	45052.22	25501.5	64794.09	36531.28	52610.8	46890.64	77666.59	115195.7	126186.1	57683.48	50987.29	50895.04	74970.27	116508.9
Q63317	1250801	1492933	2023338	1070325	833489.1	710291.6	786735.3	759360	467523.8	368784.8	370581.3	450687.3	709563.7	594543.9	558773.6	284941.5
Q63355	30155.38	10486.74	28180.49	18635.8	45528.22	22774.19	67993.2	34793.87	32351.04	27736.8	89765.53	34422.39	24193.57	58007.2	64482.27	45740.39
Q63357	31526.59	15180.36	8406.307	23569.34	13442.19	26439.88	23416.74	22759.38	14500.21	21912.35	46178.52	36085.48	11807.49	9661.449	9107.561	10806.04
Q63416	417007.4	327415.2	336277.8	385219.2	357241.3	304191.7	361252.7	333908.4	276149.3	275520.4	305135.4	368919.6	372410.5	399651.9	497376.8	352368
Q63424	163233	141887.5	116635	202217.6	291352.7	104529.1	284540	100976.8	201526	317138.6	326792.2	161280	68639.8	81765.97	164656.2	272088.6
Q63467	2741796	3378746	1115650	1295368	1766409	1219342	3196837	1301153	1760391	1095034	724433.9	1408223	1908471	1714053	1478599	1865673
Q63474	15403.32	30588.74	23788.52	33263.96	23290.52	23707.11	18157.49	22152.54	12399.46	36234.6	26021.65	18078.34	10023.2	19536.78	15503.81	16611.2
Q63475	50633.77	77509.86	44607.66	105808.8	41662.49	29946.26	54268.92	37860.38	24490.74	34386.44	30494.05	37624.36	12874.53	26278.48	27284.5	16173.63
Q63493	22833.81	12122.77	7859.152	13413.52	9190.185	20775.05	25459.17	20257.01	3957.652	8164.696	20691.5	14171.28	7312.196	8993.946	1653.039	2524
Q63514	37092.53	69941.76	39838.64	110508.2	65814.57	64321.23	45973.18	60222.3	60952.2	105028.8	77700.69	84689.23	84263.81	74916.84	93248.48	111303.7
Q63515	88014.09	131007.9	141846.5	155624.3	124116.3	94350.91	177233.6	131765.8	123670.3	133091.2	143514.4	155263.3	174674.5	200106.9	188815.2	238482.8
Q63530	176355.2	114452	267934.2	109750.2	338124.5	192452.2	193540.3	209737.8	260598.3	377321.4	259610.5	167681.4	194216.5	189101.8	242998.5	250331.4
Q63532	246328.5	758026.8	130119.9	215001.8	89534.69	269110.2	447696.7	82274.52	82137.85	168985.7	141819.8	187657	112418.6	388554.1	137957.5	303363.8
Q63556	396502.8	492474	426885.8	305727.8	364397.1	210367	163512.5	364342.3	324825.1	204114.8	219997.6	419231.8	387309.5	347113.1	432402.9	326667.8
Q63598	5369.51	8977.086	3196.779	11111.6	5177.393	5055.475	3059.144	7667.902	2419.898	8931.768	7793.218	4523.971	3236.354	3707.078	1631.339	2671.257
Q63610	19321.05	37075.49	18494.64	54962.13	53778.27	22987.98	30250.27	30638.29	25076.9	52294.59	37912.62	29109.85	16847.19	25115.36	30805.62	11595.8
Q63617	43836.61	9560.438	9459.603	85230.64	12606.4	54590.68	117862.4	129519.8	22121.57	6313.008	19260.43	6518.806	14296.17	16627.12	4434.927	12758.47
Q63618	28753.59	32202.1	14373.92	24044.73	28937.01	12791.36	24111.05	8530.521	50019.49	53042.83	57022	27226.77	35189.95	14753.36	31072.21	34202.07
Q63621	258906.3	514174.1	360153.7	405312.3	342354.3	382930.3	308453.9	446294.1	283720.9	308731.3	271921.5	345308.1	335532.7	394674.7	367373.1	364055.7
Q63661	7393.618	11835.18	4936.638	4380.406	3072.219	2625.598	2689.082	1518.615	9308.157	8351.813	8557.78	5099.551	3922.039	4853.891	4506.848	3911.76
Q63678	128827.1	139890.3	133030.6	152520.6	83179.79	124919.3	87486.36	119387.3	69851.69	85415.49	56334.84	99216.32	66200.69	56611.3	77513.73	56802.83
Q63691	204382.2	224984.2	232423.5	268959.5	252746.1	244152.8	233262.1	234054.1	218412.4	217260.3	196231.8	247066.1	207042.6	244367.1	202192.8	243102.2
Q63716	240966.2	207861.9	225649.5	196606.3	246668.1	303092.4	232261.9	218745.4	317492.1	261711.8	349089.4	207013.1	252966.7	204644.6	239150.4	234752.5
Q63751	303431.3	216268.1	990886.6	425150.6	349528	450850.1	396873.6	319077.4	39347.57	52693.32	208239.3	195617.6	113989.8	38070.47	96674.19	47125.94
Q63772	137569.3	114049.2	97042.59	121698.9	115920.3	81343.14	104907.8	47434.2	82812.3	169075.6	84368.83	114989	118496.6	129085.2	112676.4	170207.7
Q63797	11863.37	18864.23	12554.36	26801.29	20398.58	6765.442	21762.74	16375.17	17474.89	8703.038	16471.21	13802	8752.397	21222.09	18828.27	15953.85
Q63942	105749.1	112497.7	34772.65	30941.64	82153.3	80193.21	18205.11	73166.93	153122	38027.61	24951.44	9937.377	30241.72	43507.04	43684.74	52723.53
Q64057	71123.14	135181.4	70053.8	235461.6	48169.91	53874.16	102175.1	104876.6	67500.34	76460.72	99926.3	81803.77	49874.91	47311.3	56662.55	54183.84
Q64093	23966.85	27862.81	4475.593	30157.54	48687.4	24322.27	74112.26	19248.57	63430.14	54590.04	37149.83	29624.29	5778.62	6799.178	25186.4	46155.4
Q64119	55466.89	59223.28	49611.33	45582.39	84117.3	36831.81	47269.65	42537.72	69379.2	54085.42	90816.21	56799.68	65072.9	40892.9	52855.76	58505.02
Q64194	15519.85	18123.64	24547.71	23659.63	21147.33	9628.712	12573.03	20371.51	9261.789	14863.88	67738.22	11347.48	8663.283	18905.69	18334.79	13152.75
Q641X3	344107.7	383691.1	489217.4	386091.1	351099	241096	236604.9	457274.2	171656.7	220147.2	310420.3	261320.2	279832.6	415325.3	317402.9	327798.3
Q641Z6	81671.87	81945.64	66143.27	63092.12	91941.16	63847.82	124476.3	44868.11	152052.3	82165.3	155978.4	86958.69	67953.94	54744.07	98259.08	53452.8
Q641Z7	55531.23	29466.94	55894.61	56645.17	64995.62	57839.23	60795.57	63298.74	38964	60261.08	36882.52	30172.87	38841.8	49480.53	51759.02	49603.82
Q641Z8	32837.67	47662.66	49895.78	55759.48	43958.56	44131.81	56245.31	50134.63	55133	56132.65	45988.69	32762.24	45920.67	51609.23	40410.67	47091.38
Q64230	5969956	6728317	7281973	6941259	4809064	4566655	7653161	5422926	4837934	4213258	5774448	4623600	4815869	5998636	5271551	8105480
Q64240	33969444	50698264	39044600	37125804	24895406	41715784	49000272	36884412	25645460	22271562	33258362	33707088	37733648	39685076	48011140	36093020
Q64268	30479.39	12240.14	25205.76	33958.39	10937.38	12059.54	13340.01	33644.04	13955.42	18408.9	14414.56	14419.17	12243.21	14242.96	15938.36	9375.782

Q642A7	563459.1	708195.3	654392.6	483002.6	879606.8	670335.4	632985.5	519401.8	720138.2	770251	552262.1	629055.8	916146.9	1086347	979244.6	1396845
Q64319	3249077	2588979	2299540	2159170	5627998	2710996	6005152	1633744	4179472	4450492	5654359	3020607	1699660	1700468	3244648	3846459
Q64335	42983.59	29453.53	106201.2	37661.81	61580.29	27540.45	90136.17	52258.59	47064.07	34137.21	55835.44	88064.94	75949.8	82432.69	78655.88	111638.6
Q64361	214257.8	244412.7	181210.1	289126.4	205702.6	276470.4	149979.5	223495.9	56244.24	136004	70553.31	130447.8	52314.73	55636.35	77471.21	42021.5
Q64537	21029.41	16550.03	13442.69	22452.71	25460.3	23299.9	24609.41	38103.45	14038.74	14403.42	18780.74	19937.84	14921.38	11612.45	13937.09	12637.63
Q64573	178650	172307.9	200745.9	162990.2	111754.9	114607.6	144006.8	189841.4	109225.2	81747.21	68726.62	70260.87	174228.5	115311.5	158892.6	97488.22
Q64602	140264.9	183000.6	133456.2	208341.1	180366.7	160518.9	178613.4	105917.9	321134.2	309325.4	414724.1	230288	270021.8	163291.4	235735.7	306745.5
Q64604	55984.28	149328.4	110195.5	134633.5	93876.97	152299.9	165411.7	130347	133387.1	116505.3	132851.5	164818.3	137011.4	155756.8	158509.5	154091.3
Q64605	11068.78	17000.49	12576.39	23732.31	24025.08	8342.485	14866.88	9090.632	7326.58	16560.86	13817.2	14374.79	11457.59	16119.5	13647.75	12455.75
Q64611	29983.29	16381.17	10922.27	15585.05	22391.92	21439.53	5993.49	18559.7	42632.16	34127.76	42102.01	19348.66	33138.02	22594.72	11569.62	23941.85
Q64640	17492.63	14479.07	10824.57	18037.7	10888.65	8166.542	13466.04	23695.13	27273.71	17666.02	24774.66	17968.04	16063.79	17478.56	19869.05	15793.69
Q66H12	268704.9	298077.8	303012.2	223870.3	354683.1	201375.7	220584	192278.8	187944.4	196705.1	221175.5	253881.4	251150	355507.8	227997.6	323640
Q66H69	55354.18	40219.85	32992.68	22355.03	10678.29	40102.77	13150.44	33257.49	22002.79	25804.09	34282.95	17502.32	19818.07	20355.44	35498.03	27381.16
Q66H94	8992.968	31562.14	16484.98	10910.19	19653.43	16467.35	24715.84	19766.41	24910.36	20101.62	25354.57	24877.29	21328.26	22332.91	26685.02	14420.01
Q66HD0	14869.17	25100.31	16599.74	34339.98	20943.39	35216.2	17856.91	22593.37	16505.78	22139.36	22871.91	15323.53	18545.52	17570.52	7171.662	7613.952
Q66HG4	40620.88	28338.46	52102.34	61033.8	52018.93	42732.09	35689.79	59942.73	52981.25	113465.1	89093.35	33762.1	32769.66	45480.72	62455.74	62046.02
Q675A5	68793.83	100503.5	86998.63	143676	148036.8	76082.89	105321.9	109779.4	88845.27	62013.07	93291.35	65972.09	80618.77	145890.7	110190.1	52128.29
Q68FP1	9619363	12995259	10818342	20403004	6866547	13288536	12774693	9995395	7847501	8173828	10445221	10772961	7687022	8145027	12894998	6662200
Q68FQ2	252314	412653.4	425544.8	418339.1	376806.6	357673.5	442575	428812.4	398870.3	285271.7	303237.8	325072.3	408113.4	228649.7	432156.3	370480.2
Q68FS4	17653.53	20825.6	29861.53	4386.843	19655.4	30391.6	10095.58	8838.422	38974.13	40711.34	30096	19706.36	32875.17	20220.86	34232.5	30641.32
Q68FT5	54204.84	14416.61	26743.51	63700.68	45983.92	34276.06	34217.34	31978.08	36320.91	29594.79	46870.18	34146.46	37979.45	25172.26	36782.82	28550.19
Q68FV8	24841.72	19627.64	12277.7	17057.61	10215.49	6322.76	5095.313	15640.25	84070.97	4278.543	9263.47	8623.173	6744.853	4610.065	15437.75	14624.89
Q6AXR4	166508.7	251309.8	364144	214179.3	265049.2	174342.9	119325.9	341191.2	140835.3	156487.4	167895	175625.8	172931.4	315517.3	234545.9	233204.4
Q6AXS4	276331.3	237202	120489.8	107798.1	71346.12	107295	139287.1	75236.42	100883.3	98196.02	144254.9	127963.1	74027.84	69556.86	90955.06	87658.34
Q6AY33	49629.16	18905.64	17509.88	28137.49	23691.54	25741.44	21751.68	24913.5	10409.79	16639.67	18956.16	10130.42	14449.7	20269.14	13273.59	16097.19
Q6AY41	54942.44	36033.85	38291.82	56433.5	65493.9	41825.33	59005.54	22394.12	53887.45	71009.56	84320.63	43287.6	43611.4	42410.05	53943.02	85397.56
Q6AY61	2413.113	4288.724	4983.132	5770.31	2456.675	2145.979	1827.656	4245.696	2856.081	3980.152	2455.708	1477.948	2761.282	3758.308	2195.836	2388.734
Q6AYD4	500677.2	685550.9	569061.6	525068.4	531363.6	527859.9	622972.8	510352.5	531421.9	441813.7	356157	377880.8	514046.6	550100.6	548726.9	398457.1
Q6AYE5	58736.93	80379.11	123711	71198.87	148108.9	73046.8	98881.67	68891.98	64054.52	98155.24	85127.59	66310.82	80392.51	99950.03	78901.86	79096.86
Q6AYL6	63907.56	26324.14	12555.99	26464.6	9289.113	40055.7	47609.5	32676.38	25644.33	30479.52	45506.09	38828.05	2316.018	24438.57	14232.19	26696.59
Q6AYP5	140028.9	186786.4	191913.1	162709.6	276442.8	156434.5	180825	162205.7	136757.4	126754.5	147578.1	112090.5	143552.9	158511	126869.7	210695.9
Q6AYQ8	6744.549	13724.45	4611.92	12554.65	4241.181	5948.764	6859.793	6742.679	5895.548	3961.398	10176.05	44790.31	2657.958	9801.756	6341.905	2303.132
Q6AYR6	25353.04	19661.19	41484.95	31295.09	20073.48	16456.36	23932.68	18184.08	13857.11	22739.11	28553.65	15206.18	19328	19348.55	19636.96	12934.94
Q6AYR8	19948.11	22444.04	103386.9	27269.73	48239.51	19597.41	20808.44	12975.91	18625.38	18257.43	24049.61	19506.38	21707.08	16343.94	30568.51	30772.14
Q6AYR9	108228.7	80163.82	23042	147015.8	23678.86	148650.3	92560.61	118607.4	40357.69	69060.76	50984.32	342951.6	100932.5	65648.45	7789.053	10651.98
Q6AYS4	12216.58	36915.92	35394.26	90828.87	95286.17	19225.05	40984.78	111398	30692.66	34482.68	63632.93	27539.23	24345.96	26511.76	20267.53	17343.66
Q6AYS7	213611.1	196349.1	212943.1	277051.8	237994.4	198709	190601.2	156122.2	640552.8	495149.6	901186.1	298270.3	480882.1	342795	443361.4	453089.9
Q6AYT0	117795.7	122189.2	138240	122489.1	178363	81177.8	146248.2	101711	290885.8	163503.3	265529.7	90736.44	215230.3	118919.5	213108.3	219798.5
Q6B345	43395.3	36930.4	72718.09	43571.26	50734.19	63280.84	46807.25	76480.03	73923.09	82038.49	94673.73	59431.84	31982.91	62202.35	51742.51	31795.38
Q6BEA2	19534.26	11582.49	20313.85	25483.72	19305.5	14011.43	10842.03	8339.102	12905.93	16089.7	12388.24	12900.78	19052.27	15766.22	19773.79	14686.23
Q6DGG1	451776.3	776874.3	2361183	1223921	793150.8	899770.4	589584.9	807401.1	711989.9	784611.1	683584.9	686261.7	671158.8	657830.4	904233.6	808476.8
Q6GMN2	70735.97	32714.43	20965.87	30459.22	45145.6	41799.26	45541.66	39014.57	33048.92	37073.88	81925.34	52170.75	35890.16	28081.22	13862.68	18996
Q6IE51	108802.2	167863.7	81382.16	119305.7	80343.69	98978.4	180860.4	19720.74	66431.46	56571.26	55472.91	53356.96	65974.16	123559.7	125076.6	71203.23
Q6IE52	247099.5	333917	335645.7	243683.1	161588.1	202286.3	221368	313289.3	223322.1	160732.5	173361.1	127553.4	246162.8	182551.7	181614.1	223372.4
Q6IE64	4651.978	8216.229	7862.112	10890.18	7989.948	4992.361	7146.427	5120.597	5421.985	6344.985	6579.697	6827.62	8213.843	8175.13	8800.949	13350.45
Q6IFU7	42260.46	23273.57	7302.26	27285.32	28371.27	43824	5589.889	9766.511	15502.96	28105.83	42217.18	52125.09	9370.417	13416.54	12299.47	25849.89
Q6IFU8	1135742	741186.6	786826.4	788325.7	813845.6	837668.5	269342.1	401266.8	408663.7	489423.7	1114481	2884499	218064.2	433975.1	255302.1	398327.3
Q6IFV1	7386.893	2921.728	1664.905	1409.8	3012.7	4823.468	1447.377	3813.688	1322.321	2967.232	1180.561	2981.341	3576.808	332.2289	3731.653	1069.31
Q6IFW2	23375.4	1020095	43793.8	47425.05	21026.67	56404.18	36687.43	29610.61	83881.82	17515.28	38471.7	31314.7	22455.63	19772.48	19350.59	18682.94

Q6IFW6	876525.3	277149.4	733444.9	784398.1	754131.6	703008.4	198976.1	306257.2	189872.4	361030.4	564605.6	1098493	115862.5	233631.3	131560.6	272850.4
Q6IG02	321424.2	19958.79	299805.4	194612.9	233819.2	149976.7	72595.4	172321.1	92925.64	152661.1	172797	513546.4	49890.48	88394.31	56906.04	80577.86
Q6IMF3	1678499	634973.9	1100035	1273984	1274097	1408770	410778.1	594738.9	433389.6	869969.9	943261.6	2472724	239734	499712.6	278242.2	606414.6
Q6IRE4	90585.42	5946.549	90797.03	5320.988	55669.28	19946.48	22604.05	13699.27	21463.6	12827.61	23765.14	14274.4	17513.86	18672.31	15912.97	25937.9
Q6IRK9	4070901	6554484	5033862	5213763	4596302	4410719	6554710	3300706	4174010	3134621	3652252	3602425	3905955	6019744	3927917	6201286
Q6IUU3	1248727	984702.3	998911.4	1102666	488247.9	1090360	974735.7	1003633	565474.1	723591	1072437	1416909	721008.3	581763.4	496568.8	464492.4
Q6JE36	23819.24	14760.57	13222.77	5080.363	11503.94	7685.926	18157.21	6624.563	27146.04	20225.28	33327.24	19307.71	22254.43	16839.96	21058.23	15485.85
Q6MG61	374585.2	474595.9	252494.4	343557	350316.6	290676.9	388701.3	267752.1	562503.9	502183.3	965274.5	555303.3	463737.9	281480.7	348514.1	611505.6
Q6MG71	906656.1	733626.3	631908.4	820444.6	638781.7	714893.1	814140.8	753757.8	578776.6	925265.3	1693011	803711.8	519884.3	832206.1	599810.6	688956.5
Q6NX65	1792.689	6620.682	5113.636	6857.267	4723.821	5869.083	3814.935	6269.04	4024.924	3655.406	3492.468	4475.469	3618.788	4111.157	3625.01	3894.464
Q6NYB7	65535.27	54373.25	44081.55	69085.67	62495.22	61618.42	67424.58	53687.05	67088.51	100834.9	146836.6	71447.55	48794.98	40035.66	32064.11	68562.63
Q6P6Q2	988781.9	261071	519389.7	582413.7	628882.1	580445.6	172370.2	409627.8	170103.3	435882.3	726703.5	914714.7	107476.8	193262.5	93752.45	310340.3
Q6P6R2	7636.648	18017.19	7902.721	11525.07	4087.791	33088.21	10531.15	15476.19	11190.05	11911.97	5263.644	7419.292	2773.255	6192.718	3215.219	11190.3
Q6P6S9	19428.81	22595.32	21493.91	25204.86	19112.33	22908.27	14300.76	21252.76	12663.39	12514.76	19701.75	23691.58	12273.01	19970.48	19804.54	14981.26
Q6P6T1	33021.55	32229.03	30713.44	38546.66	22788.6	29957.21	33178.26	34023.13	14658.48	38292.82	17823.33	54717.79	34326.21	26909.04	25475.54	104637.2
Q6P6V0	65911	35786.75	36507.23	30272.38	50498.39	37225.35	38746.75	60749.09	34642.32	72789.78	63529.7	30303.43	28435.25	28183.4	35456.08	33231.19
Q6P734	247513.3	378422.4	209394.7	331753.2	210883.3	220246.2	195653.4	267644.6	159156.1	224714.6	246497.2	300609.9	157551.2	173720.8	210494.1	202375.8
Q6P767	689514.8	721142.8	1004872	911441.9	1392109	905088.8	616582.4	590337.5	421390	398854.9	636706.3	354944.4	538962.1	1361578	522104	1000606
Q6P777	35178.29	18532.87	63390.56	22880.53	28249.27	19012.92	10679.77	15246.86	17246.38	14586.84	22529.31	18036.17	16689.24	24831.11	10918.07	25153.84
Q6P7A9	732236.6	716713.1	776064.6	747942.6	697093.4	633139.9	546139.2	730873.8	612313.9	658123.2	602491.8	728533.6	705022.9	1199763	741427.6	727613.4
Q6P7Q4	36779.27	26512.72	26109.08	32592.14	24964.96	38980.66	26167.66	33231.35	37445.35	50139.38	44418.64	39170.77	35818.11	36919.17	36821.17	40717.44
Q6P7S1	113610	143447.2	182313.9	115888	223908.9	106413.4	88037.05	87881.27	76485.74	101884.6	62652.9	74879.41	152239.6	187557.5	159494	174832.8
Q6P9T8	128081	53169.37	40527.34	44832.24	40352.36	85420.09	114406.7	89052.79	41279.6	42217.41	52590.96	26348.75	42607.96	42611.7	43843.67	40028.02
Q6PCU2	10747.81	10604.97	21843.7	21121.96	23245.42	27420.13	31153.08	16730.8	54293.01	66787.64	67403.2	37542.69	22463.04	24412.24	36162.15	20617.57
Q6PEC4	28418.36	28694.32	36279.13	18619.76	49814.89	41430.22	22463.45	31095.75	24704.36	36406.84	56862.4	35271.4	26414.22	36124.7	37349.08	43512.79
Q6Q0N1	88320.77	100321.8	81293.52	96888.02	253918.7	106905.8	180114.5	97713.46	219829.9	229164	326311.6	124871	167792.8	89600.41	162112.3	125843.1
Q6Q7Y5	210552.3	189925.7	122167.5	273475.7	209173.9	268721.5	250327.2	232090.5	266928.3	244948.7	370684.4	289073.7	179602.3	201263.9	211110.4	226199.5
Q6RUV5	83202.84	96810.51	88395.77	190501.3	88278.3	198277.7	142936.8	109334.1	113929.9	132359.6	128336.2	110311.5	57166.04	54535.73	53021.59	41575.11
Q6RY07	27782.56	42411.74	22769.36	42090.68	35725.79	33722.8	31058.62	45894.42	29719.53	25452.92	26840.21	195802.5	15871.26	26179.52	25231.44	24508.55
Q6TMA8	23468.63	112330.4	53906.6	161159.8	31155.35	48355.79	27393.87	71100.08	39396.8	22997.96	36814.79	34604.57	43567.94	32012.96	28798.44	19377.46
Q6TUD4	17415772	29005524	19084712	15087184	5800728	20565688	32584628	22588664	21954510	22074204	25648110	24439138	35690456	54945772	36567412	45553556
Q6VBQ5	40085.25	44014.93	24617.05	15130.97	51684.76	35400.92	31714.23	38034.14	25724.98	47271.2	40334.16	21338.55	28581.66	40808.16	59404.18	69885.49
Q6X936	23022.01	48060.86	35639.6	39911.08	36139.43	36419.46	39828.65	29942.69	28711.19	29963.53	29215.01	25394.36	33468.55	35606.19	39395.04	38840.59
Q711G3	86053.26	43982.41	95322.69	49960.22	122210.8	61468.98	45712.18	60196.03	137034.5	196855.9	114829.1	71628.88	96746.8	74277.52	101780.4	87177.59
Q71MB6	29121.73	51151.74	32504.11	37060.59	68694.27	22232.48	42042.22	17678.7	30286.28	47921.2	43420.95	26372.44	20420.48	17227.59	39112.85	44832.39
Q71UF4	5644.635	11239.91	9748.498	12144.94	13332.91	9465.727	13927.95	8239.7	6535.295	8172.271	10894.92	7826.267	6341.838	9949.59	6919.771	5081.154
Q76HN1	64250.11	80162.24	91876.16	107084.1	78270.62	126341.1	59732.38	59050.86	79840.91	64565.29	76124.66	98476.92	92693.24	99043.72	113704.4	47600.85
Q793F9	72040.23	82691.3	86894.4	98025.61	30670.22	40841.93	63333.55	76045.48	89468.94	117199.6	125241.2	66391.8	77421.75	128942.6	104729.9	128661.6
Q794F9	16380.44	50998.45	23721.78	43567.13	17391.89	14931.6	23056.2	25297.55	13965.42	19799.61	26080.64	14006.32	19281.86	18343.94	11967.16	19825.2
Q7M0E3	58335.46	82547.95	35296.19	89414.8	38031.4	65839.64	45058.79	75494.81	86882.78	67033.65	101016	65958.61	80802.09	40316.58	46878.18	62981.78
Q7TP52	139312.1	147090.6	41908.69	117676.5	64540.14	82570.45	52598.12	37447.48	41500.32	95195.01	68084.66	35427.74	32653.93	34868.71	39473.54	48682.66
Q7TPB1	3261.302	2499.387	1715.771	1508.061	1899.897	1788.451	2140.615	1977.084	2574.855	2885.549	21065.87	2015.348	9550.538	7046.074	1767.485	4732.747
Q7TPB4	60699.99	112978.4	101192.4	66584.38	76076.36	69093.05	73546.45	86612.01	99791.2	65666.77	87115.25	76343.09	69689.85	105866.4	102874.2	106223.6
Q7TQ94	33422.07	18989.44	27014.54	16224.48	68098.42	22717.24	27895.32	26354.63	41051.7	35980.05	56001.85	27767.92	35575.05	32456.75	40943.34	47553.2
Q80W57	112611.5	130334.6	71414.18	124419.8	175418	92668.35	167980	73478.55	152969.9	252433.5	329310.2	83400.57	67287.23	84386.33	139601	173229.1
Q80WD0	64854.36	101098.3	78549.2	104149.1	116213.7	119973	70503.03	76787	83429.34	87668.7	90447.63	81448.95	103233.1	131631.7	118157.6	124814.1
Q80WD1	114140.2	191038.3	187113.9	140850	157383	175920.1	169667.7	123029.7	158650.5	132909	145637.5	130833.5	171147.1	222765.5	194985.5	208591.5
Q80WF4	34787.34	48105.04	40707.57	35098.8	37978.64	34764.43	38566.91	42344.82	51466.86	43073.08	40480.68	41318.13	59095.69	69712.57	62378.91	70123.07
Q80WL1	4905.131	26526.56	8495.394	28155.05	3673.604	3395.373	14531.08	24103.24	8267.707	9886.656	9231.116	20507.16	9477.109	6898.299	873.3964	6070.203

Q80WY6	55282.99	37380.89	48430.1	41484.65	44235.81	43996.61	55576.06	23897.96	13044.84	14553.06	17638.77	30298.09	26745.05	30236.01	26454.11	18528.18
Q80YN4	22932.03	20537.2	17356.98	79199.61	27922.85	33713.75	26810.96	23317.26	16892.4	22000.29	28139.79	25130.67	16879.96	26195.7	18044.87	22381.38
Q810F4	145189.5	177839.5	148724.6	156466.8	295312.1	213043.7	342603.4	156509.8	185046	155818.9	218288.8	159148.4	278464.9	188931.1	208441.8	192590.2
Q811A3	39029.71	16945.5	23776.87	17252.77	20301.24	11755.85	14514.84	24394.04	11751.33	21572.9	26807.88	13374.77	10309.73	529917.8	23918.51	22345.07
Q811M5	18878.2	28642.4	36049.07	44212.27	36949.69	19876.27	45122.64	17081.61	33386.26	36952.02	33178.96	29441.57	42329.47	50057.13	44375.29	41546.44
Q811X6	49302.71	38391.23	44712.06	34019.29	86602.33	59629.61	34677.74	38628.5	81593.62	113259.5	78090.8	52760.91	72986.45	66619.18	58409.37	94771.86
Q812E4	22524.71	6783.931	11702.85	12561.44	3608.655	12950.04	10733.46	11589.8	4843.884	10664.68	10860.29	16717.79	7744.121	5591.78	9767.519	9127.727
Q812E9	282255	250612.2	173697.2	317240.8	344409	137782.7	347558.5	135207.6	200995.2	254578.9	235529.8	190250.8	133669.2	121121.9	245122.4	281054.4
Q8CFN2	60753.47	66286.02	55224.96	84813.89	64220.76	108619.2	78303.78	78972.8	108826.8	120685.3	215943.1	114376	78862.73	56216.48	41527.11	21619.48
Q8CG08	90150.91	107054.2	103725.5	115226.8	100214.3	104624	95322.94	72525.37	136248.8	106268.8	87060.35	87572.99	148163.8	102250.3	106405.4	110410.4
Q8CG45	80165.63	54501.38	83368.78	52351.13	127706.8	62949.01	68020.07	87960.59	162808.5	140847	138410.5	89818.6	88067.1	82838.3	99406.91	105857.6
Q8CGS4	31646	31902.14	25248.06	31166.71	1939.539	26665.04	28434.7	33556.24	20221.16	33072.9	134582.5	30864.63	24018.75	11255.24	35880.94	8600.729
Q8CHN3	4544084	4733484	4009247	3210039	5008516	3902393	4456387	2520471	3658889	3270080	2739826	3132472	3396539	4569171	3775999	5501591
Q8CHN8	52837.87	58128.89	28171.73	27626.11	49918.34	31580.7	60275.48	16406.33	41989.48	57745.57	28392.3	42238.03	37196.44	23990.39	29599.75	44766.34
Q8CIZ5	26966.04	109113.5	49472	73023.05	33152.79	127236.2	63793.48	65303.65	26151.9	45705.64	39063.29	644842.4	30553.85	49144.89	36008.84	35847.79
Q8CJ52	72386.32	23185.9	10910.58	51817.02	29056.62	46630.58	68947.16	83861.38	5976.621	16238.57	35221.48	59780	29114.88	18875.77	4032.483	21313.12
Q8CJD3	11505.11	10127.59	32714.54	13642.98	64541.73	11338.68	20945.06	13838.67	12709.69	13233.87	13110.85	21657.59	5513.063	23234.7	20515.61	10894.1
Q8JZQ0	121328.6	129559.6	140989.9	106563.9	111424.7	95185.66	160887.8	132254	133233.8	104658.6	112938.6	133996.5	126810.2	165365.2	130938.9	142258.9
Q8K1G0	58224.41	57886.39	63073.69	85638.41	39288.34	45217.61	44701.73	59395.49	25488	35302.53	25253.73	48326.2	19064.38	20368.95	31252.37	11813.94
Q8K3V3	26717.21	33385.65	33039.88	26357.85	24882.78	25481.41	26327.39	33410.37	29401.2	33999.57	53890.46	25708.86	27942.46	32807.31	31446.53	27596.35
Q8K4G9	12894.83	8397.061	28020.03	44517.59	33157.57	17001.05	29324.7	4231.593	25257.76	11970.82	25389.02	11738.04	10751.65	14454.75	28298.32	20362.89
Q8N7M5	24244.53	33650.89	31601.83	16047.12	37919.22	33266.3	39417.84	27559.38	22557.36	17805.81	20534.41	15335.65	24041.43	20271.9	26872.4	26891.82
Q8R431	10586.55	14915.12	18659.05	10389.93	6399.599	9928.078	15335.9	7570.272	17726.09	21645.63	43864.62	12147.65	9788.896	110757	10574.9	13764.72
Q8R491	74504.8	164308	102709.8	147120.8	82897.8	122227.4	185509.7	65190.79	111213	164330.7	331198.9	172336.1	100776.6	119114.5	120654.3	119114
Q8R4E1	76057.34	80249.68	50611.1	55851.04	49499.54	60097.92	65939.86	62565.91	34377.51	62911.36	71107.36	57298.6	24834.14	33645.11	33378.26	49111.59
Q8R5M3	83204.44	45347.89	59228.73	83220.52	72303.16	49001.08	69669.8	15914.6	111889.5	95954.07	116948.5	93215.81	144005	85088.9	89785.86	126828.6
Q8R5M5	25093.44	26629.41	50576.43	30703.49	50874.66	33131.82	33376.84	26928.13	43620.58	37922.89	40552.95	36630.8	57932.24	29468.66	26721.92	27326.96
Q8VD89	82701.55	232280.5	138198.2	86657.13	143987.7	141612.6	54444.1	157406.6	327032.4	186261.9	116699.4	409699.7	163805.4	337101.8	185100.9	139072.3
Q8VIF7	151649	141789.8	149527.8	129272.2	209341	138871.4	153572.9	161265.6	212897.3	232720	214620.2	151843.3	177576	175653.8	238315.6	220697.9
Q91XN4	67035.55	104989.8	84779	97496.39	62383.93	83132.94	99627.73	107436.8	71027.86	52048.52	75846.35	64501.79	57764.14	69094.56	92492.59	59883.38
Q91XT9	27334.08	32415.23	31131.59	39987.76	30677.23	25085.24	26302.84	30306.4	20038.91	17994.95	22455	21591.36	25908.58	29987.71	24300.81	25319.21
Q91ZS3	37109.64	56960.37	18584.02	10421.68	8593.856	31335.16	22743.46	15096.51	35461.21	27208.67	29538.25	18149.41	17754.27	12515.64	10173.04	12174.12
Q920A6	1054537	1087771	1244364	943203.8	1608475	757899.1	1564954	1069132	766054.7	997434.9	1438039	1132022	845089.5	1642677	828965.3	1413849
Q920G2	9570.896	6400.156	7141.996	11511.9	8828.225	6215.11	5871.546	6713.97	6133.827	10403.06	3526.12	2249.648	3620.515	3610.743	6805.969	5682.383
Q920P0	32638.5	15707.7	28052.42	8821.334	55188.89	24309.75	12452.13	25747.96	56909.96	56949.66	41210.2	22601.94	33199.95	21292.11	28583.53	24655.51
Q923M1	25217.84	22347.12	23675.37	13665.04	47787.25	25960.02	31709.57	18459.45	57970.38	63384.91	68365.23	24103.54	40207.61	14354.2	47244.62	51409.31
Q923S2	122357.7	118024	98628.29	104836.4	139759.7	53342.9	119106.2	70055.02	232010.4	265139	277407.6	189508.5	163088.4	88171.26	146982.8	266881
Q923V8	15354.82	21399.88	14062.94	16111.82	6303.387	19184.98	12200.71	12898.29	14239.07	14652.18	13942.28	19104.96	11482.09	11680.81	20325.62	9898.35
Q924C3	6346.987	7035.571	3627.609	7828.662	5236.377	5738.951	5896.766	5904.755	6495.258	6161.411	7072.158	4446.002	7621.767	4856.642	3576.792	6971.49
Q99041	1548798	342934.1	1103457	4958348	1982765	3482012	5651548	4703396	263834.9	388854.6	1336707	2983775	707063.4	84629.95	543978.8	628559.3
Q99068	10508.19	16164.12	16601.46	47882.37	15233.42	9388.91	13543.25	4970.794	10433.85	12559.72	7600.396	8710.976	13800.25	14995.43	12241.26	11880.47
Q99376	8563.738	15890.71	15770.09	11024.55	16473.03	14517.71	8228.319	7024.045	9467.946	9951.551	18994.25	10849.45	7842.965	7855.61	9653.737	9021.165
Q99J86	199614.4	289001.3	267433.8	260634.9	256837.2	177539.8	193107.9	236489.6	223594.1	260790.1	242648.4	208813.9	270102	343261.2	242021.3	281876
Q99M75	30761.45	37215.45	33842.07	30315.68	47385.61	18885.27	14893.99	36858.85	46010.01	41742.71	40809.22	34999.49	51011.01	52386.77	41228.66	46593.07
Q99MA2	612211.4	603174.8	572315.6	600485.6	723299.6	451087.2	739342.3	430671.3	1056048	697508.6	917747.9	866133.7	625742.4	542830.1	742631.1	979528.6
Q99MF4	77743.65	100836.3	105096.2	63106.91	82466.8	71609.5	76771.66	73036.45	71180.27	93535.76	47369.75	53181.1	68461.79	86985.58	40456.49	50574.77
Q99MH3	421929.9	2641896	280959.5	1152422	724146.1	274043	980188	1081579	2196468	453452.3	2045464	491888.9	694481.5	2476645	1108774	2046014
Q99PP0	12913.62	23483.64	14826.86	19708.35	9003.219	14923.73	49189.84	11590.09	13667.96	17039.26	16996.26	10197.42	20708.29	9113.011	25829.27	25581.26
Q99PS8	408470.3	904266	1670476	508352.1	1517349	740554.5	889939	1042110	1231511	929565.2	698368.9	766002	1397559	1251792	1124332	1357414

Q99PW3	205596.2	234284.6	293102.3	196952.9	174811.2	306745.4	252537.7	191432	124876.5	110429.3	115903.2	128748.1	173519	223250.7	116204	166371
Q99PW7	19308.5	24504.07	19743.82	25626.99	29696.67	25326.72	23102.26	17928.56	18141.07	14517.49	12833.53	13899.13	16077.78	20706.53	18419.05	15100.81
Q9EPB1	5778425	6649823	6557358	6156832	7221372	4109293	4785156	5338772	9463602	9548730	42412584	8328545	9297145	17393608	12973765	11741971
Q9EPF2	64179.55	91240.27	96545.11	53145.23	91068.38	68743.13	86644.52	80138.14	71794.04	92257.19	59497.8	64834.97	86855.65	76717.58	91694.11	128051.1
Q9EQS0	6177.029	16910.87	7850.834	10446.64	3956.204	5964.171	8799.432	9375.658	3841.669	4031.316	4906.666	7229.695	1356.297	4814.211	1204.515	1689.803
Q9EQV6	122954.4	95053.92	89333.74	147832.4	53553.9	79615.77	65283.73	87245.29	113675.4	96967.97	106330.1	121231.4	120816.6	145925.8	132199.7	86709.34
Q9EQV9	123958.4	119811	72528.95	151853.6	50039.55	71180.45	73532.73	97662.61	50294.99	64023.17	50224.44	81829.52	22722.98	50438.4	77877.41	24658.8
Q9EQX9	23254.26	18704.04	14061.39	19938.04	17801.73	15210.31	16984.64	23818.17	29898.32	20688.91	26068.96	17500.07	19176.88	17512.93	21952.44	20820.2
Q9ERA7	11455.41	27756.18	15218.72	23808.92	15242.09	13112.2	20738.66	15552.91	16256.09	17620.8	23858.08	15873.14	15687.34	13885.42	18934.54	14953.57
Q9ES87	730506.4	809257.1	848653.3	536844.1	585055.8	502921.6	786643.4	648512.9	686980.6	748937.3	751121.6	607697.9	527286.1	785486.3	874688	914130
Q9ESG3	33153.26	44856.86	33476.29	49117.66	68369.05	42687.11	88894.59	35972.09	82266.89	127293.9	87417.02	70408.52	49770.19	37563.78	57492.79	88554.68
Q9ESS6	164144.6	315038.1	218505.2	285419.4	316927.8	181783	276677.9	296756.5	211788	206818	177829.2	186704.2	188901	266249.4	241615.3	203927.6
Q9ESW0	26692.29	12466.76	23453.54	37140.89	28649.3	7732.436	19683.88	15305.65	4160.204	7195.925	22210.48	21555.21	3201.085	9094.442	11209.73	5666.01
Q9ET32	23095.21	8538.136	14788.4	3321.875	15891.72	14633.45	20529.93	15368.23	6835.203	11404.06	9313.787	20979.3	5541.04	14523.8	6209.125	11644.78
Q9JHB9	907214.2	560832.7	118993.8	582127.6	359368.6	1285990	586771.9	717624.2	252415.6	263920.2	711025.8	1140118	678107.3	302113.8	46893.3	27393.99
Q9JHW1	25297.79	29896.04	20246.96	31502.7	19433.74	27705.26	19803.54	23290.31	11030.23	16671.79	20343.24	21646.75	12796.36	16308.8	15909.12	14992.65
Q9JHY1	1401662	1613736	1795628	1373013	1106246	1164398	1502732	1508253	1258699	1210930	1167264	1314217	1909662	1945444	1810615	2106636
Q9JI85	680774.2	315362.8	118966.3	429297.1	118914.8	551063	576086.9	367507.2	140021.2	122335	333533.6	319640.3	209498	139278.6	78891.29	45276.66
Q9JI92	236547.5	109661.8	169507.1	340454.3	190943.8	169600.6	131709.5	159282.3	110861.1	99143.71	213353.9	152286.2	146638.5	159015.2	101773.6	107501.6
Q9JID2	24752.85	15475.99	17368.17	34809.84	17988.05	15261.85	21337.62	21492.85	28007.08	31665.62	59337.02	33805.98	15673.89	26886.49	26163.82	18781.74
Q9JIK1	920038.9	700527.1	907508.6	672419	652738.1	741258.9	613597.9	955254.9	606864.3	371298.1	592779.6	653927.4	912860.6	763878	986960.1	1096237
Q9JJ19	650098.6	638418.3	510961.1	644110.8	719133.1	541987	607225.3	358874.3	1162355	974076.2	1327592	1003325	890250.2	551620.4	789857.7	1107798
Q9JJ22	17297.81	14945.16	8759.821	9051.805	7120.067	6814.846	6855.738	7107.286	8146.149	4967.46	4454.698	9630.275	5114.779	5665.382	7255.139	4441.063
Q9JJ40	776118.1	774003.4	499262.3	781870.5	1033590	614714.8	864194.3	383050.1	869410.4	1142349	1292325	676447.2	444761.8	499054.3	713334.1	1164999
Q9JJ50	17371.63	22696.79	16187.92	16846.5	21767.66	7261.214	11732.64	10833.03	25614.49	25037.03	29576.98	14788.81	9704.885	30434.38	19575.05	22726.37
Q9JJ73	16645.06	36488.89	49760.63	26189.47	42777.39	24798.59	24902.51	24711.08	21429.12	19780.48	22908.59	26451.84	24639.45	40616.59	20727.91	33734.07
Q9JJS8	10773.98	23432.31	31089.84	19945.16	10029.11	11553.84	4048.074	9917.663	8869.731	12103.82	5294.957	22342.13	6200.079	10165.1	5027.975	12129.87
Q9JLJ3	60008.39	69586.07	60213.65	43106	85379.15	67903.53	73999.61	53240.24	108188.3	104870	132416.8	119031.2	76802.8	85197.85	70651.8	86475.95
Q9JLS4	155176.8	268137.7	264717	178533.2	242415.6	199573.5	240938.5	206257	117533	193716.8	212845.2	126068.1	162137.7	154086.5	135457.8	277767.2
Q9QUL6	1134226	671018.6	3388407	7300277	364767	835477.1	1533033	3355635	552861.2	1265761	445516.1	4588011	4654222	2695751	3504756	3537936
Q9QW07	48432.42	6673.979	87218.77	12274.73	37098.77	9026.523	8233.941	10512.38	2083.393	3970.602	3868.812	8954.079	54723.55	65556.66	11972.99	15403.93
Q9QW30	24232.47	31712.85	22657.94	23725.49	25245.39	25832.2	33677.02	39463.35	36200.73	18054.75	29520.92	33123.38	36383.19	34296.4	39005.46	20838.34
Q9QWJ9	14363.2	30538.05	29508.94	19404.26	45471.55	11844.76	11392.68	32497.56	20497.25	26667.45	25720.73	30377.6	21297.16	168731.9	17620.01	34796.13
Q9QX79	2785265	1499610	2847933	2001760	1781355	1103556	1970980	2986152	1146716	814780.2	859513.4	767905.9	746569.8	1021477	996452.3	695972.7
Q9QXQ0	20497.1	35310.7	14214.8	38588.63	28513.01	21998.62	19571.59	41126.88	43821.5	51002.79	63347.62	35121.44	43257.69	38553.83	35964.32	56676.84
Q9QY17	30146.6	14213.63	13600.11	19708.92	21426.81	17149.62	35225.13	20901	23464.66	20671.09	31892.59	40183.26	19812.19	19107.33	9341.297	16463.61
Q9QYU4	36194.68	43010.24	21955.71	40860.95	49615.39	24005.31	32101.42	31935.49	61757.6	64955.52	129503.8	28829.76	45174.59	21967.59	45082.7	48016.84
Q9QZ76	15526.77	10252.33	19984.29	10566.44	7905.052	21389.02	12393.33	10994.15	24080.17	12512.86	14783	16583.03	16888.12	13775.83	26072.04	14988.01
Q9QZA2	302051.3	255468.9	248818.6	259675.7	295411.1	265049.8	267869.8	236516.8	224195.6	257714.8	295852.4	243771.8	216918.3	229095.4	218049.6	225927.3
Q9QZA6	12855.8	10736.72	4549.373	11604.51	7478.867	5612.815	10854.58	21950.82	4312.993	4133.661	7047.771	10922.53	1835.211	5158.426	7986.906	3727.458
Q9QZH0	179347.7	120670.8	183787.7	511333.2	139350.8	201954.7	223042.9	174473	144060.7	49917.46	61997.38	162433.9	223482.5	234167.6	227612.1	243171.3
Q9QZK8	53027.9	36631.97	29401.02	62738.22	39621.09	56252.7	46211.06	53968.04	18128.38	21195.01	29062.75	41315.3	24904.83	26087.45	19834.47	19226.75
Q9QZK9	29491.94	14766.81	32943.76	36547.27	27299.21	83825.09	58753.34	44311.87	4391.229	43016.6	40249.13	124001.3	53831.84	13893.54	12748.87	1442.526
Q9QZQ5	46835.64	186161.9	92769.33	163683.3	100143.7	188314.8	296014.7	126888.6	106928.3	123436.3	133621.5	124861.8	86188.31	90819.43	135030	102471.2
Q9R044	17596.32	22293.9	22587.88	38223.02	24512.22	22880.59	106092.1	33479.32	17340.51	18080.62	25278.23	19432.46	21060.91	27425.26	28816.88	26395.87
Q9R063	139381.2	104944.9	93951.99	79628.7	88634.8	100264	123472.4	153999.7	117134.5	118300.3	191740.2	168594.8	91390.95	93507.65	76438.88	61272.68
Q9R066	105684.2	135105.3	128105.2	81140.64	150329.9	87571.7	138196.8	131423.6	72123.31	102969	77175.27	66196.18	103284.7	84924.62	76748.83	103591.9
Q9R0D6	169720.2	176484.2	135236.3	204373.8	182977.9	155620.4	179683.7	177481.2	133731.4	186792.8	132913.3	165787.7	148983.5	152542.3	137430	124502.6
Q9R0J8	85581.37	141564.6	122054.6	115609.3	75084.75	58935.38	85033.23	61053.67	72639.8	94200.21	276237.7	106001.9	55973.66	115434.6	98633.2	95375.16

Q9R0T3	38278.03	37282.77	20431.59	68079.05	17032.23	42581.86	62974.02	61516.12	12786.24	19491.39	17908.47	25993.38	22901.99	8620.049	2335.08	21245.75
Q9R0T4	8978921	20963068	11048058	12510219	11989258	3749927	10675380	12424805	5747888	4126560	8402504	8679344	9621849	13480900	8311478	10573261
Q9R141	10619.26	13060.68	8455.326	11741.3	14910.78	8970.396	13979.63	6944.188	19027.73	24293.28	125192.2	15435.25	7597.284	11792.12	20508.6	17814.88
Q9R1T1	30318.35	25449.74	34270.18	24585.8	22157.59	10545.52	28680.07	21031.58	12820.13	16245.01	31154.62	25018.56	10845.71	12705.84	12643.11	10540.28
Q9R1T3	165029.2	173538.7	145109.8	170177.5	94108.67	187565.8	155888.8	118892.7	94345.38	91812.16	62357.6	113967	122674.8	85674.05	137025.2	65747.98
Q9R1T5	13115.52	19607.2	12692.29	13729.18	16262.05	15946.31	23278.8	11213.4	27885.76	28309.63	26866.91	7221.2	22853.56	17780.72	18640.68	28342.36
Q9WTQ2	349463.4	321450.8	378944.5	267097.8	466565.8	164159.3	244783.1	229077.1	123879.8	171736.6	204721.3	151167.7	148355.8	229727.6	232656.9	279415.8
Q9WTT6	14719.3	19193.25	13545.8	21999.69	20608.32	51210.41	13951.54	58680.24	13410.48	13872.48	5021.948	13871.58	12370.79	14656.49	10285.62	10184.44
Q9WTW7	139867.5	158407.4	94038.74	208269.4	300165.3	120961.9	177270.5	79460.15	212219.7	361889.7	314670.3	155691.9	165526.3	124381.9	247580.4	291677.5
Q9WUC4	1022278	1060951	1002882	958786.1	668261.2	553156.1	1206152	416251.4	564304.3	810058.4	789571.6	742890.9	807259.9	1256688	977273.7	880167.1
Q9WUD9	37664.82	22866.03	12412.31	25263.27	22104.75	37226.91	26988.82	34720.61	19143.37	18602.03	31477.04	26321.73	16538.22	23372.79	12165.41	10739.45
Q9WUF4	21341.59	15921.02	10442.85	18630.2	8025.884	14608.71	21231.19	16875.93	15533.46	12898.68	10077.84	14473.31	12191.24	14445.72	9522.592	13793.01
Q9WUK5	260928	273156.9	457963.4	232983.9	311474.2	133710.9	372098.4	396975.4	438868.4	266801.6	387058.5	451300	494455.3	486039.4	441501.6	633342.9
Q9WUW3	151768.7	58219.48	131076.1	121689.6	65406.52	95945.51	104986.8	165591.8	74292.79	59859.86	79613.18	81503.99	88053.8	99832.88	97026.75	92902.25
Q9WUW8	15768.87	14628.49	10502.04	13343.97	8860.885	9289.858	7843.891	12807.06	16817.74	31837.08	15402.48	17001.04	5939.339	10231.32	9088.273	41071.18
Q9WUW9	58397.64	68813.47	35913.34	84583	72131.98	61044.79	64713.32	52057.06	92137.36	108510.4	134453.1	52274.8	40346.2	16872.72	51520.52	67495.01
Q9WVH8	44512.98	60907.47	48354.84	51921.2	32689.92	50371.52	50869.41	46874.8	32212	38955.09	66560.96	38534.55	34753.96	32984.41	44967.18	42180.59
Q9Z0J6	14423.28	12126.09	20910.51	14251.75	12473.52	7554.138	11410.53	16167.28	13167.46	9123.537	19615.77	15068.9	16740.92	22115.07	18838.72	18577.63
Q9Z0T0	24631.88	27948.56	21347.62	20311.54	48677.16	20510.03	13224.63	18585.82	24081.87	18604.86	27771.04	21720.93	21831.9	19544.09	24800.93	22800.34
Q9Z0V6	46923.27	109058.2	92840.55	217656.7	135199.7	303983.8	65774.34	150726.6	20250.84	32899.99	24043.48	42552.85	15103.04	40128.52	29295.3	21912.42
Q9Z0W7	86850.46	121509.8	66916.91	100290	103311.3	65956.13	68267.21	79480.55	177749.2	231627.2	193705.3	113557.8	118840.2	62738.07	119886.7	153019.3
Q9Z1F2	5416.464	6917.603	11221.48	51332.14	8890.597	4512.072	16374.21	6091.852	17627.72	26033.51	22504.46	10100.91	6681.101	12717.89	25306.42	24822.68
Q9Z1Y3	764051.8	808271.1	720918.9	488922.3	1021748	380385	827598.9	789592.8	419620.5	295071.7	486714.2	368743	341783.1	688930.4	570879.6	484002.2
Q9Z2Y9	72119.07	76805.27	77009.1	51414.07	59398.75	62936.57	62846.53	70776.8	90814.5	113173.3	95839.5	75315.72	61298.37	106962.4	84405.98	73777.23
Q9Z339	185382.2	202707.3	209113	243577.9	71447.31	227073.8	145242.8	167338.2	178045.5	245469.5	245946.3	208628.4	151868.8	134369.9	194328.3	92039.33

17	18	19	20	21	22	23	24	25	26	27	28	29	30	31	32	33
D5-RAT9	D7-RAT1	D7-RAT2	D7-RAT3	D7-RAT4	D7-RAT5	D7-RAT6	D7-RAT7	D7-RAT8	D7-RAT9	D14-RAT1	D14-RAT2	D14-RAT3	D14-RAT4	D14-RAT5	D14-RAT6	D14-RAT7
16409.45	40589.12	2141.118	37576.02	13033.28	13716.8	58501.69	26713.88	24140.14	33812.65	64789.26	27535.17	18832.64	13814.6	59696.07	23590.44	13013.9
14286.39	7344.103	17865.4	18523.71	12305.39	17762.75	19601.82	13526.2	11451.17	9604.707	14297.91	17291.13	9033.076	9330.402	10136.51	14520.95	13315.13
113124.4	89433.42	92411.85	87298.63	67301.41	87418.37	73479.37	105684.8	89757.45	128014.8	92918.11	98061.97	78825.59	85872.91	114612.4	101006	123234.5
30708.06	34558.35	23731.73	29057.57	50572.02	29900.36	31497.6	24261.83	32927.95	41216.46	30318.05	26290.13	25593.36	75819.54	34790.47	34183.2	28082.34
61633.55	85519.85	67520.53	60941.14	48076.27	63983.53	58514.8	53533.32	65006.48	58975.52	78914.07	68590.84	37589.41	68242.02	75032.71	74378.56	57204.02
59581.73	31511.96	23703.23	89621.48	64417.37	29578.78	12468.06	85426.31	17968.29	506773.1	34008.63	29027.6	42224.63	53838.54	37504.82	6546.228	52620.46
5520.021	5405.481	5451.005	6895.621	9616.894	6408.81	8254.396	5886.378	8354.117	6811.54	9882.305	16425.59	17804.35	11182.35	9848.104	9754.882	8475.594
43216.66	43558.24	52089.6	61934.24	41482.23	54537.77	46729.7	51151.99	41025.54	37796.33	35870.59	35826.62	34956.41	35854.78	45817.39	43017.93	31018.4
10112.32	11457.15	13721.82	12815.75	6998.285	8029.446	7578.182	10391.31	5521.094	11583.02	12786.77	16481.96	8901.125	11386.72	10664.86	5777.747	10324.61
8030.705	17490.16	7864.747	6417.9	10698.86	9496.132	8500.532	14155.74	20262.97	24939.29	19669.79	29092.16	22351.91	12915.96	13919.75	13508.26	21772.37
8604.83	16593.3	13255.91	12502.14	10746.85	12579.38	14889.91	11322.64	11075.41	11893.75	19204.62	15887.34	15749.11	8235.604	11498.41	15223.53	13562.47
266954.1	523678.1	594696.5	570464.7	426001.2	478320.6	392730.3	377828.4	516228	209165.9	424897.8	165241.1	351557	358454.9	320086.5	397200.1	370448.9
12617.67	15497.32	10661.92	10635.38	13654.43	8722.39	14800.6	11773.47	19382	15082.27	14209.02	19047.66	15666.47	22521.96	18401.27	12172.34	19405.27
5942.279	22653.29	22217.79	22114.63	31095.99	16047.89	25229.53	48207.08	21940.86	35318.67	24397.9	42754.01	27174.98	42634.55	37342.16	15072.86	34293.61
15299.88	16616.25	15377.5	19169.6	16800.17	15990.21	25355.76	20161.68	16294.3	16980.08	18180.31	11982.91	15723.82	16895.5	16166.55	17765.73	16028.91
231454.1	264518.2	223655.9	264987	364408.3	221253.5	270751.9	205424.1	148020.2	289750.5	268132.8	218276	325454.1	356417.5	269616.9	294796.8	213358
18124.59	19126.01	24407.64	27724.87	17099.06	27305.02	25917.32	26923.24	26628.44	19165.02	14527.05	9964.531	19037.56	11801.84	21301.64	19502.62	25053.24
12739.06	22590.79	12735.7	12898.74	17876.03	16929.19	23757.84	10863.25	24411.86	99907.27	25401.48	13224.48	14792.03	7302.9	17243.78	18923.87	17389.46
352512.6	520288.7	420213.9	112973.1	806840	240163.4	413696.6	408960.9	117545.5	374193.5	753344	403539.9	298815.1	538055.6	321854.2	240973.3	203167.3
39272.26	40420.23	39380.13	53322.19	46012.56	68985.11	64961.41	34676.19	45372.27	48879.53	62617.02	25886.4	17021.13	40198.31	29720.12	34981.65	50339.7
28900.87	24915.89	24031.41	60105.71	21268.52	21183.79	80461.17	43957.04	26605.39	71027.56	31025.35	22513.13	17363.14	23860.91	28575.19	34328.66	50631.95
20300.61	24204.99	16607.3	18656.73	16195.32	18355.74	21453.29	18134.61	20233.57	20034.29	20806.8	26800.19	19579.1	25385.95	20240.78	20701.71	18029.15
27605.96	10130.93	15503.58	9163.746	18769.95	17835.47	35088.91	14664.36	17493.26	104570.8	16764.21	4524.325	20201.21	14368.51	16089.38	31002.43	18942.11
8031.242	7228.785	9705.91	9302.823	9158.746	7281.458	5914.833	4465.369	6246.215	8610.237	9129.702	13288.46	16936.93	8841.938	8617.375	10070.88	6503.398
26881.31	17671.05	17471.54	25658.22	65458.93	18231.71	31832.85	21900.45	23479.97	36998.94	45436.02	113698.6	90451.09	46035.39	17929.42	42634.2	37233.67
6165.384	19519.55	8422.743	10403.29	11193.7	10856.86	7761.612	6454.188	9924.569	6968.948	25915.65	13573.91	8487.023	10174.04	9164.275	15808.13	10109.7
102002.4	159046.3	114913.7	157142.2	127495.9	279446.6	275280.3	146444.7	204512	153441.2	249965	69840.99	109815.9	69984.05	114562.1	181582.6	122838.9
204769.3	268785.4	166229	188316.5	163315.8	211785.4	219088	182477.9	169233.3	186069.9	289091.6	220553.2	227645.7	208922.1	241977.2	300560.4	234855.4
87351.03	161865.7	54411.75	97554.02	83994.84	80515.82	102054.3	109964.1	95772.34	90118.44	135485.8	163795.1	85541.83	124056.9	136764.2	106084.9	161499.5
42844.1	26066.46	27645.89	35670.68	22316.92	17854.1	16620.48	32099.58	22064.25	30375.36	30143.5	31128.67	24339.2	21181.92	23040.9	22538.26	23123.73
71719.5	151368.1	138363.2	103007.6	91452.92	59415.2	242196.8	91958.42	140579.4	75596.25	128222.8	92598.64	56799.48	84364.73	50699.09	215503.9	120785.6
78593.96	17426.02	12041.01	12411.13	21983.13	7402.255	9924.226	6562.431	10479.42	10803.68	22490.92	39463.84	35698.39	17658.43	9943.374	16621.82	14065.2
13813.69	25209.09	20590.32	19385.92	24659.38	16869.6	18608.65	19878.62	18719.24	15350.71	18428.9	28342.91	19661.45	19748.58	31176.65	12836.86	15149.07
38573.54	28563.84	20931.05	22598.1	17859.88	24278.96	10269.39	15230.7	21181.97	26799.04	32899.61	26599.28	37959.88	19501.5	21504.67	17993.73	21301.05
110296.8	116227.2	111513.3	121823.1	83153.86	111906.4	128514.3	105373	130738.1	79638.08	134167.5	91838.3	128548	104512.5	103457.4	150907.3	136778.6
36017.06	78188.84	60860.38	72188.27	95122.2	67518.45	82433.92	65928.65	86558.27	58203.84	86051.27	58779.54	57349.2	78396.6	70661.71	60801.83	72003.73
480650.1	585811.3	664805.1	663743.4	502957.9	517562.5	809139.9	593337.6	515210	587775.9	540155.7	548755.6	634510.1	517471.3	497566.6	460554.7	512954.4
254137.9	276583.6	231701.7	191256.7	235513.3	178159	284972.9	198663.7	227611.6	245402.1	287023.1	264265.3	158711.3	226589.8	215310.6	292924.3	286194.9
39799.46	38121.86	48417.24	35543.01	21216.23	30008.41	30734.68	34316.1	45681.87	55335.28	32260.79	27977.82	33232.85	25921.48	30758.95	33732.78	39232.27
17654.02	30401.97	27279.09	62354.74	22955.29	20651.76	22946.01	20687.59	29580.75	20839.1	24406.43	13943.75	31609.73	22029.02	22758.29	20590.12	21079.96
56969.48	95021.52	61236.05	87802	116367.5	58321.16	62210.47	55075.19	91425.25	93430.94	79677.22	144955.4	97761.11	103766.1	94720.17	66282.84	63874.26
13953.26	28074.63	21173.29	15293.07	44667.39	19398.97	16718.54	7122.662	9926.847	5322.711	31067.46	108598.4	68356.63	17150.47	20767.51	16066.91	22837.65
427793.2	913598.8	628417.1	611836.9	695030.6	989977	664730.1	613560.1	1042073	580621.7	675966.4	357057.1	446313	590301.6	604404.6	622688.5	684724.4
28841.15	35475.07	30112.1	29631.66	40871.52	31759.25	22734.54	33963.11	62778.28	35704.21	24774.43	34401.38	18870.95	24635.44	23056.32	11718.43	17168.93
212428.3	320852.8	213630.9	210135.4	181609.8	204871.5	247856.8	207055.5	234845.1	351693.3	269632.2	212119.9	204811.8	249007.1	301880	323801.8	186451.4

3454.755	5122.269	994.0897	4145.578	5721.109	1729.642	2897.516	2387.956	4461.434	5767.614	3297.199	2800.418	4648.53	4527.162	3287.482	8154.285	7558.656
54511.96	54384.79	45521.55	35661.2	31560.82	33207.73	33942.66	39826.92	38133.07	49515.63	40685.24	40640.27	29577.75	51215.72	39157.52	45315.1	43896.64
7461	16033.58	6056.116	2670.082	4604.742	8907.607	17831.32	7230.302	8495.391	15232.18	5552.188	9319.682	8662.006	4799.119	13235.45	8371.368	8605.067
58839.99	123586.8	103042.1	129797.6	111185.5	67517.75	65763.16	89863.3	59649.51	68974.44	173427.9	147334.3	112960.3	100266.8	75054.47	107463	107576.6
249277.4	90774.08	5972383	4328526	17336.01	8125751	5342056	1589192	82449.98	11488245	51420.66	4008110	22481.8	136222.9	2911048	25511.63	8259.557
214993.8	192960.9	72406.8	119883.7	288710.2	151917.6	64970.65	125672.6	179233.4	225383.2	168543.5	124389.2	165045.8	249421.5	155438.8	150065	271424.5
288134.8	357757.5	333520.4	146191.9	229824.2	231403.4	286819.3	227723.3	235021.8	172816.4	387493.4	79340.86	91339.33	243010.7	208120	173114.7	222323.4
75485.67	79180.66	86874.82	38461.25	57915.18	127926.1	135810.8	89478.87	107923.1	84373.9	71959.63	57129.08	27337.98	66164.19	119575.5	124162.3	85514.21
16629.28	12342.08	12991.12	10520.21	12399.06	9572.342	13213.09	13247.24	11721.32	9931.431	19568.19	14183.77	15430.38	15392.75	9665.49	12194.52	12418.06
346938.3	365529.6	347652.6	620129.6	618740.9	437681.3	157763.7	359449.5	141886	502891.6	307566.3	518487.6	423262.6	436747.3	445563.9	168460.9	280537
972951.4	709298.9	733712.6	838464.3	1093469	1118063	1317351	761310.9	1173249	1176385	765291.9	485501.9	799811.4	738809.8	498692.3	769404.8	754341.8
26760.83	21846.9	23462.09	16203.56	16766.11	21134.2	15338.69	17554.31	21108.55	18354.84	26861.77	28355.91	19816.77	21868.02	19042.74	18038.5	20916.45
66548.07	13296.38	19900.52	25218.25	17321.2	12998.28	23328.94	24517.79	12306.5	8434.337	23435.09	31523.5	29549.62	22646.74	20886.81	26425.52	139996
388103.2	314413.8	344249.2	367661	279837.1	294426.6	597888.2	374103.4	431049.2	487739.3	387444.5	295368.9	381775.6	360965.4	300195.6	619691.4	319517.2
30393.27	87538.5	45816.1	52191.25	61776.14	56918.56	52830	51454.01	42092.49	62736.59	89343.55	94421.63	102415.6	58072.46	73689.56	56641.64	53787.66
12874.72	12723.37	17029.46	18789.13	21955.06	46095.98	10936.14	20274.52	7525.877	22939.57	18479.87	29752.05	22921.93	15939.64	19210.39	12402.62	13100.56
2159402	1931012	1028835	1327633	1490578	1640900	1753028	1266886	1143494	1424964	3048161	1372770	1071375	1537889	2062092	1765237	1122659
311058.3	334076.3	280030.9	407555.9	290574.5	416307.9	453208.5	365814.7	415573.3	307303.4	431543.8	445443.1	396171.1	258957.9	359035.5	457847.8	425190.1
1968546	897260.9	761230.3	821655.4	786082.7	869182.9	1744751	1536333	1145939	1500191	1045457	879829.6	923797.3	1087935	855897.3	1277936	1928861
128369.3	216172.9	201858.8	231913.9	188054.8	197145.9	285429.5	226625.5	247840.7	153064.6	211242.3	173831.5	205459.2	172791.6	167078.2	223107.7	220742.5
2205.504	47392.66	33043.64	32216.97	9610.803	33106.6	30429.63	29032.92	39471.77	64710.06	39433.92	28912.04	22348.33	26829.44	26030.96	26153.13	27275.45
29224.84	44460.35	29924.24	52415.84	34408.98	55235.57	11449.03	54218.37	39035.55	95405.87	60903.84	46874.55	43002.3	47085	74012.69	22770.14	25979.5
37820.43	53133.98	46416.66	43120.5	30234.79	53110.08	53042.99	47934.96	43215.44	31214.68	52941.95	21757.56	31146.33	24715.45	41120.84	45376.26	31692.28
25909.75	26837.32	24020.63	28151.52	17723	13995	17435.64	16880.27	25339.63	21137.69	28299.43	32038.84	25783.17	18687.49	24264.23	19815.74	38964.88
7894.962	10943.65	9278.419	10195.94	18399.75	7051.344	7547.733	8162.724	8611.984	6353.865	14638.12	12538.63	10247.47	5730.228	10222.72	8600.902	9810.514
244711.3	185212.5	204848	292570.1	473196	257417.7	230236.9	194432.7	260686	402822.2	162065	190800.4	235479.7	259157	212633.5	154235.4	209853
97655.73	161467.1	129097.4	68587.9	100358.5	104324.5	112485.1	106931.8	160327.9	146846.9	184239.2	269294.1	119847.8	133240.6	178646	156395.2	183733.4
5539.836	10214.56	4071.702	2828.157	1523.435	5298.313	4121.46	3258.96	2142.793	7065.372	7657.307	6060.182	2825.824	4020.169	5001.757	19005.97	5438.631
59326.64	109985.1	94431.32	107820.4	82353.83	89442.16	90141.66	96327.99	129473.1	46342.73	75194.36	71840.95	76933.11	70127.5	63224.96	80604.19	87836.47
328902.3	377273.4	292018.2	310480.8	282093.9	189729.5	211547.5	253533.1	257201.4	421333.7	482729.4	496504.2	344249.1	322133.1	348979.8	434787.7	432305.2
17039.4	21123.05	18577.2	14455.67	14624.41	12474.06	21795.01	15483.14	18404.17	25140.52	24570.49	19589.08	13483.86	19358.5	19229.67	23507.15	17211.4
75263.95	139114.6	135398.7	49075.47	158414.3	75097	78465.05	110363	54491.42	85779.62	133626.4	82630.69	91452.41	118997.4	100740.1	87410.05	143689.9
485288.4	500517.4	375015.6	377387.3	346647.6	333743.7	352041.8	369645.3	463928.5	657287	528701.5	504816.7	352599.7	515224.3	504725.3	417990.5	437154.3
3754.587	6596.15	6498.385	9906.641	5307.82	8708.906	5508.131	3596.05	9277.288	30029.85	4946.664	21487.55	6868.787	5713.32	5561.101	5895.179	5338.163
24833176	22178334	21523186	22598218	25674460	23505452	21123780	23079798	24707346	20867062	24031712	25413634	27650286	24431700	20905432	28060262	28157964
53697.1	579089.8	183121.5	91761.64	39865.13	26346.88	427301.2	48845.1	832940.3	395266.1	472960.3	324497	140062.8	180070.9	69763.59	570526.3	110011.7
1498.709	7563.232	14987	37462.29	12857.15	109936.9	67403.21	92931.6	40457.01	102840.6	23509.85	9721.613	14871.73	6143.755	16543.72	14088.02	11433.35
18953730	14051439	13550457	17675060	10993960	13762350	19570556	18820050	18181886	16498043	13672925	12171926	16502042	12905515	13678156	17362916	17632002
9560049	6899943	8642256	6503365	13009048	5819692	13609300	6920969	6447363	8513606	7570485	13248232	10471589	14804671	9407778	10449226	11771222
262268.7	38548.75	1648.614	768.4922	19717.09	1355.971	15156.62	9855.689	24743.21	30785.41	20259.79	15839.17	18891.96	83597.95	20056.03	17454.13	26393.35
306632.4	369640.1	363771.2	567980.6	460584.6	434768.3	479903.4	479011.2	502945.3	423786.8	370481.3	367151.3	524762.7	393159.5	377596.4	396407.2	505194.2
2297640	2023410	1543629	2246528	3305984	1718206	2241599	2095560	1709689	3501487	2222514	2297183	2261748	2802920	2000884	2334733	2387455
507047	519682	505361.9	376046.2	275506.1	366470.8	317393.1	335849.6	383951.9	643628.7	490928.5	385459.7	350797.6	364707.4	501498.8	555854.8	563367.4
1327373	808930.6	1139984	2167539	1715236	946875.3	678417.9	1636026	408756.6	3104188	945789.1	2040872	1739312	1786580	1764218	721616.6	1495417
580405.4	419237.7	335433.9	372129.9	309093.6	235058.8	342540.1	409819.1	338173.6	2911686	315501.8	197196.1	233618.7	312085.8	187993.4	160215.8	277667
114959.3	127259.8	63285.93	152306	133664.8	80586.63	26809.38	75784.31	63099.13	217915.1	81244.98	65223.55	105404.2	107792.8	57875.4	137304.5	61014.32
9051840	2833832	2352370	1887555	4274717	2768800	3937472	1971432	3590995	31822900	2051206	1852033	1530979	2098744	1762781	1901068	1622116
1519802	578682	765983.3	1727449	1579239	544997.6	667228.3	207820	377696	1621497	1009302	2701755	1796828	1365085	335568.2	747356.4	216966.5
183640.8	220556.5	255436	329942.9	331018.7	350293.7	237182	322454.8	209586.3	203060.5	226662.5	179121.6	224347.8	333783.1	346150.9	223938.9	281517.5

22605840	13112615	14023.26	21644372	32731486	55872.24	13326896	57721.17	4883546	10692512	18261274	20091388	19968662	34991300	52995.02	11810644	40715.49
606350.6	1554506	617914.9	5578249	6414119	3650419	1013924	4149593	1169588	6150326	1212845	5659525	5356775	6168946	4350635	7657749	754328.4
307656.6	29296.88	47485.69	29166.13	34410.21	23452.5	236345.2	110240.1	135455.3	556172.9	304510.8	29036.06	332075.9	19125.75	13649.79	10331.44	13403.66
620311.9	15227	63574.5	33639.91	9458.206	19830.09	378210.3	178989.7	176286.7	1261024	356320.6	35771.83	468667.7	11621.26	20888.76	1560.917	15521.4
38665.56	319021.1	134536	154960	96858.02	76047.92	55836.13	90281.09	190610.1	22312.24	106005.1	43927.43	64403.16	132005.1	96281.38	66692.06	88827.84
188240.4	439601.6	444725.1	156029.8	208014.5	421937.8	506914.8	240698.1	77118.02	217714.9	382000	128530.2	140825.5	304507.3	671548.8	133967.1	217477.5
23118.16	53168.48	63192.04	29367.97	27142.79	24016.4	53475.84	37258.05	25966.38	5600.811	78144.15	46561.11	56897.07	25054.25	11591.03	39331.08	34177.09
4937222	3570804	2670346	3631970	2480804	3750675	5454650	3854560	5939745	2252972	3451325	3036874	2790515	3079092	4019868	4665427	2433014
190130.7	455503.3	397229.2	413538.3	279487.3	353696.3	236049.7	360512.1	293544.1	268174.2	483100.4	261290.6	301425.4	294737	267792	269388.4	363772.8
38001.55	14789.52	19291.05	21122.02	21970.95	24342.37	14348.38	33096.39	25825.3	102314	14550	15851.08	22096.29	26926.17	15205.02	11240.24	9997.863
24671.84	17147.57	18316.18	20227.01	22434.52	23774.84	7677.204	13159.08	20180.95	19832.5	27596.56	17496.2	11612.94	43233.32	13982.83	17595.28	24916.57
8.08E+08	6.34E+08	6.02E+08	8.35E+08	1.39E+09	4.08E+08	4.05E+08	7.73E+08	2.29E+08	6.64E+08	7.93E+08	1.86E+09	8.06E+08	1.4E+09	1.09E+09	4.99E+08	9.07E+08
11842238	4546048	5563774	5264617	8285532	6039870	6484497	7908757	11778194	95184856	5766928	3410766	3910543	3563599	4258228	3691427	4829129
1562604	1892219	1153515	1347247	1414158	1195302	1291451	1301649	1421397	3806717	1390785	1419530	1303730	1757387	1455309	1134936	1395282
1.18E+08	54251328	43963780	39275136	45472444	40145384	37291740	48989740	27812412	2.72E+08	40373508	42149088	36229740	45094332	42401364	29189832	36435084
19955032	93376632	50343228	59921780	85872496	28701542	76483248	31170416	42107892	53644528	42947504	1.59E+08	1.67E+08	83010184	64936984	80598328	82491936
6090998	23006872	17257882	22669980	39281664	21565758	20237512	15793345	14980469	18269918	56158652	82536480	60457136	32370326	24266080	23962298	25937972
8948493	13259205	14382230	12356621	26064356	16427112	11636877	12632519	6029461	11290093	37631772	48514208	39586256	23817366	23832040	14484070	13893933
7637.26	8019.108	6775.165	5401.868	6346.777	7225.367	15164.89	7951.529	6308.379	21296.35	6370.268	7229.108	4849.594	20854.63	15278.35	2622.389	2709.852
20298.18	12214.16	15155.67	42543.11	24399.45	70587.41	24748.99	62679.12	23866.94	40130.32	33523.14	5061.341	13332.24	21583.47	4769.323	21915.17	7894.582
24205.65	16275.76	23537.28	24849.71	26477.13	24665.91	9863.101	11295.97	23784.41	19942.54	12673.37	15941.99	19406.22	10614	11751.55	11573.02	18111.78
23646.88	35270.34	20812.66	22133.97	17904.09	19340.24	18827.08	18682.91	19313.31	51520.95	27489.73	28692.74	14956.34	29911.89	25159.88	29920.82	25349.92
63325.64	107278	166811.1	77136.62	237828.5	111308.1	165008.5	107792.7	165307.8	40292.87	102042.6	101308.7	73240.56	168715.7	72092.8	143504.1	119864.1
8237.298	6669.188	4727.193	5559.108	2365.663	1622.322	6307.249	3727.002	9570.329	6179.067	5569.885	3998.107	2746.571	6689.723	5806.961	6509.688	4539.996
6576.282	10991.66	14393.02	11202.59	4549.495	6707.218	12209.92	11690.16	12630.04	11615.17	77056.38	57487.84	8175.226	11789.74	5662.111	7722.524	9145.394
38957.9	39406.79	55386.25	56296.9	51954.02	64547.35	50989.08	50818.45	50094.8	81579.75	120987.8	53988.43	99136.03	53430.64	63198.98	20161.13	68157.91
1531813	1666193	1476052	1699626	1276664	1232981	1083960	1614488	1261347	4185104	1130185	926881.3	1290949	1176611	1122191	783716.2	987961.3
45546.19	23277.82	30630.04	24692.46	32636.59	37095.82	35256.42	36179.65	9934.196	79681.17	35569.07	12487.48	32570.13	23531.92	47328.57	27847.34	20121.62
30820.81	59193.52	71206.42	68406.73	50579.83	34970.27	96484.73	60238.68	93871.03	19811.24	59165.88	25267.31	45517.09	54647.94	35144.41	46821.22	35883.28
332173.8	97225.49	56427.98	78758.38	67759.13	80816.49	90827.61	87849.38	96808.29	1267214	81395.67	55735.52	70438.77	98137.41	100302.3	68630.56	72651.65
137545.4	165767.8	244067.7	216455	149090.2	146001.1	137962.8	149677.3	201823.1	525522.8	177393.2	243605.9	168012.3	171604.8	186438.9	110342.5	149433.3
12325.56	13159.98	9657.21	6068.071	7027.464	7681.871	12328.02	12931.1	8286.594	12362.67	11382.66	13134.49	6275.31	11717.07	6570.491	8424.89	11952.27
579480.8	899038.4	819891.9	592173.7	525521.3	573707.3	593692.5	597985.5	736306.8	561322.9	690414.3	579990.4	498448.7	518946.8	753586	566442	747751.5
68259.03	109881.7	99154.98	110120.2	77076.45	98509.92	118001.1	87418.81	111176.7	73123.47	131816.1	126299.7	130397.6	84088.36	89591.11	106873.4	106146.8
628591	720426.6	666969.4	564096.4	775641.4	617328.9	427604.2	659634.4	474023.5	713751.8	698879.9	876718.6	515218.2	712913.1	744815.4	762021.1	713963.9
157349.7	274159.4	200597	215472.1	310007.2	163651.4	157064.6	173759.2	204422.1	176623.8	243976.5	548284.9	377177.2	292351.8	222539.7	201885.2	231379
12228.34	20620.84	20386.95	18836.87	17562.67	19441.44	14511.45	16555.17	13382.68	19846.03	20889.4	22170.5	15390.61	18745	18388.72	17920.31	13233.36
1235540	1078306	1142182	973811.6	782540.6	849513.4	765721.4	861111.6	903691.9	1223708	1154923	956321.4	774617.3	929216	1276376	879239.3	1058204
12940.54	32204.17	21597.25	27987.01	61775.22	19923.22	34409.45	14418.83	32955.99	31484.31	27695.23	90001.84	71930.63	27218.71	28366.31	44288.79	40247.98
48529.52	35995.33	26017.24	32625.39	51817.99	19736.65	68100.66	62375.9	27974.66	49050.89	32845.66	38832.14	21919.05	29375.97	25076.88	60773.35	44064.64
166515.5	189209.3	125554.9	194490.1	184220.3	115965	113165.3	183273.4	144394.2	150089	150889.9	191947.9	181667.6	260022.6	116701.3	126803.3	190212.3
11685376	13559358	13532746	12438762	9540416	12851670	12309059	9922544	9347394	10725840	12963153	7479205	9576603	11050859	9727049	11020158	11229860
90454.47	125900.3	104330.5	100729.2	93123.91	100436.5	95065.65	100895.6	102688	133964.5	118800.3	183769.6	131510.3	105969.2	175187.3	138171.7	109636.7
16419.64	48547.38	36429.46	28586.21	33681.16	20877.08	37099.03	20601.1	29108.84	25761.52	38921.21	52167.36	39734.32	27681.18	23034.19	29781.99	32426.8
23979.82	30926.11	14349.9	8165.486	14728.74	8631.935	38815.18	10182.37	9140.275	11044.52	51591.3	41855.29	31499.64	21969.47	13509.73	20951.74	16178
252554.5	383539.8	297005.3	301643.1	260414.6	324617.7	475637.1	345005.2	573697.9	313997.2	512637.3	425942.7	649220.5	329116.6	374224.6	548307.3	641783.3
16521.35	9398.499	22974.01	34021.21	12816.7	38058.19	27328.83	5398.363	27643.87	101049.1	29489.88	12612.76	15595.57	6652.757	21811.06	37401.75	15679.86
62437272	68482264	42481220	34037680	38700136	51135664	64183012	39647744	41929204	97023704	66634836	57480756	38536864	41520292	58418304	54232732	48053836
29717894	30698948	17432026	15904374	18613576	23549964	29529542	17216450	19396848	40715372	31244400	28550048	18348068	20230984	26257600	24267004	22402838

5968.756	15935.2	8179.181	9855.604	13637.52	8253.899	11482.28	12183.61	11628.31	10086.77	18242.81	41249.13	28847.72	15836.33	14839.01	10281.13	11739.2
35777	24636.44	47273.9	20515.78	19823.39	23021.95	16927.39	20676.59	13060.74	39725.49	33135.18	6604.214	27012.78	40489.85	28307.81	18536.75	12752.8
290969.1	516389.5	392747.6	84611.38	352054.7	86346.26	334316.4	103034.7	171402.5	195292.8	374105.6	288361.2	371762.8	356622.1	216452.8	192711.1	200099.2
38417.98	32100.02	26661.83	29731.42	30356.76	30294.69	27051.46	35003.97	38097.09	112411.2	28756.08	27370.77	30318.08	29741.07	17688.08	18855.51	19047.41
32775.64	37630.79	35863.44	44969.82	29922.93	26225.3	23703.88	55406.98	37412.56	39061.02	40515	55836.69	56074.18	45376.29	48451	40008.21	61484.07
23860.64	37393.95	18643.59	14956.68	29653.92	8573.916	10016.4	11647.13	43496.81	13719.95	26571.46	33413.29	28009.23	25386.54	16191.54	24431.74	25387.49
4521216	602783.3	1210100	1559702	4631309	1640717	3977029	1848388	8897583	861526.1	6628974	349509.8	2144386	2171171	809840.6	6307388	1679670
49442.91	74705	78470.04	68977.99	97573.48	98793.83	80852.59	63642.16	81861.87	79122.92	133805.7	134240.1	152951.6	76433.47	68426.44	78372.41	93961.35
10838991	2363379	2982321	4277538	10853451	3194380	1294787	4241659	4310299	33865424	2463174	3445041	2335071	3823227	2190480	947159.7	2191006
11905.3	23714.96	196381.7	56757.99	64785	562601.3	414520.3	133210.3	1152104	30768.87	367727	1318308	742741.8	177238.9	985142.9	661910.9	378584.6
49240.07	67733.31	45255.07	35685.57	216498.4	38061.66	124731.7	61381.92	138044.7	92604.91	133642.5	47573.21	63763.46	70815.11	35524.09	77713.42	63468.73
17930908	6033079	8800958	5371405	13773583	9136158	6023252	10838323	5313530	16896690	9482472	12297484	6881744	10546833	11265272	5134966	11528044
133283.3	183454.6	161902.1	116258.6	155937.4	169670	174217.2	150951.8	192009.8	123279	171790.6	154009.2	161889.9	149297.1	126254.9	152871.5	159337
189772.2	1939065	924438.5	1367265	359634.7	609698.1	1664685	813320.5	1030142	535996.8	1088757	786497.6	623393.8	405325.7	588717.1	344972.3	352432.1
6525723	7078493	5169561	6546713	5327915	4747451	4625062	6043160	4721532	6927464	5662442	6435204	4796358	6929584	6192658	5051492	5046995
87621.76	109590	121675.8	138699.8	109866.5	114870.5	107143	123915.8	102817.7	65608.85	122026.6	110578.3	130188.7	113843.8	110527.5	114198.7	117936.1
18008.49	28434.54	43579.29	30441.24	18434.17	29562.61	7391.793	30603.71	19695.18	12949.25	44263.82	27863.97	26336.15	19751.62	30672.18	36680.89	41631.09
102110	266581.9	220065.8	206132.4	116604.1	228287.4	226299.8	178247.8	244665.2	166052.3	174418	137724.9	126489	119101.6	179270.4	130886.5	205898.7
34800640	55203960	48992892	43100196	41986608	45543568	66537944	37356672	52120464	42693164	50318240	42995664	40742764	45014784	46259348	61363384	42680864
7101234	6684872	6201288	4678415	6493638	11666079	8451946	9029183	6666933	12279389	7410488	4920705	4211002	7181730	7521837	6082056	7001494
4699543	12560958	11701440	10368777	18982310	14215154	7077323	6205147	4684306	5484585	21377522	26547136	24721620	11728107	11830152	7374452	17433576
1130986	1915514	1179124	1738822	2423597	1060933	1459984	1026538	955167.7	1394551	1949561	3971388	2960712	1814878	1179234	1701182	1366335
498375.5	544315.9	552325.6	565767.8	403502.3	710249.1	659182.9	535880.6	556408.9	405035.1	520852.1	246968.2	401482.8	415439.5	443121.5	492459.9	416065.9
30930.94	72954.91	62270.11	45443.66	40726	55397.93	45921.86	29365.08	69810.53	39871.23	50852	42027.09	34872.71	40040.69	41071.05	44854.36	32800.62
30070.43	76219.91	70476.27	53351.87	108700.2	38998.82	84657.48	28100.04	79790.76	49163.98	97702.34	94853.73	123791	70895.28	48296.92	74958.88	57805.66
47757.85	443160.5	147946	94497.34	107075.7	67535	232579.8	94873.87	165403.9	219788.1	236673.2	183302.6	108283.1	123637.1	107402	135321.4	123085.7
26024.05	35279.55	21902.37	25729.48	27133.7	23606.79	14678.23	15979.4	13400.3	17206.43	48422.36	11997.43	18481.56	28953.91	17713.56	23971.14	21017.07
462851.3	687604.2	902853.1	727967.7	510469.8	730695.8	537118.8	607726.1	722139.9	394597.8	647552.1	375696.2	565610.2	446976.5	550928.7	478024.5	541133
15941.26	28299.85	6156.635	16471.81	14494.54	17856.12	11862.25	26517.2	21139.93	60204.2	20082.06	24529.32	14647	16155.87	35795.91	5272.83	17086.94
277046.3	540337.8	487540.1	531998.8	418067	473108.8	580730.1	407321.6	379918.3	335776.7	478707.1	378462.5	438160.3	345319.1	368389.3	588163.3	408345
68286.67	110762.8	22991.15	43964.98	73489.04	37191.53	60436.92	46832.54	36438.86	40638.18	94298.1	123960.7	108258.9	60648.05	45174.79	51350.58	49592.29
2511399	1482231	2786377	1013313	2616728	2657152	1418109	1292178	1231014	2155188	2113841	516937.5	814952.8	800312.6	652674.9	1083083	1252755
91403.58	147364.8	154341.8	196229.6	133688	169099	185432	196832.6	172334.6	198030.7	159839.5	171475.1	156505.9	144928.8	150706.8	120582.1	170106
3315.523	9013.943	8056.02	12310.66	5930.043	8016.806	10669.11	9250.818	9649.122	6405.708	12775.49	9548.531	19087.62	6118.742	15553.9	9379.425	13567.12
1691591	4861240	742517.6	1100310	3920468	2071000	1438991	3203890	8038335	3475301	4463737	2369480	1177418	3275947	2999160	1979629	3013175
2488552	12130267	9473760	8179346	24889288	11905919	7767085	5108367	6429009	3353711	21726196	38596796	24642832	11375977	9370022	11804144	12715828
22347.36	60568.44	39240.38	41849.01	67863.08	30942.34	36546.2	39592.8	34400.11	39079.8	54293.13	117881.6	112287.8	62926.41	52986.45	27491.05	53648.52
15578525	3083740	3395758	2749515	4896047	3079115	3843754	2587673	4767344	35100000	2285830	2011644	1633738	1745472	2226764	2212980	1835768
21744.21	31170.22	27895.42	22193.08	24022.53	32284.43	30179.38	26889.77	23670.21	62252.61	27322.9	21415.63	17987.52	23574.58	19602.69	25132.14	19718.59
495810	587549.9	535091.6	524502.5	643248.6	549240.5	1465040	1138173	711871.6	262931.5	1236095	835866.4	1043992	599735.7	732589.4	1051636	1164860
1340166	463255.6	515980.8	597168.7	643432.4	616398.2	426588.5	770443.1	839089	10478106	404492.8	480136.5	436932.4	395609.6	414366.8	247980.1	406746.8
103826.2	166465.1	112639.5	110963	119720.9	111421.2	74526.9	97007.16	146805.6	140174.5	110531.5	169966.2	88602.16	109308.7	154240.1	157559.3	185172.1
4104.653	17556.4	11657.17	14843.99	22945.31	14286.01	12296	8062.406	10911.63	14590.61	18297.66	47772.1	45902.33	16054.75	12994.46	20096.26	18871.25
20795.54	23726.05	15216.64	30468.87	42682.38	10807.58	19522.24	17972.45	26736.36	32156.46	19921.43	85096.91	46916.54	53059.57	30854.73	38586.26	36395.64
27577.04	7342.002	7527.768	13775.82	41475.35	16161.41	7883.037	9360.873	26656.71	8520.644	5219.621	3260.006	12171.18	15729.86	11456.96	8535.902	5751.604
77482.83	74406.13	50263.27	69459.95	37191.46	59514.52	88046.21	70247.57	49929.73	89293.91	60311.88	61285.53	46803.23	63467.27	101499	119921.4	72630.31
20000.44	73074.41	65305.02	35946.98	103905.4	39022.02	45524.48	21375.24	20736.34	47257.77	155070.6	192321.5	177638.3	89733.3	50292.2	60904.56	62881.49
6372.185	12649.52	11949.51	12361.31	11417.91	12596.35	6672.089	10711.23	10589.73	9383.939	6153.979	9826.065	4940.984	8953.561	9586.209	7735.492	6029.942
761774.9	1211810	798519.5	927317.5	1327443	777820.4	1035503	980188.3	676747.8	1100963	1408429	1768299	1144888	1445981	1230919	1121189	1011010

11334.58	40328.18	23749.63	34381.93	49464.45	25998.5	32056.3	23562.42	24824.91	39741.51	35686.98	107360.1	69309.64	38964.81	34281.91	41177.39	33480.86
98133.88	180343.9	105317.7	123884	99958.29	89568.51	179155.8	95799.02	149628.1	118436.8	226228.6	232081.8	163183.4	162631.3	128851.1	289371.3	133704.5
67872.16	172093.6	108222.6	111489.1	111263.7	82873.82	125711.9	71151.55	107764.4	132242	142979.1	170439	111125.9	91846.45	97389.03	185774.6	123269.8
158247.2	189493.8	154662.8	157954.9	196505.5	212105	205558.6	215009.3	152677	118844.1	201814.8	119494.6	184519.5	163452.9	210301.2	268456.5	173529.4
1187358	1110951	1328131	1371779	1049623	1580366	1453357	1440372	1633022	909247.4	1231378	953672.2	1014170	1121188	1265306	1404867	1263278
7383.788	19321.24	10109.36	14145.49	16815.74	6600.608	14532.19	10513.47	16010.43	14492.7	13738.42	28873.1	14528.97	11380.4	12864.59	7852.212	7730.263
17964.18	24341.7	2436.102	25553.27	1647.466	6872.28	4353.837	18798.86	51324.78	11801.71	45159	169132.5	39601.74	30381.78	68215.14	44972.95	46618.79
39054.62	73721.75	62762.39	73981.8	29136.8	30740.17	62740.12	34554.29	47015.99	17299.19	63323.05	33332.77	55207.14	62901.37	39126.97	38769.23	25864.06
82686.67	922841.1	586823.4	452394.6	1324904	447159.6	442983.5	329389.6	130625.4	302602.5	648687.4	524283.4	520477.9	961523.1	1415694	861347.3	650299
49304.36	41122.75	39690.38	55213.37	38264.59	41386.02	40174.71	53229.82	42477.89	60379.34	52977.29	61200.99	43870.74	48122.11	51126.16	55486.68	54363.37
3060209	1952208	1631375	1425378	1140645	1396973	1727994	1809853	1068893	8124333	1747421	1767448	1462225	1601244	2351091	1311048	1781599
6146562	5813362	7369073	4587996	3020093	6159035	11246112	5237943	6239236	5279755	9314235	3814936	4426101	4963228	6065419	7769367	6284431
42731.25	100775.5	102611.3	21631.36	53380.47	54969.79	105201.4	62505.46	46982	56765.41	133512.8	32293.06	30751.08	54711.82	69810.48	47936.58	67760.5
369602.3	1572248	860638	666009.6	863510.4	658750.1	1183702	584286.7	880714.4	735904.1	1300713	906767.3	1209634	686738	737261.3	830922.3	825394.5
69350.92	110698.9	84382.2	73386.98	92509.27	63993.7	75608.65	67884.89	88511.47	127722.8	98958.38	79940.07	51793.02	72359.81	89056.09	82424.04	102596.7
6130.701	12052.98	8271.726	12537.28	3941.955	5693.195	10104.6	6990.843	13301.32	9872.801	6342.868	6768.963	10154.77	6410.04	7440.418	6150.458	6359.935
39114.92	62418.02	33678.76	34007.51	46278.94	35619.63	31247.84	22850.16	30630.48	38573.13	62971.8	29451.46	58314.22	35284.42	36604.46	28582.26	38969.95
9528.423	7184.004	17436.19	9471.096	10768.95	9732.044	13461.06	13358.01	10352.64	11534.98	14308.53	13140.19	6550.106	12897.28	9112.839	10484.23	17099.28
11405.34	6141.782	15875.81	15426.12	25316.19	16592.69	16823.37	17258.41	22697.64	112690.4	8972.392	17587.42	15818.86	3278.116	13531.55	23242.04	13931.53
45519.78	85036.44	68186.72	63645.7	69811.64	45466.4	52270.39	48136.5	50823.88	56672.37	78916.3	62942.07	67492.84	57859.97	58133.32	50345.2	43223.11
104046.5	66683.34	512898.3	256549.7	169277.2	652505.7	766976.2	240728.5	2138949	182295.2	672753.3	1172943	558779.2	376901.4	1195972	1098836	564734.4
44787212	12390833	9616179	10393860	11571314	8290366	8691070	10331784	7316032	85978320	10675349	9774102	10197818	9520210	7688862	6954001	10490321
14863.21	32567.39	13807.05	16274.39	23308.84	17273.71	14232.8	15048.47	19738.81	24466.31	21675.13	44616.26	30563.13	21816.41	26907.08	20497.6	9216.586
2512.483	5796.713	2066.551	1828.277	1862.378	1004.488	7523.483	1587.909	1840.525	2961.946	2624.771	3053.378	2036.281	1084.642	2083.433	1487.38	1687.126
59215.1	107312.2	112424.1	79688.55	52226.27	65989.2	89455.44	75562.21	103234.1	59583.27	93235.14	70862.6	59600.5	57026.37	75248.33	73374.86	90580.5
39130.53	42983.17	44260.45	46303.22	37335.06	74525.41	82734.14	64344.66	42551.52	27580.88	65796.26	33218.25	45776.69	48711.36	84967.36	44114.52	67796.59
565146.8	312726.2	64492.89	317038.6	1434891	505604.1	105301.8	647824.6	49932.68	119875.6	387525.7	516235.8	101009.1	430137.3	619287	510212.7	488509.8
23004.63	123341.3	132732.3	96478.61	93842.34	131821.1	135628.2	108451.8	161780	21166.46	154684	85060.95	118895.2	93134.25	66057.3	104602.8	86395.67
2766145	484004.8	618772.3	560258.9	696466.7	632831.9	725739.3	702193.3	717250.4	14532400	481069.4	518668.9	461865.1	514590.9	530396.5	401345	453639.3
96460.28	101576	122649.5	71457.17	54047.79	87100.46	79429.86	64954.71	56609.73	75348.38	115236.8	53292.3	83184.48	44926.27	44964.03	82452	105982.6
7719290	2064212	1470135	2696161	1187727	2413837	2383739	2137578	1501895	16053146	1273375	1582331	1701837	1971516	2698896	1281949	1981822
4284.325	7424.627	8289.061	2680.467	5189.887	3402.737	2442.533	4296.671	7408.355	3976.833	5975.657	4330.265	6289.862	5645.326	4815.99	4901.089	6125.331
13575.83	13628.27	15546.67	22141.04	8497.57	9005.454	18362.65	9277.023	15320.17	15865.16	16943.02	12768.56	12506.49	6670.021	13518.33	7983.663	10563.3
61796.26	15854.1	51397.65	9937.932	16539.37	17575.26	34161.08	60219.39	55898.87	196670.1	20291.54	12932.78	35906.95	37281.64	7625.911	8919.877	6393.747
153502.1	174450.1	95645	129915.4	94998.82	113288.5	287703.1	141819.6	176132.8	234576.3	169243.5	128579.4	161273.1	156433.7	161450.4	232718.2	156281.3
1158498	804125	880600.3	699229.4	950351.7	1368232	1304110	1274739	1292926	966673.5	953311.6	568869.2	712986.4	907666.1	1044643	1114078	1239907
4868.635	47024.02	67933.52	86785.71	15551.6	85711.31	26709.1	125872.9	92769.66	110428.5	86795.95	83291.33	63622.61	38205.04	98492.47	46165.26	49457.63
21816.88	56067.13	25962.46	15815.42	98230.8	14280.76	81628.12	25931.78	71597.48	64440.87	72958.5	17089.09	36310.11	29612.04	15661.45	35466.93	21170.22
3347207	2783649	1947115	2203987	2581106	2839781	2175996	3242883	2628819	3414779	2113027	2371961	1980518	3408177	3030170	2248833	2483467
8493442	7711174	7412697	9209367	9222394	10453949	7901047	6510257	6800542	9287225	8102116	7431319	8867728	7917490	8685120	6296488	6619304
119573.4	108523.2	119301.9	136742.6	127315.4	141816	142355.4	129464	157607.9	92779.06	126968.1	94040.39	132066.4	114717.8	111729.3	140159.3	127219.3
114182.2	92765.9	103417.4	87150.73	69826.26	62521.59	68664.73	76835.63	92549.38	94606.06	114438	78585.92	58324.21	83945.91	87004.55	69854.52	94482.94
4007582	4346930	4638334	3008684	4960898	3490620	4415920	3374702	2938928	4046913	6584990	6837971	5902024	3480792	2950724	5496375	4430577
6495.402	7344.225	8187.961	13552.25	7377.396	12366.93	15289.71	9230.638	18714.42	9520.147	12000.04	14162.9	9809.021	4937.142	9093.389	14635.88	10392.31
9948981	4516830	34449080	4790071	8501985	2164826	34305000	2835146	6111862	2112302	5682016	11249102	11835511	5621393	10081988	10648544	19119496
104790.1	152931.5	98848.77	78809.5	67686.73	110526.7	96471.7	61061.09	76571.14	60262.35	90534.55	46395.93	75285.1	53061.82	58841.73	73551.08	62760.96
2141051	5024282	2962588	2918682	3313243	2777251	4211190	3204386	3283894	3840802	4042194	3538701	2401426	5105565	3898853	2838764	3497723
2702.244	6540.531	5176.681	5662.398	6556.366	5837.581	6283.798	4557.952	7255.102	3175.2	8011.455	6144.447	7809.799	3578.045	3496.322	6038.597	4946.381
22513.46	32229.33	30468.85	30412.54	34854.44	34917.4	33003.02	29325.82	26140.59	16270.9	30778.17	32619.45	34933.61	22478.91	23595.06	34574.3	32226.71

1086728	796097.5	1202288	905106.1	782122.1	312551.6	286298.5	288217.9	915420.3	1649548	776433.4	603491.2	679991.8	1140648	674495.8	261742.8	280135.6
26431.45	35111.45	37038.16	37171.55	33367.25	22515.44	43941.01	27814.44	260966.3	22770.25	43782.52	25813.24	43057.56	64644.51	35757.2	25829.9	366102.4
4221.188	10933.2	21499.13	9291.594	13669.99	10703.46	18042.79	4251.172	5264.777	2239	11332.57	2047.52	5104.802	15102.49	3250.439	12764.35	2601.615
61864.36	53367.27	62145.67	40557.28	68326.11	54240.65	45293.49	51324.96	38549.96	40664.86	55375.8	64100.94	41946.53	78553.64	64179.1	44646.73	52340.24
27046.47	21401.64	17453.4	16108.12	19092.71	16125.08	11532.6	25980.26	25310.1	94510.48	11704.03	13248.7	12220.32	6672.445	14636.14	9895.827	6747.386
35637.23	37982.7	33902.68	33068.19	52436.27	31041.69	52823.92	33628.77	43977.74	27193.08	40331.31	38258.5	37662.14	32263.74	35781.4	39559.77	39698.39
801322.4	722414.2	1129524	849295.9	902831.1	237224.3	152018.9	232629.7	835581.8	970456.9	727194.3	616042.9	828290.8	1029629	387139	228058.9	142984.5
3847970	11539439	5025861	7982297	9967507	7236297	14331637	9759648	13212709	9601861	10025288	5988832	8559529	8634257	7606798	10038763	10700290
38341.75	88115.04	68696.2	50187.99	40420.47	50800.45	33054.1	47580.19	40688.43	44401.64	61842.1	52564.01	42582.22	46169.48	55585.68	50251.43	51683.74
18093.96	51893.24	28781.3	13616.22	12116.1	19027.52	20310.51	27297.43	15027.73	41074.29	29590.12	14262.57	14654.74	10287.41	12926.03	13375.62	23740.5
131154.3	59732.56	203892.4	63491.73	108149.8	199486	135416.2	118419.3	263212	54509.57	135431.3	123843.3	103625.6	117093.7	64775.75	226153.3	106943.7
388090.4	241938.5	238907.2	229763	399943.5	330938	565678.8	247783.5	792957.3	390964.7	257746	289258.3	199603	256619.9	292293.3	397273.1	288477.1
24129842	6732318	6843787	5966170	6200220	6767628	5317197	6766818	5242906	74801984	6609344	6207856	5219905	6761464	6832438	3962931	6434721
55945.87	80782.16	98554.28	49692.24	73080.05	37684.14	4770.723	25389.86	59459.98	32452.11	29213.08	43665.97	23770.08	74473.97	52340.34	29387.31	45151.09
8273314	7791544	5498750	9272732	5585026	7718314	8881419	7725861	8256854	6514594	6124349	2684503	3970000	4716894	5604861	4820079	6105928
7567.112	10085.82	9168.101	9596.006	4059.67	8911.394	8906.331	7615.611	11362.64	14046.56	6248.596	7318.802	7756.577	6907.296	9160.519	7511.238	6544.88
24273.21	69584.57	51252.21	36450.95	42296.49	44876.41	31867.9	48851.17	42567.79	30219.11	107292.8	110863.3	61331.41	62232.98	42154.81	44463.18	90176.42
12909.62	19795.76	12740.94	21403.38	21264.85	33624.88	21836.61	14949.5	17671.63	20558.81	15323.28	12496.62	16238.14	16530.55	26255.58	16441.88	12296.46
31992.38	19958.27	15451.12	17766.76	20534.04	11777.26	11276.85	19809.3	18106.42	77282.45	14039.04	13710.32	15584.7	16879.59	13226.12	8046.819	19132.37
101026	87808.95	64433.81	86181.36	59585.79	57054.35	49708.76	67477.13	67850.88	87968.95	70906.41	80270.38	74201.8	99152.65	88163.63	74560.28	72800.16
80188.72	86321.77	71685.8	69181.52	44411.27	53577.56	56867.56	56809.91	80576.57	105845.4	77268.76	59504.05	51657.01	62054.27	68926.4	65011.89	56572.2
7309.236	5901.738	3790.226	6494.08	5716.966	5225.158	54841.46	9506.181	10595.67	8503.344	1830.457	7823.791	5318.877	32663.88	7652.759	1847.49	3296.17
91173.92	104036.9	90945.88	189482.1	34076.65	45898.1	480548.2	112436.9	107522.9	222851.9	120108.1	177710.5	251625.4	45915.19	57447.19	552835.3	127814.1
39359.85	18912.77	12542.04	18378.03	28333.63	11977.92	5296.531	8480.99	27559.08	103029.5	12606.19	33529.9	17250.97	51905.63	26371.11	5789.625	10755.86
626244.9	752970.3	432778.6	689815.2	441563.2	442190.2	441224	546385.2	492765.8	973893.8	681973.7	639508.1	551349.3	708745.3	709694.5	601378.1	537622.4
30411.03	20227.31	11633.68	10351.47	31903.05	34724.23	46498.5	44953.58	185386.2	25265.73	101392.9	619691.8	135686.8	133863.6	305713.4	65776.34	153496.9
141473.1	228159.1	224337.8	126503.5	153251.2	144346.3	140695.2	128789.6	164327.4	183673	182341	217303.3	148839.9	121692.3	154956.3	179384	228581.3
15459.89	31872.16	23085.21	24836.41	26553.63	30759.43	31598.29	47107.04	30375.24	12031.04	17843.42	19850.31	21536.65	21552.23	29231.7	29326.75	24288.55
303219.2	387225.5	159621.2	116690	182308.5	241172.1	350051	162984.4	177841	305558.5	619747.6	205156.8	263853.4	182720.3	290575.3	300493.6	195943.4
25113176	20890810	20718868	22280118	23179490	24271688	22261228	22567006	18707028	55769580	19641622	17087956	15155616	23059688	21611892	15429051	18256520
7473.593	10971.39	11767.88	9144.552	21178.75	8981.313	15157.44	10368.12	10546.09	12747.14	13637.68	25815.82	15805.78	15841.08	15401.85	16410.13	7922.413
2005338	1549430	1620111	1590539	1385130	1991929	3154649	1334966	2385196	1505759	1862972	1303083	1628458	1315464	1548037	3058498	1962570
13769.35	13654.37	3483.306	10958.92	33858.22	2708.813	9416.002	4907.073	24379.52	8276.698	18046.51	10307.06	8351.118	9553.744	9078.124	14136.11	6381.25
12314.12	19457.35	13284	10507.08	12941.97	9702.807	5463.365	11761.47	10001.59	99944.74	13952.91	19264.48	19655.07	10779.03	15439.13	15036.84	15024.44
653328.8	444171.6	577838.6	427002.1	435701.6	572980.3	680572.8	604527.8	336193.2	1413327	618088.4	543441.8	645423.9	728196.4	781640.3	743793.1	640824.4
911019.3	367013.4	453863.7	507022.3	437683.5	533873.3	511764.9	752388.9	267823.9	2193914	524391.3	1380597	859288.7	779816.1	883785.9	690786.4	1204341
390564.2	71049.3	71045.81	97710.27	181980.5	128535.8	127433.1	171505.4	61938.33	1216378	86978.22	183341.5	148974	215522.8	185552.7	138661.8	225821.3
586501.3	705749.1	389558.7	1088409	867638.9	520527.6	1134240	398623.5	195738.5	1208562	753705.1	415510.7	1286654	1021528	790059.2	964046.9	774258.6
44516.73	21173.84	25032.54	9998.406	2857.665	67602.66	26021.85	9904.109	36022.06	38272.16	7079.621	24784.43	13163.99	11475.75	56408.72	40113.17	14477.11
27395096	17849102	25314518	26873948	30399254	17696020	28413594	10301376	26802288	20372544	22746232	17153902	28407064	29258356	13694896	29369066	12108223
232561	212250.7	221933.3	256819.4	185182.2	341513.1	212643.5	172694.7	379819.4	206530	217868.4	145089.8	229137.9	163962.5	219172.4	251371.3	226928.3
24870.81	29039.88	34657.45	26506.2	24026.26	23531.85	43975.13	26126.69	41186.2	37066.79	30351.38	23269.81	23220.83	22032.75	20173.53	30859.54	30079.22
10069.41	5464.541	11437.02	11618.99	19318.2	14752.68	12231.48	7874.408	13387.21	7643.675	15476.15	5737.221	10022.22	9715.201	13994.61	13174.77	11632.57
5797.984	6493.6	6254.67	3929.805	7996.298	6037.139	5915.76	5877.812	9431.94	4527.233	3308.145	6272.339	7337.497	9771.064	7015.034	7156.162	5648.026
32917370	33414578	45963460	46301048	37001856	38805280	45042248	45448008	35443712	31235738	54852528	41491168	48009112	38633728	33491768	48055128	65624684
68639.85	10563.82	62697.44	86264.17	84003.38	73195.3	103657.1	67976.73	106536	95905.63	43615.07	5794.097	49558.71	49135.54	84440.66	61194.67	63603.85
99827.83	108236.8	154205.6	29488.68	764012.9	161979.9	225779.9	471042.9	594949.6	419776.6	225827.8	2047514	217706.2	110758	430356.8	106525.9	618215.6
2586222	6014092	3569691	4203240	5360843	3624241	6953627	3859845	3280725	3597689	5677677	5429135	7648929	6660877	5701218	3833798	4584951
18119.8	20365.03	13171.78	19462.77	14567.38	20759.86	17334.76	21134.94	17730.12	17334.44	16118.65	21368.08	20287.74	18847.68	16026.68	12250.01	18841.13

33611844	32618522	36092124	24940516	54963588	50298160	44416840	20455968	17155086	42008384	63556704	78954704	75159120	37838264	43932848	44819416	36896216
25930116	61749300	35017008	31331050	54999288	44762308	42791432	19096926	16319268	39533652	73181880	90908368	72207320	39146528	38836536	36187184	43320452
85338.77	134379	175238.8	37630.01	102427.8	90948.34	100653.4	82967.44	89082.46	80692.39	124971.7	92355.59	36087.67	96542.62	131292	116160	139039
9037.3	7906.315	5968.131	5449.673	14536.86	4291.722	6785.034	6530.879	9662.779	10721.81	6877.103	5950.401	3199.164	10911.44	5026.708	5427.777	8191.927
137922.1	130685.8	132943.5	131233.2	189653	156655.6	114517.8	99705.65	107302.3	96732.95	181392.7	184274.8	184186.1	144076.7	121673.5	172158.1	142293.2
11344.16	122694.2	11274.01	20620.04	29392.64	9308.056	36960.83	12192.99	8221.443	9125.54	58820.88	200150.8	5811.48	13791.79	25748.7	14855.32	14364.05
3708714	2620017	2605035	2528218	2929279	1893886	3080213	2248361	3197027	4229728	2602770	1681435	1852653	3013400	2099317	2738527	2658680
26733.74	35302.22	13543.9	39067.2	24031.64	29408.47	22104.7	25651.33	24335.4	45001.8	25295.47	38162.5	17060.89	38204.83	39167.43	21099	17282.93
257076.1	326985.6	267388.4	314136.9	389443.6	272656.2	336713.5	312760.4	279913.6	399655.7	331189.8	379633.2	466647	323925.8	256788.3	299304.4	360103.5
891389.3	582401	693693.6	443856.7	524828.7	469422.7	703722.1	742044.8	665687.3	792510.6	370467.4	241876.9	422618.4	399562.3	492109.9	427001.1	545489
4882.762	6913.159	6057.266	9630.68	4729.95	8056.546	11067.29	12592.62	5836.511	9700.437	11104.63	9890.442	8805.271	11319.6	9988.992	22441.27	10942.87
32454504	62609048	50033408	58254584	25282302	42172748	33192702	25088052	21976124	35230328	41277884	18292000	26473034	31902334	34814708	24594718	33665160
1900786	1282685	1655830	1267742	1913101	1370461	3557035	2080139	3147803	2130922	2658066	1547932	1418004	1604864	1165954	2721024	1865158
51574.34	331679.4	192672.1	246995.3	336983.3	115289	267283.5	97841.27	165973.4	89549.95	401689.9	605141.7	531190.5	172236.5	127323.2	335433.5	210085.3
312441.6	177956.4	234305.6	304410	250537.7	231594.3	160263.9	311560.4	218590.4	181106.3	147425.3	179613.2	347695.1	317665.2	282073.5	173708.8	233795.7
171121.3	365842.4	108110.4	96067.7	186660.3	112040.3	214911.3	111933.5	263295.5	378577.4	243488.6	196141.2	92094.2	144274.7	156032.4	173555.3	220265.9
69098.34	389368.7	35324.61	51940.57	246532.8	30795.6	34638.05	13181.4	62060.33	81056.56	22093.72	227056.2	44803.31	191762.3	160412.9	79808.14	24954.35
148319.6	128069.3	113728.8	103563.5	80810.27	80205.97	107671.9	113675.9	131938.1	154787.8	121651.5	106608	85711.92	112974	111516.4	124396.3	123244.1
119152	131884	150784.3	109222.6	98638.72	87742.69	74451.88	107969.6	122967.1	113370.9	153685	182154.2	118276.3	109127.3	124267.3	117869.3	141309.3
2717.947	10854.29	11642.21	12437.89	27709.61	10293.85	15955.05	8820.332	12124.64	17890.57	10052.36	3467.294	30327.08	14548.11	32382.38	12833.04	3564.655
22090.95	18148.1	23210.92	13813.97	17739.56	35865.99	10498.55	15647.01	17835.9	8325.001	15068.67	19902.82	31520.51	19538	18338	7173.443	36055.74
3612186	2836035	2655476	2890274	2615580	3838125	3331539	3090399	3339565	1670980	2485821	2629904	2317227	2361469	2566801	3092397	2927008
8078.606	14961.27	9365.905	9671.017	12546.83	11584.56	16890.79	13681.73	13794.82	9664.133	18246.36	13892.39	17210.29	17563.81	12721.92	16567.68	14734.98
21703.63	56735.32	44244.38	32249.6	17496.72	30458.58	76806.97	36612.87	53722.53	35234.2	53350.61	33461.89	27273.87	25695.71	29600.41	39009.13	44129.5
870451.3	1790448	1471190	1491972	1102338	1612388	1508478	1301075	1152280	1309044	1487443	892718.8	1085209	1150652	1126588	1179328	1161491
17565.54	18639.85	20440.3	17192.02	23061.38	10889.19	35875.67	15073.61	25174.29	13003.82	29307.19	9561.35	15005.19	17420.21	10227.69	28379.41	16141.22
224689.3	273064.9	195033.5	204540.7	258782.8	182309.8	279103.3	255103.9	472529.1	233984	221027.4	232684.9	196604.3	223082.1	269895.9	253814.5	272898.6
26892.6	10095.31	12080.01	5739.99	15191.48	5866.487	15492.71	9776.735	27156.51	16374.58	10069.44	3230.008	6921.721	8811.279	15849.71	10992.59	16674.08
4727873	5167372	6079916	4948830	5728328	5646872	4835185	5274391	4200939	5466558	6143318	5601226	5892453	5473782	5318892	4622143	4997701
20584384	33456064	20436056	29990882	19395016	28318918	67036156	65157916	35425752	78411296	18234034	45734584	20687000	67410008	71130776	34030952	38429700
12008.91	13440.59	14959.54	8925.872	8620.217	9281.263	5601.902	10754.37	9592.791	12630.38	12043.16	11777.2	9812.383	14540.05	10227.19	9909.946	13717.42
4060.93	5715.18	2207.319	5508.774	4105.043	1912.964	7407.725	4099.91	2605.913	3233.488	3086.615	17017.45	6704.263	4208.079	5483.854	4007.264	5809.047
98225.14	73929	96330.49	70657.83	61457.14	64870.01	43923.39	63037.91	76052.61	100830.2	105535	157779.8	77932.93	89186.94	121794.6	98713.8	96269.08
23904.57	23251.47	15232.08	22529.2	15674.38	18719.93	14469.88	15144.21	19866.28	23204.87	24083.79	15098.2	11431.78	20903.59	22468.56	17440.86	10322.24
36333.39	45693.71	42550.46	48267.16	40796.21	47855.41	53626.44	47585.23	54230.06	41862.81	47077.62	44927.71	42545.3	40549.1	45103.38	38933.19	52141.01
57759.43	136700	77458.7	57860.9	41827.47	51466.23	249499.1	55699.88	61070.24	99715.14	237573.6	78040.37	79822.14	102166	58935.72	196564	86308.02
1815874	1868797	1304333	1721703	1958767	1313454	1633407	1702232	1763516	1662958	1472201	1618263	1413984	2370074	1484465	1458134	1328634
22138.48	54920.29	27663.22	21069.47	35094.81	20495.8	65245.63	23884.51	41494.98	39825.44	42815.13	19694.19	21291.87	13023.61	26285.16	48512.4	35609.32
114955.3	100373.8	112654.6	119786.2	126890.4	122544	91763.7	121441	119676.5	259901.6	115908	76685.89	93622.08	107548.7	116179.3	97623.97	108949.3
2068902	3027628	2533624	2088469	2622383	2479858	3795263	2923179	3164886	2825973	2816124	2460834	2016590	2377949	2225622	3109913	3016497
704168.6	742578.6	507401.9	650350	673087.6	503831.8	470620.7	719278.1	552394.4	624953.3	636962.3	545777.2	568797.1	860695.9	765160.9	544754.9	569385.8
34519.94	94463.53	125820.4	155581.8	340533.4	109295.4	99388.41	82091.9	135693.4	39444.18	193346.3	220303.8	424593.3	169729	92223.44	109159.4	99433.94
62545.66	73996.16	64636.52	56095.66	61744.93	76667.97	98403.83	55620.18	65764.29	73724.88	77000.15	76064.59	52548.59	65163.04	71771.65	87762.64	63666.51
1833721	991152.1	2757458	1445733	2527824	1716996	1620117	2482869	2895603	1.24E+08	1311383	1242683	1601911	1423510	1227731	1798850	1642370
74095.3	63250.17	46388.94	44213.02	53896.25	54255.76	54822.68	63347.74	48882.03	104566.8	68530.09	81729.68	55075.75	65575.44	75047.02	67578.4	66506.16
21296.85	15219.13	6898.783	28656	61347.82	35414.57	21533.42	12725.78	23383.92	41548.93	18605.7	19938.23	24089.07	32712.9	33019.39	23731.54	7848.445
296210.9	223517.1	263050.1	222620.4	205900.5	178596.2	143007.7	261832.1	217773	286023	320824.9	315769	177957.1	400422.9	348645.4	220946.8	355034.1
72370.15	15639.05	18762.3	88409.63	23930.41	88902.26	22275.96	21620.59	22636.32	4557.115	32140.47	37003.52	90882.91	18498.08	17049.05	21595.37	15845.17
422064.3	956998.7	938343.7	704782.8	594743.2	765094.1	879500.5	615405.3	1309688	635745.2	1687374	3979499	994353.9	1236690	2589459	1808572	1206706

1536848	1987168	1397093	1174453	647035.9	1678832	2570203	1271357	2646792	3394650	1785587	1259253	1040686	984272.6	1445484	1781054	1481115
154014.7	101180.7	8293.345	14876.2	72146.57	12838.99	13108.72	15061.13	97820.59	26512.46	26914.45	33992.65	24621.31	133093.6	33564.71	22828.95	11084.74
1678954	1995238	1582111	1616626	2205074	1375725	1584763	1305831	1422722	1874182	2011241	3502330	2734707	2000458	1767526	2067853	1508470
1078909	1512861	1562483	1490745	1281166	1676204	1562639	1249538	1570812	1777621	1740049	1171449	1259331	1154663	925386	1699725	1388680
25715.64	39534.74	41916.8	22770.09	23518.63	21578.93	24164.81	28091.77	51714.54	44520.14	43933.77	39630.34	23048.26	31004	30596.37	37046.11	62114.62
10920.6	16632	9850.412	11558.48	15685.12	8726.854	8339.566	12955.7	13356.88	24775.38	16991.76	14308.75	13283.13	15052.57	15475.11	11514.05	10754.75
48042.39	43792.04	3334.235	38412.2	34045.98	30590.95	52664.63	56902.28	50488.3	54698.08	51052.66	47565.93	63970.29	49420.43	93060.7	57135.49	54898.27
48904.38	63105.3	29357.37	36086.22	91307.45	73502.65	223843.6	130377.2	39085.99	55322.78	132143.6	70255.26	59208.14	94573.85	83780.73	160081	145510.6
16574.88	18270.82	18485.24	24613.63	47691.54	32913.76	35018.24	26614.59	21982.08	10156.72	39311.43	21584.24	56367.38	32219.29	28009.65	32438.77	40240.62
14335.9	19012.64	14871.9	19099.44	19548.78	13327.14	9421.471	12931.58	14459.96	14396.05	19271.2	29954.89	26462.74	18548.8	18069.23	16941.67	17542.31
9612996	57732.56	14695.59	67433.08	5174879	5241068	6040214	5872631	1858323	102799.3	1104736	122136.8	93240.18	5413609	57843.28	66992.77	38544.75
9709.584	27718.13	124684.5	28216.19	49326.34	16592.59	22657.22	16011.47	15027.68	18247.24	31219.72	85291.51	61283.89	35525.51	24386.93	100702.6	52754.82
10118.64	14600.73	24090.8	18904.18	10563.66	18182.16	14169.23	13341.9	16564.53	15767.48	21728.52	15682.78	19498.38	25215.28	21982.79	20418.28	16489.38
44005.95	63221.63	45384.21	61177.41	68288.57	44913.44	37767.16	34484.68	29556.2	46428.05	34561.42	20614.5	40078.82	42753.3	36839.24	36858.64	34141.16
8320.371	8413.954	8012.42	8903.297	11168.83	7563.645	6583.944	11989.71	14676.59	5053.86	2956.771	2935.998	4988.425	13631.09	7403.379	6062.946	11907.23
2404.631	6216.027	2965.527	1536.235	652.1683	677.0096	7406.199	780.1038	956.1459	4995.871	3124.037	1172.048	1198.31	1406.779	1374.347	3532.982	1453.8
71820.3	99762.65	94767.45	72436.48	79646.04	54865.14	125592.8	80421.34	75575.74	78801.21	116494.1	112121.6	97775.19	79902.7	82776.65	83291.51	76792.72
27917.94	28331.55	15241.83	31691.66	11786.75	24116.73	19160.76	15184.88	15666.09	45414.75	20767.04	23738.62	25690.13	24929.31	16800.46	17007.08	27364.63
29729.1	70054.46	77286.98	79759.34	63381.42	55295.46	43671.24	45254.56	57278.96	12583.08	63015.38	43228.36	68759.39	84538.34	45908.97	44868.66	55200.83
61722580	60767368	60491824	74584136	88981232	61065528	69965824	59876072	59484756	59304380	1.07E+08	1.16E+08	1.22E+08	74730928	54117676	68552432	74970576
3161413	6383655	5304236	4684152	10244696	5620866	2096854	2717102	1763485	5971160	14887841	35505148	20488372	8323910	6673843	5004531	7829832
930757.3	666934.6	763079.2	910679.8	410661.8	1132745	1968263	828377.2	1118627	1867554	1517506	703161.3	1140416	487335.6	1330009	1622645	786482.2
2053064	557664.3	1114643	1284919	1060943	1724221	1444668	1975619	1326479	4890053	1200605	1045399	1124005	1626128	1834518	1238623	1725172
60624.31	48777.54	32773.72	37116.02	38975.63	34535.77	26939.56	33251.45	44370.44	48714.13	33428.65	54570.33	38452.14	41837.79	29347.55	70926.33	34351.27
183492	264983	269781.7	212143.9	230720.8	234446	264474.2	252877.5	200188.5	144999.8	232020.8	115308.9	171507.4	205819.5	167851.6	232678.8	217637
5686.796	9073.686	3935.962	3727.057	6255.975	6535.693	10346.1	5555.273	12554.34	7147.079	7041.512	2010.944	5422.711	5415.553	5962.963	9099.25	4269.611
12198.42	12760.3	8218.883	15505.45	17696.84	10785.18	6077.988	13489.65	17617.79	19996.82	16491.07	20066.9	17772.47	12935.39	10613.04	7607.805	16890.46
46603.14	59730.03	56286.38	52494.16	48538.51	69010.39	64032.27	40980.98	51056.24	46679.09	71027.09	42149.77	44663.82	43138.69	37822.2	62064.02	42945.38
346934.3	312487.4	278455.8	354781.5	211054.9	245609	316873	289931.1	242095.9	377824.9	262969.3	227453.3	259516.8	287656.3	288364.6	333198.8	306092.5
17596.35	36534.21	44132.59	24628.32	20768.9	28159.03	22444.95	20710.38	27210.36	25476.54	33270.12	24258.64	16825.17	17661.16	32446.08	29241.31	29946.87
30594.27	65418.98	80540.27	65535.4	131937.4	82119.92	71790.55	63638.07	55977.96	36199.66	96050.05	198454.3	202887.2	98566.91	73049.19	83002.25	89682.78
27536.16	47553.36	26835.88	35402.58	3648.214	30254.76	29362.76	31385.02	36296.11	45792.77	39974.61	47496.05	29604.97	41184.56	31122.07	31111.08	42422.43
132888.2	160936	145396.1	88916.22	95434.98	132729.3	100245.1	77944.02	183930.4	148593.1	170457.7	111602.4	94043.03	109942.7	152800.9	155253.1	165926.2
181769	366910.8	433897.8	327940.7	239085.3	230023.9	197091.6	246239.4	411284.9	240648.9	336546.2	264413.7	214712.1	229227	308911.4	189247	331915.5
6858230	12300580	8977248	15206516	24808712	6002002	4064029	1552140	4086412	4857277	3843434	8339093	3483077	16721070	18197856	9172110	6863314
279082.8	341011.1	361924.9	332256.2	273484.2	297022.7	508918.4	422787.7	330837.2	485197.9	406471.3	282244.1	411493.1	358645.6	377657.3	414448.7	436501.3
33981.49	44572.08	43541.37	49480.18	40048.07	36996.01	32089.51	39285.64	44150.69	66167.75	58110.09	45656.76	50295.49	78322.8	51838.33	41684.59	36727.46
51092.38	65956.03	43509.82	37708.97	41198.55	58927.33	108702.8	64826	53366.59	67904.42	90130.44	71665.06	49282.11	49291.22	71658.69	103963.6	65023.4
47010.91	38985.09	18814.48	20265.52	40947.64	23560.75	17650.35	24358.06	26608.83	42068.01	44910.65	45822.36	29292	46236.07	41803.53	34584.7	22314.42
263119.9	330200.1	247205.4	227285.2	120281.5	123050.6	245806.4	190898.8	150357.6	276365.2	256737.6	197679.1	189509.1	186872.4	156065.2	190459.5	168532.8
45384.67	107594.4	88447.08	99378.16	151619.6	66015.49	86646.75	48476.87	78231.6	70425.82	119501.8	252731.4	242277.1	93410.16	88594.69	112189.9	97012.74
157684.6	178138.2	136093	82206.05	90804.38	174094.8	46622.99	178126	100239.5	187475.4	115911.6	116765.6	89894.27	158314.8	166026	84942.64	113592.7
15676.7	9290.886	13654.72	29011.9	28628.69	23255.22	20946.65	22509.12	19733.84	19811.81	17952.63	5509.845	30060.5	30770.72	32634.83	29994.44	19276.91
164964.2	156329.5	120068.5	117828.8	93226.03	127403.4	116991.1	136974.9	109987.5	172356.8	137154.9	149202.9	125648.7	127894.8	131989.9	134482.5	97804.63
12644.07	28617.87	26566.58	46938.74	45919.44	32243.01	37073.71	15386.28	55811.38	15845.62	54939.6	126586.5	86538.88	35302.27	51421.38	98970.56	75028.44
110145.8	46441.68	146365.5	69830.03	77347.55	92584.24	130581.7	87377.51	103561.5	100992.3	53640.93	58848.08	87143.45	89600.13	89883.48	134006.3	100203.2
380526.7	310931.6	211411.8	461358.3	284684.1	412788.4	242559.8	271202.1	210539.8	186200.7	326574.2	215487.2	432507.7	299924.9	396327.7	362301.3	445173.3
8095.726	1842698	3486774	1421657	3207056	1479184	423979.2	393695.8	4186607	11763.1	1801802	1571658	7499.462	2930353	1261722	502620.2	392738.5
5178.345	14120.36	12132.68	14508.96	11419.69	6269.665	15449.83	14564.47	13723.2	10003.09	17253.73	24232.25	11508.41	11959.95	13321.73	11885.79	5832.549

24561.39	44617.38	29209.6	26701.6	40910.91	35109.7	30670.69	25322.84	27695.4	23698.25	40451.51	60582.8	23502.04	39556.81	35747.38	46004.3	19195.56
5637270	1284527	1748802	1680285	1663267	2619775	1970789	2393171	2750946	10248411	1280652	1636546	2331745	1712904	2018553	1344977	1339100
25010.22	21846.43	20458.31	21966.46	22898.04	25888.34	19472.19	26628.68	39444.43	17925.9	22987.63	27284.84	21673.29	27495.59	29911.31	22742.1	31058.66
259669.3	334603.2	413354.2	267034.5	253861.2	210748.4	225284.1	259487.9	357563.9	252767.4	296209.2	220772.8	170220.1	199443	232688.3	257634.2	276467.7
301892	308435.5	212708.4	280615.3	175313.4	173717	227057.4	205445.3	179758.2	351728.1	250398.5	244633	218732.9	244661.7	282375.7	300704.5	206055
20208.29	47808.67	47505.9	46978.96	42010.69	34254.12	68303	30197.2	39239.22	27727.45	36692.18	32408.74	37719.74	39565.4	31774.74	36205.14	19463.59
31926.99	46579.44	27473.71	31937	21449.19	19372.62	37125.54	31920.65	39002.95	36551.76	51952.39	36054.02	32307.21	29692.35	26350.12	37122.39	43229.33
7444.417	11140.25	11502.35	11051.08	7545.122	5924.219	7785.664	6015.298	7758.372	8296.696	11488.93	4206.278	6351.164	9251.008	6805.955	8975.24	4203.758
109902.6	11991.61	46227.84	63326.41	1442330	7322.306	24696.09	98047.63	49767.43	61994.93	28037.24	12845.48	66454.27	253990	4384.857	15660.03	144897.3
166520.7	10692.67	72393.98	65322.16	1296137	12509.75	34504.76	92714.8	129964.4	116896.6	51393.14	36126.8	75395.09	313895.5	3183.624	17544.59	204620.4
1533594	1333991	876954.6	1456427	910734.8	1538440	2592028	1556059	1598470	2845332	1249837	1218276	1022334	1478723	1794204	1451774	1085760
12975.06	16765.54	9501.975	12838.04	11635.04	8679.907	36233.68	5998.487	9636.616	10330.58	11521.26	15206.44	13061.65	9958.981	13683.47	10362.34	13464.49
15378.62	13074.84	24116.17	9914.69	17337.27	21523.52	65041.2	16090.42	30698.15	11152.92	17146.67	22653.83	55535.25	17226.93	17402.74	24283.43	18527.31
17635.35	33669.91	32163.24	31702.03	5978.908	19554.3	26220.19	20330.61	29007.56	20403.73	18931.74	61750.25	37928.44	23131.63	26706.95	33004.73	33222.82
102321	103509.6	103588.5	96905.39	104618.6	79836.31	65143.89	91892.11	106861.9	97597.34	125187.6	214510.9	138587.2	120277.3	103127.2	92831.94	119127.7
580736.4	367033.2	359342.2	524249.6	507948.8	498870.1	391382.7	473735.4	418837.7	552382.4	410611.5	370502.6	474503.8	470975.7	497174.8	405468.7	550954.6
16634.57	16275.71	9725.796	11006.32	17209.18	11805.73	8947.501	11860.26	17127.03	11859.57	12011.71	23379.62	21794.79	17291.86	13676.04	12675.21	12766.67
37000.71	35553.28	44139.91	27023.2	32455.48	46554.07	23241.87	29117.45	19780.64	47024.41	42760.06	25517.55	38876.09	29326.86	32794.58	19787.92	24080.14
874384.3	780497.7	1044698	711294.3	603689.4	729287.8	310330.8	596590.8	664496.3	729971.5	842724.6	721507.5	646926.1	578816.5	903485.2	729500.4	948245.3
16355.18	33889.6	35805.06	47931.95	28850.24	29344.63	35158.61	34232.84	47516.83	13338.87	36064.4	33079.56	39178.89	24927.9	26177.26	25779.49	31209.34
28360.03	68615.02	39405.27	32854.87	24840.94	31616.18	20669.13	25660.48	25716.9	63595.68	62938.13	18780.05	30345.92	36863.14	46932.48	32127.44	29517.44
20365.34	139569.6	132206.6	268181	206171.3	168809.4	32693.63	164837.7	164173.3	176322.5	139731	142207.1	18294.76	170553.9	171720.9	15677.89	204997.8
358.1067	4309.732	2435.475	1461.186	3038.525	2395.483	8040.03	2633.016	2425.807	3223.24	1392.384	4405.676	2755.27	2871.958	1800.379	3696.287	1816.805
5188650	809797.4	208552.1	305721.9	1636829	306519.7	508842.5	437543	544053.9	170679.4	218798.3	817684.8	752354.7	1021521	3808414	603615.8	1349168
1512611	1252689	1416278	796898.8	797661.4	890599.2	1056661	1070352	932723.9	1452707	1329619	1139832	736340.7	975620.9	1299378	1153983	1349331
52854.83	43419.33	53711.42	47754.09	48691.96	51328.67	44401.98	58414.52	31915.14	48137.85	37976.42	30981.69	50016.64	58159.89	59171.9	51389.01	53560.16
17259.95	14916.17	9611.338	18352.44	11739.33	9174.281	13196.44	4143.527	10077.8	12427.4	14743.99	12371.91	12210.73	16630.31	20549.83	15785.12	10681.31
80957.77	107915.8	82311.88	99961.91	101068.3	88710.23	156165.6	123086.9	197165.3	120756.9	108694	77407.66	83780.9	87480.04	79771.61	108745.2	88367.78
46244.68	63924.21	37163.02	56361.18	61323.74	37500.77	23342.7	54208.8	60273.66	64736.52	49608.95	53834.87	51083.44	84181.05	54347.15	27073.92	40538.18
10292.46	22019.75	7769.391	22701.17	12200.98	38951.16	41266.07	24958.3	21237.72	23055.63	22446.47	42269.5	13044.52	17406.91	21789.13	15614.39	14559.19
90293.56	131142.8	127932.4	100396.1	132338.8	138566.2	146176.9	137275.5	149190.1	104161.7	168588.8	226374.4	193489.3	127925.9	112970.2	126814.3	163878.7
38362.76	66171.09	66959.52	61959.89	97081.59	52114.82	62090.76	41547.89	36339.63	47953.9	67730.84	150813.2	111916.2	68672.88	55499.05	65683.88	56409.66
98175.13	135540.2	115513.9	115330.6	153764.7	108198.7	85329.3	110002.9	106524.3	119808.1	124042.6	268664.1	178628.4	118448.6	118374.8	119124.4	132056.1
9948.421	10812.93	10589.78	9371.325	14824.41	6659.999	6313.094	6525.586	6285.308	9422.066	11443.67	19931.63	23114.95	13094.32	9185.444	9634.538	11710.01
17234.79	26176.23	28836.15	10475.74	9720.687	18655.93	25076.45	16908.55	22078.14	17525.51	34042.87	9660.859	27262.71	24356.3	9782.203	21454.17	24325.4
6791.926	17851.2	32355.6	12155.17	8781.307	17267.58	14487.06	12065.32	12344.12	16050.87	21196.96	20067.16	7135.285	18544.09	21245.84	10755.14	9508.706
10530.01	18500.05	9497.748	16584.23	13364.11	4586.536	23825.37	5923.024	12266	17836.7	17864.04	13855.75	25226.96	14955.92	20480.88	53201.66	8804.479
155242.4	116289.1	8174.892	6857.639	55375.7	13567	25816.14	54897.8	95420.72	112539.1	125936.7	314077.9	61561.76	350123.3	46681.64	42300.54	35961.57
63429.38	126760.1	88891.63	85404.37	105358.7	86363.57	101352.6	78685.09	87035.11	49085.76	106473.6	89806.09	102622.4	82433.06	86052.13	131945.6	72683.36
25609.01	14038.88	17057.4	15909.15	7699.828	26449	13425.05	8240.551	4400.806	34263.12	18759.05	11628.05	16555.31	9655.32	15000.82	9521.158	6535.919
32996.41	53679.8	33759.59	36748.76	37682.66	38297.06	47802.93	33391.21	40257.19	38905.87	42460.01	44273.8	39203.75	42574.63	41139.68	36003.15	27302.61
20513.36	7026.329	12279.04	10360.5	14021.69	9826.933	8997.323	6222.225	9460.466	13872.79	13258.35	13549.98	10741.22	23176.51	12012.89	9839.074	10843.36
10442.21	8982.868	4474.655	17473.78	7189.338	11226.71	269825.6	4316.765	397436.7	37615.4	180948.7	5706.771	4088.924	46349.49	8433.543	1550984	641415.4
61123.91	87484.63	94798.41	82993.73	75479.7	98476.02	118112.9	91770.51	131260.4	70723.88	78737.45	56377.54	55533.73	60879.52	65754.41	103575.4	79433.5
4260.005	6024.635	5886.434	6289.046	4469.158	2754.313	6685.268	4134.357	2881.795	7842.863	5723.238	5335.025	4728.191	8306.078	4028.446	8618.845	4961.074
5623.846	15559.83	15461.37	13157.21	9843.855	13119.62	11701.78	16382.63	18645.03	100712.4	13856.19	15642.52	12516.69	16445.51	12522.57	16479.09	12963.96
102681.6	474485	313137.1	170531.2	79664.36	186917.9	461708.5	201161.3	392119.1	342953.2	267758.3	133498.3	200429.5	135209.8	155184	409793.1	321128.4
2117874	1501817	1754392	1872606	1689799	1676646	1463866	1436901	1570070	2351979	1782451	1702678	1853704	2074908	1948476	1855548	1457915
13671.46	24106.16	21379.72	24196.67	43420.66	26636.38	19191.39	15692	12566.8	13853.44	34302.35	67895.71	83863.68	36448.47	27177.38	35550.28	25615.11

16560.54	11937.33	13803.88	15659.16	19626.55	19442.27	11689.17	18952.01	15689.46	16465.15	14705.87	17813.68	13086.04	17098.53	17207.42	9817.307	54471.62
15211.97	73546.09	20786.66	39687.94	42519.25	17356.54	18180.55	20964.79	69610.55	54490.42	58875.4	103692.6	55776.74	62980.19	42367.81	31278.66	39774.72
99463.66	141066.5	113943.9	83039.07	76694.26	94580.11	98971.97	107178.4	123721.4	117694.8	120413.9	93331.63	59940.28	82646.08	111962.3	90831.68	131564.5
83685.14	141445.8	109508.9	78043.09	137633	83043.07	99174.72	90703.99	86342.62	150739.9	97858.99	254959.4	147839.6	87975.98	96989.26	116467.3	105693.7
24119.68	28282.75	41625.26	39921.84	37260	22182.69	18031.42	30372.46	29608.65	21270.68	33876.94	37084.48	31939.97	35261.18	27711.62	24037.63	36095.83
403627.2	529672.9	555612.1	292509.4	410315.2	344757.8	771573.4	602011.1	623658.2	630308.6	1286569	529421.6	291058.1	442063.4	309832.1	320040.2	380536
28648.29	54269.52	43099.06	42108.68	83932.79	30523.23	33213.87	32881.49	38551.38	23469.56	51885.9	109093.8	104535.4	42098.61	64584.74	39867.56	42189.64
55326.05	111413.9	91937.58	78299.33	83347.37	61504.52	89343.36	58081.64	86627.56	102374.3	103008.6	167297.1	128818	84643.59	82031.86	101121.4	76405.95
133640	246288.7	141689.6	163850.5	212112.8	146406.9	122013.7	130258.1	137295.6	176069.7	198278.4	285216.1	245695.4	233723.7	189972.8	197396	169009.9
61546.52	48204.24	48495.35	119734.3	84348.39	19032.15	24109.84	16651.69	20680.05	69061.46	82201.97	25982.02	115593.9	85384.78	56867.9	101420.4	59371.09
23275.27	39468.77	48096.11	26371.79	16737.5	16244.16	16607.63	15867.59	42154.08	17152.17	33101.87	32162.5	27772.88	30809.95	26068.26	30629.52	40717.65
69299.45	85972.04	74536.04	63293.48	81473.33	58403.92	70139.53	63954.09	52043.43	94547.63	75526.91	160823.4	116283	83342.68	86664.37	69830.84	76701.19
92384.59	84601.42	84824.44	74232.77	216235.5	62433.89	159842.5	79756.42	224918.7	101110.3	154322.9	134606	93585.89	109928.1	92852.3	144363.6	106637.5
44156.41	118795.1	63417.14	32186.88	61182.05	35753.39	39840.79	41824.82	38587.09	33420.84	73455.07	92892.16	42976.12	61329.42	58433.37	65751.46	37207.55
35241.71	93797.93	74513.87	45553.45	54762.96	41674.36	52939.08	36192.58	56522.35	43642.74	64479.09	69131.63	59081.46	50380.8	45899.55	65619.88	47833.22
343748.1	430169.2	400282.8	369914.5	385440.8	326217.5	331092.4	276892.4	316618.8	394971.8	525955.1	678711.1	614733.1	429131.3	363388.2	379097.1	420255.1
10224.72	20616.88	25415	16275.26	12233.11	13885.51	15789.03	16390.24	15300.92	14502.19	22128.51	20718.74	15565.58	12443.8	16409.64	13071.94	13331.58
4710.559	3337.127	3375.818	2389.23	4460.145	5383.567	1977.639	2663.038	1279.121	5180.047	1510.969	6699.697	4677.791	3036.987	3495.272	2723.267	2124.54
17554.07	52086.8	32250.28	35685.31	52677.18	31071.59	33402.29	40420.97	28150.3	27730.31	43644.5	93322.14	58688.7	56137.64	46219.54	42499.57	36309.4
104015.4	152393.2	156661.8	78390.1	183277.4	123625.5	113252.5	117069.9	168839.3	184187.5	224299.7	344750.8	247479.4	81050.38	88642.7	173686.4	136417.4
18241.94	63072.75	46886.62	37970.37	50375.98	42038.73	53245.81	36156.95	36242.59	59079.93	27767.88	147497.2	89402.63	55201.8	49671.47	65734.54	46026.1
23817.61	32627.59	19611	24353.79	18082.22	18914.34	24822.02	19822.39	20507.14	27820.9	31825.14	28063.25	34356.86	26346.56	29810.42	21552.35	22332.8
3897134	2867235	2722302	3314915	2497660	2673537	1986718	2304392	2691101	4114625	3094466	3270874	2854506	3264293	3690510	3443646	2206002
172325.6	358990.5	244856.1	230708.4	281987.2	182092.7	300472.7	162735.9	210047.5	255517.3	342666.2	460039.8	401523.2	231911.6	285106.8	265684.8	255121.8
34299.71	57102.04	61873.11	71102.55	73075.96	62506.7	59133.11	54036.67	141533.8	41108.92	81425.28	228678.7	119221.5	97224.64	141918.8	111511.1	115181.3
6440.003	12029.15	8033.75	11369.39	6183.813	7633.096	11887.91	14936.69	5720.194	9263.053	9119.699	5388.302	5651.81	6691.479	6818.745	4743.188	9138.624
37200.57	46491.86	49625.65	62219.7	53752.11	52050.67	40582.04	44013.52	100384.3	44109.34	70009.39	191557.3	91337.2	70181.62	107588.3	99180.98	92993.36
42556.23	47020.92	49112.3	26586.56	84831.48	41024.25	38302.14	40356.52	43656.08	67215.95	8242.469	79707.44	42039.25	78681.17	46901.31	40322.62	47550.01
67170.49	62508.27	108677.5	81328.9	70557.59	81481.59	83361.08	75815.19	127062.1	89344.95	81528.79	67994.22	83351.72	61831.19	78203.74	121093.1	113710.6
107337.1	183627.8	90833.3	107362	124480.3	143047	78090.16	110341.4	103015.6	203830.1	100545.4	123679.8	113810.8	124205.4	148551.2	95668.69	90331.59
42712.44	69514.78	32458.84	59448.02	49830	54364.22	34567.69	26641.31	55971.52	52640.85	71012.9	64139.44	57070.84	41144.78	53987.1	48801.49	50928.79
93315.93	90652.77	53895.01	76935.87	66092.58	66098.66	68584.2	75654.36	101103.3	91145.2	84026.17	71085.22	65585.02	73511.7	73966.77	62072.74	96949.26
67323.68	32169.86	54407.87	83811.48	113813.1	93426.63	152689.3	84724.8	187933.1	41301.48	97797.44	49408	74115.7	54818.68	83746.1	80251.72	57575.42
132785.6	35985.77	42633.41	69820.66	230214.5	49959.05	31803.85	90116.45	19152.41	231037	57408.5	107175.6	97182.3	168570.4	75675.09	20706.7	47367.55
215390.6	273042.6	356570.9	315834.9	205192.2	306539.7	334511.2	246345.5	485700.7	324560.8	266577.5	214143.3	298169.7	193527.4	290313.3	288041	279881.5
130694.8	141788.8	87979.51	60645.91	142566.3	196855.8	156155.2	180836.8	84276.77	95873.86	149577.3	66890.57	93003.09	100361.1	150557.6	156440	177055
267177.2	360956.6	373508.4	175716.3	211261.8	288874	455841.8	240776.8	300262.8	185660.3	654219.1	124682.2	168193.9	210249.9	311028.8	279996.6	260491.6
42572.77	39511.39	58850.2	35519.83	21218.33	44008.98	38521.03	32520.39	64305.13	37946.47	79283.74	31506.12	57266.69	23435.05	45919.72	44485.4	67648.19
96288.1	202804.4	189359.8	154041	189885.1	94657.36	82753	177591.8	144124.6	173391.8	169967	191322.5	111304.7	133587.7	180949.3	147844	177461.6
1.77E+08	1.54E+08	1.35E+08	1.64E+08	1.98E+08	1.61E+08	1.31E+08	1.86E+08	1.01E+08	1.2E+08	2.81E+08	1.15E+08	2.3E+08	2.07E+08	2.24E+08	1.62E+08	2.78E+08
1.38E+08	1.18E+08	1.26E+08	1.29E+08	2.16E+08	31930870	71183232	1.05E+08	63945760	89469912	1.49E+08	1.32E+08	1.32E+08	1.61E+08	1.29E+08	1.89E+08	1.31E+08
491551	495960.4	428776.1	289538	255051.2	483116.5	646046.9	351424	565916.6	535393.4	519869.9	363157.4	264666.6	309738	441704.8	538074.9	325983.1
6529.535	24995.18	14386	33958.08	25768.04	4860.804	2875.51	7666.254	13312.66	14851.63	31676.14	64029.81	52417.55	20275.22	18958.38	19657.93	18713.72
106544.5	107733.1	52050.21	25400.23	80342.55	36732.48	29377.52	31552.8	77147.12	175735.9	88069.33	77073	87957.41	60707.09	45762.6	47355.38	47852.71
75788768	62812828	62625804	56525708	17055418	66282752	7543777	72328768	4117089	3683899	92695160	54396256	12525200	68345240	5968503	78201704	8113124
13572.67	24523.04	13658.75	7537.246	38332.07	18350.72	2739.67	4415.745	8065.291	115034.3	15300.16	23040.2	27945.4	5618	32527.69	15935.5	18928.22
49452.14	48698.41	57076.05	68927.3	65485.55	92166.48	65245.31	58545.49	56020.89	43578.11	82558.74	80166.56	78193.64	64225.87	71285.34	58973.37	69269.92
34400.1	56277.79	47894.84	40238.21	34390.29	32071.62	40091.5	35805.45	42755.51	41927.85	43804.11	44346.97	40487.99	43884.41	43532.48	40874.07	45384.49
843528.2	1062634	682348.6	341503.2	901669.8	608761.3	1040858	1028478	1304741	938953.1	1458360	487320.8	266854.2	960986.5	515216.9	694434.3	823427.8

59277.13	22724.46	51352.09	43053.41	42973.89	34827.14	54599.05	36642.43	48001.01	34982.9	49108.8	37480.73	34560.34	32786.69	33599.16	37823.89	38866.43
12427.14	20602.21	14546.07	17362	20795.49	24606.08	8679.661	15510.07	16471.22	22150.88	14600.43	16028.45	14011.79	20151.09	14040.98	10054.04	14509.7
783323.3	931995.3	938004.7	926296.7	721895.8	964571.1	925397.4	789342.4	1081188	745032.3	1015132	728653.4	720669.4	748099.1	909498.6	1032196	934046.2
117253.8	134515.1	120035.4	104974.2	95285.53	111589.8	94430.28	124475.4	90044.42	168370.2	80672.27	106194.3	89303.13	109581.9	89639.63	80874.3	79898.77
14709.27	23224.12	15560.28	15973.36	103628.7	22344.94	10733.43	15008.61	3790.28	18209.05	39124.17	65530.9	27435.55	23461.33	23004.38	11619.1	20383.49
7787.954	9594.548	13093.81	9684.919	17347.2	6441.722	18169.21	9410.176	12627.06	9215.357	12346.86	6765.98	9582.537	12071.27	9577.294	6486.552	11162.34
56398.15	204222.7	195456.8	150410.5	84941	95650.61	308191.3	187746.6	378009.4	148676.8	156856	150938.4	190625.4	88361.41	82747.26	197373.8	159011.6
1551762	7635.984	3344490	6782.261	47193.48	32166.02	3929346	96214.76	279736.3	1	286491.3	397583.8	10032.11	149618.8	866885.8	1942602	2526499
25122.96	9741.083	15789.49	7244.373	13196.1	12883.69	20105.31	10262.95	19197.47	8982.375	31543.59	20939.68	14572.6	9046.818	11424.19	18135.04	19883.46
93012.92	135446.9	127998.9	116232.3	91191.41	129188.1	189301.6	137402.4	210360.4	96144.26	152644.9	107772	114341.1	112281.1	110394.2	158196.7	147930.8
33088.74	52834.65	66414.86	60685.62	54223.1	76408.59	76383.69	79587.77	66772.16	40737	7901.646	25307.72	12114.62	34934.59	51686.81	89154.33	66743.1
13279.54	9318.582	7555.988	10599.2	4757.25	6740.705	3743.28	8813.834	4914.395	6196.583	11096.67	14638.06	7740.759	10973.13	10193.07	9641.556	9854.167
2655.057	5486.058	3613.598	2646.345	3873.316	6555.052	6397.142	3505.422	4309.231	4116.647	4841.875	9107.912	2427.519	4161.414	2077.54	4445.679	2488.326
273347.2	206705.4	98835.56	99312.49	137023.1	82693.63	96363.92	105314.2	163686.5	89088.93	127343.9	122767.8	139310.4	193854.3	131874.8	132356	129717.9
24421.34	42124.66	34375.31	22914.76	24915.96	14595.06	21529.32	14510.62	38284.46	18385.18	37617.04	36190.6	27436.46	25118.69	30152.09	32583.38	35382.83
96375.98	95239.52	100185.1	97320.18	97761.22	106813.4	110010.1	105777.5	107916.2	97683.74	107746.1	73458.91	109790.4	92714.46	98184.38	112744.5	121949.2
110070.3	147275.1	199028.2	102292.1	96020.32	107829.4	124870.4	96004.66	83468.66	95776.93	144451.4	83872.72	90477.75	112443.8	115856	88851.93	81404.65
17085.83	494096.7	1083551	26697.99	1012935	579470.8	123358.9	96008.09	1240549	21525.6	404868.9	475310.6	37589.02	876354.9	352515.4	149195.2	102115.9
484451.3	294723.9	11346.87	4418.79	135033.6	7682.856	13157.21	19836.64	193137.5	19391.27	68983.23	67912.94	40550.66	369821.9	108417.4	38583.02	31011.2
20502.92	24196.31	22850.09	16356.4	15294.71	19197.83	21480.9	16811.36	18819.59	37532.16	15387.72	24890.64	14255.47	12282.88	26738.77	18933.09	14941.24
1892743	2403069	2454549	2389196	1576640	2275390	3655489	2635003	2860971	1774459	2237825	1723920	1988495	1763889	2014035	2703807	2167038
211532.5	220805	270634.7	294876.3	183280.2	245295	328734.2	257305.3	302154.3	267848.2	229111.8	168243.2	203735.3	186196	162453.7	279046.5	229572.1
117234.5	195287.7	167954	147029.7	72540.21	130777.8	137671.3	105305.4	137652.2	193338.3	165034.8	75736.38	116762	76192.26	83325.6	102870	117278.8
156856.2	213995.5	187996.1	220328.4	158016	203635.3	199544.4	193636.1	275551.9	155473.6	212495.8	144625.4	150072	141218.3	159234.1	193039.1	183953.2
197540.3	120366.2	123900.3	176722.6	227719.5	94761.15	92077.4	47980.74	94566.33	218444.3	258641.6	83518.13	209592.3	190548.8	74521.28	217775	166489.9
7341258	8852226	5496963	5703614	4886607	4723700	5185923	4009635	3439510	11832403	7042020	3013630	4258405	4254199	5200613	3852441	4656554
597116.2	1383275	793556	334944.8	519449.3	415981.5	1299783	601305.4	1121253	808357.3	1580254	333391.3	377854.1	759480.8	663315.6	864686.8	742679.2
1468164	1015310	1267365	1098397	1246971	1496056	1410026	1439134	1346466	1530064	1362716	1115323	1205437	1181802	1266025	1329993	1500051
21143.48	28594.47	38910.71	33334.65	21316.09	18753.99	15340.69	10709.51	19512.84	10899.28	23065.63	14118.47	32207.91	26912.03	16277.02	32462.76	21070.31
107094.9	85790.75	109335.3	149026.5	166119	124643.8	126612	109629.7	117899.1	222394.3	123421.8	101195.8	157648	127451.1	129571.5	141556.1	111992.7
124101.5	137810.4	116020.2	114100.1	72033.77	104008.3	82375.45	115185.2	103204.2	106206.1	105371.8	104101.2	92292.08	100189.2	124749	101544.9	120350
752042.2	3090450	3020379	230768	876801.7	343174.8	2537598	956302.1	407476.7	1098084	6865983	415658.2	462076.7	183230.3	440225.3	668454.5	454302.7
409003.3	309050.7	209480.7	240817.6	157677.9	217434.9	262013.2	247419.5	134503.3	377785.7	251846.3	247046.1	222518.1	244120.9	260940.1	266482.5	236002
858308.6	346460.3	207075.7	301100.9	273546.2	433534.3	488824.4	328162	402501.2	1944121	308446.3	289109.3	331210.4	341110.1	424851.8	325979.8	343825.6
42870.36	42857.42	46859.43	50695.83	53381.33	50530.25	60006.75	43117.59	39838.61	35686.07	59739.83	46836.09	75364.8	54311.7	50280.29	77588.05	37840.98
1814617	3870368	2932188	3947034	6829719	6119644	4921432	4252603	3089596	1633692	5348491	2882134	3792200	6464664	4821402	6038466	4782191
18051	37072.77	26778.93	30402.67	18754.21	23467.48	45243.8	20517.27	47073.46	42832.61	32505.33	18958.8	22926.31	17013.53	21052.85	34761.57	28360.57
83596.67	94469.8	65061.77	4626.258	40678.95	64387.29	104125.3	70634.98	61975.64	159975.3	19757.22	41183.3	63991.63	75785.45	115325.8	104705.5	54512.59
13107.61	17515.82	16131.7	10277.84	15116.91	13798.26	16027.77	14960.25	14432.43	16611.32	13987.16	15857.05	13288.09	14442.13	11854.95	15117.23	16102.23
206574.5	170764.1	111444.2	91043.98	117720.8	94695.34	173541.6	142827.9	278541.8	114884.7	154982.5	210910.7	122362.1	337704.4	143880.4	142997.7	195850.8
716116.1	727645.8	958115.3	872614.8	1273364	774711.3	600872.9	614247.5	684061.9	524384.3	880574.4	779770.5	1181354	798847.4	713756.1	578240.6	852487
5608.84	95028.39	84006.9	49928.44	76732.06	55169.71	82479.66	95911.16	87655.59	18117.61	120073.4	127783.4	60922.73	51926.46	59559.94	123289.8	120553.1
92920.93	81932.94	80850.84	91896.49	52504.21	110994.6	52590.26	104950	86266.13	91395.13	90551.11	61319.63	56906.18	89462.45	113820.5	46728.1	81126.4
207409.3	275532.1	261326.7	272583.8	252287	263459.4	387268.9	261848.9	348328.4	183321.8	331396.4	196392.2	284486.3	206309	314884.6	457848.7	366512.4
38091.42	34857.68	39056.38	8855.286	27595.75	30412.06	37238.24	33974.3	34149.91	33309.22	74776.96	26073.29	9142.575	25710.34	21680.77	36704.2	37856.29
18807.24	18455.98	16397.2	9751.71	12028.51	8953.066	31967.7	14067.99	10800.72	29188.59	12168.72	14333.8	6118.094	23162.56	16918.96	8802.634	14049.53
27171.81	29053.46	17397.36	30324.29	57201.06	28266.44	27089.17	30586.43	57054.04	37724.8	39735.67	22432.92	30197.99	34109.47	25390.49	37854.51	17589.73
28825.09	43295.02	49432.7	54514.8	51978.82	54960.12	43068.6	53242.01	59808.98	49485.33	57837.19	43763.88	39760.35	39489.11	42875.3	42997.01	51548.72
27968.91	30477.98	31630.77	23266.04	19634.48	20648.38	20556.62	26919.27	26561.32	28370.94	27189.64	25081.68	27147.35	15306.19	28153.71	21838.88	31759.72

261337.9	217451	258901.3	223874.7	168237.9	193367.6	201064.9	192827.3	236298.8	197243.4	178456.1	188846.8	183245.6	177837.7	158840.9	230638.1	247439.8
272627.5	257968	283978	327971.5	147245.3	268081.2	408379.1	247336.5	445880.9	226891.4	188151.2	157512.4	238891.2	98575.03	194518.4	290529.4	224134.7
33420.55	24007.56	57476.23	56675.75	35599.46	38192.39	49978.81	59887.77	32164.89	42635.57	17187.67	29326.42	18174.11	31341.62	23778.94	34657.01	34705.28
26544.55	29607.47	22158.42	8791.347	51227.94	23444.72	16397.34	35054.24	23874.01	23156.85	10425.97	47668.75	52415.4	59360.59	28167.56	6963.158	23246.93
253456.5	438153.8	450951.3	144039.2	360787.6	187822.8	450060.2	346464.4	406448.6	334639.7	445452.7	208056.1	34429.77	340182.3	164849.5	369088.7	154525.5
17873.29	25210.72	29775.76	38528.44	18835.34	27936.47	17499.49	30795.64	20482.12	22765.21	28434.04	16570.84	18969.09	24643.93	20629.53	7352.449	24313.41
463248.6	927368.3	872713.2	941922.2	632908.3	800806.7	963293	890806.4	1389661	516726.3	694210.4	532709.6	601551.9	690883.1	762269.1	608271.6	760628.8
56733.6	92484.59	73574.43	50700.98	75000.66	22093.99	100490	33233.37	28668.3	62793.36	110021.3	77927.38	47882.07	49734.45	51307.61	98409.84	63195.43
95563.92	128060.4	123301.4	157728.4	126789.1	144229	168021.1	153860.2	166879.7	74789.2	120372.1	70457.38	115288.8	109160.1	125499.6	148718.9	131982.5
14535.26	14194.2	13726.55	12666.13	16156.79	15854.65	12212.95	11159.82	8381.392	26464.1	23655.02	15835.36	19446.15	17288.17	20204.17	11632.82	20806.89
255806.6	12637.42	835680.9	15808.11	23570.5	16121.96	660755.3	26463.8	40865.53	112173.6	84793.47	126936.3	14608.39	38056.16	234468.2	261152.9	495414.7
184459.9	88037	127629.6	107829.8	129468.5	129405.9	177976.2	110013.2	207387.6	153130.5	127001.1	71354.07	126879.1	112852.4	117119	147463.2	138528
38419.5	68772.19	50276.3	34491.01	47541.63	28674.02	45115.24	28451.48	32660.97	44294.47	79994.79	103744.6	74170.76	37486.16	38938.46	48790.07	56257.88
215810.4	398871.2	291746.8	293023.8	255631.2	302759.9	268816.5	319682.7	337591.4	281081.4	352373	365543.1	315499.7	326050.4	289413.9	262331.3	290308.6
124278	113360.6	82684.02	106930.2	146095.1	145235	72894.59	65119.42	61508.39	125393.2	133316.5	259141.9	192186.2	148488.6	126621.6	101890.1	80206.5
17249.25	22876.34	18455.46	18240.98	21088.69	29679.85	14959.77	19416.86	22359.33	18460.54	20885.89	43726.4	21916.74	21780.7	19082.5	27211.93	16401.63
11430.32	14742.73	12003.03	10947.36	12822.13	7813.63	8838.707	11845.08	13767.83	16097.82	18778.26	13530.49	16583.47	15516.95	15547.86	17931.27	11287.38
18015.69	24715.84	26700.04	22234.25	16844.72	23512.74	25201.18	17895.58	20194.34	16223.92	24676.05	14943.99	32887.21	11162.41	17162.13	18722.54	15730.85
40167.45	60481.32	33865.17	39477.73	29403.89	35696.88	16251.9	33094.23	34511.29	53325.93	40085.22	41428.7	30416.11	52501.87	59003.75	22499.87	22432.41
62463.81	62973.11	59027.09	44880.18	36077.78	39486.55	37952.34	26519.53	49652.29	54919.95	64720.19	64109.89	44628.56	45415.92	59281.71	52659.79	39096.61
9604.725	8849.237	6315.753	5409.962	2670.851	2941.197	8098.021	2133.182	2459.08	2597.428	6367.678	13004.02	7115.132	6008.583	3916.794	11928.79	8499.333
12890.3	26224.51	13211.96	19323.28	34729.46	23215.83	24686.45	23502.29	13532.55	18548.86	17327.07	25708.36	16373.28	25112.99	18544.44	11172.05	14877.22
83553.6	108385.4	133713.3	124077.7	97671.23	111920.3	101457.4	94308.09	101817.9	50515.98	93208.08	58904.98	93601.32	100904	74924.56	95569	81830.75
580628.4	477282.9	572741.6	569454	483181.8	700897	649847.3	773180.1	575418.6	549750.8	582451.4	460949.6	527294.5	528780.4	578311.4	581255.1	672714.6
21900.74	25895.08	26852.54	19817.01	25751.89	31590.37	27765.63	27647.93	42780.12	17255.21	26387.53	14645.04	29701.72	26765.43	21873.26	22336.37	27493.28
134473.9	553778.1	113189.4	396363.2	290265.3	101902.4	601598.1	114162.6	187410.9	766195	767278.3	348191.6	233576.8	130819.3	222994.8	465167.1	637394.6
3747.377	3785.884	5954.535	1100.13	3538.708	6175.534	4306.491	6244.43	12776.38	1983.576	5690.443	5274.754	3007.257	1284.649	1040.381	3416.23	3311.344
208162	150688	135988.7	177150	248978.6	160332	271343.2	163303.8	304770.7	265943.4	209925	201934.4	293038.3	226659.1	193102.3	217979.2	195552.2
256202.2	77541.34	119446.6	77192.62	185512.8	46141.97	51939.8	73162.41	162199.4	106175.3	103416	21015.08	75990.99	61797.37	47858.76	105979.9	79479.02
80235.17	84039.77	61326.04	93582.4	59375	54070.57	21394.28	62173.11	57595.02	80871.81	106338	120677.1	81857.59	91861.52	109444.3	73863.4	88074.99
70253.6	69796.14	67961.88	34332.8	54395.01	23707.76	56979.01	51510.81	63152.07	55060.64	37652.84	59569.76	35899.31	52334.48	33672.21	46545.36	58631.14
128620.4	75953.03	63382.86	71813.18	114868.1	74984.48	119155.3	94184.56	128934.3	79917.26	89704.84	74543.09	77126.55	70318.72	79844.19	136434.2	91763.11
645137.4	621325.8	489896.6	594898.6	495827.4	582815.9	692236.9	649816.1	504824.4	552207.3	600812.7	628360.3	581198.3	504439.2	554427.7	594811.4	650601
11618.63	5573.178	10053.89	11828.91	7223.226	9368.974	6372.768	13529.2	13527.92	8592.351	4055.101	4580.09	9553.042	9197.94	10233.09	8017	9429.463
273266	284532	295720.5	258620.9	384861.6	271488.9	224114.5	214750.6	232853.8	156349.5	277756.7	265280.5	226577.7	372135.1	264944.4	226691.5	222771.6
24423.28	15204.8	9686.485	42028.61	25702.94	33988.41	28564.32	30290.88	22922.16	18080.61	26413.09	40830.44	30283.65	29149.78	26456.45	56298.73	17566.22
15746.21	28496.44	17879.74	25027.99	20462.86	15109.7	23331.14	17471.2	22574.27	16688.68	23530.27	27312.06	24603.36	16757.21	15791.34	31103.01	19270.41
2811.715	23244.89	16267.74	9914.579	14175.97	12739.26	10501.08	10244.98	17245.65	104532.6	23385.44	12023.82	16221.52	13381.62	13517.51	2417.199	16668.9
113009.8	219425.4	183219.3	182315.4	214884.5	371299.2	249689	220143.9	267755	104252	229428.3	308313.8	269854.3	200212.3	173551.8	224594.4	221520.2
10871.15	11436.01	6663.705	14203.55	11940.77	13172.95	34245.11	10441.41	25257.38	9146.721	5207.143	11538.96	9547.542	11966.78	9182.151	21607.31	10075.27
162535.2	154821.2	144159.2	146371.2	158786.4	97455.68	244817.1	171619.6	197343	176137.2	155485.2	146715.3	187751.6	138636.5	173705.2	196225	177444.7
22393.69	46315.8	39593.4	21169.21	27160.91	22282.82	38518.15	15695.13	12314.46	42040.91	68428.02	50813.54	48050.93	23773.43	27279.7	19668.19	22354.66
2849.303	16810.02	32432.47	8350.922	6575.654	3683.325	23060.81	7776.244	9111.976	6263.405	11115.24	9048.761	7319.377	9726.534	4833.88	6423.254	1076.025
82880.42	45293.44	63160.71	43802.06	51040.48	44504.07	6761.322	34299.45	43484.86	26004.75	124408.1	51255.79	89894.48	89914.13	27689.62	54842.42	50258.34
56580.73	15439	15341.73	12515.06	9578.945	13941.3	26541.02	19422.2	14845.6	5329.56	11823.79	13873.95	11637.96	13310.07	13143.34	22215.41	12299.52
219212.4	328303.3	310223.7	272152.2	220981.8	300411.6	404325.3	324137.6	344485.8	336304.4	320504.1	265084	321367	282456.2	264810.7	357716.2	348783.6
200755.8	209250	177620.3	199344.3	193094.4	243120.4	377833.1	165814.6	250690.4	231102.5	223139.7	155198.7	193216.5	178423	237635.3	328008.1	200227.4
5516.706	11541.31	21635.54	12175.15	10112.98	5274.033	11348.05	6569.333	13115.63	20646.54	19347.57	13911.18	14599.17	14635.14	9028.185	3866.258	15993.59
1635611	1710742	1371447	1314751	1539902	1718752	1978829	1532458	2142501	2382642	1322807	1238393	1057539	1418624	1389285	1731051	2121249

31734.64	42935.41	49208.95	37610.87	34742.17	42905.66	47951.7	40344.24	42767.13	35722.65	58987.86	32476.56	34004.03	32938.6	42546.85	49095.6	33106.71
6844.163	11142.1	52183.33	38909.04	31373.89	147159.8	115015.9	37721.73	529947.2	22773.35	182663.9	495096.2	189302.4	108909.8	344563.3	168471.6	113917.3
130407.7	12427.02	273122.9	1788.178	18605.87	20197.16	15015.74	15206.81	4170.09	27632.58	10923.38	51122.98	2474.828	9546.399	10370.84	20608.57	6694.211
283857.2	301501.5	340653.7	340692.6	286177.6	345495.4	352533	335899.9	401412.8	254092.6	303874	206495.4	336310.6	281020.9	307218	370752.4	297869.6
8398.801	16819.37	4711.352	7305.258	10193.45	8915.946	29107.78	13700.3	19166.83	28082.02	14136.61	8434.143	8534.416	6702.299	11845.55	21341.33	12983.29
121024.3	135442.5	153326.9	168488.5	128162.8	483853.1	141656.7	150842.5	263594.7	108664.4	203372.1	81945.76	104738.3	111405.5	127799.6	141999.1	138090.4
106168.4	228618.6	103966.4	66468.42	76592.55	69518.27	84391.77	102131	272613.8	151079.4	146445.8	82609.83	83712.47	76793.68	92057.06	112960.1	113750.9
116060.9	192771.6	95676.2	131970.6	127554.2	99860.97	80681.48	131119.7	120726.6	155464.2	150206.4	155828	120542.1	173046.3	151131.4	71121.05	103386.4
40215.66	52663.3	42463.2	44798.75	37676.77	33027.23	24098.28	37458.47	40947.19	48929.57	43676.97	49339.66	33974.16	49203.7	56524.57	32711.25	45089.88
25165.94	31663.23	29216.23	9549.05	20701.28	29791.68	19118.49	27247.77	14047.4	38505.14	30723.71	30963.28	27051.67	2067.284	15075.22	19595.74	12865.63
75482.4	175255.5	120096.8	62921.1	126934.4	74278.94	188701.1	61208.39	159373.8	103766.4	147004.9	75952.38	130544.2	47805.93	126636.7	148480.6	127665.2
3196.877	13471.36	19881.45	21281.79	6753.437	13497.36	13590.93	27072.92	12716.75	6622.938	14602.35	15401.74	22564.08	9543.61	11031.98	5595.654	16292.3
359154	230876.4	199874.9	197803.9	182780.3	161979.8	211785.7	261982.4	178815	333449.7	203993.7	245171.5	176869.1	213224.5	192244	246331.8	287524.9
50868.35	111120	79636.94	70633.63	143511.6	64592.43	60922.8	80828.17	49285.92	68056.73	159986.1	172490.7	166209.7	73887.77	45192.32	54007.73	67868.86
17753.32	15968.32	1645.201	15141.62	26284.42	20323.67	21693.93	14294.57	17562.88	159572.8	22592.47	35312.97	30524.53	20124.65	7503.102	25438.21	20482.83
16713.69	18454.22	21776.81	24260.61	19487.63	26822.19	31618.64	18666.87	14523.75	11253.91	24383.07	14365.98	22257.51	10637.25	17407.87	27420.77	16487.43
161460	121685.2	98286.37	151540.8	86839.83	107982.1	42234.75	117511.8	102370.3	164675.8	101308.1	135460.7	98247.79	121020.6	138960.8	117401.9	103863.3
19290.26	15606.81	16914.03	21787.82	36384.96	38011.81	12569.57	18059.62	14937.83	17454.48	16962.54	35652.09	30668.33	18418.33	29385.01	15240.09	20830.38
37854.47	28920.3	34035.9	29492.64	27084.55	23946.2	18166.84	23062.5	22300.7	34446.71	25404.1	30903.67	26423.28	22867.12	32362.28	27209.41	31958.18
143974.6	262726.9	126104.4	231055.1	274932.1	112831.7	99585.13	256948.7	173376.1	195467.4	210891.3	266334.9	177392.4	463612.7	243100	99338.96	233647
70084.59	86426.27	40392.23	54760.75	115411.1	33988.47	51989.65	56071.76	68854.25	72120.67	75224.33	77118.64	31465.27	111946.1	25222.45	41314.82	59212.05
140285.8	141794.9	175093.8	138790.4	86682.75	117403.7	100919.1	137639.1	155999.3	155135.4	146182.4	148291.3	122684.5	153045.8	155192.8	141503	163313.4
38105.82	16445.82	13614.72	16183.47	59570.88	14008.65	30747.17	27886.58	33927.65	18312.01	6065.413	17482.58	21427.95	32889.51	17975.07	23326.29	22604.97
9646.622	20557.63	7754.538	10403.9	13371.72	8895.749	5757.787	7218.541	11838.2	10906.55	13837.85	19987.53	14217.36	14189.65	12640.86	17415.68	10641.21
31705.97	38297.8	31097.13	46179.68	51831.84	45060.04	34682.98	40990.29	45176.52	29634.21	31849.59	31649.52	42613.97	42381.56	47025.23	35733.84	43365.15
5108.81	29684.69	28157.79	7852.146	6311.769	8245.463	32159.44	4133.232	15177.2	26009.12	16798.59	20440.58	10922.21	18019.83	14808	17142.31	7055.26
2955.321	10230.07	4275.437	3188.931	2061.628	2051.107	4431.344	4261.896	5222.212	6932.782	5134.807	1994.805	3039.572	4663.939	3132.134	4612.602	5106.517
1146.596	5170.103	2297.41	2152.135	2175.648	1328.249	8923.931	1533.807	1180.316	881.7726	3494.705	6940.023	2128.219	2542.456	1933.394	1602.761	1950.049
7037.442	23931.57	18495.05	15687.61	15245.2	12152.98	8773.464	13252.57	16180.85	10651.93	12551.11	20485.37	16668.08	6379.371	19078.35	9890.254	18978.92
4805481	6477518	5735424	7317574	6009313	6968098	6590374	3635190	8777853	4308628	6325006	2819234	5756617	5969346	6557265	6155943	6078001
90974.82	96012.42	82228.49	75410.19	107809.9	62441.34	154239.1	85187.34	43766.8	143245.2	102633.7	165010.5	120525.9	121431.5	100494.1	101177.4	83285.3
8072.324	11998.12	16600.61	21372.41	13279.25	21546.33	11269.79	15137.99	11455.3	7854.388	7145.406	16001.33	17644.17	16869.14	18664.57	14319.02	14758.22
22919.99	49349.2	27090.36	22465.63	35854.01	28858.65	43390	35995.87	34222.41	31047.62	45700.98	59584.34	31532.23	28779.58	29261.49	53122.86	42722.02
19424.16	20128.3	24060.28	16279.2	15186.06	12518.96	19798.09	9389.956	13442.77	14146.68	10556.81	10830.04	11084.62	13547.96	12255.06	17654.91	11996.37
4093.353	10008.89	7607.195	7365.906	2631.101	5373.053	9974.552	4380.709	13060.53	11682.48	5711.171	5665.343	7050.039	5526.962	6881.124	7640.8	3990.328
199716.3	260353.8	282325.4	308610.5	203057.3	275454.7	374169.8	328390.9	408330.3	155569	261506.7	133669.1	266645.7	173204	197971.2	270758.9	275371.5
296747.4	201595.1	826732.3	12766.42	112007.9	1399.315	86144.55	19567.6	192628.5	54443.1	82255.71	101920.5	63975.23	286313.9	69467.16	82044.16	60278.89
17550.21	44218.27	10075.37	12604.64	29904.56	10381.85	35025.2	3207.559	17033.4	24403.13	29310.31	47664.16	18628.61	22847.79	20755.33	26630.93	16099.88
50150.72	98055.95	96065.72	98505.84	87324.66	77803.09	129710.8	89086.85	113861.1	59208.23	121753.6	105590.5	135279	85127.94	93819.23	108133.4	101925.4
60511.4	67057.66	37648.5	64143.65	54841.93	50994.52	18041.46	65869.69	62697.7	84475.05	60113.48	69409.67	46311.45	96049.45	78209.63	26435.09	44519.37
370166.6	492590.4	479832.1	710175.2	444949	567990.8	400648.8	504936.9	521732.9	234019.3	373820.2	148264.9	553758.2	281587.6	265152.7	357110	554534.3
53058.41	93063.91	79752.46	83523.47	88137.15	88870.42	101144.6	111417.2	105029.4	58872.65	111561.6	101880.3	101738.8	83534.28	73772.23	82701.97	105688.1
9003.771	29239.71	22452.85	23918.39	42287.43	21572.41	13947.91	23234.6	15436.17	29993.34	21954.88	64232.05	56007.23	36533.92	26886.26	18173.01	25810.79
95612.08	156484.7	170353	112877	113759.7	155688.3	173192.3	121333.5	146435.2	49398.52	137519.2	77944.96	102678.8	99647.55	103140.3	137876.8	103866
54934.15	72621.23	44247.85	55863.95	52473.73	42523.94	28740.65	50648.54	73027.52	75454.05	59373.55	50713.49	48718.53	65208.8	54827.32	30691.85	44519.9
6062.638	10548.14	7639.284	5440.852	7684.906	4359.646	12433.05	7522.518	9171.798	8701.019	4193.336	6602.748	5016.755	6286.204	6941.918	7035.219	6003.164
4519626	4945466	5613542	5261419	2624722	6012541	4576530	5688443	3784922	4408044	4248067	3505781	3595108	3357706	4432268	4642744	6206681
5456.152	5474.559	13871.28	8198.31	5838.596	6697.701	5270.013	5804.535	4116.444	6168.423	5086.182	7273.778	2121.362	6217.851	4153.219	5824.068	8223.005
20837.86	73276.64	81722.7	104752.1	111716.5	99671.09	75410.31	31301.84	34980.66	27714.1	95730.38	110923.2	315569.9	60591.97	84933.88	70720.55	65505.23

445626.1	223534.9	266774.2	284704.7	339839.8	238814.2	207267.6	279407.9	303291.6	2760429	208066.4	215876.4	181374.5	204309.1	178928.4	115145.1	200302.9
114279.4	91146.88	84939.1	158418	170777.5	140246.4	153571.6	123822.2	178005.5	95205.23	123234.4	75825.83	223837.7	121657.7	136010.1	197433.3	158807.5
25878.88	14518.19	13330.67	11911.74	16919.55	11048.81	14653.46	14269.35	14586.01	72984.91	21124.6	10360.92	2875.255	13473.11	13004.83	6387.134	19547.31
24404.44	15821.07	27950.52	48353.68	4428.555	85822.28	23605.35	11176.27	13744.73	22435.48	5542.864	7428.246	6571.508	11976.1	62289.95	13768.74	13874.77
10896538	13694721	13413209	13111808	7436227	12660321	14374901	12498447	13928497	13488685	14161606	10860960	9049786	10425517	11409345	12918230	15537210
61599.24	34810.56	21393.07	17220.65	17763.19	14331	19404.61	19479.39	42909.29	15833.73	22458.02	14758.89	18349.93	38771.88	19154.62	27258.21	18542.72
348869.1	911285.7	853342.7	1371889	783183	655058.6	898564.3	882320.8	1044889	315061.6	879487.3	772303	1459883	803170.9	581354.3	823125.8	972946.1
15749.2	23096.7	22536.07	17261.27	7688.511	7651.875	11578.59	9932.222	14930.33	13912.07	25118.24	16594.53	10892.08	8929.504	24648.95	16150.29	13792.76
22127.34	25632.66	22197.38	21281.47	8514.543	11721.39	27516.24	13801.56	8269.04	52768.18	32814.21	12560.29	11509.2	9599.759	12854.83	28321.22	20587.26
5858532	6931102	7324527	7554390	6218254	7530209	11694779	8247241	10970256	9713835	8074038	4642716	7284158	5728438	6010065	6983023	8755680
45341.5	70773.98	54373.84	55617.7	48586.36	43246.7	44982.39	50788.45	59638.96	59403.29	66159.45	48642.6	64338.81	56993.29	45791.01	64383.84	61721.72
403456.9	476457.1	560637.8	527203.1	511404.4	585151.4	613684.9	598977.1	349489.2	289127.7	504397.9	591950.8	420855.1	568855.1	642640.9	730131.8	635926.8
11847.74	20201.44	29469.83	24515.85	61425.63	63302.12	47972.49	58761.36	64551.92	20739.92	35262.82	73412.16	63687.23	68726.66	24293.46	71092.6	18589.77
8427.437	25913.15	14638.9	21353.66	48401.7	15583.53	11463.91	14199.14	13580.61	15742.68	22978.43	81703.69	56845.26	35287.31	23973.73	21438.08	23535.11
542426.9	254018.2	464351	216320.8	285912.5	397117.2	305354.3	258377.4	200770.7	613933.1	277969.8	160309.7	210376.4	284247.1	273986.3	245131.1	200720.9
145956.9	209769.5	105946.6	149731.8	180246	114294.8	61328.14	147767.7	147047.5	230307.8	143977.6	188120.4	123497.7	300265	188435.9	64563.83	114487.9
770468.4	1638229	1144970	1129343	1546350	944627.7	1374595	636895.4	694466	866997.8	1256881	317382.6	1760473	622832.3	1108067	1072628	751009.1
18865.6	24430.67	15473.86	17967.92	15842.55	12773.55	36284.22	14830.98	12934.9	9975.137	21832.68	12223.44	7630.214	30996.01	25834.51	16999.33	23437.97
12784.76	23998.49	18004.54	25040.42	26177.79	13315.7	25506.84	25921.04	19925.4	13739.32	34595.08	19786.15	24498.89	40720.36	34039	36918.36	31268.87
9445.766	15731.21	13399.06	9478.19	24142.38	8736.872	14049.76	9375.484	12814.37	15505.72	26856.68	27382.6	24629.71	11101.58	12850.01	18065.45	10904.09
66623.45	79134.71	33621.77	39878.55	45260.07	61509.29	61425.96	43477.36	62403.77	85282.33	23860.93	14790.91	28008.29	29465.55	18837.1	7407.075	23558.82
79953.02	101911.9	131011	140336.3	128262	157911.5	156972.6	116977.4	162619.5	141776.8	132434.6	58413.77	96571.93	74621.55	75590.59	108579.2	126239.1
220929.2	249351.8	200235.8	181818.5	173161	137479	181549.9	156492	206218	270910.3	230746.1	193353	138495.1	190862.4	225876.7	229033	274321.4
42659.36	89799.73	749918.7	28593.49	21973.98	64105.86	118919.5	131000.4	49979.37	52668.5	197572	15010.1	42321.09	74497.34	37858.78	247538	83349.27
705498.6	279464.7	368606.8	254278.5	286130.6	549165.8	378752.2	381597.6	251523	1442876	377767.1	176220	353051	210212.2	289025.2	317030.2	251737
3344.815	8781.475	4066.547	2174.695	4157.481	3544.513	9566.298	2980.016	6196.904	3545.044	3568.912	6227.465	4479.003	4619.433	2876.187	5156.754	4888.691
27516.38	23635.02	18210.36	12832.55	25513.75	26704.8	42034.36	19175.97	29850.37	21278.04	24261.7	24017.56	26470.2	26238.7	16751.78	40611.67	34668.89
12370.26	17298.48	15447.34	36472.05	11076.09	23755.33	29850.52	24525.54	18846.54	10454.87	30265.28	23818.45	69570.12	5853.269	14669.82	23022.1	46230.06
39898.8	21085.52	22126.92	22643.56	22122.18	19131.88	18430.49	15237.29	17385.48	48385.12	20258.58	17607.52	17024.47	30363.14	31803.88	19300.09	15769.06
456882.7	312409.1	328331.4	279517.3	357286.3	361293	365362.8	300664.1	309216.2	594842.1	353494.3	347433.9	311216	264360.2	329020.7	315061.2	328858.1
2087.15	4605.65	3525.885	1042.644	3904.026	1737	3515.911	1430.611	2088.923	5765.626	2988.535	1622.325	2921.265	2934.098	1028.14	5656.428	4232.334
139271.5	79895.18	88201.81	87318.8	108054.1	95605.78	80476.11	97570.67	78024.94	300550.1	78529.35	92057.33	77547.87	103627.5	84218.96	72995.54	100136.6
118441	225033.1	268547.1	180453.3	234838.2	211393.7	217196.1	149673.3	275060	261928.6	272341.7	339793.1	298505.8	158160.9	177280.8	237434.6	192346.4
207255.4	254207.2	207046.8	194207.5	209743	154702.6	262551.2	197962.5	232294.8	228511.8	269969.3	200721	178979.7	227613.2	205440.9	244203.1	214448.9
313829.9	202647.7	73320.49	154820.9	264368.9	224482.9	985342.7	448647.3	518546.9	154885.7	821257.3	388893.9	462940.7	166142.6	283737.3	633968.6	601588.3
65943.12	87634.86	84921.46	84346.98	66978.26	61806.04	122540	124580	156687.6	54965.4	81870.25	67795.7	65334.55	84646.05	86712.07	82197.14	96847.58
20343.47	20236.93	12615.93	19696.54	12160.96	8578.516	10518.92	21565.94	21075.61	21169.86	10834.88	10299.86	16095.79	14155.61	15931.07	11661.31	17108.19
24370.85	205847.4	39789.29	5831.271	7403.182	8940.537	44598.45	16567.73	4716.485	98165.51	30088.03	100507.1	46523.77	10249.26	10574.29	17322.36	10796.26
61598.05	70423.97	56828.71	44950.34	70155.91	36110.15	60903.67	8975.979	53508.69	46960.1	69654.2	54975.47	40261.79	92669.95	60493.45	45948.07	8780.651
33895	33864.12	18246.34	46004.7	41071.05	24587.93	211220.7	35174.98	36358.61	40840.3	27012.26	37479.13	23750.65	49648.65	36747.34	24283.45	23819.79
48795.34	46812.18	48404.78	48610.79	35333.91	35949.99	37085.63	33018.72	38093.91	42003.8	46310.4	40386.04	22721.32	47310.23	44276.93	54869.97	32409.21
7820.807	33658.85	17395.72	10383.76	9074.7	11865.53	46319.93	5778.56	6972.785	18717.23	10480.75	10410.48	12872.64	15532.81	3902.072	3190.187	13917.2
241336.2	271721.2	244203.7	292865.5	223766.9	291912.7	465506.9	232979.6	342756.5	271641.5	304450.9	244486.1	293052.6	221357.8	228350.1	383092.9	298028.5
66206.95	98265.81	53010.29	92261.23	85519.2	59625.41	62316.28	92040.98	46139.12	71625.09	30745.53	105019	92946.45	155639.9	84336.84	70497.41	66891.66
32077.83	50052.81	51488.98	46706.58	40738.96	46221.59	59856.49	81266.27	70808.13	57530.8	61415.59	46577.97	41577.24	55998.37	45946.9	63275.89	77312.72
36472.54	50133.31	41746.14	23514.13	28252.38	31655.44	39425.57	28113.46	23621.7	31311.94	55425.61	35411.25	37209.66	46085.78	46448.15	41449.09	35085.84
5073509	4468582	4274711	4034562	3886404	3823186	6923380	4374210	6740260	4767591	4483315	3876098	3629911	4543874	3767388	6032469	4311034
26285180	27628798	24797100	33936896	35750740	44056576	22550798	32631316	29188856	21389116	28664250	26941286	31383126	27713674	31067060	30748010	38176592
20641.5	23969.44	29380.28	18794.27	18602.99	16362.06	11417.83	13967.93	14350.24	30220.97	13258.8	14251.02	13217.38	20944.34	15656.77	10806.83	20691.05

399217.7	904448.7	819481.8	651082.2	663400.5	846448.8	1145276	753257.5	1259692	358590.3	1143346	624396.6	581474.8	653942.7	792774.8	988951.3	883399.1
2299364	4053033	2011862	2616251	3000644	1742177	1524894	3288218	2634159	2941556	3288268	3331482	2425620	4900998	2346785	2022908	2488406
46737.18	46094.45	44226.28	45314.27	40370.47	57010.01	35050.79	36262.41	49012.02	25163.53	51768.32	17878.69	68162.69	24096.68	25000.56	48490.76	71740.88
87858.55	182469.9	64388.34	84888.98	114084.3	77481.77	145180.7	85038.3	160444.5	137009	173244.6	75414.13	71657.24	90863.87	74081.46	137400.6	124167.9
18914.93	20636.1	26329.69	17763.65	15843.44	14265.7	36190.7	14963.74	32753.14	21483.9	25601.44	29223.47	22422.6	19727.4	16838.43	19317.84	23574.19
367207.2	85994.74	138925.9	169685.3	119407.3	183024	94022.95	213051.7	101210.9	644963.8	80876.22	151466.6	140271.4	139786.1	256432	75850.29	132193.3
251328.8	206581	163530.7	221154.3	185460.1	172573.9	112720.6	191520.1	186510.7	336029.9	191013	208030	184077.4	241564.8	246383.6	136927.5	196002.3
156114.9	148724.9	96049.08	168929.9	131620.1	157746.9	150370.2	157558.7	159913.4	103903.5	170774.2	178706.2	166094.8	158886.1	119688.6	148606	177812.5
12656.52	14978.59	16949.39	11807.32	9401.858	10435.01	17276.94	11260.52	17901.96	13204.05	19926.82	9002.491	13904.39	13665.77	11543.86	19747.03	15958.08
28445.88	32102.88	11911.4	29307.32	17706.26	21254.03	20123.03	20026.4	13497.75	33732.5	20986.27	24864.81	28834.33	35590.35	35112.13	23034.64	19479.05
19687.05	22800.03	22039.81	20870.7	21660.1	23112.29	13743.89	12221.68	14913.88	16030.99	21367.93	26929.66	27623.84	29240.1	18464.74	18120.29	16914.76
252838.7	185864.7	197721.8	176544.3	219715.5	231975.7	320216.5	181808.2	257651.7	200738.6	179944.7	219247.2	179451.7	174830.1	178351.9	248818.4	185979.2
23690.18	23107.71	68081.29	42535.65	20026.21	19217.98	26837.48	23422	22301.54	28697.18	26460.28	18984.11	35671.64	16281.9	11157.63	40516.95	26317.25
10633.4	23093.02	26138.58	23453.06	18729.04	23554.49	15254.59	19241.99	15469.43	100282.4	18415.76	21583.42	12348.19	18746.84	17873.58	13833.82	20632.17
7971.503	17680.22	18434.32	15091.37	21068.35	15731.57	16834.03	16070.76	42433.73	103274.9	17735.35	15807.88	25632.74	11061.69	11528.12	11471.62	19557.32
49777.25	77752.25	69079.39	51992.51	37978.96	37856.65	38523.62	54435.8	49948.48	70466.13	63036.23	44798.54	61358.55	52490.2	51164.7	40159.23	71474.34
53275.23	94988.45	69399.43	81066.05	67439.76	90775.36	171356.1	91801.66	75778.76	110585	91706.45	70906.58	60141.8	95317.52	78182.46	138551.1	105213.1
10329625	8652034	9174702	10830838	9185695	10863155	6817039	9557445	5637331	8877192	9089995	10665924	11621992	9577718	12123828	8216101	9445912
101811.9	141294.4	305755.3	317626.6	283601.4	352925.5	416887.4	309892.7	402629.1	207683.5	320330.2	209976.4	304680.1	298087.9	340249	336462.5	294162.7
37440.05	26990.86	27013.01	25557.46	17080.66	22594.25	16808.49	27048.6	28892.14	32513.1	29731.98	23279.02	18066.54	18975.33	21462.05	25155.22	24082.47
33796.28	47307.46	20169.85	38167.59	28852.95	22262.56	39341.45	32745.5	24826.71	35291.53	39632.63	50278.89	43822.88	55611.54	28561.83	31089.17	35997.11
19503.1	6536.045	6327.45	4028.688	13275.39	6226.841	10844.89	97828.47	14821.11	29766.28	21119.84	16748.83	18907.66	155545.6	16179.11	10085.99	148330.8
188118.5	156775.1	171276	220678.7	145004.2	211732.2	269608.4	161498.4	209539.3	143372.8	161003	138523.4	187842.8	137962.8	153577.3	213614.3	177303.3
65393.93	132405	121893.4	143099.5	128884.1	97309.13	58239.82	131891.8	88354.32	102256.5	176050.6	167480.8	140280.7	111905.7	103373.4	120415	161404.7
9217.117	22603.87	7377.307	13496.09	13877.6	12367.26	10658.65	14672.91	20033.3	101820.6	42659.06	40148.12	12555.22	13095.48	21890.88	9771.178	18341.68
44625.89	45940.38	36831.9	45379.57	47886.96	39238.38	29182.73	55183.19	44430	48104.88	48158.06	59950.68	47361.01	69709.54	65337.95	29359.28	43437.21
2130.375	5170.103	2892.461	1924.836	2781.119	2214.575	16160.42	1469.761	1985.219	4995.871	2154.988	5209.567	2397.578	3124.661	2223.073	1731.544	1879.221
319089.7	477286.6	438505.6	415740	378311.3	535747.8	518431.8	389749.9	426489.8	294534.7	349538.1	238108.7	367553	344632.3	339130.1	449095.1	361596.4
45161.34	77096.18	88468.55	79720.24	58203.01	66356.47	135944.6	84975.28	100935	47665.41	88605.84	62193.66	78043.88	60646.28	65081.86	91192.64	83469.02
14953.71	36475.33	22932.73	28484.38	48079.53	4871.854	25562.24	22176.24	22485.59	6476.018	35905.24	70332.19	53805.1	17709.31	54340.55	43645.2	26593.89
92261.14	175256.7	147674.9	148301.3	115070.8	151716	199199.9	118524.2	191738.5	84139.88	148023.9	84322.17	129489.7	109659.1	121730.5	132218.7	153310.6
18301.08	4451.456	2228.736	3486.82	2136.247	2328.956	3561.231	3551.404	4244.534	7785.022	11407.64	11568.4	132.1305	17601.65	3565.459	5884.56	2235.85
12448.54	17385.63	19248.47	15616.7	13117.73	22944.06	23768.4	16139.19	21129.83	28587.4	18617.05	11526.15	17150.36	13669.85	25443.49	23790.71	16953.68
19052.88	20809.02	33954.45	19886.48	16398.68	51537.9	32967.02	29813.56	38018.39	26050.67	22888.34	23920.41	16606.44	22087.2	18165.02	22580.22	23984.13
58790.8	174315.9	110218.4	115400.3	204695.5	65162.99	74405.98	46305.15	70452.32	65698.07	207434.9	175664.1	505429.2	321094.8	152410.3	99141.63	75968.77
36165.34	42502.65	18630.46	19012.02	45840.02	32229.98	33719.49	19622.25	50100.25	29518.21	44454.38	37395.35	42463.98	32340.45	45260.96	55064.53	36292.4
444174.2	426806	361664.9	333863.7	196899.3	270218.5	367777.8	308855.3	256084.8	526738.1	376888.9	341845.1	248121.3	311990.4	375749.5	341909.3	341995.7
163516	195114.4	118077.4	132299.4	93986.69	147717.3	129946.6	164773.7	116811.5	157735.3	175618.4	162302.1	97743.45	131244.6	191449.2	124563.4	155408
87729.06	93189.56	66365.04	70497.37	68483.38	42010.51	153089.3	56218.5	91640.17	61499.39	84564.03	48456.3	44176.57	71815.44	87038.72	92736.08	52144.41
7024.821	13584.25	22898.95	10877.98	6570.768	10066.16	19953.61	12743.89	14897.04	2186.894	8667.759	10790.2	15712.02	10849.4	6571.175	9283.678	8845.973
752285	932217.1	769113.3	838066.1	749302.7	2393640	1004762	924721.6	903140.7	922629	1048306	891772.8	931836.4	792453.3	921291.1	703476.5	881417.6
13490.25	60505.05	39420.21	33810.54	52259.76	33975.87	54997.04	25312.36	28759.31	32486.62	69516.92	102971.2	97426.08	55836.51	40138.23	56829.22	46917.59
64054.65	61745.99	88616.67	38697.04	34004.78	24762.84	65420.92	55711.11	38733.74	39014.69	89752.55	32103.9	21616.18	16463.06	7688.9	24032.57	31845.69
431085.5	196282.3	151709.7	185415.8	147730.3	215172.2	274227.1	181827.9	176410.5	1021831	145414.5	159963.3	175311.9	133311.3	217037	172788	175281.3
4363.784	4976.903	7926.247	5053.716	5528.75	7406.072	7876.457	11197.7	8010.515	6063.343	8632.175	5896.906	7328.57	11028.91	5227.279	4422.8	7342.715
4765.529	43793.36	19020.36	22505.36	17031.7	28763.65	105159.9	36289.07	14626.53	40727.46	57735.09	53576.79	15508.07	27944.19	11277.87	63687.8	37076.95
359920.6	1093500	323906.8	565812.4	581280.2	248103.7	1878408	327217.4	457120.6	2040000	1441557	655464.1	458267.2	451964	410401	1326198	1144205
3720.745	4041.987	2031.383	2893.302	5123.542	2374.015	8979.879	3152.264	1528.075	7113.705	6459.508	3919.048	2581.405	4248.908	2185.736	7186.8	3812.205
13018.43	31349.37	139567.9	27228.87	11172.04	17154.75	63103.68	19398.67	11458.21	440536.8	76267.74	28084.05	27499.27	85768.45	21582.42	53097.41	14472.47

169529.5	818131.9	167270.7	377263.4	212228.3	168723.4	985028.4	202007.3	237273.3	969871.4	972627.9	509104.2	241574.2	184195.9	281477.6	743327.5	755783.5
85998.8	335645.6	59843.97	229598.1	74508.07	47055.23	393671.3	61002.95	80572.84	365769.4	416814.7	195815.5	78061.23	60063.36	116337.5	285699.1	325841.5
381862.3	1494431	333920.8	830451	847456.9	428587.7	2767373	456998.2	572443.5	2273624	2013624	1044739	686790.6	399715.1	886763.5	1881535	1786153
9379.347	12085.3	8177.057	22538.86	17124.49	8059.895	12761.59	13681.83	12443.46	10790.44	5237.424	27414.45	19784.81	16951.8	15882.91	14346.2	14523.28
4141369	2299624	2204733	3522357	3116025	4586399	3758996	1961562	3904247	3154517	2807938	3570288	2820651	2042485	2748398	3778692	2194888
1497492	745994.1	707161.8	611377.4	658916	661817.1	877967.4	614419.9	1373857	528994.9	789583.4	781880.4	1229629	688199.4	676324.3	676976.1	699852.3
14849.34	12048.12	17156.69	16252.04	13221.79	18114.14	14074.48	15105.12	15087.66	10987.58	15773.45	28334.66	15338.7	8385.632	8590.143	14985.29	18043.91
449940	312294.3	371285.4	386834.7	487033.7	313511.3	234138.5	271301.3	343566.7	415968.5	307423.7	386482.9	372480.3	547115.6	407820.8	358486.4	188378.6
453372.6	1163952	831484.8	739864.8	1064215	640535.3	898421.3	630075.8	839594.1	604321.7	1203789	1478505	1837745	876333.1	575595	634507.9	761108.6
3339.276	5690.693	4465.006	4474.109	3502.198	3065.21	7346.952	4808.74	4426.604	3932.111	3965.486	10476.71	6519.8	6770.638	5833.019	1815.439	2894.314
35559.49	118310.6	48382.49	80392.45	81257.11	31592.91	66589.02	43839.34	59270.25	83381.64	80587.59	132598.9	89145.18	79663	64282.4	55965.14	43979.72
130222.1	1138177	161449.1	335256.9	376324.3	178927.9	1755455	215093.8	295034	1521794	1251196	467712.6	327013.5	161178.8	352489.8	989842.8	883063.9
15807.37	6945.835	5686.527	7947.694	21466.56	2583.37	21570.92	9483.072	46917.24	7191.769	10471.71	12481.32	5652.56	8774.621	5962.135	12387.86	5704.111
11197.14	27736.54	18653.85	21525.06	13706.28	17589.32	29981.68	12609.78	6290.558	16189.27	19747.68	22463.51	23730.62	16456.94	17940.52	24950.21	23417.66
61751.21	22145.24	45753.04	26494.45	27876.29	23138.24	19268.7	28904.51	33321.7	160887.6	33390.79	73430.65	27203.58	23512.17	23461.02	25770.33	14873.95
22275.01	46137.38	39034.77	39921.21	26700.97	23670.96	24558.65	28988.64	40515.58	30226.68	39616.2	61007.93	32607.32	35816.78	42215.34	32119.88	43248.7
274075.6	216380.1	237699.1	267342.4	316824.6	217936.9	211362.3	268851.3	249053.7	525078.6	201271.4	250329.7	247337.3	252305.8	171864.2	179711.6	237388.6
284019.7	483365.5	197447.5	546637.3	177132.7	874799.6	1203399	498341.1	751888.9	380164.7	453919.6	663546.8	390603.1	515050.4	883485.6	1420690	356556.7
16429.71	11673.12	13564.17	13546.9	19398.66	11668.91	19042	14297.69	18468.81	14735.89	14322.63	26505.96	16685.6	8354.552	9096.812	17654.91	14436.97
811681.8	612341.6	544937.8	705530.9	764705.4	605930.1	1250364	691238.5	675327.8	684694.5	614135	508059.2	760426.9	784352.3	544492.9	974609.6	816735.8
43412.59	44099.57	51867.66	35140.79	39751.77	32590.88	37704.36	35469.48	44006.13	39850.57	53052.15	59228.45	45618.43	40731.48	36980.3	46929.18	41635.59
68519.24	75285.8	90298.42	81937.08	89655.16	139162.3	197242.8	109278.1	189260.7	110598.7	78237.27	89003.86	60693.86	88294.08	145821.1	132601.6	113820.5
27854.04	44426.96	37950.16	35310.54	50710.29	33664.19	55538.25	19716.46	85641.88	55298.02	86651.31	179852.5	63598.77	63245	90393.35	89971.16	73930.58
9796.545	47234.39	33865.55	20765.11	24545.11	22566.98	9459.352	11552.48	16005.1	24933.15	40328.48	59944.84	29199.65	24031.59	38948.98	42945.21	35523.34
26002.02	21163.8	28271.93	27558.57	40897.1	30746.66	18483.71	30136.7	31603.03	27333.94	48703.99	58482.36	42333.7	41768.59	19478.74	25272.52	39131.16
160779.3	152837.5	120206.3	147851.2	100817.7	85638.48	86845.89	119461.8	72506.55	158159.1	131807	102600.4	108434.3	136874.3	152038.5	128674.5	137494.3
318043	267529	205214.8	225103.5	296813.7	186326.7	169696.8	203022.3	223719.9	190814.2	294560	592206.5	371931	295834.5	240031.8	222783.5	244010.8
65992.6	136312.3	89226.52	61890.95	133012.6	45580.89	116667.5	65723.05	61660.84	173169.8	81566.05	333566	181593.7	105693.5	111941.7	109935.8	88874.31
30971.53	19120.46	31761.87	33966.77	35996.36	19866.75	79882.13	26415.72	37761.29	34091.66	35056.32	22142.83	23295.32	28333.02	14836.25	44203.25	26372.97
11680.54	77047.1	39453.5	80020.44	58371.77	34225.42	91177.91	35187.82	47625.25	50909.34	22079.04	25064.8	125254.3	53935.54	37610.46	92911.7	59802.97
20287698	32381824	23305054	17135654	21579148	20837908	35611508	22953746	23738886	18483068	43584456	12106685	18861524	21071598	22954334	26458076	26029164
30944.77	38273.01	34538.18	50260.76	37259.52	34659.5	32645.55	52165.3	50823.1	38947.47	41407.75	41758.98	49180.23	30462.07	41062.73	50448.11	64458.93
21029.27	35502.29	27612.72	26720.38	26832.27	32039.08	33220.35	29679.52	37187.06	14962.98	36639.15	23949.81	24777.5	25713.99	29695.09	29147.23	31994.22
97412.55	113049.9	112295.3	70311.05	72111.95	68486.31	61258.19	74447.33	85507.63	85100.15	84424.77	80157.86	67271	76577.83	91853.17	81640.63	103373.8
39899.3	43980.96	17495.88	31781.67	42255.75	17784.01	11235.88	35774.03	21842.27	59817.7	33704.32	37856.06	29413.26	48854.17	42067.39	18360.66	31078.21
9688.752	10535.28	11247.22	13665.98	9017.09	8951.447	9079.191	8856.966	10463.22	5931.173	12172.64	9919.766	8643.663	4571.813	7108.387	6900.247	7271.504
52980.26	49553.77	86695.28	81765.84	62168.63	54756.16	56806.37	84241.48	52891.8	35880.73	60535.8	51766.6	83889.65	75780.53	64685.37	67094.65	102044.9
60745.15	75132.42	54755.66	67550.23	57829.93	53312.41	88158.23	70894.14	123420.2	66025.65	67861.2	61567.78	69737.27	51836.37	69703.52	115268.8	83781.44
22302.54	20646.56	17417.38	15839.88	14083.39	21353.29	30882.46	14187.07	21886.11	21016.18	39065.09	14544.09	20672.55	15829.78	14858.44	18057.09	16229.13
44235.07	95627.5	63061.93	34852.22	41161.14	50841.78	62430.22	48829.41	58518.55	68920.31	64255.63	97155.06	73985.59	62060.54	45698.8	62146.95	62854.81
54621.98	43362.65	41650.52	36702.04	19258.45	58982.66	25271.07	34274.25	36527.58	49337.26	46714.05	22936.46	20125.45	29930.73	42390.81	31781.65	28881.66
1144.456	2884.884	2346.497	1669.411	1355.702	1695.671	4241.451	9657.297	10769.74	5663.623	2449.417	19272.9	3352.774	2305.841	14213.7	2375.089	2854.21
57133.84	68328.52	49381.75	81514.77	62199.46	75771.2	68131.18	49916.13	82600.4	43250.09	56147.06	41370.44	45451.31	53963.71	50502.63	60346.07	51143.48
44688.1	35142.91	28303.86	28026.31	23366.18	25317.86	40558.5	35379.03	33204.85	50917.63	37086.39	38698.87	25756.37	36759.36	37560.3	40809.05	35590.38
82429.59	154874.4	70540.98	107537.2	73547.67	69655.82	57785.01	94627.05	115074	113836.1	94642.39	128014.7	83256.31	142696.5	116881.9	58093.84	84013.26
60527.05	77866.82	101548.1	95430.57	57674.82	81974.19	104955.5	94454.07	115998.2	48586.02	80581.65	61241.87	86361.46	67485.98	85767.35	76306.65	92034.6
123774.3	130837.2	135008.8	160296.7	116568.3	134197.5	122464	123246.1	159841.1	143313.1	104830.6	90296.72	103486.5	117831.5	111980.4	91648.52	121588
47055.18	38619.72	52125.1	54144.06	39613.27	45700.6	54411.4	54084.71	53441.3	36271.5	38323.97	37662.82	55835.25	44218.02	46772.69	43013.79	51622.2
9678.516	11257.01	7794.672	2223.076	21862.93	17501.14	7486.204	1703.31	7816.492	10816.48	7570.313	7172.28	6855.319	26282.93	22469.83	8106.028	1146.905

45533.32	33267.83	40513.99	32628.3	35514.18	28236.16	30208.72	22826.75	13363.46	47792.59	21639.07	19848.96	18194.02	23692.61	24496	20003.55	23362.95
12766.25	21699.14	20146.28	13800.12	35320.22	9126.146	26870.45	20524.14	22240.3	14070.11	21210.3	19556.75	23000.84	17863.34	9564.768	22253.55	20253.21
138904.1	173440	232474.6	254947.6	173573.3	291208.6	222362.7	257262.4	176383.5	104380.8	231966.9	178828.7	222848.3	137169.9	252074.9	193367.6	260459.8
10430.29	24296.39	18158.36	32190.64	14129.98	13996.67	25817.53	30940.42	24471.63	16919.11	24463.07	15840.03	30236.32	19805.4	19759.35	21008.95	34815.12
34285.21	31875.71	27776.29	18632.66	20643.33	19878.61	23692.99	17803.83	11902.1	31321.21	40043.65	13944.93	17109.94	16249.4	21042.29	24528.02	18780.21
49082.94	80871.29	56576.46	43102.38	34294.01	48073.33	54642.55	47263.78	68251.96	64461.32	74361.99	45311.31	33956.92	45985.13	56185.81	65157.37	62259.71
1654.096	14644.39	10183.91	8354.261	14111.99	8751.078	6404.036	6397.82	6550.483	5373.123	18629.6	31598.81	24630.57	14612.77	10763.85	14488.98	14936.82
185827.4	271667.2	122942.9	185017.4	173901	183005.9	145718.6	214325.1	214894.4	303929.4	152186.6	190136.6	124306	231218	194176.1	93686.01	179019.6
57711.68	118560.1	90779.7	56678.18	70651.65	50066.87	106427.9	57539.77	55346.06	127501.5	95340.28	186846.1	162883	86655.6	92069.24	104158.1	71497.68
56888.21	99569.88	90147.12	98136.46	75085.38	90326.4	64994.85	50259.14	55939.89	66160.96	127346.4	39529.48	98414.17	54695.11	69724.07	53148.47	44454.37
95041.21	103512	89138.13	76152.55	67689.18	68683.47	67667.95	78049.49	78763.76	88934.94	99550.91	94861.88	70565.18	82186.41	81273.65	94006.8	89257.35
31866.19	38226.44	23417.66	19147.46	47114.69	24153.16	23324.51	24148.16	30296.05	23786	42459.78	52668.55	42746.07	35433.87	21204.28	23096.9	28386.78
3144311	2866629	3826700	2536971	2638654	2655395	4960078	3710348	3468733	2340095	3384035	1857861	3005627	2246233	1952779	5113937	3577769
35785.42	18451.73	35280.41	51028.6	31045.49	28447.73	5471.671	31184.99	31124.82	37152.36	62146.47	24341.65	21665.27	16848.04	11616.59	16079.13	21800.34
131389.9	71128.98	127817.8	41693.31	105401.8	41570.91	37252.44	46143.89	134346.9	37915.39	112411.4	129819.4	66071.91	297978.8	54667.56	104891	84878.94
10605.95	37207.42	25233.87	27003.81	69048.45	37860.23	27641.34	26893.14	17117.86	33669.71	61575.19	137574.5	105811.5	53735.94	42383	35710.27	50550.01
15839.1	11616.53	8798.371	17509.21	23132.17	13148.12	3340.273	62428.54	25877.42	109409.1	62934.73	220703.1	29005.09	217470.1	33881.72	9014.67	19986.85
74866.55	102442.8	109907.2	128285	127954.3	117755.2	109316.5	107166.1	153639.3	102657.8	98542.34	64018.15	82995.38	114920.5	88235.02	92638.53	95163.84
32368.55	32831.36	23600.39	51984	47514.85	34617.51	24688.94	33466.58	17887.3	5715.597	36218.34	50539.75	40650.56	41502.82	37037.36	21946.02	32073.57
20090.39	35342.53	27793.65	38712.93	24759.25	32654.1	51089.06	26605.94	40923.57	27763.2	34263.66	21494.03	43722.02	21541.73	21134.76	38229.04	25453.67
16153.77	15173.02	8776.332	6722.311	13577.1	16417.85	21484.2	21470.13	14303	10359.69	20722.66	16988.41	7564.244	20404.57	11170.9	22114.54	15986.04
21001.87	9982.123	21900.25	6954.264	15222.12	19342.49	10935.07	20326.63	17078.36	4467.405	9545.632	13210.26	6156.237	22237.88	9402.442	20514.92	19869.24
41109.78	15895.86	7053.026	10037.72	10196.33	6106.4	14439.6	9445.305	6089.044	12352.33	13514.63	11495.61	10303.39	13015.66	9667.894	7472.645	8245.396
64729.45	160569.2	117977.2	62422.51	135092.9	69511.66	124493.3	64570.35	71045.95	124584.9	130337.8	88061.95	97189	73937.68	59726.1	71741.96	61801.08
37984.03	67271.28	55970.47	53677.44	55707.96	55485.99	48976.29	43289.17	64529.5	50670.38	75022.62	79072.48	54326.23	54311.61	36120.09	54354.84	56856.25
76538.13	70238.89	87964.95	90190.6	72097.03	90593.07	50650.18	48616.93	101459.5	42175.78	69514.4	37355.1	63400.39	58475.53	61329.14	37211.48	48415.02
25011.19	23545.87	26563.48	14716.1	23334.61	35083.91	30217.46	23310.63	26684.32	24841.14	30405.92	37294.17	25727.96	23499.25	16937.03	16023.69	20087.8
512590.5	69226.14	301517.6	310331.1	431905.8	162147	160694.5	147978.5	311941.5	106745.1	241284	124900.5	316620.5	151691.9	56822.55	290806	51869.75
162103.7	340321.9	192377.5	108623.1	156387.2	130523.4	185647.8	170987.8	285822.7	191170.4	194927.5	120958.5	113382.1	129908.4	147803.3	184874.1	234363.9
53661.95	63658.26	37335.68	44707.71	58523.35	39346.85	115343.1	86401.97	90570.36	65404.86	134161.8	33663.47	44172.97	55237.57	51907.73	48499.03	87738.2
19240.86	25120.65	25392.11	25031.21	22741.08	24605.53	37183.27	32420.05	30269.96	20642.97	28082.37	34073.84	24548.63	46123.83	26461.54	28774.01	27436.75
9734.112	14834.25	6456.29	17445.13	17913.04	6469.446	8145.345	6771.848	21953.95	12393.82	27450.25	25709	31092.9	21903.26	7058.002	26711.91	16162.1
1297327	1171039	924502.9	1151653	1047178	1162283	2063655	838003.8	1300726	1310077	1327942	1133280	1333757	1107323	943971.4	1857085	1272433
5389.94	6641.931	5080.942	3279.728	4043.696	4868.198	15087.07	2260.753	4484.699	6215.422	3952.722	8488.163	5103.985	11940.42	1872.83	5237.064	4999.24
35027.68	41689.41	41203.84	20169.68	22002.88	21501.11	16725.89	25754.95	22218.1	14303.18	30876.01	28424.88	20836.09	7910.72	32380.96	23040.69	39590.52
39763.27	41969.86	30465.36	26526.66	21897.85	25261.07	17697.44	36812.07	28204.99	23710.1	41103.1	24317.36	27467.55	33939.91	39822.38	7953.953	28107.96
144613.9	148522.8	125758.6	82859.72	98907.99	90074.95	75682.86	112857.7	103094.5	197901	151886.1	142373.6	82841.84	138092.3	145019.1	104597.5	102450.1
12379.65	19772.41	16952.55	11119.06	36004.72	17170.87	18063.73	12467.56	12408.14	5284.041	27865.8	30596.63	25919.87	20486.75	11048.11	15066.7	21557.27
3910.853	4375.091	7077.566	8342.079	5349.685	4671.515	13589.41	4031.385	7311.779	5118.065	4685.33	5776.295	11162.31	3164.105	7218.502	8721.578	7485.155
2772298	478092.4	3778837	671515.8	2019566	673091.5	9044420	791303.8	3813753	171595.7	1819618	1414662	1100643	631075.4	1012671	1199233	3515683
5955.65	20591.31	11206.33	9839.771	7611.121	9720.759	18867.94	13172.03	12630.84	6501.832	18031.55	8834.029	10379.01	9384.151	11990.35	15405.64	10800.76
10524.75	15816.1	9867.305	20914.75	10184.69	9492.215	10996.07	9825.152	8677.456	11371.12	11129.39	13372.96	11565.56	6625.514	9380.972	6424.353	13839.11
211032.3	206094.1	218942.8	254878.1	178505.2	237830.6	228474.1	236557	352045.4	192942.1	200994.4	131357.1	205124.6	190203.5	174963.7	225670.5	242793.4
31550.01	27178.82	44692.62	45500.37	21329.02	42693.63	29504.24	36939.82	42342.41	21628.44	39935.54	31142.05	39965.08	27917.1	37067	39405.35	27222.44
675315.4	699739.4	500923.7	647630	735953	476661.6	455446.2	650207.7	692648.2	534649.3	645674.3	697401.8	517707.9	959097.6	627354.6	675407.6	607395.5
62381.87	80689.88	44681.03	70570.71	76963.55	48485.82	50943.34	32491.69	66416.46	36287.71	26071.06	35493.98	44888.51	68312.34	29035.95	64936.08	52061.5
140317.7	3045892	1417931	91732.49	969346.8	187043.8	2656139	325917.8	1734298	310903.5	3432669	908690.6	245972.4	590750.8	671476.9	442538	906160.6
7580.811	16108.76	19948.89	13772.47	8546.223	12669.08	11763.93	5145.005	8628.142	99762.57	11959.18	19509.72	4787.597	12274.46	12569.38	13145.94	10518.04
874101.4	1179545	1326272	833645.1	697329.8	965701.4	805121.8	693766.3	473554.5	659178.8	1081679	458759.7	656445.5	716541.2	675866.6	1049591	710429.2

129168.7	160010.3	122582.7	100695.6	120013.2	185222.6	273468.8	159848.9	266109.4	168290.9	191155.6	137775.7	155527.1	164301.7	168715	241114.6	191612.9
10617.21	17006.98	21736.68	12019.41	10153.04	13428.27	23588.91	16259.04	15052.86	9397.634	13741.43	10278.62	9122.91	13357.39	12175.4	20740.32	19176.53
5567274	10815105	8893546	6151181	4416577	5638968	23627200	7136234	9396045	8505663	10801836	4415494	7497807	6083591	4902517	9475042	7817662
97375.73	115369.6	84273.15	83552.09	98209.87	86334.96	144669.5	115203	100490	86477.9	118526.2	83561.8	61128.25	68817.84	76586.81	90042.92	82134.7
1292.341	6167.378	4587.322	5147.244	6993.602	26836.02	7500.672	2155.666	7054.64	130532.2	6305.25	66902.26	2979.082	9413.354	2033.182	4493.175	5979.49
135994.9	127805.3	124294.5	172746.7	166048.8	175245.3	122290.5	199538.8	131861.7	145955.8	160782.5	200507.5	211561.1	147424.2	141271.1	139859.2	183326.7
141829.2	69721.48	60282.72	63080.49	137617.1	45498.31	54278.08	82079.27	44897.42	330590.9	68552.59	106771.2	69667.06	122354.6	89794.13	38389.12	85833.31
17635.72	22725.94	21591.57	12992.07	14770.5	14744	14344.35	17177	19324.62	23776.38	24031.07	22847.21	22565.74	17044.52	21264.45	21951.04	16538.11
11206.55	18573.72	15897.25	18264.92	9132.483	16845.22	15048.15	13293.92	13376.15	440492.8	10042.63	16920.58	19165.45	12531.69	15279.25	16588.18	15605.58
477418.3	950184.6	556937.5	600821.4	474255.9	644580.1	903960.9	615044.5	624337.1	741060.4	852627.6	426534.4	566700.4	609899.7	591042.2	706591.1	584034.4
55406.44	77305.78	50764.05	51942.78	65023.55	38465.22	36453.08	56008.08	51040.15	67307.17	59856.9	61837.33	47448.29	91185.9	69556.73	36957.09	40135.5
183032.2	207387.1	224284.7	197098.2	199263.7	243855.5	212777.8	240851.5	245874.4	241722.3	260250.8	191386.2	245441	215674.6	265876.8	222389	271039.6
12145.92	21083.12	5744.561	11644.8	15617.66	5140.123	9630.309	4784.86	5168.28	11763.33	8587.378	16970.86	6592.129	13634	8421.449	11928.36	12278.83
6701.574	10005.17	3774.954	10259.33	9119.777	5885.256	9332.71	6560.8	8531.987	5701.861	12878.94	20728.08	28062.46	8573.564	4966.123	16796.58	10923.92
183793.3	983286.2	582823.5	448141	917461.6	511801.4	697744.3	303441.6	424534.8	516392.8	2010469	1619214	1672331	712924	555956.6	929268.7	680732.1
13038.91	14676.02	21922.36	17551.24	16825.89	19309.28	28691.86	26161.31	13898.99	14077.34	20067.46	15098.27	23825.85	17058.69	19586.56	20104.65	23853.17
1372459	1217729	1549153	1879189	1269063	1796486	1612153	1602229	2409491	1256878	1716159	1286462	1904833	1472045	1672414	1638931	1420472
94335.78	354549.4	257489.4	295098.3	396814.8	266026.4	208982.1	176144.7	147357.6	54573.28	757971.4	790720.6	732346.5	255020.3	219264.8	247390.4	355528.6
119873.8	121020.4	137387.1	121560.6	156612.4	179944.9	173311.8	148053.9	115629.3	131540.7	146065.3	182743.4	154202.7	128299.2	122099.6	145272.3	144092
164977.4	33227.71	12082.3	11401.19	40500.02	21521.94	20672.95	23251.01	15801.36	102552.9	14842.5	51515.99	59392.6	37131.95	18996.1	15965.74	25062.13
909522.3	643670.4	563445.7	608442.9	506710.4	905713.8	812156.8	1094615	1128230	765184.8	618586.6	456649.1	632189.9	555508.3	740508.9	733797.9	1161323
881370.1	690526.5	592621.6	679644.5	862249	699638.5	494060.2	662413.1	568075.9	751322.8	695728.1	806276.6	724214.8	1064182	846146.3	724088.5	524257.3
9809.966	7557.77	3525.08	5173.682	6136.473	6229.587	2187.901	7835.69	7499.897	4575.004	4384.243	6076.576	8737.096	6397.078	4886.636	4165.869	7625.377
618177.1	709893.1	408657.6	651461.1	708973	668566.2	233518.3	690213.7	756976.6	734403.6	656959.4	641755	491233.9	905833.6	775417.9	454495.7	490554.2
46393.69	30318.6	15036.65	23104.01	14782.88	13371.56	22080.82	16022.14	11077.94	56036.27	23909.51	29662.36	19264.2	19799.85	25498.72	35764.8	16601.81
27338.83	21974.82	23580.32	26111.23	27650.49	28423.64	37219.43	28202.6	32757.18	20600.31	13578.13	21115.87	21207.75	23936.57	22619.23	34450.94	27072.6
7472.891	22020.37	5773.541	28643.72	20804.02	14810.68	25525.52	21440.19	15054.21	17687.43	224779.3	32755.22	15382.13	5208.827	26547.75	14161.99	22794.93
94361.69	87387.27	59870.77	60755.12	60062.05	49012.69	46774.3	62706.25	73069.52	77612.65	84156.65	65402.47	88413.09	72780.62	91547.4	87538.59	73506.28
117897.9	185736.8	170839.4	159174.7	81619.25	181475.4	146569.6	167014.2	293626.5	77539.17	108063.8	72782.46	59713.69	114436.9	77676.34	122006.4	176499.2
1510133	189001	2730990	1514660	1102714	1970774	1207511	1970869	2936722	624317.6	811064.1	754161.4	904558.8	1622246	1524576	1629720	4041973
182749.3	6669.974	3685.537	5308.035	11617.84	38857.81	4177.196	1367.453	3643.787	5092.503	10832.59	28124.21	29664.82	6361.753	16949.88	8279.234	3453.571
10185.52	32401.35	25583.4	30378.42	24195.98	38784.23	38484.02	27558.9	24495.45	25952.91	25546.93	7129.458	12774.64	21327.51	23246.45	35987.89	26060.98
22147.54	32552.89	23362.08	25805.24	20531.76	21496.18	42782.83	31606.96	28907.54	18692.22	37775.01	16958.78	27694.53	22745.38	31155.57	30682.87	29279.2
1034883	1559676	1060392	926581.8	857697.8	1020617	1361814	1252606	821506.9	2441467	1378584	1213625	1007976	943757.8	739851.4	1251196	1157418
35773.94	34046.19	41587.21	30273.78	26497.3	38973.8	46061.26	32785.98	37373.53	29791.39	34303.72	15918.18	33745.66	38948.71	28726.58	43715.7	21652.03
21222.67	19045.27	15327.88	26081.62	44014.68	18997.83	5947.619	14097.55	20496.7	22563.94	21129.46	34366.54	37160.84	34074.09	29928.03	22656.34	13250.83
24863.42	51992.06	33916.04	22684.49	31131.11	16374.81	24239.67	31812.29	30986.82	53649.53	44648.35	48468.55	26962.72	29877.66	36814.54	26446.63	29884.54
15261.46	15475.01	19226.6	14185.47	18392.8	24287.75	13283.77	49119.74	14209.42	20310.58	23033.47	15256.63	20233.01	25669.97	25684.58	17089.52	26539.94
216328.5	293837.6	229059.5	235230	275426.2	216031.5	262919.3	203210.6	242447.6	227274.3	309994.6	337727.4	306229.4	250052.4	264516.2	252260.8	269695.8
16708.34	11090.56	3689.91	7520.206	8268.234	2271.717	4994.917	4614.695	6067.526	5661.563	9195.462	19273.29	17248.15	11831.45	6683.432	7646.704	11097.54
196872.2	103668.8	140352.5	194244.5	96783.34	279255.4	112033.6	203380.2	222630.7	146638.3	164269.7	197424	125968.3	147935.3	151174.7	148820.3	179028.5
28359.12	33477.28	33333.61	32770.7	56361.8	34366.77	39386.37	32904.12	44810.29	38055.64	54729.8	61321.7	49307.59	34330.77	26503.4	39461.35	39203.13
114777	17476.07	43504.88	4560.7	53584.07	34100.47	27944.09	23292.07	140072.2	56523.8	25003.46	47877.01	78054.88	44080.84	50292.68	22946.08	58956.65
78919.1	104166.3	118052.5	100585.7	70300.81	88723.98	27780.4	71030.73	85859.89	36484.9	71689.09	34191.68	63236.13	51798.08	38497.29	45421.92	58061.83
16392.15	36020.46	19828.85	46855.22	41115.73	23122.54	28786.37	25903.78	28480.59	13523.02	20072.52	16519.5	26514.19	29502.41	14440.32	22365.48	24646.61
85864.49	129203.5	124753.3	76939.44	176242.6	107428.5	113657.2	93000.93	86322.09	117015.8	170134.7	237256	208551.5	128939.6	114328.2	169664.8	162510.8
56060.81	107461.1	77495.48	89133.07	81523.58	96491.32	125093.1	83034.66	112661.7	57855.41	107455.8	65646.01	77841.98	61667.29	93325.8	94480.83	72368.25
112991.8	131574	163276.3	167719.8	204524.9	133366.3	180533.5	143937.2	173156.1	114981.3	142746.1	166216	190564.7	173087.8	128301.2	141597.1	173506.5
80718.31	118079	68559.46	119534.8	96547.05	74750.48	118547	85329.98	78260.72	105938	105271.3	90866.69	133310.2	60251.05	64458.55	132696.8	96682.2

17814.37	30723.27	22144.38	22069.24	30646.86	27418.51	20847.74	16184.91	12809.52	26594.37	47589.75	50316.14	41736.25	9496.36	20230.57	20039.65	32047.46
6927949	6223514	8463826	8141038	6620712	7696792	8724828	7449627	5894083	8401303	9177219	5741700	9404905	7339696	8249849	9346826	7123726
9425.876	21043.54	11740.22	10106.06	11289.57	12224.96	9977.839	12798.82	8947.41	29676.29	18048.95	11325.83	12747.85	19453.06	17183.79	5075.033	7715.44
13228.28	20783.21	6263.84	5160.603	14226.56	14816.84	16750.86	12868.32	15221.06	37274.99	19706.72	15004.79	7898.087	14514.72	16474.43	16513.57	19826.43
87700.38	90085.76	96181.67	91404.5	169228.4	131446.8	100739.7	135808.1	128529.4	194954.6	94106.17	62727.49	113475.4	108184	118962.4	128356.2	148310.7
24226.4	20502.61	17360.65	11504.2	8518.949	13689.22	7507.211	10270.57	14463.62	18280.21	7925.176	12239.18	10558.32	13028.77	16374.92	23380.8	10317.62
159300.8	216011.5	207655	237878.1	221121.6	221538.2	306830	368993.3	344384.3	299148.6	331677.1	298791.4	323026	287340.4	264701.3	441455.1	438049.1
13922.88	11003.23	3067.637	13472.66	41538.8	5808.667	24478.61	10331.73	51617.77	23610.08	28756.13	22236.08	15161.47	17702.75	12002.61	13448.77	7385.713
162421.6	152637.8	134177	196200.5	180883.7	257984.7	63876.16	207818.2	199068.4	185085	111722.8	144488.8	157346	268995	323986.2	74128.67	124211.9
531733.3	1100011	597162.2	411200.2	834831.5	480803.9	1197821	793317.3	630081.4	1022595	957509.1	466296.8	340497	502495.9	442526.3	735645.3	765045.9
11285.12	27555.14	16967.61	30694.34	36632.65	20163.56	17864.86	16519.42	22542.57	24053.56	31020.36	54181.41	58058.88	35738.68	33195.91	25764.44	24064.64
13604.14	14183.7	12564.81	7382.049	20064.84	9500.807	7080.939	4587.615	7861.518	22995.81	19236.63	41017.6	23874.66	19814.68	19204.27	13969.07	13782.4
305797.3	203208.8	296631.8	209963	238947.6	287626.8	337533.5	285993.6	389969.4	436571.2	255926.3	136710.5	181875.4	235235.7	228578.8	292493.8	193493.1
95324.71	112702.3	109467.5	102456	71214.39	99875.71	110424.2	123742.4	99331.51	174341.3	69742.28	50913.52	59402.05	60246.04	61333.19	67637.63	85383.72
3569.324	9419.096	9785.672	8302.189	5106.143	20881.47	8881.713	2468.67	15107.38	13927.28	15866.41	9058.198	12891.59	19674.54	21503.07	6277.843	13480.93
57125.08	83442.52	41289.82	51254.44	39460.88	29759.36	20883.61	30335.56	47517.5	95809.68	58732	36732.56	34410.65	53910.39	40027.31	32774.98	35684.71
27546.02	39663.36	39307.59	40598.46	29010.2	38897.27	46673.56	25728.12	33994.07	30937.05	38474.55	35886.51	40069.75	24367.66	23495.58	21359.6	42158.61
9500.954	21264.54	11387.34	14883.71	9438.347	27144.62	35122.94	22572.27	33510.57	12803.54	8540.184	16402.59	13071.03	9101.051	11269.08	8779.093	11427.65
37298.66	26745.84	20563.43	32461.89	14171.65	14936.57	17678.28	24959.86	10889.19	28670.06	25125.43	18358.58	28372.74	25308.11	39725.54	13654.39	28254.02
64387.98	41698.3	40647.68	40884.11	92599.7	54885.98	98495.4	65589.88	124758.6	87206.07	77601.13	42998.67	32986.96	87955.44	61485.78	80869.42	46428.19
158036.2	108347.3	90673.9	145022	114460.8	86007.63	86295.56	93399.63	86442.91	256625.5	106193.7	105639.7	109948.3	158923.3	175576.7	89957.39	94333.19
19488.31	9510.844	9860.66	14739.68	13502.44	13206.28	8424.219	17803.86	11715.24	20063.72	7037.271	12644.03	11231.48	23203.95	13978.74	6202.929	12583.14
520496.5	363692.8	454811.5	538392	440547.3	371293.2	498884.3	547828.3	315239	570356.4	590373.6	390953.2	409610.9	430829.8	339386.2	571500.6	472228.4
55423.35	89945.59	79935.66	70068.47	73086.16	52884.11	101154.8	51748.9	76193.08	56510.29	93404.09	75932.45	63231.96	71047.45	63887.17	75333.55	80745.53
204213.9	188008.2	201640.5	192455.5	214903.7	234368.2	120841.8	174369.1	95947.62	233327.2	178474.8	229714.5	194833.4	198639.1	210284	177120.6	192268.5

34	35	36	37	38	39	40	41	42	43	44
D14-RAT8	D14-RAT9	D28-RAT1	D28-RAT2	D28-RAT3	D28-RAT4	D28-RAT5	D28-RAT6	D28-RAT7	D28-RAT8	D28-RAT9
155788.1	20204.29	21336.48	30427.57	23676.25	40176.18	12990.77	15347.64	10537.96	24048.95	32673.98
14123.66	7989.45	13226.53	6200.314	9278.951	6754.75	8308.238	8363.703	10620.64	9046.325	8228.332
86253.53	136202.4	140598.8	104960	77887.41	97992.01	92607.1	88201.2	103857.5	70922.68	96176.72
19742.19	43904.27	20173.95	24722.84	67337.19	30377	30465.66	23884.95	25661.61	36155.29	28465.7
77344.48	58605.24	77373.64	57755.68	66175.89	57461.89	60878.83	51144.44	116432.7	49526.02	49638.64
49627.23	71784.88	20617.01	55655.93	229098	94788.01	60050.2	84497.25	106384.7	268933.3	57219.07
6809.479	7596.529	12667.86	12622.99	15891.76	13283.4	18732.56	5081.171	5697.1	6251.495	9770.96
43727.63	26800.33	52217.92	33233.14	42423.1	37521.04	34589.1	35258.89	41003.49	30877.75	23245.51
67791.44	11156.92	12616.66	13505.34	10050.33	11648.2	8957.851	8345.189	11221.57	6352.279	2119.855
19215.83	11185.4	16926.78	22349.06	12392.04	18084.36	16659.14	15963.26	15699.3	20378.71	14813.79
54153.37	5414.913	13673.45	16390.6	9101.563	10756.81	15124.03	12639.64	13499.2	7165.229	14780.59
410551	164979.8	417607.6	218544.5	257580.8	249482.8	260343.7	281452.7	368282.6	166615.1	320748.3
12174.99	13119.78	15884.58	15409.66	9676.676	10605.71	20276.13	12017.35	4522.757	14089.93	19503.14
20590.6	31487.26	25840.64	50674.42	35787.95	34302.2	38413.86	63085.39	30314.4	26327.92	26884.22
30847.39	12212.52	13188.2	9723.611	15278.48	15981.04	13013.39	15783.41	12929.09	27330.82	24602.79
281153.6	212288.7	266032.2	163214.2	264709.3	233251.9	121296.9	165165.6	203208.5	143114.3	230469.9
20153.23	20286.55	21954.71	13615.11	9011.063	14969.84	13254.67	16292.47	28527.43	17583.19	13702.91
24464.15	10578.26	16313.04	11355.53	13181.67	12076.86	11438.66	18827.77	16668.29	16094.71	20515.17
194908.9	412776.6	588292.2	318247.7	175788.8	382448.2	185787.7	439336	200947.1	767918.2	291168.2
64868.28	33167.56	51463.95	30401.65	31525.27	31028.43	28006.65	39632.06	17887.89	37380.1	41223.15
31103.6	25647.4	31301.54	23407.03	18566.6	23886.9	24106.56	15461.57	99546.41	21333.19	21372.26
62435.27	27922.42	18424.24	30214.38	29291.61	26477.63	24014.89	20630.03	28524.79	4575.064	27519.04
152332.4	15741.15	18466.34	10543.12	17658.85	10791.38	22999.01	25953.59	20960.25	78165.31	24862.89
27972.36	8417.016	12570.1	17047.5	11382.7	10619.1	15314.49	5459.387	7626.478	7356.269	9181.49
20424.56	18991.97	72405.45	115199.6	58686.47	76075.76	97207.2	46093.19	8086.643	64404.9	62293.33
22601.67	6341.124	12672.8	6864.183	11493.45	7182.016	12213.73	17421.31	12249.25	7075.405	6887.55
356593.3	40879.53	162186	65472.19	16781.39	49070.99	15198.48	126103.1	126690.6	73658.64	103305.7
282434.3	258301	231383.2	198116.6	175273.2	233417.3	124081.7	151468.3	123527.4	259333.7	194397.4
139389.5	84816.74	144962.7	136848.3	107462.1	142597.6	139833.1	96177.37	102848.5	60374	170235.2
13806.39	45751.17	21628.52	37384.91	19136.46	28725.6	31319.79	29250.71	56678.57	53218.23	23551.32
78893.13	51311.54	127862.4	76510.3	64035.09	43993.07	116595.2	83530.14	94772.84	49588.62	105345.4
9360.226	6417.771	25401.57	41840.5	23056.02	23747.79	31377.75	18237.99	4011.427	25728.79	26377.99
16406.16	15603.16	24001.7	22621.2	20027.39	21028.89	16709.19	18724.73	17819.1	19750.66	31570.15
10958.45	29341.58	27410.47	36619.96	25337.94	32999.52	42379.96	24541.58	31835.43	25977.02	21150.22
166700.8	103665.4	110752.5	79247.52	81852.91	92177.88	94452.61	129386.9	97125.02	100023	91248.53
55459.54	33671.12	46338.26	56588.31	44742.13	78522.56	79428.16	51866.52	80661.23	31868.79	62453.95
405103.1	499803.5	575158.9	509502.8	503618.8	541517.4	735489	456586.2	841791	529666.6	641951.3
253848.6	212836.4	275824	293573	234084.5	233562.1	209362.6	320612	344626.5	229111.7	260924.4
9698.107	37698.21	58014.87	40964.34	21047.12	28142.83	33209.32	25826.59	35188.18	23007.4	45660.63
22699.25	18420.7	25961.19	21464.33	9567.948	18338.96	21321.34	22302.62	30013.66	13461.31	23090.29
111607.3	100961	98854.56	135362	120572.5	123663.4	101488.2	81001.08	68945.77	91790.74	124936.9
12556.99	6961.425	60450	103305.2	22667.86	54957.25	76393.82	21959.32	8175.47	38030.01	56299.27
571253.3	211955.2	493562.3	348813.8	576745.3	608121.6	369034.5	480609	468565.6	391484.1	741423.5
53634.78	21691.39	24091.58	29076.09	23152.35	22781.36	17167.92	32035.57	32167.55	28913.17	20190.89
208390.6	208950	241452.7	261393.4	211094.5	213998.4	229647.8	199389.8	189695.6	177579.2	262634

7126	3365.674	2582.307	3685.167	3081.103	1211.463	3528.473	3228.616	5977.339	7412.273	4940.635
133964	50417.61	41320.08	40495.55	54800.36	38442.05	36272.73	42796.76	91994.12	45795.34	41537.53
22226.37	9211.516	9384.242	9313.284	9222.809	12177.99	9026.525	5791.409	8204.051	13791.04	16432.73
52774.48	95153.79	131785.8	144947.3	89034.22	94355.05	55321.72	157564.2	48334.93	84113.15	102636.7
1842646	4134.286	8103607	2351323	2835127	9716421	2322021	44709.01	6058145	2931687	94849.95
226523.1	232702.5	265362.8	160516.7	218880.8	81937.11	75816.57	179546.4	122767.4	221360.3	114928.9
226455.5	150986	227703.5	93457.52	108014.1	82881.78	8972.438	124042.5	33430.5	162591.1	71904.83
105150	76163.73	74156.8	60585.88	62646.03	119085.8	105734.3	66966.07	62308.23	61804.31	119469.2
26103.8	11330.97	16102.79	12818.21	12679.17	10364.8	13797.91	4607.329	9611.772	10448.85	9372.041
136111.7	392998.5	363659	460869.9	625942.6	607688.3	289814.8	705538.8	462497.1	336175.4	259124.5
369898.1	604925.7	594341.3	414449.4	659502.4	620379.5	743106.3	727276.6	568106.6	651338.3	817668.3
23671.16	18998.95	24808.48	18513.16	21914.16	20477.14	23674.4	22058.59	21324.65	32313.18	19880.93
53493.78	138430.9	18814.34	28658.3	25331.26	24224	21933.09	33708.93	45075.02	6455.742	26833.05
523137.3	268555.9	301374.1	230349	277273.9	306512.6	441987.9	291867.3	303846.8	253807.9	398090.3
55159.9	33631.67	69524.86	69157.83	61167.29	83527.08	57468.57	53971.87	32121.09	50424.57	110803
7890.614	21836.9	13984.82	20763.89	28035.29	15570.07	17530.54	17039.58	35305.18	21952.84	16587.42
1636751	2322679	1008887	1221219	1498260	1750925	948007.5	1549978	1355419	2022982	1365114
339643.4	440497.7	389462	349231.1	283327.8	364611.9	333629.9	580523.3	405934.4	212437.7	282738.9
941622.8	1159907	646256.8	555344.8	988745.8	1068829	1353738	2057170	900539.7	1411552	1165682
190303.7	164904	211770.1	158225.5	150939.2	178736.4	212700.3	219313.1	184968.4	166605	205715.8
689413.1	49048.05	25280.3	29897.86	56239.56	33637.59	28659.1	26690.38	29013.44	43300.54	48259.13
29755.25	41638.71	44138.02	20847.15	48264.06	37322.52	41379.2	32040.41	23672.09	69644.91	26453.5
46751.61	23409.06	42236.61	19846.28	31174.32	34903.1	17773.87	49963.39	39801.29	38582.14	32541.09
23018.01	20155.82	31167.62	18755.59	18180.89	11216.38	23737.69	11619.8	51689.04	18305.42	15877.08
20520.12	11936.81	12016.19	7473.978	11419.58	23048.68	13770.92	6499.424	19492.23	4750.657	3518.26
243692.3	223346.6	179368.2	182588.5	222325	229498.5	194783.3	162477.1	321609.5	247578.3	187093.3
81501.37	116657.1	203671	167584.8	126135.4	167278.6	197641	276974.4	110453.6	84328.17	185686.3
4347.784	8038.721	5644.125	7537.392	4338.011	4857.809	7986.481	7828.12	2586.142	4345.121	5196.463
91916.84	41814.88	72126.81	68891.2	46466.28	60756.97	47787.68	71639.65	92816.41	48016.17	70930.43
353754.5	347296.4	523930.6	341214.3	350685	381326.4	241736.2	319218.3	384973.1	479518.5	374066.1
39359.96	18539.07	23059.26	14396.01	15217.66	14250.66	14482.35	24453	32090	23297.62	13529.22
151028.8	96370.2	128738.7	82008.63	87036.06	113373.5	65916.98	101074.6	54007.67	152543.3	114654
524005.1	531996.3	504797.9	415991.4	570226.4	411234.9	388500.3	382051.3	843365.6	429604.9	484287
16167.45	27901.7	5747.729	6082.584	6389.07	5983.264	5799.18	6642.211	22514.67	7903.963	4949.223
51929856	22715356	26611386	22274772	19863974	25867366	17072224	14073966	22851824	23424650	19664766
123094.1	104010.8	224639.4	230919	262190.2	75757.56	342301.6	122780.6	702018.9	176801.9	663047.3
6809.597	25523.71	1585.053	15965.81	23413.81	24459.52	8230.537	40886.26	25094.91	56249.99	20093.67
25039162	16017596	13476519	8861215	11429348	13491654	16240049	22197434	14966160	16237820	15919437
10842148	14351158	3218151	10140484	15799754	11871116	10847370	14424971	8052134	27937752	10130993
820833.4	35737.51	4574.729	10924.86	96790.97	10695.61	6879.222	70592.07	8940.557	31331.55	52982.99
328592.9	372680.2	383772.3	352842.6	456895.3	423791.9	418024.9	557319.7	481813.6	462430.3	402318.1
1858285	2254485	1827000	2094759	3816281	2745011	2546883	3695416	3137201	3230675	2536661
410860.9	521957.6	667744.5	396712.8	385074.1	418440.4	430996.6	339986.1	620794.7	485383.8	468370.1
580871.8	1685135	1401177	2734747	3190629	2865809	2071932	1780956	2280295	3675917	1586992
615861.9	903540.3	252603	351851.4	266631.7	221451.6	156206.5	245860.4	266060.1	4765737	241609
129589.5	69802	94597.56	112707.7	147519.2	139170.1	103606.6	98502.17	282588.3	191450.1	124234.8
4212360	6838807	1831256	2153439	2155067	1973460	2241082	1775708	4120311	13313571	2895202
417254.8	1936669	872803.1	741425.6	1626348	418444.8	1084322	305607.3	572142.4	6814228	1453849
179521.1	266252.2	250915.7	235409	331896.6	305854	227323.1	425402	295524.4	169438.3	223302.4

61693.58	14924610	40220.88	24075.46	33750188	95723.01	13598511	86711.96	46133.23	24260244	16228211
5501080	490758.6	6436399	3731724	5293566	3589859	4492322	5910019	5099081	6128689	635797.5
26552.58	849418.6	37604.83	27945.62	50202.39	165847.2	11565.58	37991.71	14719.56	123690.7	57262.39
36230.56	2338687	23684.23	23499.94	25626.66	175956.6	7195.914	21820.66	19455.93	282364.6	79881.07
53188.5	27075.66	149661.4	96327.48	98872.48	70859.53	43413.49	117720	37184.34	96103.33	125243.5
238382.9	81003.23	213193.9	233970	259943.9	461944.3	25216.61	286934.4	83408.16	319936.1	209946.6
42873.13	21247.23	45631.35	70021.11	34222.71	22179.91	20688.45	44255.62	19642.03	84657.81	50320.87
4384613	4394768	3165177	2420993	2634222	3718181	4299694	3583340	3789835	4342804	4107497
321429.6	348756.4	349980.6	287784.7	200978.1	185499.4	76153.77	304237.4	104407.8	456722.8	191880.1
11378.88	46275.45	16618.22	26244.79	24944.53	20591.66	12197.15	11563.27	14695.42	84928.46	7902.526
40994.81	16835.43	39896.73	21909.58	37134.66	19790.19	8922.909	17959.69	28070.01	34971.5	17912.32
2.84E+08	1.19E+09	6.89E+08	1.43E+09	2.01E+09	2.22E+09	1.17E+09	1.35E+09	1.1E+09	2.05E+09	1.06E+09
6480135	6660577	4064406	3391653	5216951	3792579	3448882	5373736	7498884	5736216	4157671
1489983	5785315	1335218	2057717	2200582	1961122	1101263	2101227	1045678	7785329	1547545
38484760	2.35E+08	42069952	79231992	62100668	57343476	35782368	46852936	36062720	9.39E+08	38542480
35490092	30604730	1.36E+08	1.51E+08	1.03E+08	67710632	72885624	1.28E+08	15672292	95908208	1.25E+08
14849984	11674917	43580788	66599616	47245568	51228220	36724080	42299024	4987759	53597588	59860292
7754912	10644314	23032012	34953840	33221706	36400272	16723766	38278992	4898195	26382472	30017994
33131.82	43712.78	1117.708	7609.681	8082.69	6382.804	10032.99	12163.7	5557.59	27839.34	8857.296
18763.01	4755.913	30368.94	15255.89	16844.74	26127.77	4205.783	36468.84	27176.67	33436.49	35473.03
18405.26	8229.903	18706.72	9844.069	13106.94	8724.885	6085.601	14898.34	10281.81	19403.15	11687.16
26833.66	29303.22	27703.54	39507.65	41130.52	24372.57	29398.41	33966.95	22468.33	45762.03	28990.49
171729.6	61825.52	126063.3	123778.3	148250.8	100018.9	219243.1	78471.57	147960.1	116008.7	149775.5
7833.213	5860.69	7384.896	4223.748	4500.776	4838.555	2629.784	8178.216	4377.915	7152.915	5553.268
40821.09	5827.417	8909.846	110628.2	13872.69	15352.57	7411.49	9136.019	287917.4	26406.68	26559.43
84110.23	25292.31	66191.36	58132.61	63327.79	81183.81	33556.74	51700.22	55444.26	48676.64	90732.95
964044	2616002	936204.9	1455744	1071879	1051479	899787.8	1173929	1370752	8038020	1099251
16380.47	13910.14	39554.74	16872.37	15628.73	27463.28	3975.685	40789.45	21261.86	36088.12	48527.13
105260.6	22741.84	36920.96	21840.78	23645.8	18307.73	46295.25	34663.88	337642.5	26844.4	47716.39
91169.26	1052936	102069.2	180464.3	93723.44	76287.55	44445.69	90110.59	44768.38	2905679	61204.78
194146.7	123762.4	225537.9	167441.9	145787.2	152973.7	186875.6	143558.1	193760.7	115421.8	187649.8
24094.54	6932.366	11360.33	11727.3	21626.17	9622.503	23384.87	5505.156	256865.5	41645.66	28885.75
560809.5	509583.5	820576.9	494545.9	428155.8	552527	480741.8	631674.4	600335.6	423912.7	578207.3
143792.8	65230.84	144293.7	96214.87	90182.28	110673.3	96359.81	132501.7	102287.5	58987.37	117586.4
835013.8	727215.4	833234.7	660027.9	661302	711411.4	610435.7	784264.8	639284.3	661483.5	545247.2
186615.5	196333.5	368622.4	546365.5	350364.1	317489.6	499577.1	209792	94808.02	217060.3	309559.1
26708.11	25849.27	20432.28	19552.28	16263.05	28124.22	12638.46	15977.85	37017.17	32155.03	17269.65
706105.6	973888	1317051	1102599	936933.1	887389.1	873461.4	712597.4	1657249	957949.1	999153.3
10289.43	20719.35	47460.02	97796.94	59355.57	59525.45	118256.8	60701.46	10638.44	73364.51	73592.83
40134.72	66632.76	37899.48	76215.64	70183.52	57797.84	42103.55	46439.16	64107.96	71108.84	55146.79
126278.6	342124.3	155530.2	207824.4	379229.4	174966.3	176136.6	244043	474197.5	436054.7	186394.6
10329052	7253919	12832311	5974442	9120280	6758220	7406253	10439038	8413030	4353906	9143308
105872.8	118992.4	142746.9	132376.6	101508.9	139617.6	216913.2	149336.5	116838.4	121307.5	239130.3
41166.65	17281.14	53726.69	35832.14	25673.74	31150.26	42125.96	21356.21	28927.21	30252.8	42380.47
20481.87	9470.595	26295.24	42982.79	12362.29	18964.51	24900.29	25837.33	10463.76	34139.75	36811.14
352872.4	297016.8	365880.9	300407.1	250465	368867	449637.9	852591.2	306440.4	404497.1	534589.5
21269.63	2330.5	28716.42	1296.038	3189.525	12006	17339.33	19406.19	26533	14146.36	9294.668
49355232	1.02E+08	55482004	42922476	44427980	52659064	41480328	57145908	27425830	1.85E+08	54908840
22503164	51469992	22383564	20651656	21149482	28484942	23319698	29246462	16305853	93490248	24311898

19410.38	12553.9	12886.64	19972.83	16197.11	16537.47	18318.91	17369.11	9402.826	19108.82	35730.16
11508.18	24950.63	28492.1	25827.21	16703.31	17541.78	6655.731	12257.32	12396.25	21535.49	25403.66
780711.3	115824.3	279450.3	795782	312939.4	199649.6	119201.6	270743.3	54828.98	270264.8	346925.4
50957.17	55360.31	25993.58	30744.81	27482.41	24365.39	22174.06	19296.05	26099.94	126205.6	16401.72
55156.25	48027.46	49888.44	53003.19	46058.35	26400.24	45529.52	39179.67	37688.47	42472.42	39384.04
131108.8	17854.97	35410.84	16844.08	21133.34	20380.63	8337.433	24795.75	12469.83	25787.3	24216.91
3251506	487013	369420.3	3090310	4693414	2358182	6842569	3392767	4680661	395287.7	5530990
66151.59	54127.61	92606.42	120866.3	74100.58	102959	117733.4	133104.5	72022.89	124492.8	112883.1
3094831	6899498	3230875	5997892	8685102	3977018	1507520	4910224	9262087	20921514	1259096
118571.3	69829.11	182998.4	309853	268643.5	518171.7	883644.2	859186	72579.77	50276.46	680063.4
48606.28	32024.29	55570.1	52983.62	75033.17	65211.71	100796.4	94538.5	64253.38	44215.03	117973.7
3563185	10162450	8843509	13043791	11389558	12294663	4345083	20276274	9177259	17896444	3931810
170616	142584.2	142226.4	139187	136129	145183	175613	169159.9	210019.6	185637.7	147389.3
393877.8	240911	1076592	406081.9	379553.6	397419.3	305810.5	547388.4	287689.8	278948.7	854278.4
5759688	7635266	5090430	5251022	7310121	5428128	5344945	6098793	4776538	5157206	5893027
168368.9	107001.9	111834.5	82603.56	90271.48	88166.84	108961.7	138626.9	122456.6	87046.55	83980.05
27993.13	23707.59	61949.22	35895.76	25763.55	32853.08	29141.52	21931.34	26979.86	23507.36	27078.37
115655.6	124425	221436.6	147693	113558.1	142006.2	136495.8	207097.6	137941.4	73302.05	131925.7
64191064	39493028	48225352	33811108	39626484	46854896	47387372	37983232	38404768	33003594	44361508
5729539	6527460	6187457	3396435	8641972	6642918	4313007	11987437	7180061	7110592	7843215
4578879	5666269	17292620	31823234	20268484	19442330	23327368	25539248	3408521	18981226	16406828
1110813	1217636	2027667	3061212	3052837	2652542	4358125	1562627	695614.3	2061123	3287027
557024.3	264589.1	500758.3	203786.9	267490.2	234967.2	215369.7	336478.3	341515.8	127591.3	377404.1
47864.74	26120.49	40085.88	21781.21	25698.84	46510.17	26450.23	31753.75	21371.63	33938.74	37788.82
36891.45	29372.71	84102.95	201138.5	103095.9	77381.81	182289.8	56721.33	36568.22	171521.4	133688.6
131058.5	122206.5	154993.5	114244.8	111628	110751.4	133056.8	177070.3	83187.45	346365.6	188883.1
25550.43	16075.52	20600.05	11399.23	20101.58	14873.02	8394.658	19551	85990.59	13010.34	15578.27
554845.5	404226.3	648323.3	384599.6	420480.9	480040.8	346602.5	433969.6	482972.9	301774.2	441331.6
24535.05	40106.5	19123.04	8125.473	12474.43	27232.76	3413.123	24636.7	15942.67	59314.03	18401.05
388583.8	302856.1	453079.1	288104.8	319135.7	317045.3	318078.1	479658.3	300005.5	225767.8	375926.4
40127.39	73633.06	51181.96	99545.24	141293.8	91733.12	106839.9	71133.63	25652.64	138388.6	73836.01
1130372	274980.3	2210000	515693.9	688695.8	686958.9	230273.8	1436071	1803277	467513.9	634523.1
133913.1	171011.1	174686.2	159344.4	153987.1	165389.1	149651.1	139286.9	199709.1	160797.1	136249.9
8841.756	10181.23	10589.88	7588.206	8301.83	8486.513	13850.53	7050.591	6154.047	4800.085	11243.13
557548.6	2910932	4535052	495070.2	2416980	1672456	1281220	2996944	3482831	1590745	3223050
2646474	3607799	23796520	33516248	19189012	28376938	31624490	27804504	3452334	11845307	20762276
17750.39	28919.31	70731.65	115455.3	68985.49	83575.89	117636	53098.08	6666.389	101243.2	93774.17
3175983	8281821	2505236	2645408	2301158	2198371	2163854	2150554	6403371	12527619	2632101
7690.153	35589.14	24900.19	24982.34	24773.59	21356.08	18440.95	23207.14	18652.25	83812.02	23049.37
1186613	685139.3	886265.5	1399281	820882.9	1026326	712476.9	1363392	723528.9	560302.2	758996.6
2468692	1651036	454196.3	883796.8	789064.3	590989.5	374949.7	556046.6	847839.3	4466312	410255.1
91795	128312.4	166172.8	98659.44	96105.13	158545.8	102009.4	91489.59	139080.9	103562.6	102202.3
13676.04	9049.874	26270.7	42093.38	26873.7	23935.73	37612.11	22270.66	6226.834	26572.49	27334.68
35330.5	24101.26	43444.42	48029.08	59082.99	44705.15	47788.68	46992.74	17296.99	30269.71	43023.42
27404.65	5851.997	4948.687	3107.147	17267.77	11327.59	11934.87	18340.07	14357.21	11270.04	11277.34
71595.13	97374.29	45008.99	63141.88	60786.39	72188.14	73217.98	60196.75	158578.6	63817.74	81752.42
21829.08	40884.74	118251.4	285017	121549.5	103868.6	152630	83698.38	8096.779	95342.24	96714.88
18926.84	7367.294	8644.892	9809.421	9578.337	8953.134	5204.568	9707.799	12943.65	5814.725	8797.592
1285717	1066947	1138872	1471811	1553305	1472674	1544791	1309669	1037611	1287520	1327364

85021.63	25739.72	32819.28	104568.4	74724.58	48712.36	63326.58	47470.52	32606.49	64824.29	50757.58
359301.8	136121.1	166798.9	165224.6	139555.9	166119.9	151709.5	229446	62362.97	95564.18	236173.4
98158.76	65841.32	158459.6	160388.3	117276.4	125823	135119.8	144050.1	80968.27	90151.67	174757.4
267313.2	157130.4	170354.7	112239.6	167347.1	185867.3	121461.5	371808.1	151161.9	153837.7	167456.3
1194173	1147593	1347244	873000.5	1024132	1150682	1039146	1493353	1304247	534876.9	1057897
20530.49	8189.41	12134.82	15038.24	15306.66	20581.15	15096.44	14819.27	10485.18	19041.36	22801.31
23951.68	29455.48	16848.95	42584.8	32635.35	104802.2	163928.5	140709.4	8336.558	18104.16	105748.4
158834.9	15243.17	42723.88	18650.66	103661.3	19757.43	35031.91	26692.22	384323.3	31185.23	41372.09
346577.2	234064.4	1002931	509871.1	1220315	1290642	865614.3	734935.1	705259.5	157781	513185.5
40952.65	54954.69	45748.58	46720.02	51254.23	50112.12	59144.84	37825.59	185179.8	57166.65	56794.89
1565774	7927041	2092748	2718651	1953315	2536365	1632876	1482376	1335541	19583392	1943939
7210946	6028439	5711667	4502000	3770733	4501472	1790279	4390483	2429885	4411807	4400922
61408.45	38543.18	74545.05	57941.41	5345.694	30695.38	41153.94	50938.56	6682.243	17384.56	25939.72
881522	313312.1	1094479	887994.6	810791.9	604503.2	803265.1	1057360	592533.4	534035.3	1213314
111510.7	95933.13	109390.8	64540.85	66226.21	74811.43	81449.63	53323.63	147505.6	71209.53	98993.03
9472.295	7048.093	10038.5	3822.262	5395.148	7628.595	5916.854	5001.105	27472	5298.11	11421.55
39126.88	30199.15	36511.65	32333.9	30725.47	37070.25	22770.1	32085.17	7442.595	15213.5	41205.86
44461.62	8993.773	12887.02	18401.79	9699.97	3523.159	6704.767	10436.43	1131.249	7790.958	5776.245
31753.96	27398.11	4882.453	3354.066	11655.96	13302.38	13539.07	16285.73	16326.58	77959.82	13804.67
87122.88	8995.816	54471.56	51035.18	45110.7	62522	56875.09	48286.42	71481.1	77520.73	53235.54
173289.2	96711.72	362386.6	433150.9	397500.6	794021.4	1601147	1435088	141934.6	219771.1	1347002
8929592	89451992	10507518	23167476	16227557	11302608	6999139	11255448	10463510	2.83E+08	8243577
28119.97	19460.96	24852.37	34756.3	30767.47	30610.35	45336.8	31771.83	13358.95	30078.42	29290.2
14659.29	4384.658	1823.034	2313.197	5118.428	1147.109	1689.775	1065.089	2791.273	1339.353	2379.35
67611.95	74143.78	112208.3	61992.79	37027.04	57237.22	67932.09	42791.21	64593.73	36663.18	71198.23
31808.82	31757.38	39424.94	41032.9	73457.91	72319.21	104496.8	24933.43	66373.72	19607.98	70345.07
582792.6	172643.8	33587.32	676442	1223464	2130736	708130	2515943	962720.1	344579	110512.3
43848.91	29187.08	103796.5	24704.46	59486.39	37691.57	75395.06	86260.05	100847.1	9206.805	53596.9
589543.8	4106948	481217.2	1023026	734473.7	691417.7	483851.2	387704.6	609021.1	10476794	506997.8
46003.77	42892.17	85944.27	68466.16	69517.13	85655.55	54049.27	84517.45	101966.9	177779.5	87320.84
1816782	15964433	1272729	3079488	2915083	2352313	2662327	1533634	1815538	36785008	2301136
11652.24	4837.916	10519.26	4463.412	4794.234	5781.273	4545.865	5357.605	3535.426	4378.14	7232.601
27172.95	12596.34	19872.1	13361.95	14740.96	8552.719	16368.43	11175.95	31874.69	10289.81	15939.34
24287.15	69804.09	19638.1	43535.77	32088.39	33477.49	7015.958	11156.09	10787.64	153124.4	15330.18
133907.6	308413	144027.8	118865.6	214180.3	162856.7	258738.5	249626	160871	177297.2	270844.8
1292612	758874.6	1016284	630647.1	753638.6	898968	204834.1	1077957	946197.6	895097.3	694071.3
28261.02	45838.87	66846.62	32662.66	58480.09	92239.63	83544.44	102872.6	99772.46	48912.19	82405.75
10020.53	15449.91	24913.22	27523.25	23256.77	24450.09	47311.19	43963.49	31408.78	24042.99	62183.3
4540725	3979279	1766375	1806637	3441257	2837762	2843860	2257248	2910517	2738860	3074981
5319172	8633410	7919139	8430157	7840345	8008400	7162183	9903477	8377068	8969363	7634747
187844.9	101427.6	128905.8	86480.96	119389.8	125943.8	116322.1	147555.8	152602.4	89858.08	122404.9
68311.82	79523.47	100818.1	109418.4	75982.36	72519.34	70768.67	56752.41	127766.9	84847.05	70951.34
4444311	3231776	5108269	5367766	4407745	4611543	6209415	5591034	2821388	4559162	4898384
22460.41	6630.715	9845.598	8799.068	8728.369	10477.88	21852.96	10321.92	34242	12297.75	11946.68
4933438	20842756	6477532	17556256	23334120	7138770	12037763	6188452	1975732	7078930	8878839
38147.57	59470.74	89823.02	40303	65307.22	62049.07	32499.19	52789.75	55603.84	59877.67	66138.34
6860339	2693199	2779115	2456021	4019062	3411006	3589123	3158902	2770297	2630908	4068194
6605.405	2008.096	5299.182	4831.626	4033.195	6572.525	5911.896	4743.805	2382.271	3254.409	7035.448
58814.5	21383.81	27624.5	30975.45	25554.41	29365.32	33988.52	39983.18	28314.29	13579.72	26234.44

617437.6	852113.9	1291200	921764.5	1127401	452030.6	346846.3	338873.7	1076277	439515.2	450409.6
106915.8	31647.87	261244.7	21033.9	118773	19229.25	35616.74	29130.95	91609.2	46750.19	50821.8
10229.45	1868.397	15255.92	2333.611	9270.668	14738.55	13924.69	22278.49	39604.09	14098.92	5932.967
53142.04	30801.33	53440.39	48184.51	79703.52	71007.48	49313.87	54309.79	61050.66	46955.66	43499.2
23276.81	77342.2	9500.393	24127.73	10824.28	18327.94	8849.152	13689.92	16734.98	305517.6	10329.41
52004.91	57084.02	29962.92	24308.59	57579.77	60896.8	40814.2	63636.95	50500.41	85211.22	48400.9
874976.9	664371.1	1014380	782826.6	905799.3	310420.3	234983.7	246236	801845.8	3616725	404658.7
8828620	5720413	8743797	4553273	7474928	6476597	6538837	10907026	9453132	3802064	9825654
31376.06	52106.32	68353.09	54084.13	37085.25	49856.75	40962.35	53679.61	52448.09	64055.95	54650.73
19782.6	33305.1	37542.77	6066.825	8682.173	17273.85	12106.93	20300.55	30972.81	75147.92	10432.74
259277.2	103159.7	66152.73	103454.4	109171.3	62645.43	110863.9	93895.23	211629.9	95025.84	125025.5
767725.9	237368.9	267360.1	206660.2	260191.3	253338.7	427524.4	207722.7	433595.3	310581.3	356068.7
18715132	43982840	7900021	14839755	10767596	9060354	4237436	5650322	6215767	1.2E+08	5915009
345364.6	10152.81	64479.44	52503.27	90606.1	28423.15	26112.51	22475.38	57211.23	87619	45037
7645334	5176946	5160622	3478891	5265774	2729094	5149599	5235497	5786298	4352978	4408529
55973.17	7898.701	10766.3	7816.012	6646.603	3467.707	4683.003	7190.604	6929.358	4244.639	5911.521
77816.13	56043.17	76070.46	118125.7	46346.3	73961.78	85102.45	148229.8	52597.18	29585.85	83790.96
19007.29	34315.55	32699.13	7630.01	12403.51	14643.25	15774.69	13344.59	11019.52	22745.08	12935.53
22206.15	23919.93	12587.77	20472.09	20138.37	18903.58	17771.29	14331.43	38555.3	38579.63	6546.96
197491.6	86133.61	57045.89	63922.37	87953.88	63399.36	68965.8	47670.24	243879.3	64735.89	95877.45
77471.84	75186.97	77952.96	44553.13	53361.5	58774.7	53562.16	33787.32	114734.5	62384.4	71031.23
68992.17	24147.22	6596.416	5790.692	18811.89	5370.105	7082.012	10949.19	7225.004	12302	4594.992
123233.9	232106.6	123194.3	148746.8	37185	75308.21	441969.8	196970.8	101757.7	210938.8	436549.3
266205.2	19775.24	23372.19	38861.87	48970.75	42419.25	20521.92	16501.55	24385.43	30287.87	47092.44
590933.6	692494.3	531929.3	464697.5	654861.5	580589.2	531333.3	392928.1	1304082	517478.4	702985.1
42965.36	54855.43	64009.99	90725.73	144125.6	342210.9	555823.6	393297.3	29709.85	58005.16	333895.1
142890.1	127466.3	268563.9	190551.6	126140.9	172503.8	207706.1	198472.5	186436.3	153203.4	174687.3
33862.21	30034.37	30183.28	20471.66	33624.95	65770.23	25478.02	35177.97	31471.73	29652.44	27631.47
311176.4	169549.6	262100.3	191575.8	146081.1	204059.3	69078.19	162090.9	117939.1	272189.4	185154.5
18460740	27119312	21820608	17094810	23191160	19189476	14241954	26309252	20639402	30676412	19555396
19257.28	7607.356	14604.53	23547.45	12127.02	17628.81	23802.62	14655.93	9317.789	11774.25	21517.98
3446873	1446496	2221578	1371629	1396084	1446657	1542023	1202530	1807896	1351091	1789465
21415.26	8237.518	10603.31	9734.452	5900.078	9590.883	13546.31	13498.57	11355.33	10675.14	16319.22
20451.27	13059.05	21975.17	8904.027	7192.146	22038.15	15384.78	12037.53	15007.93	9232.066	15406.42
447408.8	998163.9	863614.3	919666	981827.4	1001321	561329.6	841016.9	196541.2	2499292	630440.1
505447	2977078	1234233	1787655	1117405	1471206	1238772	2258824	580255.5	7832636	962340.6
84689.17	2052067	152479.9	417483.3	346301.8	348016.4	240578.4	351833.1	215451.5	6517378	215991.1
543628.9	1157524	748830.4	396324.9	686754.9	613433.3	629724.6	385096.7	853100.3	1856235	625381.8
52632.62	25753.68	31609.97	18916.92	14844.36	51701.1	41364.01	32236.02	36901.15	34648.3	42243.51
28887112	11404263	28871170	15109030	26551362	12080010	19965480	13505121	21791238	17871292	30343736
22351.63	219544.1	215556.5	163159.8	148796	194583	191406.3	213727.7	269782.1	171630.1	95726.17
38681.82	21824.87	30526.24	17468.36	15118.53	22998.59	16654.97	24798.23	21172.04	20743.53	23723.57
19985.29	9114.82	5730.993	6109.172	8632.497	12958.62	7450.046	10536.76	6331.257	4800.085	11950.28
6307.826	6707.259	5347.724	4372.59	6961.818	8591.97	4510.35	7570.888	11998.81	5361.115	6430.381
35998288	49969928	59231152	46075372	38687300	34146256	38229136	31368076	24355734	31476796	30646964
50174.65	72447.13	71548.59	46621.34	54950.84	46372.21	23981.04	46924.07	46056.56	47440.32	57661.54
25234.03	225392.7	345351.9	315986	233421.8	996786.3	147368.8	2026605	308247.9	1711572	139848.9
4114241	3017594	3462237	4027049	5881394	5752702	5100756	6822208	3870197	3919794	7146796
21081.8	12588.14	20997.63	20929.49	17764.89	20169.02	14250.8	16890.03	29571.74	19661.41	16807.05

17636170	34416012	52502996	72579640	49380224	85636464	1.03E+08	61603200	10800365	1.3E+08	80665712
15825423	25839442	50708948	78015568	62946956	71691776	1.07E+08	67317352	9732446	1.57E+08	79609528
152469.5	89320.34	227769.6	112717.2	47689.48	111065.2	41592.61	38023	32924.34	111544.1	52144.8
32242.93	9477.207	6860.436	9172.298	7555.46	4708.515	3589.055	8084.623	7865.71	6495.642	5026.871
145115.2	129374.4	145104.4	146559	142756.1	172601.1	145736.4	167561.5	122559.3	144265.3	138179.3
18401.99	9262.908	6319.086	27186.46	170403	144806.3	2212.349	11656.17	6166.777	65988.09	34110.42
2713418	3897738	3362172	2052871	2287984	1370194	2010522	2609384	2072738	3965399	2327369
56953.16	21968	26878.88	30681.54	40758.85	22698.35	24675.27	40003.79	29010.37	42524.15	14026.59
251589.1	397128.8	335372.3	362942.8	435350.9	394529.4	375371.9	527934.4	669402.1	733076.1	359676.9
587262.3	565014.9	640561.6	245085.2	459856.8	416189.7	131631.7	717909.5	233243.5	210064.6	379480.6
15547.93	8354.994	6491.769	8348.637	8740.925	10526.46	2369.201	8162.879	5989.839	8096.181	7819.631
22681558	23082304	30930914	18282336	41585024	17062154	24430274	24595290	27435892	24707564	31850120
1907687	1359730	2005851	1742411	1710752	1559656	2372799	1372384	1704788	1518974	2464504
118238.8	19830.91	263928.9	359830	339762.6	294012.8	596767.5	289320.2	78229.01	174653	544127.3
464051.5	296018	240811.9	181662.7	256222.7	208682.7	183930.3	150237.8	191330.2	207810.1	187646.6
126595.9	132766.7	170886.8	86286.29	140964.8	157801.9	72310.94	228081.7	105944.1	139903.7	132193
1024309	78431.73	34364.58	40407.01	187141.4	59364.46	45205.35	79406.07	90060	132959.6	60282.62
134559.6	168652.1	125669.7	101572.8	84491.56	78388.73	110644.1	54484.14	316452.6	115526.8	111298.8
101651.8	91626.2	168589.1	117629.7	92398.21	121249	119096.3	139398.6	110279.8	86032.33	121025
11126.75	15088.55	8744.255	12556.24	10250.69	12436.53	14724.71	6387.842	14372.52	16820.55	14624.82
37321.34	14467.98	15386.81	25558.7	11370.05	25124.31	18491.73	26341.59	21700.37	19736.32	7586.788
2506452	2352523	3710740	2334989	2614861	3309076	1945014	4468065	2195346	1666023	2540091
27191.99	8599.422	11073.43	10703.67	11762.73	11991.88	14727.35	13724.3	16922.07	10521.82	10124.21
74829.95	29542.96	32359.2	33937.76	14272.47	27008.45	22307.25	43454.88	24804.53	41732.43	49759.43
1105578	748236.2	1566546	850086.9	1051989	901593.3	822776.1	1410957	1107500	563830.8	1216754
38658.57	9511.834	15441.79	8141.399	11158.91	10676.77	14902.43	18746.67	166815.4	114224.9	23645.77
241400.4	193796.3	377701.8	186536.4	257490.9	233231.7	182496.2	255539.8	155627.4	141073.5	214154.7
125240.5	7195.343	11853.57	12413.45	18313.07	1471.816	10885.3	24258.31	17981.09	29511.67	15970.52
4047845	2562076	6621746	5693424	5635790	4704508	5147609	6791969	7552943	5098030	5584881
24789104	66869540	23544396	36501540	26325852	65725108	34341660	66789812	51217940	69996232	37796912
10285.13	11269.39	19344.33	13423.36	10863.18	11896.1	12200.44	8705.223	12192.59	13929.94	20306.58
17821.67	2498.03	5274.939	10888.69	6086.124	10079.14	13468.5	10711.43	6516.366	7412.273	9022.531
92492.96	85737.67	128597	88754.95	85237.84	119881.1	140557.7	85206.26	137505.6	46747.27	113166.2
36736.54	28746.56	13605.03	8194.231	13836.61	15587.51	15223.24	16579.28	26399.17	21248.59	10216.47
60170.73	37320.62	44204.6	33462.02	32304.3	42234.75	45254.83	35292.57	45961.22	42362.23	41007.15
188103.3	130181.8	99108.12	98332.29	106294.8	71306.94	115014.8	230291.1	76314.25	309609.7	280806.2
5123728	2208908	1138059	1313409	2398561	1432794	1647370	1681573	1834512	2122965	1735316
26946.41	32117.44	34401.06	27484.18	32990.49	51687.24	38374.59	36959.53	26800.63	20417.14	33913.86
85430.83	98313.66	110768	97236.41	94997.34	113899	78383.8	123323.7	134504.9	120238.3	87517.36
2841292	1997592	2357924	2024301	2632950	2554953	2660672	2964244	2533180	3194860	2907143
824672.4	681891.4	505038.6	415910.6	793868.1	583761.9	532651.2	489595.9	565501.8	720198.3	650563.1
86393.38	36079.2	190757.8	273260.4	210098.2	176171.7	205322.5	141543.1	31597.04	56677.41	156704.6
86335.83	63548.44	66026.11	53925.23	59933.38	77114.58	64932.77	72456.08	48716.47	57283.3	73951.23
2267844	1056116	1660290	1185932	942181.1	1239752	1095582	1542285	1961892	519838.1	1541611
35471.97	74398.47	68065.09	72633.38	52880.21	66373.58	84444.48	49325.75	70245.04	118489.7	63696.46
50519.34	36159.22	13209.39	26204.5	11135.8	9246.631	21505.07	32437.46	13133.9	34481.86	19967.62
336383.9	317241.1	349067	411931.6	451495.4	387130.9	271533.5	506588.5	718903.8	429898.2	261309.7
110219.3	20354.91	17137.2	103054.1	66485.04	24943.13	137975.5	29984.98	47593.86	75750.21	34925.07
514698	650633.1	1110373	1504568	1424374	2322700	3222377	4799898	683073.5	637326.2	2732032

1692041	3680049	1737186	1293737	888232.1	1114029	1725597	1398323	1558034	5276399	2214934
709580.9	92315.73	7988.463	10646.42	283768.6	17414.75	18225.86	44274.49	22022.76	55127.38	112070.2
1462710	1898157	2545606	3784890	2291245	2489977	3005900	1906473	999443.9	2494145	2615860
1930747	1044890	1985667	1500675	1334160	1098507	1215563	1853604	1250498	1214961	1635245
42207.83	32277.96	74552.16	32883.32	30327.84	23253.1	29304.23	27973.96	44329.79	32892.74	38089.61
5665.175	19123.46	16753.17	13338.56	12529.27	12516.77	14742.66	13265.15	20864.17	11474.77	25196.41
47244.33	66980.61	47598.55	67506.85	45835.49	78738.77	26669.7	94016.15	35696.01	52364.58	73708.04
788563	35366.85	117826.3	30013.29	116652.4	66717.6	29719.02	10675.46	27968.57	114468.2	139866.1
35140.79	17905.75	34584.61	31220.81	28894.7	40881.8	41628.82	11942.75	19757.79	20621.95	28683.93
11799.95	14259.69	21672.31	36170.08	23090.77	21940.02	24867.91	17770.34	16896.08	20533.05	25467.16
3321175	92380.05	75208.75	70408.72	85452.16	8515408	79692.61	94769.09	25863.37	130082.1	99825.05
43800.66	24049.6	36650.1	102485.6	62821.65	58987.67	78135.03	29307.35	21695.42	32550.66	16153.58
31467.54	7603.16	19491.63	6754.681	11925.58	13680.27	4936.386	12968.55	105826.8	33177.79	14450.06
39873.54	20753.5	28877.37	14493.43	29199.32	22260.72	18550.44	29074.17	28431.12	25122.93	34060.86
4296.6	4103.752	9107.21	3710.821	4279.344	8830.339	2862.642	7357.129	9796.557	4568.737	5957.599
14659.29	5590.783	2388.973	2289.452	5270.635	1082.532	2792.868	1552.848	2692.838	6607.921	2901.325
78711.91	54057.49	99639	119235	64580.42	88253.81	68717.92	104956	73846.09	203032.1	56795.77
21551.58	50725.52	30118.33	13989.09	28959.26	15319.88	21262.77	25571.09	25908.11	112495.5	25428.03
62571.39	22332.71	61679.84	37714.49	68889.27	38555.05	42649.16	52065.78	53034.22	18156.43	48986.05
48372936	63606068	93590352	1.12E+08	82173144	86606504	93865776	1.18E+08	39908724	92417312	76656272
1307033	5006925	15752807	27387636	18293638	23618200	14166875	23238240	1406969	19194474	13646196
1210546	1092499	802272.3	686965.9	290117.8	1260611	2188600	937256.2	707479.6	962266.4	979612.1
1430304	5453275	1444288	1532678	1728820	1713704	1382648	1669605	1593255	13883026	1263376
46757.77	53913.92	39451.97	52306.61	64642.27	28959.91	42840.48	45527.57	38470.02	55436.53	52809.61
187186.3	253528.9	213499	234403.6	289682.9	150709.6	112954	245475.8	204344.5	140321.2	218812.3
14281.39	5398.219	3442.243	5954.799	3633.532	6040.894	2794.858	9061.57	4988.592	4378.14	6100.509
21337.13	14473.41	19934.06	15900.17	16594.86	17936.18	15890.75	7857.003	25780.14	17291.48	17955.21
68267.14	36841.52	50852.61	31584.39	29612.05	33585.43	30907.26	56817.18	30944.18	33279.11	45516.11
257926.4	293931.5	265940.8	233044.8	282288.9	275625.6	299674.8	177086.3	718513	155272.8	277096.4
46442.57	23692.56	52132.68	21291.39	16887.77	27729.74	26674.99	33780.15	23699.81	17545.85	14286.6
89195.06	36399.82	120774.9	188688.5	150503.7	148601.7	198654.1	95731.1	55059.89	85054.55	125309.2
16904.52	18226.26	44062.16	43387.57	44522.23	39510.51	25610.17	35567.15	41176.94	56793.91	61637.2
205365.1	109980.7	215507.6	104664	88258.52	128645.8	114105	96640.48	161659.8	80245.17	115785.3
244496.2	204723.5	479317.1	212449.1	164770.8	221599.2	206760	143135.5	197495.5	119157.6	218772.2
6998012	4633601	6762331	7642927	16650224	12263090	7374374	10985883	15571175	7296175	5576969
398459.7	315375.1	395855.7	305587.8	369787	386730.7	340256.7	392252.6	304001.2	239307.7	382527.2
51252.13	71187.62	57612.42	44904.72	64738.08	74227.42	36574.61	45118.68	36008.88	55166.47	51324.69
113404.3	64137.28	59639.99	62894.84	60059.25	73109.3	74272.16	71058.78	44875.6	81221.76	55417.7
23595.54	61772.91	22394.88	40174.83	25279.89	38570.06	27249.85	35726.23	21241.75	52583.8	37794.64
257170.5	309945.4	204039	207170.7	148986.1	116098.4	137180.4	152989.3	444681.9	183727	211515.5
54804.23	62235.32	144965.3	235229.6	112740.1	154060.5	233482	131609.4	104080.2	184044.4	183406
94642.4	170440	122984.8	94072.17	199378.2	126278	91317.43	72439.87	121880.5	124978.2	100544.8
23797.73	20392.58	12510.13	12429.35	32328.67	20459.44	30065.52	10908.53	36366.68	25821.2	25566.96
137139.8	175739.1	133418.6	103829	107934.1	114335.8	84923.59	121610.2	242218.7	92287.73	119871.1
19249.89	16970.86	64122.27	118081.5	43264.27	66852.81	130583.3	102790.9	10100.54	66083.96	94429.7
111374.2	93328.34	83213.79	64252.29	96733.69	110996.2	116502.6	87017.82	88649.98	69711.2	131292
296379.4	297292.8	315873.8	148664.3	378914.5	432599.9	309613.8	514695.6	452253.7	135395	267282.5
3786485	48068.44	3289183	2495735	2609464	1284150	340301.5	421316.8	2974842	1348107	1380543
23157.94	12674.79	8396.852	12473.34	17572.3	14588.01	19187.65	17565.91	11498.12	6357.567	10945.7

22071.26	32951.16	34265.85	38834.1	45715.55	37862.38	63961.06	25841.4	24684.51	31202.14	51226.85
1685796	7001597	1711487	2260404	2418276	2152022	2008749	2214413	1840459	12531705	1800155
111503.9	23342.38	26131.8	26487.59	27285.04	26318.47	23909.77	41677.78	33166.57	11979	19371.11
166815.9	229423.6	279653.9	274863.1	183637.3	222491	263072.5	294849.9	359017.2	221621.3	283015.5
227476.1	259465.5	158514.6	182285.5	203607.7	216035.4	193236.6	203795.8	559856.1	203674.8	281872.3
28343.24	19496.34	26631.47	30056.26	38058.89	30673.64	28067.14	26582.1	14038.09	26973.81	33783.67
187700.8	18584.32	50936.69	31813.49	42167.39	12980.99	37457.08	43363.91	25846.13	21627.12	28199.29
28250.39	6353.78	7523.099	2219.088	8391.185	1848.827	2578.282	8723.991	4952.154	7094.025	6996.305
1168390	22854.57	5927.846	8931.558	190616.5	5404.759	17972.38	172685.6	15211.23	19474.98	24779.25
2537570	16757.55	26551.05	3808.034	212044.6	8567.636	7734.958	224096.3	32661.8	57317.55	25958.72
1933054	3234984	986169.1	832222.6	1398243	1388934	1938759	1157809	1682451	2646605	1849832
29720.67	8849.609	15082.92	12201.69	10207.19	11850.49	11697.56	7307.419	28313.92	14219.56	13997.62
18400.56	12303.84	23362.06	24083.57	61851.23	21901.68	21421.76	43012.6	9454.655	16014.54	21606.38
34389.47	55396.68	41066.51	57389.38	6561.24	41640.92	63346.37	20036	22449.25	57518.47	35463.21
106534.5	110924.4	168308.7	184842	140790.8	143582.7	192454.4	87585.13	96752.95	112757	135477.1
567957.4	637080.7	480626.3	364927.4	575822.3	508128.1	397124.6	547720.4	546723.9	562315.7	364485.4
10086.66	12576.81	16644.72	20579.86	18497.8	12154.81	18122.1	19941.11	9571.71	12795.39	13233.57
13500.09	24625.8	36523.21	18896.41	12752.05	23173.18	16907.36	40844.96	26437.12	19313	41322.8
498053.7	819139.6	1166990	851649.9	596240	758830.6	730985.2	476969.6	747187.1	618040.3	624757.8
23095.7	18973.46	35985.7	32429.39	22477.07	26194.34	35819.73	14559.16	20136.11	23414.18	31982.53
25299.36	33862.86	45700.93	17569.07	28049.89	26608.05	8357.123	35002.36	16420.74	45672.38	30747.37
134110	40420.28	154029.1	16488.36	176632.8	169972.2	17826.29	14595.59	168098.4	32301.29	166572.9
14659.29	2843.138	3826.205	4382.452	5118.428	2539.05	1952.762	5366.604	5138.432	7840.936	3776.741
575573.5	2526459	190356.2	743193	2058248	755556.1	259294.5	1437236	763501.2	2233126	882254.7
1048770	1462770	1216861	1103553	721397.8	815238.6	805845.2	1249944	1695501	1460782	920674.6
52723.06	37390.04	51181.38	50588.1	64650.13	61175.87	34287.64	64539.14	63441.04	46992.44	42390.97
22612.53	22293.45	8732.855	7481.927	14777.04	10622.5	6962.541	10121.46	18587.28	15883.56	14858.53
128543.3	54578.66	77069.92	55486.11	67526.59	72766.38	78521.63	79074.3	91026.95	74270.26	91974.45
67600.84	64067.6	37979.93	27976.66	82349.84	51151.86	59255.86	42290.79	53199.32	56194.27	73488.84
40010.39	36097.25	17902.13	30513.56	35615.46	62537.38	35190.07	122486.6	37843.19	55862.13	28867.93
106619.8	103186.1	164380.4	216699.7	174619.9	189159.5	217480.3	176701.1	111760.7	170692.5	164583.5
42294.66	46457.33	84516.45	143603.4	94905.94	96111.7	115974.9	63677.34	40030.23	73474.95	87136.2
95013.68	110625.7	172615.5	305682.6	170907.1	186326	205127.2	126841.5	78949.88	170541.3	182855.6
17290.01	8571.734	16317.85	23173.98	12287.71	16825.74	24340.1	4113.359	1896.568	8342.56	22028.41
24160.87	15370.66	25635.32	17065.81	6449.209	22536.14	14967.17	8259.758	30471.35	15046.26	32149.52
10931.94	9818.806	40003.87	29336.7	14779.04	24557.41	19741.37	16670.79	14045.96	22206.88	16671.98
20792.17	12261.7	6308.923	10394.2	15730.02	29405.98	21383.05	28930.36	30403.31	22814.28	27955.81
2671217	119147.8	13071.62	8220.121	350368.3	16891.5	16337.33	207610.2	51494.03	77221.47	111755.7
132643.3	64641.28	79279.44	59675.99	66752.25	97358.15	77539.97	100591.4	77658.48	87480.33	81849.35
19361.01	104488.5	19159.59	23085.14	11035.58	4750.449	10816.35	7273.684	9444.284	190705.8	11811.75
26395.69	39724.76	31707.78	40150.75	39455.75	41976.13	51641.88	45700	28382.22	42225.19	61780.09
146300	14015.25	18604.88	18833.71	14696.25	11913.18	8614.188	2015.555	10768.89	9522.469	5802.99
233725.7	13396.67	913724.4	8356.588	17211.69	129926.5	54172.25	9428.053	8934.26	89786.66	376104
133263	61290.78	84823.73	53517.74	55850.12	59260.45	68046.07	88043.03	99966.96	42895.18	90870.96
6207.196	7381.014	5762.208	6086.825	4293.193	6342.52	2637.574	7744.035	4747.53	7412.273	5913.928
38865.09	18156.5	17064.29	10753.61	6515.413	13420.68	12434.26	14245.66	13546.91	14862.77	13498.69
372386	120482.2	322183.5	138822.4	132168.2	127258.6	177837.6	123559.7	177969.5	222234.3	285214.3
1647963	1900688	1594382	1787118	1814879	1682146	2161518	937627.4	2047554	1993244	2022948
24838.46	20497.82	47279.26	114483.7	65092.52	93624.1	84199.74	41033.98	9078.629	55678.2	53356

15385.27	14277.16	10408.99	12541.63	21213.89	14357.97	8978.951	12568.62	16555.45	13980.44	14203.36
58976.76	41890.58	65983.14	57688.52	70978.86	55373.64	51734.05	57825.66	33717.19	47048.66	121283.7
85838.53	118982.3	130856.4	91896.01	72360.1	87557.36	87909.73	95900.9	94117.48	72512.16	73434.15
105164.1	89350.46	167194.8	249979.6	92211.28	126749.1	188463.5	130931.9	47254.73	202987.7	140201.1
27372.19	25565.83	34259.84	31349.5	42302.96	28844.36	34341.69	31574.53	25305.32	22924.8	18495.03
738115.1	341490.3	276572.8	439647.3	223252.5	337433.6	230038.8	341324	188038	714728.8	336147.4
47737.95	54195.61	51601.55	57539.13	47376.3	82251.7	70062.91	46877.85	59242.18	27266.23	86264.64
72460.18	54938.86	127712.7	173840.2	98703.49	99902.65	156898.1	87614.74	99989.66	58559.39	155055.1
191738.5	161575.5	188466.7	299349.6	241531.3	225624.4	308846.1	137239.8	142864.6	287920.4	308000.6
66063.2	16332.81	87248.72	29487.71	56631.97	56995.27	87519.91	37510.68	176171.6	65274.71	72237.39
37193.66	17586.98	47908.03	30942.8	8815.147	24666.27	27654.56	21083.81	39071.3	23270.59	21904.52
88994.4	86985.77	75683.28	107997.9	99356.5	97536.6	104877.5	154874.2	39704.35	126272.8	88731.73
149361.3	45319.73	69743.29	108520.7	91577.09	115592.4	215224.3	225932.4	346821.3	109623.8	173907.6
52199.3	56230.53	67059.92	91263.68	74372.37	55988.3	46497.01	77865.8	40308.61	77927.31	66468.68
51529.24	42646.85	65200.14	77546.06	45399.79	53833.45	63378.35	44357.64	39868.12	50660.65	66755.48
348870.7	446340.1	498115	820626.8	551108.1	390993	604352.9	490076.1	454517.3	618165.4	511370
21320.83	11913.24	17819.09	14799.74	11514.3	15759.03	12285.86	20709.54	8705.799	8536.24	13507.25
53127.44	3575.945	4113.804	7951.901	5562.74	4203.058	6588.817	4362.189	2752.879	4300.76	4776.596
53762.84	31598.64	52545.25	50312.35	42211.68	38136.15	74739.01	27564.89	16649.77	57171.23	78121.46
137920.8	59643.7	225041.1	302012.9	114987.4	258347.6	194924.2	232791.4	183719	73027.61	129423.9
66892.1	43069.87	84296.12	112400.5	71351.8	84681.39	67049.95	70977	26791.67	109502.5	76886.38
26693.95	19824.6	28476.94	32093.34	22325.02	30264.48	32026.02	37914.58	18458.45	26423.97	12233.74
3168142	4239056	2692195	3717409	3107586	2920719	3193585	1600500	3074742	3686413	3412919
186629	205377.3	391392.5	439598.3	292163.3	357087.4	388940.4	257210	227093	266897.9	383294.4
56744.98	44321.84	69295.38	65149.73	89433.08	160922.4	203983.2	130780.8	108658.9	61706.45	151334.9
11787.45	9188.558	7438.908	4681.288	9697.474	6321.075	12609.53	11140.52	4181.967	6694.133	7542.159
35155.89	51263.18	59007.04	75581.27	74830.93	121981.7	153039.1	149367.8	96346.8	45271.66	126308.7
55749.39	29041.46	68499.31	49391.59	58873.59	48093.5	33538.18	46232.53	74548.45	39568.83	50554.27
66757.57	77609.16	95909.69	70570.34	75196.17	97279.04	74146.9	90274.52	78260.61	85163.16	90333.59
191391.5	192805.4	90124.55	174946.6	129817.9	196125.1	127602.7	82268.88	130564	152787.1	149489.4
60825.3	57390.96	42245.65	28913.4	43890.71	55474.84	31881.46	23560.23	97093.03	51744.01	66565.1
130059.2	80160.31	103894.1	68623.25	64215.66	74156.96	78298.35	56075.78	91629.03	38635.01	73760.49
184668.2	36654.97	31379.59	27912.73	82678.36	43095.84	117167.4	87989.93	76102.98	65900.16	61281.4
190908.8	151099.8	74249.96	284719.1	318248.2	117124.7	102284.6	148524.2	146654.2	242773	80583.55
403789.2	228884.6	419363.8	251082.8	206394.4	270011	259067.8	284364.4	325564	265302.2	265231.8
95416.97	100270.1	77819.34	83187.35	83725.04	101351.6	51653.29	162542.8	105238.6	67000.8	88965.26
270968.4	168423.5	316385.1	202224.8	108369.3	247344	105138.5	285683.8	44884.29	183148.6	110105.8
40566.77	28902.92	46124.36	47095.98	41804.36	38562.73	16773.71	59353.24	50924.45	34892.19	60215.62
65038.85	87937.69	182824.3	138446.9	159275.5	94411.36	114088.8	173583.9	112607.6	356887.4	85520.09
2.06E+08	1.73E+08	1.76E+08	1.25E+08	1.55E+08	2.14E+08	29179176	2.63E+08	47016528	1.64E+08	91053760
1.92E+08	1.51E+08	1.33E+08	70859904	1.9E+08	94525920	57609164	1.81E+08	62152008	1.21E+08	83294664
565391.1	482138.5	438171.8	378463.3	333599.2	362210.3	406098.2	352321	380595.2	515009.8	387817.6
6955.77	5815.799	35915.57	60136.29	34400.14	25620.13	54221.4	5995.195	10068.22	11767.59	30941.02
34923.32	26358.66	143482.9	29507.65	28123.45	52430.94	36871.59	69890.02	93673.98	47374.79	67896.38
1.09E+08	89382592	69406856	57192576	12074344	11942027	4086063	85193592	25065090	76801272	39807872
44025.08	24752.49	21528.87	18450.26	14251.91	27737.44	18862.51	23474.29	3923.78	75860.34	14339.05
45921.93	27575.67	73612.06	76001.75	63989.96	71954.73	84629.65	107401.6	110940.5	24178.3	84076.69
18515.42	31918.05	58551.93	43218.02	39936.98	36220.71	41178.79	39870.9	56776.68	24793.37	44692.46
1411651	733956.7	577462.4	334717.2	356982	337678	136291.5	386019.5	507082	637008.6	391661.5

57633.5	27745.85	44225.2	35814.2	34931.14	29698.27	42920.05	42631.7	61911.54	35636.46	22005.53
15008.64	15209.65	18415.38	17165.13	18343.74	13562.75	7392.229	15855.18	19898.02	13621.8	10206.11
924468.9	786721	891122.9	635732.1	660167.9	745036.8	867913	1140079	848553.8	552987	777231.4
93483.73	78998.8	81765.38	130218.4	79545.97	100875.7	98607.89	108688.8	71739.85	64960.98	77260.47
24085.57	13922.92	14356.9	34611.34	18327.92	56186.18	39802.08	34665.11	3518.002	56300.83	26086.02
21927.3	7341.798	8808.033	12520.41	10951.2	9501.343	7723.068	10940.21	8081.276	4815.989	10902.91
138859.3	115899.7	200211	121524.1	71825.38	76848.08	184894.4	110936.4	109590	114496.4	193411.2
428292	2278621	115553.4	520047.2	3643256	318909.3	134502.9	252338.8	101275.9	360645.2	281988.7
62251.62	6868.055	14447.83	13789.05	15499.13	11224.54	15303.05	19079.32	4688.452	15662.43	18668.75
138134.1	82000.27	134711.4	93632.23	110770.1	107986.9	108043.8	123856.6	99631.28	75451.3	122779.1
46758.11	51412.03	24847.45	9072.215	16805.28	48664.83	16620.1	92605.52	57995.96	5478.951	39875.82
54510.72	9441.729	6417.317	10118.97	12842.22	19470.13	12173.25	12646.39	27066.91	7278.279	3404.667
14659.29	3408.061	3824.815	7572.08	5118.428	5021.75	5873.981	4720.589	987.9618	6811.12	5509.954
1250748	135000.4	99242.8	114697.5	285377.8	118597.2	93732.41	138870.3	118096.8	112192.2	203942.8
42505.74	15922.66	41672.92	30498.91	27851.95	19577.34	38804.57	34351.46	17311.22	23806.32	34033.91
96451.78	88903.7	106034.3	70314.76	84025.15	95953.36	87183.84	118421.5	83340.73	100939.5	109448.9
230655.4	104611.9	134047.6	177649.8	96847.28	119175.8	94700.48	139429	71602.4	106153.5	107904.6
1149168	11030.59	629345.6	664176.3	720238.8	334279.6	105791.8	155468.6	654685.6	23188.55	420108.8
4072115	102294.4	11190.66	11928.6	574077.2	12548.71	23941.86	207713.8	35201.38	95298.55	345820.8
25481.65	13437.47	23432.02	12610.65	18367.56	15912.91	18875.58	15937.13	12029.77	19978.82	19391.69
2285288	1721316	2044689	1296668	1582535	1769244	2051118	1674475	1778875	1427523	1999727
225819.1	123979	215698.1	174939	168850.2	184371.4	241439.3	251133.6	252885.3	66472.65	199822.3
76564.55	90253.78	177132.2	69991.63	69768.25	65450.92	64552.29	110081.3	52290.32	53796.53	116234.4
195635	154275.6	208969.8	106830.2	131051.1	123324.4	146245.3	150429.9	189990.6	87942.58	166951.6
320093.1	47993.65	200911.3	56351.66	122095.5	90105.5	132145.2	80963.66	101045.3	137260.3	109234.5
4705867	7625018	6899650	4571155	5149872	3235934	3128627	4814103	4145977	20693448	4309731
1210023	508463.5	908549	346543.5	385517.2	294631.9	78452.98	672064.4	184372.2	233655.8	433334.3
1693190	1231915	1164735	966329.8	1146578	1377917	1251791	1598538	1517211	1139905	927178.4
24506.75	7603.16	23905.56	15829.73	13799.85	12377.76	25889.82	5699.855	648256.1	25487.98	15596.95
302434.2	149754.2	130921.6	139167.5	180368.5	160318.1	141797.2	187780.8	190283.8	162639.5	130098.7
112828.6	138214.6	124535.7	122452.7	115749.3	100639.4	108900.9	89040.86	312379.3	85573.83	113319.5
1735794	536823.2	1066752	1116655	511664.2	595823.7	884035.2	947959.9	204143.3	1339417	1004736
245883.9	435310.6	170700.1	228148	262424.9	217066.9	219202.9	255560.2	650231.8	379630	181687.6
2323914	1683971	404284.8	360110.7	345368.1	439972.2	296562.6	331289.4	234139	3286504	438346.7
52355.86	29702.19	33349.15	37872.08	54959.22	52014.63	35545.96	80742.77	40164.84	52924.94	74305.58
1428416	2105708	3480728	2384442	5630357	5616475	4050587	7218935	3974292	1151057	6171916
34069.09	51525.78	27670.32	17904.57	15515.38	13298.4	27027.45	22444.47	19749.61	37585.68	31706.74
56982.23	158884.7	70787.73	72752.12	41048.18	50205.49	36506.57	75562.75	26464.61	96423.91	58907.2
7946.629	6496.207	15787.69	12988.5	8808.574	14307.17	9609.881	14431.45	15767.1	10340.75	10935.51
208862.4	184317.7	147254.4	108687	775712.7	127824.7	95948.92	936663.3	135970	118275.7	491344.6
526705.3	389611.7	724837.6	980830.4	804906.9	654583.4	509453.6	875868.6	409108.8	734961.8	772486.3
72608.48	10670.66	138112.9	90286.48	56423.47	107568.1	82544.82	132828.1	83784.14	36617.4	70183.87
101735.3	97943.5	93706.34	21362.6	80185.81	84857.41	62549.59	51795.8	73760.33	72603.15	78844.99
222986.3	290150.6	391848.3	226730.5	196235.5	281524.5	162989.4	390137	194021	91859.11	307577.6
105105.6	16489.8	44191.89	19294.64	9194.407	22605.47	3305.521	20123.95	21600.15	20299.62	17996.34
13231.94	12570.29	17398.17	7545.69	7616.095	11672.57	14707.85	12413.16	429470.9	23086.15	20949.88
49036.61	29971.66	17219.72	12778.3	25725.13	33786.88	41102.55	43550.35	34499.29	39437.02	55836.16
36829.86	34117.28	63255.81	50549.54	45219.62	70526.8	47677.67	52072.8	45361.3	40768.45	48672.51
36818.19	27550.22	33558.2	24417.68	22176.36	37574.29	22858.24	20213.54	40351.36	26166.77	21146.26

130227.1	138923.8	235150.7	223373.3	249260.8	160310.1	180784.6	369111.6	346642.5	168561	218111
467917.2	158020.2	340709.6	145802.6	115789.1	168818	41009.89	247892.2	161059.4	142178.9	242311.5
59352.93	36852.67	33420.43	39636.74	31399.42	26457.43	34927.06	42910.48	45427.2	47513.13	37936.04
32969.43	31432.64	33820.72	42467.25	46531.05	38897.59	47617.66	53780.2	21141.74	46858.73	37114.92
372204.9	149680.8	367665.8	222530.9	80076.76	38360.54	230569.8	242665.2	283663.2	167848.5	90223.39
36329.93	24401.62	31668.92	16375.64	12904.21	19833.29	17186.12	21747.35	21111.6	25258.57	1599.627
776223	484844.3	727953.1	455226.1	643884.4	624556.3	629879	564907.8	197591.7	444097.1	826292.6
280278.6	49615.49	39788.34	85719.55	107478.1	82768.36	58504.42	33956.22	56849.29	650040.4	46647.96
132057.9	81291.84	112353.9	70093.88	90118.29	110411	99022.52	147446.1	100990.3	117688.9	104886.6
20618.61	13916.31	17458.01	22814.88	22677.01	23708.78	20401.83	21106.85	23488.64	31672	16854.64
34125.11	670666	20695.65	109092.3	317817.9	71071.88	49371.63	20167.69	8937.504	90651.21	65292.45
305354.6	109586.6	160760.8	100221.9	93952.25	81082.71	97494.55	62486.31	151213.2	77798.78	123267.6
48603.63	32818.3	39865.19	65272.79	60684.37	61109.52	69724.59	76911.64	50067.49	44229.5	73904.39
287405.8	337960	281366.3	268326.8	369766.3	319018.7	341440.9	237206.1	353316.6	304117.1	321503.5
84085.05	176123.3	152261.5	260316.9	183956.7	194958.9	216093.5	99370.89	51160.55	161376.5	158730.7
18074.97	27361.17	26489.4	48159.65	20889.07	30794.04	23954.98	28872.05	18620.57	33813.09	24087.44
9343.153	14960.3	17530.45	13767.57	11259.99	10024.23	9812.268	7617.251	5223.711	9181.36	13088.61
16976.82	17026.57	17242.11	16979.6	9370.116	13206.44	22263.18	15978.75	19083.79	16592.98	19369.96
61175.96	64129.09	37141.27	30989.67	69672.06	52551.88	28831.38	28038.02	46323.74	70435.67	40158.51
85811.75	60291.66	60059.77	52497.41	57027.05	44646.86	43850.63	33810.5	91405	57531.53	46528.98
12688.47	8721.643	7264.796	13539.2	8027.892	6722.396	8464.531	6601.873	5766.858	5313.682	6150.798
7947.056	16253.78	13278.47	21062.86	23007.63	24847.45	16122.2	25728.6	26846.73	35469.45	13350.23
111753.7	62399.27	84353.87	63343.29	63546.31	63860.94	46851.83	107549.3	73811.75	82792.23	68809.38
598949.8	593753.7	557769.7	428420.3	445632	544429.5	416786	787662.3	519993.1	458822	423485.4
34175.22	12724.65	29520.06	16662.49	15813.74	15006.79	14719.4	10773.08	37004.63	8803.082	22249.12
436075.8	319767.6	305821.3	686742.6	509286.8	353131.3	223233.7	140945.1	167727.6	4847373	116564.3
15207.59	2168.43	4192.742	6624.76	5690.44	4647.893	722.5555	938.4665	3495.678	6811.12	2589.506
257239.3	262272.9	215379.4	271878	287034.2	232118.4	293945.9	211010.3	293455	324554.5	284801.1
158124.7	67426.66	77231.21	96714.78	48158.84	38867.91	97524.7	21313.9	85850.25	81055.02	59879.38
68250.09	97336.53	81358.64	67166.16	98039.91	108720	81980.25	70307.42	117613	75687.52	104997
34278.43	79058.17	46418.92	37419.94	24670.46	41497.28	59371.76	32708.23	50154.12	23359.46	61695.61
163768.2	111837.2	93610.52	56871.7	82233.66	69116.1	91471.03	149456.6	85086.71	95184.92	81421.95
519799.8	613411.4	569990.1	435554.4	589304.8	636215.8	694502	647459.9	692514.1	597383.3	600182.7
7571.313	11832.24	9582.551	7547.051	7205.191	5473.983	1555.581	9499.677	5998.021	5389.109	15356.36
224215.5	177008.3	249036.5	254132.3	322353.2	249994.5	219727.9	335587.7	355706.5	97450.88	245014.6
55976.82	27765.01	32073.77	20449.11	31922.28	28213.07	46279	38100.96	32514.2	20448.55	28068.04
29036.54	21597.53	17150.1	19207.68	19852.45	21162.16	31736.12	6814.951	16786.88	39639.75	27224.58
23728.36	27512.15	11571.23	10554.52	1897.472	15292.58	17544.19	16988.79	35250.52	11855.82	17085.7
219503.4	102172.4	244807	282189.8	225944.7	243846.2	228589.5	241816.8	141807.1	120868.8	198725.3
19570.89	9146.581	5209.886	2915.548	10768.04	10687.83	19631.06	6713.105	4851.637	15749	12871.78
207025.5	138257.1	151469.7	140902.5	141424.3	158586.5	174228.4	107962.6	162282.3	148738.7	185498.8
30610.83	11245	40061.75	90083.77	27278.57	39966.18	23369.24	27783.81	12654.56	57288.96	32896.87
28054.46	14655.96	7143.679	8596.226	7123.607	5693.371	2141.385	2693.64	7300.26	9884.755	6580.528
61528.02	18813.7	49194.76	84916.7	62793.58	51085.21	114940.5	74674.57	44650.41	46191.63	60596.43
10185.95	16027.09	13882	11301.14	15218.95	7662.642	22830.61	22091.49	12108.57	19642.69	12662.15
307902.7	234697.5	266776.4	217853.2	235299.6	275618.7	289761.9	258494.8	322658.4	297521.7	281021.4
306713.6	232604.9	206025.7	185819.2	206142.3	211543.8	255364.6	135844.2	231705.3	220463.9	274273.2
23664	19800.05	16776.39	18043.48	14849.96	13380.98	15167.39	12699.97	17317.06	11953.64	15997.76
2011096	1377041	1158470	923640.1	1289700	1253393	1109613	2640725	1514515	1095615	1513706

26659.55	38601.91	56368.83	32102.81	32242.48	37139.64	28990.28	40011.6	44871.43	25193.86	33866.45
28659.05	16879.52	91032.76	150363.2	112748	210344.5	470443.8	329223.9	20418.61	39117.49	367047
47589.64	27248.36	6903.859	7836.921	19826.6	8466.545	13366.51	20789.02	15458.78	56339.72	4524.17
220786.9	258004.8	326072.6	243075.6	217703.3	294133.1	238939.4	323123	250988.6	158260.2	310070.8
10364.24	10149.51	5193.047	2985.586	7873.2	16091.41	14238.81	16042.31	10423.51	13753.79	20012.46
146498.9	144299.4	128239	81133.16	101230.7	89458.24	66421.98	111445.3	95746.01	71896.51	132848.8
1643904	108325.2	131734	90484.63	61946.73	71192.02	81563.97	110571.2	110345.4	106183.5	82659.62
181347	126305.1	118296.7	94147.43	190380.4	151916.7	118346.4	105062.6	112169.3	105738	149237.1
39969.56	50782.25	45656.45	39751.7	40218.32	52445.43	42269.78	26142.69	89065.09	42635.11	50400.16
31448.62	51706.29	56777.4	40901.23	46139.98	33706.84	18091.53	66745.73	19603.36	23201.16	14441.69
82816.8	102235.2	65660.41	44475.36	117522.8	59427.24	50272.41	147931.9	42223.78	92917.03	100460.4
7400.913	7832.849	20772.49	18093.61	5155.09	12466.75	10894.96	18952.13	6071.304	9310.454	9933.783
276551	286958.2	192927.4	244774.8	248326.2	196238.7	205256.9	163309.4	576352.5	483237.2	275974.3
116541.9	32531.49	108027.5	151099.1	79012	107135.1	73779.23	77074.82	19122.98	87816.63	65914.8
38804.48	22238.72	27266.35	19135.56	19207.03	35396.74	42650.97	16537.72	24368.21	55107.71	25889.32
17950.3	12653.49	20878.75	14901.5	14891.59	20526.1	17473.15	11819.65	18170.82	22244.2	22132.66
99831.84	191644	81152.64	133528.6	124027.5	128431.8	37193.64	115852.8	189763.9	112670.2	97863.4
37163.12	19637.79	19690.84	28358.56	30606.09	35511.1	47711.96	60631.87	24642.11	22660.33	30681.98
31974.28	31218.87	34819.95	32789.16	21294.09	23877.23	31878.28	14406.76	23220.49	28159.13	32727.36
412400.3	169063.2	151991.4	117437.4	488055	237949.3	149738.9	219535.3	216044	217700.9	248969.3
37265.23	113229.4	90121.66	46939	100365.4	23185.1	52245.05	42644.22	40037.96	66275.05	52443.02
192370	199131.3	156030.9	149876	135973.8	122206.3	135275.9	147847.4	223184.5	131236.5	114298.2
162013.5	15758.38	7338.388	10115.52	38164.63	14824.01	11343.87	31501.05	16764.61	19488.37	21536
14717.82	11383.95	17257.65	26521	12141.55	20691.11	21162.1	9675.385	9842.486	7707.109	13318.81
47206.61	28533.39	29173.8	28587.81	40638.34	52461.69	36963.21	22405.25	42575.75	45694.21	27282.93
14469.46	3591.274	7890.681	11048.54	9614.8	6667.906	16237.72	13232.04	2772.027	9967.007	21405.74
17415.29	2770.466	3279.147	7577.416	3046.175	3587.529	3214.988	2528.388	1952.971	4378.14	4702.013
1566.764	3485.11	2869.872	5213.523	4615.114	4143.557	4173.243	2326.394	1673.741	6811.12	1288.833
16432.69	21209.7	15362.89	14621.34	17467.72	9558.702	12512.44	37474.48	15564.26	18651.04	19482.31
7272300	2869384	5115623	3552897	3184289	5840191	4657539	5579009	6322464	2379719	5400219
120644.1	163125.6	106096.2	141003.9	114995.4	112723	135148.3	135801.9	23183.54	385070.3	116588.4
19420.55	14665.74	13230.49	13546.06	14557.75	17948.68	14264.66	11738.94	16417.19	10827.03	11958.56
38262.38	19822.54	47669.37	42640.89	34104.78	35404.39	22073.29	55948.79	19343.49	23387.84	43653.8
11046.64	10027.18	21494.54	15589.15	9871.036	14669.2	9748.436	12380.01	22114.41	105115.7	13938.37
11598.62	4807.185	8282.063	3314.08	4767.728	6512.538	3356.76	7229.879	3790.006	1777.139	5057.552
211990.4	172047.2	257246.7	128938.1	151932.8	171793.7	175090.5	264227.6	178202	79604.9	233585.2
1508342	47988.8	24061.94	64060.05	336298.2	39751.13	105645.2	219566.8	32905.64	46411.76	272413.8
21853.63	22752.35	19616.87	42532.24	17963.16	24035.81	17421.82	24553.12	14239.74	47114.6	57930.23
116545.2	65786.5	107167.2	127672	92482.71	114421	127391.9	131289.8	79452.46	89459.11	117035.8
83422.99	103237.9	50389.96	47723.7	96740.02	81204.72	47183.96	42074.64	64237.42	61055.44	75354.28
373560.6	212633.2	404918.7	79252.3	252837	140161.3	197309.3	381860.3	407275.8	86795.71	323853.4
95598.77	54762.15	78967.2	57992.66	77709.68	93041.87	79383.39	61632.23	74661.3	71350.9	97088.66
18920.97	33281.24	40956.48	69428.68	45943.37	44840.99	52466	22280.11	12641.39	144891.6	41710.64
121714.2	36199.91	131032.2	68936.61	84862.85	81142.13	93725.68	91599.08	88159.78	21836.45	118516.5
70408.06	75657.31	44010.53	28649.93	56310.95	49048.7	44340.32	37299.47	65918.1	50106.04	70190.36
15301.86	6085.469	9068.406	4883.486	5978.97	4023.521	7174.676	3394.74	15001.43	4568.737	6614.502
3311521	5089641	5684742	5335244	4949832	5487343	4953978	8219228	3659002	3042092	4357266
25996.47	5335.801	4807.846	6452.969	6253.498	5334.046	1228.389	7298.354	7932.516	6898.677	2125.762
32547.26	14050.6	90100.55	152098.3	53922.56	165386.5	167188.4	74189.05	38381.96	105963.3	119470.9

218575	438020.9	200655.5	295607.4	277324.8	212347.3	175699.5	268037.3	457966	1303014	154350.1
202216.7	99564.4	149818.1	114829.8	135700.3	188878.2	95303.02	247318.8	110144.4	107410.2	143105
21367.25	43117.96	10871.44	10886.08	10318.39	3273.129	3842.817	4604.393	24987.36	60453.36	10043
39301.39	11780.27	13543.32	30564.03	25254.17	22086.17	17617.6	8278.294	21867.48	33541.19	14589.66
13314429	14863973	13118237	9901422	10173353	10390059	11666159	12892558	10970102	11167961	11743430
234278.8	28030.78	18878.33	12991.08	47480.08	11797.74	15801.67	27806.07	17290.43	13596.15	39837.78
956203.6	493657.4	883829.3	647819.7	744421.5	627924.3	292208.5	1063243	739882.6	297626.7	611019.1
17156.33	14173.14	18471.5	21218.7	10206.82	15339.5	12267.9	5845.457	41402.09	14325.05	13085.18
23705.35	12448.95	26674.55	12256.31	16108.17	14401.24	10085.88	18078.74	36059.04	22008.62	16012.34
8570692	5178178	7437387	4531895	6045438	6716842	5355064	6861159	6239909	3795150	5719596
74852.08	68374.92	67803.67	46151.28	62052.53	48946.95	53059.82	44548.28	103012.8	30641.13	53826.07
313033.4	411482.6	526056.8	427332.1	639434.7	716553.3	458597.9	832492.8	372428	506548.5	543522.2
19818.13	19719.66	51696.51	44335.4	34744.88	65783.67	31975.85	47252.73	17209.87	33489.32	64269.26
19664.72	18970.52	37650.46	64142.36	49230.64	58894.13	69962.39	29305.44	11482.88	48336.96	66159.46
216118.8	347045.3	294617	294448.4	241341.2	234290.4	145680.5	43120.74	204329.1	1118864	215187.7
216043.7	228400.2	114891.5	97922.94	310090.6	194027.9	125042.1	109472.7	163262.6	181081.4	187954.7
1626762	558687.7	1716743	371510.5	616787.4	659909.1	103118	1129931	212960.8	532225	1153877
19905.49	24385.01	14201.83	5649.392	24851.89	9204.999	23178.31	18305.84	18441.13	26510.71	38411.52
58038.23	19590.87	41956.19	22988.03	23999.73	33704	3224.661	50977.28	5218.472	18454.25	35832.38
12609.24	10169.17	20804.15	25573.53	17669.68	23441.95	25989.8	21160.25	5475.782	21350.38	38259.2
34404.53	34303.46	34012.75	8071.934	38780.87	8983.522	21087.93	26575.32	54255.27	28999.18	46890.27
134083.3	44248.68	109024.4	62693.68	74961.08	64796.07	66060.84	98495.15	114814.5	39101.5	82217.83
270053.6	271609.3	245799.1	189266.3	186564.3	205628.7	214054.7	170681.7	396060.6	302063.2	230289.3
315553.9	61080.08	95504.01	39901.79	47947.81	46827.49	12057.63	76101.84	25142.2	89066.65	26257.19
603147.1	1186426	352651.3	438788.7	176740.4	224352.1	262171.6	137658.8	207078.2	2475726	141733.1
7474.599	4087.95	3038.732	4278.056	3811.293	2035.546	3307.819	5948.195	1396.001	6811.12	5175.5
50279.2	18299.15	25714.58	19945.15	31224.54	25588.21	25232.59	38564.46	19700.75	20633.98	33148.63
4505.07	18614.79	15172.71	24110.55	5637.263	13428.49	40483.33	49875.21	10386.16	14611.24	25947.32
15384.6	52666.53	17316.86	27961.25	18988.32	17259.33	30117.57	15385.33	18853.93	33105.59	33134.36
283190.3	473112.7	336044.5	383113.7	286913.2	384299.6	320235.7	354062.6	211797.5	733977.2	315350.3
8041.51	4290.523	4241.021	2142.531	2819.706	1270.706	2808.193	2291.163	1410.88	6607.921	4653.241
113457.9	240340.1	97801.09	159181.2	133092.2	116825.8	92455.16	108055	111152.3	819954.1	83339.67
204192	114465.6	323802.7	393337.8	227773.1	290837.7	382628.2	225457.2	136979.1	192822.9	329374.7
276870.4	185976.8	208796.1	199221.7	187037.5	181175.5	225631.1	202516.3	402868.7	188658.1	232281.5
853521.8	406640.8	475394.3	437772.7	283078.3	496973.4	299805.1	646101.9	253085.8	325935.8	359050.7
100930	80214.75	106996.4	47949.86	69402.31	80838.14	81896.51	98811.26	97345.24	66240.67	79766.19
8480.773	16376.71	12155.15	15151.91	10872.02	11055.07	16041.91	16529.28	17782.01	14123.22	7022.743
14003.51	7817.591	37049.55	23196.06	40535.9	38124.62	16498.22	13088.86	26082.49	74184.73	19489.65
50269.18	66909.04	43154.39	103347.5	77047.15	152811.3	16186.33	98090.79	67118.94	134714.5	191606.6
30494.08	49074.31	17398.6	20912.29	49615.46	30143.65	20449.93	44341.1	24022.6	30952.7	30365.76
33171.55	56093.35	45140.77	47490.17	44581.19	43426	37035.84	60389.95	48300.17	19776.07	46284.85
11298.34	17094.42	13478.59	9119.36	13355.95	16865.67	4875.083	16699.48	5208.725	17484.13	15425.72
352497.8	216637.4	294173.9	259123.5	262250.7	234629.5	332323.3	168754.7	319567.9	261410.8	342355.6
55636.36	72264.09	56530.52	81942.03	148459.8	95119.14	102467.5	66982.19	54208.3	56680.88	93214.09
41873.9	43653.41	60999.47	22579.14	37926.62	51129.06	38041.79	50241.34	44436.94	33684.31	53742.15
52687.2	40822.98	38062.78	40988.16	26835.7	31357.87	14057.43	15179.37	15352.36	49590.47	26673.13
9515740	4480609	3870728	3109937	4563030	3991751	4683374	4599808	5379027	4584651	5187770
34107920	20492448	30244590	29248978	32539316	30702400	22844052	47464308	37167776	23847130	33493776
8728.468	23573.04	15945.4	16125.45	25281.98	13255.96	8479.496	9704.065	12450.98	66193.45	10741.81

1141028	379432.8	900351.3	540532.5	647016.9	844718.4	636243.6	720923.6	936788	323611.5	639057.6
4510697	3112021	2463971	1974763	4855695	2571670	2167398	1925658	2128989	1794857	2882472
58402.25	26391.48	36082.02	24875.83	38534.57	28654.3	16216.58	40391.04	60181.03	33642.22	27603.61
286500.4	125513.7	158781.9	73574.95	187757.9	130769.2	120440	77207.43	104079.8	138959	160430.9
44750.13	8903.665	25723.42	24316.91	22194.75	19116.04	20773.33	24808.28	13479.98	21482.09	20638.05
93274.46	765776	193739.4	308756.9	230855.3	223384.6	163792.5	177930.4	231870.8	1359754	135397.7
207663.5	242613.5	156399.5	149207.6	203464.4	195545.9	201769.8	132771.6	806813.9	241760.4	209286
137414.5	73519.91	156142.3	105545.5	123973.3	145038	173890.3	158829.1	152953.8	132203.7	132026.2
22100.94	7182.34	21027.33	5269.797	9695.147	7996.906	7795.039	13018.71	18370.56	5392.769	16236.54
22063.28	34072.5	12280.51	28887.05	33930.51	33748.88	31671.01	15144.86	124337.4	40405.35	19330.76
24431.19	18046.94	18046.75	23029.28	23049.14	35663.16	23146.75	17840.51	27978.32	29812.63	20060.93
246842.1	200904.3	211117.6	187469.8	189655.6	192865.4	277107.3	117272.1	287617.6	237736.7	236847.2
22067.13	27563.92	18897.75	10938.08	13431.93	12205.01	10989.27	17330.03	13960.38	51632.14	24702.39
19564.54	13167.08	19073.66	11111.35	17031.98	17649.02	10380.29	11908.49	18656.26	20859.13	20559.9
27376.43	14339.76	17572.61	8607.924	17115.11	16658.8	14175.05	17775.9	25261.54	16654.52	19851.2
86140.41	49738.6	58267.16	47329.08	52991.69	55942.35	50235.1	40773.59	83806.42	46220.3	60062.54
107189.2	76216.81	109822	69049.09	58156.31	70392.97	122796.2	92973.54	112504.5	39119.69	92716.64
6871733	9302745	9897002	10913310	13959971	13335151	10267418	14035189	11767288	6716505	9107521
523601.6	84561.36	256396	140155.4	193929.3	206847.1	222993.5	293252.5	235177.2	143070.3	287261.6
6519.116	25704.45	27071.46	17502.08	21693.44	19121.59	21293.74	6121.763	73367.7	25904.69	22639.82
31640.3	29581.01	16031.88	38329.23	36775.02	31259.32	42387.32	30967.97	64442.54	49574.54	48666.26
13906.3	27624.69	11192.21	19540.15	11341.61	12626.53	16013.31	20266.99	11219.61	32615.77	8790.412
256381.3	156718.6	168814.9	143065.8	166084.2	147552.3	207994	96125.63	202478.1	191048.3	180591.3
78668.1	109199	178821.6	162867.8	127149.3	141567.5	83500.95	120235.8	123026.8	168375.4	98774.45
27473.01	16503.38	3023.857	5049.754	15890.02	20847.51	18396.04	24972.91	17924.27	37357.82	17997.26
56094.28	71957.66	45581.21	56536.98	73750.07	56036.71	54763.19	43870.88	41304.63	42724.06	59588.21
14659.29	4384.658	2597.302	1691.028	2160.641	2953.623	2961.978	2037.554	2449.862	6811.12	3683.02
398265.6	236532.3	354541.4	233966	362489.1	363656.3	352269.1	408084.9	307926.8	264436.6	371863.2
67064.52	43640.69	81544.38	54161.44	58428.72	59926.13	71205.31	80036.56	67211.22	40276.29	69171.47
24832.49	23402.62	3565.561	35649.64	10347.59	76448.52	98026.33	49459.76	1801.146	53166.48	77971.54
124117	64771.45	121961.8	81515.3	91934.55	94220.73	98192.62	103888.4	106652.8	73114.13	146182
2904.331	6385.891	2640.559	11131.23	21007.05	9622.503	3749.496	6120.742	93965.8	6674.617	8113.964
29233.1	14378.82	20233.82	13540.15	13296.21	24180.62	17946.05	13349.48	12771.17	13504.43	22767.5
20713.17	21054.81	12317.89	11797.76	17158.13	12700.77	13428.6	14480.73	57590.11	23210.87	22390.24
151561.6	54919.13	113911.6	313232.7	312551.5	92992.43	158368.3	101005.8	18720.08	132602	191754.5
130143.1	15178.94	54787.31	21534.02	33134.71	43673.91	38777.32	15390.45	15414.58	13519.66	31474.32
178688.7	484762.2	377612.2	330511.8	283532	285508.9	278776.8	224402.6	834975.1	207809.5	331079.1
147806.1	130370.7	126733.9	110842.5	108306.5	168597.7	90394.78	133102	462848.6	100502.3	131153.2
72578.44	57960.14	60190.42	35069.47	33622.42	47940.21	45489.7	84458.74	38023.64	64851.58	86912.61
15769.71	6017.46	9840.26	7880.801	7455.044	9830.068	3190.55	11673.33	5231.99	7967.586	11764.7
943687.2	785318.9	891386.6	702399.6	919139.6	798041.6	723865	568374.4	972911.3	774434.1	811306
35898.02	26114.06	83409.83	115605	69444.41	78199.08	92169.27	51717.96	27270	50528.18	89762.52
9630.422	32307.68	23465.28	14269.91	30328.74	34820.43	20707.99	22794.45	9833.408	26917.15	17704.86
161205.1	947520	202331.3	283822.3	134718	238471.5	140568.1	238776.7	156450.5	2529462	180813.3
9307.772	6757.859	9923.149	6375.604	4172.489	7514.122	3450.515	7051.638	6327.523	4980.369	8721.554
37808.8	21037.9	22371.07	16465.06	54419.1	42973.46	24609.58	24287.86	56867.5	138875.5	20361.58
938634.4	473319.3	649895.4	548557.3	1174480	748676.5	493717.9	289322.5	449523.9	5658876	517099.1
3086.113	3097.544	2989.666	4766.357	11015.86	4143.941	3186.583	2594.23	1768.537	16947.81	2285.105
34624.98	17556.5	52709.27	27637.32	31653.57	38478.68	27747.63	21603.82	44457.7	48649.37	25984.47

404370.6	391243.1	451564.7	565778.6	619639	623063.3	289712.3	168212.9	283685.8	3856408	175232.6
130929.2	196707.1	197142.7	220969.6	181694.7	280838.4	108591.6	37914.67	57831.89	3097458	99408.43
946484	910365.6	905930.7	1381125	1391462	1334968	554764.6	342489.8	590887.8	9444096	399435.1
65057.52	13695.73	18498.39	20479.81	19929.11	19728.8	30569.25	28624.1	21773.59	5626.38	16800.72
4183169	3443516	2986486	3648241	3309589	3281341	4449049	2320018	5938205	5215289	3894399
670269.5	356410	894537.1	711794.6	690820.1	846701.3	879943.3	746195.9	563915.1	575630.5	709684.4
18923.63	12778.11	20725.44	15046.94	16240.06	17181.89	12657.44	15034.71	11998.67	18439.61	14789.8
368051	457968.3	335296.3	382210.7	359816.6	388406.8	304731.1	209156.6	385052	297400.9	426014.9
1015235	491199.6	1281500	1279822	1068472	851991.1	1050501	638618.3	422267.3	636215.8	1207500
17936.54	4434.069	6136.125	9039.926	4315.081	4871.344	4385.16	4382.25	4114.357	6811.12	8685.069
73795.93	54383.55	93737.05	105402.1	113422.3	102508.9	96353.59	64822.59	47988.84	68736.92	122438
516795.8	346320.8	479025.7	605912.3	640589.8	533068.8	316015.3	149347.3	262168.5	4914604	233455.1
47626.5	5391.761	4430.277	9739.677	5902.99	4842.744	13823.37	20851.92	53999.93	8799.877	24603.61
19741.26	11434.16	19874.7	12536.28	20835.34	12164.53	16760.67	10315.84	23599.85	23189.64	16688.82
65094.95	29353.2	30823.95	58467.63	93694.55	29812.15	13914.28	37803.67	67876.41	78488.58	6885.443
29325.63	27714.83	56247.58	38204.11	36119.07	49422.79	61483.54	45851.12	53922.44	17240.39	54778.47
286205	223032.6	273154.3	353071.1	392244.6	285748.7	274933.2	300804.6	585325.8	408712.7	238757.5
685023.8	549573.9	701798.7	320116.1	464519.5	891237.9	1392442	400248.7	831280.8	608456.1	1104721
15515.32	7653.891	16484.93	23346.02	13647.53	17669.48	19349.36	12903.14	7801.027	15363.36	12316.41
977049.6	840246.7	499657.7	450383.1	732838.4	507001.1	593220.1	461812.5	607537.9	785682.8	802927.3
75800.55	46954.91	74440.98	71865.32	56112.55	44151.74	53637.16	53321.59	53304.28	50123.4	61333.84
169387.1	84572.2	98429.69	68253.95	79168.95	119199.1	87867.67	107567.2	100070.1	62416.45	135102.3
39925.19	62805.92	39615.85	45110.48	56198.53	112840.8	126452.6	123951.7	58779.82	63260.33	102898.1
14703.5	11618.63	49223.27	43121.08	31910.49	35654.93	12438.07	17107.41	11913.7	14223.39	12780.33
45911.57	17858	40829.48	47335.31	43104.13	39779.26	52835.79	56014.85	32033.41	26083.16	22801.84
100894.2	154757.1	103074.1	86316.73	115931.8	111984.9	79841.15	94388.5	152294.7	131687.5	107146.8
216621.1	432172.9	428922.8	571902	621385.1	323159.8	476105.8	449302.6	107930.4	198318.9	241045.9
77261.24	93471.95	196274.5	181176.5	183183.3	170633.6	149765.9	139581.5	35626.87	283313.8	228496.2
17169.48	19558.48	40923.3	31212.73	30573.02	12656.59	46173.16	16466.35	30431.81	30397.86	32269.4
74079.33	54955.04	38856.86	42768.31	94908.75	41115.93	105682.2	59295.68	95380.85	33300.78	82027.49
30185902	16360250	23619324	16224261	15328608	20728534	6771060	27193928	8614233	11007210	16732646
53326.66	33170.64	48939.36	33415.42	34117.4	27063.49	30408.81	38417.66	36149.88	23163.59	32981.56
36854.82	16984.23	29424.5	14716.35	19879.69	25005.66	22915.48	32908.69	26973.6	17951.21	29655.61
84672.7	78192.56	106691.7	81531.73	66777.77	72248.01	76944.16	44615.14	129431.7	76843.98	78204.63
34983.65	34828.71	25214.9	27567.99	30416.89	31257.52	26052.09	23745.99	41321.88	83331.27	30777.72
36826.39	5282.692	6788.864	8215.774	8243.883	6629.62	6800.382	9574.257	11451.44	4797.057	8583.554
51207.78	54102.04	91282.73	62952.52	66436.39	71120.76	50742.36	96287.5	48608.92	82129.45	50334.18
94125.98	43766.1	75487.51	55680.53	58054.92	64574.54	66554.38	56608.8	65894.92	67910.38	77404.73
27566.83	18361.25	17650.66	12407.89	15280.13	15949.75	19088.3	18460.58	16382.36	33645.79	15195.31
91128.31	33181.41	74939.99	91878.85	67260.7	19424.89	93552.84	79618.7	29869.31	43141.26	87838.01
38261.14	30953.05	59627.78	22283.38	29472.55	26176.2	19585.11	37525.72	40202.89	34871.14	32300.04
2118.758	1936.949	13736.22	2643.672	2696.255	4677.659	15854.84	2554.646	27418.49	1842.391	14194.88
52343.66	31284.13	46356.34	32341.94	36241.24	34885.26	37874.6	57786.45	49853.84	35833.62	53447.87
47673.69	44583.45	31063.84	36588.76	30221.68	27179.77	25219.9	21337.86	83191.42	37371.64	36134.38
103443.6	136458	94796.65	82257.11	113674	89432.32	101890.6	71636.11	88766.51	67194.55	68539.2
98332.7	64760.94	81307.19	37764.71	56196.92	64112.32	67713.22	64977.36	60184.17	49996.05	49310.68
122178.9	99928.7	95275.69	89333.02	112262.6	100581.4	113178.9	103918.3	105146.2	140658.4	114142.1
60050.74	46431.99	44882.66	35580.16	41476.3	46749.11	36406.88	36045.84	46004.45	49290.38	35573.55
12586.35	11507.16	6878.378	6664.061	23453.08	18938.12	1375.354	3015.165	16814.45	18249.91	5831.605

11710.36	27461.29	26317.35	7337.092	19455.67	23944.38	13063.81	25928.97	33259.64	29598.24	17509.67
32943.9	12880.97	13930.09	18462.15	13806.75	18724.87	15965	23730.24	4507.979	17876.54	12360.83
140993.5	100171.6	226999.3	166069.7	153838.2	274300.2	174055.5	290330.4	176984.9	101512.5	159493.9
19661.91	19309.7	21816.92	12581.24	16864.01	14225.18	19379.75	24073	18674.42	30853.86	19378.47
23643.77	16273.7	23887.75	13938.75	16194.78	12453.31	22034	19361.09	13305.1	23583.31	22230.06
91130.64	46837.55	90135.45	41694.8	36264.14	41705.95	47271.24	22836.14	97558.71	27326.73	48225.3
3674.056	5438.134	13223.7	69050.09	24831.11	37584.7	27399.48	12039	1787.377	32248.9	24113.3
215529.2	330432.2	128758.1	124982.9	241087.5	158019.5	170440.9	156402	237779.7	268923.1	236406
41019.99	46515.9	128021.2	166998.9	150828.6	156689.5	127003.3	108111.8	21500.1	162598.6	160961.9
48912.31	31102.82	59968.6	17083.72	59629.77	38586.55	33567.19	36946.73	38113.46	45179.21	52144.83
99188.54	93481.6	107745.9	84305.05	79730.61	80548.02	68114.13	51940.7	149283.2	95467.53	79503.59
35532.5	17143.28	47675.64	60066.15	47407.62	44994.86	39011.95	39198.89	77137.52	30966.14	44663.11
4064353	1867566	2921567	1564915	2828579	2258916	1577781	4194383	2316740	1576993	2594544
22037.61	25119.43	37095.25	26412.85	28672.55	16697.4	20324.22	16418.17	30559.81	42029.51	9265.074
3306328	126118.6	49743.3	20208.24	278849.1	17942.14	29498.64	187588.3	99612.2	6247.539	101362.2
18539.9	20667.19	70273.36	116551.1	76871.97	105107	115490.5	55053.23	11923.99	104423.6	82968.8
216843.4	105843.8	9037.422	9118.698	207987.4	5495.972	9763.33	116335.9	44373.53	125308.5	10156.28
116835	62704.88	82007.11	52694.13	90166.16	71022.23	69191.96	85501.23	89163.48	60931.52	86727.34
102687.4	23078.06	35807.33	51781.82	48694.37	46883.38	46239.68	52859.46	58321.5	90600.54	110151.6
30307.92	21057.25	24675.71	21740.8	24527.68	28408.93	27640.02	28436.55	26431.78	12950.49	39505.65
16654.14	12452.54	8567.604	12881.97	10603.01	13392.9	11943.7	14845.44	16112.59	16615.9	17957.02
20791.81	11707.9	23304.15	12109.75	2832.559	18239.86	13783.19	21126.49	14948.87	19279.66	8120.729
9643.268	11675.17	10045.4	12064.55	19319.8	12251.89	8669.379	8309.637	10569.06	15320.14	15362.35
90473.72	22458.43	115062.3	85357.52	62454.29	75138.48	69348.94	54603.23	54867.45	72238.99	105290.7
33574.85	39832.41	70938.53	72904.57	52981.91	55000.46	61673.21	57813.16	50187.09	37627.89	73750.22
74648.48	48469.08	70879.95	33041.44	44645.3	44058.85	25761.79	24225	48449.39	37990.67	46322.09
44829.59	41069.86	33810.93	39900.94	22307.04	28068.97	26736.17	26074.63	29640.9	16891.94	27752.11
241125.5	141497.3	81561.86	262521	220432.3	93069.21	157492.2	67142.23	313913.1	327726.1	137840.8
2301160	154326.4	213753.3	121490.4	113765.1	126476.6	132299.8	139981	224134.8	123351.3	117158
104504.1	40106.01	73465.52	33951.77	46117.61	48196.89	57482.99	53369.98	11778.78	47897.32	35154.85
45659.96	28314.19	24607.14	21697.02	61084.44	26354.18	33983.32	36240.87	38169.05	19068.13	33277.08
23310.48	13071.02	16532.51	30719.61	16389.35	11909.8	6065.333	20113.58	13610.96	4755.832	7243.569
1433510	1238443	1117411	1102401	1207840	1055438	1356722	808649.3	1545178	1673173	1569877
25225.12	9846.707	6491.241	6152.3	8351.082	4955.369	3082.676	16286.2	5859.704	9003.648	2222.279
17390.39	19442.16	33682.52	27583.54	14366.45	11305.19	20309.01	14858.64	38921.89	22250.85	12381.64
31895.83	25212.72	20424.53	26869.08	42359.66	26844.68	24404.68	28355.12	34621.54	15840.65	25348.4
107444.8	182654.3	108157.2	170614.4	138766.5	114778.7	111922.9	85495.03	87168.63	120286.6	139094.7
7619.23	15721.74	20486.49	6024.521	14730.79	18375.7	19852.13	23055.78	14086.22	23801.94	20979.45
5733.455	4392.015	7944.53	9007.766	2857.012	10419.59	14893.18	6354.239	4930.32	5875.24	14711.17
2122048	170742.9	2421005	2699838	753880.1	2037757	1087478	4167675	510465.3	1129429	821807.5
4959.611	5396.844	10146.31	7832.47	4920.212	10980.33	11197.94	12061.21	15759.87	7589.961	13620.45
208567.7	11485.92	7382.875	9235.503	2579.525	5911.606	8619.599	6120.926	7548.832	2446.556	12604.53
273400.6	226879.2	195729.6	170663.7	203951	236310	176691.9	197524.3	176725.6	232920.4	214497.7
35889.45	26251.76	41770.94	31864.42	30567.36	32494.62	33145.34	40629.23	29251.72	29769.66	18867.14
1404804	761128.4	490229.2	562553.1	982631.7	549309.8	610260.9	766911.4	586298.3	425354.8	734220.8
48165.96	49181.86	45953.25	50156.19	74199.52	34902.12	75294.02	40538.83	36055.73	44809.82	69619.58
181231.5	851663.4	1323302	433669.8	420319.8	187958.1	20362.61	1018252	287906.1	162486.8	830089.1
17765.07	13963.43	10638.1	14253.47	9764.283	13148.33	11306.64	9202.671	13116.51	74735.12	5173.869
684937.8	612592.4	1177513	505363.6	590401.1	568701.9	455779.6	730052.4	646070.1	787439.5	797642.3

338635.7	212051.4	211105.8	102464.9	93266.05	108716.8	140644.6	120477.6	216500.5	219746.6	183791.5
8803.929	9020.776	14404.96	11136.17	8379.303	15015.52	14097	13393.2	9882.335	18552.94	37099.87
8419250	5656445	9612660	3607122	4432478	3849758	5084868	3627225	5748898	5152870	6966084
95097.61	57886.61	78174.16	46450.56	66352.15	81439.11	61505.66	99517.69	77292.02	124104.4	78879.38
21589.46	121086.7	4301.48	7025.965	6643.481	2534.077	4377.638	5545.381	17423.32	128689.5	4086.77
86895.47	203391.4	172381.9	211236.5	191838.3	186528	185843.6	175489.8	206462.1	180226.3	170137
54918.83	250048.8	93356.41	158035.5	208696.3	142038.5	81311.15	100392.4	97965.9	635042.8	82471.91
13490.38	17315.6	33755.34	28101.78	16849.23	16790.31	23856.78	19619.33	14200.59	11667.67	25812.53
18302.09	15024.85	7424.147	10730.3	9175.35	13527.18	13049.26	18898.37	14591.36	17194.57	13132.32
771054.6	575327.3	733089.5	424059.1	737371.1	628433.8	542007.6	675049.6	471292.3	445461.3	561962
84017.55	70914.82	41521.59	44311.5	83943	64980.33	46875.55	44818.68	45678.75	71655.2	59722.64
209245.2	182267.2	266033.6	207845.3	179050	241788.3	207552.6	237516.8	231064.2	152459.6	249253.8
33068.16	16186.26	8079.594	13631.72	6981.801	23307.11	5393.147	16256.24	8301.827	6648.689	15269.23
19864.32	7672.535	6910.088	11262.22	8657.77	11459.2	36650.75	20946.5	8511.445	11383.78	26364.18
309263.8	227885.3	1609827	1721028	894625	1140966	666212.1	1466518	97047.84	483117.3	1432987
12568.03	15135	13705.58	21319.01	20199.31	19405.02	24577.43	27177.13	30296.76	13550.3	21425.55
1559291	1202518	2229632	1523638	1465674	1702001	1153217	2041413	1690777	874487	1464236
130772.6	118923.2	387222.8	562961.7	220863.4	398465.9	248971	531978.9	158756.3	153411.8	396475.1
162727.4	156182.2	149460.1	142376.6	136741.4	165822.8	211642	143013.5	131286.7	176763.9	179157.3
12369.12	20355.96	49976.01	64665.74	50984.9	56128.47	49749.99	25367.34	14899.1	41190.17	19267.78
2897705	856768.2	574228.9	387481.8	501842.1	689555.7	597402.8	1146679	795498.3	662760.3	858790.5
665242.4	1231776	571884	904872.4	921824.4	717805.6	744813.4	783499.5	491087.6	719306.2	730225.3
31698.75	7006.691	10917.1	8058.423	7154.276	9050.872	4803.856	9547.961	4782.922	4980.369	6102.25
517457.8	843169.1	600684.8	369721	753205.9	679552.1	482535.2	651174.1	555874.9	511267.1	649490.8
13201.79	36731.65	18974.3	22389.28	11061.23	17734.92	22151.6	22045.96	19774.31	25828.79	14123.26
27050.9	22575.92	17992.84	10853.15	25807.83	24786.38	29825.18	29333.4	24378.91	17936.15	31801.97
23173.12	34722.99	21162.93	36375.88	48659.74	109201.6	5325.178	11684.51	13525.43	29869.28	61504.22
159253.9	106382.7	92459.78	54310.08	59570.63	53248.58	44595.13	45456.46	123750.4	73335.45	74159.82
184875	102567	115226.5	107576.7	139291.3	103471	108395	181648.7	133398.8	60034.65	181308.8
747407.1	23284.37	3252012	2742859	1355118	1044108	622141.7	4133542	2840692	5236889	912864.9
28213.97	13408.08	18567.59	27969.63	18686.45	21309.62	23890.42	3939.154	1082.775	32645.24	26829.66
46408.35	7908.901	31009.14	8631.158	17323.07	14482.29	22155.71	26603.92	22810.33	15359.72	17710.21
68547.76	22511.5	26751.93	15237.47	24858.21	24290.07	30320.54	12941.64	24918.28	23981.02	33046.14
547656.5	2547998	1129818	1527474	1104056	813233.3	930540.6	1645874	848023.1	6602645	1121030
71567.65	18599.11	37342.21	22993.19	31714.82	19879.34	39661.14	9933.579	48109.54	20717.85	47654.91
21595.69	22119.17	23858.25	38217.84	32916.79	41199.95	43145.4	18937.58	8761.666	26413.74	22297.95
32400.7	34603.38	35921.9	31521.08	28959.42	38473.7	31303.75	14249.04	42304.4	31149.43	46880.89
14235	30451.15	29167.35	33695.59	27857.86	50450.67	7696.467	32127.66	9609.006	33264.4	22749.63
330828.1	272329.2	286951.7	292005.3	259208.9	319828.1	369828.1	194580.7	229730.1	335279	319949.4
27804.73	7719.243	10447.23	21114.54	15588.21	15863.13	15340.64	8438.015	5849.697	2400.854	11209.32
138096	184983.2	165983.9	250357	279635.3	121232.1	187596	353746.5	140260.2	104315.6	208689.6
23053.23	18420.84	40634.94	58405.59	46410.83	43454.49	58530.04	60243.24	39989.39	28071.98	44750.02
24960.55	13876.92	84132.27	114459.4	55454.67	100762.3	123143.3	70085.13	17393.93	110886.9	80561.18
63501.05	26175.48	93150.84	79748.69	42142.02	47635.97	37422.34	58637.73	100714.3	22059.63	54736.21
24710.51	23531.38	15321.86	24910.05	27487.86	16356.69	19727.74	18653.59	16733.11	14735.08	41054.29
113853	96043.02	198613.6	243791.9	168854.8	186733.3	188135.1	204952.8	78371.72	173453.9	193363.3
126607.6	56852.79	115383.3	74549.64	62001.2	106798.8	68550.91	102336.2	70021.72	78479.72	80019.55
164228.1	124411.2	151323.6	157316.8	171396.8	159073.2	201859.8	138539.7	139640.5	141778.7	191731.1
19481.41	100004.4	86972.06	84756.07	107674.6	96129.89	92027.2	95869.44	174581.1	162211.4	101985.2

4690.854	13673.22	34709.88	38103.96	26017.4	40123.68	44116.64	46016.57	20426.58	10522.24	32186.73
8719680	5817402	9601989	6495109	6879085	6924819	7410366	7973227	7318923	3948695	11437204
13030.98	15819.92	13768.1	8979.183	19681.28	14526.33	7633.005	7474.977	7030.173	11590.74	28350.81
19142.79	4900.805	13687.72	15124.3	14890.74	11005.25	16203.06	10705.5	11491.59	15183.59	17161.61
117860.3	104985	128039	94693.59	105655.9	126763.7	129256.9	119712.3	102528.6	132694.7	119537.1
22204.94	18453.36	16221.11	8737.503	6025.144	9029.209	51041.98	14400.55	32874.98	14629.39	22002.5
420763.4	304167.6	317133.6	269884.3	298569.7	279030.9	381865.2	433552.9	307758.6	251389.7	341582
63029.64	7959.417	15026.4	12015.67	18792.98	13076.53	21838.32	19259.22	13873.47	24598.67	22392.55
222593.6	201917.7	127121.9	124233.1	237946.7	277986.9	140566.6	112484.9	180216	138313.2	237312.2
597536.5	452180.8	534232.6	344580.6	453528.5	538322.3	233346	630442.6	292019.6	811492.4	702173.9
18374.21	12773.44	33779.88	56264.67	40333.42	37277.05	53998.81	23356.1	8360.649	55757.54	59267.65
21431.9	19652.15	20130.84	39099.5	11484.55	19102.51	21968.34	9489.051	6228.472	36488.34	22865.98
329313	103722.1	279455.4	128505.1	169130.2	152123.5	168683.5	218139.8	350891.4	135833.8	195742.5
83461.78	93676.25	58042.89	58597.35	60122.52	43159.95	48802.78	80653.73	80205.97	251660.8	79470.59
11507.97	21954.69	13462.1	8509.378	8813.293	10007	14177.19	9691.097	27949.93	13361.57	11362.51
42146.82	71691.3	50114.53	27706.38	64094.66	36976.99	15326.41	12764.95	63462.25	30984.16	44032.08
39568.66	20019.73	50951.38	50483.58	31542.62	24111.46	18193.92	25221.49	30133.82	28682.04	50399.59
20482.52	9861.799	12438.06	7831.242	11581.09	12076.27	10387.65	15211.81	3868.548	14503.56	14374.64
22909.04	36245.27	18356.93	26220.62	41375.07	40722.35	26006.72	25235.23	39801.84	20568.44	29166.86
94763.52	85434.66	38532.53	29165.48	102243.9	61509.92	101026.6	62692.64	138994.4	66352.77	112974.4
125275.1	197634	95637.02	112064.2	164248.8	96192.55	167954.5	73228.58	131789.2	208251.3	129984.8
11024.96	21063.02	13168.35	12817.18	24102	16870.27	12189.95	10987.07	44867.62	13483.55	14923.79
576086.8	406406.8	672456.8	390340.2	409918	365217.5	372913	449390.4	463103.5	225745	423546
76651.21	57188.92	86433.67	68305.13	70350.52	73602.14	112763.1	64722.64	63390.48	54931.12	77651.29
82020.84	194417.9	253992.4	209803.2	223297.1	234203.3	179447.6	230225.8	240228.4	198437.7	171982.1
