# Supplementary material for: Dynamic Changes of Urine Proteome in Rat Models Inoculated with Two Different Hepatoma Cell Lines
Source: J Oncol. 2021 Jan 7;2021:8895330. doi: 10.1155/2021/8895330 (PMC7810548; doi:10.1155/2021/8895330)
Supplement: Supplementary Materials — Supplementary Figure 1. Functional analysis of differentially expressed proteins at days 5, 7, 14, and 28 in two models. (a) Cell component for the CBRH-7919 model. (b) Molecular function for the CBRH-7919 model. (c) Cell component for the RH-35 model. (d) Molecular function for the RH-35 model. Supplementary Table 1. All urinary proteins identified in the CBRH-7919 model. Supplementary Table 2. All urinary proteins identified in the RH-35 model. Supplementary Table 3. The details of 6435 random allocations in the CBRH-7919 model. Supplementary Table 4. The occurrence of the protein in 6435 random allocations in the CBRH-7919 model. Supplementary Table 5. The details of 12155 random allocations in the RH-35 model. Supplementary Table 6. The occurrence of the protein in 12155 random allocations in the RH-35 model. [file 8895330.f1.zip › 8895330.f1/Supplementary Table 3.pdf]

Title: Dynamic changes of urine proteome in rat models inoculated with two different hepatoma cell lines. Full author: Yameng Zhang, Yufei Gao, Jing Wei & Youhe

Table S3 The details of 6435 random allocations in CBRH-7919 model.

Day 5

Random allocation	Group1	Group2	Numbers of differential urinary proteins	Differential protein ID
1	1,2,3,4,5,6,7,8	9,10,11,12,13,14,15,16	132	A2RUW1,B0BNA5,B0LT89,B1H234,D3ZHA0,D4A5U3,O08557,O35244,O35763,O55004,O70257,O70377,O70594,O88339;Q4V882,P00774,P00884,P01681,P01836,P02631,P02780,P02781,P04904,P05371,P06760,P06761,P06911,P07647,P08649,P08721,P08937,P10247,P13432,P15399,P18418,P18757,P19112,P19223,P19468,P19629,P20646,P20760,P20761,P20762,P22282,P23928,P24268,P29975,P30904,P31044,P31430,P36860,P38438,P38918,P46720,P46844,P48037,P48508,P50115,P50116,P50280,P52590,P52847,P53790,P55091,P57113,P60711;P63259,P63081,P68035;P68136,P80299,P97580,P98089,Q03248,Q05175,Q05702,Q30KJ2,Q3T1J9,Q3ZAV1,Q4G063,Q4G075,Q4KLZ6,Q5GRG2,Q5I0D7,Q5I0E9,Q5M7T9,Q5M8C6,Q5QE79,Q5RKI0,Q5RLM2,Q5U2Q3,Q5U2V4,Q62635,Q62714,Q62812,Q62946,Q63270,Q63317,Q63424,Q63618,Q63751,Q64602,Q66H69,Q66HG3,Q6AYS7,Q6AYT0,Q6IG05,Q6MG61,Q6PCU2,Q6Q0N0,Q6Q0N1,Q6TMA8,Q71MB6,Q80W57,Q8CIZ5,Q8R431,Q8R5M3,Q91ZS3,Q920G2,Q923S2,Q99MZ8,Q9JI85,Q9JJ19,Q9JJ40,Q9JLJ3,Q9QX74,Q9QYU4,Q9QZK8,Q9R0T3,Q9WTW7,Q9WUW8,Q9WUW9,Q9Z0V6,Q9Z0W7
2	1,2,3,4,5,6,7,9	8,10,11,12,13,14,15,16	18	B1H234,O70594,P08649,P08721,P18418,P19223,P23928,P36860,P50116,P52590,P55091,P80299,Q09030,Q4KLZ6,Q5QE79,Q63317,Q6TMA8,Q9R0T3
3	1,2,3,4,5,6,7,10	8,9,11,12,13,14,15,16	79	A2RUW1,B0LT89,B1H234,O08557,O35763,O70377,O70594,O88339;Q4V882,P01836,P04904,P08721,P10536,P10760,P17988,P18297,P18418,P18757,P19223,P19468,P23928,P29975,P30904,P36860,P38918,P46844,P48037,P48508,P50116,P51907,P52590,P52847,P53790,P54921,P55091,P57113,P60711;P63259,P62630,P68035;P68136,P80299,Q03248,Q05175,Q3T1J9,Q3ZAV1,Q4KLZ6,Q5I0D7,Q5I0E9,Q5M7T9,Q5RKI0,Q5RLM2,Q63270,Q63317,Q63355,Q63424,Q63618,Q64602,Q66HG3,Q68FT5,Q6AYS7,Q6MG61,Q6P6T4,Q6Q0N1,Q6TMA8,Q71MB6,Q7M0E3,Q80W57,Q8K3P7,Q8R431,Q8VI04,Q923S2,Q99MZ8,Q9JJ19,Q9JJ40,Q9JLJ3,Q9QYU4,Q9R0T3,Q9WTW7,Q9WUW8,Q9WUW9,Q9Z0W7
4	1,2,3,4,5,6,7,11	8,9,10,12,13,14,15,16	32	B1H234,O08557,P01039,P02780,P02781,P07647,P08649,P08721,P11598,P17988,P18418,P19223,P22283,P31430,P36860,P47967,P48037,P50116,P52590,P55091,P70619,P80299,P97840,Q4KLZ6,Q5GRG2,Q63317,Q63493,Q63617,Q6TMA8,Q8R5M3,Q9JI85,Q9R0T3
5	1,2,3,4,5,6,7,12	8,9,10,11,13,14,15,16	29	B0LT89,B1H234,O08557,P08721,P11762,P19223,P19814,P30904,P31430,P36860,P46844,P47967,P48508,P50116,P52590,P55091,P80299,P97840,Q4KLZ6,Q5I0D7,Q5RLM2,Q63270,Q63317,Q66HG3,Q6Q0N1,Q6TMA8,Q9QYU4,Q9R0T3,Q9WUW8
6	1,2,3,4,5,6,7,13	8,9,10,11,12,14,15,16	28	A2RUW1,B1H234,O08557,O70594,P01836,P02761,P08649,P08721,P19223,P36860,P46844,P47967,P48508,P50116,P52590,P55091,P63081,P80299,P97840,Q4KLZ6,Q5I0D7,Q62714,Q63317,Q63618,Q6Q0N1,Q6TMA8,Q9R0T3,Q9WUW8
7	1,2,3,4,5,6,7,14	8,9,10,11,12,13,15,16	33	A2RUW1,B0LT89,B1H234,O08557,O70594,P02761,P08649,P08721,P19223,P23928,P46844,P48037,P48508,P50116,P52590,P55091,P57113,P63081,P80299,Q4KLZ6,Q5I0D7,Q5QE79,Q5RLM2,Q62714,Q62812,Q63317,Q63751,Q64602,Q6TMA8,Q71MB6,Q9R0T3,Q9WUW8,Q9WUW9

8	1,2,3,4,5,6,7,15	8,9,10,11,12,13,14,16	49	A2RUW1,B0LT89,B1H234,O08557,O70594,O88339;Q4V882,P01946,P02091,P08649,P08721,P17988,P18297,P18757,P19223,P19468,P23928,P30904,P36860,P38918,P46844,P48508,P50116,P52590,P53790,P55091,P70619,P80299,Q03248,Q4KLZ6,Q5I0D7,Q5I0E9,Q5M7T9,Q62714,Q62812,Q63270,Q63317,Q63618,Q64602,Q6MG61,Q6PCU2,Q6Q0N1,Q6TMA8,Q71MB6,Q7M0E3,Q8K3P7,Q99MZ8,Q9QYU4,Q9WUW8,Q9Z0W7
9	1,2,3,4,5,6,7,16	8,9,10,11,12,13,14,15	63	B0LT89,B1H234,O08557,O70594,P01836,P02761,P02783,P04904,P07647,P08649,P08721,P17988,P18297,P18418,P18757,P19223,P19468,P23928,P29975,P36860,P36970,P38918,P46844,P48037,P48508,P50116,P52590,P52847,P53790,P55091,P57113,P80299,Q03248,Q3ZAV1,Q4KLZ6,Q5I0D7,Q5I0E9,Q5M7T9,Q5RLM2,Q5U2Q3,Q62812,Q63270,Q63317,Q63424,Q63618,Q64602,Q68FT5,Q6MG61,Q6Q0N1,Q6TMA8,Q71MB6,Q80W57,Q8R431,Q8R5M3,Q923S2,Q9JJ40,Q9JLJ3,Q9QYU4,Q9R0T3,Q9WTW7,Q9WUW8,Q9WUW9,Q9Z0W7
10	1,2,3,4,5,6,8,9	7,10,11,12,13,14,15,16	22	B1H234,D4A5U3,O70594,P00774,P01681,P08937,P10247,P18418,P19223,P20760,P20761,P31430,P36860,P52590,P55091,P97580,Q05702,Q09030,Q30KJ2,Q4KLZ6,Q63317,Q8CIZ5
11	1,2,3,4,5,6,8,10	7,9,11,12,13,14,15,16	76	A2RUW1,O08557,O35763,O55004,O70257,O70377,O70594,O88267,O88339;Q4V882,P01681,P01836,P02631,P05371,P08937,P10247,P15399,P18418,P18757,P19223,P19468,P19629,P20760,P20761,P20762,P23928,P25031,P30904,P31430,P35952,P36860,P38918,P46844,P48037,P48508,P52590,P53790,P54921,P55091,P57113,P60711;P63259,P68035;P68136,P97580,P98089,Q03248,Q05175,Q05702,Q30KJ2,Q3T1J9,Q3ZAV1,Q498D9,Q4KLZ6,Q5I0E9,Q5M7T9,Q5RLM2,Q63270,Q63317,Q63424,Q63618,Q64602,Q66HG3,Q6MG61,Q6PCU2,Q6Q0N1,Q71MB6,Q80W57,Q8CIZ5,Q8R431,Q923S2,Q9JJ19,Q9JJ40,Q9JLJ3,Q9QYU4,Q9WTW7,Q9WUW8,Q9WUW9,Q9Z0W7
12	1,2,3,4,5,6,8,11	7,9,10,12,13,14,15,16	43	B1H234,D4A5U3,P00774,P01681,P02780,P02781,P06911,P07647,P08649,P08937,P10247,P12020,P15399,P18418,P19223,P20760,P20761,P22282,P31430,P40241,P50280,P52590,P55091,P63029,P97580,P98089,Q05702,Q10758,Q30KJ2,Q4FZU2,Q4KLZ6,Q5GRG2,Q63493,Q6IFU7,Q6IFU8,Q6IFW6,Q6IG02,Q6IG05,Q6IMF3,Q6P6Q2,Q8CIZ5,Q9JI85,Q9R0T3
13	1,2,3,4,5,6,8,12	7,9,10,11,13,14,15,16	32	P00774,P01681,P06911,P08937,P10247,P11762,P18418,P19223,P19814,P20760,P30904,P31430,P36860,P46844,P48508,P52590,P55091,P97580,P98089,Q05702,Q30KJ2,Q498D9,Q4KLZ6,Q5I0D7,Q5RLM2,Q63270,Q63317,Q66HG3,Q6IG05,Q6Q0N1,Q8CIZ5,Q9JI85
14	1,2,3,4,5,6,8,13	7,9,10,11,12,14,15,16	35	B1H234,D4A5U3,O55004,O70257,O70594,P00774,P08937,P10247,P15399,P18418,P19223,P20760,P20761,P31430,P36860,P46844,P48508,P52590,P55091,P97580,P98089,Q05702,Q30KJ2,Q498D9,Q4KLZ6,Q5I0D7,Q62714,Q63317,Q63618,Q6PCU2,Q6Q0N1,Q6TMA8,Q8CIZ5,Q9JI85,Q9WUW8
15	1,2,3,4,5,6,8,14	7,9,10,11,12,13,15,16	35	B1H234,D3ZHA0,D4A5U3,O70594,P01681,P08937,P10247,P15399,P18418,P19223,P20760,P20761,P20762,P31430,P46844,P48037,P48508,P50280,P52590,P54921,P55091,P97580,P98089,Q05702,Q30KJ2,Q4KLZ6,Q5RLM2,Q62635,Q62714,Q63317,Q63751,Q6PCU2,Q6TMA8,Q8CIZ5,Q9WUW8
16	1,2,3,4,5,6,8,15	7,9,10,11,12,13,14,16	48	O08557,O70377,O70594,O88339;Q4V882,P00774,P01681,P01946,P02091,P10247,P15399,P18418,P19132,P19223,P19629,P20760,P20761,P20762,P30904,P31430,P36860,P38918,P46844,P48508,P50280,P52590,P53790,P54921,P55091,P97580,P98089,Q05702,Q30KJ2,Q498D9,Q4KLZ6,Q5I0D7,Q62714,Q63270,Q63317,Q63618,Q6IFU7,Q6IG05,Q6MG61,Q6PCU2,Q6Q0N1,Q6TMA8,Q8CGS4,Q8CIZ5,Q9WUW8

17	1,2,3,4,5,6,8,16	7,9,10,11,12,13,14,15	55	B1H234,D4A5U3,O55004,O70377,O70594,P00774,P02781,P02783,P07151,P07647,P10247,P15399,P18418,P18757,P19223,P19468,P20760,P20761,P20762,P23928,P38652,P38918,P46844,P48037,P48508,P50280,P52590,P52847,P53790,P55091,P97580,Q05702,Q30KJ2,Q3ZAV1,Q4KLZ6,Q5I0D7,Q5M7T9,Q5U2Q3,Q62761;Q62762;Q62763,Q63270,Q63317,Q63618,Q64602,Q6IG05,Q6MG61,Q6Q0N1,Q6TMA8,Q71MB6,Q8CIZ5,Q8R431,Q923S2,Q9JI85,Q9WUW8,Q9WUW9,Q9Z0W7
18	1,2,3,4,5,6,9,10	7,8,11,12,13,14,15,16	8	O70594,P36860,P52590,Q06000,Q09030,Q4KLZ6,Q63618,Q811M5
19	1,2,3,4,5,6,9,11	7,8,10,12,13,14,15,16	3	B1H234,P52590,Q4KLZ6
20	1,2,3,4,5,6,9,12	7,8,10,11,13,14,15,16	5	P36860,P52590,Q03191,Q09030,Q4KLZ6
21	1,2,3,4,5,6,9,13	7,8,10,11,12,14,15,16	5	B1H234,P36860,P52590,Q09030,Q4KLZ6
22	1,2,3,4,5,6,9,14	7,8,10,11,12,13,15,16	5	B1H234,P52590,Q09030,Q4KLZ6,Q63751
23	1,2,3,4,5,6,9,15	7,8,10,11,12,13,14,16	6	P01946,P02091,P36860,P52590,P62804,Q4KLZ6
24	1,2,3,4,5,6,9,16	7,8,10,11,12,13,14,15	8	B1H234,P02783,P22006,P23593,P52590,Q09030,Q498R7,Q4KLZ6
25	1,2,3,4,5,6,10,11	7,8,9,12,13,14,15,16	5	B1H234,P36860,P48037,P52590,Q4KLZ6
26	1,2,3,4,5,6,10,12	7,8,9,11,13,14,15,16	9	P11762,P36860,P48508,P52590,Q498D9,Q4KLZ6,Q5RLM2,Q63618,Q66HG3
27	1,2,3,4,5,6,10,13	7,8,9,11,12,14,15,16	14	A2RUW1,B1H234,O70594,P36860,P46844,P48508,P52590,P80299,P97584,Q06000,Q498D9,Q4KLZ6,Q63618,Q9WUW8
28	1,2,3,4,5,6,10,14	7,8,9,11,12,13,15,16	12	A2RUW1,B1H234,O70594,P48037,P48508,P52590,P54921,Q06000,Q4KLZ6,Q5RLM2,Q71MB6,Q9WUW8
29	1,2,3,4,5,6,10,15	7,8,9,11,12,13,14,16	26	A2RUW1,O08557,O70594,O88339;Q4V882,P01946,P02091,P17988,P19468,P21674,P23928,P36860,P38918,P46844,P48508,P52590,P53790,P54921,Q498D9,Q4KLZ6,Q5I0E9,Q63270,Q63618,Q6MG61,Q6Q0N1,Q71MB6,Q812E4
30	1,2,3,4,5,6,10,16	7,8,9,11,12,13,14,15	30	O70594,P02783,P17988,P18297,P19468,P23928,P46844,P48037,P48508,P52590,P53790,P57113,Q03248,Q3ZAV1,Q4KLZ6,Q5I0D7,Q5I0E9,Q63270,Q63424,Q63618,Q64602,Q6MG61,Q6Q0N1,Q71MB6,Q8R431,Q923S2,Q9JJ40,Q9QYU4,Q9WUW8,Q9Z0W7

31	1,2,3,4,5,6,11,12	7,8,9,10,13,14,15,16	6	B1H234,P11762,P31430,P47967,P52590,Q4KLZ6
32	1,2,3,4,5,6,11,13	7,8,9,10,12,14,15,16	4	B1H234,P36860,P52590,Q4KLZ6
33	1,2,3,4,5,6,11,14	7,8,9,10,12,13,15,16	3	B1H234,P52590,Q4KLZ6
34	1,2,3,4,5,6,11,15	7,8,9,10,12,13,14,16	14	B1H234,P01946,P02091,P08721,P17988,P21674,P52590,Q4FZU2,Q4KLZ6,Q6IFU8,Q6IFW6,Q6IG02,Q6IMF3,Q6P6Q2
35	1,2,3,4,5,6,11,16	7,8,9,10,12,13,14,15	9	B1H234,P01039,P02783,P07647,P17988,P35745,P52590,Q4KLZ6,Q5I0D7
36	1,2,3,4,5,6,12,13	7,8,9,10,11,14,15,16	5	P36860,P47967,P52590,P80299,Q4KLZ6
37	1,2,3,4,5,6,12,14	7,8,9,10,11,13,15,16	4	P11762,P52590,Q4KLZ6,Q5RLM2
38	1,2,3,4,5,6,12,15	7,8,9,10,11,13,14,16	7	P01946,P02091,P11762,P36860,P52590,Q498D9,Q4KLZ6
39	1,2,3,4,5,6,12,16	7,8,9,10,11,13,14,15	4	P02783,P52590,Q4KLZ6,Q5I0D7
40	1,2,3,4,5,6,13,14	7,8,9,10,11,12,15,16	5	A2RUW1,B1H234,P52590,Q4KLZ6,Q9WUW8
41	1,2,3,4,5,6,13,15	7,8,9,10,11,12,14,16	11	A2RUW1,B1H234,P01946,P02091,P21674,P36860,P52590,P62804,P97584,Q498D9,Q4KLZ6
42	1,2,3,4,5,6,13,16	7,8,9,10,11,12,14,15	9	B1H234,P02783,P46844,P52590,P97584,Q4KLZ6,Q5I0D7,Q9EQS0,Q9WUW8
43	1,2,3,4,5,6,14,15	7,8,9,10,11,12,13,16	8	A2RUW1,B1H234,P01946,P02091,P52590,P54921,Q4KLZ6,Q62714
44	1,2,3,4,5,6,14,16	7,8,9,10,11,12,13,15	8	B1H234,P02783,P48037,P52590,Q4KLZ6,Q5I0D7,Q71MB6,Q9WUW8
45	1,2,3,4,5,6,15,16	7,8,9,10,11,12,13,14	15	B0LT89,B1H234,O70594,P01946,P02091,P02783,P17988,P46844,P48508,P52590,P97584,Q4KLZ6,Q5I0D7,Q63270,Q71MB6
46	1,2,3,4,5,7,8,9	6,10,11,12,13,14,15,16	27	D3ZHA0,D4A5U3,O70594,P00774,P01681,P01836,P06760,P08649,P08937,P19223,P20646,P20760,P20761,P48508,Q4FZU6,Q5QE79,Q5U2V4,Q62946,Q63317,Q63618,Q66H69,Q6Q0N1,Q6TMA8,Q8CIZ5,Q9QX74,Q9WUW8,Q9Z0V6

47	1,2,3,4,5,7,8,10	6,9,11,12,13,14,15,16	90	D3ZHA0,D4A5U3,O08557,O35763,O55004,O70257,O70377,O70594,O88339;Q4V882,P00774,P01681,P01836,P04904,P06760,P08937,P10536,P10760,P18418,P18757,P19223,P19468,P20646,P20760,P20761,P20762,P23928,P24268,P29975,P30904,P36860,P38918,P46413,P46720,P46844,P48508,P51907,P53790,P54921,P55091,P57113,P60711;P63259,P62630,P68035;P68136,P80299,P97580,Q03248,Q05175,Q30KJ2,Q3T1J9,Q3ZAV1,Q4G075,Q5I0E9,Q5M7T9,Q5RLM2,Q62687,Q62753,Q62946,Q63270,Q63317,Q63355,Q63424,Q63598,Q63618,Q64093,Q64319,Q64602,Q66H69,Q66HG3,Q6AY41,Q6AYS7,Q6MG61,Q6PCU2,Q6Q0N1,Q6TMA8,Q71MB6,Q80W57,Q8CIZ5,Q8R431,Q923S2,Q99MZ8,Q9JJ19,Q9JJ40,Q9JLJ3,Q9QX74,Q9QYU4,Q9WTW7,Q9WUW8,Q9WUW9,Q9Z0V6,Q9Z0W7
48	1,2,3,4,5,7,8,11	6,9,10,12,13,14,15,16	49	D3ZHA0,D4A5U3,P00774,P01681,P02780,P02781,P02782,P06760,P08649,P08723,P08937,P19223,P20646,P20760,P20761,P22282,P27590,P30904,P31430,P40241,P47967,P50115,P50280,P97840,Q10758,Q4FZU2,Q4G075,Q4KLZ6,Q5M8C6,Q62946,Q63317,Q63493,Q66H69,Q6B345,Q6IFU7,Q6IFU8,Q6IFW6,Q6IG02,Q6IG05,Q6IMF3,Q6P6Q2,Q6TMA8,Q8CIZ5,Q9JHB9,Q9QX74,Q9R0T3,Q9WTT6,Q9WUW8,Q9Z0V6
49	1,2,3,4,5,7,8,12	6,9,10,11,13,14,15,16	34	D3ZHA0,D4A5U3,iRT-Kit_WR_fusion,P00774,P01681,P01836,P06760,P08649,P19223,P20646,P20760,P20761,P30904,P46844,P47967,P48508,P80299,P97840,P98089,Q4G075,Q5PQL7,Q5RLM2,Q62946,Q63270,Q63317,Q63618,Q66H69,Q6IG05,Q6MG61,Q6Q0N1,Q6TMA8,Q8CIZ5,Q9QX74,Q9WUW8
50	1,2,3,4,5,7,8,13	6,9,10,11,12,14,15,16	37	B0BNN3,D3ZHA0,D4A5U3,O70257,O70594,P00774,P01681,P01836,P06760,P08649,P19223,P19468,P20646,P20760,P20761,P25809,P30904,P36860,P46844,P47967,P48508,P80299,P97840,Q4G075,Q62714,Q62946,Q63317,Q63618,Q66H69,Q6PCU2,Q6Q0N1,Q6TMA8,Q8CIZ5,Q9QX74,Q9WUW8,Q9WUW9,Q9Z0W7
51	1,2,3,4,5,7,8,14	6,9,10,11,12,13,15,16	35	B0BNN3,B0LT89,D3ZHA0,D4A5U3,O70594,P01681,P06760,P08649,P08937,P19223,P20646,P20760,P20761,P20762,P46844,P48508,P97580,Q4G075,Q5QE79,Q5RLM2,Q62635,Q62714,Q62946,Q63317,Q63751,Q64602,Q66H69,Q6PCU2,Q6Q0N1,Q6TMA8,Q8CIZ5,Q923S2,Q9QX74,Q9WUW8,Q9WUW9
52	1,2,3,4,5,7,8,15	6,9,10,11,12,13,14,16	58	B0LT89,D3ZHA0,D4A5U3,O08557,O35763,O70377,O70594,O88339;Q4V882,P00774,P01681,P06760,P08649,P08721,P18757,P19132,P19223,P19468,P20646,P20760,P20761,P20762,P30904,P36860,P38918,P46844,P48508,P53790,Q05175,Q3ZAV1,Q4G075,Q5I0E9,Q5M7T9,Q5RLM2,Q62714,Q63270,Q63317,Q63355,Q63424,Q63618,Q64093,Q64602,Q66H69,Q6IG05,Q6MG61,Q6PCU2,Q6Q0N1,Q6TMA8,Q80W57,Q8CIZ5,Q8R431,Q923S2,Q99MZ8,Q9JJ19,Q9QX74,Q9WTW7,Q9WUW8,Q9Z0V6,Q9Z0W7
53	1,2,3,4,5,7,8,16	6,9,10,11,12,13,14,15	69	B0BNN3,B0LT89,D3ZHA0,D4A5U3,O35763,O70377,O70594,P00774,P01681,P01836,P02761,P02781,P04904,P06760,P07151,P07647,P08649,P18757,P19223,P19468,P20646,P20760,P20761,P20762,P23928,P38918,P46720,P46844,P48508,P53790,P57113,P97580,Q03248,Q05175,Q30KJ2,Q3ZAV1,Q4G075,Q5I0D7,Q5I0E9,Q5M7T9,Q5RLM2,Q63270,Q63317,Q63355,Q63424,Q63618,Q64093,Q64602,Q66H69,Q6AYQ8,Q6MG61,Q6PCU2,Q6Q0N1,Q6TMA8,Q71MB6,Q80W57,Q8CIZ5,Q8R431,Q8R5M3,Q923S2,Q9JHB9,Q9JJ19,Q9JJ40,Q9QX74,Q9WTW7,Q9WUW8,Q9WUW9,Q9Z0V6,Q9Z0W7

54	1,2,3,4,5,7,9,10	6,8,11,12,13,14,15,16	5	O70594,P36860,P70545,P80299,Q63618
55	1,2,3,4,5,7,9,11	6,8,10,12,13,14,15,16	2	P08649,Q4KLZ6
56	1,2,3,4,5,7,9,12	6,8,10,11,13,14,15,16	2	P80299,Q03191
57	1,2,3,4,5,7,9,13	6,8,10,11,12,14,15,16	3	P08649,P36860,P80299
58	1,2,3,4,5,7,9,14	6,8,10,11,12,13,15,16	2	P08649,Q63751
59	1,2,3,4,5,7,9,15	6,8,10,11,12,13,14,16	4	P01681,P08649,P08721,P80299
60	1,2,3,4,5,7,9,16	6,8,10,11,12,13,14,15	1	P08649
61	1,2,3,4,5,7,10,11	6,8,9,12,13,14,15,16	5	O08557,P47967,P80299,P97840,Q4KLZ6
62	1,2,3,4,5,7,10,12	6,8,9,11,13,14,15,16	13	P18297,P30904,P36860,P46844,P47967,P48508,P80299,P97840,Q5RLM2,Q63618,Q6Q0N1,Q71MB6,Q9WUW8
63	1,2,3,4,5,7,10,13	6,8,9,11,12,14,15,16	19	A2RUW1,O70594,P18297,P19223,P19468,P36860,P46844,P47967,P48508,P80299,P97840,Q5I0E9,Q63355,Q63424,Q63618,Q64093,Q6Q0N1,Q9WUW8,Q9WUW9
64	1,2,3,4,5,7,10,14	6,8,9,11,12,13,15,16	16	O70594,P08753,P18297,P46844,P48037,P48508,P54921,P80299,Q5I0E9,Q5RLM2,Q63355,Q63618,Q71MB6,Q923S2,Q9WUW8,Q9WUW9
65	1,2,3,4,5,7,10,15	6,8,9,11,12,13,14,16	40	A2RUW1,B0LT89,O08557,O70377,O70594,O88339;Q4V882,P08721,P10536,P17988,P18297,P19468,P30904,P36860,P38918,P46844,P48508,P53790,P54921,P63095,P80299,Q05175,Q5I0E9,Q5RKI1,Q5RLM2,Q63270,Q63355,Q63424,Q63618,Q64093,Q6MG61,Q6Q0N1,Q71MB6,Q80W57,Q8R431,Q923S2,Q99MZ8,Q9JJ19,Q9WTW7,Q9WUW8,Q9Z0W7
66	1,2,3,4,5,7,10,16	6,8,9,11,12,13,14,15	48	B0LT89,O70377,O70594,P02761,P17988,P18297,P18757,P19468,P23928,P29975,P46413,P46720,P46844,P48037,P48508,P53790,P57113,P62630,P80299,P97840,Q03248,Q3ZAV1,Q5I0D7,Q5I0E9,Q5M7T9,Q5RLM2,Q63270,Q63317,Q63355,Q63424,Q63618,Q64093,Q64602,Q6AYQ8,Q6MG61,Q6Q0N1,Q6TMA8,Q71MB6,Q80W57,Q8R431,Q923S2,Q9JJ19,Q9JJ40,Q9QYU4,Q9WTW7,Q9WUW8,Q9WUW9,Q9Z0W7
67	1,2,3,4,5,7,11,12	6,8,9,10,13,14,15,16	8	P08649,P35053,P47967,P70709,P80299,P97840,Q4KLZ6,Q5I0D1

68	1,2,3,4,5,7,11,13	6,8,9,10,12,14,15,16	7	B0BNN3,P08649,P47967,P80299,P97840,Q4KLZ6,Q5I0D1
69	1,2,3,4,5,7,11,14	6,8,9,10,12,13,15,16	4	P08649,Q4KLZ6,Q63751,Q9WUW8
70	1,2,3,4,5,7,11,15	6,8,9,10,12,13,14,16	13	O08557,P08649,P08721,P17988,P97840,Q4FZU2,Q4KLZ6,Q6IFU8,Q6IFW6,Q6IG02,Q6IMF3,Q6P6Q2,Q8CJ52
71	1,2,3,4,5,7,11,16	6,8,9,10,12,13,14,15	13	P02761,P02781,P07647,P08649,P17988,P47967,P97840,Q08463,Q4FZU2,Q4KLZ6,Q6AYQ8,Q8R5M3,Q9JHB9
72	1,2,3,4,5,7,12,13	6,8,9,10,11,14,15,16	5	P36860,P47967,P80299,P97840,Q9WUW8
73	1,2,3,4,5,7,12,14	6,8,9,10,11,13,15,16	4	P08649,P80299,Q5RLM2,Q9WUW8
74	1,2,3,4,5,7,12,15	6,8,9,10,11,13,14,16	7	O89117,P08649,P08721,P10758,P80299,P97840,Q6AY61
75	1,2,3,4,5,7,12,16	6,8,9,10,11,13,14,15	11	B0LT89,P08649,P18297,P46844,P47967,P80299,P97840,Q5I0D7,Q5RLM2,Q71MB6,Q9WUW8
76	1,2,3,4,5,7,13,14	6,8,9,10,11,12,15,16	5	B0BNN3,P02761,P08649,P80299,Q9WUW8
77	1,2,3,4,5,7,13,15	6,8,9,10,11,12,14,16	11	O70594,P08649,P08721,P21674,P36860,P80299,P97840,Q63618,Q6AYQ8,Q6Q0N1,Q9WUW8
78	1,2,3,4,5,7,13,16	6,8,9,10,11,12,14,15	15	B0BNN3,P02761,P08649,P18297,P19223,P19468,P46844,P47967,P80299,P97840,Q08463,Q5I0D7,Q6AYQ8,Q6TMA8,Q9WUW8
79	1,2,3,4,5,7,14,15	6,8,9,10,11,12,13,16	5	B0LT89,P08649,Q5RLM2,Q62714,Q9WUW8
80	1,2,3,4,5,7,14,16	6,8,9,10,11,12,13,15	13	B0BNN3,B0LT89,P02761,P08649,P18297,P46844,Q4KLZ6,Q5I0D7,Q5RLM2,Q63317,Q6TMA8,Q71MB6,Q9WUW8
81	1,2,3,4,5,7,15,16	6,8,9,10,11,12,13,14	23	B0LT89,O70594,P02761,P08649,P08721,P17988,P18297,P19468,P46844,P48508,Q5I0D7,Q5I0E9,Q63270,Q63317,Q64093,Q6AYQ8,Q6Q0N1,Q6TMA8,Q71MB6,Q8R431,Q9WTW7,Q9WUW8,Q9Z0W7
82	1,2,3,4,5,8,9,10	6,7,11,12,13,14,15,16	8	O70594,P00774,P01681,P20760,P70545,P97580,Q30KJ2,Q63618
83	1,2,3,4,5,8,9,11	6,7,10,12,13,14,15,16	14	O88797,P00774,P01681,P08649,P20760,P27590,Q10758,Q4FZU2,Q4KLZ6,Q6IFU7,Q6IFU8,Q6IFW6,Q6IMF3,Q6P6Q2
84	1,2,3,4,5,8,9,12	6,7,10,11,13,14,15,16	4	P00774,P01681,P20760,P36376
85	1,2,3,4,5,8,9,13	6,7,10,11,12,14,15,16	5	D4A5U3,P00774,P01681,P20760,Q8CIZ5

86	1,2,3,4,5,8,9,14	6,7,10,11,12,13,15,16	6	P00774,P01681,P08649,P20760,P97580,Q63751
87	1,2,3,4,5,8,9,15	6,7,10,11,12,13,14,16	5	P00774,P01681,P19132,P20760,P36376
88	1,2,3,4,5,8,9,16	6,7,10,11,12,13,14,15	6	P00774,P01681,P08649,P20760,P23593,Q30KJ2
89	1,2,3,4,5,8,10,11	6,7,9,12,13,14,15,16	12	P01681,P27590,P97580,Q10758,Q4FZU2,Q4KLZ6,Q6IFU7,Q6IFU8,Q6IFW6,Q6IG02,Q6IMF3,Q6P6Q2
90	1,2,3,4,5,8,10,12	6,7,9,11,13,14,15,16	12	P01681,P0DMW0;P0DMW1,P30904,P34901,P46844,P48508,P97580,Q498D9,Q5RLM2,Q63618,Q6Q0N1,Q8CIZ5
91	1,2,3,4,5,8,10,13	6,7,9,11,12,14,15,16	16	O70594,P01681,P19223,P30904,P36860,P46844,P48508,P97580,Q30KJ2,Q498D9,Q63618,Q66H69,Q6Q0N1,Q8CIZ5,Q9WUW8,Q9WUW9
92	1,2,3,4,5,8,10,14	6,7,9,11,12,13,15,16	16	O70594,P01681,P08937,P20760,P20762,P46844,P48508,P54921,P97580,Q30KJ2,Q5RLM2,Q63618,Q8CIZ5,Q923S2,Q9WUW8,Q9WUW9
93	1,2,3,4,5,8,10,15	6,7,9,11,12,13,14,16	33	O35763,O70377,O70594,O88339;Q4V882,P01681,P10536,P19132,P19468,P20762,P30904,P34901,P46844,P48508,P53790,P54921,P97580,Q05175,Q30KJ2,Q498D9,Q5I0E9,Q62753,Q63270,Q63355,Q63424,Q63618,Q64093,Q6IG05,Q6MG61,Q6Q0N1,Q80W57,Q8R431,Q923S2,Q9Z0W7
94	1,2,3,4,5,8,10,16	6,7,9,11,12,13,14,15	35	O35763,O70377,O70594,P01681,P07151,P18757,P19468,P20760,P20762,P46844,P48508,P53790,P97580,Q05175,Q30KJ2,Q3ZAV1,Q5I0E9,Q5RLM2,Q63270,Q63355,Q63424,Q63618,Q64093,Q64602,Q6MG61,Q6Q0N1,Q71MB6,Q80W57,Q8R431,Q923S2,Q9JJ40,Q9WTW7,Q9WUW8,Q9WUW9,Q9Z0W7
95	1,2,3,4,5,8,11,12	6,7,9,10,13,14,15,16	16	P00774,P01681,P27590,P47967,Q10758,Q4FZU2,Q4KLZ6,Q5I0D1,Q5PQL7,Q6IFU7,Q6IFU8,Q6IFW6,Q6IG02,Q6IG05,Q6IMF3,Q6P6Q2
96	1,2,3,4,5,8,11,13	6,7,9,10,12,14,15,16	17	B0BNN3,P00774,P01681,P08649,P47967,Q10758,Q4FZU2,Q4KLZ6,Q66H69,Q6IFU7,Q6IFU8,Q6IFW6,Q6IG02,Q6IG05,Q6IMF3,Q6P6Q2,Q8CIZ5
97	1,2,3,4,5,8,11,14	6,7,9,10,12,13,15,16	16	B0BNN3,P01681,P08649,P08937,P97580,Q10758,Q4FZU2,Q4KLZ6,Q6IFU7,Q6IFU8,Q6IFW6,Q6IG02,Q6IG05,Q6IMF3,Q6P6Q2,Q8CIZ5
98	1,2,3,4,5,8,11,15	6,7,9,10,12,13,14,16	13	P00774,P01681,P08649,Q10758,Q4FZU2,Q4KLZ6,Q6IFU7,Q6IFU8,Q6IFW6,Q6IG02,Q6IG05,Q6IMF3,Q6P6Q2
99	1,2,3,4,5,8,11,16	6,7,9,10,12,13,14,15	24	O88797,P00762,P00774,P01681,P02780,P02781,P02782,P05539,P07647,P08649,P30120,P97580,Q10758,Q30KJ2,Q4FZU2,Q4KLZ6,Q6IFU7,Q6IFU8,Q6IFW6,Q6IG02,Q6IG05,Q6IMF3,Q6P6Q2,Q9JHB9
100	1,2,3,4,5,8,12,13	6,7,9,10,11,14,15,16	4	P00774,P47967,Q66H69,Q8CIZ5
101	1,2,3,4,5,8,12,14	6,7,9,10,11,13,15,16	4	P01681,P97580,Q5RLM2,Q8CIZ5
102	1,2,3,4,5,8,12,15	6,7,9,10,11,13,14,16	7	P00774,P01681,P19132,P34901,P36376,Q498D9,Q6IG05

103	1,2,3,4,5,8,12,16	6,7,9,10,11,13,14,15	7	P00774,P01681,P20760,P46844,Q30KJ2,Q5RLM2,Q6IG05
104	1,2,3,4,5,8,13,14	6,7,9,10,11,12,15,16	6	B0BNN3,D4A5U3,P01681,P97580,Q8CIZ5,Q9WUW8
105	1,2,3,4,5,8,13,15	6,7,9,10,11,12,14,16	10	P00774,P01681,P19132,P21674,Q498D9,Q63618,Q6IG05,Q6Q0N1,Q8CIZ5,Q9WUW8
106	1,2,3,4,5,8,13,16	6,7,9,10,11,12,14,15	10	B0BNN3,P00762,P00774,P19223,P20760,P46844,P97580,Q30KJ2,Q8CIZ5,Q9WUW8
107	1,2,3,4,5,8,14,15	6,7,9,10,11,12,13,16	7	P01681,P08649,P19132,P54921,P97580,Q6IG05,Q9WUW8
108	1,2,3,4,5,8,14,16	6,7,9,10,11,12,13,15	10	B0BNN3,P01681,P08649,P20760,P20762,P97580,Q30KJ2,Q4KLZ6,Q5RLM2,Q9WUW8
109	1,2,3,4,5,8,15,16	6,7,9,10,11,12,13,14	13	O70594,P01681,P08649,P20760,P20762,P46844,P48508,P97580,Q30KJ2,Q63270,Q6IG05,Q6Q0N1,Q9WUW8
110	1,2,3,4,5,9,10,11	6,7,8,12,13,14,15,16	3	P70549,Q4KLZ6,Q9Z2L0
111	1,2,3,4,5,9,10,12	6,7,8,11,13,14,15,16	2	Q03191,Q99041
112	1,2,3,4,5,9,10,13	6,7,8,11,12,14,15,16	2	Q9QW07,Q9QZK9
113	1,2,3,4,5,9,10,14	6,7,8,11,12,13,15,16	1	P30120
114	1,2,3,4,5,9,10,15	6,7,8,11,12,13,14,16	5	P01681,P30120,P46462,Q8CFN2,Q99041
115	1,2,3,4,5,9,10,16	6,7,8,11,12,13,14,15	0	
116	1,2,3,4,5,9,11,12	6,7,8,10,13,14,15,16	2	Q03191,Q4KLZ6
117	1,2,3,4,5,9,11,13	6,7,8,10,12,14,15,16	1	Q4KLZ6
118	1,2,3,4,5,9,11,14	6,7,8,10,12,13,15,16	3	P08649,P70549,Q4KLZ6
119	1,2,3,4,5,9,11,15	6,7,8,10,12,13,14,16	8	P01681,Q4FZU2,Q4KLZ6,Q6IFU8,Q6IFW6,Q6IG02,Q6IMF3,Q6P6Q2
120	1,2,3,4,5,9,11,16	6,7,8,10,12,13,14,15	3	P08649,P70549,Q4KLZ6
121	1,2,3,4,5,9,12,13	6,7,8,10,11,14,15,16	2	Q03191,Q99041
122	1,2,3,4,5,9,12,14	6,7,8,10,11,13,15,16	1	Q03191

123	1,2,3,4,5,9,12,15	6,7,8,10,11,13,14,16	4	P30120,P34901,P36376,Q99041
124	1,2,3,4,5,9,12,16	6,7,8,10,11,13,14,15	2	Q03191,Q99041
125	1,2,3,4,5,9,13,14	6,7,8,10,11,12,15,16	1	Q9QW07
126	1,2,3,4,5,9,13,15	6,7,8,10,11,12,14,16	2	P21674,P62804
127	1,2,3,4,5,9,13,16	6,7,8,10,11,12,14,15	0	
128	1,2,3,4,5,9,14,15	6,7,8,10,11,12,13,16	2	P01681,P30120
129	1,2,3,4,5,9,14,16	6,7,8,10,11,12,13,15	2	P23593,Q4KLZ6
130	1,2,3,4,5,9,15,16	6,7,8,10,11,12,13,14	1	P29315
131	1,2,3,4,5,10,11,12	6,7,8,9,13,14,15,16	4	P47967,P97840,Q4KLZ6,Q5I0D1
132	1,2,3,4,5,10,11,13	6,7,8,9,12,14,15,16	2	P47967,Q4KLZ6
133	1,2,3,4,5,10,11,14	6,7,8,9,12,13,15,16	1	Q4KLZ6
134	1,2,3,4,5,10,11,15	6,7,8,9,12,13,14,16	7	Q4FZU2,Q4KLZ6,Q6IFU8,Q6IFW6,Q6IG02,Q6IMF3,Q6P6Q2
135	1,2,3,4,5,10,11,16	6,7,8,9,12,13,14,15	2	P70549,Q4KLZ6
136	1,2,3,4,5,10,12,13	6,7,8,9,11,14,15,16	3	P47967,P97840,Q812E4
137	1,2,3,4,5,10,12,14	6,7,8,9,11,13,15,16	1	Q5RLM2
138	1,2,3,4,5,10,12,15	6,7,8,9,11,13,14,16	5	P10758,P34901,Q498D9,Q812E4,Q99041
139	1,2,3,4,5,10,12,16	6,7,8,9,11,13,14,15	1	P0DMW0;P0DMW1
140	1,2,3,4,5,10,13,14	6,7,8,9,11,12,15,16	2	Q9QW07,Q9WUW8
141	1,2,3,4,5,10,13,15	6,7,8,9,11,12,14,16	5	P21674,Q498D9,Q63618,Q812E4,Q8CFN2
142	1,2,3,4,5,10,13,16	6,7,8,9,11,12,14,15	2	Q63618,Q9WUW8

143	1,2,3,4,5,10,14,15	6,7,8,9,11,12,13,16	2	P30120,P54921
144	1,2,3,4,5,10,14,16	6,7,8,9,11,12,13,15	4	Q4KLZ6,Q5RLM2,Q71MB6,Q9WUW8
145	1,2,3,4,5,10,15,16	6,7,8,9,11,12,13,14	5	P17988,P18297,P19468,P48508,Q71MB6
146	1,2,3,4,5,11,12,13	6,7,8,9,10,14,15,16	4	P47967,P97840,Q4KLZ6,Q5I0D1
147	1,2,3,4,5,11,12,14	6,7,8,9,10,13,15,16	1	Q4KLZ6
148	1,2,3,4,5,11,12,15	6,7,8,9,10,13,14,16	7	Q4FZU2,Q4KLZ6,Q6IFU8,Q6IFW6,Q6IG02,Q6IMF3,Q6P6Q2
149	1,2,3,4,5,11,12,16	6,7,8,9,10,13,14,15	2	P97840,Q4KLZ6
150	1,2,3,4,5,11,13,14	6,7,8,9,10,12,15,16	3	B0BNN3,P35745,Q4KLZ6
151	1,2,3,4,5,11,13,15	6,7,8,9,10,12,14,16	8	P21674,Q4FZU2,Q4KLZ6,Q6IFU8,Q6IFW6,Q6IG02,Q6IMF3,Q6P6Q2
152	1,2,3,4,5,11,13,16	6,7,8,9,10,12,14,15	2	P35745,Q4KLZ6
153	1,2,3,4,5,11,14,15	6,7,8,9,10,12,13,16	8	P08649,Q4FZU2,Q4KLZ6,Q6IFU8,Q6IFW6,Q6IG02,Q6IMF3,Q6P6Q2
154	1,2,3,4,5,11,14,16	6,7,8,9,10,12,13,15	4	P08649,P35745,P70549,Q4KLZ6
155	1,2,3,4,5,11,15,16	6,7,8,9,10,12,13,14	10	P08649,P17988,P29315,Q4FZU2,Q4KLZ6,Q6IFU8,Q6IFW6,Q6IG02,Q6IMF3,Q6P6Q2
156	1,2,3,4,5,12,13,14	6,7,8,9,10,11,15,16	0	
157	1,2,3,4,5,12,13,15	6,7,8,9,10,11,14,16	1	P21674
158	1,2,3,4,5,12,13,16	6,7,8,9,10,11,14,15	1	P97840
159	1,2,3,4,5,12,14,15	6,7,8,9,10,11,13,16	0	
160	1,2,3,4,5,12,14,16	6,7,8,9,10,11,13,15	1	Q4KLZ6
161	1,2,3,4,5,12,15,16	6,7,8,9,10,11,13,14	1	P10758
162	1,2,3,4,5,13,14,15	6,7,8,9,10,11,12,16	1	P21674

163	1,2,3,4,5,13,14,16	6,7,8,9,10,11,12,15	4	B0BNN3,P35745,Q4KLZ6,Q9WUW8
164	1,2,3,4,5,13,15,16	6,7,8,9,10,11,12,14	1	P21674
165	1,2,3,4,5,14,15,16	6,7,8,9,10,11,12,13	1	Q4KLZ6
166	1,2,3,4,6,7,8,9	5,10,11,12,13,14,15,16	48	B1H234,D3ZHA0,D4A5U3,iRT-Kit_WR_fusion,O35763,O55004,O70417,O70594,P00774,P01681,P01836,P02631,P06761,P06911,P18418,P19223,P19629,P20646,P20761,P23739,P31430,P48508,P50115,P50280,P55091,P80299,P97580,P98089,Q05702,Q30KJ2,Q5GRG2,Q5QE79,Q5U2V4,Q62635,Q62946,Q63317,Q63598,Q63618,Q63751,Q6P6R2,Q6Q0N0,Q6Q0N1,Q6TMA8,Q78P75,Q8CIZ5,Q9JI85,Q9QX74,Q9R0T3
167	1,2,3,4,6,7,8,10	5,9,11,12,13,14,15,16	115	D3ZHA0,D4A5U3,O08557,O35763,O55004,O70257,O70377,O70417,O70594,O88339;Q4V882,P00774,P01836,P02454,P02631,P04904,P05371,P06761,P06911,P08937,P11762,P15399,P18418,P18757,P19112,P19223,P19468,P19629,P20646,P20761,P20762,P20766,P23739,P23928,P28570,P29975,P30904,P31044,P31430,P36860,P38918,P46720,P46844,P48037,P48508,P50115,P50280,P51907,P53790,P54921,P55091,P57113,P60711;P63259,P63081,P68035;P68136,P80299,P97580,P98089,Q03248,Q05175,Q05702,Q30KJ2,Q3MIE4,Q3T1J9,Q3ZAV1,Q5BJY9,Q5GRG2,Q5I0E9,Q5M7T9,Q5QE79,Q5RKI0,Q5RLM2,Q5U2Q3,Q62635,Q62687,Q62946,Q63270,Q63317,Q63355,Q63424,Q63598,Q63618,Q64093,Q64319,Q64602,Q66H69,Q66HG3,Q68FT5,Q6AY41,Q6AYS7,Q6AYT0,Q6MG61,Q6Q0N0,Q6Q0N1,Q6TMA8,Q71MB6,Q7TQ94,Q80W57,Q8CIZ5,Q8K3P7,Q8R431,Q923S2,Q99MZ8,Q9JI85,Q9JJ19,Q9JJ40,Q9JLJ3,Q9QX74,Q9QYU4,Q9R0T3,Q9R1T5,Q9WTW7,Q9WUW8,Q9WUW9,Q9Z0W7,Q9Z1F2
168	1,2,3,4,6,7,8,11	5,9,10,12,13,14,15,16	77	B1H234,D3ZHA0,D4A5U3,iRT-Kit_WR_fusion,O35547,O55004,P00774,P02631,P02780,P02781,P02782,P05371,P06761,P06911,P07150,P07647,P08649,P08723,P09456,P0C0A9,P11598,P11762,P12020,P15399,P18418,P18757,P19223,P19629,P20646,P20761,P22273,P22282,P22283,P30904,P31044,P31430,P36374,P40241,P46462,P50115,P50280,P55091,P80299,P97580,P98089,Q05702,Q10758,Q30KJ2,Q4G063,Q4G075,Q5GRG2,Q5M7T9,Q5M8C6,Q5QE79,Q5U2V4,Q62635,Q62902,Q62946,Q63317,Q63493,Q63617,Q66H69,Q6AYR9,Q6IFU8,Q6P6S4,Q6Q0N0,Q6TMA8,Q78P75,Q8CIZ5,Q99041,Q9JHB9,Q9JI85,Q9QW07,Q9QX74,Q9QZK8,Q9R0T3,Q9WTW7
169	1,2,3,4,6,7,8,12	5,9,10,11,13,14,15,16	59	D3ZHA0,D4A5U3,iRT-Kit_WR_fusion,O35763,O55004,O70417,P00774,P01836,P02631,P02781,P05371,P06761,P06911,P11762,P12020,P18418,P18757,P19223,P19629,P20761,P30904,P31044,P31430,P38918,P46844,P48508,P50115,P50280,P55091,P57113,P80299,P97580,P98089,Q05702,Q30KJ2,Q3ZAV1,Q5GRG2,Q5M7T9,Q5QE79,Q5RLM2,Q62635,Q62946,Q63270,Q63317,Q63598,Q63618,Q64602,Q66H69,Q66HG3,Q6MG61,Q6Q0N1,Q6TMA8,Q8CIZ5,Q9JI85,Q9QX74,Q9QYU4,Q9R0T3,Q9WTW7,Q9Z0W7

170	1,2,3,4,6,7,8,13	5,9,10,11,12,14,15,16	64	B0BNN3,B1H234,D3ZHA0,D4A5U3,O55004,O70257,O70377,O70594,P00774,P01836,P02631,P02761,P02781,P05371,P06761,P06911,P15399,P18418,P19223,P19468,P19629,P20761,P30904,P31044,P31430,P36860,P46844,P48508,P50115,P50280,P55091,P57113,P63081,P80299,P97580,P98089,Q05702,Q30KJ2,Q3ZAV1,Q5GRG2,Q5M7T9,Q5QE79,Q62635,Q62714,Q62902,Q62946,Q63317,Q63424,Q63598,Q63618,Q66H69,Q66HG3,Q6AYS7,Q6Q0N0,Q6Q0N1,Q6TMA8,Q8CIZ5,Q9JI85,Q9QX74,Q9R0T3,Q9WTW7,Q9WUW8,Q9WUW9,Q9Z0W7
171	1,2,3,4,6,7,8,14	5,9,10,11,12,13,15,16	64	B0BNN3,B1H234,D3ZHA0,D4A5U3,O55004,O70377,O70417,O70594,P02631,P02761,P06761,P06911,P08937,P11762,P12020,P15399,P18418,P18757,P19223,P19629,P20646,P20761,P20762,P31044,P31430,P46844,P48037,P48508,P50115,P50280,P54921,P55091,P57113,P63081,P80299,P97580,P98089,Q05702,Q30KJ2,Q4G075,Q5GRG2,Q5M7T9,Q5QE79,Q5RLM2,Q62635,Q62714,Q62812,Q62946,Q63317,Q63598,Q63618,Q63751,Q64602,Q66H69,Q66HG3,Q6Q0N1,Q6TMA8,Q8CIZ5,Q923S2,Q9JI85,Q9QX74,Q9R0T3,Q9WUW8,Q9WUW9
172	1,2,3,4,6,7,8,15	5,9,10,11,12,13,14,16	95	B0LT89,B1H234,D3ZHA0,D4A5U3,iRT-Kit_WR_fusion,O08557,O35763,O55004,O70257,O70377,O70594,O88339;Q4V882,P00774,P02091,P02631,P04904,P04916,P06761,P06911,P08721,P09606,P11762,P15399,P17988,P18418,P18757,P19112,P19223,P19468,P19629,P20646,P20761,P20762,P29975,P30904,P31044,P31430,P36860,P38918,P46844,P48508,P50115,P50280,P51907,P53790,P54921,P55091,P57113,P68035;P68136,P80299,P97580,P98089,Q03248,Q05175,Q05702,Q30KJ2,Q3ZAV1,Q498D9,Q5GRG2,Q5I0E9,Q5M7T9,Q5U2Q3,Q62635,Q62714,Q62812,Q62946,Q63270,Q63317,Q63424,Q63598,Q63618,Q64093,Q64602,Q66H69,Q6AYS7,Q6AYT0,Q6MG61,Q6PCU2,Q6Q0N0,Q6Q0N1,Q6TMA8,Q80W57,Q8CIZ5,Q8R431,Q923S2,Q99MZ8,Q9JI85,Q9JLJ3,Q9QX74,Q9QYU4,Q9R0T3,Q9R1T5,Q9WTW7,Q9Z0V6,Q9Z0W7
173	1,2,3,4,6,7,8,16	5,9,10,11,12,13,14,15	114	B0BNA5,B0BNN3,B0LT89,B1H234,D3ZHA0,D4A5U3,O35763,O55004,O70257,O70377,O70594,P00774,P01836,P02631,P02761,P02780,P02781,P02782,P02783,P04904,P05371,P06761,P06911,P07151,P07647,P08723,P09606,P11598,P15399,P18418,P18757,P19112,P19223,P19468,P19629,P20646,P20761,P20762,P22006,P22282,P22283,P23928,P28570,P29975,P31430,P38918,P41562,P46720,P46844,P48037,P48508,P50115,P50280,P52847,P53790,P55091,P57113,P63081,P68035;P68136,P80299,P97580,P98089,Q03248,Q05175,Q05702,Q1WIM3,Q30KJ2,Q3T1J9,Q3ZAV1,Q5GRG2,Q5I0D7,Q5I0E9,Q5M7T9,Q5M8C6,Q5U2Q3,Q62635,Q62812,Q62902,Q62946,Q63270,Q63317,Q63424,Q63598,Q63618,Q64093,Q64602,Q66H69,Q66HG3,Q68FT5,Q6AY33,Q6AY41,Q6AYS7,Q6MG61,Q6Q0N0,Q6Q0N1,Q6TMA8,Q71MB6,Q80W57,Q8CIZ5,Q8R431,Q923S2,Q9JHB9,Q9JI85,Q9JJ40,Q9JLJ3,Q9QX74,Q9QYU4,Q9QZK8,Q9R0T3,Q9R1T5,Q9WTW7,Q9WUW8,Q9WUW9,Q9Z0W7
174	1,2,3,4,6,7,9,10	5,8,11,12,13,14,15,16	10	O70594,P20766,P23739,P36860,P55091,P80299,Q63598,Q63618,Q8K1G0,Q9WVH8
175	1,2,3,4,6,7,9,11	5,8,10,12,13,14,15,16	9	B1H234,P11598,P22283,P55091,P80299,Q5GRG2,Q63493,Q63617,Q9R0T3

176	1,2,3,4,6,7,9,12	5,8,10,11,13,14,15,16	1	P80299
177	1,2,3,4,6,7,9,13	5,8,10,11,12,14,15,16	6	B1H234,P19223,P36860,P55091,P80299,Q5QE79
178	1,2,3,4,6,7,9,14	5,8,10,11,12,13,15,16	4	B1H234,P80299,Q5QE79,Q63751
179	1,2,3,4,6,7,9,15	5,8,10,11,12,13,14,16	4	B1H234,P02091,P08721,P80299
180	1,2,3,4,6,7,9,16	5,8,10,11,12,13,14,15	7	B1H234,P02783,P07647,P22006,P23739,P80299,Q498R7
181	1,2,3,4,6,7,10,11	5,8,9,12,13,14,15,16	13	B1H234,O08557,P08721,P11598,P18757,P20766,P22283,P31430,P48037,P55091,P80299,Q9R0T3,Q9WTW7
182	1,2,3,4,6,7,10,12	5,8,9,11,13,14,15,16	22	P10758,P11762,P18297,P19468,P30904,P36860,P46844,P48508,P55091,P57113,P80299,Q03248,Q5RLM2,Q63270,Q63598,Q63618,Q66HG3,Q6Q0N1,Q71MB6,Q9JJ40,Q9QYU4,Q9WTW7
183	1,2,3,4,6,7,10,13	5,8,9,11,12,14,15,16	29	B1H234,O55004,O70377,O70594,P18297,P19223,P19468,P29975,P36860,P46844,P48508,P51907,P55091,P57113,P80299,Q5I0E9,Q5M7T9,Q63424,Q63598,Q63618,Q64093,Q66HG3,Q6Q0N1,Q80W57,Q923M1,Q9WTW7,Q9WUW8,Q9WUW9,Q9Z0W7
184	1,2,3,4,6,7,10,14	5,8,9,11,12,13,15,16	28	B1H234,O70377,O70594,P18297,P18757,P19223,P19468,P46844,P48037,P48508,P54921,P55091,P57113,P80299,Q5I0E9,Q5QE79,Q5RLM2,Q63598,Q63618,Q63751,Q64602,Q66HG3,Q6Q0N1,Q71MB6,Q80WL1,Q923S2,Q9WUW8,Q9WUW9
185	1,2,3,4,6,7,10,15	5,8,9,11,12,13,14,16	53	O08557,O35763,O70377,O70594,O88339;Q4V882,P02091,P08721,P10758,P17988,P18297,P18757,P19468,P19629,P20766,P29975,P30904,P36860,P38918,P46844,P48508,P51907,P53790,P54921,P55091,P57113,P68035;P68136,P80299,Q03248,Q05175,Q3MIE4,Q5I0E9,Q5M7T9,Q5RKI1,Q63270,Q63424,Q63598,Q63618,Q64093,Q64602,Q6MG61,Q6Q0N1,Q71MB6,Q80W57,Q8K3P7,Q8R431,Q923M1,Q923S2,Q9JJ40,Q9JLJ3,Q9QYU4,Q9R1T5,Q9WTW7,Q9Z0W7
186	1,2,3,4,6,7,10,16	5,8,9,11,12,13,14,15	64	B0LT89,O35763,O55004,O70377,O70594,P02761,P02783,P07647,P17988,P18297,P18757,P19223,P19468,P19629,P20766,P23739,P23928,P28570,P29975,P38918,P46720,P46844,P48037,P48508,P51907,P52847,P53790,P57113,P60711;P63259,P68035;P68136,P80299,Q03248,Q05175,Q1WIM3,Q3T1J9,Q3ZAV1,Q5I0D7,Q5I0E9,Q5M7T9,Q63270,Q63317,Q63424,Q63598,Q63618,Q64093,Q64602,Q66HG3,Q68FT5,Q6MG61,Q6Q0N1,Q71MB6,Q80W57,Q8R431,Q8VI04,Q923M1,Q923S2,Q9JJ40,Q9JLJ3,Q9QYU4,Q9R1T5,Q9WTW7,Q9WUW8,Q9WUW9,Q9Z0W7
187	1,2,3,4,6,7,11,12	5,8,9,10,13,14,15,16	15	B1H234,P07647,P11598,P11762,P22283,P31430,P47967,P55091,P70709,P80299,Q5GRG2,Q63493,Q63617,Q9JI85,Q9R0T3
188	1,2,3,4,6,7,11,13	5,8,9,10,12,14,15,16	13	B0BNN3,B1H234,P05369,P07647,P11598,P22283,P55091,P80299,Q5GRG2,Q63493,Q63617,Q9JI85,Q9R0T3

189	1,2,3,4,6,7,11,14	5,8,9,10,12,13,15,16	8	B1H234,P07647,P11598,P22283,P80299,Q4KLZ6,Q5GRG2,Q63751
190	1,2,3,4,6,7,11,15	5,8,9,10,12,13,14,16	12	B1H234,O08557,P02091,P08721,P11598,P17988,P22283,P80299,Q5GRG2,Q6IFW6,Q6IG02,Q8CJ52
191	1,2,3,4,6,7,11,16	5,8,9,10,12,13,14,15	25	B1H234,O35547,P02761,P02780,P02781,P02782,P02783,P05369,P07647,P08723,P0C0A9,P11598,P17988,P22006,P22283,P80299,P81556,Q4KLZ6,Q5GRG2,Q63493,Q63617,Q9JHB9,Q9JI85,Q9R0T3,Q9WTW7
192	1,2,3,4,6,7,12,13	5,8,9,10,11,14,15,16	6	B1H234,P01835,P11762,P36860,P47967,P80299
193	1,2,3,4,6,7,12,14	5,8,9,10,11,13,15,16	5	P11762,P80299,Q5RLM2,Q63751,Q80WL1
194	1,2,3,4,6,7,12,15	5,8,9,10,11,13,14,16	7	P08721,P10758,P11762,P17988,P19629,P80299,Q63598
195	1,2,3,4,6,7,12,16	5,8,9,10,11,13,14,15	11	P02783,P07647,P11762,P18297,P46844,P80299,Q03248,Q5I0D7,Q63598,Q71MB6,Q9WTW7
196	1,2,3,4,6,7,13,14	5,8,9,10,11,12,15,16	8	B0BNN3,B1H234,P01835,P02761,P19223,P80299,Q5QE79,Q9WUW8
197	1,2,3,4,6,7,13,15	5,8,9,10,11,12,14,16	9	B1H234,O70594,P01835,P08721,P19468,P36860,P80299,Q63618,Q6Q0N1
198	1,2,3,4,6,7,13,16	5,8,9,10,11,12,14,15	21	B0BNN3,B1H234,O70594,P01835,P02761,P02783,P07647,P18297,P19223,P19468,P22006,P28570,P46844,P57113,P80299,Q1WIM3,Q5I0D7,Q63424,Q6AYQ8,Q6Q0N1,Q9WUW8
199	1,2,3,4,6,7,14,15	5,8,9,10,11,12,13,16	9	B0LT89,B1H234,P02091,P08721,P11762,P17988,P54921,P80299,Q62714
200	1,2,3,4,6,7,14,16	5,8,9,10,11,12,13,15	18	B0BNN3,B0LT89,B1H234,P02761,P02783,P07647,P18297,P19223,P22006,P46844,P48037,P57113,P80299,Q4KLZ6,Q62812,Q63317,Q71MB6,Q9WUW8
201	1,2,3,4,6,7,15,16	5,8,9,10,11,12,13,14	36	B0LT89,B1H234,O70594,P02091,P02761,P02783,P07647,P08721,P10758,P17988,P18297,P19468,P19629,P22006,P29975,P38918,P46844,P48508,P53790,P57113,P80299,Q03248,Q5I0D7,Q5I0E9,Q5M7T9,Q63270,Q63317,Q63424,Q63598,Q6AYQ8,Q6Q0N1,Q71MB6,Q80W57,Q9R1T5,Q9WTW7,Q9Z0W7
202	1,2,3,4,6,8,9,10	5,7,11,12,13,14,15,16	20	O55004,O70594,P00774,P01681,P02454,P02631,P15399,P17559,P18418,P19629,P23739,P55091,P70545,P97580,Q05702,Q06000,Q30KJ2,Q63598,Q63618,Q8CIZ5
203	1,2,3,4,6,8,9,11	5,7,10,12,13,14,15,16	17	D4A5U3,P00774,P01681,P06911,P15399,P31430,P50280,P55091,P97580,Q05702,Q10758,Q30KJ2,Q5GRG2,Q63493,Q6P6S4,Q8CIZ5,Q9JI85
204	1,2,3,4,6,8,9,12	5,7,10,11,13,14,15,16	9	P00774,P06911,P97580,P98089,Q05702,Q09030,Q30KJ2,Q8CIZ5,Q9JI85
205	1,2,3,4,6,8,9,13	5,7,10,11,12,14,15,16	10	D4A5U3,P00774,P19223,P55091,P97580,Q05702,Q09030,Q30KJ2,Q8CIZ5,Q9JI85

206	1,2,3,4,6,8,9,14	5,7,10,11,12,13,15,16	13	D4A5U3,O70417,P00774,P01681,P15399,P23593,P97580,Q05702,Q09030,Q30KJ2,Q62635,Q63751,Q8CIZ5
207	1,2,3,4,6,8,9,15	5,7,10,11,12,13,14,16	11	P00774,P01681,P02091,P15399,P19629,P23593,P36376,P97580,Q05702,Q30KJ2,Q99MH3
208	1,2,3,4,6,8,9,16	5,7,10,11,12,13,14,15	14	D4A5U3,P00774,P02783,P07151,P15399,P20761,P22006,P23593,P50280,P97580,Q05702,Q09030,Q30KJ2,Q498R7
209	1,2,3,4,6,8,10,11	5,7,9,12,13,14,15,16	21	O55004,P05371,P06911,P15399,P18418,P19629,P31430,P50280,P55091,P63029,P97580,Q05702,Q10758,Q30KJ2,Q5BJY9,Q5GRG2,Q63493,Q68G31,Q6P6S4,Q8CIZ5,Q9JI85
210	1,2,3,4,6,8,10,12	5,7,9,11,13,14,15,16	21	O55004,O70377,P02631,P11762,P15399,P19629,P30904,P46844,P48508,P55091,P97580,P98089,Q05702,Q30KJ2,Q498D9,Q63598,Q63618,Q66HG3,Q6Q0N1,Q8CIZ5,Q9JI85
211	1,2,3,4,6,8,10,13	5,7,9,11,12,14,15,16	29	O55004,O70257,O70377,O70594,O88339;Q4V882,P02631,P05371,P15399,P19223,P19468,P30904,P36860,P46844,P48508,P51907,P55091,P97580,Q05702,Q30KJ2,Q498D9,Q63424,Q63598,Q63618,Q66HG3,Q68G31,Q6Q0N1,Q8CIZ5,Q9JI85,Q9WUW8
212	1,2,3,4,6,8,10,14	5,7,9,11,12,13,15,16	25	O55004,O70377,O70417,O70594,O88339;Q4V882,P15399,P20762,P46844,P48508,P54921,P55091,P57113,P97580,Q05702,Q06000,Q30KJ2,Q5RLM2,Q63618,Q63751,Q68G31,Q6Q0N1,Q8CIZ5,Q923S2,Q9WUW8,Q9WUW9
213	1,2,3,4,6,8,10,15	5,7,9,11,12,13,14,16	43	O35763,O55004,O70377,O70594,O88339;Q4V882,P02091,P15399,P19468,P19629,P20762,P30904,P38918,P46844,P48508,P50280,P51907,P53790,P54921,P55091,P68035;P68136,P97580,Q03248,Q05175,Q05702,Q30KJ2,Q3ZAV1,Q498D9,Q5I0E9,Q5M7T9,Q63270,Q63424,Q63598,Q63618,Q6MG61,Q6Q0N1,Q80W57,Q8CIZ5,Q8R431,Q923S2,Q9JLJ3,Q9R1T5,Q9WTW7,Q9Z0W7
214	1,2,3,4,6,8,10,16	5,7,9,11,12,13,14,15	61	O35763,O55004,O70257,O70377,O70594,O88339;Q4V882,P02631,P02783,P05371,P07151,P15399,P18418,P18757,P19223,P19468,P19629,P20761,P20762,P28570,P29975,P38918,P46844,P48508,P50280,P51907,P53790,P57113,P68035;P68136,P97580,Q03248,Q05175,Q05702,Q30KJ2,Q3T1J9,Q3ZAV1,Q5BJY9,Q5I0E9,Q5M7T9,Q5U2Q3,Q63270,Q63424,Q63598,Q63618,Q64093,Q64602,Q6AY41,Q6MG61,Q6Q0N1,Q71MB6,Q80W57,Q8R431,Q923S2,Q9JI85,Q9JJ40,Q9JLJ3,Q9QYU4,Q9R1T5,Q9WTW7,Q9WUW8,Q9WUW9,Q9Z0W7
215	1,2,3,4,6,8,11,12	5,7,9,10,13,14,15,16	18	P00774,P06911,P11762,P12020,P15399,P31430,P40241,P50280,P55091,P97580,P98089,Q05702,Q10758,Q30KJ2,Q5GRG2,Q63493,Q8CIZ5,Q9JI85
216	1,2,3,4,6,8,11,13	5,7,9,10,12,14,15,16	22	B0BNN3,D4A5U3,P00774,P02781,P06911,P08723,P12020,P15399,P31430,P40241,P50280,P55091,P97580,Q05702,Q10758,Q30KJ2,Q5GRG2,Q62902,Q63493,Q68G31,Q8CIZ5,Q9JI85
217	1,2,3,4,6,8,11,14	5,7,9,10,12,13,15,16	17	B0BNN3,P06911,P12020,P15399,P31430,P50280,P63029,P97580,Q10758,Q30KJ2,Q4KLZ6,Q5GRG2,Q62635,Q63493,Q63751,Q8CIZ5,Q9JI85
218	1,2,3,4,6,8,11,15	5,7,9,10,12,13,14,16	24	P00774,P02091,P06911,P15399,P19629,P31430,P50280,P97580,Q05702,Q10758,Q30KJ2,Q4FZU2,Q5GRG2,Q63493,Q6IFU7,Q6IFU8,Q6IFW6,Q6IG02,Q6IMF3,Q6P6Q2,Q6P6S4,Q8CIZ5,Q8CJ52,Q9JI85

219	1,2,3,4,6,8,11,16	5,7,9,10,12,13,14,15	35	O35547,P00762,P00774,P02780,P02781,P02782,P02783,P05369,P06911,P07151,P07647,P08723,P09456,P0C0A9,P12020,P15399,P20761,P22282,P30120,P31430,P36374,P40241,P46462,P50280,P97580,Q05702,Q10758,Q30KJ2,Q4KLZ6,Q5GRG2,Q5M8C6,Q62902,Q63493,Q9JHB9,Q9JI85
220	1,2,3,4,6,8,12,13	5,7,9,10,11,14,15,16	10	P00774,P02631,P06911,P11762,P97580,P98089,Q05702,Q30KJ2,Q8CIZ5,Q9JI85
221	1,2,3,4,6,8,12,14	5,7,9,10,11,13,15,16	14	O70417,P06911,P11762,P15399,P97580,P98089,Q05702,Q30KJ2,Q5GRG2,Q5RLM2,Q62635,Q63751,Q8CIZ5,Q9JI85
222	1,2,3,4,6,8,12,15	5,7,9,10,11,13,14,16	13	P00774,P02091,P11762,P15399,P19629,P36376,P97580,P98089,Q05702,Q30KJ2,Q498D9,Q63598,Q8CIZ5
223	1,2,3,4,6,8,12,16	5,7,9,10,11,13,14,15	17	O55004,P00774,P02783,P06911,P07151,P07647,P11762,P15399,P19629,P46844,P50280,P97580,P98089,Q05702,Q30KJ2,Q63598,Q9JI85
224	1,2,3,4,6,8,13,14	5,7,9,10,11,12,15,16	12	B0BNN3,D4A5U3,P15399,P19223,P97580,Q30KJ2,Q62635,Q68G31,Q8CIZ5,Q9JI85,Q9QWN8,Q9WUW8
225	1,2,3,4,6,8,13,15	5,7,9,10,11,12,14,16	14	O55004,P00774,P02091,P15399,P19629,P97580,Q05702,Q30KJ2,Q498D9,Q63618,Q68G31,Q6Q0N1,Q8CIZ5,Q9JI85
226	1,2,3,4,6,8,13,16	5,7,9,10,11,12,14,15	20	B0BNN3,D4A5U3,O55004,O70257,P00762,P00774,P02783,P07151,P07647,P15399,P19223,P20761,P46844,P97580,Q05702,Q30KJ2,Q6Q0N1,Q8CIZ5,Q9JI85,Q9WUW8
227	1,2,3,4,6,8,14,15	5,7,9,10,11,12,13,16	8	P02091,P15399,P50280,P54921,P97580,Q05702,Q30KJ2,Q8CIZ5
228	1,2,3,4,6,8,14,16	5,7,9,10,11,12,13,15	15	B0BNN3,P02783,P07151,P07647,P15399,P20761,P46844,P50280,P97580,Q05702,Q30KJ2,Q4KLZ6,Q62635,Q9JI85,Q9WUW8
229	1,2,3,4,6,8,15,16	5,7,9,10,11,12,13,14	26	O55004,O70377,O70594,P02091,P02783,P07151,P15399,P17988,P19468,P19629,P20761,P46844,P48508,P50280,P53790,P97580,Q05702,Q30KJ2,Q5M7T9,Q5U2Q3,Q63270,Q63598,Q6Q0N1,Q9R1T5,Q9WTW7,Q9Z0W7
230	1,2,3,4,6,9,10,11	5,7,8,12,13,14,15,16	0	
231	1,2,3,4,6,9,10,12	5,7,8,11,13,14,15,16	0	
232	1,2,3,4,6,9,10,13	5,7,8,11,12,14,15,16	3	P80299,Q06000,Q8K1G0
233	1,2,3,4,6,9,10,14	5,7,8,11,12,13,15,16	2	Q06000,Q8K1G0
234	1,2,3,4,6,9,10,15	5,7,8,11,12,13,14,16	4	P02091,Q811M5,Q8K1G0,Q99MH3
235	1,2,3,4,6,9,10,16	5,7,8,11,12,13,14,15	6	D3ZUC6,P02783,P22006,Q498R7,Q811M5,Q8K1G0
236	1,2,3,4,6,9,11,12	5,7,8,10,13,14,15,16	1	O35077
237	1,2,3,4,6,9,11,13	5,7,8,10,12,14,15,16	0	

238	1,2,3,4,6,9,11,14	5,7,8,10,12,13,15,16	2	P0DMW0;P0DMW1,Q4KLZ6
239	1,2,3,4,6,9,11,15	5,7,8,10,12,13,14,16	2	P02091,Q99MH3
240	1,2,3,4,6,9,11,16	5,7,8,10,12,13,14,15	5	D3ZUC6,P02783,P07647,P22006,Q4KLZ6
241	1,2,3,4,6,9,12,13	5,7,8,10,11,14,15,16	0	
242	1,2,3,4,6,9,12,14	5,7,8,10,11,13,15,16	0	
243	1,2,3,4,6,9,12,15	5,7,8,10,11,13,14,16	1	Q99MH3
244	1,2,3,4,6,9,12,16	5,7,8,10,11,13,14,15	5	D3ZUC6,P02783,P22006,Q09030,Q498R7
245	1,2,3,4,6,9,13,14	5,7,8,10,11,12,15,16	1	Q09030
246	1,2,3,4,6,9,13,15	5,7,8,10,11,12,14,16	1	P62804
247	1,2,3,4,6,9,13,16	5,7,8,10,11,12,14,15	6	D3ZUC6,P02783,P22006,P23593,Q09030,Q498R7
248	1,2,3,4,6,9,14,15	5,7,8,10,11,12,13,16	1	P02091
249	1,2,3,4,6,9,14,16	5,7,8,10,11,12,13,15	6	P02783,P22006,P23593,Q09030,Q498R7,Q4KLZ6
250	1,2,3,4,6,9,15,16	5,7,8,10,11,12,13,14	6	D3ZUC6,P02091,P02783,P22006,P23593,Q498R7
251	1,2,3,4,6,10,11,12	5,7,8,9,13,14,15,16	1	P11762
252	1,2,3,4,6,10,11,13	5,7,8,9,12,14,15,16	1	Q68G31
253	1,2,3,4,6,10,11,14	5,7,8,9,12,13,15,16	1	Q4KLZ6
254	1,2,3,4,6,10,11,15	5,7,8,9,12,13,14,16	2	P02091,Q6IG02
255	1,2,3,4,6,10,11,16	5,7,8,9,12,13,14,15	3	P02783,P07647,Q4KLZ6
256	1,2,3,4,6,10,12,13	5,7,8,9,11,14,15,16	2	P11762,P80299
257	1,2,3,4,6,10,12,14	5,7,8,9,11,13,15,16	1	P11762

258	1,2,3,4,6,10,12,15	5,7,8,9,11,13,14,16	5	P02091,P10758,P11762,Q498D9,Q63598
259	1,2,3,4,6,10,12,16	5,7,8,9,11,13,14,15	5	P02783,P18297,P33436,P46844,Q71MB6
260	1,2,3,4,6,10,13,14	5,7,8,9,11,12,15,16	2	O54800;Q5DWV2,Q06000
261	1,2,3,4,6,10,13,15	5,7,8,9,11,12,14,16	4	P02091,Q498D9,Q63618,Q923M1
262	1,2,3,4,6,10,13,16	5,7,8,9,11,12,14,15	7	P02783,P18297,P19468,P28570,P46844,Q63424,Q63618
263	1,2,3,4,6,10,14,15	5,7,8,9,11,12,13,16	3	P01946,P02091,P54921
264	1,2,3,4,6,10,14,16	5,7,8,9,11,12,13,15	5	P02783,P18297,Q4KLZ6,Q71MB6,Q9QYP1
265	1,2,3,4,6,10,15,16	5,7,8,9,11,12,13,14	18	O70377,O70594,P02091,P02783,P17988,P18297,P19468,P46844,P48508,P53790,Q03248,Q63424,Q63598,Q6Q0N1,Q71MB6,Q923M1,Q9QYP1,Q9WTW7
266	1,2,3,4,6,11,12,13	5,7,8,9,10,14,15,16	1	P11762
267	1,2,3,4,6,11,12,14	5,7,8,9,10,13,15,16	3	P11762,Q4KLZ6,Q5GRG2
268	1,2,3,4,6,11,12,15	5,7,8,9,10,13,14,16	2	P11762,Q6IG02
269	1,2,3,4,6,11,12,16	5,7,8,9,10,13,14,15	5	O35547,P02783,P05369,P07647,Q4KLZ6
270	1,2,3,4,6,11,13,14	5,7,8,9,10,12,15,16	3	B0BNN3,P35745,Q4KLZ6
271	1,2,3,4,6,11,13,15	5,7,8,9,10,12,14,16	2	P21674,Q6IG02
272	1,2,3,4,6,11,13,16	5,7,8,9,10,12,14,15	5	P02783,P05369,P07647,P35745,Q4KLZ6
273	1,2,3,4,6,11,14,15	5,7,8,9,10,12,13,16	3	P02091,Q4KLZ6,Q6IG02
274	1,2,3,4,6,11,14,16	5,7,8,9,10,12,13,15	4	P02783,P07647,P35745,Q4KLZ6
275	1,2,3,4,6,11,15,16	5,7,8,9,10,12,13,14	7	P02091,P02783,P07647,P17988,P29315,Q4KLZ6,Q6IG02
276	1,2,3,4,6,12,13,14	5,7,8,9,10,11,15,16	2	P11762,P49134
277	1,2,3,4,6,12,13,15	5,7,8,9,10,11,14,16	1	P11762

278	1,2,3,4,6,12,13,16	5,7,8,9,10,11,14,15	1	P02783
279	1,2,3,4,6,12,14,15	5,7,8,9,10,11,13,16	1	P11762
280	1,2,3,4,6,12,14,16	5,7,8,9,10,11,13,15	2	P02783,Q4KLZ6
281	1,2,3,4,6,12,15,16	5,7,8,9,10,11,13,14	3	P02783,P10758,P17988
282	1,2,3,4,6,13,14,15	5,7,8,9,10,11,12,16	1	P49134
283	1,2,3,4,6,13,14,16	5,7,8,9,10,11,12,15	4	B0BNN3,P02783,P35745,Q4KLZ6
284	1,2,3,4,6,13,15,16	5,7,8,9,10,11,12,14	1	P02783
285	1,2,3,4,6,14,15,16	5,7,8,9,10,11,12,13	4	P02091,P02783,P17988,Q4KLZ6
286	1,2,3,4,7,8,9,10	5,6,11,12,13,14,15,16	25	D4A5U3,O35763,O55004,O70377,O70417,O70594,P00774,P01681,P19223,P20646,P20761,P20766,P23739,P70545,P80299,P97580,Q30KJ2,Q63598,Q63618,Q64093,Q66H69,Q6P6R2,Q6Q0N1,Q8CIZ5,Q9Z1F2
287	1,2,3,4,7,8,9,11	5,6,10,12,13,14,15,16	18	D4A5U3,P00774,P01681,P02780,P08649,P08723,P20646,P35280,P40241,P50280,Q63493,Q66H69,Q6IFU8,Q6P6R2,Q6P6S4,Q812E4,Q8CIZ5,Q9JHB9
288	1,2,3,4,7,8,9,12	5,6,10,11,13,14,15,16	8	D4A5U3,iRT-Kit_WR_fusion,O70417,P00774,P01681,P20646,Q66H69,Q8CIZ5
289	1,2,3,4,7,8,9,13	5,6,10,11,12,14,15,16	10	B0BNN3,D4A5U3,P00774,P01681,P19223,P20646,P20761,P80299,Q66H69,Q8CIZ5
290	1,2,3,4,7,8,9,14	5,6,10,11,12,13,15,16	14	B0BNN3,D4A5U3,O70417,P00774,P01681,P08649,P19223,P20646,P20761,P97580,Q5QE79,Q62635,Q63751,Q8CIZ5
291	1,2,3,4,7,8,9,15	5,6,10,11,12,13,14,16	8	D4A5U3,P00774,P01681,P20646,P20761,P35280,Q8CIZ5,Q9Z0V6
292	1,2,3,4,7,8,9,16	5,6,10,11,12,13,14,15	13	B0BNN3,D4A5U3,P00774,P01681,P19223,P20646,P20761,P23593,P97580,Q30KJ2,Q63317,Q6TMA8,Q8CIZ5
293	1,2,3,4,7,8,10,11	5,6,9,12,13,14,15,16	27	D4A5U3,O55004,P00774,P02780,P02781,P08723,P18757,P19223,P20646,P20761,P20766,P30904,P40241,P50280,P97580,Q10758,Q30KJ2,Q5BJY9,Q63424,Q63493,Q64093,Q66H69,Q6B345,Q6IFU8,Q8CIZ5,Q9JHB9,Q9WTW7

294	1,2,3,4,7,8,10,12	5,6,9,11,13,14,15,16	36	D4A5U3,O35763,O55004,O70377,O70417,P00774,P10758,P19468,P20766,P30904,P46844,P48508,P53790,P80299,P97580,Q05175,Q30KJ2,Q3ZAV1,Q5M7T9,Q5RLM2,Q63424,Q63598,Q63618,Q64093,Q66H69,Q66HG3,Q6MG61,Q6Q0N1,Q80W57,Q8CIZ5,Q923S2,Q923V8,Q9JJ40,Q9WTW7,Q9Z0W7,Q9Z1F2
295	1,2,3,4,7,8,10,13	5,6,9,11,12,14,15,16	47	B0BNN3,D4A5U3,O35763,O55004,O70257,O70377,O70594,O88339;Q4V882,P00774,P19223,P19468,P20646,P20761,P25809,P28570,P30904,P46844,P48508,P51907,P53790,P57113,P80299,P97580,Q05175,Q30KJ2,Q3ZAV1,Q5I0E9,Q5M7T9,Q62687,Q63355,Q63424,Q63598,Q63618,Q64093,Q66H69,Q6AYQ8,Q6MG61,Q6Q0N1,Q80W57,Q8CIZ5,Q8R431,Q9JJ40,Q9WTW7,Q9WUW8,Q9WUW9,Q9Z0W7,Q9Z1F2
296	1,2,3,4,7,8,10,14	5,6,9,11,12,13,15,16	40	B0BNN3,D4A5U3,O55004,O70377,O70417,O70594,P18757,P19223,P19468,P20646,P20761,P20762,P46844,P48508,P53790,P54921,P57113,P97580,Q05175,Q30KJ2,Q5I0E9,Q5RLM2,Q62635,Q63355,Q63424,Q63598,Q63618,Q63751,Q64093,Q64319,Q64602,Q66H69,Q6Q0N1,Q80W57,Q8CIZ5,Q923S2,Q9WTW7,Q9WUW8,Q9WUW9,Q9Z1F2
297	1,2,3,4,7,8,10,15	5,6,9,11,12,13,14,16	59	O35763,O55004,O70377,O70594,O88339;Q4V882,P01681,P10536,P10758,P18757,P19223,P19468,P20646,P20761,P20762,P20766,P29975,P30904,P38918,P46844,P48508,P51907,P53790,P54921,P57113,P68035;P68136,P97580,Q03248,Q05175,Q30KJ2,Q3MIE4,Q3ZAV1,Q5I0E9,Q5M7T9,Q5RKI1,Q62687,Q63270,Q63355,Q63424,Q63598,Q63618,Q64093,Q64319,Q64602,Q66H69,Q6AY41,Q6MG61,Q6Q0N1,Q80W57,Q8CIZ5,Q8R431,Q923S2,Q9JJ19,Q9JJ40,Q9R1T5,Q9WTW7,Q9WUW9,Q9Z0V6,Q9Z0W7,Q9Z1F2
298	1,2,3,4,7,8,10,16	5,6,9,11,12,13,14,15	68	B0BNN3,D4A5U3,O35763,O55004,O70257,O70377,O70594,P02761,P07151,P18757,P19223,P19468,P20646,P20761,P20762,P20766,P28570,P29975,P38918,P46413,P46720,P46844,P48508,P51907,P53790,P57113,P68035;P68136,P97580,P97608,Q03248,Q05175,Q30KJ2,Q3T1J9,Q3ZAV1,Q5BJY9,Q5I0E9,Q5M7T9,Q5RLM2,Q62687,Q63270,Q63317,Q63355,Q63424,Q63598,Q63618,Q64093,Q64319,Q64602,Q66H69,Q6AY41,Q6AYQ8,Q6MG61,Q6Q0N1,Q6TMA8,Q71MB6,Q80W57,Q8CIZ5,Q8R431,Q923S2,Q9JJ19,Q9JJ40,Q9JLJ3,Q9QYU4,Q9R1T5,Q9WTW7,Q9WUW8,Q9WUW9,Q9Z0W7
299	1,2,3,4,7,8,11,12	5,6,9,10,13,14,15,16	26	D4A5U3,iRT-Kit_WR_fusion,P00774,P02780,P02781,P02782,P06911,P08723,P22282,P31430,P36374,P40241,P47967,P50280,P70709,Q5GRG2,Q5I0D1,Q5M8C6,Q5PQL7,Q63493,Q66H69,Q6IFU8,Q811M5,Q8CIZ5,Q9JHB9,Q9JI85
300	1,2,3,4,7,8,11,13	5,6,9,10,12,14,15,16	27	B0BNN3,D4A5U3,P00774,P02780,P02781,P02782,P06911,P08649,P08723,P19223,P20646,P20761,P22282,P40241,P47967,P80299,Q10758,Q4G075,Q5M8C6,Q62902,Q63493,Q66H69,Q6AYQ8,Q6IFU8,Q811M5,Q8CIZ5,Q9JI85
301	1,2,3,4,7,8,11,14	5,6,9,10,12,13,15,16	25	B0BNN3,D4A5U3,O70417,P02780,P02781,P06911,P08649,P08723,P19223,P20646,P20761,P50280,P97580,Q10758,Q4G075,Q5GRG2,Q5M8C6,Q62635,Q63493,Q63751,Q66H69,Q6IFU8,Q811M5,Q8CIZ5,Q9JHB9
302	1,2,3,4,7,8,11,15	5,6,9,10,12,13,14,16	26	B0BNN3,D4A5U3,P00774,P01681,P02780,P02781,P08649,P08723,P20646,P20761,P40241,P50280,Q10758,Q4FZU2,Q4G075,Q63493,Q66H69,Q6IFU7,Q6IFU8,Q6IFW6,Q6IG02,Q6IMF3,Q6P6Q2,Q8CIZ5,Q8CJ52,Q9WTW7

303	1,2,3,4,7,8,11,16	5,6,9,10,12,13,14,15	47	B0BNN3,D4A5U3,O35547,P00762,P00774,P02761,P02780,P02781,P02782,P04905,P06911,P07647,P08010,P08649,P08723,P09456,P0C0A9,P16636,P19223,P20646,P20761,P22282,P22283,P24368,P30120,P36374,P40241,P46462,P50280,P97580,Q00715,Q10758,Q30KJ2,Q4FZU2,Q4G075,Q5M8C6,Q62902,Q63493,Q66H69,Q6AYQ8,Q6IFU8,Q6TMA8,Q812E4,Q8CIZ5,Q9JHB9,Q9JI85,Q9WTW7
304	1,2,3,4,7,8,12,13	5,6,9,10,11,14,15,16	11	B0BNN3,D4A5U3,iRT-Kit_WR_fusion,P00774,P19223,P47967,P80299,Q66H69,Q811M5,Q8CIZ5,Q923V8
305	1,2,3,4,7,8,12,14	5,6,9,10,11,13,15,16	11	B0BNN3,D4A5U3,O70417,P20761,P97580,Q5RLM2,Q62635,Q63751,Q66H69,Q811M5,Q8CIZ5
306	1,2,3,4,7,8,12,15	5,6,9,10,11,13,14,16	10	iRT-Kit_WR_fusion,P00774,P10758,P20646,P30904,Q63598,Q66H69,Q6Q0N1,Q8CIZ5,Q9WTW7
307	1,2,3,4,7,8,12,16	5,6,9,10,11,13,14,15	22	B0BNN3,D4A5U3,iRT-Kit_WR_fusion,P00762,P00774,P02781,P10758,P20646,P20761,P46844,P97580,Q30KJ2,Q63317,Q63598,Q64093,Q66H69,Q6Q0N1,Q6TMA8,Q8CIZ5,Q9JHB9,Q9JJ40,Q9WTW7
308	1,2,3,4,7,8,13,14	5,6,9,10,11,12,15,16	16	B0BNN3,D4A5U3,P02761,P19223,P20646,P20761,P25809,P80299,P97580,Q62635,Q66H69,Q6TMA8,Q811M5,Q8CIZ5,Q9WUW8,Q9Z2L0
309	1,2,3,4,7,8,13,15	5,6,9,10,11,12,14,16	22	B0BNN3,D4A5U3,O70377,O70594,P00774,P04916,P19223,P19468,P20646,P20761,P46844,P80299,P97580,Q63598,Q63618,Q64093,Q66H69,Q6AYQ8,Q6Q0N1,Q8CIZ5,Q9WTW7,Q9WUW8
310	1,2,3,4,7,8,13,16	5,6,9,10,11,12,14,15	35	B0BNN3,D4A5U3,O55004,O70257,O70594,P00762,P00774,P02761,P02781,P02782,P07151,P19223,P19468,P20646,P20761,P28570,P46844,P80299,P97580,Q30KJ2,Q3ZAV1,Q62902,Q63317,Q63424,Q63618,Q64093,Q66H69,Q6AYQ8,Q6Q0N1,Q6TMA8,Q8CIZ5,Q9WTW7,Q9WUW8,Q9WUW9,Q9Z0W7
311	1,2,3,4,7,8,14,15	5,6,9,10,11,12,13,16	16	B0BNN3,D4A5U3,O70377,P01681,P08649,P20646,P20761,P20762,P37996,P54921,P97580,Q62635,Q6Q0N1,Q6TMA8,Q8CIZ5,Q9WUW8
312	1,2,3,4,7,8,14,16	5,6,9,10,11,12,13,15	26	B0BNN3,D3ZHA0,D4A5U3,O70417,P02761,P02781,P08649,P19223,P19468,P20646,P20761,P20762,P46844,P97580,Q30KJ2,Q5RLM2,Q62635,Q63317,Q64093,Q6AYQ8,Q6Q0N1,Q6TMA8,Q8CIZ5,Q923S2,Q9WUW8,Q9WUW9
313	1,2,3,4,7,8,15,16	5,6,9,10,11,12,13,14	46	B0BNN3,B0LT89,D4A5U3,O70377,O70594,P00774,P02761,P04916,P07151,P08649,P09606,P10758,P17988,P19223,P19468,P20646,P20761,P20762,P46844,P48508,P53790,P97580,Q03248,Q05175,Q30KJ2,Q3ZAV1,Q5I0E9,Q5M7T9,Q63270,Q63317,Q63424,Q63598,Q64093,Q6AYQ8,Q6MG61,Q6Q0N1,Q6TMA8,Q80W57,Q8CIZ5,Q8R431,Q923S2,Q9JJ40,Q9R1T5,Q9WTW7,Q9Z0V6,Q9Z0W7
314	1,2,3,4,7,9,10,11	5,6,8,12,13,14,15,16	2	P20766,P80299
315	1,2,3,4,7,9,10,12	5,6,8,11,13,14,15,16	2	P10758,P80299
316	1,2,3,4,7,9,10,13	5,6,8,11,12,14,15,16	1	P80299
317	1,2,3,4,7,9,10,14	5,6,8,11,12,13,15,16	2	P80299,Q8K1G0

318	1,2,3,4,7,9,10,15	5,6,8,11,12,13,14,16	4	P10758,P80299,Q5RKI1,Q8K1G0
319	1,2,3,4,7,9,10,16	5,6,8,11,12,13,14,15	4	P18297,P20766,P80299,Q8K1G0
320	1,2,3,4,7,9,11,12	5,6,8,10,13,14,15,16	1	P70709
321	1,2,3,4,7,9,11,13	5,6,8,10,12,14,15,16	1	P80299
322	1,2,3,4,7,9,11,14	5,6,8,10,12,13,15,16	1	P0DMW0;P0DMW1
323	1,2,3,4,7,9,11,15	5,6,8,10,12,13,14,16	3	P35280,Q6IFW6,Q8CJ52
324	1,2,3,4,7,9,11,16	5,6,8,10,12,13,14,15	1	Q9R168
325	1,2,3,4,7,9,12,13	5,6,8,10,11,14,15,16	1	P80299
326	1,2,3,4,7,9,12,14	5,6,8,10,11,13,15,16	0	
327	1,2,3,4,7,9,12,15	5,6,8,10,11,13,14,16	1	P10758
328	1,2,3,4,7,9,12,16	5,6,8,10,11,13,14,15	1	P10758
329	1,2,3,4,7,9,13,14	5,6,8,10,11,12,15,16	2	B0BNN3,P80299
330	1,2,3,4,7,9,13,15	5,6,8,10,11,12,14,16	1	P80299
331	1,2,3,4,7,9,13,16	5,6,8,10,11,12,14,15	3	B0BNN3,P80299,Q6AYQ8
332	1,2,3,4,7,9,14,15	5,6,8,10,11,12,13,16	0	
333	1,2,3,4,7,9,14,16	5,6,8,10,11,12,13,15	2	B0BNN3,Q9R168
334	1,2,3,4,7,9,15,16	5,6,8,10,11,12,13,14	2	P10758,Q6AYQ8
335	1,2,3,4,7,10,11,12	5,6,8,9,13,14,15,16	3	P47967,P80299,P97840
336	1,2,3,4,7,10,11,13	5,6,8,9,12,14,15,16	4	B0BNN3,P47967,P80299,Q6AYQ8
337	1,2,3,4,7,10,11,14	5,6,8,9,12,13,15,16	0	

338	1,2,3,4,7,10,11,15	5,6,8,9,12,13,14,16	4	Q5RKI1,Q6IFW6,Q6IG02,Q9WTW7
339	1,2,3,4,7,10,11,16	5,6,8,9,12,13,14,15	6	P07647,P20766,Q63424,Q64093,Q6AYQ8,Q9WTW7
340	1,2,3,4,7,10,12,13	5,6,8,9,11,14,15,16	4	P10758,P47967,P80299,P97840
341	1,2,3,4,7,10,12,14	5,6,8,9,11,13,15,16	2	P80299,Q5RLM2
342	1,2,3,4,7,10,12,15	5,6,8,9,11,13,14,16	7	P10758,P18297,P80299,Q5RKI1,Q63598,Q64093,Q9WTW7
343	1,2,3,4,7,10,12,16	5,6,8,9,11,13,14,15	11	P10758,P18297,P19468,P46844,P80299,Q63424,Q63598,Q64093,Q71MB6,Q9JJ40,Q9WTW7
344	1,2,3,4,7,10,13,14	5,6,8,9,11,12,15,16	3	B0BNN3,P80299,Q9WUW8
345	1,2,3,4,7,10,13,15	5,6,8,9,11,12,14,16	15	P10758,P18297,P19468,P51907,P63095,P80299,Q5RKI1,Q63424,Q63598,Q63618,Q64093,Q6AYQ8,Q6Q0N1,Q923M1,Q9WTW7
346	1,2,3,4,7,10,13,16	5,6,8,9,11,12,14,15	18	B0BNN3,P02761,P18297,P19468,P28570,P46844,P63095,P80299,Q63424,Q63618,Q64093,Q6AYQ8,Q6Q0N1,Q80W57,Q923M1,Q9JJ40,Q9WTW7,Q9WUW8
347	1,2,3,4,7,10,14,15	5,6,8,9,11,12,13,16	5	P18297,P19468,P54921,P63095,Q5RKI1
348	1,2,3,4,7,10,14,16	5,6,8,9,11,12,13,15	14	B0BNN3,P02761,P18297,P19468,P46844,P57113,Q63424,Q64093,Q6AYQ8,Q71MB6,Q923S2,Q9QYP1,Q9WUW8,Q9WUW9
349	1,2,3,4,7,10,15,16	5,6,8,9,11,12,13,14	32	O70377,O70594,P10758,P17988,P18297,P19468,P29975,P46844,P48508,P51907,P53790,P63095,Q03248,Q5I0E9,Q5RKI1,Q63270,Q63355,Q63424,Q63598,Q63618,Q64093,Q6AYQ8,Q6MG61,Q6Q0N1,Q71MB6,Q80W57,Q8R431,Q923M1,Q9JJ40,Q9QYP1,Q9WTW7,Q9Z0W7
350	1,2,3,4,7,11,12,13	5,6,8,9,10,14,15,16	5	P47967,P80299,P97840,Q5I0D1,Q811M5
351	1,2,3,4,7,11,12,14	5,6,8,9,10,13,15,16	1	Q811M5
352	1,2,3,4,7,11,12,15	5,6,8,9,10,13,14,16	3	P10758,Q6IFW6,Q6IG02
353	1,2,3,4,7,11,12,16	5,6,8,9,10,13,14,15	4	P05369,P07647,P97840,Q9JHB9
354	1,2,3,4,7,11,13,14	5,6,8,9,10,12,15,16	3	B0BNN3,P80299,Q811M5
355	1,2,3,4,7,11,13,15	5,6,8,9,10,12,14,16	6	B0BNN3,P25031,P80299,Q6AYQ8,Q6IFW6,Q6IG02
356	1,2,3,4,7,11,13,16	5,6,8,9,10,12,14,15	9	B0BNN3,P02761,P02781,P02782,P05369,P07647,P80299,Q08463,Q6AYQ8
357	1,2,3,4,7,11,14,15	5,6,8,9,10,12,13,16	6	B0BNN3,P08649,P25031,Q6IFW6,Q6IG02,Q8CJ52

358	1,2,3,4,7,11,14,16	5,6,8,9,10,12,13,15	6 B0BNN3,P02761,P07647,P08649,Q4KLZ6,Q6AYQ8
359	1,2,3,4,7,11,15,16	5,6,8,9,10,12,13,14	9 P02761,P08649,P17988,P29315,Q4FZU2,Q6AYQ8,Q6IFW6,Q6IG02,Q8CJ52
360	1,2,3,4,7,12,13,14	5,6,8,9,10,11,15,16	3 B0BNN3,P80299,Q811M5
361	1,2,3,4,7,12,13,15	5,6,8,9,10,11,14,16	2 P10758,P80299
362	1,2,3,4,7,12,13,16	5,6,8,9,10,11,14,15	5 B0BNN3,P10758,P80299,P97840,Q6AYQ8
363	1,2,3,4,7,12,14,15	5,6,8,9,10,11,13,16	0
364	1,2,3,4,7,12,14,16	5,6,8,9,10,11,13,15	1 B0BNN3
365	1,2,3,4,7,12,15,16	5,6,8,9,10,11,13,14	2 P10758,P17988
366	1,2,3,4,7,13,14,15	5,6,8,9,10,11,12,16	2 B0BNN3,P80299
367	1,2,3,4,7,13,14,16	5,6,8,9,10,11,12,15	5 B0BNN3,P02761,P80299,Q6AYQ8,Q9WUW8
368	1,2,3,4,7,13,15,16	5,6,8,9,10,11,12,14	8 B0BNN3,P02761,P10758,P18297,P19468,P80299,Q64093,Q6AYQ8
369	1,2,3,4,7,14,15,16	5,6,8,9,10,11,12,13	6 B0BNN3,B0LT89,P02761,P17988,P18297,Q6AYQ8
370	1,2,3,4,8,9,10,11	5,6,7,12,13,14,15,16	4 P00774,P01681,P97580,Q6P6S4
371	1,2,3,4,8,9,10,12	5,6,7,11,13,14,15,16	2 P00774,P01681
372	1,2,3,4,8,9,10,13	5,6,7,11,12,14,15,16	4 P00774,P97580,Q30KJ2,Q63618
373	1,2,3,4,8,9,10,14	5,6,7,11,12,13,15,16	4 O70417,P01681,P97580,Q30KJ2
374	1,2,3,4,8,9,10,15	5,6,7,11,12,13,14,16	5 P00774,P01681,P36376,P97580,Q30KJ2
375	1,2,3,4,8,9,10,16	5,6,7,11,12,13,14,15	4 P00774,P01681,P97580,Q30KJ2
376	1,2,3,4,8,9,11,12	5,6,7,10,13,14,15,16	5 O88797,P00774,P01681,Q64335,Q6Q7Y5
377	1,2,3,4,8,9,11,13	5,6,7,10,12,14,15,16	4 B0BNN3,P00774,P01681,Q64335

378	1,2,3,4,8,9,11,14	5,6,7,10,12,13,15,16	2	P00774,P01681
379	1,2,3,4,8,9,11,15	5,6,7,10,12,13,14,16	12	P00774,P01681,P36376,Q10758,Q4FZU2,Q6IFU7,Q6IFU8,Q6IFW6,Q6IG02,Q6IMF3,Q6P6Q2,Q6P6S4
380	1,2,3,4,8,9,11,16	5,6,7,10,12,13,14,15	8	O88797,P00762,P00774,P01681,P02782,Q10758,Q30KJ2,Q812E4
381	1,2,3,4,8,9,12,13	5,6,7,10,11,14,15,16	1	P00774
382	1,2,3,4,8,9,12,14	5,6,7,10,11,13,15,16	2	O70417,P01681
383	1,2,3,4,8,9,12,15	5,6,7,10,11,13,14,16	4	P00774,P01681,P34901,P36376
384	1,2,3,4,8,9,12,16	5,6,7,10,11,13,14,15	2	P00774,Q30KJ2
385	1,2,3,4,8,9,13,14	5,6,7,10,11,12,15,16	2	B0BNN3,P97580
386	1,2,3,4,8,9,13,15	5,6,7,10,11,12,14,16	2	P00774,P01681
387	1,2,3,4,8,9,13,16	5,6,7,10,11,12,14,15	5	B0BNN3,P00774,P23593,P97580,Q30KJ2
388	1,2,3,4,8,9,14,15	5,6,7,10,11,12,13,16	4	P01681,P23593,P36376,P97580
389	1,2,3,4,8,9,14,16	5,6,7,10,11,12,13,15	5	B0BNN3,P01681,P23593,P97580,Q30KJ2
390	1,2,3,4,8,9,15,16	5,6,7,10,11,12,13,14	6	P00774,P01681,P23593,P29315,P97580,Q30KJ2
391	1,2,3,4,8,10,11,12	5,6,7,9,13,14,15,16	2	P97580,Q10758
392	1,2,3,4,8,10,11,13	5,6,7,9,12,14,15,16	5	B0BNN3,P97580,Q10758,Q30KJ2,Q66H69
393	1,2,3,4,8,10,11,14	5,6,7,9,12,13,15,16	4	B0BNN3,P97580,Q10758,Q30KJ2
394	1,2,3,4,8,10,11,15	5,6,7,9,12,13,14,16	10	P97580,Q10758,Q30KJ2,Q4FZU2,Q6IFU7,Q6IFU8,Q6IFW6,Q6IG02,Q6IMF3,Q6P6Q2
395	1,2,3,4,8,10,11,16	5,6,7,9,12,13,14,15	8	P00762,P02782,P15399,P97580,Q10758,Q30KJ2,Q5BJY9,Q6IFU8
396	1,2,3,4,8,10,12,13	5,6,7,9,11,14,15,16	4	P97580,Q30KJ2,Q63618,Q66H69
397	1,2,3,4,8,10,12,14	5,6,7,9,11,13,15,16	4	O70417,P97580,Q30KJ2,Q5RLM2

398	1,2,3,4,8,10,12,15	5,6,7,9,11,13,14,16	6	P10758,P34901,P97580,Q30KJ2,Q498D9,Q63598
399	1,2,3,4,8,10,12,16	5,6,7,9,11,13,14,15	8	P0DMW0;P0DMW1,P10758,P46844,P97580,Q30KJ2,Q63598,Q64093,Q9WTW7
400	1,2,3,4,8,10,13,14	5,6,7,9,11,12,15,16	4	B0BNN3,P97580,Q30KJ2,Q9WUW8
401	1,2,3,4,8,10,13,15	5,6,7,9,11,12,14,16	9	O70377,O88339;Q4V882,P97580,Q30KJ2,Q498D9,Q63598,Q63618,Q64093,Q6Q0N1
402	1,2,3,4,8,10,13,16	5,6,7,9,11,12,14,15	16	B0BNN3,O55004,O70377,P00762,P07151,P15399,P19468,P28570,P46844,P97580,Q30KJ2,Q63424,Q63618,Q64093,Q6Q0N1,Q9WUW8
403	1,2,3,4,8,10,14,15	5,6,7,9,11,12,13,16	4	O70377,P54921,P97580,Q30KJ2
404	1,2,3,4,8,10,14,16	5,6,7,9,11,12,13,15	7	B0BNN3,O70377,P15399,P46844,P97580,Q30KJ2,Q9WUW8
405	1,2,3,4,8,10,15,16	5,6,7,9,11,12,13,14	22	O70377,O70594,P07151,P10758,P15399,P19468,P46844,P48508,P53790,P97580,Q05175,Q30KJ2,Q3ZAV1,Q63424,Q63598,Q63618,Q64093,Q6Q0N1,Q80W57,Q8R431,Q9WTW7,Q9Z0W7
406	1,2,3,4,8,11,12,13	5,6,7,9,10,14,15,16	5	B0BNN3,P00774,Q64335,Q66H69,Q811M5
407	1,2,3,4,8,11,12,14	5,6,7,9,10,13,15,16	2	P11883,Q811M5
408	1,2,3,4,8,11,12,15	5,6,7,9,10,13,14,16	9	P00774,Q10758,Q4FZU2,Q6IFU7,Q6IFU8,Q6IFW6,Q6IG02,Q6IMF3,Q6P6Q2
409	1,2,3,4,8,11,12,16	5,6,7,9,10,13,14,15	9	P00762,P00774,P02781,P02782,P08723,P30120,Q10758,Q6IFU8,Q9JHB9
410	1,2,3,4,8,11,13,14	5,6,7,9,10,12,15,16	6	B0BNN3,O54728,P97580,Q10758,Q64335,Q811M5
411	1,2,3,4,8,11,13,15	5,6,7,9,10,12,14,16	10	B0BNN3,P00774,Q10758,Q4FZU2,Q6IFU7,Q6IFU8,Q6IFW6,Q6IG02,Q6IMF3,Q6P6Q2
412	1,2,3,4,8,11,13,16	5,6,7,9,10,12,14,15	11	B0BNN3,P00762,P00774,P02781,P02782,P08723,P30120,P97580,Q10758,Q30KJ2,Q6IFU8
413	1,2,3,4,8,11,14,15	5,6,7,9,10,12,13,16	10	B0BNN3,P97580,Q10758,Q4FZU2,Q6IFU7,Q6IFU8,Q6IFW6,Q6IG02,Q6IMF3,Q6P6Q2
414	1,2,3,4,8,11,14,16	5,6,7,9,10,12,13,15	10	B0BNN3,O54728,P00762,P02782,P30120,P97580,Q10758,Q30KJ2,Q4KLZ6,Q6IFU8
415	1,2,3,4,8,11,15,16	5,6,7,9,10,12,13,14	15	P00762,P00774,P02782,P29315,P50280,P97580,Q10758,Q30KJ2,Q4FZU2,Q6IFU7,Q6IFU8,Q6IFW6,Q6IG02,Q6IMF3,Q6P6Q2
416	1,2,3,4,8,12,13,14	5,6,7,9,10,11,15,16	3	B0BNN3,P97580,Q811M5
417	1,2,3,4,8,12,13,15	5,6,7,9,10,11,14,16	1	P00774

418	1,2,3,4,8,12,13,16	5,6,7,9,10,11,14,15	5	B0BNN3,P00762,P00774,P97580,Q30KJ2
419	1,2,3,4,8,12,14,15	5,6,7,9,10,11,13,16	1	P97580
420	1,2,3,4,8,12,14,16	5,6,7,9,10,11,13,15	4	B0BNN3,P00762,P97580,Q30KJ2
421	1,2,3,4,8,12,15,16	5,6,7,9,10,11,13,14	4	P00762,P10758,P97580,Q30KJ2
422	1,2,3,4,8,13,14,15	5,6,7,9,10,11,12,16	2	B0BNN3,P97580
423	1,2,3,4,8,13,14,16	5,6,7,9,10,11,12,15	6	B0BNN3,O54728,P00762,P97580,Q30KJ2,Q9WUW8
424	1,2,3,4,8,13,15,16	5,6,7,9,10,11,12,14	5	B0BNN3,P00762,P97580,Q30KJ2,Q6AYQ8
425	1,2,3,4,8,14,15,16	5,6,7,9,10,11,12,13	4	B0BNN3,P00762,P97580,Q30KJ2
426	1,2,3,4,9,10,11,12	5,6,7,8,13,14,15,16	1	Q62714
427	1,2,3,4,9,10,11,13	5,6,7,8,12,14,15,16	0	
428	1,2,3,4,9,10,11,14	5,6,7,8,12,13,15,16	0	
429	1,2,3,4,9,10,11,15	5,6,7,8,12,13,14,16	0	
430	1,2,3,4,9,10,11,16	5,6,7,8,12,13,14,15	1	D3ZUC6
431	1,2,3,4,9,10,12,13	5,6,7,8,11,14,15,16	2	Q99041,Q9QZK9
432	1,2,3,4,9,10,12,14	5,6,7,8,11,13,15,16	2	P01041,Q99041
433	1,2,3,4,9,10,12,15	5,6,7,8,11,13,14,16	6	P09456,P10758,P30120,P34901,P46462,Q99041
434	1,2,3,4,9,10,12,16	5,6,7,8,11,13,14,15	3	D3ZUC6,P10758,Q99041
435	1,2,3,4,9,10,13,14	5,6,7,8,11,12,15,16	0	
436	1,2,3,4,9,10,13,15	5,6,7,8,11,12,14,16	1	Q8CFN2
437	1,2,3,4,9,10,13,16	5,6,7,8,11,12,14,15	1	D3ZUC6

438	1,2,3,4,9,10,14,15	5,6,7,8,11,12,13,16	3	P09456,P30120,P46462
439	1,2,3,4,9,10,14,16	5,6,7,8,11,12,13,15	0	
440	1,2,3,4,9,10,15,16	5,6,7,8,11,12,13,14	4	D3ZUC6,P10758,P29315,Q8K1G0
441	1,2,3,4,9,11,12,13	5,6,7,8,10,14,15,16	3	O35077,Q62761;Q62762;Q62763,Q64335
442	1,2,3,4,9,11,12,14	5,6,7,8,10,13,15,16	2	O35077,Q64335
443	1,2,3,4,9,11,12,15	5,6,7,8,10,13,14,16	1	Q62761;Q62762;Q62763
444	1,2,3,4,9,11,12,16	5,6,7,8,10,13,14,15	1	D3ZUC6
445	1,2,3,4,9,11,13,14	5,6,7,8,10,12,15,16	3	B0BNN3,P0DMW0;P0DMW1,Q64335
446	1,2,3,4,9,11,13,15	5,6,7,8,10,12,14,16	1	P25031
447	1,2,3,4,9,11,13,16	5,6,7,8,10,12,14,15	1	D3ZUC6
448	1,2,3,4,9,11,14,15	5,6,7,8,10,12,13,16	2	P0DMW0;P0DMW1,P25031
449	1,2,3,4,9,11,14,16	5,6,7,8,10,12,13,15	1	Q4KLZ6
450	1,2,3,4,9,11,15,16	5,6,7,8,10,12,13,14	2	D3ZUC6,P29315
451	1,2,3,4,9,12,13,14	5,6,7,8,10,11,15,16	0	
452	1,2,3,4,9,12,13,15	5,6,7,8,10,11,14,16	1	Q99041
453	1,2,3,4,9,12,13,16	5,6,7,8,10,11,14,15	0	
454	1,2,3,4,9,12,14,15	5,6,7,8,10,11,13,16	1	Q99041
455	1,2,3,4,9,12,14,16	5,6,7,8,10,11,13,15	0	
456	1,2,3,4,9,12,15,16	5,6,7,8,10,11,13,14	3	P10758,P29315,Q99041
457	1,2,3,4,9,13,14,15	5,6,7,8,10,11,12,16	0	

458	1,2,3,4,9,13,14,16	5,6,7,8,10,11,12,15	2	B0BNN3,P23593
459	1,2,3,4,9,13,15,16	5,6,7,8,10,11,12,14	1	P29315
460	1,2,3,4,9,14,15,16	5,6,7,8,10,11,12,13	2	P23593,P29315
461	1,2,3,4,10,11,12,13	5,6,7,8,9,14,15,16	0	
462	1,2,3,4,10,11,12,14	5,6,7,8,9,13,15,16	0	
463	1,2,3,4,10,11,12,15	5,6,7,8,9,13,14,16	1	Q6IG02
464	1,2,3,4,10,11,12,16	5,6,7,8,9,13,14,15	0	
465	1,2,3,4,10,11,13,14	5,6,7,8,9,12,15,16	2	B0BNN3,P35745
466	1,2,3,4,10,11,13,15	5,6,7,8,9,12,14,16	1	Q6IG02
467	1,2,3,4,10,11,13,16	5,6,7,8,9,12,14,15	2	P35745,Q6AYQ8
468	1,2,3,4,10,11,14,15	5,6,7,8,9,12,13,16	1	Q6IG02
469	1,2,3,4,10,11,14,16	5,6,7,8,9,12,13,15	2	P35745,Q4KLZ6
470	1,2,3,4,10,11,15,16	5,6,7,8,9,12,13,14	3	P29315,Q6IFW6,Q6IG02
471	1,2,3,4,10,12,13,14	5,6,7,8,9,11,15,16	0	
472	1,2,3,4,10,12,13,15	5,6,7,8,9,11,14,16	3	P06760,P10758,Q812E4
473	1,2,3,4,10,12,13,16	5,6,7,8,9,11,14,15	2	P06760,P10758
474	1,2,3,4,10,12,14,15	5,6,7,8,9,11,13,16	1	P09456
475	1,2,3,4,10,12,14,16	5,6,7,8,9,11,13,15	0	
476	1,2,3,4,10,12,15,16	5,6,7,8,9,11,13,14	3	P06760,P10758,P29315
477	1,2,3,4,10,13,14,15	5,6,7,8,9,11,12,16	1	Q8CFN2

478	1,2,3,4,10,13,14,16	5,6,7,8,9,11,12,15	1	B0BNN3
479	1,2,3,4,10,13,15,16	5,6,7,8,9,11,12,14	5	P06760,P10758,P29315,Q64093,Q6AYQ8
480	1,2,3,4,10,14,15,16	5,6,7,8,9,11,12,13	2	P29315,Q9QYP1
481	1,2,3,4,11,12,13,14	5,6,7,8,9,10,15,16	4	B0BNN3,P35745,Q64335,Q811M5
482	1,2,3,4,11,12,13,15	5,6,7,8,9,10,14,16	2	P25031,Q6IG02
483	1,2,3,4,11,12,13,16	5,6,7,8,9,10,14,15	2	P05369,P35745
484	1,2,3,4,11,12,14,15	5,6,7,8,9,10,13,16	2	P25031,Q6IG02
485	1,2,3,4,11,12,14,16	5,6,7,8,9,10,13,15	2	P35745,Q4KLZ6
486	1,2,3,4,11,12,15,16	5,6,7,8,9,10,13,14	3	P29315,Q6IFW6,Q6IG02
487	1,2,3,4,11,13,14,15	5,6,7,8,9,10,12,16	3	B0BNN3,P25031,Q6IG02
488	1,2,3,4,11,13,14,16	5,6,7,8,9,10,12,15	4	B0BNN3,O54728,P35745,Q4KLZ6
489	1,2,3,4,11,13,15,16	5,6,7,8,9,10,12,14	3	P29315,Q6AYQ8,Q6IG02
490	1,2,3,4,11,14,15,16	5,6,7,8,9,10,12,13	5	P25031,P29315,Q4KLZ6,Q6IFW6,Q6IG02
491	1,2,3,4,12,13,14,15	5,6,7,8,9,10,11,16	0	
492	1,2,3,4,12,13,14,16	5,6,7,8,9,10,11,15	1	B0BNN3
493	1,2,3,4,12,13,15,16	5,6,7,8,9,10,11,14	3	P06760,P10758,P29315
494	1,2,3,4,12,14,15,16	5,6,7,8,9,10,11,13	1	P29315
495	1,2,3,4,13,14,15,16	5,6,7,8,9,10,11,12	2	B0BNN3,P29315
496	1,2,3,5,6,7,8,9	4,10,11,12,13,14,15,16	24	iRT-Kit_WR_fusion,O70594,P06760,P08649,P08937,P10247,P19629,P23928,P36860,P48508,P50115,P50116,P52590,P55091,Q09030,Q4FZU6,Q5QE79,Q5U2V4,Q62714,Q62812,Q63317,Q6IG05,Q920G2,Q9QX74

497	1,2,3,5,6,7,8,10	4,9,11,12,13,14,15,16	86	iRT-Kit_WR_fusion,O08557,O35763,O70257,O70377,O70594,O88339;Q4V882,P01836,P02631,P04904,P08937,P10247,P10536,P10760,P18757,P19468,P19629,P20766,P23928,P25031,P29975,P30904,P31044,P36860,P36970,P38918,P46720,P46844,P48037,P48508,P50115,P50116,P51907,P52590,P53790,P54921,P55091,P57113,P60711;P63259,P63081,P68035;P68136,P97580,P97605,Q03248,Q05175,Q3T1J9,Q3ZAV1,Q5I0E9,Q5M7T9,Q5RLM2,Q62687,Q62714,Q62812,Q63270,Q63317,Q63355,Q63424,Q63598,Q63618,Q64093,Q64602,Q66H69,Q66HG3,Q68FT5,Q6AY41,Q6AYS7,Q6IG05,Q6MG61,Q6Q0N1,Q71MB6,Q80W57,Q8K3P7,Q8R431,Q91ZS3,Q920G2,Q923S2,Q99MZ8,Q9JJ19,Q9JJ40,Q9JLJ3,Q9QX74,Q9QYU4,Q9WTW7,Q9WUW8,Q9WUW9,Q9Z0W7
498	1,2,3,5,6,7,8,11	4,9,10,12,13,14,15,16	39	iRT-Kit_WR_fusion,O08557,P02780,P06760,P06761,P06911,P08649,P08721,P08723,P08937,P09456,P10247,P12020,P18757,P19629,P22283,P31044,P31430,P47967,P50115,P50116,P50280,P52590,P55091,P63029,Q5GRG2,Q5M8C6,Q5U2V4,Q62812,Q63493,Q66H69,Q6IFU7,Q6IG05,Q6IMF3,Q6Q0N0,Q920G2,Q9JHB9,Q9JI85,Q9QX74
499	1,2,3,5,6,7,8,12	4,9,10,11,13,14,15,16	30	iRT-Kit_WR_fusion,P06911,P08937,P10247,P19629,P19814,P30904,P31044,P36860,P38918,P46844,P47967,P48508,P50115,P50116,P52590,P55091,P57113,Q5RLM2,Q62714,Q63270,Q64602,Q66H69,Q66HG3,Q6IG05,Q6Q0N1,Q91ZS3,Q920G2,Q9QX74,Q9WUW8
500	1,2,3,5,6,7,8,13	4,9,10,11,12,14,15,16	29	iRT-Kit_WR_fusion,O70594,P01836,P02631,P08937,P10247,P19629,P25809,P30904,P31044,P36860,P46844,P47967,P48508,P50115,P50116,P52590,P55091,P57113,P63081,Q62714,Q62812,Q63618,Q66H69,Q6IG05,Q6Q0N1,Q9JI85,Q9QX74,Q9WUW8
501	1,2,3,5,6,7,8,14	4,9,10,11,12,13,15,16	28	O70594,P08649,P08937,P18757,P19629,P23928,P31044,P46844,P48037,P48508,P50115,P50116,P52590,P57113,P63081,P97580,Q5QE79,Q5RLM2,Q62714,Q62812,Q63317,Q63751,Q64602,Q6IG05,Q923S2,Q9QX74,Q9WUW8,Q9WUW9
502	1,2,3,5,6,7,8,15	4,9,10,11,12,13,14,16	53	iRT-Kit_WR_fusion,O08557,O35763,O70377,O70594,O88339;Q4V882,P01946,P02091,P08649,P08721,P08937,P10247,P18757,P19132,P19468,P19629,P23928,P30904,P31044,P36860,P38918,P46844,P48508,P50115,P50116,P50280,P52590,P53790,P57113,Q05175,Q3ZAV1,Q498D9,Q5I0E9,Q5M7T9,Q62714,Q62812,Q63270,Q63317,Q63618,Q64602,Q6IG05,Q6MG61,Q6PCU2,Q6Q0N1,Q8CGS4,Q920G2,Q923S2,Q99MZ8,Q9QX74,Q9WTW7,Q9WUW8,Q9Z0V6,Q9Z0W7
503	1,2,3,5,6,7,8,16	4,9,10,11,12,13,14,15	67	B0LT89,iRT-Kit_WR_fusion,O08557,O35763,O70377,O70594,P01836,P02783,P08649,P08937,P10247,P18757,P19468,P19629,P23928,P29975,P31044,P36970,P38918,P46720,P46844,P48037,P48508,P50115,P50116,P50280,P52590,P52847,P53790,P57113,P63081,P97580,Q03248,Q05175,Q3ZAV1,Q5I0D7,Q5I0E9,Q5M7T9,Q5RLM2,Q62714,Q62761;Q62762;Q62763,Q62812,Q63270,Q63317,Q63424,Q63618,Q64602,Q66H69,Q68FT5,Q6AY41,Q6IG05,Q6MG61,Q6Q0N1,Q71MB6,Q80W57,Q8R431,Q920G2,Q923S2,Q9JHB9,Q9JJ40,Q9JLJ3,Q9QX74,Q9QYU4,Q9WTW7,Q9WUW8,Q9WUW9,Q9Z0W7

504	1,2,3,5,6,7,9,10	4,8,11,12,13,14,15,16	9	D3ZTX0,O54728,O70594,P00762,P20766,P23928,P36860,P50116,Q9JJ50
505	1,2,3,5,6,7,9,11	4,8,10,12,13,14,15,16	3	P08649,P50116,P52590
506	1,2,3,5,6,7,9,12	4,8,10,11,13,14,15,16	6	O54728,P36860,P50116,P52590,Q09030,Q6AY61
507	1,2,3,5,6,7,9,13	4,8,10,11,12,14,15,16	5	P01835,P36860,P52590,Q09030,Q62714
508	1,2,3,5,6,7,9,14	4,8,10,11,12,13,15,16	6	P50116,P52590,Q09030,Q5QE79,Q62714,Q63751
509	1,2,3,5,6,7,9,15	4,8,10,11,12,13,14,16	7	O54728,P01946,P02091,P08721,P36860,Q62714,Q99MH3
510	1,2,3,5,6,7,9,16	4,8,10,11,12,13,14,15	2	P52590,Q09030
511	1,2,3,5,6,7,10,11	4,8,9,12,13,14,15,16	8	O08557,P08721,P20766,P36860,P47967,P48037,P50116,P63029
512	1,2,3,5,6,7,10,12	4,8,9,11,13,14,15,16	13	O54728,P19814,P36860,P38918,P46844,P47967,P48508,P50116,P57113,Q5RLM2,Q66HG3,Q6AY61,Q9QYU4
513	1,2,3,5,6,7,10,13	4,8,9,11,12,14,15,16	15	A2RUW1,O70594,P01835,P19468,P23928,P36860,P46844,P47967,P48508,P57113,P80299,Q62714,Q63618,Q6AY61,Q9WUW8
514	1,2,3,5,6,7,10,14	4,8,9,11,12,13,15,16	16	A2RUW1,O70594,P23928,P48037,P48508,P50116,P52590,P57113,Q5I0E9,Q5RLM2,Q62714,Q64602,Q6AY61,Q71MB6,Q923S2,Q9WUW8
515	1,2,3,5,6,7,10,15	4,8,9,11,12,13,14,16	44	A2RUW1,O08557,O54728,O70377,O70594,O88339;Q4V882,P01946,P02091,P08721,P10536,P17988,P18297,P18757,P19468,P20766,P23928,P29975,P36860,P38918,P46844,P48508,P53790,P54921,P57113,Q03248,Q05175,Q5I0E9,Q5RKI1,Q62714,Q63270,Q63618,Q64602,Q6AY61,Q6MG61,Q6Q0N1,Q71MB6,Q7M0E3,Q80W57,Q8K3P7,Q923S2,Q9QYU4,Q9WTW7,Q9WUW8,Q9Z0W7
516	1,2,3,5,6,7,10,16	4,8,9,11,12,13,14,15	48	O08557,O70377,O70594,P08721,P17988,P18297,P18757,P19468,P20766,P23928,P29975,P36970,P38918,P43427,P46844,P48037,P48508,P52590,P52847,P53790,P57113,P60711;P63259,Q03248,Q05175,Q3T1J9,Q3ZAV1,Q5I0D7,Q5I0E9,Q5RLM2,Q62714,Q63270,Q63424,Q63618,Q64093,Q64602,Q68FT5,Q6MG61,Q6Q0N1,Q71MB6,Q80W57,Q8R431,Q923S2,Q9JJ40,Q9QYU4,Q9WTW7,Q9WUW8,Q9WUW9,Q9Z0W7
517	1,2,3,5,6,7,11,12	4,8,9,10,13,14,15,16	3	P47967,P50116,Q5I0D1

518	1,2,3,5,6,7,11,13	4,8,9,10,12,14,15,16	5	P01835,P36860,P47967,P52590,P63029
519	1,2,3,5,6,7,11,14	4,8,9,10,12,13,15,16	5	P08649,P50116,P52590,P63029,Q63751
520	1,2,3,5,6,7,11,15	4,8,9,10,12,13,14,16	7	O08557,P01946,P02091,P08649,P08721,P17988,Q6IFW6
521	1,2,3,5,6,7,11,16	4,8,9,10,12,13,14,15	9	P01039,P02783,P07647,P08649,P08721,P17988,P52590,Q8R5M3,Q9JHB9
522	1,2,3,5,6,7,12,13	4,8,9,10,11,14,15,16	6	P01835,P19814,P36860,P47967,P52590,Q6AY61
523	1,2,3,5,6,7,12,14	4,8,9,10,11,13,15,16	6	P01835,P19814,P52590,Q5RLM2,Q62714,Q6AY61
524	1,2,3,5,6,7,12,15	4,8,9,10,11,13,14,16	8	O54728,P01946,P02091,P08721,P19814,P36860,Q62714,Q6AY61
525	1,2,3,5,6,7,12,16	4,8,9,10,11,13,14,15	6	P46844,P47967,P52590,Q5I0D7,Q5RLM2,Q6AY61
526	1,2,3,5,6,7,13,14	4,8,9,10,11,12,15,16	6	P01835,P52590,P63081,Q62714,Q6AY61,Q9WUW8
527	1,2,3,5,6,7,13,15	4,8,9,10,11,12,14,16	10	A2RUW1,O70594,P01835,P01946,P02091,P08721,P36860,Q62714,Q6AY61,Q9WUW8
528	1,2,3,5,6,7,13,16	4,8,9,10,11,12,14,15	8	O70594,P01835,P46844,P52590,P57113,Q5I0D7,Q62714,Q9WUW8
529	1,2,3,5,6,7,14,15	4,8,9,10,11,12,13,16	9	P01835,P01946,P02091,P08721,P52590,Q62714,Q62812,Q6AY61,Q9WUW8
530	1,2,3,5,6,7,14,16	4,8,9,10,11,12,13,15	12	B0LT89,P01835,P02783,P48037,P52590,P57113,Q5I0D7,Q5RLM2,Q62714,Q62812,Q71MB6,Q9WUW8
531	1,2,3,5,6,7,15,16	4,8,9,10,11,12,13,14	25	B0LT89,O08557,O70594,P01835,P01946,P02091,P02783,P08721,P17988,P18297,P19468,P38918,P46844,P48508,P52590,P53790,P57113,Q5I0D7,Q5I0E9,Q62714,Q62812,Q63270,Q71MB6,Q9WTW7,Q9WUW8
532	1,2,3,5,6,8,9,10	4,7,11,12,13,14,15,16	9	O70594,P08937,P10247,P25031,P36860,P70545,P97580,Q09030,Q99MH3
533	1,2,3,5,6,8,9,11	4,7,10,12,13,14,15,16	8	P08937,P10247,P52590,P63029,Q5I0J9,Q6IG05,Q6P6S4,Q99MH3
534	1,2,3,5,6,8,9,12	4,7,10,11,13,14,15,16	5	P10247,P36376,Q09030,Q6IG05,Q99MH3
535	1,2,3,5,6,8,9,13	4,7,10,11,12,14,15,16	4	P08937,P10247,P52590,Q09030

536	1,2,3,5,6,8,9,14	4,7,10,11,12,13,15,16	6	P00714,P08937,P52590,Q09030,Q5I0J9,Q63751
537	1,2,3,5,6,8,9,15	4,7,10,11,12,13,14,16	10	P00714,P01681,P01946,P02091,P10247,P19132,P36376,Q62714,Q6IG05,Q99MH3
538	1,2,3,5,6,8,9,16	4,7,10,11,12,13,14,15	3	P52590,Q09030,Q6IG05
539	1,2,3,5,6,8,10,11	4,7,9,12,13,14,15,16	9	P08937,P10247,P15399,P25031,P63029,P97580,Q68G31,Q6IFU7,Q6IG05
540	1,2,3,5,6,8,10,12	4,7,9,11,13,14,15,16	14	P08937,P10247,P19814,P25031,P30904,P36860,P46844,P48508,P97580,Q498D9,Q5RLM2,Q66HG3,Q6IG05,Q91ZS3
541	1,2,3,5,6,8,10,13	4,7,9,11,12,14,15,16	14	O70594,O88339;Q4V882,P08937,P10247,P25031,P36860,P46844,P48508,P97580,Q498D9,Q62714,Q63618,Q68G31,Q9WUW8
542	1,2,3,5,6,8,10,14	4,7,9,11,12,13,15,16	15	O70594,O88339;Q4V882,P08937,P10247,P25031,P48508,P52590,P54921,P57113,P97580,Q5RLM2,Q62714,Q68G31,Q923S2,Q9WUW8
543	1,2,3,5,6,8,10,15	4,7,9,11,12,13,14,16	30	O70377,O70594,O88339;Q4V882,P01946,P02091,P10247,P15399,P19132,P19468,P19629,P23928,P25031,P30904,P36860,P38918,P46844,P48508,P53790,P54921,P97580,Q05175,Q498D9,Q5I0E9,Q62714,Q63270,Q63618,Q6IG05,Q6MG61,Q6Q0N1,Q99MH3
544	1,2,3,5,6,8,10,16	4,7,9,11,12,13,14,15	38	O70377,O70594,O88339;Q4V882,P10247,P15399,P18757,P19468,P23928,P25031,P38918,P46844,P48508,P52590,P53790,P57113,P97580,P97605,Q05175,Q30KJ2,Q3T1J9,Q3ZAV1,Q5I0E9,Q62761;Q62762;Q62763,Q63270,Q63424,Q63618,Q64602,Q6AY41,Q6IG05,Q6MG61,Q6Q0N1,Q71MB6,Q80W57,Q8R431,Q923S2,Q9WTW7,Q9WUW8,Q9Z0W7
545	1,2,3,5,6,8,11,12	4,7,9,10,13,14,15,16	8	P08937,P10247,P12020,P63029,Q5GRG2,Q6IFU7,Q6IG05,Q9JI85
546	1,2,3,5,6,8,11,13	4,7,9,10,12,14,15,16	9	P08937,P10247,P52590,P63029,Q5GRG2,Q68G31,Q6IFU7,Q6IG05,Q9JI85
547	1,2,3,5,6,8,11,14	4,7,9,10,12,13,15,16	8	P08937,P52590,P63029,P97580,Q5GRG2,Q63751,Q6IFU7,Q6IG05
548	1,2,3,5,6,8,11,15	4,7,9,10,12,13,14,16	14	P01946,P02091,P10247,P50280,P63029,Q4FZU2,Q6IFU7,Q6IFU8,Q6IFW6,Q6IG02,Q6IG05,Q6IMF3,Q6P6Q2,Q6P6S4
549	1,2,3,5,6,8,11,16	4,7,9,10,12,13,14,15	10	P02782,P02783,P50280,P52590,P63029,Q5GRG2,Q6IFU7,Q6IG05,Q9JHB9,Q9JI85
550	1,2,3,5,6,8,12,13	4,7,9,10,11,14,15,16	4	P10247,P19814,P52590,Q6IG05
551	1,2,3,5,6,8,12,14	4,7,9,10,11,13,15,16	5	P00714,P08937,P52590,Q5RLM2,Q6IG05
552	1,2,3,5,6,8,12,15	4,7,9,10,11,13,14,16	9	P01946,P02091,P10247,P19132,P19814,P36376,Q498D9,Q6IG05,Q9R168

553	1,2,3,5,6,8,12,16	4,7,9,10,11,13,14,15	3	P46844,P52590,Q6IG05
554	1,2,3,5,6,8,13,14	4,7,9,10,11,12,15,16	6	P08937,P52590,P97580,Q62714,Q68G31,Q9WUW8
555	1,2,3,5,6,8,13,15	4,7,9,10,11,12,14,16	10	O70594,P01946,P02091,P10247,P19132,Q498D9,Q62714,Q68G31,Q6IG05,Q8CGS4
556	1,2,3,5,6,8,13,16	4,7,9,10,11,12,14,15	7	O70594,P46844,P52590,P97580,Q62714,Q6IG05,Q9WUW8
557	1,2,3,5,6,8,14,15	4,7,9,10,11,12,13,16	10	P00714,P01946,P02091,P08937,P19132,P52590,P54921,P97580,Q62714,Q6IG05
558	1,2,3,5,6,8,14,16	4,7,9,10,11,12,13,15	7	P02783,P08937,P52590,P97580,Q62714,Q6IG05,Q9WUW8
559	1,2,3,5,6,8,15,16	4,7,9,10,11,12,13,14	13	O70594,P01946,P02091,P02783,P10247,P46844,P48508,P52590,P53790,P97580,Q62714,Q63270,Q6IG05
560	1,2,3,5,6,9,10,11	4,7,8,12,13,14,15,16	4	P63029,Q5I0J9,Q99MH3,Q9Z2L0
561	1,2,3,5,6,9,10,12	4,7,8,11,13,14,15,16	6	O54728,P0C0A9,P30120,Q09030,Q99041,Q99MH3
562	1,2,3,5,6,9,10,13	4,7,8,11,12,14,15,16	5	P0C0A9,P36860,Q06000,Q09030,Q99MH3
563	1,2,3,5,6,9,10,14	4,7,8,11,12,13,15,16	6	P00714,P30120,P52590,Q06000,Q09030,Q5I0J9
564	1,2,3,5,6,9,10,15	4,7,8,11,12,13,14,16	9	O54728,P00714,P01946,P02091,P30120,P36860,Q811M5,Q99041,Q99MH3
565	1,2,3,5,6,9,10,16	4,7,8,11,12,13,14,15	1	Q09030
566	1,2,3,5,6,9,11,12	4,7,8,10,13,14,15,16	3	Q03191,Q5I0J9,Q99MH3
567	1,2,3,5,6,9,11,13	4,7,8,10,12,14,15,16	0	
568	1,2,3,5,6,9,11,14	4,7,8,10,12,13,15,16	4	P0DMW0;P0DMW1,P52590,P63029,Q5I0J9
569	1,2,3,5,6,9,11,15	4,7,8,10,12,13,14,16	3	P01946,P02091,Q99MH3
570	1,2,3,5,6,9,11,16	4,7,8,10,12,13,14,15	2	P52590,Q5I0J9
571	1,2,3,5,6,9,12,13	4,7,8,10,11,14,15,16	3	P0C0A9,Q09030,Q99041
572	1,2,3,5,6,9,12,14	4,7,8,10,11,13,15,16	5	P00714,P52590,Q09030,Q5I0J9,Q99041

573	1,2,3,5,6,9,12,15	4,7,8,10,11,13,14,16	9	O54728,P00714,P01946,P02091,P0C0A9,P30120,Q6AY61,Q99041,Q99MH3
574	1,2,3,5,6,9,12,16	4,7,8,10,11,13,14,15	3	P52590,Q09030,Q99041
575	1,2,3,5,6,9,13,14	4,7,8,10,11,12,15,16	2	P52590,Q09030
576	1,2,3,5,6,9,13,15	4,7,8,10,11,12,14,16	4	P01946,P02091,P62804,Q99MH3
577	1,2,3,5,6,9,13,16	4,7,8,10,11,12,14,15	3	P52590,Q09030,Q9EQS0
578	1,2,3,5,6,9,14,15	4,7,8,10,11,12,13,16	7	P00714,P01946,P02091,P22273,P30120,P52590,Q99MH3
579	1,2,3,5,6,9,14,16	4,7,8,10,11,12,13,15	3	P52590,Q09030,Q5I0J9
580	1,2,3,5,6,9,15,16	4,7,8,10,11,12,13,14	2	P01946,P02091
581	1,2,3,5,6,10,11,12	4,7,8,9,13,14,15,16	0	
582	1,2,3,5,6,10,11,13	4,7,8,9,12,14,15,16	2	P63029,Q68G31
583	1,2,3,5,6,10,11,14	4,7,8,9,12,13,15,16	2	P52590,P63029
584	1,2,3,5,6,10,11,15	4,7,8,9,12,13,14,16	3	P01946,P02091,Q99MH3
585	1,2,3,5,6,10,11,16	4,7,8,9,12,13,14,15	0	
586	1,2,3,5,6,10,12,13	4,7,8,9,11,14,15,16	3	P36860,Q6AY61,Q812E4
587	1,2,3,5,6,10,12,14	4,7,8,9,11,13,15,16	4	P00714,P52590,Q5RLM2,Q6AY61
588	1,2,3,5,6,10,12,15	4,7,8,9,11,13,14,16	11	O54728,P01946,P02091,P19814,P30120,P36860,Q498D9,Q6AY61,Q812E4,Q99041,Q99MH3
589	1,2,3,5,6,10,12,16	4,7,8,9,11,13,14,15	0	
590	1,2,3,5,6,10,13,14	4,7,8,9,11,12,15,16	4	P52590,Q06000,Q62714,Q9WUW8
591	1,2,3,5,6,10,13,15	4,7,8,9,11,12,14,16	7	P01946,P02091,P21674,P36860,Q498D9,Q62714,Q812E4
592	1,2,3,5,6,10,13,16	4,7,8,9,11,12,14,15	2	P52590,Q9WUW8

593	1,2,3,5,6,10,14,15	4,7,8,9,11,12,13,16	8 P00714,P01946,P02091,P30120,P52590,P54921,P63322,Q62714
594	1,2,3,5,6,10,14,16	4,7,8,9,11,12,13,15	4 P14668,P52590,Q71MB6,Q9WUW8
595	1,2,3,5,6,10,15,16	4,7,8,9,11,12,13,14	9 O70594,P01946,P02091,P17988,P19468,P48508,P53790,P82471,Q71MB6
596	1,2,3,5,6,11,12,13	4,7,8,9,10,14,15,16	1 P47967
597	1,2,3,5,6,11,12,14	4,7,8,9,10,13,15,16	3 P52590,P63029,Q5I0J9
598	1,2,3,5,6,11,12,15	4,7,8,9,10,13,14,16	3 P01946,P02091,Q99MH3
599	1,2,3,5,6,11,12,16	4,7,8,9,10,13,14,15	0
600	1,2,3,5,6,11,13,14	4,7,8,9,10,12,15,16	2 P52590,P63029
601	1,2,3,5,6,11,13,15	4,7,8,9,10,12,14,16	3 P01946,P02091,P21674
602	1,2,3,5,6,11,13,16	4,7,8,9,10,12,14,15	1 P52590
603	1,2,3,5,6,11,14,15	4,7,8,9,10,12,13,16	3 P01946,P02091,P52590
604	1,2,3,5,6,11,14,16	4,7,8,9,10,12,13,15	3 P02783,P52590,Q4KLZ6
605	1,2,3,5,6,11,15,16	4,7,8,9,10,12,13,14	4 P01946,P02091,P02783,P17988
606	1,2,3,5,6,12,13,14	4,7,8,9,10,11,15,16	4 P01835,P49134,P52590,Q6AY61
607	1,2,3,5,6,12,13,15	4,7,8,9,10,11,14,16	5 P01946,P02091,P19814,Q6AY61,Q812E4
608	1,2,3,5,6,12,13,16	4,7,8,9,10,11,14,15	1 P52590
609	1,2,3,5,6,12,14,15	4,7,8,9,10,11,13,16	5 P00714,P01946,P02091,P52590,Q6AY61
610	1,2,3,5,6,12,14,16	4,7,8,9,10,11,13,15	1 P52590
611	1,2,3,5,6,12,15,16	4,7,8,9,10,11,13,14	3 P01946,P02091,Q6AY61
612	1,2,3,5,6,13,14,15	4,7,8,9,10,11,12,16	6 P01835,P01946,P02091,P21674,P52590,Q62714

613	1,2,3,5,6,13,14,16	4,7,8,9,10,11,12,15	4	P01835,P52590,Q62714,Q9WUW8
614	1,2,3,5,6,13,15,16	4,7,8,9,10,11,12,14	5	P01835,P01946,P02091,P52590,Q62714
615	1,2,3,5,6,14,15,16	4,7,8,9,10,11,12,13	5	P01946,P02091,P02783,P52590,Q62714
616	1,2,3,5,7,8,9,10	4,6,11,12,13,14,15,16	6	O35763,O70594,P20766,P70545,Q4FZU6,Q63618
617	1,2,3,5,7,8,9,11	4,6,10,12,13,14,15,16	6	iRT-Kit_WR_fusion,P06760,P08649,P35280,Q6IG05,Q6IMF3
618	1,2,3,5,7,8,9,12	4,6,10,11,13,14,15,16	2	iRT-Kit_WR_fusion,Q6IG05
619	1,2,3,5,7,8,9,13	4,6,10,11,12,14,15,16	3	iRT-Kit_WR_fusion,P08649,Q66H69
620	1,2,3,5,7,8,9,14	4,6,10,11,12,13,15,16	5	P00714,P06760,P08649,P08937,Q63751
621	1,2,3,5,7,8,9,15	4,6,10,11,12,13,14,16	11	iRT-Kit_WR_fusion,P00714,P01681,P08649,P19132,P35280,P36376,Q4FZU6,Q62714,Q6IG05,Q9Z0V6
622	1,2,3,5,7,8,9,16	4,6,10,11,12,13,14,15	2	iRT-Kit_WR_fusion,P08649
623	1,2,3,5,7,8,10,11	4,6,9,12,13,14,15,16	7	P08937,P20766,P47967,Q66H69,Q6IFU7,Q6IG05,Q6IMF3
624	1,2,3,5,7,8,10,12	4,6,9,11,13,14,15,16	16	iRT-Kit_WR_fusion,P19814,P20766,P30904,P46844,P47967,P48508,Q05175,Q5RLM2,Q63618,Q64093,Q66H69,Q6IG05,Q6MG61,Q6Q0N1,Q9WUW8
625	1,2,3,5,7,8,10,13	4,6,9,11,12,14,15,16	24	O70377,O70594,P19468,P25809,P30904,P36860,P46844,P47967,P48508,P57113,Q05175,Q3ZAV1,Q62714,Q63355,Q63424,Q63618,Q64093,Q66H69,Q6AY41,Q6MG61,Q6Q0N1,Q80W57,Q9WUW8,Q9WUW9
626	1,2,3,5,7,8,10,14	4,6,9,11,12,13,15,16	16	O70377,O70594,P08937,P46844,P48508,P57113,Q05175,Q5RLM2,Q62714,Q63355,Q63751,Q64602,Q66H69,Q923S2,Q9WUW8,Q9WUW9

627	1,2,3,5,7,8,10,15	4,6,9,11,12,13,14,16	45	O35763,O70377,O70594,O88339;Q4V882,P10536,P12368,P18757,P19132,P19468,P20766,P23928,P30904,P38918,P46844,P48508,P51907,P53790,P54921,P57113,Q05175,Q3ZAV1,Q5I0E9,Q5RKI1,Q5RLM2,Q62714,Q62753,Q63270,Q63355,Q63424,Q63618,Q64093,Q64602,Q66H69,Q6AY41,Q6IG05,Q6MG61,Q6Q0N1,Q80W57,Q8R431,Q923S2,Q9JJ19,Q9WTW7,Q9WUW8,Q9Z0V6,Q9Z0W7
628	1,2,3,5,7,8,10,16	4,6,9,11,12,13,14,15	42	O35077,O35763,O70377,O70594,P18757,P19468,P20766,P23928,P29975,P38918,P46720,P46844,P48508,P53790,P57113,Q03248,Q05175,Q3ZAV1,Q5I0E9,Q5M7T9,Q5RLM2,Q62687,Q63270,Q63355,Q63424,Q63618,Q64093,Q64602,Q66H69,Q6AY41,Q6MG61,Q6Q0N1,Q71MB6,Q80W57,Q8R431,Q923S2,Q9JJ19,Q9JJ40,Q9WTW7,Q9WUW8,Q9WUW9,Q9Z0W7
629	1,2,3,5,7,8,11,12	4,6,9,10,13,14,15,16	9	iRT-Kit_WR_fusion,P08649,P47967,P70709,Q5I0D1,Q66H69,Q6IG05,Q6IMF3,Q811M5
630	1,2,3,5,7,8,11,13	4,6,9,10,12,14,15,16	7	P08649,P47967,Q66H69,Q6IFU7,Q6IG05,Q6IMF3,Q811M5
631	1,2,3,5,7,8,11,14	4,6,9,10,12,13,15,16	10	P06760,P08649,P08937,Q63751,Q66H69,Q6IFU7,Q6IFU8,Q6IG05,Q6IMF3,Q811M5
632	1,2,3,5,7,8,11,15	4,6,9,10,12,13,14,16	12	iRT-Kit_WR_fusion,P08649,P19132,Q4FZU2,Q66H69,Q6IFU7,Q6IFU8,Q6IFW6,Q6IG02,Q6IG05,Q6IMF3,Q6P6Q2
633	1,2,3,5,7,8,11,16	4,6,9,10,12,13,14,15	17	iRT-Kit_WR_fusion,P02781,P02782,P08010,P08649,P08723,P09456,P30120,P47967,Q00715,Q4FZU2,Q66H69,Q6IFU7,Q6IFU8,Q6IG05,Q6IMF3,Q9JHB9
634	1,2,3,5,7,8,12,13	4,6,9,10,11,14,15,16	7	iRT-Kit_WR_fusion,P19814,P47967,Q66H69,Q6IG05,Q811M5,Q9WUW8
635	1,2,3,5,7,8,12,14	4,6,9,10,11,13,15,16	9	iRT-Kit_WR_fusion,P00714,P08937,Q5RLM2,Q63751,Q66H69,Q6IG05,Q811M5,Q9WUW8
636	1,2,3,5,7,8,12,15	4,6,9,10,11,13,14,16	6	iRT-Kit_WR_fusion,P19132,P19814,P34901,Q66H69,Q6IG05
637	1,2,3,5,7,8,12,16	4,6,9,10,11,13,14,15	7	iRT-Kit_WR_fusion,P46844,P47967,Q5RLM2,Q66H69,Q6IG05,Q9WUW8
638	1,2,3,5,7,8,13,14	4,6,9,10,11,12,15,16	7	P08649,P08937,P25809,Q62714,Q66H69,Q811M5,Q9WUW8
639	1,2,3,5,7,8,13,15	4,6,9,10,11,12,14,16	8	O70594,P08649,P19132,Q62714,Q66H69,Q6IG05,Q6Q0N1,Q9WUW8
640	1,2,3,5,7,8,13,16	4,6,9,10,11,12,14,15	8	O70594,P08649,P46844,P47967,Q62714,Q64093,Q66H69,Q9WUW8
641	1,2,3,5,7,8,14,15	4,6,9,10,11,12,13,16	7	P00714,P08649,P19132,Q5RLM2,Q62714,Q6IG05,Q9WUW8
642	1,2,3,5,7,8,14,16	4,6,9,10,11,12,13,15	6	P08649,P46844,Q5RLM2,Q62714,Q62812,Q9WUW8

643	1,2,3,5,7,8,15,16	4,6,9,10,11,12,13,14	19	iRT-Kit_WR_fusion,O70594,P08649,P19132,P19468,P46844,P48508,P53790,Q05175,Q5I0E9,Q62714,Q63270,Q64093,Q6IG05,Q6Q0N1,Q8R431,Q9WTW7,Q9WUW8,Q9Z0W7
644	1,2,3,5,7,9,10,11	4,6,8,12,13,14,15,16	1	P20766
645	1,2,3,5,7,9,10,12	4,6,8,11,13,14,15,16	4	O54728,Q03191,Q6AY61,Q99041
646	1,2,3,5,7,9,10,13	4,6,8,11,12,14,15,16	1	Q6AY61
647	1,2,3,5,7,9,10,14	4,6,8,11,12,13,15,16	2	P00714,Q6AY61
648	1,2,3,5,7,9,10,15	4,6,8,11,12,13,14,16	6	O54728,P00714,P30120,Q5RKI1,Q6AY61,Q99041
649	1,2,3,5,7,9,10,16	4,6,8,11,12,13,14,15	1	P20766
650	1,2,3,5,7,9,11,12	4,6,8,10,13,14,15,16	3	P47967,Q03191,Q6AY61
651	1,2,3,5,7,9,11,13	4,6,8,10,12,14,15,16	1	P47967
652	1,2,3,5,7,9,11,14	4,6,8,10,12,13,15,16	2	P08649,P0DMW0;P0DMW1
653	1,2,3,5,7,9,11,15	4,6,8,10,12,13,14,16	2	P08649,P35280
654	1,2,3,5,7,9,11,16	4,6,8,10,12,13,14,15	1	P08649
655	1,2,3,5,7,9,12,13	4,6,8,10,11,14,15,16	4	P47967,Q03191,Q6AY61,Q99041
656	1,2,3,5,7,9,12,14	4,6,8,10,11,13,15,16	3	P00714,Q6AY61,Q99041
657	1,2,3,5,7,9,12,15	4,6,8,10,11,13,14,16	4	O54728,P00714,Q6AY61,Q99041
658	1,2,3,5,7,9,12,16	4,6,8,10,11,13,14,15	3	Q03191,Q6AY61,Q99041
659	1,2,3,5,7,9,13,14	4,6,8,10,11,12,15,16	2	P0DMW0;P0DMW1,Q6AY61
660	1,2,3,5,7,9,13,15	4,6,8,10,11,12,14,16	1	Q6AY61
661	1,2,3,5,7,9,13,16	4,6,8,10,11,12,14,15	0	
662	1,2,3,5,7,9,14,15	4,6,8,10,11,12,13,16	2	P00714,Q6AY61

663	1,2,3,5,7,9,14,16	4,6,8,10,11,12,13,15	1	P08649
664	1,2,3,5,7,9,15,16	4,6,8,10,11,12,13,14	0	
665	1,2,3,5,7,10,11,12	4,6,8,9,13,14,15,16	5	P23593,P47967,P97840,Q5I0D1,Q6AY61
666	1,2,3,5,7,10,11,13	4,6,8,9,12,14,15,16	1	P47967
667	1,2,3,5,7,10,11,14	4,6,8,9,12,13,15,16	0	
668	1,2,3,5,7,10,11,15	4,6,8,9,12,13,14,16	2	Q5RKI1,Q6IFW6
669	1,2,3,5,7,10,11,16	4,6,8,9,12,13,14,15	2	P20766,P47967
670	1,2,3,5,7,10,12,13	4,6,8,9,11,14,15,16	3	P47967,P97840,Q6AY61
671	1,2,3,5,7,10,12,14	4,6,8,9,11,13,15,16	3	P00714,Q5RLM2,Q6AY61
672	1,2,3,5,7,10,12,15	4,6,8,9,11,13,14,16	7	O54728,P19814,P34901,Q5RKI1,Q6AY61,Q6RUV5,Q99041
673	1,2,3,5,7,10,12,16	4,6,8,9,11,13,14,15	5	P18297,P47967,Q5RLM2,Q6AY61,Q71MB6
674	1,2,3,5,7,10,13,14	4,6,8,9,11,12,15,16	3	Q62714,Q6AY61,Q9WUW8
675	1,2,3,5,7,10,13,15	4,6,8,9,11,12,14,16	8	O70594,P63095,Q5RKI1,Q62714,Q63618,Q64093,Q6AY61,Q9WUW8
676	1,2,3,5,7,10,13,16	4,6,8,9,11,12,14,15	10	O70594,P18297,P19468,P46844,P47967,P63095,Q63424,Q64093,Q6AY61,Q9WUW8
677	1,2,3,5,7,10,14,15	4,6,8,9,11,12,13,16	7	P00714,P54921,P63095,Q5RKI1,Q62714,Q6AY61,Q9WUW8
678	1,2,3,5,7,10,14,16	4,6,8,9,11,12,13,15	7	P18297,P57113,P63095,Q5RLM2,Q6AY61,Q71MB6,Q9WUW8
679	1,2,3,5,7,10,15,16	4,6,8,9,11,12,13,14	21	O70594,P17988,P18297,P19468,P20766,P46844,P48508,P53790,P63095,Q05175,Q5I0E9,Q5RKI1,Q63355,Q63424,Q64093,Q6AY61,Q71MB6,Q80W57,Q8R431,Q9WTW7,Q9Z0W7
680	1,2,3,5,7,11,12,13	4,6,8,9,10,14,15,16	5	P47967,P97840,Q5I0D1,Q6AY61,Q811M5
681	1,2,3,5,7,11,12,14	4,6,8,9,10,13,15,16	3	P47967,Q6AY61,Q811M5
682	1,2,3,5,7,11,12,15	4,6,8,9,10,13,14,16	3	P47967,Q6AY61,Q6IFW6

683	1,2,3,5,7,11,12,16	4,6,8,9,10,13,14,15	3	P47967,P97840,Q6AY61
684	1,2,3,5,7,11,13,14	4,6,8,9,10,12,15,16	2	P08649,Q811M5
685	1,2,3,5,7,11,13,15	4,6,8,9,10,12,14,16	3	P08649,P47967,Q6IFW6
686	1,2,3,5,7,11,13,16	4,6,8,9,10,12,14,15	2	P08649,P47967
687	1,2,3,5,7,11,14,15	4,6,8,9,10,12,13,16	2	P08649,Q6IFW6
688	1,2,3,5,7,11,14,16	4,6,8,9,10,12,13,15	1	P08649
689	1,2,3,5,7,11,15,16	4,6,8,9,10,12,13,14	4	P08649,P17988,Q4FZU2,Q6IFW6
690	1,2,3,5,7,12,13,14	4,6,8,9,10,11,15,16	4	P01835,P47967,Q6AY61,Q811M5
691	1,2,3,5,7,12,13,15	4,6,8,9,10,11,14,16	3	P19814,P47967,Q6AY61
692	1,2,3,5,7,12,13,16	4,6,8,9,10,11,14,15	3	P47967,P97840,Q6AY61
693	1,2,3,5,7,12,14,15	4,6,8,9,10,11,13,16	2	P00714,Q6AY61
694	1,2,3,5,7,12,14,16	4,6,8,9,10,11,13,15	2	Q5RLM2,Q6AY61
695	1,2,3,5,7,12,15,16	4,6,8,9,10,11,13,14	1	Q6AY61
696	1,2,3,5,7,13,14,15	4,6,8,9,10,11,12,16	4	P01835,Q62714,Q6AY61,Q9WUW8
697	1,2,3,5,7,13,14,16	4,6,8,9,10,11,12,15	4	P01835,Q62714,Q6AY61,Q9WUW8
698	1,2,3,5,7,13,15,16	4,6,8,9,10,11,12,14	4	P01835,Q62714,Q6AY61,Q9WUW8
699	1,2,3,5,7,14,15,16	4,6,8,9,10,11,12,13	4	P08649,Q62714,Q6AY61,Q9WUW8
700	1,2,3,5,8,9,10,11	4,6,7,12,13,14,15,16	2	Q99MH3,Q9Z2L0
701	1,2,3,5,8,9,10,12	4,6,7,11,13,14,15,16	6	P00714,P11598,P34901,P36376,Q99041,Q99MH3
702	1,2,3,5,8,9,10,13	4,6,7,11,12,14,15,16	0	

703	1,2,3,5,8,9,10,14	4,6,7,11,12,13,15,16	2	P00714,P11598
704	1,2,3,5,8,9,10,15	4,6,7,11,12,13,14,16	8	P00714,P01681,P11598,P19132,P34901,P36376,Q99041,Q99MH3
705	1,2,3,5,8,9,10,16	4,6,7,11,12,13,14,15	0	
706	1,2,3,5,8,9,11,12	4,6,7,10,13,14,15,16	5	O88797,P36376,Q03191,Q6IG05,Q99MH3
707	1,2,3,5,8,9,11,13	4,6,7,10,12,14,15,16	1	Q6IG05
708	1,2,3,5,8,9,11,14	4,6,7,10,12,13,15,16	1	Q6IG05
709	1,2,3,5,8,9,11,15	4,6,7,10,12,13,14,16	11	P01681,P36376,Q4FZU2,Q6IFU7,Q6IFU8,Q6IFW6,Q6IG05,Q6IMF3,Q6P6Q2,Q6P6S4,Q99MH3
710	1,2,3,5,8,9,11,16	4,6,7,10,12,13,14,15	2	O88797,Q6IG05
711	1,2,3,5,8,9,12,13	4,6,7,10,11,14,15,16	1	Q99041
712	1,2,3,5,8,9,12,14	4,6,7,10,11,13,15,16	4	P00714,P11598,P36376,Q99041
713	1,2,3,5,8,9,12,15	4,6,7,10,11,13,14,16	9	P00714,P01681,P11598,P19132,P34901,P36376,Q6IG05,Q99041,Q99MH3
714	1,2,3,5,8,9,12,16	4,6,7,10,11,13,14,15	2	Q6IG05,Q99041
715	1,2,3,5,8,9,13,14	4,6,7,10,11,12,15,16	1	P00714
716	1,2,3,5,8,9,13,15	4,6,7,10,11,12,14,16	6	P00714,P11598,P19132,P36376,Q6IG05,Q99041
717	1,2,3,5,8,9,13,16	4,6,7,10,11,12,14,15	0	
718	1,2,3,5,8,9,14,15	4,6,7,10,11,12,13,16	6	P00714,P01681,P11598,P19132,P36376,Q6IG05
719	1,2,3,5,8,9,14,16	4,6,7,10,11,12,13,15	1	P00714
720	1,2,3,5,8,9,15,16	4,6,7,10,11,12,13,14	5	P01681,P19132,P36376,Q6IG05,Q99041
721	1,2,3,5,8,10,11,12	4,6,7,9,13,14,15,16	3	P47967,Q6IFU7,Q6IG05
722	1,2,3,5,8,10,11,13	4,6,7,9,12,14,15,16	2	Q6IFU7,Q6IG05

723	1,2,3,5,8,10,11,14	4,6,7,9,12,13,15,16	2	Q6IFU7,Q6IG05
724	1,2,3,5,8,10,11,15	4,6,7,9,12,13,14,16	8	Q4FZU2,Q6IFU7,Q6IFU8,Q6IFW6,Q6IG02,Q6IG05,Q6IMF3,Q6P6Q2
725	1,2,3,5,8,10,11,16	4,6,7,9,12,13,14,15	2	Q6IFU7,Q6IG05
726	1,2,3,5,8,10,12,13	4,6,7,9,11,14,15,16	1	P47967
727	1,2,3,5,8,10,12,14	4,6,7,9,11,13,15,16	4	P00714,Q5RLM2,Q63617,Q6IG05
728	1,2,3,5,8,10,12,15	4,6,7,9,11,13,14,16	9	P00714,P19132,P34901,P36376,Q498D9,Q63617,Q6IG05,Q99041,Q9R168
729	1,2,3,5,8,10,12,16	4,6,7,9,11,13,14,15	2	P0DMW0;P0DMW1,Q6IG05
730	1,2,3,5,8,10,13,14	4,6,7,9,11,12,15,16	1	Q9WUW8
731	1,2,3,5,8,10,13,15	4,6,7,9,11,12,14,16	6	P19132,Q498D9,Q62714,Q63618,Q6IG05,Q99376
732	1,2,3,5,8,10,13,16	4,6,7,9,11,12,14,15	2	P46844,Q9WUW8
733	1,2,3,5,8,10,14,15	4,6,7,9,11,12,13,16	7	P00714,P19132,P54921,P63322,Q62714,Q63617,Q6IG05
734	1,2,3,5,8,10,14,16	4,6,7,9,11,12,13,15	3	P97580,Q5RLM2,Q9WUW8
735	1,2,3,5,8,10,15,16	4,6,7,9,11,12,13,14	10	O70377,O70594,P19468,P48508,P53790,Q05175,Q64093,Q6IG05,Q8R431,Q9WTW7
736	1,2,3,5,8,11,12,13	4,6,7,9,10,14,15,16	5	P47967,Q5I0D1,Q6IFU7,Q6IG05,Q811M5
737	1,2,3,5,8,11,12,14	4,6,7,9,10,13,15,16	4	P11883,Q6IFU7,Q6IG05,Q811M5
738	1,2,3,5,8,11,12,15	4,6,7,9,10,13,14,16	9	P36376,Q4FZU2,Q6IFU7,Q6IFU8,Q6IFW6,Q6IG02,Q6IG05,Q6IMF3,Q6P6Q2
739	1,2,3,5,8,11,12,16	4,6,7,9,10,13,14,15	3	O88797,Q6IFU7,Q6IG05
740	1,2,3,5,8,11,13,14	4,6,7,9,10,12,15,16	3	Q6IFU7,Q6IG05,Q811M5
741	1,2,3,5,8,11,13,15	4,6,7,9,10,12,14,16	8	Q4FZU2,Q6IFU7,Q6IFU8,Q6IFW6,Q6IG02,Q6IG05,Q6IMF3,Q6P6Q2
742	1,2,3,5,8,11,13,16	4,6,7,9,10,12,14,15	3	P30120,Q6IFU7,Q6IG05

743	1,2,3,5,8,11,14,15	4,6,7,9,10,12,13,16	9	P00714,Q4FZU2,Q6IFU7,Q6IFU8,Q6IFW6,Q6IG02,Q6IG05,Q6IMF3,Q6P6Q2
744	1,2,3,5,8,11,14,16	4,6,7,9,10,12,13,15	2	Q6IFU7,Q6IG05
745	1,2,3,5,8,11,15,16	4,6,7,9,10,12,13,14	8	Q4FZU2,Q6IFU7,Q6IFU8,Q6IFW6,Q6IG02,Q6IG05,Q6IMF3,Q6P6Q2
746	1,2,3,5,8,12,13,14	4,6,7,9,10,11,15,16	3	P00714,Q6IG05,Q811M5
747	1,2,3,5,8,12,13,15	4,6,7,9,10,11,14,16	3	P19132,P36376,Q6IG05
748	1,2,3,5,8,12,13,16	4,6,7,9,10,11,14,15	1	Q6IG05
749	1,2,3,5,8,12,14,15	4,6,7,9,10,11,13,16	5	P00714,P19132,P36376,Q63617,Q6IG05
750	1,2,3,5,8,12,14,16	4,6,7,9,10,11,13,15	1	Q6IG05
751	1,2,3,5,8,12,15,16	4,6,7,9,10,11,13,14	2	Q6IG05,Q99041
752	1,2,3,5,8,13,14,15	4,6,7,9,10,11,12,16	3	P00714,Q62714,Q6IG05
753	1,2,3,5,8,13,14,16	4,6,7,9,10,11,12,15	2	Q62714,Q9WUW8
754	1,2,3,5,8,13,15,16	4,6,7,9,10,11,12,14	2	Q62714,Q6IG05
755	1,2,3,5,8,14,15,16	4,6,7,9,10,11,12,13	3	P00714,Q62714,Q6IG05
756	1,2,3,5,9,10,11,12	4,6,7,8,13,14,15,16	3	Q03191,Q99MH3,Q9Z2L0
757	1,2,3,5,9,10,11,13	4,6,7,8,12,14,15,16	1	Q9Z2L0
758	1,2,3,5,9,10,11,14	4,6,7,8,12,13,15,16	1	Q9Z2L0
759	1,2,3,5,9,10,11,15	4,6,7,8,12,13,14,16	2	Q99MH3,Q9Z2L0
760	1,2,3,5,9,10,11,16	4,6,7,8,12,13,14,15	2	P70549,Q9Z2L0
761	1,2,3,5,9,10,12,13	4,6,7,8,11,14,15,16	19	P02780,P02781,P06761,P07150,P08723,P0C0A9,P11598,P22273,P22282,P22283,P46462,Q03191,Q5M8C6,Q63617,Q6AY61,Q8CJ52,Q99041,Q9QW07,Q9QZK9
762	1,2,3,5,9,10,12,14	4,6,7,8,11,13,15,16	21	P00714,P02780,P02781,P02782,P06761,P08723,P09456,P0C0A9,P11598,P22273,P22282,P22283,P24368,P30120,P36374,P46462,Q5M8C6,Q63617,Q6AY61,Q8CJ52,Q99041

763	1,2,3,5,9,10,12,15	4,6,7,8,11,13,14,16	23	O54728,P00714,P02780,P02781,P02782,P06761,P07647,P08723,P09456,P0C0A9,P11598,P22273,P22282,P22283,P30120,P34901,P46462,Q5M8C6,Q63617,Q6AY61,Q8CFN2,Q99041,Q99MH3
764	1,2,3,5,9,10,12,16	4,6,7,8,11,13,14,15	7	P06761,P11598,P22273,P22282,P46462,Q63617,Q99041
765	1,2,3,5,9,10,13,14	4,6,7,8,11,12,15,16	20	P00714,P02780,P02781,P02782,P06761,P08723,P0C0A9,P11598,P22273,P22282,P22283,P30120,P36374,P46462,Q5M8C6,Q63617,Q8CJ52,Q99041,Q9QW07,Q9QZK9
766	1,2,3,5,9,10,13,15	4,6,7,8,11,12,14,16	24	P00714,P02780,P02781,P02782,P06761,P07150,P08723,P0C0A9,P11598,P12020,P22273,P22282,P22283,P30120,P36374,P46462,Q5M8C6,Q63617,Q8CFN2,Q99041,Q99376,Q99MH3,Q9QW07,Q9QZK9
767	1,2,3,5,9,10,13,16	4,6,7,8,11,12,14,15	8	P06761,P07150,P11598,P22273,P22282,P46462,Q63617,Q99041
768	1,2,3,5,9,10,14,15	4,6,7,8,11,12,13,16	22	P00714,P02780,P02781,P02782,P06761,P07647,P08723,P09456,P0C0A9,P11598,P12020,P22273,P22282,P22283,P24368,P30120,P36374,P46462,Q5M8C6,Q63617,Q8CFN2,Q99041
769	1,2,3,5,9,10,14,16	4,6,7,8,11,12,13,15	8	P00714,P06761,P11598,P22273,P22282,P46462,Q63617,Q99041
770	1,2,3,5,9,10,15,16	4,6,7,8,11,12,13,14	10	P06761,P11598,P12020,P22273,P22282,P22283,P46462,Q5M8C6,Q63617,Q99041
771	1,2,3,5,9,11,12,13	4,6,7,8,10,14,15,16	2	P54921,Q03191
772	1,2,3,5,9,11,12,14	4,6,7,8,10,13,15,16	4	O70257,P00714,P07151,Q03191
773	1,2,3,5,9,11,12,15	4,6,7,8,10,13,14,16	2	Q03191,Q99MH3
774	1,2,3,5,9,11,12,16	4,6,7,8,10,13,14,15	1	Q03191
775	1,2,3,5,9,11,13,14	4,6,7,8,10,12,15,16	1	P0DMW0;P0DMW1
776	1,2,3,5,9,11,13,15	4,6,7,8,10,12,14,16	1	Q99MH3
777	1,2,3,5,9,11,13,16	4,6,7,8,10,12,14,15	0	
778	1,2,3,5,9,11,14,15	4,6,7,8,10,12,13,16	2	P00714,P0DMW0;P0DMW1
779	1,2,3,5,9,11,14,16	4,6,7,8,10,12,13,15	0	
780	1,2,3,5,9,11,15,16	4,6,7,8,10,12,13,14	0	
781	1,2,3,5,9,12,13,14	4,6,7,8,10,11,15,16	15	P00714,P02780,P06761,P08723,P0C0A9,P11598,P22273,P22282,P22283,P46462,Q5M8C6,Q63617,Q6AY61,Q99041,Q9QW07
782	1,2,3,5,9,12,13,15	4,6,7,8,10,11,14,16	18	P00714,P02780,P02781,P06761,P07150,P08723,P0C0A9,P11598,P22273,P22282,P22283,P30120,P46462,Q5M8C6,Q63617,Q6AY61,Q99041,Q99MH3

783	1,2,3,5,9,12,13,16	4,6,7,8,10,11,14,15	7	P06761,P07150,P0C0A9,P11598,P22273,P22282,Q99041
784	1,2,3,5,9,12,14,15	4,6,7,8,10,11,13,16	20	P00714,P02780,P02781,P02782,P06761,P07647,P08723,P09456,P0C0A9,P11598,P22273,P22282,P22283,P24368,P30120,P46462,Q5M8C6,Q63617,Q6AY61,Q99041
785	1,2,3,5,9,12,14,16	4,6,7,8,10,11,13,15	8	P00714,P06761,P11598,P22273,P22282,Q63617,Q6AY61,Q99041
786	1,2,3,5,9,12,15,16	4,6,7,8,10,11,13,14	9	P00714,P06761,P11598,P22273,P22282,P46462,Q63617,Q6AY61,Q99041
787	1,2,3,5,9,13,14,15	4,6,7,8,10,11,12,16	19	P00714,P02780,P02781,P04905,P06761,P08723,P0C0A9,P0DMW0;P0DMW1,P11598,P22273,P22282,P22283,P30120,P36374,P46462,Q5M8C6,Q63617,Q99041,Q9QW07
788	1,2,3,5,9,13,14,16	4,6,7,8,10,11,12,15	6	P06761,P11598,P22273,P22282,Q63617,Q99041
789	1,2,3,5,9,13,15,16	4,6,7,8,10,11,12,14	8	P06761,P07150,P11598,P22273,P22282,P46462,Q63617,Q99041
790	1,2,3,5,9,14,15,16	4,6,7,8,10,11,12,13	10	P00714,P06761,P11598,P22273,P22282,P22283,P46462,Q5M8C6,Q63617,Q99041
791	1,2,3,5,10,11,12,13	4,6,7,8,9,14,15,16	1	P47967
792	1,2,3,5,10,11,12,14	4,6,7,8,9,13,15,16	0	
793	1,2,3,5,10,11,12,15	4,6,7,8,9,13,14,16	0	
794	1,2,3,5,10,11,12,16	4,6,7,8,9,13,14,15	0	
795	1,2,3,5,10,11,13,14	4,6,7,8,9,12,15,16	0	
796	1,2,3,5,10,11,13,15	4,6,7,8,9,12,14,16	1	P21674
797	1,2,3,5,10,11,13,16	4,6,7,8,9,12,14,15	0	
798	1,2,3,5,10,11,14,15	4,6,7,8,9,12,13,16	1	P00714
799	1,2,3,5,10,11,14,16	4,6,7,8,9,12,13,15	0	
800	1,2,3,5,10,11,15,16	4,6,7,8,9,12,13,14	0	
801	1,2,3,5,10,12,13,14	4,6,7,8,9,11,15,16	17	P00714,P02780,P06761,P07150,P08723,P0C0A9,P11598,P22273,P22282,P22283,P46462,Q5M8C6,Q63617,Q6AY61,Q8CJ52,Q99041,Q9QW07
802	1,2,3,5,10,12,13,15	4,6,7,8,9,11,14,16	19	P02780,P06761,P07150,P08723,P09456,P0C0A9,P11598,P22273,P22282,P22283,P46462,Q5M8C6,Q63617,Q6AY61,Q6RUV5,Q812E4,Q8CFN2,Q99041,Q99376

803	1,2,3,5,10,12,13,16	4,6,7,8,9,11,14,15	7	P07150,P22273,P22282,Q63617,Q6AY61,Q8CJ52,Q99041
804	1,2,3,5,10,12,14,15	4,6,7,8,9,11,13,16	21	P00714,P02780,P02781,P02782,P06761,P08723,P09456,P0C0A9,P11598,P22273,P22282,P22283,P30120,P46462,Q5M8C6,Q62902,Q63617,Q6AY61,Q6RUV5,Q812E4,Q99041
805	1,2,3,5,10,12,14,16	4,6,7,8,9,11,13,15	6	P06761,P22273,P22282,Q63617,Q6AY61,Q99041
806	1,2,3,5,10,12,15,16	4,6,7,8,9,11,13,14	10	P06761,P07150,P11598,P22273,P22282,P46462,Q63617,Q6AY61,Q812E4,Q99041
807	1,2,3,5,10,13,14,15	4,6,7,8,9,11,12,16	24	P00714,P02780,P02781,P04905,P06761,P07150,P08723,P09456,P0C0A9,P11598,P22273,P22282,P22283,P30120,P36374,P46462,P63322,P97523,Q5M8C6,Q63617,Q812E4,Q8CFN2,Q99041,Q9QW07
808	1,2,3,5,10,13,14,16	4,6,7,8,9,11,12,15	5	P07150,P22273,P22282,Q63617,Q9WUW8
809	1,2,3,5,10,13,15,16	4,6,7,8,9,11,12,14	8	P06761,P07150,P22273,P22282,P46462,Q63617,Q812E4,Q99041
810	1,2,3,5,10,14,15,16	4,6,7,8,9,11,12,13	8	P00714,P06761,P11598,P22273,P22282,P46462,Q63617,Q99041
811	1,2,3,5,11,12,13,14	4,6,7,8,9,10,15,16	1	Q811M5
812	1,2,3,5,11,12,13,15	4,6,7,8,9,10,14,16	0	
813	1,2,3,5,11,12,13,16	4,6,7,8,9,10,14,15	1	P47967
814	1,2,3,5,11,12,14,15	4,6,7,8,9,10,13,16	1	P00714
815	1,2,3,5,11,12,14,16	4,6,7,8,9,10,13,15	0	
816	1,2,3,5,11,12,15,16	4,6,7,8,9,10,13,14	0	
817	1,2,3,5,11,13,14,15	4,6,7,8,9,10,12,16	0	
818	1,2,3,5,11,13,14,16	4,6,7,8,9,10,12,15	0	
819	1,2,3,5,11,13,15,16	4,6,7,8,9,10,12,14	1	O70417
820	1,2,3,5,11,14,15,16	4,6,7,8,9,10,12,13	0	
821	1,2,3,5,12,13,14,15	4,6,7,8,9,10,11,16	16	P00714,P02780,P06761,P07150,P08723,P0C0A9,P11598,P22273,P22282,P22283,P46462,Q5M8C6,Q63617,Q6AY61,Q6P6R2,Q99041
822	1,2,3,5,12,13,14,16	4,6,7,8,9,10,11,15	7	P07150,P22273,P22282,Q63617,Q6AY61,Q6P6R2,Q99041

823	1,2,3,5,12,13,15,16	4,6,7,8,9,10,11,14	8	P06761,P07150,P22273,P22282,Q63617,Q6AY61,Q6P6R2,Q99041
824	1,2,3,5,12,14,15,16	4,6,7,8,9,10,11,13	9	P00714,P06761,P11598,P22273,P22282,Q63617,Q6AY61,Q6P6R2,Q99041
825	1,2,3,5,13,14,15,16	4,6,7,8,9,10,11,12	7	P06761,P07150,P22273,P22282,Q62714,Q63617,Q6P6R2
826	1,2,3,6,7,8,9,10	4,5,11,12,13,14,15,16	16	iRT-Kit_WR_fusion,O35763,O70377,O70594,P02631,P19629,P20766,P23739,P55091,P70545,P97580,Q05175,Q63598,Q63618,Q6P6R2,Q99MH3
827	1,2,3,6,7,8,9,11	4,5,10,12,13,14,15,16	18	iRT-Kit_WR_fusion,P06911,P08723,P12020,P19629,P35280,P50280,P55091,P63029,Q5GRG2,Q63493,Q6P6R2,Q6P6S4,Q78P75,Q812E4,Q99MH3,Q9JHB9,Q9JI85
828	1,2,3,6,7,8,9,12	4,5,10,11,13,14,15,16	5	iRT-Kit_WR_fusion,P06911,P19629,Q63598,Q99MH3
829	1,2,3,6,7,8,9,13	4,5,10,11,12,14,15,16	3	iRT-Kit_WR_fusion,P02631,P19629
830	1,2,3,6,7,8,9,14	4,5,10,11,12,13,15,16	5	iRT-Kit_WR_fusion,P08937,P97580,Q5QE79,Q63751
831	1,2,3,6,7,8,9,15	4,5,10,11,12,13,14,16	6	iRT-Kit_WR_fusion,P02091,P19629,P35280,Q63598,Q99MH3
832	1,2,3,6,7,8,9,16	4,5,10,11,12,13,14,15	5	iRT-Kit_WR_fusion,P19629,P23739,Q09030,Q63598
833	1,2,3,6,7,8,10,11	4,5,9,12,13,14,15,16	23	iRT-Kit_WR_fusion,P02631,P06911,P08723,P12020,P15399,P18757,P19629,P20766,P22283,P31044,P50280,P55091,P63029,P97580,Q5GRG2,Q63493,Q66H69,Q68G31,Q6P6S4,Q9JHB9,Q9JI85,Q9WTW7
834	1,2,3,6,7,8,10,12	4,5,9,11,13,14,15,16	33	iRT-Kit_WR_fusion,O35763,O70377,P02631,P06911,P18757,P19468,P19629,P20766,P30904,P31044,P38918,P46844,P48508,P53790,P57113,P97580,Q05175,Q3ZAV1,Q5RLM2,Q63598,Q63618,Q64602,Q66H69,Q66HG3,Q6AY41,Q6MG61,Q6Q0N1,Q80W57,Q91ZS3,Q923S2,Q9QYU4,Q9WTW7
835	1,2,3,6,7,8,10,13	4,5,9,11,12,14,15,16	42	O35763,O70257,O70377,O70594,O88339;Q4V882,P02631,P19468,P19629,P20766,P25809,P29975,P30904,P31044,P36860,P38918,P46844,P48508,P51907,P53790,P55091,P57113,P97580,Q05175,Q3T1J9,Q3ZAV1,Q5I0E9,Q62714,Q63424,Q63598,Q63618,Q64093,Q66H69,Q66HG3,Q68G31,Q6AY41,Q6MG61,Q6Q0N1,Q80W57,Q9WTW7,Q9WUW8,Q9WUW9,Q9Z0W7

836	1,2,3,6,7,8,10,14	4,5,9,11,12,13,15,16	33	O70377,O70594,O88339;Q4V882,P02631,P08937,P18757,P19468,P19629,P20766,P31044,P46844,P48508,P53790,P54921,P57113,P63081,P97580,Q05175,Q5I0E9,Q5QE79,Q5RLM2,Q62714,Q63598,Q63618,Q63751,Q64602,Q66HG3,Q68G31,Q80W57,Q923S2,Q9WTW7,Q9WUW8,Q9WUW9
837	1,2,3,6,7,8,10,15	4,5,9,11,12,13,14,16	52	iRT-Kit_WR_fusion,O35763,O70377,O70594,O88339;Q4V882,P02091,P02631,P15399,P18757,P19468,P19629,P20766,P23928,P29975,P30904,P31044,P38918,P46844,P48508,P51907,P53790,P54921,P57113,P68035;P68136,P97580,Q03248,Q05175,Q3T1J9,Q3ZAV1,Q498D9,Q5I0E9,Q5M7T9,Q5RKI1,Q62714,Q63270,Q63424,Q63598,Q63618,Q64093,Q64602,Q6AY41,Q6MG61,Q6Q0N1,Q80W57,Q8R431,Q923S2,Q9JJ19,Q9JLJ3,Q9QYU4,Q9R1T5,Q9WTW7,Q9Z0W7
838	1,2,3,6,7,8,10,16	4,5,9,11,12,13,14,15	61	iRT-Kit_WR_fusion,O35763,O70257,O70377,O70594,O88339;Q4V882,P02631,P02783,P07151,P15399,P18757,P19468,P19629,P20766,P23928,P29975,P31044,P38918,P46720,P46844,P48508,P51907,P53790,P57113,P60711;P63259,P68035;P68136,P97580,Q03248,Q05175,Q30KJ2,Q3T1J9,Q3ZAV1,Q5I0E9,Q5M7T9,Q62687,Q62761;Q62762;Q62763,Q63270,Q63424,Q63598,Q63618,Q64093,Q64602,Q66HG3,Q68FT5,Q6AY33,Q6AY41,Q6MG61,Q6Q0N1,Q71MB6,Q80W57,Q8R431,Q923S2,Q9JJ19,Q9JJ40,Q9JLJ3,Q9QYU4,Q9R1T5,Q9WTW7,Q9WUW8,Q9WUW9,Q9Z0W7
839	1,2,3,6,7,8,11,12	4,5,9,10,13,14,15,16	20	iRT-Kit_WR_fusion,P06911,P08723,P12020,P19629,P22283,P31044,P31430,P46462,P50280,P63029,P70709,Q5GRG2,Q63493,Q66H69,Q6IG05,Q78P75,Q9JHB9,Q9JI85,Q9QW07
840	1,2,3,6,7,8,11,13	4,5,9,10,12,14,15,16	17	iRT-Kit_WR_fusion,P06911,P08723,P12020,P19629,P22283,P31044,P46462,P50280,P63029,Q5GRG2,Q63493,Q66H69,Q68G31,Q78P75,Q9JHB9,Q9JI85
841	1,2,3,6,7,8,11,14	4,5,9,10,12,13,15,16	16	iRT-Kit_WR_fusion,P06911,P08723,P08937,P12020,P19629,P22283,P31044,P50280,P63029,P97580,Q5GRG2,Q63493,Q63751,Q9JHB9,Q9JI85
842	1,2,3,6,7,8,11,15	4,5,9,10,12,13,14,16	20	iRT-Kit_WR_fusion,P02091,P06911,P08723,P12020,P19629,P31044,P50280,Q5GRG2,Q63493,Q6IFU7,Q6IFW6,Q6IG05,Q6IMF3,Q6P6S4,Q6Q0N0,Q8CJ52,Q9JHB9,Q9JI85,Q9QW07
843	1,2,3,6,7,8,11,16	4,5,9,10,12,13,14,15	34	iRT-Kit_WR_fusion,O35547,P02780,P02781,P02782,P02783,P05369,P06761,P06911,P07647,P08010,P08723,P09456,P0C0A9,P12020,P19629,P22283,P24368,P30120,P31044,P46462,P50280,P63029,P97580,Q00715,Q5GRG2,Q5M8C6,Q63493,Q66H69,Q6Q0N0,Q9JHB9,Q9JI85,Q9QW07,Q9WTW7
844	1,2,3,6,7,8,12,13	4,5,9,10,11,14,15,16	9	iRT-Kit_WR_fusion,P01835,P02631,P06911,P19629,P31044,Q63598,Q66H69,Q9JI85
845	1,2,3,6,7,8,12,14	4,5,9,10,11,13,15,16	10	iRT-Kit_WR_fusion,P06911,P08937,P12020,P19629,P31044,P97580,Q5GRG2,Q5RLM2,Q63751
846	1,2,3,6,7,8,12,15	4,5,9,10,11,13,14,16	8	iRT-Kit_WR_fusion,P02091,P06911,P19629,P31044,Q63598,Q6IG05,Q9WTW7

847	1,2,3,6,7,8,12,16	4,5,9,10,11,13,14,15	11	iRT-Kit_WR_fusion,P02783,P06911,P12020,P19629,P46844,P57113,Q63598,Q66H69,Q9JHB9,Q9WTW7
848	1,2,3,6,7,8,13,14	4,5,9,10,11,12,15,16	11	P01835,P06911,P08937,P31044,P63081,P97580,Q5QE79,Q62714,Q66H69,Q68G31,Q9WUW8
849	1,2,3,6,7,8,13,15	4,5,9,10,11,12,14,16	10	iRT-Kit_WR_fusion,O70594,P01835,P02091,P19629,Q62714,Q63598,Q66H69,Q68G31,Q6Q0N1
850	1,2,3,6,7,8,13,16	4,5,9,10,11,12,14,15	19	iRT-Kit_WR_fusion,O70257,O70594,P01835,P02631,P19468,P19629,P46844,P57113,P97580,Q3ZAV1,Q62714,Q63424,Q63598,Q66H69,Q6AY41,Q9JI85,Q9WTW7,Q9WUW8
851	1,2,3,6,7,8,14,15	4,5,9,10,11,12,13,16	8	iRT-Kit_WR_fusion,P02091,P19629,P50280,P54921,P97580,Q62714,Q62812
852	1,2,3,6,7,8,14,16	4,5,9,10,11,12,13,15	13	iRT-Kit_WR_fusion,P02783,P06911,P19629,P46844,P50280,P57113,P63081,P97580,Q62714,Q62812,Q923S2,Q9WUW8
853	1,2,3,6,7,8,15,16	4,5,9,10,11,12,13,14	35	iRT-Kit_WR_fusion,O70377,O70594,P02091,P02783,P09606,P15399,P17988,P19468,P19629,P29975,P38918,P46844,P48508,P50280,P53790,P57113,P97580,Q03248,Q05175,Q3ZAV1,Q5I0E9,Q5M7T9,Q62714,Q62812,Q63270,Q63424,Q63598,Q64093,Q6AY41,Q6Q0N1,Q80W57,Q9R1T5,Q9WTW7,Q9Z0W7
854	1,2,3,6,7,9,10,11	4,5,8,12,13,14,15,16	2	P20766,Q99MH3
855	1,2,3,6,7,9,10,12	4,5,8,11,13,14,15,16	3	O54728,P20766,Q99MH3
856	1,2,3,6,7,9,10,13	4,5,8,11,12,14,15,16	2	P01835,P20766
857	1,2,3,6,7,9,10,14	4,5,8,11,12,13,15,16	0	
858	1,2,3,6,7,9,10,15	4,5,8,11,12,13,14,16	6	O54728,P02091,P20766,Q5RKI1,Q63598,Q99MH3
859	1,2,3,6,7,9,10,16	4,5,8,11,12,13,14,15	3	P20766,P23739,Q63598
860	1,2,3,6,7,9,11,12	4,5,8,10,13,14,15,16	1	Q99MH3
861	1,2,3,6,7,9,11,13	4,5,8,10,12,14,15,16	1	P0DMW0;P0DMW1
862	1,2,3,6,7,9,11,14	4,5,8,10,12,13,15,16	1	P0DMW0;P0DMW1
863	1,2,3,6,7,9,11,15	4,5,8,10,12,13,14,16	3	P02091,P35280,Q99MH3

864	1,2,3,6,7,9,11,16	4,5,8,10,12,13,14,15	2	P02783,Q9JHB9
865	1,2,3,6,7,9,12,13	4,5,8,10,11,14,15,16	1	P01835
866	1,2,3,6,7,9,12,14	4,5,8,10,11,13,15,16	0	
867	1,2,3,6,7,9,12,15	4,5,8,10,11,13,14,16	4	O54728,P02091,Q6AY61,Q99MH3
868	1,2,3,6,7,9,12,16	4,5,8,10,11,13,14,15	0	
869	1,2,3,6,7,9,13,14	4,5,8,10,11,12,15,16	2	P01835,P0DMW0;P0DMW1
870	1,2,3,6,7,9,13,15	4,5,8,10,11,12,14,16	2	P01835,P02091
871	1,2,3,6,7,9,13,16	4,5,8,10,11,12,14,15	1	P01835
872	1,2,3,6,7,9,14,15	4,5,8,10,11,12,13,16	2	P02091,P0DMW0;P0DMW1
873	1,2,3,6,7,9,14,16	4,5,8,10,11,12,13,15	2	P02783,Q09030
874	1,2,3,6,7,9,15,16	4,5,8,10,11,12,13,14	1	P02091
875	1,2,3,6,7,10,11,12	4,5,8,9,13,14,15,16	1	P20766
876	1,2,3,6,7,10,11,13	4,5,8,9,12,14,15,16	3	P01835,P20766,Q68G31
877	1,2,3,6,7,10,11,14	4,5,8,9,12,13,15,16	1	P63029
878	1,2,3,6,7,10,11,15	4,5,8,9,12,13,14,16	4	P02091,P08721,P20766,Q5RKI1
879	1,2,3,6,7,10,11,16	4,5,8,9,12,13,14,15	4	P02783,P20766,Q9JHB9,Q9WTW7
880	1,2,3,6,7,10,12,13	4,5,8,9,11,14,15,16	5	P01835,P20766,Q63598,Q66HG3,Q6AY61
881	1,2,3,6,7,10,12,14	4,5,8,9,11,13,15,16	4	P01835,P57113,Q5RLM2,Q6AY61
882	1,2,3,6,7,10,12,15	4,5,8,9,11,13,14,16	8	O54728,P02091,P19629,P20766,Q5RKI1,Q63598,Q6AY61,Q9WTW7
883	1,2,3,6,7,10,12,16	4,5,8,9,11,13,14,15	8	P18297,P19468,P20766,P46844,P57113,Q63598,Q71MB6,Q9WTW7

884	1,2,3,6,7,10,13,14	4,5,8,9,11,12,15,16	5	O54800;Q5DWV2,P01835,P57113,Q62714,Q9WUW8
885	1,2,3,6,7,10,13,15	4,5,8,9,11,12,14,16	11	O70594,O88339;Q4V882,P01835,P02091,P19468,P20766,P57113,Q5RKI1,Q62714,Q63598,Q63618
886	1,2,3,6,7,10,13,16	4,5,8,9,11,12,14,15	13	O70594,P01835,P18297,P19468,P20766,P29975,P46844,P57113,Q63424,Q63598,Q64093,Q9WTW7,Q9WUW8
887	1,2,3,6,7,10,14,15	4,5,8,9,11,12,13,16	8	O88339;Q4V882,P01835,P01946,P02091,P54921,P57113,Q5RKI1,Q62714
888	1,2,3,6,7,10,14,16	4,5,8,9,11,12,13,15	8	P01835,P02783,P18297,P19468,P46844,P57113,Q71MB6,Q9WUW8
889	1,2,3,6,7,10,15,16	4,5,8,9,11,12,13,14	30	O70377,O70594,P01835,P02091,P02783,P17988,P18297,P19468,P19629,P20766,P29975,P38918,P46844,P48508,P53790,P57113,P63095,Q03248,Q05175,Q5I0E9,Q5RKI1,Q63270,Q63424,Q63598,Q64093,Q6Q0N1,Q71MB6,Q80W57,Q9WTW7,Q9Z0W7
890	1,2,3,6,7,11,12,13	4,5,8,9,10,14,15,16	2	P01835,P47967
891	1,2,3,6,7,11,12,14	4,5,8,9,10,13,15,16	1	Q5GRG2
892	1,2,3,6,7,11,12,15	4,5,8,9,10,13,14,16	1	P02091
893	1,2,3,6,7,11,12,16	4,5,8,9,10,13,14,15	4	P02783,P05369,Q00715,Q9JHB9
894	1,2,3,6,7,11,13,14	4,5,8,9,10,12,15,16	2	P01835,P63029
895	1,2,3,6,7,11,13,15	4,5,8,9,10,12,14,16	2	P01835,P02091
896	1,2,3,6,7,11,13,16	4,5,8,9,10,12,14,15	3	P01835,P02783,P05369
897	1,2,3,6,7,11,14,15	4,5,8,9,10,12,13,16	1	P02091
898	1,2,3,6,7,11,14,16	4,5,8,9,10,12,13,15	2	P02783,Q9JHB9
899	1,2,3,6,7,11,15,16	4,5,8,9,10,12,13,14	5	P02091,P02783,P08721,P17988,Q8CJ52
900	1,2,3,6,7,12,13,14	4,5,8,9,10,11,15,16	2	P01835,Q6AY61
901	1,2,3,6,7,12,13,15	4,5,8,9,10,11,14,16	2	P01835,Q6AY61
902	1,2,3,6,7,12,13,16	4,5,8,9,10,11,14,15	1	P01835
903	1,2,3,6,7,12,14,15	4,5,8,9,10,11,13,16	3	P01835,P02091,Q6AY61

904	1,2,3,6,7,12,14,16	4,5,8,9,10,11,13,15	2	P01835,P02783
905	1,2,3,6,7,12,15,16	4,5,8,9,10,11,13,14	6	P01835,P02091,P02783,P17988,Q63598,Q6AY61
906	1,2,3,6,7,13,14,15	4,5,8,9,10,11,12,16	3	P01835,P02091,Q62714
907	1,2,3,6,7,13,14,16	4,5,8,9,10,11,12,15	4	P01835,P02783,Q62714,Q9WUW8
908	1,2,3,6,7,13,15,16	4,5,8,9,10,11,12,14	3	P01835,P02091,Q62714
909	1,2,3,6,7,14,15,16	4,5,8,9,10,11,12,13	4	P01835,P02091,P02783,Q62714
910	1,2,3,6,8,9,10,11	4,5,7,12,13,14,15,16	4	P63029,Q68G31,Q6P6S4,Q99MH3
911	1,2,3,6,8,9,10,12	4,5,7,11,13,14,15,16	1	Q99MH3
912	1,2,3,6,8,9,10,13	4,5,7,11,12,14,15,16	3	P97580,Q68G31,Q99MH3
913	1,2,3,6,8,9,10,14	4,5,7,11,12,13,15,16	6	P97580,P97840,Q06000,Q09030,Q68G31,Q99MH3
914	1,2,3,6,8,9,10,15	4,5,7,11,12,13,14,16	7	P02091,P19629,P36376,P97580,Q63598,Q68G31,Q99MH3
915	1,2,3,6,8,9,10,16	4,5,7,11,12,13,14,15	5	P15399,P97580,Q09030,Q30KJ2,Q63598
916	1,2,3,6,8,9,11,12	4,5,7,10,13,14,15,16	5	P09527,P63029,Q5GRG2,Q6P6S4,Q99MH3
917	1,2,3,6,8,9,11,13	4,5,7,10,12,14,15,16	5	P63029,Q68G31,Q6P6S4,Q99MH3,Q9JI85
918	1,2,3,6,8,9,11,14	4,5,7,10,12,13,15,16	4	P0DMW0;P0DMW1,P63029,Q5GRG2,Q6P6S4
919	1,2,3,6,8,9,11,15	4,5,7,10,12,13,14,16	4	P02091,P36376,Q6P6S4,Q99MH3
920	1,2,3,6,8,9,11,16	4,5,7,10,12,13,14,15	3	P02783,P50280,Q6P6S4
921	1,2,3,6,8,9,12,13	4,5,7,10,11,14,15,16	2	Q09030,Q99MH3
922	1,2,3,6,8,9,12,14	4,5,7,10,11,13,15,16	2	Q09030,Q99MH3
923	1,2,3,6,8,9,12,15	4,5,7,10,11,13,14,16	3	P02091,P36376,Q99MH3

924	1,2,3,6,8,9,12,16	4,5,7,10,11,13,14,15	1	Q09030
925	1,2,3,6,8,9,13,14	4,5,7,10,11,12,15,16	4	P0DMW0;P0DMW1,P97580,Q09030,Q68G31
926	1,2,3,6,8,9,13,15	4,5,7,10,11,12,14,16	3	P02091,Q68G31,Q99MH3
927	1,2,3,6,8,9,13,16	4,5,7,10,11,12,14,15	2	P97580,Q09030
928	1,2,3,6,8,9,14,15	4,5,7,10,11,12,13,16	5	P00714,P02091,P97580,P97840,Q99MH3
929	1,2,3,6,8,9,14,16	4,5,7,10,11,12,13,15	5	P02783,P23593,P97580,P97840,Q09030
930	1,2,3,6,8,9,15,16	4,5,7,10,11,12,13,14	3	P02091,P23593,Q99MH3
931	1,2,3,6,8,10,11,12	4,5,7,9,13,14,15,16	5	P12020,P63029,Q5GRG2,Q68G31,Q9JI85
932	1,2,3,6,8,10,11,13	4,5,7,9,12,14,15,16	5	P15399,P63029,P97580,Q68G31,Q9JI85
933	1,2,3,6,8,10,11,14	4,5,7,9,12,13,15,16	5	P15399,P63029,P97580,Q5GRG2,Q68G31
934	1,2,3,6,8,10,11,15	4,5,7,9,12,13,14,16	11	P01946,P02091,P15399,P19629,P63029,P97580,Q68G31,Q6IFU7,Q6IFW6,Q6P6S4,Q99MH3
935	1,2,3,6,8,10,11,16	4,5,7,9,12,13,14,15	9	P02783,P15399,P19629,P50280,P63029,P97580,Q30KJ2,Q68G31,Q9JI85
936	1,2,3,6,8,10,12,13	4,5,7,9,11,14,15,16	4	P02631,P97580,Q63598,Q68G31
937	1,2,3,6,8,10,12,14	4,5,7,9,11,13,15,16	2	P97580,Q68G31
938	1,2,3,6,8,10,12,15	4,5,7,9,11,13,14,16	6	P02091,P19629,P97580,Q498D9,Q63598,Q99MH3
939	1,2,3,6,8,10,12,16	4,5,7,9,11,13,14,15	7	P15399,P19629,P46844,P97580,Q30KJ2,Q63598,Q9WTW7
940	1,2,3,6,8,10,13,14	4,5,7,9,11,12,15,16	5	O54800;Q5DWV2,P14668,P15399,P97580,Q68G31
941	1,2,3,6,8,10,13,15	4,5,7,9,11,12,14,16	13	O70377,O70594,O88339;Q4V882,P01946,P02091,P15399,P19629,P97580,Q498D9,Q62714,Q63598,Q63618,Q68G31
942	1,2,3,6,8,10,13,16	4,5,7,9,11,12,14,15	9	O70377,P15399,P19468,P46844,P97580,Q30KJ2,Q63424,Q63598,Q68G31
943	1,2,3,6,8,10,14,15	4,5,7,9,11,12,13,16	10	O70377,O88339;Q4V882,P01946,P02091,P15399,P19629,P54921,P97580,Q62714,Q68G31

944	1,2,3,6,8,10,14,16	4,5,7,9,11,12,13,15	9	O70377,P02783,P14668,P15399,P46844,P57113,P97580,Q30KJ2,Q68G31
945	1,2,3,6,8,10,15,16	4,5,7,9,11,12,13,14	27	O70377,O70594,O88339;Q4V882,P01946,P02091,P02783,P15399,P19468,P19629,P38918,P46844,P48508,P53790,P57113,P97580,Q05175,Q30KJ2,Q3ZAV1,Q63424,Q63598,Q64093,Q68G31,Q6AY41,Q6Q0N1,Q80W57,Q9WTW7,Q9Z0W7
946	1,2,3,6,8,11,12,13	4,5,7,9,10,14,15,16	5	P12020,P63029,Q5GRG2,Q68G31,Q9JI85
947	1,2,3,6,8,11,12,14	4,5,7,9,10,13,15,16	5	P06911,P12020,P63029,Q5GRG2,Q9JI85
948	1,2,3,6,8,11,12,15	4,5,7,9,10,13,14,16	8	P02091,P12020,Q5GRG2,Q6IFU7,Q6IFW6,Q6IG05,Q6P6S4,Q99MH3
949	1,2,3,6,8,11,12,16	4,5,7,9,10,13,14,15	9	P02783,P12020,P30120,P50280,Q00715,Q5GRG2,Q6IG05,Q9JHB9,Q9JI85
950	1,2,3,6,8,11,13,14	4,5,7,9,10,12,15,16	5	P63029,P97580,Q5GRG2,Q68G31,Q9JI85
951	1,2,3,6,8,11,13,15	4,5,7,9,10,12,14,16	5	P02091,Q68G31,Q6IFU7,Q6IFW6,Q9JI85
952	1,2,3,6,8,11,13,16	4,5,7,9,10,12,14,15	10	P02782,P02783,P05369,P15399,P30120,P63029,P97580,Q5GRG2,Q68G31,Q9JI85
953	1,2,3,6,8,11,14,15	4,5,7,9,10,12,13,16	9	P02091,P50280,P63029,P97580,Q5GRG2,Q68G31,Q6IFU7,Q6IFW6,Q6IG05
954	1,2,3,6,8,11,14,16	4,5,7,9,10,12,13,15	7	P02783,P15399,P50280,P63029,P97580,Q5GRG2,Q9JHB9
955	1,2,3,6,8,11,15,16	4,5,7,9,10,12,13,14	10	P02091,P02783,P15399,P19629,P50280,P97580,Q6IFU7,Q6IFW6,Q6IG05,Q6P6S4
956	1,2,3,6,8,12,13,14	4,5,7,9,10,11,15,16	2	P97580,Q68G31
957	1,2,3,6,8,12,13,15	4,5,7,9,10,11,14,16	2	P02091,Q68G31
958	1,2,3,6,8,12,13,16	4,5,7,9,10,11,14,15	1	P97580
959	1,2,3,6,8,12,14,15	4,5,7,9,10,11,13,16	3	P00714,P02091,P97580
960	1,2,3,6,8,12,14,16	4,5,7,9,10,11,13,15	2	P02783,P97580
961	1,2,3,6,8,12,15,16	4,5,7,9,10,11,13,14	4	P02091,P02783,P19629,Q6IG05
962	1,2,3,6,8,13,14,15	4,5,7,9,10,11,12,16	4	P02091,P97580,Q62714,Q68G31
963	1,2,3,6,8,13,14,16	4,5,7,9,10,11,12,15	4	P02783,P97580,Q62714,Q68G31

964	1,2,3,6,8,13,15,16	4,5,7,9,10,11,12,14	5	P02091,P15399,P97580,Q62714,Q68G31
965	1,2,3,6,8,14,15,16	4,5,7,9,10,11,12,13	6	P02091,P02783,P15399,P97580,Q30KJ2,Q62714
966	1,2,3,6,9,10,11,12	4,5,7,8,13,14,15,16	1	Q99MH3
967	1,2,3,6,9,10,11,13	4,5,7,8,12,14,15,16	2	Q68G31,Q99MH3
968	1,2,3,6,9,10,11,14	4,5,7,8,12,13,15,16	1	Q99MH3
969	1,2,3,6,9,10,11,15	4,5,7,8,12,13,14,16	3	P02091,Q99MH3,Q9Z2L0
970	1,2,3,6,9,10,11,16	4,5,7,8,12,13,14,15	2	D3ZUC6,Q9Z2L0
971	1,2,3,6,9,10,12,13	4,5,7,8,11,14,15,16	5	P06760,P0C0A9,Q4G075,Q99041,Q99MH3
972	1,2,3,6,9,10,12,14	4,5,7,8,11,13,15,16	4	P22273,P30120,Q99041,Q99MH3
973	1,2,3,6,9,10,12,15	4,5,7,8,11,13,14,16	9	O54728,P02091,P02780,P06760,P22273,P30120,Q4G075,Q99041,Q99MH3
974	1,2,3,6,9,10,12,16	4,5,7,8,11,13,14,15	4	D3ZUC6,P06760,Q4G075,Q99041
975	1,2,3,6,9,10,13,14	4,5,7,8,11,12,15,16	4	O54800;Q5DWV2,P02780,P22273,Q06000
976	1,2,3,6,9,10,13,15	4,5,7,8,11,12,14,16	7	P02091,P02780,P06760,P22273,Q4G075,Q5M8C6,Q99MH3
977	1,2,3,6,9,10,13,16	4,5,7,8,11,12,14,15	3	D3ZUC6,O54800;Q5DWV2,P06760
978	1,2,3,6,9,10,14,15	4,5,7,8,11,12,13,16	10	P00714,P01946,P02091,P02780,P22273,P22282,P30120,P97840,Q5M8C6,Q99MH3
979	1,2,3,6,9,10,14,16	4,5,7,8,11,12,13,15	3	O54800;Q5DWV2,P22273,Q09030
980	1,2,3,6,9,10,15,16	4,5,7,8,11,12,13,14	7	D3ZUC6,P02091,P06760,P22273,Q4G075,Q811M5,Q99MH3
981	1,2,3,6,9,11,12,13	4,5,7,8,10,14,15,16	2	O35077,Q99MH3
982	1,2,3,6,9,11,12,14	4,5,7,8,10,13,15,16	2	O35077,Q99MH3
983	1,2,3,6,9,11,12,15	4,5,7,8,10,13,14,16	3	O35077,P02091,Q99MH3

984	1,2,3,6,9,11,12,16	4,5,7,8,10,13,14,15	0
985	1,2,3,6,9,11,13,14	4,5,7,8,10,12,15,16	1 P0DMW0;P0DMW1
986	1,2,3,6,9,11,13,15	4,5,7,8,10,12,14,16	3 P02091,P0DMW0;P0DMW1,Q99MH3
987	1,2,3,6,9,11,13,16	4,5,7,8,10,12,14,15	1 D3ZUC6
988	1,2,3,6,9,11,14,15	4,5,7,8,10,12,13,16	3 P02091,P0DMW0;P0DMW1,Q99MH3
989	1,2,3,6,9,11,14,16	4,5,7,8,10,12,13,15	2 P02783,P0DMW0;P0DMW1
990	1,2,3,6,9,11,15,16	4,5,7,8,10,12,13,14	3 P02091,P02783,Q99MH3
991	1,2,3,6,9,12,13,14	4,5,7,8,10,11,15,16	1 Q09030
992	1,2,3,6,9,12,13,15	4,5,7,8,10,11,14,16	6 P06760,P22273,Q4G075,Q6B345,Q99041,Q99MH3
993	1,2,3,6,9,12,13,16	4,5,7,8,10,11,14,15	3 P06760,Q09030,Q6B345
994	1,2,3,6,9,12,14,15	4,5,7,8,10,11,13,16	7 P00714,P02091,P02780,P22273,P30120,Q99041,Q99MH3
995	1,2,3,6,9,12,14,16	4,5,7,8,10,11,13,15	1 Q09030
996	1,2,3,6,9,12,15,16	4,5,7,8,10,11,13,14	6 P02091,P06760,P22273,Q4G075,Q99041,Q99MH3
997	1,2,3,6,9,13,14,15	4,5,7,8,10,11,12,16	4 P02091,P02780,P0DMW0;P0DMW1,P22273
998	1,2,3,6,9,13,14,16	4,5,7,8,10,11,12,15	1 Q09030
999	1,2,3,6,9,13,15,16	4,5,7,8,10,11,12,14	2 P02091,P06760
1000	1,2,3,6,9,14,15,16	4,5,7,8,10,11,12,13	6 P02091,P02783,P22273,P47967,P97840,Q5I0D1
1001	1,2,3,6,10,11,12,13	4,5,7,8,9,14,15,16	2 P20646,Q68G31
1002	1,2,3,6,10,11,12,14	4,5,7,8,9,13,15,16	0
1003	1,2,3,6,10,11,12,15	4,5,7,8,9,13,14,16	2 P02091,Q99MH3

1004	1,2,3,6,10,11,12,16	4,5,7,8,9,13,14,15	0	
1005	1,2,3,6,10,11,13,14	4,5,7,8,9,12,15,16	2	P63029,Q68G31
1006	1,2,3,6,10,11,13,15	4,5,7,8,9,12,14,16	2	P02091,Q68G31
1007	1,2,3,6,10,11,13,16	4,5,7,8,9,12,14,15	1	Q68G31
1008	1,2,3,6,10,11,14,15	4,5,7,8,9,12,13,16	2	P01946,P02091
1009	1,2,3,6,10,11,14,16	4,5,7,8,9,12,13,15	1	P02783
1010	1,2,3,6,10,11,15,16	4,5,7,8,9,12,13,14	4	P01946,P02091,P02783,P06760
1011	1,2,3,6,10,12,13,14	4,5,7,8,9,11,15,16	5	O54800;Q5DWV2,P01835,P06760,P14668,P49134
1012	1,2,3,6,10,12,13,15	4,5,7,8,9,11,14,16	4	P02091,P06760,P07150,Q812E4
1013	1,2,3,6,10,12,13,16	4,5,7,8,9,11,14,15	3	O54800;Q5DWV2,P06760,P07150
1014	1,2,3,6,10,12,14,15	4,5,7,8,9,11,13,16	8	P00714,P01946,P02091,P02780,P06760,P22273,P30120,P82471
1015	1,2,3,6,10,12,14,16	4,5,7,8,9,11,13,15	3	O54800;Q5DWV2,P06760,P14668
1016	1,2,3,6,10,12,15,16	4,5,7,8,9,11,13,14	3	P02091,P06760,P82471
1017	1,2,3,6,10,13,14,15	4,5,7,8,9,11,12,16	8	O54800;Q5DWV2,P01835,P01946,P02091,P02780,P06760,P22273,Q68G31
1018	1,2,3,6,10,13,14,16	4,5,7,8,9,11,12,15	4	O54800;Q5DWV2,P01835,P06760,P14668
1019	1,2,3,6,10,13,15,16	4,5,7,8,9,11,12,14	4	P01835,P02091,P06760,P07150
1020	1,2,3,6,10,14,15,16	4,5,7,8,9,11,12,13	7	P01946,P02091,P02783,P06760,P14668,P22273,P82471
1021	1,2,3,6,11,12,13,14	4,5,7,8,9,10,15,16	2	P20646,Q9Z0V6
1022	1,2,3,6,11,12,13,15	4,5,7,8,9,10,14,16	1	P20760
1023	1,2,3,6,11,12,13,16	4,5,7,8,9,10,14,15	2	P05369,P06760

1024	1,2,3,6,11,12,14,15	4,5,7,8,9,10,13,16	1 P02091
1025	1,2,3,6,11,12,14,16	4,5,7,8,9,10,13,15	1 P02783
1026	1,2,3,6,11,12,15,16	4,5,7,8,9,10,13,14	3 P02091,P02783,P06760
1027	1,2,3,6,11,13,14,15	4,5,7,8,9,10,12,16	2 P02091,Q68G31
1028	1,2,3,6,11,13,14,16	4,5,7,8,9,10,12,15	1 P02783
1029	1,2,3,6,11,13,15,16	4,5,7,8,9,10,12,14	2 P02091,P06760
1030	1,2,3,6,11,14,15,16	4,5,7,8,9,10,12,13	2 P02091,P02783
1031	1,2,3,6,12,13,14,15	4,5,7,8,9,10,11,16	5 P01835,P06760,P22273,P49134,Q6B345
1032	1,2,3,6,12,13,14,16	4,5,7,8,9,10,11,15	6 P01835,P06760,P49134,Q6B345,Q6P6R2,Q9Z0V6
1033	1,2,3,6,12,13,15,16	4,5,7,8,9,10,11,14	5 P01835,P02091,P06760,P07150,Q6B345
1034	1,2,3,6,12,14,15,16	4,5,7,8,9,10,11,13	4 P02091,P02783,P06760,P22273
1035	1,2,3,6,13,14,15,16	4,5,7,8,9,10,11,12	6 P01835,P02091,P02783,P06760,P49134,Q62714
1036	1,2,3,7,8,9,10,11	4,5,6,12,13,14,15,16	4 iRT-Kit_WR_fusion,P20766,P35280,Q6P6S4
1037	1,2,3,7,8,9,10,12	4,5,6,11,13,14,15,16	3 iRT-Kit_WR_fusion,P20766,Q63598
1038	1,2,3,7,8,9,10,13	4,5,6,11,12,14,15,16	1 P20766
1039	1,2,3,7,8,9,10,14	4,5,6,11,12,13,15,16	0
1040	1,2,3,7,8,9,10,15	4,5,6,11,12,13,14,16	6 iRT-Kit_WR_fusion,P20766,P35280,Q5RKI1,Q63598,Q99MH3
1041	1,2,3,7,8,9,10,16	4,5,6,11,12,13,14,15	3 P20766,Q63598,Q64093
1042	1,2,3,7,8,9,11,12	4,5,6,10,13,14,15,16	3 iRT-Kit_WR_fusion,P35280,P70709
1043	1,2,3,7,8,9,11,13	4,5,6,10,12,14,15,16	3 iRT-Kit_WR_fusion,P35280,Q66H69

1044	1,2,3,7,8,9,11,14	4,5,6,10,12,13,15,16	5	iRT-Kit_WR_fusion,P0DMW0;P0DMW1,P35280,Q63751,Q812E4
1045	1,2,3,7,8,9,11,15	4,5,6,10,12,13,14,16	6	iRT-Kit_WR_fusion,P35280,Q6IFW6,Q6IMF3,Q6P6S4,Q99MH3
1046	1,2,3,7,8,9,11,16	4,5,6,10,12,13,14,15	8	iRT-Kit_WR_fusion,P02782,P08723,P09456,P30120,P35280,Q812E4,Q9JHB9
1047	1,2,3,7,8,9,12,13	4,5,6,10,11,14,15,16	2	iRT-Kit_WR_fusion,Q66H69
1048	1,2,3,7,8,9,12,14	4,5,6,10,11,13,15,16	1	iRT-Kit_WR_fusion
1049	1,2,3,7,8,9,12,15	4,5,6,10,11,13,14,16	4	iRT-Kit_WR_fusion,P35280,P36376,Q99MH3
1050	1,2,3,7,8,9,12,16	4,5,6,10,11,13,14,15	1	iRT-Kit_WR_fusion
1051	1,2,3,7,8,9,13,14	4,5,6,10,11,12,15,16	1	P0DMW0;P0DMW1
1052	1,2,3,7,8,9,13,15	4,5,6,10,11,12,14,16	2	iRT-Kit_WR_fusion,P35280
1053	1,2,3,7,8,9,13,16	4,5,6,10,11,12,14,15	1	iRT-Kit_WR_fusion
1054	1,2,3,7,8,9,14,15	4,5,6,10,11,12,13,16	2	P00714,P35280
1055	1,2,3,7,8,9,14,16	4,5,6,10,11,12,13,15	0	
1056	1,2,3,7,8,9,15,16	4,5,6,10,11,12,13,14	2	iRT-Kit_WR_fusion,P35280
1057	1,2,3,7,8,10,11,12	4,5,6,9,13,14,15,16	4	iRT-Kit_WR_fusion,P20766,P47967,Q66H69
1058	1,2,3,7,8,10,11,13	4,5,6,9,12,14,15,16	2	P20766,Q66H69
1059	1,2,3,7,8,10,11,14	4,5,6,9,12,13,15,16	1	Q66H69
1060	1,2,3,7,8,10,11,15	4,5,6,9,12,13,14,16	8	P20766,Q5RKI1,Q64093,Q66H69,Q6IFU7,Q6IFW6,Q6IMF3,Q9WTW7
1061	1,2,3,7,8,10,11,16	4,5,6,9,12,13,14,15	10	P02782,P08723,P20766,P30120,Q00715,Q63424,Q64093,Q66H69,Q9JHB9,Q9WTW7
1062	1,2,3,7,8,10,12,13	4,5,6,9,11,14,15,16	7	iRT-Kit_WR_fusion,P20766,P47967,Q63598,Q64093,Q66H69,Q923V8
1063	1,2,3,7,8,10,12,14	4,5,6,9,11,13,15,16	2	Q5RLM2,Q66H69

1064	1,2,3,7,8,10,12,15	4,5,6,9,11,13,14,16	11	iRT-Kit_WR_fusion,O70377,P20766,P34901,Q05175,Q5RKI1,Q63598,Q64093,Q66H69,Q6AY41,Q9WTW7
1065	1,2,3,7,8,10,12,16	4,5,6,9,11,13,14,15	16	iRT-Kit_WR_fusion,P19468,P20766,P46844,P53790,P57113,Q3ZAV1,Q63424,Q63598,Q64093,Q66H69,Q6AY41,Q80W57,Q923V8,Q9JJ40,Q9WTW7
1066	1,2,3,7,8,10,13,14	4,5,6,9,11,12,15,16	3	P25809,Q66H69,Q9WUW8
1067	1,2,3,7,8,10,13,15	4,5,6,9,11,12,14,16	17	O70377,O70594,O88339;Q4V882,P19468,P20766,P51907,Q05175,Q5RKI1,Q63424,Q63598,Q63618,Q64093,Q66H69,Q6AY41,Q6Q0N1,Q80W57,Q9WTW7
1068	1,2,3,7,8,10,13,16	4,5,6,9,11,12,14,15	18	O70377,O70594,P19468,P20766,P46844,P53790,P57113,Q3ZAV1,Q63424,Q63598,Q63618,Q64093,Q66H69,Q6AY41,Q80W57,Q9WTW7,Q9WUW8,Q9Z0W7
1069	1,2,3,7,8,10,14,15	4,5,6,9,11,12,13,16	8	O70377,O88339;Q4V882,P54921,Q05175,Q5RKI1,Q62714,Q63598,Q64093
1070	1,2,3,7,8,10,14,16	4,5,6,9,11,12,13,15	16	O70377,P19468,P46844,P53790,P57113,P97580,Q05175,Q63424,Q63598,Q64093,Q6AY41,Q80W57,Q923S2,Q9WTW7,Q9WUW8,Q9WUW9
1071	1,2,3,7,8,10,15,16	4,5,6,9,11,12,13,14	31	O70377,O70594,O88339;Q4V882,P19468,P20766,P29975,P38918,P46844,P48508,P51907,P53790,P57113,P97608,Q05175,Q3ZAV1,Q5I0E9,Q5RKI1,Q63355,Q63424,Q63598,Q64093,Q6AY41,Q6MG61,Q6Q0N1,Q80W57,Q8R431,Q923S2,Q9JJ40,Q9R1T5,Q9WTW7,Q9Z0W7
1072	1,2,3,7,8,11,12,13	4,5,6,9,10,14,15,16	4	iRT-Kit_WR_fusion,P47967,Q66H69,Q811M5
1073	1,2,3,7,8,11,12,14	4,5,6,9,10,13,15,16	4	iRT-Kit_WR_fusion,P11883,Q66H69,Q811M5
1074	1,2,3,7,8,11,12,15	4,5,6,9,10,13,14,16	5	iRT-Kit_WR_fusion,Q66H69,Q6IFW6,Q6IG05,Q6IMF3
1075	1,2,3,7,8,11,12,16	4,5,6,9,10,13,14,15	8	iRT-Kit_WR_fusion,P02782,P08723,P30120,Q00715,Q66H69,Q811M5,Q9JHB9
1076	1,2,3,7,8,11,13,14	4,5,6,9,10,12,15,16	2	Q66H69,Q811M5
1077	1,2,3,7,8,11,13,15	4,5,6,9,10,12,14,16	6	iRT-Kit_WR_fusion,Q66H69,Q6IFU7,Q6IFW6,Q6IMF3,Q811M5
1078	1,2,3,7,8,11,13,16	4,5,6,9,10,12,14,15	10	P02782,P05369,P08723,P09456,P24368,P30120,Q66H69,Q6AYQ8,Q811M5,Q9JHB9
1079	1,2,3,7,8,11,14,15	4,5,6,9,10,12,13,16	5	Q6IFU7,Q6IFU8,Q6IFW6,Q6IMF3,Q811M5
1080	1,2,3,7,8,11,14,16	4,5,6,9,10,12,13,15	7	P02782,P08723,P30120,Q00715,Q811M5,Q812E4,Q9JHB9
1081	1,2,3,7,8,11,15,16	4,5,6,9,10,12,13,14	11	iRT-Kit_WR_fusion,P02782,P08723,P30120,Q4FZU2,Q6IFU8,Q6IFW6,Q6IMF3,Q8CJ52,Q9JHB9,Q9WTW7

1082	1,2,3,7,8,12,13,14	4,5,6,9,10,11,15,16	3	iRT-Kit_WR_fusion,Q66H69,Q811M5
1083	1,2,3,7,8,12,13,15	4,5,6,9,10,11,14,16	2	iRT-Kit_WR_fusion,Q66H69
1084	1,2,3,7,8,12,13,16	4,5,6,9,10,11,14,15	3	iRT-Kit_WR_fusion,Q66H69,Q811M5
1085	1,2,3,7,8,12,14,15	4,5,6,9,10,11,13,16	2	iRT-Kit_WR_fusion,P00714
1086	1,2,3,7,8,12,14,16	4,5,6,9,10,11,13,15	2	iRT-Kit_WR_fusion,Q811M5
1087	1,2,3,7,8,12,15,16	4,5,6,9,10,11,13,14	4	iRT-Kit_WR_fusion,Q63598,Q64093,Q9WTW7
1088	1,2,3,7,8,13,14,15	4,5,6,9,10,11,12,16	2	Q62714,Q811M5
1089	1,2,3,7,8,13,14,16	4,5,6,9,10,11,12,15	3	Q62714,Q811M5,Q9WUW8
1090	1,2,3,7,8,13,15,16	4,5,6,9,10,11,12,14	5	P19468,Q62714,Q64093,Q6AYQ8,Q9WTW7
1091	1,2,3,7,8,14,15,16	4,5,6,9,10,11,12,13	1	Q62714
1092	1,2,3,7,9,10,11,12	4,5,6,8,13,14,15,16	1	P20766
1093	1,2,3,7,9,10,11,13	4,5,6,8,12,14,15,16	1	P20766
1094	1,2,3,7,9,10,11,14	4,5,6,8,12,13,15,16	0	
1095	1,2,3,7,9,10,11,15	4,5,6,8,12,13,14,16	4	P20766,P35280,Q5RKI1,Q99MH3
1096	1,2,3,7,9,10,11,16	4,5,6,8,12,13,14,15	1	P20766
1097	1,2,3,7,9,10,12,13	4,5,6,8,11,14,15,16	2	Q6AY61,Q99041
1098	1,2,3,7,9,10,12,14	4,5,6,8,11,13,15,16	2	Q6AY61,Q99041
1099	1,2,3,7,9,10,12,15	4,5,6,8,11,13,14,16	6	O54728,P20766,Q5RKI1,Q6AY61,Q99041,Q99MH3
1100	1,2,3,7,9,10,12,16	4,5,6,8,11,13,14,15	2	P20766,Q99041
1101	1,2,3,7,9,10,13,14	4,5,6,8,11,12,15,16	0	

1102	1,2,3,7,9,10,13,15	4,5,6,8,11,12,14,16	3 P20766,Q5RKI1,Q8CFN2
1103	1,2,3,7,9,10,13,16	4,5,6,8,11,12,14,15	1 P20766
1104	1,2,3,7,9,10,14,15	4,5,6,8,11,12,13,16	4 P00714,P22273,P30120,Q5RKI1
1105	1,2,3,7,9,10,14,16	4,5,6,8,11,12,13,15	0
1106	1,2,3,7,9,10,15,16	4,5,6,8,11,12,13,14	2 P20766,Q5RKI1
1107	1,2,3,7,9,11,12,13	4,5,6,8,10,14,15,16	2 P54921,Q62761;Q62762;Q62763
1108	1,2,3,7,9,11,12,14	4,5,6,8,10,13,15,16	1 P0DMW0;P0DMW1
1109	1,2,3,7,9,11,12,15	4,5,6,8,10,13,14,16	4 P25031,P35280,Q62761;Q62762;Q62763,Q99MH3
1110	1,2,3,7,9,11,12,16	4,5,6,8,10,13,14,15	0
1111	1,2,3,7,9,11,13,14	4,5,6,8,10,12,15,16	3 P0DMW0;P0DMW1,P25031,Q9R168
1112	1,2,3,7,9,11,13,15	4,5,6,8,10,12,14,16	3 P0DMW0;P0DMW1,P25031,P35280
1113	1,2,3,7,9,11,13,16	4,5,6,8,10,12,14,15	2 P54921,Q9R168
1114	1,2,3,7,9,11,14,15	4,5,6,8,10,12,13,16	3 P0DMW0;P0DMW1,P25031,P35280
1115	1,2,3,7,9,11,14,16	4,5,6,8,10,12,13,15	2 P0DMW0;P0DMW1,Q9R168
1116	1,2,3,7,9,11,15,16	4,5,6,8,10,12,13,14	1 P35280
1117	1,2,3,7,9,12,13,14	4,5,6,8,10,11,15,16	1 Q6AY61
1118	1,2,3,7,9,12,13,15	4,5,6,8,10,11,14,16	2 Q6AY61,Q99041
1119	1,2,3,7,9,12,13,16	4,5,6,8,10,11,14,15	0
1120	1,2,3,7,9,12,14,15	4,5,6,8,10,11,13,16	4 P00714,P22273,Q6AY61,Q99041
1121	1,2,3,7,9,12,14,16	4,5,6,8,10,11,13,15	0

1122	1,2,3,7,9,12,15,16	4,5,6,8,10,11,13,14	2	Q6AY61,Q99041
1123	1,2,3,7,9,13,14,15	4,5,6,8,10,11,12,16	2	P0DMW0;P0DMW1,P22273
1124	1,2,3,7,9,13,14,16	4,5,6,8,10,11,12,15	1	Q9R168
1125	1,2,3,7,9,13,15,16	4,5,6,8,10,11,12,14	0	
1126	1,2,3,7,9,14,15,16	4,5,6,8,10,11,12,13	1	P22273
1127	1,2,3,7,10,11,12,13	4,5,6,8,9,14,15,16	3	P20760,P23593,P47967
1128	1,2,3,7,10,11,12,14	4,5,6,8,9,13,15,16	0	
1129	1,2,3,7,10,11,12,15	4,5,6,8,9,13,14,16	3	P20760,P20766,Q5RKI1
1130	1,2,3,7,10,11,12,16	4,5,6,8,9,13,14,15	1	P20766
1131	1,2,3,7,10,11,13,14	4,5,6,8,9,12,15,16	0	
1132	1,2,3,7,10,11,13,15	4,5,6,8,9,12,14,16	3	P20760,P20766,Q5RKI1
1133	1,2,3,7,10,11,13,16	4,5,6,8,9,12,14,15	2	P20766,Q6AYQ8
1134	1,2,3,7,10,11,14,15	4,5,6,8,9,12,13,16	1	Q5RKI1
1135	1,2,3,7,10,11,14,16	4,5,6,8,9,12,13,15	0	
1136	1,2,3,7,10,11,15,16	4,5,6,8,9,12,13,14	3	P20766,Q5RKI1,Q9WTW7
1137	1,2,3,7,10,12,13,14	4,5,6,8,9,11,15,16	2	P01835,Q6AY61
1138	1,2,3,7,10,12,13,15	4,5,6,8,9,11,14,16	3	P20760,Q5RKI1,Q6AY61
1139	1,2,3,7,10,12,13,16	4,5,6,8,9,11,14,15	3	P20766,Q64093,Q6AY61
1140	1,2,3,7,10,12,14,15	4,5,6,8,9,11,13,16	4	P00714,Q5RKI1,Q6AY61,Q6RUV5
1141	1,2,3,7,10,12,14,16	4,5,6,8,9,11,13,15	1	Q6AY61

1142	1,2,3,7,10,12,15,16	4,5,6,8,9,11,13,14	7	P18297,P20766,Q5RKI1,Q63598,Q64093,Q6AY61,Q9WTW7
1143	1,2,3,7,10,13,14,15	4,5,6,8,9,11,12,16	3	P01835,P63095,Q5RKI1
1144	1,2,3,7,10,13,14,16	4,5,6,8,9,11,12,15	3	P01835,P63095,Q9WUW8
1145	1,2,3,7,10,13,15,16	4,5,6,8,9,11,12,14	11	P01835,P18297,P19468,P20766,P61206;P84079,P63095,Q5RKI1,Q63424,Q64093,Q6AYQ8,Q9WTW7
1146	1,2,3,7,10,14,15,16	4,5,6,8,9,11,12,13	5	P18297,P61206;P84079,P63095,Q5RKI1,Q64093
1147	1,2,3,7,11,12,13,14	4,5,6,8,9,10,15,16	3	P20760,P25031,Q811M5
1148	1,2,3,7,11,12,13,15	4,5,6,8,9,10,14,16	2	P20760,P25031
1149	1,2,3,7,11,12,13,16	4,5,6,8,9,10,14,15	3	P05369,P47967,Q811M5
1150	1,2,3,7,11,12,14,15	4,5,6,8,9,10,13,16	3	P20760,P25031,Q811M5
1151	1,2,3,7,11,12,14,16	4,5,6,8,9,10,13,15	1	Q811M5
1152	1,2,3,7,11,12,15,16	4,5,6,8,9,10,13,14	0	
1153	1,2,3,7,11,13,14,15	4,5,6,8,9,10,12,16	2	P25031,Q811M5
1154	1,2,3,7,11,13,14,16	4,5,6,8,9,10,12,15	1	Q811M5
1155	1,2,3,7,11,13,15,16	4,5,6,8,9,10,12,14	2	P25031,Q6AYQ8
1156	1,2,3,7,11,14,15,16	4,5,6,8,9,10,12,13	1	P25031
1157	1,2,3,7,12,13,14,15	4,5,6,8,9,10,11,16	2	P01835,Q6AY61
1158	1,2,3,7,12,13,14,16	4,5,6,8,9,10,11,15	3	P01835,Q6AY61,Q811M5
1159	1,2,3,7,12,13,15,16	4,5,6,8,9,10,11,14	2	P01835,Q6AY61
1160	1,2,3,7,12,14,15,16	4,5,6,8,9,10,11,13	1	Q6AY61
1161	1,2,3,7,13,14,15,16	4,5,6,8,9,10,11,12	2	P01835,Q62714

1162	1,2,3,8,9,10,11,12	4,5,6,7,13,14,15,16	1 Q99MH3
1163	1,2,3,8,9,10,11,13	4,5,6,7,12,14,15,16	0
1164	1,2,3,8,9,10,11,14	4,5,6,7,12,13,15,16	0
1165	1,2,3,8,9,10,11,15	4,5,6,7,12,13,14,16	3 Q6IFW6,Q6P6S4,Q99MH3
1166	1,2,3,8,9,10,11,16	4,5,6,7,12,13,14,15	1 O88797
1167	1,2,3,8,9,10,12,13	4,5,6,7,11,14,15,16	1 Q99041
1168	1,2,3,8,9,10,12,14	4,5,6,7,11,13,15,16	4 P00714,P11598,Q99041,Q9Z0J6
1169	1,2,3,8,9,10,12,15	4,5,6,7,11,13,14,16	6 P00714,P11598,P34901,P36376,Q99041,Q99MH3
1170	1,2,3,8,9,10,12,16	4,5,6,7,11,13,14,15	1 Q99041
1171	1,2,3,8,9,10,13,14	4,5,6,7,11,12,15,16	0
1172	1,2,3,8,9,10,13,15	4,5,6,7,11,12,14,16	4 P11598,Q99041,Q99376,Q99MH3
1173	1,2,3,8,9,10,13,16	4,5,6,7,11,12,14,15	0
1174	1,2,3,8,9,10,14,15	4,5,6,7,11,12,13,16	5 P00714,P11598,P97840,Q99041,Q99MH3
1175	1,2,3,8,9,10,14,16	4,5,6,7,11,12,13,15	0
1176	1,2,3,8,9,10,15,16	4,5,6,7,11,12,13,14	1 Q99041
1177	1,2,3,8,9,11,12,13	4,5,6,7,10,14,15,16	1 Q64335
1178	1,2,3,8,9,11,12,14	4,5,6,7,10,13,15,16	1 P11883
1179	1,2,3,8,9,11,12,15	4,5,6,7,10,13,14,16	4 P36376,Q6IFW6,Q6P6S4,Q99MH3
1180	1,2,3,8,9,11,12,16	4,5,6,7,10,13,14,15	1 O88797
1181	1,2,3,8,9,11,13,14	4,5,6,7,10,12,15,16	2 P0DMW0;P0DMW1,Q64335

1182	1,2,3,8,9,11,13,15	4,5,6,7,10,12,14,16	2 P0DMW0;P0DMW1,Q99MH3
1183	1,2,3,8,9,11,13,16	4,5,6,7,10,12,14,15	0
1184	1,2,3,8,9,11,14,15	4,5,6,7,10,12,13,16	2 P0DMW0;P0DMW1,Q99MH3
1185	1,2,3,8,9,11,14,16	4,5,6,7,10,12,13,15	2 O88797,Q812E4
1186	1,2,3,8,9,11,15,16	4,5,6,7,10,12,13,14	2 Q6IFW6,Q6P6S4
1187	1,2,3,8,9,12,13,14	4,5,6,7,10,11,15,16	1 P17988
1188	1,2,3,8,9,12,13,15	4,5,6,7,10,11,14,16	4 P11598,P36376,Q99041,Q99MH3
1189	1,2,3,8,9,12,13,16	4,5,6,7,10,11,14,15	1 Q99041
1190	1,2,3,8,9,12,14,15	4,5,6,7,10,11,13,16	6 P00714,P11598,P36376,Q99041,Q99MH3,Q9Z0J6
1191	1,2,3,8,9,12,14,16	4,5,6,7,10,11,13,15	0
1192	1,2,3,8,9,12,15,16	4,5,6,7,10,11,13,14	2 P36376,Q99041
1193	1,2,3,8,9,13,14,15	4,5,6,7,10,11,12,16	3 P00714,P0DMW0;P0DMW1,P11598
1194	1,2,3,8,9,13,14,16	4,5,6,7,10,11,12,15	0
1195	1,2,3,8,9,13,15,16	4,5,6,7,10,11,12,14	0
1196	1,2,3,8,9,14,15,16	4,5,6,7,10,11,12,13	5 P00714,P23593,P47967,P97840,Q5I0D1
1197	1,2,3,8,10,11,12,13	4,5,6,7,9,14,15,16	0
1198	1,2,3,8,10,11,12,14	4,5,6,7,9,13,15,16	2 P11883,Q9Z0J6
1199	1,2,3,8,10,11,12,15	4,5,6,7,9,13,14,16	3 Q6IFU7,Q6IFW6,Q99MH3
1200	1,2,3,8,10,11,12,16	4,5,6,7,9,13,14,15	0
1201	1,2,3,8,10,11,13,14	4,5,6,7,9,12,15,16	1 Q68G31

1202	1,2,3,8,10,11,13,15	4,5,6,7,9,12,14,16	3	Q68G31,Q6IFU7,Q6IFW6
1203	1,2,3,8,10,11,13,16	4,5,6,7,9,12,14,15	0	
1204	1,2,3,8,10,11,14,15	4,5,6,7,9,12,13,16	2	Q6IFU7,Q6IFW6
1205	1,2,3,8,10,11,14,16	4,5,6,7,9,12,13,15	1	P97580
1206	1,2,3,8,10,11,15,16	4,5,6,7,9,12,13,14	2	Q6IFU7,Q6IFW6
1207	1,2,3,8,10,12,13,14	4,5,6,7,9,11,15,16	1	Q9Z0J6
1208	1,2,3,8,10,12,13,15	4,5,6,7,9,11,14,16	1	Q99376
1209	1,2,3,8,10,12,13,16	4,5,6,7,9,11,14,15	0	
1210	1,2,3,8,10,12,14,15	4,5,6,7,9,11,13,16	3	P00714,Q63617,Q9Z0J6
1211	1,2,3,8,10,12,14,16	4,5,6,7,9,11,13,15	0	
1212	1,2,3,8,10,12,15,16	4,5,6,7,9,11,13,14	2	Q63598,Q99041
1213	1,2,3,8,10,13,14,15	4,5,6,7,9,11,12,16	0	
1214	1,2,3,8,10,13,14,16	4,5,6,7,9,11,12,15	1	P97580
1215	1,2,3,8,10,13,15,16	4,5,6,7,9,11,12,14	1	Q64093
1216	1,2,3,8,10,14,15,16	4,5,6,7,9,11,12,13	2	P97580,Q63617
1217	1,2,3,8,11,12,13,14	4,5,6,7,9,10,15,16	2	Q64335,Q811M5
1218	1,2,3,8,11,12,13,15	4,5,6,7,9,10,14,16	3	Q6IFU7,Q6IFW6,Q6IG05
1219	1,2,3,8,11,12,13,16	4,5,6,7,9,10,14,15	2	P30120,Q811M5
1220	1,2,3,8,11,12,14,15	4,5,6,7,9,10,13,16	6	P11883,Q6IFU7,Q6IFW6,Q6IG05,Q811M5,Q9Z0J6
1221	1,2,3,8,11,12,14,16	4,5,6,7,9,10,13,15	1	Q811M5

1222	1,2,3,8,11,12,15,16	4,5,6,7,9,10,13,14	3	Q6IFU7,Q6IFW6,Q6IG05
1223	1,2,3,8,11,13,14,15	4,5,6,7,9,10,12,16	3	Q6IFU7,Q6IFW6,Q811M5
1224	1,2,3,8,11,13,14,16	4,5,6,7,9,10,12,15	2	P30120,Q811M5
1225	1,2,3,8,11,13,15,16	4,5,6,7,9,10,12,14	2	Q6IFU7,Q6IFW6
1226	1,2,3,8,11,14,15,16	4,5,6,7,9,10,12,13	2	Q6IFU7,Q6IFW6
1227	1,2,3,8,12,13,14,15	4,5,6,7,9,10,11,16	2	P00714,Q9Z0J6
1228	1,2,3,8,12,13,14,16	4,5,6,7,9,10,11,15	1	Q811M5
1229	1,2,3,8,12,13,15,16	4,5,6,7,9,10,11,14	0	
1230	1,2,3,8,12,14,15,16	4,5,6,7,9,10,11,13	0	
1231	1,2,3,8,13,14,15,16	4,5,6,7,9,10,11,12	0	
1232	1,2,3,9,10,11,12,13	4,5,6,7,8,14,15,16	3	P20760,Q62761;Q62762;Q62763,Q99MH3
1233	1,2,3,9,10,11,12,14	4,5,6,7,8,13,15,16	1	Q9Z0J6
1234	1,2,3,9,10,11,12,15	4,5,6,7,8,13,14,16	5	P06761,P20760,Q62761;Q62762;Q62763,Q99MH3,Q9Z2L0
1235	1,2,3,9,10,11,12,16	4,5,6,7,8,13,14,15	2	P07171,Q9Z2L0
1236	1,2,3,9,10,11,13,14	4,5,6,7,8,12,15,16	1	P0DMW0;P0DMW1
1237	1,2,3,9,10,11,13,15	4,5,6,7,8,12,14,16	3	P06761,P20760,Q99MH3
1238	1,2,3,9,10,11,13,16	4,5,6,7,8,12,14,15	1	D3ZUC6
1239	1,2,3,9,10,11,14,15	4,5,6,7,8,12,13,16	2	P06761,Q99MH3
1240	1,2,3,9,10,11,14,16	4,5,6,7,8,12,13,15	0	
1241	1,2,3,9,10,11,15,16	4,5,6,7,8,12,13,14	3	P07171,Q99MH3,Q9Z2L0

1242	1,2,3,9,10,12,13,14	4,5,6,7,8,11,15,16	23	P02780,P02781,P02782,P06760,P06761,P07150,P07647,P08723,P09456,P0C0A9,P11598,P22273,P22282,P22283,P36374,P46462,Q4G063,Q4G075,Q5M8C6,Q63617,Q8CJ52,Q99041,Q9Z0J6
1243	1,2,3,9,10,12,13,15	4,5,6,7,8,11,14,16	26	P02780,P02781,P02782,P04905,P06760,P06761,P07150,P07647,P08723,P09456,P0C0A9,P11598,P20760,P22273,P22282,P22283,P30120,P46462,Q4G075,Q5M8C6,Q63617,Q8CFN2,Q99041,Q99376,Q99MH3,Q9R0T3
1244	1,2,3,9,10,12,13,16	4,5,6,7,8,11,14,15	9	P02780,P06760,P06761,P07150,P11598,P22273,P22282,Q4G075,Q99041
1245	1,2,3,9,10,12,14,15	4,5,6,7,8,11,13,16	25	P00714,P02780,P02781,P02782,P06760,P06761,P07647,P08723,P09456,P0C0A9,P11598,P22273,P22282,P22283,P30120,P36374,P46462,Q4G075,Q5M8C6,Q62902,Q63617,Q99041,Q99MH3,Q9R0T3,Q9Z0J6
1246	1,2,3,9,10,12,14,16	4,5,6,7,8,11,13,15	11	P02780,P06760,P06761,P11598,P22273,P22282,P22283,P46462,Q4G075,Q63617,Q99041
1247	1,2,3,9,10,12,15,16	4,5,6,7,8,11,13,14	14	P02780,P06760,P06761,P07150,P11598,P22273,P22282,P22283,P46462,Q4G075,Q5M8C6,Q63617,Q99041,Q99MH3
1248	1,2,3,9,10,13,14,15	4,5,6,7,8,11,12,16	25	P00714,P02780,P02781,P02782,P04905,P06760,P06761,P07150,P07647,P08723,P09456,P0C0A9,P11598,P22273,P22282,P22283,P30120,P36374,P46462,Q4G075,Q5M8C6,Q63617,Q8CFN2,Q99041,Q9R0T3
1249	1,2,3,9,10,13,14,16	4,5,6,7,8,11,12,15	13	P02780,P06760,P06761,P07150,P11598,P22273,P22282,P36374,P46462,Q4G075,Q5M8C6,Q63617,Q99041
1250	1,2,3,9,10,13,15,16	4,5,6,7,8,11,12,14	15	P02780,P06760,P06761,P07150,P11598,P12020,P19218,P22273,P22282,P46462,Q4G075,Q5M8C6,Q63617,Q99041,Q9R0T3
1251	1,2,3,9,10,14,15,16	4,5,6,7,8,11,12,13	17	P00714,P02780,P06760,P06761,P11598,P22273,P22282,P22283,P36374,P46462,P47967,Q4G075,Q5I0D1,Q5M8C6,Q63617,Q99041,Q9R0T3
1252	1,2,3,9,11,12,13,14	4,5,6,7,8,10,15,16	13	O35077,O70594,O88267,P0DMW0;P0DMW1,P20760,P25031,P53790,Q03248,Q4QQT4,Q62761;Q62762;Q62763,Q63270,Q64335,Q8R431
1253	1,2,3,9,11,12,13,15	4,5,6,7,8,10,14,16	6	P06761,P20760,P25031,P57113,Q62761;Q62762;Q62763,Q99MH3
1254	1,2,3,9,11,12,13,16	4,5,6,7,8,10,14,15	1	P54921
1255	1,2,3,9,11,12,14,15	4,5,6,7,8,10,13,16	8	O70257,P00714,P06761,P0DMW0;P0DMW1,P20760,P25031,Q62761;Q62762;Q62763,Q99MH3
1256	1,2,3,9,11,12,14,16	4,5,6,7,8,10,13,15	3	P07171,P36860,Q63618
1257	1,2,3,9,11,12,15,16	4,5,6,7,8,10,13,14	2	P07171,P25031
1258	1,2,3,9,11,13,14,15	4,5,6,7,8,10,12,16	4	O88267,P06761,P0DMW0;P0DMW1,P25031
1259	1,2,3,9,11,13,14,16	4,5,6,7,8,10,12,15	3	O88267,P0DMW0;P0DMW1,P25031
1260	1,2,3,9,11,13,15,16	4,5,6,7,8,10,12,14	2	P25031,Q5RLM2

1261	1,2,3,9,11,14,15,16	4,5,6,7,8,10,12,13	3	P07171,P0DMW0;P0DMW1,P25031
1262	1,2,3,9,12,13,14,15	4,5,6,7,8,10,11,16	26	P00714,P02780,P02781,P02782,P04905,P06760,P06761,P07150,P07647,P08723,P09456,P0C0A9,P11598,P22273,P22282,P22283,P25031,P30120,P46462,Q4G075,Q5M8C6,Q63617,Q6B345,Q99041,Q9R0T3,Q9Z0J6
1263	1,2,3,9,12,13,14,16	4,5,6,7,8,10,11,15	11	P02780,P06760,P06761,P07150,P11598,P22273,P22282,Q4G075,Q63617,Q6B345,Q99041
1264	1,2,3,9,12,13,15,16	4,5,6,7,8,10,11,14	13	P02780,P06760,P06761,P07150,P11598,P22273,P22282,Q4G075,Q5M8C6,Q63617,Q6B345,Q99041,Q9R0T3
1265	1,2,3,9,12,14,15,16	4,5,6,7,8,10,11,13	16	P00714,P02780,P06760,P06761,P07171,P11598,P22273,P22282,P22283,P46462,Q4G075,Q5M8C6,Q63617,Q6B345,Q99041,Q9R0T3
1266	1,2,3,9,13,14,15,16	4,5,6,7,8,10,11,12	14	P02780,P06760,P06761,P07150,P0DMW0;P0DMW1,P11598,P22273,P22282,P46462,Q5I0D1,Q5M8C6,Q63617,Q99041,Q9R0T3
1267	1,2,3,10,11,12,13,14	4,5,6,7,8,9,15,16	3	P20760,Q9Z0J6,Q9Z0V6
1268	1,2,3,10,11,12,13,15	4,5,6,7,8,9,14,16	2	P06760,P20760
1269	1,2,3,10,11,12,13,16	4,5,6,7,8,9,14,15	2	P06760,P20760
1270	1,2,3,10,11,12,14,15	4,5,6,7,8,9,13,16	4	P06760,P06761,P20760,Q9Z0J6
1271	1,2,3,10,11,12,14,16	4,5,6,7,8,9,13,15	1	P06760
1272	1,2,3,10,11,12,15,16	4,5,6,7,8,9,13,14	3	P06760,P20760,Q5QE79
1273	1,2,3,10,11,13,14,15	4,5,6,7,8,9,12,16	1	P20760
1274	1,2,3,10,11,13,14,16	4,5,6,7,8,9,12,15	1	P06760
1275	1,2,3,10,11,13,15,16	4,5,6,7,8,9,12,14	3	P06760,P20760,Q5QE79
1276	1,2,3,10,11,14,15,16	4,5,6,7,8,9,12,13	1	P06760
1277	1,2,3,10,12,13,14,15	4,5,6,7,8,9,11,16	25	P00714,P02780,P02781,P02782,P04905,P06760,P06761,P07150,P07647,P08723,P09456,P0C0A9,P11598,P20760,P22273,P22282,P22283,P46462,Q4G075,Q5M8C6,Q63617,Q812E4,Q99041,Q9R0T3,Q9Z0J6
1278	1,2,3,10,12,13,14,16	4,5,6,7,8,9,11,15	11	P02780,P06760,P06761,P07150,P22273,P22282,Q63617,Q6P6R2,Q99041,Q9QX74,Q9Z0V6
1279	1,2,3,10,12,13,15,16	4,5,6,7,8,9,11,14	16	P02780,P06760,P06761,P07150,P20760,P22273,P22282,P46462,Q4G075,Q5M8C6,Q5QE79,Q63617,Q6B345,Q6P6R2,Q812E4,Q99041

1280	1,2,3,10,12,14,15,16	4,5,6,7,8,9,11,13	15	P02780,P06760,P06761,P07150,P11598,P22273,P22282,P46462,P82471,Q4G075,Q5M8C6,Q63617,Q99041,Q9R0T3,Q9Z0J6
1281	1,2,3,10,13,14,15,16	4,5,6,7,8,9,11,12	11	P02780,P06760,P06761,P07150,P22273,P22282,P46462,P63095,Q5M8C6,Q63617,Q9R0T3
1282	1,2,3,11,12,13,14,15	4,5,6,7,8,9,10,16	6	P06760,P20760,P25031,P70545,Q811M5,Q9Z0J6
1283	1,2,3,11,12,13,14,16	4,5,6,7,8,9,10,15	6	P06760,P20760,P25031,Q6P6R2,Q811M5,Q9Z0V6
1284	1,2,3,11,12,13,15,16	4,5,6,7,8,9,10,14	5	P06760,P20760,P25031,Q5QE79,Q6P6R2
1285	1,2,3,11,12,14,15,16	4,5,6,7,8,9,10,13	4	P06760,P07171,P20760,P25031
1286	1,2,3,11,13,14,15,16	4,5,6,7,8,9,10,12	3	P06760,P20760,P25031
1287	1,2,3,12,13,14,15,16	4,5,6,7,8,9,10,11	16	P02780,P06760,P06761,P07150,P20760,P22273,P22282,P49134,P50116,P70545,Q63617,Q6B345,Q6P6R2,Q99041,Q9QX74,Q9R0T3
1288	1,2,4,5,6,7,8,9	3,10,11,12,13,14,15,16	46	B1H234,D3ZHA0,D4A5U3,iRT-Kit_WR_fusion,O55004,O70594,P02454,P02631,P06760,P06761,P06911,P13432,P18418,P19223,P19629,P20646,P20760,P20761,P20762,P23928,P31430,P36860,P50115,P50116,P50280,P52590,P55091,P70545,P80299,P98089,Q4G075,Q5GRG2,Q5QE79,Q62635,Q62946,Q63618,Q6PCU2,Q6TMA8,Q78P75,Q8CIZ5,Q9JI85,Q9QX74,Q9R0T3,Q9Z0J6,Q9Z0V6,Q9Z1F2
1289	1,2,4,5,6,7,8,10	3,9,11,12,13,14,15,16	104	A2RUW1,B1H234,D3ZHA0,D4A5U3,O08557,O35763,O55004,O70257,O70377,O70594,O88267,O88339;Q4V882,P01836,P02454,P02631,P04904,P05371,P06760,P06761,P06911,P10247,P13432,P15399,P18418,P18757,P19112,P19223,P19468,P19629,P19814,P20646,P20760,P20761,P20762,P23928,P24268,P25031,P30904,P31044,P31430,P35952,P36860,P38438,P38918,P46844,P48037,P48508,P50115,P50116,P50280,P51907,P52590,P53790,P54921,P55091,P57113,P80299,P97580,P98089,Q03248,Q05175,Q30KJ2,Q3ZAV1,Q4G075,Q5GRG2,Q5I0E9,Q5M7T9,Q5RLM2,Q62635,Q63270,Q63424,Q63598,Q63618,Q64602,Q66H69,Q66HG3,Q68FT5,Q6AYS7,Q6MG61,Q6PCU2,Q6Q0N1,Q6TMA8,Q71MB6,Q80W57,Q8CIZ5,Q8K3P7,Q8R431,Q91ZS3,Q920G2,Q923S2,Q99MA2,Q9JI85,Q9JJ19,Q9JJ40,Q9JLJ3,Q9QX74,Q9QYU4,Q9R0T3,Q9WTW7,Q9WUW8,Q9WUW9,Q9Z0V6,Q9Z0W7,Q9Z1F2
1290	1,2,4,5,6,7,8,11	3,9,10,12,13,14,15,16	83	B1H234,D3ZHA0,D4A5U3,iRT-Kit_WR_fusion,O08557,O35547,O55004,O88917,P01039,P02631,P02780,P02781,P02782,P04905,P05371,P06760,P06761,P06911,P07150,P07647,P08649,P08723,P09456,P0C0A9,P11598,P12020,P13432,P18418,P19223,P19629,P20646,P20673,P20760,P20762,P22273,P22282,P22283,P31044,P31430,P35952,P36374,P36860,P40241,P46462,P50115,P50116,P50280,P52590,P55091,P80299,P98089,Q09326,Q10758,Q3T1J1,Q4FZU2,Q4G063,Q4G075,Q4KLZ6,Q5GRG2,Q5M8C6,Q62635,Q62946,Q63493,Q63617,Q66H69,Q6AYR9,Q6B345,Q6IFU8,Q6IMF3,Q6P6Q2,Q6TMA8,Q78P75,Q8CIZ5,Q8R5M3,Q99041,Q9JHB9,Q9JI85,Q9QX74,Q9QZK8,Q9R0T3,Q9WTT6,Q9Z0J6,Q9Z0V6

1291	1,2,4,5,6,7,8,12	3,9,10,11,13,14,15,16	53	B1H234,D3ZHA0,D4A5U3,iRT-Kit_WR_fusion,O55004,O88267,P02631,P02780,P02781,P06760,P06761,P06911,P12020,P13432,P18418,P19223,P19629,P19814,P20760,P20762,P22282,P30904,P31044,P31430,P35952,P36860,P46844,P48508,P50115,P50116,P50280,P52590,P55091,P80299,P98089,Q4G075,Q5GRG2,Q5RLM2,Q62635,Q62946,Q63270,Q63618,Q66H69,Q66HG3,Q6Q0N1,Q6TMA8,Q8CIZ5,Q91ZS3,Q9JI85,Q9QX74,Q9R0T3,Q9Z0V6,Q9Z1F2
1292	1,2,4,5,6,7,8,13	3,9,10,11,12,14,15,16	58	A2RUW1,B1H234,D3ZHA0,D4A5U3,O55004,O70257,O70594,P01836,P02631,P02781,P05371,P06760,P06761,P06911,P07171,P07647,P13432,P18418,P19223,P19629,P20760,P20761,P20762,P22282,P25809,P30904,P31044,P31430,P35952,P36860,P46844,P48508,P50115,P50116,P50280,P52590,P55091,P80299,P98089,Q30KJ2,Q4G075,Q5GRG2,Q62635,Q62714,Q62946,Q63618,Q66H69,Q6PCU2,Q6Q0N1,Q6TMA8,Q78P75,Q8CIZ5,Q9JI85,Q9QX74,Q9R0T3,Q9WUW8,Q9Z0J6,Q9Z1F2
1293	1,2,4,5,6,7,8,14	3,9,10,11,12,13,15,16	54	A2RUW1,B1H234,D3ZHA0,D4A5U3,O55004,O70594,P02631,P02781,P06760,P06761,P06911,P08649,P13432,P18418,P19223,P19629,P20646,P20760,P20761,P20762,P23928,P31044,P31430,P35952,P48508,P50115,P50116,P50280,P52590,P54921,P55091,P57113,P63081,P80299,P97580,P98089,Q30KJ2,Q4G075,Q5GRG2,Q5QE79,Q5RLM2,Q62635,Q62714,Q62946,Q66H69,Q6PCU2,Q6TMA8,Q8CIZ5,Q9JI85,Q9QX74,Q9R0T3,Q9WUW8,Q9WUW9,Q9Z1F2
1294	1,2,4,5,6,7,8,15	3,9,10,11,12,13,14,16	71	A2RUW1,B1H234,D3ZHA0,D4A5U3,iRT-Kit_WR_fusion,O08557,O55004,O70594,O88267,O88339;Q4V882,P02631,P06760,P06761,P06911,P08721,P10247,P13432,P15999,P17988,P18418,P18757,P19112,P19132,P19223,P19629,P19814,P20646,P20760,P20761,P20762,P23928,P30904,P31044,P31430,P35952,P36860,P38918,P46844,P48508,P50115,P50116,P50280,P52590,P54921,P55091,P80299,P98089,Q03248,Q30KJ2,Q4G075,Q5GRG2,Q5I0E9,Q5M7T9,Q62635,Q62714,Q63270,Q63618,Q66H69,Q6MG61,Q6PCU2,Q6Q0N1,Q6TMA8,Q8CIZ5,Q91Y81,Q9JI85,Q9QX74,Q9R0T3,Q9Z0J6,Q9Z0V6,Q9Z0W7,Q9Z1F2
1295	1,2,4,5,6,7,8,16	3,9,10,11,12,13,14,15	99	B1H234,D3ZHA0,D4A5U3,iRT-Kit_WR_fusion,O08557,O55004,O70257,O70594,O88267,P01836,P02631,P02761,P02780,P02781,P02782,P04904,P05371,P06760,P06761,P06911,P07151,P07647,P08649,P11598,P13432,P15399,P17988,P18418,P18757,P19112,P19223,P19468,P19629,P20646,P20673,P20760,P20761,P20762,P22282,P22283,P23928,P31044,P31430,P35952,P36860,P38918,P46844,P48508,P50115,P50116,P50280,P52590,P52847,P53790,P55091,P57113,P80299,P97580,P98089,Q03248,Q30KJ2,Q3ZAV1,Q4G075,Q5GRG2,Q5I0D7,Q5I0E9,Q5M7T9,Q5M8C6,Q5RLM2,Q5U2Q3,Q62635,Q63270,Q63618,Q64602,Q66H69,Q68FT5,Q6MG61,Q6PCU2,Q6Q0N1,Q6TMA8,Q71MB6,Q8CIZ5,Q8R431,Q8R5M3,Q91Y81,Q923S2,Q99MA2,Q9JHB9,Q9JI85,Q9JJ40,Q9JLJ3,Q9QX74,Q9QZK8,Q9R0T3,Q9WUW8,Q9WUW9,Q9Z0J6,Q9Z0V6,Q9Z0W7
1296	1,2,4,5,6,7,9,10	3,8,11,12,13,14,15,16	18	B1H234,D3ZTX0,O70594,P00762,P18418,P19223,P23928,P36860,P50116,P52590,P55091,P70545,P80299,Q63618,Q9R0T3,Q9WVH8,Q9Z0J6,Q9Z1F2
1297	1,2,4,5,6,7,9,11	3,8,10,12,13,14,15,16	18	B1H234,P01039,P07647,P11598,P18418,P22283,P31430,P36860,P50116,P52590,P55091,P80299,Q4KLZ6,Q63617,Q9EQS0,Q9R0T3,Q9R168,Q9Z0J6

1298	1,2,4,5,6,7,9,12	3,8,10,11,13,14,15,16	10	B1H234,P18418,P19814,P36860,P50116,P52590,P55091,P80299,Q03191,Q9R0T3
1299	1,2,4,5,6,7,9,13	3,8,10,11,12,14,15,16	11	B1H234,P15978,P18418,P19223,P36860,P50116,P52590,P55091,P80299,Q9EQS0,Q9Z0J6
1300	1,2,4,5,6,7,9,14	3,8,10,11,12,13,15,16	8	B1H234,P00762,P18418,P50116,P52590,P55091,P80299,Q9R168
1301	1,2,4,5,6,7,9,15	3,8,10,11,12,13,14,16	10	B1H234,P00762,P08721,P18418,P36860,P50116,P52590,P55091,P80299,Q9Z0J6
1302	1,2,4,5,6,7,9,16	3,8,10,11,12,13,14,15	13	B1H234,P07647,P15978,P18418,P22006,P50116,P52590,P55091,P80299,Q6TMA8,Q9EQS0,Q9R168,Q9Z0J6
1303	1,2,4,5,6,7,10,11	3,8,9,12,13,14,15,16	18	B1H234,O08557,P01039,P07647,P08721,P11598,P17988,P18418,P22283,P31430,P36860,P50116,P52590,P55091,P80299,Q4KLZ6,Q9R0T3,Q9Z0J6
1304	1,2,4,5,6,7,10,12	3,8,9,11,13,14,15,16	14	B1H234,O88267,P18418,P19814,P36860,P48508,P50116,P55091,P57113,P80299,Q5RLM2,Q63618,Q66HG3,Q9R0T3
1305	1,2,4,5,6,7,10,13	3,8,9,11,12,14,15,16	18	A2RUW1,B1H234,O70594,P01835,P18418,P19223,P23928,P36860,P48508,P50116,P52590,P55091,P57113,P80299,Q63618,Q9R0T3,Q9WUW8,Q9Z1F2
1306	1,2,4,5,6,7,10,14	3,8,9,11,12,13,15,16	22	A2RUW1,B1H234,O08557,O70594,P09527,P18418,P19223,P23928,P36860,P48037,P48508,P50116,P52590,P54921,P55091,P57113,P80299,Q5RLM2,Q63618,Q80WL1,Q9WUW8,Q9Z1F2
1307	1,2,4,5,6,7,10,15	3,8,9,11,12,13,14,16	31	A2RUW1,B1H234,O08557,O70594,O88267,O88339;Q4V882,P08721,P09527,P17988,P18297,P18418,P19468,P23928,P36860,P38918,P48508,P50116,P51907,P54921,P55091,P57113,P80299,Q03248,Q5I0E9,Q63270,Q63618,Q6MG61,Q6Q0N1,Q8K3P7,Q99MA2,Q9Z1F2
1308	1,2,4,5,6,7,10,16	3,8,9,11,12,13,14,15	46	B1H234,O08557,O70594,O88267,P07647,P09527,P17988,P18297,P18418,P18757,P19223,P19468,P23928,P36860,P46844,P48037,P48508,P50116,P52590,P53790,P55091,P57113,P80299,Q03248,Q3ZAV1,Q5I0E9,Q5M7T9,Q5RLM2,Q63270,Q63424,Q63618,Q64602,Q68FT5,Q6MG61,Q6Q0N1,Q6TMA8,Q71MB6,Q8R431,Q923S2,Q99MA2,Q9JJ40,Q9QYU4,Q9R0T3,Q9WUW8,Q9Z0J6,Q9Z0W7
1309	1,2,4,5,6,7,11,12	3,8,9,10,13,14,15,16	22	B1H234,O35547,P01039,P02780,P07647,P11598,P18418,P22283,P31430,P36374,P36860,P47967,P50116,P52590,P55091,P80299,Q4KLZ6,Q5GRG2,Q5I0D1,Q63617,Q9JI85,Q9R0T3
1310	1,2,4,5,6,7,11,13	3,8,9,10,12,14,15,16	20	B1H234,P01039,P02781,P07647,P11598,P18418,P22283,P31430,P36860,P50116,P52590,P55091,P80299,Q4KLZ6,Q5GRG2,Q63617,Q9EQS0,Q9JI85,Q9R0T3,Q9Z0J6
1311	1,2,4,5,6,7,11,14	3,8,9,10,12,13,15,16	15	B1H234,P01039,P07647,P08649,P11598,P18418,P22283,P31430,P50116,P52590,P55091,P80299,Q4KLZ6,Q5GRG2,Q9R0T3
1312	1,2,4,5,6,7,11,15	3,8,9,10,12,13,14,16	22	B1H234,O08557,P01039,P07647,P08721,P11598,P17988,P18418,P22283,P31430,P36860,P50116,P52590,P55091,P80299,Q4FZU2,Q4KLZ6,Q5GRG2,Q6P6Q2,Q8CJ52,Q9R0T3,Q9Z0J6

1313	1,2,4,5,6,7,11,16	3,8,9,10,12,13,14,15	33	B1H234,O35547,P01039,P02761,P02780,P02781,P02782,P07647,P08649,P08721,P08723,P09456,P0C0A9,P11598,P17988,P18418,P22283,P31430,P36374,P50116,P52590,P55091,P80299,Q4KLZ6,Q5GRG2,Q5M872,Q63617,Q6TMA8,Q8R5M3,Q9EQS0,Q9JHB9,Q9R0T3,Q9Z0J6
1314	1,2,4,5,6,7,12,13	3,8,9,10,11,14,15,16	10	B1H234,P01835,P19814,P36860,P47967,P50116,P52590,P55091,P80299,Q9R0T3
1315	1,2,4,5,6,7,12,14	3,8,9,10,11,13,15,16	6	B1H234,P19814,P50116,P52590,P80299,Q5RLM2
1316	1,2,4,5,6,7,12,15	3,8,9,10,11,13,14,16	8	B1H234,P08721,P17988,P19814,P36860,P50116,P52590,P80299
1317	1,2,4,5,6,7,12,16	3,8,9,10,11,13,14,15	11	B1H234,P07647,P17988,P18418,P50116,P52590,P80299,Q5I0D7,Q6TMA8,Q9R0T3,Q9Z0J6
1318	1,2,4,5,6,7,13,14	3,8,9,10,11,12,15,16	10	A2RUW1,B1H234,P01835,P02761,P19223,P50116,P52590,P80299,Q9EQS0,Q9WUW8
1319	1,2,4,5,6,7,13,15	3,8,9,10,11,12,14,16	11	A2RUW1,B1H234,P01835,P08721,P36860,P50116,P52590,P80299,Q62714,Q63618,Q9Z0J6
1320	1,2,4,5,6,7,13,16	3,8,9,10,11,12,14,15	17	B1H234,P01835,P02761,P07647,P18418,P19223,P36860,P50116,P52590,P80299,Q5I0D7,Q6TMA8,Q8R5M3,Q9EQS0,Q9R0T3,Q9WUW8,Q9Z0J6
1321	1,2,4,5,6,7,14,15	3,8,9,10,11,12,13,16	9	A2RUW1,B1H234,P08721,P17988,P50116,P52590,P54921,P80299,Q62714
1322	1,2,4,5,6,7,14,16	3,8,9,10,11,12,13,15	15	B1H234,P02761,P07647,P17988,P18418,P19223,P48037,P50116,P52590,P80299,Q4KLZ6,Q5RLM2,Q6TMA8,Q9EQS0,Q9WUW8
1323	1,2,4,5,6,7,15,16	3,8,9,10,11,12,13,14	20	B1H234,B2RYW9,O08557,O70594,P02761,P07647,P08721,P10719,P17988,P18418,P36860,P48508,P50116,P52590,P80299,Q03248,Q5I0D7,Q63270,Q6TMA8,Q9Z0J6
1324	1,2,4,5,6,8,9,10	3,7,11,12,13,14,15,16	17	D3ZTX0,O70594,P02454,P02631,P15399,P17559,P18418,P20760,P25031,P35952,P36860,P52590,P55091,P70545,P97580,Q30KJ2,Q63618
1325	1,2,4,5,6,8,9,11	3,7,10,12,13,14,15,16	19	B1H234,P18418,P20760,P31430,P50280,P52590,P55091,P60905,P61206;P84079,Q09326,Q10758,Q3T1J1,Q4KLZ6,Q5GRG2,Q5RKI1,Q63493,Q6IFU8,Q6P6S4,Q9JI85
1326	1,2,4,5,6,8,9,12	3,7,10,11,13,14,15,16	9	P18418,P20760,P31430,P36860,P52590,P55091,P98089,Q03191,Q5RKI1
1327	1,2,4,5,6,8,9,13	3,7,10,11,12,14,15,16	9	B1H234,P18418,P19223,P20760,P36860,P52590,P55091,Q5RKI1,Q9JI85
1328	1,2,4,5,6,8,9,14	3,7,10,11,12,13,15,16	7	P18418,P20760,P52590,P55091,P97580,Q30KJ2,Q5RKI1
1329	1,2,4,5,6,8,9,15	3,7,10,11,12,13,14,16	7	P13432,P18418,P19132,P20760,P36860,P52590,P55091

1330	1,2,4,5,6,8,9,16	3,7,10,11,12,13,14,15	12	P07647,P13432,P15399,P18418,P20760,P23593,P52590,P55091,P97580,Q30KJ2,Q5RKI1,Q9Z0J6
1331	1,2,4,5,6,8,10,11	3,7,9,12,13,14,15,16	19	B1H234,P15399,P18418,P20760,P25031,P31430,P35952,P36860,P50280,P52590,P55091,P97580,Q10758,Q30KJ2,Q4KLZ6,Q5GRG2,Q68G31,Q6IFU8,Q9JI85
1332	1,2,4,5,6,8,10,12	3,7,9,11,13,14,15,16	24	O55004,O88267,P02454,P02631,P0DMW0;P0DMW1,P15399,P18418,P19814,P20760,P25031,P30904,P31430,P35952,P36860,P48508,P55091,P97580,P98089,Q30KJ2,Q498D9,Q5RLM2,Q63618,Q66HG3,Q91ZS3
1333	1,2,4,5,6,8,10,13	3,7,9,11,12,14,15,16	21	A2RUW1,O55004,O70257,O70594,P02631,P15399,P18418,P19223,P20760,P25031,P35952,P36860,P52590,P55091,P97580,Q30KJ2,Q498D9,Q63618,Q68G31,Q8CIZ5,Q9JI85
1334	1,2,4,5,6,8,10,14	3,7,9,11,12,13,15,16	18	A2RUW1,P15399,P18418,P19223,P20760,P20762,P25031,P35952,P48508,P52590,P54921,P55091,P97580,Q30KJ2,Q5RLM2,Q63618,Q68G31,Q9WUW8
1335	1,2,4,5,6,8,10,15	3,7,9,11,12,13,14,16	30	A2RUW1,O55004,O70594,O88267,O88339;Q4V882,P10247,P13432,P15399,P18418,P19132,P19629,P20760,P20762,P23928,P25031,P30904,P35952,P36860,P48508,P54921,P55091,P97580,Q30KJ2,Q498D9,Q63270,Q63474,Q63618,Q68G31,Q6PCU2,Q6Q0N1
1336	1,2,4,5,6,8,10,16	3,7,9,11,12,13,14,15	36	O55004,O70257,O70594,O88267,P02631,P07151,P07647,P15399,P18418,P19223,P19468,P19629,P20760,P20762,P23928,P25031,P35952,P46844,P48508,P50280,P52590,P53790,P55091,P57113,P97580,Q03248,Q30KJ2,Q3ZAV1,Q5M7T9,Q63270,Q63618,Q6Q0N1,Q71MB6,Q8R431,Q923S2,Q99MA2
1337	1,2,4,5,6,8,11,12	3,7,9,10,13,14,15,16	26	B1H234,O35547,P02780,P02781,P06911,P12020,P14046,P18418,P22282,P31430,P36374,P40241,P50280,P52590,P55091,P98089,Q09326,Q10758,Q3T1J1,Q4KLZ6,Q5GRG2,Q5RKI1,Q63493,Q6IFU8,Q6IG05,Q9JI85
1338	1,2,4,5,6,8,11,13	3,7,9,10,12,14,15,16	27	B1H234,P02781,P06911,P07647,P12020,P18418,P22282,P31430,P35952,P36860,P40241,P50280,P52590,P55091,P60905,Q09326,Q10758,Q3T1J1,Q4KLZ6,Q5GRG2,Q5RKI1,Q63493,Q66H69,Q68G31,Q6IFU8,Q8CIZ5,Q9JI85
1339	1,2,4,5,6,8,11,14	3,7,9,10,12,13,15,16	21	B1H234,P06911,P12020,P15399,P18418,P19218,P20760,P31430,P50280,P52590,P55091,P97580,Q09326,Q10758,Q3T1J1,Q4KLZ6,Q5GRG2,Q5RKI1,Q62635,Q6IFU8,Q9JI85
1340	1,2,4,5,6,8,11,15	3,7,9,10,12,13,14,16	20	B1H234,P15399,P18418,P31430,P50280,P52590,P55091,Q10758,Q4FZU2,Q4KLZ6,Q5GRG2,Q63493,Q6IFU7,Q6IFU8,Q6IFW6,Q6IG05,Q6IMF3,Q6P6Q2,Q8CJ52,Q9JI85
1341	1,2,4,5,6,8,11,16	3,7,9,10,12,13,14,15	41	B1H234,O35547,O88917,P02780,P02781,P02782,P04905,P06911,P07647,P08723,P09456,P0C0A9,P12020,P15399,P18418,P20760,P22282,P22283,P30120,P31430,P36374,P40241,P46462,P50280,P52590,P55091,P60905,P97580,Q10758,Q30KJ2,Q4KLZ6,Q5GRG2,Q5M8C6,Q5RKI1,Q63493,Q6GMN2,Q6IFU8,Q6P6Q2,Q9JHB9,Q9JI85,Q9Z0J6
1342	1,2,4,5,6,8,12,13	3,7,9,10,11,14,15,16	12	P02631,P18418,P19814,P31430,P36860,P52590,P55091,P98089,Q5RKI1,Q66H69,Q8CIZ5,Q9JI85
1343	1,2,4,5,6,8,12,14	3,7,9,10,11,13,15,16	12	P06911,P18418,P19814,P20760,P31430,P52590,P97580,P98089,Q5GRG2,Q5RKI1,Q5RLM2,Q62635
1344	1,2,4,5,6,8,12,15	3,7,9,10,11,13,14,16	9	P13432,P18418,P19132,P19814,P31430,P36860,P98089,Q498D9,Q6IG05
1345	1,2,4,5,6,8,12,16	3,7,9,10,11,13,14,15	11	O88267,P07647,P15399,P18418,P20760,P52590,P97580,P98089,Q30KJ2,Q5RKI1,Q9JI85

1346	1,2,4,5,6,8,13,14	3,7,9,10,11,12,15,16	15	A2RUW1,B1H234,P18418,P19223,P20760,P52590,P97580,Q30KJ2,Q5RKI1,Q62635,Q68G31,Q8CIZ5,Q9JI85,Q9QWN8,Q9WUW8
1347	1,2,4,5,6,8,13,15	3,7,9,10,11,12,14,16	17	A2RUW1,B1H234,P13432,P15399,P18418,P19132,P20760,P35952,P36860,P52590,Q30KJ2,Q498D9,Q63618,Q68G31,Q6PCU2,Q8CIZ5,Q9JI85
1348	1,2,4,5,6,8,13,16	3,7,9,10,11,12,14,15	16	B1H234,O55004,P02781,P07151,P07647,P15399,P18418,P19223,P20760,P52590,P97580,Q30KJ2,Q5RKI1,Q6TMA8,Q9JI85,Q9Z0J6
1349	1,2,4,5,6,8,14,15	3,7,9,10,11,12,13,16	13	A2RUW1,P13432,P15399,P18418,P19132,P20760,P20762,P50280,P52590,P54921,P97580,Q30KJ2,Q6PCU2
1350	1,2,4,5,6,8,14,16	3,7,9,10,11,12,13,15	15	B1H234,P07647,P15399,P18418,P19223,P20760,P20762,P50280,P52590,P97580,Q30KJ2,Q4KLZ6,Q5RKI1,Q6TMA8,Q9WUW8
1351	1,2,4,5,6,8,15,16	3,7,9,10,11,12,13,14	19	B2RYW9,O88267,P07151,P07647,P13432,P15399,P17988,P18418,P19629,P20760,P20762,P48508,P50280,P52590,P97580,Q30KJ2,Q63270,Q6PCU2,Q6TMA8
1352	1,2,4,5,6,9,10,11	3,7,8,12,13,14,15,16	3	B1H234,P36860,Q4KLZ6
1353	1,2,4,5,6,9,10,12	3,7,8,11,13,14,15,16	2	P36860,Q03191
1354	1,2,4,5,6,9,10,13	3,7,8,11,12,14,15,16	4	D3ZTX0,P11883,P36860,Q9EQS0
1355	1,2,4,5,6,9,10,14	3,7,8,11,12,13,15,16	2	P00762,P52590
1356	1,2,4,5,6,9,10,15	3,7,8,11,12,13,14,16	3	P00762,P36860,Q811M5
1357	1,2,4,5,6,9,10,16	3,7,8,11,12,13,14,15	5	D3ZUC6,P11883,P52590,Q811M5,Q9EQS0
1358	1,2,4,5,6,9,11,12	3,7,8,10,13,14,15,16	6	B1H234,O35077,P61206;P84079,Q03191,Q4KLZ6,Q5RKI1
1359	1,2,4,5,6,9,11,13	3,7,8,10,12,14,15,16	5	B1H234,P52590,Q4KLZ6,Q5RKI1,Q9EQS0
1360	1,2,4,5,6,9,11,14	3,7,8,10,12,13,15,16	5	B1H234,P52590,Q4KLZ6,Q5I0J9,Q5RKI1
1361	1,2,4,5,6,9,11,15	3,7,8,10,12,13,14,16	2	B1H234,Q4KLZ6
1362	1,2,4,5,6,9,11,16	3,7,8,10,12,13,14,15	10	B1H234,D3ZUC6,P07647,P22006,P52590,P70549,Q4KLZ6,Q5RKI1,Q9EQS0,Q9Z0J6
1363	1,2,4,5,6,9,12,13	3,7,8,10,11,14,15,16	5	P36860,P52590,Q03191,Q5RKI1,Q9EQS0
1364	1,2,4,5,6,9,12,14	3,7,8,10,11,13,15,16	3	P52590,Q03191,Q5RKI1
1365	1,2,4,5,6,9,12,15	3,7,8,10,11,13,14,16	2	P36860,Q03191

1366	1,2,4,5,6,9,12,16	3,7,8,10,11,13,14,15	4	D3ZUC6,P52590,Q03191,Q5RKI1
1367	1,2,4,5,6,9,13,14	3,7,8,10,11,12,15,16	4	B1H234,P52590,Q5RKI1,Q9EQS0
1368	1,2,4,5,6,9,13,15	3,7,8,10,11,12,14,16	5	B1H234,P11883,P36860,P62804,Q9EQS0
1369	1,2,4,5,6,9,13,16	3,7,8,10,11,12,14,15	7	B1H234,D3ZUC6,P11883,P52590,Q5RKI1,Q9EQS0,Q9Z0J6
1370	1,2,4,5,6,9,14,15	3,7,8,10,11,12,13,16	1	P52590
1371	1,2,4,5,6,9,14,16	3,7,8,10,11,12,13,15	6	B1H234,P23593,P52590,Q4KLZ6,Q5RKI1,Q9EQS0
1372	1,2,4,5,6,9,15,16	3,7,8,10,11,12,13,14	4	D3ZUC6,P11883,P52590,Q9EQS0
1373	1,2,4,5,6,10,11,12	3,7,8,9,13,14,15,16	3	P35280,P36860,Q4KLZ6
1374	1,2,4,5,6,10,11,13	3,7,8,9,12,14,15,16	5	B1H234,P35280,P36860,Q4KLZ6,Q68G31
1375	1,2,4,5,6,10,11,14	3,7,8,9,12,13,15,16	4	B1H234,P35280,P52590,Q4KLZ6
1376	1,2,4,5,6,10,11,15	3,7,8,9,12,13,14,16	4	B1H234,P17988,P36860,Q4KLZ6
1377	1,2,4,5,6,10,11,16	3,7,8,9,12,13,14,15	6	B1H234,P07647,P17988,P35280,P52590,Q4KLZ6
1378	1,2,4,5,6,10,12,13	3,7,8,9,11,14,15,16	3	P19814,P35280,P36860
1379	1,2,4,5,6,10,12,14	3,7,8,9,11,13,15,16	4	P19814,P35280,P52590,Q5RLM2
1380	1,2,4,5,6,10,12,15	3,7,8,9,11,13,14,16	4	P19814,P35280,P36860,Q498D9
1381	1,2,4,5,6,10,12,16	3,7,8,9,11,13,14,15	4	O88267,P0DMW0;P0DMW1,P35280,P52590
1382	1,2,4,5,6,10,13,14	3,7,8,9,11,12,15,16	4	A2RUW1,B1H234,P35280,P52590
1383	1,2,4,5,6,10,13,15	3,7,8,9,11,12,14,16	7	A2RUW1,P11883,P21674,P35280,P36860,Q498D9,Q63618
1384	1,2,4,5,6,10,13,16	3,7,8,9,11,12,14,15	5	P11883,P35280,P52590,Q63618,Q9EQS0
1385	1,2,4,5,6,10,14,15	3,7,8,9,11,12,13,16	4	A2RUW1,P35280,P52590,P54921

1386	1,2,4,5,6,10,14,16	3,7,8,9,11,12,13,15	3	P35280,P52590,Q4KLZ6
1387	1,2,4,5,6,10,15,16	3,7,8,9,11,12,13,14	4	O88267,P11883,P17988,P35280
1388	1,2,4,5,6,11,12,13	3,7,8,9,10,14,15,16	4	B1H234,P35280,Q4KLZ6,Q5RKI1
1389	1,2,4,5,6,11,12,14	3,7,8,9,10,13,15,16	6	B1H234,P35280,P52590,Q4KLZ6,Q5GRG2,Q5RKI1
1390	1,2,4,5,6,11,12,15	3,7,8,9,10,13,14,16	2	B1H234,Q4KLZ6
1391	1,2,4,5,6,11,12,16	3,7,8,9,10,13,14,15	7	B1H234,O35547,P07647,P35280,P52590,Q4KLZ6,Q5RKI1
1392	1,2,4,5,6,11,13,14	3,7,8,9,10,12,15,16	6	B1H234,P35280,P52590,Q4KLZ6,Q5RKI1,Q9EQS0
1393	1,2,4,5,6,11,13,15	3,7,8,9,10,12,14,16	6	B1H234,O70417,P21674,P36860,Q4KLZ6,Q9EQS0
1394	1,2,4,5,6,11,13,16	3,7,8,9,10,12,14,15	9	B1H234,O35547,P07647,P35280,P52590,Q4KLZ6,Q5RKI1,Q9EQS0,Q9Z0J6
1395	1,2,4,5,6,11,14,15	3,7,8,9,10,12,13,16	3	B1H234,P52590,Q4KLZ6
1396	1,2,4,5,6,11,14,16	3,7,8,9,10,12,13,15	7	B1H234,P07647,P35280,P52590,Q4KLZ6,Q5RKI1,Q9EQS0
1397	1,2,4,5,6,11,15,16	3,7,8,9,10,12,13,14	6	B1H234,P07647,P17988,P52590,Q4KLZ6,Q8CJ52
1398	1,2,4,5,6,12,13,14	3,7,8,9,10,11,15,16	3	P35280,P52590,Q5RKI1
1399	1,2,4,5,6,12,13,15	3,7,8,9,10,11,14,16	3	P19814,P35280,P36860
1400	1,2,4,5,6,12,13,16	3,7,8,9,10,11,14,15	4	P35280,P52590,Q5RKI1,Q9EQS0
1401	1,2,4,5,6,12,14,15	3,7,8,9,10,11,13,16	3	P19814,P35280,P52590
1402	1,2,4,5,6,12,14,16	3,7,8,9,10,11,13,15	4	P35280,P52590,Q4KLZ6,Q5RKI1
1403	1,2,4,5,6,12,15,16	3,7,8,9,10,11,13,14	3	P17988,P35280,P52590
1404	1,2,4,5,6,13,14,15	3,7,8,9,10,11,12,16	5	A2RUW1,B1H234,P21674,P35280,P52590
1405	1,2,4,5,6,13,14,16	3,7,8,9,10,11,12,15	6	B1H234,P35280,P52590,Q4KLZ6,Q5RKI1,Q9EQS0

1406	1,2,4,5,6,13,15,16	3,7,8,9,10,11,12,14	6	B1H234,O70417,P11883,P35280,P52590,Q9EQS0
1407	1,2,4,5,6,14,15,16	3,7,8,9,10,11,12,13	4	P17988,P35280,P52590,Q4KLZ6
1408	1,2,4,5,7,8,9,10	3,6,11,12,13,14,15,16	17	D4A5U3,O70594,P06760,P18418,P19223,P20646,P20760,P20762,P36860,P70545,Q63618,Q66H69,Q6TMA8,Q8CIZ5,Q9QX74,Q9Z0V6,Q9Z1F2
1409	1,2,4,5,7,8,9,11	3,6,10,12,13,14,15,16	29	D4A5U3,iRT-Kit_WR_fusion,P02780,P02781,P06760,P07150,P08649,P08723,P09456,P18418,P20646,P20760,P27590,P31430,P40241,P97697,Q10758,Q4G075,Q63493,Q66H69,Q6IFU8,Q6IMF3,Q6P6Q2,Q6TMA8,Q812E4,Q9JHB9,Q9QX74,Q9WTT6,Q9Z0V6
1410	1,2,4,5,7,8,9,12	3,6,10,11,13,14,15,16	12	D4A5U3,iRT-Kit_WR_fusion,P06760,P20646,P20760,Q03191,Q66H69,Q6TMA8,Q8CIZ5,Q9QX74,Q9Z0V6,Q9Z1F2
1411	1,2,4,5,7,8,9,13	3,6,10,11,12,14,15,16	14	D4A5U3,P06760,P19223,P20646,P20760,P36860,P80299,P97697,Q66H69,Q6TMA8,Q8CIZ5,Q9QX74,Q9Z0V6,Q9Z1F2
1412	1,2,4,5,7,8,9,14	3,6,10,11,12,13,15,16	14	D3ZHA0,D4A5U3,P06760,P08649,P19223,P20646,P20760,Q4G075,Q62635,Q6TMA8,Q8CIZ5,Q9QX74,Q9Z0V6,Q9Z1F2
1413	1,2,4,5,7,8,9,15	3,6,10,11,12,13,14,16	10	P06760,P08649,P15999,P19132,P20646,P20760,Q6TMA8,Q9QX74,Q9Z0V6,Q9Z1F2
1414	1,2,4,5,7,8,9,16	3,6,10,11,12,13,14,15	13	D3ZHA0,D4A5U3,P06760,P08649,P18418,P19223,P20646,P20760,P20762,Q6TMA8,Q9QX74,Q9Z0J6,Q9Z0V6
1415	1,2,4,5,7,8,10,11	3,6,9,12,13,14,15,16	30	P02780,P02781,P06760,P07150,P08723,P18418,P19223,P20646,P20762,P22282,P27590,P31430,P36860,P40241,Q10758,Q4FZU2,Q4G075,Q63493,Q66H69,Q6B345,Q6IFU8,Q6IMF3,Q6P6Q2,Q6TMA8,Q8CIZ5,Q9JHB9,Q9QX74,Q9R0T3,Q9Z0V6,Q9Z1F2
1416	1,2,4,5,7,8,10,12	3,6,9,11,13,14,15,16	17	O88267,P06760,P18418,P19814,P20762,P30904,P36860,P48508,Q5RLM2,Q63618,Q66H69,Q6Q0N1,Q6TMA8,Q8CIZ5,Q9QX74,Q9Z0V6,Q9Z1F2
1417	1,2,4,5,7,8,10,13	3,6,9,11,12,14,15,16	30	D4A5U3,O55004,O70257,O70594,P06760,P18418,P19223,P19468,P20646,P20760,P20762,P25809,P30904,P36860,P46844,P48508,P51907,P80299,Q3ZAV1,Q4G075,Q63618,Q66H69,Q6PCU2,Q6Q0N1,Q6TMA8,Q8CIZ5,Q9QX74,Q9WUW8,Q9WUW9,Q9Z1F2
1418	1,2,4,5,7,8,10,14	3,6,9,11,12,13,15,16	23	O70594,P06760,P18418,P19223,P20646,P20760,P20762,P25809,P48508,P54921,Q4G075,Q5RLM2,Q62635,Q63618,Q66H69,Q6PCU2,Q6TMA8,Q8CIZ5,Q923S2,Q9QX74,Q9WUW8,Q9WUW9,Q9Z1F2

1419	1,2,4,5,7,8,10,15	3,6,9,11,12,13,14,16	44	O08557,O70594,O88267,O88339;Q4V882,P06760,P10536,P18418,P19132,P19223,P19468,P20646,P20760,P20762,P23928,P30904,P36860,P46844,P48508,P51907,P53790,P54921,Q05175,Q3ZAV1,Q5I0E9,Q5M7T9,Q63270,Q63355,Q63424,Q63618,Q64093,Q66H69,Q6MG61,Q6PCU2,Q6Q0N1,Q6TMA8,Q80W57,Q8CIZ5,Q8R431,Q923S2,Q9QX74,Q9WTW7,Q9Z0V6,Q9Z0W7,Q9Z1F2
1420	1,2,4,5,7,8,10,16	3,6,9,11,12,13,14,15	50	O55004,O70257,O70594,O88267,P02781,P06760,P07151,P18418,P18757,P19223,P19468,P20646,P20760,P20762,P23928,P46844,P48508,P51907,P53790,P57113,Q03248,Q05175,Q30KJ2,Q3ZAV1,Q4G075,Q5I0E9,Q5M7T9,Q5RLM2,Q63270,Q63355,Q63424,Q63618,Q64093,Q64602,Q66H69,Q6MG61,Q6Q0N1,Q6TMA8,Q71MB6,Q80W57,Q8R431,Q923S2,Q99MA2,Q9JJ40,Q9QX74,Q9WTW7,Q9WUW8,Q9WUW9,Q9Z0V6,Q9Z0W7
1421	1,2,4,5,7,8,11,12	3,6,9,10,13,14,15,16	32	iRT-Kit_WR_fusion,P02780,P02781,P02782,P04905,P06760,P06911,P07150,P08649,P08723,P22282,P27590,P31430,P36374,P40241,P47967,Q10758,Q4FZU2,Q4G075,Q5GRG2,Q5I0D1,Q5M8C6,Q63493,Q66H69,Q6IFU8,Q6IMF3,Q6P6Q2,Q6TMA8,Q8CIZ5,Q9JHB9,Q9QX74,Q9R0T3
1422	1,2,4,5,7,8,11,13	3,6,9,10,12,14,15,16	31	D4A5U3,P02780,P02781,P02782,P06760,P08649,P08723,P09456,P18418,P19223,P22273,P22282,P22283,P31430,P36374,P40241,P47967,Q10758,Q4FZU2,Q4G075,Q63493,Q66H69,Q6IFU8,Q6IMF3,Q6P6Q2,Q6TMA8,Q8CIZ5,Q9JHB9,Q9JI85,Q9QX74,Q9WTT6
1423	1,2,4,5,7,8,11,14	3,6,9,10,12,13,15,16	31	D4A5U3,P02780,P02781,P02782,P06760,P07150,P08649,P08723,P09456,P19223,P20646,P20760,P31430,P36374,P40241,P50280,Q10758,Q4FZU2,Q4G075,Q5GRG2,Q62635,Q63493,Q66H69,Q6IFU8,Q6IMF3,Q6P6Q2,Q6TMA8,Q8CIZ5,Q9JHB9,Q9QX74,Q9WTT6
1424	1,2,4,5,7,8,11,15	3,6,9,10,12,13,14,16	26	P02780,P02781,P06760,P08649,P08723,P20646,P36374,P40241,P50280,Q10758,Q4FZU2,Q4G075,Q63493,Q66H69,Q6IFU8,Q6IFW6,Q6IMF3,Q6P6Q2,Q6TMA8,Q8CIZ5,Q8CJ52,Q9JHB9,Q9QX74,Q9WTT6,Q9Z0V6,Q9Z1F2
1425	1,2,4,5,7,8,11,16	3,6,9,10,12,13,14,15	44	D4A5U3,O35547,P02761,P02780,P02781,P02782,P04905,P06760,P07150,P07647,P08010,P08649,P08723,P09456,P0C0A9,P18418,P19223,P20646,P20760,P20762,P22273,P22282,P22283,P30120,P36374,P40241,P46462,P50280,Q00715,Q10758,Q4FZU2,Q4G075,Q5M8C6,Q63493,Q66H69,Q6IFU8,Q6IMF3,Q6P6Q2,Q6TMA8,Q812E4,Q8R5M3,Q9JHB9,Q9QX74,Q9Z0J6
1426	1,2,4,5,7,8,12,13	3,6,9,10,11,14,15,16	14	D4A5U3,iRT-Kit_WR_fusion,P06760,P19223,P19814,P36860,P47967,P80299,P98089,Q4G075,Q66H69,Q6TMA8,Q8CIZ5,Q9Z1F2
1427	1,2,4,5,7,8,12,14	3,6,9,10,11,13,15,16	11	D3ZHA0,P06760,P19814,P20760,Q4G075,Q5RLM2,Q62635,Q66H69,Q6TMA8,Q8CIZ5,Q9Z1F2
1428	1,2,4,5,7,8,12,15	3,6,9,10,11,13,14,16	10	iRT-Kit_WR_fusion,P19132,P19814,P20646,Q66H69,Q6TMA8,Q8CIZ5,Q9QX74,Q9Z0V6,Q9Z1F2
1429	1,2,4,5,7,8,12,16	3,6,9,10,11,13,14,15	12	iRT-Kit_WR_fusion,P02781,P07647,P18418,P20646,P20760,P20762,Q4G075,Q5RLM2,Q66H69,Q6TMA8,Q9JHB9
1430	1,2,4,5,7,8,13,14	3,6,9,10,11,12,15,16	17	B0BNN3,D3ZHA0,D4A5U3,P06760,P08649,P19223,P20646,P20760,P25809,Q4G075,Q62635,Q66H69,Q6PCU2,Q6TMA8,Q8CIZ5,Q9WUW8,Q9Z1F2
1431	1,2,4,5,7,8,13,15	3,6,9,10,11,12,14,16	15	P06760,P19132,P19223,P20646,P36860,Q4G075,Q63618,Q66H69,Q6PCU2,Q6Q0N1,Q6TMA8,Q8CIZ5,Q9QX74,Q9Z0V6,Q9Z1F2

1432	1,2,4,5,7,8,13,16	3,6,9,10,11,12,14,15	20	B0BNN3,D3ZHA0,D4A5U3,P02761,P02781,P02782,P07647,P08649,P18418,P19223,P20646,P20760,P46844,Q4G075,Q63618,Q66H69,Q6TMA8,Q8CIZ5,Q9WUW8,Q9Z0J6
1433	1,2,4,5,7,8,14,15	3,6,9,10,11,12,13,16	15	P06760,P08649,P19132,P20646,P20760,P20762,P54921,Q4G075,Q62635,Q6PCU2,Q6TMA8,Q8CIZ5,Q9QX74,Q9Z0V6,Q9Z1F2
1434	1,2,4,5,7,8,14,16	3,6,9,10,11,12,13,15	16	D3ZHA0,D4A5U3,P02761,P02781,P06760,P08649,P19223,P20646,P20760,P20762,Q4G075,Q5RLM2,Q62635,Q6TMA8,Q9QX74,Q9WUW8
1435	1,2,4,5,7,8,15,16	3,6,9,10,11,12,13,14	23	B2RYW9,O70594,P02761,P02781,P08649,P17988,P19132,P19223,P19468,P20646,P20760,P20762,P46844,P48508,Q4G075,Q63270,Q66H69,Q6PCU2,Q6Q0N1,Q6TMA8,Q8R431,Q9QX74,Q9Z0V6
1436	1,2,4,5,7,9,10,11	3,6,8,12,13,14,15,16	2	P70549,Q9R168
1437	1,2,4,5,7,9,10,12	3,6,8,11,13,14,15,16	4	P36860,P80299,Q03191,Q9Z1F2
1438	1,2,4,5,7,9,10,13	3,6,8,11,12,14,15,16	4	P36860,P80299,Q9QZK9,Q9Z1F2
1439	1,2,4,5,7,9,10,14	3,6,8,11,12,13,15,16	3	P00762,Q9R168,Q9Z1F2
1440	1,2,4,5,7,9,10,15	3,6,8,11,12,13,14,16	3	P00762,P36860,Q9Z1F2
1441	1,2,4,5,7,9,10,16	3,6,8,11,12,13,14,15	1	Q9R168
1442	1,2,4,5,7,9,11,12	3,6,8,10,13,14,15,16	1	Q03191
1443	1,2,4,5,7,9,11,13	3,6,8,10,12,14,15,16	2	P80299,Q9R168
1444	1,2,4,5,7,9,11,14	3,6,8,10,12,13,15,16	3	P08649,P70549,Q9R168
1445	1,2,4,5,7,9,11,15	3,6,8,10,12,13,14,16	3	P08649,Q6P6Q2,Q8CJ52
1446	1,2,4,5,7,9,11,16	3,6,8,10,12,13,14,15	5	P07647,P08649,P70549,Q9R168,Q9Z0J6
1447	1,2,4,5,7,9,12,13	3,6,8,10,11,14,15,16	2	P80299,Q03191
1448	1,2,4,5,7,9,12,14	3,6,8,10,11,13,15,16	1	Q03191
1449	1,2,4,5,7,9,12,15	3,6,8,10,11,13,14,16	1	Q03191
1450	1,2,4,5,7,9,12,16	3,6,8,10,11,13,14,15	1	Q03191
1451	1,2,4,5,7,9,13,14	3,6,8,10,11,12,15,16	2	P80299,Q9R168

1452	1,2,4,5,7,9,13,15	3,6,8,10,11,12,14,16	2	P36860,P80299
1453	1,2,4,5,7,9,13,16	3,6,8,10,11,12,14,15	4	P80299,Q9EQS0,Q9R168,Q9Z0J6
1454	1,2,4,5,7,9,14,15	3,6,8,10,11,12,13,16	1	P02625
1455	1,2,4,5,7,9,14,16	3,6,8,10,11,12,13,15	1	Q9R168
1456	1,2,4,5,7,9,15,16	3,6,8,10,11,12,13,14	0	
1457	1,2,4,5,7,10,11,12	3,6,8,9,13,14,15,16	3	P47967,P97840,Q5I0D1
1458	1,2,4,5,7,10,11,13	3,6,8,9,12,14,15,16	3	P36860,P47967,P80299
1459	1,2,4,5,7,10,11,14	3,6,8,9,12,13,15,16	0	
1460	1,2,4,5,7,10,11,15	3,6,8,9,12,13,14,16	3	P17988,Q4FZU2,Q6P6Q2
1461	1,2,4,5,7,10,11,16	3,6,8,9,12,13,14,15	4	P07647,P17988,P70549,Q9JHB9
1462	1,2,4,5,7,10,12,13	3,6,8,9,11,14,15,16	6	P19814,P36860,P47967,P80299,P97840,Q9Z1F2
1463	1,2,4,5,7,10,12,14	3,6,8,9,11,13,15,16	2	Q5RLM2,Q9Z1F2
1464	1,2,4,5,7,10,12,15	3,6,8,9,11,13,14,16	5	P09527,P10758,P19814,P36860,Q9Z1F2
1465	1,2,4,5,7,10,12,16	3,6,8,9,11,13,14,15	4	P09527,P0DMW0;P0DMW1,P10758,Q5RLM2
1466	1,2,4,5,7,10,13,14	3,6,8,9,11,12,15,16	5	A2RUW1,P09527,P80299,Q9WUW8,Q9Z1F2
1467	1,2,4,5,7,10,13,15	3,6,8,9,11,12,14,16	6	A2RUW1,P09527,P36860,P80299,Q63618,Q9Z1F2
1468	1,2,4,5,7,10,13,16	3,6,8,9,11,12,14,15	6	P09527,P19468,P80299,Q63618,Q6AYQ8,Q9WUW8
1469	1,2,4,5,7,10,14,15	3,6,8,9,11,12,13,16	4	A2RUW1,P09527,P54921,Q9Z1F2
1470	1,2,4,5,7,10,14,16	3,6,8,9,11,12,13,15	6	P02761,P09527,Q5RLM2,Q71MB6,Q9QYP1,Q9WUW8
1471	1,2,4,5,7,10,15,16	3,6,8,9,11,12,13,14	13	P09527,P10758,P17988,P18297,P19468,P48508,Q5I0E9,Q63424,Q63618,Q64093,Q71MB6,Q8R431,Q9QYP1

1472	1,2,4,5,7,11,12,13	3,6,8,9,10,14,15,16	4 P47967,P80299,P97840,Q5I0D1
1473	1,2,4,5,7,11,12,14	3,6,8,9,10,13,15,16	0
1474	1,2,4,5,7,11,12,15	3,6,8,9,10,13,14,16	2 Q4FZU2,Q6P6Q2
1475	1,2,4,5,7,11,12,16	3,6,8,9,10,13,14,15	5 P02781,P07647,P36374,P97840,Q9JHB9
1476	1,2,4,5,7,11,13,14	3,6,8,9,10,12,15,16	1 P08649
1477	1,2,4,5,7,11,13,15	3,6,8,9,10,12,14,16	2 Q4FZU2,Q6P6Q2
1478	1,2,4,5,7,11,13,16	3,6,8,9,10,12,14,15	8 P02761,P02781,P02782,P07647,P08649,Q6AYQ8,Q9EQS0,Q9Z0J6
1479	1,2,4,5,7,11,14,15	3,6,8,9,10,12,13,16	5 P08649,Q4FZU2,Q6IFU8,Q6P6Q2,Q8CJ52
1480	1,2,4,5,7,11,14,16	3,6,8,9,10,12,13,15	7 P02761,P07647,P08649,P70549,Q4KLZ6,Q9JHB9,Q9R168
1481	1,2,4,5,7,11,15,16	3,6,8,9,10,12,13,14	8 P07647,P08649,P17988,Q4FZU2,Q6IFU8,Q6P6Q2,Q8CJ52,Q9Z0J6
1482	1,2,4,5,7,12,13,14	3,6,8,9,10,11,15,16	1 P80299
1483	1,2,4,5,7,12,13,15	3,6,8,9,10,11,14,16	3 P19814,P36860,P80299
1484	1,2,4,5,7,12,13,16	3,6,8,9,10,11,14,15	2 P80299,P97840
1485	1,2,4,5,7,12,14,15	3,6,8,9,10,11,13,16	1 P19814
1486	1,2,4,5,7,12,14,16	3,6,8,9,10,11,13,15	1 Q5RLM2
1487	1,2,4,5,7,12,15,16	3,6,8,9,10,11,13,14	2 P10758,P17988
1488	1,2,4,5,7,13,14,15	3,6,8,9,10,11,12,16	1 A2RUW1
1489	1,2,4,5,7,13,14,16	3,6,8,9,10,11,12,15	3 B0BNN3,P02761,Q9WUW8
1490	1,2,4,5,7,13,15,16	3,6,8,9,10,11,12,14	2 P02761,Q6AYQ8
1491	1,2,4,5,7,14,15,16	3,6,8,9,10,11,12,13	3 P02761,P09527,P17988

1492	1,2,4,5,8,9<br>,10,11	3,6,7,12,1<br>3,14,15,16	4	P70545,P70549,Q10758,Q6IFU8
1493	1,2,4,5,8,9<br>,10,12	3,6,7,11,1<br>3,14,15,16	1	Q03191
1494	1,2,4,5,8,9<br>,10,13	3,6,7,11,1<br>2,14,15,16	1	P20760
1495	1,2,4,5,8,9<br>,10,14	3,6,7,11,1<br>2,13,15,16	1	P20760
1496	1,2,4,5,8,9<br>,10,15	3,6,7,11,1<br>2,13,14,16	2	P19132,P20760
1497	1,2,4,5,8,9<br>,10,16	3,6,7,11,1<br>2,13,14,15	3	P20760,P70545,Q30KJ2
1498	1,2,4,5,8,9<br>,11,12	3,6,7,10,1<br>3,14,15,16	3	Q03191,Q5RKI1,Q6IFU8
1499	1,2,4,5,8,9<br>,11,13	3,6,7,10,1<br>2,14,15,16	3	Q10758,Q5RKI1,Q6IFU8
1500	1,2,4,5,8,9<br>,11,14	3,6,7,10,1<br>2,13,15,16	4	P20760,P70549,Q5RKI1,Q6IFU8
1501	1,2,4,5,8,9<br>,11,15	3,6,7,10,1<br>2,13,14,16	6	Q10758,Q4FZU2,Q6IFU8,Q6IFW6,Q6IMF3,Q6P6Q2
1502	1,2,4,5,8,9<br>,11,16	3,6,7,10,1<br>2,13,14,15	9	O88797,P02782,P20760,P60905,P70549,Q10758,Q5RKI1,Q6IFU8,Q812E4
1503	1,2,4,5,8,9<br>,12,13	3,6,7,10,1<br>1,14,15,16	2	Q03191,Q5RKI1
1504	1,2,4,5,8,9<br>,12,14	3,6,7,10,1<br>1,13,15,16	3	P20760,Q03191,Q5RKI1
1505	1,2,4,5,8,9<br>,12,15	3,6,7,10,1<br>1,13,14,16	2	P19132,P36376
1506	1,2,4,5,8,9<br>,12,16	3,6,7,10,1<br>1,13,14,15	3	P20760,Q03191,Q5RKI1
1507	1,2,4,5,8,9<br>,13,14	3,6,7,10,1<br>1,12,15,16	2	P20760,Q5RKI1
1508	1,2,4,5,8,9<br>,13,15	3,6,7,10,1<br>1,12,14,16	1	P19132
1509	1,2,4,5,8,9<br>,13,16	3,6,7,10,1<br>1,12,14,15	2	P20760,Q5RKI1
1510	1,2,4,5,8,9<br>,14,15	3,6,7,10,1<br>1,12,13,16	2	P19132,P20760
1511	1,2,4,5,8,9<br>,14,16	3,6,7,10,1<br>1,12,13,15	3	P20760,P23593,Q5RKI1

1512	1,2,4,5,8,9,15,16	3,6,7,10,11,12,13,14	2	P20760,P23593
1513	1,2,4,5,8,10,11,12	3,6,7,9,13,14,15,16	3	Q10758,Q66H69,Q6IFU8
1514	1,2,4,5,8,10,11,13	3,6,7,9,12,14,15,16	3	Q10758,Q66H69,Q6IFU8
1515	1,2,4,5,8,10,11,14	3,6,7,9,12,13,15,16	2	Q10758,Q6IFU8
1516	1,2,4,5,8,10,11,15	3,6,7,9,12,13,14,16	7	Q10758,Q4FZU2,Q6IFU7,Q6IFU8,Q6IFW6,Q6IMF3,Q6P6Q2
1517	1,2,4,5,8,10,11,16	3,6,7,9,12,13,14,15	7	P02782,P04905,P70549,Q10758,Q30KJ2,Q6IFU8,Q6P6Q2
1518	1,2,4,5,8,10,12,13	3,6,7,9,11,14,15,16	2	P19814,Q66H69
1519	1,2,4,5,8,10,12,14	3,6,7,9,11,13,15,16	1	Q5RLM2
1520	1,2,4,5,8,10,12,15	3,6,7,9,11,13,14,16	4	P19132,P19814,P34901,Q498D9
1521	1,2,4,5,8,10,12,16	3,6,7,9,11,13,14,15	3	O88267,P0DMW0;P0DMW1,Q30KJ2
1522	1,2,4,5,8,10,13,14	3,6,7,9,11,12,15,16	1	Q9WUW8
1523	1,2,4,5,8,10,13,15	3,6,7,9,11,12,14,16	4	P19132,P36860,Q498D9,Q63618
1524	1,2,4,5,8,10,13,16	3,6,7,9,11,12,14,15	2	Q30KJ2,Q63618
1525	1,2,4,5,8,10,14,15	3,6,7,9,11,12,13,16	3	P19132,P20762,P54921
1526	1,2,4,5,8,10,14,16	3,6,7,9,11,12,13,15	5	P20760,P20762,P97580,Q30KJ2,Q9WUW8
1527	1,2,4,5,8,10,15,16	3,6,7,9,11,12,13,14	5	O88267,P20762,Q30KJ2,Q63618,Q8R431
1528	1,2,4,5,8,11,12,13	3,6,7,9,10,14,15,16	4	Q10758,Q5RKI1,Q66H69,Q6IFU8
1529	1,2,4,5,8,11,12,14	3,6,7,9,10,13,15,16	2	Q5RKI1,Q6IFU8
1530	1,2,4,5,8,11,12,15	3,6,7,9,10,13,14,16	7	Q10758,Q4FZU2,Q6IFU8,Q6IFW6,Q6IG05,Q6IMF3,Q6P6Q2
1531	1,2,4,5,8,11,12,16	3,6,7,9,10,13,14,15	12	O35547,P02781,P02782,P04905,P07647,P30120,P36374,Q10758,Q5RKI1,Q6IFU8,Q6P6Q2,Q9JHB9

1532	1,2,4,5,8,11,13,14	3,6,7,9,10,12,15,16	4	O54728,Q10758,Q5RKI1,Q6IFU8
1533	1,2,4,5,8,11,13,15	3,6,7,9,10,12,14,16	8	Q10758,Q4FZU2,Q6IFU7,Q6IFU8,Q6IFW6,Q6IG02,Q6IMF3,Q6P6Q2
1534	1,2,4,5,8,11,13,16	3,6,7,9,10,12,14,15	10	P02781,P02782,P07647,P09456,P30120,P60905,Q10758,Q5RKI1,Q6IFU8,Q6P6Q2
1535	1,2,4,5,8,11,14,15	3,6,7,9,10,12,13,16	7	Q10758,Q4FZU2,Q6IFU7,Q6IFU8,Q6IFW6,Q6IMF3,Q6P6Q2
1536	1,2,4,5,8,11,14,16	3,6,7,9,10,12,13,15	10	O54728,P02781,P02782,P30120,P70549,Q10758,Q4KLZ6,Q5RKI1,Q6IFU8,Q6P6Q2
1537	1,2,4,5,8,11,15,16	3,6,7,9,10,12,13,14	8	P02782,Q10758,Q4FZU2,Q6IFU7,Q6IFU8,Q6IFW6,Q6IMF3,Q6P6Q2
1538	1,2,4,5,8,12,13,14	3,6,7,9,10,11,15,16	1	Q5RKI1
1539	1,2,4,5,8,12,13,15	3,6,7,9,10,11,14,16	2	P19132,P19814
1540	1,2,4,5,8,12,13,16	3,6,7,9,10,11,14,15	1	Q5RKI1
1541	1,2,4,5,8,12,14,15	3,6,7,9,10,11,13,16	1	P19132
1542	1,2,4,5,8,12,14,16	3,6,7,9,10,11,13,15	1	Q5RKI1
1543	1,2,4,5,8,12,15,16	3,6,7,9,10,11,13,14	0	
1544	1,2,4,5,8,13,14,15	3,6,7,9,10,11,12,16	0	
1545	1,2,4,5,8,13,14,16	3,6,7,9,10,11,12,15	5	B0BNN3,O54728,P20760,Q5RKI1,Q9WUW8
1546	1,2,4,5,8,13,15,16	3,6,7,9,10,11,12,14	1	P01015
1547	1,2,4,5,8,14,15,16	3,6,7,9,10,11,12,13	2	P20760,P20762
1548	1,2,4,5,9,10,11,12	3,6,7,8,13,14,15,16	3	P70549,Q03191,Q62714
1549	1,2,4,5,9,10,11,13	3,6,7,8,12,14,15,16	0	
1550	1,2,4,5,9,10,11,14	3,6,7,8,12,13,15,16	1	P70549
1551	1,2,4,5,9,10,11,15	3,6,7,8,12,13,14,16	0	

1552	1,2,4,5,9,10,11,16	3,6,7,8,12,13,14,15	3	D3ZUC6,P70549,Q9Z2L0
1553	1,2,4,5,9,10,12,13	3,6,7,8,11,14,15,16	2	Q03191,Q9QZK9
1554	1,2,4,5,9,10,12,14	3,6,7,8,11,13,15,16	1	Q03191
1555	1,2,4,5,9,10,12,15	3,6,7,8,11,13,14,16	3	P30120,Q03191,Q99041
1556	1,2,4,5,9,10,12,16	3,6,7,8,11,13,14,15	2	D3ZUC6,Q03191
1557	1,2,4,5,9,10,13,14	3,6,7,8,11,12,15,16	2	Q9QW07,Q9QZK9
1558	1,2,4,5,9,10,13,15	3,6,7,8,11,12,14,16	2	Q8CFN2,Q9QZK9
1559	1,2,4,5,9,10,13,16	3,6,7,8,11,12,14,15	2	D3ZUC6,P11883
1560	1,2,4,5,9,10,14,15	3,6,7,8,11,12,13,16	1	P30120
1561	1,2,4,5,9,10,14,16	3,6,7,8,11,12,13,15	0	
1562	1,2,4,5,9,10,15,16	3,6,7,8,11,12,13,14	1	D3ZUC6
1563	1,2,4,5,9,11,12,13	3,6,7,8,10,14,15,16	4	O35077,Q03191,Q5RKI1,Q62761;Q62762;Q62763
1564	1,2,4,5,9,11,12,14	3,6,7,8,10,13,15,16	5	O35077,P70549,Q03191,Q5RKI1,Q62761;Q62762;Q62763
1565	1,2,4,5,9,11,12,15	3,6,7,8,10,13,14,16	3	O35077,Q03191,Q62761;Q62762;Q62763
1566	1,2,4,5,9,11,12,16	3,6,7,8,10,13,14,15	3	P70549,Q03191,Q5RKI1
1567	1,2,4,5,9,11,13,14	3,6,7,8,10,12,15,16	2	P0DMW0;P0DMW1,Q5RKI1
1568	1,2,4,5,9,11,13,15	3,6,7,8,10,12,14,16	1	O70417
1569	1,2,4,5,9,11,13,16	3,6,7,8,10,12,14,15	4	D3ZUC6,O70417,Q5RKI1,Q9EQS0
1570	1,2,4,5,9,11,14,15	3,6,7,8,10,12,13,16	0	
1571	1,2,4,5,9,11,14,16	3,6,7,8,10,12,13,15	4	P70549,Q4KLZ6,Q5RKI1,Q9R168

1572	1,2,4,5,9,11,15,16	3,6,7,8,10,12,13,14	2	O70417,P70549
1573	1,2,4,5,9,12,13,14	3,6,7,8,10,11,15,16	3	Q03191,Q5RKI1,Q9QZK9
1574	1,2,4,5,9,12,13,15	3,6,7,8,10,11,14,16	1	Q03191
1575	1,2,4,5,9,12,13,16	3,6,7,8,10,11,14,15	2	Q03191,Q5RKI1
1576	1,2,4,5,9,12,14,15	3,6,7,8,10,11,13,16	1	Q03191
1577	1,2,4,5,9,12,14,16	3,6,7,8,10,11,13,15	2	Q03191,Q5RKI1
1578	1,2,4,5,9,12,15,16	3,6,7,8,10,11,13,14	2	Q03191,Q99041
1579	1,2,4,5,9,13,14,15	3,6,7,8,10,11,12,16	0	
1580	1,2,4,5,9,13,14,16	3,6,7,8,10,11,12,15	2	Q5RKI1,Q9EQS0
1581	1,2,4,5,9,13,15,16	3,6,7,8,10,11,12,14	1	O70417
1582	1,2,4,5,9,14,15,16	3,6,7,8,10,11,12,13	0	
1583	1,2,4,5,10,11,12,13	3,6,7,8,9,14,15,16	1	P35280
1584	1,2,4,5,10,11,12,14	3,6,7,8,9,13,15,16	1	P35280
1585	1,2,4,5,10,11,12,15	3,6,7,8,9,13,14,16	0	
1586	1,2,4,5,10,11,12,16	3,6,7,8,9,13,14,15	2	P35280,P70549
1587	1,2,4,5,10,11,13,14	3,6,7,8,9,12,15,16	1	P35280
1588	1,2,4,5,10,11,13,15	3,6,7,8,9,12,14,16	2	O70417,P21674
1589	1,2,4,5,10,11,13,16	3,6,7,8,9,12,14,15	2	O70417,P35280
1590	1,2,4,5,10,11,14,15	3,6,7,8,9,12,13,16	0	
1591	1,2,4,5,10,11,14,16	3,6,7,8,9,12,13,15	3	P35280,P70549,Q4KLZ6

1592	1,2,4,5,10,<br>11,15,16	3,6,7,8,9,1<br>2,13,14	2	O70417,P17988
1593	1,2,4,5,10,<br>12,13,14	3,6,7,8,9,1<br>1,15,16	2	P35280,Q6P6S4
1594	1,2,4,5,10,<br>12,13,15	3,6,7,8,9,1<br>1,14,16	2	P35280,Q812E4
1595	1,2,4,5,10,<br>12,13,16	3,6,7,8,9,1<br>1,14,15	1	P35280
1596	1,2,4,5,10,<br>12,14,15	3,6,7,8,9,1<br>1,13,16	1	P35280
1597	1,2,4,5,10,<br>12,14,16	3,6,7,8,9,1<br>1,13,15	1	P35280
1598	1,2,4,5,10,<br>12,15,16	3,6,7,8,9,1<br>1,13,14	3	P0DMW0;P0DMW1,P10758,P35280
1599	1,2,4,5,10,<br>13,14,15	3,6,7,8,9,1<br>1,12,16	1	P35280
1600	1,2,4,5,10,<br>13,14,16	3,6,7,8,9,1<br>1,12,15	3	P09527,P35280,Q6P6S4
1601	1,2,4,5,10,<br>13,15,16	3,6,7,8,9,1<br>1,12,14	4	O70417,P09527,P11883,P35280
1602	1,2,4,5,10,<br>14,15,16	3,6,7,8,9,1<br>1,12,13	3	P09527,P35280,Q9QYP1
1603	1,2,4,5,11,<br>12,13,14	3,6,7,8,9,1<br>0,15,16	2	P35280,Q5RKI1
1604	1,2,4,5,11,<br>12,13,15	3,6,7,8,9,1<br>0,14,16	1	O70417
1605	1,2,4,5,11,<br>12,13,16	3,6,7,8,9,1<br>0,14,15	3	O70417,P35280,Q5RKI1
1606	1,2,4,5,11,<br>12,14,15	3,6,7,8,9,1<br>0,13,16	0	
1607	1,2,4,5,11,<br>12,14,16	3,6,7,8,9,1<br>0,13,15	3	P35280,Q4KLZ6,Q5RKI1
1608	1,2,4,5,11,<br>12,15,16	3,6,7,8,9,1<br>0,13,14	2	O70417,Q5QE79
1609	1,2,4,5,11,<br>13,14,15	3,6,7,8,9,1<br>0,12,16	0	
1610	1,2,4,5,11,<br>13,14,16	3,6,7,8,9,1<br>0,12,15	4	O54728,P35280,Q4KLZ6,Q5RKI1
1611	1,2,4,5,11,<br>13,15,16	3,6,7,8,9,1<br>0,12,14	1	O70417

1612	1,2,4,5,11,14,15,16	3,6,7,8,9,10,12,13	2	Q4KLZ6,Q6P6Q2
1613	1,2,4,5,12,13,14,15	3,6,7,8,9,10,11,16	2	P35280,Q6P6R2
1614	1,2,4,5,12,13,14,16	3,6,7,8,9,10,11,15	4	P35280,Q5RKI1,Q6P6R2,Q6P6S4
1615	1,2,4,5,12,13,15,16	3,6,7,8,9,10,11,14	3	O70417,P35280,Q6P6R2
1616	1,2,4,5,12,14,15,16	3,6,7,8,9,10,11,13	2	P35280,Q6P6R2
1617	1,2,4,5,13,14,15,16	3,6,7,8,9,10,11,12	2	P35280,Q6P6R2
1618	1,2,4,6,7,8,9,10	3,5,11,12,13,14,15,16	39	D4A5U3,iRT-Kit_WR_fusion,O55004,O70594,P02454,P02631,P05371,P06761,P06911,P15399,P17559,P18418,P19223,P19629,P20646,P20762,P23739,P31044,P31430,P36860,P50280,P55091,P70545,P80299,P97580,P98089,Q30KJ2,Q62635,Q63474,Q63598,Q63618,Q6P6R2,Q78P75,Q8CIZ5,Q9JI85,Q9R0T3,Q9WVH8,Q9Z0V6,Q9Z1F2
1619	1,2,4,6,7,8,9,11	3,5,10,12,13,14,15,16	53	B1H234,D4A5U3,iRT-Kit_WR_fusion,P02631,P02780,P02781,P02782,P04905,P06761,P06911,P07150,P07647,P08723,P09456,P0C0A9,P11598,P12020,P18418,P19223,P19629,P20646,P22273,P22282,P22283,P31430,P36374,P40241,P46462,P50280,P55091,P60905,P80299,P98089,Q09326,Q4G063,Q4G075,Q5GRG2,Q5M8C6,Q62635,Q63493,Q63617,Q6AYR9,Q6P6R2,Q6P6S4,Q6Q7Y5,Q78P75,Q812E4,Q8CIZ5,Q9JHB9,Q9JI85,Q9QW07,Q9R0T3,Q9Z1F2
1620	1,2,4,6,7,8,9,12	3,5,10,11,13,14,15,16	20	D4A5U3,iRT-Kit_WR_fusion,P02631,P06761,P06911,P12020,P18418,P19629,P31430,P50280,P55091,P80299,P98089,Q5GRG2,Q62635,Q78P75,Q8CIZ5,Q9JI85,Q9R0T3,Q9Z1F2
1621	1,2,4,6,7,8,9,13	3,5,10,11,12,14,15,16	25	B1H234,D4A5U3,iRT-Kit_WR_fusion,O55004,P02631,P06761,P06911,P18418,P19223,P19629,P31430,P36860,P50280,P55091,P80299,P98089,Q30KJ2,Q5GRG2,Q62635,Q66H69,Q78P75,Q8CIZ5,Q9JI85,Q9R0T3,Q9Z1F2
1622	1,2,4,6,7,8,9,14	3,5,10,11,12,13,15,16	25	B1H234,D3ZHA0,D4A5U3,P02631,P06761,P06911,P18418,P19223,P19629,P20646,P31430,P50280,P55091,P80299,P97580,P98089,Q30KJ2,Q5GRG2,Q5QE79,Q62635,Q6TMA8,Q78P75,Q8CIZ5,Q9JI85,Q9Z1F2
1623	1,2,4,6,7,8,9,15	3,5,10,11,12,13,14,16	25	D4A5U3,iRT-Kit_WR_fusion,O55004,P02631,P06761,P06911,P15999,P18418,P19223,P19629,P20646,P50280,P55091,P80299,P98089,Q30KJ2,Q5GRG2,Q62635,Q63474,Q78P75,Q8CIZ5,Q8CJ52,Q9JI85,Q9Z0V6,Q9Z1F2
1624	1,2,4,6,7,8,9,16	3,5,10,11,12,13,14,15	33	D3ZUC6,D4A5U3,iRT-Kit_WR_fusion,O55004,P02631,P02780,P02781,P02782,P06761,P06911,P07151,P07647,P15399,P18418,P19223,P19629,P20646,P20761,P22006,P23593,P50280,P55091,P80299,P97580,P98089,Q30KJ2,Q5GRG2,Q62635,Q6TMA8,Q9JHB9,Q9JI85,Q9R0T3,Q9Z0J6

1625	1,2,4,6,7,8,10,11	3,5,9,12,13,14,15,16	57	B1H234,D4A5U3,O55004,P02631,P02780,P02781,P02782,P04905,P05371,P06761,P06911,P07150,P07647,P08723,P09456,P0C0A9,P11598,P12020,P15399,P18418,P19223,P19629,P20762,P22273,P22282,P22283,P31044,P31430,P35952,P36374,P40241,P46462,P50280,P55091,P80299,P97580,P98089,Q10758,Q30KJ2,Q4G063,Q4G075,Q5GRG2,Q5M8C6,Q62635,Q63493,Q63617,Q66H69,Q68G31,Q6B345,Q78P75,Q8CIZ5,Q99041,Q9JHB9,Q9JI85,Q9QZK8,Q9R0T3,Q9Z1F2
1626	1,2,4,6,7,8,10,12	3,5,9,11,13,14,15,16	45	iRT-Kit_WR_fusion,O55004,O88267,P02454,P02631,P05371,P06761,P06911,P12020,P15399,P18418,P19223,P19629,P20762,P30904,P31044,P31430,P36860,P46844,P48508,P50280,P55091,P57113,P80299,P97580,P98089,Q03248,Q30KJ2,Q3ZAV1,Q5GRG2,Q5M7T9,Q5RLM2,Q62635,Q63474,Q63598,Q63618,Q66H69,Q66HG3,Q6Q0N1,Q8CIZ5,Q91ZS3,Q9JI85,Q9R0T3,Q9WTW7,Q9Z1F2
1627	1,2,4,6,7,8,10,13	3,5,9,11,12,14,15,16	50	D4A5U3,O55004,O70257,O70594,P02454,P02631,P05371,P06761,P06911,P07171,P15399,P18418,P19223,P19468,P19629,P25809,P30904,P31044,P31430,P35952,P36860,P46844,P48508,P50280,P51907,P55091,P57113,P80299,P97580,P98089,Q30KJ2,Q3ZAV1,Q5GRG2,Q5M7T9,Q62635,Q63424,Q63474,Q63598,Q63618,Q66H69,Q66HG3,Q68G31,Q6Q0N1,Q6TMA8,Q78P75,Q8CIZ5,Q9JI85,Q9R0T3,Q9WUW9,Q9Z1F2
1628	1,2,4,6,7,8,10,14	3,5,9,11,12,13,15,16	39	D4A5U3,O55004,O70594,P02631,P06761,P06911,P15399,P18418,P19223,P19629,P20762,P31044,P31430,P46844,P48508,P50280,P54921,P55091,P57113,P80299,P97580,P98089,Q30KJ2,Q5GRG2,Q5RLM2,Q62635,Q63474,Q63598,Q63618,Q66H69,Q66HG3,Q68G31,Q6TMA8,Q80WL1,Q8CIZ5,Q923S2,Q9JI85,Q9WUW9,Q9Z1F2
1629	1,2,4,6,7,8,10,15	3,5,9,11,12,13,14,16	68	O08557,O35763,O55004,O70257,O70377,O70594,O88267,O88339;Q4V882,P02631,P05371,P06761,P06911,P15399,P18418,P18757,P19112,P19223,P19468,P19629,P20646,P20762,P23928,P30904,P31044,P31430,P36860,P38918,P46844,P48508,P50280,P51907,P53790,P54921,P55091,P57113,P80299,P97580,P98089,Q03248,Q05175,Q30KJ2,Q3ZAV1,Q5GRG2,Q5I0E9,Q5M7T9,Q62635,Q63270,Q63424,Q63474,Q63598,Q63618,Q64093,Q66H69,Q6MG61,Q6Q0N1,Q6TMA8,Q80W57,Q8CIZ5,Q8K3P7,Q8R431,Q923S2,Q9JI85,Q9JLJ3,Q9R1T5,Q9WTW7,Q9Z0V6,Q9Z0W7,Q9Z1F2
1630	1,2,4,6,7,8,10,16	3,5,9,11,12,13,14,15	84	D4A5U3,O35763,O55004,O70257,O70377,O70594,O88267,P02454,P02631,P02780,P02781,P02782,P05371,P06761,P06911,P07151,P07647,P11598,P15399,P18418,P18427,P18757,P19112,P19223,P19468,P19629,P20761,P20762,P22283,P23928,P28570,P29975,P31044,P38918,P46844,P48508,P50280,P51907,P53790,P55091,P57113,P80299,P97580,P98089,Q03248,Q05175,Q30KJ2,Q3T1J9,Q3ZAV1,Q5GRG2,Q5I0E9,Q5M7T9,Q5U2Q3,Q62635,Q63270,Q63424,Q63598,Q63618,Q64093,Q64602,Q66H69,Q66HG3,Q68FT5,Q6AY33,Q6AY41,Q6MG61,Q6Q0N1,Q6TMA8,Q71MB6,Q80W57,Q8CIZ5,Q8R431,Q923S2,Q99MA2,Q9JHB9,Q9JI85,Q9JJ40,Q9JLJ3,Q9QYU4,Q9R0T3,Q9R1T5,Q9WTW7,Q9WUW9,Q9Z0W7
1631	1,2,4,6,7,8,11,12	3,5,9,10,13,14,15,16	52	B1H234,iRT-Kit_WR_fusion,O35547,P02631,P02780,P02781,P02782,P04905,P06761,P06911,P07150,P07647,P08723,P09456,P0C0A9,P11598,P11762,P12020,P18418,P19629,P22273,P22282,P22283,P31044,P31430,P36374,P40241,P46462,P50280,P55091,P60905,P80299,P98089,Q00715,Q09326,Q4G063,Q4G075,Q5GRG2,Q5M8C6,Q62635,Q63493,Q63617,Q66H69,Q6AYR9,Q78P75,Q8CIZ5,Q9JHB9,Q9JI85,Q9QW07,Q9QZK8,Q9R0T3,Q9Z1F2

1632	1,2,4,6,7,8,11,13	3,5,9,10,12,14,15,16	62	B1H234,D4A5U3,iRT-Kit_WR_fusion,O35547,O55004,P02631,P02780,P02781,P02782,P04905,P05371,P06761,P06911,P07150,P07647,P08723,P09456,P0C0A9,P11598,P12020,P15399,P18418,P19223,P19629,P22273,P22282,P22283,P24368,P30120,P31044,P31430,P36374,P40241,P46462,P50280,P55091,P60905,P80299,P98089,Q09326,Q10758,Q30KJ2,Q4G063,Q4G075,Q5GRG2,Q5M8C6,Q62635,Q62902,Q63493,Q63617,Q66H69,Q68G31,Q6AYR9,Q6TMA8,Q78P75,Q8CIZ5,Q99041,Q9JHB9,Q9JI85,Q9QZK8,Q9R0T3,Q9Z1F2
1633	1,2,4,6,7,8,11,14	3,5,9,10,12,13,15,16	54	B1H234,D4A5U3,P02780,P02781,P02782,P04905,P06761,P06911,P07150,P07647,P08723,P09456,P0C0A9,P11598,P12020,P15399,P18418,P19223,P19629,P22273,P22282,P22283,P31044,P31430,P36374,P40241,P46462,P50280,P55091,P80299,P97580,P98089,Q09326,Q30KJ2,Q4G063,Q4G075,Q5GRG2,Q5M8C6,Q62635,Q63493,Q63617,Q66H69,Q6AYR9,Q6TMA8,Q78P75,Q812E4,Q8CIZ5,Q99041,Q9JHB9,Q9JI85,Q9QW07,Q9QZK8,Q9R0T3,Q9Z1F2
1634	1,2,4,6,7,8,11,15	3,5,9,10,12,13,14,16	57	B1H234,iRT-Kit_WR_fusion,O55004,P02780,P02781,P02782,P06761,P06911,P07150,P07647,P08723,P09456,P0C0A9,P11598,P12020,P15399,P18418,P19629,P22273,P22282,P22283,P31044,P31430,P36374,P40241,P46462,P50280,P55091,P80299,P98089,Q09326,Q10758,Q30KJ2,Q4FZU2,Q4G063,Q4G075,Q5GRG2,Q5M8C6,Q62635,Q63493,Q63617,Q66H69,Q6AYR9,Q6IFU8,Q6IMF3,Q6P6Q2,Q6P6S4,Q78P75,Q8CIZ5,Q8CJ52,Q99041,Q9JHB9,Q9JI85,Q9QW07,Q9R0T3,Q9Z0V6,Q9Z1F2
1635	1,2,4,6,7,8,11,16	3,5,9,10,12,13,14,15	70	B1H234,D4A5U3,iRT-Kit_WR_fusion,O35547,O55004,O88917,P02631,P02761,P02780,P02781,P02782,P04905,P05369,P05371,P06761,P06911,P07150,P07151,P07647,P08010,P08723,P09456,P0C0A9,P11598,P12020,P15399,P18418,P19223,P19629,P22006,P22273,P22282,P22283,P24368,P30120,P31044,P31430,P36374,P40241,P46462,P50280,P55091,P60905,P80299,P97523,P97580,P98089,Q00715,Q10758,Q30KJ2,Q4G063,Q4G075,Q5GRG2,Q5M8C6,Q62635,Q62902,Q63493,Q63617,Q66H69,Q6AYR9,Q6TMA8,Q78P75,Q812E4,Q99041,Q9JHB9,Q9JI85,Q9QW07,Q9QZK8,Q9R0T3,Q9Z0J6
1636	1,2,4,6,7,8,12,13	3,5,9,10,11,14,15,16	30	D4A5U3,iRT-Kit_WR_fusion,O55004,P02631,P02781,P06761,P06911,P12020,P18418,P19223,P19629,P22282,P31044,P31430,P36860,P50280,P55091,P80299,P98089,Q30KJ2,Q5GRG2,Q62635,Q66H69,Q6TMA8,Q78P75,Q8CIZ5,Q9JI85,Q9R0T3,Q9Z1F2,Q9Z2L0
1637	1,2,4,6,7,8,12,14	3,5,9,10,11,13,15,16	26	D4A5U3,iRT-Kit_WR_fusion,P02631,P06761,P06911,P11762,P12020,P18418,P19223,P19629,P31044,P31430,P50280,P55091,P80299,P97580,P98089,Q30KJ2,Q5GRG2,Q5RLM2,Q62635,Q66H69,Q6TMA8,Q8CIZ5,Q9JI85,Q9Z1F2
1638	1,2,4,6,7,8,12,15	3,5,9,10,11,13,14,16	26	iRT-Kit_WR_fusion,O55004,P02631,P06761,P06911,P11762,P12020,P18418,P19629,P19814,P31044,P31430,P50280,P55091,P80299,P98089,Q30KJ2,Q5GRG2,Q62635,Q63598,Q66H69,Q6TMA8,Q8CIZ5,Q9JI85,Q9Z0V6,Q9Z1F2

1639	1,2,4,6,7,8,12,16	3,5,9,10,11,13,14,15	37	D4A5U3,iRT-Kit_WR_fusion,O55004,P02631,P02780,P02781,P02782,P06761,P06911,P07151,P07647,P11598,P12020,P15399,P18418,P19223,P19629,P22282,P22283,P31430,P46844,P50280,P55091,P80299,P97580,P98089,Q30KJ2,Q5GRG2,Q5M8C6,Q62635,Q63598,Q66H69,Q6TMA8,Q8CIZ5,Q9JHB9,Q9JI85,Q9R0T3
1640	1,2,4,6,7,8,13,14	3,5,9,10,11,12,15,16	33	B0BNN3,B1H234,D3ZHA0,D4A5U3,O55004,P02631,P06761,P06911,P12020,P18418,P19223,P19629,P25809,P31044,P31430,P50280,P55091,P80299,P97580,P98089,Q30KJ2,Q4G075,Q5GRG2,Q62635,Q66H69,Q68G31,Q6TMA8,Q78P75,Q8CIZ5,Q9JI85,Q9WUW8,Q9Z1F2,Q9Z2L0
1641	1,2,4,6,7,8,13,15	3,5,9,10,11,12,14,16	31	B1H234,D4A5U3,O55004,P02631,P04916,P06761,P06911,P15399,P18418,P19223,P19629,P31044,P36860,P50280,P55091,P80299,P97580,P98089,Q30KJ2,Q5GRG2,Q62635,Q63598,Q63618,Q66H69,Q68G31,Q6Q0N1,Q6TMA8,Q78P75,Q8CIZ5,Q9JI85,Q9Z1F2
1642	1,2,4,6,7,8,13,16	3,5,9,10,11,12,14,15	48	B0BNN3,B1H234,D4A5U3,O35956,O55004,O70257,P02631,P02761,P02780,P02781,P02782,P05371,P06761,P06911,P07151,P07647,P11598,P15399,P18418,P19223,P19468,P19629,P20761,P22282,P22283,P30120,P46844,P50280,P55091,P80299,P97580,P98089,Q30KJ2,Q3ZAV1,Q5GRG2,Q5M7T9,Q5M8C6,Q62635,Q63598,Q63618,Q66H69,Q6Q0N1,Q6TMA8,Q8CIZ5,Q9JHB9,Q9JI85,Q9R0T3,Q9Z0J6
1643	1,2,4,6,7,8,14,15	3,5,9,10,11,12,13,16	24	O55004,P06761,P06911,P15399,P15999,P18418,P19223,P19629,P20762,P31044,P50280,P54921,P55091,P80299,P97580,P98089,Q30KJ2,Q5GRG2,Q62635,Q63474,Q6TMA8,Q8CIZ5,Q9JI85,Q9Z1F2
1644	1,2,4,6,7,8,14,16	3,5,9,10,11,12,13,15	37	B1H234,D3ZHA0,D4A5U3,O55004,P02631,P02761,P02780,P02781,P02782,P06761,P06911,P07151,P07647,P11598,P12020,P15399,P18418,P19223,P19629,P20761,P20762,P46844,P50280,P55091,P57113,P80299,P97580,P98089,Q30KJ2,Q4G075,Q5GRG2,Q62635,Q6TMA8,Q8CIZ5,Q9JHB9,Q9JI85,Q9WUW8
1645	1,2,4,6,7,8,15,16	3,5,9,10,11,12,13,14	51	B2RYW9,iRT-Kit_WR_fusion,O55004,O70594,P02631,P02761,P02780,P02781,P02782,P06761,P06911,P07151,P07647,P15399,P15999,P17988,P18418,P19112,P19223,P19468,P19629,P20646,P20761,P20762,P46844,P48508,P50280,P53790,P55091,P57113,P80299,P97580,P98089,Q03248,Q30KJ2,Q3ZAV1,Q5GRG2,Q5M7T9,Q5U2Q3,Q62635,Q63270,Q63598,Q6Q0N1,Q6TMA8,Q8CJ52,Q9JHB9,Q9JI85,Q9R1T5,Q9WTW7,Q9Z0V6,Q9Z0W7
1646	1,2,4,6,7,9,10,11	3,5,8,12,13,14,15,16	6	B1H234,P11598,P55091,P80299,Q63617,Q9R168
1647	1,2,4,6,7,9,10,12	3,5,8,11,13,14,15,16	5	P36860,P55091,P80299,Q80WL1,Q9Z1F2
1648	1,2,4,6,7,9,10,13	3,5,8,11,12,14,15,16	6	P36860,P55091,P80299,Q9R168,Q9WVH8,Q9Z1F2
1649	1,2,4,6,7,9,10,14	3,5,8,11,12,13,15,16	6	P00762,P80299,Q80WL1,Q9R168,Q9WVH8,Q9Z1F2
1650	1,2,4,6,7,9,10,15	3,5,8,11,12,13,14,16	7	P00762,P36860,P80299,Q63474,Q811M5,Q9WVH8,Q9Z1F2
1651	1,2,4,6,7,9,10,16	3,5,8,11,12,13,14,15	6	D3ZUC6,P07647,P22006,P80299,Q9R168,Q9WVH8

1652	1,2,4,6,7,9,11,12	3,5,8,10,13,14,15,16	7	B1H234,O35077,P11598,P22283,P80299,Q5GRG2,Q63617
1653	1,2,4,6,7,9,11,13	3,5,8,10,12,14,15,16	10	B1H234,P11598,P22283,P55091,P80299,Q5GRG2,Q63617,Q9EQS0,Q9JI85,Q9R168
1654	1,2,4,6,7,9,11,14	3,5,8,10,12,13,15,16	7	B1H234,P0DMW0;P0DMW1,P11598,P80299,Q5GRG2,Q63617,Q9R168
1655	1,2,4,6,7,9,11,15	3,5,8,10,12,13,14,16	5	B1H234,P11598,P80299,Q63617,Q8CJ52
1656	1,2,4,6,7,9,11,16	3,5,8,10,12,13,14,15	21	B1H234,D3ZUC6,P02780,P02781,P02782,P07647,P08723,P09456,P0C0A9,P11598,P22006,P22283,P80299,P81556,Q5GRG2,Q63617,Q812E4,Q9EQS0,Q9JHB9,Q9R168,Q9Z0J6
1657	1,2,4,6,7,9,12,13	3,5,8,10,11,14,15,16	1	P80299
1658	1,2,4,6,7,9,12,14	3,5,8,10,11,13,15,16	2	P80299,Q80WL1
1659	1,2,4,6,7,9,12,15	3,5,8,10,11,13,14,16	1	P80299
1660	1,2,4,6,7,9,12,16	3,5,8,10,11,13,14,15	4	D3ZUC6,P07647,P22006,P80299
1661	1,2,4,6,7,9,13,14	3,5,8,10,11,12,15,16	4	B1H234,P01835,P80299,Q9R168
1662	1,2,4,6,7,9,13,15	3,5,8,10,11,12,14,16	4	B1H234,P01835,P36860,P80299
1663	1,2,4,6,7,9,13,16	3,5,8,10,11,12,14,15	8	B1H234,D3ZUC6,P07647,P22006,P80299,Q9EQS0,Q9R168,Q9Z0J6
1664	1,2,4,6,7,9,14,15	3,5,8,10,11,12,13,16	1	P80299
1665	1,2,4,6,7,9,14,16	3,5,8,10,11,12,13,15	6	B1H234,P07647,P22006,P80299,Q80WL1,Q9R168
1666	1,2,4,6,7,9,15,16	3,5,8,10,11,12,13,14	4	D3ZUC6,P22006,P80299,Q9R168
1667	1,2,4,6,7,10,11,12	3,5,8,9,13,14,15,16	6	P11598,P22283,P31430,P55091,P80299,Q5GRG2
1668	1,2,4,6,7,10,11,13	3,5,8,9,12,14,15,16	10	B1H234,P11598,P22283,P36860,P55091,P80299,Q63617,Q68G31,Q9JI85,Q9Z1F2
1669	1,2,4,6,7,10,11,14	3,5,8,9,12,13,15,16	7	B1H234,P11598,P55091,P80299,Q5GRG2,Q80WL1,Q9Z1F2
1670	1,2,4,6,7,10,11,15	3,5,8,9,12,13,14,16	8	B1H234,P08721,P11598,P17988,P19629,P80299,Q8CJ52,Q9Z1F2
1671	1,2,4,6,7,10,11,16	3,5,8,9,12,13,14,15	15	B1H234,P02780,P02781,P02782,P07647,P08723,P0C0A9,P11598,P17988,P22283,P80299,Q5GRG2,Q5M872,Q63617,Q9JHB9

1672	1,2,4,6,7,10,12,13	3,5,8,9,11,14,15,16	7	O88797,P01835,P02631,P36860,P80299,Q66HG3,Q9Z1F2
1673	1,2,4,6,7,10,12,14	3,5,8,9,11,13,15,16	4	P80299,Q5RLM2,Q80WL1,Q9Z1F2
1674	1,2,4,6,7,10,12,15	3,5,8,9,11,13,14,16	6	P10758,P19629,P36860,P80299,Q63598,Q9Z1F2
1675	1,2,4,6,7,10,12,16	3,5,8,9,11,13,14,15	7	P07647,P10758,P18297,P57113,P80299,Q03248,Q63598
1676	1,2,4,6,7,10,13,14	3,5,8,9,11,12,15,16	6	B1H234,O88797,P01835,P80299,Q80WL1,Q9Z1F2
1677	1,2,4,6,7,10,13,15	3,5,8,9,11,12,14,16	11	O88797,P01835,P19468,P36860,P51907,P80299,Q63474,Q63598,Q63618,Q923M1,Q9Z1F2
1678	1,2,4,6,7,10,13,16	3,5,8,9,11,12,14,15	17	B1H234,O55004,P01835,P02761,P07647,P09527,P18297,P19468,P28570,P36376,P46844,P51907,P57113,P80299,Q63424,Q63598,Q63618
1679	1,2,4,6,7,10,14,15	3,5,8,9,11,12,13,16	8	O88797,P09527,P54921,P80299,Q63474,Q80WL1,Q9QYP1,Q9Z1F2
1680	1,2,4,6,7,10,14,16	3,5,8,9,11,12,13,15	9	P02761,P07647,P09527,P18297,P57113,P80299,Q71MB6,Q80WL1,Q9QYP1
1681	1,2,4,6,7,10,15,16	3,5,8,9,11,12,13,14	25	P09527,P10758,P17988,P18297,P19468,P19629,P46844,P48508,P51907,P53790,P57113,P80299,Q03248,Q5I0E9,Q5M7T9,Q63270,Q63424,Q63598,Q63618,Q6Q0N1,Q71MB6,Q923M1,Q9QYP1,Q9R1T5,Q9WTW7
1682	1,2,4,6,7,11,12,13	3,5,8,9,10,14,15,16	10	B1H234,P05369,P07647,P11598,P22283,P36374,P80299,Q5GRG2,Q63617,Q9JI85
1683	1,2,4,6,7,11,12,14	3,5,8,9,10,13,15,16	6	B1H234,P11598,P22283,P80299,Q5GRG2,Q80WL1
1684	1,2,4,6,7,11,12,15	3,5,8,9,10,13,14,16	7	B1H234,P11598,P22283,P36374,P80299,Q5GRG2,Q8CJ52
1685	1,2,4,6,7,11,12,16	3,5,8,9,10,13,14,15	19	B1H234,O35547,P02780,P02781,P02782,P05369,P07647,P08723,P0C0A9,P11598,P22283,P36374,P80299,Q00715,Q5GRG2,Q5M872,Q63617,Q9JHB9,Q9JI85
1686	1,2,4,6,7,11,13,14	3,5,8,9,10,12,15,16	10	B1H234,P01835,P07647,P11598,P22283,P80299,Q5GRG2,Q63617,Q99041,Q9JI85
1687	1,2,4,6,7,11,13,15	3,5,8,9,10,12,14,16	9	B1H234,P01835,P11598,P22283,P80299,Q5GRG2,Q63617,Q8CJ52,Q9JI85
1688	1,2,4,6,7,11,13,16	3,5,8,9,10,12,14,15	26	B1H234,O35547,P00714,P02761,P02780,P02781,P02782,P05369,P07647,P08723,P09456,P0C0A9,P11598,P22283,P24368,P36374,P80299,Q5GRG2,Q5M872,Q63617,Q99041,Q9EQS0,Q9JHB9,Q9JI85,Q9R168,Q9Z0J6
1689	1,2,4,6,7,11,14,15	3,5,8,9,10,12,13,16	5	B1H234,P11598,P80299,Q5GRG2,Q8CJ52
1690	1,2,4,6,7,11,14,16	3,5,8,9,10,12,13,15	21	B1H234,O35547,P02761,P02780,P02781,P02782,P07647,P08723,P09456,P0C0A9,P11598,P22006,P22283,P80299,Q4KLZ6,Q5GRG2,Q5M872,Q63617,Q99041,Q9JHB9,Q9R168

1691	1,2,4,6,7,11,15,16	3,5,8,9,10,12,13,14	16	B1H234,P02780,P02781,P02782,P07647,P08723,P0C0A9,P11598,P17988,P22283,P36374,P80299,Q5GRG2,Q63617,Q8CJ52,Q9JHB9
1692	1,2,4,6,7,12,13,14	3,5,8,9,10,11,15,16	3	O88797,P01835,P80299
1693	1,2,4,6,7,12,13,15	3,5,8,9,10,11,14,16	4	O88797,P01835,P36860,P80299
1694	1,2,4,6,7,12,13,16	3,5,8,9,10,11,14,15	3	P01835,P07647,P80299
1695	1,2,4,6,7,12,14,15	3,5,8,9,10,11,13,16	2	P80299,Q80WL1
1696	1,2,4,6,7,12,14,16	3,5,8,9,10,11,13,15	3	P07647,P80299,Q80WL1
1697	1,2,4,6,7,12,15,16	3,5,8,9,10,11,13,14	5	P07647,P10758,P17988,P19629,P80299
1698	1,2,4,6,7,13,14,15	3,5,8,9,10,11,12,16	4	B1H234,O88797,P01835,P80299
1699	1,2,4,6,7,13,14,16	3,5,8,9,10,11,12,15	8	B0BNN3,B1H234,P01835,P02761,P07647,P36376,P80299,Q9EQS0
1700	1,2,4,6,7,13,15,16	3,5,8,9,10,11,12,14	7	B1H234,P01835,P02761,P07647,P10719,P80299,Q9EQS0
1701	1,2,4,6,7,14,15,16	3,5,8,9,10,11,12,13	7	B1H234,P01835,P02761,P07647,P17988,P80299,Q9QYP1
1702	1,2,4,6,8,9,10,11	3,5,7,12,13,14,15,16	13	P02454,P15399,P18418,P31430,P50280,P55091,P97580,Q30KJ2,Q5GRG2,Q68G31,Q6AY61,Q6P6S4,Q9JI85
1703	1,2,4,6,8,9,10,12	3,5,7,11,13,14,15,16	7	P02454,P02631,P15399,P17559,P18418,P55091,Q30KJ2
1704	1,2,4,6,8,9,10,13	3,5,7,11,12,14,15,16	11	O55004,P02454,P02631,P15399,P17559,P18418,P55091,P97580,Q30KJ2,Q68G31,Q9Z1F2
1705	1,2,4,6,8,9,10,14	3,5,7,11,12,13,15,16	9	P15399,P17559,P18418,P47967,P97580,P97840,Q30KJ2,Q68G31,Q9Z1F2
1706	1,2,4,6,8,9,10,15	3,5,7,11,12,13,14,16	10	P02454,P15399,P18418,P19629,P97580,Q30KJ2,Q63474,Q68G31,Q811M5,Q9Z1F2
1707	1,2,4,6,8,9,10,16	3,5,7,11,12,13,14,15	12	D3ZUC6,O55004,P02454,P02631,P07151,P15399,P18418,P19629,P22006,P70545,P97580,Q30KJ2
1708	1,2,4,6,8,9,11,12	3,5,7,10,13,14,15,16	12	O35077,P06911,P12020,P31430,P50280,P60905,P61206;P84079,Q5GRG2,Q5RKI1,Q6P6S4,Q6Q7Y5,Q9JI85
1709	1,2,4,6,8,9,11,13	3,5,7,10,12,14,15,16	13	P06911,P31430,P50280,P55091,P60905,Q09326,Q5GRG2,Q5RKI1,Q68G31,Q6AY61,Q6AYR9,Q6P6S4,Q9JI85
1710	1,2,4,6,8,9,11,14	3,5,7,10,12,13,15,16	12	P06911,P12020,P31430,P50280,P97580,Q5GRG2,Q5RKI1,Q62635,Q6AY61,Q6P6S4,Q812E4,Q9JI85

1711	1,2,4,6,8,9,11,15	3,5,7,10,12,13,14,16	8	P15399,P50280,Q10758,Q5GRG2,Q6IFU8,Q6P6S4,Q8CJ52,Q9JI85
1712	1,2,4,6,8,9,11,16	3,5,7,10,12,13,14,15	29	D3ZUC6,O35547,P02780,P02781,P02782,P04905,P06911,P07647,P08723,P09456,P0C0A9,P15399,P18418,P22006,P30120,P36374,P46462,P50280,P60905,Q10758,Q30KJ2,Q5GRG2,Q5RKI1,Q6AY61,Q6P6S4,Q812E4,Q9JHB9,Q9JI85,Q9QW07
1713	1,2,4,6,8,9,12,13	3,5,7,10,11,14,15,16	2	Q5RKI1,Q9JI85
1714	1,2,4,6,8,9,12,14	3,5,7,10,11,13,15,16	3	Q5GRG2,Q5RKI1,Q62635
1715	1,2,4,6,8,9,12,15	3,5,7,10,11,13,14,16	0	
1716	1,2,4,6,8,9,12,16	3,5,7,10,11,13,14,15	4	D3ZUC6,P22006,Q30KJ2,Q5RKI1
1717	1,2,4,6,8,9,13,14	3,5,7,10,11,12,15,16	5	P97580,Q30KJ2,Q5RKI1,Q62635,Q68G31
1718	1,2,4,6,8,9,13,15	3,5,7,10,11,12,14,16	2	Q30KJ2,Q68G31
1719	1,2,4,6,8,9,13,16	3,5,7,10,11,12,14,15	9	D3ZUC6,P15399,P18418,P22006,P23593,P97580,Q30KJ2,Q5RKI1,Q9JI85
1720	1,2,4,6,8,9,14,15	3,5,7,10,11,12,13,16	5	P23593,P47967,P97580,P97840,Q30KJ2
1721	1,2,4,6,8,9,14,16	3,5,7,10,11,12,13,15	8	P15399,P22006,P23593,P47967,P97580,P97840,Q30KJ2,Q5RKI1
1722	1,2,4,6,8,9,15,16	3,5,7,10,11,12,13,14	9	D3ZUC6,P15399,P19629,P22006,P23593,P47967,P50280,P97580,Q30KJ2
1723	1,2,4,6,8,10,11,12	3,5,7,9,13,14,15,16	13	P02631,P06911,P12020,P15399,P18418,P31430,P50280,P55091,P97580,Q30KJ2,Q5GRG2,Q68G31,Q9JI85
1724	1,2,4,6,8,10,11,13	3,5,7,9,12,14,15,16	13	O55004,P02631,P15399,P18418,P31430,P50280,P55091,P97580,Q10758,Q30KJ2,Q5GRG2,Q68G31,Q9JI85
1725	1,2,4,6,8,10,11,14	3,5,7,9,12,13,15,16	14	P06911,P12020,P15399,P18418,P31430,P50280,P55091,P97580,Q10758,Q30KJ2,Q5GRG2,Q62635,Q68G31,Q9JI85
1726	1,2,4,6,8,10,11,15	3,5,7,9,12,13,14,16	13	P15399,P18418,P19629,P31430,P50280,P97580,Q10758,Q30KJ2,Q5GRG2,Q63474,Q68G31,Q6IFU8,Q9JI85
1727	1,2,4,6,8,10,11,16	3,5,7,9,12,13,14,15	26	O55004,P02780,P02781,P02782,P04905,P07151,P07647,P08723,P09456,P0C0A9,P12020,P15399,P18418,P19629,P30120,P36374,P46462,P50280,P97580,Q10758,Q30KJ2,Q5GRG2,Q68G31,Q6AY61,Q9JHB9,Q9JI85
1728	1,2,4,6,8,10,12,13	3,5,7,9,11,14,15,16	8	O55004,P02631,P15399,P97580,Q30KJ2,Q66H69,Q68G31,Q9JI85
1729	1,2,4,6,8,10,12,14	3,5,7,9,11,13,15,16	7	P02631,P15399,P54921,P97580,Q30KJ2,Q68G31,Q9Z1F2

1730	1,2,4,6,8,10,12,15	3,5,7,9,11,13,14,16	11	O55004,P02631,P15399,P19629,P54921,P97580,Q30KJ2,Q498D9,Q63474,Q63598,Q9Z1F2
1731	1,2,4,6,8,10,12,16	3,5,7,9,11,13,14,15	12	O55004,O88267,P02631,P07151,P0DMW0;P0DMW1,P15399,P18418,P19629,P97580,Q30KJ2,Q63598,Q9JI85
1732	1,2,4,6,8,10,13,14	3,5,7,9,11,12,15,16	8	O55004,P02631,P15399,P97580,Q30KJ2,Q68G31,Q9JI85,Q9Z1F2
1733	1,2,4,6,8,10,13,15	3,5,7,9,11,12,14,16	14	O55004,P02631,P15399,P19629,P36860,P51907,P97580,Q30KJ2,Q498D9,Q63474,Q63598,Q63618,Q68G31,Q9Z1F2
1734	1,2,4,6,8,10,13,16	3,5,7,9,11,12,14,15	12	O55004,O70257,P02631,P07151,P15399,P18418,P46844,P97580,Q30KJ2,Q63618,Q68G31,Q9JI85
1735	1,2,4,6,8,10,14,15	3,5,7,9,11,12,13,16	10	O55004,P15399,P19629,P20762,P54921,P97580,Q30KJ2,Q63474,Q68G31,Q9Z1F2
1736	1,2,4,6,8,10,14,16	3,5,7,9,11,12,13,15	9	O55004,P07151,P15399,P18418,P20762,P54921,P97580,Q30KJ2,Q68G31
1737	1,2,4,6,8,10,15,16	3,5,7,9,11,12,13,14	20	O55004,P07151,P15399,P18418,P19468,P19629,P20762,P50280,P51907,P53790,P54921,P97580,Q03248,Q30KJ2,Q63598,Q63618,Q68G31,Q6Q0N1,Q9R1T5,Q9WTW7
1738	1,2,4,6,8,11,12,13	3,5,7,9,10,14,15,16	16	O35547,O54861,P06911,P12020,P31430,P36374,P40241,P46462,P50280,P60905,Q09326,Q5GRG2,Q5RKI1,Q66H69,Q68G31,Q9JI85
1739	1,2,4,6,8,11,12,14	3,5,7,9,10,13,15,16	10	P06911,P12020,P31430,P36374,P50280,P97580,Q5GRG2,Q5RKI1,Q62635,Q9JI85
1740	1,2,4,6,8,11,12,15	3,5,7,9,10,13,14,16	12	P06911,P12020,P15399,P19629,P31430,P36374,P50280,Q10758,Q5GRG2,Q6IFU8,Q9JI85,Q9QW07
1741	1,2,4,6,8,11,12,16	3,5,7,9,10,13,14,15	30	O35547,P02780,P02781,P02782,P04905,P06911,P07647,P08723,P09456,P0C0A9,P12020,P15399,P22282,P22283,P30120,P31430,P36374,P40241,P46462,P50280,P60905,Q00715,Q10758,Q30KJ2,Q5GRG2,Q5M8C6,Q5RKI1,Q9JHB9,Q9JI85,Q9QW07
1742	1,2,4,6,8,11,13,14	3,5,7,9,10,12,15,16	18	O54728,O54861,P06911,P12020,P15399,P31430,P46462,P50280,P97580,Q09326,Q10758,Q30KJ2,Q5GRG2,Q5RKI1,Q62635,Q68G31,Q9JI85,Q9QWN8
1743	1,2,4,6,8,11,13,15	3,5,7,9,10,12,14,16	10	O54861,P15399,P50280,Q09326,Q10758,Q30KJ2,Q5GRG2,Q68G31,Q6IFU8,Q9JI85
1744	1,2,4,6,8,11,13,16	3,5,7,9,10,12,14,15	33	O35547,O54861,P02780,P02781,P02782,P04905,P05369,P06911,P07647,P08723,P09456,P0C0A9,P12020,P15399,P18418,P22282,P22283,P24368,P30120,P36374,P40241,P46462,P50280,P60905,P97580,Q10758,Q30KJ2,Q5GRG2,Q5RKI1,Q68G31,Q6AY61,Q9JHB9,Q9JI85
1745	1,2,4,6,8,11,14,15	3,5,7,9,10,12,13,16	14	O54861,P06911,P12020,P15399,P50280,P97580,Q10758,Q30KJ2,Q5GRG2,Q62635,Q68G31,Q6IFU8,Q8CJ52,Q9JI85
1746	1,2,4,6,8,11,14,16	3,5,7,9,10,12,13,15	30	O35547,O54728,O54861,P02780,P02781,P02782,P04905,P06911,P07647,P08723,P09456,P0C0A9,P12020,P15399,P30120,P36374,P46462,P50280,P60905,P97580,Q10758,Q30KJ2,Q4KLZ6,Q5GRG2,Q5RKI1,Q62635,Q6AY61,Q812E4,Q9JHB9,Q9JI85
1747	1,2,4,6,8,11,15,16	3,5,7,9,10,12,13,14	26	O35547,O54861,P02780,P02781,P02782,P07647,P08723,P09456,P0C0A9,P12020,P15399,P19629,P30120,P36374,P46462,P50280,P60905,P97580,Q10758,Q30KJ2,Q5GRG2,Q6IFU8,Q8CJ52,Q9JHB9,Q9JI85,Q9QW07

1748	1,2,4,6,8,12,13,14	3,5,7,9,10,11,15,16	8	P06911,P97580,Q30KJ2,Q5GRG2,Q5RKI1,Q62635,Q68G31,Q9JI85
1749	1,2,4,6,8,12,13,15	3,5,7,9,10,11,14,16	3	Q30KJ2,Q68G31,Q9JI85
1750	1,2,4,6,8,12,13,16	3,5,7,9,10,11,14,15	7	P02631,P07647,P15399,P97580,Q30KJ2,Q5RKI1,Q9JI85
1751	1,2,4,6,8,12,14,15	3,5,7,9,10,11,13,16	5	P54921,P97580,Q30KJ2,Q5GRG2,Q62635
1752	1,2,4,6,8,12,14,16	3,5,7,9,10,11,13,15	8	P06911,P12020,P15399,P97580,Q30KJ2,Q5GRG2,Q5RKI1,Q62635
1753	1,2,4,6,8,12,15,16	3,5,7,9,10,11,13,14	5	P15399,P19629,P50280,P97580,Q30KJ2
1754	1,2,4,6,8,13,14,15	3,5,7,9,10,11,12,16	4	P15399,P97580,Q30KJ2,Q68G31
1755	1,2,4,6,8,13,14,16	3,5,7,9,10,11,12,15	10	B0BNN3,O54728,P07647,P15399,P97580,Q30KJ2,Q5RKI1,Q62635,Q68G31,Q9JI85
1756	1,2,4,6,8,13,15,16	3,5,7,9,10,11,12,14	10	O55004,P01015,P07151,P15399,P19629,P50280,P97580,Q30KJ2,Q68G31,Q9JI85
1757	1,2,4,6,8,14,15,16	3,5,7,9,10,11,12,13	7	P15399,P20762,P47967,P50280,P54921,P97580,Q30KJ2
1758	1,2,4,6,9,10,11,12	3,5,7,8,13,14,15,16	2	O35077,Q62714
1759	1,2,4,6,9,10,11,13	3,5,7,8,12,14,15,16	2	D3ZUC6,Q68G31
1760	1,2,4,6,9,10,11,14	3,5,7,8,12,13,15,16	0	
1761	1,2,4,6,9,10,11,15	3,5,7,8,12,13,14,16	2	Q811M5,Q99MH3
1762	1,2,4,6,9,10,11,16	3,5,7,8,12,13,14,15	2	D3ZUC6,P22006
1763	1,2,4,6,9,10,12,13	3,5,7,8,11,14,15,16	0	
1764	1,2,4,6,9,10,12,14	3,5,7,8,11,13,15,16	1	P01041
1765	1,2,4,6,9,10,12,15	3,5,7,8,11,13,14,16	2	Q811M5,Q99MH3
1766	1,2,4,6,9,10,12,16	3,5,7,8,11,13,14,15	1	D3ZUC6
1767	1,2,4,6,9,10,13,14	3,5,7,8,11,12,15,16	0	

1768	1,2,4,6,9,10,13,15	3,5,7,8,11,12,14,16	2 P11883,Q811M5
1769	1,2,4,6,9,10,13,16	3,5,7,8,11,12,14,15	3 D3ZUC6,P11883,Q9EQS0
1770	1,2,4,6,9,10,14,15	3,5,7,8,11,12,13,16	3 P47967,P97840,Q811M5
1771	1,2,4,6,9,10,14,16	3,5,7,8,11,12,13,15	2 D3ZUC6,P47967
1772	1,2,4,6,9,10,15,16	3,5,7,8,11,12,13,14	4 D3ZUC6,P11883,P47967,Q811M5
1773	1,2,4,6,9,11,12,13	3,5,7,8,10,14,15,16	3 O35077,Q5RKI1,Q62761;Q62762;Q62763
1774	1,2,4,6,9,11,12,14	3,5,7,8,10,13,15,16	2 O35077,Q5RKI1
1775	1,2,4,6,9,11,12,15	3,5,7,8,10,13,14,16	2 O35077,Q99MH3
1776	1,2,4,6,9,11,12,16	3,5,7,8,10,13,14,15	4 D3ZUC6,P07647,P22006,Q5RKI1
1777	1,2,4,6,9,11,13,14	3,5,7,8,10,12,15,16	3 P0DMW0;P0DMW1,P12368,Q5RKI1
1778	1,2,4,6,9,11,13,15	3,5,7,8,10,12,14,16	0
1779	1,2,4,6,9,11,13,16	3,5,7,8,10,12,14,15	6 D3ZUC6,P07647,P22006,Q5RKI1,Q9EQS0,Q9R168
1780	1,2,4,6,9,11,14,15	3,5,7,8,10,12,13,16	2 P0DMW0;P0DMW1,P47967
1781	1,2,4,6,9,11,14,16	3,5,7,8,10,12,13,15	6 D3ZUC6,P07647,P22006,Q4KLZ6,Q5RKI1,Q9R168
1782	1,2,4,6,9,11,15,16	3,5,7,8,10,12,13,14	3 D3ZUC6,P22006,Q8CJ52
1783	1,2,4,6,9,12,13,14	3,5,7,8,10,11,15,16	2 O35077,Q5RKI1
1784	1,2,4,6,9,12,13,15	3,5,7,8,10,11,14,16	0
1785	1,2,4,6,9,12,13,16	3,5,7,8,10,11,14,15	3 D3ZUC6,Q5RKI1,Q9EQS0
1786	1,2,4,6,9,12,14,15	3,5,7,8,10,11,13,16	1 P47967
1787	1,2,4,6,9,12,14,16	3,5,7,8,10,11,13,15	2 D3ZUC6,Q5RKI1

1788	1,2,4,6,9,12,15,16	3,5,7,8,10,11,13,14	1 D3ZUC6
1789	1,2,4,6,9,13,14,15	3,5,7,8,10,11,12,16	2 P0DMW0;P0DMW1,P47967
1790	1,2,4,6,9,13,14,16	3,5,7,8,10,11,12,15	6 D3ZUC6,P22006,P23593,Q5RKI1,Q9EQS0,Q9R168
1791	1,2,4,6,9,13,15,16	3,5,7,8,10,11,12,14	4 D3ZUC6,P11883,P22006,Q9EQS0
1792	1,2,4,6,9,14,15,16	3,5,7,8,10,11,12,13	6 D3ZUC6,P22006,P23593,P47967,P97840,Q5I0D1
1793	1,2,4,6,10,11,12,13	3,5,7,8,9,14,15,16	2 P35280,Q68G31
1794	1,2,4,6,10,11,12,14	3,5,7,8,9,13,15,16	1 P35280
1795	1,2,4,6,10,11,12,15	3,5,7,8,9,13,14,16	0
1796	1,2,4,6,10,11,12,16	3,5,7,8,9,13,14,15	3 D3ZUC6,P07647,P35280
1797	1,2,4,6,10,11,13,14	3,5,7,8,9,12,15,16	2 P35280,Q68G31
1798	1,2,4,6,10,11,13,15	3,5,7,8,9,12,14,16	1 Q68G31
1799	1,2,4,6,10,11,13,16	3,5,7,8,9,12,14,15	4 D3ZUC6,P07647,P35280,Q68G31
1800	1,2,4,6,10,11,14,15	3,5,7,8,9,12,13,16	0
1801	1,2,4,6,10,11,14,16	3,5,7,8,9,12,13,15	3 P07647,P35280,Q4KLZ6
1802	1,2,4,6,10,11,15,16	3,5,7,8,9,12,13,14	2 D3ZUC6,P17988
1803	1,2,4,6,10,12,13,14	3,5,7,8,9,11,15,16	1 P35280
1804	1,2,4,6,10,12,13,15	3,5,7,8,9,11,14,16	1 P35280
1805	1,2,4,6,10,12,13,16	3,5,7,8,9,11,14,15	2 D3ZUC6,P35280
1806	1,2,4,6,10,12,14,15	3,5,7,8,9,11,13,16	2 P35280,P54921
1807	1,2,4,6,10,12,14,16	3,5,7,8,9,11,13,15	2 P35280,Q9QYP1

1808	1,2,4,6,10,12,15,16	3,5,7,8,9,11,13,14	4	D3ZUC6,P10758,P35280,Q9QYP1
1809	1,2,4,6,10,13,14,15	3,5,7,8,9,11,12,16	3	O88797,P35280,Q68G31
1810	1,2,4,6,10,13,14,16	3,5,7,8,9,11,12,15	3	P14668,P35280,Q9QYP1
1811	1,2,4,6,10,13,15,16	3,5,7,8,9,11,12,14	4	D3ZUC6,P11883,P35280,Q9QYP1
1812	1,2,4,6,10,14,15,16	3,5,7,8,9,11,12,13	4	P35280,P47967,P54921,Q9QYP1
1813	1,2,4,6,11,12,13,14	3,5,7,8,9,10,15,16	3	P35280,Q5GRG2,Q5RKI1
1814	1,2,4,6,11,12,13,15	3,5,7,8,9,10,14,16	0	
1815	1,2,4,6,11,12,13,16	3,5,7,8,9,10,14,15	5	O35547,P05369,P07647,P35280,Q5RKI1
1816	1,2,4,6,11,12,14,15	3,5,7,8,9,10,13,16	0	
1817	1,2,4,6,11,12,14,16	3,5,7,8,9,10,13,15	5	P07647,P35280,Q4KLZ6,Q5GRG2,Q5RKI1
1818	1,2,4,6,11,12,15,16	3,5,7,8,9,10,13,14	1	P07647
1819	1,2,4,6,11,13,14,15	3,5,7,8,9,10,12,16	1	Q68G31
1820	1,2,4,6,11,13,14,16	3,5,7,8,9,10,12,15	7	O54728,P07647,P12368,P35280,Q4KLZ6,Q5RKI1,Q9EQS0
1821	1,2,4,6,11,13,15,16	3,5,7,8,9,10,12,14	2	P07647,Q9EQS0
1822	1,2,4,6,11,14,15,16	3,5,7,8,9,10,12,13	3	P07647,Q4KLZ6,Q8CJ52
1823	1,2,4,6,12,13,14,15	3,5,7,8,9,10,11,16	1	P35280
1824	1,2,4,6,12,13,14,16	3,5,7,8,9,10,11,15	2	P35280,Q5RKI1
1825	1,2,4,6,12,13,15,16	3,5,7,8,9,10,11,14	1	P35280
1826	1,2,4,6,12,14,15,16	3,5,7,8,9,10,11,13	1	P35280
1827	1,2,4,6,13,14,15,16	3,5,7,8,9,10,11,12	2	P01835,P35280

1828	1,2,4,7,8,9,10,11	3,5,6,12,13,14,15,16	8	P08723,P20646,P70545,Q63493,Q66H69,Q6P6R2,Q812E4,Q9Z1F2
1829	1,2,4,7,8,9,10,12	3,5,6,11,13,14,15,16	3	iRT-Kit_WR_fusion,Q66H69,Q9Z1F2
1830	1,2,4,7,8,9,10,13	3,5,6,11,12,14,15,16	7	D4A5U3,O55004,P20646,P80299,Q63618,Q66H69,Q9Z1F2
1831	1,2,4,7,8,9,10,14	3,5,6,11,12,13,15,16	2	P20646,Q9Z1F2
1832	1,2,4,7,8,9,10,15	3,5,6,11,12,13,14,16	5	P20646,Q63474,Q63598,Q9Z0V6,Q9Z1F2
1833	1,2,4,7,8,9,10,16	3,5,6,11,12,13,14,15	6	D3ZUC6,O55004,P20646,P70545,Q30KJ2,Q63598
1834	1,2,4,7,8,9,11,12	3,5,6,10,13,14,15,16	13	iRT-Kit_WR_fusion,P02780,P06911,P08723,P36374,P40241,Q5GRG2,Q63493,Q66H69,Q6Q7Y5,Q812E4,Q9JHB9,Q9Z1F2
1835	1,2,4,7,8,9,11,13	3,5,6,10,12,14,15,16	10	D4A5U3,P08723,P09456,P20646,P40241,Q63493,Q66H69,Q6AYR9,Q812E4,Q9Z1F2
1836	1,2,4,7,8,9,11,14	3,5,6,10,12,13,15,16	10	D4A5U3,P07150,P08723,P20646,Q5GRG2,Q62635,Q63493,Q6AYR9,Q812E4,Q9Z1F2
1837	1,2,4,7,8,9,11,15	3,5,6,10,12,13,14,16	15	iRT-Kit_WR_fusion,P08723,P20646,P50280,Q10758,Q63493,Q6AYR9,Q6IFU8,Q6IMF3,Q6P6Q2,Q6P6S4,Q812E4,Q8CJ52,Q9Z0V6,Q9Z1F2
1838	1,2,4,7,8,9,11,16	3,5,6,10,12,13,14,15	22	iRT-Kit_WR_fusion,P02780,P02781,P02782,P04905,P07647,P08723,P09456,P0C0A9,P20646,P22283,P24368,P30120,P36374,P40241,P50280,P60905,P97523,Q5M8C6,Q63493,Q812E4,Q9JHB9
1839	1,2,4,7,8,9,12,13	3,5,6,10,11,14,15,16	3	iRT-Kit_WR_fusion,Q66H69,Q9Z1F2
1840	1,2,4,7,8,9,12,14	3,5,6,10,11,13,15,16	3	iRT-Kit_WR_fusion,Q62635,Q9Z1F2
1841	1,2,4,7,8,9,12,15	3,5,6,10,11,13,14,16	4	iRT-Kit_WR_fusion,P20646,Q9Z0V6,Q9Z1F2
1842	1,2,4,7,8,9,12,16	3,5,6,10,11,13,14,15	2	iRT-Kit_WR_fusion,P20646
1843	1,2,4,7,8,9,13,14	3,5,6,10,11,12,15,16	5	B0BNN3,D4A5U3,P20646,Q62635,Q9Z1F2
1844	1,2,4,7,8,9,13,15	3,5,6,10,11,12,14,16	3	P20646,Q9Z0V6,Q9Z1F2
1845	1,2,4,7,8,9,13,16	3,5,6,10,11,12,14,15	3	B0BNN3,D4A5U3,P20646
1846	1,2,4,7,8,9,14,15	3,5,6,10,11,12,13,16	4	P20646,Q62635,Q9Z0V6,Q9Z1F2

1847	1,2,4,7,8,9,14,16	3,5,6,10,11,12,13,15	3	D4A5U3,P20646,Q62635
1848	1,2,4,7,8,9,15,16	3,5,6,10,11,12,13,14	3	iRT-Kit_WR_fusion,P20646,Q9Z0V6
1849	1,2,4,7,8,10,11,12	3,5,6,9,13,14,15,16	10	iRT-Kit_WR_fusion,P02780,P08723,P36374,P40241,Q5GRG2,Q63493,Q66H69,Q9JHB9,Q9Z1F2
1850	1,2,4,7,8,10,11,13	3,5,6,9,12,14,15,16	6	P08723,P40241,Q63493,Q66H69,Q8CIZ5,Q9Z1F2
1851	1,2,4,7,8,10,11,14	3,5,6,9,12,13,15,16	6	P08723,Q4G075,Q5GRG2,Q62635,Q66H69,Q9Z1F2
1852	1,2,4,7,8,10,11,15	3,5,6,9,12,13,14,16	13	P08723,P20646,P50280,Q10758,Q4FZU2,Q63493,Q66H69,Q6IFU8,Q6IMF3,Q6P6Q2,Q8CJ52,Q9Z0V6,Q9Z1F2
1853	1,2,4,7,8,10,11,16	3,5,6,9,12,13,14,15	23	O55004,P02780,P02781,P02782,P04905,P07647,P08723,P09456,P0C0A9,P22282,P22283,P24368,P30120,P36374,P40241,P50280,Q00715,Q30KJ2,Q5M8C6,Q63493,Q66H69,Q9JHB9,Q9WTW7
1854	1,2,4,7,8,10,12,13	3,5,6,9,11,14,15,16	7	O55004,P80299,Q63598,Q63618,Q66H69,Q8CIZ5,Q9Z1F2
1855	1,2,4,7,8,10,12,14	3,5,6,9,11,13,15,16	4	Q5RLM2,Q62635,Q66H69,Q9Z1F2
1856	1,2,4,7,8,10,12,15	3,5,6,9,11,13,14,16	7	iRT-Kit_WR_fusion,P10758,Q63474,Q63598,Q66H69,Q9Z0V6,Q9Z1F2
1857	1,2,4,7,8,10,12,16	3,5,6,9,11,13,14,15	11	iRT-Kit_WR_fusion,O55004,P10758,P46844,Q30KJ2,Q3ZAV1,Q63598,Q64093,Q66H69,Q9JJ40,Q9WTW7
1858	1,2,4,7,8,10,13,14	3,5,6,9,11,12,15,16	11	B0BNN3,O55004,P19223,P25809,P80299,P97580,Q62635,Q63618,Q66H69,Q8CIZ5,Q9Z1F2
1859	1,2,4,7,8,10,13,15	3,5,6,9,11,12,14,16	12	O55004,P19468,P51907,P80299,Q63424,Q63474,Q63598,Q63618,Q64093,Q66H69,Q6Q0N1,Q9Z1F2
1860	1,2,4,7,8,10,13,16	3,5,6,9,11,12,14,15	19	B0BNN3,O55004,O70257,P07151,P19223,P19468,P28570,P46844,P51907,Q30KJ2,Q3ZAV1,Q63424,Q63598,Q63618,Q64093,Q66H69,Q6AYQ8,Q6Q0N1,Q9WTW7
1861	1,2,4,7,8,10,14,15	3,5,6,9,11,12,13,16	5	P20646,P20762,P54921,Q63474,Q9Z1F2
1862	1,2,4,7,8,10,14,16	3,5,6,9,11,12,13,15	10	O55004,P07151,P20646,P20762,P57113,P97580,Q30KJ2,Q63424,Q64093,Q9QYP1
1863	1,2,4,7,8,10,15,16	3,5,6,9,11,12,13,14	35	B2RYW9,O55004,O70377,O70594,P07151,P10758,P19468,P20646,P20762,P46844,P48508,P51907,P53790,Q03248,Q05175,Q30KJ2,Q3ZAV1,Q5I0E9,Q5M7T9,Q63355,Q63424,Q63598,Q63618,Q64093,Q66H69,Q6AYQ8,Q6Q0N1,Q80W57,Q8R431,Q9JJ40,Q9QYP1,Q9R1T5,Q9WTW7,Q9Z0V6,Q9Z0W7
1864	1,2,4,7,8,11,12,13	3,5,6,9,10,14,15,16	17	iRT-Kit_WR_fusion,P02780,P02781,P02782,P06911,P08723,P22282,P36374,P40241,Q5GRG2,Q63493,Q66H69,Q811M5,Q8CIZ5,Q9JHB9,Q9JI85,Q9Z1F2

1865	1,2,4,7,8,11,12,14	3,5,6,9,10,13,15,16	18	iRT-Kit_WR_fusion,P02780,P02781,P06911,P08723,P11883,P12020,P36374,P40241,Q4G075,Q5GRG2,Q62635,Q63493,Q66H69,Q811M5,Q812E4,Q9JHB9,Q9Z1F2
1866	1,2,4,7,8,11,12,15	3,5,6,9,10,13,14,16	16	iRT-Kit_WR_fusion,P02780,P08723,P36374,P40241,P50280,Q10758,Q5GRG2,Q63493,Q66H69,Q6IFU8,Q6IMF3,Q6P6Q2,Q8CJ52,Q9JHB9,Q9Z1F2
1867	1,2,4,7,8,11,12,16	3,5,6,9,10,13,14,15	28	iRT-Kit_WR_fusion,O35547,P02780,P02781,P02782,P04905,P06911,P07647,P08723,P09456,P0C0A9,P22282,P22283,P24368,P30120,P36374,P40241,P46462,P50280,P60905,P97523,Q00715,Q5GRG2,Q5M8C6,Q63493,Q66H69,Q812E4,Q9JHB9
1868	1,2,4,7,8,11,13,14	3,5,6,9,10,12,15,16	16	B0BNN3,D4A5U3,O54728,P02781,P02782,P08723,Q4G075,Q5GRG2,Q62635,Q63493,Q66H69,Q6AYR9,Q811M5,Q812E4,Q8CIZ5,Q9Z1F2
1869	1,2,4,7,8,11,13,15	3,5,6,9,10,12,14,16	15	P08723,P36374,P40241,P50280,Q10758,Q4FZU2,Q4G075,Q63493,Q66H69,Q6AYR9,Q6IFU8,Q6IMF3,Q6P6Q2,Q8CJ52,Q9Z1F2
1870	1,2,4,7,8,11,13,16	3,5,6,9,10,12,14,15	29	B0BNN3,O35547,P02761,P02780,P02781,P02782,P04905,P05369,P07647,P08723,P09456,P0C0A9,P22282,P22283,P24368,P30120,P36374,P40241,P46462,P50280,P60905,Q4G075,Q5M8C6,Q63493,Q66H69,Q6AYQ8,Q812E4,Q9JHB9,Q9JI85
1871	1,2,4,7,8,11,14,15	3,5,6,9,10,12,13,16	14	P08723,P0C0A9,P50280,Q10758,Q4FZU2,Q4G075,Q5GRG2,Q62635,Q63493,Q6IFU8,Q6IMF3,Q6P6Q2,Q8CJ52,Q9Z1F2
1872	1,2,4,7,8,11,14,16	3,5,6,9,10,12,13,15	26	B0BNN3,O54728,P02761,P02780,P02781,P02782,P04905,P07647,P08723,P09456,P0C0A9,P22282,P22283,P30120,P36374,P40241,P46462,P50280,Q00715,Q4G075,Q5GRG2,Q5M8C6,Q62635,Q63493,Q812E4,Q9JHB9
1873	1,2,4,7,8,11,15,16	3,5,6,9,10,12,13,14	28	B2RYW9,P02780,P02781,P02782,P04905,P07647,P08723,P09456,P0C0A9,P20646,P22283,P24368,P30120,P36374,P40241,P50280,Q10758,Q4FZU2,Q4G075,Q5M8C6,Q63493,Q66H69,Q6AYQ8,Q6IFU8,Q6IMF3,Q6P6Q2,Q8CJ52,Q9JHB9
1874	1,2,4,7,8,12,13,14	3,5,6,9,10,11,15,16	7	B0BNN3,Q62635,Q66H69,Q811M5,Q8CIZ5,Q9Z1F2,Q9Z2L0
1875	1,2,4,7,8,12,13,15	3,5,6,9,10,11,14,16	4	iRT-Kit_WR_fusion,Q66H69,Q9Z1F2,Q9Z2L0
1876	1,2,4,7,8,12,13,16	3,5,6,9,10,11,14,15	6	B0BNN3,iRT-Kit_WR_fusion,P02781,P02782,P30120,Q66H69
1877	1,2,4,7,8,12,14,15	3,5,6,9,10,11,13,16	3	iRT-Kit_WR_fusion,Q62635,Q9Z1F2
1878	1,2,4,7,8,12,14,16	3,5,6,9,10,11,13,15	2	iRT-Kit_WR_fusion,Q62635
1879	1,2,4,7,8,12,15,16	3,5,6,9,10,11,13,14	3	iRT-Kit_WR_fusion,P10758,Q66H69
1880	1,2,4,7,8,13,14,15	3,5,6,9,10,11,12,16	6	B0BNN3,Q62635,Q66H69,Q8CIZ5,Q9Z1F2,Q9Z2L0
1881	1,2,4,7,8,13,14,16	3,5,6,9,10,11,12,15	11	B0BNN3,D4A5U3,O54728,P02761,P02781,Q30KJ2,Q62635,Q66H69,Q6TMA8,Q9WUW8,Q9Z2L0

1882	1,2,4,7,8,13,15,16	3,5,6,9,10,11,12,14	8	B0BNN3,B2RYW9,P01015,P02761,P04916,P20646,Q66H69,Q6AYQ8
1883	1,2,4,7,8,14,15,16	3,5,6,9,10,11,12,13	7	B0BNN3,P02761,P20646,P20762,Q30KJ2,Q62635,Q6TMA8
1884	1,2,4,7,9,10,11,12	3,5,6,8,13,14,15,16	3	Q03191,Q62714,Q62761;Q62762;Q62763
1885	1,2,4,7,9,10,11,13	3,5,6,8,12,14,15,16	3	P80299,Q9R168,Q9Z1F2
1886	1,2,4,7,9,10,11,14	3,5,6,8,12,13,15,16	2	Q9R168,Q9Z1F2
1887	1,2,4,7,9,10,11,15	3,5,6,8,12,13,14,16	2	Q9R168,Q9Z1F2
1888	1,2,4,7,9,10,11,16	3,5,6,8,12,13,14,15	2	D3ZUC6,Q9R168
1889	1,2,4,7,9,10,12,13	3,5,6,8,11,14,15,16	2	P80299,Q9Z1F2
1890	1,2,4,7,9,10,12,14	3,5,6,8,11,13,15,16	1	Q9Z1F2
1891	1,2,4,7,9,10,12,15	3,5,6,8,11,13,14,16	2	P10758,Q9Z1F2
1892	1,2,4,7,9,10,12,16	3,5,6,8,11,13,14,15	2	D3ZUC6,P10758
1893	1,2,4,7,9,10,13,14	3,5,6,8,11,12,15,16	3	P80299,Q9R168,Q9Z1F2
1894	1,2,4,7,9,10,13,15	3,5,6,8,11,12,14,16	2	P80299,Q9Z1F2
1895	1,2,4,7,9,10,13,16	3,5,6,8,11,12,14,15	3	D3ZUC6,P80299,Q9R168
1896	1,2,4,7,9,10,14,15	3,5,6,8,11,12,13,16	2	Q9R168,Q9Z1F2
1897	1,2,4,7,9,10,14,16	3,5,6,8,11,12,13,15	2	Q9QYP1,Q9R168
1898	1,2,4,7,9,10,15,16	3,5,6,8,11,12,13,14	4	D3ZUC6,P10758,Q9QYP1,Q9R168
1899	1,2,4,7,9,11,12,13	3,5,6,8,10,14,15,16	3	O35077,Q03191,Q62761;Q62762;Q62763
1900	1,2,4,7,9,11,12,14	3,5,6,8,10,13,15,16	2	O35077,Q62761;Q62762;Q62763
1901	1,2,4,7,9,11,12,15	3,5,6,8,10,13,14,16	2	O35077,Q62761;Q62762;Q62763

1902	1,2,4,7,9,11,12,16	3,5,6,8,10,13,14,15	3	D3ZUC6,P63322,Q03191
1903	1,2,4,7,9,11,13,14	3,5,6,8,10,12,15,16	2	P0DMW0;P0DMW1,Q9R168
1904	1,2,4,7,9,11,13,15	3,5,6,8,10,12,14,16	2	Q8CJ52,Q9R168
1905	1,2,4,7,9,11,13,16	3,5,6,8,10,12,14,15	2	D3ZUC6,Q9R168
1906	1,2,4,7,9,11,14,15	3,5,6,8,10,12,13,16	4	P0DMW0;P0DMW1,P25031,Q8CJ52,Q9R168
1907	1,2,4,7,9,11,14,16	3,5,6,8,10,12,13,15	2	Q812E4,Q9R168
1908	1,2,4,7,9,11,15,16	3,5,6,8,10,12,13,14	3	D3ZUC6,Q8CJ52,Q9R168
1909	1,2,4,7,9,12,13,14	3,5,6,8,10,11,15,16	0	
1910	1,2,4,7,9,12,13,15	3,5,6,8,10,11,14,16	0	
1911	1,2,4,7,9,12,13,16	3,5,6,8,10,11,14,15	1	D3ZUC6
1912	1,2,4,7,9,12,14,15	3,5,6,8,10,11,13,16	0	
1913	1,2,4,7,9,12,14,16	3,5,6,8,10,11,13,15	0	
1914	1,2,4,7,9,12,15,16	3,5,6,8,10,11,13,14	3	D3ZUC6,P08937,P10758
1915	1,2,4,7,9,13,14,15	3,5,6,8,10,11,12,16	2	P0DMW0;P0DMW1,Q9R168
1916	1,2,4,7,9,13,14,16	3,5,6,8,10,11,12,15	2	B0BNN3,Q9R168
1917	1,2,4,7,9,13,15,16	3,5,6,8,10,11,12,14	3	D3ZUC6,P08937,Q9R168
1918	1,2,4,7,9,14,15,16	3,5,6,8,10,11,12,13	1	Q9R168
1919	1,2,4,7,10,11,12,13	3,5,6,8,9,14,15,16	2	P80299,Q9Z1F2
1920	1,2,4,7,10,11,12,14	3,5,6,8,9,13,15,16	1	Q9Z1F2
1921	1,2,4,7,10,11,12,15	3,5,6,8,9,13,14,16	2	P10758,Q9Z1F2

1922	1,2,4,7,10,11,12,16	3,5,6,8,9,13,14,15	1 Q9JHB9
1923	1,2,4,7,10,11,13,14	3,5,6,8,9,12,15,16	3 P80299,Q9R168,Q9Z1F2
1924	1,2,4,7,10,11,13,15	3,5,6,8,9,12,14,16	2 P80299,Q9Z1F2
1925	1,2,4,7,10,11,13,16	3,5,6,8,9,12,14,15	4 P07647,P36376,Q6AYQ8,Q9R168
1926	1,2,4,7,10,11,14,15	3,5,6,8,9,12,13,16	1 Q9Z1F2
1927	1,2,4,7,10,11,14,16	3,5,6,8,9,12,13,15	3 P36376,Q9QYP1,Q9R168
1928	1,2,4,7,10,11,15,16	3,5,6,8,9,12,13,14	4 P17988,Q6AYQ8,Q8CJ52,Q9QYP1
1929	1,2,4,7,10,12,13,14	3,5,6,8,9,11,15,16	2 P80299,Q9Z1F2
1930	1,2,4,7,10,12,13,15	3,5,6,8,9,11,14,16	4 O88797,P10758,P80299,Q9Z1F2
1931	1,2,4,7,10,12,13,16	3,5,6,8,9,11,14,15	3 P09527,P10758,P80299
1932	1,2,4,7,10,12,14,15	3,5,6,8,9,11,13,16	2 P10758,Q9Z1F2
1933	1,2,4,7,10,12,14,16	3,5,6,8,9,11,13,15	2 P09527,Q9QYP1
1934	1,2,4,7,10,12,15,16	3,5,6,8,9,11,13,14	3 P09527,P10758,Q9QYP1
1935	1,2,4,7,10,13,14,15	3,5,6,8,9,11,12,16	4 O88797,P09527,P80299,Q9Z1F2
1936	1,2,4,7,10,13,14,16	3,5,6,8,9,11,12,15	8 B0BNN3,P02761,P09527,P36376,P80299,Q6AYQ8,Q9QYP1,Q9R168
1937	1,2,4,7,10,13,15,16	3,5,6,8,9,11,12,14	7 P09527,P10758,P19468,P80299,Q64093,Q6AYQ8,Q9QYP1
1938	1,2,4,7,10,14,15,16	3,5,6,8,9,11,12,13	2 P09527,Q9QYP1
1939	1,2,4,7,11,12,13,14	3,5,6,8,9,10,15,16	1 Q811M5
1940	1,2,4,7,11,12,13,15	3,5,6,8,9,10,14,16	0
1941	1,2,4,7,11,12,13,16	3,5,6,8,9,10,14,15	2 P05369,P07647

1942	1,2,4,7,11,12,14,15	3,5,6,8,9,10,13,16	1 P25031
1943	1,2,4,7,11,12,14,16	3,5,6,8,9,10,13,15	2 P07647,Q9JHB9
1944	1,2,4,7,11,12,15,16	3,5,6,8,9,10,13,14	3 P08937,P10758,Q8CJ52
1945	1,2,4,7,11,13,14,15	3,5,6,8,9,10,12,16	2 P25031,Q8CJ52
1946	1,2,4,7,11,13,14,16	3,5,6,8,9,10,12,15	7 B0BNN3,O54728,P02761,P07647,P36376,Q6AYQ8,Q9R168
1947	1,2,4,7,11,13,15,16	3,5,6,8,9,10,12,14	3 P08937,Q6AYQ8,Q8CJ52
1948	1,2,4,7,11,14,15,16	3,5,6,8,9,10,12,13	2 Q8CJ52,Q9R168
1949	1,2,4,7,12,13,14,15	3,5,6,8,9,10,11,16	1 O88797
1950	1,2,4,7,12,13,14,16	3,5,6,8,9,10,11,15	0
1951	1,2,4,7,12,13,15,16	3,5,6,8,9,10,11,14	2 P08937,P10758
1952	1,2,4,7,12,14,15,16	3,5,6,8,9,10,11,13	0
1953	1,2,4,7,13,14,15,16	3,5,6,8,9,10,11,12	4 B0BNN3,P01835,P02761,Q6AYQ8
1954	1,2,4,8,9,10,11,12	3,5,6,7,13,14,15,16	1 Q62714
1955	1,2,4,8,9,10,11,13	3,5,6,7,12,14,15,16	0
1956	1,2,4,8,9,10,11,14	3,5,6,7,12,13,15,16	0
1957	1,2,4,8,9,10,11,15	3,5,6,7,12,13,14,16	3 Q10758,Q6IFU8,Q6P6S4
1958	1,2,4,8,9,10,11,16	3,5,6,7,12,13,14,15	3 D3ZUC6,Q30KJ2,Q6AY61
1959	1,2,4,8,9,10,12,13	3,5,6,7,11,14,15,16	1 Q9Z1F2
1960	1,2,4,8,9,10,12,14	3,5,6,7,11,13,15,16	1 Q9Z1F2
1961	1,2,4,8,9,10,12,15	3,5,6,7,11,13,14,16	1 Q9Z1F2

1962	1,2,4,8,9,10,12,16	3,5,6,7,11,13,14,15	2 D3ZUC6,Q30KJ2
1963	1,2,4,8,9,10,13,14	3,5,6,7,11,12,15,16	1 Q9Z1F2
1964	1,2,4,8,9,10,13,15	3,5,6,7,11,12,14,16	1 Q9Z1F2
1965	1,2,4,8,9,10,13,16	3,5,6,7,11,12,14,15	2 D3ZUC6,Q30KJ2
1966	1,2,4,8,9,10,14,15	3,5,6,7,11,12,13,16	3 P47967,P97840,Q9Z1F2
1967	1,2,4,8,9,10,14,16	3,5,6,7,11,12,13,15	3 P47967,P97580,Q30KJ2
1968	1,2,4,8,9,10,15,16	3,5,6,7,11,12,13,14	2 D3ZUC6,Q30KJ2
1969	1,2,4,8,9,11,12,13	3,5,6,7,10,14,15,16	1 Q5RKI1
1970	1,2,4,8,9,11,12,14	3,5,6,7,10,13,15,16	3 Q5RKI1,Q6Q7Y5,Q812E4
1971	1,2,4,8,9,11,12,15	3,5,6,7,10,13,14,16	1 Q6IFU8
1972	1,2,4,8,9,11,12,16	3,5,6,7,10,13,14,15	6 D3ZUC6,P30120,P60905,Q5RKI1,Q62714,Q812E4
1973	1,2,4,8,9,11,13,14	3,5,6,7,10,12,15,16	4 O54728,P0DMW0;P0DMW1,Q5RKI1,Q812E4
1974	1,2,4,8,9,11,13,15	3,5,6,7,10,12,14,16	2 Q10758,Q6IFU8
1975	1,2,4,8,9,11,13,16	3,5,6,7,10,12,14,15	7 D3ZUC6,P02782,P30120,P60905,Q5RKI1,Q6AY61,Q812E4
1976	1,2,4,8,9,11,14,15	3,5,6,7,10,12,13,16	3 P0DMW0;P0DMW1,P47967,Q6IFU8
1977	1,2,4,8,9,11,14,16	3,5,6,7,10,12,13,15	3 O54728,Q5RKI1,Q812E4
1978	1,2,4,8,9,11,15,16	3,5,6,7,10,12,13,14	3 D3ZUC6,Q10758,Q6IFU8
1979	1,2,4,8,9,12,13,14	3,5,6,7,10,11,15,16	1 Q5RKI1
1980	1,2,4,8,9,12,13,15	3,5,6,7,10,11,14,16	0
1981	1,2,4,8,9,12,13,16	3,5,6,7,10,11,14,15	2 D3ZUC6,Q5RKI1

1982	1,2,4,8,9,12,14,15	3,5,6,7,10,11,13,16	0
1983	1,2,4,8,9,12,14,16	3,5,6,7,10,11,13,15	1 Q5RKI1
1984	1,2,4,8,9,12,15,16	3,5,6,7,10,11,13,14	0
1985	1,2,4,8,9,13,14,15	3,5,6,7,10,11,12,16	1 P47967
1986	1,2,4,8,9,13,14,16	3,5,6,7,10,11,12,15	4 B0BNN3,O54728,P23593,Q5RKI1
1987	1,2,4,8,9,13,15,16	3,5,6,7,10,11,12,14	2 D3ZUC6,P23593
1988	1,2,4,8,9,14,15,16	3,5,6,7,10,11,12,13	4 P23593,P47967,P97840,Q5I0D1
1989	1,2,4,8,10,11,12,13	3,5,6,7,9,14,15,16	1 Q66H69
1990	1,2,4,8,10,11,12,14	3,5,6,7,9,13,15,16	0
1991	1,2,4,8,10,11,12,15	3,5,6,7,9,13,14,16	2 Q10758,Q6IFU8
1992	1,2,4,8,10,11,12,16	3,5,6,7,9,13,14,15	1 P30120
1993	1,2,4,8,10,11,13,14	3,5,6,7,9,12,15,16	2 O54728,Q68G31
1994	1,2,4,8,10,11,13,15	3,5,6,7,9,12,14,16	3 Q10758,Q68G31,Q6IFU8
1995	1,2,4,8,10,11,13,16	3,5,6,7,9,12,14,15	3 P02782,P30120,Q30KJ2
1996	1,2,4,8,10,11,14,15	3,5,6,7,9,12,13,16	2 Q10758,Q6IFU8
1997	1,2,4,8,10,11,14,16	3,5,6,7,9,12,13,15	4 O54728,P30120,P97580,Q30KJ2
1998	1,2,4,8,10,11,15,16	3,5,6,7,9,12,13,14	4 P15399,Q10758,Q30KJ2,Q6IFU8
1999	1,2,4,8,10,12,13,14	3,5,6,7,9,11,15,16	1 Q9Z1F2
2000	1,2,4,8,10,12,13,15	3,5,6,7,9,11,14,16	1 Q9Z1F2
2001	1,2,4,8,10,12,13,16	3,5,6,7,9,11,14,15	1 Q30KJ2

2002	1,2,4,8,10,<br>12,14,15	3,5,6,7,9,1<br>1,13,16	2	P54921,Q9Z1F2
2003	1,2,4,8,10,<br>12,14,16	3,5,6,7,9,1<br>1,13,15	2	P97580,Q30KJ2
2004	1,2,4,8,10,<br>12,15,16	3,5,6,7,9,1<br>1,13,14	2	P10758,Q30KJ2
2005	1,2,4,8,10,<br>13,14,15	3,5,6,7,9,1<br>1,12,16	2	P97580,Q9Z1F2
2006	1,2,4,8,10,<br>13,14,16	3,5,6,7,9,1<br>1,12,15	4	B0BNN3,O54728,P97580,Q30KJ2
2007	1,2,4,8,10,<br>13,15,16	3,5,6,7,9,1<br>1,12,14	1	Q30KJ2
2008	1,2,4,8,10,<br>14,15,16	3,5,6,7,9,1<br>1,12,13	5	P15399,P54921,P97580,Q30KJ2,Q9QYP1
2009	1,2,4,8,11,<br>12,13,14	3,5,6,7,9,1<br>0,15,16	3	O54728,Q5RKI1,Q811M5
2010	1,2,4,8,11,<br>12,13,15	3,5,6,7,9,1<br>0,14,16	2	Q10758,Q6IFU8
2011	1,2,4,8,11,<br>12,13,16	3,5,6,7,9,1<br>0,14,15	5	P02782,P30120,P36374,P60905,Q5RKI1
2012	1,2,4,8,11,<br>12,14,15	3,5,6,7,9,1<br>0,13,16	1	Q6IFU8
2013	1,2,4,8,11,<br>12,14,16	3,5,6,7,9,1<br>0,13,15	4	O54728,P02782,P30120,Q5RKI1
2014	1,2,4,8,11,<br>12,15,16	3,5,6,7,9,1<br>0,13,14	5	P02782,P30120,P36374,Q10758,Q6IFU8
2015	1,2,4,8,11,<br>13,14,15	3,5,6,7,9,1<br>0,12,16	5	B0BNN3,O54728,O54861,Q10758,Q6IFU8
2016	1,2,4,8,11,<br>13,14,16	3,5,6,7,9,1<br>0,12,15	6	B0BNN3,O54728,O54861,P02782,P30120,Q5RKI1
2017	1,2,4,8,11,<br>13,15,16	3,5,6,7,9,1<br>0,12,14	6	O54861,P02782,P30120,Q10758,Q6IFU8,Q6P6Q2
2018	1,2,4,8,11,<br>14,15,16	3,5,6,7,9,1<br>0,12,13	4	O54728,Q10758,Q6IFU8,Q6P6Q2
2019	1,2,4,8,12,<br>13,14,15	3,5,6,7,9,1<br>0,11,16	0	
2020	1,2,4,8,12,<br>13,14,16	3,5,6,7,9,1<br>0,11,15	3	B0BNN3,O54728,Q5RKI1
2021	1,2,4,8,12,<br>13,15,16	3,5,6,7,9,1<br>0,11,14	0	

2022	1,2,4,8,12,14,15,16	3,5,6,7,9,10,11,13	0
2023	1,2,4,8,13,14,15,16	3,5,6,7,9,10,11,12	4 B0BNN3,O54728,P01015,Q30KJ2
2024	1,2,4,9,10,11,12,13	3,5,6,7,8,14,15,16	3 O35077,Q62714,Q62761;Q62762;Q62763
2025	1,2,4,9,10,11,12,14	3,5,6,7,8,13,15,16	3 O35077,Q62714,Q62761;Q62762;Q62763
2026	1,2,4,9,10,11,12,15	3,5,6,7,8,13,14,16	3 P08937,Q62714,Q62761;Q62762;Q62763
2027	1,2,4,9,10,11,12,16	3,5,6,7,8,13,14,15	3 D3ZUC6,P08937,Q62714
2028	1,2,4,9,10,11,13,14	3,5,6,7,8,12,15,16	1 Q9R168
2029	1,2,4,9,10,11,13,15	3,5,6,7,8,12,14,16	1 P08937
2030	1,2,4,9,10,11,13,16	3,5,6,7,8,12,14,15	3 D3ZUC6,P08937,Q9R168
2031	1,2,4,9,10,11,14,15	3,5,6,7,8,12,13,16	0
2032	1,2,4,9,10,11,14,16	3,5,6,7,8,12,13,15	2 D3ZUC6,Q9R168
2033	1,2,4,9,10,11,15,16	3,5,6,7,8,12,13,14	2 D3ZUC6,P08937
2034	1,2,4,9,10,12,13,14	3,5,6,7,8,11,15,16	1 Q9QZK9
2035	1,2,4,9,10,12,13,15	3,5,6,7,8,11,14,16	1 P08937
2036	1,2,4,9,10,12,13,16	3,5,6,7,8,11,14,15	2 D3ZUC6,P08937
2037	1,2,4,9,10,12,14,15	3,5,6,7,8,11,13,16	3 P30120,P50115,Q5M8C6
2038	1,2,4,9,10,12,14,16	3,5,6,7,8,11,13,15	2 D3ZUC6,P01041
2039	1,2,4,9,10,12,15,16	3,5,6,7,8,11,13,14	4 D3ZUC6,P08937,P10758,Q99041
2040	1,2,4,9,10,13,14,15	3,5,6,7,8,11,12,16	3 P47967,Q5M8C6,Q8CFN2
2041	1,2,4,9,10,13,14,16	3,5,6,7,8,11,12,15	2 D3ZUC6,Q9R168

2042	1,2,4,9,10,13,15,16	3,5,6,7,8,11,12,14	3	D3ZUC6,P08937,Q63751
2043	1,2,4,9,10,14,15,16	3,5,6,7,8,11,12,13	4	D3ZUC6,P47967,Q5I0D1,Q9QYP1
2044	1,2,4,9,11,12,13,14	3,5,6,7,8,10,15,16	10	O35077,O70594,P53790,P57113,Q05175,Q5RKI1,Q62761;Q62762;Q62763,Q63270,Q6AY41,Q8R431
2045	1,2,4,9,11,12,13,15	3,5,6,7,8,10,14,16	6	O35077,P08937,P25031,P57113,Q62761;Q62762;Q62763,Q923S2
2046	1,2,4,9,11,12,13,16	3,5,6,7,8,10,14,15	6	D3ZUC6,P08937,P54921,Q05175,Q5RKI1,Q62714
2047	1,2,4,9,11,12,14,15	3,5,6,7,8,10,13,16	6	O35077,O70257,O70594,P25031,P57113,Q62761;Q62762;Q62763
2048	1,2,4,9,11,12,14,16	3,5,6,7,8,10,13,15	4	D3ZUC6,O70594,Q5RKI1,Q62714
2049	1,2,4,9,11,12,15,16	3,5,6,7,8,10,13,14	4	D3ZUC6,P08937,Q5QE79,Q62714
2050	1,2,4,9,11,13,14,15	3,5,6,7,8,10,12,16	3	P0DMW0;P0DMW1,P25031,P57113
2051	1,2,4,9,11,13,14,16	3,5,6,7,8,10,12,15	6	D3ZUC6,O54728,P0DMW0;P0DMW1,P12368,Q5RKI1,Q9R168
2052	1,2,4,9,11,13,15,16	3,5,6,7,8,10,12,14	4	D3ZUC6,O70417,P08937,Q5RLM2
2053	1,2,4,9,11,14,15,16	3,5,6,7,8,10,12,13	5	D3ZUC6,P08937,P25031,P47967,Q9R168
2054	1,2,4,9,12,13,14,15	3,5,6,7,8,10,11,16	0	
2055	1,2,4,9,12,13,14,16	3,5,6,7,8,10,11,15	1	Q5RKI1
2056	1,2,4,9,12,13,15,16	3,5,6,7,8,10,11,14	3	D3ZUC6,P08937,Q63751
2057	1,2,4,9,12,14,15,16	3,5,6,7,8,10,11,13	2	P08937,P47967
2058	1,2,4,9,13,14,15,16	3,5,6,7,8,10,11,12	4	D3ZUC6,P08937,P47967,Q5I0D1
2059	1,2,4,10,11,12,13,14	3,5,6,7,8,9,15,16	1	P35280
2060	1,2,4,10,11,12,13,15	3,5,6,7,8,9,14,16	2	P08937,Q5QE79
2061	1,2,4,10,11,12,13,16	3,5,6,7,8,9,14,15	3	P08937,P35280,Q5QE79

2062	1,2,4,10,11,12,14,15	3,5,6,7,8,9,13,16	0	
2063	1,2,4,10,11,12,14,16	3,5,6,7,8,9,13,15	1	P35280
2064	1,2,4,10,11,12,15,16	3,5,6,7,8,9,13,14	3	P08937,P10758,Q5QE79
2065	1,2,4,10,11,13,14,15	3,5,6,7,8,9,12,16	0	
2066	1,2,4,10,11,13,14,16	3,5,6,7,8,9,12,15	2	O54728,P35280
2067	1,2,4,10,11,13,15,16	3,5,6,7,8,9,12,14	4	O70417,P08937,Q5QE79,Q63751
2068	1,2,4,10,11,14,15,16	3,5,6,7,8,9,12,13	1	Q9QYP1
2069	1,2,4,10,12,13,14,15	3,5,6,7,8,9,11,16	1	P35280
2070	1,2,4,10,12,13,14,16	3,5,6,7,8,9,11,15	3	P06866,P35280,Q6P6R2
2071	1,2,4,10,12,13,15,16	3,5,6,7,8,9,11,14	6	P06760,P08937,P10758,P35280,Q5QE79,Q63751
2072	1,2,4,10,12,14,15,16	3,5,6,7,8,9,11,13	3	P08937,P35280,Q9QYP1
2073	1,2,4,10,13,14,15,16	3,5,6,7,8,9,11,12	4	P08937,P09527,P35280,Q9QYP1
2074	1,2,4,11,12,13,14,15	3,5,6,7,8,9,10,16	1	P25031
2075	1,2,4,11,12,13,14,16	3,5,6,7,8,9,10,15	3	O54728,P35280,Q5RKI1
2076	1,2,4,11,12,13,15,16	3,5,6,7,8,9,10,14	4	O70417,P08937,Q5QE79,Q63751
2077	1,2,4,11,12,14,15,16	3,5,6,7,8,9,10,13	2	P08937,P25031
2078	1,2,4,11,13,14,15,16	3,5,6,7,8,9,10,12	3	O54728,P08937,P25031
2079	1,2,4,12,13,14,15,16	3,5,6,7,8,9,10,11	3	P08937,P35280,Q6P6R2
2080	1,2,5,6,7,8,9,10	3,4,11,12,13,14,15,16	19	D3ZTX0,iRT-Kit_WR_fusion,O70594,P00762,P02454,P02631,P06760,P19629,P23928,P25031,P29315,P31044,P36860,P50115,P50116,P55091,P70545,Q63618,Q91ZS3

2081	1,2,5,6,7,8,9,11	3,4,10,12,13,14,15,16	21	iRT-Kit_WR_fusion,P06760,P06911,P07150,P09456,P29315,P31044,P31430,P50115,P50116,P50280,P55091,Q09326,Q5GRG2,Q63493,Q6P6S4,Q78P75,Q812E4,Q9JHB9,Q9JI85,Q9QX74
2082	1,2,5,6,7,8,9,12	3,4,10,11,13,14,15,16	11	iRT-Kit_WR_fusion,P06760,P19814,P29315,P36860,P50115,P50116,P55091,Q03191,Q8CJD3,Q91ZS3
2083	1,2,5,6,7,8,9,13	3,4,10,11,12,14,15,16	10	iRT-Kit_WR_fusion,P02631,P06760,P29315,P36860,P50115,P50116,P52590,P55091,Q78P75
2084	1,2,5,6,7,8,9,14	3,4,10,11,12,13,15,16	8	iRT-Kit_WR_fusion,P00762,P06760,P29315,P50115,P50116,P52590,Q62714
2085	1,2,5,6,7,8,9,15	3,4,10,11,12,13,14,16	11	iRT-Kit_WR_fusion,P00762,P15999,P19132,P19629,P35745,P36860,P50115,P50116,Q62714,Q9Z0V6
2086	1,2,5,6,7,8,9,16	3,4,10,11,12,13,14,15	5	iRT-Kit_WR_fusion,P19629,P50115,P50116,P52590
2087	1,2,5,6,7,8,10,11	3,4,9,12,13,14,15,16	26	iRT-Kit_WR_fusion,P02631,P06760,P06761,P06911,P09456,P19629,P22283,P25031,P29315,P31044,P31430,P35952,P36860,P50115,P50116,P50280,P55091,Q5GRG2,Q63493,Q66H69,Q68G31,Q91ZS3,Q9JHB9,Q9JI85,Q9QX74
2088	1,2,5,6,7,8,10,12	3,4,9,11,13,14,15,16	20	iRT-Kit_WR_fusion,P02631,P19629,P19814,P25031,P29315,P30904,P31044,P36860,P48508,P50115,P50116,P55091,P57113,Q5RLM2,Q63618,Q66H69,Q66HG3,Q91ZS3,Q99MA2
2089	1,2,5,6,7,8,10,13	3,4,9,11,12,14,15,16	25	A2RUW1,O70594,P02631,P19629,P19814,P23928,P25031,P25809,P29315,P31044,P35952,P36860,P46844,P48508,P50115,P50116,P55091,P57113,Q3ZAV1,Q62714,Q63618,Q66H69,Q68G31,Q91ZS3,Q9WUW8
2090	1,2,5,6,7,8,10,14	3,4,9,11,12,13,15,16	22	O70594,P00762,P02631,P19629,P23928,P25031,P29315,P31044,P48508,P50115,P50116,P52590,P54921,P55091,P57113,Q5RLM2,Q62714,Q68G31,Q91ZS3,Q923S2,Q9WUW8,Q9Z1F2
2091	1,2,5,6,7,8,10,15	3,4,9,11,12,13,14,16	42	A2RUW1,iRT-Kit_WR_fusion,O08557,O70594,O88339;Q4V882,P00762,P02631,P08721,P19132,P19468,P19629,P19814,P23928,P25031,P30904,P31044,P36860,P38918,P48508,P50115,P50116,P51907,P53790,P54921,P55091,P57113,Q03248,Q05175,Q3ZAV1,Q5I0E9,Q62714,Q63270,Q63474,Q63598,Q63618,Q68G31,Q6MG61,Q6PCU2,Q6Q0N1,Q99MA2,Q9Z0V6,Q9Z1F2
2092	1,2,5,6,7,8,10,16	3,4,9,11,12,13,14,15	43	iRT-Kit_WR_fusion,O70594,P02631,P18757,P19468,P19629,P23928,P25031,P31044,P38918,P46844,P48508,P50115,P50116,P51907,P52590,P53790,P55091,P57113,Q03248,Q05175,Q3ZAV1,Q5I0E9,Q5M7T9,Q5RLM2,Q62714,Q63270,Q63424,Q63618,Q64602,Q66H69,Q68FT5,Q6AY41,Q6MG61,Q6Q0N1,Q71MB6,Q8R431,Q923S2,Q99MA2,Q9JJ40,Q9WTW7,Q9WUW8,Q9Z0W7

2093	1,2,5,6,7,8,11,12	3,4,9,10,13,14,15,16	26	iRT-Kit_WR_fusion,P06760,P06761,P06911,P08723,P09456,P12020,P19629,P22283,P29315,P31044,P31430,P46462,P50115,P50116,P50280,P55091,Q00715,Q09326,Q5GRG2,Q63493,Q66H69,Q6IG05,Q78P75,Q9JHB9,Q9JI85
2094	1,2,5,6,7,8,11,13	3,4,9,10,12,14,15,16	26	iRT-Kit_WR_fusion,P02782,P06760,P06761,P06911,P08723,P09456,P12020,P22283,P29315,P31044,P31430,P36860,P46462,P50115,P50116,P50280,P55091,Q09326,Q5GRG2,Q63493,Q66H69,Q68G31,Q78P75,Q9JHB9,Q9JI85
2095	1,2,5,6,7,8,11,14	3,4,9,10,12,13,15,16	21	iRT-Kit_WR_fusion,P06760,P06761,P06911,P09456,P12020,P19218,P22283,P29315,P31044,P31430,P46462,P50115,P50116,P50280,P52590,Q09326,Q5GRG2,Q63493,Q9JHB9,Q9JI85
2096	1,2,5,6,7,8,11,15	3,4,9,10,12,13,14,16	27	iRT-Kit_WR_fusion,P02793;Q7TP54,P06760,P06911,P08721,P09456,P19132,P19629,P22283,P31044,P31430,P50115,P50116,P50280,Q09326,Q4FZU2,Q5GRG2,Q63493,Q66H69,Q6IFU8,Q6IG05,Q6IMF3,Q6P6Q2,Q8CJ52,Q9JHB9,Q9JI85,Q9QX74
2097	1,2,5,6,7,8,11,16	3,4,9,10,12,13,14,15	35	iRT-Kit_WR_fusion,O35547,P02780,P02781,P02782,P04905,P06760,P06761,P06911,P07647,P08010,P08723,P09456,P0C0A9,P12020,P19629,P22283,P30120,P31044,P46462,P50115,P50116,P50280,P52590,Q00715,Q5GRG2,Q5M8C6,Q63493,Q66H69,Q6IG05,Q812E4,Q9JHB9,Q9JI85,Q9QW07,Q9Z0J6
2098	1,2,5,6,7,8,12,13	3,4,9,10,11,14,15,16	12	iRT-Kit_WR_fusion,P01835,P02631,P06911,P19814,P29315,P31044,P36860,P50115,P50116,Q66H69,Q9JI85
2099	1,2,5,6,7,8,12,14	3,4,9,10,11,13,15,16	10	iRT-Kit_WR_fusion,P06911,P19814,P29315,P31044,P50115,P50116,P52590,Q5GRG2,Q5RLM2
2100	1,2,5,6,7,8,12,15	3,4,9,10,11,13,14,16	10	iRT-Kit_WR_fusion,P19132,P19629,P19814,P31044,P36860,P50115,P50116,Q66H69,Q6IG05
2101	1,2,5,6,7,8,12,16	3,4,9,10,11,13,14,15	9	iRT-Kit_WR_fusion,P19629,P19814,P31044,P50115,P50116,P52590,Q66H69,Q9JHB9
2102	1,2,5,6,7,8,13,14	3,4,9,10,11,12,15,16	11	P01835,P25809,P29315,P31044,P50115,P50116,P52590,Q62714,Q66H69,Q68G31,Q9WUW8
2103	1,2,5,6,7,8,13,15	3,4,9,10,11,12,14,16	14	iRT-Kit_WR_fusion,O70594,P01835,P19132,P19629,P19814,P31044,P36860,P50115,P50116,Q62714,Q66H69,Q68G31,Q6PCU2
2104	1,2,5,6,7,8,13,16	3,4,9,10,11,12,14,15	13	iRT-Kit_WR_fusion,P01835,P02631,P19629,P31044,P46844,P50115,P50116,P52590,Q62714,Q66H69,Q9JI85,Q9WUW8
2105	1,2,5,6,7,8,14,15	3,4,9,10,11,12,13,16	11	iRT-Kit_WR_fusion,P19132,P19629,P19814,P31044,P50115,P50116,P52590,P54921,Q62714,Q6PCU2

2106	1,2,5,6,7,8,14,16	3,4,9,10,11,12,13,15	11	iRT-Kit_WR_fusion,P19629,P31044,P50115,P50116,P50280,P52590,P57113,Q5RLM2,Q62714,Q9WUW8
2107	1,2,5,6,7,8,15,16	3,4,9,10,11,12,13,14	15	B2RYW9,iRT-Kit_WR_fusion,O70594,P17988,P19132,P19629,P48508,P50115,P50116,P50280,P52590,P57113,Q62714,Q63270,Q6IG05
2108	1,2,5,6,7,9,10,11	3,4,8,12,13,14,15,16	3	P00762,P36860,P50116
2109	1,2,5,6,7,9,10,12	3,4,8,11,13,14,15,16	9	D3ZTX0,O54728,P00762,P19814,P29315,P36860,P50116,Q03191,Q6AY61
2110	1,2,5,6,7,9,10,13	3,4,8,11,12,14,15,16	5	D3ZTX0,P00762,P01835,P29315,P36860
2111	1,2,5,6,7,9,10,14	3,4,8,11,12,13,15,16	4	P00762,P29315,P50116,P52590
2112	1,2,5,6,7,9,10,15	3,4,8,11,12,13,14,16	6	D3ZTX0,O54728,P00762,P36860,Q811M5,Q99MH3
2113	1,2,5,6,7,9,10,16	3,4,8,11,12,13,14,15	3	D3ZTX0,P00762,Q9R168
2114	1,2,5,6,7,9,11,12	3,4,8,10,13,14,15,16	4	O35077,P00762,P50116,Q03191
2115	1,2,5,6,7,9,11,13	3,4,8,10,12,14,15,16	3	P00762,Q9EQS0,Q9R168
2116	1,2,5,6,7,9,11,14	3,4,8,10,12,13,15,16	6	P00762,P0DMW0;P0DMW1,P50116,P52590,Q5I0J9,Q9R168
2117	1,2,5,6,7,9,11,15	3,4,8,10,12,13,14,16	3	P00762,P02625,Q8CJ52
2118	1,2,5,6,7,9,11,16	3,4,8,10,12,13,14,15	5	P52590,Q9EQS0,Q9JHB9,Q9R168,Q9Z0J6
2119	1,2,5,6,7,9,12,13	3,4,8,10,11,14,15,16	7	P00762,P01835,P19814,P29315,P36860,Q03191,Q6AY61
2120	1,2,5,6,7,9,12,14	3,4,8,10,11,13,15,16	5	P00762,P29315,P52590,Q03191,Q6AY61
2121	1,2,5,6,7,9,12,15	3,4,8,10,11,13,14,16	6	O54728,P00762,P19814,P36860,Q03191,Q6AY61
2122	1,2,5,6,7,9,12,16	3,4,8,10,11,13,14,15	1	Q03191
2123	1,2,5,6,7,9,13,14	3,4,8,10,11,12,15,16	7	P00762,P01835,P0DMW0;P0DMW1,P29315,P52590,Q9EQS0,Q9R168
2124	1,2,5,6,7,9,13,15	3,4,8,10,11,12,14,16	5	O70417,P00762,P01835,P02625,P36860

2125	1,2,5,6,7,9,13,16	3,4,8,10,11,12,14,15	4	P01835,P52590,Q9EQS0,Q9R168
2126	1,2,5,6,7,9,14,15	3,4,8,10,11,12,13,16	4	P00762,P01835,P02625,P52590
2127	1,2,5,6,7,9,14,16	3,4,8,10,11,12,13,15	2	P52590,Q9R168
2128	1,2,5,6,7,9,15,16	3,4,8,10,11,12,13,14	0	
2129	1,2,5,6,7,10,11,12	3,4,8,9,13,14,15,16	5	P00762,P23593,P29315,P36860,P50116
2130	1,2,5,6,7,10,11,13	3,4,8,9,12,14,15,16	5	P00762,P01835,P29315,P36860,Q68G31
2131	1,2,5,6,7,10,11,14	3,4,8,9,12,13,15,16	4	P00762,P29315,P50116,P52590
2132	1,2,5,6,7,10,11,15	3,4,8,9,12,13,14,16	4	P00762,P08721,P17988,P36860
2133	1,2,5,6,7,10,11,16	3,4,8,9,12,13,14,15	1	Q9JHB9
2134	1,2,5,6,7,10,12,13	3,4,8,9,11,14,15,16	6	P00762,P01835,P19814,P29315,P36860,Q6AY61
2135	1,2,5,6,7,10,12,14	3,4,8,9,11,13,15,16	7	P00762,P01835,P19814,P29315,P52590,Q5RLM2,Q6AY61
2136	1,2,5,6,7,10,12,15	3,4,8,9,11,13,14,16	6	O54728,P00762,P01835,P19814,P36860,Q6AY61
2137	1,2,5,6,7,10,12,16	3,4,8,9,11,13,14,15	3	P01835,P57113,Q6AY61
2138	1,2,5,6,7,10,13,14	3,4,8,9,11,12,15,16	8	A2RUW1,O88797,P00762,P01835,P29315,P52590,Q62714,Q9WUW8
2139	1,2,5,6,7,10,13,15	3,4,8,9,11,12,14,16	9	A2RUW1,O70417,O88797,P00762,P01835,P19814,P36860,Q62714,Q63618
2140	1,2,5,6,7,10,13,16	3,4,8,9,11,12,14,15	4	P01835,P36860,P57113,Q9EQS0
2141	1,2,5,6,7,10,14,15	3,4,8,9,11,12,13,16	6	A2RUW1,P00762,P01835,P52590,P54921,Q62714
2142	1,2,5,6,7,10,14,16	3,4,8,9,11,12,13,15	5	P01835,P09527,P52590,P57113,Q9WUW8
2143	1,2,5,6,7,10,15,16	3,4,8,9,11,12,13,14	14	B2RYW9,O70594,P01835,P08721,P09527,P17988,P19468,P23928,P48508,P57113,Q03248,Q5I0E9,Q64335,Q71MB6
2144	1,2,5,6,7,11,12,13	3,4,8,9,10,14,15,16	4	P01835,P29315,P47967,Q5I0D1

2145	1,2,5,6,7,11,12,14	3,4,8,9,10,13,15,16	4	P00762,P29315,P52590,Q5GRG2
2146	1,2,5,6,7,11,12,15	3,4,8,9,10,13,14,16	1	P00762
2147	1,2,5,6,7,11,12,16	3,4,8,9,10,13,14,15	4	O35547,P07647,Q00715,Q9JHB9
2148	1,2,5,6,7,11,13,14	3,4,8,9,10,12,15,16	4	P00762,P01835,P29315,P52590
2149	1,2,5,6,7,11,13,15	3,4,8,9,10,12,14,16	4	O70417,P00762,P01835,P36860
2150	1,2,5,6,7,11,13,16	3,4,8,9,10,12,14,15	9	O70417,P01835,P07647,P09456,P22283,P52590,Q9EQS0,Q9JHB9,Q9Z0J6
2151	1,2,5,6,7,11,14,15	3,4,8,9,10,12,13,16	4	P00762,P01835,P52590,Q8CJ52
2152	1,2,5,6,7,11,14,16	3,4,8,9,10,12,13,15	4	P01039,P01835,P52590,Q9JHB9
2153	1,2,5,6,7,11,15,16	3,4,8,9,10,12,13,14	6	B2RYW9,O70417,P08721,P17988,Q8CJ52,Q9JHB9
2154	1,2,5,6,7,12,13,14	3,4,8,9,10,11,15,16	6	P00762,P01835,P19814,P29315,P52590,Q6AY61
2155	1,2,5,6,7,12,13,15	3,4,8,9,10,11,14,16	6	O70417,P00762,P01835,P19814,P36860,Q6AY61
2156	1,2,5,6,7,12,13,16	3,4,8,9,10,11,14,15	2	P01835,Q6AY61
2157	1,2,5,6,7,12,14,15	3,4,8,9,10,11,13,16	5	P00762,P01835,P19814,P52590,Q6AY61
2158	1,2,5,6,7,12,14,16	3,4,8,9,10,11,13,15	3	P01835,P52590,Q6AY61
2159	1,2,5,6,7,12,15,16	3,4,8,9,10,11,13,14	4	P01835,P17988,P19814,Q6AY61
2160	1,2,5,6,7,13,14,15	3,4,8,9,10,11,12,16	6	A2RUW1,O88797,P00762,P01835,P52590,Q62714
2161	1,2,5,6,7,13,14,16	3,4,8,9,10,11,12,15	5	P01835,P52590,Q62714,Q9EQS0,Q9WUW8
2162	1,2,5,6,7,13,15,16	3,4,8,9,10,11,12,14	5	B2RYW9,O70417,P01835,Q62714,Q9EQS0
2163	1,2,5,6,7,14,15,16	3,4,8,9,10,11,12,13	4	P01835,P17988,P52590,Q62714
2164	1,2,5,6,8,9,10,11	3,4,7,12,13,14,15,16	5	P25031,Q5I0J9,Q68G31,Q6P6S4,Q99MH3

2165	1,2,5,6,8,9,10,12	3,4,7,11,13,14,15,16	8	P00762,P02454,P19814,P25031,P29315,P36860,Q03191,Q99MH3
2166	1,2,5,6,8,9,10,13	3,4,7,11,12,14,15,16	5	D3ZTX0,P25031,P29315,P36860,Q68G31
2167	1,2,5,6,8,9,10,14	3,4,7,11,12,13,15,16	7	P00762,P25031,P29315,P52590,P97840,Q5I0J9,Q68G31
2168	1,2,5,6,8,9,10,15	3,4,7,11,12,13,14,16	7	P00762,P19132,P25031,P36860,Q68G31,Q811M5,Q99MH3
2169	1,2,5,6,8,9,10,16	3,4,7,11,12,13,14,15	2	P25031,P70545
2170	1,2,5,6,8,9,11,12	3,4,7,10,13,14,15,16	7	P61206;P84079,Q03191,Q5GRG2,Q5I0J9,Q5RKI1,Q6IG05,Q99MH3
2171	1,2,5,6,8,9,11,13	3,4,7,10,12,14,15,16	4	Q09326,Q5RKI1,Q68G31,Q9JI85
2172	1,2,5,6,8,9,11,14	3,4,7,10,12,13,15,16	4	P52590,Q5GRG2,Q5I0J9,Q5RKI1
2173	1,2,5,6,8,9,11,15	3,4,7,10,12,13,14,16	5	P19132,Q6IG05,Q6IMF3,Q6P6S4,Q99MH3
2174	1,2,5,6,8,9,11,16	3,4,7,10,12,13,14,15	5	P09456,Q5I0J9,Q5RKI1,Q812E4,Q9JHB9
2175	1,2,5,6,8,9,12,13	3,4,7,10,11,14,15,16	3	P29315,Q5RKI1,Q8CJD3
2176	1,2,5,6,8,9,12,14	3,4,7,10,11,13,15,16	5	P29315,P52590,Q5I0J9,Q5RKI1,Q8CJD3
2177	1,2,5,6,8,9,12,15	3,4,7,10,11,13,14,16	6	P19132,P19814,P36376,Q6IG05,Q8CJD3,Q99MH3
2178	1,2,5,6,8,9,12,16	3,4,7,10,11,13,14,15	2	Q5RKI1,Q8CJD3
2179	1,2,5,6,8,9,13,14	3,4,7,10,11,12,15,16	4	P29315,P52590,Q5RKI1,Q68G31
2180	1,2,5,6,8,9,13,15	3,4,7,10,11,12,14,16	2	P19132,Q68G31
2181	1,2,5,6,8,9,13,16	3,4,7,10,11,12,14,15	2	P52590,Q5RKI1
2182	1,2,5,6,8,9,14,15	3,4,7,10,11,12,13,16	4	P00714,P19132,P52590,P97840
2183	1,2,5,6,8,9,14,16	3,4,7,10,11,12,13,15	4	P52590,P97840,Q5I0J9,Q5RKI1
2184	1,2,5,6,8,9,15,16	3,4,7,10,11,12,13,14	1	P19132

2185	1,2,5,6,8,10,11,12	3,4,7,9,13,14,15,16	5 P25031,P29315,Q5GRG2,Q68G31,Q6IG05
2186	1,2,5,6,8,10,11,13	3,4,7,9,12,14,15,16	3 P25031,Q68G31,Q9JI85
2187	1,2,5,6,8,10,11,14	3,4,7,9,12,13,15,16	4 P52590,P63029,Q5GRG2,Q68G31
2188	1,2,5,6,8,10,11,15	3,4,7,9,12,13,14,16	3 Q68G31,Q6IG05,Q6IMF3
2189	1,2,5,6,8,10,11,16	3,4,7,9,12,13,14,15	4 P15399,P25031,Q68G31,Q9JHB9
2190	1,2,5,6,8,10,12,13	3,4,7,9,11,14,15,16	6 P02631,P19814,P25031,P29315,P36860,Q68G31
2191	1,2,5,6,8,10,12,14	3,4,7,9,11,13,15,16	6 P19814,P25031,P29315,P52590,Q5RLM2,Q68G31
2192	1,2,5,6,8,10,12,15	3,4,7,9,11,13,14,16	8 P19132,P19629,P19814,P25031,P36860,Q498D9,Q68G31,Q6IG05
2193	1,2,5,6,8,10,12,16	3,4,7,9,11,13,14,15	1 P25031
2194	1,2,5,6,8,10,13,14	3,4,7,9,11,12,15,16	5 P25031,P29315,P52590,Q62714,Q68G31
2195	1,2,5,6,8,10,13,15	3,4,7,9,11,12,14,16	7 P19132,P25031,P36860,Q498D9,Q62714,Q63618,Q68G31
2196	1,2,5,6,8,10,13,16	3,4,7,9,11,12,14,15	3 P15399,P25031,Q68G31
2197	1,2,5,6,8,10,14,15	3,4,7,9,11,12,13,16	7 P00714,P19132,P25031,P52590,P54921,Q62714,Q68G31
2198	1,2,5,6,8,10,14,16	3,4,7,9,11,12,13,15	6 P14668,P15399,P25031,P52590,P97580,Q68G31
2199	1,2,5,6,8,10,15,16	3,4,7,9,11,12,13,14	4 P15399,P19629,P25031,Q68G31
2200	1,2,5,6,8,11,12,13	3,4,7,9,10,14,15,16	7 P12020,Q09326,Q5GRG2,Q5RKI1,Q68G31,Q6IG05,Q9JI85
2201	1,2,5,6,8,11,12,14	3,4,7,9,10,13,15,16	7 P12020,P19218,P52590,Q5GRG2,Q5I0J9,Q5RKI1,Q6IG05
2202	1,2,5,6,8,11,12,15	3,4,7,9,10,13,14,16	3 Q5GRG2,Q6IG05,Q6IMF3
2203	1,2,5,6,8,11,12,16	3,4,7,9,10,13,14,15	10 O35547,P02782,P12020,P30120,Q00715,Q5GRG2,Q5RKI1,Q6IG05,Q9JHB9,Q9JI85
2204	1,2,5,6,8,11,13,14	3,4,7,9,10,12,15,16	6 P52590,Q09326,Q5GRG2,Q5RKI1,Q68G31,Q9JI85

2205	1,2,5,6,8,11,13,15	3,4,7,9,10,12,14,16	4	Q68G31,Q6IG05,Q6IMF3,Q6P6Q2
2206	1,2,5,6,8,11,13,16	3,4,7,9,10,12,14,15	9	P02782,P09456,P30120,P52590,Q5GRG2,Q5RKI1,Q68G31,Q9JHB9,Q9JI85
2207	1,2,5,6,8,11,14,15	3,4,7,9,10,12,13,16	8	P50280,P52590,Q5GRG2,Q68G31,Q6IFU8,Q6IG05,Q6IMF3,Q6P6Q2
2208	1,2,5,6,8,11,14,16	3,4,7,9,10,12,13,15	6	P09456,P50280,P52590,Q5GRG2,Q5RKI1,Q9JHB9
2209	1,2,5,6,8,11,15,16	3,4,7,9,10,12,13,14	5	P50280,Q6IFU8,Q6IG05,Q6IMF3,Q6P6Q2
2210	1,2,5,6,8,12,13,14	3,4,7,9,10,11,15,16	5	P19814,P29315,P52590,Q5RKI1,Q68G31
2211	1,2,5,6,8,12,13,15	3,4,7,9,10,11,14,16	4	P19132,P19814,Q68G31,Q6IG05
2212	1,2,5,6,8,12,13,16	3,4,7,9,10,11,14,15	1	Q5RKI1
2213	1,2,5,6,8,12,14,15	3,4,7,9,10,11,13,16	5	P00714,P19132,P19814,P52590,Q6IG05
2214	1,2,5,6,8,12,14,16	3,4,7,9,10,11,13,15	2	P52590,Q5RKI1
2215	1,2,5,6,8,12,15,16	3,4,7,9,10,11,13,14	2	P19132,Q6IG05
2216	1,2,5,6,8,13,14,15	3,4,7,9,10,11,12,16	4	P19132,P52590,Q62714,Q68G31
2217	1,2,5,6,8,13,14,16	3,4,7,9,10,11,12,15	4	P52590,Q5RKI1,Q62714,Q68G31
2218	1,2,5,6,8,13,15,16	3,4,7,9,10,11,12,14	2	Q62714,Q68G31
2219	1,2,5,6,8,14,15,16	3,4,7,9,10,11,12,13	2	P52590,Q62714
2220	1,2,5,6,9,10,11,12	3,4,7,8,13,14,15,16	6	B0BNN3,P00762,Q03191,Q5I0J9,Q99MH3,Q9Z2L0
2221	1,2,5,6,9,10,11,13	3,4,7,8,12,14,15,16	3	O70417,P00762,Q68G31
2222	1,2,5,6,9,10,11,14	3,4,7,8,12,13,15,16	2	P00762,Q5I0J9
2223	1,2,5,6,9,10,11,15	3,4,7,8,12,13,14,16	4	O70417,P00762,Q99MH3,Q9Z2L0
2224	1,2,5,6,9,10,11,16	3,4,7,8,12,13,14,15	3	D3ZUC6,Q5I0J9,Q9Z2L0

2225	1,2,5,6,9,10,12,13	3,4,7,8,11,14,15,16	6	D3ZTX0,P00762,P0C0A9,P29315,P36860,Q03191
2226	1,2,5,6,9,10,12,14	3,4,7,8,11,13,15,16	5	P00762,P29315,Q03191,Q5I0J9,Q62902
2227	1,2,5,6,9,10,12,15	3,4,7,8,11,13,14,16	10	B0BNN3,O54728,P00762,P19814,P30120,Q03191,Q5M8C6,Q811M5,Q99041,Q99MH3
2228	1,2,5,6,9,10,12,16	3,4,7,8,11,13,14,15	2	D3ZUC6,Q03191
2229	1,2,5,6,9,10,13,14	3,4,7,8,11,12,15,16	3	P00762,P29315,P52590
2230	1,2,5,6,9,10,13,15	3,4,7,8,11,12,14,16	7	O70417,P00762,P11883,P36860,Q5M8C6,Q811M5,Q99MH3
2231	1,2,5,6,9,10,13,16	3,4,7,8,11,12,14,15	4	D3ZUC6,O70417,P11883,Q9EQS0
2232	1,2,5,6,9,10,14,15	3,4,7,8,11,12,13,16	8	P00714,P00762,P02780,P22273,P30120,P97840,Q5M8C6,Q811M5
2233	1,2,5,6,9,10,14,16	3,4,7,8,11,12,13,15	1	P52590
2234	1,2,5,6,9,10,15,16	3,4,7,8,11,12,13,14	5	D3ZUC6,O70417,P00762,P11883,Q811M5
2235	1,2,5,6,9,11,12,13	3,4,7,8,10,14,15,16	6	O35077,O70417,P00762,Q03191,Q5I0J9,Q5RKI1
2236	1,2,5,6,9,11,12,14	3,4,7,8,10,13,15,16	8	O35077,P00762,P52590,Q03191,Q5I0J9,Q5RKI1,Q63424,Q64093
2237	1,2,5,6,9,11,12,15	3,4,7,8,10,13,14,16	7	B0BNN3,O35077,O70417,P00762,Q03191,Q5I0J9,Q99MH3
2238	1,2,5,6,9,11,12,16	3,4,7,8,10,13,14,15	4	O70417,Q03191,Q5I0J9,Q5RKI1
2239	1,2,5,6,9,11,13,14	3,4,7,8,10,12,15,16	7	P00762,P0DMW0;P0DMW1,P52590,Q5I0J9,Q5RKI1,Q64093,Q9EQS0
2240	1,2,5,6,9,11,13,15	3,4,7,8,10,12,14,16	3	O70417,P00762,Q99MH3
2241	1,2,5,6,9,11,13,16	3,4,7,8,10,12,14,15	4	D3ZUC6,O70417,Q5RKI1,Q9EQS0
2242	1,2,5,6,9,11,14,15	3,4,7,8,10,12,13,16	5	P00762,P02625,P0DMW0;P0DMW1,Q5I0J9,Q923V8
2243	1,2,5,6,9,11,14,16	3,4,7,8,10,12,13,15	3	P52590,Q5I0J9,Q5RKI1
2244	1,2,5,6,9,11,15,16	3,4,7,8,10,12,13,14	1	O70417

2245	1,2,5,6,9,12,13,14	3,4,7,8,10,11,15,16	6	P00762,P29315,P52590,Q03191,Q5I0J9,Q5RKI1
2246	1,2,5,6,9,12,13,15	3,4,7,8,10,11,14,16	5	O70417,P00762,P0C0A9,Q99041,Q99MH3
2247	1,2,5,6,9,12,13,16	3,4,7,8,10,11,14,15	4	O70417,Q03191,Q5RKI1,Q9EQS0
2248	1,2,5,6,9,12,14,15	3,4,7,8,10,11,13,16	4	P00714,P00762,P30120,Q5I0J9
2249	1,2,5,6,9,12,14,16	3,4,7,8,10,11,13,15	4	P52590,Q03191,Q5I0J9,Q5RKI1
2250	1,2,5,6,9,12,15,16	3,4,7,8,10,11,13,14	2	O70417,Q99041
2251	1,2,5,6,9,13,14,15	3,4,7,8,10,11,12,16	7	O70417,P00762,P01835,P02625,P0DMW0;P0DMW1,P52590,Q5M8C6
2252	1,2,5,6,9,13,14,16	3,4,7,8,10,11,12,15	3	P52590,Q5RKI1,Q9EQS0
2253	1,2,5,6,9,13,15,16	3,4,7,8,10,11,12,14	3	O70417,P11883,Q9EQS0
2254	1,2,5,6,9,14,15,16	3,4,7,8,10,11,12,13	4	P47967,P52590,P97840,Q5I0D1
2255	1,2,5,6,10,11,12,13	3,4,7,8,9,14,15,16	5	O70417,P20646,P23593,P35280,Q68G31
2256	1,2,5,6,10,11,12,14	3,4,7,8,9,13,15,16	2	P00762,P35280
2257	1,2,5,6,10,11,12,15	3,4,7,8,9,13,14,16	2	O70417,P00762
2258	1,2,5,6,10,11,12,16	3,4,7,8,9,13,14,15	1	P35280
2259	1,2,5,6,10,11,13,14	3,4,7,8,9,12,15,16	3	P00762,P35280,Q68G31
2260	1,2,5,6,10,11,13,15	3,4,7,8,9,12,14,16	3	O70417,P00762,Q68G31
2261	1,2,5,6,10,11,13,16	3,4,7,8,9,12,14,15	4	O70417,P35280,Q68G31,Q9EQS0
2262	1,2,5,6,10,11,14,15	3,4,7,8,9,12,13,16	1	P00762
2263	1,2,5,6,10,11,14,16	3,4,7,8,9,12,13,15	2	P35280,P52590
2264	1,2,5,6,10,11,15,16	3,4,7,8,9,12,13,14	2	O70417,P17988

2265	1,2,5,6,10,12,13,14	3,4,7,8,9,11,15,16	6	P00762,P01835,P19814,P20646,P29315,P35280
2266	1,2,5,6,10,12,13,15	3,4,7,8,9,11,14,16	7	O70417,P00762,P01835,P19814,P35280,P36860,Q812E4
2267	1,2,5,6,10,12,13,16	3,4,7,8,9,11,14,15	3	O70417,P01835,P35280
2268	1,2,5,6,10,12,14,15	3,4,7,8,9,11,13,16	5	P00714,P00762,P19814,P35280,Q62902
2269	1,2,5,6,10,12,14,16	3,4,7,8,9,11,13,15	3	P14668,P35280,P52590
2270	1,2,5,6,10,12,15,16	3,4,7,8,9,11,13,14	2	O70417,P35280
2271	1,2,5,6,10,13,14,15	3,4,7,8,9,11,12,16	5	O70417,P00762,P01835,P35280,Q68G31
2272	1,2,5,6,10,13,14,16	3,4,7,8,9,11,12,15	4	P01835,P14668,P35280,P52590
2273	1,2,5,6,10,13,15,16	3,4,7,8,9,11,12,14	4	O70417,P01835,P11883,P35280
2274	1,2,5,6,10,14,15,16	3,4,7,8,9,11,12,13	3	P35280,P52590,Q5U316
2275	1,2,5,6,11,12,13,14	3,4,7,8,9,10,15,16	4	P20646,P35280,P52590,Q5RKI1
2276	1,2,5,6,11,12,13,15	3,4,7,8,9,10,14,16	1	O70417
2277	1,2,5,6,11,12,13,16	3,4,7,8,9,10,14,15	4	O70417,P35280,Q5RKI1,Q9EQS0
2278	1,2,5,6,11,12,14,15	3,4,7,8,9,10,13,16	0	
2279	1,2,5,6,11,12,14,16	3,4,7,8,9,10,13,15	4	P35280,P52590,Q5I0J9,Q5RKI1
2280	1,2,5,6,11,12,15,16	3,4,7,8,9,10,13,14	1	O70417
2281	1,2,5,6,11,13,14,15	3,4,7,8,9,10,12,16	4	O70417,P01835,Q68G31,Q923V8
2282	1,2,5,6,11,13,14,16	3,4,7,8,9,10,12,15	6	O70417,P01835,P35280,P52590,Q5RKI1,Q9EQS0
2283	1,2,5,6,11,13,15,16	3,4,7,8,9,10,12,14	2	O70417,Q9EQS0
2284	1,2,5,6,11,14,15,16	3,4,7,8,9,10,12,13	3	O70417,P52590,Q923V8

2285	1,2,5,6,12,13,14,15	3,4,7,8,9,10,11,16	5 O70417,P01835,P19814,P35280,Q6P6R2
2286	1,2,5,6,12,13,14,16	3,4,7,8,9,10,11,15	5 P01835,P35280,P52590,Q5RKI1,Q6P6R2
2287	1,2,5,6,12,13,15,16	3,4,7,8,9,10,11,14	5 O70417,P01835,P35280,Q6B345,Q6P6R2
2288	1,2,5,6,12,14,15,16	3,4,7,8,9,10,11,13	3 P35280,P52590,Q6P6R2
2289	1,2,5,6,13,14,15,16	3,4,7,8,9,10,11,12	6 O70417,P01835,P35280,P52590,Q62714,Q6P6R2
2290	1,2,5,7,8,9,10,11	3,4,6,12,13,14,15,16	3 iRT-Kit_WR_fusion,P06760,P70545
2291	1,2,5,7,8,9,10,12	3,4,6,11,13,14,15,16	4 iRT-Kit_WR_fusion,P29315,P35745,Q03191
2292	1,2,5,7,8,9,10,13	3,4,6,11,12,14,15,16	3 P29315,Q66H69,Q9Z1F2
2293	1,2,5,7,8,9,10,14	3,4,6,11,12,13,15,16	3 P00762,P29315,Q9Z1F2
2294	1,2,5,7,8,9,10,15	3,4,6,11,12,13,14,16	6 iRT-Kit_WR_fusion,P00762,P19132,P35745,Q9Z0V6,Q9Z1F2
2295	1,2,5,7,8,9,10,16	3,4,6,11,12,13,14,15	2 iRT-Kit_WR_fusion,P70545
2296	1,2,5,7,8,9,11,12	3,4,6,10,13,14,15,16	4 iRT-Kit_WR_fusion,P06760,Q03191,Q812E4
2297	1,2,5,7,8,9,11,13	3,4,6,10,12,14,15,16	3 iRT-Kit_WR_fusion,P06760,Q66H69
2298	1,2,5,7,8,9,11,14	3,4,6,10,12,13,15,16	4 iRT-Kit_WR_fusion,P06760,P0DMW0;P0DMW1,Q812E4
2299	1,2,5,7,8,9,11,15	3,4,6,10,12,13,14,16	7 iRT-Kit_WR_fusion,P06760,P19132,Q6IFU8,Q6IMF3,Q6P6Q2,Q9Z0V6
2300	1,2,5,7,8,9,11,16	3,4,6,10,12,13,14,15	7 iRT-Kit_WR_fusion,P02782,P06760,P09456,P30120,Q812E4,Q9JHB9
2301	1,2,5,7,8,9,12,13	3,4,6,10,11,14,15,16	5 iRT-Kit_WR_fusion,P29315,Q03191,Q66H69,Q8CJD3
2302	1,2,5,7,8,9,12,14	3,4,6,10,11,13,15,16	3 iRT-Kit_WR_fusion,P29315,Q8CJD3
2303	1,2,5,7,8,9,12,15	3,4,6,10,11,13,14,16	6 iRT-Kit_WR_fusion,P19132,P19814,P35745,Q8CJD3,Q9Z0V6
2304	1,2,5,7,8,9,12,16	3,4,6,10,11,13,14,15	3 iRT-Kit_WR_fusion,Q03191,Q8CJD3

2305	1,2,5,7,8,9,13,14	3,4,6,10,11,12,15,16	1 P29315
2306	1,2,5,7,8,9,13,15	3,4,6,10,11,12,14,16	2 iRT-Kit_WR_fusion,P19132
2307	1,2,5,7,8,9,13,16	3,4,6,10,11,12,14,15	1 iRT-Kit_WR_fusion
2308	1,2,5,7,8,9,14,15	3,4,6,10,11,12,13,16	3 iRT-Kit_WR_fusion,P00714,P19132
2309	1,2,5,7,8,9,14,16	3,4,6,10,11,12,13,15	0
2310	1,2,5,7,8,9,15,16	3,4,6,10,11,12,13,14	3 iRT-Kit_WR_fusion,P19132,Q9Z0V6
2311	1,2,5,7,8,10,11,12	3,4,6,9,13,14,15,16	6 iRT-Kit_WR_fusion,P23593,P29315,P47967,Q5I0D1,Q66H69
2312	1,2,5,7,8,10,11,13	3,4,6,9,12,14,15,16	3 P25809,P29315,Q66H69
2313	1,2,5,7,8,10,11,14	3,4,6,9,12,13,15,16	3 P06760,P29315,Q66H69
2314	1,2,5,7,8,10,11,15	3,4,6,9,12,13,14,16	7 P19132,Q4FZU2,Q66H69,Q6IFU8,Q6IG05,Q6IMF3,Q6P6Q2
2315	1,2,5,7,8,10,11,16	3,4,6,9,12,13,14,15	6 P02782,P09456,P30120,Q00715,Q66H69,Q9JHB9
2316	1,2,5,7,8,10,12,13	3,4,6,9,11,14,15,16	6 iRT-Kit_WR_fusion,P19814,P25809,P29315,P47967,Q66H69
2317	1,2,5,7,8,10,12,14	3,4,6,9,11,13,15,16	6 iRT-Kit_WR_fusion,P19814,P29315,Q5RLM2,Q66H69,Q9Z1F2
2318	1,2,5,7,8,10,12,15	3,4,6,9,11,13,14,16	6 iRT-Kit_WR_fusion,P19132,P19814,P35745,Q66H69,Q9Z1F2
2319	1,2,5,7,8,10,12,16	3,4,6,9,11,13,14,15	3 iRT-Kit_WR_fusion,Q5RLM2,Q66H69
2320	1,2,5,7,8,10,13,14	3,4,6,9,11,12,15,16	6 P25809,P29315,Q62714,Q66H69,Q9WUW8,Q9Z1F2
2321	1,2,5,7,8,10,13,15	3,4,6,9,11,12,14,16	6 P19132,P36860,Q62714,Q63618,Q66H69,Q9Z1F2
2322	1,2,5,7,8,10,13,16	3,4,6,9,11,12,14,15	4 Q63618,Q64093,Q66H69,Q9WUW8
2323	1,2,5,7,8,10,14,15	3,4,6,9,11,12,13,16	5 P00714,P19132,P54921,Q62714,Q9Z1F2
2324	1,2,5,7,8,10,14,16	3,4,6,9,11,12,13,15	3 P57113,Q5RLM2,Q9WUW8

2325	1,2,5,7,8,10,15,16	3,4,6,9,11,12,13,14	12	B2RYW9,O70594,P19132,P19468,P48508,P53790,Q05175,Q3ZAV1,Q63424,Q64093,Q8R431,Q9WTW7
2326	1,2,5,7,8,11,12,13	3,4,6,9,10,14,15,16	6	iRT-Kit_WR_fusion,P29315,P47967,Q5I0D1,Q66H69,Q811M5
2327	1,2,5,7,8,11,12,14	3,4,6,9,10,13,15,16	7	iRT-Kit_WR_fusion,P06760,P11883,P29315,Q66H69,Q6IG05,Q811M5
2328	1,2,5,7,8,11,12,15	3,4,6,9,10,13,14,16	8	iRT-Kit_WR_fusion,P19132,Q4FZU2,Q66H69,Q6IFU8,Q6IG05,Q6IMF3,Q6P6Q2
2329	1,2,5,7,8,11,12,16	3,4,6,9,10,13,14,15	9	iRT-Kit_WR_fusion,P02782,P08723,P09456,P30120,Q00715,Q66H69,Q6IG05,Q9JHB9
2330	1,2,5,7,8,11,13,14	3,4,6,9,10,12,15,16	4	P06760,P29315,Q66H69,Q811M5
2331	1,2,5,7,8,11,13,15	3,4,6,9,10,12,14,16	7	iRT-Kit_WR_fusion,Q4FZU2,Q66H69,Q6IFU8,Q6IG05,Q6IMF3,Q6P6Q2
2332	1,2,5,7,8,11,13,16	3,4,6,9,10,12,14,15	7	iRT-Kit_WR_fusion,P02782,P08723,P09456,P30120,Q66H69,Q9JHB9
2333	1,2,5,7,8,11,14,15	3,4,6,9,10,12,13,16	7	P06760,P08649,Q4FZU2,Q6IFU8,Q6IG05,Q6IMF3,Q6P6Q2
2334	1,2,5,7,8,11,14,16	3,4,6,9,10,12,13,15	7	P02782,P08649,P09456,P30120,Q00715,Q812E4,Q9JHB9
2335	1,2,5,7,8,11,15,16	3,4,6,9,10,12,13,14	14	B2RYW9,iRT-Kit_WR_fusion,P02782,P08649,P08723,P09456,P30120,Q4FZU2,Q6IFU8,Q6IG05,Q6IMF3,Q6P6Q2,Q8CJ52,Q9JHB9
2336	1,2,5,7,8,12,13,14	3,4,6,9,10,11,15,16	5	iRT-Kit_WR_fusion,P19814,P29315,Q66H69,Q811M5
2337	1,2,5,7,8,12,13,15	3,4,6,9,10,11,14,16	4	iRT-Kit_WR_fusion,P19132,P19814,Q66H69
2338	1,2,5,7,8,12,13,16	3,4,6,9,10,11,14,15	2	iRT-Kit_WR_fusion,Q66H69
2339	1,2,5,7,8,12,14,15	3,4,6,9,10,11,13,16	5	iRT-Kit_WR_fusion,P00714,P19132,P19814,Q6IG05
2340	1,2,5,7,8,12,14,16	3,4,6,9,10,11,13,15	2	iRT-Kit_WR_fusion,Q5RLM2
2341	1,2,5,7,8,12,15,16	3,4,6,9,10,11,13,14	3	iRT-Kit_WR_fusion,P19132,Q6IG05
2342	1,2,5,7,8,13,14,15	3,4,6,9,10,11,12,16	2	P19132,Q62714
2343	1,2,5,7,8,13,14,16	3,4,6,9,10,11,12,15	2	Q62714,Q9WUW8
2344	1,2,5,7,8,13,15,16	3,4,6,9,10,11,12,14	3	B2RYW9,Q62714,Q66H69

2345	1,2,5,7,8,14,15,16	3,4,6,9,10,11,12,13	3	B2RYW9,P19132,Q62714
2346	1,2,5,7,9,10,11,12	3,4,6,8,13,14,15,16	2	P00762,Q03191
2347	1,2,5,7,9,10,11,13	3,4,6,8,12,14,15,16	2	P00762,Q9R168
2348	1,2,5,7,9,10,11,14	3,4,6,8,12,13,15,16	2	P00762,Q9R168
2349	1,2,5,7,9,10,11,15	3,4,6,8,12,13,14,16	1	P00762
2350	1,2,5,7,9,10,11,16	3,4,6,8,12,13,14,15	2	P70549,Q9R168
2351	1,2,5,7,9,10,12,13	3,4,6,8,11,14,15,16	5	P00762,P29315,Q03191,Q6AY61,Q9QZK9
2352	1,2,5,7,9,10,12,14	3,4,6,8,11,13,15,16	4	P00762,P29315,Q03191,Q6AY61
2353	1,2,5,7,9,10,12,15	3,4,6,8,11,13,14,16	6	O54728,P00762,P35745,Q03191,Q6AY61,Q99041
2354	1,2,5,7,9,10,12,16	3,4,6,8,11,13,14,15	4	Q03191,Q10743,Q6AY61,Q99041
2355	1,2,5,7,9,10,13,14	3,4,6,8,11,12,15,16	4	P00762,P29315,Q9QZK9,Q9R168
2356	1,2,5,7,9,10,13,15	3,4,6,8,11,12,14,16	3	O70417,P00762,Q8CFN2
2357	1,2,5,7,9,10,13,16	3,4,6,8,11,12,14,15	1	Q9R168
2358	1,2,5,7,9,10,14,15	3,4,6,8,11,12,13,16	4	P00714,P00762,P02625,P30120
2359	1,2,5,7,9,10,14,16	3,4,6,8,11,12,13,15	1	Q9R168
2360	1,2,5,7,9,10,15,16	3,4,6,8,11,12,13,14	0	
2361	1,2,5,7,9,11,12,13	3,4,6,8,10,14,15,16	4	O70417,P54921,Q03191,Q62761;Q62762;Q62763
2362	1,2,5,7,9,11,12,14	3,4,6,8,10,13,15,16	3	P00762,Q03191,Q62761;Q62762;Q62763
2363	1,2,5,7,9,11,12,15	3,4,6,8,10,13,14,16	3	P00762,Q03191,Q62761;Q62762;Q62763
2364	1,2,5,7,9,11,12,16	3,4,6,8,10,13,14,15	1	Q03191

2365	1,2,5,7,9,11,13,14	3,4,6,8,10,12,15,16	3 P00762,P0DMW0;P0DMW1,Q9R168
2366	1,2,5,7,9,11,13,15	3,4,6,8,10,12,14,16	4 O70417,P00762,P02625,P0DMW0;P0DMW1
2367	1,2,5,7,9,11,13,16	3,4,6,8,10,12,14,15	3 O70417,P54921,Q9R168
2368	1,2,5,7,9,11,14,15	3,4,6,8,10,12,13,16	4 P00762,P02625,P0DMW0;P0DMW1,Q9R168
2369	1,2,5,7,9,11,14,16	3,4,6,8,10,12,13,15	2 P70549,Q9R168
2370	1,2,5,7,9,11,15,16	3,4,6,8,10,12,13,14	2 O70417,Q9R168
2371	1,2,5,7,9,12,13,14	3,4,6,8,10,11,15,16	4 P00762,P29315,Q03191,Q6AY61
2372	1,2,5,7,9,12,13,15	3,4,6,8,10,11,14,16	5 O70417,P00762,Q03191,Q6AY61,Q99041
2373	1,2,5,7,9,12,13,16	3,4,6,8,10,11,14,15	2 Q03191,Q6AY61
2374	1,2,5,7,9,12,14,15	3,4,6,8,10,11,13,16	4 P00714,P00762,P02625,Q6AY61
2375	1,2,5,7,9,12,14,16	3,4,6,8,10,11,13,15	2 Q03191,Q6AY61
2376	1,2,5,7,9,12,15,16	3,4,6,8,10,11,13,14	3 Q03191,Q6AY61,Q99041
2377	1,2,5,7,9,13,14,15	3,4,6,8,10,11,12,16	4 P00762,P01835,P02625,P0DMW0;P0DMW1
2378	1,2,5,7,9,13,14,16	3,4,6,8,10,11,12,15	1 Q9R168
2379	1,2,5,7,9,13,15,16	3,4,6,8,10,11,12,14	2 O70417,Q9R168
2380	1,2,5,7,9,14,15,16	3,4,6,8,10,11,12,13	1 Q9R168
2381	1,2,5,7,10,11,12,13	3,4,6,8,9,14,15,16	3 P23593,P47967,Q5I0D1
2382	1,2,5,7,10,11,12,14	3,4,6,8,9,13,15,16	2 P00762,P23593
2383	1,2,5,7,10,11,12,15	3,4,6,8,9,13,14,16	2 P00762,P23593
2384	1,2,5,7,10,11,12,16	3,4,6,8,9,13,14,15	0

2385	1,2,5,7,10,11,13,14	3,4,6,8,9,12,15,16	1	P00762
2386	1,2,5,7,10,11,13,15	3,4,6,8,9,12,14,16	2	O70417,P00762
2387	1,2,5,7,10,11,13,16	3,4,6,8,9,12,14,15	1	O70417
2388	1,2,5,7,10,11,14,15	3,4,6,8,9,12,13,16	1	P00762
2389	1,2,5,7,10,11,14,16	3,4,6,8,9,12,13,15	1	Q9R168
2390	1,2,5,7,10,11,15,16	3,4,6,8,9,12,13,14	2	O70417,P17988
2391	1,2,5,7,10,12,13,14	3,4,6,8,9,11,15,16	6	P00762,P01835,P19814,P29315,Q6AY61,Q6P6S4
2392	1,2,5,7,10,12,13,15	3,4,6,8,9,11,14,16	5	O70417,P00762,P01835,P19814,Q6AY61
2393	1,2,5,7,10,12,13,16	3,4,6,8,9,11,14,15	2	P01835,Q6AY61
2394	1,2,5,7,10,12,14,15	3,4,6,8,9,11,13,16	5	P00714,P00762,P19814,Q62902,Q6AY61
2395	1,2,5,7,10,12,14,16	3,4,6,8,9,11,13,15	1	Q6AY61
2396	1,2,5,7,10,12,15,16	3,4,6,8,9,11,13,14	1	Q6AY61
2397	1,2,5,7,10,13,14,15	3,4,6,8,9,11,12,16	3	O88797,P00762,P01835
2398	1,2,5,7,10,13,14,16	3,4,6,8,9,11,12,15	4	P01835,P09527,Q6P6S4,Q9WUW8
2399	1,2,5,7,10,13,15,16	3,4,6,8,9,11,12,14	3	O70417,P01835,P09527
2400	1,2,5,7,10,14,15,16	3,4,6,8,9,11,12,13	1	P09527
2401	1,2,5,7,11,12,13,14	3,4,6,8,9,10,15,16	1	Q811M5
2402	1,2,5,7,11,12,13,15	3,4,6,8,9,10,14,16	1	O70417
2403	1,2,5,7,11,12,13,16	3,4,6,8,9,10,14,15	2	O70417,P47967
2404	1,2,5,7,11,12,14,15	3,4,6,8,9,10,13,16	0	

2405	1,2,5,7,11,12,14,16	3,4,6,8,9,10,13,15	0
2406	1,2,5,7,11,12,15,16	3,4,6,8,9,10,13,14	1 O70417
2407	1,2,5,7,11,13,14,15	3,4,6,8,9,10,12,16	2 O70417,P01835
2408	1,2,5,7,11,13,14,16	3,4,6,8,9,10,12,15	2 P01835,Q9R168
2409	1,2,5,7,11,13,15,16	3,4,6,8,9,10,12,14	1 O70417
2410	1,2,5,7,11,14,15,16	3,4,6,8,9,10,12,13	0
2411	1,2,5,7,12,13,14,15	3,4,6,8,9,10,11,16	3 P01835,P19814,Q6AY61
2412	1,2,5,7,12,13,14,16	3,4,6,8,9,10,11,15	3 P01835,Q6AY61,Q6P6S4
2413	1,2,5,7,12,13,15,16	3,4,6,8,9,10,11,14	3 O70417,P01835,Q6AY61
2414	1,2,5,7,12,14,15,16	3,4,6,8,9,10,11,13	1 Q6AY61
2415	1,2,5,7,13,14,15,16	3,4,6,8,9,10,11,12	3 O70417,P01835,Q62714
2416	1,2,5,8,9,10,11,12	3,4,6,7,13,14,15,16	1 Q03191
2417	1,2,5,8,9,10,11,13	3,4,6,7,12,14,15,16	0
2418	1,2,5,8,9,10,11,14	3,4,6,7,12,13,15,16	0
2419	1,2,5,8,9,10,11,15	3,4,6,7,12,13,14,16	3 P07943,Q6IMF3,Q99MH3
2420	1,2,5,8,9,10,11,16	3,4,6,7,12,13,14,15	1 P70549
2421	1,2,5,8,9,10,12,13	3,4,6,7,11,14,15,16	3 P29315,Q03191,Q99041
2422	1,2,5,8,9,10,12,14	3,4,6,7,11,13,15,16	3 P00714,P29315,Q99041
2423	1,2,5,8,9,10,12,15	3,4,6,7,11,13,14,16	7 P00714,P11598,P19132,P34901,P35745,Q99041,Q99MH3
2424	1,2,5,8,9,10,12,16	3,4,6,7,11,13,14,15	2 Q03191,Q99041

2425	1,2,5,8,9,10,13,14	3,4,6,7,11,12,15,16	1	P29315
2426	1,2,5,8,9,10,13,15	3,4,6,7,11,12,14,16	1	P19132
2427	1,2,5,8,9,10,13,16	3,4,6,7,11,12,14,15	0	
2428	1,2,5,8,9,10,14,15	3,4,6,7,11,12,13,16	5	P00714,P05369,P11598,P19132,P97840
2429	1,2,5,8,9,10,14,16	3,4,6,7,11,12,13,15	0	
2430	1,2,5,8,9,10,15,16	3,4,6,7,11,12,13,14	1	P19132
2431	1,2,5,8,9,11,12,13	3,4,6,7,10,14,15,16	2	Q03191,Q5RKI1
2432	1,2,5,8,9,11,12,14	3,4,6,7,10,13,15,16	2	Q03191,Q5RKI1
2433	1,2,5,8,9,11,12,15	3,4,6,7,10,13,14,16	6	P19132,P36376,Q03191,Q6IG05,Q6IMF3,Q99MH3
2434	1,2,5,8,9,11,12,16	3,4,6,7,10,13,14,15	3	O88797,Q03191,Q5RKI1
2435	1,2,5,8,9,11,13,14	3,4,6,7,10,12,15,16	2	P0DMW0;P0DMW1,Q5RKI1
2436	1,2,5,8,9,11,13,15	3,4,6,7,10,12,14,16	1	Q6IMF3
2437	1,2,5,8,9,11,13,16	3,4,6,7,10,12,14,15	1	Q5RKI1
2438	1,2,5,8,9,11,14,15	3,4,6,7,10,12,13,16	5	P00714,P07943,P0DMW0;P0DMW1,Q6IFU8,Q6IMF3
2439	1,2,5,8,9,11,14,16	3,4,6,7,10,12,13,15	2	Q5RKI1,Q812E4
2440	1,2,5,8,9,11,15,16	3,4,6,7,10,12,13,14	3	Q6IFU8,Q6IMF3,Q6P6Q2
2441	1,2,5,8,9,12,13,14	3,4,6,7,10,11,15,16	4	P17988,P29315,Q5RKI1,Q8CJD3
2442	1,2,5,8,9,12,13,15	3,4,6,7,10,11,14,16	4	P11598,P19132,Q8CJD3,Q99041
2443	1,2,5,8,9,12,13,16	3,4,6,7,10,11,14,15	3	Q03191,Q5RKI1,Q8CJD3
2444	1,2,5,8,9,12,14,15	3,4,6,7,10,11,13,16	5	P00714,P11598,P19132,Q8CJD3,Q99041

2445	1,2,5,8,9,12,14,16	3,4,6,7,10,11,13,15	2 Q5RKI1,Q8CJD3
2446	1,2,5,8,9,12,15,16	3,4,6,7,10,11,13,14	3 P19132,Q8CJD3,Q99041
2447	1,2,5,8,9,13,14,15	3,4,6,7,10,11,12,16	2 P00714,P19132
2448	1,2,5,8,9,13,14,16	3,4,6,7,10,11,12,15	1 Q5RKI1
2449	1,2,5,8,9,13,15,16	3,4,6,7,10,11,12,14	0
2450	1,2,5,8,9,14,15,16	3,4,6,7,10,11,12,13	3 P00714,P47967,P97840
2451	1,2,5,8,10,11,12,13	3,4,6,7,9,14,15,16	1 Q66H69
2452	1,2,5,8,10,11,12,14	3,4,6,7,9,13,15,16	0
2453	1,2,5,8,10,11,12,15	3,4,6,7,9,13,14,16	2 Q6IG05,Q6IMF3
2454	1,2,5,8,10,11,12,16	3,4,6,7,9,13,14,15	0
2455	1,2,5,8,10,11,13,14	3,4,6,7,9,12,15,16	1 Q68G31
2456	1,2,5,8,10,11,13,15	3,4,6,7,9,12,14,16	2 Q68G31,Q6IMF3
2457	1,2,5,8,10,11,13,16	3,4,6,7,9,12,14,15	0
2458	1,2,5,8,10,11,14,15	3,4,6,7,9,12,13,16	3 Q6IFU8,Q6IMF3,Q6P6Q2
2459	1,2,5,8,10,11,14,16	3,4,6,7,9,12,13,15	0
2460	1,2,5,8,10,11,15,16	3,4,6,7,9,12,13,14	3 Q6IFU8,Q6IMF3,Q6P6Q2
2461	1,2,5,8,10,12,13,14	3,4,6,7,9,11,15,16	1 P29315
2462	1,2,5,8,10,12,13,15	3,4,6,7,9,11,14,16	3 P19132,P19814,P22006
2463	1,2,5,8,10,12,13,16	3,4,6,7,9,11,14,15	0
2464	1,2,5,8,10,12,14,15	3,4,6,7,9,11,13,16	4 P00714,P19132,P19814,Q63617

2465	1,2,5,8,10, 12,14,16	3,4,6,7,9,1 1,13,15	0
2466	1,2,5,8,10, 12,15,16	3,4,6,7,9,1 1,13,14	2 P19132,Q5QE79
2467	1,2,5,8,10, 13,14,15	3,4,6,7,9,1 1,12,16	3 P00714,P19132,Q63617
2468	1,2,5,8,10, 13,14,16	3,4,6,7,9,1 1,12,15	0
2469	1,2,5,8,10, 13,15,16	3,4,6,7,9,1 1,12,14	0
2470	1,2,5,8,10, 14,15,16	3,4,6,7,9,1 1,12,13	1 Q63617
2471	1,2,5,8,11, 12,13,14	3,4,6,7,9,1 0,15,16	2 Q5RKI1,Q811M5
2472	1,2,5,8,11, 12,13,15	3,4,6,7,9,1 0,14,16	2 Q6IG05,Q6IMF3
2473	1,2,5,8,11, 12,13,16	3,4,6,7,9,1 0,14,15	2 P30120,Q5RKI1
2474	1,2,5,8,11, 12,14,15	3,4,6,7,9,1 0,13,16	4 Q6IFU8,Q6IG05,Q6IMF3,Q6P6Q2
2475	1,2,5,8,11, 12,14,16	3,4,6,7,9,1 0,13,15	1 Q5RKI1
2476	1,2,5,8,11, 12,15,16	3,4,6,7,9,1 0,13,14	5 Q5QE79,Q6IFU8,Q6IG05,Q6IMF3,Q6P6Q2
2477	1,2,5,8,11, 13,14,15	3,4,6,7,9,1 0,12,16	3 Q6IFU8,Q6IMF3,Q6P6Q2
2478	1,2,5,8,11, 13,14,16	3,4,6,7,9,1 0,12,15	2 P30120,Q5RKI1
2479	1,2,5,8,11, 13,15,16	3,4,6,7,9,1 0,12,14	4 O70417,Q6IFU8,Q6IMF3,Q6P6Q2
2480	1,2,5,8,11, 14,15,16	3,4,6,7,9,1 0,12,13	3 Q6IFU8,Q6IMF3,Q6P6Q2
2481	1,2,5,8,12, 13,14,15	3,4,6,7,9,1 0,11,16	3 P00714,P19132,P19814
2482	1,2,5,8,12, 13,14,16	3,4,6,7,9,1 0,11,15	1 Q5RKI1
2483	1,2,5,8,12, 13,15,16	3,4,6,7,9,1 0,11,14	0
2484	1,2,5,8,12, 14,15,16	3,4,6,7,9,1 0,11,13	1 P00714

2485	1,2,5,8,13,14,15,16	3,4,6,7,9,10,11,12	1	Q62714
2486	1,2,5,9,10,11,12,13	3,4,6,7,8,14,15,16	5	O70417,P00762,P20761,Q03191,Q62761;Q62762;Q62763
2487	1,2,5,9,10,11,12,14	3,4,6,7,8,13,15,16	4	P00762,P20761,Q03191,Q62761;Q62762;Q62763
2488	1,2,5,9,10,11,12,15	3,4,6,7,8,13,14,16	9	B0BNN3,O70417,P00762,P20761,Q03191,Q5QE79,Q62761;Q62762;Q62763,Q99MH3,Q9Z2L0
2489	1,2,5,9,10,11,12,16	3,4,6,7,8,13,14,15	5	O70417,P70549,Q03191,Q5QE79,Q9Z2L0
2490	1,2,5,9,10,11,13,14	3,4,6,7,8,12,15,16	2	O70417,P00762
2491	1,2,5,9,10,11,13,15	3,4,6,7,8,12,14,16	2	O70417,P00762
2492	1,2,5,9,10,11,13,16	3,4,6,7,8,12,14,15	2	D3ZUC6,O70417
2493	1,2,5,9,10,11,14,15	3,4,6,7,8,12,13,16	2	P00714,P00762
2494	1,2,5,9,10,11,14,16	3,4,6,7,8,12,13,15	3	P70549,Q9R168,Q9Z2L0
2495	1,2,5,9,10,11,15,16	3,4,6,7,8,12,13,14	3	O70417,Q5QE79,Q9Z2L0
2496	1,2,5,9,10,12,13,14	3,4,6,7,8,11,15,16	20	P00762,P02780,P02781,P02782,P06761,P08723,P0C0A9,P11598,P22273,P22282,P22283,P46462,Q03191,Q5M8C6,Q62902,Q63617,Q8CJ52,Q99041,Q9QW07,Q9QZK9
2497	1,2,5,9,10,12,13,15	3,4,6,7,8,11,14,16	24	O70417,P00762,P02780,P02781,P02782,P06761,P07150,P07647,P08723,P0C0A9,P11598,P20761,P22273,P22282,P22283,P30120,P46462,Q03191,Q5M8C6,Q62902,Q63617,Q8CFN2,Q99041,Q9QZK9
2498	1,2,5,9,10,12,13,16	3,4,6,7,8,11,14,15	9	O70417,P06761,P07150,P22273,P22282,Q03191,Q5M8C6,Q63617,Q99041
2499	1,2,5,9,10,12,14,15	3,4,6,7,8,11,13,16	21	P00714,P00762,P02780,P02781,P02782,P06761,P07647,P08723,P09456,P0C0A9,P11598,P22273,P22282,P22283,P24368,P30120,P46462,Q5M8C6,Q62902,Q63617,Q99041
2500	1,2,5,9,10,12,14,16	3,4,6,7,8,11,13,15	9	P06761,P11598,P22273,P22282,Q03191,Q5M8C6,Q62902,Q63617,Q99041
2501	1,2,5,9,10,12,15,16	3,4,6,7,8,11,13,14	13	O70417,P02780,P06761,P11598,P22273,P22282,P46462,Q03191,Q5M8C6,Q5QE79,Q62902,Q63617,Q99041
2502	1,2,5,9,10,13,14,15	3,4,6,7,8,11,12,16	26	O70417,P00714,P00762,P02780,P02781,P02782,P06761,P07647,P08723,P0C0A9,P11598,P12020,P22273,P22282,P22283,P30120,P36374,P46462,Q5M8C6,Q62902,Q63617,Q8CFN2,Q99041,Q9QW07,Q9QZK9,Q9R0T3
2503	1,2,5,9,10,13,14,16	3,4,6,7,8,11,12,15	7	O70417,P02780,P06761,P22273,P22282,Q5M8C6,Q63617

2504	1,2,5,9,10,13,15,16	3,4,6,7,8,11,12,14	15	O70417,P02780,P06761,P06911,P07150,P11598,P12020,P22273,P22282,P46462,Q5M8C6,Q5QE79,Q63617,Q63751,Q99041
2505	1,2,5,9,10,14,15,16	3,4,6,7,8,11,12,13	15	P00714,P02780,P06761,P11598,P12020,P22273,P22282,P22283,P46462,P47967,Q5M8C6,Q62902,Q63617,Q99041,Q9R0T3
2506	1,2,5,9,11,12,13,14	3,4,6,7,8,10,15,16	32	O35077,O70257,O70377,O70417,O70594,P07151,P0DMW0;P0DMW1,P19468,P20761,P46844,P53790,P57113,Q03191,Q03248,Q05175,Q3ZAV1,Q5I0E9,Q5M7T9,Q5RKI1,Q62761;Q62762;Q62763,Q63270,Q63424,Q63598,Q64093,Q6AY41,Q6MG61,Q6Q0N1,Q80W57,Q8R431,Q923S2,Q9WTW7,Q9Z0W7
2507	1,2,5,9,11,12,13,15	3,4,6,7,8,10,14,16	10	O35077,O70417,P07151,P20761,P46844,P57113,Q03191,Q5QE79,Q62761;Q62762;Q62763,Q923S2
2508	1,2,5,9,11,12,13,16	3,4,6,7,8,10,14,15	5	O70417,P54921,Q03191,Q05175,Q5RKI1
2509	1,2,5,9,11,12,14,15	3,4,6,7,8,10,13,16	19	O35077,O55004,O70257,O70594,P00714,P07151,P19468,P20761,P28570,P46844,P53790,P57113,Q03191,Q3ZAV1,Q5M7T9,Q62761;Q62762;Q62763,Q63424,Q64093,Q8R431
2510	1,2,5,9,11,12,14,16	3,4,6,7,8,10,13,15	5	O70257,O70594,Q03191,Q5RKI1,Q63618
2511	1,2,5,9,11,12,15,16	3,4,6,7,8,10,13,14	4	O70257,O70417,Q03191,Q5QE79
2512	1,2,5,9,11,13,14,15	3,4,6,7,8,10,12,16	9	O70417,P02625,P07151,P0DMW0;P0DMW1,P46844,P53790,P57113,Q03248,Q9WTW7
2513	1,2,5,9,11,13,14,16	3,4,6,7,8,10,12,15	6	O70417,P0DMW0;P0DMW1,P30904,Q5RKI1,Q63598,Q9R168
2514	1,2,5,9,11,13,15,16	3,4,6,7,8,10,12,14	3	O70417,Q5QE79,Q63751
2515	1,2,5,9,11,14,15,16	3,4,6,7,8,10,12,13	2	O70417,Q923V8
2516	1,2,5,9,12,13,14,15	3,4,6,7,8,10,11,16	22	O70417,P00714,P00762,P02780,P02781,P02782,P06761,P07151,P07647,P08723,P0C0A9,P11598,P22273,P22282,P22283,P28570,P46462,Q5M8C6,Q62902,Q63617,Q99041,Q9R0T3
2517	1,2,5,9,12,13,14,16	3,4,6,7,8,10,11,15	9	O70417,P06761,P22273,P22282,Q03191,Q5RKI1,Q63617,Q6P6R2,Q99041
2518	1,2,5,9,12,13,15,16	3,4,6,7,8,10,11,14	15	O70417,P06761,P07150,P08937,P11598,P22273,P22282,Q03191,Q5M8C6,Q5QE79,Q63617,Q63751,Q6B345,Q6P6R2,Q99041
2519	1,2,5,9,12,14,15,16	3,4,6,7,8,10,11,13	13	P00714,P02780,P06761,P11598,P22273,P22282,P22283,Q5M8C6,Q62902,Q63617,Q6P6R2,Q99041,Q9R0T3
2520	1,2,5,9,13,14,15,16	3,4,6,7,8,10,11,12	10	O70417,P02780,P06761,P11598,P22273,P22282,Q5M8C6,Q63617,Q99041,Q9R0T3
2521	1,2,5,10,11,12,13,14	3,4,6,7,8,9,15,16	4	O70417,P20646,P23593,P35280
2522	1,2,5,10,11,12,13,15	3,4,6,7,8,9,14,16	4	O70417,P20761,P23593,Q5QE79

2523	1,2,5,10,11,12,13,16	3,4,6,7,8,9,14,15	3	O70417,P35280,Q5QE79
2524	1,2,5,10,11,12,14,15	3,4,6,7,8,9,13,16	1	Q62902
2525	1,2,5,10,11,12,14,16	3,4,6,7,8,9,13,15	1	P35280
2526	1,2,5,10,11,12,15,16	3,4,6,7,8,9,13,14	3	O70417,Q5QE79,Q63751
2527	1,2,5,10,11,13,14,15	3,4,6,7,8,9,12,16	1	O70417
2528	1,2,5,10,11,13,14,16	3,4,6,7,8,9,12,15	2	O70417,P35280
2529	1,2,5,10,11,13,15,16	3,4,6,7,8,9,12,14	3	O70417,Q5QE79,Q63751
2530	1,2,5,10,11,14,15,16	3,4,6,7,8,9,12,13	2	O70417,Q5QE79
2531	1,2,5,10,12,13,14,15	3,4,6,7,8,9,11,16	22	O70417,P00714,P02780,P02781,P02782,P06761,P07150,P08723,P0C0A9,P11598,P22006,P22273,P22282,P22283,P35280,P46462,Q5M8C6,Q62902,Q63617,Q6P6R2,Q812E4,Q99041
2532	1,2,5,10,12,13,14,16	3,4,6,7,8,9,11,15	8	P06761,P07150,P22273,P22282,P35280,Q63617,Q6P6R2,Q6P6S4
2533	1,2,5,10,12,13,15,16	3,4,6,7,8,9,11,14	12	O70417,P06761,P07150,P22273,P22282,P35280,Q5M8C6,Q5QE79,Q63617,Q63751,Q6P6R2,Q99041
2534	1,2,5,10,12,14,15,16	3,4,6,7,8,9,11,13	14	P00714,P02780,P06761,P11598,P22273,P22282,P35280,P46462,Q5M8C6,Q5QE79,Q62902,Q63617,Q6P6R2,Q99041
2535	1,2,5,10,13,14,15,16	3,4,6,7,8,9,11,12	12	O70417,P02780,P06761,P07150,P22273,P22282,P35280,P70709,Q5M8C6,Q63617,Q6P6R2,Q6Q7Y5
2536	1,2,5,11,12,13,14,15	3,4,6,7,8,9,10,16	3	O70417,P23739,Q6P6R2
2537	1,2,5,11,12,13,14,16	3,4,6,7,8,9,10,15	4	O70417,P35280,Q5RKI1,Q6P6R2
2538	1,2,5,11,12,13,15,16	3,4,6,7,8,9,10,14	5	O70417,P08937,Q5QE79,Q63751,Q6P6R2
2539	1,2,5,11,12,14,15,16	3,4,6,7,8,9,10,13	3	O70417,Q5QE79,Q6P6R2
2540	1,2,5,11,13,14,15,16	3,4,6,7,8,9,10,12	4	O70417,P26772,Q5QE79,Q6P6R2
2541	1,2,5,12,13,14,15,16	3,4,6,7,8,9,10,11	12	O70417,P06761,P07150,P22273,P22282,P35280,Q5M8C6,Q5QE79,Q63617,Q6B345,Q6P6R2,Q6P6S4
2542	1,2,6,7,8,9,10,11	3,4,5,12,13,14,15,16	17	iRT-Kit_WR_fusion,P02631,P08723,P18421,P19629,P29315,P31044,P50280,P55091,P70545,Q5GRG2,Q68G31,Q6P6R2,Q6P6S4,Q78P75,Q812E4,Q9JI85

2543	1,2,6,7,8,9,10,12	3,4,5,11,13,14,15,16	8	iRT-Kit_WR_fusion,P02454,P02631,P19629,P29315,P35745,Q63598,Q9Z1F2
2544	1,2,6,7,8,9,10,13	3,4,5,11,12,14,15,16	8	iRT-Kit_WR_fusion,P02454,P02631,P19629,P29315,Q68G31,Q78P75,Q9Z1F2
2545	1,2,6,7,8,9,10,14	3,4,5,11,12,13,15,16	8	iRT-Kit_WR_fusion,P00762,P02631,P19629,P29315,P97840,Q68G31,Q9Z1F2
2546	1,2,6,7,8,9,10,15	3,4,5,11,12,13,14,16	10	iRT-Kit_WR_fusion,P00762,P02631,P19629,P35745,Q63474,Q63598,Q68G31,Q99MH3,Q9Z1F2
2547	1,2,6,7,8,9,10,16	3,4,5,11,12,13,14,15	6	D3ZUC6,iRT-Kit_WR_fusion,P02631,P19629,P70545,Q63598
2548	1,2,6,7,8,9,11,12	3,4,5,10,13,14,15,16	15	iRT-Kit_WR_fusion,P06911,P08723,P12020,P19629,P50280,Q09326,Q5GRG2,Q63493,Q6P6S4,Q78P75,Q812E4,Q9JHB9,Q9JI85,Q9QW07
2549	1,2,6,7,8,9,11,13	3,4,5,10,12,14,15,16	16	iRT-Kit_WR_fusion,P06911,P08723,P09456,P12020,P22283,P50280,Q09326,Q5GRG2,Q63493,Q63617,Q68G31,Q6P6S4,Q78P75,Q812E4,Q9JI85
2550	1,2,6,7,8,9,11,14	3,4,5,10,12,13,15,16	12	iRT-Kit_WR_fusion,P06911,P09456,P0DMW0;P0DMW1,P12020,P50280,Q09326,Q5GRG2,Q6P6S4,Q78P75,Q812E4,Q9JI85
2551	1,2,6,7,8,9,11,15	3,4,5,10,12,13,14,16	13	iRT-Kit_WR_fusion,P08723,P19629,P50280,Q09326,Q5GRG2,Q63493,Q6P6S4,Q78P75,Q812E4,Q8CJ52,Q99MH3,Q9QW07
2552	1,2,6,7,8,9,11,16	3,4,5,10,12,13,14,15	25	iRT-Kit_WR_fusion,P02780,P02781,P02782,P06911,P07647,P08723,P09456,P12020,P19629,P22283,P24368,P30120,P46462,P50280,P97523,Q5GRG2,Q63493,Q63617,Q6P6S4,Q78P75,Q812E4,Q9JHB9,Q9JI85,Q9QW07
2553	1,2,6,7,8,9,12,13	3,4,5,10,11,14,15,16	4	iRT-Kit_WR_fusion,P02631,P29315,Q78P75
2554	1,2,6,7,8,9,12,14	3,4,5,10,11,13,15,16	4	iRT-Kit_WR_fusion,P06911,P29315,Q5GRG2
2555	1,2,6,7,8,9,12,15	3,4,5,10,11,13,14,16	4	iRT-Kit_WR_fusion,P19629,P35745,Q99MH3
2556	1,2,6,7,8,9,12,16	3,4,5,10,11,13,14,15	3	iRT-Kit_WR_fusion,P19629,P21674
2557	1,2,6,7,8,9,13,14	3,4,5,10,11,12,15,16	6	iRT-Kit_WR_fusion,P01835,P0DMW0;P0DMW1,P29315,Q68G31,Q78P75
2558	1,2,6,7,8,9,13,15	3,4,5,10,11,12,14,16	4	iRT-Kit_WR_fusion,P19629,Q68G31,Q78P75
2559	1,2,6,7,8,9,13,16	3,4,5,10,11,12,14,15	3	iRT-Kit_WR_fusion,P02631,P19629

2560	1,2,6,7,8,9,14,15	3,4,5,10,11,12,13,16	3	iRT-Kit_WR_fusion,P19629,P97840
2561	1,2,6,7,8,9,14,16	3,4,5,10,11,12,13,15	2	iRT-Kit_WR_fusion,P97840
2562	1,2,6,7,8,9,15,16	3,4,5,10,11,12,13,14	2	iRT-Kit_WR_fusion,P19629
2563	1,2,6,7,8,10,11,12	3,4,5,9,13,14,15,16	18	iRT-Kit_WR_fusion,P02631,P06911,P08723,P12020,P19629,P22283,P29315,P31044,P50280,Q00715,Q5GRG2,Q63493,Q66H69,Q68G31,Q78P75,Q9JHB9,Q9JI85
2564	1,2,6,7,8,10,11,13	3,4,5,9,12,14,15,16	17	iRT-Kit_WR_fusion,P02631,P06911,P08723,P12020,P19629,P22283,P29315,P31044,P50280,P55091,Q5GRG2,Q63493,Q66H69,Q68G31,Q78P75,Q9JI85
2565	1,2,6,7,8,10,11,14	3,4,5,9,12,13,15,16	10	P02631,P06911,P12020,P19629,P29315,P31044,P50280,Q5GRG2,Q68G31,Q9JI85
2566	1,2,6,7,8,10,11,15	3,4,5,9,12,13,14,16	12	iRT-Kit_WR_fusion,P08723,P15399,P19629,P31044,P50280,Q5GRG2,Q63474,Q68G31,Q8CJ52,Q9JI85,Q9Z1F2
2567	1,2,6,7,8,10,11,16	3,4,5,9,12,13,14,15	27	iRT-Kit_WR_fusion,P02631,P02780,P02781,P02782,P06911,P07647,P08723,P09456,P0C0A9,P12020,P15399,P19629,P22283,P24368,P30120,P31044,P46462,P50280,Q00715,Q5GRG2,Q63493,Q66H69,Q68G31,Q9JHB9,Q9JI85,Q9QW07
2568	1,2,6,7,8,10,12,13	3,4,5,9,11,14,15,16	11	iRT-Kit_WR_fusion,P01835,P02631,P19629,P19814,P29315,P31044,Q63598,Q66H69,Q68G31,Q9Z1F2
2569	1,2,6,7,8,10,12,14	3,4,5,9,11,13,15,16	10	iRT-Kit_WR_fusion,P02631,P19629,P29315,P31044,P57113,Q5GRG2,Q5RLM2,Q68G31,Q9Z1F2
2570	1,2,6,7,8,10,12,15	3,4,5,9,11,13,14,16	10	iRT-Kit_WR_fusion,P02631,P19629,P19814,P31044,P35745,Q63474,Q63598,Q66H69,Q9Z1F2
2571	1,2,6,7,8,10,12,16	3,4,5,9,11,13,14,15	10	iRT-Kit_WR_fusion,P02631,P19629,P31044,P46844,P57113,Q3ZAV1,Q63598,Q66H69,Q9WTW7
2572	1,2,6,7,8,10,13,14	3,4,5,9,11,12,15,16	11	P01835,P02631,P19629,P25809,P29315,P31044,P57113,Q62714,Q66H69,Q68G31,Q9Z1F2
2573	1,2,6,7,8,10,13,15	3,4,5,9,11,12,14,16	15	iRT-Kit_WR_fusion,P01835,P02631,P19629,P31044,P36860,P51907,P57113,Q62714,Q63474,Q63598,Q63618,Q66H69,Q68G31,Q9Z1F2
2574	1,2,6,7,8,10,13,16	3,4,5,9,11,12,14,15	18	iRT-Kit_WR_fusion,O70257,P01835,P02631,P15399,P19468,P19629,P31044,P46844,P51907,P57113,Q3ZAV1,Q63424,Q63598,Q63618,Q64093,Q66H69,Q68G31
2575	1,2,6,7,8,10,14,15	3,4,5,9,11,12,13,16	9	P19629,P31044,P54921,P57113,Q62714,Q63474,Q63598,Q68G31,Q9Z1F2

2576	1,2,6,7,8,10,14,16	3,4,5,9,11,12,13,15	9	P02631,P15399,P19629,P31044,P57113,P97580,Q30KJ2,Q63598,Q68G31
2577	1,2,6,7,8,10,15,16	3,4,5,9,11,12,13,14	29	B2RYW9,iRT-Kit_WR_fusion,O70377,O70594,P02631,P15399,P19468,P19629,P31044,P38918,P46844,P48508,P50280,P51907,P53790,P57113,Q03248,Q05175,Q3ZAV1,Q5I0E9,Q5M7T9,Q63424,Q63598,Q64093,Q68G31,Q6AY41,Q9R1T5,Q9WTW7,Q9Z0W7
2578	1,2,6,7,8,11,12,13	3,4,5,9,10,14,15,16	19	iRT-Kit_WR_fusion,P02631,P06911,P08723,P09456,P12020,P22283,P29315,P31044,P46462,P50280,Q09326,Q5GRG2,Q63493,Q66H69,Q68G31,Q78P75,Q9JHB9,Q9JI85
2579	1,2,6,7,8,11,12,14	3,4,5,9,10,13,15,16	16	iRT-Kit_WR_fusion,P06911,P08723,P12020,P22283,P29315,P31044,P46462,P50280,Q09326,Q5GRG2,Q63493,Q78P75,Q9JHB9,Q9JI85,Q9QW07
2580	1,2,6,7,8,11,12,15	3,4,5,9,10,13,14,16	17	iRT-Kit_WR_fusion,P06911,P08723,P12020,P19629,P22283,P31044,P50280,Q09326,Q5GRG2,Q63493,Q66H69,Q78P75,Q8CJ52,Q9JHB9,Q9JI85,Q9QW07
2581	1,2,6,7,8,11,12,16	3,4,5,9,10,13,14,15	31	iRT-Kit_WR_fusion,O35547,P02780,P02781,P02782,P04905,P05369,P06761,P06911,P07647,P08723,P09456,P0C0A9,P12020,P19629,P22283,P24368,P30120,P31044,P46462,P50280,Q00715,Q5GRG2,Q5M8C6,Q63493,Q66H69,Q812E4,Q8CFN2,Q9JHB9,Q9JI85,Q9QW07
2582	1,2,6,7,8,11,13,14	3,4,5,9,10,12,15,16	18	iRT-Kit_WR_fusion,P01835,P06911,P08723,P09456,P12020,P22283,P29315,P31044,P46462,P50280,Q09326,Q5GRG2,Q63493,Q66H69,Q68G31,Q78P75,Q9JI85
2583	1,2,6,7,8,11,13,15	3,4,5,9,10,12,14,16	17	iRT-Kit_WR_fusion,P01835,P06911,P08723,P12020,P19629,P22283,P31044,P50280,Q09326,Q5GRG2,Q63493,Q66H69,Q68G31,Q78P75,Q8CJ52,Q9JI85
2584	1,2,6,7,8,11,13,16	3,4,5,9,10,12,14,15	31	iRT-Kit_WR_fusion,O35547,P02780,P02781,P02782,P05369,P06761,P06911,P07647,P08723,P09456,P0C0A9,P12020,P19629,P22283,P24368,P30120,P31044,P46462,P50280,Q00715,Q5GRG2,Q5M8C6,Q63493,Q63617,Q66H69,Q68G31,Q78P75,Q812E4,Q9JHB9,Q9JI85
2585	1,2,6,7,8,11,14,15	3,4,5,9,10,12,13,16	13	iRT-Kit_WR_fusion,P06911,P12020,P19629,P31044,P50280,Q09326,Q5GRG2,Q68G31,Q78P75,Q8CJ52,Q9JI85,Q9QW07
2586	1,2,6,7,8,11,14,16	3,4,5,9,10,12,13,15	21	iRT-Kit_WR_fusion,P02780,P02781,P02782,P06911,P07647,P08723,P09456,P0C0A9,P12020,P19629,P22283,P30120,P46462,P50280,Q00715,Q5GRG2,Q812E4,Q9JHB9,Q9JI85,Q9QW07
2587	1,2,6,7,8,11,15,16	3,4,5,9,10,12,13,14	23	B2RYW9,iRT-Kit_WR_fusion,P02780,P02781,P02782,P06911,P07647,P08723,P09456,P0C0A9,P12020,P19629,P22283,P24368,P30120,P46462,P50280,Q5GRG2,Q63493,Q8CJ52,Q9JHB9,Q9JI85,Q9QW07
2588	1,2,6,7,8,12,13,14	3,4,5,9,10,11,15,16	11	iRT-Kit_WR_fusion,P01835,P02631,P06911,P12020,P29315,P31044,Q5GRG2,Q66H69,Q68G31,Q9Z2L0

2589	1,2,6,7,8,12,13,15	3,4,5,9,10,11,14,16	8	iRT-Kit_WR_fusion,P01835,P02631,P19629,P19814,P31044,Q66H69,Q68G31
2590	1,2,6,7,8,12,13,16	3,4,5,9,10,11,14,15	8	iRT-Kit_WR_fusion,P01835,P02631,P06911,P12020,P19629,Q66H69,Q9JI85
2591	1,2,6,7,8,12,14,15	3,4,5,9,10,11,13,16	3	iRT-Kit_WR_fusion,P19629,Q5GRG2
2592	1,2,6,7,8,12,14,16	3,4,5,9,10,11,13,15	5	iRT-Kit_WR_fusion,P06911,P12020,P19629,Q5GRG2
2593	1,2,6,7,8,12,15,16	3,4,5,9,10,11,13,14	3	iRT-Kit_WR_fusion,P19629,Q63598
2594	1,2,6,7,8,13,14,15	3,4,5,9,10,11,12,16	5	iRT-Kit_WR_fusion,P01835,P19629,Q62714,Q68G31
2595	1,2,6,7,8,13,14,16	3,4,5,9,10,11,12,15	3	P01835,Q62714,Q68G31
2596	1,2,6,7,8,13,15,16	3,4,5,9,10,11,12,14	6	B2RYW9,iRT-Kit_WR_fusion,P01835,P19629,Q62714,Q68G31
2597	1,2,6,7,8,14,15,16	3,4,5,9,10,11,12,13	5	B2RYW9,iRT-Kit_WR_fusion,P19629,P50280,Q62714
2598	1,2,6,7,9,10,11,12	3,4,5,8,13,14,15,16	2	O35077,P00762
2599	1,2,6,7,9,10,11,13	3,4,5,8,12,14,15,16	3	P00762,Q68G31,Q9R168
2600	1,2,6,7,9,10,11,14	3,4,5,8,12,13,15,16	2	P00762,Q9R168
2601	1,2,6,7,9,10,11,15	3,4,5,8,12,13,14,16	2	P00762,Q99MH3
2602	1,2,6,7,9,10,11,16	3,4,5,8,12,13,14,15	2	D3ZUC6,Q9R168
2603	1,2,6,7,9,10,12,13	3,4,5,8,11,14,15,16	5	P00762,P01835,P29315,Q6IFW6,Q6IG02
2604	1,2,6,7,9,10,12,14	3,4,5,8,11,13,15,16	4	P00762,P29315,Q6IFW6,Q6IG02
2605	1,2,6,7,9,10,12,15	3,4,5,8,11,13,14,16	3	O54728,P00762,Q99MH3
2606	1,2,6,7,9,10,12,16	3,4,5,8,11,13,14,15	2	D3ZUC6,Q6IG02
2607	1,2,6,7,9,10,13,14	3,4,5,8,11,12,15,16	6	P00762,P01835,P29315,Q6IFW6,Q6IG02,Q9R168
2608	1,2,6,7,9,10,13,15	3,4,5,8,11,12,14,16	2	P00762,P01835

2609	1,2,6,7,9,10,13,16	3,4,5,8,11,12,14,15	4 D3ZUC6,P01835,Q6IG02,Q9R168
2610	1,2,6,7,9,10,14,15	3,4,5,8,11,12,13,16	3 P00762,P01835,P97840
2611	1,2,6,7,9,10,14,16	3,4,5,8,11,12,13,15	2 Q6IG02,Q9R168
2612	1,2,6,7,9,10,15,16	3,4,5,8,11,12,13,14	2 D3ZUC6,Q811M5
2613	1,2,6,7,9,11,12,13	3,4,5,8,10,14,15,16	2 O35077,P01835
2614	1,2,6,7,9,11,12,14	3,4,5,8,10,13,15,16	4 O35077,P00762,P0DMW0;P0DMW1,Q5GRG2
2615	1,2,6,7,9,11,12,15	3,4,5,8,10,13,14,16	3 O35077,P00762,Q99MH3
2616	1,2,6,7,9,11,12,16	3,4,5,8,10,13,14,15	2 D3ZUC6,Q9JHB9
2617	1,2,6,7,9,11,13,14	3,4,5,8,10,12,15,16	4 P00762,P01835,P0DMW0;P0DMW1,Q9R168
2618	1,2,6,7,9,11,13,15	3,4,5,8,10,12,14,16	4 P00762,P01835,P0DMW0;P0DMW1,Q9R168
2619	1,2,6,7,9,11,13,16	3,4,5,8,10,12,14,15	4 D3ZUC6,P01835,Q9EQS0,Q9R168
2620	1,2,6,7,9,11,14,15	3,4,5,8,10,12,13,16	4 P00762,P0DMW0;P0DMW1,Q8CJ52,Q9R168
2621	1,2,6,7,9,11,14,16	3,4,5,8,10,12,13,15	3 P0DMW0;P0DMW1,Q812E4,Q9R168
2622	1,2,6,7,9,11,15,16	3,4,5,8,10,12,13,14	3 D3ZUC6,Q8CJ52,Q9R168
2623	1,2,6,7,9,12,13,14	3,4,5,8,10,11,15,16	5 P00762,P01835,P29315,Q6IFW6,Q6IG02
2624	1,2,6,7,9,12,13,15	3,4,5,8,10,11,14,16	2 P00762,P01835
2625	1,2,6,7,9,12,13,16	3,4,5,8,10,11,14,15	3 D3ZUC6,P01835,Q6IG02
2626	1,2,6,7,9,12,14,15	3,4,5,8,10,11,13,16	2 P00762,P01835
2627	1,2,6,7,9,12,14,16	3,4,5,8,10,11,13,15	2 P01835,Q6IG02
2628	1,2,6,7,9,12,15,16	3,4,5,8,10,11,13,14	2 D3ZUC6,P01835

2629	1,2,6,7,9,1 3,14,15	3,4,5,8,10, 11,12,16	4	P00762,P01835,P0DMW0;P0DMW1,Q9R168
2630	1,2,6,7,9,1 3,14,16	3,4,5,8,10, 11,12,15	4	P01835,Q6IG02,Q9EQS0,Q9R168
2631	1,2,6,7,9,1 3,15,16	3,4,5,8,10, 11,12,14	3	D3ZUC6,P01835,Q9R168
2632	1,2,6,7,9,1 4,15,16	3,4,5,8,10, 11,12,13	5	P01835,P47967,P97840,Q5I0D1,Q9R168
2633	1,2,6,7,10, 11,12,13	3,4,5,8,9,1 4,15,16	3	P01835,P23593,Q68G31
2634	1,2,6,7,10, 11,12,14	3,4,5,8,9,1 3,15,16	2	P00762,Q5GRG2
2635	1,2,6,7,10, 11,12,15	3,4,5,8,9,1 3,14,16	1	P00762
2636	1,2,6,7,10, 11,12,16	3,4,5,8,9,1 3,14,15	2	Q00715,Q9JHB9
2637	1,2,6,7,10, 11,13,14	3,4,5,8,9,1 2,15,16	4	P00762,P01835,P36376,Q68G31
2638	1,2,6,7,10, 11,13,15	3,4,5,8,9,1 2,14,16	3	P00762,P01835,Q68G31
2639	1,2,6,7,10, 11,13,16	3,4,5,8,9,1 2,14,15	3	P01835,P36376,Q68G31
2640	1,2,6,7,10, 11,14,15	3,4,5,8,9,1 2,13,16	2	P00762,P01835
2641	1,2,6,7,10, 11,14,16	3,4,5,8,9,1 2,13,15	2	P36376,Q9R168
2642	1,2,6,7,10, 11,15,16	3,4,5,8,9,1 2,13,14	2	P17988,Q8CJ52
2643	1,2,6,7,10, 12,13,14	3,4,5,8,9,1 1,15,16	5	O88797,P00762,P01835,P29315,Q6IG02
2644	1,2,6,7,10, 12,13,15	3,4,5,8,9,1 1,14,16	3	O88797,P00762,P01835
2645	1,2,6,7,10, 12,13,16	3,4,5,8,9,1 1,14,15	2	P01835,Q6IG02
2646	1,2,6,7,10, 12,14,15	3,4,5,8,9,1 1,13,16	3	O88797,P00762,P01835
2647	1,2,6,7,10, 12,14,16	3,4,5,8,9,1 1,13,15	2	P01835,Q6IG02
2648	1,2,6,7,10, 12,15,16	3,4,5,8,9,1 1,13,14	3	P01835,P19629,Q63598

2649	1,2,6,7,10,13,14,15	3,4,5,8,9,11,12,16	4	O88797,P00762,P01835,Q68G31
2650	1,2,6,7,10,13,14,16	3,4,5,8,9,11,12,15	6	O88797,P01835,P14668,P36376,P57113,Q6IG02
2651	1,2,6,7,10,13,15,16	3,4,5,8,9,11,12,14	2	O88797,P01835
2652	1,2,6,7,10,14,15,16	3,4,5,8,9,11,12,13	3	P01835,P09527,P57113
2653	1,2,6,7,11,12,13,14	3,4,5,8,9,10,15,16	2	P01835,Q5GRG2
2654	1,2,6,7,11,12,13,15	3,4,5,8,9,10,14,16	1	P01835
2655	1,2,6,7,11,12,13,16	3,4,5,8,9,10,14,15	4	P01835,P05369,P22283,Q9JHB9
2656	1,2,6,7,11,12,14,15	3,4,5,8,9,10,13,16	2	P01835,Q5GRG2
2657	1,2,6,7,11,12,14,16	3,4,5,8,9,10,13,15	4	P01835,Q00715,Q5GRG2,Q9JHB9
2658	1,2,6,7,11,12,15,16	3,4,5,8,9,10,13,14	3	P01835,Q8CJ52,Q9JHB9
2659	1,2,6,7,11,13,14,15	3,4,5,8,9,10,12,16	2	P01835,Q68G31
2660	1,2,6,7,11,13,14,16	3,4,5,8,9,10,12,15	3	P01835,P36376,Q9R168
2661	1,2,6,7,11,13,15,16	3,4,5,8,9,10,12,14	3	O70417,P01835,Q8CJ52
2662	1,2,6,7,11,14,15,16	3,4,5,8,9,10,12,13	2	P01835,Q8CJ52
2663	1,2,6,7,12,13,14,15	3,4,5,8,9,10,11,16	2	O88797,P01835
2664	1,2,6,7,12,13,14,16	3,4,5,8,9,10,11,15	1	P01835
2665	1,2,6,7,12,13,15,16	3,4,5,8,9,10,11,14	1	P01835
2666	1,2,6,7,12,14,15,16	3,4,5,8,9,10,11,13	1	P01835
2667	1,2,6,7,13,14,15,16	3,4,5,8,9,10,11,12	3	O88797,P01835,Q62714
2668	1,2,6,8,9,10,11,12	3,4,5,7,13,14,15,16	4	Q5GRG2,Q68G31,Q6P6S4,Q99MH3

2669	1,2,6,8,9,10,11,13	3,4,5,7,12,14,15,16	2 Q68G31,Q6P6S4
2670	1,2,6,8,9,10,11,14	3,4,5,7,12,13,15,16	3 P97840,Q5GRG2,Q68G31
2671	1,2,6,8,9,10,11,15	3,4,5,7,12,13,14,16	3 Q68G31,Q6P6S4,Q99MH3
2672	1,2,6,8,9,10,11,16	3,4,5,7,12,13,14,15	5 D3ZUC6,O89117,P15399,Q68G31,Q6P6S4
2673	1,2,6,8,9,10,12,13	3,4,5,7,11,14,15,16	2 P29315,Q68G31
2674	1,2,6,8,9,10,12,14	3,4,5,7,11,13,15,16	3 P29315,P97840,Q68G31
2675	1,2,6,8,9,10,12,15	3,4,5,7,11,13,14,16	4 P35745,Q63474,Q68G31,Q99MH3
2676	1,2,6,8,9,10,12,16	3,4,5,7,11,13,14,15	1 D3ZUC6
2677	1,2,6,8,9,10,13,14	3,4,5,7,11,12,15,16	3 P29315,P97840,Q68G31
2678	1,2,6,8,9,10,13,15	3,4,5,7,11,12,14,16	3 Q63474,Q68G31,Q99MH3
2679	1,2,6,8,9,10,13,16	3,4,5,7,11,12,14,15	2 D3ZUC6,Q68G31
2680	1,2,6,8,9,10,14,15	3,4,5,7,11,12,13,16	5 P47967,P97840,Q63474,Q68G31,Q99MH3
2681	1,2,6,8,9,10,14,16	3,4,5,7,11,12,13,15	4 P47967,P97840,Q5I0D1,Q68G31
2682	1,2,6,8,9,10,15,16	3,4,5,7,11,12,13,14	8 D3ZUC6,P15399,P19629,P47967,P97840,Q5I0D1,Q68G31,Q811M5
2683	1,2,6,8,9,11,12,13	3,4,5,7,10,14,15,16	5 O35077,Q5GRG2,Q5RKI1,Q68G31,Q9JI85
2684	1,2,6,8,9,11,12,14	3,4,5,7,10,13,15,16	5 O35077,P12020,P97840,Q5GRG2,Q5RKI1
2685	1,2,6,8,9,11,12,15	3,4,5,7,10,13,14,16	3 Q5GRG2,Q6P6S4,Q99MH3
2686	1,2,6,8,9,11,12,16	3,4,5,7,10,13,14,15	8 D3ZUC6,P12020,Q5GRG2,Q5RKI1,Q6P6S4,Q812E4,Q9JHB9,Q9QW07
2687	1,2,6,8,9,11,13,14	3,4,5,7,10,12,15,16	5 P0DMW0;P0DMW1,P97840,Q09326,Q5GRG2,Q68G31
2688	1,2,6,8,9,11,13,15	3,4,5,7,10,12,14,16	4 P0DMW0;P0DMW1,Q68G31,Q6P6S4,Q99MH3

2689	1,2,6,8,9,11,13,16	3,4,5,7,10,12,14,15	8 D3ZUC6,O89117,P09456,P30120,Q5RKI1,Q68G31,Q812E4,Q9JI85
2690	1,2,6,8,9,11,14,15	3,4,5,7,10,12,13,16	7 P0DMW0;P0DMW1,P47967,P97840,Q5GRG2,Q68G31,Q6P6S4,Q99MH3
2691	1,2,6,8,9,11,14,16	3,4,5,7,10,12,13,15	5 O89117,P97840,Q5GRG2,Q5RKI1,Q812E4
2692	1,2,6,8,9,11,15,16	3,4,5,7,10,12,13,14	5 D3ZUC6,P50280,P97840,Q6P6S4,Q9QW07
2693	1,2,6,8,9,12,13,14	3,4,5,7,10,11,15,16	3 P29315,P97840,Q68G31
2694	1,2,6,8,9,12,13,15	3,4,5,7,10,11,14,16	2 Q68G31,Q99MH3
2695	1,2,6,8,9,12,13,16	3,4,5,7,10,11,14,15	2 D3ZUC6,Q5RKI1
2696	1,2,6,8,9,12,14,15	3,4,5,7,10,11,13,16	3 P47967,P97840,Q99MH3
2697	1,2,6,8,9,12,14,16	3,4,5,7,10,11,13,15	3 P21674,P97840,Q5RKI1
2698	1,2,6,8,9,12,15,16	3,4,5,7,10,11,13,14	0
2699	1,2,6,8,9,13,14,15	3,4,5,7,10,11,12,16	4 P0DMW0;P0DMW1,P47967,P97840,Q68G31
2700	1,2,6,8,9,13,14,16	3,4,5,7,10,11,12,15	3 P97840,Q5RKI1,Q68G31
2701	1,2,6,8,9,13,15,16	3,4,5,7,10,11,12,14	3 D3ZUC6,P97840,Q68G31
2702	1,2,6,8,9,14,15,16	3,4,5,7,10,11,12,13	4 P23593,P47967,P97840,Q5I0D1
2703	1,2,6,8,10,11,12,13	3,4,5,7,9,14,15,16	4 P12020,Q5GRG2,Q68G31,Q9JI85
2704	1,2,6,8,10,11,12,14	3,4,5,7,9,13,15,16	3 P12020,Q5GRG2,Q68G31
2705	1,2,6,8,10,11,12,15	3,4,5,7,9,13,14,16	4 P19629,Q5GRG2,Q68G31,Q99MH3
2706	1,2,6,8,10,11,12,16	3,4,5,7,9,13,14,15	7 P12020,P15399,P30120,Q00715,Q5GRG2,Q68G31,Q9JHB9
2707	1,2,6,8,10,11,13,14	3,4,5,7,9,12,15,16	2 Q5GRG2,Q68G31
2708	1,2,6,8,10,11,13,15	3,4,5,7,9,12,14,16	2 P15399,Q68G31

2709	1,2,6,8,10,11,13,16	3,4,5,7,9,12,14,15	4	P15399,P30120,Q68G31,Q9JI85
2710	1,2,6,8,10,11,14,15	3,4,5,7,9,12,13,16	3	P15399,Q5GRG2,Q68G31
2711	1,2,6,8,10,11,14,16	3,4,5,7,9,12,13,15	5	O89117,P15399,P97580,Q5GRG2,Q68G31
2712	1,2,6,8,10,11,15,16	3,4,5,7,9,12,13,14	4	P15399,P19629,P50280,Q68G31
2713	1,2,6,8,10,12,13,14	3,4,5,7,9,11,15,16	3	P14668,P29315,Q68G31
2714	1,2,6,8,10,12,13,15	3,4,5,7,9,11,14,16	1	Q68G31
2715	1,2,6,8,10,12,13,16	3,4,5,7,9,11,14,15	2	P02631,Q68G31
2716	1,2,6,8,10,12,14,15	3,4,5,7,9,11,13,16	2	P54921,Q68G31
2717	1,2,6,8,10,12,14,16	3,4,5,7,9,11,13,15	2	P14668,Q68G31
2718	1,2,6,8,10,12,15,16	3,4,5,7,9,11,13,14	4	P15399,P19629,Q63598,Q68G31
2719	1,2,6,8,10,13,14,15	3,4,5,7,9,11,12,16	1	Q68G31
2720	1,2,6,8,10,13,14,16	3,4,5,7,9,11,12,15	4	P14668,P15399,P97580,Q68G31
2721	1,2,6,8,10,13,15,16	3,4,5,7,9,11,12,14	3	P15399,P19629,Q68G31
2722	1,2,6,8,10,14,15,16	3,4,5,7,9,11,12,13	10	P14668,P15399,P19629,P47967,P54921,P97580,P97840,Q30KJ2,Q5I0D1,Q68G31
2723	1,2,6,8,11,12,13,14	3,4,5,7,9,10,15,16	6	P12020,Q09326,Q5GRG2,Q5RKI1,Q68G31,Q9JI85
2724	1,2,6,8,11,12,13,15	3,4,5,7,9,10,14,16	4	P12020,Q5GRG2,Q68G31,Q9JI85
2725	1,2,6,8,11,12,13,16	3,4,5,7,9,10,14,15	9	O35547,P02782,P05369,P12020,P30120,Q5GRG2,Q5RKI1,Q68G31,Q9JI85
2726	1,2,6,8,11,12,14,15	3,4,5,7,9,10,13,16	3	P12020,Q5GRG2,Q68G31
2727	1,2,6,8,11,12,14,16	3,4,5,7,9,10,13,15	6	P12020,P30120,Q00715,Q5GRG2,Q5RKI1,Q9JHB9
2728	1,2,6,8,11,12,15,16	3,4,5,7,9,10,13,14	4	P12020,P50280,Q5GRG2,Q9QW07

2729	1,2,6,8,11,13,14,15	3,4,5,7,9,10,12,16	2	Q5GRG2,Q68G31
2730	1,2,6,8,11,13,14,16	3,4,5,7,9,10,12,15	6	P02782,P30120,Q5GRG2,Q5RKI1,Q68G31,Q9JI85
2731	1,2,6,8,11,13,15,16	3,4,5,7,9,10,12,14	3	P30120,Q68G31,Q9JI85
2732	1,2,6,8,11,14,15,16	3,4,5,7,9,10,12,13	5	P15399,P50280,P97840,Q5GRG2,Q68G31
2733	1,2,6,8,12,13,14,15	3,4,5,7,9,10,11,16	1	Q68G31
2734	1,2,6,8,12,13,14,16	3,4,5,7,9,10,11,15	2	Q5RKI1,Q68G31
2735	1,2,6,8,12,13,15,16	3,4,5,7,9,10,11,14	1	Q68G31
2736	1,2,6,8,12,14,15,16	3,4,5,7,9,10,11,13	0	
2737	1,2,6,8,13,14,15,16	3,4,5,7,9,10,11,12	3	P97840,Q62714,Q68G31
2738	1,2,6,9,10,11,12,13	3,4,5,7,8,14,15,16	6	B0LT89,O35077,P00762,P20646,Q68G31,Q99MH3
2739	1,2,6,9,10,11,12,14	3,4,5,7,8,13,15,16	2	O35077,P00762
2740	1,2,6,9,10,11,12,15	3,4,5,7,8,13,14,16	5	B0BNN3,O35077,P00762,Q99MH3,Q9WUW8
2741	1,2,6,9,10,11,12,16	3,4,5,7,8,13,14,15	1	D3ZUC6
2742	1,2,6,9,10,11,13,14	3,4,5,7,8,12,15,16	4	P00762,P0DMW0;P0DMW1,P97840,Q68G31
2743	1,2,6,9,10,11,13,15	3,4,5,7,8,12,14,16	4	O70417,P00762,Q68G31,Q99MH3
2744	1,2,6,9,10,11,13,16	3,4,5,7,8,12,14,15	4	D3ZUC6,O70417,O89117,Q68G31
2745	1,2,6,9,10,11,14,15	3,4,5,7,8,12,13,16	5	P00762,P0DMW0;P0DMW1,P47967,P97840,Q99MH3
2746	1,2,6,9,10,11,14,16	3,4,5,7,8,12,13,15	4	D3ZUC6,O89117,P97840,Q9R168
2747	1,2,6,9,10,11,15,16	3,4,5,7,8,12,13,14	3	D3ZUC6,Q811M5,Q99MH3
2748	1,2,6,9,10,12,13,14	3,4,5,7,8,11,15,16	11	P00762,P02780,P08649,P20646,P22273,Q4FZU2,Q5M8C6,Q6IFW6,Q6IG02,Q6IMF3,Q6P6Q2

2749	1,2,6,9,10,12,13,15	3,4,5,7,8,11,14,16	9	P00762,P02780,P06760,P08649,P22273,Q4G075,Q5M8C6,Q99041,Q99MH3
2750	1,2,6,9,10,12,13,16	3,4,5,7,8,11,14,15	7	D3ZUC6,P06760,P08649,Q4FZU2,Q4G075,Q6IFW6,Q6IG02
2751	1,2,6,9,10,12,14,15	3,4,5,7,8,11,13,16	11	P00762,P02780,P02782,P22273,P30120,P47967,P97840,Q4G075,Q5M8C6,Q62902,Q99MH3
2752	1,2,6,9,10,12,14,16	3,4,5,7,8,11,13,15	6	D3ZUC6,P22273,P97840,Q4FZU2,Q6IFW6,Q6IG02
2753	1,2,6,9,10,12,15,16	3,4,5,7,8,11,13,14	7	D3ZUC6,P06760,P22273,Q4G075,Q811M5,Q99041,Q99MH3
2754	1,2,6,9,10,13,14,15	3,4,5,7,8,11,12,16	9	P00762,P01835,P02780,P22273,P47967,P97840,Q5M8C6,Q5PQL7,Q68G31
2755	1,2,6,9,10,13,14,16	3,4,5,7,8,11,12,15	7	D3ZUC6,O54800;Q5DWV2,P14668,P97840,Q4FZU2,Q6IFW6,Q6IG02
2756	1,2,6,9,10,13,15,16	3,4,5,7,8,11,12,14	8	D3ZUC6,O70417,P06760,P11883,P22273,Q5I0D1,Q63751,Q811M5
2757	1,2,6,9,10,14,15,16	3,4,5,7,8,11,12,13	7	D3ZUC6,P06760,P22273,P47967,P97840,Q5I0D1,Q811M5
2758	1,2,6,9,11,12,13,14	3,4,5,7,8,10,15,16	22	O35077,O70594,P0DMW0;P0DMW1,P12368,P19468,P20646,P48508,P53790,Q05175,Q5I0E9,Q5RKI1,Q62761;Q62762;Q62763,Q63270,Q63355,Q63424,Q64093,Q6MG61,Q6Q0N1,Q8R431,Q923S2,Q9WTW7,Q9Z0W7
2759	1,2,6,9,11,12,13,15	3,4,5,7,8,10,14,16	6	O35077,O70417,Q62761;Q62762;Q62763,Q923S2,Q99MH3,Q9WUW8
2760	1,2,6,9,11,12,13,16	3,4,5,7,8,10,14,15	5	D3ZUC6,O35077,O70417,P54921,Q5RKI1
2761	1,2,6,9,11,12,14,15	3,4,5,7,8,10,13,16	12	O35077,O70257,P0DMW0;P0DMW1,P28570,P46844,P97840,Q62761;Q62762;Q62763,Q63424,Q64093,Q8R431,Q99MH3,Q9WUW8
2762	1,2,6,9,11,12,14,16	3,4,5,7,8,10,13,15	6	D3ZUC6,O35077,O70594,Q5RKI1,Q63618,Q8R431
2763	1,2,6,9,11,12,15,16	3,4,5,7,8,10,13,14	4	D3ZUC6,O70417,Q99MH3,Q9WUW8
2764	1,2,6,9,11,13,14,15	3,4,5,7,8,10,12,16	3	P0DMW0;P0DMW1,P97840,Q68G31
2765	1,2,6,9,11,13,14,16	3,4,5,7,8,10,12,15	8	D3ZUC6,O89117,P0DMW0;P0DMW1,P12368,P97840,Q5RKI1,Q9EQS0,Q9R168
2766	1,2,6,9,11,13,15,16	3,4,5,7,8,10,12,14	3	D3ZUC6,O70417,Q5RLM2
2767	1,2,6,9,11,14,15,16	3,4,5,7,8,10,12,13	4	D3ZUC6,P0DMW0;P0DMW1,P47967,P97840
2768	1,2,6,9,12,13,14,15	3,4,5,7,8,10,11,16	7	P00762,P01835,P02780,P22273,P97840,Q5M8C6,Q6B345

2769	1,2,6,9,12,13,14,16	3,4,5,7,8,10,11,15	6	P01835,Q4FZU2,Q5RKI1,Q6B345,Q6IFW6,Q6IG02
2770	1,2,6,9,12,13,15,16	3,4,5,7,8,10,11,14	8	D3ZUC6,O70417,P01835,P06760,P08937,Q4G075,Q63751,Q6B345
2771	1,2,6,9,12,14,15,16	3,4,5,7,8,10,11,13	5	P06760,P22273,P47967,P97840,Q6B345
2772	1,2,6,9,13,14,15,16	3,4,5,7,8,10,11,12	7	P01835,P06760,P0DMW0;P0DMW1,P22273,P47967,P97840,Q5I0D1
2773	1,2,6,10,11,12,13,14	3,4,5,7,8,9,15,16	3	P20646,P35280,Q68G31
2774	1,2,6,10,11,12,13,15	3,4,5,7,8,9,14,16	5	O70417,P06760,P20646,P20760,Q68G31
2775	1,2,6,10,11,12,13,16	3,4,5,7,8,9,14,15	5	O70417,P06760,P20646,P35280,Q68G31
2776	1,2,6,10,11,12,14,15	3,4,5,7,8,9,13,16	1	P20646
2777	1,2,6,10,11,12,14,16	3,4,5,7,8,9,13,15	2	P20646,P35280
2778	1,2,6,10,11,12,15,16	3,4,5,7,8,9,13,14	2	P06760,Q5QE79
2779	1,2,6,10,11,13,14,15	3,4,5,7,8,9,12,16	2	P01835,Q68G31
2780	1,2,6,10,11,13,14,16	3,4,5,7,8,9,12,15	4	O89117,P35280,P36376,Q68G31
2781	1,2,6,10,11,13,15,16	3,4,5,7,8,9,12,14	5	O70417,P06760,Q5QE79,Q63751,Q68G31
2782	1,2,6,10,11,14,15,16	3,4,5,7,8,9,12,13	0	
2783	1,2,6,10,12,13,14,15	3,4,5,7,8,9,11,16	9	P01835,P02780,P06760,P20646,P22273,P35280,Q5M8C6,Q62902,Q68G31
2784	1,2,6,10,12,13,14,16	3,4,5,7,8,9,11,15	9	O54800;Q5DWV2,P01835,P06760,P14668,P20646,P35280,Q6IFW6,Q6IG02,Q6P6R2
2785	1,2,6,10,12,13,15,16	3,4,5,7,8,9,11,14	12	O70417,P01835,P06760,P07150,P08649,P08937,P20646,P35280,Q5QE79,Q63751,Q6B345,Q6P6R2
2786	1,2,6,10,12,14,15,16	3,4,5,7,8,9,11,13	4	P06760,P14668,P22273,P35280
2787	1,2,6,10,13,14,15,16	3,4,5,7,8,9,11,12	7	P01835,P06760,P14668,P22273,P35280,P70709,Q68G31
2788	1,2,6,11,12,13,14,15	3,4,5,7,8,9,10,16	5	P01835,P20646,P20760,P70545,Q68G31

2789	1,2,6,11,12,13,14,16	3,4,5,7,8,9,10,15	6	P01835,P20646,P35280,Q5RKI1,Q6P6R2,Q9Z0V6
2790	1,2,6,11,12,13,15,16	3,4,5,7,8,9,10,14	8	O70417,P01835,P06760,P08937,P20646,P20760,Q5QE79,Q63751
2791	1,2,6,11,12,14,15,16	3,4,5,7,8,9,10,13	0	
2792	1,2,6,11,13,14,15,16	3,4,5,7,8,9,10,12	3	O70417,P01835,Q68G31
2793	1,2,6,12,13,14,15,16	3,4,5,7,8,9,10,11	6	P01835,P06760,P20646,P35280,Q6B345,Q6P6R2
2794	1,2,7,8,9,10,11,12	3,4,5,6,13,14,15,16	1	iRT-Kit_WR_fusion
2795	1,2,7,8,9,10,11,13	3,4,5,6,12,14,15,16	2	iRT-Kit_WR_fusion,Q66H69
2796	1,2,7,8,9,10,11,14	3,4,5,6,12,13,15,16	2	Q812E4,Q9Z1F2
2797	1,2,7,8,9,10,11,15	3,4,5,6,12,13,14,16	2	iRT-Kit_WR_fusion,Q9Z1F2
2798	1,2,7,8,9,10,11,16	3,4,5,6,12,13,14,15	5	iRT-Kit_WR_fusion,P08723,P09456,Q812E4,Q9JHB9
2799	1,2,7,8,9,10,12,13	3,4,5,6,11,14,15,16	4	iRT-Kit_WR_fusion,P29315,Q66H69,Q9Z1F2
2800	1,2,7,8,9,10,12,14	3,4,5,6,11,13,15,16	4	iRT-Kit_WR_fusion,P21674,P29315,Q9Z1F2
2801	1,2,7,8,9,10,12,15	3,4,5,6,11,13,14,16	3	iRT-Kit_WR_fusion,P35745,Q9Z1F2
2802	1,2,7,8,9,10,12,16	3,4,5,6,11,13,14,15	2	iRT-Kit_WR_fusion,P21674
2803	1,2,7,8,9,10,13,14	3,4,5,6,11,12,15,16	2	P29315,Q9Z1F2
2804	1,2,7,8,9,10,13,15	3,4,5,6,11,12,14,16	2	P35745,Q9Z1F2
2805	1,2,7,8,9,10,13,16	3,4,5,6,11,12,14,15	0	
2806	1,2,7,8,9,10,14,15	3,4,5,6,11,12,13,16	2	P35745,Q9Z1F2
2807	1,2,7,8,9,10,14,16	3,4,5,6,11,12,13,15	1	P21674
2808	1,2,7,8,9,10,15,16	3,4,5,6,11,12,13,14	1	iRT-Kit_WR_fusion

2809	1,2,7,8,9,11,12,13	3,4,5,6,10,14,15,16	3 iRT-Kit_WR_fusion,Q66H69,Q812E4
2810	1,2,7,8,9,11,12,14	3,4,5,6,10,13,15,16	4 iRT-Kit_WR_fusion,P11883,P21674,Q812E4
2811	1,2,7,8,9,11,12,15	3,4,5,6,10,13,14,16	1 iRT-Kit_WR_fusion
2812	1,2,7,8,9,11,12,16	3,4,5,6,10,13,14,15	9 iRT-Kit_WR_fusion,P02782,P08723,P09456,P21674,P30120,P62804,Q812E4,Q9JHB9
2813	1,2,7,8,9,11,13,14	3,4,5,6,10,12,15,16	3 iRT-Kit_WR_fusion,P0DMW0;P0DMW1,Q812E4
2814	1,2,7,8,9,11,13,15	3,4,5,6,10,12,14,16	2 iRT-Kit_WR_fusion,P0DMW0;P0DMW1
2815	1,2,7,8,9,11,13,16	3,4,5,6,10,12,14,15	7 iRT-Kit_WR_fusion,P02782,P08723,P09456,P30120,Q812E4,Q9JHB9
2816	1,2,7,8,9,11,14,15	3,4,5,6,10,12,13,16	3 iRT-Kit_WR_fusion,P0DMW0;P0DMW1,Q812E4
2817	1,2,7,8,9,11,14,16	3,4,5,6,10,12,13,15	6 iRT-Kit_WR_fusion,P08723,P09456,P30120,Q812E4,Q9JHB9
2818	1,2,7,8,9,11,15,16	3,4,5,6,10,12,13,14	6 iRT-Kit_WR_fusion,P08723,P09456,Q812E4,Q8CJ52,Q9JHB9
2819	1,2,7,8,9,12,13,14	3,4,5,6,10,11,15,16	2 iRT-Kit_WR_fusion,P29315
2820	1,2,7,8,9,12,13,15	3,4,5,6,10,11,14,16	2 iRT-Kit_WR_fusion,P35745
2821	1,2,7,8,9,12,13,16	3,4,5,6,10,11,14,15	1 iRT-Kit_WR_fusion
2822	1,2,7,8,9,12,14,15	3,4,5,6,10,11,13,16	2 iRT-Kit_WR_fusion,P35745
2823	1,2,7,8,9,12,14,16	3,4,5,6,10,11,13,15	2 iRT-Kit_WR_fusion,P21674
2824	1,2,7,8,9,12,15,16	3,4,5,6,10,11,13,14	1 iRT-Kit_WR_fusion
2825	1,2,7,8,9,13,14,15	3,4,5,6,10,11,12,16	1 P0DMW0;P0DMW1
2826	1,2,7,8,9,13,14,16	3,4,5,6,10,11,12,15	0
2827	1,2,7,8,9,13,15,16	3,4,5,6,10,11,12,14	1 iRT-Kit_WR_fusion
2828	1,2,7,8,9,14,15,16	3,4,5,6,10,11,12,13	3 iRT-Kit_WR_fusion,P47967,P97840

2829	1,2,7,8,10,11,12,13	3,4,5,6,9,14,15,16	3	iRT-Kit_WR_fusion,P23593,Q66H69
2830	1,2,7,8,10,11,12,14	3,4,5,6,9,13,15,16	4	iRT-Kit_WR_fusion,P11883,P62804,Q66H69
2831	1,2,7,8,10,11,12,15	3,4,5,6,9,13,14,16	3	iRT-Kit_WR_fusion,Q66H69,Q9Z1F2
2832	1,2,7,8,10,11,12,16	3,4,5,6,9,13,14,15	8	iRT-Kit_WR_fusion,P02782,P08723,P30120,P62804,Q00715,Q66H69,Q9JHB9
2833	1,2,7,8,10,11,13,14	3,4,5,6,9,12,15,16	3	Q66H69,Q68G31,Q9Z1F2
2834	1,2,7,8,10,11,13,15	3,4,5,6,9,12,14,16	3	Q66H69,Q68G31,Q9Z1F2
2835	1,2,7,8,10,11,13,16	3,4,5,6,9,12,14,15	7	P02782,P08723,P09456,P24368,P30120,Q66H69,Q9JHB9
2836	1,2,7,8,10,11,14,15	3,4,5,6,9,12,13,16	1	Q9Z1F2
2837	1,2,7,8,10,11,14,16	3,4,5,6,9,12,13,15	5	P08723,P30120,P62804,Q00715,Q9JHB9
2838	1,2,7,8,10,11,15,16	3,4,5,6,9,12,13,14	2	P08723,Q9JHB9
2839	1,2,7,8,10,12,13,14	3,4,5,6,9,11,15,16	3	P29315,Q66H69,Q9Z1F2
2840	1,2,7,8,10,12,13,15	3,4,5,6,9,11,14,16	4	iRT-Kit_WR_fusion,Q63598,Q66H69,Q9Z1F2
2841	1,2,7,8,10,12,13,16	3,4,5,6,9,11,14,15	2	iRT-Kit_WR_fusion,Q66H69
2842	1,2,7,8,10,12,14,15	3,4,5,6,9,11,13,16	2	iRT-Kit_WR_fusion,Q9Z1F2
2843	1,2,7,8,10,12,14,16	3,4,5,6,9,11,13,15	2	iRT-Kit_WR_fusion,P21674
2844	1,2,7,8,10,12,15,16	3,4,5,6,9,11,13,14	3	iRT-Kit_WR_fusion,Q63598,Q64093
2845	1,2,7,8,10,13,14,15	3,4,5,6,9,11,12,16	1	Q9Z1F2
2846	1,2,7,8,10,13,14,16	3,4,5,6,9,11,12,15	0	
2847	1,2,7,8,10,13,15,16	3,4,5,6,9,11,12,14	3	P19468,Q63598,Q64093
2848	1,2,7,8,10,14,15,16	3,4,5,6,9,11,12,13	0	

2849	1,2,7,8,11,12,13,14	3,4,5,6,9,10,15,16	4	iRT-Kit_WR_fusion,P11883,Q66H69,Q811M5
2850	1,2,7,8,11,12,13,15	3,4,5,6,9,10,14,16	3	iRT-Kit_WR_fusion,Q66H69,Q811M5
2851	1,2,7,8,11,12,13,16	3,4,5,6,9,10,14,15	10	iRT-Kit_WR_fusion,P02782,P08723,P09456,P24368,P30120,Q00715,Q66H69,Q811M5,Q9JHB9
2852	1,2,7,8,11,12,14,15	3,4,5,6,9,10,13,16	3	iRT-Kit_WR_fusion,P11883,Q811M5
2853	1,2,7,8,11,12,14,16	3,4,5,6,9,10,13,15	12	iRT-Kit_WR_fusion,P02782,P08723,P09456,P11883,P21674,P30120,P62804,Q00715,Q811M5,Q812E4,Q9JHB9
2854	1,2,7,8,11,12,15,16	3,4,5,6,9,10,13,14	6	iRT-Kit_WR_fusion,P02782,P08723,P30120,Q00715,Q9JHB9
2855	1,2,7,8,11,13,14,15	3,4,5,6,9,10,12,16	1	Q811M5
2856	1,2,7,8,11,13,14,16	3,4,5,6,9,10,12,15	7	P02782,P08723,P09456,P30120,Q811M5,Q812E4,Q9JHB9
2857	1,2,7,8,11,13,15,16	3,4,5,6,9,10,12,14	8	iRT-Kit_WR_fusion,P02782,P08723,P09456,P30120,Q66H69,Q8CJ52,Q9JHB9
2858	1,2,7,8,11,14,15,16	3,4,5,6,9,10,12,13	4	P08723,P30120,Q8CJ52,Q9JHB9
2859	1,2,7,8,12,13,14,15	3,4,5,6,9,10,11,16	3	iRT-Kit_WR_fusion,Q811M5,Q9Z2L0
2860	1,2,7,8,12,13,14,16	3,4,5,6,9,10,11,15	2	iRT-Kit_WR_fusion,Q811M5
2861	1,2,7,8,12,13,15,16	3,4,5,6,9,10,11,14	2	iRT-Kit_WR_fusion,Q66H69
2862	1,2,7,8,12,14,15,16	3,4,5,6,9,10,11,13	1	iRT-Kit_WR_fusion
2863	1,2,7,8,13,14,15,16	3,4,5,6,9,10,11,12	0	
2864	1,2,7,9,10,11,12,13	3,4,5,6,8,14,15,16	1	Q62761;Q62762;Q62763
2865	1,2,7,9,10,11,12,14	3,4,5,6,8,13,15,16	2	P00762,Q62761;Q62762;Q62763
2866	1,2,7,9,10,11,12,15	3,4,5,6,8,13,14,16	3	P00762,Q62761;Q62762;Q62763,Q99MH3
2867	1,2,7,9,10,11,12,16	3,4,5,6,8,13,14,15	1	D3ZUC6
2868	1,2,7,9,10,11,13,14	3,4,5,6,8,12,15,16	3	P00762,P0DMW0;P0DMW1,Q9R168

2869	1,2,7,9,10,11,13,15	3,4,5,6,8,12,14,16	2	P00762,Q9R168
2870	1,2,7,9,10,11,13,16	3,4,5,6,8,12,14,15	2	D3ZUC6,Q9R168
2871	1,2,7,9,10,11,14,15	3,4,5,6,8,12,13,16	3	P00762,P0DMW0;P0DMW1,Q9R168
2872	1,2,7,9,10,11,14,16	3,4,5,6,8,12,13,15	1	Q9R168
2873	1,2,7,9,10,11,15,16	3,4,5,6,8,12,13,14	2	D3ZUC6,Q9R168
2874	1,2,7,9,10,12,13,14	3,4,5,6,8,11,15,16	5	P00762,P29315,Q4FZU2,Q6IFW6,Q6IG02
2875	1,2,7,9,10,12,13,15	3,4,5,6,8,11,14,16	2	P00762,Q99041
2876	1,2,7,9,10,12,13,16	3,4,5,6,8,11,14,15	3	D3ZUC6,Q6IFU7,Q6IG02
2877	1,2,7,9,10,12,14,15	3,4,5,6,8,11,13,16	4	P00762,P22273,Q62902,Q99041
2878	1,2,7,9,10,12,14,16	3,4,5,6,8,11,13,15	3	P21674,Q6IFU7,Q6IG02
2879	1,2,7,9,10,12,15,16	3,4,5,6,8,11,13,14	3	D3ZUC6,P08937,Q99041
2880	1,2,7,9,10,13,14,15	3,4,5,6,8,11,12,16	6	P00762,P01835,P02780,P22273,Q5M8C6,Q9R168
2881	1,2,7,9,10,13,14,16	3,4,5,6,8,11,12,15	2	Q6IG02,Q9R168
2882	1,2,7,9,10,13,15,16	3,4,5,6,8,11,12,14	3	D3ZUC6,P08937,Q9R168
2883	1,2,7,9,10,14,15,16	3,4,5,6,8,11,12,13	4	P22273,P47967,Q5I0D1,Q9R168
2884	1,2,7,9,11,12,13,14	3,4,5,6,8,10,15,16	8	O35077,O70594,P0DMW0;P0DMW1,P25031,P53790,Q62761;Q62762;Q62763,Q63270,Q8R431
2885	1,2,7,9,11,12,13,15	3,4,5,6,8,10,14,16	5	O35077,P08937,P20760,P25031,Q62761;Q62762;Q62763
2886	1,2,7,9,11,12,13,16	3,4,5,6,8,10,14,15	1	P54921
2887	1,2,7,9,11,12,14,15	3,4,5,6,8,10,13,16	5	O35077,O70257,P0DMW0;P0DMW1,P25031,Q62761;Q62762;Q62763
2888	1,2,7,9,11,12,14,16	3,4,5,6,8,10,13,15	2	P21674,Q498D9

2889	1,2,7,9,11,12,15,16	3,4,5,6,8,10,13,14	1 P08937
2890	1,2,7,9,11,13,14,15	3,4,5,6,8,10,12,16	3 P0DMW0;P0DMW1,P25031,Q9R168
2891	1,2,7,9,11,13,14,16	3,4,5,6,8,10,12,15	2 P0DMW0;P0DMW1,Q9R168
2892	1,2,7,9,11,13,15,16	3,4,5,6,8,10,12,14	4 O70417,P08937,P25031,Q9R168
2893	1,2,7,9,11,14,15,16	3,4,5,6,8,10,12,13	3 P0DMW0;P0DMW1,P25031,Q9R168
2894	1,2,7,9,12,13,14,15	3,4,5,6,8,10,11,16	3 P01835,P0DMW0;P0DMW1,P22273
2895	1,2,7,9,12,13,14,16	3,4,5,6,8,10,11,15	3 P01835,Q6IFU7,Q6IG02
2896	1,2,7,9,12,13,15,16	3,4,5,6,8,10,11,14	3 P01835,P08937,Q6B345
2897	1,2,7,9,12,14,15,16	3,4,5,6,8,10,11,13	0
2898	1,2,7,9,13,14,15,16	3,4,5,6,8,10,11,12	3 P01835,P0DMW0;P0DMW1,Q9R168
2899	1,2,7,10,11,12,13,14	3,4,5,6,8,9,15,16	2 P20760,P23593
2900	1,2,7,10,11,12,13,15	3,4,5,6,8,9,14,16	2 P20760,P23593
2901	1,2,7,10,11,12,13,16	3,4,5,6,8,9,14,15	1 P20760
2902	1,2,7,10,11,12,14,15	3,4,5,6,8,9,13,16	2 P20760,P23593
2903	1,2,7,10,11,12,14,16	3,4,5,6,8,9,13,15	1 P62804
2904	1,2,7,10,11,12,15,16	3,4,5,6,8,9,13,14	2 P08937,P20760
2905	1,2,7,10,11,13,14,15	3,4,5,6,8,9,12,16	2 P01835,P20760
2906	1,2,7,10,11,13,14,16	3,4,5,6,8,9,12,15	2 P36376,Q9R168
2907	1,2,7,10,11,13,15,16	3,4,5,6,8,9,12,14	4 O70417,P08937,P20760,Q63751
2908	1,2,7,10,11,14,15,16	3,4,5,6,8,9,12,13	0

2909	1,2,7,10,12,13,14,15	3,4,5,6,8,9,11,16	3 O88797,P01835,P20760
2910	1,2,7,10,12,13,14,16	3,4,5,6,8,9,11,15	3 P01835,Q6IG02,Q6P6S4
2911	1,2,7,10,12,13,15,16	3,4,5,6,8,9,11,14	3 P01835,P08937,Q63751
2912	1,2,7,10,12,14,15,16	3,4,5,6,8,9,11,13	0
2913	1,2,7,10,13,14,15,16	3,4,5,6,8,9,11,12	4 O88797,P01835,P09527,P61206;P84079
2914	1,2,7,11,12,13,14,15	3,4,5,6,8,9,10,16	4 P01835,P20760,P25031,Q811M5
2915	1,2,7,11,12,13,14,16	3,4,5,6,8,9,10,15	2 P01835,Q811M5
2916	1,2,7,11,12,13,15,16	3,4,5,6,8,9,10,14	6 O70417,P01835,P08937,P20760,P25031,Q63751
2917	1,2,7,11,12,14,15,16	3,4,5,6,8,9,10,13	1 P25031
2918	1,2,7,11,13,14,15,16	3,4,5,6,8,9,10,12	3 P01835,P25031,Q9R168
2919	1,2,7,12,13,14,15,16	3,4,5,6,8,9,10,11	2 P01835,Q6B345
2920	1,2,8,9,10,11,12,13	3,4,5,6,7,14,15,16	2 B0LT89,P17988
2921	1,2,8,9,10,11,12,14	3,4,5,6,7,13,15,16	0
2922	1,2,8,9,10,11,12,15	3,4,5,6,7,13,14,16	1 Q99MH3
2923	1,2,8,9,10,11,12,16	3,4,5,6,7,13,14,15	2 D3ZUC6,P21674
2924	1,2,8,9,10,11,13,14	3,4,5,6,7,12,15,16	3 P0DMW0;P0DMW1,P17988,Q68G31
2925	1,2,8,9,10,11,13,15	3,4,5,6,7,12,14,16	2 P07943,Q68G31
2926	1,2,8,9,10,11,13,16	3,4,5,6,7,12,14,15	1 D3ZUC6
2927	1,2,8,9,10,11,14,15	3,4,5,6,7,12,13,16	3 P07943,P47967,P97840
2928	1,2,8,9,10,11,14,16	3,4,5,6,7,12,13,15	2 O89117,P97840

2929	1,2,8,9,10, 11,15,16	3,4,5,6,7,1 2,13,14	2	D3ZUC6,P07943
2930	1,2,8,9,10, 12,13,14	3,4,5,6,7,1 1,15,16	1	P17988
2931	1,2,8,9,10, 12,13,15	3,4,5,6,7,1 1,14,16	3	P11598,Q99041,Q99MH3
2932	1,2,8,9,10, 12,13,16	3,4,5,6,7,1 1,14,15	1	D3ZUC6
2933	1,2,8,9,10, 12,14,15	3,4,5,6,7,1 1,13,16	5	P00714,P11598,P47967,P97840,Q99041
2934	1,2,8,9,10, 12,14,16	3,4,5,6,7,1 1,13,15	2	P21674,P97840
2935	1,2,8,9,10, 12,15,16	3,4,5,6,7,1 1,13,14	1	Q99041
2936	1,2,8,9,10, 13,14,15	3,4,5,6,7,1 1,12,16	2	P47967,P97840
2937	1,2,8,9,10, 13,14,16	3,4,5,6,7,1 1,12,15	1	P97840
2938	1,2,8,9,10, 13,15,16	3,4,5,6,7,1 1,12,14	1	D3ZUC6
2939	1,2,8,9,10, 14,15,16	3,4,5,6,7,1 1,12,13	3	P47967,P97840,Q5I0D1
2940	1,2,8,9,11, 12,13,14	3,4,5,6,7,1 0,15,16	13	B0LT89,O35077,O70594,P0DMW0;P0DMW1,P17988,P18297,P19468,P53790,Q03248,Q5I0E9,Q 63270,Q71MB6,Q8R431
2941	1,2,8,9,11, 12,13,15	3,4,5,6,7,1 0,14,16	4	B0LT89,P18297,P57113,Q71MB6
2942	1,2,8,9,11, 12,13,16	3,4,5,6,7,1 0,14,15	3	P30120,P54921,Q5RKI1
2943	1,2,8,9,11, 12,14,15	3,4,5,6,7,1 0,13,16	2	P18297,P97840
2944	1,2,8,9,11, 12,14,16	3,4,5,6,7,1 0,13,15	3	P21674,Q5RKI1,Q812E4
2945	1,2,8,9,11, 12,15,16	3,4,5,6,7,1 0,13,14	1	Q5QE79
2946	1,2,8,9,11, 13,14,15	3,4,5,6,7,1 0,12,16	2	P0DMW0;P0DMW1,P97840
2947	1,2,8,9,11, 13,14,16	3,4,5,6,7,1 0,12,15	4	P0DMW0;P0DMW1,P97840,Q5RKI1,Q812E4
2948	1,2,8,9,11, 13,15,16	3,4,5,6,7,1 0,12,14	1	Q5RLM2

2949	1,2,8,9,11,14,15,16	3,4,5,6,7,10,12,13	3	P0DMW0;P0DMW1,P47967,P97840
2950	1,2,8,9,12,13,14,15	3,4,5,6,7,10,11,16	2	P11598,P97840
2951	1,2,8,9,12,13,14,16	3,4,5,6,7,10,11,15	1	Q5RKI1
2952	1,2,8,9,12,13,15,16	3,4,5,6,7,10,11,14	0	
2953	1,2,8,9,12,14,15,16	3,4,5,6,7,10,11,13	2	P47967,P97840
2954	1,2,8,9,13,14,15,16	3,4,5,6,7,10,11,12	3	P47967,P97840,Q5I0D1
2955	1,2,8,10,11,12,13,14	3,4,5,6,7,9,15,16	1	Q68G31
2956	1,2,8,10,11,12,13,15	3,4,5,6,7,9,14,16	2	Q5QE79,Q68G31
2957	1,2,8,10,11,12,13,16	3,4,5,6,7,9,14,15	2	P30120,Q5QE79
2958	1,2,8,10,11,12,14,15	3,4,5,6,7,9,13,16	0	
2959	1,2,8,10,11,12,14,16	3,4,5,6,7,9,13,15	0	
2960	1,2,8,10,11,12,15,16	3,4,5,6,7,9,13,14	1	Q5QE79
2961	1,2,8,10,11,13,14,15	3,4,5,6,7,9,12,16	1	Q68G31
2962	1,2,8,10,11,13,14,16	3,4,5,6,7,9,12,15	1	Q68G31
2963	1,2,8,10,11,13,15,16	3,4,5,6,7,9,12,14	3	Q5QE79,Q63751,Q68G31
2964	1,2,8,10,11,14,15,16	3,4,5,6,7,9,12,13	0	
2965	1,2,8,10,12,13,14,15	3,4,5,6,7,9,11,16	0	
2966	1,2,8,10,12,13,14,16	3,4,5,6,7,9,11,15	0	
2967	1,2,8,10,12,13,15,16	3,4,5,6,7,9,11,14	2	Q5QE79,Q63751
2968	1,2,8,10,12,14,15,16	3,4,5,6,7,9,11,13	1	Q63617

2969	1,2,8,10,13,14,15,16	3,4,5,6,7,9,11,12	0	
2970	1,2,8,11,12,13,14,15	3,4,5,6,7,9,10,16	1	Q811M5
2971	1,2,8,11,12,13,14,16	3,4,5,6,7,9,10,15	3	P30120,Q5RKI1,Q811M5
2972	1,2,8,11,12,13,15,16	3,4,5,6,7,9,10,14	3	P30120,Q5QE79,Q63751
2973	1,2,8,11,12,14,15,16	3,4,5,6,7,9,10,13	0	
2974	1,2,8,11,13,14,15,16	3,4,5,6,7,9,10,12	0	
2975	1,2,8,12,13,14,15,16	3,4,5,6,7,9,10,11	0	
2976	1,2,9,10,11,12,13,14	3,4,5,6,7,8,15,16	18	B0LT89,O35077,O70594,P06761,P17988,P20760,P20761,P46844,P48508,P50115,P53790,Q03248,Q5M7T9,Q62761;Q62762;Q62763,Q63270,Q6TMA8,Q8R431,Q9QX74
2977	1,2,9,10,11,12,13,15	3,4,5,6,7,8,14,16	16	B0LT89,O35077,O70417,P06761,P08937,P20760,P20761,P50115,Q5M8C6,Q5QE79,Q62761;Q62762;Q62763,Q63751,Q6TMA8,Q99MH3,Q9QX74,Q9WUW8
2978	1,2,9,10,11,12,13,16	3,4,5,6,7,8,14,15	12	B0LT89,D3ZUC6,O70417,P08937,P20760,P20761,P50115,P54921,Q5QE79,Q62714,Q63751,Q9QX74
2979	1,2,9,10,11,12,14,15	3,4,5,6,7,8,13,16	16	O35077,O70257,O70594,P06761,P20760,P20761,P46844,P50115,Q5I0D7,Q5M8C6,Q62761;Q62762;Q62763,Q62902,Q6TMA8,Q9QX74,Q9R0T3,Q9WUW8
2980	1,2,9,10,11,12,14,16	3,4,5,6,7,8,13,15	4	O70594,P20761,P50115,Q9QX74
2981	1,2,9,10,11,12,15,16	3,4,5,6,7,8,13,14	12	D3ZUC6,O70417,P06761,P08937,P20760,P20761,P50115,P63081,Q5QE79,Q63751,Q9QX74,Q9WUW8
2982	1,2,9,10,11,13,14,15	3,4,5,6,7,8,12,16	13	O70417,P06761,P0DMW0;P0DMW1,P20760,P20761,P26772,P46844,P50115,P97840,Q5M8C6,Q6TMA8,Q9QX74,Q9R0T3
2983	1,2,9,10,11,13,14,16	3,4,5,6,7,8,12,15	6	D3ZUC6,O89117,P50115,Q63270,Q9QX74,Q9R168
2984	1,2,9,10,11,13,15,16	3,4,5,6,7,8,12,14	8	D3ZUC6,O70417,P08937,P50115,Q5QE79,Q5RLM2,Q63751,Q9QX74
2985	1,2,9,10,11,14,15,16	3,4,5,6,7,8,12,13	9	D3ZUC6,P06761,P08937,P47967,P50115,P97840,Q5QE79,Q9QX74,Q9R0T3
2986	1,2,9,10,12,13,14,15	3,4,5,6,7,8,11,16	33	P00762,P02780,P02781,P02782,P06760,P06761,P07150,P07647,P08010,P08723,P08937,P09456,P0C0A9,P11598,P20760,P20761,P22273,P22282,P22283,P30120,P46462,P50115,Q4G063,Q4G075,Q5M8C6,Q62902,Q63493,Q63617,Q6B345,Q6TMA8,Q99041,Q9QX74,Q9R0T3
2987	1,2,9,10,12,13,14,16	3,4,5,6,7,8,11,15	17	P02780,P06760,P06761,P07150,P22273,P22282,P50115,Q4FZU2,Q4G063,Q4G075,Q5M8C6,Q63617,Q6IFW6,Q6IG02,Q99041,Q9QX74,Q9R0T3
2988	1,2,9,10,12,13,15,16	3,4,5,6,7,8,11,14	22	D3ZUC6,O70417,P02780,P06760,P06761,P07150,P08937,P11598,P20761,P22273,P22282,P46462,P50115,Q4G075,Q5M8C6,Q5QE79,Q63617,Q63751,Q6B345,Q99041,Q9QX74,Q9R0T3

2989	1,2,9,10,12,14,15,16	3,4,5,6,7,8,11,13	22	P02780,P06760,P06761,P08937,P11598,P22273,P22282,P22283,P46462,P47967,P50115,P97840,Q4G063,Q4G075,Q5M8C6,Q5QE79,Q62902,Q63493,Q63617,Q99041,Q9QX74,Q9R0T3
2990	1,2,9,10,13,14,15,16	3,4,5,6,7,8,11,12	23	O70417,P02780,P06760,P06761,P07150,P08937,P11598,P22273,P22282,P22283,P46462,P47967,P50115,P70709,P97840,Q4G075,Q5I0D1,Q5M8C6,Q63493,Q63617,Q99041,Q9QX74,Q9R0T3
2991	1,2,9,11,12,13,14,15	3,4,5,6,7,8,10,16	47	B0LT89,O35077,O70257,O70377,O70417,O70594,P06761,P07151,P08937,P0DMW0;P0DMW1,P18757,P19468,P20760,P20761,P25031,P28570,P46844,P48508,P50115,P53790,P57113,Q03248,Q05175,Q3T1J9,Q3ZAV1,Q5I0E9,Q5M7T9,Q62761;Q62762;Q62763,Q63270,Q63424,Q63618,Q64602,Q68FT5,Q6MG61,Q6Q0N1,Q6TMA8,Q71MB6,Q80W57,Q8R431,Q923S2,Q9JJ40,Q9QX74,Q9R0T3,Q9WTW7,Q9WUW8,Q9WUW9,Q9Z0W7
2992	1,2,9,11,12,13,14,16	3,4,5,6,7,8,10,15	32	O35763,O70257,O70377,O70594,O88339;Q4V882,P08937,P19468,P20761,P30904,P38918,P46844,P48508,P50115,P53790,P54921,P57113,Q03248,Q05175,Q3MIE4,Q3ZAV1,Q5I0E9,Q5M7T9,Q5RKI1,Q63270,Q63424,Q63618,Q6MG61,Q6Q0N1,Q8R431,Q923S2,Q9QX74,Q9Z0W7
2993	1,2,9,11,12,13,15,16	3,4,5,6,7,8,10,14	17	D3ZUC6,O70417,O70594,P08937,P20760,P20761,P25031,P48508,P50115,P54921,P57113,Q5QE79,Q5RLM2,Q63751,Q923S2,Q9QX74,Q9WUW8
2994	1,2,9,11,12,14,15,16	3,4,5,6,7,8,10,13	20	O70257,O70594,P06761,P07171,P08937,P20761,P25031,P30904,P36860,P46844,P48508,P50115,P57113,Q5QE79,Q63618,Q6MG61,Q8R431,Q9QX74,Q9R0T3,Q9WUW8
2995	1,2,9,11,13,14,15,16	3,4,5,6,7,8,10,12	17	O70417,P08937,P0DMW0;P0DMW1,P25031,P26772,P30904,P46844,P48508,P50115,P97840,Q5QE79,Q5RLM2,Q63270,Q6MG61,Q9QX74,Q9R0T3,Q9R168
2996	1,2,9,12,13,14,15,16	3,4,5,6,7,8,10,11	22	O70417,P02780,P06760,P06761,P07150,P08937,P11598,P22273,P22282,P30904,P50115,P50116,Q4G063,Q4G075,Q5M8C6,Q5QE79,Q63617,Q6B345,Q6P6R2,Q99041,Q9QX74,Q9R0T3
2997	1,2,10,11,12,13,14,15	3,4,5,6,7,8,9,16	12	O70417,P06760,P06761,P08937,P20646,P20760,P20761,P26772,P50115,Q5QE79,Q9QX74,Q9R0T3
2998	1,2,10,11,12,13,14,16	3,4,5,6,7,8,9,15	10	P06760,P20646,P20760,P20761,P35280,P50115,Q5QE79,Q6P6R2,Q9QX74,Q9Z0V6
2999	1,2,10,11,12,13,15,16	3,4,5,6,7,8,9,14	11	O70417,P06760,P08937,P20646,P20760,P20761,P50115,Q5QE79,Q63751,Q6P6R2,Q9QX74
3000	1,2,10,11,12,14,15,16	3,4,5,6,7,8,9,13	7	P06760,P08937,P20760,P50115,Q5QE79,Q9QX74,Q9R0T3
3001	1,2,10,11,13,14,15,16	3,4,5,6,7,8,9,12	9	O70417,P06760,P08937,P20760,P26772,P50115,Q5QE79,Q9QX74,Q9R0T3
3002	1,2,10,12,13,14,15,16	3,4,5,6,7,8,9,11	21	P02780,P06760,P06761,P07150,P08937,P20760,P22273,P22282,P35280,P50115,P50116,Q4G075,Q5M8C6,Q5QE79,Q62902,Q63493,Q63617,Q6B345,Q6P6R2,Q9QX74,Q9R0T3
3003	1,2,11,12,13,14,15,16	3,4,5,6,7,8,9,10	16	O70417,O70594,P06760,P08937,P20646,P20760,P20761,P25031,P26772,P50115,P50116,P70545,Q5QE79,Q6P6R2,Q9QX74,Q9R0T3

3004	1,3,4,5,6,7,8,9	2,10,11,12,13,14,15,16	27	D3ZHA0,O70417,O70594,P01681,P06760,P06761,P06911,P08649,P08721,P08937,P13432,P18418,P20646,P20760,P20761,P36860,P50115,P50116,Q05702,Q5QE79,Q62946,Q63751,Q6P6R2,Q6TMA8,Q9QX74,Q9R0T3,Q9Z0V6
3005	1,3,4,5,6,7,8,10	2,9,11,12,13,14,15,16	87	A2RUW1,D3ZHA0,O08557,O35763,O70257,O70377,O70417,O70594,O88339;Q4V882,P01681,P01836,P04904,P06761,P06911,P08721,P08937,P10760,P13432,P18418,P18757,P19468,P19629,P20646,P20760,P20761,P22282,P23928,P24268,P25031,P29975,P30904,P31044,P36860,P38918,P46720,P46844,P48037,P48508,P50115,P50116,P51907,P53790,P54921,P57113,P60711;P63259,P63081,P68035;P68136,P97840,Q03248,Q05175,Q05702,Q3T1J9,Q3ZAV1,Q5I0E9,Q5M7T9,Q5QE79,Q5RLM2,Q62946,Q63270,Q63424,Q63618,Q63751,Q64093,Q64602,Q66HG3,Q68FT5,Q6AY41,Q6AYS7,Q6MG61,Q6P6R2,Q6Q0N1,Q6TMA8,Q71MB6,Q80W57,Q8R431,Q923S2,Q99MZ8,Q9JJ19,Q9JJ40,Q9JLJ3,Q9QX74,Q9QYU4,Q9R0T3,Q9WTW7,Q9WUW8,Q9WUW9,Q9Z0W7
3006	1,3,4,5,6,7,8,11	2,9,10,12,13,14,15,16	67	B1H234,O35547,P01681,P02780,P02781,P02782,P04905,P06760,P06761,P06911,P07150,P07647,P08010,P08649,P08721,P08723,P08937,P09456,P0C0A9,P11598,P13432,P18418,P20646,P20760,P20761,P22273,P22282,P22283,P31430,P36374,P40241,P46462,P47967,P50115,P50116,P50280,P97840,Q05702,Q4FZU2,Q4G063,Q4G075,Q5GRG2,Q5M8C6,Q5QE79,Q62902,Q62946,Q63493,Q63617,Q63751,Q6B345,Q6IFU7,Q6IFU8,Q6IFW6,Q6IG02,Q6IMF3,Q6P6Q2,Q6P6R2,Q6Q0N0,Q6TMA8,Q99041,Q9JHB9,Q9JI85,Q9QW07,Q9QX74,Q9QZK8,Q9R0T3,Q9WTT6
3007	1,3,4,5,6,7,8,12	2,9,10,11,13,14,15,16	32	D3ZHA0,O70417,P01681,P06761,P06911,P08937,P13432,P18418,P20760,P20761,P22282,P30904,P36860,P46844,P47967,P48508,P50115,P97840,Q05702,Q5M8C6,Q5QE79,Q5RLM2,Q62946,Q63270,Q63751,Q66HG3,Q6MG61,Q6Q0N1,Q6TMA8,Q9JI85,Q9QX74,Q9R0T3
3008	1,3,4,5,6,7,8,13	2,9,10,11,12,14,15,16	36	D3ZHA0,O70257,O70594,P01681,P02781,P06761,P06911,P08721,P08937,P13432,P18418,P20646,P20760,P20761,P22282,P30904,P36860,P46844,P47967,P48508,P50115,P57113,P63081,P97840,Q5M8C6,Q5QE79,Q62714,Q62902,Q62946,Q63618,Q6Q0N1,Q6TMA8,Q9JI85,Q9QX74,Q9R0T3,Q9WUW8
3009	1,3,4,5,6,7,8,14	2,9,10,11,12,13,15,16	37	D3ZHA0,D3ZUC6,O70417,O70594,P01681,P06760,P06761,P06911,P08649,P08937,P13432,P18418,P20646,P20760,P20761,P46844,P48508,P50115,P52590,P54921,P57113,P63081,Q4G075,Q5M8C6,Q5QE79,Q5RLM2,Q62714,Q62812,Q62946,Q63751,Q64602,Q6TMA8,Q923S2,Q9QX74,Q9R0T3,Q9WUW8,Q9WUW9
3010	1,3,4,5,6,7,8,15	2,9,10,11,12,13,14,16	54	O08557,O35763,O70594,O88339;Q4V882,P01681,P01946,P02091,P06761,P06911,P08649,P08721,P08937,P09606,P13432,P17988,P18418,P18757,P19468,P19629,P20646,P20760,P20761,P23928,P30904,P36860,P38918,P46844,P48508,P50115,P53790,P54921,P57113,Q03248,Q05175,Q05702,Q5I0E9,Q5M7T9,Q62714,Q62946,Q63270,Q63618,Q64602,Q6AYS7,Q6MG61,Q6PCU2,Q6Q0N1,Q6TMA8,Q8R431,Q923S2,Q99MZ8,Q9QX74,Q9R0T3,Q9Z0V6,Q9Z0W7
3011	1,3,4,5,6,7,8,16	2,9,10,11,12,13,14,15	72	D3ZHA0,O35077,O35763,O70257,O70594,P01681,P02780,P02781,P02782,P02783,P04904,P06761,P06911,P07151,P07647,P08649,P08721,P08937,P13432,P18418,P18757,P19468,P19629,P20646,P20760,P20761,P22282,P22283,P23928,P38918,P46720,P46844,P48508,P50115,P52590,P52847,P53790,P57113,P63081,Q03248,Q05175,Q05702,Q3ZAV1,Q5I0D7,Q5I0E9,Q5M7T9,Q5M8C6,Q5RLM2,Q62812,Q62902,Q62946,Q63270,Q63424,Q64602,Q68FT5,Q6MG61,Q6Q0N1,Q6TMA8,Q71MB6,Q80W57,Q8R431,Q923S2,Q9JHB9,Q9JI85,Q9JJ40,Q9JLJ3,Q9QX74,Q9R0T3,Q9WTW7,Q9WUW8,Q9WUW9,Q9Z0W7

3012	1,3,4,5,6,7,9,10	2,8,11,12,13,14,15,16	4	O70594,P08721,P23928,P36860
3013	1,3,4,5,6,7,9,11	2,8,10,12,13,14,15,16	8	B1H234,P08649,P08721,P11598,P22283,Q63493,Q63617,Q9R0T3
3014	1,3,4,5,6,7,9,12	2,8,10,11,13,14,15,16	1	P36860
3015	1,3,4,5,6,7,9,13	2,8,10,11,12,14,15,16	2	P36860,Q5QE79
3016	1,3,4,5,6,7,9,14	2,8,10,11,12,13,15,16	3	P52590,Q5QE79,Q63751
3017	1,3,4,5,6,7,9,15	2,8,10,11,12,13,14,16	6	P01681,P01946,P02091,P08721,P36860,P62804
3018	1,3,4,5,6,7,9,16	2,8,10,11,12,13,14,15	3	P02783,P08721,P22006
3019	1,3,4,5,6,7,10,11	2,8,9,12,13,14,15,16	9	B1H234,P08721,P11598,P22283,P36860,P47967,P97840,Q63493,Q9R0T3
3020	1,3,4,5,6,7,10,12	2,8,9,11,13,14,15,16	14	P08721,P0DMW0;P0DMW1,P30904,P36860,P46844,P47967,P48508,P57113,P97840,Q03248,Q5RLM2,Q63270,Q66HG3,Q9R0T3
3021	1,3,4,5,6,7,10,13	2,8,9,11,12,14,15,16	12	A2RUW1,O70594,P08721,P19468,P36860,P46844,P47967,P48508,P57113,P97840,Q63618,Q9WUW8
3022	1,3,4,5,6,7,10,14	2,8,9,11,12,13,15,16	15	A2RUW1,O70594,P08721,P23928,P46844,P48037,P48508,P54921,P57113,Q5QE79,Q5RLM2,Q63751,Q71MB6,Q923S2,Q9WUW8
3023	1,3,4,5,6,7,10,15	2,8,9,11,12,13,14,16	37	A2RUW1,O08557,O70594,O88339;Q4V882,P01946,P02091,P08721,P17988,P18297,P19468,P23928,P30904,P36860,P38918,P46844,P48508,P53790,P54921,P57113,P62804,P97840,Q03248,Q05175,Q5I0E9,Q5RKI1,Q63270,Q63424,Q63618,Q6MG61,Q6Q0N1,Q71MB6,Q80W57,Q8K3P7,Q8R431,Q923S2,Q99MZ8,Q9Z0W7
3024	1,3,4,5,6,7,10,16	2,8,9,11,12,13,14,15	41	O35077,O70594,P02783,P07647,P08721,P17988,P18297,P18757,P19468,P23928,P29975,P46844,P48037,P48508,P52847,P53790,P57113,P97840,Q03248,Q3ZAV1,Q5I0D7,Q5I0E9,Q5M7T9,Q63270,Q63424,Q63618,Q64602,Q68FT5,Q6MG61,Q6Q0N1,Q71MB6,Q80W57,Q8R431,Q923S2,Q9JJ40,Q9QYU4,Q9R0T3,Q9WTW7,Q9WUW8,Q9WUW9,Q9Z0W7
3025	1,3,4,5,6,7,11,12	2,8,9,10,13,14,15,16	10	P08721,P11598,P22283,P35053,P47967,P97840,Q5I0D1,Q63493,Q63617,Q9R0T3
3026	1,3,4,5,6,7,11,13	2,8,9,10,12,14,15,16	11	B1H234,P08721,P11598,P22283,P36860,P47967,P97840,Q5I0D1,Q63493,Q63617,Q9R0T3

3027	1,3,4,5,6,7,11,14	2,8,9,10,12,13,15,16	11	B1H234,P08649,P08721,P11598,P22283,P52590,Q4KLZ6,Q63493,Q63751,Q99041,Q9R0T3
3028	1,3,4,5,6,7,11,15	2,8,9,10,12,13,14,16	16	B1H234,P01946,P02091,P08649,P08721,P11598,P17988,P22283,P97840,Q4FZU2,Q63493,Q6IFW6,Q6IG02,Q6IMF3,Q6P6Q2,Q8CJ52
3029	1,3,4,5,6,7,11,16	2,8,9,10,12,13,14,15	23	B1H234,O35547,P02780,P02781,P02782,P02783,P07647,P08649,P08721,P08723,P0C0A9,P11598,P17988,P22283,P36374,P97840,Q08463,Q4KLZ6,Q63493,Q63617,Q8R5M3,Q9JHB9,Q9R0T3
3030	1,3,4,5,6,7,12,13	2,8,9,10,11,14,15,16	3	P36860,P47967,P97840
3031	1,3,4,5,6,7,12,14	2,8,9,10,11,13,15,16	3	P52590,Q5RLM2,Q63751
3032	1,3,4,5,6,7,12,15	2,8,9,10,11,13,14,16	6	O89117,P01946,P02091,P08721,P36860,P97840
3033	1,3,4,5,6,7,12,16	2,8,9,10,11,13,14,15	8	P02783,P07647,P08721,P46844,P47967,P97840,Q5I0D7,Q9R0T3
3034	1,3,4,5,6,7,13,14	2,8,9,10,11,12,15,16	8	A2RUW1,B1H234,P52590,P63081,Q5QE79,Q62714,Q63751,Q9WUW8
3035	1,3,4,5,6,7,13,15	2,8,9,10,11,12,14,16	8	A2RUW1,P01946,P02091,P08721,P36860,P62804,P97840,Q62714
3036	1,3,4,5,6,7,13,16	2,8,9,10,11,12,14,15	7	P02783,P07647,P08721,P46844,P97840,Q5I0D7,Q9WUW8
3037	1,3,4,5,6,7,14,15	2,8,9,10,11,12,13,16	6	A2RUW1,P01946,P02091,P08721,P54921,Q62714
3038	1,3,4,5,6,7,14,16	2,8,9,10,11,12,13,15	10	P02783,P07647,P46844,P48037,P52590,P57113,Q5I0D7,Q71MB6,Q99MH3,Q9WUW8
3039	1,3,4,5,6,7,15,16	2,8,9,10,11,12,13,14	17	O70594,P01946,P02091,P02783,P08721,P17988,P18297,P19468,P46844,P48508,P57113,Q03248,Q5I0D7,Q63270,Q6Q0N1,Q71MB6,Q8R431
3040	1,3,4,5,6,8,9,10	2,7,11,12,13,14,15,16	9	O70594,P01681,P08937,P18418,P20760,P25031,P36860,P70545,Q05702
3041	1,3,4,5,6,8,9,11	2,7,10,12,13,14,15,16	11	O88797,P01681,P08937,P20760,Q05702,Q4FZU2,Q63493,Q6IMF3,Q6P6Q2,Q6P6S4,Q9JI85
3042	1,3,4,5,6,8,9,12	2,7,10,11,13,14,15,16	4	P01681,P08937,P20760,Q05702
3043	1,3,4,5,6,8,9,13	2,7,10,11,12,14,15,16	5	P01681,P08937,P20760,P62804,Q05702
3044	1,3,4,5,6,8,9,14	2,7,10,11,12,13,15,16	7	O70417,P01681,P08937,P20760,P52590,Q09030,Q63751

3045	1,3,4,5,6,8,9,15	2,7,10,11,12,13,14,16	7	P01681,P01946,P02091,P13432,P20760,P62804,Q05702
3046	1,3,4,5,6,8,9,16	2,7,10,11,12,13,14,15	8	P01681,P02783,P18418,P20760,P22006,P23593,Q05702,Q09030
3047	1,3,4,5,6,8,10,11	2,7,9,12,13,14,15,16	12	P01681,P08937,P18418,P25031,Q4FZU2,Q63493,Q6IFU7,Q6IFW6,Q6IG02,Q6IMF3,Q6P6Q2,Q9JI85
3048	1,3,4,5,6,8,10,12	2,7,9,11,13,14,15,16	13	P01681,P08937,P0DMW0;P0DMW1,P18418,P25031,P30904,P36860,P46844,P48508,Q05702,Q5RLM2,Q66HG3,Q9R168
3049	1,3,4,5,6,8,10,13	2,7,9,11,12,14,15,16	12	O70257,O70594,P01681,P08937,P18418,P25031,P36860,P46844,P48508,Q63618,Q6Q0N1,Q9WUW8
3050	1,3,4,5,6,8,10,14	2,7,9,11,12,13,15,16	14	O70417,O70594,P01681,P08937,P18418,P20760,P25031,P48508,P54921,P97580,Q5RLM2,Q63751,Q923S2,Q9WUW8
3051	1,3,4,5,6,8,10,15	2,7,9,11,12,13,14,16	25	O70377,O70594,O88339;Q4V882,P01681,P01946,P02091,P13432,P18418,P25031,P30904,P36860,P38918,P46844,P48508,P53790,P54921,P62804,Q05175,Q498D9,Q63270,Q63618,Q6MG61,Q6Q0N1,Q8R431,Q923S2
3052	1,3,4,5,6,8,10,16	2,7,9,11,12,13,14,15	35	O35077,O70257,O70377,O70594,P01681,P02783,P07151,P0DMW0;P0DMW1,P15399,P18418,P18757,P19468,P20760,P23928,P25031,P46844,P48508,P53790,P57113,Q03248,Q05702,Q30KJ2,Q3ZAV1,Q5M7T9,Q63270,Q63424,Q63618,Q64602,Q6MG61,Q6Q0N1,Q71MB6,Q8R431,Q923S2,Q9WUW8,Q9Z0W7
3053	1,3,4,5,6,8,11,12	2,7,9,10,13,14,15,16	21	P01681,P02780,P06911,P08937,P14046,P22282,P36374,P97840,Q05702,Q4FZU2,Q5I0D1,Q5M8C6,Q63493,Q6IFU7,Q6IFW6,Q6IG02,Q6IG05,Q6IMF3,Q6P6Q2,Q9JI85,Q9R168
3054	1,3,4,5,6,8,11,13	2,7,9,10,12,14,15,16	12	P01681,P08937,P22282,Q4FZU2,Q62902,Q63493,Q6IFU7,Q6IFW6,Q6IG02,Q6IMF3,Q6P6Q2,Q9JI85
3055	1,3,4,5,6,8,11,14	2,7,9,10,12,13,15,16	15	P01681,P06911,P08937,P19218,P52590,Q4FZU2,Q4KLZ6,Q63493,Q63751,Q6IFU7,Q6IFW6,Q6IG02,Q6IMF3,Q6P6Q2,Q9JI85
3056	1,3,4,5,6,8,11,15	2,7,9,10,12,13,14,16	14	P01681,P01946,P02091,Q10758,Q4FZU2,Q63493,Q6IFU7,Q6IFU8,Q6IFW6,Q6IG02,Q6IG05,Q6IMF3,Q6P6Q2,Q9JI85
3057	1,3,4,5,6,8,11,16	2,7,9,10,12,13,14,15	32	O35547,O88797,P01681,P02780,P02781,P02782,P02783,P04905,P07647,P08010,P08723,P0C0A9,P18418,P20760,P22282,P22283,P30120,P36374,P46462,P60905,Q4FZU2,Q4KLZ6,Q5M8C6,Q62902,Q63493,Q6IFU7,Q6IFW6,Q6IG02,Q6IMF3,Q6P6Q2,Q9JHB9,Q9JI85
3058	1,3,4,5,6,8,12,13	2,7,9,10,11,14,15,16	2	P97840,Q9R168
3059	1,3,4,5,6,8,12,14	2,7,9,10,11,13,15,16	7	O70417,P01681,P08937,P52590,Q5RLM2,Q63751,Q9R168
3060	1,3,4,5,6,8,12,15	2,7,9,10,11,13,14,16	6	P01681,P01946,P02091,Q05702,Q6IG05,Q9R168
3061	1,3,4,5,6,8,12,16	2,7,9,10,11,13,14,15	7	P01681,P02783,P0DMW0;P0DMW1,P20760,P46844,Q05702,Q9R168

3062	1,3,4,5,6,8,13,14	2,7,9,10,11,12,15,16	6	P01681,P08937,P20760,P52590,Q63751,Q9WUW8
3063	1,3,4,5,6,8,13,15	2,7,9,10,11,12,14,16	6	P01681,P01946,P02091,P13432,P62804,Q498D9
3064	1,3,4,5,6,8,13,16	2,7,9,10,11,12,14,15	5	P02783,P18418,P20760,P46844,Q9WUW8
3065	1,3,4,5,6,8,14,15	2,7,9,10,11,12,13,16	9	P01681,P01946,P02091,P08937,P13432,P20760,P54921,P62804,Q62714
3066	1,3,4,5,6,8,14,16	2,7,9,10,11,12,13,15	6	P01681,P02783,P08937,P20760,P52590,Q9WUW8
3067	1,3,4,5,6,8,15,16	2,7,9,10,11,12,13,14	14	O35077,O70594,P01681,P01946,P02091,P02783,P13432,P17988,P20760,P46844,P48508,Q63270,Q6Q0N1,Q8R431
3068	1,3,4,5,6,9,10,11	2,7,8,12,13,14,15,16	0	
3069	1,3,4,5,6,9,10,12	2,7,8,11,13,14,15,16	0	
3070	1,3,4,5,6,9,10,13	2,7,8,11,12,14,15,16	5	P11883,P36860,P62804,Q00715,Q06000
3071	1,3,4,5,6,9,10,14	2,7,8,11,12,13,15,16	1	Q06000
3072	1,3,4,5,6,9,10,15	2,7,8,11,12,13,14,16	8	P01681,P01946,P02091,P30120,P36860,P62804,Q00715,Q811M5
3073	1,3,4,5,6,9,10,16	2,7,8,11,12,13,14,15	3	P02783,P11883,Q811M5
3074	1,3,4,5,6,9,11,12	2,7,8,10,13,14,15,16	2	P61206;P84079,Q03191
3075	1,3,4,5,6,9,11,13	2,7,8,10,12,14,15,16	0	
3076	1,3,4,5,6,9,11,14	2,7,8,10,12,13,15,16	2	Q4KLZ6,Q63751
3077	1,3,4,5,6,9,11,15	2,7,8,10,12,13,14,16	8	P01681,P01946,P02091,P62804,Q4FZU2,Q6IFW6,Q6IG02,Q6P6Q2
3078	1,3,4,5,6,9,11,16	2,7,8,10,12,13,14,15	3	P02783,P22006,Q4KLZ6
3079	1,3,4,5,6,9,12,13	2,7,8,10,11,14,15,16	1	P62804
3080	1,3,4,5,6,9,12,14	2,7,8,10,11,13,15,16	0	
3081	1,3,4,5,6,9,12,15	2,7,8,10,11,13,14,16	3	P01946,P02091,P62804

3082	1,3,4,5,6,9,12,16	2,7,8,10,11,13,14,15	0
3083	1,3,4,5,6,9,13,14	2,7,8,10,11,12,15,16	2 P62804,Q00715
3084	1,3,4,5,6,9,13,15	2,7,8,10,11,12,14,16	5 P01946,P02091,P11883,P62804,Q00715
3085	1,3,4,5,6,9,13,16	2,7,8,10,11,12,14,15	2 P11883,P62804
3086	1,3,4,5,6,9,14,15	2,7,8,10,11,12,13,16	4 P01946,P02091,P62804,Q00715
3087	1,3,4,5,6,9,14,16	2,7,8,10,11,12,13,15	4 P02783,P23593,P52590,Q09030
3088	1,3,4,5,6,9,15,16	2,7,8,10,11,12,13,14	6 P01946,P02091,P02783,P11883,P23593,P62804
3089	1,3,4,5,6,10,11,12	2,7,8,9,13,14,15,16	2 P47967,P97840
3090	1,3,4,5,6,10,11,13	2,7,8,9,12,14,15,16	1 P97840
3091	1,3,4,5,6,10,11,14	2,7,8,9,12,13,15,16	1 Q4KLZ6
3092	1,3,4,5,6,10,11,15	2,7,8,9,12,13,14,16	6 P01946,P02091,Q4FZU2,Q6IFW6,Q6IG02,Q6P6Q2
3093	1,3,4,5,6,10,11,16	2,7,8,9,12,13,14,15	3 P02783,P07647,Q4KLZ6
3094	1,3,4,5,6,10,12,13	2,7,8,9,11,14,15,16	2 P36860,P97840
3095	1,3,4,5,6,10,12,14	2,7,8,9,11,13,15,16	1 Q5RLM2
3096	1,3,4,5,6,10,12,15	2,7,8,9,11,13,14,16	5 P01946,P02091,P36860,P62804,Q812E4
3097	1,3,4,5,6,10,12,16	2,7,8,9,11,13,14,15	2 P02783,P0DMW0;P0DMW1
3098	1,3,4,5,6,10,13,14	2,7,8,9,11,12,15,16	2 P62804,Q06000
3099	1,3,4,5,6,10,13,15	2,7,8,9,11,12,14,16	8 A2RUW1,P01946,P02091,P11883,P36860,P62804,Q00715,Q812E4
3100	1,3,4,5,6,10,13,16	2,7,8,9,11,12,14,15	1 P11883
3101	1,3,4,5,6,10,14,15	2,7,8,9,11,12,13,16	4 P01946,P02091,P54921,P62804

3102	1,3,4,5,6,10,14,16	2,7,8,9,11,12,13,15	2	P02783,P52590
3103	1,3,4,5,6,10,15,16	2,7,8,9,11,12,13,14	7	P01946,P02091,P02783,P11883,P17988,P62804,Q63270
3104	1,3,4,5,6,11,12,13	2,7,8,9,10,14,15,16	3	P47967,P97840,Q5I0D1
3105	1,3,4,5,6,11,12,14	2,7,8,9,10,13,15,16	1	Q4KLZ6
3106	1,3,4,5,6,11,12,15	2,7,8,9,10,13,14,16	6	P01946,P02091,Q4FZU2,Q6IFW6,Q6IG02,Q6P6Q2
3107	1,3,4,5,6,11,12,16	2,7,8,9,10,13,14,15	5	O35547,P02783,P07647,P97840,Q4KLZ6
3108	1,3,4,5,6,11,13,14	2,7,8,9,10,12,15,16	1	Q4KLZ6
3109	1,3,4,5,6,11,13,15	2,7,8,9,10,12,14,16	8	P01946,P02091,P21674,P62804,Q4FZU2,Q6IFW6,Q6IG02,Q6P6Q2
3110	1,3,4,5,6,11,13,16	2,7,8,9,10,12,14,15	3	P02783,P07647,Q4KLZ6
3111	1,3,4,5,6,11,14,15	2,7,8,9,10,12,13,16	7	P01946,P02091,Q4FZU2,Q4KLZ6,Q6IFW6,Q6IG02,Q6P6Q2
3112	1,3,4,5,6,11,14,16	2,7,8,9,10,12,13,15	4	P02783,P07647,P52590,Q4KLZ6
3113	1,3,4,5,6,11,15,16	2,7,8,9,10,12,13,14	10	P01946,P02091,P02783,P17988,Q4FZU2,Q4KLZ6,Q6IFW6,Q6IG02,Q6IMF3,Q6P6Q2
3114	1,3,4,5,6,12,13,14	2,7,8,9,10,11,15,16	1	P20766
3115	1,3,4,5,6,12,13,15	2,7,8,9,10,11,14,16	3	P01946,P02091,P62804
3116	1,3,4,5,6,12,13,16	2,7,8,9,10,11,14,15	1	P97840
3117	1,3,4,5,6,12,14,15	2,7,8,9,10,11,13,16	2	P01946,P02091
3118	1,3,4,5,6,12,14,16	2,7,8,9,10,11,13,15	2	P02783,P52590
3119	1,3,4,5,6,12,15,16	2,7,8,9,10,11,13,14	3	P01946,P02091,P02783
3120	1,3,4,5,6,13,14,15	2,7,8,9,10,11,12,16	4	P01946,P02091,P62804,Q00715
3121	1,3,4,5,6,13,14,16	2,7,8,9,10,11,12,15	2	P02783,P52590

3122	1,3,4,5,6,13,15,16	2,7,8,9,10,11,12,14	6	P01946,P02091,P02783,P11883,P62804,P97584
3123	1,3,4,5,6,14,15,16	2,7,8,9,10,11,12,13	4	P01946,P02091,P02783,P52590
3124	1,3,4,5,7,8,9,10	2,6,11,12,13,14,15,16	11	O70417,O70594,P01681,P06760,P08937,P20646,P20760,P70545,Q6P6R2,Q9QX74,Q9Z0V6
3125	1,3,4,5,7,8,9,11	2,6,10,12,13,14,15,16	19	P01681,P02780,P06760,P08649,P20646,P22282,P27590,P35280,P49134,Q4FZU2,Q5M8C6,Q63493,Q6IFU8,Q6IFW6,Q6IMF3,Q6P6Q2,Q6P6R2,Q812E4,Q9QX74
3126	1,3,4,5,7,8,9,12	2,6,10,11,13,14,15,16	5	O70417,P01681,P06760,P20646,Q9QX74
3127	1,3,4,5,7,8,9,13	2,6,10,11,12,14,15,16	6	P01681,P06760,P08649,P20646,P20760,Q9QX74
3128	1,3,4,5,7,8,9,14	2,6,10,11,12,13,15,16	10	O70417,P01681,P06760,P08649,P08937,P20646,P20760,Q5QE79,Q63751,Q9QX74
3129	1,3,4,5,7,8,9,15	2,6,10,11,12,13,14,16	7	P01681,P06760,P08649,P20646,P35280,Q9QX74,Q9Z0V6
3130	1,3,4,5,7,8,9,16	2,6,10,11,12,13,14,15	6	P01681,P06760,P08649,P20646,P20760,Q9QX74
3131	1,3,4,5,7,8,10,11	2,6,9,12,13,14,15,16	22	P01681,P02780,P06760,P08937,P20646,P22282,P27590,P30904,P47967,P97840,Q4FZU2,Q4G075,Q5M8C6,Q63493,Q6B345,Q6IFU7,Q6IFU8,Q6IFW6,Q6IG02,Q6IMF3,Q6P6Q2,Q9QX74
3132	1,3,4,5,7,8,10,12	2,6,9,11,13,14,15,16	15	O70417,P01681,P0DMW0;P0DMW1,P20646,P30904,P46844,P47967,P48508,P97840,Q5RLM2,Q63270,Q6MG61,Q6Q0N1,Q8R431,Q9QX74
3133	1,3,4,5,7,8,10,13	2,6,9,11,12,14,15,16	24	O35077,O70257,O70594,P01681,P19468,P20646,P30904,P36860,P46844,P47967,P48508,P57113,P97840,Q63355,Q63424,Q63618,Q64093,Q6MG61,Q6Q0N1,Q80W57,Q8R431,Q9QX74,Q9WUW8,Q9WUW9
3134	1,3,4,5,7,8,10,14	2,6,9,11,12,13,15,16	20	D3ZUC6,O35077,O70417,O70594,P01681,P08937,P20646,P20760,P46844,P48508,P54921,P57113,Q5RLM2,Q63355,Q63751,Q8R431,Q923S2,Q9QX74,Q9WUW8,Q9WUW9
3135	1,3,4,5,7,8,10,15	2,6,9,11,12,13,14,16	41	O35077,O35763,O70377,O70594,O88339;Q4V882,P01681,P08721,P10536,P18757,P19468,P20646,P30904,P38918,P46844,P48508,P53790,P54921,P57113,P97840,Q03248,Q05175,Q3ZAV1,Q5I0E9,Q5M7T9,Q5RKI1,Q63270,Q63355,Q63424,Q63618,Q64093,Q6MG61,Q6Q0N1,Q80W57,Q8R431,Q923S2,Q99MZ8,Q9JJ19,Q9QX74,Q9WTW7,Q9Z0V6,Q9Z0W7

3136	1,3,4,5,7,8,10,16	2,6,9,11,12,13,14,15	47	O35077,O35763,O70257,O70377,O70594,P01681,P0DMW0;P0DMW1,P18757,P19468,P20646,P20760,P29975,P46413,P46720,P46844,P48508,P53790,P57113,P97840,Q03248,Q05175,Q3ZAV1,Q5I0E9,Q5M7T9,Q5RLM2,Q63270,Q63355,Q63424,Q63618,Q64093,Q64319,Q64602,Q6AY41,Q6MG61,Q6Q0N1,Q6TMA8,Q71MB6,Q80W57,Q8R431,Q923S2,Q9JJ19,Q9JJ40,Q9QX74,Q9WTW7,Q9WUW8,Q9WUW9,Q9Z0W7
3137	1,3,4,5,7,8,11,12	2,6,9,10,13,14,15,16	24	P01681,P02780,P02781,P06760,P08649,P22282,P27590,P36374,P40241,P47967,P70709,P97840,Q4FZU2,Q4G075,Q5I0D1,Q5M8C6,Q63493,Q6IFU8,Q6IFW6,Q6IG02,Q6IMF3,Q6P6Q2,Q811M5,Q9QX74
3138	1,3,4,5,7,8,11,13	2,6,9,10,12,14,15,16	29	B0BNN3,P01681,P02780,P02781,P02782,P06760,P08649,P20646,P22273,P22282,P22283,P36374,P40241,P47967,P97840,Q4FZU2,Q4G075,Q5I0D1,Q5M8C6,Q62902,Q63493,Q6IFU7,Q6IFU8,Q6IFW6,Q6IG02,Q6IMF3,Q6P6Q2,Q811M5,Q9QX74
3139	1,3,4,5,7,8,11,14	2,6,9,10,12,13,15,16	23	B0BNN3,D3ZUC6,P01681,P02780,P02781,P06760,P08649,P08937,P20646,P22282,Q4FZU2,Q4G075,Q5M8C6,Q63493,Q63751,Q6IFU7,Q6IFU8,Q6IFW6,Q6IG02,Q6IMF3,Q6P6Q2,Q811M5,Q9QX74
3140	1,3,4,5,7,8,11,15	2,6,9,10,12,13,14,16	20	P01681,P02780,P06760,P08649,P08721,P20646,P97840,Q4FZU2,Q4G075,Q5M8C6,Q63493,Q6IFU7,Q6IFU8,Q6IFW6,Q6IG02,Q6IMF3,Q6P6Q2,Q8CJ52,Q9QX74,Q9Z0V6
3141	1,3,4,5,7,8,11,16	2,6,9,10,12,13,14,15	38	O35547,P00762,P01681,P02780,P02781,P02782,P04905,P06760,P07647,P08010,P08649,P08723,P09456,P0C0A9,P16636,P20646,P22273,P22282,P22283,P30120,P36374,P40241,P46462,P47967,P97840,Q4FZU2,Q4G075,Q5M8C6,Q62902,Q63493,Q6IFU7,Q6IFU8,Q6IFW6,Q6IG02,Q6IMF3,Q6P6Q2,Q9JHB9,Q9QX74
3142	1,3,4,5,7,8,12,13	2,6,9,10,11,14,15,16	4	P01681,P47967,P97840,Q9QX74
3143	1,3,4,5,7,8,12,14	2,6,9,10,11,13,15,16	9	D3ZUC6,O70417,P01681,P08649,P08937,Q5RLM2,Q63751,Q811M5,Q9QX74
3144	1,3,4,5,7,8,12,15	2,6,9,10,11,13,14,16	7	D3ZUC6,O89117,P01681,P10758,P20646,P97840,Q9QX74
3145	1,3,4,5,7,8,12,16	2,6,9,10,11,13,14,15	9	P01681,P02781,P08649,P20646,P46844,P47967,P97840,Q5RLM2,Q9QX74
3146	1,3,4,5,7,8,13,14	2,6,9,10,11,12,15,16	12	B0BNN3,D3ZUC6,P01681,P08649,P08937,P20646,Q63751,Q811M5,Q99MH3,Q9QX74,Q9WUW8,Q9Z2L0
3147	1,3,4,5,7,8,13,15	2,6,9,10,11,12,14,16	7	O70594,P01681,P08649,P20646,P97840,Q6Q0N1,Q9QX74
3148	1,3,4,5,7,8,13,16	2,6,9,10,11,12,14,15	16	B0BNN3,O35077,P00762,P01681,P02781,P08649,P20646,P46844,P47967,P97840,Q62902,Q6TMA8,Q8R431,Q99MH3,Q9QX74,Q9WUW8
3149	1,3,4,5,7,8,14,15	2,6,9,10,11,12,13,16	8	D3ZUC6,P01681,P08649,P20646,P37996,P54921,Q9QX74,Q9WUW8
3150	1,3,4,5,7,8,14,16	2,6,9,10,11,12,13,15	15	B0BNN3,O35077,O70417,P01681,P08649,P08937,P20646,P20760,P46844,Q5RLM2,Q63751,Q6TMA8,Q99MH3,Q9QX74,Q9WUW8
3151	1,3,4,5,7,8,15,16	2,6,9,10,11,12,13,14	21	O35077,O70594,P01681,P08649,P17988,P19468,P20646,P46844,P48508,P53790,Q63270,Q63424,Q64093,Q6MG61,Q6Q0N1,Q6TMA8,Q8R431,Q9QX74,Q9WTW7,Q9Z0V6,Q9Z0W7

3152	1,3,4,5,7,9,10,11	2,6,8,12,13,14,15,16	0
3153	1,3,4,5,7,9,10,12	2,6,8,11,13,14,15,16	2 P97840,Q03191
3154	1,3,4,5,7,9,10,13	2,6,8,11,12,14,15,16	0
3155	1,3,4,5,7,9,10,14	2,6,8,11,12,13,15,16	0
3156	1,3,4,5,7,9,10,15	2,6,8,11,12,13,14,16	2 P01681,Q5RKI1
3157	1,3,4,5,7,9,10,16	2,6,8,11,12,13,14,15	0
3158	1,3,4,5,7,9,11,12	2,6,8,10,13,14,15,16	4 P47967,P97840,Q03191,Q5I0D1
3159	1,3,4,5,7,9,11,13	2,6,8,10,12,14,15,16	3 P08649,P47967,P97840
3160	1,3,4,5,7,9,11,14	2,6,8,10,12,13,15,16	2 P08649,Q63751
3161	1,3,4,5,7,9,11,15	2,6,8,10,12,13,14,16	7 P01681,P08649,Q4FZU2,Q6IFW6,Q6IG02,Q6IMF3,Q6P6Q2
3162	1,3,4,5,7,9,11,16	2,6,8,10,12,13,14,15	1 P08649
3163	1,3,4,5,7,9,12,13	2,6,8,10,11,14,15,16	2 P47967,P97840
3164	1,3,4,5,7,9,12,14	2,6,8,10,11,13,15,16	2 Q63751,Q68G31
3165	1,3,4,5,7,9,12,15	2,6,8,10,11,13,14,16	4 O89117,P01681,P10758,Q68G31
3166	1,3,4,5,7,9,12,16	2,6,8,10,11,13,14,15	2 P97840,Q68G31
3167	1,3,4,5,7,9,13,14	2,6,8,10,11,12,15,16	0
3168	1,3,4,5,7,9,13,15	2,6,8,10,11,12,14,16	3 P01681,P62804,Q00715
3169	1,3,4,5,7,9,13,16	2,6,8,10,11,12,14,15	0
3170	1,3,4,5,7,9,14,15	2,6,8,10,11,12,13,16	2 P01681,P08649
3171	1,3,4,5,7,9,14,16	2,6,8,10,11,12,13,15	2 P08649,Q68G31

3172	1,3,4,5,7,9,15,16	2,6,8,10,11,12,13,14	2	P01681,Q68G31
3173	1,3,4,5,7,10,11,12	2,6,8,9,13,14,15,16	3	P47967,P97840,Q5I0D1
3174	1,3,4,5,7,10,11,13	2,6,8,9,12,14,15,16	3	P47967,P97840,Q5I0D1
3175	1,3,4,5,7,10,11,14	2,6,8,9,12,13,15,16	0	
3176	1,3,4,5,7,10,11,15	2,6,8,9,12,13,14,16	8	P08721,P97840,Q4FZU2,Q5RKI1,Q6IFW6,Q6IG02,Q6IMF3,Q6P6Q2
3177	1,3,4,5,7,10,11,16	2,6,8,9,12,13,14,15	2	P47967,P97840
3178	1,3,4,5,7,10,12,13	2,6,8,9,11,14,15,16	2	P47967,P97840
3179	1,3,4,5,7,10,12,14	2,6,8,9,11,13,15,16	2	P97840,Q5RLM2
3180	1,3,4,5,7,10,12,15	2,6,8,9,11,13,14,16	4	O89117,P10758,P97840,Q5RKI1
3181	1,3,4,5,7,10,12,16	2,6,8,9,11,13,14,15	5	P0DMW0;P0DMW1,P10758,P18297,P47967,P97840
3182	1,3,4,5,7,10,13,14	2,6,8,9,11,12,15,16	1	Q9WUW8
3183	1,3,4,5,7,10,13,15	2,6,8,9,11,12,14,16	2	P97840,Q5RKI1
3184	1,3,4,5,7,10,13,16	2,6,8,9,11,12,14,15	11	O35077,P18297,P19468,P46844,P47967,P97840,Q63424,Q64093,Q8R431,Q99MH3,Q9WUW8
3185	1,3,4,5,7,10,14,15	2,6,8,9,11,12,13,16	2	P54921,Q5RKI1
3186	1,3,4,5,7,10,14,16	2,6,8,9,11,12,13,15	7	O35077,P18297,P57113,Q5RLM2,Q71MB6,Q99MH3,Q9WUW8
3187	1,3,4,5,7,10,15,16	2,6,8,9,11,12,13,14	22	O35077,O70594,P08721,P10758,P17988,P18297,P19468,P46844,P48508,P53790,P97840,Q03248,Q5I0E9,Q63270,Q63355,Q63424,Q64093,Q6Q0N1,Q71MB6,Q80W57,Q8R431,Q9WTW7
3188	1,3,4,5,7,11,12,13	2,6,8,9,10,14,15,16	4	P47967,P97840,Q5I0D1,Q811M5
3189	1,3,4,5,7,11,12,14	2,6,8,9,10,13,15,16	4	P47967,P97840,Q5I0D1,Q811M5
3190	1,3,4,5,7,11,12,15	2,6,8,9,10,13,14,16	8	P47967,P97840,Q4FZU2,Q5I0D1,Q6IFW6,Q6IG02,Q6IMF3,Q6P6Q2
3191	1,3,4,5,7,11,12,16	2,6,8,9,10,13,14,15	3	P47967,P97840,Q5I0D1

3192	1,3,4,5,7,11,13,14	2,6,8,9,10,12,15,16	4	B0BNN3,P08649,P97840,Q811M5
3193	1,3,4,5,7,11,13,15	2,6,8,9,10,12,14,16	8	P08649,P47967,P97840,Q4FZU2,Q6IFW6,Q6IG02,Q6IMF3,Q6P6Q2
3194	1,3,4,5,7,11,13,16	2,6,8,9,10,12,14,15	6	P07647,P08649,P47967,P97840,Q08463,Q99MH3
3195	1,3,4,5,7,11,14,15	2,6,8,9,10,12,13,16	6	P08649,Q4FZU2,Q6IFW6,Q6IG02,Q6IMF3,Q6P6Q2
3196	1,3,4,5,7,11,14,16	2,6,8,9,10,12,13,15	2	P08649,Q99MH3
3197	1,3,4,5,7,11,15,16	2,6,8,9,10,12,13,14	10	P08649,P08721,P17988,P97840,Q4FZU2,Q6IFW6,Q6IG02,Q6IMF3,Q6P6Q2,Q8CJ52
3198	1,3,4,5,7,12,13,14	2,6,8,9,10,11,15,16	3	P47967,P97840,Q811M5
3199	1,3,4,5,7,12,13,15	2,6,8,9,10,11,14,16	3	O89117,P47967,P97840
3200	1,3,4,5,7,12,13,16	2,6,8,9,10,11,14,15	3	P47967,P97840,Q99MH3
3201	1,3,4,5,7,12,14,15	2,6,8,9,10,11,13,16	2	D3ZUC6,O89117
3202	1,3,4,5,7,12,14,16	2,6,8,9,10,11,13,15	4	P97840,Q5RLM2,Q68G31,Q99MH3
3203	1,3,4,5,7,12,15,16	2,6,8,9,10,11,13,14	3	P10758,P97840,Q68G31
3204	1,3,4,5,7,13,14,15	2,6,8,9,10,11,12,16	0	
3205	1,3,4,5,7,13,14,16	2,6,8,9,10,11,12,15	3	B0BNN3,Q99MH3,Q9WUW8
3206	1,3,4,5,7,13,15,16	2,6,8,9,10,11,12,14	1	P97840
3207	1,3,4,5,7,14,15,16	2,6,8,9,10,11,12,13	1	P08649
3208	1,3,4,5,8,9,10,11	2,6,7,12,13,14,15,16	8	O88797,P01681,P01835,P27590,Q4FZU2,Q6IFW6,Q6IMF3,Q6P6Q2
3209	1,3,4,5,8,9,10,12	2,6,7,11,13,14,15,16	2	P01681,P01835
3210	1,3,4,5,8,9,10,13	2,6,7,11,12,14,15,16	1	P01681
3211	1,3,4,5,8,9,10,14	2,6,7,11,12,13,15,16	3	P01681,P01835,P20760

3212	1,3,4,5,8,9,10,15	2,6,7,11,12,13,14,16	3	P01681,P01835,P62804
3213	1,3,4,5,8,9,10,16	2,6,7,11,12,13,14,15	3	P01681,P01835,P20760
3214	1,3,4,5,8,9,11,12	2,6,7,10,13,14,15,16	9	O88797,P01681,P01835,P27590,Q4FZU2,Q6IFW6,Q6IMF3,Q6P6Q2,Q6Q7Y5
3215	1,3,4,5,8,9,11,13	2,6,7,10,12,14,15,16	4	P01681,Q4FZU2,Q6IMF3,Q6P6Q2
3216	1,3,4,5,8,9,11,14	2,6,7,10,12,13,15,16	8	O88797,P01681,P01835,Q4FZU2,Q63751,Q6IFW6,Q6IMF3,Q6P6Q2
3217	1,3,4,5,8,9,11,15	2,6,7,10,12,13,14,16	10	O88797,P01681,P01835,Q4FZU2,Q6IFU7,Q6IFU8,Q6IFW6,Q6IG02,Q6IMF3,Q6P6Q2
3218	1,3,4,5,8,9,11,16	2,6,7,10,12,13,14,15	7	O88797,P01681,P01835,Q4FZU2,Q6IFW6,Q6IMF3,Q6P6Q2
3219	1,3,4,5,8,9,12,13	2,6,7,10,11,14,15,16	1	P01681
3220	1,3,4,5,8,9,12,14	2,6,7,10,11,13,15,16	1	P01681
3221	1,3,4,5,8,9,12,15	2,6,7,10,11,13,14,16	3	P01681,P01835,P36376
3222	1,3,4,5,8,9,12,16	2,6,7,10,11,13,14,15	2	P01681,P01835
3223	1,3,4,5,8,9,13,14	2,6,7,10,11,12,15,16	1	P01681
3224	1,3,4,5,8,9,13,15	2,6,7,10,11,12,14,16	3	P01681,P62804,Q00715
3225	1,3,4,5,8,9,13,16	2,6,7,10,11,12,14,15	1	P01681
3226	1,3,4,5,8,9,14,15	2,6,7,10,11,12,13,16	1	P01681
3227	1,3,4,5,8,9,14,16	2,6,7,10,11,12,13,15	3	P01681,P20760,P23593
3228	1,3,4,5,8,9,15,16	2,6,7,10,11,12,13,14	2	P01681,P23593
3229	1,3,4,5,8,10,11,12	2,6,7,9,13,14,15,16	12	P01681,P01835,P27590,P47967,P97840,Q4FZU2,Q5I0D1,Q6IFU7,Q6IFW6,Q6IG02,Q6IMF3,Q6P6Q2
3230	1,3,4,5,8,10,11,13	2,6,7,9,12,14,15,16	9	P01681,P47967,P97840,Q4FZU2,Q6IFU7,Q6IFW6,Q6IG02,Q6IMF3,Q6P6Q2
3231	1,3,4,5,8,10,11,14	2,6,7,9,12,13,15,16	8	P01681,P01835,Q4FZU2,Q6IFU7,Q6IFW6,Q6IG02,Q6IMF3,Q6P6Q2

3232	1,3,4,5,8,10,11,15	2,6,7,9,12,13,14,16	9	P01681,P01835,Q4FZU2,Q6IFU7,Q6IFU8,Q6IFW6,Q6IG02,Q6IMF3,Q6P6Q2
3233	1,3,4,5,8,10,11,16	2,6,7,9,12,13,14,15	9	O88797,P01681,P01835,Q4FZU2,Q6IFU7,Q6IFW6,Q6IG02,Q6IMF3,Q6P6Q2
3234	1,3,4,5,8,10,12,13	2,6,7,9,11,14,15,16	4	P01681,P47967,P97840,Q9R168
3235	1,3,4,5,8,10,12,14	2,6,7,9,11,13,15,16	2	P01681,Q5RLM2
3236	1,3,4,5,8,10,12,15	2,6,7,9,11,13,14,16	5	P01681,P0DMW0;P0DMW1,P10758,P34901,Q9R168
3237	1,3,4,5,8,10,12,16	2,6,7,9,11,13,14,15	3	P01681,P0DMW0;P0DMW1,P97840
3238	1,3,4,5,8,10,13,14	2,6,7,9,11,12,15,16	2	P01681,Q9WUW8
3239	1,3,4,5,8,10,13,15	2,6,7,9,11,12,14,16	2	P01681,P62804
3240	1,3,4,5,8,10,13,16	2,6,7,9,11,12,14,15	5	O35077,P01681,P46844,Q8R431,Q9WUW8
3241	1,3,4,5,8,10,14,15	2,6,7,9,11,12,13,16	2	P01681,P54921
3242	1,3,4,5,8,10,14,16	2,6,7,9,11,12,13,15	3	O35077,P01681,Q9WUW8
3243	1,3,4,5,8,10,15,16	2,6,7,9,11,12,13,14	10	O35077,P01681,P19468,P48508,P53790,Q63270,Q63424,Q64093,Q6Q0N1,Q8R431
3244	1,3,4,5,8,11,12,13	2,6,7,9,10,14,15,16	11	P01681,P47967,P97840,Q4FZU2,Q5I0D1,Q6IFU7,Q6IFW6,Q6IG02,Q6IMF3,Q6P6Q2,Q811M5
3245	1,3,4,5,8,11,12,14	2,6,7,9,10,13,15,16	9	D3ZUC6,P01681,Q4FZU2,Q6IFU7,Q6IFW6,Q6IG02,Q6IMF3,Q6P6Q2,Q811M5
3246	1,3,4,5,8,11,12,15	2,6,7,9,10,13,14,16	11	P01681,P97840,Q4FZU2,Q6IFU7,Q6IFU8,Q6IFW6,Q6IG02,Q6IG05,Q6IMF3,Q6P6Q2,Q9R168
3247	1,3,4,5,8,11,12,16	2,6,7,9,10,13,14,15	14	O88797,P00762,P01681,P01835,P02782,P08010,P36374,P97840,Q4FZU2,Q6IFU7,Q6IFW6,Q6IG02,Q6IMF3,Q6P6Q2
3248	1,3,4,5,8,11,13,14	2,6,7,9,10,12,15,16	10	B0BNN3,O54728,P01681,Q4FZU2,Q6IFU7,Q6IFW6,Q6IG02,Q6IMF3,Q6P6Q2,Q811M5
3249	1,3,4,5,8,11,13,15	2,6,7,9,10,12,14,16	8	P01681,Q4FZU2,Q6IFU7,Q6IFU8,Q6IFW6,Q6IG02,Q6IMF3,Q6P6Q2
3250	1,3,4,5,8,11,13,16	2,6,7,9,10,12,14,15	11	B0BNN3,P00762,P01681,P02782,P30120,Q4FZU2,Q6IFU7,Q6IFW6,Q6IG02,Q6IMF3,Q6P6Q2
3251	1,3,4,5,8,11,14,15	2,6,7,9,10,12,13,16	9	D3ZUC6,P01681,Q4FZU2,Q6IFU7,Q6IFU8,Q6IFW6,Q6IG02,Q6IMF3,Q6P6Q2

3252	1,3,4,5,8,11,14,16	2,6,7,9,10,12,13,15	13	O54728,O88797,P00762,P01681,P02782,P08649,Q4FZU2,Q6IFU7,Q6IFU8,Q6IFW6,Q6IG02,Q6IMF3,Q6P6Q2
3253	1,3,4,5,8,11,15,16	2,6,7,9,10,12,13,14	12	O88797,P00762,P01681,P29315,Q10758,Q4FZU2,Q6IFU7,Q6IFU8,Q6IFW6,Q6IG02,Q6IMF3,Q6P6Q2
3254	1,3,4,5,8,12,13,14	2,6,7,9,10,11,15,16	4	D3ZUC6,P01681,P20766,Q811M5
3255	1,3,4,5,8,12,13,15	2,6,7,9,10,11,14,16	3	P01681,P97840,Q9R168
3256	1,3,4,5,8,12,13,16	2,6,7,9,10,11,14,15	2	P00762,P97840
3257	1,3,4,5,8,12,14,15	2,6,7,9,10,11,13,16	3	D3ZUC6,P01681,Q9R168
3258	1,3,4,5,8,12,14,16	2,6,7,9,10,11,13,15	2	P00762,P01681
3259	1,3,4,5,8,12,15,16	2,6,7,9,10,11,13,14	3	P00762,P01681,Q9R168
3260	1,3,4,5,8,13,14,15	2,6,7,9,10,11,12,16	3	B0BNN3,D3ZUC6,P01681
3261	1,3,4,5,8,13,14,16	2,6,7,9,10,11,12,15	6	B0BNN3,O35077,O54728,P00762,Q99MH3,Q9WUW8
3262	1,3,4,5,8,13,15,16	2,6,7,9,10,11,12,14	3	O35077,P00762,P01681
3263	1,3,4,5,8,14,15,16	2,6,7,9,10,11,12,13	2	O35077,P01681
3264	1,3,4,5,9,10,11,12	2,6,7,8,13,14,15,16	3	P01835,Q03191,Q62714
3265	1,3,4,5,9,10,11,13	2,6,7,8,12,14,15,16	0	
3266	1,3,4,5,9,10,11,14	2,6,7,8,12,13,15,16	0	
3267	1,3,4,5,9,10,11,15	2,6,7,8,12,13,14,16	5	P01681,Q4FZU2,Q6IFW6,Q6IG02,Q6P6Q2
3268	1,3,4,5,9,10,11,16	2,6,7,8,12,13,14,15	0	
3269	1,3,4,5,9,10,12,13	2,6,7,8,11,14,15,16	2	P50280,Q9QZK9
3270	1,3,4,5,9,10,12,14	2,6,7,8,11,13,15,16	2	P09456,P30120
3271	1,3,4,5,9,10,12,15	2,6,7,8,11,13,14,16	7	P01681,P09456,P10758,P30120,P46462,Q00715,Q99041

3272	1,3,4,5,9,10,12,16	2,6,7,8,11,13,14,15	0
3273	1,3,4,5,9,10,13,14	2,6,7,8,11,12,15,16	2 P12020,Q00715
3274	1,3,4,5,9,10,13,15	2,6,7,8,11,12,14,16	6 P12020,P30120,P46462,P62804,Q00715,Q8CFN2
3275	1,3,4,5,9,10,13,16	2,6,7,8,11,12,14,15	2 P11883,P12020
3276	1,3,4,5,9,10,14,15	2,6,7,8,11,12,13,16	7 P01681,P09456,P12020,P30120,P46462,P62804,Q00715
3277	1,3,4,5,9,10,14,16	2,6,7,8,11,12,13,15	1 P12020
3278	1,3,4,5,9,10,15,16	2,6,7,8,11,12,13,14	1 P12020
3279	1,3,4,5,9,11,12,13	2,6,7,8,10,14,15,16	2 Q03191,Q62761;Q62762;Q62763
3280	1,3,4,5,9,11,12,14	2,6,7,8,10,13,15,16	3 P02631,Q03191,Q62761;Q62762;Q62763
3281	1,3,4,5,9,11,12,15	2,6,7,8,10,13,14,16	7 P01681,P02631,Q4FZU2,Q62761;Q62762;Q62763,Q6IFW6,Q6IG02,Q6P6Q2
3282	1,3,4,5,9,11,12,16	2,6,7,8,10,13,14,15	2 P02631,Q03191
3283	1,3,4,5,9,11,13,14	2,6,7,8,10,12,15,16	3 P02631,P20766,Q63598
3284	1,3,4,5,9,11,13,15	2,6,7,8,10,12,14,16	6 P02631,P62804,Q4FZU2,Q6IFW6,Q6IG02,Q6P6Q2
3285	1,3,4,5,9,11,13,16	2,6,7,8,10,12,14,15	0
3286	1,3,4,5,9,11,14,15	2,6,7,8,10,12,13,16	6 P01681,P02631,Q4FZU2,Q6IFW6,Q6IG02,Q6P6Q2
3287	1,3,4,5,9,11,14,16	2,6,7,8,10,12,13,15	1 P02631
3288	1,3,4,5,9,11,15,16	2,6,7,8,10,12,13,14	8 P01681,P02631,P25809,P29315,Q4FZU2,Q6IFW6,Q6IG02,Q6P6Q2
3289	1,3,4,5,9,12,13,14	2,6,7,8,10,11,15,16	2 P20766,P50280
3290	1,3,4,5,9,12,13,15	2,6,7,8,10,11,14,16	2 P62804,Q00715
3291	1,3,4,5,9,12,13,16	2,6,7,8,10,11,14,15	0

3292	1,3,4,5,9,12,14,15	2,6,7,8,10,11,13,16	5 P01681,P02631,P09456,P30120,Q00715
3293	1,3,4,5,9,12,14,16	2,6,7,8,10,11,13,15	1 Q68G31
3294	1,3,4,5,9,12,15,16	2,6,7,8,10,11,13,14	3 P02631,P25809,Q68G31
3295	1,3,4,5,9,13,14,15	2,6,7,8,10,11,12,16	5 P02631,P12020,P20766,P62804,Q00715
3296	1,3,4,5,9,13,14,16	2,6,7,8,10,11,12,15	0
3297	1,3,4,5,9,13,15,16	2,6,7,8,10,11,12,14	3 P12020,P62804,Q00715
3298	1,3,4,5,9,14,15,16	2,6,7,8,10,11,12,13	2 P02631,P12020
3299	1,3,4,5,10,11,12,13	2,6,7,8,9,14,15,16	3 P47967,P97840,Q5I0D1
3300	1,3,4,5,10,11,12,14	2,6,7,8,9,13,15,16	0
3301	1,3,4,5,10,11,12,15	2,6,7,8,9,13,14,16	5 P97840,Q4FZU2,Q6IFW6,Q6IG02,Q6P6Q2
3302	1,3,4,5,10,11,12,16	2,6,7,8,9,13,14,15	1 P97840
3303	1,3,4,5,10,11,13,14	2,6,7,8,9,12,15,16	0
3304	1,3,4,5,10,11,13,15	2,6,7,8,9,12,14,16	5 P97840,Q4FZU2,Q6IFW6,Q6IG02,Q6P6Q2
3305	1,3,4,5,10,11,13,16	2,6,7,8,9,12,14,15	1 P97840
3306	1,3,4,5,10,11,14,15	2,6,7,8,9,12,13,16	4 Q4FZU2,Q6IFW6,Q6IG02,Q6P6Q2
3307	1,3,4,5,10,11,14,16	2,6,7,8,9,12,13,15	0
3308	1,3,4,5,10,11,15,16	2,6,7,8,9,12,13,14	6 P29315,Q4FZU2,Q6IFW6,Q6IG02,Q6IMF3,Q6P6Q2
3309	1,3,4,5,10,12,13,14	2,6,7,8,9,11,15,16	0
3310	1,3,4,5,10,12,13,15	2,6,7,8,9,11,14,16	4 P09456,P62804,P97840,Q812E4
3311	1,3,4,5,10,12,13,16	2,6,7,8,9,11,14,15	2 P0DMW0;P0DMW1,P97840

3312	1,3,4,5,10,<br>12,14,15	2,6,7,8,9,1<br>1,13,16	3 P09456,P30120,Q812E4
3313	1,3,4,5,10,<br>12,14,16	2,6,7,8,9,1<br>1,13,15	0
3314	1,3,4,5,10,<br>12,15,16	2,6,7,8,9,1<br>1,13,14	3 P0DMW0;P0DMW1,P10758,P97840
3315	1,3,4,5,10,<br>13,14,15	2,6,7,8,9,1<br>1,12,16	5 P09456,P12020,P62804,Q00715,Q812E4
3316	1,3,4,5,10,<br>13,14,16	2,6,7,8,9,1<br>1,12,15	1 Q99MH3
3317	1,3,4,5,10,<br>13,15,16	2,6,7,8,9,1<br>1,12,14	3 O35077,P11883,P12020
3318	1,3,4,5,10,<br>14,15,16	2,6,7,8,9,1<br>1,12,13	0
3319	1,3,4,5,11,<br>12,13,14	2,6,7,8,9,1<br>0,15,16	2 P20766,Q811M5
3320	1,3,4,5,11,<br>12,13,15	2,6,7,8,9,1<br>0,14,16	5 P97840,Q4FZU2,Q6IFW6,Q6IG02,Q6P6Q2
3321	1,3,4,5,11,<br>12,13,16	2,6,7,8,9,1<br>0,14,15	2 P47967,P97840
3322	1,3,4,5,11,<br>12,14,15	2,6,7,8,9,1<br>0,13,16	6 P02631,P20766,Q4FZU2,Q6IFW6,Q6IG02,Q6P6Q2
3323	1,3,4,5,11,<br>12,14,16	2,6,7,8,9,1<br>0,13,15	1 P02631
3324	1,3,4,5,11,<br>12,15,16	2,6,7,8,9,1<br>0,13,14	8 P02631,P29315,P97840,Q4FZU2,Q6IFW6,Q6IG02,Q6IMF3,Q6P6Q2
3325	1,3,4,5,11,<br>13,14,15	2,6,7,8,9,1<br>0,12,16	6 P02631,P20766,Q4FZU2,Q6IFW6,Q6IG02,Q6P6Q2
3326	1,3,4,5,11,<br>13,14,16	2,6,7,8,9,1<br>0,12,15	3 B0BNN3,O54728,Q99MH3
3327	1,3,4,5,11,<br>13,15,16	2,6,7,8,9,1<br>0,12,14	5 P29315,Q4FZU2,Q6IFW6,Q6IG02,Q6P6Q2
3328	1,3,4,5,11,<br>14,15,16	2,6,7,8,9,1<br>0,12,13	7 P02631,P29315,Q4FZU2,Q6IFW6,Q6IG02,Q6IMF3,Q6P6Q2
3329	1,3,4,5,12,<br>13,14,15	2,6,7,8,9,1<br>0,11,16	1 P20766
3330	1,3,4,5,12,<br>13,14,16	2,6,7,8,9,1<br>0,11,15	3 P20766,Q6P6S4,Q99MH3
3331	1,3,4,5,12,<br>13,15,16	2,6,7,8,9,1<br>0,11,14	1 P97840

3332	1,3,4,5,12,14,15,16	2,6,7,8,9,10,11,13	1	P02631
3333	1,3,4,5,13,14,15,16	2,6,7,8,9,10,11,12	1	P20766
3334	1,3,4,6,7,8,9,10	2,5,11,12,13,14,15,16	16	O35763,O70417,O70594,P01681,P06761,P18418,P19629,P20646,P23739,P70545,Q5QE79,Q63598,Q63618,Q6P6R2,Q923S2,Q9R0T3
3335	1,3,4,6,7,8,9,11	2,5,10,12,13,14,15,16	38	O70417,P01681,P02780,P02781,P02782,P06761,P06911,P07150,P07647,P08723,P0C0A9,P11598,P20646,P22273,P22282,P22283,P35280,P36374,P40241,P46462,P50280,Q5GRG2,Q5M8C6,Q5QE79,Q62902,Q62946,Q63493,Q63617,Q63751,Q6AYR9,Q6P6R2,Q6P6S4,Q6Q7Y5,Q812E4,Q9JHB9,Q9JI85,Q9QW07,Q9R0T3
3336	1,3,4,6,7,8,9,12	2,5,10,11,13,14,15,16	7	O70417,P01681,P06761,P06911,Q5QE79,Q62946,Q9R0T3
3337	1,3,4,6,7,8,9,13	2,5,10,11,12,14,15,16	11	O70417,P01681,P06761,P06911,P20646,Q5QE79,Q62902,Q62946,Q6P6R2,Q9JI85,Q9R0T3
3338	1,3,4,6,7,8,9,14	2,5,10,11,12,13,15,16	9	O70417,P01681,P06761,P06911,P08937,P20646,Q5QE79,Q63751,Q6P6R2
3339	1,3,4,6,7,8,9,15	2,5,10,11,12,13,14,16	8	O70417,P01681,P02091,P19629,P20646,P35280,Q6P6R2,Q9Z0V6
3340	1,3,4,6,7,8,9,16	2,5,10,11,12,13,14,15	19	O70417,P01681,P02780,P02781,P02782,P02783,P06761,P06911,P07647,P18418,P19629,P20646,P20761,P22006,P23593,Q5M8C6,Q62902,Q9JHB9,Q9R0T3
3341	1,3,4,6,7,8,10,11	2,5,9,12,13,14,15,16	36	O70417,P02780,P02781,P02782,P06761,P06911,P07647,P08723,P0C0A9,P11598,P18418,P18757,P19629,P22273,P22282,P22283,P30904,P36374,P40241,P46462,P50280,Q4G063,Q5GRG2,Q5M7T9,Q5M8C6,Q62902,Q63493,Q63617,Q6B345,Q6P6R2,Q99041,Q9JHB9,Q9JI85,Q9QW07,Q9R0T3,Q9WTW7
3342	1,3,4,6,7,8,10,12	2,5,9,11,13,14,15,16	30	O35763,O70377,O70417,P06761,P06911,P18418,P19629,P30904,P46844,P48508,P53790,P57113,Q03248,Q05175,Q3ZAV1,Q5M7T9,Q5RLM2,Q63270,Q63598,Q63618,Q66HG3,Q6MG61,Q6Q0N1,Q80W57,Q8R431,Q923S2,Q923V8,Q9JJ40,Q9R0T3,Q9WTW7
3343	1,3,4,6,7,8,10,13	2,5,9,11,12,14,15,16	35	O55004,O70257,O70377,O70417,O70594,P02631,P06761,P06911,P18418,P19468,P19629,P30904,P36860,P46844,P48508,P51907,P53790,P57113,P70549,Q3ZAV1,Q5M7T9,Q63424,Q63598,Q63618,Q64093,Q66HG3,Q6MG61,Q6Q0N1,Q80W57,Q8R431,Q9JI85,Q9R0T3,Q9WTW7,Q9WUW9,Q9Z0W7
3344	1,3,4,6,7,8,10,14	2,5,9,11,12,13,15,16	32	O70257,O70377,O70417,O70594,P06761,P06911,P08937,P18418,P18757,P19629,P20761,P46844,P48508,P53790,P54921,P57113,P63081,P97580,Q03248,Q05175,Q5M7T9,Q5QE79,Q5RLM2,Q63424,Q63598,Q63751,Q64602,Q66HG3,Q6Q0N1,Q923S2,Q9WUW8,Q9WUW9

3345	1,3,4,6,7,8,10,15	2,5,9,11,12,13,14,16	52	O35763,O70257,O70377,O70417,O70594,O88339;Q4V882,P01681,P02091,P06761,P08721,P10758,P18418,P18757,P19468,P19629,P20646,P20761,P29975,P30904,P38918,P46844,P48508,P51907,P53790,P54921,P57113,P68035;P68136,Q03248,Q05175,Q3MIE4,Q3ZAV1,Q5I0E9,Q5M7T9,Q5RKI1,Q63270,Q63424,Q63598,Q63618,Q64093,Q64602,Q6AY41,Q6MG61,Q6Q0N1,Q80W57,Q8R431,Q923S2,Q99MZ8,Q9JJ40,Q9JLJ3,Q9R1T5,Q9WTW7,Q9Z0W7
3346	1,3,4,6,7,8,10,16	2,5,9,11,12,13,14,15	72	O35077,O35763,O55004,O70257,O70377,O70417,O70594,P02780,P02781,P02782,P02783,P06761,P06911,P07151,P07647,P15399,P18418,P18757,P19468,P19629,P20646,P20761,P22282,P22283,P23928,P28570,P29975,P38918,P46720,P46844,P48508,P51907,P53790,P57113,P60711;P63259,P68035;P68136,P97580,Q03248,Q05175,Q30KJ2,Q3T1J9,Q3ZAV1,Q5I0E9,Q5M7T9,Q5M8C6,Q62687,Q63270,Q63424,Q63598,Q63618,Q64093,Q64319,Q64602,Q66HG3,Q68FT5,Q6AY33,Q6AY41,Q6MG61,Q6Q0N1,Q71MB6,Q80W57,Q8R431,Q923S2,Q9JHB9,Q9JJ40,Q9JLJ3,Q9QYU4,Q9R0T3,Q9R1T5,Q9WTW7,Q9WUW9,Q9Z0W7
3347	1,3,4,6,7,8,11,12	2,5,9,10,13,14,15,16	35	O35547,O70417,P02780,P02781,P02782,P04905,P06761,P06911,P07647,P08723,P0C0A9,P11598,P12020,P19629,P22273,P22282,P22283,P36374,P40241,P46462,P50280,P70709,P97840,Q5GRG2,Q5I0D1,Q5M8C6,Q62902,Q62946,Q63493,Q63617,Q99041,Q9JHB9,Q9JI85,Q9QW07,Q9R0T3
3348	1,3,4,6,7,8,11,13	2,5,9,10,12,14,15,16	30	B0BNN3,P02780,P02781,P02782,P06761,P06911,P07647,P08723,P0C0A9,P11598,P12020,P22273,P22282,P22283,P36374,P40241,P46462,P50280,Q4G063,Q5GRG2,Q5M8C6,Q62902,Q62946,Q63493,Q63617,Q6AYR9,Q99041,Q9JHB9,Q9JI85,Q9R0T3
3349	1,3,4,6,7,8,11,14	2,5,9,10,12,13,15,16	38	B0BNN3,O70417,P02780,P02781,P02782,P02783,P06761,P06911,P07150,P07647,P08723,P08937,P0C0A9,P11598,P12020,P22273,P22282,P22283,P36374,P40241,P46462,P50280,Q4G063,Q4G075,Q5GRG2,Q5M8C6,Q5QE79,Q62902,Q63493,Q63617,Q63751,Q6AYR9,Q812E4,Q99041,Q9JHB9,Q9JI85,Q9QW07,Q9R0T3
3350	1,3,4,6,7,8,11,15	2,5,9,10,12,13,14,16	42	P01681,P02091,P02780,P02781,P02782,P06761,P06911,P07647,P08721,P08723,P0C0A9,P11598,P19629,P22273,P22282,P22283,P36374,P40241,P46462,P50280,Q4FZU2,Q4G063,Q5GRG2,Q5M8C6,Q62902,Q63493,Q63617,Q6AYR9,Q6IFU7,Q6IFU8,Q6IFW6,Q6IG02,Q6IMF3,Q6P6Q2,Q6P6S4,Q6Q0N0,Q8CJ52,Q99041,Q9JHB9,Q9JI85,Q9QW07,Q9R0T3
3351	1,3,4,6,7,8,11,16	2,5,9,10,12,13,14,15	47	O35547,P00762,P02780,P02781,P02782,P02783,P04905,P05369,P06761,P06911,P07647,P08010,P08723,P09456,P0C0A9,P11598,P12020,P18418,P19629,P20761,P22006,P22273,P22282,P22283,P24368,P30120,P36374,P40241,P46462,P50280,P60905,Q4G063,Q5GRG2,Q5M8C6,Q62902,Q63493,Q63617,Q6Q0N0,Q812E4,Q8CFN2,Q99041,Q9JHB9,Q9JI85,Q9QW07,Q9QZK8,Q9QZK9,Q9R0T3
3352	1,3,4,6,7,8,12,13	2,5,9,10,11,14,15,16	10	O70417,P06761,P06911,P22282,P70549,P97840,Q62946,Q9JI85,Q9R0T3,Q9Z2L0
3353	1,3,4,6,7,8,12,14	2,5,9,10,11,13,15,16	8	O70417,P06761,P06911,P08937,Q5QE79,Q5RLM2,Q63751,Q9Z2L0
3354	1,3,4,6,7,8,12,15	2,5,9,10,11,13,14,16	8	O70417,P02091,P06761,P06911,P10758,P19629,P70549,Q63598
3355	1,3,4,6,7,8,12,16	2,5,9,10,11,13,14,15	22	O70417,P02780,P02781,P02782,P02783,P06761,P06911,P07151,P07647,P19629,P20761,P22282,P22283,P46844,P57113,Q03248,Q5M8C6,Q63598,Q9JHB9,Q9JI85,Q9R0T3,Q9WTW7

3356	1,3,4,6,7,8,13,14	2,5,9,10,11,12,15,16	13	B0BNN3,O70417,P06761,P06911,P08937,P20761,P63081,P70549,Q5QE79,Q63751,Q9JI85,Q9WUW8,Q9Z2L0
3357	1,3,4,6,7,8,13,15	2,5,9,10,11,12,14,16	10	O70257,O70594,P02091,P06761,P06911,P19629,P70549,Q6Q0N1,Q9JI85,Q9Z2L0
3358	1,3,4,6,7,8,13,16	2,5,9,10,11,12,14,15	32	B0BNN3,O70257,O70594,P00762,P02780,P02781,P02782,P02783,P06761,P06911,P07151,P07647,P11598,P18418,P19468,P20761,P22006,P22282,P22283,P30120,P46844,P57113,Q5M7T9,Q5M8C6,Q62902,Q63424,Q6Q0N1,Q9JHB9,Q9JI85,Q9R0T3,Q9WUW8,Q9Z2L0
3359	1,3,4,6,7,8,14,15	2,5,9,10,11,12,13,16	12	O70417,P02091,P02783,P06761,P06911,P19629,P20646,P20761,P54921,Q5QE79,Q63751,Q9Z2L0
3360	1,3,4,6,7,8,14,16	2,5,9,10,11,12,13,15	23	B0BNN3,O70417,P02780,P02781,P02782,P02783,P06761,P06911,P07151,P07647,P20761,P22006,P22283,P46844,P57113,P63081,Q5M8C6,Q5QE79,Q63751,Q923S2,Q9JHB9,Q9WUW8,Q9Z2L0
3361	1,3,4,6,7,8,15,16	2,5,9,10,11,12,13,14	44	O35077,O70257,O70377,O70594,P02091,P02781,P02782,P02783,P06761,P06911,P07151,P07647,P08721,P09606,P17988,P19468,P19629,P20646,P20761,P22006,P46844,P48508,P50280,P53790,P57113,Q03248,Q05175,Q3ZAV1,Q5M7T9,Q5M8C6,Q63270,Q63424,Q63598,Q64093,Q6MG61,Q6Q0N1,Q80W57,Q8CJ52,Q8R431,Q923S2,Q9JHB9,Q9R1T5,Q9WTW7,Q9Z0W7
3362	1,3,4,6,7,9,10,11	2,5,8,12,13,14,15,16	2	P11598,Q63617
3363	1,3,4,6,7,9,10,12	2,5,8,11,13,14,15,16	0	
3364	1,3,4,6,7,9,10,13	2,5,8,11,12,14,15,16	0	
3365	1,3,4,6,7,9,10,14	2,5,8,11,12,13,15,16	3	O70417,Q5QE79,Q63751
3366	1,3,4,6,7,9,10,15	2,5,8,11,12,13,14,16	5	P02091,P08721,P10758,Q5RKI1,Q8K1G0
3367	1,3,4,6,7,9,10,16	2,5,8,11,12,13,14,15	3	P02783,P22006,Q8K1G0
3368	1,3,4,6,7,9,11,12	2,5,8,10,13,14,15,16	4	P11598,P22283,Q63493,Q63617
3369	1,3,4,6,7,9,11,13	2,5,8,10,12,14,15,16	3	P11598,P22283,Q63617
3370	1,3,4,6,7,9,11,14	2,5,8,10,12,13,15,16	4	P11598,Q5QE79,Q63617,Q63751
3371	1,3,4,6,7,9,11,15	2,5,8,10,12,13,14,16	4	P02091,P08721,Q63617,Q8CJ52
3372	1,3,4,6,7,9,11,16	2,5,8,10,12,13,14,15	13	P02780,P02781,P02783,P07647,P08723,P0C0A9,P11598,P22006,P22283,Q62902,Q63617,Q9JHB9,Q9QW07
3373	1,3,4,6,7,9,12,13	2,5,8,10,11,14,15,16	0	

3374	1,3,4,6,7,9,12,14	2,5,8,10,11,13,15,16	2	O70417,Q63751
3375	1,3,4,6,7,9,12,15	2,5,8,10,11,13,14,16	2	P02091,P10758
3376	1,3,4,6,7,9,12,16	2,5,8,10,11,13,14,15	2	P02783,P22006
3377	1,3,4,6,7,9,13,14	2,5,8,10,11,12,15,16	1	Q5QE79
3378	1,3,4,6,7,9,13,15	2,5,8,10,11,12,14,16	4	P02091,P62804,P70549,Q00715
3379	1,3,4,6,7,9,13,16	2,5,8,10,11,12,14,15	2	P02783,P22006
3380	1,3,4,6,7,9,14,15	2,5,8,10,11,12,13,16	1	P02091
3381	1,3,4,6,7,9,14,16	2,5,8,10,11,12,13,15	2	P02783,P22006
3382	1,3,4,6,7,9,15,16	2,5,8,10,11,12,13,14	4	P02091,P02783,P08721,P22006
3383	1,3,4,6,7,10,11,12	2,5,8,9,13,14,15,16	4	P11598,P22283,P47967,P97840
3384	1,3,4,6,7,10,11,13	2,5,8,9,12,14,15,16	5	P11598,P22283,P97840,Q63617,Q8CJD3
3385	1,3,4,6,7,10,11,14	2,5,8,9,12,13,15,16	3	P11598,Q63751,Q8CJD3
3386	1,3,4,6,7,10,11,15	2,5,8,9,12,13,14,16	9	P02091,P08721,P11598,P17988,Q5RKI1,Q6IFW6,Q6IG02,Q8CJ52,Q8CJD3
3387	1,3,4,6,7,10,11,16	2,5,8,9,12,13,14,15	12	P02780,P02781,P02783,P07647,P08723,P0C0A9,P11598,P22006,P22283,Q63617,Q8CJD3,Q9JHB9
3388	1,3,4,6,7,10,12,13	2,5,8,9,11,14,15,16	3	P70549,P97840,Q66HG3
3389	1,3,4,6,7,10,12,14	2,5,8,9,11,13,15,16	2	O70417,P57113
3390	1,3,4,6,7,10,12,15	2,5,8,9,11,13,14,16	4	P02091,P10758,Q5RKI1,Q63598
3391	1,3,4,6,7,10,12,16	2,5,8,9,11,13,14,15	12	P02783,P0DMW0;P0DMW1,P10758,P18297,P46844,P57113,P97840,Q03248,Q63598,Q71MB6,Q9JJ40,Q9WTW7
3392	1,3,4,6,7,10,13,14	2,5,8,9,11,12,15,16	2	P57113,P70549
3393	1,3,4,6,7,10,13,15	2,5,8,9,11,12,14,16	9	P02091,P08721,P19468,P51792;P51794;P51796,P57113,P70549,Q5RKI1,Q63618,Q923M1

3394	1,3,4,6,7,10,13,16	2,5,8,9,11,12,14,15	9	P02783,P18297,P19468,P28570,P46844,P51792;P51794;P51796,P57113,Q63424,Q64093
3395	1,3,4,6,7,10,14,15	2,5,8,9,11,12,13,16	5	P01946,P02091,P54921,P57113,Q5RKI1
3396	1,3,4,6,7,10,14,16	2,5,8,9,11,12,13,15	7	P02783,P18297,P46844,P57113,Q03248,Q71MB6,Q923S2
3397	1,3,4,6,7,10,15,16	2,5,8,9,11,12,13,14	30	O70377,O70594,P02091,P02783,P08721,P10758,P17988,P18297,P19468,P29975,P46844,P48508,P53790,P57113,Q03248,Q5I0E9,Q5M7T9,Q5RKI1,Q63270,Q63424,Q63598,Q64093,Q6Q0N1,Q71MB6,Q80W57,Q8R431,Q923M1,Q9JJ40,Q9WTW7,Q9Z0W7
3398	1,3,4,6,7,11,12,13	2,5,8,9,10,14,15,16	7	P11598,P22283,P47967,P97840,Q5I0D1,Q63493,Q63617
3399	1,3,4,6,7,11,12,14	2,5,8,9,10,13,15,16	3	P11598,P22283,Q63751
3400	1,3,4,6,7,11,12,15	2,5,8,9,10,13,14,16	8	P02091,P08721,P11598,P22283,P97840,Q6IFW6,Q6IG02,Q8CJ52
3401	1,3,4,6,7,11,12,16	2,5,8,9,10,13,14,15	18	O35547,P02780,P02781,P02782,P02783,P05369,P07647,P08723,P0C0A9,P11598,P22006,P22283,P36374,P97840,Q63493,Q63617,Q9JHB9,Q9QW07
3402	1,3,4,6,7,11,13,14	2,5,8,9,10,12,15,16	5	B0BNN3,P11598,P22283,Q63617,Q99041
3403	1,3,4,6,7,11,13,15	2,5,8,9,10,12,14,16	8	P02091,P08721,P11598,P22283,Q63617,Q6IG02,Q8CJ52,Q8CJD3
3404	1,3,4,6,7,11,13,16	2,5,8,9,10,12,14,15	17	O35547,P00714,P02780,P02781,P02782,P02783,P05369,P07647,P08723,P0C0A9,P11598,P22006,P22283,Q62902,Q63617,Q99041,Q9JHB9
3405	1,3,4,6,7,11,14,15	2,5,8,9,10,12,13,16	5	P02091,P02783,P11598,Q6IG02,Q8CJ52
3406	1,3,4,6,7,11,14,16	2,5,8,9,10,12,13,15	13	P02780,P02781,P02782,P02783,P07647,P08723,P0C0A9,P11598,P22006,P22283,Q63617,Q99041,Q9JHB9
3407	1,3,4,6,7,11,15,16	2,5,8,9,10,12,13,14	18	P02091,P02780,P02781,P02783,P07647,P08721,P08723,P0C0A9,P11598,P17988,P22006,P22283,Q63617,Q6IFW6,Q6IG02,Q8CJ52,Q9JHB9,Q9QW07
3408	1,3,4,6,7,12,13,14	2,5,8,9,10,11,15,16	2	P70549,Q9Z2L0
3409	1,3,4,6,7,12,13,15	2,5,8,9,10,11,14,16	4	P02091,P10758,P70549,P97840
3410	1,3,4,6,7,12,13,16	2,5,8,9,10,11,14,15	5	P02783,P07647,P07943,P70549,P97840
3411	1,3,4,6,7,12,14,15	2,5,8,9,10,11,13,16	1	P02091
3412	1,3,4,6,7,12,14,16	2,5,8,9,10,11,13,15	1	P02783
3413	1,3,4,6,7,12,15,16	2,5,8,9,10,11,13,14	4	P02091,P02783,P10758,P17988

3414	1,3,4,6,7,13,14,15	2,5,8,9,10,11,12,16	3	P02091,P70549,Q9Z2L0
3415	1,3,4,6,7,13,14,16	2,5,8,9,10,11,12,15	4	B0BNN3,P02783,Q99MH3,Q9Z2L0
3416	1,3,4,6,7,13,15,16	2,5,8,9,10,11,12,14	4	P02091,P02783,P08721,P70549
3417	1,3,4,6,7,14,15,16	2,5,8,9,10,11,12,13	3	P02091,P02783,P17988
3418	1,3,4,6,8,9,10,11	2,5,7,12,13,14,15,16	4	P01681,Q63493,Q6AY61,Q6P6S4
3419	1,3,4,6,8,9,10,12	2,5,7,11,13,14,15,16	2	O70417,P01681
3420	1,3,4,6,8,9,10,13	2,5,7,11,12,14,15,16	0	
3421	1,3,4,6,8,9,10,14	2,5,7,11,12,13,15,16	4	O70417,P01681,Q06000,Q63751
3422	1,3,4,6,8,9,10,15	2,5,7,11,12,13,14,16	3	P01681,P02091,P62804
3423	1,3,4,6,8,9,10,16	2,5,7,11,12,13,14,15	4	P01681,P02783,P22006,Q30KJ2
3424	1,3,4,6,8,9,11,12	2,5,7,10,13,14,15,16	10	O88797,P01681,P06911,P09527,P61206;P84079,Q63493,Q6P6S4,Q6Q7Y5,Q9JI85,Q9QW07
3425	1,3,4,6,8,9,11,13	2,5,7,10,12,14,15,16	5	Q62902,Q63493,Q6AY61,Q6P6S4,Q9JI85
3426	1,3,4,6,8,9,11,14	2,5,7,10,12,13,15,16	7	O70417,P01681,Q63751,Q6AY61,Q6P6S4,Q6Q7Y5,Q812E4
3427	1,3,4,6,8,9,11,15	2,5,7,10,12,13,14,16	11	P01681,P02091,Q4FZU2,Q63493,Q6AY61,Q6IFW6,Q6IG02,Q6IMF3,Q6P6Q2,Q6P6S4,Q9QW07
3428	1,3,4,6,8,9,11,16	2,5,7,10,12,13,14,15	21	O88797,P01681,P02780,P02781,P02782,P02783,P07647,P08723,P0C0A9,P22006,P30120,P60905,Q5M8C6,Q62902,Q63493,Q6AY61,Q6P6S4,Q812E4,Q9JHB9,Q9JI85,Q9QW07
3429	1,3,4,6,8,9,12,13	2,5,7,10,11,14,15,16	1	P70549
3430	1,3,4,6,8,9,12,14	2,5,7,10,11,13,15,16	3	O70417,P01681,Q63751
3431	1,3,4,6,8,9,12,15	2,5,7,10,11,13,14,16	2	P01681,P02091
3432	1,3,4,6,8,9,12,16	2,5,7,10,11,13,14,15	2	P02783,P22006
3433	1,3,4,6,8,9,13,14	2,5,7,10,11,12,15,16	1	O70417

3434	1,3,4,6,8,9,13,15	2,5,7,10,11,12,14,16	5	P01681,P02091,P62804,P70549,Q00715
3435	1,3,4,6,8,9,13,16	2,5,7,10,11,12,14,15	3	P02783,P22006,P23593
3436	1,3,4,6,8,9,14,15	2,5,7,10,11,12,13,16	4	O70417,P01681,P02091,P23593
3437	1,3,4,6,8,9,14,16	2,5,7,10,11,12,13,15	4	O70417,P02783,P22006,P23593
3438	1,3,4,6,8,9,15,16	2,5,7,10,11,12,13,14	5	P01681,P02091,P02783,P22006,P23593
3439	1,3,4,6,8,10,11,12	2,5,7,9,13,14,15,16	3	P06911,Q63493,Q9JI85
3440	1,3,4,6,8,10,11,13	2,5,7,9,12,14,15,16	3	Q63493,Q6AY61,Q9JI85
3441	1,3,4,6,8,10,11,14	2,5,7,9,12,13,15,16	4	O70417,P15399,Q63751,Q9JI85
3442	1,3,4,6,8,10,11,15	2,5,7,9,12,13,14,16	9	P02091,P15399,Q4FZU2,Q6IFU7,Q6IFW6,Q6IG02,Q6IMF3,Q6P6Q2,Q6P6S4
3443	1,3,4,6,8,10,11,16	2,5,7,9,12,13,14,15	16	P02780,P02781,P02782,P02783,P07151,P07647,P08723,P0C0A9,P15399,P30120,Q5M8C6,Q63493,Q6AY61,Q9JHB9,Q9JI85,Q9QW07
3444	1,3,4,6,8,10,12,13	2,5,7,9,11,14,15,16	2	P70549,Q9R168
3445	1,3,4,6,8,10,12,14	2,5,7,9,11,13,15,16	2	O70417,P54921
3446	1,3,4,6,8,10,12,15	2,5,7,9,11,13,14,16	5	P02091,P10758,P54921,Q63598,Q9R168
3447	1,3,4,6,8,10,12,16	2,5,7,9,11,13,14,15	7	P02625,P02783,P07151,P0DMW0;P0DMW1,P15399,P46844,Q63598
3448	1,3,4,6,8,10,13,14	2,5,7,9,11,12,15,16	3	O70417,P70549,P97580
3449	1,3,4,6,8,10,13,15	2,5,7,9,11,12,14,16	6	P02091,P15399,P62804,P70549,Q498D9,Q63618
3450	1,3,4,6,8,10,13,16	2,5,7,9,11,12,14,15	8	O70257,P02783,P07151,P15399,P46844,P97580,Q30KJ2,Q63424
3451	1,3,4,6,8,10,14,15	2,5,7,9,11,12,13,16	6	O70417,P01946,P02091,P15399,P54921,P97580
3452	1,3,4,6,8,10,14,16	2,5,7,9,11,12,13,15	9	O70417,P02783,P07151,P15399,P46844,P54921,P57113,P97580,Q30KJ2
3453	1,3,4,6,8,10,15,16	2,5,7,9,11,12,13,14	23	O35077,O70377,O70594,P01946,P02091,P02783,P07151,P15399,P19468,P19629,P46844,P48508,P53790,P54921,Q03248,Q05175,Q30KJ2,Q63270,Q63424,Q63598,Q6Q0N1,Q8R431,Q9WTW7

3454	1,3,4,6,8,11,12,13	2,5,7,9,10,14,15,16	6	P06911,P12020,P22282,P36374,Q63493,Q9JI85
3455	1,3,4,6,8,11,12,14	2,5,7,9,10,13,15,16	7	O70417,P06911,P12020,Q5GRG2,Q63493,Q63751,Q9JI85
3456	1,3,4,6,8,11,12,15	2,5,7,9,10,13,14,16	12	P02091,P06911,Q4FZU2,Q63493,Q6IFU7,Q6IFW6,Q6IG02,Q6IMF3,Q6P6Q2,Q9JI85,Q9QW07,Q9R168
3457	1,3,4,6,8,11,12,16	2,5,7,9,10,13,14,15	25	O35547,P00762,P02780,P02781,P02782,P02783,P04905,P06911,P07647,P08723,P0C0A9,P12020,P22282,P22283,P30120,P36374,P40241,P46462,P60905,Q5GRG2,Q5M8C6,Q63493,Q9JHB9,Q9JI85,Q9QW07
3458	1,3,4,6,8,11,13,14	2,5,7,9,10,12,15,16	7	B0BNN3,O54728,P06911,Q63493,Q6AY61,Q99041,Q9JI85
3459	1,3,4,6,8,11,13,15	2,5,7,9,10,12,14,16	9	P02091,Q4FZU2,Q63493,Q6IFU7,Q6IFW6,Q6IG02,Q6IMF3,Q6P6Q2,Q9JI85
3460	1,3,4,6,8,11,13,16	2,5,7,9,10,12,14,15	25	B0BNN3,O35547,P00762,P02780,P02781,P02782,P02783,P05369,P07647,P08723,P0C0A9,P22282,P22283,P24368,P30120,P36374,P46462,P60905,Q5M8C6,Q62902,Q63493,Q6AY61,Q99041,Q9JHB9,Q9JI85
3461	1,3,4,6,8,11,14,15	2,5,7,9,10,12,13,16	8	P02091,P02783,Q4FZU2,Q6IFU7,Q6IFW6,Q6IG02,Q6IMF3,Q6P6Q2
3462	1,3,4,6,8,11,14,16	2,5,7,9,10,12,13,15	19	O54728,P00762,P02780,P02781,P02782,P02783,P06911,P07647,P08723,P0C0A9,P30120,P46462,Q5GRG2,Q5M8C6,Q6AY61,Q99041,Q9JHB9,Q9JI85,Q9QW07
3463	1,3,4,6,8,11,15,16	2,5,7,9,10,12,13,14	27	P00762,P02091,P02780,P02781,P02782,P02783,P07647,P08723,P0C0A9,P15399,P30120,P36374,P50280,Q4FZU2,Q5M8C6,Q63493,Q6AY61,Q6IFU7,Q6IFW6,Q6IG02,Q6IMF3,Q6P6Q2,Q8CJ52,Q9JHB9,Q9JI85,Q9QW07,Q9QZK9
3464	1,3,4,6,8,12,13,14	2,5,7,9,10,11,15,16	3	O70417,P70549,Q9Z2L0
3465	1,3,4,6,8,12,13,15	2,5,7,9,10,11,14,16	3	P02091,P70549,Q9R168
3466	1,3,4,6,8,12,13,16	2,5,7,9,10,11,14,15	3	P00762,P02783,P70549
3467	1,3,4,6,8,12,14,15	2,5,7,9,10,11,13,16	5	O70417,P02091,P54921,P70549,Q9R168
3468	1,3,4,6,8,12,14,16	2,5,7,9,10,11,13,15	3	O70417,P00762,P02783
3469	1,3,4,6,8,12,15,16	2,5,7,9,10,11,13,14	5	P00762,P02091,P02783,P10758,Q9R168
3470	1,3,4,6,8,13,14,15	2,5,7,9,10,11,12,16	5	B0BNN3,P02091,P70549,Q03191,Q9Z2L0
3471	1,3,4,6,8,13,14,16	2,5,7,9,10,11,12,15	6	B0BNN3,O54728,P00762,P02783,P97580,Q9Z2L0
3472	1,3,4,6,8,13,15,16	2,5,7,9,10,11,12,14	4	P00762,P02091,P02783,P70549

3473	1,3,4,6,8,14,15,16	2,5,7,9,10,11,12,13	4	P02091,P02783,P54921,Q03191
3474	1,3,4,6,9,10,11,12	2,5,7,8,13,14,15,16	1	Q62714
3475	1,3,4,6,9,10,11,13	2,5,7,8,12,14,15,16	0	
3476	1,3,4,6,9,10,11,14	2,5,7,8,12,13,15,16	1	Q06000
3477	1,3,4,6,9,10,11,15	2,5,7,8,12,13,14,16	1	P02091
3478	1,3,4,6,9,10,11,16	2,5,7,8,12,13,14,15	2	P02783,P22006
3479	1,3,4,6,9,10,12,13	2,5,7,8,11,14,15,16	0	
3480	1,3,4,6,9,10,12,14	2,5,7,8,11,13,15,16	0	
3481	1,3,4,6,9,10,12,15	2,5,7,8,11,13,14,16	3	P02091,P10758,Q00715
3482	1,3,4,6,9,10,12,16	2,5,7,8,11,13,14,15	1	P02783
3483	1,3,4,6,9,10,13,14	2,5,7,8,11,12,15,16	2	Q00715,Q06000
3484	1,3,4,6,9,10,13,15	2,5,7,8,11,12,14,16	5	P02091,P11883,P62804,P70549,Q00715
3485	1,3,4,6,9,10,13,16	2,5,7,8,11,12,14,15	2	P11883,P22006
3486	1,3,4,6,9,10,14,15	2,5,7,8,11,12,13,16	5	P01946,P02091,P30120,P62804,Q00715
3487	1,3,4,6,9,10,14,16	2,5,7,8,11,12,13,15	3	P02783,P22006,Q06000
3488	1,3,4,6,9,10,15,16	2,5,7,8,11,12,13,14	5	P02091,P02783,P11883,P22006,Q811M5
3489	1,3,4,6,9,11,12,13	2,5,7,8,10,14,15,16	0	
3490	1,3,4,6,9,11,12,14	2,5,7,8,10,13,15,16	0	
3491	1,3,4,6,9,11,12,15	2,5,7,8,10,13,14,16	2	P02091,P25809
3492	1,3,4,6,9,11,12,16	2,5,7,8,10,13,14,15	3	P02783,P22006,P25809

3493	1,3,4,6,9,11,13,14	2,5,7,8,10,12,15,16	1 P0DMW0;P0DMW1
3494	1,3,4,6,9,11,13,15	2,5,7,8,10,12,14,16	2 P02091,P62804
3495	1,3,4,6,9,11,13,16	2,5,7,8,10,12,14,15	2 P02783,P22006
3496	1,3,4,6,9,11,14,15	2,5,7,8,10,12,13,16	1 P02091
3497	1,3,4,6,9,11,14,16	2,5,7,8,10,12,13,15	2 P02783,P22006
3498	1,3,4,6,9,11,15,16	2,5,7,8,10,12,13,14	5 P02091,P02783,P22006,P25809,P29315
3499	1,3,4,6,9,12,13,14	2,5,7,8,10,11,15,16	0
3500	1,3,4,6,9,12,13,15	2,5,7,8,10,11,14,16	4 P02091,P62804,P70549,Q00715
3501	1,3,4,6,9,12,13,16	2,5,7,8,10,11,14,15	1 P22006
3502	1,3,4,6,9,12,14,15	2,5,7,8,10,11,13,16	2 P02091,Q00715
3503	1,3,4,6,9,12,14,16	2,5,7,8,10,11,13,15	2 P02783,P22006
3504	1,3,4,6,9,12,15,16	2,5,7,8,10,11,13,14	5 P02091,P02783,P10758,P22006,P25809
3505	1,3,4,6,9,13,14,15	2,5,7,8,10,11,12,16	4 P02091,P62804,P70549,Q00715
3506	1,3,4,6,9,13,14,16	2,5,7,8,10,11,12,15	3 P02783,P22006,P23593
3507	1,3,4,6,9,13,15,16	2,5,7,8,10,11,12,14	7 P02091,P02783,P11883,P22006,P23593,P62804,Q00715
3508	1,3,4,6,9,14,15,16	2,5,7,8,10,11,12,13	5 P02091,P02783,P22006,P23593,P47967
3509	1,3,4,6,10,11,12,13	2,5,7,8,9,14,15,16	0
3510	1,3,4,6,10,11,12,14	2,5,7,8,9,13,15,16	0
3511	1,3,4,6,10,11,12,15	2,5,7,8,9,13,14,16	2 P02091,Q6IG02
3512	1,3,4,6,10,11,12,16	2,5,7,8,9,13,14,15	1 P02783

3513	1,3,4,6,10,11,13,14	2,5,7,8,9,12,15,16	1	Q8CJD3
3514	1,3,4,6,10,11,13,15	2,5,7,8,9,12,14,16	3	P02091,Q6IG02,Q8CJD3
3515	1,3,4,6,10,11,13,16	2,5,7,8,9,12,14,15	2	P02783,Q8CJD3
3516	1,3,4,6,10,11,14,15	2,5,7,8,9,12,13,16	4	P01946,P02091,Q6IG02,Q8CJD3
3517	1,3,4,6,10,11,14,16	2,5,7,8,9,12,13,15	2	P02783,Q8CJD3
3518	1,3,4,6,10,11,15,16	2,5,7,8,9,12,13,14	6	P02091,P02783,P17988,P29315,Q6IG02,Q8CJD3
3519	1,3,4,6,10,12,13,14	2,5,7,8,9,11,15,16	1	P70549
3520	1,3,4,6,10,12,13,15	2,5,7,8,9,11,14,16	6	P02091,P06760,P10758,P62804,P70549,Q812E4
3521	1,3,4,6,10,12,13,16	2,5,7,8,9,11,14,15	2	P02783,P06760
3522	1,3,4,6,10,12,14,15	2,5,7,8,9,11,13,16	4	P01946,P02091,P06760,P54921
3523	1,3,4,6,10,12,14,16	2,5,7,8,9,11,13,15	2	P02783,P06760
3524	1,3,4,6,10,12,15,16	2,5,7,8,9,11,13,14	5	P02091,P02783,P06760,P0DMW0;P0DMW1,P10758
3525	1,3,4,6,10,13,14,15	2,5,7,8,9,11,12,16	6	P01946,P02091,P06760,P62804,P70549,Q00715
3526	1,3,4,6,10,13,14,16	2,5,7,8,9,11,12,15	2	P02783,P06760
3527	1,3,4,6,10,13,15,16	2,5,7,8,9,11,12,14	6	P02091,P02783,P06760,P11883,P62804,P70549
3528	1,3,4,6,10,14,15,16	2,5,7,8,9,11,12,13	6	P01946,P02091,P02783,P06760,P54921,Q9QYP1
3529	1,3,4,6,11,12,13,14	2,5,7,8,9,10,15,16	1	Q6AYE5
3530	1,3,4,6,11,12,13,15	2,5,7,8,9,10,14,16	1	Q6IG02
3531	1,3,4,6,11,12,13,16	2,5,7,8,9,10,14,15	2	P02783,P05369
3532	1,3,4,6,11,12,14,15	2,5,7,8,9,10,13,16	2	P02091,Q6IG02

3533	1,3,4,6,11,12,14,16	2,5,7,8,9,10,13,15	1	P02783
3534	1,3,4,6,11,12,15,16	2,5,7,8,9,10,13,14	5	P02091,P02783,P25809,P29315,Q6IG02
3535	1,3,4,6,11,13,14,15	2,5,7,8,9,10,12,16	2	P02091,Q6IG02
3536	1,3,4,6,11,13,14,16	2,5,7,8,9,10,12,15	4	B0BNN3,O54728,P02783,P19132
3537	1,3,4,6,11,13,15,16	2,5,7,8,9,10,12,14	4	P02091,P02783,P29315,Q6IG02
3538	1,3,4,6,11,14,15,16	2,5,7,8,9,10,12,13	4	P02091,P02783,P29315,Q6IG02
3539	1,3,4,6,12,13,14,15	2,5,7,8,9,10,11,16	3	P02091,P06760,P70549
3540	1,3,4,6,12,13,14,16	2,5,7,8,9,10,11,15	2	P02783,P06760
3541	1,3,4,6,12,13,15,16	2,5,7,8,9,10,11,14	5	P02091,P02783,P06760,P10758,P70549
3542	1,3,4,6,12,14,15,16	2,5,7,8,9,10,11,13	3	P02091,P02783,P06760
3543	1,3,4,6,13,14,15,16	2,5,7,8,9,10,11,12	4	P02091,P02783,P06760,P70549
3544	1,3,4,7,8,9,10,11	2,5,6,12,13,14,15,16	8	O70417,P01681,P08723,P20646,P35280,P49134,Q63493,Q6P6R2
3545	1,3,4,7,8,9,10,12	2,5,6,11,13,14,15,16	5	O70417,P01681,P10758,P20646,Q923V8
3546	1,3,4,7,8,9,10,13	2,5,6,11,12,14,15,16	2	P01681,P20646
3547	1,3,4,7,8,9,10,14	2,5,6,11,12,13,15,16	4	O70417,P01681,P20646,Q63751
3548	1,3,4,7,8,9,10,15	2,5,6,11,12,13,14,16	8	O70417,P01681,P10758,P20646,P35280,Q5RKI1,Q6P6R2,Q9Z0V6
3549	1,3,4,7,8,9,10,16	2,5,6,11,12,13,14,15	4	O70417,P01681,P20646,Q64093
3550	1,3,4,7,8,9,11,12	2,5,6,10,13,14,15,16	10	O70417,P01681,P08723,P35280,P36374,P70709,Q5M8C6,Q63493,Q6Q7Y5,Q812E4
3551	1,3,4,7,8,9,11,13	2,5,6,10,12,14,15,16	9	B0BNN3,P01681,P08723,P20646,P35280,Q62902,Q63493,Q6Q7Y5,Q812E4
3552	1,3,4,7,8,9,11,14	2,5,6,10,12,13,15,16	10	B0BNN3,O70417,P01681,P20646,P35280,Q63493,Q63751,Q6P6R2,Q6Q7Y5,Q812E4

3553	1,3,4,7,8,9,11,15	2,5,6,10,12,13,14,16	14	P01681,P08723,P20646,P35280,Q4FZU2,Q63493,Q6IFU8,Q6IFW6,Q6IG02,Q6IMF3,Q6P6Q2,Q6P6R2,Q6P6S4,Q8CJ52
3554	1,3,4,7,8,9,11,16	2,5,6,10,12,13,14,15	21	P01681,P02780,P02781,P02782,P04905,P07647,P08723,P09456,P0C0A9,P20646,P22282,P22283,P30120,P35280,P36374,Q5M8C6,Q62902,Q63493,Q6Q7Y5,Q812E4,Q9JHB9
3555	1,3,4,7,8,9,12,13	2,5,6,10,11,14,15,16	1	P01681
3556	1,3,4,7,8,9,12,14	2,5,6,10,11,13,15,16	3	O70417,P01681,Q63751
3557	1,3,4,7,8,9,12,15	2,5,6,10,11,13,14,16	5	O70417,P01681,P10758,P20646,P35280
3558	1,3,4,7,8,9,12,16	2,5,6,10,11,13,14,15	4	O70417,P01681,P10758,P20646
3559	1,3,4,7,8,9,13,14	2,5,6,10,11,12,15,16	6	B0BNN3,O70417,P01681,P20646,Q5QE79,Q63751
3560	1,3,4,7,8,9,13,15	2,5,6,10,11,12,14,16	3	P01681,P20646,P35280
3561	1,3,4,7,8,9,13,16	2,5,6,10,11,12,14,15	4	B0BNN3,P01681,P20646,Q62902
3562	1,3,4,7,8,9,14,15	2,5,6,10,11,12,13,16	4	O70417,P01681,P20646,P35280
3563	1,3,4,7,8,9,14,16	2,5,6,10,11,12,13,15	5	B0BNN3,O70417,P01681,P20646,P23593
3564	1,3,4,7,8,9,15,16	2,5,6,10,11,12,13,14	5	P01681,P10758,P20646,P23593,P35280
3565	1,3,4,7,8,10,11,12	2,5,6,9,13,14,15,16	8	O70417,P08723,P36374,P47967,P97840,Q5I0D1,Q5M8C6,Q63493
3566	1,3,4,7,8,10,11,13	2,5,6,9,12,14,15,16	5	B0BNN3,P08723,P47967,P97840,Q63493
3567	1,3,4,7,8,10,11,14	2,5,6,9,12,13,15,16	4	B0BNN3,O70417,Q63493,Q63751
3568	1,3,4,7,8,10,11,15	2,5,6,9,12,13,14,16	12	P01681,P08723,P20646,Q4FZU2,Q5RKI1,Q63493,Q6IFU8,Q6IFW6,Q6IG02,Q6IMF3,Q6P6Q2,Q8CJ52
3569	1,3,4,7,8,10,11,16	2,5,6,9,12,13,14,15	21	P00762,P02780,P02781,P02782,P04905,P07647,P08010,P08723,P0C0A9,P20646,P22282,P22283,P24368,P30120,P36374,Q5M8C6,Q63424,Q63493,Q64093,Q9JHB9,Q9WTW7
3570	1,3,4,7,8,10,12,13	2,5,6,9,11,14,15,16	5	P10758,P47967,P97840,Q5I0J9,Q923V8
3571	1,3,4,7,8,10,12,14	2,5,6,9,11,13,15,16	2	O70417,Q5RLM2
3572	1,3,4,7,8,10,12,15	2,5,6,9,11,13,14,16	8	O70417,P01681,P10758,Q5I0J9,Q5RKI1,Q63598,Q64093,Q9WTW7

3573	1,3,4,7,8,10,12,16	2,5,6,9,11,13,14,15	16	O70417,P0DMW0;P0DMW1,P10758,P19468,P46844,P97840,Q3ZAV1,Q63424,Q63598,Q64093,Q6AY41,Q80W57,Q8R431,Q923V8,Q9JJ40,Q9WTW7
3574	1,3,4,7,8,10,13,14	2,5,6,9,11,12,15,16	3	B0BNN3,O70417,Q9WUW8
3575	1,3,4,7,8,10,13,15	2,5,6,9,11,12,14,16	14	O70377,P10758,P19468,P20646,P70549,Q5I0J9,Q5RKI1,Q63424,Q63598,Q63618,Q64093,Q6Q0N1,Q80W57,Q8R431
3576	1,3,4,7,8,10,13,16	2,5,6,9,11,12,14,15	21	B0BNN3,O35077,O70257,O70377,P00762,P19468,P28570,P46844,P57113,Q3ZAV1,Q5I0J9,Q63424,Q63598,Q63618,Q64093,Q6AY41,Q6Q0N1,Q80W57,Q8R431,Q9JJ40,Q9WTW7
3577	1,3,4,7,8,10,14,15	2,5,6,9,11,12,13,16	9	O70377,O70417,P01681,P20646,P54921,Q05175,Q5RKI1,Q64093,Q923S2
3578	1,3,4,7,8,10,14,16	2,5,6,9,11,12,13,15	16	B0BNN3,O35077,O70377,O70417,P19468,P20646,P46844,P57113,P97608,Q63424,Q64093,Q8R431,Q923S2,Q99MH3,Q9WUW8,Q9WUW9
3579	1,3,4,7,8,10,15,16	2,5,6,9,11,12,13,14	33	O35077,O70377,O70594,P10758,P19468,P20646,P46844,P48508,P53790,P57113,P97608,Q03248,Q05175,Q3ZAV1,Q5I0E9,Q5I0J9,Q5M7T9,Q5RKI1,Q63270,Q63355,Q63424,Q63598,Q64093,Q64268,Q6AY41,Q6MG61,Q6Q0N1,Q80W57,Q8R431,Q923S2,Q9JJ40,Q9WTW7,Q9Z0W7
3580	1,3,4,7,8,11,12,13	2,5,6,9,10,14,15,16	13	B0BNN3,P02780,P02782,P08723,P22282,P36374,P40241,P47967,P97840,Q5I0D1,Q5M8C6,Q63493,Q811M5
3581	1,3,4,7,8,11,12,14	2,5,6,9,10,13,15,16	8	O70417,P06911,P11883,P36374,Q5M8C6,Q63493,Q63751,Q811M5
3582	1,3,4,7,8,11,12,15	2,5,6,9,10,13,14,16	12	P01681,P08723,P36374,P97840,Q4FZU2,Q63493,Q6IFU8,Q6IFW6,Q6IG02,Q6IMF3,Q6P6Q2,Q8CJ52
3583	1,3,4,7,8,11,12,16	2,5,6,9,10,13,14,15	23	O35547,P00762,P02780,P02781,P02782,P04905,P07647,P08010,P08723,P0C0A9,P22282,P22283,P24368,P30120,P36374,P40241,P46462,P97840,Q5M8C6,Q63493,Q811M5,Q9JHB9,Q9QW07
3584	1,3,4,7,8,11,13,14	2,5,6,9,10,12,15,16	7	B0BNN3,O54728,P08723,Q5M8C6,Q63493,Q811M5,Q99041
3585	1,3,4,7,8,11,13,15	2,5,6,9,10,12,14,16	10	B0BNN3,P08723,Q4FZU2,Q63493,Q6IFU8,Q6IFW6,Q6IG02,Q6IMF3,Q6P6Q2,Q8CJ52
3586	1,3,4,7,8,11,13,16	2,5,6,9,10,12,14,15	26	B0BNN3,P00762,P02780,P02781,P02782,P05369,P07647,P08010,P08723,P09456,P0C0A9,P16636,P22282,P22283,P24368,P30120,P36374,P40241,P46462,P97840,Q5M8C6,Q62902,Q63493,Q811M5,Q99MH3,Q9JHB9
3587	1,3,4,7,8,11,14,15	2,5,6,9,10,12,13,16	12	B0BNN3,D3ZTX0,P01681,P20646,Q4FZU2,Q63493,Q6IFU8,Q6IFW6,Q6IG02,Q6IMF3,Q6P6Q2,Q8CJ52
3588	1,3,4,7,8,11,14,16	2,5,6,9,10,12,13,15	26	B0BNN3,D3ZTX0,O54728,O70417,P00762,P02780,P02781,P02782,P07647,P08010,P08649,P08723,P0C0A9,P22282,P22283,P30120,P36374,P46462,Q5M8C6,Q62902,Q63493,Q811M5,Q812E4,Q99041,Q99MH3,Q9JHB9
3589	1,3,4,7,8,11,15,16	2,5,6,9,10,12,13,14	27	P00762,P01681,P02780,P02781,P02782,P07647,P08010,P08723,P0C0A9,P16636,P20646,P22282,P22283,P30120,P36374,Q4FZU2,Q5M8C6,Q62902,Q63493,Q6IFU8,Q6IFW6,Q6IG02,Q6IMF3,Q6P6Q2,Q8CJ52,Q9JHB9,Q9QW07
3590	1,3,4,7,8,12,13,14	2,5,6,9,10,11,15,16	4	B0BNN3,O70417,Q811M5,Q9Z2L0

3591	1,3,4,7,8,12,13,15	2,5,6,9,10,11,14,16	5	P10758,P70549,P97840,Q5I0J9,Q9Z2L0
3592	1,3,4,7,8,12,13,16	2,5,6,9,10,11,14,15	5	B0BNN3,P00762,P97840,Q923V8,Q9Z2L0
3593	1,3,4,7,8,12,14,15	2,5,6,9,10,11,13,16	3	O70417,P01681,Q9Z2L0
3594	1,3,4,7,8,12,14,16	2,5,6,9,10,11,13,15	5	B0BNN3,O70417,P00762,Q99MH3,Q9Z2L0
3595	1,3,4,7,8,12,15,16	2,5,6,9,10,11,13,14	2	P00762,P10758
3596	1,3,4,7,8,13,14,15	2,5,6,9,10,11,12,16	3	B0BNN3,P70549,Q9Z2L0
3597	1,3,4,7,8,13,14,16	2,5,6,9,10,11,12,15	7	B0BNN3,O54728,P00762,Q811M5,Q99MH3,Q9WUW8,Q9Z2L0
3598	1,3,4,7,8,13,15,16	2,5,6,9,10,11,12,14	10	B0BNN3,O35077,P00762,P10758,P16636,P20646,Q5I0J9,Q64093,Q6AYQ8,Q9Z2L0
3599	1,3,4,7,8,14,15,16	2,5,6,9,10,11,12,13	6	B0BNN3,O35077,O70417,P00762,P20646,Q9Z2L0
3600	1,3,4,7,9,10,11,12	2,5,6,8,13,14,15,16	1	Q62714
3601	1,3,4,7,9,10,11,13	2,5,6,8,12,14,15,16	0	
3602	1,3,4,7,9,10,11,14	2,5,6,8,12,13,15,16	0	
3603	1,3,4,7,9,10,11,15	2,5,6,8,12,13,14,16	1	Q5RKI1
3604	1,3,4,7,9,10,11,16	2,5,6,8,12,13,14,15	0	
3605	1,3,4,7,9,10,12,13	2,5,6,8,11,14,15,16	1	P10758
3606	1,3,4,7,9,10,12,14	2,5,6,8,11,13,15,16	0	
3607	1,3,4,7,9,10,12,15	2,5,6,8,11,13,14,16	2	P10758,Q5RKI1
3608	1,3,4,7,9,10,12,16	2,5,6,8,11,13,14,15	1	P10758
3609	1,3,4,7,9,10,13,14	2,5,6,8,11,12,15,16	0	
3610	1,3,4,7,9,10,13,15	2,5,6,8,11,12,14,16	3	P10758,Q00715,Q5RKI1

3611	1,3,4,7,9,10,13,16	2,5,6,8,11,12,14,15	0
3612	1,3,4,7,9,10,14,15	2,5,6,8,11,12,13,16	1 Q5RKI1
3613	1,3,4,7,9,10,14,16	2,5,6,8,11,12,13,15	0
3614	1,3,4,7,9,10,15,16	2,5,6,8,11,12,13,14	3 P10758,Q5RKI1,Q64268
3615	1,3,4,7,9,11,12,13	2,5,6,8,10,14,15,16	2 P97840,Q62761;Q62762;Q62763
3616	1,3,4,7,9,11,12,14	2,5,6,8,10,13,15,16	1 Q62761;Q62762;Q62763
3617	1,3,4,7,9,11,12,15	2,5,6,8,10,13,14,16	2 P25031,Q62761;Q62762;Q62763
3618	1,3,4,7,9,11,12,16	2,5,6,8,10,13,14,15	0
3619	1,3,4,7,9,11,13,14	2,5,6,8,10,12,15,16	3 B0BNN3,P0DMW0;P0DMW1,P25031
3620	1,3,4,7,9,11,13,15	2,5,6,8,10,12,14,16	2 P25031,Q6IG02
3621	1,3,4,7,9,11,13,16	2,5,6,8,10,12,14,15	0
3622	1,3,4,7,9,11,14,15	2,5,6,8,10,12,13,16	2 P0DMW0;P0DMW1,P25031
3623	1,3,4,7,9,11,14,16	2,5,6,8,10,12,13,15	0
3624	1,3,4,7,9,11,15,16	2,5,6,8,10,12,13,14	4 P25031,P29315,Q6IFW6,Q8CJ52
3625	1,3,4,7,9,12,13,14	2,5,6,8,10,11,15,16	0
3626	1,3,4,7,9,12,13,15	2,5,6,8,10,11,14,16	1 P10758
3627	1,3,4,7,9,12,13,16	2,5,6,8,10,11,14,15	0
3628	1,3,4,7,9,12,14,15	2,5,6,8,10,11,13,16	0
3629	1,3,4,7,9,12,14,16	2,5,6,8,10,11,13,15	1 Q68G31
3630	1,3,4,7,9,12,15,16	2,5,6,8,10,11,13,14	2 P10758,Q68G31

3631	1,3,4,7,9,13,14,15	2,5,6,8,10,11,12,16	2 P25031,Q00715
3632	1,3,4,7,9,13,14,16	2,5,6,8,10,11,12,15	1 B0BNN3
3633	1,3,4,7,9,13,15,16	2,5,6,8,10,11,12,14	1 P10758
3634	1,3,4,7,9,14,15,16	2,5,6,8,10,11,12,13	0
3635	1,3,4,7,10,11,12,13	2,5,6,8,9,14,15,16	3 P47967,P97840,Q5I0D1
3636	1,3,4,7,10,11,12,14	2,5,6,8,9,13,15,16	0
3637	1,3,4,7,10,11,12,15	2,5,6,8,9,13,14,16	5 P10758,P97840,Q5RKI1,Q6IFW6,Q6IG02
3638	1,3,4,7,10,11,12,16	2,5,6,8,9,13,14,15	2 P10758,P97840
3639	1,3,4,7,10,11,13,14	2,5,6,8,9,12,15,16	2 B0BNN3,Q8CJD3
3640	1,3,4,7,10,11,13,15	2,5,6,8,9,12,14,16	5 P97840,Q5RKI1,Q6IFW6,Q6IG02,Q8CJD3
3641	1,3,4,7,10,11,13,16	2,5,6,8,9,12,14,15	3 P97840,Q6AYQ8,Q8CJD3
3642	1,3,4,7,10,11,14,15	2,5,6,8,9,12,13,16	4 Q5RKI1,Q6IFW6,Q6IG02,Q8CJD3
3643	1,3,4,7,10,11,14,16	2,5,6,8,9,12,13,15	1 Q8CJD3
3644	1,3,4,7,10,11,15,16	2,5,6,8,9,12,13,14	7 P10758,P17988,P29315,Q5RKI1,Q6IFW6,Q6IG02,Q8CJD3
3645	1,3,4,7,10,12,13,14	2,5,6,8,9,11,15,16	0
3646	1,3,4,7,10,12,13,15	2,5,6,8,9,11,14,16	5 P10758,P70549,P97840,Q5I0J9,Q5RKI1
3647	1,3,4,7,10,12,13,16	2,5,6,8,9,11,14,15	3 P10758,P97840,Q5I0J9
3648	1,3,4,7,10,12,14,15	2,5,6,8,9,11,13,16	2 P10758,Q5RKI1
3649	1,3,4,7,10,12,14,16	2,5,6,8,9,11,13,15	1 P10758
3650	1,3,4,7,10,12,15,16	2,5,6,8,9,11,13,14	7 P10758,P18297,P97840,Q5I0J9,Q5RKI1,Q64093,Q64268

3651	1,3,4,7,10,13,14,15	2,5,6,8,9,11,12,16	1	Q5RKI1
3652	1,3,4,7,10,13,14,16	2,5,6,8,9,11,12,15	3	B0BNN3,P36376,Q99MH3
3653	1,3,4,7,10,13,15,16	2,5,6,8,9,11,12,14	10	P10758,P18297,P19468,P51792;P51794;P51796,Q5I0J9,Q5RKI1,Q63424,Q64093,Q64268,Q6AYQ8
3654	1,3,4,7,10,14,15,16	2,5,6,8,9,11,12,13	5	P10758,P18297,Q5RKI1,Q64268,Q9QYP1
3655	1,3,4,7,11,12,13,14	2,5,6,8,9,10,15,16	5	B0BNN3,P25031,P97840,Q6AYE5,Q811M5
3656	1,3,4,7,11,12,13,15	2,5,6,8,9,10,14,16	4	P25031,P97840,Q6IFW6,Q6IG02
3657	1,3,4,7,11,12,13,16	2,5,6,8,9,10,14,15	4	P05369,P47967,P97840,Q811M5
3658	1,3,4,7,11,12,14,15	2,5,6,8,9,10,13,16	4	D3ZTX0,P25031,Q6IFW6,Q6IG02
3659	1,3,4,7,11,12,14,16	2,5,6,8,9,10,13,15	3	D3ZTX0,Q811M5,Q99MH3
3660	1,3,4,7,11,12,15,16	2,5,6,8,9,10,13,14	8	P10758,P17559,P25031,P29315,P97840,Q6IFW6,Q6IG02,Q8CJ52
3661	1,3,4,7,11,13,14,15	2,5,6,8,9,10,12,16	4	B0BNN3,D3ZTX0,P25031,Q6IG02
3662	1,3,4,7,11,13,14,16	2,5,6,8,9,10,12,15	7	B0BNN3,D3ZTX0,O54728,P25031,P36376,Q811M5,Q99MH3
3663	1,3,4,7,11,13,15,16	2,5,6,8,9,10,12,14	7	B0BNN3,P25031,P29315,Q6AYQ8,Q6IFW6,Q6IG02,Q8CJ52
3664	1,3,4,7,11,14,15,16	2,5,6,8,9,10,12,13	7	D3ZTX0,P17559,P25031,P29315,Q6IFW6,Q6IG02,Q8CJ52
3665	1,3,4,7,12,13,14,15	2,5,6,8,9,10,11,16	3	P25031,P70549,Q9Z2L0
3666	1,3,4,7,12,13,14,16	2,5,6,8,9,10,11,15	4	B0BNN3,Q811M5,Q99MH3,Q9Z2L0
3667	1,3,4,7,12,13,15,16	2,5,6,8,9,10,11,14	3	P10758,P97840,Q5I0J9
3668	1,3,4,7,12,14,15,16	2,5,6,8,9,10,11,13	1	P10758
3669	1,3,4,7,13,14,15,16	2,5,6,8,9,10,11,12	3	B0BNN3,Q99MH3,Q9Z2L0
3670	1,3,4,8,9,10,11,12	2,5,6,7,13,14,15,16	5	O88797,P01681,P01835,Q62714,Q6Q7Y5

3671	1,3,4,8,9,10,11,13	2,5,6,7,12,14,15,16	2	P01681,Q6AY61
3672	1,3,4,8,9,10,11,14	2,5,6,7,12,13,15,16	3	O70417,P01681,P01835
3673	1,3,4,8,9,10,11,15	2,5,6,7,12,13,14,16	8	P01681,P01835,Q4FZU2,Q6IFW6,Q6IG02,Q6IMF3,Q6P6Q2,Q6P6S4
3674	1,3,4,8,9,10,11,16	2,5,6,7,12,13,14,15	4	O88797,P01681,P01835,Q6AY61
3675	1,3,4,8,9,10,12,13	2,5,6,7,11,14,15,16	1	P01681
3676	1,3,4,8,9,10,12,14	2,5,6,7,11,13,15,16	2	O70417,P01681
3677	1,3,4,8,9,10,12,15	2,5,6,7,11,13,14,16	3	P01681,P01835,P10758
3678	1,3,4,8,9,10,12,16	2,5,6,7,11,13,14,15	3	P01681,P01835,P10758
3679	1,3,4,8,9,10,13,14	2,5,6,7,11,12,15,16	1	P01681
3680	1,3,4,8,9,10,13,15	2,5,6,7,11,12,14,16	3	P01681,P62804,Q00715
3681	1,3,4,8,9,10,13,16	2,5,6,7,11,12,14,15	0	
3682	1,3,4,8,9,10,14,15	2,5,6,7,11,12,13,16	1	P01681
3683	1,3,4,8,9,10,14,16	2,5,6,7,11,12,13,15	2	O70417,P01681
3684	1,3,4,8,9,10,15,16	2,5,6,7,11,12,13,14	2	P01681,P10758
3685	1,3,4,8,9,11,12,13	2,5,6,7,10,14,15,16	4	P01681,P09527,Q64335,Q6Q7Y5
3686	1,3,4,8,9,11,12,14	2,5,6,7,10,13,15,16	6	O70417,O88797,P01681,P01835,Q64335,Q6Q7Y5
3687	1,3,4,8,9,11,12,15	2,5,6,7,10,13,14,16	9	O88797,P01681,P01835,Q4FZU2,Q6IFW6,Q6IG02,Q6IMF3,Q6P6Q2,Q6Q7Y5
3688	1,3,4,8,9,11,12,16	2,5,6,7,10,13,14,15	5	O88797,P00762,P01681,P01835,Q6Q7Y5
3689	1,3,4,8,9,11,13,14	2,5,6,7,10,12,15,16	5	B0BNN3,O54728,P01681,Q64335,Q6AY61
3690	1,3,4,8,9,11,13,15	2,5,6,7,10,12,14,16	6	P01681,Q4FZU2,Q6IFW6,Q6IG02,Q6IMF3,Q6P6Q2

3691	1,3,4,8,9,11,13,16	2,5,6,7,10,12,14,15	6	O88797,P00762,P02782,P30120,Q62902,Q6AY61
3692	1,3,4,8,9,11,14,15	2,5,6,7,10,12,13,16	7	P01681,P01835,Q4FZU2,Q6IFW6,Q6IG02,Q6IMF3,Q6P6Q2
3693	1,3,4,8,9,11,14,16	2,5,6,7,10,12,13,15	7	O54728,O88797,P00762,P01681,P01835,Q6AY61,Q812E4
3694	1,3,4,8,9,11,15,16	2,5,6,7,10,12,13,14	11	O88797,P00762,P01681,P01835,P29315,Q4FZU2,Q6AY61,Q6IFW6,Q6IG02,Q6IMF3,Q6P6Q2
3695	1,3,4,8,9,12,13,14	2,5,6,7,10,11,15,16	1	P17988
3696	1,3,4,8,9,12,13,15	2,5,6,7,10,11,14,16	1	P01681
3697	1,3,4,8,9,12,13,16	2,5,6,7,10,11,14,15	1	P00762
3698	1,3,4,8,9,12,14,15	2,5,6,7,10,11,13,16	1	P01681
3699	1,3,4,8,9,12,14,16	2,5,6,7,10,11,13,15	3	O70417,P00762,P01681
3700	1,3,4,8,9,12,15,16	2,5,6,7,10,11,13,14	3	P00762,P01681,P10758
3701	1,3,4,8,9,13,14,15	2,5,6,7,10,11,12,16	2	P01681,Q00715
3702	1,3,4,8,9,13,14,16	2,5,6,7,10,11,12,15	4	B0BNN3,O54728,P00762,P23593
3703	1,3,4,8,9,13,15,16	2,5,6,7,10,11,12,14	3	P00762,P01681,P23593
3704	1,3,4,8,9,14,15,16	2,5,6,7,10,11,12,13	3	P01681,P23593,P47967
3705	1,3,4,8,10,11,12,13	2,5,6,7,9,14,15,16	0	
3706	1,3,4,8,10,11,12,14	2,5,6,7,9,13,15,16	1	O70417
3707	1,3,4,8,10,11,12,15	2,5,6,7,9,13,14,16	6	Q4FZU2,Q6IFW6,Q6IG02,Q6IMF3,Q6P6Q2,Q9R168
3708	1,3,4,8,10,11,12,16	2,5,6,7,9,13,14,15	3	P00762,P01835,P02625
3709	1,3,4,8,10,11,13,14	2,5,6,7,9,12,15,16	2	B0BNN3,O54728
3710	1,3,4,8,10,11,13,15	2,5,6,7,9,12,14,16	6	Q4FZU2,Q6IFU7,Q6IFW6,Q6IG02,Q6IMF3,Q6P6Q2

3711	1,3,4,8,10,11,13,16	2,5,6,7,9,12,14,15	5	B0BNN3,P00762,P02782,P30120,Q6AY61
3712	1,3,4,8,10,11,14,15	2,5,6,7,9,12,13,16	6	Q4FZU2,Q6IFU7,Q6IFW6,Q6IG02,Q6IMF3,Q6P6Q2
3713	1,3,4,8,10,11,14,16	2,5,6,7,9,12,13,15	2	O54728,P00762
3714	1,3,4,8,10,11,15,16	2,5,6,7,9,12,13,14	8	P00762,P29315,Q4FZU2,Q6IFU7,Q6IFW6,Q6IG02,Q6IMF3,Q6P6Q2
3715	1,3,4,8,10,12,13,14	2,5,6,7,9,11,15,16	0	
3716	1,3,4,8,10,12,13,15	2,5,6,7,9,11,14,16	3	P10758,P70549,Q9R168
3717	1,3,4,8,10,12,13,16	2,5,6,7,9,11,14,15	2	P00762,P02625
3718	1,3,4,8,10,12,14,15	2,5,6,7,9,11,13,16	2	P54921,Q9R168
3719	1,3,4,8,10,12,14,16	2,5,6,7,9,11,13,15	2	O70417,P00762
3720	1,3,4,8,10,12,15,16	2,5,6,7,9,11,13,14	3	P00762,P0DMW0;P0DMW1,P10758
3721	1,3,4,8,10,13,14,15	2,5,6,7,9,11,12,16	1	B0BNN3
3722	1,3,4,8,10,13,14,16	2,5,6,7,9,11,12,15	4	B0BNN3,O35077,O54728,P00762
3723	1,3,4,8,10,13,15,16	2,5,6,7,9,11,12,14	4	O35077,P00762,Q5I0J9,Q64093
3724	1,3,4,8,10,14,15,16	2,5,6,7,9,11,12,13	3	O35077,P00762,P54921
3725	1,3,4,8,11,12,13,14	2,5,6,7,9,10,15,16	5	B0BNN3,D3ZTX0,O54728,Q64335,Q811M5
3726	1,3,4,8,11,12,13,15	2,5,6,7,9,10,14,16	5	Q4FZU2,Q6IFW6,Q6IG02,Q6IMF3,Q6P6Q2
3727	1,3,4,8,11,12,13,16	2,5,6,7,9,10,14,15	4	P00762,P02782,P30120,Q811M5
3728	1,3,4,8,11,12,14,15	2,5,6,7,9,10,13,16	7	D3ZTX0,Q4FZU2,Q6IFU7,Q6IFW6,Q6IG02,Q6IMF3,Q6P6Q2
3729	1,3,4,8,11,12,14,16	2,5,6,7,9,10,13,15	4	D3ZTX0,O54728,P00762,Q811M5
3730	1,3,4,8,11,12,15,16	2,5,6,7,9,10,13,14	8	D3ZTX0,P00762,P29315,Q4FZU2,Q6IFW6,Q6IG02,Q6IMF3,Q6P6Q2

3731	1,3,4,8,11,13,14,15	2,5,6,7,9,10,12,16	9	B0BNN3,D3ZTX0,O54728,Q4FZU2,Q6IFU7,Q6IFW6,Q6IG02,Q6IMF3,Q6P6Q2
3732	1,3,4,8,11,13,14,16	2,5,6,7,9,10,12,15	9	B0BNN3,D3ZTX0,O54728,P00762,P02782,P30120,Q6AY61,Q811M5,Q99MH3
3733	1,3,4,8,11,13,15,16	2,5,6,7,9,10,12,14	11	B0BNN3,P00762,P02782,P29315,P30120,Q4FZU2,Q6IFU7,Q6IFW6,Q6IG02,Q6IMF3,Q6P6Q2
3734	1,3,4,8,11,14,15,16	2,5,6,7,9,10,12,13	12	B0BNN3,D3ZTX0,O54728,P00762,P29315,Q4FZU2,Q6IFU7,Q6IFU8,Q6IFW6,Q6IG02,Q6IMF3,Q6P6Q2
3735	1,3,4,8,12,13,14,15	2,5,6,7,9,10,11,16	2	P70549,Q9Z2L0
3736	1,3,4,8,12,13,14,16	2,5,6,7,9,10,11,15	5	B0BNN3,O54728,P00762,Q99MH3,Q9Z2L0
3737	1,3,4,8,12,13,15,16	2,5,6,7,9,10,11,14	3	P00762,P10758,P50116
3738	1,3,4,8,12,14,15,16	2,5,6,7,9,10,11,13	2	D3ZTX0,P00762
3739	1,3,4,8,13,14,15,16	2,5,6,7,9,10,11,12	4	B0BNN3,O54728,P00762,P50116
3740	1,3,4,9,10,11,12,13	2,5,6,7,8,14,15,16	3	P50115,Q62714,Q62761;Q62762;Q62763
3741	1,3,4,9,10,11,12,14	2,5,6,7,8,13,15,16	3	P50115,Q62714,Q62761;Q62762;Q62763
3742	1,3,4,9,10,11,12,15	2,5,6,7,8,13,14,16	3	P50115,Q62714,Q62761;Q62762;Q62763
3743	1,3,4,9,10,11,12,16	2,5,6,7,8,13,14,15	2	P50115,Q62714
3744	1,3,4,9,10,11,13,14	2,5,6,7,8,12,15,16	1	P50115
3745	1,3,4,9,10,11,13,15	2,5,6,7,8,12,14,16	2	P50115,P50116
3746	1,3,4,9,10,11,13,16	2,5,6,7,8,12,14,15	1	P50116
3747	1,3,4,9,10,11,14,15	2,5,6,7,8,12,13,16	1	P50115
3748	1,3,4,9,10,11,14,16	2,5,6,7,8,12,13,15	1	P50115
3749	1,3,4,9,10,11,15,16	2,5,6,7,8,12,13,14	3	P25809,P29315,P50116
3750	1,3,4,9,10,12,13,14	2,5,6,7,8,11,15,16	5	P09456,P50115,P50116,P50280,Q00715

3751	1,3,4,9,10,12,13,15	2,5,6,7,8,11,14,16	6	P09456,P10758,P50115,P50116,P50280,Q00715
3752	1,3,4,9,10,12,13,16	2,5,6,7,8,11,14,15	2	P50115,P50116
3753	1,3,4,9,10,12,14,15	2,5,6,7,8,11,13,16	6	P09456,P30120,P46462,P50115,P50116,Q00715
3754	1,3,4,9,10,12,14,16	2,5,6,7,8,11,13,15	2	P50115,P50116
3755	1,3,4,9,10,12,15,16	2,5,6,7,8,11,13,14	5	P10758,P25809,P29315,P50115,P50116
3756	1,3,4,9,10,13,14,15	2,5,6,7,8,11,12,16	7	P09456,P12020,P46462,P50115,P50116,P62804,Q00715
3757	1,3,4,9,10,13,14,16	2,5,6,7,8,11,12,15	2	P50115,P50116
3758	1,3,4,9,10,13,15,16	2,5,6,7,8,11,12,14	5	P12020,P29315,P50115,P50116,Q00715
3759	1,3,4,9,10,14,15,16	2,5,6,7,8,11,12,13	5	P12020,P29315,P47967,P50115,P50116
3760	1,3,4,9,11,12,13,14	2,5,6,7,8,10,15,16	10	O70594,P25031,P50115,P50116,P53790,Q05175,Q3ZAV1,Q62761;Q62762;Q62763,Q63270,Q64335
3761	1,3,4,9,11,12,13,15	2,5,6,7,8,10,14,16	6	P25031,P50115,P50116,P57113,Q62761;Q62762;Q62763,Q6IG02
3762	1,3,4,9,11,12,13,16	2,5,6,7,8,10,14,15	4	P50115,P50116,P54921,Q62714
3763	1,3,4,9,11,12,14,15	2,5,6,7,8,10,13,16	7	O70594,P02631,P25031,P25809,P50115,P50116,Q62761;Q62762;Q62763
3764	1,3,4,9,11,12,14,16	2,5,6,7,8,10,13,15	8	O70594,P02631,P25809,P36860,P50115,P50116,Q62714,Q63618
3765	1,3,4,9,11,12,15,16	2,5,6,7,8,10,13,14	7	P02631,P25031,P25809,P29315,P50115,P50116,Q62714
3766	1,3,4,9,11,13,14,15	2,5,6,7,8,10,12,16	7	P02631,P0DMW0;P0DMW1,P25031,P36970,P50115,P50116,Q6IG02
3767	1,3,4,9,11,13,14,16	2,5,6,7,8,10,12,15	6	O54728,P19132,P19814,P25031,P50115,P50116
3768	1,3,4,9,11,13,15,16	2,5,6,7,8,10,12,14	6	P02631,P25031,P29315,P50116,Q5RLM2,Q6IG02
3769	1,3,4,9,11,14,15,16	2,5,6,7,8,10,12,13	6	P02631,P25031,P25809,P29315,P50115,P50116
3770	1,3,4,9,12,13,14,15	2,5,6,7,8,10,11,16	5	P09456,P25031,P50115,P50116,Q00715

3771	1,3,4,9,12,13,14,16	2,5,6,7,8,10,11,15	2	P50115,P50116
3772	1,3,4,9,12,13,15,16	2,5,6,7,8,10,11,14	4	P10758,P29315,P50115,P50116
3773	1,3,4,9,12,14,15,16	2,5,6,7,8,10,11,13	5	P02631,P25809,P29315,P50115,P50116
3774	1,3,4,9,13,14,15,16	2,5,6,7,8,10,11,12	6	P02631,P23593,P29315,P50115,P50116,Q00715
3775	1,3,4,10,11,12,13,14	2,5,6,7,8,9,15,16	2	P50115,P50116
3776	1,3,4,10,11,12,13,15	2,5,6,7,8,9,14,16	3	P50115,P50116,Q6IG02
3777	1,3,4,10,11,12,13,16	2,5,6,7,8,9,14,15	3	P50115,P50116,P97840
3778	1,3,4,10,11,12,14,15	2,5,6,7,8,9,13,16	3	P50115,P50116,Q6IG02
3779	1,3,4,10,11,12,14,16	2,5,6,7,8,9,13,15	2	P50115,P50116
3780	1,3,4,10,11,12,15,16	2,5,6,7,8,9,13,14	5	P10758,P29315,P50115,P50116,Q6IG02
3781	1,3,4,10,11,13,14,15	2,5,6,7,8,9,12,16	4	P50115,P50116,Q6IG02,Q8CJD3
3782	1,3,4,10,11,13,14,16	2,5,6,7,8,9,12,15	6	B0BNN3,O54728,P19132,P50115,P50116,Q8CJD3
3783	1,3,4,10,11,13,15,16	2,5,6,7,8,9,12,14	4	P29315,P50116,Q6IG02,Q8CJD3
3784	1,3,4,10,11,14,15,16	2,5,6,7,8,9,12,13	5	P29315,P50115,P50116,Q6IG02,Q8CJD3
3785	1,3,4,10,12,13,14,15	2,5,6,7,8,9,11,16	6	P06760,P07150,P09456,P50115,P50116,P70549
3786	1,3,4,10,12,13,14,16	2,5,6,7,8,9,11,15	3	P06760,P50115,P50116
3787	1,3,4,10,12,13,15,16	2,5,6,7,8,9,11,14	5	P06760,P07150,P10758,P50115,P50116
3788	1,3,4,10,12,14,15,16	2,5,6,7,8,9,11,13	4	P06760,P10758,P50115,P50116
3789	1,3,4,10,13,14,15,16	2,5,6,7,8,9,11,12	3	P06760,P50115,P50116
3790	1,3,4,11,12,13,14,15	2,5,6,7,8,9,10,16	9	D3ZTX0,P02454,P02631,P25031,P50115,P50116,P70545,Q6AYE5,Q6IG02

3791	1,3,4,11,12,13,14,16	2,5,6,7,8,9,10,15	10	D3ZTX0,O54728,P00762,P02454,P19132,P25031,P50115,P50116,Q6AYE5,Q811M5
3792	1,3,4,11,12,13,15,16	2,5,6,7,8,9,10,14	8	D3ZTX0,P00762,P02454,P25031,P29315,P50115,P50116,Q6IG02
3793	1,3,4,11,12,14,15,16	2,5,6,7,8,9,10,13	11	D3ZTX0,P02454,P02631,P17559,P25031,P25809,P29315,P50115,P50116,Q6AYE5,Q6IG02
3794	1,3,4,11,13,14,15,16	2,5,6,7,8,9,10,12	11	B0BNN3,D3ZTX0,O54728,P00762,P02454,P02631,P25031,P29315,P50115,P50116,Q6IG02
3795	1,3,4,12,13,14,15,16	2,5,6,7,8,9,10,11	9	D3ZTX0,P02454,P06760,P07150,P25031,P50115,P50116,P70545,Q6P6R2
3796	1,3,5,6,7,8,9,10	2,4,11,12,13,14,15,16	8	O70594,P08937,P23928,P25031,P36860,P70545,Q6P6R2,Q9QX74
3797	1,3,5,6,7,8,9,11	2,4,10,12,13,14,15,16	11	P06760,P08937,P22283,P35280,P50115,Q63493,Q6P6R2,Q6P6S4,Q812E4,Q9QX74,Q9QYP2
3798	1,3,5,6,7,8,9,12	2,4,10,11,13,14,15,16	2	P08937,Q9QX74
3799	1,3,5,6,7,8,9,13	2,4,10,11,12,14,15,16	3	P08937,Q62714,Q9QX74
3800	1,3,5,6,7,8,9,14	2,4,10,11,12,13,15,16	5	P08937,Q5QE79,Q62714,Q63751,Q9QX74
3801	1,3,5,6,7,8,9,15	2,4,10,11,12,13,14,16	9	P01681,P01946,P02091,P08721,P08937,P19132,P35280,Q62714,Q9QX74
3802	1,3,5,6,7,8,9,16	2,4,10,11,12,13,14,15	2	P08937,Q9QX74
3803	1,3,5,6,7,8,10,11	2,4,9,12,13,14,15,16	8	P06761,P08937,P22283,P31044,P47967,P50115,Q63493,Q9QX74
3804	1,3,5,6,7,8,10,12	2,4,9,11,13,14,15,16	14	P08937,P19629,P25031,P30904,P36860,P46844,P47967,P48508,P57113,Q05175,Q5RLM2,Q66HG3,Q6MG61,Q9QX74
3805	1,3,5,6,7,8,10,13	2,4,9,11,12,14,15,16	17	O70257,O70594,P08937,P25031,P36860,P46844,P47967,P48508,P57113,P63081,Q3ZAV1,Q62714,Q63618,Q6MG61,Q8R431,Q9QX74,Q9WUW8
3806	1,3,5,6,7,8,10,14	2,4,9,11,12,13,15,16	19	D3ZUC6,O70594,P08937,P23928,P25031,P46844,P48508,P54921,P57113,P63081,Q05175,Q5QE79,Q5RLM2,Q62714,Q63751,Q64602,Q923S2,Q9QX74,Q9WUW8

3807	1,3,5,6,7,8,10,15	2,4,9,11,12,13,14,16	41	O35077,O35763,O70377,O70594,O88339;Q4V882,P01946,P02091,P02793;Q7TP54,P08721,P08937,P18757,P19468,P19629,P23928,P25031,P30904,P36860,P38918,P46844,P48508,P53790,P54921,P57113,Q03248,Q05175,Q3ZAV1,Q5I0E9,Q5RKI1,Q62714,Q63270,Q63618,Q64602,Q6AY41,Q6MG61,Q6Q0N1,Q80W57,Q8R431,Q923S2,Q9QX74,Q9WTW7,Q9Z0W7
3808	1,3,5,6,7,8,10,16	2,4,9,11,12,13,14,15	44	O35077,O35763,O70257,O70377,O70594,P08937,P18757,P19468,P19629,P23928,P25031,P29975,P38918,P46720,P46844,P48508,P53790,P57113,P60711;P63259,Q03248,Q05175,Q3T1J9,Q3ZAV1,Q5I0E9,Q5M7T9,Q62714,Q63270,Q63424,Q64093,Q64602,Q68FT5,Q6AY41,Q6MG61,Q6Q0N1,Q71MB6,Q80W57,Q8R431,Q923S2,Q9JJ40,Q9QX74,Q9WTW7,Q9WUW8,Q9WUW9,Q9Z0W7
3809	1,3,5,6,7,8,11,12	2,4,9,10,13,14,15,16	17	P06761,P06911,P08010,P08937,P12020,P22283,P47967,P97840,Q5GRG2,Q5I0D1,Q5M8C6,Q63493,Q6IG05,Q9JHB9,Q9JI85,Q9QW07,Q9QX74
3810	1,3,5,6,7,8,11,13	2,4,9,10,12,14,15,16	9	P06761,P08937,P22283,P47967,P50115,Q5M8C6,Q63493,Q9JI85,Q9QX74
3811	1,3,5,6,7,8,11,14	2,4,9,10,12,13,15,16	12	D3ZUC6,P06761,P06911,P08649,P08937,P19218,P22283,Q5GRG2,Q5M8C6,Q63493,Q63751,Q9QX74
3812	1,3,5,6,7,8,11,15	2,4,9,10,12,13,14,16	18	P01946,P02091,P02793;Q7TP54,P08721,P08937,P22283,P50115,Q4FZU2,Q63493,Q6IFU7,Q6IFW6,Q6IG02,Q6IG05,Q6IMF3,Q6P6Q2,Q8CJ52,Q9QW07,Q9QX74
3813	1,3,5,6,7,8,11,16	2,4,9,10,12,13,14,15	22	O35547,P02780,P02781,P02782,P02783,P06761,P07647,P08010,P08649,P08723,P08937,P09456,P22283,P30120,P46462,P50115,Q5M8C6,Q63493,Q9JHB9,Q9JI85,Q9QW07,Q9QX74
3814	1,3,5,6,7,8,12,13	2,4,9,10,11,14,15,16	3	P08937,P47967,P97840
3815	1,3,5,6,7,8,12,14	2,4,9,10,11,13,15,16	5	D3ZUC6,P08937,P19218,Q5RLM2,Q63751
3816	1,3,5,6,7,8,12,15	2,4,9,10,11,13,14,16	9	D3ZUC6,P01946,P02091,P08721,P19629,Q62714,Q6IG05,Q9QX74,Q9R168
3817	1,3,5,6,7,8,12,16	2,4,9,10,11,13,14,15	3	P08937,P46844,P57113
3818	1,3,5,6,7,8,13,14	2,4,9,10,11,12,15,16	7	D3ZUC6,P08937,P63081,Q5QE79,Q62714,Q63751,Q9WUW8
3819	1,3,5,6,7,8,13,15	2,4,9,10,11,12,14,16	7	O70594,P01946,P02091,P08721,P70549,Q62714,Q9QX74
3820	1,3,5,6,7,8,13,16	2,4,9,10,11,12,14,15	8	O35077,O70594,P08937,P46844,P57113,P63081,Q62714,Q9WUW8
3821	1,3,5,6,7,8,14,15	2,4,9,10,11,12,13,16	8	D3ZUC6,P01946,P02091,P08937,P54921,Q62714,Q63751,Q9QX74
3822	1,3,5,6,7,8,14,16	2,4,9,10,11,12,13,15	12	O35077,P02783,P08937,P46844,P52590,P57113,P63081,Q62714,Q62812,Q63751,Q9QX74,Q9WUW8
3823	1,3,5,6,7,8,15,16	2,4,9,10,11,12,13,14	18	O35077,O70594,P01946,P02091,P02783,P08721,P09606,P19468,P19629,P46844,P48508,P53790,P57113,Q05175,Q62714,Q63270,Q8R431,Q9QX74
3824	1,3,5,6,7,9,10,11	2,4,8,12,13,14,15,16	0	

3825	1,3,5,6,7,9,10,12	2,4,8,11,13,14,15,16	1 O54728
3826	1,3,5,6,7,9,10,13	2,4,8,11,12,14,15,16	1 P36860
3827	1,3,5,6,7,9,10,14	2,4,8,11,12,13,15,16	1 Q63751
3828	1,3,5,6,7,9,10,15	2,4,8,11,12,13,14,16	6 O54728,P01946,P02091,P08721,P36860,Q5RKI1
3829	1,3,5,6,7,9,10,16	2,4,8,11,12,13,14,15	0
3830	1,3,5,6,7,9,11,12	2,4,8,10,13,14,15,16	1 Q03191
3831	1,3,5,6,7,9,11,13	2,4,8,10,12,14,15,16	0
3832	1,3,5,6,7,9,11,14	2,4,8,10,12,13,15,16	1 Q63751
3833	1,3,5,6,7,9,11,15	2,4,8,10,12,13,14,16	3 P01946,P02091,P08721
3834	1,3,5,6,7,9,11,16	2,4,8,10,12,13,14,15	0
3835	1,3,5,6,7,9,12,13	2,4,8,10,11,14,15,16	0
3836	1,3,5,6,7,9,12,14	2,4,8,10,11,13,15,16	1 Q63751
3837	1,3,5,6,7,9,12,15	2,4,8,10,11,13,14,16	3 O54728,P01946,P02091
3838	1,3,5,6,7,9,12,16	2,4,8,10,11,13,14,15	0
3839	1,3,5,6,7,9,13,14	2,4,8,10,11,12,15,16	0
3840	1,3,5,6,7,9,13,15	2,4,8,10,11,12,14,16	4 P01946,P02091,P08721,P62804
3841	1,3,5,6,7,9,13,16	2,4,8,10,11,12,14,15	0
3842	1,3,5,6,7,9,14,15	2,4,8,10,11,12,13,16	2 P01946,P02091
3843	1,3,5,6,7,9,14,16	2,4,8,10,11,12,13,15	0
3844	1,3,5,6,7,9,15,16	2,4,8,10,11,12,13,14	3 P01946,P02091,P08721

3845	1,3,5,6,7,10,11,12	2,4,8,9,13,14,15,16	4	P23593,P47967,P97840,Q5I0D1
3846	1,3,5,6,7,10,11,13	2,4,8,9,12,14,15,16	2	P47967,P97840
3847	1,3,5,6,7,10,11,14	2,4,8,9,12,13,15,16	0	
3848	1,3,5,6,7,10,11,15	2,4,8,9,12,13,14,16	4	P01946,P02091,P08721,Q5RKI1
3849	1,3,5,6,7,10,11,16	2,4,8,9,12,13,14,15	1	P02783
3850	1,3,5,6,7,10,12,13	2,4,8,9,11,14,15,16	4	P36860,P47967,P97840,Q6AY61
3851	1,3,5,6,7,10,12,14	2,4,8,9,11,13,15,16	3	P57113,Q5RLM2,Q6AY61
3852	1,3,5,6,7,10,12,15	2,4,8,9,11,13,14,16	7	O54728,P01946,P02091,P08721,P36860,Q5RKI1,Q6AY61
3853	1,3,5,6,7,10,12,16	2,4,8,9,11,13,14,15	3	P0DMW0;P0DMW1,P57113,P97840
3854	1,3,5,6,7,10,13,14	2,4,8,9,11,12,15,16	3	P57113,Q62714,Q9WUW8
3855	1,3,5,6,7,10,13,15	2,4,8,9,11,12,14,16	7	P01946,P02091,P08721,P36860,P57113,Q5RKI1,Q62714
3856	1,3,5,6,7,10,13,16	2,4,8,9,11,12,14,15	3	P46844,P57113,Q9WUW8
3857	1,3,5,6,7,10,14,15	2,4,8,9,11,12,13,16	7	P01946,P02091,P08721,P54921,P57113,Q5RKI1,Q62714
3858	1,3,5,6,7,10,14,16	2,4,8,9,11,12,13,15	4	P02783,P57113,Q71MB6,Q9WUW8
3859	1,3,5,6,7,10,15,16	2,4,8,9,11,12,13,14	17	O35077,O70594,P01946,P02091,P08721,P17988,P18297,P19468,P48508,P53790,P57113,Q03248,Q5I0E9,Q5RKI1,Q63270,Q71MB6,Q8R431
3860	1,3,5,6,7,11,12,13	2,4,8,9,10,14,15,16	3	P47967,P97840,Q5I0D1
3861	1,3,5,6,7,11,12,14	2,4,8,9,10,13,15,16	1	P19218
3862	1,3,5,6,7,11,12,15	2,4,8,9,10,13,14,16	5	P01946,P02091,P08721,P47967,P97840
3863	1,3,5,6,7,11,12,16	2,4,8,9,10,13,14,15	2	P47967,P97840
3864	1,3,5,6,7,11,13,14	2,4,8,9,10,12,15,16	0	

3865	1,3,5,6,7,11,13,15	2,4,8,9,10,12,14,16	4	P01946,P02091,P08721,P47967
3866	1,3,5,6,7,11,13,16	2,4,8,9,10,12,14,15	2	P22283,P47967
3867	1,3,5,6,7,11,14,15	2,4,8,9,10,12,13,16	3	P01946,P02091,P08721
3868	1,3,5,6,7,11,14,16	2,4,8,9,10,12,13,15	1	P02783
3869	1,3,5,6,7,11,15,16	2,4,8,9,10,12,13,14	8	P01946,P02091,P02783,P08721,P17988,Q4FZU2,Q6IFW6,Q8CJ52
3870	1,3,5,6,7,12,13,14	2,4,8,9,10,11,15,16	3	D3ZUC6,P01835,Q6AY61
3871	1,3,5,6,7,12,13,15	2,4,8,9,10,11,14,16	6	P01946,P02091,P47967,P70549,P97840,Q6AY61
3872	1,3,5,6,7,12,13,16	2,4,8,9,10,11,14,15	2	P47967,P97840
3873	1,3,5,6,7,12,14,15	2,4,8,9,10,11,13,16	4	D3ZUC6,P01946,P02091,Q6AY61
3874	1,3,5,6,7,12,14,16	2,4,8,9,10,11,13,15	1	P02783
3875	1,3,5,6,7,12,15,16	2,4,8,9,10,11,13,14	4	P01946,P02091,P08721,Q6AY61
3876	1,3,5,6,7,13,14,15	2,4,8,9,10,11,12,16	6	D3ZUC6,P01835,P01946,P02091,P70549,Q62714
3877	1,3,5,6,7,13,14,16	2,4,8,9,10,11,12,15	3	P01835,Q62714,Q9WUW8
3878	1,3,5,6,7,13,15,16	2,4,8,9,10,11,12,14	5	P01835,P01946,P02091,P08721,Q62714
3879	1,3,5,6,7,14,15,16	2,4,8,9,10,11,12,13	5	P01946,P02091,P02783,P08721,Q62714
3880	1,3,5,6,8,9,10,11	2,4,7,12,13,14,15,16	2	P08937,Q6P6S4
3881	1,3,5,6,8,9,10,12	2,4,7,11,13,14,15,16	1	P25031
3882	1,3,5,6,8,9,10,13	2,4,7,11,12,14,15,16	1	P25031
3883	1,3,5,6,8,9,10,14	2,4,7,11,12,13,15,16	3	P08937,P25031,Q63751
3884	1,3,5,6,8,9,10,15	2,4,7,11,12,13,14,16	5	P01681,P01946,P02091,P25031,P62804

3885	1,3,5,6,8,9,10,16	2,4,7,11,12,13,14,15	1	P25031
3886	1,3,5,6,8,9,11,12	2,4,7,10,13,14,15,16	3	O88797,P09527,Q6IG05
3887	1,3,5,6,8,9,11,13	2,4,7,10,12,14,15,16	1	P09527
3888	1,3,5,6,8,9,11,14	2,4,7,10,12,13,15,16	3	P08937,Q5I0J9,Q63751
3889	1,3,5,6,8,9,11,15	2,4,7,10,12,13,14,16	10	P01681,P01946,P02091,Q4FZU2,Q6IFU7,Q6IFW6,Q6IG05,Q6IMF3,Q6P6Q2,Q6P6S4
3890	1,3,5,6,8,9,11,16	2,4,7,10,12,13,14,15	1	O88797
3891	1,3,5,6,8,9,12,13	2,4,7,10,11,14,15,16	0	
3892	1,3,5,6,8,9,12,14	2,4,7,10,11,13,15,16	3	P08937,Q09030,Q63751
3893	1,3,5,6,8,9,12,15	2,4,7,10,11,13,14,16	6	P01681,P01946,P02091,P36376,Q6IG05,Q9R168
3894	1,3,5,6,8,9,12,16	2,4,7,10,11,13,14,15	1	Q09030
3895	1,3,5,6,8,9,13,14	2,4,7,10,11,12,15,16	2	P08937,Q09030
3896	1,3,5,6,8,9,13,15	2,4,7,10,11,12,14,16	4	P01946,P02091,P62804,Q00715
3897	1,3,5,6,8,9,13,16	2,4,7,10,11,12,14,15	1	Q09030
3898	1,3,5,6,8,9,14,15	2,4,7,10,11,12,13,16	5	P00714,P01681,P01946,P02091,P08937
3899	1,3,5,6,8,9,14,16	2,4,7,10,11,12,13,15	2	P08937,Q09030
3900	1,3,5,6,8,9,15,16	2,4,7,10,11,12,13,14	2	P01946,P02091
3901	1,3,5,6,8,10,11,12	2,4,7,9,13,14,15,16	2	Q6IG05,Q9R168
3902	1,3,5,6,8,10,11,13	2,4,7,9,12,14,15,16	1	Q6IFU7
3903	1,3,5,6,8,10,11,14	2,4,7,9,12,13,15,16	2	P08937,Q6IFU7
3904	1,3,5,6,8,10,11,15	2,4,7,9,12,13,14,16	10	P01946,P02091,P02793;Q7TP54,Q4FZU2,Q6IFU7,Q6IFW6,Q6IG02,Q6IG05,Q6IMF3,Q6P6Q2

3905	1,3,5,6,8,10,11,16	2,4,7,9,12,13,14,15	4	P02783,P08010,P25031,Q6IFU7
3906	1,3,5,6,8,10,12,13	2,4,7,9,11,14,15,16	2	P25031,Q9R168
3907	1,3,5,6,8,10,12,14	2,4,7,9,11,13,15,16	4	P08937,P25031,Q5RLM2,Q9R168
3908	1,3,5,6,8,10,12,15	2,4,7,9,11,13,14,16	5	P01946,P02091,P25031,Q6IG05,Q9R168
3909	1,3,5,6,8,10,12,16	2,4,7,9,11,13,14,15	3	P0DMW0;P0DMW1,P25031,Q9R168
3910	1,3,5,6,8,10,13,14	2,4,7,9,11,12,15,16	3	P08937,P25031,Q62714
3911	1,3,5,6,8,10,13,15	2,4,7,9,11,12,14,16	6	P01946,P02091,P62804,P70549,Q498D9,Q62714
3912	1,3,5,6,8,10,13,16	2,4,7,9,11,12,14,15	2	O35077,P25031
3913	1,3,5,6,8,10,14,15	2,4,7,9,11,12,13,16	6	P00714,P01946,P02091,P08937,P54921,Q62714
3914	1,3,5,6,8,10,14,16	2,4,7,9,11,12,13,15	5	O35077,P02783,P08937,P25031,P57113
3915	1,3,5,6,8,10,15,16	2,4,7,9,11,12,13,14	8	O35077,O70594,P01946,P02091,P25031,P53790,Q05175,Q8R431
3916	1,3,5,6,8,11,12,13	2,4,7,9,10,14,15,16	4	P47967,Q5I0D1,Q6IG05,Q9R168
3917	1,3,5,6,8,11,12,14	2,4,7,9,10,13,15,16	4	D3ZUC6,P08937,P19218,Q6IG05
3918	1,3,5,6,8,11,12,15	2,4,7,9,10,13,14,16	10	P01946,P02091,Q4FZU2,Q6IFU7,Q6IFW6,Q6IG02,Q6IG05,Q6IMF3,Q6P6Q2,Q9R168
3919	1,3,5,6,8,11,12,16	2,4,7,9,10,13,14,15	4	O88797,P08010,Q6IG05,Q9QW07
3920	1,3,5,6,8,11,13,14	2,4,7,9,10,12,15,16	3	P08937,P19218,Q6IFU7
3921	1,3,5,6,8,11,13,15	2,4,7,9,10,12,14,16	9	P01946,P02091,Q4FZU2,Q6IFU7,Q6IFW6,Q6IG02,Q6IG05,Q6IMF3,Q6P6Q2
3922	1,3,5,6,8,11,13,16	2,4,7,9,10,12,14,15	3	P02782,P30120,Q6IFU7
3923	1,3,5,6,8,11,14,15	2,4,7,9,10,12,13,16	12	D3ZUC6,P01946,P02091,P08937,P19218,Q4FZU2,Q6IFU7,Q6IFW6,Q6IG02,Q6IG05,Q6IMF3,Q6P6Q2
3924	1,3,5,6,8,11,14,16	2,4,7,9,10,12,13,15	4	P02783,P08937,P19218,Q6IFU7

3925	1,3,5,6,8,11,15,16	2,4,7,9,10,12,13,14	10	P01946,P02091,P02783,Q4FZU2,Q6IFU7,Q6IFW6,Q6IG02,Q6IG05,Q6IMF3,Q6P6Q2
3926	1,3,5,6,8,12,13,14	2,4,7,9,10,11,15,16	3	D3ZUC6,P08937,Q9R168
3927	1,3,5,6,8,12,13,15	2,4,7,9,10,11,14,16	5	P01946,P02091,P70549,Q6IG05,Q9R168
3928	1,3,5,6,8,12,13,16	2,4,7,9,10,11,14,15	0	
3929	1,3,5,6,8,12,14,15	2,4,7,9,10,11,13,16	6	D3ZUC6,P00714,P01946,P02091,Q6IG05,Q9R168
3930	1,3,5,6,8,12,14,16	2,4,7,9,10,11,13,15	1	P02783
3931	1,3,5,6,8,12,15,16	2,4,7,9,10,11,13,14	4	P01946,P02091,Q6IG05,Q9R168
3932	1,3,5,6,8,13,14,15	2,4,7,9,10,11,12,16	6	D3ZUC6,P01946,P02091,P62804,P70549,Q62714
3933	1,3,5,6,8,13,14,16	2,4,7,9,10,11,12,15	1	Q62714
3934	1,3,5,6,8,13,15,16	2,4,7,9,10,11,12,14	4	O35077,P01946,P02091,Q62714
3935	1,3,5,6,8,14,15,16	2,4,7,9,10,11,12,13	5	O35077,P01946,P02091,P02783,Q62714
3936	1,3,5,6,9,10,11,12	2,4,7,8,13,14,15,16	1	Q03191
3937	1,3,5,6,9,10,11,13	2,4,7,8,12,14,15,16	0	
3938	1,3,5,6,9,10,11,14	2,4,7,8,12,13,15,16	0	
3939	1,3,5,6,9,10,11,15	2,4,7,8,12,13,14,16	2	P01946,P02091
3940	1,3,5,6,9,10,11,16	2,4,7,8,12,13,14,15	1	Q9Z2L0
3941	1,3,5,6,9,10,12,13	2,4,7,8,11,14,15,16	2	P0C0A9,Q4G075
3942	1,3,5,6,9,10,12,14	2,4,7,8,11,13,15,16	2	P0C0A9,P30120
3943	1,3,5,6,9,10,12,15	2,4,7,8,11,13,14,16	9	B0BNN3,O54728,P01946,P02091,P09456,P0C0A9,P30120,Q4G075,Q99041
3944	1,3,5,6,9,10,12,16	2,4,7,8,11,13,14,15	1	Q4G075

3945	1,3,5,6,9,10,13,14	2,4,7,8,11,12,15,16	4	P02780,P0C0A9,Q00715,Q06000
3946	1,3,5,6,9,10,13,15	2,4,7,8,11,12,14,16	8	P01946,P02091,P02780,P0C0A9,P11883,P30120,P62804,Q00715
3947	1,3,5,6,9,10,13,16	2,4,7,8,11,12,14,15	1	P11883
3948	1,3,5,6,9,10,14,15	2,4,7,8,11,12,13,16	9	P00714,P01946,P02091,P02780,P02782,P22273,P30120,P62804,Q00715
3949	1,3,5,6,9,10,14,16	2,4,7,8,11,12,13,15	0	
3950	1,3,5,6,9,10,15,16	2,4,7,8,11,12,13,14	3	P01946,P02091,Q811M5
3951	1,3,5,6,9,11,12,13	2,4,7,8,10,14,15,16	1	P20762
3952	1,3,5,6,9,11,12,14	2,4,7,8,10,13,15,16	4	O55004,P51907,Q5I0J9,Q64093
3953	1,3,5,6,9,11,12,15	2,4,7,8,10,13,14,16	2	P01946,P02091
3954	1,3,5,6,9,11,12,16	2,4,7,8,10,13,14,15	1	Q9Z1F2
3955	1,3,5,6,9,11,13,14	2,4,7,8,10,12,15,16	2	P0DMW0;P0DMW1,P20762
3956	1,3,5,6,9,11,13,15	2,4,7,8,10,12,14,16	4	P01946,P02091,P20762,P62804
3957	1,3,5,6,9,11,13,16	2,4,7,8,10,12,14,15	1	Q9Z1F2
3958	1,3,5,6,9,11,14,15	2,4,7,8,10,12,13,16	3	P01946,P02091,P0DMW0;P0DMW1
3959	1,3,5,6,9,11,14,16	2,4,7,8,10,12,13,15	2	Q5I0J9,Q9Z1F2
3960	1,3,5,6,9,11,15,16	2,4,7,8,10,12,13,14	4	P01946,P02091,P25809,Q9Z1F2
3961	1,3,5,6,9,12,13,14	2,4,7,8,10,11,15,16	2	P0C0A9,P20762
3962	1,3,5,6,9,12,13,15	2,4,7,8,10,11,14,16	5	P01946,P02091,P0C0A9,P62804,Q00715
3963	1,3,5,6,9,12,13,16	2,4,7,8,10,11,14,15	0	
3964	1,3,5,6,9,12,14,15	2,4,7,8,10,11,13,16	5	P00714,P01946,P02091,P22273,P30120

3965	1,3,5,6,9,12,14,16	2,4,7,8,10,11,13,15	1	Q09030
3966	1,3,5,6,9,12,15,16	2,4,7,8,10,11,13,14	3	P01946,P02091,P25809
3967	1,3,5,6,9,13,14,15	2,4,7,8,10,11,12,16	6	P01946,P02091,P02780,P30120,P62804,Q00715
3968	1,3,5,6,9,13,14,16	2,4,7,8,10,11,12,15	1	Q09030
3969	1,3,5,6,9,13,15,16	2,4,7,8,10,11,12,14	4	P01946,P02091,P11883,P62804
3970	1,3,5,6,9,14,15,16	2,4,7,8,10,11,12,13	2	P01946,P02091
3971	1,3,5,6,10,11,12,13	2,4,7,8,9,14,15,16	1	P47967
3972	1,3,5,6,10,11,12,14	2,4,7,8,9,13,15,16	0	
3973	1,3,5,6,10,11,12,15	2,4,7,8,9,13,14,16	2	P01946,P02091
3974	1,3,5,6,10,11,12,16	2,4,7,8,9,13,14,15	0	
3975	1,3,5,6,10,11,13,14	2,4,7,8,9,12,15,16	0	
3976	1,3,5,6,10,11,13,15	2,4,7,8,9,12,14,16	2	P01946,P02091
3977	1,3,5,6,10,11,13,16	2,4,7,8,9,12,14,15	0	
3978	1,3,5,6,10,11,14,15	2,4,7,8,9,12,13,16	2	P01946,P02091
3979	1,3,5,6,10,11,14,16	2,4,7,8,9,12,13,15	1	P02783
3980	1,3,5,6,10,11,15,16	2,4,7,8,9,12,13,14	2	P01946,P02091
3981	1,3,5,6,10,12,13,14	2,4,7,8,9,11,15,16	0	
3982	1,3,5,6,10,12,13,15	2,4,7,8,9,11,14,16	6	P01946,P02091,P07150,P62804,P70549,Q812E4
3983	1,3,5,6,10,12,13,16	2,4,7,8,9,11,14,15	1	P07150
3984	1,3,5,6,10,12,14,15	2,4,7,8,9,11,13,16	7	P00714,P01946,P02091,P09456,P30120,P82471,Q812E4

3985	1,3,5,6,10,<br>12,14,16	2,4,7,8,9,1<br>1,13,15	0
3986	1,3,5,6,10,<br>12,15,16	2,4,7,8,9,1<br>1,13,14	3 P01946,P02091,P82471
3987	1,3,5,6,10,<br>13,14,15	2,4,7,8,9,1<br>1,12,16	6 P01946,P02091,P02780,P62804,Q00715,Q812E4
3988	1,3,5,6,10,<br>13,14,16	2,4,7,8,9,1<br>1,12,15	1 P14668
3989	1,3,5,6,10,<br>13,15,16	2,4,7,8,9,1<br>1,12,14	6 P01946,P02091,P07150,P11883,P62804,P82471
3990	1,3,5,6,10,<br>14,15,16	2,4,7,8,9,1<br>1,12,13	4 P01946,P02091,P02783,P82471
3991	1,3,5,6,11,<br>12,13,14	2,4,7,8,9,1<br>0,15,16	2 P20646,P20762
3992	1,3,5,6,11,<br>12,13,15	2,4,7,8,9,1<br>0,14,16	3 P01946,P02091,P20762
3993	1,3,5,6,11,<br>12,13,16	2,4,7,8,9,1<br>0,14,15	2 P47967,Q9Z1F2
3994	1,3,5,6,11,<br>12,14,15	2,4,7,8,9,1<br>0,13,16	2 P01946,P02091
3995	1,3,5,6,11,<br>12,14,16	2,4,7,8,9,1<br>0,13,15	2 P02783,Q9Z1F2
3996	1,3,5,6,11,<br>12,15,16	2,4,7,8,9,1<br>0,13,14	4 P01946,P02091,P25809,Q9Z1F2
3997	1,3,5,6,11,<br>13,14,15	2,4,7,8,9,1<br>0,12,16	2 P01946,P02091
3998	1,3,5,6,11,<br>13,14,16	2,4,7,8,9,1<br>0,12,15	1 Q9Z1F2
3999	1,3,5,6,11,<br>13,15,16	2,4,7,8,9,1<br>0,12,14	3 P01946,P02091,Q9Z1F2
4000	1,3,5,6,11,<br>14,15,16	2,4,7,8,9,1<br>0,12,13	4 P01946,P02091,P02783,Q9Z1F2
4001	1,3,5,6,12,<br>13,14,15	2,4,7,8,9,1<br>0,11,16	3 P01946,P02091,P70549
4002	1,3,5,6,12,<br>13,14,16	2,4,7,8,9,1<br>0,11,15	0
4003	1,3,5,6,12,<br>13,15,16	2,4,7,8,9,1<br>0,11,14	3 P01946,P02091,P07150
4004	1,3,5,6,12,<br>14,15,16	2,4,7,8,9,1<br>0,11,13	3 P01946,P02091,P02783

4005	1,3,5,6,13,14,15,16	2,4,7,8,9,10,11,12	3	P01946,P02091,Q62714
4006	1,3,5,7,8,9,10,11	2,4,6,12,13,14,15,16	5	P01681,P06760,P35280,P49134,Q6P6R2
4007	1,3,5,7,8,9,10,12	2,4,6,11,13,14,15,16	0	
4008	1,3,5,7,8,9,10,13	2,4,6,11,12,14,15,16	0	
4009	1,3,5,7,8,9,10,14	2,4,6,11,12,13,15,16	3	P01681,P08937,Q63751
4010	1,3,5,7,8,9,10,15	2,4,6,11,12,13,14,16	3	P01681,P35280,Q5RKI1
4011	1,3,5,7,8,9,10,16	2,4,6,11,12,13,14,15	0	
4012	1,3,5,7,8,9,11,12	2,4,6,10,13,14,15,16	4	P01681,P06760,P35280,P47967
4013	1,3,5,7,8,9,11,13	2,4,6,10,12,14,15,16	2	P06760,P35280
4014	1,3,5,7,8,9,11,14	2,4,6,10,12,13,15,16	7	P01681,P06760,P08649,P08937,P35280,Q63751,Q812E4
4015	1,3,5,7,8,9,11,15	2,4,6,10,12,13,14,16	9	P01681,P06760,P08649,P35280,Q4FZU2,Q6IFW6,Q6IG02,Q6IMF3,Q6P6Q2
4016	1,3,5,7,8,9,11,16	2,4,6,10,12,13,14,15	7	O88797,P06760,P08010,P08649,P35280,Q812E4,Q9JHB9
4017	1,3,5,7,8,9,12,13	2,4,6,10,11,14,15,16	0	
4018	1,3,5,7,8,9,12,14	2,4,6,10,11,13,15,16	2	P08937,Q63751
4019	1,3,5,7,8,9,12,15	2,4,6,10,11,13,14,16	3	P01681,P19132,P35280
4020	1,3,5,7,8,9,12,16	2,4,6,10,11,13,14,15	0	
4021	1,3,5,7,8,9,13,14	2,4,6,10,11,12,15,16	1	P08937
4022	1,3,5,7,8,9,13,15	2,4,6,10,11,12,14,16	2	P01681,P35280
4023	1,3,5,7,8,9,13,16	2,4,6,10,11,12,14,15	0	
4024	1,3,5,7,8,9,14,15	2,4,6,10,11,12,13,16	3	P00714,P01681,P35280

4025	1,3,5,7,8,9,14,16	2,4,6,10,11,12,13,15	0	
4026	1,3,5,7,8,9,15,16	2,4,6,10,11,12,13,14	2	P01681,P35280
4027	1,3,5,7,8,10,11,12	2,4,6,9,13,14,15,16	3	P47967,P97840,Q5I0D1
4028	1,3,5,7,8,10,11,13	2,4,6,9,12,14,15,16	2	P47967,P97840
4029	1,3,5,7,8,10,11,14	2,4,6,9,12,13,15,16	3	D3ZUC6,P08937,Q63751
4030	1,3,5,7,8,10,11,15	2,4,6,9,12,13,14,16	7	Q4FZU2,Q5RKI1,Q6IFU7,Q6IFW6,Q6IG02,Q6IMF3,Q6P6Q2
4031	1,3,5,7,8,10,11,16	2,4,6,9,12,13,14,15	5	O35077,P02782,P08010,P47967,Q9JHB9
4032	1,3,5,7,8,10,12,13	2,4,6,9,11,14,15,16	2	P47967,P97840
4033	1,3,5,7,8,10,12,14	2,4,6,9,11,13,15,16	3	D3ZUC6,P08937,Q5RLM2
4034	1,3,5,7,8,10,12,15	2,4,6,9,11,13,14,16	2	D3ZUC6,Q5RKI1
4035	1,3,5,7,8,10,12,16	2,4,6,9,11,13,14,15	5	P0DMW0;P0DMW1,P46844,P47967,P97840,Q6AY41
4036	1,3,5,7,8,10,13,14	2,4,6,9,11,12,15,16	4	D3ZUC6,P08937,Q62714,Q9WUW8
4037	1,3,5,7,8,10,13,15	2,4,6,9,11,12,14,16	5	O35077,O70594,Q5RKI1,Q62714,Q64093
4038	1,3,5,7,8,10,13,16	2,4,6,9,11,12,14,15	10	O35077,O70594,P19468,P46844,P57113,Q63424,Q64093,Q6AY41,Q8R431,Q9WUW8
4039	1,3,5,7,8,10,14,15	2,4,6,9,11,12,13,16	7	D3ZUC6,O35077,P00714,P54921,Q05175,Q5RKI1,Q62714
4040	1,3,5,7,8,10,14,16	2,4,6,9,11,12,13,15	5	O35077,P57113,Q5RLM2,Q923S2,Q9WUW8
4041	1,3,5,7,8,10,15,16	2,4,6,9,11,12,13,14	23	O35077,O70377,O70594,P19468,P46844,P48508,P53790,P57113,P97608,Q05175,Q3ZAV1,Q5I0E9,Q5RKI1,Q63270,Q63355,Q63424,Q64093,Q6AY41,Q6MG61,Q80W57,Q8R431,Q9WTW7,Q9Z0W7
4042	1,3,5,7,8,11,12,13	2,4,6,9,10,14,15,16	5	D3ZUC6,P47967,P97840,Q5I0D1,Q811M5
4043	1,3,5,7,8,11,12,14	2,4,6,9,10,13,15,16	6	D3ZUC6,P08937,P11883,Q5I0D1,Q63751,Q811M5
4044	1,3,5,7,8,11,12,15	2,4,6,9,10,13,14,16	10	D3ZUC6,P47967,P97840,Q4FZU2,Q6IFU7,Q6IFW6,Q6IG02,Q6IG05,Q6IMF3,Q6P6Q2

4045	1,3,5,7,8,11,12,16	2,4,6,9,10,13,14,15	8	P02782,P08010,P30120,P47967,P97840,Q6IG05,Q811M5,Q9JHB9
4046	1,3,5,7,8,11,13,14	2,4,6,9,10,12,15,16	4	D3ZUC6,P08649,P08937,Q811M5
4047	1,3,5,7,8,11,13,15	2,4,6,9,10,12,14,16	9	D3ZUC6,P01041,P47967,Q4FZU2,Q6IFU7,Q6IFW6,Q6IG02,Q6IMF3,Q6P6Q2
4048	1,3,5,7,8,11,13,16	2,4,6,9,10,12,14,15	8	P02782,P08010,P08649,P09456,P30120,P47967,Q811M5,Q9JHB9
4049	1,3,5,7,8,11,14,15	2,4,6,9,10,12,13,16	10	D3ZUC6,P08649,Q4FZU2,Q6IFU7,Q6IFU8,Q6IFW6,Q6IG02,Q6IG05,Q6IMF3,Q6P6Q2
4050	1,3,5,7,8,11,14,16	2,4,6,9,10,12,13,15	5	P02782,P08010,P08649,Q811M5,Q9JHB9
4051	1,3,5,7,8,11,15,16	2,4,6,9,10,12,13,14	12	P01041,P02782,P08010,P08649,Q4FZU2,Q6IFU7,Q6IFU8,Q6IFW6,Q6IG02,Q6IG05,Q6IMF3,Q6P6Q2
4052	1,3,5,7,8,12,13,14	2,4,6,9,10,11,15,16	2	D3ZUC6,Q811M5
4053	1,3,5,7,8,12,13,15	2,4,6,9,10,11,14,16	3	D3ZUC6,P47967,P97840
4054	1,3,5,7,8,12,13,16	2,4,6,9,10,11,14,15	3	P47967,P97840,Q811M5
4055	1,3,5,7,8,12,14,15	2,4,6,9,10,11,13,16	2	D3ZUC6,P00714
4056	1,3,5,7,8,12,14,16	2,4,6,9,10,11,13,15	2	Q5RLM2,Q811M5
4057	1,3,5,7,8,12,15,16	2,4,6,9,10,11,13,14	1	Q6IG05
4058	1,3,5,7,8,13,14,15	2,4,6,9,10,11,12,16	2	D3ZUC6,Q62714
4059	1,3,5,7,8,13,14,16	2,4,6,9,10,11,12,15	6	O35077,O55145,Q62714,Q811M5,Q99MH3,Q9WUW8
4060	1,3,5,7,8,13,15,16	2,4,6,9,10,11,12,14	2	O35077,Q62714
4061	1,3,5,7,8,14,15,16	2,4,6,9,10,11,12,13	3	O35077,O55145,Q62714
4062	1,3,5,7,9,10,11,12	2,4,6,8,13,14,15,16	2	P47967,Q03191
4063	1,3,5,7,9,10,11,13	2,4,6,8,12,14,15,16	0	
4064	1,3,5,7,9,10,11,14	2,4,6,8,12,13,15,16	0	

4065	1,3,5,7,9,10,11,15	2,4,6,8,12,13,14,16	1	Q5RKI1
4066	1,3,5,7,9,10,11,16	2,4,6,8,12,13,14,15	0	
4067	1,3,5,7,9,10,12,13	2,4,6,8,11,14,15,16	3	P47967,P50280,Q6AY61
4068	1,3,5,7,9,10,12,14	2,4,6,8,11,13,15,16	3	P30120,P50280,Q6AY61
4069	1,3,5,7,9,10,12,15	2,4,6,8,11,13,14,16	5	O54728,P30120,Q5RKI1,Q6AY61,Q99041
4070	1,3,5,7,9,10,12,16	2,4,6,8,11,13,14,15	0	
4071	1,3,5,7,9,10,13,14	2,4,6,8,11,12,15,16	1	P50280
4072	1,3,5,7,9,10,13,15	2,4,6,8,11,12,14,16	3	P12020,Q00715,Q5RKI1
4073	1,3,5,7,9,10,13,16	2,4,6,8,11,12,14,15	1	P12020
4074	1,3,5,7,9,10,14,15	2,4,6,8,11,12,13,16	4	P00714,P12020,P30120,Q5RKI1
4075	1,3,5,7,9,10,14,16	2,4,6,8,11,12,13,15	0	
4076	1,3,5,7,9,10,15,16	2,4,6,8,11,12,13,14	2	P12020,Q5RKI1
4077	1,3,5,7,9,11,12,13	2,4,6,8,10,14,15,16	7	P20762,P47967,P54921,P97840,Q03191,Q5I0D1,Q62761;Q62762;Q62763
4078	1,3,5,7,9,11,12,14	2,4,6,8,10,13,15,16	3	O55004,P07151,Q03191
4079	1,3,5,7,9,11,12,15	2,4,6,8,10,13,14,16	4	O55004,P02631,P07151,Q62761;Q62762;Q62763
4080	1,3,5,7,9,11,12,16	2,4,6,8,10,13,14,15	1	Q03191
4081	1,3,5,7,9,11,13,14	2,4,6,8,10,12,15,16	1	P0DMW0;P0DMW1
4082	1,3,5,7,9,11,13,15	2,4,6,8,10,12,14,16	0	
4083	1,3,5,7,9,11,13,16	2,4,6,8,10,12,14,15	1	P54921
4084	1,3,5,7,9,11,14,15	2,4,6,8,10,12,13,16	3	O55004,P02631,P0DMW0;P0DMW1

4085	1,3,5,7,9,11,14,16	2,4,6,8,10,12,13,15	1 P02631
4086	1,3,5,7,9,11,15,16	2,4,6,8,10,12,13,14	1 P02631
4087	1,3,5,7,9,12,13,14	2,4,6,8,10,11,15,16	2 P50280,Q6AY61
4088	1,3,5,7,9,12,13,15	2,4,6,8,10,11,14,16	1 Q6AY61
4089	1,3,5,7,9,12,13,16	2,4,6,8,10,11,14,15	0
4090	1,3,5,7,9,12,14,15	2,4,6,8,10,11,13,16	5 O55004,P00714,P02631,P30120,Q6AY61
4091	1,3,5,7,9,12,14,16	2,4,6,8,10,11,13,15	1 Q68G31
4092	1,3,5,7,9,12,15,16	2,4,6,8,10,11,13,14	2 Q68G31,Q6AY61
4093	1,3,5,7,9,13,14,15	2,4,6,8,10,11,12,16	1 Q00715
4094	1,3,5,7,9,13,14,16	2,4,6,8,10,11,12,15	0
4095	1,3,5,7,9,13,15,16	2,4,6,8,10,11,12,14	1 P12020
4096	1,3,5,7,9,14,15,16	2,4,6,8,10,11,12,13	1 P02631
4097	1,3,5,7,10,11,12,13	2,4,6,8,9,14,15,16	4 P23593,P47967,P97840,Q5I0D1
4098	1,3,5,7,10,11,12,14	2,4,6,8,9,13,15,16	2 P23593,P47967
4099	1,3,5,7,10,11,12,15	2,4,6,8,9,13,14,16	4 P23593,P47967,P97840,Q5RKI1
4100	1,3,5,7,10,11,12,16	2,4,6,8,9,13,14,15	2 P47967,P97840
4101	1,3,5,7,10,11,13,14	2,4,6,8,9,12,15,16	0
4102	1,3,5,7,10,11,13,15	2,4,6,8,9,12,14,16	3 P47967,P97840,Q5RKI1
4103	1,3,5,7,10,11,13,16	2,4,6,8,9,12,14,15	2 P47967,P97840
4104	1,3,5,7,10,11,14,15	2,4,6,8,9,12,13,16	1 Q5RKI1

4105	1,3,5,7,10,11,14,16	2,4,6,8,9,12,13,15	0
4106	1,3,5,7,10,11,15,16	2,4,6,8,9,12,13,14	2 Q5RKI1,Q6IFW6
4107	1,3,5,7,10,12,13,14	2,4,6,8,9,11,15,16	5 D3ZUC6,P47967,P50280,Q6AY61,Q6P6S4
4108	1,3,5,7,10,12,13,15	2,4,6,8,9,11,14,16	4 P47967,P97840,Q5RKI1,Q6AY61
4109	1,3,5,7,10,12,13,16	2,4,6,8,9,11,14,15	3 P47967,P97840,Q6AY61
4110	1,3,5,7,10,12,14,15	2,4,6,8,9,11,13,16	5 D3ZUC6,P00714,P30120,Q5RKI1,Q6AY61
4111	1,3,5,7,10,12,14,16	2,4,6,8,9,11,13,15	1 Q6AY61
4112	1,3,5,7,10,12,15,16	2,4,6,8,9,11,13,14	3 P97840,Q5RKI1,Q6AY61
4113	1,3,5,7,10,13,14,15	2,4,6,8,9,11,12,16	2 D3ZUC6,Q5RKI1
4114	1,3,5,7,10,13,14,16	2,4,6,8,9,11,12,15	3 O35077,Q6P6S4,Q9WUW8
4115	1,3,5,7,10,13,15,16	2,4,6,8,9,11,12,14	3 O35077,P12020,Q5RKI1
4116	1,3,5,7,10,14,15,16	2,4,6,8,9,11,12,13	2 O35077,Q5RKI1
4117	1,3,5,7,11,12,13,14	2,4,6,8,9,10,15,16	4 D3ZUC6,P47967,Q5I0D1,Q811M5
4118	1,3,5,7,11,12,13,15	2,4,6,8,9,10,14,16	4 P17559,P47967,P97840,Q5I0D1
4119	1,3,5,7,11,12,13,16	2,4,6,8,9,10,14,15	4 P17559,P47967,P97840,Q811M5
4120	1,3,5,7,11,12,14,15	2,4,6,8,9,10,13,16	4 D3ZUC6,O55004,P02631,P17559
4121	1,3,5,7,11,12,14,16	2,4,6,8,9,10,13,15	2 P17559,Q811M5
4122	1,3,5,7,11,12,15,16	2,4,6,8,9,10,13,14	3 P17559,P97840,Q6IFW6
4123	1,3,5,7,11,13,14,15	2,4,6,8,9,10,12,16	2 D3ZUC6,P17559
4124	1,3,5,7,11,13,14,16	2,4,6,8,9,10,12,15	2 P17559,Q811M5

4125	1,3,5,7,11,13,15,16	2,4,6,8,9,10,12,14	2 P17559,Q6IFW6
4126	1,3,5,7,11,14,15,16	2,4,6,8,9,10,12,13	3 P02631,P17559,Q6IFW6
4127	1,3,5,7,12,13,14,15	2,4,6,8,9,10,11,16	2 D3ZUC6,Q6AY61
4128	1,3,5,7,12,13,14,16	2,4,6,8,9,10,11,15	3 Q6AY61,Q6P6S4,Q811M5
4129	1,3,5,7,12,13,15,16	2,4,6,8,9,10,11,14	2 P97840,Q6AY61
4130	1,3,5,7,12,14,15,16	2,4,6,8,9,10,11,13	2 P17559,Q6AY61
4131	1,3,5,7,13,14,15,16	2,4,6,8,9,10,11,12	2 P17559,Q62714
4132	1,3,5,8,9,10,11,12	2,4,6,7,13,14,15,16	2 O88797,P01835
4133	1,3,5,8,9,10,11,13	2,4,6,7,12,14,15,16	1 Q80WL1
4134	1,3,5,8,9,10,11,14	2,4,6,7,12,13,15,16	0
4135	1,3,5,8,9,10,11,15	2,4,6,7,12,13,14,16	6 P01681,Q4FZU2,Q6IFU7,Q6IFW6,Q6IMF3,Q6P6Q2
4136	1,3,5,8,9,10,11,16	2,4,6,7,12,13,14,15	2 O88797,P01835
4137	1,3,5,8,9,10,12,13	2,4,6,7,11,14,15,16	3 P11598,Q80WL1,Q8CJ52
4138	1,3,5,8,9,10,12,14	2,4,6,7,11,13,15,16	2 P00714,P11598
4139	1,3,5,8,9,10,12,15	2,4,6,7,11,13,14,16	6 P00714,P01681,P11598,P34901,P36376,Q99041
4140	1,3,5,8,9,10,12,16	2,4,6,7,11,13,14,15	0
4141	1,3,5,8,9,10,13,14	2,4,6,7,11,12,15,16	1 P11598
4142	1,3,5,8,9,10,13,15	2,4,6,7,11,12,14,16	5 P01681,P11598,P62804,Q00715,Q80WL1
4143	1,3,5,8,9,10,13,16	2,4,6,7,11,12,14,15	1 Q80WL1
4144	1,3,5,8,9,10,14,15	2,4,6,7,11,12,13,16	5 P00714,P01681,P05369,P07647,P11598

4145	1,3,5,8,9,10,14,16	2,4,6,7,11,12,13,15	0	
4146	1,3,5,8,9,10,15,16	2,4,6,7,11,12,13,14	2	P01681,P11598
4147	1,3,5,8,9,11,12,13	2,4,6,7,10,14,15,16	3	O88797,P09527,Q80WL1
4148	1,3,5,8,9,11,12,14	2,4,6,7,10,13,15,16	2	O88797,P09527
4149	1,3,5,8,9,11,12,15	2,4,6,7,10,13,14,16	12	O88797,P01681,P09527,P36376,Q4FZU2,Q6IFU7,Q6IFW6,Q6IG02,Q6IG05,Q6IMF3,Q6P6Q2,Q80WL1
4150	1,3,5,8,9,11,12,16	2,4,6,7,10,13,14,15	2	O88797,Q80WL1
4151	1,3,5,8,9,11,13,14	2,4,6,7,10,12,15,16	2	P0DMW0;P0DMW1,Q80WL1
4152	1,3,5,8,9,11,13,15	2,4,6,7,10,12,14,16	8	P01681,Q4FZU2,Q6IFU7,Q6IFW6,Q6IG02,Q6IMF3,Q6P6Q2,Q80WL1
4153	1,3,5,8,9,11,13,16	2,4,6,7,10,12,14,15	2	O88797,Q80WL1
4154	1,3,5,8,9,11,14,15	2,4,6,7,10,12,13,16	9	P00714,P01681,P80299,Q4FZU2,Q6IFU7,Q6IFW6,Q6IG02,Q6IMF3,Q6P6Q2
4155	1,3,5,8,9,11,14,16	2,4,6,7,10,12,13,15	2	O88797,P80299
4156	1,3,5,8,9,11,15,16	2,4,6,7,10,12,13,14	10	O88797,P01681,P80299,Q4FZU2,Q6IFU7,Q6IFW6,Q6IG02,Q6IMF3,Q6P6Q2,Q80WL1
4157	1,3,5,8,9,12,13,14	2,4,6,7,10,11,15,16	2	P11598,P17988
4158	1,3,5,8,9,12,13,15	2,4,6,7,10,11,14,16	4	P11598,P36376,Q80WL1,Q99041
4159	1,3,5,8,9,12,13,16	2,4,6,7,10,11,14,15	1	Q80WL1
4160	1,3,5,8,9,12,14,15	2,4,6,7,10,11,13,16	4	P00714,P01681,P11598,P36376
4161	1,3,5,8,9,12,14,16	2,4,6,7,10,11,13,15	0	
4162	1,3,5,8,9,12,15,16	2,4,6,7,10,11,13,14	4	P01681,P11598,Q80WL1,Q99041
4163	1,3,5,8,9,13,14,15	2,4,6,7,10,11,12,16	6	P00714,P01681,P11598,Q00715,Q5M872,Q80WL1
4164	1,3,5,8,9,13,14,16	2,4,6,7,10,11,12,15	1	Q80WL1

4165	1,3,5,8,9,13,15,16	2,4,6,7,10,11,12,14	1 Q80WL1
4166	1,3,5,8,9,14,15,16	2,4,6,7,10,11,12,13	4 P00714,P01681,P11598,P80299
4167	1,3,5,8,10,11,12,13	2,4,6,7,9,14,15,16	3 P47967,Q5I0D1,Q80WL1
4168	1,3,5,8,10,11,12,14	2,4,6,7,9,13,15,16	1 D3ZUC6
4169	1,3,5,8,10,11,12,15	2,4,6,7,9,13,14,16	8 Q4FZU2,Q6IFU7,Q6IFW6,Q6IG02,Q6IG05,Q6IMF3,Q6P6Q2,Q9R168
4170	1,3,5,8,10,11,12,16	2,4,6,7,9,13,14,15	1 O88797
4171	1,3,5,8,10,11,13,14	2,4,6,7,9,12,15,16	1 Q6IFU7
4172	1,3,5,8,10,11,13,15	2,4,6,7,9,12,14,16	7 Q4FZU2,Q6IFU7,Q6IFW6,Q6IG02,Q6IMF3,Q6P6Q2,Q80WL1
4173	1,3,5,8,10,11,13,16	2,4,6,7,9,12,14,15	2 Q6IFU7,Q80WL1
4174	1,3,5,8,10,11,14,15	2,4,6,7,9,12,13,16	7 D3ZUC6,Q4FZU2,Q6IFU7,Q6IFW6,Q6IG02,Q6IMF3,Q6P6Q2
4175	1,3,5,8,10,11,14,16	2,4,6,7,9,12,13,15	1 Q6IFU7
4176	1,3,5,8,10,11,15,16	2,4,6,7,9,12,13,14	6 Q4FZU2,Q6IFU7,Q6IFW6,Q6IG02,Q6IMF3,Q6P6Q2
4177	1,3,5,8,10,12,13,14	2,4,6,7,9,11,15,16	2 D3ZUC6,Q8CJ52
4178	1,3,5,8,10,12,13,15	2,4,6,7,9,11,14,16	3 P11598,Q80WL1,Q9R168
4179	1,3,5,8,10,12,13,16	2,4,6,7,9,11,14,15	1 Q80WL1
4180	1,3,5,8,10,12,14,15	2,4,6,7,9,11,13,16	6 D3ZUC6,P00714,P11598,Q63617,Q9R168,Q9Z0J6
4181	1,3,5,8,10,12,14,16	2,4,6,7,9,11,13,15	0
4182	1,3,5,8,10,12,15,16	2,4,6,7,9,11,13,14	2 P0DMW0;P0DMW1,Q9R168
4183	1,3,5,8,10,13,14,15	2,4,6,7,9,11,12,16	3 D3ZUC6,P00714,P63322
4184	1,3,5,8,10,13,14,16	2,4,6,7,9,11,12,15	1 O35077

4185	1,3,5,8,10,13,15,16	2,4,6,7,9,11,12,14	2	O35077,Q80WL1
4186	1,3,5,8,10,14,15,16	2,4,6,7,9,11,12,13	2	O35077,Q63617
4187	1,3,5,8,11,12,13,14	2,4,6,7,9,10,15,16	3	D3ZUC6,Q811M5,Q9WVH8
4188	1,3,5,8,11,12,13,15	2,4,6,7,9,10,14,16	10	Q4FZU2,Q6IFU7,Q6IFW6,Q6IG02,Q6IG05,Q6IMF3,Q6P6Q2,Q80WL1,Q9R168,Q9WVH8
4189	1,3,5,8,11,12,13,16	2,4,6,7,9,10,14,15	3	P47967,Q80WL1,Q811M5
4190	1,3,5,8,11,12,14,15	2,4,6,7,9,10,13,16	10	D3ZUC6,P80299,Q4FZU2,Q6IFU7,Q6IFW6,Q6IG02,Q6IG05,Q6IMF3,Q6P6Q2,Q9WVH8
4191	1,3,5,8,11,12,14,16	2,4,6,7,9,10,13,15	4	O88797,P80299,Q811M5,Q9WVH8
4192	1,3,5,8,11,12,15,16	2,4,6,7,9,10,13,14	11	O88797,P80299,Q4FZU2,Q6IFU7,Q6IFW6,Q6IG02,Q6IG05,Q6IMF3,Q6P6Q2,Q80WL1,Q9WVH8
4193	1,3,5,8,11,13,14,15	2,4,6,7,9,10,12,16	8	D3ZUC6,Q4FZU2,Q6IFU7,Q6IFW6,Q6IG02,Q6IMF3,Q6P6Q2,Q80WL1
4194	1,3,5,8,11,13,14,16	2,4,6,7,9,10,12,15	3	Q6IFU7,Q80WL1,Q811M5
4195	1,3,5,8,11,13,15,16	2,4,6,7,9,10,12,14	7	Q4FZU2,Q6IFU7,Q6IFW6,Q6IG02,Q6IMF3,Q6P6Q2,Q80WL1
4196	1,3,5,8,11,14,15,16	2,4,6,7,9,10,12,13	7	P80299,Q4FZU2,Q6IFU7,Q6IFW6,Q6IG02,Q6IMF3,Q6P6Q2
4197	1,3,5,8,12,13,14,15	2,4,6,7,9,10,11,16	4	D3ZUC6,P00714,P11598,Q9R168
4198	1,3,5,8,12,13,14,16	2,4,6,7,9,10,11,15	1	Q811M5
4199	1,3,5,8,12,13,15,16	2,4,6,7,9,10,11,14	2	Q80WL1,Q9R168
4200	1,3,5,8,12,14,15,16	2,4,6,7,9,10,11,13	1	P80299
4201	1,3,5,8,13,14,15,16	2,4,6,7,9,10,11,12	3	O35077,Q62714,Q80WL1
4202	1,3,5,9,10,11,12,13	2,4,6,7,8,14,15,16	8	P20761,P20762,P50280,P98089,Q03191,Q62635,Q62761;Q62762;Q62763,Q6TMA8
4203	1,3,5,9,10,11,12,14	2,4,6,7,8,13,15,16	5	O55004,P02631,P07151,P98089,Q6TMA8
4204	1,3,5,9,10,11,12,15	2,4,6,7,8,13,14,16	8	D4A5U3,O55004,P02631,P20761,P98089,Q62635,Q62761;Q62762;Q62763,Q6TMA8

4205	1,3,5,9,10,11,12,16	2,4,6,7,8,13,14,15	4	P98089,Q03191,Q62635,Q8CIZ5
4206	1,3,5,9,10,11,13,14	2,4,6,7,8,12,15,16	4	O55004,P20762,P50280,P98089
4207	1,3,5,9,10,11,13,15	2,4,6,7,8,12,14,16	5	P02631,P12020,P20762,P98089,Q62635
4208	1,3,5,9,10,11,13,16	2,4,6,7,8,12,14,15	2	P98089,Q62635
4209	1,3,5,9,10,11,14,15	2,4,6,7,8,12,13,16	4	O55004,P00714,P02631,P98089
4210	1,3,5,9,10,11,14,16	2,4,6,7,8,12,13,15	3	P02631,P98089,Q8CIZ5
4211	1,3,5,9,10,11,15,16	2,4,6,7,8,12,13,14	5	P02631,P98089,Q62635,Q8CIZ5,Q9Z2L0
4212	1,3,5,9,10,12,13,14	2,4,6,7,8,11,15,16	25	P02780,P02781,P02782,P06761,P07150,P07647,P08723,P09456,P0C0A9,P11598,P12020,P20762,P22273,P22282,P22283,P30120,P36374,P46462,P50280,P98089,Q4G075,Q5M8C6,Q63617,Q8CJ52,Q99041
4213	1,3,5,9,10,12,13,15	2,4,6,7,8,11,14,16	26	P02780,P02781,P02782,P06761,P07150,P07647,P08723,P09456,P0C0A9,P11598,P12020,P20762,P22273,P22282,P22283,P30120,P46462,P50280,P98089,Q00715,Q4G075,Q5GRG2,Q5M8C6,Q62635,Q63617,Q99041
4214	1,3,5,9,10,12,13,16	2,4,6,7,8,11,14,15	16	P06761,P07150,P0C0A9,P11598,P12020,P22273,P22282,P46462,P50280,P98089,Q4G075,Q5GRG2,Q62635,Q63617,Q8CJ52,Q99041
4215	1,3,5,9,10,12,14,15	2,4,6,7,8,11,13,16	31	D4A5U3,O55004,P00714,P02631,P02780,P02781,P02782,P05369,P06761,P07647,P08723,P09456,P0C0A9,P11598,P12020,P22273,P22282,P22283,P24368,P30120,P36374,P46462,P50280,P98089,Q00715,Q4G075,Q5M8C6,Q62902,Q63617,Q99041,Q9R0T3
4216	1,3,5,9,10,12,14,16	2,4,6,7,8,11,13,15	13	P02780,P06761,P11598,P22273,P22282,P46462,P50280,P55091,P98089,Q4G075,Q63617,Q8CIZ5,Q99041
4217	1,3,5,9,10,12,15,16	2,4,6,7,8,11,13,14	20	D4A5U3,P02631,P02780,P06761,P07150,P0C0A9,P11598,P12020,P22273,P22282,P46462,P50280,P55091,P98089,Q4G075,Q5GRG2,Q62635,Q63617,Q8CIZ5,Q99041
4218	1,3,5,9,10,13,14,15	2,4,6,7,8,11,12,16	34	P00714,P02631,P02780,P02781,P02782,P04905,P06761,P06911,P07150,P07647,P08723,P09456,P0C0A9,P11598,P12020,P22273,P22282,P22283,P30120,P36374,P46462,P50280,P62804,P97523,P98089,Q00715,Q4G075,Q5GRG2,Q5M8C6,Q63617,Q8CFN2,Q99041,Q9JHB9,Q9R0T3
4219	1,3,5,9,10,13,14,16	2,4,6,7,8,11,12,15	12	P02780,P06761,P07150,P11598,P12020,P22273,P22282,P46462,P50280,P98089,Q5GRG2,Q63617
4220	1,3,5,9,10,13,15,16	2,4,6,7,8,11,12,14	18	P02780,P06761,P06911,P07150,P11598,P12020,P22273,P22282,P46462,P50280,P98089,Q00715,Q4G075,Q5GRG2,Q5M8C6,Q62635,Q63617,Q99041
4221	1,3,5,9,10,14,15,16	2,4,6,7,8,11,12,13	23	P00714,P02631,P02780,P06761,P06911,P11598,P12020,P22273,P22282,P22283,P46462,P50280,P55091,P98089,Q4G075,Q5GRG2,Q5M8C6,Q62635,Q63617,Q8CIZ5,Q99041,Q9JI85,Q9R0T3
4222	1,3,5,9,11,12,13,14	2,4,6,7,8,10,15,16	28	O55004,O70257,O70594,P02631,P07151,P17988,P19468,P20761,P20762,P50280,P51907,P53790,P98089,Q03248,Q05175,Q3ZAV1,Q4QQT4,Q5I0E9,Q5M7T9,Q62761;Q62762;Q62763,Q63270,Q63424,Q63598,Q63618,Q6Q0N1,Q9R1T5,Q9WTW7,Q9Z0W7

4223	1,3,5,9,11,12,13,15	2,4,6,7,8,10,14,16	11	O55004,P02631,P06399,P07151,P20761,P20762,P57113,P98089,Q62635,Q62761;Q62762;Q62763,Q80WL1
4224	1,3,5,9,11,12,13,16	2,4,6,7,8,10,14,15	9	O55004,P02631,P20762,P54921,P98089,Q03191,Q62635,Q80WL1,Q9Z1F2
4225	1,3,5,9,11,12,14,15	2,4,6,7,8,10,13,16	16	O55004,O70257,P00714,P02631,P07151,P20762,P28570,P46844,P51907,P55091,P98089,Q3ZAV1,Q5M7T9,Q62761;Q62762;Q62763,Q63424,Q63618
4226	1,3,5,9,11,12,14,16	2,4,6,7,8,10,13,15	11	O55004,O70257,P02631,P20762,P51907,P55091,P98089,Q63598,Q63618,Q8CIZ5,Q9Z1F2
4227	1,3,5,9,11,12,15,16	2,4,6,7,8,10,13,14	10	O55004,P02631,P07171,P20762,P25809,P55091,P98089,Q62635,Q8CIZ5,Q9Z1F2
4228	1,3,5,9,11,13,14,15	2,4,6,7,8,10,12,16	8	O55004,P02631,P07151,P0DMW0;P0DMW1,P20762,P55091,P98089,Q63598
4229	1,3,5,9,11,13,14,16	2,4,6,7,8,10,12,15	7	O55004,P02631,P20762,P55091,P98089,Q63598,Q9Z1F2
4230	1,3,5,9,11,13,15,16	2,4,6,7,8,10,12,14	8	O55004,P02631,P20762,P55091,P98089,Q62635,Q80WL1,Q9Z1F2
4231	1,3,5,9,11,14,15,16	2,4,6,7,8,10,12,13	9	O55004,P02631,P25809,P55091,P80299,P98089,Q63618,Q8CIZ5,Q9Z1F2
4232	1,3,5,9,12,13,14,15	2,4,6,7,8,10,11,16	32	O55004,P00714,P02631,P02780,P02781,P02782,P06761,P07150,P07151,P07647,P08723,P09456,P0C0A9,P11598,P12020,P20762,P22273,P22282,P22283,P24368,P28570,P30120,P46462,P50280,P55091,P98089,Q00715,Q4G075,Q5M8C6,Q63617,Q99041,Q9R0T3
4233	1,3,5,9,12,13,14,16	2,4,6,7,8,10,11,15	13	O55004,P02631,P06761,P07150,P11598,P20762,P22273,P22282,P50280,P55091,P98089,Q63617,Q99041
4234	1,3,5,9,12,13,15,16	2,4,6,7,8,10,11,14	20	O55004,P02631,P06761,P07150,P0C0A9,P11598,P12020,P20762,P22273,P22282,P50280,P55091,P98089,Q4G075,Q5GRG2,Q62635,Q63617,Q6B345,Q80WL1,Q99041
4235	1,3,5,9,12,14,15,16	2,4,6,7,8,10,11,13	18	O55004,P00714,P02631,P02780,P06761,P11598,P22273,P22282,P25809,P46462,P50280,P55091,P98089,Q4G075,Q63617,Q8CIZ5,Q99041,Q9R0T3
4236	1,3,5,9,13,14,15,16	2,4,6,7,8,10,11,12	19	O55004,P02631,P02780,P06761,P07150,P11598,P12020,P22273,P22282,P31430,P46462,P50280,P55091,P98089,Q00715,Q5GRG2,Q5M8C6,Q63617,Q9R0T3
4237	1,3,5,10,11,12,13,14	2,4,6,7,8,9,15,16	3	P20762,P50280,P98089
4238	1,3,5,10,11,12,13,15	2,4,6,7,8,9,14,16	5	D4A5U3,P20762,P23593,P98089,Q62635
4239	1,3,5,10,11,12,13,16	2,4,6,7,8,9,14,15	4	P47967,P97840,P98089,Q62635
4240	1,3,5,10,11,12,14,15	2,4,6,7,8,9,13,16	4	D4A5U3,O55004,P02631,P98089
4241	1,3,5,10,11,12,14,16	2,4,6,7,8,9,13,15	3	P02631,P98089,Q8CIZ5
4242	1,3,5,10,11,12,15,16	2,4,6,7,8,9,13,14	6	D4A5U3,P02631,P98089,Q5QE79,Q62635,Q8CIZ5

4243	1,3,5,10,11,13,14,15	2,4,6,7,8,9,12,16	2	P02631,P98089
4244	1,3,5,10,11,13,14,16	2,4,6,7,8,9,12,15	1	P98089
4245	1,3,5,10,11,13,15,16	2,4,6,7,8,9,12,14	3	P98089,Q5QE79,Q62635
4246	1,3,5,10,11,14,15,16	2,4,6,7,8,9,12,13	4	P02631,P55091,P98089,Q8CIZ5
4247	1,3,5,10,12,13,14,15	2,4,6,7,8,9,11,16	26	P00714,P02780,P02781,P02782,P06761,P07150,P07647,P08723,P09456,P0C0A9,P11598,P22273,P22282,P22283,P30120,P46462,P50280,P55091,P97523,P98089,Q5M8C6,Q63617,Q812E4,Q99041,Q9R0T3,Q9Z0J6
4248	1,3,5,10,12,13,14,16	2,4,6,7,8,9,11,15	11	P06761,P07150,P22273,P22282,P50280,P55091,P98089,Q63617,Q6P6R2,Q6P6S4,Q8CJ52
4249	1,3,5,10,12,13,15,16	2,4,6,7,8,9,11,14	19	D4A5U3,P06761,P07150,P11598,P12020,P22273,P22282,P46462,P50280,P55091,P98089,Q4G075,Q5GRG2,Q5QE79,Q62635,Q63617,Q6P6R2,Q812E4,Q99041
4250	1,3,5,10,12,14,15,16	2,4,6,7,8,9,11,13	16	D4A5U3,P02631,P02780,P06761,P07150,P11598,P22273,P22282,P46462,P50280,P55091,P82471,P98089,Q63617,Q8CIZ5,Q99041
4251	1,3,5,10,13,14,15,16	2,4,6,7,8,9,11,12	16	P02780,P06761,P07150,P11598,P12020,P22273,P22282,P46462,P50280,P55091,P70709,P82471,P98089,Q5GRG2,Q63617,Q9R0T3
4252	1,3,5,11,12,13,14,15	2,4,6,7,8,9,10,16	10	O55004,P02454,P02631,P07151,P20762,P23739,P55091,P70545,P98089,Q9WVH8
4253	1,3,5,11,12,13,14,16	2,4,6,7,8,9,10,15	11	O55004,P02454,P02631,P17559,P20762,P55091,P98089,Q6P6R2,Q811M5,Q9Z0V6,Q9Z1F2
4254	1,3,5,11,12,13,15,16	2,4,6,7,8,9,10,14	11	O55004,P02454,P02631,P17559,P20762,P55091,P98089,Q5QE79,Q62635,Q80WL1,Q9Z1F2
4255	1,3,5,11,12,14,15,16	2,4,6,7,8,9,10,13	12	D4A5U3,O55004,P02454,P02631,P17559,P55091,P80299,P98089,Q63618,Q8CIZ5,Q9WVH8,Q9Z1F2
4256	1,3,5,11,13,14,15,16	2,4,6,7,8,9,10,12	7	O55004,P02454,P02631,P17559,P55091,P98089,Q9Z1F2
4257	1,3,5,12,13,14,15,16	2,4,6,7,8,9,10,11	16	P02454,P02631,P06761,P07150,P11598,P22273,P22282,P50280,P55091,P70545,P98089,Q63617,Q6B345,Q6P6R2,Q6P6S4,Q9R0T3
4258	1,3,6,7,8,9,10,11	2,4,5,12,13,14,15,16	4	P35280,Q63493,Q6P6R2,Q6P6S4
4259	1,3,6,7,8,9,10,12	2,4,5,11,13,14,15,16	2	O70417,Q63598
4260	1,3,6,7,8,9,10,13	2,4,5,11,12,14,15,16	1	P70549
4261	1,3,6,7,8,9,10,14	2,4,5,11,12,13,15,16	4	O70417,P08937,Q5QE79,Q63751
4262	1,3,6,7,8,9,10,15	2,4,5,11,12,13,14,16	6	P02091,P19629,P35280,Q5RKI1,Q63598,Q6P6R2

4263	1,3,6,7,8,9,10,16	2,4,5,11,12,13,14,15	2	P19629,Q63598
4264	1,3,6,7,8,9,11,12	2,4,5,10,13,14,15,16	10	iRT-Kit_WR_fusion,P06911,P09527,P35280,Q5GRG2,Q63493,Q63617,Q6P6S4,Q812E4,Q9QW07
4265	1,3,6,7,8,9,11,13	2,4,5,10,12,14,15,16	7	P22283,P35280,Q63493,Q63617,Q6P6S4,Q812E4,Q9JI85
4266	1,3,6,7,8,9,11,14	2,4,5,10,12,13,15,16	11	O70417,P06911,P08937,P0DMW0;P0DMW1,P35280,Q5GRG2,Q63493,Q63751,Q6P6S4,Q812E4,Q9QW07
4267	1,3,6,7,8,9,11,15	2,4,5,10,12,13,14,16	8	P02091,P35280,Q63493,Q6IFW6,Q6P6R2,Q6P6S4,Q8CJ52,Q9QW07
4268	1,3,6,7,8,9,11,16	2,4,5,10,12,13,14,15	15	P02782,P02783,P08723,P09456,P22283,P30120,P35280,P46462,Q5M8C6,Q63493,Q63617,Q6P6S4,Q812E4,Q9JHB9,Q9QW07
4269	1,3,6,7,8,9,12,13	2,4,5,10,11,14,15,16	1	P70549
4270	1,3,6,7,8,9,12,14	2,4,5,10,11,13,15,16	3	O70417,P21674,Q63751
4271	1,3,6,7,8,9,12,15	2,4,5,10,11,13,14,16	5	iRT-Kit_WR_fusion,P02091,P19629,P35280,P70549
4272	1,3,6,7,8,9,12,16	2,4,5,10,11,13,14,15	1	P21674
4273	1,3,6,7,8,9,13,14	2,4,5,10,11,12,15,16	3	P70549,Q5QE79,Q63751
4274	1,3,6,7,8,9,13,15	2,4,5,10,11,12,14,16	3	P02091,P35280,P70549
4275	1,3,6,7,8,9,13,16	2,4,5,10,11,12,14,15	1	P70549
4276	1,3,6,7,8,9,14,15	2,4,5,10,11,12,13,16	2	P02091,P35280
4277	1,3,6,7,8,9,14,16	2,4,5,10,11,12,13,15	3	O70417,P02783,P21674
4278	1,3,6,7,8,9,15,16	2,4,5,10,11,12,13,14	4	P02091,P02783,P19629,P35280
4279	1,3,6,7,8,10,11,12	2,4,5,9,13,14,15,16	5	P06911,P19629,P22283,Q63493,Q9QW07
4280	1,3,6,7,8,10,11,13	2,4,5,9,12,14,15,16	3	P22283,Q63493,Q9JI85
4281	1,3,6,7,8,10,11,14	2,4,5,9,12,13,15,16	5	O70417,P08937,Q5GRG2,Q63493,Q63751
4282	1,3,6,7,8,10,11,15	2,4,5,9,12,13,14,16	8	P01946,P02091,P19629,Q5RKI1,Q63493,Q6IFW6,Q6P6S4,Q9QW07

4283	1,3,6,7,8,10,11,16	2,4,5,9,12,13,14,15	17	P02781,P02782,P02783,P08010,P08723,P0C0A9,P19629,P22283,P24368,P30120,P46462,P57113,Q5M8C6,Q63493,Q9JHB9,Q9QW07,Q9WTW7
4284	1,3,6,7,8,10,12,13	2,4,5,9,11,14,15,16	3	P57113,P70549,Q63598
4285	1,3,6,7,8,10,12,14	2,4,5,9,11,13,15,16	5	O70417,P08937,P57113,P70549,Q63751
4286	1,3,6,7,8,10,12,15	2,4,5,9,11,13,14,16	8	P02091,P19629,P57113,P70549,Q05175,Q5RKI1,Q63598,Q9WTW7
4287	1,3,6,7,8,10,12,16	2,4,5,9,11,13,14,15	8	P19629,P46844,P53790,P57113,Q3ZAV1,Q63598,Q6AY41,Q9WTW7
4288	1,3,6,7,8,10,13,14	2,4,5,9,11,12,15,16	5	P08937,P57113,P63081,P70549,Q62714
4289	1,3,6,7,8,10,13,15	2,4,5,9,11,12,14,16	13	O70377,O70594,P01946,P02091,P19468,P19629,P57113,P70549,Q05175,Q5RKI1,Q62714,Q63598,Q64093
4290	1,3,6,7,8,10,13,16	2,4,5,9,11,12,14,15	15	O35077,O70257,O70594,P19468,P19629,P46844,P53790,P57113,P70549,Q3ZAV1,Q63424,Q63598,Q64093,Q6AY41,Q9WTW7
4291	1,3,6,7,8,10,14,15	2,4,5,9,11,12,13,16	12	O70377,P01946,P02091,P19629,P54921,P57113,P70549,Q03191,Q05175,Q5RKI1,Q62714,Q63598
4292	1,3,6,7,8,10,14,16	2,4,5,9,11,12,13,15	8	O35077,O70417,P02783,P19629,P46844,P53790,P57113,Q923S2
4293	1,3,6,7,8,10,15,16	2,4,5,9,11,12,13,14	34	O35077,O70377,O70594,P01946,P02091,P02783,P19468,P19629,P29975,P38918,P46844,P48508,P53790,P57113,Q03191,Q03248,Q05175,Q3ZAV1,Q5I0E9,Q5M7T9,Q5RKI1,Q63270,Q63424,Q63598,Q64093,Q6AY41,Q6MG61,Q6Q0N1,Q80W57,Q8R431,Q923S2,Q9R1T5,Q9WTW7,Q9Z0W7
4294	1,3,6,7,8,11,12,13	2,4,5,9,10,14,15,16	8	P06911,P12020,P22283,P46462,P47967,Q5GRG2,Q63493,Q9JI85
4295	1,3,6,7,8,11,12,14	2,4,5,9,10,13,15,16	8	O70417,P06911,P12020,P22283,Q5GRG2,Q63493,Q63751,Q9QW07
4296	1,3,6,7,8,11,12,15	2,4,5,9,10,13,14,16	8	P02091,P06911,P19629,P22283,Q5GRG2,Q63493,Q6IFW6,Q9QW07
4297	1,3,6,7,8,11,12,16	2,4,5,9,10,13,14,15	22	O35547,P02780,P02781,P02782,P02783,P06911,P07647,P08010,P08723,P09456,P12020,P22283,P30120,P46462,Q5GRG2,Q5M8C6,Q63493,Q8CFN2,Q9JHB9,Q9JI85,Q9QW07,Q9QZK9
4298	1,3,6,7,8,11,13,14	2,4,5,9,10,12,15,16	7	P06911,P22283,P46462,Q5GRG2,Q63493,Q99041,Q9JI85
4299	1,3,6,7,8,11,13,15	2,4,5,9,10,12,14,16	6	P02091,P22283,Q63493,Q6IFW6,Q8CJ52,Q9JI85
4300	1,3,6,7,8,11,13,16	2,4,5,9,10,12,14,15	22	P02780,P02781,P02782,P02783,P05369,P06761,P06911,P07647,P08010,P08723,P09456,P22283,P24368,P30120,P46462,Q5M8C6,Q62902,Q63493,Q63617,Q9JHB9,Q9JI85,Q9QW07
4301	1,3,6,7,8,11,14,15	2,4,5,9,10,12,13,16	6	P02091,Q5GRG2,Q63493,Q6IFW6,Q8CJ52,Q9QW07

4302	1,3,6,7,8,11,14,16	2,4,5,9,10,12,13,15	20	P02781,P02782,P02783,P06911,P08010,P08723,P09456,P0C0A9,P22283,P30120,P46462,Q5GRG2,Q5M8C6,Q63493,Q812E4,Q8CFN2,Q99041,Q9JHB9,Q9QW07,Q9QZK9
4303	1,3,6,7,8,11,15,16	2,4,5,9,10,12,13,14	18	P02091,P02781,P02782,P02783,P08010,P08723,P0C0A9,P19629,P22283,P30120,P46462,Q5M8C6,Q63493,Q6IFW6,Q8CJ52,Q9JHB9,Q9QW07,Q9QZK9
4304	1,3,6,7,8,12,13,14	2,4,5,9,10,11,15,16	2	P70549,Q9Z2L0
4305	1,3,6,7,8,12,13,15	2,4,5,9,10,11,14,16	3	P02091,P70549,Q9Z2L0
4306	1,3,6,7,8,12,13,16	2,4,5,9,10,11,14,15	1	P70549
4307	1,3,6,7,8,12,14,15	2,4,5,9,10,11,13,16	2	P02091,P70549
4308	1,3,6,7,8,12,14,16	2,4,5,9,10,11,13,15	3	O70417,P02783,P21674
4309	1,3,6,7,8,12,15,16	2,4,5,9,10,11,13,14	5	P02091,P02783,P19629,P70549,Q63598
4310	1,3,6,7,8,13,14,15	2,4,5,9,10,11,12,16	5	P02091,P70549,Q03191,Q62714,Q9Z2L0
4311	1,3,6,7,8,13,14,16	2,4,5,9,10,11,12,15	4	P02783,P70549,Q62714,Q9Z2L0
4312	1,3,6,7,8,13,15,16	2,4,5,9,10,11,12,14	7	O35077,P02091,P02783,P19629,P70549,Q03191,Q62714
4313	1,3,6,7,8,14,15,16	2,4,5,9,10,11,12,13	7	P02091,P02783,P19629,P57113,P70549,Q03191,Q62714
4314	1,3,6,7,9,10,11,12	2,4,5,8,13,14,15,16	0	
4315	1,3,6,7,9,10,11,13	2,4,5,8,12,14,15,16	0	
4316	1,3,6,7,9,10,11,14	2,4,5,8,12,13,15,16	0	
4317	1,3,6,7,9,10,11,15	2,4,5,8,12,13,14,16	2	P02091,Q5RKI1
4318	1,3,6,7,9,10,11,16	2,4,5,8,12,13,14,15	0	
4319	1,3,6,7,9,10,12,13	2,4,5,8,11,14,15,16	2	P70549,Q6IFU8
4320	1,3,6,7,9,10,12,14	2,4,5,8,11,13,15,16	0	
4321	1,3,6,7,9,10,12,15	2,4,5,8,11,13,14,16	3	O54728,P02091,Q5RKI1

4322	1,3,6,7,9,10,12,16	2,4,5,8,11,13,14,15	1	P21674
4323	1,3,6,7,9,10,13,14	2,4,5,8,11,12,15,16	1	P70549
4324	1,3,6,7,9,10,13,15	2,4,5,8,11,12,14,16	4	P02091,P70549,Q00715,Q5RKI1
4325	1,3,6,7,9,10,13,16	2,4,5,8,11,12,14,15	0	
4326	1,3,6,7,9,10,14,15	2,4,5,8,11,12,13,16	3	P01946,P02091,Q5RKI1
4327	1,3,6,7,9,10,14,16	2,4,5,8,11,12,13,15	0	
4328	1,3,6,7,9,10,15,16	2,4,5,8,11,12,13,14	2	P02091,Q5RKI1
4329	1,3,6,7,9,11,12,13	2,4,5,8,10,14,15,16	1	P20762
4330	1,3,6,7,9,11,12,14	2,4,5,8,10,13,15,16	0	
4331	1,3,6,7,9,11,12,15	2,4,5,8,10,13,14,16	1	P02091
4332	1,3,6,7,9,11,12,16	2,4,5,8,10,13,14,15	1	Q9QW07
4333	1,3,6,7,9,11,13,14	2,4,5,8,10,12,15,16	1	P0DMW0;P0DMW1
4334	1,3,6,7,9,11,13,15	2,4,5,8,10,12,14,16	2	P02091,P0DMW0;P0DMW1
4335	1,3,6,7,9,11,13,16	2,4,5,8,10,12,14,15	0	
4336	1,3,6,7,9,11,14,15	2,4,5,8,10,12,13,16	3	P02091,P0DMW0;P0DMW1,P35280
4337	1,3,6,7,9,11,14,16	2,4,5,8,10,12,13,15	1	P02783
4338	1,3,6,7,9,11,15,16	2,4,5,8,10,12,13,14	4	P02091,P02783,Q8CJ52,Q9QW07
4339	1,3,6,7,9,12,13,14	2,4,5,8,10,11,15,16	1	P70549
4340	1,3,6,7,9,12,13,15	2,4,5,8,10,11,14,16	2	P02091,P70549
4341	1,3,6,7,9,12,13,16	2,4,5,8,10,11,14,15	1	P70549

4342	1,3,6,7,9,12,14,15	2,4,5,8,10,11,13,16	1 P02091
4343	1,3,6,7,9,12,14,16	2,4,5,8,10,11,13,15	1 P21674
4344	1,3,6,7,9,12,15,16	2,4,5,8,10,11,13,14	1 P02091
4345	1,3,6,7,9,13,14,15	2,4,5,8,10,11,12,16	4 P02091,P0DMW0;P0DMW1,P70549,Q00715
4346	1,3,6,7,9,13,14,16	2,4,5,8,10,11,12,15	0
4347	1,3,6,7,9,13,15,16	2,4,5,8,10,11,12,14	2 P02091,P70549
4348	1,3,6,7,9,14,15,16	2,4,5,8,10,11,12,13	2 P02091,P02783
4349	1,3,6,7,10,11,12,13	2,4,5,8,9,14,15,16	3 P20760,P23593,P47967
4350	1,3,6,7,10,11,12,14	2,4,5,8,9,13,15,16	0
4351	1,3,6,7,10,11,12,15	2,4,5,8,9,13,14,16	3 P02091,P20760,Q5RKI1
4352	1,3,6,7,10,11,12,16	2,4,5,8,9,13,14,15	1 P02783
4353	1,3,6,7,10,11,13,14	2,4,5,8,9,12,15,16	0
4354	1,3,6,7,10,11,13,15	2,4,5,8,9,12,14,16	3 P02091,Q5RKI1,Q8CJD3
4355	1,3,6,7,10,11,13,16	2,4,5,8,9,12,14,15	1 P36376
4356	1,3,6,7,10,11,14,15	2,4,5,8,9,12,13,16	3 P01946,P02091,Q5RKI1
4357	1,3,6,7,10,11,14,16	2,4,5,8,9,12,13,15	2 P02783,P36376
4358	1,3,6,7,10,11,15,16	2,4,5,8,9,12,13,14	4 P01946,P02091,P02783,Q5RKI1
4359	1,3,6,7,10,12,13,14	2,4,5,8,9,11,15,16	2 P01835,P70549
4360	1,3,6,7,10,12,13,15	2,4,5,8,9,11,14,16	3 P02091,P70549,Q5RKI1
4361	1,3,6,7,10,12,13,16	2,4,5,8,9,11,14,15	2 P57113,P70549

4362	1,3,6,7,10,12,14,15	2,4,5,8,9,11,13,16	4	P01946,P02091,P70549,Q5RKI1
4363	1,3,6,7,10,12,14,16	2,4,5,8,9,11,13,15	2	P02783,P57113
4364	1,3,6,7,10,12,15,16	2,4,5,8,9,11,13,14	4	P02091,P57113,Q5RKI1,Q63598
4365	1,3,6,7,10,13,14,15	2,4,5,8,9,11,12,16	6	P01835,P01946,P02091,P05539,P70549,Q5RKI1
4366	1,3,6,7,10,13,14,16	2,4,5,8,9,11,12,15	4	P01835,P36376,P57113,P70549
4367	1,3,6,7,10,13,15,16	2,4,5,8,9,11,12,14	7	P01835,P02091,P19468,P51792;P51794;P51796,P57113,P70549,Q5RKI1
4368	1,3,6,7,10,14,15,16	2,4,5,8,9,11,12,13	5	P01946,P02091,P02783,P57113,Q5RKI1
4369	1,3,6,7,11,12,13,14	2,4,5,8,9,10,15,16	0	
4370	1,3,6,7,11,12,13,15	2,4,5,8,9,10,14,16	2	P02091,P20760
4371	1,3,6,7,11,12,13,16	2,4,5,8,9,10,14,15	3	P05369,P22283,P47967
4372	1,3,6,7,11,12,14,15	2,4,5,8,9,10,13,16	1	P02091
4373	1,3,6,7,11,12,14,16	2,4,5,8,9,10,13,15	1	P02783
4374	1,3,6,7,11,12,15,16	2,4,5,8,9,10,13,14	3	P02091,P02783,Q9QW07
4375	1,3,6,7,11,13,14,15	2,4,5,8,9,10,12,16	1	P02091
4376	1,3,6,7,11,13,14,16	2,4,5,8,9,10,12,15	3	P02783,P34901,P36376
4377	1,3,6,7,11,13,15,16	2,4,5,8,9,10,12,14	3	P02091,P02783,Q8CJ52
4378	1,3,6,7,11,14,15,16	2,4,5,8,9,10,12,13	3	P02091,P02783,Q8CJ52
4379	1,3,6,7,12,13,14,15	2,4,5,8,9,10,11,16	3	P01835,P02091,P70549
4380	1,3,6,7,12,13,14,16	2,4,5,8,9,10,11,15	2	P01835,P70549
4381	1,3,6,7,12,13,15,16	2,4,5,8,9,10,11,14	3	P01835,P02091,P70549

4382	1,3,6,7,12,14,15,16	2,4,5,8,9,10,11,13	3	P02091,P02783,P70549
4383	1,3,6,7,13,14,15,16	2,4,5,8,9,10,11,12	5	P01835,P02091,P02783,P70549,Q62714
4384	1,3,6,8,9,10,11,12	2,4,5,7,13,14,15,16	1	Q6P6S4
4385	1,3,6,8,9,10,11,13	2,4,5,7,12,14,15,16	1	Q6P6S4
4386	1,3,6,8,9,10,11,14	2,4,5,7,12,13,15,16	0	
4387	1,3,6,8,9,10,11,15	2,4,5,7,12,13,14,16	2	P02091,Q6P6S4
4388	1,3,6,8,9,10,11,16	2,4,5,7,12,13,14,15	1	Q6P6S4
4389	1,3,6,8,9,10,12,13	2,4,5,7,11,14,15,16	1	P70549
4390	1,3,6,8,9,10,12,14	2,4,5,7,11,13,15,16	1	O70417
4391	1,3,6,8,9,10,12,15	2,4,5,7,11,13,14,16	1	P02091
4392	1,3,6,8,9,10,12,16	2,4,5,7,11,13,14,15	1	P21674
4393	1,3,6,8,9,10,13,14	2,4,5,7,11,12,15,16	1	P70549
4394	1,3,6,8,9,10,13,15	2,4,5,7,11,12,14,16	4	P02091,P62804,P70549,Q00715
4395	1,3,6,8,9,10,13,16	2,4,5,7,11,12,14,15	0	
4396	1,3,6,8,9,10,14,15	2,4,5,7,11,12,13,16	2	P01946,P02091
4397	1,3,6,8,9,10,14,16	2,4,5,7,11,12,13,15	0	
4398	1,3,6,8,9,10,15,16	2,4,5,7,11,12,13,14	1	P02091
4399	1,3,6,8,9,11,12,13	2,4,5,7,10,14,15,16	1	P09527
4400	1,3,6,8,9,11,12,14	2,4,5,7,10,13,15,16	1	P09527
4401	1,3,6,8,9,11,12,15	2,4,5,7,10,13,14,16	4	P02091,P09527,Q6P6S4,Q9QW07

4402	1,3,6,8,9,11,12,16	2,4,5,7,10,13,14,15	4	O88797,P09527,Q6P6S4,Q9QW07
4403	1,3,6,8,9,11,13,14	2,4,5,7,10,12,15,16	2	P09527,P0DMW0;P0DMW1
4404	1,3,6,8,9,11,13,15	2,4,5,7,10,12,14,16	3	P02091,P09527,Q6P6S4
4405	1,3,6,8,9,11,13,16	2,4,5,7,10,12,14,15	1	P30120
4406	1,3,6,8,9,11,14,15	2,4,5,7,10,12,13,16	4	P02091,P09527,P0DMW0;P0DMW1,Q6P6S4
4407	1,3,6,8,9,11,14,16	2,4,5,7,10,12,13,15	2	P02783,Q812E4
4408	1,3,6,8,9,11,15,16	2,4,5,7,10,12,13,14	4	P02091,P02783,Q6P6S4,Q9QW07
4409	1,3,6,8,9,12,13,14	2,4,5,7,10,11,15,16	2	P17988,P70549
4410	1,3,6,8,9,12,13,15	2,4,5,7,10,11,14,16	2	P02091,P70549
4411	1,3,6,8,9,12,13,16	2,4,5,7,10,11,14,15	1	P70549
4412	1,3,6,8,9,12,14,15	2,4,5,7,10,11,13,16	2	P02091,P70549
4413	1,3,6,8,9,12,14,16	2,4,5,7,10,11,13,15	1	P21674
4414	1,3,6,8,9,12,15,16	2,4,5,7,10,11,13,14	1	P02091
4415	1,3,6,8,9,13,14,15	2,4,5,7,10,11,12,16	3	P02091,P70549,Q00715
4416	1,3,6,8,9,13,14,16	2,4,5,7,10,11,12,15	0	
4417	1,3,6,8,9,13,15,16	2,4,5,7,10,11,12,14	2	P02091,P70549
4418	1,3,6,8,9,14,15,16	2,4,5,7,10,11,12,13	6	P02091,P02783,P23593,P47967,P97840,Q5I0D1
4419	1,3,6,8,10,11,12,13	2,4,5,7,9,14,15,16	0	
4420	1,3,6,8,10,11,12,14	2,4,5,7,9,13,15,16	0	
4421	1,3,6,8,10,11,12,15	2,4,5,7,9,13,14,16	3	P02091,Q6IFW6,Q9R168

4422	1,3,6,8,10,11,12,16	2,4,5,7,9,13,14,15	2 P02783,Q9QW07
4423	1,3,6,8,10,11,13,14	2,4,5,7,9,12,15,16	0
4424	1,3,6,8,10,11,13,15	2,4,5,7,9,12,14,16	3 P01946,P02091,Q6IFU7
4425	1,3,6,8,10,11,13,16	2,4,5,7,9,12,14,15	1 P30120
4426	1,3,6,8,10,11,14,15	2,4,5,7,9,12,13,16	3 P01946,P02091,Q6IFU7
4427	1,3,6,8,10,11,14,16	2,4,5,7,9,12,13,15	1 P02783
4428	1,3,6,8,10,11,15,16	2,4,5,7,9,12,13,14	5 P01946,P02091,P02783,Q6IFW6,Q9QW07
4429	1,3,6,8,10,12,13,14	2,4,5,7,9,11,15,16	1 P70549
4430	1,3,6,8,10,12,13,15	2,4,5,7,9,11,14,16	3 P02091,P70549,Q9R168
4431	1,3,6,8,10,12,13,16	2,4,5,7,9,11,14,15	1 P70549
4432	1,3,6,8,10,12,14,15	2,4,5,7,9,11,13,16	6 P01946,P02091,P54921,P70549,Q9R168,Q9Z0J6
4433	1,3,6,8,10,12,14,16	2,4,5,7,9,11,13,15	1 P02783
4434	1,3,6,8,10,12,15,16	2,4,5,7,9,11,13,14	2 P02091,Q9R168
4435	1,3,6,8,10,13,14,15	2,4,5,7,9,11,12,16	4 P01946,P02091,P70549,Q03191
4436	1,3,6,8,10,13,14,16	2,4,5,7,9,11,12,15	2 P14668,P70549
4437	1,3,6,8,10,13,15,16	2,4,5,7,9,11,12,14	5 O35077,P01946,P02091,P70549,Q03191
4438	1,3,6,8,10,14,15,16	2,4,5,7,9,11,12,13	6 O35077,P01946,P02091,P02783,P54921,Q03191
4439	1,3,6,8,11,12,13,14	2,4,5,7,9,10,15,16	0
4440	1,3,6,8,11,12,13,15	2,4,5,7,9,10,14,16	3 P02091,P70549,Q9R168
4441	1,3,6,8,11,12,13,16	2,4,5,7,9,10,14,15	1 P30120

4442	1,3,6,8,11,12,14,15	2,4,5,7,9,10,13,16	1	P02091
4443	1,3,6,8,11,12,14,16	2,4,5,7,9,10,13,15	3	P02783,Q8CFN2,Q9QW07
4444	1,3,6,8,11,12,15,16	2,4,5,7,9,10,13,14	5	P02091,P02783,Q6IFW6,Q9QW07,Q9QZK9
4445	1,3,6,8,11,13,14,15	2,4,5,7,9,10,12,16	2	P02091,Q6IFU7
4446	1,3,6,8,11,13,14,16	2,4,5,7,9,10,12,15	2	P02783,P30120
4447	1,3,6,8,11,13,15,16	2,4,5,7,9,10,12,14	4	P02091,P02783,P30120,Q6IFW6
4448	1,3,6,8,11,14,15,16	2,4,5,7,9,10,12,13	6	P01946,P02091,P02783,Q6IFW6,Q9QW07,Q9QZK9
4449	1,3,6,8,12,13,14,15	2,4,5,7,9,10,11,16	3	P02091,P70549,Q9R168
4450	1,3,6,8,12,13,14,16	2,4,5,7,9,10,11,15	1	P70549
4451	1,3,6,8,12,13,15,16	2,4,5,7,9,10,11,14	3	P02091,P70549,Q9R168
4452	1,3,6,8,12,14,15,16	2,4,5,7,9,10,11,13	3	P02091,P02783,P70549
4453	1,3,6,8,13,14,15,16	2,4,5,7,9,10,11,12	6	P01946,P02091,P02783,P70549,Q03191,Q62714
4454	1,3,6,9,10,11,12,13	2,4,5,7,8,14,15,16	5	B0LT89,P20760,P20762,Q4G075,Q6TMA8
4455	1,3,6,9,10,11,12,14	2,4,5,7,8,13,15,16	3	P20762,Q4G075,Q6TMA8
4456	1,3,6,9,10,11,12,15	2,4,5,7,8,13,14,16	7	P02091,P20760,P20762,P25809,Q4G075,Q6TMA8,Q9WUW8
4457	1,3,6,9,10,11,12,16	2,4,5,7,8,13,14,15	4	P25809,Q4G075,Q6TMA8,Q8CIZ5
4458	1,3,6,9,10,11,13,14	2,4,5,7,8,12,15,16	2	P20762,Q6TMA8
4459	1,3,6,9,10,11,13,15	2,4,5,7,8,12,14,16	5	P02091,P20762,Q4G075,Q5RLM2,Q6TMA8
4460	1,3,6,9,10,11,13,16	2,4,5,7,8,12,14,15	2	P20762,Q6TMA8
4461	1,3,6,9,10,11,14,15	2,4,5,7,8,12,13,16	3	P01946,P02091,Q6TMA8

4462	1,3,6,9,10,11,14,16	2,4,5,7,8,12,13,15	3	P02783,Q6TMA8,Q8CIZ5
4463	1,3,6,9,10,11,15,16	2,4,5,7,8,12,13,14	5	P02091,P25809,Q4G075,Q6TMA8,Q8CIZ5
4464	1,3,6,9,10,12,13,14	2,4,5,7,8,11,15,16	9	P02780,P06760,P0C0A9,P20762,P22273,P70549,Q4G075,Q6IFU8,Q6TMA8
4465	1,3,6,9,10,12,13,15	2,4,5,7,8,11,14,16	13	P02091,P02780,P02781,P06760,P07150,P09456,P0C0A9,P20762,P22273,P70549,Q00715,Q4G075,Q6TMA8
4466	1,3,6,9,10,12,13,16	2,4,5,7,8,11,14,15	6	P06760,P07150,P20762,Q4G075,Q6IFU8,Q6TMA8
4467	1,3,6,9,10,12,14,15	2,4,5,7,8,11,13,16	13	P01946,P02091,P02780,P02781,P02782,P06760,P09456,P22273,P30120,Q00715,Q4G075,Q6TMA8,Q9Z0J6
4468	1,3,6,9,10,12,14,16	2,4,5,7,8,11,13,15	5	P06760,P22273,Q4G075,Q6IFU8,Q6TMA8
4469	1,3,6,9,10,12,15,16	2,4,5,7,8,11,13,14	7	P02091,P06760,P22273,P25809,Q4G075,Q6TMA8,Q8CIZ5
4470	1,3,6,9,10,13,14,15	2,4,5,7,8,11,12,16	15	P01946,P02091,P02780,P02781,P06760,P09456,P22273,P30120,P62804,P70549,Q00715,Q4G075,Q5M8C6,Q5PQL7,Q6TMA8
4471	1,3,6,9,10,13,14,16	2,4,5,7,8,11,12,15	3	P06760,Q4G075,Q6IFU8
4472	1,3,6,9,10,13,15,16	2,4,5,7,8,11,12,14	8	P02091,P06760,P07150,P11883,P22273,P70549,Q00715,Q4G075
4473	1,3,6,9,10,14,15,16	2,4,5,7,8,11,12,13	9	P01946,P02091,P06760,P22273,P47967,P82471,Q4G075,Q5I0D1,Q8CIZ5
4474	1,3,6,9,11,12,13,14	2,4,5,7,8,10,15,16	17	O70594,O88267,P0DMW0;P0DMW1,P19468,P20646,P20762,P51907,P53790,Q62761;Q62762;Q62763,Q63270,Q63355,Q63424,Q63618,Q64093,Q6TMA8,Q8R431,Q9Z0V6
4475	1,3,6,9,11,12,13,15	2,4,5,7,8,10,14,16	8	P02091,P20760,P20762,Q4G075,Q5RLM2,Q62761;Q62762;Q62763,Q6TMA8,Q9WUW8
4476	1,3,6,9,11,12,13,16	2,4,5,7,8,10,14,15	4	P20762,P54921,Q6TMA8,Q9Z1F2
4477	1,3,6,9,11,12,14,15	2,4,5,7,8,10,13,16	9	O55004,P02091,P20762,P25809,P28570,Q62761;Q62762;Q62763,Q63618,Q6TMA8,Q9WUW8
4478	1,3,6,9,11,12,14,16	2,4,5,7,8,10,13,15	6	P20762,P25809,Q63618,Q6TMA8,Q8CIZ5,Q9Z0V6
4479	1,3,6,9,11,12,15,16	2,4,5,7,8,10,13,14	7	P02091,P20762,P25809,Q4G075,Q6TMA8,Q8CIZ5,Q9WUW8
4480	1,3,6,9,11,13,14,15	2,4,5,7,8,10,12,16	5	O88267,P02091,P0DMW0;P0DMW1,P20762,Q6TMA8
4481	1,3,6,9,11,13,14,16	2,4,5,7,8,10,12,15	3	P0DMW0;P0DMW1,P20762,Q9Z0V6

4482	1,3,6,9,11,13,15,16	2,4,5,7,8,10,12,14	3	P02091,P20762,Q5RLM2
4483	1,3,6,9,11,14,15,16	2,4,5,7,8,10,12,13	4	P02091,P02783,P25809,Q8CIZ5
4484	1,3,6,9,12,13,14,15	2,4,5,7,8,10,11,16	11	P02091,P02780,P06760,P07150,P20762,P22273,P70549,Q00715,Q4G075,Q6B345,Q6TMA8
4485	1,3,6,9,12,13,14,16	2,4,5,7,8,10,11,15	7	P06760,P20762,P70549,Q4G075,Q6B345,Q6IFU8,Q9Z0V6
4486	1,3,6,9,12,13,15,16	2,4,5,7,8,10,11,14	7	P02091,P06760,P07150,P20762,P70549,Q4G075,Q6B345
4487	1,3,6,9,12,14,15,16	2,4,5,7,8,10,11,13	7	P02091,P06760,P22273,P25809,Q4G075,Q6B345,Q8CIZ5
4488	1,3,6,9,13,14,15,16	2,4,5,7,8,10,11,12	7	P02091,P06760,P22273,P70549,Q00715,Q4G075,Q5I0D1
4489	1,3,6,10,11,12,13,14	2,4,5,7,8,9,15,16	6	P06760,P20646,P20760,P20762,Q6TMA8,Q9Z0V6
4490	1,3,6,10,11,12,13,15	2,4,5,7,8,9,14,16	6	P02091,P06760,P20646,P20760,P20762,Q6TMA8
4491	1,3,6,10,11,12,13,16	2,4,5,7,8,9,14,15	6	P06760,P20646,P20760,P20762,Q6TMA8,Q9Z0V6
4492	1,3,6,10,11,12,14,15	2,4,5,7,8,9,13,16	7	D4A5U3,P01946,P02091,P06760,P20760,Q6TMA8,Q9Z0J6
4493	1,3,6,10,11,12,14,16	2,4,5,7,8,9,13,15	5	P02783,P06760,P20646,Q6TMA8,Q9Z0V6
4494	1,3,6,10,11,12,15,16	2,4,5,7,8,9,13,14	6	D4A5U3,P02091,P06760,P20760,P25809,Q6TMA8
4495	1,3,6,10,11,13,14,15	2,4,5,7,8,9,12,16	5	P01946,P02091,P06760,P20760,Q6TMA8
4496	1,3,6,10,11,13,14,16	2,4,5,7,8,9,12,15	3	P02783,P06760,Q9Z0V6
4497	1,3,6,10,11,13,15,16	2,4,5,7,8,9,12,14	4	P01946,P02091,P06760,P20760
4498	1,3,6,10,11,14,15,16	2,4,5,7,8,9,12,13	4	P01946,P02091,P02783,P06760
4499	1,3,6,10,12,13,14,15	2,4,5,7,8,9,11,16	14	P01946,P02091,P02780,P06760,P07150,P09456,P20646,P20760,P22273,P70549,P82471,Q4G075,Q6TMA8,Q9Z0J6
4500	1,3,6,10,12,13,14,16	2,4,5,7,8,9,11,15	7	P06760,P07150,P14668,P20646,P70549,Q4G075,Q9Z0V6
4501	1,3,6,10,12,13,15,16	2,4,5,7,8,9,11,14	8	P02091,P06760,P07150,P20760,P70549,P82471,Q4G075,Q6B345

4502	1,3,6,10,12,14,15,16	2,4,5,7,8,9,11,13	9	D4A5U3,P01946,P02091,P02783,P06760,P22273,P82471,P97697,Q4G075
4503	1,3,6,10,13,14,15,16	2,4,5,7,8,9,11,12	11	P01946,P02091,P06760,P07150,P14668,P22273,P70549,P70709,P82471,Q4G075,Q5PQL7
4504	1,3,6,11,12,13,14,15	2,4,5,7,8,9,10,16	7	P02091,P06760,P20646,P20760,P20762,P70545,Q6TMA8
4505	1,3,6,11,12,13,14,16	2,4,5,7,8,9,10,15	5	P06760,P20646,P20760,P20762,Q9Z0V6
4506	1,3,6,11,12,13,15,16	2,4,5,7,8,9,10,14	5	P02091,P06760,P20646,P20760,P20762
4507	1,3,6,11,12,14,15,16	2,4,5,7,8,9,10,13	5	P02091,P02783,P06760,P20760,P25809
4508	1,3,6,11,13,14,15,16	2,4,5,7,8,9,10,12	3	P02091,P02783,P06760
4509	1,3,6,12,13,14,15,16	2,4,5,7,8,9,10,11	9	P02091,P06760,P07150,P20646,P70545,P70549,Q4G075,Q6B345,Q6P6R2
4510	1,3,7,8,9,10,11,12	2,4,5,6,13,14,15,16	2	P21674,P35280
4511	1,3,7,8,9,10,11,13	2,4,5,6,12,14,15,16	1	P35280
4512	1,3,7,8,9,10,11,14	2,4,5,6,12,13,15,16	1	P35280
4513	1,3,7,8,9,10,11,15	2,4,5,6,12,13,14,16	4	P35280,Q5RKI1,Q6IFW6,Q6P6S4
4514	1,3,7,8,9,10,11,16	2,4,5,6,12,13,14,15	3	P21674,P35280,Q812E4
4515	1,3,7,8,9,10,12,13	2,4,5,6,11,14,15,16	2	Q4KLZ6,Q923V8
4516	1,3,7,8,9,10,12,14	2,4,5,6,11,13,15,16	2	O70417,P21674
4517	1,3,7,8,9,10,12,15	2,4,5,6,11,13,14,16	3	P35280,Q4KLZ6,Q5RKI1
4518	1,3,7,8,9,10,12,16	2,4,5,6,11,13,14,15	1	P21674
4519	1,3,7,8,9,10,13,14	2,4,5,6,11,12,15,16	0	
4520	1,3,7,8,9,10,13,15	2,4,5,6,11,12,14,16	4	P35280,P70549,Q4KLZ6,Q5RKI1
4521	1,3,7,8,9,10,13,16	2,4,5,6,11,12,14,15	0	

4522	1,3,7,8,9,10,14,15	2,4,5,6,11,12,13,16	2	P35280,Q5RKI1
4523	1,3,7,8,9,10,14,16	2,4,5,6,11,12,13,15	1	P21674
4524	1,3,7,8,9,10,15,16	2,4,5,6,11,12,13,14	2	P35280,Q5RKI1
4525	1,3,7,8,9,11,12,13	2,4,5,6,10,14,15,16	1	P35280
4526	1,3,7,8,9,11,12,14	2,4,5,6,10,13,15,16	4	P11883,P21674,P35280,Q812E4
4527	1,3,7,8,9,11,12,15	2,4,5,6,10,13,14,16	2	P35280,Q6IFW6
4528	1,3,7,8,9,11,12,16	2,4,5,6,10,13,14,15	4	P21674,P35280,Q812E4,Q9JHB9
4529	1,3,7,8,9,11,13,14	2,4,5,6,10,12,15,16	3	P0DMW0;P0DMW1,P35280,Q812E4
4530	1,3,7,8,9,11,13,15	2,4,5,6,10,12,14,16	2	P35280,Q6IFW6
4531	1,3,7,8,9,11,13,16	2,4,5,6,10,12,14,15	4	P02782,P30120,P35280,Q812E4
4532	1,3,7,8,9,11,14,15	2,4,5,6,10,12,13,16	3	P0DMW0;P0DMW1,P35280,Q6IFW6
4533	1,3,7,8,9,11,14,16	2,4,5,6,10,12,13,15	3	P21674,P35280,Q812E4
4534	1,3,7,8,9,11,15,16	2,4,5,6,10,12,13,14	4	P35280,Q6IFW6,Q812E4,Q8CJ52
4535	1,3,7,8,9,12,13,14	2,4,5,6,10,11,15,16	2	P17988,P70549
4536	1,3,7,8,9,12,13,15	2,4,5,6,10,11,14,16	3	P35280,P70549,Q4KLZ6
4537	1,3,7,8,9,12,13,16	2,4,5,6,10,11,14,15	0	
4538	1,3,7,8,9,12,14,15	2,4,5,6,10,11,13,16	1	P35280
4539	1,3,7,8,9,12,14,16	2,4,5,6,10,11,13,15	1	P21674
4540	1,3,7,8,9,12,15,16	2,4,5,6,10,11,13,14	1	P35280
4541	1,3,7,8,9,13,14,15	2,4,5,6,10,11,12,16	3	P0DMW0;P0DMW1,P35280,P70549

4542	1,3,7,8,9,13,14,16	2,4,5,6,10,11,12,15	0
4543	1,3,7,8,9,13,15,16	2,4,5,6,10,11,12,14	1 P35280
4544	1,3,7,8,9,14,15,16	2,4,5,6,10,11,12,13	1 P35280
4545	1,3,7,8,10,11,12,13	2,4,5,6,9,14,15,16	2 P47967,Q5I0D1
4546	1,3,7,8,10,11,12,14	2,4,5,6,9,13,15,16	1 P11883
4547	1,3,7,8,10,11,12,15	2,4,5,6,9,13,14,16	2 Q5RKI1,Q6IFW6
4548	1,3,7,8,10,11,12,16	2,4,5,6,9,13,14,15	3 P08010,P21674,Q9JHB9
4549	1,3,7,8,10,11,13,14	2,4,5,6,9,12,15,16	0
4550	1,3,7,8,10,11,13,15	2,4,5,6,9,12,14,16	2 Q5RKI1,Q6IFW6
4551	1,3,7,8,10,11,13,16	2,4,5,6,9,12,14,15	2 P02782,P30120
4552	1,3,7,8,10,11,14,15	2,4,5,6,9,12,13,16	2 Q5RKI1,Q6IFW6
4553	1,3,7,8,10,11,14,16	2,4,5,6,9,12,13,15	0
4554	1,3,7,8,10,11,15,16	2,4,5,6,9,12,13,14	4 Q5RKI1,Q64093,Q6IFW6,Q9WTW7
4555	1,3,7,8,10,12,13,14	2,4,5,6,9,11,15,16	1 P70549
4556	1,3,7,8,10,12,13,15	2,4,5,6,9,11,14,16	3 P70549,Q4KLZ6,Q5RKI1
4557	1,3,7,8,10,12,13,16	2,4,5,6,9,11,14,15	2 Q64093,Q923V8
4558	1,3,7,8,10,12,14,15	2,4,5,6,9,11,13,16	3 Q5RKI1,Q9EQS0,Q9Z0J6
4559	1,3,7,8,10,12,14,16	2,4,5,6,9,11,13,15	1 P21674
4560	1,3,7,8,10,12,15,16	2,4,5,6,9,11,13,14	5 Q5RKI1,Q63598,Q64093,Q6AY41,Q9WTW7
4561	1,3,7,8,10,13,14,15	2,4,5,6,9,11,12,16	3 P70549,Q03191,Q5RKI1

4562	1,3,7,8,10,13,14,16	2,4,5,6,9,11,12,15	1	O35077
4563	1,3,7,8,10,13,15,16	2,4,5,6,9,11,12,14	8	O35077,P19468,P70549,Q5RKI1,Q63424,Q64093,Q6AY41,Q9WTW7
4564	1,3,7,8,10,14,15,16	2,4,5,6,9,11,12,13	6	O35077,P61206;P84079,P97608,Q03191,Q5RKI1,Q64093
4565	1,3,7,8,11,12,13,14	2,4,5,6,9,10,15,16	3	D3ZUC6,P11883,Q811M5
4566	1,3,7,8,11,12,13,15	2,4,5,6,9,10,14,16	2	Q6IFW6,Q811M5
4567	1,3,7,8,11,12,13,16	2,4,5,6,9,10,14,15	6	P02782,P08723,P30120,P47967,Q811M5,Q9JHB9
4568	1,3,7,8,11,12,14,15	2,4,5,6,9,10,13,16	5	D3ZTX0,D3ZUC6,P11883,Q6IFW6,Q811M5
4569	1,3,7,8,11,12,14,16	2,4,5,6,9,10,13,15	7	D3ZTX0,P02782,P11883,P21674,P30120,Q811M5,Q9JHB9
4570	1,3,7,8,11,12,15,16	2,4,5,6,9,10,13,14	2	Q6IFW6,Q9QW07
4571	1,3,7,8,11,13,14,15	2,4,5,6,9,10,12,16	3	D3ZUC6,Q6IFW6,Q811M5
4572	1,3,7,8,11,13,14,16	2,4,5,6,9,10,12,15	3	P02782,P30120,Q811M5
4573	1,3,7,8,11,13,15,16	2,4,5,6,9,10,12,14	4	P02782,P16636,P30120,Q6IFW6
4574	1,3,7,8,11,14,15,16	2,4,5,6,9,10,12,13	3	D3ZTX0,Q6IFW6,Q9QZK9
4575	1,3,7,8,12,13,14,15	2,4,5,6,9,10,11,16	4	D3ZUC6,P70549,Q811M5,Q9Z2L0
4576	1,3,7,8,12,13,14,16	2,4,5,6,9,10,11,15	3	P70549,Q811M5,Q9Z2L0
4577	1,3,7,8,12,13,15,16	2,4,5,6,9,10,11,14	1	P70549
4578	1,3,7,8,12,14,15,16	2,4,5,6,9,10,11,13	0	
4579	1,3,7,8,13,14,15,16	2,4,5,6,9,10,11,12	5	O35077,P70549,Q03191,Q62714,Q9Z2L0
4580	1,3,7,9,10,11,12,13	2,4,5,6,8,14,15,16	3	P20760,P20762,Q62761;Q62762;Q62763
4581	1,3,7,9,10,11,12,14	2,4,5,6,8,13,15,16	1	Q62761;Q62762;Q62763

4582	1,3,7,9,10,<br>11,12,15	2,4,5,6,8,1<br>3,14,16	4 P20760,P52590,Q5RKI1,Q62761;Q62762;Q62763
4583	1,3,7,9,10,<br>11,12,16	2,4,5,6,8,1<br>3,14,15	1 P21674
4584	1,3,7,9,10,<br>11,13,14	2,4,5,6,8,1<br>2,15,16	1 P0DMW0;P0DMW1
4585	1,3,7,9,10,<br>11,13,15	2,4,5,6,8,1<br>2,14,16	2 P20760,Q5RKI1
4586	1,3,7,9,10,<br>11,13,16	2,4,5,6,8,1<br>2,14,15	0
4587	1,3,7,9,10,<br>11,14,15	2,4,5,6,8,1<br>2,13,16	1 Q5RKI1
4588	1,3,7,9,10,<br>11,14,16	2,4,5,6,8,1<br>2,13,15	0
4589	1,3,7,9,10,<br>11,15,16	2,4,5,6,8,1<br>2,13,14	1 Q5RKI1
4590	1,3,7,9,10,<br>12,13,14	2,4,5,6,8,1<br>1,15,16	1 P50280
4591	1,3,7,9,10,<br>12,13,15	2,4,5,6,8,1<br>1,14,16	5 P20760,P50280,P70549,Q4KLZ6,Q5RKI1
4592	1,3,7,9,10,<br>12,13,16	2,4,5,6,8,1<br>1,14,15	1 P50280
4593	1,3,7,9,10,<br>12,14,15	2,4,5,6,8,1<br>1,13,16	4 P22273,P30120,Q5RKI1,Q9Z0J6
4594	1,3,7,9,10,<br>12,14,16	2,4,5,6,8,1<br>1,13,15	1 P21674
4595	1,3,7,9,10,<br>12,15,16	2,4,5,6,8,1<br>1,13,14	1 Q5RKI1
4596	1,3,7,9,10,<br>13,14,15	2,4,5,6,8,1<br>1,12,16	5 P02780,P12020,P22273,Q00715,Q5RKI1
4597	1,3,7,9,10,<br>13,14,16	2,4,5,6,8,1<br>1,12,15	0
4598	1,3,7,9,10,<br>13,15,16	2,4,5,6,8,1<br>1,12,14	2 P12020,Q5RKI1
4599	1,3,7,9,10,<br>14,15,16	2,4,5,6,8,1<br>1,12,13	2 P22273,Q5RKI1
4600	1,3,7,9,11,<br>12,13,14	2,4,5,6,8,1<br>0,15,16	8 O55004,O88267,P07151,P0DMW0;P0DMW1,P20760,P20762,P25031,Q62761;Q62762;Q62763
4601	1,3,7,9,11,<br>12,13,15	2,4,5,6,8,1<br>0,14,16	4 P20760,P20762,P25031,Q62761;Q62762;Q62763

4602	1,3,7,9,11,<br>12,13,16	2,4,5,6,8,1<br>0,14,15	3	P20760,P20762,P54921
4603	1,3,7,9,11,<br>12,14,15	2,4,5,6,8,1<br>0,13,16	8	O55004,P02631,P07151,P20760,P20762,P25031,Q62761;Q62762;Q62763,Q63618
4604	1,3,7,9,11,<br>12,14,16	2,4,5,6,8,1<br>0,13,15	5	P21674,P25031,P36860,Q498D9,Q63618
4605	1,3,7,9,11,<br>12,15,16	2,4,5,6,8,1<br>0,13,14	3	P20760,P25031,P25809
4606	1,3,7,9,11,<br>13,14,15	2,4,5,6,8,1<br>0,12,16	6	O88267,P02631,P0DMW0;P0DMW1,P20760,P20762,P25031
4607	1,3,7,9,11,<br>13,14,16	2,4,5,6,8,1<br>0,12,15	5	O88267,P0DMW0;P0DMW1,P20762,P25031,Q498D9
4608	1,3,7,9,11,<br>13,15,16	2,4,5,6,8,1<br>0,12,14	4	P20760,P20762,P25031,Q5RLM2
4609	1,3,7,9,11,<br>14,15,16	2,4,5,6,8,1<br>0,12,13	3	P02631,P0DMW0;P0DMW1,P25031
4610	1,3,7,9,12,<br>13,14,15	2,4,5,6,8,1<br>0,11,16	5	P20760,P20762,P22273,P25031,P70549
4611	1,3,7,9,12,<br>13,14,16	2,4,5,6,8,1<br>0,11,15	1	P20762
4612	1,3,7,9,12,<br>13,15,16	2,4,5,6,8,1<br>0,11,14	1	Q6B345
4613	1,3,7,9,12,<br>14,15,16	2,4,5,6,8,1<br>0,11,13	1	P22273
4614	1,3,7,9,13,<br>14,15,16	2,4,5,6,8,1<br>0,11,12	1	P25031
4615	1,3,7,10,1<br>1,12,13,14	2,4,5,6,8,9<br>,15,16	2	P20760,P23593
4616	1,3,7,10,1<br>1,12,13,15	2,4,5,6,8,9<br>,14,16	3	P20760,P23593,Q5RKI1
4617	1,3,7,10,1<br>1,12,13,16	2,4,5,6,8,9<br>,14,15	3	P20760,P47967,P97840
4618	1,3,7,10,1<br>1,12,14,15	2,4,5,6,8,9<br>,13,16	3	P20760,Q5RKI1,Q9Z0J6
4619	1,3,7,10,1<br>1,12,14,16	2,4,5,6,8,9<br>,13,15	1	P20760
4620	1,3,7,10,1<br>1,12,15,16	2,4,5,6,8,9<br>,13,14	2	P20760,Q5RKI1
4621	1,3,7,10,1<br>1,13,14,15	2,4,5,6,8,9<br>,12,16	2	P20760,Q5RKI1

4622	1,3,7,10,11,13,14,16	2,4,5,6,8,9,12,15	1 P36376
4623	1,3,7,10,11,13,15,16	2,4,5,6,8,9,12,14	2 P20760,Q5RKI1
4624	1,3,7,10,11,14,15,16	2,4,5,6,8,9,12,13	2 P20760,Q5RKI1
4625	1,3,7,10,12,13,14,15	2,4,5,6,8,9,11,16	4 P20760,P70549,Q5RKI1,Q9Z0J6
4626	1,3,7,10,12,13,14,16	2,4,5,6,8,9,11,15	0
4627	1,3,7,10,12,13,15,16	2,4,5,6,8,9,11,14	3 P20760,P70549,Q5RKI1
4628	1,3,7,10,12,14,15,16	2,4,5,6,8,9,11,13	1 Q5RKI1
4629	1,3,7,10,13,14,15,16	2,4,5,6,8,9,11,12	4 P61206;P84079,P63095,P70549,Q5RKI1
4630	1,3,7,11,12,13,14,15	2,4,5,6,8,9,10,16	8 D3ZTX0,P02454,P17559,P20760,P20762,P25031,P70545,Q811M5
4631	1,3,7,11,12,13,14,16	2,4,5,6,8,9,10,15	7 D3ZTX0,P02454,P17559,P20760,P20762,P25031,Q811M5
4632	1,3,7,11,12,13,15,16	2,4,5,6,8,9,10,14	5 P02454,P17559,P20760,P20762,P25031
4633	1,3,7,11,12,14,15,16	2,4,5,6,8,9,10,13	6 D3ZTX0,P02454,P02631,P17559,P20760,P25031
4634	1,3,7,11,13,14,15,16	2,4,5,6,8,9,10,12	5 D3ZTX0,P02454,P17559,P20760,P25031
4635	1,3,7,12,13,14,15,16	2,4,5,6,8,9,10,11	7 P02454,P17559,P20760,P25031,P70545,P70549,Q6B345
4636	1,3,8,9,10,11,12,13	2,4,5,6,7,14,15,16	3 B0LT89,P17988,P50116
4637	1,3,8,9,10,11,12,14	2,4,5,6,7,13,15,16	3 P21674,P50116,Q9Z0J6
4638	1,3,8,9,10,11,12,15	2,4,5,6,7,13,14,16	2 P50116,P52590
4639	1,3,8,9,10,11,12,16	2,4,5,6,7,13,14,15	4 A2RUW1,O88797,P21674,P50116
4640	1,3,8,9,10,11,13,14	2,4,5,6,7,12,15,16	2 P17988,P50116
4641	1,3,8,9,10,11,13,15	2,4,5,6,7,12,14,16	2 P50116,Q80WL1

4642	1,3,8,9,10,11,13,16	2,4,5,6,7,12,14,15	2	P50116,Q80WL1
4643	1,3,8,9,10,11,14,15	2,4,5,6,7,12,13,16	2	P50116,Q9Z0J6
4644	1,3,8,9,10,11,14,16	2,4,5,6,7,12,13,15	3	O88797,P36860,P50116
4645	1,3,8,9,10,11,15,16	2,4,5,6,7,12,13,14	2	P50116,Q6IFW6
4646	1,3,8,9,10,12,13,14	2,4,5,6,7,11,15,16	5	P01039,P11598,P17988,P50116,Q9Z0J6
4647	1,3,8,9,10,12,13,15	2,4,5,6,7,11,14,16	7	P01039,P07647,P11598,P50116,P70549,Q4KLZ6,Q99041
4648	1,3,8,9,10,12,13,16	2,4,5,6,7,11,14,15	2	P01039,P50116
4649	1,3,8,9,10,12,14,15	2,4,5,6,7,11,13,16	6	P00714,P01039,P07647,P11598,P50116,Q9Z0J6
4650	1,3,8,9,10,12,14,16	2,4,5,6,7,11,13,15	3	P01039,P21674,P50116
4651	1,3,8,9,10,12,15,16	2,4,5,6,7,11,13,14	4	P01039,P11598,P50116,Q99041
4652	1,3,8,9,10,13,14,15	2,4,5,6,7,11,12,16	8	P01039,P02780,P07647,P11598,P50116,P70549,Q00715,Q9Z0J6
4653	1,3,8,9,10,13,14,16	2,4,5,6,7,11,12,15	2	P01039,P50116
4654	1,3,8,9,10,13,15,16	2,4,5,6,7,11,12,14	3	P01039,P50116,Q80WL1
4655	1,3,8,9,10,14,15,16	2,4,5,6,7,11,12,13	5	P01039,P11598,P47967,P50116,Q5I0D1
4656	1,3,8,9,11,12,13,14	2,4,5,6,7,10,15,16	10	B0LT89,O70594,P09527,P17988,P18297,P50116,Q03248,Q5I0E9,Q63270,Q64335
4657	1,3,8,9,11,12,13,15	2,4,5,6,7,10,14,16	4	B0LT89,P09527,P50116,Q80WL1
4658	1,3,8,9,11,12,13,16	2,4,5,6,7,10,14,15	6	A2RUW1,O88797,P09527,P50116,P54921,Q80WL1
4659	1,3,8,9,11,12,14,15	2,4,5,6,7,10,13,16	5	P09527,P50116,P80299,Q63618,Q9Z0J6
4660	1,3,8,9,11,12,14,16	2,4,5,6,7,10,13,15	7	A2RUW1,O88797,P21674,P36860,P50116,P80299,Q63618
4661	1,3,8,9,11,12,15,16	2,4,5,6,7,10,13,14	5	A2RUW1,O88797,P50116,P80299,Q6IFW6

4662	1,3,8,9,11,13,14,15	2,4,5,6,7,10,12,16	3	P0DMW0;P0DMW1,P36970,P50116
4663	1,3,8,9,11,13,14,16	2,4,5,6,7,10,12,15	3	P0DMW0;P0DMW1,P36860,P50116
4664	1,3,8,9,11,13,15,16	2,4,5,6,7,10,12,14	3	P50116,Q5RLM2,Q80WL1
4665	1,3,8,9,11,14,15,16	2,4,5,6,7,10,12,13	3	P36860,P50116,P80299
4666	1,3,8,9,12,13,14,15	2,4,5,6,7,10,11,16	7	P01039,P07647,P11598,P17988,P50116,P70549,Q9Z0J6
4667	1,3,8,9,12,13,14,16	2,4,5,6,7,10,11,15	3	P01039,P17988,P50116
4668	1,3,8,9,12,13,15,16	2,4,5,6,7,10,11,14	3	P01039,P50116,Q80WL1
4669	1,3,8,9,12,14,15,16	2,4,5,6,7,10,11,13	5	P01039,P11598,P36860,P50116,P80299
4670	1,3,8,9,13,14,15,16	2,4,5,6,7,10,11,12	2	P01039,P50116
4671	1,3,8,10,11,12,13,14	2,4,5,6,7,9,15,16	2	P50116,Q9Z0J6
4672	1,3,8,10,11,12,13,15	2,4,5,6,7,9,14,16	3	P50116,P52590,Q6IFW6
4673	1,3,8,10,11,12,13,16	2,4,5,6,7,9,14,15	1	P50116
4674	1,3,8,10,11,12,14,15	2,4,5,6,7,9,13,16	4	P50116,Q6IFW6,Q9EQS0,Q9Z0J6
4675	1,3,8,10,11,12,14,16	2,4,5,6,7,9,13,15	2	P36860,P50116
4676	1,3,8,10,11,12,15,16	2,4,5,6,7,9,13,14	2	P50116,Q6IFW6
4677	1,3,8,10,11,13,14,15	2,4,5,6,7,9,12,16	2	P50116,Q9Z0J6
4678	1,3,8,10,11,13,14,16	2,4,5,6,7,9,12,15	1	P50116
4679	1,3,8,10,11,13,15,16	2,4,5,6,7,9,12,14	3	P50116,Q6IFW6,Q80WL1
4680	1,3,8,10,11,14,15,16	2,4,5,6,7,9,12,13	3	P50116,P80299,Q6IFW6
4681	1,3,8,10,12,13,14,15	2,4,5,6,7,9,11,16	5	P01039,P11598,P50116,P70549,Q9Z0J6

4682	1,3,8,10,12,13,14,16	2,4,5,6,7,9,11,15	2	P50116,Q9Z0J6
4683	1,3,8,10,12,13,15,16	2,4,5,6,7,9,11,14	3	P01039,P50116,P70549
4684	1,3,8,10,12,14,15,16	2,4,5,6,7,9,11,13	4	P01039,P50116,Q63617,Q9Z0J6
4685	1,3,8,10,13,14,15,16	2,4,5,6,7,9,11,12	4	O35077,P50116,P70549,Q03191
4686	1,3,8,11,12,13,14,15	2,4,5,6,7,9,10,16	6	D3ZTX0,P50116,P70545,Q811M5,Q9WVH8,Q9Z0J6
4687	1,3,8,11,12,13,14,16	2,4,5,6,7,9,10,15	5	D3ZTX0,P00762,P36860,P50116,Q811M5
4688	1,3,8,11,12,13,15,16	2,4,5,6,7,9,10,14	4	P00762,P50116,Q6IFW6,Q80WL1
4689	1,3,8,11,12,14,15,16	2,4,5,6,7,9,10,13	6	D3ZTX0,P36860,P50116,P80299,Q6IFW6,Q9WVH8
4690	1,3,8,11,13,14,15,16	2,4,5,6,7,9,10,12	4	D3ZTX0,P36860,P50116,Q6IFW6
4691	1,3,8,12,13,14,15,16	2,4,5,6,7,9,10,11	2	P50116,P70549
4692	1,3,9,10,11,12,13,14	2,4,5,6,7,8,15,16	26	B0LT89,O55004,O70594,O88267,P06761,P07151,P17988,P20673,P20760,P20761,P20762,P48508,P50115,P50116,P50280,P55091,P98089,Q4G075,Q5M7T9,Q62761;Q62762;Q62763,Q63270,Q6PCU2,Q6TMA8,Q9QX74,Q9Z0J6,Q9Z0V6
4693	1,3,9,10,11,12,13,15	2,4,5,6,7,8,14,16	21	B0LT89,D3ZHA0,D4A5U3,P06760,P06761,P20760,P20761,P20762,P50115,P50116,P50280,P52590,P55091,P98089,Q4G075,Q62635,Q62761;Q62762;Q62763,Q6TMA8,Q8CIZ5,Q9QX74,Q9WUW8
4694	1,3,9,10,11,12,13,16	2,4,5,6,7,8,14,15	16	B0LT89,P06760,P20760,P20761,P20762,P50115,P50116,P54921,P55091,P98089,Q4G075,Q62635,Q6PCU2,Q6TMA8,Q8CIZ5,Q9QX74
4695	1,3,9,10,11,12,14,15	2,4,5,6,7,8,13,16	23	D3ZHA0,D4A5U3,O55004,O70257,P02631,P02781,P06761,P07151,P20760,P20761,P20762,P50115,P50116,P55091,P98089,Q4G075,Q62761;Q62762;Q62763,Q6TMA8,Q8CIZ5,Q9QX74,Q9R0T3,Q9WUW8,Q9Z0J6
4696	1,3,9,10,11,12,14,16	2,4,5,6,7,8,13,15	19	O55004,P02631,P07171,P20760,P20761,P20762,P21674,P36860,P50115,P50116,P55091,P98089,Q4G075,Q63618,Q6PCU2,Q6TMA8,Q8CIZ5,Q9QX74,Q9Z0V6
4697	1,3,9,10,11,12,15,16	2,4,5,6,7,8,13,14	22	D3ZHA0,D4A5U3,P02631,P06760,P06761,P07171,P20760,P20761,P20762,P25809,P50115,P50116,P55091,P98089,Q4G075,Q5QE79,Q62635,Q6PCU2,Q6TMA8,Q8CIZ5,Q9QX74,Q9WUW8
4698	1,3,9,10,11,13,14,15	2,4,5,6,7,8,12,16	18	O88267,P02631,P02780,P06761,P0DMW0;P0DMW1,P20760,P20761,P20762,P50115,P50116,P55091,P98089,Q4G075,Q6TMA8,Q8CIZ5,Q9QX74,Q9R0T3,Q9Z0J6
4699	1,3,9,10,11,13,14,16	2,4,5,6,7,8,12,15	10	P20762,P50115,P50116,P55091,P98089,Q4G075,Q6TMA8,Q8CIZ5,Q9QX74,Q9Z0V6
4700	1,3,9,10,11,13,15,16	2,4,5,6,7,8,12,14	16	D3ZHA0,P02631,P06760,P06761,P20760,P20762,P50115,P50116,P55091,P98089,Q4G075,Q5RLM2,Q62635,Q6TMA8,Q8CIZ5,Q9QX74

4701	1,3,9,10,11,14,15,16	2,4,5,6,7,8,12,13	14	D4A5U3,P02631,P06761,P07171,P25809,P36860,P50115,P50116,P55091,P98089,Q4G075,Q6TMA8,Q8CIZ5,Q9R0T3
4702	1,3,9,10,12,13,14,15	2,4,5,6,7,8,11,16	44	D4A5U3,P01039,P02780,P02781,P02782,P04905,P06760,P06761,P07150,P07647,P08723,P09456,P0C0A9,P11598,P12020,P20673,P20760,P20761,P20762,P22273,P22282,P22283,P30120,P31430,P36374,P46462,P50115,P50116,P50280,P55091,P70549,P98089,Q00715,Q4G063,Q4G075,Q5M8C6,Q63617,Q6TMA8,Q8CIZ5,Q99041,Q9JHB9,Q9QX74,Q9R0T3,Q9Z0J6
4703	1,3,9,10,12,13,14,16	2,4,5,6,7,8,11,15	28	P01039,P02780,P06760,P06761,P07150,P11598,P20762,P22273,P22282,P31430,P46462,P50115,P50116,P50280,P55091,P98089,Q4G063,Q4G075,Q63617,Q6IFU8,Q6TMA8,Q8CIZ5,Q8CJ52,Q99041,Q9QX74,Q9R0T3,Q9WTT6,Q9Z0V6
4704	1,3,9,10,12,13,15,16	2,4,5,6,7,8,11,14	32	D4A5U3,P01039,P02780,P06760,P06761,P07150,P11598,P12020,P20760,P20762,P22273,P22282,P31430,P46462,P50115,P50116,P50280,P55091,P98089,Q4G063,Q4G075,Q5GRG2,Q5M8C6,Q5QE79,Q62635,Q63617,Q6B345,Q6TMA8,Q8CIZ5,Q99041,Q9QX74,Q9R0T3
4705	1,3,9,10,12,14,15,16	2,4,5,6,7,8,11,13	34	D4A5U3,P01039,P02631,P02780,P02781,P06760,P06761,P07150,P07171,P09456,P11598,P12020,P22273,P22282,P22283,P25809,P31430,P46462,P50115,P50116,P50280,P55091,P98089,Q4G063,Q4G075,Q5M8C6,Q63617,Q6TMA8,Q8CIZ5,Q99041,Q9QX74,Q9R0T3,Q9WTT6,Q9Z0J6
4706	1,3,9,10,13,14,15,16	2,4,5,6,7,8,11,12	32	P01039,P02631,P02780,P02781,P06760,P06761,P06911,P07150,P11598,P12020,P22273,P22282,P22283,P31430,P36374,P46462,P50115,P50116,P50280,P55091,P70709,P98089,Q00715,Q4G075,Q5GRG2,Q5M8C6,Q62635,Q63617,Q6TMA8,Q8CIZ5,Q9QX74,Q9R0T3
4707	1,3,9,11,12,13,14,15	2,4,5,6,7,8,10,16	53	B0LT89,O55004,O70257,O70594,O88267,P02454,P02631,P06761,P07151,P0DMW0;P0DMW1,P18418,P18757,P19468,P20760,P20761,P20762,P25031,P28570,P36970,P46844,P48508,P50115,P50116,P51907,P53790,P55091,P57113,P98089,Q03248,Q05175,Q3ZAV1,Q4G075,Q5I0E9,Q5M7T9,Q62761;Q62762;Q62763,Q63270,Q63424,Q63618,Q64602,Q68FT5,Q6MG61,Q6Q0N1,Q6TMA8,Q71MB6,Q8CIZ5,Q8R431,Q923S2,Q99MA2,Q9JJ40,Q9QX74,Q9R0T3,Q9Z0J6,Q9Z0W7
4708	1,3,9,11,12,13,14,16	2,4,5,6,7,8,10,15	40	A2RUW1,O08557,O55004,O70594,O88267,O88339;Q4V882,P02454,P02631,P20760,P20761,P20762,P25031,P30904,P36860,P38918,P48508,P50115,P50116,P51907,P53790,P54921,P55091,P98089,Q03248,Q05175,Q3ZAV1,Q4G075,Q5I0E9,Q5M7T9,Q63270,Q63618,Q6MG61,Q6PCU2,Q6Q0N1,Q6TMA8,Q8CIZ5,Q8R431,Q923S2,Q9QX74,Q9Z0V6
4709	1,3,9,11,12,13,15,16	2,4,5,6,7,8,10,14	30	D3ZHA0,D4A5U3,O55004,O70594,P02454,P02631,P06760,P07171,P20760,P20761,P20762,P25031,P25809,P48508,P50115,P50116,P54921,P55091,P98089,Q4G075,Q5QE79,Q5RLM2,Q62635,Q63618,Q6PCU2,Q6TMA8,Q8CIZ5,Q923S2,Q9QX74,Q9WUW8
4710	1,3,9,11,12,14,15,16	2,4,5,6,7,8,10,13	29	A2RUW1,D4A5U3,O55004,O70257,O70594,P02454,P02631,P06761,P07171,P20760,P20762,P25031,P25809,P30904,P36860,P50115,P50116,P51907,P55091,P80299,P98089,Q4G075,Q63618,Q6PCU2,Q6TMA8,Q8CIZ5,Q9QX74,Q9R0T3,Q9WUW8
4711	1,3,9,11,13,14,15,16	2,4,5,6,7,8,10,12	23	O55004,O88267,P02454,P02631,P0DMW0;P0DMW1,P20760,P20762,P25031,P30904,P36860,P38438,P48508,P50115,P50116,P55091,P98089,Q5RLM2,Q63270,Q63618,Q6TMA8,Q8CIZ5,Q9QX74,Q9R0T3
4712	1,3,9,12,13,14,15,16	2,4,5,6,7,8,10,11	30	O55004,P01039,P02454,P02631,P02780,P06760,P06761,P07150,P11598,P20760,P20762,P22273,P22282,P25031,P31430,P50115,P50116,P50280,P55091,P98089,Q4G063,Q4G075,Q5M8C6,Q63617,Q6B345,Q6TMA8,Q8CIZ5,Q99041,Q9QX74,Q9R0T3

4713	1,3,10,11,12,13,14,15	2,4,5,6,7,8,9,16	17	D3ZHA0,D4A5U3,P06760,P06761,P20646,P20760,P20761,P20762,P50115,P50116,P55091,P98089,Q4G075,Q6TMA8,Q9QX74,Q9R0T3,Q9Z0J6
4714	1,3,10,11,12,13,14,16	2,4,5,6,7,8,9,15	13	P06760,P15999,P20646,P20760,P20762,P50115,P50116,P55091,P98089,Q6TMA8,Q8CIZ5,Q9QX74,Q9Z0V6
4715	1,3,10,11,12,13,15,16	2,4,5,6,7,8,9,14	15	D3ZHA0,D4A5U3,P06760,P20760,P20762,P50115,P50116,P55091,P98089,Q4G075,Q5QE79,Q62635,Q6TMA8,Q8CIZ5,Q9QX74
4716	1,3,10,11,12,14,15,16	2,4,5,6,7,8,9,13	18	D4A5U3,P02631,P06760,P07171,P20760,P25809,P36860,P50115,P50116,P55091,P98089,Q4G075,Q5QE79,Q6TMA8,Q8CIZ5,Q9QX74,Q9R0T3,Q9Z0J6
4717	1,3,10,11,13,14,15,16	2,4,5,6,7,8,9,12	12	D4A5U3,P02631,P06760,P20760,P50115,P50116,P55091,P98089,Q6TMA8,Q8CIZ5,Q9QX74,Q9R0T3
4718	1,3,10,12,13,14,15,16	2,4,5,6,7,8,9,11	29	D4A5U3,P02780,P06760,P06761,P07150,P11598,P20760,P22273,P22282,P31430,P46462,P50115,P50116,P50280,P55091,P70549,P82471,P98089,Q4G075,Q5M8C6,Q63617,Q6B345,Q6P6R2,Q6TMA8,Q8CIZ5,Q9QX74,Q9R0T3,Q9WTT6,Q9Z0J6
4719	1,3,11,12,13,14,15,16	2,4,5,6,7,8,9,10	26	D3ZTX0,D4A5U3,O55004,O70594,P02454,P02631,P06760,P17559,P20646,P20760,P20762,P25031,P36860,P50115,P50116,P55091,P70545,P98089,Q5QE79,Q63618,Q6P6R2,Q6TMA8,Q8CIZ5,Q9QX74,Q9R0T3,Q9Z0V6
4720	1,4,5,6,7,8,9,10	2,3,11,12,13,14,15,16	19	O70594,P02454,P06760,P06761,P13432,P18418,P20646,P20760,P23928,P25031,P36860,P49134,P50116,P70545,Q6P6R2,Q9QX74,Q9R0T3,Q9Z0V6,Q9Z1F2
4721	1,4,5,6,7,8,9,11	2,3,10,12,13,14,15,16	50	B1H234,P02780,P02781,P02782,P04905,P06760,P06761,P06911,P07150,P07647,P08723,P09456,P0C0A9,P11598,P13432,P18418,P20646,P20760,P22273,P22282,P22283,P36374,P40241,P46462,P49134,P50115,P50116,P50280,P60905,Q09326,Q3T1J1,Q4G063,Q4G075,Q5GRG2,Q5M8C6,Q63493,Q63617,Q6AYR9,Q6P6R2,Q6P6S4,Q812E4,Q99041,Q9JHB9,Q9JI85,Q9QW07,Q9QX74,Q9QYP2,Q9R0T3,Q9WTT6,Q9Z0J6
4722	1,4,5,6,7,8,9,12	2,3,10,11,13,14,15,16	10	P06760,P06761,P06911,P13432,P18418,P20760,P22282,P36860,Q9QX74,Q9R0T3
4723	1,4,5,6,7,8,9,13	2,3,10,11,12,14,15,16	11	P06760,P06761,P13432,P18418,P20646,P20760,P22282,P36860,Q9QX74,Q9R0T3,Q9Z0J6
4724	1,4,5,6,7,8,9,14	2,3,10,11,12,13,15,16	11	P06760,P06761,P06911,P13432,P18418,P20646,P20760,P52590,Q5QE79,Q9QX74,Q9R0T3
4725	1,4,5,6,7,8,9,15	2,3,10,11,12,13,14,16	10	P06760,P06761,P08721,P13432,P18418,P20646,P20760,P36860,Q9QX74,Q9Z0V6

4726	1,4,5,6,7,8,9,16	2,3,10,11,12,13,14,15	19	P02780,P02781,P02782,P06760,P06761,P07647,P13432,P18418,P20646,P20760,P22006,P22282,P22283,Q5M8C6,Q6TMA8,Q9JHB9,Q9QX74,Q9R0T3,Q9Z0J6
4727	1,4,5,6,7,8,10,11	2,3,9,12,13,14,15,16	41	O88917,P02780,P02781,P02782,P04905,P06760,P06761,P06911,P07150,P07647,P08723,P09456,P0C0A9,P11598,P13432,P18418,P22273,P22282,P22283,P25031,P31044,P35952,P36374,P36860,P40241,P46462,P50115,P50116,P50280,Q4G063,Q4G075,Q5GRG2,Q5M8C6,Q63493,Q63617,Q6B345,Q99041,Q9JHB9,Q9JI85,Q9QX74,Q9R0T3
4728	1,4,5,6,7,8,10,12	2,3,9,11,13,14,15,16	21	O88267,P06761,P06911,P0DMW0;P0DMW1,P13432,P18418,P19629,P19814,P20760,P22282,P25031,P30904,P31044,P36860,P48508,P57113,Q5RLM2,Q66HG3,Q9QX74,Q9R0T3,Q9Z1F2
4729	1,4,5,6,7,8,10,13	2,3,9,11,12,14,15,16	24	A2RUW1,O70257,O70594,P02631,P06761,P07171,P13432,P18418,P20760,P22282,P25031,P30904,P31044,P35952,P36860,P46844,P48508,P57113,Q63618,Q8R431,Q9JI85,Q9QX74,Q9R0T3,Q9Z1F2
4730	1,4,5,6,7,8,10,14	2,3,9,11,12,13,15,16	17	A2RUW1,O70594,P06760,P06761,P13432,P18418,P20760,P23928,P25031,P48508,P54921,P57113,Q5RLM2,Q923S2,Q9QX74,Q9R0T3,Q9Z1F2
4731	1,4,5,6,7,8,10,15	2,3,9,11,12,13,14,16	39	A2RUW1,O70594,O88267,O88339;Q4V882,P06761,P08721,P13432,P18418,P19468,P19629,P20646,P20760,P23928,P25031,P30904,P36860,P46844,P48508,P51907,P53790,P54921,P57113,Q03248,Q05175,Q3ZAV1,Q5I0E9,Q5M7T9,Q63270,Q63474,Q63618,Q6MG61,Q6PCU2,Q6Q0N1,Q8R431,Q923S2,Q9QX74,Q9R0T3,Q9Z0V6,Q9Z1F2
4732	1,4,5,6,7,8,10,16	2,3,9,11,12,13,14,15	49	O35077,O70257,O70594,O88267,P02780,P02781,P02782,P06761,P07151,P07647,P0DMW0;P0DMW1,P13432,P18418,P18757,P19468,P19629,P20646,P20760,P22282,P22283,P23928,P25031,P46844,P48508,P53790,P57113,Q03248,Q3ZAV1,Q5I0E9,Q5M7T9,Q5M8C6,Q63270,Q63424,Q63618,Q64602,Q68FT5,Q6MG61,Q6Q0N1,Q6TMA8,Q71MB6,Q8R431,Q923S2,Q99MA2,Q9JHB9,Q9JJ40,Q9QX74,Q9R0T3,Q9Z0J6,Q9Z0W7
4733	1,4,5,6,7,8,11,12	2,3,9,10,13,14,15,16	42	O35547,P02780,P02781,P02782,P04905,P06760,P06761,P06911,P07150,P07647,P08723,P09456,P0C0A9,P11598,P12020,P13432,P18418,P19218,P22273,P22282,P22283,P31430,P36374,P40241,P46462,P50280,P60905,P97840,Q09326,Q4G063,Q4G075,Q5GRG2,Q5I0D1,Q5M8C6,Q63493,Q63617,Q99041,Q9JHB9,Q9JI85,Q9QW07,Q9QX74,Q9R0T3
4734	1,4,5,6,7,8,11,13	2,3,9,10,12,14,15,16	42	B1H234,O35547,O88917,P02780,P02781,P02782,P04905,P06760,P06761,P06911,P07150,P07647,P08723,P09456,P0C0A9,P11598,P13432,P18418,P22273,P22282,P22283,P36374,P36860,P40241,P46462,P50115,P60905,Q09326,Q4G063,Q4G075,Q5GRG2,Q5M8C6,Q62902,Q63493,Q63617,Q6AYR9,Q99041,Q9JHB9,Q9JI85,Q9QX74,Q9R0T3,Q9Z0J6
4735	1,4,5,6,7,8,11,14	2,3,9,10,12,13,15,16	43	B1H234,P02780,P02781,P02782,P04905,P06760,P06761,P06911,P07150,P07647,P08723,P09456,P0C0A9,P11598,P12020,P13432,P18418,P19218,P20760,P22273,P22282,P22283,P36374,P40241,P46462,P50280,Q09326,Q4FZU2,Q4G063,Q4G075,Q5GRG2,Q5M8C6,Q63493,Q63617,Q6AYR9,Q6P6Q2,Q812E4,Q99041,Q9JHB9,Q9JI85,Q9QW07,Q9QX74,Q9R0T3
4736	1,4,5,6,7,8,11,15	2,3,9,10,12,13,14,16	47	B1H234,P02780,P02781,P02782,P04905,P06760,P06761,P06911,P07150,P07647,P08721,P08723,P09456,P0C0A9,P11598,P13432,P18418,P19629,P22273,P22282,P22283,P36374,P40241,P46462,P50280,Q09326,Q4FZU2,Q4G063,Q4G075,Q5GRG2,Q5M8C6,Q63493,Q63617,Q6AYR9,Q6IFU8,Q6IFW6,Q6IMF3,Q6P6Q2,Q8CJ52,Q99041,Q9JHB9,Q9JI85,Q9QW07,Q9QX74,Q9R0T3,Q9WTT6,Q9Z0V6

4737	1,4,5,6,7,8,11,16	2,3,9,10,12,13,14,15	54	B1H234,O35547,O88917,P02780,P02781,P02782,P04905,P06760,P06761,P06911,P07150,P07647,P08010,P08723,P09456,P0C0A9,P11598,P12020,P13432,P18418,P20760,P22273,P22282,P22283,P24368,P30120,P36374,P40241,P46462,P50115,P50280,P60905,P97523,Q4FZU2,Q4G063,Q4G075,Q5GRG2,Q5M8C6,Q62902,Q63493,Q63617,Q6GMN2,Q6P6Q2,Q6TMA8,Q812E4,Q8CFN2,Q99041,Q9JHB9,Q9JI85,Q9QW07,Q9QX74,Q9QZK8,Q9R0T3,Q9Z0J6
4738	1,4,5,6,7,8,12,13	2,3,9,10,11,14,15,16	12	P06761,P06911,P13432,P18418,P19814,P22282,P36860,P97840,Q9JI85,Q9QX74,Q9R0T3,Q9Z2L0
4739	1,4,5,6,7,8,12,14	2,3,9,10,11,13,15,16	12	P06760,P06761,P06911,P13432,P18418,P19218,P20760,P22282,Q5RLM2,Q9QX74,Q9R0T3,Q9Z2L0
4740	1,4,5,6,7,8,12,15	2,3,9,10,11,13,14,16	11	P06761,P06911,P13432,P18418,P19629,P19814,P22282,P36860,Q9QX74,Q9R0T3,Q9Z0V6
4741	1,4,5,6,7,8,12,16	2,3,9,10,11,13,14,15	18	O88267,P02780,P02781,P02782,P06761,P06911,P07647,P13432,P18418,P20760,P22282,P22283,Q5M8C6,Q6TMA8,Q9JHB9,Q9JI85,Q9QX74,Q9R0T3
4742	1,4,5,6,7,8,13,14	2,3,9,10,11,12,15,16	17	A2RUW1,P06760,P06761,P06911,P07171,P13432,P18418,P20760,P22282,P52590,Q4G075,Q99MH3,Q9JI85,Q9QX74,Q9R0T3,Q9WUW8,Q9Z2L0
4743	1,4,5,6,7,8,13,15	2,3,9,10,11,12,14,16	12	A2RUW1,P06761,P08721,P13432,P18418,P22282,P36860,Q62714,Q6PCU2,Q9QX74,Q9Z0V6,Q9Z2L0
4744	1,4,5,6,7,8,13,16	2,3,9,10,11,12,14,15	24	O70257,P02780,P02781,P02782,P06761,P06911,P07647,P11598,P13432,P18418,P20760,P22282,P22283,P30120,P46844,Q4G075,Q5M8C6,Q6TMA8,Q99MH3,Q9JHB9,Q9JI85,Q9QX74,Q9R0T3,Q9Z0J6
4745	1,4,5,6,7,8,14,15	2,3,9,10,11,12,13,16	9	A2RUW1,P06761,P13432,P18418,P20760,P54921,Q4G075,Q62714,Q9QX74
4746	1,4,5,6,7,8,14,16	2,3,9,10,11,12,13,15	20	P02780,P02781,P02782,P06761,P06911,P07647,P13432,P18418,P20760,P22282,P22283,P52590,P57113,Q4G075,Q5M8C6,Q6TMA8,Q99MH3,Q9JHB9,Q9QX74,Q9R0T3
4747	1,4,5,6,7,8,15,16	2,3,9,10,11,12,13,14	29	B2RYW9,O70594,O88267,P02780,P02781,P02782,P06761,P07647,P08721,P13432,P17988,P18418,P19629,P20646,P20760,P22282,P22283,P48508,P57113,Q03248,Q5M7T9,Q63270,Q6TMA8,Q8CJ52,Q8R431,Q9JHB9,Q9QX74,Q9Z0J6,Q9Z0V6
4748	1,4,5,6,7,9,10,11	2,3,8,12,13,14,15,16	4	P11598,P36860,Q63617,Q9R0T3
4749	1,4,5,6,7,9,10,12	2,3,8,11,13,14,15,16	3	P00762,P36860,Q03191
4750	1,4,5,6,7,9,10,13	2,3,8,11,12,14,15,16	2	P11883,P36860
4751	1,4,5,6,7,9,10,14	2,3,8,11,12,13,15,16	1	P00762
4752	1,4,5,6,7,9,10,15	2,3,8,11,12,13,14,16	4	P00762,P08721,P36860,Q811M5
4753	1,4,5,6,7,9,10,16	2,3,8,11,12,13,14,15	5	P11883,P18418,P22006,Q811M5,Q9Z0J6
4754	1,4,5,6,7,9,11,12	2,3,8,10,13,14,15,16	6	P11598,P22283,P61206;P84079,Q03191,Q63617,Q9R0T3

4755	1,4,5,6,7,9,11,13	2,3,8,10,12,14,15,16	5	B1H234,P11598,P22283,Q63617,Q9Z0J6
4756	1,4,5,6,7,9,11,14	2,3,8,10,12,13,15,16	3	B1H234,P11598,Q63617
4757	1,4,5,6,7,9,11,15	2,3,8,10,12,13,14,16	4	B1H234,P08721,Q63617,Q8CJ52
4758	1,4,5,6,7,9,11,16	2,3,8,10,12,13,14,15	14	B1H234,P02780,P02781,P02782,P07647,P0C0A9,P11598,P22006,P22283,P36374,Q63617,Q9JHB9,Q9R0T3,Q9Z0J6
4759	1,4,5,6,7,9,12,13	2,3,8,10,11,14,15,16	2	P36860,Q03191
4760	1,4,5,6,7,9,12,14	2,3,8,10,11,13,15,16	1	Q03191
4761	1,4,5,6,7,9,12,15	2,3,8,10,11,13,14,16	1	P36860
4762	1,4,5,6,7,9,12,16	2,3,8,10,11,13,14,15	2	P22006,Q03191
4763	1,4,5,6,7,9,13,14	2,3,8,10,11,12,15,16	0	
4764	1,4,5,6,7,9,13,15	2,3,8,10,11,12,14,16	2	P36860,P62804
4765	1,4,5,6,7,9,13,16	2,3,8,10,11,12,14,15	4	P11883,P22006,Q9EQS0,Q9Z0J6
4766	1,4,5,6,7,9,14,15	2,3,8,10,11,12,13,16	1	P02625
4767	1,4,5,6,7,9,14,16	2,3,8,10,11,12,13,15	2	P22006,P52590
4768	1,4,5,6,7,9,15,16	2,3,8,10,11,12,13,14	3	P08721,P22006,Q9Z0J6
4769	1,4,5,6,7,10,11,12	2,3,8,9,13,14,15,16	8	P11598,P22283,P36374,P36860,P47967,P97840,Q5I0D1,Q9R0T3
4770	1,4,5,6,7,10,11,13	2,3,8,9,12,14,15,16	9	B1H234,P11598,P22283,P36376,P36860,P97840,Q63617,Q99041,Q9R0T3
4771	1,4,5,6,7,10,11,14	2,3,8,9,12,13,15,16	5	B1H234,P11598,P22283,P36376,Q99041
4772	1,4,5,6,7,10,11,15	2,3,8,9,12,13,14,16	6	P08721,P11598,P17988,P22283,P36860,Q8CJ52
4773	1,4,5,6,7,10,11,16	2,3,8,9,12,13,14,15	16	P02780,P02781,P02782,P07647,P0C0A9,P11598,P17988,P18418,P22283,P36374,P36376,Q63617,Q99041,Q9JHB9,Q9R0T3,Q9Z0J6
4774	1,4,5,6,7,10,12,13	2,3,8,9,11,14,15,16	2	P36860,P97840

4775	1,4,5,6,7,10,12,14	2,3,8,9,11,13,15,16	1	Q5RLM2
4776	1,4,5,6,7,10,12,15	2,3,8,9,11,13,14,16	4	P08721,P10758,P19814,P36860
4777	1,4,5,6,7,10,12,16	2,3,8,9,11,13,14,15	4	P0DMW0;P0DMW1,P57113,P97840,Q03248
4778	1,4,5,6,7,10,13,14	2,3,8,9,11,12,15,16	2	A2RUW1,P36376
4779	1,4,5,6,7,10,13,15	2,3,8,9,11,12,14,16	4	A2RUW1,P08721,P11883,P36860
4780	1,4,5,6,7,10,13,16	2,3,8,9,11,12,14,15	8	P07647,P11883,P18418,P36376,P36860,P57113,Q99MH3,Q9Z0J6
4781	1,4,5,6,7,10,14,15	2,3,8,9,11,12,13,16	3	A2RUW1,P08721,P54921
4782	1,4,5,6,7,10,14,16	2,3,8,9,11,12,13,15	4	P09527,P36376,P57113,Q99MH3
4783	1,4,5,6,7,10,15,16	2,3,8,9,11,12,13,14	10	P08721,P09527,P11883,P17988,P19468,P57113,Q03248,Q63270,Q64335,Q8R431
4784	1,4,5,6,7,11,12,13	2,3,8,9,10,14,15,16	8	P11598,P22283,P36374,P47967,P97840,Q5I0D1,Q63617,Q9R0T3
4785	1,4,5,6,7,11,12,14	2,3,8,9,10,13,15,16	4	P11598,P19218,P22283,P36374
4786	1,4,5,6,7,11,12,15	2,3,8,9,10,13,14,16	6	P08721,P11598,P22283,P36374,P97840,Q8CJ52
4787	1,4,5,6,7,11,12,16	2,3,8,9,10,13,14,15	15	O35547,P02780,P02781,P02782,P07647,P0C0A9,P11598,P22283,P36374,P97840,Q63617,Q9JHB9,Q9QW07,Q9R0T3,Q9Z0J6
4788	1,4,5,6,7,11,13,14	2,3,8,9,10,12,15,16	6	B1H234,P11598,P22283,P36376,Q63617,Q99041
4789	1,4,5,6,7,11,13,15	2,3,8,9,10,12,14,16	8	B1H234,P08721,P11598,P22283,P36860,Q63617,Q8CJ52,Q99041
4790	1,4,5,6,7,11,13,16	2,3,8,9,10,12,14,15	19	B1H234,O35547,P00714,P02780,P02781,P02782,P07647,P09456,P0C0A9,P11598,P22283,P36374,P36376,Q63617,Q99041,Q99MH3,Q9JHB9,Q9R0T3,Q9Z0J6
4791	1,4,5,6,7,11,14,15	2,3,8,9,10,12,13,16	6	B1H234,P08721,P11598,Q4FZU2,Q8CJ52,Q99041
4792	1,4,5,6,7,11,14,16	2,3,8,9,10,12,13,15	16	B1H234,O35547,P02780,P02781,P02782,P07647,P0C0A9,P11598,P22283,P36374,P36376,P52590,Q63617,Q99041,Q99MH3,Q9JHB9
4793	1,4,5,6,7,11,15,16	2,3,8,9,10,12,13,14	16	B1H234,P02780,P02781,P02782,P07647,P08721,P0C0A9,P11598,P17988,P22283,P36374,Q4FZU2,Q6P6Q2,Q8CJ52,Q9JHB9,Q9Z0J6
4794	1,4,5,6,7,12,13,14	2,3,8,9,10,11,15,16	1	Q9Z2L0

4795	1,4,5,6,7,12,13,15	2,3,8,9,10,11,14,16	3	P19814,P36860,P97840
4796	1,4,5,6,7,12,13,16	2,3,8,9,10,11,14,15	3	P07647,P97840,Q99MH3
4797	1,4,5,6,7,12,14,15	2,3,8,9,10,11,13,16	0	
4798	1,4,5,6,7,12,14,16	2,3,8,9,10,11,13,15	1	Q99MH3
4799	1,4,5,6,7,12,15,16	2,3,8,9,10,11,13,14	3	P08721,P10758,P17988
4800	1,4,5,6,7,13,14,15	2,3,8,9,10,11,12,16	1	A2RUW1
4801	1,4,5,6,7,13,14,16	2,3,8,9,10,11,12,15	4	P07647,P36376,P52590,Q99MH3
4802	1,4,5,6,7,13,15,16	2,3,8,9,10,11,12,14	4	P08721,P10719,P11883,Q9Z0J6
4803	1,4,5,6,7,14,15,16	2,3,8,9,10,11,12,13	2	P10719,P17988
4804	1,4,5,6,8,9,10,11	2,3,7,12,13,14,15,16	5	P18418,P20760,P25031,P49134,Q6AY61
4805	1,4,5,6,8,9,10,12	2,3,7,11,13,14,15,16	4	P18418,P20760,P25031,P36860
4806	1,4,5,6,8,9,10,13	2,3,7,11,12,14,15,16	4	P18418,P20760,P25031,P36860
4807	1,4,5,6,8,9,10,14	2,3,7,11,12,13,15,16	4	P13432,P18418,P20760,P25031
4808	1,4,5,6,8,9,10,15	2,3,7,11,12,13,14,16	7	P13432,P18418,P20760,P25031,P36860,Q63474,Q811M5
4809	1,4,5,6,8,9,10,16	2,3,7,11,12,13,14,15	5	P13432,P18418,P20760,P25031,Q811M5
4810	1,4,5,6,8,9,11,12	2,3,7,10,13,14,15,16	11	P18418,P36374,P60905,P61206;P84079,P63095,Q03191,Q3T1J1,Q5RKI1,Q63493,Q6Q7Y5,Q9JI85
4811	1,4,5,6,8,9,11,13	2,3,7,10,12,14,15,16	6	P18418,P60905,Q5RKI1,Q63493,Q6AY61,Q9JI85
4812	1,4,5,6,8,9,11,14	2,3,7,10,12,13,15,16	6	P20760,P20766,Q3T1J1,Q5RKI1,Q6AY61,Q812E4
4813	1,4,5,6,8,9,11,15	2,3,7,10,12,13,14,16	8	P13432,P60905,Q4FZU2,Q6IFU8,Q6IMF3,Q6P6Q2,Q6P6S4,Q8CJ52
4814	1,4,5,6,8,9,11,16	2,3,7,10,12,13,14,15	23	O35547,P02780,P02781,P02782,P04905,P07647,P08723,P09456,P18418,P20760,P22006,P22282,P30120,P36374,P46462,P60905,Q5RKI1,Q63493,Q6AY61,Q812E4,Q9JHB9,Q9QW07,Q9Z0J6

4815	1,4,5,6,8,9,12,13	2,3,7,10,11,14,15,16	2	P20766,Q5RKI1
4816	1,4,5,6,8,9,12,14	2,3,7,10,11,13,15,16	3	P20760,P20766,Q5RKI1
4817	1,4,5,6,8,9,12,15	2,3,7,10,11,13,14,16	1	P13432
4818	1,4,5,6,8,9,12,16	2,3,7,10,11,13,14,15	4	P13432,P18418,P20760,Q5RKI1
4819	1,4,5,6,8,9,13,14	2,3,7,10,11,12,15,16	3	P20760,P20766,Q5RKI1
4820	1,4,5,6,8,9,13,15	2,3,7,10,11,12,14,16	2	P13432,P62804
4821	1,4,5,6,8,9,13,16	2,3,7,10,11,12,14,15	4	P13432,P18418,P20760,Q5RKI1
4822	1,4,5,6,8,9,14,15	2,3,7,10,11,12,13,16	3	P13432,P20760,P20766
4823	1,4,5,6,8,9,14,16	2,3,7,10,11,12,13,15	7	P13432,P18418,P20760,P20766,P23593,P52590,Q5RKI1
4824	1,4,5,6,8,9,15,16	2,3,7,10,11,12,13,14	4	P13432,P18418,P20760,P23593
4825	1,4,5,6,8,10,11,12	2,3,7,9,13,14,15,16	5	P18418,P22282,P25031,P36374,Q9JI85
4826	1,4,5,6,8,10,11,13	2,3,7,9,12,14,15,16	4	P18418,P22282,P25031,Q9JI85
4827	1,4,5,6,8,10,11,14	2,3,7,9,12,13,15,16	3	P18418,P19218,Q99041
4828	1,4,5,6,8,10,11,15	2,3,7,9,12,13,14,16	6	P18418,Q4FZU2,Q6IFU8,Q6IFW6,Q6IMF3,Q6P6Q2
4829	1,4,5,6,8,10,11,16	2,3,7,9,12,13,14,15	19	O88917,P02780,P02781,P02782,P04905,P07647,P08723,P0C0A9,P15399,P18418,P22282,P25031,P30120,P36374,P60905,Q5M8C6,Q6AY61,Q9JHB9,Q9JI85
4830	1,4,5,6,8,10,12,13	2,3,7,9,11,14,15,16	4	P18418,P25031,P35280,P36860
4831	1,4,5,6,8,10,12,14	2,3,7,9,11,13,15,16	4	P18418,P25031,P35280,P54921
4832	1,4,5,6,8,10,12,15	2,3,7,9,11,13,14,16	7	O88267,P0DMW0;P0DMW1,P13432,P18418,P25031,P36860,P54921
4833	1,4,5,6,8,10,12,16	2,3,7,9,11,13,14,15	6	O88267,P0DMW0;P0DMW1,P13432,P18418,P25031,P35280
4834	1,4,5,6,8,10,13,14	2,3,7,9,11,12,15,16	3	P18418,P25031,P35280

4835	1,4,5,6,8,10,13,15	2,3,7,9,11,12,14,16	5	P13432,P18418,P25031,P36860,P62804
4836	1,4,5,6,8,10,13,16	2,3,7,9,11,12,14,15	7	O70257,P11883,P13432,P18418,P20760,P25031,P35280
4837	1,4,5,6,8,10,14,15	2,3,7,9,11,12,13,16	4	P13432,P18418,P25031,P54921
4838	1,4,5,6,8,10,14,16	2,3,7,9,11,12,13,15	6	P13432,P18418,P20760,P25031,P35280,P54921
4839	1,4,5,6,8,10,15,16	2,3,7,9,11,12,13,14	9	O35077,O88267,P13432,P15399,P18418,P20760,P25031,P54921,Q8R431
4840	1,4,5,6,8,11,12,13	2,3,7,9,10,14,15,16	11	O35547,P06911,P14046,P20766,P22282,P36374,P46462,P60905,Q5RKI1,Q63493,Q9JI85
4841	1,4,5,6,8,11,12,14	2,3,7,9,10,13,15,16	8	P06911,P12020,P19218,P20766,P36374,Q5GRG2,Q5RKI1,Q9JI85
4842	1,4,5,6,8,11,12,15	2,3,7,9,10,13,14,16	8	P36374,Q4FZU2,Q63493,Q6IFU8,Q6IFW6,Q6IMF3,Q6P6Q2,Q9QW07
4843	1,4,5,6,8,11,12,16	2,3,7,9,10,13,14,15	26	O35547,P02780,P02781,P02782,P04905,P06911,P07647,P08010,P08723,P0C0A9,P12020,P18418,P22282,P22283,P30120,P36374,P40241,P46462,P60905,Q5M8C6,Q5RKI1,Q63493,Q8CFN2,Q9JHB9,Q9JI85,Q9QW07
4844	1,4,5,6,8,11,13,14	2,3,7,9,10,12,15,16	8	O54728,P19218,P20766,P22282,Q09326,Q5RKI1,Q99041,Q9JI85
4845	1,4,5,6,8,11,13,15	2,3,7,9,10,12,14,16	10	P20766,P36374,P60905,Q4FZU2,Q6IFU8,Q6IFW6,Q6IG02,Q6IMF3,Q6P6Q2,Q9JI85
4846	1,4,5,6,8,11,13,16	2,3,7,9,10,12,14,15	24	O35547,P02780,P02781,P02782,P04905,P07647,P08723,P09456,P0C0A9,P18418,P22282,P22283,P30120,P36374,P46462,P60905,Q5M8C6,Q5RKI1,Q63493,Q6AY61,Q6GMN2,Q99041,Q9JHB9,Q9JI85
4847	1,4,5,6,8,11,14,15	2,3,7,9,10,12,13,16	8	P19218,P20766,Q4FZU2,Q6IFU8,Q6IFW6,Q6IMF3,Q6P6Q2,Q8CJ52
4848	1,4,5,6,8,11,14,16	2,3,7,9,10,12,13,15	26	O35547,O54728,P02780,P02781,P02782,P04905,P07647,P08723,P09456,P0C0A9,P18418,P19218,P20760,P20766,P22282,P30120,P36374,P46462,P60905,Q5M8C6,Q5RKI1,Q6AY61,Q6GMN2,Q99041,Q9JHB9,Q9JI85
4849	1,4,5,6,8,11,15,16	2,3,7,9,10,12,13,14	24	O35547,P02780,P02781,P02782,P04905,P07647,P08723,P0C0A9,P13432,P18418,P22282,P30120,P36374,P46462,P60905,Q10758,Q4FZU2,Q6IFU8,Q6IFW6,Q6IMF3,Q6P6Q2,Q8CJ52,Q9JHB9,Q9QW07
4850	1,4,5,6,8,12,13,14	2,3,7,9,10,11,15,16	4	P19218,P20766,P35280,Q5RKI1
4851	1,4,5,6,8,12,13,15	2,3,7,9,10,11,14,16	2	P13432,P20766
4852	1,4,5,6,8,12,13,16	2,3,7,9,10,11,14,15	4	P18418,P20766,P35280,Q5RKI1

4853	1,4,5,6,8,12,14,15	2,3,7,9,10,11,13,16	3	P13432,P20766,P54921
4854	1,4,5,6,8,12,14,16	2,3,7,9,10,11,13,15	5	P13432,P20760,P20766,P35280,Q5RKI1
4855	1,4,5,6,8,12,15,16	2,3,7,9,10,11,13,14	1	P13432
4856	1,4,5,6,8,13,14,15	2,3,7,9,10,11,12,16	2	P13432,P20766
4857	1,4,5,6,8,13,14,16	2,3,7,9,10,11,12,15	9	O54728,P13432,P18418,P20760,P20766,P35280,P52590,Q5RKI1,Q99MH3
4858	1,4,5,6,8,13,15,16	2,3,7,9,10,11,12,14	2	P13432,P18418
4859	1,4,5,6,8,14,15,16	2,3,7,9,10,11,12,13	4	P13432,P20760,P20766,P54921
4860	1,4,5,6,9,10,11,12	2,3,7,8,13,14,15,16	1	Q03191
4861	1,4,5,6,9,10,11,13	2,3,7,8,12,14,15,16	0	
4862	1,4,5,6,9,10,11,14	2,3,7,8,12,13,15,16	0	
4863	1,4,5,6,9,10,11,15	2,3,7,8,12,13,14,16	1	Q811M5
4864	1,4,5,6,9,10,11,16	2,3,7,8,12,13,14,15	0	
4865	1,4,5,6,9,10,12,13	2,3,7,8,11,14,15,16	2	P36860,Q03191
4866	1,4,5,6,9,10,12,14	2,3,7,8,11,13,15,16	1	Q03191
4867	1,4,5,6,9,10,12,15	2,3,7,8,11,13,14,16	1	Q811M5
4868	1,4,5,6,9,10,12,16	2,3,7,8,11,13,14,15	2	Q03191,Q811M5
4869	1,4,5,6,9,10,13,14	2,3,7,8,11,12,15,16	1	Q00715
4870	1,4,5,6,9,10,13,15	2,3,7,8,11,12,14,16	5	P11883,P36860,P62804,Q00715,Q811M5
4871	1,4,5,6,9,10,13,16	2,3,7,8,11,12,14,15	2	P11883,Q811M5
4872	1,4,5,6,9,10,14,15	2,3,7,8,11,12,13,16	4	P30120,P62804,Q00715,Q811M5

4873	1,4,5,6,9,10,14,16	2,3,7,8,11,12,13,15	0	
4874	1,4,5,6,9,10,15,16	2,3,7,8,11,12,13,14	2	P11883,Q811M5
4875	1,4,5,6,9,11,12,13	2,3,7,8,10,14,15,16	6	P20766,P61206;P84079,Q03191,Q5RKI1,Q62761;Q62762;Q62763,Q64093
4876	1,4,5,6,9,11,12,14	2,3,7,8,10,13,15,16	8	P19468,P20766,P61206;P84079,Q03191,Q5RKI1,Q63424,Q64093,Q9WTW7
4877	1,4,5,6,9,11,12,15	2,3,7,8,10,13,14,16	3	P20766,Q03191,Q62761;Q62762;Q62763
4878	1,4,5,6,9,11,12,16	2,3,7,8,10,13,14,15	2	Q03191,Q5RKI1
4879	1,4,5,6,9,11,13,14	2,3,7,8,10,12,15,16	5	P20766,Q5RKI1,Q63598,Q64093,Q9WTW7
4880	1,4,5,6,9,11,13,15	2,3,7,8,10,12,14,16	1	P20766
4881	1,4,5,6,9,11,13,16	2,3,7,8,10,12,14,15	4	P20766,Q5RKI1,Q9EQS0,Q9Z0J6
4882	1,4,5,6,9,11,14,15	2,3,7,8,10,12,13,16	3	P20766,Q64093,Q923V8
4883	1,4,5,6,9,11,14,16	2,3,7,8,10,12,13,15	2	P20766,Q5RKI1
4884	1,4,5,6,9,11,15,16	2,3,7,8,10,12,13,14	1	Q8CJ52
4885	1,4,5,6,9,12,13,14	2,3,7,8,10,11,15,16	4	P20766,Q03191,Q5RKI1,Q9WTW7
4886	1,4,5,6,9,12,13,15	2,3,7,8,10,11,14,16	3	P20766,P62804,Q00715
4887	1,4,5,6,9,12,13,16	2,3,7,8,10,11,14,15	3	P20766,Q03191,Q5RKI1
4888	1,4,5,6,9,12,14,15	2,3,7,8,10,11,13,16	1	P20766
4889	1,4,5,6,9,12,14,16	2,3,7,8,10,11,13,15	3	P20766,Q03191,Q5RKI1
4890	1,4,5,6,9,12,15,16	2,3,7,8,10,11,13,14	0	
4891	1,4,5,6,9,13,14,15	2,3,7,8,10,11,12,16	3	P20766,P62804,Q00715
4892	1,4,5,6,9,13,14,16	2,3,7,8,10,11,12,15	2	P20766,Q5RKI1

4893	1,4,5,6,9,13,15,16	2,3,7,8,10,11,12,14	3	P11883,P20766,P62804
4894	1,4,5,6,9,14,15,16	2,3,7,8,10,11,12,13	2	P20766,P47967
4895	1,4,5,6,10,11,12,13	2,3,7,8,9,14,15,16	1	P35280
4896	1,4,5,6,10,11,12,14	2,3,7,8,9,13,15,16	1	P35280
4897	1,4,5,6,10,11,12,15	2,3,7,8,9,13,14,16	0	
4898	1,4,5,6,10,11,12,16	2,3,7,8,9,13,14,15	1	P35280
4899	1,4,5,6,10,11,13,14	2,3,7,8,9,12,15,16	2	P35280,P36376
4900	1,4,5,6,10,11,13,15	2,3,7,8,9,12,14,16	0	
4901	1,4,5,6,10,11,13,16	2,3,7,8,9,12,14,15	3	P07647,P35280,P36376
4902	1,4,5,6,10,11,14,15	2,3,7,8,9,12,13,16	0	
4903	1,4,5,6,10,11,14,16	2,3,7,8,9,12,13,15	2	P35280,P36376
4904	1,4,5,6,10,11,15,16	2,3,7,8,9,12,13,14	1	P17988
4905	1,4,5,6,10,12,13,14	2,3,7,8,9,11,15,16	1	P35280
4906	1,4,5,6,10,12,13,15	2,3,7,8,9,11,14,16	2	P35280,P36860
4907	1,4,5,6,10,12,13,16	2,3,7,8,9,11,14,15	2	P0DMW0;P0DMW1,P35280
4908	1,4,5,6,10,12,14,15	2,3,7,8,9,11,13,16	2	P35280,P54921
4909	1,4,5,6,10,12,14,16	2,3,7,8,9,11,13,15	1	P35280
4910	1,4,5,6,10,12,15,16	2,3,7,8,9,11,13,14	2	P0DMW0;P0DMW1,P35280
4911	1,4,5,6,10,13,14,15	2,3,7,8,9,11,12,16	4	A2RUW1,P35280,P62804,Q00715
4912	1,4,5,6,10,13,14,16	2,3,7,8,9,11,12,15	3	P35280,P36376,Q99MH3

4913	1,4,5,6,10,13,15,16	2,3,7,8,9,11,12,14	2	P11883,P35280
4914	1,4,5,6,10,14,15,16	2,3,7,8,9,11,12,13	2	P35280,P54921
4915	1,4,5,6,11,12,13,14	2,3,7,8,9,10,15,16	4	P19218,P20766,P35280,Q5RKI1
4916	1,4,5,6,11,12,13,15	2,3,7,8,9,10,14,16	1	P20766
4917	1,4,5,6,11,12,13,16	2,3,7,8,9,10,14,15	5	O35547,P07647,P20766,P35280,Q5RKI1
4918	1,4,5,6,11,12,14,15	2,3,7,8,9,10,13,16	1	P20766
4919	1,4,5,6,11,12,14,16	2,3,7,8,9,10,13,15	3	P20766,P35280,Q5RKI1
4920	1,4,5,6,11,12,15,16	2,3,7,8,9,10,13,14	1	P20766
4921	1,4,5,6,11,13,14,15	2,3,7,8,9,10,12,16	1	P20766
4922	1,4,5,6,11,13,14,16	2,3,7,8,9,10,12,15	7	O54728,P07647,P20766,P35280,P36376,Q5RKI1,Q99MH3
4923	1,4,5,6,11,13,15,16	2,3,7,8,9,10,12,14	2	O70417,P20766
4924	1,4,5,6,11,14,15,16	2,3,7,8,9,10,12,13	2	P20766,Q8CJ52
4925	1,4,5,6,12,13,14,15	2,3,7,8,9,10,11,16	2	P20766,P35280
4926	1,4,5,6,12,13,14,16	2,3,7,8,9,10,11,15	4	P20766,P35280,Q5RKI1,Q99MH3
4927	1,4,5,6,12,13,15,16	2,3,7,8,9,10,11,14	2	P20766,P35280
4928	1,4,5,6,12,14,15,16	2,3,7,8,9,10,11,13	2	P20766,P35280
4929	1,4,5,6,13,14,15,16	2,3,7,8,9,10,11,12	2	P20766,P35280
4930	1,4,5,7,8,9,10,11	2,3,6,12,13,14,15,16	8	P06760,P20646,P27590,P49134,P70545,Q63493,Q6B345,Q6P6R2
4931	1,4,5,7,8,9,10,12	2,3,6,11,13,14,15,16	5	P06760,P20646,P49134,Q03191,Q9Z1F2
4932	1,4,5,7,8,9,10,13	2,3,6,11,12,14,15,16	3	P06760,P20646,Q9Z1F2

4933	1,4,5,7,8,9,10,14	2,3,6,11,12,13,15,16	4	P06760,P20646,P20760,Q9Z1F2
4934	1,4,5,7,8,9,10,15	2,3,6,11,12,13,14,16	6	P06760,P20646,P35745,P49134,Q9Z0V6,Q9Z1F2
4935	1,4,5,7,8,9,10,16	2,3,6,11,12,13,14,15	5	P06760,P20646,P20760,P49134,P70545
4936	1,4,5,7,8,9,11,12	2,3,6,10,13,14,15,16	10	P02780,P06760,P22282,P27590,P36374,P49134,Q03191,Q63493,Q6Q7Y5,Q812E4
4937	1,4,5,7,8,9,11,13	2,3,6,10,12,14,15,16	9	P02780,P06760,P20646,P22282,P36374,P60905,P97697,Q63493,Q812E4
4938	1,4,5,7,8,9,11,14	2,3,6,10,12,13,15,16	7	P06760,P07150,P08649,P20646,Q4G075,Q63493,Q812E4
4939	1,4,5,7,8,9,11,15	2,3,6,10,12,13,14,16	12	P06760,P20646,P36374,P49134,Q4FZU2,Q63493,Q6IFU8,Q6IFW6,Q6IMF3,Q6P6Q2,Q8CJ52,Q9Z0V6
4940	1,4,5,7,8,9,11,16	2,3,6,10,12,13,14,15	23	P02780,P02781,P02782,P04905,P06760,P07647,P08649,P08723,P09456,P0C0A9,P20646,P22282,P22283,P30120,P36374,P49134,P60905,Q5M8C6,Q63493,Q6P6Q2,Q812E4,Q9JHB9,Q9Z0J6
4941	1,4,5,7,8,9,12,13	2,3,6,10,11,14,15,16	2	P06760,Q03191
4942	1,4,5,7,8,9,12,14	2,3,6,10,11,13,15,16	1	P06760
4943	1,4,5,7,8,9,12,15	2,3,6,10,11,13,14,16	4	P06760,P20646,P35745,Q9Z0V6
4944	1,4,5,7,8,9,12,16	2,3,6,10,11,13,14,15	4	P06760,P20646,P20760,Q03191
4945	1,4,5,7,8,9,13,14	2,3,6,10,11,12,15,16	2	P06760,P20646
4946	1,4,5,7,8,9,13,15	2,3,6,10,11,12,14,16	3	P06760,P20646,Q9Z0V6
4947	1,4,5,7,8,9,13,16	2,3,6,10,11,12,14,15	3	P06760,P20646,P20760
4948	1,4,5,7,8,9,14,15	2,3,6,10,11,12,13,16	3	P06760,P20646,Q9Z0V6
4949	1,4,5,7,8,9,14,16	2,3,6,10,11,12,13,15	3	P06760,P20646,P20760
4950	1,4,5,7,8,9,15,16	2,3,6,10,11,12,13,14	5	P06760,P20646,P20760,P49134,Q9Z0V6
4951	1,4,5,7,8,10,11,12	2,3,6,9,13,14,15,16	11	P02780,P04905,P06760,P22282,P27590,P36374,P47967,P49134,P97840,Q5I0D1,Q63493
4952	1,4,5,7,8,10,11,13	2,3,6,9,12,14,15,16	8	P02780,P06760,P22282,P36374,P47967,P97840,Q4G075,Q63493

4953	1,4,5,7,8,10,11,14	2,3,6,9,12,13,15,16	6	P06760,Q4G075,Q63493,Q6B345,Q6P6Q2,Q99041
4954	1,4,5,7,8,10,11,15	2,3,6,9,12,13,14,16	12	P06760,P20646,P36374,P49134,Q4FZU2,Q63493,Q6IFU8,Q6IFW6,Q6IMF3,Q6P6Q2,Q8CJ52,Q9Z0V6
4955	1,4,5,7,8,10,11,16	2,3,6,9,12,13,14,15	22	P02780,P02781,P02782,P04905,P06760,P07647,P08010,P08723,P09456,P0C0A9,P20646,P22282,P22283,P30120,P36374,P49134,Q4FZU2,Q4G075,Q5M8C6,Q63493,Q6P6Q2,Q9JHB9
4956	1,4,5,7,8,10,12,13	2,3,6,9,11,14,15,16	3	P47967,P97840,Q9Z1F2
4957	1,4,5,7,8,10,12,14	2,3,6,9,11,13,15,16	2	Q5RLM2,Q9Z1F2
4958	1,4,5,7,8,10,12,15	2,3,6,9,11,13,14,16	4	P0DMW0;P0DMW1,P10758,Q9Z0V6,Q9Z1F2
4959	1,4,5,7,8,10,12,16	2,3,6,9,11,13,14,15	3	O88267,P0DMW0;P0DMW1,P97840
4960	1,4,5,7,8,10,13,14	2,3,6,9,11,12,15,16	2	Q99MH3,Q9Z1F2
4961	1,4,5,7,8,10,13,15	2,3,6,9,11,12,14,16	4	P36860,Q63618,Q9Z0V6,Q9Z1F2
4962	1,4,5,7,8,10,13,16	2,3,6,9,11,12,14,15	4	O35077,O70257,Q8R431,Q99MH3
4963	1,4,5,7,8,10,14,15	2,3,6,9,11,12,13,16	3	P20646,P54921,Q9Z1F2
4964	1,4,5,7,8,10,14,16	2,3,6,9,11,12,13,15	5	O35077,P20646,P20760,P57113,Q99MH3
4965	1,4,5,7,8,10,15,16	2,3,6,9,11,12,13,14	11	O35077,O88267,P19468,P20646,P48508,Q03248,Q63270,Q63424,Q64093,Q8R431,Q9Z0V6
4966	1,4,5,7,8,11,12,13	2,3,6,9,10,14,15,16	17	P02780,P02781,P02782,P06760,P22273,P22282,P22283,P36374,P40241,P47967,P97840,Q4G075,Q5I0D1,Q5M8C6,Q63493,Q66H69,Q811M5
4967	1,4,5,7,8,11,12,14	2,3,6,9,10,13,15,16	10	P02780,P06760,P19218,P22282,P36374,Q4G075,Q5M8C6,Q63493,Q6P6Q2,Q811M5
4968	1,4,5,7,8,11,12,15	2,3,6,9,10,13,14,16	14	P02780,P06760,P22282,P36374,P40241,P97840,Q4FZU2,Q63493,Q6IFU8,Q6IFW6,Q6IG02,Q6IMF3,Q6P6Q2,Q8CJ52
4969	1,4,5,7,8,11,12,16	2,3,6,9,10,13,14,15	26	O35547,P02780,P02781,P02782,P04905,P06760,P07647,P08010,P08723,P09456,P0C0A9,P22273,P22282,P22283,P30120,P36374,P40241,P46462,P60905,P97840,Q4FZU2,Q4G075,Q5M8C6,Q63493,Q6P6Q2,Q9JHB9
4970	1,4,5,7,8,11,13,14	2,3,6,9,10,12,15,16	13	B0BNN3,O54728,P02780,P02782,P06760,P22282,P36374,Q4G075,Q63493,Q6P6Q2,Q811M5,Q99041,Q99MH3
4971	1,4,5,7,8,11,13,15	2,3,6,9,10,12,14,16	12	P06760,P22282,P36374,Q4FZU2,Q4G075,Q63493,Q6IFU8,Q6IFW6,Q6IG02,Q6IMF3,Q6P6Q2,Q8CJ52

4972	1,4,5,7,8,11,13,16	2,3,6,9,10,12,14,15	28	O35547,P02780,P02781,P02782,P04905,P06760,P07647,P08010,P08723,P09456,P0C0A9,P22273,P22282,P22283,P24368,P30120,P36374,P40241,P46462,P60905,Q4FZU2,Q4G075,Q5M8C6,Q63493,Q6P6Q2,Q99041,Q99MH3,Q9JHB9
4973	1,4,5,7,8,11,14,15	2,3,6,9,10,12,13,16	12	P06760,P08649,P36374,Q4FZU2,Q4G075,Q63493,Q6IFU8,Q6IFW6,Q6IG02,Q6IMF3,Q6P6Q2,Q8CJ52
4974	1,4,5,7,8,11,14,16	2,3,6,9,10,12,13,15	26	O54728,P02780,P02781,P02782,P04905,P06760,P07647,P08010,P08649,P08723,P09456,P0C0A9,P22282,P22283,P30120,P36374,P46462,Q4FZU2,Q4G075,Q5M8C6,Q63493,Q6P6Q2,Q812E4,Q99041,Q99MH3,Q9JHB9
4975	1,4,5,7,8,11,15,16	2,3,6,9,10,12,13,14	30	P02780,P02781,P02782,P04905,P06760,P07647,P08010,P08649,P08723,P09456,P0C0A9,P20646,P22282,P22283,P30120,P36374,P40241,P60905,Q4FZU2,Q4G075,Q5M8C6,Q63493,Q6IFU8,Q6IFW6,Q6IG02,Q6IMF3,Q6P6Q2,Q8CJ52,Q9JHB9,Q9Z0V6
4976	1,4,5,7,8,12,13,14	2,3,6,9,10,11,15,16	3	Q811M5,Q99MH3,Q9Z2L0
4977	1,4,5,7,8,12,13,15	2,3,6,9,10,11,14,16	2	P97840,Q9Z2L0
4978	1,4,5,7,8,12,13,16	2,3,6,9,10,11,14,15	4	P02781,P97840,Q99MH3,Q9Z2L0
4979	1,4,5,7,8,12,14,15	2,3,6,9,10,11,13,16	1	Q9Z2L0
4980	1,4,5,7,8,12,14,16	2,3,6,9,10,11,13,15	1	Q99MH3
4981	1,4,5,7,8,12,15,16	2,3,6,9,10,11,13,14	1	P10758
4982	1,4,5,7,8,13,14,15	2,3,6,9,10,11,12,16	1	Q9Z2L0
4983	1,4,5,7,8,13,14,16	2,3,6,9,10,11,12,15	4	B0BNN3,O54728,Q99MH3,Q9Z2L0
4984	1,4,5,7,8,13,15,16	2,3,6,9,10,11,12,14	3	O35077,P20646,Q99MH3
4985	1,4,5,7,8,14,15,16	2,3,6,9,10,11,12,13	3	O35077,P20646,Q99MH3
4986	1,4,5,7,9,10,11,12	2,3,6,8,13,14,15,16	2	Q03191,Q62761;Q62762;Q62763
4987	1,4,5,7,9,10,11,13	2,3,6,8,12,14,15,16	0	
4988	1,4,5,7,9,10,11,14	2,3,6,8,12,13,15,16	0	
4989	1,4,5,7,9,10,11,15	2,3,6,8,12,13,14,16	0	
4990	1,4,5,7,9,10,11,16	2,3,6,8,12,13,14,15	1	P49134

4991	1,4,5,7,9,10,12,13	2,3,6,8,11,14,15,16	1	Q03191
4992	1,4,5,7,9,10,12,14	2,3,6,8,11,13,15,16	1	Q03191
4993	1,4,5,7,9,10,12,15	2,3,6,8,11,13,14,16	2	P10758,Q03191
4994	1,4,5,7,9,10,12,16	2,3,6,8,11,13,14,15	1	Q03191
4995	1,4,5,7,9,10,13,14	2,3,6,8,11,12,15,16	0	
4996	1,4,5,7,9,10,13,15	2,3,6,8,11,12,14,16	1	Q00715
4997	1,4,5,7,9,10,13,16	2,3,6,8,11,12,14,15	0	
4998	1,4,5,7,9,10,14,15	2,3,6,8,11,12,13,16	0	
4999	1,4,5,7,9,10,14,16	2,3,6,8,11,12,13,15	0	
5000	1,4,5,7,9,10,15,16	2,3,6,8,11,12,13,14	1	P10758
5001	1,4,5,7,9,11,12,13	2,3,6,8,10,14,15,16	4	P97840,Q03191,Q5I0D1,Q62761;Q62762;Q62763
5002	1,4,5,7,9,11,12,14	2,3,6,8,10,13,15,16	2	Q03191,Q62761;Q62762;Q62763
5003	1,4,5,7,9,11,12,15	2,3,6,8,10,13,14,16	2	Q03191,Q62761;Q62762;Q62763
5004	1,4,5,7,9,11,12,16	2,3,6,8,10,13,14,15	2	P63322,Q03191
5005	1,4,5,7,9,11,13,14	2,3,6,8,10,12,15,16	0	
5006	1,4,5,7,9,11,13,15	2,3,6,8,10,12,14,16	0	
5007	1,4,5,7,9,11,13,16	2,3,6,8,10,12,14,15	2	Q9R168,Q9Z0J6
5008	1,4,5,7,9,11,14,15	2,3,6,8,10,12,13,16	2	P02625,Q8CJ52
5009	1,4,5,7,9,11,14,16	2,3,6,8,10,12,13,15	1	Q9R168
5010	1,4,5,7,9,11,15,16	2,3,6,8,10,12,13,14	1	Q8CJ52

5011	1,4,5,7,9,12,13,14	2,3,6,8,10,11,15,16	1	Q03191
5012	1,4,5,7,9,12,13,15	2,3,6,8,10,11,14,16	1	Q03191
5013	1,4,5,7,9,12,13,16	2,3,6,8,10,11,14,15	1	Q03191
5014	1,4,5,7,9,12,14,15	2,3,6,8,10,11,13,16	0	
5015	1,4,5,7,9,12,14,16	2,3,6,8,10,11,13,15	2	Q03191,Q68G31
5016	1,4,5,7,9,12,15,16	2,3,6,8,10,11,13,14	3	P10758,Q03191,Q68G31
5017	1,4,5,7,9,13,14,15	2,3,6,8,10,11,12,16	2	P02625,Q00715
5018	1,4,5,7,9,13,14,16	2,3,6,8,10,11,12,15	2	Q99MH3,Q9R168
5019	1,4,5,7,9,13,15,16	2,3,6,8,10,11,12,14	0	
5020	1,4,5,7,9,14,15,16	2,3,6,8,10,11,12,13	0	
5021	1,4,5,7,10,11,12,13	2,3,6,8,9,14,15,16	4	P23593,P47967,P97840,Q5I0D1
5022	1,4,5,7,10,11,12,14	2,3,6,8,9,13,15,16	0	
5023	1,4,5,7,10,11,12,15	2,3,6,8,9,13,14,16	1	P97840
5024	1,4,5,7,10,11,12,16	2,3,6,8,9,13,14,15	1	P97840
5025	1,4,5,7,10,11,13,14	2,3,6,8,9,12,15,16	2	P36376,Q99MH3
5026	1,4,5,7,10,11,13,15	2,3,6,8,9,12,14,16	1	P97840
5027	1,4,5,7,10,11,13,16	2,3,6,8,9,12,14,15	3	P36376,P97840,Q99MH3
5028	1,4,5,7,10,11,14,15	2,3,6,8,9,12,13,16	0	
5029	1,4,5,7,10,11,14,16	2,3,6,8,9,12,13,15	2	P36376,Q99MH3
5030	1,4,5,7,10,11,15,16	2,3,6,8,9,12,13,14	4	P17988,Q4FZU2,Q6P6Q2,Q8CJ52

5031	1,4,5,7,10,12,13,14	2,3,6,8,9,11,15,16	2	Q6P6S4,Q99MH3
5032	1,4,5,7,10,12,13,15	2,3,6,8,9,11,14,16	2	P10758,P97840
5033	1,4,5,7,10,12,13,16	2,3,6,8,9,11,14,15	3	P97840,Q6P6S4,Q99MH3
5034	1,4,5,7,10,12,14,15	2,3,6,8,9,11,13,16	0	
5035	1,4,5,7,10,12,14,16	2,3,6,8,9,11,13,15	2	Q6P6S4,Q99MH3
5036	1,4,5,7,10,12,15,16	2,3,6,8,9,11,13,14	3	P0DMW0;P0DMW1,P10758,P97840
5037	1,4,5,7,10,13,14,15	2,3,6,8,9,11,12,16	0	
5038	1,4,5,7,10,13,14,16	2,3,6,8,9,11,12,15	4	P09527,P36376,Q6P6S4,Q99MH3
5039	1,4,5,7,10,13,15,16	2,3,6,8,9,11,12,14	3	P09527,P11883,Q99MH3
5040	1,4,5,7,10,14,15,16	2,3,6,8,9,11,12,13	3	P09527,Q99MH3,Q9QYP1
5041	1,4,5,7,11,12,13,14	2,3,6,8,9,10,15,16	3	Q5I0D1,Q811M5,Q99MH3
5042	1,4,5,7,11,12,13,15	2,3,6,8,9,10,14,16	1	P97840
5043	1,4,5,7,11,12,13,16	2,3,6,8,9,10,14,15	4	P36374,P47967,P97840,Q99MH3
5044	1,4,5,7,11,12,14,15	2,3,6,8,9,10,13,16	0	
5045	1,4,5,7,11,12,14,16	2,3,6,8,9,10,13,15	1	Q99MH3
5046	1,4,5,7,11,12,15,16	2,3,6,8,9,10,13,14	5	P36374,P97840,Q4FZU2,Q6P6Q2,Q8CJ52
5047	1,4,5,7,11,13,14,15	2,3,6,8,9,10,12,16	0	
5048	1,4,5,7,11,13,14,16	2,3,6,8,9,10,12,15	3	O54728,P36376,Q99MH3
5049	1,4,5,7,11,13,15,16	2,3,6,8,9,10,12,14	4	Q4FZU2,Q6P6Q2,Q8CJ52,Q99MH3
5050	1,4,5,7,11,14,15,16	2,3,6,8,9,10,12,13	5	P17559,Q4FZU2,Q6P6Q2,Q8CJ52,Q99MH3

5051	1,4,5,7,12,13,14,15	2,3,6,8,9,10,11,16	2	Q6P6S4,Q9Z2L0
5052	1,4,5,7,12,13,14,16	2,3,6,8,9,10,11,15	2	Q6P6S4,Q99MH3
5053	1,4,5,7,12,13,15,16	2,3,6,8,9,10,11,14	3	P10758,P97840,Q99MH3
5054	1,4,5,7,12,14,15,16	2,3,6,8,9,10,11,13	1	Q99MH3
5055	1,4,5,7,13,14,15,16	2,3,6,8,9,10,11,12	2	Q6P6S4,Q99MH3
5056	1,4,5,8,9,10,11,12	2,3,6,7,13,14,15,16	4	P01835,P49134,Q03191,Q62714
5057	1,4,5,8,9,10,11,13	2,3,6,7,12,14,15,16	0	
5058	1,4,5,8,9,10,11,14	2,3,6,7,12,13,15,16	1	P01835
5059	1,4,5,8,9,10,11,15	2,3,6,7,12,13,14,16	7	P01835,P49134,Q4FZU2,Q6IFU8,Q6IFW6,Q6IMF3,Q6P6Q2
5060	1,4,5,8,9,10,11,16	2,3,6,7,12,13,14,15	4	O88797,P01835,P49134,Q6AY61
5061	1,4,5,8,9,10,12,13	2,3,6,7,11,14,15,16	1	Q03191
5062	1,4,5,8,9,10,12,14	2,3,6,7,11,13,15,16	0	
5063	1,4,5,8,9,10,12,15	2,3,6,7,11,13,14,16	0	
5064	1,4,5,8,9,10,12,16	2,3,6,7,11,13,14,15	0	
5065	1,4,5,8,9,10,13,14	2,3,6,7,11,12,15,16	0	
5066	1,4,5,8,9,10,13,15	2,3,6,7,11,12,14,16	1	Q00715
5067	1,4,5,8,9,10,13,16	2,3,6,7,11,12,14,15	0	
5068	1,4,5,8,9,10,14,15	2,3,6,7,11,12,13,16	1	P05369
5069	1,4,5,8,9,10,14,16	2,3,6,7,11,12,13,15	1	P20760
5070	1,4,5,8,9,10,15,16	2,3,6,7,11,12,13,14	0	

5071	1,4,5,8,9,11,12,13	2,3,6,7,10,14,15,16	6	P18297,P20766,P60905,P63095,Q03191,Q5RKI1
5072	1,4,5,8,9,11,12,14	2,3,6,7,10,13,15,16	7	P01835,P18297,P20766,P63095,Q03191,Q5RKI1,Q6Q7Y5
5073	1,4,5,8,9,11,12,15	2,3,6,7,10,13,14,16	8	P01835,P20766,Q03191,Q4FZU2,Q6IFU8,Q6IFW6,Q6IMF3,Q6P6Q2
5074	1,4,5,8,9,11,12,16	2,3,6,7,10,13,14,15	7	O88797,P01835,P04905,P36374,P60905,Q03191,Q5RKI1
5075	1,4,5,8,9,11,13,14	2,3,6,7,10,12,15,16	3	O54728,P20766,Q5RKI1
5076	1,4,5,8,9,11,13,15	2,3,6,7,10,12,14,16	5	P20766,Q4FZU2,Q6IFU8,Q6IMF3,Q6P6Q2
5077	1,4,5,8,9,11,13,16	2,3,6,7,10,12,14,15	5	P02782,P30120,P60905,Q5RKI1,Q6AY61
5078	1,4,5,8,9,11,14,15	2,3,6,7,10,12,13,16	6	P20766,Q4FZU2,Q6IFU8,Q6IFW6,Q6IMF3,Q6P6Q2
5079	1,4,5,8,9,11,14,16	2,3,6,7,10,12,13,15	7	O54728,O88797,P20766,P60905,Q5RKI1,Q6AY61,Q812E4
5080	1,4,5,8,9,11,15,16	2,3,6,7,10,12,13,14	8	P01835,P49134,P60905,Q4FZU2,Q6IFU8,Q6IFW6,Q6IMF3,Q6P6Q2
5081	1,4,5,8,9,12,13,14	2,3,6,7,10,11,15,16	2	P20766,Q5RKI1
5082	1,4,5,8,9,12,13,15	2,3,6,7,10,11,14,16	1	P20766
5083	1,4,5,8,9,12,13,16	2,3,6,7,10,11,14,15	2	P20766,Q5RKI1
5084	1,4,5,8,9,12,14,15	2,3,6,7,10,11,13,16	1	P20766
5085	1,4,5,8,9,12,14,16	2,3,6,7,10,11,13,15	2	P20766,Q5RKI1
5086	1,4,5,8,9,12,15,16	2,3,6,7,10,11,13,14	0	
5087	1,4,5,8,9,13,14,15	2,3,6,7,10,11,12,16	2	P20766,Q00715
5088	1,4,5,8,9,13,14,16	2,3,6,7,10,11,12,15	3	O54728,P20766,Q5RKI1
5089	1,4,5,8,9,13,15,16	2,3,6,7,10,11,12,14	1	P20766
5090	1,4,5,8,9,14,15,16	2,3,6,7,10,11,12,13	3	P20766,P23593,P47967

5091	1,4,5,8,10,<br>11,12,13	2,3,6,7,9,1<br>4,15,16	1 Q5I0D1
5092	1,4,5,8,10,<br>11,12,14	2,3,6,7,9,1<br>3,15,16	0
5093	1,4,5,8,10,<br>11,12,15	2,3,6,7,9,1<br>3,14,16	5 Q4FZU2,Q6IFU8,Q6IFW6,Q6IMF3,Q6P6Q2
5094	1,4,5,8,10,<br>11,12,16	2,3,6,7,9,1<br>3,14,15	3 P04905,P0DMW0;P0DMW1,P36374
5095	1,4,5,8,10,<br>11,13,14	2,3,6,7,9,1<br>2,15,16	1 O54728
5096	1,4,5,8,10,<br>11,13,15	2,3,6,7,9,1<br>2,14,16	5 Q4FZU2,Q6IFU8,Q6IFW6,Q6IMF3,Q6P6Q2
5097	1,4,5,8,10,<br>11,13,16	2,3,6,7,9,1<br>2,14,15	2 P02782,P30120
5098	1,4,5,8,10,<br>11,14,15	2,3,6,7,9,1<br>2,13,16	5 Q4FZU2,Q6IFU8,Q6IFW6,Q6IMF3,Q6P6Q2
5099	1,4,5,8,10,<br>11,14,16	2,3,6,7,9,1<br>2,13,15	1 O54728
5100	1,4,5,8,10,<br>11,15,16	2,3,6,7,9,1<br>2,13,14	5 Q4FZU2,Q6IFU8,Q6IFW6,Q6IMF3,Q6P6Q2
5101	1,4,5,8,10,<br>12,13,14	2,3,6,7,9,1<br>1,15,16	1 P35280
5102	1,4,5,8,10,<br>12,13,15	2,3,6,7,9,1<br>1,14,16	0
5103	1,4,5,8,10,<br>12,13,16	2,3,6,7,9,1<br>1,14,15	2 P0DMW0;P0DMW1,P35280
5104	1,4,5,8,10,<br>12,14,15	2,3,6,7,9,1<br>1,13,16	1 P54921
5105	1,4,5,8,10,<br>12,14,16	2,3,6,7,9,1<br>1,13,15	1 P35280
5106	1,4,5,8,10,<br>12,15,16	2,3,6,7,9,1<br>1,13,14	2 P0DMW0;P0DMW1,P10758
5107	1,4,5,8,10,<br>13,14,15	2,3,6,7,9,1<br>1,12,16	0
5108	1,4,5,8,10,<br>13,14,16	2,3,6,7,9,1<br>1,12,15	3 O54728,P35280,Q99MH3
5109	1,4,5,8,10,<br>13,15,16	2,3,6,7,9,1<br>1,12,14	1 O35077
5110	1,4,5,8,10,<br>14,15,16	2,3,6,7,9,1<br>1,12,13	2 O35077,P54921

5111	1,4,5,8,11,12,13,14	2,3,6,7,9,10,15,16	5	O54728,P20766,P23739,Q5RKI1,Q811M5
5112	1,4,5,8,11,12,13,15	2,3,6,7,9,10,14,16	7	P20766,Q4FZU2,Q6IFU8,Q6IFW6,Q6IG02,Q6IMF3,Q6P6Q2
5113	1,4,5,8,11,12,13,16	2,3,6,7,9,10,14,15	6	P02782,P20766,P30120,P36374,P60905,Q5RKI1
5114	1,4,5,8,11,12,14,15	2,3,6,7,9,10,13,16	6	P20766,Q4FZU2,Q6IFU8,Q6IFW6,Q6IMF3,Q6P6Q2
5115	1,4,5,8,11,12,14,16	2,3,6,7,9,10,13,15	7	O54728,P02782,P04905,P20766,P36374,Q5RKI1,Q99MH3
5116	1,4,5,8,11,12,15,16	2,3,6,7,9,10,13,14	7	P36374,Q4FZU2,Q6IFU8,Q6IFW6,Q6IG02,Q6IMF3,Q6P6Q2
5117	1,4,5,8,11,13,14,15	2,3,6,7,9,10,12,16	9	O54728,P20766,P23739,Q4FZU2,Q6IFU8,Q6IFW6,Q6IG02,Q6IMF3,Q6P6Q2
5118	1,4,5,8,11,13,14,16	2,3,6,7,9,10,12,15	7	O54728,P02782,P20766,P30120,P60905,Q5RKI1,Q99MH3
5119	1,4,5,8,11,13,15,16	2,3,6,7,9,10,12,14	10	P02782,P20766,P30120,P60905,Q4FZU2,Q6IFU8,Q6IFW6,Q6IG02,Q6IMF3,Q6P6Q2
5120	1,4,5,8,11,14,15,16	2,3,6,7,9,10,12,13	8	O54728,P20766,Q4FZU2,Q6IFU8,Q6IFW6,Q6IG02,Q6IMF3,Q6P6Q2
5121	1,4,5,8,12,13,14,15	2,3,6,7,9,10,11,16	2	P20766,P23739
5122	1,4,5,8,12,13,14,16	2,3,6,7,9,10,11,15	5	O54728,P20766,P35280,Q5RKI1,Q99MH3
5123	1,4,5,8,12,13,15,16	2,3,6,7,9,10,11,14	1	P20766
5124	1,4,5,8,12,14,15,16	2,3,6,7,9,10,11,13	1	P20766
5125	1,4,5,8,13,14,15,16	2,3,6,7,9,10,11,12	3	O54728,P20766,Q99MH3
5126	1,4,5,9,10,11,12,13	2,3,6,7,8,14,15,16	4	B0LT89,Q03191,Q62714,Q62761;Q62762;Q62763
5127	1,4,5,9,10,11,12,14	2,3,6,7,8,13,15,16	3	Q03191,Q62714,Q62761;Q62762;Q62763
5128	1,4,5,9,10,11,12,15	2,3,6,7,8,13,14,16	3	Q03191,Q62714,Q62761;Q62762;Q62763
5129	1,4,5,9,10,11,12,16	2,3,6,7,8,13,14,15	2	Q03191,Q62714
5130	1,4,5,9,10,11,13,14	2,3,6,7,8,12,15,16	0	

5131	1,4,5,9,10,11,13,15	2,3,6,7,8,12,14,16	0	
5132	1,4,5,9,10,11,13,16	2,3,6,7,8,12,14,15	0	
5133	1,4,5,9,10,11,14,15	2,3,6,7,8,12,13,16	0	
5134	1,4,5,9,10,11,14,16	2,3,6,7,8,12,13,15	0	
5135	1,4,5,9,10,11,15,16	2,3,6,7,8,12,13,14	0	
5136	1,4,5,9,10,12,13,14	2,3,6,7,8,11,15,16	3	P50280,Q03191,Q9QZK9
5137	1,4,5,9,10,12,13,15	2,3,6,7,8,11,14,16	3	P50280,Q00715,Q03191
5138	1,4,5,9,10,12,13,16	2,3,6,7,8,11,14,15	2	P50280,Q03191
5139	1,4,5,9,10,12,14,15	2,3,6,7,8,11,13,16	3	P09456,P30120,Q00715
5140	1,4,5,9,10,12,14,16	2,3,6,7,8,11,13,15	1	Q03191
5141	1,4,5,9,10,12,15,16	2,3,6,7,8,11,13,14	1	P10758
5142	1,4,5,9,10,13,14,15	2,3,6,7,8,11,12,16	3	P12020,P62804,Q00715
5143	1,4,5,9,10,13,14,16	2,3,6,7,8,11,12,15	1	P12020
5144	1,4,5,9,10,13,15,16	2,3,6,7,8,11,12,14	3	P11883,P12020,Q00715
5145	1,4,5,9,10,14,15,16	2,3,6,7,8,11,12,13	2	P12020,P47967
5146	1,4,5,9,11,12,13,14	2,3,6,7,8,10,15,16	34	B0LT89,O70377,O70594,O88339;Q4V882,P19468,P20766,P29975,P38918,P46844,P48508,P51907,P53790,P57113,P97605,Q03191,Q03248,Q05175,Q3T1J9,Q3ZAV1,Q5I0E9,Q5M7T9,Q5RKI1,Q62761;Q62762;Q62763,Q63270,Q63424,Q63598,Q64093,Q6AY41,Q6Q0N1,Q80W57,Q923S2,Q9R1T5,Q9WTW7,Q9Z0W7
5147	1,4,5,9,11,12,13,15	2,3,6,7,8,10,14,16	8	P20766,P46844,P53790,P57113,Q03191,Q05175,Q62761;Q62762;Q62763,Q923S2
5148	1,4,5,9,11,12,13,16	2,3,6,7,8,10,14,15	8	O70377,O88339;Q4V882,P20766,P54921,Q03191,Q05175,Q5RKI1,Q62714
5149	1,4,5,9,11,12,14,15	2,3,6,7,8,10,13,16	18	O70257,O70594,P02631,P19468,P20766,P28570,P46844,P53790,P57113,Q03191,Q3ZAV1,Q62761;Q62762;Q62763,Q63424,Q63598,Q63618,Q64093,Q6AY41,Q9WTW7

5150	1,4,5,9,11,12,14,16	2,3,6,7,8,10,13,15	12	O70377,O70594,O88339;Q4V882,P02631,P20766,P51907,Q03191,Q05175,Q5RKI1,Q62714,Q63598,Q63618
5151	1,4,5,9,11,12,15,16	2,3,6,7,8,10,13,14	5	P02631,P20766,P25809,Q03191,Q5QE79
5152	1,4,5,9,11,13,14,15	2,3,6,7,8,10,12,16	10	P02631,P0DMW0;P0DMW1,P20766,P46844,P53790,P57113,Q3ZAV1,Q63598,Q6AY41,Q9WTW7
5153	1,4,5,9,11,13,14,16	2,3,6,7,8,10,12,15	7	O54728,P02631,P20766,Q05175,Q5RKI1,Q63598,Q9WTW7
5154	1,4,5,9,11,13,15,16	2,3,6,7,8,10,12,14	4	O70417,P02631,P20766,Q5RLM2
5155	1,4,5,9,11,14,15,16	2,3,6,7,8,10,12,13	3	P02631,P20766,Q63598
5156	1,4,5,9,12,13,14,15	2,3,6,7,8,10,11,16	3	P20766,Q00715,Q9WTW7
5157	1,4,5,9,12,13,14,16	2,3,6,7,8,10,11,15	3	P20766,Q03191,Q5RKI1
5158	1,4,5,9,12,13,15,16	2,3,6,7,8,10,11,14	1	P20766
5159	1,4,5,9,12,14,15,16	2,3,6,7,8,10,11,13	2	P02631,P20766
5160	1,4,5,9,13,14,15,16	2,3,6,7,8,10,11,12	4	P02631,P12020,P20766,Q00715
5161	1,4,5,10,11,12,13,14	2,3,6,7,8,9,15,16	1	P35280
5162	1,4,5,10,11,12,13,15	2,3,6,7,8,9,14,16	1	Q5QE79
5163	1,4,5,10,11,12,13,16	2,3,6,7,8,9,14,15	2	P35280,P97840
5164	1,4,5,10,11,12,14,15	2,3,6,7,8,9,13,16	0	
5165	1,4,5,10,11,12,14,16	2,3,6,7,8,9,13,15	1	P35280
5166	1,4,5,10,11,12,15,16	2,3,6,7,8,9,13,14	1	Q5QE79
5167	1,4,5,10,11,13,14,15	2,3,6,7,8,9,12,16	0	
5168	1,4,5,10,11,13,14,16	2,3,6,7,8,9,12,15	4	O54728,P35280,P36376,Q99MH3
5169	1,4,5,10,11,13,15,16	2,3,6,7,8,9,12,14	2	O70417,Q5QE79

5170	1,4,5,10,11,14,15,16	2,3,6,7,8,9,12,13	0	
5171	1,4,5,10,12,13,14,15	2,3,6,7,8,9,11,16	2	P35280,Q6P6S4
5172	1,4,5,10,12,13,14,16	2,3,6,7,8,9,11,15	3	P35280,Q6P6S4,Q99MH3
5173	1,4,5,10,12,13,15,16	2,3,6,7,8,9,11,14	4	P10758,P35280,Q5QE79,Q63751
5174	1,4,5,10,12,14,15,16	2,3,6,7,8,9,11,13	2	P35280,Q6P6S4
5175	1,4,5,10,13,14,15,16	2,3,6,7,8,9,11,12	4	P35280,P70709,Q6P6S4,Q99MH3
5176	1,4,5,11,12,13,14,15	2,3,6,7,8,9,10,16	4	P02631,P20766,P23739,P70545
5177	1,4,5,11,12,13,14,16	2,3,6,7,8,9,10,15	9	O54728,O88339;Q4V882,P20766,P23739,P35280,Q05175,Q5RKI1,Q63598,Q99MH3
5178	1,4,5,11,12,13,15,16	2,3,6,7,8,9,10,14	4	O70417,P20766,P23739,Q5QE79
5179	1,4,5,11,12,14,15,16	2,3,6,7,8,9,10,13	2	P02631,P20766
5180	1,4,5,11,13,14,15,16	2,3,6,7,8,9,10,12	5	O54728,P02631,P20766,P23739,Q99MH3
5181	1,4,5,12,13,14,15,16	2,3,6,7,8,9,10,11	6	P20766,P23739,P35280,Q6P6R2,Q6P6S4,Q99MH3
5182	1,4,6,7,8,9,10,11	2,3,5,12,13,14,15,16	31	P02780,P02781,P04905,P06761,P06911,P07150,P08723,P0C0A9,P11598,P18418,P22273,P22282,P22283,P36374,P46462,P49134,Q5GRG2,Q5M8C6,Q63493,Q63617,Q6AY61,Q6AYR9,Q6P6R2,Q6P6S4,Q812E4,Q99041,Q9JHB9,Q9JI85,Q9QW07,Q9R0T3,Q9Z1F2
5183	1,4,6,7,8,9,10,12	2,3,5,11,13,14,15,16	6	P06761,P06911,P18418,P19629,P21674,Q9Z1F2
5184	1,4,6,7,8,9,10,13	2,3,5,11,12,14,15,16	4	P02631,P06761,P18418,Q9Z1F2
5185	1,4,6,7,8,9,10,14	2,3,5,11,12,13,15,16	4	O70417,P18418,Q6P6R2,Q9Z1F2
5186	1,4,6,7,8,9,10,15	2,3,5,11,12,13,14,16	8	P18418,P19629,P20646,Q63474,Q63598,Q6P6R2,Q9Z0V6,Q9Z1F2
5187	1,4,6,7,8,9,10,16	2,3,5,11,12,13,14,15	7	P06761,P07647,P18418,P19629,P20646,P22006,P70545
5188	1,4,6,7,8,9,11,12	2,3,5,10,13,14,15,16	33	P02780,P02781,P02782,P04905,P06761,P06911,P07150,P08723,P0C0A9,P11598,P12020,P21674,P22273,P22282,P22283,P36374,P40241,P46462,P60905,Q09326,Q5GRG2,Q5M8C6,Q63493,Q63617,Q6AYR9,Q6P6S4,Q6Q7Y5,Q78P75,Q812E4,Q9JHB9,Q9JI85,Q9QW07,Q9R0T3

5189	1,4,6,7,8,9,11,13	2,3,5,10,12,14,15,16	34	P02780,P02781,P02782,P06761,P06911,P07647,P08723,P09456,P0C0A9,P11598,P18418,P22273,P22282,P22283,P36374,P40241,P46462,P60905,Q09326,Q5GRG2,Q5M8C6,Q62902,Q63493,Q63617,Q6AY61,Q6AYR9,Q6Q7Y5,Q78P75,Q812E4,Q99041,Q9JHB9,Q9JI85,Q9QW07,Q9R0T3
5190	1,4,6,7,8,9,11,14	2,3,5,10,12,13,15,16	33	P02780,P02781,P02782,P06761,P06911,P07150,P08723,P09456,P0C0A9,P11598,P12020,P22273,P22282,P22283,P36374,P46462,P50280,P60905,Q09326,Q5GRG2,Q5M8C6,Q63493,Q63617,Q6AY61,Q6AYR9,Q6P6R2,Q6Q7Y5,Q78P75,Q812E4,Q99041,Q9JHB9,Q9JI85,Q9QW07
5191	1,4,6,7,8,9,11,15	2,3,5,10,12,13,14,16	30	P02780,P02781,P06761,P06911,P07150,P08723,P0C0A9,P11598,P22273,P22282,P22283,P36374,P46462,P50280,P60905,Q5GRG2,Q5M8C6,Q63493,Q63617,Q6AY61,Q6AYR9,Q6P6R2,Q6P6S4,Q78P75,Q812E4,Q8CJ52,Q99041,Q9JHB9,Q9JI85,Q9QW07
5192	1,4,6,7,8,9,11,16	2,3,5,10,12,13,14,15	43	O35547,P02780,P02781,P02782,P04905,P06761,P06911,P07150,P07647,P08723,P09456,P0C0A9,P11598,P18418,P22006,P22273,P22282,P22283,P24368,P30120,P36374,P40241,P46462,P50280,P60905,P97523,Q5GRG2,Q5M8C6,Q62902,Q63493,Q63617,Q6AY61,Q6AYR9,Q6P6S4,Q6Q7Y5,Q6RUV5,Q812E4,Q8CFN2,Q99041,Q9JHB9,Q9JI85,Q9QW07,Q9R0T3
5193	1,4,6,7,8,9,12,13	2,3,5,10,11,14,15,16	3	P06761,P06911,Q9JI85
5194	1,4,6,7,8,9,12,14	2,3,5,10,11,13,15,16	4	O70417,P06761,P06911,P21674
5195	1,4,6,7,8,9,12,15	2,3,5,10,11,13,14,16	2	P06911,P19629
5196	1,4,6,7,8,9,12,16	2,3,5,10,11,13,14,15	10	P02781,P06761,P06911,P07647,P18418,P21674,P22006,P22282,Q9JHB9,Q9QW07
5197	1,4,6,7,8,9,13,14	2,3,5,10,11,12,15,16	3	P06761,P06911,Q5QE79
5198	1,4,6,7,8,9,13,15	2,3,5,10,11,12,14,16	1	P70549
5199	1,4,6,7,8,9,13,16	2,3,5,10,11,12,14,15	10	P02781,P02782,P06761,P07647,P18418,P22006,P22282,P22283,Q63617,Q9JI85
5200	1,4,6,7,8,9,14,15	2,3,5,10,11,12,13,16	1	P20646
5201	1,4,6,7,8,9,14,16	2,3,5,10,11,12,13,15	8	P02781,P06761,P06911,P07647,P18418,P21674,P22006,P23593
5202	1,4,6,7,8,9,15,16	2,3,5,10,11,12,13,14	9	P06761,P07647,P18418,P19629,P20646,P22006,P23593,Q8CJ52,Q9QW07
5203	1,4,6,7,8,10,11,12	2,3,5,9,13,14,15,16	30	P02780,P02781,P02782,P04905,P06761,P06911,P07647,P08723,P0C0A9,P11598,P12020,P18418,P19629,P22273,P22282,P22283,P31044,P36374,P40241,P46462,Q5GRG2,Q5M8C6,Q63493,Q63617,Q99041,Q9JHB9,Q9JI85,Q9QW07,Q9R0T3,Q9Z1F2
5204	1,4,6,7,8,10,11,13	2,3,5,9,12,14,15,16	27	P00714,P02780,P02781,P02782,P06761,P06911,P07647,P08723,P0C0A9,P11598,P18418,P22273,P22282,P22283,P36374,P40241,P46462,Q5GRG2,Q5M8C6,Q63493,Q63617,Q6AYR9,Q99041,Q9JHB9,Q9JI85,Q9R0T3,Q9Z1F2

5205	1,4,6,7,8,10,11,14	2,3,5,9,12,13,15,16	28	P02780,P02781,P02782,P04905,P06761,P06911,P07150,P08723,P0C0A9,P11598,P12020,P18418,P22273,P22282,P22283,P36374,P46462,P50280,Q5GRG2,Q5M8C6,Q63493,Q63617,Q99041,Q9JHB9,Q9JI85,Q9QW07,Q9R0T3,Q9Z1F2
5206	1,4,6,7,8,10,11,15	2,3,5,9,12,13,14,16	26	P02780,P02781,P06761,P06911,P08723,P0C0A9,P11598,P18418,P19629,P22273,P22282,P22283,P36374,P46462,P50280,Q5GRG2,Q5M8C6,Q63474,Q63493,Q63617,Q8CJ52,Q99041,Q9JHB9,Q9JI85,Q9QW07,Q9Z1F2
5207	1,4,6,7,8,10,11,16	2,3,5,9,12,13,14,15	41	O35547,O88917,P00714,P02780,P02781,P02782,P04905,P06761,P06911,P07151,P07647,P08010,P08723,P09456,P0C0A9,P11598,P15399,P18418,P19629,P22273,P22282,P22283,P24368,P30120,P36374,P40241,P46462,P50280,P60905,Q4G063,Q5GRG2,Q5M8C6,Q63493,Q63617,Q6AY61,Q812E4,Q99041,Q9JHB9,Q9JI85,Q9QW07,Q9R0T3
5208	1,4,6,7,8,10,12,13	2,3,5,9,11,14,15,16	8	P02631,P06761,P06911,P18418,P19629,P70549,Q9JI85,Q9Z1F2
5209	1,4,6,7,8,10,12,14	2,3,5,9,11,13,15,16	6	O70417,P06761,P06911,P18418,P57113,Q9Z1F2
5210	1,4,6,7,8,10,12,15	2,3,5,9,11,13,14,16	7	P06911,P10758,P18418,P19629,Q63474,Q63598,Q9Z1F2
5211	1,4,6,7,8,10,12,16	2,3,5,9,11,13,14,15	18	P02781,P06761,P06911,P07151,P07647,P0DMW0;P0DMW1,P10758,P18418,P19629,P21674,P22282,P22283,P46844,P57113,Q03248,Q5M8C6,Q63598,Q9JHB9
5212	1,4,6,7,8,10,13,14	2,3,5,9,11,12,15,16	7	P06761,P06911,P18418,P57113,P70549,Q9Z1F2,Q9Z2L0
5213	1,4,6,7,8,10,13,15	2,3,5,9,11,12,14,16	12	O70257,P06761,P18418,P19629,P36860,P51907,P57113,P70549,Q63474,Q63598,Q63618,Q9Z1F2
5214	1,4,6,7,8,10,13,16	2,3,5,9,11,12,14,15	21	O35956,O55004,O70257,P02631,P02781,P02782,P06761,P07151,P07647,P11598,P18418,P19468,P19629,P22282,P22283,P46844,P57113,Q3ZAV1,Q63424,Q63598,Q9JI85
5215	1,4,6,7,8,10,14,15	2,3,5,9,11,12,13,16	6	P18418,P19629,P54921,P57113,Q63474,Q9Z1F2
5216	1,4,6,7,8,10,14,16	2,3,5,9,11,12,13,15	10	P02781,P06761,P06911,P07151,P07647,P18418,P19629,P57113,Q03248,Q99MH3
5217	1,4,6,7,8,10,15,16	2,3,5,9,11,12,13,14	31	O55004,O70257,O70594,P02781,P06761,P07151,P07647,P10758,P15399,P17988,P18418,P19468,P19629,P46844,P48508,P51907,P53790,P57113,Q03248,Q05175,Q3ZAV1,Q4QQT4,Q5M7T9,Q63270,Q63424,Q63598,Q64093,Q6Q0N1,Q8R431,Q9R1T5,Q9WTW7
5218	1,4,6,7,8,11,12,13	2,3,5,9,10,14,15,16	33	O35547,P02780,P02781,P02782,P04905,P06761,P06911,P07647,P08723,P09456,P0C0A9,P11598,P12020,P22273,P22282,P22283,P30120,P36374,P40241,P46462,P60905,Q09326,Q5GRG2,Q5M8C6,Q63493,Q63617,Q6AYR9,Q78P75,Q99041,Q9JHB9,Q9JI85,Q9QW07,Q9R0T3
5219	1,4,6,7,8,11,12,14	2,3,5,9,10,13,15,16	34	O35547,P02780,P02781,P02782,P04905,P06761,P06911,P07150,P07647,P08723,P0C0A9,P11598,P12020,P19218,P22273,P22282,P22283,P36374,P40241,P46462,P50280,P60905,Q09326,Q5GRG2,Q5M8C6,Q63493,Q63617,Q6AYR9,Q812E4,Q99041,Q9JHB9,Q9JI85,Q9QW07,Q9R0T3
5220	1,4,6,7,8,11,12,15	2,3,5,9,10,13,14,16	28	P02780,P02781,P02782,P06761,P06911,P08723,P0C0A9,P11598,P12020,P19629,P22273,P22282,P22283,P36374,P40241,P46462,P50280,P60905,Q5GRG2,Q5M8C6,Q63493,Q63617,Q6AYR9,Q8CJ52,Q99041,Q9JHB9,Q9JI85,Q9QW07

5221	1,4,6,7,8,11,12,16	2,3,5,9,10,13,14,15	39	O08815,O35547,P00714,P02780,P02781,P02782,P04905,P06761,P06911,P07647,P08010,P08723,P09456,P0C0A9,P11598,P12020,P18418,P22273,P22282,P22283,P24368,P30120,P36374,P40241,P46462,P50280,P60905,P97523,Q5GRG2,Q5M8C6,Q63493,Q63617,Q812E4,Q8CFN2,Q99041,Q9JHB9,Q9JI85,Q9QW07,Q9R0T3
5222	1,4,6,7,8,11,13,14	2,3,5,9,10,12,15,16	33	B0BNN3,O54728,P02780,P02781,P02782,P06761,P06911,P07647,P08723,P09456,P0C0A9,P11598,P12020,P22273,P22282,P22283,P36374,P40241,P46462,P48032,P60905,Q09326,Q4G075,Q5GRG2,Q5M8C6,Q63493,Q63617,Q6AYR9,Q78P75,Q812E4,Q99041,Q9JHB9,Q9JI85
5223	1,4,6,7,8,11,13,15	2,3,5,9,10,12,14,16	28	P02780,P02781,P02782,P06761,P06911,P07647,P08723,P0C0A9,P11598,P22273,P22282,P22283,P36374,P40241,P46462,P50280,P60905,Q09326,Q5GRG2,Q5M8C6,Q63493,Q63617,Q6AYR9,Q8CJ52,Q99041,Q9JHB9,Q9JI85,Q9QW07
5224	1,4,6,7,8,11,13,16	2,3,5,9,10,12,14,15	44	O35547,O88917,P00714,P02780,P02781,P02782,P04905,P05369,P06761,P06911,P07647,P08010,P08723,P09456,P0C0A9,P11598,P12020,P18418,P22273,P22282,P22283,P24368,P30120,P36374,P40241,P46462,P50280,P60905,P97523,Q4G063,Q5GRG2,Q5M8C6,Q62902,Q63493,Q63617,Q6AY61,Q6AYR9,Q812E4,Q8CFN2,Q99041,Q9JHB9,Q9JI85,Q9QW07,Q9R0T3
5225	1,4,6,7,8,11,14,15	2,3,5,9,10,12,13,16	27	P02780,P02781,P02782,P06761,P06911,P07150,P08723,P0C0A9,P11598,P12020,P22273,P22282,P22283,P36374,P46462,P50280,Q09326,Q5GRG2,Q5M8C6,Q63493,Q63617,Q6AYR9,Q8CJ52,Q99041,Q9JHB9,Q9JI85,Q9QW07
5226	1,4,6,7,8,11,14,16	2,3,5,9,10,12,13,15	45	O35547,O54728,P02780,P02781,P02782,P04905,P06761,P06911,P07150,P07647,P08010,P08723,P09456,P0C0A9,P11598,P12020,P18418,P22006,P22273,P22282,P22283,P24368,P30120,P36374,P40241,P46462,P50280,P60905,P97523,Q4G075,Q5GRG2,Q5M8C6,Q63493,Q63617,Q6AY61,Q6AYR9,Q812E4,Q8CFN2,Q99041,Q99MH3,Q9JHB9,Q9JI85,Q9QW07,Q9QZK9,Q9R0T3
5227	1,4,6,7,8,11,15,16	2,3,5,9,10,12,13,14	39	O35547,P02780,P02781,P02782,P04905,P06761,P06911,P07647,P08723,P09456,P0C0A9,P11598,P18418,P19629,P22273,P22282,P22283,P24368,P30120,P36374,P40241,P46462,P50280,P60905,Q4FZU2,Q4G063,Q5GRG2,Q5M8C6,Q63493,Q63617,Q6AYR9,Q6P6Q2,Q812E4,Q8CJ52,Q99041,Q9JHB9,Q9JI85,Q9QW07,Q9QZK9
5228	1,4,6,7,8,12,13,14	2,3,5,9,10,11,15,16	5	P06761,P06911,P70549,Q9JI85,Q9Z2L0
5229	1,4,6,7,8,12,13,15	2,3,5,9,10,11,14,16	6	P06761,P06911,P19629,P70549,Q9JI85,Q9Z2L0
5230	1,4,6,7,8,12,13,16	2,3,5,9,10,11,14,15	16	P02780,P02781,P02782,P06761,P06911,P07647,P11598,P18418,P22282,P22283,P30120,P70549,Q5M8C6,Q9JHB9,Q9JI85,Q9Z2L0
5231	1,4,6,7,8,12,14,15	2,3,5,9,10,11,13,16	3	P06911,P19629,Q9Z2L0
5232	1,4,6,7,8,12,14,16	2,3,5,9,10,11,13,15	12	P02781,P02782,P06761,P06911,P07647,P21674,P22282,P22283,Q5M8C6,Q99MH3,Q9JHB9,Q9Z2L0
5233	1,4,6,7,8,12,15,16	2,3,5,9,10,11,13,14	11	P02781,P06761,P06911,P07647,P10758,P19629,P22282,P22283,Q9JHB9,Q9QW07,Q9Z2L0
5234	1,4,6,7,8,13,14,15	2,3,5,9,10,11,12,16	3	P06761,P70549,Q9Z2L0
5235	1,4,6,7,8,13,14,16	2,3,5,9,10,11,12,15	15	B0BNN3,O54728,P02781,P02782,P06761,P06911,P07647,P11598,P18418,P22282,P22283,P30120,Q99MH3,Q9JI85,Q9Z2L0

5236	1,4,6,7,8,13,15,16	2,3,5,9,10,11,12,14	12	P02781,P02782,P06761,P07647,P18418,P19629,P22282,P22283,P70549,Q8CJ52,Q9JI85,Q9Z2L0
5237	1,4,6,7,8,14,15,16	2,3,5,9,10,11,12,13	6	P02781,P06761,P07647,P19629,Q8CJ52,Q9Z2L0
5238	1,4,6,7,9,10,11,12	2,3,5,8,13,14,15,16	2	P11598,Q63617
5239	1,4,6,7,9,10,11,13	2,3,5,8,12,14,15,16	3	P11598,P22283,Q63617
5240	1,4,6,7,9,10,11,14	2,3,5,8,12,13,15,16	2	P11598,Q63617
5241	1,4,6,7,9,10,11,15	2,3,5,8,12,13,14,16	2	Q63617,Q8CJ52
5242	1,4,6,7,9,10,11,16	2,3,5,8,12,13,14,15	7	P07647,P0C0A9,P11598,P22006,P22283,Q63617,Q9JHB9
5243	1,4,6,7,9,10,12,13	2,3,5,8,11,14,15,16	0	
5244	1,4,6,7,9,10,12,14	2,3,5,8,11,13,15,16	0	
5245	1,4,6,7,9,10,12,15	2,3,5,8,11,13,14,16	1	P10758
5246	1,4,6,7,9,10,12,16	2,3,5,8,11,13,14,15	3	P10758,P21674,P22006
5247	1,4,6,7,9,10,13,14	2,3,5,8,11,12,15,16	0	
5248	1,4,6,7,9,10,13,15	2,3,5,8,11,12,14,16	2	P11883,Q00715
5249	1,4,6,7,9,10,13,16	2,3,5,8,11,12,14,15	3	P11883,P22006,P36376
5250	1,4,6,7,9,10,14,15	2,3,5,8,11,12,13,16	0	
5251	1,4,6,7,9,10,14,16	2,3,5,8,11,12,13,15	2	P22006,P36376
5252	1,4,6,7,9,10,15,16	2,3,5,8,11,12,13,14	4	P10758,P11883,P22006,Q811M5
5253	1,4,6,7,9,11,12,13	2,3,5,8,10,14,15,16	4	P11598,P22283,Q62761;Q62762;Q62763,Q63617
5254	1,4,6,7,9,11,12,14	2,3,5,8,10,13,15,16	3	P11598,Q62761;Q62762;Q62763,Q63617
5255	1,4,6,7,9,11,12,15	2,3,5,8,10,13,14,16	4	Q62761;Q62762;Q62763,Q63617,Q8CJ52,Q9QW07

5256	1,4,6,7,9,11,12,16	2,3,5,8,10,13,14,15	15	O08815,O35547,P02781,P07647,P08723,P0C0A9,P11598,P21674,P22006,P22283,P36374,P63322,Q63617,Q9JHB9,Q9QW07
5257	1,4,6,7,9,11,13,14	2,3,5,8,10,12,15,16	5	P0DMW0;P0DMW1,P11598,Q63617,Q99041,Q9R168
5258	1,4,6,7,9,11,13,15	2,3,5,8,10,12,14,16	3	P11598,Q63617,Q8CJ52
5259	1,4,6,7,9,11,13,16	2,3,5,8,10,12,14,15	14	P00714,P02781,P02782,P07647,P08723,P0C0A9,P11598,P22006,P22283,P34901,P36376,Q63617,Q9JHB9,Q9R168
5260	1,4,6,7,9,11,14,15	2,3,5,8,10,12,13,16	2	P0DMW0;P0DMW1,Q8CJ52
5261	1,4,6,7,9,11,14,16	2,3,5,8,10,12,13,15	11	P07647,P0C0A9,P11598,P22006,P22283,Q63617,Q812E4,Q99041,Q9JHB9,Q9QW07,Q9R168
5262	1,4,6,7,9,11,15,16	2,3,5,8,10,12,13,14	10	P07647,P08723,P0C0A9,P11598,P22006,P22283,Q63617,Q8CJ52,Q9JHB9,Q9QW07
5263	1,4,6,7,9,12,13,14	2,3,5,8,10,11,15,16	0	
5264	1,4,6,7,9,12,13,15	2,3,5,8,10,11,14,16	1	P70549
5265	1,4,6,7,9,12,13,16	2,3,5,8,10,11,14,15	1	P22006
5266	1,4,6,7,9,12,14,15	2,3,5,8,10,11,13,16	0	
5267	1,4,6,7,9,12,14,16	2,3,5,8,10,11,13,15	2	P21674,P22006
5268	1,4,6,7,9,12,15,16	2,3,5,8,10,11,13,14	2	P10758,P22006
5269	1,4,6,7,9,13,14,15	2,3,5,8,10,11,12,16	2	P70549,Q00715
5270	1,4,6,7,9,13,14,16	2,3,5,8,10,11,12,15	4	P22006,P34901,P36376,Q9R168
5271	1,4,6,7,9,13,15,16	2,3,5,8,10,11,12,14	2	P11883,P22006
5272	1,4,6,7,9,14,15,16	2,3,5,8,10,11,12,13	2	P22006,P47967
5273	1,4,6,7,10,11,12,13	2,3,5,8,9,14,15,16	4	P11598,P22283,P36376,Q63617
5274	1,4,6,7,10,11,12,14	2,3,5,8,9,13,15,16	3	P11598,P22283,P36376
5275	1,4,6,7,10,11,12,15	2,3,5,8,9,13,14,16	2	P11598,Q8CJ52

5276	1,4,6,7,10,11,12,16	2,3,5,8,9,13,14,15	12	P00714,P02781,P07647,P08723,P0C0A9,P11598,P22283,P36374,P36376,Q63617,Q9JHB9,Q9QW07
5277	1,4,6,7,10,11,13,14	2,3,5,8,9,12,15,16	5	P11598,P22283,P36376,Q63617,Q99041
5278	1,4,6,7,10,11,13,15	2,3,5,8,9,12,14,16	5	P11598,P22283,Q8CJ52,Q8CJD3,Q99041
5279	1,4,6,7,10,11,13,16	2,3,5,8,9,12,14,15	13	P00714,P02781,P02782,P07647,P08723,P0C0A9,P11598,P22283,P24368,P36376,Q63617,Q99041,Q9JHB9
5280	1,4,6,7,10,11,14,15	2,3,5,8,9,12,13,16	3	P11598,Q8CJ52,Q99041
5281	1,4,6,7,10,11,14,16	2,3,5,8,9,12,13,15	7	P07647,P0C0A9,P11598,P22283,P36376,Q99041,Q9JHB9
5282	1,4,6,7,10,11,15,16	2,3,5,8,9,12,13,14	9	P07647,P08723,P0C0A9,P11598,P17988,P22283,Q8CJ52,Q9JHB9,Q9QW07
5283	1,4,6,7,10,12,13,14	2,3,5,8,9,11,15,16	2	P36376,P70549
5284	1,4,6,7,10,12,13,15	2,3,5,8,9,11,14,16	3	O88797,P10758,P70549
5285	1,4,6,7,10,12,13,16	2,3,5,8,9,11,14,15	2	P10758,P36376
5286	1,4,6,7,10,12,14,15	2,3,5,8,9,11,13,16	0	
5287	1,4,6,7,10,12,14,16	2,3,5,8,9,11,13,15	1	P36376
5288	1,4,6,7,10,12,15,16	2,3,5,8,9,11,13,14	1	P10758
5289	1,4,6,7,10,13,14,15	2,3,5,8,9,11,12,16	2	O88797,P70549
5290	1,4,6,7,10,13,14,16	2,3,5,8,9,11,12,15	3	P36376,P57113,Q99MH3
5291	1,4,6,7,10,13,15,16	2,3,5,8,9,11,12,14	4	P10758,P11883,P36376,P51792;P51794;P51796
5292	1,4,6,7,10,14,15,16	2,3,5,8,9,11,12,13	2	P57113,Q9QYP1
5293	1,4,6,7,11,12,13,14	2,3,5,8,9,10,15,16	5	P11598,P22283,P36376,Q63617,Q99041
5294	1,4,6,7,11,12,13,15	2,3,5,8,9,10,14,16	5	P11598,P22283,P36374,Q63617,Q8CJ52
5295	1,4,6,7,11,12,13,16	2,3,5,8,9,10,14,15	17	O08815,O35547,P00714,P02780,P02781,P02782,P05369,P07647,P08723,P0C0A9,P11598,P22283,P36374,P36376,Q63617,Q99041,Q9JHB9

5296	1,4,6,7,11,12,14,15	2,3,5,8,9,10,13,16	2	P11598,Q8CJ52
5297	1,4,6,7,11,12,14,16	2,3,5,8,9,10,13,15	14	O35547,P02781,P02782,P07647,P0C0A9,P11598,P22283,P36374,P36376,Q63617,Q99041,Q99376,Q9JHB9,Q9QW07
5298	1,4,6,7,11,12,15,16	2,3,5,8,9,10,13,14	13	O08815,O35547,P02781,P07647,P08723,P0C0A9,P11598,P22283,P36374,Q63617,Q8CJ52,Q9JHB9,Q9QW07
5299	1,4,6,7,11,13,14,15	2,3,5,8,9,10,12,16	4	P11598,P22283,Q8CJ52,Q99041
5300	1,4,6,7,11,13,14,16	2,3,5,8,9,10,12,15	15	O54728,P00714,P02781,P02782,P07647,P0C0A9,P11598,P22283,P34901,P36374,P36376,Q63617,Q99041,Q99MH3,Q9JHB9
5301	1,4,6,7,11,13,15,16	2,3,5,8,9,10,12,14	12	P00714,P02781,P02782,P07647,P08723,P0C0A9,P11598,P22283,P36374,Q63617,Q8CJ52,Q99041
5302	1,4,6,7,11,14,15,16	2,3,5,8,9,10,12,13	9	P07647,P0C0A9,P11598,P22283,P36374,Q8CJ52,Q99041,Q9JHB9,Q9QW07
5303	1,4,6,7,12,13,14,15	2,3,5,8,9,10,11,16	3	O88797,P70549,Q9Z2L0
5304	1,4,6,7,12,13,14,16	2,3,5,8,9,10,11,15	3	P36376,Q99MH3,Q9Z2L0
5305	1,4,6,7,12,13,15,16	2,3,5,8,9,10,11,14	2	P10758,P70549
5306	1,4,6,7,12,14,15,16	2,3,5,8,9,10,11,13	0	
5307	1,4,6,7,13,14,15,16	2,3,5,8,9,10,11,12	5	P01835,P36376,P70549,Q99MH3,Q9Z2L0
5308	1,4,6,8,9,10,11,12	2,3,5,7,13,14,15,16	3	Q62714,Q6AY61,Q6Q7Y5
5309	1,4,6,8,9,10,11,13	2,3,5,7,12,14,15,16	2	Q6AY61,Q9JI85
5310	1,4,6,8,9,10,11,14	2,3,5,7,12,13,15,16	1	Q6AY61
5311	1,4,6,8,9,10,11,15	2,3,5,7,12,13,14,16	3	Q63474,Q6AY61,Q6P6S4
5312	1,4,6,8,9,10,11,16	2,3,5,7,12,13,14,15	12	P02782,P04905,P07647,P08723,P0C0A9,P22006,P30120,P60905,Q6AY61,Q812E4,Q9JHB9,Q9QW07
5313	1,4,6,8,9,10,12,13	2,3,5,7,11,14,15,16	0	
5314	1,4,6,8,9,10,12,14	2,3,5,7,11,13,15,16	0	
5315	1,4,6,8,9,10,12,15	2,3,5,7,11,13,14,16	1	Q63474

5316	1,4,6,8,9,10,12,16	2,3,5,7,11,13,14,15	1	P21674
5317	1,4,6,8,9,10,13,14	2,3,5,7,11,12,15,16	0	
5318	1,4,6,8,9,10,13,15	2,3,5,7,11,12,14,16	2	Q00715,Q63474
5319	1,4,6,8,9,10,13,16	2,3,5,7,11,12,14,15	1	Q6AY61
5320	1,4,6,8,9,10,14,15	2,3,5,7,11,12,13,16	2	P47967,Q63474
5321	1,4,6,8,9,10,14,16	2,3,5,7,11,12,13,15	2	P47967,Q6AY61
5322	1,4,6,8,9,10,15,16	2,3,5,7,11,12,13,14	3	P47967,Q6AY61,Q811M5
5323	1,4,6,8,9,11,12,13	2,3,5,7,10,14,15,16	9	B0LT89,P36374,P60905,P61206;P84079,P63095,Q5RKI1,Q6AY61,Q6Q7Y5,Q9JI85
5324	1,4,6,8,9,11,12,14	2,3,5,7,10,13,15,16	10	P06911,P20766,P60905,P61206;P84079,P63095,Q5GRG2,Q5RKI1,Q6Q7Y5,Q812E4,Q9QW07
5325	1,4,6,8,9,11,12,15	2,3,5,7,10,13,14,16	7	P36374,P60905,P63095,Q6P6S4,Q6Q7Y5,Q9QW07,Q9WUW8
5326	1,4,6,8,9,11,12,16	2,3,5,7,10,13,14,15	24	O08815,O35547,P02780,P02781,P02782,P04905,P07647,P08723,P21674,P22006,P30120,P36374,P46462,P60905,P63095,P97523,Q5RKI1,Q63357,Q6AY61,Q6Q7Y5,Q812E4,Q9JHB9,Q9JI85,Q9QW07
5327	1,4,6,8,9,11,13,14	2,3,5,7,10,12,15,16	9	O54728,P0DMW0;P0DMW1,P20766,P60905,Q5RKI1,Q6AY61,Q6AYR9,Q812E4,Q9JI85
5328	1,4,6,8,9,11,13,15	2,3,5,7,10,12,14,16	6	P60905,Q5RLM2,Q6AY61,Q6AYR9,Q6P6S4,Q9JI85
5329	1,4,6,8,9,11,13,16	2,3,5,7,10,12,14,15	17	P02781,P02782,P07647,P08723,P09456,P0C0A9,P22006,P22282,P30120,P36374,P46462,P60905,Q5RKI1,Q6AY61,Q6RUV5,Q812E4,Q9JI85
5330	1,4,6,8,9,11,14,15	2,3,5,7,10,12,13,16	5	P20766,P47967,Q6AY61,Q8CJ52,Q9QW07
5331	1,4,6,8,9,11,14,16	2,3,5,7,10,12,13,15	15	O54728,P02782,P04905,P07647,P08723,P0C0A9,P22006,P30120,P46462,P60905,Q5RKI1,Q6AY61,Q812E4,Q9JHB9,Q9QW07
5332	1,4,6,8,9,11,15,16	2,3,5,7,10,12,13,14	12	P02782,P07647,P08723,P0C0A9,P22006,P36374,P60905,Q6AY61,Q6P6S4,Q812E4,Q8CJ52,Q9QW07
5333	1,4,6,8,9,12,13,14	2,3,5,7,10,11,15,16	1	P20766
5334	1,4,6,8,9,12,13,15	2,3,5,7,10,11,14,16	1	P70549
5335	1,4,6,8,9,12,13,16	2,3,5,7,10,11,14,15	1	Q5RKI1

5336	1,4,6,8,9,12,14,15	2,3,5,7,10,11,13,16	2	P20766,P47967
5337	1,4,6,8,9,12,14,16	2,3,5,7,10,11,13,15	3	P20766,P21674,Q5RKI1
5338	1,4,6,8,9,12,15,16	2,3,5,7,10,11,13,14	0	
5339	1,4,6,8,9,13,14,15	2,3,5,7,10,11,12,16	4	P20766,P47967,P70549,Q00715
5340	1,4,6,8,9,13,14,16	2,3,5,7,10,11,12,15	6	O54728,P20766,P22006,P23593,Q5RKI1,Q6AY61
5341	1,4,6,8,9,13,15,16	2,3,5,7,10,11,12,14	3	P22006,P23593,Q6AY61
5342	1,4,6,8,9,14,15,16	2,3,5,7,10,11,12,13	5	P22006,P23593,P47967,P97840,Q5I0D1
5343	1,4,6,8,10,11,12,13	2,3,5,7,9,14,15,16	2	P36374,Q9JI85
5344	1,4,6,8,10,11,12,14	2,3,5,7,9,13,15,16	3	P06911,P12020,Q5GRG2
5345	1,4,6,8,10,11,12,15	2,3,5,7,9,13,14,16	2	P36374,Q9QW07
5346	1,4,6,8,10,11,12,16	2,3,5,7,9,13,14,15	16	O35547,P02780,P02781,P02782,P04905,P07647,P08723,P0C0A9,P22282,P30120,P36374,P46462,P60905,Q9JHB9,Q9JI85,Q9QW07
5347	1,4,6,8,10,11,13,14	2,3,5,7,9,12,15,16	4	O54728,Q6AY61,Q99041,Q9JI85
5348	1,4,6,8,10,11,13,15	2,3,5,7,9,12,14,16	2	Q6AY61,Q9JI85
5349	1,4,6,8,10,11,13,16	2,3,5,7,9,12,14,15	16	P00714,P02781,P02782,P07647,P08723,P0C0A9,P15399,P22282,P24368,P30120,P36374,P46462,P60905,Q6AY61,Q99041,Q9JI85
5350	1,4,6,8,10,11,14,15	2,3,5,7,9,12,13,16	1	Q6AY61
5351	1,4,6,8,10,11,14,16	2,3,5,7,9,12,13,15	13	O54728,P02781,P02782,P04905,P07647,P08723,P0C0A9,P15399,P30120,Q6AY61,Q99041,Q9JHB9,Q9QW07
5352	1,4,6,8,10,11,15,16	2,3,5,7,9,12,13,14	10	P02782,P07647,P08723,P0C0A9,P15399,P30120,P36374,Q6AY61,Q8CJ52,Q9QW07
5353	1,4,6,8,10,12,13,14	2,3,5,7,9,11,15,16	2	P35280,P70549
5354	1,4,6,8,10,12,13,15	2,3,5,7,9,11,14,16	1	P70549
5355	1,4,6,8,10,12,13,16	2,3,5,7,9,11,14,15	1	P35280

5356	1,4,6,8,10,12,14,15	2,3,5,7,9,11,13,16	2	P54921,Q63474
5357	1,4,6,8,10,12,14,16	2,3,5,7,9,11,13,15	1	P35280
5358	1,4,6,8,10,12,15,16	2,3,5,7,9,11,13,14	2	P0DMW0;P0DMW1,P10758
5359	1,4,6,8,10,13,14,15	2,3,5,7,9,11,12,16	3	P54921,P70549,Q63474
5360	1,4,6,8,10,13,14,16	2,3,5,7,9,11,12,15	3	O54728,P15399,P35280
5361	1,4,6,8,10,13,15,16	2,3,5,7,9,11,12,14	4	P07151,P11883,P15399,P70549
5362	1,4,6,8,10,14,15,16	2,3,5,7,9,11,12,13	3	P15399,P47967,P54921
5363	1,4,6,8,11,12,13,14	2,3,5,7,9,10,15,16	11	O54728,P06911,P12020,P20766,P36374,P46462,P60905,Q5GRG2,Q5RKI1,Q99041,Q9JI85
5364	1,4,6,8,11,12,13,15	2,3,5,7,9,10,14,16	3	P36374,P60905,Q9JI85
5365	1,4,6,8,11,12,13,16	2,3,5,7,9,10,14,15	27	O08815,O35547,P00714,P02780,P02781,P02782,P04905,P05369,P06911,P07647,P08723,P0C0A9,P12020,P22282,P22283,P24368,P30120,P36374,P46462,P60905,Q5GRG2,Q5M8C6,Q5RKI1,Q6AY61,Q9JHB9,Q9JI85,Q9QW07
5366	1,4,6,8,11,12,14,15	2,3,5,7,9,10,13,16	6	P06911,P12020,P20766,P36374,Q5GRG2,Q9QW07
5367	1,4,6,8,11,12,14,16	2,3,5,7,9,10,13,15	22	O35547,O54728,P02780,P02781,P02782,P04905,P06911,P07647,P08723,P0C0A9,P12020,P20766,P30120,P36374,P46462,P60905,Q5GRG2,Q5RKI1,Q8CFN2,Q9JHB9,Q9JI85,Q9QW07
5368	1,4,6,8,11,12,15,16	2,3,5,7,9,10,13,14	17	O08815,O35547,P02780,P02781,P02782,P04905,P07647,P08723,P0C0A9,P30120,P36374,P46462,P60905,Q8CJ52,Q9JHB9,Q9JI85,Q9QW07
5369	1,4,6,8,11,13,14,15	2,3,5,7,9,10,12,16	5	O54728,P20766,Q6AY61,Q99041,Q9JI85
5370	1,4,6,8,11,13,14,16	2,3,5,7,9,10,12,15	21	O35547,O54728,P02781,P02782,P07647,P08723,P09456,P0C0A9,P20766,P22282,P30120,P34901,P36374,P46462,P60905,Q5GRG2,Q5RKI1,Q6AY61,Q99041,Q9JHB9,Q9JI85
5371	1,4,6,8,11,13,15,16	2,3,5,7,9,10,12,14	14	P02781,P02782,P07647,P08723,P0C0A9,P30120,P36374,P46462,P60905,Q6AY61,Q8CJ52,Q99041,Q9JI85,Q9QW07
5372	1,4,6,8,11,14,15,16	2,3,5,7,9,10,12,13	16	O54728,P02781,P02782,P07647,P08723,P0C0A9,P30120,P36374,P46462,P60905,Q6AY61,Q8CJ52,Q99041,Q9JHB9,Q9QW07,Q9QZK9
5373	1,4,6,8,12,13,14,15	2,3,5,7,9,10,11,16	3	P20766,P70549,Q9Z2L0
5374	1,4,6,8,12,13,14,16	2,3,5,7,9,10,11,15	5	O54728,P20766,P35280,Q5RKI1,Q9Z2L0
5375	1,4,6,8,12,13,15,16	2,3,5,7,9,10,11,14	1	P70549

5376	1,4,6,8,12,14,15,16	2,3,5,7,9,10,11,13	1 P20766
5377	1,4,6,8,13,14,15,16	2,3,5,7,9,10,11,12	4 O54728,P20766,P70549,Q9Z2L0
5378	1,4,6,9,10,11,12,13	2,3,5,7,8,14,15,16	3 B0LT89,Q62714,Q62761;Q62762;Q62763
5379	1,4,6,9,10,11,12,14	2,3,5,7,8,13,15,16	2 Q62714,Q62761;Q62762;Q62763
5380	1,4,6,9,10,11,12,15	2,3,5,7,8,13,14,16	3 Q62714,Q62761;Q62762;Q62763,Q9WUW8
5381	1,4,6,9,10,11,12,16	2,3,5,7,8,13,14,15	1 Q62714
5382	1,4,6,9,10,11,13,14	2,3,5,7,8,12,15,16	0
5383	1,4,6,9,10,11,13,15	2,3,5,7,8,12,14,16	1 Q5RLM2
5384	1,4,6,9,10,11,13,16	2,3,5,7,8,12,14,15	1 P22006
5385	1,4,6,9,10,11,14,15	2,3,5,7,8,12,13,16	1 P47967
5386	1,4,6,9,10,11,14,16	2,3,5,7,8,12,13,15	1 P22006
5387	1,4,6,9,10,11,15,16	2,3,5,7,8,12,13,14	2 P22006,Q811M5
5388	1,4,6,9,10,12,13,14	2,3,5,7,8,11,15,16	0
5389	1,4,6,9,10,12,13,15	2,3,5,7,8,11,14,16	3 P08649,P70549,Q00715
5390	1,4,6,9,10,12,13,16	2,3,5,7,8,11,14,15	0
5391	1,4,6,9,10,12,14,15	2,3,5,7,8,11,13,16	2 P47967,Q00715
5392	1,4,6,9,10,12,14,16	2,3,5,7,8,11,13,15	1 P21674
5393	1,4,6,9,10,12,15,16	2,3,5,7,8,11,13,14	3 P10758,P25809,Q811M5
5394	1,4,6,9,10,13,14,15	2,3,5,7,8,11,12,16	4 P47967,P62804,Q00715,Q5PQL7
5395	1,4,6,9,10,13,14,16	2,3,5,7,8,11,12,15	1 Q5PQL7

5396	1,4,6,9,10,13,15,16	2,3,5,7,8,11,12,14	2	P11883,Q811M5
5397	1,4,6,9,10,14,15,16	2,3,5,7,8,11,12,13	3	P47967,Q5I0D1,Q811M5
5398	1,4,6,9,11,12,13,14	2,3,5,7,8,10,15,16	23	B0LT89,O35077,O70377,O70594,P12368,P19468,P20766,P48508,P53790,Q05175,Q3ZAV1,Q5I0E9,Q5RKI1,Q62761;Q62762;Q62763,Q63270,Q63355,Q63424,Q64093,Q6AY41,Q80W57,Q8R431,Q9WTW7,Q9Z0W7
5399	1,4,6,9,11,12,13,15	2,3,5,7,8,10,14,16	6	B0LT89,P20766,Q5RLM2,Q62761;Q62762;Q62763,Q923S2,Q9WUW8
5400	1,4,6,9,11,12,13,16	2,3,5,7,8,10,14,15	9	O08815,O88339;Q4V882,P12368,P22006,P54921,Q05175,Q5RKI1,Q62714,Q62753
5401	1,4,6,9,11,12,14,15	2,3,5,7,8,10,13,16	9	O70594,P20766,P25809,P46844,Q62761;Q62762;Q62763,Q63355,Q63424,Q64093,Q9WUW8
5402	1,4,6,9,11,12,14,16	2,3,5,7,8,10,13,15	10	O70594,O88339;Q4V882,P20766,P22006,P25809,Q5RKI1,Q62714,Q62753,Q63355,Q63618
5403	1,4,6,9,11,12,15,16	2,3,5,7,8,10,13,14	4	O08815,P22006,P25809,Q9WUW8
5404	1,4,6,9,11,13,14,15	2,3,5,7,8,10,12,16	3	P0DMW0;P0DMW1,P20766,Q5RLM2
5405	1,4,6,9,11,13,14,16	2,3,5,7,8,10,12,15	7	O54728,P12368,P20766,P22006,P34901,Q05175,Q5RKI1
5406	1,4,6,9,11,13,15,16	2,3,5,7,8,10,12,14	2	P22006,Q5RLM2
5407	1,4,6,9,11,14,15,16	2,3,5,7,8,10,12,13	5	P20766,P22006,P25809,P47967,Q8CJ52
5408	1,4,6,9,12,13,14,15	2,3,5,7,8,10,11,16	3	P20766,P70549,Q00715
5409	1,4,6,9,12,13,14,16	2,3,5,7,8,10,11,15	2	P20766,Q5RKI1
5410	1,4,6,9,12,13,15,16	2,3,5,7,8,10,11,14	1	P08937
5411	1,4,6,9,12,14,15,16	2,3,5,7,8,10,11,13	3	P20766,P25809,P47967
5412	1,4,6,9,13,14,15,16	2,3,5,7,8,10,11,12	6	P20766,P23593,P47967,Q00715,Q5I0D1,Q5PQL7
5413	1,4,6,10,11,12,13,14	2,3,5,7,8,9,15,16	1	P35280
5414	1,4,6,10,11,12,13,15	2,3,5,7,8,9,14,16	0	
5415	1,4,6,10,11,12,13,16	2,3,5,7,8,9,14,15	3	P00714,P35280,P36376

5416	1,4,6,10,11,12,14,15	2,3,5,7,8,9,13,16	0	
5417	1,4,6,10,11,12,14,16	2,3,5,7,8,9,13,15	2	P35280,P36376
5418	1,4,6,10,11,12,15,16	2,3,5,7,8,9,13,14	0	
5419	1,4,6,10,11,13,14,15	2,3,5,7,8,9,12,16	0	
5420	1,4,6,10,11,13,14,16	2,3,5,7,8,9,12,15	5	O54728,P19132,P34901,P35280,P36376
5421	1,4,6,10,11,13,15,16	2,3,5,7,8,9,12,14	0	
5422	1,4,6,10,11,14,15,16	2,3,5,7,8,9,12,13	0	
5423	1,4,6,10,12,13,14,15	2,3,5,7,8,9,11,16	3	P06760,P35280,P70549
5424	1,4,6,10,12,13,14,16	2,3,5,7,8,9,11,15	3	P06760,P35280,P36376
5425	1,4,6,10,12,13,15,16	2,3,5,7,8,9,11,14	5	P06760,P08937,P10758,P35280,P70549
5426	1,4,6,10,12,14,15,16	2,3,5,7,8,9,11,13	3	P06760,P35280,Q9QYP1
5427	1,4,6,10,13,14,15,16	2,3,5,7,8,9,11,12	5	P06760,P35280,P70709,Q5PQL7,Q9QYP1
5428	1,4,6,11,12,13,14,15	2,3,5,7,8,9,10,16	2	P20766,P70545
5429	1,4,6,11,12,13,14,16	2,3,5,7,8,9,10,15	7	O54728,P12368,P19132,P20766,P35280,P36376,Q5RKI1
5430	1,4,6,11,12,13,15,16	2,3,5,7,8,9,10,14	2	O08815,P08937
5431	1,4,6,11,12,14,15,16	2,3,5,7,8,9,10,13	2	P20766,P25809
5432	1,4,6,11,13,14,15,16	2,3,5,7,8,9,10,12	2	O54728,P20766
5433	1,4,6,12,13,14,15,16	2,3,5,7,8,9,10,11	5	P06760,P20766,P35280,P70545,P70549
5434	1,4,7,8,9,10,11,12	2,3,5,6,13,14,15,16	8	P21674,P36374,P49134,Q62714,Q63493,Q6Q7Y5,Q812E4,Q9Z1F2
5435	1,4,7,8,9,10,11,13	2,3,5,6,12,14,15,16	3	Q63493,Q812E4,Q9Z1F2

5436	1,4,7,8,9,10,11,14	2,3,5,6,12,13,15,16	2	Q812E4,Q9Z1F2
5437	1,4,7,8,9,10,11,15	2,3,5,6,12,13,14,16	5	P20646,P49134,Q6P6R2,Q8CJ52,Q9Z1F2
5438	1,4,7,8,9,10,11,16	2,3,5,6,12,13,14,15	16	P02780,P02781,P02782,P04905,P08723,P09456,P0C0A9,P20646,P21674,P30120,P36374,P49134,Q63493,Q6AY61,Q812E4,Q9JHB9
5439	1,4,7,8,9,10,12,13	2,3,5,6,11,14,15,16	1	Q9Z1F2
5440	1,4,7,8,9,10,12,14	2,3,5,6,11,13,15,16	2	P21674,Q9Z1F2
5441	1,4,7,8,9,10,12,15	2,3,5,6,11,13,14,16	3	P10758,P35745,Q9Z1F2
5442	1,4,7,8,9,10,12,16	2,3,5,6,11,13,14,15	2	P10758,P21674
5443	1,4,7,8,9,10,13,14	2,3,5,6,11,12,15,16	1	Q9Z1F2
5444	1,4,7,8,9,10,13,15	2,3,5,6,11,12,14,16	1	Q9Z1F2
5445	1,4,7,8,9,10,13,16	2,3,5,6,11,12,14,15	0	
5446	1,4,7,8,9,10,14,15	2,3,5,6,11,12,13,16	2	P20646,Q9Z1F2
5447	1,4,7,8,9,10,14,16	2,3,5,6,11,12,13,15	2	P20646,P21674
5448	1,4,7,8,9,10,15,16	2,3,5,6,11,12,13,14	3	P10758,P20646,P49134
5449	1,4,7,8,9,11,12,13	2,3,5,6,10,14,15,16	5	P36374,P60905,Q63493,Q6Q7Y5,Q812E4
5450	1,4,7,8,9,11,12,14	2,3,5,6,10,13,15,16	5	P21674,P36374,Q63493,Q6Q7Y5,Q812E4
5451	1,4,7,8,9,11,12,15	2,3,5,6,10,13,14,16	5	P36374,Q63493,Q6Q7Y5,Q812E4,Q8CJ52
5452	1,4,7,8,9,11,12,16	2,3,5,6,10,13,14,15	26	O08815,P02780,P02781,P02782,P04905,P07647,P08723,P09456,P0C0A9,P21674,P22282,P22283,P30120,P36374,P46462,P49134,P60905,P63322,P97523,Q5M8C6,Q63357,Q63493,Q6Q7Y5,Q812E4,Q9JHB9,Q9QW07
5453	1,4,7,8,9,11,13,14	2,3,5,6,10,12,15,16	5	O54728,P0DMW0;P0DMW1,Q63493,Q6AYR9,Q812E4
5454	1,4,7,8,9,11,13,15	2,3,5,6,10,12,14,16	4	Q63493,Q6AYR9,Q812E4,Q8CJ52
5455	1,4,7,8,9,11,13,16	2,3,5,6,10,12,14,15	22	P02780,P02781,P02782,P04905,P07647,P08723,P09456,P0C0A9,P22282,P22283,P24368,P30120,P36374,P46462,P60905,Q5M8C6,Q62902,Q63493,Q63617,Q6AY61,Q812E4,Q9JHB9

5456	1,4,7,8,9,11,14,15	2,3,5,6,10,12,13,16	3	P20646,Q812E4,Q8CJ52
5457	1,4,7,8,9,11,14,16	2,3,5,6,10,12,13,15	17	O54728,P02780,P02781,P02782,P04905,P08723,P09456,P0C0A9,P21674,P30120,P36374,P60905,Q5M8C6,Q63493,Q6AY61,Q812E4,Q9JHB9
5458	1,4,7,8,9,11,15,16	2,3,5,6,10,12,13,14	16	P02780,P02781,P02782,P08723,P09456,P0C0A9,P20646,P30120,P36374,P49134,P60905,Q63493,Q812E4,Q8CJ52,Q9JHB9,Q9QW07
5459	1,4,7,8,9,12,13,14	2,3,5,6,10,11,15,16	0	
5460	1,4,7,8,9,12,13,15	2,3,5,6,10,11,14,16	0	
5461	1,4,7,8,9,12,13,16	2,3,5,6,10,11,14,15	0	
5462	1,4,7,8,9,12,14,15	2,3,5,6,10,11,13,16	0	
5463	1,4,7,8,9,12,14,16	2,3,5,6,10,11,13,15	1	P21674
5464	1,4,7,8,9,12,15,16	2,3,5,6,10,11,13,14	1	P10758
5465	1,4,7,8,9,13,14,15	2,3,5,6,10,11,12,16	1	Q9Z1F2
5466	1,4,7,8,9,13,14,16	2,3,5,6,10,11,12,15	3	B0BNN3,O54728,Q99MH3
5467	1,4,7,8,9,13,15,16	2,3,5,6,10,11,12,14	1	P20646
5468	1,4,7,8,9,14,15,16	2,3,5,6,10,11,12,13	2	P20646,P47967
5469	1,4,7,8,10,11,12,13	2,3,5,6,9,14,15,16	5	P22282,P36374,Q5I0D1,Q63493,Q9Z1F2
5470	1,4,7,8,10,11,12,14	2,3,5,6,9,13,15,16	3	P21674,P36374,Q9Z1F2
5471	1,4,7,8,10,11,12,15	2,3,5,6,9,13,14,16	4	P10758,P36374,Q63493,Q9Z1F2
5472	1,4,7,8,10,11,12,16	2,3,5,6,9,13,14,15	18	P02780,P02781,P02782,P04905,P07647,P08723,P0C0A9,P21674,P22282,P22283,P24368,P30120,P36374,P40241,Q5M8C6,Q63493,Q9JHB9,Q9QW07
5473	1,4,7,8,10,11,13,14	2,3,5,6,9,12,15,16	4	B0BNN3,O54728,Q99041,Q9Z1F2
5474	1,4,7,8,10,11,13,15	2,3,5,6,9,12,14,16	2	Q63493,Q9Z1F2
5475	1,4,7,8,10,11,13,16	2,3,5,6,9,12,14,15	19	P00714,P02780,P02781,P02782,P04905,P07647,P08723,P09456,P0C0A9,P22282,P22283,P24368,P30120,P36374,Q5M8C6,Q63493,Q99041,Q99MH3,Q9JHB9

5476	1,4,7,8,10,11,14,15	2,3,5,6,9,12,13,16	2	Q8CJ52,Q9Z1F2
5477	1,4,7,8,10,11,14,16	2,3,5,6,9,12,13,15	16	O54728,P02780,P02781,P02782,P04905,P07647,P08723,P0C0A9,P22283,P30120,P36374,Q5M8C6,Q812E4,Q99041,Q99MH3,Q9JHB9
5478	1,4,7,8,10,11,15,16	2,3,5,6,9,12,13,14	15	P02780,P02781,P02782,P04905,P08723,P0C0A9,P24368,P30120,P36374,P49134,Q4FZU2,Q6P6Q2,Q8CJ52,Q9JHB9,Q9QW07
5479	1,4,7,8,10,12,13,14	2,3,5,6,9,11,15,16	2	Q9Z1F2,Q9Z2L0
5480	1,4,7,8,10,12,13,15	2,3,5,6,9,11,14,16	4	P10758,P70549,Q5I0J9,Q9Z1F2
5481	1,4,7,8,10,12,13,16	2,3,5,6,9,11,14,15	3	P10758,Q5I0J9,Q99MH3
5482	1,4,7,8,10,12,14,15	2,3,5,6,9,11,13,16	1	Q9Z1F2
5483	1,4,7,8,10,12,14,16	2,3,5,6,9,11,13,15	2	P21674,Q99MH3
5484	1,4,7,8,10,12,15,16	2,3,5,6,9,11,13,14	3	P0DMW0;P0DMW1,P10758,Q5I0J9
5485	1,4,7,8,10,13,14,15	2,3,5,6,9,11,12,16	2	Q9Z1F2,Q9Z2L0
5486	1,4,7,8,10,13,14,16	2,3,5,6,9,11,12,15	4	B0BNN3,O54728,Q99MH3,Q9Z2L0
5487	1,4,7,8,10,13,15,16	2,3,5,6,9,11,12,14	4	O35077,P10758,Q5I0J9,Q64093
5488	1,4,7,8,10,14,15,16	2,3,5,6,9,11,12,13	3	O35077,P97608,Q9QYP1
5489	1,4,7,8,11,12,13,14	2,3,5,6,9,10,15,16	5	O54728,P36374,Q63493,Q811M5,Q99MH3
5490	1,4,7,8,11,12,13,15	2,3,5,6,9,10,14,16	3	P36374,Q63493,Q8CJ52
5491	1,4,7,8,11,12,13,16	2,3,5,6,9,10,14,15	25	O08815,O35547,P00714,P02780,P02781,P02782,P04905,P07647,P08723,P09456,P0C0A9,P22282,P22283,P24368,P30120,P36374,P40241,P46462,P60905,Q5M8C6,Q63493,Q811M5,Q812E4,Q99MH3,Q9JHB9
5492	1,4,7,8,11,12,14,15	2,3,5,6,9,10,13,16	4	D3ZTX0,P36374,Q63493,Q8CJ52
5493	1,4,7,8,11,12,14,16	2,3,5,6,9,10,13,15	24	D3ZTX0,O54728,P02780,P02781,P02782,P04905,P07647,P08723,P09456,P0C0A9,P21674,P22282,P22283,P30120,P36374,P46462,P60905,Q5M8C6,Q63493,Q811M5,Q812E4,Q99MH3,Q9JHB9,Q9QW07
5494	1,4,7,8,11,12,15,16	2,3,5,6,9,10,13,14	22	O08815,P02780,P02781,P02782,P04905,P07647,P08723,P0C0A9,P10758,P22282,P22283,P30120,P36374,P40241,P60905,Q4FZU2,Q5M8C6,Q63493,Q6P6Q2,Q8CJ52,Q9JHB9,Q9QW07

5495	1,4,7,8,11,13,14,15	2,3,5,6,9,10,12,16	5	B0BNN3,O54728,Q63493,Q8CJ52,Q99041
5496	1,4,7,8,11,13,14,16	2,3,5,6,9,10,12,15	24	B0BNN3,O54728,P02780,P02781,P02782,P04905,P07647,P08723,P09456,P0C0A9,P22282,P22283,P24368,P30120,P36374,P46462,P60905,Q5M8C6,Q63493,Q811M5,Q812E4,Q99041,Q99MH3,Q9JHB9
5497	1,4,7,8,11,13,15,16	2,3,5,6,9,10,12,14	21	P02780,P02781,P02782,P07647,P08723,P09456,P0C0A9,P22282,P22283,P24368,P30120,P36374,P46462,P60905,Q4FZU2,Q5M8C6,Q63493,Q6P6Q2,Q8CJ52,Q99041,Q9JHB9
5498	1,4,7,8,11,14,15,16	2,3,5,6,9,10,12,13	17	D3ZTX0,O54728,P02780,P02781,P02782,P08723,P0C0A9,P30120,P36374,Q4FZU2,Q6P6Q2,Q812E4,Q8CJ52,Q99041,Q99MH3,Q9JHB9,Q9QW07
5499	1,4,7,8,12,13,14,15	2,3,5,6,9,10,11,16	2	P70549,Q9Z2L0
5500	1,4,7,8,12,13,14,16	2,3,5,6,9,10,11,15	5	B0BNN3,O54728,Q811M5,Q99MH3,Q9Z2L0
5501	1,4,7,8,12,13,15,16	2,3,5,6,9,10,11,14	3	P10758,Q5I0J9,Q9Z2L0
5502	1,4,7,8,12,14,15,16	2,3,5,6,9,10,11,13	2	Q99MH3,Q9Z2L0
5503	1,4,7,8,13,14,15,16	2,3,5,6,9,10,11,12	4	B0BNN3,O54728,Q99MH3,Q9Z2L0
5504	1,4,7,9,10,11,12,13	2,3,5,6,8,14,15,16	2	Q62714,Q62761;Q62762;Q62763
5505	1,4,7,9,10,11,12,14	2,3,5,6,8,13,15,16	3	P21674,Q62714,Q62761;Q62762;Q62763
5506	1,4,7,9,10,11,12,15	2,3,5,6,8,13,14,16	4	P10758,Q62714,Q62761;Q62762;Q62763,Q9WUW8
5507	1,4,7,9,10,11,12,16	2,3,5,6,8,13,14,15	2	P21674,Q62714
5508	1,4,7,9,10,11,13,14	2,3,5,6,8,12,15,16	2	P36376,Q9R168
5509	1,4,7,9,10,11,13,15	2,3,5,6,8,12,14,16	0	
5510	1,4,7,9,10,11,13,16	2,3,5,6,8,12,14,15	2	P36376,Q9R168
5511	1,4,7,9,10,11,14,15	2,3,5,6,8,12,13,16	0	
5512	1,4,7,9,10,11,14,16	2,3,5,6,8,12,13,15	2	P36376,Q9R168
5513	1,4,7,9,10,11,15,16	2,3,5,6,8,12,13,14	1	Q8CJ52
5514	1,4,7,9,10,12,13,14	2,3,5,6,8,11,15,16	1	Q6IFU7

5515	1,4,7,9,10,<br>12,13,15	2,3,5,6,8,1<br>1,14,16	1 P10758
5516	1,4,7,9,10,<br>12,13,16	2,3,5,6,8,1<br>1,14,15	2 P10758,Q6IFU7
5517	1,4,7,9,10,<br>12,14,15	2,3,5,6,8,1<br>1,13,16	0
5518	1,4,7,9,10,<br>12,14,16	2,3,5,6,8,1<br>1,13,15	2 P21674,Q6IFU7
5519	1,4,7,9,10,<br>12,15,16	2,3,5,6,8,1<br>1,13,14	2 P08937,P10758
5520	1,4,7,9,10,<br>13,14,15	2,3,5,6,8,1<br>1,12,16	1 Q00715
5521	1,4,7,9,10,<br>13,14,16	2,3,5,6,8,1<br>1,12,15	2 P36376,Q9R168
5522	1,4,7,9,10,<br>13,15,16	2,3,5,6,8,1<br>1,12,14	2 P08937,P10758
5523	1,4,7,9,10,<br>14,15,16	2,3,5,6,8,1<br>1,12,13	2 P47967,Q9QYP1
5524	1,4,7,9,11,<br>12,13,14	2,3,5,6,8,1<br>0,15,16	8 O70594,O88339;Q4V882,P25031,P53790,P97605,Q05175,Q62761;Q62762;Q62763,Q63270
5525	1,4,7,9,11,<br>12,13,15	2,3,5,6,8,1<br>0,14,16	2 P25031,Q62761;Q62762;Q62763
5526	1,4,7,9,11,<br>12,13,16	2,3,5,6,8,1<br>0,14,15	5 O08815,O88339;Q4V882,P54921,P63322,Q62714
5527	1,4,7,9,11,<br>12,14,15	2,3,5,6,8,1<br>0,13,16	4 O70594,P25031,P97605,Q62761;Q62762;Q62763
5528	1,4,7,9,11,<br>12,14,16	2,3,5,6,8,1<br>0,13,15	9 O70594,O88339;Q4V882,P10247,P21674,P63322,Q498D9,Q62714,Q63618,Q812E4
5529	1,4,7,9,11,<br>12,15,16	2,3,5,6,8,1<br>0,13,14	5 O08815,P08937,P10758,P25809,Q8CJ52
5530	1,4,7,9,11,<br>13,14,15	2,3,5,6,8,1<br>0,12,16	3 P0DMW0;P0DMW1,P25031,P36970
5531	1,4,7,9,11,<br>13,14,16	2,3,5,6,8,1<br>0,12,15	6 O54728,P36376,Q498D9,Q812E4,Q99MH3,Q9R168
5532	1,4,7,9,11,<br>13,15,16	2,3,5,6,8,1<br>0,12,14	4 P08937,P25031,Q5RLM2,Q8CJ52
5533	1,4,7,9,11,<br>14,15,16	2,3,5,6,8,1<br>0,12,13	3 P25031,Q8CJ52,Q9R168
5534	1,4,7,9,12,<br>13,14,15	2,3,5,6,8,1<br>0,11,16	0

5535	1,4,7,9,12,13,14,16	2,3,5,6,8,10,11,15	2	Q6IFU7,Q99MH3
5536	1,4,7,9,12,13,15,16	2,3,5,6,8,10,11,14	2	P08937,P10758
5537	1,4,7,9,12,14,15,16	2,3,5,6,8,10,11,13	0	
5538	1,4,7,9,13,14,15,16	2,3,5,6,8,10,11,12	0	
5539	1,4,7,10,11,12,13,14	2,3,5,6,8,9,15,16	1	P36376
5540	1,4,7,10,11,12,13,15	2,3,5,6,8,9,14,16	1	P10758
5541	1,4,7,10,11,12,13,16	2,3,5,6,8,9,14,15	4	P00714,P36376,P97840,Q99MH3
5542	1,4,7,10,11,12,14,15	2,3,5,6,8,9,13,16	0	
5543	1,4,7,10,11,12,14,16	2,3,5,6,8,9,13,15	3	P21674,P36376,Q99MH3
5544	1,4,7,10,11,12,15,16	2,3,5,6,8,9,13,14	2	P08937,P10758
5545	1,4,7,10,11,13,14,15	2,3,5,6,8,9,12,16	0	
5546	1,4,7,10,11,13,14,16	2,3,5,6,8,9,12,15	3	O54728,P36376,Q99MH3
5547	1,4,7,10,11,13,15,16	2,3,5,6,8,9,12,14	2	P08937,P36376
5548	1,4,7,10,11,14,15,16	2,3,5,6,8,9,12,13	3	P36376,Q8CJ52,Q9QYP1
5549	1,4,7,10,12,13,14,15	2,3,5,6,8,9,11,16	1	O88797
5550	1,4,7,10,12,13,14,16	2,3,5,6,8,9,11,15	3	P36376,Q6P6S4,Q99MH3
5551	1,4,7,10,12,13,15,16	2,3,5,6,8,9,11,14	3	P08937,P10758,Q5I0J9
5552	1,4,7,10,12,14,15,16	2,3,5,6,8,9,11,13	2	P10758,Q9QYP1
5553	1,4,7,10,13,14,15,16	2,3,5,6,8,9,11,12	4	P09527,P36376,Q99MH3,Q9QYP1
5554	1,4,7,11,12,13,14,15	2,3,5,6,8,9,10,16	2	D3ZTX0,P25031

5555	1,4,7,11,12,13,14,16	2,3,5,6,8,9,10,15	5	O54728,P25031,P36376,Q811M5,Q99MH3
5556	1,4,7,11,12,13,15,16	2,3,5,6,8,9,10,14	4	O08815,P08937,P17559,P25031
5557	1,4,7,11,12,14,15,16	2,3,5,6,8,9,10,13	4	D3ZTX0,P17559,P25031,Q8CJ52
5558	1,4,7,11,13,14,15,16	2,3,5,6,8,9,10,12	7	D3ZTX0,O54728,P17559,P25031,P36376,Q8CJ52,Q99MH3
5559	1,4,7,12,13,14,15,16	2,3,5,6,8,9,10,11	3	Q6P6S4,Q99MH3,Q9Z2L0
5560	1,4,8,9,10,11,12,13	2,3,5,6,7,14,15,16	2	B0LT89,Q62714
5561	1,4,8,9,10,11,12,14	2,3,5,6,7,13,15,16	3	P01835,P21674,Q62714
5562	1,4,8,9,10,11,12,15	2,3,5,6,7,13,14,16	3	P01835,Q62714,Q9WUW8
5563	1,4,8,9,10,11,12,16	2,3,5,6,7,13,14,15	3	P01835,P21674,Q62714
5564	1,4,8,9,10,11,13,14	2,3,5,6,7,12,15,16	3	B0LT89,O54728,Q6AY61
5565	1,4,8,9,10,11,13,15	2,3,5,6,7,12,14,16	2	B0LT89,Q6AY61
5566	1,4,8,9,10,11,13,16	2,3,5,6,7,12,14,15	3	P30120,P60905,Q6AY61
5567	1,4,8,9,10,11,14,15	2,3,5,6,7,12,13,16	2	P47967,Q6AY61
5568	1,4,8,9,10,11,14,16	2,3,5,6,7,12,13,15	4	O54728,P01835,Q6AY61,Q812E4
5569	1,4,8,9,10,11,15,16	2,3,5,6,7,12,13,14	3	P01835,P49134,Q6AY61
5570	1,4,8,9,10,12,13,14	2,3,5,6,7,11,15,16	1	P17988
5571	1,4,8,9,10,12,13,15	2,3,5,6,7,11,14,16	0	
5572	1,4,8,9,10,12,13,16	2,3,5,6,7,11,14,15	0	
5573	1,4,8,9,10,12,14,15	2,3,5,6,7,11,13,16	1	P47967
5574	1,4,8,9,10,12,14,16	2,3,5,6,7,11,13,15	1	P21674

5575	1,4,8,9,10,12,15,16	2,3,5,6,7,11,13,14	1	P10758
5576	1,4,8,9,10,13,14,15	2,3,5,6,7,11,12,16	2	P47967,Q00715
5577	1,4,8,9,10,13,14,16	2,3,5,6,7,11,12,15	1	O54728
5578	1,4,8,9,10,13,15,16	2,3,5,6,7,11,12,14	0	
5579	1,4,8,9,10,14,15,16	2,3,5,6,7,11,12,13	2	P47967,Q5I0D1
5580	1,4,8,9,11,12,13,14	2,3,5,6,7,10,15,16	17	B0LT89,O54728,O70594,P17988,P18297,P19468,P20766,P48508,P53790,P57113,P63095,Q05175,Q5I0E9,Q63270,Q6Q7Y5,Q71MB6,Q9WTW7
5581	1,4,8,9,11,12,13,15	2,3,5,6,7,10,14,16	6	B0LT89,P18297,P36970,P57113,Q71MB6,Q9WUW8
5582	1,4,8,9,11,12,13,16	2,3,5,6,7,10,14,15	12	B0LT89,O08815,P02782,P30120,P54921,P60905,Q5RKI1,Q62714,Q63357,Q6AY61,Q6Q7Y5,Q812E4
5583	1,4,8,9,11,12,14,15	2,3,5,6,7,10,13,16	5	O70594,P18297,P20766,P36970,Q9WUW8
5584	1,4,8,9,11,12,14,16	2,3,5,6,7,10,13,15	9	O54728,O70594,P20766,P21674,P60905,Q5RKI1,Q62714,Q6Q7Y5,Q812E4
5585	1,4,8,9,11,12,15,16	2,3,5,6,7,10,13,14	4	O08815,P60905,Q62714,Q9WUW8
5586	1,4,8,9,11,13,14,15	2,3,5,6,7,10,12,16	7	B0LT89,O54728,P0DMW0;P0DMW1,P18297,P20766,P36970,Q6AY61
5587	1,4,8,9,11,13,14,16	2,3,5,6,7,10,12,15	7	O54728,P20766,P30120,P60905,Q5RKI1,Q6AY61,Q812E4
5588	1,4,8,9,11,13,15,16	2,3,5,6,7,10,12,14	4	P30120,P60905,Q5RLM2,Q6AY61
5589	1,4,8,9,11,14,15,16	2,3,5,6,7,10,12,13	5	O54728,P20766,P47967,Q6AY61,Q812E4
5590	1,4,8,9,12,13,14,15	2,3,5,6,7,10,11,16	1	P20766
5591	1,4,8,9,12,13,14,16	2,3,5,6,7,10,11,15	3	O54728,P20766,Q5RKI1
5592	1,4,8,9,12,13,15,16	2,3,5,6,7,10,11,14	1	P50116
5593	1,4,8,9,12,14,15,16	2,3,5,6,7,10,11,13	2	P20766,P47967
5594	1,4,8,9,13,14,15,16	2,3,5,6,7,10,11,12	6	O54728,P20766,P23593,P47967,P50116,Q63199

5595	1,4,8,10,11,12,13,14	2,3,5,6,7,9,15,16	1	O54728
5596	1,4,8,10,11,12,13,15	2,3,5,6,7,9,14,16	0	
5597	1,4,8,10,11,12,13,16	2,3,5,6,7,9,14,15	2	P02782,P30120
5598	1,4,8,10,11,12,14,15	2,3,5,6,7,9,13,16	0	
5599	1,4,8,10,11,12,14,16	2,3,5,6,7,9,13,15	2	O54728,P21674
5600	1,4,8,10,11,12,15,16	2,3,5,6,7,9,13,14	2	P10758,Q5QE79
5601	1,4,8,10,11,13,14,15	2,3,5,6,7,9,12,16	2	O54728,Q6AY61
5602	1,4,8,10,11,13,14,16	2,3,5,6,7,9,12,15	4	O54728,P30120,Q6AY61,Q99MH3
5603	1,4,8,10,11,13,15,16	2,3,5,6,7,9,12,14	2	P30120,Q6AY61
5604	1,4,8,10,11,14,15,16	2,3,5,6,7,9,12,13	2	O54728,Q6AY61
5605	1,4,8,10,12,13,14,15	2,3,5,6,7,9,11,16	1	P70549
5606	1,4,8,10,12,13,14,16	2,3,5,6,7,9,11,15	3	O54728,P35280,Q99MH3
5607	1,4,8,10,12,13,15,16	2,3,5,6,7,9,11,14	2	P10758,P50116
5608	1,4,8,10,12,14,15,16	2,3,5,6,7,9,11,13	0	
5609	1,4,8,10,13,14,15,16	2,3,5,6,7,9,11,12	2	O54728,P50116
5610	1,4,8,11,12,13,14,15	2,3,5,6,7,9,10,16	3	D3ZTX0,O54728,P20766
5611	1,4,8,11,12,13,14,16	2,3,5,6,7,9,10,15	9	D3ZTX0,O54728,P02782,P20766,P30120,P60905,Q5RKI1,Q811M5,Q99MH3
5612	1,4,8,11,12,13,15,16	2,3,5,6,7,9,10,14	7	O08815,P02782,P30120,P36374,P50116,P60905,Q5QE79
5613	1,4,8,11,12,14,15,16	2,3,5,6,7,9,10,13	3	D3ZTX0,O54728,P20766
5614	1,4,8,11,13,14,15,16	2,3,5,6,7,9,10,12	6	D3ZTX0,O54728,P20766,P30120,P50116,Q6AY61

5615	1,4,8,12,13,14,15,16	2,3,5,6,7,9,10,11	4	O54728,P20766,P50116,Q9Z2L0
5616	1,4,9,10,11,12,13,14	2,3,5,6,7,8,15,16	14	B0LT89,O70594,P38918,P46844,P48508,P50115,P50116,P53790,Q05175,Q62714,Q62761;Q62762;Q62763,Q63270,Q9WTW7,Q9Z0W7
5617	1,4,9,10,11,12,13,15	2,3,5,6,7,8,14,16	10	B0LT89,P08937,P36970,P50115,P50116,P52590,Q5RLM2,Q62714,Q62761;Q62762;Q62763,Q9WUW8
5618	1,4,9,10,11,12,13,16	2,3,5,6,7,8,14,15	5	B0LT89,P08937,P50115,P50116,Q62714
5619	1,4,9,10,11,12,14,15	2,3,5,6,7,8,13,16	7	O70594,P46844,P50115,P50116,Q62714,Q62761;Q62762;Q62763,Q9WUW8
5620	1,4,9,10,11,12,14,16	2,3,5,6,7,8,13,15	7	O70594,P10247,P21674,P50115,P50116,Q62714,Q8CGS4
5621	1,4,9,10,11,12,15,16	2,3,5,6,7,8,13,14	9	P08937,P10758,P25809,P50115,P50116,P63081,Q5QE79,Q62714,Q9WUW8
5622	1,4,9,10,11,13,14,15	2,3,5,6,7,8,12,16	5	B0LT89,P36970,P46844,P50115,P50116
5623	1,4,9,10,11,13,14,16	2,3,5,6,7,8,12,15	4	O54728,P36376,P50115,P50116
5624	1,4,9,10,11,13,15,16	2,3,5,6,7,8,12,14	6	P08937,P50115,P50116,Q5QE79,Q5RLM2,Q63751
5625	1,4,9,10,11,14,15,16	2,3,5,6,7,8,12,13	4	P08937,P47967,P50115,P50116
5626	1,4,9,10,12,13,14,15	2,3,5,6,7,8,11,16	8	P02780,P08937,P09456,P50115,P50116,P50280,Q00715,Q5M8C6
5627	1,4,9,10,12,13,14,16	2,3,5,6,7,8,11,15	3	P50115,P50116,P50280
5628	1,4,9,10,12,13,15,16	2,3,5,6,7,8,11,14	6	P08937,P10758,P50115,P50116,Q5QE79,Q63751
5629	1,4,9,10,12,14,15,16	2,3,5,6,7,8,11,13	4	P08937,P47967,P50115,P50116
5630	1,4,9,10,13,14,15,16	2,3,5,6,7,8,11,12	8	P08937,P12020,P47967,P50115,P50116,P70709,Q00715,Q5I0D1
5631	1,4,9,11,12,13,14,15	2,3,5,6,7,8,10,16	49	B0LT89,O35763,O70257,O70377,O70594,O88339;Q4V882,P02631,P08937,P18757,P19468,P20766,P25031,P29975,P36970,P38918,P46720,P46844,P48508,P50115,P50116,P53790,P57113,P97605,Q03248,Q05175,Q3T1J9,Q3ZAV1,Q5I0E9,Q5M7T9,Q5RLM2,Q62761;Q62762;Q62763,Q63270,Q63424,Q63618,Q64093,Q64602,Q68FT5,Q6AY41,Q6MG61,Q6Q0N1,Q71MB6,Q80W57,Q8R431,Q923S2,Q9JJ40,Q9WTW7,Q9WUW8,Q9WUW9,Q9Z0W7
5632	1,4,9,11,12,13,14,16	2,3,5,6,7,8,10,15	42	B0LT89,O08557,O35763,O54728,O70377,O70594,O88339;Q4V882,P10247,P12368,P19468,P20766,P25031,P30904,P38918,P46844,P48508,P50115,P50116,P51907,P53790,P54921,P57113,P97605,Q05175,Q3T1J9,Q3ZAV1,Q498D9,Q5I0E9,Q5M7T9,Q5RKI1,Q62714,Q62753,Q63270,Q63618,Q6MG61,Q6Q0N1,Q80W57,Q8CGS4,Q8R431,Q923S2,Q9WTW7,Q9Z0W7

5633	1,4,9,11,12,13,15,16	2,3,5,6,7,8,10,14	22	B0LT89,O08815,O70594,O88339;Q4V882,P08937,P20766,P25031,P25809,P48508,P50115,P50116,P53790,P54921,P57113,P63081,Q05175,Q5QE79,Q5RLM2,Q62714,Q63751,Q923S2,Q9WUW8
5634	1,4,9,11,12,14,15,16	2,3,5,6,7,8,10,13	23	O70257,O70594,O88339;Q4V882,P02631,P08937,P10247,P20766,P25031,P25809,P30904,P36860,P46844,P48508,P50115,P50116,P53790,P57113,P97605,Q05175,Q3ZAV1,Q62714,Q63618,Q9WUW8
5635	1,4,9,11,13,14,15,16	2,3,5,6,7,8,10,12	17	O54728,O70594,P02631,P08937,P0DMW0;P0DMW1,P20766,P25031,P30904,P36970,P46844,P48508,P50115,P50116,P53790,Q05175,Q5RLM2,Q63270
5636	1,4,9,12,13,14,15,16	2,3,5,6,7,8,10,11	4	P08937,P20766,P50115,P50116
5637	1,4,10,11,12,13,14,15	2,3,5,6,7,8,9,16	2	P50115,P50116
5638	1,4,10,11,12,13,14,16	2,3,5,6,7,8,9,15	9	O54728,O70594,P19132,P35280,P36376,P50115,P50116,Q62714,Q99MH3
5639	1,4,10,11,12,13,15,16	2,3,5,6,7,8,9,14	5	P08937,P50115,P50116,Q5QE79,Q63751
5640	1,4,10,11,12,14,15,16	2,3,5,6,7,8,9,13	4	P08937,P50115,P50116,Q5QE79
5641	1,4,10,11,13,14,15,16	2,3,5,6,7,8,9,12	5	O54728,P08937,P50115,P50116,Q5QE79
5642	1,4,10,12,13,14,15,16	2,3,5,6,7,8,9,11	8	P06760,P08937,P35280,P50115,P50116,Q5QE79,Q6P6R2,Q6P6S4
5643	1,4,11,12,13,14,15,16	2,3,5,6,7,8,9,10	15	D3ZTX0,O54728,O70594,P02454,P02631,P08937,P17559,P20766,P25031,P50115,P50116,P70545,Q4FZU6,Q5QE79,Q6P6R2
5644	1,5,6,7,8,9,10,11	2,3,4,12,13,14,15,16	8	P06760,P18421,P25031,P29315,P35745,P49134,Q63493,Q6P6R2
5645	1,5,6,7,8,9,10,12	2,3,4,11,13,14,15,16	5	P00762,P25031,P29315,P35745,P36860
5646	1,5,6,7,8,9,10,13	2,3,4,11,12,14,15,16	4	P25031,P29315,P35745,P36860
5647	1,5,6,7,8,9,10,14	2,3,4,11,12,13,15,16	4	P00762,P25031,P29315,P35745
5648	1,5,6,7,8,9,10,15	2,3,4,11,12,13,14,16	8	P00762,P19132,P19629,P25031,P35745,P36860,Q63474,Q9Z0V6

5649	1,5,6,7,8,9,10,16	2,3,4,11,12,13,14,15	3	P25031,P35745,P70545
5650	1,5,6,7,8,9,11,12	2,3,4,10,13,14,15,16	10	iRT-Kit_WR_fusion,P06760,P06911,P22283,P29315,P35745,Q03191,Q63493,Q812E4,Q9QW07
5651	1,5,6,7,8,9,11,13	2,3,4,10,12,14,15,16	8	P06760,P09456,P22283,P29315,Q09326,Q63493,Q63617,Q812E4
5652	1,5,6,7,8,9,11,14	2,3,4,10,12,13,15,16	5	P06760,P29315,Q09326,Q63493,Q812E4
5653	1,5,6,7,8,9,11,15	2,3,4,10,12,13,14,16	8	P06760,P35745,P49134,Q63493,Q6IMF3,Q6P6S4,Q8CJ52,Q9QW07
5654	1,5,6,7,8,9,11,16	2,3,4,10,12,13,14,15	18	P02780,P02781,P02782,P04905,P06760,P06761,P08723,P09456,P22283,P30120,P46462,P49134,Q5M8C6,Q63493,Q63617,Q812E4,Q9JHB9,Q9QW07
5655	1,5,6,7,8,9,12,13	2,3,4,10,11,14,15,16	2	P29315,P35745
5656	1,5,6,7,8,9,12,14	2,3,4,10,11,13,15,16	2	P29315,P35745
5657	1,5,6,7,8,9,12,15	2,3,4,10,11,13,14,16	3	iRT-Kit_WR_fusion,P19132,P35745
5658	1,5,6,7,8,9,12,16	2,3,4,10,11,13,14,15	3	iRT-Kit_WR_fusion,P21674,P35745
5659	1,5,6,7,8,9,13,14	2,3,4,10,11,12,15,16	1	P29315
5660	1,5,6,7,8,9,13,15	2,3,4,10,11,12,14,16	1	P35745
5661	1,5,6,7,8,9,13,16	2,3,4,10,11,12,14,15	0	
5662	1,5,6,7,8,9,14,15	2,3,4,10,11,12,13,16	1	P35745
5663	1,5,6,7,8,9,14,16	2,3,4,10,11,12,13,15	0	
5664	1,5,6,7,8,9,15,16	2,3,4,10,11,12,13,14	2	P13432,P35745
5665	1,5,6,7,8,10,11,12	2,3,4,9,13,14,15,16	5	P22283,P29315,P31044,Q5I0D1,Q63493
5666	1,5,6,7,8,10,11,13	2,3,4,9,12,14,15,16	5	P06761,P22283,P29315,P31044,Q63493
5667	1,5,6,7,8,10,11,14	2,3,4,9,12,13,15,16	3	P19218,P29315,P31044
5668	1,5,6,7,8,10,11,15	2,3,4,9,12,13,14,16	8	P02793;Q7TP54,P19629,P31044,P35745,Q63493,Q6IMF3,Q6P6Q2,Q8CJ52

5669	1,5,6,7,8,10,11,16	2,3,4,9,12,13,14,15	17	P02780,P02781,P02782,P04905,P06761,P07647,P08010,P08723,P09456,P22283,P25031,P30120,P46462,Q5M8C6,Q63493,Q9JHB9,Q9QW07
5670	1,5,6,7,8,10,12,13	2,3,4,9,11,14,15,16	6	P19814,P25031,P29315,P31044,P35745,P36860
5671	1,5,6,7,8,10,12,14	2,3,4,9,11,13,15,16	6	P19814,P25031,P29315,P35745,P57113,Q5RLM2
5672	1,5,6,7,8,10,12,15	2,3,4,9,11,13,14,16	7	P19132,P19629,P19814,P25031,P29315,P35745,P36860
5673	1,5,6,7,8,10,12,16	2,3,4,9,11,13,14,15	5	P0DMW0;P0DMW1,P19629,P25031,P35745,P57113
5674	1,5,6,7,8,10,13,14	2,3,4,9,11,12,15,16	4	P25031,P29315,P57113,Q62714
5675	1,5,6,7,8,10,13,15	2,3,4,9,11,12,14,16	5	P29315,P35745,P36860,P57113,Q62714
5676	1,5,6,7,8,10,13,16	2,3,4,9,11,12,14,15	3	O35077,P25031,P57113
5677	1,5,6,7,8,10,14,15	2,3,4,9,11,12,13,16	5	P29315,P35745,P54921,P57113,Q62714
5678	1,5,6,7,8,10,14,16	2,3,4,9,11,12,13,15	3	O35077,P25031,P57113
5679	1,5,6,7,8,10,15,16	2,3,4,9,11,12,13,14	13	B2RYW9,O35077,O70594,P13432,P19629,P25031,P35745,P53790,P57113,Q03248,Q05175,Q64335,Q8R431
5680	1,5,6,7,8,11,12,13	2,3,4,9,10,14,15,16	12	P06761,P06911,P12020,P22283,P29315,P46462,P47967,Q09326,Q5GRG2,Q5I0D1,Q63493,Q9JI85
5681	1,5,6,7,8,11,12,14	2,3,4,9,10,13,15,16	10	P06911,P12020,P19218,P22283,P29315,P46462,Q09326,Q5GRG2,Q63493,Q9QW07
5682	1,5,6,7,8,11,12,15	2,3,4,9,10,13,14,16	9	P22283,P35745,Q5GRG2,Q63493,Q6IG05,Q6IMF3,Q6P6Q2,Q8CJ52,Q9QW07
5683	1,5,6,7,8,11,12,16	2,3,4,9,10,13,14,15	22	O35547,P02780,P02781,P02782,P04905,P06761,P06911,P07647,P08010,P08723,P09456,P12020,P22283,P30120,P36374,P46462,Q5GRG2,Q5M8C6,Q63493,Q8CFN2,Q9JHB9,Q9QW07
5684	1,5,6,7,8,11,13,14	2,3,4,9,10,12,15,16	9	P06761,P19218,P22283,P29315,P46462,Q09326,Q5GRG2,Q63493,Q99041
5685	1,5,6,7,8,11,13,15	2,3,4,9,10,12,14,16	7	P01041,P22283,Q09326,Q63493,Q6IMF3,Q6P6Q2,Q8CJ52
5686	1,5,6,7,8,11,13,16	2,3,4,9,10,12,14,15	19	O35547,P02780,P02781,P02782,P06761,P07647,P08010,P08723,P09456,P22283,P24368,P30120,P46462,Q5M8C6,Q63493,Q63617,Q99041,Q9JHB9,Q9JI85
5687	1,5,6,7,8,11,14,15	2,3,4,9,10,12,13,16	8	P19218,Q4FZU2,Q5GRG2,Q63493,Q6IMF3,Q6P6Q2,Q8CJ52,Q9QW07
5688	1,5,6,7,8,11,14,16	2,3,4,9,10,12,13,15	22	P02780,P02781,P02782,P04905,P06761,P07647,P08010,P08723,P09456,P0C0A9,P19218,P22283,P30120,P46462,Q5GRG2,Q5M8C6,Q63493,Q812E4,Q8CFN2,Q99041,Q9JHB9,Q9QW07

5689	1,5,6,7,8,11,15,16	2,3,4,9,10,12,13,14	20	B2RYW9,P02780,P02781,P02782,P06761,P08010,P08723,P09456,P0C0A9,P22283,P30120,P46462,Q4FZU2,Q63493,Q6IMF3,Q6P6Q2,Q8CJ52,Q9JHB9,Q9QW07,Q9QZK9
5690	1,5,6,7,8,12,13,14	2,3,4,9,10,11,15,16	3	P19218,P29315,Q9Z2L0
5691	1,5,6,7,8,12,13,15	2,3,4,9,10,11,14,16	4	P19814,P29315,P35745,P70549
5692	1,5,6,7,8,12,13,16	2,3,4,9,10,11,14,15	0	
5693	1,5,6,7,8,12,14,15	2,3,4,9,10,11,13,16	5	D3ZUC6,P19218,P19814,P29315,P35745
5694	1,5,6,7,8,12,14,16	2,3,4,9,10,11,13,15	1	P19218
5695	1,5,6,7,8,12,15,16	2,3,4,9,10,11,13,14	2	P19629,P35745
5696	1,5,6,7,8,13,14,15	2,3,4,9,10,11,12,16	4	P29315,P70549,Q62714,Q9Z2L0
5697	1,5,6,7,8,13,14,16	2,3,4,9,10,11,12,15	1	Q62714
5698	1,5,6,7,8,13,15,16	2,3,4,9,10,11,12,14	1	Q62714
5699	1,5,6,7,8,14,15,16	2,3,4,9,10,11,12,13	2	P13432,Q62714
5700	1,5,6,7,9,10,11,12	2,3,4,8,13,14,15,16	4	B0BNN3,P00762,P29315,Q03191
5701	1,5,6,7,9,10,11,13	2,3,4,8,12,14,15,16	2	P00762,P29315
5702	1,5,6,7,9,10,11,14	2,3,4,8,12,13,15,16	3	P00762,P00774,P29315
5703	1,5,6,7,9,10,11,15	2,3,4,8,12,13,14,16	2	P00762,P35745
5704	1,5,6,7,9,10,11,16	2,3,4,8,12,13,14,15	0	
5705	1,5,6,7,9,10,12,13	2,3,4,8,11,14,15,16	5	P00762,P29315,P35745,P36860,Q03191
5706	1,5,6,7,9,10,12,14	2,3,4,8,11,13,15,16	5	P00762,P00774,P05539,P29315,P35745
5707	1,5,6,7,9,10,12,15	2,3,4,8,11,13,14,16	4	B0BNN3,O54728,P00762,P35745
5708	1,5,6,7,9,10,12,16	2,3,4,8,11,13,14,15	1	Q03191

5709	1,5,6,7,9,10,13,14	2,3,4,8,11,12,15,16	5	P00762,P00774,P05539,P29315,Q5PQL7
5710	1,5,6,7,9,10,13,15	2,3,4,8,11,12,14,16	3	P00762,P35745,P36860
5711	1,5,6,7,9,10,13,16	2,3,4,8,11,12,14,15	1	P11883
5712	1,5,6,7,9,10,14,15	2,3,4,8,11,12,13,16	5	P00762,P00774,P02625,P05539,P35745
5713	1,5,6,7,9,10,14,16	2,3,4,8,11,12,13,15	2	P00762,P00774
5714	1,5,6,7,9,10,15,16	2,3,4,8,11,12,13,14	4	P00762,P35745,Q64335,Q811M5
5715	1,5,6,7,9,11,12,13	2,3,4,8,10,14,15,16	5	P00762,P20762,P29315,Q03191,Q62761;Q62762;Q62763
5716	1,5,6,7,9,11,12,14	2,3,4,8,10,13,15,16	4	P00762,P29315,Q03191,Q64093
5717	1,5,6,7,9,11,12,15	2,3,4,8,10,13,14,16	4	P00762,P35745,Q03191,Q62761;Q62762;Q62763
5718	1,5,6,7,9,11,12,16	2,3,4,8,10,13,14,15	1	Q03191
5719	1,5,6,7,9,11,13,14	2,3,4,8,10,12,15,16	3	P00762,P0DMW0;P0DMW1,P29315
5720	1,5,6,7,9,11,13,15	2,3,4,8,10,12,14,16	1	P00762
5721	1,5,6,7,9,11,13,16	2,3,4,8,10,12,14,15	0	
5722	1,5,6,7,9,11,14,15	2,3,4,8,10,12,13,16	4	P00762,P02625,P0DMW0;P0DMW1,Q8CJ52
5723	1,5,6,7,9,11,14,16	2,3,4,8,10,12,13,15	1	Q9R168
5724	1,5,6,7,9,11,15,16	2,3,4,8,10,12,13,14	1	Q8CJ52
5725	1,5,6,7,9,12,13,14	2,3,4,8,10,11,15,16	3	P00762,P05539,P29315
5726	1,5,6,7,9,12,13,15	2,3,4,8,10,11,14,16	2	P00762,P35745
5727	1,5,6,7,9,12,13,16	2,3,4,8,10,11,14,15	1	Q03191
5728	1,5,6,7,9,12,14,15	2,3,4,8,10,11,13,16	6	P00762,P00774,P02625,P05539,P35745,Q5BJY9

5729	1,5,6,7,9,12,14,16	2,3,4,8,10,11,13,15	1	P00774
5730	1,5,6,7,9,12,15,16	2,3,4,8,10,11,13,14	1	P35745
5731	1,5,6,7,9,13,14,15	2,3,4,8,10,11,12,16	6	P00762,P00774,P01835,P02625,P05539,Q5PQL7
5732	1,5,6,7,9,13,14,16	2,3,4,8,10,11,12,15	1	P00774
5733	1,5,6,7,9,13,15,16	2,3,4,8,10,11,12,14	0	
5734	1,5,6,7,9,14,15,16	2,3,4,8,10,11,12,13	1	P00774
5735	1,5,6,7,10,11,12,13	2,3,4,8,9,14,15,16	5	P00774,P23593,P29315,P47967,Q5I0D1
5736	1,5,6,7,10,11,12,14	2,3,4,8,9,13,15,16	4	P00762,P00774,P23593,P29315
5737	1,5,6,7,10,11,12,15	2,3,4,8,9,13,14,16	3	P00762,P00774,P23593
5738	1,5,6,7,10,11,12,16	2,3,4,8,9,13,14,15	1	P00774
5739	1,5,6,7,10,11,13,14	2,3,4,8,9,12,15,16	5	P00762,P00774,P05539,P29315,P36376
5740	1,5,6,7,10,11,13,15	2,3,4,8,9,12,14,16	2	P00762,P00774
5741	1,5,6,7,10,11,13,16	2,3,4,8,9,12,14,15	2	P00774,P36376
5742	1,5,6,7,10,11,14,15	2,3,4,8,9,12,13,16	2	P00762,P00774
5743	1,5,6,7,10,11,14,16	2,3,4,8,9,12,13,15	2	P00774,P36376
5744	1,5,6,7,10,11,15,16	2,3,4,8,9,12,13,14	2	P00774,P17988
5745	1,5,6,7,10,12,13,14	2,3,4,8,9,11,15,16	5	P00762,P00774,P01835,P05539,P29315
5746	1,5,6,7,10,12,13,15	2,3,4,8,9,11,14,16	6	P00762,P00774,P01835,P19814,P35745,P36860
5747	1,5,6,7,10,12,13,16	2,3,4,8,9,11,14,15	3	P00774,P01835,P36376
5748	1,5,6,7,10,12,14,15	2,3,4,8,9,11,13,16	4	P00762,P00774,P05539,P35745

5749	1,5,6,7,10,<br>12,14,16	2,3,4,8,9,1<br>1,13,15	1 P00774
5750	1,5,6,7,10,<br>12,15,16	2,3,4,8,9,1<br>1,13,14	2 P00774,Q64335
5751	1,5,6,7,10,<br>13,14,15	2,3,4,8,9,1<br>1,12,16	7 O88797,P00762,P00774,P01835,P05539,Q5PQL7,Q62714
5752	1,5,6,7,10,<br>13,14,16	2,3,4,8,9,1<br>1,12,15	4 P00774,P01835,P36376,P57113
5753	1,5,6,7,10,<br>13,15,16	2,3,4,8,9,1<br>1,12,14	3 P00774,P01835,P11883
5754	1,5,6,7,10,<br>14,15,16	2,3,4,8,9,1<br>1,12,13	3 P00774,P57113,Q64335
5755	1,5,6,7,11,<br>12,13,14	2,3,4,8,9,1<br>0,15,16	3 P00774,P19218,P29315
5756	1,5,6,7,11,<br>12,13,15	2,3,4,8,9,1<br>0,14,16	0
5757	1,5,6,7,11,<br>12,13,16	2,3,4,8,9,1<br>0,14,15	2 P22283,P47967
5758	1,5,6,7,11,<br>12,14,15	2,3,4,8,9,1<br>0,13,16	2 P00774,P19218
5759	1,5,6,7,11,<br>12,14,16	2,3,4,8,9,1<br>0,13,15	2 P00774,P19218
5760	1,5,6,7,11,<br>12,15,16	2,3,4,8,9,1<br>0,13,14	2 Q8CJ52,Q9QW07
5761	1,5,6,7,11,<br>13,14,15	2,3,4,8,9,1<br>0,12,16	3 P00774,P01835,P05539
5762	1,5,6,7,11,<br>13,14,16	2,3,4,8,9,1<br>0,12,15	3 P00774,P34901,P36376
5763	1,5,6,7,11,<br>13,15,16	2,3,4,8,9,1<br>0,12,14	2 O70417,Q8CJ52
5764	1,5,6,7,11,<br>14,15,16	2,3,4,8,9,1<br>0,12,13	2 P00774,Q8CJ52
5765	1,5,6,7,12,<br>13,14,15	2,3,4,8,9,1<br>0,11,16	4 P00774,P01835,P05539,P70549
5766	1,5,6,7,12,<br>13,14,16	2,3,4,8,9,1<br>0,11,15	3 P00774,P01835,P36376
5767	1,5,6,7,12,<br>13,15,16	2,3,4,8,9,1<br>0,11,14	2 P00774,P01835
5768	1,5,6,7,12,<br>14,15,16	2,3,4,8,9,1<br>0,11,13	1 P00774

5769	1,5,6,7,13,14,15,16	2,3,4,8,9,10,11,12	4	P00774,P01835,Q5PQL7,Q62714
5770	1,5,6,8,9,10,11,12	2,3,4,7,13,14,15,16	3	P29315,P35745,Q03191
5771	1,5,6,8,9,10,11,13	2,3,4,7,12,14,15,16	2	B0LT89,P29315
5772	1,5,6,8,9,10,11,14	2,3,4,7,12,13,15,16	1	P29315
5773	1,5,6,8,9,10,11,15	2,3,4,7,12,13,14,16	2	P35745,Q6P6S4
5774	1,5,6,8,9,10,11,16	2,3,4,7,12,13,14,15	1	P25031
5775	1,5,6,8,9,10,12,13	2,3,4,7,11,14,15,16	3	P25031,P29315,P35745
5776	1,5,6,8,9,10,12,14	2,3,4,7,11,13,15,16	3	P25031,P29315,P35745
5777	1,5,6,8,9,10,12,15	2,3,4,7,11,13,14,16	3	B0BNN3,P25031,P35745
5778	1,5,6,8,9,10,12,16	2,3,4,7,11,13,14,15	2	P25031,P35745
5779	1,5,6,8,9,10,13,14	2,3,4,7,11,12,15,16	2	P25031,P29315
5780	1,5,6,8,9,10,13,15	2,3,4,7,11,12,14,16	1	P35745
5781	1,5,6,8,9,10,13,16	2,3,4,7,11,12,14,15	1	P25031
5782	1,5,6,8,9,10,14,15	2,3,4,7,11,12,13,16	1	P35745
5783	1,5,6,8,9,10,14,16	2,3,4,7,11,12,13,15	1	P25031
5784	1,5,6,8,9,10,15,16	2,3,4,7,11,12,13,14	3	P25031,P35745,Q811M5
5785	1,5,6,8,9,11,12,13	2,3,4,7,10,14,15,16	7	B0LT89,P18297,P29315,P61206;P84079,P63095,Q03191,Q5RKI1
5786	1,5,6,8,9,11,12,14	2,3,4,7,10,13,15,16	9	P18297,P19468,P20766,P61206;P84079,P63095,Q5I0J9,Q5RKI1,Q63424,Q64093
5787	1,5,6,8,9,11,12,15	2,3,4,7,10,13,14,16	2	P18297,P35745
5788	1,5,6,8,9,11,12,16	2,3,4,7,10,13,14,15	5	O88797,P63095,Q03191,Q5RKI1,Q9QW07

5789	1,5,6,8,9,11,13,14	2,3,4,7,10,12,15,16	6	P0DMW0;P0DMW1,P10758,P18297,P20766,Q5RKI1,Q64093
5790	1,5,6,8,9,11,13,15	2,3,4,7,10,12,14,16	0	
5791	1,5,6,8,9,11,13,16	2,3,4,7,10,12,14,15	2	P30120,Q5RKI1
5792	1,5,6,8,9,11,14,15	2,3,4,7,10,12,13,16	0	
5793	1,5,6,8,9,11,14,16	2,3,4,7,10,12,13,15	3	Q5I0J9,Q5RKI1,Q812E4
5794	1,5,6,8,9,11,15,16	2,3,4,7,10,12,13,14	1	Q9QW07
5795	1,5,6,8,9,12,13,14	2,3,4,7,10,11,15,16	3	P17988,P20766,P29315
5796	1,5,6,8,9,12,13,15	2,3,4,7,10,11,14,16	1	P35745
5797	1,5,6,8,9,12,13,16	2,3,4,7,10,11,14,15	1	Q5RKI1
5798	1,5,6,8,9,12,14,15	2,3,4,7,10,11,13,16	4	P00714,P20766,P28570,P35745
5799	1,5,6,8,9,12,14,16	2,3,4,7,10,11,13,15	2	P21674,Q5RKI1
5800	1,5,6,8,9,12,15,16	2,3,4,7,10,11,13,14	1	P35745
5801	1,5,6,8,9,13,14,15	2,3,4,7,10,11,12,16	2	P20766,Q00715
5802	1,5,6,8,9,13,14,16	2,3,4,7,10,11,12,15	2	P20766,Q5RKI1
5803	1,5,6,8,9,13,15,16	2,3,4,7,10,11,12,14	0	
5804	1,5,6,8,9,14,15,16	2,3,4,7,10,11,12,13	2	P47967,P97840
5805	1,5,6,8,10,11,12,13	2,3,4,7,9,14,15,16	1	P29315
5806	1,5,6,8,10,11,12,14	2,3,4,7,9,13,15,16	2	P19218,P29315
5807	1,5,6,8,10,11,12,15	2,3,4,7,9,13,14,16	0	
5808	1,5,6,8,10,11,12,16	2,3,4,7,9,13,14,15	1	P25031

5809	1,5,6,8,10,11,13,14	2,3,4,7,9,12,15,16	1	P29315
5810	1,5,6,8,10,11,13,15	2,3,4,7,9,12,14,16	0	
5811	1,5,6,8,10,11,13,16	2,3,4,7,9,12,14,15	1	P30120
5812	1,5,6,8,10,11,14,15	2,3,4,7,9,12,13,16	0	
5813	1,5,6,8,10,11,14,16	2,3,4,7,9,12,13,15	0	
5814	1,5,6,8,10,11,15,16	2,3,4,7,9,12,13,14	1	Q6IMF3
5815	1,5,6,8,10,12,13,14	2,3,4,7,9,11,15,16	3	P25031,P29315,P35280
5816	1,5,6,8,10,12,13,15	2,3,4,7,9,11,14,16	3	P19814,P35745,P70549
5817	1,5,6,8,10,12,13,16	2,3,4,7,9,11,14,15	2	P25031,P35280
5818	1,5,6,8,10,12,14,15	2,3,4,7,9,11,13,16	2	P35745,P54921
5819	1,5,6,8,10,12,14,16	2,3,4,7,9,11,13,15	2	P25031,P35280
5820	1,5,6,8,10,12,15,16	2,3,4,7,9,11,13,14	3	P0DMW0;P0DMW1,P25031,P35745
5821	1,5,6,8,10,13,14,15	2,3,4,7,9,11,12,16	0	
5822	1,5,6,8,10,13,14,16	2,3,4,7,9,11,12,15	3	P14668,P25031,P35280
5823	1,5,6,8,10,13,15,16	2,3,4,7,9,11,12,14	0	
5824	1,5,6,8,10,14,15,16	2,3,4,7,9,11,12,13	2	P00774,P54921
5825	1,5,6,8,11,12,13,14	2,3,4,7,9,10,15,16	5	P19218,P20766,P29315,Q5RKI1,Q8K1G0
5826	1,5,6,8,11,12,13,15	2,3,4,7,9,10,14,16	0	
5827	1,5,6,8,11,12,13,16	2,3,4,7,9,10,14,15	4	O35547,P02782,P30120,Q5RKI1
5828	1,5,6,8,11,12,14,15	2,3,4,7,9,10,13,16	2	P19218,P20766

5829	1,5,6,8,11,12,14,16	2,3,4,7,9,10,13,15	5	P19218,P20766,Q5RKI1,Q8CFN2,Q9QW07
5830	1,5,6,8,11,12,15,16	2,3,4,7,9,10,13,14	2	Q6IMF3,Q9QW07
5831	1,5,6,8,11,13,14,15	2,3,4,7,9,10,12,16	2	P19218,P20766
5832	1,5,6,8,11,13,14,16	2,3,4,7,9,10,12,15	4	P19218,P20766,P30120,Q5RKI1
5833	1,5,6,8,11,13,15,16	2,3,4,7,9,10,12,14	1	P30120
5834	1,5,6,8,11,14,15,16	2,3,4,7,9,10,12,13	2	P19218,Q6IMF3
5835	1,5,6,8,12,13,14,15	2,3,4,7,9,10,11,16	2	P20766,P70549
5836	1,5,6,8,12,13,14,16	2,3,4,7,9,10,11,15	3	P20766,P35280,Q5RKI1
5837	1,5,6,8,12,13,15,16	2,3,4,7,9,10,11,14	0	
5838	1,5,6,8,12,14,15,16	2,3,4,7,9,10,11,13	1	P20766
5839	1,5,6,8,13,14,15,16	2,3,4,7,9,10,11,12	2	P20766,Q62714
5840	1,5,6,9,10,11,12,13	2,3,4,7,8,14,15,16	6	B0LT89,P00762,P20761,P20762,Q03191,Q62761;Q62762;Q62763
5841	1,5,6,9,10,11,12,14	2,3,4,7,8,13,15,16	7	P00762,P00774,P20761,Q03191,Q5I0J9,Q64093,Q8CIZ5
5842	1,5,6,9,10,11,12,15	2,3,4,7,8,13,14,16	8	B0BNN3,D4A5U3,P00762,P20761,Q03191,Q62761;Q62762;Q62763,Q8CIZ5,Q9WUW8
5843	1,5,6,9,10,11,12,16	2,3,4,7,8,13,14,15	4	B0BNN3,P20761,Q03191,Q8CIZ5
5844	1,5,6,9,10,11,13,14	2,3,4,7,8,12,15,16	4	B0LT89,P00762,P00774,P20761
5845	1,5,6,9,10,11,13,15	2,3,4,7,8,12,14,16	4	B0LT89,O70417,P00762,P20761
5846	1,5,6,9,10,11,13,16	2,3,4,7,8,12,14,15	2	O70417,Q8CIZ5
5847	1,5,6,9,10,11,14,15	2,3,4,7,8,12,13,16	5	D4A5U3,P00762,P00774,P20761,Q8CIZ5
5848	1,5,6,9,10,11,14,16	2,3,4,7,8,12,13,15	2	P00774,Q8CIZ5

5849	1,5,6,9,10,11,15,16	2,3,4,7,8,12,13,14	6	B0BNN3,D4A5U3,O70417,P00774,Q811M5,Q8CIZ5
5850	1,5,6,9,10,12,13,14	2,3,4,7,8,11,15,16	7	P00762,P00774,P02780,P05539,P0C0A9,P20761,P29315
5851	1,5,6,9,10,12,13,15	2,3,4,7,8,11,14,16	11	B0BNN3,D4A5U3,P00762,P00774,P02780,P0C0A9,P20761,P35745,Q00715,Q4G075,Q5M8C6
5852	1,5,6,9,10,12,13,16	2,3,4,7,8,11,14,15	4	P00774,Q03191,Q4G075,Q8CIZ5
5853	1,5,6,9,10,12,14,15	2,3,4,7,8,11,13,16	15	B0BNN3,D4A5U3,P00762,P00774,P02780,P02781,P02782,P20761,P22273,P30120,P35745,Q4G075,Q5M8C6,Q62902,Q8CIZ5
5854	1,5,6,9,10,12,14,16	2,3,4,7,8,11,13,15	2	P00774,Q8CIZ5
5855	1,5,6,9,10,12,15,16	2,3,4,7,8,11,13,14	6	B0BNN3,D4A5U3,P00774,Q4G075,Q811M5,Q8CIZ5
5856	1,5,6,9,10,13,14,15	2,3,4,7,8,11,12,16	10	P00762,P00774,P02780,P05539,P30120,P62804,P70709,Q00715,Q5M8C6,Q5PQL7
5857	1,5,6,9,10,13,14,16	2,3,4,7,8,11,12,15	3	P00774,Q5PQL7,Q8CIZ5
5858	1,5,6,9,10,13,15,16	2,3,4,7,8,11,12,14	5	O70417,P00774,P11883,Q811M5,Q8CIZ5
5859	1,5,6,9,10,14,15,16	2,3,4,7,8,11,12,13	6	D4A5U3,P00774,P47967,Q5PQL7,Q811M5,Q8CIZ5
5860	1,5,6,9,11,12,13,14	2,3,4,7,8,10,15,16	40	B0LT89,O55004,O70377,O70594,O88339;Q4V882,P00774,P07151,P19468,P20646,P20761,P20762,P20766,P46844,P48508,P51907,P53790,Q03191,Q03248,Q05175,Q3MIE4,Q3ZAV1,Q5I0E9,Q5M7T9,Q5RKI1,Q62761;Q62762;Q62763,Q63270,Q63355,Q63424,Q63598,Q63618,Q64093,Q6AY41,Q6MG61,Q6Q0N1,Q80W57,Q8R431,Q923S2,Q9JJ40,Q9WTW7,Q9Z0W7
5861	1,5,6,9,11,12,13,15	2,3,4,7,8,10,14,16	15	B0LT89,O70377,O70417,P20761,P20762,P46844,P53790,Q03191,Q62761;Q62762;Q62763,Q63355,Q63424,Q64093,Q923S2,Q9WUW8,Q9WUW9
5862	1,5,6,9,11,12,13,16	2,3,4,7,8,10,14,15	11	O70377,O70417,O88339;Q4V882,P20761,P20762,P54921,Q03191,Q05175,Q5RKI1,Q63355,Q8CIZ5
5863	1,5,6,9,11,12,14,15	2,3,4,7,8,10,13,16	28	D4A5U3,O55004,O70257,O70377,O70594,P00762,P00774,P07151,P19468,P20761,P20766,P28570,P46844,P51907,P53790,Q3ZAV1,Q5BJY9,Q62761;Q62762;Q62763,Q63355,Q63424,Q63618,Q64093,Q6Q0N1,Q8CIZ5,Q9JJ40,Q9WTW7,Q9WUW8,Q9Z0W7
5864	1,5,6,9,11,12,14,16	2,3,4,7,8,10,13,15	19	O55004,O70377,O70594,O88339;Q4V882,P00774,P19468,P20761,P20766,P51907,Q03191,Q5I0J9,Q5RKI1,Q63355,Q63424,Q63618,Q64093,Q6Q0N1,Q8CIZ5,Q9WTW7
5865	1,5,6,9,11,12,15,16	2,3,4,7,8,10,13,14	8	B0BNN3,D4A5U3,O70417,P20761,P25809,Q03191,Q8CIZ5,Q9WUW8
5866	1,5,6,9,11,13,14,15	2,3,4,7,8,10,12,16	14	P00774,P02625,P0DMW0;P0DMW1,P20761,P20766,P46844,P53790,Q3ZAV1,Q63424,Q63598,Q64093,Q923V8,Q9JJ40,Q9WTW7
5867	1,5,6,9,11,13,14,16	2,3,4,7,8,10,12,15	9	P00774,P12368,P20766,P30904,Q5RKI1,Q63598,Q64093,Q8CIZ5,Q9WTW7

5868	1,5,6,9,11,13,15,16	2,3,4,7,8,10,12,14	3	O70417,Q5RLM2,Q8CIZ5
5869	1,5,6,9,11,14,15,16	2,3,4,7,8,10,12,13	5	P00774,P20766,Q63618,Q8CIZ5,Q923V8
5870	1,5,6,9,12,13,14,15	2,3,4,7,8,10,11,16	12	P00762,P00774,P02780,P05539,P20761,P20766,P28570,Q00715,Q5BJY9,Q63424,Q64093,Q9WTW7
5871	1,5,6,9,12,13,14,16	2,3,4,7,8,10,11,15	5	P00774,P20766,Q5RKI1,Q8CIZ5,Q9WTW7
5872	1,5,6,9,12,13,15,16	2,3,4,7,8,10,11,14	3	O70417,Q6B345,Q8CIZ5
5873	1,5,6,9,12,14,15,16	2,3,4,7,8,10,11,13	5	D4A5U3,P00774,P20766,Q5BJY9,Q8CIZ5
5874	1,5,6,9,13,14,15,16	2,3,4,7,8,10,11,12	5	P00774,P20766,P70709,Q5PQL7,Q8CIZ5
5875	1,5,6,10,11,12,13,14	2,3,4,7,8,9,15,16	4	P00774,P20646,P20761,P35280
5876	1,5,6,10,11,12,13,15	2,3,4,7,8,9,14,16	6	D4A5U3,O70417,P00774,P20646,P20761,P23593
5877	1,5,6,10,11,12,13,16	2,3,4,7,8,9,14,15	4	O70417,P00774,P20646,P35280
5878	1,5,6,10,11,12,14,15	2,3,4,7,8,9,13,16	6	D4A5U3,P00762,P00774,P20646,P20761,Q8CIZ5
5879	1,5,6,10,11,12,14,16	2,3,4,7,8,9,13,15	5	D4A5U3,P00774,P20646,P35280,Q8CIZ5
5880	1,5,6,10,11,12,15,16	2,3,4,7,8,9,13,14	6	B0BNN3,D4A5U3,O70417,P00774,Q5QE79,Q8CIZ5
5881	1,5,6,10,11,13,14,15	2,3,4,7,8,9,12,16	2	D4A5U3,P00774
5882	1,5,6,10,11,13,14,16	2,3,4,7,8,9,12,15	3	P00774,P35280,P36376
5883	1,5,6,10,11,13,15,16	2,3,4,7,8,9,12,14	5	D4A5U3,O70417,P00774,Q5QE79,Q8CIZ5
5884	1,5,6,10,11,14,15,16	2,3,4,7,8,9,12,13	3	D4A5U3,P00774,Q8CIZ5
5885	1,5,6,10,12,13,14,15	2,3,4,7,8,9,11,16	9	D4A5U3,P00762,P00774,P02780,P05539,P20646,P20761,P35280,Q62902
5886	1,5,6,10,12,13,14,16	2,3,4,7,8,9,11,15	5	P00774,P14668,P20646,P35280,Q6P6R2
5887	1,5,6,10,12,13,15,16	2,3,4,7,8,9,11,14	8	D4A5U3,O70417,P00774,P07150,P35280,Q5QE79,Q63751,Q8CIZ5

5888	1,5,6,10,12,14,15,16	2,3,4,7,8,9,11,13	5	D4A5U3,P00774,P35280,P82471,Q8CIZ5
5889	1,5,6,10,13,14,15,16	2,3,4,7,8,9,11,12	5	P00774,P35280,P70709,Q5PQL7,Q8CIZ5
5890	1,5,6,11,12,13,14,15	2,3,4,7,8,9,10,16	7	D4A5U3,P00774,P20646,P20761,P20766,P23739,P70545
5891	1,5,6,11,12,13,14,16	2,3,4,7,8,9,10,15	6	P00774,P20646,P20766,P35280,Q5RKI1,Q9Z0V6
5892	1,5,6,11,12,13,15,16	2,3,4,7,8,9,10,14	6	D4A5U3,O70417,P00774,P20646,Q5QE79,Q8CIZ5
5893	1,5,6,11,12,14,15,16	2,3,4,7,8,9,10,13	5	D4A5U3,P00774,P20766,Q63618,Q8CIZ5
5894	1,5,6,11,13,14,15,16	2,3,4,7,8,9,10,12	5	O70417,P00774,P20766,Q8CIZ5,Q923V8
5895	1,5,6,12,13,14,15,16	2,3,4,7,8,9,10,11	8	D4A5U3,P00774,P20646,P20766,P35280,Q6B345,Q6P6R2,Q8CIZ5
5896	1,5,7,8,9,10,11,12	2,3,4,6,13,14,15,16	5	P06760,P29315,P35745,P49134,Q03191
5897	1,5,7,8,9,10,11,13	2,3,4,6,12,14,15,16	2	P06760,P29315
5898	1,5,7,8,9,10,11,14	2,3,4,6,12,13,15,16	2	P06760,P29315
5899	1,5,7,8,9,10,11,15	2,3,4,6,12,13,14,16	4	P06760,P35745,P49134,Q6IMF3
5900	1,5,7,8,9,10,11,16	2,3,4,6,12,13,14,15	2	P49134,Q812E4
5901	1,5,7,8,9,10,12,13	2,3,4,6,11,14,15,16	3	P29315,P35745,Q4KLZ6
5902	1,5,7,8,9,10,12,14	2,3,4,6,11,13,15,16	3	P21674,P29315,P35745
5903	1,5,7,8,9,10,12,15	2,3,4,6,11,13,14,16	3	P19132,P35745,Q4KLZ6
5904	1,5,7,8,9,10,12,16	2,3,4,6,11,13,14,15	3	P21674,P35745,P49134
5905	1,5,7,8,9,10,13,14	2,3,4,6,11,12,15,16	2	P29315,P35745
5906	1,5,7,8,9,10,13,15	2,3,4,6,11,12,14,16	2	P35745,Q4KLZ6
5907	1,5,7,8,9,10,13,16	2,3,4,6,11,12,14,15	1	P35745

5908	1,5,7,8,9,10,14,15	2,3,4,6,11,12,13,16	2	P05369,P35745
5909	1,5,7,8,9,10,14,16	2,3,4,6,11,12,13,15	2	P21674,P35745
5910	1,5,7,8,9,10,15,16	2,3,4,6,11,12,13,14	2	P35745,P49134
5911	1,5,7,8,9,11,12,13	2,3,4,6,10,14,15,16	3	P06760,P29315,Q03191
5912	1,5,7,8,9,11,12,14	2,3,4,6,10,13,15,16	5	P06760,P21674,P29315,Q03191,Q812E4
5913	1,5,7,8,9,11,12,15	2,3,4,6,10,13,14,16	3	P35745,P49134,Q6IMF3
5914	1,5,7,8,9,11,12,16	2,3,4,6,10,13,14,15	6	P09456,P21674,P49134,Q03191,Q812E4,Q9JHB9
5915	1,5,7,8,9,11,13,14	2,3,4,6,10,12,15,16	4	P06760,P0DMW0;P0DMW1,P29315,Q812E4
5916	1,5,7,8,9,11,13,15	2,3,4,6,10,12,14,16	2	P35745,Q6IMF3
5917	1,5,7,8,9,11,13,16	2,3,4,6,10,12,14,15	4	P02782,P09456,P30120,Q812E4
5918	1,5,7,8,9,11,14,15	2,3,4,6,10,12,13,16	4	P06760,P35745,Q6IMF3,Q6P6Q2
5919	1,5,7,8,9,11,14,16	2,3,4,6,10,12,13,15	3	P06760,P09456,Q812E4
5920	1,5,7,8,9,11,15,16	2,3,4,6,10,12,13,14	6	P35745,P49134,Q6IMF3,Q6P6Q2,Q812E4,Q8CJ52
5921	1,5,7,8,9,12,13,14	2,3,4,6,10,11,15,16	3	P17988,P29315,P35745
5922	1,5,7,8,9,12,13,15	2,3,4,6,10,11,14,16	2	P35745,Q4KLZ6
5923	1,5,7,8,9,12,13,16	2,3,4,6,10,11,14,15	1	P35745
5924	1,5,7,8,9,12,14,15	2,3,4,6,10,11,13,16	2	P00714,P35745
5925	1,5,7,8,9,12,14,16	2,3,4,6,10,11,13,15	2	P21674,P35745
5926	1,5,7,8,9,12,15,16	2,3,4,6,10,11,13,14	1	P35745
5927	1,5,7,8,9,13,14,15	2,3,4,6,10,11,12,16	1	P35745

5928	1,5,7,8,9,13,14,16	2,3,4,6,10,11,12,15	0	
5929	1,5,7,8,9,13,15,16	2,3,4,6,10,11,12,14	1	P35745
5930	1,5,7,8,9,14,15,16	2,3,4,6,10,11,12,13	1	P35745
5931	1,5,7,8,10,11,12,13	2,3,4,6,9,14,15,16	4	P23593,P29315,P47967,Q5I0D1
5932	1,5,7,8,10,11,12,14	2,3,4,6,9,13,15,16	2	P23593,P29315
5933	1,5,7,8,10,11,12,15	2,3,4,6,9,13,14,16	4	P23593,P35745,Q6IMF3,Q6P6Q2
5934	1,5,7,8,10,11,12,16	2,3,4,6,9,13,14,15	3	P08010,P30120,Q9JHB9
5935	1,5,7,8,10,11,13,14	2,3,4,6,9,12,15,16	1	P29315
5936	1,5,7,8,10,11,13,15	2,3,4,6,9,12,14,16	2	Q6IMF3,Q6P6Q2
5937	1,5,7,8,10,11,13,16	2,3,4,6,9,12,14,15	3	P02782,P09456,P30120
5938	1,5,7,8,10,11,14,15	2,3,4,6,9,12,13,16	2	Q6IMF3,Q6P6Q2
5939	1,5,7,8,10,11,14,16	2,3,4,6,9,12,13,15	0	
5940	1,5,7,8,10,11,15,16	2,3,4,6,9,12,13,14	4	P49134,Q4FZU2,Q6IMF3,Q6P6Q2
5941	1,5,7,8,10,12,13,14	2,3,4,6,9,11,15,16	1	P29315
5942	1,5,7,8,10,12,13,15	2,3,4,6,9,11,14,16	2	P35745,Q4KLZ6
5943	1,5,7,8,10,12,13,16	2,3,4,6,9,11,14,15	0	
5944	1,5,7,8,10,12,14,15	2,3,4,6,9,11,13,16	2	D3ZUC6,P35745
5945	1,5,7,8,10,12,14,16	2,3,4,6,9,11,13,15	1	P21674
5946	1,5,7,8,10,12,15,16	2,3,4,6,9,11,13,14	1	P35745
5947	1,5,7,8,10,13,14,15	2,3,4,6,9,11,12,16	1	P35745

5948	1,5,7,8,10,13,14,16	2,3,4,6,9,11,12,15	2	O35077,Q99MH3
5949	1,5,7,8,10,13,15,16	2,3,4,6,9,11,12,14	2	O35077,P35745
5950	1,5,7,8,10,14,15,16	2,3,4,6,9,11,12,13	2	O35077,P35745
5951	1,5,7,8,11,12,13,14	2,3,4,6,9,10,15,16	5	D3ZUC6,P19218,P29315,Q811M5,Q8K1G0
5952	1,5,7,8,11,12,13,15	2,3,4,6,9,10,14,16	2	Q6IMF3,Q6P6Q2
5953	1,5,7,8,11,12,13,16	2,3,4,6,9,10,14,15	6	P02782,P09456,P30120,P47967,Q811M5,Q9JHB9
5954	1,5,7,8,11,12,14,15	2,3,4,6,9,10,13,16	4	D3ZUC6,Q6IMF3,Q6P6Q2,Q811M5
5955	1,5,7,8,11,12,14,16	2,3,4,6,9,10,13,15	6	P02782,P21674,P30120,Q811M5,Q812E4,Q9JHB9
5956	1,5,7,8,11,12,15,16	2,3,4,6,9,10,13,14	5	Q06000,Q4FZU2,Q6IMF3,Q6P6Q2,Q9JHB9
5957	1,5,7,8,11,13,14,15	2,3,4,6,9,10,12,16	4	D3ZUC6,Q6IMF3,Q6P6Q2,Q811M5
5958	1,5,7,8,11,13,14,16	2,3,4,6,9,10,12,15	5	P02782,P09456,P30120,Q811M5,Q99MH3
5959	1,5,7,8,11,13,15,16	2,3,4,6,9,10,12,14	7	P01041,P02782,P09456,P30120,Q4FZU2,Q6IMF3,Q6P6Q2
5960	1,5,7,8,11,14,15,16	2,3,4,6,9,10,12,13	4	Q4FZU2,Q6IMF3,Q6P6Q2,Q8CJ52
5961	1,5,7,8,12,13,14,15	2,3,4,6,9,10,11,16	3	D3ZUC6,P35745,Q9Z2L0
5962	1,5,7,8,12,13,14,16	2,3,4,6,9,10,11,15	3	Q811M5,Q99MH3,Q9Z2L0
5963	1,5,7,8,12,13,15,16	2,3,4,6,9,10,11,14	1	P35745
5964	1,5,7,8,12,14,15,16	2,3,4,6,9,10,11,13	1	P35745
5965	1,5,7,8,13,14,15,16	2,3,4,6,9,10,11,12	1	Q62714
5966	1,5,7,9,10,11,12,13	2,3,4,6,8,14,15,16	6	B0LT89,P00762,P20761,P20762,Q03191,Q62761;Q62762;Q62763
5967	1,5,7,9,10,11,12,14	2,3,4,6,8,13,15,16	6	P00762,P00774,P07151,P20761,Q03191,Q62761;Q62762;Q62763

5968	1,5,7,9,10,11,12,15	2,3,4,6,8,13,14,16	5	P00762,P20761,P35745,Q03191,Q62761;Q62762;Q62763
5969	1,5,7,9,10,11,12,16	2,3,4,6,8,13,14,15	2	Q03191,Q8CIZ5
5970	1,5,7,9,10,11,13,14	2,3,4,6,8,12,15,16	2	P00762,P00774
5971	1,5,7,9,10,11,13,15	2,3,4,6,8,12,14,16	2	O70417,P00762
5972	1,5,7,9,10,11,13,16	2,3,4,6,8,12,14,15	0	
5973	1,5,7,9,10,11,14,15	2,3,4,6,8,12,13,16	3	P00762,P00774,P02625
5974	1,5,7,9,10,11,14,16	2,3,4,6,8,12,13,15	3	P00774,Q8CIZ5,Q9R168
5975	1,5,7,9,10,11,15,16	2,3,4,6,8,12,13,14	1	Q8CIZ5
5976	1,5,7,9,10,12,13,14	2,3,4,6,8,11,15,16	5	P00762,P00774,P29315,P50280,Q03191
5977	1,5,7,9,10,12,13,15	2,3,4,6,8,11,14,16	4	P00762,P35745,P50280,Q4KLZ6
5978	1,5,7,9,10,12,13,16	2,3,4,6,8,11,14,15	2	P50280,Q03191
5979	1,5,7,9,10,12,14,15	2,3,4,6,8,11,13,16	6	P00714,P00762,P00774,P30120,P35745,Q62902
5980	1,5,7,9,10,12,14,16	2,3,4,6,8,11,13,15	4	P00774,P21674,Q03191,Q8CIZ5
5981	1,5,7,9,10,12,15,16	2,3,4,6,8,11,13,14	3	P00774,P35745,Q8CIZ5
5982	1,5,7,9,10,13,14,15	2,3,4,6,8,11,12,16	7	P00762,P00774,P02625,P12020,P35745,P50280,Q00715
5983	1,5,7,9,10,13,14,16	2,3,4,6,8,11,12,15	1	P00774
5984	1,5,7,9,10,13,15,16	2,3,4,6,8,11,12,14	3	O70417,P00774,P12020
5985	1,5,7,9,10,14,15,16	2,3,4,6,8,11,12,13	3	P00774,P12020,Q8CIZ5
5986	1,5,7,9,11,12,13,14	2,3,4,6,8,10,15,16	21	O55004,O70377,O70594,O88339;Q4V882,P07151,P19468,P20761,P20762,P46844,P53790,Q03191,Q05175,Q5M7T9,Q62761;Q62762;Q62763,Q63270,Q63424,Q63598,Q63618,Q6Q0N1,Q9WTW7,Q9Z0W7
5987	1,5,7,9,11,12,13,15	2,3,4,6,8,10,14,16	7	O55004,O70417,P07151,P20761,P20762,Q03191,Q62761;Q62762;Q62763

5988	1,5,7,9,11,<br>12,13,16	2,3,4,6,8,1<br>0,14,15	4	O88339;Q4V882,P20762,P54921,Q03191
5989	1,5,7,9,11,<br>12,14,15	2,3,4,6,8,1<br>0,13,16	14	O55004,O70257,P00774,P02625,P02631,P07151,P20761,P28570,P46844,Q03191,Q5BJY9,Q62761;Q62762;Q62763,Q63424,Q63618
5990	1,5,7,9,11,<br>12,14,16	2,3,4,6,8,1<br>0,13,15	7	O55004,O88339;Q4V882,P21674,Q03191,Q498D9,Q63618,Q8CIZ5
5991	1,5,7,9,11,<br>12,15,16	2,3,4,6,8,1<br>0,13,14	4	O55004,P02631,Q03191,Q8CIZ5
5992	1,5,7,9,11,<br>13,14,15	2,3,4,6,8,1<br>0,12,16	7	O55004,P00774,P02625,P02631,P07151,P0DMW0;P0DMW1,P46844
5993	1,5,7,9,11,<br>13,14,16	2,3,4,6,8,1<br>0,12,15	3	P0DMW0;P0DMW1,Q498D9,Q9R168
5994	1,5,7,9,11,<br>13,15,16	2,3,4,6,8,1<br>0,12,14	1	O70417
5995	1,5,7,9,11,<br>14,15,16	2,3,4,6,8,1<br>0,12,13	4	O55004,P00774,P02631,Q8CIZ5
5996	1,5,7,9,12,<br>13,14,15	2,3,4,6,8,1<br>0,11,16	8	O55004,P00762,P00774,P07151,P28570,P35745,P50280,Q5BJY9
5997	1,5,7,9,12,<br>13,14,16	2,3,4,6,8,1<br>0,11,15	2	P00774,P50280
5998	1,5,7,9,12,<br>13,15,16	2,3,4,6,8,1<br>0,11,14	1	O70417
5999	1,5,7,9,12,<br>14,15,16	2,3,4,6,8,1<br>0,11,13	4	P00774,P02631,Q5BJY9,Q8CIZ5
6000	1,5,7,9,13,<br>14,15,16	2,3,4,6,8,1<br>0,11,12	1	P00774
6001	1,5,7,10,1<br>1,12,13,14	2,3,4,6,8,9<br>,15,16	3	P00774,P23593,P29315
6002	1,5,7,10,1<br>1,12,13,15	2,3,4,6,8,9<br>,14,16	3	O70417,P00774,P23593
6003	1,5,7,10,1<br>1,12,13,16	2,3,4,6,8,9<br>,14,15	5	P00774,P23593,P36376,P47967,P97840
6004	1,5,7,10,1<br>1,12,14,15	2,3,4,6,8,9<br>,13,16	2	P00774,P23593
6005	1,5,7,10,1<br>1,12,14,16	2,3,4,6,8,9<br>,13,15	2	P00774,P36376
6006	1,5,7,10,1<br>1,12,15,16	2,3,4,6,8,9<br>,13,14	2	P00774,Q8CIZ5
6007	1,5,7,10,1<br>1,13,14,15	2,3,4,6,8,9<br>,12,16	1	P00774

6008	1,5,7,10,11,13,14,16	2,3,4,6,8,9,12,15	3	P00774,P36376,Q99MH3
6009	1,5,7,10,11,13,15,16	2,3,4,6,8,9,12,14	2	O70417,P00774
6010	1,5,7,10,11,14,15,16	2,3,4,6,8,9,12,13	2	P00774,Q8CIZ5
6011	1,5,7,10,12,13,14,15	2,3,4,6,8,9,11,16	3	P00774,P50280,Q6P6S4
6012	1,5,7,10,12,13,14,16	2,3,4,6,8,9,11,15	4	P00774,P36376,Q6P6S4,Q99MH3
6013	1,5,7,10,12,13,15,16	2,3,4,6,8,9,11,14	2	O70417,P00774
6014	1,5,7,10,12,14,15,16	2,3,4,6,8,9,11,13	2	P00774,Q6P6S4
6015	1,5,7,10,13,14,15,16	2,3,4,6,8,9,11,12	2	P00774,Q6P6S4
6016	1,5,7,11,12,13,14,15	2,3,4,6,8,9,10,16	6	O55004,P00774,P07151,P17559,P23739,Q811M5
6017	1,5,7,11,12,13,14,16	2,3,4,6,8,9,10,15	5	P00774,P17559,P36376,Q811M5,Q99MH3
6018	1,5,7,11,12,13,15,16	2,3,4,6,8,9,10,14	2	O70417,P17559
6019	1,5,7,11,12,14,15,16	2,3,4,6,8,9,10,13	4	O55004,P00774,P02631,P17559
6020	1,5,7,11,13,14,15,16	2,3,4,6,8,9,10,12	3	O70417,P00774,P17559
6021	1,5,7,12,13,14,15,16	2,3,4,6,8,9,10,11	3	P00774,P17559,Q6P6S4
6022	1,5,8,9,10,11,12,13	2,3,4,6,7,14,15,16	3	B0LT89,P17988,Q03191
6023	1,5,8,9,10,11,12,14	2,3,4,6,7,13,15,16	1	Q03191
6024	1,5,8,9,10,11,12,15	2,3,4,6,7,13,14,16	1	P35745
6025	1,5,8,9,10,11,12,16	2,3,4,6,7,13,14,15	4	O88797,P49134,P80299,Q03191
6026	1,5,8,9,10,11,13,14	2,3,4,6,7,12,15,16	2	B0LT89,P17988
6027	1,5,8,9,10,11,13,15	2,3,4,6,7,12,14,16	3	B0LT89,P07943,Q80WL1

6028	1,5,8,9,10,11,13,16	2,3,4,6,7,12,14,15	2	B0LT89,Q80WL1
6029	1,5,8,9,10,11,14,15	2,3,4,6,7,12,13,16	2	P07943,P80299
6030	1,5,8,9,10,11,14,16	2,3,4,6,7,12,13,15	1	P80299
6031	1,5,8,9,10,11,15,16	2,3,4,6,7,12,13,14	3	P07943,P49134,P80299
6032	1,5,8,9,10,12,13,14	2,3,4,6,7,11,15,16	2	P17988,P29315
6033	1,5,8,9,10,12,13,15	2,3,4,6,7,11,14,16	3	P11598,P35745,Q4KLZ6
6034	1,5,8,9,10,12,13,16	2,3,4,6,7,11,14,15	0	
6035	1,5,8,9,10,12,14,15	2,3,4,6,7,11,13,16	5	P00714,P05369,P07647,P11598,P35745
6036	1,5,8,9,10,12,14,16	2,3,4,6,7,11,13,15	1	P21674
6037	1,5,8,9,10,12,15,16	2,3,4,6,7,11,13,14	1	P35745
6038	1,5,8,9,10,13,14,15	2,3,4,6,7,11,12,16	4	P07647,P11598,P35745,Q00715
6039	1,5,8,9,10,13,14,16	2,3,4,6,7,11,12,15	0	
6040	1,5,8,9,10,13,15,16	2,3,4,6,7,11,12,14	2	P35745,Q80WL1
6041	1,5,8,9,10,14,15,16	2,3,4,6,7,11,12,13	3	P35745,P47967,P80299
6042	1,5,8,9,11,12,13,14	2,3,4,6,7,10,15,16	28	B0LT89,O70377,O70594,P17988,P18297,P19468,P20766,P29975,P46844,P48508,P51907,P53790,P57113,Q03248,Q5I0E9,Q5M7T9,Q63270,Q63424,Q63598,Q63618,Q64093,Q6Q0N1,Q71MB6,Q80W57,Q923M1,Q9JJ40,Q9WTW7,Q9Z0W7
6043	1,5,8,9,11,12,13,15	2,3,4,6,7,10,14,16	7	B0LT89,P18297,P48037,P57113,Q71MB6,Q80WL1,Q923S2
6044	1,5,8,9,11,12,13,16	2,3,4,6,7,10,14,15	6	B0LT89,P18297,P54921,Q03191,Q5RKI1,Q80WL1
6045	1,5,8,9,11,12,14,15	2,3,4,6,7,10,13,16	9	O70594,P18297,P19468,P20766,P28570,P46844,P80299,Q63424,Q63618
6046	1,5,8,9,11,12,14,16	2,3,4,6,7,10,13,15	12	O70594,O88339;Q4V882,O88797,P18297,P19468,P20766,P21674,P51907,P80299,Q5RKI1,Q63618,Q923M1
6047	1,5,8,9,11,12,15,16	2,3,4,6,7,10,13,14	4	O88797,P80299,Q5QE79,Q80WL1

6048	1,5,8,9,11,13,14,15	2,3,4,6,7,10,12,16	10	B0LT89,P0DMW0;P0DMW1,P18297,P20766,P46844,Q03248,Q71MB6,Q80WL1,Q9JJ40,Q9WTW7
6049	1,5,8,9,11,13,14,16	2,3,4,6,7,10,12,15	5	P18297,P20766,P80299,Q5RKI1,Q63598
6050	1,5,8,9,11,13,15,16	2,3,4,6,7,10,12,14	3	P80299,Q5RLM2,Q80WL1
6051	1,5,8,9,11,14,15,16	2,3,4,6,7,10,12,13	2	P20766,P80299
6052	1,5,8,9,12,13,14,15	2,3,4,6,7,10,11,16	5	P11598,P20766,P28570,P35745,Q9WTW7
6053	1,5,8,9,12,13,14,16	2,3,4,6,7,10,11,15	3	P17988,P20766,Q5RKI1
6054	1,5,8,9,12,13,15,16	2,3,4,6,7,10,11,14	2	P35745,Q80WL1
6055	1,5,8,9,12,14,15,16	2,3,4,6,7,10,11,13	3	P20766,P35745,P80299
6056	1,5,8,9,13,14,15,16	2,3,4,6,7,10,11,12	2	P20766,Q80WL1
6057	1,5,8,10,11,12,13,14	2,3,4,6,7,9,15,16	1	P23739
6058	1,5,8,10,11,12,13,15	2,3,4,6,7,9,14,16	2	Q5QE79,Q80WL1
6059	1,5,8,10,11,12,13,16	2,3,4,6,7,9,14,15	2	Q5QE79,Q80WL1
6060	1,5,8,10,11,12,14,15	2,3,4,6,7,9,13,16	1	P80299
6061	1,5,8,10,11,12,14,16	2,3,4,6,7,9,13,15	1	P80299
6062	1,5,8,10,11,12,15,16	2,3,4,6,7,9,13,14	2	P80299,Q5QE79
6063	1,5,8,10,11,13,14,15	2,3,4,6,7,9,12,16	1	P23739
6064	1,5,8,10,11,13,14,16	2,3,4,6,7,9,12,15	0	
6065	1,5,8,10,11,13,15,16	2,3,4,6,7,9,12,14	2	Q5QE79,Q80WL1
6066	1,5,8,10,11,14,15,16	2,3,4,6,7,9,12,13	2	P80299,Q6IMF3
6067	1,5,8,10,12,13,14,15	2,3,4,6,7,9,11,16	2	P22006,P23739

6068	1,5,8,10,12,13,14,16	2,3,4,6,7,9,11,15	1	P35280
6069	1,5,8,10,12,13,15,16	2,3,4,6,7,9,11,14	2	Q5QE79,Q80WL1
6070	1,5,8,10,12,14,15,16	2,3,4,6,7,9,11,13	3	P00774,P80299,Q63617
6071	1,5,8,10,13,14,15,16	2,3,4,6,7,9,11,12	3	O35077,P00774,P70709
6072	1,5,8,11,12,13,14,15	2,3,4,6,7,9,10,16	6	P18297,P20766,P23739,Q811M5,Q8K1G0,Q9WVH8
6073	1,5,8,11,12,13,14,16	2,3,4,6,7,9,10,15	5	P20766,P23739,Q5RKI1,Q811M5,Q8K1G0
6074	1,5,8,11,12,13,15,16	2,3,4,6,7,9,10,14	4	P23739,Q5QE79,Q80WL1,Q8K1G0
6075	1,5,8,11,12,14,15,16	2,3,4,6,7,9,10,13	3	P20766,P80299,Q9WVH8
6076	1,5,8,11,13,14,15,16	2,3,4,6,7,9,10,12	4	P20766,P23739,P80299,Q80WL1
6077	1,5,8,12,13,14,15,16	2,3,4,6,7,9,10,11	2	P20766,P23739
6078	1,5,9,10,11,12,13,14	2,3,4,6,7,8,15,16	34	B0LT89,D4A5U3,O55004,O70377,O70594,P00774,P07151,P17988,P19468,P20761,P20762,P46844,P48508,P50115,P50280,P53790,P98089,Q03191,Q03248,Q05175,Q3ZAV1,Q5I0E9,Q5M7T9,Q62761;Q62762;Q62763,Q63270,Q63424,Q63598,Q6Q0N1,Q6TMA8,Q8CIZ5,Q923S2,Q9R1T5,Q9WTW7,Q9Z0W7
6079	1,5,9,10,11,12,13,15	2,3,4,6,7,8,14,16	20	B0LT89,D3ZHA0,D4A5U3,O55004,O70417,P07151,P20761,P20762,P46844,P50115,P50280,P98089,Q03191,Q5QE79,Q62635,Q62761;Q62762;Q62763,Q6TMA8,Q8CIZ5,Q923S2,Q9WUW8
6080	1,5,9,10,11,12,13,16	2,3,4,6,7,8,14,15	14	B0LT89,D4A5U3,O70417,P20761,P20762,P50115,P50280,P54921,P98089,Q03191,Q5QE79,Q62635,Q6TMA8,Q8CIZ5
6081	1,5,9,10,11,12,14,15	2,3,4,6,7,8,13,16	27	D4A5U3,O55004,O70257,O70594,P00762,P00774,P02631,P06761,P07151,P19468,P20761,P28570,P46844,P50115,P55091,P98089,Q03191,Q3ZAV1,Q5M7T9,Q62761;Q62762;Q62763,Q62902,Q63424,Q63618,Q6Q0N1,Q6TMA8,Q8CIZ5,Q9WUW8
6082	1,5,9,10,11,12,14,16	2,3,4,6,7,8,13,15	15	D4A5U3,O55004,O70257,O70594,P00774,P02631,P20761,P50115,P55091,P98089,Q03191,Q63618,Q6Q0N1,Q6TMA8,Q8CIZ5
6083	1,5,9,10,11,12,15,16	2,3,4,6,7,8,13,14	19	B0BNN3,D3ZHA0,D4A5U3,O55004,O70417,P00774,P02631,P20761,P25809,P50115,P55091,P63081,P98089,Q03191,Q5QE79,Q62635,Q6TMA8,Q8CIZ5,Q9WUW8
6084	1,5,9,10,11,13,14,15	2,3,4,6,7,8,12,16	18	B0LT89,D4A5U3,O55004,O70417,P00774,P02631,P06761,P07151,P20761,P20762,P46844,P50115,P98089,Q5M7T9,Q6TMA8,Q8CIZ5,Q9R0T3,Q9WTW7
6085	1,5,9,10,11,13,14,16	2,3,4,6,7,8,12,15	8	B0LT89,P00774,P20761,P30904,P50115,P98089,Q63598,Q8CIZ5
6086	1,5,9,10,11,13,15,16	2,3,4,6,7,8,12,14	13	B0LT89,D4A5U3,O70417,P00774,P02631,P20761,P50115,P98089,Q5QE79,Q5RLM2,Q62635,Q63751,Q8CIZ5

6087	1,5,9,10,11,14,15,16	2,3,4,6,7,8,12,13	11	D4A5U3,O55004,P00774,P02631,P20761,P50115,P55091,P80299,P98089,Q5QE79,Q8CIZ5
6088	1,5,9,10,12,13,14,15	2,3,4,6,7,8,11,16	39	D4A5U3,O55004,P00762,P00774,P02780,P02781,P02782,P06761,P07150,P07151,P07647,P08723,P09456,P0C0A9,P11598,P12020,P20761,P22273,P22282,P22283,P28570,P30120,P46462,P50115,P50280,P55091,P98089,Q00715,Q4G063,Q4G075,Q5M8C6,Q5U1Y4,Q62902,Q63617,Q6TMA8,Q8CIZ5,Q99041,Q9JHB9,Q9R0T3
6089	1,5,9,10,12,13,14,16	2,3,4,6,7,8,11,15	18	D4A5U3,P00774,P02780,P06761,P07150,P11598,P20761,P22273,P22282,P50115,P50280,P55091,P98089,Q4G063,Q4G075,Q5M8C6,Q63617,Q8CIZ5
6090	1,5,9,10,12,13,15,16	2,3,4,6,7,8,11,14	29	D4A5U3,O70417,P00774,P02780,P06761,P06911,P07150,P08937,P0C0A9,P11598,P12020,P20761,P22273,P22282,P46462,P50115,P50280,P55091,P98089,Q4G075,Q5GRG2,Q5M8C6,Q5QE79,Q62635,Q63617,Q63751,Q8CIZ5,Q99041,Q9R0T3
6091	1,5,9,10,12,14,15,16	2,3,4,6,7,8,11,13	27	D4A5U3,O55004,P00774,P02631,P02780,P06761,P11598,P12020,P20761,P22273,P22282,P22283,P46462,P50115,P50280,P55091,P98089,Q4G075,Q5M8C6,Q5QE79,Q62635,Q62902,Q63617,Q8CIZ5,Q99041,Q9JI85,Q9R0T3
6092	1,5,9,10,13,14,15,16	2,3,4,6,7,8,11,12	29	D4A5U3,O70417,P00774,P02631,P02780,P06761,P06911,P07150,P11598,P12020,P20761,P22273,P22282,P22283,P31430,P46462,P50115,P50280,P55091,P70709,P98089,Q00715,Q4G075,Q5GRG2,Q5M8C6,Q62635,Q63617,Q8CIZ5,Q9R0T3
6093	1,5,9,11,12,13,14,15	2,3,4,6,7,8,10,16	72	B0LT89,D4A5U3,O35763,O55004,O70257,O70377,O70417,O70594,O88339;Q4V882,P00774,P01836,P02631,P07151,P18427,P18757,P19468,P20761,P20762,P20766,P23739,P28570,P29975,P36970,P38918,P46720,P46844,P48508,P50115,P50280,P51907,P53790,P55091,P57113,P68035;P68136,P98089,Q03248,Q05175,Q3MIE4,Q3T1J9,Q3ZAV1,Q5BJY9,Q5I0E9,Q5M7T9,Q62687,Q62761;Q62762;Q62763,Q63270,Q63355,Q63424,Q63598,Q63618,Q64093,Q64602,Q68FT5,Q6AY41,Q6MG61,Q6Q0N1,Q6TMA8,Q71MB6,Q80W57,Q8CIZ5,Q8R431,Q923S2,Q9JJ19,Q9JJ40,Q9JLJ3,Q9QYU4,Q9R0T3,Q9R1T5,Q9WTW7,Q9WUW8,Q9WUW9,Q9Z0W7
6094	1,5,9,11,12,13,14,16	2,3,4,6,7,8,10,15	55	B0LT89,O35763,O55004,O70257,O70377,O70594,O88339;Q4V882,P00774,P02631,P18757,P19468,P20761,P20762,P20766,P29975,P30904,P38918,P46844,P48508,P50115,P51907,P53790,P54921,P55091,P57113,P68035;P68136,P98089,Q03191,Q03248,Q05175,Q3MIE4,Q3T1J9,Q3ZAV1,Q498D9,Q5I0E9,Q5M7T9,Q5RKI1,Q63270,Q63355,Q63424,Q63598,Q63618,Q64093,Q6MG61,Q6Q0N1,Q80W57,Q8CIZ5,Q8R431,Q923S2,Q9JJ19,Q9JJ40,Q9JLJ3,Q9R1T5,Q9WTW7,Q9Z0W7
6095	1,5,9,11,12,13,15,16	2,3,4,6,7,8,10,14	37	B0LT89,D4A5U3,O55004,O70257,O70377,O70417,O70594,O88339;Q4V882,P02631,P08937,P20761,P20762,P20766,P46844,P48508,P50115,P53790,P54921,P55091,P57113,P98089,Q03191,Q05175,Q5I0E9,Q5M7T9,Q5QE79,Q5RLM2,Q62635,Q63424,Q63618,Q63751,Q6Q0N1,Q80WL1,Q8CIZ5,Q923S2,Q9WUW8,Q9WUW9
6096	1,5,9,11,12,14,15,16	2,3,4,6,7,8,10,13	41	D4A5U3,O55004,O70257,O70377,O70594,O88339;Q4V882,P00774,P02631,P19468,P20761,P20766,P25809,P28570,P30904,P46844,P48508,P50115,P51907,P53790,P55091,P57113,P80299,P98089,Q05175,Q3T1J9,Q3ZAV1,Q5BJY9,Q5I0E9,Q5M7T9,Q5QE79,Q63424,Q63598,Q63618,Q64093,Q6MG61,Q6Q0N1,Q8CIZ5,Q9WTW7,Q9WUW8,Q9WUW9,Q9Z0W7

6097	1,5,9,11,13,14,15,16	2,3,4,6,7,8,10,12	33	D4A5U3,O55004,O70377,O70417,P00774,P02631,P0DMW0;P0DMW1,P20761,P20762,P20766,P23739,P30904,P46844,P48508,P50115,P53790,P55091,P98089,Q05175,Q3ZAV1,Q5M7T9,Q5RLM2,Q63270,Q63598,Q63618,Q66HG3,Q6MG61,Q6Q0N1,Q8CIZ5,Q923S2,Q9JJ40,Q9R0T3,Q9WTW7
6098	1,5,9,12,13,14,15,16	2,3,4,6,7,8,10,11	31	D4A5U3,O55004,O70417,P00774,P02631,P02780,P06761,P07150,P11598,P20761,P20762,P20766,P22273,P22282,P30904,P31430,P50115,P50280,P55091,P98089,Q4G075,Q5BJY9,Q5M7T9,Q5M8C6,Q5QE79,Q63617,Q6B345,Q6P6R2,Q8CIZ5,Q9R0T3,Q9WTW7
6099	1,5,10,11,12,13,14,15	2,3,4,6,7,8,9,16	18	D4A5U3,O55004,O70417,P00774,P02631,P07151,P20646,P20761,P20762,P22006,P23593,P23739,P50115,P55091,P98089,Q5QE79,Q6TMA8,Q8CIZ5
6100	1,5,10,11,12,13,14,16	2,3,4,6,7,8,9,15	12	D4A5U3,P00774,P20646,P20761,P23739,P35280,P50115,P55091,P98089,Q5QE79,Q6P6R2,Q8CIZ5
6101	1,5,10,11,12,13,15,16	2,3,4,6,7,8,9,14	15	D3ZHA0,D4A5U3,O70417,P00774,P08937,P20761,P23739,P50115,P55091,P98089,Q5QE79,Q62635,Q63751,Q6P6R2,Q8CIZ5
6102	1,5,10,11,12,14,15,16	2,3,4,6,7,8,9,13	11	D4A5U3,O55004,P00774,P02631,P20761,P50115,P55091,P80299,P98089,Q5QE79,Q8CIZ5
6103	1,5,10,11,13,14,15,16	2,3,4,6,7,8,9,12	13	D4A5U3,O70417,P00774,P02631,P20761,P23739,P26772,P50115,P55091,P98089,Q05702,Q5QE79,Q8CIZ5
6104	1,5,10,12,13,14,15,16	2,3,4,6,7,8,9,11	24	D4A5U3,O70417,P00774,P02780,P06761,P07150,P11598,P20761,P22273,P22282,P23739,P35280,P50115,P50280,P55091,P98089,Q5M8C6,Q5QE79,Q62902,Q63617,Q6P6R2,Q6P6S4,Q8CIZ5,Q9R0T3
6105	1,5,11,12,13,14,15,16	2,3,4,6,7,8,9,10	23	D4A5U3,O35763,O55004,O70417,O70594,P00774,P02454,P02631,P17559,P20646,P20761,P20762,P20766,P23739,P50115,P55091,P70545,P98089,Q5QE79,Q63598,Q63618,Q6P6R2,Q8CIZ5
6106	1,6,7,8,9,10,11,12	2,3,4,5,13,14,15,16	6	P21674,P29315,P35745,Q63493,Q812E4,Q9QW07
6107	1,6,7,8,9,10,11,13	2,3,4,5,12,14,15,16	2	P29315,Q63617
6108	1,6,7,8,9,10,11,14	2,3,4,5,12,13,15,16	3	P21674,P29315,Q812E4
6109	1,6,7,8,9,10,11,15	2,3,4,5,12,13,14,16	7	P19629,P35745,P49134,Q63474,Q6P6S4,Q8CJ52,Q9QW07
6110	1,6,7,8,9,10,11,16	2,3,4,5,12,13,14,15	11	P02782,P08723,P09456,P21674,P22283,P30120,P49134,Q63617,Q812E4,Q9JHB9,Q9QW07
6111	1,6,7,8,9,10,12,13	2,3,4,5,11,14,15,16	4	P29315,P35745,P70549,Q4KLZ6

6112	1,6,7,8,9,10,12,14	2,3,4,5,11,13,15,16	3	P21674,P29315,P35745
6113	1,6,7,8,9,10,12,15	2,3,4,5,11,13,14,16	4	P19629,P35745,Q4KLZ6,Q63474
6114	1,6,7,8,9,10,12,16	2,3,4,5,11,13,14,15	2	P21674,P35745
6115	1,6,7,8,9,10,13,14	2,3,4,5,11,12,15,16	3	P29315,P35745,P70549
6116	1,6,7,8,9,10,13,15	2,3,4,5,11,12,14,16	4	P35745,P70549,Q4KLZ6,Q63474
6117	1,6,7,8,9,10,13,16	2,3,4,5,11,12,14,15	0	
6118	1,6,7,8,9,10,14,15	2,3,4,5,11,12,13,16	2	P35745,Q63474
6119	1,6,7,8,9,10,14,16	2,3,4,5,11,12,13,15	1	P21674
6120	1,6,7,8,9,10,15,16	2,3,4,5,11,12,13,14	2	P19629,P35745
6121	1,6,7,8,9,11,12,13	2,3,4,5,10,14,15,16	13	B0LT89,iRT-Kit_WR_fusion,P06911,P22283,P29315,Q09326,Q5GRG2,Q63493,Q63617,Q78P75,Q812E4,Q9JI85,Q9QW07
6122	1,6,7,8,9,11,12,14	2,3,4,5,10,13,15,16	8	P06911,P12020,P21674,P29315,Q5GRG2,Q63493,Q812E4,Q9QW07
6123	1,6,7,8,9,11,12,15	2,3,4,5,10,13,14,16	7	iRT-Kit_WR_fusion,P35745,Q63493,Q6P6S4,Q812E4,Q8CJ52,Q9QW07
6124	1,6,7,8,9,11,12,16	2,3,4,5,10,13,14,15	19	iRT-Kit_WR_fusion,P02782,P06911,P08723,P09456,P21674,P22283,P30120,P46462,P63322,P97523,Q5GRG2,Q63357,Q63493,Q63617,Q812E4,Q8CFN2,Q9JHB9,Q9QW07
6125	1,6,7,8,9,11,13,14	2,3,4,5,10,12,15,16	7	P0DMW0;P0DMW1,P29315,Q09326,Q5GRG2,Q63617,Q78P75,Q812E4
6126	1,6,7,8,9,11,13,15	2,3,4,5,10,12,14,16	6	P35745,Q63493,Q63617,Q6P6S4,Q8CJ52,Q9QW07
6127	1,6,7,8,9,11,13,16	2,3,4,5,10,12,14,15	14	P02782,P08723,P09456,P22283,P24368,P30120,P46462,P60905,Q63493,Q63617,Q812E4,Q9JHB9,Q9JI85,Q9QW07
6128	1,6,7,8,9,11,14,15	2,3,4,5,10,12,13,16	5	P0DMW0;P0DMW1,P35745,Q812E4,Q8CJ52,Q9QW07
6129	1,6,7,8,9,11,14,16	2,3,4,5,10,12,13,15	12	P02782,P08723,P09456,P21674,P22283,P30120,P46462,Q5GRG2,Q812E4,Q8CFN2,Q9JHB9,Q9QW07
6130	1,6,7,8,9,11,15,16	2,3,4,5,10,12,13,14	11	P02782,P08723,P09456,P22283,P30120,Q63493,Q6P6S4,Q812E4,Q8CJ52,Q9JHB9,Q9QW07

6131	1,6,7,8,9,12,13,14	2,3,4,5,10,11,15,16	4	P21674,P29315,P35745,P70549
6132	1,6,7,8,9,12,13,15	2,3,4,5,10,11,14,16	3	P35745,P70549,Q4KLZ6
6133	1,6,7,8,9,12,13,16	2,3,4,5,10,11,14,15	2	P21674,P70549
6134	1,6,7,8,9,12,14,15	2,3,4,5,10,11,13,16	1	P35745
6135	1,6,7,8,9,12,14,16	2,3,4,5,10,11,13,15	1	P21674
6136	1,6,7,8,9,12,15,16	2,3,4,5,10,11,13,14	4	iRT-Kit_WR_fusion,P19629,P35745,Q9QW07
6137	1,6,7,8,9,13,14,15	2,3,4,5,10,11,12,16	3	P0DMW0;P0DMW1,P35745,P70549
6138	1,6,7,8,9,13,14,16	2,3,4,5,10,11,12,15	0	
6139	1,6,7,8,9,13,15,16	2,3,4,5,10,11,12,14	2	P35745,P70549
6140	1,6,7,8,9,14,15,16	2,3,4,5,10,11,12,13	3	P35745,P47967,P97840
6141	1,6,7,8,10,11,12,13	2,3,4,5,9,14,15,16	5	P06911,P22283,P29315,Q63493,Q9JI85
6142	1,6,7,8,10,11,12,14	2,3,4,5,9,13,15,16	6	P06911,P12020,P21674,P29315,Q5GRG2,Q9QW07
6143	1,6,7,8,10,11,12,15	2,3,4,5,9,13,14,16	3	P19629,P35745,Q9QW07
6144	1,6,7,8,10,11,12,16	2,3,4,5,9,13,14,15	17	P02782,P04905,P06911,P08010,P08723,P09456,P12020,P19629,P21674,P22283,P24368,P30120,P46462,Q5GRG2,Q63493,Q9JHB9,Q9QW07
6145	1,6,7,8,10,11,13,14	2,3,4,5,9,12,15,16	4	P22283,P29315,Q5GRG2,Q99041
6146	1,6,7,8,10,11,13,15	2,3,4,5,9,12,14,16	2	P19629,P22283
6147	1,6,7,8,10,11,13,16	2,3,4,5,9,12,14,15	13	P02781,P02782,P08723,P09456,P0C0A9,P22283,P24368,P30120,P46462,Q99041,Q9JHB9,Q9JI85,Q9QW07
6148	1,6,7,8,10,11,14,15	2,3,4,5,9,12,13,16	2	Q8CJ52,Q9QW07
6149	1,6,7,8,10,11,14,16	2,3,4,5,9,12,13,15	13	P02782,P08723,P09456,P0C0A9,P21674,P22283,P30120,P46462,Q5GRG2,Q812E4,Q99041,Q9JHB9,Q9QW07
6150	1,6,7,8,10,11,15,16	2,3,4,5,9,12,13,14	9	P02782,P08723,P0C0A9,P19629,P22283,P30120,Q8CJ52,Q9JHB9,Q9QW07

6151	1,6,7,8,10,12,13,14	2,3,4,5,9,11,15,16	2	P29315,P70549
6152	1,6,7,8,10,12,13,15	2,3,4,5,9,11,14,16	4	P19629,P35745,P70549,Q4KLZ6
6153	1,6,7,8,10,12,13,16	2,3,4,5,9,11,14,15	1	P70549
6154	1,6,7,8,10,12,14,15	2,3,4,5,9,11,13,16	4	P19629,P35745,P70549,Q63474
6155	1,6,7,8,10,12,14,16	2,3,4,5,9,11,13,15	2	P21674,P57113
6156	1,6,7,8,10,12,15,16	2,3,4,5,9,11,13,14	4	P19629,P35745,P57113,Q63598
6157	1,6,7,8,10,13,14,15	2,3,4,5,9,11,12,16	3	P35745,P70549,Q63474
6158	1,6,7,8,10,13,14,16	2,3,4,5,9,11,12,15	3	P36376,P57113,P70549
6159	1,6,7,8,10,13,15,16	2,3,4,5,9,11,12,14	3	P19629,P57113,P70549
6160	1,6,7,8,10,14,15,16	2,3,4,5,9,11,12,13	2	P19629,P57113
6161	1,6,7,8,11,12,13,14	2,3,4,5,9,10,15,16	9	P06911,P12020,P22283,P29315,P46462,Q09326,Q5GRG2,Q63493,Q9JI85
6162	1,6,7,8,11,12,13,15	2,3,4,5,9,10,14,16	5	P22283,Q5GRG2,Q63493,Q9JI85,Q9QW07
6163	1,6,7,8,11,12,13,16	2,3,4,5,9,10,14,15	23	O35547,P02780,P02781,P02782,P05369,P06911,P07647,P08723,P09456,P12020,P22283,P24368,P30120,P46462,Q5GRG2,Q5M8C6,Q63493,Q63617,Q812E4,Q8CFN2,Q9JHB9,Q9JI85,Q9QW07
6164	1,6,7,8,11,12,14,15	2,3,4,5,9,10,13,16	5	P06911,P12020,Q5GRG2,Q8CJ52,Q9QW07
6165	1,6,7,8,11,12,14,16	2,3,4,5,9,10,13,15	20	O35547,P02781,P02782,P06911,P08723,P09456,P0C0A9,P12020,P21674,P22283,P30120,P46462,Q5GRG2,Q5M8C6,Q63493,Q812E4,Q8CFN2,Q9JHB9,Q9QW07,Q9QZK9
6166	1,6,7,8,11,12,15,16	2,3,4,5,9,10,13,14	16	iRT-Kit_WR_fusion,P02782,P08723,P09456,P0C0A9,P12020,P19629,P22283,P30120,P46462,Q5GRG2,Q63493,Q8CJ52,Q9JHB9,Q9QW07,Q9QZK9
6167	1,6,7,8,11,13,14,15	2,3,4,5,9,10,12,16	4	Q09326,Q5GRG2,Q8CJ52,Q99041
6168	1,6,7,8,11,13,14,16	2,3,4,5,9,10,12,15	18	P02781,P02782,P08723,P09456,P0C0A9,P22283,P24368,P30120,P34901,P36376,P46462,Q5GRG2,Q812E4,Q8CFN2,Q99041,Q9JHB9,Q9JI85,Q9QW07
6169	1,6,7,8,11,13,15,16	2,3,4,5,9,10,12,14	14	P02781,P02782,P08723,P09456,P0C0A9,P22283,P24368,P30120,P46462,Q63493,Q8CJ52,Q99041,Q9JHB9,Q9QW07

6170	1,6,7,8,11,14,15,16	2,3,4,5,9,10,12,13	14	P02782,P08723,P09456,P0C0A9,P22283,P30120,P46462,Q5GRG2,Q812E4,Q8CJ52,Q99041,Q9JHB9,Q9QW07,Q9QZK9
6171	1,6,7,8,12,13,14,15	2,3,4,5,9,10,11,16	3	P35745,P70549,Q9Z2L0
6172	1,6,7,8,12,13,14,16	2,3,4,5,9,10,11,15	2	P70549,Q9Z2L0
6173	1,6,7,8,12,13,15,16	2,3,4,5,9,10,11,14	2	P70549,Q9Z2L0
6174	1,6,7,8,12,14,15,16	2,3,4,5,9,10,11,13	2	P70549,Q9Z2L0
6175	1,6,7,8,13,14,15,16	2,3,4,5,9,10,11,12	3	P70549,Q62714,Q9Z2L0
6176	1,6,7,9,10,11,12,13	2,3,4,5,8,14,15,16	4	B0LT89,P00762,P20762,Q62761;Q62762;Q62763
6177	1,6,7,9,10,11,12,14	2,3,4,5,8,13,15,16	4	P00762,P00774,P05539,P21674
6178	1,6,7,9,10,11,12,15	2,3,4,5,8,13,14,16	4	P00762,P35745,Q62761;Q62762;Q62763,Q9WUW8
6179	1,6,7,9,10,11,12,16	2,3,4,5,8,13,14,15	1	P21674
6180	1,6,7,9,10,11,13,14	2,3,4,5,8,12,15,16	6	P00762,P00774,P05539,P0DMW0;P0DMW1,P36376,Q5PQL7
6181	1,6,7,9,10,11,13,15	2,3,4,5,8,12,14,16	1	P00762
6182	1,6,7,9,10,11,13,16	2,3,4,5,8,12,14,15	2	P36376,Q9R168
6183	1,6,7,9,10,11,14,15	2,3,4,5,8,12,13,16	3	P00762,P00774,P05539
6184	1,6,7,9,10,11,14,16	2,3,4,5,8,12,13,15	4	P00774,P21674,P36376,Q9R168
6185	1,6,7,9,10,11,15,16	2,3,4,5,8,12,13,14	1	Q8CJ52
6186	1,6,7,9,10,12,13,14	2,3,4,5,8,11,15,16	11	P00762,P00774,P05539,P29315,P70549,Q4FZU2,Q6IFU7,Q6IFU8,Q6IFW6,Q6IG02,Q6IMF3
6187	1,6,7,9,10,12,13,15	2,3,4,5,8,11,14,16	5	P00762,P05539,P35745,P70549,Q4KLZ6
6188	1,6,7,9,10,12,13,16	2,3,4,5,8,11,14,15	4	Q6IFU7,Q6IFU8,Q6IFW6,Q6IG02
6189	1,6,7,9,10,12,14,15	2,3,4,5,8,11,13,16	4	P00762,P00774,P05539,P35745

6190	1,6,7,9,10,12,14,16	2,3,4,5,8,11,13,15	6	P00774,P21674,Q6IFU7,Q6IFU8,Q6IFW6,Q6IG02
6191	1,6,7,9,10,12,15,16	2,3,4,5,8,11,13,14	2	P00774,P35745
6192	1,6,7,9,10,13,14,15	2,3,4,5,8,11,12,16	7	P00762,P00774,P01835,P05539,P70549,Q00715,Q5PQL7
6193	1,6,7,9,10,13,14,16	2,3,4,5,8,11,12,15	9	P00774,P05539,P36376,Q5PQL7,Q6IFU7,Q6IFU8,Q6IFW6,Q6IG02,Q9R168
6194	1,6,7,9,10,13,15,16	2,3,4,5,8,11,12,14	4	P00774,P11883,P70549,Q5PQL7
6195	1,6,7,9,10,14,15,16	2,3,4,5,8,11,12,13	5	P00774,P05539,P47967,Q5I0D1,Q5PQL7
6196	1,6,7,9,11,12,13,14	2,3,4,5,8,10,15,16	12	B0LT89,O70594,P05539,P0DMW0;P0DMW1,P20762,P53790,Q62761;Q62762;Q62763,Q63270,Q63355,Q63424,Q63618,Q8R431
6197	1,6,7,9,11,12,13,15	2,3,4,5,8,10,14,16	4	P20760,P20762,Q62761;Q62762;Q62763,Q9WUW8
6198	1,6,7,9,11,12,13,16	2,3,4,5,8,10,14,15	4	P20762,P54921,P63322,Q62753
6199	1,6,7,9,11,12,14,15	2,3,4,5,8,10,13,16	7	P00762,P00774,P05539,P28570,Q62761;Q62762;Q62763,Q63618,Q9WUW8
6200	1,6,7,9,11,12,14,16	2,3,4,5,8,10,13,15	4	P21674,Q498D9,Q63618,Q9QW07
6201	1,6,7,9,11,12,15,16	2,3,4,5,8,10,13,14	4	P25809,Q8CJ52,Q9QW07,Q9WUW8
6202	1,6,7,9,11,13,14,15	2,3,4,5,8,10,12,16	4	P00774,P05539,P0DMW0;P0DMW1,Q5PQL7
6203	1,6,7,9,11,13,14,16	2,3,4,5,8,10,12,15	6	P0DMW0;P0DMW1,P12368,P34901,P36376,Q498D9,Q9R168
6204	1,6,7,9,11,13,15,16	2,3,4,5,8,10,12,14	2	Q5RLM2,Q8CJ52
6205	1,6,7,9,11,14,15,16	2,3,4,5,8,10,12,13	4	P00774,P0DMW0;P0DMW1,Q8CJ52,Q9QW07
6206	1,6,7,9,12,13,14,15	2,3,4,5,8,10,11,16	6	P00762,P00774,P01835,P05539,P70549,Q5BJY9
6207	1,6,7,9,12,13,14,16	2,3,4,5,8,10,11,15	8	P00774,P01835,P05539,P21674,Q6IFU7,Q6IFU8,Q6IFW6,Q6IG02
6208	1,6,7,9,12,13,15,16	2,3,4,5,8,10,11,14	3	P01835,P70549,Q6B345
6209	1,6,7,9,12,14,15,16	2,3,4,5,8,10,11,13	1	P00774

6210	1,6,7,9,13,<br>14,15,16	2,3,4,5,8,1<br>0,11,12	5	P00774,P01835,P05539,P70549,Q5PQL7
6211	1,6,7,10,1<br>1,12,13,14	2,3,4,5,8,9<br>,15,16	6	P00774,P05539,P20760,P23593,P29315,P36376
6212	1,6,7,10,1<br>1,12,13,15	2,3,4,5,8,9<br>,14,16	3	P00774,P20760,P23593
6213	1,6,7,10,1<br>1,12,13,16	2,3,4,5,8,9<br>,14,15	2	P00774,P36376
6214	1,6,7,10,1<br>1,12,14,15	2,3,4,5,8,9<br>,13,16	3	P00774,P05539,P20760
6215	1,6,7,10,1<br>1,12,14,16	2,3,4,5,8,9<br>,13,15	3	P00774,P21674,P36376
6216	1,6,7,10,1<br>1,12,15,16	2,3,4,5,8,9<br>,13,14	3	P00774,P20760,Q9QW07
6217	1,6,7,10,1<br>1,13,14,15	2,3,4,5,8,9<br>,12,16	6	P00774,P01835,P05539,P20760,P36376,Q5PQL7
6218	1,6,7,10,1<br>1,13,14,16	2,3,4,5,8,9<br>,12,15	3	P00774,P34901,P36376
6219	1,6,7,10,1<br>1,13,15,16	2,3,4,5,8,9<br>,12,14	2	P00774,P36376
6220	1,6,7,10,1<br>1,14,15,16	2,3,4,5,8,9<br>,12,13	2	P00774,P36376
6221	1,6,7,10,1<br>2,13,14,15	2,3,4,5,8,9<br>,11,16	5	O88797,P00774,P01835,P05539,P70549
6222	1,6,7,10,1<br>2,13,14,16	2,3,4,5,8,9<br>,11,15	7	P00774,P01835,P05539,P36376,P70549,Q6IFU8,Q6IG02
6223	1,6,7,10,1<br>2,13,15,16	2,3,4,5,8,9<br>,11,14	3	P00774,P01835,P70549
6224	1,6,7,10,1<br>2,14,15,16	2,3,4,5,8,9<br>,11,13	2	P00774,P05539
6225	1,6,7,10,1<br>3,14,15,16	2,3,4,5,8,9<br>,11,12	7	O88797,P00774,P01835,P05539,P36376,P70549,Q5PQL7
6226	1,6,7,11,1<br>2,13,14,15	2,3,4,5,8,9<br>,10,16	5	P00774,P01835,P05539,P20760,P70545
6227	1,6,7,11,1<br>2,13,14,16	2,3,4,5,8,9<br>,10,15	4	P00774,P01835,P34901,P36376
6228	1,6,7,11,1<br>2,13,15,16	2,3,4,5,8,9<br>,10,14	2	P01835,P20760
6229	1,6,7,11,1<br>2,14,15,16	2,3,4,5,8,9<br>,10,13	3	P00774,Q8CJ52,Q9QW07

6230	1,6,7,11,13,14,15,16	2,3,4,5,8,9,10,12	4	P00774,P01835,P36376,Q8CJ52
6231	1,6,7,12,13,14,15,16	2,3,4,5,8,9,10,11	6	P00774,P01835,P05539,P70549,Q6B345,Q9Z2L0
6232	1,6,8,9,10,11,12,13	2,3,4,5,7,14,15,16	1	B0LT89
6233	1,6,8,9,10,11,12,14	2,3,4,5,7,13,15,16	2	B0LT89,P21674
6234	1,6,8,9,10,11,12,15	2,3,4,5,7,13,14,16	4	B0LT89,P35745,Q6P6S4,Q9WUW8
6235	1,6,8,9,10,11,12,16	2,3,4,5,7,13,14,15	2	P21674,Q9QW07
6236	1,6,8,9,10,11,13,14	2,3,4,5,7,12,15,16	1	B0LT89
6237	1,6,8,9,10,11,13,15	2,3,4,5,7,12,14,16	2	B0LT89,Q5RLM2
6238	1,6,8,9,10,11,13,16	2,3,4,5,7,12,14,15	2	B0LT89,O89117
6239	1,6,8,9,10,11,14,15	2,3,4,5,7,12,13,16	2	P47967,P97840
6240	1,6,8,9,10,11,14,16	2,3,4,5,7,12,13,15	4	O89117,P21674,P97840,Q812E4
6241	1,6,8,9,10,11,15,16	2,3,4,5,7,12,13,14	2	Q6P6S4,Q9QW07
6242	1,6,8,9,10,12,13,14	2,3,4,5,7,11,15,16	4	P08649,P17988,P29315,P70549
6243	1,6,8,9,10,12,13,15	2,3,4,5,7,11,14,16	4	P08649,P35745,P70549,Q4KLZ6
6244	1,6,8,9,10,12,13,16	2,3,4,5,7,11,14,15	1	P08649
6245	1,6,8,9,10,12,14,15	2,3,4,5,7,11,13,16	3	P35745,P47967,P97840
6246	1,6,8,9,10,12,14,16	2,3,4,5,7,11,13,15	1	P21674
6247	1,6,8,9,10,12,15,16	2,3,4,5,7,11,13,14	1	P35745
6248	1,6,8,9,10,13,14,15	2,3,4,5,7,11,12,16	6	P35745,P47967,P70549,P97840,Q00715,Q5PQL7
6249	1,6,8,9,10,13,14,16	2,3,4,5,7,11,12,15	2	P47967,P97840

6250	1,6,8,9,10,13,15,16	2,3,4,5,7,11,12,14	1	P70549
6251	1,6,8,9,10,14,15,16	2,3,4,5,7,11,12,13	3	P47967,P97840,Q5I0D1
6252	1,6,8,9,11,12,13,14	2,3,4,5,7,10,15,16	17	B0LT89,O70594,P17988,P18297,P19468,P20766,P48508,P53790,P63095,Q5I0E9,Q63270,Q63355,Q63424,Q64093,Q71MB6,Q8R431,Q9WTW7
6253	1,6,8,9,11,12,13,15	2,3,4,5,7,10,14,16	5	B0LT89,P18297,Q5RLM2,Q71MB6,Q9WUW8
6254	1,6,8,9,11,12,13,16	2,3,4,5,7,10,14,15	7	B0LT89,P30120,P54921,Q5RKI1,Q63355,Q63357,Q812E4
6255	1,6,8,9,11,12,14,15	2,3,4,5,7,10,13,16	9	P18297,P19468,P28570,Q63355,Q63424,Q63618,Q64093,Q9QW07,Q9WUW8
6256	1,6,8,9,11,12,14,16	2,3,4,5,7,10,13,15	9	O70594,P21674,P63095,Q5RKI1,Q63355,Q63357,Q63618,Q812E4,Q9QW07
6257	1,6,8,9,11,12,15,16	2,3,4,5,7,10,13,14	2	Q9QW07,Q9WUW8
6258	1,6,8,9,11,13,14,15	2,3,4,5,7,10,12,16	6	B0LT89,P0DMW0;P0DMW1,P18297,P97840,Q5RLM2,Q71MB6
6259	1,6,8,9,11,13,14,16	2,3,4,5,7,10,12,15	9	B0LT89,O89117,P0DMW0;P0DMW1,P12368,P30120,P34901,P97840,Q5RKI1,Q812E4
6260	1,6,8,9,11,13,15,16	2,3,4,5,7,10,12,14	1	Q5RLM2
6261	1,6,8,9,11,14,15,16	2,3,4,5,7,10,12,13	4	P47967,P97840,Q812E4,Q9QW07
6262	1,6,8,9,12,13,14,15	2,3,4,5,7,10,11,16	3	P20766,P35745,P70549
6263	1,6,8,9,12,13,14,16	2,3,4,5,7,10,11,15	1	P21674
6264	1,6,8,9,12,13,15,16	2,3,4,5,7,10,11,14	1	P70549
6265	1,6,8,9,12,14,15,16	2,3,4,5,7,10,11,13	2	P47967,P97840
6266	1,6,8,9,13,14,15,16	2,3,4,5,7,10,11,12	4	P47967,P70549,P97840,Q5I0D1
6267	1,6,8,10,11,12,13,14	2,3,4,5,7,9,15,16	0	
6268	1,6,8,10,11,12,13,15	2,3,4,5,7,9,14,16	0	
6269	1,6,8,10,11,12,13,16	2,3,4,5,7,9,14,15	1	P30120

6270	1,6,8,10,11,12,14,15	2,3,4,5,7,9,13,16	0	
6271	1,6,8,10,11,12,14,16	2,3,4,5,7,9,13,15	2	P21674,Q9QW07
6272	1,6,8,10,11,12,15,16	2,3,4,5,7,9,13,14	1	Q9QW07
6273	1,6,8,10,11,13,14,15	2,3,4,5,7,9,12,16	0	
6274	1,6,8,10,11,13,14,16	2,3,4,5,7,9,12,15	2	O89117,P30120
6275	1,6,8,10,11,13,15,16	2,3,4,5,7,9,12,14	0	
6276	1,6,8,10,11,14,15,16	2,3,4,5,7,9,12,13	1	Q9QW07
6277	1,6,8,10,12,13,14,15	2,3,4,5,7,9,11,16	2	P08649,P70549
6278	1,6,8,10,12,13,14,16	2,3,4,5,7,9,11,15	4	P08649,P14668,P35280,P70549
6279	1,6,8,10,12,13,15,16	2,3,4,5,7,9,11,14	2	P08649,P70549
6280	1,6,8,10,12,14,15,16	2,3,4,5,7,9,11,13	1	P00774
6281	1,6,8,10,13,14,15,16	2,3,4,5,7,9,11,12	4	P00774,P14668,P70549,P70709
6282	1,6,8,11,12,13,14,15	2,3,4,5,7,9,10,16	1	P20766
6283	1,6,8,11,12,13,14,16	2,3,4,5,7,9,10,15	2	P30120,Q5RKI1
6284	1,6,8,11,12,13,15,16	2,3,4,5,7,9,10,14	2	P30120,Q9QW07
6285	1,6,8,11,12,14,15,16	2,3,4,5,7,9,10,13	1	Q9QW07
6286	1,6,8,11,13,14,15,16	2,3,4,5,7,9,10,12	1	P30120
6287	1,6,8,12,13,14,15,16	2,3,4,5,7,9,10,11	2	P20766,P70549
6288	1,6,9,10,11,12,13,14	2,3,4,5,7,8,15,16	29	B0LT89,O70594,P00774,P05539,P08649,P17988,P19468,P20646,P20760,P20761,P20762,P46844,P48508,P53790,Q05175,Q5I0E9,Q5M7T9,Q62761;Q62762;Q62763,Q63270,Q63424,Q64093,Q6Q0N1,Q6TMA8,Q8CIZ5,Q8R431,Q9WTW7,Q9WUW8,Q9Z0V6,Q9Z0W7
6289	1,6,9,10,11,12,13,15	2,3,4,5,7,8,14,16	15	B0LT89,D3ZHA0,D4A5U3,P00774,P08649,P20646,P20760,P20761,P20762,Q4G075,Q5RLM2,Q62761;Q62762;Q62763,Q6TMA8,Q8CIZ5,Q9WUW8

6290	1,6,9,10,11,12,13,16	2,3,4,5,7,8,14,15	12	B0LT89,D4A5U3,P08649,P20646,P20761,P20762,P45592,Q4G075,Q6PCU2,Q6TMA8,Q8CIZ5,Q9WUW8
6291	1,6,9,10,11,12,14,15	2,3,4,5,7,8,13,16	19	B0LT89,D4A5U3,O70594,P00762,P00774,P05539,P08649,P20646,P20760,P20761,P20762,P28570,P46844,Q4G075,Q5I0D7,Q62761;Q62762;Q62763,Q6TMA8,Q8CIZ5,Q9WUW8
6292	1,6,9,10,11,12,14,16	2,3,4,5,7,8,13,15	12	D4A5U3,O70594,P00774,P20646,P20761,P21674,Q63618,Q6PCU2,Q6Q0N1,Q6TMA8,Q8CIZ5,Q9WUW8
6293	1,6,9,10,11,12,15,16	2,3,4,5,7,8,13,14	13	B0BNN3,D3ZHA0,D4A5U3,P00774,P08649,P08937,P20761,P25809,Q4G075,Q5QE79,Q6TMA8,Q8CIZ5,Q9WUW8
6294	1,6,9,10,11,13,14,15	2,3,4,5,7,8,12,16	13	B0LT89,D4A5U3,P00774,P05539,P0DMW0;P0DMW1,P20760,P20761,P20762,P46844,Q5PQL7,Q5RLM2,Q6TMA8,Q8CIZ5
6295	1,6,9,10,11,13,14,16	2,3,4,5,7,8,12,15	9	B0LT89,O89117,P00774,P20761,P34901,P36376,Q5PQL7,Q6TMA8,Q8CIZ5
6296	1,6,9,10,11,13,15,16	2,3,4,5,7,8,12,14	12	B0LT89,D4A5U3,O70417,P00774,P08937,P20761,Q4G075,Q5RLM2,Q63751,Q6TMA8,Q8CIZ5,Q9WUW8
6297	1,6,9,10,11,14,15,16	2,3,4,5,7,8,12,13	8	D4A5U3,P00774,P20761,P25809,P47967,P97840,Q6TMA8,Q8CIZ5
6298	1,6,9,10,12,13,14,15	2,3,4,5,7,8,11,16	19	D4A5U3,P00762,P00774,P02780,P02781,P05539,P06760,P08649,P20646,P20761,P20762,P22273,P70549,Q00715,Q4G075,Q5M8C6,Q5PQL7,Q6TMA8,Q8CIZ5
6299	1,6,9,10,12,13,14,16	2,3,4,5,7,8,11,15	17	P00774,P06760,P08649,P20646,P20761,Q4FZU2,Q4G075,Q6IFU7,Q6IFU8,Q6IFW6,Q6IG02,Q6IMF3,Q6P6Q2,Q6TMA8,Q8CIZ5,Q9WTT6,Q9Z0V6
6300	1,6,9,10,12,13,15,16	2,3,4,5,7,8,11,14	14	D4A5U3,P00774,P06760,P07150,P08649,P08937,P20761,P70549,Q4G075,Q5QE79,Q63751,Q6B345,Q6TMA8,Q8CIZ5
6301	1,6,9,10,12,14,15,16	2,3,4,5,7,8,11,13	11	D4A5U3,P00774,P06760,P08649,P20761,P22273,P25809,P47967,Q4G075,Q6TMA8,Q8CIZ5
6302	1,6,9,10,13,14,15,16	2,3,4,5,7,8,11,12	15	D4A5U3,P00774,P02780,P06760,P08649,P22273,P47967,P70709,P97840,Q00715,Q4G075,Q5I0D1,Q5PQL7,Q6TMA8,Q8CIZ5
6303	1,6,9,11,12,13,14,15	2,3,4,5,7,8,10,16	56	B0LT89,D4A5U3,O35077,O35763,O55004,O70257,O70377,O70594,P00774,P05539,P07151,P0DMW0;P0DMW1,P18757,P19468,P20646,P20760,P20761,P20762,P20766,P28570,P36970,P46413,P46720,P46844,P48508,P53790,Q03248,Q05175,Q3ZAV1,Q5BJY9,Q5I0E9,Q5M7T9,Q5RLM2,Q62687,Q62761;Q62762;Q62763,Q63270,Q63355,Q63424,Q63618,Q64093,Q64602,Q6AY41,Q6MG61,Q6Q0N1,Q6TMA8,Q71MB6,Q80W57,Q8CIZ5,Q8R431,Q923S2,Q9JJ19,Q9JJ40,Q9WTW7,Q9WUW8,Q9WUW9,Q9Z0W7
6304	1,6,9,11,12,13,14,16	2,3,4,5,7,8,10,15	45	B0LT89,O35763,O70377,O70594,O88339;Q4V882,P00774,P10536,P12368,P19468,P20646,P20761,P20762,P20766,P30904,P38918,P46844,P48508,P51907,P53790,P54921,Q05175,Q3MIE4,Q3ZAV1,Q5I0E9,Q5M7T9,Q5RKI1,Q62753,Q63270,Q63355,Q63424,Q63618,Q64093,Q6MG61,Q6PCU2,Q6Q0N1,Q6TMA8,Q80W57,Q8CIZ5,Q8R431,Q923S2,Q9JJ19,Q9WTW7,Q9WUW8,Q9Z0V6,Q9Z0W7
6305	1,6,9,11,12,13,15,16	2,3,4,5,7,8,10,14	26	B0LT89,D4A5U3,O70417,O70594,P08937,P20646,P20760,P20761,P20762,P25809,P46844,P48508,P54921,Q05175,Q4G075,Q5QE79,Q5RLM2,Q62753,Q63355,Q63618,Q63751,Q6TMA8,Q8CIZ5,Q923S2,Q9WUW8,Q9WUW9

6306	1,6,9,11,12,14,15,16	2,3,4,5,7,8,10,13	25	D4A5U3,O55004,O70257,O70594,P00774,P19468,P20761,P20762,P25809,P30904,P46844,P47967,P48508,Q3ZAV1,Q63355,Q63424,Q63618,Q64093,Q6MG61,Q6Q0N1,Q6TMA8,Q8CIZ5,Q8R431,Q9WUW8,Q9WUW9
6307	1,6,9,11,13,14,15,16	2,3,4,5,7,8,10,12	20	B0LT89,P00774,P0DMW0;P0DMW1,P12368,P20761,P20762,P20766,P30904,P46844,P47967,P48508,P97840,Q5PQL7,Q5RLM2,Q63270,Q63618,Q6MG61,Q6Q0N1,Q6TMA8,Q8CIZ5
6308	1,6,9,12,13,14,15,16	2,3,4,5,7,8,10,11	15	D4A5U3,P00774,P06760,P20646,P20761,P20762,P20766,P22273,P30904,P47967,P70549,Q4G075,Q6B345,Q6TMA8,Q8CIZ5
6309	1,6,10,11,12,13,14,15	2,3,4,5,7,8,9,16	11	D4A5U3,P00774,P05539,P06760,P08649,P20646,P20760,P20761,P20762,Q6TMA8,Q8CIZ5
6310	1,6,10,11,12,13,14,16	2,3,4,5,7,8,9,15	12	D4A5U3,P00774,P06760,P08649,P20646,P20760,P20761,P35280,P36376,Q6TMA8,Q8CIZ5,Q9Z0V6
6311	1,6,10,11,12,13,15,16	2,3,4,5,7,8,9,14	14	D3ZHA0,D4A5U3,O70417,P00774,P06760,P08649,P08937,P20646,P20760,P20761,Q5QE79,Q63751,Q6TMA8,Q8CIZ5
6312	1,6,10,11,12,14,15,16	2,3,4,5,7,8,9,13	9	D4A5U3,P00774,P06760,P20646,P20760,P20761,Q5QE79,Q6TMA8,Q8CIZ5
6313	1,6,10,11,13,14,15,16	2,3,4,5,7,8,9,12	9	D4A5U3,P00774,P06760,P20646,P20760,P36376,Q5PQL7,Q6TMA8,Q8CIZ5
6314	1,6,10,12,13,14,15,16	2,3,4,5,7,8,9,11	18	D4A5U3,P00774,P06760,P07150,P08649,P14668,P20646,P20760,P20761,P35280,P70549,P82471,Q4G075,Q6B345,Q6P6R2,Q6TMA8,Q8CIZ5,Q9QX74
6315	1,6,11,12,13,14,15,16	2,3,4,5,7,8,9,10	17	D4A5U3,O70594,P00774,P06760,P20646,P20760,P20761,P20762,P20766,P70545,Q4FZU6,Q5QE79,Q63618,Q6P6R2,Q6TMA8,Q8CIZ5,Q9Z0V6
6316	1,7,8,9,10,11,12,13	2,3,4,5,6,14,15,16	3	B0LT89,P35745,Q4KLZ6
6317	1,7,8,9,10,11,12,14	2,3,4,5,6,13,15,16	4	P21674,P35745,Q5I0D7,Q812E4
6318	1,7,8,9,10,11,12,15	2,3,4,5,6,13,14,16	5	P35745,P49134,P52590,Q4KLZ6,Q9WUW8
6319	1,7,8,9,10,11,12,16	2,3,4,5,6,13,14,15	4	P21674,P35745,P49134,Q812E4
6320	1,7,8,9,10,11,13,14	2,3,4,5,6,12,15,16	3	B0LT89,Q5I0D7,Q812E4
6321	1,7,8,9,10,11,13,15	2,3,4,5,6,12,14,16	4	B0LT89,P35745,P52590,Q4KLZ6

6322	1,7,8,9,10,<br>11,13,16	2,3,4,5,6,1<br>2,14,15	2	P30120,Q812E4
6323	1,7,8,9,10,<br>11,14,15	2,3,4,5,6,1<br>2,13,16	2	P35745,Q5I0D7
6324	1,7,8,9,10,<br>11,14,16	2,3,4,5,6,1<br>2,13,15	2	P21674,Q812E4
6325	1,7,8,9,10,<br>11,15,16	2,3,4,5,6,1<br>2,13,14	2	P35745,P49134
6326	1,7,8,9,10,<br>12,13,14	2,3,4,5,6,1<br>1,15,16	5	P17988,P21674,P29315,P35745,Q4KLZ6
6327	1,7,8,9,10,<br>12,13,15	2,3,4,5,6,1<br>1,14,16	4	P35745,P52590,P70549,Q4KLZ6
6328	1,7,8,9,10,<br>12,13,16	2,3,4,5,6,1<br>1,14,15	3	P21674,P35745,Q4KLZ6
6329	1,7,8,9,10,<br>12,14,15	2,3,4,5,6,1<br>1,13,16	2	P35745,Q4KLZ6
6330	1,7,8,9,10,<br>12,14,16	2,3,4,5,6,1<br>1,13,15	2	P21674,P35745
6331	1,7,8,9,10,<br>12,15,16	2,3,4,5,6,1<br>1,13,14	3	P21674,P35745,Q4KLZ6
6332	1,7,8,9,10,<br>13,14,15	2,3,4,5,6,1<br>1,12,16	2	P35745,Q4KLZ6
6333	1,7,8,9,10,<br>13,14,16	2,3,4,5,6,1<br>1,12,15	1	P21674
6334	1,7,8,9,10,<br>13,15,16	2,3,4,5,6,1<br>1,12,14	2	P35745,Q4KLZ6
6335	1,7,8,9,10,<br>14,15,16	2,3,4,5,6,1<br>1,12,13	2	P35745,P47967
6336	1,7,8,9,11,<br>12,13,14	2,3,4,5,6,1<br>0,15,16	8	B0LT89,O70594,P17988,P21674,P53790,Q63270,Q71MB6,Q812E4
6337	1,7,8,9,11,<br>12,13,15	2,3,4,5,6,1<br>0,14,16	4	B0LT89,P35745,Q4KLZ6,Q71MB6
6338	1,7,8,9,11,<br>12,13,16	2,3,4,5,6,1<br>0,14,15	7	B0LT89,P02782,P21674,P30120,P54921,Q63357,Q812E4
6339	1,7,8,9,11,<br>12,14,15	2,3,4,5,6,1<br>0,13,16	2	P35745,Q812E4
6340	1,7,8,9,11,<br>12,14,16	2,3,4,5,6,1<br>0,13,15	4	P21674,Q498D9,Q63618,Q812E4
6341	1,7,8,9,11,<br>12,15,16	2,3,4,5,6,1<br>0,13,14	4	P35745,Q812E4,Q9QW07,Q9WUW8

6342	1,7,8,9,11,13,14,15	2,3,4,5,6,10,12,16	3	P0DMW0;P0DMW1,P36970,Q812E4
6343	1,7,8,9,11,13,14,16	2,3,4,5,6,10,12,15	6	P09456,P0DMW0;P0DMW1,P21674,P30120,Q498D9,Q812E4
6344	1,7,8,9,11,13,15,16	2,3,4,5,6,10,12,14	3	P30120,Q5RLM2,Q812E4
6345	1,7,8,9,11,14,15,16	2,3,4,5,6,10,12,13	2	Q812E4,Q8CJ52
6346	1,7,8,9,12,13,14,15	2,3,4,5,6,10,11,16	3	P35745,P70549,Q4KLZ6
6347	1,7,8,9,12,13,14,16	2,3,4,5,6,10,11,15	1	P21674
6348	1,7,8,9,12,13,15,16	2,3,4,5,6,10,11,14	2	P35745,Q4KLZ6
6349	1,7,8,9,12,14,15,16	2,3,4,5,6,10,11,13	2	P21674,P35745
6350	1,7,8,9,13,14,15,16	2,3,4,5,6,10,11,12	0	
6351	1,7,8,10,11,12,13,14	2,3,4,5,6,9,15,16	2	P23593,P29315
6352	1,7,8,10,11,12,13,15	2,3,4,5,6,9,14,16	4	P23593,P35745,P52590,Q4KLZ6
6353	1,7,8,10,11,12,13,16	2,3,4,5,6,9,14,15	2	P02782,P30120
6354	1,7,8,10,11,12,14,15	2,3,4,5,6,9,13,16	1	P35745
6355	1,7,8,10,11,12,14,16	2,3,4,5,6,9,13,15	2	P21674,P30120
6356	1,7,8,10,11,12,15,16	2,3,4,5,6,9,13,14	1	Q9QW07
6357	1,7,8,10,11,13,14,15	2,3,4,5,6,9,12,16	0	
6358	1,7,8,10,11,13,14,16	2,3,4,5,6,9,12,15	2	P30120,P36376
6359	1,7,8,10,11,13,15,16	2,3,4,5,6,9,12,14	1	P30120
6360	1,7,8,10,11,14,15,16	2,3,4,5,6,9,12,13	0	
6361	1,7,8,10,12,13,14,15	2,3,4,5,6,9,11,16	3	P35745,P70549,Q4KLZ6

6362	1,7,8,10,12,13,14,16	2,3,4,5,6,9,11,15	1	P21674
6363	1,7,8,10,12,13,15,16	2,3,4,5,6,9,11,14	3	P35745,P70549,Q4KLZ6
6364	1,7,8,10,12,14,15,16	2,3,4,5,6,9,11,13	1	P35745
6365	1,7,8,10,13,14,15,16	2,3,4,5,6,9,11,12	1	P70549
6366	1,7,8,11,12,13,14,15	2,3,4,5,6,9,10,16	1	Q811M5
6367	1,7,8,11,12,13,14,16	2,3,4,5,6,9,10,15	6	P02782,P21674,P30120,Q811M5,Q812E4,Q99MH3
6368	1,7,8,11,12,13,15,16	2,3,4,5,6,9,10,14	2	P02782,P30120
6369	1,7,8,11,12,14,15,16	2,3,4,5,6,9,10,13	4	D3ZTX0,P17559,Q811M5,Q9QW07
6370	1,7,8,11,13,14,15,16	2,3,4,5,6,9,10,12	2	P30120,Q811M5
6371	1,7,8,12,13,14,15,16	2,3,4,5,6,9,10,11	2	P70549,Q9Z2L0
6372	1,7,9,10,11,12,13,14	2,3,4,5,6,8,15,16	13	B0LT89,O70594,P00774,P17988,P20760,P20761,P20762,P48508,P53790,Q05702,Q5I0D7,Q62761;Q62762;Q62763,Q63270
6373	1,7,9,10,11,12,13,15	2,3,4,5,6,8,14,16	10	B0LT89,P08937,P20760,P20761,P20762,P52590,Q4KLZ6,Q62761;Q62762;Q62763,Q6TMA8,Q9WUW8
6374	1,7,9,10,11,12,13,16	2,3,4,5,6,8,14,15	7	B0LT89,P20760,P20761,P20762,P21674,P54921,Q8CIZ5
6375	1,7,9,10,11,12,14,15	2,3,4,5,6,8,13,16	8	P00774,P20760,P20761,P46844,Q5I0D7,Q62761;Q62762;Q62763,Q8CIZ5,Q9WUW8
6376	1,7,9,10,11,12,14,16	2,3,4,5,6,8,13,15	9	O70594,P00774,P10247,P20761,P21674,Q498D9,Q63618,Q8CGS4,Q8CIZ5
6377	1,7,9,10,11,12,15,16	2,3,4,5,6,8,13,14	7	D4A5U3,P08937,P20760,P20761,P52590,Q8CIZ5,Q9WUW8
6378	1,7,9,10,11,13,14,15	2,3,4,5,6,8,12,16	7	B0LT89,P00774,P0DMW0;P0DMW1,P20760,P20761,Q05702,Q5I0D7
6379	1,7,9,10,11,13,14,16	2,3,4,5,6,8,12,15	8	P00774,P10247,P36376,P98089,Q05702,Q498D9,Q8CIZ5,Q9R168
6380	1,7,9,10,11,13,15,16	2,3,4,5,6,8,12,14	5	P08937,P20760,Q05702,Q5RLM2,Q8CIZ5
6381	1,7,9,10,11,14,15,16	2,3,4,5,6,8,12,13	6	P00774,P10247,P98089,Q05702,Q8CIZ5,Q9R168

6382	1,7,9,10,12,13,14,15	2,3,4,5,6,8,11,16	11	P00762,P00774,P02780,P20760,P20761,P22273,P35745,P50280,P70549,Q05702,Q4KLZ6
6383	1,7,9,10,12,13,14,16	2,3,4,5,6,8,11,15	10	P00774,P10247,P21674,P50280,Q10758,Q6IFU7,Q6IFU8,Q6IFW6,Q6IG02,Q8CIZ5
6384	1,7,9,10,12,13,15,16	2,3,4,5,6,8,11,14	8	P00774,P08937,P20760,P35745,P50280,Q4KLZ6,Q63751,Q8CIZ5
6385	1,7,9,10,12,14,15,16	2,3,4,5,6,8,11,13	5	P00774,P10247,P22273,P35745,Q8CIZ5
6386	1,7,9,10,13,14,15,16	2,3,4,5,6,8,11,12	6	P00774,P10247,P31430,Q05702,Q5PQL7,Q8CIZ5
6387	1,7,9,11,12,13,14,15	2,3,4,5,6,8,10,16	38	B0LT89,O55004,O70257,O70377,O70594,P00774,P07151,P0DMW0;P0DMW1,P19468,P20760,P20761,P20762,P25031,P28570,P36970,P46844,P48508,P53790,Q03248,Q05175,Q05702,Q3T1J9,Q3ZAV1,Q5BJY9,Q5I0D7,Q5I0E9,Q5M7T9,Q62761;Q62762;Q62763,Q63270,Q63424,Q63618,Q6MG61,Q6Q0N1,Q71MB6,Q8R431,Q923S2,Q9WUW8,Q9Z0W7
6388	1,7,9,11,12,13,14,16	2,3,4,5,6,8,10,15	26	B0LT89,O70377,O70594,O88339;Q4V882,P10247,P20760,P20761,P20762,P21674,P25031,P30904,P38918,P48508,P53790,P54921,Q05175,Q05702,Q498D9,Q5I0E9,Q62753,Q63270,Q63618,Q6MG61,Q6Q0N1,Q8CIZ5,Q923S2
6389	1,7,9,11,12,13,15,16	2,3,4,5,6,8,10,14	14	O70594,O88339;Q4V882,P08937,P20760,P20761,P20762,P25031,P48508,P54921,Q5RLM2,Q63618,Q8CIZ5,Q923S2,Q9WUW8
6390	1,7,9,11,12,14,15,16	2,3,4,5,6,8,10,13	19	O55004,O70257,O70594,O88339;Q4V882,P00774,P02631,P10247,P20760,P20761,P25031,P25809,P30904,P36860,P46844,Q05702,Q498D9,Q63618,Q8CIZ5,Q9WUW8
6391	1,7,9,11,13,14,15,16	2,3,4,5,6,8,10,12	14	P00774,P02631,P0DMW0;P0DMW1,P10247,P20760,P25031,P30904,P48508,Q05702,Q498D9,Q5RLM2,Q63618,Q8CIZ5,Q9R168
6392	1,7,9,12,13,14,15,16	2,3,4,5,6,8,10,11	9	P00774,P08937,P10247,P20760,P25031,Q05702,Q5BJY9,Q6B345,Q8CIZ5
6393	1,7,10,11,12,13,14,15	2,3,4,5,6,8,9,16	5	P00774,P20760,P20761,P23593,Q05702
6394	1,7,10,11,12,13,14,16	2,3,4,5,6,8,9,15	5	P00774,P10247,P20760,P36376,Q05702
6395	1,7,10,11,12,13,15,16	2,3,4,5,6,8,9,14	8	D4A5U3,P00774,P08937,P20760,P52590,Q05702,Q63751,Q8CIZ5
6396	1,7,10,11,12,14,15,16	2,3,4,5,6,8,9,13	7	D4A5U3,P00774,P10247,P17559,P20760,Q05702,Q8CIZ5
6397	1,7,10,11,13,14,15,16	2,3,4,5,6,8,9,12	5	P00774,P20760,P36376,Q05702,Q8CIZ5

6398	1,7,10,12,13,14,15,16	2,3,4,5,6,8,9,11	7	P00774,P08937,P20760,P70549,Q05702,Q6P6S4,Q8CIZ5
6399	1,7,11,12,13,14,15,16	2,3,4,5,6,8,9,10	14	D3ZTX0,O70594,P00774,P02454,P08937,P10247,P17559,P20760,P25031,P70545,Q05702,Q63618,Q811M5,Q8CIZ5
6400	1,8,9,10,11,12,13,14	2,3,4,5,6,7,15,16	15	B0LT89,O70594,P17988,P18297,P19468,P46844,P48508,P50116,P53790,Q03248,Q5I0D7,Q5I0E9,Q63270,Q6TMA8,Q71MB6
6401	1,8,9,10,11,12,13,15	2,3,4,5,6,7,14,16	13	B0LT89,P35745,P36970,P48037,P50116,P52590,Q4KLZ6,Q5I0D7,Q5QE79,Q5RLM2,Q6TMA8,Q71MB6,Q9WUW8
6402	1,8,9,10,11,12,13,16	2,3,4,5,6,7,14,15	4	B0LT89,P21674,P50116,Q62714
6403	1,8,9,10,11,12,14,15	2,3,4,5,6,7,13,16	9	B0LT89,O70594,P35745,P46844,P50116,P80299,Q5I0D7,Q6TMA8,Q9WUW8
6404	1,8,9,10,11,12,14,16	2,3,4,5,6,7,13,15	8	B0LT89,O70594,P21674,P36860,P50116,P80299,Q5I0D7,Q62714
6405	1,8,9,10,11,12,15,16	2,3,4,5,6,7,13,14	6	B0LT89,P50116,P52590,P80299,Q5QE79,Q9WUW8
6406	1,8,9,10,11,13,14,15	2,3,4,5,6,7,12,16	5	B0LT89,P36970,P50116,Q5I0D7,Q6TMA8
6407	1,8,9,10,11,13,14,16	2,3,4,5,6,7,12,15	3	B0LT89,O89117,P50116
6408	1,8,9,10,11,13,15,16	2,3,4,5,6,7,12,14	5	B0LT89,P50116,Q5QE79,Q5RLM2,Q80WL1
6409	1,8,9,10,11,14,15,16	2,3,4,5,6,7,12,13	6	P36860,P47967,P50116,P80299,P97840,Q5I0D7
6410	1,8,9,10,12,13,14,15	2,3,4,5,6,7,11,16	13	P01039,P02780,P07647,P11598,P17988,P35745,P50116,P70549,Q4KLZ6,Q5I0D7,Q6TMA8,Q8R5M3,Q9R0T3
6411	1,8,9,10,12,13,14,16	2,3,4,5,6,7,11,15	5	O08557,P01039,P17988,P21674,P50116
6412	1,8,9,10,12,13,15,16	2,3,4,5,6,7,11,14	8	P01039,P08649,P35745,P50116,P52590,Q4KLZ6,Q5QE79,Q63751
6413	1,8,9,10,12,14,15,16	2,3,4,5,6,7,11,13	6	P01039,P35745,P47967,P50116,P80299,Q9R0T3
6414	1,8,9,10,13,14,15,16	2,3,4,5,6,7,11,12	7	P01039,P47967,P50116,P70709,P97840,Q5I0D1,Q9R0T3
6415	1,8,9,11,12,13,14,15	2,3,4,5,6,7,10,16	47	B0LT89,O08557,O70594,P0DMW0;P0DMW1,P17988,P18297,P18757,P19468,P20766,P28570,P29975,P36970,P38918,P46844,P48037,P48508,P50116,P52847,P53790,P57113,P80299,Q03248,Q3T1J9,Q3ZAV1,Q5I0D7,Q5I0E9,Q5M7T9,Q5RLM2,Q63270,Q63424,Q63618,Q64602,Q68FT5,Q6MG61,Q6Q0N1,Q6TMA8,Q71MB6,Q80W57,Q8R431,Q8VI04,Q923S2,Q9JJ40,Q9QYU4,Q9WTW7,Q9WUW8,Q9WUW9,Q9Z0W7

6416	1,8,9,11,12,13,14,16	2,3,4,5,6,7,10,15	37	A2RUW1,B0LT89,O08557,O70594,O88339;Q4V882,P17988,P18297,P19468,P20766,P21674,P30904,P36860,P38918,P46844,P48508,P50116,P53790,P54921,P80299,Q03248,Q05175,Q3MIE4,Q5I0E9,Q5M7T9,Q5RKI1,Q63270,Q63357,Q63424,Q63618,Q6MG61,Q6Q0N1,Q71MB6,Q7M0E3,Q812E4,Q8K3P7,Q923S2,Q9Z0W7
6417	1,8,9,11,12,13,15,16	2,3,4,5,6,7,10,14	17	A2RUW1,B0LT89,O70594,P18297,P48037,P48508,P50116,P54921,P57113,P80299,Q5I0E9,Q5QE79,Q5RLM2,Q71MB6,Q80WL1,Q923S2,Q9WUW8
6418	1,8,9,11,12,14,15,16	2,3,4,5,6,7,10,13	10	A2RUW1,O70594,P18297,P36860,P46844,P48508,P50116,P80299,Q63618,Q9WUW8
6419	1,8,9,11,13,14,15,16	2,3,4,5,6,7,10,12	15	B0LT89,P0DMW0;P0DMW1,P18297,P30904,P36860,P36970,P46844,P47967,P48508,P50116,P80299,Q5RLM2,Q63270,Q63618,Q66HG3
6420	1,8,9,12,13,14,15,16	2,3,4,5,6,7,10,11	8	O08557,P01039,P20766,P48037,P50116,P80299,Q6B345,Q9R0T3
6421	1,8,10,11,12,13,14,15	2,3,4,5,6,7,9,16	1	P50116
6422	1,8,10,11,12,13,14,16	2,3,4,5,6,7,9,15	1	P50116
6423	1,8,10,11,12,13,15,16	2,3,4,5,6,7,9,14	4	P50116,P52590,Q5QE79,Q63751
6424	1,8,10,11,12,14,15,16	2,3,4,5,6,7,9,13	4	P36860,P50116,P80299,Q5QE79
6425	1,8,10,11,13,14,15,16	2,3,4,5,6,7,9,12	3	P50116,P80299,Q5QE79
6426	1,8,10,12,13,14,15,16	2,3,4,5,6,7,9,11	6	P00774,P01039,P50116,P70549,Q5QE79,Q9R0T3
6427	1,8,11,12,13,14,15,16	2,3,4,5,6,7,9,10	11	D3ZTX0,O70594,P20766,P36860,P50116,P70545,P80299,Q5QE79,Q63618,Q811M5,Q9WVH8
6428	1,9,10,11,12,13,14,15	2,3,4,5,6,7,8,16	76	B0LT89,D3ZHA0,D4A5U3,O55004,O70257,O70594,P00774,P01836,P02631,P02780,P02781,P04904,P06761,P07151,P08649,P08937,P18757,P19223,P19468,P20646,P20673,P20760,P20761,P20762,P28570,P36970,P38438,P38918,P46720,P46844,P48037,P48508,P50115,P50116,P50280,P52847,P53790,P55091,P57113,P98089,Q03248,Q05175,Q05702,Q3ZAV1,Q4G075,Q5I0D7,Q5I0E9,Q5M7T9,Q5M8C6,Q5QE79,Q5RLM2,Q5U2Q3,Q62761;Q62762;Q62763,Q63270,Q63317,Q63424,Q63618,Q64602,Q6MG61,Q6PCU2,Q6Q0N1,Q6TMA8,Q71MB6,Q8CIZ5,Q8R431,Q8R5M3,Q923S2,Q9JJ40,Q9JLJ3,Q9QX74,Q9QYU4,Q9R0T3,Q9WTW7,Q9WUW8,Q9WUW9,Q9Z0W7

6429	1,9,10,11,12,13,14,16	2,3,4,5,6,7,8,15	54	B0LT89,D3ZHA0,D4A5U3,O08557,O35763,O55004,O70377,O70594,O88339;Q4V882,P00774,P01836,P08937,P10247,P17988,P19468,P20646,P20760,P20761,P20762,P30904,P36860,P38918,P46844,P48508,P50115,P50116,P50280,P53790,P55091,P98089,Q05175,Q05702,Q498D9,Q4G075,Q5I0D7,Q5I0E9,Q5M7T9,Q5QE79,Q62714,Q63270,Q63618,Q6AYS7,Q6MG61,Q6PCU2,Q6Q0N1,Q6TMA8,Q8CGS4,Q8CIZ5,Q923S2,Q9QX74,Q9R0T3,Q9WUW8,Q9Z0V6,Q9Z0W7
6430	1,9,10,11,12,13,15,16	2,3,4,5,6,7,8,14	37	B0LT89,D3ZHA0,D4A5U3,O70417,O70594,P00774,P06760,P06761,P08649,P08937,P19223,P20760,P20761,P20762,P46844,P48037,P48508,P50115,P50116,P52590,P55091,P63081,P98089,Q4G075,Q5QE79,Q5RLM2,Q62635,Q62714,Q63751,Q6PCU2,Q6TMA8,Q8CIZ5,Q923S2,Q9QX74,Q9R0T3,Q9WUW8,Q9WUW9
6431	1,9,10,11,12,14,15,16	2,3,4,5,6,7,8,13	44	B0LT89,D3ZHA0,D4A5U3,O55004,O70257,O70594,P00774,P01836,P02631,P06761,P07171,P08937,P10247,P19223,P20760,P20761,P20762,P22734,P25809,P30904,P36860,P46844,P48508,P50115,P50116,P55091,P63081,P80299,P98089,Q05702,Q4G075,Q5I0D7,Q5M7T9,Q5QE79,Q62714,Q62946,Q63618,Q6PCU2,Q6Q0N1,Q6TMA8,Q8CIZ5,Q9QX74,Q9R0T3,Q9WUW8
6432	1,9,10,11,13,14,15,16	2,3,4,5,6,7,8,12	35	B0LT89,D3ZHA0,D4A5U3,P00774,P01836,P02631,P06761,P08937,P10247,P20760,P20761,P20762,P26772,P30904,P31430,P38438,P46844,P48508,P50115,P50116,P55091,P98089,Q05702,Q4G075,Q5I0D7,Q5QE79,Q5RLM2,Q62946,Q63270,Q6Q0N1,Q6TMA8,Q8CIZ5,Q920G2,Q9QX74,Q9R0T3
6433	1,9,10,12,13,14,15,16	2,3,4,5,6,7,8,11	46	D3ZHA0,D4A5U3,O08557,P00774,P01039,P02780,P02781,P06760,P06761,P07150,P08649,P08937,P10247,P11598,P12020,P20760,P20761,P20762,P22273,P22282,P22283,P30904,P31430,P46462,P50115,P50116,P50280,P55091,P98089,Q05702,Q4G063,Q4G075,Q5M8C6,Q5QE79,Q5U2V4,Q62902,Q62946,Q63493,Q63617,Q6B345,Q6TMA8,Q8CIZ5,Q99041,Q9QX74,Q9R0T3,Q9WTT6
6434	1,9,11,12,13,14,15,16	2,3,4,5,6,7,8,10	94	B0LT89,D3ZHA0,D4A5U3,O08557,O35763,O55004,O70257,O70377,O70594,O88339;Q4V882,P00774,P01836,P02454,P02631,P04904,P06761,P08937,P10247,P10760,P18757,P19223,P19468,P20646,P20760,P20761,P20762,P20766,P23928,P25031,P25809,P26772,P29975,P30904,P36860,P36970,P38438,P38918,P46720,P46844,P48037,P48508,P50115,P50116,P51907,P52847,P53790,P54921,P55091,P57113,P60711;P63259,P68035;P68136,P80299,P98089,Q03248,Q05175,Q05702,Q3MIE4,Q3T1J9,Q3ZAV1,Q4G075,Q5I0E9,Q5M7T9,Q5QE79,Q5RLM2,Q62753,Q62761;Q62762;Q62763,Q63270,Q63317,Q63355,Q63424,Q63618,Q64602,Q66HG3,Q6AYS7,Q6MG61,Q6PCU2,Q6Q0N1,Q6TMA8,Q71MB6,Q80W57,Q8CIZ5,Q8R431,Q920G2,Q923S2,Q9JJ19,Q9JJ40,Q9JLJ3,Q9QX74,Q9QYU4,Q9R0T3,Q9WTW7,Q9WUW8,Q9WUW9,Q9Z0W7
6435	1,10,11,12,13,14,15,16	2,3,4,5,6,7,8,9	31	D3ZHA0,D4A5U3,O70594,P00774,P01836,P06760,P08937,P10247,P19223,P20646,P20760,P20761,P20762,P26772,P50115,P50116,P55091,P98089,Q05702,Q4FZU6,Q4G075,Q5QE79,Q5U2V4,Q62946,Q6P6R2,Q6TMA8,Q8CIZ5,Q920G2,Q9QX74,Q9R0T3,Q9Z0V6

Day 7

Random allocation	Group1	Group2	Numbers of differential urinary proteins	Differential protein ID

1	1,2,3,4,5,6,7,8	17,18,19,20,21,22,23,24	52	B1H234,D3ZHA0,iRT-Kit_WR_fusion,O54858,O70594,P01681,P01836,P11762,P15399,P18418,P18757,P19629,P20760,P20761,P23593,P30919,P31044,P33436,P36860,P42854,P46844,P48508,P50115,P50116,P50280,P52590,P80202,P80299,P97580,Q05702,Q09030,Q30KJ2,Q4AEF8,Q4FZU2,Q4FZU4,Q4KLZ6,Q5BK81,Q5I0E9,Q62946,Q64240,Q6IFU7,Q6IFU8,Q6IFW6,Q6IG02,Q6IG05,Q6IMF3,Q6P6Q2,Q91XN4,Q91ZS3,Q99041,Q9QZQ5,Q9Z2L0
2	1,2,3,4,5,6,7,17	8,18,19,20,21,22,23,24	15	B1H234,O70594,P11762,P23593,P33436,P36860,P42854,P50116,P52590,P80299,Q09030,Q4AEF8,Q4KLZ6,Q99PP0,Q9Z2L0
3	1,2,3,4,5,6,7,18	8,17,19,20,21,22,23,24	21	B1H234,O54800;Q5DWV2,O70594,P11762,P23593,P33436,P36860,P42854,P50116,P52590,P80299,Q03191,Q09030,Q4AEF8,Q4KLZ6,Q5I0E9,Q6P6R2,Q91XN4,Q91ZS3,Q9QZQ5,Q9Z2L0
4	1,2,3,4,5,6,7,19	8,17,18,20,21,22,23,24	13	B1H234,O70594,P11762,P42854,P47967,P50116,P52590,P80299,Q09030,Q4AEF8,Q4FZU4,Q4KLZ6,Q9Z2L0
5	1,2,3,4,5,6,7,20	8,17,18,19,21,22,23,24	21	B1H234,P0DP29;P0DP30;P0DP31,P11598,P11762,P19629,P23593,P33436,P42854,P47967,P50116,P52590,P80299,Q03191,Q09030,Q4AEF8,Q4KLZ6,Q5GRG2,Q5I0D1,Q6IG02,Q91ZS3,Q9Z2L0
6	1,2,3,4,5,6,7,21	8,17,18,19,20,22,23,24	17	B1H234,O70594,P04762,P11762,P30919,P36860,P42854,P47967,P48037,P50116,P52590,P70549,P80299,Q03191,Q09030,Q4AEF8,Q4KLZ6
7	1,2,3,4,5,6,7,22	8,17,18,19,20,21,23,24	15	B1H234,P11762,P23593,P30919,P42854,P50116,P52590,P80299,Q03191,Q09030,Q4FZU4,Q4KLZ6,Q6P6R2,Q91XN4,Q9Z2L0
8	1,2,3,4,5,6,7,23	8,17,18,19,20,21,22,24	24	B0LT89,B1H234,O70594,P02783,P09656,P11762,P23593,P30120,P36860,P42854,P46844,P47727,P50116,P52590,P80299,Q03191,Q09030,Q4KLZ6,Q63532,Q6P6R2,Q91XN4,Q99MH3,Q9JJ50,Q9Z2L0
9	1,2,3,4,5,6,7,24	8,17,18,19,20,21,22,23	19	B1H234,O08557,O70594,P02761,P11762,P23593,P30919,P36860,P42854,P46844,P50116,P52590,P80299,Q09030,Q4KLZ6,Q6AY61,Q6P6R2,Q91XN4,Q9Z2L0
10	1,2,3,4,5,6,8,17	7,18,19,20,21,22,23,24	28	O70594,P01681,P11762,P15399,P18418,P23593,P33436,P42854,P52590,P97580,Q05702,Q09030,Q30KJ2,Q4FZU2,Q4KLZ6,Q5BK81,Q64240,Q6IFU7,Q6IFU8,Q6IFW6,Q6IG02,Q6IG05,Q6IMF3,Q6P6Q2,Q91XN4,Q99PP0,Q9QZQ5,Q9Z2L0
11	1,2,3,4,5,6,8,18	7,17,19,20,21,22,23,24	28	O54800;Q5DWV2,O70594,P01681,P11762,P15399,P18418,P20762,P23593,P33436,P42854,P52590,P97580,Q05702,Q09030,Q30KJ2,Q4FZU2,Q4KLZ6,Q5BK81,Q64240,Q6IFU7,Q6IG02,Q6IG05,Q6IMF3,Q6P6Q2,Q91XN4,Q91ZS3,Q9QZQ5,Q9Z2L0
12	1,2,3,4,5,6,8,19	7,17,18,20,21,22,23,24	23	O70594,P01681,P11762,P15399,P18418,P30919,P42854,P52590,P97580,Q05702,Q09030,Q10743,Q30KJ2,Q4FZU2,Q4KLZ6,Q6IFU7,Q6IFW6,Q6IG02,Q6IG05,Q6IMF3,Q6P6Q2,Q9QZQ5,Q9Z2L0

13	1,2,3,4,5,6,8,20	7,17,18,19,21,22,23,24	37	D4A5U3,P01681,P06911,P11762,P12020,P15399,P17559,P18418,P19629,P23593,P25031,P33436,P36375,P42854,P50280,P52590,P97580,Q05702,Q09030,Q10743,Q10758,Q30KJ2,Q4FZU2,Q4KLZ6,Q5GRG2,Q6IFU7,Q6IFU8,Q6IFW6,Q6IG02,Q6IG05,Q6IMF3,Q6P6Q2,Q91XN4,Q91ZS3,Q9JI85,Q9QZQ5,Q9Z2L0
14	1,2,3,4,5,6,8,21	7,17,18,19,20,22,23,24	32	B1H234,O70594,P01681,P02625,P11762,P18418,P23593,P30919,P36860,P42854,P52590,P70549,P97580,Q05702,Q09030,Q10743,Q30KJ2,Q4FZU2,Q4KLZ6,Q5BK81,Q6AYC4,Q6IFU7,Q6IFU8,Q6IFW6,Q6IG02,Q6IG05,Q6IMF3,Q6P6Q2,Q91XN4,Q91ZS3,Q9QZQ5,Q9Z2L0
15	1,2,3,4,5,6,8,22	7,17,18,19,20,21,23,24	27	P00507,P01681,P11762,P15399,P20760,P23593,P30919,P42854,P52590,P80202,P97580,Q05702,Q09030,Q10743,Q30KJ2,Q4FZU2,Q4KLZ6,Q6IFU7,Q6IFW6,Q6IG02,Q6IG05,Q6IMF3,Q6P6Q2,Q6P6R2,Q91XN4,Q9QZQ5,Q9Z2L0
16	1,2,3,4,5,6,8,23	7,17,18,19,20,21,22,24	34	O70594,P01681,P02783,P09656,P11762,P20760,P20761,P23593,P23785,P30919,P36376,P42854,P52590,P80202,P97580,Q05702,Q09030,Q30KJ2,Q4FZU2,Q4KLZ6,Q5BK81,Q63532,Q6IFU7,Q6IFU8,Q6IFW6,Q6IG02,Q6IG05,Q6IMF3,Q6P6Q2,Q6P6R2,Q91XN4,Q99MH3,Q9JJ50,Q9Z2L0
17	1,2,3,4,5,6,8,24	7,17,18,19,20,21,22,23	30	O70594,P01681,P11762,P15399,P18418,P20760,P23593,P30919,P36860,P42854,P52590,P97580,Q05702,Q09030,Q10743,Q30KJ2,Q4FZU2,Q4KLZ6,Q6IFU7,Q6IFU8,Q6IFW6,Q6IG02,Q6IG05,Q6IMF3,Q6P6Q2,Q6P6R2,Q91XN4,Q91ZS3,Q9QZQ5,Q9Z2L0
18	1,2,3,4,5,6,17,18	7,8,19,20,21,22,23,24	10	O54800;Q5DWV2,O70594,P23593,P33436,P52590,Q06000,Q09030,Q4KLZ6,Q99PP0,Q9Z2L0
19	1,2,3,4,5,6,17,19	7,8,18,20,21,22,23,24	6	P11762,P33436,P42854,P52590,Q09030,Q4KLZ6
20	1,2,3,4,5,6,17,20	7,8,18,19,21,22,23,24	8	P19223,P33436,P42854,P52590,P55281,Q09030,Q4KLZ6,Q6IG02
21	1,2,3,4,5,6,17,21	7,8,18,19,20,22,23,24	7	B1H234,O70594,P33436,P36860,P52590,Q09030,Q4KLZ6
22	1,2,3,4,5,6,17,22	7,8,18,19,20,21,23,24	8	P01946,P02091,P23593,P52590,Q09030,Q4KLZ6,Q9WTT6,Q9Z2L0
23	1,2,3,4,5,6,17,23	7,8,18,19,20,21,22,24	10	O70594,P30120,P33436,P42854,P52590,Q09030,Q4KLZ6,Q99MH3,Q9WTT6,Q9Z2L0
24	1,2,3,4,5,6,17,24	7,8,18,19,20,21,22,23	9	P01039,P23593,P33436,P36860,P52590,Q09030,Q4KLZ6,Q6AY61,Q9WTT6
25	1,2,3,4,5,6,18,19	7,8,17,20,21,22,23,24	7	B1H234,O54800;Q5DWV2,P11762,P42854,P52590,Q4KLZ6,Q9Z2L0

26	1,2,3,4,5,6,18,20	7,8,17,19,21,22,23,24	9	O54800;Q5DWV2,P11762,P33436,P42854,P52590,Q03191,Q4KLZ6,Q91ZS3,Q9Z2L0
27	1,2,3,4,5,6,18,21	7,8,17,19,20,22,23,24	10	B1H234,O54800;Q5DWV2,O89117,P36860,P52590,Q09030,Q4KLZ6,Q9JJ50,Q9WTT6,Q9Z2L0
28	1,2,3,4,5,6,18,22	7,8,17,19,20,21,23,24	13	O54800;Q5DWV2,P11762,P23593,P30120,P52590,Q03191,Q09030,Q4KLZ6,Q6P6R2,Q91XN4,Q9EQS0,Q9WTT6,Q9Z2L0
29	1,2,3,4,5,6,18,23	7,8,17,19,20,21,22,24	18	O54800;Q5DWV2,O70594,P16636,P30120,P33436,P42854,P47727,P52590,Q03191,Q09030,Q4KLZ6,Q6P6R2,Q91XN4,Q99MH3,Q9EQS0,Q9JJ50,Q9WTT6,Q9Z2L0
30	1,2,3,4,5,6,18,24	7,8,17,19,20,21,22,23	12	B1H234,O54800;Q5DWV2,P23593,P33436,P52590,Q09030,Q4KLZ6,Q6P6R2,Q91XN4,Q9QX74,Q9WTT6,Q9Z2L0
31	1,2,3,4,5,6,19,20	7,8,17,18,21,22,23,24	6	P11762,P42854,P52590,Q4KLZ6,Q5I0D1,Q9Z2L0
32	1,2,3,4,5,6,19,21	7,8,17,18,20,22,23,24	7	B1H234,P11762,P30919,P42854,P52590,Q09030,Q4KLZ6
33	1,2,3,4,5,6,19,22	7,8,17,18,20,21,23,24	11	P00507,P01946,P11762,P30919,P42854,P52590,Q09030,Q4KLZ6,Q6P6R2,Q9WTT6,Q9Z2L0
34	1,2,3,4,5,6,19,23	7,8,17,18,20,21,22,24	9	P02783,P11762,P42854,P47727,P52590,Q09030,Q4KLZ6,Q9WTT6,Q9Z2L0
35	1,2,3,4,5,6,19,24	7,8,17,18,20,21,22,23	9	B1H234,P11762,P30919,P42854,P52590,Q09030,Q4KLZ6,Q6P6R2,Q9WTT6
36	1,2,3,4,5,6,20,21	7,8,17,18,19,22,23,24	10	B1H234,O55145,P11762,P42854,P47967,P52590,P70549,Q09030,Q4KLZ6,Q6IG02
37	1,2,3,4,5,6,20,22	7,8,17,18,19,21,23,24	10	P11762,P23593,P42854,P52590,Q03191,Q09030,Q4KLZ6,Q6P6R2,Q9QX74,Q9Z2L0
38	1,2,3,4,5,6,20,23	7,8,17,18,19,21,22,24	14	P02783,P09656,P10758,P23739,P42854,P52590,Q03191,Q09030,Q4KLZ6,Q6IG02,Q6P6R2,Q99MH3,Q9QX74,Q9Z2L0

39	1,2,3,4,5,6,20,24	7,8,17,18,19,21,22,23	8	P11762,P42854,P52590,Q09030,Q4KLZ6,Q6IG02,Q6P6R2,Q9QX74
40	1,2,3,4,5,6,21,22	7,8,17,18,19,20,23,24	12	B1H234,P01946,P23593,P30919,P36860,P42854,P52590,Q09030,Q4KLZ6,Q6AYC4,Q9WTT6,Q9Z2L0
41	1,2,3,4,5,6,21,23	7,8,17,18,19,20,22,24	14	B1H234,P02783,P09656,P30919,P36860,P42854,P47727,P52590,Q09030,Q4KLZ6,Q63532,Q9JJ50,Q9WTT6,Q9Z2L0
42	1,2,3,4,5,6,21,24	7,8,17,18,19,20,22,23	8	B1H234,P30919,P36860,P52590,Q09030,Q10743,Q4KLZ6,Q9WTT6
43	1,2,3,4,5,6,22,23	7,8,17,18,19,20,21,24	20	P00507,P02783,P09656,P13676,P23593,P30120,P30919,P42854,P47727,P52590,Q03191,Q09030,Q4KLZ6,Q63532,Q6P6R2,Q99MH3,Q9EQS0,Q9QX74,Q9WTT6,Q9Z2L0
44	1,2,3,4,5,6,22,24	7,8,17,18,19,20,21,23	12	P00507,P11762,P21674,P23593,P30919,P52590,Q09030,Q4KLZ6,Q6P6R2,Q9QX74,Q9WTT6,Q9Z2L0
45	1,2,3,4,5,6,23,24	7,8,17,18,19,20,21,22	13	P09656,P30919,P36860,P42854,P47727,P52590,Q09030,Q4KLZ6,Q6P6R2,Q9JJ50,Q9QX74,Q9WTT6,Q9Z2L0
46	1,2,3,4,5,7,8,17	6,18,19,20,21,22,23,24	16	O70594,P01681,P22006,P23593,P28648,P97580,Q30KJ2,Q4FZU2,Q6IFU7,Q6IFU8,Q6IFW6,Q6IG02,Q6IG05,Q6IMF3,Q6P6Q2,Q811M5
47	1,2,3,4,5,7,8,18	6,17,19,20,21,22,23,24	22	O70594,P01681,P20762,P20766,P23593,P46844,P97580,Q30KJ2,Q4FZU2,Q4KLZ6,Q5I0E9,Q63317,Q6IFU7,Q6IFU8,Q6IFW6,Q6IG02,Q6IG05,Q6IMF3,Q6P6Q2,Q811M5,Q91ZS3,Q9WUW8
48	1,2,3,4,5,7,8,19	6,17,18,20,21,22,23,24	15	A0JPJ7,iRT-Kit_WR_fusion,O70594,P01681,P47967,P97580,Q30KJ2,Q4FZU2,Q66H69,Q6IFU8,Q6IFW6,Q6IG02,Q6IG05,Q6IMF3,Q6P6Q2
49	1,2,3,4,5,7,8,20	6,17,18,19,21,22,23,24	28	D4A5U3,iRT-Kit_WR_fusion,P01681,P01836,P02780,P06911,P23593,P28648,P47967,P70549,P97697,Q03191,Q05702,Q10758,Q4FZU2,Q4G075,Q5I0D1,Q62946,Q6IFU7,Q6IFU8,Q6IFW6,Q6IG02,Q6IG05,Q6IMF3,Q6P6Q2,Q811M5,Q91ZS3,Q9WUW8
50	1,2,3,4,5,7,8,21	6,17,18,19,20,22,23,24	24	iRT-Kit_WR_fusion,O70594,P01681,P11883,P18757,P22006,P23593,P30919,P36860,P46844,P47967,P70549,Q00715,Q4FZU2,Q6IFU7,Q6IFU8,Q6IFW6,Q6IG02,Q6IG05,Q6IMF3,Q6P6Q2,Q811M5,Q91ZS3,Q9WUW8
51	1,2,3,4,5,7,8,22	6,17,18,19,20,21,23,24	19	O70594,P01681,P23593,P27590,P35280,P97580,Q09030,Q30KJ2,Q4FZU2,Q5RLM2,Q6IFU7,Q6IFU8,Q6IFW6,Q6IG02,Q6IG05,Q6IMF3,Q6P6Q2,Q6P6R2,Q9WUW8

52	1,2,3,4,5,7,8,23	6,17,18,19,20,21,22,24	18	O70594,P01681,P20761,P20762,P23593,P34901,P46844,Q09030,Q4FZU2,Q6AYQ8,Q6IFU7,Q6IFU8,Q6IFW6,Q6IG02,Q6IG05,Q6IMF3,Q6P6Q2,Q9Z2L0
53	1,2,3,4,5,7,8,24	6,17,18,19,20,21,22,23	17	O70594,P01681,P23593,P30919,P46844,Q4FZU2,Q4KLZ6,Q5RLM2,Q6IFU7,Q6IFU8,Q6IFW6,Q6IG02,Q6IG05,Q6IMF3,Q6P6Q2,Q6P6R2,Q9WUW8
54	1,2,3,4,5,7,17,18	6,8,19,20,21,22,23,24	3	O70594,Q4KLZ6,Q811M5
55	1,2,3,4,5,7,17,19	6,8,18,20,21,22,23,24	2	O70594,P80299
56	1,2,3,4,5,7,17,20	6,8,18,19,21,22,23,24	7	P19223,P47967,P80299,Q03191,Q4FZU2,Q6IG02,Q811M5
57	1,2,3,4,5,7,17,21	6,8,18,19,20,22,23,24	11	O70594,P19939,P22006,P36860,P70549,Q4FZU2,Q6AY61,Q6IFW6,Q6IG02,Q811M5,Q9R168
58	1,2,3,4,5,7,17,22	6,8,18,19,20,21,23,24	4	P23593,P80299,Q9R168,Q9WUW8
59	1,2,3,4,5,7,17,23	6,8,18,19,20,21,22,24	1	O70594
60	1,2,3,4,5,7,17,24	6,8,18,19,20,21,22,23	5	P19939,P36860,Q4FZU2,Q4KLZ6,Q6AY61
61	1,2,3,4,5,7,18,19	6,8,17,20,21,22,23,24	5	P20766,P47967,P80299,P97840,Q4KLZ6
62	1,2,3,4,5,7,18,20	6,8,17,19,21,22,23,24	9	P47967,P70549,P80299,P97840,Q03191,Q4KLZ6,Q5I0D1,Q811M5,Q9WUW8
63	1,2,3,4,5,7,18,21	6,8,17,19,20,22,23,24	8	O89117,P36860,P47967,P70549,P80299,P97840,Q03191,Q4KLZ6
64	1,2,3,4,5,7,18,22	6,8,17,19,20,21,23,24	7	P23593,P80299,Q03191,Q4KLZ6,Q6P6R2,Q9WUW8,Q9Z2L0

65	1,2,3,4,5,7,18,23	6,8,17,19,20,21,22,24	10	O70594,O89117,P30120,P80299,Q03191,Q4KLZ6,Q6AYQ8,Q6P6R2,Q9JJ50,Q9Z2L0
66	1,2,3,4,5,7,18,24	6,8,17,19,20,21,22,23	5	O89117,Q03191,Q4KLZ6,Q5RLM2,Q6P6R2
67	1,2,3,4,5,7,19,20	6,8,17,18,21,22,23,24	5	P10758,P47967,P80299,P97840,Q5I0D1
68	1,2,3,4,5,7,19,21	6,8,17,18,20,22,23,24	6	P08649,P22006,P47967,P80299,P97840,Q62761;Q62762;Q62763
69	1,2,3,4,5,7,19,22	6,8,17,18,20,21,23,24	1	P80299
70	1,2,3,4,5,7,19,23	6,8,17,18,20,21,22,24	4	P10758,P80299,Q6AYQ8,Q9Z2L0
71	1,2,3,4,5,7,19,24	6,8,17,18,20,21,22,23	2	Q4KLZ6,Q62761;Q62762;Q62763
72	1,2,3,4,5,7,20,21	6,8,17,18,19,22,23,24	11	P08649,P47967,P70549,P80299,P97840,Q03191,Q5I0D1,Q6IFW6,Q6IG02,Q811M5,Q9WUW8
73	1,2,3,4,5,7,20,22	6,8,17,18,19,21,23,24	6	P23593,P47967,P80299,P97840,Q03191,Q9WUW8
74	1,2,3,4,5,7,20,23	6,8,17,18,19,21,22,24	6	P08649,P10758,P23739,P80299,Q03191,Q6IG02
75	1,2,3,4,5,7,20,24	6,8,17,18,19,21,22,23	8	P08649,P47967,Q03191,Q4FZU2,Q4KLZ6,Q6IFW6,Q6IG02,Q9WUW8
76	1,2,3,4,5,7,21,22	6,8,17,18,19,20,23,24	7	P08649,P17046,P47967,P70549,P80299,Q9R168,Q9WUW8
77	1,2,3,4,5,7,21,23	6,8,17,18,19,20,22,24	6	O89117,P08649,P36860,P70549,P80299,Q03191

78	1,2,3,4,5,7,21,24	6,8,17,18,19,20,22,23	12	O89117,P02761,P04762,P08649,P19939,P30919,P36860,Q4FZU2,Q4KLZ6,Q6AY61,Q6IFW6,Q6IG02
79	1,2,3,4,5,7,22,23	6,8,17,18,19,20,21,24	11	B0LT89,P08649,P23593,P30120,P47727,P80299,Q03191,Q63556,Q6AYQ8,Q6P6R2,Q9Z2L0
80	1,2,3,4,5,7,22,24	6,8,17,18,19,20,21,23	8	P08649,P23593,P30919,P80299,Q4KLZ6,Q5RLM2,Q6P6R2,Q9WUW8
81	1,2,3,4,5,7,23,24	6,8,17,18,19,20,21,22	7	B0LT89,P08649,P47727,Q4KLZ6,Q5RLM2,Q6AYQ8,Q6P6R2
82	1,2,3,4,5,8,17,18	6,7,19,20,21,22,23,24	16	O70594,P01681,P23593,P97580,Q30KJ2,Q4FZU2,Q4KLZ6,Q6IFU7,Q6IFU8,Q6IFW6,Q6IG02,Q6IG05,Q6IMF3,Q6P6Q2,Q811M5,Q9JJS8
83	1,2,3,4,5,8,17,19	6,7,18,20,21,22,23,24	13	P01681,P01835,P22006,P97580,Q30KJ2,Q4FZU2,Q66H69,Q6IFU8,Q6IFW6,Q6IG02,Q6IG05,Q6IMF3,Q6P6Q2
84	1,2,3,4,5,8,17,20	6,7,18,19,21,22,23,24	16	D4A5U3,P01681,P19223,P28648,P36375,P36376,Q10758,Q4FZU2,Q6IFU7,Q6IFU8,Q6IFW6,Q6IG02,Q6IG05,Q6IMF3,Q6P6Q2,Q811M5
85	1,2,3,4,5,8,17,21	6,7,18,19,20,22,23,24	13	O70594,P01681,P22006,P70549,Q4FZU2,Q6IFU7,Q6IFU8,Q6IFW6,Q6IG02,Q6IG05,Q6IMF3,Q6P6Q2,Q811M5
86	1,2,3,4,5,8,17,22	6,7,18,19,20,21,23,24	12	P01681,P23593,P27590,P97580,Q30KJ2,Q4FZU2,Q6IFU7,Q6IFU8,Q6IFW6,Q6IG02,Q6IMF3,Q6P6Q2
87	1,2,3,4,5,8,17,23	6,7,18,19,20,21,22,24	15	O70594,P01681,P22006,P23593,P34901,P36376,Q4FZU2,Q5PQL7,Q6IFU7,Q6IFU8,Q6IFW6,Q6IG02,Q6IG05,Q6IMF3,Q6P6Q2
88	1,2,3,4,5,8,17,24	6,7,18,19,20,21,22,23	11	P01681,P23593,Q4FZU2,Q4KLZ6,Q6IFU7,Q6IFU8,Q6IFW6,Q6IG02,Q6IG05,Q6IMF3,Q6P6Q2
89	1,2,3,4,5,8,18,19	6,7,17,20,21,22,23,24	7	P01681,P97580,Q30KJ2,Q4KLZ6,Q6IG05,Q6IMF3,Q6P6Q2
90	1,2,3,4,5,8,18,20	6,7,17,19,21,22,23,24	14	P01681,Q10758,Q4FZU2,Q4KLZ6,Q6IFU7,Q6IFU8,Q6IFW6,Q6IG02,Q6IG05,Q6IMF3,Q6P6Q2,Q811M5,Q91ZS3,Q9R168

91	1,2,3,4,5,8,18,21	6,7,17,19,20,22,23,24	15	O89117,P01681,P02625,P22006,P70549,Q4FZU2,Q4KLZ6,Q6IFU7,Q6IFU8,Q6IFW6,Q6IG02,Q6IG05,Q6IMF3,Q6P6Q2,Q91ZS3
92	1,2,3,4,5,8,18,22	6,7,17,19,20,21,23,24	8	P01681,P23593,P27590,P97580,Q30KJ2,Q4KLZ6,Q6IFU7,Q9Z2L0
93	1,2,3,4,5,8,18,23	6,7,17,19,20,21,22,24	12	O70594,P01681,P20762,Q4KLZ6,Q6IFU7,Q6IFW6,Q6IG02,Q6IG05,Q6IMF3,Q6P6Q2,Q99MH3,Q9Z2L0
94	1,2,3,4,5,8,18,24	6,7,17,19,20,21,22,23	11	P01681,P23593,Q4FZU2,Q4KLZ6,Q6IFU7,Q6IFU8,Q6IFW6,Q6IG02,Q6IMF3,Q6P6Q2,Q6P6R2
95	1,2,3,4,5,8,19,20	6,7,17,18,21,22,23,24	14	D4A5U3,P01681,P47967,P97580,Q10758,Q4FZU2,Q5I0D1,Q6IFU7,Q6IFU8,Q6IFW6,Q6IG02,Q6IG05,Q6IMF3,Q6P6Q2
96	1,2,3,4,5,8,19,21	6,7,17,18,20,22,23,24	13	P01681,P22006,P30919,P97580,Q10743,Q4FZU2,Q66H69,Q6IFU8,Q6IFW6,Q6IG02,Q6IG05,Q6IMF3,Q6P6Q2
97	1,2,3,4,5,8,19,22	6,7,17,18,20,21,23,24	9	P00507,P01681,P27590,P30919,P97580,Q30KJ2,Q6IFW6,Q6IMF3,Q6P6Q2
98	1,2,3,4,5,8,19,23	6,7,17,18,20,21,22,24	9	P01681,P97580,Q4FZU2,Q6IFW6,Q6IG02,Q6IG05,Q6IMF3,Q6P6Q2,Q9Z2L0
99	1,2,3,4,5,8,19,24	6,7,17,18,20,21,22,23	11	O54728,P01681,P30919,Q10743,Q4FZU2,Q4KLZ6,Q6IFU8,Q6IFW6,Q6IG02,Q6IMF3,Q6P6Q2
100	1,2,3,4,5,8,20,21	6,7,17,18,19,22,23,24	18	D4A5U3,P00731,P01681,P36375,P47967,P70549,Q10743,Q10758,Q4FZU2,Q5I0D1,Q6IFU7,Q6IFU8,Q6IFW6,Q6IG02,Q6IG05,Q6IMF3,Q6P6Q2,Q811M5
101	1,2,3,4,5,8,20,22	6,7,17,18,19,21,23,24	12	P01681,P23593,P97580,Q30KJ2,Q4FZU2,Q6IFU7,Q6IFU8,Q6IFW6,Q6IG02,Q6IG05,Q6IMF3,Q6P6Q2
102	1,2,3,4,5,8,20,23	6,7,17,18,19,21,22,24	16	P01681,P10758,P19132,P23739,P34901,P36376,Q10758,Q4FZU2,Q6IFU7,Q6IFU8,Q6IFW6,Q6IG02,Q6IG05,Q6IMF3,Q6P6Q2,Q9Z2L0
103	1,2,3,4,5,8,20,24	6,7,17,18,19,21,22,23	12	P01681,Q10743,Q10758,Q4FZU2,Q4KLZ6,Q6IFU7,Q6IFU8,Q6IFW6,Q6IG02,Q6IG05,Q6IMF3,Q6P6Q2

104	1,2,3,4,5,8,21,22	6,7,17,18,19,20,23,24	14	P01681,P23593,P30919,P70549,P97580,Q10743,Q30KJ2,Q4FZU2,Q6IFU7,Q6IFU8,Q6IFW6,Q6IG02,Q6IMF3,Q6P6Q2
105	1,2,3,4,5,8,21,23	6,7,17,18,19,20,22,24	12	P01681,P22006,P30919,P70549,Q4FZU2,Q6IFU7,Q6IFU8,Q6IFW6,Q6IG02,Q6IG05,Q6IMF3,Q6P6Q2
106	1,2,3,4,5,8,21,24	6,7,17,18,19,20,22,23	13	P01681,P30919,P36860,Q10743,Q4FZU2,Q4KLZ6,Q6IFU7,Q6IFU8,Q6IFW6,Q6IG02,Q6IG05,Q6IMF3,Q6P6Q2
107	1,2,3,4,5,8,22,23	6,7,17,18,19,20,21,24	17	P00507,P01681,P23593,P27590,P30919,P34901,P97580,Q09030,Q30KJ2,Q6IFU7,Q6IFW6,Q6IG02,Q6IMF3,Q6P6Q2,Q6P6R2,Q99MH3,Q9Z2L0
108	1,2,3,4,5,8,22,24	6,7,17,18,19,20,21,23	16	P00507,P01681,P23593,P30919,P97580,Q10743,Q30KJ2,Q4FZU2,Q4KLZ6,Q6IFU7,Q6IFU8,Q6IFW6,Q6IG02,Q6IMF3,Q6P6Q2,Q6P6R2
109	1,2,3,4,5,8,23,24	6,7,17,18,19,20,21,22	15	P01681,P23593,P30919,P34901,Q4FZU2,Q4KLZ6,Q5PQL7,Q6IFU7,Q6IFU8,Q6IFW6,Q6IG02,Q6IG05,Q6IMF3,Q6P6Q2,Q6P6R2
110	1,2,3,4,5,17,18,19	6,7,8,20,21,22,23,24	1	Q4KLZ6
111	1,2,3,4,5,17,18,20	6,7,8,19,21,22,23,24	3	P19223,Q4KLZ6,Q811M5
112	1,2,3,4,5,17,18,21	6,7,8,19,20,22,23,24	3	P22006,Q4KLZ6,Q811M5
113	1,2,3,4,5,17,18,22	6,7,8,19,20,21,23,24	3	Q4KLZ6,Q63617,Q812E4
114	1,2,3,4,5,17,18,23	6,7,8,19,20,21,22,24	5	P08723,P30120,Q4KLZ6,Q63617,Q9Z2L0
115	1,2,3,4,5,17,18,24	6,7,8,19,20,21,22,23	3	Q4KLZ6,Q63617,Q9WTT6
116	1,2,3,4,5,17,19,20	6,7,8,18,21,22,23,24	2	P19223,Q6IG02
117	1,2,3,4,5,17,19,21	6,7,8,18,20,22,23,24	2	P22006,Q9WVK7
118	1,2,3,4,5,17,19,22	6,7,8,18,20,21,23,24	0	
119	1,2,3,4,5,17,19,23	6,7,8,18,20,21,22,24	0	
120	1,2,3,4,5,17,19,24	6,7,8,18,20,21,22,23	1	Q4KLZ6

121	1,2,3,4,5,17,20,21	6,7,8,18,19,22,23,24	5	P19223,P70549,Q4FZU2,Q6IG02,Q811M5
122	1,2,3,4,5,17,20,22	6,7,8,18,19,21,23,24	1	P19223
123	1,2,3,4,5,17,20,23	6,7,8,18,19,21,22,24	4	P19223,P23739,P36376,Q6IG02
124	1,2,3,4,5,17,20,24	6,7,8,18,19,21,22,23	4	P19223,Q4FZU2,Q4KLZ6,Q6IG02
125	1,2,3,4,5,17,21,22	6,7,8,18,19,20,23,24	1	Q9R168
126	1,2,3,4,5,17,21,23	6,7,8,18,19,20,22,24	1	P22006
127	1,2,3,4,5,17,21,24	6,7,8,18,19,20,22,23	7	P19939,P36860,Q4FZU2,Q4KLZ6,Q6AY61,Q6IFW6,Q6IG02
128	1,2,3,4,5,17,22,23	6,7,8,18,19,20,21,24	8	P08723,P22283,P30120,P47727,Q63617,Q9QW07,Q9WTT6,Q9Z2L0
129	1,2,3,4,5,17,22,24	6,7,8,18,19,20,21,23	5	P23593,Q4KLZ6,Q63617,Q6P6R2,Q9WTT6
130	1,2,3,4,5,17,23,24	6,7,8,18,19,20,21,22	4	Q4KLZ6,Q5PQL7,Q63617,Q9WTT6
131	1,2,3,4,5,18,19,20	6,7,8,17,21,22,23,24	3	P47967,Q4KLZ6,Q5I0D1
132	1,2,3,4,5,18,19,21	6,7,8,17,20,22,23,24	1	Q4KLZ6
133	1,2,3,4,5,18,19,22	6,7,8,17,20,21,23,24	2	Q4KLZ6,Q9Z2L0
134	1,2,3,4,5,18,19,23	6,7,8,17,20,21,22,24	3	P30120,Q4KLZ6,Q9Z2L0
135	1,2,3,4,5,18,19,24	6,7,8,17,20,21,22,23	2	Q4KLZ6,Q9WTT6
136	1,2,3,4,5,18,20,21	6,7,8,17,19,22,23,24	7	P47967,P70549,P97840,Q03191,Q4KLZ6,Q6IG02,Q811M5
137	1,2,3,4,5,18,20,22	6,7,8,17,19,21,23,24	2	Q03191,Q4KLZ6
138	1,2,3,4,5,18,20,23	6,7,8,17,19,21,22,24	4	P10758,Q03191,Q4KLZ6,Q9Z2L0
139	1,2,3,4,5,18,20,24	6,7,8,17,19,21,22,23	2	Q4KLZ6,Q9QX74
140	1,2,3,4,5,18,21,22	6,7,8,17,19,20,23,24	3	P70549,Q4KLZ6,Q9WTT6

141	1,2,3,4,5,18,21,23	6,7,8,17,19,20,22,24	7	O89117,P70549,Q4KLZ6,Q9JJ50,Q9QZK9,Q9WTT6,Q9Z2L0
142	1,2,3,4,5,18,21,24	6,7,8,17,19,20,22,23	3	O89117,Q4KLZ6,Q9WTT6
143	1,2,3,4,5,18,22,23	6,7,8,17,19,20,21,24	18	P08723,P09456,P13676,P22283,P24368,P30120,P36374,P47727,Q03191,Q4KLZ6,Q63617,Q6P6R2,Q812E4,Q99MH3,Q9EQS0,Q9QX74,Q9WTT6,Q9Z2L0
144	1,2,3,4,5,18,22,24	6,7,8,17,19,20,21,23	6	P21674,Q4KLZ6,Q63617,Q6P6R2,Q9QX74,Q9WTT6
145	1,2,3,4,5,18,23,24	6,7,8,17,19,20,21,22	7	O89117,Q4KLZ6,Q63617,Q6P6R2,Q9QX74,Q9WTT6,Q9Z2L0
146	1,2,3,4,5,19,20,21	6,7,8,17,18,22,23,24	3	P47967,Q5I0D1,Q6IG02
147	1,2,3,4,5,19,20,22	6,7,8,17,18,21,23,24	0	
148	1,2,3,4,5,19,20,23	6,7,8,17,18,21,22,24	2	P10758,P23739
149	1,2,3,4,5,19,20,24	6,7,8,17,18,21,22,23	2	Q4KLZ6,Q6IG02
150	1,2,3,4,5,19,21,22	6,7,8,17,18,20,23,24	1	P30919
151	1,2,3,4,5,19,21,23	6,7,8,17,18,20,22,24	0	
152	1,2,3,4,5,19,21,24	6,7,8,17,18,20,22,23	3	P30919,Q4KLZ6,Q62761;Q62762;Q62763
153	1,2,3,4,5,19,22,23	6,7,8,17,18,20,21,24	7	P00507,P30120,P47727,Q6P6R2,Q9QX74,Q9WTT6,Q9Z2L0
154	1,2,3,4,5,19,22,24	6,7,8,17,18,20,21,23	6	P00507,P30919,Q4KLZ6,Q6P6R2,Q9QX74,Q9WTT6
155	1,2,3,4,5,19,23,24	6,7,8,17,18,20,21,22	4	Q4KLZ6,Q6P6R2,Q9QX74,Q9WTT6
156	1,2,3,4,5,20,21,22	6,7,8,17,18,19,23,24	2	P70549,Q6IG02
157	1,2,3,4,5,20,21,23	6,7,8,17,18,19,22,24	6	P08649,P10758,P23739,P70549,Q03191,Q6IG02
158	1,2,3,4,5,20,21,24	6,7,8,17,18,19,22,23	6	P08649,P70549,Q4KLZ6,Q6IFW6,Q6IG02,Q6P6Q2
159	1,2,3,4,5,20,22,23	6,7,8,17,18,19,21,24	5	P23739,Q03191,Q6P6R2,Q9QX74,Q9Z2L0
160	1,2,3,4,5,20,22,24	6,7,8,17,18,19,21,23	5	P21674,Q4KLZ6,Q6IG02,Q6P6R2,Q9QX74

161	1,2,3,4,5,20,23,24	6,7,8,17,18,19,21,22	8	P10758,P23739,Q4KLZ6,Q5QE79,Q6IG02,Q6P6R2,Q9QX74,Q9QYP1
162	1,2,3,4,5,21,22,23	6,7,8,17,18,19,20,24	4	P30919,P47727,Q9WTT6,Q9Z2L0
163	1,2,3,4,5,21,22,24	6,7,8,17,18,19,20,23	4	P21674,P30919,Q4KLZ6,Q9WTT6
164	1,2,3,4,5,21,23,24	6,7,8,17,18,19,20,22	6	O89117,P08649,P30919,P36860,Q4KLZ6,Q9WTT6
165	1,2,3,4,5,22,23,24	6,7,8,17,18,19,20,21	15	P00507,P06761,P07150,P13676,P22283,P24368,P30919,P47727,P62804,Q4KLZ6,Q63617,Q6P6R2,Q9QX74,Q9WTT6,Q9Z2L0
166	1,2,3,4,6,7,8,17	5,18,19,20,21,22,23,24	28	iRT-Kit_WR_fusion,O54800;Q5DWV2,O70417,O70594,P01681,P06911,P11762,P15399,P18418,P19629,P23593,P33436,P42854,P46844,P50115,P50280,P80299,P97580,Q05702,Q09030,Q30KJ2,Q4V885,Q5BK81,Q5GRG2,Q62946,Q63598,Q99PP0,Q9QZQ5
167	1,2,3,4,6,7,8,18	5,17,19,20,21,22,23,24	36	O54800;Q5DWV2,O55004,O70417,O70594,P02625,P07151,P11762,P15399,P18418,P18757,P19629,P20762,P20766,P23593,P31044,P33436,P42854,P46844,P50280,P80299,P97580,P97615,Q05702,Q09030,Q30KJ2,Q3ZAV1,Q4V885,Q5BK81,Q5I0E9,Q63598,Q64240,Q91XN4,Q91ZS3,Q9QZQ5,Q9WTW7,Q9Z0W7
168	1,2,3,4,6,7,8,19	5,17,18,20,21,22,23,24	30	A0JPJ7,iRT-Kit_WR_fusion,O54800;Q5DWV2,O70417,O70594,P01681,P07151,P11598,P11762,P15399,P18418,P19629,P20761,P22282,P31044,P42854,P50115,P50280,P80299,P97580,P97615,Q05702,Q09030,Q30KJ2,Q4FZU4,Q4V885,Q66H69,Q99041,Q9JI85,Q9WTW7
169	1,2,3,4,6,7,8,20	5,17,18,19,21,22,23,24	41	D4A5U3,iRT-Kit_WR_fusion,O54800;Q5DWV2,O70417,P01836,P02631,P02780,P05964,P06761,P06911,P11598,P11762,P12020,P15399,P18418,P19629,P22273,P22282,P23593,P31044,P42854,P50280,P80299,P97580,Q05702,Q09030,Q30KJ2,Q4G075,Q5BK81,Q5GRG2,Q62946,Q6IG02,Q6P9T8,Q78P75,Q91ZS3,Q99041,Q9JHB9,Q9JI85,Q9QW07,Q9QZQ5,Q9R0T3
170	1,2,3,4,6,7,8,21	5,17,18,19,20,22,23,24	39	B1H234,iRT-Kit_WR_fusion,O54800;Q5DWV2,O70594,P00714,P02625,P02631,P11598,P11762,P15399,P16228,P18418,P18757,P19629,P20761,P23593,P30919,P36860,P42854,P46844,P50115,P50280,P80299,P97580,Q00715,Q05702,Q09030,Q10743,Q30KJ2,Q4V885,Q5BK81,Q63493,Q63598,Q6AYC4,Q6IFW6,Q91ZS3,Q99041,Q9JI85,Q9QZQ5
171	1,2,3,4,6,7,8,22	5,17,18,19,20,21,23,24	24	iRT-Kit_WR_fusion,O54800;Q5DWV2,O70417,O70594,P11762,P15399,P19629,P20761,P23593,P35280,P42854,P46844,P50280,P80202,P80299,P97580,Q05702,Q09030,Q30KJ2,Q4FZU4,Q4V885,Q63598,Q91XN4,Q99041
172	1,2,3,4,6,7,8,23	5,17,18,19,20,21,22,24	29	iRT-Kit_WR_fusion,O70594,P02783,P09656,P11762,P15399,P19629,P20761,P20762,P23593,P38918,P42854,P46720,P46844,P50115,P50280,P53790,P80202,P80299,P97580,Q05702,Q09030,Q30KJ2,Q3ZAV1,Q5BK81,Q63532,Q63598,Q99MH3,Q9WTW7

173	1,2,3,4,6,7,8,24	5,17,18,19,20,21,22,23	29	iRT-Kit_WR_fusion,O54800;Q5DWV2,O70417,O70594,P02761,P07151,P11762,P15399,P18418,P19629,P20761,P23593,P30919,P42854,P46844,P50280,P53790,P80299,P97580,Q05702,Q09030,Q10743,Q30KJ2,Q3ZAV1,Q4KLZ6,Q63598,Q6IFW6,Q6P6R2,Q91ZS3
174	1,2,3,4,6,7,17,18	5,8,19,20,21,22,23,24	10	O54800;Q5DWV2,O70594,P11762,P20766,P33436,P80299,P97615,Q63598,Q99PP0,Q9WVH8
175	1,2,3,4,6,7,17,19	5,8,18,20,21,22,23,24	4	O54800;Q5DWV2,O70594,P11762,P80299
176	1,2,3,4,6,7,17,20	5,8,18,19,21,22,23,24	9	O54800;Q5DWV2,P05964,P11598,P11762,P19223,P33436,P55091,P80299,Q5GRG2
177	1,2,3,4,6,7,17,21	5,8,18,19,20,22,23,24	8	B1H234,O54800;Q5DWV2,O70594,P11762,P18297,P36860,P80299,Q9R168
178	1,2,3,4,6,7,17,22	5,8,18,19,20,21,23,24	8	O54800;Q5DWV2,P01946,P02091,P11762,P23593,P80299,Q09030,Q9R168
179	1,2,3,4,6,7,17,23	5,8,18,19,20,21,22,24	10	O54800;Q5DWV2,O70594,P02783,P09656,P11762,P33436,P42854,P80299,Q09030,Q63598
180	1,2,3,4,6,7,17,24	5,8,18,19,20,21,22,23	10	O54800;Q5DWV2,O70594,P01039,P11762,P23593,P36860,P80299,Q4KLZ6,Q63598,Q6AY61
181	1,2,3,4,6,7,18,19	5,8,17,20,21,22,23,24	7	B1H234,O54800;Q5DWV2,P11762,P20766,P42854,P80299,P97615
182	1,2,3,4,6,7,18,20	5,8,17,19,21,22,23,24	10	O54800;Q5DWV2,P11598,P11762,P19629,P33436,P80299,P97615,Q5GRG2,Q80WL1,Q91ZS3
183	1,2,3,4,6,7,18,21	5,8,17,19,20,22,23,24	6	B1H234,O54800;Q5DWV2,O89117,P11762,P23739,P80299
184	1,2,3,4,6,7,18,22	5,8,17,19,20,21,23,24	6	O54800;Q5DWV2,P11762,P23593,P80299,Q1WIM3,Q6P6R2
185	1,2,3,4,6,7,18,23	5,8,17,19,20,21,22,24	13	O54800;Q5DWV2,O70594,P02783,P09656,P11762,P20766,P42854,P46844,P80299,Q09030,Q63598,Q6AYQ8,Q99MH3

186	1,2,3,4,6,7,18,24	5,8,17,19,20,21,22,23	9	B1H234,O54800;Q5DWV2,P06760,P11762,P20766,P80299,Q4KLZ6,Q63598,Q6P6R2
187	1,2,3,4,6,7,19,20	5,8,17,18,21,22,23,24	12	P05964,P10758,P11598,P11762,P19629,P42854,P80299,Q5GRG2,Q5I0D1,Q63617,Q9JI85,Q9R0T3
188	1,2,3,4,6,7,19,21	5,8,17,18,20,22,23,24	5	B1H234,P11598,P11762,P42854,P80299
189	1,2,3,4,6,7,19,22	5,8,17,18,20,21,23,24	3	P11762,P42854,P80299
190	1,2,3,4,6,7,19,23	5,8,17,18,20,21,22,24	5	P02783,P10758,P11762,P42854,P80299
191	1,2,3,4,6,7,19,24	5,8,17,18,20,21,22,23	4	B1H234,P11762,P80299,Q4KLZ6
192	1,2,3,4,6,7,20,21	5,8,17,18,19,22,23,24	12	B1H234,O55145,P01835,P05964,P11598,P11762,P19629,P42854,P47967,P80299,Q5GRG2,Q63617
193	1,2,3,4,6,7,20,22	5,8,17,18,19,21,23,24	7	O54800;Q5DWV2,P11762,P19629,P23593,P42854,P80299,Q5GRG2
194	1,2,3,4,6,7,20,23	5,8,17,18,19,21,22,24	11	P02783,P09656,P10758,P11598,P11762,P19629,P42854,P80299,Q09030,Q62894,Q9QYP1
195	1,2,3,4,6,7,20,24	5,8,17,18,19,21,22,23	7	O54800;Q5DWV2,P11598,P11762,P80299,Q4KLZ6,Q5GRG2,Q9QYP1
196	1,2,3,4,6,7,21,22	5,8,17,18,19,20,23,24	7	B1H234,P11762,P17046,P23593,P80299,Q09030,Q9R168
197	1,2,3,4,6,7,21,23	5,8,17,18,19,20,22,24	11	B1H234,P02783,P09656,P11762,P18297,P36860,P42854,P46844,P80299,Q09030,Q63532
198	1,2,3,4,6,7,21,24	5,8,17,18,19,20,22,23	11	B1H234,P01835,P02761,P04762,P11762,P18297,P30919,P36860,P46844,P80299,Q4KLZ6

199	1,2,3,4,6,7,22,23	5,8,17,18,19,20,21,24	13	B0LT89,P02783,P06760,P09656,P11762,P23593,P30120,P42854,P80299,Q09030,Q63532,Q6P6R2,Q99MH3
200	1,2,3,4,6,7,22,24	5,8,17,18,19,20,21,23	12	O54800;Q5DWV2,P02761,P06760,P11762,P22006,P23593,P30919,P80299,Q09030,Q4KLZ6,Q6P6R2,Q9QX74
201	1,2,3,4,6,7,23,24	5,8,17,18,19,20,21,22	14	B0LT89,P02783,P06760,P09656,P11762,P42854,P46844,P80299,Q09030,Q4KLZ6,Q63598,Q6AYQ8,Q6P6R2,Q9QX74
202	1,2,3,4,6,8,17,18	5,7,19,20,21,22,23,24	17	G3V686,O54800;Q5DWV2,O70594,P08649,P11762,P15399,P23593,P33436,P97580,P97615,Q05702,Q06000,Q09030,Q30KJ2,Q63598,Q99PP0,Q9QZQ5
203	1,2,3,4,6,8,17,19	5,7,18,20,21,22,23,24	8	P01681,P11762,P15399,P42854,P97580,Q05702,Q30KJ2,Q9JI85
204	1,2,3,4,6,8,17,20	5,7,18,19,21,22,23,24	21	D4A5U3,O70417,P06911,P11762,P12020,P15399,P17559,P19223,P19629,P33436,P42854,P50280,P55091,P63029,P97580,Q05702,Q10758,Q30KJ2,Q5GRG2,Q6IG02,Q9JI85
205	1,2,3,4,6,8,17,21	5,7,18,19,20,22,23,24	11	O70594,P02625,P02631,P15399,P97580,Q05702,Q09030,Q30KJ2,Q6IFW6,Q6IG02,Q9JI85
206	1,2,3,4,6,8,17,22	5,7,18,19,20,21,23,24	10	O70417,P01946,P02091,P11762,P15399,P23593,P97580,Q05702,Q09030,Q30KJ2
207	1,2,3,4,6,8,17,23	5,7,18,19,20,21,22,24	16	O70594,P02783,P09656,P15399,P20761,P23593,P33436,P36376,P42854,P97580,Q05702,Q09030,Q30KJ2,Q63532,Q63598,Q99MH3
208	1,2,3,4,6,8,17,24	5,7,18,19,20,21,22,23	9	P11762,P15399,P23593,P97580,Q05702,Q09030,Q30KJ2,Q4KLZ6,Q63598
209	1,2,3,4,6,8,18,19	5,7,17,20,21,22,23,24	11	O54800;Q5DWV2,P11762,P15399,P42854,P50280,P97580,P97615,Q05702,Q30KJ2,Q5PPH0,Q9JI85
210	1,2,3,4,6,8,18,20	5,7,17,19,21,22,23,24	19	D4A5U3,O54800;Q5DWV2,P06911,P11762,P12020,P15399,P19629,P33436,P42854,P50280,P97580,P97615,Q05702,Q10758,Q30KJ2,Q5GRG2,Q91ZS3,Q9JI85,Q9R168
211	1,2,3,4,6,8,18,21	5,7,17,19,20,22,23,24	13	O54800;Q5DWV2,P02625,P02631,P11762,P15399,P54921,P97580,Q05702,Q09030,Q10743,Q30KJ2,Q91ZS3,Q9QZQ5

212	1,2,3,4,6,8,18,22	5,7,17,19,20,21,23,24	9	O54800;Q5DWV2,P11762,P15399,P23593,P54921,P97580,Q05702,Q09030,Q30KJ2
213	1,2,3,4,6,8,18,23	5,7,17,19,20,21,22,24	20	O54800;Q5DWV2,O70594,P02783,P09656,P11762,P15399,P20761,P20762,P23593,P42854,P54921,P80202,P97580,Q05702,Q09030,Q30KJ2,Q5BK81,Q63532,Q63598,Q99MH3
214	1,2,3,4,6,8,18,24	5,7,17,19,20,21,22,23	14	O54800;Q5DWV2,P02625,P07151,P11762,P15399,P23593,P97580,P97615,Q05702,Q09030,Q30KJ2,Q4KLZ6,Q63598,Q91ZS3
215	1,2,3,4,6,8,19,20	5,7,17,18,21,22,23,24	17	D4A5U3,P02780,P06911,P11762,P12020,P15399,P19629,P42854,P50280,P97580,Q05702,Q30KJ2,Q5GRG2,Q68G31,Q6IG02,Q6P9T8,Q9JI85
216	1,2,3,4,6,8,19,21	5,7,17,18,20,22,23,24	10	P11762,P15399,P30919,P42854,P97580,Q05702,Q10743,Q30KJ2,Q66H69,Q9JI85
217	1,2,3,4,6,8,19,22	5,7,17,18,20,21,23,24	10	P00507,P11762,P15399,P30919,P42854,P97580,Q05702,Q09030,Q30KJ2,Q5PPH0
218	1,2,3,4,6,8,19,23	5,7,17,18,20,21,22,24	11	P01681,P02783,P11762,P15399,P20761,P42854,P97580,Q05702,Q09030,Q30KJ2,Q63532
219	1,2,3,4,6,8,19,24	5,7,17,18,20,21,22,23	12	O54728,P11762,P15399,P30919,P42854,P97580,Q05702,Q10743,Q30KJ2,Q4KLZ6,Q68G31,Q9JI85
220	1,2,3,4,6,8,20,21	5,7,17,18,19,22,23,24	21	D4A5U3,O55145,P02631,P02780,P06911,P11762,P12020,P15399,P19629,P42854,P50280,P97580,Q05702,Q10743,Q10758,Q30KJ2,Q5GRG2,Q6IFW6,Q6IG02,Q91ZS3,Q9JI85
221	1,2,3,4,6,8,20,22	5,7,17,18,19,21,23,24	14	P06911,P11762,P12020,P15399,P19629,P23593,P42854,P50280,P97580,Q05702,Q09030,Q10743,Q30KJ2,Q5GRG2
222	1,2,3,4,6,8,20,23	5,7,17,18,19,21,22,24	21	P02783,P06911,P09656,P10758,P11762,P12020,P15399,P17559,P19629,P36376,P42854,P50280,P97580,Q05702,Q09030,Q10758,Q30KJ2,Q5GRG2,Q63532,Q6IG02,Q99MH3
223	1,2,3,4,6,8,20,24	5,7,17,18,19,21,22,23	16	P06911,P11762,P12020,P15399,P19629,P42854,P50280,P97580,Q05702,Q10743,Q30KJ2,Q4KLZ6,Q5GRG2,Q6IFW6,Q6IG02,Q9JI85
224	1,2,3,4,6,8,21,22	5,7,17,18,19,20,23,24	13	P02625,P11762,P15399,P19629,P23593,P30919,P54921,P97580,Q05702,Q09030,Q10743,Q30KJ2,Q6AYC4

225	1,2,3,4,6,8,21,23	5,7,17,18,19,20,22,24	13	P02783,P09656,P19629,P20761,P30919,P42854,P54921,P97580,Q05702,Q09030,Q30KJ2,Q63532,Q99MH3
226	1,2,3,4,6,8,21,24	5,7,17,18,19,20,22,23	13	P02625,P11762,P15399,P30919,P36860,P97580,Q05702,Q09030,Q10743,Q30KJ2,Q4KLZ6,Q6IFW6,Q6IG02
227	1,2,3,4,6,8,22,23	5,7,17,18,19,20,21,24	18	P00507,P02783,P09656,P11762,P15399,P20761,P23593,P30919,P42854,P54921,P80202,P97580,Q05702,Q09030,Q30KJ2,Q63532,Q6P6R2,Q99MH3
228	1,2,3,4,6,8,22,24	5,7,17,18,19,20,21,23	13	P00507,P06760,P11762,P15399,P23593,P30919,P97580,Q05702,Q09030,Q10743,Q30KJ2,Q4KLZ6,Q6P6R2
229	1,2,3,4,6,8,23,24	5,7,17,18,19,20,21,22	18	P02783,P06760,P09656,P11762,P15399,P20761,P23593,P30919,P42854,P46844,P97580,Q05702,Q09030,Q30KJ2,Q4KLZ6,Q63532,Q63598,Q6P6R2
230	1,2,3,4,6,17,18,19	5,7,8,20,21,22,23,24	4	O54800;Q5DWV2,P33436,Q06000,Q8K1G0
231	1,2,3,4,6,17,18,20	5,7,8,19,21,22,23,24	7	O54800;Q5DWV2,P19223,P33436,P55091,Q06000,Q5GRG2,Q8K1G0
232	1,2,3,4,6,17,18,21	5,7,8,19,20,22,23,24	2	O54800;Q5DWV2,P33436
233	1,2,3,4,6,17,18,22	5,7,8,19,20,21,23,24	7	O54800;Q5DWV2,P01946,P02091,P06760,P23593,Q06000,Q9WTT6
234	1,2,3,4,6,17,18,23	5,7,8,19,20,21,22,24	5	O54800;Q5DWV2,O70594,P06760,P33436,Q99MH3
235	1,2,3,4,6,17,18,24	5,7,8,19,20,21,22,23	5	O54800;Q5DWV2,P06760,P33436,Q4KLZ6,Q9WTT6
236	1,2,3,4,6,17,19,20	5,7,8,18,21,22,23,24	3	P11762,P19223,Q8K1G0
237	1,2,3,4,6,17,19,21	5,7,8,18,20,22,23,24	1	Q9WVK7
238	1,2,3,4,6,17,19,22	5,7,8,18,20,21,23,24	3	P01946,P02091,P06760
239	1,2,3,4,6,17,19,23	5,7,8,18,20,21,22,24	3	P02783,P06760,P42854
240	1,2,3,4,6,17,19,24	5,7,8,18,20,21,22,23	2	P06760,Q4KLZ6
241	1,2,3,4,6,17,20,21	5,7,8,18,19,22,23,24	4	O55145,P01041,P19223,P63029
242	1,2,3,4,6,17,20,22	5,7,8,18,19,21,23,24	4	P01946,P02091,P06760,P19223

243	1,2,3,4,6,17,20,23	5,7,8,18,19,21,22,24	7	P02783,P06760,P09656,P10758,P19223,P33436,P63029
244	1,2,3,4,6,17,20,24	5,7,8,18,19,21,22,23	4	P06760,P19223,Q4KLZ6,Q9QYP1
245	1,2,3,4,6,17,21,22	5,7,8,18,19,20,23,24	3	P01946,P02091,Q9R168
246	1,2,3,4,6,17,21,23	5,7,8,18,19,20,22,24	2	P02783,P09656
247	1,2,3,4,6,17,21,24	5,7,8,18,19,20,22,23	3	P01039,P36860,Q4KLZ6
248	1,2,3,4,6,17,22,23	5,7,8,18,19,20,21,24	10	P01946,P02091,P02783,P06760,P09656,P23593,Q09030,Q63532,Q99MH3,Q9WTT6
249	1,2,3,4,6,17,22,24	5,7,8,18,19,20,21,23	7	D3ZUC6,P01946,P02091,P06760,P23593,Q4KLZ6,Q9WTT6
250	1,2,3,4,6,17,23,24	5,7,8,18,19,20,21,22	4	P06760,P09656,Q4KLZ6,Q9WTT6
251	1,2,3,4,6,18,19,20	5,7,8,17,21,22,23,24	3	O54800;Q5DWV2,P11762,P39069
252	1,2,3,4,6,18,19,21	5,7,8,17,20,22,23,24	1	O54800;Q5DWV2
253	1,2,3,4,6,18,19,22	5,7,8,17,20,21,23,24	5	O54800;Q5DWV2,P06760,P11762,Q5PPH0,Q9WTT6
254	1,2,3,4,6,18,19,23	5,7,8,17,20,21,22,24	4	O54800;Q5DWV2,P06760,P42854,Q9WTT6
255	1,2,3,4,6,18,19,24	5,7,8,17,20,21,22,23	5	O54800;Q5DWV2,P06760,P11762,Q4KLZ6,Q9WTT6
256	1,2,3,4,6,18,20,21	5,7,8,17,19,22,23,24	2	O54800;Q5DWV2,O55145
257	1,2,3,4,6,18,20,22	5,7,8,17,19,21,23,24	4	O54800;Q5DWV2,P06760,P11762,Q9QX74
258	1,2,3,4,6,18,20,23	5,7,8,17,19,21,22,24	9	O54800;Q5DWV2,P02783,P06760,P09656,P10758,P42854,P54921,Q99MH3,Q9QX74
259	1,2,3,4,6,18,20,24	5,7,8,17,19,21,22,23	5	O54800;Q5DWV2,P06760,Q4KLZ6,Q9QX74,Q9QYP1
260	1,2,3,4,6,18,21,22	5,7,8,17,19,20,23,24	4	O54800;Q5DWV2,P23739,P54921,Q9WTT6
261	1,2,3,4,6,18,21,23	5,7,8,17,19,20,22,24	5	O54800;Q5DWV2,P02783,P09656,P54921,Q9WTT6
262	1,2,3,4,6,18,21,24	5,7,8,17,19,20,22,23	3	O54800;Q5DWV2,Q4KLZ6,Q9WTT6

263	1,2,3,4,6,18,22,23	5,7,8,17,19,20,21,24	17	O54800;Q5DWV2,P02783,P06760,P09456,P09656,P13676,P30120,P54921,P55159,Q09030,Q63532,Q6P6R2,Q99MH3,Q9EQS0,Q9QX74,Q9WTT6,Q9Z2L0
264	1,2,3,4,6,18,22,24	5,7,8,17,19,20,21,23	10	D3ZUC6,O54800;Q5DWV2,P06760,P13676,P22006,P23593,Q4KLZ6,Q6P6R2,Q9QX74,Q9WTT6
265	1,2,3,4,6,18,23,24	5,7,8,17,19,20,21,22	8	O54800;Q5DWV2,P06760,P09656,P13676,Q4KLZ6,Q6P6R2,Q9QX74,Q9WTT6
266	1,2,3,4,6,19,20,21	5,7,8,17,18,22,23,24	5	O55145,P05369,P11762,P42854,Q9JI85
267	1,2,3,4,6,19,20,22	5,7,8,17,18,21,23,24	4	P06760,P11762,P42854,Q9QX74
268	1,2,3,4,6,19,20,23	5,7,8,17,18,21,22,24	7	P02783,P06760,P09656,P10758,P11762,P42854,Q9QX74
269	1,2,3,4,6,19,20,24	5,7,8,17,18,21,22,23	5	P06760,P11762,Q4KLZ6,Q68G31,Q9QX74
270	1,2,3,4,6,19,21,22	5,7,8,17,18,20,23,24	3	P01946,P11762,P30919
271	1,2,3,4,6,19,21,23	5,7,8,17,18,20,22,24	3	P02783,P09656,P42854
272	1,2,3,4,6,19,21,24	5,7,8,17,18,20,22,23	3	P11762,P30919,Q4KLZ6
273	1,2,3,4,6,19,22,23	5,7,8,17,18,20,21,24	10	P00507,P02783,P06760,P09656,P42854,Q63532,Q811M5,Q99MH3,Q9QX74,Q9WTT6
274	1,2,3,4,6,19,22,24	5,7,8,17,18,20,21,23	8	P00507,P06760,P11762,P30919,Q4KLZ6,Q6P6R2,Q9QX74,Q9WTT6
275	1,2,3,4,6,19,23,24	5,7,8,17,18,20,21,22	5	P06760,P42854,Q4KLZ6,Q9QX74,Q9WTT6
276	1,2,3,4,6,20,21,22	5,7,8,17,18,19,23,24	2	O55145,P09656
277	1,2,3,4,6,20,21,23	5,7,8,17,18,19,22,24	5	O55145,P02783,P09656,P10758,P42854
278	1,2,3,4,6,20,21,24	5,7,8,17,18,19,22,23	2	O55145,Q4KLZ6
279	1,2,3,4,6,20,22,23	5,7,8,17,18,19,21,24	8	P02783,P06760,P09656,P10758,P42854,P54921,Q99MH3,Q9QX74
280	1,2,3,4,6,20,22,24	5,7,8,17,18,19,21,23	7	P06760,P11762,P22006,Q4KLZ6,Q6P6R2,Q9QX74,Q9Z0V6
281	1,2,3,4,6,20,23,24	5,7,8,17,18,19,21,22	8	P02783,P06760,P09656,P10758,Q4KLZ6,Q6P6R2,Q9QX74,Q9QYP1
282	1,2,3,4,6,21,22,23	5,7,8,17,18,19,20,24	8	P02783,P09656,P30919,P54921,Q09030,Q63532,Q99MH3,Q9WTT6

283	1,2,3,4,6,21,22,24	5,7,8,17,18,19,20,23	4	P30919,Q4KLZ6,Q9QX74,Q9WTT6
284	1,2,3,4,6,21,23,24	5,7,8,17,18,19,20,22	7	P02783,P09656,P30919,P36860,Q4KLZ6,Q9QX74,Q9WTT6
285	1,2,3,4,6,22,23,24	5,7,8,17,18,19,20,21	20	D3ZUC6,P00507,P02783,P06760,P07150,P09656,P13676,P22006,P23593,P30919,P47727,P55159,P62804,Q09030,Q4KLZ6,Q63532,Q6P6R2,Q99MH3,Q9QX74,Q9WTT6
286	1,2,3,4,7,8,17,18	5,6,19,20,21,22,23,24	11	G3V686,O70417,O70594,P01681,P20766,P97580,P97615,Q30KJ2,Q63598,Q64093,Q811M5
287	1,2,3,4,7,8,17,19	5,6,18,20,21,22,23,24	8	iRT-Kit_WR_fusion,O70417,O70594,P01681,P20646,P97580,Q30KJ2,Q66H69
288	1,2,3,4,7,8,17,20	5,6,18,19,21,22,23,24	14	D4A5U3,O70417,P06911,P13676,P19223,P97580,Q10758,Q4FZU2,Q4G075,Q5GRG2,Q62946,Q6IFW6,Q6IG02,Q811M5
289	1,2,3,4,7,8,17,21	5,6,18,19,20,22,23,24	12	iRT-Kit_WR_fusion,O70594,P22006,P97580,Q00715,Q4FZU2,Q63598,Q6IFU8,Q6IFW6,Q6IG02,Q6IMF3,Q811M5
290	1,2,3,4,7,8,17,22	5,6,18,19,20,21,23,24	6	O70417,P01681,P23593,P35280,P97580,Q30KJ2
291	1,2,3,4,7,8,17,23	5,6,18,19,20,21,22,24	8	O70594,P01681,P20761,P46720,P46844,P97580,Q63598,Q64093
292	1,2,3,4,7,8,17,24	5,6,18,19,20,21,22,23	12	O70417,O70594,P01681,P23593,P46844,P97580,Q4FZU2,Q63598,Q64093,Q6IFW6,Q6IG02,Q811M5
293	1,2,3,4,7,8,18,19	5,6,17,20,21,22,23,24	8	A0JPJ7,P01681,P20766,P97580,P97615,Q30KJ2,Q66H69,Q9WTW7
294	1,2,3,4,7,8,18,20	5,6,17,19,21,22,23,24	11	D4A5U3,O70417,P02780,P06911,P50280,P97580,P97615,Q4G075,Q5GRG2,Q811M5,Q91ZS3
295	1,2,3,4,7,8,18,21	5,6,17,19,20,22,23,24	7	O89117,P02625,P46844,P97580,Q00715,Q811M5,Q91ZS3
296	1,2,3,4,7,8,18,22	5,6,17,19,20,21,23,24	6	P23593,P35280,P97580,Q30KJ2,Q63618,Q6AYQ8

297	1,2,3,4,7,8,18,23	5,6,17,19,20,21,22,24	13	O70594,P20761,P20762,P20766,P46844,P97580,Q30KJ2,Q3ZAV1,Q63424,Q63598,Q64093,Q6AYQ8,Q9WTW7
298	1,2,3,4,7,8,18,24	5,6,17,19,20,21,22,23	9	P02625,P20766,P46844,P97580,P97615,Q3ZAV1,Q63598,Q64093,Q6AYQ8
299	1,2,3,4,7,8,19,20	5,6,17,18,21,22,23,24	15	D4A5U3,iRT-Kit_WR_fusion,O70417,P01681,P02780,P06911,P10758,P47967,P50280,P80299,P97580,Q4G075,Q5I0D1,Q66H69,Q6IG02
300	1,2,3,4,7,8,19,21	5,6,17,18,20,22,23,24	9	B0BNN3,iRT-Kit_WR_fusion,P01681,P04355,P11883,P97580,Q00715,Q66H69,Q6IFW6
301	1,2,3,4,7,8,19,22	5,6,17,18,20,21,23,24	7	iRT-Kit_WR_fusion,P01681,P35280,P80299,P97580,Q30KJ2,Q66H69
302	1,2,3,4,7,8,19,23	5,6,17,18,20,21,22,24	7	iRT-Kit_WR_fusion,P01681,P10758,P20761,P97580,Q66H69,Q6AYQ8
303	1,2,3,4,7,8,19,24	5,6,17,18,20,21,22,23	7	iRT-Kit_WR_fusion,O54728,P01681,P97580,Q66H69,Q6AYQ8,Q6IFW6
304	1,2,3,4,7,8,20,21	5,6,17,18,19,22,23,24	18	D4A5U3,iRT-Kit_WR_fusion,P02780,P05964,P06911,P19629,P47967,Q00715,Q10758,Q4G075,Q62946,Q63493,Q6IFU8,Q6IFW6,Q6IG02,Q811M5,Q812E4,Q9JHB9
305	1,2,3,4,7,8,20,22	5,6,17,18,19,21,23,24	10	D4A5U3,O70417,P02780,P06911,P23593,P35280,P80299,P97580,Q30KJ2,Q4G075
306	1,2,3,4,7,8,20,23	5,6,17,18,19,21,22,24	6	D4A5U3,P06911,P10758,P20761,Q4G075,Q6IG02
307	1,2,3,4,7,8,20,24	5,6,17,18,19,21,22,23	11	D4A5U3,O70417,P02780,P06911,Q4G075,Q6IFU8,Q6IFW6,Q6IG02,Q811M5,Q812E4,Q9JHB9
308	1,2,3,4,7,8,21,22	5,6,17,18,19,20,23,24	7	iRT-Kit_WR_fusion,P11883,P23593,P35280,P97580,Q30KJ2,Q6IFW6
309	1,2,3,4,7,8,21,23	5,6,17,18,19,20,22,24	6	iRT-Kit_WR_fusion,O70594,P20761,P46844,Q63598,Q6IFW6

310	1,2,3,4,7,8,21,24	5,6,17,18,19,20,22,23	11	iRT-Kit_WR_fusion,P02625,P02761,P11883,P46844,Q4FZU2,Q63598,Q6IFU8,Q6IFW6,Q6IG02,Q6IMF3
311	1,2,3,4,7,8,22,23	5,6,17,18,19,20,21,24	8	P01681,P20761,P23593,P35280,P80299,P97580,Q30KJ2,Q6AYQ8
312	1,2,3,4,7,8,22,24	5,6,17,18,19,20,21,23	7	P23593,P35280,P97580,Q30KJ2,Q6AYQ8,Q6IFW6,Q6P6R2
313	1,2,3,4,7,8,23,24	5,6,17,18,19,20,21,22	10	P20761,P46720,P46844,Q3ZAV1,Q5PQL7,Q63598,Q64093,Q6AYQ8,Q6IFW6,Q6P6R2
314	1,2,3,4,7,17,18,19	5,6,8,20,21,22,23,24	1	P20766
315	1,2,3,4,7,17,18,20	5,6,8,19,21,22,23,24	3	P19223,Q811M5,Q8K1G0
316	1,2,3,4,7,17,18,21	5,6,8,19,20,22,23,24	1	Q811M5
317	1,2,3,4,7,17,18,22	5,6,8,19,20,21,23,24	1	P80299
318	1,2,3,4,7,17,18,23	5,6,8,19,20,21,22,24	1	O70594
319	1,2,3,4,7,17,18,24	5,6,8,19,20,21,22,23	2	P19218,P20766
320	1,2,3,4,7,17,19,20	5,6,8,18,21,22,23,24	5	P10758,P19223,P20646,P80299,Q811M5
321	1,2,3,4,7,17,19,21	5,6,8,18,20,22,23,24	3	P80299,Q9R168,Q9WVK7
322	1,2,3,4,7,17,19,22	5,6,8,18,20,21,23,24	2	P80299,Q9R168
323	1,2,3,4,7,17,19,23	5,6,8,18,20,21,22,24	1	P80299
324	1,2,3,4,7,17,19,24	5,6,8,18,20,21,22,23	1	P54921
325	1,2,3,4,7,17,20,21	5,6,8,18,19,22,23,24	3	P19223,P80299,Q811M5
326	1,2,3,4,7,17,20,22	5,6,8,18,19,21,23,24	2	P19223,P80299
327	1,2,3,4,7,17,20,23	5,6,8,18,19,21,22,24	4	P10758,P19223,P80299,Q9QYP1

328	1,2,3,4,7,17,20,24	5,6,8,18,19,21,22,23	3	P19223,Q811M5,Q9QYP1
329	1,2,3,4,7,17,21,22	5,6,8,18,19,20,23,24	2	P80299,Q9R168
330	1,2,3,4,7,17,21,23	5,6,8,18,19,20,22,24	1	Q9R168
331	1,2,3,4,7,17,21,24	5,6,8,18,19,20,22,23	4	P36860,Q6AY61,Q811M5,Q9R168
332	1,2,3,4,7,17,22,23	5,6,8,18,19,20,21,24	2	P80299,Q9R168
333	1,2,3,4,7,17,22,24	5,6,8,18,19,20,21,23	1	Q9R168
334	1,2,3,4,7,17,23,24	5,6,8,18,19,20,21,22	1	Q5PQL7
335	1,2,3,4,7,18,19,20	5,6,8,17,21,22,23,24	5	P10758,P47967,P80299,P97840,Q5I0D1
336	1,2,3,4,7,18,19,21	5,6,8,17,20,22,23,24	1	P80299
337	1,2,3,4,7,18,19,22	5,6,8,17,20,21,23,24	2	P80299,Q6AYQ8
338	1,2,3,4,7,18,19,23	5,6,8,17,20,21,22,24	4	P10758,P20766,P80299,Q6AYQ8
339	1,2,3,4,7,18,19,24	5,6,8,17,20,21,22,23	2	P20766,Q6AYQ8
340	1,2,3,4,7,18,20,21	5,6,8,17,19,22,23,24	4	P47967,P80299,P97840,Q811M5
341	1,2,3,4,7,18,20,22	5,6,8,17,19,21,23,24	1	P80299
342	1,2,3,4,7,18,20,23	5,6,8,17,19,21,22,24	3	P10758,P80299,Q9QYP1
343	1,2,3,4,7,18,20,24	5,6,8,17,19,21,22,23	1	Q9QYP1
344	1,2,3,4,7,18,21,22	5,6,8,17,19,20,23,24	2	P23739,P80299
345	1,2,3,4,7,18,21,23	5,6,8,17,19,20,22,24	2	O89117,P80299
346	1,2,3,4,7,18,21,24	5,6,8,17,19,20,22,23	1	O89117
347	1,2,3,4,7,18,22,23	5,6,8,17,19,20,21,24	4	P30120,P80299,Q6AYQ8,Q6P6R2

348	1,2,3,4,7,18,22,24	5,6,8,17,19,20,21,23	3	P06760,Q6AYQ8,Q6P6R2
349	1,2,3,4,7,18,23,24	5,6,8,17,19,20,21,22	5	O89117,P06760,P20766,Q6AYQ8,Q6P6R2
350	1,2,3,4,7,19,20,21	5,6,8,17,18,22,23,24	5	P05369,P10758,P47967,P80299,Q5I0D1
351	1,2,3,4,7,19,20,22	5,6,8,17,18,21,23,24	2	P10758,P80299
352	1,2,3,4,7,19,20,23	5,6,8,17,18,21,22,24	2	P10758,P80299
353	1,2,3,4,7,19,20,24	5,6,8,17,18,21,22,23	2	P10758,P80299
354	1,2,3,4,7,19,21,22	5,6,8,17,18,20,23,24	3	B0BNN3,P80299,Q9R168
355	1,2,3,4,7,19,21,23	5,6,8,17,18,20,22,24	2	P10758,P80299
356	1,2,3,4,7,19,21,24	5,6,8,17,18,20,22,23	2	B0BNN3,Q62761;Q62762;Q62763
357	1,2,3,4,7,19,22,23	5,6,8,17,18,20,21,24	2	P80299,Q6AYQ8
358	1,2,3,4,7,19,22,24	5,6,8,17,18,20,21,23	2	P80299,Q6AYQ8
359	1,2,3,4,7,19,23,24	5,6,8,17,18,20,21,22	2	P10758,Q6AYQ8
360	1,2,3,4,7,20,21,22	5,6,8,17,18,19,23,24	2	P17046,P80299
361	1,2,3,4,7,20,21,23	5,6,8,17,18,19,22,24	3	P08649,P10758,P80299
362	1,2,3,4,7,20,21,24	5,6,8,17,18,19,22,23	3	P02761,P08649,Q811M5
363	1,2,3,4,7,20,22,23	5,6,8,17,18,19,21,24	2	P10758,P80299
364	1,2,3,4,7,20,22,24	5,6,8,17,18,19,21,23	2	P80299,Q9QX74
365	1,2,3,4,7,20,23,24	5,6,8,17,18,19,21,22	2	P10758,Q9QYP1
366	1,2,3,4,7,21,22,23	5,6,8,17,18,19,20,24	2	P80299,Q9R168
367	1,2,3,4,7,21,22,24	5,6,8,17,18,19,20,23	3	P02761,P80299,Q9R168

368	1,2,3,4,7,21,23,24	5,6,8,17,18,19,20,22	3	P02761,P08649,P18297
369	1,2,3,4,7,22,23,24	5,6,8,17,18,19,20,21	6	B0LT89,P06760,P80299,Q6AYQ8,Q6P6R2,Q9QX74
370	1,2,3,4,8,17,18,19	5,6,7,20,21,22,23,24	7	G3V686,P01681,P01835,P97580,P97615,Q30KJ2,Q66H69
371	1,2,3,4,8,17,18,20	5,6,7,19,21,22,23,24	7	D4A5U3,G3V686,P19223,P97580,Q10758,Q6IG02,Q811M5
372	1,2,3,4,8,17,18,21	5,6,7,19,20,22,23,24	3	P02625,P97580,Q811M5
373	1,2,3,4,8,17,18,22	5,6,7,19,20,21,23,24	4	G3V686,P23593,P97580,Q30KJ2
374	1,2,3,4,8,17,18,23	5,6,7,19,20,21,22,24	4	O70594,P01681,P97580,Q30KJ2
375	1,2,3,4,8,17,18,24	5,6,7,19,20,21,22,23	1	P97580
376	1,2,3,4,8,17,19,20	5,6,7,18,21,22,23,24	8	D4A5U3,P01681,P13676,P19223,P97580,Q10758,Q6IG02,Q811M5
377	1,2,3,4,8,17,19,21	5,6,7,18,20,22,23,24	6	B0BNN3,P01681,P22006,P97580,Q66H69,Q9WVK7
378	1,2,3,4,8,17,19,22	5,6,7,18,20,21,23,24	3	P01681,P97580,Q30KJ2
379	1,2,3,4,8,17,19,23	5,6,7,18,20,21,22,24	2	P01681,P97580
380	1,2,3,4,8,17,19,24	5,6,7,18,20,21,22,23	3	O54728,P01681,P97580
381	1,2,3,4,8,17,20,21	5,6,7,18,19,22,23,24	8	D4A5U3,P00762,P19223,Q10758,Q6IFU8,Q6IFW6,Q6IG02,Q811M5
382	1,2,3,4,8,17,20,22	5,6,7,18,19,21,23,24	6	D4A5U3,O70417,P19223,P97580,Q30KJ2,Q6IG02
383	1,2,3,4,8,17,20,23	5,6,7,18,19,21,22,24	6	D4A5U3,P10758,P19223,P36376,Q10758,Q6IG02
384	1,2,3,4,8,17,20,24	5,6,7,18,19,21,22,23	7	D4A5U3,P19223,Q10758,Q4FZU2,Q6IFW6,Q6IG02,Q811M5
385	1,2,3,4,8,17,21,22	5,6,7,18,19,20,23,24	2	P97580,Q30KJ2
386	1,2,3,4,8,17,21,23	5,6,7,18,19,20,22,24	2	P22006,Q6IFW6
387	1,2,3,4,8,17,21,24	5,6,7,18,19,20,22,23	6	P00762,Q4FZU2,Q6IFU8,Q6IFW6,Q6IG02,Q811M5

388	1,2,3,4,8,17,22,23	5,6,7,18,19,20,21,24	4	P01681,P23593,P97580,Q30KJ2
389	1,2,3,4,8,17,22,24	5,6,7,18,19,20,21,23	3	P23593,P97580,Q30KJ2
390	1,2,3,4,8,17,23,24	5,6,7,18,19,20,21,22	2	Q5PQL7,Q6IFW6
391	1,2,3,4,8,18,19,20	5,6,7,17,21,22,23,24	4	P97580,P97615,Q63475,Q9R168
392	1,2,3,4,8,18,19,21	5,6,7,17,20,22,23,24	2	P97580,Q66H69
393	1,2,3,4,8,18,19,22	5,6,7,17,20,21,23,24	3	P15399,P97580,Q30KJ2
394	1,2,3,4,8,18,19,23	5,6,7,17,20,21,22,24	4	P01681,P97580,Q30KJ2,Q9Z0J6
395	1,2,3,4,8,18,19,24	5,6,7,17,20,21,22,23	2	O54728,P97580
396	1,2,3,4,8,18,20,21	5,6,7,17,19,22,23,24	6	D4A5U3,P00762,P25809,Q10758,Q6IG02,Q811M5
397	1,2,3,4,8,18,20,22	5,6,7,17,19,21,23,24	3	P15399,P97580,Q30KJ2
398	1,2,3,4,8,18,20,23	5,6,7,17,19,21,22,24	6	P05539,P10758,P36376,P54921,Q10758,Q9R168
399	1,2,3,4,8,18,20,24	5,6,7,17,19,21,22,23	2	Q10758,Q6IG02
400	1,2,3,4,8,18,21,22	5,6,7,17,19,20,23,24	4	P02625,P54921,P97580,Q30KJ2
401	1,2,3,4,8,18,21,23	5,6,7,17,19,20,22,24	1	P54921
402	1,2,3,4,8,18,21,24	5,6,7,17,19,20,22,23	3	O89117,P02625,Q6IFW6
403	1,2,3,4,8,18,22,23	5,6,7,17,19,20,21,24	4	P54921,P97580,Q30KJ2,Q99MH3
404	1,2,3,4,8,18,22,24	5,6,7,17,19,20,21,23	4	P23593,P97580,Q30KJ2,Q6P6R2
405	1,2,3,4,8,18,23,24	5,6,7,17,19,20,21,22	1	P05539
406	1,2,3,4,8,19,20,21	5,6,7,17,18,22,23,24	7	B0BNN3,D4A5U3,P00762,Q10758,Q66H69,Q6IG02,Q9JI85
407	1,2,3,4,8,19,20,22	5,6,7,17,18,21,23,24	3	P15399,P97580,Q30KJ2

408	1,2,3,4,8,19,20,23	5,6,7,17,18,21,22,24	2	P10758,P36376
409	1,2,3,4,8,19,20,24	5,6,7,17,18,21,22,23	2	O54728,Q6IG02
410	1,2,3,4,8,19,21,22	5,6,7,17,18,20,23,24	4	B0BNN3,P30919,P97580,Q30KJ2
411	1,2,3,4,8,19,21,23	5,6,7,17,18,20,22,24	1	Q9Z0J6
412	1,2,3,4,8,19,21,24	5,6,7,17,18,20,22,23	6	B0BNN3,O54728,P30919,Q10743,Q66H69,Q6IFW6
413	1,2,3,4,8,19,22,23	5,6,7,17,18,20,21,24	4	P00507,P01681,P97580,Q30KJ2
414	1,2,3,4,8,19,22,24	5,6,7,17,18,20,21,23	6	O54728,P00507,P15399,P30919,P97580,Q30KJ2
415	1,2,3,4,8,19,23,24	5,6,7,17,18,20,21,22	3	O54728,P01681,Q9Z0J6
416	1,2,3,4,8,20,21,22	5,6,7,17,18,19,23,24	8	D4A5U3,P00762,P97580,Q10743,Q10758,Q30KJ2,Q6IFW6,Q6IG02
417	1,2,3,4,8,20,21,23	5,6,7,17,18,19,22,24	6	P00762,P10758,P36376,Q10758,Q6IFW6,Q6IG02
418	1,2,3,4,8,20,21,24	5,6,7,17,18,19,22,23	9	D4A5U3,P00762,Q10743,Q10758,Q6IFU8,Q6IFW6,Q6IG02,Q6P6Q2,Q811M5
419	1,2,3,4,8,20,22,23	5,6,7,17,18,19,21,24	6	P05539,P10758,P36376,P97580,Q30KJ2,Q99MH3
420	1,2,3,4,8,20,22,24	5,6,7,17,18,19,21,23	6	P00762,P15399,P97580,Q10743,Q30KJ2,Q6IG02
421	1,2,3,4,8,20,23,24	5,6,7,17,18,19,21,22	7	P00762,P05539,P10758,P36376,Q6IFW6,Q6IG02,Q9QYP1
422	1,2,3,4,8,21,22,23	5,6,7,17,18,19,20,24	4	P30919,P54921,P97580,Q30KJ2
423	1,2,3,4,8,21,22,24	5,6,7,17,18,19,20,23	7	P00762,P02625,P30919,P97580,Q10743,Q30KJ2,Q6IFW6
424	1,2,3,4,8,21,23,24	5,6,7,17,18,19,20,22	4	P00762,P30919,Q6IFW6,Q6IG02
425	1,2,3,4,8,22,23,24	5,6,7,17,18,19,20,21	8	P00507,P05539,P06760,P23593,P30919,P97580,Q30KJ2,Q6P6R2
426	1,2,3,4,17,18,19,20	5,6,7,8,21,22,23,24	4	G3V686,P19223,Q62714,Q8K1G0
427	1,2,3,4,17,18,19,21	5,6,7,8,20,22,23,24	1	Q9WVK7

428	1,2,3,4,17, 18,19,22	5,6,7,8,20, 21,23,24	1	Q06000
429	1,2,3,4,17, 18,19,23	5,6,7,8,20, 21,22,24	0	
430	1,2,3,4,17, 18,19,24	5,6,7,8,20, 21,22,23	0	
431	1,2,3,4,17, 18,20,21	5,6,7,8,19, 22,23,24	2	P19223,Q811M5
432	1,2,3,4,17, 18,20,22	5,6,7,8,19, 21,23,24	1	P19223
433	1,2,3,4,17, 18,20,23	5,6,7,8,19, 21,22,24	2	P19223,Q9QYP1
434	1,2,3,4,17, 18,20,24	5,6,7,8,19, 21,22,23	2	P19223,Q9QYP1
435	1,2,3,4,17, 18,21,22	5,6,7,8,19, 20,23,24	0	
436	1,2,3,4,17, 18,21,23	5,6,7,8,19, 20,22,24	0	
437	1,2,3,4,17, 18,21,24	5,6,7,8,19, 20,22,23	1	P19218
438	1,2,3,4,17, 18,22,23	5,6,7,8,19, 20,21,24	5	P06760,P09456,P30120,P55159,Q63617
439	1,2,3,4,17, 18,22,24	5,6,7,8,19, 20,21,23	4	D3ZUC6,P06760,Q63617,Q9WTT6
440	1,2,3,4,17, 18,23,24	5,6,7,8,19, 20,21,22	4	P06760,P19218,Q63617,Q9WTT6
441	1,2,3,4,17, 19,20,21	5,6,7,8,18, 22,23,24	2	P19223,Q9WVK7
442	1,2,3,4,17, 19,20,22	5,6,7,8,18, 21,23,24	0	
443	1,2,3,4,17, 19,20,23	5,6,7,8,18, 21,22,24	2	P10758,P19223
444	1,2,3,4,17, 19,20,24	5,6,7,8,18, 21,22,23	0	
445	1,2,3,4,17, 19,21,22	5,6,7,8,18, 20,23,24	2	Q9R168,Q9WVK7
446	1,2,3,4,17, 19,21,23	5,6,7,8,18, 20,22,24	0	
447	1,2,3,4,17, 19,21,24	5,6,7,8,18, 20,22,23	1	Q9WVK7

448	1,2,3,4,17, 19,22,23	5,6,7,8,18, 20,21,24	1	P06760
449	1,2,3,4,17, 19,22,24	5,6,7,8,18, 20,21,23	2	D3ZUC6,P06760
450	1,2,3,4,17, 19,23,24	5,6,7,8,18, 20,21,22	1	P06760
451	1,2,3,4,17, 20,21,22	5,6,7,8,18, 19,23,24	1	P19223
452	1,2,3,4,17, 20,21,23	5,6,7,8,18, 19,22,24	1	P19223
453	1,2,3,4,17, 20,21,24	5,6,7,8,18, 19,22,23	2	P19223,Q811M5
454	1,2,3,4,17, 20,22,23	5,6,7,8,18, 19,21,24	1	P06760
455	1,2,3,4,17, 20,22,24	5,6,7,8,18, 19,21,23	1	P06760
456	1,2,3,4,17, 20,23,24	5,6,7,8,18, 19,21,22	2	P06760,Q9QYP1
457	1,2,3,4,17, 21,22,23	5,6,7,8,18, 19,20,24	1	Q9R168
458	1,2,3,4,17, 21,22,24	5,6,7,8,18, 19,20,23	1	Q9R168
459	1,2,3,4,17, 21,23,24	5,6,7,8,18, 19,20,22	0	
460	1,2,3,4,17, 22,23,24	5,6,7,8,18, 19,20,21	8	D3ZUC6,P06760,P06761,P22283,P55159,Q5I0D1,Q63617,Q9WTT6
461	1,2,3,4,18, 19,20,21	5,6,7,8,17, 22,23,24	0	
462	1,2,3,4,18, 19,20,22	5,6,7,8,17, 21,23,24	0	
463	1,2,3,4,18, 19,20,23	5,6,7,8,17, 21,22,24	1	P10758
464	1,2,3,4,18, 19,20,24	5,6,7,8,17, 21,22,23	0	
465	1,2,3,4,18, 19,21,22	5,6,7,8,17, 20,23,24	0	
466	1,2,3,4,18, 19,21,23	5,6,7,8,17, 20,22,24	1	Q9Z0J6
467	1,2,3,4,18, 19,21,24	5,6,7,8,17, 20,22,23	0	

468	1,2,3,4,18, 19,22,23	5,6,7,8,17, 20,21,24	3	P06760,P09456,Q9WTT6
469	1,2,3,4,18, 19,22,24	5,6,7,8,17, 20,21,23	3	P06760,Q9QX74,Q9WTT6
470	1,2,3,4,18, 19,23,24	5,6,7,8,17, 20,21,22	4	P06760,Q9QX74,Q9WTT6,Q9Z0J6
471	1,2,3,4,18, 20,21,22	5,6,7,8,17, 19,23,24	0	
472	1,2,3,4,18, 20,21,23	5,6,7,8,17, 19,22,24	1	P10758
473	1,2,3,4,18, 20,21,24	5,6,7,8,17, 19,22,23	0	
474	1,2,3,4,18, 20,22,23	5,6,7,8,17, 19,21,24	3	P06760,P54921,Q9QX74
475	1,2,3,4,18, 20,22,24	5,6,7,8,17, 19,21,23	4	P06760,P21674,P50115,Q9QX74
476	1,2,3,4,18, 20,23,24	5,6,7,8,17, 19,21,22	5	P06760,P10758,Q5QE79,Q9QX74,Q9QYP1
477	1,2,3,4,18, 21,22,23	5,6,7,8,17, 19,20,24	2	P54921,Q9WTT6
478	1,2,3,4,18, 21,22,24	5,6,7,8,17, 19,20,23	2	Q8CJD3,Q9WTT6
479	1,2,3,4,18, 21,23,24	5,6,7,8,17, 19,20,22	2	O89117,Q9WTT6
480	1,2,3,4,18, 22,23,24	5,6,7,8,17, 19,20,21	9	P06760,P06761,P13676,P55159,Q63617,Q6AYQ8,Q6P6R2,Q9QX74,Q9WTT6
481	1,2,3,4,19, 20,21,22	5,6,7,8,17, 18,23,24	0	
482	1,2,3,4,19, 20,21,23	5,6,7,8,17, 18,22,24	1	P10758
483	1,2,3,4,19, 20,21,24	5,6,7,8,17, 18,22,23	1	Q64335
484	1,2,3,4,19, 20,22,23	5,6,7,8,17, 18,21,24	3	P06760,P10758,Q9QX74
485	1,2,3,4,19, 20,22,24	5,6,7,8,17, 18,21,23	2	P06760,Q9QX74
486	1,2,3,4,19, 20,23,24	5,6,7,8,17, 18,21,22	4	P06760,P10758,Q9QX74,Q9QYP1
487	1,2,3,4,19, 21,22,23	5,6,7,8,17, 18,20,24	0	

488	1,2,3,4,19,21,22,24	5,6,7,8,17,18,20,23	3	B0BNN3,P30919,Q64335
489	1,2,3,4,19,21,23,24	5,6,7,8,17,18,20,22	2	Q64335,Q9Z0J6
490	1,2,3,4,19,22,23,24	5,6,7,8,17,18,20,21	5	P00507,P06760,Q6P6R2,Q9QX74,Q9WTT6
491	1,2,3,4,20,21,22,23	5,6,7,8,17,18,19,24	1	P09656
492	1,2,3,4,20,21,22,24	5,6,7,8,17,18,19,23	1	Q9QX74
493	1,2,3,4,20,21,23,24	5,6,7,8,17,18,19,22	3	P10758,Q9QX74,Q9QYP1
494	1,2,3,4,20,22,23,24	5,6,7,8,17,18,19,21	6	P06760,P07150,Q5QE79,Q6P6R2,Q9QX74,Q9QYP1
495	1,2,3,4,21,22,23,24	5,6,7,8,17,18,19,20	5	P07150,P13676,P30919,Q9QX74,Q9WTT6
496	1,2,3,5,6,7,8,17	4,18,19,20,21,22,23,24	14	iRT-Kit_WR_fusion,O70594,P23593,P50115,P50116,P52590,P97580,Q05702,Q09030,Q6IFU7,Q6IG05,Q6IMF3,Q91XN4,Q9QZQ5
497	1,2,3,5,6,7,8,18	4,17,19,20,21,22,23,24	20	iRT-Kit_WR_fusion,O54800;Q5DWV2,O70594,P20766,P23593,P31044,P38918,P50115,P50116,P52590,P80202,P97580,Q05702,Q09030,Q6IG05,Q91XN4,Q91ZS3,Q9JJ50,Q9QZQ5,Q9Z2L0
498	1,2,3,5,6,7,8,19	4,17,18,20,21,22,23,24	16	iRT-Kit_WR_fusion,O70594,P19629,P31044,P42854,P50115,P50116,P52590,P97580,Q05702,Q09030,Q4FZU4,Q66H69,Q6IG05,Q6IMF3,Q9Z2L0
499	1,2,3,5,6,7,8,20	4,17,18,19,21,22,23,24	23	iRT-Kit_WR_fusion,P06911,P12020,P19629,P23593,P25031,P31044,P42854,P47967,P50115,P50116,P50280,P52590,Q05702,Q09030,Q4G075,Q5GRG2,Q5I0D1,Q6IFU7,Q6IG05,Q6IMF3,Q91XN4,Q91ZS3
500	1,2,3,5,6,7,8,21	4,17,18,19,20,22,23,24	26	iRT-Kit_WR_fusion,O70594,P02631,P04762,P08937,P11883,P18757,P19629,P30919,P36860,P46844,P47967,P50115,P50116,P52590,Q00715,Q05702,Q09030,Q62714,Q6IFU7,Q6IFW6,Q6IG05,Q6IMF3,Q91XN4,Q91ZS3,Q9JJ50
501	1,2,3,5,6,7,8,22	4,17,18,19,20,21,23,24	21	iRT-Kit_WR_fusion,O54858,O70594,P19629,P23593,P35280,P50115,P50116,P52590,P80202,P97580,Q05702,Q09030,Q30KJ2,Q4FZU4,Q62714,Q6IG05,Q6P6R2,Q91XN4,Q91ZS3,Q9Z2L0
502	1,2,3,5,6,7,8,23	4,17,18,19,20,21,22,24	23	iRT-Kit_WR_fusion,O70594,P19629,P23593,P23785,P38918,P42854,P46844,P50115,P50116,P52590,P80202,Q05702,Q09030,Q62714,Q63532,Q6IG05,Q6IMF3,Q91XN4,Q91ZS3,Q99MH3,Q9JJ50,Q9Z2L0

503	1,2,3,5,6,7,8,24	4,17,18,19,20,21,22,23	19	iRT-Kit_WR_fusion,O70594,P23593,P30919,P36860,P46844,P50115,P50116,P52590,P80202,Q05702,Q09030,Q62714,Q6IFW6,Q6IG05,Q6IMF3,Q6P6R2,Q91XN4,Q91ZS3
504	1,2,3,5,6,7,17,18	4,8,19,20,21,22,23,24	8	O54800;Q5DWV2,O70594,P20766,P50116,P52590,Q09030,Q6AY61,Q9Z2L0
505	1,2,3,5,6,7,17,19	4,8,18,20,21,22,23,24	4	O70594,P50116,P52590,Q6AY61
506	1,2,3,5,6,7,17,20	4,8,18,19,21,22,23,24	5	P50116,P52590,P55281,P63029,Q6AY61
507	1,2,3,5,6,7,17,21	4,8,18,19,20,22,23,24	9	O70594,P04762,P05539,P36860,P50116,P52590,Q09030,Q6AY61,Q9R168
508	1,2,3,5,6,7,17,22	4,8,18,19,20,21,23,24	7	P01946,P02091,P23593,P52590,Q09030,Q9R168,Q9Z2L0
509	1,2,3,5,6,7,17,23	4,8,18,19,20,21,22,24	6	O70594,P50116,P52590,Q09030,Q99MH3,Q9Z2L0
510	1,2,3,5,6,7,17,24	4,8,18,19,20,21,22,23	6	O70594,P01039,P36860,P52590,Q09030,Q6AY61
511	1,2,3,5,6,7,18,19	4,8,17,20,21,22,23,24	6	O54800;Q5DWV2,P00762,P20766,P47967,P52590,Q9Z2L0
512	1,2,3,5,6,7,18,20	4,8,17,19,21,22,23,24	6	O54800;Q5DWV2,P47967,P52590,Q03191,Q91ZS3,Q9Z2L0
513	1,2,3,5,6,7,18,21	4,8,17,19,20,22,23,24	8	O54800;Q5DWV2,P01835,P36860,P47967,P52590,Q09030,Q91ZS3,Q9JJ50
514	1,2,3,5,6,7,18,22	4,8,17,19,20,21,23,24	8	O54800;Q5DWV2,P23593,P36374,P52590,Q09030,Q6P6R2,Q91XN4,Q9Z2L0
515	1,2,3,5,6,7,18,23	4,8,17,19,20,21,22,24	13	O54800;Q5DWV2,O70594,P16636,P20766,P30120,P36374,P52590,Q09030,Q6P6R2,Q91XN4,Q99MH3,Q9JJ50,Q9Z2L0

516	1,2,3,5,6,7,18,24	4,8,17,19,20,21,22,23	9	O54800;Q5DWV2,P20766,P52590,Q09030,Q6AY61,Q6P6R2,Q91XN4,Q9JJ50,Q9Z2L0
517	1,2,3,5,6,7,19,20	4,8,17,18,21,22,23,24	4	P0DP29;P0DP30;P0DP31,P47967,P52590,Q5I0D1
518	1,2,3,5,6,7,19,21	4,8,17,18,20,22,23,24	3	P47967,P52590,Q6AY61
519	1,2,3,5,6,7,19,22	4,8,17,18,20,21,23,24	5	P01946,P52590,Q09030,Q62714,Q9Z2L0
520	1,2,3,5,6,7,19,23	4,8,17,18,20,21,22,24	6	P42854,P52590,Q09030,Q99MH3,Q9JJ50,Q9Z2L0
521	1,2,3,5,6,7,19,24	4,8,17,18,20,21,22,23	2	P52590,Q6AY61
522	1,2,3,5,6,7,20,21	4,8,17,18,19,22,23,24	8	P01835,P19629,P47967,P50116,P52590,Q5I0D1,Q5I0J9,Q6AY61
523	1,2,3,5,6,7,20,22	4,8,17,18,19,21,23,24	4	P01835,P52590,Q09030,Q9Z2L0
524	1,2,3,5,6,7,20,23	4,8,17,18,19,21,22,24	7	P01835,P09656,P52590,Q03191,Q09030,Q99MH3,Q9Z2L0
525	1,2,3,5,6,7,20,24	4,8,17,18,19,21,22,23	3	P01835,P52590,Q6AY61
526	1,2,3,5,6,7,21,22	4,8,17,18,19,20,23,24	7	P01835,P01946,P36860,P52590,Q09030,Q62714,Q9R168
527	1,2,3,5,6,7,21,23	4,8,17,18,19,20,22,24	10	O70594,P01835,P09656,P36860,P52590,Q09030,Q62714,Q99MH3,Q9JJ50,Q9Z2L0
528	1,2,3,5,6,7,21,24	4,8,17,18,19,20,22,23	9	P01835,P04762,P30919,P36860,P52590,Q09030,Q62714,Q6AY61,Q9JJ50

529	1,2,3,5,6,7,22,23	4,8,17,18,19,20,21,24	13	P02783,P23593,P30120,P36374,P47727,P52590,P60905,Q09030,Q62714,Q6P6R2,Q99MH3,Q9EQS0,Q9Z2L0
530	1,2,3,5,6,7,22,24	4,8,17,18,19,20,21,23	6	P23593,P30919,Q09030,Q62714,Q6P6R2,Q9Z2L0
531	1,2,3,5,6,7,23,24	4,8,17,18,19,20,21,22	9	P36860,P47727,P52590,Q09030,Q62714,Q6P6R2,Q99MH3,Q9JJ50,Q9Z2L0
532	1,2,3,5,6,8,17,18	4,7,19,20,21,22,23,24	9	O54800;Q5DWV2,O70594,P52590,P97580,Q05702,Q09030,Q6IFU7,Q6IG05,Q9Z2L0
533	1,2,3,5,6,8,17,19	4,7,18,20,21,22,23,24	5	O70594,P52590,P97580,Q09030,Q6IG05
534	1,2,3,5,6,8,17,20	4,7,18,19,21,22,23,24	15	P06911,P17559,P25031,P36375,P36376,P52590,P55281,P63029,Q05702,Q09030,Q5GRG2,Q5I0J9,Q6IFU7,Q6IG05,Q6IMF3
535	1,2,3,5,6,8,17,21	4,7,18,19,20,22,23,24	8	O70594,P36860,P52590,Q05702,Q09030,Q6IFU7,Q6IG05,Q6IMF3
536	1,2,3,5,6,8,17,22	4,7,18,19,20,21,23,24	10	P01946,P02091,P23593,P52590,P97580,Q09030,Q30KJ2,Q6IFU7,Q6IG05,Q9Z2L0
537	1,2,3,5,6,8,17,23	4,7,18,19,20,21,22,24	10	O70594,P23785,P36376,P52590,Q05702,Q09030,Q6IFU7,Q6IG05,Q99MH3,Q9Z2L0
538	1,2,3,5,6,8,17,24	4,7,18,19,20,21,22,23	10	O70594,P01039,P10247,P23593,P52590,Q05702,Q09030,Q6IFU7,Q6IG05,Q6IMF3
539	1,2,3,5,6,8,18,19	4,7,17,20,21,22,23,24	7	O54800;Q5DWV2,P52590,P97580,Q05702,Q09030,Q6IG05,Q9Z2L0
540	1,2,3,5,6,8,18,20	4,7,17,19,21,22,23,24	12	O54800;Q5DWV2,P12020,P25031,P52590,Q05702,Q09030,Q5GRG2,Q6IFU7,Q6IG05,Q91ZS3,Q9R168,Q9Z2L0
541	1,2,3,5,6,8,18,21	4,7,17,19,20,22,23,24	11	O54800;Q5DWV2,P52590,Q01984,Q05702,Q09030,Q6IFU7,Q6IG05,Q91XN4,Q91ZS3,Q9JJ50,Q9Z2L0

542	1,2,3,5,6,8,18,22	4,7,17,19,20,21,23,24	14	O54800;Q5DWV2,P15399,P23593,P52590,P80202,P97580,Q09030,Q30KJ2,Q6IFU7,Q6IG05,Q91XN4,Q91ZS3,Q99MH3,Q9Z2L0
543	1,2,3,5,6,8,18,23	4,7,17,19,20,21,22,24	17	O54800;Q5DWV2,O70594,P16636,P23785,P36374,P52590,P80202,Q05702,Q09030,Q63532,Q6IFU7,Q6IG05,Q91XN4,Q91ZS3,Q99MH3,Q9JJ50,Q9Z2L0
544	1,2,3,5,6,8,18,24	4,7,17,19,20,21,22,23	12	O54800;Q5DWV2,P20646,P52590,Q05702,Q09030,Q6IFU7,Q6IG05,Q6P6R2,Q91XN4,Q91ZS3,Q9JJ50,Q9Z2L0
545	1,2,3,5,6,8,19,20	4,7,17,18,21,22,23,24	8	P25031,P42854,P52590,Q05702,Q5GRG2,Q5I0J9,Q68G31,Q6IG05
546	1,2,3,5,6,8,19,21	4,7,17,18,20,22,23,24	6	P08937,P30919,P52590,Q05702,Q09030,Q6IG05
547	1,2,3,5,6,8,19,22	4,7,17,18,20,21,23,24	11	P00507,P01946,P15399,P19218,P30919,P52590,P97580,Q09030,Q30KJ2,Q6IG05,Q9Z2L0
548	1,2,3,5,6,8,19,23	4,7,17,18,20,21,22,24	8	P42854,P52590,Q05702,Q09030,Q6IG05,Q8CJD3,Q99MH3,Q9Z2L0
549	1,2,3,5,6,8,19,24	4,7,17,18,20,21,22,23	5	P30919,P52590,Q09030,Q68G31,Q6IG05
550	1,2,3,5,6,8,20,21	4,7,17,18,19,22,23,24	12	P19629,P25031,P36375,P47967,P52590,Q05702,Q09030,Q5I0J9,Q6IFU7,Q6IG05,Q6IMF3,Q91ZS3
551	1,2,3,5,6,8,20,22	4,7,17,18,19,21,23,24	15	P15399,P19218,P19629,P23593,P25031,P52590,P97580,Q05702,Q09030,Q5I0J9,Q6IFU7,Q6IG05,Q91ZS3,Q99MH3,Q9Z2L0
552	1,2,3,5,6,8,20,23	4,7,17,18,19,21,22,24	14	P09656,P19132,P19629,P23739,P25031,P36376,P42854,P52590,Q05702,Q09030,Q6IFU7,Q6IG05,Q99MH3,Q9Z2L0
553	1,2,3,5,6,8,20,24	4,7,17,18,19,21,22,23	8	P52590,Q05702,Q09030,Q5I0J9,Q6IFU7,Q6IG05,Q6IMF3,Q91ZS3
554	1,2,3,5,6,8,21,22	4,7,17,18,19,20,23,24	11	P01946,P23593,P30919,P52590,P97580,Q09030,Q62714,Q6AYC4,Q6IFU7,Q6IG05,Q9Z2L0

555	1,2,3,5,6,8,21,23	4,7,17,18,19,20,22,24	13	P09656,P19132,P30919,P52590,Q05702,Q09030,Q62714,Q63532,Q6IFU7,Q6IG05,Q99MH3,Q9JJ50,Q9Z2L0
556	1,2,3,5,6,8,21,24	4,7,17,18,19,20,22,23	11	P30919,P36860,P52590,Q05702,Q09030,Q62714,Q6IFU7,Q6IFW6,Q6IG05,Q6IMF3,Q9JJ50
557	1,2,3,5,6,8,22,23	4,7,17,18,19,20,21,24	17	P00507,P02783,P20646,P23593,P30919,P36374,P52590,P80202,P97580,Q09030,Q62714,Q63532,Q6IG05,Q6P6R2,Q91XN4,Q99MH3,Q9Z2L0
558	1,2,3,5,6,8,22,24	4,7,17,18,19,20,21,23	12	P00507,P20646,P23593,P30919,P97580,Q09030,Q62714,Q6IFU7,Q6IG05,Q6P6R2,Q91XN4,Q9Z2L0
559	1,2,3,5,6,8,23,24	4,7,17,18,19,20,21,22	14	P20646,P23785,P30919,P80202,Q05702,Q09030,Q62714,Q63532,Q6IG05,Q6P6R2,Q91XN4,Q99MH3,Q9JJ50,Q9Z2L0
560	1,2,3,5,6,17,18,19	4,7,8,20,21,22,23,24	3	O54800;Q5DWV2,P52590,Q06000
561	1,2,3,5,6,17,18,20	4,7,8,19,21,22,23,24	3	O54800;Q5DWV2,P52590,P63029
562	1,2,3,5,6,17,18,21	4,7,8,19,20,22,23,24	3	O54800;Q5DWV2,P36860,P52590
563	1,2,3,5,6,17,18,22	4,7,8,19,20,21,23,24	9	O54800;Q5DWV2,P01946,P02091,P0C0A9,P36374,Q06000,Q63617,Q812E4,Q9Z2L0
564	1,2,3,5,6,17,18,23	4,7,8,19,20,21,22,24	12	O54800;Q5DWV2,O70594,P08723,P0C0A9,P16636,P30120,P36374,Q09030,Q63617,Q99MH3,Q9JJ50,Q9Z2L0
565	1,2,3,5,6,17,18,24	4,7,8,19,20,21,22,23	5	O54800;Q5DWV2,P01039,Q63617,Q6AY61,Q9WTT6
566	1,2,3,5,6,17,19,20	4,7,8,18,21,22,23,24	3	P52590,P63029,Q5I0J9
567	1,2,3,5,6,17,19,21	4,7,8,18,20,22,23,24	3	P05539,P52590,Q6AY61
568	1,2,3,5,6,17,19,22	4,7,8,18,20,21,23,24	3	P01946,P02091,P52590
569	1,2,3,5,6,17,19,23	4,7,8,18,20,21,22,24	3	P52590,Q99MH3,Q9Z2L0
570	1,2,3,5,6,17,19,24	4,7,8,18,20,21,22,23	3	P01039,P52590,Q6AY61
571	1,2,3,5,6,17,20,21	4,7,8,18,19,22,23,24	4	P52590,P63029,Q5I0J9,Q6AY61
572	1,2,3,5,6,17,20,22	4,7,8,18,19,21,23,24	5	O89117,P01946,P02091,P63029,Q5I0J9

573	1,2,3,5,6,17,20,23	4,7,8,18,19,21,22,24	4	P63029,Q09030,Q99MH3,Q9Z2L0
574	1,2,3,5,6,17,20,24	4,7,8,18,19,21,22,23	4	P01039,P63029,Q5I0J9,Q6AY61
575	1,2,3,5,6,17,21,22	4,7,8,18,19,20,23,24	5	P01946,P02091,P05539,Q09030,Q9R168
576	1,2,3,5,6,17,21,23	4,7,8,18,19,20,22,24	3	P36860,Q09030,Q99MH3
577	1,2,3,5,6,17,21,24	4,7,8,18,19,20,22,23	4	P01039,P04762,P36860,Q6AY61
578	1,2,3,5,6,17,22,23	4,7,8,18,19,20,21,24	16	P01946,P02091,P02783,P08723,P0C0A9,P22273,P22282,P30120,P36374,P47727,Q09030,Q63617,Q812E4,Q99MH3,Q9WTT6,Q9Z2L0
579	1,2,3,5,6,17,22,24	4,7,8,18,19,20,21,23	8	P01039,P01946,P02091,P22273,Q09030,Q63617,Q6P6R2,Q9WTT6
580	1,2,3,5,6,17,23,24	4,7,8,18,19,20,21,22	7	P01039,P22273,Q09030,Q63617,Q99MH3,Q9WTT6,Q9Z2L0
581	1,2,3,5,6,18,19,20	4,7,8,17,21,22,23,24	3	O54800;Q5DWV2,P52590,Q9Z2L0
582	1,2,3,5,6,18,19,21	4,7,8,17,20,22,23,24	1	P52590
583	1,2,3,5,6,18,19,22	4,7,8,17,20,21,23,24	5	O54800;Q5DWV2,P01946,P52590,Q9WTT6,Q9Z2L0
584	1,2,3,5,6,18,19,23	4,7,8,17,20,21,22,24	5	P30120,P52590,Q99MH3,Q9JJ50,Q9Z2L0
585	1,2,3,5,6,18,19,24	4,7,8,17,20,21,22,23	2	P52590,Q9WTT6
586	1,2,3,5,6,18,20,21	4,7,8,17,19,22,23,24	3	O54800;Q5DWV2,P47967,Q5I0J9
587	1,2,3,5,6,18,20,22	4,7,8,17,19,21,23,24	3	O54800;Q5DWV2,Q9QX74,Q9Z2L0
588	1,2,3,5,6,18,20,23	4,7,8,17,19,21,22,24	5	O54800;Q5DWV2,P16636,Q99MH3,Q9QX74,Q9Z2L0
589	1,2,3,5,6,18,20,24	4,7,8,17,19,21,22,23	3	O54800;Q5DWV2,P20646,Q9QX74
590	1,2,3,5,6,18,21,22	4,7,8,17,19,20,23,24	5	O54800;Q5DWV2,P01946,P36374,Q9WTT6,Q9Z2L0
591	1,2,3,5,6,18,21,23	4,7,8,17,19,20,22,24	7	P20646,P36374,Q09030,Q99MH3,Q9JJ50,Q9WTT6,Q9Z2L0
592	1,2,3,5,6,18,21,24	4,7,8,17,19,20,22,23	5	P20646,P36860,Q4G075,Q9JJ50,Q9WTT6

593	1,2,3,5,6,18,22,23	4,7,8,17,19,20,21,24	26	O54800;Q5DWV2,P02781,P02782,P02783,P08723,P09456,P13676,P16636,P20646,P22273,P22282,P24368,P30120,P36374,P47727,P60905,Q09030,Q5M8C6,Q63617,Q6P6R2,Q812E4,Q99MH3,Q9EQS0,Q9QX74,Q9WTT6,Q9Z2L0
594	1,2,3,5,6,18,22,24	4,7,8,17,19,20,21,23	9	O54800;Q5DWV2,P13676,P20646,P36374,Q63617,Q6P6R2,Q9QX74,Q9WTT6,Q9Z2L0
595	1,2,3,5,6,18,23,24	4,7,8,17,19,20,21,22	10	P20646,P36374,Q09030,Q63617,Q6P6R2,Q99MH3,Q9JJ50,Q9QX74,Q9WTT6,Q9Z2L0
596	1,2,3,5,6,19,20,21	4,7,8,17,18,22,23,24	4	P47967,P52590,Q5I0D1,Q5I0J9
597	1,2,3,5,6,19,20,22	4,7,8,17,18,21,23,24	4	P01946,P52590,Q5I0J9,Q9Z2L0
598	1,2,3,5,6,19,20,23	4,7,8,17,18,21,22,24	5	P23739,P42854,P52590,Q99MH3,Q9Z2L0
599	1,2,3,5,6,19,20,24	4,7,8,17,18,21,22,23	4	P52590,Q5I0J9,Q68G31,Q9QX74
600	1,2,3,5,6,19,21,22	4,7,8,17,18,20,23,24	4	P01946,P02091,P30919,P52590
601	1,2,3,5,6,19,21,23	4,7,8,17,18,20,22,24	3	P52590,Q9JJ50,Q9Z2L0
602	1,2,3,5,6,19,21,24	4,7,8,17,18,20,22,23	4	P30919,P52590,Q4G075,Q6AY61
603	1,2,3,5,6,19,22,23	4,7,8,17,18,20,21,24	16	P00507,P01946,P02091,P02783,P22273,P30120,P47727,P52590,Q09030,Q6P6R2,Q811M5,Q99MH3,Q9EQS0,Q9QX74,Q9WTT6,Q9Z2L0
604	1,2,3,5,6,19,22,24	4,7,8,17,18,20,21,23	7	P00507,P01946,P30919,P52590,Q6P6R2,Q9QX74,Q9WTT6
605	1,2,3,5,6,19,23,24	4,7,8,17,18,20,21,22	6	P22273,P52590,Q6P6R2,Q9QX74,Q9WTT6,Q9Z2L0
606	1,2,3,5,6,20,21,22	4,7,8,17,18,19,23,24	3	P01835,P01946,Q5I0J9
607	1,2,3,5,6,20,21,23	4,7,8,17,18,19,22,24	5	P01835,P09656,Q09030,Q99MH3,Q9Z2L0
608	1,2,3,5,6,20,21,24	4,7,8,17,18,19,22,23	4	P01835,P20646,Q5I0J9,Q6AY61
609	1,2,3,5,6,20,22,23	4,7,8,17,18,19,21,24	8	P02783,P09656,P20646,Q09030,Q6P6R2,Q99MH3,Q9QX74,Q9Z2L0
610	1,2,3,5,6,20,22,24	4,7,8,17,18,19,21,23	7	P07150,P20646,P21674,Q5I0J9,Q6P6R2,Q9QX74,Q9Z0V6
611	1,2,3,5,6,20,23,24	4,7,8,17,18,19,21,22	8	P09656,P20646,P23739,Q09030,Q6P6R2,Q99MH3,Q9QX74,Q9Z2L0
612	1,2,3,5,6,21,22,23	4,7,8,17,18,19,20,24	13	P01946,P02783,P08723,P09656,P20646,P30919,P36374,P47727,Q09030,Q62714,Q99MH3,Q9WTT6,Q9Z2L0

613	1,2,3,5,6,21,22,24	4,7,8,17,18,19,20,23	11	P01946,P07150,P20646,P30919,P36860,Q09030,Q4G075,Q62714,Q6P6R2,Q9QX74,Q9WTT6
614	1,2,3,5,6,21,23,24	4,7,8,17,18,19,20,22	9	P09656,P20646,P30919,P36860,Q09030,Q4G075,Q62714,Q9JJ50,Q9WTT6
615	1,2,3,5,6,22,23,24	4,7,8,17,18,19,20,21	24	P00507,P01946,P02783,P06760,P07150,P13676,P20646,P22273,P22282,P24368,P30120,P30919,P36374,P47727,P62804,Q09030,Q62714,Q63617,Q6P6R2,Q99MH3,Q9EQS0,Q9QX74,Q9WTT6,Q9Z2L0
616	1,2,3,5,7,8,17,18	4,6,19,20,21,22,23,24	5	O70594,P20766,P22006,Q6IG05,Q811M5
617	1,2,3,5,7,8,17,19	4,6,18,20,21,22,23,24	8	iRT-Kit_WR_fusion,O70594,P01681,P22006,Q66H69,Q6IG05,Q6IMF3,Q811M5
618	1,2,3,5,7,8,17,20	4,6,18,19,21,22,23,24	11	D4A5U3,iRT-Kit_WR_fusion,P13676,Q4FZU2,Q4G075,Q6IFU7,Q6IFW6,Q6IG05,Q6IMF3,Q6P6Q2,Q811M5
619	1,2,3,5,7,8,17,21	4,6,18,19,20,22,23,24	13	iRT-Kit_WR_fusion,O70594,P04762,P06760,P11883,P22006,Q00715,Q4FZU2,Q6AY61,Q6IFW6,Q6IG05,Q6IMF3,Q811M5
620	1,2,3,5,7,8,17,22	4,6,18,19,20,21,23,24	4	P23593,P35280,Q6IG05,Q6IMF3
621	1,2,3,5,7,8,17,23	4,6,18,19,20,21,22,24	6	iRT-Kit_WR_fusion,O70594,P19132,P22006,Q6IG05,Q6IMF3
622	1,2,3,5,7,8,17,24	4,6,18,19,20,21,22,23	7	O70594,Q4FZU2,Q6AY61,Q6IFW6,Q6IG05,Q6IMF3,Q811M5
623	1,2,3,5,7,8,18,19	4,6,17,20,21,22,23,24	6	iRT-Kit_WR_fusion,P20766,P22006,P47967,Q66H69,Q6IG05
624	1,2,3,5,7,8,18,20	4,6,17,19,21,22,23,24	4	P47967,Q6IG05,Q811M5,Q91ZS3
625	1,2,3,5,7,8,18,21	4,6,17,19,20,22,23,24	10	iRT-Kit_WR_fusion,O70594,P11883,P22006,P47967,Q00715,Q6IG05,Q6IMF3,Q811M5,Q91ZS3
626	1,2,3,5,7,8,18,22	4,6,17,19,20,21,23,24	4	P23593,P35280,Q6IG05,Q9WUW8

627	1,2,3,5,7,8,18,23	4,6,17,19,20,21,22,24	7	O70594,P20766,P22006,Q6IG05,Q99MH3,Q9JJ50,Q9Z2L0
628	1,2,3,5,7,8,18,24	4,6,17,19,20,21,22,23	2	P20766,Q6IG05
629	1,2,3,5,7,8,19,20	4,6,17,18,21,22,23,24	7	iRT-Kit_WR_fusion,P47967,Q4G075,Q5I0D1,Q66H69,Q6IG05,Q6IMF3
630	1,2,3,5,7,8,19,21	4,6,17,18,20,22,23,24	10	iRT-Kit_WR_fusion,P06760,P08937,P11883,P22006,P47967,Q00715,Q66H69,Q6IG05,Q6IMF3
631	1,2,3,5,7,8,19,22	4,6,17,18,20,21,23,24	4	iRT-Kit_WR_fusion,P11883,P35280,Q6IG05
632	1,2,3,5,7,8,19,23	4,6,17,18,20,21,22,24	7	iRT-Kit_WR_fusion,O70594,P22006,Q66H69,Q6IG05,Q6IMF3,Q8CJD3
633	1,2,3,5,7,8,19,24	4,6,17,18,20,21,22,23	4	iRT-Kit_WR_fusion,Q66H69,Q6IG05,Q6IMF3
634	1,2,3,5,7,8,20,21	4,6,17,18,19,22,23,24	19	D4A5U3,iRT-Kit_WR_fusion,P00731,P06760,P08649,P11883,P22006,P47967,P70549,Q00715,Q5I0D1,Q6IFU7,Q6IFU8,Q6IFW6,Q6IG05,Q6IMF3,Q6P6Q2,Q811M5,Q91ZS3
635	1,2,3,5,7,8,20,22	4,6,17,18,19,21,23,24	7	iRT-Kit_WR_fusion,P35280,P47967,Q4G075,Q6IG05,Q6IMF3,Q9WUW8
636	1,2,3,5,7,8,20,23	4,6,17,18,19,21,22,24	8	iRT-Kit_WR_fusion,P19132,P23739,P36376,Q4G075,Q6IG05,Q6IMF3,Q99MH3
637	1,2,3,5,7,8,20,24	4,6,17,18,19,21,22,23	7	iRT-Kit_WR_fusion,Q4FZU2,Q6IFW6,Q6IG05,Q6IMF3,Q6P6Q2,Q811M5
638	1,2,3,5,7,8,21,22	4,6,17,18,19,20,23,24	8	iRT-Kit_WR_fusion,P11883,P35280,Q62714,Q6IFW6,Q6IG05,Q6IMF3,Q9WUW8
639	1,2,3,5,7,8,21,23	4,6,17,18,19,20,22,24	11	iRT-Kit_WR_fusion,O70594,P08649,P11883,P19132,P22006,Q62714,Q6IFW6,Q6IG05,Q6IMF3,Q9JJ50

640	1,2,3,5,7,8,21,24	4,6,17,18,19,20,22,23	15	iRT-Kit_WR_fusion,P04762,P08649,P11883,P30919,P36860,Q4FZU2,Q62714,Q6AY61,Q6IFU8,Q6IFW6,Q6IG05,Q6IMF3,Q6P6Q2,Q811M5
641	1,2,3,5,7,8,22,23	4,6,17,18,19,20,21,24	8	iRT-Kit_WR_fusion,P23593,P35280,Q62714,Q6IG05,Q6P6R2,Q99MH3,Q9Z2L0
642	1,2,3,5,7,8,22,24	4,6,17,18,19,20,21,23	8	P23593,P35280,Q5RLM2,Q62714,Q6IG05,Q6IMF3,Q6P6R2,Q9WUW8
643	1,2,3,5,7,8,23,24	4,6,17,18,19,20,21,22	7	iRT-Kit_WR_fusion,Q62714,Q6IFW6,Q6IG05,Q6IMF3,Q6P6R2,Q9JJ50
644	1,2,3,5,7,17,18,19	4,6,8,20,21,22,23,24	2	P20766,P22006
645	1,2,3,5,7,17,18,20	4,6,8,19,21,22,23,24	2	P47967,Q811M5
646	1,2,3,5,7,17,18,21	4,6,8,19,20,22,23,24	3	P22006,Q6AY61,Q811M5
647	1,2,3,5,7,17,18,22	4,6,8,19,20,21,23,24	0	
648	1,2,3,5,7,17,18,23	4,6,8,19,20,21,22,24	4	O70594,P22006,P30120,P36374
649	1,2,3,5,7,17,18,24	4,6,8,19,20,21,22,23	2	P20766,Q6AY61
650	1,2,3,5,7,17,19,20	4,6,8,18,21,22,23,24	3	P47967,Q6AY61,Q811M5
651	1,2,3,5,7,17,19,21	4,6,8,18,20,22,23,24	3	P22006,P47967,Q6AY61
652	1,2,3,5,7,17,19,22	4,6,8,18,20,21,23,24	1	Q9R168
653	1,2,3,5,7,17,19,23	4,6,8,18,20,21,22,24	1	P22006
654	1,2,3,5,7,17,19,24	4,6,8,18,20,21,22,23	2	P54921,Q6AY61
655	1,2,3,5,7,17,20,21	4,6,8,18,19,22,23,24	4	P22006,P47967,Q6AY61,Q811M5
656	1,2,3,5,7,17,20,22	4,6,8,18,19,21,23,24	0	
657	1,2,3,5,7,17,20,23	4,6,8,18,19,21,22,24	1	P23739

658	1,2,3,5,7,17,20,24	4,6,8,18,19,21,22,23	2 Q6AY61,Q811M5
659	1,2,3,5,7,17,21,22	4,6,8,18,19,20,23,24	1 Q9R168
660	1,2,3,5,7,17,21,23	4,6,8,18,19,20,22,24	2 P22006,Q6AY61
661	1,2,3,5,7,17,21,24	4,6,8,18,19,20,22,23	5 P04762,P36860,Q6AY61,Q811M5,Q9R168
662	1,2,3,5,7,17,22,23	4,6,8,18,19,20,21,24	5 P22273,P22282,P30120,P36374,Q9R168
663	1,2,3,5,7,17,22,24	4,6,8,18,19,20,21,23	1 Q9R168
664	1,2,3,5,7,17,23,24	4,6,8,18,19,20,21,22	1 Q6AY61
665	1,2,3,5,7,18,19,20	4,6,8,17,21,22,23,24	4 P20766,P47967,P97840,Q5I0D1
666	1,2,3,5,7,18,19,21	4,6,8,17,20,22,23,24	2 P22006,P47967
667	1,2,3,5,7,18,19,22	4,6,8,17,20,21,23,24	0
668	1,2,3,5,7,18,19,23	4,6,8,17,20,21,22,24	2 P20766,Q9Z2L0
669	1,2,3,5,7,18,19,24	4,6,8,17,20,21,22,23	1 P20766
670	1,2,3,5,7,18,20,21	4,6,8,17,19,22,23,24	4 P47967,P97840,Q5I0D1,Q811M5
671	1,2,3,5,7,18,20,22	4,6,8,17,19,21,23,24	3 P47967,Q03191,Q9WUW8
672	1,2,3,5,7,18,20,23	4,6,8,17,19,21,22,24	1 Q03191
673	1,2,3,5,7,18,20,24	4,6,8,17,19,21,22,23	1 P47967
674	1,2,3,5,7,18,21,22	4,6,8,17,19,20,23,24	2 P47967,Q9WUW8
675	1,2,3,5,7,18,21,23	4,6,8,17,19,20,22,24	4 O89117,P22006,P36374,Q9JJ50
676	1,2,3,5,7,18,21,24	4,6,8,17,19,20,22,23	3 O89117,Q6AY61,Q9JJ50
677	1,2,3,5,7,18,22,23	4,6,8,17,19,20,21,24	9 P08723,P22282,P30120,P36374,P47727,P60905,Q6P6R2,Q99MH3,Q9Z2L0

678	1,2,3,5,7,18,22,24	4,6,8,17,19,20,21,23	3	P36374,Q6P6R2,Q9WUW8
679	1,2,3,5,7,18,23,24	4,6,8,17,19,20,21,22	4	P20766,P36374,Q6P6R2,Q9JJ50
680	1,2,3,5,7,19,20,21	4,6,8,17,18,22,23,24	4	P08649,P47967,Q5I0D1,Q6AY61
681	1,2,3,5,7,19,20,22	4,6,8,17,18,21,23,24	1	P47967
682	1,2,3,5,7,19,20,23	4,6,8,17,18,21,22,24	3	P08649,P23739,P47967
683	1,2,3,5,7,19,20,24	4,6,8,17,18,21,22,23	3	P08649,P47967,Q6AY61
684	1,2,3,5,7,19,21,22	4,6,8,17,18,20,23,24	2	P08649,P47967
685	1,2,3,5,7,19,21,23	4,6,8,17,18,20,22,24	2	P08649,P22006
686	1,2,3,5,7,19,21,24	4,6,8,17,18,20,22,23	4	P08649,P47967,Q62761;Q62762;Q62763,Q6AY61
687	1,2,3,5,7,19,22,23	4,6,8,17,18,20,21,24	3	P30120,P47727,Q9Z2L0
688	1,2,3,5,7,19,22,24	4,6,8,17,18,20,21,23	0	
689	1,2,3,5,7,19,23,24	4,6,8,17,18,20,21,22	1	P08649
690	1,2,3,5,7,20,21,22	4,6,8,17,18,19,23,24	4	P01835,P08649,P47967,Q9WUW8
691	1,2,3,5,7,20,21,23	4,6,8,17,18,19,22,24	3	P01835,P08649,P47967
692	1,2,3,5,7,20,21,24	4,6,8,17,18,19,22,23	5	P01835,P08649,P47967,Q6AY61,Q811M5
693	1,2,3,5,7,20,22,23	4,6,8,17,18,19,21,24	3	P08649,Q03191,Q99MH3
694	1,2,3,5,7,20,22,24	4,6,8,17,18,19,21,23	3	P08649,Q6P6R2,Q9WUW8
695	1,2,3,5,7,20,23,24	4,6,8,17,18,19,21,22	2	P08649,P23739
696	1,2,3,5,7,21,22,23	4,6,8,17,18,19,20,24	5	P08649,P36374,P47727,Q62714,Q9R168
697	1,2,3,5,7,21,22,24	4,6,8,17,18,19,20,23	4	P08649,Q62714,Q9R168,Q9WUW8

698	1,2,3,5,7,21,23,24	4,6,8,17,18,19,20,22	5	P08649,P36860,Q62714,Q6AY61,Q9JJ50
699	1,2,3,5,7,22,23,24	4,6,8,17,18,19,20,21	9	B0LT89,P08649,P22273,P22282,P36374,P47727,Q62714,Q6P6R2,Q9QX74
700	1,2,3,5,8,17,18,19	4,6,7,20,21,22,23,24	2	P22006,Q6IG05
701	1,2,3,5,8,17,18,20	4,6,7,19,21,22,23,24	3	Q6IFU7,Q6IG05,Q811M5
702	1,2,3,5,8,17,18,21	4,6,7,19,20,22,23,24	4	P22006,Q6IFU7,Q6IG05,Q811M5
703	1,2,3,5,8,17,18,22	4,6,7,19,20,21,23,24	3	P00714,P11598,Q63617
704	1,2,3,5,8,17,18,23	4,6,7,19,20,21,22,24	7	O70594,P00714,P22006,P36376,Q63617,Q6IG05,Q99MH3
705	1,2,3,5,8,17,18,24	4,6,7,19,20,21,22,23	3	Q63617,Q6IFU7,Q6IG05
706	1,2,3,5,8,17,19,20	4,6,7,18,21,22,23,24	5	P13676,Q6IG05,Q6IMF3,Q811M5,Q8CJD3
707	1,2,3,5,8,17,19,21	4,6,7,18,20,22,23,24	5	P22006,Q66H69,Q6IG05,Q6IMF3,Q9WVK7
708	1,2,3,5,8,17,19,22	4,6,7,18,20,21,23,24	1	Q6IG05
709	1,2,3,5,8,17,19,23	4,6,7,18,20,21,22,24	4	P22006,P36376,Q6IG05,Q8CJD3
710	1,2,3,5,8,17,19,24	4,6,7,18,20,21,22,23	2	Q6IG05,Q6IMF3
711	1,2,3,5,8,17,20,21	4,6,7,18,19,22,23,24	12	P13676,P22006,P25809,P36375,P63029,Q4FZU2,Q6IFU7,Q6IFW6,Q6IG05,Q6IMF3,Q6P6Q2,Q811M5
712	1,2,3,5,8,17,20,22	4,6,7,18,19,21,23,24	2	Q6IFU7,Q6IG05
713	1,2,3,5,8,17,20,23	4,6,7,18,19,21,22,24	8	P19132,P23739,P36376,P63029,Q6IFU7,Q6IG05,Q6IMF3,Q99MH3
714	1,2,3,5,8,17,20,24	4,6,7,18,19,21,22,23	7	Q4FZU2,Q6IFU7,Q6IFW6,Q6IG05,Q6IMF3,Q6P6Q2,Q811M5
715	1,2,3,5,8,17,21,22	4,6,7,18,19,20,23,24	2	Q6IFU7,Q6IG05
716	1,2,3,5,8,17,21,23	4,6,7,18,19,20,22,24	6	P19132,P22006,P36376,Q6IFU7,Q6IG05,Q6IMF3
717	1,2,3,5,8,17,21,24	4,6,7,18,19,20,22,23	8	Q4FZU2,Q6AY61,Q6IFU7,Q6IFW6,Q6IG05,Q6IMF3,Q6P6Q2,Q811M5

718	1,2,3,5,8,17,22,23	4,6,7,18,19,20,21,24	8	P00714,P11598,P22283,P36374,P36376,Q63617,Q6IG05,Q99MH3
719	1,2,3,5,8,17,22,24	4,6,7,18,19,20,21,23	2	P11598,Q63617
720	1,2,3,5,8,17,23,24	4,6,7,18,19,20,21,22	7	P11598,P36376,Q5PQL7,Q63617,Q6IG05,Q6IMF3,Q80WL1
721	1,2,3,5,8,18,19,20	4,6,7,17,21,22,23,24	3	P47967,Q6IG05,Q9R168
722	1,2,3,5,8,18,19,21	4,6,7,17,20,22,23,24	3	P22006,Q66H69,Q6IG05
723	1,2,3,5,8,18,19,22	4,6,7,17,20,21,23,24	0	
724	1,2,3,5,8,18,19,23	4,6,7,17,20,21,22,24	4	P22006,Q6IG05,Q9Z0J6,Q9Z2L0
725	1,2,3,5,8,18,19,24	4,6,7,17,20,21,22,23	1	Q6IG05
726	1,2,3,5,8,18,20,21	4,6,7,17,19,22,23,24	6	P25809,P47967,Q6IFU7,Q6IG05,Q811M5,Q91ZS3
727	1,2,3,5,8,18,20,22	4,6,7,17,19,21,23,24	2	Q6IFU7,Q6IG05
728	1,2,3,5,8,18,20,23	4,6,7,17,19,21,22,24	8	P19132,P23739,P36376,Q6IFU7,Q6IG05,Q99MH3,Q9R168,Q9Z2L0
729	1,2,3,5,8,18,20,24	4,6,7,17,19,21,22,23	3	Q6IFU7,Q6IG05,Q9R168
730	1,2,3,5,8,18,21,22	4,6,7,17,19,20,23,24	0	
731	1,2,3,5,8,18,21,23	4,6,7,17,19,20,22,24	5	P19132,P22006,Q6IG05,Q99MH3,Q9JJ50
732	1,2,3,5,8,18,21,24	4,6,7,17,19,20,22,23	2	Q6IFW6,Q6IG05
733	1,2,3,5,8,18,22,23	4,6,7,17,19,20,21,24	8	P00714,P11598,P22283,P36374,Q63617,Q6IG05,Q99MH3,Q9Z2L0
734	1,2,3,5,8,18,22,24	4,6,7,17,19,20,21,23	2	Q63617,Q6P6R2
735	1,2,3,5,8,18,23,24	4,6,7,17,19,20,21,22	5	P36374,Q63617,Q6IG05,Q6P6R2,Q9JJ50
736	1,2,3,5,8,19,20,21	4,6,7,17,18,22,23,24	5	P22006,P47967,Q5I0D1,Q6IG05,Q6IMF3
737	1,2,3,5,8,19,20,22	4,6,7,17,18,21,23,24	2	O88797,Q6IG05

738	1,2,3,5,8,19,20,23	4,6,7,17,18,21,22,24	4	P23739,P36376,Q6IG05,Q8CJD3
739	1,2,3,5,8,19,20,24	4,6,7,17,18,21,22,23	2	Q6IG05,Q6IMF3
740	1,2,3,5,8,19,21,22	4,6,7,17,18,20,23,24	3	P30919,Q6IG05,Q9QYP1
741	1,2,3,5,8,19,21,23	4,6,7,17,18,20,22,24	3	P22006,Q6IG05,Q9Z0J6
742	1,2,3,5,8,19,21,24	4,6,7,17,18,20,22,23	4	P30919,Q6IFW6,Q6IG05,Q6IMF3
743	1,2,3,5,8,19,22,23	4,6,7,17,18,20,21,24	5	P00507,Q6IG05,Q99MH3,Q9Z0J6,Q9Z2L0
744	1,2,3,5,8,19,22,24	4,6,7,17,18,20,21,23	2	P00507,P30919
745	1,2,3,5,8,19,23,24	4,6,7,17,18,20,21,22	2	Q6IG05,Q9Z0J6
746	1,2,3,5,8,20,21,22	4,6,7,17,18,19,23,24	3	F1M3L7,Q6IFU7,Q6IG05
747	1,2,3,5,8,20,21,23	4,6,7,17,18,19,22,24	7	P19132,P23739,P36376,Q6IFU7,Q6IFW6,Q6IG05,Q6IMF3
748	1,2,3,5,8,20,21,24	4,6,7,17,18,19,22,23	6	Q6IFU7,Q6IFW6,Q6IG05,Q6IMF3,Q6P6Q2,Q811M5
749	1,2,3,5,8,20,22,23	4,6,7,17,18,19,21,24	5	P23739,P36376,Q6IG05,Q99MH3,Q9Z2L0
750	1,2,3,5,8,20,22,24	4,6,7,17,18,19,21,23	1	Q6IG05
751	1,2,3,5,8,20,23,24	4,6,7,17,18,19,21,22	5	P23739,P36376,Q5QE79,Q6IG05,Q6IMF3
752	1,2,3,5,8,21,22,23	4,6,7,17,18,19,20,24	5	P30919,P36374,Q62714,Q6IG05,Q99MH3
753	1,2,3,5,8,21,22,24	4,6,7,17,18,19,20,23	4	P30919,Q62714,Q6IFW6,Q6IMF3
754	1,2,3,5,8,21,23,24	4,6,7,17,18,19,20,22	8	P30919,Q62714,Q6IFW6,Q6IG05,Q6IMF3,Q80WL1,Q9JJ50,Q9Z0J6
755	1,2,3,5,8,22,23,24	4,6,7,17,18,19,20,21	10	P00507,P11598,P20646,P22283,P30919,P36374,Q62714,Q63617,Q6P6R2,Q99MH3
756	1,2,3,5,17,18,19,20	4,6,7,8,21,22,23,24	1	Q99041
757	1,2,3,5,17,18,19,21	4,6,7,8,20,22,23,24	3	P22006,P22273,Q99041

758	1,2,3,5,17,18,19,22	4,6,7,8,20,21,23,24	6	P00714,P11598,P22273,P22282,Q63617,Q812E4
759	1,2,3,5,17,18,19,23	4,6,7,8,20,21,22,24	10	P00714,P02780,P08723,P11598,P22006,P22273,P22282,Q5M8C6,Q63617,Q99041
760	1,2,3,5,17,18,19,24	4,6,7,8,20,21,22,23	4	P22273,Q4G075,Q63617,Q99041
761	1,2,3,5,17,18,20,21	4,6,7,8,19,22,23,24	2	Q811M5,Q99041
762	1,2,3,5,17,18,20,22	4,6,7,8,19,21,23,24	4	P00714,P0C0A9,P22282,Q63474
763	1,2,3,5,17,18,20,23	4,6,7,8,19,21,22,24	4	P00714,P0C0A9,P22282,Q99041
764	1,2,3,5,17,18,20,24	4,6,7,8,19,21,22,23	2	Q811M5,Q99041
765	1,2,3,5,17,18,21,22	4,6,7,8,19,20,23,24	10	P00714,P02780,P08723,P0C0A9,P11598,P22273,P22282,P36374,Q63617,Q812E4
766	1,2,3,5,17,18,21,23	4,6,7,8,19,20,22,24	13	P00714,P02780,P06761,P08723,P0C0A9,P22006,P22273,P22282,P36374,Q63617,Q99041,Q9QW07,Q9QZK9
767	1,2,3,5,17,18,21,24	4,6,7,8,19,20,22,23	7	P02780,P22273,P22282,Q4G075,Q63617,Q6AY61,Q99041
768	1,2,3,5,17,18,22,23	4,6,7,8,19,20,21,24	25	P00714,P02780,P02781,P02782,P04905,P06761,P08723,P09456,P0C0A9,P11598,P22273,P22282,P22283,P24368,P30120,P36374,P46462,Q5M8C6,Q63617,Q812E4,Q99041,Q99MH3,Q9QW07,Q9R0T3,Q9Z2L0
769	1,2,3,5,17,18,22,24	4,6,7,8,19,20,21,23	14	P00714,P02780,P06761,P0C0A9,P11598,P22273,P22282,P22283,P36374,P46462,Q63617,Q812E4,Q99041,Q9WTT6
770	1,2,3,5,17,18,23,24	4,6,7,8,19,20,21,22	14	P00714,P02780,P06761,P0C0A9,P11598,P22273,P22282,P22283,P36374,P46462,Q4G075,Q63617,Q99041,Q9WTT6
771	1,2,3,5,17,19,20,21	4,6,7,8,18,22,23,24	4	P22006,Q6AY61,Q99041,Q9WVK7
772	1,2,3,5,17,19,20,22	4,6,7,8,18,21,23,24	1	O89117
773	1,2,3,5,17,19,20,23	4,6,7,8,18,21,22,24	2	P23739,Q99041
774	1,2,3,5,17,19,20,24	4,6,7,8,18,21,22,23	3	P54921,Q6AY61,Q99041
775	1,2,3,5,17,19,21,22	4,6,7,8,18,20,23,24	3	P01946,P02091,P22273
776	1,2,3,5,17,19,21,23	4,6,7,8,18,20,22,24	5	P02780,P12020,P22006,P22273,Q99041
777	1,2,3,5,17,19,21,24	4,6,7,8,18,20,22,23	5	P22273,P54921,Q4G075,Q6AY61,Q99041

778	1,2,3,5,17, 19,22,23	4,6,7,8,18, 20,21,24	12	P00714,P02091,P02780,P06761,P08723,P11598,P22273,P22282,P22283,Q5M8C6,Q63617,Q9QW07
779	1,2,3,5,17, 19,22,24	4,6,7,8,18, 20,21,23	6	P02780,P06761,P11598,P22273,P22282,Q63617
780	1,2,3,5,17, 19,23,24	4,6,7,8,18, 20,21,22	7	P02780,P06761,P11598,P22273,P22282,Q63617,Q99041
781	1,2,3,5,17, 20,21,22	4,6,7,8,18, 19,23,24	1	P63029
782	1,2,3,5,17, 20,21,23	4,6,7,8,18, 19,22,24	3	P22006,P63029,Q99041
783	1,2,3,5,17, 20,21,24	4,6,7,8,18, 19,22,23	3	Q6AY61,Q811M5,Q99041
784	1,2,3,5,17, 20,22,23	4,6,7,8,18, 19,21,24	9	P00714,P06761,P0C0A9,P22273,P22282,P23739,P63029,Q63617,Q99MH3
785	1,2,3,5,17, 20,22,24	4,6,7,8,18, 19,21,23	1	P22282
786	1,2,3,5,17, 20,23,24	4,6,7,8,18, 19,21,22	4	P00714,P22282,P23739,Q99041
787	1,2,3,5,17, 21,22,23	4,6,7,8,18, 19,20,24	13	P00714,P02780,P06761,P08723,P0C0A9,P11598,P22273,P22282,P22283,P36374,P46462,Q63617,Q9QW07
788	1,2,3,5,17, 21,22,24	4,6,7,8,18, 19,20,23	9	P02780,P06761,P0C0A9,P11598,P22273,P22282,Q4G075,Q63617,Q9R168
789	1,2,3,5,17, 21,23,24	4,6,7,8,18, 19,20,22	11	P02780,P06761,P0C0A9,P11598,P12020,P22273,P22282,Q4G075,Q63617,Q6AY61,Q99041
790	1,2,3,5,17, 22,23,24	4,6,7,8,18, 19,20,21	21	P00714,P02780,P04905,P06761,P07150,P0C0A9,P11598,P22273,P22282,P22283,P24368,P36374,P46462,P47727,Q5M8C6,Q63617,Q6P6R2,Q99041,Q9QW07,Q9R0T3,Q9WTT6
791	1,2,3,5,18, 19,20,21	4,6,7,8,17, 22,23,24	1	P47967
792	1,2,3,5,18, 19,20,22	4,6,7,8,17, 21,23,24	1	Q63474
793	1,2,3,5,18, 19,20,23	4,6,7,8,17, 21,22,24	1	Q99041
794	1,2,3,5,18, 19,20,24	4,6,7,8,17, 21,22,23	1	Q99041
795	1,2,3,5,18, 19,21,22	4,6,7,8,17, 20,23,24	2	P22273,Q4G075
796	1,2,3,5,18, 19,21,23	4,6,7,8,17, 20,22,24	6	P06911,P22006,P22273,Q4G075,Q99041,Q9Z0J6
797	1,2,3,5,18, 19,21,24	4,6,7,8,17, 20,22,23	3	P22273,Q4G075,Q99041

798	1,2,3,5,18,19,22,23	4,6,7,8,17,20,21,24	19	P00714,P02780,P02781,P02782,P06761,P08723,P09456,P11598,P22273,P22282,P22283,P30120,P46462,P47727,Q5M8C6,Q63617,Q99MH3,Q9WTT6,Q9Z2L0
799	1,2,3,5,18,19,22,24	4,6,7,8,17,20,21,23	6	P11598,P22273,P22282,Q4G075,Q63617,Q9WTT6
800	1,2,3,5,18,19,23,24	4,6,7,8,17,20,21,22	10	P02780,P06761,P11598,P22273,P22282,Q4G075,Q63617,Q99041,Q9WTT6,Q9Z0J6
801	1,2,3,5,18,20,21,22	4,6,7,8,17,19,23,24	0	
802	1,2,3,5,18,20,21,23	4,6,7,8,17,19,22,24	1	Q99041
803	1,2,3,5,18,20,21,24	4,6,7,8,17,19,22,23	1	Q99041
804	1,2,3,5,18,20,22,23	4,6,7,8,17,19,21,24	8	P00714,P06761,P22282,Q5QE79,Q63474,Q99MH3,Q9QX74,Q9Z2L0
805	1,2,3,5,18,20,22,24	4,6,7,8,17,19,21,23	3	P21674,P22282,Q9QX74
806	1,2,3,5,18,20,23,24	4,6,7,8,17,19,21,22	6	P00714,P20646,P22282,Q5QE79,Q99041,Q9QX74
807	1,2,3,5,18,21,22,23	4,6,7,8,17,19,20,24	19	P00714,P02780,P06761,P08723,P09456,P11598,P22273,P22282,P22283,P36374,P46462,P47727,Q4G075,Q5M8C6,Q63617,Q6RUV5,Q99MH3,Q9WTT6,Q9Z2L0
808	1,2,3,5,18,21,22,24	4,6,7,8,17,19,20,23	8	P06761,P20646,P22273,P22282,P36374,Q4G075,Q63617,Q9WTT6
809	1,2,3,5,18,21,23,24	4,6,7,8,17,19,20,22	13	O89117,P02780,P06761,P20646,P22273,P22282,P36374,Q4G075,Q63617,Q99041,Q9JJ50,Q9QZK9,Q9WTT6
810	1,2,3,5,18,22,23,24	4,6,7,8,17,19,20,21	31	D4A5U3,P00714,P02780,P04905,P06761,P07150,P08723,P0C0A9,P11598,P13676,P20646,P22273,P22282,P22283,P24368,P30120,P36374,P46462,P47727,Q4G075,Q5M8C6,Q5QE79,Q63617,Q6P6R2,Q6RUV5,Q99041,Q99MH3,Q9QX74,Q9R0T3,Q9WTT6,Q9Z2L0
811	1,2,3,5,19,20,21,22	4,6,7,8,17,18,23,24	1	P47967
812	1,2,3,5,19,20,21,23	4,6,7,8,17,18,22,24	0	
813	1,2,3,5,19,20,21,24	4,6,7,8,17,18,22,23	2	Q6AY61,Q99041
814	1,2,3,5,19,20,22,23	4,6,7,8,17,18,21,24	2	P23739,Q9QX74
815	1,2,3,5,19,20,22,24	4,6,7,8,17,18,21,23	1	Q9QX74
816	1,2,3,5,19,20,23,24	4,6,7,8,17,18,21,22	3	P23739,Q99041,Q9QX74
817	1,2,3,5,19,21,22,23	4,6,7,8,17,18,20,24	6	P06761,P06911,P12020,P22273,P22282,P47727

818	1,2,3,5,19,21,22,24	4,6,7,8,17,18,20,23	4	P06911,P22273,P30919,Q4G075
819	1,2,3,5,19,21,23,24	4,6,7,8,17,18,20,22	8	O70417,P06911,P08649,P12020,P22273,Q4G075,Q99041,Q9Z0J6
820	1,2,3,5,19,22,23,24	4,6,7,8,17,18,20,21	15	P00507,P02631,P02780,P06761,P06911,P11598,P22273,P22282,P22283,P47727,Q5M8C6,Q63617,Q6P6R2,Q9QX74,Q9WTT6
821	1,2,3,5,20,21,22,23	4,6,7,8,17,18,19,24	2	P08649,P22282
822	1,2,3,5,20,21,22,24	4,6,7,8,17,18,19,23	4	P07150,P08649,P21674,Q9QX74
823	1,2,3,5,20,21,23,24	4,6,7,8,17,18,19,22	3	P08649,P23739,Q99041
824	1,2,3,5,20,22,23,24	4,6,7,8,17,18,19,21	10	P00714,P06761,P07150,P20646,P22273,P22282,P23739,Q5QE79,Q6P6R2,Q9QX74
825	1,2,3,5,21,22,23,24	4,6,7,8,17,18,19,20	23	P02780,P06761,P06911,P07150,P08649,P11598,P12020,P20646,P22273,P22282,P22283,P30919,P36374,P46462,P47727,Q4G075,Q62714,Q63617,Q6P6R2,Q6RUV5,Q9QX74,Q9R0T3,Q9WTT6
826	1,2,3,6,7,8,17,18	4,5,19,20,21,22,23,24	7	O54800;Q5DWV2,O70594,P20766,P38918,P97580,P97615,Q63598
827	1,2,3,6,7,8,17,19	4,5,18,20,21,22,23,24	6	iRT-Kit_WR_fusion,O54800;Q5DWV2,O70594,P50115,P97580,Q66H69
828	1,2,3,6,7,8,17,20	4,5,18,19,21,22,23,24	15	D4A5U3,iRT-Kit_WR_fusion,O54800;Q5DWV2,O70417,P05964,P06911,P12020,P13676,P19629,P31044,P50280,P55091,P63029,Q5GRG2,Q9JHB9
829	1,2,3,6,7,8,17,21	4,5,18,19,20,22,23,24	9	iRT-Kit_WR_fusion,O54800;Q5DWV2,O70594,P02631,P04762,P05539,P19629,Q00715,Q63598
830	1,2,3,6,7,8,17,22	4,5,18,19,20,21,23,24	11	iRT-Kit_WR_fusion,O54800;Q5DWV2,P01946,P02091,P15399,P23593,P35280,P97580,Q09030,Q30KJ2,Q63598
831	1,2,3,6,7,8,17,23	4,5,18,19,20,21,22,24	8	iRT-Kit_WR_fusion,O70594,P19629,P38918,P46720,Q09030,Q63598,Q99MH3
832	1,2,3,6,7,8,17,24	4,5,18,19,20,21,22,23	5	iRT-Kit_WR_fusion,O54800;Q5DWV2,O70594,P23593,Q63598
833	1,2,3,6,7,8,18,19	4,5,17,20,21,22,23,24	8	iRT-Kit_WR_fusion,O54800;Q5DWV2,P19629,P20766,P31044,P97580,P97615,Q66H69

834	1,2,3,6,7,8,18,20	4,5,17,19,21,22,23,24	13	iRT-Kit_WR_fusion,O54800;Q5DWV2,P06911,P12020,P19629,P20766,P31044,P50280,P69897,P97615,Q5GRG2,Q91ZS3,Q9JHB9
835	1,2,3,6,7,8,18,21	4,5,17,19,20,22,23,24	12	iRT-Kit_WR_fusion,O54800;Q5DWV2,O70594,P02625,P02631,P18757,P19629,P20766,P46844,Q00715,Q63598,Q91ZS3
836	1,2,3,6,7,8,18,22	4,5,17,19,20,21,23,24	12	iRT-Kit_WR_fusion,O54800;Q5DWV2,P15399,P19629,P20766,P23593,P35280,P80202,P97580,Q09030,Q30KJ2,Q91ZS3
837	1,2,3,6,7,8,18,23	4,5,17,19,20,21,22,24	16	iRT-Kit_WR_fusion,O54800;Q5DWV2,O70594,P19629,P20766,P38918,P46844,P80202,Q09030,Q3ZAV1,Q62894,Q63532,Q63598,Q91ZS3,Q99MH3,Q9WTW7
838	1,2,3,6,7,8,18,24	4,5,17,19,20,21,22,23	7	O54800;Q5DWV2,P20766,P46844,P97615,Q3ZAV1,Q63598,Q91ZS3
839	1,2,3,6,7,8,19,20	4,5,17,18,21,22,23,24	12	iRT-Kit_WR_fusion,P06911,P12020,P19629,P31044,P50280,Q5GRG2,Q66H69,Q68G31,Q6P9T8,Q9JI85,Q9QW07
840	1,2,3,6,7,8,19,21	4,5,17,18,20,22,23,24	8	iRT-Kit_WR_fusion,P02631,P11883,P19629,P97580,Q00715,Q66H69,Q9JI85
841	1,2,3,6,7,8,19,22	4,5,17,18,20,21,23,24	6	iRT-Kit_WR_fusion,P15399,P19629,P35280,P97580,Q30KJ2
842	1,2,3,6,7,8,19,23	4,5,17,18,20,21,22,24	8	iRT-Kit_WR_fusion,O70594,P19629,P42854,P97580,Q09030,Q99MH3,Q9Z0J6
843	1,2,3,6,7,8,19,24	4,5,17,18,20,21,22,23	3	iRT-Kit_WR_fusion,Q66H69,Q68G31
844	1,2,3,6,7,8,20,21	4,5,17,18,19,22,23,24	19	D4A5U3,iRT-Kit_WR_fusion,P01835,P02631,P05964,P06911,P12020,P19629,P31044,P47967,P50280,Q00715,Q5GRG2,Q5RKI1,Q78P75,Q812E4,Q91ZS3,Q9JHB9,Q9JI85
845	1,2,3,6,7,8,20,22	4,5,17,18,19,21,23,24	14	iRT-Kit_WR_fusion,O54800;Q5DWV2,P06911,P12020,P15399,P19629,P23593,P31044,P35280,P50280,P97580,Q5GRG2,Q6P9T8,Q91ZS3
846	1,2,3,6,7,8,20,23	4,5,17,18,19,21,22,24	12	iRT-Kit_WR_fusion,P06911,P09656,P12020,P19629,P31044,P50280,Q05702,Q09030,Q5GRG2,Q62894,Q99MH3

847	1,2,3,6,7,8,20,24	4,5,17,18,19,21,22,23	10	iRT-Kit_WR_fusion,P06911,P12020,P19629,P31044,P50280,Q5GRG2,Q91ZS3,Q9JHB9,Q9QW07
848	1,2,3,6,7,8,21,22	4,5,17,18,19,20,23,24	8	iRT-Kit_WR_fusion,P02631,P11883,P19629,P35280,P97580,Q09030,Q62714
849	1,2,3,6,7,8,21,23	4,5,17,18,19,20,22,24	12	iRT-Kit_WR_fusion,O70594,P02631,P02783,P09656,P19629,P46844,Q09030,Q62714,Q63532,Q63598,Q99MH3
850	1,2,3,6,7,8,21,24	4,5,17,18,19,20,22,23	11	iRT-Kit_WR_fusion,P02625,P02631,P04762,P19629,P30919,P36860,P46844,Q09030,Q62714,Q63598
851	1,2,3,6,7,8,22,23	4,5,17,18,19,20,21,24	14	iRT-Kit_WR_fusion,P02783,P09656,P19629,P23593,P35280,P80202,P97580,Q09030,Q62714,Q62894,Q63532,Q63598,Q99MH3
852	1,2,3,6,7,8,22,24	4,5,17,18,19,20,21,23	10	iRT-Kit_WR_fusion,O54800;Q5DWV2,P15399,P23593,P35280,P97580,Q09030,Q62714,Q63598,Q6P6R2
853	1,2,3,6,7,8,23,24	4,5,17,18,19,20,21,22	11	iRT-Kit_WR_fusion,P19629,P46844,P80202,Q09030,Q3ZAV1,Q62714,Q63532,Q63598,Q6P6R2,Q99MH3
854	1,2,3,6,7,17,18,19	4,5,8,20,21,22,23,24	2	O54800;Q5DWV2,P20766
855	1,2,3,6,7,17,18,20	4,5,8,19,21,22,23,24	4	O54800;Q5DWV2,P55091,P63029,Q5GRG2
856	1,2,3,6,7,17,18,21	4,5,8,19,20,22,23,24	4	O54800;Q5DWV2,P01835,P05539,P23739
857	1,2,3,6,7,17,18,22	4,5,8,19,20,21,23,24	4	O54800;Q5DWV2,P01946,P02091,P23739
858	1,2,3,6,7,17,18,23	4,5,8,19,20,21,22,24	5	O54800;Q5DWV2,O70594,P20766,Q63598,Q99MH3
859	1,2,3,6,7,17,18,24	4,5,8,19,20,21,22,23	5	O54800;Q5DWV2,P01039,P20766,Q63598,Q6AY61
860	1,2,3,6,7,17,19,20	4,5,8,18,21,22,23,24	2	O89117,P63029
861	1,2,3,6,7,17,19,21	4,5,8,18,20,22,23,24	4	P05539,Q5QE79,Q6AY61,Q9R168
862	1,2,3,6,7,17,19,22	4,5,8,18,20,21,23,24	4	O89117,P01946,P02091,Q9R168
863	1,2,3,6,7,17,19,23	4,5,8,18,20,21,22,24	0	

864	1,2,3,6,7,17,19,24	4,5,8,18,20,21,22,23	2	P54921,Q6AY61
865	1,2,3,6,7,17,20,21	4,5,8,18,19,22,23,24	6	P01835,P05539,P05964,P19629,P63029,Q6AY61
866	1,2,3,6,7,17,20,22	4,5,8,18,19,21,23,24	5	O54800;Q5DWV2,O89117,P01946,P02091,P63029
867	1,2,3,6,7,17,20,23	4,5,8,18,19,21,22,24	3	P63029,Q62894,Q99MH3
868	1,2,3,6,7,17,20,24	4,5,8,18,19,21,22,23	3	O54800;Q5DWV2,P63029,Q6AY61
869	1,2,3,6,7,17,21,22	4,5,8,18,19,20,23,24	5	P01835,P01946,P02091,P05539,Q9R168
870	1,2,3,6,7,17,21,23	4,5,8,18,19,20,22,24	3	P01835,P09656,Q9R168
871	1,2,3,6,7,17,21,24	4,5,8,18,19,20,22,23	7	P01039,P01835,P04762,P05539,P36860,Q6AY61,Q9R168
872	1,2,3,6,7,17,22,23	4,5,8,18,19,20,21,24	5	P01946,P02091,P02783,Q99MH3,Q9R168
873	1,2,3,6,7,17,22,24	4,5,8,18,19,20,21,23	4	O54800;Q5DWV2,P01946,P02091,Q9R168
874	1,2,3,6,7,17,23,24	4,5,8,18,19,20,21,22	1	Q63598
875	1,2,3,6,7,18,19,20	4,5,8,17,21,22,23,24	2	O54800;Q5DWV2,P20766
876	1,2,3,6,7,18,19,21	4,5,8,17,20,22,23,24	3	O54800;Q5DWV2,P20766,P23739
877	1,2,3,6,7,18,19,22	4,5,8,17,20,21,23,24	3	O54800;Q5DWV2,P20766,P23739
878	1,2,3,6,7,18,19,23	4,5,8,17,20,21,22,24	3	O54800;Q5DWV2,P20766,Q99MH3
879	1,2,3,6,7,18,19,24	4,5,8,17,20,21,22,23	2	O54800;Q5DWV2,P20766
880	1,2,3,6,7,18,20,21	4,5,8,17,19,22,23,24	4	O54800;Q5DWV2,P01835,P19629,P47967
881	1,2,3,6,7,18,20,22	4,5,8,17,19,21,23,24	2	O54800;Q5DWV2,P01835
882	1,2,3,6,7,18,20,23	4,5,8,17,19,21,22,24	5	O54800;Q5DWV2,P01835,P20766,Q62894,Q99MH3
883	1,2,3,6,7,18,20,24	4,5,8,17,19,21,22,23	3	O54800;Q5DWV2,P01835,P20766

884	1,2,3,6,7,18,21,22	4,5,8,17,19,20,23,24	3 O54800;Q5DWV2,P01835,P23739
885	1,2,3,6,7,18,21,23	4,5,8,17,19,20,22,24	5 O54800;Q5DWV2,P01835,P09656,P20766,Q99MH3
886	1,2,3,6,7,18,21,24	4,5,8,17,19,20,22,23	4 O54800;Q5DWV2,P01835,P20766,P23739
887	1,2,3,6,7,18,22,23	4,5,8,17,19,20,21,24	9 O54800;Q5DWV2,P01835,P02783,P06760,P30120,P36374,Q6P6R2,Q99MH3,Q9EQS0
888	1,2,3,6,7,18,22,24	4,5,8,17,19,20,21,23	6 O54800;Q5DWV2,P01835,P06760,P20766,P23739,Q6P6R2
889	1,2,3,6,7,18,23,24	4,5,8,17,19,20,21,22	7 O54800;Q5DWV2,P01835,P06760,P20766,Q63598,Q6P6R2,Q99MH3
890	1,2,3,6,7,19,20,21	4,5,8,17,18,22,23,24	5 P01835,P05369,P19629,P47967,Q63617
891	1,2,3,6,7,19,20,22	4,5,8,17,18,21,23,24	0
892	1,2,3,6,7,19,20,23	4,5,8,17,18,21,22,24	1 P19629
893	1,2,3,6,7,19,20,24	4,5,8,17,18,21,22,23	1 Q68G31
894	1,2,3,6,7,19,21,22	4,5,8,17,18,20,23,24	3 P01835,P01946,Q9R168
895	1,2,3,6,7,19,21,23	4,5,8,17,18,20,22,24	1 P01835
896	1,2,3,6,7,19,21,24	4,5,8,17,18,20,22,23	2 P01835,Q6AY61
897	1,2,3,6,7,19,22,23	4,5,8,17,18,20,21,24	2 P02783,Q99MH3
898	1,2,3,6,7,19,22,24	4,5,8,17,18,20,21,23	0
899	1,2,3,6,7,19,23,24	4,5,8,17,18,20,21,22	0
900	1,2,3,6,7,20,21,22	4,5,8,17,18,19,23,24	2 P01835,P19629
901	1,2,3,6,7,20,21,23	4,5,8,17,18,19,22,24	3 P01835,P09656,P19629
902	1,2,3,6,7,20,21,24	4,5,8,17,18,19,22,23	3 P01835,P04762,Q6AY61
903	1,2,3,6,7,20,22,23	4,5,8,17,18,19,21,24	6 P01835,P02783,P09656,P19629,Q62894,Q99MH3

904	1,2,3,6,7,20,22,24	4,5,8,17,18,19,21,23	3	P01835,P22006,Q9QX74
905	1,2,3,6,7,20,23,24	4,5,8,17,18,19,21,22	4	P01835,P09656,Q62894,Q9QX74
906	1,2,3,6,7,21,22,23	4,5,8,17,18,19,20,24	7	P01835,P02783,P09656,Q09030,Q62714,Q99MH3,Q9R168
907	1,2,3,6,7,21,22,24	4,5,8,17,18,19,20,23	3	P01835,Q62714,Q9R168
908	1,2,3,6,7,21,23,24	4,5,8,17,18,19,20,22	4	P01835,P09656,P36860,Q62714
909	1,2,3,6,7,22,23,24	4,5,8,17,18,19,20,21	9	P01835,P02783,P06760,P09656,Q09030,Q62714,Q6P6R2,Q99MH3,Q9QX74
910	1,2,3,6,8,17,18,19	4,5,7,20,21,22,23,24	5	O54800;Q5DWV2,P08649,P97580,P97615,Q06000
911	1,2,3,6,8,17,18,20	4,5,7,19,21,22,23,24	7	O54800;Q5DWV2,P06911,P08649,P12020,P55091,P63029,Q5GRG2
912	1,2,3,6,8,17,18,21	4,5,7,19,20,22,23,24	2	O54800;Q5DWV2,P97580
913	1,2,3,6,8,17,18,22	4,5,7,19,20,21,23,24	7	O54800;Q5DWV2,P01946,P02091,P08649,P15399,P97580,Q30KJ2
914	1,2,3,6,8,17,18,23	4,5,7,19,20,21,22,24	5	O54800;Q5DWV2,O70594,P97580,Q63598,Q99MH3
915	1,2,3,6,8,17,18,24	4,5,7,19,20,21,22,23	2	O54800;Q5DWV2,Q63598
916	1,2,3,6,8,17,19,20	4,5,7,18,21,22,23,24	7	O89117,P06911,P63029,P97580,Q5GRG2,Q68G31,Q9JI85
917	1,2,3,6,8,17,19,21	4,5,7,18,20,22,23,24	4	P97580,Q66H69,Q68G31,Q9WVK7
918	1,2,3,6,8,17,19,22	4,5,7,18,20,21,23,24	6	O89117,P01946,P02091,P15399,P97580,Q30KJ2
919	1,2,3,6,8,17,19,23	4,5,7,18,20,21,22,24	2	P97580,Q99MH3
920	1,2,3,6,8,17,19,24	4,5,7,18,20,21,22,23	3	P97580,P97840,Q68G31
921	1,2,3,6,8,17,20,21	4,5,7,18,19,22,23,24	6	D4A5U3,P06911,P19629,P63029,Q5GRG2,Q9JI85
922	1,2,3,6,8,17,20,22	4,5,7,18,19,21,23,24	9	O89117,P01946,P02091,P06911,P12020,P15399,P63029,P97580,Q5GRG2
923	1,2,3,6,8,17,20,23	4,5,7,18,19,21,22,24	7	P06911,P17559,P19629,P36376,P63029,Q5GRG2,Q99MH3

924	1,2,3,6,8,17,20,24	4,5,7,18,19,21,22,23	4	P06911,P63029,Q5GRG2,Q68G31
925	1,2,3,6,8,17,21,22	4,5,7,18,19,20,23,24	3	P01946,P02091,P97580
926	1,2,3,6,8,17,21,23	4,5,7,18,19,20,22,24	3	P09656,Q09030,Q99MH3
927	1,2,3,6,8,17,21,24	4,5,7,18,19,20,22,23	0	
928	1,2,3,6,8,17,22,23	4,5,7,18,19,20,21,24	8	P01946,P02091,P02783,P97580,P97840,Q09030,Q63532,Q99MH3
929	1,2,3,6,8,17,22,24	4,5,7,18,19,20,21,23	6	P01946,P02091,P15399,P23593,P97580,P97840
930	1,2,3,6,8,17,23,24	4,5,7,18,19,20,21,22	4	P97840,Q09030,Q63598,Q99MH3
931	1,2,3,6,8,18,19,20	4,5,7,17,21,22,23,24	10	O54800;Q5DWV2,P12020,P15399,P50280,P97580,P97615,Q5GRG2,Q68G31,Q9JI85,Q9R168
932	1,2,3,6,8,18,19,21	4,5,7,17,20,22,23,24	4	O54800;Q5DWV2,P97580,Q66H69,Q68G31
933	1,2,3,6,8,18,19,22	4,5,7,17,20,21,23,24	4	O54800;Q5DWV2,P15399,P97580,Q30KJ2
934	1,2,3,6,8,18,19,23	4,5,7,17,20,21,22,24	4	O54800;Q5DWV2,P97580,Q99MH3,Q9Z0J6
935	1,2,3,6,8,18,19,24	4,5,7,17,20,21,22,23	3	O54800;Q5DWV2,P97580,Q68G31
936	1,2,3,6,8,18,20,21	4,5,7,17,19,22,23,24	9	O54800;Q5DWV2,P02631,P12020,P19629,P25809,Q5GRG2,Q68G31,Q91ZS3,Q9JI85
937	1,2,3,6,8,18,20,22	4,5,7,17,19,21,23,24	8	O54800;Q5DWV2,P12020,P15399,P97580,Q30KJ2,Q5GRG2,Q91ZS3,Q99MH3
938	1,2,3,6,8,18,20,23	4,5,7,17,19,21,22,24	6	O54800;Q5DWV2,P19629,P36376,Q5GRG2,Q99MH3,Q9R168
939	1,2,3,6,8,18,20,24	4,5,7,17,19,21,22,23	6	O54800;Q5DWV2,P12020,Q5GRG2,Q68G31,Q91ZS3,Q9R168
940	1,2,3,6,8,18,21,22	4,5,7,17,19,20,23,24	6	O54800;Q5DWV2,P02625,P23739,P54921,P97580,Q30KJ2
941	1,2,3,6,8,18,21,23	4,5,7,17,19,20,22,24	6	O54800;Q5DWV2,P09656,P54921,Q63532,Q99MH3,Q9Z0J6
942	1,2,3,6,8,18,21,24	4,5,7,17,19,20,22,23	3	O54800;Q5DWV2,P02625,Q68G31
943	1,2,3,6,8,18,22,23	4,5,7,17,19,20,21,24	10	O54800;Q5DWV2,P02783,P36374,P54921,P80202,P97580,Q09030,Q30KJ2,Q63532,Q99MH3

944	1,2,3,6,8,18,22,24	4,5,7,17,19,20,21,23	7	O54800;Q5DWV2,P06760,P15399,P20646,P97580,Q30KJ2,Q6P6R2
945	1,2,3,6,8,18,23,24	4,5,7,17,19,20,21,22	6	O54800;Q5DWV2,P06760,P20646,Q63598,Q99MH3,Q9Z0J6
946	1,2,3,6,8,19,20,21	4,5,7,17,18,22,23,24	5	P05369,P19629,Q5GRG2,Q68G31,Q9JI85
947	1,2,3,6,8,19,20,22	4,5,7,17,18,21,23,24	5	P15399,P19629,P97580,Q5GRG2,Q68G31
948	1,2,3,6,8,19,20,23	4,5,7,17,18,21,22,24	4	P19629,P36376,Q68G31,Q99MH3
949	1,2,3,6,8,19,20,24	4,5,7,17,18,21,22,23	4	P15399,Q5GRG2,Q68G31,Q9JI85
950	1,2,3,6,8,19,21,22	4,5,7,17,18,20,23,24	5	P01946,P02091,P15399,P30919,P97580
951	1,2,3,6,8,19,21,23	4,5,7,17,18,20,22,24	1	Q9Z0J6
952	1,2,3,6,8,19,21,24	4,5,7,17,18,20,22,23	2	P30919,Q68G31
953	1,2,3,6,8,19,22,23	4,5,7,17,18,20,21,24	8	P00507,P01946,P02783,P15399,P97580,Q63532,Q99MH3,Q9Z0J6
954	1,2,3,6,8,19,22,24	4,5,7,17,18,20,21,23	5	P00507,P15399,P30919,P97580,Q68G31
955	1,2,3,6,8,19,23,24	4,5,7,17,18,20,21,22	2	Q68G31,Q9Z0J6
956	1,2,3,6,8,20,21,22	4,5,7,17,18,19,23,24	3	P15399,P19629,P97580
957	1,2,3,6,8,20,21,23	4,5,7,17,18,19,22,24	3	P09656,P19629,Q99MH3
958	1,2,3,6,8,20,21,24	4,5,7,17,18,19,22,23	4	P19629,Q5GRG2,Q68G31,Q9JI85
959	1,2,3,6,8,20,22,23	4,5,7,17,18,19,21,24	9	P02783,P09656,P15399,P19629,P36376,P97580,Q09030,Q63532,Q99MH3
960	1,2,3,6,8,20,22,24	4,5,7,17,18,19,21,23	4	P12020,P15399,P97580,Q5GRG2
961	1,2,3,6,8,20,23,24	4,5,7,17,18,19,21,22	3	P09656,P19629,Q99MH3
962	1,2,3,6,8,21,22,23	4,5,7,17,18,19,20,24	9	P02783,P09656,P30919,P54921,P97580,Q09030,Q62714,Q63532,Q99MH3
963	1,2,3,6,8,21,22,24	4,5,7,17,18,19,20,23	3	P30919,P97580,Q62714

964	1,2,3,6,8,21,23,24	4,5,7,17,18,19,20,22	7	P09656,P20646,P30919,Q09030,Q62714,Q63532,Q9Z0J6
965	1,2,3,6,8,22,23,24	4,5,7,17,18,19,20,21	14	P00507,P02783,P06760,P09656,P20646,P30919,P80202,P97580,P97840,Q09030,Q62714,Q63532,Q6P6R2,Q99MH3
966	1,2,3,6,17,18,19,20	4,5,7,8,21,22,23,24	4	O54800;Q5DWV2,P39069,P63029,Q06000
967	1,2,3,6,17,18,19,21	4,5,7,8,20,22,23,24	3	O54800;Q5DWV2,P23739,Q4G075
968	1,2,3,6,17,18,19,22	4,5,7,8,20,21,23,24	6	O54800;Q5DWV2,P01946,P02091,P06760,P22273,Q06000
969	1,2,3,6,17,18,19,23	4,5,7,8,20,21,22,24	4	O54800;Q5DWV2,P06760,P22273,Q99MH3
970	1,2,3,6,17,18,19,24	4,5,7,8,20,21,22,23	3	O54800;Q5DWV2,P06760,Q4G075
971	1,2,3,6,17,18,20,21	4,5,7,8,19,22,23,24	2	O54800;Q5DWV2,P63029
972	1,2,3,6,17,18,20,22	4,5,7,8,19,21,23,24	5	O54800;Q5DWV2,P01946,P02091,P06760,P63029
973	1,2,3,6,17,18,20,23	4,5,7,8,19,21,22,24	4	O54800;Q5DWV2,P06760,P63029,Q99MH3
974	1,2,3,6,17,18,20,24	4,5,7,8,19,21,22,23	3	O54800;Q5DWV2,P06760,P63029
975	1,2,3,6,17,18,21,22	4,5,7,8,19,20,23,24	5	O54800;Q5DWV2,P01946,P02091,P23739,Q4G075
976	1,2,3,6,17,18,21,23	4,5,7,8,19,20,22,24	3	O54800;Q5DWV2,Q4G075,Q99MH3
977	1,2,3,6,17,18,21,24	4,5,7,8,19,20,22,23	2	O54800;Q5DWV2,Q4G075
978	1,2,3,6,17,18,22,23	4,5,7,8,19,20,21,24	16	O54800;Q5DWV2,P01946,P02091,P02780,P02783,P06760,P22273,P22282,P30120,P36374,P55159,Q63617,Q812E4,Q99MH3,Q9EQS0,Q9WTT6
979	1,2,3,6,17,18,22,24	4,5,7,8,19,20,21,23	9	D3ZUC6,O54800;Q5DWV2,P01946,P02091,P06760,P22273,Q4G075,Q63617,Q9WTT6
980	1,2,3,6,17,18,23,24	4,5,7,8,19,20,21,22	8	O54800;Q5DWV2,P02780,P06760,P22273,Q4G075,Q63617,Q99MH3,Q9WTT6
981	1,2,3,6,17,19,20,21	4,5,7,8,18,22,23,24	4	P49134,P63029,Q68G31,Q9WVK7
982	1,2,3,6,17,19,20,22	4,5,7,8,18,21,23,24	4	O89117,P01946,P02091,P63029
983	1,2,3,6,17,19,20,23	4,5,7,8,18,21,22,24	2	O89117,P63029

984	1,2,3,6,17,19,20,24	4,5,7,8,18,21,22,23	3	O89117,P63029,Q68G31
985	1,2,3,6,17,19,21,22	4,5,7,8,18,20,23,24	3	P01946,P02091,Q4G075
986	1,2,3,6,17,19,21,23	4,5,7,8,18,20,22,24	1	Q4G075
987	1,2,3,6,17,19,21,24	4,5,7,8,18,20,22,23	3	Q4G075,Q68G31,Q6AY61
988	1,2,3,6,17,19,22,23	4,5,7,8,18,20,21,24	7	O89117,P01946,P02091,P02783,P06760,P22273,Q99MH3
989	1,2,3,6,17,19,22,24	4,5,7,8,18,20,21,23	7	D3ZUC6,O89117,P01946,P02091,P06760,P22273,Q4G075
990	1,2,3,6,17,19,23,24	4,5,7,8,18,20,21,22	3	P06760,P22273,Q4G075
991	1,2,3,6,17,20,21,22	4,5,7,8,18,19,23,24	3	P01946,P02091,P63029
992	1,2,3,6,17,20,21,23	4,5,7,8,18,19,22,24	2	P09656,P63029
993	1,2,3,6,17,20,21,24	4,5,7,8,18,19,22,23	2	P63029,Q6AY61
994	1,2,3,6,17,20,22,23	4,5,7,8,18,19,21,24	8	O89117,P01946,P02091,P02783,P06760,P09656,P63029,Q99MH3
995	1,2,3,6,17,20,22,24	4,5,7,8,18,19,21,23	5	O89117,P01946,P02091,P06760,P63029
996	1,2,3,6,17,20,23,24	4,5,7,8,18,19,21,22	2	P06760,P63029
997	1,2,3,6,17,21,22,23	4,5,7,8,18,19,20,24	8	P01946,P02091,P02783,P09656,P22282,Q4G075,Q99MH3,Q9R168
998	1,2,3,6,17,21,22,24	4,5,7,8,18,19,20,23	4	P01946,P02091,Q4G075,Q9R168
999	1,2,3,6,17,21,23,24	4,5,7,8,18,19,20,22	2	P09656,Q4G075
1000	1,2,3,6,17,22,23,24	4,5,7,8,18,19,20,21	17	D3ZUC6,P01946,P02091,P02780,P02783,P06760,P07150,P22273,P22282,P47967,P55159,P97840,Q4G075,Q5I0D1,Q63617,Q99MH3,Q9WTT6
1001	1,2,3,6,18,19,20,21	4,5,7,8,17,22,23,24	3	O54800;Q5DWV2,P39069,Q68G31
1002	1,2,3,6,18,19,20,22	4,5,7,8,17,21,23,24	3	O54800;Q5DWV2,P06760,P39069
1003	1,2,3,6,18,19,20,23	4,5,7,8,17,21,22,24	4	O54800;Q5DWV2,P06760,P39069,Q99MH3

1004	1,2,3,6,18, 19,20,24	4,5,7,8,17, 21,22,23	4	O54800;Q5DWV2,P06760,Q68G31,Q9QX74
1005	1,2,3,6,18, 19,21,22	4,5,7,8,17, 20,23,24	4	O54800;Q5DWV2,P01946,P23739,Q4G075
1006	1,2,3,6,18, 19,21,23	4,5,7,8,17, 20,22,24	3	O54800;Q5DWV2,Q4G075,Q9Z0J6
1007	1,2,3,6,18, 19,21,24	4,5,7,8,17, 20,22,23	3	O54800;Q5DWV2,Q4G075,Q68G31
1008	1,2,3,6,18, 19,22,23	4,5,7,8,17, 20,21,24	10	O54800;Q5DWV2,P02783,P06760,P22273,Q4G075,Q811M5,Q99MH3,Q9EQS0,Q9QX74,Q9WT T6
1009	1,2,3,6,18, 19,22,24	4,5,7,8,17, 20,21,23	6	O54800;Q5DWV2,P06760,P22273,Q4G075,Q9QX74,Q9WTT6
1010	1,2,3,6,18, 19,23,24	4,5,7,8,17, 20,21,22	7	O54800;Q5DWV2,P06760,P22273,Q4G075,Q9QX74,Q9WTT6,Q9Z0J6
1011	1,2,3,6,18, 20,21,22	4,5,7,8,17, 19,23,24	2	O54800;Q5DWV2,P01835
1012	1,2,3,6,18, 20,21,23	4,5,7,8,17, 19,22,24	4	O54800;Q5DWV2,P01835,P09656,Q99MH3
1013	1,2,3,6,18, 20,21,24	4,5,7,8,17, 19,22,23	3	O54800;Q5DWV2,P01835,Q68G31
1014	1,2,3,6,18, 20,22,23	4,5,7,8,17, 19,21,24	7	O54800;Q5DWV2,P02783,P04355,P06760,P09656,Q99MH3,Q9QX74
1015	1,2,3,6,18, 20,22,24	4,5,7,8,17, 19,21,23	6	O54800;Q5DWV2,P06760,P20646,P22006,Q9QX74,Q9Z0V6
1016	1,2,3,6,18, 20,23,24	4,5,7,8,17, 19,21,22	6	O54800;Q5DWV2,P06760,P20646,Q5QE79,Q99MH3,Q9QX74
1017	1,2,3,6,18, 21,22,23	4,5,7,8,17, 19,20,24	11	O54800;Q5DWV2,P01835,P02783,P09656,P13676,P20646,P36374,P54921,Q4G075,Q99MH3,Q 9WTT6
1018	1,2,3,6,18, 21,22,24	4,5,7,8,17, 19,20,23	6	O54800;Q5DWV2,P01835,P20646,P23739,Q4G075,Q9WTT6
1019	1,2,3,6,18, 21,23,24	4,5,7,8,17, 19,20,22	7	O54800;Q5DWV2,P01835,P09656,P20646,Q4G075,Q9WTT6,Q9Z0J6
1020	1,2,3,6,18, 22,23,24	4,5,7,8,17, 19,20,21	18	D4A5U3,O54800;Q5DWV2,P02783,P06760,P07150,P13676,P20646,P22273,P22282,P36374,P55 159,Q4G075,Q63617,Q6P6R2,Q99MH3,Q9EQS0,Q9QX74,Q9WTT6
1021	1,2,3,6,19, 20,21,22	4,5,7,8,17, 18,23,24	1	P01946
1022	1,2,3,6,19, 20,21,23	4,5,7,8,17, 18,22,24	1	P09656
1023	1,2,3,6,19, 20,21,24	4,5,7,8,17, 18,22,23	1	Q68G31

1024	1,2,3,6,19,20,22,23	4,5,7,8,17,18,21,24	5	P02783,P06760,P09656,Q99MH3,Q9QX74
1025	1,2,3,6,19,20,22,24	4,5,7,8,17,18,21,23	5	P06760,P22006,Q68G31,Q9QX74,Q9Z0V6
1026	1,2,3,6,19,20,23,24	4,5,7,8,17,18,21,22	3	P06760,Q68G31,Q9QX74
1027	1,2,3,6,19,21,22,23	4,5,7,8,17,18,20,24	7	P01946,P02091,P02783,P09656,Q4G075,Q99MH3,Q9Z0J6
1028	1,2,3,6,19,21,22,24	4,5,7,8,17,18,20,23	5	P01946,P02091,P30919,Q4G075,Q9WTT6
1029	1,2,3,6,19,21,23,24	4,5,7,8,17,18,20,22	3	P09656,Q4G075,Q9Z0J6
1030	1,2,3,6,19,22,23,24	4,5,7,8,17,18,20,21	13	P00507,P01946,P02783,P06760,P13676,P20646,P22273,Q4G075,Q6P6R2,Q811M5,Q9QX74,Q9WTT6,Q9Z0J6
1031	1,2,3,6,20,21,22,23	4,5,7,8,17,18,19,24	5	P01835,P02783,P09656,Q99MH3,Q9QX74
1032	1,2,3,6,20,21,22,24	4,5,7,8,17,18,19,23	3	P01835,P20646,Q9QX74
1033	1,2,3,6,20,21,23,24	4,5,7,8,17,18,19,22	4	P01835,P09656,P20646,Q9QX74
1034	1,2,3,6,20,22,23,24	4,5,7,8,17,18,19,21	11	P02783,P06760,P07150,P09656,P20646,P22006,Q5QE79,Q6P6R2,Q99MH3,Q9QX74,Q9WTT6
1035	1,2,3,6,21,22,23,24	4,5,7,8,17,18,19,20	16	P01835,P01946,P02783,P06760,P07150,P09656,P13676,P20646,P22282,P30919,P36374,Q4G075,Q62714,Q99MH3,Q9QX74,Q9WTT6
1036	1,2,3,7,8,17,18,19	4,5,6,20,21,22,23,24	4	P20766,P22006,Q66H69,Q811M5
1037	1,2,3,7,8,17,18,20	4,5,6,19,21,22,23,24	7	D4A5U3,P06911,P13676,P20766,P25809,Q5GRG2,Q811M5
1038	1,2,3,7,8,17,18,21	4,5,6,19,20,22,23,24	5	iRT-Kit_WR_fusion,P20766,P22006,Q00715,Q811M5
1039	1,2,3,7,8,17,18,22	4,5,6,19,20,21,23,24	1	P35280
1040	1,2,3,7,8,17,18,23	4,5,6,19,20,21,22,24	5	O70594,P20766,P22006,Q63598,Q64093
1041	1,2,3,7,8,17,18,24	4,5,6,19,20,21,22,23	4	P20766,Q63598,Q64093,Q811M5
1042	1,2,3,7,8,17,19,20	4,5,6,18,21,22,23,24	6	D4A5U3,iRT-Kit_WR_fusion,P13676,Q66H69,Q811M5,Q9WTT6
1043	1,2,3,7,8,17,19,21	4,5,6,18,20,22,23,24	10	iRT-Kit_WR_fusion,P04355,P11883,P22006,P62804,Q00715,Q66H69,Q811M5,Q9QX74,Q9WVK7

1044	1,2,3,7,8,17,19,22	4,5,6,18,20,21,23,24	3	iRT-Kit_WR_fusion,P35280,Q66H69
1045	1,2,3,7,8,17,19,23	4,5,6,18,20,21,22,24	4	iRT-Kit_WR_fusion,P21674,P22006,Q66H69
1046	1,2,3,7,8,17,19,24	4,5,6,18,20,21,22,23	3	iRT-Kit_WR_fusion,P54921,Q66H69
1047	1,2,3,7,8,17,20,21	4,5,6,18,19,22,23,24	9	D4A5U3,iRT-Kit_WR_fusion,P13676,P25809,P62804,P63029,Q00715,Q811M5,Q9WTT6
1048	1,2,3,7,8,17,20,22	4,5,6,18,19,21,23,24	3	iRT-Kit_WR_fusion,O89117,P35280
1049	1,2,3,7,8,17,20,23	4,5,6,18,19,21,22,24	2	iRT-Kit_WR_fusion,P36376
1050	1,2,3,7,8,17,20,24	4,5,6,18,19,21,22,23	2	P13676,Q811M5
1051	1,2,3,7,8,17,21,22	4,5,6,18,19,20,23,24	3	iRT-Kit_WR_fusion,P11883,P35280
1052	1,2,3,7,8,17,21,23	4,5,6,18,19,20,22,24	3	iRT-Kit_WR_fusion,P22006,Q63598
1053	1,2,3,7,8,17,21,24	4,5,6,18,19,20,22,23	5	iRT-Kit_WR_fusion,P04762,Q63598,Q6AY61,Q811M5
1054	1,2,3,7,8,17,22,23	4,5,6,18,19,20,21,24	2	P35280,Q99MH3
1055	1,2,3,7,8,17,22,24	4,5,6,18,19,20,21,23	1	P35280
1056	1,2,3,7,8,17,23,24	4,5,6,18,19,20,21,22	4	P46720,Q5PQL7,Q63598,Q64093
1057	1,2,3,7,8,18,19,20	4,5,6,17,21,22,23,24	4	iRT-Kit_WR_fusion,P20766,P47967,Q66H69
1058	1,2,3,7,8,18,19,21	4,5,6,17,20,22,23,24	7	iRT-Kit_WR_fusion,P11883,P20766,P22006,P62804,Q00715,Q66H69
1059	1,2,3,7,8,18,19,22	4,5,6,17,20,21,23,24	3	iRT-Kit_WR_fusion,P20766,P35280
1060	1,2,3,7,8,18,19,23	4,5,6,17,20,21,22,24	4	iRT-Kit_WR_fusion,P20766,Q66H69,Q9Z0J6
1061	1,2,3,7,8,18,19,24	4,5,6,17,20,21,22,23	2	P20766,Q66H69
1062	1,2,3,7,8,18,20,21	4,5,6,17,19,22,23,24	8	iRT-Kit_WR_fusion,P25809,P47967,P62804,Q00715,Q5RKI1,Q811M5,Q91ZS3
1063	1,2,3,7,8,18,20,22	4,5,6,17,19,21,23,24	1	P35280

1064	1,2,3,7,8,18,20,23	4,5,6,17,19,21,22,24	2	P20766,Q99MH3
1065	1,2,3,7,8,18,20,24	4,5,6,17,19,21,22,23	2	P20766,Q811M5
1066	1,2,3,7,8,18,21,22	4,5,6,17,19,20,23,24	3	iRT-Kit_WR_fusion,P11883,P35280
1067	1,2,3,7,8,18,21,23	4,5,6,17,19,20,22,24	4	iRT-Kit_WR_fusion,P20766,P22006,Q9Z0J6
1068	1,2,3,7,8,18,21,24	4,5,6,17,19,20,22,23	4	iRT-Kit_WR_fusion,P02625,P20766,Q811M5
1069	1,2,3,7,8,18,22,23	4,5,6,17,19,20,21,24	4	P20766,P35280,P36374,Q99MH3
1070	1,2,3,7,8,18,22,24	4,5,6,17,19,20,21,23	2	P20766,P35280
1071	1,2,3,7,8,18,23,24	4,5,6,17,19,20,21,22	5	P20766,Q3ZAV1,Q63598,Q64093,Q9Z0J6
1072	1,2,3,7,8,19,20,21	4,5,6,17,18,22,23,24	12	D4A5U3,iRT-Kit_WR_fusion,P11883,P47967,P62804,Q00715,Q5I0D1,Q5RKI1,Q66H69,Q811M5,Q812E4,Q9WTT6
1073	1,2,3,7,8,19,20,22	4,5,6,17,18,21,23,24	2	iRT-Kit_WR_fusion,P35280
1074	1,2,3,7,8,19,20,23	4,5,6,17,18,21,22,24	1	iRT-Kit_WR_fusion
1075	1,2,3,7,8,19,20,24	4,5,6,17,18,21,22,23	4	iRT-Kit_WR_fusion,P08723,Q66H69,Q812E4
1076	1,2,3,7,8,19,21,22	4,5,6,17,18,20,23,24	4	iRT-Kit_WR_fusion,P11883,P35280,Q66H69
1077	1,2,3,7,8,19,21,23	4,5,6,17,18,20,22,24	5	iRT-Kit_WR_fusion,P11883,P22006,Q66H69,Q9Z0J6
1078	1,2,3,7,8,19,21,24	4,5,6,17,18,20,22,23	4	iRT-Kit_WR_fusion,P04355,P11883,Q66H69
1079	1,2,3,7,8,19,22,23	4,5,6,17,18,20,21,24	3	iRT-Kit_WR_fusion,P35280,Q9Z0J6
1080	1,2,3,7,8,19,22,24	4,5,6,17,18,20,21,23	2	iRT-Kit_WR_fusion,P35280
1081	1,2,3,7,8,19,23,24	4,5,6,17,18,20,21,22	2	iRT-Kit_WR_fusion,Q9Z0J6
1082	1,2,3,7,8,20,21,22	4,5,6,17,18,19,23,24	3	iRT-Kit_WR_fusion,P11883,P35280
1083	1,2,3,7,8,20,21,23	4,5,6,17,18,19,22,24	2	iRT-Kit_WR_fusion,Q5RKI1

1084	1,2,3,7,8,20,21,24	4,5,6,17,18,19,22,23	4	iRT-Kit_WR_fusion,Q811M5,Q812E4,Q9JHB9
1085	1,2,3,7,8,20,22,23	4,5,6,17,18,19,21,24	3	iRT-Kit_WR_fusion,P35280,Q99MH3
1086	1,2,3,7,8,20,22,24	4,5,6,17,18,19,21,23	2	iRT-Kit_WR_fusion,P35280
1087	1,2,3,7,8,20,23,24	4,5,6,17,18,19,21,22	1	iRT-Kit_WR_fusion
1088	1,2,3,7,8,21,22,23	4,5,6,17,18,19,20,24	4	iRT-Kit_WR_fusion,P11883,P35280,Q62714
1089	1,2,3,7,8,21,22,24	4,5,6,17,18,19,20,23	4	iRT-Kit_WR_fusion,P11883,P35280,Q62714
1090	1,2,3,7,8,21,23,24	4,5,6,17,18,19,20,22	3	iRT-Kit_WR_fusion,Q62714,Q9Z0J6
1091	1,2,3,7,8,22,23,24	4,5,6,17,18,19,20,21	3	P35280,Q62714,Q6P6R2
1092	1,2,3,7,17,18,19,20	4,5,6,8,21,22,23,24	3	P20766,P30919,Q811M5
1093	1,2,3,7,17,18,19,21	4,5,6,8,20,22,23,24	2	P20766,P22006
1094	1,2,3,7,17,18,19,22	4,5,6,8,20,21,23,24	0	
1095	1,2,3,7,17,18,19,23	4,5,6,8,20,21,22,24	2	P20766,Q10743
1096	1,2,3,7,17,18,19,24	4,5,6,8,20,21,22,23	2	P20766,P54921
1097	1,2,3,7,17,18,20,21	4,5,6,8,19,22,23,24	2	P00507,Q811M5
1098	1,2,3,7,17,18,20,22	4,5,6,8,19,21,23,24	0	
1099	1,2,3,7,17,18,20,23	4,5,6,8,19,21,22,24	1	P30919
1100	1,2,3,7,17,18,20,24	4,5,6,8,19,21,22,23	1	Q811M5
1101	1,2,3,7,17,18,21,22	4,5,6,8,19,20,23,24	1	P23739
1102	1,2,3,7,17,18,21,23	4,5,6,8,19,20,22,24	1	P22006
1103	1,2,3,7,17,18,21,24	4,5,6,8,19,20,22,23	3	P19218,Q6AY61,Q811M5

1104	1,2,3,7,17,<br>18,22,23	4,5,6,8,19,<br>20,21,24	3	P22273,P22282,P36374
1105	1,2,3,7,17,<br>18,22,24	4,5,6,8,19,<br>20,21,23	1	P22273
1106	1,2,3,7,17,<br>18,23,24	4,5,6,8,19,<br>20,21,22	3	P19218,P20766,P22273
1107	1,2,3,7,17,<br>19,20,21	4,5,6,8,18,<br>22,23,24	3	Q6AY61,Q811M5,Q9WVK7
1108	1,2,3,7,17,<br>19,20,22	4,5,6,8,18,<br>21,23,24	1	O89117
1109	1,2,3,7,17,<br>19,20,23	4,5,6,8,18,<br>21,22,24	0	
1110	1,2,3,7,17,<br>19,20,24	4,5,6,8,18,<br>21,22,23	2	P54921,Q6AY61
1111	1,2,3,7,17,<br>19,21,22	4,5,6,8,18,<br>20,23,24	1	Q9R168
1112	1,2,3,7,17,<br>19,21,23	4,5,6,8,18,<br>20,22,24	1	P22006
1113	1,2,3,7,17,<br>19,21,24	4,5,6,8,18,<br>20,22,23	3	P54921,Q6AY61,Q9R168
1114	1,2,3,7,17,<br>19,22,23	4,5,6,8,18,<br>20,21,24	2	P22273,Q9R168
1115	1,2,3,7,17,<br>19,22,24	4,5,6,8,18,<br>20,21,23	3	P22273,P54921,Q9R168
1116	1,2,3,7,17,<br>19,23,24	4,5,6,8,18,<br>20,21,22	2	P22273,P54921
1117	1,2,3,7,17,<br>20,21,22	4,5,6,8,18,<br>19,23,24	1	P63029
1118	1,2,3,7,17,<br>20,21,23	4,5,6,8,18,<br>19,22,24	1	P63029
1119	1,2,3,7,17,<br>20,21,24	4,5,6,8,18,<br>19,22,23	2	Q6AY61,Q811M5
1120	1,2,3,7,17,<br>20,22,23	4,5,6,8,18,<br>19,21,24	0	
1121	1,2,3,7,17,<br>20,22,24	4,5,6,8,18,<br>19,21,23	0	
1122	1,2,3,7,17,<br>20,23,24	4,5,6,8,18,<br>19,21,22	0	
1123	1,2,3,7,17,<br>21,22,23	4,5,6,8,18,<br>19,20,24	1	Q9R168

1124	1,2,3,7,17,<br>21,22,24	4,5,6,8,18,<br>19,20,23	1	Q9R168
1125	1,2,3,7,17,<br>21,23,24	4,5,6,8,18,<br>19,20,22	2	Q6AY61,Q9R168
1126	1,2,3,7,17,<br>22,23,24	4,5,6,8,18,<br>19,20,21	3	P22273,P22282,Q9R168
1127	1,2,3,7,18,<br>19,20,21	4,5,6,8,17,<br>22,23,24	2	P20760,P47967
1128	1,2,3,7,18,<br>19,20,22	4,5,6,8,17,<br>21,23,24	0	
1129	1,2,3,7,18,<br>19,20,23	4,5,6,8,17,<br>21,22,24	1	P20766
1130	1,2,3,7,18,<br>19,20,24	4,5,6,8,17,<br>21,22,23	1	P20766
1131	1,2,3,7,18,<br>19,21,22	4,5,6,8,17,<br>20,23,24	1	P23739
1132	1,2,3,7,18,<br>19,21,23	4,5,6,8,17,<br>20,22,24	2	P20766,Q9Z0J6
1133	1,2,3,7,18,<br>19,21,24	4,5,6,8,17,<br>20,22,23	1	P20766
1134	1,2,3,7,18,<br>19,22,23	4,5,6,8,17,<br>20,21,24	2	P20766,P22273
1135	1,2,3,7,18,<br>19,22,24	4,5,6,8,17,<br>20,21,23	1	P20766
1136	1,2,3,7,18,<br>19,23,24	4,5,6,8,17,<br>20,21,22	3	P20766,Q6AYQ8,Q9Z0J6
1137	1,2,3,7,18,<br>20,21,22	4,5,6,8,17,<br>19,23,24	1	P01835
1138	1,2,3,7,18,<br>20,21,23	4,5,6,8,17,<br>19,22,24	1	P01835
1139	1,2,3,7,18,<br>20,21,24	4,5,6,8,17,<br>19,22,23	2	P01835,Q811M5
1140	1,2,3,7,18,<br>20,22,23	4,5,6,8,17,<br>19,21,24	1	Q99MH3
1141	1,2,3,7,18,<br>20,22,24	4,5,6,8,17,<br>19,21,23	0	
1142	1,2,3,7,18,<br>20,23,24	4,5,6,8,17,<br>19,21,22	1	P20766
1143	1,2,3,7,18,<br>21,22,23	4,5,6,8,17,<br>19,20,24	3	P01835,P36374,Q6RUV5

1144	1,2,3,7,18,<br>21,22,24	4,5,6,8,17,<br>19,20,23	2	P01835,P23739
1145	1,2,3,7,18,<br>21,23,24	4,5,6,8,17,<br>19,20,22	2	P01835,P20766
1146	1,2,3,7,18,<br>22,23,24	4,5,6,8,17,<br>19,20,21	8	P06760,P22273,P22282,P36374,Q64268,Q6AYQ8,Q6P6R2,Q9QX74
1147	1,2,3,7,19,<br>20,21,22	4,5,6,8,17,<br>18,23,24	1	P47967
1148	1,2,3,7,19,<br>20,21,23	4,5,6,8,17,<br>18,22,24	0	
1149	1,2,3,7,19,<br>20,21,24	4,5,6,8,17,<br>18,22,23	1	Q6AY61
1150	1,2,3,7,19,<br>20,22,23	4,5,6,8,17,<br>18,21,24	0	
1151	1,2,3,7,19,<br>20,22,24	4,5,6,8,17,<br>18,21,23	0	
1152	1,2,3,7,19,<br>20,23,24	4,5,6,8,17,<br>18,21,22	0	
1153	1,2,3,7,19,<br>21,22,23	4,5,6,8,17,<br>18,20,24	1	Q9Z0J6
1154	1,2,3,7,19,<br>21,22,24	4,5,6,8,17,<br>18,20,23	1	Q9R168
1155	1,2,3,7,19,<br>21,23,24	4,5,6,8,17,<br>18,20,22	1	Q9Z0J6
1156	1,2,3,7,19,<br>22,23,24	4,5,6,8,17,<br>18,20,21	4	P06760,P22273,Q6AYQ8,Q9Z0J6
1157	1,2,3,7,20,<br>21,22,23	4,5,6,8,17,<br>18,19,24	1	P01835
1158	1,2,3,7,20,<br>21,22,24	4,5,6,8,17,<br>18,19,23	1	P01835
1159	1,2,3,7,20,<br>21,23,24	4,5,6,8,17,<br>18,19,22	2	P01835,P08649
1160	1,2,3,7,20,<br>22,23,24	4,5,6,8,17,<br>18,19,21	1	Q9QX74
1161	1,2,3,7,21,<br>22,23,24	4,5,6,8,17,<br>18,19,20	3	P01835,Q62714,Q9R168
1162	1,2,3,8,17,<br>18,19,20	4,5,6,7,21,<br>22,23,24	2	P25809,Q811M5
1163	1,2,3,8,17,<br>18,19,21	4,5,6,7,20,<br>22,23,24	3	P22006,Q66H69,Q9WVK7

1164	1,2,3,8,17,18,19,22	4,5,6,7,20,21,23,24	1 P97580
1165	1,2,3,8,17,18,19,23	4,5,6,7,20,21,22,24	2 P22006,Q9Z0J6
1166	1,2,3,8,17,18,19,24	4,5,6,7,20,21,22,23	0
1167	1,2,3,8,17,18,20,21	4,5,6,7,19,22,23,24	3 P25809,P63029,Q811M5
1168	1,2,3,8,17,18,20,22	4,5,6,7,19,21,23,24	0
1169	1,2,3,8,17,18,20,23	4,5,6,7,19,21,22,24	4 P00714,P36376,P63029,Q99MH3
1170	1,2,3,8,17,18,20,24	4,5,6,7,19,21,22,23	1 Q811M5
1171	1,2,3,8,17,18,21,22	4,5,6,7,19,20,23,24	0
1172	1,2,3,8,17,18,21,23	4,5,6,7,19,20,22,24	1 P22006
1173	1,2,3,8,17,18,21,24	4,5,6,7,19,20,22,23	1 Q811M5
1174	1,2,3,8,17,18,22,23	4,5,6,7,19,20,21,24	7 P00714,P11598,P22283,P36374,Q63617,Q99MH3,Q9R0T3
1175	1,2,3,8,17,18,22,24	4,5,6,7,19,20,21,23	2 P11598,Q63617
1176	1,2,3,8,17,18,23,24	4,5,6,7,19,20,21,22	2 Q63617,Q99041
1177	1,2,3,8,17,19,20,21	4,5,6,7,18,22,23,24	9 D4A5U3,P13676,P25809,P63029,P97675,Q66H69,Q6P6R2,Q811M5,Q9WVK7
1178	1,2,3,8,17,19,20,22	4,5,6,7,18,21,23,24	1 O89117
1179	1,2,3,8,17,19,20,23	4,5,6,7,18,21,22,24	3 O89117,P36376,P63029
1180	1,2,3,8,17,19,20,24	4,5,6,7,18,21,22,23	2 O89117,Q68G31
1181	1,2,3,8,17,19,21,22	4,5,6,7,18,20,23,24	2 P02091,Q9WVK7
1182	1,2,3,8,17,19,21,23	4,5,6,7,18,20,22,24	2 P22006,Q9Z0J6
1183	1,2,3,8,17,19,21,24	4,5,6,7,18,20,22,23	2 Q66H69,Q9WVK7

1184	1,2,3,8,17,<br>19,22,23	4,5,6,7,18,<br>20,21,24	4	O89117,P02091,P11598,Q9Z0J6
1185	1,2,3,8,17,<br>19,22,24	4,5,6,7,18,<br>20,21,23	1	O89117
1186	1,2,3,8,17,<br>19,23,24	4,5,6,7,18,<br>20,21,22	1	Q9Z0J6
1187	1,2,3,8,17,<br>20,21,22	4,5,6,7,18,<br>19,23,24	2	P25809,P63029
1188	1,2,3,8,17,<br>20,21,23	4,5,6,7,18,<br>19,22,24	2	P36376,P63029
1189	1,2,3,8,17,<br>20,21,24	4,5,6,7,18,<br>19,22,23	2	P63029,Q811M5
1190	1,2,3,8,17,<br>20,22,23	4,5,6,7,18,<br>19,21,24	5	O89117,P00714,P36376,P63029,Q99MH3
1191	1,2,3,8,17,<br>20,22,24	4,5,6,7,18,<br>19,21,23	1	O89117
1192	1,2,3,8,17,<br>20,23,24	4,5,6,7,18,<br>19,21,22	2	P36376,P63029
1193	1,2,3,8,17,<br>21,22,23	4,5,6,7,18,<br>19,20,24	0	
1194	1,2,3,8,17,<br>21,22,24	4,5,6,7,18,<br>19,20,23	0	
1195	1,2,3,8,17,<br>21,23,24	4,5,6,7,18,<br>19,20,22	1	Q9Z0J6
1196	1,2,3,8,17,<br>22,23,24	4,5,6,7,18,<br>19,20,21	8	P00714,P11598,P22283,P47967,P97840,Q5I0D1,Q63617,Q9R0T3
1197	1,2,3,8,18,<br>19,20,21	4,5,6,7,17,<br>22,23,24	2	P25809,Q68G31
1198	1,2,3,8,18,<br>19,20,22	4,5,6,7,17,<br>21,23,24	1	P36860
1199	1,2,3,8,18,<br>19,20,23	4,5,6,7,17,<br>21,22,24	3	P36860,Q9R168,Q9Z0J6
1200	1,2,3,8,18,<br>19,20,24	4,5,6,7,17,<br>21,22,23	2	Q68G31,Q9R168
1201	1,2,3,8,18,<br>19,21,22	4,5,6,7,17,<br>20,23,24	0	
1202	1,2,3,8,18,<br>19,21,23	4,5,6,7,17,<br>20,22,24	2	P22006,Q9Z0J6
1203	1,2,3,8,18,<br>19,21,24	4,5,6,7,17,<br>20,22,23	2	Q68G31,Q9Z0J6

1204	1,2,3,8,18, 19,22,23	4,5,6,7,17, 20,21,24	2	Q99MH3,Q9Z0J6
1205	1,2,3,8,18, 19,22,24	4,5,6,7,17, 20,21,23	0	
1206	1,2,3,8,18, 19,23,24	4,5,6,7,17, 20,21,22	1	Q9Z0J6
1207	1,2,3,8,18, 20,21,22	4,5,6,7,17, 19,23,24	1	P25809
1208	1,2,3,8,18, 20,21,23	4,5,6,7,17, 19,22,24	0	
1209	1,2,3,8,18, 20,21,24	4,5,6,7,17, 19,22,23	1	Q811M5
1210	1,2,3,8,18, 20,22,23	4,5,6,7,17, 19,21,24	2	P00714,Q99MH3
1211	1,2,3,8,18, 20,22,24	4,5,6,7,17, 19,21,23	0	
1212	1,2,3,8,18, 20,23,24	4,5,6,7,17, 19,21,22	2	Q5QE79,Q9R168
1213	1,2,3,8,18, 21,22,23	4,5,6,7,17, 19,20,24	4	P36374,P54921,Q99MH3,Q9Z0J6
1214	1,2,3,8,18, 21,22,24	4,5,6,7,17, 19,20,23	0	
1215	1,2,3,8,18, 21,23,24	4,5,6,7,17, 19,20,22	1	Q9Z0J6
1216	1,2,3,8,18, 22,23,24	4,5,6,7,17, 19,20,21	10	P00714,P06760,P11598,P22283,P36374,Q63617,Q6P6R2,Q99MH3,Q9R0T3,Q9Z0J6
1217	1,2,3,8,19, 20,21,22	4,5,6,7,17, 18,23,24	0	
1218	1,2,3,8,19, 20,21,23	4,5,6,7,17, 18,22,24	1	Q9Z0J6
1219	1,2,3,8,19, 20,21,24	4,5,6,7,17, 18,22,23	1	Q68G31
1220	1,2,3,8,19, 20,22,23	4,5,6,7,17, 18,21,24	1	P36860
1221	1,2,3,8,19, 20,22,24	4,5,6,7,17, 18,21,23	0	
1222	1,2,3,8,19, 20,23,24	4,5,6,7,17, 18,21,22	1	Q9Z0J6
1223	1,2,3,8,19, 21,22,23	4,5,6,7,17, 18,20,24	1	Q9Z0J6

1224	1,2,3,8,19,21,22,24	4,5,6,7,17,18,20,23	1	Q9Z0J6
1225	1,2,3,8,19,21,23,24	4,5,6,7,17,18,20,22	1	Q9Z0J6
1226	1,2,3,8,19,22,23,24	4,5,6,7,17,18,20,21	3	P00507,P11598,Q9Z0J6
1227	1,2,3,8,20,21,22,23	4,5,6,7,17,18,19,24	1	Q99MH3
1228	1,2,3,8,20,21,22,24	4,5,6,7,17,18,19,23	0	
1229	1,2,3,8,20,21,23,24	4,5,6,7,17,18,19,22	0	
1230	1,2,3,8,20,22,23,24	4,5,6,7,17,18,19,21	1	Q5QE79
1231	1,2,3,8,21,22,23,24	4,5,6,7,17,18,19,20	2	P30919,Q9Z0J6
1232	1,2,3,17,18,19,20,21	4,5,6,7,8,22,23,24	4	O54858,P20760,Q99041,Q9WVK7
1233	1,2,3,17,18,19,20,22	4,5,6,7,8,21,23,24	3	P00714,P36860,Q63474
1234	1,2,3,17,18,19,20,23	4,5,6,7,8,21,22,24	2	P00714,Q99041
1235	1,2,3,17,18,19,20,24	4,5,6,7,8,21,22,23	2	O54858,Q99041
1236	1,2,3,17,18,19,21,22	4,5,6,7,8,20,23,24	3	P22273,P23739,Q4G075
1237	1,2,3,17,18,19,21,23	4,5,6,7,8,20,22,24	6	P02780,P22006,P22273,Q4G075,Q99041,Q9Z0J6
1238	1,2,3,17,18,19,21,24	4,5,6,7,8,20,22,23	3	P22273,Q4G075,Q99041
1239	1,2,3,17,18,19,22,23	4,5,6,7,8,20,21,24	13	P00714,P02780,P06760,P06761,P11598,P22273,P22282,P22283,Q4G075,Q5M8C6,Q63617,Q99041,Q9R0T3
1240	1,2,3,17,18,19,22,24	4,5,6,7,8,20,21,23	9	P02780,P06760,P06761,P11598,P22273,P22282,Q4G075,Q63617,Q99041
1241	1,2,3,17,18,19,23,24	4,5,6,7,8,20,21,22	11	P00714,P02780,P06760,P06761,P11598,P22273,P22282,Q4G075,Q63617,Q99041,Q9Z0J6
1242	1,2,3,17,18,20,21,22	4,5,6,7,8,19,23,24	1	P63029
1243	1,2,3,17,18,20,21,23	4,5,6,7,8,19,22,24	4	P00714,P22282,P63029,Q99041

1244	1,2,3,17,18,20,21,24	4,5,6,7,8,19,22,23	5	O54858,P19218,P50115,Q811M5,Q99041
1245	1,2,3,17,18,20,22,23	4,5,6,7,8,19,21,24	7	P00714,P06760,P06761,P22273,P22282,Q99041,Q99MH3
1246	1,2,3,17,18,20,22,24	4,5,6,7,8,19,21,23	6	P00714,P06760,P22273,P22282,P50115,Q99041
1247	1,2,3,17,18,20,23,24	4,5,6,7,8,19,21,22	6	P00714,P06760,P19218,P22273,P22282,Q99041
1248	1,2,3,17,18,21,22,23	4,5,6,7,8,19,20,24	15	P00714,P02780,P06761,P11598,P22273,P22282,P36374,P46462,P82471,Q4G075,Q5M8C6,Q63617,Q6RUV5,Q99041,Q9R0T3
1249	1,2,3,17,18,21,22,24	4,5,6,7,8,19,20,23	8	P02780,P06761,P11598,P22273,P22282,Q4G075,Q63617,Q99041
1250	1,2,3,17,18,21,23,24	4,5,6,7,8,19,20,22	8	P02780,P06761,P19218,P22273,P22282,Q4G075,Q63617,Q99041
1251	1,2,3,17,18,22,23,24	4,5,6,7,8,19,20,21	21	D3ZUC6,P00714,P02780,P04905,P06760,P06761,P0C0A9,P11598,P22273,P22282,P22283,P36374,P46462,P55159,Q4G075,Q5I0D1,Q5M8C6,Q63617,Q99041,Q9R0T3,Q9WTT6
1252	1,2,3,17,19,20,21,22	4,5,6,7,8,18,23,24	4	O89117,P49134,P63029,Q9WVK7
1253	1,2,3,17,19,20,21,23	4,5,6,7,8,18,22,24	4	O54858,P49134,P63029,Q99041
1254	1,2,3,17,19,20,21,24	4,5,6,7,8,18,22,23	6	O54858,P49134,P54921,Q6AY61,Q99041,Q9WVK7
1255	1,2,3,17,19,20,22,23	4,5,6,7,8,18,21,24	4	O89117,P00714,P22273,P36860
1256	1,2,3,17,19,20,22,24	4,5,6,7,8,18,21,23	2	O89117,P22273
1257	1,2,3,17,19,20,23,24	4,5,6,7,8,18,21,22	2	P22273,Q99041
1258	1,2,3,17,19,21,22,23	4,5,6,7,8,18,20,24	7	P02091,P02780,P06761,P11598,P22273,P22282,Q4G075
1259	1,2,3,17,19,21,22,24	4,5,6,7,8,18,20,23	5	P02091,P02780,P22273,Q4G075,Q9R168
1260	1,2,3,17,19,21,23,24	4,5,6,7,8,18,20,22	6	P02780,P12020,P22273,Q4G075,Q99041,Q9Z0J6
1261	1,2,3,17,19,22,23,24	4,5,6,7,8,18,20,21	14	P00714,P02091,P02780,P06760,P06761,P11598,P22273,P22282,P22283,Q4G075,Q5M8C6,Q63617,Q99041,Q9R0T3
1262	1,2,3,17,20,21,22,23	4,5,6,7,8,18,19,24	4	P00714,P22282,P63029,Q99041
1263	1,2,3,17,20,21,22,24	4,5,6,7,8,18,19,23	2	P63029,Q99041

1264	1,2,3,17,20,21,23,24	4,5,6,7,8,18,19,22	3	P22282,P63029,Q99041
1265	1,2,3,17,20,22,23,24	4,5,6,7,8,18,19,21	7	P00714,P06760,P06761,P07150,P22273,P22282,Q99041
1266	1,2,3,17,21,22,23,24	4,5,6,7,8,18,19,20	13	P00714,P02780,P06761,P07150,P11598,P22273,P22282,Q4G075,Q5I0D1,Q63617,Q99041,Q9R0T3,Q9R168
1267	1,2,3,18,19,20,21,22	4,5,6,7,8,17,23,24	1	P20760
1268	1,2,3,18,19,20,21,23	4,5,6,7,8,17,22,24	2	Q99041,Q9Z0J6
1269	1,2,3,18,19,20,21,24	4,5,6,7,8,17,22,23	5	O54858,P20760,P50115,Q68G31,Q99041
1270	1,2,3,18,19,20,22,23	4,5,6,7,8,17,21,24	7	P00714,P06760,P22273,P36860,P50116,Q63474,Q9QX74
1271	1,2,3,18,19,20,22,24	4,5,6,7,8,17,21,23	4	P06760,P50115,P50116,Q9QX74
1272	1,2,3,18,19,20,23,24	4,5,6,7,8,17,21,22	7	P06760,P22273,P50115,P50116,Q99041,Q9QX74,Q9Z0J6
1273	1,2,3,18,19,21,22,23	4,5,6,7,8,17,20,24	6	P02780,P06911,P22273,P22282,Q4G075,Q9Z0J6
1274	1,2,3,18,19,21,22,24	4,5,6,7,8,17,20,23	4	P22273,P50115,P50116,Q4G075
1275	1,2,3,18,19,21,23,24	4,5,6,7,8,17,20,22	5	P02780,P22273,Q4G075,Q99041,Q9Z0J6
1276	1,2,3,18,19,22,23,24	4,5,6,7,8,17,20,21	21	D4A5U3,P00714,P02780,P06760,P06761,P11598,P13676,P19223,P22273,P22282,P50115,P50116,P97697,Q4G075,Q5M8C6,Q63617,Q99041,Q9QX74,Q9R0T3,Q9WTT6,Q9Z0J6
1277	1,2,3,18,20,21,22,23	4,5,6,7,8,17,19,24	3	P00714,P22282,P50115
1278	1,2,3,18,20,21,22,24	4,5,6,7,8,17,19,23	1	P50115
1279	1,2,3,18,20,21,23,24	4,5,6,7,8,17,19,22	2	P50115,Q99041
1280	1,2,3,18,20,22,23,24	4,5,6,7,8,17,19,21	12	P00714,P06760,P06761,P07150,P20646,P22273,P22282,P50115,P50116,Q5QE79,Q99041,Q9QX74
1281	1,2,3,18,21,22,23,24	4,5,6,7,8,17,19,20	22	D4A5U3,P00714,P02780,P06761,P07150,P11598,P13676,P20646,P22273,P22282,P36374,P46462,P50115,P50116,Q4G075,Q63617,Q6RUV5,Q99041,Q9QX74,Q9R0T3,Q9WTT6,Q9Z0J6
1282	1,2,3,19,20,21,22,23	4,5,6,7,8,17,18,24	0	
1283	1,2,3,19,20,21,22,24	4,5,6,7,8,17,18,23	2	O70594,Q64335

1284	1,2,3,19,20,21,23,24	4,5,6,7,8,17,18,22	3	Q64335,Q99041,Q9Z0J6
1285	1,2,3,19,20,22,23,24	4,5,6,7,8,17,18,21	4	P06760,P22273,P50116,Q9QX74
1286	1,2,3,19,21,22,23,24	4,5,6,7,8,17,18,20	13	P02780,P06761,P06911,P11598,P12020,P22273,P22282,P50116,Q4G075,Q64335,Q9QX74,Q9WTT6,Q9Z0J6
1287	1,2,3,20,21,22,23,24	4,5,6,7,8,17,18,19	5	P07150,P22282,P50115,Q99041,Q9QX74
1288	1,2,4,5,6,7,8,17	3,18,19,20,21,22,23,24	25	B1H234,iRT-Kit_WR_fusion,O54858,O70594,P02454,P13432,P18418,P20760,P23593,P31044,P36860,P50115,P50116,P52590,P80299,P97580,Q05702,Q30KJ2,Q4FZU2,Q5GRG2,Q6IFU8,Q6IMF3,Q6P6Q2,Q99041,Q9QZQ5
1289	1,2,4,5,6,7,8,18	3,17,19,20,21,22,23,24	27	B1H234,O54858,O70594,P02454,P11598,P16636,P18418,P19629,P20762,P23593,P31044,P36860,P42854,P50115,P50116,P50280,P52590,P80299,P97580,Q03191,Q05702,Q30KJ2,Q4FZU4,Q4KLZ6,Q91ZS3,Q99041,Q9QZQ5
1290	1,2,4,5,6,7,8,19	3,17,18,20,21,22,23,24	29	B1H234,iRT-Kit_WR_fusion,O54858,O70594,P06761,P11598,P11762,P15399,P18418,P19629,P22282,P31044,P35952,P42854,P50115,P50116,P50280,P52590,P80299,P97580,Q30KJ2,Q4FZU4,Q66H69,Q6IMF3,Q6TMA8,Q91ZS3,Q99041,Q9JI85,Q9QZQ5
1291	1,2,4,5,6,7,8,20	3,17,18,19,21,22,23,24	50	B1H234,D4A5U3,iRT-Kit_WR_fusion,O54858,P02454,P02631,P02780,P06761,P06911,P0DP29;P0DP30;P0DP31,P11598,P11762,P12020,P15399,P17559,P18418,P19629,P22273,P22282,P23593,P25031,P31044,P35952,P42854,P50115,P50116,P50280,P52590,P80299,P97697,Q03191,Q05702,Q30KJ2,Q4AEF8,Q4G075,Q5GRG2,Q5I0D1,Q62946,Q6IFU8,Q6IG05,Q6IMF3,Q6P6Q2,Q78P75,Q91ZS3,Q99041,Q9JHB9,Q9JI85,Q9QZQ5,Q9R0T3,Q9Z0J6
1292	1,2,4,5,6,7,8,21	3,17,18,19,20,22,23,24	34	B1H234,iRT-Kit_WR_fusion,O54858,O70594,P02454,P02631,P11598,P16228,P18418,P19629,P22282,P23593,P30919,P35952,P36860,P42854,P50115,P50116,P52590,P70549,P80299,Q00715,Q03191,Q05702,Q30KJ2,Q4FZU2,Q63474,Q6IFU8,Q6IMF3,Q6P6Q2,Q91ZS3,Q99041,Q9JI85,Q9QZQ5
1293	1,2,4,5,6,7,8,22	3,17,18,19,20,21,23,24	24	B1H234,iRT-Kit_WR_fusion,O54858,P15399,P18418,P19218,P19629,P20760,P23593,P27590,P42854,P50115,P50116,P50280,P52590,P80299,P97580,Q03191,Q09030,Q30KJ2,Q4FZU4,Q6P6R2,Q91ZS3,Q99041
1294	1,2,4,5,6,7,8,23	3,17,18,19,20,21,22,24	27	B1H234,iRT-Kit_WR_fusion,O54858,O70594,P18418,P19629,P20760,P20761,P20762,P23593,P23785,P36860,P42854,P50115,P50116,P52590,P80299,Q03191,Q05702,Q09030,Q30KJ2,Q4FZU4,Q63532,Q6IMF3,Q91ZS3,Q99041,Q9JJ50
1295	1,2,4,5,6,7,8,24	3,17,18,19,20,21,22,23	25	B1H234,iRT-Kit_WR_fusion,O54858,O70594,P18418,P20760,P23593,P30919,P36860,P50115,P50116,P50280,P52590,Q03191,Q05702,Q30KJ2,Q4FZU2,Q4KLZ6,Q63474,Q6IFU8,Q6IMF3,Q6P6Q2,Q6P6R2,Q91ZS3,Q99041

1296	1,2,4,5,6,7,17,18	3,8,19,20,21,22,23,24	13	B1H234,O70594,P00762,P16636,P18418,P36860,P50116,P52590,P80299,Q03191,Q4KLZ6,Q9WVH8,Q9Z0J6
1297	1,2,4,5,6,7,17,19	3,8,18,20,21,22,23,24	7	B1H234,P00762,P18418,P50116,P52590,P80299,Q9R168
1298	1,2,4,5,6,7,17,20	3,8,18,19,21,22,23,24	14	B1H234,P0DP29;P0DP30;P0DP31,P11598,P18418,P19223,P50116,P52590,P55091,P55281,P80299,P97697,Q03191,Q5GRG2,Q9Z0J6
1299	1,2,4,5,6,7,17,21	3,8,18,19,20,22,23,24	11	B1H234,P05539,P19939,P36860,P50116,P52590,P80299,Q03191,Q63474,Q9R168,Q9Z0J6
1300	1,2,4,5,6,7,17,22	3,8,18,19,20,21,23,24	10	B1H234,P23593,P36860,P50116,P52590,P80299,Q03191,Q9EQS0,Q9R168,Q9Z0J6
1301	1,2,4,5,6,7,17,23	3,8,18,19,20,21,22,24	9	B1H234,O70594,P36860,P50116,P52590,P80299,Q03191,Q9QZ76,Q9R168
1302	1,2,4,5,6,7,17,24	3,8,18,19,20,21,22,23	13	B1H234,P01039,P18418,P19939,P36860,P50116,P52590,P80299,Q03191,Q4KLZ6,Q6AY61,Q9R168,Q9Z0J6
1303	1,2,4,5,6,7,18,19	3,8,17,20,21,22,23,24	8	B1H234,P00762,P18418,P50116,P52590,P80299,Q03191,Q4KLZ6
1304	1,2,4,5,6,7,18,20	3,8,17,19,21,22,23,24	13	B1H234,P0DP29;P0DP30;P0DP31,P11598,P18418,P50116,P52590,P80299,Q03191,Q4KLZ6,Q5GRG2,Q80WL1,Q91ZS3,Q9Z0J6
1305	1,2,4,5,6,7,18,21	3,8,17,19,20,22,23,24	13	B1H234,O89117,P11598,P18418,P36860,P50116,P52590,P80299,Q00715,Q03191,Q4KLZ6,Q91ZS3,Q9JJ50
1306	1,2,4,5,6,7,18,22	3,8,17,19,20,21,23,24	11	B1H234,P00762,P16636,P23593,P50116,P52590,P80299,Q03191,Q4KLZ6,Q6P6R2,Q9EQS0
1307	1,2,4,5,6,7,18,23	3,8,17,19,20,21,22,24	13	B1H234,O70594,P16636,P36860,P50116,P52590,P80299,Q03191,Q4KLZ6,Q6P6R2,Q9EQS0,Q9JJ50,Q9Z2L0
1308	1,2,4,5,6,7,18,24	3,8,17,19,20,21,22,23	9	B1H234,P18418,P36860,P50116,P52590,P80299,Q03191,Q4KLZ6,Q6P6R2

1309	1,2,4,5,6,7,19,20	3,8,17,18,21,22,23,24	14	B1H234,P02780,P0DP29;P0DP30;P0DP31,P11598,P18418,P42854,P50116,P52590,P80299,Q03191,Q5GRG2,Q5I0D1,Q63617,Q9R0T3
1310	1,2,4,5,6,7,19,21	3,8,17,18,20,22,23,24	8	B1H234,P11598,P15978,P36860,P50116,P52590,P80299,Q63474
1311	1,2,4,5,6,7,19,22	3,8,17,18,20,21,23,24	11	B1H234,P00762,P42854,P50116,P52590,P80299,Q03191,Q4FZU4,Q99041,Q9EQS0,Q9R168
1312	1,2,4,5,6,7,19,23	3,8,17,18,20,21,22,24	8	B1H234,P00762,P42854,P50116,P52590,P80299,Q03191,Q9EQS0
1313	1,2,4,5,6,7,19,24	3,8,17,18,20,21,22,23	9	B1H234,P15978,P17988,P50116,P52590,P80299,Q4KLZ6,Q5M872,Q62761;Q62762;Q62763
1314	1,2,4,5,6,7,20,21	3,8,17,18,19,22,23,24	22	B1H234,O55145,P01835,P02780,P0DP29;P0DP30;P0DP31,P11598,P15978,P19629,P22283,P36860,P47967,P50116,P52590,P70549,P80299,Q03191,Q5GRG2,Q5I0D1,Q63617,Q91ZS3,Q9R0T3,Q9Z0J6
1315	1,2,4,5,6,7,20,22	3,8,17,18,19,21,23,24	11	B1H234,P0DP29;P0DP30;P0DP31,P11598,P23593,P42854,P50116,P52590,P80299,Q03191,Q5GRG2,Q9Z0J6
1316	1,2,4,5,6,7,20,23	3,8,17,18,19,21,22,24	11	B1H234,P0DP29;P0DP30;P0DP31,P10758,P11598,P19629,P23739,P42854,P50116,P52590,P80299,Q03191
1317	1,2,4,5,6,7,20,24	3,8,17,18,19,21,22,23	12	B1H234,P0DP29;P0DP30;P0DP31,P11598,P18418,P50116,P52590,P80299,Q03191,Q4KLZ6,Q5GRG2,Q9JHB9,Q9Z0J6
1318	1,2,4,5,6,7,21,22	3,8,17,18,19,20,23,24	9	B1H234,P01835,P17046,P36860,P50116,P52590,P80299,Q03191,Q9R168
1319	1,2,4,5,6,7,21,23	3,8,17,18,19,20,22,24	12	B1H234,P01835,P36860,P42854,P50116,P52590,P80299,Q03191,Q63474,Q63532,Q9JJ50,Q9R168
1320	1,2,4,5,6,7,21,24	3,8,17,18,19,20,22,23	15	B1H234,P01835,P02761,P15978,P19939,P30919,P36860,P50116,P52590,P80299,Q03191,Q4KLZ6,Q63474,Q6AY61,Q9R168
1321	1,2,4,5,6,7,22,23	3,8,17,18,19,20,21,24	13	B1H234,P10719,P23593,P42854,P50116,P52590,P80299,Q03191,Q63532,Q6P6R2,Q9EQS0,Q9R168,Q9Z2L0

1322	1,2,4,5,6,7,22,24	3,8,17,18,19,20,21,23	13	B1H234,P10719,P23593,P30919,P36860,P52590,P70619,P80299,Q03191,Q4KLZ6,Q6P6R2,Q9EQS0,Q9R168
1323	1,2,4,5,6,7,23,24	3,8,17,18,19,20,21,22	12	B1H234,O08557,P36860,P50116,P52590,P70619,P80299,Q03191,Q4KLZ6,Q6P6R2,Q9EQS0,Q9JJ50
1324	1,2,4,5,6,8,17,18	3,7,19,20,21,22,23,24	11	O70594,P02454,P13432,P16636,P18418,P23593,P52590,P97580,Q30KJ2,Q4KLZ6,Q9QZQ5
1325	1,2,4,5,6,8,17,19	3,7,18,20,21,22,23,24	6	P15399,P18418,P52590,P97580,Q30KJ2,Q9QZQ5
1326	1,2,4,5,6,8,17,20	3,7,18,19,21,22,23,24	23	D4A5U3,P02454,P06911,P12020,P13432,P15399,P17559,P18418,P19223,P25031,P36375,P50280,P52590,P55091,P97697,Q10758,Q30KJ2,Q5GRG2,Q6IMF3,Q6P6Q2,Q9JI85,Q9QZQ5,Q9Z0J6
1327	1,2,4,5,6,8,17,21	3,7,18,19,20,22,23,24	14	B1H234,P02631,P18418,P35952,P36860,P52590,P97580,Q30KJ2,Q4FZU2,Q63474,Q6IFU8,Q6IMF3,Q6P6Q2,Q9QZQ5
1328	1,2,4,5,6,8,17,22	3,7,18,19,20,21,23,24	8	O54858,P15399,P18418,P20760,P23593,P52590,P97580,Q30KJ2
1329	1,2,4,5,6,8,17,23	3,7,18,19,20,21,22,24	11	O54858,O70594,P18418,P20760,P23593,P42854,P52590,P97580,Q09030,Q30KJ2,Q63532
1330	1,2,4,5,6,8,17,24	3,7,18,19,20,21,22,23	12	P01039,P18418,P20760,P23593,P36860,P52590,Q30KJ2,Q4FZU2,Q4KLZ6,Q63474,Q6IMF3,Q6P6Q2
1331	1,2,4,5,6,8,18,19	3,7,17,20,21,22,23,24	9	P15399,P18418,P42854,P52590,P97580,Q30KJ2,Q4KLZ6,Q91ZS3,Q9QZQ5
1332	1,2,4,5,6,8,18,20	3,7,17,19,21,22,23,24	21	P02454,P06911,P12020,P13432,P15399,P17559,P18418,P25031,P36375,P50280,P52590,Q03191,Q05702,Q10758,Q30KJ2,Q4KLZ6,Q5GRG2,Q6RY07,Q91ZS3,Q9JI85,Q9QZQ5
1333	1,2,4,5,6,8,18,21	3,7,17,19,20,22,23,24	11	B1H234,P02631,P18418,P35952,P36860,P52590,P97580,Q30KJ2,Q4KLZ6,Q91ZS3,Q9QZQ5
1334	1,2,4,5,6,8,18,22	3,7,17,19,20,21,23,24	13	O54858,P15399,P16636,P18418,P20762,P23593,P52590,P54921,P97580,Q03191,Q30KJ2,Q4KLZ6,Q91ZS3

1335	1,2,4,5,6,8,18,23	3,7,17,19,20,21,22,24	19	O54858,O70594,P16636,P18418,P20760,P20762,P23785,P42854,P52590,P54921,P97580,Q03191,Q30KJ2,Q4KLZ6,Q63532,Q91ZS3,Q99MH3,Q9JJ50,Q9Z2L0
1336	1,2,4,5,6,8,18,24	3,7,17,19,20,21,22,23	8	O54858,P18418,P23593,P36860,P52590,Q30KJ2,Q4KLZ6,Q91ZS3
1337	1,2,4,5,6,8,19,20	3,7,17,18,21,22,23,24	20	D4A5U3,P02780,P06911,P12020,P14046,P15399,P18418,P19629,P22282,P25031,P35952,P36374,P42854,P50280,P52590,P97580,Q30KJ2,Q5GRG2,Q68G31,Q9JI85
1338	1,2,4,5,6,8,19,21	3,7,17,18,20,22,23,24	13	B1H234,P14046,P18418,P30919,P35952,P42854,P52590,P97580,Q10743,Q30KJ2,Q63474,Q66H69,Q9JI85
1339	1,2,4,5,6,8,19,22	3,7,17,18,20,21,23,24	9	O54858,P00507,P15399,P19218,P30919,P42854,P52590,P97580,Q30KJ2
1340	1,2,4,5,6,8,19,23	3,7,17,18,20,21,22,24	10	O54858,P00507,P18418,P20760,P42854,P52590,P97580,Q30KJ2,Q63532,Q8CJD3
1341	1,2,4,5,6,8,19,24	3,7,17,18,20,21,22,23	10	O54728,P15399,P18418,P30919,P52590,P97580,Q10743,Q30KJ2,Q4KLZ6,Q68G31
1342	1,2,4,5,6,8,20,21	3,7,17,18,19,22,23,24	28	B1H234,D4A5U3,O55145,P02631,P02780,P06911,P12020,P14046,P18418,P19629,P25031,P35952,P36375,P42854,P50280,P52590,P70549,Q03191,Q05702,Q10743,Q10758,Q5GRG2,Q6IFU8,Q6IMF3,Q6P6Q2,Q91ZS3,Q9JI85,Q9QZQ5
1343	1,2,4,5,6,8,20,22	3,7,17,18,19,21,23,24	17	O54858,P06911,P12020,P15399,P18418,P19218,P19629,P23593,P25031,P42854,P50280,P52590,P97580,Q03191,Q30KJ2,Q5GRG2,Q91ZS3
1344	1,2,4,5,6,8,20,23	3,7,17,18,19,21,22,24	20	P06911,P12020,P17559,P18418,P19132,P19629,P20760,P23739,P25031,P36375,P42854,P50280,P52590,Q03191,Q05702,Q10758,Q30KJ2,Q5GRG2,Q63532,Q91ZS3
1345	1,2,4,5,6,8,20,24	3,7,17,18,19,21,22,23	18	P06911,P12020,P15399,P18418,P25031,P36375,P50280,P52590,Q03191,Q10743,Q10758,Q4KLZ6,Q5GRG2,Q6IFU8,Q6IMF3,Q6P6Q2,Q91ZS3,Q9JI85
1346	1,2,4,5,6,8,21,22	3,7,17,18,19,20,23,24	12	B1H234,O54858,P15399,P23593,P30919,P36860,P52590,P97580,Q10743,Q30KJ2,Q6AYC4,Q91ZS3
1347	1,2,4,5,6,8,21,23	3,7,17,18,19,20,22,24	12	O54858,P18418,P30919,P35952,P36860,P42854,P52590,P54921,Q09030,Q63474,Q63532,Q9JJ50

1348	1,2,4,5,6,8,21,24	3,7,17,18,19,20,22,23	15	B1H234,P07174,P18418,P30919,P35952,P36860,P52590,Q10743,Q4FZU2,Q4KLZ6,Q63474,Q6IFU8,Q6IMF3,Q6P6Q2,Q91ZS3
1349	1,2,4,5,6,8,22,23	3,7,17,18,19,20,21,24	17	O54858,P00507,P15399,P20760,P23593,P30919,P42854,P52590,P54921,P97580,Q03191,Q09030,Q30KJ2,Q63532,Q6P6R2,Q99MH3,Q9Z2L0
1350	1,2,4,5,6,8,22,24	3,7,17,18,19,20,21,23	12	O54858,P00507,P15399,P20760,P23593,P30919,P52590,P97580,Q10743,Q30KJ2,Q4KLZ6,Q6P6R2
1351	1,2,4,5,6,8,23,24	3,7,17,18,19,20,21,22	14	O54858,P00507,P18418,P20760,P23593,P30919,P36860,P42854,P52590,Q09030,Q4KLZ6,Q63532,Q6P6R2,Q9JJ50
1352	1,2,4,5,6,17,18,19	3,7,8,20,21,22,23,24	4	B1H234,P35280,P52590,Q4KLZ6
1353	1,2,4,5,6,17,18,20	3,7,8,19,21,22,23,24	8	P19223,P35280,P52590,P55091,Q03191,Q4KLZ6,Q5GRG2,Q9Z0J6
1354	1,2,4,5,6,17,18,21	3,7,8,19,20,22,23,24	5	B1H234,P35280,P36860,P52590,Q4KLZ6
1355	1,2,4,5,6,17,18,22	3,7,8,19,20,21,23,24	6	P16636,P52590,Q03191,Q4KLZ6,Q9EQS0,Q9Z0J6
1356	1,2,4,5,6,17,18,23	3,7,8,19,20,21,22,24	6	P16636,P35280,P52590,Q03191,Q4KLZ6,Q9EQS0
1357	1,2,4,5,6,17,18,24	3,7,8,19,20,21,22,23	6	B1H234,P01039,P35280,P36860,P52590,Q4KLZ6
1358	1,2,4,5,6,17,19,20	3,7,8,18,21,22,23,24	3	P19223,P35280,P52590
1359	1,2,4,5,6,17,19,21	3,7,8,18,20,22,23,24	4	B1H234,P35280,P36860,P52590
1360	1,2,4,5,6,17,19,22	3,7,8,18,20,21,23,24	2	P52590,Q9EQS0
1361	1,2,4,5,6,17,19,23	3,7,8,18,20,21,22,24	2	P35280,P52590
1362	1,2,4,5,6,17,19,24	3,7,8,18,20,21,22,23	5	B1H234,P01039,P35280,P52590,Q4KLZ6
1363	1,2,4,5,6,17,20,21	3,7,8,18,19,22,23,24	9	B1H234,P19223,P35280,P36860,P52590,P55091,Q03191,Q5I0J9,Q9Z0J6
1364	1,2,4,5,6,17,20,22	3,7,8,18,19,21,23,24	4	P19223,P52590,Q03191,Q9Z0J6
1365	1,2,4,5,6,17,20,23	3,7,8,18,19,21,22,24	7	P19223,P23739,P35280,P52590,P55091,Q03191,Q63467

1366	1,2,4,5,6,17,20,24	3,7,8,18,19,21,22,23	7	P01039,P19223,P35280,P52590,Q03191,Q4KLZ6,Q9Z0J6
1367	1,2,4,5,6,17,21,22	3,7,8,18,19,20,23,24	4	B1H234,P36860,P52590,Q9R168
1368	1,2,4,5,6,17,21,23	3,7,8,18,19,20,22,24	4	B1H234,P35280,P36860,P52590
1369	1,2,4,5,6,17,21,24	3,7,8,18,19,20,22,23	10	B1H234,P01039,P19939,P35280,P36860,P52590,Q4KLZ6,Q63474,Q6AY61,Q9R168
1370	1,2,4,5,6,17,22,23	3,7,8,18,19,20,21,24	4	P52590,Q03191,Q9EQS0,Q9WTT6
1371	1,2,4,5,6,17,22,24	3,7,8,18,19,20,21,23	10	D3ZUC6,P01039,P23593,P36860,P52590,Q4KLZ6,Q6P6R2,Q9EQS0,Q9R168,Q9WTT6
1372	1,2,4,5,6,17,23,24	3,7,8,18,19,20,21,22	6	P01039,P35280,P36860,P52590,Q4KLZ6,Q9WTT6
1373	1,2,4,5,6,18,19,20	3,7,8,17,21,22,23,24	5	B1H234,P35280,P52590,Q03191,Q4KLZ6
1374	1,2,4,5,6,18,19,21	3,7,8,17,20,22,23,24	4	B1H234,P35280,P52590,Q4KLZ6
1375	1,2,4,5,6,18,19,22	3,7,8,17,20,21,23,24	4	B1H234,P52590,Q4KLZ6,Q9EQS0
1376	1,2,4,5,6,18,19,23	3,7,8,17,20,21,22,24	6	P16636,P35280,P52590,Q4KLZ6,Q9EQS0,Q9Z2L0
1377	1,2,4,5,6,18,19,24	3,7,8,17,20,21,22,23	5	B1H234,P35280,P52590,Q4KLZ6,Q9WTT6
1378	1,2,4,5,6,18,20,21	3,7,8,17,19,22,23,24	8	B1H234,O55145,P35280,P52590,P70549,Q03191,Q4KLZ6,Q91ZS3
1379	1,2,4,5,6,18,20,22	3,7,8,17,19,21,23,24	5	P52590,Q03191,Q4KLZ6,Q9EQS0,Q9Z0J6
1380	1,2,4,5,6,18,20,23	3,7,8,17,19,21,22,24	7	P04355,P16636,P35280,P52590,Q03191,Q4KLZ6,Q9EQS0
1381	1,2,4,5,6,18,20,24	3,7,8,17,19,21,22,23	4	B1H234,P35280,Q03191,Q4KLZ6
1382	1,2,4,5,6,18,21,22	3,7,8,17,19,20,23,24	9	B1H234,P13676,P36860,P52590,P54921,Q03191,Q4KLZ6,Q9EQS0,Q9WTT6
1383	1,2,4,5,6,18,21,23	3,7,8,17,19,20,22,24	11	B1H234,O89117,P16636,P35280,P36860,P52590,P54921,Q03191,Q4KLZ6,Q9EQS0,Q9JJ50
1384	1,2,4,5,6,18,21,24	3,7,8,17,19,20,22,23	8	B1H234,O89117,P35280,P36860,Q03191,Q4KLZ6,Q9JJ50,Q9WTT6
1385	1,2,4,5,6,18,22,23	3,7,8,17,19,20,21,24	13	P13676,P16636,P30120,P54921,Q03191,Q4KLZ6,Q63532,Q6P6R2,Q99MH3,Q9EQS0,Q9QX74,Q9WTT6,Q9Z2L0

1386	1,2,4,5,6,18,22,24	3,7,8,17,19,20,21,23	9	B1H234,D3ZUC6,P13676,Q03191,Q4KLZ6,Q6P6R2,Q9EQS0,Q9QX74,Q9WTT6
1387	1,2,4,5,6,18,23,24	3,7,8,17,19,20,21,22	10	P13676,P16636,P35280,Q03191,Q4KLZ6,Q6P6R2,Q9EQS0,Q9JJ50,Q9QX74,Q9WTT6
1388	1,2,4,5,6,19,20,21	3,7,8,17,18,22,23,24	5	B1H234,P0DP29;P0DP30;P0DP31,P35280,P52590,Q5I0J9
1389	1,2,4,5,6,19,20,22	3,7,8,17,18,21,23,24	4	P19218,P42854,P52590,Q03191
1390	1,2,4,5,6,19,20,23	3,7,8,17,18,21,22,24	6	P10758,P23739,P35280,P42854,P52590,Q03191
1391	1,2,4,5,6,19,20,24	3,7,8,17,18,21,22,23	5	B1H234,P35280,P52590,Q4KLZ6,Q68G31
1392	1,2,4,5,6,19,21,22	3,7,8,17,18,20,23,24	3	B1H234,P30919,P52590
1393	1,2,4,5,6,19,21,23	3,7,8,17,18,20,22,24	4	B1H234,P35280,P42854,P52590
1394	1,2,4,5,6,19,21,24	3,7,8,17,18,20,22,23	8	B1H234,P07174,P30919,P35280,P36860,P52590,Q4KLZ6,Q63474
1395	1,2,4,5,6,19,22,23	3,7,8,17,18,20,21,24	8	P00507,P13676,P42854,P52590,Q6P6R2,Q811M5,Q9EQS0,Q9WTT6
1396	1,2,4,5,6,19,22,24	3,7,8,17,18,20,21,23	10	B1H234,P00507,P13676,P30919,P52590,Q4KLZ6,Q6P6R2,Q9EQS0,Q9QX74,Q9WTT6
1397	1,2,4,5,6,19,23,24	3,7,8,17,18,20,21,22	8	P00507,P35280,P52590,Q4KLZ6,Q6P6R2,Q9EQS0,Q9QX74,Q9WTT6
1398	1,2,4,5,6,20,21,22	3,7,8,17,18,19,23,24	5	B1H234,O55145,P52590,Q03191,Q9Z0J6
1399	1,2,4,5,6,20,21,23	3,7,8,17,18,19,22,24	4	B1H234,P35280,P52590,Q03191
1400	1,2,4,5,6,20,21,24	3,7,8,17,18,19,22,23	6	B1H234,O55145,P35280,P36860,Q03191,Q4KLZ6
1401	1,2,4,5,6,20,22,23	3,7,8,17,18,19,21,24	6	P23739,P42854,Q03191,Q6P6R2,Q9EQS0,Q9QX74
1402	1,2,4,5,6,20,22,24	3,7,8,17,18,19,21,23	7	P21674,P22006,Q03191,Q4KLZ6,Q6P6R2,Q9QX74,Q9Z0J6
1403	1,2,4,5,6,20,23,24	3,7,8,17,18,19,21,22	8	P23739,P35280,Q03191,Q4KLZ6,Q5QE79,Q6P6R2,Q9QX74,Q9QYP1
1404	1,2,4,5,6,21,22,23	3,7,8,17,18,19,20,24	9	B1H234,P13676,P30919,P36860,P54921,Q03191,Q63532,Q9EQS0,Q9WTT6
1405	1,2,4,5,6,21,22,24	3,7,8,17,18,19,20,23	7	B1H234,P13676,P30919,P36860,Q4KLZ6,Q9R168,Q9WTT6

1406	1,2,4,5,6,21,23,24	3,7,8,17,18,19,20,22	10	B1H234,P13676,P30919,P35280,P36860,Q03191,Q4KLZ6,Q63474,Q9JJ50,Q9WTT6
1407	1,2,4,5,6,22,23,24	3,7,8,17,18,19,20,21	13	D3ZUC6,P00507,P13676,P30919,P47727,Q03191,Q4KLZ6,Q63532,Q6P6R2,Q811M5,Q9EQS0,Q9QX74,Q9WTT6
1408	1,2,4,5,7,8,17,18	3,6,19,20,21,22,23,24	5	O70594,P18418,Q03191,Q811M5,Q9Z1F2
1409	1,2,4,5,7,8,17,19	3,6,18,20,21,22,23,24	7	iRT-Kit_WR_fusion,P20646,P22006,Q66H69,Q6IFU8,Q6IMF3,Q6P6Q2
1410	1,2,4,5,7,8,17,20	3,6,18,19,21,22,23,24	17	D4A5U3,P02780,P06911,P19223,P97697,Q03191,Q10758,Q4FZU2,Q4G075,Q5GRG2,Q62946,Q6IFU8,Q6IMF3,Q6P6Q2,Q811M5,Q9JHB9,Q9Z0J6
1411	1,2,4,5,7,8,17,21	3,6,18,19,20,22,23,24	16	iRT-Kit_WR_fusion,O70594,P06760,P19939,P22006,P36860,P70549,Q00715,Q4FZU2,Q63474,Q6IFU8,Q6IMF3,Q6P6Q2,Q811M5,Q9QX74,Q9Z1F2
1412	1,2,4,5,7,8,17,22	3,6,18,19,20,21,23,24	6	P23593,P27590,Q30KJ2,Q6IMF3,Q6P6Q2,Q9Z1F2
1413	1,2,4,5,7,8,17,23	3,6,18,19,20,21,22,24	7	O70594,P19132,P20760,Q03191,Q6IFU8,Q6IMF3,Q6P6Q2
1414	1,2,4,5,7,8,17,24	3,6,18,19,20,21,22,23	7	P19939,P36860,Q4FZU2,Q6IFU8,Q6IMF3,Q6P6Q2,Q9Z1F2
1415	1,2,4,5,7,8,18,19	3,6,17,20,21,22,23,24	3	P18418,P20762,Q66H69
1416	1,2,4,5,7,8,18,20	3,6,17,19,21,22,23,24	15	D4A5U3,P02780,P06911,P18418,P50280,P70549,P97697,Q03191,Q10758,Q4G075,Q5GRG2,Q6P6Q2,Q811M5,Q91ZS3,Q9JHB9
1417	1,2,4,5,7,8,18,21	3,6,17,19,20,22,23,24	12	iRT-Kit_WR_fusion,O89117,P36860,P70549,Q00715,Q03191,Q6IFU8,Q6IMF3,Q6P6Q2,Q811M5,Q91ZS3,Q9Z1F2
1418	1,2,4,5,7,8,18,22	3,6,17,19,20,21,23,24	6	P20762,P23593,P27590,Q03191,Q30KJ2,Q9Z1F2
1419	1,2,4,5,7,8,18,23	3,6,17,19,20,21,22,24	3	O70594,P20762,Q03191

1420	1,2,4,5,7,8,18,24	3,6,17,19,20,21,22,23	9	O89117,P18418,P20762,Q03191,Q4KLZ6,Q6IMF3,Q6P6Q2,Q91ZS3,Q9Z1F2
1421	1,2,4,5,7,8,19,20	3,6,17,18,21,22,23,24	17	D4A5U3,iRT-Kit_WR_fusion,P02780,P22282,P36374,P47967,P50280,P97697,Q4G075,Q5I0D1,Q66H69,Q6IFU8,Q6IMF3,Q6P6Q2,Q6TMA8,Q812E4,Q8CJD3
1422	1,2,4,5,7,8,19,21	3,6,17,18,20,22,23,24	9	iRT-Kit_WR_fusion,P06760,P22006,Q00715,Q63474,Q66H69,Q6IFU8,Q6IMF3,Q6P6Q2
1423	1,2,4,5,7,8,19,22	3,6,17,18,20,21,23,24	4	iRT-Kit_WR_fusion,P27590,Q30KJ2,Q66H69
1424	1,2,4,5,7,8,19,23	3,6,17,18,20,21,22,24	4	iRT-Kit_WR_fusion,Q66H69,Q6IMF3,Q8CJD3
1425	1,2,4,5,7,8,19,24	3,6,17,18,20,21,22,23	7	iRT-Kit_WR_fusion,O54728,Q66H69,Q6IFU8,Q6IMF3,Q6P6Q2,Q6TMA8
1426	1,2,4,5,7,8,20,21	3,6,17,18,19,22,23,24	22	D4A5U3,iRT-Kit_WR_fusion,P02780,P06760,P22273,P47967,P55159,P70549,P97697,Q00715,Q03191,Q10758,Q4FZU2,Q4G075,Q5I0D1,Q6IFU8,Q6IMF3,Q6P6Q2,Q811M5,Q812E4,Q91ZS3,Q9JHB9
1427	1,2,4,5,7,8,20,22	3,6,17,18,19,21,23,24	9	D4A5U3,iRT-Kit_WR_fusion,P02780,P23593,Q03191,Q4G075,Q6IMF3,Q6P6Q2,Q9Z0J6
1428	1,2,4,5,7,8,20,23	3,6,17,18,19,21,22,24	11	D4A5U3,iRT-Kit_WR_fusion,P10758,P19132,P23739,P97697,Q03191,Q4G075,Q6IFU8,Q6IMF3,Q6P6Q2
1429	1,2,4,5,7,8,20,24	3,6,17,18,19,21,22,23	12	D4A5U3,P02780,P97697,Q03191,Q4FZU2,Q4G075,Q6IFU8,Q6IMF3,Q6P6Q2,Q6TMA8,Q812E4,Q9JHB9
1430	1,2,4,5,7,8,21,22	3,6,17,18,19,20,23,24	7	iRT-Kit_WR_fusion,P70549,Q03191,Q6IFU8,Q6IMF3,Q6P6Q2,Q9Z1F2
1431	1,2,4,5,7,8,21,23	3,6,17,18,19,20,22,24	10	iRT-Kit_WR_fusion,P19132,P22006,P36860,P70549,Q03191,Q63474,Q6IFU8,Q6IMF3,Q6P6Q2
1432	1,2,4,5,7,8,21,24	3,6,17,18,19,20,22,23	9	iRT-Kit_WR_fusion,P19939,P36860,Q4FZU2,Q63474,Q6IFU8,Q6IMF3,Q6P6Q2,Q9Z1F2

1433	1,2,4,5,7,8,22,23	3,6,17,18,19,20,21,24	6	P20760,P20762,P23593,P27590,Q03191,Q6P6R2
1434	1,2,4,5,7,8,22,24	3,6,17,18,19,20,21,23	5	P23593,Q03191,Q6IMF3,Q6P6Q2,Q6P6R2
1435	1,2,4,5,7,8,23,24	3,6,17,18,19,20,21,22	6	P20760,Q03191,Q6IFU8,Q6IMF3,Q6P6Q2,Q6P6R2
1436	1,2,4,5,7,17,18,19	3,6,8,20,21,22,23,24	1	P13265
1437	1,2,4,5,7,17,18,20	3,6,8,19,21,22,23,24	4	P19223,Q03191,Q811M5,Q9Z0J6
1438	1,2,4,5,7,17,18,21	3,6,8,19,20,22,23,24	5	P19939,P22006,P36860,Q03191,Q811M5
1439	1,2,4,5,7,17,18,22	3,6,8,19,20,21,23,24	2	Q03191,Q9Z0J6
1440	1,2,4,5,7,17,18,23	3,6,8,19,20,21,22,24	1	Q03191
1441	1,2,4,5,7,17,18,24	3,6,8,19,20,21,22,23	3	P19939,Q03191,Q4KLZ6
1442	1,2,4,5,7,17,19,20	3,6,8,18,21,22,23,24	3	P19223,P20646,P36374
1443	1,2,4,5,7,17,19,21	3,6,8,18,20,22,23,24	4	P19939,P22006,Q62761;Q62762;Q62763,Q9R168
1444	1,2,4,5,7,17,19,22	3,6,8,18,20,21,23,24	2	P80299,Q9R168
1445	1,2,4,5,7,17,19,23	3,6,8,18,20,21,22,24	0	
1446	1,2,4,5,7,17,19,24	3,6,8,18,20,21,22,23	3	P54921,Q62761;Q62762;Q62763,Q9R168
1447	1,2,4,5,7,17,20,21	3,6,8,18,19,22,23,24	6	P19223,P70549,P97697,Q03191,Q811M5,Q9Z0J6
1448	1,2,4,5,7,17,20,22	3,6,8,18,19,21,23,24	4	P19223,P80299,Q03191,Q9Z0J6
1449	1,2,4,5,7,17,20,23	3,6,8,18,19,21,22,24	4	P10758,P19223,P23739,Q03191
1450	1,2,4,5,7,17,20,24	3,6,8,18,19,21,22,23	5	P19223,Q03191,Q811M5,Q9QYP1,Q9Z0J6
1451	1,2,4,5,7,17,21,22	3,6,8,18,19,20,23,24	3	P36860,Q03191,Q9R168

1452	1,2,4,5,7,17,21,23	3,6,8,18,19,20,22,24	4	P22006,P36860,Q03191,Q9R168
1453	1,2,4,5,7,17,21,24	3,6,8,18,19,20,22,23	6	P01039,P19939,P36860,Q63474,Q6AY61,Q9R168
1454	1,2,4,5,7,17,22,23	3,6,8,18,19,20,21,24	4	P80299,Q03191,Q9EQS0,Q9R168
1455	1,2,4,5,7,17,22,24	3,6,8,18,19,20,21,23	2	Q03191,Q9R168
1456	1,2,4,5,7,17,23,24	3,6,8,18,19,20,21,22	3	P36860,Q03191,Q9R168
1457	1,2,4,5,7,18,19,20	3,6,8,17,21,22,23,24	5	P47967,P80299,P97840,Q03191,Q5I0D1
1458	1,2,4,5,7,18,19,21	3,6,8,17,20,22,23,24	1	O89117
1459	1,2,4,5,7,18,19,22	3,6,8,17,20,21,23,24	3	P80299,Q03191,Q9EQS0
1460	1,2,4,5,7,18,19,23	3,6,8,17,20,21,22,24	2	P80299,Q03191
1461	1,2,4,5,7,18,19,24	3,6,8,17,20,21,22,23	1	Q4KLZ6
1462	1,2,4,5,7,18,20,21	3,6,8,17,19,22,23,24	5	P47967,P70549,P97840,Q03191,Q811M5
1463	1,2,4,5,7,18,20,22	3,6,8,17,19,21,23,24	3	P80299,Q03191,Q9Z0J6
1464	1,2,4,5,7,18,20,23	3,6,8,17,19,21,22,24	2	P10758,Q03191
1465	1,2,4,5,7,18,20,24	3,6,8,17,19,21,22,23	3	Q03191,Q4KLZ6,Q9QYP1
1466	1,2,4,5,7,18,21,22	3,6,8,17,19,20,23,24	4	O89117,P70549,P80299,Q03191
1467	1,2,4,5,7,18,21,23	3,6,8,17,19,20,22,24	5	O89117,P36860,P70549,Q03191,Q9JJ50
1468	1,2,4,5,7,18,21,24	3,6,8,17,19,20,22,23	5	O89117,P19939,P36860,Q03191,Q4KLZ6
1469	1,2,4,5,7,18,22,23	3,6,8,17,19,20,21,24	5	P30120,P80299,Q03191,Q6P6R2,Q9EQS0
1470	1,2,4,5,7,18,22,24	3,6,8,17,19,20,21,23	4	Q03191,Q4KLZ6,Q6P6R2,Q9EQS0
1471	1,2,4,5,7,18,23,24	3,6,8,17,19,20,21,22	7	O89117,Q03191,Q4KLZ6,Q6AYQ8,Q6P6R2,Q9JJ50,Q9WVK7

1472	1,2,4,5,7,19,20,21	3,6,8,17,18,22,23,24	7	P08649,P47967,P55159,P70549,P80299,Q03191,Q5I0D1
1473	1,2,4,5,7,19,20,22	3,6,8,17,18,21,23,24	2	P80299,Q03191
1474	1,2,4,5,7,19,20,23	3,6,8,17,18,21,22,24	4	P10758,P23739,P80299,Q03191
1475	1,2,4,5,7,19,20,24	3,6,8,17,18,21,22,23	0	
1476	1,2,4,5,7,19,21,22	3,6,8,17,18,20,23,24	2	P80299,Q9R168
1477	1,2,4,5,7,19,21,23	3,6,8,17,18,20,22,24	2	P08649,P80299
1478	1,2,4,5,7,19,21,24	3,6,8,17,18,20,22,23	4	P08649,Q62761;Q62762;Q62763,Q63474,Q9R168
1479	1,2,4,5,7,19,22,23	3,6,8,17,18,20,21,24	3	P80299,Q03191,Q9EQS0
1480	1,2,4,5,7,19,22,24	3,6,8,17,18,20,21,23	1	Q9R168
1481	1,2,4,5,7,19,23,24	3,6,8,17,18,20,21,22	1	P14173
1482	1,2,4,5,7,20,21,22	3,6,8,17,18,19,23,24	6	P08649,P17046,P70549,P80299,Q03191,Q9Z0J6
1483	1,2,4,5,7,20,21,23	3,6,8,17,18,19,22,24	6	P08649,P10758,P23739,P70549,P80299,Q03191
1484	1,2,4,5,7,20,21,24	3,6,8,17,18,19,22,23	3	P08649,P70549,Q03191
1485	1,2,4,5,7,20,22,23	3,6,8,17,18,19,21,24	4	P10758,P23739,P80299,Q03191
1486	1,2,4,5,7,20,22,24	3,6,8,17,18,19,21,23	3	Q03191,Q6P6R2,Q9Z0J6
1487	1,2,4,5,7,20,23,24	3,6,8,17,18,19,21,22	5	P08649,P10758,P23739,Q03191,Q9QYP1
1488	1,2,4,5,7,21,22,23	3,6,8,17,18,19,20,24	4	P08649,P80299,Q03191,Q9R168
1489	1,2,4,5,7,21,22,24	3,6,8,17,18,19,20,23	4	P08649,P36860,Q03191,Q9R168
1490	1,2,4,5,7,21,23,24	3,6,8,17,18,19,20,22	6	O89117,P08649,P36860,Q03191,Q63474,Q9R168
1491	1,2,4,5,7,22,23,24	3,6,8,17,18,19,20,21	5	P13676,Q03191,Q6P6R2,Q9EQS0,Q9R168

1492	1,2,4,5,8,17,18,19	3,6,7,20,21,22,23,24	1	Q66H69
1493	1,2,4,5,8,17,18,20	3,6,7,19,21,22,23,24	6	D4A5U3,P19223,P36375,Q10758,Q6P6Q2,Q811M5
1494	1,2,4,5,8,17,18,21	3,6,7,19,20,22,23,24	3	P22006,Q6P6Q2,Q811M5
1495	1,2,4,5,8,17,18,22	3,6,7,19,20,21,23,24	1	Q30KJ2
1496	1,2,4,5,8,17,18,23	3,6,7,19,20,21,22,24	1	Q9JJS8
1497	1,2,4,5,8,17,18,24	3,6,7,19,20,21,22,23	2	Q4KLZ6,Q6P6Q2
1498	1,2,4,5,8,17,19,20	3,6,7,18,21,22,23,24	7	D4A5U3,P19223,P36374,Q10758,Q6IMF3,Q6P6Q2,Q8CJD3
1499	1,2,4,5,8,17,19,21	3,6,7,18,20,22,23,24	5	P22006,Q66H69,Q6IFU8,Q6IMF3,Q6P6Q2
1500	1,2,4,5,8,17,19,22	3,6,7,18,20,21,23,24	2	P97580,Q30KJ2
1501	1,2,4,5,8,17,19,23	3,6,7,18,20,21,22,24	1	Q8CJD3
1502	1,2,4,5,8,17,19,24	3,6,7,18,20,21,22,23	3	O54728,Q6IMF3,Q6P6Q2
1503	1,2,4,5,8,17,20,21	3,6,7,18,19,22,23,24	12	D4A5U3,P19223,P25809,P36375,P70549,P97697,Q10758,Q4FZU2,Q6IFU8,Q6IMF3,Q6P6Q2,Q811M5
1504	1,2,4,5,8,17,20,22	3,6,7,18,19,21,23,24	4	D4A5U3,P19223,Q6P6Q2,Q9Z0J6
1505	1,2,4,5,8,17,20,23	3,6,7,18,19,21,22,24	7	P19132,P19223,P23739,P36376,Q10758,Q6IMF3,Q6P6Q2
1506	1,2,4,5,8,17,20,24	3,6,7,18,19,21,22,23	8	D4A5U3,P19223,Q10758,Q4FZU2,Q6IFU8,Q6IMF3,Q6P6Q2,Q811M5
1507	1,2,4,5,8,17,21,22	3,6,7,18,19,20,23,24	1	Q6P6Q2
1508	1,2,4,5,8,17,21,23	3,6,7,18,19,20,22,24	6	P19132,P22006,Q63474,Q6IFU8,Q6IMF3,Q6P6Q2
1509	1,2,4,5,8,17,21,24	3,6,7,18,19,20,22,23	7	P19939,P36860,Q4FZU2,Q63474,Q6IFU8,Q6IMF3,Q6P6Q2
1510	1,2,4,5,8,17,22,23	3,6,7,18,19,20,21,24	2	P00507,P20760
1511	1,2,4,5,8,17,22,24	3,6,7,18,19,20,21,23	2	P23593,Q6P6Q2

1512	1,2,4,5,8,17,23,24	3,6,7,18,19,20,21,22	5	P20760,Q4FZU2,Q5PQL7,Q6IMF3,Q6P6Q2
1513	1,2,4,5,8,18,19,20	3,6,7,17,21,22,23,24	1	Q10758
1514	1,2,4,5,8,18,19,21	3,6,7,17,20,22,23,24	1	Q66H69
1515	1,2,4,5,8,18,19,22	3,6,7,17,20,21,23,24	2	P97580,Q30KJ2
1516	1,2,4,5,8,18,19,23	3,6,7,17,20,21,22,24	1	P20762
1517	1,2,4,5,8,18,19,24	3,6,7,17,20,21,22,23	2	O54728,Q4KLZ6
1518	1,2,4,5,8,18,20,21	3,6,7,17,19,22,23,24	9	P25809,P36375,P70549,Q03191,Q10758,Q6IFU8,Q6P6Q2,Q811M5,Q91ZS3
1519	1,2,4,5,8,18,20,22	3,6,7,17,19,21,23,24	1	Q03191
1520	1,2,4,5,8,18,20,23	3,6,7,17,19,21,22,24	4	P19132,P23739,Q03191,Q10758
1521	1,2,4,5,8,18,20,24	3,6,7,17,19,21,22,23	4	Q03191,Q10758,Q4KLZ6,Q6P6Q2
1522	1,2,4,5,8,18,21,22	3,6,7,17,19,20,23,24	1	P70549
1523	1,2,4,5,8,18,21,23	3,6,7,17,19,20,22,24	3	O89117,P54921,P70549
1524	1,2,4,5,8,18,21,24	3,6,7,17,19,20,22,23	5	O89117,P36860,Q4KLZ6,Q6IFU8,Q6P6Q2
1525	1,2,4,5,8,18,22,23	3,6,7,17,19,20,21,24	4	P20762,P54921,Q03191,Q30KJ2
1526	1,2,4,5,8,18,22,24	3,6,7,17,19,20,21,23	2	Q4KLZ6,Q6P6R2
1527	1,2,4,5,8,18,23,24	3,6,7,17,19,20,21,22	3	P20762,Q4KLZ6,Q6P6R2
1528	1,2,4,5,8,19,20,21	3,6,7,17,18,22,23,24	9	D4A5U3,P36374,P70549,Q10758,Q5I0D1,Q66H69,Q6IFU8,Q6IMF3,Q6P6Q2
1529	1,2,4,5,8,19,20,22	3,6,7,17,18,21,23,24	1	P19218
1530	1,2,4,5,8,19,20,23	3,6,7,17,18,21,22,24	3	P10758,P23739,Q8CJD3
1531	1,2,4,5,8,19,20,24	3,6,7,17,18,21,22,23	5	O54728,P36374,Q6IFU8,Q6IMF3,Q6P6Q2

1532	1,2,4,5,8,19,21,22	3,6,7,17,18,20,23,24	0
1533	1,2,4,5,8,19,21,23	3,6,7,17,18,20,22,24	1 P22006
1534	1,2,4,5,8,19,21,24	3,6,7,17,18,20,22,23	8 O54728,P07174,P30919,Q63474,Q66H69,Q6IFU8,Q6IMF3,Q6P6Q2
1535	1,2,4,5,8,19,22,23	3,6,7,17,18,20,21,24	1 P00507
1536	1,2,4,5,8,19,22,24	3,6,7,17,18,20,21,23	3 O54728,P00507,P30919
1537	1,2,4,5,8,19,23,24	3,6,7,17,18,20,21,22	2 O54728,P00507
1538	1,2,4,5,8,20,21,22	3,6,7,17,18,19,23,24	4 P70549,Q03191,Q10758,Q6P6Q2
1539	1,2,4,5,8,20,21,23	3,6,7,17,18,19,22,24	8 P19132,P23739,P70549,Q03191,Q10758,Q6IFU8,Q6IMF3,Q6P6Q2
1540	1,2,4,5,8,20,21,24	3,6,7,17,18,19,22,23	7 P70549,Q10743,Q10758,Q4FZU2,Q6IFU8,Q6IMF3,Q6P6Q2
1541	1,2,4,5,8,20,22,23	3,6,7,17,18,19,21,24	2 P23739,Q03191
1542	1,2,4,5,8,20,22,24	3,6,7,17,18,19,21,23	1 Q6P6Q2
1543	1,2,4,5,8,20,23,24	3,6,7,17,18,19,21,22	7 P23739,Q03191,Q5QE79,Q6IFU8,Q6IMF3,Q6P6Q2,Q9QYP1
1544	1,2,4,5,8,21,22,23	3,6,7,17,18,19,20,24	2 P30919,P54921
1545	1,2,4,5,8,21,22,24	3,6,7,17,18,19,20,23	4 P30919,Q10743,Q6IMF3,Q6P6Q2
1546	1,2,4,5,8,21,23,24	3,6,7,17,18,19,20,22	6 P30919,P36860,Q63474,Q6IFU8,Q6IMF3,Q6P6Q2
1547	1,2,4,5,8,22,23,24	3,6,7,17,18,19,20,21	4 P00507,P20760,P30919,Q6P6R2
1548	1,2,4,5,17,18,19,20	3,6,7,8,21,22,23,24	4 P13265,P19223,P35280,Q62714
1549	1,2,4,5,17,18,19,21	3,6,7,8,20,22,23,24	2 P22006,P35280
1550	1,2,4,5,17,18,19,22	3,6,7,8,20,21,23,24	0
1551	1,2,4,5,17,18,19,23	3,6,7,8,20,21,22,24	1 P35280

1552	1,2,4,5,17, 18,19,24	3,6,7,8,20, 21,22,23	3	P13265,P35280,Q4KLZ6
1553	1,2,4,5,17, 18,20,21	3,6,7,8,19, 22,23,24	5	P19223,P35280,P70549,Q03191,Q811M5
1554	1,2,4,5,17, 18,20,22	3,6,7,8,19, 21,23,24	3	P19223,Q03191,Q9Z0J6
1555	1,2,4,5,17, 18,20,23	3,6,7,8,19, 21,22,24	4	P19223,P23739,P35280,Q03191
1556	1,2,4,5,17, 18,20,24	3,6,7,8,19, 21,22,23	4	P19223,P35280,Q03191,Q4KLZ6
1557	1,2,4,5,17, 18,21,22	3,6,7,8,19, 20,23,24	0	
1558	1,2,4,5,17, 18,21,23	3,6,7,8,19, 20,22,24	3	P19814,P35280,Q9QZK9
1559	1,2,4,5,17, 18,21,24	3,6,7,8,19, 20,22,23	4	P19939,P35280,P36860,Q4KLZ6
1560	1,2,4,5,17, 18,22,23	3,6,7,8,19, 20,21,24	5	P08723,P30120,Q03191,Q63617,Q9EQS0
1561	1,2,4,5,17, 18,22,24	3,6,7,8,19, 20,21,23	5	D3ZUC6,Q4KLZ6,Q5RKI1,Q63617,Q9WTT6
1562	1,2,4,5,17, 18,23,24	3,6,7,8,19, 20,21,22	3	P35280,Q4KLZ6,Q63617
1563	1,2,4,5,17, 19,20,21	3,6,7,8,18, 22,23,24	2	P19223,P35280
1564	1,2,4,5,17, 19,20,22	3,6,7,8,18, 21,23,24	1	P19223
1565	1,2,4,5,17, 19,20,23	3,6,7,8,18, 21,22,24	6	P02625,P10758,P19223,P23739,P35280,Q8CJD3
1566	1,2,4,5,17, 19,20,24	3,6,7,8,18, 21,22,23	1	P35280
1567	1,2,4,5,17, 19,21,22	3,6,7,8,18, 20,23,24	1	Q9R168
1568	1,2,4,5,17, 19,21,23	3,6,7,8,18, 20,22,24	2	P22006,P35280
1569	1,2,4,5,17, 19,21,24	3,6,7,8,18, 20,22,23	5	P19939,P35280,Q62761;Q62762;Q62763,Q62894,Q9R168
1570	1,2,4,5,17, 19,22,23	3,6,7,8,18, 20,21,24	2	P00507,Q9EQS0
1571	1,2,4,5,17, 19,22,24	3,6,7,8,18, 20,21,23	3	D3ZUC6,P00507,Q9R168

1572	1,2,4,5,17, 19,23,24	3,6,7,8,18, 20,21,22	1	P35280
1573	1,2,4,5,17, 20,21,22	3,6,7,8,18, 19,23,24	3	P19223,Q03191,Q9Z0J6
1574	1,2,4,5,17, 20,21,23	3,6,7,8,18, 19,22,24	4	P19223,P23739,P35280,Q03191
1575	1,2,4,5,17, 20,21,24	3,6,7,8,18, 19,22,23	2	P19223,P35280
1576	1,2,4,5,17, 20,22,23	3,6,7,8,18, 19,21,24	2	P23739,Q03191
1577	1,2,4,5,17, 20,22,24	3,6,7,8,18, 19,21,23	1	Q9Z0J6
1578	1,2,4,5,17, 20,23,24	3,6,7,8,18, 19,21,22	6	P08937,P23739,P35280,Q03191,Q63751,Q9QYP1
1579	1,2,4,5,17, 21,22,23	3,6,7,8,18, 19,20,24	1	Q9R168
1580	1,2,4,5,17, 21,22,24	3,6,7,8,18, 19,20,23	2	P36860,Q9R168
1581	1,2,4,5,17, 21,23,24	3,6,7,8,18, 19,20,22	3	P35280,P36860,Q9R168
1582	1,2,4,5,17, 22,23,24	3,6,7,8,18, 19,20,21	9	D3ZUC6,P00507,P08937,Q5RKI1,Q63617,Q6P6R2,Q9EQS0,Q9R168,Q9WTT6
1583	1,2,4,5,18, 19,20,21	3,6,7,8,17, 22,23,24	1	P35280
1584	1,2,4,5,18, 19,20,22	3,6,7,8,17, 21,23,24	1	Q03191
1585	1,2,4,5,18, 19,20,23	3,6,7,8,17, 21,22,24	4	P10758,P23739,P35280,Q03191
1586	1,2,4,5,18, 19,20,24	3,6,7,8,17, 21,22,23	2	P35280,Q4KLZ6
1587	1,2,4,5,18, 19,21,22	3,6,7,8,17, 20,23,24	0	
1588	1,2,4,5,18, 19,21,23	3,6,7,8,17, 20,22,24	2	O70417,P35280
1589	1,2,4,5,18, 19,21,24	3,6,7,8,17, 20,22,23	3	O70417,P35280,Q4KLZ6
1590	1,2,4,5,18, 19,22,23	3,6,7,8,17, 20,21,24	3	P00507,P13676,Q9EQS0
1591	1,2,4,5,18, 19,22,24	3,6,7,8,17, 20,21,23	5	P00507,P13676,Q4KLZ6,Q9EQS0,Q9WTT6

1592	1,2,4,5,18, 19,23,24	3,6,7,8,17, 20,21,22	5	O70417,P13676,P35280,Q4KLZ6,Q9WTT6
1593	1,2,4,5,18, 20,21,22	3,6,7,8,17, 19,23,24	2	P70549,Q03191
1594	1,2,4,5,18, 20,21,23	3,6,7,8,17, 19,22,24	4	P35280,P70549,Q03191,Q9QZK9
1595	1,2,4,5,18, 20,21,24	3,6,7,8,17, 19,22,23	3	P35280,Q03191,Q4KLZ6
1596	1,2,4,5,18, 20,22,23	3,6,7,8,17, 19,21,24	5	P04355,Q03191,Q5QE79,Q9EQS0,Q9QX74
1597	1,2,4,5,18, 20,22,24	3,6,7,8,17, 19,21,23	5	P21674,Q03191,Q4KLZ6,Q5QE79,Q9QX74
1598	1,2,4,5,18, 20,23,24	3,6,7,8,17, 19,21,22	7	P23739,P35280,Q03191,Q4KLZ6,Q5QE79,Q9QX74,Q9QYP1
1599	1,2,4,5,18, 21,22,23	3,6,7,8,17, 19,20,24	5	O70417,P13676,P54921,Q03191,Q9EQS0
1600	1,2,4,5,18, 21,22,24	3,6,7,8,17, 19,20,23	3	P13676,Q4KLZ6,Q9WTT6
1601	1,2,4,5,18, 21,23,24	3,6,7,8,17, 19,20,22	9	O70417,O89117,P13676,P35280,Q03191,Q4KLZ6,Q9JJ50,Q9QZK9,Q9WTT6
1602	1,2,4,5,18, 22,23,24	3,6,7,8,17, 19,20,21	11	P00507,P13676,Q03191,Q4KLZ6,Q5QE79,Q63617,Q6P6R2,Q9EQS0,Q9QX74,Q9WTT6,Q9WVK 7
1603	1,2,4,5,19, 20,21,22	3,6,7,8,17, 18,23,24	0	
1604	1,2,4,5,19, 20,21,23	3,6,7,8,17, 18,22,24	3	P10758,P23739,P35280
1605	1,2,4,5,19, 20,21,24	3,6,7,8,17, 18,22,23	1	P35280
1606	1,2,4,5,19, 20,22,23	3,6,7,8,17, 18,21,24	3	P10758,P23739,Q03191
1607	1,2,4,5,19, 20,22,24	3,6,7,8,17, 18,21,23	1	Q9QX74
1608	1,2,4,5,19, 20,23,24	3,6,7,8,17, 18,21,22	5	P10758,P23739,P35280,Q9QX74,Q9QYP1
1609	1,2,4,5,19, 21,22,23	3,6,7,8,17, 18,20,24	1	O70417
1610	1,2,4,5,19, 21,22,24	3,6,7,8,17, 18,20,23	2	P30919,Q9R168
1611	1,2,4,5,19, 21,23,24	3,6,7,8,17, 18,20,22	4	O70417,P07174,P14173,P35280

1612	1,2,4,5,19,22,23,24	3,6,7,8,17,18,20,21	7	P00507,P13676,P26772,Q6P6R2,Q9EQS0,Q9QX74,Q9WTT6
1613	1,2,4,5,20,21,22,23	3,6,7,8,17,18,19,24	2	P70549,Q03191
1614	1,2,4,5,20,21,22,24	3,6,7,8,17,18,19,23	1	Q03191
1615	1,2,4,5,20,21,23,24	3,6,7,8,17,18,19,22	6	O70417,P08649,P23739,P35280,Q03191,Q9QYP1
1616	1,2,4,5,20,22,23,24	3,6,7,8,17,18,19,21	7	P23739,Q03191,Q5QE79,Q63751,Q6P6R2,Q9QX74,Q9QYP1
1617	1,2,4,5,21,22,23,24	3,6,7,8,17,18,19,20	5	O70417,P13676,P30919,Q9R168,Q9WTT6
1618	1,2,4,6,7,8,17,18	3,5,19,20,21,22,23,24	16	G3V686,O70594,P02454,P06911,P13432,P18418,P31044,P50280,P80299,P97580,Q30KJ2,Q5GRG2,Q63598,Q99PP0,Q9QZQ5,Q9Z1F2
1619	1,2,4,6,7,8,17,19	3,5,18,20,21,22,23,24	15	iRT-Kit_WR_fusion,P11598,P15399,P18418,P22282,P31044,P50115,P50280,P80299,P97580,Q30KJ2,Q5GRG2,Q66H69,Q99041,Q9JI85
1620	1,2,4,6,7,8,17,20	3,5,18,19,21,22,23,24	35	D4A5U3,iRT-Kit_WR_fusion,P02454,P02631,P02780,P05964,P06761,P06911,P11598,P12020,P15399,P17559,P18418,P19223,P19629,P22273,P22282,P31044,P46462,P50280,P55091,P80299,P97580,Q10758,Q30KJ2,Q4G075,Q5GRG2,Q62946,Q6P9T8,Q78P75,Q812E4,Q9JHB9,Q9JI85,Q9R0T3,Q9Z0J6
1621	1,2,4,6,7,8,17,21	3,5,18,19,20,22,23,24	20	B1H234,iRT-Kit_WR_fusion,O70594,P02631,P11598,P18418,P19629,P22282,P36860,P80299,P97580,Q00715,Q30KJ2,Q5GRG2,Q63474,Q63493,Q63598,Q78P75,Q9JI85,Q9Z1F2
1622	1,2,4,6,7,8,17,22	3,5,18,19,20,21,23,24	12	iRT-Kit_WR_fusion,O54858,P06911,P15399,P19629,P23593,P80299,P97580,Q30KJ2,Q5GRG2,Q99041,Q9Z1F2
1623	1,2,4,6,7,8,17,23	3,5,18,19,20,21,22,24	12	iRT-Kit_WR_fusion,O54858,O70594,P18418,P19629,P20761,P80299,P97580,Q30KJ2,Q63474,Q63532,Q63598
1624	1,2,4,6,7,8,17,24	3,5,18,19,20,21,22,23	13	P01039,P06911,P07379,P18418,P23593,P36860,P97580,Q30KJ2,Q5GRG2,Q63474,Q63598,Q9JHB9,Q9Z1F2
1625	1,2,4,6,7,8,18,19	3,5,17,20,21,22,23,24	19	iRT-Kit_WR_fusion,O55004,P06761,P11598,P11762,P15399,P18418,P19629,P22282,P31044,P50280,P80299,P97580,Q30KJ2,Q5GRG2,Q66H69,Q91ZS3,Q99041,Q9JI85

1626	1,2,4,6,7,8,18,20	3,5,17,19,21,22,23,24	35	D4A5U3,iRT-Kit_WR_fusion,O55004,P02454,P02631,P02780,P05964,P06761,P06911,P11598,P12020,P15399,P18418,P19629,P22273,P22282,P22283,P31044,P50280,P69897,P80299,P97580,Q30KJ2,Q4G075,Q5GRG2,Q63617,Q6P9T8,Q6RY07,Q78P75,Q80WL1,Q91ZS3,Q99041,Q9JHB9,Q9JI85,Q9QW07
1627	1,2,4,6,7,8,18,21	3,5,17,19,20,22,23,24	19	B1H234,iRT-Kit_WR_fusion,O55004,P02631,P11598,P18418,P19629,P22282,P36860,P50280,P80299,P97580,Q00715,Q30KJ2,Q63474,Q91ZS3,Q99041,Q9JI85,Q9Z1F2
1628	1,2,4,6,7,8,18,22	3,5,17,19,20,21,23,24	13	O54858,P15399,P18418,P19629,P20762,P23593,P50280,P80299,P97580,Q30KJ2,Q91ZS3,Q99041,Q9Z1F2
1629	1,2,4,6,7,8,18,23	3,5,17,19,20,21,22,24	15	O54858,O70594,P18418,P19629,P20761,P20762,P50280,P54921,P80299,P97580,Q30KJ2,Q3ZAV1,Q63532,Q63598,Q91ZS3
1630	1,2,4,6,7,8,18,24	3,5,17,19,20,21,22,23	12	O55004,P07151,P18418,P20762,P50280,P97580,Q30KJ2,Q3ZAV1,Q63598,Q91ZS3,Q9JHB9,Q9Z1F2
1631	1,2,4,6,7,8,19,20	3,5,17,18,21,22,23,24	43	D4A5U3,iRT-Kit_WR_fusion,P00714,P02631,P02780,P05964,P06761,P06911,P08723,P0C0A9,P10758,P11598,P11762,P12020,P15399,P18418,P19629,P22273,P22282,P22283,P31044,P36374,P42854,P46462,P50280,P80299,P97580,Q30KJ2,Q4G075,Q5GRG2,Q63493,Q63617,Q66H69,Q68G31,Q6P9T8,Q78P75,Q812E4,Q99041,Q9JHB9,Q9JI85,Q9QW07,Q9QZK8,Q9R0T3
1632	1,2,4,6,7,8,19,21	3,5,17,18,20,22,23,24	25	B1H234,iRT-Kit_WR_fusion,P00714,P02631,P06761,P0C0A9,P11598,P15399,P18418,P19629,P22282,P22283,P35952,P50280,P80299,P97580,Q00715,Q30KJ2,Q63474,Q63493,Q63617,Q66H69,Q6Q7Y5,Q99041,Q9JI85
1633	1,2,4,6,7,8,19,22	3,5,17,18,20,21,23,24	15	iRT-Kit_WR_fusion,O54858,P11762,P15399,P19629,P22282,P42854,P50280,P80299,P97580,Q30KJ2,Q4FZU4,Q66H69,Q99041,Q9JI85
1634	1,2,4,6,7,8,19,23	3,5,17,18,20,21,22,24	16	iRT-Kit_WR_fusion,O54858,P15399,P18418,P19629,P20761,P22282,P42854,P50280,P80299,P97580,Q30KJ2,Q63532,Q66H69,Q99041,Q9JI85
1635	1,2,4,6,7,8,19,24	3,5,17,18,20,21,22,23	20	iRT-Kit_WR_fusion,O54728,P00714,P07151,P08723,P0C0A9,P11598,P11762,P15399,P18418,P22282,P50280,P80299,P97580,Q30KJ2,Q63474,Q66H69,Q68G31,Q99041,Q9JI85
1636	1,2,4,6,7,8,20,21	3,5,17,18,19,22,23,24	48	B1H234,D4A5U3,iRT-Kit_WR_fusion,P00714,P02631,P02780,P02781,P05964,P06761,P06911,P07647,P08723,P0C0A9,P11598,P12020,P15399,P16228,P18418,P19629,P22273,P22282,P22283,P31044,P35952,P46462,P50280,P69897,P80299,Q00715,Q10758,Q30KJ2,Q4G075,Q5GRG2,Q5M8C6,Q62946,Q63474,Q63493,Q63617,Q6AYR9,Q78P75,Q812E4,Q8CJ52,Q91ZS3,Q99041,Q9JHB9,Q9JI85,Q9QW07,Q9R0T3

1637	1,2,4,6,7,8,20,22	3,5,17,18,19,21,23,24	26	D4A5U3,iRT-Kit_WR_fusion,P02780,P06911,P11598,P12020,P15399,P19629,P22273,P22282,P23593,P31044,P42854,P50280,P80299,P97580,Q30KJ2,Q4G075,Q5GRG2,Q6P9T8,Q78P75,Q91ZS3,Q99041,Q9JHB9,Q9JI85,Q9QW07
1638	1,2,4,6,7,8,20,23	3,5,17,18,19,21,22,24	26	D4A5U3,iRT-Kit_WR_fusion,P02780,P06911,P10758,P11598,P12020,P15399,P17559,P18418,P19629,P22273,P22282,P31044,P42854,P50280,P80299,Q30KJ2,Q4G075,Q5GRG2,Q63532,Q78P75,Q812E4,Q91ZS3,Q9JHB9,Q9JI85
1639	1,2,4,6,7,8,20,24	3,5,17,18,19,21,22,23	31	D4A5U3,iRT-Kit_WR_fusion,P00714,P02780,P02781,P06761,P06911,P08723,P09456,P11598,P12020,P15399,P18418,P19629,P22273,P22282,P22283,P31044,P46462,P50280,P80299,Q30KJ2,Q4G075,Q5GRG2,Q63617,Q78P75,Q812E4,Q91ZS3,Q9JHB9,Q9JI85,Q9QW07
1640	1,2,4,6,7,8,21,22	3,5,17,18,19,20,23,24	17	B1H234,iRT-Kit_WR_fusion,O54858,P02631,P11598,P15399,P19629,P23593,P50280,P80299,P97580,Q30KJ2,Q63474,Q91ZS3,Q99041,Q9JI85,Q9Z1F2
1641	1,2,4,6,7,8,21,23	3,5,17,18,19,20,22,24	13	iRT-Kit_WR_fusion,O54858,P02631,P11598,P18418,P19629,P20761,P36860,P80299,Q30KJ2,Q63474,Q63532,Q63598
1642	1,2,4,6,7,8,21,24	3,5,17,18,19,20,22,23	19	B1H234,iRT-Kit_WR_fusion,P00714,P02631,P02782,P11598,P16228,P18418,P19629,P22282,P36860,Q30KJ2,Q63474,Q63598,Q6AYR9,Q91ZS3,Q9JHB9,Q9JI85,Q9Z1F2
1643	1,2,4,6,7,8,22,23	3,5,17,18,19,20,21,24	13	iRT-Kit_WR_fusion,O54858,P15399,P19629,P20761,P20762,P23593,P42854,P80299,P97580,Q30KJ2,Q63532,Q99041
1644	1,2,4,6,7,8,22,24	3,5,17,18,19,20,21,23	11	iRT-Kit_WR_fusion,O54858,P15399,P23593,P50280,P80299,P97580,Q30KJ2,Q6P6R2,Q99041,Q9Z1F2
1645	1,2,4,6,7,8,23,24	3,5,17,18,19,20,21,22	12	iRT-Kit_WR_fusion,O54858,P18418,P19629,P20761,P80299,Q30KJ2,Q3ZAV1,Q63474,Q63532,Q63598,Q6P6R2
1646	1,2,4,6,7,17,18,19	3,5,8,20,21,22,23,24	3	B1H234,G3V686,P80299
1647	1,2,4,6,7,17,18,20	3,5,8,19,21,22,23,24	8	G3V686,P11598,P19223,P55091,P80299,Q5GRG2,Q80WL1,Q9Z0J6
1648	1,2,4,6,7,17,18,21	3,5,8,19,20,22,23,24	5	B1H234,P36860,P80299,Q9WVH8,Q9Z1F2
1649	1,2,4,6,7,17,18,22	3,5,8,19,20,21,23,24	4	D3ZUC6,P80299,Q9EQS0,Q9Z1F2
1650	1,2,4,6,7,17,18,23	3,5,8,19,20,21,22,24	2	P80299,Q9EQS0

1651	1,2,4,6,7,17,18,24	3,5,8,19,20,21,22,23	5	B1H234,D3ZUC6,P01039,P80299,Q9Z1F2
1652	1,2,4,6,7,17,19,20	3,5,8,18,21,22,23,24	9	P11598,P19223,P36374,P55091,P80299,Q5GRG2,Q63617,Q9JI85,Q9R0T3
1653	1,2,4,6,7,17,19,21	3,5,8,18,20,22,23,24	6	B1H234,P05539,P11598,P80299,Q63474,Q9R168
1654	1,2,4,6,7,17,19,22	3,5,8,18,20,21,23,24	3	D3ZUC6,P80299,Q9R168
1655	1,2,4,6,7,17,19,23	3,5,8,18,20,21,22,24	2	P80299,Q9R168
1656	1,2,4,6,7,17,19,24	3,5,8,18,20,21,22,23	5	B1H234,D3ZUC6,P01039,P80299,Q9R168
1657	1,2,4,6,7,17,20,21	3,5,8,18,19,22,23,24	12	B1H234,P05964,P07647,P11598,P19223,P19629,P22283,P55091,P80299,Q5GRG2,Q63617,Q9Z0J6
1658	1,2,4,6,7,17,20,22	3,5,8,18,19,21,23,24	5	P19223,P55091,P80299,Q5GRG2,Q9Z0J6
1659	1,2,4,6,7,17,20,23	3,5,8,18,19,21,22,24	8	P10758,P11598,P19223,P55091,P80299,Q5GRG2,Q63467,Q9QYP1
1660	1,2,4,6,7,17,20,24	3,5,8,18,19,21,22,23	8	P01039,P11598,P19223,P80299,Q5GRG2,Q9JHB9,Q9QYP1,Q9Z0J6
1661	1,2,4,6,7,17,21,22	3,5,8,18,19,20,23,24	5	B1H234,P36860,P80299,Q9R168,Q9Z1F2
1662	1,2,4,6,7,17,21,23	3,5,8,18,19,20,22,24	5	B1H234,P36860,P80299,Q63474,Q9R168
1663	1,2,4,6,7,17,21,24	3,5,8,18,19,20,22,23	8	B1H234,P01039,P36860,P80299,Q63474,Q6AY61,Q9R168,Q9Z1F2
1664	1,2,4,6,7,17,22,23	3,5,8,18,19,20,21,24	4	D3ZUC6,P80299,Q9EQS0,Q9R168
1665	1,2,4,6,7,17,22,24	3,5,8,18,19,20,21,23	4	D3ZUC6,P01039,P80299,Q9R168
1666	1,2,4,6,7,17,23,24	3,5,8,18,19,20,21,22	6	D3ZUC6,P01039,P36860,P80299,Q63598,Q9R168
1667	1,2,4,6,7,18,19,20	3,5,8,17,21,22,23,24	7	B1H234,P11598,P22283,P80299,Q5GRG2,Q63617,Q80WL1
1668	1,2,4,6,7,18,19,21	3,5,8,17,20,22,23,24	3	B1H234,P11598,P80299
1669	1,2,4,6,7,18,19,22	3,5,8,17,20,21,23,24	4	B1H234,P80299,Q80WL1,Q9EQS0
1670	1,2,4,6,7,18,19,23	3,5,8,17,20,21,22,24	2	P80299,Q9EQS0

1671	1,2,4,6,7,18,19,24	3,5,8,17,20,21,22,23	3	B1H234,P80299,Q5M872
1672	1,2,4,6,7,18,20,21	3,5,8,17,19,22,23,24	13	B1H234,O55145,P01835,P02631,P07647,P11598,P19629,P22283,P80299,Q5GRG2,Q63617,Q80WL1,Q91ZS3
1673	1,2,4,6,7,18,20,22	3,5,8,17,19,21,23,24	5	P11598,P80299,Q5GRG2,Q80WL1,Q9Z0J6
1674	1,2,4,6,7,18,20,23	3,5,8,17,19,21,22,24	7	P10758,P11598,P19629,P80299,Q5GRG2,Q80WL1,Q9QYP1
1675	1,2,4,6,7,18,20,24	3,5,8,17,19,21,22,23	6	B1H234,P11598,P80299,Q5GRG2,Q9JHB9,Q9QYP1
1676	1,2,4,6,7,18,21,22	3,5,8,17,19,20,23,24	5	B1H234,P01835,P23739,P80299,Q9Z1F2
1677	1,2,4,6,7,18,21,23	3,5,8,17,19,20,22,24	4	B1H234,O89117,P01835,P80299
1678	1,2,4,6,7,18,21,24	3,5,8,17,19,20,22,23	7	B1H234,O89117,P01835,P36376,P36860,P80299,Q9Z1F2
1679	1,2,4,6,7,18,22,23	3,5,8,17,19,20,21,24	6	P13676,P54921,P80299,Q63532,Q6P6R2,Q9EQS0
1680	1,2,4,6,7,18,22,24	3,5,8,17,19,20,21,23	8	B1H234,D3ZUC6,P06760,P13676,P22006,P80299,Q6P6R2,Q9EQS0
1681	1,2,4,6,7,18,23,24	3,5,8,17,19,20,21,22	4	P06760,P80299,Q6P6R2,Q9EQS0
1682	1,2,4,6,7,19,20,21	3,5,8,17,18,22,23,24	18	B1H234,O35547,P00714,P02780,P05369,P05964,P07647,P0C0A9,P11598,P19629,P22283,P36374,P80299,Q5GRG2,Q63617,Q8CJ52,Q9JI85,Q9R0T3
1683	1,2,4,6,7,19,20,22	3,5,8,17,18,21,23,24	5	P11598,P80299,Q5GRG2,Q63617,Q80WL1
1684	1,2,4,6,7,19,20,23	3,5,8,17,18,21,22,24	6	P10758,P11598,P19629,P42854,P80299,Q63617
1685	1,2,4,6,7,19,20,24	3,5,8,17,18,21,22,23	9	B1H234,P08723,P11598,P22283,P36374,P80299,Q5GRG2,Q63617,Q68G31
1686	1,2,4,6,7,19,21,22	3,5,8,17,18,20,23,24	3	B1H234,P80299,Q9R168
1687	1,2,4,6,7,19,21,23	3,5,8,17,18,20,22,24	3	B1H234,P80299,Q63474
1688	1,2,4,6,7,19,21,24	3,5,8,17,18,20,22,23	8	B1H234,P00714,P11598,P80299,Q5M872,Q62761;Q62762;Q62763,Q63474,Q9R168
1689	1,2,4,6,7,19,22,23	3,5,8,17,18,20,21,24	5	P42854,P80299,Q811M5,Q9EQS0,Q9R168
1690	1,2,4,6,7,19,22,24	3,5,8,17,18,20,21,23	5	B1H234,D3ZUC6,P22006,P80299,Q9R168

1691	1,2,4,6,7,19,23,24	3,5,8,17,18,20,21,22	1	P80299
1692	1,2,4,6,7,20,21,22	3,5,8,17,18,19,23,24	9	B1H234,O55145,P01835,P11598,P17046,P19629,P80299,Q5GRG2,Q63617
1693	1,2,4,6,7,20,21,23	3,5,8,17,18,19,22,24	6	B1H234,P01835,P10758,P11598,P19629,P80299
1694	1,2,4,6,7,20,21,24	3,5,8,17,18,19,22,23	13	B1H234,P01835,P07647,P11598,P19629,P22283,P80299,Q5GRG2,Q63474,Q63617,Q8CJ52,Q9JHB9,Q9QYP1
1695	1,2,4,6,7,20,22,23	3,5,8,17,18,19,21,24	4	P10758,P19629,P80299,Q9EQS0
1696	1,2,4,6,7,20,22,24	3,5,8,17,18,19,21,23	5	P01835,P11598,P22006,P80299,Q5GRG2
1697	1,2,4,6,7,20,23,24	3,5,8,17,18,19,21,22	5	P01835,P10758,P11598,P80299,Q9QYP1
1698	1,2,4,6,7,21,22,23	3,5,8,17,18,19,20,24	6	B1H234,P01835,P80299,Q63532,Q9EQS0,Q9R168
1699	1,2,4,6,7,21,22,24	3,5,8,17,18,19,20,23	6	B1H234,P01835,P36860,P80299,Q63474,Q9R168
1700	1,2,4,6,7,21,23,24	3,5,8,17,18,19,20,22	6	B1H234,P01835,P36860,P80299,Q63474,Q9R168
1701	1,2,4,6,7,22,23,24	3,5,8,17,18,19,20,21	10	D3ZUC6,P06760,P13676,P22006,P80299,Q63532,Q6P6R2,Q9EQS0,Q9QX74,Q9R168
1702	1,2,4,6,8,17,18,19	3,5,7,20,21,22,23,24	10	G3V686,P08649,P13432,P15399,P18418,P97580,Q30KJ2,Q66H69,Q68G31,Q9JI85
1703	1,2,4,6,8,17,18,20	3,5,7,19,21,22,23,24	17	D4A5U3,G3V686,P06911,P08649,P12020,P13432,P15399,P17559,P18418,P19223,P50280,P55091,P97580,Q10758,Q30KJ2,Q5GRG2,Q9JI85
1704	1,2,4,6,8,17,18,21	3,5,7,19,20,22,23,24	5	P02631,P13432,P18418,P97580,Q30KJ2
1705	1,2,4,6,8,17,18,22	3,5,7,19,20,21,23,24	8	D3ZUC6,G3V686,P08649,P13432,P15399,P23593,P97580,Q30KJ2
1706	1,2,4,6,8,17,18,23	3,5,7,19,20,21,22,24	4	P08649,P97580,Q30KJ2,Q63532
1707	1,2,4,6,8,17,18,24	3,5,7,19,20,21,22,23	5	P08649,P13432,P18418,P97580,Q30KJ2
1708	1,2,4,6,8,17,19,20	3,5,7,18,21,22,23,24	15	D4A5U3,O89117,P06911,P12020,P15399,P19223,P36374,P50280,P55091,P97580,Q10758,Q30KJ2,Q5GRG2,Q68G31,Q9JI85
1709	1,2,4,6,8,17,19,21	3,5,7,18,20,22,23,24	8	P02631,P15399,P97580,Q30KJ2,Q63474,Q66H69,Q68G31,Q9JI85
1710	1,2,4,6,8,17,19,22	3,5,7,18,20,21,23,24	5	O89117,P00507,P15399,P97580,Q30KJ2

1711	1,2,4,6,8,17,19,23	3,5,7,18,20,21,22,24	4	P15399,P97580,Q30KJ2,Q63532
1712	1,2,4,6,8,17,19,24	3,5,7,18,20,21,22,23	8	O54728,P15399,P97580,P97840,Q30KJ2,Q63474,Q68G31,Q9JI85
1713	1,2,4,6,8,17,20,21	3,5,7,18,19,22,23,24	16	D4A5U3,P02631,P06911,P12020,P15399,P17559,P19223,P19629,P25809,P50280,P55091,P63029,Q10758,Q30KJ2,Q5GRG2,Q9JI85
1714	1,2,4,6,8,17,20,22	3,5,7,18,19,21,23,24	11	D4A5U3,O89117,P06911,P12020,P15399,P19223,P50280,P55091,P97580,Q30KJ2,Q5GRG2
1715	1,2,4,6,8,17,20,23	3,5,7,18,19,21,22,24	12	D4A5U3,P06911,P12020,P15399,P17559,P19223,P19629,P50280,P55091,Q10758,Q30KJ2,Q5GRG2
1716	1,2,4,6,8,17,20,24	3,5,7,18,19,21,22,23	12	D4A5U3,P06911,P12020,P15399,P19223,P50280,Q10758,Q30KJ2,Q5GRG2,Q68G31,Q9JHB9,Q9JI85
1717	1,2,4,6,8,17,21,22	3,5,7,18,19,20,23,24	3	P15399,P97580,Q30KJ2
1718	1,2,4,6,8,17,21,23	3,5,7,18,19,20,22,24	3	Q30KJ2,Q63474,Q63532
1719	1,2,4,6,8,17,21,24	3,5,7,18,19,20,22,23	4	P36860,Q30KJ2,Q63474,Q6AYR9
1720	1,2,4,6,8,17,22,23	3,5,7,18,19,20,21,24	9	D3ZUC6,P00507,P15399,P23593,P47967,P97580,P97840,Q30KJ2,Q63532
1721	1,2,4,6,8,17,22,24	3,5,7,18,19,20,21,23	7	D3ZUC6,P15399,P23593,P47967,P97580,P97840,Q30KJ2
1722	1,2,4,6,8,17,23,24	3,5,7,18,19,20,21,22	6	D3ZUC6,P47967,P97840,Q30KJ2,Q63474,Q63532
1723	1,2,4,6,8,18,19,20	3,5,7,17,21,22,23,24	15	P02780,P06911,P12020,P15399,P18418,P19629,P22282,P36374,P50280,P97580,Q30KJ2,Q5GRG2,Q68G31,Q6P9T8,Q9JI85
1724	1,2,4,6,8,18,19,21	3,5,7,17,20,22,23,24	7	P02631,P15399,P97580,Q30KJ2,Q66H69,Q68G31,Q9JI85
1725	1,2,4,6,8,18,19,22	3,5,7,17,20,21,23,24	5	P00507,P15399,P50280,P97580,Q30KJ2
1726	1,2,4,6,8,18,19,23	3,5,7,17,20,21,22,24	4	P15399,P97580,Q30KJ2,Q63532
1727	1,2,4,6,8,18,19,24	3,5,7,17,20,21,22,23	6	O54728,P15399,P18418,P97580,Q30KJ2,Q68G31
1728	1,2,4,6,8,18,20,21	3,5,7,17,19,22,23,24	16	O55145,P02631,P06911,P12020,P15399,P18418,P19629,P25809,P50280,P54921,Q10758,Q30KJ2,Q5GRG2,Q68G31,Q91ZS3,Q9JI85
1729	1,2,4,6,8,18,20,22	3,5,7,17,19,21,23,24	11	P06911,P12020,P15399,P19629,P50280,P54921,P97580,Q30KJ2,Q5GRG2,Q6AY61,Q91ZS3
1730	1,2,4,6,8,18,20,23	3,5,7,17,19,21,22,24	12	P06911,P12020,P15399,P17559,P19629,P50280,P54921,Q10758,Q30KJ2,Q5GRG2,Q63532,Q91ZS3

1731	1,2,4,6,8,18,20,24	3,5,7,17,19,21,22,23	12	P06911,P12020,P15399,P18418,P50280,Q30KJ2,Q5GRG2,Q68G31,Q91ZS3,Q9JHB9,Q9JI85,Q9QW07
1732	1,2,4,6,8,18,21,22	3,5,7,17,19,20,23,24	5	P15399,P54921,P97580,Q30KJ2,Q91ZS3
1733	1,2,4,6,8,18,21,23	3,5,7,17,19,20,22,24	4	P02631,P54921,Q30KJ2,Q63532
1734	1,2,4,6,8,18,21,24	3,5,7,17,19,20,22,23	6	P02631,P18418,Q30KJ2,Q63474,Q68G31,Q91ZS3
1735	1,2,4,6,8,18,22,23	3,5,7,17,19,20,21,24	10	O54858,P00507,P15399,P20762,P54921,P97580,Q30KJ2,Q63532,Q6AY61,Q99MH3
1736	1,2,4,6,8,18,22,24	3,5,7,17,19,20,21,23	6	D3ZUC6,P15399,P22006,P23593,P97580,Q30KJ2
1737	1,2,4,6,8,18,23,24	3,5,7,17,19,20,21,22	3	P20762,Q30KJ2,Q63532
1738	1,2,4,6,8,19,20,21	3,5,7,17,18,22,23,24	24	D4A5U3,O35547,P02631,P02780,P04905,P05369,P06911,P07647,P12020,P15399,P19629,P22282,P22283,P35952,P36374,P46462,P50280,Q10758,Q30KJ2,Q5GRG2,Q63493,Q68G31,Q812E4,Q9JI85
1739	1,2,4,6,8,19,20,22	3,5,7,17,18,21,23,24	12	P06911,P12020,P15399,P19629,P42854,P50280,P97580,Q30KJ2,Q5GRG2,Q68G31,Q6P9T8,Q9JI85
1740	1,2,4,6,8,19,20,23	3,5,7,17,18,21,22,24	11	P06911,P10758,P12020,P15399,P19629,P42854,P50280,Q30KJ2,Q5GRG2,Q68G31,Q9JI85
1741	1,2,4,6,8,19,20,24	3,5,7,17,18,21,22,23	16	O35547,O54728,P06911,P08723,P12020,P15399,P22282,P36374,P50280,P60905,Q30KJ2,Q5GRG2,Q68G31,Q812E4,Q9JI85,Q9QW07
1742	1,2,4,6,8,19,21,22	3,5,7,17,18,20,23,24	6	O54861,P15399,P30919,P97580,Q30KJ2,Q9JI85
1743	1,2,4,6,8,19,21,23	3,5,7,17,18,20,22,24	6	O54861,P42854,Q30KJ2,Q63474,Q63532,Q9JI85
1744	1,2,4,6,8,19,21,24	3,5,7,17,18,20,22,23	9	O54728,P07174,P15399,P30919,Q10743,Q30KJ2,Q63474,Q68G31,Q9JI85
1745	1,2,4,6,8,19,22,23	3,5,7,17,18,20,21,24	9	O54861,P00507,P15399,P42854,P97580,Q30KJ2,Q63532,Q6AY61,Q811M5
1746	1,2,4,6,8,19,22,24	3,5,7,17,18,20,21,23	8	D3ZUC6,O54728,P00507,P15399,P30919,P97580,Q30KJ2,Q68G31
1747	1,2,4,6,8,19,23,24	3,5,7,17,18,20,21,22	7	O54728,P00507,P15399,Q30KJ2,Q63474,Q63532,Q68G31
1748	1,2,4,6,8,20,21,22	3,5,7,17,18,19,23,24	13	O55145,P02631,P06911,P12020,P15399,P19629,P50280,P54921,P97580,Q10743,Q30KJ2,Q5GRG2,Q9JI85
1749	1,2,4,6,8,20,21,23	3,5,7,17,18,19,22,24	10	P02631,P06911,P12020,P19629,P50280,P54921,Q10758,Q5GRG2,Q63532,Q9JI85
1750	1,2,4,6,8,20,21,24	3,5,7,17,18,19,22,23	19	P02631,P02782,P06911,P07647,P08723,P12020,P15399,P19629,P30120,P50280,Q10743,Q10758,Q5GRG2,Q63474,Q68G31,Q6AYR9,Q812E4,Q9JHB9,Q9JI85

1751	1,2,4,6,8,20,22,23	3,5,7,17,18,19,21,24	13	P06911,P12020,P15399,P19629,P42854,P50280,P54921,P97580,Q30KJ2,Q5GRG2,Q63532,Q6AY61,Q99MH3
1752	1,2,4,6,8,20,22,24	3,5,7,17,18,19,21,23	9	P06911,P12020,P15399,P22006,P50280,P97580,Q10743,Q30KJ2,Q5GRG2
1753	1,2,4,6,8,20,23,24	3,5,7,17,18,19,21,22	8	P06911,P12020,P15399,P19629,P50280,Q5GRG2,Q63532,Q9QYP1
1754	1,2,4,6,8,21,22,23	3,5,7,17,18,19,20,24	6	P15399,P30919,P54921,P97580,Q30KJ2,Q63532
1755	1,2,4,6,8,21,22,24	3,5,7,17,18,19,20,23	6	P15399,P30919,P97580,Q10743,Q30KJ2,Q63474
1756	1,2,4,6,8,21,23,24	3,5,7,17,18,19,20,22	4	P30919,P36860,Q63474,Q63532
1757	1,2,4,6,8,22,23,24	3,5,7,17,18,19,20,21	13	D3ZUC6,O54858,P00507,P06760,P15399,P23593,P30919,P47967,P97580,P97840,Q30KJ2,Q63532,Q6P6R2
1758	1,2,4,6,17,18,19,20	3,5,7,8,21,22,23,24	7	G3V686,P08649,P19223,P35280,P55091,Q5GRG2,Q62714
1759	1,2,4,6,17,18,19,21	3,5,7,8,20,22,23,24	1	P35280
1760	1,2,4,6,17,18,19,22	3,5,7,8,20,21,23,24	4	D3ZUC6,G3V686,P08649,Q9EQS0
1761	1,2,4,6,17,18,19,23	3,5,7,8,20,21,22,24	2	P35280,Q9EQS0
1762	1,2,4,6,17,18,19,24	3,5,7,8,20,21,22,23	2	D3ZUC6,P35280
1763	1,2,4,6,17,18,20,21	3,5,7,8,19,22,23,24	5	P01041,P19223,P35280,P55091,Q5GRG2
1764	1,2,4,6,17,18,20,22	3,5,7,8,19,21,23,24	6	D3ZUC6,G3V686,P19223,P55091,Q5GRG2,Q9Z0J6
1765	1,2,4,6,17,18,20,23	3,5,7,8,19,21,22,24	5	P19223,P35280,P55091,Q63467,Q9QYP1
1766	1,2,4,6,17,18,20,24	3,5,7,8,19,21,22,23	5	O35077,P19223,P35280,Q5GRG2,Q9QYP1
1767	1,2,4,6,17,18,21,22	3,5,7,8,19,20,23,24	2	D3ZUC6,P23739
1768	1,2,4,6,17,18,21,23	3,5,7,8,19,20,22,24	1	P35280
1769	1,2,4,6,17,18,21,24	3,5,7,8,19,20,22,23	3	P01039,P35280,P36860
1770	1,2,4,6,17,18,22,23	3,5,7,8,19,20,21,24	4	D3ZUC6,P06760,P47967,Q9EQS0

1771	1,2,4,6,17,<br>18,22,24	3,5,7,8,19,<br>20,21,23	6	D3ZUC6,P06760,P22006,P47967,Q9EQS0,Q9WTT6
1772	1,2,4,6,17,<br>18,23,24	3,5,7,8,19,<br>20,21,22	7	D3ZUC6,P06760,P08937,P35280,P47967,Q9EQS0,Q9WTT6
1773	1,2,4,6,17,<br>19,20,21	3,5,7,8,18,<br>22,23,24	5	P19223,P35280,P36374,Q68G31,Q9JI85
1774	1,2,4,6,17,<br>19,20,22	3,5,7,8,18,<br>21,23,24	2	O89117,P19223
1775	1,2,4,6,17,<br>19,20,23	3,5,7,8,18,<br>21,22,24	3	P10758,P19223,P35280
1776	1,2,4,6,17,<br>19,20,24	3,5,7,8,18,<br>21,22,23	4	P19223,P35280,P36374,Q68G31
1777	1,2,4,6,17,<br>19,21,22	3,5,7,8,18,<br>20,23,24	1	Q9R168
1778	1,2,4,6,17,<br>19,21,23	3,5,7,8,18,<br>20,22,24	2	P35280,Q63474
1779	1,2,4,6,17,<br>19,21,24	3,5,7,8,18,<br>20,22,23	4	P35280,Q63474,Q68G31,Q9R168
1780	1,2,4,6,17,<br>19,22,23	3,5,7,8,18,<br>20,21,24	6	D3ZUC6,P00507,P06760,P47967,Q811M5,Q9EQS0
1781	1,2,4,6,17,<br>19,22,24	3,5,7,8,18,<br>20,21,23	4	D3ZUC6,P00507,P06760,Q9R168
1782	1,2,4,6,17,<br>19,23,24	3,5,7,8,18,<br>20,21,22	4	D3ZUC6,P06760,P35280,P47967
1783	1,2,4,6,17,<br>20,21,22	3,5,7,8,18,<br>19,23,24	2	P19223,Q9Z0J6
1784	1,2,4,6,17,<br>20,21,23	3,5,7,8,18,<br>19,22,24	4	P19223,P35280,P55091,P63029
1785	1,2,4,6,17,<br>20,21,24	3,5,7,8,18,<br>19,22,23	3	O35077,P19223,P35280
1786	1,2,4,6,17,<br>20,22,23	3,5,7,8,18,<br>19,21,24	3	D3ZUC6,P19223,Q9EQS0
1787	1,2,4,6,17,<br>20,22,24	3,5,7,8,18,<br>19,21,23	6	D3ZUC6,O35077,P06760,P19223,P22006,Q9Z0J6
1788	1,2,4,6,17,<br>20,23,24	3,5,7,8,18,<br>19,21,22	7	D3ZUC6,O35077,P06760,P08937,P19223,P35280,Q9QYP1
1789	1,2,4,6,17,<br>21,22,23	3,5,7,8,18,<br>19,20,24	2	D3ZUC6,Q9R168
1790	1,2,4,6,17,<br>21,22,24	3,5,7,8,18,<br>19,20,23	3	D3ZUC6,P36860,Q9R168

1791	1,2,4,6,17,<br>21,23,24	3,5,7,8,18,<br>19,20,22	6	D3ZUC6,P35280,P36860,P47967,Q63474,Q9R168
1792	1,2,4,6,17,<br>22,23,24	3,5,7,8,18,<br>19,20,21	11	D3ZUC6,P00507,P06760,P07335,P08937,P47967,P97840,Q5I0D1,Q9EQS0,Q9R168,Q9WTT6
1793	1,2,4,6,18,<br>19,20,21	3,5,7,8,17,<br>22,23,24	3	P35280,Q68G31,Q9JI85
1794	1,2,4,6,18,<br>19,20,22	3,5,7,8,17,<br>21,23,24	2	Q80WL1,Q9EQS0
1795	1,2,4,6,18,<br>19,20,23	3,5,7,8,17,<br>21,22,24	2	P10758,P35280
1796	1,2,4,6,18,<br>19,20,24	3,5,7,8,17,<br>21,22,23	2	P35280,Q68G31
1797	1,2,4,6,18,<br>19,21,22	3,5,7,8,17,<br>20,23,24	1	P23739
1798	1,2,4,6,18,<br>19,21,23	3,5,7,8,17,<br>20,22,24	2	P06866,P35280
1799	1,2,4,6,18,<br>19,21,24	3,5,7,8,17,<br>20,22,23	3	B1H234,P35280,Q68G31
1800	1,2,4,6,18,<br>19,22,23	3,5,7,8,17,<br>20,21,24	7	D3ZUC6,P00507,P06760,P13676,Q811M5,Q9EQS0,Q9WTT6
1801	1,2,4,6,18,<br>19,22,24	3,5,7,8,17,<br>20,21,23	8	D3ZUC6,P00507,P06760,P13676,P22006,Q9EQS0,Q9QX74,Q9WTT6
1802	1,2,4,6,18,<br>19,23,24	3,5,7,8,17,<br>20,21,22	7	D3ZUC6,P06760,P06866,P13676,P35280,Q9EQS0,Q9WTT6
1803	1,2,4,6,18,<br>20,21,22	3,5,7,8,17,<br>19,23,24	3	O55145,P54921,Q5PQL7
1804	1,2,4,6,18,<br>20,21,23	3,5,7,8,17,<br>19,22,24	2	P35280,P54921
1805	1,2,4,6,18,<br>20,21,24	3,5,7,8,17,<br>19,22,23	2	P35280,Q68G31
1806	1,2,4,6,18,<br>20,22,23	3,5,7,8,17,<br>19,21,24	5	P04355,P06760,P54921,Q9EQS0,Q9QX74
1807	1,2,4,6,18,<br>20,22,24	3,5,7,8,17,<br>19,21,23	4	D3ZUC6,P06760,P22006,Q9QX74
1808	1,2,4,6,18,<br>20,23,24	3,5,7,8,17,<br>19,21,22	5	P06760,P35280,Q5QE79,Q9QX74,Q9QYP1
1809	1,2,4,6,18,<br>21,22,23	3,5,7,8,17,<br>19,20,24	5	P13676,P54921,Q63532,Q9EQS0,Q9WTT6
1810	1,2,4,6,18,<br>21,22,24	3,5,7,8,17,<br>19,20,23	3	D3ZUC6,P13676,Q9WTT6

1811	1,2,4,6,18,21,23,24	3,5,7,8,17,19,20,22	3	P13676,P35280,Q9WTT6
1812	1,2,4,6,18,22,23,24	3,5,7,8,17,19,20,21	12	D3ZUC6,P00507,P06760,P13676,P22006,P47967,Q5I0D1,Q6P6R2,Q811M5,Q9EQS0,Q9QX74,Q9WTT6
1813	1,2,4,6,19,20,21,22	3,5,7,8,17,18,23,24	1	Q5PQL7
1814	1,2,4,6,19,20,21,23	3,5,7,8,17,18,22,24	2	P10758,P35280
1815	1,2,4,6,19,20,21,24	3,5,7,8,17,18,22,23	7	O35547,P05369,P07647,P35280,P36374,Q68G31,Q9JI85
1816	1,2,4,6,19,20,22,23	3,5,7,8,17,18,21,24	5	P06760,P10758,Q811M5,Q9EQS0,Q9QX74
1817	1,2,4,6,19,20,22,24	3,5,7,8,17,18,21,23	5	D3ZUC6,P06760,P22006,Q68G31,Q9QX74
1818	1,2,4,6,19,20,23,24	3,5,7,8,17,18,21,22	6	P06760,P10758,P35280,Q68G31,Q9QX74,Q9QYP1
1819	1,2,4,6,19,21,22,23	3,5,7,8,17,18,20,24	3	P13676,Q811M5,Q9EQS0
1820	1,2,4,6,19,21,22,24	3,5,7,8,17,18,20,23	3	D3ZUC6,P30919,Q9R168
1821	1,2,4,6,19,21,23,24	3,5,7,8,17,18,20,22	3	P07174,P35280,Q63474
1822	1,2,4,6,19,22,23,24	3,5,7,8,17,18,20,21	12	D3ZUC6,P00507,P06760,P13676,P22006,P26772,P47967,Q6P6R2,Q811M5,Q9EQS0,Q9QX74,Q9WTT6
1823	1,2,4,6,20,21,22,23	3,5,7,8,17,18,19,24	1	P54921
1824	1,2,4,6,20,21,22,24	3,5,7,8,17,18,19,23	4	O55145,P01835,P22006,Q9QX74
1825	1,2,4,6,20,21,23,24	3,5,7,8,17,18,19,22	3	P01835,P35280,Q9QYP1
1826	1,2,4,6,20,22,23,24	3,5,7,8,17,18,19,21	8	D3ZUC6,P06760,P22006,Q5QE79,Q6P6R2,Q9EQS0,Q9QX74,Q9QYP1
1827	1,2,4,6,21,22,23,24	3,5,7,8,17,18,19,20	8	D3ZUC6,P13676,P30919,Q63532,Q811M5,Q9QX74,Q9R168,Q9WTT6
1828	1,2,4,7,8,17,18,19	3,5,6,20,21,22,23,24	4	G3V686,P20646,Q66H69,Q9Z1F2
1829	1,2,4,7,8,17,18,20	3,5,6,19,21,22,23,24	13	D4A5U3,G3V686,P06911,P19223,P25809,P47727,P55091,Q10758,Q4G075,Q5GRG2,Q811M5,Q9JHB9,Q9Z1F2
1830	1,2,4,7,8,17,18,21	3,5,6,19,20,22,23,24	5	P62804,Q00715,Q811M5,Q9QX74,Q9Z1F2

1831	1,2,4,7,8,17,18,22	3,5,6,19,20,21,23,24	4	G3V686,P97580,Q30KJ2,Q9Z1F2
1832	1,2,4,7,8,17,18,23	3,5,6,19,20,21,22,24	3	O70594,Q63598,Q9Z1F2
1833	1,2,4,7,8,17,18,24	3,5,6,19,20,21,22,23	3	G3V686,Q63598,Q9Z1F2
1834	1,2,4,7,8,17,19,20	3,5,6,18,21,22,23,24	17	D4A5U3,iRT-Kit_WR_fusion,P02780,P06911,P08723,P13676,P19223,P20646,P22282,P36374,P47727,Q4G075,Q5GRG2,Q66H69,Q811M5,Q812E4,Q9WTT6
1835	1,2,4,7,8,17,19,21	3,5,6,18,20,22,23,24	9	iRT-Kit_WR_fusion,P20646,P22006,P62804,Q00715,Q63474,Q66H69,Q9QX74,Q9Z1F2
1836	1,2,4,7,8,17,19,22	3,5,6,18,20,21,23,24	5	iRT-Kit_WR_fusion,P20646,P97580,Q30KJ2,Q66H69
1837	1,2,4,7,8,17,19,23	3,5,6,18,20,21,22,24	3	iRT-Kit_WR_fusion,P21674,Q66H69
1838	1,2,4,7,8,17,19,24	3,5,6,18,20,21,22,23	3	O54728,Q66H69,Q9Z1F2
1839	1,2,4,7,8,17,20,21	3,5,6,18,19,22,23,24	22	D4A5U3,iRT-Kit_WR_fusion,P02780,P05964,P06911,P13676,P19223,P25809,P47727,P55091,P62804,Q00715,Q10758,Q4G075,Q5GRG2,Q63493,Q6AYR9,Q811M5,Q812E4,Q9JHB9,Q9WTT6,Q9Z1F2
1840	1,2,4,7,8,17,20,22	3,5,6,18,19,21,23,24	5	D4A5U3,P06911,P19223,Q4G075,Q5GRG2
1841	1,2,4,7,8,17,20,23	3,5,6,18,19,21,22,24	8	D4A5U3,P06911,P10758,P19223,P55091,Q4G075,Q5GRG2,Q9QYP1
1842	1,2,4,7,8,17,20,24	3,5,6,18,19,21,22,23	11	D4A5U3,P06911,P08723,P19223,P47727,Q4G075,Q5GRG2,Q811M5,Q812E4,Q9JHB9,Q9QYP1
1843	1,2,4,7,8,17,21,22	3,5,6,18,19,20,23,24	2	iRT-Kit_WR_fusion,Q9Z1F2
1844	1,2,4,7,8,17,21,23	3,5,6,18,19,20,22,24	4	iRT-Kit_WR_fusion,Q63474,Q9Z0V6,Q9Z1F2
1845	1,2,4,7,8,17,21,24	3,5,6,18,19,20,22,23	6	P02782,P36860,Q63474,Q6AYR9,Q811M5,Q9Z1F2
1846	1,2,4,7,8,17,22,23	3,5,6,18,19,20,21,24	2	Q30KJ2,Q9Z1F2
1847	1,2,4,7,8,17,22,24	3,5,6,18,19,20,21,23	2	P23593,Q9Z1F2
1848	1,2,4,7,8,17,23,24	3,5,6,18,19,20,21,22	4	Q5PQL7,Q63474,Q63598,Q9Z1F2
1849	1,2,4,7,8,18,19,20	3,5,6,17,21,22,23,24	11	D4A5U3,P02780,P06911,P22282,P36374,P47727,P50280,Q4G075,Q5GRG2,Q66H69,Q812E4

1850	1,2,4,7,8,18,19,21	3,5,6,17,20,22,23,24	5	iRT-Kit_WR_fusion,P62804,Q00715,Q66H69,Q9Z1F2
1851	1,2,4,7,8,18,19,22	3,5,6,17,20,21,23,24	4	P97580,Q30KJ2,Q66H69,Q9Z1F2
1852	1,2,4,7,8,18,19,23	3,5,6,17,20,21,22,24	2	P20762,Q66H69
1853	1,2,4,7,8,18,19,24	3,5,6,17,20,21,22,23	3	O54728,Q66H69,Q9Z1F2
1854	1,2,4,7,8,18,20,21	3,5,6,17,19,22,23,24	17	D4A5U3,iRT-Kit_WR_fusion,P02780,P06911,P25809,P47727,P50280,P62804,Q00715,Q10758,Q4G075,Q5GRG2,Q811M5,Q812E4,Q91ZS3,Q9JHB9,Q9Z1F2
1855	1,2,4,7,8,18,20,22	3,5,6,17,19,21,23,24	6	P02780,P06911,P50280,Q30KJ2,Q4G075,Q5GRG2
1856	1,2,4,7,8,18,20,23	3,5,6,17,19,21,22,24	4	P06911,P10758,P20762,Q4G075
1857	1,2,4,7,8,18,20,24	3,5,6,17,19,21,22,23	10	P02780,P06911,P08723,P50280,Q4G075,Q5GRG2,Q812E4,Q9JHB9,Q9QYP1,Q9Z1F2
1858	1,2,4,7,8,18,21,22	3,5,6,17,19,20,23,24	2	Q30KJ2,Q9Z1F2
1859	1,2,4,7,8,18,21,23	3,5,6,17,19,20,22,24	2	O89117,Q9Z1F2
1860	1,2,4,7,8,18,21,24	3,5,6,17,19,20,22,23	4	O89117,Q00715,Q63474,Q9Z1F2
1861	1,2,4,7,8,18,22,23	3,5,6,17,19,20,21,24	5	P20762,P54921,Q30KJ2,Q6AYQ8,Q9Z1F2
1862	1,2,4,7,8,18,22,24	3,5,6,17,19,20,21,23	2	Q30KJ2,Q9Z1F2
1863	1,2,4,7,8,18,23,24	3,5,6,17,19,20,21,22	5	P20762,Q3ZAV1,Q63598,Q6AYQ8,Q9Z1F2
1864	1,2,4,7,8,19,20,21	3,5,6,17,18,22,23,24	27	D4A5U3,iRT-Kit_WR_fusion,O08815,P02780,P02781,P04905,P05369,P08723,P0C0A9,P22282,P22283,P24368,P30120,P36374,P40241,P50280,P62804,Q00715,Q4G075,Q5I0D1,Q63493,Q63617,Q66H69,Q812E4,Q9JHB9,Q9JI85,Q9WTT6
1865	1,2,4,7,8,19,20,22	3,5,6,17,18,21,23,24	7	D4A5U3,iRT-Kit_WR_fusion,P02780,P36374,P50280,P80299,Q4G075
1866	1,2,4,7,8,19,20,23	3,5,6,17,18,21,22,24	6	D4A5U3,iRT-Kit_WR_fusion,P02780,P10758,Q4G075,Q812E4
1867	1,2,4,7,8,19,20,24	3,5,6,17,18,21,22,23	15	D4A5U3,iRT-Kit_WR_fusion,O54728,P02780,P08723,P09456,P0C0A9,P22282,P36374,P50280,Q4G075,Q66H69,Q6TMA8,Q812E4,Q9JHB9

1868	1,2,4,7,8,19,21,22	3,5,6,17,18,20,23,24	2	iRT-Kit_WR_fusion,Q66H69
1869	1,2,4,7,8,19,21,23	3,5,6,17,18,20,22,24	3	iRT-Kit_WR_fusion,Q63474,Q66H69
1870	1,2,4,7,8,19,21,24	3,5,6,17,18,20,22,23	6	iRT-Kit_WR_fusion,O54728,P02782,Q63474,Q66H69,Q9Z1F2
1871	1,2,4,7,8,19,22,23	3,5,6,17,18,20,21,24	2	iRT-Kit_WR_fusion,Q30KJ2
1872	1,2,4,7,8,19,22,24	3,5,6,17,18,20,21,23	2	iRT-Kit_WR_fusion,O54728
1873	1,2,4,7,8,19,23,24	3,5,6,17,18,20,21,22	4	iRT-Kit_WR_fusion,O54728,Q66H69,Q6AYQ8
1874	1,2,4,7,8,20,21,22	3,5,6,17,18,19,23,24	6	D4A5U3,iRT-Kit_WR_fusion,P02780,Q4G075,Q812E4,Q9JHB9
1875	1,2,4,7,8,20,21,23	3,5,6,17,18,19,22,24	8	D4A5U3,iRT-Kit_WR_fusion,P02780,P10758,P19629,Q10758,Q4G075,Q812E4
1876	1,2,4,7,8,20,21,24	3,5,6,17,18,19,22,23	13	D4A5U3,iRT-Kit_WR_fusion,P02780,P02781,P02782,P08723,P09456,P30120,Q63474,Q6AYR9,Q811M5,Q812E4,Q9JHB9
1877	1,2,4,7,8,20,22,23	3,5,6,17,18,19,21,24	2	P10758,Q4G075
1878	1,2,4,7,8,20,22,24	3,5,6,17,18,19,21,23	2	Q4G075,Q9JHB9
1879	1,2,4,7,8,20,23,24	3,5,6,17,18,19,21,22	4	P10758,Q4G075,Q812E4,Q9QYP1
1880	1,2,4,7,8,21,22,23	3,5,6,17,18,19,20,24	2	iRT-Kit_WR_fusion,Q9Z1F2
1881	1,2,4,7,8,21,22,24	3,5,6,17,18,19,20,23	4	iRT-Kit_WR_fusion,Q63474,Q6AYR9,Q9Z1F2
1882	1,2,4,7,8,21,23,24	3,5,6,17,18,19,20,22	4	iRT-Kit_WR_fusion,Q63474,Q6AYR9,Q9Z1F2
1883	1,2,4,7,8,22,23,24	3,5,6,17,18,19,20,21	3	Q6AYQ8,Q6P6R2,Q9Z1F2
1884	1,2,4,7,17,18,19,20	3,5,6,8,21,22,23,24	6	G3V686,P19223,P20646,P30919,P36374,Q62714
1885	1,2,4,7,17,18,19,21	3,5,6,8,20,22,23,24	1	P20646
1886	1,2,4,7,17,18,19,22	3,5,6,8,20,21,23,24	2	G3V686,P80299
1887	1,2,4,7,17,18,19,23	3,5,6,8,20,21,22,24	0	

1888	1,2,4,7,17, 18,19,24	3,5,6,8,20, 21,22,23	0	
1889	1,2,4,7,17, 18,20,21	3,5,6,8,19, 22,23,24	5	P00507,P19223,P25809,P55091,Q811M5
1890	1,2,4,7,17, 18,20,22	3,5,6,8,19, 21,23,24	3	G3V686,P19223,Q9Z0J6
1891	1,2,4,7,17, 18,20,23	3,5,6,8,19, 21,22,24	5	P10758,P19223,P30919,P55091,Q9QYP1
1892	1,2,4,7,17, 18,20,24	3,5,6,8,19, 21,22,23	4	P08937,P19223,Q811M5,Q9QYP1
1893	1,2,4,7,17, 18,21,22	3,5,6,8,19, 20,23,24	1	Q9Z1F2
1894	1,2,4,7,17, 18,21,23	3,5,6,8,19, 20,22,24	1	Q9Z1F2
1895	1,2,4,7,17, 18,21,24	3,5,6,8,19, 20,22,23	2	P19218,Q9Z1F2
1896	1,2,4,7,17, 18,22,23	3,5,6,8,19, 20,21,24	1	Q9EQS0
1897	1,2,4,7,17, 18,22,24	3,5,6,8,19, 20,21,23	2	D3ZUC6,Q9Z1F2
1898	1,2,4,7,17, 18,23,24	3,5,6,8,19, 20,21,22	2	P08937,Q64268
1899	1,2,4,7,17, 19,20,21	3,5,6,8,18, 22,23,24	3	P19223,P20646,P36374
1900	1,2,4,7,17, 19,20,22	3,5,6,8,18, 21,23,24	3	P19223,P20646,P80299
1901	1,2,4,7,17, 19,20,23	3,5,6,8,18, 21,22,24	3	P10758,P19223,P80299
1902	1,2,4,7,17, 19,20,24	3,5,6,8,18, 21,22,23	4	P19223,P36374,P54921,Q9QYP1
1903	1,2,4,7,17, 19,21,22	3,5,6,8,18, 20,23,24	2	P80299,Q9R168
1904	1,2,4,7,17, 19,21,23	3,5,6,8,18, 20,22,24	1	Q9R168
1905	1,2,4,7,17, 19,21,24	3,5,6,8,18, 20,22,23	3	Q62761;Q62762;Q62763,Q63474,Q9R168
1906	1,2,4,7,17, 19,22,23	3,5,6,8,18, 20,21,24	2	P80299,Q9R168
1907	1,2,4,7,17, 19,22,24	3,5,6,8,18, 20,21,23	2	D3ZUC6,Q9R168

1908	1,2,4,7,17,<br>19,23,24	3,5,6,8,18,<br>20,21,22	1	Q9R168
1909	1,2,4,7,17,<br>20,21,22	3,5,6,8,18,<br>19,23,24	4	P19223,P80299,Q9R168,Q9Z0J6
1910	1,2,4,7,17,<br>20,21,23	3,5,6,8,18,<br>19,22,24	2	P19223,Q9QYP1
1911	1,2,4,7,17,<br>20,21,24	3,5,6,8,18,<br>19,22,23	3	P19223,Q811M5,Q9QYP1
1912	1,2,4,7,17,<br>20,22,23	3,5,6,8,18,<br>19,21,24	4	P08937,P19223,P80299,Q9QYP1
1913	1,2,4,7,17,<br>20,22,24	3,5,6,8,18,<br>19,21,23	4	P08937,P19223,Q9QYP1,Q9Z0J6
1914	1,2,4,7,17,<br>20,23,24	3,5,6,8,18,<br>19,21,22	4	P08937,P10758,P19223,Q9QYP1
1915	1,2,4,7,17,<br>21,22,23	3,5,6,8,18,<br>19,20,24	2	P80299,Q9R168
1916	1,2,4,7,17,<br>21,22,24	3,5,6,8,18,<br>19,20,23	2	Q9R168,Q9Z1F2
1917	1,2,4,7,17,<br>21,23,24	3,5,6,8,18,<br>19,20,22	3	P36860,Q63474,Q9R168
1918	1,2,4,7,17,<br>22,23,24	3,5,6,8,18,<br>19,20,21	3	D3ZUC6,P08937,Q9R168
1919	1,2,4,7,18,<br>19,20,21	3,5,6,8,17,<br>22,23,24	2	O08815,P80299
1920	1,2,4,7,18,<br>19,20,22	3,5,6,8,17,<br>21,23,24	2	P80299,Q80WL1
1921	1,2,4,7,18,<br>19,20,23	3,5,6,8,17,<br>21,22,24	2	P10758,P80299
1922	1,2,4,7,18,<br>19,20,24	3,5,6,8,17,<br>21,22,23	1	Q9QYP1
1923	1,2,4,7,18,<br>19,21,22	3,5,6,8,17,<br>20,23,24	2	P36376,P80299
1924	1,2,4,7,18,<br>19,21,23	3,5,6,8,17,<br>20,22,24	2	P06866,P80299
1925	1,2,4,7,18,<br>19,21,24	3,5,6,8,17,<br>20,22,23	1	P36376
1926	1,2,4,7,18,<br>19,22,23	3,5,6,8,17,<br>20,21,24	3	P80299,Q6AYQ8,Q9EQS0
1927	1,2,4,7,18,<br>19,22,24	3,5,6,8,17,<br>20,21,23	1	P36376

1928	1,2,4,7,18,<br>19,23,24	3,5,6,8,17,<br>20,21,22	1 Q6AYQ8
1929	1,2,4,7,18,<br>20,21,22	3,5,6,8,17,<br>19,23,24	1 P80299
1930	1,2,4,7,18,<br>20,21,23	3,5,6,8,17,<br>19,22,24	2 P10758,Q9QYP1
1931	1,2,4,7,18,<br>20,21,24	3,5,6,8,17,<br>19,22,23	1 Q9QYP1
1932	1,2,4,7,18,<br>20,22,23	3,5,6,8,17,<br>19,21,24	3 P10758,P80299,Q9EQS0
1933	1,2,4,7,18,<br>20,22,24	3,5,6,8,17,<br>19,21,23	1 Q9QYP1
1934	1,2,4,7,18,<br>20,23,24	3,5,6,8,17,<br>19,21,22	3 P08937,P10758,Q9QYP1
1935	1,2,4,7,18,<br>21,22,23	3,5,6,8,17,<br>19,20,24	1 P80299
1936	1,2,4,7,18,<br>21,22,24	3,5,6,8,17,<br>19,20,23	2 P36376,Q9Z1F2
1937	1,2,4,7,18,<br>21,23,24	3,5,6,8,17,<br>19,20,22	1 O89117
1938	1,2,4,7,18,<br>22,23,24	3,5,6,8,17,<br>19,20,21	8 D3ZUC6,P06760,P08937,P13676,Q64268,Q6AYQ8,Q6P6R2,Q9EQS0
1939	1,2,4,7,19,<br>20,21,22	3,5,6,8,17,<br>18,23,24	1 P80299
1940	1,2,4,7,19,<br>20,21,23	3,5,6,8,17,<br>18,22,24	2 P10758,P80299
1941	1,2,4,7,19,<br>20,21,24	3,5,6,8,17,<br>18,22,23	4 O35547,P08723,P36374,Q63617
1942	1,2,4,7,19,<br>20,22,23	3,5,6,8,17,<br>18,21,24	2 P10758,P80299
1943	1,2,4,7,19,<br>20,22,24	3,5,6,8,17,<br>18,21,23	1 P80299
1944	1,2,4,7,19,<br>20,23,24	3,5,6,8,17,<br>18,21,22	2 P10758,Q9QYP1
1945	1,2,4,7,19,<br>21,22,23	3,5,6,8,17,<br>18,20,24	2 P80299,Q9R168
1946	1,2,4,7,19,<br>21,22,24	3,5,6,8,17,<br>18,20,23	2 P36376,Q9R168
1947	1,2,4,7,19,<br>21,23,24	3,5,6,8,17,<br>18,20,22	4 P14173,Q62761;Q62762;Q62763,Q63474,Q9R168

1948	1,2,4,7,19, 22,23,24	3,5,6,8,17, 18,20,21	5	D3ZUC6,P26772,P80299,Q6AYQ8,Q9R168
1949	1,2,4,7,20, 21,22,23	3,5,6,8,17, 18,19,24	1	P80299
1950	1,2,4,7,20, 21,22,24	3,5,6,8,17, 18,19,23	1	P01835
1951	1,2,4,7,20, 21,23,24	3,5,6,8,17, 18,19,22	3	P01835,P10758,Q9QYP1
1952	1,2,4,7,20, 22,23,24	3,5,6,8,17, 18,19,21	4	P08937,P10758,Q9QX74,Q9QYP1
1953	1,2,4,7,21, 22,23,24	3,5,6,8,17, 18,19,20	1	Q9R168
1954	1,2,4,8,17, 18,19,20	3,5,6,7,21, 22,23,24	8	D4A5U3,G3V686,P19223,P25809,P36374,P47727,Q10758,Q62714
1955	1,2,4,8,17, 18,19,21	3,5,6,7,20, 22,23,24	1	Q66H69
1956	1,2,4,8,17, 18,19,22	3,5,6,7,20, 21,23,24	3	G3V686,P97580,Q30KJ2
1957	1,2,4,8,17, 18,19,23	3,5,6,7,20, 21,22,24	1	G3V686
1958	1,2,4,8,17, 18,19,24	3,5,6,7,20, 21,22,23	3	G3V686,O54728,Q66H69
1959	1,2,4,8,17, 18,20,21	3,5,6,7,19, 22,23,24	7	D4A5U3,P19223,P25809,P47727,P55091,Q10758,Q811M5
1960	1,2,4,8,17, 18,20,22	3,5,6,7,19, 21,23,24	4	G3V686,P19223,P25809,Q30KJ2
1961	1,2,4,8,17, 18,20,23	3,5,6,7,19, 21,22,24	4	G3V686,P19223,P55091,Q10758
1962	1,2,4,8,17, 18,20,24	3,5,6,7,19, 21,22,23	6	G3V686,P19223,P47727,Q10758,Q811M5,Q9QYP1
1963	1,2,4,8,17, 18,21,22	3,5,6,7,19, 20,23,24	2	Q30KJ2,Q9Z1F2
1964	1,2,4,8,17, 18,21,23	3,5,6,7,19, 20,22,24	0	
1965	1,2,4,8,17, 18,21,24	3,5,6,7,19, 20,22,23	1	Q9Z1F2
1966	1,2,4,8,17, 18,22,23	3,5,6,7,19, 20,21,24	2	P47967,Q30KJ2
1967	1,2,4,8,17, 18,22,24	3,5,6,7,19, 20,21,23	5	D3ZUC6,G3V686,P47967,Q30KJ2,Q9Z1F2

1968	1,2,4,8,17,18,23,24	3,5,6,7,19,20,21,22	1	P47967
1969	1,2,4,8,17,19,20,21	3,5,6,7,18,22,23,24	13	B0LT89,D4A5U3,P04905,P19223,P25809,P30120,P36374,P47727,P97675,Q10758,Q66H69,Q6P6R2,Q9JI85
1970	1,2,4,8,17,19,20,22	3,5,6,7,18,21,23,24	4	D4A5U3,O89117,P19223,P36374
1971	1,2,4,8,17,19,20,23	3,5,6,7,18,21,22,24	3	P10758,P19223,Q8CJD3
1972	1,2,4,8,17,19,20,24	3,5,6,7,18,21,22,23	8	D4A5U3,O54728,P08723,P19223,P36374,P47727,P60905,Q68G31
1973	1,2,4,8,17,19,21,22	3,5,6,7,18,20,23,24	1	Q66H69
1974	1,2,4,8,17,19,21,23	3,5,6,7,18,20,22,24	2	Q63474,Q66H69
1975	1,2,4,8,17,19,21,24	3,5,6,7,18,20,22,23	3	O54728,Q63474,Q66H69
1976	1,2,4,8,17,19,22,23	3,5,6,7,18,20,21,24	3	P00507,P47967,Q30KJ2
1977	1,2,4,8,17,19,22,24	3,5,6,7,18,20,21,23	3	D3ZUC6,O54728,P00507
1978	1,2,4,8,17,19,23,24	3,5,6,7,18,20,21,22	2	O54728,P47967
1979	1,2,4,8,17,20,21,22	3,5,6,7,18,19,23,24	4	D4A5U3,P19223,P25809,Q10758
1980	1,2,4,8,17,20,21,23	3,5,6,7,18,19,22,24	3	D4A5U3,P19223,Q10758
1981	1,2,4,8,17,20,21,24	3,5,6,7,18,19,22,23	9	D4A5U3,P02782,P19223,P30120,P47727,P97675,Q10758,Q6AYR9,Q811M5
1982	1,2,4,8,17,20,22,23	3,5,6,7,18,19,21,24	1	P19223
1983	1,2,4,8,17,20,22,24	3,5,6,7,18,19,21,23	1	P19223
1984	1,2,4,8,17,20,23,24	3,5,6,7,18,19,21,22	2	P19223,Q9QYP1
1985	1,2,4,8,17,21,22,23	3,5,6,7,18,19,20,24	0	
1986	1,2,4,8,17,21,22,24	3,5,6,7,18,19,20,23	1	Q9Z1F2
1987	1,2,4,8,17,21,23,24	3,5,6,7,18,19,20,22	1	Q63474

1988	1,2,4,8,17,<br>22,23,24	3,5,6,7,18,<br>19,20,21	5 D3ZUC6,P00507,P47967,P97840,Q5I0D1
1989	1,2,4,8,18,<br>19,20,21	3,5,6,7,17,<br>22,23,24	7 O08815,P25809,P36374,P47727,Q10758,Q66H69,Q68G31
1990	1,2,4,8,18,<br>19,20,22	3,5,6,7,17,<br>21,23,24	4 P04762,P15399,Q30KJ2,Q6AY61
1991	1,2,4,8,18,<br>19,20,23	3,5,6,7,17,<br>21,22,24	1 P10758
1992	1,2,4,8,18,<br>19,20,24	3,5,6,7,17,<br>21,22,23	3 O54728,P36374,Q68G31
1993	1,2,4,8,18,<br>19,21,22	3,5,6,7,17,<br>20,23,24	1 Q30KJ2
1994	1,2,4,8,18,<br>19,21,23	3,5,6,7,17,<br>20,22,24	1 P06866
1995	1,2,4,8,18,<br>19,21,24	3,5,6,7,17,<br>20,22,23	3 O54728,Q66H69,Q68G31
1996	1,2,4,8,18,<br>19,22,23	3,5,6,7,17,<br>20,21,24	4 P00507,P04762,Q30KJ2,Q6AY61
1997	1,2,4,8,18,<br>19,22,24	3,5,6,7,17,<br>20,21,23	4 O54728,P00507,Q07523,Q30KJ2
1998	1,2,4,8,18,<br>19,23,24	3,5,6,7,17,<br>20,21,22	1 O54728
1999	1,2,4,8,18,<br>20,21,22	3,5,6,7,17,<br>19,23,24	3 P25809,P54921,Q10758
2000	1,2,4,8,18,<br>20,21,23	3,5,6,7,17,<br>19,22,24	2 P54921,Q10758
2001	1,2,4,8,18,<br>20,21,24	3,5,6,7,17,<br>19,22,23	1 Q10758
2002	1,2,4,8,18,<br>20,22,23	3,5,6,7,17,<br>19,21,24	4 P05539,P54921,Q5QE79,Q6AY61
2003	1,2,4,8,18,<br>20,22,24	3,5,6,7,17,<br>19,21,23	0
2004	1,2,4,8,18,<br>20,23,24	3,5,6,7,17,<br>19,21,22	3 P05539,Q5QE79,Q9QYP1
2005	1,2,4,8,18,<br>21,22,23	3,5,6,7,17,<br>19,20,24	1 P54921
2006	1,2,4,8,18,<br>21,22,24	3,5,6,7,17,<br>19,20,23	1 Q9Z1F2
2007	1,2,4,8,18,<br>21,23,24	3,5,6,7,17,<br>19,20,22	1 O89117

2008	1,2,4,8,18,22,23,24	3,5,6,7,17,19,20,21	5	D3ZUC6,P00507,P47967,Q5QE79,Q6P6R2
2009	1,2,4,8,19,20,21,22	3,5,6,7,17,18,23,24	0	
2010	1,2,4,8,19,20,21,23	3,5,6,7,17,18,22,24	2	P10758,Q812E4
2011	1,2,4,8,19,20,21,24	3,5,6,7,17,18,22,23	9	O35547,O54728,P02782,P08723,P30120,P36374,P60905,Q68G31,Q812E4
2012	1,2,4,8,19,20,22,23	3,5,6,7,17,18,21,24	3	P04762,P10758,Q6AY61
2013	1,2,4,8,19,20,22,24	3,5,6,7,17,18,21,23	2	O54728,P15399
2014	1,2,4,8,19,20,23,24	3,5,6,7,17,18,21,22	3	O54728,P10758,Q9QYP1
2015	1,2,4,8,19,21,22,23	3,5,6,7,17,18,20,24	0	
2016	1,2,4,8,19,21,22,24	3,5,6,7,17,18,20,23	1	O54728
2017	1,2,4,8,19,21,23,24	3,5,6,7,17,18,20,22	2	O54728,Q63474
2018	1,2,4,8,19,22,23,24	3,5,6,7,17,18,20,21	4	O54728,P00507,P26772,P47967
2019	1,2,4,8,20,21,22,23	3,5,6,7,17,18,19,24	1	P54921
2020	1,2,4,8,20,21,22,24	3,5,6,7,17,18,19,23	0	
2021	1,2,4,8,20,21,23,24	3,5,6,7,17,18,19,22	3	Q10758,Q812E4,Q9QYP1
2022	1,2,4,8,20,22,23,24	3,5,6,7,17,18,19,21	3	P05539,Q5QE79,Q9QYP1
2023	1,2,4,8,21,22,23,24	3,5,6,7,17,18,19,20	1	P30919
2024	1,2,4,17,18,19,20,21	3,5,6,7,8,22,23,24	5	P19223,P25809,P35280,Q62714,Q6P6R2
2025	1,2,4,17,18,19,20,22	3,5,6,7,8,21,23,24	2	G3V686,Q62714
2026	1,2,4,17,18,19,20,23	3,5,6,7,8,21,22,24	4	P10758,P30919,P35280,Q62714
2027	1,2,4,17,18,19,20,24	3,5,6,7,8,21,22,23	4	G3V686,P35280,Q62714,Q9QYP1

2028	1,2,4,17,18,19,21,22	3,5,6,7,8,20,23,24	0	
2029	1,2,4,17,18,19,21,23	3,5,6,7,8,20,22,24	1	P35280
2030	1,2,4,17,18,19,21,24	3,5,6,7,8,20,22,23	1	P35280
2031	1,2,4,17,18,19,22,23	3,5,6,7,8,20,21,24	2	D3ZUC6,Q9EQS0
2032	1,2,4,17,18,19,22,24	3,5,6,7,8,20,21,23	1	D3ZUC6
2033	1,2,4,17,18,19,23,24	3,5,6,7,8,20,21,22	3	D3ZUC6,P08937,P35280
2034	1,2,4,17,18,20,21,22	3,5,6,7,8,19,23,24	1	P19223
2035	1,2,4,17,18,20,21,23	3,5,6,7,8,19,22,24	2	P19223,P35280
2036	1,2,4,17,18,20,21,24	3,5,6,7,8,19,22,23	4	P19223,P35280,P50115,Q9QYP1
2037	1,2,4,17,18,20,22,23	3,5,6,7,8,19,21,24	1	P08937
2038	1,2,4,17,18,20,22,24	3,5,6,7,8,19,21,23	5	D3ZUC6,O35077,P08937,P50115,Q9QYP1
2039	1,2,4,17,18,20,23,24	3,5,6,7,8,19,21,22	4	P08937,P35280,P50115,Q9QYP1
2040	1,2,4,17,18,21,22,23	3,5,6,7,8,19,20,24	0	
2041	1,2,4,17,18,21,22,24	3,5,6,7,8,19,20,23	2	D3ZUC6,P50115
2042	1,2,4,17,18,21,23,24	3,5,6,7,8,19,20,22	2	P19218,P35280
2043	1,2,4,17,18,22,23,24	3,5,6,7,8,19,20,21	8	D3ZUC6,P06760,P08937,P47967,Q5I0D1,Q63617,Q9EQS0,Q9WTT6
2044	1,2,4,17,19,20,21,22	3,5,6,7,8,18,23,24	1	P19223
2045	1,2,4,17,19,20,21,23	3,5,6,7,8,18,22,24	3	P10758,P19223,P35280
2046	1,2,4,17,19,20,21,24	3,5,6,7,8,18,22,23	3	P19223,P35280,P36374
2047	1,2,4,17,19,20,22,23	3,5,6,7,8,18,21,24	1	P10758

2048	1,2,4,17,19,20,22,24	3,5,6,7,8,18,21,23	1 D3ZUC6
2049	1,2,4,17,19,20,23,24	3,5,6,7,8,18,21,22	4 P08937,P10758,P35280,Q9QYP1
2050	1,2,4,17,19,21,22,23	3,5,6,7,8,18,20,24	1 Q9R168
2051	1,2,4,17,19,21,22,24	3,5,6,7,8,18,20,23	2 D3ZUC6,Q9R168
2052	1,2,4,17,19,21,23,24	3,5,6,7,8,18,20,22	3 P35280,Q63474,Q9R168
2053	1,2,4,17,19,22,23,24	3,5,6,7,8,18,20,21	6 D3ZUC6,P00507,P06760,P08937,P47967,Q9R168
2054	1,2,4,17,20,21,22,23	3,5,6,7,8,18,19,24	1 P19223
2055	1,2,4,17,20,21,22,24	3,5,6,7,8,18,19,23	1 P19223
2056	1,2,4,17,20,21,23,24	3,5,6,7,8,18,19,22	4 P08937,P19223,P35280,Q9QYP1
2057	1,2,4,17,20,22,23,24	3,5,6,7,8,18,19,21	6 D3ZUC6,P06760,P08937,P47967,Q63751,Q9QYP1
2058	1,2,4,17,21,22,23,24	3,5,6,7,8,18,19,20	5 D3ZUC6,P08937,P47967,Q5I0D1,Q9R168
2059	1,2,4,18,19,20,21,22	3,5,6,7,8,17,23,24	0
2060	1,2,4,18,19,20,21,23	3,5,6,7,8,17,22,24	3 P06866,P10758,P35280
2061	1,2,4,18,19,20,21,24	3,5,6,7,8,17,22,23	3 P35280,P50115,Q68G31
2062	1,2,4,18,19,20,22,23	3,5,6,7,8,17,21,24	4 P04762,P10758,Q6AY61,Q9EQS0
2063	1,2,4,18,19,20,22,24	3,5,6,7,8,17,21,23	2 P50115,Q9QX74
2064	1,2,4,18,19,20,23,24	3,5,6,7,8,17,21,22	4 P10758,P35280,P50115,Q9QYP1
2065	1,2,4,18,19,21,22,23	3,5,6,7,8,17,20,24	2 P06866,P13676
2066	1,2,4,18,19,21,22,24	3,5,6,7,8,17,20,23	1 P50115
2067	1,2,4,18,19,21,23,24	3,5,6,7,8,17,20,22	3 O70417,P06866,P35280

2068	1,2,4,18,19,22,23,24	3,5,6,7,8,17,20,21	9	D3ZUC6,P00507,P06760,P13676,P26772,P50115,Q9EQS0,Q9QX74,Q9WTT6
2069	1,2,4,18,20,21,22,23	3,5,6,7,8,17,19,24	2	P50115,P54921
2070	1,2,4,18,20,21,22,24	3,5,6,7,8,17,19,23	1	P50115
2071	1,2,4,18,20,21,23,24	3,5,6,7,8,17,19,22	3	P35280,P50115,Q9QYP1
2072	1,2,4,18,20,22,23,24	3,5,6,7,8,17,19,21	8	D3ZUC6,P06760,P08937,P50115,Q5QE79,Q9EQS0,Q9QX74,Q9QYP1
2073	1,2,4,18,21,22,23,24	3,5,6,7,8,17,19,20	4	D3ZUC6,P13676,P50115,Q9WTT6
2074	1,2,4,19,20,21,22,23	3,5,6,7,8,17,18,24	1	P10758
2075	1,2,4,19,20,21,22,24	3,5,6,7,8,17,18,23	1	O70594
2076	1,2,4,19,20,21,23,24	3,5,6,7,8,17,18,22	3	P10758,P35280,Q9QYP1
2077	1,2,4,19,20,22,23,24	3,5,6,7,8,17,18,21	6	P06760,P08937,P10758,P26772,Q9QX74,Q9QYP1
2078	1,2,4,19,21,22,23,24	3,5,6,7,8,17,18,20	3	O70417,P13676,Q9R168
2079	1,2,4,20,21,22,23,24	3,5,6,7,8,17,18,19	3	P50115,Q9QX74,Q9QYP1
2080	1,2,5,6,7,8,17,18	3,4,19,20,21,22,23,24	10	iRT-Kit_WR_fusion,O70594,P00762,P13432,P31044,P50115,P50116,P52590,Q64335,Q91ZS3
2081	1,2,5,6,7,8,17,19	3,4,18,20,21,22,23,24	9	iRT-Kit_WR_fusion,O70594,P00762,P31044,P50115,P50116,P52590,Q66H69,Q8CJD3
2082	1,2,5,6,7,8,17,20	3,4,18,19,21,22,23,24	19	D4A5U3,iRT-Kit_WR_fusion,P06911,P12020,P13432,P19629,P25031,P31044,P36375,P50115,P50116,P50280,P52590,P55091,Q4G075,Q5GRG2,Q6IG05,Q91ZS3,Q9JHB9
2083	1,2,5,6,7,8,17,21	3,4,18,19,20,22,23,24	14	iRT-Kit_WR_fusion,O70594,P02631,P05539,P06760,P19629,P36860,P50115,P50116,P52590,Q00715,Q63474,Q6IG05,Q9QX74
2084	1,2,5,6,7,8,17,22	3,4,18,19,20,21,23,24	7	iRT-Kit_WR_fusion,O54858,P00762,P23593,P50115,P50116,P52590

2085	1,2,5,6,7,8,17,23	3,4,18,19,20,21,22,24	9	iRT-Kit_WR_fusion,O54858,O70594,P00762,P19132,P50115,P50116,P52590,Q6IG05
2086	1,2,5,6,7,8,17,24	3,4,18,19,20,21,22,23	8	iRT-Kit_WR_fusion,P01039,P36860,P50115,P50116,P52590,Q63474,Q6AY61
2087	1,2,5,6,7,8,18,19	3,4,17,20,21,22,23,24	8	iRT-Kit_WR_fusion,P00762,P31044,P50115,P50116,P52590,Q66H69,Q91ZS3
2088	1,2,5,6,7,8,18,20	3,4,17,19,21,22,23,24	17	iRT-Kit_WR_fusion,O54715,P06911,P12020,P19629,P25031,P31044,P50116,P50280,P52590,P69897,Q03191,Q4G075,Q5GRG2,Q6IG05,Q91ZS3,Q9JHB9
2089	1,2,5,6,7,8,18,21	3,4,17,19,20,22,23,24	10	iRT-Kit_WR_fusion,P02631,P19629,P31044,P36860,P50116,P52590,Q00715,Q91ZS3,Q9JJ50
2090	1,2,5,6,7,8,18,22	3,4,17,19,20,21,23,24	6	iRT-Kit_WR_fusion,O54858,P00762,P16636,P23593,Q91ZS3
2091	1,2,5,6,7,8,18,23	3,4,17,19,20,21,22,24	13	iRT-Kit_WR_fusion,O54858,O70594,P00762,P16636,P19132,P23785,P31044,P50116,Q03191,Q91ZS3,Q99MH3,Q9JJ50
2092	1,2,5,6,7,8,18,24	3,4,17,19,20,21,22,23	5	iRT-Kit_WR_fusion,O54858,P36860,Q91ZS3,Q9JJ50
2093	1,2,5,6,7,8,19,20	3,4,17,18,21,22,23,24	22	iRT-Kit_WR_fusion,P02780,P06911,P0DP29;P0DP30;P0DP31,P12020,P19218,P19629,P25031,P31044,P50115,P50116,P50280,P52590,Q4G075,Q5GRG2,Q5I0D1,Q66H69,Q68G31,Q6IG05,Q8CJD3,Q91ZS3,Q9JI85
2094	1,2,5,6,7,8,19,21	3,4,17,18,20,22,23,24	14	iRT-Kit_WR_fusion,P02631,P06760,P19629,P31044,P35952,P50115,P50116,P52590,Q00715,Q63474,Q66H69,Q6IG05,Q9JI85
2095	1,2,5,6,7,8,19,22	3,4,17,18,20,21,23,24	7	iRT-Kit_WR_fusion,O54858,P00762,P19218,P50115,P52590,Q4FZU4
2096	1,2,5,6,7,8,19,23	3,4,17,18,20,21,22,24	10	iRT-Kit_WR_fusion,O54858,P00762,P19629,P31044,P50115,P50116,P52590,Q6IG05,Q8CJD3
2097	1,2,5,6,7,8,19,24	3,4,17,18,20,21,22,23	7	iRT-Kit_WR_fusion,O54858,P50115,P50116,P52590,Q66H69,Q68G31

2098	1,2,5,6,7,8,20,21	3,4,17,18,19,22,23,24	26	iRT-Kit_WR_fusion,P01835,P02631,P02780,P06760,P06911,P12020,P19629,P25031,P31044,P35952,P36375,P47967,P50116,P50280,P52590,Q00715,Q4G075,Q5GRG2,Q5I0J9,Q6IG05,Q78P75,Q812E4,Q91ZS3,Q9JHB9,Q9JI85
2099	1,2,5,6,7,8,20,22	3,4,17,18,19,21,23,24	14	iRT-Kit_WR_fusion,O54858,P06911,P12020,P19218,P19629,P25031,P31044,P50116,P50280,Q03191,Q4G075,Q5GRG2,Q91ZS3
2100	1,2,5,6,7,8,20,23	3,4,17,18,19,21,22,24	17	iRT-Kit_WR_fusion,O54858,P06911,P12020,P19132,P19629,P23739,P25031,P31044,P50116,P50280,Q03191,Q4G075,Q5GRG2,Q6IG05,Q91ZS3,Q99MH3
2101	1,2,5,6,7,8,20,24	3,4,17,18,19,21,22,23	12	iRT-Kit_WR_fusion,P06911,P12020,P19629,P25031,P31044,P50116,P50280,Q5GRG2,Q6IG05,Q91ZS3,Q9JHB9
2102	1,2,5,6,7,8,21,22	3,4,17,18,19,20,23,24	9	iRT-Kit_WR_fusion,O54858,P01835,P02631,P19629,P36860,P50116,Q62714,Q91ZS3
2103	1,2,5,6,7,8,21,23	3,4,17,18,19,20,22,24	16	iRT-Kit_WR_fusion,O54858,P01835,P02631,P19132,P19629,P36860,P50115,P50116,P52590,Q62714,Q63474,Q63532,Q6IG05,Q91ZS3,Q9JJ50
2104	1,2,5,6,7,8,21,24	3,4,17,18,19,20,22,23	12	iRT-Kit_WR_fusion,O54858,P01835,P02631,P19629,P30919,P36860,P50116,Q62714,Q63474,Q91ZS3,Q9JJ50
2105	1,2,5,6,7,8,22,23	3,4,17,18,19,20,21,24	10	iRT-Kit_WR_fusion,O54858,P19629,P23593,P50115,Q09030,Q62714,Q63532,Q6P6R2,Q99MH3
2106	1,2,5,6,7,8,22,24	3,4,17,18,19,20,21,23	5	iRT-Kit_WR_fusion,O54858,P23593,Q62714,Q6P6R2
2107	1,2,5,6,7,8,23,24	3,4,17,18,19,20,21,22	6	iRT-Kit_WR_fusion,O54858,P36860,Q62714,Q6P6R2,Q9JJ50
2108	1,2,5,6,7,17,18,19	3,4,8,20,21,22,23,24	3	P00762,P50116,P52590
2109	1,2,5,6,7,17,18,20	3,4,8,19,21,22,23,24	6	P00762,P50116,P55091,Q03191,Q5GRG2,Q9Z0J6
2110	1,2,5,6,7,17,18,21	3,4,8,19,20,22,23,24	5	P00762,P01835,P05539,P36860,P50116
2111	1,2,5,6,7,17,18,22	3,4,8,19,20,21,23,24	3	P00762,Q64335,Q9EQS0
2112	1,2,5,6,7,17,18,23	3,4,8,19,20,21,22,24	7	O70594,P00762,P16636,P19814,Q03191,Q9EQS0,Q9JJ50

2113	1,2,5,6,7,17,18,24	3,4,8,19,20,21,22,23	4	P00762,P01039,P36860,Q6AY61
2114	1,2,5,6,7,17,19,20	3,4,8,18,21,22,23,24	4	P00762,P0DP29;P0DP30;P0DP31,P50116,P52590
2115	1,2,5,6,7,17,19,21	3,4,8,18,20,22,23,24	7	P00762,P05539,P36860,P50116,P52590,Q6AY61,Q9R168
2116	1,2,5,6,7,17,19,22	3,4,8,18,20,21,23,24	3	P00762,P52590,Q9R168
2117	1,2,5,6,7,17,19,23	3,4,8,18,20,21,22,24	4	P00762,P50116,P52590,Q8CJD3
2118	1,2,5,6,7,17,19,24	3,4,8,18,20,21,22,23	5	P00762,P01039,P52590,P54921,Q6AY61
2119	1,2,5,6,7,17,20,21	3,4,8,18,19,22,23,24	9	P01835,P05539,P0DP29;P0DP30;P0DP31,P36860,P50116,P63029,Q5I0J9,Q6AY61,Q9Z0J6
2120	1,2,5,6,7,17,20,22	3,4,8,18,19,21,23,24	4	P00762,P50116,Q03191,Q9Z0J6
2121	1,2,5,6,7,17,20,23	3,4,8,18,19,21,22,24	3	P00762,P50116,Q03191
2122	1,2,5,6,7,17,20,24	3,4,8,18,19,21,22,23	5	P00762,P01039,P50116,Q6AY61,Q9Z0J6
2123	1,2,5,6,7,17,21,22	3,4,8,18,19,20,23,24	6	P00762,P01835,P05539,P36860,P50116,Q9R168
2124	1,2,5,6,7,17,21,23	3,4,8,18,19,20,22,24	9	P00762,P01835,P05539,P19814,P36860,P50116,Q63474,Q9JJ50,Q9R168
2125	1,2,5,6,7,17,21,24	3,4,8,18,19,20,22,23	9	P00762,P01039,P01835,P05539,P36860,P50116,Q63474,Q6AY61,Q9R168
2126	1,2,5,6,7,17,22,23	3,4,8,18,19,20,21,24	3	P00762,Q9EQS0,Q9R168
2127	1,2,5,6,7,17,22,24	3,4,8,18,19,20,21,23	4	P00762,P01039,P36860,Q9R168
2128	1,2,5,6,7,17,23,24	3,4,8,18,19,20,21,22	4	P00762,P01039,P36860,Q6AY61
2129	1,2,5,6,7,18,19,20	3,4,8,17,21,22,23,24	3	P00762,P0DP29;P0DP30;P0DP31,P52590
2130	1,2,5,6,7,18,19,21	3,4,8,17,20,22,23,24	4	P00762,P01835,P52590,Q00715
2131	1,2,5,6,7,18,19,22	3,4,8,17,20,21,23,24	3	P00762,P52590,Q9EQS0
2132	1,2,5,6,7,18,19,23	3,4,8,17,20,21,22,24	4	P00762,P52590,Q9EQS0,Q9JJ50

2133	1,2,5,6,7,18,19,24	3,4,8,17,20,21,22,23	2	P00762,P52590
2134	1,2,5,6,7,18,20,21	3,4,8,17,19,22,23,24	6	P00762,P01835,P47967,Q00715,Q03191,Q91ZS3
2135	1,2,5,6,7,18,20,22	3,4,8,17,19,21,23,24	4	P00762,P01835,Q03191,Q9EQS0
2136	1,2,5,6,7,18,20,23	3,4,8,17,19,21,22,24	6	P00762,P01835,P16636,Q03191,Q9EQS0,Q9JJ50
2137	1,2,5,6,7,18,20,24	3,4,8,17,19,21,22,23	3	P00762,P01835,Q03191
2138	1,2,5,6,7,18,21,22	3,4,8,17,19,20,23,24	4	P00762,P01835,P36860,Q03191
2139	1,2,5,6,7,18,21,23	3,4,8,17,19,20,22,24	8	O70417,P00762,P01835,P16636,P19814,P36860,Q03191,Q9JJ50
2140	1,2,5,6,7,18,21,24	3,4,8,17,19,20,22,23	5	P00762,P01835,P36860,Q6AY61,Q9JJ50
2141	1,2,5,6,7,18,22,23	3,4,8,17,19,20,21,24	12	P00762,P01835,P13676,P16636,P30120,P36374,Q03191,Q6P6R2,Q99MH3,Q9EQS0,Q9WVK7,Q9Z2L0
2142	1,2,5,6,7,18,22,24	3,4,8,17,19,20,21,23	6	P00762,P01835,Q03191,Q6P6R2,Q9EQS0,Q9WVK7
2143	1,2,5,6,7,18,23,24	3,4,8,17,19,20,21,22	8	P00762,P01835,P16636,Q03191,Q6P6R2,Q9EQS0,Q9JJ50,Q9WVK7
2144	1,2,5,6,7,19,20,21	3,4,8,17,18,22,23,24	9	P00762,P01835,P0DP29;P0DP30;P0DP31,P47967,P50116,P52590,Q5I0D1,Q5I0J9,Q63617
2145	1,2,5,6,7,19,20,22	3,4,8,17,18,21,23,24	3	P00762,P19218,P52590
2146	1,2,5,6,7,19,20,23	3,4,8,17,18,21,22,24	4	P00762,P23739,P52590,Q8CJD3
2147	1,2,5,6,7,19,20,24	3,4,8,17,18,21,22,23	3	P00762,P52590,Q68G31
2148	1,2,5,6,7,19,21,22	3,4,8,17,18,20,23,24	4	P00762,P01835,P52590,Q9R168
2149	1,2,5,6,7,19,21,23	3,4,8,17,18,20,22,24	6	O70417,P00762,P01835,P14173,P52590,Q9JJ50
2150	1,2,5,6,7,19,21,24	3,4,8,17,18,20,22,23	7	P00762,P01835,P14173,P36860,P52590,Q63474,Q6AY61
2151	1,2,5,6,7,19,22,23	3,4,8,17,18,20,21,24	4	P00762,P52590,Q811M5,Q9EQS0
2152	1,2,5,6,7,19,22,24	3,4,8,17,18,20,21,23	2	P00762,P52590

2153	1,2,5,6,7,19,23,24	3,4,8,17,18,20,21,22	4 P00762,P14173,P52590,Q9JJ50
2154	1,2,5,6,7,20,21,22	3,4,8,17,18,19,23,24	4 P01835,Q03191,Q5I0J9,Q5PQL7
2155	1,2,5,6,7,20,21,23	3,4,8,17,18,19,22,24	4 P01835,P19629,P50116,Q03191
2156	1,2,5,6,7,20,21,24	3,4,8,17,18,19,22,23	3 P01835,P36860,Q6AY61
2157	1,2,5,6,7,20,22,23	3,4,8,17,18,19,21,24	5 P00762,P01835,Q03191,Q99MH3,Q9EQS0
2158	1,2,5,6,7,20,22,24	3,4,8,17,18,19,21,23	4 P00762,P01835,Q03191,Q6P6R2
2159	1,2,5,6,7,20,23,24	3,4,8,17,18,19,21,22	4 P00762,P01835,P23739,Q03191
2160	1,2,5,6,7,21,22,23	3,4,8,17,18,19,20,24	7 P00762,P01835,P36860,Q03191,Q62714,Q9EQS0,Q9R168
2161	1,2,5,6,7,21,22,24	3,4,8,17,18,19,20,23	5 P00762,P01835,P36860,Q62714,Q9R168
2162	1,2,5,6,7,21,23,24	3,4,8,17,18,19,20,22	7 O70417,P00762,P01835,P36860,Q62714,Q63474,Q9JJ50
2163	1,2,5,6,7,22,23,24	3,4,8,17,18,19,20,21	8 P00762,P01835,P13676,Q03191,Q62714,Q6P6R2,Q9EQS0,Q9WVK7
2164	1,2,5,6,8,17,18,19	3,4,7,20,21,22,23,24	4 P00762,P13432,P52590,Q68G31
2165	1,2,5,6,8,17,18,20	3,4,7,19,21,22,23,24	8 P06911,P12020,P13432,P36375,P55091,P63029,Q5GRG2,Q91ZS3
2166	1,2,5,6,8,17,18,21	3,4,7,19,20,22,23,24	2 P13432,P36860
2167	1,2,5,6,8,17,18,22	3,4,7,19,20,21,23,24	1 P13432
2168	1,2,5,6,8,17,18,23	3,4,7,19,20,21,22,24	5 P16636,P19132,P19814,P23785,Q99MH3
2169	1,2,5,6,8,17,18,24	3,4,7,19,20,21,22,23	2 P01039,P13432
2170	1,2,5,6,8,17,19,20	3,4,7,18,21,22,23,24	8 O89117,P52590,P63029,Q5GRG2,Q5I0J9,Q68G31,Q6IG05,Q8CJD3
2171	1,2,5,6,8,17,19,21	3,4,7,18,20,22,23,24	4 P52590,Q63474,Q66H69,Q68G31
2172	1,2,5,6,8,17,19,22	3,4,7,18,20,21,23,24	3 O89117,P00507,P52590

2173	1,2,5,6,8,17,19,23	3,4,7,18,20,21,22,24	2	P52590,Q8CJD3
2174	1,2,5,6,8,17,19,24	3,4,7,18,20,21,22,23	4	P01039,P52590,P97840,Q68G31
2175	1,2,5,6,8,17,20,21	3,4,7,18,19,22,23,24	6	P25809,P36375,P63029,Q5GRG2,Q5I0J9,Q6IG05
2176	1,2,5,6,8,17,20,22	3,4,7,18,19,21,23,24	6	O89117,P06911,P12020,P63029,Q5GRG2,Q5I0J9
2177	1,2,5,6,8,17,20,23	3,4,7,18,19,21,22,24	10	P17559,P19132,P23739,P25031,P36375,P36376,P63029,Q5GRG2,Q6IG05,Q99MH3
2178	1,2,5,6,8,17,20,24	3,4,7,18,19,21,22,23	7	P01039,P06911,P36375,P63029,Q5GRG2,Q5I0J9,Q68G31
2179	1,2,5,6,8,17,21,22	3,4,7,18,19,20,23,24	1	P10758
2180	1,2,5,6,8,17,21,23	3,4,7,18,19,20,22,24	4	P19132,P19814,P36860,Q63474
2181	1,2,5,6,8,17,21,24	3,4,7,18,19,20,22,23	4	P01039,P36860,P97840,Q63474
2182	1,2,5,6,8,17,22,23	3,4,7,18,19,20,21,24	5	O54858,P00507,P97840,Q63532,Q99MH3
2183	1,2,5,6,8,17,22,24	3,4,7,18,19,20,21,23	3	P01039,P10758,P97840
2184	1,2,5,6,8,17,23,24	3,4,7,18,19,20,21,22	2	P01039,P97840
2185	1,2,5,6,8,18,19,20	3,4,7,17,21,22,23,24	6	P12020,P50280,P52590,Q5GRG2,Q68G31,Q91ZS3
2186	1,2,5,6,8,18,19,21	3,4,7,17,20,22,23,24	3	P52590,Q66H69,Q68G31
2187	1,2,5,6,8,18,19,22	3,4,7,17,20,21,23,24	2	P15399,P52590
2188	1,2,5,6,8,18,19,23	3,4,7,17,20,21,22,24	3	P52590,Q99MH3,Q9JJ50
2189	1,2,5,6,8,18,19,24	3,4,7,17,20,21,22,23	2	P52590,Q68G31
2190	1,2,5,6,8,18,20,21	3,4,7,17,19,22,23,24	9	P02631,P19629,P25809,P36375,Q00715,Q5GRG2,Q5I0J9,Q68G31,Q91ZS3
2191	1,2,5,6,8,18,20,22	3,4,7,17,19,21,23,24	4	P12020,P15399,Q5GRG2,Q91ZS3
2192	1,2,5,6,8,18,20,23	3,4,7,17,19,21,22,24	6	P16636,P19132,P25031,Q5GRG2,Q91ZS3,Q99MH3

2193	1,2,5,6,8,18,20,24	3,4,7,17,19,21,22,23	4	P12020,Q5GRG2,Q68G31,Q91ZS3
2194	1,2,5,6,8,18,21,22	3,4,7,17,19,20,23,24	1	Q91ZS3
2195	1,2,5,6,8,18,21,23	3,4,7,17,19,20,22,24	8	P16636,P19132,P19814,P54921,Q63532,Q91ZS3,Q99MH3,Q9JJ50
2196	1,2,5,6,8,18,21,24	3,4,7,17,19,20,22,23	4	P36860,Q68G31,Q91ZS3,Q9JJ50
2197	1,2,5,6,8,18,22,23	3,4,7,17,19,20,21,24	7	O54858,P00507,P16636,P54921,Q63532,Q99MH3,Q9Z2L0
2198	1,2,5,6,8,18,22,24	3,4,7,17,19,20,21,23	3	O54858,P20646,Q6P6R2
2199	1,2,5,6,8,18,23,24	3,4,7,17,19,20,21,22	6	O54858,P16636,P20646,Q6P6R2,Q99MH3,Q9JJ50
2200	1,2,5,6,8,19,20,21	3,4,7,17,18,22,23,24	8	P19629,P52590,Q5GRG2,Q5I0J9,Q64268,Q68G31,Q6IG05,Q9JI85
2201	1,2,5,6,8,19,20,22	3,4,7,17,18,21,23,24	6	P15399,P19218,P52590,Q5GRG2,Q5I0J9,Q68G31
2202	1,2,5,6,8,19,20,23	3,4,7,17,18,21,22,24	6	P23739,P25031,P52590,Q68G31,Q6IG05,Q8CJD3
2203	1,2,5,6,8,19,20,24	3,4,7,17,18,21,22,23	4	P52590,Q5GRG2,Q5I0J9,Q68G31
2204	1,2,5,6,8,19,21,22	3,4,7,17,18,20,23,24	3	P19218,P30919,P52590
2205	1,2,5,6,8,19,21,23	3,4,7,17,18,20,22,24	3	P52590,Q63474,Q8CJD3
2206	1,2,5,6,8,19,21,24	3,4,7,17,18,20,22,23	4	P30919,P52590,Q63474,Q68G31
2207	1,2,5,6,8,19,22,23	3,4,7,17,18,20,21,24	8	O54858,P00507,P19218,P52590,Q63532,Q811M5,Q8CJD3,Q99MH3
2208	1,2,5,6,8,19,22,24	3,4,7,17,18,20,21,23	5	P00507,P15399,P30919,P52590,Q68G31
2209	1,2,5,6,8,19,23,24	3,4,7,17,18,20,21,22	3	P00507,P52590,Q68G31
2210	1,2,5,6,8,20,21,22	3,4,7,17,18,19,23,24	4	F1M3L7,P19629,Q5I0J9,Q91ZS3
2211	1,2,5,6,8,20,21,23	3,4,7,17,18,19,22,24	7	P19132,P19629,P23739,P25031,P36375,Q6IG05,Q91ZS3
2212	1,2,5,6,8,20,21,24	3,4,7,17,18,19,22,23	5	P36375,Q5GRG2,Q5I0J9,Q68G31,Q91ZS3

2213	1,2,5,6,8,20,22,23	3,4,7,17,18,19,21,24	5	P19132,P19218,P23739,P25031,Q99MH3
2214	1,2,5,6,8,20,22,24	3,4,7,17,18,19,21,23	2	Q5GRG2,Q5I0J9
2215	1,2,5,6,8,20,23,24	3,4,7,17,18,19,21,22	2	P23739,Q5QE79
2216	1,2,5,6,8,21,22,23	3,4,7,17,18,19,20,24	6	O54858,P30919,P54921,Q62714,Q63532,Q99MH3
2217	1,2,5,6,8,21,22,24	3,4,7,17,18,19,20,23	3	P30919,P36860,Q62714
2218	1,2,5,6,8,21,23,24	3,4,7,17,18,19,20,22	6	P20646,P30919,P36860,Q62714,Q63474,Q9JJ50
2219	1,2,5,6,8,22,23,24	3,4,7,17,18,19,20,21	9	O54858,P00507,P20646,P30919,P97840,Q62714,Q63532,Q6P6R2,Q99MH3
2220	1,2,5,6,17,18,19,20	3,4,7,8,21,22,23,24	5	P00762,P20761,P35280,P52590,Q5I0J9
2221	1,2,5,6,17,18,19,21	3,4,7,8,20,22,23,24	5	O70417,P00762,P05539,P35280,P52590
2222	1,2,5,6,17,18,19,22	3,4,7,8,20,21,23,24	3	P00762,P52590,Q9EQS0
2223	1,2,5,6,17,18,19,23	3,4,7,8,20,21,22,24	4	P00762,P35280,P52590,Q9EQS0
2224	1,2,5,6,17,18,19,24	3,4,7,8,20,21,22,23	4	P00762,P01039,P35280,P52590
2225	1,2,5,6,17,18,20,21	3,4,7,8,19,22,23,24	4	P20761,P35280,P63029,Q5I0J9
2226	1,2,5,6,17,18,20,22	3,4,7,8,19,21,23,24	2	P00762,Q9Z0J6
2227	1,2,5,6,17,18,20,23	3,4,7,8,19,21,22,24	5	P00762,P04355,P16636,P35280,P63029
2228	1,2,5,6,17,18,20,24	3,4,7,8,19,21,22,23	3	P00762,P01039,P35280
2229	1,2,5,6,17,18,21,22	3,4,7,8,19,20,23,24	3	P00762,P05539,P10758
2230	1,2,5,6,17,18,21,23	3,4,7,8,19,20,22,24	7	O70417,P00762,P16636,P19814,P35280,P36860,Q9JJ50
2231	1,2,5,6,17,18,21,24	3,4,7,8,19,20,22,23	7	O70417,P01039,P10758,P35280,P36860,Q4G075,Q6AY61
2232	1,2,5,6,17,18,22,23	3,4,7,8,19,20,21,24	13	P00714,P00762,P08723,P16636,P19814,P22273,P22282,P30120,P36374,Q5M8C6,Q63617,Q99MH3,Q9EQS0

2233	1,2,5,6,17,<br>18,22,24	3,4,7,8,19,<br>20,21,23	7 D3ZUC6,P00762,P01039,P10758,Q63617,Q9EQS0,Q9WTT6
2234	1,2,5,6,17,<br>18,23,24	3,4,7,8,19,<br>20,21,22	9 O70417,P00762,P01039,P16636,P35280,Q63617,Q9EQS0,Q9JJ50,Q9WTT6
2235	1,2,5,6,17,<br>19,20,21	3,4,7,8,18,<br>22,23,24	7 P05539,P20761,P35280,P52590,P63029,Q5I0J9,Q68G31
2236	1,2,5,6,17,<br>19,20,22	3,4,7,8,18,<br>21,23,24	4 O89117,P00762,P52590,Q5I0J9
2237	1,2,5,6,17,<br>19,20,23	3,4,7,8,18,<br>21,22,24	7 P00762,P02625,P23739,P35280,P52590,P63029,Q8CJD3
2238	1,2,5,6,17,<br>19,20,24	3,4,7,8,18,<br>21,22,23	6 P00762,P01039,P35280,P52590,Q5I0J9,Q68G31
2239	1,2,5,6,17,<br>19,21,22	3,4,7,8,18,<br>20,23,24	4 P00762,P05539,P52590,Q9R168
2240	1,2,5,6,17,<br>19,21,23	3,4,7,8,18,<br>20,22,24	4 O70417,P00762,P35280,P52590
2241	1,2,5,6,17,<br>19,21,24	3,4,7,8,18,<br>20,22,23	9 O70417,P00762,P01039,P05539,P35280,P52590,Q63474,Q68G31,Q6AY61
2242	1,2,5,6,17,<br>19,22,23	3,4,7,8,18,<br>20,21,24	5 P00507,P00762,P22273,P52590,Q9EQS0
2243	1,2,5,6,17,<br>19,22,24	3,4,7,8,18,<br>20,21,23	5 D3ZUC6,P00507,P00762,P01039,P52590
2244	1,2,5,6,17,<br>19,23,24	3,4,7,8,18,<br>20,21,22	6 O70417,P00762,P01039,P22273,P35280,P52590
2245	1,2,5,6,17,<br>20,21,22	3,4,7,8,18,<br>19,23,24	2 P63029,Q5I0J9
2246	1,2,5,6,17,<br>20,21,23	3,4,7,8,18,<br>19,22,24	3 P35280,P63029,Q5I0J9
2247	1,2,5,6,17,<br>20,21,24	3,4,7,8,18,<br>19,22,23	6 P01039,P35280,P36860,P63029,Q5I0J9,Q6AY61
2248	1,2,5,6,17,<br>20,22,23	3,4,7,8,18,<br>19,21,24	5 O89117,P00762,P63029,Q99MH3,Q9EQS0
2249	1,2,5,6,17,<br>20,22,24	3,4,7,8,18,<br>19,21,23	3 O35077,P01039,Q5I0J9
2250	1,2,5,6,17,<br>20,23,24	3,4,7,8,18,<br>19,21,22	4 P01039,P23739,P35280,P63029
2251	1,2,5,6,17,<br>21,22,23	3,4,7,8,18,<br>19,20,24	4 O70417,P00762,Q9EQS0,Q9R168
2252	1,2,5,6,17,<br>21,22,24	3,4,7,8,18,<br>19,20,23	5 P01039,P05539,P10758,P36860,Q9R168

2253	1,2,5,6,17,21,23,24	3,4,7,8,18,19,20,22	5	O70417,P01039,P35280,P36860,Q63474
2254	1,2,5,6,17,22,23,24	3,4,7,8,18,19,20,21	14	D3ZUC6,P00507,P00762,P01039,P02780,P22273,P22282,P47967,P97840,Q5I0D1,Q63617,Q6P6R2,Q9EQS0,Q9WTT6
2255	1,2,5,6,18,19,20,21	3,4,7,8,17,22,23,24	6	O70417,P35280,P52590,Q5I0J9,Q5PQL7,Q68G31
2256	1,2,5,6,18,19,20,22	3,4,7,8,17,21,23,24	4	P00762,P52590,Q5PQL7,Q9EQS0
2257	1,2,5,6,18,19,20,23	3,4,7,8,17,21,22,24	5	O70417,P00762,P35280,P52590,Q9EQS0
2258	1,2,5,6,18,19,20,24	3,4,7,8,17,21,22,23	3	P00762,P35280,Q68G31
2259	1,2,5,6,18,19,21,22	3,4,7,8,17,20,23,24	4	O70417,P00762,P52590,Q5PQL7
2260	1,2,5,6,18,19,21,23	3,4,7,8,17,20,22,24	5	O70417,P00762,P35280,P52590,Q9JJ50
2261	1,2,5,6,18,19,21,24	3,4,7,8,17,20,22,23	5	O70417,P00762,P35280,Q4G075,Q68G31
2262	1,2,5,6,18,19,22,23	3,4,7,8,17,20,21,24	8	O70417,P00507,P00762,P13676,Q5M8C6,Q811M5,Q99MH3,Q9EQS0
2263	1,2,5,6,18,19,22,24	3,4,7,8,17,20,21,23	6	O70417,P00507,P00762,P13676,Q9EQS0,Q9WTT6
2264	1,2,5,6,18,19,23,24	3,4,7,8,17,20,21,22	7	O70417,P00762,P13676,P35280,Q9EQS0,Q9JJ50,Q9WTT6
2265	1,2,5,6,18,20,21,22	3,4,7,8,17,19,23,24	4	P01835,Q03191,Q5I0J9,Q5PQL7
2266	1,2,5,6,18,20,21,23	3,4,7,8,17,19,22,24	5	O70417,P01835,P35280,Q03191,Q9JJ50
2267	1,2,5,6,18,20,21,24	3,4,7,8,17,19,22,23	5	O70417,P01835,P35280,Q5I0J9,Q68G31
2268	1,2,5,6,18,20,22,23	3,4,7,8,17,19,21,24	9	P00762,P04355,P16636,P20646,Q03191,Q5QE79,Q99MH3,Q9EQS0,Q9QX74
2269	1,2,5,6,18,20,22,24	3,4,7,8,17,19,21,23	4	P20646,P22006,Q9EQS0,Q9QX74
2270	1,2,5,6,18,20,23,24	3,4,7,8,17,19,21,22	8	O70417,P04355,P20646,P35280,Q03191,Q5QE79,Q9EQS0,Q9QX74
2271	1,2,5,6,18,21,22,23	3,4,7,8,17,19,20,24	11	O70417,P00762,P01835,P13676,P16636,P20646,P36374,P54921,Q99MH3,Q9EQS0,Q9WTT6
2272	1,2,5,6,18,21,22,24	3,4,7,8,17,19,20,23	7	O70417,P01835,P10758,P13676,P20646,Q4G075,Q9WTT6

2273	1,2,5,6,18,21,23,24	3,4,7,8,17,19,20,22	9	O70417,P01835,P13676,P20646,P35280,P36860,Q4G075,Q9JJ50,Q9WTT6
2274	1,2,5,6,18,22,23,24	3,4,7,8,17,19,20,21	19	D4A5U3,O70417,P00507,P00762,P13676,P16636,P20646,P22273,P22282,P36374,Q5QE79,Q63617,Q6P6R2,Q811M5,Q99MH3,Q9EQS0,Q9QX74,Q9WTT6,Q9WVK7
2275	1,2,5,6,19,20,21,22	3,4,7,8,17,18,23,24	4	O70417,P52590,Q5I0J9,Q5PQL7
2276	1,2,5,6,19,20,21,23	3,4,7,8,17,18,22,24	4	O70417,P35280,P52590,Q5I0J9
2277	1,2,5,6,19,20,21,24	3,4,7,8,17,18,22,23	5	O35547,O70417,P35280,Q5I0J9,Q68G31
2278	1,2,5,6,19,20,22,23	3,4,7,8,17,18,21,24	3	P00762,P02625,Q9EQS0
2279	1,2,5,6,19,20,22,24	3,4,7,8,17,18,21,23	3	Q5I0J9,Q68G31,Q9QX74
2280	1,2,5,6,19,20,23,24	3,4,7,8,17,18,21,22	5	O70417,P23739,P35280,Q68G31,Q9QX74
2281	1,2,5,6,19,21,22,23	3,4,7,8,17,18,20,24	4	O70417,P00762,Q811M5,Q9EQS0
2282	1,2,5,6,19,21,22,24	3,4,7,8,17,18,20,23	2	O70417,P30919
2283	1,2,5,6,19,21,23,24	3,4,7,8,17,18,20,22	5	O70417,P14173,P35280,Q4G075,Q9JJ50
2284	1,2,5,6,19,22,23,24	3,4,7,8,17,18,20,21	13	D4A5U3,O70417,P00507,P00762,P13676,P20646,P22273,P26772,Q6P6R2,Q811M5,Q9EQS0,Q9QX74,Q9WTT6
2285	1,2,5,6,20,21,22,23	3,4,7,8,17,18,19,24	3	O70417,P01835,Q03191
2286	1,2,5,6,20,21,22,24	3,4,7,8,17,18,19,23	4	O70417,P01835,P20646,Q5I0J9
2287	1,2,5,6,20,21,23,24	3,4,7,8,17,18,19,22	4	O70417,P01835,P20646,P35280
2288	1,2,5,6,20,22,23,24	3,4,7,8,17,18,19,21	6	O70417,P20646,Q5QE79,Q6P6R2,Q9EQS0,Q9QX74
2289	1,2,5,6,21,22,23,24	3,4,7,8,17,18,19,20	11	O70417,P01835,P13676,P20646,P30919,P36860,Q62714,Q6P6R2,Q9EQS0,Q9QX74,Q9WTT6
2290	1,2,5,7,8,17,18,19	3,4,6,20,21,22,23,24	6	iRT-Kit_WR_fusion,P02783,P09656,P22006,Q66H69,Q9QX74
2291	1,2,5,7,8,17,18,20	3,4,6,19,21,22,23,24	4	P25809,Q4G075,Q5GRG2,Q811M5
2292	1,2,5,7,8,17,18,21	3,4,6,19,20,22,23,24	7	iRT-Kit_WR_fusion,P06760,P22006,P62804,Q00715,Q811M5,Q9QX74

2293	1,2,5,7,8,17,18,22	3,4,6,19,20,21,23,24	0	
2294	1,2,5,7,8,17,18,23	3,4,6,19,20,21,22,24	4	O70594,P19132,P19814,P22006
2295	1,2,5,7,8,17,18,24	3,4,6,19,20,21,22,23	3	P02783,P09656,Q811M5
2296	1,2,5,7,8,17,19,20	3,4,6,18,21,22,23,24	8	D4A5U3,iRT-Kit_WR_fusion,P13676,Q4G075,Q66H69,Q811M5,Q8CJD3,Q9WTT6
2297	1,2,5,7,8,17,19,21	3,4,6,18,20,22,23,24	8	iRT-Kit_WR_fusion,P06760,P22006,P34080,P62804,Q00715,Q66H69,Q9QX74
2298	1,2,5,7,8,17,19,22	3,4,6,18,20,21,23,24	2	iRT-Kit_WR_fusion,Q66H69
2299	1,2,5,7,8,17,19,23	3,4,6,18,20,21,22,24	4	iRT-Kit_WR_fusion,P22006,Q66H69,Q8CJD3
2300	1,2,5,7,8,17,19,24	3,4,6,18,20,21,22,23	4	iRT-Kit_WR_fusion,P09656,P54921,Q66H69
2301	1,2,5,7,8,17,20,21	3,4,6,18,19,22,23,24	11	D4A5U3,iRT-Kit_WR_fusion,P06760,P13676,P22006,P25809,P62804,Q00715,Q811M5,Q9QX74,Q9WTT6
2302	1,2,5,7,8,17,20,22	3,4,6,18,19,21,23,24	2	iRT-Kit_WR_fusion,Q4G075
2303	1,2,5,7,8,17,20,23	3,4,6,18,19,21,22,24	4	iRT-Kit_WR_fusion,P19132,P23739,Q4G075
2304	1,2,5,7,8,17,20,24	3,4,6,18,19,21,22,23	1	Q811M5
2305	1,2,5,7,8,17,21,22	3,4,6,18,19,20,23,24	2	iRT-Kit_WR_fusion,P06760
2306	1,2,5,7,8,17,21,23	3,4,6,18,19,20,22,24	7	iRT-Kit_WR_fusion,P06760,P19132,P19814,P22006,Q63474,Q9Z0V6
2307	1,2,5,7,8,17,21,24	3,4,6,18,19,20,22,23	6	iRT-Kit_WR_fusion,P36860,Q63474,Q6AY61,Q6IMF3,Q811M5
2308	1,2,5,7,8,17,22,23	3,4,6,18,19,20,21,24	0	
2309	1,2,5,7,8,17,22,24	3,4,6,18,19,20,21,23	0	
2310	1,2,5,7,8,17,23,24	3,4,6,18,19,20,21,22	0	
2311	1,2,5,7,8,18,19,20	3,4,6,17,21,22,23,24	4	iRT-Kit_WR_fusion,P47967,Q5I0D1,Q66H69
2312	1,2,5,7,8,18,19,21	3,4,6,17,20,22,23,24	6	iRT-Kit_WR_fusion,P06760,P22006,P62804,Q00715,Q66H69

2313	1,2,5,7,8,18,19,22	3,4,6,17,20,21,23,24	2	iRT-Kit_WR_fusion,Q66H69
2314	1,2,5,7,8,18,19,23	3,4,6,17,20,21,22,24	2	iRT-Kit_WR_fusion,Q66H69
2315	1,2,5,7,8,18,19,24	3,4,6,17,20,21,22,23	3	P02783,P09656,Q66H69
2316	1,2,5,7,8,18,20,21	3,4,6,17,19,22,23,24	8	iRT-Kit_WR_fusion,P06760,P25809,P47967,P62804,Q00715,Q811M5,Q91ZS3
2317	1,2,5,7,8,18,20,22	3,4,6,17,19,21,23,24	2	Q03191,Q91ZS3
2318	1,2,5,7,8,18,20,23	3,4,6,17,19,21,22,24	3	P19132,P23739,Q03191
2319	1,2,5,7,8,18,20,24	3,4,6,17,19,21,22,23	2	Q811M5,Q91ZS3
2320	1,2,5,7,8,18,21,22	3,4,6,17,19,20,23,24	2	iRT-Kit_WR_fusion,Q00715
2321	1,2,5,7,8,18,21,23	3,4,6,17,19,20,22,24	5	iRT-Kit_WR_fusion,P19132,P19814,P22006,Q9JJ50
2322	1,2,5,7,8,18,21,24	3,4,6,17,19,20,22,23	3	iRT-Kit_WR_fusion,P36860,Q00715
2323	1,2,5,7,8,18,22,23	3,4,6,17,19,20,21,24	0	
2324	1,2,5,7,8,18,22,24	3,4,6,17,19,20,21,23	0	
2325	1,2,5,7,8,18,23,24	3,4,6,17,19,20,21,22	2	Q9JJ50,Q9WVK7
2326	1,2,5,7,8,19,20,21	3,4,6,17,18,22,23,24	10	iRT-Kit_WR_fusion,P06760,P47967,P55159,P62804,Q00715,Q5I0D1,Q66H69,Q812E4,Q9WTT6
2327	1,2,5,7,8,19,20,22	3,4,6,17,18,21,23,24	3	iRT-Kit_WR_fusion,P19218,Q4G075
2328	1,2,5,7,8,19,20,23	3,4,6,17,18,21,22,24	4	iRT-Kit_WR_fusion,P23739,Q4G075,Q8CJD3
2329	1,2,5,7,8,19,20,24	3,4,6,17,18,21,22,23	3	iRT-Kit_WR_fusion,Q66H69,Q812E4
2330	1,2,5,7,8,19,21,22	3,4,6,17,18,20,23,24	3	iRT-Kit_WR_fusion,P06760,Q66H69
2331	1,2,5,7,8,19,21,23	3,4,6,17,18,20,22,24	5	iRT-Kit_WR_fusion,P06760,P22006,Q66H69,Q8CJD3
2332	1,2,5,7,8,19,21,24	3,4,6,17,18,20,22,23	3	iRT-Kit_WR_fusion,Q63474,Q66H69

2333	1,2,5,7,8,19,22,23	3,4,6,17,18,20,21,24	2 iRT-Kit_WR_fusion,Q8CJD3
2334	1,2,5,7,8,19,22,24	3,4,6,17,18,20,21,23	1 iRT-Kit_WR_fusion
2335	1,2,5,7,8,19,23,24	3,4,6,17,18,20,21,22	2 iRT-Kit_WR_fusion,Q8CJD3
2336	1,2,5,7,8,20,21,22	3,4,6,17,18,19,23,24	2 iRT-Kit_WR_fusion,P06760
2337	1,2,5,7,8,20,21,23	3,4,6,17,18,19,22,24	5 iRT-Kit_WR_fusion,P06760,P08649,P19132,P23739
2338	1,2,5,7,8,20,21,24	3,4,6,17,18,19,22,23	7 iRT-Kit_WR_fusion,P06760,P08649,P09456,Q811M5,Q812E4,Q9JHB9
2339	1,2,5,7,8,20,22,23	3,4,6,17,18,19,21,24	5 iRT-Kit_WR_fusion,P19132,P23739,Q03191,Q4G075
2340	1,2,5,7,8,20,22,24	3,4,6,17,18,19,21,23	1 iRT-Kit_WR_fusion
2341	1,2,5,7,8,20,23,24	3,4,6,17,18,19,21,22	3 iRT-Kit_WR_fusion,P19132,P23739
2342	1,2,5,7,8,21,22,23	3,4,6,17,18,19,20,24	2 iRT-Kit_WR_fusion,Q62714
2343	1,2,5,7,8,21,22,24	3,4,6,17,18,19,20,23	2 iRT-Kit_WR_fusion,Q62714
2344	1,2,5,7,8,21,23,24	3,4,6,17,18,19,20,22	6 iRT-Kit_WR_fusion,P08649,P36860,Q62714,Q63474,Q9JJ50
2345	1,2,5,7,8,22,23,24	3,4,6,17,18,19,20,21	2 Q62714,Q6P6R2
2346	1,2,5,7,17,18,19,20	3,4,6,8,21,22,23,24	4 P00762,P02783,P09656,P30919
2347	1,2,5,7,17,18,19,21	3,4,6,8,20,22,23,24	3 P00762,P02783,P22006
2348	1,2,5,7,17,18,19,22	3,4,6,8,20,21,23,24	2 P00762,P09656
2349	1,2,5,7,17,18,19,23	3,4,6,8,20,21,22,24	3 P00762,P22006,Q10743
2350	1,2,5,7,17,18,19,24	3,4,6,8,20,21,22,23	5 P00762,P02783,P09656,P54921,Q6AY61
2351	1,2,5,7,17,18,20,21	3,4,6,8,19,22,23,24	3 P00507,P25809,Q811M5
2352	1,2,5,7,17,18,20,22	3,4,6,8,19,21,23,24	3 P00762,Q03191,Q9Z0J6

2353	1,2,5,7,17, 18,20,23	3,4,6,8,19, 21,22,24	4	P00762,P30919,Q03191,Q10743
2354	1,2,5,7,17, 18,20,24	3,4,6,8,19, 21,22,23	2	P02783,Q811M5
2355	1,2,5,7,17, 18,21,22	3,4,6,8,19, 20,23,24	1	P00762
2356	1,2,5,7,17, 18,21,23	3,4,6,8,19, 20,22,24	4	O70417,P00762,P19814,P22006
2357	1,2,5,7,17, 18,21,24	3,4,6,8,19, 20,22,23	3	P02783,P36860,Q6AY61
2358	1,2,5,7,17, 18,22,23	3,4,6,8,19, 20,21,24	4	P00714,P00762,Q10743,Q9EQS0
2359	1,2,5,7,17, 18,22,24	3,4,6,8,19, 20,21,23	1	P00762
2360	1,2,5,7,17, 18,23,24	3,4,6,8,19, 20,21,22	1	P00762
2361	1,2,5,7,17, 19,20,21	3,4,6,8,18, 22,23,24	2	Q6AY61,Q811M5
2362	1,2,5,7,17, 19,20,22	3,4,6,8,18, 21,23,24	3	O89117,P00762,P02625
2363	1,2,5,7,17, 19,20,23	3,4,6,8,18, 21,22,24	4	P00762,P02625,P23739,Q8CJD3
2364	1,2,5,7,17, 19,20,24	3,4,6,8,18, 21,22,23	3	P02783,P54921,Q6AY61
2365	1,2,5,7,17, 19,21,22	3,4,6,8,18, 20,23,24	2	P00762,Q9R168
2366	1,2,5,7,17, 19,21,23	3,4,6,8,18, 20,22,24	4	O70417,P00762,P22006,Q9R168
2367	1,2,5,7,17, 19,21,24	3,4,6,8,18, 20,22,23	4	P54921,Q62761;Q62762;Q62763,Q6AY61,Q9R168
2368	1,2,5,7,17, 19,22,23	3,4,6,8,18, 20,21,24	3	P00762,P02625,Q9R168
2369	1,2,5,7,17, 19,22,24	3,4,6,8,18, 20,21,23	3	P00762,P54921,Q9R168
2370	1,2,5,7,17, 19,23,24	3,4,6,8,18, 20,21,22	2	P00762,P54921
2371	1,2,5,7,17, 20,21,22	3,4,6,8,18, 19,23,24	0	
2372	1,2,5,7,17, 20,21,23	3,4,6,8,18, 19,22,24	0	

2373	1,2,5,7,17, 20,21,24	3,4,6,8,18, 19,22,23	2 Q6AY61,Q811M5
2374	1,2,5,7,17, 20,22,23	3,4,6,8,18, 19,21,24	3 P00762,P02625,Q03191
2375	1,2,5,7,17, 20,22,24	3,4,6,8,18, 19,21,23	0
2376	1,2,5,7,17, 20,23,24	3,4,6,8,18, 19,21,22	1 P23739
2377	1,2,5,7,17, 21,22,23	3,4,6,8,18, 19,20,24	1 Q9R168
2378	1,2,5,7,17, 21,22,24	3,4,6,8,18, 19,20,23	2 P36860,Q9R168
2379	1,2,5,7,17, 21,23,24	3,4,6,8,18, 19,20,22	4 O70417,P36860,Q6AY61,Q9R168
2380	1,2,5,7,17, 22,23,24	3,4,6,8,18, 19,20,21	3 P00762,P22273,Q9R168
2381	1,2,5,7,18, 19,20,21	3,4,6,8,17, 22,23,24	2 P47967,Q5I0D1
2382	1,2,5,7,18, 19,20,22	3,4,6,8,17, 21,23,24	1 P00762
2383	1,2,5,7,18, 19,20,23	3,4,6,8,17, 21,22,24	1 P00762
2384	1,2,5,7,18, 19,20,24	3,4,6,8,17, 21,22,23	2 P00762,P02783
2385	1,2,5,7,18, 19,21,22	3,4,6,8,17, 20,23,24	2 O70417,P00762
2386	1,2,5,7,18, 19,21,23	3,4,6,8,17, 20,22,24	3 O70417,P00762,P22006
2387	1,2,5,7,18, 19,21,24	3,4,6,8,17, 20,22,23	2 O70417,P02783
2388	1,2,5,7,18, 19,22,23	3,4,6,8,17, 20,21,24	3 O70417,P00762,Q9EQS0
2389	1,2,5,7,18, 19,22,24	3,4,6,8,17, 20,21,23	1 P00762
2390	1,2,5,7,18, 19,23,24	3,4,6,8,17, 20,21,22	2 O70417,P00762
2391	1,2,5,7,18, 20,21,22	3,4,6,8,17, 19,23,24	2 P01835,Q03191
2392	1,2,5,7,18, 20,21,23	3,4,6,8,17, 19,22,24	3 O70417,P01835,Q03191

2393	1,2,5,7,18,<br>20,21,24	3,4,6,8,17,<br>19,22,23	1 P01835
2394	1,2,5,7,18,<br>20,22,23	3,4,6,8,17,<br>19,21,24	3 P00762,Q03191,Q9EQS0
2395	1,2,5,7,18,<br>20,22,24	3,4,6,8,17,<br>19,21,23	1 Q03191
2396	1,2,5,7,18,<br>20,23,24	3,4,6,8,17,<br>19,21,22	1 Q03191
2397	1,2,5,7,18,<br>21,22,23	3,4,6,8,17,<br>19,20,24	4 O70417,P01835,Q03191,Q9EQS0
2398	1,2,5,7,18,<br>21,22,24	3,4,6,8,17,<br>19,20,23	2 O70417,P01835
2399	1,2,5,7,18,<br>21,23,24	3,4,6,8,17,<br>19,20,22	4 O70417,O89117,P01835,Q9JJ50
2400	1,2,5,7,18,<br>22,23,24	3,4,6,8,17,<br>19,20,21	8 O70417,P00762,P13676,P36374,Q03191,Q6P6R2,Q9EQS0,Q9WVK7
2401	1,2,5,7,19,<br>20,21,22	3,4,6,8,17,<br>18,23,24	1 P47967
2402	1,2,5,7,19,<br>20,21,23	3,4,6,8,17,<br>18,22,24	3 O70417,P08649,P14173
2403	1,2,5,7,19,<br>20,21,24	3,4,6,8,17,<br>18,22,23	4 O70417,P08649,P14173,Q6AY61
2404	1,2,5,7,19,<br>20,22,23	3,4,6,8,17,<br>18,21,24	3 P00762,P02625,P23739
2405	1,2,5,7,19,<br>20,22,24	3,4,6,8,17,<br>18,21,23	0
2406	1,2,5,7,19,<br>20,23,24	3,4,6,8,17,<br>18,21,22	2 P14173,P23739
2407	1,2,5,7,19,<br>21,22,23	3,4,6,8,17,<br>18,20,24	4 O70417,P00762,P08649,Q9R168
2408	1,2,5,7,19,<br>21,22,24	3,4,6,8,17,<br>18,20,23	3 O70417,P08649,Q9R168
2409	1,2,5,7,19,<br>21,23,24	3,4,6,8,17,<br>18,20,22	3 O70417,P08649,P14173
2410	1,2,5,7,19,<br>22,23,24	3,4,6,8,17,<br>18,20,21	3 O70417,P00762,P26772
2411	1,2,5,7,20,<br>21,22,23	3,4,6,8,17,<br>18,19,24	4 O70417,P01835,P08649,Q03191
2412	1,2,5,7,20,<br>21,22,24	3,4,6,8,17,<br>18,19,23	2 P01835,P08649

2413	1,2,5,7,20, 21,23,24	3,4,6,8,17, 18,19,22	4	O70417,P01835,P08649,P23739
2414	1,2,5,7,20, 22,23,24	3,4,6,8,17, 18,19,21	4	P08649,P23739,Q03191,Q6P6R2
2415	1,2,5,7,21, 22,23,24	3,4,6,8,17, 18,19,20	5	O70417,P01835,P08649,Q62714,Q9R168
2416	1,2,5,8,17, 18,19,20	3,4,6,7,21, 22,23,24	3	P02783,P09656,P25809
2417	1,2,5,8,17, 18,19,21	3,4,6,7,20, 22,23,24	3	P02783,P22006,Q66H69
2418	1,2,5,8,17, 18,19,22	3,4,6,7,20, 21,23,24	1	P09656
2419	1,2,5,8,17, 18,19,23	3,4,6,7,20, 21,22,24	1	P22006
2420	1,2,5,8,17, 18,19,24	3,4,6,7,20, 21,22,23	2	P02783,P09656
2421	1,2,5,8,17, 18,20,21	3,4,6,7,19, 22,23,24	4	P22006,P25809,P36375,Q811M5
2422	1,2,5,8,17, 18,20,22	3,4,6,7,19, 21,23,24	2	P00714,P25809
2423	1,2,5,8,17, 18,20,23	3,4,6,7,19, 21,22,24	3	P00714,P19132,P23739
2424	1,2,5,8,17, 18,20,24	3,4,6,7,19, 21,22,23	2	P02783,Q811M5
2425	1,2,5,8,17, 18,21,22	3,4,6,7,19, 20,23,24	1	P10758
2426	1,2,5,8,17, 18,21,23	3,4,6,7,19, 20,22,24	3	P19132,P19814,P22006
2427	1,2,5,8,17, 18,21,24	3,4,6,7,19, 20,22,23	2	P02783,P10758
2428	1,2,5,8,17, 18,22,23	3,4,6,7,19, 20,21,24	4	P00714,P11598,P22283,Q63617
2429	1,2,5,8,17, 18,22,24	3,4,6,7,19, 20,21,23	4	P00714,P10758,P11598,Q63617
2430	1,2,5,8,17, 18,23,24	3,4,6,7,19, 20,21,22	2	P00714,Q63617
2431	1,2,5,8,17, 19,20,21	3,4,6,7,18, 22,23,24	9	B0LT89,P06760,P22006,P25809,P97675,Q66H69,Q6P6R2,Q811M5,Q8CJD3
2432	1,2,5,8,17, 19,20,22	3,4,6,7,18, 21,23,24	1	O89117

2433	1,2,5,8,17, 19,20,23	3,4,6,7,18, 21,22,24	2	P23739,Q8CJD3
2434	1,2,5,8,17, 19,20,24	3,4,6,7,18, 21,22,23	2	P02783,Q68G31
2435	1,2,5,8,17, 19,21,22	3,4,6,7,18, 20,23,24	0	
2436	1,2,5,8,17, 19,21,23	3,4,6,7,18, 20,22,24	2	P22006,Q8CJD3
2437	1,2,5,8,17, 19,21,24	3,4,6,7,18, 20,22,23	2	Q63474,Q66H69
2438	1,2,5,8,17, 19,22,23	3,4,6,7,18, 20,21,24	4	P00507,P00714,P11598,Q8CJD3
2439	1,2,5,8,17, 19,22,24	3,4,6,7,18, 20,21,23	1	P00507
2440	1,2,5,8,17, 19,23,24	3,4,6,7,18, 20,21,22	1	Q8CJD3
2441	1,2,5,8,17, 20,21,22	3,4,6,7,18, 19,23,24	2	F1M3L7,P25809
2442	1,2,5,8,17, 20,21,23	3,4,6,7,18, 19,22,24	5	P19132,P22006,P23739,P36375,P63029
2443	1,2,5,8,17, 20,21,24	3,4,6,7,18, 19,22,23	1	Q811M5
2444	1,2,5,8,17, 20,22,23	3,4,6,7,18, 19,21,24	3	P00714,P19132,P23739
2445	1,2,5,8,17, 20,22,24	3,4,6,7,18, 19,21,23	0	
2446	1,2,5,8,17, 20,23,24	3,4,6,7,18, 19,21,22	2	P19132,P23739
2447	1,2,5,8,17, 21,22,23	3,4,6,7,18, 19,20,24	1	P68511
2448	1,2,5,8,17, 21,22,24	3,4,6,7,18, 19,20,23	1	P10758
2449	1,2,5,8,17, 21,23,24	3,4,6,7,18, 19,20,22	2	Q63474,Q80WL1
2450	1,2,5,8,17, 22,23,24	3,4,6,7,18, 19,20,21	7	P00507,P00714,P11598,P22283,P47967,P97840,Q63617
2451	1,2,5,8,18, 19,20,21	3,4,6,7,17, 22,23,24	4	F1M3L7,P25809,Q66H69,Q68G31
2452	1,2,5,8,18, 19,20,22	3,4,6,7,17, 21,23,24	0	

2453	1,2,5,8,18, 19,20,23	3,4,6,7,17, 21,22,24	1	P23739
2454	1,2,5,8,18, 19,20,24	3,4,6,7,17, 21,22,23	2	P02783,Q68G31
2455	1,2,5,8,18, 19,21,22	3,4,6,7,17, 20,23,24	0	
2456	1,2,5,8,18, 19,21,23	3,4,6,7,17, 20,22,24	1	P22006
2457	1,2,5,8,18, 19,21,24	3,4,6,7,17, 20,22,23	3	P02783,Q66H69,Q68G31
2458	1,2,5,8,18, 19,22,23	3,4,6,7,17, 20,21,24	2	P00507,P00714
2459	1,2,5,8,18, 19,22,24	3,4,6,7,17, 20,21,23	1	P00507
2460	1,2,5,8,18, 19,23,24	3,4,6,7,17, 20,21,22	0	
2461	1,2,5,8,18, 20,21,22	3,4,6,7,17, 19,23,24	2	F1M3L7,P25809
2462	1,2,5,8,18, 20,21,23	3,4,6,7,17, 19,22,24	1	P19132
2463	1,2,5,8,18, 20,21,24	3,4,6,7,17, 19,22,23	0	
2464	1,2,5,8,18, 20,22,23	3,4,6,7,17, 19,21,24	4	P00714,P19132,Q5QE79,Q99MH3
2465	1,2,5,8,18, 20,22,24	3,4,6,7,17, 19,21,23	1	Q5QE79
2466	1,2,5,8,18, 20,23,24	3,4,6,7,17, 19,21,22	2	P23739,Q5QE79
2467	1,2,5,8,18, 21,22,23	3,4,6,7,17, 19,20,24	1	P54921
2468	1,2,5,8,18, 21,22,24	3,4,6,7,17, 19,20,23	0	
2469	1,2,5,8,18, 21,23,24	3,4,6,7,17, 19,20,22	1	Q9JJ50
2470	1,2,5,8,18, 22,23,24	3,4,6,7,17, 19,20,21	9	P00507,P00714,P11598,P20646,Q5QE79,Q63617,Q6P6R2,Q9R0T3,Q9WVK7
2471	1,2,5,8,19, 20,21,22	3,4,6,7,17, 18,23,24	1	F1M3L7
2472	1,2,5,8,19, 20,21,23	3,4,6,7,17, 18,22,24	3	P19132,P23739,Q8CJD3

2473	1,2,5,8,19,<br>20,21,24	3,4,6,7,17,<br>18,22,23	2	P30120,Q68G31
2474	1,2,5,8,19,<br>20,22,23	3,4,6,7,17,<br>18,21,24	2	P23739,Q8CJD3
2475	1,2,5,8,19,<br>20,22,24	3,4,6,7,17,<br>18,21,23	0	
2476	1,2,5,8,19,<br>20,23,24	3,4,6,7,17,<br>18,21,22	2	P23739,Q8CJD3
2477	1,2,5,8,19,<br>21,22,23	3,4,6,7,17,<br>18,20,24	0	
2478	1,2,5,8,19,<br>21,22,24	3,4,6,7,17,<br>18,20,23	0	
2479	1,2,5,8,19,<br>21,23,24	3,4,6,7,17,<br>18,20,22	2	O70417,Q63474
2480	1,2,5,8,19,<br>22,23,24	3,4,6,7,17,<br>18,20,21	2	P00507,P26772
2481	1,2,5,8,20,<br>21,22,23	3,4,6,7,17,<br>18,19,24	3	F1M3L7,P19132,P23739
2482	1,2,5,8,20,<br>21,22,24	3,4,6,7,17,<br>18,19,23	1	F1M3L7
2483	1,2,5,8,20,<br>21,23,24	3,4,6,7,17,<br>18,19,22	2	P19132,P23739
2484	1,2,5,8,20,<br>22,23,24	3,4,6,7,17,<br>18,19,21	2	P23739,Q5QE79
2485	1,2,5,8,21,<br>22,23,24	3,4,6,7,17,<br>18,19,20	3	P30919,Q62714,Q8K1G0
2486	1,2,5,17,1<br>8,19,20,21	3,4,6,7,8,2<br>2,23,24	7	B0LT89,O70417,P02783,P20761,P25809,P35280,Q99041
2487	1,2,5,17,1<br>8,19,20,22	3,4,6,7,8,2<br>1,23,24	3	P00714,P00762,P20761
2488	1,2,5,17,1<br>8,19,20,23	3,4,6,7,8,2<br>1,22,24	6	O70417,P00714,P00762,P02625,P35280,Q99041
2489	1,2,5,17,1<br>8,19,20,24	3,4,6,7,8,2<br>1,22,23	5	P02783,P09656,P20761,P35280,Q99041
2490	1,2,5,17,1<br>8,19,21,22	3,4,6,7,8,2<br>0,23,24	3	O70417,P20761,P22273
2491	1,2,5,17,1<br>8,19,21,23	3,4,6,7,8,2<br>0,22,24	6	O70417,P02780,P22006,P22273,P35280,Q99041
2492	1,2,5,17,1<br>8,19,21,24	3,4,6,7,8,2<br>0,22,23	7	O70417,P02783,P20761,P22273,P35280,Q4G075,Q99041

2493	1,2,5,17,18,19,22,23	3,4,6,7,8,20,21,24	11	O70417,P00714,P00762,P02780,P06761,P11598,P22273,P22282,Q5M8C6,Q63617,Q9EQS0
2494	1,2,5,17,18,19,22,24	3,4,6,7,8,20,21,23	9	O70417,P00714,P00762,P02780,P09656,P11598,P20761,P22273,Q63617
2495	1,2,5,17,18,19,23,24	3,4,6,7,8,20,21,22	10	O70417,P00714,P00762,P02780,P11598,P22273,P22282,P35280,Q63617,Q99041
2496	1,2,5,17,18,20,21,22	3,4,6,7,8,19,23,24	3	P00714,P20761,P25809
2497	1,2,5,17,18,20,21,23	3,4,6,7,8,19,22,24	5	O70417,P00714,P35280,Q99041,Q9QZK9
2498	1,2,5,17,18,20,21,24	3,4,6,7,8,19,22,23	5	O70417,P02783,P20761,P35280,Q99041
2499	1,2,5,17,18,20,22,23	3,4,6,7,8,19,21,24	6	P00714,P04355,P22282,Q63617,Q99041,Q9EQS0
2500	1,2,5,17,18,20,22,24	3,4,6,7,8,19,21,23	4	P00714,P20761,P22282,Q99041
2501	1,2,5,17,18,20,23,24	3,4,6,7,8,19,21,22	6	O70417,P00714,P22282,P23739,P35280,Q99041
2502	1,2,5,17,18,21,22,23	3,4,6,7,8,19,20,24	18	O70417,P00714,P02780,P06761,P08723,P0C0A9,P11598,P19814,P22273,P22282,P46462,P68511,Q5M8C6,Q62902,Q63617,Q99041,Q9QW07,Q9QZK9
2503	1,2,5,17,18,21,22,24	3,4,6,7,8,19,20,23	11	O70417,P00714,P02780,P10758,P11598,P20761,P22273,P22282,Q4G075,Q63617,Q99041
2504	1,2,5,17,18,21,23,24	3,4,6,7,8,19,20,22	11	O70417,P00714,P02780,P06761,P22273,P22282,P35280,Q4G075,Q63617,Q99041,Q9QZK9
2505	1,2,5,17,18,22,23,24	3,4,6,7,8,19,20,21	20	D3ZUC6,D4A5U3,O70417,P00714,P02780,P06761,P0C0A9,P11598,P22273,P22282,P22283,P36374,P46462,Q5I0D1,Q5M8C6,Q63617,Q99041,Q9EQS0,Q9R0T3,Q9WTT6
2506	1,2,5,17,19,20,21,22	3,4,6,7,8,18,23,24	2	O70257,P20761
2507	1,2,5,17,19,20,21,23	3,4,6,7,8,18,22,24	6	O70257,O70417,P23739,P35280,Q8CJD3,Q99041
2508	1,2,5,17,19,20,21,24	3,4,6,7,8,18,22,23	7	O70417,P02783,P20761,P35280,P54921,Q6AY61,Q99041
2509	1,2,5,17,19,20,22,23	3,4,6,7,8,18,21,24	4	O89117,P00714,P02625,P23739
2510	1,2,5,17,19,20,22,24	3,4,6,7,8,18,21,23	3	O89117,P02625,P20761
2511	1,2,5,17,19,20,23,24	3,4,6,7,8,18,21,22	5	O70417,P02625,P23739,P35280,Q99041
2512	1,2,5,17,19,21,22,23	3,4,6,7,8,18,20,24	7	O70417,P00714,P02780,P11598,P22273,P22282,Q9R168

2513	1,2,5,17,19,21,22,24	3,4,6,7,8,18,20,23	5	O70417,P20761,P22273,Q4G075,Q9R168
2514	1,2,5,17,19,21,23,24	3,4,6,7,8,18,20,22	7	O70417,P02780,P12020,P22273,P35280,Q4G075,Q99041
2515	1,2,5,17,19,22,23,24	3,4,6,7,8,18,20,21	14	O70417,P00507,P00714,P00762,P02625,P02780,P06761,P11598,P22273,P22282,P26772,Q5M8C6,Q63617,Q99041
2516	1,2,5,17,20,21,22,23	3,4,6,7,8,18,19,24	3	O70417,P00714,P22282
2517	1,2,5,17,20,21,22,24	3,4,6,7,8,18,19,23	2	O70417,P20761
2518	1,2,5,17,20,21,23,24	3,4,6,7,8,18,19,22	4	O70417,P23739,P35280,Q99041
2519	1,2,5,17,20,22,23,24	3,4,6,7,8,18,19,21	10	P00714,P02625,P06761,P08937,P22273,P22282,P23739,Q63617,Q63751,Q99041
2520	1,2,5,17,21,22,23,24	3,4,6,7,8,18,19,20	11	O70417,P00714,P02780,P06761,P11598,P22273,P22282,Q4G075,Q63617,Q99041,Q9R168
2521	1,2,5,18,19,20,21,22	3,4,6,7,8,17,23,24	3	F1M3L7,O70417,P20761
2522	1,2,5,18,19,20,21,23	3,4,6,7,8,17,22,24	4	O70417,P23593,P35280,Q99041
2523	1,2,5,18,19,20,21,24	3,4,6,7,8,17,22,23	6	O70417,P02783,P20761,P35280,Q68G31,Q99041
2524	1,2,5,18,19,20,22,23	3,4,6,7,8,17,21,24	5	O70417,P00714,P02625,P04762,Q9EQS0
2525	1,2,5,18,19,20,22,24	3,4,6,7,8,17,21,23	2	O70417,P20761
2526	1,2,5,18,19,20,23,24	3,4,6,7,8,17,21,22	5	O70417,P23739,P35280,Q5QE79,Q99041
2527	1,2,5,18,19,21,22,23	3,4,6,7,8,17,20,24	8	O70417,P00714,P06911,P22273,P22282,Q4G075,Q5M8C6,Q9EQS0
2528	1,2,5,18,19,21,22,24	3,4,6,7,8,17,20,23	4	O70417,P06911,P22273,Q4G075
2529	1,2,5,18,19,21,23,24	3,4,6,7,8,17,20,22	6	O70417,P06911,P22273,P35280,Q4G075,Q99041
2530	1,2,5,18,19,22,23,24	3,4,6,7,8,17,20,21	21	D4A5U3,O70417,P00507,P00714,P00762,P02780,P06761,P06911,P11598,P13676,P22273,P22282,P26772,Q4G075,Q5M8C6,Q62946,Q63617,Q9EQS0,Q9QX74,Q9R0T3,Q9WTT6
2531	1,2,5,18,20,21,22,23	3,4,6,7,8,17,19,24	4	O70417,P00714,P22282,Q03191
2532	1,2,5,18,20,21,22,24	3,4,6,7,8,17,19,23	3	O70417,P20761,P50115

2533	1,2,5,18,20,21,23,24	3,4,6,7,8,17,19,22	5	O70417,P35280,Q5QE79,Q99041,Q9QZK9
2534	1,2,5,18,20,22,23,24	3,4,6,7,8,17,19,21	9	O70417,P00714,P04355,P20646,P22282,Q5QE79,Q99041,Q9EQS0,Q9QX74
2535	1,2,5,18,21,22,23,24	3,4,6,7,8,17,19,20	18	D4A5U3,O70417,P00714,P02780,P06761,P06911,P11598,P13676,P20646,P22273,P22282,P36374,Q4G075,Q63617,Q99041,Q9EQS0,Q9R0T3,Q9WTT6
2536	1,2,5,19,20,21,22,23	3,4,6,7,8,17,18,24	1	O70417
2537	1,2,5,19,20,21,22,24	3,4,6,7,8,17,18,23	3	O70417,O70594,P20761
2538	1,2,5,19,20,21,23,24	3,4,6,7,8,17,18,22	5	O70417,P14173,P23739,P35280,Q99041
2539	1,2,5,19,20,22,23,24	3,4,6,7,8,17,18,21	7	O70417,P02625,P22273,P23739,P26772,Q5QE79,Q9QX74
2540	1,2,5,19,21,22,23,24	3,4,6,7,8,17,18,20	8	O70417,P02780,P06911,P12020,P13676,P22273,P22282,Q4G075
2541	1,2,5,20,21,22,23,24	3,4,6,7,8,17,18,19	5	O70417,P20646,P22282,Q5QE79,Q9QX74
2542	1,2,6,7,8,17,18,19	3,4,5,20,21,22,23,24	6	iRT-Kit_WR_fusion,P08649,P20766,P31044,Q66H69,Q68G31
2543	1,2,6,7,8,17,18,20	3,4,5,19,21,22,23,24	14	iRT-Kit_WR_fusion,P06911,P12020,P13432,P19629,P25809,P31044,P47727,P50280,P55091,Q5GRG2,Q78P75,Q91ZS3,Q9JHB9
2544	1,2,6,7,8,17,18,21	3,4,5,19,20,22,23,24	7	iRT-Kit_WR_fusion,P02631,P05539,P62804,Q00715,Q9QX74,Q9Z1F2
2545	1,2,6,7,8,17,18,22	3,4,5,19,20,21,23,24	0	
2546	1,2,6,7,8,17,18,23	3,4,5,19,20,21,22,24	2	O70594,Q63598
2547	1,2,6,7,8,17,18,24	3,4,5,19,20,21,22,23	3	P13432,Q63598,Q9Z1F2
2548	1,2,6,7,8,17,19,20	3,4,5,18,21,22,23,24	19	D4A5U3,iRT-Kit_WR_fusion,O89117,P06911,P12020,P13676,P19629,P31044,P47727,P50280,P55091,Q5GRG2,Q66H69,Q68G31,Q6P9T8,Q812E4,Q9JHB9,Q9JI85,Q9WTT6
2549	1,2,6,7,8,17,19,21	3,4,5,18,20,22,23,24	12	iRT-Kit_WR_fusion,P02631,P05539,P19629,P21674,P62804,Q00715,Q63474,Q66H69,Q68G31,Q9JI85,Q9QX74
2550	1,2,6,7,8,17,19,22	3,4,5,18,20,21,23,24	5	iRT-Kit_WR_fusion,O89117,P15399,P97580,Q66H69
2551	1,2,6,7,8,17,19,23	3,4,5,18,20,21,22,24	4	iRT-Kit_WR_fusion,P21674,Q66H69,Q8CJD3

2552	1,2,6,7,8,17,19,24	3,4,5,18,20,21,22,23	5	iRT-Kit_WR_fusion,P97840,Q63474,Q66H69,Q68G31
2553	1,2,6,7,8,17,20,21	3,4,5,18,19,22,23,24	23	D4A5U3,iRT-Kit_WR_fusion,P02631,P05964,P06911,P09456,P12020,P19629,P22283,P25809,P31044,P47727,P55091,P62804,P63029,Q00715,Q5GRG2,Q63474,Q78P75,Q812E4,Q9JHB9,Q9JI85,Q9WTT6
2554	1,2,6,7,8,17,20,22	3,4,5,18,19,21,23,24	12	iRT-Kit_WR_fusion,O89117,P06911,P12020,P19629,P31044,P50280,P55091,P63029,Q5GRG2,Q78P75,Q9JHB9
2555	1,2,6,7,8,17,20,23	3,4,5,18,19,21,22,24	8	iRT-Kit_WR_fusion,P06911,P12020,P19629,P31044,P55091,P63029,Q5GRG2
2556	1,2,6,7,8,17,20,24	3,4,5,18,19,21,22,23	13	iRT-Kit_WR_fusion,P06911,P08723,P09456,P12020,P19629,P31044,P47727,Q5GRG2,Q68G31,Q78P75,Q812E4,Q9JHB9
2557	1,2,6,7,8,17,21,22	3,4,5,18,19,20,23,24	4	iRT-Kit_WR_fusion,P02631,P05539,P19629
2558	1,2,6,7,8,17,21,23	3,4,5,18,19,20,22,24	6	iRT-Kit_WR_fusion,P02631,P19629,P21674,Q63474,Q63598
2559	1,2,6,7,8,17,21,24	3,4,5,18,19,20,22,23	7	iRT-Kit_WR_fusion,P01039,P02631,P05539,P36860,Q63474,Q63598
2560	1,2,6,7,8,17,22,23	3,4,5,18,19,20,21,24	5	iRT-Kit_WR_fusion,O54858,P97840,Q63532,Q99MH3
2561	1,2,6,7,8,17,22,24	3,4,5,18,19,20,21,23	2	iRT-Kit_WR_fusion,P97840
2562	1,2,6,7,8,17,23,24	3,4,5,18,19,20,21,22	4	iRT-Kit_WR_fusion,P97840,Q63474,Q63598
2563	1,2,6,7,8,18,19,20	3,4,5,17,21,22,23,24	17	iRT-Kit_WR_fusion,P06911,P12020,P19629,P31044,P47727,P50280,P69897,Q5GRG2,Q66H69,Q68G31,Q6P9T8,Q812E4,Q91ZS3,Q9JHB9,Q9JI85,Q9QW07
2564	1,2,6,7,8,18,19,21	3,4,5,17,20,22,23,24	10	iRT-Kit_WR_fusion,P02631,P19629,P31044,P62804,Q00715,Q66H69,Q68G31,Q91ZS3,Q9JI85
2565	1,2,6,7,8,18,19,22	3,4,5,17,20,21,23,24	3	iRT-Kit_WR_fusion,P15399,P97580
2566	1,2,6,7,8,18,19,23	3,4,5,17,20,21,22,24	3	iRT-Kit_WR_fusion,P20766,P31044
2567	1,2,6,7,8,18,19,24	3,4,5,17,20,21,22,23	4	iRT-Kit_WR_fusion,P20766,Q66H69,Q68G31
2568	1,2,6,7,8,18,20,21	3,4,5,17,19,22,23,24	22	iRT-Kit_WR_fusion,P01835,P02631,P06911,P12020,P19629,P22283,P25809,P31044,P47727,P50280,P62804,P69897,Q00715,Q5GRG2,Q68G31,Q78P75,Q812E4,Q91ZS3,Q9JHB9,Q9JI85,Q9QW07

2569	1,2,6,7,8,18,20,22	3,4,5,17,19,21,23,24	11	iRT-Kit_WR_fusion,P06911,P12020,P15399,P19629,P31044,P50280,Q5GRG2,Q6P9T8,Q91ZS3,Q9JHB9
2570	1,2,6,7,8,18,20,23	3,4,5,17,19,21,22,24	10	iRT-Kit_WR_fusion,P06911,P12020,P19629,P31044,P50280,Q5GRG2,Q62894,Q91ZS3,Q99MH3
2571	1,2,6,7,8,18,20,24	3,4,5,17,19,21,22,23	13	iRT-Kit_WR_fusion,P06911,P12020,P19629,P31044,P50280,Q5GRG2,Q68G31,Q78P75,Q812E4,Q91ZS3,Q9JHB9,Q9QW07
2572	1,2,6,7,8,18,21,22	3,4,5,17,19,20,23,24	7	iRT-Kit_WR_fusion,P01835,P02631,P19629,P23739,Q00715,Q91ZS3
2573	1,2,6,7,8,18,21,23	3,4,5,17,19,20,22,24	7	iRT-Kit_WR_fusion,P01835,P02631,P19629,Q00715,Q63532,Q91ZS3
2574	1,2,6,7,8,18,21,24	3,4,5,17,19,20,22,23	9	iRT-Kit_WR_fusion,P01835,P02631,P36860,Q00715,Q63474,Q68G31,Q91ZS3,Q9Z1F2
2575	1,2,6,7,8,18,22,23	3,4,5,17,19,20,21,24	5	iRT-Kit_WR_fusion,O54858,P19629,Q63532,Q99MH3
2576	1,2,6,7,8,18,22,24	3,4,5,17,19,20,21,23	1	O54858
2577	1,2,6,7,8,18,23,24	3,4,5,17,19,20,21,22	3	P20766,Q3ZAV1,Q63598
2578	1,2,6,7,8,19,20,21	3,4,5,17,18,22,23,24	31	iRT-Kit_WR_fusion,O35547,P02631,P02780,P05369,P05964,P06761,P06911,P08723,P09456,P12020,P19629,P22283,P24368,P31044,P46462,P47727,P50280,P62804,Q00715,Q5GRG2,Q63493,Q63617,Q66H69,Q68G31,Q78P75,Q812E4,Q9JHB9,Q9JI85,Q9QW07,Q9WTT6
2579	1,2,6,7,8,19,20,22	3,4,5,17,18,21,23,24	13	iRT-Kit_WR_fusion,P06911,P12020,P15399,P19218,P19629,P31044,P50280,Q5GRG2,Q68G31,Q6P9T8,Q9JI85,Q9QW07
2580	1,2,6,7,8,19,20,23	3,4,5,17,18,21,22,24	12	iRT-Kit_WR_fusion,P06911,P12020,P19629,P31044,P50280,Q5GRG2,Q68G31,Q812E4,Q8CJD3,Q9JI85,Q9QW07
2581	1,2,6,7,8,19,20,24	3,4,5,17,18,21,22,23	15	iRT-Kit_WR_fusion,P06911,P08723,P09456,P12020,P19629,P31044,P50280,Q5GRG2,Q63617,Q68G31,Q812E4,Q9JHB9,Q9JI85,Q9QW07
2582	1,2,6,7,8,19,21,22	3,4,5,17,18,20,23,24	4	iRT-Kit_WR_fusion,P02631,P19629,Q66H69
2583	1,2,6,7,8,19,21,23	3,4,5,17,18,20,22,24	6	iRT-Kit_WR_fusion,P02631,P19629,P21674,Q63474,Q66H69
2584	1,2,6,7,8,19,21,24	3,4,5,17,18,20,22,23	7	iRT-Kit_WR_fusion,P02631,P19629,Q63474,Q66H69,Q68G31,Q9JI85
2585	1,2,6,7,8,19,22,23	3,4,5,17,18,20,21,24	4	iRT-Kit_WR_fusion,O54858,P19629,Q63532

2586	1,2,6,7,8,19,22,24	3,4,5,17,18,20,21,23	3	iRT-Kit_WR_fusion,P15399,Q68G31
2587	1,2,6,7,8,19,23,24	3,4,5,17,18,20,21,22	3	iRT-Kit_WR_fusion,Q63474,Q68G31
2588	1,2,6,7,8,20,21,22	3,4,5,17,18,19,23,24	12	iRT-Kit_WR_fusion,P01835,P02631,P06911,P12020,P19629,P50280,Q5GRG2,Q78P75,Q91ZS3,Q9JHB9,Q9JI85
2589	1,2,6,7,8,20,21,23	3,4,5,17,18,19,22,24	11	iRT-Kit_WR_fusion,P01835,P02631,P06911,P12020,P19629,Q5GRG2,Q78P75,Q812E4,Q91ZS3,Q9JI85
2590	1,2,6,7,8,20,21,24	3,4,5,17,18,19,22,23	22	iRT-Kit_WR_fusion,P01835,P02631,P02782,P06911,P08723,P09456,P12020,P19629,P22283,P30120,P50280,Q5GRG2,Q63474,Q68G31,Q6AYR9,Q78P75,Q812E4,Q91ZS3,Q9JHB9,Q9JI85,Q9QW07
2591	1,2,6,7,8,20,22,23	3,4,5,17,18,19,21,24	8	iRT-Kit_WR_fusion,P06911,P12020,P19629,P50280,Q5GRG2,Q62894,Q99MH3
2592	1,2,6,7,8,20,22,24	3,4,5,17,18,19,21,23	11	iRT-Kit_WR_fusion,P01835,P06911,P12020,P15399,P19629,P22006,P50280,Q5GRG2,Q9JHB9,Q9QW07
2593	1,2,6,7,8,20,23,24	3,4,5,17,18,19,21,22	9	iRT-Kit_WR_fusion,P01835,P06911,P12020,P19629,Q5GRG2,Q812E4,Q9JHB9,Q9QW07
2594	1,2,6,7,8,21,22,23	3,4,5,17,18,19,20,24	6	iRT-Kit_WR_fusion,O54858,P01835,P19629,Q62714,Q63532
2595	1,2,6,7,8,21,22,24	3,4,5,17,18,19,20,23	5	iRT-Kit_WR_fusion,P01835,P19629,Q62714,Q63474
2596	1,2,6,7,8,21,23,24	3,4,5,17,18,19,20,22	7	iRT-Kit_WR_fusion,P01835,P19629,P36860,Q62714,Q63474,Q63598
2597	1,2,6,7,8,22,23,24	3,4,5,17,18,19,20,21	6	iRT-Kit_WR_fusion,O54858,P97840,Q62714,Q63532,Q6P6R2
2598	1,2,6,7,17,18,19,20	3,4,5,8,21,22,23,24	4	P00762,P30919,P55091,Q5GRG2
2599	1,2,6,7,17,18,19,21	3,4,5,8,20,22,23,24	3	P00762,P05539,P23739
2600	1,2,6,7,17,18,19,22	3,4,5,8,20,21,23,24	2	P00762,P23739
2601	1,2,6,7,17,18,19,23	3,4,5,8,20,21,22,24	2	P00762,Q10743
2602	1,2,6,7,17,18,19,24	3,4,5,8,20,21,22,23	1	P00762
2603	1,2,6,7,17,18,20,21	3,4,5,8,19,22,23,24	7	P00507,P01835,P05539,P25809,P55091,P63029,Q5GRG2

2604	1,2,6,7,17, 18,20,22	3,4,5,8,19, 21,23,24	4	P00762,P01835,P55091,Q5GRG2
2605	1,2,6,7,17, 18,20,23	3,4,5,8,19, 21,22,24	5	P00762,P01835,P30919,P55091,Q5GRG2
2606	1,2,6,7,17, 18,20,24	3,4,5,8,19, 21,22,23	2	P01835,Q5GRG2
2607	1,2,6,7,17, 18,21,22	3,4,5,8,19, 20,23,24	4	P00762,P01835,P05539,P23739
2608	1,2,6,7,17, 18,21,23	3,4,5,8,19, 20,22,24	3	P00762,P01835,P19814
2609	1,2,6,7,17, 18,21,24	3,4,5,8,19, 20,22,23	5	P01039,P01835,P05539,P36860,Q6AY61
2610	1,2,6,7,17, 18,22,23	3,4,5,8,19, 20,21,24	2	P00762,Q9EQS0
2611	1,2,6,7,17, 18,22,24	3,4,5,8,19, 20,21,23	2	D3ZUC6,P00762
2612	1,2,6,7,17, 18,23,24	3,4,5,8,19, 20,21,22	2	O88797,P00762
2613	1,2,6,7,17, 19,20,21	3,4,5,8,18, 22,23,24	7	P01835,P05539,P22283,P63029,Q5GRG2,Q63617,Q68G31
2614	1,2,6,7,17, 19,20,22	3,4,5,8,18, 21,23,24	3	O89117,P00762,Q5GRG2
2615	1,2,6,7,17, 19,20,23	3,4,5,8,18, 21,22,24	2	O89117,P00762
2616	1,2,6,7,17, 19,20,24	3,4,5,8,18, 21,22,23	4	O89117,P54921,Q5GRG2,Q68G31
2617	1,2,6,7,17, 19,21,22	3,4,5,8,18, 20,23,24	3	P00762,P05539,Q9R168
2618	1,2,6,7,17, 19,21,23	3,4,5,8,18, 20,22,24	5	P00762,P05539,P21674,Q63474,Q9R168
2619	1,2,6,7,17, 19,21,24	3,4,5,8,18, 20,22,23	7	P01039,P05539,P54921,Q63474,Q68G31,Q6AY61,Q9R168
2620	1,2,6,7,17, 19,22,23	3,4,5,8,18, 20,21,24	4	O89117,P00762,Q9EQS0,Q9R168
2621	1,2,6,7,17, 19,22,24	3,4,5,8,18, 20,21,23	4	D3ZUC6,O89117,P00762,Q9R168
2622	1,2,6,7,17, 19,23,24	3,4,5,8,18, 20,21,22	1	P00762
2623	1,2,6,7,17, 20,21,22	3,4,5,8,18, 19,23,24	3	P01835,P05539,P63029

2624	1,2,6,7,17,<br>20,21,23	3,4,5,8,18,<br>19,22,24	3	P01835,P55091,P63029
2625	1,2,6,7,17,<br>20,21,24	3,4,5,8,18,<br>19,22,23	4	P01835,P05539,P63029,Q6AY61
2626	1,2,6,7,17,<br>20,22,23	3,4,5,8,18,<br>19,21,24	4	O89117,P00762,P01835,P63029
2627	1,2,6,7,17,<br>20,22,24	3,4,5,8,18,<br>19,21,23	4	O35077,O89117,P01835,P22006
2628	1,2,6,7,17,<br>20,23,24	3,4,5,8,18,<br>19,21,22	1	P01835
2629	1,2,6,7,17,<br>21,22,23	3,4,5,8,18,<br>19,20,24	3	P01835,P05539,Q9R168
2630	1,2,6,7,17,<br>21,22,24	3,4,5,8,18,<br>19,20,23	5	P01039,P01835,P05539,P36860,Q9R168
2631	1,2,6,7,17,<br>21,23,24	3,4,5,8,18,<br>19,20,22	4	P01835,P36860,Q63474,Q9R168
2632	1,2,6,7,17,<br>22,23,24	3,4,5,8,18,<br>19,20,21	8	D3ZUC6,P00762,P01835,P47967,P97840,Q5I0D1,Q9EQS0,Q9R168
2633	1,2,6,7,18,<br>19,20,21	3,4,5,8,17,<br>22,23,24	5	P01835,P22283,Q5PQL7,Q63617,Q68G31
2634	1,2,6,7,18,<br>19,20,22	3,4,5,8,17,<br>21,23,24	2	P00762,Q5PQL7
2635	1,2,6,7,18,<br>19,20,23	3,4,5,8,17,<br>21,22,24	1	P00762
2636	1,2,6,7,18,<br>19,20,24	3,4,5,8,17,<br>21,22,23	3	P00762,P01835,Q68G31
2637	1,2,6,7,18,<br>19,21,22	3,4,5,8,17,<br>20,23,24	5	P00762,P01835,P23739,P36376,Q5PQL7
2638	1,2,6,7,18,<br>19,21,23	3,4,5,8,17,<br>20,22,24	2	P00762,P01835
2639	1,2,6,7,18,<br>19,21,24	3,4,5,8,17,<br>20,22,23	3	P01835,P36376,Q68G31
2640	1,2,6,7,18,<br>19,22,23	3,4,5,8,17,<br>20,21,24	4	P00762,Q6IG02,Q811M5,Q9EQS0
2641	1,2,6,7,18,<br>19,22,24	3,4,5,8,17,<br>20,21,23	3	P00762,P36376,Q9EQS0
2642	1,2,6,7,18,<br>19,23,24	3,4,5,8,17,<br>20,21,22	2	P00762,P20766
2643	1,2,6,7,18,<br>20,21,22	3,4,5,8,17,<br>19,23,24	2	P01835,Q5PQL7

2644	1,2,6,7,18, 20,21,23	3,4,5,8,17, 19,22,24	2	P01835,P19629
2645	1,2,6,7,18, 20,21,24	3,4,5,8,17, 19,22,23	2	P01835,Q68G31
2646	1,2,6,7,18, 20,22,23	3,4,5,8,17, 19,21,24	5	P00762,P01835,P04355,Q99MH3,Q9EQS0
2647	1,2,6,7,18, 20,22,24	3,4,5,8,17, 19,21,23	2	P01835,P22006
2648	1,2,6,7,18, 20,23,24	3,4,5,8,17, 19,21,22	1	P01835
2649	1,2,6,7,18, 21,22,23	3,4,5,8,17, 19,20,24	2	P01835,Q9EQS0
2650	1,2,6,7,18, 21,22,24	3,4,5,8,17, 19,20,23	3	P01835,P23739,P36376
2651	1,2,6,7,18, 21,23,24	3,4,5,8,17, 19,20,22	1	P01835
2652	1,2,6,7,18, 22,23,24	3,4,5,8,17, 19,20,21	8	D3ZUC6,P00762,P01835,P06760,P13676,Q6P6R2,Q9EQS0,Q9WVK7
2653	1,2,6,7,19, 20,21,22	3,4,5,8,17, 18,23,24	3	P01835,Q5PQL7,Q63617
2654	1,2,6,7,19, 20,21,23	3,4,5,8,17, 18,22,24	2	P01835,P19629
2655	1,2,6,7,19, 20,21,24	3,4,5,8,17, 18,22,23	7	O35547,P01835,P05369,P08723,P22283,Q63617,Q68G31
2656	1,2,6,7,19, 20,22,23	3,4,5,8,17, 18,21,24	2	P00762,P01835
2657	1,2,6,7,19, 20,22,24	3,4,5,8,17, 18,21,23	3	P01835,P22006,Q68G31
2658	1,2,6,7,19, 20,23,24	3,4,5,8,17, 18,21,22	3	P01835,P14173,Q68G31
2659	1,2,6,7,19, 21,22,23	3,4,5,8,17, 18,20,24	3	P00762,P01835,Q9R168
2660	1,2,6,7,19, 21,22,24	3,4,5,8,17, 18,20,23	3	P01835,P36376,Q9R168
2661	1,2,6,7,19, 21,23,24	3,4,5,8,17, 18,20,22	4	P01835,P14173,Q63474,Q6DGG1
2662	1,2,6,7,19, 22,23,24	3,4,5,8,17, 18,20,21	5	P00762,P01835,P26772,Q811M5,Q9EQS0
2663	1,2,6,7,20, 21,22,23	3,4,5,8,17, 18,19,24	2	P01835,P19629

2664	1,2,6,7,20,<br>21,22,24	3,4,5,8,17,<br>18,19,23	1	P01835
2665	1,2,6,7,20,<br>21,23,24	3,4,5,8,17,<br>18,19,22	1	P01835
2666	1,2,6,7,20,<br>22,23,24	3,4,5,8,17,<br>18,19,21	3	P01835,P22006,Q9QX74
2667	1,2,6,7,21,<br>22,23,24	3,4,5,8,17,<br>18,19,20	4	P01835,P13676,Q62714,Q9R168
2668	1,2,6,8,17,<br>18,19,20	3,4,5,7,21,<br>22,23,24	12	B0LT89,O89117,P06911,P08649,P12020,P13432,P25809,P47727,P55091,Q5GRG2,Q68G31,Q9JI<br>85
2669	1,2,6,8,17,<br>18,19,21	3,4,5,7,20,<br>22,23,24	3	P08649,Q66H69,Q68G31
2670	1,2,6,8,17,<br>18,19,22	3,4,5,7,20,<br>21,23,24	5	P08649,P15399,P97580,P97840,Q68G31
2671	1,2,6,8,17,<br>18,19,23	3,4,5,7,20,<br>21,22,24	3	P08649,P97840,Q68G31
2672	1,2,6,8,17,<br>18,19,24	3,4,5,7,20,<br>21,22,23	3	P08649,P97840,Q68G31
2673	1,2,6,8,17,<br>18,20,21	3,4,5,7,19,<br>22,23,24	10	P02631,P06911,P12020,P25809,P47727,P55091,P63029,Q5GRG2,Q68G31,Q9JI85
2674	1,2,6,8,17,<br>18,20,22	3,4,5,7,19,<br>21,23,24	8	O89117,P06911,P08649,P12020,P15399,P55091,P63029,Q5GRG2
2675	1,2,6,8,17,<br>18,20,23	3,4,5,7,19,<br>21,22,24	7	P06911,P08649,P12020,P55091,P63029,Q5GRG2,Q99MH3
2676	1,2,6,8,17,<br>18,20,24	3,4,5,7,19,<br>21,22,23	8	P06911,P08649,P12020,P13432,P47727,P97840,Q5GRG2,Q68G31
2677	1,2,6,8,17,<br>18,21,22	3,4,5,7,19,<br>20,23,24	3	P08649,P10758,P97840
2678	1,2,6,8,17,<br>18,21,23	3,4,5,7,19,<br>20,22,24	1	P97840
2679	1,2,6,8,17,<br>18,21,24	3,4,5,7,19,<br>20,22,23	2	P97840,Q68G31
2680	1,2,6,8,17,<br>18,22,23	3,4,5,7,19,<br>20,21,24	5	P08649,P47967,P97840,Q5I0D1,Q99MH3
2681	1,2,6,8,17,<br>18,22,24	3,4,5,7,19,<br>20,21,23	6	D3ZUC6,P08649,P10758,P47967,P97840,Q5I0D1
2682	1,2,6,8,17,<br>18,23,24	3,4,5,7,19,<br>20,21,22	4	P08649,P47967,P97840,Q5I0D1
2683	1,2,6,8,17,<br>19,20,21	3,4,5,7,18,<br>22,23,24	9	B0LT89,O89117,P25809,P47727,P63029,Q5GRG2,Q68G31,Q6P6R2,Q9JI85

2684	1,2,6,8,17, 19,20,22	3,4,5,7,18, 21,23,24	6	O89117,P12020,P15399,P63029,Q5GRG2,Q68G31
2685	1,2,6,8,17, 19,20,23	3,4,5,7,18, 21,22,24	6	O89117,P63029,P97840,Q5GRG2,Q68G31,Q8CJD3
2686	1,2,6,8,17, 19,20,24	3,4,5,7,18, 21,22,23	6	O89117,P47727,P97840,Q5GRG2,Q68G31,Q9JI85
2687	1,2,6,8,17, 19,21,22	3,4,5,7,18, 20,23,24	3	O89117,P97840,Q68G31
2688	1,2,6,8,17, 19,21,23	3,4,5,7,18, 20,22,24	4	P21674,P97840,Q63474,Q68G31
2689	1,2,6,8,17, 19,21,24	3,4,5,7,18, 20,22,23	4	P97840,Q63474,Q66H69,Q68G31
2690	1,2,6,8,17, 19,22,23	3,4,5,7,18, 20,21,24	4	O89117,P00507,P47967,P97840
2691	1,2,6,8,17, 19,22,24	3,4,5,7,18, 20,21,23	7	D3ZUC6,O89117,P00507,P15399,P47967,P97840,Q68G31
2692	1,2,6,8,17, 19,23,24	3,4,5,7,18, 20,21,22	3	P47967,P97840,Q68G31
2693	1,2,6,8,17, 20,21,22	3,4,5,7,18, 19,23,24	5	O89117,P19629,P25809,P63029,Q5GRG2
2694	1,2,6,8,17, 20,21,23	3,4,5,7,18, 19,22,24	5	P19629,P55091,P63029,P97840,Q5GRG2
2695	1,2,6,8,17, 20,21,24	3,4,5,7,18, 19,22,23	8	P30120,P47727,P63029,P97840,Q5GRG2,Q63474,Q68G31,Q9JI85
2696	1,2,6,8,17, 20,22,23	3,4,5,7,18, 19,21,24	5	O89117,P63029,P97840,Q5GRG2,Q99MH3
2697	1,2,6,8,17, 20,22,24	3,4,5,7,18, 19,21,23	8	O89117,P06911,P12020,P15399,P63029,P97840,Q5GRG2,Q68G31
2698	1,2,6,8,17, 20,23,24	3,4,5,7,18, 19,21,22	4	P63029,P97840,Q5GRG2,Q68G31
2699	1,2,6,8,17, 21,22,23	3,4,5,7,18, 19,20,24	3	P47967,P97840,Q63532
2700	1,2,6,8,17, 21,22,24	3,4,5,7,18, 19,20,23	3	P10758,P97840,Q63474
2701	1,2,6,8,17, 21,23,24	3,4,5,7,18, 19,20,22	3	P47967,P97840,Q63474
2702	1,2,6,8,17, 22,23,24	3,4,5,7,18, 19,20,21	6	D3ZUC6,P00507,P07335,P47967,P97840,Q5I0D1
2703	1,2,6,8,18, 19,20,21	3,4,5,7,17, 22,23,24	8	P02631,P19629,P25809,P47727,P50280,Q5GRG2,Q68G31,Q9JI85

2704	1,2,6,8,18,<br>19,20,22	3,4,5,7,17,<br>21,23,24	5	P12020,P15399,P50280,Q5GRG2,Q68G31
2705	1,2,6,8,18,<br>19,20,23	3,4,5,7,17,<br>21,22,24	2	Q5GRG2,Q68G31
2706	1,2,6,8,18,<br>19,20,24	3,4,5,7,17,<br>21,22,23	6	P12020,P50280,Q5GRG2,Q68G31,Q9JI85,Q9QW07
2707	1,2,6,8,18,<br>19,21,22	3,4,5,7,17,<br>20,23,24	2	P15399,Q68G31
2708	1,2,6,8,18,<br>19,21,23	3,4,5,7,17,<br>20,22,24	1	Q68G31
2709	1,2,6,8,18,<br>19,21,24	3,4,5,7,17,<br>20,22,23	1	Q68G31
2710	1,2,6,8,18,<br>19,22,23	3,4,5,7,17,<br>20,21,24	5	P00507,P15399,Q6AY61,Q811M5,Q99MH3
2711	1,2,6,8,18,<br>19,22,24	3,4,5,7,17,<br>20,21,23	4	P00507,P15399,P97840,Q68G31
2712	1,2,6,8,18,<br>19,23,24	3,4,5,7,17,<br>20,21,22	2	P97840,Q68G31
2713	1,2,6,8,18,<br>20,21,22	3,4,5,7,17,<br>19,23,24	7	P12020,P19629,P25809,P54921,Q5GRG2,Q68G31,Q91ZS3
2714	1,2,6,8,18,<br>20,21,23	3,4,5,7,17,<br>19,22,24	5	P19629,P54921,Q5GRG2,Q68G31,Q91ZS3
2715	1,2,6,8,18,<br>20,21,24	3,4,5,7,17,<br>19,22,23	5	P12020,Q5GRG2,Q68G31,Q91ZS3,Q9JI85
2716	1,2,6,8,18,<br>20,22,23	3,4,5,7,17,<br>19,21,24	6	P04355,P12020,P54921,Q5GRG2,Q6AY61,Q99MH3
2717	1,2,6,8,18,<br>20,22,24	3,4,5,7,17,<br>19,21,23	5	P12020,P15399,P22006,Q5GRG2,Q68G31
2718	1,2,6,8,18,<br>20,23,24	3,4,5,7,17,<br>19,21,22	4	P97840,Q5GRG2,Q5QE79,Q68G31
2719	1,2,6,8,18,<br>21,22,23	3,4,5,7,17,<br>19,20,24	3	P54921,Q63532,Q99MH3
2720	1,2,6,8,18,<br>21,22,24	3,4,5,7,17,<br>19,20,23	2	P97840,Q68G31
2721	1,2,6,8,18,<br>21,23,24	3,4,5,7,17,<br>19,20,22	3	P20646,P97840,Q68G31
2722	1,2,6,8,18,<br>22,23,24	3,4,5,7,17,<br>19,20,21	10	P00507,P06760,P13676,P20646,P47967,P97840,Q5I0D1,Q63532,Q6P6R2,Q99MH3
2723	1,2,6,8,19,<br>20,21,22	3,4,5,7,17,<br>18,23,24	5	P15399,P19629,Q5GRG2,Q68G31,Q9JI85

2724	1,2,6,8,19,20,21,23	3,4,5,7,17,18,22,24	3	P19629,Q68G31,Q9JI85
2725	1,2,6,8,19,20,21,24	3,4,5,7,17,18,22,23	9	O35547,P08723,P09456,P30120,Q5GRG2,Q68G31,Q812E4,Q9JI85,Q9QW07
2726	1,2,6,8,19,20,22,23	3,4,5,7,17,18,21,24	4	P15399,P19629,Q68G31,Q99MH3
2727	1,2,6,8,19,20,22,24	3,4,5,7,17,18,21,23	4	P15399,P22006,Q5GRG2,Q68G31
2728	1,2,6,8,19,20,23,24	3,4,5,7,17,18,21,22	2	P97840,Q68G31
2729	1,2,6,8,19,21,22,23	3,4,5,7,17,18,20,24	3	P97840,Q63532,Q68G31
2730	1,2,6,8,19,21,22,24	3,4,5,7,17,18,20,23	3	P30919,P97840,Q68G31
2731	1,2,6,8,19,21,23,24	3,4,5,7,17,18,20,22	3	P97840,Q63474,Q68G31
2732	1,2,6,8,19,22,23,24	3,4,5,7,17,18,20,21	6	P00507,P26772,P47967,P97840,Q68G31,Q811M5
2733	1,2,6,8,20,21,22,23	3,4,5,7,17,18,19,24	4	P19629,P54921,Q63532,Q99MH3
2734	1,2,6,8,20,21,22,24	3,4,5,7,17,18,19,23	4	P01835,P19629,Q5GRG2,Q68G31
2735	1,2,6,8,20,21,23,24	3,4,5,7,17,18,19,22	6	P01835,P19629,P97840,Q63474,Q68G31,Q812E4
2736	1,2,6,8,20,22,23,24	3,4,5,7,17,18,19,21	4	P22006,P97840,Q5QE79,Q99MH3
2737	1,2,6,8,21,22,23,24	3,4,5,7,17,18,19,20	5	P20646,P30919,P47967,P97840,Q63532
2738	1,2,6,17,18,19,20,21	3,4,5,7,8,22,23,24	7	B0LT89,P20761,P25809,P35280,P63029,Q68G31,Q6P6R2
2739	1,2,6,17,18,19,20,22	3,4,5,7,8,21,23,24	4	O89117,P00762,P08649,P20761
2740	1,2,6,17,18,19,20,23	3,4,5,7,8,21,22,24	3	P00762,P35280,Q68G31
2741	1,2,6,17,18,19,20,24	3,4,5,7,8,21,22,23	3	P20761,P35280,Q68G31
2742	1,2,6,17,18,19,21,22	3,4,5,7,8,20,23,24	3	P05539,P23739,Q4G075
2743	1,2,6,17,18,19,21,23	3,4,5,7,8,20,22,24	3	O70417,P35280,Q4G075

2744	1,2,6,17,18,19,21,24	3,4,5,7,8,20,22,23	3	P35280,Q4G075,Q68G31
2745	1,2,6,17,18,19,22,23	3,4,5,7,8,20,21,24	7	D3ZUC6,P00762,P06760,P08649,P22273,P47967,Q9EQS0
2746	1,2,6,17,18,19,22,24	3,4,5,7,8,20,21,23	7	D3ZUC6,P00762,P06760,P08649,P22273,P97840,Q4G075
2747	1,2,6,17,18,19,23,24	3,4,5,7,8,20,21,22	8	D3ZUC6,P00762,P06760,P22273,P35280,P47967,P97840,Q4G075
2748	1,2,6,17,18,20,21,22	3,4,5,7,8,19,23,24	3	P01835,P20761,P63029
2749	1,2,6,17,18,20,21,23	3,4,5,7,8,19,22,24	4	P01835,P35280,P55091,P63029
2750	1,2,6,17,18,20,21,24	3,4,5,7,8,19,22,23	5	O35077,P01835,P35280,P63029,Q68G31
2751	1,2,6,17,18,20,22,23	3,4,5,7,8,19,21,24	5	P04355,P06760,P63029,Q99MH3,Q9EQS0
2752	1,2,6,17,18,20,22,24	3,4,5,7,8,19,21,23	4	D3ZUC6,O35077,P06760,P22006
2753	1,2,6,17,18,20,23,24	3,4,5,7,8,19,21,22	4	O35077,P06760,P08937,P35280
2754	1,2,6,17,18,21,22,23	3,4,5,7,8,19,20,24	3	Q4G075,Q5I0D1,Q9EQS0
2755	1,2,6,17,18,21,22,24	3,4,5,7,8,19,20,23	5	D3ZUC6,P10758,P23739,Q4G075,Q5I0D1
2756	1,2,6,17,18,21,23,24	3,4,5,7,8,19,20,22	5	O70417,P35280,P97840,Q4G075,Q5I0D1
2757	1,2,6,17,18,22,23,24	3,4,5,7,8,19,20,21	13	D3ZUC6,P02780,P06760,P22273,P22282,P47967,P55159,P97840,Q4G075,Q5I0D1,Q63617,Q9EQS0,Q9WTT6
2758	1,2,6,17,19,20,21,22	3,4,5,7,8,18,23,24	4	O89117,P49134,P63029,Q68G31
2759	1,2,6,17,19,20,21,23	3,4,5,7,8,18,22,24	4	P35280,P49134,P63029,Q68G31
2760	1,2,6,17,19,20,21,24	3,4,5,7,8,18,22,23	5	O35547,P27590,P35280,P63029,Q68G31
2761	1,2,6,17,19,20,22,23	3,4,5,7,8,18,21,24	3	O89117,P02625,P63029
2762	1,2,6,17,19,20,22,24	3,4,5,7,8,18,21,23	5	D3ZUC6,O35077,O89117,P22006,Q68G31
2763	1,2,6,17,19,20,23,24	3,4,5,7,8,18,21,22	4	O89117,P35280,P97840,Q68G31

2764	1,2,6,17,19,21,22,23	3,4,5,7,8,18,20,24	3	P97840,Q4G075,Q9R168
2765	1,2,6,17,19,21,22,24	3,4,5,7,8,18,20,23	5	D3ZUC6,P97840,Q4G075,Q68G31,Q9R168
2766	1,2,6,17,19,21,23,24	3,4,5,7,8,18,20,22	6	O70417,P35280,P97840,Q4G075,Q63474,Q68G31
2767	1,2,6,17,19,22,23,24	3,4,5,7,8,18,20,21	12	D3ZUC6,O89117,P00507,P00762,P06760,P22273,P47967,P97840,Q4G075,Q5I0D1,Q811M5,Q9EQS0
2768	1,2,6,17,20,21,22,23	3,4,5,7,8,18,19,24	2	P01835,P63029
2769	1,2,6,17,20,21,22,24	3,4,5,7,8,18,19,23	3	O35077,P01835,P63029
2770	1,2,6,17,20,21,23,24	3,4,5,7,8,18,19,22	4	P01835,P35280,P63029,P97840
2771	1,2,6,17,20,22,23,24	3,4,5,7,8,18,19,21	9	D3ZUC6,O35077,O89117,P06760,P08937,P22006,P47967,P63029,P97840
2772	1,2,6,17,21,22,23,24	3,4,5,7,8,18,19,20	8	D3ZUC6,P01835,P02780,P47967,P97840,Q4G075,Q5I0D1,Q9R168
2773	1,2,6,18,19,20,21,22	3,4,5,7,8,17,23,24	2	Q5PQL7,Q68G31
2774	1,2,6,18,19,20,21,23	3,4,5,7,8,17,22,24	3	O70417,P35280,Q68G31
2775	1,2,6,18,19,20,21,24	3,4,5,7,8,17,22,23	3	P01835,P35280,Q68G31
2776	1,2,6,18,19,20,22,23	3,4,5,7,8,17,21,24	3	P04355,P06760,Q9EQS0
2777	1,2,6,18,19,20,22,24	3,4,5,7,8,17,21,23	4	P06760,P22006,Q68G31,Q9QX74
2778	1,2,6,18,19,20,23,24	3,4,5,7,8,17,21,22	4	P06760,P35280,Q68G31,Q9QX74
2779	1,2,6,18,19,21,22,23	3,4,5,7,8,17,20,24	5	O70417,P13676,Q4G075,Q811M5,Q9EQS0
2780	1,2,6,18,19,21,22,24	3,4,5,7,8,17,20,23	6	O70417,P13676,P23739,P36376,Q4G075,Q68G31
2781	1,2,6,18,19,21,23,24	3,4,5,7,8,17,20,22	4	O70417,P35280,Q4G075,Q68G31
2782	1,2,6,18,19,22,23,24	3,4,5,7,8,17,20,21	15	D3ZUC6,D4A5U3,O70417,P00507,P00762,P06760,P13676,P22273,P26772,P47967,Q4G075,Q811M5,Q9EQS0,Q9QX74,Q9WTT6
2783	1,2,6,18,20,21,22,23	3,4,5,7,8,17,19,24	2	P01835,P54921

2784	1,2,6,18,20,21,22,24	3,4,5,7,8,17,19,23	3	P01835,P22006,Q68G31
2785	1,2,6,18,20,21,23,24	3,4,5,7,8,17,19,22	5	O70417,P01835,P20646,P35280,Q68G31
2786	1,2,6,18,20,22,23,24	3,4,5,7,8,17,19,21	8	P01835,P04355,P06760,P20646,P22006,Q5QE79,Q9EQS0,Q9QX74
2787	1,2,6,18,21,22,23,24	3,4,5,7,8,17,19,20	10	D4A5U3,O70417,P01835,P13676,P20646,Q4G075,Q5I0D1,Q9EQS0,Q9QX74,Q9WTT6
2788	1,2,6,19,20,21,22,23	3,4,5,7,8,17,18,24	2	O70417,P01835
2789	1,2,6,19,20,21,22,24	3,4,5,7,8,17,18,23	3	O70594,P01835,Q68G31
2790	1,2,6,19,20,21,23,24	3,4,5,7,8,17,18,22	5	O70417,P01835,P14173,P35280,Q68G31
2791	1,2,6,19,20,22,23,24	3,4,5,7,8,17,18,21	6	P06760,P22006,P26772,Q68G31,Q811M5,Q9QX74
2792	1,2,6,19,21,22,23,24	3,4,5,7,8,17,18,20	6	O70417,P01835,P13676,P97840,Q4G075,Q811M5
2793	1,2,6,20,21,22,23,24	3,4,5,7,8,17,18,19	4	O70417,P01835,P20646,Q9QX74
2794	1,2,7,8,17,18,19,20	3,4,5,6,21,22,23,24	9	iRT-Kit_WR_fusion,P13676,P25809,P30919,P47727,Q5GRG2,Q66H69,Q811M5,Q9WTT6
2795	1,2,7,8,17,18,19,21	3,4,5,6,20,22,23,24	7	iRT-Kit_WR_fusion,P22006,P62804,Q00715,Q66H69,Q6P6R2,Q9QX74
2796	1,2,7,8,17,18,19,22	3,4,5,6,20,21,23,24	2	P09656,Q66H69
2797	1,2,7,8,17,18,19,23	3,4,5,6,20,21,22,24	2	P21674,Q66H69
2798	1,2,7,8,17,18,19,24	3,4,5,6,20,21,22,23	3	P02783,P09656,Q66H69
2799	1,2,7,8,17,18,20,21	3,4,5,6,19,22,23,24	11	iRT-Kit_WR_fusion,P00507,P25809,P47727,P55091,P62804,Q00715,Q5GRG2,Q811M5,Q9QX74,Q9WTT6
2800	1,2,7,8,17,18,20,22	3,4,5,6,19,21,23,24	3	P25809,P30919,Q5GRG2
2801	1,2,7,8,17,18,20,23	3,4,5,6,19,21,22,24	2	P30919,P55091
2802	1,2,7,8,17,18,20,24	3,4,5,6,19,21,22,23	4	P47727,Q5GRG2,Q811M5,Q9JHB9
2803	1,2,7,8,17,18,21,22	3,4,5,6,19,20,23,24	1	Q9Z1F2

2804	1,2,7,8,17,18,21,23	3,4,5,6,19,20,22,24	5	iRT-Kit_WR_fusion,P21674,P22006,Q9Z0V6,Q9Z1F2
2805	1,2,7,8,17,18,21,24	3,4,5,6,19,20,22,23	2	Q811M5,Q9Z1F2
2806	1,2,7,8,17,18,22,23	3,4,5,6,19,20,21,24	0	
2807	1,2,7,8,17,18,22,24	3,4,5,6,19,20,21,23	1	Q9Z1F2
2808	1,2,7,8,17,18,23,24	3,4,5,6,19,20,21,22	1	Q63598
2809	1,2,7,8,17,19,20,21	3,4,5,6,18,22,23,24	17	D4A5U3,iRT-Kit_WR_fusion,P09456,P13676,P24368,P25809,P30120,P47727,P62804,P97675,Q00715,Q66H69,Q6P6R2,Q811M5,Q812E4,Q9QX74,Q9WTT6
2810	1,2,7,8,17,19,20,22	3,4,5,6,18,21,23,24	3	iRT-Kit_WR_fusion,O89117,Q9WTT6
2811	1,2,7,8,17,19,20,23	3,4,5,6,18,21,22,24	6	iRT-Kit_WR_fusion,P21674,Q66H69,Q812E4,Q8CJD3,Q9WTT6
2812	1,2,7,8,17,19,20,24	3,4,5,6,18,21,22,23	10	iRT-Kit_WR_fusion,P08723,P09456,P30120,P47727,P54921,Q66H69,Q68G31,Q812E4,Q9WTT6
2813	1,2,7,8,17,19,21,22	3,4,5,6,18,20,23,24	3	iRT-Kit_WR_fusion,Q66H69,Q9QX74
2814	1,2,7,8,17,19,21,23	3,4,5,6,18,20,22,24	6	iRT-Kit_WR_fusion,P21674,P22006,Q63474,Q66H69,Q9QX74
2815	1,2,7,8,17,19,21,24	3,4,5,6,18,20,22,23	4	iRT-Kit_WR_fusion,P54921,Q63474,Q66H69
2816	1,2,7,8,17,19,22,23	3,4,5,6,18,20,21,24	1	iRT-Kit_WR_fusion
2817	1,2,7,8,17,19,22,24	3,4,5,6,18,20,21,23	2	iRT-Kit_WR_fusion,Q66H69
2818	1,2,7,8,17,19,23,24	3,4,5,6,18,20,21,22	2	iRT-Kit_WR_fusion,Q66H69
2819	1,2,7,8,17,20,21,22	3,4,5,6,18,19,23,24	3	iRT-Kit_WR_fusion,P25809,Q9WTT6
2820	1,2,7,8,17,20,21,23	3,4,5,6,18,19,22,24	4	iRT-Kit_WR_fusion,P25809,Q812E4,Q9WTT6
2821	1,2,7,8,17,20,21,24	3,4,5,6,18,19,22,23	10	iRT-Kit_WR_fusion,P02782,P08723,P09456,P25809,P30120,P47727,Q811M5,Q812E4,Q9JHB9
2822	1,2,7,8,17,20,22,23	3,4,5,6,18,19,21,24	2	iRT-Kit_WR_fusion,O89117
2823	1,2,7,8,17,20,22,24	3,4,5,6,18,19,21,23	1	O89117

2824	1,2,7,8,17,<br>20,23,24	3,4,5,6,18,<br>19,21,22	1	Q812E4
2825	1,2,7,8,17,<br>21,22,23	3,4,5,6,18,<br>19,20,24	1	iRT-Kit_WR_fusion
2826	1,2,7,8,17,<br>21,22,24	3,4,5,6,18,<br>19,20,23	2	iRT-Kit_WR_fusion,Q9Z1F2
2827	1,2,7,8,17,<br>21,23,24	3,4,5,6,18,<br>19,20,22	2	iRT-Kit_WR_fusion,Q63474
2828	1,2,7,8,17,<br>22,23,24	3,4,5,6,18,<br>19,20,21	2	P47967,P97840
2829	1,2,7,8,18,<br>19,20,21	3,4,5,6,17,<br>22,23,24	9	iRT-Kit_WR_fusion,P25809,P47727,P62804,Q00715,Q66H69,Q68G31,Q812E4,Q9WTT6
2830	1,2,7,8,18,<br>19,20,22	3,4,5,6,17,<br>21,23,24	1	iRT-Kit_WR_fusion
2831	1,2,7,8,18,<br>19,20,23	3,4,5,6,17,<br>21,22,24	1	iRT-Kit_WR_fusion
2832	1,2,7,8,18,<br>19,20,24	3,4,5,6,17,<br>21,22,23	6	iRT-Kit_WR_fusion,P08723,P47727,Q66H69,Q68G31,Q812E4
2833	1,2,7,8,18,<br>19,21,22	3,4,5,6,17,<br>20,23,24	3	iRT-Kit_WR_fusion,P62804,Q66H69
2834	1,2,7,8,18,<br>19,21,23	3,4,5,6,17,<br>20,22,24	2	iRT-Kit_WR_fusion,Q66H69
2835	1,2,7,8,18,<br>19,21,24	3,4,5,6,17,<br>20,22,23	3	iRT-Kit_WR_fusion,Q66H69,Q68G31
2836	1,2,7,8,18,<br>19,22,23	3,4,5,6,17,<br>20,21,24	1	iRT-Kit_WR_fusion
2837	1,2,7,8,18,<br>19,22,24	3,4,5,6,17,<br>20,21,23	0	
2838	1,2,7,8,18,<br>19,23,24	3,4,5,6,17,<br>20,21,22	1	P20766
2839	1,2,7,8,18,<br>20,21,22	3,4,5,6,17,<br>19,23,24	3	iRT-Kit_WR_fusion,P25809,P62804
2840	1,2,7,8,18,<br>20,21,23	3,4,5,6,17,<br>19,22,24	3	iRT-Kit_WR_fusion,P25809,Q812E4
2841	1,2,7,8,18,<br>20,21,24	3,4,5,6,17,<br>19,22,23	8	iRT-Kit_WR_fusion,P25809,P30120,P47727,Q00715,Q811M5,Q812E4,Q9JHB9
2842	1,2,7,8,18,<br>20,22,23	3,4,5,6,17,<br>19,21,24	0	
2843	1,2,7,8,18,<br>20,22,24	3,4,5,6,17,<br>19,21,23	0	

2844	1,2,7,8,18, 20,23,24	3,4,5,6,17, 19,21,22	0	
2845	1,2,7,8,18, 21,22,23	3,4,5,6,17, 19,20,24	1	iRT-Kit_WR_fusion
2846	1,2,7,8,18, 21,22,24	3,4,5,6,17, 19,20,23	1	Q9Z1F2
2847	1,2,7,8,18, 21,23,24	3,4,5,6,17, 19,20,22	1	iRT-Kit_WR_fusion
2848	1,2,7,8,18, 22,23,24	3,4,5,6,17, 19,20,21	1	Q64268
2849	1,2,7,8,19, 20,21,22	3,4,5,6,17, 18,23,24	5	iRT-Kit_WR_fusion,P62804,Q66H69,Q812E4,Q9WTT6
2850	1,2,7,8,19, 20,21,23	3,4,5,6,17, 18,22,24	5	iRT-Kit_WR_fusion,P62804,Q66H69,Q812E4,Q9WTT6
2851	1,2,7,8,19, 20,21,24	3,4,5,6,17, 18,22,23	12	iRT-Kit_WR_fusion,O35547,P02782,P08723,P09456,P24368,P30120,P47727,Q66H69,Q68G31,Q812E4,Q9JHB9
2852	1,2,7,8,19, 20,22,23	3,4,5,6,17, 18,21,24	1	iRT-Kit_WR_fusion
2853	1,2,7,8,19, 20,22,24	3,4,5,6,17, 18,21,23	3	iRT-Kit_WR_fusion,P08723,Q812E4
2854	1,2,7,8,19, 20,23,24	3,4,5,6,17, 18,21,22	2	iRT-Kit_WR_fusion,Q812E4
2855	1,2,7,8,19, 21,22,23	3,4,5,6,17, 18,20,24	1	iRT-Kit_WR_fusion
2856	1,2,7,8,19, 21,22,24	3,4,5,6,17, 18,20,23	2	iRT-Kit_WR_fusion,Q66H69
2857	1,2,7,8,19, 21,23,24	3,4,5,6,17, 18,20,22	3	iRT-Kit_WR_fusion,Q63474,Q66H69
2858	1,2,7,8,19, 22,23,24	3,4,5,6,17, 18,20,21	2	iRT-Kit_WR_fusion,P26772
2859	1,2,7,8,20, 21,22,23	3,4,5,6,17, 18,19,24	1	iRT-Kit_WR_fusion
2860	1,2,7,8,20, 21,22,24	3,4,5,6,17, 18,19,23	4	iRT-Kit_WR_fusion,P01835,Q812E4,Q9JHB9
2861	1,2,7,8,20, 21,23,24	3,4,5,6,17, 18,19,22	3	iRT-Kit_WR_fusion,P01835,Q812E4
2862	1,2,7,8,20, 22,23,24	3,4,5,6,17, 18,19,21	1	iRT-Kit_WR_fusion
2863	1,2,7,8,21, 22,23,24	3,4,5,6,17, 18,19,20	1	iRT-Kit_WR_fusion

2864	1,2,7,17,18,19,20,21	3,4,5,6,8,22,23,24	7	P00507,P20760,P25809,P30919,P62804,Q6P6R2,Q811M5
2865	1,2,7,17,18,19,20,22	3,4,5,6,8,21,23,24	1	P30919
2866	1,2,7,17,18,19,20,23	3,4,5,6,8,21,22,24	2	P30919,Q10743
2867	1,2,7,17,18,19,20,24	3,4,5,6,8,21,22,23	3	P02783,P30919,P54921
2868	1,2,7,17,18,19,21,22	3,4,5,6,8,20,23,24	1	P23739
2869	1,2,7,17,18,19,21,23	3,4,5,6,8,20,22,24	3	P21674,P22006,Q10743
2870	1,2,7,17,18,19,21,24	3,4,5,6,8,20,22,23	1	P02783
2871	1,2,7,17,18,19,22,23	3,4,5,6,8,20,21,24	4	P00762,P22273,Q10743,Q9EQS0
2872	1,2,7,17,18,19,22,24	3,4,5,6,8,20,21,23	3	D3ZUC6,P00762,P09656
2873	1,2,7,17,18,19,23,24	3,4,5,6,8,20,21,22	2	P00762,Q10743
2874	1,2,7,17,18,20,21,22	3,4,5,6,8,19,23,24	3	P01835,P25809,P30919
2875	1,2,7,17,18,20,21,23	3,4,5,6,8,19,22,24	2	P01835,P30919
2876	1,2,7,17,18,20,21,24	3,4,5,6,8,19,22,23	3	P00507,P01835,Q811M5
2877	1,2,7,17,18,20,22,23	3,4,5,6,8,19,21,24	2	P30919,Q10743
2878	1,2,7,17,18,20,22,24	3,4,5,6,8,19,21,23	0	
2879	1,2,7,17,18,20,23,24	3,4,5,6,8,19,21,22	2	P08937,P30919
2880	1,2,7,17,18,21,22,23	3,4,5,6,8,19,20,24	0	
2881	1,2,7,17,18,21,22,24	3,4,5,6,8,19,20,23	1	P42854
2882	1,2,7,17,18,21,23,24	3,4,5,6,8,19,20,22	1	P19218
2883	1,2,7,17,18,22,23,24	3,4,5,6,8,19,20,21	7	D3ZUC6,P08937,P22273,P22282,Q10743,Q5I0D1,Q64268

2884	1,2,7,17,19,20,21,22	3,4,5,6,8,18,23,24	0	
2885	1,2,7,17,19,20,21,23	3,4,5,6,8,18,22,24	1	P23593
2886	1,2,7,17,19,20,21,24	3,4,5,6,8,18,22,23	2	P54921,Q6AY61
2887	1,2,7,17,19,20,22,23	3,4,5,6,8,18,21,24	3	O89117,P02625,Q10743
2888	1,2,7,17,19,20,22,24	3,4,5,6,8,18,21,23	2	O89117,P54921
2889	1,2,7,17,19,20,23,24	3,4,5,6,8,18,21,22	2	P02625,P54921
2890	1,2,7,17,19,21,22,23	3,4,5,6,8,18,20,24	1	Q9R168
2891	1,2,7,17,19,21,22,24	3,4,5,6,8,18,20,23	2	P54921,Q9R168
2892	1,2,7,17,19,21,23,24	3,4,5,6,8,18,20,22	3	P14173,Q63474,Q9R168
2893	1,2,7,17,19,22,23,24	3,4,5,6,8,18,20,21	3	D3ZUC6,P22273,Q9R168
2894	1,2,7,17,20,21,22,23	3,4,5,6,8,18,19,24	1	P01835
2895	1,2,7,17,20,21,22,24	3,4,5,6,8,18,19,23	2	P01835,Q9R168
2896	1,2,7,17,20,21,23,24	3,4,5,6,8,18,19,22	1	P01835
2897	1,2,7,17,20,22,23,24	3,4,5,6,8,18,19,21	1	P08937
2898	1,2,7,17,21,22,23,24	3,4,5,6,8,18,19,20	2	P01835,Q9R168
2899	1,2,7,18,19,20,21,22	3,4,5,6,8,17,23,24	0	
2900	1,2,7,18,19,20,21,23	3,4,5,6,8,17,22,24	1	P23593
2901	1,2,7,18,19,20,21,24	3,4,5,6,8,17,22,23	2	P01835,Q68G31
2902	1,2,7,18,19,20,22,23	3,4,5,6,8,17,21,24	0	
2903	1,2,7,18,19,20,22,24	3,4,5,6,8,17,21,23	0	

2904	1,2,7,18,19,20,23,24	3,4,5,6,8,17,21,22	0
2905	1,2,7,18,19,21,22,23	3,4,5,6,8,17,20,24	1 O70417
2906	1,2,7,18,19,21,22,24	3,4,5,6,8,17,20,23	1 P36376
2907	1,2,7,18,19,21,23,24	3,4,5,6,8,17,20,22	1 O70417
2908	1,2,7,18,19,22,23,24	3,4,5,6,8,17,20,21	3 P22273,P26772,Q9EQS0
2909	1,2,7,18,20,21,22,23	3,4,5,6,8,17,19,24	1 P01835
2910	1,2,7,18,20,21,22,24	3,4,5,6,8,17,19,23	1 P01835
2911	1,2,7,18,20,21,23,24	3,4,5,6,8,17,19,22	1 P01835
2912	1,2,7,18,20,22,23,24	3,4,5,6,8,17,19,21	2 P01835,P08937
2913	1,2,7,18,21,22,23,24	3,4,5,6,8,17,19,20	3 O70417,P01835,P13676
2914	1,2,7,19,20,21,22,23	3,4,5,6,8,17,18,24	1 P01835
2915	1,2,7,19,20,21,22,24	3,4,5,6,8,17,18,23	2 O70594,P01835
2916	1,2,7,19,20,21,23,24	3,4,5,6,8,17,18,22	4 O70417,P01835,P14173,P23593
2917	1,2,7,19,20,22,23,24	3,4,5,6,8,17,18,21	1 P26772
2918	1,2,7,19,21,22,23,24	3,4,5,6,8,17,18,20	3 O70417,P01835,Q9R168
2919	1,2,7,20,21,22,23,24	3,4,5,6,8,17,18,19	1 P01835
2920	1,2,8,17,18,19,20,21	3,4,5,6,7,22,23,24	8 B0LT89,P25809,P47727,P97675,Q66H69,Q68G31,Q6P6R2,Q811M5
2921	1,2,8,17,18,19,20,22	3,4,5,6,7,21,23,24	4 O89117,P25809,P36860,P47727
2922	1,2,8,17,18,19,20,23	3,4,5,6,7,21,22,24	1 P30919
2923	1,2,8,17,18,19,20,24	3,4,5,6,7,21,22,23	3 P02783,P47727,Q68G31

2924	1,2,8,17,18,19,21,22	3,4,5,6,7,20,23,24	0	
2925	1,2,8,17,18,19,21,23	3,4,5,6,7,20,22,24	3	P21674,P22006,Q66H69
2926	1,2,8,17,18,19,21,24	3,4,5,6,7,20,22,23	3	P02783,Q66H69,Q68G31
2927	1,2,8,17,18,19,22,23	3,4,5,6,7,20,21,24	1	P00714
2928	1,2,8,17,18,19,22,24	3,4,5,6,7,20,21,23	1	D3ZUC6
2929	1,2,8,17,18,19,23,24	3,4,5,6,7,20,21,22	2	P47967,P97840
2930	1,2,8,17,18,20,21,22	3,4,5,6,7,19,23,24	2	P25809,P47727
2931	1,2,8,17,18,20,21,23	3,4,5,6,7,19,22,24	1	P25809
2932	1,2,8,17,18,20,21,24	3,4,5,6,7,19,22,23	5	P25809,P30120,P47727,Q68G31,Q811M5
2933	1,2,8,17,18,20,22,23	3,4,5,6,7,19,21,24	1	P00714
2934	1,2,8,17,18,20,22,24	3,4,5,6,7,19,21,23	0	
2935	1,2,8,17,18,20,23,24	3,4,5,6,7,19,21,22	1	Q99041
2936	1,2,8,17,18,21,22,23	3,4,5,6,7,19,20,24	2	P00714,P68511
2937	1,2,8,17,18,21,22,24	3,4,5,6,7,19,20,23	2	P10758,P42854
2938	1,2,8,17,18,21,23,24	3,4,5,6,7,19,20,22	2	P97840,Q99041
2939	1,2,8,17,18,22,23,24	3,4,5,6,7,19,20,21	8	D3ZUC6,P00714,P11598,P47967,P97840,Q5I0D1,Q63617,Q9R0T3
2940	1,2,8,17,19,20,21,22	3,4,5,6,7,18,23,24	5	B0LT89,O89117,P25809,P97675,Q6P6R2
2941	1,2,8,17,19,20,21,23	3,4,5,6,7,18,22,24	4	P25809,P63029,Q68G31,Q6P6R2
2942	1,2,8,17,19,20,21,24	3,4,5,6,7,18,22,23	7	B0LT89,P25809,P30120,P47727,P60905,P97675,Q68G31
2943	1,2,8,17,19,20,22,23	3,4,5,6,7,18,21,24	1	O89117

2944	1,2,8,17,19,20,22,24	3,4,5,6,7,18,21,23	2	O89117,Q68G31
2945	1,2,8,17,19,20,23,24	3,4,5,6,7,18,21,22	3	O89117,P97840,Q68G31
2946	1,2,8,17,19,21,22,23	3,4,5,6,7,18,20,24	0	
2947	1,2,8,17,19,21,22,24	3,4,5,6,7,18,20,23	1	P97840
2948	1,2,8,17,19,21,23,24	3,4,5,6,7,18,20,22	3	P97840,Q63474,Q68G31
2949	1,2,8,17,19,22,23,24	3,4,5,6,7,18,20,21	7	D3ZUC6,O89117,P00507,P11598,P47967,P97840,Q5I0D1
2950	1,2,8,17,20,21,22,23	3,4,5,6,7,18,19,24	1	P63029
2951	1,2,8,17,20,21,22,24	3,4,5,6,7,18,19,23	0	
2952	1,2,8,17,20,21,23,24	3,4,5,6,7,18,19,22	1	P63029
2953	1,2,8,17,20,22,23,24	3,4,5,6,7,18,19,21	3	O89117,P47967,P97840
2954	1,2,8,17,21,22,23,24	3,4,5,6,7,18,19,20	3	P47967,P97840,Q5I0D1
2955	1,2,8,18,19,20,21,22	3,4,5,6,7,17,23,24	2	P25809,Q68G31
2956	1,2,8,18,19,20,21,23	3,4,5,6,7,17,22,24	2	P25809,Q68G31
2957	1,2,8,18,19,20,21,24	3,4,5,6,7,17,22,23	4	P25809,P30120,P47727,Q68G31
2958	1,2,8,18,19,20,22,23	3,4,5,6,7,17,21,24	3	P04762,P36860,Q6AY61
2959	1,2,8,18,19,20,22,24	3,4,5,6,7,17,21,23	1	Q68G31
2960	1,2,8,18,19,20,23,24	3,4,5,6,7,17,21,22	1	Q68G31
2961	1,2,8,18,19,21,22,23	3,4,5,6,7,17,20,24	0	
2962	1,2,8,18,19,21,22,24	3,4,5,6,7,17,20,23	1	Q68G31
2963	1,2,8,18,19,21,23,24	3,4,5,6,7,17,20,22	1	Q68G31

2964	1,2,8,18,19,22,23,24	3,4,5,6,7,17,20,21	1	P00507
2965	1,2,8,18,20,21,22,23	3,4,5,6,7,17,19,24	2	P25809,P54921
2966	1,2,8,18,20,21,22,24	3,4,5,6,7,17,19,23	0	
2967	1,2,8,18,20,21,23,24	3,4,5,6,7,17,19,22	0	
2968	1,2,8,18,20,22,23,24	3,4,5,6,7,17,19,21	1	Q5QE79
2969	1,2,8,18,21,22,23,24	3,4,5,6,7,17,19,20	0	
2970	1,2,8,19,20,21,22,23	3,4,5,6,7,17,18,24	0	
2971	1,2,8,19,20,21,22,24	3,4,5,6,7,17,18,23	3	O70594,P30120,Q68G31
2972	1,2,8,19,20,21,23,24	3,4,5,6,7,17,18,22	2	Q68G31,Q812E4
2973	1,2,8,19,20,22,23,24	3,4,5,6,7,17,18,21	1	P26772
2974	1,2,8,19,21,22,23,24	3,4,5,6,7,17,18,20	0	
2975	1,2,8,20,21,22,23,24	3,4,5,6,7,17,18,19	1	Q80WY6
2976	1,2,17,18,19,20,21,22	3,4,5,6,7,8,23,24	6	B0LT89,P20760,P20761,P25809,P50115,Q05702
2977	1,2,17,18,19,20,21,23	3,4,5,6,7,8,22,24	6	O70417,P23593,P30919,P35280,P49134,Q99041
2978	1,2,17,18,19,20,21,24	3,4,5,6,7,8,22,23	9	B0LT89,P02783,P20760,P20761,P23593,P35280,P50115,Q68G31,Q99041
2979	1,2,17,18,19,20,22,23	3,4,5,6,7,8,21,24	9	P00714,P02625,P04762,P22273,P30919,P36860,P50115,Q10743,Q99041
2980	1,2,17,18,19,20,22,24	3,4,5,6,7,8,21,23	6	D3ZUC6,O70594,P20761,P50115,Q05702,Q99041

2981	1,2,17,18,19,20,23,24	3,4,5,6,7,8,21,22	5	P08937,P30919,P35280,P50115,Q99041
2982	1,2,17,18,19,21,22,23	3,4,5,6,7,8,20,24	8	O70417,P00714,P02780,P22273,P22282,Q05702,Q4G075,Q99041
2983	1,2,17,18,19,21,22,24	3,4,5,6,7,8,20,23	11	D3ZUC6,O70417,P02780,P20761,P22273,P25031,P42854,P50115,Q05702,Q4G075,Q99041
2984	1,2,17,18,19,21,23,24	3,4,5,6,7,8,20,22	7	O70417,P02780,P22273,P23593,P35280,Q4G075,Q99041
2985	1,2,17,18,19,22,23,24	3,4,5,6,7,8,20,21	21	D3ZUC6,D4A5U3,O70417,P00714,P02780,P06760,P06761,P08937,P11598,P22273,P22282,P47967,P50115,P50116,Q05702,Q4G075,Q5I0D1,Q5M8C6,Q63617,Q99041,Q9R0T3
2986	1,2,17,18,20,21,22,23	3,4,5,6,7,8,19,24	4	P00714,P22282,P50115,Q99041
2987	1,2,17,18,20,21,22,24	3,4,5,6,7,8,19,23	4	P20761,P42854,P50115,Q99041
2988	1,2,17,18,20,21,23,24	3,4,5,6,7,8,19,22	5	O70417,P19218,P35280,P50115,Q99041
2989	1,2,17,18,20,22,23,24	3,4,5,6,7,8,19,21	10	D3ZUC6,P00714,P06760,P06761,P08937,P22273,P22282,P47967,P50115,Q99041
2990	1,2,17,18,21,22,23,24	3,4,5,6,7,8,19,20	16	D3ZUC6,O70417,P00714,P02780,P06761,P11598,P22273,P22282,P42854,P47967,P50115,Q4G075,Q5I0D1,Q63617,Q99041,Q9R0T3
2991	1,2,17,19,20,21,22,23	3,4,5,6,7,8,18,24	3	O70417,O89117,P49134
2992	1,2,17,19,20,21,22,24	3,4,5,6,7,8,18,23	6	O70594,O89117,P20761,P49134,P50115,Q05702
2993	1,2,17,19,20,21,23,24	3,4,5,6,7,8,18,22	5	O70417,P23593,P35280,P49134,Q99041

2994	1,2,17,19,20,22,23,24	3,4,5,6,7,8,18,21	8	D3ZUC6,O89117,P02625,P08937,P22273,P50115,Q05702,Q99041
2995	1,2,17,19,21,22,23,24	3,4,5,6,7,8,18,20	13	D3ZUC6,O70417,P02780,P06761,P11598,P22273,P22282,P47967,Q05702,Q4G075,Q5I0D1,Q99041,Q9R168
2996	1,2,17,20,21,22,23,24	3,4,5,6,7,8,18,19	5	O70417,P08937,P22282,P50115,Q99041
2997	1,2,18,19,20,21,22,23	3,4,5,6,7,8,17,24	4	O70417,O70594,P23593,P50115
2998	1,2,18,19,20,21,22,24	3,4,5,6,7,8,17,23	6	O70417,O70594,P20761,P50115,Q05702,Q68G31
2999	1,2,18,19,20,21,23,24	3,4,5,6,7,8,17,22	6	O70417,P23593,P35280,P50115,Q68G31,Q99041
3000	1,2,18,19,20,22,23,24	3,4,5,6,7,8,17,21	11	O70417,O70594,P06760,P22273,P26772,P50115,P50116,Q05702,Q5QE79,Q99041,Q9QX74
3001	1,2,18,19,21,22,23,24	3,4,5,6,7,8,17,20	13	D4A5U3,O70417,P02780,P06911,P13676,P22273,P22282,P50115,P50116,Q05702,Q4G075,Q62946,Q99041
3002	1,2,18,20,21,22,23,24	3,4,5,6,7,8,17,19	9	O70417,O70594,P01835,P22282,P50115,P50116,Q5QE79,Q99041,Q9QX74
3003	1,2,19,20,21,22,23,24	3,4,5,6,7,8,17,18	4	O70417,O70594,P50115,Q99PP0
3004	1,3,4,5,6,7,8,17	2,18,19,20,21,22,23,24	19	O70417,O70594,P01681,P13432,P18418,P23593,P49134,P50115,P50116,Q05702,Q09030,Q4FZU2,Q62946,Q6IFU7,Q6IFW6,Q6IG02,Q6IMF3,Q6P6Q2,Q99041
3005	1,3,4,5,6,7,8,18	2,17,19,20,21,22,23,24	15	O70417,O70594,P01681,P18418,P23593,P46844,P49134,Q05702,Q09030,Q4FZU2,Q6IFW6,Q6IG02,Q6IMF3,Q6P6Q2,Q99041
3006	1,3,4,5,6,7,8,19	2,17,18,20,21,22,23,24	17	O70417,O70594,P01681,P18418,P19218,P22282,P42854,P50115,P52590,Q05702,Q09030,Q4FZU2,Q6IFW6,Q6IG02,Q6IMF3,Q6P6Q2,Q99041

3007	1,3,4,5,6,7,8,20	2,17,18,19,21,22,23,24	31	O70417,P01681,P02780,P06761,P06911,P11598,P12020,P18418,P19629,P22273,P22282,P23593,P25031,P42854,P47967,Q05702,Q09030,Q4FZU2,Q4G075,Q5GRG2,Q5I0D1,Q62946,Q6IFU7,Q6IFW6,Q6IG02,Q6IMF3,Q6P6Q2,Q99041,Q9JHB9,Q9QW07,Q9R0T3
3008	1,3,4,5,6,7,8,21	2,17,18,19,20,22,23,24	25	O70594,P01681,P04762,P08937,P11598,P18418,P18757,P19629,P22282,P23593,P30919,P36860,P46844,P47967,P49134,P50115,Q05702,Q09030,Q4FZU2,Q63493,Q6IFW6,Q6IG02,Q6IMF3,Q6P6Q2,Q99041
3009	1,3,4,5,6,7,8,22	2,17,18,19,20,21,23,24	19	O70417,P01681,P19218,P23593,P27590,P30919,P35280,P42854,P49134,P50115,P97580,Q05702,Q09030,Q4FZU2,Q6IFW6,Q6IG02,Q6IMF3,Q6P6Q2,Q99041
3010	1,3,4,5,6,7,8,23	2,17,18,19,20,21,22,24	20	O70594,P01681,P02783,P18418,P20760,P20761,P23593,P25809,P42854,P46844,P49134,P50115,Q05702,Q09030,Q4FZU2,Q6IFW6,Q6IG02,Q6IMF3,Q6P6Q2,Q99041
3011	1,3,4,5,6,7,8,24	2,17,18,19,20,21,22,23	18	O70417,O70594,P01681,P18418,P23593,P25809,P30919,P36860,P46844,P49134,Q05702,Q09030,Q4FZU2,Q6IFW6,Q6IG02,Q6IMF3,Q6P6Q2,Q99041
3012	1,3,4,5,6,7,17,18	2,8,19,20,21,22,23,24	1	O70594
3013	1,3,4,5,6,7,17,19	2,8,18,20,21,22,23,24	1	P52590
3014	1,3,4,5,6,7,17,20	2,8,18,19,21,22,23,24	1	Q6IG02
3015	1,3,4,5,6,7,17,21	2,8,18,19,20,22,23,24	6	O70594,P04762,P05539,P36860,Q4FZU2,Q6IG02
3016	1,3,4,5,6,7,17,22	2,8,18,19,20,21,23,24	5	P01946,P02091,P23593,P25809,Q68G31
3017	1,3,4,5,6,7,17,23	2,8,18,19,20,21,22,24	3	O70594,P25809,Q09030
3018	1,3,4,5,6,7,17,24	2,8,18,19,20,21,22,23	4	P25809,P36860,Q4FZU2,Q6AY61
3019	1,3,4,5,6,7,18,19	2,8,17,20,21,22,23,24	3	P47967,P52590,P97840

3020	1,3,4,5,6,7,18,20	2,8,17,19,21,22,23,24	4	P11598,P47967,P97840,Q5I0D1
3021	1,3,4,5,6,7,18,21	2,8,17,19,20,22,23,24	5	B1H234,O89117,P36860,P47967,P97840
3022	1,3,4,5,6,7,18,22	2,8,17,19,20,21,23,24	3	P23593,P97840,Q6P6R2
3023	1,3,4,5,6,7,18,23	2,8,17,19,20,21,22,24	6	O70594,O89117,P25809,P30120,Q09030,Q9QX79
3024	1,3,4,5,6,7,18,24	2,8,17,19,20,21,22,23	4	O89117,P25809,Q4KLZ6,Q6P6R2
3025	1,3,4,5,6,7,19,20	2,8,17,18,21,22,23,24	10	P02780,P10758,P11598,P42854,P47967,P52590,P97840,Q5I0D1,Q63617,Q9R0T3
3026	1,3,4,5,6,7,19,21	2,8,17,18,20,22,23,24	4	B1H234,P47967,P52590,P97840
3027	1,3,4,5,6,7,19,22	2,8,17,18,20,21,23,24	5	P01946,P19218,P25809,P52590,Q99041
3028	1,3,4,5,6,7,19,23	2,8,17,18,20,21,22,24	4	P02783,P25809,P42854,P52590
3029	1,3,4,5,6,7,19,24	2,8,17,18,20,21,22,23	2	P25809,P52590
3030	1,3,4,5,6,7,20,21	2,8,17,18,19,22,23,24	9	P02780,P11598,P22283,P47967,P97840,Q5I0D1,Q63617,Q6IG02,Q9R0T3
3031	1,3,4,5,6,7,20,22	2,8,17,18,19,21,23,24	5	P19218,P23593,P47967,P97840,Q99041
3032	1,3,4,5,6,7,20,23	2,8,17,18,19,21,22,24	7	P02783,P09656,P10758,P23739,P42854,P97840,Q6IG02

3033	1,3,4,5,6,7,20,24	2,8,17,18,19,21,22,23	2	P11598,Q6IG02
3034	1,3,4,5,6,7,21,22	2,8,17,18,19,20,23,24	5	P01946,P30919,P36860,P97840,Q99041
3035	1,3,4,5,6,7,21,23	2,8,17,18,19,20,22,24	5	P02783,P09656,P25809,P36860,Q09030
3036	1,3,4,5,6,7,21,24	2,8,17,18,19,20,22,23	9	B1H234,O89117,P04762,P25809,P30919,P36860,Q4FZU2,Q6IFW6,Q6IG02
3037	1,3,4,5,6,7,22,23	2,8,17,18,19,20,21,24	9	P02783,P09656,P23593,P25809,P30120,P47727,Q09030,Q68G31,Q6P6R2
3038	1,3,4,5,6,7,22,24	2,8,17,18,19,20,21,23	4	P23593,P25809,P30919,Q6P6R2
3039	1,3,4,5,6,7,23,24	2,8,17,18,19,20,21,22	6	P25809,P36860,P47727,P62804,Q09030,Q6P6R2
3040	1,3,4,5,6,8,17,18	2,7,19,20,21,22,23,24	11	O70594,P01681,P13432,P18418,P23593,Q05702,Q4FZU2,Q6IFU7,Q6IG02,Q6IMF3,Q6P6Q2
3041	1,3,4,5,6,8,17,19	2,7,18,20,21,22,23,24	9	P01681,P01835,P52590,Q05702,Q4FZU2,Q6IFW6,Q6IG02,Q6IMF3,Q6P6Q2
3042	1,3,4,5,6,8,17,20	2,7,18,19,21,22,23,24	13	O70417,P01681,P06911,P13432,P25031,Q05702,Q4FZU2,Q5GRG2,Q6IFU7,Q6IFW6,Q6IG02,Q6IMF3,Q6P6Q2
3043	1,3,4,5,6,8,17,21	2,7,18,19,20,22,23,24	11	O70594,P01681,P36860,Q05702,Q09030,Q4FZU2,Q6IFU7,Q6IFW6,Q6IG02,Q6IMF3,Q6P6Q2
3044	1,3,4,5,6,8,17,22	2,7,18,19,20,21,23,24	14	O70417,P01681,P01946,P02091,P23593,P97580,Q05702,Q09030,Q4FZU2,Q6IFU7,Q6IFW6,Q6IG02,Q6IMF3,Q6P6Q2
3045	1,3,4,5,6,8,17,23	2,7,18,19,20,21,22,24	12	O70594,P01681,P02783,P23593,Q05702,Q09030,Q4FZU2,Q6IFU7,Q6IFW6,Q6IG02,Q6IMF3,Q6P6Q2

3046	1,3,4,5,6,8,17,24	2,7,18,19,20,21,22,23	10	P01681,P23593,Q05702,Q09030,Q4FZU2,Q6IFU7,Q6IFW6,Q6IG02,Q6IMF3,Q6P6Q2
3047	1,3,4,5,6,8,18,19	2,7,17,20,21,22,23,24	3	P01681,P52590,Q05702
3048	1,3,4,5,6,8,18,20	2,7,17,19,21,22,23,24	11	P01681,P06911,P18418,Q05702,Q4FZU2,Q6IFU7,Q6IFW6,Q6IG02,Q6IMF3,Q6P6Q2,Q9R168
3049	1,3,4,5,6,8,18,21	2,7,17,19,20,22,23,24	11	O89117,P01681,P02625,P08937,Q05702,Q4FZU2,Q6IFU7,Q6IFW6,Q6IG02,Q6IMF3,Q6P6Q2
3050	1,3,4,5,6,8,18,22	2,7,17,19,20,21,23,24	8	P01681,P23593,P49134,P54921,P97580,Q05702,Q09030,Q6IFU7
3051	1,3,4,5,6,8,18,23	2,7,17,19,20,21,22,24	7	O70594,P01681,P49134,P54921,Q05702,Q09030,Q6IG02
3052	1,3,4,5,6,8,18,24	2,7,17,19,20,21,22,23	11	P01681,P23593,P49134,Q05702,Q4FZU2,Q4KLZ6,Q6IFU7,Q6IFW6,Q6IG02,Q6IMF3,Q6P6Q2
3053	1,3,4,5,6,8,19,20	2,7,17,18,21,22,23,24	17	P01681,P02780,P06911,P14046,P19218,P22282,P25031,P42854,P52590,Q05702,Q4FZU2,Q5I0D1,Q6IFW6,Q6IG02,Q6IMF3,Q6P6Q2,Q9JI85
3054	1,3,4,5,6,8,19,21	2,7,17,18,20,22,23,24	12	P01681,P08937,P14046,P30919,P52590,Q05702,Q10743,Q4FZU2,Q6IFW6,Q6IG02,Q6IMF3,Q6P6Q2
3055	1,3,4,5,6,8,19,22	2,7,17,18,20,21,23,24	11	P00507,P01681,P01946,P15399,P19218,P30919,P42854,P52590,P97580,Q05702,Q99041
3056	1,3,4,5,6,8,19,23	2,7,17,18,20,21,22,24	10	P01681,P02783,P25809,P42854,P52590,Q05702,Q09030,Q4FZU2,Q6IG02,Q6IMF3
3057	1,3,4,5,6,8,19,24	2,7,17,18,20,21,22,23	11	O54728,P01681,P25809,P30919,Q05702,Q10743,Q4FZU2,Q6IFW6,Q6IG02,Q6IMF3,Q6P6Q2
3058	1,3,4,5,6,8,20,21	2,7,17,18,19,22,23,24	16	P01681,P02780,P14046,P19629,P25031,P47967,Q05702,Q10743,Q4FZU2,Q5I0D1,Q6IFU7,Q6IFW6,Q6IG02,Q6IMF3,Q6P6Q2,Q9JI85

3059	1,3,4,5,6,8,20,22	2,7,17,18,19,21,23,24	15	P01681,P06911,P15399,P19218,P23593,P25031,Q05702,Q09030,Q10743,Q4FZU2,Q6IFU7,Q6IFW6,Q6IG02,Q6IMF3,Q6P6Q2
3060	1,3,4,5,6,8,20,23	2,7,17,18,19,21,22,24	15	P01681,P02783,P09656,P10758,P23739,P25031,P42854,Q05702,Q09030,Q4FZU2,Q6IFU7,Q6IFW6,Q6IG02,Q6IMF3,Q6P6Q2
3061	1,3,4,5,6,8,20,24	2,7,17,18,19,21,22,23	10	P01681,P06911,Q05702,Q10743,Q4FZU2,Q6IFU7,Q6IFW6,Q6IG02,Q6IMF3,Q6P6Q2
3062	1,3,4,5,6,8,21,22	2,7,17,18,19,20,23,24	13	P01681,P01946,P23593,P30919,Q05702,Q09030,Q10743,Q4FZU2,Q6IFU7,Q6IFW6,Q6IG02,Q6IMF3,Q6P6Q2
3063	1,3,4,5,6,8,21,23	2,7,17,18,19,20,22,24	13	P01681,P02783,P09656,P30919,P54921,Q05702,Q09030,Q4FZU2,Q6IFU7,Q6IFW6,Q6IG02,Q6IMF3,Q6P6Q2
3064	1,3,4,5,6,8,21,24	2,7,17,18,19,20,22,23	13	P01681,P02625,P30919,P36860,Q05702,Q09030,Q10743,Q4FZU2,Q6IFU7,Q6IFW6,Q6IG02,Q6IMF3,Q6P6Q2
3065	1,3,4,5,6,8,22,23	2,7,17,18,19,20,21,24	17	P00507,P01681,P02783,P09656,P19218,P20760,P23593,P25809,P30919,P42854,P54921,P62804,Q05702,Q09030,Q63532,Q6IG02,Q6P6R2
3066	1,3,4,5,6,8,22,24	2,7,17,18,19,20,21,23	16	P00507,P01681,P23593,P25809,P30919,P49134,P62804,Q05702,Q09030,Q10743,Q4FZU2,Q6IFW6,Q6IG02,Q6IMF3,Q6P6Q2,Q6P6R2
3067	1,3,4,5,6,8,23,24	2,7,17,18,19,20,21,22	14	P01681,P20760,P23593,P25809,P30919,P62804,Q05702,Q09030,Q4FZU2,Q6IFW6,Q6IG02,Q6IMF3,Q6P6Q2,Q6P6R2
3068	1,3,4,5,6,17,18,19	2,7,8,20,21,22,23,24	1	Q06000
3069	1,3,4,5,6,17,18,20	2,7,8,19,21,22,23,24	1	Q06000
3070	1,3,4,5,6,17,18,21	2,7,8,19,20,22,23,24	1	P36860
3071	1,3,4,5,6,17,18,22	2,7,8,19,20,21,23,24	5	P01946,P02091,P62804,Q06000,Q812E4
3072	1,3,4,5,6,17,18,23	2,7,8,19,20,21,22,24	2	P30120,P62804
3073	1,3,4,5,6,17,18,24	2,7,8,19,20,21,22,23	3	P62804,Q4KLZ6,Q9WTT6
3074	1,3,4,5,6,17,19,20	2,7,8,18,21,22,23,24	1	Q6IG02

3075	1,3,4,5,6,17,19,21	2,7,8,18,20,22,23,24	1	P52590
3076	1,3,4,5,6,17,19,22	2,7,8,18,20,21,23,24	3	P01946,P02091,P25809
3077	1,3,4,5,6,17,19,23	2,7,8,18,20,21,22,24	1	P25809
3078	1,3,4,5,6,17,19,24	2,7,8,18,20,21,22,23	2	P25809,P62804
3079	1,3,4,5,6,17,20,21	2,7,8,18,19,22,23,24	1	Q6IG02
3080	1,3,4,5,6,17,20,22	2,7,8,18,19,21,23,24	3	P01946,P02091,P20766
3081	1,3,4,5,6,17,20,23	2,7,8,18,19,21,22,24	3	P09656,P23739,Q6IG02
3082	1,3,4,5,6,17,20,24	2,7,8,18,19,21,22,23	2	P62804,Q6IG02
3083	1,3,4,5,6,17,21,22	2,7,8,18,19,20,23,24	3	P01946,P02091,P20766
3084	1,3,4,5,6,17,21,23	2,7,8,18,19,20,22,24	2	P09656,P36860
3085	1,3,4,5,6,17,21,24	2,7,8,18,19,20,22,23	3	P36860,Q4FZU2,Q6IG02
3086	1,3,4,5,6,17,22,23	2,7,8,18,19,20,21,24	10	P01946,P02091,P02783,P25809,P30120,P47727,P62804,Q00715,Q09030,Q9WTT6
3087	1,3,4,5,6,17,22,24	2,7,8,18,19,20,21,23	7	P01946,P02091,P23593,P25809,P62804,Q00715,Q9WTT6
3088	1,3,4,5,6,17,23,24	2,7,8,18,19,20,21,22	4	P25809,P62804,Q00715,Q9WTT6
3089	1,3,4,5,6,18,19,20	2,7,8,17,21,22,23,24	2	P97840,Q5I0D1
3090	1,3,4,5,6,18,19,21	2,7,8,17,20,22,23,24	0	
3091	1,3,4,5,6,18,19,22	2,7,8,17,20,21,23,24	1	P01946
3092	1,3,4,5,6,18,19,23	2,7,8,17,20,21,22,24	1	P25809
3093	1,3,4,5,6,18,19,24	2,7,8,17,20,21,22,23	4	P25809,P62804,Q4KLZ6,Q9WTT6
3094	1,3,4,5,6,18,20,21	2,7,8,17,19,22,23,24	3	P47967,P97840,Q6IG02

3095	1,3,4,5,6,18,20,22	2,7,8,17,19,21,23,24	0	
3096	1,3,4,5,6,18,20,23	2,7,8,17,19,21,22,24	0	
3097	1,3,4,5,6,18,20,24	2,7,8,17,19,21,22,23	2	P62804,Q4KLZ6
3098	1,3,4,5,6,18,21,22	2,7,8,17,19,20,23,24	2	P01946,Q9WTT6
3099	1,3,4,5,6,18,21,23	2,7,8,17,19,20,22,24	4	O89117,P09656,P54921,Q9WTT6
3100	1,3,4,5,6,18,21,24	2,7,8,17,19,20,22,23	4	O89117,P36860,Q4KLZ6,Q9WTT6
3101	1,3,4,5,6,18,22,23	2,7,8,17,19,20,21,24	11	P02783,P09456,P13676,P25809,P30120,P47727,P54921,P62804,Q6P6R2,Q9EQS0,Q9WTT6
3102	1,3,4,5,6,18,22,24	2,7,8,17,19,20,21,23	7	P13676,P25809,P62804,Q4KLZ6,Q6P6R2,Q9QX74,Q9WTT6
3103	1,3,4,5,6,18,23,24	2,7,8,17,19,20,21,22	8	O89117,P13676,P25809,P62804,Q4KLZ6,Q6P6R2,Q9QX74,Q9WTT6
3104	1,3,4,5,6,19,20,21	2,7,8,17,18,22,23,24	4	P47967,P97840,Q5I0D1,Q6IG02
3105	1,3,4,5,6,19,20,22	2,7,8,17,18,21,23,24	2	P01946,P19218
3106	1,3,4,5,6,19,20,23	2,7,8,17,18,21,22,24	4	P02783,P10758,P23739,P42854
3107	1,3,4,5,6,19,20,24	2,7,8,17,18,21,22,23	1	Q6IG02
3108	1,3,4,5,6,19,21,22	2,7,8,17,18,20,23,24	3	P01946,P02091,P30919
3109	1,3,4,5,6,19,21,23	2,7,8,17,18,20,22,24	3	P02783,P09656,P25809
3110	1,3,4,5,6,19,21,24	2,7,8,17,18,20,22,23	2	P25809,P30919
3111	1,3,4,5,6,19,22,23	2,7,8,17,18,20,21,24	11	P00507,P01946,P02091,P02783,P25809,P30120,P47727,P62804,Q00715,Q811M5,Q9WTT6
3112	1,3,4,5,6,19,22,24	2,7,8,17,18,20,21,23	9	P00507,P01946,P25809,P30919,P55091,P62804,Q00715,Q6P6R2,Q9WTT6
3113	1,3,4,5,6,19,23,24	2,7,8,17,18,20,21,22	5	P25809,P30919,P62804,Q00715,Q9WTT6
3114	1,3,4,5,6,20,21,22	2,7,8,17,18,19,23,24	3	P01946,P20766,Q6IG02

3115	1,3,4,5,6,20,21,23	2,7,8,17,18,19,22,24	3	P02783,P09656,Q6IG02
3116	1,3,4,5,6,20,21,24	2,7,8,17,18,19,22,23	1	Q6IG02
3117	1,3,4,5,6,20,22,23	2,7,8,17,18,19,21,24	7	P02783,P09656,P23739,P62804,Q00715,Q66H69,Q9QX74
3118	1,3,4,5,6,20,22,24	2,7,8,17,18,19,21,23	4	P22006,P62804,Q6P6R2,Q9QX74
3119	1,3,4,5,6,20,23,24	2,7,8,17,18,19,21,22	6	P09656,P23739,P62804,Q6IG02,Q6P6R2,Q9QX74
3120	1,3,4,5,6,21,22,23	2,7,8,17,18,19,20,24	9	P01946,P02783,P09656,P25809,P30919,P47727,P54921,Q09030,Q9WTT6
3121	1,3,4,5,6,21,22,24	2,7,8,17,18,19,20,23	5	P01946,P25809,P30919,P36860,Q9WTT6
3122	1,3,4,5,6,21,23,24	2,7,8,17,18,19,20,22	5	P09656,P25809,P30919,P36860,Q9WTT6
3123	1,3,4,5,6,22,23,24	2,7,8,17,18,19,20,21	16	P00507,P01946,P02783,P06760,P09656,P13676,P25809,P30919,P47727,P62804,Q00715,Q09030,Q6P6R2,Q811M5,Q9QX74,Q9WTT6
3124	1,3,4,5,7,8,17,18	2,6,19,20,21,22,23,24	10	O70417,O70594,P01681,P49134,Q4FZU2,Q6IFW6,Q6IG02,Q6IMF3,Q6P6Q2,Q811M5
3125	1,3,4,5,7,8,17,19	2,6,18,20,21,22,23,24	10	O70417,O70594,P01681,P01835,P20646,Q4FZU2,Q6IFW6,Q6IG02,Q6IMF3,Q6P6Q2
3126	1,3,4,5,7,8,17,20	2,6,18,19,21,22,23,24	17	D4A5U3,O70417,P01681,P02780,P06911,P13676,P28648,P97697,P97840,Q4FZU2,Q4G075,Q62946,Q6IFW6,Q6IG02,Q6IMF3,Q6P6Q2,Q811M5
3127	1,3,4,5,7,8,17,21	2,6,18,19,20,22,23,24	12	O70594,P01681,P06760,P22006,Q4FZU2,Q6IFU8,Q6IFW6,Q6IG02,Q6IMF3,Q6P6Q2,Q811M5,Q9QX74
3128	1,3,4,5,7,8,17,22	2,6,18,19,20,21,23,24	12	O70417,P01681,P23593,P27590,P35280,P49134,Q4FZU2,Q68G31,Q6IFW6,Q6IG02,Q6IMF3,Q6P6Q2
3129	1,3,4,5,7,8,17,23	2,6,18,19,20,21,22,24	9	O70594,P01681,P49134,Q4FZU2,Q68G31,Q6IFW6,Q6IG02,Q6IMF3,Q6P6Q2
3130	1,3,4,5,7,8,17,24	2,6,18,19,20,21,22,23	10	O70417,P01681,P23593,P49134,Q4FZU2,Q6IFW6,Q6IG02,Q6IMF3,Q6P6Q2,Q811M5

3131	1,3,4,5,7,8,18,19	2,6,17,20,21,22,23,24	9	O35077,P01681,P47967,P63029,P97840,Q4FZU2,Q6IFW6,Q6IMF3,Q6P6Q2
3132	1,3,4,5,7,8,18,20	2,6,17,19,21,22,23,24	14	O70417,P01681,P02780,P47967,P97840,Q4FZU2,Q4G075,Q5I0D1,Q6IFW6,Q6IG02,Q6IMF3,Q6P6Q2,Q811M5,Q9R168
3133	1,3,4,5,7,8,18,21	2,6,17,19,20,22,23,24	12	O89117,P01681,P02625,P47967,P49134,P97840,Q4FZU2,Q6IFW6,Q6IG02,Q6IMF3,Q6P6Q2,Q811M5
3134	1,3,4,5,7,8,18,22	2,6,17,19,20,21,23,24	10	P01681,P23593,P27590,P35280,P49134,P97840,Q4FZU2,Q6IFW6,Q6IMF3,Q6P6Q2
3135	1,3,4,5,7,8,18,23	2,6,17,19,20,21,22,24	12	O35077,O70594,O89117,P01681,P49134,P97840,Q4FZU2,Q6IFW6,Q6IG02,Q6IMF3,Q6P6Q2,Q8R431
3136	1,3,4,5,7,8,18,24	2,6,17,19,20,21,22,23	9	O89117,P01681,P49134,P63029,Q4FZU2,Q6IFW6,Q6IG02,Q6IMF3,Q6P6Q2
3137	1,3,4,5,7,8,19,20	2,6,17,18,21,22,23,24	16	D3ZUC6,O70417,P01681,P02780,P10758,P22282,P36374,P47967,P97840,Q4FZU2,Q4G075,Q5I0D1,Q6IFW6,Q6IG02,Q6IMF3,Q6P6Q2
3138	1,3,4,5,7,8,19,21	2,6,17,18,20,22,23,24	15	D3ZUC6,O35077,P01681,P06760,P08937,P22006,P47967,P97840,Q4FZU2,Q63493,Q66H69,Q6IFW6,Q6IG02,Q6IMF3,Q6P6Q2
3139	1,3,4,5,7,8,19,22	2,6,17,18,20,21,23,24	11	P01681,P19218,P27590,P35280,P97840,Q4FZU2,Q6IFW6,Q6IG02,Q6IMF3,Q6P6Q2,Q99041
3140	1,3,4,5,7,8,19,23	2,6,17,18,20,21,22,24	8	O35077,P01681,P10758,Q4FZU2,Q6IFW6,Q6IG02,Q6IMF3,Q6P6Q2
3141	1,3,4,5,7,8,19,24	2,6,17,18,20,21,22,23	8	O54728,P01681,P63029,Q4FZU2,Q6IFW6,Q6IG02,Q6IMF3,Q6P6Q2
3142	1,3,4,5,7,8,20,21	2,6,17,18,19,22,23,24	19	D3ZUC6,P01681,P02780,P06760,P08649,P22273,P47967,P97840,Q4FZU2,Q4G075,Q5I0D1,Q62946,Q63493,Q6IFU8,Q6IFW6,Q6IG02,Q6IMF3,Q6P6Q2,Q811M5
3143	1,3,4,5,7,8,20,22	2,6,17,18,19,21,23,24	14	O70417,P01681,P02780,P23593,P27590,P35280,P47967,P97840,Q4FZU2,Q4G075,Q6IFW6,Q6IG02,Q6IMF3,Q6P6Q2

3144	1,3,4,5,7,8,20,23	2,6,17,18,19,21,22,24	10	P01681,P10758,P23739,P97840,Q4FZU2,Q4G075,Q6IFW6,Q6IG02,Q6IMF3,Q6P6Q2
3145	1,3,4,5,7,8,20,24	2,6,17,18,19,21,22,23	11	P01681,P02780,P97840,Q4FZU2,Q4G075,Q6IFU8,Q6IFW6,Q6IG02,Q6IMF3,Q6P6Q2,Q811M5
3146	1,3,4,5,7,8,21,22	2,6,17,18,19,20,23,24	11	P01681,P08649,P27590,P35280,P49134,P97840,Q4FZU2,Q6IFW6,Q6IG02,Q6IMF3,Q6P6Q2
3147	1,3,4,5,7,8,21,23	2,6,17,18,19,20,22,24	10	O35077,O89117,P01681,P08649,P49134,Q4FZU2,Q6IFW6,Q6IG02,Q6IMF3,Q6P6Q2
3148	1,3,4,5,7,8,21,24	2,6,17,18,19,20,22,23	13	O89117,P01681,P04762,P08649,P30919,P36860,P49134,Q4FZU2,Q6IFU8,Q6IFW6,Q6IG02,Q6IMF3,Q6P6Q2
3149	1,3,4,5,7,8,22,23	2,6,17,18,19,20,21,24	11	P01681,P23593,P27590,P35280,P49134,Q4FZU2,Q68G31,Q6IFW6,Q6IG02,Q6IMF3,Q6P6Q2
3150	1,3,4,5,7,8,22,24	2,6,17,18,19,20,21,23	11	P01681,P23593,P27590,P35280,P49134,Q4FZU2,Q6IFW6,Q6IG02,Q6IMF3,Q6P6Q2,Q6P6R2
3151	1,3,4,5,7,8,23,24	2,6,17,18,19,20,21,22	10	P01681,P20761,P25809,P49134,Q4FZU2,Q6IFW6,Q6IG02,Q6IMF3,Q6P6Q2,Q6P6R2
3152	1,3,4,5,7,17,18,19	2,6,8,20,21,22,23,24	1	P97840
3153	1,3,4,5,7,17,18,20	2,6,8,19,21,22,23,24	3	P47967,P97840,Q811M5
3154	1,3,4,5,7,17,18,21	2,6,8,19,20,22,23,24	2	P97840,Q811M5
3155	1,3,4,5,7,17,18,22	2,6,8,19,20,21,23,24	2	P97840,Q68G31
3156	1,3,4,5,7,17,18,23	2,6,8,19,20,21,22,24	3	O70594,P97840,Q68G31
3157	1,3,4,5,7,17,18,24	2,6,8,19,20,21,22,23	0	
3158	1,3,4,5,7,17,19,20	2,6,8,18,21,22,23,24	5	P20646,P47967,P97840,Q5I0D1,Q6IG02
3159	1,3,4,5,7,17,19,21	2,6,8,18,20,22,23,24	6	P22006,P35053,P47967,P97840,Q4FZU2,Q62761;Q62762;Q62763

3160	1,3,4,5,7,17,19,22	2,6,8,18,20,21,23,24	1	P97840
3161	1,3,4,5,7,17,19,23	2,6,8,18,20,21,22,24	2	P01681,P25809
3162	1,3,4,5,7,17,19,24	2,6,8,18,20,21,22,23	4	P25809,P54921,Q4FZU2,Q62761;Q62762;Q62763
3163	1,3,4,5,7,17,20,21	2,6,8,18,19,22,23,24	9	P19223,P47967,P97840,Q4FZU2,Q5I0D1,Q6IFW6,Q6IG02,Q6P6Q2,Q811M5
3164	1,3,4,5,7,17,20,22	2,6,8,18,19,21,23,24	3	P97840,Q68G31,Q6IG02
3165	1,3,4,5,7,17,20,23	2,6,8,18,19,21,22,24	5	P10758,P23739,P97840,Q68G31,Q6IG02
3166	1,3,4,5,7,17,20,24	2,6,8,18,19,21,22,23	6	P97840,Q4FZU2,Q6IFW6,Q6IG02,Q6P6Q2,Q811M5
3167	1,3,4,5,7,17,21,22	2,6,8,18,19,20,23,24	3	P97840,Q68G31,Q9R168
3168	1,3,4,5,7,17,21,23	2,6,8,18,19,20,22,24	4	P08649,P22006,Q4FZU2,Q68G31
3169	1,3,4,5,7,17,21,24	2,6,8,18,19,20,22,23	7	P04762,P08649,P36860,Q4FZU2,Q6AY61,Q6IFW6,Q6IG02
3170	1,3,4,5,7,17,22,23	2,6,8,18,19,20,21,24	2	P25809,Q68G31
3171	1,3,4,5,7,17,22,24	2,6,8,18,19,20,21,23	2	P25809,Q68G31
3172	1,3,4,5,7,17,23,24	2,6,8,18,19,20,21,22	3	P25809,Q4FZU2,Q68G31
3173	1,3,4,5,7,18,19,20	2,6,8,17,21,22,23,24	4	P10758,P47967,P97840,Q5I0D1
3174	1,3,4,5,7,18,19,21	2,6,8,17,20,22,23,24	3	O89117,P47967,P97840
3175	1,3,4,5,7,18,19,22	2,6,8,17,20,21,23,24	2	P63029,P97840
3176	1,3,4,5,7,18,19,23	2,6,8,17,20,21,22,24	2	P63029,P97840
3177	1,3,4,5,7,18,19,24	2,6,8,17,20,21,22,23	3	P25809,P63029,P97840
3178	1,3,4,5,7,18,20,21	2,6,8,17,19,22,23,24	6	O89117,P47967,P97840,Q5I0D1,Q6IG02,Q811M5
3179	1,3,4,5,7,18,20,22	2,6,8,17,19,21,23,24	2	P47967,P97840

3180	1,3,4,5,7,18,20,23	2,6,8,17,19,21,22,24	2	P10758,P97840
3181	1,3,4,5,7,18,20,24	2,6,8,17,19,21,22,23	3	P47967,P97840,Q6IG02
3182	1,3,4,5,7,18,21,22	2,6,8,17,19,20,23,24	4	O89117,P47967,P97840,Q68G31
3183	1,3,4,5,7,18,21,23	2,6,8,17,19,20,22,24	3	O89117,P97840,Q68G31
3184	1,3,4,5,7,18,21,24	2,6,8,17,19,20,22,23	2	O89117,P97840
3185	1,3,4,5,7,18,22,23	2,6,8,17,19,20,21,24	6	P25809,P30120,P47727,P97840,Q68G31,Q6P6R2
3186	1,3,4,5,7,18,22,24	2,6,8,17,19,20,21,23	4	P25809,P63029,P97840,Q6P6R2
3187	1,3,4,5,7,18,23,24	2,6,8,17,19,20,21,22	5	O89117,P25809,P63029,P97840,Q6P6R2
3188	1,3,4,5,7,19,20,21	2,6,8,17,18,22,23,24	6	D3ZUC6,P08649,P47967,P97840,Q5I0D1,Q6IG02
3189	1,3,4,5,7,19,20,22	2,6,8,17,18,21,23,24	3	P47967,P97840,Q5I0D1
3190	1,3,4,5,7,19,20,23	2,6,8,17,18,21,22,24	6	P08649,P10758,P23739,P47967,P97840,Q5I0D1
3191	1,3,4,5,7,19,20,24	2,6,8,17,18,21,22,23	6	P08649,P10758,P47967,P97840,Q5I0D1,Q6IG02
3192	1,3,4,5,7,19,21,22	2,6,8,17,18,20,23,24	3	P08649,P47967,P97840
3193	1,3,4,5,7,19,21,23	2,6,8,17,18,20,22,24	2	P08649,P97840
3194	1,3,4,5,7,19,21,24	2,6,8,17,18,20,22,23	6	P08649,P47967,P97840,Q62761;Q62762;Q62763,Q6IFW6,Q99MH3
3195	1,3,4,5,7,19,22,23	2,6,8,17,18,20,21,24	4	P25809,P47727,P97840,Q68G31
3196	1,3,4,5,7,19,22,24	2,6,8,17,18,20,21,23	4	P25809,P55091,P63029,P97840
3197	1,3,4,5,7,19,23,24	2,6,8,17,18,20,21,22	3	P08649,P25809,P63029
3198	1,3,4,5,7,20,21,22	2,6,8,17,18,19,23,24	4	P08649,P47967,P97840,Q6IG02
3199	1,3,4,5,7,20,21,23	2,6,8,17,18,19,22,24	6	P08649,P10758,P23739,P47967,P97840,Q6IG02

3200	1,3,4,5,7,20,21,24	2,6,8,17,18,19,22,23	7	P08649,P47967,P97840,Q4FZU2,Q6IFW6,Q6IG02,Q6P6Q2
3201	1,3,4,5,7,20,22,23	2,6,8,17,18,19,21,24	5	P08649,P10758,P23739,P97840,Q68G31
3202	1,3,4,5,7,20,22,24	2,6,8,17,18,19,21,23	3	P08649,P97840,Q6IG02
3203	1,3,4,5,7,20,23,24	2,6,8,17,18,19,21,22	6	P08649,P10758,P23739,P97840,Q6IG02,Q9QYP1
3204	1,3,4,5,7,21,22,23	2,6,8,17,18,19,20,24	4	P08649,P47727,P97840,Q68G31
3205	1,3,4,5,7,21,22,24	2,6,8,17,18,19,20,23	4	P08649,P25809,P97840,Q6IFW6
3206	1,3,4,5,7,21,23,24	2,6,8,17,18,19,20,22	6	O89117,P08649,P25809,P36860,Q4FZU2,Q6IFW6
3207	1,3,4,5,7,22,23,24	2,6,8,17,18,19,20,21	7	P08649,P25809,P47727,P62804,Q00715,Q68G31,Q6P6R2
3208	1,3,4,5,8,17,18,19	2,6,7,20,21,22,23,24	6	P01681,P01835,Q4FZU2,Q6IG02,Q6IMF3,Q6P6Q2
3209	1,3,4,5,8,17,18,20	2,6,7,19,21,22,23,24	10	P01681,P01835,Q4FZU2,Q6IFU7,Q6IFW6,Q6IG02,Q6IMF3,Q6P6Q2,Q811M5,Q9R168
3210	1,3,4,5,8,17,18,21	2,6,7,19,20,22,23,24	9	P01681,P22006,Q4FZU2,Q6IFU7,Q6IFW6,Q6IG02,Q6IMF3,Q6P6Q2,Q811M5
3211	1,3,4,5,8,17,18,22	2,6,7,19,20,21,23,24	4	P01681,P01835,P27590,Q4FZU2
3212	1,3,4,5,8,17,18,23	2,6,7,19,20,21,22,24	7	P01681,P01835,Q4FZU2,Q6IFW6,Q6IG02,Q6IMF3,Q6P6Q2
3213	1,3,4,5,8,17,18,24	2,6,7,19,20,21,22,23	7	P01681,P01835,Q4FZU2,Q6IFW6,Q6IG02,Q6IMF3,Q6P6Q2
3214	1,3,4,5,8,17,19,20	2,6,7,18,21,22,23,24	7	P01681,P01835,Q4FZU2,Q6IFW6,Q6IG02,Q6IMF3,Q6P6Q2
3215	1,3,4,5,8,17,19,21	2,6,7,18,20,22,23,24	8	P01681,P01835,P22006,Q4FZU2,Q6IFW6,Q6IG02,Q6IMF3,Q6P6Q2
3216	1,3,4,5,8,17,19,22	2,6,7,18,20,21,23,24	7	P01681,P01835,Q4FZU2,Q6IFW6,Q6IG02,Q6IMF3,Q6P6Q2
3217	1,3,4,5,8,17,19,23	2,6,7,18,20,21,22,24	7	P01681,P01835,Q4FZU2,Q6IFW6,Q6IG02,Q6IMF3,Q6P6Q2
3218	1,3,4,5,8,17,19,24	2,6,7,18,20,21,22,23	8	O54728,P01681,P01835,Q4FZU2,Q6IFW6,Q6IG02,Q6IMF3,Q6P6Q2
3219	1,3,4,5,8,17,20,21	2,6,7,18,19,22,23,24	12	P00762,P01681,P19223,Q10758,Q4FZU2,Q6IFU7,Q6IFU8,Q6IFW6,Q6IG02,Q6IMF3,Q6P6Q2,Q811M5

3220	1,3,4,5,8,17,20,22	2,6,7,18,19,21,23,24	7	P01681,Q4FZU2,Q6IFU7,Q6IFW6,Q6IG02,Q6IMF3,Q6P6Q2
3221	1,3,4,5,8,17,20,23	2,6,7,18,19,21,22,24	10	P01681,P23739,P28648,P36376,Q4FZU2,Q6IFU7,Q6IFW6,Q6IG02,Q6IMF3,Q6P6Q2
3222	1,3,4,5,8,17,20,24	2,6,7,18,19,21,22,23	8	P01681,Q4FZU2,Q6IFU7,Q6IFW6,Q6IG02,Q6IMF3,Q6P6Q2,Q811M5
3223	1,3,4,5,8,17,21,22	2,6,7,18,19,20,23,24	6	P01681,Q4FZU2,Q6IFW6,Q6IG02,Q6IMF3,Q6P6Q2
3224	1,3,4,5,8,17,21,23	2,6,7,18,19,20,22,24	7	P01681,P22006,Q4FZU2,Q6IFW6,Q6IG02,Q6IMF3,Q6P6Q2
3225	1,3,4,5,8,17,21,24	2,6,7,18,19,20,22,23	8	P01681,Q4FZU2,Q6IFU7,Q6IFU8,Q6IFW6,Q6IG02,Q6IMF3,Q6P6Q2
3226	1,3,4,5,8,17,22,23	2,6,7,18,19,20,21,24	9	P01681,P01835,P27590,Q4FZU2,Q68G31,Q6IFW6,Q6IG02,Q6IMF3,Q6P6Q2
3227	1,3,4,5,8,17,22,24	2,6,7,18,19,20,21,23	7	P01681,P23593,Q4FZU2,Q6IFW6,Q6IG02,Q6IMF3,Q6P6Q2
3228	1,3,4,5,8,17,23,24	2,6,7,18,19,20,21,22	7	P01681,Q4FZU2,Q5PQL7,Q6IFW6,Q6IG02,Q6IMF3,Q6P6Q2
3229	1,3,4,5,8,18,19,20	2,6,7,17,21,22,23,24	9	P01681,P01835,P47967,P97840,Q5I0D1,Q6IG02,Q6IMF3,Q6P6Q2,Q9R168
3230	1,3,4,5,8,18,19,21	2,6,7,17,20,22,23,24	6	P01681,Q4FZU2,Q6IFW6,Q6IG02,Q6IMF3,Q6P6Q2
3231	1,3,4,5,8,18,19,22	2,6,7,17,20,21,23,24	3	P01681,P01835,P27590
3232	1,3,4,5,8,18,19,23	2,6,7,17,20,21,22,24	3	O35077,P01681,P01835
3233	1,3,4,5,8,18,19,24	2,6,7,17,20,21,22,23	9	O54728,P01681,P01835,P63029,Q4FZU2,Q6IFW6,Q6IG02,Q6IMF3,Q6P6Q2
3234	1,3,4,5,8,18,20,21	2,6,7,17,19,22,23,24	13	D3ZUC6,P00762,P01681,P47967,P97840,Q4FZU2,Q6IFU7,Q6IFW6,Q6IG02,Q6IMF3,Q6P6Q2,Q811M5,Q9R168
3235	1,3,4,5,8,18,20,22	2,6,7,17,19,21,23,24	4	P01681,Q6IG02,Q6P6Q2,Q9R168
3236	1,3,4,5,8,18,20,23	2,6,7,17,19,21,22,24	8	P01681,P10758,P23739,Q6IFW6,Q6IG02,Q6IMF3,Q6P6Q2,Q9R168
3237	1,3,4,5,8,18,20,24	2,6,7,17,19,21,22,23	8	P01681,Q4FZU2,Q6IFU7,Q6IFW6,Q6IG02,Q6IMF3,Q6P6Q2,Q9R168
3238	1,3,4,5,8,18,21,22	2,6,7,17,19,20,23,24	4	P01681,Q6IFW6,Q6IG02,Q6P6Q2
3239	1,3,4,5,8,18,21,23	2,6,7,17,19,20,22,24	8	O89117,P01681,P54921,Q4FZU2,Q6IFW6,Q6IG02,Q6IMF3,Q6P6Q2

3240	1,3,4,5,8,18,21,24	2,6,7,17,19,20,22,23	8	O89117,P01681,P02625,Q4FZU2,Q6IFW6,Q6IG02,Q6IMF3,Q6P6Q2
3241	1,3,4,5,8,18,22,23	2,6,7,17,19,20,21,24	4	P01681,P27590,P54921,Q6AY61
3242	1,3,4,5,8,18,22,24	2,6,7,17,19,20,21,23	5	P01681,P49134,Q4FZU2,Q6IFW6,Q6P6Q2
3243	1,3,4,5,8,18,23,24	2,6,7,17,19,20,21,22	8	O89117,P01681,P49134,Q4FZU2,Q6IFW6,Q6IG02,Q6IMF3,Q6P6Q2
3244	1,3,4,5,8,19,20,21	2,6,7,17,18,22,23,24	11	D3ZUC6,O88797,P01681,P47967,P97840,Q4FZU2,Q5I0D1,Q6IFW6,Q6IG02,Q6IMF3,Q6P6Q2
3245	1,3,4,5,8,19,20,22	2,6,7,17,18,21,23,24	7	O88797,P01681,P19218,Q6IFW6,Q6IG02,Q6IMF3,Q6P6Q2
3246	1,3,4,5,8,19,20,23	2,6,7,17,18,21,22,24	8	P01681,P10758,P23739,Q4FZU2,Q6IFW6,Q6IG02,Q6IMF3,Q6P6Q2
3247	1,3,4,5,8,19,20,24	2,6,7,17,18,21,22,23	7	O54728,P01681,Q4FZU2,Q6IFW6,Q6IG02,Q6IMF3,Q6P6Q2
3248	1,3,4,5,8,19,21,22	2,6,7,17,18,20,23,24	8	O88797,P01681,P30919,Q4FZU2,Q6IFW6,Q6IG02,Q6IMF3,Q6P6Q2
3249	1,3,4,5,8,19,21,23	2,6,7,17,18,20,22,24	7	O35077,P01681,Q4FZU2,Q6IFW6,Q6IG02,Q6IMF3,Q6P6Q2
3250	1,3,4,5,8,19,21,24	2,6,7,17,18,20,22,23	9	O54728,P01681,P30919,Q10743,Q4FZU2,Q6IFW6,Q6IG02,Q6IMF3,Q6P6Q2
3251	1,3,4,5,8,19,22,23	2,6,7,17,18,20,21,24	4	P00507,P01681,P01835,P27590
3252	1,3,4,5,8,19,22,24	2,6,7,17,18,20,21,23	11	O54728,P00507,P01681,P01835,P30919,P55091,Q4FZU2,Q6IFW6,Q6IG02,Q6IMF3,Q6P6Q2
3253	1,3,4,5,8,19,23,24	2,6,7,17,18,20,21,22	9	O54728,P01681,P01835,P25809,Q4FZU2,Q6IFW6,Q6IG02,Q6IMF3,Q6P6Q2
3254	1,3,4,5,8,20,21,22	2,6,7,17,18,19,23,24	10	O88797,P00762,P01681,P20766,Q4FZU2,Q6IFU7,Q6IFW6,Q6IG02,Q6IMF3,Q6P6Q2
3255	1,3,4,5,8,20,21,23	2,6,7,17,18,19,22,24	10	D3ZUC6,P00762,P01681,P23739,Q4FZU2,Q6IFU7,Q6IFW6,Q6IG02,Q6IMF3,Q6P6Q2
3256	1,3,4,5,8,20,21,24	2,6,7,17,18,19,22,23	10	P00762,P01681,Q10743,Q4FZU2,Q6IFU7,Q6IFU8,Q6IFW6,Q6IG02,Q6IMF3,Q6P6Q2
3257	1,3,4,5,8,20,22,23	2,6,7,17,18,19,21,24	6	P01681,P23739,Q6IFW6,Q6IG02,Q6IMF3,Q6P6Q2
3258	1,3,4,5,8,20,22,24	2,6,7,17,18,19,21,23	6	P01681,Q4FZU2,Q6IFW6,Q6IG02,Q6IMF3,Q6P6Q2
3259	1,3,4,5,8,20,23,24	2,6,7,17,18,19,21,22	8	P01681,P10758,P23739,Q4FZU2,Q6IFW6,Q6IG02,Q6IMF3,Q6P6Q2

3260	1,3,4,5,8,21,22,23	2,6,7,17,18,19,20,24	8	P01681,P30919,P54921,Q4FZU2,Q6IFW6,Q6IG02,Q6IMF3,Q6P6Q2
3261	1,3,4,5,8,21,22,24	2,6,7,17,18,19,20,23	9	P00762,P01681,P30919,Q10743,Q4FZU2,Q6IFW6,Q6IG02,Q6IMF3,Q6P6Q2
3262	1,3,4,5,8,21,23,24	2,6,7,17,18,19,20,22	9	O89117,P00762,P01681,P30919,Q4FZU2,Q6IFW6,Q6IG02,Q6IMF3,Q6P6Q2
3263	1,3,4,5,8,22,23,24	2,6,7,17,18,19,20,21	12	P00507,P01681,P25809,P30919,P62804,Q00715,Q4FZU2,Q6IFW6,Q6IG02,Q6IMF3,Q6P6Q2,Q6P6R2
3264	1,3,4,5,17,18,19,20	2,6,7,8,21,22,23,24	3	O54858,P97840,Q62714
3265	1,3,4,5,17,18,19,21	2,6,7,8,20,22,23,24	1	P97840
3266	1,3,4,5,17,18,19,22	2,6,7,8,20,21,23,24	2	P01835,P02631
3267	1,3,4,5,17,18,19,23	2,6,7,8,20,21,22,24	2	P01835,P02631
3268	1,3,4,5,17,18,19,24	2,6,7,8,20,21,22,23	1	P02631
3269	1,3,4,5,17,18,20,21	2,6,7,8,19,22,23,24	4	O54858,P97840,Q6IG02,Q811M5
3270	1,3,4,5,17,18,20,22	2,6,7,8,19,21,23,24	2	P97840,Q63474
3271	1,3,4,5,17,18,20,23	2,6,7,8,19,21,22,24	1	P23739
3272	1,3,4,5,17,18,20,24	2,6,7,8,19,21,22,23	1	Q6IG02
3273	1,3,4,5,17,18,21,22	2,6,7,8,19,20,23,24	0	
3274	1,3,4,5,17,18,21,23	2,6,7,8,19,20,22,24	0	
3275	1,3,4,5,17,18,21,24	2,6,7,8,19,20,22,23	0	
3276	1,3,4,5,17,18,22,23	2,6,7,8,19,20,21,24	9	P02631,P08723,P09456,P30120,P62804,Q00715,Q63617,Q68G31,Q812E4
3277	1,3,4,5,17,18,22,24	2,6,7,8,19,20,21,23	4	P02631,P62804,Q63617,Q9WTT6
3278	1,3,4,5,17,18,23,24	2,6,7,8,19,20,21,22	5	P02631,P25809,P62804,Q63617,Q9WTT6
3279	1,3,4,5,17,19,20,21	2,6,7,8,18,22,23,24	3	O54858,P97840,Q6IG02

3280	1,3,4,5,17, 19,20,22	2,6,7,8,18, 21,23,24	0
3281	1,3,4,5,17, 19,20,23	2,6,7,8,18, 21,22,24	2 P10758,P23739
3282	1,3,4,5,17, 19,20,24	2,6,7,8,18, 21,22,23	1 Q6IG02
3283	1,3,4,5,17, 19,21,22	2,6,7,8,18, 20,23,24	2 P01946,P02091
3284	1,3,4,5,17, 19,21,23	2,6,7,8,18, 20,22,24	2 P12020,P22006
3285	1,3,4,5,17, 19,21,24	2,6,7,8,18, 20,22,23	4 P12020,Q4FZU2,Q62761;Q62762;Q62763,Q99MH3
3286	1,3,4,5,17, 19,22,23	2,6,7,8,18, 20,21,24	5 P02091,P02631,P25809,P62804,Q00715
3287	1,3,4,5,17, 19,22,24	2,6,7,8,18, 20,21,23	4 P02631,P25809,P62804,Q00715
3288	1,3,4,5,17, 19,23,24	2,6,7,8,18, 20,21,22	5 P02631,P12020,P25809,P62804,Q00715
3289	1,3,4,5,17, 20,21,22	2,6,7,8,18, 19,23,24	2 P20766,Q6IG02
3290	1,3,4,5,17, 20,21,23	2,6,7,8,18, 19,22,24	3 P20766,P23739,Q6IG02
3291	1,3,4,5,17, 20,21,24	2,6,7,8,18, 19,22,23	5 Q4FZU2,Q6IFW6,Q6IG02,Q6P6Q2,Q811M5
3292	1,3,4,5,17, 20,22,23	2,6,7,8,18, 19,21,24	5 P20766,P23739,P62804,Q00715,Q68G31
3293	1,3,4,5,17, 20,22,24	2,6,7,8,18, 19,21,23	4 P20766,P62804,Q00715,Q6IG02
3294	1,3,4,5,17, 20,23,24	2,6,7,8,18, 19,21,22	5 P23739,P62804,Q00715,Q6IG02,Q9QYP1
3295	1,3,4,5,17, 21,22,23	2,6,7,8,18, 19,20,24	3 P12020,P20766,Q68G31
3296	1,3,4,5,17, 21,22,24	2,6,7,8,18, 19,20,23	1 P20766
3297	1,3,4,5,17, 21,23,24	2,6,7,8,18, 19,20,22	4 P12020,P50280,Q4FZU2,Q6IG02
3298	1,3,4,5,17, 22,23,24	2,6,7,8,18, 19,20,21	8 P02631,P25809,P47727,P62804,Q00715,Q63617,Q68G31,Q9WTT6
3299	1,3,4,5,18, 19,20,21	2,6,7,8,17, 22,23,24	4 O54858,P47967,P97840,Q5I0D1

3300	1,3,4,5,18,19,20,22	2,6,7,8,17,21,23,24	2	P97840,Q63474
3301	1,3,4,5,18,19,20,23	2,6,7,8,17,21,22,24	3	P10758,P23739,P97840
3302	1,3,4,5,18,19,20,24	2,6,7,8,17,21,22,23	1	P97840
3303	1,3,4,5,18,19,21,22	2,6,7,8,17,20,23,24	1	P97840
3304	1,3,4,5,18,19,21,23	2,6,7,8,17,20,22,24	3	O89117,P12020,P97840
3305	1,3,4,5,18,19,21,24	2,6,7,8,17,20,22,23	1	O89117
3306	1,3,4,5,18,19,22,23	2,6,7,8,17,20,21,24	7	P02631,P09456,P25809,P30120,P47727,P62804,Q00715
3307	1,3,4,5,18,19,22,24	2,6,7,8,17,20,21,23	6	P02631,P25809,P55091,P62804,P63029,Q9WTT6
3308	1,3,4,5,18,19,23,24	2,6,7,8,17,20,21,22	5	P02631,P25809,P62804,P63029,Q9WTT6
3309	1,3,4,5,18,20,21,22	2,6,7,8,17,19,23,24	1	P97840
3310	1,3,4,5,18,20,21,23	2,6,7,8,17,19,22,24	2	O89117,P97840
3311	1,3,4,5,18,20,21,24	2,6,7,8,17,19,22,23	3	O89117,P97840,Q6IG02
3312	1,3,4,5,18,20,22,23	2,6,7,8,17,19,21,24	3	P62804,P97840,Q63474
3313	1,3,4,5,18,20,22,24	2,6,7,8,17,19,21,23	3	P21674,P62804,Q9QX74
3314	1,3,4,5,18,20,23,24	2,6,7,8,17,19,21,22	5	P23739,P62804,Q5QE79,Q9QX74,Q9QYP1
3315	1,3,4,5,18,21,22,23	2,6,7,8,17,19,20,24	6	O89117,P09456,P47727,P54921,Q68G31,Q9WTT6
3316	1,3,4,5,18,21,22,24	2,6,7,8,17,19,20,23	3	O89117,Q8CJD3,Q9WTT6
3317	1,3,4,5,18,21,23,24	2,6,7,8,17,19,20,22	3	O89117,P12020,Q9WTT6
3318	1,3,4,5,18,22,23,24	2,6,7,8,17,19,20,21	11	D4A5U3,P02631,P13676,P25809,P47727,P62804,Q00715,Q63617,Q6P6R2,Q9QX74,Q9WTT6
3319	1,3,4,5,19,20,21,22	2,6,7,8,17,18,23,24	3	P20766,P47967,P97840

3320	1,3,4,5,19,20,21,23	2,6,7,8,17,18,22,24	5	P08649,P10758,P23739,P97840,Q6IG02
3321	1,3,4,5,19,20,21,24	2,6,7,8,17,18,22,23	3	P08649,P97840,Q6IG02
3322	1,3,4,5,19,20,22,23	2,6,7,8,17,18,21,24	4	P02631,P10758,P23739,Q00715
3323	1,3,4,5,19,20,22,24	2,6,7,8,17,18,21,23	3	P02631,P62804,Q00715
3324	1,3,4,5,19,20,23,24	2,6,7,8,17,18,21,22	5	P02631,P10758,P23739,P62804,Q00715
3325	1,3,4,5,19,21,22,23	2,6,7,8,17,18,20,24	3	P12020,P47727,P55091
3326	1,3,4,5,19,21,22,24	2,6,7,8,17,18,20,23	4	P12020,P25809,P30919,P55091
3327	1,3,4,5,19,21,23,24	2,6,7,8,17,18,20,22	4	P08649,P12020,P25809,P55091
3328	1,3,4,5,19,22,23,24	2,6,7,8,17,18,20,21	11	P00507,P02631,P12020,P25809,P47727,P55091,P62804,Q00715,Q6P6R2,Q9QX74,Q9WTT6
3329	1,3,4,5,20,21,22,23	2,6,7,8,17,18,19,24	5	P08649,P20766,P23739,P97840,Q6IG02
3330	1,3,4,5,20,21,22,24	2,6,7,8,17,18,19,23	3	P08649,P20766,Q6IG02
3331	1,3,4,5,20,21,23,24	2,6,7,8,17,18,19,22	4	P08649,P23739,Q6IFW6,Q6IG02
3332	1,3,4,5,20,22,23,24	2,6,7,8,17,18,19,21	9	P02631,P07150,P23739,P62804,Q00715,Q5QE79,Q66H69,Q6P6R2,Q9QX74
3333	1,3,4,5,21,22,23,24	2,6,7,8,17,18,19,20	9	P07150,P08649,P12020,P13676,P25809,P30919,P47727,P55091,Q9WTT6
3334	1,3,4,6,7,8,17,18	2,5,19,20,21,22,23,24	7	O70417,O70594,P01681,P13432,P23593,Q05702,Q63598
3335	1,3,4,6,7,8,17,19	2,5,18,20,21,22,23,24	7	O70417,O70594,P01681,P22282,Q05702,Q63493,Q99041
3336	1,3,4,6,7,8,17,20	2,5,18,19,21,22,23,24	23	D4A5U3,O70417,P02780,P06761,P06911,P11598,P12020,P13676,P19223,P19629,P22273,P22282,Q05702,Q4G075,Q5GRG2,Q5M8C6,Q62946,Q63493,Q6IG02,Q99041,Q9JHB9,Q9JI85,Q9QW07
3337	1,3,4,6,7,8,17,21	2,5,18,19,20,22,23,24	12	O70417,O70594,P04762,P22282,Q05702,Q4FZU2,Q62946,Q63493,Q6IFW6,Q6IG02,Q99041,Q9QX74

3338	1,3,4,6,7,8,17,22	2,5,18,19,20,21,23,24	8	O70417,P01681,P01946,P02091,P23593,P35280,P97580,Q99041
3339	1,3,4,6,7,8,17,23	2,5,18,19,20,21,22,24	8	O70417,O70594,P01681,P02783,P20761,P23593,Q05702,Q63598
3340	1,3,4,6,7,8,17,24	2,5,18,19,20,21,22,23	9	O70417,O70594,P01681,P23593,Q05702,Q4FZU2,Q63598,Q6IFW6,Q6IG02
3341	1,3,4,6,7,8,18,19	2,5,17,20,21,22,23,24	7	O70417,P01681,P06761,P11598,P22282,Q05702,Q99041
3342	1,3,4,6,7,8,18,20	2,5,17,19,21,22,23,24	19	O70417,P02780,P06761,P06911,P11598,P12020,P19629,P22273,P22282,P22283,P50280,Q05702,Q4G075,Q5GRG2,Q63617,Q99041,Q9JHB9,Q9JI85,Q9QW07
3343	1,3,4,6,7,8,18,21	2,5,17,19,20,22,23,24	10	O70417,P02625,P11598,P18757,P19629,P22282,P46844,Q05702,Q63493,Q99041
3344	1,3,4,6,7,8,18,22	2,5,17,19,20,21,23,24	7	O70417,P23593,P35280,P49134,P97580,Q30KJ2,Q99041
3345	1,3,4,6,7,8,18,23	2,5,17,19,20,21,22,24	9	O70417,O70594,P02783,P20761,P46844,P49134,Q05702,Q3ZAV1,Q63598
3346	1,3,4,6,7,8,18,24	2,5,17,19,20,21,22,23	9	O70257,O70417,P02625,P23593,P46844,P49134,Q05702,Q3ZAV1,Q63598
3347	1,3,4,6,7,8,19,20	2,5,17,18,21,22,23,24	35	O70417,P00714,P02780,P06761,P06911,P08723,P0C0A9,P10758,P11598,P12020,P19629,P22273,P22282,P22283,P36374,P42854,P46462,P50280,Q05702,Q4G075,Q5GRG2,Q5I0D1,Q5M8C6,Q62902,Q62946,Q63493,Q63617,Q6IG02,Q6Q7Y5,Q812E4,Q99041,Q9JHB9,Q9JI85,Q9QW07,Q9R0T3
3348	1,3,4,6,7,8,19,21	2,5,17,18,20,22,23,24	15	O70417,P00714,P01681,P06761,P0C0A9,P11598,P19629,P22282,P22283,Q05702,Q63493,Q63617,Q6Q7Y5,Q99041,Q9JI85
3349	1,3,4,6,7,8,19,22	2,5,17,18,20,21,23,24	9	O70417,P01681,P15399,P19218,P22282,P35280,P97580,Q99041,Q9QZK9
3350	1,3,4,6,7,8,19,23	2,5,17,18,20,21,22,24	9	O70417,P01681,P02783,P10758,P20761,P22282,P42854,Q05702,Q99041

3351	1,3,4,6,7,8,19,24	2,5,17,18,20,21,22,23	7	O54728,O70417,P00714,P01681,P22282,Q62902,Q99041
3352	1,3,4,6,7,8,20,21	2,5,17,18,19,22,23,24	34	D4A5U3,O70417,P00714,P02631,P02780,P06761,P06911,P0C0A9,P11598,P12020,P19629,P22273,P22282,P22283,P46462,P47967,Q05702,Q4G075,Q5GRG2,Q5M8C6,Q5RKI1,Q62946,Q63493,Q63617,Q6AYR9,Q6IFW6,Q6IG02,Q6Q7Y5,Q812E4,Q99041,Q9JHB9,Q9JI85,Q9QW07,Q9R0T3
3353	1,3,4,6,7,8,20,22	2,5,17,18,19,21,23,24	18	O70417,P02780,P06911,P11598,P12020,P15399,P19629,P22273,P22282,P23593,P35280,P50280,Q05702,Q4G075,Q5GRG2,Q99041,Q9JHB9,Q9QW07
3354	1,3,4,6,7,8,20,23	2,5,17,18,19,21,22,24	20	O70417,P02780,P02783,P06911,P09656,P10758,P11598,P12020,P19629,P20761,P22273,P22282,P42854,Q05702,Q4G075,Q5GRG2,Q62894,Q6IG02,Q99041,Q9QW07
3355	1,3,4,6,7,8,20,24	2,5,17,18,19,21,22,23	24	O70417,P00714,P02780,P06761,P06911,P08723,P11598,P12020,P19629,P22273,P22282,P22283,Q05702,Q4G075,Q5GRG2,Q5M8C6,Q63617,Q6IFW6,Q6IG02,Q812E4,Q99041,Q9JHB9,Q9JI85,Q9QW07
3356	1,3,4,6,7,8,21,22	2,5,17,18,19,20,23,24	6	O70417,P02625,P19629,P23593,P35280,Q99041
3357	1,3,4,6,7,8,21,23	2,5,17,18,19,20,22,24	9	P02783,P09656,P19629,P20761,P46844,Q05702,Q63493,Q63532,Q99041
3358	1,3,4,6,7,8,21,24	2,5,17,18,19,20,22,23	16	P00714,P02625,P02782,P04762,P11598,P22282,P30919,P36860,P46844,Q05702,Q10743,Q4FZU2,Q6AYR9,Q6IFW6,Q6IG02,Q99041
3359	1,3,4,6,7,8,22,23	2,5,17,18,19,20,21,24	13	O70417,P01681,P02783,P09656,P19629,P20761,P23593,P25809,P35280,P49134,Q09030,Q63532,Q99041
3360	1,3,4,6,7,8,22,24	2,5,17,18,19,20,21,23	7	O70417,P22006,P23593,P25809,P35280,P49134,Q99041
3361	1,3,4,6,7,8,23,24	2,5,17,18,19,20,21,22	11	O70417,P02783,P09656,P20761,P23593,P25809,P46844,P49134,Q05702,Q3ZAV1,Q63598
3362	1,3,4,6,7,17,18,19	2,5,8,20,21,22,23,24	0	
3363	1,3,4,6,7,17,18,20	2,5,8,19,21,22,23,24	1	P11598
3364	1,3,4,6,7,17,18,21	2,5,8,19,20,22,23,24	1	P05539

3365	1,3,4,6,7,17,18,22	2,5,8,19,20,21,23,24	2	P01946,P02091
3366	1,3,4,6,7,17,18,23	2,5,8,19,20,21,22,24	1	O70594
3367	1,3,4,6,7,17,18,24	2,5,8,19,20,21,22,23	0	
3368	1,3,4,6,7,17,19,20	2,5,8,18,21,22,23,24	4	P11598,P22283,P36374,Q63617
3369	1,3,4,6,7,17,19,21	2,5,8,18,20,22,23,24	2	P05539,Q5QE79
3370	1,3,4,6,7,17,19,22	2,5,8,18,20,21,23,24	2	P01946,P02091
3371	1,3,4,6,7,17,19,23	2,5,8,18,20,21,22,24	2	P02783,P25809
3372	1,3,4,6,7,17,19,24	2,5,8,18,20,21,22,23	2	P08721,P25809
3373	1,3,4,6,7,17,20,21	2,5,8,18,19,22,23,24	4	P11598,P19223,P22283,Q63617
3374	1,3,4,6,7,17,20,22	2,5,8,18,19,21,23,24	3	O70417,P01946,P02091
3375	1,3,4,6,7,17,20,23	2,5,8,18,19,21,22,24	3	P02783,P09656,P10758
3376	1,3,4,6,7,17,20,24	2,5,8,18,19,21,22,23	0	
3377	1,3,4,6,7,17,21,22	2,5,8,18,19,20,23,24	4	P01946,P02091,P05539,Q9R168
3378	1,3,4,6,7,17,21,23	2,5,8,18,19,20,22,24	2	P02783,P09656
3379	1,3,4,6,7,17,21,24	2,5,8,18,19,20,22,23	3	P04762,P05539,P36860
3380	1,3,4,6,7,17,22,23	2,5,8,18,19,20,21,24	5	P01946,P02091,P02783,P09656,P25809
3381	1,3,4,6,7,17,22,24	2,5,8,18,19,20,21,23	4	P01946,P02091,P23593,P25809
3382	1,3,4,6,7,17,23,24	2,5,8,18,19,20,21,22	1	P25809
3383	1,3,4,6,7,18,19,20	2,5,8,17,21,22,23,24	6	P10758,P11598,P22283,P97840,Q5I0D1,Q63617
3384	1,3,4,6,7,18,19,21	2,5,8,17,20,22,23,24	1	P11598

3385	1,3,4,6,7,18,19,22	2,5,8,17,20,21,23,24	0	
3386	1,3,4,6,7,18,19,23	2,5,8,17,20,21,22,24	2	P02783,P25809
3387	1,3,4,6,7,18,19,24	2,5,8,17,20,21,22,23	1	P25809
3388	1,3,4,6,7,18,20,21	2,5,8,17,19,22,23,24	5	P11598,P22283,P47967,P97840,Q63617
3389	1,3,4,6,7,18,20,22	2,5,8,17,19,21,23,24	0	
3390	1,3,4,6,7,18,20,23	2,5,8,17,19,21,22,24	4	P02783,P09656,P10758,P11598
3391	1,3,4,6,7,18,20,24	2,5,8,17,19,21,22,23	1	P11598
3392	1,3,4,6,7,18,21,22	2,5,8,17,19,20,23,24	2	P23739,Q8CJD3
3393	1,3,4,6,7,18,21,23	2,5,8,17,19,20,22,24	3	O89117,P02783,P09656
3394	1,3,4,6,7,18,21,24	2,5,8,17,19,20,22,23	2	O89117,Q8CJD3
3395	1,3,4,6,7,18,22,23	2,5,8,17,19,20,21,24	5	P02783,P06760,P09656,P25809,P54921
3396	1,3,4,6,7,18,22,24	2,5,8,17,19,20,21,23	5	P06760,P22006,P25809,Q6P6R2,Q8CJD3
3397	1,3,4,6,7,18,23,24	2,5,8,17,19,20,21,22	3	P06760,P25809,Q6P6R2
3398	1,3,4,6,7,19,20,21	2,5,8,17,18,22,23,24	14	P00714,P02780,P05369,P10758,P11598,P22283,P36374,P47967,P97840,Q5I0D1,Q63493,Q63617,Q9JI85,Q9R0T3
3399	1,3,4,6,7,19,20,22	2,5,8,17,18,21,23,24	3	P11598,Q63617,Q99041
3400	1,3,4,6,7,19,20,23	2,5,8,17,18,21,22,24	5	P02783,P09656,P10758,P11598,Q63617
3401	1,3,4,6,7,19,20,24	2,5,8,17,18,21,22,23	7	P08723,P10758,P11598,P22283,P36374,Q63617,Q9QW07
3402	1,3,4,6,7,19,21,22	2,5,8,17,18,20,23,24	2	P01946,Q99041
3403	1,3,4,6,7,19,21,23	2,5,8,17,18,20,22,24	2	P02783,P09656
3404	1,3,4,6,7,19,21,24	2,5,8,17,18,20,22,23	3	P00714,P25809,Q62761;Q62762;Q62763

3405	1,3,4,6,7,19,22,23	2,5,8,17,18,20,21,24	2	P02783,P25809
3406	1,3,4,6,7,19,22,24	2,5,8,17,18,20,21,23	3	P19132,P22006,P25809
3407	1,3,4,6,7,19,23,24	2,5,8,17,18,20,21,22	2	P02783,P25809
3408	1,3,4,6,7,20,21,22	2,5,8,17,18,19,23,24	2	P11598,Q63617
3409	1,3,4,6,7,20,21,23	2,5,8,17,18,19,22,24	6	P02783,P09656,P10758,P11598,P19629,Q63617
3410	1,3,4,6,7,20,21,24	2,5,8,17,18,19,22,23	4	P00714,P11598,P22283,Q63617
3411	1,3,4,6,7,20,22,23	2,5,8,17,18,19,21,24	3	P02783,P09656,P10758
3412	1,3,4,6,7,20,22,24	2,5,8,17,18,19,21,23	1	P22006
3413	1,3,4,6,7,20,23,24	2,5,8,17,18,19,21,22	4	P02783,P09656,P10758,Q9QYP1
3414	1,3,4,6,7,21,22,23	2,5,8,17,18,19,20,24	3	P02783,P09656,P25809
3415	1,3,4,6,7,21,22,24	2,5,8,17,18,19,20,23	2	P25809,P30919
3416	1,3,4,6,7,21,23,24	2,5,8,17,18,19,20,22	4	P02783,P09656,P25809,P36860
3417	1,3,4,6,7,22,23,24	2,5,8,17,18,19,20,21	7	P02783,P06760,P09656,P22006,P25809,P62804,Q6P6R2
3418	1,3,4,6,8,17,18,19	2,5,7,20,21,22,23,24	6	O70417,P01681,P01835,P08649,P13432,Q06000
3419	1,3,4,6,8,17,18,20	2,5,7,19,21,22,23,24	8	O70417,P06911,P12020,P13432,Q05702,Q5GRG2,Q6IG02,Q9R168
3420	1,3,4,6,8,17,18,21	2,5,7,19,20,22,23,24	1	P02625
3421	1,3,4,6,8,17,18,22	2,5,7,19,20,21,23,24	8	O70417,P01946,P02091,P08649,P13432,P23593,P97580,Q30KJ2
3422	1,3,4,6,8,17,18,23	2,5,7,19,20,21,22,24	2	O70594,Q05702
3423	1,3,4,6,8,17,18,24	2,5,7,19,20,21,22,23	2	O70417,P13432
3424	1,3,4,6,8,17,19,20	2,5,7,18,21,22,23,24	7	O70417,P01681,P06911,P36374,Q5GRG2,Q6IG02,Q9JI85

3425	1,3,4,6,8,17,19,21	2,5,7,18,20,22,23,24	2	P01681,Q9JI85
3426	1,3,4,6,8,17,19,22	2,5,7,18,20,21,23,24	6	O70417,P01681,P01946,P02091,P15399,P97580
3427	1,3,4,6,8,17,19,23	2,5,7,18,20,21,22,24	2	P01681,P02783
3428	1,3,4,6,8,17,19,24	2,5,7,18,20,21,22,23	3	O54728,O70417,P01681
3429	1,3,4,6,8,17,20,21	2,5,7,18,19,22,23,24	11	D4A5U3,O70417,P00762,P06911,P19223,Q05702,Q10758,Q5GRG2,Q6IFW6,Q6IG02,Q9JI85
3430	1,3,4,6,8,17,20,22	2,5,7,18,19,21,23,24	7	O70417,P01946,P02091,P06911,P15399,Q5GRG2,Q6IG02
3431	1,3,4,6,8,17,20,23	2,5,7,18,19,21,22,24	6	O70417,P02783,P06911,P09656,Q05702,Q6IG02
3432	1,3,4,6,8,17,20,24	2,5,7,18,19,21,22,23	6	O70417,P06911,Q05702,Q5GRG2,Q6IFW6,Q6IG02
3433	1,3,4,6,8,17,21,22	2,5,7,18,19,20,23,24	2	P01946,P02091
3434	1,3,4,6,8,17,21,23	2,5,7,18,19,20,22,24	3	P02783,P09656,Q05702
3435	1,3,4,6,8,17,21,24	2,5,7,18,19,20,22,23	4	Q10743,Q4FZU2,Q6IFW6,Q6IG02
3436	1,3,4,6,8,17,22,23	2,5,7,18,19,20,21,24	7	O70417,P01681,P01946,P02091,P02783,P09656,P23593
3437	1,3,4,6,8,17,22,24	2,5,7,18,19,20,21,23	4	O70417,P01946,P02091,P23593
3438	1,3,4,6,8,17,23,24	2,5,7,18,19,20,21,22	0	
3439	1,3,4,6,8,18,19,20	2,5,7,17,21,22,23,24	6	O70417,P06911,P22282,Q9JI85,Q9QW07,Q9R168
3440	1,3,4,6,8,18,19,21	2,5,7,17,20,22,23,24	1	Q9JI85
3441	1,3,4,6,8,18,19,22	2,5,7,17,20,21,23,24	4	P15399,P97580,Q30KJ2,Q6AY61
3442	1,3,4,6,8,18,19,23	2,5,7,17,20,21,22,24	2	P01681,P02783
3443	1,3,4,6,8,18,19,24	2,5,7,17,20,21,22,23	1	O54728
3444	1,3,4,6,8,18,20,21	2,5,7,17,19,22,23,24	9	P00762,P02625,P06911,P19629,P54921,Q05702,Q6IG02,Q9JI85,Q9R168

3445	1,3,4,6,8,18,20,22	2,5,7,17,19,21,23,24	7	O70417,P06911,P12020,P15399,P54921,Q6AY61,Q9R168
3446	1,3,4,6,8,18,20,23	2,5,7,17,19,21,22,24	7	P02783,P06911,P09656,P10758,P54921,Q05702,Q9R168
3447	1,3,4,6,8,18,20,24	2,5,7,17,19,21,22,23	6	O70417,P06911,Q05702,Q6IG02,Q9QW07,Q9R168
3448	1,3,4,6,8,18,21,22	2,5,7,17,19,20,23,24	2	P02625,P54921
3449	1,3,4,6,8,18,21,23	2,5,7,17,19,20,22,24	4	P02783,P09656,P54921,Q05702
3450	1,3,4,6,8,18,21,24	2,5,7,17,19,20,22,23	2	P02625,Q10743
3451	1,3,4,6,8,18,22,23	2,5,7,17,19,20,21,24	5	P02783,P09656,P54921,Q63532,Q6AY61
3452	1,3,4,6,8,18,22,24	2,5,7,17,19,20,21,23	3	P22006,P23593,Q8CJD3
3453	1,3,4,6,8,18,23,24	2,5,7,17,19,20,21,22	0	
3454	1,3,4,6,8,19,20,21	2,5,7,17,18,22,23,24	12	P02780,P05369,P19629,P22282,P22283,P36374,Q10743,Q63493,Q6IG02,Q6Q7Y5,Q9JI85,Q9QW07
3455	1,3,4,6,8,19,20,22	2,5,7,17,18,21,23,24	5	O70417,P06911,P15399,P19218,Q9JI85
3456	1,3,4,6,8,19,20,23	2,5,7,17,18,21,22,24	4	P02783,P09656,P10758,P42854
3457	1,3,4,6,8,19,20,24	2,5,7,17,18,21,22,23	9	O54728,P06911,P08723,P22282,P36374,Q10743,Q6IG02,Q9JI85,Q9QW07
3458	1,3,4,6,8,19,21,22	2,5,7,17,18,20,23,24	4	P01946,P02091,P15399,P30919
3459	1,3,4,6,8,19,21,23	2,5,7,17,18,20,22,24	3	P02783,P09656,Q9Z0J6
3460	1,3,4,6,8,19,21,24	2,5,7,17,18,20,22,23	6	O54728,P30919,Q10743,Q6IFW6,Q6Q7Y5,Q9JI85
3461	1,3,4,6,8,19,22,23	2,5,7,17,18,20,21,24	8	P00507,P01681,P01946,P02091,P02783,P15399,P25809,Q6AY61
3462	1,3,4,6,8,19,22,24	2,5,7,17,18,20,21,23	6	O54728,P00507,P15399,P22006,P25809,P30919
3463	1,3,4,6,8,19,23,24	2,5,7,17,18,20,21,22	5	O54728,P01681,P02783,P25809,Q9Z0J6
3464	1,3,4,6,8,20,21,22	2,5,7,17,18,19,23,24	6	P00762,P15399,P19629,P54921,Q10743,Q6IG02

3465	1,3,4,6,8,20,21,23	2,5,7,17,18,19,22,24	8	P00762,P02783,P09656,P10758,P19629,P54921,Q05702,Q6IG02
3466	1,3,4,6,8,20,21,24	2,5,7,17,18,19,22,23	9	P00762,P08723,Q05702,Q10743,Q6AYR9,Q6IFW6,Q6IG02,Q9JI85,Q9QW07
3467	1,3,4,6,8,20,22,23	2,5,7,17,18,19,21,24	5	P02783,P09656,P15399,P54921,Q6AY61
3468	1,3,4,6,8,20,22,24	2,5,7,17,18,19,21,23	7	O70417,P06911,P15399,P22006,Q10743,Q6IG02,Q9QW07
3469	1,3,4,6,8,20,23,24	2,5,7,17,18,19,21,22	6	P02783,P09656,P10758,Q05702,Q6IG02,Q9QYP1
3470	1,3,4,6,8,21,22,23	2,5,7,17,18,19,20,24	5	P02783,P09656,P30919,P54921,Q63532
3471	1,3,4,6,8,21,22,24	2,5,7,17,18,19,20,23	4	P02625,P30919,Q10743,Q6IFW6
3472	1,3,4,6,8,21,23,24	2,5,7,17,18,19,20,22	6	P00762,P02783,P09656,P30919,Q05702,Q6IFW6
3473	1,3,4,6,8,22,23,24	2,5,7,17,18,19,20,21	11	P00507,P02783,P06760,P09656,P22006,P23593,P25809,P30919,P62804,Q63532,Q6P6R2
3474	1,3,4,6,17,18,19,20	2,5,7,8,21,22,23,24	3	Q06000,Q62714,Q8K1G0
3475	1,3,4,6,17,18,19,21	2,5,7,8,20,22,23,24	0	
3476	1,3,4,6,17,18,19,22	2,5,7,8,20,21,23,24	3	P01946,P02091,Q06000
3477	1,3,4,6,17,18,19,23	2,5,7,8,20,21,22,24	0	
3478	1,3,4,6,17,18,19,24	2,5,7,8,20,21,22,23	2	P70549,Q06000
3479	1,3,4,6,17,18,20,21	2,5,7,8,19,22,23,24	0	
3480	1,3,4,6,17,18,20,22	2,5,7,8,19,21,23,24	3	P01946,P02091,Q06000
3481	1,3,4,6,17,18,20,23	2,5,7,8,19,21,22,24	0	
3482	1,3,4,6,17,18,20,24	2,5,7,8,19,21,22,23	0	
3483	1,3,4,6,17,18,21,22	2,5,7,8,19,20,23,24	4	P01946,P02091,P23739,Q8CJD3
3484	1,3,4,6,17,18,21,23	2,5,7,8,19,20,22,24	1	P09656

3485	1,3,4,6,17,18,21,24	2,5,7,8,19,20,22,23	2	Q4G075,Q8CJD3
3486	1,3,4,6,17,18,22,23	2,5,7,8,19,20,21,24	7	P01946,P02091,P02783,P06760,P55159,P62804,Q9WTT6
3487	1,3,4,6,17,18,22,24	2,5,7,8,19,20,21,23	7	P01946,P02091,P06760,P22006,P62804,Q8CJD3,Q9WTT6
3488	1,3,4,6,17,18,23,24	2,5,7,8,19,20,21,22	4	P06760,P25809,P62804,Q9WTT6
3489	1,3,4,6,17,19,20,21	2,5,7,8,18,22,23,24	1	P36374
3490	1,3,4,6,17,19,20,22	2,5,7,8,18,21,23,24	4	O89117,P01946,P02091,Q9JJ50
3491	1,3,4,6,17,19,20,23	2,5,7,8,18,21,22,24	2	P02783,P10758
3492	1,3,4,6,17,19,20,24	2,5,7,8,18,21,22,23	1	P36374
3493	1,3,4,6,17,19,21,22	2,5,7,8,18,20,23,24	2	P01946,P02091
3494	1,3,4,6,17,19,21,23	2,5,7,8,18,20,22,24	1	P02783
3495	1,3,4,6,17,19,21,24	2,5,7,8,18,20,22,23	0	
3496	1,3,4,6,17,19,22,23	2,5,7,8,18,20,21,24	7	P01946,P02091,P02783,P06760,P25809,P62804,Q00715
3497	1,3,4,6,17,19,22,24	2,5,7,8,18,20,21,23	8	P01946,P02091,P06760,P19132,P25809,P62804,P70549,Q00715
3498	1,3,4,6,17,19,23,24	2,5,7,8,18,20,21,22	5	P06760,P25809,P62804,P70549,Q00715
3499	1,3,4,6,17,20,21,22	2,5,7,8,18,19,23,24	3	P01946,P02091,P20766
3500	1,3,4,6,17,20,21,23	2,5,7,8,18,19,22,24	2	P02783,P09656
3501	1,3,4,6,17,20,21,24	2,5,7,8,18,19,22,23	0	
3502	1,3,4,6,17,20,22,23	2,5,7,8,18,19,21,24	7	P01946,P02091,P02783,P06760,P09656,P62804,Q00715
3503	1,3,4,6,17,20,22,24	2,5,7,8,18,19,21,23	6	P01946,P02091,P06760,P22006,P62804,Q00715
3504	1,3,4,6,17,20,23,24	2,5,7,8,18,19,21,22	5	P06760,P09656,P62804,Q00715,Q9QYP1

3505	1,3,4,6,17,21,22,23	2,5,7,8,18,19,20,24	4	P01946,P02091,P02783,P09656
3506	1,3,4,6,17,21,22,24	2,5,7,8,18,19,20,23	2	P01946,P02091
3507	1,3,4,6,17,21,23,24	2,5,7,8,18,19,20,22	2	P09656,P25809
3508	1,3,4,6,17,22,23,24	2,5,7,8,18,19,20,21	13	P01946,P02091,P02783,P06760,P07335,P09656,P25809,P47967,P55159,P62804,Q00715,Q5I0D1,Q9WTT6
3509	1,3,4,6,18,19,20,21	2,5,7,8,17,22,23,24	0	
3510	1,3,4,6,18,19,20,22	2,5,7,8,17,21,23,24	1	P39069
3511	1,3,4,6,18,19,20,23	2,5,7,8,17,21,22,24	2	P02783,P10758
3512	1,3,4,6,18,19,20,24	2,5,7,8,17,21,22,23	0	
3513	1,3,4,6,18,19,21,22	2,5,7,8,17,20,23,24	1	P01946
3514	1,3,4,6,18,19,21,23	2,5,7,8,17,20,22,24	1	P02783
3515	1,3,4,6,18,19,21,24	2,5,7,8,17,20,22,23	1	Q4G075
3516	1,3,4,6,18,19,22,23	2,5,7,8,17,20,21,24	8	P02783,P06760,P13676,P25809,P62804,Q6AY61,Q811M5,Q9WTT6
3517	1,3,4,6,18,19,22,24	2,5,7,8,17,20,21,23	7	P06760,P13676,P19132,P22006,P25809,P62804,Q9WTT6
3518	1,3,4,6,18,19,23,24	2,5,7,8,17,20,21,22	4	P06760,P25809,P62804,Q9WTT6
3519	1,3,4,6,18,20,21,22	2,5,7,8,17,19,23,24	3	P54921,Q5PQL7,Q8CJD3
3520	1,3,4,6,18,20,21,23	2,5,7,8,17,19,22,24	3	P02783,P09656,P54921
3521	1,3,4,6,18,20,21,24	2,5,7,8,17,19,22,23	1	Q8CJD3
3522	1,3,4,6,18,20,22,23	2,5,7,8,17,19,21,24	9	P02783,P04355,P06760,P09656,P34080,P54921,P62804,Q6AY61,Q9QX74
3523	1,3,4,6,18,20,22,24	2,5,7,8,17,19,21,23	5	P06760,P22006,P62804,Q8CJD3,Q9QX74
3524	1,3,4,6,18,20,23,24	2,5,7,8,17,19,21,22	5	P06760,P09656,P62804,Q9QX74,Q9QYP1

3525	1,3,4,6,18, 21,22,23	2,5,7,8,17, 19,20,24	5	P02783,P09656,P13676,P54921,Q9WTT6
3526	1,3,4,6,18, 21,22,24	2,5,7,8,17, 19,20,23	4	P13676,Q4G075,Q8CJD3,Q9WTT6
3527	1,3,4,6,18, 21,23,24	2,5,7,8,17, 19,20,22	5	O89117,P09656,P13676,Q4G075,Q9WTT6
3528	1,3,4,6,18, 22,23,24	2,5,7,8,17, 19,20,21	15	D4A5U3,P02783,P06760,P09656,P13676,P22006,P25809,P55159,P62804,Q00715,Q6P6R2,Q811 M5,Q8CJD3,Q9QX74,Q9WTT6
3529	1,3,4,6,19, 20,21,22	2,5,7,8,17, 18,23,24	2	P01946,Q5PQL7
3530	1,3,4,6,19, 20,21,23	2,5,7,8,17, 18,22,24	3	P02783,P09656,P10758
3531	1,3,4,6,19, 20,21,24	2,5,7,8,17, 18,22,23	2	O35547,P36374
3532	1,3,4,6,19, 20,22,23	2,5,7,8,17, 18,21,24	6	P02783,P06760,P09656,P10758,Q00715,Q9QX74
3533	1,3,4,6,19, 20,22,24	2,5,7,8,17, 18,21,23	6	P06760,P22006,P62804,Q00715,Q9QX74,Q9Z0V6
3534	1,3,4,6,19, 20,23,24	2,5,7,8,17, 18,21,22	8	P02783,P06760,P09656,P10758,P62804,Q00715,Q9QX74,Q9QYP1
3535	1,3,4,6,19, 21,22,23	2,5,7,8,17, 18,20,24	6	P01946,P02091,P02783,P09656,P25809,Q811M5
3536	1,3,4,6,19, 21,22,24	2,5,7,8,17, 18,20,23	5	P01946,P02091,P19132,P25809,P30919
3537	1,3,4,6,19, 21,23,24	2,5,7,8,17, 18,20,22	3	P02783,P09656,P25809
3538	1,3,4,6,19, 22,23,24	2,5,7,8,17, 18,20,21	14	P00507,P01946,P02091,P02783,P06760,P13676,P22006,P25809,P62804,P70549,Q00715,Q811 M5,Q9QX74,Q9WTT6
3539	1,3,4,6,20, 21,22,23	2,5,7,8,17, 18,19,24	4	P02783,P09656,P20766,P54921
3540	1,3,4,6,20, 21,22,24	2,5,7,8,17, 18,19,23	1	P22006
3541	1,3,4,6,20, 21,23,24	2,5,7,8,17, 18,19,22	3	P02783,P09656,Q9QYP1
3542	1,3,4,6,20, 22,23,24	2,5,7,8,17, 18,19,21	10	P02783,P06760,P09656,P22006,P62804,Q00715,Q66H69,Q6P6R2,Q9QX74,Q9QYP1
3543	1,3,4,6,21, 22,23,24	2,5,7,8,17, 18,19,20	9	P01946,P02783,P06760,P09656,P13676,P25809,P30919,Q9QX74,Q9WTT6
3544	1,3,4,7,8,1 7,18,19	2,5,6,20,2 1,22,23,24	5	O70417,P01681,P01835,P20646,Q9QX74

3545	1,3,4,7,8,17,18,20	2,5,6,19,21,22,23,24	6	O70417,P06911,P13676,Q4G075,Q6IG02,Q811M5
3546	1,3,4,7,8,17,18,21	2,5,6,19,20,22,23,24	5	O70417,P01681,Q6IFW6,Q811M5,Q9QX74
3547	1,3,4,7,8,17,18,22	2,5,6,19,20,21,23,24	3	O70417,P01681,P35280
3548	1,3,4,7,8,17,18,23	2,5,6,19,20,21,22,24	4	O70417,O70594,P01681,Q5I0J9
3549	1,3,4,7,8,17,18,24	2,5,6,19,20,21,22,23	3	O70417,P01681,Q811M5
3550	1,3,4,7,8,17,19,20	2,5,6,18,21,22,23,24	16	D4A5U3,O70417,P01681,P02780,P06911,P10758,P13676,P20646,P22282,P36374,Q4G075,Q62946,Q63493,Q6IG02,Q811M5,Q9WTT6
3551	1,3,4,7,8,17,19,21	2,5,6,18,20,22,23,24	12	B0BNN3,O70417,P01681,P04355,P20646,Q5QE79,Q63493,Q66H69,Q6IFW6,Q811M5,Q9EQS0,Q9QX74
3552	1,3,4,7,8,17,19,22	2,5,6,18,20,21,23,24	4	O70417,P01681,P20646,P35280
3553	1,3,4,7,8,17,19,23	2,5,6,18,20,21,22,24	4	O70417,P01681,P20646,P21674
3554	1,3,4,7,8,17,19,24	2,5,6,18,20,21,22,23	5	O54728,O70417,P01681,Q4FZU2,Q6IFW6
3555	1,3,4,7,8,17,20,21	2,5,6,18,19,22,23,24	19	D4A5U3,O70417,P00762,P02780,P06911,P13676,P19223,P20646,Q4FZU2,Q5M8C6,Q62946,Q63493,Q6IFW6,Q6IG02,Q6P6Q2,Q811M5,Q9EQS0,Q9QX74,Q9WTT6
3556	1,3,4,7,8,17,20,22	2,5,6,18,19,21,23,24	7	O70417,P01681,P06911,P35280,Q4G075,Q6IG02,Q9JJ50
3557	1,3,4,7,8,17,20,23	2,5,6,18,19,21,22,24	6	O70417,P01681,P06911,P10758,Q4G075,Q6IG02
3558	1,3,4,7,8,17,20,24	2,5,6,18,19,21,22,23	9	O70417,P06911,P08723,Q4FZU2,Q6IFW6,Q6IG02,Q6P6Q2,Q811M5,Q9JHB9
3559	1,3,4,7,8,17,21,22	2,5,6,18,19,20,23,24	4	O70417,P01681,P35280,Q6IFW6
3560	1,3,4,7,8,17,21,23	2,5,6,18,19,20,22,24	4	P01681,Q4FZU2,Q6IFW6,Q6IG02
3561	1,3,4,7,8,17,21,24	2,5,6,18,19,20,22,23	7	P01681,P02782,Q4FZU2,Q6IFW6,Q6IG02,Q811M5,Q9EQS0
3562	1,3,4,7,8,17,22,23	2,5,6,18,19,20,21,24	4	O70417,P01681,P35280,Q68G31
3563	1,3,4,7,8,17,22,24	2,5,6,18,19,20,21,23	5	O70417,P01681,P23593,P35280,Q6IFW6
3564	1,3,4,7,8,17,23,24	2,5,6,18,19,20,21,22	6	O70417,P01681,Q4FZU2,Q5PQL7,Q63598,Q6IFW6

3565	1,3,4,7,8,18,19,20	2,5,6,17,21,22,23,24	11	O70417,P01039,P01681,P02780,P10758,P22282,P36374,P47967,P97840,Q5I0D1,Q9R168
3566	1,3,4,7,8,18,19,21	2,5,6,17,20,22,23,24	5	P01039,P01681,P97840,Q63751,Q66H69
3567	1,3,4,7,8,18,19,22	2,5,6,17,20,21,23,24	4	O70417,P01039,P01681,P35280
3568	1,3,4,7,8,18,19,23	2,5,6,17,20,21,22,24	4	P01039,P01681,P10758,Q5I0J9
3569	1,3,4,7,8,18,19,24	2,5,6,17,20,21,22,23	3	O54728,P01681,P63029
3570	1,3,4,7,8,18,20,21	2,5,6,17,19,22,23,24	9	P00762,P01039,P02780,P47967,P97840,Q63493,Q6IFW6,Q6IG02,Q811M5
3571	1,3,4,7,8,18,20,22	2,5,6,17,19,21,23,24	5	O70417,P01039,P35280,P97840,Q4G075
3572	1,3,4,7,8,18,20,23	2,5,6,17,19,21,22,24	4	O70417,P01039,P10758,Q9R168
3573	1,3,4,7,8,18,20,24	2,5,6,17,19,21,22,23	5	O70417,Q6IFW6,Q6IG02,Q811M5,Q9JHB9
3574	1,3,4,7,8,18,21,22	2,5,6,17,19,20,23,24	3	P01039,P02625,P35280
3575	1,3,4,7,8,18,21,23	2,5,6,17,19,20,22,24	2	O89117,P01039
3576	1,3,4,7,8,18,21,24	2,5,6,17,19,20,22,23	3	O89117,P02625,Q6IFW6
3577	1,3,4,7,8,18,22,23	2,5,6,17,19,20,21,24	6	P01039,P01681,P35280,P49134,Q5I0J9,Q6AY61
3578	1,3,4,7,8,18,22,24	2,5,6,17,19,20,21,23	4	O70417,P35280,P49134,Q8CJD3
3579	1,3,4,7,8,18,23,24	2,5,6,17,19,20,21,22	4	P49134,Q3ZAV1,Q5I0J9,Q64268
3580	1,3,4,7,8,19,20,21	2,5,6,17,18,22,23,24	27	B0BNN3,D4A5U3,P00762,P01039,P02780,P04905,P08723,P0C0A9,P10758,P16636,P22282,P22283,P24368,P36374,P47967,P97840,Q5I0D1,Q5M8C6,Q5RKI1,Q63493,Q63617,Q6IFW6,Q6IG02,Q6Q7Y5,Q812E4,Q9EQS0,Q9WTT6
3581	1,3,4,7,8,19,20,22	2,5,6,17,18,21,23,24	7	O70417,P01039,P01681,P02780,P10758,P35280,Q4G075
3582	1,3,4,7,8,19,20,23	2,5,6,17,18,21,22,24	6	O70417,P01039,P01681,P10758,Q4G075,Q812E4
3583	1,3,4,7,8,19,20,24	2,5,6,17,18,21,22,23	13	O54728,O70417,P01681,P02780,P08723,P10758,P16636,P22282,P36374,Q6IFW6,Q6IG02,Q812E4,Q9QW07
3584	1,3,4,7,8,19,21,22	2,5,6,17,18,20,23,24	4	B0BNN3,P01039,P01681,P35280

3585	1,3,4,7,8,19,21,23	2,5,6,17,18,20,22,24	3	O35077,P01039,P01681
3586	1,3,4,7,8,19,21,24	2,5,6,17,18,20,22,23	8	B0BNN3,O54728,P01681,P02782,P04355,P16636,Q6IFW6,Q6Q7Y5
3587	1,3,4,7,8,19,22,23	2,5,6,17,18,20,21,24	3	P01039,P01681,P35280
3588	1,3,4,7,8,19,22,24	2,5,6,17,18,20,21,23	4	O54728,O70417,P01681,P35280
3589	1,3,4,7,8,19,23,24	2,5,6,17,18,20,21,22	4	O54728,P01681,P10758,P25809
3590	1,3,4,7,8,20,21,22	2,5,6,17,18,19,23,24	7	O70417,P00762,P01039,P02780,P35280,Q6IFW6,Q6IG02
3591	1,3,4,7,8,20,21,23	2,5,6,17,18,19,22,24	7	P00762,P01039,P10758,Q63493,Q6IFW6,Q6IG02,Q812E4
3592	1,3,4,7,8,20,21,24	2,5,6,17,18,19,22,23	15	P00762,P02780,P02781,P02782,P08723,Q4FZU2,Q5M8C6,Q6AYR9,Q6IFW6,Q6IG02,Q6P6Q2,Q811M5,Q812E4,Q9EQS0,Q9JHB9
3593	1,3,4,7,8,20,22,23	2,5,6,17,18,19,21,24	5	O70417,P01039,P10758,P35280,Q4G075
3594	1,3,4,7,8,20,22,24	2,5,6,17,18,19,21,23	5	O70417,P00762,P35280,Q6IFW6,Q6IG02
3595	1,3,4,7,8,20,23,24	2,5,6,17,18,19,21,22	6	P00762,P10758,Q6IFW6,Q6IG02,Q812E4,Q9QYP1
3596	1,3,4,7,8,21,22,23	2,5,6,17,18,19,20,24	3	P01039,P02783,P35280
3597	1,3,4,7,8,21,22,24	2,5,6,17,18,19,20,23	3	P00762,P35280,Q6IFW6
3598	1,3,4,7,8,21,23,24	2,5,6,17,18,19,20,22	3	P00762,Q6IFW6,Q6IG02
3599	1,3,4,7,8,22,23,24	2,5,6,17,18,19,20,21	4	P01681,P20761,P25809,P35280
3600	1,3,4,7,17,18,19,20	2,5,6,8,21,22,23,24	6	O54858,P20646,P30919,P97840,Q62714,Q8K1G0
3601	1,3,4,7,17,18,19,21	2,5,6,8,20,22,23,24	2	P20646,P97840
3602	1,3,4,7,17,18,19,22	2,5,6,8,20,21,23,24	0	
3603	1,3,4,7,17,18,19,23	2,5,6,8,20,21,22,24	0	
3604	1,3,4,7,17,18,19,24	2,5,6,8,20,21,22,23	0	

3605	1,3,4,7,17,<br>18,20,21	2,5,6,8,19,<br>22,23,24	4	O54858,P00507,P97840,Q811M5
3606	1,3,4,7,17,<br>18,20,22	2,5,6,8,19,<br>21,23,24	1	P97840
3607	1,3,4,7,17,<br>18,20,23	2,5,6,8,19,<br>21,22,24	2	P10758,P30919
3608	1,3,4,7,17,<br>18,20,24	2,5,6,8,19,<br>21,22,23	2	Q811M5,Q9QYP1
3609	1,3,4,7,17,<br>18,21,22	2,5,6,8,19,<br>20,23,24	1	Q8CJD3
3610	1,3,4,7,17,<br>18,21,23	2,5,6,8,19,<br>20,22,24	0	
3611	1,3,4,7,17,<br>18,21,24	2,5,6,8,19,<br>20,22,23	1	Q8CJD3
3612	1,3,4,7,17,<br>18,22,23	2,5,6,8,19,<br>20,21,24	2	Q5I0J9,Q68G31
3613	1,3,4,7,17,<br>18,22,24	2,5,6,8,19,<br>20,21,23	1	Q8CJD3
3614	1,3,4,7,17,<br>18,23,24	2,5,6,8,19,<br>20,21,22	2	Q5I0J9,Q64268
3615	1,3,4,7,17,<br>19,20,21	2,5,6,8,18,<br>22,23,24	7	O54858,P16636,P20646,P36374,P97840,Q6P6R2,Q811M5
3616	1,3,4,7,17,<br>19,20,22	2,5,6,8,18,<br>21,23,24	2	P20646,Q9JJ50
3617	1,3,4,7,17,<br>19,20,23	2,5,6,8,18,<br>21,22,24	1	P10758
3618	1,3,4,7,17,<br>19,20,24	2,5,6,8,18,<br>21,22,23	4	P16636,P36374,P54921,Q99MH3
3619	1,3,4,7,17,<br>19,21,22	2,5,6,8,18,<br>20,23,24	2	B0BNN3,Q9R168
3620	1,3,4,7,17,<br>19,21,23	2,5,6,8,18,<br>20,22,24	0	
3621	1,3,4,7,17,<br>19,21,24	2,5,6,8,18,<br>20,22,23	4	P16636,P54921,Q62761;Q62762;Q62763,Q99MH3
3622	1,3,4,7,17,<br>19,22,23	2,5,6,8,18,<br>20,21,24	1	P25809
3623	1,3,4,7,17,<br>19,22,24	2,5,6,8,18,<br>20,21,23	1	P25809
3624	1,3,4,7,17,<br>19,23,24	2,5,6,8,18,<br>20,21,22	1	P25809

3625	1,3,4,7,17,<br>20,21,22	2,5,6,8,18,<br>19,23,24	0
3626	1,3,4,7,17,<br>20,21,23	2,5,6,8,18,<br>19,22,24	1 P10758
3627	1,3,4,7,17,<br>20,21,24	2,5,6,8,18,<br>19,22,23	1 Q811M5
3628	1,3,4,7,17,<br>20,22,23	2,5,6,8,18,<br>19,21,24	2 P10758,Q68G31
3629	1,3,4,7,17,<br>20,22,24	2,5,6,8,18,<br>19,21,23	0
3630	1,3,4,7,17,<br>20,23,24	2,5,6,8,18,<br>19,21,22	2 P10758,Q9QYP1
3631	1,3,4,7,17,<br>21,22,23	2,5,6,8,18,<br>19,20,24	2 Q68G31,Q9R168
3632	1,3,4,7,17,<br>21,22,24	2,5,6,8,18,<br>19,20,23	1 Q9R168
3633	1,3,4,7,17,<br>21,23,24	2,5,6,8,18,<br>19,20,22	0
3634	1,3,4,7,17,<br>22,23,24	2,5,6,8,18,<br>19,20,21	4 P25809,Q00715,Q64268,Q68G31
3635	1,3,4,7,18,<br>19,20,21	2,5,6,8,17,<br>22,23,24	6 O54858,P01039,P47967,P97840,Q5I0D1,Q63617
3636	1,3,4,7,18,<br>19,20,22	2,5,6,8,17,<br>21,23,24	2 P01039,P97840
3637	1,3,4,7,18,<br>19,20,23	2,5,6,8,17,<br>21,22,24	3 P01039,P10758,P97840
3638	1,3,4,7,18,<br>19,20,24	2,5,6,8,17,<br>21,22,23	2 P10758,P97840
3639	1,3,4,7,18,<br>19,21,22	2,5,6,8,17,<br>20,23,24	2 P01039,P97840
3640	1,3,4,7,18,<br>19,21,23	2,5,6,8,17,<br>20,22,24	3 O89117,P01039,P97840
3641	1,3,4,7,18,<br>19,21,24	2,5,6,8,17,<br>20,22,23	5 O89117,P17559,P36376,P63029,P97840
3642	1,3,4,7,18,<br>19,22,23	2,5,6,8,17,<br>20,21,24	3 P01039,P36375,Q5I0J9
3643	1,3,4,7,18,<br>19,22,24	2,5,6,8,17,<br>20,21,23	5 P17559,P19132,P25809,P36375,P63029
3644	1,3,4,7,18,<br>19,23,24	2,5,6,8,17,<br>20,21,22	4 P25809,P63029,Q5I0J9,Q6AYQ8

3645	1,3,4,7,18,<br>20,21,22	2,5,6,8,17,<br>19,23,24	3	P01039,P97840,Q8CJD3
3646	1,3,4,7,18,<br>20,21,23	2,5,6,8,17,<br>19,22,24	3	P01039,P10758,P97840
3647	1,3,4,7,18,<br>20,21,24	2,5,6,8,17,<br>19,22,23	2	P97840,Q8CJD3
3648	1,3,4,7,18,<br>20,22,23	2,5,6,8,17,<br>19,21,24	3	P01039,P10758,P97840
3649	1,3,4,7,18,<br>20,22,24	2,5,6,8,17,<br>19,21,23	2	P97840,Q8CJD3
3650	1,3,4,7,18,<br>20,23,24	2,5,6,8,17,<br>19,21,22	2	P10758,Q9QYP1
3651	1,3,4,7,18,<br>21,22,23	2,5,6,8,17,<br>19,20,24	2	P01039,Q68G31
3652	1,3,4,7,18,<br>21,22,24	2,5,6,8,17,<br>19,20,23	2	O89117,Q8CJD3
3653	1,3,4,7,18,<br>21,23,24	2,5,6,8,17,<br>19,20,22	2	O89117,Q64268
3654	1,3,4,7,18,<br>22,23,24	2,5,6,8,17,<br>19,20,21	8	P06760,P08934,P25809,Q5I0J9,Q64268,Q6AYQ8,Q6P6R2,Q8CJD3
3655	1,3,4,7,19,<br>20,21,22	2,5,6,8,17,<br>18,23,24	5	B0BNN3,P01039,P16636,P47967,P97840
3656	1,3,4,7,19,<br>20,21,23	2,5,6,8,17,<br>18,22,24	3	P01039,P10758,P97840
3657	1,3,4,7,19,<br>20,21,24	2,5,6,8,17,<br>18,22,23	5	P16636,P36374,P97840,Q63617,Q99MH3
3658	1,3,4,7,19,<br>20,22,23	2,5,6,8,17,<br>18,21,24	3	P01039,P10758,P97840
3659	1,3,4,7,19,<br>20,22,24	2,5,6,8,17,<br>18,21,23	1	P16636
3660	1,3,4,7,19,<br>20,23,24	2,5,6,8,17,<br>18,21,22	2	P10758,Q9QYP1
3661	1,3,4,7,19,<br>21,22,23	2,5,6,8,17,<br>18,20,24	1	P01039
3662	1,3,4,7,19,<br>21,22,24	2,5,6,8,17,<br>18,20,23	4	B0BNN3,P16636,P17559,P55091
3663	1,3,4,7,19,<br>21,23,24	2,5,6,8,17,<br>18,20,22	3	P08649,P25809,Q62761;Q62762;Q62763
3664	1,3,4,7,19,<br>22,23,24	2,5,6,8,17,<br>18,20,21	4	P25809,P36375,P55091,Q00715

3665	1,3,4,7,20, 21,22,23	2,5,6,8,17, 18,19,24	4	P01039,P08649,P10758,P97840
3666	1,3,4,7,20, 21,22,24	2,5,6,8,17, 18,19,23	1	P08649
3667	1,3,4,7,20, 21,23,24	2,5,6,8,17, 18,19,22	3	P08649,P10758,Q9QYP1
3668	1,3,4,7,20, 22,23,24	2,5,6,8,17, 18,19,21	3	P10758,Q00715,Q9QYP1
3669	1,3,4,7,21, 22,23,24	2,5,6,8,17, 18,19,20	2	P08649,P25809
3670	1,3,4,8,17, 18,19,20	2,5,6,7,21, 22,23,24	5	O70417,P01681,P01835,Q62714,Q9R168
3671	1,3,4,8,17, 18,19,21	2,5,6,7,20, 22,23,24	2	P01681,P01835
3672	1,3,4,8,17, 18,19,22	2,5,6,7,20, 21,23,24	2	P01681,P01835
3673	1,3,4,8,17, 18,19,23	2,5,6,7,20, 21,22,24	2	P01681,P01835
3674	1,3,4,8,17, 18,19,24	2,5,6,7,20, 21,22,23	3	O54728,P01681,P01835
3675	1,3,4,8,17, 18,20,21	2,5,6,7,19, 22,23,24	4	P00762,Q10758,Q6IG02,Q811M5
3676	1,3,4,8,17, 18,20,22	2,5,6,7,19, 21,23,24	1	O70417
3677	1,3,4,8,17, 18,20,23	2,5,6,7,19, 21,22,24	1	Q9R168
3678	1,3,4,8,17, 18,20,24	2,5,6,7,19, 21,22,23	4	O70417,P00762,Q6IG02,Q811M5
3679	1,3,4,8,17, 18,21,22	2,5,6,7,19, 20,23,24	0	
3680	1,3,4,8,17, 18,21,23	2,5,6,7,19, 20,22,24	0	
3681	1,3,4,8,17, 18,21,24	2,5,6,7,19, 20,22,23	3	P00762,P02625,Q6IFW6
3682	1,3,4,8,17, 18,22,23	2,5,6,7,19, 20,21,24	2	P01681,Q6AY61
3683	1,3,4,8,17, 18,22,24	2,5,6,7,19, 20,21,23	1	P50116
3684	1,3,4,8,17, 18,23,24	2,5,6,7,19, 20,21,22	1	P01681

3685	1,3,4,8,17, 19,20,21	2,5,6,7,18, 22,23,24	10	B0LT89,P00762,P13676,P16636,P36374,Q6IFW6,Q6IG02,Q6P6R2,Q811M5,Q9EQS0
3686	1,3,4,8,17, 19,20,22	2,5,6,7,18, 21,23,24	5	O70417,O89117,P01681,P01835,Q9JJ50
3687	1,3,4,8,17, 19,20,23	2,5,6,7,18, 21,22,24	4	P01681,P01835,P10758,Q6IG02
3688	1,3,4,8,17, 19,20,24	2,5,6,7,18, 21,22,23	8	O54728,P00762,P01681,P01835,P16636,P36374,P60905,Q6IG02
3689	1,3,4,8,17, 19,21,22	2,5,6,7,18, 20,23,24	3	B0BNN3,P01681,P02091
3690	1,3,4,8,17, 19,21,23	2,5,6,7,18, 20,22,24	1	P01681
3691	1,3,4,8,17, 19,21,24	2,5,6,7,18, 20,22,23	9	B0BNN3,O54728,P00762,P01681,P16636,Q4FZU2,Q6IFW6,Q6IG02,Q9EQS0
3692	1,3,4,8,17, 19,22,23	2,5,6,7,18, 20,21,24	3	P01681,P01835,P02091
3693	1,3,4,8,17, 19,22,24	2,5,6,7,18, 20,21,23	3	O54728,P01681,P01835
3694	1,3,4,8,17, 19,23,24	2,5,6,7,18, 20,21,22	3	O54728,P01681,P01835
3695	1,3,4,8,17, 20,21,22	2,5,6,7,18, 19,23,24	5	P00762,P20766,Q6IFW6,Q6IG02,Q9JJ50
3696	1,3,4,8,17, 20,21,23	2,5,6,7,18, 19,22,24	3	P00762,Q6IFW6,Q6IG02
3697	1,3,4,8,17, 20,21,24	2,5,6,7,18, 19,22,23	8	P00762,P30120,Q4FZU2,Q6IFW6,Q6IG02,Q6P6Q2,Q811M5,Q9EQS0
3698	1,3,4,8,17, 20,22,23	2,5,6,7,18, 19,21,24	1	P00762
3699	1,3,4,8,17, 20,22,24	2,5,6,7,18, 19,21,23	5	O70417,P00762,Q6IFW6,Q6IG02,Q9JJ50
3700	1,3,4,8,17, 20,23,24	2,5,6,7,18, 19,21,22	4	P00762,Q6IFW6,Q6IG02,Q9QYP1
3701	1,3,4,8,17, 21,22,23	2,5,6,7,18, 19,20,24	2	P00762,P01681
3702	1,3,4,8,17, 21,22,24	2,5,6,7,18, 19,20,23	2	P00762,Q6IFW6
3703	1,3,4,8,17, 21,23,24	2,5,6,7,18, 19,20,22	4	P00762,Q4FZU2,Q6IFW6,Q6IG02
3704	1,3,4,8,17, 22,23,24	2,5,6,7,18, 19,20,21	4	P01681,P47967,P62804,Q00715

3705	1,3,4,8,18, 19,20,21	2,5,6,7,17, 22,23,24	3	P00762,P01039,Q9R168
3706	1,3,4,8,18, 19,20,22	2,5,6,7,17, 21,23,24	5	P01039,P36860,P50116,Q6AY61,Q9R168
3707	1,3,4,8,18, 19,20,23	2,5,6,7,17, 21,22,24	4	P01039,P10758,P36860,Q9R168
3708	1,3,4,8,18, 19,20,24	2,5,6,7,17, 21,22,23	4	O54728,P00762,P50116,Q9R168
3709	1,3,4,8,18, 19,21,22	2,5,6,7,17, 20,23,24	1	P01039
3710	1,3,4,8,18, 19,21,23	2,5,6,7,17, 20,22,24	3	P01039,P01681,Q9Z0J6
3711	1,3,4,8,18, 19,21,24	2,5,6,7,17, 20,22,23	2	O54728,P00762
3712	1,3,4,8,18, 19,22,23	2,5,6,7,17, 20,21,24	4	P01039,P01681,P50116,Q6AY61
3713	1,3,4,8,18, 19,22,24	2,5,6,7,17, 20,21,23	2	O54728,P50116
3714	1,3,4,8,18, 19,23,24	2,5,6,7,17, 20,21,22	4	O54728,P01681,P50116,Q9Z0J6
3715	1,3,4,8,18, 20,21,22	2,5,6,7,17, 19,23,24	3	P00762,P01039,P54921
3716	1,3,4,8,18, 20,21,23	2,5,6,7,17, 19,22,24	5	P00762,P01039,P54921,Q6IG02,Q9R168
3717	1,3,4,8,18, 20,21,24	2,5,6,7,17, 19,22,23	3	P00762,Q6IFW6,Q6IG02
3718	1,3,4,8,18, 20,22,23	2,5,6,7,17, 19,21,24	5	P01039,P50116,P54921,Q6AY61,Q9R168
3719	1,3,4,8,18, 20,22,24	2,5,6,7,17, 19,21,23	2	P00762,P50116
3720	1,3,4,8,18, 20,23,24	2,5,6,7,17, 19,21,22	5	P00762,P10758,P50116,Q9QYP1,Q9R168
3721	1,3,4,8,18, 21,22,23	2,5,6,7,17, 19,20,24	5	P00762,P01039,P50116,P54921,Q6AY61
3722	1,3,4,8,18, 21,22,24	2,5,6,7,17, 19,20,23	4	P00762,P02625,P50116,Q8CJD3
3723	1,3,4,8,18, 21,23,24	2,5,6,7,17, 19,20,22	4	O89117,P00762,P50116,Q9Z0J6
3724	1,3,4,8,18, 22,23,24	2,5,6,7,17, 19,20,21	2	P50116,P62804

3725	1,3,4,8,19,20,21,22	2,5,6,7,17,18,23,24	4	B0BNN3,P00762,P01039,P16636
3726	1,3,4,8,19,20,21,23	2,5,6,7,17,18,22,24	4	P00762,P01039,P10758,Q6IG02
3727	1,3,4,8,19,20,21,24	2,5,6,7,17,18,22,23	14	B0BNN3,O35547,O54728,P00762,P08723,P16636,P30120,P36374,P60905,Q64335,Q6IFW6,Q6IG02,Q812E4,Q9EQS0
3728	1,3,4,8,19,20,22,23	2,5,6,7,17,18,21,24	5	P01039,P01681,P10758,P36860,Q6AY61
3729	1,3,4,8,19,20,22,24	2,5,6,7,17,18,21,23	5	O54728,P00762,P16636,P50116,Q9JJ50
3730	1,3,4,8,19,20,23,24	2,5,6,7,17,18,21,22	4	O54728,P00762,P10758,Q6IG02
3731	1,3,4,8,19,21,22,23	2,5,6,7,17,18,20,24	3	P01039,P01681,Q9Z0J6
3732	1,3,4,8,19,21,22,24	2,5,6,7,17,18,20,23	8	B0BNN3,O54728,P00762,P16636,P30919,P50116,P55091,Q64335
3733	1,3,4,8,19,21,23,24	2,5,6,7,17,18,20,22	5	O54728,P00762,Q64335,Q6IFW6,Q9Z0J6
3734	1,3,4,8,19,22,23,24	2,5,6,7,17,18,20,21	8	O54728,P00507,P01681,P25809,P50116,P55091,Q00715,Q9Z0J6
3735	1,3,4,8,20,21,22,23	2,5,6,7,17,18,19,24	4	P00762,P01039,P54921,Q6IG02
3736	1,3,4,8,20,21,22,24	2,5,6,7,17,18,19,23	4	P00762,Q10743,Q6IFW6,Q6IG02
3737	1,3,4,8,20,21,23,24	2,5,6,7,17,18,19,22	3	P00762,Q6IFW6,Q6IG02
3738	1,3,4,8,20,22,23,24	2,5,6,7,17,18,19,21	3	P00762,P50116,Q00715
3739	1,3,4,8,21,22,23,24	2,5,6,7,17,18,19,20	4	P00762,P30919,P50116,Q6IFW6
3740	1,3,4,17,18,19,20,21	2,5,6,7,8,22,23,24	3	O54858,Q62714,Q6P6R2
3741	1,3,4,17,18,19,20,22	2,5,6,7,8,21,23,24	7	O54858,P36860,P50115,P50116,Q62714,Q63474,Q9JJ50
3742	1,3,4,17,18,19,20,23	2,5,6,7,8,21,22,24	3	O54858,P10758,Q62714
3743	1,3,4,17,18,19,20,24	2,5,6,7,8,21,22,23	5	O54858,P50115,P50116,Q62714,Q63532
3744	1,3,4,17,18,19,21,22	2,5,6,7,8,20,23,24	1	P50116

3745	1,3,4,17,18,19,21,23	2,5,6,7,8,20,22,24	1	O54858
3746	1,3,4,17,18,19,21,24	2,5,6,7,8,20,22,23	4	O54858,P50116,Q4G075,Q99MH3
3747	1,3,4,17,18,19,22,23	2,5,6,7,8,20,21,24	3	P02631,P50116,Q00715
3748	1,3,4,17,18,19,22,24	2,5,6,7,8,20,21,23	7	P02631,P06760,P19132,P50115,P50116,P70549,Q00715
3749	1,3,4,17,18,19,23,24	2,5,6,7,8,20,21,22	5	P02631,P06760,P50116,P70549,Q00715
3750	1,3,4,17,18,20,21,22	2,5,6,7,8,19,23,24	2	O54858,P50115
3751	1,3,4,17,18,20,21,23	2,5,6,7,8,19,22,24	2	O54858,P50115
3752	1,3,4,17,18,20,21,24	2,5,6,7,8,19,22,23	4	O54858,P50115,Q811M5,Q8CJD3
3753	1,3,4,17,18,20,22,23	2,5,6,7,8,19,21,24	3	P50115,P50116,Q00715
3754	1,3,4,17,18,20,22,24	2,5,6,7,8,19,21,23	3	P50115,P50116,Q8CJD3
3755	1,3,4,17,18,20,23,24	2,5,6,7,8,19,21,22	3	P50115,P50116,Q9QYP1
3756	1,3,4,17,18,21,22,23	2,5,6,7,8,19,20,24	2	P50115,P50116
3757	1,3,4,17,18,21,22,24	2,5,6,7,8,19,20,23	5	P25031,P50115,P50116,Q4G075,Q8CJD3
3758	1,3,4,17,18,21,23,24	2,5,6,7,8,19,20,22	4	P19218,P50115,P50116,Q4G075
3759	1,3,4,17,18,22,23,24	2,5,6,7,8,19,20,21	12	P02631,P06760,P25809,P50115,P50116,P55159,P62804,Q00715,Q5I0D1,Q63617,Q64268,Q9WTT6
3760	1,3,4,17,19,20,21,22	2,5,6,7,8,18,23,24	4	O54858,P16636,P20766,Q9JJ50
3761	1,3,4,17,19,20,21,23	2,5,6,7,8,18,22,24	2	O54858,P10758
3762	1,3,4,17,19,20,21,24	2,5,6,7,8,18,22,23	4	O54858,P16636,P36374,Q99MH3
3763	1,3,4,17,19,20,22,23	2,5,6,7,8,18,21,24	5	P10758,P36860,P50116,Q00715,Q9JJ50
3764	1,3,4,17,19,20,22,24	2,5,6,7,8,18,21,23	5	P16636,P50115,P50116,Q00715,Q9JJ50

3765	1,3,4,17,19,20,23,24	2,5,6,7,8,18,21,22	5	O54858,P10758,P50116,Q00715,Q9QYP1
3766	1,3,4,17,19,21,22,23	2,5,6,7,8,18,20,24	3	P02091,P12020,P50116
3767	1,3,4,17,19,21,22,24	2,5,6,7,8,18,20,23	4	P02091,P16636,P50116,Q4G075
3768	1,3,4,17,19,21,23,24	2,5,6,7,8,18,20,22	2	P12020,Q4G075
3769	1,3,4,17,19,22,23,24	2,5,6,7,8,18,20,21	8	P02091,P02631,P06760,P25809,P50116,P62804,P70549,Q00715
3770	1,3,4,17,20,21,22,23	2,5,6,7,8,18,19,24	2	P20766,P50115
3771	1,3,4,17,20,21,22,24	2,5,6,7,8,18,19,23	2	P20766,P50115
3772	1,3,4,17,20,21,23,24	2,5,6,7,8,18,19,22	3	O54858,P50115,Q9QYP1
3773	1,3,4,17,20,22,23,24	2,5,6,7,8,18,19,21	7	P02631,P06760,P50115,P50116,P62804,Q00715,Q9QYP1
3774	1,3,4,17,21,22,23,24	2,5,6,7,8,18,19,20	4	P50115,P50116,P50280,Q4G075
3775	1,3,4,18,19,20,21,22	2,5,6,7,8,17,23,24	5	O54858,P01039,P50115,P50116,P97840
3776	1,3,4,18,19,20,21,23	2,5,6,7,8,17,22,24	5	O54858,P01039,P10758,P50115,P50116
3777	1,3,4,18,19,20,21,24	2,5,6,7,8,17,22,23	3	O54858,P50115,P50116
3778	1,3,4,18,19,20,22,23	2,5,6,7,8,17,21,24	8	P01039,P10758,P36860,P50115,P50116,Q00715,Q63474,Q6AY61
3779	1,3,4,18,19,20,22,24	2,5,6,7,8,17,21,23	3	P06760,P50115,P50116
3780	1,3,4,18,19,20,23,24	2,5,6,7,8,17,21,22	5	P06760,P10758,P50115,P50116,Q9QYP1
3781	1,3,4,18,19,21,22,23	2,5,6,7,8,17,20,24	3	P01039,P50115,P50116
3782	1,3,4,18,19,21,22,24	2,5,6,7,8,17,20,23	7	P17559,P19132,P25031,P50115,P50116,P55091,Q4G075
3783	1,3,4,18,19,21,23,24	2,5,6,7,8,17,20,22	5	P12020,P50115,P50116,Q4G075,Q9Z0J6
3784	1,3,4,18,19,22,23,24	2,5,6,7,8,17,20,21	14	D4A5U3,P02631,P06760,P13676,P25809,P36375,P50115,P50116,P55091,P62804,Q00715,Q4G075,Q9QX74,Q9WTT6

3785	1,3,4,18,20,21,22,23	2,5,6,7,8,17,19,24	4	P01039,P50115,P50116,P54921
3786	1,3,4,18,20,21,22,24	2,5,6,7,8,17,19,23	3	P50115,P50116,Q8CJD3
3787	1,3,4,18,20,21,23,24	2,5,6,7,8,17,19,22	3	P50115,P50116,Q9QYP1
3788	1,3,4,18,20,22,23,24	2,5,6,7,8,17,19,21	9	P02631,P06760,P50115,P50116,P62804,Q00715,Q5QE79,Q9QX74,Q9QYP1
3789	1,3,4,18,21,22,23,24	2,5,6,7,8,17,19,20	7	D4A5U3,P13676,P50115,P50116,Q4G075,Q8CJD3,Q9WTT6
3790	1,3,4,19,20,21,22,23	2,5,6,7,8,17,18,24	4	P01039,P10758,P50115,P50116
3791	1,3,4,19,20,21,22,24	2,5,6,7,8,17,18,23	5	O70594,P16636,P50115,P50116,Q64335
3792	1,3,4,19,20,21,23,24	2,5,6,7,8,17,18,22	5	O54858,P10758,P50115,P50116,Q64335
3793	1,3,4,19,20,22,23,24	2,5,6,7,8,17,18,21	9	P02631,P06760,P10758,P50115,P50116,P62804,Q00715,Q64335,Q9QX74
3794	1,3,4,19,21,22,23,24	2,5,6,7,8,17,18,20	8	P12020,P25809,P50115,P50116,P55091,Q4G075,Q64335,Q9Z0J6
3795	1,3,4,20,21,22,23,24	2,5,6,7,8,17,18,19	5	P00762,P20766,P50115,P50116,Q9QX74
3796	1,3,5,6,7,8,17,18	2,4,19,20,21,22,23,24	3	O70594,P13432,P49134
3797	1,3,5,6,7,8,17,19	2,4,18,20,21,22,23,24	2	O70594,P50115
3798	1,3,5,6,7,8,17,20	2,4,18,19,21,22,23,24	6	O70417,P06911,P13676,Q5GRG2,Q6IG05,Q6IMF3
3799	1,3,5,6,7,8,17,21	2,4,18,19,20,22,23,24	11	O70594,P04762,P05539,P08937,P36860,P50115,Q4FZU2,Q6IFW6,Q6IG05,Q6IMF3,Q9QX74
3800	1,3,5,6,7,8,17,22	2,4,18,19,20,21,23,24	5	P01946,P02091,P23593,P35280,Q09030
3801	1,3,5,6,7,8,17,23	2,4,18,19,20,21,22,24	4	O70594,P50115,Q09030,Q6IG05

3802	1,3,5,6,7,8,17,24	2,4,18,19,20,21,22,23	4	O70594,Q4FZU2,Q6IFW6,Q6IMF3
3803	1,3,5,6,7,8,18,19	2,4,17,20,21,22,23,24	1	P08937
3804	1,3,5,6,7,8,18,20	2,4,17,19,21,22,23,24	6	P06911,P19629,P31044,P47967,Q6IG05,Q9R168
3805	1,3,5,6,7,8,18,21	2,4,17,19,20,22,23,24	4	O70594,P08937,P47967,P49134
3806	1,3,5,6,7,8,18,22	2,4,17,19,20,21,23,24	3	P23593,P35280,P49134
3807	1,3,5,6,7,8,18,23	2,4,17,19,20,21,22,24	3	O70594,P49134,Q09030
3808	1,3,5,6,7,8,18,24	2,4,17,19,20,21,22,23	1	P49134
3809	1,3,5,6,7,8,19,20	2,4,17,18,21,22,23,24	11	D3ZUC6,P02780,P06911,P08937,P19218,P19629,P31044,P47967,Q5I0D1,Q6IG05,Q9QW07
3810	1,3,5,6,7,8,19,21	2,4,17,18,20,22,23,24	7	D3ZUC6,iRT-Kit_WR_fusion,P08937,P47967,P50115,Q63493,Q6IMF3
3811	1,3,5,6,7,8,19,22	2,4,17,18,20,21,23,24	4	P01946,P08937,P19218,P35280
3812	1,3,5,6,7,8,19,23	2,4,17,18,20,21,22,24	2	O35077,P50115
3813	1,3,5,6,7,8,19,24	2,4,17,18,20,21,22,23	0	
3814	1,3,5,6,7,8,20,21	2,4,17,18,19,22,23,24	11	D3ZUC6,P06911,P08937,P19629,P47967,Q5I0D1,Q5RKI1,Q63493,Q6IFW6,Q6IG05,Q6IMF3

3815	1,3,5,6,7,8,20,22	2,4,17,18,19,21,23,24	4	P06911,P19218,P19629,P35280
3816	1,3,5,6,7,8,20,23	2,4,17,18,19,21,22,24	6	P06911,P19629,P23739,P25031,Q09030,Q6IG05
3817	1,3,5,6,7,8,20,24	2,4,17,18,19,21,22,23	6	P06911,Q6IFW6,Q6IG05,Q6IMF3,Q9JHB9,Q9QW07
3818	1,3,5,6,7,8,21,22	2,4,17,18,19,20,23,24	6	P01946,P08937,P19218,P35280,Q09030,Q62714
3819	1,3,5,6,7,8,21,23	2,4,17,18,19,20,22,24	5	O70594,P19629,Q09030,Q62714,Q6IG05
3820	1,3,5,6,7,8,21,24	2,4,17,18,19,20,22,23	7	P04762,P30919,P36860,Q4FZU2,Q62714,Q6IFW6,Q6IMF3
3821	1,3,5,6,7,8,22,23	2,4,17,18,19,20,21,24	7	P02783,P19218,P23593,P35280,P49134,Q09030,Q62714
3822	1,3,5,6,7,8,22,24	2,4,17,18,19,20,21,23	7	P23593,P30919,P35280,P49134,Q09030,Q62714,Q6P6R2
3823	1,3,5,6,7,8,23,24	2,4,17,18,19,20,21,22	5	P25809,P49134,Q09030,Q62714,Q6P6R2
3824	1,3,5,6,7,17,18,19	2,4,8,20,21,22,23,24	0	
3825	1,3,5,6,7,17,18,20	2,4,8,19,21,22,23,24	0	
3826	1,3,5,6,7,17,18,21	2,4,8,19,20,22,23,24	3	P04762,P05539,P36860
3827	1,3,5,6,7,17,18,22	2,4,8,19,20,21,23,24	2	P01946,P02091
3828	1,3,5,6,7,17,18,23	2,4,8,19,20,21,22,24	1	O70594
3829	1,3,5,6,7,17,18,24	2,4,8,19,20,21,22,23	1	Q6AY61
3830	1,3,5,6,7,17,19,20	2,4,8,18,21,22,23,24	1	P47967

3831	1,3,5,6,7,17,19,21	2,4,8,18,20,22,23,24	3	P05539,Q5QE79,Q6AY61
3832	1,3,5,6,7,17,19,22	2,4,8,18,20,21,23,24	2	P01946,P02091
3833	1,3,5,6,7,17,19,23	2,4,8,18,20,21,22,24	1	P25809
3834	1,3,5,6,7,17,19,24	2,4,8,18,20,21,22,23	4	P08721,P25809,P54921,Q6AY61
3835	1,3,5,6,7,17,20,21	2,4,8,18,19,22,23,24	4	P04762,P05539,P47967,Q6AY61
3836	1,3,5,6,7,17,20,22	2,4,8,18,19,21,23,24	2	P01946,P02091
3837	1,3,5,6,7,17,20,23	2,4,8,18,19,21,22,24	1	P23739
3838	1,3,5,6,7,17,20,24	2,4,8,18,19,21,22,23	1	Q6AY61
3839	1,3,5,6,7,17,21,22	2,4,8,18,19,20,23,24	4	P01946,P02091,P05539,Q9R168
3840	1,3,5,6,7,17,21,23	2,4,8,18,19,20,22,24	4	P04762,P05539,P36860,Q07523
3841	1,3,5,6,7,17,21,24	2,4,8,18,19,20,22,23	4	P04762,P05539,P36860,Q6AY61
3842	1,3,5,6,7,17,22,23	2,4,8,18,19,20,21,24	5	P01946,P02091,P02783,P25809,P30120
3843	1,3,5,6,7,17,22,24	2,4,8,18,19,20,21,23	3	P01946,P02091,P25809
3844	1,3,5,6,7,17,23,24	2,4,8,18,19,20,21,22	1	P25809
3845	1,3,5,6,7,18,19,20	2,4,8,17,21,22,23,24	3	P47967,P97840,Q5I0D1
3846	1,3,5,6,7,18,19,21	2,4,8,17,20,22,23,24	1	P47967
3847	1,3,5,6,7,18,19,22	2,4,8,17,20,21,23,24	2	P01946,P19223
3848	1,3,5,6,7,18,19,23	2,4,8,17,20,21,22,24	2	P19223,P25809
3849	1,3,5,6,7,18,19,24	2,4,8,17,20,21,22,23	2	P19223,P25809
3850	1,3,5,6,7,18,20,21	2,4,8,17,19,22,23,24	3	P47967,P97840,Q5I0D1

3851	1,3,5,6,7,18,20,22	2,4,8,17,19,21,23,24	1 P47967
3852	1,3,5,6,7,18,20,23	2,4,8,17,19,21,22,24	0
3853	1,3,5,6,7,18,20,24	2,4,8,17,19,21,22,23	0
3854	1,3,5,6,7,18,21,22	2,4,8,17,19,20,23,24	1 P01946
3855	1,3,5,6,7,18,21,23	2,4,8,17,19,20,22,24	2 O89117,Q9JJ50
3856	1,3,5,6,7,18,21,24	2,4,8,17,19,20,22,23	3 O89117,P04762,P36860
3857	1,3,5,6,7,18,22,23	2,4,8,17,19,20,21,24	9 D4A5U3,P02783,P19223,P25809,P30120,P36374,P47727,P97675,Q6P6R2
3858	1,3,5,6,7,18,22,24	2,4,8,17,19,20,21,23	4 D4A5U3,P19223,P25809,Q6P6R2
3859	1,3,5,6,7,18,23,24	2,4,8,17,19,20,21,22	5 D4A5U3,P19223,P25809,P97675,Q6P6R2
3860	1,3,5,6,7,19,20,21	2,4,8,17,18,22,23,24	6 D3ZUC6,P22283,P47967,P97840,Q5I0D1,Q63617
3861	1,3,5,6,7,19,20,22	2,4,8,17,18,21,23,24	3 P01946,P19218,P47967
3862	1,3,5,6,7,19,20,23	2,4,8,17,18,21,22,24	1 P23739
3863	1,3,5,6,7,19,20,24	2,4,8,17,18,21,22,23	1 P47967
3864	1,3,5,6,7,19,21,22	2,4,8,17,18,20,23,24	3 P01946,P02091,P47967
3865	1,3,5,6,7,19,21,23	2,4,8,17,18,20,22,24	0
3866	1,3,5,6,7,19,21,24	2,4,8,17,18,20,22,23	1 Q6AY61
3867	1,3,5,6,7,19,22,23	2,4,8,17,18,20,21,24	7 P01946,P02091,P02783,P19218,P19223,P25809,P47727
3868	1,3,5,6,7,19,22,24	2,4,8,17,18,20,21,23	4 P01946,P19223,P25809,P55091
3869	1,3,5,6,7,19,23,24	2,4,8,17,18,20,21,22	2 P19223,P25809
3870	1,3,5,6,7,20,21,22	2,4,8,17,18,19,23,24	4 P01835,P01946,P47967,Q5PQL7

3871	1,3,5,6,7,20,21,23	2,4,8,17,18,19,22,24	3	P01835,P09656,P47967
3872	1,3,5,6,7,20,21,24	2,4,8,17,18,19,22,23	4	P01835,P04762,P47967,Q6AY61
3873	1,3,5,6,7,20,22,23	2,4,8,17,18,19,21,24	2	P02783,P09656
3874	1,3,5,6,7,20,22,24	2,4,8,17,18,19,21,23	1	P22006
3875	1,3,5,6,7,20,23,24	2,4,8,17,18,19,21,22	1	P23739
3876	1,3,5,6,7,21,22,23	2,4,8,17,18,19,20,24	6	P01946,P02783,P09656,P25809,P47727,Q62714
3877	1,3,5,6,7,21,22,24	2,4,8,17,18,19,20,23	6	P01946,P04762,P25809,P30919,P36860,Q62714
3878	1,3,5,6,7,21,23,24	2,4,8,17,18,19,20,22	4	P04762,P25809,P36860,Q62714
3879	1,3,5,6,7,22,23,24	2,4,8,17,18,19,20,21	10	D4A5U3,P01946,P02783,P19223,P25809,P47727,P62804,P97675,Q62714,Q6P6R2
3880	1,3,5,6,8,17,18,19	2,4,7,20,21,22,23,24	1	P13432
3881	1,3,5,6,8,17,18,20	2,4,7,19,21,22,23,24	2	P13432,Q9R168
3882	1,3,5,6,8,17,18,21	2,4,7,19,20,22,23,24	1	P13432
3883	1,3,5,6,8,17,18,22	2,4,7,19,20,21,23,24	3	P01946,P02091,P13432
3884	1,3,5,6,8,17,18,23	2,4,7,19,20,21,22,24	1	O70594
3885	1,3,5,6,8,17,18,24	2,4,7,19,20,21,22,23	1	P13432
3886	1,3,5,6,8,17,19,20	2,4,7,18,21,22,23,24	1	O89117
3887	1,3,5,6,8,17,19,21	2,4,7,18,20,22,23,24	1	P08937
3888	1,3,5,6,8,17,19,22	2,4,7,18,20,21,23,24	2	P01946,P02091
3889	1,3,5,6,8,17,19,23	2,4,7,18,20,21,22,24	0	
3890	1,3,5,6,8,17,19,24	2,4,7,18,20,21,22,23	0	

3891	1,3,5,6,8,17,20,21	2,4,7,18,19,22,23,24	4	P63029,Q6IFU7,Q6IG05,Q6IMF3
3892	1,3,5,6,8,17,20,22	2,4,7,18,19,21,23,24	3	O89117,P01946,P02091
3893	1,3,5,6,8,17,20,23	2,4,7,18,19,21,22,24	3	P23739,P36376,Q6IG05
3894	1,3,5,6,8,17,20,24	2,4,7,18,19,21,22,23	1	Q6IMF3
3895	1,3,5,6,8,17,21,22	2,4,7,18,19,20,23,24	2	P01946,P02091
3896	1,3,5,6,8,17,21,23	2,4,7,18,19,20,22,24	0	
3897	1,3,5,6,8,17,21,24	2,4,7,18,19,20,22,23	6	P04762,P36860,Q4FZU2,Q6IFW6,Q6IMF3,Q80WL1
3898	1,3,5,6,8,17,22,23	2,4,7,18,19,20,21,24	4	P01946,P02091,P02783,Q09030
3899	1,3,5,6,8,17,22,24	2,4,7,18,19,20,21,23	3	P01946,P02091,P23593
3900	1,3,5,6,8,17,23,24	2,4,7,18,19,20,21,22	1	Q80WL1
3901	1,3,5,6,8,18,19,20	2,4,7,17,21,22,23,24	1	Q9R168
3902	1,3,5,6,8,18,19,21	2,4,7,17,20,22,23,24	1	P08937
3903	1,3,5,6,8,18,19,22	2,4,7,17,20,21,23,24	3	P01946,P19223,Q9QYP1
3904	1,3,5,6,8,18,19,23	2,4,7,17,20,21,22,24	1	P19223
3905	1,3,5,6,8,18,19,24	2,4,7,17,20,21,22,23	1	P19223
3906	1,3,5,6,8,18,20,21	2,4,7,17,19,22,23,24	4	D3ZUC6,P08937,P47967,Q9R168
3907	1,3,5,6,8,18,20,22	2,4,7,17,19,21,23,24	1	Q9R168
3908	1,3,5,6,8,18,20,23	2,4,7,17,19,21,22,24	1	Q9R168
3909	1,3,5,6,8,18,20,24	2,4,7,17,19,21,22,23	1	Q9R168
3910	1,3,5,6,8,18,21,22	2,4,7,17,19,20,23,24	2	P01946,P08937

3911	1,3,5,6,8,18,21,23	2,4,7,17,19,20,22,24	1	P54921
3912	1,3,5,6,8,18,21,24	2,4,7,17,19,20,22,23	0	
3913	1,3,5,6,8,18,22,23	2,4,7,17,19,20,21,24	5	P02783,P19223,P36374,P54921,Q09030
3914	1,3,5,6,8,18,22,24	2,4,7,17,19,20,21,23	1	P19223
3915	1,3,5,6,8,18,23,24	2,4,7,17,19,20,21,22	2	P19223,P20646
3916	1,3,5,6,8,19,20,21	2,4,7,17,18,22,23,24	5	D3ZUC6,P08937,P47967,Q5I0D1,Q9JI85
3917	1,3,5,6,8,19,20,22	2,4,7,17,18,21,23,24	2	P01946,P19218
3918	1,3,5,6,8,19,20,23	2,4,7,17,18,21,22,24	2	P19218,P23739
3919	1,3,5,6,8,19,20,24	2,4,7,17,18,21,22,23	0	
3920	1,3,5,6,8,19,21,22	2,4,7,17,18,20,23,24	6	P01946,P02091,P08937,P19218,P30919,Q9QYP1
3921	1,3,5,6,8,19,21,23	2,4,7,17,18,20,22,24	2	P08937,Q9Z0J6
3922	1,3,5,6,8,19,21,24	2,4,7,17,18,20,22,23	2	P08937,P30919
3923	1,3,5,6,8,19,22,23	2,4,7,17,18,20,21,24	6	P00507,P01946,P02091,P02783,P19218,P19223
3924	1,3,5,6,8,19,22,24	2,4,7,17,18,20,21,23	7	P00507,P01946,P02091,P19218,P19223,P30919,P55091
3925	1,3,5,6,8,19,23,24	2,4,7,17,18,20,21,22	4	P19223,P25809,Q80WL1,Q9Z0J6
3926	1,3,5,6,8,20,21,22	2,4,7,17,18,19,23,24	2	P01946,P19218
3927	1,3,5,6,8,20,21,23	2,4,7,17,18,19,22,24	4	D3ZUC6,P09656,P23739,Q6IG05
3928	1,3,5,6,8,20,21,24	2,4,7,17,18,19,22,23	2	Q6IFW6,Q6IMF3
3929	1,3,5,6,8,20,22,23	2,4,7,17,18,19,21,24	4	P02783,P09656,P19218,P23739
3930	1,3,5,6,8,20,22,24	2,4,7,17,18,19,21,23	0	

3931	1,3,5,6,8,20,23,24	2,4,7,17,18,19,21,22	1	P23739
3932	1,3,5,6,8,21,22,23	2,4,7,17,18,19,20,24	7	P01946,P02783,P09656,P30919,P54921,Q09030,Q62714
3933	1,3,5,6,8,21,22,24	2,4,7,17,18,19,20,23	3	P01946,P30919,Q62714
3934	1,3,5,6,8,21,23,24	2,4,7,17,18,19,20,22	6	P20646,P30919,Q09030,Q62714,Q6IFW6,Q80WL1
3935	1,3,5,6,8,22,23,24	2,4,7,17,18,19,20,21	12	P00507,P01946,P02783,P19223,P20646,P25809,P30919,P62804,Q09030,Q62714,Q6P6R2,Q80WL1
3936	1,3,5,6,17,18,19,20	2,4,7,8,21,22,23,24	2	P20761,Q06000
3937	1,3,5,6,17,18,19,21	2,4,7,8,20,22,23,24	2	P05539,Q4G075
3938	1,3,5,6,17,18,19,22	2,4,7,8,20,21,23,24	3	P01946,P02091,Q06000
3939	1,3,5,6,17,18,19,23	2,4,7,8,20,21,22,24	0	
3940	1,3,5,6,17,18,19,24	2,4,7,8,20,21,22,23	1	Q4G075
3941	1,3,5,6,17,18,20,21	2,4,7,8,19,22,23,24	1	P05539
3942	1,3,5,6,17,18,20,22	2,4,7,8,19,21,23,24	2	P01946,P02091
3943	1,3,5,6,17,18,20,23	2,4,7,8,19,21,22,24	0	
3944	1,3,5,6,17,18,20,24	2,4,7,8,19,21,22,23	0	
3945	1,3,5,6,17,18,21,22	2,4,7,8,19,20,23,24	4	P01946,P02091,P05539,Q4G075
3946	1,3,5,6,17,18,21,23	2,4,7,8,19,20,22,24	1	Q4G075
3947	1,3,5,6,17,18,21,24	2,4,7,8,19,20,22,23	3	P05539,P36860,Q4G075
3948	1,3,5,6,17,18,22,23	2,4,7,8,19,20,21,24	15	D4A5U3,P01946,P02091,P02780,P02783,P08723,P09456,P22273,P22282,P30120,P36374,P62804,P82471,Q63617,Q812E4
3949	1,3,5,6,17,18,22,24	2,4,7,8,19,20,21,23	7	D4A5U3,P01946,P02091,P62804,Q4G075,Q63617,Q9WTT6
3950	1,3,5,6,17,18,23,24	2,4,7,8,19,20,21,22	7	D4A5U3,P02780,P25809,P62804,Q4G075,Q63617,Q9WTT6

3951	1,3,5,6,17,<br>19,20,21	2,4,7,8,18,<br>22,23,24	2	P05539,P20762
3952	1,3,5,6,17,<br>19,20,22	2,4,7,8,18,<br>21,23,24	3	O89117,P01946,P02091
3953	1,3,5,6,17,<br>19,20,23	2,4,7,8,18,<br>21,22,24	2	P23739,Q9Z1F2
3954	1,3,5,6,17,<br>19,20,24	2,4,7,8,18,<br>21,22,23	1	P20762
3955	1,3,5,6,17,<br>19,21,22	2,4,7,8,18,<br>20,23,24	3	P01946,P02091,P05539
3956	1,3,5,6,17,<br>19,21,23	2,4,7,8,18,<br>20,22,24	1	Q4G075
3957	1,3,5,6,17,<br>19,21,24	2,4,7,8,18,<br>20,22,23	4	P05539,P20762,Q4G075,Q6AY61
3958	1,3,5,6,17,<br>19,22,23	2,4,7,8,18,<br>20,21,24	7	P01946,P02091,P02783,P22273,P25809,P62804,Q00715
3959	1,3,5,6,17,<br>19,22,24	2,4,7,8,18,<br>20,21,23	6	P01946,P02091,P25809,P62804,Q00715,Q4G075
3960	1,3,5,6,17,<br>19,23,24	2,4,7,8,18,<br>20,21,22	5	P22273,P25809,P62804,Q00715,Q4G075
3961	1,3,5,6,17,<br>20,21,22	2,4,7,8,18,<br>19,23,24	5	P01946,P02091,P05539,P20762,P20766
3962	1,3,5,6,17,<br>20,21,23	2,4,7,8,18,<br>19,22,24	2	P09656,P63029
3963	1,3,5,6,17,<br>20,21,24	2,4,7,8,18,<br>19,22,23	3	P05539,P20762,Q6AY61
3964	1,3,5,6,17,<br>20,22,23	2,4,7,8,18,<br>19,21,24	5	P01946,P02091,P02783,P62804,Q00715
3965	1,3,5,6,17,<br>20,22,24	2,4,7,8,18,<br>19,21,23	4	P01946,P02091,P62804,Q00715
3966	1,3,5,6,17,<br>20,23,24	2,4,7,8,18,<br>19,21,22	3	P23739,P62804,Q00715
3967	1,3,5,6,17,<br>21,22,23	2,4,7,8,18,<br>19,20,24	3	P01946,P02091,P02783
3968	1,3,5,6,17,<br>21,22,24	2,4,7,8,18,<br>19,20,23	5	P01946,P02091,P05539,P10758,Q4G075
3969	1,3,5,6,17,<br>21,23,24	2,4,7,8,18,<br>19,20,22	2	P36860,Q4G075
3970	1,3,5,6,17,<br>22,23,24	2,4,7,8,18,<br>19,20,21	15	D4A5U3,P01946,P02091,P02780,P02783,P07150,P22273,P22282,P25809,P47727,P62804,Q0071<br>5,Q4G075,Q63617,Q9WTT6

3971	1,3,5,6,18, 19,20,21	2,4,7,8,17, 22,23,24	2	P47967,Q5PQL7
3972	1,3,5,6,18, 19,20,22	2,4,7,8,17, 21,23,24	3	P01946,Q5PQL7,Q63474
3973	1,3,5,6,18, 19,20,23	2,4,7,8,17, 21,22,24	1	Q9Z1F2
3974	1,3,5,6,18, 19,20,24	2,4,7,8,17, 21,22,23	0	
3975	1,3,5,6,18, 19,21,22	2,4,7,8,17, 20,23,24	6	D4A5U3,P01946,P02091,P19223,Q4G075,Q5PQL7
3976	1,3,5,6,18, 19,21,23	2,4,7,8,17, 20,22,24	2	D4A5U3,Q4G075
3977	1,3,5,6,18, 19,21,24	2,4,7,8,17, 20,22,23	3	D4A5U3,P19223,Q4G075
3978	1,3,5,6,18, 19,22,23	2,4,7,8,17, 20,21,24	13	D4A5U3,P01946,P02091,P02783,P09456,P19223,P22273,P25809,P30120,P47727,P62804,Q811M5,Q9WTT6
3979	1,3,5,6,18, 19,22,24	2,4,7,8,17, 20,21,23	8	D4A5U3,P01946,P19223,P25809,P55091,P62804,Q4G075,Q9WTT6
3980	1,3,5,6,18, 19,23,24	2,4,7,8,17, 20,21,22	7	D4A5U3,P19223,P25809,P62804,P98089,Q4G075,Q9WTT6
3981	1,3,5,6,18, 20,21,22	2,4,7,8,17, 19,23,24	2	P01946,Q5PQL7
3982	1,3,5,6,18, 20,21,23	2,4,7,8,17, 19,22,24	0	
3983	1,3,5,6,18, 20,21,24	2,4,7,8,17, 19,22,23	0	
3984	1,3,5,6,18, 20,22,23	2,4,7,8,17, 19,21,24	5	P02783,P04355,P62804,Q66H69,Q9QX74
3985	1,3,5,6,18, 20,22,24	2,4,7,8,17, 19,21,23	5	P20646,P22006,P62804,Q9QX74,Q9Z0V6
3986	1,3,5,6,18, 20,23,24	2,4,7,8,17, 19,21,22	3	P20646,P62804,Q9QX74
3987	1,3,5,6,18, 21,22,23	2,4,7,8,17, 19,20,24	14	D4A5U3,P01946,P02783,P08723,P13676,P19223,P20646,P36374,P47727,P54921,P82471,Q4G075,Q6TMA8,Q9WTT6
3988	1,3,5,6,18, 21,22,24	2,4,7,8,17, 19,20,23	6	D4A5U3,P01946,P19223,P20646,Q4G075,Q9WTT6
3989	1,3,5,6,18, 21,23,24	2,4,7,8,17, 19,20,22	5	D4A5U3,P19223,P20646,Q4G075,Q9WTT6
3990	1,3,5,6,18, 22,23,24	2,4,7,8,17, 19,20,21	23	D4A5U3,P01946,P02783,P06760,P07150,P13676,P19223,P20646,P22273,P22282,P24368,P25809,P36374,P47727,P62804,P97675,Q00715,Q4G075,Q63617,Q66H69,Q6P6R2,Q9QX74,Q9WTT6

3991	1,3,5,6,19,20,21,22	2,4,7,8,17,18,23,24	4	P01946,P02091,P19218,Q5PQL7
3992	1,3,5,6,19,20,21,23	2,4,7,8,17,18,22,24	1	Q9Z1F2
3993	1,3,5,6,19,20,21,24	2,4,7,8,17,18,22,23	1	P20762
3994	1,3,5,6,19,20,22,23	2,4,7,8,17,18,21,24	7	P01946,P02091,P02783,P19218,P62804,Q00715,Q9Z1F2
3995	1,3,5,6,19,20,22,24	2,4,7,8,17,18,21,23	5	P01946,P22006,P62804,Q9QX74,Q9Z0V6
3996	1,3,5,6,19,20,23,24	2,4,7,8,17,18,21,22	5	P23739,P62804,Q00715,Q9QX74,Q9Z1F2
3997	1,3,5,6,19,21,22,23	2,4,7,8,17,18,20,24	9	D4A5U3,P01946,P02091,P02783,P19223,P25809,P47727,Q4G075,Q811M5
3998	1,3,5,6,19,21,22,24	2,4,7,8,17,18,20,23	8	D4A5U3,P01946,P02091,P19223,P25809,P30919,P55091,Q4G075
3999	1,3,5,6,19,21,23,24	2,4,7,8,17,18,20,22	5	D4A5U3,P19223,P25809,Q4G075,Q9Z0J6
4000	1,3,5,6,19,22,23,24	2,4,7,8,17,18,20,21	18	D4A5U3,P00507,P01946,P02091,P02783,P13676,P19223,P22273,P25809,P47727,P55091,P62804,Q00715,Q4G075,Q6P6R2,Q811M5,Q9QX74,Q9WTT6
4001	1,3,5,6,20,21,22,23	2,4,7,8,17,18,19,24	3	P01946,P02783,P09656
4002	1,3,5,6,20,21,22,24	2,4,7,8,17,18,19,23	1	P01946
4003	1,3,5,6,20,21,23,24	2,4,7,8,17,18,19,22	2	P09656,P20646
4004	1,3,5,6,20,22,23,24	2,4,7,8,17,18,19,21	12	P01946,P02783,P07150,P09656,P20646,P22006,P23739,P62804,Q00715,Q66H69,Q6P6R2,Q9QX74
4005	1,3,5,6,21,22,23,24	2,4,7,8,17,18,19,20	14	D4A5U3,P01946,P02783,P07150,P09656,P13676,P19223,P20646,P25809,P30919,P47727,Q4G075,Q62714,Q9WTT6
4006	1,3,5,7,8,17,18,19	2,4,6,20,21,22,23,24	3	P01681,P22006,Q9QX74
4007	1,3,5,7,8,17,18,20	2,4,6,19,21,22,23,24	3	P13676,P47967,Q811M5
4008	1,3,5,7,8,17,18,21	2,4,6,19,20,22,23,24	4	P06760,P22006,Q811M5,Q9QX74
4009	1,3,5,7,8,17,18,22	2,4,6,19,20,21,23,24	1	P35280
4010	1,3,5,7,8,17,18,23	2,4,6,19,20,21,22,24	2	O70594,P22006

4011	1,3,5,7,8,17,18,24	2,4,6,19,20,21,22,23	2	P49134,Q811M5
4012	1,3,5,7,8,17,19,20	2,4,6,18,21,22,23,24	6	P13676,P47967,Q5I0D1,Q6IMF3,Q811M5,Q9WTT6
4013	1,3,5,7,8,17,19,21	2,4,6,18,20,22,23,24	8	P06760,P22006,Q4FZU2,Q66H69,Q6IFW6,Q6IMF3,Q811M5,Q9QX74
4014	1,3,5,7,8,17,19,22	2,4,6,18,20,21,23,24	2	P01681,P35280
4015	1,3,5,7,8,17,19,23	2,4,6,18,20,21,22,24	3	P01681,P21674,P22006
4016	1,3,5,7,8,17,19,24	2,4,6,18,20,21,22,23	4	P01681,P54921,Q4FZU2,Q6IMF3
4017	1,3,5,7,8,17,20,21	2,4,6,18,19,22,23,24	13	D3ZUC6,P06760,P13676,P22006,P47967,Q4FZU2,Q6IFW6,Q6IMF3,Q6P6Q2,Q811M5,Q9EQS0,Q9QX74,Q9WTT6
4018	1,3,5,7,8,17,20,22	2,4,6,18,19,21,23,24	1	P35280
4019	1,3,5,7,8,17,20,23	2,4,6,18,19,21,22,24	2	P23739,Q6IMF3
4020	1,3,5,7,8,17,20,24	2,4,6,18,19,21,22,23	6	P13676,Q4FZU2,Q6IFW6,Q6IMF3,Q6P6Q2,Q811M5
4021	1,3,5,7,8,17,21,22	2,4,6,18,19,20,23,24	3	P35280,Q6IFW6,Q6IMF3
4022	1,3,5,7,8,17,21,23	2,4,6,18,19,20,22,24	4	P22006,Q4FZU2,Q6IFW6,Q6IMF3
4023	1,3,5,7,8,17,21,24	2,4,6,18,19,20,22,23	8	P04762,Q4FZU2,Q6AY61,Q6IFW6,Q6IMF3,Q6P6Q2,Q80WL1,Q811M5
4024	1,3,5,7,8,17,22,23	2,4,6,18,19,20,21,24	2	P35280,Q68G31
4025	1,3,5,7,8,17,22,24	2,4,6,18,19,20,21,23	2	P35280,Q6IFW6
4026	1,3,5,7,8,17,23,24	2,4,6,18,19,20,21,22	4	Q4FZU2,Q6IFW6,Q6IMF3,Q80WL1
4027	1,3,5,7,8,18,19,20	2,4,6,17,21,22,23,24	5	D3ZUC6,P47967,P97840,Q5I0D1,Q9R168
4028	1,3,5,7,8,18,19,21	2,4,6,17,20,22,23,24	6	D3ZUC6,O35077,P06760,P08937,P22006,P47967
4029	1,3,5,7,8,18,19,22	2,4,6,17,20,21,23,24	3	O35077,P19223,P35280
4030	1,3,5,7,8,18,19,23	2,4,6,17,20,21,22,24	1	O35077

4031	1,3,5,7,8,18,19,24	2,4,6,17,20,21,22,23	1	P19223
4032	1,3,5,7,8,18,20,21	2,4,6,17,19,22,23,24	6	D3ZUC6,P06760,P47967,P97840,Q5I0D1,Q811M5
4033	1,3,5,7,8,18,20,22	2,4,6,17,19,21,23,24	2	P35280,P47967
4034	1,3,5,7,8,18,20,23	2,4,6,17,19,21,22,24	3	D3ZUC6,P23739,Q9R168
4035	1,3,5,7,8,18,20,24	2,4,6,17,19,21,22,23	3	P47967,Q811M5,Q9R168
4036	1,3,5,7,8,18,21,22	2,4,6,17,19,20,23,24	1	P35280
4037	1,3,5,7,8,18,21,23	2,4,6,17,19,20,22,24	4	D3ZUC6,O35077,O89117,P22006
4038	1,3,5,7,8,18,21,24	2,4,6,17,19,20,22,23	3	O89117,P49134,Q6IFW6
4039	1,3,5,7,8,18,22,23	2,4,6,17,19,20,21,24	3	P19223,P35280,P49134
4040	1,3,5,7,8,18,22,24	2,4,6,17,19,20,21,23	3	P19223,P35280,P49134
4041	1,3,5,7,8,18,23,24	2,4,6,17,19,20,21,22	2	P19223,P49134
4042	1,3,5,7,8,19,20,21	2,4,6,17,18,22,23,24	13	D3ZUC6,P06760,P08649,P08937,P47967,P97840,Q5I0D1,Q5RKI1,Q6IFW6,Q6IMF3,Q811M5,Q9EQS0,Q9WTT6
4043	1,3,5,7,8,19,20,22	2,4,6,17,18,21,23,24	4	P19218,P35280,P47967,Q5I0D1
4044	1,3,5,7,8,19,20,23	2,4,6,17,18,21,22,24	4	D3ZUC6,P23739,P47967,Q5I0D1
4045	1,3,5,7,8,19,20,24	2,4,6,17,18,21,22,23	4	P47967,Q5I0D1,Q6IFW6,Q6IMF3
4046	1,3,5,7,8,19,21,22	2,4,6,17,18,20,23,24	5	O35077,P08937,P35280,P47967,Q9QYP1
4047	1,3,5,7,8,19,21,23	2,4,6,17,18,20,22,24	6	D3ZUC6,O35077,P08649,P22006,Q6IMF3,Q9Z0J6
4048	1,3,5,7,8,19,21,24	2,4,6,17,18,20,22,23	5	P08649,P55091,Q6IFW6,Q6IMF3,Q9WU74
4049	1,3,5,7,8,19,22,23	2,4,6,17,18,20,21,24	3	O35077,P19223,P35280
4050	1,3,5,7,8,19,22,24	2,4,6,17,18,20,21,23	3	P19223,P35280,P55091

4051	1,3,5,7,8,19,23,24	2,4,6,17,18,20,21,22	4	P19223,P55091,Q80WL1,Q9Z0J6
4052	1,3,5,7,8,20,21,22	2,4,6,17,18,19,23,24	5	D3ZUC6,P08649,P35280,P47967,Q6IFW6
4053	1,3,5,7,8,20,21,23	2,4,6,17,18,19,22,24	7	D3ZUC6,P08649,P19132,P23739,P47967,Q6IFW6,Q6IMF3
4054	1,3,5,7,8,20,21,24	2,4,6,17,18,19,22,23	10	D3ZUC6,P08649,P47967,Q4FZU2,Q6IFW6,Q6IMF3,Q6P6Q2,Q811M5,Q9EQS0,Q9WU74
4055	1,3,5,7,8,20,22,23	2,4,6,17,18,19,21,24	2	P23739,P35280
4056	1,3,5,7,8,20,22,24	2,4,6,17,18,19,21,23	2	P35280,Q6IFW6
4057	1,3,5,7,8,20,23,24	2,4,6,17,18,19,21,22	4	P08649,P23739,Q6IFW6,Q6IMF3
4058	1,3,5,7,8,21,22,23	2,4,6,17,18,19,20,24	4	O35077,P08649,P35280,Q62714
4059	1,3,5,7,8,21,22,24	2,4,6,17,18,19,20,23	6	P08649,P35280,P55091,Q62714,Q6IFW6,Q6IMF3
4060	1,3,5,7,8,21,23,24	2,4,6,17,18,19,20,22	6	P08649,Q4FZU2,Q62714,Q6IFW6,Q6IMF3,Q80WL1
4061	1,3,5,7,8,22,23,24	2,4,6,17,18,19,20,21	8	P19223,P25809,P35280,P49134,P55091,Q62714,Q6P6R2,Q80WL1
4062	1,3,5,7,17,18,19,20	2,4,6,8,21,22,23,24	5	P30919,P47967,P97840,Q63532,Q811M5
4063	1,3,5,7,17,18,19,21	2,4,6,8,20,22,23,24	3	P22006,P47967,P97840
4064	1,3,5,7,17,18,19,22	2,4,6,8,20,21,23,24	0	
4065	1,3,5,7,17,18,19,23	2,4,6,8,20,21,22,24	1	Q10743
4066	1,3,5,7,17,18,19,24	2,4,6,8,20,21,22,23	2	P54921,Q63532
4067	1,3,5,7,17,18,20,21	2,4,6,8,19,22,23,24	5	P00507,P47967,P97840,Q63532,Q811M5
4068	1,3,5,7,17,18,20,22	2,4,6,8,19,21,23,24	2	P97840,Q63474
4069	1,3,5,7,17,18,20,23	2,4,6,8,19,21,22,24	1	P30919
4070	1,3,5,7,17,18,20,24	2,4,6,8,19,21,22,23	2	Q63532,Q811M5

4071	1,3,5,7,17, 18,21,22	2,4,6,8,19, 20,23,24	0	
4072	1,3,5,7,17, 18,21,23	2,4,6,8,19, 20,22,24	1	P22006
4073	1,3,5,7,17, 18,21,24	2,4,6,8,19, 20,22,23	3	P04762,Q6AY61,Q811M5
4074	1,3,5,7,17, 18,22,23	2,4,6,8,19, 20,21,24	3	P30120,P36374,Q68G31
4075	1,3,5,7,17, 18,22,24	2,4,6,8,19, 20,21,23	1	P02631
4076	1,3,5,7,17, 18,23,24	2,4,6,8,19, 20,21,22	0	
4077	1,3,5,7,17, 19,20,21	2,4,6,8,18, 22,23,24	8	O54858,P20762,P47967,P97840,Q5I0D1,Q63532,Q6AY61,Q811M5
4078	1,3,5,7,17, 19,20,22	2,4,6,8,18, 21,23,24	0	
4079	1,3,5,7,17, 19,20,23	2,4,6,8,18, 21,22,24	1	P23739
4080	1,3,5,7,17, 19,20,24	2,4,6,8,18, 21,22,23	3	P54921,Q63532,Q6AY61
4081	1,3,5,7,17, 19,21,22	2,4,6,8,18, 20,23,24	3	P01946,P02091,Q9R168
4082	1,3,5,7,17, 19,21,23	2,4,6,8,18, 20,22,24	2	P12020,P22006
4083	1,3,5,7,17, 19,21,24	2,4,6,8,18, 20,22,23	3	P12020,P54921,Q6AY61
4084	1,3,5,7,17, 19,22,23	2,4,6,8,18, 20,21,24	2	P02091,P02631
4085	1,3,5,7,17, 19,22,24	2,4,6,8,18, 20,21,23	2	P02631,P54921
4086	1,3,5,7,17, 19,23,24	2,4,6,8,18, 20,21,22	5	O55145,P02631,P12020,P25809,P54921
4087	1,3,5,7,17, 20,21,22	2,4,6,8,18, 19,23,24	1	P47967
4088	1,3,5,7,17, 20,21,23	2,4,6,8,18, 19,22,24	1	P08649
4089	1,3,5,7,17, 20,21,24	2,4,6,8,18, 19,22,23	6	P04762,P08649,P20762,Q63532,Q6AY61,Q811M5
4090	1,3,5,7,17, 20,22,23	2,4,6,8,18, 19,21,24	2	P23739,Q68G31

4091	1,3,5,7,17, 20,22,24	2,4,6,8,18, 19,21,23	0	
4092	1,3,5,7,17, 20,23,24	2,4,6,8,18, 19,21,22	1	P23739
4093	1,3,5,7,17, 21,22,23	2,4,6,8,18, 19,20,24	3	P50280,Q68G31,Q9R168
4094	1,3,5,7,17, 21,22,24	2,4,6,8,18, 19,20,23	2	P50280,Q9R168
4095	1,3,5,7,17, 21,23,24	2,4,6,8,18, 19,20,22	5	P04762,P08649,P12020,P50280,Q6AY61
4096	1,3,5,7,17, 22,23,24	2,4,6,8,18, 19,20,21	6	P02631,P22273,P25809,P50280,Q00715,Q68G31
4097	1,3,5,7,18, 19,20,21	2,4,6,8,17, 22,23,24	6	D3ZUC6,P07335,P47967,P97840,Q5I0D1,Q63532
4098	1,3,5,7,18, 19,20,22	2,4,6,8,17, 21,23,24	3	P47967,P97840,Q63474
4099	1,3,5,7,18, 19,20,23	2,4,6,8,17, 21,22,24	2	P47967,P97840
4100	1,3,5,7,18, 19,20,24	2,4,6,8,17, 21,22,23	3	P47967,P97840,Q63532
4101	1,3,5,7,18, 19,21,22	2,4,6,8,17, 20,23,24	3	P17559,P47967,P97840
4102	1,3,5,7,18, 19,21,23	2,4,6,8,17, 20,22,24	3	P12020,P22006,P97840
4103	1,3,5,7,18, 19,21,24	2,4,6,8,17, 20,22,23	4	P17559,P47967,P55091,P97840
4104	1,3,5,7,18, 19,22,23	2,4,6,8,17, 20,21,24	3	D4A5U3,P02631,P19223
4105	1,3,5,7,18, 19,22,24	2,4,6,8,17, 20,21,23	5	D4A5U3,P02631,P17559,P19223,P55091
4106	1,3,5,7,18, 19,23,24	2,4,6,8,17, 20,21,22	6	D4A5U3,P02631,P19223,P25809,P55091,P98089
4107	1,3,5,7,18, 20,21,22	2,4,6,8,17, 19,23,24	2	P47967,P97840
4108	1,3,5,7,18, 20,21,23	2,4,6,8,17, 19,22,24	3	D3ZUC6,P47967,P97840
4109	1,3,5,7,18, 20,21,24	2,4,6,8,17, 19,22,23	3	P47967,P97840,Q811M5
4110	1,3,5,7,18, 20,22,23	2,4,6,8,17, 19,21,24	2	P97840,Q63474

4111	1,3,5,7,18, 20,22,24	2,4,6,8,17, 19,21,23	1	P97840
4112	1,3,5,7,18, 20,23,24	2,4,6,8,17, 19,21,22	0	
4113	1,3,5,7,18, 21,22,23	2,4,6,8,17, 19,20,24	3	D4A5U3,P36374,Q68G31
4114	1,3,5,7,18, 21,22,24	2,4,6,8,17, 19,20,23	2	P17559,P55091
4115	1,3,5,7,18, 21,23,24	2,4,6,8,17, 19,20,22	2	D4A5U3,O89117
4116	1,3,5,7,18, 22,23,24	2,4,6,8,17, 19,20,21	8	D4A5U3,P02631,P19223,P25809,P36374,P55091,P97675,Q6P6R2
4117	1,3,5,7,19, 20,21,22	2,4,6,8,17, 18,23,24	4	P08649,P47967,P97840,Q5I0D1
4118	1,3,5,7,19, 20,21,23	2,4,6,8,17, 18,22,24	5	D3ZUC6,P08649,P47967,P97840,Q5I0D1
4119	1,3,5,7,19, 20,21,24	2,4,6,8,17, 18,22,23	7	P08649,P47967,P97840,Q5I0D1,Q63532,Q6AY61,Q9WU74
4120	1,3,5,7,19, 20,22,23	2,4,6,8,17, 18,21,24	2	P08649,P23739
4121	1,3,5,7,19, 20,22,24	2,4,6,8,17, 18,21,23	1	P08649
4122	1,3,5,7,19, 20,23,24	2,4,6,8,17, 18,21,22	2	P08649,P23739
4123	1,3,5,7,19, 21,22,23	2,4,6,8,17, 18,20,24	3	P08649,P12020,P55091
4124	1,3,5,7,19, 21,22,24	2,4,6,8,17, 18,20,23	5	P08649,P12020,P17559,P19223,P55091
4125	1,3,5,7,19, 21,23,24	2,4,6,8,17, 18,20,22	6	P08649,P12020,P17559,P25809,P55091,Q9Z0J6
4126	1,3,5,7,19, 22,23,24	2,4,6,8,17, 18,20,21	11	D4A5U3,P02631,P08649,P12020,P17559,P19223,P22273,P25809,P55091,P98089,Q00715
4127	1,3,5,7,20, 21,22,23	2,4,6,8,17, 18,19,24	2	P08649,P97840
4128	1,3,5,7,20, 21,22,24	2,4,6,8,17, 18,19,23	2	P08649,P47967
4129	1,3,5,7,20, 21,23,24	2,4,6,8,17, 18,19,22	2	P08649,P23739
4130	1,3,5,7,20, 22,23,24	2,4,6,8,17, 18,19,21	2	P08649,P23739

4131	1,3,5,7,21,22,23,24	2,4,6,8,17,18,19,20	8	D4A5U3,P08649,P12020,P19223,P25809,P50280,P55091,Q62714
4132	1,3,5,8,17,18,19,20	2,4,6,7,21,22,23,24	2	Q811M5,Q9R168
4133	1,3,5,8,17,18,19,21	2,4,6,7,20,22,23,24	2	P22006,Q9QX74
4134	1,3,5,8,17,18,19,22	2,4,6,7,20,21,23,24	1	P01835
4135	1,3,5,8,17,18,19,23	2,4,6,7,20,21,22,24	2	P01835,P22006
4136	1,3,5,8,17,18,19,24	2,4,6,7,20,21,22,23	1	P80299
4137	1,3,5,8,17,18,20,21	2,4,6,7,19,22,23,24	1	Q811M5
4138	1,3,5,8,17,18,20,22	2,4,6,7,19,21,23,24	1	P00714
4139	1,3,5,8,17,18,20,23	2,4,6,7,19,21,22,24	4	P00714,P23739,P36376,Q9R168
4140	1,3,5,8,17,18,20,24	2,4,6,7,19,21,22,23	3	P80299,Q811M5,Q9R168
4141	1,3,5,8,17,18,21,22	2,4,6,7,19,20,23,24	0	
4142	1,3,5,8,17,18,21,23	2,4,6,7,19,20,22,24	1	P22006
4143	1,3,5,8,17,18,21,24	2,4,6,7,19,20,22,23	4	P80299,Q6IFW6,Q80WL1,Q811M5
4144	1,3,5,8,17,18,22,23	2,4,6,7,19,20,21,24	4	P00714,P11598,P22283,Q63617
4145	1,3,5,8,17,18,22,24	2,4,6,7,19,20,21,23	3	P11598,P80299,Q63617
4146	1,3,5,8,17,18,23,24	2,4,6,7,19,20,21,22	5	P00714,P11598,P80299,Q63617,Q80WL1
4147	1,3,5,8,17,19,20,21	2,4,6,7,18,22,23,24	9	B0LT89,D3ZUC6,P06760,P13676,P22006,Q6IMF3,Q6P6R2,Q811M5,Q9EQS0
4148	1,3,5,8,17,19,20,22	2,4,6,7,18,21,23,24	1	O89117
4149	1,3,5,8,17,19,20,23	2,4,6,7,18,21,22,24	2	P23739,P36376
4150	1,3,5,8,17,19,20,24	2,4,6,7,18,21,22,23	1	Q6IMF3

4151	1,3,5,8,17, 19,21,22	2,4,6,7,18, 20,23,24	3	P01946,P02091,Q9QYP1
4152	1,3,5,8,17, 19,21,23	2,4,6,7,18, 20,22,24	3	P22006,Q80WL1,Q9Z0J6
4153	1,3,5,8,17, 19,21,24	2,4,6,7,18, 20,22,23	5	P80299,Q4FZU2,Q6IFW6,Q6IMF3,Q80WL1
4154	1,3,5,8,17, 19,22,23	2,4,6,7,18, 20,21,24	4	P01681,P02091,P11598,Q80WL1
4155	1,3,5,8,17, 19,22,24	2,4,6,7,18, 20,21,23	3	P02091,P11598,Q80WL1
4156	1,3,5,8,17, 19,23,24	2,4,6,7,18, 20,21,22	5	O55145,P11598,P80299,Q80WL1,Q9Z0J6
4157	1,3,5,8,17, 20,21,22	2,4,6,7,18, 19,23,24	1	P20766
4158	1,3,5,8,17, 20,21,23	2,4,6,7,18, 19,22,24	5	P19132,P22006,P23739,Q6IFW6,Q6IMF3
4159	1,3,5,8,17, 20,21,24	2,4,6,7,18, 19,22,23	9	P80299,Q4FZU2,Q6IFW6,Q6IG02,Q6IMF3,Q6P6Q2,Q80WL1,Q811M5,Q9EQS0
4160	1,3,5,8,17, 20,22,23	2,4,6,7,18, 19,21,24	3	P00714,P23739,P36376
4161	1,3,5,8,17, 20,22,24	2,4,6,7,18, 19,21,23	0	
4162	1,3,5,8,17, 20,23,24	2,4,6,7,18, 19,21,22	5	P23739,P80299,Q6IFW6,Q6IMF3,Q80WL1
4163	1,3,5,8,17, 21,22,23	2,4,6,7,18, 19,20,24	2	P11598,Q80WL1
4164	1,3,5,8,17, 21,22,24	2,4,6,7,18, 19,20,23	3	P80299,Q6IFW6,Q80WL1
4165	1,3,5,8,17, 21,23,24	2,4,6,7,18, 19,20,22	5	P80299,Q4FZU2,Q6IFW6,Q6IMF3,Q80WL1
4166	1,3,5,8,17, 22,23,24	2,4,6,7,18, 19,20,21	7	P00714,P11598,P22283,P62804,Q00715,Q63617,Q80WL1
4167	1,3,5,8,18, 19,20,21	2,4,6,7,17, 22,23,24	5	D3ZUC6,P01039,P47967,Q5I0D1,Q9R168
4168	1,3,5,8,18, 19,20,22	2,4,6,7,17, 21,23,24	2	P01039,Q9R168
4169	1,3,5,8,18, 19,20,23	2,4,6,7,17, 21,22,24	3	P01039,P23739,Q9R168
4170	1,3,5,8,18, 19,20,24	2,4,6,7,17, 21,22,23	1	Q9R168

4171	1,3,5,8,18, 19,21,22	2,4,6,7,17, 20,23,24	1	Q9QYP1
4172	1,3,5,8,18, 19,21,23	2,4,6,7,17, 20,22,24	4	O35077,P01039,P22006,Q9Z0J6
4173	1,3,5,8,18, 19,21,24	2,4,6,7,17, 20,22,23	2	P55091,P80299
4174	1,3,5,8,18, 19,22,23	2,4,6,7,17, 20,21,24	3	P01039,P19223,Q9Z0J6
4175	1,3,5,8,18, 19,22,24	2,4,6,7,17, 20,21,23	2	P19223,P55091
4176	1,3,5,8,18, 19,23,24	2,4,6,7,17, 20,21,22	3	P19223,P55091,Q9Z0J6
4177	1,3,5,8,18, 20,21,22	2,4,6,7,17, 19,23,24	1	P01039
4178	1,3,5,8,18, 20,21,23	2,4,6,7,17, 19,22,24	3	D3ZUC6,P01039,Q9R168
4179	1,3,5,8,18, 20,21,24	2,4,6,7,17, 19,22,23	4	P80299,Q6IFW6,Q811M5,Q9R168
4180	1,3,5,8,18, 20,22,23	2,4,6,7,17, 19,21,24	3	P00714,P01039,Q9R168
4181	1,3,5,8,18, 20,22,24	2,4,6,7,17, 19,21,23	1	Q9R168
4182	1,3,5,8,18, 20,23,24	2,4,6,7,17, 19,21,22	3	P23739,Q5QE79,Q9R168
4183	1,3,5,8,18, 21,22,23	2,4,6,7,17, 19,20,24	2	P01039,P54921
4184	1,3,5,8,18, 21,22,24	2,4,6,7,17, 19,20,23	1	P55091
4185	1,3,5,8,18, 21,23,24	2,4,6,7,17, 19,20,22	4	O89117,P80299,Q80WL1,Q9Z0J6
4186	1,3,5,8,18, 22,23,24	2,4,6,7,17, 19,20,21	9	P00714,P11598,P19223,P36374,P55091,P62804,Q63617,Q6P6R2,Q9R0T3
4187	1,3,5,8,19, 20,21,22	2,4,6,7,17, 18,23,24	3	O88797,P01039,P19218
4188	1,3,5,8,19, 20,21,23	2,4,6,7,17, 18,22,24	3	D3ZUC6,P01039,P23739
4189	1,3,5,8,19, 20,21,24	2,4,6,7,17, 18,22,23	3	Q6IFW6,Q6IMF3,Q9EQS0
4190	1,3,5,8,19, 20,22,23	2,4,6,7,17, 18,21,24	4	P01039,P19218,P23739,Q9WVH8

4191	1,3,5,8,19,<br>20,22,24	2,4,6,7,17,<br>18,21,23	0	
4192	1,3,5,8,19,<br>20,23,24	2,4,6,7,17,<br>18,21,22	2	P23739,Q80WL1
4193	1,3,5,8,19,<br>21,22,23	2,4,6,7,17,<br>18,20,24	6	O35077,P01039,P19223,P55091,Q80WL1,Q9Z0J6
4194	1,3,5,8,19,<br>21,22,24	2,4,6,7,17,<br>18,20,23	4	P19223,P30919,P55091,Q80WL1
4195	1,3,5,8,19,<br>21,23,24	2,4,6,7,17,<br>18,20,22	4	P19223,P55091,Q80WL1,Q9Z0J6
4196	1,3,5,8,19,<br>22,23,24	2,4,6,7,17,<br>18,20,21	8	P00507,P11598,P19223,P25809,P55091,Q00715,Q80WL1,Q9Z0J6
4197	1,3,5,8,20,<br>21,22,23	2,4,6,7,17,<br>18,19,24	2	P01039,P23739
4198	1,3,5,8,20,<br>21,22,24	2,4,6,7,17,<br>18,19,23	1	Q6IFW6
4199	1,3,5,8,20,<br>21,23,24	2,4,6,7,17,<br>18,19,22	5	P08649,P23739,Q6IFW6,Q6IMF3,Q80WL1
4200	1,3,5,8,20,<br>22,23,24	2,4,6,7,17,<br>18,19,21	4	P23739,Q00715,Q5QE79,Q80WL1
4201	1,3,5,8,21,<br>22,23,24	2,4,6,7,17,<br>18,19,20	6	P11598,P19223,P30919,P55091,Q62714,Q80WL1
4202	1,3,5,17,1<br>8,19,20,21	2,4,6,7,8,2<br>2,23,24	5	B0LT89,O54858,P20761,Q63532,Q6P6R2
4203	1,3,5,17,1<br>8,19,20,22	2,4,6,7,8,2<br>1,23,24	6	O54858,P00714,P02631,P20761,Q63474,Q63532
4204	1,3,5,17,1<br>8,19,20,23	2,4,6,7,8,2<br>1,22,24	4	O54858,P00714,P02631,Q99041
4205	1,3,5,17,1<br>8,19,20,24	2,4,6,7,8,2<br>1,22,23	5	O54858,P02631,P20761,Q63532,Q99041
4206	1,3,5,17,1<br>8,19,21,22	2,4,6,7,8,2<br>0,23,24	7	P01946,P02091,P12020,P20761,P22273,P50280,Q4G075
4207	1,3,5,17,1<br>8,19,21,23	2,4,6,7,8,2<br>0,22,24	7	P02780,P12020,P22006,P22273,P50280,Q4G075,Q99041
4208	1,3,5,17,1<br>8,19,21,24	2,4,6,7,8,2<br>0,22,23	8	O54858,P12020,P20761,P22273,P50280,Q4G075,Q63532,Q99041
4209	1,3,5,17,1<br>8,19,22,23	2,4,6,7,8,2<br>0,21,24	19	D4A5U3,P00714,P02091,P02631,P02780,P06761,P08723,P09456,P11598,P12020,P16228,P2227<br>3,P22282,P50280,Q00715,Q4G075,Q5M8C6,Q63617,Q812E4
4210	1,3,5,17,1<br>8,19,22,24	2,4,6,7,8,2<br>0,21,23	10	D4A5U3,P00714,P02631,P02780,P11598,P20761,P22273,P50280,Q4G075,Q63617

4211	1,3,5,17,18,19,23,24	2,4,6,7,8,20,21,22	12	D4A5U3,P00714,P02631,P02780,P11598,P12020,P22273,P22282,P50280,Q4G075,Q63617,Q99041
4212	1,3,5,17,18,20,21,22	2,4,6,7,8,19,23,24	3	O54858,P00714,P20761
4213	1,3,5,17,18,20,21,23	2,4,6,7,8,19,22,24	3	O54858,P00714,Q99041
4214	1,3,5,17,18,20,21,24	2,4,6,7,8,19,22,23	6	O54858,P20761,P42854,Q63532,Q811M5,Q99041
4215	1,3,5,17,18,20,22,23	2,4,6,7,8,19,21,24	5	P00714,P02631,P22282,Q63474,Q63617
4216	1,3,5,17,18,20,22,24	2,4,6,7,8,19,21,23	5	P00714,P02631,P20761,P22282,P50115
4217	1,3,5,17,18,20,23,24	2,4,6,7,8,19,21,22	5	P00714,P02631,P22282,P23739,Q99041
4218	1,3,5,17,18,21,22,23	2,4,6,7,8,19,20,24	18	D4A5U3,P00714,P02780,P06761,P08723,P0C0A9,P11598,P12020,P22273,P22282,P36374,P46462,P50280,P82471,Q4G075,Q63617,Q6TMA8,Q9R0T3
4219	1,3,5,17,18,21,22,24	2,4,6,7,8,19,20,23	11	D4A5U3,P02780,P10758,P11598,P12020,P22273,P22282,P42854,P50280,Q4G075,Q63617
4220	1,3,5,17,18,21,23,24	2,4,6,7,8,19,20,22	12	D4A5U3,P00714,P02780,P06761,P11598,P12020,P22273,P22282,P50280,Q4G075,Q63617,Q99041
4221	1,3,5,17,18,22,23,24	2,4,6,7,8,19,20,21	24	D4A5U3,P00714,P02631,P02780,P06761,P0C0A9,P11598,P12020,P22273,P22282,P22283,P36374,P46462,P50280,P62804,Q00715,Q4G075,Q5M8C6,Q63617,Q812E4,Q99041,Q9JI85,Q9R0T3,Q9WTT6
4222	1,3,5,17,19,20,21,22	2,4,6,7,8,18,23,24	6	O54858,P01946,P02091,P20761,P20762,Q3ZAV1
4223	1,3,5,17,19,20,21,23	2,4,6,7,8,18,22,24	4	O54858,P20762,P23739,Q99041
4224	1,3,5,17,19,20,21,24	2,4,6,7,8,18,22,23	7	O54858,P20761,P20762,P54921,Q63532,Q6AY61,Q99041
4225	1,3,5,17,19,20,22,23	2,4,6,7,8,18,21,24	6	O55004,P00714,P02091,P02631,P23739,Q00715
4226	1,3,5,17,19,20,22,24	2,4,6,7,8,18,21,23	5	P02631,P20761,P20762,Q00715,Q63532
4227	1,3,5,17,19,20,23,24	2,4,6,7,8,18,21,22	4	P02631,P23739,Q00715,Q99041
4228	1,3,5,17,19,21,22,23	2,4,6,7,8,18,20,24	12	O55004,P01946,P02091,P02780,P06911,P11598,P12020,P22273,P22282,P50280,Q4G075,Q5GRG2
4229	1,3,5,17,19,21,22,24	2,4,6,7,8,18,20,23	10	P01946,P02091,P11598,P12020,P17559,P20762,P22273,P50280,Q4G075,Q80WL1
4230	1,3,5,17,19,21,23,24	2,4,6,7,8,18,20,22	10	P02780,P06911,P11598,P12020,P22273,P50280,Q4G075,Q5GRG2,Q80WL1,Q99041

4231	1,3,5,17,19,22,23,24	2,4,6,7,8,18,20,21	20	D4A5U3,O55004,P00714,P01946,P02091,P02631,P02780,P06761,P11598,P12020,P22273,P22282,P25809,P50280,P62804,Q00715,Q4G075,Q5GRG2,Q63617,Q80WL1
4232	1,3,5,17,20,21,22,23	2,4,6,7,8,18,19,24	3	P00714,P20766,P22282
4233	1,3,5,17,20,21,22,24	2,4,6,7,8,18,19,23	3	P20761,P20762,P20766
4234	1,3,5,17,20,21,23,24	2,4,6,7,8,18,19,22	3	P20762,P23739,Q99041
4235	1,3,5,17,20,22,23,24	2,4,6,7,8,18,19,21	12	O55004,P00714,P02631,P06761,P07150,P11598,P22273,P22282,P23739,P62804,Q00715,Q66H69
4236	1,3,5,17,21,22,23,24	2,4,6,7,8,18,19,20	19	D4A5U3,O55004,P00714,P01946,P02780,P06761,P06911,P07150,P0C0A9,P11598,P12020,P22273,P22282,P50280,Q4G075,Q5GRG2,Q63617,Q80WL1,Q9R0T3
4237	1,3,5,18,19,20,21,22	2,4,6,7,8,17,23,24	4	P01039,P20761,P47967,P97840
4238	1,3,5,18,19,20,21,23	2,4,6,7,8,17,22,24	3	O54858,P01039,P23593
4239	1,3,5,18,19,20,21,24	2,4,6,7,8,17,22,23	3	O54858,P20761,Q63532
4240	1,3,5,18,19,20,22,23	2,4,6,7,8,17,21,24	5	P00714,P01039,P02631,P36860,Q63474
4241	1,3,5,18,19,20,22,24	2,4,6,7,8,17,21,23	4	P02631,P20761,P50115,Q63474
4242	1,3,5,18,19,20,23,24	2,4,6,7,8,17,21,22	3	P02631,P23739,Q99041
4243	1,3,5,18,19,21,22,23	2,4,6,7,8,17,20,24	12	D4A5U3,P01039,P02780,P06911,P12020,P19223,P22273,P22282,P50280,P55091,Q4G075,Q5GRG2
4244	1,3,5,18,19,21,22,24	2,4,6,7,8,17,20,23	9	D4A5U3,P06911,P12020,P17559,P19223,P22273,P55091,Q4G075,Q5GRG2
4245	1,3,5,18,19,21,23,24	2,4,6,7,8,17,20,22	13	D4A5U3,P02780,P06911,P12020,P17559,P19223,P22273,P50280,P55091,Q4G075,Q5GRG2,Q99041,Q9Z0J6
4246	1,3,5,18,19,22,23,24	2,4,6,7,8,17,20,21	24	D4A5U3,P00714,P02631,P02780,P06761,P06911,P11598,P12020,P13676,P17559,P19223,P22273,P22282,P25809,P50280,P55091,P62804,P98089,Q00715,Q4G075,Q5GRG2,Q63617,Q9R0T3,Q9WTT6
4247	1,3,5,18,20,21,22,23	2,4,6,7,8,17,19,24	4	P00714,P01039,P22282,P50115
4248	1,3,5,18,20,21,22,24	2,4,6,7,8,17,19,23	2	P20761,P50115
4249	1,3,5,18,20,21,23,24	2,4,6,7,8,17,19,22	2	P50115,Q99041
4250	1,3,5,18,20,22,23,24	2,4,6,7,8,17,19,21	12	P00714,P02631,P06761,P07150,P20646,P22282,P50115,P62804,Q00715,Q5QE79,Q66H69,Q9QX74

4251	1,3,5,18,21,22,23,24	2,4,6,7,8,17,19,20	24	D4A5U3,P00714,P02780,P06761,P06911,P07150,P11598,P12020,P13676,P19223,P20646,P22273,P22282,P36374,P46462,P50115,P50280,P55091,Q4G075,Q5GRG2,Q63617,Q6TMA8,Q9R0T3,Q9WTT6
4252	1,3,5,19,20,21,22,23	2,4,6,7,8,17,18,24	2	P01039,P08649
4253	1,3,5,19,20,21,22,24	2,4,6,7,8,17,18,23	3	O70594,P08649,P20762
4254	1,3,5,19,20,21,23,24	2,4,6,7,8,17,18,22	3	P08649,P23739,Q99041
4255	1,3,5,19,20,22,23,24	2,4,6,7,8,17,18,21	8	P02631,P22273,P23739,P62804,Q00715,Q9QX74,Q9WVH8,Q9Z1F2
4256	1,3,5,19,21,22,23,24	2,4,6,7,8,17,18,20	20	D4A5U3,P02454,P02631,P02780,P06761,P06911,P08649,P11598,P12020,P17559,P19223,P22273,P22282,P25809,P50280,P55091,Q4G075,Q5GRG2,Q80WL1,Q9Z0J6
4257	1,3,5,20,21,22,23,24	2,4,6,7,8,17,18,19	5	P07150,P08649,P20766,P22282,P50115
4258	1,3,6,7,8,17,18,19	2,4,5,20,21,22,23,24	2	P21674,P70549
4259	1,3,6,7,8,17,18,20	2,4,5,19,21,22,23,24	5	O70417,P06911,P12020,P13432,Q5GRG2
4260	1,3,6,7,8,17,18,21	2,4,5,19,20,22,23,24	2	P05539,Q9QX74
4261	1,3,6,7,8,17,18,22	2,4,5,19,20,21,23,24	4	O70417,P01946,P02091,P35280
4262	1,3,6,7,8,17,18,23	2,4,5,19,20,21,22,24	3	O70594,P21674,Q63598
4263	1,3,6,7,8,17,18,24	2,4,5,19,20,21,22,23	1	Q63598
4264	1,3,6,7,8,17,19,20	2,4,5,18,21,22,23,24	8	O70417,O89117,P06911,P13676,Q5GRG2,Q9JI85,Q9QW07,Q9WTT6
4265	1,3,6,7,8,17,19,21	2,4,5,18,20,22,23,24	5	P05539,P21674,Q5QE79,Q63493,Q9QX74
4266	1,3,6,7,8,17,19,22	2,4,5,18,20,21,23,24	7	O70417,O89117,P01946,P02091,P35280,P70549,Q9QZK9
4267	1,3,6,7,8,17,19,23	2,4,5,18,20,21,22,24	2	P21674,P70549
4268	1,3,6,7,8,17,19,24	2,4,5,18,20,21,22,23	1	P70549
4269	1,3,6,7,8,17,20,21	2,4,5,18,19,22,23,24	10	P05539,P06911,P13676,P19629,P22283,Q5GRG2,Q5RKI1,Q63493,Q9JI85,Q9WTT6
4270	1,3,6,7,8,17,20,22	2,4,5,18,19,21,23,24	9	O70417,O89117,P01946,P02091,P06911,P12020,P19629,P35280,Q5GRG2

4271	1,3,6,7,8,17,20,23	2,4,5,18,19,21,22,24	4 O70417,P06911,P19629,Q5GRG2
4272	1,3,6,7,8,17,20,24	2,4,5,18,19,21,22,23	5 O70417,P06911,Q5GRG2,Q9JHB9,Q9QW07
4273	1,3,6,7,8,17,21,22	2,4,5,18,19,20,23,24	4 P01946,P02091,P05539,P35280
4274	1,3,6,7,8,17,21,23	2,4,5,18,19,20,22,24	1 P21674
4275	1,3,6,7,8,17,21,24	2,4,5,18,19,20,22,23	2 P04762,P05539
4276	1,3,6,7,8,17,22,23	2,4,5,18,19,20,21,24	4 P01946,P02091,P02783,P35280
4277	1,3,6,7,8,17,22,24	2,4,5,18,19,20,21,23	5 O70417,P01946,P02091,P35280,P70549
4278	1,3,6,7,8,17,23,24	2,4,5,18,19,20,21,22	2 P70549,Q63598
4279	1,3,6,7,8,18,19,20	2,4,5,17,21,22,23,24	9 P06911,P12020,P19629,P31044,Q5GRG2,Q63617,Q9JI85,Q9QW07,Q9R168
4280	1,3,6,7,8,18,19,21	2,4,5,17,20,22,23,24	3 P08937,P20759,Q63751
4281	1,3,6,7,8,18,19,22	2,4,5,17,20,21,23,24	1 P35280
4282	1,3,6,7,8,18,19,23	2,4,5,17,20,21,22,24	2 P21674,Q9Z0J6
4283	1,3,6,7,8,18,19,24	2,4,5,17,20,21,22,23	1 P70549
4284	1,3,6,7,8,18,20,21	2,4,5,17,19,22,23,24	7 P06911,P19629,P22283,P47967,Q5GRG2,Q5RKI1,Q9QW07
4285	1,3,6,7,8,18,20,22	2,4,5,17,19,21,23,24	7 O70417,P06911,P12020,P19629,P35280,Q5GRG2,Q9QW07
4286	1,3,6,7,8,18,20,23	2,4,5,17,19,21,22,24	5 P06911,P19629,Q62894,Q9QW07,Q9R168
4287	1,3,6,7,8,18,20,24	2,4,5,17,19,21,22,23	6 P06911,P12020,Q5GRG2,Q9JHB9,Q9QW07,Q9R168
4288	1,3,6,7,8,18,21,22	2,4,5,17,19,20,23,24	2 P23739,P35280
4289	1,3,6,7,8,18,21,23	2,4,5,17,19,20,22,24	0
4290	1,3,6,7,8,18,21,24	2,4,5,17,19,20,22,23	1 P02625

4291	1,3,6,7,8,18,22,23	2,4,5,17,19,20,21,24	2	P02783,P35280
4292	1,3,6,7,8,18,22,24	2,4,5,17,19,20,21,23	1	P35280
4293	1,3,6,7,8,18,23,24	2,4,5,17,19,20,21,22	2	Q3ZAV1,Q63598
4294	1,3,6,7,8,19,20,21	2,4,5,17,18,22,23,24	20	iRT-Kit_WR_fusion,P02780,P05369,P06761,P06911,P08723,P08937,P19629,P20759,P22283,P24368,P46462,P47967,Q5I0D1,Q5RKI1,Q63493,Q63617,Q812E4,Q9JI85,Q9QW07
4295	1,3,6,7,8,19,20,22	2,4,5,17,18,21,23,24	6	P06911,P19218,P19629,P35280,Q9QW07,Q9QZK9
4296	1,3,6,7,8,19,20,23	2,4,5,17,18,21,22,24	4	P06911,P19629,Q812E4,Q9QW07
4297	1,3,6,7,8,19,20,24	2,4,5,17,18,21,22,23	8	P06911,P08723,Q63617,Q812E4,Q9JHB9,Q9JI85,Q9QW07,Q9QZK9
4298	1,3,6,7,8,19,21,22	2,4,5,17,18,20,23,24	5	iRT-Kit_WR_fusion,P01946,P02091,P08937,P35280
4299	1,3,6,7,8,19,21,23	2,4,5,17,18,20,22,24	5	iRT-Kit_WR_fusion,P02783,P19629,P21674,Q9Z0J6
4300	1,3,6,7,8,19,21,24	2,4,5,17,18,20,22,23	0	
4301	1,3,6,7,8,19,22,23	2,4,5,17,18,20,21,24	6	P01946,P02091,P02783,P19223,P35280,P70549
4302	1,3,6,7,8,19,22,24	2,4,5,17,18,20,21,23	4	P19223,P35280,P70549,Q9QZK9
4303	1,3,6,7,8,19,23,24	2,4,5,17,18,20,21,22	4	P19223,P25809,P70549,Q9Z0J6
4304	1,3,6,7,8,20,21,22	2,4,5,17,18,19,23,24	5	P06911,P19629,P35280,Q5RKI1,Q9QW07
4305	1,3,6,7,8,20,21,23	2,4,5,17,18,19,22,24	6	P02783,P09656,P19629,Q5RKI1,Q812E4,Q9QW07
4306	1,3,6,7,8,20,21,24	2,4,5,17,18,19,22,23	10	P02782,P04762,P06911,P08723,P19629,P22283,Q5RKI1,Q812E4,Q9JHB9,Q9QW07
4307	1,3,6,7,8,20,22,23	2,4,5,17,18,19,21,24	6	P02783,P06911,P09656,P19629,P35280,Q62894
4308	1,3,6,7,8,20,22,24	2,4,5,17,18,19,21,23	5	P06911,P19629,P22006,P35280,Q9QW07
4309	1,3,6,7,8,20,23,24	2,4,5,17,18,19,21,22	5	P06911,P19629,Q62894,Q812E4,Q9QW07
4310	1,3,6,7,8,21,22,23	2,4,5,17,18,19,20,24	6	P02783,P09656,P13265,P19629,P35280,Q62714

4311	1,3,6,7,8,21,22,24	2,4,5,17,18,19,20,23	2	P35280,Q62714
4312	1,3,6,7,8,21,23,24	2,4,5,17,18,19,20,22	2	P09656,Q62714
4313	1,3,6,7,8,22,23,24	2,4,5,17,18,19,20,21	6	P02783,P19223,P25809,P35280,Q62714,Q6P6R2
4314	1,3,6,7,17,18,19,20	2,4,5,8,21,22,23,24	1	P30919
4315	1,3,6,7,17,18,19,21	2,4,5,8,20,22,23,24	3	P05539,P23739,Q5QE79
4316	1,3,6,7,17,18,19,22	2,4,5,8,20,21,23,24	3	P01946,P02091,P70549
4317	1,3,6,7,17,18,19,23	2,4,5,8,20,21,22,24	2	P21674,P70549
4318	1,3,6,7,17,18,19,24	2,4,5,8,20,21,22,23	1	P70549
4319	1,3,6,7,17,18,20,21	2,4,5,8,19,22,23,24	2	P00507,P05539
4320	1,3,6,7,17,18,20,22	2,4,5,8,19,21,23,24	2	P01946,P02091
4321	1,3,6,7,17,18,20,23	2,4,5,8,19,21,22,24	1	P30919
4322	1,3,6,7,17,18,20,24	2,4,5,8,19,21,22,23	0	
4323	1,3,6,7,17,18,21,22	2,4,5,8,19,20,23,24	4	P01946,P02091,P05539,P23739
4324	1,3,6,7,17,18,21,23	2,4,5,8,19,20,22,24	1	P05539
4325	1,3,6,7,17,18,21,24	2,4,5,8,19,20,22,23	2	P04762,P05539
4326	1,3,6,7,17,18,22,23	2,4,5,8,19,20,21,24	3	P01946,P02091,P02783
4327	1,3,6,7,17,18,22,24	2,4,5,8,19,20,21,23	4	P01946,P02091,P70549,Q8CJD3
4328	1,3,6,7,17,18,23,24	2,4,5,8,19,20,21,22	1	P70549
4329	1,3,6,7,17,19,20,21	2,4,5,8,18,22,23,24	5	P05539,P20762,P22283,Q63617,Q6P6R2
4330	1,3,6,7,17,19,20,22	2,4,5,8,18,21,23,24	4	O89117,P01946,P02091,P70549

4331	1,3,6,7,17,<br>19,20,23	2,4,5,8,18,<br>21,22,24	1 P70549
4332	1,3,6,7,17,<br>19,20,24	2,4,5,8,18,<br>21,22,23	2 P54921,P70549
4333	1,3,6,7,17,<br>19,21,22	2,4,5,8,18,<br>20,23,24	5 P01946,P02091,P05539,Q5QE79,Q9R168
4334	1,3,6,7,17,<br>19,21,23	2,4,5,8,18,<br>20,22,24	2 P05539,P21674
4335	1,3,6,7,17,<br>19,21,24	2,4,5,8,18,<br>20,22,23	4 P05539,P20762,P54921,Q6AY61
4336	1,3,6,7,17,<br>19,22,23	2,4,5,8,18,<br>20,21,24	5 P01946,P02091,P02783,P25809,P70549
4337	1,3,6,7,17,<br>19,22,24	2,4,5,8,18,<br>20,21,23	4 P01946,P02091,P25809,P70549
4338	1,3,6,7,17,<br>19,23,24	2,4,5,8,18,<br>20,21,22	2 P25809,P70549
4339	1,3,6,7,17,<br>20,21,22	2,4,5,8,18,<br>19,23,24	3 P01946,P02091,P05539
4340	1,3,6,7,17,<br>20,21,23	2,4,5,8,18,<br>19,22,24	2 P05539,P09656
4341	1,3,6,7,17,<br>20,21,24	2,4,5,8,18,<br>19,22,23	4 P04762,P05539,P20762,Q6AY61
4342	1,3,6,7,17,<br>20,22,23	2,4,5,8,18,<br>19,21,24	3 P01946,P02091,P02783
4343	1,3,6,7,17,<br>20,22,24	2,4,5,8,18,<br>19,21,23	3 P01946,P02091,P22006
4344	1,3,6,7,17,<br>20,23,24	2,4,5,8,18,<br>19,21,22	0
4345	1,3,6,7,17,<br>21,22,23	2,4,5,8,18,<br>19,20,24	6 P01946,P02091,P02783,P05539,P09656,Q9R168
4346	1,3,6,7,17,<br>21,22,24	2,4,5,8,18,<br>19,20,23	5 P01946,P02091,P04762,P05539,Q9R168
4347	1,3,6,7,17,<br>21,23,24	2,4,5,8,18,<br>19,20,22	2 P04762,P05539
4348	1,3,6,7,17,<br>22,23,24	2,4,5,8,18,<br>19,20,21	6 P01946,P02091,P02783,P06760,P25809,P70549
4349	1,3,6,7,18,<br>19,20,21	2,4,5,8,17,<br>22,23,24	5 P20760,P22283,P47967,Q5PQL7,Q63617
4350	1,3,6,7,18,<br>19,20,22	2,4,5,8,17,<br>21,23,24	1 Q5PQL7

4351	1,3,6,7,18, 19,20,23	2,4,5,8,17, 21,22,24	0	
4352	1,3,6,7,18, 19,20,24	2,4,5,8,17, 21,22,23	2	P70549,Q9QW07
4353	1,3,6,7,18, 19,21,22	2,4,5,8,17, 20,23,24	3	P01946,P23739,Q5PQL7
4354	1,3,6,7,18, 19,21,23	2,4,5,8,17, 20,22,24	0	
4355	1,3,6,7,18, 19,21,24	2,4,5,8,17, 20,22,23	1	P36376
4356	1,3,6,7,18, 19,22,23	2,4,5,8,17, 20,21,24	4	D4A5U3,P02783,P19223,P70549
4357	1,3,6,7,18, 19,22,24	2,4,5,8,17, 20,21,23	4	D4A5U3,P19223,P25809,P70549
4358	1,3,6,7,18, 19,23,24	2,4,5,8,17, 20,21,22	4	D4A5U3,P19223,P25809,P70549
4359	1,3,6,7,18, 20,21,22	2,4,5,8,17, 19,23,24	2	P01835,Q5PQL7
4360	1,3,6,7,18, 20,21,23	2,4,5,8,17, 19,22,24	2	P01835,P09656
4361	1,3,6,7,18, 20,21,24	2,4,5,8,17, 19,22,23	1	P01835
4362	1,3,6,7,18, 20,22,23	2,4,5,8,17, 19,21,24	2	P02783,Q62894
4363	1,3,6,7,18, 20,22,24	2,4,5,8,17, 19,21,23	1	P22006
4364	1,3,6,7,18, 20,23,24	2,4,5,8,17, 19,21,22	0	
4365	1,3,6,7,18, 21,22,23	2,4,5,8,17, 19,20,24	3	P01835,P02783,P09656
4366	1,3,6,7,18, 21,22,24	2,4,5,8,17, 19,20,23	4	P01835,P23739,P36376,Q8CJD3
4367	1,3,6,7,18, 21,23,24	2,4,5,8,17, 19,20,22	1	P01835
4368	1,3,6,7,18, 22,23,24	2,4,5,8,17, 19,20,21	10	D4A5U3,P02783,P06760,P13676,P19223,P25809,P70549,P97675,Q64268,Q6P6R2
4369	1,3,6,7,19, 20,21,22	2,4,5,8,17, 18,23,24	3	P01946,Q5PQL7,Q63617
4370	1,3,6,7,19, 20,21,23	2,4,5,8,17, 18,22,24	1	P09656

4371	1,3,6,7,19, 20,21,24	2,4,5,8,17, 18,22,23	4	O35547,P05369,P22283,Q63617
4372	1,3,6,7,19, 20,22,23	2,4,5,8,17, 18,21,24	1	P02783
4373	1,3,6,7,19, 20,22,24	2,4,5,8,17, 18,21,23	2	P22006,P70549
4374	1,3,6,7,19, 20,23,24	2,4,5,8,17, 18,21,22	1	P70549
4375	1,3,6,7,19, 21,22,23	2,4,5,8,17, 18,20,24	3	P01946,P02091,P02783
4376	1,3,6,7,19, 21,22,24	2,4,5,8,17, 18,20,23	3	P01946,P02091,P36376
4377	1,3,6,7,19, 21,23,24	2,4,5,8,17, 18,20,22	2	P25809,Q9Z0J6
4378	1,3,6,7,19, 22,23,24	2,4,5,8,17, 18,20,21	11	D4A5U3,P01946,P02091,P02783,P06760,P19223,P25809,P36375,P70549,Q10758,Q811M5
4379	1,3,6,7,20, 21,22,23	2,4,5,8,17, 18,19,24	3	P01835,P02783,P09656
4380	1,3,6,7,20, 21,22,24	2,4,5,8,17, 18,19,23	2	P01835,P22006
4381	1,3,6,7,20, 21,23,24	2,4,5,8,17, 18,19,22	2	P01835,P09656
4382	1,3,6,7,20, 22,23,24	2,4,5,8,17, 18,19,21	5	P02783,P06760,P09656,P22006,Q9QX74
4383	1,3,6,7,21, 22,23,24	2,4,5,8,17, 18,19,20	7	D4A5U3,P01835,P01946,P02783,P09656,P25809,Q62714
4384	1,3,6,8,17, 18,19,20	2,4,5,7,21, 22,23,24	3	B0LT89,P08649,Q9R168
4385	1,3,6,8,17, 18,19,21	2,4,5,7,20, 22,23,24	0	
4386	1,3,6,8,17, 18,19,22	2,4,5,7,20, 21,23,24	4	P01946,P02091,P08649,P70549
4387	1,3,6,8,17, 18,19,23	2,4,5,7,20, 21,22,24	3	P21674,P70549,Q9Z0J6
4388	1,3,6,8,17, 18,19,24	2,4,5,7,20, 21,22,23	1	P70549
4389	1,3,6,8,17, 18,20,21	2,4,5,7,19, 22,23,24	0	
4390	1,3,6,8,17, 18,20,22	2,4,5,7,19, 21,23,24	2	P01946,P02091

4391	1,3,6,8,17,<br>18,20,23	2,4,5,7,19,<br>21,22,24	1	Q9R168
4392	1,3,6,8,17,<br>18,20,24	2,4,5,7,19,<br>21,22,23	2	P13432,Q9R168
4393	1,3,6,8,17,<br>18,21,22	2,4,5,7,19,<br>20,23,24	2	P01946,P02091
4394	1,3,6,8,17,<br>18,21,23	2,4,5,7,19,<br>20,22,24	0	
4395	1,3,6,8,17,<br>18,21,24	2,4,5,7,19,<br>20,22,23	0	
4396	1,3,6,8,17,<br>18,22,23	2,4,5,7,19,<br>20,21,24	4	P01946,P02091,P02783,P70549
4397	1,3,6,8,17,<br>18,22,24	2,4,5,7,19,<br>20,21,23	3	P01946,P02091,P70549
4398	1,3,6,8,17,<br>18,23,24	2,4,5,7,19,<br>20,21,22	1	P70549
4399	1,3,6,8,17,<br>19,20,21	2,4,5,7,18,<br>22,23,24	3	B0LT89,Q6P6R2,Q9JI85
4400	1,3,6,8,17,<br>19,20,22	2,4,5,7,18,<br>21,23,24	4	O89117,P01946,P02091,P70549
4401	1,3,6,8,17,<br>19,20,23	2,4,5,7,18,<br>21,22,24	2	O89117,P70549
4402	1,3,6,8,17,<br>19,20,24	2,4,5,7,18,<br>21,22,23	2	O89117,P70549
4403	1,3,6,8,17,<br>19,21,22	2,4,5,7,18,<br>20,23,24	2	P01946,P02091
4404	1,3,6,8,17,<br>19,21,23	2,4,5,7,18,<br>20,22,24	2	P21674,Q9Z0J6
4405	1,3,6,8,17,<br>19,21,24	2,4,5,7,18,<br>20,22,23	1	P70549
4406	1,3,6,8,17,<br>19,22,23	2,4,5,7,18,<br>20,21,24	5	O89117,P01946,P02091,P02783,P70549
4407	1,3,6,8,17,<br>19,22,24	2,4,5,7,18,<br>20,21,23	4	O89117,P01946,P02091,P70549
4408	1,3,6,8,17,<br>19,23,24	2,4,5,7,18,<br>20,21,22	2	P70549,Q9Z0J6
4409	1,3,6,8,17,<br>20,21,22	2,4,5,7,18,<br>19,23,24	3	P01946,P02091,P63029
4410	1,3,6,8,17,<br>20,21,23	2,4,5,7,18,<br>19,22,24	2	P09656,P63029

4411	1,3,6,8,17, 20,21,24	2,4,5,7,18, 19,22,23	0	
4412	1,3,6,8,17, 20,22,23	2,4,5,7,18, 19,21,24	5	O89117,P01946,P02091,P02783,P63029
4413	1,3,6,8,17, 20,22,24	2,4,5,7,18, 19,21,23	3	O89117,P01946,P02091
4414	1,3,6,8,17, 20,23,24	2,4,5,7,18, 19,21,22	1	P70549
4415	1,3,6,8,17, 21,22,23	2,4,5,7,18, 19,20,24	4	P01946,P02091,P02783,P09656
4416	1,3,6,8,17, 21,22,24	2,4,5,7,18, 19,20,23	2	P01946,P02091
4417	1,3,6,8,17, 21,23,24	2,4,5,7,18, 19,20,22	1	Q80WL1
4418	1,3,6,8,17, 22,23,24	2,4,5,7,18, 19,20,21	7	P01946,P02091,P02783,P47967,P62804,P70549,Q5I0D1
4419	1,3,6,8,18, 19,20,21	2,4,5,7,17, 22,23,24	3	Q9JI85,Q9QW07,Q9R168
4420	1,3,6,8,18, 19,20,22	2,4,5,7,17, 21,23,24	2	P36860,Q9R168
4421	1,3,6,8,18, 19,20,23	2,4,5,7,17, 21,22,24	1	Q9R168
4422	1,3,6,8,18, 19,20,24	2,4,5,7,17, 21,22,23	3	P70549,Q9QW07,Q9R168
4423	1,3,6,8,18, 19,21,22	2,4,5,7,17, 20,23,24	2	P01946,P02091
4424	1,3,6,8,18, 19,21,23	2,4,5,7,17, 20,22,24	1	Q9Z0J6
4425	1,3,6,8,18, 19,21,24	2,4,5,7,17, 20,22,23	0	
4426	1,3,6,8,18, 19,22,23	2,4,5,7,17, 20,21,24	7	P01946,P02091,P02783,P19223,P70549,Q6AY61,Q9Z0J6
4427	1,3,6,8,18, 19,22,24	2,4,5,7,17, 20,21,23	2	P19223,P70549
4428	1,3,6,8,18, 19,23,24	2,4,5,7,17, 20,21,22	3	P19223,P70549,Q9Z0J6
4429	1,3,6,8,18, 20,21,22	2,4,5,7,17, 19,23,24	1	P54921
4430	1,3,6,8,18, 20,21,23	2,4,5,7,17, 19,22,24	3	P09656,P54921,Q9R168

4431	1,3,6,8,18, 20,21,24	2,4,5,7,17, 19,22,23	2	Q9QW07,Q9R168
4432	1,3,6,8,18, 20,22,23	2,4,5,7,17, 19,21,24	4	P02783,P54921,Q6AY61,Q9R168
4433	1,3,6,8,18, 20,22,24	2,4,5,7,17, 19,21,23	2	P22006,Q9R168
4434	1,3,6,8,18, 20,23,24	2,4,5,7,17, 19,21,22	1	Q9R168
4435	1,3,6,8,18, 21,22,23	2,4,5,7,17, 19,20,24	3	P02783,P09656,P54921
4436	1,3,6,8,18, 21,22,24	2,4,5,7,17, 19,20,23	1	Q8CJD3
4437	1,3,6,8,18, 21,23,24	2,4,5,7,17, 19,20,22	1	Q9Z0J6
4438	1,3,6,8,18, 22,23,24	2,4,5,7,17, 19,20,21	6	P02783,P06760,P19223,P20646,P62804,P70549
4439	1,3,6,8,19, 20,21,22	2,4,5,7,17, 18,23,24	2	P01946,P02091
4440	1,3,6,8,19, 20,21,23	2,4,5,7,17, 18,22,24	2	P09656,Q9Z0J6
4441	1,3,6,8,19, 20,21,24	2,4,5,7,17, 18,22,23	5	O35547,P08723,P30120,Q9JI85,Q9QW07
4442	1,3,6,8,19, 20,22,23	2,4,5,7,17, 18,21,24	4	P01946,P02091,P02783,P19218
4443	1,3,6,8,19, 20,22,24	2,4,5,7,17, 18,21,23	3	P22006,P70549,Q9QW07
4444	1,3,6,8,19, 20,23,24	2,4,5,7,17, 18,21,22	3	P70549,Q9QW07,Q9Z0J6
4445	1,3,6,8,19, 21,22,23	2,4,5,7,17, 18,20,24	5	P01946,P02091,P02783,P09656,Q9Z0J6
4446	1,3,6,8,19, 21,22,24	2,4,5,7,17, 18,20,23	3	P01946,P02091,P30919
4447	1,3,6,8,19, 21,23,24	2,4,5,7,17, 18,20,22	1	Q9Z0J6
4448	1,3,6,8,19, 22,23,24	2,4,5,7,17, 18,20,21	10	P00507,P01946,P02091,P02783,P19223,P25809,P62804,P70549,Q811M5,Q9Z0J6
4449	1,3,6,8,20, 21,22,23	2,4,5,7,17, 18,19,24	5	P02783,P09656,P13265,P19629,P54921
4450	1,3,6,8,20, 21,22,24	2,4,5,7,17, 18,19,23	0	

4451	1,3,6,8,20,21,23,24	2,4,5,7,17,18,19,22	1 P09656
4452	1,3,6,8,20,22,23,24	2,4,5,7,17,18,19,21	4 P02783,P09656,P22006,P62804
4453	1,3,6,8,21,22,23,24	2,4,5,7,17,18,19,20	8 P01946,P02783,P09656,P13265,P20646,P30919,Q62714,Q9Z0J6
4454	1,3,6,17,18,19,20,21	2,4,5,7,8,22,23,24	7 B0LT89,P05539,P20760,P20761,P20762,P39069,Q6P6R2
4455	1,3,6,17,18,19,20,22	2,4,5,7,8,21,23,24	6 O89117,P01946,P02091,P39069,P70549,Q06000
4456	1,3,6,17,18,19,20,23	2,4,5,7,8,21,22,24	2 P39069,P70549
4457	1,3,6,17,18,19,20,24	2,4,5,7,8,21,22,23	1 P70549
4458	1,3,6,17,18,19,21,22	2,4,5,7,8,20,23,24	5 P01946,P02091,P05539,P23739,Q4G075
4459	1,3,6,17,18,19,21,23	2,4,5,7,8,20,22,24	1 Q4G075
4460	1,3,6,17,18,19,21,24	2,4,5,7,8,20,22,23	3 P05539,P70549,Q4G075
4461	1,3,6,17,18,19,22,23	2,4,5,7,8,20,21,24	9 D4A5U3,P01946,P02091,P02783,P06760,P22273,P70549,P82471,Q4G075
4462	1,3,6,17,18,19,22,24	2,4,5,7,8,20,21,23	7 D4A5U3,P01946,P02091,P06760,P22273,P70549,Q4G075
4463	1,3,6,17,18,19,23,24	2,4,5,7,8,20,21,22	5 D4A5U3,P06760,P22273,P70549,Q4G075
4464	1,3,6,17,18,20,21,22	2,4,5,7,8,19,23,24	3 P01946,P02091,Q5PQL7
4465	1,3,6,17,18,20,21,23	2,4,5,7,8,19,22,24	0
4466	1,3,6,17,18,20,21,24	2,4,5,7,8,19,22,23	0
4467	1,3,6,17,18,20,22,23	2,4,5,7,8,19,21,24	5 P01946,P02091,P02783,P04355,P06760
4468	1,3,6,17,18,20,22,24	2,4,5,7,8,19,21,23	5 P01946,P02091,P06760,P22006,P70549
4469	1,3,6,17,18,20,23,24	2,4,5,7,8,19,21,22	3 P06760,P62804,P70549
4470	1,3,6,17,18,21,22,23	2,4,5,7,8,19,20,24	8 D4A5U3,P01946,P02091,P02780,P02783,P82471,Q4G075,Q6TMA8

4471	1,3,6,17,18,21,22,24	2,4,5,7,8,19,20,23	8	P01946,P02091,P05539,P10758,P23739,Q4G075,Q6TMA8,Q8CJD3
4472	1,3,6,17,18,21,23,24	2,4,5,7,8,19,20,22	4	D4A5U3,P02780,Q4G075,Q6TMA8
4473	1,3,6,17,18,22,23,24	2,4,5,7,8,19,20,21	19	D4A5U3,P01946,P02091,P02780,P02783,P06760,P22273,P22282,P25809,P47967,P55159,P62804,P70549,Q00715,Q4G075,Q5I0D1,Q63617,Q6TMA8,Q9WTT6
4474	1,3,6,17,19,20,21,22	2,4,5,7,8,18,23,24	6	O89117,P01946,P02091,P05539,P20762,Q5PQL7
4475	1,3,6,17,19,20,21,23	2,4,5,7,8,18,22,24	1	P20762
4476	1,3,6,17,19,20,21,24	2,4,5,7,8,18,22,23	2	P05539,P20762
4477	1,3,6,17,19,20,22,23	2,4,5,7,8,18,21,24	6	O89117,P01946,P02091,P02783,P70549,Q00715
4478	1,3,6,17,19,20,22,24	2,4,5,7,8,18,21,23	7	O89117,P01946,P02091,P20762,P22006,P70549,Q00715
4479	1,3,6,17,19,20,23,24	2,4,5,7,8,18,21,22	2	P70549,Q00715
4480	1,3,6,17,19,21,22,23	2,4,5,7,8,18,20,24	5	P01946,P02091,P02783,P70549,Q4G075
4481	1,3,6,17,19,21,22,24	2,4,5,7,8,18,20,23	6	P01946,P02091,P05539,P20762,P70549,Q4G075
4482	1,3,6,17,19,21,23,24	2,4,5,7,8,18,20,22	4	P20762,P70549,Q4G075,Q9Z0J6
4483	1,3,6,17,19,22,23,24	2,4,5,7,8,18,20,21	13	D4A5U3,P01946,P02091,P02780,P02783,P06760,P22273,P25809,P62804,P70549,Q00715,Q4G075,Q811M5
4484	1,3,6,17,20,21,22,23	2,4,5,7,8,18,19,24	5	P01946,P02091,P02783,P09656,P63029
4485	1,3,6,17,20,21,22,24	2,4,5,7,8,18,19,23	3	P01946,P02091,P20762
4486	1,3,6,17,20,21,23,24	2,4,5,7,8,18,19,22	2	P09656,P20762
4487	1,3,6,17,20,22,23,24	2,4,5,7,8,18,19,21	11	O89117,P01946,P02091,P02783,P06760,P09656,P22006,P62804,P70549,Q00715,Q66H69
4488	1,3,6,17,21,22,23,24	2,4,5,7,8,18,19,20	9	D4A5U3,P01946,P02091,P02780,P02783,P09656,Q4G075,Q5I0D1,Q6TMA8
4489	1,3,6,18,19,20,21,22	2,4,5,7,8,17,23,24	4	P01946,P20760,P39069,Q5PQL7
4490	1,3,6,18,19,20,21,23	2,4,5,7,8,17,22,24	1	P39069

4491	1,3,6,18,19,20,21,24	2,4,5,7,8,17,22,23	1	P20760
4492	1,3,6,18,19,20,22,23	2,4,5,7,8,17,21,24	5	P02783,P06760,P36860,P39069,P70549
4493	1,3,6,18,19,20,22,24	2,4,5,7,8,17,21,23	6	P06760,P22006,P39069,P70549,Q9QX74,Q9Z0V6
4494	1,3,6,18,19,20,23,24	2,4,5,7,8,17,21,22	2	P06760,P70549
4495	1,3,6,18,19,21,22,23	2,4,5,7,8,17,20,24	11	D4A5U3,P01946,P02091,P02783,P19223,P82471,P97697,Q4G075,Q6TMA8,Q811M5,Q9Z0J6
4496	1,3,6,18,19,21,22,24	2,4,5,7,8,17,20,23	8	D4A5U3,P01946,P02091,P19223,P23739,P70549,P97697,Q4G075
4497	1,3,6,18,19,21,23,24	2,4,5,7,8,17,20,22	6	D4A5U3,P19223,P70549,P97697,Q4G075,Q9Z0J6
4498	1,3,6,18,19,22,23,24	2,4,5,7,8,17,20,21	18	D4A5U3,P01946,P02091,P02783,P06760,P13676,P19223,P22273,P25809,P62804,P70549,P97697,Q00715,Q4G075,Q811M5,Q9QX74,Q9WTT6,Q9Z0J6
4499	1,3,6,18,20,21,22,23	2,4,5,7,8,17,19,24	4	P02783,P09656,P54921,Q6TMA8
4500	1,3,6,18,20,21,22,24	2,4,5,7,8,17,19,23	2	P22006,Q8CJD3
4501	1,3,6,18,20,21,23,24	2,4,5,7,8,17,19,22	2	P09656,P20646
4502	1,3,6,18,20,22,23,24	2,4,5,7,8,17,19,21	11	P02783,P04355,P06760,P20646,P22006,P62804,P70549,Q00715,Q5QE79,Q66H69,Q9QX74
4503	1,3,6,18,21,22,23,24	2,4,5,7,8,17,19,20	13	D4A5U3,P01946,P02783,P06760,P09656,P13676,P19223,P20646,P97697,Q4G075,Q6TMA8,Q8CJD3,Q9WTT6
4504	1,3,6,19,20,21,22,23	2,4,5,7,8,17,18,24	4	P01946,P02091,P02783,P09656
4505	1,3,6,19,20,21,22,24	2,4,5,7,8,17,18,23	6	O70594,P01946,P02091,P20762,P22006,Q9Z0V6
4506	1,3,6,19,20,21,23,24	2,4,5,7,8,17,18,22	1	P09656
4507	1,3,6,19,20,22,23,24	2,4,5,7,8,17,18,21	10	P01946,P02091,P02783,P06760,P22006,P62804,P70549,Q00715,Q811M5,Q9QX74
4508	1,3,6,19,21,22,23,24	2,4,5,7,8,17,18,20	14	D4A5U3,P01946,P02091,P02783,P09656,P19223,P25809,P55091,P70549,P97697,Q4G075,Q811M5,Q9WTT6,Q9Z0J6
4509	1,3,6,20,21,22,23,24	2,4,5,7,8,17,18,19	7	P01946,P02783,P07150,P09656,P20646,P22006,Q9QX74
4510	1,3,7,8,17,18,19,20	2,4,5,6,21,22,23,24	5	P13676,P30919,P47727,Q811M5,Q9WTT6

4511	1,3,7,8,17,18,19,21	2,4,5,6,20,22,23,24	6	P21674,P22006,Q66H69,Q6P6R2,Q811M5,Q9QX74
4512	1,3,7,8,17,18,19,22	2,4,5,6,20,21,23,24	1	P35280
4513	1,3,7,8,17,18,19,23	2,4,5,6,20,21,22,24	2	O55145,P21674
4514	1,3,7,8,17,18,19,24	2,4,5,6,20,21,22,23	1	P70549
4515	1,3,7,8,17,18,20,21	2,4,5,6,19,22,23,24	7	P00507,P13676,Q6P6R2,Q811M5,Q9EQS0,Q9QX74,Q9WTT6
4516	1,3,7,8,17,18,20,22	2,4,5,6,19,21,23,24	2	O70417,P35280
4517	1,3,7,8,17,18,20,23	2,4,5,6,19,21,22,24	1	P30919
4518	1,3,7,8,17,18,20,24	2,4,5,6,19,21,22,23	1	Q811M5
4519	1,3,7,8,17,18,21,22	2,4,5,6,19,20,23,24	1	P35280
4520	1,3,7,8,17,18,21,23	2,4,5,6,19,20,22,24	3	P21674,P22006,Q9QX74
4521	1,3,7,8,17,18,21,24	2,4,5,6,19,20,22,23	1	Q811M5
4522	1,3,7,8,17,18,22,23	2,4,5,6,19,20,21,24	1	P35280
4523	1,3,7,8,17,18,22,24	2,4,5,6,19,20,21,23	1	P35280
4524	1,3,7,8,17,18,23,24	2,4,5,6,19,20,21,22	1	Q64268
4525	1,3,7,8,17,19,20,21	2,4,5,6,18,22,23,24	11	P13676,P16636,P24368,Q5RKI1,Q63493,Q6P6R2,Q811M5,Q812E4,Q9EQS0,Q9QX74,Q9WTT6
4526	1,3,7,8,17,19,20,22	2,4,5,6,18,21,23,24	6	O70417,O89117,P13676,P35280,Q9JJ50,Q9WTT6
4527	1,3,7,8,17,19,20,23	2,4,5,6,18,21,22,24	3	P13676,P21674,Q9WTT6
4528	1,3,7,8,17,19,20,24	2,4,5,6,18,21,22,23	7	P08723,P13676,P16636,P54921,Q812E4,Q9EQS0,Q9WTT6
4529	1,3,7,8,17,19,21,22	2,4,5,6,18,20,23,24	3	P02091,P35280,Q9QX74
4530	1,3,7,8,17,19,21,23	2,4,5,6,18,20,22,24	4	P21674,P22006,Q9QX74,Q9Z0J6

4531	1,3,7,8,17,<br>19,21,24	2,4,5,6,18,<br>20,22,23	5	P04355,P16636,P54921,Q9EQS0,Q9QX74
4532	1,3,7,8,17,<br>19,22,23	2,4,5,6,18,<br>20,21,24	4	O55145,P02091,P35280,P70549
4533	1,3,7,8,17,<br>19,22,24	2,4,5,6,18,<br>20,21,23	2	P35280,P70549
4534	1,3,7,8,17,<br>19,23,24	2,4,5,6,18,<br>20,21,22	3	O55145,P70549,Q9Z0J6
4535	1,3,7,8,17,<br>20,21,22	2,4,5,6,18,<br>19,23,24	2	P35280,Q9WTT6
4536	1,3,7,8,17,<br>20,21,23	2,4,5,6,18,<br>19,22,24	2	P13676,Q9WTT6
4537	1,3,7,8,17,<br>20,21,24	2,4,5,6,18,<br>19,22,23	6	P13676,P16636,P30120,Q811M5,Q812E4,Q9EQS0
4538	1,3,7,8,17,<br>20,22,23	2,4,5,6,18,<br>19,21,24	1	P35280
4539	1,3,7,8,17,<br>20,22,24	2,4,5,6,18,<br>19,21,23	1	P35280
4540	1,3,7,8,17,<br>20,23,24	2,4,5,6,18,<br>19,21,22	0	
4541	1,3,7,8,17,<br>21,22,23	2,4,5,6,18,<br>19,20,24	1	P35280
4542	1,3,7,8,17,<br>21,22,24	2,4,5,6,18,<br>19,20,23	1	P35280
4543	1,3,7,8,17,<br>21,23,24	2,4,5,6,18,<br>19,20,22	1	Q80WL1
4544	1,3,7,8,17,<br>22,23,24	2,4,5,6,18,<br>19,20,21	1	P35280
4545	1,3,7,8,18,<br>19,20,21	2,4,5,6,17,<br>22,23,24	8	D3ZUC6,P01039,P07335,P47967,Q5I0D1,Q5RKI1,Q811M5,Q9WTT6
4546	1,3,7,8,18,<br>19,20,22	2,4,5,6,17,<br>21,23,24	3	P01039,P35280,P36860
4547	1,3,7,8,18,<br>19,20,23	2,4,5,6,17,<br>21,22,24	3	P01039,P36860,Q9R168
4548	1,3,7,8,18,<br>19,20,24	2,4,5,6,17,<br>21,22,23	2	P08723,Q9R168
4549	1,3,7,8,18,<br>19,21,22	2,4,5,6,17,<br>20,23,24	2	P01039,P35280
4550	1,3,7,8,18,<br>19,21,23	2,4,5,6,17,<br>20,22,24	4	O35077,P01039,P21674,Q9Z0J6

4551	1,3,7,8,18,<br>19,21,24	2,4,5,6,17,<br>20,22,23	0	
4552	1,3,7,8,18,<br>19,22,23	2,4,5,6,17,<br>20,21,24	4	P01039,P19223,P35280,Q9Z0J6
4553	1,3,7,8,18,<br>19,22,24	2,4,5,6,17,<br>20,21,23	3	P19223,P35280,P70549
4554	1,3,7,8,18,<br>19,23,24	2,4,5,6,17,<br>20,21,22	3	P19223,P70549,Q9Z0J6
4555	1,3,7,8,18,<br>20,21,22	2,4,5,6,17,<br>19,23,24	2	P01039,P35280
4556	1,3,7,8,18,<br>20,21,23	2,4,5,6,17,<br>19,22,24	3	D3ZUC6,P01039,Q5RKI1
4557	1,3,7,8,18,<br>20,21,24	2,4,5,6,17,<br>19,22,23	2	Q811M5,Q812E4
4558	1,3,7,8,18,<br>20,22,23	2,4,5,6,17,<br>19,21,24	2	P01039,P35280
4559	1,3,7,8,18,<br>20,22,24	2,4,5,6,17,<br>19,21,23	1	P35280
4560	1,3,7,8,18,<br>20,23,24	2,4,5,6,17,<br>19,21,22	1	Q9R168
4561	1,3,7,8,18,<br>21,22,23	2,4,5,6,17,<br>19,20,24	2	P01039,P35280
4562	1,3,7,8,18,<br>21,22,24	2,4,5,6,17,<br>19,20,23	2	P35280,Q8CJD3
4563	1,3,7,8,18,<br>21,23,24	2,4,5,6,17,<br>19,20,22	1	Q9Z0J6
4564	1,3,7,8,18,<br>22,23,24	2,4,5,6,17,<br>19,20,21	3	P19223,P35280,Q64268
4565	1,3,7,8,19,<br>20,21,22	2,4,5,6,17,<br>18,23,24	4	P01039,P16636,P35280,Q5RKI1
4566	1,3,7,8,19,<br>20,21,23	2,4,5,6,17,<br>18,22,24	5	D3ZUC6,P01039,Q5RKI1,Q812E4,Q9WTT6
4567	1,3,7,8,19,<br>20,21,24	2,4,5,6,17,<br>18,22,23	7	P02782,P08723,P16636,P30120,Q5RKI1,Q812E4,Q9EQS0
4568	1,3,7,8,19,<br>20,22,23	2,4,5,6,17,<br>18,21,24	3	P01039,P35280,P36860
4569	1,3,7,8,19,<br>20,22,24	2,4,5,6,17,<br>18,21,23	3	P08723,P16636,P35280
4570	1,3,7,8,19,<br>20,23,24	2,4,5,6,17,<br>18,21,22	1	Q812E4

4571	1,3,7,8,19,<br>21,22,23	2,4,5,6,17,<br>18,20,24	4	O35077,P01039,P35280,Q9Z0J6
4572	1,3,7,8,19,<br>21,22,24	2,4,5,6,17,<br>18,20,23	4	P16636,P17559,P35280,P55091
4573	1,3,7,8,19,<br>21,23,24	2,4,5,6,17,<br>18,20,22	1	Q9Z0J6
4574	1,3,7,8,19,<br>22,23,24	2,4,5,6,17,<br>18,20,21	5	P19223,P35280,P55091,P70549,Q9Z0J6
4575	1,3,7,8,20,<br>21,22,23	2,4,5,6,17,<br>18,19,24	2	P01039,P35280
4576	1,3,7,8,20,<br>21,22,24	2,4,5,6,17,<br>18,19,23	2	P16636,P35280
4577	1,3,7,8,20,<br>21,23,24	2,4,5,6,17,<br>18,19,22	1	Q812E4
4578	1,3,7,8,20,<br>22,23,24	2,4,5,6,17,<br>18,19,21	1	P35280
4579	1,3,7,8,21,<br>22,23,24	2,4,5,6,17,<br>18,19,20	2	P35280,Q62714
4580	1,3,7,17,1<br>8,19,20,21	2,4,5,6,8,2<br>2,23,24	7	O54858,P00507,P20760,P30919,Q63532,Q6P6R2,Q811M5
4581	1,3,7,17,1<br>8,19,20,22	2,4,5,6,8,2<br>1,23,24	5	O54858,P30919,P36860,Q63474,Q63532
4582	1,3,7,17,1<br>8,19,20,23	2,4,5,6,8,2<br>1,22,24	4	O54858,P30919,Q10743,Q6AYC4
4583	1,3,7,17,1<br>8,19,20,24	2,4,5,6,8,2<br>1,22,23	5	O54858,P30919,P54921,P70549,Q63532
4584	1,3,7,17,1<br>8,19,21,22	2,4,5,6,8,2<br>0,23,24	2	P17559,P23739
4585	1,3,7,17,1<br>8,19,21,23	2,4,5,6,8,2<br>0,22,24	3	O54858,P21674,Q10743
4586	1,3,7,17,1<br>8,19,21,24	2,4,5,6,8,2<br>0,22,23	6	O54858,P17559,P25031,P42854,P54921,Q63532
4587	1,3,7,17,1<br>8,19,22,23	2,4,5,6,8,2<br>0,21,24	4	P22273,P36375,P70549,Q10743
4588	1,3,7,17,1<br>8,19,22,24	2,4,5,6,8,2<br>0,21,23	4	P17559,P25031,P36375,P70549
4589	1,3,7,17,1<br>8,19,23,24	2,4,5,6,8,2<br>0,21,22	3	O55145,P70549,Q10743
4590	1,3,7,17,1<br>8,20,21,22	2,4,5,6,8,1<br>9,23,24	2	O54858,P20760

4591	1,3,7,17,18,20,21,23	2,4,5,6,8,19,22,24	3 O54858,P00507,P30919
4592	1,3,7,17,18,20,21,24	2,4,5,6,8,19,22,23	6 O54858,P00507,P20760,P42854,Q63532,Q811M5
4593	1,3,7,17,18,20,22,23	2,4,5,6,8,19,21,24	2 P30919,Q10743
4594	1,3,7,17,18,20,22,24	2,4,5,6,8,19,21,23	1 P42854
4595	1,3,7,17,18,20,23,24	2,4,5,6,8,19,21,22	2 P30919,Q6AYC4
4596	1,3,7,17,18,21,22,23	2,4,5,6,8,19,20,24	0
4597	1,3,7,17,18,21,22,24	2,4,5,6,8,19,20,23	4 P17559,P25031,P42854,Q8CJD3
4598	1,3,7,17,18,21,23,24	2,4,5,6,8,19,20,22	2 P42854,Q64268
4599	1,3,7,17,18,22,23,24	2,4,5,6,8,19,20,21	5 D4A5U3,P02631,P22273,P70549,Q64268
4600	1,3,7,17,19,20,21,22	2,4,5,6,8,18,23,24	5 O54858,P16636,P20760,P20762,Q9JJ50
4601	1,3,7,17,19,20,21,23	2,4,5,6,8,18,22,24	2 O54858,P20762
4602	1,3,7,17,19,20,21,24	2,4,5,6,8,18,22,23	8 O54858,P16636,P20760,P20762,P54921,Q63532,Q6AY61,Q9Z2L0
4603	1,3,7,17,19,20,22,23	2,4,5,6,8,18,21,24	2 O89117,P36860
4604	1,3,7,17,19,20,22,24	2,4,5,6,8,18,21,23	7 O89117,P16636,P20762,P54921,P70549,Q63532,Q9JJ50
4605	1,3,7,17,19,20,23,24	2,4,5,6,8,18,21,22	2 P54921,P70549
4606	1,3,7,17,19,21,22,23	2,4,5,6,8,18,20,24	2 P02091,Q9R168
4607	1,3,7,17,19,21,22,24	2,4,5,6,8,18,20,23	7 P02091,P16636,P17559,P20762,P25031,P54921,Q9R168
4608	1,3,7,17,19,21,23,24	2,4,5,6,8,18,20,22	2 P12020,Q9Z0J6
4609	1,3,7,17,19,22,23,24	2,4,5,6,8,18,20,21	9 O55145,P02091,P02631,P22273,P25809,P36375,P70549,Q00715,Q9QZQ5
4610	1,3,7,17,20,21,22,23	2,4,5,6,8,18,19,24	0

4611	1,3,7,17,20,21,22,24	2,4,5,6,8,18,19,23	2	P16636,P20762
4612	1,3,7,17,20,21,23,24	2,4,5,6,8,18,19,22	0	
4613	1,3,7,17,20,22,23,24	2,4,5,6,8,18,19,21	1	Q00715
4614	1,3,7,17,21,22,23,24	2,4,5,6,8,18,19,20	2	P50280,Q9R168
4615	1,3,7,18,19,20,21,22	2,4,5,6,8,17,23,24	6	O54858,P01039,P20760,P47967,P97840,Q5PQL7
4616	1,3,7,18,19,20,21,23	2,4,5,6,8,17,22,24	4	O54858,P01039,P20760,P23593
4617	1,3,7,18,19,20,21,24	2,4,5,6,8,17,22,23	3	O54858,P20760,Q63532
4618	1,3,7,18,19,20,22,23	2,4,5,6,8,17,21,24	3	P01039,P36860,Q63474
4619	1,3,7,18,19,20,22,24	2,4,5,6,8,17,21,23	0	
4620	1,3,7,18,19,20,23,24	2,4,5,6,8,17,21,22	0	
4621	1,3,7,18,19,21,22,23	2,4,5,6,8,17,20,24	5	D4A5U3,P01039,P17559,P19223,Q9Z0J6
4622	1,3,7,18,19,21,22,24	2,4,5,6,8,17,20,23	6	P17559,P19223,P25031,P36375,P36376,P55091
4623	1,3,7,18,19,21,23,24	2,4,5,6,8,17,20,22	4	D4A5U3,P17559,P19223,Q9Z0J6
4624	1,3,7,18,19,22,23,24	2,4,5,6,8,17,20,21	12	D4A5U3,P02631,P17559,P19223,P22273,P25809,P36375,P55091,P70549,Q64268,Q9QZQ5,Q9Z0J6
4625	1,3,7,18,20,21,22,23	2,4,5,6,8,17,19,24	1	P01039
4626	1,3,7,18,20,21,22,24	2,4,5,6,8,17,19,23	2	P20760,Q8CJD3
4627	1,3,7,18,20,21,23,24	2,4,5,6,8,17,19,22	0	
4628	1,3,7,18,20,22,23,24	2,4,5,6,8,17,19,21	1	Q64268
4629	1,3,7,18,21,22,23,24	2,4,5,6,8,17,19,20	5	D4A5U3,P17559,P19223,Q64268,Q8CJD3
4630	1,3,7,19,20,21,22,23	2,4,5,6,8,17,18,24	1	P01039

4631	1,3,7,19,20,21,22,24	2,4,5,6,8,17,18,23	3	P16636,P20760,P20762
4632	1,3,7,19,20,21,23,24	2,4,5,6,8,17,18,22	2	P08649,P16636
4633	1,3,7,19,20,22,23,24	2,4,5,6,8,17,18,21	3	P70549,Q00715,Q9QZQ5
4634	1,3,7,19,21,22,23,24	2,4,5,6,8,17,18,20	10	D4A5U3,P02454,P12020,P17559,P19223,P25809,P36375,P55091,Q9QZQ5,Q9Z0J6
4635	1,3,7,20,21,22,23,24	2,4,5,6,8,17,18,19	2	P08649,Q9QZQ5
4636	1,3,8,17,18,19,20,21	2,4,5,6,7,22,23,24	6	B0LT89,P47727,P70619,Q6P6R2,Q811M5,Q9EQS0
4637	1,3,8,17,18,19,20,22	2,4,5,6,7,21,23,24	4	O89117,P36860,P50116,Q9JJ50
4638	1,3,8,17,18,19,20,23	2,4,5,6,7,21,22,24	4	P30919,P36860,P50116,Q9R168
4639	1,3,8,17,18,19,20,24	2,4,5,6,7,21,22,23	4	P50116,P70549,P80299,Q9R168
4640	1,3,8,17,18,19,21,22	2,4,5,6,7,20,23,24	2	P02091,P50116
4641	1,3,8,17,18,19,21,23	2,4,5,6,7,20,22,24	4	P21674,P22006,P50116,Q9Z0J6
4642	1,3,8,17,18,19,21,24	2,4,5,6,7,20,22,23	2	P50116,P80299
4643	1,3,8,17,18,19,22,23	2,4,5,6,7,20,21,24	6	P00714,P02091,P11598,P50116,P70549,Q9Z0J6
4644	1,3,8,17,18,19,22,24	2,4,5,6,7,20,21,23	2	P50116,P70549
4645	1,3,8,17,18,19,23,24	2,4,5,6,7,20,21,22	5	O55145,P50116,P70549,P80299,Q9Z0J6
4646	1,3,8,17,18,20,21,22	2,4,5,6,7,19,23,24	1	P50116
4647	1,3,8,17,18,20,21,23	2,4,5,6,7,19,22,24	0	
4648	1,3,8,17,18,20,21,24	2,4,5,6,7,19,22,23	5	P42854,P50116,P80299,Q811M5,Q9EQS0
4649	1,3,8,17,18,20,22,23	2,4,5,6,7,19,21,24	2	P00714,P50116
4650	1,3,8,17,18,20,22,24	2,4,5,6,7,19,21,23	1	P50116

4651	1,3,8,17,18,20,23,24	2,4,5,6,7,19,21,22	3	P50116,P80299,Q9R168
4652	1,3,8,17,18,21,22,23	2,4,5,6,7,19,20,24	2	P00714,P50116
4653	1,3,8,17,18,21,22,24	2,4,5,6,7,19,20,23	3	P42854,P50116,P80299
4654	1,3,8,17,18,21,23,24	2,4,5,6,7,19,20,22	4	P50116,P80299,Q80WL1,Q9Z0J6
4655	1,3,8,17,18,22,23,24	2,4,5,6,7,19,20,21	8	P00714,P11598,P50116,P70549,P80299,Q5I0D1,Q63617,Q9R0T3
4656	1,3,8,17,19,20,21,22	2,4,5,6,7,18,23,24	6	B0LT89,O89117,P02091,P16636,Q6P6R2,Q9JJ50
4657	1,3,8,17,19,20,21,23	2,4,5,6,7,18,22,24	1	Q6P6R2
4658	1,3,8,17,19,20,21,24	2,4,5,6,7,18,22,23	6	B0LT89,P16636,P30120,Q6P6R2,Q9EQS0,Q9Z2L0
4659	1,3,8,17,19,20,22,23	2,4,5,6,7,18,21,24	4	O89117,P02091,P36860,P50116
4660	1,3,8,17,19,20,22,24	2,4,5,6,7,18,21,23	5	O89117,P16636,P50116,P70549,Q9JJ50
4661	1,3,8,17,19,20,23,24	2,4,5,6,7,18,21,22	2	P50116,P70549
4662	1,3,8,17,19,21,22,23	2,4,5,6,7,18,20,24	3	P02091,P50116,Q9Z0J6
4663	1,3,8,17,19,21,22,24	2,4,5,6,7,18,20,23	4	P02091,P16636,P50116,Q80WL1
4664	1,3,8,17,19,21,23,24	2,4,5,6,7,18,20,22	4	P50116,P80299,Q80WL1,Q9Z0J6
4665	1,3,8,17,19,22,23,24	2,4,5,6,7,18,20,21	8	O55145,P02091,P11598,P50116,P70549,Q00715,Q80WL1,Q9Z0J6
4666	1,3,8,17,20,21,22,23	2,4,5,6,7,18,19,24	1	P50116
4667	1,3,8,17,20,21,22,24	2,4,5,6,7,18,19,23	2	P16636,P50116
4668	1,3,8,17,20,21,23,24	2,4,5,6,7,18,19,22	3	P50116,P80299,Q80WL1
4669	1,3,8,17,20,22,23,24	2,4,5,6,7,18,19,21	2	P50116,Q00715
4670	1,3,8,17,21,22,23,24	2,4,5,6,7,18,19,20	3	P11598,P50116,Q80WL1

4671	1,3,8,18,19,20,21,22	2,4,5,6,7,17,23,24	3 P01039,P36860,P50116
4672	1,3,8,18,19,20,21,23	2,4,5,6,7,17,22,24	4 P01039,P50116,Q9R168,Q9Z0J6
4673	1,3,8,18,19,20,21,24	2,4,5,6,7,17,22,23	3 P30120,P50116,Q9R168
4674	1,3,8,18,19,20,22,23	2,4,5,6,7,17,21,24	5 P01039,P36860,P50116,Q6AY61,Q9R168
4675	1,3,8,18,19,20,22,24	2,4,5,6,7,17,21,23	3 P36860,P50116,Q9R168
4676	1,3,8,18,19,20,23,24	2,4,5,6,7,17,21,22	4 P36860,P50116,Q9R168,Q9Z0J6
4677	1,3,8,18,19,21,22,23	2,4,5,6,7,17,20,24	4 P01039,P19223,P50116,Q9Z0J6
4678	1,3,8,18,19,21,22,24	2,4,5,6,7,17,20,23	5 P17559,P19223,P50116,P55091,Q9Z0J6
4679	1,3,8,18,19,21,23,24	2,4,5,6,7,17,20,22	3 P19223,P50116,Q9Z0J6
4680	1,3,8,18,19,22,23,24	2,4,5,6,7,17,20,21	6 P11598,P19223,P50116,P55091,P70549,Q9Z0J6
4681	1,3,8,18,20,21,22,23	2,4,5,6,7,17,19,24	3 P01039,P50116,P54921
4682	1,3,8,18,20,21,22,24	2,4,5,6,7,17,19,23	1 P50116
4683	1,3,8,18,20,21,23,24	2,4,5,6,7,17,19,22	3 P50116,Q9R168,Q9Z0J6
4684	1,3,8,18,20,22,23,24	2,4,5,6,7,17,19,21	3 P50116,Q5QE79,Q9R168
4685	1,3,8,18,21,22,23,24	2,4,5,6,7,17,19,20	4 P19223,P50116,Q9R0T3,Q9Z0J6
4686	1,3,8,19,20,21,22,23	2,4,5,6,7,17,18,24	3 P01039,P50116,Q9Z0J6
4687	1,3,8,19,20,21,22,24	2,4,5,6,7,17,18,23	3 O70594,P16636,P50116
4688	1,3,8,19,20,21,23,24	2,4,5,6,7,17,18,22	4 P16636,P50116,Q64335,Q9Z0J6
4689	1,3,8,19,20,22,23,24	2,4,5,6,7,17,18,21	6 P36860,P50116,P70549,Q00715,Q9WVH8,Q9Z0J6
4690	1,3,8,19,21,22,23,24	2,4,5,6,7,17,18,20	6 P19223,P50116,P55091,Q64335,Q80WL1,Q9Z0J6

4691	1,3,8,20,21,22,23,24	2,4,5,6,7,17,18,19	1	P50116
4692	1,3,17,18,19,20,21,22	2,4,5,6,7,8,23,24	9	B0LT89,O54858,P20760,P20761,P20762,P50115,P50116,Q63532,Q6P6R2
4693	1,3,17,18,19,20,21,23	2,4,5,6,7,8,22,24	8	O54858,P20760,P23593,P30919,P50115,P50116,Q6P6R2,Q99041
4694	1,3,17,18,19,20,21,24	2,4,5,6,7,8,22,23	11	B0LT89,O54858,P20760,P20761,P20762,P23593,P42854,P50115,P50116,Q63532,Q99041
4695	1,3,17,18,19,20,22,23	2,4,5,6,7,8,21,24	11	O54858,P00714,P02631,P22273,P30919,P36860,P50115,P50116,P70549,Q00715,Q63474
4696	1,3,17,18,19,20,22,24	2,4,5,6,7,8,21,23	10	O54858,P02631,P20761,P20762,P36860,P42854,P50115,P50116,P70549,Q63532
4697	1,3,17,18,19,20,23,24	2,4,5,6,7,8,21,22	8	O54858,P02631,P50115,P50116,P70549,Q00715,Q6AYC4,Q99041
4698	1,3,17,18,19,21,22,23	2,4,5,6,7,8,20,24	15	D4A5U3,O54858,P00714,P02091,P02780,P11598,P12020,P22273,P22282,P50115,P50116,P50280,P82471,Q4G075,Q6TMA8
4699	1,3,17,18,19,21,22,24	2,4,5,6,7,8,20,23	16	D4A5U3,O54858,P02091,P02780,P12020,P17559,P20761,P20762,P22273,P25031,P42854,P50115,P50116,P50280,P70549,Q4G075
4700	1,3,17,18,19,21,23,24	2,4,5,6,7,8,20,22	15	D4A5U3,O54858,P02780,P12020,P22273,P23593,P25031,P42854,P50115,P50116,P50280,P70549,Q4G075,Q99041,Q9Z0J6
4701	1,3,17,18,19,22,23,24	2,4,5,6,7,8,20,21	26	D4A5U3,P00714,P02091,P02454,P02631,P02780,P06760,P06761,P11598,P12020,P22273,P22282,P25031,P50115,P50116,P50280,P62804,P70549,P97697,Q00715,Q4G075,Q63617,Q99041,Q9QZQ5,Q9R0T3,Q9Z0J6
4702	1,3,17,18,20,21,22,23	2,4,5,6,7,8,19,24	6	O54858,P00714,P22282,P50115,P50116,Q6TMA8
4703	1,3,17,18,20,21,22,24	2,4,5,6,7,8,19,23	9	O54858,P20760,P20761,P20762,P42854,P50115,P50116,Q8CJD3,Q99041
4704	1,3,17,18,20,21,23,24	2,4,5,6,7,8,19,22	5	O54858,P42854,P50115,P50116,Q99041

4705	1,3,17,18,20,22,23,24	2,4,5,6,7,8,19,21	13	P00714,P02631,P06760,P06761,P22273,P22282,P50115,P50116,P62804,P70549,Q00715,Q99041,Q9QZQ5
4706	1,3,17,18,21,22,23,24	2,4,5,6,7,8,19,20	19	D4A5U3,P00714,P02780,P06761,P11598,P12020,P22273,P22282,P25031,P42854,P50115,P50116,P50280,Q4G075,Q5I0D1,Q63617,Q6TMA8,Q99041,Q9R0T3
4707	1,3,17,19,20,21,22,23	2,4,5,6,7,8,18,24	6	O54858,P02091,P20762,P50115,P50116,Q9QZQ5
4708	1,3,17,19,20,21,22,24	2,4,5,6,7,8,18,23	11	O54858,O70594,P02091,P16636,P20760,P20761,P20762,P50115,P50116,Q63532,Q9JJ50
4709	1,3,17,19,20,21,23,24	2,4,5,6,7,8,18,22	8	O54858,P16636,P20762,P23593,P50115,P50116,Q99041,Q9QZQ5
4710	1,3,17,19,20,22,23,24	2,4,5,6,7,8,18,21	14	O89117,P02091,P02454,P02631,P20762,P22273,P36860,P50115,P50116,P62804,P70549,Q00715,Q99041,Q9QZQ5
4711	1,3,17,19,21,22,23,24	2,4,5,6,7,8,18,20	23	D4A5U3,P01946,P02091,P02454,P02780,P06761,P06911,P11598,P12020,P17559,P20762,P22273,P22282,P25031,P50115,P50116,P50280,P70549,Q4G075,Q5GRG2,Q6RY07,Q9QZQ5,Q9Z0J6
4712	1,3,17,20,21,22,23,24	2,4,5,6,7,8,18,19	6	P20762,P22282,P50115,P50116,Q99041,Q9QZQ5
4713	1,3,18,19,20,21,22,23	2,4,5,6,7,8,17,24	7	O54858,P01039,P20760,P36860,P50115,P50116,Q9QZQ5
4714	1,3,18,19,20,21,22,24	2,4,5,6,7,8,17,23	7	O54858,O70594,P20760,P20761,P20762,P50115,P50116
4715	1,3,18,19,20,21,23,24	2,4,5,6,7,8,17,22	8	O54858,P20760,P23593,P50115,P50116,Q99041,Q9QZQ5,Q9Z0J6
4716	1,3,18,19,20,22,23,24	2,4,5,6,7,8,17,21	12	O70594,P02454,P02631,P06760,P22273,P36860,P50115,P50116,P70549,Q00715,Q9QX74,Q9QZQ5
4717	1,3,18,19,21,22,23,24	2,4,5,6,7,8,17,20	23	D4A5U3,P02454,P02780,P06911,P11598,P12020,P17559,P19223,P22273,P22282,P25031,P50115,P50116,P50280,P55091,P55281,P97697,Q01205,Q4G075,Q5GRG2,Q9QZQ5,Q9R0T3,Q9Z0J6

4718	1,3,18,20,21,22,23,24	2,4,5,6,7,8,17,19	7	O70594,P22282,P50115,P50116,P52590,Q6TMA8,Q9QZQ5
4719	1,3,19,20,21,22,23,24	2,4,5,6,7,8,17,18	10	O70594,P02454,P16636,P20762,P50115,P50116,Q64335,Q99PP0,Q9QZQ5,Q9Z0J6
4720	1,4,5,6,7,8,17,18	2,3,19,20,21,22,23,24	7	O70594,P13432,P18418,P22282,P49134,Q64335,Q99041
4721	1,4,5,6,7,8,17,19	2,3,18,20,21,22,23,24	9	P06761,P13432,P18418,P22282,P50115,P50116,P52590,Q99041,Q9QX74
4722	1,4,5,6,7,8,17,20	2,3,18,19,21,22,23,24	28	D4A5U3,P02780,P06761,P06911,P11598,P12020,P13432,P18418,P19223,P19629,P22273,P22282,P22283,P25031,P46462,P50116,P97697,Q4FZU2,Q4G075,Q5GRG2,Q62946,Q6IMF3,Q6P6Q2,Q99041,Q9JHB9,Q9JI85,Q9R0T3,Q9Z0J6
4723	1,4,5,6,7,8,17,21	2,3,18,19,20,22,23,24	16	P06760,P06761,P11598,P13432,P18418,P22282,P36860,P50115,P50116,Q4FZU2,Q63474,Q63493,Q6IMF3,Q6P6Q2,Q99041,Q9QX74
4724	1,4,5,6,7,8,17,22	2,3,18,19,20,21,23,24	6	P13432,P18418,P23593,P49134,Q99041,Q9Z0J6
4725	1,4,5,6,7,8,17,23	2,3,18,19,20,21,22,24	7	O70594,P13432,P18418,P20760,P49134,P50115,Q99041
4726	1,4,5,6,7,8,17,24	2,3,18,19,20,21,22,23	10	P13432,P18418,P22282,P23593,P36860,P49134,Q4FZU2,Q63474,Q6IMF3,Q6P6Q2
4727	1,4,5,6,7,8,18,19	2,3,17,20,21,22,23,24	6	P06761,P11598,P13432,P18418,P22282,Q99041
4728	1,4,5,6,7,8,18,20	2,3,17,19,21,22,23,24	22	P02780,P06761,P06911,P11598,P12020,P13432,P18418,P19629,P22273,P22282,P22283,P25031,P31044,P50280,Q03191,Q4G075,Q5GRG2,Q99041,Q9JHB9,Q9QW07,Q9R0T3,Q9Z0J6
4729	1,4,5,6,7,8,18,21	2,3,17,19,20,22,23,24	10	O89117,P06761,P11598,P13432,P18418,P22273,P22282,P36860,P49134,Q99041
4730	1,4,5,6,7,8,18,22	2,3,17,19,20,21,23,24	8	O54858,P13432,P18418,P23593,P27590,P49134,Q99041,Q9WVK7

4731	1,4,5,6,7,8,18,23	2,3,17,19,20,21,22,24	5	O70594,P18418,P49134,Q99041,Q9WVK7
4732	1,4,5,6,7,8,18,24	2,3,17,19,20,21,22,23	7	P13432,P18418,P22282,P36860,P49134,Q99041,Q9WVK7
4733	1,4,5,6,7,8,19,20	2,3,17,18,21,22,23,24	33	P00714,P02780,P06761,P06911,P08723,P0C0A9,P11598,P12020,P18418,P19218,P19629,P22273,P22282,P22283,P25031,P31044,P36374,P46462,P50280,Q4G075,Q5GRG2,Q5I0D1,Q5M8C6,Q63493,Q63617,Q6P6Q2,Q6RUV5,Q812E4,Q99041,Q9JHB9,Q9JI85,Q9QW07,Q9R0T3
4734	1,4,5,6,7,8,19,21	2,3,17,18,20,22,23,24	20	P00714,P06760,P06761,P0C0A9,P11598,P18418,P19218,P22282,P22283,P35952,P50115,P52590,Q63474,Q63493,Q63617,Q6IMF3,Q6Q7Y5,Q99041,Q9JI85,Q9R0T3
4735	1,4,5,6,7,8,19,22	2,3,17,18,20,21,23,24	4	P18418,P19218,P22282,Q99041
4736	1,4,5,6,7,8,19,23	2,3,17,18,20,21,22,24	7	P18418,P19218,P20760,P22282,P42854,P50115,Q99041
4737	1,4,5,6,7,8,19,24	2,3,17,18,20,21,22,23	6	O54728,P00714,P06761,P18418,P22282,Q99041
4738	1,4,5,6,7,8,20,21	2,3,17,18,19,22,23,24	39	O88917,P00714,P02631,P02780,P06760,P06761,P06911,P07647,P0C0A9,P11598,P12020,P18418,P19629,P22273,P22282,P22283,P25031,P35952,P46462,P47967,P50116,P55159,Q4FZU2,Q4G075,Q5GRG2,Q5I0D1,Q5M8C6,Q63493,Q63617,Q6AYR9,Q6IMF3,Q6P6Q2,Q812E4,Q8CJ52,Q99041,Q9JHB9,Q9JI85,Q9QW07,Q9R0T3
4739	1,4,5,6,7,8,20,22	2,3,17,18,19,21,23,24	18	P02780,P06761,P06911,P11598,P12020,P18418,P19218,P19629,P22273,P22282,P23593,P25031,Q4G075,Q5GRG2,Q99041,Q9JHB9,Q9QW07,Q9Z0J6
4740	1,4,5,6,7,8,20,23	2,3,17,18,19,21,22,24	16	P02780,P06911,P10758,P11598,P18418,P19629,P22273,P22282,P23739,P25031,Q03191,Q4G075,Q5GRG2,Q6P6Q2,Q99041,Q9WVK7
4741	1,4,5,6,7,8,20,24	2,3,17,18,19,21,22,23	24	P02780,P06761,P06911,P08723,P11598,P12020,P18418,P19629,P22273,P22282,P22283,P46462,Q4FZU2,Q4G075,Q5GRG2,Q5M8C6,Q63617,Q6IMF3,Q6P6Q2,Q812E4,Q99041,Q9JHB9,Q9QW07,Q9R0T3
4742	1,4,5,6,7,8,21,22	2,3,17,18,19,20,23,24	6	P18418,P19218,P22282,P36860,P49134,Q99041
4743	1,4,5,6,7,8,21,23	2,3,17,18,19,20,22,24	6	P18418,P19629,P36860,P49134,Q63474,Q99041

4744	1,4,5,6,7,8,21,24	2,3,17,18,19,20,22,23	14	P00714,P02782,P11598,P18418,P22282,P30919,P36860,P49134,Q4FZU2,Q63474,Q6AYR9,Q6IMF3,Q6P6Q2,Q99041
4745	1,4,5,6,7,8,22,23	2,3,17,18,19,20,21,24	8	O54858,P19218,P20760,P23593,P49134,Q63532,Q99041,Q9WVK7
4746	1,4,5,6,7,8,22,24	2,3,17,18,19,20,21,23	7	O54858,P18418,P23593,P49134,Q6P6R2,Q99041,Q9WVK7
4747	1,4,5,6,7,8,23,24	2,3,17,18,19,20,21,22	10	O54858,P18418,P20760,P25809,P36860,P49134,Q63474,Q6P6Q2,Q99041,Q9WVK7
4748	1,4,5,6,7,17,18,19	2,3,8,20,21,22,23,24	1	P00762
4749	1,4,5,6,7,17,18,20	2,3,8,19,21,22,23,24	3	P11598,Q03191,Q9Z0J6
4750	1,4,5,6,7,17,18,21	2,3,8,19,20,22,23,24	3	B1H234,P05539,P36860
4751	1,4,5,6,7,17,18,22	2,3,8,19,20,21,23,24	1	Q9Z0J6
4752	1,4,5,6,7,17,18,23	2,3,8,19,20,21,22,24	0	
4753	1,4,5,6,7,17,18,24	2,3,8,19,20,21,22,23	1	P36860
4754	1,4,5,6,7,17,19,20	2,3,8,18,21,22,23,24	6	P11598,P22283,P36374,Q63617,Q9R0T3,Q9Z0J6
4755	1,4,5,6,7,17,19,21	2,3,8,18,20,22,23,24	5	B1H234,P05539,P36860,Q5QE79,Q9R168
4756	1,4,5,6,7,17,19,22	2,3,8,18,20,21,23,24	1	Q9R168
4757	1,4,5,6,7,17,19,23	2,3,8,18,20,21,22,24	1	P25809
4758	1,4,5,6,7,17,19,24	2,3,8,18,20,21,22,23	2	P08721,P25809
4759	1,4,5,6,7,17,20,21	2,3,8,18,19,22,23,24	8	P05539,P11598,P19223,P22283,P36860,Q63617,Q9R0T3,Q9Z0J6
4760	1,4,5,6,7,17,20,22	2,3,8,18,19,21,23,24	1	Q9Z0J6
4761	1,4,5,6,7,17,20,23	2,3,8,18,19,21,22,24	2	P23739,Q03191

4762	1,4,5,6,7,17,20,24	2,3,8,18,19,21,22,23	2 P11598,Q9Z0J6
4763	1,4,5,6,7,17,21,22	2,3,8,18,19,20,23,24	4 P05539,P36860,Q9R168,Q9Z0J6
4764	1,4,5,6,7,17,21,23	2,3,8,18,19,20,22,24	3 P36860,Q63474,Q9R168
4765	1,4,5,6,7,17,21,24	2,3,8,18,19,20,22,23	5 B1H234,P05539,P36860,Q63474,Q9R168
4766	1,4,5,6,7,17,22,23	2,3,8,18,19,20,21,24	2 P25809,Q9R168
4767	1,4,5,6,7,17,22,24	2,3,8,18,19,20,21,23	4 P25809,P36860,Q9R168,Q9Z0J6
4768	1,4,5,6,7,17,23,24	2,3,8,18,19,20,21,22	2 P25809,P36860
4769	1,4,5,6,7,18,19,20	2,3,8,17,21,22,23,24	8 P02780,P11598,P22283,P36374,P97840,Q5I0D1,Q63617,Q9R0T3
4770	1,4,5,6,7,18,19,21	2,3,8,17,20,22,23,24	3 B1H234,P11598,P36376
4771	1,4,5,6,7,18,19,22	2,3,8,17,20,21,23,24	2 P36376,Q99041
4772	1,4,5,6,7,18,19,23	2,3,8,17,20,21,22,24	0
4773	1,4,5,6,7,18,19,24	2,3,8,17,20,21,22,23	2 P36376,P63029
4774	1,4,5,6,7,18,20,21	2,3,8,17,19,22,23,24	8 B1H234,P11598,P22283,P47967,P97840,Q03191,Q63617,Q9Z0J6
4775	1,4,5,6,7,18,20,22	2,3,8,17,19,21,23,24	5 P11598,Q03191,Q5PQL7,Q9WVK7,Q9Z0J6
4776	1,4,5,6,7,18,20,23	2,3,8,17,19,21,22,24	4 P10758,P11598,Q03191,Q9WVK7
4777	1,4,5,6,7,18,20,24	2,3,8,17,19,21,22,23	4 P11598,Q03191,Q9WVK7,Q9Z0J6
4778	1,4,5,6,7,18,21,22	2,3,8,17,19,20,23,24	3 B1H234,P36376,P36860
4779	1,4,5,6,7,18,21,23	2,3,8,17,19,20,22,24	3 O89117,P36860,Q03191
4780	1,4,5,6,7,18,21,24	2,3,8,17,19,20,22,23	4 B1H234,O89117,P36376,P36860
4781	1,4,5,6,7,18,22,23	2,3,8,17,19,20,21,24	7 P13676,P25809,P97675,Q03191,Q6P6R2,Q9EQS0,Q9WVK7

4782	1,4,5,6,7,18,22,24	2,3,8,17,19,20,21,23	7	P13676,P22006,P25809,P36376,P49134,Q6P6R2,Q9WVK7
4783	1,4,5,6,7,18,23,24	2,3,8,17,19,20,21,22	4	O89117,P25809,Q6P6R2,Q9WVK7
4784	1,4,5,6,7,19,20,21	2,3,8,17,18,22,23,24	14	B1H234,O35547,P00714,P02780,P07647,P11598,P22283,P36374,P47967,P55159,P97840,Q5I0D1,Q63617,Q9R0T3
4785	1,4,5,6,7,19,20,22	2,3,8,17,18,21,23,24	6	P11598,P19218,Q5PQL7,Q63617,Q99041,Q9Z0J6
4786	1,4,5,6,7,19,20,23	2,3,8,17,18,21,22,24	4	P10758,P11598,P23739,Q63617
4787	1,4,5,6,7,19,20,24	2,3,8,17,18,21,22,23	5	P11598,P22283,P36374,Q63617,Q9R0T3
4788	1,4,5,6,7,19,21,22	2,3,8,17,18,20,23,24	5	B1H234,P19218,P36376,Q99041,Q9R168
4789	1,4,5,6,7,19,21,23	2,3,8,17,18,20,22,24	0	
4790	1,4,5,6,7,19,21,24	2,3,8,17,18,20,22,23	6	B1H234,P00714,P36376,P36860,Q62761;Q62762;Q62763,Q63474
4791	1,4,5,6,7,19,22,23	2,3,8,17,18,20,21,24	5	P19218,P25809,Q811M5,Q99041,Q9EQS0
4792	1,4,5,6,7,19,22,24	2,3,8,17,18,20,21,23	2	P25809,P36376
4793	1,4,5,6,7,19,23,24	2,3,8,17,18,20,21,22	1	P25809
4794	1,4,5,6,7,20,21,22	2,3,8,17,18,19,23,24	7	P11598,P19218,Q03191,Q5PQL7,Q63617,Q99041,Q9Z0J6
4795	1,4,5,6,7,20,21,23	2,3,8,17,18,19,22,24	3	P10758,P11598,Q03191
4796	1,4,5,6,7,20,21,24	2,3,8,17,18,19,22,23	6	B1H234,P07647,P11598,P22283,P36860,Q63617
4797	1,4,5,6,7,20,22,23	2,3,8,17,18,19,21,24	4	P19218,P23739,Q03191,Q9WVK7
4798	1,4,5,6,7,20,22,24	2,3,8,17,18,19,21,23	4	P22006,Q03191,Q9WVK7,Q9Z0J6
4799	1,4,5,6,7,20,23,24	2,3,8,17,18,19,21,22	5	P10758,P23739,Q03191,Q9QYP1,Q9WVK7
4800	1,4,5,6,7,21,22,23	2,3,8,17,18,19,20,24	2	P36860,Q9R168
4801	1,4,5,6,7,21,22,24	2,3,8,17,18,19,20,23	4	B1H234,P36376,P36860,Q9R168

4802	1,4,5,6,7,21,23,24	2,3,8,17,18,19,20,22	4	O89117,P25809,P36860,Q63474
4803	1,4,5,6,7,22,23,24	2,3,8,17,18,19,20,21	7	P10719,P13676,P25809,P62804,Q6P6R2,Q9EQS0,Q9WVK7
4804	1,4,5,6,8,17,18,19	2,3,7,20,21,22,23,24	2	P13432,P18418
4805	1,4,5,6,8,17,18,20	2,3,7,19,21,22,23,24	6	P06911,P12020,P13432,P18418,Q5GRG2,Q9Z0J6
4806	1,4,5,6,8,17,18,21	2,3,7,19,20,22,23,24	3	P13432,P18418,P36860
4807	1,4,5,6,8,17,18,22	2,3,7,19,20,21,23,24	1	P13432
4808	1,4,5,6,8,17,18,23	2,3,7,19,20,21,22,24	2	P13432,P18418
4809	1,4,5,6,8,17,18,24	2,3,7,19,20,21,22,23	2	P13432,P18418
4810	1,4,5,6,8,17,19,20	2,3,7,18,21,22,23,24	9	B0LT89,P06911,P13432,P18418,P22282,P36374,Q5GRG2,Q6RUV5,Q9JI85
4811	1,4,5,6,8,17,19,21	2,3,7,18,20,22,23,24	3	P13432,P18418,Q63474
4812	1,4,5,6,8,17,19,22	2,3,7,18,20,21,23,24	2	P00507,P13432
4813	1,4,5,6,8,17,19,23	2,3,7,18,20,21,22,24	0	
4814	1,4,5,6,8,17,19,24	2,3,7,18,20,21,22,23	3	O54728,P13432,P18418
4815	1,4,5,6,8,17,20,21	2,3,7,18,19,22,23,24	12	P06911,P13432,P18418,P19223,P20766,P36375,Q10758,Q4FZU2,Q5GRG2,Q6IMF3,Q6P6Q2,Q9JI85
4816	1,4,5,6,8,17,20,22	2,3,7,18,19,21,23,24	5	P06911,P13432,P20766,Q5GRG2,Q9Z0J6
4817	1,4,5,6,8,17,20,23	2,3,7,18,19,21,22,24	6	P06911,P13432,P20766,P23739,P25031,Q6P6Q2
4818	1,4,5,6,8,17,20,24	2,3,7,18,19,21,22,23	7	P06911,P13432,P18418,Q4FZU2,Q5GRG2,Q6IMF3,Q6P6Q2
4819	1,4,5,6,8,17,21,22	2,3,7,18,19,20,23,24	1	P20766
4820	1,4,5,6,8,17,21,23	2,3,7,18,19,20,22,24	3	P20766,P36860,Q63474
4821	1,4,5,6,8,17,21,24	2,3,7,18,19,20,22,23	7	P13432,P18418,P36860,Q4FZU2,Q63474,Q6IMF3,Q6P6Q2

4822	1,4,5,6,8,17,22,23	2,3,7,18,19,20,21,24	3	P00507,P20760,P20766
4823	1,4,5,6,8,17,22,24	2,3,7,18,19,20,21,23	3	P13432,P20766,P23593
4824	1,4,5,6,8,17,23,24	2,3,7,18,19,20,21,22	2	P20760,Q6P6Q2
4825	1,4,5,6,8,18,19,20	2,3,7,17,21,22,23,24	8	P02780,P13432,P18418,P22282,P36374,Q9JI85,Q9QW07,Q9R168
4826	1,4,5,6,8,18,19,21	2,3,7,17,20,22,23,24	1	P18418
4827	1,4,5,6,8,18,19,22	2,3,7,17,20,21,23,24	2	P00507,Q6AY61
4828	1,4,5,6,8,18,19,23	2,3,7,17,20,21,22,24	0	
4829	1,4,5,6,8,18,19,24	2,3,7,17,20,21,22,23	2	O54728,P18418
4830	1,4,5,6,8,18,20,21	2,3,7,17,19,22,23,24	6	P02780,P13432,P18418,P22273,P54921,Q9JI85
4831	1,4,5,6,8,18,20,22	2,3,7,17,19,21,23,24	5	P06911,P12020,P13432,P54921,Q6AY61
4832	1,4,5,6,8,18,20,23	2,3,7,17,19,21,22,24	7	P13432,P18418,P23739,P25031,P54921,Q9R168,Q9WVK7
4833	1,4,5,6,8,18,20,24	2,3,7,17,19,21,22,23	5	P06911,P13432,P18418,Q9QW07,Q9R168
4834	1,4,5,6,8,18,21,22	2,3,7,17,19,20,23,24	1	P54921
4835	1,4,5,6,8,18,21,23	2,3,7,17,19,20,22,24	2	O89117,P54921
4836	1,4,5,6,8,18,21,24	2,3,7,17,19,20,22,23	3	O89117,P18418,P36860
4837	1,4,5,6,8,18,22,23	2,3,7,17,19,20,21,24	6	P00507,P49134,P54921,Q63532,Q6AY61,Q9WVK7
4838	1,4,5,6,8,18,22,24	2,3,7,17,19,20,21,23	2	P49134,Q9WVK7
4839	1,4,5,6,8,18,23,24	2,3,7,17,19,20,21,22	2	P49134,Q9WVK7
4840	1,4,5,6,8,19,20,21	2,3,7,17,18,22,23,24	17	O35547,P02780,P04905,P14046,P18418,P19218,P20766,P22282,P22283,P35952,P36374,P46462,P61589,Q5I0D1,Q63493,Q6P6Q2,Q9JI85
4841	1,4,5,6,8,19,20,22	2,3,7,17,18,21,23,24	6	P02780,P15399,P19218,P20766,P36374,Q99041

4842	1,4,5,6,8,19,20,23	2,3,7,17,18,21,22,24	4	P10758,P19218,P23739,P25031
4843	1,4,5,6,8,19,20,24	2,3,7,17,18,21,22,23	12	O35547,O54728,P02780,P08723,P18418,P22282,P36374,P60905,Q6P6Q2,Q6RUV5,Q9JI85,Q9QW07
4844	1,4,5,6,8,19,21,22	2,3,7,17,18,20,23,24	4	P19218,P20766,P30919,Q99041
4845	1,4,5,6,8,19,21,23	2,3,7,17,18,20,22,24	1	Q63474
4846	1,4,5,6,8,19,21,24	2,3,7,17,18,20,22,23	6	O54728,P18418,P30919,Q10743,Q63474,Q6P6Q2
4847	1,4,5,6,8,19,22,23	2,3,7,17,18,20,21,24	4	P00507,P19218,Q6AY61,Q811M5
4848	1,4,5,6,8,19,22,24	2,3,7,17,18,20,21,23	4	O54728,P00507,P19218,P30919
4849	1,4,5,6,8,19,23,24	2,3,7,17,18,20,21,22	3	O54728,P00507,P25809
4850	1,4,5,6,8,20,21,22	2,3,7,17,18,19,23,24	4	P19218,P20766,P54921,Q99041
4851	1,4,5,6,8,20,21,23	2,3,7,17,18,19,22,24	6	P19629,P20766,P23739,P25031,P54921,Q6P6Q2
4852	1,4,5,6,8,20,21,24	2,3,7,17,18,19,22,23	9	P02782,P18418,P20766,Q10743,Q4FZU2,Q6AYR9,Q6IMF3,Q6P6Q2,Q9JI85
4853	1,4,5,6,8,20,22,23	2,3,7,17,18,19,21,24	6	P19218,P20766,P23739,P25031,P54921,Q6AY61
4854	1,4,5,6,8,20,22,24	2,3,7,17,18,19,21,23	4	P19218,P20766,P22006,Q6P6Q2
4855	1,4,5,6,8,20,23,24	2,3,7,17,18,19,21,22	3	P23739,Q6P6Q2,Q9WVK7
4856	1,4,5,6,8,21,22,23	2,3,7,17,18,19,20,24	5	P19218,P20766,P30919,P54921,Q63532
4857	1,4,5,6,8,21,22,24	2,3,7,17,18,19,20,23	4	P20766,P30919,P36860,Q10743
4858	1,4,5,6,8,21,23,24	2,3,7,17,18,19,20,22	4	P30919,P36860,Q63474,Q6P6Q2
4859	1,4,5,6,8,22,23,24	2,3,7,17,18,19,20,21	8	O54858,P00507,P20760,P25809,P30919,P62804,Q6P6R2,Q9WVK7
4860	1,4,5,6,17,18,19,20	2,3,7,8,21,22,23,24	2	P11883,P35280
4861	1,4,5,6,17,18,19,21	2,3,7,8,20,22,23,24	1	P35280

4862	1,4,5,6,17, 18,19,22	2,3,7,8,20, 21,23,24	0
4863	1,4,5,6,17, 18,19,23	2,3,7,8,20, 21,22,24	2 P11883,P35280
4864	1,4,5,6,17, 18,19,24	2,3,7,8,20, 21,22,23	1 P35280
4865	1,4,5,6,17, 18,20,21	2,3,7,8,19, 22,23,24	2 P35280,Q9Z0J6
4866	1,4,5,6,17, 18,20,22	2,3,7,8,19, 21,23,24	2 P11883,Q9Z0J6
4867	1,4,5,6,17, 18,20,23	2,3,7,8,19, 21,22,24	3 P04355,P11883,P35280
4868	1,4,5,6,17, 18,20,24	2,3,7,8,19, 21,22,23	3 P11883,P35280,Q9Z0J6
4869	1,4,5,6,17, 18,21,22	2,3,7,8,19, 20,23,24	0
4870	1,4,5,6,17, 18,21,23	2,3,7,8,19, 20,22,24	2 P35280,P36860
4871	1,4,5,6,17, 18,21,24	2,3,7,8,19, 20,22,23	2 P35280,P36860
4872	1,4,5,6,17, 18,22,23	2,3,7,8,19, 20,21,24	3 P11883,P62804,Q9EQS0
4873	1,4,5,6,17, 18,22,24	2,3,7,8,19, 20,21,23	3 P11883,P62804,Q9WTT6
4874	1,4,5,6,17, 18,23,24	2,3,7,8,19, 20,21,22	3 P11883,P35280,P62804
4875	1,4,5,6,17, 19,20,21	2,3,7,8,18, 22,23,24	4 B0LT89,P20766,P35280,P36374
4876	1,4,5,6,17, 19,20,22	2,3,7,8,18, 21,23,24	2 P20766,Q9Z0J6
4877	1,4,5,6,17, 19,20,23	2,3,7,8,18, 21,22,24	4 P10758,P20766,P23739,P35280
4878	1,4,5,6,17, 19,20,24	2,3,7,8,18, 21,22,23	2 P35280,P36374
4879	1,4,5,6,17, 19,21,22	2,3,7,8,18, 20,23,24	1 P20766
4880	1,4,5,6,17, 19,21,23	2,3,7,8,18, 20,22,24	2 P20766,P35280
4881	1,4,5,6,17, 19,21,24	2,3,7,8,18, 20,22,23	2 P35280,Q63474

4882	1,4,5,6,17, 19,22,23	2,3,7,8,18, 20,21,24	6	P00507,P20766,P25809,P62804,Q00715,Q811M5
4883	1,4,5,6,17, 19,22,24	2,3,7,8,18, 20,21,23	5	P00507,P20766,P25809,P62804,Q00715
4884	1,4,5,6,17, 19,23,24	2,3,7,8,18, 20,21,22	4	P25809,P35280,P62804,Q00715
4885	1,4,5,6,17, 20,21,22	2,3,7,8,18, 19,23,24	2	P20766,Q9Z0J6
4886	1,4,5,6,17, 20,21,23	2,3,7,8,18, 19,22,24	3	P20766,P23739,P35280
4887	1,4,5,6,17, 20,21,24	2,3,7,8,18, 19,22,23	4	P20766,P35280,P36860,Q9Z0J6
4888	1,4,5,6,17, 20,22,23	2,3,7,8,18, 19,21,24	4	P20766,P23739,P62804,Q00715
4889	1,4,5,6,17, 20,22,24	2,3,7,8,18, 19,21,23	4	P20766,P22006,P62804,Q9Z0J6
4890	1,4,5,6,17, 20,23,24	2,3,7,8,18, 19,21,22	7	P11883,P20766,P23739,P35280,P62804,Q00715,Q9QYP1
4891	1,4,5,6,17, 21,22,23	2,3,7,8,18, 19,20,24	1	P20766
4892	1,4,5,6,17, 21,22,24	2,3,7,8,18, 19,20,23	3	P20766,P36860,Q9R168
4893	1,4,5,6,17, 21,23,24	2,3,7,8,18, 19,20,22	4	P20766,P35280,P36860,Q63474
4894	1,4,5,6,17, 22,23,24	2,3,7,8,18, 19,20,21	8	P00507,P07335,P20766,P25809,P47967,P62804,Q00715,Q9WTT6
4895	1,4,5,6,18, 19,20,21	2,3,7,8,17, 22,23,24	2	P35280,Q5PQL7
4896	1,4,5,6,18, 19,20,22	2,3,7,8,17, 21,23,24	1	Q5PQL7
4897	1,4,5,6,18, 19,20,23	2,3,7,8,17, 21,22,24	3	P10758,P11883,P35280
4898	1,4,5,6,18, 19,20,24	2,3,7,8,17, 21,22,23	1	P35280
4899	1,4,5,6,18, 19,21,22	2,3,7,8,17, 20,23,24	2	P36376,Q5PQL7
4900	1,4,5,6,18, 19,21,23	2,3,7,8,17, 20,22,24	1	P35280
4901	1,4,5,6,18, 19,21,24	2,3,7,8,17, 20,22,23	2	P35280,P36376

4902	1,4,5,6,18, 19,22,23	2,3,7,8,17, 20,21,24	6	D4A5U3,P00507,P13676,P25809,Q811M5,Q9EQS0
4903	1,4,5,6,18, 19,22,24	2,3,7,8,17, 20,21,23	7	P00507,P13676,P22006,P25809,P36376,P62804,Q9WTT6
4904	1,4,5,6,18, 19,23,24	2,3,7,8,17, 20,21,22	6	D4A5U3,P13676,P25809,P35280,P62804,Q9WTT6
4905	1,4,5,6,18, 20,21,22	2,3,7,8,17, 19,23,24	3	P54921,Q5PQL7,Q9Z0J6
4906	1,4,5,6,18, 20,21,23	2,3,7,8,17, 19,22,24	2	P35280,P54921
4907	1,4,5,6,18, 20,21,24	2,3,7,8,17, 19,22,23	1	P35280
4908	1,4,5,6,18, 20,22,23	2,3,7,8,17, 19,21,24	6	P04355,P11883,P54921,Q03191,Q9EQS0,Q9WVK7
4909	1,4,5,6,18, 20,22,24	2,3,7,8,17, 19,21,23	3	P22006,P62804,Q9WVK7
4910	1,4,5,6,18, 20,23,24	2,3,7,8,17, 19,21,22	8	P04355,P11883,P23739,P35280,P62804,Q5QE79,Q9QYP1,Q9WVK7
4911	1,4,5,6,18, 21,22,23	2,3,7,8,17, 19,20,24	4	P13676,P54921,Q9EQS0,Q9WTT6
4912	1,4,5,6,18, 21,22,24	2,3,7,8,17, 19,20,23	3	P13676,P36376,Q9WTT6
4913	1,4,5,6,18, 21,23,24	2,3,7,8,17, 19,20,22	5	O89117,P13676,P35280,P36860,Q9WTT6
4914	1,4,5,6,18, 22,23,24	2,3,7,8,17, 19,20,21	14	D4A5U3,P00507,P11883,P13676,P22006,P25809,P62804,Q00715,Q6P6R2,Q811M5,Q9EQS0,Q9 QX74,Q9WTT6,Q9WVK7
4915	1,4,5,6,19, 20,21,22	2,3,7,8,17, 18,23,24	3	P19218,P20766,Q5PQL7
4916	1,4,5,6,19, 20,21,23	2,3,7,8,17, 18,22,24	4	P10758,P20766,P23739,P35280
4917	1,4,5,6,19, 20,21,24	2,3,7,8,17, 18,22,23	4	O35547,P20766,P35280,P36374
4918	1,4,5,6,19, 20,22,23	2,3,7,8,17, 18,21,24	6	P10758,P19218,P20766,P23739,Q00715,Q811M5
4919	1,4,5,6,19, 20,22,24	2,3,7,8,17, 18,21,23	3	P20766,P22006,P62804
4920	1,4,5,6,19, 20,23,24	2,3,7,8,17, 18,21,22	4	P10758,P23739,P35280,P62804
4921	1,4,5,6,19, 21,22,23	2,3,7,8,17, 18,20,24	3	P13676,P20766,Q811M5

4922	1,4,5,6,19,21,22,24	2,3,7,8,17,18,20,23	3	P20766,P30919,P36376
4923	1,4,5,6,19,21,23,24	2,3,7,8,17,18,20,22	3	P25809,P35280,Q63474
4924	1,4,5,6,19,22,23,24	2,3,7,8,17,18,20,21	11	D4A5U3,P00507,P13676,P25809,P62804,Q00715,Q6P6R2,Q811M5,Q9EQS0,Q9QX74,Q9WTT6
4925	1,4,5,6,20,21,22,23	2,3,7,8,17,18,19,24	2	P20766,P54921
4926	1,4,5,6,20,21,22,24	2,3,7,8,17,18,19,23	2	P20766,P22006
4927	1,4,5,6,20,21,23,24	2,3,7,8,17,18,19,22	3	P20766,P23739,P35280
4928	1,4,5,6,20,22,23,24	2,3,7,8,17,18,19,21	11	P20766,P22006,P23739,P62804,Q00715,Q05175,Q5QE79,Q66H69,Q6P6R2,Q9QX74,Q9WVK7
4929	1,4,5,6,21,22,23,24	2,3,7,8,17,18,19,20	7	P13676,P20766,P25809,P30919,P36860,Q811M5,Q9WTT6
4930	1,4,5,7,8,17,18,19	2,3,6,20,21,22,23,24	5	P09656,P13432,P20646,Q66H69,Q9QX74
4931	1,4,5,7,8,17,18,20	2,3,6,19,21,22,23,24	9	P02780,P06911,P13432,P97697,Q4G075,Q6P6Q2,Q811M5,Q9WTT6,Q9Z0J6
4932	1,4,5,7,8,17,18,21	2,3,6,19,20,22,23,24	7	P06760,P22006,P49134,Q6P6Q2,Q811M5,Q9QX74,Q9Z1F2
4933	1,4,5,7,8,17,18,22	2,3,6,19,20,21,23,24	3	P13432,P27590,P49134
4934	1,4,5,7,8,17,18,23	2,3,6,19,20,21,22,24	2	P49134,Q9Z0V6
4935	1,4,5,7,8,17,18,24	2,3,6,19,20,21,22,23	4	P13432,P49134,Q4FZU2,Q6P6Q2
4936	1,4,5,7,8,17,19,20	2,3,6,18,21,22,23,24	15	P02780,P06760,P06911,P13676,P20646,P22282,P36374,P97697,Q4G075,Q5I0D1,Q6IMF3,Q6P6Q2,Q811M5,Q9QX74,Q9WTT6
4937	1,4,5,7,8,17,19,21	2,3,6,18,20,22,23,24	12	P06760,P20646,P22006,P22282,Q4FZU2,Q63474,Q63493,Q66H69,Q6IMF3,Q6P6Q2,Q99MH3,Q9QX74
4938	1,4,5,7,8,17,19,22	2,3,6,18,20,21,23,24	1	P20646
4939	1,4,5,7,8,17,19,23	2,3,6,18,20,21,22,24	2	P20646,P21674
4940	1,4,5,7,8,17,19,24	2,3,6,18,20,21,22,23	5	O54728,Q4FZU2,Q6IMF3,Q6P6Q2,Q99MH3
4941	1,4,5,7,8,17,20,21	2,3,6,18,19,22,23,24	18	D4A5U3,P02780,P06760,P13676,P19223,P22273,P55159,P97697,Q4FZU2,Q4G075,Q63493,Q6IMF3,Q6P6Q2,Q811M5,Q812E4,Q9QX74,Q9WTT6,Q9Z0J6

4942	1,4,5,7,8,17,20,22	2,3,6,18,19,21,23,24	4	P06911,Q4G075,Q6P6Q2,Q9Z0J6
4943	1,4,5,7,8,17,20,23	2,3,6,18,19,21,22,24	6	P10758,P23739,P97697,Q4G075,Q6IMF3,Q6P6Q2
4944	1,4,5,7,8,17,20,24	2,3,6,18,19,21,22,23	8	P06911,P97697,Q4FZU2,Q4G075,Q6IMF3,Q6P6Q2,Q811M5,Q9JHB9
4945	1,4,5,7,8,17,21,22	2,3,6,18,19,20,23,24	5	P06760,Q4FZU2,Q6IMF3,Q6P6Q2,Q9QX74
4946	1,4,5,7,8,17,21,23	2,3,6,18,19,20,22,24	8	P06760,P22006,Q4FZU2,Q63474,Q6IMF3,Q6P6Q2,Q9QX74,Q9Z0V6
4947	1,4,5,7,8,17,21,24	2,3,6,18,19,20,22,23	10	P02782,P06760,P36860,Q4FZU2,Q63474,Q6IMF3,Q6P6Q2,Q811M5,Q99MH3,Q9QX74
4948	1,4,5,7,8,17,22,23	2,3,6,18,19,20,21,24	3	P27590,P49134,Q68G31
4949	1,4,5,7,8,17,22,24	2,3,6,18,19,20,21,23	3	P49134,Q4FZU2,Q6P6Q2
4950	1,4,5,7,8,17,23,24	2,3,6,18,19,20,21,22	4	P49134,Q4FZU2,Q6IMF3,Q6P6Q2
4951	1,4,5,7,8,18,19,20	2,3,6,17,21,22,23,24	7	P02780,P22282,P36374,P47967,P97840,Q4G075,Q5I0D1
4952	1,4,5,7,8,18,19,21	2,3,6,17,20,22,23,24	5	O89117,P06760,P22282,P63029,Q66H69
4953	1,4,5,7,8,18,19,22	2,3,6,17,20,21,23,24	2	P27590,P63029
4954	1,4,5,7,8,18,19,23	2,3,6,17,20,21,22,24	1	P63029
4955	1,4,5,7,8,18,19,24	2,3,6,17,20,21,22,23	3	O54728,P09656,P63029
4956	1,4,5,7,8,18,20,21	2,3,6,17,19,22,23,24	10	P02780,P06760,P22273,P22282,P47967,P97840,Q4G075,Q5I0D1,Q6P6Q2,Q811M5
4957	1,4,5,7,8,18,20,22	2,3,6,17,19,21,23,24	4	P02780,P97840,Q03191,Q4G075
4958	1,4,5,7,8,18,20,23	2,3,6,17,19,21,22,24	5	P10758,P23739,Q03191,Q4G075,Q9WVK7
4959	1,4,5,7,8,18,20,24	2,3,6,17,19,21,22,23	4	P02780,Q4G075,Q6P6Q2,Q9JHB9
4960	1,4,5,7,8,18,21,22	2,3,6,17,19,20,23,24	1	P49134
4961	1,4,5,7,8,18,21,23	2,3,6,17,19,20,22,24	2	O89117,P49134

4962	1,4,5,7,8,18,21,24	2,3,6,17,19,20,22,23	5	O89117,P36860,P49134,P63029,Q6P6Q2
4963	1,4,5,7,8,18,22,23	2,3,6,17,19,20,21,24	4	P27590,P49134,P63029,Q9WVK7
4964	1,4,5,7,8,18,22,24	2,3,6,17,19,20,21,23	3	P49134,P63029,Q9WVK7
4965	1,4,5,7,8,18,23,24	2,3,6,17,19,20,21,22	4	O89117,P49134,P63029,Q9WVK7
4966	1,4,5,7,8,19,20,21	2,3,6,17,18,22,23,24	22	P02780,P04905,P06760,P0C0A9,P22273,P22282,P22283,P36374,P40241,P47967,P55159,P97840,Q4G075,Q5I0D1,Q5M8C6,Q63493,Q63617,Q6IMF3,Q6P6Q2,Q812E4,Q9R0T3,Q9WTT6
4967	1,4,5,7,8,19,20,22	2,3,6,17,18,21,23,24	7	P02780,P19218,P22282,P36374,Q4G075,Q5I0D1,Q99041
4968	1,4,5,7,8,19,20,23	2,3,6,17,18,21,22,24	7	P02780,P10758,P23739,P36374,Q4G075,Q5I0D1,Q812E4
4969	1,4,5,7,8,19,20,24	2,3,6,17,18,21,22,23	12	O54728,P02780,P08723,P22282,P36374,P60905,Q4G075,Q5I0D1,Q6IMF3,Q6P6Q2,Q812E4,Q9JHB9
4970	1,4,5,7,8,19,21,22	2,3,6,17,18,20,23,24	3	P06760,P19218,Q99041
4971	1,4,5,7,8,19,21,23	2,3,6,17,18,20,22,24	2	P06760,Q6IMF3
4972	1,4,5,7,8,19,21,24	2,3,6,17,18,20,22,23	10	O54728,P02782,P06760,P22282,P63029,Q4FZU2,Q63474,Q6IMF3,Q6P6Q2,Q99MH3
4973	1,4,5,7,8,19,22,23	2,3,6,17,18,20,21,24	3	P19218,P27590,P63029
4974	1,4,5,7,8,19,22,24	2,3,6,17,18,20,21,23	2	O54728,P63029
4975	1,4,5,7,8,19,23,24	2,3,6,17,18,20,21,22	2	O54728,P63029
4976	1,4,5,7,8,20,21,22	2,3,6,17,18,19,23,24	4	P02780,P06760,Q4G075,Q6P6Q2
4977	1,4,5,7,8,20,21,23	2,3,6,17,18,19,22,24	9	P02780,P06760,P08649,P10758,P23739,Q4G075,Q6IMF3,Q6P6Q2,Q812E4
4978	1,4,5,7,8,20,21,24	2,3,6,17,18,19,22,23	20	P02780,P02781,P02782,P06760,P08649,P08723,P09456,P22273,P22282,P30120,P55159,Q4FZU2,Q4G075,Q5M8C6,Q6AYR9,Q6IMF3,Q6P6Q2,Q811M5,Q812E4,Q9JHB9
4979	1,4,5,7,8,20,22,23	2,3,6,17,18,19,21,24	6	P10758,P19218,P23739,Q03191,Q4G075,Q9WVK7
4980	1,4,5,7,8,20,22,24	2,3,6,17,18,19,21,23	3	P02780,Q4G075,Q6P6Q2
4981	1,4,5,7,8,20,23,24	2,3,6,17,18,19,21,22	8	P10758,P23739,Q4G075,Q6IMF3,Q6P6Q2,Q812E4,Q9QYP1,Q9WVK7

4982	1,4,5,7,8,21,22,23	2,3,6,17,18,19,20,24	1 P49134
4983	1,4,5,7,8,21,22,24	2,3,6,17,18,19,20,23	4 P49134,Q4FZU2,Q6IMF3,Q6P6Q2
4984	1,4,5,7,8,21,23,24	2,3,6,17,18,19,20,22	8 O89117,P08649,P36860,P49134,Q4FZU2,Q63474,Q6IMF3,Q6P6Q2
4985	1,4,5,7,8,22,23,24	2,3,6,17,18,19,20,21	5 P25809,P49134,P63029,Q6P6R2,Q9WVK7
4986	1,4,5,7,17,18,19,20	2,3,6,8,21,22,23,24	4 P20646,P30919,P36374,P97840
4987	1,4,5,7,17,18,19,21	2,3,6,8,20,22,23,24	3 P20646,P97840,Q99MH3
4988	1,4,5,7,17,18,19,22	2,3,6,8,20,21,23,24	0
4989	1,4,5,7,17,18,19,23	2,3,6,8,20,21,22,24	0
4990	1,4,5,7,17,18,19,24	2,3,6,8,20,21,22,23	2 P09656,Q99MH3
4991	1,4,5,7,17,18,20,21	2,3,6,8,19,22,23,24	5 P00507,P19223,P97840,Q811M5,Q9Z0J6
4992	1,4,5,7,17,18,20,22	2,3,6,8,19,21,23,24	3 P97840,Q03191,Q9Z0J6
4993	1,4,5,7,17,18,20,23	2,3,6,8,19,21,22,24	3 P23739,P30919,Q03191
4994	1,4,5,7,17,18,20,24	2,3,6,8,19,21,22,23	1 Q9Z0J6
4995	1,4,5,7,17,18,21,22	2,3,6,8,19,20,23,24	0
4996	1,4,5,7,17,18,21,23	2,3,6,8,19,20,22,24	0
4997	1,4,5,7,17,18,21,24	2,3,6,8,19,20,22,23	3 P36376,P36860,Q99MH3
4998	1,4,5,7,17,18,22,23	2,3,6,8,19,20,21,24	1 Q68G31
4999	1,4,5,7,17,18,22,24	2,3,6,8,19,20,21,23	0
5000	1,4,5,7,17,18,23,24	2,3,6,8,19,20,21,22	0
5001	1,4,5,7,17,19,20,21	2,3,6,8,18,22,23,24	7 P06760,P20646,P36374,P55159,P97840,Q5I0D1,Q99MH3

5002	1,4,5,7,17,<br>19,20,22	2,3,6,8,18,<br>21,23,24	3 P20646,P36374,Q9Z0J6
5003	1,4,5,7,17,<br>19,20,23	2,3,6,8,18,<br>21,22,24	3 P02625,P10758,P23739
5004	1,4,5,7,17,<br>19,20,24	2,3,6,8,18,<br>21,22,23	3 P36374,P54921,Q99MH3
5005	1,4,5,7,17,<br>19,21,22	2,3,6,8,18,<br>20,23,24	2 Q99MH3,Q9R168
5006	1,4,5,7,17,<br>19,21,23	2,3,6,8,18,<br>20,22,24	0
5007	1,4,5,7,17,<br>19,21,24	2,3,6,8,18,<br>20,22,23	4 P54921,Q62761;Q62762;Q62763,Q99MH3,Q9R168
5008	1,4,5,7,17,<br>19,22,23	2,3,6,8,18,<br>20,21,24	1 Q9R168
5009	1,4,5,7,17,<br>19,22,24	2,3,6,8,18,<br>20,21,23	2 Q99MH3,Q9R168
5010	1,4,5,7,17,<br>19,23,24	2,3,6,8,18,<br>20,21,22	1 P25809
5011	1,4,5,7,17,<br>20,21,22	2,3,6,8,18,<br>19,23,24	1 Q9Z0J6
5012	1,4,5,7,17,<br>20,21,23	2,3,6,8,18,<br>19,22,24	1 P23739
5013	1,4,5,7,17,<br>20,21,24	2,3,6,8,18,<br>19,22,23	3 Q811M5,Q99MH3,Q9Z0J6
5014	1,4,5,7,17,<br>20,22,23	2,3,6,8,18,<br>19,21,24	3 P23739,Q03191,Q68G31
5015	1,4,5,7,17,<br>20,22,24	2,3,6,8,18,<br>19,21,23	1 Q9Z0J6
5016	1,4,5,7,17,<br>20,23,24	2,3,6,8,18,<br>19,21,22	2 P23739,Q9QYP1
5017	1,4,5,7,17,<br>21,22,23	2,3,6,8,18,<br>19,20,24	2 Q68G31,Q9R168
5018	1,4,5,7,17,<br>21,22,24	2,3,6,8,18,<br>19,20,23	3 P36860,Q99MH3,Q9R168
5019	1,4,5,7,17,<br>21,23,24	2,3,6,8,18,<br>19,20,22	3 P36860,Q63474,Q9R168
5020	1,4,5,7,17,<br>22,23,24	2,3,6,8,18,<br>19,20,21	4 P25809,Q00715,Q68G31,Q9R168
5021	1,4,5,7,18,<br>19,20,21	2,3,6,8,17,<br>22,23,24	5 P36374,P47967,P97840,Q5I0D1,Q99MH3

5022	1,4,5,7,18, 19,20,22	2,3,6,8,17, 21,23,24	1 P97840
5023	1,4,5,7,18, 19,20,23	2,3,6,8,17, 21,22,24	3 P10758,P23739,P97840
5024	1,4,5,7,18, 19,20,24	2,3,6,8,17, 21,22,23	3 P36374,P97840,Q99MH3
5025	1,4,5,7,18, 19,21,22	2,3,6,8,17, 20,23,24	3 P36376,P63029,P97840
5026	1,4,5,7,18, 19,21,23	2,3,6,8,17, 20,22,24	3 O89117,P63029,P97840
5027	1,4,5,7,18, 19,21,24	2,3,6,8,17, 20,22,23	5 O89117,P36376,P63029,P97840,Q99MH3
5028	1,4,5,7,18, 19,22,23	2,3,6,8,17, 20,21,24	1 P63029
5029	1,4,5,7,18, 19,22,24	2,3,6,8,17, 20,21,23	3 P17559,P36376,P63029
5030	1,4,5,7,18, 19,23,24	2,3,6,8,17, 20,21,22	2 P25809,P63029
5031	1,4,5,7,18, 20,21,22	2,3,6,8,17, 19,23,24	3 P97840,Q03191,Q9Z0J6
5032	1,4,5,7,18, 20,21,23	2,3,6,8,17, 19,22,24	3 O89117,P97840,Q03191
5033	1,4,5,7,18, 20,21,24	2,3,6,8,17, 19,22,23	3 O89117,P97840,Q99MH3
5034	1,4,5,7,18, 20,22,23	2,3,6,8,17, 19,21,24	3 P97840,Q03191,Q9WVK7
5035	1,4,5,7,18, 20,22,24	2,3,6,8,17, 19,21,23	4 P97840,Q03191,Q9WVK7,Q9Z0J6
5036	1,4,5,7,18, 20,23,24	2,3,6,8,17, 19,21,22	5 P10758,P23739,Q03191,Q9QYP1,Q9WVK7
5037	1,4,5,7,18, 21,22,23	2,3,6,8,17, 19,20,24	3 O89117,Q03191,Q68G31
5038	1,4,5,7,18, 21,22,24	2,3,6,8,17, 19,20,23	3 O89117,P36376,P63029
5039	1,4,5,7,18, 21,23,24	2,3,6,8,17, 19,20,22	2 O89117,P63029
5040	1,4,5,7,18, 22,23,24	2,3,6,8,17, 19,20,21	6 D4A5U3,P13676,P25809,P63029,Q6P6R2,Q9WVK7
5041	1,4,5,7,19, 20,21,22	2,3,6,8,17, 18,23,24	4 P08649,P47967,P97840,Q5I0D1

5042	1,4,5,7,19, 20,21,23	2,3,6,8,17, 18,22,24	5 P08649,P10758,P23739,P97840,Q5I0D1
5043	1,4,5,7,19, 20,21,24	2,3,6,8,17, 18,22,23	7 O35547,P08649,P36374,P55159,P97840,Q5I0D1,Q99MH3
5044	1,4,5,7,19, 20,22,23	2,3,6,8,17, 18,21,24	3 P02625,P10758,P23739
5045	1,4,5,7,19, 20,22,24	2,3,6,8,17, 18,21,23	0
5046	1,4,5,7,19, 20,23,24	2,3,6,8,17, 18,21,22	4 P08649,P10758,P23739,Q9QYP1
5047	1,4,5,7,19, 21,22,23	2,3,6,8,17, 18,20,24	1 P08649
5048	1,4,5,7,19, 21,22,24	2,3,6,8,17, 18,20,23	7 P08649,P17559,P36376,P55091,P63029,Q99MH3,Q9R168
5049	1,4,5,7,19, 21,23,24	2,3,6,8,17, 18,20,22	4 P08649,P25809,P63029,Q62761;Q62762;Q62763
5050	1,4,5,7,19, 22,23,24	2,3,6,8,17, 18,20,21	5 P25809,P26772,P55091,P63029,Q00715
5051	1,4,5,7,20, 21,22,23	2,3,6,8,17, 18,19,24	3 P08649,P97840,Q03191
5052	1,4,5,7,20, 21,22,24	2,3,6,8,17, 18,19,23	1 P08649
5053	1,4,5,7,20, 21,23,24	2,3,6,8,17, 18,19,22	3 P08649,P23739,Q9QYP1
5054	1,4,5,7,20, 22,23,24	2,3,6,8,17, 18,19,21	5 P08649,P23739,Q03191,Q9QYP1,Q9WVK7
5055	1,4,5,7,21, 22,23,24	2,3,6,8,17, 18,19,20	3 P08649,P25809,Q9R168
5056	1,4,5,8,17, 18,19,20	2,3,6,7,21, 22,23,24	5 B0LT89,P01835,P13432,P36374,Q62714
5057	1,4,5,8,17, 18,19,21	2,3,6,7,20, 22,23,24	3 P06760,P22006,Q9QX74
5058	1,4,5,8,17, 18,19,22	2,3,6,7,20, 21,23,24	1 P01835
5059	1,4,5,8,17, 18,19,23	2,3,6,7,20, 21,22,24	1 P01835
5060	1,4,5,8,17, 18,19,24	2,3,6,7,20, 21,22,23	3 O54728,P01835,P09656
5061	1,4,5,8,17, 18,20,21	2,3,6,7,19, 22,23,24	5 P06760,P13432,Q10758,Q6P6Q2,Q811M5

5062	1,4,5,8,17,<br>18,20,22	2,3,6,7,19,<br>21,23,24	2	P13432,Q9Z0J6
5063	1,4,5,8,17,<br>18,20,23	2,3,6,7,19,<br>21,22,24	1	P23739
5064	1,4,5,8,17,<br>18,20,24	2,3,6,7,19,<br>21,22,23	2	P13432,Q6P6Q2
5065	1,4,5,8,17,<br>18,21,22	2,3,6,7,19,<br>20,23,24	0	
5066	1,4,5,8,17,<br>18,21,23	2,3,6,7,19,<br>20,22,24	0	
5067	1,4,5,8,17,<br>18,21,24	2,3,6,7,19,<br>20,22,23	2	Q4FZU2,Q6P6Q2
5068	1,4,5,8,17,<br>18,22,23	2,3,6,7,19,<br>20,21,24	0	
5069	1,4,5,8,17,<br>18,22,24	2,3,6,7,19,<br>20,21,23	0	
5070	1,4,5,8,17,<br>18,23,24	2,3,6,7,19,<br>20,21,22	0	
5071	1,4,5,8,17,<br>19,20,21	2,3,6,7,18,<br>22,23,24	8	B0LT89,P04905,P06760,P20766,P36374,Q6IMF3,Q6P6Q2,Q6P6R2
5072	1,4,5,8,17,<br>19,20,22	2,3,6,7,18,<br>21,23,24	3	P01835,P20766,P36374
5073	1,4,5,8,17,<br>19,20,23	2,3,6,7,18,<br>21,22,24	4	P01835,P10758,P23739,P36374
5074	1,4,5,8,17,<br>19,20,24	2,3,6,7,18,<br>21,22,23	7	O54728,P01835,P36374,P60905,Q6IMF3,Q6P6Q2,Q99MH3
5075	1,4,5,8,17,<br>19,21,22	2,3,6,7,18,<br>20,23,24	1	P20766
5076	1,4,5,8,17,<br>19,21,23	2,3,6,7,18,<br>20,22,24	2	P20766,P22006
5077	1,4,5,8,17,<br>19,21,24	2,3,6,7,18,<br>20,22,23	7	O54728,Q4FZU2,Q62894,Q63474,Q6IMF3,Q6P6Q2,Q99MH3
5078	1,4,5,8,17,<br>19,22,23	2,3,6,7,18,<br>20,21,24	3	P00507,P01835,P20766
5079	1,4,5,8,17,<br>19,22,24	2,3,6,7,18,<br>20,21,23	3	O54728,P00507,P01835
5080	1,4,5,8,17,<br>19,23,24	2,3,6,7,18,<br>20,21,22	2	O54728,P01835
5081	1,4,5,8,17,<br>20,21,22	2,3,6,7,18,<br>19,23,24	2	P20766,Q6P6Q2

5082	1,4,5,8,17, 20,21,23	2,3,6,7,18, 19,22,24	4	P20766,P23739,Q6IMF3,Q6P6Q2
5083	1,4,5,8,17, 20,21,24	2,3,6,7,18, 19,22,23	7	P02782,P20766,P30120,Q4FZU2,Q6IMF3,Q6P6Q2,Q811M5
5084	1,4,5,8,17, 20,22,23	2,3,6,7,18, 19,21,24	2	P20766,P23739
5085	1,4,5,8,17, 20,22,24	2,3,6,7,18, 19,21,23	2	P20766,Q6P6Q2
5086	1,4,5,8,17, 20,23,24	2,3,6,7,18, 19,21,22	5	P20766,P23739,Q4FZU2,Q6IMF3,Q6P6Q2
5087	1,4,5,8,17, 21,22,23	2,3,6,7,18, 19,20,24	1	P20766
5088	1,4,5,8,17, 21,22,24	2,3,6,7,18, 19,20,23	3	P20766,Q4FZU2,Q6P6Q2
5089	1,4,5,8,17, 21,23,24	2,3,6,7,18, 19,20,22	5	P20766,Q4FZU2,Q63474,Q6IMF3,Q6P6Q2
5090	1,4,5,8,17, 22,23,24	2,3,6,7,18, 19,20,21	4	P00507,P20766,P47967,Q00715
5091	1,4,5,8,18, 19,20,21	2,3,6,7,17, 22,23,24	6	B0LT89,O08815,P04905,P06760,P36374,Q5I0D1
5092	1,4,5,8,18, 19,20,22	2,3,6,7,17, 21,23,24	1	Q6AY61
5093	1,4,5,8,18, 19,20,23	2,3,6,7,17, 21,22,24	4	P01039,P10758,P23739,Q9R168
5094	1,4,5,8,18, 19,20,24	2,3,6,7,17, 21,22,23	4	O54728,P36374,P60905,Q9R168
5095	1,4,5,8,18, 19,21,22	2,3,6,7,17, 20,23,24	0	
5096	1,4,5,8,18, 19,21,23	2,3,6,7,17, 20,22,24	0	
5097	1,4,5,8,18, 19,21,24	2,3,6,7,17, 20,22,23	3	O54728,P63029,Q99MH3
5098	1,4,5,8,18, 19,22,23	2,3,6,7,17, 20,21,24	3	P00507,P63029,Q6AY61
5099	1,4,5,8,18, 19,22,24	2,3,6,7,17, 20,21,23	3	O54728,P00507,P63029
5100	1,4,5,8,18, 19,23,24	2,3,6,7,17, 20,21,22	2	O54728,P63029
5101	1,4,5,8,18, 20,21,22	2,3,6,7,17, 19,23,24	1	P54921

5102	1,4,5,8,18,<br>20,21,23	2,3,6,7,17,<br>19,22,24	2	P23739,P54921
5103	1,4,5,8,18,<br>20,21,24	2,3,6,7,17,<br>19,22,23	1	Q6P6Q2
5104	1,4,5,8,18,<br>20,22,23	2,3,6,7,17,<br>19,21,24	6	P01039,P23739,P54921,Q5QE79,Q6AY61,Q9WVK7
5105	1,4,5,8,18,<br>20,22,24	2,3,6,7,17,<br>19,21,23	0	
5106	1,4,5,8,18,<br>20,23,24	2,3,6,7,17,<br>19,21,22	4	P23739,Q5QE79,Q9R168,Q9WVK7
5107	1,4,5,8,18,<br>21,22,23	2,3,6,7,17,<br>19,20,24	2	P54921,Q6AY61
5108	1,4,5,8,18,<br>21,22,24	2,3,6,7,17,<br>19,20,23	0	
5109	1,4,5,8,18,<br>21,23,24	2,3,6,7,17,<br>19,20,22	1	O89117
5110	1,4,5,8,18,<br>22,23,24	2,3,6,7,17,<br>19,20,21	4	P00507,P49134,P63029,Q9WVK7
5111	1,4,5,8,19,<br>20,21,22	2,3,6,7,17,<br>18,23,24	3	P19218,P20766,P36374
5112	1,4,5,8,19,<br>20,21,23	2,3,6,7,17,<br>18,22,24	5	P10758,P20766,P23739,P36374,Q6P6Q2
5113	1,4,5,8,19,<br>20,21,24	2,3,6,7,17,<br>18,22,23	12	O35547,O54728,P02782,P04905,P20766,P30120,P36374,P60905,Q6IMF3,Q6P6Q2,Q812E4,Q99<br>MH3
5114	1,4,5,8,19,<br>20,22,23	2,3,6,7,17,<br>18,21,24	6	P01039,P10758,P19218,P20766,P23739,Q6AY61
5115	1,4,5,8,19,<br>20,22,24	2,3,6,7,17,<br>18,21,23	3	O54728,P20766,P36374
5116	1,4,5,8,19,<br>20,23,24	2,3,6,7,17,<br>18,21,22	4	O54728,P10758,P23739,Q6P6Q2
5117	1,4,5,8,19,<br>21,22,23	2,3,6,7,17,<br>18,20,24	1	P20766
5118	1,4,5,8,19,<br>21,22,24	2,3,6,7,17,<br>18,20,23	4	O54728,P20766,P30919,P55091
5119	1,4,5,8,19,<br>21,23,24	2,3,6,7,17,<br>18,20,22	2	O54728,Q63474
5120	1,4,5,8,19,<br>22,23,24	2,3,6,7,17,<br>18,20,21	6	O54728,P00507,P25809,P55091,P63029,Q00715
5121	1,4,5,8,20,<br>21,22,23	2,3,6,7,17,<br>18,19,24	3	P20766,P23739,P54921

5122	1,4,5,8,20,21,22,24	2,3,6,7,17,18,19,23	2	P20766,Q6P6Q2
5123	1,4,5,8,20,21,23,24	2,3,6,7,17,18,19,22	4	P20766,P23739,Q6IMF3,Q6P6Q2
5124	1,4,5,8,20,22,23,24	2,3,6,7,17,18,19,21	4	P20766,P23739,Q5QE79,Q9WVK7
5125	1,4,5,8,21,22,23,24	2,3,6,7,17,18,19,20	2	P20766,P30919
5126	1,4,5,17,18,19,20,21	2,3,6,7,8,22,23,24	6	B0LT89,P35280,P36374,Q62714,Q6P6R2,Q99MH3
5127	1,4,5,17,18,19,20,22	2,3,6,7,8,21,23,24	1	Q62714
5128	1,4,5,17,18,19,20,23	2,3,6,7,8,21,22,24	4	P10758,P23739,P35280,Q62714
5129	1,4,5,17,18,19,20,24	2,3,6,7,8,21,22,23	4	P35280,Q62714,Q63532,Q99MH3
5130	1,4,5,17,18,19,21,22	2,3,6,7,8,20,23,24	1	Q62894
5131	1,4,5,17,18,19,21,23	2,3,6,7,8,20,22,24	1	P35280
5132	1,4,5,17,18,19,21,24	2,3,6,7,8,20,22,23	3	P35280,Q62894,Q99MH3
5133	1,4,5,17,18,19,22,23	2,3,6,7,8,20,21,24	2	P02631,Q00715
5134	1,4,5,17,18,19,22,24	2,3,6,7,8,20,21,23	1	P02631
5135	1,4,5,17,18,19,23,24	2,3,6,7,8,20,21,22	2	P02631,P35280
5136	1,4,5,17,18,20,21,22	2,3,6,7,8,19,23,24	2	P20766,Q9Z0J6
5137	1,4,5,17,18,20,21,23	2,3,6,7,8,19,22,24	1	P35280
5138	1,4,5,17,18,20,21,24	2,3,6,7,8,19,22,23	2	P35280,Q99MH3
5139	1,4,5,17,18,20,22,23	2,3,6,7,8,19,21,24	0	
5140	1,4,5,17,18,20,22,24	2,3,6,7,8,19,21,23	2	P50115,Q9Z0J6
5141	1,4,5,17,18,20,23,24	2,3,6,7,8,19,21,22	4	P11883,P23739,P35280,Q9QYP1

5142	1,4,5,17,18,21,22,23	2,3,6,7,8,19,20,24	0
5143	1,4,5,17,18,21,22,24	2,3,6,7,8,19,20,23	0
5144	1,4,5,17,18,21,23,24	2,3,6,7,8,19,20,22	1 P35280
5145	1,4,5,17,18,22,23,24	2,3,6,7,8,19,20,21	7 D4A5U3,P02631,P62804,Q00715,Q5RKI1,Q63617,Q9WTT6
5146	1,4,5,17,19,20,21,22	2,3,6,7,8,18,23,24	3 B0LT89,P20766,Q3ZAV1
5147	1,4,5,17,19,20,21,23	2,3,6,7,8,18,22,24	3 P20766,P23739,P35280
5148	1,4,5,17,19,20,21,24	2,3,6,7,8,18,22,23	6 B0LT89,P20766,P35280,P36374,P80202,Q99MH3
5149	1,4,5,17,19,20,22,23	2,3,6,7,8,18,21,24	4 P02625,P20766,P23739,Q00715
5150	1,4,5,17,19,20,22,24	2,3,6,7,8,18,21,23	2 P20766,Q00715
5151	1,4,5,17,19,20,23,24	2,3,6,7,8,18,21,22	6 P02625,P10758,P20766,P23739,P35280,Q00715
5152	1,4,5,17,19,21,22,23	2,3,6,7,8,18,20,24	2 P12020,P20766
5153	1,4,5,17,19,21,22,24	2,3,6,7,8,18,20,23	4 P20766,Q62894,Q99MH3,Q9R168
5154	1,4,5,17,19,21,23,24	2,3,6,7,8,18,20,22	3 P12020,P20766,P35280
5155	1,4,5,17,19,22,23,24	2,3,6,7,8,18,20,21	6 P00507,P02631,P20766,P25809,P62804,Q00715
5156	1,4,5,17,20,21,22,23	2,3,6,7,8,18,19,24	2 P20766,P23739
5157	1,4,5,17,20,21,22,24	2,3,6,7,8,18,19,23	1 P20766
5158	1,4,5,17,20,21,23,24	2,3,6,7,8,18,19,22	4 P20766,P23739,P35280,Q9QYP1
5159	1,4,5,17,20,22,23,24	2,3,6,7,8,18,19,21	5 P02631,P20766,P23739,P62804,Q00715
5160	1,4,5,17,21,22,23,24	2,3,6,7,8,18,19,20	4 P12020,P20766,P50280,Q9R168
5161	1,4,5,18,19,20,21,22	2,3,6,7,8,17,23,24	3 P97840,Q5PQL7,Q63598

5162	1,4,5,18,19,20,21,23	2,3,6,7,8,17,22,24	2	P10758,P35280
5163	1,4,5,18,19,20,21,24	2,3,6,7,8,17,22,23	2	P35280,Q99MH3
5164	1,4,5,18,19,20,22,23	2,3,6,7,8,17,21,24	4	P01039,P04762,P10758,Q6AY61
5165	1,4,5,18,19,20,22,24	2,3,6,7,8,17,21,23	2	O70594,P50115
5166	1,4,5,18,19,20,23,24	2,3,6,7,8,17,21,22	3	P10758,P23739,P35280
5167	1,4,5,18,19,21,22,23	2,3,6,7,8,17,20,24	1	P13676
5168	1,4,5,18,19,21,22,24	2,3,6,7,8,17,20,23	5	P13676,P17559,P36376,P63029,Q62894
5169	1,4,5,18,19,21,23,24	2,3,6,7,8,17,20,22	5	O70417,O89117,P12020,P35280,P63029
5170	1,4,5,18,19,22,23,24	2,3,6,7,8,17,20,21	9	D4A5U3,P00507,P02631,P13676,P25809,P62804,P63029,Q00715,Q9WTT6
5171	1,4,5,18,20,21,22,23	2,3,6,7,8,17,19,24	3	P20766,P50115,P54921
5172	1,4,5,18,20,21,22,24	2,3,6,7,8,17,19,23	1	P50115
5173	1,4,5,18,20,21,23,24	2,3,6,7,8,17,19,22	5	O89117,P23739,P35280,P50115,Q9QYP1
5174	1,4,5,18,20,22,23,24	2,3,6,7,8,17,19,21	9	P02631,P04355,P23739,P50115,P62804,Q00715,Q5QE79,Q9QX74,Q9WVK7
5175	1,4,5,18,21,22,23,24	2,3,6,7,8,17,19,20	7	D4A5U3,O70417,O89117,P13676,P50115,Q5U2V4,Q9WTT6
5176	1,4,5,19,20,21,22,23	2,3,6,7,8,17,18,24	3	P20766,P23739,Q63598
5177	1,4,5,19,20,21,22,24	2,3,6,7,8,17,18,23	4	O70594,P20766,Q63598,Q99MH3
5178	1,4,5,19,20,21,23,24	2,3,6,7,8,17,18,22	5	P08649,P10758,P20766,P23739,P35280
5179	1,4,5,19,20,22,23,24	2,3,6,7,8,17,18,21	7	P02631,P10758,P20766,P23739,P26772,P62804,Q00715
5180	1,4,5,19,21,22,23,24	2,3,6,7,8,17,18,20	8	D4A5U3,O70417,P06911,P12020,P13676,P20766,P25809,P55091
5181	1,4,5,20,21,22,23,24	2,3,6,7,8,17,18,19	4	P08649,P20766,P23739,P50115

5182	1,4,6,7,8,17,18,19	2,3,5,20,21,22,23,24	10	P06761,P07150,P11598,P13432,P18418,P20646,P21674,P22282,Q99041,Q9QX74
5183	1,4,6,7,8,17,18,20	2,3,5,19,21,22,23,24	19	P02780,P06761,P06911,P11598,P12020,P13432,P18418,P19629,P22273,P22282,P22283,P47727,P50280,Q4G075,Q5GRG2,Q9JHB9,Q9JI85,Q9QW07,Q9Z0J6
5184	1,4,6,7,8,17,18,21	2,3,5,19,20,22,23,24	9	P06761,P11598,P13432,P18418,P22282,P22283,Q63493,Q9QX74,Q9Z1F2
5185	1,4,6,7,8,17,18,22	2,3,5,19,20,21,23,24	3	P13432,Q99041,Q9Z1F2
5186	1,4,6,7,8,17,18,23	2,3,5,19,20,21,22,24	3	P13432,P18418,P21674
5187	1,4,6,7,8,17,18,24	2,3,5,19,20,21,22,23	4	P13432,P18418,P49134,Q9Z1F2
5188	1,4,6,7,8,17,19,20	2,3,5,18,21,22,23,24	33	D4A5U3,P02780,P04905,P06761,P06911,P08723,P0C0A9,P11598,P12020,P13432,P18418,P19629,P20646,P22273,P22282,P22283,P36374,P46462,P47727,P50280,Q4G075,Q5GRG2,Q5M8C6,Q63493,Q63617,Q6RUV5,Q812E4,Q99041,Q9JHB9,Q9JI85,Q9QW07,Q9R0T3,Q9WTT6
5189	1,4,6,7,8,17,19,21	2,3,5,18,20,22,23,24	17	P00714,P06761,P0C0A9,P11598,P18418,P20646,P21674,P22282,P22283,Q5QE79,Q63474,Q63493,Q63617,Q66H69,Q99041,Q9JI85,Q9QX74
5190	1,4,6,7,8,17,19,22	2,3,5,18,20,21,23,24	2	P22282,Q99041
5191	1,4,6,7,8,17,19,23	2,3,5,18,20,21,22,24	3	P21674,P22282,Q99041
5192	1,4,6,7,8,17,19,24	2,3,5,18,20,21,22,23	5	O54728,P08723,P18418,P22282,Q63474
5193	1,4,6,7,8,17,20,21	2,3,5,18,19,22,23,24	41	D4A5U3,P02631,P02780,P02781,P02782,P06761,P06911,P07647,P08723,P09456,P11598,P12020,P13432,P18418,P19223,P19629,P22273,P22282,P22283,P30120,P36374,P46462,P47727,Q4G075,Q5GRG2,Q5M8C6,Q62946,Q63474,Q63493,Q63617,Q6AYR9,Q78P75,Q812E4,Q8CJ52,Q99041,Q9JHB9,Q9JI85,Q9QW07,Q9QX74,Q9R0T3,Q9WTT6
5194	1,4,6,7,8,17,20,22	2,3,5,18,19,21,23,24	16	O70417,P02780,P06761,P06911,P11598,P12020,P13432,P19629,P22273,P22282,Q4G075,Q5GRG2,Q99041,Q9JHB9,Q9QW07,Q9Z0J6
5195	1,4,6,7,8,17,20,23	2,3,5,18,19,21,22,24	11	P02780,P06911,P10758,P11598,P12020,P19629,P22273,P22282,Q4G075,Q5GRG2,Q812E4
5196	1,4,6,7,8,17,20,24	2,3,5,18,19,21,22,23	25	P02780,P02781,P06761,P06911,P08723,P09456,P11598,P12020,P13432,P18418,P22273,P22282,P22283,P36374,P46462,P47727,Q4G075,Q5GRG2,Q5M8C6,Q63617,Q6AYR9,Q812E4,Q9JHB9,Q9JI85,Q9QW07
5197	1,4,6,7,8,17,21,22	2,3,5,18,19,20,23,24	2	Q99041,Q9Z1F2
5198	1,4,6,7,8,17,21,23	2,3,5,18,19,20,22,24	3	P21674,Q63474,Q63493
5199	1,4,6,7,8,17,21,24	2,3,5,18,19,20,22,23	9	P02782,P18418,P22282,P22283,P36860,Q63474,Q6AYR9,Q9JHB9,Q9Z1F2

5200	1,4,6,7,8,17,22,23	2,3,5,18,19,20,21,24	1	Q99041
5201	1,4,6,7,8,17,22,24	2,3,5,18,19,20,21,23	2	P23593,Q99041
5202	1,4,6,7,8,17,23,24	2,3,5,18,19,20,21,22	2	Q63474,Q63598
5203	1,4,6,7,8,18,19,20	2,3,5,17,21,22,23,24	28	O08815,P02780,P06761,P06911,P08723,P0C0A9,P11598,P12020,P18418,P19629,P22273,P22282,P22283,P31044,P36374,P46462,P47727,P50280,Q4G075,Q5GRG2,Q63493,Q63617,Q812E4,Q99041,Q9JHB9,Q9JI85,Q9QW07,Q9R0T3
5204	1,4,6,7,8,18,19,21	2,3,5,17,20,22,23,24	12	P00714,P06761,P0C0A9,P11598,P18418,P22282,P22283,Q63493,Q63617,Q66H69,Q99041,Q9JI85
5205	1,4,6,7,8,18,19,22	2,3,5,17,20,21,23,24	4	P0C0A9,P22282,Q6AY61,Q99041
5206	1,4,6,7,8,18,19,23	2,3,5,17,20,21,22,24	4	P0C0A9,P21674,P22282,Q99041
5207	1,4,6,7,8,18,19,24	2,3,5,17,20,21,22,23	9	O54728,P06761,P08723,P0C0A9,P11598,P18418,P22282,Q99041,Q9QW07
5208	1,4,6,7,8,18,20,21	2,3,5,17,19,22,23,24	28	P02631,P02780,P02781,P06761,P06911,P07647,P08723,P0C0A9,P11598,P12020,P18418,P19629,P22273,P22282,P22283,P46462,P50280,Q5GRG2,Q5M8C6,Q63493,Q63617,Q78P75,Q812E4,Q99041,Q9JHB9,Q9JI85,Q9QW07,Q9R0T3
5209	1,4,6,7,8,18,20,22	2,3,5,17,19,21,23,24	16	P02780,P06761,P06911,P11598,P12020,P19629,P22273,P22282,P50280,P54921,Q4G075,Q5GRG2,Q6AY61,Q99041,Q9JHB9,Q9QW07
5210	1,4,6,7,8,18,20,23	2,3,5,17,19,21,22,24	17	P02780,P06761,P06911,P10758,P11598,P12020,P18418,P19629,P22273,P22282,P50280,P54921,Q4G075,Q5GRG2,Q812E4,Q99041,Q9QW07
5211	1,4,6,7,8,18,20,24	2,3,5,17,19,21,22,23	20	P02780,P06761,P06911,P08723,P0C0A9,P11598,P12020,P13432,P18418,P22273,P22282,P22283,P50280,Q5GRG2,Q5M8C6,Q63617,Q812E4,Q9JHB9,Q9JI85,Q9QW07
5212	1,4,6,7,8,18,21,22	2,3,5,17,19,20,23,24	4	P11598,P54921,Q99041,Q9Z1F2
5213	1,4,6,7,8,18,21,23	2,3,5,17,19,20,22,24	4	P11598,P19629,P54921,Q99041
5214	1,4,6,7,8,18,21,24	2,3,5,17,19,20,22,23	11	P02782,P11598,P18418,P22282,P22283,P36860,P49134,Q63474,Q6AYR9,Q9JHB9,Q9Z1F2
5215	1,4,6,7,8,18,22,23	2,3,5,17,19,20,21,24	6	P49134,P54921,Q63532,Q6AY61,Q99041,Q9WVK7
5216	1,4,6,7,8,18,22,24	2,3,5,17,19,20,21,23	5	P22006,P49134,Q99041,Q9WVK7,Q9Z1F2
5217	1,4,6,7,8,18,23,24	2,3,5,17,19,20,21,22	3	P49134,Q3ZAV1,Q9WVK7

5218	1,4,6,7,8,19,20,21	2,3,5,17,18,22,23,24	46	iRT-Kit_WR_fusion,O08815,O35547,P00714,P02631,P02780,P02781,P02782,P04905,P05369,P06761,P06911,P07647,P08723,P09456,P0C0A9,P11598,P12020,P18418,P19629,P22273,P22282,P22283,P24368,P30120,P36374,P40241,P46462,P50280,Q4G075,Q5GRG2,Q5I0D1,Q5M8C6,Q63493,Q63617,Q6AYR9,Q6Q7Y5,Q812E4,Q8CJ52,Q99041,Q9JHB9,Q9JI85,Q9QW07,Q9QZK8,Q9R0T3,Q9WTT6
5219	1,4,6,7,8,19,20,22	2,3,5,17,18,21,23,24	26	P02780,P06761,P06911,P08723,P0C0A9,P11598,P12020,P15399,P19218,P19629,P22273,P22282,P22283,P36374,P46462,P50280,Q4G075,Q5GRG2,Q63493,Q63617,Q812E4,Q99041,Q9JHB9,Q9JI85,Q9QW07,Q9QZK9
5220	1,4,6,7,8,19,20,23	2,3,5,17,18,21,22,24	23	P02780,P06761,P06911,P08723,P0C0A9,P10758,P11598,P12020,P19629,P22273,P22282,P22283,P36374,P46462,P50280,Q4G075,Q5GRG2,Q63493,Q63617,Q812E4,Q99041,Q9JI85,Q9QW07
5221	1,4,6,7,8,19,20,24	2,3,5,17,18,21,22,23	35	O35547,O54728,P00714,P02780,P02781,P06761,P06911,P07647,P08723,P09456,P0C0A9,P11598,P12020,P18418,P19629,P22273,P22282,P22283,P36374,P46462,P50280,P60905,Q4G075,Q5GRG2,Q5M8C6,Q63493,Q63617,Q6RUV5,Q812E4,Q8CJ52,Q99041,Q9JHB9,Q9JI85,Q9QW07,Q9R0T3
5222	1,4,6,7,8,19,21,22	2,3,5,17,18,20,23,24	10	P00714,P06761,P0C0A9,P11598,P22282,P22283,Q63493,Q63617,Q99041,Q9JI85
5223	1,4,6,7,8,19,21,23	2,3,5,17,18,20,22,24	13	P00714,P06761,P0C0A9,P11598,P19629,P21674,P22282,P22283,Q63474,Q63493,Q63617,Q99041,Q9JI85
5224	1,4,6,7,8,19,21,24	2,3,5,17,18,20,22,23	20	O54728,P00714,P02782,P06761,P07647,P08723,P0C0A9,P11598,P18418,P22282,P22283,Q63474,Q63493,Q63617,Q6AYR9,Q6Q7Y5,Q8CJ52,Q99041,Q9JI85,Q9QW07
5225	1,4,6,7,8,19,22,23	2,3,5,17,18,20,21,24	3	P19218,Q6AY61,Q99041
5226	1,4,6,7,8,19,22,24	2,3,5,17,18,20,21,23	5	O54728,P0C0A9,P22006,P22282,Q99041
5227	1,4,6,7,8,19,23,24	2,3,5,17,18,20,21,22	6	O54728,P0C0A9,P22282,P25809,Q63474,Q99041
5228	1,4,6,7,8,20,21,22	2,3,5,17,18,19,23,24	24	P02780,P06761,P06911,P08723,P0C0A9,P11598,P12020,P19629,P22273,P22282,P22283,P46462,Q4G075,Q5GRG2,Q5M8C6,Q63493,Q63617,Q6AYR9,Q78P75,Q812E4,Q99041,Q9JHB9,Q9JI85,Q9QW07
5229	1,4,6,7,8,20,21,23	2,3,5,17,18,19,22,24	23	P02780,P06761,P06911,P0C0A9,P10758,P11598,P19629,P22273,P22282,P22283,Q4G075,Q5GRG2,Q5M8C6,Q63493,Q63617,Q6AYR9,Q78P75,Q812E4,Q8CJ52,Q99041,Q9JHB9,Q9JI85,Q9QW07
5230	1,4,6,7,8,20,21,24	2,3,5,17,18,19,22,23	36	O35547,O88917,P00714,P02780,P02781,P02782,P06761,P06911,P07647,P08723,P09456,P0C0A9,P11598,P12020,P18418,P19629,P22273,P22282,P22283,P30120,P36374,P46462,Q5GRG2,Q5M8C6,Q63474,Q63493,Q63617,Q6AYR9,Q78P75,Q812E4,Q8CJ52,Q99041,Q9JHB9,Q9JI85,Q9QW07,Q9R0T3
5231	1,4,6,7,8,20,22,23	2,3,5,17,18,19,21,24	13	P02780,P06911,P10758,P11598,P12020,P19629,P22273,P54921,Q4G075,Q5GRG2,Q6AY61,Q99041,Q9QW07
5232	1,4,6,7,8,20,22,24	2,3,5,17,18,19,21,23	16	P02780,P06911,P08723,P11598,P12020,P19629,P22006,P22273,P22282,Q4G075,Q5GRG2,Q63617,Q812E4,Q99041,Q9JHB9,Q9QW07

5233	1,4,6,7,8,20,23,24	2,3,5,17,18,19,21,22	15	P02780,P06911,P08723,P10758,P11598,P12020,P19629,P22273,P22282,Q4G075,Q5GRG2,Q812E4,Q9JHB9,Q9QW07,Q9QYP1
5234	1,4,6,7,8,21,22,23	2,3,5,17,18,19,20,24	4	P19629,P54921,Q63532,Q99041
5235	1,4,6,7,8,21,22,24	2,3,5,17,18,19,20,23	4	P02782,Q63474,Q6AYR9,Q99041
5236	1,4,6,7,8,21,23,24	2,3,5,17,18,19,20,22	3	P36860,Q63474,Q6AYR9
5237	1,4,6,7,8,22,23,24	2,3,5,17,18,19,20,21	6	O54858,P22006,P25809,P49134,Q99041,Q9WVK7
5238	1,4,6,7,17,18,19,20	2,3,5,8,21,22,23,24	6	P11598,P20646,P22283,P30919,P36374,Q63617
5239	1,4,6,7,17,18,19,21	2,3,5,8,20,22,23,24	4	P05539,P11598,P22283,P36376
5240	1,4,6,7,17,18,19,22	2,3,5,8,20,21,23,24	1	P36376
5241	1,4,6,7,17,18,19,23	2,3,5,8,20,21,22,24	1	P21674
5242	1,4,6,7,17,18,19,24	2,3,5,8,20,21,22,23	2	P36376,P70549
5243	1,4,6,7,17,18,20,21	2,3,5,8,19,22,23,24	8	P00507,P05539,P07647,P11598,P19223,P22283,Q63617,Q9Z0J6
5244	1,4,6,7,17,18,20,22	2,3,5,8,19,21,23,24	1	Q9Z0J6
5245	1,4,6,7,17,18,20,23	2,3,5,8,19,21,22,24	3	P11598,P30919,Q9QYP1
5246	1,4,6,7,17,18,20,24	2,3,5,8,19,21,22,23	3	P11598,Q9QYP1,Q9Z0J6
5247	1,4,6,7,17,18,21,22	2,3,5,8,19,20,23,24	3	P05539,P23739,P36376
5248	1,4,6,7,17,18,21,23	2,3,5,8,19,20,22,24	0	
5249	1,4,6,7,17,18,21,24	2,3,5,8,19,20,22,23	3	P05539,P36376,P36860
5250	1,4,6,7,17,18,22,23	2,3,5,8,19,20,21,24	1	Q9EQS0
5251	1,4,6,7,17,18,22,24	2,3,5,8,19,20,21,23	2	P22006,P36376
5252	1,4,6,7,17,18,23,24	2,3,5,8,19,20,21,22	1	Q64268

5253	1,4,6,7,17, 19,20,21	2,3,5,8,18, 22,23,24	14	O08815,O35547,P05539,P07647,P11598,P20646,P22283,P24368,P36374,Q63493,Q63617,Q6P6R2,Q9JI85,Q9R0T3
5254	1,4,6,7,17, 19,20,22	2,3,5,8,18, 21,23,24	6	O89117,P11598,P36374,Q63617,Q9JJ50,Q9Z0J6
5255	1,4,6,7,17, 19,20,23	2,3,5,8,18, 21,22,24	4	P10758,P11598,P36374,Q63617
5256	1,4,6,7,17, 19,20,24	2,3,5,8,18, 21,22,23	6	O35547,P08723,P11598,P22283,P36374,Q63617
5257	1,4,6,7,17, 19,21,22	2,3,5,8,18, 20,23,24	3	P05539,P36376,Q9R168
5258	1,4,6,7,17, 19,21,23	2,3,5,8,18, 20,22,24	3	P21674,Q63474,Q9R168
5259	1,4,6,7,17, 19,21,24	2,3,5,8,18, 20,22,23	7	P05539,P22283,P36376,Q62761;Q62762;Q62763,Q63474,Q99MH3,Q9R168
5260	1,4,6,7,17, 19,22,23	2,3,5,8,18, 20,21,24	2	P25809,Q9R168
5261	1,4,6,7,17, 19,22,24	2,3,5,8,18, 20,21,23	5	P22006,P25809,P36376,P70549,Q9R168
5262	1,4,6,7,17, 19,23,24	2,3,5,8,18, 20,21,22	2	P25809,P70549
5263	1,4,6,7,17, 20,21,22	2,3,5,8,18, 19,23,24	5	P05539,P11598,P22283,Q63617,Q9Z0J6
5264	1,4,6,7,17, 20,21,23	2,3,5,8,18, 19,22,24	1	P11598
5265	1,4,6,7,17, 20,21,24	2,3,5,8,18, 19,22,23	10	P02781,P02782,P07647,P08723,P11598,P22283,P36374,Q63617,Q9JHB9,Q9Z2L0
5266	1,4,6,7,17, 20,22,23	2,3,5,8,18, 19,21,24	0	
5267	1,4,6,7,17, 20,22,24	2,3,5,8,18, 19,21,23	2	P22006,Q9Z0J6
5268	1,4,6,7,17, 20,23,24	2,3,5,8,18, 19,21,22	1	Q9QYP1
5269	1,4,6,7,17, 21,22,23	2,3,5,8,18, 19,20,24	1	Q9R168
5270	1,4,6,7,17, 21,22,24	2,3,5,8,18, 19,20,23	4	P05539,P36376,P36860,Q9R168
5271	1,4,6,7,17, 21,23,24	2,3,5,8,18, 19,20,22	3	P36860,Q63474,Q9R168
5272	1,4,6,7,17, 22,23,24	2,3,5,8,18, 19,20,21	5	P07335,P22006,P25809,P47967,Q9R168

5273	1,4,6,7,18, 19,20,21	2,3,5,8,17, 22,23,24	12	O08815,P02780,P07647,P0C0A9,P11598,P22283,P36374,Q5PQL7,Q63617,Q9JI85,Q9QW07,Q9R0T3
5274	1,4,6,7,18, 19,20,22	2,3,5,8,17, 21,23,24	4	P11598,P22283,Q5PQL7,Q63617
5275	1,4,6,7,18, 19,20,23	2,3,5,8,17, 21,22,24	3	P10758,P11598,Q63617
5276	1,4,6,7,18, 19,20,24	2,3,5,8,17, 21,22,23	6	P08723,P11598,P22283,P36374,Q63617,Q9QW07
5277	1,4,6,7,18, 19,21,22	2,3,5,8,17, 20,23,24	3	P23739,P36376,Q5PQL7
5278	1,4,6,7,18, 19,21,23	2,3,5,8,17, 20,22,24	0	
5279	1,4,6,7,18, 19,21,24	2,3,5,8,17, 20,22,23	3	P11598,P22283,P36376
5280	1,4,6,7,18, 19,22,23	2,3,5,8,17, 20,21,24	2	Q811M5,Q9EQS0
5281	1,4,6,7,18, 19,22,24	2,3,5,8,17, 20,21,23	4	P19132,P22006,P36376,P63029
5282	1,4,6,7,18, 19,23,24	2,3,5,8,17, 20,21,22	1	P25809
5283	1,4,6,7,18, 20,21,22	2,3,5,8,17, 19,23,24	4	P11598,P22283,Q5PQL7,Q63617
5284	1,4,6,7,18, 20,21,23	2,3,5,8,17, 19,22,24	4	P10758,P11598,P22283,Q63617
5285	1,4,6,7,18, 20,21,24	2,3,5,8,17, 19,22,23	7	P07647,P11598,P22283,P36376,Q63617,Q9JHB9,Q9QW07
5286	1,4,6,7,18, 20,22,23	2,3,5,8,17, 19,21,24	3	P54921,Q6AY61,Q9WVK7
5287	1,4,6,7,18, 20,22,24	2,3,5,8,17, 19,21,23	3	P11598,P22006,Q9WVK7
5288	1,4,6,7,18, 20,23,24	2,3,5,8,17, 19,21,22	5	P10758,P11598,P22006,Q9QYP1,Q9WVK7
5289	1,4,6,7,18, 21,22,23	2,3,5,8,17, 19,20,24	2	P13676,P54921
5290	1,4,6,7,18, 21,22,24	2,3,5,8,17, 19,20,23	3	P22006,P36376,Q8CJD3
5291	1,4,6,7,18, 21,23,24	2,3,5,8,17, 19,20,22	1	O89117
5292	1,4,6,7,18, 22,23,24	2,3,5,8,17, 19,20,21	8	P06760,P13676,P22006,P25809,Q64268,Q6P6R2,Q9EQS0,Q9WVK7

5293	1,4,6,7,19, 20,21,22	2,3,5,8,17, 18,23,24	7	P0C0A9,P11598,P22283,P36374,Q5PQL7,Q63617,Q99041
5294	1,4,6,7,19, 20,21,23	2,3,5,8,17, 18,22,24	8	O35547,P0C0A9,P10758,P11598,P22283,P36374,Q63617,Q8CJ52
5295	1,4,6,7,19, 20,21,24	2,3,5,8,17, 18,22,23	20	O08815,O35547,P00714,P02781,P02782,P05369,P07647,P08723,P0C0A9,P11598,P22283,P36374,P36376,Q63617,Q812E4,Q8CJ52,Q99MH3,Q9JI85,Q9QW07,Q9R0T3
5296	1,4,6,7,19, 20,22,23	2,3,5,8,17, 18,21,24	2	P10758,Q63617
5297	1,4,6,7,19, 20,22,24	2,3,5,8,17, 18,21,23	7	P08723,P11598,P22006,P22283,P36374,Q63617,Q9QW07
5298	1,4,6,7,19, 20,23,24	2,3,5,8,17, 18,21,22	6	P10758,P11598,P36374,Q63617,Q9QW07,Q9QYP1
5299	1,4,6,7,19, 21,22,23	2,3,5,8,17, 18,20,24	2	Q811M5,Q9R168
5300	1,4,6,7,19, 21,22,24	2,3,5,8,17, 18,20,23	2	P36376,Q9R168
5301	1,4,6,7,19, 21,23,24	2,3,5,8,17, 18,20,22	2	P25809,Q63474
5302	1,4,6,7,19, 22,23,24	2,3,5,8,17, 18,20,21	5	P13676,P22006,P25809,P70549,Q811M5
5303	1,4,6,7,20, 21,22,23	2,3,5,8,17, 18,19,24	1	P11598
5304	1,4,6,7,20, 21,22,24	2,3,5,8,17, 18,19,23	6	P01835,P11598,P22006,P22283,P36376,Q63617
5305	1,4,6,7,20, 21,23,24	2,3,5,8,17, 18,19,22	7	P01835,P10758,P11598,P22283,Q63617,Q8CJ52,Q9QYP1
5306	1,4,6,7,20, 22,23,24	2,3,5,8,17, 18,19,21	3	P22006,Q9QYP1,Q9WVK7
5307	1,4,6,7,21, 22,23,24	2,3,5,8,17, 18,19,20	3	P13676,P25809,Q9R168
5308	1,4,6,8,17, 18,19,20	2,3,5,7,21, 22,23,24	11	B0LT89,P06911,P08649,P12020,P13432,P22282,P36374,P47727,Q5GRG2,Q62714,Q9JI85
5309	1,4,6,8,17, 18,19,21	2,3,5,7,20, 22,23,24	3	P13432,Q9JI85,Q9QX74
5310	1,4,6,8,17, 18,19,22	2,3,5,7,20, 21,23,24	3	P08649,P13432,Q6AY61
5311	1,4,6,8,17, 18,19,23	2,3,5,7,20, 21,22,24	3	P08649,P13432,P21674
5312	1,4,6,8,17, 18,19,24	2,3,5,7,20, 21,22,23	4	O54728,P08649,P13432,P70549

5313	1,4,6,8,17,18,20,21	2,3,5,7,19,22,23,24	7	P06911,P13432,P19223,P47727,Q10758,Q5GRG2,Q9JI85
5314	1,4,6,8,17,18,20,22	2,3,5,7,19,21,23,24	6	P06911,P08649,P12020,P13432,Q5GRG2,Q6AY61
5315	1,4,6,8,17,18,20,23	2,3,5,7,19,21,22,24	3	P06911,P13432,Q5GRG2
5316	1,4,6,8,17,18,20,24	2,3,5,7,19,21,22,23	6	P06911,P12020,P13432,P47727,Q5GRG2,Q9QW07
5317	1,4,6,8,17,18,21,22	2,3,5,7,19,20,23,24	1	P13432
5318	1,4,6,8,17,18,21,23	2,3,5,7,19,20,22,24	1	P13432
5319	1,4,6,8,17,18,21,24	2,3,5,7,19,20,22,23	2	P13432,Q63474
5320	1,4,6,8,17,18,22,23	2,3,5,7,19,20,21,24	4	P08649,P13432,P47967,Q6AY61
5321	1,4,6,8,17,18,22,24	2,3,5,7,19,20,21,23	4	P08649,P13432,P22006,P47967
5322	1,4,6,8,17,18,23,24	2,3,5,7,19,20,21,22	2	P13432,P47967
5323	1,4,6,8,17,19,20,21	2,3,5,7,18,22,23,24	19	B0LT89,O08815,O35547,P04905,P06911,P07647,P08723,P22282,P22283,P24368,P30120,P36374,P46462,P47727,Q5GRG2,Q63493,Q6P6R2,Q812E4,Q9JI85
5324	1,4,6,8,17,19,20,22	2,3,5,7,18,21,23,24	9	B0LT89,O89117,P06911,P15399,P20766,P36374,Q5GRG2,Q9JI85,Q9JJ50
5325	1,4,6,8,17,19,20,23	2,3,5,7,18,21,22,24	5	P06911,P10758,P36374,Q5GRG2,Q9JI85
5326	1,4,6,8,17,19,20,24	2,3,5,7,18,21,22,23	15	B0LT89,O35547,O54728,P06911,P08723,P13432,P22282,P30120,P36374,P47727,P60905,Q5GRG2,Q6RUV5,Q9JI85,Q9QW07
5327	1,4,6,8,17,19,21,22	2,3,5,7,18,20,23,24	1	P20766
5328	1,4,6,8,17,19,21,23	2,3,5,7,18,20,22,24	2	P21674,Q63474
5329	1,4,6,8,17,19,21,24	2,3,5,7,18,20,22,23	4	O54728,P02782,Q63474,Q9JI85
5330	1,4,6,8,17,19,22,23	2,3,5,7,18,20,21,24	3	P00507,P47967,Q6AY61
5331	1,4,6,8,17,19,22,24	2,3,5,7,18,20,21,23	4	O54728,P00507,P47967,P70549
5332	1,4,6,8,17,19,23,24	2,3,5,7,18,20,21,22	3	O54728,P47967,P70549

5333	1,4,6,8,17, 20,21,22	2,3,5,7,18, 19,23,24	4	P06911,P20766,Q5GRG2,Q9JI85
5334	1,4,6,8,17, 20,21,23	2,3,5,7,18, 19,22,24	2	P06911,P20766
5335	1,4,6,8,17, 20,21,24	2,3,5,7,18, 19,22,23	17	P02781,P02782,P06911,P07647,P08723,P20766,P30120,P36374,P47727,P60905,Q5GRG2,Q63474,Q6AYR9,Q812E4,Q9JHB9,Q9JI85,Q9Z2L0
5336	1,4,6,8,17, 20,22,23	2,3,5,7,18, 19,21,24	3	P06911,P20766,Q6AY61
5337	1,4,6,8,17, 20,22,24	2,3,5,7,18, 19,21,23	4	P06911,P20766,P22006,Q5GRG2
5338	1,4,6,8,17, 20,23,24	2,3,5,7,18, 19,21,22	2	P06911,Q9QYP1
5339	1,4,6,8,17, 21,22,23	2,3,5,7,18, 19,20,24	2	P20766,P47967
5340	1,4,6,8,17, 21,22,24	2,3,5,7,18, 19,20,23	3	P20766,Q63474,Q6AYR9
5341	1,4,6,8,17, 21,23,24	2,3,5,7,18, 19,20,22	3	P47967,Q63474,Q6AYR9
5342	1,4,6,8,17, 22,23,24	2,3,5,7,18, 19,20,21	5	P00507,P07335,P47967,P62804,Q5I0D1
5343	1,4,6,8,18, 19,20,21	2,3,5,7,17, 22,23,24	13	B0LT89,O08815,O35547,P02780,P04905,P0C0A9,P22282,P22283,P36374,P47727,Q63493,Q9JI85,Q9QW07
5344	1,4,6,8,18, 19,20,22	2,3,5,7,17, 21,23,24	6	P12020,P15399,P36374,Q6AY61,Q9JI85,Q9QW07
5345	1,4,6,8,18, 19,20,23	2,3,5,7,17, 21,22,24	3	P10758,Q9QW07,Q9R168
5346	1,4,6,8,18, 19,20,24	2,3,5,7,17, 21,22,23	9	O54728,P08723,P22282,P36374,P60905,Q5GRG2,Q9JI85,Q9QW07,Q9R168
5347	1,4,6,8,18, 19,21,22	2,3,5,7,17, 20,23,24	1	Q6AY61
5348	1,4,6,8,18, 19,21,23	2,3,5,7,17, 20,22,24	0	
5349	1,4,6,8,18, 19,21,24	2,3,5,7,17, 20,22,23	2	O54728,Q9JI85
5350	1,4,6,8,18, 19,22,23	2,3,5,7,17, 20,21,24	3	P00507,Q6AY61,Q811M5
5351	1,4,6,8,18, 19,22,24	2,3,5,7,17, 20,21,23	4	O54728,P00507,P15399,P22006
5352	1,4,6,8,18, 19,23,24	2,3,5,7,17, 20,21,22	1	O54728

5353	1,4,6,8,18, 20,21,22	2,3,5,7,17, 19,23,24	4	P12020,P54921,Q5PQL7,Q6AY61
5354	1,4,6,8,18, 20,21,23	2,3,5,7,17, 19,22,24	1	P54921
5355	1,4,6,8,18, 20,21,24	2,3,5,7,17, 19,22,23	7	P02782,P08723,P30120,Q6AYR9,Q9JHB9,Q9JI85,Q9QW07
5356	1,4,6,8,18, 20,22,23	2,3,5,7,17, 19,21,24	2	P54921,Q6AY61
5357	1,4,6,8,18, 20,22,24	2,3,5,7,17, 19,21,23	4	P06911,P12020,P22006,Q9QW07
5358	1,4,6,8,18, 20,23,24	2,3,5,7,17, 19,21,22	4	Q9QW07,Q9QYP1,Q9R168,Q9WVK7
5359	1,4,6,8,18, 21,22,23	2,3,5,7,17, 19,20,24	3	P54921,Q63532,Q6AY61
5360	1,4,6,8,18, 21,22,24	2,3,5,7,17, 19,20,23	0	
5361	1,4,6,8,18, 21,23,24	2,3,5,7,17, 19,20,22	0	
5362	1,4,6,8,18, 22,23,24	2,3,5,7,17, 19,20,21	6	P00507,P13676,P22006,P47967,Q6AY61,Q9WVK7
5363	1,4,6,8,19, 20,21,22	2,3,5,7,17, 18,23,24	11	O35547,P02780,P15399,P19218,P20766,P22282,P36374,Q5PQL7,Q99041,Q9JI85,Q9QW07
5364	1,4,6,8,19, 20,21,23	2,3,5,7,17, 18,22,24	9	O35547,P10758,P19629,P22282,P36374,Q63493,Q812E4,Q9JI85,Q9QW07
5365	1,4,6,8,19, 20,21,24	2,3,5,7,17, 18,22,23	23	O08815,O35547,O54728,P02780,P02781,P02782,P04905,P07647,P08723,P09456,P0C0A9,P22282,P22283,P24368,P30120,P36374,P46462,P60905,Q63474,Q6AYR9,Q812E4,Q9JI85,Q9QW07
5366	1,4,6,8,19, 20,22,23	2,3,5,7,17, 18,21,24	4	P10758,P15399,P19218,Q6AY61
5367	1,4,6,8,19, 20,22,24	2,3,5,7,17, 18,21,23	8	O54728,P08723,P15399,P22006,P36374,P60905,Q9JI85,Q9QW07
5368	1,4,6,8,19, 20,23,24	2,3,5,7,17, 18,21,22	6	O54728,P08723,P10758,P36374,Q812E4,Q9QW07
5369	1,4,6,8,19, 21,22,23	2,3,5,7,17, 18,20,24	2	Q6AY61,Q811M5
5370	1,4,6,8,19, 21,22,24	2,3,5,7,17, 18,20,23	2	O54728,P30919
5371	1,4,6,8,19, 21,23,24	2,3,5,7,17, 18,20,22	2	O54728,Q63474
5372	1,4,6,8,19, 22,23,24	2,3,5,7,17, 18,20,21	8	O54728,P00507,P22006,P25809,P47967,P70549,Q6AY61,Q811M5

5373	1,4,6,8,20,21,22,23	2,3,5,7,17,18,19,24	4	P19629,P20766,P54921,Q6AY61
5374	1,4,6,8,20,21,22,24	2,3,5,7,17,18,19,23	7	P02782,P08723,P20766,P22006,Q10743,Q6AYR9,Q9QW07
5375	1,4,6,8,20,21,23,24	2,3,5,7,17,18,19,22	4	Q63474,Q6AYR9,Q812E4,Q9QW07
5376	1,4,6,8,20,22,23,24	2,3,5,7,17,18,19,21	3	P20766,P22006,Q6AY61
5377	1,4,6,8,21,22,23,24	2,3,5,7,17,18,19,20	4	P20766,P30919,Q63474,Q63532
5378	1,4,6,17,18,19,20,21	2,3,5,7,8,22,23,24	6	B0LT89,O08815,P35280,P36374,Q62714,Q6P6R2
5379	1,4,6,17,18,19,20,22	2,3,5,7,8,21,23,24	1	Q62714
5380	1,4,6,17,18,19,20,23	2,3,5,7,8,21,22,24	4	P10758,P11883,P35280,Q62714
5381	1,4,6,17,18,19,20,24	2,3,5,7,8,21,22,23	4	P11883,P35280,P36374,Q62714
5382	1,4,6,17,18,19,21,22	2,3,5,7,8,20,23,24	1	P36376
5383	1,4,6,17,18,19,21,23	2,3,5,7,8,20,22,24	1	P35280
5384	1,4,6,17,18,19,21,24	2,3,5,7,8,20,22,23	2	P35280,P36376
5385	1,4,6,17,18,19,22,23	2,3,5,7,8,20,21,24	5	P11883,P47967,P70549,Q811M5,Q9EQS0
5386	1,4,6,17,18,19,22,24	2,3,5,7,8,20,21,23	4	P19132,P22006,P36376,P70549
5387	1,4,6,17,18,19,23,24	2,3,5,7,8,20,21,22	4	P11883,P35280,P47967,P70549
5388	1,4,6,17,18,20,21,22	2,3,5,7,8,19,23,24	1	Q5PQL7
5389	1,4,6,17,18,20,21,23	2,3,5,7,8,19,22,24	2	P11883,P35280
5390	1,4,6,17,18,20,21,24	2,3,5,7,8,19,22,23	1	P35280
5391	1,4,6,17,18,20,22,23	2,3,5,7,8,19,21,24	2	P04355,P11883
5392	1,4,6,17,18,20,22,24	2,3,5,7,8,19,21,23	2	P11883,P22006

5393	1,4,6,17,18,20,23,24	2,3,5,7,8,19,21,22	3	P11883,P35280,Q9QYP1
5394	1,4,6,17,18,21,22,23	2,3,5,7,8,19,20,24	0	
5395	1,4,6,17,18,21,22,24	2,3,5,7,8,19,20,23	2	P36376,Q8CJD3
5396	1,4,6,17,18,21,23,24	2,3,5,7,8,19,20,22	1	P35280
5397	1,4,6,17,18,22,23,24	2,3,5,7,8,19,20,21	11	D4A5U3,P06760,P11883,P22006,P47967,P62804,P70549,Q00715,Q5I0D1,Q9EQS0,Q9WTT6
5398	1,4,6,17,19,20,21,22	2,3,5,7,8,18,23,24	5	B0LT89,P20766,P36374,Q5PQL7,Q9JJ50
5399	1,4,6,17,19,20,21,23	2,3,5,7,8,18,22,24	4	P10758,P20766,P35280,P36374
5400	1,4,6,17,19,20,21,24	2,3,5,7,8,18,22,23	9	B0LT89,O35547,P07647,P20766,P35280,P36374,P60905,Q99MH3,Q9Z2L0
5401	1,4,6,17,19,20,22,23	2,3,5,7,8,18,21,24	3	O89117,P20766,Q00715
5402	1,4,6,17,19,20,22,24	2,3,5,7,8,18,21,23	7	O89117,P20766,P22006,P36374,P70549,Q00715,Q9JJ50
5403	1,4,6,17,19,20,23,24	2,3,5,7,8,18,21,22	7	P10758,P11883,P35280,P36374,P70549,Q00715,Q9QYP1
5404	1,4,6,17,19,21,22,23	2,3,5,7,8,18,20,24	1	P20766
5405	1,4,6,17,19,21,22,24	2,3,5,7,8,18,20,23	3	P20766,P36376,Q9R168
5406	1,4,6,17,19,21,23,24	2,3,5,7,8,18,20,22	2	P35280,Q63474
5407	1,4,6,17,19,22,23,24	2,3,5,7,8,18,20,21	10	P00507,P06760,P07335,P22006,P25809,P47967,P62804,P70549,Q00715,Q811M5
5408	1,4,6,17,20,21,22,23	2,3,5,7,8,18,19,24	1	P20766
5409	1,4,6,17,20,21,22,24	2,3,5,7,8,18,19,23	2	P20766,P22006
5410	1,4,6,17,20,21,23,24	2,3,5,7,8,18,19,22	3	P20766,P35280,Q9QYP1
5411	1,4,6,17,20,22,23,24	2,3,5,7,8,18,19,21	9	P06760,P08937,P11883,P20766,P22006,P47967,P62804,Q00715,Q9QYP1
5412	1,4,6,17,21,22,23,24	2,3,5,7,8,18,19,20	4	P20766,P47967,Q5I0D1,Q9R168

5413	1,4,6,18,19,20,21,22	2,3,5,7,8,17,23,24	1	Q5PQL7
5414	1,4,6,18,19,20,21,23	2,3,5,7,8,17,22,24	2	P10758,P35280
5415	1,4,6,18,19,20,21,24	2,3,5,7,8,17,22,23	5	O08815,O35547,P07647,P35280,P36374
5416	1,4,6,18,19,20,22,23	2,3,5,7,8,17,21,24	5	P04355,P04762,P10758,Q6AY61,Q811M5
5417	1,4,6,18,19,20,22,24	2,3,5,7,8,17,21,23	1	P22006
5418	1,4,6,18,19,20,23,24	2,3,5,7,8,17,21,22	5	P10758,P11883,P22006,P35280,Q9QYP1
5419	1,4,6,18,19,21,22,23	2,3,5,7,8,17,20,24	3	P13676,Q6AY61,Q811M5
5420	1,4,6,18,19,21,22,24	2,3,5,7,8,17,20,23	4	P13676,P22006,P36376,Q4G075
5421	1,4,6,18,19,21,23,24	2,3,5,7,8,17,20,22	3	P13676,P35280,Q4G075
5422	1,4,6,18,19,22,23,24	2,3,5,7,8,17,20,21	13	D4A5U3,P00507,P06760,P13676,P22006,P25809,P47967,P62804,P70549,Q00715,Q811M5,Q9EQS0,Q9WTT6
5423	1,4,6,18,20,21,22,23	2,3,5,7,8,17,19,24	2	P54921,Q6AY61
5424	1,4,6,18,20,21,22,24	2,3,5,7,8,17,19,23	2	P22006,Q8CJD3
5425	1,4,6,18,20,21,23,24	2,3,5,7,8,17,19,22	2	P35280,Q9QYP1
5426	1,4,6,18,20,22,23,24	2,3,5,7,8,17,19,21	11	P04355,P06760,P11883,P22006,P62804,Q00715,Q5QE79,Q66H69,Q9QX74,Q9QYP1,Q9WVK7
5427	1,4,6,18,21,22,23,24	2,3,5,7,8,17,19,20	7	D4A5U3,P13676,P22006,Q4G075,Q5U2V4,Q811M5,Q9WTT6
5428	1,4,6,19,20,21,22,23	2,3,5,7,8,17,18,24	2	P10758,P20766
5429	1,4,6,19,20,21,22,24	2,3,5,7,8,17,18,23	6	O35547,O70594,P12368,P20766,P22006,P36374
5430	1,4,6,19,20,21,23,24	2,3,5,7,8,17,18,22	4	O35547,P10758,P35280,P36374
5431	1,4,6,19,20,22,23,24	2,3,5,7,8,17,18,21	8	P06760,P10758,P20766,P22006,P62804,Q00715,Q811M5,Q9QX74
5432	1,4,6,19,21,22,23,24	2,3,5,7,8,17,18,20	4	P13676,P20766,P25809,Q811M5

5433	1,4,6,20,21,22,23,24	2,3,5,7,8,17,18,19	2	P20766,P22006
5434	1,4,7,8,17,18,19,20	2,3,5,6,21,22,23,24	16	B0LT89,P02780,P06911,P07150,P08723,P20646,P22282,P30919,P36374,P47727,Q4G075,Q62714,Q811M5,Q812E4,Q9QX74,Q9WTT6
5435	1,4,7,8,17,18,19,21	2,3,5,6,20,22,23,24	8	P20646,P21674,P22282,Q66H69,Q6P6R2,Q99MH3,Q9QX74,Q9Z2L0
5436	1,4,7,8,17,18,19,22	2,3,5,6,20,21,23,24	1	P20646
5437	1,4,7,8,17,18,19,23	2,3,5,6,20,21,22,24	2	P20646,P21674
5438	1,4,7,8,17,18,19,24	2,3,5,6,20,21,22,23	4	O54728,P09656,P20646,Q99MH3
5439	1,4,7,8,17,18,20,21	2,3,5,6,19,22,23,24	14	P00507,P02780,P06911,P19223,P20646,P47727,Q63493,Q6P6R2,Q811M5,Q812E4,Q9JHB9,Q9QX74,Q9WTT6,Q9Z2L0
5440	1,4,7,8,17,18,20,22	2,3,5,6,19,21,23,24	2	P06911,Q4G075
5441	1,4,7,8,17,18,20,23	2,3,5,6,19,21,22,24	4	P06911,P10758,P30919,Q4G075
5442	1,4,7,8,17,18,20,24	2,3,5,6,19,21,22,23	7	P06911,P08723,P47727,Q811M5,Q812E4,Q9JHB9,Q9Z2L0
5443	1,4,7,8,17,18,21,22	2,3,5,6,19,20,23,24	2	Q9QX74,Q9Z1F2
5444	1,4,7,8,17,18,21,23	2,3,5,6,19,20,22,24	4	P21674,Q9QX74,Q9Z0V6,Q9Z1F2
5445	1,4,7,8,17,18,21,24	2,3,5,6,19,20,22,23	6	P02782,Q811M5,Q99MH3,Q9QX74,Q9Z1F2,Q9Z2L0
5446	1,4,7,8,17,18,22,23	2,3,5,6,19,20,21,24	2	Q5I0J9,Q9Z1F2
5447	1,4,7,8,17,18,22,24	2,3,5,6,19,20,21,23	1	Q9Z1F2
5448	1,4,7,8,17,18,23,24	2,3,5,6,19,20,21,22	3	Q5I0J9,Q64268,Q9Z1F2
5449	1,4,7,8,17,19,20,21	2,3,5,6,18,22,23,24	34	B0LT89,D4A5U3,O08815,P02780,P02781,P02782,P04905,P06760,P08723,P09456,P0C0A9,P13676,P19223,P20646,P22282,P22283,P24368,P30120,P36374,P40241,P46462,P47727,Q4G075,Q5M8C6,Q63493,Q63617,Q66H69,Q6P6R2,Q811M5,Q812E4,Q99MH3,Q9QX74,Q9WTT6,Q9Z2L0
5450	1,4,7,8,17,19,20,22	2,3,5,6,18,21,23,24	7	P02780,P06911,P20646,P36374,Q4G075,Q9JJ50,Q9WTT6
5451	1,4,7,8,17,19,20,23	2,3,5,6,18,21,22,24	7	P10758,P20646,P21674,P36374,Q4G075,Q812E4,Q9WTT6
5452	1,4,7,8,17,19,20,24	2,3,5,6,18,21,22,23	17	O54728,P02780,P02781,P06911,P08723,P09456,P20646,P22282,P30120,P36374,P47727,P60905,Q812E4,Q99MH3,Q9JHB9,Q9WTT6,Q9Z2L0

5453	1,4,7,8,17, 19,21,22	2,3,5,6,18, 20,23,24	3	P20646,Q9QX74,Q9Z2L0
5454	1,4,7,8,17, 19,21,23	2,3,5,6,18, 20,22,24	5	P20646,P21674,Q63474,Q9QX74,Q9Z0V6
5455	1,4,7,8,17, 19,21,24	2,3,5,6,18, 20,22,23	12	O54728,P02782,P04355,P08723,P16636,P20646,P22282,Q63474,Q66H69,Q99MH3,Q9QX74,Q9Z2L0
5456	1,4,7,8,17, 19,22,23	2,3,5,6,18, 20,21,24	0	
5457	1,4,7,8,17, 19,22,24	2,3,5,6,18, 20,21,23	1	O54728
5458	1,4,7,8,17, 19,23,24	2,3,5,6,18, 20,21,22	1	O54728
5459	1,4,7,8,17, 20,21,22	2,3,5,6,18, 19,23,24	4	P19223,Q4G075,Q9WTT6,Q9Z2L0
5460	1,4,7,8,17, 20,21,23	2,3,5,6,18, 19,22,24	7	P19223,P21674,Q4G075,Q63493,Q6AYR9,Q812E4,Q9WTT6
5461	1,4,7,8,17, 20,21,24	2,3,5,6,18, 19,22,23	17	P02781,P02782,P08723,P09456,P19223,P30120,P36374,P47727,Q5M8C6,Q63474,Q6AYR9,Q811M5,Q812E4,Q99MH3,Q9JHB9,Q9WTT6,Q9Z2L0
5462	1,4,7,8,17, 20,22,23	2,3,5,6,18, 19,21,24	1	Q4G075
5463	1,4,7,8,17, 20,22,24	2,3,5,6,18, 19,21,23	5	P06911,P08723,Q9JHB9,Q9JJ50,Q9Z2L0
5464	1,4,7,8,17, 20,23,24	2,3,5,6,18, 19,21,22	3	P10758,Q812E4,Q9QYP1
5465	1,4,7,8,17, 21,22,23	2,3,5,6,18, 19,20,24	0	
5466	1,4,7,8,17, 21,22,24	2,3,5,6,18, 19,20,23	4	P02782,Q6AYR9,Q9Z1F2,Q9Z2L0
5467	1,4,7,8,17, 21,23,24	2,3,5,6,18, 19,20,22	3	Q63474,Q6AYR9,Q9Z1F2
5468	1,4,7,8,17, 22,23,24	2,3,5,6,18, 19,20,21	1	P47967
5469	1,4,7,8,18, 19,20,21	2,3,5,6,17, 22,23,24	19	O08815,P01039,P02780,P04905,P08723,P0C0A9,P22273,P22282,P22283,P24368,P30120,P36374,P47727,Q5I0D1,Q63493,Q63617,Q812E4,Q9QW07,Q9WTT6
5470	1,4,7,8,18, 19,20,22	2,3,5,6,17, 21,23,24	7	P01039,P02780,P0C0A9,P22282,P36374,Q4G075,Q6AY61
5471	1,4,7,8,18, 19,20,23	2,3,5,6,17, 21,22,24	6	P01039,P0C0A9,P10758,P36374,Q4G075,Q812E4
5472	1,4,7,8,18, 19,20,24	2,3,5,6,17, 21,22,23	12	O08815,O54728,P02780,P08723,P0C0A9,P22282,P36374,P47727,P60905,Q812E4,Q9JHB9,Q9QW07

5473	1,4,7,8,18, 19,21,22	2,3,5,6,17, 20,23,24	1	P01039
5474	1,4,7,8,18, 19,21,23	2,3,5,6,17, 20,22,24	2	P01039,P21674
5475	1,4,7,8,18, 19,21,24	2,3,5,6,17, 20,22,23	8	O54728,P02782,P0C0A9,P22282,P63029,Q66H69,Q99MH3,Q9Z2L0
5476	1,4,7,8,18, 19,22,23	2,3,5,6,17, 20,21,24	4	P01039,P63029,Q5I0J9,Q6AY61
5477	1,4,7,8,18, 19,22,24	2,3,5,6,17, 20,21,23	2	O54728,P63029
5478	1,4,7,8,18, 19,23,24	2,3,5,6,17, 20,21,22	3	O54728,P63029,Q5I0J9
5479	1,4,7,8,18, 20,21,22	2,3,5,6,17, 19,23,24	2	P01039,P02780
5480	1,4,7,8,18, 20,21,23	2,3,5,6,17, 19,22,24	3	P01039,P10758,Q812E4
5481	1,4,7,8,18, 20,21,24	2,3,5,6,17, 19,22,23	15	P02780,P02781,P02782,P08723,P22282,P22283,P30120,P47727,Q5M8C6,Q6AYR9,Q811M5,Q812E4,Q9JHB9,Q9QW07,Q9Z2L0
5482	1,4,7,8,18, 20,22,23	2,3,5,6,17, 19,21,24	5	P01039,P10758,P54921,Q4G075,Q6AY61
5483	1,4,7,8,18, 20,22,24	2,3,5,6,17, 19,21,23	1	Q9JHB9
5484	1,4,7,8,18, 20,23,24	2,3,5,6,17, 19,21,22	4	P10758,Q812E4,Q9QYP1,Q9WVK7
5485	1,4,7,8,18, 21,22,23	2,3,5,6,17, 19,20,24	4	P01039,P54921,Q6AY61,Q9Z1F2
5486	1,4,7,8,18, 21,22,24	2,3,5,6,17, 19,20,23	1	Q9Z1F2
5487	1,4,7,8,18, 21,23,24	2,3,5,6,17, 19,20,22	2	O89117,Q9Z1F2
5488	1,4,7,8,18, 22,23,24	2,3,5,6,17, 19,20,21	5	P49134,P63029,Q5I0J9,Q64268,Q9WVK7
5489	1,4,7,8,19, 20,21,22	2,3,5,6,17, 18,23,24	13	P01039,P02780,P04905,P08723,P0C0A9,P22282,P22283,P36374,Q63493,Q63617,Q812E4,Q9WTT6,Q9Z2L0
5490	1,4,7,8,19, 20,21,23	2,3,5,6,17, 18,22,24	11	P01039,P02780,P0C0A9,P10758,P22282,P22283,P36374,Q63493,Q63617,Q812E4,Q9WTT6
5491	1,4,7,8,19, 20,21,24	2,3,5,6,17, 18,22,23	32	O08815,O35547,O54728,P02780,P02781,P02782,P04905,P07647,P08723,P09456,P0C0A9,P16636,P22282,P22283,P24368,P30120,P36374,P40241,P46462,P47727,P60905,Q5M8C6,Q63493,Q63617,Q6AYR9,Q6Q7Y5,Q812E4,Q8CJ52,Q99MH3,Q9JHB9,Q9QW07,Q9Z2L0
5492	1,4,7,8,19, 20,22,23	2,3,5,6,17, 18,21,24	5	P01039,P10758,Q4G075,Q6AY61,Q812E4

5493	1,4,7,8,19,<br>20,22,24	2,3,5,6,17,<br>18,21,23	8	O54728,P02780,P08723,P0C0A9,P22282,P36374,Q812E4,Q9QW07
5494	1,4,7,8,19,<br>20,23,24	2,3,5,6,17,<br>18,21,22	7	O54728,P08723,P0C0A9,P10758,P36374,Q812E4,Q9QYP1
5495	1,4,7,8,19,<br>21,22,23	2,3,5,6,17,<br>18,20,24	1	P01039
5496	1,4,7,8,19,<br>21,22,24	2,3,5,6,17,<br>18,20,23	5	O54728,P02782,P16636,Q99MH3,Q9Z2L0
5497	1,4,7,8,19,<br>21,23,24	2,3,5,6,17,<br>18,20,22	2	O54728,Q63474
5498	1,4,7,8,19,<br>22,23,24	2,3,5,6,17,<br>18,20,21	2	O54728,P63029
5499	1,4,7,8,20,<br>21,22,23	2,3,5,6,17,<br>18,19,24	2	P01039,Q812E4
5500	1,4,7,8,20,<br>21,22,24	2,3,5,6,17,<br>18,19,23	8	P02780,P02782,P08723,P30120,Q6AYR9,Q812E4,Q9JHB9,Q9Z2L0
5501	1,4,7,8,20,<br>21,23,24	2,3,5,6,17,<br>18,19,22	6	P02782,P08723,P10758,Q6AYR9,Q812E4,Q9QYP1
5502	1,4,7,8,20,<br>22,23,24	2,3,5,6,17,<br>18,19,21	4	P10758,Q812E4,Q9QYP1,Q9WVK7
5503	1,4,7,8,21,<br>22,23,24	2,3,5,6,17,<br>18,19,20	0	
5504	1,4,7,17,1<br>8,19,20,21	2,3,5,6,8,2<br>2,23,24	10	B0LT89,O08815,P20646,P22283,P30919,P36374,Q62714,Q6P6R2,Q99MH3,Q9Z2L0
5505	1,4,7,17,1<br>8,19,20,22	2,3,5,6,8,2<br>1,23,24	5	P20646,P30919,P36374,Q62714,Q9JJ50
5506	1,4,7,17,1<br>8,19,20,23	2,3,5,6,8,2<br>1,22,24	5	P10758,P20646,P30919,Q62714,Q6AYC4
5507	1,4,7,17,1<br>8,19,20,24	2,3,5,6,8,2<br>1,22,23	6	P30919,P36374,Q62714,Q63532,Q99MH3,Q9Z2L0
5508	1,4,7,17,1<br>8,19,21,22	2,3,5,6,8,2<br>0,23,24	3	P20646,P36376,Q99MH3
5509	1,4,7,17,1<br>8,19,21,23	2,3,5,6,8,2<br>0,22,24	1	P21674
5510	1,4,7,17,1<br>8,19,21,24	2,3,5,6,8,2<br>0,22,23	3	P36376,Q99MH3,Q9Z2L0
5511	1,4,7,17,1<br>8,19,22,23	2,3,5,6,8,2<br>0,21,24	0	
5512	1,4,7,17,1<br>8,19,22,24	2,3,5,6,8,2<br>0,21,23	2	P36376,Q99MH3

5513	1,4,7,17,18,19,23,24	2,3,5,6,8,20,21,22	0	
5514	1,4,7,17,18,20,21,22	2,3,5,6,8,19,23,24	2	Q9Z0J6,Q9Z2L0
5515	1,4,7,17,18,20,21,23	2,3,5,6,8,19,22,24	1	P30919
5516	1,4,7,17,18,20,21,24	2,3,5,6,8,19,22,23	4	P00507,Q811M5,Q99MH3,Q9Z2L0
5517	1,4,7,17,18,20,22,23	2,3,5,6,8,19,21,24	1	P30919
5518	1,4,7,17,18,20,22,24	2,3,5,6,8,19,21,23	1	Q9Z0J6
5519	1,4,7,17,18,20,23,24	2,3,5,6,8,19,21,22	4	P08937,P30919,Q6AYC4,Q9QYP1
5520	1,4,7,17,18,21,22,23	2,3,5,6,8,19,20,24	0	
5521	1,4,7,17,18,21,22,24	2,3,5,6,8,19,20,23	5	P36376,Q8CJD3,Q99MH3,Q9Z1F2,Q9Z2L0
5522	1,4,7,17,18,21,23,24	2,3,5,6,8,19,20,22	1	Q64268
5523	1,4,7,17,18,22,23,24	2,3,5,6,8,19,20,21	3	P08937,Q5I0J9,Q64268
5524	1,4,7,17,19,20,21,22	2,3,5,6,8,18,23,24	5	P20646,P36374,Q99MH3,Q9JJ50,Q9Z2L0
5525	1,4,7,17,19,20,21,23	2,3,5,6,8,18,22,24	6	P10758,P20646,P21674,P36374,Q6P6R2,Q9Z2L0
5526	1,4,7,17,19,20,21,24	2,3,5,6,8,18,22,23	12	O08815,O35547,P02781,P08723,P16636,P20646,P22283,P24368,P36374,P60905,Q99MH3,Q9Z2L0
5527	1,4,7,17,19,20,22,23	2,3,5,6,8,18,21,24	3	P02625,P10758,Q9JJ50
5528	1,4,7,17,19,20,22,24	2,3,5,6,8,18,21,23	4	P36374,Q99MH3,Q9JJ50,Q9Z2L0
5529	1,4,7,17,19,20,23,24	2,3,5,6,8,18,21,22	3	P10758,P36374,Q9QYP1
5530	1,4,7,17,19,21,22,23	2,3,5,6,8,18,20,24	1	Q9R168
5531	1,4,7,17,19,21,22,24	2,3,5,6,8,18,20,23	6	P16636,P17559,P36376,Q99MH3,Q9R168,Q9Z2L0
5532	1,4,7,17,19,21,23,24	2,3,5,6,8,18,20,22	3	Q63474,Q9R168,Q9Z2L0

5533	1,4,7,17,19,22,23,24	2,3,5,6,8,18,20,21	4 P25809,P70549,Q00715,Q9R168
5534	1,4,7,17,20,21,22,23	2,3,5,6,8,18,19,24	0
5535	1,4,7,17,20,21,22,24	2,3,5,6,8,18,19,23	2 Q99MH3,Q9Z2L0
5536	1,4,7,17,20,21,23,24	2,3,5,6,8,18,19,22	2 Q9QYP1,Q9Z2L0
5537	1,4,7,17,20,22,23,24	2,3,5,6,8,18,19,21	3 P08937,Q00715,Q9QYP1
5538	1,4,7,17,21,22,23,24	2,3,5,6,8,18,19,20	1 Q9R168
5539	1,4,7,18,19,20,21,22	2,3,5,6,8,17,23,24	4 P01039,P36376,P97840,Q5PQL7
5540	1,4,7,18,19,20,21,23	2,3,5,6,8,17,22,24	3 P01039,P10758,P23593
5541	1,4,7,18,19,20,21,24	2,3,5,6,8,17,22,23	9 O08815,O35547,P08723,P22283,P36374,P36376,Q63617,Q99MH3,Q9Z2L0
5542	1,4,7,18,19,20,22,23	2,3,5,6,8,17,21,24	3 P01039,P10758,Q6AY61
5543	1,4,7,18,19,20,22,24	2,3,5,6,8,17,21,23	1 P36376
5544	1,4,7,18,19,20,23,24	2,3,5,6,8,17,21,22	3 P10758,Q6AYC4,Q9QYP1
5545	1,4,7,18,19,21,22,23	2,3,5,6,8,17,20,24	1 P01039
5546	1,4,7,18,19,21,22,24	2,3,5,6,8,17,20,23	4 P17559,P36376,P63029,Q99MH3
5547	1,4,7,18,19,21,23,24	2,3,5,6,8,17,20,22	3 O89117,P36376,P63029
5548	1,4,7,18,19,22,23,24	2,3,5,6,8,17,20,21	8 D4A5U3,P13676,P17559,P25809,P36375,P63029,Q5I0J9,Q64268
5549	1,4,7,18,20,21,22,23	2,3,5,6,8,17,19,24	1 P01039
5550	1,4,7,18,20,21,22,24	2,3,5,6,8,17,19,23	2 P36376,Q8CJD3
5551	1,4,7,18,20,21,23,24	2,3,5,6,8,17,19,22	1 Q9QYP1
5552	1,4,7,18,20,22,23,24	2,3,5,6,8,17,19,21	4 P22006,Q64268,Q9QYP1,Q9WVK7

5553	1,4,7,18,21,22,23,24	2,3,5,6,8,17,19,20	4	O89117,P13676,P36376,Q64268
5554	1,4,7,19,20,21,22,23	2,3,5,6,8,17,18,24	2	P01039,P10758
5555	1,4,7,19,20,21,22,24	2,3,5,6,8,17,18,23	8	O35547,O70594,P08723,P16636,P36374,P36376,Q99MH3,Q9Z2L0
5556	1,4,7,19,20,21,23,24	2,3,5,6,8,17,18,22	5	O35547,P10758,P36374,Q9QYP1,Q9Z2L0
5557	1,4,7,19,20,22,23,24	2,3,5,6,8,17,18,21	3	P10758,Q00715,Q9QYP1
5558	1,4,7,19,21,22,23,24	2,3,5,6,8,17,18,20	3	P17559,P25809,Q9R168
5559	1,4,7,20,21,22,23,24	2,3,5,6,8,17,18,19	1	Q9QYP1
5560	1,4,8,17,18,19,20,21	2,3,5,6,7,22,23,24	11	B0LT89,O08815,P04905,P24368,P30120,P36374,P47727,Q62714,Q6P6R2,Q9QX74,Q9Z2L0
5561	1,4,8,17,18,19,20,22	2,3,5,6,7,21,23,24	7	B0LT89,P36374,P36860,P47727,Q62714,Q6AY61,Q9JJ50
5562	1,4,8,17,18,19,20,23	2,3,5,6,7,21,22,24	5	B0LT89,P10758,P30919,P36374,Q62714
5563	1,4,8,17,18,19,20,24	2,3,5,6,7,21,22,23	8	B0LT89,O54728,P08723,P36374,P47727,P60905,Q62714,Q9Z2L0
5564	1,4,8,17,18,19,21,22	2,3,5,6,7,20,23,24	0	
5565	1,4,8,17,18,19,21,23	2,3,5,6,7,20,22,24	1	P21674
5566	1,4,8,17,18,19,21,24	2,3,5,6,7,20,22,23	3	O54728,Q99MH3,Q9Z2L0
5567	1,4,8,17,18,19,22,23	2,3,5,6,7,20,21,24	2	P01835,Q6AY61
5568	1,4,8,17,18,19,22,24	2,3,5,6,7,20,21,23	3	O54728,P01835,P50116
5569	1,4,8,17,18,19,23,24	2,3,5,6,7,20,21,22	3	O54728,P01835,P47967
5570	1,4,8,17,18,20,21,22	2,3,5,6,7,19,23,24	0	
5571	1,4,8,17,18,20,21,23	2,3,5,6,7,19,22,24	0	
5572	1,4,8,17,18,20,21,24	2,3,5,6,7,19,22,23	4	P30120,P47727,Q811M5,Q9Z2L0

5573	1,4,8,17,18,20,22,23	2,3,5,6,7,19,21,24	1	Q6AY61
5574	1,4,8,17,18,20,22,24	2,3,5,6,7,19,21,23	0	
5575	1,4,8,17,18,20,23,24	2,3,5,6,7,19,21,22	1	Q9QYP1
5576	1,4,8,17,18,21,22,23	2,3,5,6,7,19,20,24	1	Q6AY61
5577	1,4,8,17,18,21,22,24	2,3,5,6,7,19,20,23	1	Q9Z2L0
5578	1,4,8,17,18,21,23,24	2,3,5,6,7,19,20,22	0	
5579	1,4,8,17,18,22,23,24	2,3,5,6,7,19,20,21	3	P47967,P50116,Q5I0D1
5580	1,4,8,17,19,20,21,22	2,3,5,6,7,18,23,24	7	B0LT89,P20766,P30120,P36374,Q6P6R2,Q9JJ50,Q9Z2L0
5581	1,4,8,17,19,20,21,23	2,3,5,6,7,18,22,24	7	B0LT89,P10758,P20766,P21674,P36374,Q6P6R2,Q9Z2L0
5582	1,4,8,17,19,20,21,24	2,3,5,6,7,18,22,23	18	B0LT89,O08815,O35547,O54728,P02781,P02782,P04905,P08723,P16636,P24368,P30120,P36374,P47727,P60905,Q6P6R2,Q812E4,Q99MH3,Q9Z2L0
5583	1,4,8,17,19,20,22,23	2,3,5,6,7,18,21,24	5	O89117,P10758,P20766,Q6AY61,Q9JJ50
5584	1,4,8,17,19,20,22,24	2,3,5,6,7,18,21,23	7	O54728,O89117,P20766,P36374,P60905,Q9JJ50,Q9Z2L0
5585	1,4,8,17,19,20,23,24	2,3,5,6,7,18,21,22	5	O54728,P10758,P36374,P60905,Q9QYP1
5586	1,4,8,17,19,21,22,23	2,3,5,6,7,18,20,24	1	P20766
5587	1,4,8,17,19,21,22,24	2,3,5,6,7,18,20,23	5	O54728,P16636,P20766,Q99MH3,Q9Z2L0
5588	1,4,8,17,19,21,23,24	2,3,5,6,7,18,20,22	3	O54728,Q63474,Q9Z2L0
5589	1,4,8,17,19,22,23,24	2,3,5,6,7,18,20,21	5	O54728,P00507,P47967,P70549,Q00715
5590	1,4,8,17,20,21,22,23	2,3,5,6,7,18,19,24	1	P20766
5591	1,4,8,17,20,21,22,24	2,3,5,6,7,18,19,23	5	P20766,P30120,Q6AYR9,Q9JJ50,Q9Z2L0
5592	1,4,8,17,20,21,23,24	2,3,5,6,7,18,19,22	4	P20766,Q6AYR9,Q9QYP1,Q9Z2L0

5593	1,4,8,17,20,22,23,24	2,3,5,6,7,18,19,21	4	P20766,P47967,Q00715,Q9QYP1
5594	1,4,8,17,21,22,23,24	2,3,5,6,7,18,19,20	2	P20766,P47967
5595	1,4,8,18,19,20,21,22	2,3,5,6,7,17,23,24	4	B0LT89,P01039,P36374,Q6AY61
5596	1,4,8,18,19,20,21,23	2,3,5,6,7,17,22,24	3	P01039,P10758,P36374
5597	1,4,8,18,19,20,21,24	2,3,5,6,7,17,22,23	14	B0LT89,O08815,O35547,O54728,P02782,P04905,P08723,P30120,P36374,P47727,P60905,Q812E4,Q99MH3,Q9Z2L0
5598	1,4,8,18,19,20,22,23	2,3,5,6,7,17,21,24	6	P01039,P04762,P10758,P36860,P50116,Q6AY61
5599	1,4,8,18,19,20,22,24	2,3,5,6,7,17,21,23	4	O54728,P36374,P50116,Q6AY61
5600	1,4,8,18,19,20,23,24	2,3,5,6,7,17,21,22	4	O54728,P10758,P50116,Q9R168
5601	1,4,8,18,19,21,22,23	2,3,5,6,7,17,20,24	3	P01039,P50116,Q6AY61
5602	1,4,8,18,19,21,22,24	2,3,5,6,7,17,20,23	2	O54728,P50116
5603	1,4,8,18,19,21,23,24	2,3,5,6,7,17,20,22	2	O54728,P50116
5604	1,4,8,18,19,22,23,24	2,3,5,6,7,17,20,21	5	O54728,P00507,P50116,P63029,Q6AY61
5605	1,4,8,18,20,21,22,23	2,3,5,6,7,17,19,24	3	P01039,P54921,Q6AY61
5606	1,4,8,18,20,21,22,24	2,3,5,6,7,17,19,23	1	P50116
5607	1,4,8,18,20,21,23,24	2,3,5,6,7,17,19,22	1	Q9QYP1
5608	1,4,8,18,20,22,23,24	2,3,5,6,7,17,19,21	4	P50116,Q5QE79,Q6AY61,Q9WVK7
5609	1,4,8,18,21,22,23,24	2,3,5,6,7,17,19,20	2	P50116,P55281
5610	1,4,8,19,20,21,22,23	2,3,5,6,7,17,18,24	4	P01039,P10758,P20766,Q6AY61
5611	1,4,8,19,20,21,22,24	2,3,5,6,7,17,18,23	13	O35547,O54728,O70594,P02782,P08723,P16636,P20766,P30120,P36374,P60905,Q812E4,Q99MH3,Q9Z2L0
5612	1,4,8,19,20,21,23,24	2,3,5,6,7,17,18,22	8	O35547,O54728,P08723,P10758,P30120,P36374,P60905,Q812E4

5613	1,4,8,19,20,22,23,24	2,3,5,6,7,17,18,21	5	O54728,P10758,P50116,Q00715,Q6AY61
5614	1,4,8,19,21,22,23,24	2,3,5,6,7,17,18,20	3	O54728,P50116,P55281
5615	1,4,8,20,21,22,23,24	2,3,5,6,7,17,18,19	2	P20766,P50116
5616	1,4,17,18,19,20,21,22	2,3,5,6,7,8,23,24	7	B0LT89,O70594,P50115,Q62714,Q6P6R2,Q9JJ50,Q9Z2L0
5617	1,4,17,18,19,20,21,23	2,3,5,6,7,8,22,24	8	B0LT89,O54858,P23593,P30919,P35280,P50115,Q62714,Q6P6R2
5618	1,4,17,18,19,20,21,24	2,3,5,6,7,8,22,23	13	B0LT89,O08815,O54858,P23593,P35280,P36374,P50115,P60905,P80202,Q62714,Q63532,Q99MH3,Q9Z2L0
5619	1,4,17,18,19,20,22,23	2,3,5,6,7,8,21,24	8	P04762,P30919,P36860,P50115,P50116,Q00715,Q62714,Q6AY61
5620	1,4,17,18,19,20,22,24	2,3,5,6,7,8,21,23	6	O70594,P50115,P50116,Q62714,Q9JJ50,Q9Z2L0
5621	1,4,17,18,19,20,23,24	2,3,5,6,7,8,21,22	9	P08937,P10758,P11883,P35280,P50115,P50116,Q62714,Q6AYC4,Q9QYP1
5622	1,4,17,18,19,21,22,23	2,3,5,6,7,8,20,24	2	P50115,P50116
5623	1,4,17,18,19,21,22,24	2,3,5,6,7,8,20,23	8	P17559,P25031,P36376,P50115,P50116,Q4G075,Q99MH3,Q9Z2L0
5624	1,4,17,18,19,21,23,24	2,3,5,6,7,8,20,22	4	P35280,P50115,P50116,Q4G075
5625	1,4,17,18,19,22,23,24	2,3,5,6,7,8,20,21	9	D4A5U3,P02631,P06760,P08937,P47967,P50115,P50116,P70549,Q00715
5626	1,4,17,18,20,21,22,23	2,3,5,6,7,8,19,24	2	P50115,P50116

5627	1,4,17,18,20,21,22,24	2,3,5,6,7,8,19,23	6	O70594,P42854,P50115,P50116,Q8CJD3,Q9Z2L0
5628	1,4,17,18,20,21,23,24	2,3,5,6,7,8,19,22	5	P35280,P50115,P50116,Q9QYP1,Q9Z2L0
5629	1,4,17,18,20,22,23,24	2,3,5,6,7,8,19,21	7	P08937,P11883,P47967,P50115,P50116,Q00715,Q9QYP1
5630	1,4,17,18,21,22,23,24	2,3,5,6,7,8,19,20	5	P47967,P50115,P50116,Q4G075,Q5I0D1
5631	1,4,17,19,20,21,22,23	2,3,5,6,7,8,18,24	2	P20766,P50115
5632	1,4,17,19,20,21,22,24	2,3,5,6,7,8,18,23	11	B0LT89,O70594,P16636,P20766,P36374,P50115,P50116,P80202,Q99MH3,Q9JJ50,Q9Z2L0
5633	1,4,17,19,20,21,23,24	2,3,5,6,7,8,18,22	7	P20766,P23593,P35280,P36374,P50115,Q9QYP1,Q9Z2L0
5634	1,4,17,19,20,22,23,24	2,3,5,6,7,8,18,21	10	O70594,P02625,P02631,P08937,P20766,P50115,P50116,Q00715,Q9JJ50,Q9QYP1
5635	1,4,17,19,21,22,23,24	2,3,5,6,7,8,18,20	6	P12020,P20766,P47967,P50115,P50116,Q9R168
5636	1,4,17,20,21,22,23,24	2,3,5,6,7,8,18,19	6	O70594,P20766,P50115,P50116,Q9QYP1,Q9Z2L0
5637	1,4,18,19,20,21,22,23	2,3,5,6,7,8,17,24	5	O70594,P01039,P50115,P50116,Q6AY61
5638	1,4,18,19,20,21,22,24	2,3,5,6,7,8,17,23	6	O70594,P36376,P50115,P50116,Q99MH3,Q9Z2L0
5639	1,4,18,19,20,21,23,24	2,3,5,6,7,8,17,22	7	O70594,P10758,P23593,P35280,P50115,P50116,Q9QYP1

5640	1,4,18,19,20,22,23,24	2,3,5,6,7,8,17,21	9	O70594,P02631,P04762,P10758,P22006,P50115,P50116,Q00715,Q6AY61
5641	1,4,18,19,21,22,23,24	2,3,5,6,7,8,17,20	7	D4A5U3,P13676,P17559,P50115,P50116,P55281,Q4G075
5642	1,4,18,20,21,22,23,24	2,3,5,6,7,8,17,19	4	O70594,P50115,P50116,Q9QYP1
5643	1,4,19,20,21,22,23,24	2,3,5,6,7,8,17,18	6	O70594,P20766,P50115,P50116,Q99PP0,Q9QZQ5
5644	1,5,6,7,8,17,18,19	2,3,4,20,21,22,23,24	3	P00762,P13432,Q9QX74
5645	1,5,6,7,8,17,18,20	2,3,4,19,21,22,23,24	7	P06911,P12020,P13432,P31044,P35745,Q5GRG2,Q64335
5646	1,5,6,7,8,17,18,21	2,3,4,19,20,22,23,24	6	P05539,P06760,P13432,P35745,P36860,Q9QX74
5647	1,5,6,7,8,17,18,22	2,3,4,19,20,21,23,24	4	P13432,P35745,P49134,Q64335
5648	1,5,6,7,8,17,18,23	2,3,4,19,20,21,22,24	4	O70594,P13432,P35745,Q64335
5649	1,5,6,7,8,17,18,24	2,3,4,19,20,21,22,23	3	P13432,P35745,P49134
5650	1,5,6,7,8,17,19,20	2,3,4,18,21,22,23,24	8	P06911,P13432,P13676,P31044,P36374,Q5GRG2,Q9QX74,Q9WTT6
5651	1,5,6,7,8,17,19,21	2,3,4,18,20,22,23,24	8	iRT-Kit_WR_fusion,P05539,P06760,P21674,Q63474,Q63493,Q66H69,Q9QX74
5652	1,5,6,7,8,17,19,22	2,3,4,18,20,21,23,24	0	
5653	1,5,6,7,8,17,19,23	2,3,4,18,20,21,22,24	3	P21674,P50115,Q8CJD3
5654	1,5,6,7,8,17,19,24	2,3,4,18,20,21,22,23	0	
5655	1,5,6,7,8,17,20,21	2,3,4,18,19,22,23,24	12	P05539,P06760,P06911,P13432,P19629,P22283,P35745,Q5GRG2,Q63493,Q9JHB9,Q9QX74,Q9WTT6
5656	1,5,6,7,8,17,20,22	2,3,4,18,19,21,23,24	6	P06911,P13432,P35745,Q4G075,Q5GRG2,Q9Z0J6
5657	1,5,6,7,8,17,20,23	2,3,4,18,19,21,22,24	7	P06911,P13432,P19132,P23739,P25031,P35745,Q5GRG2

5658	1,5,6,7,8,17,20,24	2,3,4,18,19,21,22,23	6	P06911,P09456,P13432,P35745,Q5GRG2,Q9JHB9
5659	1,5,6,7,8,17,21,22	2,3,4,18,19,20,23,24	3	P05539,P35745,Q9QX74
5660	1,5,6,7,8,17,21,23	2,3,4,18,19,20,22,24	5	P21674,P35745,P36860,Q63474,Q9QX74
5661	1,5,6,7,8,17,21,24	2,3,4,18,19,20,22,23	4	P05539,P35745,P36860,Q63474
5662	1,5,6,7,8,17,22,23	2,3,4,18,19,20,21,24	1	P35745
5663	1,5,6,7,8,17,22,24	2,3,4,18,19,20,21,23	1	P35745
5664	1,5,6,7,8,17,23,24	2,3,4,18,19,20,21,22	1	P35745
5665	1,5,6,7,8,18,19,20	2,3,4,17,21,22,23,24	8	P06761,P06911,P13432,P22283,P31044,Q5GRG2,Q5I0D1,Q9QW07
5666	1,5,6,7,8,18,19,21	2,3,4,17,20,22,23,24	1	P06760
5667	1,5,6,7,8,18,19,22	2,3,4,17,20,21,23,24	0	
5668	1,5,6,7,8,18,19,23	2,3,4,17,20,21,22,24	0	
5669	1,5,6,7,8,18,19,24	2,3,4,17,20,21,22,23	0	
5670	1,5,6,7,8,18,20,21	2,3,4,17,19,22,23,24	9	P06760,P06761,P06911,P19629,P22283,P35745,P47967,Q9JHB9,Q9QW07
5671	1,5,6,7,8,18,20,22	2,3,4,17,19,21,23,24	4	P06911,P12020,P35745,Q9WVK7
5672	1,5,6,7,8,18,20,23	2,3,4,17,19,21,22,24	4	P06911,P19629,P35745,Q9WVK7
5673	1,5,6,7,8,18,20,24	2,3,4,17,19,21,22,23	6	P06911,P13432,Q5GRG2,Q9JHB9,Q9QW07,Q9WVK7
5674	1,5,6,7,8,18,21,22	2,3,4,17,19,20,23,24	2	P35745,P49134
5675	1,5,6,7,8,18,21,23	2,3,4,17,19,20,22,24	1	P35745
5676	1,5,6,7,8,18,21,24	2,3,4,17,19,20,22,23	2	P36860,P49134
5677	1,5,6,7,8,18,22,23	2,3,4,17,19,20,21,24	2	P49134,Q9WVK7

5678	1,5,6,7,8,18,22,24	2,3,4,17,19,20,21,23	2	P49134,Q9WVK7
5679	1,5,6,7,8,18,23,24	2,3,4,17,19,20,21,22	2	P49134,Q9WVK7
5680	1,5,6,7,8,19,20,21	2,3,4,17,18,22,23,24	20	iRT-Kit_WR_fusion,P02780,P04905,P06760,P06761,P09456,P19218,P19629,P22282,P22283,P46462,P47967,P55159,Q5I0D1,Q63493,Q63617,Q812E4,Q9JI85,Q9QW07,Q9WTT6
5681	1,5,6,7,8,19,20,22	2,3,4,17,18,21,23,24	3	P19218,Q99041,Q9QW07
5682	1,5,6,7,8,19,20,23	2,3,4,17,18,21,22,24	7	P19218,P19629,P23739,P25031,Q812E4,Q8CJD3,Q9QW07
5683	1,5,6,7,8,19,20,24	2,3,4,17,18,21,22,23	6	P08723,P09456,P22283,Q812E4,Q9JHB9,Q9QW07
5684	1,5,6,7,8,19,21,22	2,3,4,17,18,20,23,24	4	iRT-Kit_WR_fusion,P19218,Q99041,Q9QYP1
5685	1,5,6,7,8,19,21,23	2,3,4,17,18,20,22,24	3	iRT-Kit_WR_fusion,P21674,Q63474
5686	1,5,6,7,8,19,21,24	2,3,4,17,18,20,22,23	2	iRT-Kit_WR_fusion,Q63474
5687	1,5,6,7,8,19,22,23	2,3,4,17,18,20,21,24	2	P19218,P19223
5688	1,5,6,7,8,19,22,24	2,3,4,17,18,20,21,23	2	P19218,P19223
5689	1,5,6,7,8,19,23,24	2,3,4,17,18,20,21,22	1	P19223
5690	1,5,6,7,8,20,21,22	2,3,4,17,18,19,23,24	5	P06760,P19218,P19629,P35745,Q5PQL7
5691	1,5,6,7,8,20,21,23	2,3,4,17,18,19,22,24	7	P06760,P19132,P19629,P23739,P25031,P35745,Q812E4
5692	1,5,6,7,8,20,21,24	2,3,4,17,18,19,22,23	10	P02782,P08723,P09456,P19629,P22283,P35745,Q63474,Q812E4,Q9JHB9,Q9QW07
5693	1,5,6,7,8,20,22,23	2,3,4,17,18,19,21,24	5	P19218,P19629,P23739,P35745,Q9WVK7
5694	1,5,6,7,8,20,22,24	2,3,4,17,18,19,21,23	5	P19218,P35745,Q9JHB9,Q9QW07,Q9WVK7
5695	1,5,6,7,8,20,23,24	2,3,4,17,18,19,21,22	5	P23739,P35745,Q812E4,Q9QW07,Q9WVK7
5696	1,5,6,7,8,21,22,23	2,3,4,17,18,19,20,24	3	P19218,P35745,Q62714
5697	1,5,6,7,8,21,22,24	2,3,4,17,18,19,20,23	3	P35745,P36860,Q62714

5698	1,5,6,7,8,21,23,24	2,3,4,17,18,19,20,22	5	P35745,P36860,Q62714,Q63474,Q8K1G0
5699	1,5,6,7,8,22,23,24	2,3,4,17,18,19,20,21	9	O54858,P19223,P25809,P29315,P35745,P49134,Q62714,Q6P6R2,Q9WVK7
5700	1,5,6,7,17,18,19,20	2,3,4,8,21,22,23,24	3	P00762,P20761,P30919
5701	1,5,6,7,17,18,19,21	2,3,4,8,20,22,23,24	2	P00762,P05539
5702	1,5,6,7,17,18,19,22	2,3,4,8,20,21,23,24	1	P00762
5703	1,5,6,7,17,18,19,23	2,3,4,8,20,21,22,24	2	P00762,Q10743
5704	1,5,6,7,17,18,19,24	2,3,4,8,20,21,22,23	1	P00762
5705	1,5,6,7,17,18,20,21	2,3,4,8,19,22,23,24	2	P00507,P05539
5706	1,5,6,7,17,18,20,22	2,3,4,8,19,21,23,24	2	P00762,Q9Z0J6
5707	1,5,6,7,17,18,20,23	2,3,4,8,19,21,22,24	2	P00762,P30919
5708	1,5,6,7,17,18,20,24	2,3,4,8,19,21,22,23	1	P00762
5709	1,5,6,7,17,18,21,22	2,3,4,8,19,20,23,24	2	P00762,P05539
5710	1,5,6,7,17,18,21,23	2,3,4,8,19,20,22,24	4	P00762,P05539,P19814,P36860
5711	1,5,6,7,17,18,21,24	2,3,4,8,19,20,22,23	4	P05539,P36376,P36860,Q6AY61
5712	1,5,6,7,17,18,22,23	2,3,4,8,19,20,21,24	2	P00762,Q9EQS0
5713	1,5,6,7,17,18,22,24	2,3,4,8,19,20,21,23	1	P00762
5714	1,5,6,7,17,18,23,24	2,3,4,8,19,20,21,22	1	P00762
5715	1,5,6,7,17,19,20,21	2,3,4,8,18,22,23,24	4	B0LT89,P05539,P22283,Q63617
5716	1,5,6,7,17,19,20,22	2,3,4,8,18,21,23,24	3	O89117,P00762,P02625
5717	1,5,6,7,17,19,20,23	2,3,4,8,18,21,22,24	3	P00762,P02625,P23739

5718	1,5,6,7,17,<br>19,20,24	2,3,4,8,18,<br>21,22,23	2 P00762,P54921
5719	1,5,6,7,17,<br>19,21,22	2,3,4,8,18,<br>20,23,24	3 P00762,P05539,Q9R168
5720	1,5,6,7,17,<br>19,21,23	2,3,4,8,18,<br>20,22,24	3 P00762,P05539,P21674
5721	1,5,6,7,17,<br>19,21,24	2,3,4,8,18,<br>20,22,23	7 P00762,P05539,P36376,P54921,Q5BJY9,Q63474,Q6AY61
5722	1,5,6,7,17,<br>19,22,23	2,3,4,8,18,<br>20,21,24	2 P00762,P02625
5723	1,5,6,7,17,<br>19,22,24	2,3,4,8,18,<br>20,21,23	2 P00762,Q5BJY9
5724	1,5,6,7,17,<br>19,23,24	2,3,4,8,18,<br>20,21,22	2 P00762,P25809
5725	1,5,6,7,17,<br>20,21,22	2,3,4,8,18,<br>19,23,24	3 P05539,Q5PQL7,Q9Z0J6
5726	1,5,6,7,17,<br>20,21,23	2,3,4,8,18,<br>19,22,24	2 P05539,P35745
5727	1,5,6,7,17,<br>20,21,24	2,3,4,8,18,<br>19,22,23	3 P05539,P36860,Q6AY61
5728	1,5,6,7,17,<br>20,22,23	2,3,4,8,18,<br>19,21,24	2 P00762,P02625
5729	1,5,6,7,17,<br>20,22,24	2,3,4,8,18,<br>19,21,23	1 Q9Z0J6
5730	1,5,6,7,17,<br>20,23,24	2,3,4,8,18,<br>19,21,22	1 P23739
5731	1,5,6,7,17,<br>21,22,23	2,3,4,8,18,<br>19,20,24	3 P00762,P05539,Q9R168
5732	1,5,6,7,17,<br>21,22,24	2,3,4,8,18,<br>19,20,23	5 P05539,P36376,P36860,Q5BJY9,Q9R168
5733	1,5,6,7,17,<br>21,23,24	2,3,4,8,18,<br>19,20,22	3 P05539,P36860,Q63474
5734	1,5,6,7,17,<br>22,23,24	2,3,4,8,18,<br>19,20,21	4 D4A5U3,P00762,P25809,Q9R168
5735	1,5,6,7,18,<br>19,20,21	2,3,4,8,17,<br>22,23,24	5 P22283,P47967,Q5I0D1,Q5PQL7,Q63617
5736	1,5,6,7,18,<br>19,20,22	2,3,4,8,17,<br>21,23,24	2 P00762,Q5PQL7
5737	1,5,6,7,18,<br>19,20,23	2,3,4,8,17,<br>21,22,24	1 P00762

5738	1,5,6,7,18,<br>19,20,24	2,3,4,8,17,<br>21,22,23	1 P00762
5739	1,5,6,7,18,<br>19,21,22	2,3,4,8,17,<br>20,23,24	3 P00762,P36376,Q5PQL7
5740	1,5,6,7,18,<br>19,21,23	2,3,4,8,17,<br>20,22,24	1 P00762
5741	1,5,6,7,18,<br>19,21,24	2,3,4,8,17,<br>20,22,23	2 P00762,P36376
5742	1,5,6,7,18,<br>19,22,23	2,3,4,8,17,<br>20,21,24	5 D4A5U3,P00762,P19223,Q811M5,Q9EQS0
5743	1,5,6,7,18,<br>19,22,24	2,3,4,8,17,<br>20,21,23	4 D4A5U3,P00762,P19223,P36376
5744	1,5,6,7,18,<br>19,23,24	2,3,4,8,17,<br>20,21,22	5 D4A5U3,P00762,P19223,P25809,Q9WVK7
5745	1,5,6,7,18,<br>20,21,22	2,3,4,8,17,<br>19,23,24	2 P01835,Q5PQL7
5746	1,5,6,7,18,<br>20,21,23	2,3,4,8,17,<br>19,22,24	1 P01835
5747	1,5,6,7,18,<br>20,21,24	2,3,4,8,17,<br>19,22,23	1 P01835
5748	1,5,6,7,18,<br>20,22,23	2,3,4,8,17,<br>19,21,24	4 P00762,P04355,Q9EQS0,Q9WVK7
5749	1,5,6,7,18,<br>20,22,24	2,3,4,8,17,<br>19,21,23	2 P22006,Q9WVK7
5750	1,5,6,7,18,<br>20,23,24	2,3,4,8,17,<br>19,21,22	1 Q9WVK7
5751	1,5,6,7,18,<br>21,22,23	2,3,4,8,17,<br>19,20,24	3 D4A5U3,P00762,P01835
5752	1,5,6,7,18,<br>21,22,24	2,3,4,8,17,<br>19,20,23	3 D4A5U3,P01835,P36376
5753	1,5,6,7,18,<br>21,23,24	2,3,4,8,17,<br>19,20,22	5 D4A5U3,O89117,P01835,P36860,Q9JJ50
5754	1,5,6,7,18,<br>22,23,24	2,3,4,8,17,<br>19,20,21	9 D4A5U3,P00762,P13676,P19223,P25809,P97675,Q6P6R2,Q9EQS0,Q9WVK7
5755	1,5,6,7,19,<br>20,21,22	2,3,4,8,17,<br>18,23,24	3 P19218,Q5PQL7,Q63617
5756	1,5,6,7,19,<br>20,21,23	2,3,4,8,17,<br>18,22,24	0
5757	1,5,6,7,19,<br>20,21,24	2,3,4,8,17,<br>18,22,23	4 O35547,P22283,Q63617,Q9WUC4

5758	1,5,6,7,19,<br>20,22,23	2,3,4,8,17,<br>18,21,24	3	P00762,P02625,P19218
5759	1,5,6,7,19,<br>20,22,24	2,3,4,8,17,<br>18,21,23	3	P00762,P19218,P22006
5760	1,5,6,7,19,<br>20,23,24	2,3,4,8,17,<br>18,21,22	1	P23739
5761	1,5,6,7,19,<br>21,22,23	2,3,4,8,17,<br>18,20,24	2	P00762,P19218
5762	1,5,6,7,19,<br>21,22,24	2,3,4,8,17,<br>18,20,23	3	P00774,P36376,Q5BJY9
5763	1,5,6,7,19,<br>21,23,24	2,3,4,8,17,<br>18,20,22	2	P25809,Q63474
5764	1,5,6,7,19,<br>22,23,24	2,3,4,8,17,<br>18,20,21	7	D4A5U3,P00762,P00774,P19223,P25809,P26772,Q811M5
5765	1,5,6,7,20,<br>21,22,23	2,3,4,8,17,<br>18,19,24	1	P01835
5766	1,5,6,7,20,<br>21,22,24	2,3,4,8,17,<br>18,19,23	1	P01835
5767	1,5,6,7,20,<br>21,23,24	2,3,4,8,17,<br>18,19,22	1	P01835
5768	1,5,6,7,20,<br>22,23,24	2,3,4,8,17,<br>18,19,21	2	P22006,Q9WVK7
5769	1,5,6,7,21,<br>22,23,24	2,3,4,8,17,<br>18,19,20	6	D4A5U3,P01835,P13676,P25809,P36860,Q62714
5770	1,5,6,8,17,<br>18,19,20	2,3,4,7,21,<br>22,23,24	2	B0LT89,P13432
5771	1,5,6,8,17,<br>18,19,21	2,3,4,7,20,<br>22,23,24	2	P13432,Q9QX74
5772	1,5,6,8,17,<br>18,19,22	2,3,4,7,20,<br>21,23,24	1	P13432
5773	1,5,6,8,17,<br>18,19,23	2,3,4,7,20,<br>21,22,24	1	P13432
5774	1,5,6,8,17,<br>18,19,24	2,3,4,7,20,<br>21,22,23	1	P13432
5775	1,5,6,8,17,<br>18,20,21	2,3,4,7,19,<br>22,23,24	1	P13432
5776	1,5,6,8,17,<br>18,20,22	2,3,4,7,19,<br>21,23,24	1	P13432
5777	1,5,6,8,17,<br>18,20,23	2,3,4,7,19,<br>21,22,24	2	P13432,P23739

5778	1,5,6,8,17, 18,20,24	2,3,4,7,19, 21,22,23	1	P13432
5779	1,5,6,8,17, 18,21,22	2,3,4,7,19, 20,23,24	2	P10758,P13432
5780	1,5,6,8,17, 18,21,23	2,3,4,7,19, 20,22,24	2	P13432,P19814
5781	1,5,6,8,17, 18,21,24	2,3,4,7,19, 20,22,23	3	P10758,P13432,P36860
5782	1,5,6,8,17, 18,22,23	2,3,4,7,19, 20,21,24	2	P13432,P68511
5783	1,5,6,8,17, 18,22,24	2,3,4,7,19, 20,21,23	2	P10758,P13432
5784	1,5,6,8,17, 18,23,24	2,3,4,7,19, 20,21,22	1	P13432
5785	1,5,6,8,17, 19,20,21	2,3,4,7,18, 22,23,24	4	B0LT89,P06760,Q6P6R2,Q9JI85
5786	1,5,6,8,17, 19,20,22	2,3,4,7,18, 21,23,24	4	B0LT89,O89117,P19218,P20766
5787	1,5,6,8,17, 19,20,23	2,3,4,7,18, 21,22,24	3	O89117,P23739,Q8CJD3
5788	1,5,6,8,17, 19,20,24	2,3,4,7,18, 21,22,23	3	B0LT89,P13432,P60905
5789	1,5,6,8,17, 19,21,22	2,3,4,7,18, 20,23,24	3	P05539,P20766,Q9QYP1
5790	1,5,6,8,17, 19,21,23	2,3,4,7,18, 20,22,24	1	P21674
5791	1,5,6,8,17, 19,21,24	2,3,4,7,18, 20,22,23	1	Q63474
5792	1,5,6,8,17, 19,22,23	2,3,4,7,18, 20,21,24	1	P00507
5793	1,5,6,8,17, 19,22,24	2,3,4,7,18, 20,21,23	1	P00507
5794	1,5,6,8,17, 19,23,24	2,3,4,7,18, 20,21,22	0	
5795	1,5,6,8,17, 20,21,22	2,3,4,7,18, 19,23,24	2	P20766,P35745
5796	1,5,6,8,17, 20,21,23	2,3,4,7,18, 19,22,24	4	P19132,P20766,P23739,P35745
5797	1,5,6,8,17, 20,21,24	2,3,4,7,18, 19,22,23	1	P20766

5798	1,5,6,8,17,<br>20,22,23	2,3,4,7,18,<br>19,21,24	3 O89117,P20766,P23739
5799	1,5,6,8,17,<br>20,22,24	2,3,4,7,18,<br>19,21,23	3 O89117,P13432,P20766
5800	1,5,6,8,17,<br>20,23,24	2,3,4,7,18,<br>19,21,22	1 P23739
5801	1,5,6,8,17,<br>21,22,23	2,3,4,7,18,<br>19,20,24	2 P20766,P68511
5802	1,5,6,8,17,<br>21,22,24	2,3,4,7,18,<br>19,20,23	2 P10758,P20766
5803	1,5,6,8,17,<br>21,23,24	2,3,4,7,18,<br>19,20,22	3 P36860,Q63474,Q80WL1
5804	1,5,6,8,17,<br>22,23,24	2,3,4,7,18,<br>19,20,21	3 P00507,P47967,P62804
5805	1,5,6,8,18,<br>19,20,21	2,3,4,7,17,<br>22,23,24	2 B0LT89,Q5PQL7
5806	1,5,6,8,18,<br>19,20,22	2,3,4,7,17,<br>21,23,24	1 Q5PQL7
5807	1,5,6,8,18,<br>19,20,23	2,3,4,7,17,<br>21,22,24	2 P23739,Q9R168
5808	1,5,6,8,18,<br>19,20,24	2,3,4,7,17,<br>21,22,23	2 Q9QW07,Q9R168
5809	1,5,6,8,18,<br>19,21,22	2,3,4,7,17,<br>20,23,24	2 Q5PQL7,Q9QYP1
5810	1,5,6,8,18,<br>19,21,23	2,3,4,7,17,<br>20,22,24	0
5811	1,5,6,8,18,<br>19,21,24	2,3,4,7,17,<br>20,22,23	0
5812	1,5,6,8,18,<br>19,22,23	2,3,4,7,17,<br>20,21,24	4 P00507,P19223,Q6AY61,Q811M5
5813	1,5,6,8,18,<br>19,22,24	2,3,4,7,17,<br>20,21,23	2 P00507,P19223
5814	1,5,6,8,18,<br>19,23,24	2,3,4,7,17,<br>20,21,22	1 P19223
5815	1,5,6,8,18,<br>20,21,22	2,3,4,7,17,<br>19,23,24	2 P54921,Q5PQL7
5816	1,5,6,8,18,<br>20,21,23	2,3,4,7,17,<br>19,22,24	1 P54921
5817	1,5,6,8,18,<br>20,21,24	2,3,4,7,17,<br>19,22,23	0

5818	1,5,6,8,18,<br>20,22,23	2,3,4,7,17,<br>19,21,24	4	P04355,P54921,Q6AY61,Q9WVK7
5819	1,5,6,8,18,<br>20,22,24	2,3,4,7,17,<br>19,21,23	2	P22006,Q9WVK7
5820	1,5,6,8,18,<br>20,23,24	2,3,4,7,17,<br>19,21,22	4	P23739,Q5QE79,Q9R168,Q9WVK7
5821	1,5,6,8,18,<br>21,22,23	2,3,4,7,17,<br>19,20,24	1	P54921
5822	1,5,6,8,18,<br>21,22,24	2,3,4,7,17,<br>19,20,23	1	P10758
5823	1,5,6,8,18,<br>21,23,24	2,3,4,7,17,<br>19,20,22	1	P20646
5824	1,5,6,8,18,<br>22,23,24	2,3,4,7,17,<br>19,20,21	5	P00507,P13676,P19223,P20646,Q9WVK7
5825	1,5,6,8,19,<br>20,21,22	2,3,4,7,17,<br>18,23,24	4	B0LT89,P19218,P20766,Q5PQL7
5826	1,5,6,8,19,<br>20,21,23	2,3,4,7,17,<br>18,22,24	2	P19218,P23739
5827	1,5,6,8,19,<br>20,21,24	2,3,4,7,17,<br>18,22,23	6	B0LT89,O35547,P30120,P60905,Q812E4,Q9QW07
5828	1,5,6,8,19,<br>20,22,23	2,3,4,7,17,<br>18,21,24	2	P19218,P23739
5829	1,5,6,8,19,<br>20,22,24	2,3,4,7,17,<br>18,21,23	1	P19218
5830	1,5,6,8,19,<br>20,23,24	2,3,4,7,17,<br>18,21,22	1	P23739
5831	1,5,6,8,19,<br>21,22,23	2,3,4,7,17,<br>18,20,24	1	P19218
5832	1,5,6,8,19,<br>21,22,24	2,3,4,7,17,<br>18,20,23	1	P30919
5833	1,5,6,8,19,<br>21,23,24	2,3,4,7,17,<br>18,20,22	2	Q63474,Q80WL1
5834	1,5,6,8,19,<br>22,23,24	2,3,4,7,17,<br>18,20,21	5	P00507,P19218,P19223,P25809,Q811M5
5835	1,5,6,8,20,<br>21,22,23	2,3,4,7,17,<br>18,19,24	3	P19218,P20766,P54921
5836	1,5,6,8,20,<br>21,22,24	2,3,4,7,17,<br>18,19,23	1	P20766
5837	1,5,6,8,20,<br>21,23,24	2,3,4,7,17,<br>18,19,22	1	P23739

5838	1,5,6,8,20,<br>22,23,24	2,3,4,7,17,<br>18,19,21	4	P20766,P23739,Q5QE79,Q9WVK7
5839	1,5,6,8,21,<br>22,23,24	2,3,4,7,17,<br>18,19,20	5	P20646,P20766,P30919,Q62714,Q8K1G0
5840	1,5,6,17,1<br>8,19,20,21	2,3,4,7,8,2<br>2,23,24	6	B0LT89,P05539,P20761,P35280,Q5PQL7,Q6P6R2
5841	1,5,6,17,1<br>8,19,20,22	2,3,4,7,8,2<br>1,23,24	4	B0LT89,P00762,P20761,Q5PQL7
5842	1,5,6,17,1<br>8,19,20,23	2,3,4,7,8,2<br>1,22,24	4	P00762,P02625,P11883,P35280
5843	1,5,6,17,1<br>8,19,20,24	2,3,4,7,8,2<br>1,22,23	4	B0LT89,P11883,P20761,P35280
5844	1,5,6,17,1<br>8,19,21,22	2,3,4,7,8,2<br>0,23,24	5	P00762,P05539,P20761,Q4G075,Q5PQL7
5845	1,5,6,17,1<br>8,19,21,23	2,3,4,7,8,2<br>0,22,24	4	O70417,P00762,P35280,Q4G075
5846	1,5,6,17,1<br>8,19,21,24	2,3,4,7,8,2<br>0,22,23	4	P05539,P20761,P35280,Q4G075
5847	1,5,6,17,1<br>8,19,22,23	2,3,4,7,8,2<br>0,21,24	4	D4A5U3,P00762,Q811M5,Q9EQS0
5848	1,5,6,17,1<br>8,19,22,24	2,3,4,7,8,2<br>0,21,23	4	D4A5U3,P00762,P20761,Q4G075
5849	1,5,6,17,1<br>8,19,23,24	2,3,4,7,8,2<br>0,21,22	5	D4A5U3,P00762,P11883,P35280,Q4G075
5850	1,5,6,17,1<br>8,20,21,22	2,3,4,7,8,1<br>9,23,24	3	P05539,P20761,Q5PQL7
5851	1,5,6,17,1<br>8,20,21,23	2,3,4,7,8,1<br>9,22,24	1	P35280
5852	1,5,6,17,1<br>8,20,21,24	2,3,4,7,8,1<br>9,22,23	3	P05539,P20761,P35280
5853	1,5,6,17,1<br>8,20,22,23	2,3,4,7,8,1<br>9,21,24	2	P04355,P11883
5854	1,5,6,17,1<br>8,20,22,24	2,3,4,7,8,1<br>9,21,23	3	P11883,P20761,P22006
5855	1,5,6,17,1<br>8,20,23,24	2,3,4,7,8,1<br>9,21,22	3	P04355,P11883,P35280
5856	1,5,6,17,1<br>8,21,22,23	2,3,4,7,8,1<br>9,20,24	5	D4A5U3,P68511,P82471,Q4G075,Q6TMA8
5857	1,5,6,17,1<br>8,21,22,24	2,3,4,7,8,1<br>9,20,23	5	D4A5U3,P05539,P10758,P20761,Q4G075

5858	1,5,6,17,18,21,23,24	2,3,4,7,8,19,20,22	5	D4A5U3,O70417,P35280,P36860,Q4G075
5859	1,5,6,17,18,22,23,24	2,3,4,7,8,19,20,21	13	D4A5U3,P00762,P02780,P11883,P22273,P47967,P62804,Q00715,Q4G075,Q5I0D1,Q63617,Q9EQS0,Q9WTT6
5860	1,5,6,17,19,20,21,22	2,3,4,7,8,18,23,24	8	B0LT89,P05539,P20761,P20762,P20766,Q3ZAV1,Q5I0J9,Q5PQL7
5861	1,5,6,17,19,20,21,23	2,3,4,7,8,18,22,24	3	B0LT89,P20766,P35280
5862	1,5,6,17,19,20,21,24	2,3,4,7,8,18,22,23	7	B0LT89,O35547,P05539,P20761,P20762,P20766,P35280
5863	1,5,6,17,19,20,22,23	2,3,4,7,8,18,21,24	6	O89117,P00762,P02625,P20766,P23739,Q00715
5864	1,5,6,17,19,20,22,24	2,3,4,7,8,18,21,23	5	O89117,P02625,P20761,P20762,P20766
5865	1,5,6,17,19,20,23,24	2,3,4,7,8,18,21,22	5	P02625,P11883,P23739,P35280,Q00715
5866	1,5,6,17,19,21,22,23	2,3,4,7,8,18,20,24	1	P20766
5867	1,5,6,17,19,21,22,24	2,3,4,7,8,18,20,23	6	P05539,P20761,P20762,P20766,Q4G075,Q5BJY9
5868	1,5,6,17,19,21,23,24	2,3,4,7,8,18,20,22	3	O70417,P35280,Q4G075
5869	1,5,6,17,19,22,23,24	2,3,4,7,8,18,20,21	10	D4A5U3,P00507,P00762,P02625,P22273,P25809,P62804,Q00715,Q4G075,Q811M5
5870	1,5,6,17,20,21,22,23	2,3,4,7,8,18,19,24	1	P20766
5871	1,5,6,17,20,21,22,24	2,3,4,7,8,18,19,23	4	P05539,P20761,P20762,P20766
5872	1,5,6,17,20,21,23,24	2,3,4,7,8,18,19,22	3	P20766,P23739,P35280
5873	1,5,6,17,20,22,23,24	2,3,4,7,8,18,19,21	7	P02625,P11883,P20766,P23739,P62804,Q00715,Q66H69
5874	1,5,6,17,21,22,23,24	2,3,4,7,8,18,19,20	4	D4A5U3,O70417,P20766,Q4G075
5875	1,5,6,18,19,20,21,22	2,3,4,7,8,17,23,24	2	P20761,Q5PQL7
5876	1,5,6,18,19,20,21,23	2,3,4,7,8,17,22,24	3	O70417,P23593,P35280
5877	1,5,6,18,19,20,21,24	2,3,4,7,8,17,22,23	2	P20761,P35280

5878	1,5,6,18,19,20,22,23	2,3,4,7,8,17,21,24	4	P00762,P04355,Q811M5,Q9EQS0
5879	1,5,6,18,19,20,22,24	2,3,4,7,8,17,21,23	3	P20761,P22006,Q5PQL7
5880	1,5,6,18,19,20,23,24	2,3,4,7,8,17,21,22	2	P11883,P35280
5881	1,5,6,18,19,21,22,23	2,3,4,7,8,17,20,24	6	D4A5U3,O70417,P13676,P19223,Q4G075,Q811M5
5882	1,5,6,18,19,21,22,24	2,3,4,7,8,17,20,23	7	D4A5U3,O70417,P13676,P19223,P20761,P36376,Q4G075
5883	1,5,6,18,19,21,23,24	2,3,4,7,8,17,20,22	6	D4A5U3,O70417,P13676,P19223,P35280,Q4G075
5884	1,5,6,18,19,22,23,24	2,3,4,7,8,17,20,21	14	D4A5U3,O70417,P00507,P00762,P13676,P19223,P22273,P25809,P62804,Q00715,Q4G075,Q811M5,Q9EQS0,Q9WTT6
5885	1,5,6,18,20,21,22,23	2,3,4,7,8,17,19,24	2	O70417,P54921
5886	1,5,6,18,20,21,22,24	2,3,4,7,8,17,19,23	2	P20761,Q5PQL7
5887	1,5,6,18,20,21,23,24	2,3,4,7,8,17,19,22	3	O70417,P20646,P35280
5888	1,5,6,18,20,22,23,24	2,3,4,7,8,17,19,21	11	P04355,P11883,P20646,P22006,P62804,Q00715,Q5QE79,Q66H69,Q9EQS0,Q9QX74,Q9WVK7
5889	1,5,6,18,21,22,23,24	2,3,4,7,8,17,19,20	9	D4A5U3,O70417,P13676,P19223,P20646,Q4G075,Q5U2V4,Q811M5,Q9WTT6
5890	1,5,6,19,20,21,22,23	2,3,4,7,8,17,18,24	3	O70417,P19218,P20766
5891	1,5,6,19,20,21,22,24	2,3,4,7,8,17,18,23	4	O70594,P20761,P20766,Q5PQL7
5892	1,5,6,19,20,21,23,24	2,3,4,7,8,17,18,22	3	O70417,P23739,P35280
5893	1,5,6,19,20,22,23,24	2,3,4,7,8,17,18,21	9	P02625,P20766,P22006,P23739,P26772,P62804,Q00715,Q811M5,Q9QX74
5894	1,5,6,19,21,22,23,24	2,3,4,7,8,17,18,20	9	D4A5U3,O70417,P00774,P13676,P19223,P20766,P25809,Q4G075,Q811M5
5895	1,5,6,20,21,22,23,24	2,3,4,7,8,17,18,19	3	O70417,P20646,P20766
5896	1,5,7,8,17,18,19,20	2,3,4,6,21,22,23,24	8	B0LT89,P06760,P09656,P30919,P47727,Q811M5,Q9QX74,Q9WTT6
5897	1,5,7,8,17,18,19,21	2,3,4,6,20,22,23,24	6	P06760,P09656,P22006,Q66H69,Q6P6R2,Q9QX74

5898	1,5,7,8,17, 18,19,22	2,3,4,6,20, 21,23,24	2	P09656,Q9QX74
5899	1,5,7,8,17, 18,19,23	2,3,4,6,20, 21,22,24	4	O55145,P21674,P22006,Q9QX74
5900	1,5,7,8,17, 18,19,24	2,3,4,6,20, 21,22,23	3	O55145,P02783,P09656
5901	1,5,7,8,17, 18,20,21	2,3,4,6,19, 22,23,24	6	P00507,P06760,P35745,Q811M5,Q9QX74,Q9WTT6
5902	1,5,7,8,17, 18,20,22	2,3,4,6,19, 21,23,24	1	P35745
5903	1,5,7,8,17, 18,20,23	2,3,4,6,19, 21,22,24	4	P19132,P23739,P30919,P35745
5904	1,5,7,8,17, 18,20,24	2,3,4,6,19, 21,22,23	5	P02783,P09656,P13432,P35745,Q811M5
5905	1,5,7,8,17, 18,21,22	2,3,4,6,19, 20,23,24	3	P06760,P35745,Q9QX74
5906	1,5,7,8,17, 18,21,23	2,3,4,6,19, 20,22,24	7	P06760,P19814,P21674,P22006,P35745,Q9QX74,Q9Z0V6
5907	1,5,7,8,17, 18,21,24	2,3,4,6,19, 20,22,23	6	P02783,P06760,P35745,P42854,Q811M5,Q9QX74
5908	1,5,7,8,17, 18,22,23	2,3,4,6,19, 20,21,24	1	P35745
5909	1,5,7,8,17, 18,22,24	2,3,4,6,19, 20,21,23	2	P09656,P49134
5910	1,5,7,8,17, 18,23,24	2,3,4,6,19, 20,21,22	2	O55145,P35745
5911	1,5,7,8,17, 19,20,21	2,3,4,6,18, 22,23,24	11	B0LT89,P06760,P13676,P35745,P36374,P55159,Q6P6R2,Q811M5,Q812E4,Q9QX74,Q9WTT6
5912	1,5,7,8,17, 19,20,22	2,3,4,6,18, 21,23,24	2	O89117,Q9WTT6
5913	1,5,7,8,17, 19,20,23	2,3,4,6,18, 21,22,24	4	P21674,P23739,Q8CJD3,Q9WTT6
5914	1,5,7,8,17, 19,20,24	2,3,4,6,18, 21,22,23	6	P09456,P09656,P36374,P54921,Q812E4,Q9WTT6
5915	1,5,7,8,17, 19,21,22	2,3,4,6,18, 20,23,24	2	P06760,Q9QX74
5916	1,5,7,8,17, 19,21,23	2,3,4,6,18, 20,22,24	4	P06760,P21674,P22006,Q9QX74
5917	1,5,7,8,17, 19,21,24	2,3,4,6,18, 20,22,23	5	P06760,P54921,Q63474,Q99MH3,Q9QX74

5918	1,5,7,8,17,<br>19,22,23	2,3,4,6,18,<br>20,21,24	1 O55145
5919	1,5,7,8,17,<br>19,22,24	2,3,4,6,18,<br>20,21,23	2 O55145,P09656
5920	1,5,7,8,17,<br>19,23,24	2,3,4,6,18,<br>20,21,22	1 O55145
5921	1,5,7,8,17,<br>20,21,22	2,3,4,6,18,<br>19,23,24	4 P06760,P35745,Q9QX74,Q9WTT6
5922	1,5,7,8,17,<br>20,21,23	2,3,4,6,18,<br>19,22,24	6 P06760,P19132,P23739,P35745,Q9QX74,Q9WTT6
5923	1,5,7,8,17,<br>20,21,24	2,3,4,6,18,<br>19,22,23	8 P02782,P06760,P09456,P30120,P35745,Q811M5,Q812E4,Q9WTT6
5924	1,5,7,8,17,<br>20,22,23	2,3,4,6,18,<br>19,21,24	2 P23739,P35745
5925	1,5,7,8,17,<br>20,22,24	2,3,4,6,18,<br>19,21,23	1 P35745
5926	1,5,7,8,17,<br>20,23,24	2,3,4,6,18,<br>19,21,22	2 P23739,P35745
5927	1,5,7,8,17,<br>21,22,23	2,3,4,6,18,<br>19,20,24	2 P35745,P68511
5928	1,5,7,8,17,<br>21,22,24	2,3,4,6,18,<br>19,20,23	1 P35745
5929	1,5,7,8,17,<br>21,23,24	2,3,4,6,18,<br>19,20,22	3 P35745,Q63474,Q80WL1
5930	1,5,7,8,17,<br>22,23,24	2,3,4,6,18,<br>19,20,21	2 O55145,P35745
5931	1,5,7,8,18,<br>19,20,21	2,3,4,6,17,<br>22,23,24	8 D3ZUC6,P06760,P07335,P47967,P55159,Q5I0D1,Q812E4,Q9WTT6
5932	1,5,7,8,18,<br>19,20,22	2,3,4,6,17,<br>21,23,24	0
5933	1,5,7,8,18,<br>19,20,23	2,3,4,6,17,<br>21,22,24	1 P23739
5934	1,5,7,8,18,<br>19,20,24	2,3,4,6,17,<br>21,22,23	1 P09656
5935	1,5,7,8,18,<br>19,21,22	2,3,4,6,17,<br>20,23,24	1 P06760
5936	1,5,7,8,18,<br>19,21,23	2,3,4,6,17,<br>20,22,24	2 P06760,P22006
5937	1,5,7,8,18,<br>19,21,24	2,3,4,6,17,<br>20,22,23	2 P06760,P09656

5938	1,5,7,8,18,<br>19,22,23	2,3,4,6,17,<br>20,21,24	1	P19223
5939	1,5,7,8,18,<br>19,22,24	2,3,4,6,17,<br>20,21,23	3	P09656,P19223,P63029
5940	1,5,7,8,18,<br>19,23,24	2,3,4,6,17,<br>20,21,22	2	O55145,P19223
5941	1,5,7,8,18,<br>20,21,22	2,3,4,6,17,<br>19,23,24	2	P06760,P35745
5942	1,5,7,8,18,<br>20,21,23	2,3,4,6,17,<br>19,22,24	4	D3ZUC6,P06760,P19132,P35745
5943	1,5,7,8,18,<br>20,21,24	2,3,4,6,17,<br>19,22,23	4	P06760,P35745,Q811M5,Q812E4
5944	1,5,7,8,18,<br>20,22,23	2,3,4,6,17,<br>19,21,24	2	P35745,Q9WVK7
5945	1,5,7,8,18,<br>20,22,24	2,3,4,6,17,<br>19,21,23	1	Q9WVK7
5946	1,5,7,8,18,<br>20,23,24	2,3,4,6,17,<br>19,21,22	2	P23739,Q9WVK7
5947	1,5,7,8,18,<br>21,22,23	2,3,4,6,17,<br>19,20,24	1	P35745
5948	1,5,7,8,18,<br>21,22,24	2,3,4,6,17,<br>19,20,23	1	P49134
5949	1,5,7,8,18,<br>21,23,24	2,3,4,6,17,<br>19,20,22	1	O89117
5950	1,5,7,8,18,<br>22,23,24	2,3,4,6,17,<br>19,20,21	3	P19223,P49134,Q9WVK7
5951	1,5,7,8,19,<br>20,21,22	2,3,4,6,17,<br>18,23,24	3	P06760,P19218,Q9WTT6
5952	1,5,7,8,19,<br>20,21,23	2,3,4,6,17,<br>18,22,24	5	D3ZUC6,P06760,P23739,Q812E4,Q9WTT6
5953	1,5,7,8,19,<br>20,21,24	2,3,4,6,17,<br>18,22,23	9	O35547,P02782,P06760,P08723,P09456,P30120,P55159,Q812E4,Q9WUC4
5954	1,5,7,8,19,<br>20,22,23	2,3,4,6,17,<br>18,21,24	3	P01039,P19218,P23739
5955	1,5,7,8,19,<br>20,22,24	2,3,4,6,17,<br>18,21,23	1	P19218
5956	1,5,7,8,19,<br>20,23,24	2,3,4,6,17,<br>18,21,22	2	P23739,Q812E4
5957	1,5,7,8,19,<br>21,22,23	2,3,4,6,17,<br>18,20,24	0	

5958	1,5,7,8,19,21,22,24	2,3,4,6,17,18,20,23	1	P55091
5959	1,5,7,8,19,21,23,24	2,3,4,6,17,18,20,22	3	P08649,Q63474,Q80WL1
5960	1,5,7,8,19,22,23,24	2,3,4,6,17,18,20,21	4	O55145,P01041,P19223,P55091
5961	1,5,7,8,20,21,22,23	2,3,4,6,17,18,19,24	3	P08649,P23739,P35745
5962	1,5,7,8,20,21,22,24	2,3,4,6,17,18,19,23	2	P08649,P35745
5963	1,5,7,8,20,21,23,24	2,3,4,6,17,18,19,22	5	P08649,P23739,P35745,Q06000,Q812E4
5964	1,5,7,8,20,22,23,24	2,3,4,6,17,18,19,21	3	P23739,P35745,Q9WVK7
5965	1,5,7,8,21,22,23,24	2,3,4,6,17,18,19,20	4	P08649,P35745,Q62714,Q8K1G0
5966	1,5,7,17,18,19,20,21	2,3,4,6,8,22,23,24	8	B0LT89,P00507,P06760,P20761,P23593,P30919,Q63532,Q6P6R2
5967	1,5,7,17,18,19,20,22	2,3,4,6,8,21,23,24	6	P00762,P09656,P20761,P30919,Q05702,Q63532
5968	1,5,7,17,18,19,20,23	2,3,4,6,8,21,22,24	5	P00762,P02625,P30919,Q10743,Q6AYC4
5969	1,5,7,17,18,19,20,24	2,3,4,6,8,21,22,23	7	P02783,P09656,P20761,P30919,P54921,Q63532,Q6AYC4
5970	1,5,7,17,18,19,21,22	2,3,4,6,8,20,23,24	5	P00762,P17559,P20761,P36376,Q05702
5971	1,5,7,17,18,19,21,23	2,3,4,6,8,20,22,24	3	P21674,P22006,Q10743
5972	1,5,7,17,18,19,21,24	2,3,4,6,8,20,22,23	9	P02783,P09656,P17559,P20761,P36376,P42854,P54921,Q63532,Q99MH3
5973	1,5,7,17,18,19,22,23	2,3,4,6,8,20,21,24	6	D4A5U3,O55145,P00762,P02625,Q05702,Q10743
5974	1,5,7,17,18,19,22,24	2,3,4,6,8,20,21,23	10	D4A5U3,O55145,P00762,P02631,P09656,P17559,P20761,P36376,P42854,Q05702
5975	1,5,7,17,18,19,23,24	2,3,4,6,8,20,21,22	5	D4A5U3,O55145,P00762,Q10743,Q6AYC4
5976	1,5,7,17,18,20,21,22	2,3,4,6,8,19,23,24	2	P20761,P42854
5977	1,5,7,17,18,20,21,23	2,3,4,6,8,19,22,24	2	P30919,Q10743

5978	1,5,7,17,18,20,21,24	2,3,4,6,8,19,22,23	6	P00507,P02783,P20761,P42854,Q63532,Q811M5
5979	1,5,7,17,18,20,22,23	2,3,4,6,8,19,21,24	2	P30919,Q10743
5980	1,5,7,17,18,20,22,24	2,3,4,6,8,19,21,23	3	P20761,P42854,Q63532
5981	1,5,7,17,18,20,23,24	2,3,4,6,8,19,21,22	4	P23739,P30919,Q10743,Q6AYC4
5982	1,5,7,17,18,21,22,23	2,3,4,6,8,19,20,24	2	P68511,Q10743
5983	1,5,7,17,18,21,22,24	2,3,4,6,8,19,20,23	3	P36376,P42854,Q05702
5984	1,5,7,17,18,21,23,24	2,3,4,6,8,19,20,22	2	P42854,P50280
5985	1,5,7,17,18,22,23,24	2,3,4,6,8,19,20,21	7	D4A5U3,O55145,P00762,P02631,P42854,Q05702,Q10743
5986	1,5,7,17,19,20,21,22	2,3,4,6,8,18,23,24	3	P20761,P20762,Q05702
5987	1,5,7,17,19,20,21,23	2,3,4,6,8,18,22,24	3	P02625,P23593,P23739
5988	1,5,7,17,19,20,21,24	2,3,4,6,8,18,22,23	6	P20761,P20762,P54921,Q63532,Q6AY61,Q99MH3
5989	1,5,7,17,19,20,22,23	2,3,4,6,8,18,21,24	4	P02625,P23739,Q05702,Q10743
5990	1,5,7,17,19,20,22,24	2,3,4,6,8,18,21,23	5	P02625,P20761,P54921,Q05702,Q63532
5991	1,5,7,17,19,20,23,24	2,3,4,6,8,18,21,22	4	P02625,P23739,P54921,Q6AYC4
5992	1,5,7,17,19,21,22,23	2,3,4,6,8,18,20,24	3	P12020,Q05702,Q9R168
5993	1,5,7,17,19,21,22,24	2,3,4,6,8,18,20,23	8	P17559,P36376,P42854,P54921,Q05702,Q5BJY9,Q99MH3,Q9R168
5994	1,5,7,17,19,21,23,24	2,3,4,6,8,18,20,22	1	P12020
5995	1,5,7,17,19,22,23,24	2,3,4,6,8,18,20,21	8	D4A5U3,O55145,P00762,P02625,P02631,P25809,Q00715,Q05702
5996	1,5,7,17,20,21,22,23	2,3,4,6,8,18,19,24	0	
5997	1,5,7,17,20,21,22,24	2,3,4,6,8,18,19,23	2	P20761,P42854

5998	1,5,7,17,20,21,23,24	2,3,4,6,8,18,19,22	2	P08649,P23739
5999	1,5,7,17,20,22,23,24	2,3,4,6,8,18,19,21	2	P02625,P23739
6000	1,5,7,17,21,22,23,24	2,3,4,6,8,18,19,20	3	P50280,Q05702,Q9R168
6001	1,5,7,18,19,20,21,22	2,3,4,6,8,17,23,24	5	P20761,P47967,P97840,Q05702,Q5PQL7
6002	1,5,7,18,19,20,21,23	2,3,4,6,8,17,22,24	1	P23593
6003	1,5,7,18,19,20,21,24	2,3,4,6,8,17,22,23	4	P20761,P23593,Q63532,Q99MH3
6004	1,5,7,18,19,20,22,23	2,3,4,6,8,17,21,24	3	P02625,Q05702,Q10743
6005	1,5,7,18,19,20,22,24	2,3,4,6,8,17,21,23	3	P20761,Q05702,Q63532
6006	1,5,7,18,19,20,23,24	2,3,4,6,8,17,21,22	2	P23739,Q6AYC4
6007	1,5,7,18,19,21,22,23	2,3,4,6,8,17,20,24	5	D4A5U3,O70417,P17559,P19223,Q05702
6008	1,5,7,18,19,21,22,24	2,3,4,6,8,17,20,23	6	D4A5U3,P17559,P19223,P36376,P55091,Q05702
6009	1,5,7,18,19,21,23,24	2,3,4,6,8,17,20,22	5	D4A5U3,O70417,P12020,P17559,P19223
6010	1,5,7,18,19,22,23,24	2,3,4,6,8,17,20,21	11	D4A5U3,P00762,P02631,P17559,P19223,P25809,P26772,P55091,P63029,Q05702,Q9WVK7
6011	1,5,7,18,20,21,22,23	2,3,4,6,8,17,19,24	0	
6012	1,5,7,18,20,21,22,24	2,3,4,6,8,17,19,23	2	P20761,P42854
6013	1,5,7,18,20,21,23,24	2,3,4,6,8,17,19,22	1	O70417
6014	1,5,7,18,20,22,23,24	2,3,4,6,8,17,19,21	1	Q9WVK7
6015	1,5,7,18,21,22,23,24	2,3,4,6,8,17,19,20	6	D4A5U3,O70417,P13676,P17559,P19223,Q05702
6016	1,5,7,19,20,21,22,23	2,3,4,6,8,17,18,24	2	P08649,Q99PP0
6017	1,5,7,19,20,21,22,24	2,3,4,6,8,17,18,23	5	O70594,P08649,P20761,Q05702,Q99PP0

6018	1,5,7,19,20,21,23,24	2,3,4,6,8,17,18,22	5	O70417,P08649,P23593,P23739,Q99PP0
6019	1,5,7,19,20,22,23,24	2,3,4,6,8,17,18,21	7	P02625,P08649,P23739,P26772,Q05702,Q99PP0,Q9QZQ5
6020	1,5,7,19,21,22,23,24	2,3,4,6,8,17,18,20	10	D4A5U3,O70417,P08649,P12020,P17559,P19223,P55091,Q05702,Q99PP0,Q9QZQ5
6021	1,5,7,20,21,22,23,24	2,3,4,6,8,17,18,19	2	P08649,Q99PP0
6022	1,5,8,17,18,19,20,21	2,3,4,6,7,22,23,24	6	B0LT89,P06760,P20761,P47727,Q6P6R2,Q9QX74
6023	1,5,8,17,18,19,20,22	2,3,4,6,7,21,23,24	2	B0LT89,P09656
6024	1,5,8,17,18,19,20,23	2,3,4,6,7,21,22,24	3	B0LT89,P23739,P30919
6025	1,5,8,17,18,19,20,24	2,3,4,6,7,21,22,23	4	B0LT89,P02783,P09656,P80299
6026	1,5,8,17,18,19,21,22	2,3,4,6,7,20,23,24	0	
6027	1,5,8,17,18,19,21,23	2,3,4,6,7,20,22,24	4	P21674,P22006,P80299,Q9QX74
6028	1,5,8,17,18,19,21,24	2,3,4,6,7,20,22,23	5	P02783,P09656,P42854,P80299,Q62894
6029	1,5,8,17,18,19,22,23	2,3,4,6,7,20,21,24	3	O55145,P00714,P11598
6030	1,5,8,17,18,19,22,24	2,3,4,6,7,20,21,23	3	O55145,P09656,P80299
6031	1,5,8,17,18,19,23,24	2,3,4,6,7,20,21,22	2	O55145,P80299
6032	1,5,8,17,18,20,21,22	2,3,4,6,7,19,23,24	0	
6033	1,5,8,17,18,20,21,23	2,3,4,6,7,19,22,24	3	P19132,P23739,P80299
6034	1,5,8,17,18,20,21,24	2,3,4,6,7,19,22,23	4	P02783,P42854,P80299,Q811M5
6035	1,5,8,17,18,20,22,23	2,3,4,6,7,19,21,24	2	P00714,P23739
6036	1,5,8,17,18,20,22,24	2,3,4,6,7,19,21,23	2	P42854,P80299
6037	1,5,8,17,18,20,23,24	2,3,4,6,7,19,21,22	3	P23739,P80299,Q6AYC4

6038	1,5,8,17,18,21,22,23	2,3,4,6,7,19,20,24	2	P00714,P68511
6039	1,5,8,17,18,21,22,24	2,3,4,6,7,19,20,23	3	P10758,P42854,P80299
6040	1,5,8,17,18,21,23,24	2,3,4,6,7,19,20,22	3	P42854,P80299,Q80WL1
6041	1,5,8,17,18,22,23,24	2,3,4,6,7,19,20,21	8	O55145,P00714,P05369,P11598,P47967,P80299,Q63617,Q9R0T3
6042	1,5,8,17,19,20,21,22	2,3,4,6,7,18,23,24	4	B0LT89,P06760,P20766,Q6P6R2
6043	1,5,8,17,19,20,21,23	2,3,4,6,7,18,22,24	5	B0LT89,P06760,P20766,P23739,Q6P6R2
6044	1,5,8,17,19,20,21,24	2,3,4,6,7,18,22,23	6	B0LT89,P06760,P30120,P60905,P80299,Q99MH3
6045	1,5,8,17,19,20,22,23	2,3,4,6,7,18,21,24	3	O89117,P20766,P23739
6046	1,5,8,17,19,20,22,24	2,3,4,6,7,18,21,23	2	O89117,P20766
6047	1,5,8,17,19,20,23,24	2,3,4,6,7,18,21,22	2	P23739,P80299
6048	1,5,8,17,19,21,22,23	2,3,4,6,7,18,20,24	1	P20766
6049	1,5,8,17,19,21,22,24	2,3,4,6,7,18,20,23	4	P20766,P80299,Q62894,Q80WL1
6050	1,5,8,17,19,21,23,24	2,3,4,6,7,18,20,22	2	P80299,Q80WL1
6051	1,5,8,17,19,22,23,24	2,3,4,6,7,18,20,21	6	O55145,P00507,P11598,P80299,Q00715,Q80WL1
6052	1,5,8,17,20,21,22,23	2,3,4,6,7,18,19,24	3	P20766,P23739,P35745
6053	1,5,8,17,20,21,22,24	2,3,4,6,7,18,19,23	2	P20766,P80299
6054	1,5,8,17,20,21,23,24	2,3,4,6,7,18,19,22	4	P20766,P23739,P80299,Q80WL1
6055	1,5,8,17,20,22,23,24	2,3,4,6,7,18,19,21	4	P20766,P23739,P80299,Q00715
6056	1,5,8,17,21,22,23,24	2,3,4,6,7,18,19,20	4	P11598,P20766,P80299,Q80WL1
6057	1,5,8,18,19,20,21,22	2,3,4,6,7,17,23,24	2	B0LT89,P01039

6058	1,5,8,18,19,20,21,23	2,3,4,6,7,17,22,24	3 P01039,P23593,P23739
6059	1,5,8,18,19,20,21,24	2,3,4,6,7,17,22,23	3 B0LT89,P30120,P80299
6060	1,5,8,18,19,20,22,23	2,3,4,6,7,17,21,24	4 P01039,P04762,P23739,Q6AY61
6061	1,5,8,18,19,20,22,24	2,3,4,6,7,17,21,23	0
6062	1,5,8,18,19,20,23,24	2,3,4,6,7,17,21,22	3 P23739,P80299,Q9R168
6063	1,5,8,18,19,21,22,23	2,3,4,6,7,17,20,24	2 P01039,P19223
6064	1,5,8,18,19,21,22,24	2,3,4,6,7,17,20,23	2 P19223,P55091
6065	1,5,8,18,19,21,23,24	2,3,4,6,7,17,20,22	2 P19223,P80299
6066	1,5,8,18,19,22,23,24	2,3,4,6,7,17,20,21	3 P00507,P19223,P55091
6067	1,5,8,18,20,21,22,23	2,3,4,6,7,17,19,24	2 P01039,P54921
6068	1,5,8,18,20,21,22,24	2,3,4,6,7,17,19,23	1 P80299
6069	1,5,8,18,20,21,23,24	2,3,4,6,7,17,19,22	2 P23739,P80299
6070	1,5,8,18,20,22,23,24	2,3,4,6,7,17,19,21	3 P23739,Q5QE79,Q9WVK7
6071	1,5,8,18,21,22,23,24	2,3,4,6,7,17,19,20	2 P19223,P80299
6072	1,5,8,19,20,21,22,23	2,3,4,6,7,17,18,24	5 P01039,P19218,P20766,P23739,Q99PP0
6073	1,5,8,19,20,21,22,24	2,3,4,6,7,17,18,23	3 O70594,P20766,Q99PP0
6074	1,5,8,19,20,21,23,24	2,3,4,6,7,17,18,22	3 P23739,Q80WL1,Q99PP0
6075	1,5,8,19,20,22,23,24	2,3,4,6,7,17,18,21	3 P23739,Q00715,Q9WVH8
6076	1,5,8,19,21,22,23,24	2,3,4,6,7,17,18,20	4 P19223,P55091,Q80WL1,Q8K1G0
6077	1,5,8,20,21,22,23,24	2,3,4,6,7,17,18,19	2 P20766,P23739

6078	1,5,17,18,19,20,21,22	2,3,4,6,7,8,23,24	8	B0LT89,P20761,P42854,P46844,Q05702,Q3ZAV1,Q63532,Q63598
6079	1,5,17,18,19,20,21,23	2,3,4,6,7,8,22,24	8	B0LT89,O70417,P20761,P23593,P30919,P35280,Q6P6R2,Q99041
6080	1,5,17,18,19,20,21,24	2,3,4,6,7,8,22,23	13	B0LT89,O54858,P02783,P20761,P20762,P23593,P35280,P42854,P50115,Q05702,Q63532,Q99041,Q99MH3
6081	1,5,17,18,19,20,22,23	2,3,4,6,7,8,21,24	8	P00714,P02625,P02631,P20761,P30919,Q05702,Q10743,Q6AYC4
6082	1,5,17,18,19,20,22,24	2,3,4,6,7,8,21,23	8	O70594,P02631,P09656,P20761,P42854,P50115,Q05702,Q63532
6083	1,5,17,18,19,20,23,24	2,3,4,6,7,8,21,22	9	P02625,P02631,P20761,P23593,P23739,P35280,Q05702,Q6AYC4,Q99041
6084	1,5,17,18,19,21,22,23	2,3,4,6,7,8,20,24	12	D4A5U3,O70417,P00714,P02780,P06911,P11598,P12020,P20761,P22273,P50280,Q05702,Q4G075
6085	1,5,17,18,19,21,22,24	2,3,4,6,7,8,20,23	13	D4A5U3,O70417,P06911,P12020,P17559,P20761,P22273,P36376,P42854,P50280,Q05702,Q4G075,Q62894
6086	1,5,17,18,19,21,23,24	2,3,4,6,7,8,20,22	14	D4A5U3,O70417,P02780,P06911,P12020,P20761,P22273,P23593,P35280,P42854,P50280,Q05702,Q4G075,Q99041
6087	1,5,17,18,19,22,23,24	2,3,4,6,7,8,20,21	20	D4A5U3,O55145,O70417,P00714,P02631,P02780,P06761,P06911,P11598,P12020,P22273,P22282,P42854,P50280,P62804,Q00715,Q05702,Q4G075,Q63617,Q9R0T3
6088	1,5,17,18,20,21,22,23	2,3,4,6,7,8,19,24	5	O70417,P00714,P20761,P50115,Q05702
6089	1,5,17,18,20,21,22,24	2,3,4,6,7,8,19,23	6	O70594,P20761,P42854,P50115,Q05702,Q63532
6090	1,5,17,18,20,21,23,24	2,3,4,6,7,8,19,22	6	O70417,P20761,P35280,P42854,P50115,Q99041

6091	1,5,17,18,20,22,23,24	2,3,4,6,7,8,19,21	12	P00714,P02631,P11883,P20761,P22282,P42854,P50115,P62804,Q00715,Q05702,Q6AYC4,Q99041
6092	1,5,17,18,21,22,23,24	2,3,4,6,7,8,19,20	18	D4A5U3,O70417,P00714,P02780,P06761,P06911,P11598,P12020,P22273,P22282,P42854,P50115,P50280,Q05702,Q4G075,Q63617,Q99041,Q9R0T3
6093	1,5,17,19,20,21,22,23	2,3,4,6,7,8,18,24	11	O70417,P02625,P07151,P20761,P20762,P20766,P23593,P97615,Q05702,Q3ZAV1,Q63598
6094	1,5,17,19,20,21,22,24	2,3,4,6,7,8,18,23	10	B0LT89,O70594,P20761,P20762,P20766,P42854,Q05702,Q3ZAV1,Q63532,Q63598
6095	1,5,17,19,20,21,23,24	2,3,4,6,7,8,18,22	10	O70417,P02625,P20761,P20762,P20766,P23593,P23739,P35280,Q05702,Q99041
6096	1,5,17,19,20,22,23,24	2,3,4,6,7,8,18,21	9	O70594,P02625,P02631,P20761,P20766,P23739,Q00715,Q05702,Q9QZQ5
6097	1,5,17,19,21,22,23,24	2,3,4,6,7,8,18,20	17	D4A5U3,O55004,O70417,P02780,P06911,P11598,P12020,P17559,P20766,P22273,P50280,P97615,Q05702,Q4G075,Q5GRG2,Q80WL1,Q9QZQ5
6098	1,5,17,20,21,22,23,24	2,3,4,6,7,8,18,19	9	O70417,P20761,P20766,P23739,P42854,P50115,P50280,P97615,Q05702
6099	1,5,18,19,20,21,22,23	2,3,4,6,7,8,17,24	9	O70417,O70594,P01039,P20761,P23593,P50115,Q05702,Q63598,Q99PP0
6100	1,5,18,19,20,21,22,24	2,3,4,6,7,8,17,23	9	O70417,O70594,P20761,P42854,P50115,Q05702,Q63532,Q63598,Q99PP0
6101	1,5,18,19,20,21,23,24	2,3,4,6,7,8,17,22	7	O70417,P20761,P23593,P35280,P50115,Q99041,Q99PP0
6102	1,5,18,19,20,22,23,24	2,3,4,6,7,8,17,21	13	O70417,O70594,P00714,P02625,P02631,P20761,P26772,P50115,Q00715,Q05702,Q5QE79,Q99PP0,Q9QZQ5
6103	1,5,18,19,21,22,23,24	2,3,4,6,7,8,17,20	18	D4A5U3,O70417,P02780,P06911,P12020,P13676,P17559,P19223,P22273,P50115,P50280,P55091,Q05702,Q4G075,Q5GRG2,Q62946,Q99PP0,Q9QZQ5

6104	1,5,18,20,21,22,23,24	2,3,4,6,7,8,17,19	6	O70417,O70594,P50115,Q05702,Q5QE79,Q99PP0
6105	1,5,19,20,21,22,23,24	2,3,4,6,7,8,17,18	11	O70417,O70594,P20766,P23593,P23739,P50115,P97615,Q05702,Q63598,Q99PP0,Q9QZQ5
6106	1,6,7,8,17,18,19,20	2,3,4,5,21,22,23,24	16	B0LT89,P06761,P06911,P12020,P13432,P21674,P22283,P30919,P31044,P36374,P47727,Q5GRG2,Q9JI85,Q9QW07,Q9QX74,Q9WTT6
6107	1,6,7,8,17,18,19,21	2,3,4,5,20,22,23,24	6	P05539,P13432,P21674,Q66H69,Q6P6R2,Q9QX74
6108	1,6,7,8,17,18,19,22	2,3,4,5,20,21,23,24	2	P13432,P70549
6109	1,6,7,8,17,18,19,23	2,3,4,5,20,21,22,24	2	P21674,P70549
6110	1,6,7,8,17,18,19,24	2,3,4,5,20,21,22,23	2	P13432,P70549
6111	1,6,7,8,17,18,20,21	2,3,4,5,19,22,23,24	17	P00507,P05539,P06911,P12020,P13432,P19629,P22283,P35745,P47727,Q5GRG2,Q6P6R2,Q78P75,Q812E4,Q9JHB9,Q9QW07,Q9QX74,Q9WTT6
6112	1,6,7,8,17,18,20,22	2,3,4,5,19,21,23,24	5	P06911,P12020,P13432,P35745,Q5GRG2
6113	1,6,7,8,17,18,20,23	2,3,4,5,19,21,22,24	7	P06911,P12020,P13432,P21674,P30919,P35745,Q5GRG2
6114	1,6,7,8,17,18,20,24	2,3,4,5,19,21,22,23	9	P06911,P08723,P12020,P13432,P35745,P47727,Q5GRG2,Q9JHB9,Q9QW07
6115	1,6,7,8,17,18,21,22	2,3,4,5,19,20,23,24	3	P05539,P35745,Q9QX74
6116	1,6,7,8,17,18,21,23	2,3,4,5,19,20,22,24	3	P21674,P35745,Q9QX74
6117	1,6,7,8,17,18,21,24	2,3,4,5,19,20,22,23	3	P05539,P13432,Q63474
6118	1,6,7,8,17,18,22,23	2,3,4,5,19,20,21,24	0	
6119	1,6,7,8,17,18,22,24	2,3,4,5,19,20,21,23	2	P13432,P70549
6120	1,6,7,8,17,18,23,24	2,3,4,5,19,20,21,22	1	P70549
6121	1,6,7,8,17,19,20,21	2,3,4,5,18,22,23,24	29	B0LT89,iRT-Kit_WR_fusion,O35547,P04905,P05539,P06761,P06911,P08723,P09456,P13676,P19629,P21674,P22283,P24368,P30120,P36374,P46462,P47727,Q5GRG2,Q63493,Q63617,Q6P6R2,Q812E4,Q9JHB9,Q9JI85,Q9QW07,Q9QX74,Q9WTT6,Q9Z2L0

6122	1,6,7,8,17,19,20,22	2,3,4,5,18,21,23,24	8	O89117,P06911,P12020,P70549,Q5GRG2,Q9QW07,Q9QZK9,Q9WTT6
6123	1,6,7,8,17,19,20,23	2,3,4,5,18,21,22,24	8	O89117,P06911,P21674,P70549,Q5GRG2,Q812E4,Q9QW07,Q9WTT6
6124	1,6,7,8,17,19,20,24	2,3,4,5,18,21,22,23	19	O89117,P06911,P08723,P09456,P22283,P30120,P36374,P46462,P47727,P60905,P70549,Q5GRG2,Q63617,Q812E4,Q9JHB9,Q9JI85,Q9QW07,Q9WTT6,Q9Z2L0
6125	1,6,7,8,17,19,21,22	2,3,4,5,18,20,23,24	3	P05539,P21674,Q9QX74
6126	1,6,7,8,17,19,21,23	2,3,4,5,18,20,22,24	3	P21674,Q63474,Q9QX74
6127	1,6,7,8,17,19,21,24	2,3,4,5,18,20,22,23	6	P02782,P05539,P08723,Q63474,Q9QX74,Q9Z2L0
6128	1,6,7,8,17,19,22,23	2,3,4,5,18,20,21,24	3	O89117,P21674,P70549
6129	1,6,7,8,17,19,22,24	2,3,4,5,18,20,21,23	2	O89117,P70549
6130	1,6,7,8,17,19,23,24	2,3,4,5,18,20,21,22	2	P21674,P70549
6131	1,6,7,8,17,20,21,22	2,3,4,5,18,19,23,24	6	P05539,P06911,P19629,P35745,Q5GRG2,Q9WTT6
6132	1,6,7,8,17,20,21,23	2,3,4,5,18,19,22,24	7	P06911,P19629,P21674,P35745,Q5GRG2,Q812E4,Q9WTT6
6133	1,6,7,8,17,20,21,24	2,3,4,5,18,19,22,23	17	P02782,P05539,P06911,P08723,P09456,P22283,P30120,P35745,P47727,Q5GRG2,Q63474,Q6AYR9,Q78P75,Q812E4,Q9JHB9,Q9QW07,Q9Z2L0
6134	1,6,7,8,17,20,22,23	2,3,4,5,18,19,21,24	5	O89117,P06911,P19629,P35745,Q5GRG2
6135	1,6,7,8,17,20,22,24	2,3,4,5,18,19,21,23	9	O89117,P06911,P08723,P12020,P22006,P35745,Q5GRG2,Q9JHB9,Q9QW07
6136	1,6,7,8,17,20,23,24	2,3,4,5,18,19,21,22	5	P06911,P35745,Q5GRG2,Q812E4,Q9QW07
6137	1,6,7,8,17,21,22,23	2,3,4,5,18,19,20,24	2	P21674,P35745
6138	1,6,7,8,17,21,22,24	2,3,4,5,18,19,20,23	3	P05539,P35745,Q63474
6139	1,6,7,8,17,21,23,24	2,3,4,5,18,19,20,22	2	P35745,Q63474
6140	1,6,7,8,17,22,23,24	2,3,4,5,18,19,20,21	3	P35745,P47967,P70549
6141	1,6,7,8,18,19,20,21	2,3,4,5,17,22,23,24	19	B0LT89,O08815,P04905,P06761,P08723,P19629,P22283,P24368,P31044,P46462,P47727,Q5GRG2,Q5PQL7,Q63493,Q63617,Q812E4,Q9JI85,Q9QW07,Q9WTT6

6142	1,6,7,8,18, 19,20,22	2,3,4,5,17, 21,23,24	5	P06911,P12020,Q5GRG2,Q5PQL7,Q9QW07
6143	1,6,7,8,18, 19,20,23	2,3,4,5,17, 21,22,24	5	P19629,P21674,P31044,Q812E4,Q9QW07
6144	1,6,7,8,18, 19,20,24	2,3,4,5,17, 21,22,23	11	P06911,P08723,P12020,P22283,P47727,P70549,Q5GRG2,Q63617,Q812E4,Q9JHB9,Q9QW07
6145	1,6,7,8,18, 19,21,22	2,3,4,5,17, 20,23,24	1	Q5PQL7
6146	1,6,7,8,18, 19,21,23	2,3,4,5,17, 20,22,24	1	P21674
6147	1,6,7,8,18, 19,21,24	2,3,4,5,17, 20,22,23	2	P22283,P36376
6148	1,6,7,8,18, 19,22,23	2,3,4,5,17, 20,21,24	3	P19223,P70549,Q6AY61
6149	1,6,7,8,18, 19,22,24	2,3,4,5,17, 20,21,23	2	P19223,P70549
6150	1,6,7,8,18, 19,23,24	2,3,4,5,17, 20,21,22	2	P19223,P70549
6151	1,6,7,8,18, 20,21,22	2,3,4,5,17, 19,23,24	8	P06911,P12020,P19629,P22283,P35745,Q5GRG2,Q5PQL7,Q9QW07
6152	1,6,7,8,18, 20,21,23	2,3,4,5,17, 19,22,24	4	P19629,P35745,Q812E4,Q9QW07
6153	1,6,7,8,18, 20,21,24	2,3,4,5,17, 19,22,23	12	P02782,P06911,P08723,P19629,P22283,P30120,P47727,Q5GRG2,Q63617,Q812E4,Q9JHB9,Q9QW07
6154	1,6,7,8,18, 20,22,23	2,3,4,5,17, 19,21,24	7	P06911,P12020,P19629,P54921,Q6AY61,Q9QW07,Q9WVK7
6155	1,6,7,8,18, 20,22,24	2,3,4,5,17, 19,21,23	7	P06911,P12020,P22006,Q5GRG2,Q9JHB9,Q9QW07,Q9WVK7
6156	1,6,7,8,18, 20,23,24	2,3,4,5,17, 19,21,22	4	P06911,Q812E4,Q9QW07,Q9WVK7
6157	1,6,7,8,18, 21,22,23	2,3,4,5,17, 19,20,24	1	P54921
6158	1,6,7,8,18, 21,22,24	2,3,4,5,17, 19,20,23	0	
6159	1,6,7,8,18, 21,23,24	2,3,4,5,17, 19,20,22	0	
6160	1,6,7,8,18, 22,23,24	2,3,4,5,17, 19,20,21	4	P19223,P29315,P70549,Q9WVK7
6161	1,6,7,8,19, 20,21,22	2,3,4,5,17, 18,23,24	13	iRT-Kit_WR_fusion,P08723,P19218,P19629,P22283,P46462,Q5PQL7,Q63493,Q63617,Q812E4,Q9JI85,Q9QW07,Q9WTT6

6162	1,6,7,8,19,20,21,23	2,3,4,5,17,18,22,24	10	iRT-Kit_WR_fusion,P19629,P21674,P22283,Q63493,Q63617,Q812E4,Q9JI85,Q9QW07,Q9WTT6
6163	1,6,7,8,19,20,21,24	2,3,4,5,17,18,22,23	28	iRT-Kit_WR_fusion,O08815,O35547,P02781,P02782,P06761,P08723,P09456,P0C0A9,P19629,P22283,P24368,P30120,P36374,P46462,P47727,P60905,Q5M8C6,Q63474,Q63493,Q63617,Q6AYR9,Q812E4,Q9JHB9,Q9JI85,Q9QW07,Q9WUC4,Q9Z2L0
6164	1,6,7,8,19,20,22,23	2,3,4,5,17,18,21,24	3	P19218,P19629,Q9QW07
6165	1,6,7,8,19,20,22,24	2,3,4,5,17,18,21,23	8	P08723,P22006,P70549,Q63617,Q812E4,Q9JHB9,Q9QW07,Q9QZK9
6166	1,6,7,8,19,20,23,24	2,3,4,5,17,18,21,22	4	P08723,P70549,Q812E4,Q9QW07
6167	1,6,7,8,19,21,22,23	2,3,4,5,17,18,20,24	2	iRT-Kit_WR_fusion,P21674
6168	1,6,7,8,19,21,22,24	2,3,4,5,17,18,20,23	0	
6169	1,6,7,8,19,21,23,24	2,3,4,5,17,18,20,22	1	Q63474
6170	1,6,7,8,19,22,23,24	2,3,4,5,17,18,20,21	3	P19223,P70549,Q811M5
6171	1,6,7,8,20,21,22,23	2,3,4,5,17,18,19,24	4	P19629,P35745,Q812E4,Q9QW07
6172	1,6,7,8,20,21,22,24	2,3,4,5,17,18,19,23	11	P01835,P02782,P08723,P19629,P22283,P30120,P35745,Q6AYR9,Q812E4,Q9JHB9,Q9QW07
6173	1,6,7,8,20,21,23,24	2,3,4,5,17,18,19,22	9	P01835,P08723,P19629,P35745,Q63474,Q6AYR9,Q812E4,Q9JHB9,Q9QW07
6174	1,6,7,8,20,22,23,24	2,3,4,5,17,18,19,21	5	P22006,P35745,Q812E4,Q9QW07,Q9WVK7
6175	1,6,7,8,21,22,23,24	2,3,4,5,17,18,19,20	2	Q62714,Q63474
6176	1,6,7,17,18,19,20,21	2,3,4,5,8,22,23,24	8	B0LT89,P05539,P20761,P22283,P30919,Q5PQL7,Q63617,Q6P6R2
6177	1,6,7,17,18,19,20,22	2,3,4,5,8,21,23,24	6	O89117,P00762,P20761,P30919,P70549,Q5PQL7
6178	1,6,7,17,18,19,20,23	2,3,4,5,8,21,22,24	5	P00762,P21674,P30919,P70549,Q10743
6179	1,6,7,17,18,19,20,24	2,3,4,5,8,21,22,23	2	P30919,P70549
6180	1,6,7,17,18,19,21,22	2,3,4,5,8,20,23,24	5	P00762,P05539,P23739,P36376,Q5PQL7

6181	1,6,7,17,18,19,21,23	2,3,4,5,8,20,22,24	3	P05539,P21674,Q10743
6182	1,6,7,17,18,19,21,24	2,3,4,5,8,20,22,23	4	P05539,P36376,P70549,Q9Z2L0
6183	1,6,7,17,18,19,22,23	2,3,4,5,8,20,21,24	3	P00762,P70549,Q10743
6184	1,6,7,17,18,19,22,24	2,3,4,5,8,20,21,23	3	P00762,P36376,P70549
6185	1,6,7,17,18,19,23,24	2,3,4,5,8,20,21,22	3	P00762,P70549,Q10743
6186	1,6,7,17,18,20,21,22	2,3,4,5,8,19,23,24	3	P01835,P05539,Q5PQL7
6187	1,6,7,17,18,20,21,23	2,3,4,5,8,19,22,24	3	P01835,P05539,P30919
6188	1,6,7,17,18,20,21,24	2,3,4,5,8,19,22,23	4	P00507,P01835,P05539,Q9Z2L0
6189	1,6,7,17,18,20,22,23	2,3,4,5,8,19,21,24	2	P30919,Q10743
6190	1,6,7,17,18,20,22,24	2,3,4,5,8,19,21,23	2	P22006,P70549
6191	1,6,7,17,18,20,23,24	2,3,4,5,8,19,21,22	2	P30919,P70549
6192	1,6,7,17,18,21,22,23	2,3,4,5,8,19,20,24	1	P05539
6193	1,6,7,17,18,21,22,24	2,3,4,5,8,19,20,23	3	P05539,P23739,P36376
6194	1,6,7,17,18,21,23,24	2,3,4,5,8,19,20,22	1	P05539
6195	1,6,7,17,18,22,23,24	2,3,4,5,8,19,20,21	6	D4A5U3,P00762,P47967,P70549,Q5I0D1,Q64268
6196	1,6,7,17,19,20,21,22	2,3,4,5,8,18,23,24	7	B0LT89,O89117,P05539,P20762,P22283,Q5PQL7,Q63617
6197	1,6,7,17,19,20,21,23	2,3,4,5,8,18,22,24	3	P05539,P21674,Q6P6R2
6198	1,6,7,17,19,20,21,24	2,3,4,5,8,18,22,23	9	B0LT89,O35547,P05539,P08723,P20762,P22283,P36374,Q63617,Q9Z2L0
6199	1,6,7,17,19,20,22,23	2,3,4,5,8,18,21,24	3	O89117,P02625,P70549
6200	1,6,7,17,19,20,22,24	2,3,4,5,8,18,21,23	3	O89117,P22006,P70549

6201	1,6,7,17,19,20,23,24	2,3,4,5,8,18,21,22	1	P70549
6202	1,6,7,17,19,21,22,23	2,3,4,5,8,18,20,24	3	P05539,P21674,Q9R168
6203	1,6,7,17,19,21,22,24	2,3,4,5,8,18,20,23	5	P05539,P36376,P70549,Q9R168,Q9Z2L0
6204	1,6,7,17,19,21,23,24	2,3,4,5,8,18,20,22	3	P05539,P70549,Q63474
6205	1,6,7,17,19,22,23,24	2,3,4,5,8,18,20,21	5	D4A5U3,P00762,P25809,P70549,Q811M5
6206	1,6,7,17,20,21,22,23	2,3,4,5,8,18,19,24	2	P01835,P05539
6207	1,6,7,17,20,21,22,24	2,3,4,5,8,18,19,23	4	P01835,P05539,P20762,Q9Z2L0
6208	1,6,7,17,20,21,23,24	2,3,4,5,8,18,19,22	2	P01835,P05539
6209	1,6,7,17,20,22,23,24	2,3,4,5,8,18,19,21	2	P22006,P70549
6210	1,6,7,17,21,22,23,24	2,3,4,5,8,18,19,20	3	P01835,P05539,Q9R168
6211	1,6,7,18,19,20,21,22	2,3,4,5,8,17,23,24	4	P22283,P36376,Q5PQL7,Q63617
6212	1,6,7,18,19,20,21,23	2,3,4,5,8,17,22,24	2	P23593,Q63617
6213	1,6,7,18,19,20,21,24	2,3,4,5,8,17,22,23	8	O08815,O35547,P01835,P22283,P36376,Q5PQL7,Q63617,Q9QW07
6214	1,6,7,18,19,20,22,23	2,3,4,5,8,17,21,24	1	P70549
6215	1,6,7,18,19,20,22,24	2,3,4,5,8,17,21,23	5	P22006,P36376,P70549,Q5PQL7,Q9QW07
6216	1,6,7,18,19,20,23,24	2,3,4,5,8,17,21,22	1	P70549
6217	1,6,7,18,19,21,22,23	2,3,4,5,8,17,20,24	1	D4A5U3
6218	1,6,7,18,19,21,22,24	2,3,4,5,8,17,20,23	5	D4A5U3,P19223,P23739,P36376,Q5PQL7
6219	1,6,7,18,19,21,23,24	2,3,4,5,8,17,20,22	3	D4A5U3,P19223,P36376
6220	1,6,7,18,19,22,23,24	2,3,4,5,8,17,20,21	9	D4A5U3,P00762,P13676,P19223,P22006,P25809,P36375,P70549,Q811M5

6221	1,6,7,18,20,21,22,23	2,3,4,5,8,17,19,24	1	P01835
6222	1,6,7,18,20,21,22,24	2,3,4,5,8,17,19,23	4	P01835,P22006,P36376,Q5PQL7
6223	1,6,7,18,20,21,23,24	2,3,4,5,8,17,19,22	1	P01835
6224	1,6,7,18,20,22,23,24	2,3,4,5,8,17,19,21	3	P01835,P22006,Q9WVK7
6225	1,6,7,18,21,22,23,24	2,3,4,5,8,17,19,20	5	D4A5U3,P01835,P13676,P19223,P36376
6226	1,6,7,19,20,21,22,23	2,3,4,5,8,17,18,24	1	P01835
6227	1,6,7,19,20,21,22,24	2,3,4,5,8,17,18,23	8	O35547,O70594,P01835,P22006,P22283,P36376,Q5PQL7,Q63617
6228	1,6,7,19,20,21,23,24	2,3,4,5,8,17,18,22	3	O35547,P01835,Q63617
6229	1,6,7,19,20,22,23,24	2,3,4,5,8,17,18,21	3	P22006,P70549,Q811M5
6230	1,6,7,19,21,22,23,24	2,3,4,5,8,17,18,20	7	D4A5U3,P01835,P19223,P25809,P36376,P70549,Q811M5
6231	1,6,7,20,21,22,23,24	2,3,4,5,8,17,18,19	2	P01835,P22006
6232	1,6,8,17,18,19,20,21	2,3,4,5,7,22,23,24	6	B0LT89,P13432,P30120,P47727,Q6P6R2,Q9JI85
6233	1,6,8,17,18,19,20,22	2,3,4,5,7,21,23,24	6	B0LT89,O89117,P08649,P13432,P47727,P70549
6234	1,6,8,17,18,19,20,23	2,3,4,5,7,21,22,24	6	B0LT89,P08649,P13432,P21674,P30919,P70549
6235	1,6,8,17,18,19,20,24	2,3,4,5,7,21,22,23	7	B0LT89,P08649,P13432,P47727,P60905,P70549,Q9QW07
6236	1,6,8,17,18,19,21,22	2,3,4,5,7,20,23,24	1	P08649
6237	1,6,8,17,18,19,21,23	2,3,4,5,7,20,22,24	1	P21674
6238	1,6,8,17,18,19,21,24	2,3,4,5,7,20,22,23	1	P70549
6239	1,6,8,17,18,19,22,23	2,3,4,5,7,20,21,24	4	P08649,P47967,P70549,Q6AY61
6240	1,6,8,17,18,19,22,24	2,3,4,5,7,20,21,23	3	P08649,P47967,P70549

6241	1,6,8,17,18,19,23,24	2,3,4,5,7,20,21,22	4	P08649,P47967,P70549,P97840
6242	1,6,8,17,18,20,21,22	2,3,4,5,7,19,23,24	1	P13432
6243	1,6,8,17,18,20,21,23	2,3,4,5,7,19,22,24	0	
6244	1,6,8,17,18,20,21,24	2,3,4,5,7,19,22,23	4	P13432,P30120,P47727,Q9Z2L0
6245	1,6,8,17,18,20,22,23	2,3,4,5,7,19,21,24	2	P08649,Q6AY61
6246	1,6,8,17,18,20,22,24	2,3,4,5,7,19,21,23	4	P08649,P13432,P22006,P70549
6247	1,6,8,17,18,20,23,24	2,3,4,5,7,19,21,22	2	P13432,P70549
6248	1,6,8,17,18,21,22,23	2,3,4,5,7,19,20,24	1	P68511
6249	1,6,8,17,18,21,22,24	2,3,4,5,7,19,20,23	1	P10758
6250	1,6,8,17,18,21,23,24	2,3,4,5,7,19,20,22	1	P47967
6251	1,6,8,17,18,22,23,24	2,3,4,5,7,19,20,21	4	P47967,P70549,P97840,Q5I0D1
6252	1,6,8,17,19,20,21,22	2,3,4,5,7,18,23,24	5	B0LT89,O89117,P20766,Q5PQL7,Q6P6R2
6253	1,6,8,17,19,20,21,23	2,3,4,5,7,18,22,24	4	B0LT89,P08753,P21674,Q6P6R2
6254	1,6,8,17,19,20,21,24	2,3,4,5,7,18,22,23	15	B0LT89,O35547,P02782,P08723,P09456,P24368,P30120,P36374,P47727,P60905,Q6P6R2,Q812E4,Q9JI85,Q9QW07,Q9Z2L0
6255	1,6,8,17,19,20,22,23	2,3,4,5,7,18,21,24	2	O89117,P70549
6256	1,6,8,17,19,20,22,24	2,3,4,5,7,18,21,23	4	B0LT89,O89117,P22006,P70549
6257	1,6,8,17,19,20,23,24	2,3,4,5,7,18,21,22	2	O89117,P70549
6258	1,6,8,17,19,21,22,23	2,3,4,5,7,18,20,24	1	P21674
6259	1,6,8,17,19,21,22,24	2,3,4,5,7,18,20,23	1	P70549
6260	1,6,8,17,19,21,23,24	2,3,4,5,7,18,20,22	3	P70549,P97840,Q63474

6261	1,6,8,17,19,22,23,24	2,3,4,5,7,18,20,21	7	O89117,P00507,P47967,P70549,P97840,Q5I0D1,Q811M5
6262	1,6,8,17,20,21,22,23	2,3,4,5,7,18,19,24	2	P20766,P63029
6263	1,6,8,17,20,21,22,24	2,3,4,5,7,18,19,23	3	P20766,P30120,Q9Z2L0
6264	1,6,8,17,20,21,23,24	2,3,4,5,7,18,19,22	0	
6265	1,6,8,17,20,22,23,24	2,3,4,5,7,18,19,21	5	O89117,P22006,P47967,P70549,P97840
6266	1,6,8,17,21,22,23,24	2,3,4,5,7,18,19,20	3	P47967,P97840,Q5I0D1
6267	1,6,8,18,19,20,21,22	2,3,4,5,7,17,23,24	3	B0LT89,Q5PQL7,Q9QW07
6268	1,6,8,18,19,20,21,23	2,3,4,5,7,17,22,24	1	B0LT89
6269	1,6,8,18,19,20,21,24	2,3,4,5,7,17,22,23	11	B0LT89,O08815,O35547,P08723,P30120,P47727,P60905,Q68G31,Q812E4,Q9JI85,Q9QW07
6270	1,6,8,18,19,20,22,23	2,3,4,5,7,17,21,24	4	P01039,P36860,P70549,Q6AY61
6271	1,6,8,18,19,20,22,24	2,3,4,5,7,17,21,23	3	P22006,P70549,Q9QW07
6272	1,6,8,18,19,20,23,24	2,3,4,5,7,17,21,22	3	P70549,Q9QW07,Q9R168
6273	1,6,8,18,19,21,22,23	2,3,4,5,7,17,20,24	2	P19223,Q6AY61
6274	1,6,8,18,19,21,22,24	2,3,4,5,7,17,20,23	1	P19223
6275	1,6,8,18,19,21,23,24	2,3,4,5,7,17,20,22	2	P19223,P70549
6276	1,6,8,18,19,22,23,24	2,3,4,5,7,17,20,21	6	P00507,P19223,P47967,P70549,Q6AY61,Q811M5
6277	1,6,8,18,20,21,22,23	2,3,4,5,7,17,19,24	2	P54921,Q6AY61
6278	1,6,8,18,20,21,22,24	2,3,4,5,7,17,19,23	2	P22006,Q9QW07
6279	1,6,8,18,20,21,23,24	2,3,4,5,7,17,19,22	1	Q9QW07
6280	1,6,8,18,20,22,23,24	2,3,4,5,7,17,19,21	4	P22006,Q5QE79,Q6AY61,Q9WVK7

6281	1,6,8,18,21,22,23,24	2,3,4,5,7,17,19,20	2	P19223,P20646
6282	1,6,8,19,20,21,22,23	2,3,4,5,7,17,18,24	1	P20766
6283	1,6,8,19,20,21,22,24	2,3,4,5,7,17,18,23	6	B0LT89,O35547,O70594,P08723,P30120,Q9QW07
6284	1,6,8,19,20,21,23,24	2,3,4,5,7,17,18,22	3	O35547,Q812E4,Q9QW07
6285	1,6,8,19,20,22,23,24	2,3,4,5,7,17,18,21	4	P22006,P70549,Q811M5,Q9QW07
6286	1,6,8,19,21,22,23,24	2,3,4,5,7,17,18,20	3	P19223,P70549,Q811M5
6287	1,6,8,20,21,22,23,24	2,3,4,5,7,17,18,19	1	P20766
6288	1,6,17,18,19,20,21,22	2,3,4,5,7,8,23,24	6	B0LT89,P05539,P20761,P20762,Q5PQL7,Q6P6R2
6289	1,6,17,18,19,20,21,23	2,3,4,5,7,8,22,24	4	B0LT89,P23593,P35280,Q6P6R2
6290	1,6,17,18,19,20,21,24	2,3,4,5,7,8,22,23	8	B0LT89,P05539,P20761,P20762,P23593,P27590,P35280,Q9Z2L0
6291	1,6,17,18,19,20,22,23	2,3,4,5,7,8,21,24	5	O89117,P02625,P11883,P30919,P70549
6292	1,6,17,18,19,20,22,24	2,3,4,5,7,8,21,23	7	B0LT89,O70594,O89117,P11883,P20761,P22006,P70549
6293	1,6,17,18,19,20,23,24	2,3,4,5,7,8,21,22	3	P11883,P35280,P70549
6294	1,6,17,18,19,21,22,23	2,3,4,5,7,8,20,24	4	D4A5U3,P70549,P82471,Q4G075
6295	1,6,17,18,19,21,22,24	2,3,4,5,7,8,20,23	6	D4A5U3,P05539,P20761,P36376,P70549,Q4G075
6296	1,6,17,18,19,21,23,24	2,3,4,5,7,8,20,22	5	D4A5U3,P23593,P35280,P70549,Q4G075

6297	1,6,17,18,19,22,23,24	2,3,4,5,7,8,20,21	12	D4A5U3,P02780,P06760,P11883,P22273,P47967,P62804,P70549,Q00715,Q4G075,Q5I0D1,Q811M5
6298	1,6,17,18,20,21,22,23	2,3,4,5,7,8,19,24	1	Q6TMA8
6299	1,6,17,18,20,21,22,24	2,3,4,5,7,8,19,23	3	P20761,P22006,P50115
6300	1,6,17,18,20,21,23,24	2,3,4,5,7,8,19,22	2	P11883,P35280
6301	1,6,17,18,20,22,23,24	2,3,4,5,7,8,19,21	9	P04355,P06760,P11883,P22006,P47967,P62804,P70549,Q00715,Q66H69
6302	1,6,17,18,21,22,23,24	2,3,4,5,7,8,19,20	7	D4A5U3,P02780,P47967,P70549,Q4G075,Q5I0D1,Q6TMA8
6303	1,6,17,19,20,21,22,23	2,3,4,5,7,8,18,24	4	B0LT89,O89117,P20762,P20766
6304	1,6,17,19,20,21,22,24	2,3,4,5,7,8,18,23	9	B0LT89,O70594,O89117,P05539,P20761,P20762,P20766,P70549,Q9Z2L0
6305	1,6,17,19,20,21,23,24	2,3,4,5,7,8,18,22	6	B0LT89,P20762,P23593,P27590,P35280,P70549
6306	1,6,17,19,20,22,23,24	2,3,4,5,7,8,18,21	7	O70594,O89117,P02625,P22006,P47967,P70549,Q00715
6307	1,6,17,19,21,22,23,24	2,3,4,5,7,8,18,20	6	D4A5U3,P47967,P70549,Q4G075,Q5I0D1,Q811M5
6308	1,6,17,20,21,22,23,24	2,3,4,5,7,8,18,19	3	A0JPJ7,P20762,P20766
6309	1,6,18,19,20,21,22,23	2,3,4,5,7,8,17,24	2	O70594,P23593

6310	1,6,18,19,20,21,22,24	2,3,4,5,7,8,17,23	6	O70594,P20761,P22006,P36376,P50115,Q5PQL7
6311	1,6,18,19,20,21,23,24	2,3,4,5,7,8,17,22	3	O70417,P23593,P35280
6312	1,6,18,19,20,22,23,24	2,3,4,5,7,8,17,21	10	O70594,P04355,P06760,P11883,P22006,P50115,P70549,Q00715,Q811M5,Q9QX74
6313	1,6,18,19,21,22,23,24	2,3,4,5,7,8,17,20	9	D4A5U3,O70417,P13676,P19223,P28648,P70549,P97697,Q4G075,Q811M5
6314	1,6,18,20,21,22,23,24	2,3,4,5,7,8,17,19	5	O70594,P01835,P20646,P22006,P50115
6315	1,6,19,20,21,22,23,24	2,3,4,5,7,8,17,18	4	O70594,P20766,P22006,Q811M5
6316	1,7,8,17,18,19,20,21	2,3,4,5,6,22,23,24	17	B0LT89,O08815,P13676,P20646,P21674,P24368,P30120,P30919,P36374,P47727,Q4KLZ6,Q6P6R2,Q811M5,Q812E4,Q9QX74,Q9WTT6,Q9Z2L0
6317	1,7,8,17,18,19,20,22	2,3,4,5,6,21,23,24	6	B0LT89,P30919,P36860,P47727,Q4KLZ6,Q9WTT6
6318	1,7,8,17,18,19,20,23	2,3,4,5,6,21,22,24	5	P21674,P30919,Q4KLZ6,Q6AYC4,Q9WTT6
6319	1,7,8,17,18,19,20,24	2,3,4,5,6,21,22,23	11	B0LT89,P08723,P09656,P30120,P30919,P36374,P47727,P70549,Q812E4,Q9WTT6,Q9Z2L0
6320	1,7,8,17,18,19,21,22	2,3,4,5,6,20,23,24	5	P42854,Q4KLZ6,Q6P6R2,Q9QX74,Q9Z2L0
6321	1,7,8,17,18,19,21,23	2,3,4,5,6,20,22,24	5	P21674,P22006,Q4KLZ6,Q6P6R2,Q9QX74
6322	1,7,8,17,18,19,21,24	2,3,4,5,6,20,22,23	7	P09656,P42854,Q66H69,Q6P6R2,Q99MH3,Q9QX74,Q9Z2L0
6323	1,7,8,17,18,19,22,23	2,3,4,5,6,20,21,24	4	O55145,P21674,P70549,Q4KLZ6
6324	1,7,8,17,18,19,22,24	2,3,4,5,6,20,21,23	5	O55145,P09656,P42854,P70549,Q9Z2L0
6325	1,7,8,17,18,19,23,24	2,3,4,5,6,20,21,22	2	O55145,P70549
6326	1,7,8,17,18,20,21,22	2,3,4,5,6,19,23,24	8	P30919,P35745,P42854,P47727,Q4KLZ6,Q9QX74,Q9WTT6,Q9Z2L0

6327	1,7,8,17,18,20,21,23	2,3,4,5,6,19,22,24	7	P21674,P30919,P35745,Q4KLZ6,Q6P6R2,Q9QX74,Q9WTT6
6328	1,7,8,17,18,20,21,24	2,3,4,5,6,19,22,23	12	P00507,P02782,P08723,P30120,P35745,P42854,P47727,Q811M5,Q812E4,Q9JHB9,Q9WTT6,Q9Z2L0
6329	1,7,8,17,18,20,22,23	2,3,4,5,6,19,21,24	3	P30919,P35745,Q4KLZ6
6330	1,7,8,17,18,20,22,24	2,3,4,5,6,19,21,23	3	P42854,P47727,Q9Z2L0
6331	1,7,8,17,18,20,23,24	2,3,4,5,6,19,21,22	3	P30919,P35745,Q6AYC4
6332	1,7,8,17,18,21,22,23	2,3,4,5,6,19,20,24	3	P35745,P68511,Q4KLZ6
6333	1,7,8,17,18,21,22,24	2,3,4,5,6,19,20,23	3	P42854,Q9Z1F2,Q9Z2L0
6334	1,7,8,17,18,21,23,24	2,3,4,5,6,19,20,22	2	P42854,Q9Z2L0
6335	1,7,8,17,18,22,23,24	2,3,4,5,6,19,20,21	5	O55145,P42854,P70549,Q5I0D1,Q64268
6336	1,7,8,17,19,20,21,22	2,3,4,5,6,18,23,24	11	B0LT89,O89117,P30120,P47727,Q4KLZ6,Q6P6R2,Q812E4,Q9JJ50,Q9QX74,Q9WTT6,Q9Z2L0
6337	1,7,8,17,19,20,21,23	2,3,4,5,6,18,22,24	8	B0LT89,P21674,Q4KLZ6,Q6P6R2,Q812E4,Q9QX74,Q9WTT6,Q9Z2L0
6338	1,7,8,17,19,20,21,24	2,3,4,5,6,18,22,23	19	B0LT89,O35547,P02782,P08723,P09456,P13676,P16636,P24368,P30120,P36374,P47727,P60905,Q6P6R2,Q812E4,Q99MH3,Q9EQS0,Q9QX74,Q9WTT6,Q9Z2L0
6339	1,7,8,17,19,20,22,23	2,3,4,5,6,18,21,24	3	O89117,Q4KLZ6,Q9WTT6
6340	1,7,8,17,19,20,22,24	2,3,4,5,6,18,21,23	7	O89117,P08723,P47727,P70549,Q9JJ50,Q9WTT6,Q9Z2L0
6341	1,7,8,17,19,20,23,24	2,3,4,5,6,18,21,22	6	P08723,P70549,Q6AYC4,Q812E4,Q9WTT6,Q9Z2L0
6342	1,7,8,17,19,21,22,23	2,3,4,5,6,18,20,24	3	P21674,Q4KLZ6,Q9QX74
6343	1,7,8,17,19,21,22,24	2,3,4,5,6,18,20,23	3	P16636,P42854,Q9Z2L0
6344	1,7,8,17,19,21,23,24	2,3,4,5,6,18,20,22	3	P21674,Q63474,Q9Z2L0
6345	1,7,8,17,19,22,23,24	2,3,4,5,6,18,20,21	2	O55145,P70549
6346	1,7,8,17,20,21,22,23	2,3,4,5,6,18,19,24	3	P35745,Q4KLZ6,Q9WTT6

6347	1,7,8,17,20,21,22,24	2,3,4,5,6,18,19,23	7	P02782,P30120,P35745,P42854,P47727,Q812E4,Q9Z2L0
6348	1,7,8,17,20,21,23,24	2,3,4,5,6,18,19,22	4	P30120,P35745,Q812E4,Q9Z2L0
6349	1,7,8,17,20,22,23,24	2,3,4,5,6,18,19,21	1	P35745
6350	1,7,8,17,21,22,23,24	2,3,4,5,6,18,19,20	2	P35745,Q9Z2L0
6351	1,7,8,18,19,20,21,22	2,3,4,5,6,17,23,24	5	P01039,P47727,Q4KLZ6,Q9WTT6,Q9Z2L0
6352	1,7,8,18,19,20,21,23	2,3,4,5,6,17,22,24	6	P01039,P21674,P23593,Q4KLZ6,Q812E4,Q9WTT6
6353	1,7,8,18,19,20,21,24	2,3,4,5,6,17,22,23	11	O08815,P02782,P08723,P22283,P24368,P30120,P47727,Q812E4,Q9QW07,Q9WTT6,Q9Z2L0
6354	1,7,8,18,19,20,22,23	2,3,4,5,6,17,21,24	4	P01039,P36860,Q4KLZ6,Q6AY61
6355	1,7,8,18,19,20,22,24	2,3,4,5,6,17,21,23	1	P08723
6356	1,7,8,18,19,20,23,24	2,3,4,5,6,17,21,22	2	Q6AYC4,Q812E4
6357	1,7,8,18,19,21,22,23	2,3,4,5,6,17,20,24	3	P01039,P55281,Q4KLZ6
6358	1,7,8,18,19,21,22,24	2,3,4,5,6,17,20,23	4	P17559,P36376,P42854,Q9Z2L0
6359	1,7,8,18,19,21,23,24	2,3,4,5,6,17,20,22	0	
6360	1,7,8,18,19,22,23,24	2,3,4,5,6,17,20,21	3	O55145,P19223,P70549
6361	1,7,8,18,20,21,22,23	2,3,4,5,6,17,19,24	3	P01039,P35745,Q4KLZ6
6362	1,7,8,18,20,21,22,24	2,3,4,5,6,17,19,23	3	P30120,P42854,Q9Z2L0
6363	1,7,8,18,20,21,23,24	2,3,4,5,6,17,19,22	1	Q812E4
6364	1,7,8,18,20,22,23,24	2,3,4,5,6,17,19,21	1	Q9WVK7
6365	1,7,8,18,21,22,23,24	2,3,4,5,6,17,19,20	2	P55281,Q64268
6366	1,7,8,19,20,21,22,23	2,3,4,5,6,17,18,24	4	P01039,Q4KLZ6,Q812E4,Q9WTT6

6367	1,7,8,19,20,21,22,24	2,3,4,5,6,17,18,23	8	O70594,P02782,P08723,P16636,P30120,Q812E4,Q99PP0,Q9Z2L0
6368	1,7,8,19,20,21,23,24	2,3,4,5,6,17,18,22	4	P08723,P30120,Q812E4,Q9Z2L0
6369	1,7,8,19,20,22,23,24	2,3,4,5,6,17,18,21	2	P70549,Q812E4
6370	1,7,8,19,21,22,23,24	2,3,4,5,6,17,18,20	2	P19223,P55281
6371	1,7,8,20,21,22,23,24	2,3,4,5,6,17,18,19	2	P35745,Q812E4
6372	1,7,17,18,19,20,21,22	2,3,4,5,6,8,23,24	12	B0LT89,P20760,P20761,P23593,P30919,P42854,Q05702,Q4KLZ6,Q5PQL7,Q63532,Q6P6R2,Q9Z2L0
6373	1,7,17,18,19,20,21,23	2,3,4,5,6,8,22,24	9	B0LT89,P21674,P23593,P30919,Q05702,Q10743,Q4KLZ6,Q6P6R2,Q9Z2L0
6374	1,7,17,18,19,20,21,24	2,3,4,5,6,8,22,23	15	B0LT89,O54858,P20760,P20761,P20762,P23593,P30919,P36376,P42854,Q05702,Q09030,Q63532,Q6P6R2,Q99MH3,Q9Z2L0
6375	1,7,17,18,19,20,22,23	2,3,4,5,6,8,21,24	8	P02625,P30919,P36860,P70549,Q05702,Q10743,Q4KLZ6,Q6AYC4
6376	1,7,17,18,19,20,22,24	2,3,4,5,6,8,21,23	8	P20761,P30919,P42854,P70549,Q05702,Q63532,Q6AYC4,Q9Z2L0
6377	1,7,17,18,19,20,23,24	2,3,4,5,6,8,21,22	7	P23593,P30919,P42854,P70549,Q05702,Q10743,Q6AYC4
6378	1,7,17,18,19,21,22,23	2,3,4,5,6,8,20,24	5	P17559,P42854,Q05702,Q10743,Q4KLZ6
6379	1,7,17,18,19,21,22,24	2,3,4,5,6,8,20,23	8	P17559,P20761,P25031,P36376,P42854,Q05702,Q99MH3,Q9Z2L0
6380	1,7,17,18,19,21,23,24	2,3,4,5,6,8,20,22	7	P17559,P23593,P25031,P42854,Q05702,Q10743,Q9Z2L0
6381	1,7,17,18,19,22,23,24	2,3,4,5,6,8,20,21	14	D4A5U3,O55145,P02631,P17559,P22273,P25031,P36375,P42854,P70549,Q05702,Q10743,Q64268,Q6AYC4,Q9QZQ5

6382	1,7,17,18,20,21,22,23	2,3,4,5,6,8,19,24	5	P30919,P42854,Q05702,Q10743,Q4KLZ6
6383	1,7,17,18,20,21,22,24	2,3,4,5,6,8,19,23	5	P20760,P20761,P42854,Q05702,Q9Z2L0
6384	1,7,17,18,20,21,23,24	2,3,4,5,6,8,19,22	4	P30919,P42854,Q05702,Q9Z2L0
6385	1,7,17,18,20,22,23,24	2,3,4,5,6,8,19,21	9	P08937,P30919,P42854,P52590,Q05702,Q10743,Q64268,Q6AYC4,Q9QZQ5
6386	1,7,17,18,21,22,23,24	2,3,4,5,6,8,19,20	8	D4A5U3,P25031,P42854,P52590,Q05702,Q5I0D1,Q64268,Q9QZQ5
6387	1,7,17,19,20,21,22,23	2,3,4,5,6,8,18,24	5	P23593,Q05702,Q4KLZ6,Q9QZQ5,Q9Z2L0
6388	1,7,17,19,20,21,22,24	2,3,4,5,6,8,18,23	10	O70594,P16636,P20761,P20762,P42854,Q05702,Q63532,Q99MH3,Q9QZQ5,Q9Z2L0
6389	1,7,17,19,20,21,23,24	2,3,4,5,6,8,18,22	5	P20762,P23593,Q05702,Q9QZQ5,Q9Z2L0
6390	1,7,17,19,20,22,23,24	2,3,4,5,6,8,18,21	8	O89117,P02625,P70549,Q00715,Q05702,Q6AYC4,Q9QZQ5,Q9Z2L0
6391	1,7,17,19,21,22,23,24	2,3,4,5,6,8,18,20	7	P17559,P42854,P70549,Q05702,Q9QZQ5,Q9R168,Q9Z2L0
6392	1,7,17,20,21,22,23,24	2,3,4,5,6,8,18,19	5	P42854,P52590,Q05702,Q9QZQ5,Q9Z2L0
6393	1,7,18,19,20,21,22,23	2,3,4,5,6,8,17,24	6	P01039,P23593,Q05702,Q4KLZ6,Q99PP0,Q9QZQ5
6394	1,7,18,19,20,21,22,24	2,3,4,5,6,8,17,23	9	O70594,P20760,P20761,P23593,P36376,P42854,Q05702,Q99PP0,Q9Z2L0

6395	1,7,18,19,20,21,23,24	2,3,4,5,6,8,17,22	5	P23593,Q05702,Q99PP0,Q9QZQ5,Q9Z2L0
6396	1,7,18,19,20,22,23,24	2,3,4,5,6,8,17,21	5	O70594,P70549,Q05702,Q6AYC4,Q9QZQ5
6397	1,7,18,19,21,22,23,24	2,3,4,5,6,8,17,20	10	D4A5U3,P17559,P19223,P25031,P36375,P36376,P42854,P55281,Q05702,Q9QZQ5
6398	1,7,18,20,21,22,23,24	2,3,4,5,6,8,17,19	6	O70594,P01835,P42854,P52590,Q05702,Q9QZQ5
6399	1,7,19,20,21,22,23,24	2,3,4,5,6,8,17,18	7	O70594,P23593,Q05702,Q64240,Q99PP0,Q9QZQ5,Q9Z2L0
6400	1,8,17,18,19,20,21,22	2,3,4,5,6,7,23,24	8	B0LT89,P20761,P42854,P47727,P50116,Q4KLZ6,Q6P6R2,Q9Z2L0
6401	1,8,17,18,19,20,21,23	2,3,4,5,6,7,22,24	7	B0LT89,P21674,P23593,P30919,Q4KLZ6,Q6P6R2,Q9Z2L0
6402	1,8,17,18,19,20,21,24	2,3,4,5,6,7,22,23	10	B0LT89,P23593,P30120,P42854,P47727,P50116,P60905,P80299,Q6P6R2,Q9Z2L0
6403	1,8,17,18,19,20,22,23	2,3,4,5,6,7,21,24	10	B0LT89,O89117,P00714,P04762,P30919,P36860,P50116,P70549,Q4KLZ6,Q6AY61
6404	1,8,17,18,19,20,22,24	2,3,4,5,6,7,21,23	10	B0LT89,O70594,P36860,P42854,P47727,P50116,P70549,P80299,Q9JJ50,Q9Z2L0
6405	1,8,17,18,19,20,23,24	2,3,4,5,6,7,21,22	6	B0LT89,P30919,P50116,P70549,P80299,Q6AYC4
6406	1,8,17,18,19,21,22,23	2,3,4,5,6,7,20,24	2	P50116,Q4KLZ6
6407	1,8,17,18,19,21,22,24	2,3,4,5,6,7,20,23	5	P42854,P50116,P70549,P80299,Q9Z2L0

6408	1,8,17,18,19,21,23,24	2,3,4,5,6,7,20,22	6	P23593,P42854,P50116,P70549,P80299,Q9Z2L0
6409	1,8,17,18,19,22,23,24	2,3,4,5,6,7,20,21	8	O55145,P11598,P42854,P47967,P50116,P70549,P80299,Q5I0D1
6410	1,8,17,18,20,21,22,23	2,3,4,5,6,7,19,24	3	P42854,P50116,Q4KLZ6
6411	1,8,17,18,20,21,22,24	2,3,4,5,6,7,19,23	7	P30120,P42854,P47727,P50116,P52590,P80299,Q9Z2L0
6412	1,8,17,18,20,21,23,24	2,3,4,5,6,7,19,22	4	P42854,P50116,P80299,Q9Z2L0
6413	1,8,17,18,20,22,23,24	2,3,4,5,6,7,19,21	7	P00714,P42854,P47967,P50116,P52590,P70549,P80299
6414	1,8,17,18,21,22,23,24	2,3,4,5,6,7,19,20	7	P42854,P47967,P50116,P52590,P80299,Q5I0D1,Q9R0T3
6415	1,8,17,19,20,21,22,23	2,3,4,5,6,7,18,24	7	B0LT89,O89117,P20766,P50116,Q4KLZ6,Q6P6R2,Q9Z2L0
6416	1,8,17,19,20,21,22,24	2,3,4,5,6,7,18,23	12	B0LT89,O70594,O89117,P16636,P20766,P30120,P42854,P47727,P50116,P60905,Q9JJ50,Q9Z2L0
6417	1,8,17,19,20,21,23,24	2,3,4,5,6,7,18,22	8	B0LT89,P23593,P30120,P50116,P80299,Q6P6R2,Q812E4,Q9Z2L0
6418	1,8,17,19,20,22,23,24	2,3,4,5,6,7,18,21	5	O89117,P36860,P50116,P70549,Q00715
6419	1,8,17,19,21,22,23,24	2,3,4,5,6,7,18,20	6	P47967,P50116,P70549,P80299,Q80WL1,Q9Z2L0
6420	1,8,17,20,21,22,23,24	2,3,4,5,6,7,18,19	6	P20766,P42854,P50116,P52590,P80299,Q9Z2L0

6421	1,8,18,19,20,21,22,23	2,3,4,5,6,7,17,24	6	O70594,P01039,P36860,P50116,Q4KLZ6,Q6AY61
6422	1,8,18,19,20,21,22,24	2,3,4,5,6,7,17,23	7	B0LT89,O70594,P30120,P42854,P50116,Q99PP0,Q9Z2L0
6423	1,8,18,19,20,21,23,24	2,3,4,5,6,7,17,22	3	P23593,P50116,P80299
6424	1,8,18,19,20,22,23,24	2,3,4,5,6,7,17,21	5	O70594,P36860,P50116,P70549,Q6AY61
6425	1,8,18,19,21,22,23,24	2,3,4,5,6,7,17,20	3	P19223,P50116,P55281
6426	1,8,18,20,21,22,23,24	2,3,4,5,6,7,17,19	5	O70594,P42854,P50115,P50116,P52590
6427	1,8,19,20,21,22,23,24	2,3,4,5,6,7,17,18	6	O70594,P20766,P50116,Q99PP0,Q9QZQ5,Q9Z2L0
6428	1,17,18,19,20,21,22,23	2,3,4,5,6,7,8,24	14	B0LT89,O70594,P00714,P20760,P20761,P23593,P30919,P42854,P50115,P50116,Q05702,Q4KLZ6,Q6P6R2,Q9QZQ5
6429	1,17,18,19,20,21,22,24	2,3,4,5,6,7,8,23	15	B0LT89,O54858,O70594,P20760,P20761,P20762,P23593,P42854,P50115,P50116,Q05702,Q09030,Q63532,Q9QZQ5,Q9Z2L0
6430	1,17,18,19,20,21,23,24	2,3,4,5,6,7,8,22	19	B0LT89,O54858,O70594,P13852,P20760,P20761,P20762,P23593,P30919,P35280,P38438,P42854,P50115,P50116,Q05702,Q09030,Q99041,Q9QZQ5,Q9Z2L0
6431	1,17,18,19,20,22,23,24	2,3,4,5,6,7,8,21	20	O70594,P00714,P02625,P02631,P20761,P22273,P23593,P30919,P36860,P38438,P42854,P50115,P50116,P70549,Q00715,Q05702,Q10743,Q6AYC4,Q99041,Q9QZQ5
6432	1,17,18,19,21,22,23,24	2,3,4,5,6,7,8,20	24	D4A5U3,O70417,P01836,P02780,P06911,P11598,P12020,P17559,P22273,P23593,P25031,P42854,P47967,P50115,P50116,P50280,P70549,Q05702,Q4G075,Q5I0D1,Q62946,Q99041,Q9QZQ5,Q9R0T3
6433	1,17,18,20,21,22,23,24	2,3,4,5,6,7,8,19	13	A0JPJ7,O70594,P00714,P13852,P20761,P42854,P50115,P50116,P52590,Q05702,Q99041,Q9QZQ5,Q9Z2L0

6434	1,17,19,20,21,22,23,24	2,3,4,5,6,7,8,18	15	O70594,P13852,P20761,P20762,P20766,P23593,P38438,P42854,P50115,P50116,Q05702,Q64240,Q99PP0,Q9QZQ5,Q9Z2L0
6435	1,18,19,20,21,22,23,24	2,3,4,5,6,7,8,17	12	O70417,O70594,P13852,P20761,P23593,P42854,P50115,P50116,Q05702,Q64240,Q99PP0,Q9QZQ5

Day 14

Random allocation	Group1	Group2	Numbers of differential urinary proteins	Differential protein ID
1	1,2,3,4,5,6,7,8	25,26,27,28,29,30,31,32	23	D3ZUC6,O70594,P01836,P02454,P02631,P02783,P06911,P19218,P42854,P50115,P52590,P97580,Q05702,Q09030,Q30KJ2,Q4KLZ6,Q5RJR2,Q63532,Q64240,Q91XN4,Q99041,Q99PP0,Q9QZQ5
2	1,2,3,4,5,6,7,25	8,26,27,28,29,30,31,32	8	O54800;Q5DWV2,P52590,Q09030,Q4AEF8,Q4KLZ6,Q99PP0,Q9QWN8,Q9WVH8
3	1,2,3,4,5,6,7,26	8,25,27,28,29,30,31,32	9	O54800;Q5DWV2,P00762,P02783,P21588,P52590,Q4KLZ6,Q5RLM2,Q99PP0,Q9WVH8
4	1,2,3,4,5,6,7,27	8,25,26,28,29,30,31,32	12	P01039,P02783,P19218,P21588,P47967,P50116,P52590,Q03191,Q4KLZ6,Q5I0D1,Q5I0D7,Q9JJ50
5	1,2,3,4,5,6,7,28	8,25,26,27,29,30,31,32	10	P00762,P11598,P19218,P47967,P52590,P97840,Q09030,Q4KLZ6,Q5RLM2,Q99PP0
6	1,2,3,4,5,6,7,29	8,25,26,27,28,30,31,32	10	O70594,P00762,P02783,P26342,P42854,P47967,P52590,Q4KLZ6,Q5RJR2,Q9QWN8
7	1,2,3,4,5,6,7,30	8,25,26,27,28,29,31,32	15	P17046,P19218,P21588,P26342,P33436,P36374,P52590,P84039,Q09030,Q4KLZ6,Q5RJR2,Q6P6R2,Q80WL1,Q9QWN8,Q9WUW8
8	1,2,3,4,5,6,7,31	8,25,26,27,28,29,30,32	12	P20766,P26342,P33436,P52590,Q09030,Q4KLZ6,Q5RJR2,Q63532,Q6AYR9,Q8CFN2,Q9QWN8,Q9QX79
9	1,2,3,4,5,6,7,32	8,25,26,27,28,29,30,31	8	O54800;Q5DWV2,P0DP29;P0DP30;P0DP31,P26342,P42854,P52590,Q09030,Q4KLZ6,Q6P6R2
10	1,2,3,4,5,6,8,25	7,26,27,28,29,30,31,32	13	P01681,P36376,P42854,P52590,P97580,Q05702,Q09030,Q30KJ2,Q4KLZ6,Q63532,Q64240,Q99PP0,Q9QZQ5

11	1,2,3,4,5,6,8,26	7,25,27,28,29,30,31,32	15	P02783,P20762,P42854,P52590,P97580,Q05702,Q09030,Q30KJ2,Q4KLZ6,Q5RJR2,Q63532,Q64240,Q99MH3,Q99PP0,Q9QZQ5
12	1,2,3,4,5,6,8,27	7,25,26,28,29,30,31,32	14	P00731,P02783,P15399,P17559,P19218,P42854,P52590,P97580,Q03191,Q09030,Q10758,Q30KJ2,Q4KLZ6,Q9QZQ5
13	1,2,3,4,5,6,8,28	7,25,26,27,29,30,31,32	13	D3ZUC6,P19218,P36376,P42854,P52590,P97580,Q09030,Q30KJ2,Q4KLZ6,Q63532,Q99PP0,Q9JI85,Q9QZQ5
14	1,2,3,4,5,6,8,29	7,25,26,27,28,30,31,32	11	O70594,P02783,P42854,P52590,P97580,Q05702,Q09030,Q30KJ2,Q4KLZ6,Q5RJR2,Q9QZQ5
15	1,2,3,4,5,6,8,30	7,25,26,27,28,29,31,32	20	O88797,P19218,P33436,P36374,P52590,P97580,Q05702,Q09030,Q30KJ2,Q4FZU2,Q4KLZ6,Q5RJR2,Q63532,Q6IFU7,Q6IFU8,Q6IFW6,Q6IG02,Q6IG05,Q6IMF3,Q6P6Q2
16	1,2,3,4,5,6,8,31	7,25,26,27,28,29,30,32	12	P33436,P36376,P52590,P97580,Q05702,Q09030,Q30KJ2,Q4KLZ6,Q5RJR2,Q63532,Q99PP0,Q9QZQ5
17	1,2,3,4,5,6,8,32	7,25,26,27,28,29,30,31	11	P01681,P42854,P52590,P97580,Q05702,Q09030,Q30KJ2,Q4KLZ6,Q63532,Q64240,Q9QZQ5
18	1,2,3,4,5,6,25,26	7,8,27,28,29,30,31,32	8	O54800;Q5DWV2,P21588,P52590,Q06000,Q09030,Q4KLZ6,Q63150,Q812E4
19	1,2,3,4,5,6,25,27	7,8,26,28,29,30,31,32	5	P52590,Q03191,Q09030,Q4KLZ6,Q5I0D7
20	1,2,3,4,5,6,25,28	7,8,26,27,29,30,31,32	3	P52590,Q09030,Q4KLZ6
21	1,2,3,4,5,6,25,29	7,8,26,27,28,30,31,32	5	P42854,P52590,Q09030,Q4KLZ6,Q9QWN8
22	1,2,3,4,5,6,25,30	7,8,26,27,28,29,31,32	13	O54800;Q5DWV2,P36374,P52590,P97523,P97584,Q09030,Q4KLZ6,Q6AYR9,Q6P6R2,Q812E4,Q9QWN8,Q9QX74,Q9WTT6
23	1,2,3,4,5,6,25,31	7,8,26,27,28,29,30,32	12	P29315,P52590,P97584,Q09030,Q4KLZ6,Q63532,Q6AYR9,Q6P6R2,Q812E4,Q8CFN2,Q9QWN8,Q9WTT6

24	1,2,3,4,5,6,25,32	7,8,26,27,28,29,30,31	9	O54800;Q5DWV2,P01681,P52590,P97584,Q09030,Q4KLZ6,Q6P6R2,Q9QX74,Q9WTT6
25	1,2,3,4,5,6,26,27	7,8,25,28,29,30,31,32	7	P01039,P02783,P21588,P52590,Q03191,Q4KLZ6,Q9JJ50
26	1,2,3,4,5,6,26,28	7,8,25,27,29,30,31,32	3	P21588,P52590,Q4KLZ6
27	1,2,3,4,5,6,26,29	7,8,25,27,28,30,31,32	5	P02783,P21588,P42854,P52590,Q4KLZ6
28	1,2,3,4,5,6,26,30	7,8,25,27,28,29,31,32	9	P21588,P36374,P47727,P52590,Q4KLZ6,Q5RJR2,Q6AYR9,Q812E4,Q99MH3
29	1,2,3,4,5,6,26,31	7,8,25,27,28,29,30,32	10	P47727,P52590,Q4KLZ6,Q63150,Q63532,Q6AYR9,Q812E4,Q8CFN2,Q99MH3,Q9Z2L0
30	1,2,3,4,5,6,26,32	7,8,25,27,28,29,30,31	4	O54800;Q5DWV2,P52590,Q4KLZ6,Q99MH3
31	1,2,3,4,5,6,27,28	7,8,25,26,29,30,31,32	6	P09656,P19218,P21588,P52590,Q03191,Q4KLZ6
32	1,2,3,4,5,6,27,29	7,8,25,26,28,30,31,32	8	P02783,P09656,P21588,P42854,P52590,Q03191,Q4KLZ6,Q9JJ50
33	1,2,3,4,5,6,27,30	7,8,25,26,28,29,31,32	15	P01039,P19218,P21588,P30120,P33436,P52590,P84039,Q03191,Q4KLZ6,Q5I0J9,Q6IG02,Q6P6Q2,Q6P6R2,Q9QX74,Q9WTT6
34	1,2,3,4,5,6,27,31	7,8,25,26,28,29,30,32	6	P01039,P33436,P52590,Q03191,Q4KLZ6,Q63532
35	1,2,3,4,5,6,27,32	7,8,25,26,28,29,30,31	9	P01039,P52590,Q03191,Q4KLZ6,Q5I0D7,Q6P6R2,Q9JJ50,Q9QX74,Q9WTT6
36	1,2,3,4,5,6,28,29	7,8,25,26,27,30,31,32	5	P09656,P42854,P47967,P52590,Q4KLZ6

37	1,2,3,4,5,6,28,30	7,8,25,26,27,29,31,32	7	P19218,P21588,P52590,Q09030,Q4KLZ6,Q9QX74,Q9WTT6
38	1,2,3,4,5,6,28,31	7,8,25,26,27,29,30,32	4	P52590,Q09030,Q4KLZ6,Q63532
39	1,2,3,4,5,6,28,32	7,8,25,26,27,29,30,31	5	P52590,Q09030,Q4KLZ6,Q9QX74,Q9WTT6
40	1,2,3,4,5,6,29,30	7,8,25,26,27,28,31,32	11	P02783,P21588,P26342,P42854,P52590,P84039,Q4KLZ6,Q5RJR2,Q6IG02,Q9QWN8,Q9WTT6
41	1,2,3,4,5,6,29,31	7,8,25,26,27,28,30,32	6	P02783,P52590,Q4KLZ6,Q5RJR2,Q9QWN8,Q9WTT6
42	1,2,3,4,5,6,29,32	7,8,25,26,27,28,30,31	7	O55145,P02783,P26342,P42854,P52590,Q4KLZ6,Q9WTT6
43	1,2,3,4,5,6,30,31	7,8,25,26,27,28,29,32	20	P08010,P20646,P26342,P30120,P33436,P47727,P52590,P97584,Q09030,Q4KLZ6,Q5RJR2,Q63532,Q6AYR9,Q6P6R2,Q812E4,Q8CFN2,Q9QWN8,Q9QX74,Q9WTT6,Q9Z2L0
44	1,2,3,4,5,6,30,32	7,8,25,26,27,28,29,31	11	O55145,P20646,P26342,P52590,P97584,Q09030,Q4KLZ6,Q5I0J9,Q6P6R2,Q9QX74,Q9WTT6
45	1,2,3,4,5,6,31,32	7,8,25,26,27,28,29,30	11	P20646,P26342,P52590,P97584,Q09030,Q4G075,Q4KLZ6,Q63532,Q6P6R2,Q9QX74,Q9WTT6
46	1,2,3,4,5,7,8,25	6,26,27,28,29,30,31,32	4	P01681,P22006,Q4KLZ6,Q811M5
47	1,2,3,4,5,7,8,26	6,25,27,28,29,30,31,32	5	P20762,P97580,Q30KJ2,Q5RLM2,Q9WUW8
48	1,2,3,4,5,7,8,27	6,25,26,28,29,30,31,32	12	P00731,P19218,P47967,P50116,P97580,Q03191,Q10758,Q30KJ2,Q4G075,Q5I0D1,Q5RLM2,Q66H69
49	1,2,3,4,5,7,8,28	6,25,26,27,29,30,31,32	6	D3ZUC6,P47967,P97840,Q5RLM2,Q811M5,Q9WUW8

50	1,2,3,4,5,7,8,29	6,25,26,27,28,30,31,32	7	O70594,P22006,P47967,P62804,P97840,Q5RJR2,Q9WUW8
51	1,2,3,4,5,7,8,30	6,25,26,27,28,29,31,32	14	O88797,P05539,P19218,P97580,Q4FZU2,Q5RJR2,Q6IFU7,Q6IFU8,Q6IFW6,Q6IG02,Q6IG05,Q6IMF3,Q6P6Q2,Q9WUW8
52	1,2,3,4,5,7,8,31	6,25,26,27,28,29,30,32	5	P20766,P36376,Q5RJR2,Q5RLM2,Q811M5
53	1,2,3,4,5,7,8,32	6,25,26,27,28,29,30,31	4	P01681,P05539,Q811M5,Q9WUW8
54	1,2,3,4,5,7,25,26	6,8,27,28,29,30,31,32	2	P22006,Q4KLZ6
55	1,2,3,4,5,7,25,27	6,8,26,28,29,30,31,32	3	Q03191,Q4KLZ6,Q63474
56	1,2,3,4,5,7,25,28	6,8,26,27,29,30,31,32	2	Q4KLZ6,Q811M5
57	1,2,3,4,5,7,25,29	6,8,26,27,28,30,31,32	3	P22006,Q4KLZ6,Q9QWN8
58	1,2,3,4,5,7,25,30	6,8,26,27,28,29,31,32	8	Q4FZU2,Q4KLZ6,Q6AYR9,Q6IFW6,Q6P6Q2,Q6P6R2,Q8CFN2,Q9QWN8
59	1,2,3,4,5,7,25,31	6,8,26,27,28,29,30,32	6	P29315,Q4KLZ6,Q6AYR9,Q811M5,Q8CFN2,Q9QWN8
60	1,2,3,4,5,7,25,32	6,8,26,27,28,29,30,31	4	P01681,Q4KLZ6,Q6P6R2,Q811M5
61	1,2,3,4,5,7,26,27	6,8,25,28,29,30,31,32	7	P21588,P47967,P97840,Q03191,Q5I0D1,Q5RLM2,Q9JJ50
62	1,2,3,4,5,7,26,28	6,8,25,27,29,30,31,32	3	P47967,P97840,Q5RLM2

63	1,2,3,4,5,7,26,29	6,8,25,27,28,30,31,32	4	P22006,P47967,P97840,Q9WUW8
64	1,2,3,4,5,7,26,30	6,8,25,27,28,29,31,32	6	P21588,P36374,P97840,Q6AYR9,Q812E4,Q9WUW8
65	1,2,3,4,5,7,26,31	6,8,25,27,28,29,30,32	5	P20766,Q5RLM2,Q6AYR9,Q812E4,Q8CFN2
66	1,2,3,4,5,7,26,32	6,8,25,27,28,29,30,31	0	
67	1,2,3,4,5,7,27,28	6,8,25,26,29,30,31,32	5	P47967,P97840,Q03191,Q5I0D1,Q5RLM2
68	1,2,3,4,5,7,27,29	6,8,25,26,28,30,31,32	5	P19223,P47967,P97840,Q03191,Q5I0D1
69	1,2,3,4,5,7,27,30	6,8,25,26,28,29,31,32	11	P00731,P19218,P21588,P47967,P70549,P84039,P97840,Q03191,Q6IFW6,Q6IG02,Q6P6Q2
70	1,2,3,4,5,7,27,31	6,8,25,26,28,29,30,32	3	P20766,P80299,Q03191
71	1,2,3,4,5,7,27,32	6,8,25,26,28,29,30,31	1	Q03191
72	1,2,3,4,5,7,28,29	6,8,25,26,27,30,31,32	4	P47967,P54921,P97840,Q9WUW8
73	1,2,3,4,5,7,28,30	6,8,25,26,27,29,31,32	4	P19218,P47967,P97840,Q9WUW8
74	1,2,3,4,5,7,28,31	6,8,25,26,27,29,30,32	3	P97840,Q5RLM2,Q811M5
75	1,2,3,4,5,7,28,32	6,8,25,26,27,29,30,31	3	P47967,P97840,Q811M5

76	1,2,3,4,5,7,29,30	6,8,25,26,27,28,31,32	10	P17046,P26342,P47967,P84039,P97840,Q6IFW6,Q6IG02,Q6P6Q2,Q9QWN8,Q9WUW8
77	1,2,3,4,5,7,29,31	6,8,25,26,27,28,30,32	2	P26342,Q9QWN8
78	1,2,3,4,5,7,29,32	6,8,25,26,27,28,30,31	4	P26342,P47967,P97840,Q9WUW8
79	1,2,3,4,5,7,30,31	6,8,25,26,27,28,29,32	7	P26342,Q5RJR2,Q6AYR9,Q6P6R2,Q812E4,Q8CFN2,Q9QWN8
80	1,2,3,4,5,7,30,32	6,8,25,26,27,28,29,31	4	P26342,P70549,Q6P6R2,Q9WUW8
81	1,2,3,4,5,7,31,32	6,8,25,26,27,28,29,30	4	P26342,P80299,Q6P6R2,Q811M5
82	1,2,3,4,5,8,25,26	6,7,27,28,29,30,31,32	5	P01681,P22006,P97580,Q30KJ2,Q4KLZ6
83	1,2,3,4,5,8,25,27	6,7,26,28,29,30,31,32	10	P01681,P01835,P22006,P36376,P97580,Q03191,Q10758,Q30KJ2,Q4KLZ6,Q6DGG1
84	1,2,3,4,5,8,25,28	6,7,26,27,29,30,31,32	5	P01681,P36376,P36860,Q4KLZ6,Q811M5
85	1,2,3,4,5,8,25,29	6,7,26,27,28,30,31,32	3	P01681,P22006,Q4KLZ6
86	1,2,3,4,5,8,25,30	6,7,26,27,28,29,31,32	15	O88797,P01681,P05539,P22006,P97580,Q05511,Q4FZU2,Q4KLZ6,Q6IFU7,Q6IFU8,Q6IFW6,Q6IG02,Q6IG05,Q6IMF3,Q6P6Q2
87	1,2,3,4,5,8,25,31	6,7,26,27,28,29,30,32	5	P01681,P29315,P36376,Q4KLZ6,Q811M5
88	1,2,3,4,5,8,25,32	6,7,26,27,28,29,30,31	4	P01681,P05539,Q4KLZ6,Q811M5

89	1,2,3,4,5,8,26,27	6,7,25,28,29,30,31,32	5	P20762,P97580,Q10758,Q30KJ2,Q5BJY9
90	1,2,3,4,5,8,26,28	6,7,25,27,29,30,31,32	2	P97580,Q5RLM2
91	1,2,3,4,5,8,26,29	6,7,25,27,28,30,31,32	3	P22006,P97580,Q5RJR2
92	1,2,3,4,5,8,26,30	6,7,25,27,28,29,31,32	14	O88797,P05539,P20762,P97580,Q30KJ2,Q4FZU2,Q5RJR2,Q6IFU7,Q6IFU8,Q6IFW6,Q6IG02,Q6IG05,Q6IMF3,Q6P6Q2
93	1,2,3,4,5,8,26,31	6,7,25,27,28,29,30,32	5	P05539,P36376,P97580,Q5RJR2,Q5RLM2
94	1,2,3,4,5,8,26,32	6,7,25,27,28,29,30,31	3	P01681,P05539,P97580
95	1,2,3,4,5,8,27,28	6,7,25,26,29,30,31,32	6	P19218,P36376,P47967,P97580,Q10758,Q30KJ2
96	1,2,3,4,5,8,27,29	6,7,25,26,28,30,31,32	6	P00731,P19223,P22006,P97580,Q10758,Q30KJ2
97	1,2,3,4,5,8,27,30	6,7,25,26,28,29,31,32	16	O88797,P00731,P19218,P70549,P97580,Q03191,Q10758,Q30KJ2,Q4FZU2,Q6IFU7,Q6IFU8,Q6IFW6,Q6IG02,Q6IG05,Q6IMF3,Q6P6Q2
98	1,2,3,4,5,8,27,31	6,7,25,26,28,29,30,32	7	P00731,P23739,P36376,P97580,Q03191,Q10758,Q30KJ2
99	1,2,3,4,5,8,27,32	6,7,25,26,28,29,30,31	5	P01681,P97580,Q03191,Q10758,Q30KJ2
100	1,2,3,4,5,8,28,29	6,7,25,26,27,30,31,32	1	P47967
101	1,2,3,4,5,8,28,30	6,7,25,26,27,29,31,32	10	O88797,P19218,Q4FZU2,Q6IFU7,Q6IFU8,Q6IFW6,Q6IG02,Q6IG05,Q6IMF3,Q6P6Q2

102	1,2,3,4,5,8,28,31	6,7,25,26,27,29,30,32	2	P36376,Q811M5
103	1,2,3,4,5,8,28,32	6,7,25,26,27,29,30,31	2	P01681,Q811M5
104	1,2,3,4,5,8,29,30	6,7,25,26,27,28,31,32	11	O88797,P22006,Q4FZU2,Q5RJR2,Q6IFU7,Q6IFU8,Q6IFW6,Q6IG02,Q6IG05,Q6IMF3,Q6P6Q2
105	1,2,3,4,5,8,29,31	6,7,25,26,27,28,30,32	2	P36376,Q5RJR2
106	1,2,3,4,5,8,29,32	6,7,25,26,27,28,30,31	1	P01681
107	1,2,3,4,5,8,30,31	6,7,25,26,27,28,29,32	12	O88797,P05539,P36376,Q4FZU2,Q5RJR2,Q6IFU7,Q6IFU8,Q6IFW6,Q6IG02,Q6IG05,Q6IMF3,Q6P6Q2
108	1,2,3,4,5,8,30,32	6,7,25,26,27,28,29,31	15	O88797,P01681,P05539,P26342,P70549,P97615,Q4FZU2,Q6IFU7,Q6IFU8,Q6IFW6,Q6IG02,Q6IG05,Q6IMF3,Q6P6Q2,Q6P6R2
109	1,2,3,4,5,8,31,32	6,7,25,26,27,28,29,30	4	P01681,P05539,P36376,Q811M5
110	1,2,3,4,5,25,26,27	6,7,8,28,29,30,31,32	2	Q03191,Q4KLZ6
111	1,2,3,4,5,25,26,28	6,7,8,27,29,30,31,32	1	Q4KLZ6
112	1,2,3,4,5,25,26,29	6,7,8,27,28,30,31,32	3	P22006,Q4KLZ6,Q9QZK9
113	1,2,3,4,5,25,26,30	6,7,8,27,28,29,31,32	8	P08723,P47727,P97523,Q06000,Q4KLZ6,Q63617,Q6AYR9,Q812E4
114	1,2,3,4,5,25,26,31	6,7,8,27,28,29,30,32	8	P18298,P29315,Q4KLZ6,Q63617,Q6AYR9,Q812E4,Q8CFN2,Q9Z2L0
115	1,2,3,4,5,25,26,32	6,7,8,27,28,29,30,31	3	P01681,Q06000,Q4KLZ6
116	1,2,3,4,5,25,27,28	6,7,8,26,29,30,31,32	2	Q03191,Q4KLZ6
117	1,2,3,4,5,25,27,29	6,7,8,26,28,30,31,32	4	O89117,P22006,Q03191,Q4KLZ6

118	1,2,3,4,5,25,27,30	6,7,8,26,28,29,31,32	9	P70549,Q03191,Q4FZU2,Q4KLZ6,Q6IFW6,Q6IG02,Q6P6Q2,Q6P6R2,Q9QX74
119	1,2,3,4,5,25,27,31	6,7,8,26,28,29,30,32	5	P23739,P29315,Q03191,Q4KLZ6,Q9QYP1
120	1,2,3,4,5,25,27,32	6,7,8,26,28,29,30,31	6	P01681,P35745,Q03191,Q4KLZ6,Q6P6R2,Q9QX74
121	1,2,3,4,5,25,28,29	6,7,8,26,27,30,31,32	1	Q4KLZ6
122	1,2,3,4,5,25,28,30	6,7,8,26,27,29,31,32	2	Q4KLZ6,Q9QX74
123	1,2,3,4,5,25,28,31	6,7,8,26,27,29,30,32	3	P29315,Q4KLZ6,Q811M5
124	1,2,3,4,5,25,28,32	6,7,8,26,27,29,30,31	4	P01681,Q4KLZ6,Q811M5,Q9QX74
125	1,2,3,4,5,25,29,30	6,7,8,26,27,28,31,32	7	P07150,P22006,Q4KLZ6,Q6IFW6,Q6IG02,Q6P6Q2,Q9QWN8
126	1,2,3,4,5,25,29,31	6,7,8,26,27,28,30,32	4	P07150,P29315,Q4KLZ6,Q9QWN8
127	1,2,3,4,5,25,29,32	6,7,8,26,27,28,30,31	2	P01681,Q4KLZ6
128	1,2,3,4,5,25,30,31	6,7,8,26,27,28,29,32	18	P07150,P08723,P22283,P29315,P30120,P47727,Q4KLZ6,Q63617,Q6AYR9,Q6IFW6,Q6IG02,Q6P6R2,Q812E4,Q8CFN2,Q9QWN8,Q9QX74,Q9WTT6,Q9Z2L0
129	1,2,3,4,5,25,30,32	6,7,8,26,27,28,29,31	8	P01681,P26342,P70549,P97523,Q4KLZ6,Q6P6R2,Q9QX74,Q9WTT6
130	1,2,3,4,5,25,31,32	6,7,8,26,27,28,29,30	8	P01681,P29315,P97584,Q4KLZ6,Q6P6R2,Q811M5,Q9QX74,Q9WTT6
131	1,2,3,4,5,26,27,28	6,7,8,25,29,30,31,32	4	P21588,P47967,P97840,Q5RLM2
132	1,2,3,4,5,26,27,29	6,7,8,25,28,30,31,32	2	P21588,Q9JJ50
133	1,2,3,4,5,26,27,30	6,7,8,25,28,29,31,32	2	P21588,Q6IG02
134	1,2,3,4,5,26,27,31	6,7,8,25,28,29,30,32	1	Q03191
135	1,2,3,4,5,26,27,32	6,7,8,25,28,29,30,31	2	Q03191,Q9JJ50
136	1,2,3,4,5,26,28,29	6,7,8,25,27,30,31,32	2	P47967,P97840
137	1,2,3,4,5,26,28,30	6,7,8,25,27,29,31,32	1	P21588

138	1,2,3,4,5,26,28,31	6,7,8,25,27,29,30,32	1	Q5RLM2
139	1,2,3,4,5,26,28,32	6,7,8,25,27,29,30,31	0	
140	1,2,3,4,5,26,29,30	6,7,8,25,27,28,31,32	2	P21588,Q6IG02
141	1,2,3,4,5,26,29,31	6,7,8,25,27,28,30,32	0	
142	1,2,3,4,5,26,29,32	6,7,8,25,27,28,30,31	0	
143	1,2,3,4,5,26,30,31	6,7,8,25,27,28,29,32	11	P08723,P0C0A9,P22283,P30120,P47727,Q63617,Q6AYR9,Q812E4,Q8CFN2,Q9QW07,Q9Z2L0
144	1,2,3,4,5,26,30,32	6,7,8,25,27,28,29,31	1	Q812E4
145	1,2,3,4,5,26,31,32	6,7,8,25,27,28,29,30	3	Q4G075,Q812E4,Q9Z2L0
146	1,2,3,4,5,27,28,29	6,7,8,25,26,30,31,32	3	P47967,P97840,Q9WVK7
147	1,2,3,4,5,27,28,30	6,7,8,25,26,29,31,32	5	P19218,P21588,P70549,Q6IG02,Q6P6Q2
148	1,2,3,4,5,27,28,31	6,7,8,25,26,29,30,32	1	Q03191
149	1,2,3,4,5,27,28,32	6,7,8,25,26,29,30,31	3	Q03191,Q62761;Q62762;Q62763,Q9QX74
150	1,2,3,4,5,27,29,30	6,7,8,25,26,28,31,32	8	P00731,P21588,P84039,Q6IFU8,Q6IFW6,Q6IG02,Q6IMF3,Q6P6Q2
151	1,2,3,4,5,27,29,31	6,7,8,25,26,28,30,32	1	Q03191
152	1,2,3,4,5,27,29,32	6,7,8,25,26,28,30,31	1	Q03191
153	1,2,3,4,5,27,30,31	6,7,8,25,26,28,29,32	8	P30120,Q03191,Q6IFW6,Q6IG02,Q6P6Q2,Q6P6R2,Q9QW07,Q9WTT6
154	1,2,3,4,5,27,30,32	6,7,8,25,26,28,29,31	7	P70549,Q03191,Q6IG02,Q6P6Q2,Q6P6R2,Q9QX74,Q9WTT6
155	1,2,3,4,5,27,31,32	6,7,8,25,26,28,29,30	6	P23739,P80299,Q03191,Q6P6R2,Q9QX74,Q9WTT6
156	1,2,3,4,5,28,29,30	6,7,8,25,26,27,31,32	2	P97840,Q6IG02
157	1,2,3,4,5,28,29,31	6,7,8,25,26,27,30,32	0	

158	1,2,3,4,5,28,29,32	6,7,8,25,26,27,30,31	0	
159	1,2,3,4,5,28,30,31	6,7,8,25,26,27,29,32	2	Q9QX74,Q9WTT6
160	1,2,3,4,5,28,30,32	6,7,8,25,26,27,29,31	3	P70549,Q9QX74,Q9WTT6
161	1,2,3,4,5,28,31,32	6,7,8,25,26,27,29,30	3	Q811M5,Q9QX74,Q9WTT6
162	1,2,3,4,5,29,30,31	6,7,8,25,26,27,28,32	8	P07150,P26342,Q5RJR2,Q6AYR9,Q6IFW6,Q6IG02,Q9QWN8,Q9WTT6
163	1,2,3,4,5,29,30,32	6,7,8,25,26,27,28,31	4	P07150,P26342,Q6IG02,Q9WTT6
164	1,2,3,4,5,29,31,32	6,7,8,25,26,27,28,30	3	P07150,P26342,Q9WTT6
165	1,2,3,4,5,30,31,32	6,7,8,25,26,27,28,29	13	P07150,P20646,P26342,P47727,P63081,P70549,P97584,Q4G075,Q6P6R2,Q812E4,Q9QW07,Q9QX74,Q9WTT6
166	1,2,3,4,6,7,8,25	5,26,27,28,29,30,31,32	14	G3V686,O54800;Q5DWV2,O70417,P02631,P06911,P12020,P97580,Q09030,Q30KJ2,Q4KLZ6,Q4V885,Q63532,Q99PP0,Q9QZQ5
167	1,2,3,4,6,7,8,26	5,25,27,28,29,30,31,32	15	G3V686,O54800;Q5DWV2,P02631,P02783,P06911,P08649,P11598,P20761,P20762,P97580,Q30KJ2,Q63532,Q64240,Q99041,Q99PP0
168	1,2,3,4,6,7,8,27	5,25,26,28,29,30,31,32	17	O70417,P02631,P02783,P06911,P11598,P15399,P19218,P22273,P22282,P50115,P97580,Q10758,Q30KJ2,Q62894,Q66H69,Q99041,Q9QZQ5
169	1,2,3,4,6,7,8,28	5,25,26,27,29,30,31,32	18	O70417,P02631,P06911,P11598,P12020,P21674,P22273,P97580,Q09030,Q30KJ2,Q63532,Q63617,Q68FX1,Q99041,Q99PP0,Q9JI85,Q9QW07,Q9QZQ5
170	1,2,3,4,6,7,8,29	5,25,26,27,28,30,31,32	13	O70594,P02631,P02783,P06911,P11598,P12020,P21674,P42854,P62804,P97580,Q30KJ2,Q4V885,Q99041
171	1,2,3,4,6,7,8,30	5,25,26,27,28,29,31,32	17	O54800;Q5DWV2,O70417,P02631,P02783,P06911,P19218,P97580,P97615,Q09030,Q30KJ2,Q4V885,Q5RJR2,Q63532,Q6IFU7,Q6IFW6,Q6IG05,Q99041
172	1,2,3,4,6,7,8,31	5,25,26,27,28,29,30,32	13	P02783,P06911,P12020,P20766,P46844,P97580,Q05702,Q09030,Q30KJ2,Q5RJR2,Q63532,Q99PP0,Q9QX79
173	1,2,3,4,6,7,8,32	5,25,26,27,28,29,30,31	16	O54800;Q5DWV2,O55004,P02454,P02631,P02783,P06911,P11598,P97580,P97615,Q09030,Q30KJ2,Q4V885,Q63532,Q99041,Q99PP0,Q9QZQ5

174	1,2,3,4,6,7,25,26	5,8,27,28,29,30,31,32	3 O54800;Q5DWV2,Q4KLZ6,Q9WVH8
175	1,2,3,4,6,7,25,27	5,8,26,28,29,30,31,32	2 O54800;Q5DWV2,Q4KLZ6
176	1,2,3,4,6,7,25,28	5,8,26,27,29,30,31,32	4 O54800;Q5DWV2,Q09030,Q4KLZ6,Q62975
177	1,2,3,4,6,7,25,29	5,8,26,27,28,30,31,32	5 O54800;Q5DWV2,P02783,P42854,Q4KLZ6,Q9QWN8
178	1,2,3,4,6,7,25,30	5,8,26,27,28,29,31,32	6 O54800;Q5DWV2,P26342,Q09030,Q4KLZ6,Q80WL1,Q9QWN8
179	1,2,3,4,6,7,25,31	5,8,26,27,28,29,30,32	8 O54800;Q5DWV2,P20766,P29315,Q09030,Q4KLZ6,Q63532,Q9QWN8,Q9WVH8
180	1,2,3,4,6,7,25,32	5,8,26,27,28,29,30,31	4 O54800;Q5DWV2,P26342,Q09030,Q4KLZ6
181	1,2,3,4,6,7,26,27	5,8,25,28,29,30,31,32	5 O54800;Q5DWV2,P02783,P09656,P21588,Q62894
182	1,2,3,4,6,7,26,28	5,8,25,27,29,30,31,32	1 P11598
183	1,2,3,4,6,7,26,29	5,8,25,27,28,30,31,32	4 O54800;Q5DWV2,P02783,P09656,P42854
184	1,2,3,4,6,7,26,30	5,8,25,27,28,29,31,32	4 O54800;Q5DWV2,P02783,P21588,Q80WL1
185	1,2,3,4,6,7,26,31	5,8,25,27,28,29,30,32	7 O54800;Q5DWV2,P02783,P20766,P57113,Q63532,Q9QX79,Q9WVH8
186	1,2,3,4,6,7,26,32	5,8,25,27,28,29,30,31	3 O54800;Q5DWV2,P02783,P97615

187	1,2,3,4,6,7,27,28	5,8,25,26,29,30,31,32	5	P09656,P11598,P11762,Q63617,Q68FX1
188	1,2,3,4,6,7,27,29	5,8,25,26,28,30,31,32	5	P02783,P09656,P19223,P42854,P80299
189	1,2,3,4,6,7,27,30	5,8,25,26,28,29,31,32	4	P02783,P19218,P21588,Q80WL1
190	1,2,3,4,6,7,27,31	5,8,25,26,28,29,30,32	5	P02783,P20766,P80299,Q62894,Q63532
191	1,2,3,4,6,7,27,32	5,8,25,26,28,29,30,31	4	O54800;Q5DWV2,P02783,P11598,P80299
192	1,2,3,4,6,7,28,29	5,8,25,26,27,30,31,32	7	P02783,P09656,P11598,P21674,P42854,P47967,Q63617
193	1,2,3,4,6,7,28,30	5,8,25,26,27,29,31,32	1	Q80WL1
194	1,2,3,4,6,7,28,31	5,8,25,26,27,29,30,32	3	P11598,P80299,Q63532
195	1,2,3,4,6,7,28,32	5,8,25,26,27,29,30,31	1	P11598
196	1,2,3,4,6,7,29,30	5,8,25,26,27,28,31,32	6	O54800;Q5DWV2,P02783,P17046,P26342,Q80WL1,Q9QWN8
197	1,2,3,4,6,7,29,31	5,8,25,26,27,28,30,32	5	P02783,P20766,P26342,P80299,Q9QWN8
198	1,2,3,4,6,7,29,32	5,8,25,26,27,28,30,31	6	O54800;Q5DWV2,O55145,P02783,P26342,P42854,P80299
199	1,2,3,4,6,7,30,31	5,8,25,26,27,28,29,32	11	O54800;Q5DWV2,P02783,P06760,P20766,P26342,Q09030,Q63532,Q6P6R2,Q8CFN2,Q9QWN8,Q9QX79

200	1,2,3,4,6,7,30,32	5,8,25,26,27,28,29,31	6	O54800;Q5DWV2,P02783,P06760,P26342,P97615,Q6P6R2
201	1,2,3,4,6,7,31,32	5,8,25,26,27,28,29,30	10	O54800;Q5DWV2,P02783,P06760,P20766,P26342,P80299,P97615,Q63532,Q6P6R2,Q9QX79
202	1,2,3,4,6,8,25,26	5,7,27,28,29,30,31,32	11	G3V686,O54800;Q5DWV2,P08649,P15399,P97580,Q05702,Q06000,Q09030,Q30KJ2,Q4KLZ6,Q99MH3
203	1,2,3,4,6,8,25,27	5,7,26,28,29,30,31,32	9	P06911,P08649,P15399,P17559,P97580,Q09030,Q10758,Q30KJ2,Q4KLZ6
204	1,2,3,4,6,8,25,28	5,7,26,27,29,30,31,32	9	P02631,P06911,P08649,P36860,P97580,Q09030,Q30KJ2,Q4KLZ6,Q9JI85
205	1,2,3,4,6,8,25,29	5,7,26,27,28,30,31,32	7	O54800;Q5DWV2,P02783,P06911,P42854,P97580,Q30KJ2,Q4KLZ6
206	1,2,3,4,6,8,25,30	5,7,26,27,28,29,31,32	13	G3V686,O54800;Q5DWV2,O70417,O88797,P06911,P97580,Q09030,Q30KJ2,Q4KLZ6,Q6IFU7,Q6IFW6,Q6IG02,Q6IG05
207	1,2,3,4,6,8,25,31	5,7,26,27,28,29,30,32	12	G3V686,O54800;Q5DWV2,P06911,P12020,P29315,P36376,P97580,Q05702,Q09030,Q30KJ2,Q4KLZ6,Q63532
208	1,2,3,4,6,8,25,32	5,7,26,27,28,29,30,31	9	G3V686,O54800;Q5DWV2,P01681,P02631,P08649,P97580,Q09030,Q30KJ2,Q4KLZ6
209	1,2,3,4,6,8,26,27	5,7,25,28,29,30,31,32	10	P02783,P08649,P09656,P15399,P17559,P20762,P97580,Q10758,Q30KJ2,Q5BJY9
210	1,2,3,4,6,8,26,28	5,7,25,27,29,30,31,32	5	P08649,P15399,P97580,Q30KJ2,Q9JI85
211	1,2,3,4,6,8,26,29	5,7,25,27,28,30,31,32	7	O54800;Q5DWV2,P02783,P08649,P09656,P42854,P97580,Q30KJ2
212	1,2,3,4,6,8,26,30	5,7,25,27,28,29,31,32	7	O54800;Q5DWV2,P02783,P15399,P97580,P97615,Q30KJ2,Q99MH3

213	1,2,3,4,6,8,26,31	5,7,25,27,28,29,30,32	7	O54800;Q5DWV2,P02783,P97580,Q05702,Q30KJ2,Q63532,Q99MH3
214	1,2,3,4,6,8,26,32	5,7,25,27,28,29,30,31	10	O54800;Q5DWV2,O55004,P02783,P08649,P15399,P97580,P97615,Q05702,Q30KJ2,Q99MH3
215	1,2,3,4,6,8,27,28	5,7,25,26,29,30,31,32	10	P09656,P11762,P15399,P19218,P97580,Q10758,Q30KJ2,Q68FX1,Q9JI85,Q9WVK7
216	1,2,3,4,6,8,27,29	5,7,25,26,28,30,31,32	8	P02783,P09656,P15399,P19223,P42854,P97580,Q10758,Q30KJ2
217	1,2,3,4,6,8,27,30	5,7,25,26,28,29,31,32	15	O70417,P00731,P02783,P09656,P15399,P19218,P97580,Q10758,Q30KJ2,Q6IFU7,Q6IFU8,Q6IFW6,Q6IG02,Q6IG05,Q6P6Q2
218	1,2,3,4,6,8,27,31	5,7,25,26,28,29,30,32	8	P02783,P15399,P36376,P97580,Q05702,Q10758,Q30KJ2,Q63532
219	1,2,3,4,6,8,27,32	5,7,25,26,28,29,30,31	7	P02783,P15399,P97580,P98166,Q10758,Q30KJ2,Q9QZQ5
220	1,2,3,4,6,8,28,29	5,7,25,26,27,30,31,32	7	P02783,P09656,P21674,P42854,P97580,Q30KJ2,Q9JI85
221	1,2,3,4,6,8,28,30	5,7,25,26,27,29,31,32	7	O70417,P19218,P97580,Q09030,Q30KJ2,Q63532,Q6IFU7
222	1,2,3,4,6,8,28,31	5,7,25,26,27,29,30,32	9	P06911,P12020,P36376,P97580,Q05702,Q09030,Q30KJ2,Q63532,Q9JI85
223	1,2,3,4,6,8,28,32	5,7,25,26,27,29,30,31	4	P97580,Q30KJ2,Q63532,Q9JI85
224	1,2,3,4,6,8,29,30	5,7,25,26,27,28,31,32	9	O88797,P02783,P42854,P97580,Q30KJ2,Q5RJR2,Q6IFU7,Q6IFW6,Q6IG02
225	1,2,3,4,6,8,29,31	5,7,25,26,27,28,30,32	6	P02783,P97580,Q05702,Q30KJ2,Q5RJR2,Q63532

226	1,2,3,4,6,8,29,32	5,7,25,26,27,28,30,31	5	O54800;Q5DWV2,P02783,P42854,P97580,Q30KJ2
227	1,2,3,4,6,8,30,31	5,7,25,26,27,28,29,32	9	P02783,P97580,P97615,Q05702,Q09030,Q30KJ2,Q5RJR2,Q63532,Q6IG05
228	1,2,3,4,6,8,30,32	5,7,25,26,27,28,29,31	10	O54800;Q5DWV2,O88797,P02783,P20646,P26342,P97580,P97615,Q09030,Q30KJ2,Q6IFU7
229	1,2,3,4,6,8,31,32	5,7,25,26,27,28,29,30	7	O54800;Q5DWV2,P02783,P97580,P97615,Q05702,Q09030,Q63532
230	1,2,3,4,6,25,26,27	5,7,8,28,29,30,31,32	4	O54800;Q5DWV2,P09656,Q06000,Q4KLZ6
231	1,2,3,4,6,25,26,28	5,7,8,27,29,30,31,32	4	O54800;Q5DWV2,P08649,Q06000,Q4KLZ6
232	1,2,3,4,6,25,26,29	5,7,8,27,28,30,31,32	4	O54800;Q5DWV2,P02783,P09656,Q4KLZ6
233	1,2,3,4,6,25,26,30	5,7,8,27,28,29,31,32	6	O54800;Q5DWV2,P06760,Q06000,Q4KLZ6,Q812E4,Q99MH3
234	1,2,3,4,6,25,26,31	5,7,8,27,28,29,30,32	6	O54800;Q5DWV2,P06760,P18298,P29315,Q4KLZ6,Q812E4
235	1,2,3,4,6,25,26,32	5,7,8,27,28,29,30,31	4	O54800;Q5DWV2,P06760,Q06000,Q4KLZ6
236	1,2,3,4,6,25,27,28	5,7,8,26,29,30,31,32	3	P09656,P11762,Q4KLZ6
237	1,2,3,4,6,25,27,29	5,7,8,26,28,30,31,32	4	P02783,P09656,P35745,Q4KLZ6
238	1,2,3,4,6,25,27,30	5,7,8,26,28,29,31,32	5	O54800;Q5DWV2,P06760,P09656,Q4KLZ6,Q9QX74
239	1,2,3,4,6,25,27,31	5,7,8,26,28,29,30,32	4	P06760,P29315,Q4KLZ6,Q9QYP1
240	1,2,3,4,6,25,27,32	5,7,8,26,28,29,30,31	5	O54800;Q5DWV2,P06760,P35745,Q4KLZ6,Q9QX74
241	1,2,3,4,6,25,28,29	5,7,8,26,27,30,31,32	2	P09656,Q4KLZ6
242	1,2,3,4,6,25,28,30	5,7,8,26,27,29,31,32	5	P06760,Q09030,Q4KLZ6,Q62975,Q9QX74
243	1,2,3,4,6,25,28,31	5,7,8,26,27,29,30,32	5	P06760,P29315,Q09030,Q4KLZ6,Q63532

244	1,2,3,4,6,25,28,32	5,7,8,26,27,29,30,31	5	O54800;Q5DWV2,P06760,Q4KLZ6,Q62975,Q9QX74
245	1,2,3,4,6,25,29,30	5,7,8,26,27,28,31,32	5	O54800;Q5DWV2,Q4KLZ6,Q6IG02,Q9QWN8,Q9WTT6
246	1,2,3,4,6,25,29,31	5,7,8,26,27,28,30,32	3	P29315,Q4KLZ6,Q9QWN8
247	1,2,3,4,6,25,29,32	5,7,8,26,27,28,30,31	4	O54800;Q5DWV2,O55145,Q4KLZ6,Q9WTT6
248	1,2,3,4,6,25,30,31	5,7,8,26,27,28,29,32	11	O54800;Q5DWV2,P06760,P29315,Q09030,Q4KLZ6,Q6P6R2,Q812E4,Q8CFN2,Q9QWN8,Q9QX74,Q9WTT6
249	1,2,3,4,6,25,30,32	5,7,8,26,27,28,29,31	9	O54800;Q5DWV2,P06760,P26342,Q09030,Q4G075,Q4KLZ6,Q6P6R2,Q9QX74,Q9WTT6
250	1,2,3,4,6,25,31,32	5,7,8,26,27,28,29,30	11	O54800;Q5DWV2,P06760,P26342,P29315,Q09030,Q4G075,Q4KLZ6,Q63532,Q6P6R2,Q9QX74,Q9WTT6
251	1,2,3,4,6,26,27,28	5,7,8,25,29,30,31,32	2	P09656,P21588
252	1,2,3,4,6,26,27,29	5,7,8,25,28,30,31,32	3	P02783,P09656,P21588
253	1,2,3,4,6,26,27,30	5,7,8,25,28,29,31,32	6	P02783,P06760,P09656,P21588,P97580,Q80WL1
254	1,2,3,4,6,26,27,31	5,7,8,25,28,29,30,32	2	P02783,P06760
255	1,2,3,4,6,26,27,32	5,7,8,25,28,29,30,31	4	O54800;Q5DWV2,P02783,P06760,P09656
256	1,2,3,4,6,26,28,29	5,7,8,25,27,30,31,32	2	P02783,P09656
257	1,2,3,4,6,26,28,30	5,7,8,25,27,29,31,32	3	P06760,P21588,Q80WL1
258	1,2,3,4,6,26,28,31	5,7,8,25,27,29,30,32	2	P06760,Q63532
259	1,2,3,4,6,26,28,32	5,7,8,25,27,29,30,31	2	O54800;Q5DWV2,P06760
260	1,2,3,4,6,26,29,30	5,7,8,25,27,28,31,32	5	O54800;Q5DWV2,P02783,P09656,P21588,Q80WL1
261	1,2,3,4,6,26,29,31	5,7,8,25,27,28,30,32	1	P02783
262	1,2,3,4,6,26,29,32	5,7,8,25,27,28,30,31	4	O54800;Q5DWV2,P02783,P06866,P09656
263	1,2,3,4,6,26,30,31	5,7,8,25,27,28,29,32	6	O54800;Q5DWV2,P02783,P06760,Q4G075,Q812E4,Q99MH3

264	1,2,3,4,6,26,30,32	5,7,8,25,27,28,29,31	9	O54800;Q5DWV2,P02783,P06760,P26342,P97615,Q4G075,Q99MH3,Q9QX74,Q9WTT6
265	1,2,3,4,6,26,31,32	5,7,8,25,27,28,29,30	7	O54800;Q5DWV2,P02783,P06760,Q4G075,Q63532,Q99MH3,Q9WTT6
266	1,2,3,4,6,27,28,29	5,7,8,25,26,30,31,32	3	P02783,P09656,Q9WVK7
267	1,2,3,4,6,27,28,30	5,7,8,25,26,29,31,32	6	P06760,P09656,P11762,P19218,P21588,Q9QX74
268	1,2,3,4,6,27,28,31	5,7,8,25,26,29,30,32	1	P06760
269	1,2,3,4,6,27,28,32	5,7,8,25,26,29,30,31	4	P06760,P09656,Q62761;Q62762;Q62763,Q9QX74
270	1,2,3,4,6,27,29,30	5,7,8,25,26,28,31,32	4	P02783,P09656,P21588,Q6IG02
271	1,2,3,4,6,27,29,31	5,7,8,25,26,28,30,32	3	P02783,P09656,P80299
272	1,2,3,4,6,27,29,32	5,7,8,25,26,28,30,31	3	P02783,P09656,P80299
273	1,2,3,4,6,27,30,31	5,7,8,25,26,28,29,32	4	P02783,P06760,Q9QX74,Q9WTT6
274	1,2,3,4,6,27,30,32	5,7,8,25,26,28,29,31	6	P02783,P06760,P09656,Q6P6R2,Q9QX74,Q9WTT6
275	1,2,3,4,6,27,31,32	5,7,8,25,26,28,29,30	6	P02783,P06760,P22006,P80299,Q9QX74,Q9WTT6
276	1,2,3,4,6,28,29,30	5,7,8,25,26,27,31,32	1	P09656
277	1,2,3,4,6,28,29,31	5,7,8,25,26,27,30,32	0	
278	1,2,3,4,6,28,29,32	5,7,8,25,26,27,30,31	1	P09656
279	1,2,3,4,6,28,30,31	5,7,8,25,26,27,29,32	4	P06760,Q63532,Q9QX74,Q9WTT6
280	1,2,3,4,6,28,30,32	5,7,8,25,26,27,29,31	4	P06760,Q4G075,Q9QX74,Q9WTT6
281	1,2,3,4,6,28,31,32	5,7,8,25,26,27,29,30	7	P06760,P22006,P80299,Q4G075,Q63532,Q9QX74,Q9WTT6
282	1,2,3,4,6,29,30,31	5,7,8,25,26,27,28,32	5	P02783,P07150,P26342,Q9QWN8,Q9WTT6
283	1,2,3,4,6,29,30,32	5,7,8,25,26,27,28,31	7	O54800;Q5DWV2,O55145,P02783,P09656,P26342,Q9WTT6,Q9Z0V6

284	1,2,3,4,6,29,31,32	5,7,8,25,26,27,28,30	5	O55145,P02783,P26342,P80299,Q9WTT6
285	1,2,3,4,6,30,31,32	5,7,8,25,26,27,28,29	11	O54800;Q5DWV2,P06760,P20646,P22006,P26342,P97615,Q4G075,Q63532,Q6P6R2,Q9QX74,Q9WTT6
286	1,2,3,4,7,8,25,26	5,6,27,28,29,30,31,32	3	G3V686,P97580,Q30KJ2
287	1,2,3,4,7,8,25,27	5,6,26,28,29,30,31,32	7	O70417,P01681,P36860,P97580,Q30KJ2,Q4G075,Q66H69
288	1,2,3,4,7,8,25,28	5,6,26,27,29,30,31,32	4	O70417,P06911,P36860,Q811M5
289	1,2,3,4,7,8,25,29	5,6,26,27,28,30,31,32	1	P22006
290	1,2,3,4,7,8,25,30	5,6,26,27,28,29,31,32	8	G3V686,O70417,P97580,Q6IFU8,Q6IFW6,Q6IG02,Q6IMF3,Q6P6Q2
291	1,2,3,4,7,8,25,31	5,6,26,27,28,29,30,32	5	G3V686,P01681,P20766,P29315,Q811M5
292	1,2,3,4,7,8,25,32	5,6,26,27,28,29,30,31	3	G3V686,P01681,Q811M5
293	1,2,3,4,7,8,26,27	5,6,25,28,29,30,31,32	6	P15399,P20762,P97580,Q30KJ2,Q5BJY9,Q66H69
294	1,2,3,4,7,8,26,28	5,6,25,27,29,30,31,32	3	P97580,Q30KJ2,Q5RLM2
295	1,2,3,4,7,8,26,29	5,6,25,27,28,30,31,32	2	P97580,Q64093
296	1,2,3,4,7,8,26,30	5,6,25,27,28,29,31,32	6	O70417,P20762,P97580,P97615,Q30KJ2,Q6IFW6
297	1,2,3,4,7,8,26,31	5,6,25,27,28,29,30,32	2	P20766,P97580

298	1,2,3,4,7,8,26,32	5,6,25,27,28,29,30,31	2	P97580,P97615
299	1,2,3,4,7,8,27,28	5,6,25,26,29,30,31,32	8	A0JPJ7,P36860,P47967,P97580,Q10758,Q30KJ2,Q66H69,Q9WVK7
300	1,2,3,4,7,8,27,29	5,6,25,26,28,30,31,32	9	P00731,P19223,P62804,P97580,Q00715,Q10758,Q30KJ2,Q4G075,Q66H69
301	1,2,3,4,7,8,27,30	5,6,25,26,28,29,31,32	14	O70417,P00731,P19218,P97580,Q10758,Q30KJ2,Q66H69,Q6IFU7,Q6IFU8,Q6IFW6,Q6IG02,Q6IG05,Q6IMF3,Q6P6Q2
302	1,2,3,4,7,8,27,31	5,6,25,26,28,29,30,32	5	P20766,P97580,Q10758,Q30KJ2,Q66H69
303	1,2,3,4,7,8,27,32	5,6,25,26,28,29,30,31	3	P97580,Q30KJ2,Q66H69
304	1,2,3,4,7,8,28,29	5,6,25,26,27,30,31,32	3	P21674,P47967,P62804
305	1,2,3,4,7,8,28,30	5,6,25,26,27,29,31,32	1	O70417
306	1,2,3,4,7,8,28,31	5,6,25,26,27,29,30,32	3	P20766,P82471,Q811M5
307	1,2,3,4,7,8,28,32	5,6,25,26,27,29,30,31	1	Q811M5
308	1,2,3,4,7,8,29,30	5,6,25,26,27,28,31,32	7	P62804,Q6IFU7,Q6IFU8,Q6IFW6,Q6IG02,Q6IMF3,Q6P6Q2
309	1,2,3,4,7,8,29,31	5,6,25,26,27,28,30,32	2	P20766,P82471
310	1,2,3,4,7,8,29,32	5,6,25,26,27,28,30,31	0	

311	1,2,3,4,7,8,30,31	5,6,25,26,27,28,29,32	5	P20766,P97615,Q6IFU8,Q6IFW6,Q6IMF3
312	1,2,3,4,7,8,30,32	5,6,25,26,27,28,29,31	6	O70417,P05539,P26342,P35280,P97615,Q6IFW6
313	1,2,3,4,7,8,31,32	5,6,25,26,27,28,29,30	5	P01681,P20766,P82471,P97615,Q811M5
314	1,2,3,4,7,25,26,27	5,6,8,28,29,30,31,32	0	
315	1,2,3,4,7,25,26,28	5,6,8,27,29,30,31,32	0	
316	1,2,3,4,7,25,26,29	5,6,8,27,28,30,31,32	1	P22006
317	1,2,3,4,7,25,26,30	5,6,8,27,28,29,31,32	0	
318	1,2,3,4,7,25,26,31	5,6,8,27,28,29,30,32	3	P18298,P20766,P29315
319	1,2,3,4,7,25,26,32	5,6,8,27,28,29,30,31	0	
320	1,2,3,4,7,25,27,28	5,6,8,26,29,30,31,32	1	P36860
321	1,2,3,4,7,25,27,29	5,6,8,26,28,30,31,32	2	P10758,P19223
322	1,2,3,4,7,25,27,30	5,6,8,26,28,29,31,32	0	
323	1,2,3,4,7,25,27,31	5,6,8,26,28,29,30,32	3	P20766,P29315,Q9QYP1
324	1,2,3,4,7,25,27,32	5,6,8,26,28,29,30,31	1	P01681
325	1,2,3,4,7,25,28,29	5,6,8,26,27,30,31,32	0	
326	1,2,3,4,7,25,28,30	5,6,8,26,27,29,31,32	1	Q62975
327	1,2,3,4,7,25,28,31	5,6,8,26,27,29,30,32	2	P29315,Q811M5
328	1,2,3,4,7,25,28,32	5,6,8,26,27,29,30,31	2	Q62975,Q811M5
329	1,2,3,4,7,25,29,30	5,6,8,26,27,28,31,32	2	Q6IG02,Q9QWN8

330	1,2,3,4,7,25,29,31	5,6,8,26,27,28,30,32	2	P29315,Q9QWN8
331	1,2,3,4,7,25,29,32	5,6,8,26,27,28,30,31	0	
332	1,2,3,4,7,25,30,31	5,6,8,26,27,28,29,32	5	P26342,P29315,Q8CFN2,Q9QWN8,Q9QYP1
333	1,2,3,4,7,25,30,32	5,6,8,26,27,28,29,31	4	P26342,Q62975,Q6P6R2,Q9R168
334	1,2,3,4,7,25,31,32	5,6,8,26,27,28,29,30	5	P01681,P26342,P29315,Q6P6R2,Q811M5
335	1,2,3,4,7,26,27,28	5,6,8,25,29,30,31,32	2	P47967,P97840
336	1,2,3,4,7,26,27,29	5,6,8,25,28,30,31,32	1	P19223
337	1,2,3,4,7,26,27,30	5,6,8,25,28,29,31,32	2	P21588,Q80WL1
338	1,2,3,4,7,26,27,31	5,6,8,25,28,29,30,32	1	P20766
339	1,2,3,4,7,26,27,32	5,6,8,25,28,29,30,31	0	
340	1,2,3,4,7,26,28,29	5,6,8,25,27,30,31,32	3	P47967,P97840,Q63424
341	1,2,3,4,7,26,28,30	5,6,8,25,27,29,31,32	1	Q80WL1
342	1,2,3,4,7,26,28,31	5,6,8,25,27,29,30,32	1	Q5RLM2
343	1,2,3,4,7,26,28,32	5,6,8,25,27,29,30,31	0	
344	1,2,3,4,7,26,29,30	5,6,8,25,27,28,31,32	1	Q80WL1
345	1,2,3,4,7,26,29,31	5,6,8,25,27,28,30,32	1	P20766
346	1,2,3,4,7,26,29,32	5,6,8,25,27,28,30,31	0	
347	1,2,3,4,7,26,30,31	5,6,8,25,27,28,29,32	3	P20766,Q812E4,Q8CFN2
348	1,2,3,4,7,26,30,32	5,6,8,25,27,28,29,31	3	P26342,P97615,Q9R168
349	1,2,3,4,7,26,31,32	5,6,8,25,27,28,29,30	1	P20766

350	1,2,3,4,7,27,28,29	5,6,8,25,26,30,31,32	4	P00507,P47967,P97840,Q9WVK7
351	1,2,3,4,7,27,28,30	5,6,8,25,26,29,31,32	0	
352	1,2,3,4,7,27,28,31	5,6,8,25,26,29,30,32	1	P80299
353	1,2,3,4,7,27,28,32	5,6,8,25,26,29,30,31	1	Q62761;Q62762;Q62763
354	1,2,3,4,7,27,29,30	5,6,8,25,26,28,31,32	2	P19223,Q6IG02
355	1,2,3,4,7,27,29,31	5,6,8,25,26,28,30,32	2	P19223,P80299
356	1,2,3,4,7,27,29,32	5,6,8,25,26,28,30,31	1	P80299
357	1,2,3,4,7,27,30,31	5,6,8,25,26,28,29,32	3	P20766,P80299,Q9QYP1
358	1,2,3,4,7,27,30,32	5,6,8,25,26,28,29,31	0	
359	1,2,3,4,7,27,31,32	5,6,8,25,26,28,29,30	2	P20766,P80299
360	1,2,3,4,7,28,29,30	5,6,8,25,26,27,31,32	1	P97840
361	1,2,3,4,7,28,29,31	5,6,8,25,26,27,30,32	1	P80299
362	1,2,3,4,7,28,29,32	5,6,8,25,26,27,30,31	1	P54921
363	1,2,3,4,7,28,30,31	5,6,8,25,26,27,29,32	0	
364	1,2,3,4,7,28,30,32	5,6,8,25,26,27,29,31	2	P26342,Q9R168
365	1,2,3,4,7,28,31,32	5,6,8,25,26,27,29,30	2	P80299,Q811M5
366	1,2,3,4,7,29,30,31	5,6,8,25,26,27,28,32	3	P26342,P80299,Q9QWN8
367	1,2,3,4,7,29,30,32	5,6,8,25,26,27,28,31	2	P17046,P26342
368	1,2,3,4,7,29,31,32	5,6,8,25,26,27,28,30	2	P26342,P80299
369	1,2,3,4,7,30,31,32	5,6,8,25,26,27,28,29	5	P06760,P26342,P80299,P97615,Q6P6R2

370	1,2,3,4,8,25,26,27	5,6,7,28,29,30,31,32	6	G3V686,P15399,P36860,P97580,Q30KJ2,Q5BJY9
371	1,2,3,4,8,25,26,28	5,6,7,27,29,30,31,32	4	G3V686,P36860,P97580,Q30KJ2
372	1,2,3,4,8,25,26,29	5,6,7,27,28,30,31,32	3	P22006,P97580,Q30KJ2
373	1,2,3,4,8,25,26,30	5,6,7,27,28,29,31,32	5	G3V686,O88797,P97580,Q30KJ2,Q6IFW6
374	1,2,3,4,8,25,26,31	5,6,7,27,28,29,30,32	4	G3V686,P29315,P97580,Q30KJ2
375	1,2,3,4,8,25,26,32	5,6,7,27,28,29,30,31	5	G3V686,P01681,P97580,Q06000,Q30KJ2
376	1,2,3,4,8,25,27,28	5,6,7,26,29,30,31,32	4	P36860,P97580,Q10758,Q30KJ2
377	1,2,3,4,8,25,27,29	5,6,7,26,28,30,31,32	5	P19223,P22006,P97580,Q10758,Q30KJ2
378	1,2,3,4,8,25,27,30	5,6,7,26,28,29,31,32	12	O70417,P97580,Q10758,Q30KJ2,Q4FZU2,Q6IFU7,Q6IFU8,Q6IFW6,Q6IG02,Q6IG05,Q6IMF3,Q6P6Q2
379	1,2,3,4,8,25,27,31	5,6,7,26,28,29,30,32	6	P01681,P29315,P36376,P97580,Q30KJ2,Q9QYP1
380	1,2,3,4,8,25,27,32	5,6,7,26,28,29,30,31	4	P00762,P01681,P97580,Q30KJ2
381	1,2,3,4,8,25,28,29	5,6,7,26,27,30,31,32	1	P36860
382	1,2,3,4,8,25,28,30	5,6,7,26,27,29,31,32	3	O70417,Q6IFU7,Q6IFW6
383	1,2,3,4,8,25,28,31	5,6,7,26,27,29,30,32	3	P29315,P36376,Q811M5
384	1,2,3,4,8,25,28,32	5,6,7,26,27,29,30,31	3	P01681,P36860,Q811M5
385	1,2,3,4,8,25,29,30	5,6,7,26,27,28,31,32	9	O88797,P22006,P97580,Q6IFU7,Q6IFU8,Q6IFW6,Q6IG02,Q6IMF3,Q6P6Q2
386	1,2,3,4,8,25,29,31	5,6,7,26,27,28,30,32	1	P29315
387	1,2,3,4,8,25,29,32	5,6,7,26,27,28,30,31	1	P01681
388	1,2,3,4,8,25,30,31	5,6,7,26,27,28,29,32	7	P01681,P02625,P29315,P36376,Q6IFU8,Q6IFW6,Q6IG02
389	1,2,3,4,8,25,30,32	5,6,7,26,27,28,29,31	7	O88797,P01681,P02625,P05539,P97615,Q6IFU7,Q6IFW6

390	1,2,3,4,8,25,31,32	5,6,7,26,27,28,29,30	4 P01681,P21588,P29315,Q811M5
391	1,2,3,4,8,26,27,28	5,6,7,25,29,30,31,32	7 P15399,P36860,P97580,Q10758,Q30KJ2,Q5BJY9,Q9WVK7
392	1,2,3,4,8,26,27,29	5,6,7,25,28,30,31,32	5 P15399,P97580,Q10758,Q30KJ2,Q5BJY9
393	1,2,3,4,8,26,27,30	5,6,7,25,28,29,31,32	9 P15399,P97580,Q10758,Q30KJ2,Q5BJY9,Q6IFU7,Q6IFU8,Q6IFW6,Q6IG02
394	1,2,3,4,8,26,27,31	5,6,7,25,28,29,30,32	5 P15399,P97580,Q10758,Q30KJ2,Q5BJY9
395	1,2,3,4,8,26,27,32	5,6,7,25,28,29,30,31	4 P15399,P97580,Q30KJ2,Q5BJY9
396	1,2,3,4,8,26,28,29	5,6,7,25,27,30,31,32	1 P97580
397	1,2,3,4,8,26,28,30	5,6,7,25,27,29,31,32	2 P97580,Q30KJ2
398	1,2,3,4,8,26,28,31	5,6,7,25,27,29,30,32	1 P36376
399	1,2,3,4,8,26,28,32	5,6,7,25,27,29,30,31	1 O54728
400	1,2,3,4,8,26,29,30	5,6,7,25,27,28,31,32	6 O88797,P97580,Q30KJ2,Q6IFU7,Q6IFW6,Q6IG02
401	1,2,3,4,8,26,29,31	5,6,7,25,27,28,30,32	0
402	1,2,3,4,8,26,29,32	5,6,7,25,27,28,30,31	0
403	1,2,3,4,8,26,30,31	5,6,7,25,27,28,29,32	3 P05539,P97580,Q30KJ2
404	1,2,3,4,8,26,30,32	5,6,7,25,27,28,29,31	5 O88797,P05539,P97580,P97615,Q30KJ2
405	1,2,3,4,8,26,31,32	5,6,7,25,27,28,29,30	2 O54728,P05539
406	1,2,3,4,8,27,28,29	5,6,7,25,26,30,31,32	5 P25809,P97580,Q10758,Q30KJ2,Q9WVK7
407	1,2,3,4,8,27,28,30	5,6,7,25,26,29,31,32	10 P15399,P19218,P97580,Q10758,Q30KJ2,Q6IFU7,Q6IFU8,Q6IFW6,Q6IG02,Q6P6Q2
408	1,2,3,4,8,27,28,31	5,6,7,25,26,29,30,32	4 P36376,P97580,Q10758,Q30KJ2
409	1,2,3,4,8,27,28,32	5,6,7,25,26,29,30,31	5 P15399,P97580,Q10758,Q30KJ2,Q6AYR9

410	1,2,3,4,8,27,29,30	5,6,7,25,26,28,31,32	11	P00731,P19223,P97580,Q10758,Q30KJ2,Q6IFU7,Q6IFU8,Q6IFW6,Q6IG02,Q6IMF3,Q6P6Q2
411	1,2,3,4,8,27,29,31	5,6,7,25,26,28,30,32	5	P19223,P97580,Q10758,Q30KJ2,Q5PQL7
412	1,2,3,4,8,27,29,32	5,6,7,25,26,28,30,31	3	P97580,Q10758,Q30KJ2
413	1,2,3,4,8,27,30,31	5,6,7,25,26,28,29,32	10	P97580,Q10758,Q30KJ2,Q6IFU7,Q6IFU8,Q6IFW6,Q6IG02,Q6IG05,Q6IMF3,Q6P6Q2
414	1,2,3,4,8,27,30,32	5,6,7,25,26,28,29,31	8	P97580,Q10758,Q30KJ2,Q6IFU7,Q6IFU8,Q6IFW6,Q6IG02,Q6P6Q2
415	1,2,3,4,8,27,31,32	5,6,7,25,26,28,29,30	3	P01681,P97580,Q10758
416	1,2,3,4,8,28,29,30	5,6,7,25,26,27,31,32	3	Q6IFU7,Q6IFW6,Q6IG02
417	1,2,3,4,8,28,29,31	5,6,7,25,26,27,30,32	1	P36376
418	1,2,3,4,8,28,29,32	5,6,7,25,26,27,30,31	0	
419	1,2,3,4,8,28,30,31	5,6,7,25,26,27,29,32	1	P36376
420	1,2,3,4,8,28,30,32	5,6,7,25,26,27,29,31	0	
421	1,2,3,4,8,28,31,32	5,6,7,25,26,27,29,30	2	P36376,Q811M5
422	1,2,3,4,8,29,30,31	5,6,7,25,26,27,28,32	7	Q5RJR2,Q6IFU7,Q6IFU8,Q6IFW6,Q6IG02,Q6IMF3,Q6P6Q2
423	1,2,3,4,8,29,30,32	5,6,7,25,26,27,28,31	6	O88797,Q6IFU7,Q6IFW6,Q6IG02,Q6IMF3,Q6P6Q2
424	1,2,3,4,8,29,31,32	5,6,7,25,26,27,28,30	0	
425	1,2,3,4,8,30,31,32	5,6,7,25,26,27,28,29	5	P02625,P05539,P26342,P63081,P97615
426	1,2,3,4,25,26,27,28	5,6,7,8,29,30,31,32	1	P36860
427	1,2,3,4,25,26,27,29	5,6,7,8,28,30,31,32	1	P35745
428	1,2,3,4,25,26,27,30	5,6,7,8,28,29,31,32	0	
429	1,2,3,4,25,26,27,31	5,6,7,8,28,29,30,32	2	P29315,Q9QYP1

430	1,2,3,4,25,<br>26,27,32	5,6,7,8,28,<br>29,30,31	2	P35745,Q06000
431	1,2,3,4,25,<br>26,28,29	5,6,7,8,27,<br>30,31,32	0	
432	1,2,3,4,25,<br>26,28,30	5,6,7,8,27,<br>29,31,32	0	
433	1,2,3,4,25,<br>26,28,31	5,6,7,8,27,<br>29,30,32	1	P29315
434	1,2,3,4,25,<br>26,28,32	5,6,7,8,27,<br>29,30,31	2	Q06000,Q62975
435	1,2,3,4,25,<br>26,29,30	5,6,7,8,27,<br>28,31,32	1	Q9QZK9
436	1,2,3,4,25,<br>26,29,31	5,6,7,8,27,<br>28,30,32	2	P19218,P29315
437	1,2,3,4,25,<br>26,29,32	5,6,7,8,27,<br>28,30,31	1	P19218
438	1,2,3,4,25,<br>26,30,31	5,6,7,8,27,<br>28,29,32	7	P06760,P29315,Q63617,Q6AYR9,Q812E4,Q8CFN2,Q9QYP1
439	1,2,3,4,25,<br>26,30,32	5,6,7,8,27,<br>28,29,31	5	D3ZUC6,P06760,Q06000,Q4G075,Q9QX74
440	1,2,3,4,25,<br>26,31,32	5,6,7,8,27,<br>28,29,30	4	P06760,P19218,P29315,Q4G075
441	1,2,3,4,25,<br>27,28,29	5,6,7,8,26,<br>30,31,32	0	
442	1,2,3,4,25,<br>27,28,30	5,6,7,8,26,<br>29,31,32	2	P06760,Q9QX74
443	1,2,3,4,25,<br>27,28,31	5,6,7,8,26,<br>29,30,32	2	P29315,Q9QYP1
444	1,2,3,4,25,<br>27,28,32	5,6,7,8,26,<br>29,30,31	3	P06760,Q62761;Q62762;Q62763,Q9QX74
445	1,2,3,4,25,<br>27,29,30	5,6,7,8,26,<br>28,31,32	2	Q6IFW6,Q6IG02
446	1,2,3,4,25,<br>27,29,31	5,6,7,8,26,<br>28,30,32	2	P29315,Q9QYP1
447	1,2,3,4,25,<br>27,29,32	5,6,7,8,26,<br>28,30,31	1	P35745
448	1,2,3,4,25,<br>27,30,31	5,6,7,8,26,<br>28,29,32	5	P06760,P29315,Q6IG02,Q9QX74,Q9QYP1
449	1,2,3,4,25,<br>27,30,32	5,6,7,8,26,<br>28,29,31	5	P00762,P06760,Q6P6R2,Q9QX74,Q9WTT6

450	1,2,3,4,25,<br>27,31,32	5,6,7,8,26,<br>28,29,30	6	P01681,P06760,P29315,Q9QX74,Q9QYP1,Q9WTT6
451	1,2,3,4,25,<br>28,29,30	5,6,7,8,26,<br>27,31,32	1	P50116
452	1,2,3,4,25,<br>28,29,31	5,6,7,8,26,<br>27,30,32	1	P29315
453	1,2,3,4,25,<br>28,29,32	5,6,7,8,26,<br>27,30,31	0	
454	1,2,3,4,25,<br>28,30,31	5,6,7,8,26,<br>27,29,32	3	P06760,P29315,Q9QX74
455	1,2,3,4,25,<br>28,30,32	5,6,7,8,26,<br>27,29,31	6	P06760,P50115,P50116,Q62975,Q9QX74,Q9WTT6
456	1,2,3,4,25,<br>28,31,32	5,6,7,8,26,<br>27,29,30	5	P06760,P29315,Q811M5,Q9QX74,Q9WTT6
457	1,2,3,4,25,<br>29,30,31	5,6,7,8,26,<br>27,28,32	6	P07150,P29315,P50116,Q6IG02,Q9QWN8,Q9WTT6
458	1,2,3,4,25,<br>29,30,32	5,6,7,8,26,<br>27,28,31	4	P07150,P50115,P50116,Q9WTT6
459	1,2,3,4,25,<br>29,31,32	5,6,7,8,26,<br>27,28,30	4	P07150,P29315,P50116,Q9WTT6
460	1,2,3,4,25,<br>30,31,32	5,6,7,8,26,<br>27,28,29	13	D3ZUC6,P01681,P06760,P07150,P26342,P29315,P50115,P50116,Q4G075,Q6P6R2,Q9QX74,Q9<br>QYP1,Q9WTT6
461	1,2,3,4,26,<br>27,28,29	5,6,7,8,25,<br>30,31,32	1	Q9WVK7
462	1,2,3,4,26,<br>27,28,30	5,6,7,8,25,<br>29,31,32	1	P21588
463	1,2,3,4,26,<br>27,28,31	5,6,7,8,25,<br>29,30,32	0	
464	1,2,3,4,26,<br>27,28,32	5,6,7,8,25,<br>29,30,31	0	
465	1,2,3,4,26,<br>27,29,30	5,6,7,8,25,<br>28,31,32	2	P21588,Q6IG02
466	1,2,3,4,26,<br>27,29,31	5,6,7,8,25,<br>28,30,32	0	
467	1,2,3,4,26,<br>27,29,32	5,6,7,8,25,<br>28,30,31	1	P35745
468	1,2,3,4,26,<br>27,30,31	5,6,7,8,25,<br>28,29,32	2	P06760,Q9QYP1
469	1,2,3,4,26,<br>27,30,32	5,6,7,8,25,<br>28,29,31	1	P06760

470	1,2,3,4,26,<br>27,31,32	5,6,7,8,25,<br>28,29,30	1	P06760
471	1,2,3,4,26,<br>28,29,30	5,6,7,8,25,<br>27,31,32	1	P50115
472	1,2,3,4,26,<br>28,29,31	5,6,7,8,25,<br>27,30,32	0	
473	1,2,3,4,26,<br>28,29,32	5,6,7,8,25,<br>27,30,31	1	P50115
474	1,2,3,4,26,<br>28,30,31	5,6,7,8,25,<br>27,29,32	3	P06760,P50115,Q4G075
475	1,2,3,4,26,<br>28,30,32	5,6,7,8,25,<br>27,29,31	3	P06760,P50115,Q4G075
476	1,2,3,4,26,<br>28,31,32	5,6,7,8,25,<br>27,29,30	4	O54728,P06760,P50115,Q4G075
477	1,2,3,4,26,<br>29,30,31	5,6,7,8,25,<br>27,28,32	2	P50115,P50116
478	1,2,3,4,26,<br>29,30,32	5,6,7,8,25,<br>27,28,31	2	P50115,P50116
479	1,2,3,4,26,<br>29,31,32	5,6,7,8,25,<br>27,28,30	1	P50115
480	1,2,3,4,26,<br>30,31,32	5,6,7,8,25,<br>27,28,29	8	P06760,P26342,P50115,P50116,P63081,Q4G075,Q812E4,Q9WTT6
481	1,2,3,4,27,<br>28,29,30	5,6,7,8,25,<br>26,31,32	2	Q6IG02,Q9WVK7
482	1,2,3,4,27,<br>28,29,31	5,6,7,8,25,<br>26,30,32	2	P80299,Q9WVK7
483	1,2,3,4,27,<br>28,29,32	5,6,7,8,25,<br>26,30,31	2	Q62761;Q62762;Q62763,Q9WVK7
484	1,2,3,4,27,<br>28,30,31	5,6,7,8,25,<br>26,29,32	2	P06760,Q9QX74
485	1,2,3,4,27,<br>28,30,32	5,6,7,8,25,<br>26,29,31	2	P06760,Q9QX74
486	1,2,3,4,27,<br>28,31,32	5,6,7,8,25,<br>26,29,30	3	P06760,P80299,Q9QX74
487	1,2,3,4,27,<br>29,30,31	5,6,7,8,25,<br>26,28,32	2	P80299,Q6IG02
488	1,2,3,4,27,<br>29,30,32	5,6,7,8,25,<br>26,28,31	3	B0BNN3,Q6IG02,Q9WTT6
489	1,2,3,4,27,<br>29,31,32	5,6,7,8,25,<br>26,28,30	1	P80299

490	1,2,3,4,27,30,31,32	5,6,7,8,25,26,28,29	6	P06760,P80299,Q6P6R2,Q9QX74,Q9QYP1,Q9WTT6
491	1,2,3,4,28,29,30,31	5,6,7,8,25,26,27,32	2	P50115,P50116
492	1,2,3,4,28,29,30,32	5,6,7,8,25,26,27,31	3	P50115,P50116,Q9WTT6
493	1,2,3,4,28,29,31,32	5,6,7,8,25,26,27,30	3	P50115,P50116,P80299
494	1,2,3,4,28,30,31,32	5,6,7,8,25,26,27,29	6	P06760,P50115,P50116,Q4G075,Q9QX74,Q9WTT6
495	1,2,3,4,29,30,31,32	5,6,7,8,25,26,27,28	8	B0BNN3,P07150,P26342,P50115,P50116,P63081,P80299,Q9WTT6
496	1,2,3,5,6,7,8,25	4,26,27,28,29,30,31,32	7	O54800;Q5DWV2,P02631,P36374,P50115,P52590,P80202,Q09030
497	1,2,3,5,6,7,8,26	4,25,27,28,29,30,31,32	11	O54800;Q5DWV2,O70594,P00762,P36374,P50115,P52590,P97580,Q09030,Q5RLM2,Q62714,Q99MH3
498	1,2,3,5,6,7,8,27	4,25,26,28,29,30,31,32	14	D3ZUC6,P00731,P19218,P47967,P50115,P50116,P52590,P97580,Q03191,Q09030,Q62714,Q66H69,Q9JJ50,Q9QZQ5
499	1,2,3,5,6,7,8,28	4,25,26,27,29,30,31,32	9	D3ZUC6,P00762,P19218,P21674,P47967,P52590,Q09030,Q5RLM2,Q9QYP1
500	1,2,3,5,6,7,8,29	4,25,26,27,28,30,31,32	9	O70594,P00762,P21674,P47967,P52590,P62804,Q09030,Q5RJR2,Q9QYP1
501	1,2,3,5,6,7,8,30	4,25,26,27,28,29,31,32	14	P19218,P36374,P52590,P80202,Q09030,Q5I0J9,Q5RJR2,Q6IFU7,Q6IFW6,Q6IG05,Q6IMF3,Q6P6Q2,Q8CGS4,Q99MH3
502	1,2,3,5,6,7,8,31	4,25,26,27,28,29,30,32	9	O70594,P20766,P36376,P52590,P80202,Q09030,Q5RJR2,Q62714,Q63532
503	1,2,3,5,6,7,8,32	4,25,26,27,28,29,30,31	7	O54800;Q5DWV2,P52590,P80202,Q09030,Q62714,Q99MH3,Q9QZQ5
504	1,2,3,5,6,7,25,26	4,8,27,28,29,30,31,32	7	O54800;Q5DWV2,P00762,P36374,P52590,Q09030,Q6AYR9,Q99MH3

505	1,2,3,5,6,7,25,27	4,8,26,28,29,30,31,32	6	O54728,P50116,P52590,Q03191,Q09030,Q9JJ50
506	1,2,3,5,6,7,25,28	4,8,26,27,29,30,31,32	3	P00762,P52590,Q09030
507	1,2,3,5,6,7,25,29	4,8,26,27,28,30,31,32	6	O54728,O54800;Q5DWV2,P00762,P52590,Q09030,Q9QWN8
508	1,2,3,5,6,7,25,30	4,8,26,27,28,29,31,32	9	O54728,O54800;Q5DWV2,P36374,P52590,P97523,Q09030,Q6AYR9,Q6P6R2,Q9QWN8
509	1,2,3,5,6,7,25,31	4,8,26,27,28,29,30,32	8	O54728,P20766,P52590,Q09030,Q62714,Q6AYR9,Q8CFN2,Q9QWN8
510	1,2,3,5,6,7,25,32	4,8,26,27,28,29,30,31	4	O54800;Q5DWV2,P52590,Q09030,Q6P6R2
511	1,2,3,5,6,7,26,27	4,8,25,28,29,30,31,32	8	P01039,P21588,P47967,P50116,P52590,Q03191,Q62714,Q9JJ50
512	1,2,3,5,6,7,26,28	4,8,25,27,29,30,31,32	5	P00762,P47967,P52590,Q5RLM2,Q99MH3
513	1,2,3,5,6,7,26,29	4,8,25,27,28,30,31,32	6	P00762,P01835,P36374,P47967,P52590,Q9JJ50
514	1,2,3,5,6,7,26,30	4,8,25,27,28,29,31,32	8	O54800;Q5DWV2,P00762,P21588,P36374,P52590,Q6AYR9,Q812E4,Q99MH3
515	1,2,3,5,6,7,26,31	4,8,25,27,28,29,30,32	10	P00762,P20766,P52590,P57113,Q5RLM2,Q62714,Q6AYR9,Q812E4,Q99MH3,Q9Z2L0
516	1,2,3,5,6,7,26,32	4,8,25,27,28,29,30,31	4	O54800;Q5DWV2,P52590,Q62714,Q99MH3
517	1,2,3,5,6,7,27,28	4,8,25,26,29,30,31,32	7	P00762,P19218,P47967,P50116,P52590,Q03191,Q6AY61

518	1,2,3,5,6,7,27,29	4,8,25,26,28,30,31,32	8	O54728,P47967,P50116,P52590,Q03191,Q07523,Q6AY61,Q9JJ50
519	1,2,3,5,6,7,27,30	4,8,25,26,28,29,31,32	11	O54728,P00731,P01039,P19218,P21588,P50116,P52590,P84039,Q03191,Q5I0J9,Q9JJ50
520	1,2,3,5,6,7,27,31	4,8,25,26,28,29,30,32	7	O54728,P01039,P20766,P50116,P52590,Q03191,Q62714
521	1,2,3,5,6,7,27,32	4,8,25,26,28,29,30,31	7	P01039,P50116,P52590,Q03191,Q62714,Q6AY61,Q9JJ50
522	1,2,3,5,6,7,28,29	4,8,25,26,27,30,31,32	8	P00762,P01835,P21674,P47967,P52590,P54921,Q6AY61,Q9QYP1
523	1,2,3,5,6,7,28,30	4,8,25,26,27,29,31,32	5	P00762,P19218,P47967,P52590,Q09030
524	1,2,3,5,6,7,28,31	4,8,25,26,27,29,30,32	4	P00762,P20766,P52590,Q09030
525	1,2,3,5,6,7,28,32	4,8,25,26,27,29,30,31	3	P47967,P52590,Q6AY61
526	1,2,3,5,6,7,29,30	4,8,25,26,27,28,31,32	8	O54728,P00762,P01835,P26342,P36374,P52590,Q9QWN8,Q9WUW8
527	1,2,3,5,6,7,29,31	4,8,25,26,27,28,30,32	8	O54728,O70594,P00762,P01835,P20766,P52590,Q62714,Q9QWN8
528	1,2,3,5,6,7,29,32	4,8,25,26,27,28,30,31	4	P01835,P26342,P52590,Q6AY61
529	1,2,3,5,6,7,30,31	4,8,25,26,27,28,29,32	16	O54728,P00762,P0C0A9,P20646,P20766,P26342,P30120,P36374,P52590,Q09030,Q62714,Q6AYR9,Q6P6R2,Q812E4,Q8CFN2,Q9QWN8
530	1,2,3,5,6,7,30,32	4,8,25,26,27,28,29,31	10	O54800;Q5DWV2,P01835,P20646,P26342,P36374,P52590,Q09030,Q5I0J9,Q6P6R2,Q99MH3

531	1,2,3,5,6,7,31,32	4,8,25,26,27,28,29,30	7	P20646,P20766,P26342,P52590,Q09030,Q62714,Q6P6R2
532	1,2,3,5,6,8,25,26	4,7,27,28,29,30,31,32	9	O54800;Q5DWV2,P08649,P36374,P52590,P80299,P97580,Q06000,Q09030,Q99MH3
533	1,2,3,5,6,8,25,27	4,7,26,28,29,30,31,32	3	P52590,P97580,Q09030
534	1,2,3,5,6,8,25,28	4,7,26,27,29,30,31,32	3	P36376,P52590,Q09030
535	1,2,3,5,6,8,25,29	4,7,26,27,28,30,31,32	3	P22006,P52590,Q09030
536	1,2,3,5,6,8,25,30	4,7,26,27,28,29,31,32	11	O54800;Q5DWV2,O88797,P20646,P36374,P52590,Q09030,Q6IFU7,Q6IFW6,Q6IG05,Q6IMF3,Q99MH3
537	1,2,3,5,6,8,25,31	4,7,26,27,28,29,30,32	4	P36376,P52590,Q09030,Q63532
538	1,2,3,5,6,8,25,32	4,7,26,27,28,29,30,31	7	O54800;Q5DWV2,P01681,P20646,P52590,P80202,Q09030,Q99MH3
539	1,2,3,5,6,8,26,27	4,7,25,28,29,30,31,32	6	P15399,P52590,P97580,Q30KJ2,Q99MH3,Q9JJ50
540	1,2,3,5,6,8,26,28	4,7,25,27,29,30,31,32	3	P52590,Q99MH3,Q9QYP1
541	1,2,3,5,6,8,26,29	4,7,25,27,28,30,31,32	4	P52590,P97580,Q99MH3,Q9QYP1
542	1,2,3,5,6,8,26,30	4,7,25,27,28,29,31,32	11	O88797,P36374,P52590,P97580,Q09030,Q5I0J9,Q5RJR2,Q6IFU7,Q6IG05,Q8CGS4,Q99MH3
543	1,2,3,5,6,8,26,31	4,7,25,27,28,29,30,32	9	P10247,P36376,P52590,Q09030,Q5RJR2,Q62714,Q63532,Q99MH3,Q9Z2L0

544	1,2,3,5,6,8,26,32	4,7,25,27,28,29,30,31	4	O54800;Q5DWV2,P52590,Q62714,Q99MH3
545	1,2,3,5,6,8,27,28	4,7,25,26,29,30,31,32	4	P19218,P36376,P52590,P97580
546	1,2,3,5,6,8,27,29	4,7,25,26,28,30,31,32	5	P00731,P52590,P97580,Q5I0J9,Q9JJ50
547	1,2,3,5,6,8,27,30	4,7,25,26,28,29,31,32	14	P00731,P19218,P20646,P52590,P97580,Q09030,Q10758,Q30KJ2,Q5I0J9,Q6IFU7,Q6IFW6,Q6IG05,Q6IMF3,Q6P6Q2
548	1,2,3,5,6,8,27,31	4,7,25,26,28,29,30,32	8	P00731,P36376,P52590,P97580,Q09030,Q62714,Q63532,Q9R168
549	1,2,3,5,6,8,27,32	4,7,25,26,28,29,30,31	6	P20646,P52590,P97580,Q5I0J9,Q62714,Q9JJ50
550	1,2,3,5,6,8,28,29	4,7,25,26,27,30,31,32	4	P00762,P21674,P52590,Q9QYP1
551	1,2,3,5,6,8,28,30	4,7,25,26,27,29,31,32	7	P19218,P52590,Q09030,Q5I0J9,Q6IFU7,Q6IG05,Q99MH3
552	1,2,3,5,6,8,28,31	4,7,25,26,27,29,30,32	4	P36376,P52590,Q09030,Q63532
553	1,2,3,5,6,8,28,32	4,7,25,26,27,29,30,31	4	P52590,Q09030,Q5I0J9,Q99MH3
554	1,2,3,5,6,8,29,30	4,7,25,26,27,28,31,32	12	O88797,P19218,P36374,P52590,Q09030,Q5I0J9,Q5RJR2,Q6IFU7,Q6IFW6,Q6IG05,Q6IMF3,Q6P6Q2
555	1,2,3,5,6,8,29,31	4,7,25,26,27,28,30,32	5	P36376,P52590,Q09030,Q5RJR2,Q63532
556	1,2,3,5,6,8,29,32	4,7,25,26,27,28,30,31	2	P52590,Q5I0J9

557	1,2,3,5,6,8,30,31	4,7,25,26,27,28,29,32	16	P0C0A9,P20646,P36374,P36376,P52590,Q09030,Q5I0J9,Q5RJR2,Q63532,Q6AYR8,Q6IFU7,Q6IG05,Q6IMF3,Q8CGS4,Q99MH3,Q9Z2L0
558	1,2,3,5,6,8,30,32	4,7,25,26,27,28,29,31	14	O88797,O89117,P19218,P20646,P36374,P52590,P97615,Q09030,Q5I0J9,Q6IFU7,Q6IG05,Q6IMF3,Q6P6R2,Q99MH3
559	1,2,3,5,6,8,31,32	4,7,25,26,27,28,29,30	8	O89117,P20646,P36376,P52590,Q09030,Q62714,Q63532,Q99MH3
560	1,2,3,5,6,25,26,27	4,7,8,28,29,30,31,32	3	P82471,Q06000,Q9JJ50
561	1,2,3,5,6,25,26,28	4,7,8,27,29,30,31,32	4	P00714,P82471,Q06000,Q99MH3
562	1,2,3,5,6,25,26,29	4,7,8,27,28,30,31,32	3	O54800;Q5DWV2,P52590,P82471
563	1,2,3,5,6,25,26,30	4,7,8,27,28,29,31,32	14	O54800;Q5DWV2,P08723,P22282,P36374,P47727,P82471,P97523,Q06000,Q09030,Q4G075,Q6AYR9,Q812E4,Q99MH3,Q9Z2L0
564	1,2,3,5,6,25,26,31	4,7,8,27,28,29,30,32	13	O54800;Q5DWV2,P00714,P02780,P08723,P18298,P22282,Q09030,Q4G075,Q63150,Q6AYR9,Q812E4,Q99MH3,Q9Z2L0
565	1,2,3,5,6,25,26,32	4,7,8,27,28,29,30,31	5	O54800;Q5DWV2,P82471,Q06000,Q4G075,Q99MH3
566	1,2,3,5,6,25,27,28	4,7,8,26,29,30,31,32	1	P82471
567	1,2,3,5,6,25,27,29	4,7,8,26,28,30,31,32	4	O54728,P52590,P82471,Q9JJ50
568	1,2,3,5,6,25,27,30	4,7,8,26,28,29,31,32	7	O54728,P30120,P82471,Q09030,Q5I0J9,Q6P6R2,Q9QX74
569	1,2,3,5,6,25,27,31	4,7,8,26,28,29,30,32	3	O54728,Q03191,Q09030
570	1,2,3,5,6,25,27,32	4,7,8,26,28,29,30,31	6	O70257,P20646,P82471,Q03191,Q6P6R2,Q9QX74
571	1,2,3,5,6,25,28,29	4,7,8,26,27,30,31,32	2	P52590,P82471
572	1,2,3,5,6,25,28,30	4,7,8,26,27,29,31,32	6	P52590,P82471,Q09030,Q4G075,Q62975,Q9QX74
573	1,2,3,5,6,25,28,31	4,7,8,26,27,29,30,32	3	P00714,Q09030,Q4G075
574	1,2,3,5,6,25,28,32	4,7,8,26,27,29,30,31	6	P52590,P82471,Q09030,Q4G075,Q62975,Q9QX74
575	1,2,3,5,6,25,29,30	4,7,8,26,27,28,31,32	9	O54728,P07150,P22282,P36374,P52590,P82471,P97523,Q09030,Q9QWN8

576	1,2,3,5,6,25,29,31	4,7,8,26,27,28,30,32	7	O54728,P07150,P22282,P52590,Q09030,Q6AYR9,Q9QWN8
577	1,2,3,5,6,25,29,32	4,7,8,26,27,28,30,31	5	O54800;Q5DWV2,P07150,P52590,P82471,Q9WTT6
578	1,2,3,5,6,25,30,31	4,7,8,26,27,28,29,32	20	O54728,P07150,P08723,P20646,P22273,P22282,P30120,P36374,P47727,P97584,Q09030,Q4G075,Q6AYR9,Q6P6R2,Q812E4,Q8CFN2,Q9QWN8,Q9QX74,Q9WTT6,Q9Z2L0
579	1,2,3,5,6,25,30,32	4,7,8,26,27,28,29,31	15	O54800;Q5DWV2,P07150,P20646,P22273,P26342,P36374,P82471,P97523,Q09030,Q4G075,Q5I0J9,Q6P6R2,Q99MH3,Q9QX74,Q9WTT6
580	1,2,3,5,6,25,31,32	4,7,8,26,27,28,29,30	10	O54800;Q5DWV2,P07150,P20646,P22273,P97584,Q09030,Q4G075,Q6P6R2,Q9QX74,Q9WTT6
581	1,2,3,5,6,26,27,28	4,7,8,25,29,30,31,32	4	P21588,P52590,P82471,Q9JJ50
582	1,2,3,5,6,26,27,29	4,7,8,25,28,30,31,32	4	P21588,P52590,P82471,Q9JJ50
583	1,2,3,5,6,26,27,30	4,7,8,25,28,29,31,32	7	P01039,P21588,P30120,P82471,Q5I0J9,Q99MH3,Q9JJ50
584	1,2,3,5,6,26,27,31	4,7,8,25,28,29,30,32	4	P01039,Q62714,Q99MH3,Q9Z2L0
585	1,2,3,5,6,26,27,32	4,7,8,25,28,29,30,31	6	P01039,P52590,P82471,Q5I0J9,Q99MH3,Q9JJ50
586	1,2,3,5,6,26,28,29	4,7,8,25,27,30,31,32	5	P00762,P47967,P52590,P82471,Q9QYP1
587	1,2,3,5,6,26,28,30	4,7,8,25,27,29,31,32	8	P00714,P00762,P21588,P52590,P82471,Q4G075,Q5I0J9,Q99MH3
588	1,2,3,5,6,26,28,31	4,7,8,25,27,29,30,32	5	P00714,P00762,P52590,Q4G075,Q99MH3
589	1,2,3,5,6,26,28,32	4,7,8,25,27,29,30,31	4	P52590,P82471,Q4G075,Q99MH3
590	1,2,3,5,6,26,29,30	4,7,8,25,27,28,31,32	8	P0C0A9,P21588,P22282,P36374,P52590,P82471,Q5I0J9,Q99MH3
591	1,2,3,5,6,26,29,31	4,7,8,25,27,28,30,32	5	P0C0A9,P22282,P52590,Q6AYR9,Q99MH3
592	1,2,3,5,6,26,29,32	4,7,8,25,27,28,30,31	3	P52590,P82471,Q99MH3
593	1,2,3,5,6,26,30,31	4,7,8,25,27,28,29,32	12	P08723,P0C0A9,P20646,P22282,P30120,P36374,P47727,Q4G075,Q6AYR9,Q812E4,Q99MH3,Q9Z2L0
594	1,2,3,5,6,26,30,32	4,7,8,25,27,28,29,31	11	O54800;Q5DWV2,P0C0A9,P20646,P36374,P52590,P82471,Q4G075,Q5I0J9,Q812E4,Q99MH3,Q9WTT6
595	1,2,3,5,6,26,31,32	4,7,8,25,27,28,29,30	8	P0C0A9,P20646,P52590,Q4G075,Q62714,Q812E4,Q99MH3,Q9Z2L0

596	1,2,3,5,6,27,28,29	4,7,8,25,26,30,31,32	4	P47967,P52590,P82471,Q6AY61
597	1,2,3,5,6,27,28,30	4,7,8,25,26,29,31,32	6	P19218,P21588,P52590,P82471,Q5I0J9,Q9QX74
598	1,2,3,5,6,27,28,31	4,7,8,25,26,29,30,32	1	P52590
599	1,2,3,5,6,27,28,32	4,7,8,25,26,29,30,31	6	P52590,P82471,Q5I0J9,Q62761;Q62762;Q62763,Q6AY61,Q9QX74
600	1,2,3,5,6,27,29,30	4,7,8,25,26,28,31,32	7	O54728,P00731,P19218,P52590,P82471,Q5I0J9,Q9JJ50
601	1,2,3,5,6,27,29,31	4,7,8,25,26,28,30,32	2	O54728,P52590
602	1,2,3,5,6,27,29,32	4,7,8,25,26,28,30,31	5	P52590,P82471,Q5I0J9,Q6AY61,Q9JJ50
603	1,2,3,5,6,27,30,31	4,7,8,25,26,28,29,32	8	O54728,P01039,P20646,P30120,Q5I0J9,Q6P6R2,Q9QX74,Q9WTT6
604	1,2,3,5,6,27,30,32	4,7,8,25,26,28,29,31	9	P01039,P20646,P52590,P82471,Q03191,Q5I0J9,Q6P6R2,Q9QX74,Q9WTT6
605	1,2,3,5,6,27,31,32	4,7,8,25,26,28,29,30	9	P01039,P20646,P52590,Q03191,Q5I0J9,Q62714,Q6P6R2,Q9QX74,Q9WTT6
606	1,2,3,5,6,28,29,30	4,7,8,25,26,27,31,32	4	P00762,P52590,P82471,Q5I0J9
607	1,2,3,5,6,28,29,31	4,7,8,25,26,27,30,32	2	P00762,P52590
608	1,2,3,5,6,28,29,32	4,7,8,25,26,27,30,31	4	P52590,P82471,Q5I0J9,Q6AY61
609	1,2,3,5,6,28,30,31	4,7,8,25,26,27,29,32	7	P00762,P52590,Q09030,Q4G075,Q5I0J9,Q9QX74,Q9WTT6
610	1,2,3,5,6,28,30,32	4,7,8,25,26,27,29,31	9	P20646,P52590,P82471,Q4G075,Q5I0J9,Q99MH3,Q9QX74,Q9WTT6,Q9Z0V6
611	1,2,3,5,6,28,31,32	4,7,8,25,26,27,29,30	5	P52590,Q09030,Q4G075,Q9QX74,Q9WTT6
612	1,2,3,5,6,29,30,31	4,7,8,25,26,27,28,32	13	O54728,P07150,P0C0A9,P20646,P22282,P26342,P36860,P52590,Q5I0J9,Q5RJR2,Q6AYR9,Q9QWN8,Q9WTT6
613	1,2,3,5,6,29,30,32	4,7,8,25,26,27,28,31	10	P01835,P07150,P20646,P26342,P52590,P82471,Q4G075,Q5I0J9,Q9WTT6,Q9Z0V6
614	1,2,3,5,6,29,31,32	4,7,8,25,26,27,28,30	6	P07150,P20646,P26342,P52590,Q4G075,Q9WTT6
615	1,2,3,5,6,30,31,32	4,7,8,25,26,27,28,29	20	P06760,P07150,P0C0A9,P20646,P22273,P22282,P26342,P36860,P47727,P52590,P97584,Q09030,Q4G075,Q5I0J9,Q6P6R2,Q812E4,Q99MH3,Q9QX74,Q9WTT6,Q9Z2L0

616	1,2,3,5,7,8,25,26	4,6,27,28,29,30,31,32	2	P22006,P36374
617	1,2,3,5,7,8,25,27	4,6,26,28,29,30,31,32	4	P00731,P22006,Q03191,Q66H69
618	1,2,3,5,7,8,25,28	4,6,26,27,29,30,31,32	3	P22006,P36860,Q811M5
619	1,2,3,5,7,8,25,29	4,6,26,27,28,30,31,32	2	P22006,Q8VD89
620	1,2,3,5,7,8,25,30	4,6,26,27,28,29,31,32	10	P22006,P36374,Q05511,Q4FZU2,Q6IFU7,Q6IFU8,Q6IFW6,Q6IG05,Q6IMF3,Q6P6Q2
621	1,2,3,5,7,8,25,31	4,6,26,27,28,29,30,32	3	P20766,P36376,Q811M5
622	1,2,3,5,7,8,25,32	4,6,26,27,28,29,30,31	2	P01681,Q811M5
623	1,2,3,5,7,8,26,27	4,6,25,28,29,30,31,32	6	P00731,P22006,P47967,Q5RLM2,Q66H69,Q9JJ50
624	1,2,3,5,7,8,26,28	4,6,25,27,29,30,31,32	3	D3ZUC6,P47967,Q5RLM2
625	1,2,3,5,7,8,26,29	4,6,25,27,28,30,31,32	6	O70594,P06760,P22006,P47967,Q9QX74,Q9WUW8
626	1,2,3,5,7,8,26,30	4,6,25,27,28,29,31,32	6	P36374,Q6IFU7,Q6IG05,Q6IMF3,Q99MH3,Q9WUW8
627	1,2,3,5,7,8,26,31	4,6,25,27,28,29,30,32	3	P20766,Q5RLM2,Q62714
628	1,2,3,5,7,8,26,32	4,6,25,27,28,29,30,31	2	Q62714,Q99MH3

629	1,2,3,5,7,8,27,28	4,6,25,26,29,30,31,32	7	D3ZUC6,P00731,P19218,P47967,Q5I0D1,Q5RLM2,Q66H69
630	1,2,3,5,7,8,27,29	4,6,25,26,28,30,31,32	11	P00731,P06760,P22006,P25809,P47967,P62804,Q00715,Q4G075,Q5I0D1,Q66H69,Q9JJ50
631	1,2,3,5,7,8,27,30	4,6,25,26,28,29,31,32	9	P00731,P19218,Q66H69,Q6IFU7,Q6IFU8,Q6IFW6,Q6IG05,Q6IMF3,Q6P6Q2
632	1,2,3,5,7,8,27,31	4,6,25,26,28,29,30,32	5	P00731,P20766,P36376,Q62714,Q66H69
633	1,2,3,5,7,8,27,32	4,6,25,26,28,29,30,31	3	Q03191,Q62714,Q66H69
634	1,2,3,5,7,8,28,29	4,6,25,26,27,30,31,32	9	D3ZUC6,P21674,P22006,P47967,P54921,P62804,Q00715,Q9QYP1,Q9WUW8
635	1,2,3,5,7,8,28,30	4,6,25,26,27,29,31,32	6	P19218,P47967,Q6IFU7,Q6IG05,Q6IMF3,Q9WUW8
636	1,2,3,5,7,8,28,31	4,6,25,26,27,29,30,32	5	D3ZUC6,P20766,P36376,Q5RLM2,Q811M5
637	1,2,3,5,7,8,28,32	4,6,25,26,27,29,30,31	2	P47967,Q811M5
638	1,2,3,5,7,8,29,30	4,6,25,26,27,28,31,32	12	P22006,P36374,P47967,P62804,Q6IFU7,Q6IFU8,Q6IFW6,Q6IG02,Q6IG05,Q6IMF3,Q6P6Q2,Q9WUW8
639	1,2,3,5,7,8,29,31	4,6,25,26,27,28,30,32	2	O70594,P20766
640	1,2,3,5,7,8,29,32	4,6,25,26,27,28,30,31	1	Q9WUW8
641	1,2,3,5,7,8,30,31	4,6,25,26,27,28,29,32	8	P20766,Q5RJR2,Q6AYR8,Q6IFU7,Q6IFW6,Q6IG05,Q6IMF3,Q6P6Q2

642	1,2,3,5,7,8,30,32	4,6,25,26,27,28,29,31	8 P26342,P35280,P97615,Q6IFU7,Q6IG05,Q6IMF3,Q6P6Q2,Q9WUW8
643	1,2,3,5,7,8,31,32	4,6,25,26,27,28,29,30	3 P20766,Q62714,Q811M5
644	1,2,3,5,7,25,26,27	4,6,8,28,29,30,31,32	3 P22006,Q03191,Q9JJ50
645	1,2,3,5,7,25,26,28	4,6,8,27,29,30,31,32	1 P00714
646	1,2,3,5,7,25,26,29	4,6,8,27,28,30,31,32	2 P22006,Q8VD89
647	1,2,3,5,7,25,26,30	4,6,8,27,28,29,31,32	6 P00714,P22006,P22282,P36374,Q6AYR9,Q812E4
648	1,2,3,5,7,25,26,31	4,6,8,27,28,29,30,32	7 P00714,P18298,P20766,P22282,Q6AYR9,Q812E4,Q8CFN2
649	1,2,3,5,7,25,26,32	4,6,8,27,28,29,30,31	0
650	1,2,3,5,7,25,27,28	4,6,8,26,29,30,31,32	2 Q03191,Q6AY61
651	1,2,3,5,7,25,27,29	4,6,8,26,28,30,31,32	5 O54728,P22006,Q03191,Q6AY61,Q8VD89
652	1,2,3,5,7,25,27,30	4,6,8,26,28,29,31,32	4 O54728,P22006,Q03191,Q05511
653	1,2,3,5,7,25,27,31	4,6,8,26,28,29,30,32	3 O54728,P20766,Q03191
654	1,2,3,5,7,25,27,32	4,6,8,26,28,29,30,31	3 O70257,Q03191,Q6AY61
655	1,2,3,5,7,25,28,29	4,6,8,26,27,30,31,32	4 P22006,P47967,P54921,Q6AY61
656	1,2,3,5,7,25,28,30	4,6,8,26,27,29,31,32	2 P00714,Q62975
657	1,2,3,5,7,25,28,31	4,6,8,26,27,29,30,32	2 P00714,Q811M5
658	1,2,3,5,7,25,28,32	4,6,8,26,27,29,30,31	3 Q62975,Q6AY61,Q811M5
659	1,2,3,5,7,25,29,30	4,6,8,26,27,28,31,32	5 O54728,P22006,P22282,P36374,Q9QWN8
660	1,2,3,5,7,25,29,31	4,6,8,26,27,28,30,32	5 O54728,P22282,Q6AYR9,Q8VD89,Q9QWN8

661	1,2,3,5,7,25,29,32	4,6,8,26,27,28,30,31	1 Q6AY61
662	1,2,3,5,7,25,30,31	4,6,8,26,27,28,29,32	7 O54728,P22282,Q6AYR9,Q6P6R2,Q812E4,Q8CFN2,Q9QWN8
663	1,2,3,5,7,25,30,32	4,6,8,26,27,28,29,31	5 P26342,P97523,Q62975,Q6P6R2,Q9R168
664	1,2,3,5,7,25,31,32	4,6,8,26,27,28,29,30	3 P20766,Q6P6R2,Q811M5
665	1,2,3,5,7,26,27,28	4,6,8,25,29,30,31,32	3 P47967,P97840,Q5RLM2
666	1,2,3,5,7,26,27,29	4,6,8,25,28,30,31,32	3 P22006,P47967,Q9JJ50
667	1,2,3,5,7,26,27,30	4,6,8,25,28,29,31,32	2 P21588,Q9JJ50
668	1,2,3,5,7,26,27,31	4,6,8,25,28,29,30,32	3 P20766,Q5RLM2,Q62714
669	1,2,3,5,7,26,27,32	4,6,8,25,28,29,30,31	2 Q03191,Q9JJ50
670	1,2,3,5,7,26,28,29	4,6,8,25,27,30,31,32	5 P00762,P47967,P54921,P97840,Q9QYP1
671	1,2,3,5,7,26,28,30	4,6,8,25,27,29,31,32	5 P00714,P00762,P47967,Q5RLM2,Q9WUW8
672	1,2,3,5,7,26,28,31	4,6,8,25,27,29,30,32	3 P00714,P00762,Q5RLM2
673	1,2,3,5,7,26,28,32	4,6,8,25,27,29,30,31	1 P47967
674	1,2,3,5,7,26,29,30	4,6,8,25,27,28,31,32	5 P22006,P22282,P36374,P47967,Q9WUW8
675	1,2,3,5,7,26,29,31	4,6,8,25,27,28,30,32	2 P20766,Q6AYR9
676	1,2,3,5,7,26,29,32	4,6,8,25,27,28,30,31	1 P47967
677	1,2,3,5,7,26,30,31	4,6,8,25,27,28,29,32	9 P0C0A9,P20766,P22282,P36374,P47727,Q6AYR9,Q812E4,Q8CFN2,Q9Z2L0
678	1,2,3,5,7,26,30,32	4,6,8,25,27,28,29,31	3 P36374,Q99MH3,Q9WUW8
679	1,2,3,5,7,26,31,32	4,6,8,25,27,28,29,30	2 P20766,Q62714
680	1,2,3,5,7,27,28,29	4,6,8,25,26,30,31,32	5 P00507,P47967,P54921,P97840,Q6AY61

681	1,2,3,5,7,27,28,30	4,6,8,25,26,29,31,32	2 P19218,P47967
682	1,2,3,5,7,27,28,31	4,6,8,25,26,29,30,32	2 P47967,Q6AY61
683	1,2,3,5,7,27,28,32	4,6,8,25,26,29,30,31	4 P47967,Q03191,Q62761;Q62762;Q62763,Q6AY61
684	1,2,3,5,7,27,29,30	4,6,8,25,26,28,31,32	4 O54728,P00731,P22006,P47967
685	1,2,3,5,7,27,29,31	4,6,8,25,26,28,30,32	3 O54728,P08649,Q6AY61
686	1,2,3,5,7,27,29,32	4,6,8,25,26,28,30,31	4 P47967,Q03191,Q6AY61,Q9JJ50
687	1,2,3,5,7,27,30,31	4,6,8,25,26,28,29,32	5 O54728,P00731,P08649,P20766,P30120
688	1,2,3,5,7,27,30,32	4,6,8,25,26,28,29,31	3 P70549,Q03191,Q6P6R2
689	1,2,3,5,7,27,31,32	4,6,8,25,26,28,29,30	4 P20766,Q03191,Q62714,Q6AY61
690	1,2,3,5,7,28,29,30	4,6,8,25,26,27,31,32	5 P00762,P47967,P54921,P97840,Q9WUW8
691	1,2,3,5,7,28,29,31	4,6,8,25,26,27,30,32	4 P00762,P47967,P54921,Q6AY61
692	1,2,3,5,7,28,29,32	4,6,8,25,26,27,30,31	3 P47967,P54921,Q6AY61
693	1,2,3,5,7,28,30,31	4,6,8,25,26,27,29,32	0
694	1,2,3,5,7,28,30,32	4,6,8,25,26,27,29,31	0
695	1,2,3,5,7,28,31,32	4,6,8,25,26,27,29,30	2 Q6AY61,Q811M5
696	1,2,3,5,7,29,30,31	4,6,8,25,26,27,28,32	6 O54728,P08649,P22282,P26342,Q6AYR9,Q9QWN8
697	1,2,3,5,7,29,30,32	4,6,8,25,26,27,28,31	3 P01835,P26342,Q9WUW8
698	1,2,3,5,7,29,31,32	4,6,8,25,26,27,28,30	3 P08649,P26342,Q6AY61
699	1,2,3,5,7,30,31,32	4,6,8,25,26,27,28,29	5 P08649,P20646,P22273,P26342,Q6P6R2
700	1,2,3,5,8,25,26,27	4,6,7,28,29,30,31,32	3 P00714,P22006,P97580

701	1,2,3,5,8,25,26,28	4,6,7,27,29,30,31,32	3	P00714,P22006,P80299
702	1,2,3,5,8,25,26,29	4,6,7,27,28,30,31,32	3	P00714,P22006,Q8VD89
703	1,2,3,5,8,25,26,30	4,6,7,27,28,29,31,32	14	O88797,P00714,P11598,P22006,P22283,P36374,P80299,Q05511,Q63617,Q6IFU7,Q6IFW6,Q6IG05,Q6IMF3,Q99MH3
704	1,2,3,5,8,25,26,31	4,6,7,27,28,29,30,32	3	P00714,P36376,Q63617
705	1,2,3,5,8,25,26,32	4,6,7,27,28,29,30,31	3	P01681,Q63617,Q99MH3
706	1,2,3,5,8,25,27,28	4,6,7,26,29,30,31,32	3	P00714,P36376,P36860
707	1,2,3,5,8,25,27,29	4,6,7,26,28,30,31,32	2	P00731,P22006
708	1,2,3,5,8,25,27,30	4,6,7,26,28,29,31,32	12	P00731,P22006,Q05511,Q4FZU2,Q63617,Q6IFU7,Q6IFU8,Q6IFW6,Q6IG02,Q6IG05,Q6IMF3,Q6P6Q2
709	1,2,3,5,8,25,27,31	4,6,7,26,28,29,30,32	3	P23739,P36376,Q63617
710	1,2,3,5,8,25,27,32	4,6,7,26,28,29,30,31	1	P01681
711	1,2,3,5,8,25,28,29	4,6,7,26,27,30,31,32	2	P00714,P22006
712	1,2,3,5,8,25,28,30	4,6,7,26,27,29,31,32	7	O88797,P00714,P36376,Q6IFU7,Q6IFW6,Q6IG05,Q6IMF3
713	1,2,3,5,8,25,28,31	4,6,7,26,27,29,30,32	3	P00714,P36376,Q811M5
714	1,2,3,5,8,25,28,32	4,6,7,26,27,29,30,31	2	P01681,Q811M5
715	1,2,3,5,8,25,29,30	4,6,7,26,27,28,31,32	10	O88797,P11598,P22006,Q6IFU7,Q6IFU8,Q6IFW6,Q6IG02,Q6IG05,Q6IMF3,Q6P6Q2
716	1,2,3,5,8,25,29,31	4,6,7,26,27,28,30,32	2	P36376,Q8VD89
717	1,2,3,5,8,25,29,32	4,6,7,26,27,28,30,31	1	P01681
718	1,2,3,5,8,25,30,31	4,6,7,26,27,28,29,32	11	O88797,P00714,P11598,P22283,P36376,Q05511,Q63617,Q6IFU7,Q6IFW6,Q6IG05,Q6IMF3
719	1,2,3,5,8,25,30,32	4,6,7,26,27,28,29,31	10	O88797,P01681,P20646,Q05511,Q63617,Q6IFU7,Q6IFW6,Q6IG05,Q6IMF3,Q6P6R2
720	1,2,3,5,8,25,31,32	4,6,7,26,27,28,29,30	7	P01681,P20646,P21588,P36376,Q63617,Q80WL1,Q811M5

721	1,2,3,5,8,26,27,28	4,6,7,25,29,30,31,32	3	P00714,P47967,Q5RLM2
722	1,2,3,5,8,26,27,29	4,6,7,25,28,30,31,32	4	P00731,P22006,P25809,Q9JJ50
723	1,2,3,5,8,26,27,30	4,6,7,25,28,29,31,32	9	P00731,P97580,Q63617,Q6IFU7,Q6IFU8,Q6IFW6,Q6IG05,Q6IMF3,Q6P6Q2
724	1,2,3,5,8,26,27,31	4,6,7,25,28,29,30,32	1	P36376
725	1,2,3,5,8,26,27,32	4,6,7,25,28,29,30,31	1	Q9JJ50
726	1,2,3,5,8,26,28,29	4,6,7,25,27,30,31,32	4	P00714,P22006,P47967,Q9QYP1
727	1,2,3,5,8,26,28,30	4,6,7,25,27,29,31,32	4	P00714,Q6IFU7,Q6IG05,Q99MH3
728	1,2,3,5,8,26,28,31	4,6,7,25,27,29,30,32	3	P00714,P36376,Q5RLM2
729	1,2,3,5,8,26,28,32	4,6,7,25,27,29,30,31	1	Q99MH3
730	1,2,3,5,8,26,29,30	4,6,7,25,27,28,31,32	7	O88797,P22006,P36374,Q6IFU7,Q6IFW6,Q6IG05,Q6IMF3
731	1,2,3,5,8,26,29,31	4,6,7,25,27,28,30,32	0	
732	1,2,3,5,8,26,29,32	4,6,7,25,27,28,30,31	0	
733	1,2,3,5,8,26,30,31	4,6,7,25,27,28,29,32	12	P00714,P0C0A9,P22283,Q63617,Q6AYR8,Q6AYR9,Q6IG05,Q6IMF3,Q812E4,Q8CGS4,Q99MH3,Q9Z2L0
734	1,2,3,5,8,26,30,32	4,6,7,25,27,28,29,31	5	O88797,Q63617,Q6IFU7,Q6IG05,Q99MH3
735	1,2,3,5,8,26,31,32	4,6,7,25,27,28,29,30	2	Q63617,Q99MH3
736	1,2,3,5,8,27,28,29	4,6,7,25,26,30,31,32	4	P00731,P25809,P47967,Q9WVK7
737	1,2,3,5,8,27,28,30	4,6,7,25,26,29,31,32	8	P00731,P19218,Q6AYR8,Q6IFU7,Q6IFW6,Q6IG05,Q6IMF3,Q6P6Q2
738	1,2,3,5,8,27,28,31	4,6,7,25,26,29,30,32	1	P36376
739	1,2,3,5,8,27,28,32	4,6,7,25,26,29,30,31	0	
740	1,2,3,5,8,27,29,30	4,6,7,25,26,28,31,32	10	P00731,P19218,P22006,Q6IFU7,Q6IFU8,Q6IFW6,Q6IG02,Q6IG05,Q6IMF3,Q6P6Q2

741	1,2,3,5,8,27,29,31	4,6,7,25,26,28,30,32	3	P00731,P36376,Q5PQL7
742	1,2,3,5,8,27,29,32	4,6,7,25,26,28,30,31	0	
743	1,2,3,5,8,27,30,31	4,6,7,25,26,28,29,32	11	P00731,P23739,P36376,Q63617,Q6AYR8,Q6IFU7,Q6IFU8,Q6IFW6,Q6IG05,Q6IMF3,Q6P6Q2
744	1,2,3,5,8,27,30,32	4,6,7,25,26,28,29,31	8	P00731,P20646,P70549,Q6IFU7,Q6IFW6,Q6IG05,Q6IMF3,Q6P6Q2
745	1,2,3,5,8,27,31,32	4,6,7,25,26,28,29,30	2	P23739,P36376
746	1,2,3,5,8,28,29,30	4,6,7,25,26,27,31,32	6	O88797,Q6IFU7,Q6IFW6,Q6IG05,Q6IMF3,Q6P6Q2
747	1,2,3,5,8,28,29,31	4,6,7,25,26,27,30,32	1	P36376
748	1,2,3,5,8,28,29,32	4,6,7,25,26,27,30,31	1	Q9QYP1
749	1,2,3,5,8,28,30,31	4,6,7,25,26,27,29,32	6	P00714,P36376,Q6AYR8,Q6IFU7,Q6IG05,Q6IMF3
750	1,2,3,5,8,28,30,32	4,6,7,25,26,27,29,31	4	O88797,Q6IFU7,Q6IG05,Q6IMF3
751	1,2,3,5,8,28,31,32	4,6,7,25,26,27,29,30	2	P36376,Q811M5
752	1,2,3,5,8,29,30,31	4,6,7,25,26,27,28,32	10	O88797,P0C0A9,Q5RJR2,Q6IFU7,Q6IFU8,Q6IFW6,Q6IG02,Q6IG05,Q6IMF3,Q6P6Q2
753	1,2,3,5,8,29,30,32	4,6,7,25,26,27,28,31	6	O88797,Q6IFU7,Q6IFW6,Q6IG05,Q6IMF3,Q6P6Q2
754	1,2,3,5,8,29,31,32	4,6,7,25,26,27,28,30	0	
755	1,2,3,5,8,30,31,32	4,6,7,25,26,27,28,29	9	O88797,P0C0A9,P20646,P63081,Q63617,Q6IFU7,Q6IG05,Q6IMF3,Q6P6R2
756	1,2,3,5,25,26,27,28	4,6,7,8,29,30,31,32	2	P00714,P82471
757	1,2,3,5,25,26,27,29	4,6,7,8,28,30,31,32	7	P00714,P22006,P82471,Q63617,Q99041,Q9JJ50,Q9QZK9
758	1,2,3,5,25,26,27,30	4,6,7,8,28,29,31,32	10	P00714,P02780,P08723,P11598,P22273,P22282,P30120,P82471,Q63617,Q99041
759	1,2,3,5,25,26,27,31	4,6,7,8,28,29,30,32	7	P00714,P02780,P08723,P22273,P22282,Q63617,Q99041
760	1,2,3,5,25,26,27,32	4,6,7,8,28,29,30,31	5	O70257,P22273,P82471,Q63617,Q99041

761	1,2,3,5,25,<br>26,28,29	4,6,7,8,27,<br>30,31,32	6	P00714,P22006,P22282,P82471,Q99041,Q9QZK9
762	1,2,3,5,25,<br>26,28,30	4,6,7,8,27,<br>29,31,32	6	P00714,P22273,P22282,P82471,Q4G075,Q99041
763	1,2,3,5,25,<br>26,28,31	4,6,7,8,27,<br>29,30,32	5	P00714,P22273,P22282,Q4G075,Q99041
764	1,2,3,5,25,<br>26,28,32	4,6,7,8,27,<br>29,30,31	4	P22273,P82471,Q4G075,Q99041
765	1,2,3,5,25,<br>26,29,30	4,6,7,8,27,<br>28,31,32	13	P00714,P06761,P11598,P22006,P22273,P22282,P22283,P36374,P82471,Q63617,Q6AYR9,Q99041,Q9QZK9
766	1,2,3,5,25,<br>26,29,31	4,6,7,8,27,<br>28,30,32	10	P00714,P11598,P18298,P22273,P22282,Q63617,Q6AYR9,Q6RUV5,Q8VD89,Q99041
767	1,2,3,5,25,<br>26,29,32	4,6,7,8,27,<br>28,30,31	5	P22273,P22282,P82471,Q63617,Q99041
768	1,2,3,5,25,<br>26,30,31	4,6,7,8,27,<br>28,29,32	24	P00714,P02780,P04905,P06761,P08723,P0C0A9,P11598,P18298,P22273,P22282,P22283,P30120,P46462,P47727,Q4G075,Q5M8C6,Q62902,Q63617,Q6AYR9,Q812E4,Q8CFN2,Q99041,Q9QW07,Q9Z2L0
769	1,2,3,5,25,<br>26,30,32	4,6,7,8,27,<br>28,29,31	12	P06761,P11598,P22273,P22282,P22283,P82471,P97523,Q4G075,Q63617,Q812E4,Q99041,Q99MH3
770	1,2,3,5,25,<br>26,31,32	4,6,7,8,27,<br>28,29,30	10	P06761,P11598,P22273,P22282,P22283,Q4G075,Q63617,Q812E4,Q99041,Q9Z2L0
771	1,2,3,5,25,<br>27,28,29	4,6,7,8,26,<br>30,31,32	5	P00714,P22006,P82471,Q6AY61,Q99041
772	1,2,3,5,25,<br>27,28,30	4,6,7,8,26,<br>29,31,32	3	P00714,P82471,Q9QX74
773	1,2,3,5,25,<br>27,28,31	4,6,7,8,26,<br>29,30,32	2	P00714,Q99041
774	1,2,3,5,25,<br>27,28,32	4,6,7,8,26,<br>29,30,31	6	O70257,P82471,Q62761;Q62762;Q62763,Q6AY61,Q99041,Q9QX74
775	1,2,3,5,25,<br>27,29,30	4,6,7,8,26,<br>28,31,32	10	O54728,P11598,P22006,P22273,P22282,P82471,Q63617,Q6IFW6,Q6IG02,Q99041
776	1,2,3,5,25,<br>27,29,31	4,6,7,8,26,<br>28,30,32	5	O54728,P22273,P22282,Q63617,Q99041
777	1,2,3,5,25,<br>27,29,32	4,6,7,8,26,<br>28,30,31	4	P22273,P82471,Q6AY61,Q99041
778	1,2,3,5,25,<br>27,30,31	4,6,7,8,26,<br>28,29,32	13	O54728,P02780,P06761,P08723,P11598,P22273,P22282,P30120,Q63617,Q6P6R2,Q99041,Q9QW07,Q9QX74
779	1,2,3,5,25,<br>27,30,32	4,6,7,8,26,<br>28,29,31	12	P06761,P11598,P21674,P22273,P70549,P82471,Q03191,Q63617,Q6P6R2,Q99041,Q9QX74,Q9WTT6
780	1,2,3,5,25,<br>27,31,32	4,6,7,8,26,<br>28,29,30	8	O70257,P22273,P23739,Q03191,Q63617,Q6P6R2,Q99041,Q9QX74

781	1,2,3,5,25,28,29,30	4,6,7,8,26,27,31,32	6	P00714,P22006,P22273,P22282,P82471,Q99041
782	1,2,3,5,25,28,29,31	4,6,7,8,26,27,30,32	4	P00714,P22273,P22282,Q99041
783	1,2,3,5,25,28,29,32	4,6,7,8,26,27,30,31	3	P22273,Q6AY61,Q99041
784	1,2,3,5,25,28,30,31	4,6,7,8,26,27,29,32	8	P00714,P06761,P11598,P22273,P22282,Q4G075,Q99041,Q9QX74
785	1,2,3,5,25,28,30,32	4,6,7,8,26,27,29,31	8	P22273,P22282,P82471,Q4G075,Q62975,Q99041,Q9QX74,Q9WTT6
786	1,2,3,5,25,28,31,32	4,6,7,8,26,27,29,30	6	P22273,P22282,Q4G075,Q811M5,Q99041,Q9QX74
787	1,2,3,5,25,29,30,31	4,6,7,8,26,27,28,32	13	O54728,P06761,P07150,P08723,P11598,P22273,P22282,P22283,Q63617,Q6AYR9,Q99041,Q9QWN8,Q9WTT6
788	1,2,3,5,25,29,30,32	4,6,7,8,26,27,28,31	10	P06761,P07150,P11598,P22273,P22282,P82471,P97523,Q63617,Q99041,Q9WTT6
789	1,2,3,5,25,29,31,32	4,6,7,8,26,27,28,30	8	P06761,P07150,P11598,P22273,P22282,Q63617,Q99041,Q9WTT6
790	1,2,3,5,25,30,31,32	4,6,7,8,26,27,28,29	17	P06761,P07150,P11598,P20646,P22273,P22282,P22283,P26342,Q4G075,Q63617,Q6P6R2,Q812E4,Q99041,Q9QW07,Q9QX74,Q9WTT6,Q9Z2L0
791	1,2,3,5,26,27,28,29	4,6,7,8,25,30,31,32	3	P00714,P47967,P82471
792	1,2,3,5,26,27,28,30	4,6,7,8,25,29,31,32	3	P00714,P21588,P82471
793	1,2,3,5,26,27,28,31	4,6,7,8,25,29,30,32	1	P00714
794	1,2,3,5,26,27,28,32	4,6,7,8,25,29,30,31	1	P82471
795	1,2,3,5,26,27,29,30	4,6,7,8,25,28,31,32	5	P21588,P22282,P82471,Q63617,Q9JJ50
796	1,2,3,5,26,27,29,31	4,6,7,8,25,28,30,32	3	P22282,Q63617,Q99041
797	1,2,3,5,26,27,29,32	4,6,7,8,25,28,30,31	2	P82471,Q9JJ50
798	1,2,3,5,26,27,30,31	4,6,7,8,25,28,29,32	12	P06761,P08723,P0C0A9,P11598,P22273,P22282,P30120,Q5M8C6,Q63617,Q812E4,Q9QW07,Q9Z2L0
799	1,2,3,5,26,27,30,32	4,6,7,8,25,28,29,31	4	P12020,P22273,P82471,Q63617
800	1,2,3,5,26,27,31,32	4,6,7,8,25,28,29,30	4	P22273,Q63617,Q99041,Q9QW07

801	1,2,3,5,26,<br>28,29,30	4,6,7,8,25,<br>27,31,32	4	P00714,P22273,P22282,P82471
802	1,2,3,5,26,<br>28,29,31	4,6,7,8,25,<br>27,30,32	7	O70417,P00714,P22273,P22282,Q01205,Q99041,Q99376
803	1,2,3,5,26,<br>28,29,32	4,6,7,8,25,<br>27,30,31	2	P22273,P82471
804	1,2,3,5,26,<br>28,30,31	4,6,7,8,25,<br>27,29,32	5	P00714,P0C0A9,P22273,P22282,Q4G075
805	1,2,3,5,26,<br>28,30,32	4,6,7,8,25,<br>27,29,31	5	P22273,P22282,P82471,Q4G075,Q99MH3
806	1,2,3,5,26,<br>28,31,32	4,6,7,8,25,<br>27,29,30	4	P22273,P22282,Q4G075,Q99041
807	1,2,3,5,26,<br>29,30,31	4,6,7,8,25,<br>27,28,32	14	P06761,P08723,P0C0A9,P11598,P22273,P22282,P22283,Q5M8C6,Q63617,Q6AYR9,Q812E4,Q9<br>9041,Q9QW07,Q9QWN8
808	1,2,3,5,26,<br>29,30,32	4,6,7,8,25,<br>27,28,31	8	P06761,P0C0A9,P22273,P22282,P82471,Q4G075,Q63617,Q99041
809	1,2,3,5,26,<br>29,31,32	4,6,7,8,25,<br>27,28,30	8	O70417,P0C0A9,P22273,P22282,Q4G075,Q5QE79,Q63617,Q99041
810	1,2,3,5,26,<br>30,31,32	4,6,7,8,25,<br>27,28,29	16	P06761,P0C0A9,P11598,P20646,P22273,P22282,P22283,P47727,Q4G075,Q63617,Q812E4,Q990<br>41,Q99MH3,Q9QW07,Q9WTT6,Q9Z2L0
811	1,2,3,5,27,<br>28,29,30	4,6,7,8,25,<br>26,31,32	2	P47967,P82471
812	1,2,3,5,27,<br>28,29,31	4,6,7,8,25,<br>26,30,32	2	Q6AY61,Q99041
813	1,2,3,5,27,<br>28,29,32	4,6,7,8,25,<br>26,30,31	3	P47967,Q62761;Q62762;Q62763,Q6AY61
814	1,2,3,5,27,<br>28,30,31	4,6,7,8,25,<br>26,29,32	1	P22273
815	1,2,3,5,27,<br>28,30,32	4,6,7,8,25,<br>26,29,31	3	P22273,P82471,Q9QX74
816	1,2,3,5,27,<br>28,31,32	4,6,7,8,25,<br>26,29,30	3	P22273,Q6AY61,Q9QX74
817	1,2,3,5,27,<br>29,30,31	4,6,7,8,25,<br>26,28,32	10	O54728,P00731,P07150,P08649,P11598,P22273,P22282,Q63617,Q99041,Q9QW07
818	1,2,3,5,27,<br>29,30,32	4,6,7,8,25,<br>26,28,31	3	P22273,P82471,Q63617
819	1,2,3,5,27,<br>29,31,32	4,6,7,8,25,<br>26,28,30	3	P22273,Q6AY61,Q99041
820	1,2,3,5,27,<br>30,31,32	4,6,7,8,25,<br>26,28,29	9	P06761,P20646,P22273,P22282,Q63617,Q6P6R2,Q9QW07,Q9QX74,Q9WTT6

821	1,2,3,5,28,29,30,31	4,6,7,8,25,26,27,32	3	P22273,P22282,Q99041
822	1,2,3,5,28,29,30,32	4,6,7,8,25,26,27,31	3	P22273,P22282,Q9Z0V6
823	1,2,3,5,28,29,31,32	4,6,7,8,25,26,27,30	4	O70417,P22273,Q6AY61,Q99041
824	1,2,3,5,28,30,31,32	4,6,7,8,25,26,27,29	6	P06761,P22273,P22282,Q4G075,Q9QX74,Q9WTT6
825	1,2,3,5,29,30,31,32	4,6,7,8,25,26,27,28	16	P06761,P07150,P08649,P0C0A9,P11598,P20646,P22273,P22282,P26342,P36860,P63081,Q4G075,Q63617,Q99041,Q9QW07,Q9WTT6
826	1,2,3,6,7,8,25,26	4,5,27,28,29,30,31,32	6	O54800;Q5DWV2,P06911,P08649,P97580,Q63598,Q99MH3
827	1,2,3,6,7,8,25,27	4,5,26,28,29,30,31,32	8	O54800;Q5DWV2,P02631,P06911,P50115,P97580,Q30KJ2,Q62894,Q66H69
828	1,2,3,6,7,8,25,28	4,5,26,27,29,30,31,32	9	O54800;Q5DWV2,P02631,P06911,P12020,P36860,Q09030,Q5GRG2,Q63598,Q9QW07
829	1,2,3,6,7,8,25,29	4,5,26,27,28,30,31,32	3	O54800;Q5DWV2,P02631,P06911
830	1,2,3,6,7,8,25,30	4,5,26,27,28,29,31,32	9	O54800;Q5DWV2,O70417,P02631,P06911,P36374,P80202,P97580,Q09030,Q6IG05
831	1,2,3,6,7,8,25,31	4,5,26,27,28,29,30,32	8	O54800;Q5DWV2,P06911,P12020,P20766,P80202,Q09030,Q63532,Q63598
832	1,2,3,6,7,8,25,32	4,5,26,27,28,29,30,31	5	O54800;Q5DWV2,P02631,P06911,P80202,Q09030
833	1,2,3,6,7,8,26,27	4,5,25,28,29,30,31,32	8	O54800;Q5DWV2,P15399,P50115,P97580,Q30KJ2,Q62894,Q66H69,Q99MH3
834	1,2,3,6,7,8,26,28	4,5,25,27,29,30,31,32	4	P97580,Q63598,Q99MH3,Q9JI85
835	1,2,3,6,7,8,26,29	4,5,25,27,28,30,31,32	5	O54800;Q5DWV2,O70594,P02783,P20759,P97580

836	1,2,3,6,7,8,26,30	4,5,25,27,28,29,31,32	6	O54800;Q5DWV2,P36374,P97580,P97615,Q6IG05,Q99MH3
837	1,2,3,6,7,8,26,31	4,5,25,27,28,29,30,32	7	O54800;Q5DWV2,P20766,P57113,Q62714,Q63532,Q63598,Q99MH3
838	1,2,3,6,7,8,26,32	4,5,25,27,28,29,30,31	7	O54800;Q5DWV2,O89117,P57113,P97580,P97615,Q62714,Q99MH3
839	1,2,3,6,7,8,27,28	4,5,25,26,29,30,31,32	7	P06911,P15399,P19218,P50115,P97580,Q66H69,Q9JI85
840	1,2,3,6,7,8,27,29	4,5,25,26,28,30,31,32	6	P00731,P02783,P62804,P97580,Q00715,Q66H69
841	1,2,3,6,7,8,27,30	4,5,25,26,28,29,31,32	7	P00731,P19218,P97580,Q30KJ2,Q66H69,Q6IFU7,Q6IG05
842	1,2,3,6,7,8,27,31	4,5,25,26,28,29,30,32	5	P20766,P97580,Q62714,Q62894,Q63532
843	1,2,3,6,7,8,27,32	4,5,25,26,28,29,30,31	3	O54800;Q5DWV2,P97580,P98166
844	1,2,3,6,7,8,28,29	4,5,25,26,27,30,31,32	7	P06911,P21674,P62804,Q00715,Q9JI85,Q9QW07,Q9QYP1
845	1,2,3,6,7,8,28,30	4,5,25,26,27,29,31,32	1	P19218
846	1,2,3,6,7,8,28,31	4,5,25,26,27,29,30,32	8	P06911,P12020,P20766,P21674,Q5GRG2,Q63532,Q63598,Q811M5
847	1,2,3,6,7,8,28,32	4,5,25,26,27,29,30,31	3	O89117,P02631,Q9JI85
848	1,2,3,6,7,8,29,30	4,5,25,26,27,28,31,32	4	O54800;Q5DWV2,P62804,Q6IFU7,Q6IG05

849	1,2,3,6,7,8,29,31	4,5,25,26,27,28,30,32	5	O70594,P12020,P20766,P21674,Q63532
850	1,2,3,6,7,8,29,32	4,5,25,26,27,28,30,31	1	O54800;Q5DWV2
851	1,2,3,6,7,8,30,31	4,5,25,26,27,28,29,32	8	O54800;Q5DWV2,O89117,P20766,P97615,Q09030,Q63532,Q6IG05,Q99MH3
852	1,2,3,6,7,8,30,32	4,5,25,26,27,28,29,31	7	O54800;Q5DWV2,O89117,P26342,P35280,P97615,Q6IG05,Q99MH3
853	1,2,3,6,7,8,31,32	4,5,25,26,27,28,29,30	7	O54800;Q5DWV2,O89117,P20766,P57113,P97615,Q62714,Q63532
854	1,2,3,6,7,25,26,27	4,5,8,28,29,30,31,32	2	O54800;Q5DWV2,P63081
855	1,2,3,6,7,25,26,28	4,5,8,27,29,30,31,32	2	O54800;Q5DWV2,Q63598
856	1,2,3,6,7,25,26,29	4,5,8,27,28,30,31,32	1	O54800;Q5DWV2
857	1,2,3,6,7,25,26,30	4,5,8,27,28,29,31,32	3	O54800;Q5DWV2,P36374,Q99MH3
858	1,2,3,6,7,25,26,31	4,5,8,27,28,29,30,32	6	O54800;Q5DWV2,P18298,P20766,P57113,Q63598,Q99MH3
859	1,2,3,6,7,25,26,32	4,5,8,27,28,29,30,31	2	O54800;Q5DWV2,Q99MH3
860	1,2,3,6,7,25,27,28	4,5,8,26,29,30,31,32	2	P0DMW0;P0DMW1,P63081
861	1,2,3,6,7,25,27,29	4,5,8,26,28,30,31,32	1	O54728
862	1,2,3,6,7,25,27,30	4,5,8,26,28,29,31,32	2	O54728,O54800;Q5DWV2
863	1,2,3,6,7,25,27,31	4,5,8,26,28,29,30,32	3	O54728,P20766,Q62894
864	1,2,3,6,7,25,27,32	4,5,8,26,28,29,30,31	1	O54800;Q5DWV2
865	1,2,3,6,7,25,28,29	4,5,8,26,27,30,31,32	0	
866	1,2,3,6,7,25,28,30	4,5,8,26,27,29,31,32	2	O54800;Q5DWV2,Q62975

867	1,2,3,6,7,25,28,31	4,5,8,26,27,29,30,32	2	P20766,Q63598
868	1,2,3,6,7,25,28,32	4,5,8,26,27,29,30,31	2	O54800;Q5DWV2,Q62975
869	1,2,3,6,7,25,29,30	4,5,8,26,27,28,31,32	4	O54728,O54800;Q5DWV2,P01835,Q9QWN8
870	1,2,3,6,7,25,29,31	4,5,8,26,27,28,30,32	4	O54728,O54800;Q5DWV2,P20766,Q9QWN8
871	1,2,3,6,7,25,29,32	4,5,8,26,27,28,30,31	2	O54800;Q5DWV2,P01835
872	1,2,3,6,7,25,30,31	4,5,8,26,27,28,29,32	7	O54728,O54800;Q5DWV2,P20766,Q09030,Q6AYR9,Q6P6R2,Q9QWN8
873	1,2,3,6,7,25,30,32	4,5,8,26,27,28,29,31	7	O54800;Q5DWV2,P06760,P26342,Q62975,Q6P6R2,Q9QX74,Q9R168
874	1,2,3,6,7,25,31,32	4,5,8,26,27,28,29,30	4	O54800;Q5DWV2,P20766,P26342,Q6P6R2
875	1,2,3,6,7,26,27,28	4,5,8,25,29,30,31,32	1	P0DMW0;P0DMW1
876	1,2,3,6,7,26,27,29	4,5,8,25,28,30,31,32	3	P01835,P02783,Q9JJ50
877	1,2,3,6,7,26,27,30	4,5,8,25,28,29,31,32	3	O54800;Q5DWV2,P21588,Q99MH3
878	1,2,3,6,7,26,27,31	4,5,8,25,28,29,30,32	2	P20766,Q62894
879	1,2,3,6,7,26,27,32	4,5,8,25,28,29,30,31	1	O54800;Q5DWV2
880	1,2,3,6,7,26,28,29	4,5,8,25,27,30,31,32	5	P00762,P01835,P21674,P23739,P47967
881	1,2,3,6,7,26,28,30	4,5,8,25,27,29,31,32	4	O54800;Q5DWV2,P00762,P01835,Q99MH3
882	1,2,3,6,7,26,28,31	4,5,8,25,27,29,30,32	5	P00762,P01835,P20766,P57113,Q63598
883	1,2,3,6,7,26,28,32	4,5,8,25,27,29,30,31	3	O54800;Q5DWV2,P01835,Q99MH3
884	1,2,3,6,7,26,29,30	4,5,8,25,27,28,31,32	3	O54800;Q5DWV2,P01835,P23739
885	1,2,3,6,7,26,29,31	4,5,8,25,27,28,30,32	4	O54800;Q5DWV2,P01835,P20766,P57113
886	1,2,3,6,7,26,29,32	4,5,8,25,27,28,30,31	2	O54800;Q5DWV2,P01835

887	1,2,3,6,7,26,30,31	4,5,8,25,27,28,29,32	7	O54800;Q5DWV2,P01835,P20766,P57113,Q6AYR9,Q812E4,Q99MH3
888	1,2,3,6,7,26,30,32	4,5,8,25,27,28,29,31	6	O54800;Q5DWV2,P01835,P26342,P97615,Q99MH3,Q9R168
889	1,2,3,6,7,26,31,32	4,5,8,25,27,28,29,30	7	O54800;Q5DWV2,O89117,P01835,P20766,P57113,Q62714,Q99MH3
890	1,2,3,6,7,27,28,29	4,5,8,25,26,30,31,32	8	P00507,P01835,P09656,P0DMW0;P0DMW1,P47967,Q00715,Q6AY61,Q9WVK7
891	1,2,3,6,7,27,28,30	4,5,8,25,26,29,31,32	1	P19218
892	1,2,3,6,7,27,28,31	4,5,8,25,26,29,30,32	2	P20766,Q62894
893	1,2,3,6,7,27,28,32	4,5,8,25,26,29,30,31	3	P05964,Q62761;Q62762;Q62763,Q6AY61
894	1,2,3,6,7,27,29,30	4,5,8,25,26,28,31,32	2	O54728,P01835
895	1,2,3,6,7,27,29,31	4,5,8,25,26,28,30,32	4	O54728,P01835,P20766,P80299
896	1,2,3,6,7,27,29,32	4,5,8,25,26,28,30,31	2	P01835,Q6AY61
897	1,2,3,6,7,27,30,31	4,5,8,25,26,28,29,32	2	O54728,P20766
898	1,2,3,6,7,27,30,32	4,5,8,25,26,28,29,31	2	O54800;Q5DWV2,P06760
899	1,2,3,6,7,27,31,32	4,5,8,25,26,28,29,30	3	P20766,P80299,Q62714
900	1,2,3,6,7,28,29,30	4,5,8,25,26,27,31,32	3	P00762,P01835,P23739
901	1,2,3,6,7,28,29,31	4,5,8,25,26,27,30,32	3	P00762,P01835,P21674
902	1,2,3,6,7,28,29,32	4,5,8,25,26,27,30,31	3	P01835,P54921,Q6AY61
903	1,2,3,6,7,28,30,31	4,5,8,25,26,27,29,32	1	P01835
904	1,2,3,6,7,28,30,32	4,5,8,25,26,27,29,31	4	O54800;Q5DWV2,P01835,P06760,Q62975
905	1,2,3,6,7,28,31,32	4,5,8,25,26,27,29,30	3	P01835,P20766,P22006
906	1,2,3,6,7,29,30,31	4,5,8,25,26,27,28,32	5	O54728,P01835,P20766,P26342,Q9QWN8

907	1,2,3,6,7,29,30,32	4,5,8,25,26,27,28,31	3	O54800;Q5DWV2,P01835,P26342
908	1,2,3,6,7,29,31,32	4,5,8,25,26,27,28,30	5	O54800;Q5DWV2,P01835,P20766,P26342,P80299
909	1,2,3,6,7,30,31,32	4,5,8,25,26,27,28,29	9	O54800;Q5DWV2,O89117,P01835,P06760,P20646,P20766,P26342,P97615,Q6P6R2
910	1,2,3,6,8,25,26,27	4,5,7,28,29,30,31,32	8	O54800;Q5DWV2,P08649,P15399,P97580,Q06000,Q30KJ2,Q5BJY9,Q99MH3
911	1,2,3,6,8,25,26,28	4,5,7,27,29,30,31,32	6	O54800;Q5DWV2,P00714,P08649,P97580,Q63598,Q99MH3
912	1,2,3,6,8,25,26,29	4,5,7,27,28,30,31,32	3	O54800;Q5DWV2,P08649,P97580
913	1,2,3,6,8,25,26,30	4,5,7,27,28,29,31,32	8	O54800;Q5DWV2,P08649,P36374,P97580,Q06000,Q30KJ2,Q6IG05,Q99MH3
914	1,2,3,6,8,25,26,31	4,5,7,27,28,29,30,32	6	O54800;Q5DWV2,P08649,P97580,P97840,Q63532,Q99MH3
915	1,2,3,6,8,25,26,32	4,5,7,27,28,29,30,31	6	O54800;Q5DWV2,P08649,P97580,Q06000,Q68G31,Q99MH3
916	1,2,3,6,8,25,27,28	4,5,7,26,29,30,31,32	5	P08649,P15399,P19629,P36860,P97580
917	1,2,3,6,8,25,27,29	4,5,7,26,28,30,31,32	1	P97580
918	1,2,3,6,8,25,27,30	4,5,7,26,28,29,31,32	6	O54800;Q5DWV2,P15399,P97580,Q30KJ2,Q6IFU7,Q6IG05
919	1,2,3,6,8,25,27,31	4,5,7,26,28,29,30,32	2	P36376,P97580
920	1,2,3,6,8,25,27,32	4,5,7,26,28,29,30,31	4	O54800;Q5DWV2,P15399,P97580,P98166
921	1,2,3,6,8,25,28,29	4,5,7,26,27,30,31,32	0	
922	1,2,3,6,8,25,28,30	4,5,7,26,27,29,31,32	2	Q09030,Q6IG05
923	1,2,3,6,8,25,28,31	4,5,7,26,27,29,30,32	4	P36376,Q09030,Q5GRG2,Q63532
924	1,2,3,6,8,25,28,32	4,5,7,26,27,29,30,31	4	O54800;Q5DWV2,P08649,Q09030,Q68G31
925	1,2,3,6,8,25,29,30	4,5,7,26,27,28,31,32	3	O54800;Q5DWV2,Q6IFU7,Q6IG05
926	1,2,3,6,8,25,29,31	4,5,7,26,27,28,30,32	0	

927	1,2,3,6,8,25,29,32	4,5,7,26,27,28,30,31	2	O54800;Q5DWV2,Q68G31
928	1,2,3,6,8,25,30,31	4,5,7,26,27,28,29,32	7	O54800;Q5DWV2,P20646,P97840,Q09030,Q63532,Q6IG05,Q99MH3
929	1,2,3,6,8,25,30,32	4,5,7,26,27,28,29,31	9	O54800;Q5DWV2,O88797,P02625,P20646,P97615,Q09030,Q68G31,Q6IG05,Q99MH3
930	1,2,3,6,8,25,31,32	4,5,7,26,27,28,29,30	7	O54800;Q5DWV2,P20646,P21588,P97840,Q09030,Q63532,Q99MH3
931	1,2,3,6,8,26,27,28	4,5,7,25,29,30,31,32	6	P08649,P15399,P97580,Q30KJ2,Q99MH3,Q9JI85
932	1,2,3,6,8,26,27,29	4,5,7,25,28,30,31,32	5	P02783,P09656,P15399,P97580,Q30KJ2
933	1,2,3,6,8,26,27,30	4,5,7,25,28,29,31,32	5	P15399,P97580,Q30KJ2,Q6IG05,Q99MH3
934	1,2,3,6,8,26,27,31	4,5,7,25,28,29,30,32	5	P15399,P97580,Q30KJ2,Q5BJY9,Q99MH3
935	1,2,3,6,8,26,27,32	4,5,7,25,28,29,30,31	5	O54800;Q5DWV2,P15399,P97580,Q30KJ2,Q99MH3
936	1,2,3,6,8,26,28,29	4,5,7,25,27,30,31,32	2	P21674,Q9QYP1
937	1,2,3,6,8,26,28,30	4,5,7,25,27,29,31,32	3	O89117,P97580,Q99MH3
938	1,2,3,6,8,26,28,31	4,5,7,25,27,29,30,32	4	O89117,Q63532,Q63598,Q99MH3
939	1,2,3,6,8,26,28,32	4,5,7,25,27,29,30,31	5	O54800;Q5DWV2,O89117,P08649,Q99MH3,Q9JI85
940	1,2,3,6,8,26,29,30	4,5,7,25,27,28,31,32	3	O54800;Q5DWV2,P97580,Q99MH3
941	1,2,3,6,8,26,29,31	4,5,7,25,27,28,30,32	1	Q99MH3
942	1,2,3,6,8,26,29,32	4,5,7,25,27,28,30,31	3	O54800;Q5DWV2,Q68G31,Q99MH3
943	1,2,3,6,8,26,30,31	4,5,7,25,27,28,29,32	6	O54800;Q5DWV2,O89117,P97580,Q63532,Q6IG05,Q99MH3
944	1,2,3,6,8,26,30,32	4,5,7,25,27,28,29,31	6	O54800;Q5DWV2,O89117,P97580,P97615,Q68G31,Q99MH3
945	1,2,3,6,8,26,31,32	4,5,7,25,27,28,29,30	4	O54800;Q5DWV2,O89117,Q63532,Q99MH3
946	1,2,3,6,8,27,28,29	4,5,7,25,26,30,31,32	5	P09656,P15399,P97580,Q9JI85,Q9WVK7

947	1,2,3,6,8,27,28,30	4,5,7,25,26,29,31,32	7	P15399,P19218,P97580,Q30KJ2,Q6IFU7,Q6IG05,Q6P6S4
948	1,2,3,6,8,27,28,31	4,5,7,25,26,29,30,32	4	P15399,P36376,P97580,Q63532
949	1,2,3,6,8,27,28,32	4,5,7,25,26,29,30,31	4	P15399,P97580,Q6P6S4,Q9JI85
950	1,2,3,6,8,27,29,30	4,5,7,25,26,28,31,32	5	P00731,P97580,Q30KJ2,Q6IFU7,Q6IG05
951	1,2,3,6,8,27,29,31	4,5,7,25,26,28,30,32	1	P97580
952	1,2,3,6,8,27,29,32	4,5,7,25,26,28,30,31	2	P97580,P98166
953	1,2,3,6,8,27,30,31	4,5,7,25,26,28,29,32	3	P20646,P97580,Q6IG05
954	1,2,3,6,8,27,30,32	4,5,7,25,26,28,29,31	6	P15399,P20646,P97580,Q68G31,Q6IFU7,Q6IG05
955	1,2,3,6,8,27,31,32	4,5,7,25,26,28,29,30	3	P20646,P97580,Q63532
956	1,2,3,6,8,28,29,30	4,5,7,25,26,27,31,32	1	Q6IFU7
957	1,2,3,6,8,28,29,31	4,5,7,25,26,27,30,32	1	P21674
958	1,2,3,6,8,28,29,32	4,5,7,25,26,27,30,31	1	Q9JI85
959	1,2,3,6,8,28,30,31	4,5,7,25,26,27,29,32	5	O89117,P36376,Q63532,Q6IG05,Q99MH3
960	1,2,3,6,8,28,30,32	4,5,7,25,26,27,29,31	4	O89117,Q68G31,Q6P6S4,Q99MH3
961	1,2,3,6,8,28,31,32	4,5,7,25,26,27,29,30	2	O89117,Q63532
962	1,2,3,6,8,29,30,31	4,5,7,25,26,27,28,32	1	Q6IG05
963	1,2,3,6,8,29,30,32	4,5,7,25,26,27,28,31	5	O54800;Q5DWV2,O88797,P20646,Q68G31,Q6IFU7
964	1,2,3,6,8,29,31,32	4,5,7,25,26,27,28,30	0	
965	1,2,3,6,8,30,31,32	4,5,7,25,26,27,28,29	9	O54800;Q5DWV2,O89117,P06760,P20646,P26342,P97615,Q63532,Q6IG05,Q99MH3
966	1,2,3,6,25,26,27,28	4,5,7,8,29,30,31,32	3	P0DMW0;P0DMW1,P63081,P82471

967	1,2,3,6,25,26,27,29	4,5,7,8,28,30,31,32	3	O54800;Q5DWV2,P09656,P82471
968	1,2,3,6,25,26,27,30	4,5,7,8,28,29,31,32	5	O54800;Q5DWV2,P06760,P82471,Q06000,Q99MH3
969	1,2,3,6,25,26,27,31	4,5,7,8,28,29,30,32	1	O54800;Q5DWV2
970	1,2,3,6,25,26,27,32	4,5,7,8,28,29,30,31	5	O54800;Q5DWV2,P06760,P82471,Q06000,Q99MH3
971	1,2,3,6,25,26,28,29	4,5,7,8,27,30,31,32	2	P00714,P82471
972	1,2,3,6,25,26,28,30	4,5,7,8,27,29,31,32	9	O54800;Q5DWV2,P00714,P06760,P82471,Q06000,Q4G075,Q62975,Q6TMA8,Q99MH3
973	1,2,3,6,25,26,28,31	4,5,7,8,27,29,30,32	5	P00714,P06760,Q4G075,Q63598,Q99MH3
974	1,2,3,6,25,26,28,32	4,5,7,8,27,29,30,31	8	O54800;Q5DWV2,P06760,P82471,Q06000,Q4G075,Q62975,Q68G31,Q99MH3
975	1,2,3,6,25,26,29,30	4,5,7,8,27,28,31,32	4	O54800;Q5DWV2,P22282,P82471,Q99MH3
976	1,2,3,6,25,26,29,31	4,5,7,8,27,28,30,32	2	O54800;Q5DWV2,P22282
977	1,2,3,6,25,26,29,32	4,5,7,8,27,28,30,31	3	O54800;Q5DWV2,P82471,Q68G31
978	1,2,3,6,25,26,30,31	4,5,7,8,27,28,29,32	11	O54800;Q5DWV2,P06760,P18298,P22273,P22282,Q4G075,Q6AYR9,Q6P777,Q812E4,Q99MH3,Q9Z2L0
979	1,2,3,6,25,26,30,32	4,5,7,8,27,28,29,31	10	D3ZUC6,O54800;Q5DWV2,P06760,P22273,P82471,Q06000,Q4G075,Q68G31,Q99MH3,Q9QX74
980	1,2,3,6,25,26,31,32	4,5,7,8,27,28,29,30	5	O54800;Q5DWV2,P06760,P22273,Q4G075,Q99MH3
981	1,2,3,6,25,27,28,29	4,5,7,8,26,30,31,32	3	P09656,P0DMW0;P0DMW1,P82471
982	1,2,3,6,25,27,28,30	4,5,7,8,26,29,31,32	3	P06760,P82471,Q9QX74
983	1,2,3,6,25,27,28,31	4,5,7,8,26,29,30,32	1	P06760
984	1,2,3,6,25,27,28,32	4,5,7,8,26,29,30,31	4	P06760,P82471,Q5RJR2,Q9QX74
985	1,2,3,6,25,27,29,30	4,5,7,8,26,28,31,32	2	O54728,P82471
986	1,2,3,6,25,27,29,31	4,5,7,8,26,28,30,32	1	O54728

987	1,2,3,6,25, 27,29,32	4,5,7,8,26, 28,30,31	2	O54800;Q5DWV2,P82471
988	1,2,3,6,25, 27,30,31	4,5,7,8,26, 28,29,32	3	O54728,P06760,Q9QX74
989	1,2,3,6,25, 27,30,32	4,5,7,8,26, 28,29,31	8	O54800;Q5DWV2,P06760,P20646,P82471,Q68G31,Q6P6R2,Q9QX74,Q9WTT6
990	1,2,3,6,25, 27,31,32	4,5,7,8,26, 28,29,30	4	P06760,P20646,Q9QX74,Q9WTT6
991	1,2,3,6,25, 28,29,30	4,5,7,8,26, 27,31,32	1	P82471
992	1,2,3,6,25, 28,29,31	4,5,7,8,26, 27,30,32	0	
993	1,2,3,6,25, 28,29,32	4,5,7,8,26, 27,30,31	2	P82471,Q68G31
994	1,2,3,6,25, 28,30,31	4,5,7,8,26, 27,29,32	3	P06760,Q4G075,Q9QX74
995	1,2,3,6,25, 28,30,32	4,5,7,8,26, 27,29,31	8	O54800;Q5DWV2,P06760,P82471,Q4G075,Q62975,Q68G31,Q9QX74,Q9WTT6
996	1,2,3,6,25, 28,31,32	4,5,7,8,26, 27,29,30	5	P06760,Q4G075,Q62975,Q9QX74,Q9WTT6
997	1,2,3,6,25, 29,30,31	4,5,7,8,26, 27,28,32	6	O54728,O54800;Q5DWV2,P07150,P22282,Q9QWN8,Q9WTT6
998	1,2,3,6,25, 29,30,32	4,5,7,8,26, 27,28,31	7	O54800;Q5DWV2,P07150,P82471,Q4G075,Q68G31,Q9QX74,Q9WTT6
999	1,2,3,6,25, 29,31,32	4,5,7,8,26, 27,28,30	4	O54800;Q5DWV2,P07150,Q4G075,Q9WTT6
1000	1,2,3,6,25, 30,31,32	4,5,7,8,26, 27,28,29	15	D3ZUC6,O54800;Q5DWV2,P06760,P07150,P20646,P22273,P22282,P26342,P47967,P97840,Q4 G075,Q6P6R2,Q99MH3,Q9QX74,Q9WTT6
1001	1,2,3,6,26, 27,28,29	4,5,7,8,25, 30,31,32	4	P09656,P0DMW0;P0DMW1,P82471,Q9WVK7
1002	1,2,3,6,26, 27,28,30	4,5,7,8,25, 29,31,32	4	P06760,P21588,P82471,Q99MH3
1003	1,2,3,6,26, 27,28,31	4,5,7,8,25, 29,30,32	0	
1004	1,2,3,6,26, 27,28,32	4,5,7,8,25, 29,30,31	2	P06760,P82471
1005	1,2,3,6,26, 27,29,30	4,5,7,8,25, 28,31,32	4	P09656,P21588,P82471,Q811M5
1006	1,2,3,6,26, 27,29,31	4,5,7,8,25, 28,30,32	0	

1007	1,2,3,6,26,<br>27,29,32	4,5,7,8,25,<br>28,30,31	2	P09656,P82471
1008	1,2,3,6,26,<br>27,30,31	4,5,7,8,25,<br>28,29,32	2	P06760,Q99MH3
1009	1,2,3,6,26,<br>27,30,32	4,5,7,8,25,<br>28,29,31	6	O54800;Q5DWV2,P06760,P82471,Q68G31,Q99MH3,Q9QX74
1010	1,2,3,6,26,<br>27,31,32	4,5,7,8,25,<br>28,29,30	2	P06760,Q99MH3
1011	1,2,3,6,26,<br>28,29,30	4,5,7,8,25,<br>27,31,32	3	P01835,P23739,P82471
1012	1,2,3,6,26,<br>28,29,31	4,5,7,8,25,<br>27,30,32	2	P01835,Q01205
1013	1,2,3,6,26,<br>28,29,32	4,5,7,8,25,<br>27,30,31	2	P01835,P82471
1014	1,2,3,6,26,<br>28,30,31	4,5,7,8,25,<br>27,29,32	5	O89117,P06760,Q4G075,Q6TMA8,Q99MH3
1015	1,2,3,6,26,<br>28,30,32	4,5,7,8,25,<br>27,29,31	8	O54800;Q5DWV2,O89117,P06760,P82471,Q4G075,Q68G31,Q99MH3,Q9QX74
1016	1,2,3,6,26,<br>28,31,32	4,5,7,8,25,<br>27,29,30	5	O89117,P06760,P22006,Q4G075,Q99MH3
1017	1,2,3,6,26,<br>29,30,31	4,5,7,8,25,<br>27,28,32	5	P01835,P0C0A9,P22282,Q4G075,Q99MH3
1018	1,2,3,6,26,<br>29,30,32	4,5,7,8,25,<br>27,28,31	6	O54800;Q5DWV2,P01835,P82471,Q4G075,Q68G31,Q99MH3
1019	1,2,3,6,26,<br>29,31,32	4,5,7,8,25,<br>27,28,30	4	O54800;Q5DWV2,P01835,Q4G075,Q99MH3
1020	1,2,3,6,26,<br>30,31,32	4,5,7,8,25,<br>27,28,29	13	O54800;Q5DWV2,O89117,P06760,P20646,P22273,P22282,P26342,P97615,Q4G075,Q812E4,Q9<br>9MH3,Q9QX74,Q9WTT6
1021	1,2,3,6,27,<br>28,29,30	4,5,7,8,25,<br>26,31,32	4	P09656,P14562,P82471,Q9WVK7
1022	1,2,3,6,27,<br>28,29,31	4,5,7,8,25,<br>26,30,32	1	Q9WVK7
1023	1,2,3,6,27,<br>28,29,32	4,5,7,8,25,<br>26,30,31	5	P09656,P14562,P82471,Q62761;Q62762;Q62763,Q6AY61
1024	1,2,3,6,27,<br>28,30,31	4,5,7,8,25,<br>26,29,32	2	P06760,Q9QX74
1025	1,2,3,6,27,<br>28,30,32	4,5,7,8,25,<br>26,29,31	5	P06760,P82471,Q68G31,Q9QX74,Q9Z0V6
1026	1,2,3,6,27,<br>28,31,32	4,5,7,8,25,<br>26,29,30	3	P06760,P22006,Q9QX74

1027	1,2,3,6,27,29,30,31	4,5,7,8,25,26,28,32	1	O54728
1028	1,2,3,6,27,29,30,32	4,5,7,8,25,26,28,31	5	P14562,P82471,Q68G31,Q9WTT6,Q9Z0V6
1029	1,2,3,6,27,29,31,32	4,5,7,8,25,26,28,30	2	P80299,Q9WTT6
1030	1,2,3,6,27,30,31,32	4,5,7,8,25,26,28,29	5	P06760,P20646,Q6P6R2,Q9QX74,Q9WTT6
1031	1,2,3,6,28,29,30,31	4,5,7,8,25,26,27,32	2	P01835,Q4G075
1032	1,2,3,6,28,29,30,32	4,5,7,8,25,26,27,31	7	P01835,P14562,Q4G075,Q68G31,Q9QX74,Q9WTT6,Q9Z0V6
1033	1,2,3,6,28,29,31,32	4,5,7,8,25,26,27,30	3	P01835,Q4G075,Q9WTT6
1034	1,2,3,6,28,30,31,32	4,5,7,8,25,26,27,29	7	O89117,P06760,P20646,P22006,Q4G075,Q9QX74,Q9WTT6
1035	1,2,3,6,29,30,31,32	4,5,7,8,25,26,27,28	8	P01835,P07150,P20646,P22273,P26342,P36860,Q4G075,Q9WTT6
1036	1,2,3,7,8,25,26,27	4,5,6,28,29,30,31,32	4	P22006,P36860,P97580,Q66H69
1037	1,2,3,7,8,25,26,28	4,5,6,27,29,30,31,32	3	P00714,P36860,Q63598
1038	1,2,3,7,8,25,26,29	4,5,6,27,28,30,31,32	3	P22006,Q64093,Q8VD89
1039	1,2,3,7,8,25,26,30	4,5,6,27,28,29,31,32	1	P22006
1040	1,2,3,7,8,25,26,31	4,5,6,27,28,29,30,32	2	P20766,Q63598
1041	1,2,3,7,8,25,26,32	4,5,6,27,28,29,30,31	0	
1042	1,2,3,7,8,25,27,28	4,5,6,26,29,30,31,32	2	P36860,Q66H69
1043	1,2,3,7,8,25,27,29	4,5,6,26,28,30,31,32	3	P22006,Q66H69,Q8VD89
1044	1,2,3,7,8,25,27,30	4,5,6,26,28,29,31,32	4	Q05511,Q66H69,Q6IFW6,Q6IG05
1045	1,2,3,7,8,25,27,31	4,5,6,26,28,29,30,32	1	P20766
1046	1,2,3,7,8,25,27,32	4,5,6,26,28,29,30,31	2	P01681,Q66H69

1047	1,2,3,7,8,25,28,29	4,5,6,26,27,30,31,32	5	P22006,P36860,P62804,Q64093,Q8VD89
1048	1,2,3,7,8,25,28,30	4,5,6,26,27,29,31,32	0	
1049	1,2,3,7,8,25,28,31	4,5,6,26,27,29,30,32	4	P20766,P36376,Q63598,Q811M5
1050	1,2,3,7,8,25,28,32	4,5,6,26,27,29,30,31	2	P36860,Q811M5
1051	1,2,3,7,8,25,29,30	4,5,6,26,27,28,31,32	3	P22006,P62804,Q6IFW6
1052	1,2,3,7,8,25,29,31	4,5,6,26,27,28,30,32	2	P20766,Q8VD89
1053	1,2,3,7,8,25,29,32	4,5,6,26,27,28,30,31	0	
1054	1,2,3,7,8,25,30,31	4,5,6,26,27,28,29,32	2	P20766,Q6IG05
1055	1,2,3,7,8,25,30,32	4,5,6,26,27,28,29,31	3	P01681,P35280,P97615
1056	1,2,3,7,8,25,31,32	4,5,6,26,27,28,29,30	4	P01681,P20766,P21588,Q811M5
1057	1,2,3,7,8,26,27,28	4,5,6,25,29,30,31,32	6	P25809,P36860,P47967,Q63598,Q66H69,Q9WTT6
1058	1,2,3,7,8,26,27,29	4,5,6,25,28,30,31,32	4	P22006,P25809,Q66H69,Q9WTT6
1059	1,2,3,7,8,26,27,30	4,5,6,25,28,29,31,32	3	P97580,Q66H69,Q6IG05
1060	1,2,3,7,8,26,27,31	4,5,6,25,28,29,30,32	2	P20766,Q66H69
1061	1,2,3,7,8,26,27,32	4,5,6,25,28,29,30,31	1	Q66H69
1062	1,2,3,7,8,26,28,29	4,5,6,25,27,30,31,32	7	P21674,P47967,P62804,Q63424,Q64093,Q6P6R2,Q9WTT6
1063	1,2,3,7,8,26,28,30	4,5,6,25,27,29,31,32	0	
1064	1,2,3,7,8,26,28,31	4,5,6,25,27,29,30,32	4	P20766,Q5RLM2,Q63598,Q811M5
1065	1,2,3,7,8,26,28,32	4,5,6,25,27,29,30,31	0	
1066	1,2,3,7,8,26,29,30	4,5,6,25,27,28,31,32	1	P22006

1067	1,2,3,7,8,26,29,31	4,5,6,25,27,28,30,32	2	P20766,Q64093
1068	1,2,3,7,8,26,29,32	4,5,6,25,27,28,30,31	1	Q64093
1069	1,2,3,7,8,26,30,31	4,5,6,25,27,28,29,32	2	P20766,Q99MH3
1070	1,2,3,7,8,26,30,32	4,5,6,25,27,28,29,31	3	P35280,P97615,Q99MH3
1071	1,2,3,7,8,26,31,32	4,5,6,25,27,28,29,30	1	P20766
1072	1,2,3,7,8,27,28,29	4,5,6,25,26,30,31,32	8	P00507,P25809,P47967,P62804,Q00715,Q66H69,Q812E4,Q9WVK7
1073	1,2,3,7,8,27,28,30	4,5,6,25,26,29,31,32	3	P19218,Q66H69,Q6IG05
1074	1,2,3,7,8,27,28,31	4,5,6,25,26,29,30,32	1	P20766
1075	1,2,3,7,8,27,28,32	4,5,6,25,26,29,30,31	1	Q66H69
1076	1,2,3,7,8,27,29,30	4,5,6,25,26,28,31,32	6	P00731,P62804,Q66H69,Q6IFU7,Q6IFW6,Q6IG05
1077	1,2,3,7,8,27,29,31	4,5,6,25,26,28,30,32	2	P20766,Q66H69
1078	1,2,3,7,8,27,29,32	4,5,6,25,26,28,30,31	1	Q66H69
1079	1,2,3,7,8,27,30,31	4,5,6,25,26,28,29,32	3	P00731,P20766,Q6IG05
1080	1,2,3,7,8,27,30,32	4,5,6,25,26,28,29,31	2	P35280,Q6IG05
1081	1,2,3,7,8,27,31,32	4,5,6,25,26,28,29,30	1	P20766
1082	1,2,3,7,8,28,29,30	4,5,6,25,26,27,31,32	2	P21674,P62804
1083	1,2,3,7,8,28,29,31	4,5,6,25,26,27,30,32	3	P21674,Q64093,Q811M5
1084	1,2,3,7,8,28,29,32	4,5,6,25,26,27,30,31	3	P21674,P54921,Q812E4
1085	1,2,3,7,8,28,30,31	4,5,6,25,26,27,29,32	1	P20766
1086	1,2,3,7,8,28,30,32	4,5,6,25,26,27,29,31	1	P35280

1087	1,2,3,7,8,28,31,32	4,5,6,25,26,27,29,30	4	P20766,P21588,Q811M5,Q923V8
1088	1,2,3,7,8,29,30,31	4,5,6,25,26,27,28,32	2	P20766,Q9QWN8
1089	1,2,3,7,8,29,30,32	4,5,6,25,26,27,28,31	1	P35280
1090	1,2,3,7,8,29,31,32	4,5,6,25,26,27,28,30	2	P20766,P21588
1091	1,2,3,7,8,30,31,32	4,5,6,25,26,27,28,29	5	O89117,P20766,P26342,P35280,P97615
1092	1,2,3,7,25,26,27,28	4,5,6,8,29,30,31,32	2	P0DMW0;P0DMW1,P36860
1093	1,2,3,7,25,26,27,29	4,5,6,8,28,30,31,32	2	P22006,Q8VD89
1094	1,2,3,7,25,26,27,30	4,5,6,8,28,29,31,32	0	
1095	1,2,3,7,25,26,27,31	4,5,6,8,28,29,30,32	1	P20766
1096	1,2,3,7,25,26,27,32	4,5,6,8,28,29,30,31	0	
1097	1,2,3,7,25,26,28,29	4,5,6,8,27,30,31,32	2	P00714,Q64093
1098	1,2,3,7,25,26,28,30	4,5,6,8,27,29,31,32	2	P00714,Q62975
1099	1,2,3,7,25,26,28,31	4,5,6,8,27,29,30,32	2	P00714,Q63598
1100	1,2,3,7,25,26,28,32	4,5,6,8,27,29,30,31	1	Q62975
1101	1,2,3,7,25,26,29,30	4,5,6,8,27,28,31,32	2	P22006,P22282
1102	1,2,3,7,25,26,29,31	4,5,6,8,27,28,30,32	5	P19218,P20766,P22282,Q6RUV5,Q8VD89
1103	1,2,3,7,25,26,29,32	4,5,6,8,27,28,30,31	1	P19218
1104	1,2,3,7,25,26,30,31	4,5,6,8,27,28,29,32	4	P20766,P22282,Q6AYR9,Q812E4
1105	1,2,3,7,25,26,30,32	4,5,6,8,27,28,29,31	2	Q62975,Q9R168
1106	1,2,3,7,25,26,31,32	4,5,6,8,27,28,29,30	2	P19218,P20766

1107	1,2,3,7,25, 27,28,29	4,5,6,8,26, 30,31,32	2	P0DMW0;P0DMW1,Q6AY61
1108	1,2,3,7,25, 27,28,30	4,5,6,8,26, 29,31,32	0	
1109	1,2,3,7,25, 27,28,31	4,5,6,8,26, 29,30,32	0	
1110	1,2,3,7,25, 27,28,32	4,5,6,8,26, 29,30,31	3	Q5RJR2,Q62761;Q62762;Q62763,Q6AY61
1111	1,2,3,7,25, 27,29,30	4,5,6,8,26, 28,31,32	2	O54728,P22006
1112	1,2,3,7,25, 27,29,31	4,5,6,8,26, 28,30,32	2	O54728,Q8VD89
1113	1,2,3,7,25, 27,29,32	4,5,6,8,26, 28,30,31	1	Q6AY61
1114	1,2,3,7,25, 27,30,31	4,5,6,8,26, 28,29,32	2	O54728,P20766
1115	1,2,3,7,25, 27,30,32	4,5,6,8,26, 28,29,31	0	
1116	1,2,3,7,25, 27,31,32	4,5,6,8,26, 28,29,30	1	P20766
1117	1,2,3,7,25, 28,29,30	4,5,6,8,26, 27,31,32	0	
1118	1,2,3,7,25, 28,29,31	4,5,6,8,26, 27,30,32	1	Q8VD89
1119	1,2,3,7,25, 28,29,32	4,5,6,8,26, 27,30,31	2	P54921,Q6AY61
1120	1,2,3,7,25, 28,30,31	4,5,6,8,26, 27,29,32	1	Q62975
1121	1,2,3,7,25, 28,30,32	4,5,6,8,26, 27,29,31	2	Q62975,Q9R168
1122	1,2,3,7,25, 28,31,32	4,5,6,8,26, 27,29,30	3	P21588,Q62975,Q811M5
1123	1,2,3,7,25, 29,30,31	4,5,6,8,26, 27,28,32	3	O54728,P22282,Q9QWN8
1124	1,2,3,7,25, 29,30,32	4,5,6,8,26, 27,28,31	1	Q9R168
1125	1,2,3,7,25, 29,31,32	4,5,6,8,26, 27,28,30	1	P21588
1126	1,2,3,7,25, 30,31,32	4,5,6,8,26, 27,28,29	5	P06760,P22273,P22282,P26342,Q6P6R2

1127	1,2,3,7,26,<br>27,28,29	4,5,6,8,25,<br>30,31,32	3 P0DMW0;P0DMW1,P47967,Q9WVK7
1128	1,2,3,7,26,<br>27,28,30	4,5,6,8,25,<br>29,31,32	0
1129	1,2,3,7,26,<br>27,28,31	4,5,6,8,25,<br>29,30,32	3 P0DMW0;P0DMW1,P20766,Q63598
1130	1,2,3,7,26,<br>27,28,32	4,5,6,8,25,<br>29,30,31	0
1131	1,2,3,7,26,<br>27,29,30	4,5,6,8,25,<br>28,31,32	0
1132	1,2,3,7,26,<br>27,29,31	4,5,6,8,25,<br>28,30,32	1 P20766
1133	1,2,3,7,26,<br>27,29,32	4,5,6,8,25,<br>28,30,31	0
1134	1,2,3,7,26,<br>27,30,31	4,5,6,8,25,<br>28,29,32	1 P20766
1135	1,2,3,7,26,<br>27,30,32	4,5,6,8,25,<br>28,29,31	0
1136	1,2,3,7,26,<br>27,31,32	4,5,6,8,25,<br>28,29,30	1 P20766
1137	1,2,3,7,26,<br>28,29,30	4,5,6,8,25,<br>27,31,32	1 P01835
1138	1,2,3,7,26,<br>28,29,31	4,5,6,8,25,<br>27,30,32	4 P01835,Q01205,Q63424,Q64093
1139	1,2,3,7,26,<br>28,29,32	4,5,6,8,25,<br>27,30,31	2 P01835,P54921
1140	1,2,3,7,26,<br>28,30,31	4,5,6,8,25,<br>27,29,32	0
1141	1,2,3,7,26,<br>28,30,32	4,5,6,8,25,<br>27,29,31	1 Q9R168
1142	1,2,3,7,26,<br>28,31,32	4,5,6,8,25,<br>27,29,30	0
1143	1,2,3,7,26,<br>29,30,31	4,5,6,8,25,<br>27,28,32	3 P01835,P22282,Q9QWN8
1144	1,2,3,7,26,<br>29,30,32	4,5,6,8,25,<br>27,28,31	2 P01835,Q9R168
1145	1,2,3,7,26,<br>29,31,32	4,5,6,8,25,<br>27,28,30	2 P01835,P20766
1146	1,2,3,7,26,<br>30,31,32	4,5,6,8,25,<br>27,28,29	3 P20766,P22273,P26342

1147	1,2,3,7,27, 28,29,30	4,5,6,8,25, 26,31,32	2	P00507,P47967
1148	1,2,3,7,27, 28,29,31	4,5,6,8,25, 26,30,32	2	P00507,P0DMW0;P0DMW1
1149	1,2,3,7,27, 28,29,32	4,5,6,8,25, 26,30,31	5	P47967,P54921,Q62761;Q62762;Q62763,Q6AY61,Q812E4
1150	1,2,3,7,27, 28,30,31	4,5,6,8,25, 26,29,32	0	
1151	1,2,3,7,27, 28,30,32	4,5,6,8,25, 26,29,31	0	
1152	1,2,3,7,27, 28,31,32	4,5,6,8,25, 26,29,30	2	P80299,Q6AY61
1153	1,2,3,7,27, 29,30,31	4,5,6,8,25, 26,28,32	1	O54728
1154	1,2,3,7,27, 29,30,32	4,5,6,8,25, 26,28,31	0	
1155	1,2,3,7,27, 29,31,32	4,5,6,8,25, 26,28,30	2	P80299,Q6AY61
1156	1,2,3,7,27, 30,31,32	4,5,6,8,25, 26,28,29	2	P20766,P80299
1157	1,2,3,7,28, 29,30,31	4,5,6,8,25, 26,27,32	1	P01835
1158	1,2,3,7,28, 29,30,32	4,5,6,8,25, 26,27,31	2	P01835,P54921
1159	1,2,3,7,28, 29,31,32	4,5,6,8,25, 26,27,30	4	P01835,P54921,P80299,Q6AY61
1160	1,2,3,7,28, 30,31,32	4,5,6,8,25, 26,27,29	1	P06760
1161	1,2,3,7,29, 30,31,32	4,5,6,8,25, 26,27,28	3	P01835,P26342,P80299
1162	1,2,3,8,25, 26,27,28	4,5,6,7,29, 30,31,32	4	P00714,P26342,P36860,Q5BJY9
1163	1,2,3,8,25, 26,27,29	4,5,6,7,28, 30,31,32	2	P22006,Q9Z0J6
1164	1,2,3,8,25, 26,27,30	4,5,6,7,28, 29,31,32	3	P97580,Q30KJ2,Q6IG05
1165	1,2,3,8,25, 26,27,31	4,5,6,7,28, 29,30,32	1	Q5BJY9
1166	1,2,3,8,25, 26,27,32	4,5,6,7,28, 29,30,31	0	

1167	1,2,3,8,25,<br>26,28,29	4,5,6,7,27,<br>30,31,32	3 P00714,P22006,Q9Z0J6
1168	1,2,3,8,25,<br>26,28,30	4,5,6,7,27,<br>29,31,32	2 P00714,P80299
1169	1,2,3,8,25,<br>26,28,31	4,5,6,7,27,<br>29,30,32	3 P00714,P36376,Q63598
1170	1,2,3,8,25,<br>26,28,32	4,5,6,7,27,<br>29,30,31	0
1171	1,2,3,8,25,<br>26,29,30	4,5,6,7,27,<br>28,31,32	3 P22006,P50116,Q9Z0J6
1172	1,2,3,8,25,<br>26,29,31	4,5,6,7,27,<br>28,30,32	3 P00714,Q8VD89,Q9Z0J6
1173	1,2,3,8,25,<br>26,29,32	4,5,6,7,27,<br>28,30,31	1 Q9Z0J6
1174	1,2,3,8,25,<br>26,30,31	4,5,6,7,27,<br>28,29,32	6 P00714,P11598,P22283,P50116,Q63617,Q99MH3
1175	1,2,3,8,25,<br>26,30,32	4,5,6,7,27,<br>28,29,31	5 O88797,P50116,Q63617,Q68G31,Q99MH3
1176	1,2,3,8,25,<br>26,31,32	4,5,6,7,27,<br>28,29,30	3 P21588,Q63617,Q99MH3
1177	1,2,3,8,25,<br>27,28,29	4,5,6,7,26,<br>30,31,32	2 P36860,Q9Z0J6
1178	1,2,3,8,25,<br>27,28,30	4,5,6,7,26,<br>29,31,32	3 P36860,Q6IFU7,Q6IG05
1179	1,2,3,8,25,<br>27,28,31	4,5,6,7,26,<br>29,30,32	2 P36376,P36860
1180	1,2,3,8,25,<br>27,28,32	4,5,6,7,26,<br>29,30,31	2 P36860,Q9QWN8
1181	1,2,3,8,25,<br>27,29,30	4,5,6,7,26,<br>28,31,32	6 P22006,Q6IFU7,Q6IFW6,Q6IG02,Q6IG05,Q9Z0J6
1182	1,2,3,8,25,<br>27,29,31	4,5,6,7,26,<br>28,30,32	0
1183	1,2,3,8,25,<br>27,29,32	4,5,6,7,26,<br>28,30,31	1 Q9Z0J6
1184	1,2,3,8,25,<br>27,30,31	4,5,6,7,26,<br>28,29,32	3 Q63617,Q6IFW6,Q6IG05
1185	1,2,3,8,25,<br>27,30,32	4,5,6,7,26,<br>28,29,31	4 P01681,Q68G31,Q6IFU7,Q6IG05
1186	1,2,3,8,25,<br>27,31,32	4,5,6,7,26,<br>28,29,30	2 P01681,P21588

1187	1,2,3,8,25,28,29,30	4,5,6,7,26,27,31,32	3	P50116,Q6IFU7,Q9Z0J6
1188	1,2,3,8,25,28,29,31	4,5,6,7,26,27,30,32	4	P00714,P36376,P50116,Q9Z0J6
1189	1,2,3,8,25,28,29,32	4,5,6,7,26,27,30,31	2	P50116,Q9Z0J6
1190	1,2,3,8,25,28,30,31	4,5,6,7,26,27,29,32	3	P00714,P36376,P50116
1191	1,2,3,8,25,28,30,32	4,5,6,7,26,27,29,31	2	P50116,Q68G31
1192	1,2,3,8,25,28,31,32	4,5,6,7,26,27,29,30	7	P01681,P21588,P36376,P50116,Q80WL1,Q811M5,Q923V8
1193	1,2,3,8,25,29,30,31	4,5,6,7,26,27,28,32	4	P11598,P50116,Q6IFW6,Q9Z0J6
1194	1,2,3,8,25,29,30,32	4,5,6,7,26,27,28,31	4	O88797,P50116,Q68G31,Q9Z0J6
1195	1,2,3,8,25,29,31,32	4,5,6,7,26,27,28,30	3	P21588,P50116,Q9Z0J6
1196	1,2,3,8,25,30,31,32	4,5,6,7,26,27,28,29	7	P01681,P02625,P11598,P20646,P21588,P50116,Q63617
1197	1,2,3,8,26,27,28,29	4,5,6,7,25,30,31,32	3	P25809,Q6P6R2,Q9WVK7
1198	1,2,3,8,26,27,28,30	4,5,6,7,25,29,31,32	2	P15399,P97580
1199	1,2,3,8,26,27,28,31	4,5,6,7,25,29,30,32	1	Q5BJY9
1200	1,2,3,8,26,27,28,32	4,5,6,7,25,29,30,31	2	P15399,Q9QWN8
1201	1,2,3,8,26,27,29,30	4,5,6,7,25,28,31,32	2	P97580,Q6IFU7
1202	1,2,3,8,26,27,29,31	4,5,6,7,25,28,30,32	0	
1203	1,2,3,8,26,27,29,32	4,5,6,7,25,28,30,31	0	
1204	1,2,3,8,26,27,30,31	4,5,6,7,25,28,29,32	2	P97580,Q6IG05
1205	1,2,3,8,26,27,30,32	4,5,6,7,25,28,29,31	1	P97580
1206	1,2,3,8,26,27,31,32	4,5,6,7,25,28,29,30	0	

1207	1,2,3,8,26,<br>28,29,30	4,5,6,7,25,<br>27,31,32	1	P50116
1208	1,2,3,8,26,<br>28,29,31	4,5,6,7,25,<br>27,30,32	2	P00714,Q99376
1209	1,2,3,8,26,<br>28,29,32	4,5,6,7,25,<br>27,30,31	0	
1210	1,2,3,8,26,<br>28,30,31	4,5,6,7,25,<br>27,29,32	3	P00714,P50116,Q99MH3
1211	1,2,3,8,26,<br>28,30,32	4,5,6,7,25,<br>27,29,31	4	O89117,P50116,Q68G31,Q99MH3
1212	1,2,3,8,26,<br>28,31,32	4,5,6,7,25,<br>27,29,30	1	O89117
1213	1,2,3,8,26,<br>29,30,31	4,5,6,7,25,<br>27,28,32	1	P50116
1214	1,2,3,8,26,<br>29,30,32	4,5,6,7,25,<br>27,28,31	3	O88797,P50116,Q68G31
1215	1,2,3,8,26,<br>29,31,32	4,5,6,7,25,<br>27,28,30	1	P50116
1216	1,2,3,8,26,<br>30,31,32	4,5,6,7,25,<br>27,28,29	5	O89117,P50116,P97615,Q63617,Q99MH3
1217	1,2,3,8,27,<br>28,29,30	4,5,6,7,25,<br>26,31,32	2	Q6IFU7,Q9WVK7
1218	1,2,3,8,27,<br>28,29,31	4,5,6,7,25,<br>26,30,32	1	Q9WVK7
1219	1,2,3,8,27,<br>28,29,32	4,5,6,7,25,<br>26,30,31	4	P25809,Q6AYR9,Q812E4,Q9WVK7
1220	1,2,3,8,27,<br>28,30,31	4,5,6,7,25,<br>26,29,32	1	Q6IG05
1221	1,2,3,8,27,<br>28,30,32	4,5,6,7,25,<br>26,29,31	1	Q6IFU7
1222	1,2,3,8,27,<br>28,31,32	4,5,6,7,25,<br>26,29,30	0	
1223	1,2,3,8,27,<br>29,30,31	4,5,6,7,25,<br>26,28,32	5	P00731,Q6IFU7,Q6IFW6,Q6IG02,Q6IG05
1224	1,2,3,8,27,<br>29,30,32	4,5,6,7,25,<br>26,28,31	1	Q6IFU7
1225	1,2,3,8,27,<br>29,31,32	4,5,6,7,25,<br>26,28,30	0	
1226	1,2,3,8,27,<br>30,31,32	4,5,6,7,25,<br>26,28,29	2	P20646,Q6IG05

1227	1,2,3,8,28,29,30,31	4,5,6,7,25,26,27,32	1	P50116
1228	1,2,3,8,28,29,30,32	4,5,6,7,25,26,27,31	2	P50116,Q68G31
1229	1,2,3,8,28,29,31,32	4,5,6,7,25,26,27,30	2	P21588,P50116
1230	1,2,3,8,28,30,31,32	4,5,6,7,25,26,27,29	2	O89117,P50116
1231	1,2,3,8,29,30,31,32	4,5,6,7,25,26,27,28	2	P50116,P63081
1232	1,2,3,25,26,27,28,29	4,5,6,7,8,30,31,32	5	P00714,P0DMW0;P0DMW1,P82471,Q99041,Q9Z0J6
1233	1,2,3,25,26,27,28,30	4,5,6,7,8,29,31,32	3	P00714,P82471,Q99041
1234	1,2,3,25,26,27,28,31	4,5,6,7,8,29,30,32	3	P00714,P22273,Q99041
1235	1,2,3,25,26,27,28,32	4,5,6,7,8,29,30,31	3	P82471,Q5RJR2,Q99041
1236	1,2,3,25,26,27,29,30	4,5,6,7,8,28,31,32	9	P07171,P11598,P22006,P22273,P22282,P82471,Q63617,Q99041,Q9Z0J6
1237	1,2,3,25,26,27,29,31	4,5,6,7,8,28,30,32	5	P07171,P22273,P22282,Q63617,Q99041
1238	1,2,3,25,26,27,29,32	4,5,6,7,8,28,30,31	5	P07171,P22273,P82471,Q99041,Q9Z0J6
1239	1,2,3,25,26,27,30,31	4,5,6,7,8,28,29,32	9	P00714,P02780,P06760,P06761,P11598,P22273,P22282,Q63617,Q99041
1240	1,2,3,25,26,27,30,32	4,5,6,7,8,28,29,31	9	D3ZUC6,P06760,P06761,P07171,P21674,P22273,P82471,Q63617,Q99041
1241	1,2,3,25,26,27,31,32	4,5,6,7,8,28,29,30	6	P06760,P07171,P19218,P22273,Q63617,Q99041
1242	1,2,3,25,26,28,29,30	4,5,6,7,8,27,31,32	8	P00714,P22273,P22282,P50115,P50116,P82471,Q99041,Q9Z0J6
1243	1,2,3,25,26,28,29,31	4,5,6,7,8,27,30,32	8	P00714,P19218,P22273,P22282,P50116,Q01205,Q99041,Q9Z0J6
1244	1,2,3,25,26,28,29,32	4,5,6,7,8,27,30,31	7	P19218,P22273,P50115,P50116,P82471,Q99041,Q9Z0J6
1245	1,2,3,25,26,28,30,31	4,5,6,7,8,27,29,32	9	P00714,P06760,P22273,P22282,P50115,P50116,Q4G075,Q6TMA8,Q99041
1246	1,2,3,25,26,28,30,32	4,5,6,7,8,27,29,31	11	P06760,P22273,P22282,P50115,P50116,P82471,Q4G075,Q62975,Q68G31,Q99041,Q9QX74

1247	1,2,3,25,26,28,31,32	4,5,6,7,8,27,29,30	8	P06760,P19218,P22273,P22282,P50115,P50116,Q4G075,Q99041
1248	1,2,3,25,26,29,30,31	4,5,6,7,8,27,28,32	11	P00714,P06761,P11598,P22273,P22282,P50115,P50116,Q63617,Q6RUV5,Q99041,Q9Z0J6
1249	1,2,3,25,26,29,30,32	4,5,6,7,8,27,28,31	13	D3ZUC6,P06761,P11598,P22273,P22282,P50115,P50116,P82471,Q4G075,Q63617,Q68G31,Q99041,Q9Z0J6
1250	1,2,3,25,26,29,31,32	4,5,6,7,8,27,28,30	10	P06761,P19218,P22273,P22282,P50115,P50116,Q4G075,Q63617,Q99041,Q9Z0J6
1251	1,2,3,25,26,30,31,32	4,5,6,7,8,27,28,29	15	D3ZUC6,P04636,P06760,P06761,P11598,P22273,P22282,P22283,P50115,P50116,Q4G075,Q63617,Q812E4,Q99041,Q99MH3
1252	1,2,3,25,27,28,29,30	4,5,6,7,8,26,31,32	3	P82471,Q99041,Q9Z0J6
1253	1,2,3,25,27,28,29,31	4,5,6,7,8,26,30,32	1	Q99041
1254	1,2,3,25,27,28,29,32	4,5,6,7,8,26,30,31	7	P49134,P82471,Q5RJR2,Q62761;Q62762;Q62763,Q6AY61,Q99041,Q9Z0J6
1255	1,2,3,25,27,28,30,31	4,5,6,7,8,26,29,32	5	P00714,P06760,P22273,Q99041,Q9QX74
1256	1,2,3,25,27,28,30,32	4,5,6,7,8,26,29,31	7	P06760,P22273,P49134,P82471,Q5RJR2,Q99041,Q9QX74
1257	1,2,3,25,27,28,31,32	4,5,6,7,8,26,29,30	6	P06760,P22273,P49134,Q5RJR2,Q99041,Q9QX74
1258	1,2,3,25,27,29,30,31	4,5,6,7,8,26,28,32	7	O54728,P07150,P11598,P22273,P22282,Q63617,Q99041
1259	1,2,3,25,27,29,30,32	4,5,6,7,8,26,28,31	5	P07150,P22273,P82471,Q99041,Q9Z0J6
1260	1,2,3,25,27,29,31,32	4,5,6,7,8,26,28,30	3	P07150,P22273,Q99041
1261	1,2,3,25,27,30,31,32	4,5,6,7,8,26,28,29	14	D3ZUC6,P06760,P06761,P07150,P11598,P21674,P22273,P22282,P49134,Q63617,Q6P6R2,Q99041,Q9QX74,Q9WTT6
1262	1,2,3,25,28,29,30,31	4,5,6,7,8,26,27,32	7	P00714,P22273,P22282,P50115,P50116,Q99041,Q9Z0J6
1263	1,2,3,25,28,29,30,32	4,5,6,7,8,26,27,31	8	P22273,P22282,P50115,P50116,Q62975,Q68G31,Q99041,Q9Z0J6
1264	1,2,3,25,28,29,31,32	4,5,6,7,8,26,27,30	7	P21588,P22273,P22282,P50115,P50116,Q99041,Q9Z0J6
1265	1,2,3,25,28,30,31,32	4,5,6,7,8,26,27,29	11	P06760,P06761,P22273,P22282,P50115,P50116,Q4G075,Q62975,Q99041,Q9QX74,Q9WTT6
1266	1,2,3,25,29,30,31,32	4,5,6,7,8,26,27,28	13	D3ZUC6,P06761,P07150,P11598,P22273,P22282,P50115,P50116,Q4G075,Q63617,Q99041,Q9WTT6,Q9Z0J6

1267	1,2,3,26,27,28,29,30	4,5,6,7,8,25,31,32	3	O54858,P82471,Q9WVK7
1268	1,2,3,26,27,28,29,31	4,5,6,7,8,25,30,32	3	P0DMW0;P0DMW1,Q99041,Q9WVK7
1269	1,2,3,26,27,28,29,32	4,5,6,7,8,25,30,31	2	O54858,P82471
1270	1,2,3,26,27,28,30,31	4,5,6,7,8,25,29,32	3	P00714,P06760,P22273
1271	1,2,3,26,27,28,30,32	4,5,6,7,8,25,29,31	3	P06760,P22273,P82471
1272	1,2,3,26,27,28,31,32	4,5,6,7,8,25,29,30	3	P06760,P22273,Q99041
1273	1,2,3,26,27,29,30,31	4,5,6,7,8,25,28,32	4	P22273,P22282,Q63617,Q99041
1274	1,2,3,26,27,29,30,32	4,5,6,7,8,25,28,31	2	P22273,P82471
1275	1,2,3,26,27,29,31,32	4,5,6,7,8,25,28,30	2	P22273,Q99041
1276	1,2,3,26,27,30,31,32	4,5,6,7,8,25,28,29	6	P06760,P06761,P22273,P22282,Q63617,Q99041
1277	1,2,3,26,28,29,30,31	4,5,6,7,8,25,27,32	8	P00714,P22273,P22282,P50115,P50116,Q01984,Q4G075,Q99041
1278	1,2,3,26,28,29,30,32	4,5,6,7,8,25,27,31	8	P22273,P22282,P50115,P50116,P82471,Q4G075,Q68G31,Q99041
1279	1,2,3,26,28,29,31,32	4,5,6,7,8,25,27,30	6	P22273,P50115,P50116,Q4G075,Q99041,Q99376
1280	1,2,3,26,28,30,31,32	4,5,6,7,8,25,27,29	7	P06760,P22273,P22282,P50115,P50116,Q4G075,Q99041
1281	1,2,3,26,29,30,31,32	4,5,6,7,8,25,27,28	9	P06761,P11598,P22273,P22282,P50115,P50116,Q4G075,Q63617,Q99041
1282	1,2,3,27,28,29,30,31	4,5,6,7,8,25,26,32	2	P22273,Q99041
1283	1,2,3,27,28,29,30,32	4,5,6,7,8,25,26,31	3	P22273,Q63150,Q9Z0V6
1284	1,2,3,27,28,29,31,32	4,5,6,7,8,25,26,30	3	P22273,P49134,Q99041
1285	1,2,3,27,28,30,31,32	4,5,6,7,8,25,26,29	4	P06760,P22273,P49134,Q9QX74
1286	1,2,3,27,29,30,31,32	4,5,6,7,8,25,26,28	4	P07150,P22273,Q99041,Q9WTT6

1287	1,2,3,28,29,30,31,32	4,5,6,7,8,25,26,27	7	P22273,P22282,P50115,P50116,Q4G075,Q99041,Q9WTT6
1288	1,2,4,5,6,7,8,25	3,26,27,28,29,30,31,32	12	O54858,P02454,P02631,P06911,P12020,P35952,P50115,P52590,Q09030,Q4KLZ6,Q99041,Q9QZQ5
1289	1,2,4,5,6,7,8,26	3,25,27,28,29,30,31,32	17	O54858,P00762,P02454,P02631,P11598,P20762,P22273,P35952,P50115,P52590,P97580,Q30KJ2,Q5RJR2,Q5RLM2,Q99041,Q9QZQ5,Q9Z0J6
1290	1,2,4,5,6,7,8,27	3,25,26,28,29,30,31,32	24	O54858,P00731,P01039,P02454,P02631,P06911,P11598,P19218,P22273,P22282,P50115,P50116,P52590,P97580,Q03191,Q10758,Q30KJ2,Q4G075,Q66H69,Q91ZS3,Q99041,Q9JJ50,Q9QZQ5,Q9Z0J6
1291	1,2,4,5,6,7,8,28	3,25,26,27,29,30,31,32	20	O54858,P00762,P02454,P02631,P06761,P06911,P07171,P11598,P12020,P19218,P21674,P22273,P22282,P52590,Q63532,Q63617,Q99041,Q9JI85,Q9QZQ5,Q9Z0J6
1292	1,2,4,5,6,7,8,29	3,25,26,27,28,30,31,32	12	O70594,P00762,P02631,P06911,P11598,P21674,P22273,P42854,P52590,P62804,Q5RJR2,Q99041
1293	1,2,4,5,6,7,8,30	3,25,26,27,28,29,31,32	18	O54858,P00762,P02631,P06911,P19218,P35952,P49744,P52590,P97580,Q30KJ2,Q5RJR2,Q63532,Q6IFU8,Q6IG05,Q6IMF3,Q6P6Q2,Q99041,Q9Z0J6
1294	1,2,4,5,6,7,8,31	3,25,26,27,28,29,30,32	11	O54858,P00762,P02454,P06911,P12020,P52590,Q5RJR2,Q63532,Q99041,Q9QZQ5,Q9Z0J6
1295	1,2,4,5,6,7,8,32	3,25,26,27,28,29,30,31	11	O35276,O54858,P02454,P02631,P0DP29;P0DP30;P0DP31,P11598,P52590,Q63532,Q99041,Q9QZQ5,Q9Z0J6
1296	1,2,4,5,6,7,25,26	3,8,27,28,29,30,31,32	6	P00762,P21588,P52590,Q4KLZ6,Q9EQS0,Q9WVH8
1297	1,2,4,5,6,7,25,27	3,8,26,28,29,30,31,32	5	P01039,P50116,P52590,Q03191,Q4KLZ6
1298	1,2,4,5,6,7,25,28	3,8,26,27,29,30,31,32	4	P00762,P11598,P52590,Q4KLZ6
1299	1,2,4,5,6,7,25,29	3,8,26,27,28,30,31,32	4	P00762,P52590,Q4KLZ6,Q9QWN8
1300	1,2,4,5,6,7,25,30	3,8,26,27,28,29,31,32	6	P26342,P52590,Q4KLZ6,Q6P6R2,Q9EQS0,Q9QWN8

1301	1,2,4,5,6,7,25,31	3,8,26,27,28,29,30,32	7	P00762,P52590,Q4KLZ6,Q63532,Q9EQS0,Q9QWN8,Q9WVH8
1302	1,2,4,5,6,7,25,32	3,8,26,27,28,29,30,31	7	P0DP29;P0DP30;P0DP31,P26342,P52590,Q4KLZ6,Q6P6R2,Q9EQS0,Q9R168
1303	1,2,4,5,6,7,26,27	3,8,25,28,29,30,31,32	8	P01039,P11598,P21588,P50116,P52590,Q03191,Q9JJ50,Q9Z0J6
1304	1,2,4,5,6,7,26,28	3,8,25,27,29,30,31,32	7	P00762,P11598,P14173,P21588,P52590,Q5RLM2,Q9Z0J6
1305	1,2,4,5,6,7,26,29	3,8,25,27,28,30,31,32	4	P00762,P14173,P21588,P52590
1306	1,2,4,5,6,7,26,30	3,8,25,27,28,29,31,32	6	P00762,P21588,P52590,Q80WL1,Q9EQS0,Q9Z0J6
1307	1,2,4,5,6,7,26,31	3,8,25,27,28,29,30,32	8	P00762,P14173,P52590,Q63150,Q63532,Q9EQS0,Q9WVH8,Q9Z0J6
1308	1,2,4,5,6,7,26,32	3,8,25,27,28,29,30,31	5	P14173,P52590,Q5M872,Q9EQS0,Q9Z0J6
1309	1,2,4,5,6,7,27,28	3,8,25,26,29,30,31,32	9	P00762,P01039,P11598,P19218,P21588,P50116,P52590,Q03191,Q9Z0J6
1310	1,2,4,5,6,7,27,29	3,8,25,26,28,30,31,32	9	O08839,P01039,P19223,P21588,P50116,P52590,Q03191,Q923V8,Q9JJ50
1311	1,2,4,5,6,7,27,30	3,8,25,26,28,29,31,32	9	P01039,P19218,P21588,P49744,P50116,P52590,Q03191,Q80WL1,Q9Z0J6
1312	1,2,4,5,6,7,27,31	3,8,25,26,28,29,30,32	7	P01039,P50116,P52590,P80299,Q03191,Q9QZ76,Q9Z0J6
1313	1,2,4,5,6,7,27,32	3,8,25,26,28,29,30,31	9	P01039,P0DP29;P0DP30;P0DP31,P11598,P50116,P52590,P80299,Q03191,Q9JJ50,Q9Z0J6

1314	1,2,4,5,6,7,28,29	3,8,25,26,27,30,31,32	7	P00762,P11598,P14173,P21674,P47967,P52590,Q63617
1315	1,2,4,5,6,7,28,30	3,8,25,26,27,29,31,32	6	P00762,P19218,P21588,P52590,Q80WL1,Q9Z0J6
1316	1,2,4,5,6,7,28,31	3,8,25,26,27,29,30,32	6	P00762,P11598,P14173,P52590,Q63532,Q9Z0J6
1317	1,2,4,5,6,7,28,32	3,8,25,26,27,29,30,31	5	P0DP29;P0DP30;P0DP31,P11598,P14173,P52590,Q9Z0J6
1318	1,2,4,5,6,7,29,30	3,8,25,26,27,28,31,32	11	B1H234,P00762,P01835,P17046,P21588,P26342,P36860,P52590,Q5RJR2,Q80WL1,Q9QWN8
1319	1,2,4,5,6,7,29,31	3,8,25,26,27,28,30,32	9	B1H234,P00762,P01835,P26342,P36860,P52590,P80299,Q5RJR2,Q9QWN8
1320	1,2,4,5,6,7,29,32	3,8,25,26,27,28,30,31	6	P01835,P14173,P26342,P36860,P52590,P80299
1321	1,2,4,5,6,7,30,31	3,8,25,26,27,28,29,32	11	P00762,P26342,P36860,P52590,Q5RJR2,Q63532,Q6P6R2,Q8CFN2,Q9EQS0,Q9QWN8,Q9Z0J6
1322	1,2,4,5,6,7,30,32	3,8,25,26,27,28,29,31	9	P0DP29;P0DP30;P0DP31,P26342,P36860,P52590,P70549,Q6P6R2,Q9EQS0,Q9R168,Q9Z0J6
1323	1,2,4,5,6,7,31,32	3,8,25,26,27,28,29,30	9	P0DP29;P0DP30;P0DP31,P14173,P26342,P36860,P52590,P80299,Q63532,Q6P6R2,Q9Z0J6
1324	1,2,4,5,6,8,25,26	3,7,27,28,29,30,31,32	9	G3V686,O54858,P08649,P13432,P35952,P52590,P97580,Q30KJ2,Q4KLZ6
1325	1,2,4,5,6,8,25,27	3,7,26,28,29,30,31,32	9	P15399,P17559,P52590,P97580,Q03191,Q10758,Q30KJ2,Q4KLZ6,Q9QZQ5
1326	1,2,4,5,6,8,25,28	3,7,26,27,29,30,31,32	6	P02631,P06911,P08649,P52590,Q4KLZ6,Q9QZQ5

1327	1,2,4,5,6,8,25,29	3,7,26,27,28,30,31,32	3	P42854,P52590,Q4KLZ6
1328	1,2,4,5,6,8,25,30	3,7,26,27,28,29,31,32	11	O54858,P35952,P52590,P97580,Q09030,Q30KJ2,Q4KLZ6,Q6IFU8,Q6IG05,Q6IMF3,Q6P6Q2
1329	1,2,4,5,6,8,25,31	3,7,26,27,28,29,30,32	6	O54858,P06911,P52590,Q09030,Q4KLZ6,Q63532
1330	1,2,4,5,6,8,25,32	3,7,26,27,28,29,30,31	7	O54858,P02454,P02631,P13432,P52590,Q4KLZ6,Q9QZQ5
1331	1,2,4,5,6,8,26,27	3,7,25,28,29,30,31,32	10	P15399,P17559,P20762,P21588,P52590,P97580,Q03191,Q10758,Q30KJ2,Q9JJ50
1332	1,2,4,5,6,8,26,28	3,7,25,27,29,30,31,32	8	P00762,P08649,P15399,P20762,P52590,P97580,Q30KJ2,Q9JI85
1333	1,2,4,5,6,8,26,29	3,7,25,27,28,30,31,32	5	P42854,P52590,P97580,Q30KJ2,Q5RJR2
1334	1,2,4,5,6,8,26,30	3,7,25,27,28,29,31,32	9	P20762,P21588,P35952,P52590,P97580,Q30KJ2,Q5RJR2,Q6IG05,Q6P6Q2
1335	1,2,4,5,6,8,26,31	3,7,25,27,28,29,30,32	6	O54858,P52590,P97580,Q30KJ2,Q5RJR2,Q63532
1336	1,2,4,5,6,8,26,32	3,7,25,27,28,29,30,31	5	P13432,P18418,P52590,P97580,Q30KJ2
1337	1,2,4,5,6,8,27,28	3,7,25,26,29,30,31,32	10	P15399,P19218,P22282,P52590,P97580,Q03191,Q10758,Q30KJ2,Q9JI85,Q9QZQ5
1338	1,2,4,5,6,8,27,29	3,7,25,26,28,30,31,32	11	P00731,P15399,P19218,P19223,P42854,P52590,P97580,Q03191,Q10758,Q30KJ2,Q9JJ50
1339	1,2,4,5,6,8,27,30	3,7,25,26,28,29,31,32	16	P00731,P01039,P15399,P19218,P21588,P52590,P97580,Q03191,Q10758,Q30KJ2,Q5I0J9,Q6IFU7,Q6IFU8,Q6IG05,Q6IMF3,Q6P6Q2

1340	1,2,4,5,6,8,27,31	3,7,25,26,28,29,30,32	10	O54858,P01039,P15399,P23739,P52590,P97580,Q03191,Q10758,Q30KJ2,Q63532
1341	1,2,4,5,6,8,27,32	3,7,25,26,28,29,30,31	8	P15399,P19218,P52590,P97580,Q03191,Q10758,Q30KJ2,Q9QZQ5
1342	1,2,4,5,6,8,28,29	3,7,25,26,27,30,31,32	5	P00762,P21674,P42854,P52590,Q9JI85
1343	1,2,4,5,6,8,28,30	3,7,25,26,27,29,31,32	4	P19218,P52590,Q30KJ2,Q6P6Q2
1344	1,2,4,5,6,8,28,31	3,7,25,26,27,29,30,32	2	P52590,Q63532
1345	1,2,4,5,6,8,28,32	3,7,25,26,27,29,30,31	5	P02454,P52590,Q63532,Q9JI85,Q9QZQ5
1346	1,2,4,5,6,8,29,30	3,7,25,26,27,28,31,32	8	P19218,P52590,Q5RJR2,Q6IFU7,Q6IFU8,Q6IG05,Q6IMF3,Q6P6Q2
1347	1,2,4,5,6,8,29,31	3,7,25,26,27,28,30,32	3	P52590,Q5RJR2,Q63532
1348	1,2,4,5,6,8,29,32	3,7,25,26,27,28,30,31	2	P42854,P52590
1349	1,2,4,5,6,8,30,31	3,7,25,26,27,28,29,32	10	O54858,P19218,P35952,P52590,Q5RJR2,Q63532,Q6IFU8,Q6IG05,Q6IMF3,Q6P6Q2
1350	1,2,4,5,6,8,30,32	3,7,25,26,27,28,29,31	10	O54858,P19218,P20646,P26342,P52590,Q5I0J9,Q6IG05,Q6IMF3,Q6P6Q2,Q6P6R2
1351	1,2,4,5,6,8,31,32	3,7,25,26,27,28,29,30	3	O54858,P52590,Q63532
1352	1,2,4,5,6,25,26,27	3,7,8,28,29,30,31,32	7	P01039,P21588,P35280,P52590,Q03191,Q4KLZ6,Q9JJ50
1353	1,2,4,5,6,25,26,28	3,7,8,27,29,30,31,32	6	P00762,P21588,P35280,P52590,Q4KLZ6,Q9EQS0

1354	1,2,4,5,6,25,26,29	3,7,8,27,28,30,31,32	4	P35280,P52590,Q4KLZ6,Q9EQS0
1355	1,2,4,5,6,25,26,30	3,7,8,27,28,29,31,32	5	P21588,P52590,Q4KLZ6,Q812E4,Q9EQS0
1356	1,2,4,5,6,25,26,31	3,7,8,27,28,29,30,32	6	P35280,P52590,Q4KLZ6,Q63150,Q812E4,Q9EQS0
1357	1,2,4,5,6,25,26,32	3,7,8,27,28,29,30,31	5	P00507,P52590,Q06000,Q4KLZ6,Q9EQS0
1358	1,2,4,5,6,25,27,28	3,7,8,26,29,30,31,32	4	P35280,P52590,Q03191,Q4KLZ6
1359	1,2,4,5,6,25,27,29	3,7,8,26,28,30,31,32	5	P35280,P52590,Q03191,Q4KLZ6,Q923V8
1360	1,2,4,5,6,25,27,30	3,7,8,26,28,29,31,32	6	P01039,P21588,P52590,Q03191,Q4KLZ6,Q6P6R2
1361	1,2,4,5,6,25,27,31	3,7,8,26,28,29,30,32	6	P01039,P35280,P52590,Q03191,Q4KLZ6,Q9QYP1
1362	1,2,4,5,6,25,27,32	3,7,8,26,28,29,30,31	5	P01039,P52590,Q03191,Q4KLZ6,Q6P6R2
1363	1,2,4,5,6,25,28,29	3,7,8,26,27,30,31,32	4	P00762,P35280,P52590,Q4KLZ6
1364	1,2,4,5,6,25,28,30	3,7,8,26,27,29,31,32	3	P52590,Q4KLZ6,Q9EQS0
1365	1,2,4,5,6,25,28,31	3,7,8,26,27,29,30,32	3	P35280,P52590,Q4KLZ6
1366	1,2,4,5,6,25,28,32	3,7,8,26,27,29,30,31	3	P52590,Q4KLZ6,Q9QX74
1367	1,2,4,5,6,25,29,30	3,7,8,26,27,28,31,32	3	P52590,Q4KLZ6,Q9QWN8
1368	1,2,4,5,6,25,29,31	3,7,8,26,27,28,30,32	5	B1H234,P35280,P52590,Q4KLZ6,Q9QWN8
1369	1,2,4,5,6,25,29,32	3,7,8,26,27,28,30,31	2	P52590,Q4KLZ6
1370	1,2,4,5,6,25,30,31	3,7,8,26,27,28,29,32	8	P52590,Q4KLZ6,Q6P6R2,Q812E4,Q8CFN2,Q9EQS0,Q9QWN8,Q9WTT6
1371	1,2,4,5,6,25,30,32	3,7,8,26,27,28,29,31	7	P26342,P52590,Q4KLZ6,Q6P6R2,Q9EQS0,Q9QX74,Q9WTT6
1372	1,2,4,5,6,25,31,32	3,7,8,26,27,28,29,30	6	P26342,P52590,Q4KLZ6,Q6P6R2,Q9EQS0,Q9WTT6
1373	1,2,4,5,6,26,27,28	3,7,8,25,29,30,31,32	4	P21588,P35280,P52590,Q03191

1374	1,2,4,5,6,26,27,29	3,7,8,25,28,30,31,32	7	P02625,P21588,P35280,P52590,Q03191,Q923V8,Q9JJ50
1375	1,2,4,5,6,26,27,30	3,7,8,25,28,29,31,32	7	P01039,P21588,P52590,Q03191,Q80WL1,Q9EQS0,Q9JJ50
1376	1,2,4,5,6,26,27,31	3,7,8,25,28,29,30,32	5	P01039,P35280,P52590,Q03191,Q9Z0J6
1377	1,2,4,5,6,26,27,32	3,7,8,25,28,29,30,31	5	P01039,P21588,P52590,Q03191,Q9JJ50
1378	1,2,4,5,6,26,28,29	3,7,8,25,27,30,31,32	5	P00762,P02625,P21588,P35280,P52590
1379	1,2,4,5,6,26,28,30	3,7,8,25,27,29,31,32	4	P00762,P21588,P52590,Q9EQS0
1380	1,2,4,5,6,26,28,31	3,7,8,25,27,29,30,32	4	P00762,P35280,P52590,Q9EQS0
1381	1,2,4,5,6,26,28,32	3,7,8,25,27,29,30,31	3	P14173,P52590,Q9EQS0
1382	1,2,4,5,6,26,29,30	3,7,8,25,27,28,31,32	9	B1H234,P00762,P21588,P36860,P52590,Q5RJR2,Q80WL1,Q923V8,Q9EQS0
1383	1,2,4,5,6,26,29,31	3,7,8,25,27,28,30,32	7	B1H234,O70417,P00762,P35280,P36860,P52590,Q5RJR2
1384	1,2,4,5,6,26,29,32	3,7,8,25,27,28,30,31	2	P36860,P52590
1385	1,2,4,5,6,26,30,31	3,7,8,25,27,28,29,32	6	P36860,P52590,Q5RJR2,Q812E4,Q9EQS0,Q9Z2L0
1386	1,2,4,5,6,26,30,32	3,7,8,25,27,28,29,31	5	P21588,P26342,P36860,P52590,Q9EQS0
1387	1,2,4,5,6,26,31,32	3,7,8,25,27,28,29,30	3	P36860,P52590,Q9EQS0
1388	1,2,4,5,6,27,28,29	3,7,8,25,26,30,31,32	5	P02625,P21588,P35280,P52590,Q03191
1389	1,2,4,5,6,27,28,30	3,7,8,25,26,29,31,32	5	P19218,P21588,P52590,Q03191,Q5I0J9
1390	1,2,4,5,6,27,28,31	3,7,8,25,26,29,30,32	4	P35280,P52590,Q03191,Q9Z0J6
1391	1,2,4,5,6,27,28,32	3,7,8,25,26,29,30,31	3	P52590,Q03191,Q62761;Q62762;Q62763
1392	1,2,4,5,6,27,29,30	3,7,8,25,26,28,31,32	8	B1H234,P19218,P19223,P21588,P52590,Q03191,Q5I0J9,Q923V8
1393	1,2,4,5,6,27,29,31	3,7,8,25,26,28,30,32	6	B1H234,P01039,P35280,P52590,P80299,Q03191

1394	1,2,4,5,6,27,29,32	3,7,8,25,26,28,30,31	3	P52590,Q03191,Q9JJ50
1395	1,2,4,5,6,27,30,31	3,7,8,25,26,28,29,32	7	P01039,P36860,P52590,Q03191,Q6P6R2,Q9WTT6,Q9Z0J6
1396	1,2,4,5,6,27,30,32	3,7,8,25,26,28,29,31	10	P01039,P19218,P52590,P70549,Q03191,Q5I0J9,Q64093,Q6P6R2,Q9QX74,Q9WTT6
1397	1,2,4,5,6,27,31,32	3,7,8,25,26,28,29,30	7	P01039,P52590,P80299,Q03191,Q6P6R2,Q9WTT6,Q9Z0J6
1398	1,2,4,5,6,28,29,30	3,7,8,25,26,27,31,32	4	B1H234,P00762,P21588,P52590
1399	1,2,4,5,6,28,29,31	3,7,8,25,26,27,30,32	4	B1H234,P00762,P35280,P52590
1400	1,2,4,5,6,28,29,32	3,7,8,25,26,27,30,31	1	P52590
1401	1,2,4,5,6,28,30,31	3,7,8,25,26,27,29,32	4	P00762,P52590,Q63532,Q9WTT6
1402	1,2,4,5,6,28,30,32	3,7,8,25,26,27,29,31	5	P52590,Q5I0J9,Q8VD89,Q9QX74,Q9WTT6
1403	1,2,4,5,6,28,31,32	3,7,8,25,26,27,29,30	4	P22006,P52590,Q63532,Q9WTT6
1404	1,2,4,5,6,29,30,31	3,7,8,25,26,27,28,32	7	B1H234,P26342,P36860,P52590,Q5RJR2,Q9QWN8,Q9WTT6
1405	1,2,4,5,6,29,30,32	3,7,8,25,26,27,28,31	7	B1H234,O55145,P26342,P36860,P52590,Q5I0J9,Q9WTT6
1406	1,2,4,5,6,29,31,32	3,7,8,25,26,27,28,30	7	B1H234,O70417,P26342,P36860,P52590,P80299,Q9WTT6
1407	1,2,4,5,6,30,31,32	3,7,8,25,26,27,28,29	8	P20646,P26342,P36860,P52590,Q6P6R2,Q9EQS0,Q9QX74,Q9WTT6
1408	1,2,4,5,7,8,25,26	3,6,27,28,29,30,31,32	3	G3V686,P20762,P22006
1409	1,2,4,5,7,8,25,27	3,6,26,28,29,30,31,32	5	P22006,P50116,Q03191,Q4G075,Q66H69
1410	1,2,4,5,7,8,25,28	3,6,26,27,29,30,31,32	1	Q811M5
1411	1,2,4,5,7,8,25,29	3,6,26,27,28,30,31,32	4	P06760,P22006,P48032,P62804

1412	1,2,4,5,7,8,25,30	3,6,26,27,28,29,31,32	5	Q4FZU2,Q6IFU8,Q6IG05,Q6IMF3,Q6P6Q2
1413	1,2,4,5,7,8,25,31	3,6,26,27,28,29,30,32	1	Q811M5
1414	1,2,4,5,7,8,25,32	3,6,26,27,28,29,30,31	2	P01681,Q811M5
1415	1,2,4,5,7,8,26,27	3,6,25,28,29,30,31,32	10	P20762,P50116,P97580,Q03191,Q10758,Q30KJ2,Q4G075,Q5RLM2,Q66H69,Q9JJ50
1416	1,2,4,5,7,8,26,28	3,6,25,27,29,30,31,32	3	P00762,P20762,Q5RLM2
1417	1,2,4,5,7,8,26,29	3,6,25,27,28,30,31,32	6	P06760,P20762,P22006,P62804,Q5RJR2,Q9QX74
1418	1,2,4,5,7,8,26,30	3,6,25,27,28,29,31,32	5	P20762,Q5RJR2,Q6IFU8,Q6IMF3,Q6P6Q2
1419	1,2,4,5,7,8,26,31	3,6,25,27,28,29,30,32	3	P20762,Q5RJR2,Q5RLM2
1420	1,2,4,5,7,8,26,32	3,6,25,27,28,29,30,31	1	P20762
1421	1,2,4,5,7,8,27,28	3,6,25,26,29,30,31,32	10	P19218,P22282,P25809,P47967,P50116,P62804,Q03191,Q10758,Q4G075,Q66H69
1422	1,2,4,5,7,8,27,29	3,6,25,26,28,30,31,32	12	P00731,P06760,P19223,P22006,P25809,P62804,Q00715,Q03191,Q10758,Q4G075,Q5I0D1,Q66H69
1423	1,2,4,5,7,8,27,30	3,6,25,26,28,29,31,32	13	P00731,P19218,P70549,Q03191,Q10758,Q30KJ2,Q4FZU2,Q4G075,Q66H69,Q6IFU8,Q6IG05,Q6IMF3,Q6P6Q2
1424	1,2,4,5,7,8,27,31	3,6,25,26,28,29,30,32	8	P00731,P23739,P82471,Q03191,Q10758,Q4G075,Q66H69,Q9Z0J6

1425	1,2,4,5,7,8,27,32	3,6,25,26,28,29,30,31	4	Q03191,Q10758,Q66H69,Q6TMA8
1426	1,2,4,5,7,8,28,29	3,6,25,26,27,30,31,32	6	P00762,P06760,P21674,P47967,P62804,Q00715
1427	1,2,4,5,7,8,28,30	3,6,25,26,27,29,31,32	5	P19218,P62804,Q6IFU8,Q6IMF3,Q6P6Q2
1428	1,2,4,5,7,8,28,31	3,6,25,26,27,29,30,32	3	P82471,Q5RLM2,Q811M5
1429	1,2,4,5,7,8,28,32	3,6,25,26,27,29,30,31	1	Q811M5
1430	1,2,4,5,7,8,29,30	3,6,25,26,27,28,31,32	6	P62804,Q4FZU2,Q5RJR2,Q6IFU8,Q6IMF3,Q6P6Q2
1431	1,2,4,5,7,8,29,31	3,6,25,26,27,28,30,32	3	P06760,P82471,Q5RJR2
1432	1,2,4,5,7,8,29,32	3,6,25,26,27,28,30,31	0	
1433	1,2,4,5,7,8,30,31	3,6,25,26,27,28,29,32	7	P82471,Q4FZU2,Q5RJR2,Q6IFU8,Q6IG05,Q6IMF3,Q6P6Q2
1434	1,2,4,5,7,8,30,32	3,6,25,26,27,28,29,31	5	P26342,P70549,Q6IFU8,Q6IMF3,Q6P6Q2
1435	1,2,4,5,7,8,31,32	3,6,25,26,27,28,29,30	2	P82471,Q811M5
1436	1,2,4,5,7,25,26,27	3,6,8,28,29,30,31,32	2	P21588,Q03191
1437	1,2,4,5,7,25,26,28	3,6,8,27,29,30,31,32	0	
1438	1,2,4,5,7,25,26,29	3,6,8,27,28,30,31,32	2	P22006,Q9QZK9
1439	1,2,4,5,7,25,26,30	3,6,8,27,28,29,31,32	2	Q9EQS0,Q9R168

1440	1,2,4,5,7,25,26,31	3,6,8,27,28,29,30,32	0
1441	1,2,4,5,7,25,26,32	3,6,8,27,28,29,30,31	1 Q9R168
1442	1,2,4,5,7,25,27,28	3,6,8,26,29,30,31,32	1 Q03191
1443	1,2,4,5,7,25,27,29	3,6,8,26,28,30,31,32	4 O89117,P19223,P22006,Q03191
1444	1,2,4,5,7,25,27,30	3,6,8,26,28,29,31,32	2 P70549,Q03191
1445	1,2,4,5,7,25,27,31	3,6,8,26,28,29,30,32	3 P23739,Q03191,Q9QYP1
1446	1,2,4,5,7,25,27,32	3,6,8,26,28,29,30,31	1 Q03191
1447	1,2,4,5,7,25,28,29	3,6,8,26,27,30,31,32	1 P00762
1448	1,2,4,5,7,25,28,30	3,6,8,26,27,29,31,32	1 Q9R168
1449	1,2,4,5,7,25,28,31	3,6,8,26,27,29,30,32	1 Q811M5
1450	1,2,4,5,7,25,28,32	3,6,8,26,27,29,30,31	2 Q811M5,Q9R168
1451	1,2,4,5,7,25,29,30	3,6,8,26,27,28,31,32	3 P22006,Q9QWN8,Q9R168
1452	1,2,4,5,7,25,29,31	3,6,8,26,27,28,30,32	2 P09527,Q9QWN8
1453	1,2,4,5,7,25,29,32	3,6,8,26,27,28,30,31	1 Q9R168
1454	1,2,4,5,7,25,30,31	3,6,8,26,27,28,29,32	5 P09527,P26342,Q6P6R2,Q8CFN2,Q9QWN8
1455	1,2,4,5,7,25,30,32	3,6,8,26,27,28,29,31	4 P26342,P70549,Q6P6R2,Q9R168
1456	1,2,4,5,7,25,31,32	3,6,8,26,27,28,29,30	3 P26342,Q6P6R2,Q811M5
1457	1,2,4,5,7,26,27,28	3,6,8,25,29,30,31,32	7 P02625,P14173,P21588,P47967,P97840,Q03191,Q5RLM2
1458	1,2,4,5,7,26,27,29	3,6,8,25,28,30,31,32	5 P02625,P19223,P21588,Q03191,Q9JJ50
1459	1,2,4,5,7,26,27,30	3,6,8,25,28,29,31,32	3 P21588,Q03191,Q80WL1

1460	1,2,4,5,7,26,27,31	3,6,8,25,28,29,30,32	2	Q03191,Q9Z0J6
1461	1,2,4,5,7,26,27,32	3,6,8,25,28,29,30,31	3	P14173,Q03191,Q9JJ50
1462	1,2,4,5,7,26,28,29	3,6,8,25,27,30,31,32	5	P00762,P02625,P14173,P47967,P97840
1463	1,2,4,5,7,26,28,30	3,6,8,25,27,29,31,32	3	P00762,P21588,Q9R168
1464	1,2,4,5,7,26,28,31	3,6,8,25,27,29,30,32	3	P00762,P14173,Q5RLM2
1465	1,2,4,5,7,26,28,32	3,6,8,25,27,29,30,31	2	P14173,Q9R168
1466	1,2,4,5,7,26,29,30	3,6,8,25,27,28,31,32	2	P21588,Q80WL1
1467	1,2,4,5,7,26,29,31	3,6,8,25,27,28,30,32	1	P00762
1468	1,2,4,5,7,26,29,32	3,6,8,25,27,28,30,31	2	P14173,Q9R168
1469	1,2,4,5,7,26,30,31	3,6,8,25,27,28,29,32	3	P36860,Q8CFN2,Q9EQS0
1470	1,2,4,5,7,26,30,32	3,6,8,25,27,28,29,31	3	P26342,P36860,Q9R168
1471	1,2,4,5,7,26,31,32	3,6,8,25,27,28,29,30	1	P14173
1472	1,2,4,5,7,27,28,29	3,6,8,25,26,30,31,32	4	P02625,P47967,P97840,Q03191
1473	1,2,4,5,7,27,28,30	3,6,8,25,26,29,31,32	4	P19218,P21588,P70549,Q03191
1474	1,2,4,5,7,27,28,31	3,6,8,25,26,29,30,32	3	P80299,Q03191,Q9Z0J6
1475	1,2,4,5,7,27,28,32	3,6,8,25,26,29,30,31	3	P14173,Q03191,Q62761;Q62762;Q62763
1476	1,2,4,5,7,27,29,30	3,6,8,25,26,28,31,32	6	P00731,P19223,P21588,Q03191,Q6P6Q2,Q923V8
1477	1,2,4,5,7,27,29,31	3,6,8,25,26,28,30,32	3	P19223,P80299,Q03191
1478	1,2,4,5,7,27,29,32	3,6,8,25,26,28,30,31	3	P80299,Q03191,Q6TMA8
1479	1,2,4,5,7,27,30,31	3,6,8,25,26,28,29,32	4	P80299,Q03191,Q9QYP1,Q9Z0J6

1480	1,2,4,5,7,27,30,32	3,6,8,25,26,28,29,31	4	P70549,Q03191,Q6P6R2,Q9R168
1481	1,2,4,5,7,27,31,32	3,6,8,25,26,28,29,30	4	P23739,P80299,Q03191,Q9Z0J6
1482	1,2,4,5,7,28,29,30	3,6,8,25,26,27,31,32	2	P00762,P97840
1483	1,2,4,5,7,28,29,31	3,6,8,25,26,27,30,32	2	P00762,P80299
1484	1,2,4,5,7,28,29,32	3,6,8,25,26,27,30,31	2	P14173,P54921
1485	1,2,4,5,7,28,30,31	3,6,8,25,26,27,29,32	1	P00762
1486	1,2,4,5,7,28,30,32	3,6,8,25,26,27,29,31	3	P26342,P70549,Q9R168
1487	1,2,4,5,7,28,31,32	3,6,8,25,26,27,29,30	3	P14173,P80299,Q811M5
1488	1,2,4,5,7,29,30,31	3,6,8,25,26,27,28,32	5	P26342,P36860,P80299,Q5RJR2,Q9QWN8
1489	1,2,4,5,7,29,30,32	3,6,8,25,26,27,28,31	4	P17046,P26342,P36860,Q9R168
1490	1,2,4,5,7,29,31,32	3,6,8,25,26,27,28,30	3	P26342,P36860,P80299
1491	1,2,4,5,7,30,31,32	3,6,8,25,26,27,28,29	6	P26342,P36860,P63081,P70549,P80299,Q6P6R2
1492	1,2,4,5,8,25,26,27	3,6,7,28,29,30,31,32	3	P97580,Q03191,Q30KJ2
1493	1,2,4,5,8,25,26,28	3,6,7,27,29,30,31,32	0	
1494	1,2,4,5,8,25,26,29	3,6,7,27,28,30,31,32	1	P22006
1495	1,2,4,5,8,25,26,30	3,6,7,27,28,29,31,32	3	Q6IFU8,Q6IMF3,Q6P6Q2
1496	1,2,4,5,8,25,26,31	3,6,7,27,28,29,30,32	0	
1497	1,2,4,5,8,25,26,32	3,6,7,27,28,29,30,31	2	P00507,P13432
1498	1,2,4,5,8,25,27,28	3,6,7,26,29,30,31,32	2	Q03191,Q10758
1499	1,2,4,5,8,25,27,29	3,6,7,26,28,30,31,32	4	P19223,P22006,Q03191,Q10758

1500	1,2,4,5,8,25,27,30	3,6,7,26,28,29,31,32	12	P00731,P70549,Q03191,Q10758,Q30KJ2,Q4FZU2,Q6IFU7,Q6IFU8,Q6IFW6,Q6IG05,Q6IMF3,Q6P6Q2
1501	1,2,4,5,8,25,27,31	3,6,7,26,28,29,30,32	3	P23739,Q03191,Q10758
1502	1,2,4,5,8,25,27,32	3,6,7,26,28,29,30,31	2	P01681,Q03191
1503	1,2,4,5,8,25,28,29	3,6,7,26,27,30,31,32	1	P22006
1504	1,2,4,5,8,25,28,30	3,6,7,26,27,29,31,32	3	Q6IFU8,Q6IMF3,Q6P6Q2
1505	1,2,4,5,8,25,28,31	3,6,7,26,27,29,30,32	1	Q811M5
1506	1,2,4,5,8,25,28,32	3,6,7,26,27,29,30,31	2	P01681,Q811M5
1507	1,2,4,5,8,25,29,30	3,6,7,26,27,28,31,32	8	O88797,P22006,Q4FZU2,Q6IFU7,Q6IFU8,Q6IFW6,Q6IMF3,Q6P6Q2
1508	1,2,4,5,8,25,29,31	3,6,7,26,27,28,30,32	0	
1509	1,2,4,5,8,25,29,32	3,6,7,26,27,28,30,31	0	
1510	1,2,4,5,8,25,30,31	3,6,7,26,27,28,29,32	5	Q4FZU2,Q6IFU8,Q6IG05,Q6IMF3,Q6P6Q2
1511	1,2,4,5,8,25,30,32	3,6,7,26,27,28,29,31	8	O88797,P01681,P70549,Q4FZU2,Q6IFU8,Q6IMF3,Q6P6Q2,Q6P6R2
1512	1,2,4,5,8,25,31,32	3,6,7,26,27,28,29,30	2	P01681,Q811M5
1513	1,2,4,5,8,26,27,28	3,6,7,25,29,30,31,32	5	P15399,P20762,P25809,Q10758,Q30KJ2
1514	1,2,4,5,8,26,27,29	3,6,7,25,28,30,31,32	6	P19223,P25809,Q10758,Q30KJ2,Q9JJ50,Q9JJS8
1515	1,2,4,5,8,26,27,30	3,6,7,25,28,29,31,32	11	P15399,P20762,P21588,P97580,Q03191,Q10758,Q30KJ2,Q6IFU8,Q6IG05,Q6IMF3,Q6P6Q2
1516	1,2,4,5,8,26,27,31	3,6,7,25,28,29,30,32	4	P23739,Q03191,Q10758,Q30KJ2
1517	1,2,4,5,8,26,27,32	3,6,7,25,28,29,30,31	3	P15399,Q03191,Q30KJ2
1518	1,2,4,5,8,26,28,29	3,6,7,25,27,30,31,32	0	
1519	1,2,4,5,8,26,28,30	3,6,7,25,27,29,31,32	2	P20762,Q6P6Q2

1520	1,2,4,5,8,26,28,31	3,6,7,25,27,29,30,32	0	
1521	1,2,4,5,8,26,28,32	3,6,7,25,27,29,30,31	1	O54728
1522	1,2,4,5,8,26,29,30	3,6,7,25,27,28,31,32	4	Q5RJR2,Q6IFU8,Q6IMF3,Q6P6Q2
1523	1,2,4,5,8,26,29,31	3,6,7,25,27,28,30,32	1	Q5RJR2
1524	1,2,4,5,8,26,29,32	3,6,7,25,27,28,30,31	0	
1525	1,2,4,5,8,26,30,31	3,6,7,25,27,28,29,32	4	Q5RJR2,Q6IFU8,Q6IMF3,Q6P6Q2
1526	1,2,4,5,8,26,30,32	3,6,7,25,27,28,29,31	2	P05539,Q6P6Q2
1527	1,2,4,5,8,26,31,32	3,6,7,25,27,28,29,30	1	O54728
1528	1,2,4,5,8,27,28,29	3,6,7,25,26,30,31,32	3	P25809,Q10758,Q812E4
1529	1,2,4,5,8,27,28,30	3,6,7,25,26,29,31,32	9	P19218,P70549,Q03191,Q10758,Q6IFU7,Q6IFU8,Q6IG05,Q6IMF3,Q6P6Q2
1530	1,2,4,5,8,27,28,31	3,6,7,25,26,29,30,32	3	P23739,Q03191,Q10758
1531	1,2,4,5,8,27,28,32	3,6,7,25,26,29,30,31	3	Q03191,Q10758,Q6AYR9
1532	1,2,4,5,8,27,29,30	3,6,7,25,26,28,31,32	13	P00731,P19218,P19223,Q03191,Q10758,Q4FZU2,Q6IFU7,Q6IFU8,Q6IFW6,Q6IG02,Q6IG05,Q6IMF3,Q6P6Q2
1533	1,2,4,5,8,27,29,31	3,6,7,25,26,28,30,32	4	P00731,P19223,Q03191,Q10758
1534	1,2,4,5,8,27,29,32	3,6,7,25,26,28,30,31	2	Q03191,Q10758
1535	1,2,4,5,8,27,30,31	3,6,7,25,26,28,29,32	9	P00731,P23739,Q03191,Q10758,Q4FZU2,Q6IFU8,Q6IG05,Q6IMF3,Q6P6Q2
1536	1,2,4,5,8,27,30,32	3,6,7,25,26,28,29,31	10	P19218,P70549,Q03191,Q10758,Q4FZU2,Q6IFU7,Q6IFU8,Q6IG05,Q6IMF3,Q6P6Q2
1537	1,2,4,5,8,27,31,32	3,6,7,25,26,28,29,30	3	P23739,Q03191,Q10758
1538	1,2,4,5,8,28,29,30	3,6,7,25,26,27,31,32	3	Q6IFU8,Q6IMF3,Q6P6Q2
1539	1,2,4,5,8,28,29,31	3,6,7,25,26,27,30,32	0	

1540	1,2,4,5,8,28,29,32	3,6,7,25,26,27,30,31	0
1541	1,2,4,5,8,28,30,31	3,6,7,25,26,27,29,32	2 Q6IMF3,Q6P6Q2
1542	1,2,4,5,8,28,30,32	3,6,7,25,26,27,29,31	3 P70549,Q6IMF3,Q6P6Q2
1543	1,2,4,5,8,28,31,32	3,6,7,25,26,27,29,30	1 Q811M5
1544	1,2,4,5,8,29,30,31	3,6,7,25,26,27,28,32	5 P63081,Q5RJR2,Q6IFU8,Q6IMF3,Q6P6Q2
1545	1,2,4,5,8,29,30,32	3,6,7,25,26,27,28,31	4 O88797,Q6IFU8,Q6IMF3,Q6P6Q2
1546	1,2,4,5,8,29,31,32	3,6,7,25,26,27,28,30	0
1547	1,2,4,5,8,30,31,32	3,6,7,25,26,27,28,29	8 P05539,P26342,P63081,P70549,Q6IFU8,Q6IMF3,Q6P6Q2,Q6P6R2
1548	1,2,4,5,25,26,27,28	3,6,7,8,29,30,31,32	2 P35280,Q03191
1549	1,2,4,5,25,26,27,29	3,6,7,8,28,30,31,32	5 O89117,P22006,P35280,Q03191,Q9QZK9
1550	1,2,4,5,25,26,27,30	3,6,7,8,28,29,31,32	2 P21588,Q03191
1551	1,2,4,5,25,26,27,31	3,6,7,8,28,29,30,32	4 P23739,P35280,Q03191,Q9QYP1
1552	1,2,4,5,25,26,27,32	3,6,7,8,28,29,30,31	1 Q03191
1553	1,2,4,5,25,26,28,29	3,6,7,8,27,30,31,32	2 P35280,Q9QZK9
1554	1,2,4,5,25,26,28,30	3,6,7,8,27,29,31,32	0
1555	1,2,4,5,25,26,28,31	3,6,7,8,27,29,30,32	1 P35280
1556	1,2,4,5,25,26,28,32	3,6,7,8,27,29,30,31	0
1557	1,2,4,5,25,26,29,30	3,6,7,8,27,28,31,32	2 P22006,Q9QZK9
1558	1,2,4,5,25,26,29,31	3,6,7,8,27,28,30,32	2 O70417,P35280
1559	1,2,4,5,25,26,29,32	3,6,7,8,27,28,30,31	1 Q9QZK9

1560	1,2,4,5,25, 26,30,31	3,6,7,8,27, 28,29,32	5 Q63617,Q6AYR9,Q812E4,Q8CFN2,Q9EQS0
1561	1,2,4,5,25, 26,30,32	3,6,7,8,27, 28,29,31	4 D3ZUC6,P00507,Q9EQS0,Q9R168
1562	1,2,4,5,25, 26,31,32	3,6,7,8,27, 28,29,30	2 P00507,Q9EQS0
1563	1,2,4,5,25, 27,28,29	3,6,7,8,26, 30,31,32	3 O89117,P35280,Q03191
1564	1,2,4,5,25, 27,28,30	3,6,7,8,26, 29,31,32	2 P70549,Q03191
1565	1,2,4,5,25, 27,28,31	3,6,7,8,26, 29,30,32	3 P23739,P35280,Q03191
1566	1,2,4,5,25, 27,28,32	3,6,7,8,26, 29,30,31	2 Q03191,Q62761;Q62762;Q62763
1567	1,2,4,5,25, 27,29,30	3,6,7,8,26, 28,31,32	2 Q03191,Q923V8
1568	1,2,4,5,25, 27,29,31	3,6,7,8,26, 28,30,32	2 P35280,Q03191
1569	1,2,4,5,25, 27,29,32	3,6,7,8,26, 28,30,31	1 Q03191
1570	1,2,4,5,25, 27,30,31	3,6,7,8,26, 28,29,32	4 P23739,Q03191,Q6P6R2,Q9QYP1
1571	1,2,4,5,25, 27,30,32	3,6,7,8,26, 28,29,31	4 P70549,Q03191,Q6P6R2,Q9QX74
1572	1,2,4,5,25, 27,31,32	3,6,7,8,26, 28,29,30	4 P23739,Q03191,Q6P6R2,Q9QYP1
1573	1,2,4,5,25, 28,29,30	3,6,7,8,26, 27,31,32	0
1574	1,2,4,5,25, 28,29,31	3,6,7,8,26, 27,30,32	1 P35280
1575	1,2,4,5,25, 28,29,32	3,6,7,8,26, 27,30,31	0
1576	1,2,4,5,25, 28,30,31	3,6,7,8,26, 27,29,32	0
1577	1,2,4,5,25, 28,30,32	3,6,7,8,26, 27,29,31	3 P70549,Q9QX74,Q9R168
1578	1,2,4,5,25, 28,31,32	3,6,7,8,26, 27,29,30	1 Q811M5
1579	1,2,4,5,25, 29,30,31	3,6,7,8,26, 27,28,32	2 P07150,Q9QWN8

1580	1,2,4,5,25,<br>29,30,32	3,6,7,8,26,<br>27,28,31	3 Q62894,Q9R168,Q9WTT6
1581	1,2,4,5,25,<br>29,31,32	3,6,7,8,26,<br>27,28,30	1 O70417
1582	1,2,4,5,25,<br>30,31,32	3,6,7,8,26,<br>27,28,29	8 D3ZUC6,P26342,P63081,P70549,Q6P6R2,Q9QX74,Q9QYP1,Q9WTT6
1583	1,2,4,5,26,<br>27,28,29	3,6,7,8,25,<br>30,31,32	3 P02625,P21588,P35280
1584	1,2,4,5,26,<br>27,28,30	3,6,7,8,25,<br>29,31,32	2 P21588,Q03191
1585	1,2,4,5,26,<br>27,28,31	3,6,7,8,25,<br>29,30,32	2 P35280,Q03191
1586	1,2,4,5,26,<br>27,28,32	3,6,7,8,25,<br>29,30,31	2 Q03191,Q62761;Q62762;Q62763
1587	1,2,4,5,26,<br>27,29,30	3,6,7,8,25,<br>28,31,32	3 P21588,Q03191,Q923V8
1588	1,2,4,5,26,<br>27,29,31	3,6,7,8,25,<br>28,30,32	3 O70417,P35280,Q03191
1589	1,2,4,5,26,<br>27,29,32	3,6,7,8,25,<br>28,30,31	3 O70417,Q03191,Q9JJ50
1590	1,2,4,5,26,<br>27,30,31	3,6,7,8,25,<br>28,29,32	1 Q03191
1591	1,2,4,5,26,<br>27,30,32	3,6,7,8,25,<br>28,29,31	2 P70549,Q03191
1592	1,2,4,5,26,<br>27,31,32	3,6,7,8,25,<br>28,29,30	3 O70417,P23739,Q03191
1593	1,2,4,5,26,<br>28,29,30	3,6,7,8,25,<br>27,31,32	1 P21588
1594	1,2,4,5,26,<br>28,29,31	3,6,7,8,25,<br>27,30,32	2 O70417,P35280
1595	1,2,4,5,26,<br>28,29,32	3,6,7,8,25,<br>27,30,31	1 O70417
1596	1,2,4,5,26,<br>28,30,31	3,6,7,8,25,<br>27,29,32	0
1597	1,2,4,5,26,<br>28,30,32	3,6,7,8,25,<br>27,29,31	1 Q9R168
1598	1,2,4,5,26,<br>28,31,32	3,6,7,8,25,<br>27,29,30	2 O54728,O70417
1599	1,2,4,5,26,<br>29,30,31	3,6,7,8,25,<br>27,28,32	1 P36860

1600	1,2,4,5,26,29,30,32	3,6,7,8,25,27,28,31	1	P36860
1601	1,2,4,5,26,29,31,32	3,6,7,8,25,27,28,30	3	O70417,P36860,Q5QE79
1602	1,2,4,5,26,30,31,32	3,6,7,8,25,27,28,29	6	P26342,P36860,P63081,Q4G075,Q5QE79,Q9EQS0
1603	1,2,4,5,27,28,29,30	3,6,7,8,25,26,31,32	1	P21588
1604	1,2,4,5,27,28,29,31	3,6,7,8,25,26,30,32	3	O70417,P35280,Q03191
1605	1,2,4,5,27,28,29,32	3,6,7,8,25,26,30,31	2	Q03191,Q62761;Q62762;Q62763
1606	1,2,4,5,27,28,30,31	3,6,7,8,25,26,29,32	1	Q03191
1607	1,2,4,5,27,28,30,32	3,6,7,8,25,26,29,31	3	P70549,Q03191,Q9QX74
1608	1,2,4,5,27,28,31,32	3,6,7,8,25,26,29,30	4	P23739,P80299,Q03191,Q62761;Q62762;Q62763
1609	1,2,4,5,27,29,30,31	3,6,7,8,25,26,28,32	1	Q03191
1610	1,2,4,5,27,29,30,32	3,6,7,8,25,26,28,31	2	Q03191,Q923V8
1611	1,2,4,5,27,29,31,32	3,6,7,8,25,26,28,30	3	O70417,P80299,Q03191
1612	1,2,4,5,27,30,31,32	3,6,7,8,25,26,28,29	9	P23739,P36860,P70549,P80299,Q03191,Q6P6R2,Q9QX74,Q9QYP1,Q9WTT6
1613	1,2,4,5,28,29,30,31	3,6,7,8,25,26,27,32	0	
1614	1,2,4,5,28,29,30,32	3,6,7,8,25,26,27,31	1	Q62894
1615	1,2,4,5,28,29,31,32	3,6,7,8,25,26,27,30	2	O70417,P80299
1616	1,2,4,5,28,30,31,32	3,6,7,8,25,26,27,29	4	P63081,P70549,Q9QX74,Q9WTT6
1617	1,2,4,5,29,30,31,32	3,6,7,8,25,26,27,28	7	P07150,P26342,P36860,P50115,P63081,P80299,Q9WTT6
1618	1,2,4,6,7,8,25,26	3,5,27,28,29,30,31,32	10	G3V686,O54858,P02631,P06911,P08649,P12020,P20762,P97580,Q30KJ2,Q9WVH8

1619	1,2,4,6,7,8,25,27	3,5,26,28,29,30,31,32	15	P02631,P06911,P11598,P12020,P15399,P19629,P22273,P22282,P50115,P97580,Q30KJ2,Q4G075,Q5GRG2,Q66H69,Q99041
1620	1,2,4,6,7,8,25,28	3,5,26,27,29,30,31,32	12	P02631,P06911,P08649,P11598,P12020,P19629,P22273,Q5GRG2,Q63617,Q99041,Q9JI85,Q9QW07
1621	1,2,4,6,7,8,25,29	3,5,26,27,28,30,31,32	6	P02631,P06911,P12020,P48032,P62804,Q5GRG2
1622	1,2,4,6,7,8,25,30	3,5,26,27,28,29,31,32	9	G3V686,O54858,P02631,P06911,P12020,P97580,Q30KJ2,Q5GRG2,Q99041
1623	1,2,4,6,7,8,25,31	3,5,26,27,28,29,30,32	8	G3V686,O54858,P02631,P06911,P12020,Q5GRG2,Q63532,Q9WVH8
1624	1,2,4,6,7,8,25,32	3,5,26,27,28,29,30,31	10	G3V686,O54858,O55004,P02454,P02631,P06911,P11598,P12020,P13432,Q5GRG2
1625	1,2,4,6,7,8,26,27	3,5,25,28,29,30,31,32	18	P02631,P06911,P11598,P15399,P17559,P20762,P22273,P22282,P50115,P50116,P50280,P97580,Q30KJ2,Q4G075,Q5BJY9,Q62894,Q66H69,Q99041
1626	1,2,4,6,7,8,26,28	3,5,25,27,29,30,31,32	20	P00762,P02631,P06761,P06911,P07150,P08649,P11598,P12020,P15399,P20762,P21674,P22273,P22282,P97580,Q30KJ2,Q63617,Q99041,Q9JI85,Q9QW07,Q9Z1F2
1627	1,2,4,6,7,8,26,29	3,5,25,27,28,30,31,32	12	P02631,P06911,P11598,P12020,P20762,P21674,P22273,P62804,P97580,Q30KJ2,Q99041,Q9QX74
1628	1,2,4,6,7,8,26,30	3,5,25,27,28,29,31,32	8	G3V686,P02631,P06911,P20762,P97580,Q30KJ2,Q80WL1,Q99041
1629	1,2,4,6,7,8,26,31	3,5,25,27,28,29,30,32	11	O54858,P06911,P11598,P12020,P20762,P20766,P57113,P97580,Q30KJ2,Q63532,Q9WVH8
1630	1,2,4,6,7,8,26,32	3,5,25,27,28,29,30,31	10	O55004,P02454,P02631,P06911,P11598,P18418,P20762,P97580,Q30KJ2,Q99041
1631	1,2,4,6,7,8,27,28	3,5,25,26,29,30,31,32	23	P02631,P06761,P06911,P11598,P12020,P15399,P19218,P22273,P22282,P22283,P50115,P97580,Q00715,Q10758,Q30KJ2,Q4G075,Q5GRG2,Q63617,Q66H69,Q68FX1,Q99041,Q9JI85,Q9QW07

1632	1,2,4,6,7,8,27,29	3,5,25,26,28,30,31,32	20	P02631,P06761,P06911,P11598,P12020,P15399,P19223,P22273,P22282,P25809,P62804,P97580,Q00715,Q10758,Q30KJ2,Q4G075,Q63617,Q66H69,Q99041,Q9JI85
1633	1,2,4,6,7,8,27,30	3,5,25,26,28,29,31,32	15	P02631,P06911,P11598,P15399,P19218,P19223,P22273,P22282,P49744,P97580,Q10758,Q30KJ2,Q66H69,Q80WL1,Q99041
1634	1,2,4,6,7,8,27,31	3,5,25,26,28,29,30,32	15	P06911,P11598,P12020,P15399,P22273,P22282,P80299,P97580,Q10758,Q30KJ2,Q5GRG2,Q62894,Q63532,Q66H69,Q99041
1635	1,2,4,6,7,8,27,32	3,5,25,26,28,29,30,31	15	O55004,P00714,P02631,P06911,P11598,P15399,P22273,P22282,P50280,P97580,P98166,Q30KJ2,Q66H69,Q99041,Q9JI85
1636	1,2,4,6,7,8,28,29	3,5,25,26,27,30,31,32	18	P00762,P02631,P06761,P06911,P11598,P12020,P21674,P22273,P22282,P22283,P62804,Q00715,Q5GRG2,Q63493,Q63617,Q99041,Q9JI85,Q9QW07
1637	1,2,4,6,7,8,28,30	3,5,25,26,27,29,31,32	13	P02631,P06911,P11598,P12020,P19218,P62804,Q30KJ2,Q5GRG2,Q63617,Q80WL1,Q99041,Q9JI85,Q9QW07
1638	1,2,4,6,7,8,28,31	3,5,25,26,27,29,30,32	11	P02631,P06911,P11598,P12020,P21674,P82471,Q5GRG2,Q63532,Q63617,Q99041,Q9JI85
1639	1,2,4,6,7,8,28,32	3,5,25,26,27,29,30,31	14	O55004,P02454,P02631,P06911,P08723,P11598,P12020,P22282,Q5GRG2,Q63532,Q63617,Q99041,Q9JI85,Q9QW07
1640	1,2,4,6,7,8,29,30	3,5,25,26,27,28,31,32	9	P02631,P06911,P12020,P62804,P97580,Q30KJ2,Q5RJR2,Q80WL1,Q99041
1641	1,2,4,6,7,8,29,31	3,5,25,26,27,28,30,32	9	P06911,P11598,P12020,P21674,P80299,P82471,Q5GRG2,Q5RJR2,Q63532
1642	1,2,4,6,7,8,29,32	3,5,25,26,27,28,30,31	8	O55004,P00714,P02631,P06911,P11598,P12020,Q99041,Q9JI85
1643	1,2,4,6,7,8,30,31	3,5,25,26,27,28,29,32	9	O54858,P06911,P12020,P15999,P26342,Q30KJ2,Q5RJR2,Q63532,Q99041
1644	1,2,4,6,7,8,30,32	3,5,25,26,27,28,29,31	6	O55004,P02631,P06911,P15999,P26342,Q99041

1645	1,2,4,6,7,8,31,32	3,5,25,26,27,28,29,30	11	O54858,O55004,P02631,P06911,P11598,P12020,P15999,P26342,P80299,P82471,Q63532
1646	1,2,4,6,7,25,26,27	3,5,8,28,29,30,31,32	1	P21588
1647	1,2,4,6,7,25,26,28	3,5,8,27,29,30,31,32	2	P11598,Q9WVH8
1648	1,2,4,6,7,25,26,29	3,5,8,27,28,30,31,32	1	Q9WVH8
1649	1,2,4,6,7,25,26,30	3,5,8,27,28,29,31,32	4	G3V686,Q80WL1,Q9EQS0,Q9R168
1650	1,2,4,6,7,25,26,31	3,5,8,27,28,29,30,32	2	Q9EQS0,Q9WVH8
1651	1,2,4,6,7,25,26,32	3,5,8,27,28,29,30,31	3	Q9EQS0,Q9R168,Q9WVH8
1652	1,2,4,6,7,25,27,28	3,5,8,26,29,30,31,32	1	P11598
1653	1,2,4,6,7,25,27,29	3,5,8,26,28,30,31,32	1	P19223
1654	1,2,4,6,7,25,27,30	3,5,8,26,28,29,31,32	1	Q80WL1
1655	1,2,4,6,7,25,27,31	3,5,8,26,28,29,30,32	2	P80299,Q9QYP1
1656	1,2,4,6,7,25,27,32	3,5,8,26,28,29,30,31	0	
1657	1,2,4,6,7,25,28,29	3,5,8,26,27,30,31,32	3	P00762,P11598,Q63617
1658	1,2,4,6,7,25,28,30	3,5,8,26,27,29,31,32	3	Q62975,Q80WL1,Q9R168
1659	1,2,4,6,7,25,28,31	3,5,8,26,27,29,30,32	1	Q5GRG2
1660	1,2,4,6,7,25,28,32	3,5,8,26,27,29,30,31	3	P11598,Q62975,Q9R168
1661	1,2,4,6,7,25,29,30	3,5,8,26,27,28,31,32	3	Q80WL1,Q9QWN8,Q9R168
1662	1,2,4,6,7,25,29,31	3,5,8,26,27,28,30,32	3	B1H234,P80299,Q9QWN8
1663	1,2,4,6,7,25,29,32	3,5,8,26,27,28,30,31	2	Q6AYR8,Q9R168
1664	1,2,4,6,7,25,30,31	3,5,8,26,27,28,29,32	4	P26342,Q9EQS0,Q9QWN8,Q9QYP1

1665	1,2,4,6,7,25,30,32	3,5,8,26,27,28,29,31	4	P26342,Q6P6R2,Q9EQS0,Q9R168
1666	1,2,4,6,7,25,31,32	3,5,8,26,27,28,29,30	3	P26342,P80299,Q9WVH8
1667	1,2,4,6,7,26,27,28	3,5,8,25,29,30,31,32	3	P11598,P21588,Q63617
1668	1,2,4,6,7,26,27,29	3,5,8,25,28,30,31,32	4	P11598,P19223,P21588,Q80WL1
1669	1,2,4,6,7,26,27,30	3,5,8,25,28,29,31,32	2	P21588,Q80WL1
1670	1,2,4,6,7,26,27,31	3,5,8,25,28,29,30,32	2	P80299,Q62894
1671	1,2,4,6,7,26,27,32	3,5,8,25,28,29,30,31	1	P11598
1672	1,2,4,6,7,26,28,29	3,5,8,25,27,30,31,32	6	P00762,P01835,P11598,P14173,P21674,Q63617
1673	1,2,4,6,7,26,28,30	3,5,8,25,27,29,31,32	5	P00762,P11598,P21588,Q80WL1,Q9R168
1674	1,2,4,6,7,26,28,31	3,5,8,25,27,29,30,32	2	P00762,P11598
1675	1,2,4,6,7,26,28,32	3,5,8,25,27,29,30,31	4	P11598,P14173,Q5M872,Q9R168
1676	1,2,4,6,7,26,29,30	3,5,8,25,27,28,31,32	5	B1H234,P01835,P21588,Q80WL1,Q9R168
1677	1,2,4,6,7,26,29,31	3,5,8,25,27,28,30,32	4	B1H234,P00762,P01835,P80299
1678	1,2,4,6,7,26,29,32	3,5,8,25,27,28,30,31	5	P01835,P06866,P11598,P14173,Q9R168
1679	1,2,4,6,7,26,30,31	3,5,8,25,27,28,29,32	3	P36860,Q80WL1,Q9EQS0
1680	1,2,4,6,7,26,30,32	3,5,8,25,27,28,29,31	4	P26342,Q80WL1,Q9EQS0,Q9R168
1681	1,2,4,6,7,26,31,32	3,5,8,25,27,28,29,30	5	P14173,P22006,P26342,P80299,Q9WVH8
1682	1,2,4,6,7,27,28,29	3,5,8,25,26,30,31,32	6	P11598,P22283,P80299,Q00715,Q63617,Q9JI85
1683	1,2,4,6,7,27,28,30	3,5,8,25,26,29,31,32	4	P11598,P19218,P21588,Q80WL1
1684	1,2,4,6,7,27,28,31	3,5,8,25,26,29,30,32	4	O88797,P11598,P80299,Q63617

1685	1,2,4,6,7,27,28,32	3,5,8,25,26,29,30,31	8	P05964,P11598,P22283,P80299,Q62761;Q62762;Q62763,Q63617,Q6AYR9,Q9JI85
1686	1,2,4,6,7,27,29,30	3,5,8,25,26,28,31,32	5	B1H234,P19223,P21588,P80299,Q80WL1
1687	1,2,4,6,7,27,29,31	3,5,8,25,26,28,30,32	3	B1H234,P19223,P80299
1688	1,2,4,6,7,27,29,32	3,5,8,25,26,28,30,31	4	P00714,P11598,P19223,P80299
1689	1,2,4,6,7,27,30,31	3,5,8,25,26,28,29,32	4	P80299,Q80WL1,Q9QYP1,Q9Z0J6
1690	1,2,4,6,7,27,30,32	3,5,8,25,26,28,29,31	4	P00714,P80299,Q80WL1,Q9R168
1691	1,2,4,6,7,27,31,32	3,5,8,25,26,28,29,30	6	P00714,P11598,P22006,P80299,Q9QYP1,Q9Z0J6
1692	1,2,4,6,7,28,29,30	3,5,8,25,26,27,31,32	8	B1H234,P00762,P01835,P11598,P17046,P21674,Q63617,Q80WL1
1693	1,2,4,6,7,28,29,31	3,5,8,25,26,27,30,32	7	B1H234,P00762,P01835,P11598,P21674,P80299,Q63617
1694	1,2,4,6,7,28,29,32	3,5,8,25,26,27,30,31	8	P01835,P07647,P11598,P14173,P80299,P81556,Q63617,Q9JI85
1695	1,2,4,6,7,28,30,31	3,5,8,25,26,27,29,32	4	P00762,P80299,Q63532,Q80WL1
1696	1,2,4,6,7,28,30,32	3,5,8,25,26,27,29,31	4	P11598,P26342,Q80WL1,Q9R168
1697	1,2,4,6,7,28,31,32	3,5,8,25,26,27,29,30	5	P11598,P14173,P22006,P80299,Q63532
1698	1,2,4,6,7,29,30,31	3,5,8,25,26,27,28,32	8	B1H234,P01835,P17046,P26342,P36860,P80299,Q80WL1,Q9QWN8
1699	1,2,4,6,7,29,30,32	3,5,8,25,26,27,28,31	10	B1H234,O55145,P00714,P01835,P17046,P26342,P36860,P80299,Q80WL1,Q9R168
1700	1,2,4,6,7,29,31,32	3,5,8,25,26,27,28,30	6	B1H234,P00714,P01835,P26342,P36860,P80299
1701	1,2,4,6,7,30,31,32	3,5,8,25,26,27,28,29	7	P06760,P22006,P26342,P36860,P80299,Q63532,Q6P6R2
1702	1,2,4,6,8,25,26,27	3,5,7,28,29,30,31,32	9	G3V686,P06911,P08649,P15399,P17559,P54921,P97580,Q30KJ2,Q5BJY9
1703	1,2,4,6,8,25,26,28	3,5,7,27,29,30,31,32	10	G3V686,P02631,P06911,P08649,P15399,P97580,Q30KJ2,Q5GRG2,Q9JI85,Q9QW07
1704	1,2,4,6,8,25,26,29	3,5,7,27,28,30,31,32	4	P08649,P13432,P97580,Q30KJ2

1705	1,2,4,6,8,2 5,26,30	3,5,7,27,2 8,29,31,32	7	G3V686,P08649,P15399,P54921,P97580,Q30KJ2,Q6AY61
1706	1,2,4,6,8,2 5,26,31	3,5,7,27,2 8,29,30,32	10	G3V686,P06911,P08649,P12020,P47967,P54921,P97580,P97840,Q30KJ2,Q63532
1707	1,2,4,6,8,2 5,26,32	3,5,7,27,2 8,29,30,31	10	G3V686,O55004,P00507,P08649,P13432,P15399,P97580,Q06000,Q30KJ2,Q68G31
1708	1,2,4,6,8,2 5,27,28	3,5,7,26,2 9,30,31,32	10	P02631,P06911,P08649,P15399,P19629,P97580,Q10758,Q30KJ2,Q5GRG2,Q9JI85
1709	1,2,4,6,8,2 5,27,29	3,5,7,26,2 8,30,31,32	7	P06911,P15399,P19223,P97580,Q10758,Q30KJ2,Q5GRG2
1710	1,2,4,6,8,2 5,27,30	3,5,7,26,2 8,29,31,32	6	P06911,P15399,P54921,P97580,Q10758,Q30KJ2
1711	1,2,4,6,8,2 5,27,31	3,5,7,26,2 8,29,30,32	9	P06911,P12020,P15399,P54921,P97580,Q30KJ2,Q5GRG2,Q63532,Q9QYP1
1712	1,2,4,6,8,2 5,27,32	3,5,7,26,2 8,29,30,31	6	P02631,P06911,P15399,P97580,P98166,Q30KJ2
1713	1,2,4,6,8,2 5,28,29	3,5,7,26,2 7,30,31,32	7	P02631,P06911,P08649,P12020,Q5GRG2,Q9JI85,Q9QW07
1714	1,2,4,6,8,2 5,28,30	3,5,7,26,2 7,29,31,32	7	P02631,P06911,P08649,P12020,P97580,Q30KJ2,Q5GRG2
1715	1,2,4,6,8,2 5,28,31	3,5,7,26,2 7,29,30,32	6	P06911,P08649,P12020,Q5GRG2,Q63532,Q9JI85
1716	1,2,4,6,8,2 5,28,32	3,5,7,26,2 7,29,30,31	7	P02631,P06911,P08649,Q5GRG2,Q68G31,Q9JI85,Q9QW07
1717	1,2,4,6,8,2 5,29,30	3,5,7,26,2 7,28,31,32	3	P06911,P97580,Q30KJ2
1718	1,2,4,6,8,2 5,29,31	3,5,7,26,2 7,28,30,32	4	P06911,P12020,Q5GRG2,Q63532
1719	1,2,4,6,8,2 5,29,32	3,5,7,26,2 7,28,30,31	3	P02631,P13432,Q68G31
1720	1,2,4,6,8,2 5,30,31	3,5,7,26,2 7,28,29,32	10	G3V686,O54858,P06911,P12020,P47967,P54921,P97840,Q30KJ2,Q5GRG2,Q63532
1721	1,2,4,6,8,2 5,30,32	3,5,7,26,2 7,28,29,31	5	G3V686,P02631,P47967,Q30KJ2,Q68G31
1722	1,2,4,6,8,2 5,31,32	3,5,7,26,2 7,28,29,30	6	P06911,P12020,P47967,P97840,Q5GRG2,Q63532
1723	1,2,4,6,8,2 6,27,28	3,5,7,25,2 9,30,31,32	11	P08649,P15399,P17559,P20762,P22282,P50280,P97580,Q10758,Q30KJ2,Q5BJY9,Q9JI85
1724	1,2,4,6,8,2 6,27,29	3,5,7,25,2 8,30,31,32	5	P15399,P19223,P97580,Q10758,Q30KJ2

1725	1,2,4,6,8,26,27,30	3,5,7,25,28,29,31,32	6	P15399,P20762,P21588,P97580,Q10758,Q30KJ2
1726	1,2,4,6,8,26,27,31	3,5,7,25,28,29,30,32	6	P15399,P54921,P97580,Q30KJ2,Q5BJY9,Q63532
1727	1,2,4,6,8,26,27,32	3,5,7,25,28,29,30,31	4	P15399,P50280,P97580,Q30KJ2
1728	1,2,4,6,8,26,28,29	3,5,7,25,27,30,31,32	7	P08649,P15399,P21674,P97580,Q30KJ2,Q9JI85,Q9QW07
1729	1,2,4,6,8,26,28,30	3,5,7,25,27,29,31,32	5	P08649,P15399,P97580,Q30KJ2,Q9JI85
1730	1,2,4,6,8,26,28,31	3,5,7,25,27,29,30,32	7	P08649,P12020,P15399,Q30KJ2,Q5GRG2,Q63532,Q9JI85
1731	1,2,4,6,8,26,28,32	3,5,7,25,27,29,30,31	6	O54728,P08649,P15399,P18418,Q30KJ2,Q9JI85
1732	1,2,4,6,8,26,29,30	3,5,7,25,27,28,31,32	4	P15399,P97580,Q30KJ2,Q5RJR2
1733	1,2,4,6,8,26,29,31	3,5,7,25,27,28,30,32	3	Q30KJ2,Q63474,Q63532
1734	1,2,4,6,8,26,29,32	3,5,7,25,27,28,30,31	5	O55004,P06866,P97580,Q30KJ2,Q68G31
1735	1,2,4,6,8,26,30,31	3,5,7,25,27,28,29,32	7	P15399,P54921,P97580,Q30KJ2,Q5RJR2,Q63532,Q6AY61
1736	1,2,4,6,8,26,30,32	3,5,7,25,27,28,29,31	5	P15399,P97580,Q30KJ2,Q68G31,Q99MH3
1737	1,2,4,6,8,26,31,32	3,5,7,25,27,28,29,30	5	O54728,O55004,P15399,Q30KJ2,Q63532
1738	1,2,4,6,8,27,28,29	3,5,7,25,26,30,31,32	13	P06911,P15399,P22282,P25809,P47727,P97580,Q00715,Q10758,Q30KJ2,Q5GRG2,Q6AYR9,Q812E4,Q9JI85
1739	1,2,4,6,8,27,28,30	3,5,7,25,26,29,31,32	7	P15399,P19218,P97580,Q10758,Q30KJ2,Q6P6S4,Q9JI85
1740	1,2,4,6,8,27,28,31	3,5,7,25,26,29,30,32	9	P06911,P12020,P15399,P97580,Q10758,Q30KJ2,Q5GRG2,Q63532,Q9JI85
1741	1,2,4,6,8,27,28,32	3,5,7,25,26,29,30,31	10	P15399,P22282,P50280,P97580,Q10758,Q30KJ2,Q6AYR9,Q6P6S4,Q9JI85,Q9QWN8
1742	1,2,4,6,8,27,29,30	3,5,7,25,26,28,31,32	8	O54861,P15399,P19218,P19223,P97580,Q10758,Q30KJ2,Q6IFU8
1743	1,2,4,6,8,27,29,31	3,5,7,25,26,28,30,32	8	P12020,P15399,P19223,P97580,Q10758,Q30KJ2,Q5GRG2,Q63532
1744	1,2,4,6,8,27,29,32	3,5,7,25,26,28,30,31	6	O54861,P15399,P97580,Q10758,Q30KJ2,Q9JI85

1745	1,2,4,6,8,27,30,31	3,5,7,25,26,28,29,32	6	P15399,P54921,P97580,Q10758,Q30KJ2,Q63532
1746	1,2,4,6,8,27,30,32	3,5,7,25,26,28,29,31	6	P15399,P19218,P97580,Q10758,Q30KJ2,Q68G31
1747	1,2,4,6,8,27,31,32	3,5,7,25,26,28,29,30	6	P15399,P80299,P97580,Q10758,Q30KJ2,Q63532
1748	1,2,4,6,8,28,29,30	3,5,7,25,26,27,31,32	2	P21674,Q9JI85
1749	1,2,4,6,8,28,29,31	3,5,7,25,26,27,30,32	7	P06911,P12020,P21674,Q5GRG2,Q63474,Q63532,Q9JI85
1750	1,2,4,6,8,28,29,32	3,5,7,25,26,27,30,31	3	P08723,P30120,Q9JI85
1751	1,2,4,6,8,28,30,31	3,5,7,25,26,27,29,32	3	P12020,Q5GRG2,Q63532
1752	1,2,4,6,8,28,30,32	3,5,7,25,26,27,29,31	2	Q68G31,Q9JI85
1753	1,2,4,6,8,28,31,32	3,5,7,25,26,27,29,30	5	P12020,P22006,Q5GRG2,Q63532,Q9JI85
1754	1,2,4,6,8,29,30,31	3,5,7,25,26,27,28,32	4	P12020,Q5RJR2,Q63474,Q63532
1755	1,2,4,6,8,29,30,32	3,5,7,25,26,27,28,31	2	O55145,Q68G31
1756	1,2,4,6,8,29,31,32	3,5,7,25,26,27,28,30	4	P12020,P80299,Q63474,Q63532
1757	1,2,4,6,8,30,31,32	3,5,7,25,26,27,28,29	5	O89117,P20646,P22006,P26342,Q63532
1758	1,2,4,6,25,26,27,28	3,5,7,8,29,30,31,32	2	P08649,P35280
1759	1,2,4,6,25,26,27,29	3,5,7,8,28,30,31,32	1	P35280
1760	1,2,4,6,25,26,27,30	3,5,7,8,28,29,31,32	3	D3ZUC6,P21588,P54921
1761	1,2,4,6,25,26,27,31	3,5,7,8,28,29,30,32	3	P35280,P54921,Q9QYP1
1762	1,2,4,6,25,26,27,32	3,5,7,8,28,29,30,31	1	Q06000
1763	1,2,4,6,25,26,28,29	3,5,7,8,27,30,31,32	1	P35280
1764	1,2,4,6,25,26,28,30	3,5,7,8,27,29,31,32	1	Q9EQS0

1765	1,2,4,6,25,26,28,31	3,5,7,8,27,29,30,32	1	P35280
1766	1,2,4,6,25,26,28,32	3,5,7,8,27,29,30,31	2	P08649,Q68G31
1767	1,2,4,6,25,26,29,30	3,5,7,8,27,28,31,32	2	D3ZUC6,Q9EQS0
1768	1,2,4,6,25,26,29,31	3,5,7,8,27,28,30,32	1	P35280
1769	1,2,4,6,25,26,29,32	3,5,7,8,27,28,30,31	2	Q68G31,Q6AYR8
1770	1,2,4,6,25,26,30,31	3,5,7,8,27,28,29,32	7	D3ZUC6,P06760,P47967,P54921,Q812E4,Q9EQS0,Q9QYP1
1771	1,2,4,6,25,26,30,32	3,5,7,8,27,28,29,31	8	D3ZUC6,P00507,P06760,P47967,Q06000,Q68G31,Q9EQS0,Q9R168
1772	1,2,4,6,25,26,31,32	3,5,7,8,27,28,29,30	5	D3ZUC6,P00507,P06760,P47967,Q9EQS0
1773	1,2,4,6,25,27,28,29	3,5,7,8,26,30,31,32	1	P35280
1774	1,2,4,6,25,27,28,30	3,5,7,8,26,29,31,32	1	Q9QX74
1775	1,2,4,6,25,27,28,31	3,5,7,8,26,29,30,32	3	P35280,Q5GRG2,Q9QYP1
1776	1,2,4,6,25,27,28,32	3,5,7,8,26,29,30,31	2	Q62761;Q62762;Q62763,Q9QX74
1777	1,2,4,6,25,27,29,30	3,5,7,8,26,28,31,32	0	
1778	1,2,4,6,25,27,29,31	3,5,7,8,26,28,30,32	2	P35280,Q9QYP1
1779	1,2,4,6,25,27,29,32	3,5,7,8,26,28,30,31	1	Q6AYR8
1780	1,2,4,6,25,27,30,31	3,5,7,8,26,28,29,32	5	P06760,P47967,P54921,Q9QX74,Q9QYP1
1781	1,2,4,6,25,27,30,32	3,5,7,8,26,28,29,31	6	D3ZUC6,P06760,Q68G31,Q6P6R2,Q9QX74,Q9WTT6
1782	1,2,4,6,25,27,31,32	3,5,7,8,26,28,29,30	5	P06760,P47967,P80299,Q9QX74,Q9QYP1
1783	1,2,4,6,25,28,29,30	3,5,7,8,26,27,31,32	0	
1784	1,2,4,6,25,28,29,31	3,5,7,8,26,27,30,32	1	P35280

1785	1,2,4,6,25, 28,29,32	3,5,7,8,26, 27,30,31	2	Q68G31,Q6AYR8
1786	1,2,4,6,25, 28,30,31	3,5,7,8,26, 27,29,32	2	P06760,Q9QX74
1787	1,2,4,6,25, 28,30,32	3,5,7,8,26, 27,29,31	7	P06760,Q5PQL7,Q62975,Q68G31,Q9QX74,Q9R168,Q9WTT6
1788	1,2,4,6,25, 28,31,32	3,5,7,8,26, 27,29,30	3	P06760,P22006,Q9QX74
1789	1,2,4,6,25, 29,30,31	3,5,7,8,26, 27,28,32	4	B1H234,P47967,Q9QWN8,Q9WTT6
1790	1,2,4,6,25, 29,30,32	3,5,7,8,26, 27,28,31	5	D3ZUC6,O55145,Q68G31,Q9R168,Q9WTT6
1791	1,2,4,6,25, 29,31,32	3,5,7,8,26, 27,28,30	3	P47967,P80299,Q9WTT6
1792	1,2,4,6,25, 30,31,32	3,5,7,8,26, 27,28,29	11	D3ZUC6,P06760,P26342,P47967,P97840,Q4G075,Q6P6R2,Q9EQS0,Q9QX74,Q9QYP1,Q9WTT6
1793	1,2,4,6,26, 27,28,29	3,5,7,8,25, 30,31,32	2	P21588,P35280
1794	1,2,4,6,26, 27,28,30	3,5,7,8,25, 29,31,32	2	P21588,Q80WL1
1795	1,2,4,6,26, 27,28,31	3,5,7,8,25, 29,30,32	1	P35280
1796	1,2,4,6,26, 27,28,32	3,5,7,8,25, 29,30,31	1	Q9QWN8
1797	1,2,4,6,26, 27,29,30	3,5,7,8,25, 28,31,32	3	P21588,Q80WL1,Q811M5
1798	1,2,4,6,26, 27,29,31	3,5,7,8,25, 28,30,32	2	B1H234,P35280
1799	1,2,4,6,26, 27,29,32	3,5,7,8,25, 28,30,31	1	P06866
1800	1,2,4,6,26, 27,30,31	3,5,7,8,25, 28,29,32	3	P06760,P54921,Q9QYP1
1801	1,2,4,6,26, 27,30,32	3,5,7,8,25, 28,29,31	2	P06760,Q68G31
1802	1,2,4,6,26, 27,31,32	3,5,7,8,25, 28,29,30	3	P06760,P22006,P80299
1803	1,2,4,6,26, 28,29,30	3,5,7,8,25, 27,31,32	2	P21588,Q80WL1
1804	1,2,4,6,26, 28,29,31	3,5,7,8,25, 27,30,32	3	B1H234,P35280,Q63474

1805	1,2,4,6,26, 28,29,32	3,5,7,8,25, 27,30,31	1	P06866
1806	1,2,4,6,26, 28,30,31	3,5,7,8,25, 27,29,32	2	P06760,Q9EQS0
1807	1,2,4,6,26, 28,30,32	3,5,7,8,25, 27,29,31	5	P06760,Q68G31,Q8VD89,Q9EQS0,Q9R168
1808	1,2,4,6,26, 28,31,32	3,5,7,8,25, 27,29,30	3	O54728,P06760,P22006
1809	1,2,4,6,26, 29,30,31	3,5,7,8,25, 27,28,32	2	B1H234,P36860
1810	1,2,4,6,26, 29,30,32	3,5,7,8,25, 27,28,31	4	O55145,P36860,Q68G31,Q9R168
1811	1,2,4,6,26, 29,31,32	3,5,7,8,25, 27,28,30	4	B1H234,P06866,P36860,P80299
1812	1,2,4,6,26, 30,31,32	3,5,7,8,25, 27,28,29	7	P06760,P22006,P26342,P36860,Q4G075,Q9EQS0,Q9WTT6
1813	1,2,4,6,27, 28,29,30	3,5,7,8,25, 26,31,32	2	P21588,Q80WL1
1814	1,2,4,6,27, 28,29,31	3,5,7,8,25, 26,30,32	3	B1H234,P35280,P80299
1815	1,2,4,6,27, 28,29,32	3,5,7,8,25, 26,30,31	5	O35547,P80299,Q62761;Q62762;Q62763,Q6AYR9,Q9JI85
1816	1,2,4,6,27, 28,30,31	3,5,7,8,25, 26,29,32	2	P06760,Q9QX74
1817	1,2,4,6,27, 28,30,32	3,5,7,8,25, 26,29,31	4	P06760,Q68G31,Q8VD89,Q9QX74
1818	1,2,4,6,27, 28,31,32	3,5,7,8,25, 26,29,30	4	P06760,P22006,P80299,Q9QX74
1819	1,2,4,6,27, 29,30,31	3,5,7,8,25, 26,28,32	3	B1H234,P19223,P80299
1820	1,2,4,6,27, 29,30,32	3,5,7,8,25, 26,28,31	4	O55145,P14562,P80299,Q68G31
1821	1,2,4,6,27, 29,31,32	3,5,7,8,25, 26,28,30	1	P80299
1822	1,2,4,6,27, 30,31,32	3,5,7,8,25, 26,28,29	7	P06760,P22006,P80299,Q6P6R2,Q9QX74,Q9QYP1,Q9WTT6
1823	1,2,4,6,28, 29,30,31	3,5,7,8,25, 26,27,32	2	B1H234,Q63474
1824	1,2,4,6,28, 29,30,32	3,5,7,8,25, 26,27,31	2	O55145,Q68G31

1825	1,2,4,6,28,29,31,32	3,5,7,8,25,26,27,30	4	B1H234,P22006,P80299,Q63474
1826	1,2,4,6,28,30,31,32	3,5,7,8,25,26,27,29	8	P06760,P22006,P26342,P80299,Q4G075,Q8VD89,Q9QX74,Q9WTT6
1827	1,2,4,6,29,30,31,32	3,5,7,8,25,26,27,28	6	B1H234,O55145,P26342,P36860,P80299,Q9WTT6
1828	1,2,4,7,8,25,26,27	3,5,6,28,29,30,31,32	9	G3V686,P20762,P97580,Q30KJ2,Q4G075,Q5BJY9,Q66H69,Q9WTT6,Q9Z1F2
1829	1,2,4,7,8,25,26,28	3,5,6,27,29,30,31,32	4	G3V686,Q63598,Q9WTT6,Q9Z1F2
1830	1,2,4,7,8,25,26,29	3,5,6,27,28,30,31,32	2	P22006,Q9QX74
1831	1,2,4,7,8,25,26,30	3,5,6,27,28,29,31,32	3	G3V686,Q30KJ2,Q9Z1F2
1832	1,2,4,7,8,25,26,31	3,5,6,27,28,29,30,32	3	G3V686,Q9Z0V6,Q9Z1F2
1833	1,2,4,7,8,25,26,32	3,5,6,27,28,29,30,31	2	G3V686,Q9Z1F2
1834	1,2,4,7,8,25,27,28	3,5,6,26,29,30,31,32	7	P06911,P0C0A9,P22282,P36860,Q4G075,Q66H69,Q9Z1F2
1835	1,2,4,7,8,25,27,29	3,5,6,26,28,30,31,32	5	P19223,P22006,P62804,Q4G075,Q66H69
1836	1,2,4,7,8,25,27,30	3,5,6,26,28,29,31,32	7	P97580,Q10758,Q30KJ2,Q4G075,Q66H69,Q6IFU8,Q9Z1F2
1837	1,2,4,7,8,25,27,31	3,5,6,26,28,29,30,32	3	Q4G075,Q66H69,Q9QYP1
1838	1,2,4,7,8,25,27,32	3,5,6,26,28,29,30,31	1	Q66H69
1839	1,2,4,7,8,25,28,29	3,5,6,26,27,30,31,32	4	P06911,P47727,P62804,Q812E4
1840	1,2,4,7,8,25,28,30	3,5,6,26,27,29,31,32	1	Q9Z1F2
1841	1,2,4,7,8,25,28,31	3,5,6,26,27,29,30,32	5	P06911,P82471,Q5GRG2,Q811M5,Q9Z1F2
1842	1,2,4,7,8,25,28,32	3,5,6,26,27,29,30,31	2	P08723,Q811M5
1843	1,2,4,7,8,25,29,30	3,5,6,26,27,28,31,32	3	P22006,P62804,Q6IFU8
1844	1,2,4,7,8,25,29,31	3,5,6,26,27,28,30,32	1	P82471

1845	1,2,4,7,8,25,29,32	3,5,6,26,27,28,30,31	0	
1846	1,2,4,7,8,25,30,31	3,5,6,26,27,28,29,32	2	G3V686,Q9Z1F2
1847	1,2,4,7,8,25,30,32	3,5,6,26,27,28,29,31	1	G3V686
1848	1,2,4,7,8,25,31,32	3,5,6,26,27,28,29,30	3	P21588,P82471,Q811M5
1849	1,2,4,7,8,26,27,28	3,5,6,25,29,30,31,32	12	P07150,P15399,P20762,P22282,P25809,P97580,Q30KJ2,Q4G075,Q5BJY9,Q66H69,Q9WTT6,Q9Z1F2
1850	1,2,4,7,8,26,27,29	3,5,6,25,28,30,31,32	9	P19223,P25809,P62804,P97580,Q30KJ2,Q4G075,Q66H69,Q9QX74,Q9WTT6
1851	1,2,4,7,8,26,27,30	3,5,6,25,28,29,31,32	7	P15399,P20762,P97580,Q10758,Q30KJ2,Q66H69,Q9Z1F2
1852	1,2,4,7,8,26,27,31	3,5,6,25,28,29,30,32	4	Q30KJ2,Q5BJY9,Q66H69,Q9Z1F2
1853	1,2,4,7,8,26,27,32	3,5,6,25,28,29,30,31	5	P15399,P20762,Q30KJ2,Q66H69,Q9Z1F2
1854	1,2,4,7,8,26,28,29	3,5,6,25,27,30,31,32	6	P20646,P21674,P62804,Q6P6R2,Q9QX74,Q9WTT6
1855	1,2,4,7,8,26,28,30	3,5,6,25,27,29,31,32	2	P20762,Q9Z1F2
1856	1,2,4,7,8,26,28,31	3,5,6,25,27,29,30,32	3	P82471,Q63598,Q9Z1F2
1857	1,2,4,7,8,26,28,32	3,5,6,25,27,29,30,31	2	O54728,Q9Z1F2
1858	1,2,4,7,8,26,29,30	3,5,6,25,27,28,31,32	1	P62804
1859	1,2,4,7,8,26,29,31	3,5,6,25,27,28,30,32	2	P82471,Q9QX74
1860	1,2,4,7,8,26,29,32	3,5,6,25,27,28,30,31	0	
1861	1,2,4,7,8,26,30,31	3,5,6,25,27,28,29,32	1	Q9Z1F2
1862	1,2,4,7,8,26,30,32	3,5,6,25,27,28,29,31	1	Q9Z1F2
1863	1,2,4,7,8,26,31,32	3,5,6,25,27,28,29,30	3	O54728,P82471,Q9Z1F2
1864	1,2,4,7,8,27,28,29	3,5,6,25,26,30,31,32	14	P19223,P21674,P22282,P25809,P47727,P62804,Q00715,Q4G075,Q63493,Q63617,Q66H69,Q6AYR9,Q812E4,Q9Z2L0

1865	1,2,4,7,8,27,28,30	3,5,6,25,26,29,31,32	7 P19218,P62804,Q10758,Q30KJ2,Q4G075,Q66H69,Q9Z1F2
1866	1,2,4,7,8,27,28,31	3,5,6,25,26,29,30,32	3 P82471,Q66H69,Q9Z1F2
1867	1,2,4,7,8,27,28,32	3,5,6,25,26,29,30,31	7 P05964,P08723,P22282,Q66H69,Q6AYR9,Q812E4,Q9JI85
1868	1,2,4,7,8,27,29,30	3,5,6,25,26,28,31,32	9 P00731,P19223,P62804,Q10758,Q30KJ2,Q4G075,Q66H69,Q6IFU8,Q6P6Q2
1869	1,2,4,7,8,27,29,31	3,5,6,25,26,28,30,32	5 P19223,P80299,P82471,Q4G075,Q66H69
1870	1,2,4,7,8,27,29,32	3,5,6,25,26,28,30,31	6 P19223,P25809,P80299,Q4G075,Q66H69,Q6TMA8
1871	1,2,4,7,8,27,30,31	3,5,6,25,26,28,29,32	6 P82471,Q10758,Q66H69,Q6IFU8,Q9QYP1,Q9Z1F2
1872	1,2,4,7,8,27,30,32	3,5,6,25,26,28,29,31	2 Q10758,Q66H69
1873	1,2,4,7,8,27,31,32	3,5,6,25,26,28,29,30	3 P80299,P82471,Q66H69
1874	1,2,4,7,8,28,29,30	3,5,6,25,26,27,31,32	2 P21674,P62804
1875	1,2,4,7,8,28,29,31	3,5,6,25,26,27,30,32	3 P21674,P82471,Q63493
1876	1,2,4,7,8,28,29,32	3,5,6,25,26,27,30,31	6 P08723,P21674,P30120,P47727,Q63493,Q812E4
1877	1,2,4,7,8,28,30,31	3,5,6,25,26,27,29,32	2 P82471,Q9Z1F2
1878	1,2,4,7,8,28,30,32	3,5,6,25,26,27,29,31	0
1879	1,2,4,7,8,28,31,32	3,5,6,25,26,27,29,30	3 P80299,P82471,Q811M5
1880	1,2,4,7,8,29,30,31	3,5,6,25,26,27,28,32	2 P82471,Q5RJR2
1881	1,2,4,7,8,29,30,32	3,5,6,25,26,27,28,31	0
1882	1,2,4,7,8,29,31,32	3,5,6,25,26,27,28,30	2 P80299,P82471
1883	1,2,4,7,8,30,31,32	3,5,6,25,26,27,28,29	3 P15999,P26342,P82471
1884	1,2,4,7,25,26,27,28	3,5,6,8,29,30,31,32	2 P20646,Q9Z1F2

1885	1,2,4,7,25, 26,27,29	3,5,6,8,28, 30,31,32	2	P10758,P19223
1886	1,2,4,7,25, 26,27,30	3,5,6,8,28, 29,31,32	2	Q9R168,Q9Z1F2
1887	1,2,4,7,25, 26,27,31	3,5,6,8,28, 29,30,32	1	Q9QYP1
1888	1,2,4,7,25, 26,27,32	3,5,6,8,28, 29,30,31	1	Q9R168
1889	1,2,4,7,25, 26,28,29	3,5,6,8,27, 30,31,32	1	P20646
1890	1,2,4,7,25, 26,28,30	3,5,6,8,27, 29,31,32	2	Q9R168,Q9Z1F2
1891	1,2,4,7,25, 26,28,31	3,5,6,8,27, 29,30,32	2	Q63598,Q9Z1F2
1892	1,2,4,7,25, 26,28,32	3,5,6,8,27, 29,30,31	1	Q9R168
1893	1,2,4,7,25, 26,29,30	3,5,6,8,27, 28,31,32	1	Q9R168
1894	1,2,4,7,25, 26,29,31	3,5,6,8,27, 28,30,32	1	P19218
1895	1,2,4,7,25, 26,29,32	3,5,6,8,27, 28,30,31	2	Q6AYR8,Q9R168
1896	1,2,4,7,25, 26,30,31	3,5,6,8,27, 28,29,32	2	Q9QYP1,Q9Z1F2
1897	1,2,4,7,25, 26,30,32	3,5,6,8,27, 28,29,31	2	D3ZUC6,Q9R168
1898	1,2,4,7,25, 26,31,32	3,5,6,8,27, 28,29,30	2	P19218,Q9QYP1
1899	1,2,4,7,25, 27,28,29	3,5,6,8,26, 30,31,32	2	P10758,Q812E4
1900	1,2,4,7,25, 27,28,30	3,5,6,8,26, 29,31,32	0	
1901	1,2,4,7,25, 27,28,31	3,5,6,8,26, 29,30,32	1	Q9QYP1
1902	1,2,4,7,25, 27,28,32	3,5,6,8,26, 29,30,31	2	Q62761;Q62762;Q62763,Q9R168
1903	1,2,4,7,25, 27,29,30	3,5,6,8,26, 28,31,32	1	P19223
1904	1,2,4,7,25, 27,29,31	3,5,6,8,26, 28,30,32	4	P10758,P19223,P80299,Q9QYP1

1905	1,2,4,7,25,<br>27,29,32	3,5,6,8,26,<br>28,30,31	2	P80299,Q9R168
1906	1,2,4,7,25,<br>27,30,31	3,5,6,8,26,<br>28,29,32	1	Q9QYP1
1907	1,2,4,7,25,<br>27,30,32	3,5,6,8,26,<br>28,29,31	2	Q9QYP1,Q9R168
1908	1,2,4,7,25,<br>27,31,32	3,5,6,8,26,<br>28,29,30	2	P80299,Q9QYP1
1909	1,2,4,7,25,<br>28,29,30	3,5,6,8,26,<br>27,31,32	1	Q9R168
1910	1,2,4,7,25,<br>28,29,31	3,5,6,8,26,<br>27,30,32	0	
1911	1,2,4,7,25,<br>28,29,32	3,5,6,8,26,<br>27,30,31	1	Q9R168
1912	1,2,4,7,25,<br>28,30,31	3,5,6,8,26,<br>27,29,32	0	
1913	1,2,4,7,25,<br>28,30,32	3,5,6,8,26,<br>27,29,31	2	Q62975,Q9R168
1914	1,2,4,7,25,<br>28,31,32	3,5,6,8,26,<br>27,29,30	2	P80299,Q811M5
1915	1,2,4,7,25,<br>29,30,31	3,5,6,8,26,<br>27,28,32	1	Q9QWN8
1916	1,2,4,7,25,<br>29,30,32	3,5,6,8,26,<br>27,28,31	2	P26342,Q9R168
1917	1,2,4,7,25,<br>29,31,32	3,5,6,8,26,<br>27,28,30	1	P80299
1918	1,2,4,7,25,<br>30,31,32	3,5,6,8,26,<br>27,28,29	5	D3ZUC6,P26342,P80299,Q6P6R2,Q9QYP1
1919	1,2,4,7,26,<br>27,28,29	3,5,6,8,25,<br>30,31,32	2	P02625,P20646
1920	1,2,4,7,26,<br>27,28,30	3,5,6,8,25,<br>29,31,32	3	P21588,Q80WL1,Q9Z1F2
1921	1,2,4,7,26,<br>27,28,31	3,5,6,8,25,<br>29,30,32	2	O88797,Q9Z1F2
1922	1,2,4,7,26,<br>27,28,32	3,5,6,8,25,<br>29,30,31	2	P05964,Q62761;Q62762;Q62763
1923	1,2,4,7,26,<br>27,29,30	3,5,6,8,25,<br>28,31,32	3	P19223,P21588,Q80WL1
1924	1,2,4,7,26,<br>27,29,31	3,5,6,8,25,<br>28,30,32	2	P19223,P80299

1925	1,2,4,7,26, 27,29,32	3,5,6,8,25, 28,30,31	1	P80299
1926	1,2,4,7,26, 27,30,31	3,5,6,8,25, 28,29,32	2	Q9QYP1,Q9Z1F2
1927	1,2,4,7,26, 27,30,32	3,5,6,8,25, 28,29,31	1	Q9R168
1928	1,2,4,7,26, 27,31,32	3,5,6,8,25, 28,29,30	2	P80299,Q9QYP1
1929	1,2,4,7,26, 28,29,30	3,5,6,8,25, 27,31,32	2	Q80WL1,Q9R168
1930	1,2,4,7,26, 28,29,31	3,5,6,8,25, 27,30,32	0	
1931	1,2,4,7,26, 28,29,32	3,5,6,8,25, 27,30,31	2	P14173,Q9R168
1932	1,2,4,7,26, 28,30,31	3,5,6,8,25, 27,29,32	1	Q9Z1F2
1933	1,2,4,7,26, 28,30,32	3,5,6,8,25, 27,29,31	1	Q9R168
1934	1,2,4,7,26, 28,31,32	3,5,6,8,25, 27,29,30	3	O54728,P14173,P80299
1935	1,2,4,7,26, 29,30,31	3,5,6,8,25, 27,28,32	1	P36860
1936	1,2,4,7,26, 29,30,32	3,5,6,8,25, 27,28,31	2	P36376,Q9R168
1937	1,2,4,7,26, 29,31,32	3,5,6,8,25, 27,28,30	1	P80299
1938	1,2,4,7,26, 30,31,32	3,5,6,8,25, 27,28,29	4	B2RYW9,P26342,P36860,P80299
1939	1,2,4,7,27, 28,29,30	3,5,6,8,25, 26,31,32	1	Q80WL1
1940	1,2,4,7,27, 28,29,31	3,5,6,8,25, 26,30,32	2	O88797,P80299
1941	1,2,4,7,27, 28,29,32	3,5,6,8,25, 26,30,31	6	O35547,P80299,Q62761;Q62762;Q62763,Q6AYR9,Q812E4,Q8CGS4
1942	1,2,4,7,27, 28,30,31	3,5,6,8,25, 26,29,32	2	P80299,Q9QYP1
1943	1,2,4,7,27, 28,30,32	3,5,6,8,25, 26,29,31	2	P80299,Q9R168
1944	1,2,4,7,27, 28,31,32	3,5,6,8,25, 26,29,30	3	P05964,P80299,Q62761;Q62762;Q62763

1945	1,2,4,7,27,<br>29,30,31	3,5,6,8,25,<br>26,28,32	3	P19223,P80299,Q9QYP1
1946	1,2,4,7,27,<br>29,30,32	3,5,6,8,25,<br>26,28,31	3	P19223,P80299,Q9R168
1947	1,2,4,7,27,<br>29,31,32	3,5,6,8,25,<br>26,28,30	1	P80299
1948	1,2,4,7,27,<br>30,31,32	3,5,6,8,25,<br>26,28,29	2	P80299,Q9QYP1
1949	1,2,4,7,28,<br>29,30,31	3,5,6,8,25,<br>26,27,32	1	P80299
1950	1,2,4,7,28,<br>29,30,32	3,5,6,8,25,<br>26,27,31	1	Q9R168
1951	1,2,4,7,28,<br>29,31,32	3,5,6,8,25,<br>26,27,30	1	P80299
1952	1,2,4,7,28,<br>30,31,32	3,5,6,8,25,<br>26,27,29	2	P26342,P80299
1953	1,2,4,7,29,<br>30,31,32	3,5,6,8,25,<br>26,27,28	4	P26342,P36860,P63081,P80299
1954	1,2,4,8,25,<br>26,27,28	3,5,6,7,29,<br>30,31,32	5	P15399,P36860,P97580,Q30KJ2,Q5BJY9
1955	1,2,4,8,25,<br>26,27,29	3,5,6,7,28,<br>30,31,32	3	P15399,P97580,Q30KJ2
1956	1,2,4,8,25,<br>26,27,30	3,5,6,7,28,<br>29,31,32	5	G3V686,P15399,P97580,Q10758,Q30KJ2
1957	1,2,4,8,25,<br>26,27,31	3,5,6,7,28,<br>29,30,32	6	P15399,P54921,P97580,Q30KJ2,Q5BJY9,Q9QYP1
1958	1,2,4,8,25,<br>26,27,32	3,5,6,7,28,<br>29,30,31	3	P15399,P97580,Q30KJ2
1959	1,2,4,8,25,<br>26,28,29	3,5,6,7,27,<br>30,31,32	0	
1960	1,2,4,8,25,<br>26,28,30	3,5,6,7,27,<br>29,31,32	2	G3V686,Q9Z1F2
1961	1,2,4,8,25,<br>26,28,31	3,5,6,7,27,<br>29,30,32	0	
1962	1,2,4,8,25,<br>26,28,32	3,5,6,7,27,<br>29,30,31	1	O54728
1963	1,2,4,8,25,<br>26,29,30	3,5,6,7,27,<br>28,31,32	0	
1964	1,2,4,8,25,<br>26,29,31	3,5,6,7,27,<br>28,30,32	0	

1965	1,2,4,8,25, 26,29,32	3,5,6,7,27, 28,30,31	0
1966	1,2,4,8,25, 26,30,31	3,5,6,7,27, 28,29,32	4 G3V686,P47967,P54921,Q6AY61
1967	1,2,4,8,25, 26,30,32	3,5,6,7,27, 28,29,31	4 D3ZUC6,G3V686,P00507,Q68G31
1968	1,2,4,8,25, 26,31,32	3,5,6,7,27, 28,29,30	4 G3V686,O54728,P00507,P47967
1969	1,2,4,8,25, 27,28,29	3,5,6,7,26, 30,31,32	4 P25809,P47727,Q6AYR9,Q812E4
1970	1,2,4,8,25, 27,28,30	3,5,6,7,26, 29,31,32	2 Q10758,Q30KJ2
1971	1,2,4,8,25, 27,28,31	3,5,6,7,26, 29,30,32	0
1972	1,2,4,8,25, 27,28,32	3,5,6,7,26, 29,30,31	2 Q6AYR9,Q9QWN8
1973	1,2,4,8,25, 27,29,30	3,5,6,7,26, 28,31,32	4 P19223,Q10758,Q30KJ2,Q6IFU8
1974	1,2,4,8,25, 27,29,31	3,5,6,7,26, 28,30,32	1 P19223
1975	1,2,4,8,25, 27,29,32	3,5,6,7,26, 28,30,31	0
1976	1,2,4,8,25, 27,30,31	3,5,6,7,26, 28,29,32	6 P47967,P54921,Q10758,Q30KJ2,Q6IFU8,Q9QYP1
1977	1,2,4,8,25, 27,30,32	3,5,6,7,26, 28,29,31	3 Q10758,Q30KJ2,Q68G31
1978	1,2,4,8,25, 27,31,32	3,5,6,7,26, 28,29,30	2 P47967,Q9QYP1
1979	1,2,4,8,25, 28,29,30	3,5,6,7,26, 27,31,32	1 Q62714
1980	1,2,4,8,25, 28,29,31	3,5,6,7,26, 27,30,32	0
1981	1,2,4,8,25, 28,29,32	3,5,6,7,26, 27,30,31	2 P30120,Q812E4
1982	1,2,4,8,25, 28,30,31	3,5,6,7,26, 27,29,32	0
1983	1,2,4,8,25, 28,30,32	3,5,6,7,26, 27,29,31	1 Q68G31
1984	1,2,4,8,25, 28,31,32	3,5,6,7,26, 27,29,30	2 P21588,Q811M5

1985	1,2,4,8,25,<br>29,30,31	3,5,6,7,26,<br>27,28,32	0
1986	1,2,4,8,25,<br>29,30,32	3,5,6,7,26,<br>27,28,31	1 Q68G31
1987	1,2,4,8,25,<br>29,31,32	3,5,6,7,26,<br>27,28,30	1 P21588
1988	1,2,4,8,25,<br>30,31,32	3,5,6,7,26,<br>27,28,29	3 D3ZUC6,P47967,Q9QYP1
1989	1,2,4,8,26,<br>27,28,29	3,5,6,7,25,<br>30,31,32	5 P15399,P25809,Q30KJ2,Q6P6R2,Q812E4
1990	1,2,4,8,26,<br>27,28,30	3,5,6,7,25,<br>29,31,32	4 P15399,P97580,Q10758,Q30KJ2
1991	1,2,4,8,26,<br>27,28,31	3,5,6,7,25,<br>29,30,32	3 P15399,Q30KJ2,Q5BJY9
1992	1,2,4,8,26,<br>27,28,32	3,5,6,7,25,<br>29,30,31	5 O54728,P15399,Q30KJ2,Q6AYR9,Q9QWN8
1993	1,2,4,8,26,<br>27,29,30	3,5,6,7,25,<br>28,31,32	6 P15399,P19223,P97580,Q10758,Q30KJ2,Q6IFU8
1994	1,2,4,8,26,<br>27,29,31	3,5,6,7,25,<br>28,30,32	2 P19223,Q30KJ2
1995	1,2,4,8,26,<br>27,29,32	3,5,6,7,25,<br>28,30,31	4 P15399,P25809,Q30KJ2,Q9JJS8
1996	1,2,4,8,26,<br>27,30,31	3,5,6,7,25,<br>28,29,32	6 P15399,P54921,P97580,Q10758,Q30KJ2,Q6AY61
1997	1,2,4,8,26,<br>27,30,32	3,5,6,7,25,<br>28,29,31	4 P15399,P97580,Q10758,Q30KJ2
1998	1,2,4,8,26,<br>27,31,32	3,5,6,7,25,<br>28,29,30	3 O54728,P15399,Q30KJ2
1999	1,2,4,8,26,<br>28,29,30	3,5,6,7,25,<br>27,31,32	0
2000	1,2,4,8,26,<br>28,29,31	3,5,6,7,25,<br>27,30,32	1 Q63474
2001	1,2,4,8,26,<br>28,29,32	3,5,6,7,25,<br>27,30,31	2 O54728,P30120
2002	1,2,4,8,26,<br>28,30,31	3,5,6,7,25,<br>27,29,32	0
2003	1,2,4,8,26,<br>28,30,32	3,5,6,7,25,<br>27,29,31	2 O54728,Q68G31
2004	1,2,4,8,26,<br>28,31,32	3,5,6,7,25,<br>27,29,30	1 O54728

2005	1,2,4,8,26,29,30,31	3,5,6,7,25,27,28,32	1	Q63474
2006	1,2,4,8,26,29,30,32	3,5,6,7,25,27,28,31	1	Q68G31
2007	1,2,4,8,26,29,31,32	3,5,6,7,25,27,28,30	2	O54728,Q63474
2008	1,2,4,8,26,30,31,32	3,5,6,7,25,27,28,29	3	O54728,P05539,P63081
2009	1,2,4,8,27,28,29,30	3,5,6,7,25,26,31,32	2	Q10758,Q812E4
2010	1,2,4,8,27,28,29,31	3,5,6,7,25,26,30,32	1	Q10758
2011	1,2,4,8,27,28,29,32	3,5,6,7,25,26,30,31	8	O35547,P04905,P08723,P25809,P30120,P47727,Q6AYR9,Q812E4
2012	1,2,4,8,27,28,30,31	3,5,6,7,25,26,29,32	1	Q10758
2013	1,2,4,8,27,28,30,32	3,5,6,7,25,26,29,31	3	P15399,Q10758,Q6AYR9
2014	1,2,4,8,27,28,31,32	3,5,6,7,25,26,29,30	1	Q6AYR9
2015	1,2,4,8,27,29,30,31	3,5,6,7,25,26,28,32	3	P19223,Q10758,Q6IFU8
2016	1,2,4,8,27,29,30,32	3,5,6,7,25,26,28,31	2	Q10758,Q6IFU8
2017	1,2,4,8,27,29,31,32	3,5,6,7,25,26,28,30	1	P80299
2018	1,2,4,8,27,30,31,32	3,5,6,7,25,26,28,29	2	Q10758,Q9QYP1
2019	1,2,4,8,28,29,30,31	3,5,6,7,25,26,27,32	1	Q63474
2020	1,2,4,8,28,29,30,32	3,5,6,7,25,26,27,31	1	Q68G31
2021	1,2,4,8,28,29,31,32	3,5,6,7,25,26,27,30	1	Q63474
2022	1,2,4,8,28,30,31,32	3,5,6,7,25,26,27,29	1	P63081
2023	1,2,4,8,29,30,31,32	3,5,6,7,25,26,27,28	2	P63081,Q63474
2024	1,2,4,25,26,27,28,29	3,5,6,7,8,30,31,32	1	P35280

2025	1,2,4,25,2<br>6,27,28,30	3,5,6,7,8,2<br>9,31,32	0
2026	1,2,4,25,2<br>6,27,28,31	3,5,6,7,8,2<br>9,30,32	2 P35280,Q9QYP1
2027	1,2,4,25,2<br>6,27,28,32	3,5,6,7,8,2<br>9,30,31	2 Q5RJR2,Q9QWN8
2028	1,2,4,25,2<br>6,27,29,30	3,5,6,7,8,2<br>8,31,32	1 D3ZUC6
2029	1,2,4,25,2<br>6,27,29,31	3,5,6,7,8,2<br>8,30,32	2 P35280,Q9QYP1
2030	1,2,4,25,2<br>6,27,29,32	3,5,6,7,8,2<br>8,30,31	0
2031	1,2,4,25,2<br>6,27,30,31	3,5,6,7,8,2<br>8,29,32	4 D3ZUC6,P47967,P54921,Q9QYP1
2032	1,2,4,25,2<br>6,27,30,32	3,5,6,7,8,2<br>8,29,31	4 D3ZUC6,P21674,Q9QYP1,Q9R168
2033	1,2,4,25,2<br>6,27,31,32	3,5,6,7,8,2<br>8,29,30	3 D3ZUC6,P47967,Q9QYP1
2034	1,2,4,25,2<br>6,28,29,30	3,5,6,7,8,2<br>7,31,32	3 P50115,Q62714,Q9QZK9
2035	1,2,4,25,2<br>6,28,29,31	3,5,6,7,8,2<br>7,30,32	2 P19218,P35280
2036	1,2,4,25,2<br>6,28,29,32	3,5,6,7,8,2<br>7,30,31	3 P19218,P50115,Q6AYR8
2037	1,2,4,25,2<br>6,28,30,31	3,5,6,7,8,2<br>7,29,32	1 P50115
2038	1,2,4,25,2<br>6,28,30,32	3,5,6,7,8,2<br>7,29,31	5 D3ZUC6,P50115,Q62975,Q68G31,Q9R168
2039	1,2,4,25,2<br>6,28,31,32	3,5,6,7,8,2<br>7,29,30	3 O54728,P19218,P50115
2040	1,2,4,25,2<br>6,29,30,31	3,5,6,7,8,2<br>7,28,32	2 D3ZUC6,P50115
2041	1,2,4,25,2<br>6,29,30,32	3,5,6,7,8,2<br>7,28,31	4 D3ZUC6,P50115,Q68G31,Q9R168
2042	1,2,4,25,2<br>6,29,31,32	3,5,6,7,8,2<br>7,28,30	4 D3ZUC6,P00507,P19218,P50115
2043	1,2,4,25,2<br>6,30,31,32	3,5,6,7,8,2<br>7,28,29	9 D3ZUC6,P00507,P04636,P06760,P47967,P50115,Q4G075,Q9EQS0,Q9QYP1
2044	1,2,4,25,2<br>7,28,29,30	3,5,6,7,8,2<br>6,31,32	1 Q62714

2045	1,2,4,25,27,28,29,31	3,5,6,7,8,26,30,32	1 P35280
2046	1,2,4,25,27,28,29,32	3,5,6,7,8,26,30,31	6 O35547,P00758,Q62761;Q62762;Q62763,Q6AYR9,Q812E4,Q8CGS4
2047	1,2,4,25,27,28,30,31	3,5,6,7,8,26,29,32	1 Q9QYP1
2048	1,2,4,25,27,28,30,32	3,5,6,7,8,26,29,31	3 D3ZUC6,P06760,Q9QX74
2049	1,2,4,25,27,28,31,32	3,5,6,7,8,26,29,30	2 Q9QX74,Q9QYP1
2050	1,2,4,25,27,29,30,31	3,5,6,7,8,26,28,32	2 D3ZUC6,Q9QYP1
2051	1,2,4,25,27,29,30,32	3,5,6,7,8,26,28,31	1 D3ZUC6
2052	1,2,4,25,27,29,31,32	3,5,6,7,8,26,28,30	2 P80299,Q9QYP1
2053	1,2,4,25,27,30,31,32	3,5,6,7,8,26,28,29	7 D3ZUC6,P06760,P47967,Q6P6R2,Q9QX74,Q9QYP1,Q9WTT6
2054	1,2,4,25,28,29,30,31	3,5,6,7,8,26,27,32	2 P50115,P50116
2055	1,2,4,25,28,29,30,32	3,5,6,7,8,26,27,31	6 D3ZUC6,P50115,P50116,Q62714,Q68G31,Q9R168
2056	1,2,4,25,28,29,31,32	3,5,6,7,8,26,27,30	2 P50115,P50116
2057	1,2,4,25,28,30,31,32	3,5,6,7,8,26,27,29	7 D3ZUC6,P06760,P50115,P50116,Q9QX74,Q9QYP1,Q9WTT6
2058	1,2,4,25,29,30,31,32	3,5,6,7,8,26,27,28	8 D3ZUC6,P07150,P47967,P50115,P50116,P63081,Q9QYP1,Q9WTT6
2059	1,2,4,26,27,28,29,30	3,5,6,7,8,25,31,32	1 P21588
2060	1,2,4,26,27,28,29,31	3,5,6,7,8,25,30,32	1 P35280
2061	1,2,4,26,27,28,29,32	3,5,6,7,8,25,30,31	5 O35547,P00758,Q62761;Q62762;Q62763,Q6AYR9,Q8CGS4
2062	1,2,4,26,27,28,30,31	3,5,6,7,8,25,29,32	0
2063	1,2,4,26,27,28,30,32	3,5,6,7,8,25,29,31	0
2064	1,2,4,26,27,28,31,32	3,5,6,7,8,25,29,30	1 O54728

2065	1,2,4,26,27,29,30,31	3,5,6,7,8,25,28,32	0	
2066	1,2,4,26,27,29,30,32	3,5,6,7,8,25,28,31	0	
2067	1,2,4,26,27,29,31,32	3,5,6,7,8,25,28,30	2	O70417,P80299
2068	1,2,4,26,27,30,31,32	3,5,6,7,8,25,28,29	2	P06760,Q9QYP1
2069	1,2,4,26,28,29,30,31	3,5,6,7,8,25,27,32	2	P50115,Q63474
2070	1,2,4,26,28,29,30,32	3,5,6,7,8,25,27,31	3	P50115,Q68G31,Q9R168
2071	1,2,4,26,28,29,31,32	3,5,6,7,8,25,27,30	4	O54728,O70417,P50115,Q63474
2072	1,2,4,26,28,30,31,32	3,5,6,7,8,25,27,29	4	O54728,P06760,P50115,Q4G075
2073	1,2,4,26,29,30,31,32	3,5,6,7,8,25,27,28	5	P36860,P50115,P50116,P63081,Q5QE79
2074	1,2,4,27,28,29,30,31	3,5,6,7,8,25,26,32	1	P80299
2075	1,2,4,27,28,29,30,32	3,5,6,7,8,25,26,31	0	
2076	1,2,4,27,28,29,31,32	3,5,6,7,8,25,26,30	4	O35547,P36374,P80299,Q62761;Q62762;Q62763
2077	1,2,4,27,28,30,31,32	3,5,6,7,8,25,26,29	4	P06760,P80299,Q9QX74,Q9QYP1
2078	1,2,4,27,29,30,31,32	3,5,6,7,8,25,26,28	4	B0BNN3,P80299,Q9QYP1,Q9WTT6
2079	1,2,4,28,29,30,31,32	3,5,6,7,8,25,26,27	6	P50115,P50116,P63081,P80299,Q63474,Q9WTT6
2080	1,2,5,6,7,8,25,26	3,4,27,28,29,30,31,32	4	O54858,P00762,P50115,P52590
2081	1,2,5,6,7,8,25,27	3,4,26,28,29,30,31,32	9	O54858,P02631,P06911,P50115,P50116,P52590,Q03191,Q66H69,Q91ZS3
2082	1,2,5,6,7,8,25,28	3,4,26,27,29,30,31,32	6	O54858,P00762,P02631,P06911,P52590,Q5GRG2

2083	1,2,5,6,7,8,25,29	3,4,26,27,28,30,31,32	5	P00762,P02631,P06760,P52590,P62804
2084	1,2,5,6,7,8,25,30	3,4,26,27,28,29,31,32	5	O54858,P00762,P02631,P52590,Q6IG05
2085	1,2,5,6,7,8,25,31	3,4,26,27,28,29,30,32	6	O54858,P00762,P06911,P12020,P52590,Q63532
2086	1,2,5,6,7,8,25,32	3,4,26,27,28,29,30,31	4	O54858,P02631,P13432,P52590
2087	1,2,5,6,7,8,26,27	3,4,25,28,29,30,31,32	9	P15399,P50115,P50116,P52590,P97580,Q03191,Q62714,Q66H69,Q9JJ50
2088	1,2,5,6,7,8,26,28	3,4,25,27,29,30,31,32	3	P00762,P52590,Q5RLM2
2089	1,2,5,6,7,8,26,29	3,4,25,27,28,30,31,32	6	P00762,P06760,P29315,P52590,P62804,Q9QX74
2090	1,2,5,6,7,8,26,30	3,4,25,27,28,29,31,32	7	O54858,P00762,P29315,P52590,Q5RJR2,Q6IG05,Q99MH3
2091	1,2,5,6,7,8,26,31	3,4,25,27,28,29,30,32	7	O54858,P00762,P20766,P52590,P57113,Q62714,Q63532
2092	1,2,5,6,7,8,26,32	3,4,25,27,28,29,30,31	5	O54858,P29315,P52590,Q62714,Q99MH3
2093	1,2,5,6,7,8,27,28	3,4,25,26,29,30,31,32	9	P00762,P19218,P50115,P50116,P52590,Q00715,Q03191,Q66H69,Q9JI85
2094	1,2,5,6,7,8,27,29	3,4,25,26,28,30,31,32	14	P00731,P06760,P19218,P25809,P29315,P50115,P50116,P52590,P62804,Q00715,Q03191,Q4G075,Q66H69,Q9JJ50
2095	1,2,5,6,7,8,27,30	3,4,25,26,28,29,31,32	12	P00731,P01039,P19218,P29315,P50115,P50116,P52590,Q03191,Q5I0J9,Q66H69,Q6IG05,Q8CJD3

2096	1,2,5,6,7,8,27,31	3,4,25,26,28,29,30,32	10	O54858,P00731,P01039,P19218,P50115,P50116,P52590,Q03191,Q62714,Q63532
2097	1,2,5,6,7,8,27,32	3,4,25,26,28,29,30,31	10	P01039,P19218,P29315,P50115,P50116,P52590,Q03191,Q62714,Q66H69,Q9JJ50
2098	1,2,5,6,7,8,28,29	3,4,25,26,27,30,31,32	9	P00762,P06760,P21674,P29315,P52590,P62804,Q00715,Q9JI85,Q9QYP1
2099	1,2,5,6,7,8,28,30	3,4,25,26,27,29,31,32	7	P00762,P19218,P29315,P35745,P52590,P62804,Q6IG05
2100	1,2,5,6,7,8,28,31	3,4,25,26,27,29,30,32	7	O54858,P00762,P12020,P35745,P52590,Q63532,Q811M5
2101	1,2,5,6,7,8,28,32	3,4,25,26,27,29,30,31	4	P02631,P29315,P52590,Q9JI85
2102	1,2,5,6,7,8,29,30	3,4,25,26,27,28,31,32	9	P00762,P01835,P19218,P29315,P52590,P62804,Q5I0J9,Q5RJR2,Q6IG05
2103	1,2,5,6,7,8,29,31	3,4,25,26,27,28,30,32	3	P00762,P52590,Q5RJR2
2104	1,2,5,6,7,8,29,32	3,4,25,26,27,28,30,31	3	P01835,P29315,P52590
2105	1,2,5,6,7,8,30,31	3,4,25,26,27,28,29,32	8	O54858,P00762,P19218,P35745,P52590,Q5RJR2,Q63532,Q6IG05
2106	1,2,5,6,7,8,30,32	3,4,25,26,27,28,29,31	7	O54858,P19218,P26342,P29315,P52590,Q5I0J9,Q6IG05
2107	1,2,5,6,7,8,31,32	3,4,25,26,27,28,29,30	4	O54858,P52590,Q62714,Q63532
2108	1,2,5,6,7,25,26,27	3,4,8,28,29,30,31,32	4	P01039,P50116,Q03191,Q9JJ50
2109	1,2,5,6,7,25,26,28	3,4,8,27,29,30,31,32	1	P00762

2110	1,2,5,6,7,25,26,29	3,4,8,27,28,30,31,32	3	P00762,P01835,P52590
2111	1,2,5,6,7,25,26,30	3,4,8,27,28,29,31,32	3	P00762,Q9EQS0,Q9R168
2112	1,2,5,6,7,25,26,31	3,4,8,27,28,29,30,32	3	P00762,Q63150,Q9EQS0
2113	1,2,5,6,7,25,26,32	3,4,8,27,28,29,30,31	3	Q5PQL7,Q9EQS0,Q9R168
2114	1,2,5,6,7,25,27,28	3,4,8,26,29,30,31,32	3	P00762,P50116,Q03191
2115	1,2,5,6,7,25,27,29	3,4,8,26,28,30,31,32	5	O54728,P50116,P52590,Q03191,Q9JJ50
2116	1,2,5,6,7,25,27,30	3,4,8,26,28,29,31,32	4	O54728,P01039,P50116,Q03191
2117	1,2,5,6,7,25,27,31	3,4,8,26,28,29,30,32	4	O54728,P01039,P50116,Q03191
2118	1,2,5,6,7,25,27,32	3,4,8,26,28,29,30,31	4	P01039,P50116,Q03191,Q9JJ50
2119	1,2,5,6,7,25,28,29	3,4,8,26,27,30,31,32	3	P00762,P01835,P52590
2120	1,2,5,6,7,25,28,30	3,4,8,26,27,29,31,32	2	P00762,Q5PQL7
2121	1,2,5,6,7,25,28,31	3,4,8,26,27,29,30,32	1	P00762
2122	1,2,5,6,7,25,28,32	3,4,8,26,27,29,30,31	2	Q5PQL7,Q62975
2123	1,2,5,6,7,25,29,30	3,4,8,26,27,28,31,32	5	O54728,P00762,P01835,P52590,Q9QWN8
2124	1,2,5,6,7,25,29,31	3,4,8,26,27,28,30,32	5	O54728,P00762,P01835,P52590,Q9QWN8
2125	1,2,5,6,7,25,29,32	3,4,8,26,27,28,30,31	2	P01835,P52590
2126	1,2,5,6,7,25,30,31	3,4,8,26,27,28,29,32	8	O54728,P00762,P26342,Q6AYR9,Q6P6R2,Q9EQS0,Q9QWN8,Q9WVK7
2127	1,2,5,6,7,25,30,32	3,4,8,26,27,28,29,31	6	P26342,Q5PQL7,Q6P6R2,Q9EQS0,Q9R168,Q9WVK7
2128	1,2,5,6,7,25,31,32	3,4,8,26,27,28,29,30	3	P26342,Q6P6R2,Q9WVK7
2129	1,2,5,6,7,26,27,28	3,4,8,25,29,30,31,32	7	P00762,P02625,P14173,P21588,P50116,Q03191,Q9JJ50

2130	1,2,5,6,7,26,27,29	3,4,8,25,28,30,31,32	8	P00762,P01835,P02625,P21588,P50116,P52590,Q03191,Q9JJ50
2131	1,2,5,6,7,26,27,30	3,4,8,25,28,29,31,32	7	P00762,P01039,P21588,P29315,P50116,Q03191,Q9JJ50
2132	1,2,5,6,7,26,27,31	3,4,8,25,28,29,30,32	6	P00762,P01039,P50116,Q03191,Q62714,Q9JJ50
2133	1,2,5,6,7,26,27,32	3,4,8,25,28,29,30,31	6	P01039,P14173,P50116,Q03191,Q62714,Q9JJ50
2134	1,2,5,6,7,26,28,29	3,4,8,25,27,30,31,32	7	P00762,P01835,P02625,P14173,P29315,P47967,P52590
2135	1,2,5,6,7,26,28,30	3,4,8,25,27,29,31,32	5	P00762,P01835,P21588,P29315,Q9EQS0
2136	1,2,5,6,7,26,28,31	3,4,8,25,27,29,30,32	4	P00762,P01835,P14173,Q6IG02
2137	1,2,5,6,7,26,28,32	3,4,8,25,27,29,30,31	6	P00762,P01835,P14173,P29315,P52590,Q6IG02
2138	1,2,5,6,7,26,29,30	3,4,8,25,27,28,31,32	6	P00762,P01835,P21588,P29315,P36860,P52590
2139	1,2,5,6,7,26,29,31	3,4,8,25,27,28,30,32	6	O70417,P00762,P01835,P14173,P36860,P52590
2140	1,2,5,6,7,26,29,32	3,4,8,25,27,28,30,31	8	O70417,P00762,P01835,P14173,P29315,P36860,P52590,Q9JJ50
2141	1,2,5,6,7,26,30,31	3,4,8,25,27,28,29,32	6	P00762,P01835,P36860,Q6AYR9,Q812E4,Q9EQS0
2142	1,2,5,6,7,26,30,32	3,4,8,25,27,28,29,31	8	P01835,P26342,P29315,P36860,Q5I0J9,Q99MH3,Q9EQS0,Q9R168
2143	1,2,5,6,7,26,31,32	3,4,8,25,27,28,29,30	8	O70417,P01835,P14173,P36860,P57113,Q62714,Q6IG02,Q9EQS0
2144	1,2,5,6,7,27,28,29	3,4,8,25,26,30,31,32	11	P00762,P01835,P02625,P05539,P29315,P47967,P50116,P52590,Q00715,Q03191,Q6AY61
2145	1,2,5,6,7,27,28,30	3,4,8,25,26,29,31,32	8	P00762,P01039,P19218,P21588,P29315,P50116,Q03191,Q5I0J9
2146	1,2,5,6,7,27,28,31	3,4,8,25,26,29,30,32	4	P00762,P01039,P50116,Q03191
2147	1,2,5,6,7,27,28,32	3,4,8,25,26,29,30,31	7	P14173,P29315,P50116,P52590,Q03191,Q62761;Q62762;Q62763,Q6AY61
2148	1,2,5,6,7,27,29,30	3,4,8,25,26,28,31,32	14	O54728,P00731,P00762,P01039,P01835,P19218,P21588,P29315,P50116,P52590,Q03191,Q5I0J9,Q923V8,Q9JJ50
2149	1,2,5,6,7,27,29,31	3,4,8,25,26,28,30,32	9	O54728,O70417,P00762,P01039,P01835,P50116,P52590,P80299,Q03191

2150	1,2,5,6,7,27,29,32	3,4,8,25,26,28,30,31	7	P01835,P29315,P50116,P52590,Q03191,Q6AY61,Q9JJ50
2151	1,2,5,6,7,27,30,31	3,4,8,25,26,28,29,32	7	O54728,P00762,P01039,P19218,P36860,P50116,Q03191
2152	1,2,5,6,7,27,30,32	3,4,8,25,26,28,29,31	9	P01039,P19218,P29315,P50116,Q03191,Q5I0J9,Q6P6R2,Q9JJ50,Q9R168
2153	1,2,5,6,7,27,31,32	3,4,8,25,26,28,29,30	5	P01039,P50116,P80299,Q03191,Q62714
2154	1,2,5,6,7,28,29,30	3,4,8,25,26,27,31,32	4	P00762,P01835,P29315,P52590
2155	1,2,5,6,7,28,29,31	3,4,8,25,26,27,30,32	6	O70417,P00762,P01835,P14173,P21674,P52590
2156	1,2,5,6,7,28,29,32	3,4,8,25,26,27,30,31	7	P00762,P01835,P14173,P29315,P52590,P54921,Q6AY61
2157	1,2,5,6,7,28,30,31	3,4,8,25,26,27,29,32	2	P00762,P01835
2158	1,2,5,6,7,28,30,32	3,4,8,25,26,27,29,31	6	P00762,P01835,P29315,P52590,Q5I0J9,Q9R168
2159	1,2,5,6,7,28,31,32	3,4,8,25,26,27,29,30	5	P00762,P01835,P14173,P52590,Q6IG02
2160	1,2,5,6,7,29,30,31	3,4,8,25,26,27,28,32	7	O54728,P00762,P01835,P26342,P36860,P52590,Q9QWN8
2161	1,2,5,6,7,29,30,32	3,4,8,25,26,27,28,31	8	P01835,P26342,P29315,P36860,P52590,Q5BJY9,Q5I0J9,Q9R168
2162	1,2,5,6,7,29,31,32	3,4,8,25,26,27,28,30	7	O70417,P01835,P14173,P26342,P36860,P52590,P80299
2163	1,2,5,6,7,30,31,32	3,4,8,25,26,27,28,29	7	P01835,P20646,P26342,P36860,Q5I0J9,Q6P6R2,Q9EQS0
2164	1,2,5,6,8,25,26,27	3,4,7,28,29,30,31,32	5	P15399,P17559,P97580,Q30KJ2,Q9JJ50
2165	1,2,5,6,8,25,26,28	3,4,7,27,29,30,31,32	4	P00714,P00762,P08649,P80299
2166	1,2,5,6,8,25,26,29	3,4,7,27,28,30,31,32	1	P13432
2167	1,2,5,6,8,25,26,30	3,4,7,27,28,29,31,32	3	P80299,Q6IG05,Q99MH3
2168	1,2,5,6,8,25,26,31	3,4,7,27,28,29,30,32	3	O54858,P97840,Q99MH3
2169	1,2,5,6,8,25,26,32	3,4,7,27,28,29,30,31	4	P00507,P13432,Q68G31,Q99MH3

2170	1,2,5,6,8,25,27,28	3,4,7,26,29,30,31,32	0
2171	1,2,5,6,8,25,27,29	3,4,7,26,28,30,31,32	0
2172	1,2,5,6,8,25,27,30	3,4,7,26,28,29,31,32	3 Q03191,Q5I0J9,Q6IG05
2173	1,2,5,6,8,25,27,31	3,4,7,26,28,29,30,32	3 P23739,P97840,Q03191
2174	1,2,5,6,8,25,27,32	3,4,7,26,28,29,30,31	1 Q03191
2175	1,2,5,6,8,25,28,29	3,4,7,26,27,30,31,32	2 P00762,P52590
2176	1,2,5,6,8,25,28,30	3,4,7,26,27,29,31,32	1 Q6IG05
2177	1,2,5,6,8,25,28,31	3,4,7,26,27,29,30,32	2 Q5GRG2,Q63532
2178	1,2,5,6,8,25,28,32	3,4,7,26,27,29,30,31	1 Q68G31
2179	1,2,5,6,8,25,29,30	3,4,7,26,27,28,31,32	1 Q6IG05
2180	1,2,5,6,8,25,29,31	3,4,7,26,27,28,30,32	0
2181	1,2,5,6,8,25,29,32	3,4,7,26,27,28,30,31	3 P13432,P52590,Q68G31
2182	1,2,5,6,8,25,30,31	3,4,7,26,27,28,29,32	5 O54858,P20646,P97840,Q63532,Q6IG05
2183	1,2,5,6,8,25,30,32	3,4,7,26,27,28,29,31	5 P20646,Q5I0J9,Q68G31,Q6IG05,Q6P6R2
2184	1,2,5,6,8,25,31,32	3,4,7,26,27,28,29,30	5 O54858,P13432,P20646,P97840,Q63532
2185	1,2,5,6,8,26,27,28	3,4,7,25,29,30,31,32	1 P15399
2186	1,2,5,6,8,26,27,29	3,4,7,25,28,30,31,32	4 P15399,P25809,P52590,Q9JJ50
2187	1,2,5,6,8,26,27,30	3,4,7,25,28,29,31,32	9 P15399,P21588,P97580,Q30KJ2,Q5I0J9,Q6IG05,Q8CJD3,Q99MH3,Q9JJ50
2188	1,2,5,6,8,26,27,31	3,4,7,25,28,29,30,32	1 P15399
2189	1,2,5,6,8,26,27,32	3,4,7,25,28,29,30,31	3 P15399,Q5I0J9,Q9JJ50

2190	1,2,5,6,8,26,28,29	3,4,7,25,27,30,31,32	3	P00762,P52590,Q9QYP1
2191	1,2,5,6,8,26,28,30	3,4,7,25,27,29,31,32	3	P00762,Q5I0J9,Q99MH3
2192	1,2,5,6,8,26,28,31	3,4,7,25,27,29,30,32	2	P00762,Q63532
2193	1,2,5,6,8,26,28,32	3,4,7,25,27,29,30,31	1	Q99MH3
2194	1,2,5,6,8,26,29,30	3,4,7,25,27,28,31,32	6	P00762,P29315,P52590,Q5I0J9,Q5RJR2,Q6IG05
2195	1,2,5,6,8,26,29,31	3,4,7,25,27,28,30,32	3	P00762,P52590,Q5RJR2
2196	1,2,5,6,8,26,29,32	3,4,7,25,27,28,30,31	4	P13432,P29315,P52590,Q68G31
2197	1,2,5,6,8,26,30,31	3,4,7,25,27,28,29,32	7	O89117,Q5RJR2,Q63532,Q6IG05,Q8CGS4,Q99MH3,Q9Z2L0
2198	1,2,5,6,8,26,30,32	3,4,7,25,27,28,29,31	6	O89117,P29315,Q5I0J9,Q68G31,Q6IG05,Q99MH3
2199	1,2,5,6,8,26,31,32	3,4,7,25,27,28,29,30	4	O89117,Q62714,Q63532,Q99MH3
2200	1,2,5,6,8,27,28,29	3,4,7,25,26,30,31,32	4	P25809,P52590,Q00715,Q9JI85
2201	1,2,5,6,8,27,28,30	3,4,7,25,26,29,31,32	4	P15399,P19218,Q5I0J9,Q6IG05
2202	1,2,5,6,8,27,28,31	3,4,7,25,26,29,30,32	1	Q63532
2203	1,2,5,6,8,27,28,32	3,4,7,25,26,29,30,31	3	P15399,Q5I0J9,Q9JI85
2204	1,2,5,6,8,27,29,30	3,4,7,25,26,28,31,32	8	P00731,P19218,P29315,P52590,Q5I0J9,Q6IFU7,Q6IG05,Q6IMF3
2205	1,2,5,6,8,27,29,31	3,4,7,25,26,28,30,32	2	P00731,P52590
2206	1,2,5,6,8,27,29,32	3,4,7,25,26,28,30,31	4	P29315,P52590,Q5I0J9,Q9JJ50
2207	1,2,5,6,8,27,30,31	3,4,7,25,26,28,29,32	6	P00731,P01039,P19218,P20646,Q5I0J9,Q6IG05
2208	1,2,5,6,8,27,30,32	3,4,7,25,26,28,29,31	7	P19218,P20646,P29315,Q03191,Q5I0J9,Q68G31,Q6IG05
2209	1,2,5,6,8,27,31,32	3,4,7,25,26,28,29,30	5	P01039,P20646,P23739,Q03191,Q5I0J9

2210	1,2,5,6,8,28,29,30	3,4,7,25,26,27,31,32	5	P00762,P19218,P29315,P52590,Q5I0J9
2211	1,2,5,6,8,28,29,31	3,4,7,25,26,27,30,32	3	P00762,P21674,P52590
2212	1,2,5,6,8,28,29,32	3,4,7,25,26,27,30,31	5	P29315,P52590,Q5I0J9,Q9JI85,Q9QYP1
2213	1,2,5,6,8,28,30,31	3,4,7,25,26,27,29,32	5	P00762,P35745,Q5I0J9,Q63532,Q6IG05
2214	1,2,5,6,8,28,30,32	3,4,7,25,26,27,29,31	5	O89117,P19218,P29315,Q5I0J9,Q68G31
2215	1,2,5,6,8,28,31,32	3,4,7,25,26,27,29,30	2	O89117,Q63532
2216	1,2,5,6,8,29,30,31	3,4,7,25,26,27,28,32	5	P36860,P52590,Q5I0J9,Q5RJR2,Q6IG05
2217	1,2,5,6,8,29,30,32	3,4,7,25,26,27,28,31	7	P20646,P29315,P36860,P52590,Q5I0J9,Q68G31,Q6IG05
2218	1,2,5,6,8,29,31,32	3,4,7,25,26,27,28,30	2	P36860,P52590
2219	1,2,5,6,8,30,31,32	3,4,7,25,26,27,28,29	8	O89117,P20646,P26342,P36860,Q5I0J9,Q63532,Q6IG05,Q6P6R2
2220	1,2,5,6,25,26,27,28	3,4,7,8,29,30,31,32	3	P00714,P35280,P82471
2221	1,2,5,6,25,26,27,29	3,4,7,8,28,30,31,32	4	O70417,P35280,P82471,Q9JJ50
2222	1,2,5,6,25,26,27,30	3,4,7,8,28,29,31,32	6	P01039,P21588,P82471,Q03191,Q9EQS0,Q9JJ50
2223	1,2,5,6,25,26,27,31	3,4,7,8,28,29,30,32	4	O70417,P01039,P35280,Q03191
2224	1,2,5,6,25,26,27,32	3,4,7,8,28,29,30,31	4	P01039,P82471,Q03191,Q9JJ50
2225	1,2,5,6,25,26,28,29	3,4,7,8,27,30,31,32	5	O70417,P00714,P00762,P35280,P82471
2226	1,2,5,6,25,26,28,30	3,4,7,8,27,29,31,32	4	P00714,P00762,P82471,Q9EQS0
2227	1,2,5,6,25,26,28,31	3,4,7,8,27,29,30,32	5	O70417,P00714,P00762,P35280,Q9EQS0
2228	1,2,5,6,25,26,28,32	3,4,7,8,27,29,30,31	5	P82471,Q5PQL7,Q68G31,Q6IG02,Q9EQS0
2229	1,2,5,6,25,26,29,30	3,4,7,8,27,28,31,32	5	P00762,P22282,P82471,Q62902,Q9EQS0

2230	1,2,5,6,25,<br>26,29,31	3,4,7,8,27,<br>28,30,32	3	O70417,P00762,P35280
2231	1,2,5,6,25,<br>26,29,32	3,4,7,8,27,<br>28,30,31	3	O70417,P82471,Q68G31
2232	1,2,5,6,25,<br>26,30,31	3,4,7,8,27,<br>28,29,32	8	P22282,Q4G075,Q62902,Q6AYR9,Q812E4,Q99MH3,Q9EQS0,Q9Z2L0
2233	1,2,5,6,25,<br>26,30,32	3,4,7,8,27,<br>28,29,31	9	D3ZUC6,P00507,P82471,Q4G075,Q5PQL7,Q68G31,Q99MH3,Q9EQS0,Q9R168
2234	1,2,5,6,25,<br>26,31,32	3,4,7,8,27,<br>28,29,30	5	O70417,P00507,P97840,Q4G075,Q9EQS0
2235	1,2,5,6,25,<br>27,28,29	3,4,7,8,26,<br>30,31,32	3	P05539,P35280,P82471
2236	1,2,5,6,25,<br>27,28,30	3,4,7,8,26,<br>29,31,32	2	P82471,Q5I0J9
2237	1,2,5,6,25,<br>27,28,31	3,4,7,8,26,<br>29,30,32	2	P35280,Q03191
2238	1,2,5,6,25,<br>27,28,32	3,4,7,8,26,<br>29,30,31	4	P82471,Q03191,Q62761;Q62762;Q62763,Q9QX74
2239	1,2,5,6,25,<br>27,29,30	3,4,7,8,26,<br>28,31,32	4	O54728,P82471,Q5I0J9,Q923V8
2240	1,2,5,6,25,<br>27,29,31	3,4,7,8,26,<br>28,30,32	4	O54728,O70417,P35280,Q03191
2241	1,2,5,6,25,<br>27,29,32	3,4,7,8,26,<br>28,30,31	4	O70417,P82471,Q03191,Q9JJ50
2242	1,2,5,6,25,<br>27,30,31	3,4,7,8,26,<br>28,29,32	4	O54728,P01039,Q03191,Q6P6R2
2243	1,2,5,6,25,<br>27,30,32	3,4,7,8,26,<br>28,29,31	10	P01039,P20646,P82471,Q03191,Q5I0J9,Q63424,Q68G31,Q6P6R2,Q9QX74,Q9WTT6
2244	1,2,5,6,25,<br>27,31,32	3,4,7,8,26,<br>28,29,30	5	O70417,P01039,P20646,Q03191,Q6P6R2
2245	1,2,5,6,25,<br>28,29,30	3,4,7,8,26,<br>27,31,32	2	P00762,P82471
2246	1,2,5,6,25,<br>28,29,31	3,4,7,8,26,<br>27,30,32	3	O70417,P00762,P35280
2247	1,2,5,6,25,<br>28,29,32	3,4,7,8,26,<br>27,30,31	2	O70417,Q68G31
2248	1,2,5,6,25,<br>28,30,31	3,4,7,8,26,<br>27,29,32	3	P00762,Q4G075,Q9EQS0
2249	1,2,5,6,25,<br>28,30,32	3,4,7,8,26,<br>27,29,31	8	P82471,Q4G075,Q5I0J9,Q5PQL7,Q62975,Q68G31,Q9QX74,Q9WTT6

2250	1,2,5,6,25, 28,31,32	3,4,7,8,26, 27,29,30	3	O70417,Q4G075,Q9QX74
2251	1,2,5,6,25, 29,30,31	3,4,7,8,26, 27,28,32	6	O54728,P07150,P22282,P36860,Q62902,Q9QWN8
2252	1,2,5,6,25, 29,30,32	3,4,7,8,26, 27,28,31	6	P07150,P36860,P82471,Q5I0J9,Q68G31,Q9WTT6
2253	1,2,5,6,25, 29,31,32	3,4,7,8,26, 27,28,30	4	O70417,P07150,P36860,Q9WTT6
2254	1,2,5,6,25, 30,31,32	3,4,7,8,26, 27,28,29	13	P07150,P20646,P22273,P26342,P36860,P47967,P97840,Q4G075,Q6P6R2,Q9EQS0,Q9QX74,Q9WTT6,Q9WVK7
2255	1,2,5,6,26, 27,28,29	3,4,7,8,25, 30,31,32	7	O70417,P00762,P02625,P21588,P35280,P82471,Q9JJ50
2256	1,2,5,6,26, 27,28,30	3,4,7,8,25, 29,31,32	4	P00762,P21588,P82471,Q5I0J9
2257	1,2,5,6,26, 27,28,31	3,4,7,8,25, 29,30,32	3	O70417,P00762,P35280
2258	1,2,5,6,26, 27,28,32	3,4,7,8,25, 29,30,31	2	O70417,P82471
2259	1,2,5,6,26, 27,29,30	3,4,7,8,25, 28,31,32	6	P21588,P82471,Q5I0J9,Q811M5,Q923V8,Q9JJ50
2260	1,2,5,6,26, 27,29,31	3,4,7,8,25, 28,30,32	3	O70417,P35280,Q9JJ50
2261	1,2,5,6,26, 27,29,32	3,4,7,8,25, 28,30,31	3	O70417,P82471,Q9JJ50
2262	1,2,5,6,26, 27,30,31	3,4,7,8,25, 28,29,32	2	P01039,P36860
2263	1,2,5,6,26, 27,30,32	3,4,7,8,25, 28,29,31	7	P01039,P36860,P82471,Q03191,Q5I0J9,Q68G31,Q9JJ50
2264	1,2,5,6,26, 27,31,32	3,4,7,8,25, 28,29,30	4	O70417,P01039,P36860,Q03191
2265	1,2,5,6,26, 28,29,30	3,4,7,8,25, 27,31,32	5	P00762,P01835,P21588,P29315,P82471
2266	1,2,5,6,26, 28,29,31	3,4,7,8,25, 27,30,32	5	O70417,P00762,P01835,P02625,P35280
2267	1,2,5,6,26, 28,29,32	3,4,7,8,25, 27,30,31	4	O70417,P01835,P14173,P29315
2268	1,2,5,6,26, 28,30,31	3,4,7,8,25, 27,29,32	3	P00762,Q4G075,Q9EQS0
2269	1,2,5,6,26, 28,30,32	3,4,7,8,25, 27,29,31	7	P29315,P82471,Q4G075,Q5I0J9,Q68G31,Q99MH3,Q9EQS0

2270	1,2,5,6,26, 28,31,32	3,4,7,8,25, 27,29,30	4	O70417,P14173,Q4G075,Q6IG02
2271	1,2,5,6,26, 29,30,31	3,4,7,8,25, 27,28,32	7	O70417,P00762,P01835,P0C0A9,P22282,P36860,Q9EQS0
2272	1,2,5,6,26, 29,30,32	3,4,7,8,25, 27,28,31	8	O70417,P01835,P29315,P36860,P82471,Q5I0J9,Q68G31,Q9EQS0
2273	1,2,5,6,26, 29,31,32	3,4,7,8,25, 27,28,30	4	O70417,P01835,P36860,Q5QE79
2274	1,2,5,6,26, 30,31,32	3,4,7,8,25, 27,28,29	13	O70417,O89117,P0C0A9,P20646,P26342,P36860,Q4G075,Q5I0J9,Q812E4,Q99MH3,Q9EQS0,Q9 WTT6,Q9Z2L0
2275	1,2,5,6,27, 28,29,30	3,4,7,8,25, 26,31,32	7	P00762,P19218,P21588,P29315,P82471,Q5I0J9,Q923V8
2276	1,2,5,6,27, 28,29,31	3,4,7,8,25, 26,30,32	3	O70417,P00762,P35280
2277	1,2,5,6,27, 28,29,32	3,4,7,8,25, 26,30,31	6	O35547,O70417,P29315,Q5I0J9,Q62761;Q62762;Q62763,Q6AY61
2278	1,2,5,6,27, 28,30,31	3,4,7,8,25, 26,29,32	1	Q5I0J9
2279	1,2,5,6,27, 28,30,32	3,4,7,8,25, 26,29,31	6	P29315,P82471,Q03191,Q5I0J9,Q68G31,Q9QX74
2280	1,2,5,6,27, 28,31,32	3,4,7,8,25, 26,29,30	3	O70417,P22006,Q03191
2281	1,2,5,6,27, 29,30,31	3,4,7,8,25, 26,28,32	6	O54728,O70417,P01039,P36860,Q5I0J9,Q923V8
2282	1,2,5,6,27, 29,30,32	3,4,7,8,25, 26,28,31	8	P29315,P36860,P82471,Q03191,Q5I0J9,Q68G31,Q923V8,Q9JJ50
2283	1,2,5,6,27, 29,31,32	3,4,7,8,25, 26,28,30	4	O70417,P36860,P80299,Q03191
2284	1,2,5,6,27, 30,31,32	3,4,7,8,25, 26,28,29	8	P01039,P20646,P36860,Q03191,Q5I0J9,Q6P6R2,Q9QX74,Q9WTT6
2285	1,2,5,6,28, 29,30,31	3,4,7,8,25, 26,27,32	5	O70417,P00762,P01835,P36860,Q5I0J9
2286	1,2,5,6,28, 29,30,32	3,4,7,8,25, 26,27,31	5	P01835,P29315,P36860,Q5I0J9,Q68G31
2287	1,2,5,6,28, 29,31,32	3,4,7,8,25, 26,27,30	2	O70417,P01835
2288	1,2,5,6,28, 30,31,32	3,4,7,8,25, 26,27,29	8	O89117,P20646,P22006,P36860,Q4G075,Q5I0J9,Q9QX74,Q9WTT6
2289	1,2,5,6,29, 30,31,32	3,4,7,8,25, 26,27,28	9	O70417,P01835,P07150,P20646,P26342,P36860,Q4G075,Q5I0J9,Q9WTT6

2290	1,2,5,7,8,25,26,27	3,4,6,28,29,30,31,32	4 P22006,Q03191,Q66H69,Q9WTT6
2291	1,2,5,7,8,25,26,28	3,4,6,27,29,30,31,32	3 P00714,P00762,Q9WTT6
2292	1,2,5,7,8,25,26,29	3,4,6,27,28,30,31,32	4 P06760,P22006,Q8VD89,Q9QX74
2293	1,2,5,7,8,25,26,30	3,4,6,27,28,29,31,32	2 P22006,Q6IG05
2294	1,2,5,7,8,25,26,31	3,4,6,27,28,29,30,32	1 Q9Z0V6
2295	1,2,5,7,8,25,26,32	3,4,6,27,28,29,30,31	0
2296	1,2,5,7,8,25,27,28	3,4,6,26,29,30,31,32	2 Q03191,Q66H69
2297	1,2,5,7,8,25,27,29	3,4,6,26,28,30,31,32	8 P06760,P22006,P25809,P62804,Q00715,Q03191,Q4G075,Q66H69
2298	1,2,5,7,8,25,27,30	3,4,6,26,28,29,31,32	8 P00731,P22006,Q03191,Q66H69,Q6IFU8,Q6IG05,Q6IMF3,Q6P6Q2
2299	1,2,5,7,8,25,27,31	3,4,6,26,28,29,30,32	2 P23739,Q03191
2300	1,2,5,7,8,25,27,32	3,4,6,26,28,29,30,31	2 Q03191,Q66H69
2301	1,2,5,7,8,25,28,29	3,4,6,26,27,30,31,32	4 P00762,P06760,P22006,P62804
2302	1,2,5,7,8,25,28,30	3,4,6,26,27,29,31,32	1 Q6IG05
2303	1,2,5,7,8,25,28,31	3,4,6,26,27,29,30,32	1 Q811M5
2304	1,2,5,7,8,25,28,32	3,4,6,26,27,29,30,31	1 Q811M5
2305	1,2,5,7,8,25,29,30	3,4,6,26,27,28,31,32	4 P22006,P62804,Q6IG05,Q6IMF3
2306	1,2,5,7,8,25,29,31	3,4,6,26,27,28,30,32	2 P06760,Q8VD89
2307	1,2,5,7,8,25,29,32	3,4,6,26,27,28,30,31	0
2308	1,2,5,7,8,25,30,31	3,4,6,26,27,28,29,32	2 Q6IG05,Q6IMF3
2309	1,2,5,7,8,25,30,32	3,4,6,26,27,28,29,31	1 Q6IG05

2310	1,2,5,7,8,25,31,32	3,4,6,26,27,28,29,30	2	P21588,Q811M5
2311	1,2,5,7,8,26,27,28	3,4,6,25,29,30,31,32	6	P07150,P25809,P47967,Q5RLM2,Q66H69,Q9WTT6
2312	1,2,5,7,8,26,27,29	3,4,6,25,28,30,31,32	10	P06760,P22006,P25809,P62804,Q00715,Q4G075,Q66H69,Q9JJ50,Q9QX74,Q9WTT6
2313	1,2,5,7,8,26,27,30	3,4,6,25,28,29,31,32	4	P00731,Q66H69,Q6IG05,Q8CJD3
2314	1,2,5,7,8,26,27,31	3,4,6,25,28,29,30,32	1	Q66H69
2315	1,2,5,7,8,26,27,32	3,4,6,25,28,29,30,31	3	Q03191,Q66H69,Q9JJ50
2316	1,2,5,7,8,26,28,29	3,4,6,25,27,30,31,32	8	P00762,P06760,P21674,P47967,P62804,Q9QX74,Q9QYP1,Q9WTT6
2317	1,2,5,7,8,26,28,30	3,4,6,25,27,29,31,32	3	P00762,P29315,P35745
2318	1,2,5,7,8,26,28,31	3,4,6,25,27,29,30,32	3	P00762,Q5RLM2,Q811M5
2319	1,2,5,7,8,26,28,32	3,4,6,25,27,29,30,31	0	
2320	1,2,5,7,8,26,29,30	3,4,6,25,27,28,31,32	5	P00762,P06760,P22006,P29315,P62804
2321	1,2,5,7,8,26,29,31	3,4,6,25,27,28,30,32	3	P00762,P06760,Q9QX74
2322	1,2,5,7,8,26,29,32	3,4,6,25,27,28,30,31	2	P06760,P29315
2323	1,2,5,7,8,26,30,31	3,4,6,25,27,28,29,32	2	P35745,Q6IG05
2324	1,2,5,7,8,26,30,32	3,4,6,25,27,28,29,31	1	P29315
2325	1,2,5,7,8,26,31,32	3,4,6,25,27,28,29,30	1	Q62714
2326	1,2,5,7,8,27,28,29	3,4,6,25,26,30,31,32	9	P06760,P25809,P29315,P47967,P62804,Q00715,Q4G075,Q66H69,Q812E4
2327	1,2,5,7,8,27,28,30	3,4,6,25,26,29,31,32	6	P00731,P19218,P29315,P62804,Q66H69,Q6IG05
2328	1,2,5,7,8,27,28,31	3,4,6,25,26,29,30,32	1	Q66H69
2329	1,2,5,7,8,27,28,32	3,4,6,25,26,29,30,31	5	P25809,P29315,Q03191,Q66H69,Q812E4

2330	1,2,5,7,8,27,29,30	3,4,6,25,26,28,31,32	13	P00731,P06760,P19218,P22006,P25809,P29315,P62804,Q4G075,Q66H69,Q6IFU8,Q6IG05,Q6IMF3,Q6P6Q2
2331	1,2,5,7,8,27,29,31	3,4,6,25,26,28,30,32	4	P00731,P06760,Q4G075,Q66H69
2332	1,2,5,7,8,27,29,32	3,4,6,25,26,28,30,31	5	P25809,P29315,Q03191,Q66H69,Q9JJ50
2333	1,2,5,7,8,27,30,31	3,4,6,25,26,28,29,32	6	P00731,P19218,P23739,Q03191,Q6IG05,Q6IMF3
2334	1,2,5,7,8,27,30,32	3,4,6,25,26,28,29,31	7	P19218,P29315,P70549,Q03191,Q66H69,Q6IG05,Q6IMF3
2335	1,2,5,7,8,27,31,32	3,4,6,25,26,28,29,30	2	P23739,Q03191
2336	1,2,5,7,8,28,29,30	3,4,6,25,26,27,31,32	5	P00762,P21674,P29315,P35745,P62804
2337	1,2,5,7,8,28,29,31	3,4,6,25,26,27,30,32	3	P00762,P06760,P21674
2338	1,2,5,7,8,28,29,32	3,4,6,25,26,27,30,31	3	P29315,P54921,Q812E4
2339	1,2,5,7,8,28,30,31	3,4,6,25,26,27,29,32	3	P00762,P35745,Q6IG05
2340	1,2,5,7,8,28,30,32	3,4,6,25,26,27,29,31	1	P29315
2341	1,2,5,7,8,28,31,32	3,4,6,25,26,27,29,30	1	Q811M5
2342	1,2,5,7,8,29,30,31	3,4,6,25,26,27,28,32	5	P35745,Q5RJR2,Q6IG05,Q6IMF3,Q9QWN8
2343	1,2,5,7,8,29,30,32	3,4,6,25,26,27,28,31	2	P29315,Q6IMF3
2344	1,2,5,7,8,29,31,32	3,4,6,25,26,27,28,30	0	
2345	1,2,5,7,8,30,31,32	3,4,6,25,26,27,28,29	2	P26342,Q6IG05
2346	1,2,5,7,25,26,27,28	3,4,6,8,29,30,31,32	2	P00714,Q03191
2347	1,2,5,7,25,26,27,29	3,4,6,8,28,30,31,32	4	P02625,P22006,Q03191,Q9JJ50
2348	1,2,5,7,25,26,27,30	3,4,6,8,28,29,31,32	1	Q03191
2349	1,2,5,7,25,26,27,31	3,4,6,8,28,29,30,32	1	Q03191

2350	1,2,5,7,25,26,27,32	3,4,6,8,28,29,30,31	2	Q03191,Q9JJ50
2351	1,2,5,7,25,26,28,29	3,4,6,8,27,30,31,32	5	P00714,P00762,P02625,P22006,Q9QZK9
2352	1,2,5,7,25,26,28,30	3,4,6,8,27,29,31,32	3	P00714,P00762,Q9R168
2353	1,2,5,7,25,26,28,31	3,4,6,8,27,29,30,32	2	P00714,P00762
2354	1,2,5,7,25,26,28,32	3,4,6,8,27,29,30,31	2	Q6IG02,Q9R168
2355	1,2,5,7,25,26,29,30	3,4,6,8,27,28,31,32	3	P22006,Q9QZK9,Q9R168
2356	1,2,5,7,25,26,29,31	3,4,6,8,27,28,30,32	3	O70417,P00762,Q8VD89
2357	1,2,5,7,25,26,29,32	3,4,6,8,27,28,30,31	2	O70417,Q9R168
2358	1,2,5,7,25,26,30,31	3,4,6,8,27,28,29,32	6	P22282,Q62902,Q6AYR9,Q812E4,Q9EQS0,Q9WVK7
2359	1,2,5,7,25,26,30,32	3,4,6,8,27,28,29,31	3	Q9EQS0,Q9R168,Q9WVK7
2360	1,2,5,7,25,26,31,32	3,4,6,8,27,28,29,30	2	O70417,Q9WVK7
2361	1,2,5,7,25,27,28,29	3,4,6,8,26,30,31,32	4	P02625,P22006,Q03191,Q6AY61
2362	1,2,5,7,25,27,28,30	3,4,6,8,26,29,31,32	1	Q03191
2363	1,2,5,7,25,27,28,31	3,4,6,8,26,29,30,32	1	Q03191
2364	1,2,5,7,25,27,28,32	3,4,6,8,26,29,30,31	3	Q03191,Q62761;Q62762;Q62763,Q6AY61
2365	1,2,5,7,25,27,29,30	3,4,6,8,26,28,31,32	3	O54728,P22006,Q03191
2366	1,2,5,7,25,27,29,31	3,4,6,8,26,28,30,32	3	O54728,O70417,Q03191
2367	1,2,5,7,25,27,29,32	3,4,6,8,26,28,30,31	2	Q03191,Q6AY61
2368	1,2,5,7,25,27,30,31	3,4,6,8,26,28,29,32	2	O54728,Q03191
2369	1,2,5,7,25,27,30,32	3,4,6,8,26,28,29,31	4	P70549,Q03191,Q6P6R2,Q9R168

2370	1,2,5,7,25,27,31,32	3,4,6,8,26,28,29,30	2	P23739,Q03191
2371	1,2,5,7,25,28,29,30	3,4,6,8,26,27,31,32	1	P00762
2372	1,2,5,7,25,28,29,31	3,4,6,8,26,27,30,32	2	O70417,P00762
2373	1,2,5,7,25,28,29,32	3,4,6,8,26,27,30,31	2	P54921,Q6AY61
2374	1,2,5,7,25,28,30,31	3,4,6,8,26,27,29,32	1	P00762
2375	1,2,5,7,25,28,30,32	3,4,6,8,26,27,29,31	2	Q62975,Q9R168
2376	1,2,5,7,25,28,31,32	3,4,6,8,26,27,29,30	1	Q811M5
2377	1,2,5,7,25,29,30,31	3,4,6,8,26,27,28,32	3	O54728,P22282,Q9QWN8
2378	1,2,5,7,25,29,30,32	3,4,6,8,26,27,28,31	1	Q9R168
2379	1,2,5,7,25,29,31,32	3,4,6,8,26,27,28,30	1	O70417
2380	1,2,5,7,25,30,31,32	3,4,6,8,26,27,28,29	4	P22273,P26342,Q6P6R2,Q9WVK7
2381	1,2,5,7,26,27,28,29	3,4,6,8,25,30,31,32	4	O70417,P00762,P02625,P47967
2382	1,2,5,7,26,27,28,30	3,4,6,8,25,29,31,32	1	P21588
2383	1,2,5,7,26,27,28,31	3,4,6,8,25,29,30,32	4	O70417,P00762,P02625,Q5RLM2
2384	1,2,5,7,26,27,28,32	3,4,6,8,25,29,30,31	3	P14173,Q03191,Q62761;Q62762;Q62763
2385	1,2,5,7,26,27,29,30	3,4,6,8,25,28,31,32	3	P21588,P29315,Q9JJ50
2386	1,2,5,7,26,27,29,31	3,4,6,8,25,28,30,32	2	O70417,P02625
2387	1,2,5,7,26,27,29,32	3,4,6,8,25,28,30,31	5	O70417,P14173,P29315,Q03191,Q9JJ50
2388	1,2,5,7,26,27,30,31	3,4,6,8,25,28,29,32	1	Q03191
2389	1,2,5,7,26,27,30,32	3,4,6,8,25,28,29,31	4	P29315,Q03191,Q9JJ50,Q9R168

2390	1,2,5,7,26,27,31,32	3,4,6,8,25,28,29,30	2	O70417,Q03191
2391	1,2,5,7,26,28,29,30	3,4,6,8,25,27,31,32	3	P00762,P01835,P29315
2392	1,2,5,7,26,28,29,31	3,4,6,8,25,27,30,32	5	O70417,P00762,P01835,P02625,P14173
2393	1,2,5,7,26,28,29,32	3,4,6,8,25,27,30,31	7	O70417,P01835,P02625,P14173,P29315,P54921,Q6AY61
2394	1,2,5,7,26,28,30,31	3,4,6,8,25,27,29,32	1	P00762
2395	1,2,5,7,26,28,30,32	3,4,6,8,25,27,29,31	2	P29315,Q9R168
2396	1,2,5,7,26,28,31,32	3,4,6,8,25,27,29,30	3	O70417,P14173,Q6IG02
2397	1,2,5,7,26,29,30,31	3,4,6,8,25,27,28,32	5	O70417,P00762,P01835,P36860,Q9QWN8
2398	1,2,5,7,26,29,30,32	3,4,6,8,25,27,28,31	4	P01835,P29315,P36860,Q9R168
2399	1,2,5,7,26,29,31,32	3,4,6,8,25,27,28,30	4	O70417,P01835,P14173,P36860
2400	1,2,5,7,26,30,31,32	3,4,6,8,25,27,28,29	3	B2RYW9,P26342,P36860
2401	1,2,5,7,27,28,29,30	3,4,6,8,25,26,31,32	2	P29315,P47967
2402	1,2,5,7,27,28,29,31	3,4,6,8,25,26,30,32	4	O70417,P00762,P02625,Q6AY61
2403	1,2,5,7,27,28,29,32	3,4,6,8,25,26,30,31	9	O70417,P14173,P29315,P47967,P54921,Q03191,Q62761;Q62762;Q62763,Q6AY61,Q812E4
2404	1,2,5,7,27,28,30,31	3,4,6,8,25,26,29,32	0	
2405	1,2,5,7,27,28,30,32	3,4,6,8,25,26,29,31	4	P29315,P70549,Q03191,Q9R168
2406	1,2,5,7,27,28,31,32	3,4,6,8,25,26,29,30	6	O70417,P14173,P80299,Q03191,Q62761;Q62762;Q62763,Q6AY61
2407	1,2,5,7,27,29,30,31	3,4,6,8,25,26,28,32	2	O54728,P08649
2408	1,2,5,7,27,29,30,32	3,4,6,8,25,26,28,31	4	P08649,P29315,Q03191,Q9R168
2409	1,2,5,7,27,29,31,32	3,4,6,8,25,26,28,30	5	O70417,P08649,P80299,Q03191,Q6AY61

2410	1,2,5,7,27,<br>30,31,32	3,4,6,8,25,<br>26,28,29	5	P08649,P36860,P80299,Q03191,Q6P6R2
2411	1,2,5,7,28,<br>29,30,31	3,4,6,8,25,<br>26,27,32	2	P00762,P01835
2412	1,2,5,7,28,<br>29,30,32	3,4,6,8,25,<br>26,27,31	4	P01835,P29315,P54921,Q9R168
2413	1,2,5,7,28,<br>29,31,32	3,4,6,8,25,<br>26,27,30	5	O70417,P01835,P14173,P54921,Q6AY61
2414	1,2,5,7,28,<br>30,31,32	3,4,6,8,25,<br>26,27,29	0	
2415	1,2,5,7,29,<br>30,31,32	3,4,6,8,25,<br>26,27,28	6	O70417,P01835,P08649,P26342,P36860,P63081
2416	1,2,5,8,25,<br>26,27,28	3,4,6,7,29,<br>30,31,32	2	P00714,P26342
2417	1,2,5,8,25,<br>26,27,29	3,4,6,7,28,<br>30,31,32	2	P22006,P25809
2418	1,2,5,8,25,<br>26,27,30	3,4,6,7,28,<br>29,31,32	3	P22006,Q63617,Q6IG05
2419	1,2,5,8,25,<br>26,27,31	3,4,6,7,28,<br>29,30,32	2	P00714,P23739
2420	1,2,5,8,25,<br>26,27,32	3,4,6,7,28,<br>29,30,31	0	
2421	1,2,5,8,25,<br>26,28,29	3,4,6,7,27,<br>30,31,32	2	P00714,P22006
2422	1,2,5,8,25,<br>26,28,30	3,4,6,7,27,<br>29,31,32	2	P00714,P80299
2423	1,2,5,8,25,<br>26,28,31	3,4,6,7,27,<br>29,30,32	1	P00714
2424	1,2,5,8,25,<br>26,28,32	3,4,6,7,27,<br>29,30,31	0	
2425	1,2,5,8,25,<br>26,29,30	3,4,6,7,27,<br>28,31,32	2	P00714,P22006
2426	1,2,5,8,25,<br>26,29,31	3,4,6,7,27,<br>28,30,32	1	P00714
2427	1,2,5,8,25,<br>26,29,32	3,4,6,7,27,<br>28,30,31	1	P13432
2428	1,2,5,8,25,<br>26,30,31	3,4,6,7,27,<br>28,29,32	4	P00714,P11598,Q63617,Q6IG05
2429	1,2,5,8,25,<br>26,30,32	3,4,6,7,27,<br>28,29,31	3	P00507,Q63617,Q68G31

2430	1,2,5,8,25,<br>26,31,32	3,4,6,7,27,<br>28,29,30	2	P00507,Q63617
2431	1,2,5,8,25,<br>27,28,29	3,4,6,7,26,<br>30,31,32	2	P22006,P25809
2432	1,2,5,8,25,<br>27,28,30	3,4,6,7,26,<br>29,31,32	1	Q6IG05
2433	1,2,5,8,25,<br>27,28,31	3,4,6,7,26,<br>29,30,32	2	P00714,P23739
2434	1,2,5,8,25,<br>27,28,32	3,4,6,7,26,<br>29,30,31	1	Q9QWN8
2435	1,2,5,8,25,<br>27,29,30	3,4,6,7,26,<br>28,31,32	7	P00731,P22006,Q6IFU7,Q6IFU8,Q6IG05,Q6IMF3,Q6P6Q2
2436	1,2,5,8,25,<br>27,29,31	3,4,6,7,26,<br>28,30,32	0	
2437	1,2,5,8,25,<br>27,29,32	3,4,6,7,26,<br>28,30,31	0	
2438	1,2,5,8,25,<br>27,30,31	3,4,6,7,26,<br>28,29,32	4	P23739,Q63617,Q6IG05,Q6IMF3
2439	1,2,5,8,25,<br>27,30,32	3,4,6,7,26,<br>28,29,31	4	P70549,Q03191,Q68G31,Q6IG05
2440	1,2,5,8,25,<br>27,31,32	3,4,6,7,26,<br>28,29,30	3	P23739,Q03191,Q80WL1
2441	1,2,5,8,25,<br>28,29,30	3,4,6,7,26,<br>27,31,32	2	P00714,P22006
2442	1,2,5,8,25,<br>28,29,31	3,4,6,7,26,<br>27,30,32	1	P00714
2443	1,2,5,8,25,<br>28,29,32	3,4,6,7,26,<br>27,30,31	0	
2444	1,2,5,8,25,<br>28,30,31	3,4,6,7,26,<br>27,29,32	2	P00714,Q6IG05
2445	1,2,5,8,25,<br>28,30,32	3,4,6,7,26,<br>27,29,31	1	Q68G31
2446	1,2,5,8,25,<br>28,31,32	3,4,6,7,26,<br>27,29,30	3	P21588,Q80WL1,Q811M5
2447	1,2,5,8,25,<br>29,30,31	3,4,6,7,26,<br>27,28,32	3	P11598,Q6IG05,Q6IMF3
2448	1,2,5,8,25,<br>29,30,32	3,4,6,7,26,<br>27,28,31	2	Q68G31,Q6IMF3
2449	1,2,5,8,25,<br>29,31,32	3,4,6,7,26,<br>27,28,30	2	P21588,Q80WL1

2450	1,2,5,8,25,30,31,32	3,4,6,7,26,27,28,29	5	P20646,P97840,Q63617,Q6IG05,Q6P6R2
2451	1,2,5,8,26,27,28,29	3,4,6,7,25,30,31,32	1	P25809
2452	1,2,5,8,26,27,28,30	3,4,6,7,25,29,31,32	1	Q6IG05
2453	1,2,5,8,26,27,28,31	3,4,6,7,25,29,30,32	1	P00714
2454	1,2,5,8,26,27,28,32	3,4,6,7,25,29,30,31	2	P25809,Q9QWN8
2455	1,2,5,8,26,27,29,30	3,4,6,7,25,28,31,32	3	P00731,P22006,Q6IG05
2456	1,2,5,8,26,27,29,31	3,4,6,7,25,28,30,32	0	
2457	1,2,5,8,26,27,29,32	3,4,6,7,25,28,30,31	2	P25809,Q9JJ50
2458	1,2,5,8,26,27,30,31	3,4,6,7,25,28,29,32	1	Q6IG05
2459	1,2,5,8,26,27,30,32	3,4,6,7,25,28,29,31	1	Q6IG05
2460	1,2,5,8,26,27,31,32	3,4,6,7,25,28,29,30	1	P23739
2461	1,2,5,8,26,28,29,30	3,4,6,7,25,27,31,32	1	P00714
2462	1,2,5,8,26,28,29,31	3,4,6,7,25,27,30,32	2	O70417,P00714
2463	1,2,5,8,26,28,29,32	3,4,6,7,25,27,30,31	1	Q9QYP1
2464	1,2,5,8,26,28,30,31	3,4,6,7,25,27,29,32	1	P00714
2465	1,2,5,8,26,28,30,32	3,4,6,7,25,27,29,31	1	Q68G31
2466	1,2,5,8,26,28,31,32	3,4,6,7,25,27,29,30	0	
2467	1,2,5,8,26,29,30,31	3,4,6,7,25,27,28,32	1	Q5RJR2
2468	1,2,5,8,26,29,30,32	3,4,6,7,25,27,28,31	2	P29315,Q68G31
2469	1,2,5,8,26,29,31,32	3,4,6,7,25,27,28,30	2	O70417,Q5QE79

2470	1,2,5,8,26,30,31,32	3,4,6,7,25,27,28,29	3	P63081,Q5QE79,Q63617
2471	1,2,5,8,27,28,29,30	3,4,6,7,25,26,31,32	6	P00731,P25809,P29315,Q6IFU7,Q6IG05,Q6IMF3
2472	1,2,5,8,27,28,29,31	3,4,6,7,25,26,30,32	1	P25809
2473	1,2,5,8,27,28,29,32	3,4,6,7,25,26,30,31	4	P25809,P29315,Q6AYR9,Q812E4
2474	1,2,5,8,27,28,30,31	3,4,6,7,25,26,29,32	2	P23739,Q6IG05
2475	1,2,5,8,27,28,30,32	3,4,6,7,25,26,29,31	2	P29315,Q6IG05
2476	1,2,5,8,27,28,31,32	3,4,6,7,25,26,29,30	1	P23739
2477	1,2,5,8,27,29,30,31	3,4,6,7,25,26,28,32	4	P00731,Q6IFU8,Q6IG05,Q6IMF3
2478	1,2,5,8,27,29,30,32	3,4,6,7,25,26,28,31	3	P29315,Q6IG05,Q6IMF3
2479	1,2,5,8,27,29,31,32	3,4,6,7,25,26,28,30	0	
2480	1,2,5,8,27,30,31,32	3,4,6,7,25,26,28,29	3	P20646,P23739,Q6IG05
2481	1,2,5,8,28,29,30,31	3,4,6,7,25,26,27,32	0	
2482	1,2,5,8,28,29,30,32	3,4,6,7,25,26,27,31	2	P29315,Q68G31
2483	1,2,5,8,28,29,31,32	3,4,6,7,25,26,27,30	1	O70417
2484	1,2,5,8,28,30,31,32	3,4,6,7,25,26,27,29	0	
2485	1,2,5,8,29,30,31,32	3,4,6,7,25,26,27,28	3	P36860,P63081,Q6IMF3
2486	1,2,5,25,26,27,28,29	3,4,6,7,8,30,31,32	7	O70417,P00714,P02625,P35280,P82471,Q99041,Q9QZK9
2487	1,2,5,25,26,27,28,30	3,4,6,7,8,29,31,32	2	P00714,P82471
2488	1,2,5,25,26,27,28,31	3,4,6,7,8,29,30,32	4	O70417,P00714,P35280,Q99041
2489	1,2,5,25,26,27,28,32	3,4,6,7,8,29,30,31	3	O70417,P82471,Q9QWN8

2490	1,2,5,25,26,27,29,30	3,4,6,7,8,28,31,32	5	P22006,P82471,Q63617,Q99041,Q9QZK9
2491	1,2,5,25,26,27,29,31	3,4,6,7,8,28,30,32	4	O70417,P35280,Q63617,Q99041
2492	1,2,5,25,26,27,29,32	3,4,6,7,8,28,30,31	4	O70417,P82471,Q99041,Q9JJ50
2493	1,2,5,25,26,27,30,31	3,4,6,7,8,28,29,32	8	P00714,P11598,P22273,P22282,Q5M8C6,Q62902,Q63617,Q99041
2494	1,2,5,25,26,27,30,32	3,4,6,7,8,28,29,31	6	D3ZUC6,P21674,P22273,P82471,Q03191,Q63617
2495	1,2,5,25,26,27,31,32	3,4,6,7,8,28,29,30	7	O70417,P21674,P22273,P23739,Q03191,Q63617,Q99041
2496	1,2,5,25,26,28,29,30	3,4,6,7,8,27,31,32	6	P00714,P22273,P22282,P82471,Q99041,Q9QZK9
2497	1,2,5,25,26,28,29,31	3,4,6,7,8,27,30,32	6	O70417,P00714,P22273,P22282,P35280,Q99041
2498	1,2,5,25,26,28,29,32	3,4,6,7,8,27,30,31	4	O70417,P22273,Q99041,Q9QZK9
2499	1,2,5,25,26,28,30,31	3,4,6,7,8,27,29,32	5	P00714,P22273,P22282,Q4G075,Q99041
2500	1,2,5,25,26,28,30,32	3,4,6,7,8,27,29,31	6	P22273,P82471,Q4G075,Q68G31,Q99041,Q9R168
2501	1,2,5,25,26,28,31,32	3,4,6,7,8,27,29,30	5	O70417,P22273,Q4G075,Q6IG02,Q99041
2502	1,2,5,25,26,29,30,31	3,4,6,7,8,27,28,32	10	O70417,P00714,P06761,P11598,P22273,P22282,Q5M8C6,Q62902,Q63617,Q99041
2503	1,2,5,25,26,29,30,32	3,4,6,7,8,27,28,31	11	D3ZUC6,O70417,P11598,P22273,P22282,P82471,Q63617,Q68G31,Q99041,Q9QZK9,Q9R168
2504	1,2,5,25,26,29,31,32	3,4,6,7,8,27,28,30	7	O70417,P00507,P19218,P22273,P22282,Q63617,Q99041
2505	1,2,5,25,26,30,31,32	3,4,6,7,8,27,28,29	16	D3ZUC6,O70417,P00507,P04636,P06761,P11598,P22273,P22282,P22283,Q4G075,Q63617,Q812E4,Q99041,Q9EQS0,Q9WVK7,Q9Z2L0
2506	1,2,5,25,27,28,29,30	3,4,6,7,8,26,31,32	1	P82471
2507	1,2,5,25,27,28,29,31	3,4,6,7,8,26,30,32	3	O70417,P35280,Q99041
2508	1,2,5,25,27,28,29,32	3,4,6,7,8,26,30,31	4	O70417,Q62761;Q62762;Q62763,Q6AY61,Q99041
2509	1,2,5,25,27,28,30,31	3,4,6,7,8,26,29,32	4	P00714,P22273,P23739,Q99041

2510	1,2,5,25,27,28,30,32	3,4,6,7,8,26,29,31	4	P22273,P82471,Q03191,Q9QX74
2511	1,2,5,25,27,28,31,32	3,4,6,7,8,26,29,30	5	O70417,P22273,P23739,Q03191,Q99041
2512	1,2,5,25,27,29,30,31	3,4,6,7,8,26,28,32	7	O54728,O70417,P11598,P22273,P22282,Q63617,Q99041
2513	1,2,5,25,27,29,30,32	3,4,6,7,8,26,28,31	4	P22273,Q03191,Q63617,Q99041
2514	1,2,5,25,27,29,31,32	3,4,6,7,8,26,28,30	4	O70417,P22273,Q03191,Q99041
2515	1,2,5,25,27,30,31,32	3,4,6,7,8,26,28,29	12	D3ZUC6,P06761,P11598,P21674,P22273,P23739,Q03191,Q63617,Q6P6R2,Q99041,Q9QX74,Q9WTT6
2516	1,2,5,25,28,29,30,31	3,4,6,7,8,26,27,32	5	O70417,P00714,P22273,P22282,Q99041
2517	1,2,5,25,28,29,30,32	3,4,6,7,8,26,27,31	4	P22273,Q62894,Q68G31,Q99041
2518	1,2,5,25,28,29,31,32	3,4,6,7,8,26,27,30	3	O70417,P22273,Q99041
2519	1,2,5,25,28,30,31,32	3,4,6,7,8,26,27,29	6	P22273,P22282,Q4G075,Q99041,Q9QX74,Q9WTT6
2520	1,2,5,25,29,30,31,32	3,4,6,7,8,26,27,28	11	D3ZUC6,O70417,P06761,P07150,P11598,P22273,P22282,P36860,Q63617,Q99041,Q9WTT6
2521	1,2,5,26,27,28,29,30	3,4,6,7,8,25,31,32	3	O70417,P21588,P82471
2522	1,2,5,26,27,28,29,31	3,4,6,7,8,25,30,32	3	O70417,P02625,P35280
2523	1,2,5,26,27,28,29,32	3,4,6,7,8,25,30,31	2	O70417,P02625
2524	1,2,5,26,27,28,30,31	3,4,6,7,8,25,29,32	2	O70417,P00714
2525	1,2,5,26,27,28,30,32	3,4,6,7,8,25,29,31	1	P82471
2526	1,2,5,26,27,28,31,32	3,4,6,7,8,25,29,30	1	O70417
2527	1,2,5,26,27,29,30,31	3,4,6,7,8,25,28,32	6	O70417,P22273,P22282,P36860,Q63617,Q99041
2528	1,2,5,26,27,29,30,32	3,4,6,7,8,25,28,31	5	O70417,P22273,P29315,P82471,Q9JJ50
2529	1,2,5,26,27,29,31,32	3,4,6,7,8,25,28,30	3	O70417,P22273,Q99041

2530	1,2,5,26,27,30,31,32	3,4,6,7,8,25,28,29	5	O70417,P22273,P36860,Q63617,Q9QW07
2531	1,2,5,26,28,29,30,31	3,4,6,7,8,25,27,32	8	O70417,P00714,P00762,P22273,P22282,P98166,Q01984,Q99041
2532	1,2,5,26,28,29,30,32	3,4,6,7,8,25,27,31	6	O70417,P22273,P29315,P50115,Q62894,Q68G31
2533	1,2,5,26,28,29,31,32	3,4,6,7,8,25,27,30	4	O70417,P22273,Q5QE79,Q99041
2534	1,2,5,26,28,30,31,32	3,4,6,7,8,25,27,29	6	O70417,P22273,P22282,P50115,Q4G075,Q5QE79
2535	1,2,5,26,29,30,31,32	3,4,6,7,8,25,27,28	11	O70417,P06761,P22273,P22282,P36860,P50115,P63081,Q4G075,Q5QE79,Q63617,Q99041
2536	1,2,5,27,28,29,30,31	3,4,6,7,8,25,26,32	1	O70417
2537	1,2,5,27,28,29,30,32	3,4,6,7,8,25,26,31	2	O70417,P29315
2538	1,2,5,27,28,29,31,32	3,4,6,7,8,25,26,30	3	O70417,Q6AY61,Q99041
2539	1,2,5,27,28,30,31,32	3,4,6,7,8,25,26,29	4	O70417,P22273,P23739,Q9QX74
2540	1,2,5,27,29,30,31,32	3,4,6,7,8,25,26,28	5	O70417,P08649,P22273,P36860,Q99041
2541	1,2,5,28,29,30,31,32	3,4,6,7,8,25,26,27	6	O70417,P22273,P36860,P50115,P63081,Q99041
2542	1,2,6,7,8,25,26,27	3,4,5,28,29,30,31,32	7	P06911,P15399,P19629,P50115,P97580,Q30KJ2,Q66H69
2543	1,2,6,7,8,25,26,28	3,4,5,27,29,30,31,32	9	P00762,P02631,P06911,P08649,P12020,P19629,Q5GRG2,Q63598,Q9QW07
2544	1,2,6,7,8,25,26,29	3,4,5,27,28,30,31,32	2	P06911,Q9QX74
2545	1,2,6,7,8,25,26,30	3,4,5,27,28,29,31,32	2	P02631,P06911
2546	1,2,6,7,8,25,26,31	3,4,5,27,28,29,30,32	7	O54858,P06911,P12020,P20766,P57113,Q5GRG2,Q63598
2547	1,2,6,7,8,25,26,32	3,4,5,27,28,29,30,31	3	P02631,P13432,Q68G31
2548	1,2,6,7,8,25,27,28	3,4,5,26,29,30,31,32	10	P02631,P06911,P12020,P19629,P50115,P63081,Q5GRG2,Q66H69,Q9JI85,Q9QW07
2549	1,2,6,7,8,25,27,29	3,4,5,26,28,30,31,32	8	P02631,P06911,P19629,P62804,Q00715,Q4G075,Q5GRG2,Q66H69

2550	1,2,6,7,8,25,27,30	3,4,5,26,28,29,31,32	4	P02631,P06911,P19629,Q6IG05
2551	1,2,6,7,8,25,27,31	3,4,5,26,28,29,30,32	6	P06911,P12020,P19629,P31044,Q5GRG2,Q62894
2552	1,2,6,7,8,25,27,32	3,4,5,26,28,29,30,31	3	P02631,P06911,P19629
2553	1,2,6,7,8,25,28,29	3,4,5,26,27,30,31,32	11	P00762,P02631,P06911,P12020,P19629,P21674,P47727,P62804,Q5GRG2,Q9JI85,Q9QW07
2554	1,2,6,7,8,25,28,30	3,4,5,26,27,29,31,32	6	P02631,P06911,P12020,P19629,Q5GRG2,Q9QW07
2555	1,2,6,7,8,25,28,31	3,4,5,26,27,29,30,32	7	P02631,P06911,P12020,P19629,Q5GRG2,Q63532,Q63598
2556	1,2,6,7,8,25,28,32	3,4,5,26,27,29,30,31	8	P02631,P06911,P12020,P19629,Q5GRG2,Q68G31,Q9JI85,Q9QW07
2557	1,2,6,7,8,25,29,30	3,4,5,26,27,28,31,32	4	P02631,P06911,P12020,P62804
2558	1,2,6,7,8,25,29,31	3,4,5,26,27,28,30,32	3	P06911,P12020,Q5GRG2
2559	1,2,6,7,8,25,29,32	3,4,5,26,27,28,30,31	4	P02631,P06911,P13432,Q68G31
2560	1,2,6,7,8,25,30,31	3,4,5,26,27,28,29,32	6	O54858,P06911,P12020,P97840,Q5GRG2,Q63532
2561	1,2,6,7,8,25,30,32	3,4,5,26,27,28,29,31	2	P02631,Q68G31
2562	1,2,6,7,8,25,31,32	3,4,5,26,27,28,29,30	8	O54858,P02631,P06911,P12020,P21588,P97840,Q5GRG2,Q63532
2563	1,2,6,7,8,26,27,28	3,4,5,25,29,30,31,32	9	P07150,P15399,P25809,P50280,Q63598,Q66H69,Q9JI85,Q9QW07,Q9WTT6
2564	1,2,6,7,8,26,27,29	3,4,5,25,28,30,31,32	7	P15399,P25809,P62804,Q00715,Q66H69,Q9QX74,Q9WTT6
2565	1,2,6,7,8,26,27,30	3,4,5,25,28,29,31,32	5	P15399,P29315,P97580,Q30KJ2,Q66H69
2566	1,2,6,7,8,26,27,31	3,4,5,25,28,29,30,32	4	P15399,P20766,Q62894,Q63598
2567	1,2,6,7,8,26,27,32	3,4,5,25,28,29,30,31	4	P15399,P29315,P50280,Q66H69
2568	1,2,6,7,8,26,28,29	3,4,5,25,27,30,31,32	10	P00762,P01835,P21674,P29315,P62804,Q6P6R2,Q9JI85,Q9QW07,Q9QX74,Q9WTT6
2569	1,2,6,7,8,26,28,30	3,4,5,25,27,29,31,32	3	P00762,P29315,P35745

2570	1,2,6,7,8,26,28,31	3,4,5,25,27,29,30,32	8	O89117,P00762,P06911,P12020,P57113,Q5GRG2,Q63532,Q63598
2571	1,2,6,7,8,26,28,32	3,4,5,25,27,29,30,31	6	O89117,P02631,P29315,Q6IG02,Q9JI85,Q9QW07
2572	1,2,6,7,8,26,29,30	3,4,5,25,27,28,31,32	4	P00762,P01835,P29315,P62804
2573	1,2,6,7,8,26,29,31	3,4,5,25,27,28,30,32	5	P00762,P01835,P12020,Q63474,Q9QX74
2574	1,2,6,7,8,26,29,32	3,4,5,25,27,28,30,31	3	P01835,P29315,Q68G31
2575	1,2,6,7,8,26,30,31	3,4,5,25,27,28,29,32	4	O89117,P35745,Q63532,Q99MH3
2576	1,2,6,7,8,26,30,32	3,4,5,25,27,28,29,31	4	O89117,P29315,Q68G31,Q99MH3
2577	1,2,6,7,8,26,31,32	3,4,5,25,27,28,29,30	5	O89117,P20766,P57113,Q63532,Q99MH3
2578	1,2,6,7,8,27,28,29	3,4,5,25,26,30,31,32	16	P06911,P21674,P22283,P25809,P29315,P47727,P62804,Q00715,Q4G075,Q5GRG2,Q63617,Q66H69,Q6AYR9,Q812E4,Q9JI85,Q9QW07
2579	1,2,6,7,8,27,28,30	3,4,5,25,26,29,31,32	8	P06911,P15399,P19218,P29315,P62804,Q66H69,Q6P6S4,Q9JI85
2580	1,2,6,7,8,27,28,31	3,4,5,25,26,29,30,32	7	iRT-Kit_WR_fusion,P06911,P12020,Q5GRG2,Q62894,Q63532,Q9JI85
2581	1,2,6,7,8,27,28,32	3,4,5,25,26,29,30,31	12	P02631,P05964,P08723,P15399,P22283,P29315,P50280,Q6AYR9,Q6P6S4,Q812E4,Q9JI85,Q9QW07
2582	1,2,6,7,8,27,29,30	3,4,5,25,26,28,31,32	5	P00731,P19218,P29315,P62804,Q66H69
2583	1,2,6,7,8,27,29,31	3,4,5,25,26,28,30,32	2	P12020,Q5GRG2
2584	1,2,6,7,8,27,29,32	3,4,5,25,26,28,30,31	3	P25809,P29315,Q66H69
2585	1,2,6,7,8,27,30,31	3,4,5,25,26,28,29,32	1	Q6IG05
2586	1,2,6,7,8,27,30,32	3,4,5,25,26,28,29,31	3	P19218,P29315,Q68G31
2587	1,2,6,7,8,27,31,32	3,4,5,25,26,28,29,30	2	P80299,Q63532
2588	1,2,6,7,8,28,29,30	3,4,5,25,26,27,31,32	7	P00762,P01835,P12020,P21674,P29315,P62804,Q9QW07
2589	1,2,6,7,8,28,29,31	3,4,5,25,26,27,30,32	8	P00762,P01835,P06911,P12020,P21674,Q5GRG2,Q63474,Q9JI85

2590	1,2,6,7,8,28,29,32	3,4,5,25,26,27,30,31	11	P01835,P02631,P08723,P21674,P29315,P30120,P47727,Q63617,Q812E4,Q9JI85,Q9QW07
2591	1,2,6,7,8,28,30,31	3,4,5,25,26,27,29,32	7	O89117,P00762,P06911,P12020,P35745,Q5GRG2,Q63532
2592	1,2,6,7,8,28,30,32	3,4,5,25,26,27,29,31	5	O89117,P02631,P29315,Q68G31,Q9JI85
2593	1,2,6,7,8,28,31,32	3,4,5,25,26,27,29,30	5	O89117,P12020,Q5GRG2,Q63532,Q9JI85
2594	1,2,6,7,8,29,30,31	3,4,5,25,26,27,28,32	3	P01835,P12020,Q63474
2595	1,2,6,7,8,29,30,32	3,4,5,25,26,27,28,31	3	P01835,P29315,Q68G31
2596	1,2,6,7,8,29,31,32	3,4,5,25,26,27,28,30	3	P01835,P12020,Q63474
2597	1,2,6,7,8,30,31,32	3,4,5,25,26,27,28,29	5	B2RYW9,O89117,P15999,P26342,Q63532
2598	1,2,6,7,25,26,27,28	3,4,5,8,29,30,31,32	4	O88797,P0DMW0;P0DMW1,P19629,P63081
2599	1,2,6,7,25,26,27,29	3,4,5,8,28,30,31,32	0	
2600	1,2,6,7,25,26,27,30	3,4,5,8,28,29,31,32	0	
2601	1,2,6,7,25,26,27,31	3,4,5,8,28,29,30,32	2	O88797,Q62894
2602	1,2,6,7,25,26,27,32	3,4,5,8,28,29,30,31	1	Q9R168
2603	1,2,6,7,25,26,28,29	3,4,5,8,27,30,31,32	3	P00762,P01835,P05539
2604	1,2,6,7,25,26,28,30	3,4,5,8,27,29,31,32	4	P00762,Q5PQL7,Q62975,Q9R168
2605	1,2,6,7,25,26,28,31	3,4,5,8,27,29,30,32	4	P00762,Q63598,Q6IFU7,Q6IG02
2606	1,2,6,7,25,26,28,32	3,4,5,8,27,29,30,31	5	Q5PQL7,Q62975,Q68G31,Q6IG02,Q9R168
2607	1,2,6,7,25,26,29,30	3,4,5,8,27,28,31,32	2	P01835,Q9R168
2608	1,2,6,7,25,26,29,31	3,4,5,8,27,28,30,32	2	P00762,P01835
2609	1,2,6,7,25,26,29,32	3,4,5,8,27,28,30,31	3	P01835,Q68G31,Q9R168

2610	1,2,6,7,25,<br>26,30,31	3,4,5,8,27,<br>28,29,32	2 P11442,Q9EQS0
2611	1,2,6,7,25,<br>26,30,32	3,4,5,8,27,<br>28,29,31	6 D3ZUC6,P01835,Q5PQL7,Q68G31,Q9EQS0,Q9R168
2612	1,2,6,7,25,<br>26,31,32	3,4,5,8,27,<br>28,29,30	4 P57113,Q6IFU7,Q6IG02,Q9EQS0
2613	1,2,6,7,25,<br>27,28,29	3,4,5,8,26,<br>30,31,32	2 P05539,P0DMW0;P0DMW1
2614	1,2,6,7,25,<br>27,28,30	3,4,5,8,26,<br>29,31,32	0
2615	1,2,6,7,25,<br>27,28,31	3,4,5,8,26,<br>29,30,32	3 O88797,P19629,Q5GRG2
2616	1,2,6,7,25,<br>27,28,32	3,4,5,8,26,<br>29,30,31	3 P19629,Q5RJR2,Q62761;Q62762;Q62763
2617	1,2,6,7,25,<br>27,29,30	3,4,5,8,26,<br>28,31,32	2 O54728,P01835
2618	1,2,6,7,25,<br>27,29,31	3,4,5,8,26,<br>28,30,32	2 O54728,P05539
2619	1,2,6,7,25,<br>27,29,32	3,4,5,8,26,<br>28,30,31	1 P01835
2620	1,2,6,7,25,<br>27,30,31	3,4,5,8,26,<br>28,29,32	1 O54728
2621	1,2,6,7,25,<br>27,30,32	3,4,5,8,26,<br>28,29,31	2 Q68G31,Q9R168
2622	1,2,6,7,25,<br>27,31,32	3,4,5,8,26,<br>28,29,30	1 P80299
2623	1,2,6,7,25,<br>28,29,30	3,4,5,8,26,<br>27,31,32	3 P00762,P01835,Q9R168
2624	1,2,6,7,25,<br>28,29,31	3,4,5,8,26,<br>27,30,32	4 P00762,P01835,P05539,Q5GRG2
2625	1,2,6,7,25,<br>28,29,32	3,4,5,8,26,<br>27,30,31	3 P01835,Q68G31,Q9R168
2626	1,2,6,7,25,<br>28,30,31	3,4,5,8,26,<br>27,29,32	1 P00762
2627	1,2,6,7,25,<br>28,30,32	3,4,5,8,26,<br>27,29,31	5 P01835,Q5PQL7,Q62975,Q68G31,Q9R168
2628	1,2,6,7,25,<br>28,31,32	3,4,5,8,26,<br>27,29,30	1 Q6IG02
2629	1,2,6,7,25,<br>29,30,31	3,4,5,8,26,<br>27,28,32	3 O54728,P01835,Q9QWN8

2630	1,2,6,7,25,<br>29,30,32	3,4,5,8,26,<br>27,28,31	3	P01835,Q68G31,Q9R168
2631	1,2,6,7,25,<br>29,31,32	3,4,5,8,26,<br>27,28,30	1	P01835
2632	1,2,6,7,25,<br>30,31,32	3,4,5,8,26,<br>27,28,29	8	D3ZUC6,P01835,P06760,P26342,P47967,P97840,Q6P6R2,Q9WVK7
2633	1,2,6,7,26,<br>27,28,29	3,4,5,8,25,<br>30,31,32	6	O88797,P00762,P01835,P02625,P05539,P0DMW0;P0DMW1
2634	1,2,6,7,26,<br>27,28,30	3,4,5,8,25,<br>29,31,32	3	P01835,P21588,P29315
2635	1,2,6,7,26,<br>27,28,31	3,4,5,8,25,<br>29,30,32	6	O88797,P00762,P01835,P0DMW0;P0DMW1,Q63598,Q6IG02
2636	1,2,6,7,26,<br>27,28,32	3,4,5,8,25,<br>29,30,31	5	O88797,P01835,P05964,P14173,Q6IG02
2637	1,2,6,7,26,<br>27,29,30	3,4,5,8,25,<br>28,31,32	4	P01835,P21588,P29315,Q811M5
2638	1,2,6,7,26,<br>27,29,31	3,4,5,8,25,<br>28,30,32	2	O88797,P01835
2639	1,2,6,7,26,<br>27,29,32	3,4,5,8,25,<br>28,30,31	3	P01835,P29315,Q9JJ50
2640	1,2,6,7,26,<br>27,30,31	3,4,5,8,25,<br>28,29,32	1	P01835
2641	1,2,6,7,26,<br>27,30,32	3,4,5,8,25,<br>28,29,31	4	P01835,P29315,Q68G31,Q9R168
2642	1,2,6,7,26,<br>27,31,32	3,4,5,8,25,<br>28,29,30	4	O88797,P01835,P80299,Q6IG02
2643	1,2,6,7,26,<br>28,29,30	3,4,5,8,25,<br>27,31,32	4	P00762,P01835,P23739,P29315
2644	1,2,6,7,26,<br>28,29,31	3,4,5,8,25,<br>27,30,32	3	P00762,P01835,Q63474
2645	1,2,6,7,26,<br>28,29,32	3,4,5,8,25,<br>27,30,31	4	P01835,P14173,P29315,Q6IG02
2646	1,2,6,7,26,<br>28,30,31	3,4,5,8,25,<br>27,29,32	2	P00762,P01835
2647	1,2,6,7,26,<br>28,30,32	3,4,5,8,25,<br>27,29,31	5	O89117,P01835,P29315,Q68G31,Q9R168
2648	1,2,6,7,26,<br>28,31,32	3,4,5,8,25,<br>27,29,30	7	O89117,P01835,P14173,P22006,Q6IFU7,Q6IFW6,Q6IG02
2649	1,2,6,7,26,<br>29,30,31	3,4,5,8,25,<br>27,28,32	3	P00762,P01835,P36860

2650	1,2,6,7,26,29,30,32	3,4,5,8,25,27,28,31	6	P01835,P29315,P36376,P36860,Q68G31,Q9R168
2651	1,2,6,7,26,29,31,32	3,4,5,8,25,27,28,30	3	O70417,P01835,P36860
2652	1,2,6,7,26,30,31,32	3,4,5,8,25,27,28,29	7	B2RYW9,O89117,P01835,P26342,P36860,Q99MH3,Q9EQS0
2653	1,2,6,7,27,28,29,30	3,4,5,8,25,26,31,32	3	P01835,P05539,P29315
2654	1,2,6,7,27,28,29,31	3,4,5,8,25,26,30,32	6	O88797,P00762,P01835,P05539,P0DMW0;P0DMW1,P80299
2655	1,2,6,7,27,28,29,32	3,4,5,8,25,26,30,31	11	O35547,P01835,P05539,P05964,P22283,P29315,P80299,Q62761;Q62762;Q62763,Q6AY61,Q6AYR9,Q812E4
2656	1,2,6,7,27,28,30,31	3,4,5,8,25,26,29,32	1	P01835
2657	1,2,6,7,27,28,30,32	3,4,5,8,25,26,29,31	4	P01835,P29315,Q68G31,Q9R168
2658	1,2,6,7,27,28,31,32	3,4,5,8,25,26,29,30	6	O88797,P01835,P05964,P22006,P80299,Q6IG02
2659	1,2,6,7,27,29,30,31	3,4,5,8,25,26,28,32	3	O54728,P01835,P80299
2660	1,2,6,7,27,29,30,32	3,4,5,8,25,26,28,31	4	P01835,P29315,Q68G31,Q9R168
2661	1,2,6,7,27,29,31,32	3,4,5,8,25,26,28,30	2	P01835,P80299
2662	1,2,6,7,27,30,31,32	3,4,5,8,25,26,28,29	3	P01835,P22006,P80299
2663	1,2,6,7,28,29,30,31	3,4,5,8,25,26,27,32	3	P00762,P01835,Q63474
2664	1,2,6,7,28,29,30,32	3,4,5,8,25,26,27,31	4	P01835,P29315,Q68G31,Q9R168
2665	1,2,6,7,28,29,31,32	3,4,5,8,25,26,27,30	3	P01835,P80299,Q63474
2666	1,2,6,7,28,30,31,32	3,4,5,8,25,26,27,29	4	O89117,P01835,P22006,P26342
2667	1,2,6,7,29,30,31,32	3,4,5,8,25,26,27,28	4	P01835,P26342,P36860,P80299
2668	1,2,6,8,25,26,27,28	3,4,5,7,29,30,31,32	5	P08649,P15399,P19629,Q30KJ2,Q5GRG2
2669	1,2,6,8,25,26,27,29	3,4,5,7,28,30,31,32	3	P15399,P97580,Q30KJ2

2670	1,2,6,8,25,<br>26,27,30	3,4,5,7,28,<br>29,31,32	5	P15399,P97580,P97840,Q30KJ2,Q68G31
2671	1,2,6,8,25,<br>26,27,31	3,4,5,7,28,<br>29,30,32	4	P15399,P47967,P54921,P97840
2672	1,2,6,8,25,<br>26,27,32	3,4,5,7,28,<br>29,30,31	5	P08649,P13432,P15399,P97840,Q68G31
2673	1,2,6,8,25,<br>26,28,29	3,4,5,7,27,<br>30,31,32	3	P08649,Q68G31,Q9QW07
2674	1,2,6,8,25,<br>26,28,30	3,4,5,7,27,<br>29,31,32	2	P08649,Q68G31
2675	1,2,6,8,25,<br>26,28,31	3,4,5,7,27,<br>29,30,32	5	P00714,P08649,P97840,Q5GRG2,Q63598
2676	1,2,6,8,25,<br>26,28,32	3,4,5,7,27,<br>29,30,31	4	P08649,P13432,Q68G31,Q6IG02
2677	1,2,6,8,25,<br>26,29,30	3,4,5,7,27,<br>28,31,32	1	Q68G31
2678	1,2,6,8,25,<br>26,29,31	3,4,5,7,27,<br>28,30,32	1	P97840
2679	1,2,6,8,25,<br>26,29,32	3,4,5,7,27,<br>28,30,31	4	P00507,P13432,P97840,Q68G31
2680	1,2,6,8,25,<br>26,30,31	3,4,5,7,27,<br>28,29,32	4	P47967,P54921,P97840,Q99MH3
2681	1,2,6,8,25,<br>26,30,32	3,4,5,7,27,<br>28,29,31	6	D3ZUC6,P00507,P47967,P97840,Q68G31,Q99MH3
2682	1,2,6,8,25,<br>26,31,32	3,4,5,7,27,<br>28,29,30	6	P00507,P13432,P47967,P97840,Q68G31,Q99MH3
2683	1,2,6,8,25,<br>27,28,29	3,4,5,7,26,<br>30,31,32	6	P19629,P47727,Q5GRG2,Q812E4,Q9JI85,Q9QW07
2684	1,2,6,8,25,<br>27,28,30	3,4,5,7,26,<br>29,31,32	4	P15399,P19629,Q5GRG2,Q68G31
2685	1,2,6,8,25,<br>27,28,31	3,4,5,7,26,<br>29,30,32	4	P15399,P19629,P97840,Q5GRG2
2686	1,2,6,8,25,<br>27,28,32	3,4,5,7,26,<br>29,30,31	7	P15399,P19629,Q5GRG2,Q68G31,Q6AYR9,Q9JI85,Q9QWN8
2687	1,2,6,8,25,<br>27,29,30	3,4,5,7,26,<br>28,31,32	1	Q68G31
2688	1,2,6,8,25,<br>27,29,31	3,4,5,7,26,<br>28,30,32	2	P97840,Q5GRG2
2689	1,2,6,8,25,<br>27,29,32	3,4,5,7,26,<br>28,30,31	2	P97840,Q68G31

2690	1,2,6,8,25,<br>27,30,31	3,4,5,7,26,<br>28,29,32	4	P47967,P54921,P97840,Q6IG05
2691	1,2,6,8,25,<br>27,30,32	3,4,5,7,26,<br>28,29,31	3	P15399,P97840,Q68G31
2692	1,2,6,8,25,<br>27,31,32	3,4,5,7,26,<br>28,29,30	4	P19629,P47967,P97840,Q5GRG2
2693	1,2,6,8,25,<br>28,29,30	3,4,5,7,26,<br>27,31,32	2	Q5GRG2,Q68G31
2694	1,2,6,8,25,<br>28,29,31	3,4,5,7,26,<br>27,30,32	2	P12020,Q5GRG2
2695	1,2,6,8,25,<br>28,29,32	3,4,5,7,26,<br>27,30,31	5	P30120,Q5GRG2,Q68G31,Q9JI85,Q9QW07
2696	1,2,6,8,25,<br>28,30,31	3,4,5,7,26,<br>27,29,32	3	P12020,P97840,Q5GRG2
2697	1,2,6,8,25,<br>28,30,32	3,4,5,7,26,<br>27,29,31	1	Q68G31
2698	1,2,6,8,25,<br>28,31,32	3,4,5,7,26,<br>27,29,30	4	P21588,P97840,Q5GRG2,Q68G31
2699	1,2,6,8,25,<br>29,30,31	3,4,5,7,26,<br>27,28,32	2	P47967,P97840
2700	1,2,6,8,25,<br>29,30,32	3,4,5,7,26,<br>27,28,31	2	P97840,Q68G31
2701	1,2,6,8,25,<br>29,31,32	3,4,5,7,26,<br>27,28,30	5	P13432,P21588,P47967,P97840,Q68G31
2702	1,2,6,8,25,<br>30,31,32	3,4,5,7,26,<br>27,28,29	6	D3ZUC6,O89117,P20646,P47967,P97840,Q68G31
2703	1,2,6,8,26,<br>27,28,29	3,4,5,7,25,<br>30,31,32	6	P15399,P25809,P47727,Q6P6R2,Q812E4,Q9JI85
2704	1,2,6,8,26,<br>27,28,30	3,4,5,7,25,<br>29,31,32	4	P15399,P97580,Q30KJ2,Q68G31
2705	1,2,6,8,26,<br>27,28,31	3,4,5,7,25,<br>29,30,32	1	P15399
2706	1,2,6,8,26,<br>27,28,32	3,4,5,7,25,<br>29,30,31	6	P15399,P50280,Q68G31,Q6AYR9,Q9JI85,Q9QWN8
2707	1,2,6,8,26,<br>27,29,30	3,4,5,7,25,<br>28,31,32	6	P15399,P29315,P97580,Q30KJ2,Q68G31,Q811M5
2708	1,2,6,8,26,<br>27,29,31	3,4,5,7,25,<br>28,30,32	2	P15399,Q63474
2709	1,2,6,8,26,<br>27,29,32	3,4,5,7,25,<br>28,30,31	2	P15399,Q68G31

2710	1,2,6,8,26,27,30,31	3,4,5,7,25,28,29,32	4	P15399,P97580,P97840,Q30KJ2
2711	1,2,6,8,26,27,30,32	3,4,5,7,25,28,29,31	7	O89117,P15399,P29315,P97580,Q30KJ2,Q68G31,Q99MH3
2712	1,2,6,8,26,27,31,32	3,4,5,7,25,28,29,30	3	O89117,P15399,P97840
2713	1,2,6,8,26,28,29,30	3,4,5,7,25,27,31,32	2	P29315,Q68G31
2714	1,2,6,8,26,28,29,31	3,4,5,7,25,27,30,32	1	Q63474
2715	1,2,6,8,26,28,29,32	3,4,5,7,25,27,30,31	4	P29315,P30120,Q68G31,Q9JI85
2716	1,2,6,8,26,28,30,31	3,4,5,7,25,27,29,32	2	O89117,Q99MH3
2717	1,2,6,8,26,28,30,32	3,4,5,7,25,27,29,31	5	O89117,P15399,P29315,Q68G31,Q99MH3
2718	1,2,6,8,26,28,31,32	3,4,5,7,25,27,29,30	5	O89117,P22006,Q63532,Q6IG02,Q99MH3
2719	1,2,6,8,26,29,30,31	3,4,5,7,25,27,28,32	1	Q63474
2720	1,2,6,8,26,29,30,32	3,4,5,7,25,27,28,31	2	P29315,Q68G31
2721	1,2,6,8,26,29,31,32	3,4,5,7,25,27,28,30	3	P97840,Q63474,Q68G31
2722	1,2,6,8,26,30,31,32	3,4,5,7,25,27,28,29	5	B2RYW9,O89117,P97840,Q68G31,Q99MH3
2723	1,2,6,8,27,28,29,30	3,4,5,7,25,26,31,32	3	P15399,P29315,Q68G31
2724	1,2,6,8,27,28,29,31	3,4,5,7,25,26,30,32	3	Q5GRG2,Q63474,Q9JI85
2725	1,2,6,8,27,28,29,32	3,4,5,7,25,26,30,31	10	O35547,P15399,P25809,P29315,P30120,P47727,Q68G31,Q6AYR9,Q812E4,Q9JI85
2726	1,2,6,8,27,28,30,31	3,4,5,7,25,26,29,32	2	P15399,Q5GRG2
2727	1,2,6,8,27,28,30,32	3,4,5,7,25,26,29,31	7	O89117,P15399,P29315,Q68G31,Q6AYR9,Q6P6S4,Q9JI85
2728	1,2,6,8,27,28,31,32	3,4,5,7,25,26,29,30	6	O89117,P15399,P22006,Q5GRG2,Q6AYR9,Q9JI85
2729	1,2,6,8,27,29,30,31	3,4,5,7,25,26,28,32	0	

2730	1,2,6,8,27,<br>29,30,32	3,4,5,7,25,<br>26,28,31	3	P15399,P29315,Q68G31
2731	1,2,6,8,27,<br>29,31,32	3,4,5,7,25,<br>26,28,30	2	P97840,Q63474
2732	1,2,6,8,27,<br>30,31,32	3,4,5,7,25,<br>26,28,29	5	O89117,P15399,P20646,P97840,Q68G31
2733	1,2,6,8,28,<br>29,30,31	3,4,5,7,25,<br>26,27,32	1	Q63474
2734	1,2,6,8,28,<br>29,30,32	3,4,5,7,25,<br>26,27,31	2	P29315,Q68G31
2735	1,2,6,8,28,<br>29,31,32	3,4,5,7,25,<br>26,27,30	2	Q63474,Q9JI85
2736	1,2,6,8,28,<br>30,31,32	3,4,5,7,25,<br>26,27,29	4	O89117,P22006,Q63532,Q68G31
2737	1,2,6,8,29,<br>30,31,32	3,4,5,7,25,<br>26,27,28	5	P20646,P36860,P97840,Q63474,Q68G31
2738	1,2,6,25,2<br>6,27,28,29	3,4,5,7,8,3<br>0,31,32	4	P05539,P0DMW0;P0DMW1,P35280,P82471
2739	1,2,6,25,2<br>6,27,28,30	3,4,5,7,8,2<br>9,31,32	2	P82471,Q68G31
2740	1,2,6,25,2<br>6,27,28,31	3,4,5,7,8,2<br>9,30,32	2	P0DMW0;P0DMW1,P35280
2741	1,2,6,25,2<br>6,27,28,32	3,4,5,7,8,2<br>9,30,31	5	P82471,Q5RJR2,Q68G31,Q6IG02,Q9QWN8
2742	1,2,6,25,2<br>6,27,29,30	3,4,5,7,8,2<br>8,31,32	4	D3ZUC6,P82471,Q68G31,Q811M5
2743	1,2,6,25,2<br>6,27,29,31	3,4,5,7,8,2<br>8,30,32	3	O70417,P35280,P97840
2744	1,2,6,25,2<br>6,27,29,32	3,4,5,7,8,2<br>8,30,31	2	P82471,Q68G31
2745	1,2,6,25,2<br>6,27,30,31	3,4,5,7,8,2<br>8,29,32	5	D3ZUC6,P06760,P47967,P54921,P97840
2746	1,2,6,25,2<br>6,27,30,32	3,4,5,7,8,2<br>8,29,31	5	D3ZUC6,P06760,P82471,P97840,Q68G31
2747	1,2,6,25,2<br>6,27,31,32	3,4,5,7,8,2<br>8,29,30	4	D3ZUC6,P06760,P47967,P97840
2748	1,2,6,25,2<br>6,28,29,30	3,4,5,7,8,2<br>7,31,32	2	P82471,Q68G31
2749	1,2,6,25,2<br>6,28,29,31	3,4,5,7,8,2<br>7,30,32	2	O70417,P35280

2750	1,2,6,25,26,28,29,32	3,4,5,7,8,27,30,31	2	P82471,Q68G31
2751	1,2,6,25,26,28,30,31	3,4,5,7,8,27,29,32	4	P00714,P06760,Q4G075,Q9EQS0
2752	1,2,6,25,26,28,30,32	3,4,5,7,8,27,29,31	9	D3ZUC6,P06760,P82471,Q4G075,Q5PQL7,Q62975,Q68G31,Q9EQS0,Q9R168
2753	1,2,6,25,26,28,31,32	3,4,5,7,8,27,29,30	5	P06760,P97840,Q4G075,Q68G31,Q6IG02
2754	1,2,6,25,26,29,30,31	3,4,5,7,8,27,28,32	5	D3ZUC6,P01835,P22282,P47967,P97840
2755	1,2,6,25,26,29,30,32	3,4,5,7,8,27,28,31	6	D3ZUC6,P00507,P01835,P82471,Q68G31,Q9R168
2756	1,2,6,25,26,29,31,32	3,4,5,7,8,27,28,30	7	D3ZUC6,O70417,P00507,P19218,P47967,P97840,Q68G31
2757	1,2,6,25,26,30,31,32	3,4,5,7,8,27,28,29	12	D3ZUC6,P00507,P04636,P06760,P22273,P47967,P97840,Q4G075,Q5I0D1,Q68G31,Q99MH3,Q9EQS0
2758	1,2,6,25,27,28,29,30	3,4,5,7,8,26,31,32	3	P05539,P82471,Q68G31
2759	1,2,6,25,27,28,29,31	3,4,5,7,8,26,30,32	2	P05539,P35280
2760	1,2,6,25,27,28,29,32	3,4,5,7,8,26,30,31	7	O35547,P00758,P05539,P49134,Q5RJR2,Q62761;Q62762;Q62763,Q68G31
2761	1,2,6,25,27,28,30,31	3,4,5,7,8,26,29,32	2	P06760,Q9QX74
2762	1,2,6,25,27,28,30,32	3,4,5,7,8,26,29,31	5	P06760,P49134,P82471,Q68G31,Q9QX74
2763	1,2,6,25,27,28,31,32	3,4,5,7,8,26,29,30	4	P06760,P49134,P97840,Q9QX74
2764	1,2,6,25,27,29,30,31	3,4,5,7,8,26,28,32	3	D3ZUC6,O54728,P97840
2765	1,2,6,25,27,29,30,32	3,4,5,7,8,26,28,31	4	D3ZUC6,P82471,Q5RLM2,Q68G31
2766	1,2,6,25,27,29,31,32	3,4,5,7,8,26,28,30	2	O70417,P97840
2767	1,2,6,25,27,30,31,32	3,4,5,7,8,26,28,29	9	D3ZUC6,P06760,P20646,P47967,P97840,Q68G31,Q6P6R2,Q9QX74,Q9WTT6
2768	1,2,6,25,28,29,30,31	3,4,5,7,8,26,27,32	0	
2769	1,2,6,25,28,29,30,32	3,4,5,7,8,26,27,31	3	D3ZUC6,P01835,Q68G31

2770	1,2,6,25,28,29,31,32	3,4,5,7,8,26,27,30	2	O70417,Q68G31
2771	1,2,6,25,28,30,31,32	3,4,5,7,8,26,27,29	11	D3ZUC6,P06760,P22006,P47967,P97840,Q4G075,Q5I0D1,Q62975,Q68G31,Q9QX74,Q9WTT6
2772	1,2,6,25,29,30,31,32	3,4,5,7,8,26,27,28	10	D3ZUC6,P01835,P07150,P22273,P36860,P47967,P97840,Q4G075,Q68G31,Q9WTT6
2773	1,2,6,26,27,28,29,30	3,4,5,7,8,25,31,32	5	P01835,P21588,P82471,Q68G31,Q811M5
2774	1,2,6,26,27,28,29,31	3,4,5,7,8,25,30,32	5	O70417,P01835,P0DMW0;P0DMW1,P35280,Q63474
2775	1,2,6,26,27,28,29,32	3,4,5,7,8,25,30,31	6	O35547,O70417,P00758,P01835,P82471,Q68G31
2776	1,2,6,26,27,28,30,31	3,4,5,7,8,25,29,32	0	
2777	1,2,6,26,27,28,30,32	3,4,5,7,8,25,29,31	3	P06760,P82471,Q68G31
2778	1,2,6,26,27,28,31,32	3,4,5,7,8,25,29,30	3	O70417,P22006,Q6IG02
2779	1,2,6,26,27,29,30,31	3,4,5,7,8,25,28,32	3	P01835,P36860,Q811M5
2780	1,2,6,26,27,29,30,32	3,4,5,7,8,25,28,31	5	P01835,P29315,P82471,Q68G31,Q811M5
2781	1,2,6,26,27,29,31,32	3,4,5,7,8,25,28,30	2	O70417,P01835
2782	1,2,6,26,27,30,31,32	3,4,5,7,8,25,28,29	5	P06760,P22006,P36860,P97840,Q68G31
2783	1,2,6,26,28,29,30,31	3,4,5,7,8,25,27,32	5	O70417,P00762,P01835,Q01984,Q63474
2784	1,2,6,26,28,29,30,32	3,4,5,7,8,25,27,31	3	P01835,P29315,Q68G31
2785	1,2,6,26,28,29,31,32	3,4,5,7,8,25,27,30	6	O70417,P01835,Q05511,Q63474,Q6IFW6,Q6IG02
2786	1,2,6,26,28,30,31,32	3,4,5,7,8,25,27,29	7	O89117,P01835,P06760,P22006,Q4G075,Q68G31,Q99MH3
2787	1,2,6,26,29,30,31,32	3,4,5,7,8,25,27,28	6	O70417,P01835,P36860,Q4G075,Q63474,Q68G31
2788	1,2,6,27,28,29,30,31	3,4,5,7,8,25,26,32	1	P01835
2789	1,2,6,27,28,29,30,32	3,4,5,7,8,25,26,31	5	P00758,P01835,P14562,P29315,Q68G31

2790	1,2,6,27,28,29,31,32	3,4,5,7,8,25,26,30	5	O35547,O70417,P01835,P80299,Q63474
2791	1,2,6,27,28,30,31,32	3,4,5,7,8,25,26,29	4	P06760,P22006,Q68G31,Q9QX74
2792	1,2,6,27,29,30,31,32	3,4,5,7,8,25,26,28	5	P01835,P36860,P80299,Q68G31,Q9WTT6
2793	1,2,6,28,29,30,31,32	3,4,5,7,8,25,26,27	7	O70417,P01835,P36860,Q4G075,Q63474,Q68G31,Q9WTT6
2794	1,2,7,8,25,26,27,28	3,4,5,6,29,30,31,32	7	iRT-Kit_WR_fusion,P25809,P36860,Q63598,Q66H69,Q9WTT6,Q9Z1F2
2795	1,2,7,8,25,26,27,29	3,4,5,6,28,30,31,32	6	P22006,P25809,Q4G075,Q66H69,Q9QX74,Q9WTT6
2796	1,2,7,8,25,26,27,30	3,4,5,6,28,29,31,32	2	Q66H69,Q9Z1F2
2797	1,2,7,8,25,26,27,31	3,4,5,6,28,29,30,32	3	iRT-Kit_WR_fusion,Q63598,Q9Z0V6
2798	1,2,7,8,25,26,27,32	3,4,5,6,28,29,30,31	1	Q66H69
2799	1,2,7,8,25,26,28,29	3,4,5,6,27,30,31,32	4	P62804,Q6P6R2,Q9QX74,Q9WTT6
2800	1,2,7,8,25,26,28,30	3,4,5,6,27,29,31,32	1	Q9Z1F2
2801	1,2,7,8,25,26,28,31	3,4,5,6,27,29,30,32	5	iRT-Kit_WR_fusion,P00714,Q63598,Q9Z0V6,Q9Z1F2
2802	1,2,7,8,25,26,28,32	3,4,5,6,27,29,30,31	0	
2803	1,2,7,8,25,26,29,30	3,4,5,6,27,28,31,32	1	P22006
2804	1,2,7,8,25,26,29,31	3,4,5,6,27,28,30,32	2	Q8VD89,Q9QX74
2805	1,2,7,8,25,26,29,32	3,4,5,6,27,28,30,31	0	
2806	1,2,7,8,25,26,30,31	3,4,5,6,27,28,29,32	1	Q9Z1F2
2807	1,2,7,8,25,26,30,32	3,4,5,6,27,28,29,31	1	Q68G31
2808	1,2,7,8,25,26,31,32	3,4,5,6,27,28,29,30	0	
2809	1,2,7,8,25,27,28,29	3,4,5,6,26,30,31,32	8	P25809,P47727,P62804,Q00715,Q4G075,Q66H69,Q812E4,Q9WTT6

2810	1,2,7,8,25, 27,28,30	3,4,5,6,26, 29,31,32	1	Q66H69
2811	1,2,7,8,25, 27,28,31	3,4,5,6,26, 29,30,32	2	iRT-Kit_WR_fusion,Q63598
2812	1,2,7,8,25, 27,28,32	3,4,5,6,26, 29,30,31	3	Q66H69,Q6AYR9,Q812E4
2813	1,2,7,8,25, 27,29,30	3,4,5,6,26, 28,31,32	4	P22006,P62804,Q4G075,Q66H69
2814	1,2,7,8,25, 27,29,31	3,4,5,6,26, 28,30,32	2	Q66H69,Q8VD89
2815	1,2,7,8,25, 27,29,32	3,4,5,6,26, 28,30,31	1	Q66H69
2816	1,2,7,8,25, 27,30,31	3,4,5,6,26, 28,29,32	0	
2817	1,2,7,8,25, 27,30,32	3,4,5,6,26, 28,29,31	1	Q68G31
2818	1,2,7,8,25, 27,31,32	3,4,5,6,26, 28,29,30	1	P21588
2819	1,2,7,8,25, 28,29,30	3,4,5,6,26, 27,31,32	1	P62804
2820	1,2,7,8,25, 28,29,31	3,4,5,6,26, 27,30,32	0	
2821	1,2,7,8,25, 28,29,32	3,4,5,6,26, 27,30,31	4	P08723,P30120,P47727,Q812E4
2822	1,2,7,8,25, 28,30,31	3,4,5,6,26, 27,29,32	0	
2823	1,2,7,8,25, 28,30,32	3,4,5,6,26, 27,29,31	1	Q68G31
2824	1,2,7,8,25, 28,31,32	3,4,5,6,26, 27,29,30	2	P21588,Q811M5
2825	1,2,7,8,25, 29,30,31	3,4,5,6,26, 27,28,32	0	
2826	1,2,7,8,25, 29,30,32	3,4,5,6,26, 27,28,31	1	Q68G31
2827	1,2,7,8,25, 29,31,32	3,4,5,6,26, 27,28,30	1	P21588
2828	1,2,7,8,25, 30,31,32	3,4,5,6,26, 27,28,29	2	B2RYW9,P21588
2829	1,2,7,8,26, 27,28,29	3,4,5,6,25, 30,31,32	12	P0DMW0;P0DMW1,P25809,P47727,P62804,P97584,Q00715,Q4G075,Q66H69,Q6P6R2,Q812E4, Q9QX74,Q9WTT6

2830	1,2,7,8,26,<br>27,28,30	3,4,5,6,25,<br>29,31,32	5	iRT-Kit_WR_fusion,P25809,Q66H69,Q9WTT6,Q9Z1F2
2831	1,2,7,8,26,<br>27,28,31	3,4,5,6,25,<br>29,30,32	5	iRT-Kit_WR_fusion,Q63598,Q66H69,Q9WTT6,Q9Z1F2
2832	1,2,7,8,26,<br>27,28,32	3,4,5,6,25,<br>29,30,31	6	P05964,P25809,Q66H69,Q6AYR9,Q812E4,Q9QWN8
2833	1,2,7,8,26,<br>27,29,30	3,4,5,6,25,<br>28,31,32	4	P25809,P29315,P62804,Q66H69
2834	1,2,7,8,26,<br>27,29,31	3,4,5,6,25,<br>28,30,32	4	P25809,Q66H69,Q9QX74,Q9WTT6
2835	1,2,7,8,26,<br>27,29,32	3,4,5,6,25,<br>28,30,31	3	P25809,P29315,Q66H69
2836	1,2,7,8,26,<br>27,30,31	3,4,5,6,25,<br>28,29,32	1	Q9Z1F2
2837	1,2,7,8,26,<br>27,30,32	3,4,5,6,25,<br>28,29,31	2	P29315,Q66H69
2838	1,2,7,8,26,<br>27,31,32	3,4,5,6,25,<br>28,29,30	0	
2839	1,2,7,8,26,<br>28,29,30	3,4,5,6,25,<br>27,31,32	3	P21674,P29315,P62804
2840	1,2,7,8,26,<br>28,29,31	3,4,5,6,25,<br>27,30,32	5	P21674,Q63474,Q6P6R2,Q9QX74,Q9WTT6
2841	1,2,7,8,26,<br>28,29,32	3,4,5,6,25,<br>27,30,31	4	P08723,P29315,P30120,Q812E4
2842	1,2,7,8,26,<br>28,30,31	3,4,5,6,25,<br>27,29,32	3	P35745,Q63598,Q9Z1F2
2843	1,2,7,8,26,<br>28,30,32	3,4,5,6,25,<br>27,29,31	2	P29315,Q68G31
2844	1,2,7,8,26,<br>28,31,32	3,4,5,6,25,<br>27,29,30	1	Q6IG02
2845	1,2,7,8,26,<br>29,30,31	3,4,5,6,25,<br>27,28,32	1	Q63474
2846	1,2,7,8,26,<br>29,30,32	3,4,5,6,25,<br>27,28,31	2	P29315,Q68G31
2847	1,2,7,8,26,<br>29,31,32	3,4,5,6,25,<br>27,28,30	1	Q63474
2848	1,2,7,8,26,<br>30,31,32	3,4,5,6,25,<br>27,28,29	2	B2RYW9,O89117
2849	1,2,7,8,27,<br>28,29,30	3,4,5,6,25,<br>26,31,32	7	P25809,P29315,P47727,P62804,Q00715,Q66H69,Q812E4

2850	1,2,7,8,27,28,29,31	3,4,5,6,25,26,30,32	7	iRT-Kit_WR_fusion,P25809,P47727,Q00715,Q06000,Q66H69,Q812E4
2851	1,2,7,8,27,28,29,32	3,4,5,6,25,26,30,31	9	O35547,P08723,P25809,P29315,P30120,P47727,Q66H69,Q6AYR9,Q812E4
2852	1,2,7,8,27,28,30,31	3,4,5,6,25,26,29,32	1	iRT-Kit_WR_fusion
2853	1,2,7,8,27,28,30,32	3,4,5,6,25,26,29,31	3	P29315,Q6AYR9,Q812E4
2854	1,2,7,8,27,28,31,32	3,4,5,6,25,26,29,30	3	P05964,Q6AYR9,Q812E4
2855	1,2,7,8,27,29,30,31	3,4,5,6,25,26,28,32	1	P00731
2856	1,2,7,8,27,29,30,32	3,4,5,6,25,26,28,31	2	P29315,Q66H69
2857	1,2,7,8,27,29,31,32	3,4,5,6,25,26,28,30	1	P80299
2858	1,2,7,8,27,30,31,32	3,4,5,6,25,26,28,29	0	
2859	1,2,7,8,28,29,30,31	3,4,5,6,25,26,27,32	4	P21674,P35745,P62804,Q63474
2860	1,2,7,8,28,29,30,32	3,4,5,6,25,26,27,31	3	P29315,Q68G31,Q812E4
2861	1,2,7,8,28,29,31,32	3,4,5,6,25,26,27,30	4	P21588,P30120,Q63474,Q812E4
2862	1,2,7,8,28,30,31,32	3,4,5,6,25,26,27,29	2	O89117,P35745
2863	1,2,7,8,29,30,31,32	3,4,5,6,25,26,27,28	1	Q63474
2864	1,2,7,25,26,27,28,29	3,4,5,6,8,30,31,32	2	P0DMW0;P0DMW1,P20646
2865	1,2,7,25,26,27,28,30	3,4,5,6,8,29,31,32	1	Q9R168
2866	1,2,7,25,26,27,28,31	3,4,5,6,8,29,30,32	3	O88797,P0DMW0;P0DMW1,Q63598
2867	1,2,7,25,26,27,28,32	3,4,5,6,8,29,30,31	3	Q5RJR2,Q6IG02,Q9R168
2868	1,2,7,25,26,27,29,30	3,4,5,6,8,28,31,32	1	Q9R168
2869	1,2,7,25,26,27,29,31	3,4,5,6,8,28,30,32	0	

2870	1,2,7,25,26,27,29,32	3,4,5,6,8,28,30,31	1 Q9R168
2871	1,2,7,25,26,27,30,31	3,4,5,6,8,28,29,32	0
2872	1,2,7,25,26,27,30,32	3,4,5,6,8,28,29,31	2 D3ZUC6,Q9R168
2873	1,2,7,25,26,27,31,32	3,4,5,6,8,28,29,30	0
2874	1,2,7,25,26,28,29,30	3,4,5,6,8,27,31,32	1 Q9R168
2875	1,2,7,25,26,28,29,31	3,4,5,6,8,27,30,32	1 P19218
2876	1,2,7,25,26,28,29,32	3,4,5,6,8,27,30,31	2 P19218,Q9R168
2877	1,2,7,25,26,28,30,31	3,4,5,6,8,27,29,32	1 P00714
2878	1,2,7,25,26,28,30,32	3,4,5,6,8,27,29,31	3 Q62975,Q68G31,Q9R168
2879	1,2,7,25,26,28,31,32	3,4,5,6,8,27,29,30	3 P19218,Q6IFU7,Q6IG02
2880	1,2,7,25,26,29,30,31	3,4,5,6,8,27,28,32	2 P01835,P22282
2881	1,2,7,25,26,29,30,32	3,4,5,6,8,27,28,31	4 D3ZUC6,P01835,Q68G31,Q9R168
2882	1,2,7,25,26,29,31,32	3,4,5,6,8,27,28,30	2 O70417,P19218
2883	1,2,7,25,26,30,31,32	3,4,5,6,8,27,28,29	5 B2RYW9,D3ZUC6,P04636,P22273,Q9WVK7
2884	1,2,7,25,27,28,29,30	3,4,5,6,8,26,31,32	0
2885	1,2,7,25,27,28,29,31	3,4,5,6,8,26,30,32	2 O88797,P0DMW0;P0DMW1
2886	1,2,7,25,27,28,29,32	3,4,5,6,8,26,30,31	7 P00758,Q5RJR2,Q62761;Q62762;Q62763,Q6AY61,Q6AYR9,Q812E4,Q8CGS4
2887	1,2,7,25,27,28,30,31	3,4,5,6,8,26,29,32	0
2888	1,2,7,25,27,28,30,32	3,4,5,6,8,26,29,31	2 Q5RJR2,Q9R168
2889	1,2,7,25,27,28,31,32	3,4,5,6,8,26,29,30	2 O88797,Q5RJR2

2890	1,2,7,25,27,29,30,31	3,4,5,6,8,26,28,32	1 O54728
2891	1,2,7,25,27,29,30,32	3,4,5,6,8,26,28,31	2 D3ZUC6,Q9R168
2892	1,2,7,25,27,29,31,32	3,4,5,6,8,26,28,30	1 P80299
2893	1,2,7,25,27,30,31,32	3,4,5,6,8,26,28,29	1 D3ZUC6
2894	1,2,7,25,28,29,30,31	3,4,5,6,8,26,27,32	0
2895	1,2,7,25,28,29,30,32	3,4,5,6,8,26,27,31	3 P01835,Q68G31,Q9R168
2896	1,2,7,25,28,29,31,32	3,4,5,6,8,26,27,30	1 P21588
2897	1,2,7,25,28,30,31,32	3,4,5,6,8,26,27,29	1 Q62975
2898	1,2,7,25,29,30,31,32	3,4,5,6,8,26,27,28	2 D3ZUC6,P01835
2899	1,2,7,26,27,28,29,30	3,4,5,6,8,25,31,32	2 P01835,P29315
2900	1,2,7,26,27,28,29,31	3,4,5,6,8,25,30,32	5 O70417,O88797,P01835,P02625,P0DMW0;P0DMW1
2901	1,2,7,26,27,28,29,32	3,4,5,6,8,25,30,31	6 P00758,P01835,P29315,Q62761;Q62762;Q62763,Q812E4,Q8CGS4
2902	1,2,7,26,27,28,30,31	3,4,5,6,8,25,29,32	0
2903	1,2,7,26,27,28,30,32	3,4,5,6,8,25,29,31	2 P29315,Q9R168
2904	1,2,7,26,27,28,31,32	3,4,5,6,8,25,29,30	3 O88797,P05964,Q6IG02
2905	1,2,7,26,27,29,30,31	3,4,5,6,8,25,28,32	1 P01835
2906	1,2,7,26,27,29,30,32	3,4,5,6,8,25,28,31	4 P01835,P29315,P36376,Q9R168
2907	1,2,7,26,27,29,31,32	3,4,5,6,8,25,28,30	3 O70417,P01835,P80299
2908	1,2,7,26,27,30,31,32	3,4,5,6,8,25,28,29	0
2909	1,2,7,26,28,29,30,31	3,4,5,6,8,25,27,32	3 P00762,P01835,Q63474

2910	1,2,7,26,28,29,30,32	3,4,5,6,8,25,27,31	4 P01835,P29315,Q68G31,Q9R168
2911	1,2,7,26,28,29,31,32	3,4,5,6,8,25,27,30	5 O70417,P01835,P14173,Q63474,Q6IG02
2912	1,2,7,26,28,30,31,32	3,4,5,6,8,25,27,29	1 P01835
2913	1,2,7,26,29,30,31,32	3,4,5,6,8,25,27,28	2 P01835,P36860
2914	1,2,7,27,28,29,30,31	3,4,5,6,8,25,26,32	3 P01835,P80299,Q06000
2915	1,2,7,27,28,29,30,32	3,4,5,6,8,25,26,31	6 P00758,P01835,P29315,Q63150,Q812E4,Q9R168
2916	1,2,7,27,28,29,31,32	3,4,5,6,8,25,26,30	7 O70417,O88797,P01835,P80299,Q62761;Q62762;Q62763,Q6AY61,Q812E4
2917	1,2,7,27,28,30,31,32	3,4,5,6,8,25,26,29	1 P80299
2918	1,2,7,27,29,30,31,32	3,4,5,6,8,25,26,28	3 P01835,P08649,P80299
2919	1,2,7,28,29,30,31,32	3,4,5,6,8,25,26,27	3 P01835,P80299,Q63474
2920	1,2,8,25,26,27,28,29	3,4,5,6,7,30,31,32	5 P25809,P26342,P47727,Q6P6R2,Q812E4
2921	1,2,8,25,26,27,28,30	3,4,5,6,7,29,31,32	3 P00714,P15399,Q68G31
2922	1,2,8,25,26,27,28,31	3,4,5,6,7,29,30,32	3 iRT-Kit_WR_fusion,P00714,Q63598
2923	1,2,8,25,26,27,28,32	3,4,5,6,7,29,30,31	5 P15399,Q5RJR2,Q68G31,Q6AYR9,Q9QWN8
2924	1,2,8,25,26,27,29,30	3,4,5,6,7,28,31,32	2 P22006,Q68G31
2925	1,2,8,25,26,27,29,31	3,4,5,6,7,28,30,32	1 P97840
2926	1,2,8,25,26,27,29,32	3,4,5,6,7,28,30,31	1 Q68G31
2927	1,2,8,25,26,27,30,31	3,4,5,6,7,28,29,32	3 P47967,P54921,P97840
2928	1,2,8,25,26,27,30,32	3,4,5,6,7,28,29,31	3 D3ZUC6,P97840,Q68G31
2929	1,2,8,25,26,27,31,32	3,4,5,6,7,28,29,30	2 P47967,P97840

2930	1,2,8,25,26,28,29,30	3,4,5,6,7,27,31,32	2 P00714,Q68G31
2931	1,2,8,25,26,28,29,31	3,4,5,6,7,27,30,32	1 P00714
2932	1,2,8,25,26,28,29,32	3,4,5,6,7,27,30,31	2 P30120,Q68G31
2933	1,2,8,25,26,28,30,31	3,4,5,6,7,27,29,32	1 P00714
2934	1,2,8,25,26,28,30,32	3,4,5,6,7,27,29,31	1 Q68G31
2935	1,2,8,25,26,28,31,32	3,4,5,6,7,27,29,30	1 P97840
2936	1,2,8,25,26,29,30,31	3,4,5,6,7,27,28,32	3 P47967,P50116,P97840
2937	1,2,8,25,26,29,30,32	3,4,5,6,7,27,28,31	4 D3ZUC6,P00507,P50116,Q68G31
2938	1,2,8,25,26,29,31,32	3,4,5,6,7,27,28,30	6 P00507,P19218,P21588,P47967,P50116,P97840
2939	1,2,8,25,26,30,31,32	3,4,5,6,7,27,28,29	9 B2RYW9,D3ZUC6,P00507,P47967,P50116,P97840,Q5I0D1,Q63617,Q68G31
2940	1,2,8,25,27,28,29,30	3,4,5,6,7,26,31,32	2 Q68G31,Q812E4
2941	1,2,8,25,27,28,29,31	3,4,5,6,7,26,30,32	0
2942	1,2,8,25,27,28,29,32	3,4,5,6,7,26,30,31	7 P00758,P25809,P30120,P47727,Q68G31,Q6AYR9,Q812E4
2943	1,2,8,25,27,28,30,31	3,4,5,6,7,26,29,32	0
2944	1,2,8,25,27,28,30,32	3,4,5,6,7,26,29,31	2 Q68G31,Q6AYR9
2945	1,2,8,25,27,28,31,32	3,4,5,6,7,26,29,30	1 P21588
2946	1,2,8,25,27,29,30,31	3,4,5,6,7,26,28,32	1 P97840
2947	1,2,8,25,27,29,30,32	3,4,5,6,7,26,28,31	1 Q68G31
2948	1,2,8,25,27,29,31,32	3,4,5,6,7,26,28,30	2 P21588,P97840
2949	1,2,8,25,27,30,31,32	3,4,5,6,7,26,28,29	4 D3ZUC6,P47967,P97840,Q68G31

2950	1,2,8,25,28,29,30,31	3,4,5,6,7,26,27,32	1 P50116
2951	1,2,8,25,28,29,30,32	3,4,5,6,7,26,27,31	2 P50116,Q68G31
2952	1,2,8,25,28,29,31,32	3,4,5,6,7,26,27,30	2 P21588,P50116
2953	1,2,8,25,28,30,31,32	3,4,5,6,7,26,27,29	5 P21588,P47967,P50116,P97840,Q68G31
2954	1,2,8,25,29,30,31,32	3,4,5,6,7,26,27,28	6 D3ZUC6,P21588,P47967,P50116,P97840,Q68G31
2955	1,2,8,26,27,28,29,30	3,4,5,6,7,25,31,32	1 P25809
2956	1,2,8,26,27,28,29,31	3,4,5,6,7,25,30,32	3 P25809,Q63474,Q6P6R2
2957	1,2,8,26,27,28,29,32	3,4,5,6,7,25,30,31	8 P00758,P25809,P30120,P47727,Q68G31,Q6AYR9,Q812E4,Q9QWN8
2958	1,2,8,26,27,28,30,31	3,4,5,6,7,25,29,32	0
2959	1,2,8,26,27,28,30,32	3,4,5,6,7,25,29,31	4 P15399,Q68G31,Q6AYR9,Q9QWN8
2960	1,2,8,26,27,28,31,32	3,4,5,6,7,25,29,30	1 Q9QWN8
2961	1,2,8,26,27,29,30,31	3,4,5,6,7,25,28,32	0
2962	1,2,8,26,27,29,30,32	3,4,5,6,7,25,28,31	2 P29315,Q68G31
2963	1,2,8,26,27,29,31,32	3,4,5,6,7,25,28,30	1 Q63474
2964	1,2,8,26,27,30,31,32	3,4,5,6,7,25,28,29	0
2965	1,2,8,26,28,29,30,31	3,4,5,6,7,25,27,32	2 P50116,Q63474
2966	1,2,8,26,28,29,30,32	3,4,5,6,7,25,27,31	3 P29315,P50116,Q68G31
2967	1,2,8,26,28,29,31,32	3,4,5,6,7,25,27,30	4 P50116,Q5QE79,Q63474,Q99376
2968	1,2,8,26,28,30,31,32	3,4,5,6,7,25,27,29	3 O89117,P50116,Q68G31
2969	1,2,8,26,29,30,31,32	3,4,5,6,7,25,27,28	5 P50116,P63081,Q5QE79,Q63474,Q68G31

2970	1,2,8,27,28,29,30,31	3,4,5,6,7,25,26,32	1 Q63474
2971	1,2,8,27,28,29,30,32	3,4,5,6,7,25,26,31	5 P29315,Q63150,Q68G31,Q6AYR9,Q812E4
2972	1,2,8,27,28,29,31,32	3,4,5,6,7,25,26,30	4 P47727,Q63474,Q6AYR9,Q812E4
2973	1,2,8,27,28,30,31,32	3,4,5,6,7,25,26,29	0
2974	1,2,8,27,29,30,31,32	3,4,5,6,7,25,26,28	1 Q63474
2975	1,2,8,28,29,30,31,32	3,4,5,6,7,25,26,27	3 P50116,Q63474,Q68G31
2976	1,2,25,26,27,28,29,30	3,4,5,6,7,8,31,32	3 P00714,P82471,Q99041
2977	1,2,25,26,27,28,29,31	3,4,5,6,7,8,30,32	6 O70417,P00714,P0DMW0;P0DMW1,P19218,P35280,Q99041
2978	1,2,25,26,27,28,29,32	3,4,5,6,7,8,30,31	8 O70417,P00758,P49134,P82471,Q5RJR2,Q68G31,Q8CGS4,Q99041
2979	1,2,25,26,27,28,30,31	3,4,5,6,7,8,29,32	3 P00714,P22273,Q99041
2980	1,2,25,26,27,28,30,32	3,4,5,6,7,8,29,31	7 D3ZUC6,P22273,P49134,P82471,Q5RJR2,Q68G31,Q9R168
2981	1,2,25,26,27,28,31,32	3,4,5,6,7,8,29,30	7 O70417,P19218,P22273,P49134,Q5RJR2,Q6IG02,Q99041
2982	1,2,25,26,27,29,30,31	3,4,5,6,7,8,28,32	6 D3ZUC6,P11598,P22273,P22282,Q63617,Q99041
2983	1,2,25,26,27,29,30,32	3,4,5,6,7,8,28,31	6 D3ZUC6,P22273,P82471,Q68G31,Q99041,Q9R168
2984	1,2,25,26,27,29,31,32	3,4,5,6,7,8,28,30	6 D3ZUC6,O70417,P19218,P22273,P97840,Q99041

2985	1,2,25,26,27,30,31,32	3,4,5,6,7,8,28,29	9	D3ZUC6,P06760,P06761,P21674,P22273,P47967,P97840,Q63617,Q99041
2986	1,2,25,26,28,29,30,31	3,4,5,6,7,8,27,32	7	D3ZUC6,P00714,P22273,P22282,P50115,P50116,Q99041
2987	1,2,25,26,28,29,30,32	3,4,5,6,7,8,27,31	7	D3ZUC6,P22273,P50115,P50116,Q68G31,Q99041,Q9R168
2988	1,2,25,26,28,29,31,32	3,4,5,6,7,8,27,30	6	O70417,P19218,P22273,P50115,P50116,Q99041
2989	1,2,25,26,28,30,31,32	3,4,5,6,7,8,27,29	11	D3ZUC6,P06760,P22273,P22282,P47967,P50115,P50116,Q4G075,Q5I0D1,Q68G31,Q99041
2990	1,2,25,26,29,30,31,32	3,4,5,6,7,8,27,28	17	D3ZUC6,O70417,P00507,P06761,P11598,P19218,P22273,P22282,P47967,P50115,P50116,P97840,Q4G075,Q5I0D1,Q63617,Q68G31,Q99041
2991	1,2,25,27,28,29,30,31	3,4,5,6,7,8,26,32	2	P22273,Q99041
2992	1,2,25,27,28,29,30,32	3,4,5,6,7,8,26,31	7	D3ZUC6,P00758,P22273,P49134,Q5RJR2,Q68G31,Q99041
2993	1,2,25,27,28,29,31,32	3,4,5,6,7,8,26,30	5	O70417,P22273,P49134,Q5RJR2,Q99041
2994	1,2,25,27,28,30,31,32	3,4,5,6,7,8,26,29	6	D3ZUC6,P06760,P22273,P49134,Q99041,Q9QX74
2995	1,2,25,27,29,30,31,32	3,4,5,6,7,8,26,28	7	D3ZUC6,P07150,P22273,P47967,P49134,P97840,Q99041
2996	1,2,25,28,29,30,31,32	3,4,5,6,7,8,26,27	7	D3ZUC6,P22273,P22282,P50115,P50116,Q68G31,Q99041
2997	1,2,26,27,28,29,30,31	3,4,5,6,7,8,25,32	2	O70417,Q99041

2998	1,2,26,27,28,29,30,32	3,4,5,6,7,8,25,31	4	P00758,P29315,P50115,Q68G31
2999	1,2,26,27,28,29,31,32	3,4,5,6,7,8,25,30	3	O70417,Q63474,Q99041
3000	1,2,26,27,28,30,31,32	3,4,5,6,7,8,25,29	4	P06760,P22273,P49134,P50115
3001	1,2,26,27,29,30,31,32	3,4,5,6,7,8,25,28	5	O70417,P22273,P36860,P50115,Q99041
3002	1,2,26,28,29,30,31,32	3,4,5,6,7,8,25,27	10	O70417,P01835,P22273,P50115,P50116,Q4G075,Q5QE79,Q63474,Q68G31,Q99041
3003	1,2,27,28,29,30,31,32	3,4,5,6,7,8,25,26	6	O70417,P22273,P49134,P50115,P80299,Q99041
3004	1,3,4,5,6,7,8,25	2,26,27,28,29,30,31,32	6	O70417,P01681,P06911,P49134,Q09030,Q99041
3005	1,3,4,5,6,7,8,26	2,25,27,28,29,30,31,32	7	D3ZUC6,P02783,P22273,P49134,P52590,Q5RJR2,Q99041
3006	1,3,4,5,6,7,8,27	2,25,26,28,29,30,31,32	13	D3ZUC6,O70417,P02783,P11598,P19218,P22273,P22282,P47967,P50115,P50116,P52590,Q5I0D1,Q99041
3007	1,3,4,5,6,7,8,28	2,25,26,27,29,30,31,32	17	D3ZUC6,O70417,P06761,P06911,P11598,P19218,P21674,P22273,P22282,P47967,P49134,P52590,P97840,Q09030,Q63617,Q99041,Q9QW07
3008	1,3,4,5,6,7,8,29	2,25,26,27,28,30,31,32	13	D3ZUC6,O70594,P02783,P19218,P21674,P22273,P42854,P47967,P49134,P52590,P97840,Q5RJR2,Q99041
3009	1,3,4,5,6,7,8,30	2,25,26,27,28,29,31,32	15	O70417,P02783,P19218,P49134,P52590,Q09030,Q4FZU2,Q5RJR2,Q6IFU7,Q6IFW6,Q6IG02,Q6IG05,Q6IMF3,Q6P6Q2,Q99041
3010	1,3,4,5,6,7,8,31	2,25,26,27,28,29,30,32	12	D3ZUC6,P02783,P06911,P49134,P52590,Q05702,Q09030,Q5RJR2,Q63532,Q68G31,Q99041,Q9QX79

3011	1,3,4,5,6,7,8,32	2,25,26,27,28,29,30,31	8	D3ZUC6,P01681,P02783,P19218,P49134,P52590,Q09030,Q99041
3012	1,3,4,5,6,7,25,26	2,8,27,28,29,30,31,32	2	P49134,Q63150
3013	1,3,4,5,6,7,25,27	2,8,26,28,29,30,31,32	2	P50116,Q03191
3014	1,3,4,5,6,7,25,28	2,8,26,27,29,30,31,32	2	P97840,Q09030
3015	1,3,4,5,6,7,25,29	2,8,26,27,28,30,31,32	1	Q9QWN8
3016	1,3,4,5,6,7,25,30	2,8,26,27,28,29,31,32	4	P25809,Q09030,Q6IFW6,Q9QWN8
3017	1,3,4,5,6,7,25,31	2,8,26,27,28,29,30,32	6	P25809,P29315,Q09030,Q63150,Q68G31,Q9QWN8
3018	1,3,4,5,6,7,25,32	2,8,26,27,28,29,30,31	3	P01681,P25809,Q09030
3019	1,3,4,5,6,7,26,27	2,8,25,28,29,30,31,32	6	P02783,P21588,P47967,P50116,P97840,Q5I0D1
3020	1,3,4,5,6,7,26,28	2,8,25,27,29,30,31,32	6	D3ZUC6,P11598,P21588,P47967,P97840,Q5RLM2
3021	1,3,4,5,6,7,26,29	2,8,25,27,28,30,31,32	5	P02783,P21588,P47967,P49134,P97840
3022	1,3,4,5,6,7,26,30	2,8,25,27,28,29,31,32	4	P02783,P21588,P49134,P97840
3023	1,3,4,5,6,7,26,31	2,8,25,27,28,29,30,32	6	P02783,P49134,P57113,Q63150,Q68G31,Q9QX79

3024	1,3,4,5,6,7,26,32	2,8,25,27,28,29,30,31	1	P02783
3025	1,3,4,5,6,7,27,28	2,8,25,26,29,30,31,32	8	D3ZUC6,P11598,P19218,P47967,P50116,P97840,Q5I0D1,Q68G31
3026	1,3,4,5,6,7,27,29	2,8,25,26,28,30,31,32	7	D3ZUC6,P02783,P47967,P50116,P97840,Q07523,Q5I0D1
3027	1,3,4,5,6,7,27,30	2,8,25,26,28,29,31,32	9	P02783,P19218,P21588,P84039,P97840,Q03191,Q6IFW6,Q6IG02,Q6P6Q2
3028	1,3,4,5,6,7,27,31	2,8,25,26,28,29,30,32	4	P02783,P50116,Q03191,Q68G31
3029	1,3,4,5,6,7,27,32	2,8,25,26,28,29,30,31	4	P02783,P50116,P97840,Q03191
3030	1,3,4,5,6,7,28,29	2,8,25,26,27,30,31,32	7	D3ZUC6,P11598,P21674,P47967,P52590,P97840,Q63617
3031	1,3,4,5,6,7,28,30	2,8,25,26,27,29,31,32	3	P19218,P47967,P97840
3032	1,3,4,5,6,7,28,31	2,8,25,26,27,29,30,32	3	D3ZUC6,P97840,Q68G31
3033	1,3,4,5,6,7,28,32	2,8,25,26,27,29,30,31	3	P11598,P47967,P97840
3034	1,3,4,5,6,7,29,30	2,8,25,26,27,28,31,32	9	P02783,P19218,P49134,P84039,P97840,Q5RJR2,Q6IFW6,Q6IG02,Q9QWN8
3035	1,3,4,5,6,7,29,31	2,8,25,26,27,28,30,32	4	P02783,Q5RJR2,Q68G31,Q9QWN8
3036	1,3,4,5,6,7,29,32	2,8,25,26,27,28,30,31	3	P02783,P26342,P97840

3037	1,3,4,5,6,7,30,31	2,8,25,26,27,28,29,32	7	P25809,P26342,Q5RJR2,Q68G31,Q812E4,Q9QWN8,Q9QX79
3038	1,3,4,5,6,7,30,32	2,8,25,26,27,28,29,31	4	P19218,P25809,P26342,Q6P6R2
3039	1,3,4,5,6,7,31,32	2,8,25,26,27,28,29,30	3	P25809,P26342,Q9QX79
3040	1,3,4,5,6,8,25,26	2,7,27,28,29,30,31,32	5	P01681,P13432,P49134,P80299,Q06000
3041	1,3,4,5,6,8,25,27	2,7,26,28,29,30,31,32	2	P01681,P01835
3042	1,3,4,5,6,8,25,28	2,7,26,27,29,30,31,32	2	P01681,Q09030
3043	1,3,4,5,6,8,25,29	2,7,26,27,28,30,31,32	2	P01681,P49134
3044	1,3,4,5,6,8,25,30	2,7,26,27,28,29,31,32	12	O70417,O88797,P01681,Q05511,Q09030,Q4FZU2,Q6IFU7,Q6IFW6,Q6IG02,Q6IG05,Q6IMF3,Q6P6Q2
3045	1,3,4,5,6,8,25,31	2,7,26,27,28,29,30,32	6	P01681,P29315,Q05702,Q09030,Q63532,Q9R168
3046	1,3,4,5,6,8,25,32	2,7,26,27,28,29,30,31	3	P01681,P13432,Q09030
3047	1,3,4,5,6,8,26,27	2,7,25,28,29,30,31,32	4	P02783,P15399,P97580,Q30KJ2
3048	1,3,4,5,6,8,26,28	2,7,25,27,29,30,31,32	2	D3ZUC6,P49134
3049	1,3,4,5,6,8,26,29	2,7,25,27,28,30,31,32	3	P02783,P49134,Q5RJR2

3050	1,3,4,5,6,8,26,30	2,7,25,27,28,29,31,32	10	O88797,P02783,P49134,Q4FZU2,Q5RJR2,Q6IFU7,Q6IFW6,Q6IG02,Q6IMF3,Q6P6Q2
3051	1,3,4,5,6,8,26,31	2,7,25,27,28,29,30,32	6	P02783,P49134,Q05702,Q5RJR2,Q63532,Q9R168
3052	1,3,4,5,6,8,26,32	2,7,25,27,28,29,30,31	2	P01681,P02783
3053	1,3,4,5,6,8,27,28	2,7,25,26,29,30,31,32	3	D3ZUC6,P19218,P22282
3054	1,3,4,5,6,8,27,29	2,7,25,26,28,30,31,32	3	D3ZUC6,P02783,P19218
3055	1,3,4,5,6,8,27,30	2,7,25,26,28,29,31,32	14	O70417,O88797,P02783,P19218,P97580,Q10758,Q4FZU2,Q6IFU7,Q6IFU8,Q6IFW6,Q6IG02,Q6IG05,Q6IMF3,Q6P6Q2
3056	1,3,4,5,6,8,27,31	2,7,25,26,28,29,30,32	4	P02783,P19218,P23739,Q9R168
3057	1,3,4,5,6,8,27,32	2,7,25,26,28,29,30,31	3	P01681,P02783,P19218
3058	1,3,4,5,6,8,28,29	2,7,25,26,27,30,31,32	2	D3ZUC6,P21674
3059	1,3,4,5,6,8,28,30	2,7,25,26,27,29,31,32	8	O88797,P19218,Q4FZU2,Q6IFU7,Q6IFW6,Q6IG02,Q6IMF3,Q6P6Q2
3060	1,3,4,5,6,8,28,31	2,7,25,26,27,29,30,32	4	D3ZUC6,Q09030,Q63532,Q9R168
3061	1,3,4,5,6,8,28,32	2,7,25,26,27,29,30,31	1	P01681
3062	1,3,4,5,6,8,29,30	2,7,25,26,27,28,31,32	11	O88797,P02783,P19218,P49134,Q4FZU2,Q5RJR2,Q6IFU7,Q6IFW6,Q6IG02,Q6IMF3,Q6P6Q2

3063	1,3,4,5,6,8,29,31	2,7,25,26,27,28,30,32	5	P02783,P49134,Q05702,Q5RJR2,Q9R168
3064	1,3,4,5,6,8,29,32	2,7,25,26,27,28,30,31	2	P01681,P02783
3065	1,3,4,5,6,8,30,31	2,7,25,26,27,28,29,32	14	O88797,P19218,P49134,Q05702,Q09030,Q4FZU2,Q5RJR2,Q63532,Q6IFU7,Q6IFW6,Q6IG02,Q6IG05,Q6IMF3,Q6P6Q2
3066	1,3,4,5,6,8,30,32	2,7,25,26,27,28,29,31	10	O88797,P01681,P19218,P20646,Q4FZU2,Q6IFU7,Q6IFW6,Q6IG02,Q6IMF3,Q6P6Q2
3067	1,3,4,5,6,8,31,32	2,7,25,26,27,28,29,30	4	P01681,Q05702,Q09030,Q63532
3068	1,3,4,5,6,25,26,27	2,7,8,28,29,30,31,32	2	P21588,Q06000
3069	1,3,4,5,6,25,26,28	2,7,8,27,29,30,31,32	1	Q06000
3070	1,3,4,5,6,25,26,29	2,7,8,27,28,30,31,32	0	
3071	1,3,4,5,6,25,26,30	2,7,8,27,28,29,31,32	5	P21588,P25809,P47727,Q06000,Q812E4
3072	1,3,4,5,6,25,26,31	2,7,8,27,28,29,30,32	5	P25809,P29315,P62804,Q63150,Q812E4
3073	1,3,4,5,6,25,26,32	2,7,8,27,28,29,30,31	4	P01681,P25809,P62804,Q06000
3074	1,3,4,5,6,25,27,28	2,7,8,26,29,30,31,32	0	
3075	1,3,4,5,6,25,27,29	2,7,8,26,28,30,31,32	0	
3076	1,3,4,5,6,25,27,30	2,7,8,26,28,29,31,32	3	Q6IFW6,Q6IG02,Q6P6Q2
3077	1,3,4,5,6,25,27,31	2,7,8,26,28,29,30,32	2	P29315,P62804
3078	1,3,4,5,6,25,27,32	2,7,8,26,28,29,30,31	3	P01681,P62804,Q03191
3079	1,3,4,5,6,25,28,29	2,7,8,26,27,30,31,32	0	
3080	1,3,4,5,6,25,28,30	2,7,8,26,27,29,31,32	2	P25809,Q62975

3081	1,3,4,5,6,25,28,31	2,7,8,26,27,29,30,32	3	P25809,P29315,Q09030
3082	1,3,4,5,6,25,28,32	2,7,8,26,27,29,30,31	4	P01681,P25809,P62804,Q62975
3083	1,3,4,5,6,25,29,30	2,7,8,26,27,28,31,32	4	P25809,Q6IFW6,Q6IG02,Q9QWN8
3084	1,3,4,5,6,25,29,31	2,7,8,26,27,28,30,32	3	P25809,P29315,Q9QWN8
3085	1,3,4,5,6,25,29,32	2,7,8,26,27,28,30,31	2	P01681,Q9WTT6
3086	1,3,4,5,6,25,30,31	2,7,8,26,27,28,29,32	12	P25809,P29315,P47727,P62804,P97584,Q09030,Q6AYR9,Q6IG02,Q6P6R2,Q812E4,Q9QWN8,Q9WTT6
3087	1,3,4,5,6,25,30,32	2,7,8,26,27,28,29,31	8	P01681,P25809,P62804,P97584,Q00715,Q6P6R2,Q9QX74,Q9WTT6
3088	1,3,4,5,6,25,31,32	2,7,8,26,27,28,29,30	9	P01681,P25809,P29315,P62804,P97584,Q09030,Q4G075,Q6P6R2,Q9WTT6
3089	1,3,4,5,6,26,27,28	2,7,8,25,29,30,31,32	2	P21588,P97840
3090	1,3,4,5,6,26,27,29	2,7,8,25,28,30,31,32	3	P02783,P09656,P21588
3091	1,3,4,5,6,26,27,30	2,7,8,25,28,29,31,32	3	P02783,P21588,Q6IG02
3092	1,3,4,5,6,26,27,31	2,7,8,25,28,29,30,32	2	P02783,P62804
3093	1,3,4,5,6,26,27,32	2,7,8,25,28,29,30,31	2	P02783,P62804
3094	1,3,4,5,6,26,28,29	2,7,8,25,27,30,31,32	3	P21588,P47967,P97840
3095	1,3,4,5,6,26,28,30	2,7,8,25,27,29,31,32	1	P21588
3096	1,3,4,5,6,26,28,31	2,7,8,25,27,29,30,32	1	P62804
3097	1,3,4,5,6,26,28,32	2,7,8,25,27,29,30,31	1	P62804
3098	1,3,4,5,6,26,29,30	2,7,8,25,27,28,31,32	4	P02783,P21588,Q5RJR2,Q6IG02
3099	1,3,4,5,6,26,29,31	2,7,8,25,27,28,30,32	1	P02783
3100	1,3,4,5,6,26,29,32	2,7,8,25,27,28,30,31	1	P02783

3101	1,3,4,5,6,26,30,31	2,7,8,25,27,28,29,32	7	P25809,P47727,P62804,Q4G075,Q5RJR2,Q6AYR9,Q812E4
3102	1,3,4,5,6,26,30,32	2,7,8,25,27,28,29,31	6	P25809,P62804,Q00715,Q4G075,Q8VD89,Q9WTT6
3103	1,3,4,5,6,26,31,32	2,7,8,25,27,28,29,30	3	P25809,P62804,Q4G075
3104	1,3,4,5,6,27,28,29	2,7,8,25,26,30,31,32	4	D3ZUC6,P09656,P47967,P97840
3105	1,3,4,5,6,27,28,30	2,7,8,25,26,29,31,32	5	P19218,P21588,P97840,Q6IG02,Q8VD89
3106	1,3,4,5,6,27,28,31	2,7,8,25,26,29,30,32	1	Q68G31
3107	1,3,4,5,6,27,28,32	2,7,8,25,26,29,30,31	1	Q62761;Q62762;Q62763
3108	1,3,4,5,6,27,29,30	2,7,8,25,26,28,31,32	7	P02783,P19218,P21588,P84039,Q6IFW6,Q6IG02,Q6P6Q2
3109	1,3,4,5,6,27,29,31	2,7,8,25,26,28,30,32	1	P02783
3110	1,3,4,5,6,27,29,32	2,7,8,25,26,28,30,31	1	P02783
3111	1,3,4,5,6,27,30,31	2,7,8,25,26,28,29,32	4	P19218,P30120,Q6IG02,Q9WTT6
3112	1,3,4,5,6,27,30,32	2,7,8,25,26,28,29,31	6	P19218,Q6IG02,Q6P6R2,Q8VD89,Q9QX74,Q9WTT6
3113	1,3,4,5,6,27,31,32	2,7,8,25,26,28,29,30	2	P62804,Q9WTT6
3114	1,3,4,5,6,28,29,30	2,7,8,25,26,27,31,32	2	P97840,Q6IG02
3115	1,3,4,5,6,28,29,31	2,7,8,25,26,27,30,32	0	
3116	1,3,4,5,6,28,29,32	2,7,8,25,26,27,30,31	1	P97840
3117	1,3,4,5,6,28,30,31	2,7,8,25,26,27,29,32	2	P25809,Q9WTT6
3118	1,3,4,5,6,28,30,32	2,7,8,25,26,27,29,31	5	P25809,Q4G075,Q8VD89,Q9QX74,Q9WTT6
3119	1,3,4,5,6,28,31,32	2,7,8,25,26,27,29,30	5	P22006,P25809,P62804,Q4G075,Q9WTT6
3120	1,3,4,5,6,29,30,31	2,7,8,25,26,27,28,32	8	P02783,P07150,P25809,P36860,Q5RJR2,Q6IG02,Q9QWN8,Q9WTT6

3121	1,3,4,5,6,29,30,32	2,7,8,25,26,27,28,31	7	P02783,P25809,P26342,Q63598,Q6IG02,Q9WTT6,Q9Z0V6
3122	1,3,4,5,6,29,31,32	2,7,8,25,26,27,28,30	3	P25809,P62804,Q9WTT6
3123	1,3,4,5,6,30,31,32	2,7,8,25,26,27,28,29	14	P20646,P25809,P26342,P36860,P47727,P62804,P97584,Q00715,Q4G075,Q6P6R2,Q812E4,Q8VD89,Q9QX74,Q9WTT6
3124	1,3,4,5,7,8,25,26	2,6,27,28,29,30,31,32	3	P01681,P22006,P49134
3125	1,3,4,5,7,8,25,27	2,6,26,28,29,30,31,32	5	O70417,P01681,P01835,Q4G075,Q68G31
3126	1,3,4,5,7,8,25,28	2,6,26,27,29,30,31,32	6	O70417,P01681,P36860,P49134,P97840,Q811M5
3127	1,3,4,5,7,8,25,29	2,6,26,27,28,30,31,32	5	P01681,P06760,P22006,P48032,P49134
3128	1,3,4,5,7,8,25,30	2,6,26,27,28,29,31,32	13	O70417,O88797,P01681,P49134,Q05511,Q4FZU2,Q6IFU7,Q6IFU8,Q6IFW6,Q6IG02,Q6IG05,Q6IMF3,Q6P6Q2
3129	1,3,4,5,7,8,25,31	2,6,26,27,28,29,30,32	5	P01681,P29315,P49134,Q68G31,Q811M5
3130	1,3,4,5,7,8,25,32	2,6,26,27,28,29,30,31	2	P01681,Q811M5
3131	1,3,4,5,7,8,26,27	2,6,25,28,29,30,31,32	5	D3ZUC6,P47967,P97840,Q5I0D1,Q68G31
3132	1,3,4,5,7,8,26,28	2,6,25,27,29,30,31,32	5	D3ZUC6,P47967,P49134,P97840,Q5RLM2
3133	1,3,4,5,7,8,26,29	2,6,25,27,28,30,31,32	7	D3ZUC6,P06760,P22006,P47967,P49134,P97840,Q9QX74
3134	1,3,4,5,7,8,26,30	2,6,25,27,28,29,31,32	10	O70417,P49134,P97840,Q4FZU2,Q5RJR2,Q6IFU7,Q6IFW6,Q6IG02,Q6IMF3,Q6P6Q2

3135	1,3,4,5,7,8,26,31	2,6,25,27,28,29,30,32	4	D3ZUC6,P49134,Q5RLM2,Q68G31
3136	1,3,4,5,7,8,26,32	2,6,25,27,28,29,30,31	3	P01681,P49134,P97840
3137	1,3,4,5,7,8,27,28	2,6,25,26,29,30,31,32	8	D3ZUC6,P19218,P22282,P47967,P97840,Q4G075,Q5I0D1,Q68G31
3138	1,3,4,5,7,8,27,29	2,6,25,26,28,30,31,32	7	D3ZUC6,P06760,P47967,P97840,Q4G075,Q5I0D1,Q68G31
3139	1,3,4,5,7,8,27,30	2,6,25,26,28,29,31,32	12	O70417,P19218,P97840,Q10758,Q4FZU2,Q6IFU7,Q6IFU8,Q6IFW6,Q6IG02,Q6IG05,Q6IMF3,Q6P6Q2
3140	1,3,4,5,7,8,27,31	2,6,25,26,28,29,30,32	3	D3ZUC6,P23739,Q68G31
3141	1,3,4,5,7,8,27,32	2,6,25,26,28,29,30,31	3	P01681,P97840,Q03191
3142	1,3,4,5,7,8,28,29	2,6,25,26,27,30,31,32	7	D3ZUC6,P06760,P21674,P47967,P49134,P97840,Q63493
3143	1,3,4,5,7,8,28,30	2,6,25,26,27,29,31,32	12	D3ZUC6,O70417,P19218,P47967,P49134,P97840,Q4FZU2,Q6IFU7,Q6IFW6,Q6IG02,Q6IMF3,Q6P6Q2
3144	1,3,4,5,7,8,28,31	2,6,25,26,27,29,30,32	5	D3ZUC6,P49134,P97840,Q68G31,Q811M5
3145	1,3,4,5,7,8,28,32	2,6,25,26,27,29,30,31	5	D3ZUC6,P01681,P47967,P97840,Q811M5
3146	1,3,4,5,7,8,29,30	2,6,25,26,27,28,31,32	13	O88797,P19218,P47967,P49134,P97840,Q4FZU2,Q5RJR2,Q6IFU7,Q6IFU8,Q6IFW6,Q6IG02,Q6IMF3,Q6P6Q2
3147	1,3,4,5,7,8,29,31	2,6,25,26,27,28,30,32	5	D3ZUC6,P49134,P82471,Q5RJR2,Q68G31

3148	1,3,4,5,7,8,29,32	2,6,25,26,27,28,30,31	4	P01681,P47967,P49134,P97840
3149	1,3,4,5,7,8,30,31	2,6,25,26,27,28,29,32	10	P49134,Q4FZU2,Q5RJR2,Q68G31,Q6IFU7,Q6IFW6,Q6IG02,Q6IG05,Q6IMF3,Q6P6Q2
3150	1,3,4,5,7,8,30,32	2,6,25,26,27,28,29,31	11	O70417,O88797,P01681,P19218,P35280,Q4FZU2,Q6IFU7,Q6IFW6,Q6IG02,Q6IMF3,Q6P6Q2
3151	1,3,4,5,7,8,31,32	2,6,25,26,27,28,29,30	3	P01681,P82471,Q811M5
3152	1,3,4,5,7,25,26,27	2,6,8,28,29,30,31,32	1	Q68G31
3153	1,3,4,5,7,25,26,28	2,6,8,27,29,30,31,32	1	P97840
3154	1,3,4,5,7,25,26,29	2,6,8,27,28,30,31,32	3	O89117,P22006,P97840
3155	1,3,4,5,7,25,26,30	2,6,8,27,28,29,31,32	2	Q05511,Q6IFW6
3156	1,3,4,5,7,25,26,31	2,6,8,27,28,29,30,32	3	P25809,P29315,Q68G31
3157	1,3,4,5,7,25,26,32	2,6,8,27,28,29,30,31	1	P01681
3158	1,3,4,5,7,25,27,28	2,6,8,26,29,30,31,32	2	P97840,Q68G31
3159	1,3,4,5,7,25,27,29	2,6,8,26,28,30,31,32	4	O89117,P22006,P97840,Q68G31
3160	1,3,4,5,7,25,27,30	2,6,8,26,28,29,31,32	6	Q03191,Q05511,Q4FZU2,Q6IFW6,Q6IG02,Q6P6Q2
3161	1,3,4,5,7,25,27,31	2,6,8,26,28,29,30,32	4	P23739,P29315,Q03191,Q68G31
3162	1,3,4,5,7,25,27,32	2,6,8,26,28,29,30,31	2	P01681,Q03191
3163	1,3,4,5,7,25,28,29	2,6,8,26,27,30,31,32	3	O89117,P47967,P97840
3164	1,3,4,5,7,25,28,30	2,6,8,26,27,29,31,32	2	P97840,Q62975
3165	1,3,4,5,7,25,28,31	2,6,8,26,27,29,30,32	3	P29315,Q68G31,Q811M5

3166	1,3,4,5,7,25,28,32	2,6,8,26,27,29,30,31	4	P01681,P97840,Q62975,Q811M5
3167	1,3,4,5,7,25,29,30	2,6,8,26,27,28,31,32	7	P22006,P97840,Q4FZU2,Q6IFW6,Q6IG02,Q6P6Q2,Q9QWN8
3168	1,3,4,5,7,25,29,31	2,6,8,26,27,28,30,32	2	Q68G31,Q9QWN8
3169	1,3,4,5,7,25,29,32	2,6,8,26,27,28,30,31	1	P01681
3170	1,3,4,5,7,25,30,31	2,6,8,26,27,28,29,32	9	P25809,Q4FZU2,Q68G31,Q6AYR9,Q6IFW6,Q6IG02,Q812E4,Q8CFN2,Q9QWN8
3171	1,3,4,5,7,25,30,32	2,6,8,26,27,28,29,31	6	P01681,P25809,Q4FZU2,Q62975,Q6IFW6,Q6P6R2
3172	1,3,4,5,7,25,31,32	2,6,8,26,27,28,29,30	3	P01681,P25809,Q811M5
3173	1,3,4,5,7,26,27,28	2,6,8,25,29,30,31,32	7	D3ZUC6,P21588,P47967,P97840,Q5I0D1,Q5RLM2,Q68G31
3174	1,3,4,5,7,26,27,29	2,6,8,25,28,30,31,32	5	P21588,P47967,P97840,Q5I0D1,Q68G31
3175	1,3,4,5,7,26,27,30	2,6,8,25,28,29,31,32	5	P21588,P97840,Q6IFW6,Q6IG02,Q6P6Q2
3176	1,3,4,5,7,26,27,31	2,6,8,25,28,29,30,32	2	P97840,Q68G31
3177	1,3,4,5,7,26,27,32	2,6,8,25,28,29,30,31	2	P97840,Q03191
3178	1,3,4,5,7,26,28,29	2,6,8,25,27,30,31,32	4	D3ZUC6,P47967,P97840,Q68G31
3179	1,3,4,5,7,26,28,30	2,6,8,25,27,29,31,32	2	P47967,P97840
3180	1,3,4,5,7,26,28,31	2,6,8,25,27,29,30,32	3	P97840,Q5RLM2,Q68G31
3181	1,3,4,5,7,26,28,32	2,6,8,25,27,29,30,31	2	P47967,P97840
3182	1,3,4,5,7,26,29,30	2,6,8,25,27,28,31,32	3	P47967,P97840,Q6IG02
3183	1,3,4,5,7,26,29,31	2,6,8,25,27,28,30,32	2	P97840,Q68G31
3184	1,3,4,5,7,26,29,32	2,6,8,25,27,28,30,31	2	P47967,P97840
3185	1,3,4,5,7,26,30,31	2,6,8,25,27,28,29,32	5	P25809,P47727,Q68G31,Q6AYR9,Q812E4

3186	1,3,4,5,7,26,30,32	2,6,8,25,27,28,29,31	1	P97840
3187	1,3,4,5,7,26,31,32	2,6,8,25,27,28,29,30	1	Q68G31
3188	1,3,4,5,7,27,28,29	2,6,8,25,26,30,31,32	6	D3ZUC6,P00507,P47967,P97840,Q5I0D1,Q68G31
3189	1,3,4,5,7,27,28,30	2,6,8,25,26,29,31,32	5	P19218,P47967,P97840,Q6IG02,Q6P6Q2
3190	1,3,4,5,7,27,28,31	2,6,8,25,26,29,30,32	4	D3ZUC6,P47967,P97840,Q68G31
3191	1,3,4,5,7,27,28,32	2,6,8,25,26,29,30,31	3	P47967,P97840,Q62761;Q62762;Q62763
3192	1,3,4,5,7,27,29,30	2,6,8,25,26,28,31,32	10	P08649,P19218,P47967,P84039,P97840,Q4FZU2,Q6IFW6,Q6IG02,Q6IMF3,Q6P6Q2
3193	1,3,4,5,7,27,29,31	2,6,8,25,26,28,30,32	3	P08649,P97840,Q68G31
3194	1,3,4,5,7,27,29,32	2,6,8,25,26,28,30,31	2	P47967,P97840
3195	1,3,4,5,7,27,30,31	2,6,8,25,26,28,29,32	6	P08649,Q4FZU2,Q68G31,Q6IFW6,Q6IG02,Q6P6Q2
3196	1,3,4,5,7,27,30,32	2,6,8,25,26,28,29,31	7	P08649,P97840,Q03191,Q4FZU2,Q6IFW6,Q6IG02,Q6P6Q2
3197	1,3,4,5,7,27,31,32	2,6,8,25,26,28,29,30	4	P08649,P23739,Q03191,Q68G31
3198	1,3,4,5,7,28,29,30	2,6,8,25,26,27,31,32	3	P47967,P97840,Q6IG02
3199	1,3,4,5,7,28,29,31	2,6,8,25,26,27,30,32	4	D3ZUC6,P47967,P97840,Q68G31
3200	1,3,4,5,7,28,29,32	2,6,8,25,26,27,30,31	3	P47967,P54921,P97840
3201	1,3,4,5,7,28,30,31	2,6,8,25,26,27,29,32	3	P25809,P97840,Q68G31
3202	1,3,4,5,7,28,30,32	2,6,8,25,26,27,29,31	2	P47967,P97840
3203	1,3,4,5,7,28,31,32	2,6,8,25,26,27,29,30	3	P97840,Q68G31,Q811M5
3204	1,3,4,5,7,29,30,31	2,6,8,25,26,27,28,32	8	P08649,P97840,Q4FZU2,Q68G31,Q6IFW6,Q6IG02,Q6P6Q2,Q9QWN8
3205	1,3,4,5,7,29,30,32	2,6,8,25,26,27,28,31	7	P08649,P26342,P97840,Q4FZU2,Q6IFW6,Q6IG02,Q6P6Q2

3206	1,3,4,5,7,29,31,32	2,6,8,25,26,27,28,30	3	P08649,P26342,Q68G31
3207	1,3,4,5,7,30,31,32	2,6,8,25,26,27,28,29	5	P08649,P25809,P26342,Q4FZU2,Q6P6R2
3208	1,3,4,5,8,25,26,27	2,6,7,28,29,30,31,32	3	P01681,P01835,P80299
3209	1,3,4,5,8,25,26,28	2,6,7,27,29,30,31,32	3	P01681,P01835,P80299
3210	1,3,4,5,8,25,26,29	2,6,7,27,28,30,31,32	4	P01681,P22006,P49134,P80299
3211	1,3,4,5,8,25,26,30	2,6,7,27,28,29,31,32	11	O88797,P01681,P01835,P80299,Q05511,Q4FZU2,Q6IFU7,Q6IFW6,Q6IG02,Q6IMF3,Q6P6Q2
3212	1,3,4,5,8,25,26,31	2,6,7,27,28,29,30,32	3	P01681,P01835,P29315
3213	1,3,4,5,8,25,26,32	2,6,7,27,28,29,30,31	2	P01681,P01835
3214	1,3,4,5,8,25,27,28	2,6,7,26,29,30,31,32	3	P01681,P01835,P36860
3215	1,3,4,5,8,25,27,29	2,6,7,26,28,30,31,32	4	O89117,P01681,P01835,P22006
3216	1,3,4,5,8,25,27,30	2,6,7,26,28,29,31,32	13	O70417,O88797,P01681,P01835,Q05511,Q4FZU2,Q6IFU7,Q6IFU8,Q6IFW6,Q6IG02,Q6IG05,Q6IMF3,Q6P6Q2
3217	1,3,4,5,8,25,27,31	2,6,7,26,28,29,30,32	6	P01681,P01835,P23739,P29315,Q68G31,Q9R168
3218	1,3,4,5,8,25,27,32	2,6,7,26,28,29,30,31	3	P00762,P01681,P01835
3219	1,3,4,5,8,25,28,29	2,6,7,26,27,30,31,32	1	P01681
3220	1,3,4,5,8,25,28,30	2,6,7,26,27,29,31,32	10	O88797,P01681,P01835,P80299,Q4FZU2,Q6IFU7,Q6IFW6,Q6IG02,Q6IMF3,Q6P6Q2
3221	1,3,4,5,8,25,28,31	2,6,7,26,27,29,30,32	5	P01681,P01835,P29315,P36376,Q811M5
3222	1,3,4,5,8,25,28,32	2,6,7,26,27,29,30,31	3	P01681,P01835,Q811M5
3223	1,3,4,5,8,25,29,30	2,6,7,26,27,28,31,32	10	O88797,P01681,P22006,Q4FZU2,Q6IFU7,Q6IFU8,Q6IFW6,Q6IG02,Q6IMF3,Q6P6Q2
3224	1,3,4,5,8,25,29,31	2,6,7,26,27,28,30,32	3	P01681,P29315,Q9R168
3225	1,3,4,5,8,25,29,32	2,6,7,26,27,28,30,31	1	P01681

3226	1,3,4,5,8,25,30,31	2,6,7,26,27,28,29,32	12	O88797,P01681,P01835,P29315,Q05511,Q4FZU2,Q6IFU7,Q6IFW6,Q6IG02,Q6IG05,Q6IMF3,Q6P6Q2
3227	1,3,4,5,8,25,30,32	2,6,7,26,27,28,29,31	10	O88797,P01681,P01835,Q05511,Q4FZU2,Q6IFU7,Q6IFW6,Q6IG02,Q6IMF3,Q6P6Q2
3228	1,3,4,5,8,25,31,32	2,6,7,26,27,28,29,30	5	P01681,P01835,P29315,Q80WL1,Q811M5
3229	1,3,4,5,8,26,27,28	2,6,7,25,29,30,31,32	4	D3ZUC6,P01835,P47967,P97840
3230	1,3,4,5,8,26,27,29	2,6,7,25,28,30,31,32	0	
3231	1,3,4,5,8,26,27,30	2,6,7,25,28,29,31,32	9	O88797,Q4FZU2,Q6IFU7,Q6IFU8,Q6IFW6,Q6IG02,Q6IG05,Q6IMF3,Q6P6Q2
3232	1,3,4,5,8,26,27,31	2,6,7,25,28,29,30,32	4	P01835,P23739,Q68G31,Q9R168
3233	1,3,4,5,8,26,27,32	2,6,7,25,28,29,30,31	1	P01681
3234	1,3,4,5,8,26,28,29	2,6,7,25,27,30,31,32	3	D3ZUC6,P47967,P97840
3235	1,3,4,5,8,26,28,30	2,6,7,25,27,29,31,32	9	O88797,P80299,P97840,Q4FZU2,Q6IFU7,Q6IFW6,Q6IG02,Q6IMF3,Q6P6Q2
3236	1,3,4,5,8,26,28,31	2,6,7,25,27,29,30,32	1	Q9R168
3237	1,3,4,5,8,26,28,32	2,6,7,25,27,29,30,31	2	O54728,P01681
3238	1,3,4,5,8,26,29,30	2,6,7,25,27,28,31,32	9	O88797,P49134,Q4FZU2,Q5RJR2,Q6IFU7,Q6IFW6,Q6IG02,Q6IMF3,Q6P6Q2
3239	1,3,4,5,8,26,29,31	2,6,7,25,27,28,30,32	3	P49134,Q5RJR2,Q9R168
3240	1,3,4,5,8,26,29,32	2,6,7,25,27,28,30,31	1	P01681
3241	1,3,4,5,8,26,30,31	2,6,7,25,27,28,29,32	8	O88797,P49134,Q4FZU2,Q5RJR2,Q6IFW6,Q6IG02,Q6IMF3,Q6P6Q2
3242	1,3,4,5,8,26,30,32	2,6,7,25,27,28,29,31	9	O88797,P01681,P05539,Q4FZU2,Q6IFU7,Q6IFW6,Q6IG02,Q6IMF3,Q6P6Q2
3243	1,3,4,5,8,26,31,32	2,6,7,25,27,28,29,30	2	O54728,P01681
3244	1,3,4,5,8,27,28,29	2,6,7,25,26,30,31,32	3	D3ZUC6,P47967,P97840
3245	1,3,4,5,8,27,28,30	2,6,7,25,26,29,31,32	9	P19218,P97840,Q10758,Q4FZU2,Q6IFU7,Q6IFW6,Q6IG02,Q6IMF3,Q6P6Q2

3246	1,3,4,5,8,27,28,31	2,6,7,25,26,29,30,32	5	D3ZUC6,P01835,P23739,Q68G31,Q9R168
3247	1,3,4,5,8,27,28,32	2,6,7,25,26,29,30,31	2	P01681,P01835
3248	1,3,4,5,8,27,29,30	2,6,7,25,26,28,31,32	11	O88797,P19218,Q10758,Q4FZU2,Q6IFU7,Q6IFU8,Q6IFW6,Q6IG02,Q6IG05,Q6IMF3,Q6P6Q2
3249	1,3,4,5,8,27,29,31	2,6,7,25,26,28,30,32	2	Q68G31,Q9R168
3250	1,3,4,5,8,27,29,32	2,6,7,25,26,28,30,31	1	P01681
3251	1,3,4,5,8,27,30,31	2,6,7,25,26,28,29,32	11	P19218,P23739,Q10758,Q4FZU2,Q6IFU7,Q6IFU8,Q6IFW6,Q6IG02,Q6IG05,Q6IMF3,Q6P6Q2
3252	1,3,4,5,8,27,30,32	2,6,7,25,26,28,29,31	11	O88797,P01681,P19218,Q4FZU2,Q6IFU7,Q6IFU8,Q6IFW6,Q6IG02,Q6IG05,Q6IMF3,Q6P6Q2
3253	1,3,4,5,8,27,31,32	2,6,7,25,26,28,29,30	2	P01681,P23739
3254	1,3,4,5,8,28,29,30	2,6,7,25,26,27,31,32	8	O88797,P97840,Q4FZU2,Q6IFU7,Q6IFW6,Q6IG02,Q6IMF3,Q6P6Q2
3255	1,3,4,5,8,28,29,31	2,6,7,25,26,27,30,32	2	D3ZUC6,Q9R168
3256	1,3,4,5,8,28,29,32	2,6,7,25,26,27,30,31	2	P01681,P97840
3257	1,3,4,5,8,28,30,31	2,6,7,25,26,27,29,32	6	Q4FZU2,Q6IFU7,Q6IFW6,Q6IG02,Q6IMF3,Q6P6Q2
3258	1,3,4,5,8,28,30,32	2,6,7,25,26,27,29,31	8	O88797,P01681,Q4FZU2,Q6IFU7,Q6IFW6,Q6IG02,Q6IMF3,Q6P6Q2
3259	1,3,4,5,8,28,31,32	2,6,7,25,26,27,29,30	2	P01681,Q811M5
3260	1,3,4,5,8,29,30,31	2,6,7,25,26,27,28,32	9	O88797,Q4FZU2,Q5RJR2,Q6IFU7,Q6IFU8,Q6IFW6,Q6IG02,Q6IMF3,Q6P6Q2
3261	1,3,4,5,8,29,30,32	2,6,7,25,26,27,28,31	8	O88797,P01681,Q4FZU2,Q6IFU7,Q6IFW6,Q6IG02,Q6IMF3,Q6P6Q2
3262	1,3,4,5,8,29,31,32	2,6,7,25,26,27,28,30	1	P01681
3263	1,3,4,5,8,30,31,32	2,6,7,25,26,27,28,29	9	O88797,P01681,P63081,Q4FZU2,Q6IFU7,Q6IFW6,Q6IG02,Q6IMF3,Q6P6Q2
3264	1,3,4,5,25,26,27,28	2,6,7,8,29,30,31,32	0	
3265	1,3,4,5,25,26,27,29	2,6,7,8,28,30,31,32	2	O89117,P22006

3266	1,3,4,5,25,<br>26,27,30	2,6,7,8,28,<br>29,31,32	4	P21588,Q05511,Q6IFW6,Q6IG02
3267	1,3,4,5,25,<br>26,27,31	2,6,7,8,28,<br>29,30,32	4	P23739,P29315,P62804,Q68G31
3268	1,3,4,5,25,<br>26,27,32	2,6,7,8,28,<br>29,30,31	2	P01681,P62804
3269	1,3,4,5,25,<br>26,28,29	2,6,7,8,27,<br>30,31,32	0	
3270	1,3,4,5,25,<br>26,28,30	2,6,7,8,27,<br>29,31,32	1	P80299
3271	1,3,4,5,25,<br>26,28,31	2,6,7,8,27,<br>29,30,32	2	P29315,P62804
3272	1,3,4,5,25,<br>26,28,32	2,6,7,8,27,<br>29,30,31	2	P01681,P62804
3273	1,3,4,5,25,<br>26,29,30	2,6,7,8,27,<br>28,31,32	3	Q6IFW6,Q6IG02,Q9QZK9
3274	1,3,4,5,25,<br>26,29,31	2,6,7,8,27,<br>28,30,32	1	P29315
3275	1,3,4,5,25,<br>26,29,32	2,6,7,8,27,<br>28,30,31	1	P01681
3276	1,3,4,5,25,<br>26,30,31	2,6,7,8,27,<br>28,29,32	8	P25809,P29315,P47727,P62804,Q00715,Q63617,Q6AYR9,Q812E4
3277	1,3,4,5,25,<br>26,30,32	2,6,7,8,27,<br>28,29,31	5	P01681,P25809,P62804,Q00715,Q06000
3278	1,3,4,5,25,<br>26,31,32	2,6,7,8,27,<br>28,29,30	6	P01681,P25809,P29315,P62804,Q00715,Q4G075
3279	1,3,4,5,25,<br>27,28,29	2,6,7,8,26,<br>30,31,32	2	O89117,P20766
3280	1,3,4,5,25,<br>27,28,30	2,6,7,8,26,<br>29,31,32	3	Q6IFW6,Q6IG02,Q6P6Q2
3281	1,3,4,5,25,<br>27,28,31	2,6,7,8,26,<br>29,30,32	3	P23739,P29315,Q68G31
3282	1,3,4,5,25,<br>27,28,32	2,6,7,8,26,<br>29,30,31	2	P01681,Q62761;Q62762;Q62763
3283	1,3,4,5,25,<br>27,29,30	2,6,7,8,26,<br>28,31,32	5	O89117,Q4FZU2,Q6IFW6,Q6IG02,Q6P6Q2
3284	1,3,4,5,25,<br>27,29,31	2,6,7,8,26,<br>28,30,32	3	O89117,P29315,Q68G31
3285	1,3,4,5,25,<br>27,29,32	2,6,7,8,26,<br>28,30,31	1	P01681

3286	1,3,4,5,25,27,30,31	2,6,7,8,26,28,29,32	8	P23739,P29315,P62804,Q4FZU2,Q6IFW6,Q6IG02,Q6P6Q2,Q9QYP1
3287	1,3,4,5,25,27,30,32	2,6,7,8,26,28,29,31	9	P01681,P62804,Q4FZU2,Q6IFW6,Q6IG02,Q6P6Q2,Q6P6R2,Q9QX74,Q9WTT6
3288	1,3,4,5,25,27,31,32	2,6,7,8,26,28,29,30	5	P01681,P23739,P29315,P62804,Q9QYP1
3289	1,3,4,5,25,28,29,30	2,6,7,8,26,27,31,32	3	P20766,Q6IFW6,Q6IG02
3290	1,3,4,5,25,28,29,31	2,6,7,8,26,27,30,32	1	P29315
3291	1,3,4,5,25,28,29,32	2,6,7,8,26,27,30,31	2	P01681,P20766
3292	1,3,4,5,25,28,30,31	2,6,7,8,26,27,29,32	2	P25809,P29315
3293	1,3,4,5,25,28,30,32	2,6,7,8,26,27,29,31	6	P01681,P20766,P25809,Q62975,Q9QX74,Q9WTT6
3294	1,3,4,5,25,28,31,32	2,6,7,8,26,27,29,30	5	P01681,P25809,P29315,P62804,Q811M5
3295	1,3,4,5,25,29,30,31	2,6,7,8,26,27,28,32	7	P07150,P25809,P50280,Q4FZU2,Q6IFW6,Q6IG02,Q9QWN8
3296	1,3,4,5,25,29,30,32	2,6,7,8,26,27,28,31	7	P01681,P07150,P25809,Q4FZU2,Q6IFW6,Q6IG02,Q9WTT6
3297	1,3,4,5,25,29,31,32	2,6,7,8,26,27,28,30	5	P01681,P07150,P25809,P62804,Q9WTT6
3298	1,3,4,5,25,30,31,32	2,6,7,8,26,27,28,29	12	P01681,P07150,P25809,P62804,P97584,Q00715,Q4FZU2,Q4G075,Q6P6R2,Q812E4,Q9QX74,Q9WTT6
3299	1,3,4,5,26,27,28,29	2,6,7,8,25,30,31,32	3	O54858,P47967,P97840
3300	1,3,4,5,26,27,28,30	2,6,7,8,25,29,31,32	2	P21588,P97840
3301	1,3,4,5,26,27,28,31	2,6,7,8,25,29,30,32	1	Q68G31
3302	1,3,4,5,26,27,28,32	2,6,7,8,25,29,30,31	3	P62804,P97840,Q62761;Q62762;Q62763
3303	1,3,4,5,26,27,29,30	2,6,7,8,25,28,31,32	5	P21588,P97840,Q6IFW6,Q6IG02,Q6P6Q2
3304	1,3,4,5,26,27,29,31	2,6,7,8,25,28,30,32	2	P02631,Q68G31
3305	1,3,4,5,26,27,29,32	2,6,7,8,25,28,30,31	1	P12020

3306	1,3,4,5,26, 27,30,31	2,6,7,8,25, 28,29,32	3	P02631,P62804,Q6IG02
3307	1,3,4,5,26, 27,30,32	2,6,7,8,25, 28,29,31	2	P12020,P62804
3308	1,3,4,5,26, 27,31,32	2,6,7,8,25, 28,29,30	3	P02631,P23739,P62804
3309	1,3,4,5,26, 28,29,30	2,6,7,8,25, 27,31,32	3	P20766,P97840,Q6IG02
3310	1,3,4,5,26, 28,29,31	2,6,7,8,25, 27,30,32	2	P97840,Q68G31
3311	1,3,4,5,26, 28,29,32	2,6,7,8,25, 27,30,31	1	P97840
3312	1,3,4,5,26, 28,30,31	2,6,7,8,25, 27,29,32	1	P25809
3313	1,3,4,5,26, 28,30,32	2,6,7,8,25, 27,29,31	3	P50115,P97840,Q4G075
3314	1,3,4,5,26, 28,31,32	2,6,7,8,25, 27,29,30	3	O54728,P62804,Q4G075
3315	1,3,4,5,26, 29,30,31	2,6,7,8,25, 27,28,32	3	P02631,Q6IG02,Q9QWN8
3316	1,3,4,5,26, 29,30,32	2,6,7,8,25, 27,28,31	2	P50115,Q6IG02
3317	1,3,4,5,26, 29,31,32	2,6,7,8,25, 27,28,30	2	P02631,P62804
3318	1,3,4,5,26, 30,31,32	2,6,7,8,25, 27,28,29	10	P02631,P25809,P47727,P50115,P62804,P63081,Q00715,Q4G075,Q812E4,Q9WTT6
3319	1,3,4,5,27, 28,29,30	2,6,7,8,25, 26,31,32	6	P20766,P47967,P97840,Q6IFW6,Q6IG02,Q6P6Q2
3320	1,3,4,5,27, 28,29,31	2,6,7,8,25, 26,30,32	2	P97840,Q68G31
3321	1,3,4,5,27, 28,29,32	2,6,7,8,25, 26,30,31	5	O54858,P20766,P47967,P97840,Q62761;Q62762;Q62763
3322	1,3,4,5,27, 28,30,31	2,6,7,8,25, 26,29,32	2	P23739,Q6IG02
3323	1,3,4,5,27, 28,30,32	2,6,7,8,25, 26,29,31	3	P97840,Q8VD89,Q9QX74
3324	1,3,4,5,27, 28,31,32	2,6,7,8,25, 26,29,30	3	P23739,P62804,Q62761;Q62762;Q62763
3325	1,3,4,5,27, 29,30,31	2,6,7,8,25, 26,28,32	7	P02631,P08649,Q4FZU2,Q6IFW6,Q6IG02,Q6IMF3,Q6P6Q2

3326	1,3,4,5,27,29,30,32	2,6,7,8,25,26,28,31	6	P08649,Q4FZU2,Q63598,Q6IFW6,Q6IG02,Q6P6Q2
3327	1,3,4,5,27,29,31,32	2,6,7,8,25,26,28,30	3	P02631,P08649,P62804
3328	1,3,4,5,27,30,31,32	2,6,7,8,25,26,28,29	9	P08649,P23739,P62804,Q4FZU2,Q6IG02,Q6P6Q2,Q6P6R2,Q9QX74,Q9WTT6
3329	1,3,4,5,28,29,30,31	2,6,7,8,25,26,27,32	2	P50115,Q6IG02
3330	1,3,4,5,28,29,30,32	2,6,7,8,25,26,27,31	5	P20766,P50115,P97840,Q62894,Q6IG02
3331	1,3,4,5,28,29,31,32	2,6,7,8,25,26,27,30	1	P50115
3332	1,3,4,5,28,30,31,32	2,6,7,8,25,26,27,29	7	P25809,P50115,P62804,Q00715,Q4G075,Q9QX74,Q9WTT6
3333	1,3,4,5,29,30,31,32	2,6,7,8,25,26,27,28	9	P07150,P08649,P25809,P26342,P36860,P50115,P63081,Q6IG02,Q9WTT6
3334	1,3,4,6,7,8,25,26	2,5,27,28,29,30,31,32	5	O70417,P02783,P06911,P49134,Q99041
3335	1,3,4,6,7,8,25,27	2,5,26,28,29,30,31,32	7	O70417,P02783,P06911,P22273,P22282,P50115,Q99041
3336	1,3,4,6,7,8,25,28	2,5,26,27,29,30,31,32	10	O70417,P06911,P11598,P22273,P22282,P36860,Q63617,Q99041,Q9JI85,Q9QW07
3337	1,3,4,6,7,8,25,29	2,5,26,27,28,30,31,32	5	O70417,P02783,P06911,P48032,P49134
3338	1,3,4,6,7,8,25,30	2,5,26,27,28,29,31,32	6	O70417,P06911,P49134,Q6IFW6,Q6IG02,Q99041
3339	1,3,4,6,7,8,25,31	2,5,26,27,28,29,30,32	7	O70417,P01681,P06911,P12020,P49134,Q09030,Q63532
3340	1,3,4,6,7,8,25,32	2,5,26,27,28,29,30,31	4	O70417,P01681,P06911,Q99041
3341	1,3,4,6,7,8,26,27	2,5,25,28,29,30,31,32	11	O70417,P02783,P06911,P11598,P15399,P22273,P22282,P97580,Q30KJ2,Q62894,Q99041

3342	1,3,4,6,7,8,26,28	2,5,25,27,29,30,31,32	14	D3ZUC6,O70417,P02783,P06761,P06911,P11598,P21674,P22273,P22282,P49134,Q63617,Q99041,Q9JI85,Q9QW07
3343	1,3,4,6,7,8,26,29	2,5,25,27,28,30,31,32	9	P02783,P06911,P11598,P21674,P22273,P49134,P70549,Q99041,Q9QX74
3344	1,3,4,6,7,8,26,30	2,5,25,27,28,29,31,32	4	O70417,P02783,P49134,Q99041
3345	1,3,4,6,7,8,26,31	2,5,25,27,28,29,30,32	7	P02783,P06911,P49134,P57113,Q05702,Q63532,Q99041
3346	1,3,4,6,7,8,26,32	2,5,25,27,28,29,30,31	5	O70417,P02783,P11598,P57113,Q99041
3347	1,3,4,6,7,8,27,28	2,5,25,26,29,30,31,32	17	D3ZUC6,O70417,P02783,P06761,P06911,P11598,P19218,P22273,P22282,P22283,Q5M8C6,Q62902,Q63617,Q68FX1,Q99041,Q9JI85,Q9QW07
3348	1,3,4,6,7,8,27,29	2,5,25,26,28,30,31,32	11	O70417,P02783,P06761,P06911,P09656,P11598,P22273,P22282,Q4G075,Q63617,Q99041
3349	1,3,4,6,7,8,27,30	2,5,25,26,28,29,31,32	12	O70417,P02783,P06911,P19218,P22273,P22282,P97580,Q6IFU7,Q6IFW6,Q6IG02,Q6P6Q2,Q99041
3350	1,3,4,6,7,8,27,31	2,5,25,26,28,29,30,32	9	O70417,P02783,P06911,P11598,P22273,P22282,Q62894,Q99041,Q9QZK9
3351	1,3,4,6,7,8,27,32	2,5,25,26,28,29,30,31	9	O70417,P00714,P02783,P06911,P11598,P22273,P22282,P98166,Q99041
3352	1,3,4,6,7,8,28,29	2,5,25,26,27,30,31,32	17	D3ZUC6,P02783,P06761,P06911,P11598,P21674,P22273,P22282,P22283,P49134,P97840,Q5M8C6,Q63493,Q63617,Q99041,Q9JI85,Q9QW07
3353	1,3,4,6,7,8,28,30	2,5,25,26,27,29,31,32	9	O70417,P06911,P11598,P19218,P21674,P49134,Q63617,Q99041,Q9QW07
3354	1,3,4,6,7,8,28,31	2,5,25,26,27,29,30,32	10	D3ZUC6,P06911,P11598,P21674,Q63532,Q63617,Q811M5,Q99041,Q9JI85,Q9QW07

3355	1,3,4,6,7,8,28,32	2,5,25,26,27,29,30,31	11	O70417,P06911,P08723,P11598,P22282,Q5M8C6,Q62902,Q63617,Q99041,Q9JI85,Q9QW07
3356	1,3,4,6,7,8,29,30	2,5,25,26,27,28,31,32	10	O70417,P02783,P06911,P19218,P21674,P49134,Q5RJR2,Q6IFW6,Q6IG02,Q99041
3357	1,3,4,6,7,8,29,31	2,5,25,26,27,28,30,32	5	P02783,P06911,P21674,P49134,Q99041
3358	1,3,4,6,7,8,29,32	2,5,25,26,27,28,30,31	4	P00714,P02783,P11598,Q99041
3359	1,3,4,6,7,8,30,31	2,5,25,26,27,28,29,32	8	O70417,P02783,P06911,P49134,Q5RJR2,Q63532,Q6IFW6,Q99041
3360	1,3,4,6,7,8,30,32	2,5,25,26,27,28,29,31	5	O70417,P00714,P02783,P35280,Q99041
3361	1,3,4,6,7,8,31,32	2,5,25,26,27,28,29,30	7	P01681,P02783,P06911,P57113,Q63532,Q99041,Q9QZK9
3362	1,3,4,6,7,25,26,27	2,5,8,28,29,30,31,32	2	P02783,P63081
3363	1,3,4,6,7,25,26,28	2,5,8,27,29,30,31,32	1	Q62975
3364	1,3,4,6,7,25,26,29	2,5,8,27,28,30,31,32	2	P02783,P70549
3365	1,3,4,6,7,25,26,30	2,5,8,27,28,29,31,32	1	P25809
3366	1,3,4,6,7,25,26,31	2,5,8,27,28,29,30,32	3	P25809,P29315,P57113
3367	1,3,4,6,7,25,26,32	2,5,8,27,28,29,30,31	2	P25809,Q06000
3368	1,3,4,6,7,25,27,28	2,5,8,26,29,30,31,32	3	P11598,P63081,Q63617
3369	1,3,4,6,7,25,27,29	2,5,8,26,28,30,31,32	2	P02783,P09656
3370	1,3,4,6,7,25,27,30	2,5,8,26,28,29,31,32	1	O70417
3371	1,3,4,6,7,25,27,31	2,5,8,26,28,29,30,32	3	P29315,Q62894,Q9QYP1

3372	1,3,4,6,7,25,27,32	2,5,8,26,28,29,30,31	0	
3373	1,3,4,6,7,25,28,29	2,5,8,26,27,30,31,32	2	Q63617,Q9QW07
3374	1,3,4,6,7,25,28,30	2,5,8,26,27,29,31,32	3	O70417,P25809,Q62975
3375	1,3,4,6,7,25,28,31	2,5,8,26,27,29,30,32	2	P25809,P29315
3376	1,3,4,6,7,25,28,32	2,5,8,26,27,29,30,31	3	P11598,Q5PQL7,Q62975
3377	1,3,4,6,7,25,29,30	2,5,8,26,27,28,31,32	3	P02783,Q6IG02,Q9QWN8
3378	1,3,4,6,7,25,29,31	2,5,8,26,27,28,30,32	2	P25809,Q9QWN8
3379	1,3,4,6,7,25,29,32	2,5,8,26,27,28,30,31	0	
3380	1,3,4,6,7,25,30,31	2,5,8,26,27,28,29,32	2	P25809,Q9QWN8
3381	1,3,4,6,7,25,30,32	2,5,8,26,27,28,29,31	5	P25809,P26342,Q5PQL7,Q62975,Q6P6R2
3382	1,3,4,6,7,25,31,32	2,5,8,26,27,28,29,30	2	P01681,P25809
3383	1,3,4,6,7,26,27,28	2,5,8,25,29,30,31,32	6	P02783,P09656,P11598,P21588,P97840,Q63617
3384	1,3,4,6,7,26,27,29	2,5,8,25,28,30,31,32	4	P02783,P09656,P21588,P70549
3385	1,3,4,6,7,26,27,30	2,5,8,25,28,29,31,32	2	P02783,P21588
3386	1,3,4,6,7,26,27,31	2,5,8,25,28,29,30,32	2	P02783,Q62894
3387	1,3,4,6,7,26,27,32	2,5,8,25,28,29,30,31	2	P02783,P11598
3388	1,3,4,6,7,26,28,29	2,5,8,25,27,30,31,32	7	P02783,P11598,P21674,P47967,P70549,P97840,Q63617
3389	1,3,4,6,7,26,28,30	2,5,8,25,27,29,31,32	1	P97840
3390	1,3,4,6,7,26,28,31	2,5,8,25,27,29,30,32	2	P11598,P57113
3391	1,3,4,6,7,26,28,32	2,5,8,25,27,29,30,31	1	P11598

3392	1,3,4,6,7,26,29,30	2,5,8,25,27,28,31,32	1	P02783
3393	1,3,4,6,7,26,29,31	2,5,8,25,27,28,30,32	2	P02783,P70549
3394	1,3,4,6,7,26,29,32	2,5,8,25,27,28,30,31	1	P02783
3395	1,3,4,6,7,26,30,31	2,5,8,25,27,28,29,32	3	P02783,P25809,P57113
3396	1,3,4,6,7,26,30,32	2,5,8,25,27,28,29,31	2	P02783,P25809
3397	1,3,4,6,7,26,31,32	2,5,8,25,27,28,29,30	4	P02783,P22006,P25809,P57113
3398	1,3,4,6,7,27,28,29	2,5,8,25,26,30,31,32	8	P00507,P02783,P09656,P11598,P22283,P47967,P97840,Q63617
3399	1,3,4,6,7,27,28,30	2,5,8,25,26,29,31,32	3	P11598,P19218,P97840
3400	1,3,4,6,7,27,28,31	2,5,8,25,26,29,30,32	3	P11598,Q63617,Q68G31
3401	1,3,4,6,7,27,28,32	2,5,8,25,26,29,30,31	4	P11598,P22283,Q62761;Q62762;Q62763,Q63617
3402	1,3,4,6,7,27,29,30	2,5,8,25,26,28,31,32	3	P02783,P09656,Q6IG02
3403	1,3,4,6,7,27,29,31	2,5,8,25,26,28,30,32	2	P02783,Q68G31
3404	1,3,4,6,7,27,29,32	2,5,8,25,26,28,30,31	4	P00714,P02783,P09656,P11598
3405	1,3,4,6,7,27,30,31	2,5,8,25,26,28,29,32	1	P02783
3406	1,3,4,6,7,27,30,32	2,5,8,25,26,28,29,31	3	P00714,P02783,Q8VD89
3407	1,3,4,6,7,27,31,32	2,5,8,25,26,28,29,30	4	P00714,P02783,P22006,P80299
3408	1,3,4,6,7,28,29,30	2,5,8,25,26,27,31,32	4	P02783,P21674,P97840,Q63617
3409	1,3,4,6,7,28,29,31	2,5,8,25,26,27,30,32	4	P11598,P21674,P70549,Q63617
3410	1,3,4,6,7,28,29,32	2,5,8,25,26,27,30,31	3	P11598,P97840,Q63617
3411	1,3,4,6,7,28,30,31	2,5,8,25,26,27,29,32	1	P25809

3412	1,3,4,6,7,28,30,32	2,5,8,25,26,27,29,31	2	Q62975,Q8VD89
3413	1,3,4,6,7,28,31,32	2,5,8,25,26,27,29,30	3	P11598,P22006,P25809
3414	1,3,4,6,7,29,30,31	2,5,8,25,26,27,28,32	4	P02783,P25809,P26342,Q9QWN8
3415	1,3,4,6,7,29,30,32	2,5,8,25,26,27,28,31	3	P00714,P02783,P26342
3416	1,3,4,6,7,29,31,32	2,5,8,25,26,27,28,30	4	P00714,P02783,P26342,P80299
3417	1,3,4,6,7,30,31,32	2,5,8,25,26,27,28,29	6	P02783,P06760,P22006,P25809,P26342,Q6P6R2
3418	1,3,4,6,8,25,26,27	2,5,7,28,29,30,31,32	8	O70417,P02783,P08649,P15399,P54921,P97580,Q06000,Q30KJ2
3419	1,3,4,6,8,25,26,28	2,5,7,27,29,30,31,32	6	O70417,P06911,P08649,P80299,Q06000,Q9QW07
3420	1,3,4,6,8,25,26,29	2,5,7,27,28,30,31,32	2	P02783,P70549
3421	1,3,4,6,8,25,26,30	2,5,7,27,28,29,31,32	7	O70417,O88797,P54921,P80299,Q06000,Q6AY61,Q6IFW6
3422	1,3,4,6,8,25,26,31	2,5,7,27,28,29,30,32	3	P29315,P54921,Q05702
3423	1,3,4,6,8,25,26,32	2,5,7,27,28,29,30,31	4	P01681,P08649,P13432,Q06000
3424	1,3,4,6,8,25,27,28	2,5,7,26,29,30,31,32	6	O70417,P06911,P15399,P36860,Q9JI85,Q9QW07
3425	1,3,4,6,8,25,27,29	2,5,7,26,28,30,31,32	2	P02783,P09656
3426	1,3,4,6,8,25,27,30	2,5,7,26,28,29,31,32	9	O70417,P15399,P54921,P97580,Q30KJ2,Q6IFU7,Q6IFW6,Q6IG02,Q6P6Q2
3427	1,3,4,6,8,25,27,31	2,5,7,26,28,29,30,32	5	P01681,P06911,P29315,P54921,Q9R168
3428	1,3,4,6,8,25,27,32	2,5,7,26,28,29,30,31	4	O70417,P00762,P01681,P98166
3429	1,3,4,6,8,25,28,29	2,5,7,26,27,30,31,32	2	P06911,Q9QW07
3430	1,3,4,6,8,25,28,30	2,5,7,26,27,29,31,32	3	O70417,P06911,Q9QW07
3431	1,3,4,6,8,25,28,31	2,5,7,26,27,29,30,32	2	P06911,P29315

3432	1,3,4,6,8,25,28,32	2,5,7,26,27,29,30,31	3	P01681,Q9JI85,Q9QW07
3433	1,3,4,6,8,25,29,30	2,5,7,26,27,28,31,32	6	O70417,O88797,P02783,Q6IFU7,Q6IFW6,Q6IG02
3434	1,3,4,6,8,25,29,31	2,5,7,26,27,28,30,32	4	P02783,P06911,P29315,P70549
3435	1,3,4,6,8,25,29,32	2,5,7,26,27,28,30,31	3	P01681,P02783,P13432
3436	1,3,4,6,8,25,30,31	2,5,7,26,27,28,29,32	7	O70417,P02625,P54921,Q09030,Q6AY61,Q6IFW6,Q6IG02
3437	1,3,4,6,8,25,30,32	2,5,7,26,27,28,29,31	5	O70417,O88797,P01681,P02625,Q6IFW6
3438	1,3,4,6,8,25,31,32	2,5,7,26,27,28,29,30	2	P01681,P29315
3439	1,3,4,6,8,26,27,28	2,5,7,25,29,30,31,32	8	P02783,P09656,P15399,P22282,P97580,Q30KJ2,Q9JI85,Q9QW07
3440	1,3,4,6,8,26,27,29	2,5,7,25,28,30,31,32	6	P02783,P09656,P15399,P70549,P97580,Q30KJ2
3441	1,3,4,6,8,26,27,30	2,5,7,25,28,29,31,32	8	O70417,P02783,P15399,P97580,Q30KJ2,Q6AY61,Q6IFW6,Q6IG02
3442	1,3,4,6,8,26,27,31	2,5,7,25,28,29,30,32	6	P02783,P15399,P54921,P97580,Q30KJ2,Q9R168
3443	1,3,4,6,8,26,27,32	2,5,7,25,28,29,30,31	4	P02783,P15399,P97580,Q30KJ2
3444	1,3,4,6,8,26,28,29	2,5,7,25,27,30,31,32	6	P02783,P09656,P21674,P70549,Q9JI85,Q9QW07
3445	1,3,4,6,8,26,28,30	2,5,7,25,27,29,31,32	1	O70417
3446	1,3,4,6,8,26,28,31	2,5,7,25,27,29,30,32	1	P70549
3447	1,3,4,6,8,26,28,32	2,5,7,25,27,29,30,31	3	O54728,Q9JI85,Q9QW07
3448	1,3,4,6,8,26,29,30	2,5,7,25,27,28,31,32	3	O88797,P02783,Q6IG02
3449	1,3,4,6,8,26,29,31	2,5,7,25,27,28,30,32	2	P02783,P70549
3450	1,3,4,6,8,26,29,32	2,5,7,25,27,28,30,31	1	P02783
3451	1,3,4,6,8,26,30,31	2,5,7,25,27,28,29,32	4	P02783,P54921,Q5RJR2,Q6AY61

3452	1,3,4,6,8,26,30,32	2,5,7,25,27,28,29,31	2	O88797,P02783
3453	1,3,4,6,8,26,31,32	2,5,7,25,27,28,29,30	3	O54728,P02783,Q05702
3454	1,3,4,6,8,27,28,29	2,5,7,25,26,30,31,32	6	P02783,P09656,P22282,Q812E4,Q9JI85,Q9QW07
3455	1,3,4,6,8,27,28,30	2,5,7,25,26,29,31,32	8	O70417,P15399,P19218,Q10758,Q6IFU7,Q6IG02,Q6P6S4,Q8VD89
3456	1,3,4,6,8,27,28,31	2,5,7,25,26,29,30,32	1	Q9R168
3457	1,3,4,6,8,27,28,32	2,5,7,25,26,29,30,31	7	P15399,P22282,Q62902,Q6AYR9,Q6P6S4,Q9JI85,Q9QW07
3458	1,3,4,6,8,27,29,30	2,5,7,25,26,28,31,32	10	O70417,P02783,P09656,P19218,Q10758,Q6IFU7,Q6IFW6,Q6IG02,Q6IMF3,Q6P6Q2
3459	1,3,4,6,8,27,29,31	2,5,7,25,26,28,30,32	2	P02783,Q9R168
3460	1,3,4,6,8,27,29,32	2,5,7,25,26,28,30,31	3	P02783,P09656,P98166
3461	1,3,4,6,8,27,30,31	2,5,7,25,26,28,29,32	7	O70417,P02783,P19218,P54921,Q6IFW6,Q6IG02,Q6P6Q2
3462	1,3,4,6,8,27,30,32	2,5,7,25,26,28,29,31	8	O70417,P02783,P15399,P19218,Q6IFU7,Q6IFW6,Q6IG02,Q8VD89
3463	1,3,4,6,8,27,31,32	2,5,7,25,26,28,29,30	4	P01681,P02783,P22006,Q9QZK9
3464	1,3,4,6,8,28,29,30	2,5,7,25,26,27,31,32	5	P02783,P21674,Q6IFU7,Q6IG02,Q9QW07
3465	1,3,4,6,8,28,29,31	2,5,7,25,26,27,30,32	3	P21674,P70549,Q9R168
3466	1,3,4,6,8,28,29,32	2,5,7,25,26,27,30,31	3	P02783,Q9JI85,Q9QW07
3467	1,3,4,6,8,28,30,31	2,5,7,25,26,27,29,32	1	Q63532
3468	1,3,4,6,8,28,30,32	2,5,7,25,26,27,29,31	2	O70417,Q8VD89
3469	1,3,4,6,8,28,31,32	2,5,7,25,26,27,29,30	3	P22006,Q63532,Q9JI85
3470	1,3,4,6,8,29,30,31	2,5,7,25,26,27,28,32	4	P02783,Q5RJR2,Q6IFW6,Q6IG02
3471	1,3,4,6,8,29,30,32	2,5,7,25,26,27,28,31	5	O88797,P02783,Q6IFU7,Q6IFW6,Q6IG02

3472	1,3,4,6,8,29,31,32	2,5,7,25,26,27,28,30	1	P02783
3473	1,3,4,6,8,30,31,32	2,5,7,25,26,27,28,29	4	P02625,P02783,P20646,P22006
3474	1,3,4,6,25,26,27,28	2,5,7,8,29,30,31,32	2	P09656,Q06000
3475	1,3,4,6,25,26,27,29	2,5,7,8,28,30,31,32	3	P02783,P09656,P70549
3476	1,3,4,6,25,26,27,30	2,5,7,8,28,29,31,32	2	P21588,Q06000
3477	1,3,4,6,25,26,27,31	2,5,7,8,28,29,30,32	4	P29315,P54921,P62804,Q9QYP1
3478	1,3,4,6,25,26,27,32	2,5,7,8,28,29,30,31	3	P06760,P62804,Q06000
3479	1,3,4,6,25,26,28,29	2,5,7,8,27,30,31,32	2	P09656,P70549
3480	1,3,4,6,25,26,28,30	2,5,7,8,27,29,31,32	4	P06760,P25809,Q06000,Q62975
3481	1,3,4,6,25,26,28,31	2,5,7,8,27,29,30,32	4	P25809,P29315,P62804,P70549
3482	1,3,4,6,25,26,28,32	2,5,7,8,27,29,30,31	5	P00731,P06760,P62804,Q06000,Q62975
3483	1,3,4,6,25,26,29,30	2,5,7,8,27,28,31,32	1	P02783
3484	1,3,4,6,25,26,29,31	2,5,7,8,27,28,30,32	4	P02783,P25809,P29315,P70549
3485	1,3,4,6,25,26,29,32	2,5,7,8,27,28,30,31	1	P02783
3486	1,3,4,6,25,26,30,31	2,5,7,8,27,28,29,32	8	P06760,P25809,P29315,P54921,P62804,Q00715,Q4G075,Q812E4
3487	1,3,4,6,25,26,30,32	2,5,7,8,27,28,29,31	7	P06760,P25809,P62804,Q00715,Q06000,Q4G075,Q5PQL7
3488	1,3,4,6,25,26,31,32	2,5,7,8,27,28,29,30	6	P06760,P25809,P29315,P62804,Q00715,Q4G075
3489	1,3,4,6,25,27,28,29	2,5,7,8,26,30,31,32	1	P09656
3490	1,3,4,6,25,27,28,30	2,5,7,8,26,29,31,32	1	P06760
3491	1,3,4,6,25,27,28,31	2,5,7,8,26,29,30,32	1	P29315

3492	1,3,4,6,25,<br>27,28,32	2,5,7,8,26,<br>29,30,31	3	P06760,Q62761;Q62762;Q62763,Q9QX74
3493	1,3,4,6,25,<br>27,29,30	2,5,7,8,26,<br>28,31,32	3	P02783,P09656,Q6IG02
3494	1,3,4,6,25,<br>27,29,31	2,5,7,8,26,<br>28,30,32	2	P02783,P29315
3495	1,3,4,6,25,<br>27,29,32	2,5,7,8,26,<br>28,30,31	2	P02783,P09656
3496	1,3,4,6,25,<br>27,30,31	2,5,7,8,26,<br>28,29,32	5	P06760,P29315,P54921,P62804,Q9QYP1
3497	1,3,4,6,25,<br>27,30,32	2,5,7,8,26,<br>28,29,31	5	P06760,P62804,Q6P6R2,Q9QX74,Q9WTT6
3498	1,3,4,6,25,<br>27,31,32	2,5,7,8,26,<br>28,29,30	7	P01681,P06760,P29315,P62804,Q9QX74,Q9QYP1,Q9WTT6
3499	1,3,4,6,25,<br>28,29,30	2,5,7,8,26,<br>27,31,32	0	
3500	1,3,4,6,25,<br>28,29,31	2,5,7,8,26,<br>27,30,32	2	P29315,P70549
3501	1,3,4,6,25,<br>28,29,32	2,5,7,8,26,<br>27,30,31	0	
3502	1,3,4,6,25,<br>28,30,31	2,5,7,8,26,<br>27,29,32	5	P06760,P25809,P29315,Q62975,Q9JJ50
3503	1,3,4,6,25,<br>28,30,32	2,5,7,8,26,<br>27,29,31	7	P06760,P25809,Q4G075,Q5PQL7,Q62975,Q9QX74,Q9WTT6
3504	1,3,4,6,25,<br>28,31,32	2,5,7,8,26,<br>27,29,30	9	P06760,P22006,P25809,P29315,P62804,Q4G075,Q62975,Q9QX74,Q9WTT6
3505	1,3,4,6,25,<br>29,30,31	2,5,7,8,26,<br>27,28,32	5	P07150,P25809,Q6IG02,Q9QWN8,Q9WTT6
3506	1,3,4,6,25,<br>29,30,32	2,5,7,8,26,<br>27,28,31	3	P07150,P25809,Q9WTT6
3507	1,3,4,6,25,<br>29,31,32	2,5,7,8,26,<br>27,28,30	4	P07150,P25809,P62804,Q9WTT6
3508	1,3,4,6,25,<br>30,31,32	2,5,7,8,26,<br>27,28,29	11	P01681,P06760,P25809,P47967,P62804,P97584,Q00715,Q4G075,Q6P6R2,Q9QX74,Q9WTT6
3509	1,3,4,6,26,<br>27,28,29	2,5,7,8,25,<br>30,31,32	3	P02783,P09656,P70549
3510	1,3,4,6,26,<br>27,28,30	2,5,7,8,25,<br>29,31,32	3	P09656,P21588,Q8VD89
3511	1,3,4,6,26,<br>27,28,31	2,5,7,8,25,<br>29,30,32	1	P62804

3512	1,3,4,6,26,<br>27,28,32	2,5,7,8,25,<br>29,30,31	2	P09656,P62804
3513	1,3,4,6,26,<br>27,29,30	2,5,7,8,25,<br>28,31,32	5	P02783,P09656,P21588,Q6IG02,Q811M5
3514	1,3,4,6,26,<br>27,29,31	2,5,7,8,25,<br>28,30,32	2	P02783,P70549
3515	1,3,4,6,26,<br>27,29,32	2,5,7,8,25,<br>28,30,31	2	P02783,P09656
3516	1,3,4,6,26,<br>27,30,31	2,5,7,8,25,<br>28,29,32	4	P02783,P06760,P54921,P62804
3517	1,3,4,6,26,<br>27,30,32	2,5,7,8,25,<br>28,29,31	4	P02783,P06760,P62804,Q8VD89
3518	1,3,4,6,26,<br>27,31,32	2,5,7,8,25,<br>28,29,30	4	P02783,P06760,P22006,P62804
3519	1,3,4,6,26,<br>28,29,30	2,5,7,8,25,<br>27,31,32	2	P02783,P09656
3520	1,3,4,6,26,<br>28,29,31	2,5,7,8,25,<br>27,30,32	2	P02783,P70549
3521	1,3,4,6,26,<br>28,29,32	2,5,7,8,25,<br>27,30,31	2	P02783,P09656
3522	1,3,4,6,26,<br>28,30,31	2,5,7,8,25,<br>27,29,32	3	P06760,P25809,Q4G075
3523	1,3,4,6,26,<br>28,30,32	2,5,7,8,25,<br>27,29,31	4	P06760,P62804,Q4G075,Q8VD89
3524	1,3,4,6,26,<br>28,31,32	2,5,7,8,25,<br>27,29,30	7	O54728,P00731,P06760,P22006,P25809,P62804,Q4G075
3525	1,3,4,6,26,<br>29,30,31	2,5,7,8,25,<br>27,28,32	2	P02783,P25809
3526	1,3,4,6,26,<br>29,30,32	2,5,7,8,25,<br>27,28,31	1	P02783
3527	1,3,4,6,26,<br>29,31,32	2,5,7,8,25,<br>27,28,30	3	P02783,P62804,P70549
3528	1,3,4,6,26,<br>30,31,32	2,5,7,8,25,<br>27,28,29	9	P02783,P06760,P22006,P25809,P62804,Q00715,Q4G075,Q8VD89,Q9WTT6
3529	1,3,4,6,27,<br>28,29,30	2,5,7,8,25,<br>26,31,32	3	P02783,P09656,Q6IG02
3530	1,3,4,6,27,<br>28,29,31	2,5,7,8,25,<br>26,30,32	1	P02783
3531	1,3,4,6,27,<br>28,29,32	2,5,7,8,25,<br>26,30,31	5	O35547,P02783,P09656,Q62761;Q62762;Q62763,Q6AYR9

3532	1,3,4,6,27,28,30,31	2,5,7,8,25,26,29,32	2	P06760,Q8VD89
3533	1,3,4,6,27,28,30,32	2,5,7,8,25,26,29,31	4	P06760,P09656,Q8VD89,Q9QX74
3534	1,3,4,6,27,28,31,32	2,5,7,8,25,26,29,30	4	P06760,P22006,P62804,Q9QX74
3535	1,3,4,6,27,29,30,31	2,5,7,8,25,26,28,32	2	P02783,Q6IG02
3536	1,3,4,6,27,29,30,32	2,5,7,8,25,26,28,31	5	P02783,P09656,Q6IG02,Q9WTT6,Q9Z0V6
3537	1,3,4,6,27,29,31,32	2,5,7,8,25,26,28,30	2	P02783,P62804
3538	1,3,4,6,27,30,31,32	2,5,7,8,25,26,28,29	9	P02783,P06760,P22006,P62804,Q6P6R2,Q8VD89,Q9QX74,Q9QYP1,Q9WTT6
3539	1,3,4,6,28,29,30,31	2,5,7,8,25,26,27,32	0	
3540	1,3,4,6,28,29,30,32	2,5,7,8,25,26,27,31	2	Q9WTT6,Q9Z0V6
3541	1,3,4,6,28,29,31,32	2,5,7,8,25,26,27,30	1	P22006
3542	1,3,4,6,28,30,31,32	2,5,7,8,25,26,27,29	8	P06760,P22006,P25809,P62804,Q4G075,Q8VD89,Q9QX74,Q9WTT6
3543	1,3,4,6,29,30,31,32	2,5,7,8,25,26,27,28	6	P02783,P07150,P25809,P26342,P36860,Q9WTT6
3544	1,3,4,7,8,25,26,27	2,5,6,28,29,30,31,32	2	O70417,P36860
3545	1,3,4,7,8,25,26,28	2,5,6,27,29,30,31,32	4	O70417,P01039,P36860,Q5I0J9
3546	1,3,4,7,8,25,26,29	2,5,6,27,28,30,31,32	5	P01039,P22006,P49134,Q5I0J9,Q9QX74
3547	1,3,4,7,8,25,26,30	2,5,6,27,28,29,31,32	4	O70417,P49134,Q05511,Q6IFW6
3548	1,3,4,7,8,25,26,31	2,5,6,27,28,29,30,32	3	P01681,P29315,Q5I0J9
3549	1,3,4,7,8,25,26,32	2,5,6,27,28,29,30,31	2	O70417,P01681
3550	1,3,4,7,8,25,27,28	2,5,6,26,29,30,31,32	4	O70417,P01681,P22282,P36860
3551	1,3,4,7,8,25,27,29	2,5,6,26,28,30,31,32	3	O70417,P10758,Q4G075

3552	1,3,4,7,8,25,27,30	2,5,6,26,28,29,31,32	8	O70417,P01681,Q05511,Q4FZU2,Q6IFW6,Q6IG02,Q6IMF3,Q6P6Q2
3553	1,3,4,7,8,25,27,31	2,5,6,26,28,29,30,32	4	O70417,P01681,P29315,Q68G31
3554	1,3,4,7,8,25,27,32	2,5,6,26,28,29,30,31	4	O70417,P00762,P01681,Q01984
3555	1,3,4,7,8,25,28,29	2,5,6,26,27,30,31,32	4	O70417,P01039,P36860,Q9QW07
3556	1,3,4,7,8,25,28,30	2,5,6,26,27,29,31,32	3	O70417,P01039,Q6IFW6
3557	1,3,4,7,8,25,28,31	2,5,6,26,27,29,30,32	8	O70417,P01039,P01681,P29315,Q5I0J9,Q811M5,Q923V8,Q9JJ50
3558	1,3,4,7,8,25,28,32	2,5,6,26,27,29,30,31	5	O70417,P01039,P01681,P36860,Q811M5
3559	1,3,4,7,8,25,29,30	2,5,6,26,27,28,31,32	7	O70417,P01039,Q4FZU2,Q6IFW6,Q6IG02,Q6IMF3,Q6P6Q2
3560	1,3,4,7,8,25,29,31	2,5,6,26,27,28,30,32	3	P01039,P01681,Q5I0J9
3561	1,3,4,7,8,25,29,32	2,5,6,26,27,28,30,31	2	P01039,P01681
3562	1,3,4,7,8,25,30,31	2,5,6,26,27,28,29,32	6	O70417,P01681,Q05511,Q4FZU2,Q6IFW6,Q6IG02
3563	1,3,4,7,8,25,30,32	2,5,6,26,27,28,29,31	7	O70417,P01681,P02625,P35280,Q05511,Q4FZU2,Q6IFW6
3564	1,3,4,7,8,25,31,32	2,5,6,26,27,28,29,30	3	P01681,P21588,Q811M5
3565	1,3,4,7,8,26,27,28	2,5,6,25,29,30,31,32	8	D3ZUC6,O70417,P07150,P22282,P36860,P47967,P97840,Q9WTT6
3566	1,3,4,7,8,26,27,29	2,5,6,25,28,30,31,32	3	Q4G075,Q9QX74,Q9WTT6
3567	1,3,4,7,8,26,27,30	2,5,6,25,28,29,31,32	4	O70417,Q6IFW6,Q6IG02,Q6P6Q2
3568	1,3,4,7,8,26,27,31	2,5,6,25,28,29,30,32	1	Q68G31
3569	1,3,4,7,8,26,27,32	2,5,6,25,28,29,30,31	0	
3570	1,3,4,7,8,26,28,29	2,5,6,25,27,30,31,32	12	D3ZUC6,P01039,P20646,P21674,P47967,P49134,P97840,Q63424,Q64093,Q6P6R2,Q9QX74,Q9WTT6
3571	1,3,4,7,8,26,28,30	2,5,6,25,27,29,31,32	3	O70417,P01039,P97840

3572	1,3,4,7,8,26,28,31	2,5,6,25,27,29,30,32	1	Q811M5
3573	1,3,4,7,8,26,28,32	2,5,6,25,27,29,30,31	2	O54728,P01039
3574	1,3,4,7,8,26,29,30	2,5,6,25,27,28,31,32	4	P01039,P49134,Q6IFW6,Q6IG02
3575	1,3,4,7,8,26,29,31	2,5,6,25,27,28,30,32	5	P49134,P70549,Q63424,Q64093,Q9QX74
3576	1,3,4,7,8,26,29,32	2,5,6,25,27,28,30,31	1	P01039
3577	1,3,4,7,8,26,30,31	2,5,6,25,27,28,29,32	2	P49134,Q6IFW6
3578	1,3,4,7,8,26,30,32	2,5,6,25,27,28,29,31	2	O70417,P35280
3579	1,3,4,7,8,26,31,32	2,5,6,25,27,28,29,30	2	O54728,P01681
3580	1,3,4,7,8,27,28,29	2,5,6,25,26,30,31,32	12	D3ZUC6,P00507,P21674,P22282,P47967,P97840,Q4G075,Q63493,Q63617,Q812E4,Q9EQS0,Q9Z2L0
3581	1,3,4,7,8,27,28,30	2,5,6,25,26,29,31,32	6	O70417,P19218,P97840,Q6IFW6,Q6IG02,Q6P6Q2
3582	1,3,4,7,8,27,28,31	2,5,6,25,26,29,30,32	2	D3ZUC6,Q68G31
3583	1,3,4,7,8,27,28,32	2,5,6,25,26,29,30,31	7	O70417,P08723,P22282,P97840,Q62902,Q6AYR9,Q812E4
3584	1,3,4,7,8,27,29,30	2,5,6,25,26,28,31,32	9	O70417,Q4FZU2,Q4G075,Q6IFU7,Q6IFU8,Q6IFW6,Q6IG02,Q6IMF3,Q6P6Q2
3585	1,3,4,7,8,27,29,31	2,5,6,25,26,28,30,32	2	Q4G075,Q68G31
3586	1,3,4,7,8,27,29,32	2,5,6,25,26,28,30,31	0	
3587	1,3,4,7,8,27,30,31	2,5,6,25,26,28,29,32	6	O70417,Q4FZU2,Q6IFW6,Q6IG02,Q6IMF3,Q6P6Q2
3588	1,3,4,7,8,27,30,32	2,5,6,25,26,28,29,31	7	O70417,P01681,P35280,Q4FZU2,Q6IFW6,Q6IG02,Q6P6Q2
3589	1,3,4,7,8,27,31,32	2,5,6,25,26,28,29,30	1	P01681
3590	1,3,4,7,8,28,29,30	2,5,6,25,26,27,31,32	6	O70417,P01039,P21674,P97840,Q6IFW6,Q6IG02
3591	1,3,4,7,8,28,29,31	2,5,6,25,26,27,30,32	6	D3ZUC6,P01039,P21674,P82471,Q63493,Q811M5

3592	1,3,4,7,8,28,29,32	2,5,6,25,26,27,30,31	8 P01039,P08723,P21674,P30120,P97840,Q63493,Q812E4,Q9Z2L0
3593	1,3,4,7,8,28,30,31	2,5,6,25,26,27,29,32	3 O70417,P82471,Q9JJ50
3594	1,3,4,7,8,28,30,32	2,5,6,25,26,27,29,31	2 O70417,P35280
3595	1,3,4,7,8,28,31,32	2,5,6,25,26,27,29,30	4 P01681,P82471,Q811M5,Q923V8
3596	1,3,4,7,8,29,30,31	2,5,6,25,26,27,28,32	7 P82471,Q4FZU2,Q6IFW6,Q6IG02,Q6IMF3,Q6P6Q2,Q9QWN8
3597	1,3,4,7,8,29,30,32	2,5,6,25,26,27,28,31	7 B0BNN3,P35280,Q4FZU2,Q6IFW6,Q6IG02,Q6IMF3,Q6P6Q2
3598	1,3,4,7,8,29,31,32	2,5,6,25,26,27,28,30	2 P01681,P82471
3599	1,3,4,7,8,30,31,32	2,5,6,25,26,27,28,29	5 P01681,P02625,P35280,P82471,Q6IFW6
3600	1,3,4,7,25,26,27,28	2,5,6,8,29,30,31,32	2 P20646,P36860
3601	1,3,4,7,25,26,27,29	2,5,6,8,28,30,31,32	2 O89117,P10758
3602	1,3,4,7,25,26,27,30	2,5,6,8,28,29,31,32	0
3603	1,3,4,7,25,26,27,31	2,5,6,8,28,29,30,32	4 P29315,Q5I0J9,Q68G31,Q9QYP1
3604	1,3,4,7,25,26,27,32	2,5,6,8,28,29,30,31	0
3605	1,3,4,7,25,26,28,29	2,5,6,8,27,30,31,32	4 P01039,P20646,P97840,Q5I0J9
3606	1,3,4,7,25,26,28,30	2,5,6,8,27,29,31,32	1 Q62975
3607	1,3,4,7,25,26,28,31	2,5,6,8,27,29,30,32	3 P29315,Q5I0J9,Q64268
3608	1,3,4,7,25,26,28,32	2,5,6,8,27,29,30,31	2 P00731,Q62975
3609	1,3,4,7,25,26,29,30	2,5,6,8,27,28,31,32	0
3610	1,3,4,7,25,26,29,31	2,5,6,8,27,28,30,32	3 P29315,P70549,Q5I0J9
3611	1,3,4,7,25,26,29,32	2,5,6,8,27,28,30,31	0

3612	1,3,4,7,25, 26,30,31	2,5,6,8,27, 28,29,32	2	P25809,P29315
3613	1,3,4,7,25, 26,30,32	2,5,6,8,27, 28,29,31	2	P25809,Q62975
3614	1,3,4,7,25, 26,31,32	2,5,6,8,27, 28,29,30	5	P01681,P25809,P29315,P51792;P51794;P51796,Q5I0J9
3615	1,3,4,7,25, 27,28,29	2,5,6,8,26, 30,31,32	5	O89117,P10758,P97840,Q99MH3,Q9Z2L0
3616	1,3,4,7,25, 27,28,30	2,5,6,8,26, 29,31,32	0	
3617	1,3,4,7,25, 27,28,31	2,5,6,8,26, 29,30,32	2	P29315,Q68G31
3618	1,3,4,7,25, 27,28,32	2,5,6,8,26, 29,30,31	3	P01681,Q5RJR2,Q62761;Q62762;Q62763
3619	1,3,4,7,25, 27,29,30	2,5,6,8,26, 28,31,32	2	Q6IFW6,Q6IG02
3620	1,3,4,7,25, 27,29,31	2,5,6,8,26, 28,30,32	2	P10758,Q68G31
3621	1,3,4,7,25, 27,29,32	2,5,6,8,26, 28,30,31	1	P10758
3622	1,3,4,7,25, 27,30,31	2,5,6,8,26, 28,29,32	2	Q6IG02,Q9QYP1
3623	1,3,4,7,25, 27,30,32	2,5,6,8,26, 28,29,31	2	P00762,P01681
3624	1,3,4,7,25, 27,31,32	2,5,6,8,26, 28,29,30	2	P01681,Q9QYP1
3625	1,3,4,7,25, 28,29,30	2,5,6,8,26, 27,31,32	1	P01039
3626	1,3,4,7,25, 28,29,31	2,5,6,8,26, 27,30,32	3	P01039,Q5I0J9,Q9JJ50
3627	1,3,4,7,25, 28,29,32	2,5,6,8,26, 27,30,31	1	P01039
3628	1,3,4,7,25, 28,30,31	2,5,6,8,26, 27,29,32	3	P25809,Q62975,Q9JJ50
3629	1,3,4,7,25, 28,30,32	2,5,6,8,26, 27,29,31	2	P25809,Q62975
3630	1,3,4,7,25, 28,31,32	2,5,6,8,26, 27,29,30	5	P01681,P25809,Q62975,Q811M5,Q9JJ50
3631	1,3,4,7,25, 29,30,31	2,5,6,8,26, 27,28,32	3	P25809,Q6IG02,Q9QWN8

3632	1,3,4,7,25,<br>29,30,32	2,5,6,8,26,<br>27,28,31	0
3633	1,3,4,7,25,<br>29,31,32	2,5,6,8,26,<br>27,28,30	1 P01681
3634	1,3,4,7,25,<br>30,31,32	2,5,6,8,26,<br>27,28,29	6 P01681,P25809,P26342,Q00715,Q6P6R2,Q9QYP1
3635	1,3,4,7,26,<br>27,28,29	2,5,6,8,25,<br>30,31,32	5 O54858,P10758,P20646,P47967,P97840
3636	1,3,4,7,26,<br>27,28,30	2,5,6,8,25,<br>29,31,32	1 P97840
3637	1,3,4,7,26,<br>27,28,31	2,5,6,8,25,<br>29,30,32	1 Q68G31
3638	1,3,4,7,26,<br>27,28,32	2,5,6,8,25,<br>29,30,31	2 P97840,Q62761;Q62762;Q62763
3639	1,3,4,7,26,<br>27,29,30	2,5,6,8,25,<br>28,31,32	2 P97840,Q6IG02
3640	1,3,4,7,26,<br>27,29,31	2,5,6,8,25,<br>28,30,32	2 P10758,Q68G31
3641	1,3,4,7,26,<br>27,29,32	2,5,6,8,25,<br>28,30,31	1 P10758
3642	1,3,4,7,26,<br>27,30,31	2,5,6,8,25,<br>28,29,32	1 Q9QYP1
3643	1,3,4,7,26,<br>27,30,32	2,5,6,8,25,<br>28,29,31	0
3644	1,3,4,7,26,<br>27,31,32	2,5,6,8,25,<br>28,29,30	0
3645	1,3,4,7,26,<br>28,29,30	2,5,6,8,25,<br>27,31,32	2 P01039,P97840
3646	1,3,4,7,26,<br>28,29,31	2,5,6,8,25,<br>27,30,32	5 P01039,P70549,P97840,Q63424,Q68G31
3647	1,3,4,7,26,<br>28,29,32	2,5,6,8,25,<br>27,30,31	2 P01039,P97840
3648	1,3,4,7,26,<br>28,30,31	2,5,6,8,25,<br>27,29,32	0
3649	1,3,4,7,26,<br>28,30,32	2,5,6,8,25,<br>27,29,31	1 P97840
3650	1,3,4,7,26,<br>28,31,32	2,5,6,8,25,<br>27,29,30	1 O54728
3651	1,3,4,7,26,<br>29,30,31	2,5,6,8,25,<br>27,28,32	1 Q9QWN8

3652	1,3,4,7,26, 29,30,32	2,5,6,8,25, 27,28,31	0	
3653	1,3,4,7,26, 29,31,32	2,5,6,8,25, 27,28,30	0	
3654	1,3,4,7,26, 30,31,32	2,5,6,8,25, 27,28,29	2	P25809,Q00715
3655	1,3,4,7,27, 28,29,30	2,5,6,8,25, 26,31,32	5	O54858,P00507,P47967,P97840,Q6IG02
3656	1,3,4,7,27, 28,29,31	2,5,6,8,25, 26,30,32	5	P00507,P10758,P97840,Q68G31,Q99MH3
3657	1,3,4,7,27, 28,29,32	2,5,6,8,25, 26,30,31	9	O54858,P10758,P47967,P97840,Q62761;Q62762;Q62763,Q6AYR9,Q812E4,Q8CGS4,Q9Z2L0
3658	1,3,4,7,27, 28,30,31	2,5,6,8,25, 26,29,32	0	
3659	1,3,4,7,27, 28,30,32	2,5,6,8,25, 26,29,31	1	P97840
3660	1,3,4,7,27, 28,31,32	2,5,6,8,25, 26,29,30	1	Q62761;Q62762;Q62763
3661	1,3,4,7,27, 29,30,31	2,5,6,8,25, 26,28,32	2	P08649,Q6IG02
3662	1,3,4,7,27, 29,30,32	2,5,6,8,25, 26,28,31	2	B0BNN3,Q6IG02
3663	1,3,4,7,27, 29,31,32	2,5,6,8,25, 26,28,30	3	B0BNN3,P10758,P80299
3664	1,3,4,7,27, 30,31,32	2,5,6,8,25, 26,28,29	2	P08649,Q9QYP1
3665	1,3,4,7,28, 29,30,31	2,5,6,8,25, 26,27,32	2	P97840,Q9JJ50
3666	1,3,4,7,28, 29,30,32	2,5,6,8,25, 26,27,31	3	P01039,P17559,P97840
3667	1,3,4,7,28, 29,31,32	2,5,6,8,25, 26,27,30	1	P17559
3668	1,3,4,7,28, 30,31,32	2,5,6,8,25, 26,27,29	2	P25809,Q9JJ50
3669	1,3,4,7,29, 30,31,32	2,5,6,8,25, 26,27,28	5	B0BNN3,P08649,P17559,P25809,P26342
3670	1,3,4,8,25, 26,27,28	2,5,6,7,29, 30,31,32	3	P01835,P26342,P36860
3671	1,3,4,8,25, 26,27,29	2,5,6,7,28, 30,31,32	0	

3672	1,3,4,8,25,26,27,30	2,5,6,7,28,29,31,32	5	O70417,P01835,Q05511,Q6IFW6,Q6IG02
3673	1,3,4,8,25,26,27,31	2,5,6,7,28,29,30,32	4	P01681,P01835,P29315,P54921
3674	1,3,4,8,25,26,27,32	2,5,6,7,28,29,30,31	3	P00762,P01681,P01835
3675	1,3,4,8,25,26,28,29	2,5,6,7,27,30,31,32	3	P01039,P50116,P70549
3676	1,3,4,8,25,26,28,30	2,5,6,7,27,29,31,32	4	O70417,P01039,P50116,P80299
3677	1,3,4,8,25,26,28,31	2,5,6,7,27,29,30,32	4	P01039,P01835,P29315,P50116
3678	1,3,4,8,25,26,28,32	2,5,6,7,27,29,30,31	4	O54728,P01039,P01681,P50116
3679	1,3,4,8,25,26,29,30	2,5,6,7,27,28,31,32	5	O88797,P01039,P50116,Q6IFW6,Q6IG02
3680	1,3,4,8,25,26,29,31	2,5,6,7,27,28,30,32	4	P01039,P29315,P50116,P70549
3681	1,3,4,8,25,26,29,32	2,5,6,7,27,28,30,31	3	P01039,P01681,P50116
3682	1,3,4,8,25,26,30,31	2,5,6,7,27,28,29,32	5	P29315,P50116,P54921,Q6AY61,Q6IFW6
3683	1,3,4,8,25,26,30,32	2,5,6,7,27,28,29,31	4	O88797,P00762,P01681,P50116
3684	1,3,4,8,25,26,31,32	2,5,6,7,27,28,29,30	5	O54728,P00762,P01681,P29315,P50116
3685	1,3,4,8,25,27,28,29	2,5,6,7,26,30,31,32	2	P36860,Q812E4
3686	1,3,4,8,25,27,28,30	2,5,6,7,26,29,31,32	7	O70417,P01835,P36860,Q6IFU7,Q6IFW6,Q6IG02,Q6P6Q2
3687	1,3,4,8,25,27,28,31	2,5,6,7,26,29,30,32	6	D3ZTX0,P01681,P01835,P29315,P36860,Q9R168
3688	1,3,4,8,25,27,28,32	2,5,6,7,26,29,30,31	6	P00762,P01681,P01835,P36860,Q6AYR9,Q9QWN8
3689	1,3,4,8,25,27,29,30	2,5,6,7,26,28,31,32	8	O70417,Q4FZU2,Q6IFU7,Q6IFU8,Q6IFW6,Q6IG02,Q6IMF3,Q6P6Q2
3690	1,3,4,8,25,27,29,31	2,5,6,7,26,28,30,32	3	P01681,P29315,Q9R168
3691	1,3,4,8,25,27,29,32	2,5,6,7,26,28,30,31	2	P00762,P01681

3692	1,3,4,8,25, 27,30,31	2,5,6,7,26, 28,29,32	12	O70417,P01681,P01835,P29315,P54921,Q05511,Q4FZU2,Q6IFW6,Q6IG02,Q6IMF3,Q6P6Q2,Q9QYP1
3693	1,3,4,8,25, 27,30,32	2,5,6,7,26, 28,29,31	11	O70417,P00762,P01681,P01835,P02625,Q05511,Q4FZU2,Q6IFU7,Q6IFW6,Q6IG02,Q6P6Q2
3694	1,3,4,8,25, 27,31,32	2,5,6,7,26, 28,29,30	5	P00762,P01681,P01835,P29315,Q9QYP1
3695	1,3,4,8,25, 28,29,30	2,5,6,7,26, 27,31,32	4	P01039,P50116,Q6IFW6,Q6IG02
3696	1,3,4,8,25, 28,29,31	2,5,6,7,26, 27,30,32	5	D3ZTX0,P01039,P29315,P50116,Q9JJ50
3697	1,3,4,8,25, 28,29,32	2,5,6,7,26, 27,30,31	3	P01039,P01681,P50116
3698	1,3,4,8,25, 28,30,31	2,5,6,7,26, 27,29,32	6	P01039,P01681,P29315,P50116,Q6IFW6,Q9JJ50
3699	1,3,4,8,25, 28,30,32	2,5,6,7,26, 27,29,31	5	O70417,O88797,P01039,P01681,P50116
3700	1,3,4,8,25, 28,31,32	2,5,6,7,26, 27,29,30	8	P01039,P01681,P21588,P29315,P50116,Q811M5,Q923V8,Q9JJ50
3701	1,3,4,8,25, 29,30,31	2,5,6,7,26, 27,28,32	6	P01039,P50116,Q4FZU2,Q6IFW6,Q6IG02,Q6IMF3
3702	1,3,4,8,25, 29,30,32	2,5,6,7,26, 27,28,31	8	O88797,P00762,P01039,P01681,P50116,Q4FZU2,Q6IFW6,Q6IG02
3703	1,3,4,8,25, 29,31,32	2,5,6,7,26, 27,28,30	4	P01039,P01681,P21588,P50116
3704	1,3,4,8,25, 30,31,32	2,5,6,7,26, 27,28,29	8	O88797,P00762,P01681,P02625,P50116,Q00715,Q4FZU2,Q6IFW6
3705	1,3,4,8,26, 27,28,29	2,5,6,7,25, 30,31,32	2	P26342,Q6P6R2
3706	1,3,4,8,26, 27,28,30	2,5,6,7,25, 29,31,32	1	Q6IG02
3707	1,3,4,8,26, 27,28,31	2,5,6,7,25, 29,30,32	1	Q9R168
3708	1,3,4,8,26, 27,28,32	2,5,6,7,25, 29,30,31	3	O54728,Q6AYR9,Q9QWN8
3709	1,3,4,8,26, 27,29,30	2,5,6,7,25, 28,31,32	4	Q6IFU7,Q6IFW6,Q6IG02,Q6P6Q2
3710	1,3,4,8,26, 27,29,31	2,5,6,7,25, 28,30,32	1	Q9R168
3711	1,3,4,8,26, 27,29,32	2,5,6,7,25, 28,30,31	1	P00762

3712	1,3,4,8,26,<br>27,30,31	2,5,6,7,25,<br>28,29,32	3	Q6AY61,Q6IFW6,Q6IG02
3713	1,3,4,8,26,<br>27,30,32	2,5,6,7,25,<br>28,29,31	2	P00762,Q6IG02
3714	1,3,4,8,26,<br>27,31,32	2,5,6,7,25,<br>28,29,30	2	O54728,P00762
3715	1,3,4,8,26,<br>28,29,30	2,5,6,7,25,<br>27,31,32	3	P01039,P50116,Q6IG02
3716	1,3,4,8,26,<br>28,29,31	2,5,6,7,25,<br>27,30,32	3	P01039,P50116,P70549
3717	1,3,4,8,26,<br>28,29,32	2,5,6,7,25,<br>27,30,31	3	O54728,P01039,P50116
3718	1,3,4,8,26,<br>28,30,31	2,5,6,7,25,<br>27,29,32	2	P01039,P50116
3719	1,3,4,8,26,<br>28,30,32	2,5,6,7,25,<br>27,29,31	3	O54728,P01039,P50116
3720	1,3,4,8,26,<br>28,31,32	2,5,6,7,25,<br>27,29,30	3	O54728,P01039,P50116
3721	1,3,4,8,26,<br>29,30,31	2,5,6,7,25,<br>27,28,32	4	P01039,P50116,Q6IFW6,Q6IG02
3722	1,3,4,8,26,<br>29,30,32	2,5,6,7,25,<br>27,28,31	4	O88797,P01039,P50116,Q6IG02
3723	1,3,4,8,26,<br>29,31,32	2,5,6,7,25,<br>27,28,30	3	O54728,P01039,P50116
3724	1,3,4,8,26,<br>30,31,32	2,5,6,7,25,<br>27,28,29	3	O54728,P50116,Q00715
3725	1,3,4,8,27,<br>28,29,30	2,5,6,7,25,<br>26,31,32	4	Q6IFU7,Q6IFW6,Q6IG02,Q6P6Q2
3726	1,3,4,8,27,<br>28,29,31	2,5,6,7,25,<br>26,30,32	1	Q9R168
3727	1,3,4,8,27,<br>28,29,32	2,5,6,7,25,<br>26,30,31	4	O35547,Q6AYR9,Q812E4,Q9Z2L0
3728	1,3,4,8,27,<br>28,30,31	2,5,6,7,25,<br>26,29,32	2	Q6IFW6,Q6IG02
3729	1,3,4,8,27,<br>28,30,32	2,5,6,7,25,<br>26,29,31	4	P00762,Q6AYR9,Q6IG02,Q8VD89
3730	1,3,4,8,27,<br>28,31,32	2,5,6,7,25,<br>26,29,30	3	P00762,P01681,Q6AYR9
3731	1,3,4,8,27,<br>29,30,31	2,5,6,7,25,<br>26,28,32	6	Q4FZU2,Q6IFU7,Q6IFW6,Q6IG02,Q6IMF3,Q6P6Q2

3732	1,3,4,8,27,29,30,32	2,5,6,7,25,26,28,31	8 B0BNN3,P00762,Q4FZU2,Q6IFU7,Q6IFW6,Q6IG02,Q6IMF3,Q6P6Q2
3733	1,3,4,8,27,29,31,32	2,5,6,7,25,26,28,30	2 B0BNN3,P00762
3734	1,3,4,8,27,30,31,32	2,5,6,7,25,26,28,29	6 P00762,P01681,Q4FZU2,Q6IFW6,Q6IG02,Q6P6Q2
3735	1,3,4,8,28,29,30,31	2,5,6,7,25,26,27,32	5 P01039,P50116,Q6IFW6,Q6IG02,Q9JJ50
3736	1,3,4,8,28,29,30,32	2,5,6,7,25,26,27,31	4 O88797,P01039,P50116,Q6IG02
3737	1,3,4,8,28,29,31,32	2,5,6,7,25,26,27,30	3 P01039,P21588,P50116
3738	1,3,4,8,28,30,31,32	2,5,6,7,25,26,27,29	2 P50116,Q9JJ50
3739	1,3,4,8,29,30,31,32	2,5,6,7,25,26,27,28	6 B0BNN3,P50116,P63081,Q6IFW6,Q6IG02,Q6IMF3
3740	1,3,4,25,26,27,28,29	2,5,6,7,8,30,31,32	3 O54858,P10758,P20646
3741	1,3,4,25,26,27,28,30	2,5,6,7,8,29,31,32	0
3742	1,3,4,25,26,27,28,31	2,5,6,7,8,29,30,32	2 P29315,P62804
3743	1,3,4,25,26,27,28,32	2,5,6,7,8,29,30,31	2 P62804,Q5RJR2
3744	1,3,4,25,26,27,29,30	2,5,6,7,8,28,31,32	2 O54858,Q6IG02
3745	1,3,4,25,26,27,29,31	2,5,6,7,8,28,30,32	2 P10758,P29315
3746	1,3,4,25,26,27,29,32	2,5,6,7,8,28,30,31	3 O54858,P00762,P10758
3747	1,3,4,25,26,27,30,31	2,5,6,7,8,28,29,32	4 P29315,P54921,P62804,Q9QYP1
3748	1,3,4,25,26,27,30,32	2,5,6,7,8,28,29,31	3 P00762,P06760,P62804
3749	1,3,4,25,26,27,31,32	2,5,6,7,8,28,29,30	5 P00762,P01681,P29315,P62804,Q9QYP1
3750	1,3,4,25,26,28,29,30	2,5,6,7,8,27,31,32	5 O54858,P01039,P50115,P50116,Q62714
3751	1,3,4,25,26,28,29,31	2,5,6,7,8,27,30,32	8 P01039,P19218,P29315,P50115,P50116,P70549,Q08163,Q5I0J9

3752	1,3,4,25,26,28,29,32	2,5,6,7,8,27,30,31	5	O54858,P00731,P01039,P50115,P50116
3753	1,3,4,25,26,28,30,31	2,5,6,7,8,27,29,32	9	P25809,P29315,P50115,P50116,P62804,Q00715,Q08163,Q4G075,Q9JJ50
3754	1,3,4,25,26,28,30,32	2,5,6,7,8,27,29,31	8	P06760,P25809,P50115,P50116,P62804,Q00715,Q4G075,Q62975
3755	1,3,4,25,26,28,31,32	2,5,6,7,8,27,29,30	11	O54728,P00731,P19218,P25809,P29315,P50115,P50116,P62804,Q00715,Q08163,Q4G075
3756	1,3,4,25,26,29,30,31	2,5,6,7,8,27,28,32	4	P25809,P29315,P50115,P50116
3757	1,3,4,25,26,29,30,32	2,5,6,7,8,27,28,31	2	P50115,P50116
3758	1,3,4,25,26,29,31,32	2,5,6,7,8,27,28,30	5	P19218,P29315,P50115,P50116,P62804
3759	1,3,4,25,26,30,31,32	2,5,6,7,8,27,28,29	9	P06760,P25809,P29315,P50115,P50116,P62804,Q00715,Q4G075,Q9QYP1
3760	1,3,4,25,27,28,29,30	2,5,6,7,8,26,31,32	4	O54858,P20766,Q62714,Q6IG02
3761	1,3,4,25,27,28,29,31	2,5,6,7,8,26,30,32	4	D3ZTX0,O54858,P10758,P29315
3762	1,3,4,25,27,28,29,32	2,5,6,7,8,26,30,31	6	O54858,P00758,P10758,P20766,Q62761;Q62762;Q62763,Q8CGS4
3763	1,3,4,25,27,28,30,31	2,5,6,7,8,26,29,32	3	P06760,P29315,Q9QYP1
3764	1,3,4,25,27,28,30,32	2,5,6,7,8,26,29,31	3	P00762,P06760,Q9QX74
3765	1,3,4,25,27,28,31,32	2,5,6,7,8,26,29,30	6	P00762,P01681,P06760,P29315,P62804,Q9QYP1
3766	1,3,4,25,27,29,30,31	2,5,6,7,8,26,28,32	3	Q6IFW6,Q6IG02,Q9QYP1
3767	1,3,4,25,27,29,30,32	2,5,6,7,8,26,28,31	3	O54858,P00762,Q6IG02
3768	1,3,4,25,27,29,31,32	2,5,6,7,8,26,28,30	6	P00762,P01681,P10758,P29315,P62804,Q9QYP1
3769	1,3,4,25,27,30,31,32	2,5,6,7,8,26,28,29	9	P00762,P01681,P06760,P62804,Q00715,Q6P6R2,Q9QX74,Q9QYP1,Q9WTT6
3770	1,3,4,25,28,29,30,31	2,5,6,7,8,26,27,32	5	P01039,P25809,P50115,P50116,Q9JJ50
3771	1,3,4,25,28,29,30,32	2,5,6,7,8,26,27,31	6	O54858,P01039,P20766,P50115,P50116,Q62975

3772	1,3,4,25,28,29,31,32	2,5,6,7,8,26,27,30	6	P01039,P50115,P50116,P62804,Q8CJD3,Q9JJ50
3773	1,3,4,25,28,30,31,32	2,5,6,7,8,26,27,29	12	P01681,P06760,P25809,P50115,P50116,P62804,Q00715,Q4G075,Q62975,Q9JJ50,Q9QX74,Q9WTT6
3774	1,3,4,25,29,30,31,32	2,5,6,7,8,26,27,28	7	P07150,P25809,P50115,P50116,P62804,Q00715,Q9WTT6
3775	1,3,4,26,27,28,29,30	2,5,6,7,8,25,31,32	2	O54858,Q6IG02
3776	1,3,4,26,27,28,29,31	2,5,6,7,8,25,30,32	3	O54858,P02631,P70549
3777	1,3,4,26,27,28,29,32	2,5,6,7,8,25,30,31	4	O54858,P00758,Q62761;Q62762;Q62763,Q8CGS4
3778	1,3,4,26,27,28,30,31	2,5,6,7,8,25,29,32	0	
3779	1,3,4,26,27,28,30,32	2,5,6,7,8,25,29,31	2	P06760,Q8VD89
3780	1,3,4,26,27,28,31,32	2,5,6,7,8,25,29,30	2	O54728,P62804
3781	1,3,4,26,27,29,30,31	2,5,6,7,8,25,28,32	3	O54858,P02631,Q6IG02
3782	1,3,4,26,27,29,30,32	2,5,6,7,8,25,28,31	3	O54858,P50115,Q6IG02
3783	1,3,4,26,27,29,31,32	2,5,6,7,8,25,28,30	3	O54858,P02631,P62804
3784	1,3,4,26,27,30,31,32	2,5,6,7,8,25,28,29	7	P00762,P02631,P06760,P50115,P62804,Q00715,Q9QYP1
3785	1,3,4,26,28,29,30,31	2,5,6,7,8,25,27,32	4	O54858,P01039,P50115,P50116
3786	1,3,4,26,28,29,30,32	2,5,6,7,8,25,27,31	5	O54858,P01039,P20766,P50115,P50116
3787	1,3,4,26,28,29,31,32	2,5,6,7,8,25,27,30	6	O54728,O54858,P01039,P50115,P50116,P62804
3788	1,3,4,26,28,30,31,32	2,5,6,7,8,25,27,29	9	O54728,P06760,P25809,P50115,P50116,P62804,Q00715,Q08163,Q4G075
3789	1,3,4,26,29,30,31,32	2,5,6,7,8,25,27,28	7	P02631,P25809,P50115,P50116,P62804,Q00715,Q4G075
3790	1,3,4,27,28,29,30,31	2,5,6,7,8,25,26,32	3	O54858,P50115,Q6IG02
3791	1,3,4,27,28,29,30,32	2,5,6,7,8,25,26,31	4	O54858,P20766,P50115,Q6IG02

3792	1,3,4,27,28,29,31,32	2,5,6,7,8,25,26,30	3	O54858,P50115,Q62761;Q62762;Q62763
3793	1,3,4,27,28,30,31,32	2,5,6,7,8,25,26,29	6	P06760,P22006,P50115,P62804,Q8VD89,Q9QX74
3794	1,3,4,27,29,30,31,32	2,5,6,7,8,25,26,28	7	B0BNN3,O54858,P00762,P08649,P50115,Q6IG02,Q9WTT6
3795	1,3,4,28,29,30,31,32	2,5,6,7,8,25,26,27	7	O54858,P01039,P17559,P50115,P50116,Q9JJ50,Q9WTT6
3796	1,3,5,6,7,8,25,26	2,4,27,28,29,30,31,32	2	P49134,P80299
3797	1,3,5,6,7,8,25,27	2,4,26,28,29,30,31,32	2	P50115,P50116
3798	1,3,5,6,7,8,25,28	2,4,26,27,29,30,31,32	3	P06911,Q09030,Q9QW07
3799	1,3,5,6,7,8,25,29	2,4,26,27,28,30,31,32	1	P49134
3800	1,3,5,6,7,8,25,30	2,4,26,27,28,29,31,32	9	O70417,P49134,P80299,Q05511,Q09030,Q6IFU7,Q6IFW6,Q6IG05,Q6IMF3
3801	1,3,5,6,7,8,25,31	2,4,26,27,28,29,30,32	2	P49134,Q09030
3802	1,3,5,6,7,8,25,32	2,4,26,27,28,29,30,31	3	P01681,P13432,Q09030
3803	1,3,5,6,7,8,26,27	2,4,25,28,29,30,31,32	4	D3ZUC6,P50115,P50116,Q62714
3804	1,3,5,6,7,8,26,28	2,4,25,27,29,30,31,32	7	D3ZUC6,P47967,P49134,P80299,Q5RLM2,Q9QW07,Q9QYP1
3805	1,3,5,6,7,8,26,29	2,4,25,27,28,30,31,32	7	D3ZUC6,O70594,P06760,P47967,P49134,Q9QX74,Q9QYP1
3806	1,3,5,6,7,8,26,30	2,4,25,27,28,29,31,32	4	P36374,P49134,P80299,Q6IG05

3807	1,3,5,6,7,8,26,31	2,4,25,27,28,29,30,32	4	D3ZUC6,P49134,P57113,Q62714
3808	1,3,5,6,7,8,26,32	2,4,25,27,28,29,30,31	2	P57113,Q62714
3809	1,3,5,6,7,8,27,28	2,4,25,26,29,30,31,32	6	D3ZUC6,P19218,P47967,P50115,P50116,Q5I0D1
3810	1,3,5,6,7,8,27,29	2,4,25,26,28,30,31,32	5	D3ZUC6,P19218,P47967,P50115,Q5I0D1
3811	1,3,5,6,7,8,27,30	2,4,25,26,28,29,31,32	6	P19218,Q6IFU7,Q6IFW6,Q6IG05,Q6IMF3,Q6P6Q2
3812	1,3,5,6,7,8,27,31	2,4,25,26,28,29,30,32	4	D3ZUC6,P19218,P50115,Q62714
3813	1,3,5,6,7,8,27,32	2,4,25,26,28,29,30,31	2	P19218,Q62714
3814	1,3,5,6,7,8,28,29	2,4,25,26,27,30,31,32	8	D3ZUC6,P00762,P21674,P47967,P49134,P97840,Q9QW07,Q9QYP1
3815	1,3,5,6,7,8,28,30	2,4,25,26,27,29,31,32	6	D3ZUC6,P19218,P47967,P49134,Q6IFU7,Q6IG05
3816	1,3,5,6,7,8,28,31	2,4,25,26,27,29,30,32	4	D3ZUC6,P21674,P49134,Q811M5
3817	1,3,5,6,7,8,28,32	2,4,25,26,27,29,30,31	4	D3ZUC6,P47967,Q9QW07,Q9QYP1
3818	1,3,5,6,7,8,29,30	2,4,25,26,27,28,31,32	8	P19218,P49134,Q5RJR2,Q6IFU7,Q6IFW6,Q6IG05,Q6IMF3,Q6P6Q2
3819	1,3,5,6,7,8,29,31	2,4,25,26,27,28,30,32	5	D3ZUC6,O70594,P21674,P49134,Q5RJR2

3820	1,3,5,6,7,8,29,32	2,4,25,26,27,28,30,31	1	Q9QYP1
3821	1,3,5,6,7,8,30,31	2,4,25,26,27,28,29,32	9	P19218,P49134,Q09030,Q5RJR2,Q62714,Q6IFW6,Q6IG05,Q6IMF3,Q8CGS4
3822	1,3,5,6,7,8,30,32	2,4,25,26,27,28,29,31	5	P19218,P35280,Q6IFU7,Q6IG05,Q6IMF3
3823	1,3,5,6,7,8,31,32	2,4,25,26,27,28,29,30	2	P57113,Q62714
3824	1,3,5,6,7,25,26,27	2,4,8,28,29,30,31,32	1	P63081
3825	1,3,5,6,7,25,26,28	2,4,8,27,29,30,31,32	0	
3826	1,3,5,6,7,25,26,29	2,4,8,27,28,30,31,32	0	
3827	1,3,5,6,7,25,26,30	2,4,8,27,28,29,31,32	4	P25809,P36374,P80299,Q6AYR9
3828	1,3,5,6,7,25,26,31	2,4,8,27,28,29,30,32	4	P25809,P57113,Q63150,Q6AYR9
3829	1,3,5,6,7,25,26,32	2,4,8,27,28,29,30,31	1	Q5PQL7
3830	1,3,5,6,7,25,27,28	2,4,8,26,29,30,31,32	1	P63081
3831	1,3,5,6,7,25,27,29	2,4,8,26,28,30,31,32	3	O54728,P05539,Q07523
3832	1,3,5,6,7,25,27,30	2,4,8,26,28,29,31,32	1	O54728
3833	1,3,5,6,7,25,27,31	2,4,8,26,28,29,30,32	1	O54728
3834	1,3,5,6,7,25,27,32	2,4,8,26,28,29,30,31	0	
3835	1,3,5,6,7,25,28,29	2,4,8,26,27,30,31,32	1	P05539
3836	1,3,5,6,7,25,28,30	2,4,8,26,27,29,31,32	2	Q5PQL7,Q62975
3837	1,3,5,6,7,25,28,31	2,4,8,26,27,29,30,32	1	P25809

3838	1,3,5,6,7,25,28,32	2,4,8,26,27,29,30,31	2 Q5PQL7,Q62975
3839	1,3,5,6,7,25,29,30	2,4,8,26,27,28,31,32	2 O54728,Q9QWN8
3840	1,3,5,6,7,25,29,31	2,4,8,26,27,28,30,32	2 O54728,Q9QWN8
3841	1,3,5,6,7,25,29,32	2,4,8,26,27,28,30,31	0
3842	1,3,5,6,7,25,30,31	2,4,8,26,27,28,29,32	6 O54728,P25809,Q09030,Q6AYR9,Q812E4,Q9QWN8
3843	1,3,5,6,7,25,30,32	2,4,8,26,27,28,29,31	5 P25809,Q5BJY9,Q5PQL7,Q62975,Q6P6R2
3844	1,3,5,6,7,25,31,32	2,4,8,26,27,28,29,30	1 P25809
3845	1,3,5,6,7,26,27,28	2,4,8,25,29,30,31,32	4 D3ZUC6,P21588,P47967,P97840
3846	1,3,5,6,7,26,27,29	2,4,8,25,28,30,31,32	3 P21588,P47967,Q9JJ50
3847	1,3,5,6,7,26,27,30	2,4,8,25,28,29,31,32	1 P21588
3848	1,3,5,6,7,26,27,31	2,4,8,25,28,29,30,32	1 Q62714
3849	1,3,5,6,7,26,27,32	2,4,8,25,28,29,30,31	2 Q62714,Q9JJ50
3850	1,3,5,6,7,26,28,29	2,4,8,25,27,30,31,32	7 D3ZUC6,P00762,P21674,P47967,P97840,Q01205,Q9QYP1
3851	1,3,5,6,7,26,28,30	2,4,8,25,27,29,31,32	3 P00762,P47967,P97840
3852	1,3,5,6,7,26,28,31	2,4,8,25,27,29,30,32	2 P00762,P57113
3853	1,3,5,6,7,26,28,32	2,4,8,25,27,29,30,31	1 P47967
3854	1,3,5,6,7,26,29,30	2,4,8,25,27,28,31,32	0
3855	1,3,5,6,7,26,29,31	2,4,8,25,27,28,30,32	1 P57113
3856	1,3,5,6,7,26,29,32	2,4,8,25,27,28,30,31	0
3857	1,3,5,6,7,26,30,31	2,4,8,25,27,28,29,32	5 P25809,P47727,P57113,Q6AYR9,Q812E4

3858	1,3,5,6,7,26,30,32	2,4,8,25,27,28,29,31	0
3859	1,3,5,6,7,26,31,32	2,4,8,25,27,28,29,30	2 P57113,Q62714
3860	1,3,5,6,7,27,28,29	2,4,8,25,26,30,31,32	7 D3ZUC6,P00507,P05539,P47967,P97840,Q07523,Q6AY61
3861	1,3,5,6,7,27,28,30	2,4,8,25,26,29,31,32	3 P19218,P47967,P97840
3862	1,3,5,6,7,27,28,31	2,4,8,25,26,29,30,32	2 D3ZUC6,Q68G31
3863	1,3,5,6,7,27,28,32	2,4,8,25,26,29,30,31	3 P47967,Q62761;Q62762;Q62763,Q6AY61
3864	1,3,5,6,7,27,29,30	2,4,8,25,26,28,31,32	3 O54728,P19218,P47967
3865	1,3,5,6,7,27,29,31	2,4,8,25,26,28,30,32	2 O54728,Q07523
3866	1,3,5,6,7,27,29,32	2,4,8,25,26,28,30,31	3 P47967,Q07523,Q6AY61
3867	1,3,5,6,7,27,30,31	2,4,8,25,26,28,29,32	2 O54728,P19218
3868	1,3,5,6,7,27,30,32	2,4,8,25,26,28,29,31	1 P19218
3869	1,3,5,6,7,27,31,32	2,4,8,25,26,28,29,30	1 Q62714
3870	1,3,5,6,7,28,29,30	2,4,8,25,26,27,31,32	3 P00762,P47967,P97840
3871	1,3,5,6,7,28,29,31	2,4,8,25,26,27,30,32	4 D3ZUC6,P00762,P21674,P47967
3872	1,3,5,6,7,28,29,32	2,4,8,25,26,27,30,31	5 P47967,P54921,P97840,Q6AY61,Q9QYP1
3873	1,3,5,6,7,28,30,31	2,4,8,25,26,27,29,32	2 P00762,P25809
3874	1,3,5,6,7,28,30,32	2,4,8,25,26,27,29,31	2 Q5BJY9,Q62975
3875	1,3,5,6,7,28,31,32	2,4,8,25,26,27,29,30	0
3876	1,3,5,6,7,29,30,31	2,4,8,25,26,27,28,32	3 O54728,P36860,Q9QWN8
3877	1,3,5,6,7,29,30,32	2,4,8,25,26,27,28,31	3 P01835,P26342,Q5BJY9

3878	1,3,5,6,7,29,31,32	2,4,8,25,26,27,28,30	0
3879	1,3,5,6,7,30,31,32	2,4,8,25,26,27,28,29	7 P20646,P25809,P26342,P36860,Q5BJY9,Q62714,Q6P6R2
3880	1,3,5,6,8,25,26,27	2,4,7,28,29,30,31,32	1 P80299
3881	1,3,5,6,8,25,26,28	2,4,7,27,29,30,31,32	2 P00714,P80299
3882	1,3,5,6,8,25,26,29	2,4,7,27,28,30,31,32	2 P13432,P80299
3883	1,3,5,6,8,25,26,30	2,4,7,27,28,29,31,32	6 O88797,P80299,Q05511,Q06000,Q6IFU7,Q6IG05
3884	1,3,5,6,8,25,26,31	2,4,7,27,28,29,30,32	1 P80299
3885	1,3,5,6,8,25,26,32	2,4,7,27,28,29,30,31	4 P01681,P13432,P80299,Q06000
3886	1,3,5,6,8,25,27,28	2,4,7,26,29,30,31,32	2 P36860,P80299
3887	1,3,5,6,8,25,27,29	2,4,7,26,28,30,31,32	0
3888	1,3,5,6,8,25,27,30	2,4,7,26,28,29,31,32	9 P19218,P80299,Q05511,Q6IFU7,Q6IFW6,Q6IG02,Q6IG05,Q6IMF3,Q6P6Q2
3889	1,3,5,6,8,25,27,31	2,4,7,26,28,29,30,32	3 P23739,Q80WL1,Q9R168
3890	1,3,5,6,8,25,27,32	2,4,7,26,28,29,30,31	2 P01681,Q80WL1
3891	1,3,5,6,8,25,28,29	2,4,7,26,27,30,31,32	1 P80299
3892	1,3,5,6,8,25,28,30	2,4,7,26,27,29,31,32	5 O88797,P80299,Q09030,Q6IFU7,Q6IG05
3893	1,3,5,6,8,25,28,31	2,4,7,26,27,29,30,32	4 P36376,Q09030,Q80WL1,Q9R168
3894	1,3,5,6,8,25,28,32	2,4,7,26,27,29,30,31	2 P01681,Q80WL1
3895	1,3,5,6,8,25,29,30	2,4,7,26,27,28,31,32	8 O88797,P80299,Q6IFU7,Q6IFW6,Q6IG02,Q6IG05,Q6IMF3,Q6P6Q2
3896	1,3,5,6,8,25,29,31	2,4,7,26,27,28,30,32	2 Q80WL1,Q9R168
3897	1,3,5,6,8,25,29,32	2,4,7,26,27,28,30,31	3 P01681,P13432,Q80WL1

3898	1,3,5,6,8,25,30,31	2,4,7,26,27,28,29,32	7	P20646,Q09030,Q6IFU7,Q6IFW6,Q6IG05,Q6IMF3,Q8CGS4
3899	1,3,5,6,8,25,30,32	2,4,7,26,27,28,29,31	8	O88797,P01681,P20646,P80299,Q09030,Q6IFU7,Q6IG05,Q6IMF3
3900	1,3,5,6,8,25,31,32	2,4,7,26,27,28,29,30	4	P01681,P20646,Q09030,Q80WL1
3901	1,3,5,6,8,26,27,28	2,4,7,25,29,30,31,32	2	D3ZUC6,P80299
3902	1,3,5,6,8,26,27,29	2,4,7,25,28,30,31,32	0	
3903	1,3,5,6,8,26,27,30	2,4,7,25,28,29,31,32	3	P80299,Q6IFU7,Q6IG05
3904	1,3,5,6,8,26,27,31	2,4,7,25,28,29,30,32	1	Q9R168
3905	1,3,5,6,8,26,27,32	2,4,7,25,28,29,30,31	0	
3906	1,3,5,6,8,26,28,29	2,4,7,25,27,30,31,32	4	D3ZUC6,P21674,P80299,Q9QYP1
3907	1,3,5,6,8,26,28,30	2,4,7,25,27,29,31,32	2	P80299,Q6IFU7
3908	1,3,5,6,8,26,28,31	2,4,7,25,27,29,30,32	2	D3ZUC6,Q9R168
3909	1,3,5,6,8,26,28,32	2,4,7,25,27,29,30,31	2	P80299,Q9QYP1
3910	1,3,5,6,8,26,29,30	2,4,7,25,27,28,31,32	4	O88797,P80299,Q5RJR2,Q6IFU7
3911	1,3,5,6,8,26,29,31	2,4,7,25,27,28,30,32	2	P70549,Q9R168
3912	1,3,5,6,8,26,29,32	2,4,7,25,27,28,30,31	2	P13432,Q9QYP1
3913	1,3,5,6,8,26,30,31	2,4,7,25,27,28,29,32	4	P0C0A9,Q5RJR2,Q6IG05,Q8CGS4
3914	1,3,5,6,8,26,30,32	2,4,7,25,27,28,29,31	2	O88797,P80299
3915	1,3,5,6,8,26,31,32	2,4,7,25,27,28,29,30	1	Q62714
3916	1,3,5,6,8,27,28,29	2,4,7,25,26,30,31,32	3	D3ZUC6,P19218,P47967
3917	1,3,5,6,8,27,28,30	2,4,7,25,26,29,31,32	4	P19218,Q6IFU7,Q6IG05,Q6P6Q2

3918	1,3,5,6,8,27,28,31	2,4,7,25,26,29,30,32	2	D3ZUC6,Q9R168
3919	1,3,5,6,8,27,28,32	2,4,7,25,26,29,30,31	1	P19218
3920	1,3,5,6,8,27,29,30	2,4,7,25,26,28,31,32	7	P19218,Q6IFU7,Q6IFW6,Q6IG02,Q6IG05,Q6IMF3,Q6P6Q2
3921	1,3,5,6,8,27,29,31	2,4,7,25,26,28,30,32	1	Q9R168
3922	1,3,5,6,8,27,29,32	2,4,7,25,26,28,30,31	0	
3923	1,3,5,6,8,27,30,31	2,4,7,25,26,28,29,32	8	P19218,P20646,Q6IFU7,Q6IFW6,Q6IG05,Q6IMF3,Q6P6Q2,Q9R168
3924	1,3,5,6,8,27,30,32	2,4,7,25,26,28,29,31	7	P19218,P20646,Q5I0J9,Q6IFU7,Q6IG05,Q6IMF3,Q6P6Q2
3925	1,3,5,6,8,27,31,32	2,4,7,25,26,28,29,30	4	P20646,P23739,Q62714,Q80WL1
3926	1,3,5,6,8,28,29,30	2,4,7,25,26,27,31,32	5	O88797,P19218,Q6IFU7,Q6IMF3,Q9QYP1
3927	1,3,5,6,8,28,29,31	2,4,7,25,26,27,30,32	3	D3ZUC6,P21674,Q9R168
3928	1,3,5,6,8,28,29,32	2,4,7,25,26,27,30,31	1	Q9QYP1
3929	1,3,5,6,8,28,30,31	2,4,7,25,26,27,29,32	3	P19218,Q6IFU7,Q6IG05
3930	1,3,5,6,8,28,30,32	2,4,7,25,26,27,29,31	3	O88797,P19218,Q6IFU7
3931	1,3,5,6,8,28,31,32	2,4,7,25,26,27,29,30	1	Q80WL1
3932	1,3,5,6,8,29,30,31	2,4,7,25,26,27,28,32	6	Q5RJR2,Q6IFU7,Q6IFW6,Q6IG02,Q6IG05,Q6IMF3
3933	1,3,5,6,8,29,30,32	2,4,7,25,26,27,28,31	4	O88797,Q5I0J9,Q6IFU7,Q6IMF3
3934	1,3,5,6,8,29,31,32	2,4,7,25,26,27,28,30	1	Q80WL1
3935	1,3,5,6,8,30,31,32	2,4,7,25,26,27,28,29	5	O88797,O89117,P20646,Q6IG05,Q6IMF3
3936	1,3,5,6,25,26,27,28	2,4,7,8,29,30,31,32	3	P63081,P80299,P82471
3937	1,3,5,6,25,26,27,29	2,4,7,8,28,30,31,32	1	P82471

3938	1,3,5,6,25,<br>26,27,30	2,4,7,8,28,<br>29,31,32	4	P21588,P80299,P82471,Q06000
3939	1,3,5,6,25,<br>26,27,31	2,4,7,8,28,<br>29,30,32	1	P62804
3940	1,3,5,6,25,<br>26,27,32	2,4,7,8,28,<br>29,30,31	3	P62804,P82471,Q06000
3941	1,3,5,6,25,<br>26,28,29	2,4,7,8,27,<br>30,31,32	4	P00714,P80299,P82471,Q01205
3942	1,3,5,6,25,<br>26,28,30	2,4,7,8,27,<br>29,31,32	7	P00714,P80299,P82471,Q4G075,Q5PQL7,Q62975,Q6TMA8
3943	1,3,5,6,25,<br>26,28,31	2,4,7,8,27,<br>29,30,32	4	P00714,P25809,P62804,Q4G075
3944	1,3,5,6,25,<br>26,28,32	2,4,7,8,27,<br>29,30,31	7	P19223,P62804,P82471,Q06000,Q4G075,Q5PQL7,Q62975
3945	1,3,5,6,25,<br>26,29,30	2,4,7,8,27,<br>28,31,32	2	P80299,P82471
3946	1,3,5,6,25,<br>26,29,31	2,4,7,8,27,<br>28,30,32	1	P70549
3947	1,3,5,6,25,<br>26,29,32	2,4,7,8,27,<br>28,30,31	1	P82471
3948	1,3,5,6,25,<br>26,30,31	2,4,7,8,27,<br>28,29,32	9	P01946,P22282,P25809,P47727,P62804,Q00715,Q4G075,Q6AYR9,Q812E4
3949	1,3,5,6,25,<br>26,30,32	2,4,7,8,27,<br>28,29,31	8	P25809,P62804,P80299,P82471,Q00715,Q06000,Q4G075,Q5PQL7
3950	1,3,5,6,25,<br>26,31,32	2,4,7,8,27,<br>28,29,30	4	P25809,P62804,Q00715,Q4G075
3951	1,3,5,6,25,<br>27,28,29	2,4,7,8,26,<br>30,31,32	2	P05539,P82471
3952	1,3,5,6,25,<br>27,28,30	2,4,7,8,26,<br>29,31,32	1	P82471
3953	1,3,5,6,25,<br>27,28,31	2,4,7,8,26,<br>29,30,32	0	
3954	1,3,5,6,25,<br>27,28,32	2,4,7,8,26,<br>29,30,31	3	P62804,P82471,Q62761;Q62762;Q62763
3955	1,3,5,6,25,<br>27,29,30	2,4,7,8,26,<br>28,31,32	3	O54728,P82471,Q6IG02
3956	1,3,5,6,25,<br>27,29,31	2,4,7,8,26,<br>28,30,32	1	O54728
3957	1,3,5,6,25,<br>27,29,32	2,4,7,8,26,<br>28,30,31	2	P82471,Q9Z1F2

3958	1,3,5,6,25,27,30,31	2,4,7,8,26,28,29,32	3	O54728,P30120,P62804
3959	1,3,5,6,25,27,30,32	2,4,7,8,26,28,29,31	6	P20646,P62804,P82471,Q6P6R2,Q9QX74,Q9WTT6
3960	1,3,5,6,25,27,31,32	2,4,7,8,26,28,29,30	5	P20646,P23739,P62804,Q80WL1,Q9WTT6
3961	1,3,5,6,25,28,29,30	2,4,7,8,26,27,31,32	1	P82471
3962	1,3,5,6,25,28,29,31	2,4,7,8,26,27,30,32	1	P20762
3963	1,3,5,6,25,28,29,32	2,4,7,8,26,27,30,31	2	P20762,P82471
3964	1,3,5,6,25,28,30,31	2,4,7,8,26,27,29,32	2	P25809,Q4G075
3965	1,3,5,6,25,28,30,32	2,4,7,8,26,27,29,31	8	P25809,P82471,Q4G075,Q5BJY9,Q5PQL7,Q62975,Q9QX74,Q9WTT6
3966	1,3,5,6,25,28,31,32	2,4,7,8,26,27,29,30	6	P20762,P25809,P62804,Q4G075,Q80WL1,Q9WTT6
3967	1,3,5,6,25,29,30,31	2,4,7,8,26,27,28,32	8	O54728,P07150,P20762,P22282,P25809,Q6AYR9,Q9QWN8,Q9WTT6
3968	1,3,5,6,25,29,30,32	2,4,7,8,26,27,28,31	7	P07150,P25809,P82471,Q4G075,Q5BJY9,Q9WTT6,Q9Z1F2
3969	1,3,5,6,25,29,31,32	2,4,7,8,26,27,28,30	8	P07150,P20762,P25809,P62804,Q4G075,Q80WL1,Q9WTT6,Q9Z1F2
3970	1,3,5,6,25,30,31,32	2,4,7,8,26,27,28,29	13	P07150,P20646,P22273,P25809,P62804,P97584,Q00715,Q4G075,Q5BJY9,Q6P6R2,Q812E4,Q9QX74,Q9WTT6
3971	1,3,5,6,26,27,28,29	2,4,7,8,25,30,31,32	2	P47967,P82471
3972	1,3,5,6,26,27,28,30	2,4,7,8,25,29,31,32	2	P21588,P82471
3973	1,3,5,6,26,27,28,31	2,4,7,8,25,29,30,32	1	P62804
3974	1,3,5,6,26,27,28,32	2,4,7,8,25,29,30,31	2	P62804,P82471
3975	1,3,5,6,26,27,29,30	2,4,7,8,25,28,31,32	3	P21588,P82471,Q811M5
3976	1,3,5,6,26,27,29,31	2,4,7,8,25,28,30,32	0	
3977	1,3,5,6,26,27,29,32	2,4,7,8,25,28,30,31	2	P82471,Q9JJ50

3978	1,3,5,6,26,27,30,31	2,4,7,8,25,28,29,32	2	P30120,P62804
3979	1,3,5,6,26,27,30,32	2,4,7,8,25,28,29,31	2	P62804,P82471
3980	1,3,5,6,26,27,31,32	2,4,7,8,25,28,29,30	2	P62804,Q62714
3981	1,3,5,6,26,28,29,30	2,4,7,8,25,27,31,32	2	P82471,Q6TMA8
3982	1,3,5,6,26,28,29,31	2,4,7,8,25,27,30,32	2	P70549,Q01205
3983	1,3,5,6,26,28,29,32	2,4,7,8,25,27,30,31	2	P82471,Q9QYP1
3984	1,3,5,6,26,28,30,31	2,4,7,8,25,27,29,32	4	P25809,P62804,Q4G075,Q6TMA8
3985	1,3,5,6,26,28,30,32	2,4,7,8,25,27,29,31	3	P62804,P82471,Q4G075
3986	1,3,5,6,26,28,31,32	2,4,7,8,25,27,29,30	3	P22006,P62804,Q4G075
3987	1,3,5,6,26,29,30,31	2,4,7,8,25,27,28,32	6	P0C0A9,P22282,P36860,Q4G075,Q6AYR9,Q9QWN8
3988	1,3,5,6,26,29,30,32	2,4,7,8,25,27,28,31	3	P36860,P82471,Q4G075
3989	1,3,5,6,26,29,31,32	2,4,7,8,25,27,28,30	3	P36860,P62804,Q4G075
3990	1,3,5,6,26,30,31,32	2,4,7,8,25,27,28,29	11	P0C0A9,P20646,P22273,P25809,P36860,P47727,P62804,Q00715,Q4G075,Q812E4,Q9WTT6
3991	1,3,5,6,27,28,29,30	2,4,7,8,25,26,31,32	2	P19218,P82471
3992	1,3,5,6,27,28,29,31	2,4,7,8,25,26,30,32	1	P05539
3993	1,3,5,6,27,28,29,32	2,4,7,8,25,26,30,31	3	P82471,Q62761;Q62762;Q62763,Q6AY61
3994	1,3,5,6,27,28,30,31	2,4,7,8,25,26,29,32	0	
3995	1,3,5,6,27,28,30,32	2,4,7,8,25,26,29,31	5	P19218,P82471,Q5I0J9,Q8VD89,Q9QX74
3996	1,3,5,6,27,28,31,32	2,4,7,8,25,26,29,30	2	P22006,P62804
3997	1,3,5,6,27,29,30,31	2,4,7,8,25,26,28,32	1	O54728

3998	1,3,5,6,27,29,30,32	2,4,7,8,25,26,28,31	5	P82471,Q5I0J9,Q9WTT6,Q9Z0V6,Q9Z1F2
3999	1,3,5,6,27,29,31,32	2,4,7,8,25,26,28,30	2	P62804,Q9Z1F2
4000	1,3,5,6,27,30,31,32	2,4,7,8,25,26,28,29	5	P20646,P62804,Q6P6R2,Q9QX74,Q9WTT6
4001	1,3,5,6,28,29,30,31	2,4,7,8,25,26,27,32	0	
4002	1,3,5,6,28,29,30,32	2,4,7,8,25,26,27,31	5	P82471,Q4G075,Q5BJY9,Q9WTT6,Q9Z0V6
4003	1,3,5,6,28,29,31,32	2,4,7,8,25,26,27,30	2	P20762,Q4G075
4004	1,3,5,6,28,30,31,32	2,4,7,8,25,26,27,29	7	P22006,P25809,P62804,Q4G075,Q5BJY9,Q9QX74,Q9WTT6
4005	1,3,5,6,29,30,31,32	2,4,7,8,25,26,27,28	10	P07150,P20646,P25809,P26342,P36860,P62804,Q4G075,Q5BJY9,Q9WTT6,Q9Z1F2
4006	1,3,5,7,8,25,26,27	2,4,6,28,29,30,31,32	4	O55145,P22006,P80299,Q9WTT6
4007	1,3,5,7,8,25,26,28	2,4,6,27,29,30,31,32	4	O55145,P00714,P80299,Q9WTT6
4008	1,3,5,7,8,25,26,29	2,4,6,27,28,30,31,32	5	P06760,P22006,P49134,P80299,Q9QX74
4009	1,3,5,7,8,25,26,30	2,4,6,27,28,29,31,32	7	P22006,P49134,P80299,Q05511,Q6IFW6,Q6IG05,Q6IMF3
4010	1,3,5,7,8,25,26,31	2,4,6,27,28,29,30,32	2	O55145,P49134
4011	1,3,5,7,8,25,26,32	2,4,6,27,28,29,30,31	2	P01681,P80299
4012	1,3,5,7,8,25,27,28	2,4,6,26,29,30,31,32	2	O55145,P36860
4013	1,3,5,7,8,25,27,29	2,4,6,26,28,30,31,32	3	P06760,P22006,Q4G075
4014	1,3,5,7,8,25,27,30	2,4,6,26,28,29,31,32	9	P22006,Q05511,Q4FZU2,Q6IFU7,Q6IFW6,Q6IG02,Q6IG05,Q6IMF3,Q6P6Q2
4015	1,3,5,7,8,25,27,31	2,4,6,26,28,29,30,32	4	O55145,P23739,Q68G31,Q80WL1
4016	1,3,5,7,8,25,27,32	2,4,6,26,28,29,30,31	2	P01681,Q80WL1
4017	1,3,5,7,8,25,28,29	2,4,6,26,27,30,31,32	2	P06760,P22006

4018	1,3,5,7,8,25,28,30	2,4,6,26,27,29,31,32	5	P80299,Q6IFU7,Q6IFW6,Q6IG05,Q6IMF3
4019	1,3,5,7,8,25,28,31	2,4,6,26,27,29,30,32	3	O55145,Q80WL1,Q811M5
4020	1,3,5,7,8,25,28,32	2,4,6,26,27,29,30,31	3	P01681,Q80WL1,Q811M5
4021	1,3,5,7,8,25,29,30	2,4,6,26,27,28,31,32	10	O88797,P22006,Q05511,Q4FZU2,Q6IFU7,Q6IFW6,Q6IG02,Q6IG05,Q6IMF3,Q6P6Q2
4022	1,3,5,7,8,25,29,31	2,4,6,26,27,28,30,32	1	Q80WL1
4023	1,3,5,7,8,25,29,32	2,4,6,26,27,28,30,31	2	P01681,Q80WL1
4024	1,3,5,7,8,25,30,31	2,4,6,26,27,28,29,32	6	Q05511,Q4FZU2,Q6IFW6,Q6IG05,Q6IMF3,Q6P6Q2
4025	1,3,5,7,8,25,30,32	2,4,6,26,27,28,29,31	9	O88797,P01681,P35280,Q05511,Q4FZU2,Q6IFW6,Q6IG05,Q6IMF3,Q6P6Q2
4026	1,3,5,7,8,25,31,32	2,4,6,26,27,28,29,30	4	P01681,P21588,Q80WL1,Q811M5
4027	1,3,5,7,8,26,27,28	2,4,6,25,29,30,31,32	6	D3ZUC6,O55145,P47967,P97840,Q5RLM2,Q9WTT6
4028	1,3,5,7,8,26,27,29	2,4,6,25,28,30,31,32	7	D3ZUC6,P06760,P22006,P47967,Q5I0D1,Q9QX74,Q9WTT6
4029	1,3,5,7,8,26,27,30	2,4,6,25,28,29,31,32	5	Q6IFU7,Q6IFW6,Q6IG05,Q6IMF3,Q6P6Q2
4030	1,3,5,7,8,26,27,31	2,4,6,25,28,29,30,32	2	O55145,Q68G31
4031	1,3,5,7,8,26,27,32	2,4,6,25,28,29,30,31	0	
4032	1,3,5,7,8,26,28,29	2,4,6,25,27,30,31,32	10	D3ZUC6,P06760,P21674,P47967,P49134,P97840,Q6P6R2,Q9QX74,Q9QYP1,Q9WTT6
4033	1,3,5,7,8,26,28,30	2,4,6,25,27,29,31,32	4	P47967,P49134,P80299,P97840
4034	1,3,5,7,8,26,28,31	2,4,6,25,27,29,30,32	4	D3ZUC6,O55145,Q5RLM2,Q811M5
4035	1,3,5,7,8,26,28,32	2,4,6,25,27,29,30,31	2	D3ZUC6,P47967
4036	1,3,5,7,8,26,29,30	2,4,6,25,27,28,31,32	6	P22006,P49134,Q6IFU7,Q6IFW6,Q6IMF3,Q9QX74
4037	1,3,5,7,8,26,29,31	2,4,6,25,27,28,30,32	3	P06760,P49134,Q9QX74

4038	1,3,5,7,8,26,29,32	2,4,6,25,27,28,30,31	0	
4039	1,3,5,7,8,26,30,31	2,4,6,25,27,28,29,32	3	P49134,Q6IG05,Q6IMF3
4040	1,3,5,7,8,26,30,32	2,4,6,25,27,28,29,31	1	P35280
4041	1,3,5,7,8,26,31,32	2,4,6,25,27,28,29,30	1	Q62714
4042	1,3,5,7,8,27,28,29	2,4,6,25,26,30,31,32	7	D3ZUC6,P00507,P06760,P47967,P97840,Q5I0D1,Q812E4
4043	1,3,5,7,8,27,28,30	2,4,6,25,26,29,31,32	9	D3ZUC6,P19218,P47967,P97840,Q6IFU7,Q6IFW6,Q6IG05,Q6IMF3,Q6P6Q2
4044	1,3,5,7,8,27,28,31	2,4,6,25,26,29,30,32	4	D3ZUC6,O55145,P47967,Q68G31
4045	1,3,5,7,8,27,28,32	2,4,6,25,26,29,30,31	3	D3ZUC6,P47967,P97840
4046	1,3,5,7,8,27,29,30	2,4,6,25,26,28,31,32	10	P19218,P22006,P47967,Q4FZU2,Q6IFU7,Q6IFW6,Q6IG02,Q6IG05,Q6IMF3,Q6P6Q2
4047	1,3,5,7,8,27,29,31	2,4,6,25,26,28,30,32	3	D3ZUC6,P06760,Q68G31
4048	1,3,5,7,8,27,29,32	2,4,6,25,26,28,30,31	1	P47967
4049	1,3,5,7,8,27,30,31	2,4,6,25,26,28,29,32	9	P19218,P23739,Q4FZU2,Q6AYR8,Q6IFU7,Q6IFW6,Q6IG05,Q6IMF3,Q6P6Q2
4050	1,3,5,7,8,27,30,32	2,4,6,25,26,28,29,31	8	P19218,P35280,Q4FZU2,Q6IFU7,Q6IFW6,Q6IG05,Q6IMF3,Q6P6Q2
4051	1,3,5,7,8,27,31,32	2,4,6,25,26,28,29,30	3	P23739,Q62714,Q80WL1
4052	1,3,5,7,8,28,29,30	2,4,6,25,26,27,31,32	9	D3ZUC6,P21674,P47967,P97840,Q6IFU7,Q6IFW6,Q6IMF3,Q6P6Q2,Q9QYP1
4053	1,3,5,7,8,28,29,31	2,4,6,25,26,27,30,32	4	D3ZUC6,P21674,P47967,Q811M5
4054	1,3,5,7,8,28,29,32	2,4,6,25,26,27,30,31	6	D3ZUC6,P21674,P47967,P54921,P97840,Q9QYP1
4055	1,3,5,7,8,28,30,31	2,4,6,25,26,27,29,32	2	Q6IG05,Q6IMF3
4056	1,3,5,7,8,28,30,32	2,4,6,25,26,27,29,31	2	P35280,Q6IMF3
4057	1,3,5,7,8,28,31,32	2,4,6,25,26,27,29,30	3	D3ZUC6,Q80WL1,Q811M5

4058	1,3,5,7,8,29,30,31	2,4,6,25,26,27,28,32	9 P49134,Q4FZU2,Q5RJR2,Q6IFW6,Q6IG02,Q6IG05,Q6IMF3,Q6P6Q2,Q9QWN8
4059	1,3,5,7,8,29,30,32	2,4,6,25,26,27,28,31	7 O88797,P35280,Q4FZU2,Q6IFU7,Q6IFW6,Q6IMF3,Q6P6Q2
4060	1,3,5,7,8,29,31,32	2,4,6,25,26,27,28,30	2 P21588,Q80WL1
4061	1,3,5,7,8,30,31,32	2,4,6,25,26,27,28,29	4 P35280,Q4FZU2,Q6IG05,Q6IMF3
4062	1,3,5,7,25,26,27,28	2,4,6,8,29,30,31,32	2 O55145,P00714
4063	1,3,5,7,25,26,27,29	2,4,6,8,28,30,31,32	2 P22006,Q63532
4064	1,3,5,7,25,26,27,30	2,4,6,8,28,29,31,32	1 Q05511
4065	1,3,5,7,25,26,27,31	2,4,6,8,28,29,30,32	2 O55145,Q68G31
4066	1,3,5,7,25,26,27,32	2,4,6,8,28,29,30,31	0
4067	1,3,5,7,25,26,28,29	2,4,6,8,27,30,31,32	3 P00714,P22006,P47967
4068	1,3,5,7,25,26,28,30	2,4,6,8,27,29,31,32	3 P00714,P80299,Q62975
4069	1,3,5,7,25,26,28,31	2,4,6,8,27,29,30,32	2 O55145,P00714
4070	1,3,5,7,25,26,28,32	2,4,6,8,27,29,30,31	1 Q62975
4071	1,3,5,7,25,26,29,30	2,4,6,8,27,28,31,32	1 P22006
4072	1,3,5,7,25,26,29,31	2,4,6,8,27,28,30,32	0
4073	1,3,5,7,25,26,29,32	2,4,6,8,27,28,30,31	0
4074	1,3,5,7,25,26,30,31	2,4,6,8,27,28,29,32	5 P22282,P25809,P47727,Q6AYR9,Q812E4
4075	1,3,5,7,25,26,30,32	2,4,6,8,27,28,29,31	1 Q62975
4076	1,3,5,7,25,26,31,32	2,4,6,8,27,28,29,30	1 P25809
4077	1,3,5,7,25,27,28,29	2,4,6,8,26,30,31,32	3 P47967,P97840,Q6AY61

4078	1,3,5,7,25,27,28,30	2,4,6,8,26,29,31,32	0	
4079	1,3,5,7,25,27,28,31	2,4,6,8,26,29,30,32	2	O55145,Q68G31
4080	1,3,5,7,25,27,28,32	2,4,6,8,26,29,30,31	2	Q62761;Q62762;Q62763,Q6AY61
4081	1,3,5,7,25,27,29,30	2,4,6,8,26,28,31,32	6	O54728,P08649,P22006,Q63532,Q6IFW6,Q6IG02
4082	1,3,5,7,25,27,29,31	2,4,6,8,26,28,30,32	3	O54728,P08649,Q68G31
4083	1,3,5,7,25,27,29,32	2,4,6,8,26,28,30,31	2	Q63532,Q6AY61
4084	1,3,5,7,25,27,30,31	2,4,6,8,26,28,29,32	3	O54728,P08649,Q05511
4085	1,3,5,7,25,27,30,32	2,4,6,8,26,28,29,31	1	Q05511
4086	1,3,5,7,25,27,31,32	2,4,6,8,26,28,29,30	2	P23739,Q80WL1
4087	1,3,5,7,25,28,29,30	2,4,6,8,26,27,31,32	0	
4088	1,3,5,7,25,28,29,31	2,4,6,8,26,27,30,32	1	P20762
4089	1,3,5,7,25,28,29,32	2,4,6,8,26,27,30,31	3	P20762,P54921,Q6AY61
4090	1,3,5,7,25,28,30,31	2,4,6,8,26,27,29,32	2	P25809,Q62975
4091	1,3,5,7,25,28,30,32	2,4,6,8,26,27,29,31	2	Q5BJY9,Q62975
4092	1,3,5,7,25,28,31,32	2,4,6,8,26,27,29,30	3	Q62975,Q80WL1,Q811M5
4093	1,3,5,7,25,29,30,31	2,4,6,8,26,27,28,32	6	O54728,P08649,P22282,P25809,P50280,Q9QWN8
4094	1,3,5,7,25,29,30,32	2,4,6,8,26,27,28,31	1	Q5BJY9
4095	1,3,5,7,25,29,31,32	2,4,6,8,26,27,28,30	3	P08649,P20762,Q80WL1
4096	1,3,5,7,25,30,31,32	2,4,6,8,26,27,28,29	5	P08649,P22273,P25809,Q5BJY9,Q6P6R2
4097	1,3,5,7,26,27,28,29	2,4,6,8,25,30,31,32	3	D3ZUC6,P47967,P97840

4098	1,3,5,7,26,<br>27,28,30	2,4,6,8,25,<br>29,31,32	2	P47967,P97840
4099	1,3,5,7,26,<br>27,28,31	2,4,6,8,25,<br>29,30,32	3	O55145,P47967,Q68G31
4100	1,3,5,7,26,<br>27,28,32	2,4,6,8,25,<br>29,30,31	2	P47967,P97840
4101	1,3,5,7,26,<br>27,29,30	2,4,6,8,25,<br>28,31,32	3	P47967,P97840,Q63532
4102	1,3,5,7,26,<br>27,29,31	2,4,6,8,25,<br>28,30,32	3	P02631,P08649,Q68G31
4103	1,3,5,7,26,<br>27,29,32	2,4,6,8,25,<br>28,30,31	2	P47967,Q63532
4104	1,3,5,7,26,<br>27,30,31	2,4,6,8,25,<br>28,29,32	1	P08649
4105	1,3,5,7,26,<br>27,30,32	2,4,6,8,25,<br>28,29,31	1	P12020
4106	1,3,5,7,26,<br>27,31,32	2,4,6,8,25,<br>28,29,30	0	
4107	1,3,5,7,26,<br>28,29,30	2,4,6,8,25,<br>27,31,32	2	P47967,P97840
4108	1,3,5,7,26,<br>28,29,31	2,4,6,8,25,<br>27,30,32	4	P47967,P97840,Q01205,Q68G31
4109	1,3,5,7,26,<br>28,29,32	2,4,6,8,25,<br>27,30,31	3	P47967,P54921,P97840
4110	1,3,5,7,26,<br>28,30,31	2,4,6,8,25,<br>27,29,32	0	
4111	1,3,5,7,26,<br>28,30,32	2,4,6,8,25,<br>27,29,31	1	P97840
4112	1,3,5,7,26,<br>28,31,32	2,4,6,8,25,<br>27,29,30	0	
4113	1,3,5,7,26,<br>29,30,31	2,4,6,8,25,<br>27,28,32	3	P08649,Q6AYR9,Q9QWN8
4114	1,3,5,7,26,<br>29,30,32	2,4,6,8,25,<br>27,28,31	0	
4115	1,3,5,7,26,<br>29,31,32	2,4,6,8,25,<br>27,28,30	1	P08649
4116	1,3,5,7,26,<br>30,31,32	2,4,6,8,25,<br>27,28,29	2	P08649,P25809
4117	1,3,5,7,27,<br>28,29,30	2,4,6,8,25,<br>26,31,32	4	P00507,P08649,P47967,P97840

4118	1,3,5,7,27,<br>28,29,31	2,4,6,8,25,<br>26,30,32	6	D3ZUC6,P00507,P08649,P47967,P97840,Q68G31
4119	1,3,5,7,27,<br>28,29,32	2,4,6,8,25,<br>26,30,31	6	P08649,P47967,P54921,P97840,Q62761;Q62762;Q62763,Q6AY61
4120	1,3,5,7,27,<br>28,30,31	2,4,6,8,25,<br>26,29,32	1	P08649
4121	1,3,5,7,27,<br>28,30,32	2,4,6,8,25,<br>26,29,31	3	P08649,P47967,P97840
4122	1,3,5,7,27,<br>28,31,32	2,4,6,8,25,<br>26,29,30	1	P08649
4123	1,3,5,7,27,<br>29,30,31	2,4,6,8,25,<br>26,28,32	2	O54728,P08649
4124	1,3,5,7,27,<br>29,30,32	2,4,6,8,25,<br>26,28,31	1	P08649
4125	1,3,5,7,27,<br>29,31,32	2,4,6,8,25,<br>26,28,30	1	P08649
4126	1,3,5,7,27,<br>30,31,32	2,4,6,8,25,<br>26,28,29	2	P08649,P23739
4127	1,3,5,7,28,<br>29,30,31	2,4,6,8,25,<br>26,27,32	2	P08649,P17559
4128	1,3,5,7,28,<br>29,30,32	2,4,6,8,25,<br>26,27,31	6	P08649,P17559,P47967,P54921,P97840,Q5BJY9
4129	1,3,5,7,28,<br>29,31,32	2,4,6,8,25,<br>26,27,30	5	P08649,P17559,P20762,P54921,Q6AY61
4130	1,3,5,7,28,<br>30,31,32	2,4,6,8,25,<br>26,27,29	4	P08649,P17559,P25809,Q5BJY9
4131	1,3,5,7,29,<br>30,31,32	2,4,6,8,25,<br>26,27,28	5	P08649,P17559,P26342,P36860,Q5BJY9
4132	1,3,5,8,25,<br>26,27,28	2,4,6,7,29,<br>30,31,32	5	O55145,P00714,P26342,P36860,P80299
4133	1,3,5,8,25,<br>26,27,29	2,4,6,7,28,<br>30,31,32	2	P22006,P80299
4134	1,3,5,8,25,<br>26,27,30	2,4,6,7,28,<br>29,31,32	8	P80299,Q05511,Q63617,Q6IFU7,Q6IFW6,Q6IG05,Q6IMF3,Q6P6Q2
4135	1,3,5,8,25,<br>26,27,31	2,4,6,7,28,<br>29,30,32	5	O55145,P00714,P23739,Q80WL1,Q9R168
4136	1,3,5,8,25,<br>26,27,32	2,4,6,7,28,<br>29,30,31	3	P01681,P80299,Q80WL1
4137	1,3,5,8,25,<br>26,28,29	2,4,6,7,27,<br>30,31,32	5	P00714,P01039,P22006,P80299,Q9Z0J6

4138	1,3,5,8,25,<br>26,28,30	2,4,6,7,27,<br>29,31,32	3	O88797,P00714,P80299
4139	1,3,5,8,25,<br>26,28,31	2,4,6,7,27,<br>29,30,32	4	O55145,P00714,P80299,Q80WL1
4140	1,3,5,8,25,<br>26,28,32	2,4,6,7,27,<br>29,30,31	3	P01681,P80299,Q80WL1
4141	1,3,5,8,25,<br>26,29,30	2,4,6,7,27,<br>28,31,32	10	O88797,P00714,P05964,P11598,P22006,P80299,Q6IFU7,Q6IFW6,Q6IG02,Q6IMF3
4142	1,3,5,8,25,<br>26,29,31	2,4,6,7,27,<br>28,30,32	2	P00714,Q80WL1
4143	1,3,5,8,25,<br>26,29,32	2,4,6,7,27,<br>28,30,31	4	P01681,P13432,P80299,Q80WL1
4144	1,3,5,8,25,<br>26,30,31	2,4,6,7,27,<br>28,29,32	9	O88797,P00714,P11598,P80299,Q05511,Q63617,Q6IFW6,Q6IG05,Q6IMF3
4145	1,3,5,8,25,<br>26,30,32	2,4,6,7,27,<br>28,29,31	5	O88797,P01681,P80299,Q05511,Q63617
4146	1,3,5,8,25,<br>26,31,32	2,4,6,7,27,<br>28,29,30	3	P01681,Q63617,Q80WL1
4147	1,3,5,8,25,<br>27,28,29	2,4,6,7,26,<br>30,31,32	3	P22006,P36860,Q80WL1
4148	1,3,5,8,25,<br>27,28,30	2,4,6,7,26,<br>29,31,32	7	P80299,Q05511,Q6IFU7,Q6IFW6,Q6IG05,Q6IMF3,Q6P6Q2
4149	1,3,5,8,25,<br>27,28,31	2,4,6,7,26,<br>29,30,32	5	O55145,P00714,P23739,Q80WL1,Q9R168
4150	1,3,5,8,25,<br>27,28,32	2,4,6,7,26,<br>29,30,31	2	P01681,Q80WL1
4151	1,3,5,8,25,<br>27,29,30	2,4,6,7,26,<br>28,31,32	10	O88797,P22006,Q05511,Q4FZU2,Q6IFU7,Q6IFW6,Q6IG02,Q6IG05,Q6IMF3,Q6P6Q2
4152	1,3,5,8,25,<br>27,29,31	2,4,6,7,26,<br>28,30,32	2	Q80WL1,Q9R168
4153	1,3,5,8,25,<br>27,29,32	2,4,6,7,26,<br>28,30,31	2	P01681,Q80WL1
4154	1,3,5,8,25,<br>27,30,31	2,4,6,7,26,<br>28,29,32	11	P11598,P23739,Q05511,Q4FZU2,Q63617,Q6IFU7,Q6IFW6,Q6IG02,Q6IG05,Q6IMF3,Q6P6Q2
4155	1,3,5,8,25,<br>27,30,32	2,4,6,7,26,<br>28,29,31	9	O88797,P01681,Q05511,Q4FZU2,Q6IFU7,Q6IFW6,Q6IG05,Q6IMF3,Q6P6Q2
4156	1,3,5,8,25,<br>27,31,32	2,4,6,7,26,<br>28,29,30	3	P01681,P23739,Q80WL1
4157	1,3,5,8,25,<br>28,29,30	2,4,6,7,26,<br>27,31,32	9	O88797,P00714,P22006,P80299,Q6IFU7,Q6IFW6,Q6IMF3,Q6P6Q2,Q9Z0J6

4158	1,3,5,8,25,28,29,31	2,4,6,7,26,27,30,32	3	P00714,Q80WL1,Q9R168
4159	1,3,5,8,25,28,29,32	2,4,6,7,26,27,30,31	3	P01039,P01681,Q80WL1
4160	1,3,5,8,25,28,30,31	2,4,6,7,26,27,29,32	4	P00714,Q6IFW6,Q6IG05,Q6IMF3
4161	1,3,5,8,25,28,30,32	2,4,6,7,26,27,29,31	4	O88797,P01681,P80299,Q6IFU7
4162	1,3,5,8,25,28,31,32	2,4,6,7,26,27,29,30	4	P01681,P21588,Q80WL1,Q811M5
4163	1,3,5,8,25,29,30,31	2,4,6,7,26,27,28,32	10	O88797,P05964,P11598,Q4FZU2,Q6IFU7,Q6IFW6,Q6IG02,Q6IG05,Q6IMF3,Q6P6Q2
4164	1,3,5,8,25,29,30,32	2,4,6,7,26,27,28,31	8	O88797,P01681,P11598,Q4FZU2,Q6IFU7,Q6IFW6,Q6IMF3,Q6P6Q2
4165	1,3,5,8,25,29,31,32	2,4,6,7,26,27,28,30	3	P01681,P21588,Q80WL1
4166	1,3,5,8,25,30,31,32	2,4,6,7,26,27,28,29	12	O88797,P01681,P11598,P20646,P21588,Q05511,Q4FZU2,Q63617,Q6IFW6,Q6IG05,Q6IMF3,Q80WL1
4167	1,3,5,8,26,27,28,29	2,4,6,7,25,30,31,32	4	D3ZUC6,P26342,P47967,Q6P6R2
4168	1,3,5,8,26,27,28,30	2,4,6,7,25,29,31,32	2	P80299,Q6IFU7
4169	1,3,5,8,26,27,28,31	2,4,6,7,25,29,30,32	3	O55145,P00714,Q9R168
4170	1,3,5,8,26,27,28,32	2,4,6,7,25,29,30,31	1	Q9QWN8
4171	1,3,5,8,26,27,29,30	2,4,6,7,25,28,31,32	6	Q6IFU7,Q6IFW6,Q6IG02,Q6IG05,Q6IMF3,Q6P6Q2
4172	1,3,5,8,26,27,29,31	2,4,6,7,25,28,30,32	1	Q9R168
4173	1,3,5,8,26,27,29,32	2,4,6,7,25,28,30,31	0	
4174	1,3,5,8,26,27,30,31	2,4,6,7,25,28,29,32	4	P23739,Q6IFW6,Q6IG05,Q6IMF3
4175	1,3,5,8,26,27,30,32	2,4,6,7,25,28,29,31	3	Q6IFU7,Q6IG05,Q6IMF3
4176	1,3,5,8,26,27,31,32	2,4,6,7,25,28,29,30	2	P23739,Q80WL1
4177	1,3,5,8,26,28,29,30	2,4,6,7,25,27,31,32	5	O88797,P00714,P80299,Q6IFU7,Q9QYP1

4178	1,3,5,8,26, 28,29,31	2,4,6,7,25, 27,30,32	2	P00714,Q9R168
4179	1,3,5,8,26, 28,29,32	2,4,6,7,25, 27,30,31	1	Q9QYP1
4180	1,3,5,8,26, 28,30,31	2,4,6,7,25, 27,29,32	1	P00714
4181	1,3,5,8,26, 28,30,32	2,4,6,7,25, 27,29,31	2	O88797,P80299
4182	1,3,5,8,26, 28,31,32	2,4,6,7,25, 27,29,30	1	Q80WL1
4183	1,3,5,8,26, 29,30,31	2,4,6,7,25, 27,28,32	2	Q6IFW6,Q6IMF3
4184	1,3,5,8,26, 29,30,32	2,4,6,7,25, 27,28,31	3	O88797,Q6IFU7,Q6IMF3
4185	1,3,5,8,26, 29,31,32	2,4,6,7,25, 27,28,30	1	Q80WL1
4186	1,3,5,8,26, 30,31,32	2,4,6,7,25, 27,28,29	2	O88797,Q63617
4187	1,3,5,8,27, 28,29,30	2,4,6,7,25, 26,31,32	8	P19218,P47967,Q6IFU7,Q6IFW6,Q6IG02,Q6IMF3,Q6P6Q2,Q9WVH8
4188	1,3,5,8,27, 28,29,31	2,4,6,7,25, 26,30,32	3	D3ZUC6,Q80WL1,Q9R168
4189	1,3,5,8,27, 28,29,32	2,4,6,7,25, 26,30,31	3	P47967,Q80WL1,Q812E4
4190	1,3,5,8,27, 28,30,31	2,4,6,7,25, 26,29,32	8	P23739,Q6AYR8,Q6IFU7,Q6IFW6,Q6IG05,Q6IMF3,Q6P6Q2,Q9WVH8
4191	1,3,5,8,27, 28,30,32	2,4,6,7,25, 26,29,31	5	P19218,Q6IFU7,Q6IMF3,Q6P6Q2,Q9WVH8
4192	1,3,5,8,27, 28,31,32	2,4,6,7,25, 26,29,30	2	P23739,Q80WL1
4193	1,3,5,8,27, 29,30,31	2,4,6,7,25, 26,28,32	7	Q4FZU2,Q6IFU7,Q6IFW6,Q6IG02,Q6IG05,Q6IMF3,Q6P6Q2
4194	1,3,5,8,27, 29,30,32	2,4,6,7,25, 26,28,31	9	O88797,Q4FZU2,Q6IFU7,Q6IFW6,Q6IG02,Q6IG05,Q6IMF3,Q6P6Q2,Q9WVH8
4195	1,3,5,8,27, 29,31,32	2,4,6,7,25, 26,28,30	1	Q80WL1
4196	1,3,5,8,27, 30,31,32	2,4,6,7,25, 26,28,29	8	P20646,P23739,Q4FZU2,Q6IFU7,Q6IFW6,Q6IG05,Q6IMF3,Q6P6Q2
4197	1,3,5,8,28, 29,30,31	2,4,6,7,25, 26,27,32	3	Q6IFU7,Q6IFW6,Q6IMF3

4198	1,3,5,8,28,29,30,32	2,4,6,7,25,26,27,31	3	O88797,Q6IFU7,Q6IMF3
4199	1,3,5,8,28,29,31,32	2,4,6,7,25,26,27,30	2	P21588,Q80WL1
4200	1,3,5,8,28,30,31,32	2,4,6,7,25,26,27,29	0	
4201	1,3,5,8,29,30,31,32	2,4,6,7,25,26,27,28	6	O88797,P63081,Q6IFU7,Q6IFW6,Q6IMF3,Q6P6Q2
4202	1,3,5,25,26,27,28,29	2,4,6,7,8,30,31,32	6	O54858,P00714,P15999,P26342,P82471,Q63532
4203	1,3,5,25,26,27,28,30	2,4,6,7,8,29,31,32	4	O55004,P00714,P80299,P82471
4204	1,3,5,25,26,27,28,31	2,4,6,7,8,29,30,32	5	O55145,P00714,P02631,P62804,Q99041
4205	1,3,5,25,26,27,28,32	2,4,6,7,8,29,30,31	4	P12020,P62804,P82471,Q5RJR2
4206	1,3,5,25,26,27,29,30	2,4,6,7,8,28,31,32	7	P02631,P11598,P12020,P22006,P82471,Q63532,Q63617
4207	1,3,5,25,26,27,29,31	2,4,6,7,8,28,30,32	3	P02631,Q63617,Q99041
4208	1,3,5,25,26,27,29,32	2,4,6,7,8,28,30,31	5	P07171,P12020,P82471,Q63532,Q99041
4209	1,3,5,25,26,27,30,31	2,4,6,7,8,28,29,32	10	P00714,P02631,P02780,P11598,P22273,P22282,P30120,P62804,Q63617,Q99041
4210	1,3,5,25,26,27,30,32	2,4,6,7,8,28,29,31	7	P11598,P12020,P21674,P22273,P62804,P82471,Q63617
4211	1,3,5,25,26,27,31,32	2,4,6,7,8,28,29,30	7	P02631,P22273,P23739,P62804,Q63617,Q80WL1,Q99041
4212	1,3,5,25,26,28,29,30	2,4,6,7,8,27,31,32	7	P00714,P22273,P22282,P80299,P82471,Q63532,Q6TMA8
4213	1,3,5,25,26,28,29,31	2,4,6,7,8,27,30,32	6	P00714,P02631,P22273,P22282,Q01205,Q99041
4214	1,3,5,25,26,28,29,32	2,4,6,7,8,27,30,31	4	P22273,P82471,Q63532,Q99041
4215	1,3,5,25,26,28,30,31	2,4,6,7,8,27,29,32	10	P00714,P02631,P11598,P22273,P22282,P25809,P62804,Q4G075,Q6TMA8,Q99041
4216	1,3,5,25,26,28,30,32	2,4,6,7,8,27,29,31	9	P19223,P22273,P50115,P62804,P80299,P82471,Q00715,Q4G075,Q62975
4217	1,3,5,25,26,28,31,32	2,4,6,7,8,27,29,30	7	P19223,P22273,P62804,Q00715,Q4G075,Q80WL1,Q99041

4218	1,3,5,25,26,29,30,31	2,4,6,7,8,27,28,32	12	P00714,P02631,P05964,P06761,P11598,P22273,P22282,P25809,P50280,Q63617,Q6AYR9,Q99041
4219	1,3,5,25,26,29,30,32	2,4,6,7,8,27,28,31	11	P06761,P11598,P12020,P22273,P22282,P50115,P82471,Q4G075,Q63532,Q63617,Q99041
4220	1,3,5,25,26,29,31,32	2,4,6,7,8,27,28,30	10	P02631,P11598,P19218,P22273,P22282,P62804,Q4G075,Q63617,Q80WL1,Q99041
4221	1,3,5,25,26,30,31,32	2,4,6,7,8,27,28,29	15	P02631,P06761,P11598,P22273,P22282,P22283,P25809,P47727,P50115,P62804,Q00715,Q4G075,Q63617,Q812E4,Q99041
4222	1,3,5,25,27,28,29,30	2,4,6,7,8,26,31,32	5	O54858,O55004,P20766,P82471,Q63532
4223	1,3,5,25,27,28,29,31	2,4,6,7,8,26,30,32	5	O55004,P02631,P20762,Q80WL1,Q99041
4224	1,3,5,25,27,28,29,32	2,4,6,7,8,26,30,31	7	O54858,P20762,P82471,Q62761;Q62762;Q62763,Q63532,Q6AY61,Q80WL1
4225	1,3,5,25,27,28,30,31	2,4,6,7,8,26,29,32	5	O55004,P00714,P02631,P22273,P23739
4226	1,3,5,25,27,28,30,32	2,4,6,7,8,26,29,31	3	P22273,P82471,Q9QX74
4227	1,3,5,25,27,28,31,32	2,4,6,7,8,26,29,30	6	P20762,P22273,P23739,P62804,Q80WL1,Q99041
4228	1,3,5,25,27,29,30,31	2,4,6,7,8,26,28,32	13	O54728,O55004,P02631,P07150,P08649,P11598,P20762,P22273,P22282,Q63617,Q6IFW6,Q6IG02,Q99041
4229	1,3,5,25,27,29,30,32	2,4,6,7,8,26,28,31	8	P07150,P11598,P12020,P20762,P22273,P82471,Q63532,Q63617
4230	1,3,5,25,27,29,31,32	2,4,6,7,8,26,28,30	8	P02631,P07150,P08649,P20762,P22273,P62804,Q80WL1,Q99041
4231	1,3,5,25,27,30,31,32	2,4,6,7,8,26,28,29	16	P02631,P06761,P07150,P08649,P11598,P20762,P21674,P22273,P23739,P62804,Q00715,Q63617,Q6P6R2,Q99041,Q9QX74,Q9WTT6
4232	1,3,5,25,28,29,30,31	2,4,6,7,8,26,27,32	10	O55004,P00714,P02631,P11598,P20762,P22273,P22282,P50115,P50280,Q99041
4233	1,3,5,25,28,29,30,32	2,4,6,7,8,26,27,31	7	P20762,P20766,P22273,P50115,Q5BJY9,Q62894,Q62975
4234	1,3,5,25,28,29,31,32	2,4,6,7,8,26,27,30	6	P20762,P22273,P50115,P62804,Q80WL1,Q99041
4235	1,3,5,25,28,30,31,32	2,4,6,7,8,26,27,29	13	P20762,P22273,P22282,P25809,P50115,P62804,Q00715,Q4G075,Q5BJY9,Q62975,Q99041,Q9QX74,Q9WTT6
4236	1,3,5,25,29,30,31,32	2,4,6,7,8,26,27,28	19	P02631,P06761,P07150,P08649,P11598,P17559,P20762,P22273,P22282,P25809,P50115,P50116,P50280,P62804,Q4G075,Q5BJY9,Q63617,Q99041,Q9WTT6
4237	1,3,5,26,27,28,29,30	2,4,6,7,8,25,31,32	7	O54858,O55004,P02631,P12020,P47967,P82471,Q63532

4238	1,3,5,26,27,28,29,31	2,4,6,7,8,25,30,32	3	O54858,P02631,Q01205
4239	1,3,5,26,27,28,29,32	2,4,6,7,8,25,30,31	6	O54858,P02631,P12020,P47967,P82471,Q63532
4240	1,3,5,26,27,28,30,31	2,4,6,7,8,25,29,32	2	P00714,P02631
4241	1,3,5,26,27,28,30,32	2,4,6,7,8,25,29,31	3	P02631,P12020,P82471
4242	1,3,5,26,27,28,31,32	2,4,6,7,8,25,29,30	2	P02631,P62804
4243	1,3,5,26,27,29,30,31	2,4,6,7,8,25,28,32	7	P02454,P02631,P08649,P11598,P22273,P22282,Q63617
4244	1,3,5,26,27,29,30,32	2,4,6,7,8,25,28,31	7	O54858,P02631,P06911,P12020,P22273,P82471,Q63532
4245	1,3,5,26,27,29,31,32	2,4,6,7,8,25,28,30	3	P02631,P22273,P62804
4246	1,3,5,26,27,30,31,32	2,4,6,7,8,25,28,29	7	P02631,P08649,P12020,P22273,P62804,Q00715,Q63617
4247	1,3,5,26,28,29,30,31	2,4,6,7,8,25,27,32	7	P00714,P02631,P22273,P22282,P50115,Q4G075,Q6TMA8
4248	1,3,5,26,28,29,30,32	2,4,6,7,8,25,27,31	8	O54858,P02631,P12020,P22273,P50115,P82471,Q4G075,Q62894
4249	1,3,5,26,28,29,31,32	2,4,6,7,8,25,27,30	6	P02631,P22273,P50115,P62804,Q4G075,Q99041
4250	1,3,5,26,28,30,31,32	2,4,6,7,8,25,27,29	8	P02631,P22273,P22282,P25809,P50115,P62804,Q00715,Q4G075
4251	1,3,5,26,29,30,31,32	2,4,6,7,8,25,27,28	14	P02631,P06761,P06911,P08649,P0C0A9,P11598,P22273,P22282,P36860,P50115,P62804,Q4G075,Q63617,Q99041
4252	1,3,5,27,28,29,30,31	2,4,6,7,8,25,26,32	5	O54858,O55004,P02631,P08649,Q9WVH8
4253	1,3,5,27,28,29,30,32	2,4,6,7,8,25,26,31	7	O54858,P02631,P08649,P12020,P20766,Q63532,Q9WVH8
4254	1,3,5,27,28,29,31,32	2,4,6,7,8,25,26,30	5	O54858,P02631,P08649,P20762,Q80WL1
4255	1,3,5,27,28,30,31,32	2,4,6,7,8,25,26,29	7	P02631,P08649,P22273,P23739,P62804,Q9QX74,Q9WVH8
4256	1,3,5,27,29,30,31,32	2,4,6,7,8,25,26,28	6	P02631,P07150,P08649,P20762,P22273,Q9WTT6
4257	1,3,5,28,29,30,31,32	2,4,6,7,8,25,26,27	10	P02631,P08649,P17559,P20762,P22273,P50115,P50116,Q4G075,Q5BJY9,Q9WTT6

4258	1,3,6,7,8,25,26,27	2,4,5,28,29,30,31,32	2	P63081,P70549
4259	1,3,6,7,8,25,26,28	2,4,5,27,29,30,31,32	5	P06911,P70549,P80299,Q63598,Q9QW07
4260	1,3,6,7,8,25,26,29	2,4,5,27,28,30,31,32	2	P70549,Q9QX74
4261	1,3,6,7,8,25,26,30	2,4,5,27,28,29,31,32	2	O70417,P80299
4262	1,3,6,7,8,25,26,31	2,4,5,27,28,29,30,32	3	P57113,P70549,Q63598
4263	1,3,6,7,8,25,26,32	2,4,5,27,28,29,30,31	2	P13432,P57113
4264	1,3,6,7,8,25,27,28	2,4,5,26,29,30,31,32	6	O70417,P06911,P19629,P36860,P63081,Q9QW07
4265	1,3,6,7,8,25,27,29	2,4,5,26,28,30,31,32	2	P06911,P70549
4266	1,3,6,7,8,25,27,30	2,4,5,26,28,29,31,32	2	O70417,Q6IFW6
4267	1,3,6,7,8,25,27,31	2,4,5,26,28,29,30,32	2	P06911,Q62894
4268	1,3,6,7,8,25,27,32	2,4,5,26,28,29,30,31	2	P01681,P98166
4269	1,3,6,7,8,25,28,29	2,4,5,26,27,30,31,32	4	P06911,P21674,P70549,Q9QW07
4270	1,3,6,7,8,25,28,30	2,4,5,26,27,29,31,32	3	O70417,P06911,Q9QW07
4271	1,3,6,7,8,25,28,31	2,4,5,26,27,29,30,32	4	P06911,Q63598,Q811M5,Q9QW07
4272	1,3,6,7,8,25,28,32	2,4,5,26,27,29,30,31	2	P06911,Q9QW07
4273	1,3,6,7,8,25,29,30	2,4,5,26,27,28,31,32	2	O70417,Q6IFW6
4274	1,3,6,7,8,25,29,31	2,4,5,26,27,28,30,32	2	P06911,P70549
4275	1,3,6,7,8,25,29,32	2,4,5,26,27,28,30,31	1	P13432
4276	1,3,6,7,8,25,30,31	2,4,5,26,27,28,29,32	2	O70417,P06911
4277	1,3,6,7,8,25,30,32	2,4,5,26,27,28,29,31	3	O70417,P01681,P35280

4278	1,3,6,7,8,25,31,32	2,4,5,26,27,28,29,30	3	P01681,P21588,P57113
4279	1,3,6,7,8,26,27,28	2,4,5,25,29,30,31,32	5	D3ZUC6,P63081,P70549,Q9QW07,Q9WTT6
4280	1,3,6,7,8,26,27,29	2,4,5,25,28,30,31,32	4	P02783,P20759,P70549,Q9QX74
4281	1,3,6,7,8,26,27,30	2,4,5,25,28,29,31,32	2	O70417,P02783
4282	1,3,6,7,8,26,27,31	2,4,5,25,28,29,30,32	2	P70549,Q62894
4283	1,3,6,7,8,26,27,32	2,4,5,25,28,29,30,31	0	
4284	1,3,6,7,8,26,28,29	2,4,5,25,27,30,31,32	8	D3ZUC6,P20759,P21674,P70549,Q6P6R2,Q9QW07,Q9QX74,Q9QYP1
4285	1,3,6,7,8,26,28,30	2,4,5,25,27,29,31,32	1	Q9QW07
4286	1,3,6,7,8,26,28,31	2,4,5,25,27,29,30,32	3	P57113,P70549,Q63598
4287	1,3,6,7,8,26,28,32	2,4,5,25,27,29,30,31	2	P57113,Q9QW07
4288	1,3,6,7,8,26,29,30	2,4,5,25,27,28,31,32	2	P02783,P49134
4289	1,3,6,7,8,26,29,31	2,4,5,25,27,28,30,32	4	P02783,P57113,P70549,Q9QX74
4290	1,3,6,7,8,26,29,32	2,4,5,25,27,28,30,31	2	P02783,P70549
4291	1,3,6,7,8,26,30,31	2,4,5,25,27,28,29,32	1	P57113
4292	1,3,6,7,8,26,30,32	2,4,5,25,27,28,29,31	2	O89117,P35280
4293	1,3,6,7,8,26,31,32	2,4,5,25,27,28,29,30	3	O89117,P57113,Q62714
4294	1,3,6,7,8,27,28,29	2,4,5,25,26,30,31,32	12	D3ZUC6,P00507,P21674,P22283,P47967,P70549,Q63493,Q63617,Q812E4,Q9JI85,Q9QW07,Q9Z2L0
4295	1,3,6,7,8,27,28,30	2,4,5,25,26,29,31,32	4	O70417,P19218,Q6P6S4,Q9QW07
4296	1,3,6,7,8,27,28,31	2,4,5,25,26,29,30,32	3	D3ZUC6,Q62894,Q9QZK9
4297	1,3,6,7,8,27,28,32	2,4,5,25,26,29,30,31	7	P08723,P22283,Q6AYR9,Q6P6S4,Q812E4,Q9JI85,Q9QW07

4298	1,3,6,7,8,27,29,30	2,4,5,25,26,28,31,32	4	P02783,P19218,Q6IFU7,Q6IFW6
4299	1,3,6,7,8,27,29,31	2,4,5,25,26,28,30,32	2	P02783,P70549
4300	1,3,6,7,8,27,29,32	2,4,5,25,26,28,30,31	1	P02783
4301	1,3,6,7,8,27,30,31	2,4,5,25,26,28,29,32	2	P19218,Q9QZK9
4302	1,3,6,7,8,27,30,32	2,4,5,25,26,28,29,31	3	O70417,P19218,P35280
4303	1,3,6,7,8,27,31,32	2,4,5,25,26,28,29,30	2	Q62714,Q9QZK9
4304	1,3,6,7,8,28,29,30	2,4,5,25,26,27,31,32	2	P21674,Q9QW07
4305	1,3,6,7,8,28,29,31	2,4,5,25,26,27,30,32	5	D3ZUC6,P21674,P70549,Q63493,Q9QW07
4306	1,3,6,7,8,28,29,32	2,4,5,25,26,27,30,31	9	P08723,P21674,P30120,Q63493,Q63617,Q812E4,Q9JI85,Q9QW07,Q9QYP1
4307	1,3,6,7,8,28,30,31	2,4,5,25,26,27,29,32	0	
4308	1,3,6,7,8,28,30,32	2,4,5,25,26,27,29,31	3	O89117,P35280,Q9QW07
4309	1,3,6,7,8,28,31,32	2,4,5,25,26,27,29,30	5	O89117,P22006,P57113,Q811M5,Q9QZK9
4310	1,3,6,7,8,29,30,31	2,4,5,25,26,27,28,32	1	Q9QWN8
4311	1,3,6,7,8,29,30,32	2,4,5,25,26,27,28,31	1	P35280
4312	1,3,6,7,8,29,31,32	2,4,5,25,26,27,28,30	2	P21588,P70549
4313	1,3,6,7,8,30,31,32	2,4,5,25,26,27,28,29	3	O89117,P35280,Q9QZK9
4314	1,3,6,7,25,26,27,28	2,4,5,8,29,30,31,32	2	P63081,P70549
4315	1,3,6,7,25,26,27,29	2,4,5,8,28,30,31,32	3	P05539,P63081,P70549
4316	1,3,6,7,25,26,27,30	2,4,5,8,28,29,31,32	0	
4317	1,3,6,7,25,26,27,31	2,4,5,8,28,29,30,32	2	P70549,Q62894

4318	1,3,6,7,25,<br>26,27,32	2,4,5,8,28,<br>29,30,31	0
4319	1,3,6,7,25,<br>26,28,29	2,4,5,8,27,<br>30,31,32	3 P05539,P70549,Q9QW07
4320	1,3,6,7,25,<br>26,28,30	2,4,5,8,27,<br>29,31,32	2 Q5PQL7,Q62975
4321	1,3,6,7,25,<br>26,28,31	2,4,5,8,27,<br>29,30,32	3 P57113,P70549,Q63598
4322	1,3,6,7,25,<br>26,28,32	2,4,5,8,27,<br>29,30,31	3 P00731,Q5PQL7,Q62975
4323	1,3,6,7,25,<br>26,29,30	2,4,5,8,27,<br>28,31,32	0
4324	1,3,6,7,25,<br>26,29,31	2,4,5,8,27,<br>28,30,32	2 P57113,P70549
4325	1,3,6,7,25,<br>26,29,32	2,4,5,8,27,<br>28,30,31	1 P70549
4326	1,3,6,7,25,<br>26,30,31	2,4,5,8,27,<br>28,29,32	2 P25809,P57113
4327	1,3,6,7,25,<br>26,30,32	2,4,5,8,27,<br>28,29,31	3 P25809,Q5PQL7,Q62975
4328	1,3,6,7,25,<br>26,31,32	2,4,5,8,27,<br>28,29,30	3 P25809,P51792;P51794;P51796,P57113
4329	1,3,6,7,25,<br>27,28,29	2,4,5,8,26,<br>30,31,32	3 P05539,P63081,P70549
4330	1,3,6,7,25,<br>27,28,30	2,4,5,8,26,<br>29,31,32	0
4331	1,3,6,7,25,<br>27,28,31	2,4,5,8,26,<br>29,30,32	0
4332	1,3,6,7,25,<br>27,28,32	2,4,5,8,26,<br>29,30,31	2 Q5RJR2,Q62761;Q62762;Q62763
4333	1,3,6,7,25,<br>27,29,30	2,4,5,8,26,<br>28,31,32	1 O54728
4334	1,3,6,7,25,<br>27,29,31	2,4,5,8,26,<br>28,30,32	3 O54728,P05539,P70549
4335	1,3,6,7,25,<br>27,29,32	2,4,5,8,26,<br>28,30,31	0
4336	1,3,6,7,25,<br>27,30,31	2,4,5,8,26,<br>28,29,32	1 O54728
4337	1,3,6,7,25,<br>27,30,32	2,4,5,8,26,<br>28,29,31	0

4338	1,3,6,7,25,<br>27,31,32	2,4,5,8,26,<br>28,29,30	0	
4339	1,3,6,7,25,<br>28,29,30	2,4,5,8,26,<br>27,31,32	2	P05539,Q62975
4340	1,3,6,7,25,<br>28,29,31	2,4,5,8,26,<br>27,30,32	3	P05539,P20762,P70549
4341	1,3,6,7,25,<br>28,29,32	2,4,5,8,26,<br>27,30,31	4	P05539,P20762,Q62975,Q9QW07
4342	1,3,6,7,25,<br>28,30,31	2,4,5,8,26,<br>27,29,32	3	P25809,Q62975,Q9JJS8
4343	1,3,6,7,25,<br>28,30,32	2,4,5,8,26,<br>27,29,31	3	P25809,Q5PQL7,Q62975
4344	1,3,6,7,25,<br>28,31,32	2,4,5,8,26,<br>27,29,30	3	P20762,P25809,Q62975
4345	1,3,6,7,25,<br>29,30,31	2,4,5,8,26,<br>27,28,32	3	O54728,P25809,Q9QWN8
4346	1,3,6,7,25,<br>29,30,32	2,4,5,8,26,<br>27,28,31	1	Q5BJY9
4347	1,3,6,7,25,<br>29,31,32	2,4,5,8,26,<br>27,28,30	3	P20762,P25809,P70549
4348	1,3,6,7,25,<br>30,31,32	2,4,5,8,26,<br>27,28,29	4	P06760,P25809,Q62975,Q6P6R2
4349	1,3,6,7,26,<br>27,28,29	2,4,5,8,25,<br>30,31,32	4	P05539,P47967,P63081,P70549
4350	1,3,6,7,26,<br>27,28,30	2,4,5,8,25,<br>29,31,32	0	
4351	1,3,6,7,26,<br>27,28,31	2,4,5,8,25,<br>29,30,32	1	P70549
4352	1,3,6,7,26,<br>27,28,32	2,4,5,8,25,<br>29,30,31	0	
4353	1,3,6,7,26,<br>27,29,30	2,4,5,8,25,<br>28,31,32	2	P02783,Q811M5
4354	1,3,6,7,26,<br>27,29,31	2,4,5,8,25,<br>28,30,32	2	P02783,P70549
4355	1,3,6,7,26,<br>27,29,32	2,4,5,8,25,<br>28,30,31	2	P02783,P70549
4356	1,3,6,7,26,<br>27,30,31	2,4,5,8,25,<br>28,29,32	0	
4357	1,3,6,7,26,<br>27,30,32	2,4,5,8,25,<br>28,29,31	0	

4358	1,3,6,7,26,<br>27,31,32	2,4,5,8,25,<br>28,29,30	0
4359	1,3,6,7,26,<br>28,29,30	2,4,5,8,25,<br>27,31,32	2 P01835,P23739
4360	1,3,6,7,26,<br>28,29,31	2,4,5,8,25,<br>27,30,32	4 P01835,P21674,P70549,Q01205
4361	1,3,6,7,26,<br>28,29,32	2,4,5,8,25,<br>27,30,31	2 P01835,P70549
4362	1,3,6,7,26,<br>28,30,31	2,4,5,8,25,<br>27,29,32	0
4363	1,3,6,7,26,<br>28,30,32	2,4,5,8,25,<br>27,29,31	1 Q62975
4364	1,3,6,7,26,<br>28,31,32	2,4,5,8,25,<br>27,29,30	3 P22006,P57113,P70549
4365	1,3,6,7,26,<br>29,30,31	2,4,5,8,25,<br>27,28,32	2 P01835,Q9QWN8
4366	1,3,6,7,26,<br>29,30,32	2,4,5,8,25,<br>27,28,31	1 P01835
4367	1,3,6,7,26,<br>29,31,32	2,4,5,8,25,<br>27,28,30	3 P01835,P57113,P70549
4368	1,3,6,7,26,<br>30,31,32	2,4,5,8,25,<br>27,28,29	3 P22006,P25809,P57113
4369	1,3,6,7,27,<br>28,29,30	2,4,5,8,25,<br>26,31,32	2 P00507,P05539
4370	1,3,6,7,27,<br>28,29,31	2,4,5,8,25,<br>26,30,32	3 P00507,P05539,P70549
4371	1,3,6,7,27,<br>28,29,32	2,4,5,8,25,<br>26,30,31	6 O35547,P05539,P22283,Q62761;Q62762;Q62763,Q6AY61,Q812E4
4372	1,3,6,7,27,<br>28,30,31	2,4,5,8,25,<br>26,29,32	0
4373	1,3,6,7,27,<br>28,30,32	2,4,5,8,25,<br>26,29,31	0
4374	1,3,6,7,27,<br>28,31,32	2,4,5,8,25,<br>26,29,30	1 P22006
4375	1,3,6,7,27,<br>29,30,31	2,4,5,8,25,<br>26,28,32	1 O54728
4376	1,3,6,7,27,<br>29,30,32	2,4,5,8,25,<br>26,28,31	0
4377	1,3,6,7,27,<br>29,31,32	2,4,5,8,25,<br>26,28,30	0

4378	1,3,6,7,27,30,31,32	2,4,5,8,25,26,28,29	2	P06760,P22006
4379	1,3,6,7,28,29,30,31	2,4,5,8,25,26,27,32	1	P01835
4380	1,3,6,7,28,29,30,32	2,4,5,8,25,26,27,31	2	P01835,Q5BJY9
4381	1,3,6,7,28,29,31,32	2,4,5,8,25,26,27,30	3	P01835,P20762,P70549
4382	1,3,6,7,28,30,31,32	2,4,5,8,25,26,27,29	3	P06760,P22006,P25809
4383	1,3,6,7,29,30,31,32	2,4,5,8,25,26,27,28	5	P01835,P25809,P26342,P36860,Q5BJY9
4384	1,3,6,8,25,26,27,28	2,4,5,7,29,30,31,32	8	P08649,P15399,P26342,P36860,P63081,P70549,P80299,Q9QW07
4385	1,3,6,8,25,26,27,29	2,4,5,7,28,30,31,32	1	P70549
4386	1,3,6,8,25,26,27,30	2,4,5,7,28,29,31,32	2	P15399,P80299
4387	1,3,6,8,25,26,27,31	2,4,5,7,28,29,30,32	3	P54921,P70549,Q9R168
4388	1,3,6,8,25,26,27,32	2,4,5,7,28,29,30,31	3	P13432,P15399,Q06000
4389	1,3,6,8,25,26,28,29	2,4,5,7,27,30,31,32	4	P70549,P80299,Q9QW07,Q9Z0J6
4390	1,3,6,8,25,26,28,30	2,4,5,7,27,29,31,32	2	P80299,Q6TMA8
4391	1,3,6,8,25,26,28,31	2,4,5,7,27,29,30,32	2	P70549,Q63598
4392	1,3,6,8,25,26,28,32	2,4,5,7,27,29,30,31	4	P08649,P13432,P80299,Q9QW07
4393	1,3,6,8,25,26,29,30	2,4,5,7,27,28,31,32	2	P80299,Q9Z0J6
4394	1,3,6,8,25,26,29,31	2,4,5,7,27,28,30,32	1	P70549
4395	1,3,6,8,25,26,29,32	2,4,5,7,27,28,30,31	3	P13432,P70549,Q9Z0J6
4396	1,3,6,8,25,26,30,31	2,4,5,7,27,28,29,32	1	P54921
4397	1,3,6,8,25,26,30,32	2,4,5,7,27,28,29,31	2	P80299,Q06000

4398	1,3,6,8,25,<br>26,31,32	2,4,5,7,27,<br>28,29,30	3	P00507,P13432,P70549
4399	1,3,6,8,25,<br>27,28,29	2,4,5,7,26,<br>30,31,32	4	P36860,P70549,Q812E4,Q9QW07
4400	1,3,6,8,25,<br>27,28,30	2,4,5,7,26,<br>29,31,32	1	O70417
4401	1,3,6,8,25,<br>27,28,31	2,4,5,7,26,<br>29,30,32	1	Q9R168
4402	1,3,6,8,25,<br>27,28,32	2,4,5,7,26,<br>29,30,31	4	P36860,Q6AYR9,Q9QW07,Q9QWN8
4403	1,3,6,8,25,<br>27,29,30	2,4,5,7,26,<br>28,31,32	3	Q6IFU7,Q6IFW6,Q6IG02
4404	1,3,6,8,25,<br>27,29,31	2,4,5,7,26,<br>28,30,32	2	P70549,Q9R168
4405	1,3,6,8,25,<br>27,29,32	2,4,5,7,26,<br>28,30,31	1	P98166
4406	1,3,6,8,25,<br>27,30,31	2,4,5,7,26,<br>28,29,32	1	P54921
4407	1,3,6,8,25,<br>27,30,32	2,4,5,7,26,<br>28,29,31	1	P01681
4408	1,3,6,8,25,<br>27,31,32	2,4,5,7,26,<br>28,29,30	2	P01681,Q80WL1
4409	1,3,6,8,25,<br>28,29,30	2,4,5,7,26,<br>27,31,32	3	P80299,Q9QW07,Q9Z0J6
4410	1,3,6,8,25,<br>28,29,31	2,4,5,7,26,<br>27,30,32	2	P70549,Q9R168
4411	1,3,6,8,25,<br>28,29,32	2,4,5,7,26,<br>27,30,31	2	Q9QW07,Q9Z0J6
4412	1,3,6,8,25,<br>28,30,31	2,4,5,7,26,<br>27,29,32	0	
4413	1,3,6,8,25,<br>28,30,32	2,4,5,7,26,<br>27,29,31	1	Q62975
4414	1,3,6,8,25,<br>28,31,32	2,4,5,7,26,<br>27,29,30	2	P21588,Q80WL1
4415	1,3,6,8,25,<br>29,30,31	2,4,5,7,26,<br>27,28,32	0	
4416	1,3,6,8,25,<br>29,30,32	2,4,5,7,26,<br>27,28,31	2	O88797,Q9Z0J6
4417	1,3,6,8,25,<br>29,31,32	2,4,5,7,26,<br>27,28,30	3	P21588,P70549,Q80WL1

4418	1,3,6,8,25,30,31,32	2,4,5,7,26,27,28,29	5	P01681,P02625,P20646,P21588,P47967
4419	1,3,6,8,26,27,28,29	2,4,5,7,25,30,31,32	4	P26342,P70549,Q6P6R2,Q9QW07
4420	1,3,6,8,26,27,28,30	2,4,5,7,25,29,31,32	1	P15399
4421	1,3,6,8,26,27,28,31	2,4,5,7,25,29,30,32	2	P70549,Q9R168
4422	1,3,6,8,26,27,28,32	2,4,5,7,25,29,30,31	4	P15399,Q6AYR9,Q6P6S4,Q9QWN8
4423	1,3,6,8,26,27,29,30	2,4,5,7,25,28,31,32	2	P02783,Q811M5
4424	1,3,6,8,26,27,29,31	2,4,5,7,25,28,30,32	3	P02783,P70549,Q9R168
4425	1,3,6,8,26,27,29,32	2,4,5,7,25,28,30,31	2	P02783,P70549
4426	1,3,6,8,26,27,30,31	2,4,5,7,25,28,29,32	0	
4427	1,3,6,8,26,27,30,32	2,4,5,7,25,28,29,31	1	P15399
4428	1,3,6,8,26,27,31,32	2,4,5,7,25,28,29,30	0	
4429	1,3,6,8,26,28,29,30	2,4,5,7,25,27,31,32	1	P80299
4430	1,3,6,8,26,28,29,31	2,4,5,7,25,27,30,32	3	P21674,P70549,Q9R168
4431	1,3,6,8,26,28,29,32	2,4,5,7,25,27,30,31	3	P70549,Q9QW07,Q9QYP1
4432	1,3,6,8,26,28,30,31	2,4,5,7,25,27,29,32	2	O89117,Q6TMA8
4433	1,3,6,8,26,28,30,32	2,4,5,7,25,27,29,31	1	O89117
4434	1,3,6,8,26,28,31,32	2,4,5,7,25,27,29,30	3	O89117,P22006,P70549
4435	1,3,6,8,26,29,30,31	2,4,5,7,25,27,28,32	0	
4436	1,3,6,8,26,29,30,32	2,4,5,7,25,27,28,31	0	
4437	1,3,6,8,26,29,31,32	2,4,5,7,25,27,28,30	1	P70549

4438	1,3,6,8,26,30,31,32	2,4,5,7,25,27,28,29	1	O89117
4439	1,3,6,8,27,28,29,30	2,4,5,7,25,26,31,32	3	P19218,Q6IFU7,Q6P6S4
4440	1,3,6,8,27,28,29,31	2,4,5,7,25,26,30,32	2	P70549,Q9R168
4441	1,3,6,8,27,28,29,32	2,4,5,7,25,26,30,31	6	O35547,Q6AYR9,Q6P6S4,Q812E4,Q9JI85,Q9QW07
4442	1,3,6,8,27,28,30,31	2,4,5,7,25,26,29,32	0	
4443	1,3,6,8,27,28,30,32	2,4,5,7,25,26,29,31	2	Q6P6S4,Q8VD89
4444	1,3,6,8,27,28,31,32	2,4,5,7,25,26,29,30	2	P22006,Q9QZK9
4445	1,3,6,8,27,29,30,31	2,4,5,7,25,26,28,32	1	Q6IG02
4446	1,3,6,8,27,29,30,32	2,4,5,7,25,26,28,31	1	Q6IFU7
4447	1,3,6,8,27,29,31,32	2,4,5,7,25,26,28,30	0	
4448	1,3,6,8,27,30,31,32	2,4,5,7,25,26,28,29	2	P20646,Q9QZK9
4449	1,3,6,8,28,29,30,31	2,4,5,7,25,26,27,32	0	
4450	1,3,6,8,28,29,30,32	2,4,5,7,25,26,27,31	0	
4451	1,3,6,8,28,29,31,32	2,4,5,7,25,26,27,30	2	P21588,P70549
4452	1,3,6,8,28,30,31,32	2,4,5,7,25,26,27,29	2	O89117,P22006
4453	1,3,6,8,29,30,31,32	2,4,5,7,25,26,27,28	0	
4454	1,3,6,25,26,27,28,29	2,4,5,7,8,30,31,32	4	P05539,P63081,P70549,P82471
4455	1,3,6,25,26,27,28,30	2,4,5,7,8,29,31,32	1	P82471
4456	1,3,6,25,26,27,28,31	2,4,5,7,8,29,30,32	2	P62804,P70549
4457	1,3,6,25,26,27,28,32	2,4,5,7,8,29,30,31	3	P62804,P82471,Q5RJR2

4458	1,3,6,25,26,27,29,30	2,4,5,7,8,28,31,32	2	P82471,Q811M5
4459	1,3,6,25,26,27,29,31	2,4,5,7,8,28,30,32	1	P70549
4460	1,3,6,25,26,27,29,32	2,4,5,7,8,28,30,31	2	P70549,P82471
4461	1,3,6,25,26,27,30,31	2,4,5,7,8,28,29,32	4	P01946,P06760,P54921,P62804
4462	1,3,6,25,26,27,30,32	2,4,5,7,8,28,29,31	4	P06760,P62804,P82471,Q06000
4463	1,3,6,25,26,27,31,32	2,4,5,7,8,28,29,30	2	P06760,P62804
4464	1,3,6,25,26,28,29,30	2,4,5,7,8,27,31,32	3	P82471,Q6TMA8,Q9Z0J6
4465	1,3,6,25,26,28,29,31	2,4,5,7,8,27,30,32	3	P70549,Q01205,Q6TMA8
4466	1,3,6,25,26,28,29,32	2,4,5,7,8,27,30,31	3	P00731,P70549,P82471
4467	1,3,6,25,26,28,30,31	2,4,5,7,8,27,29,32	7	P00714,P01946,P06760,P25809,P62804,Q4G075,Q6TMA8
4468	1,3,6,25,26,28,30,32	2,4,5,7,8,27,29,31	8	P06760,P25809,P62804,P82471,Q06000,Q4G075,Q5PQL7,Q62975
4469	1,3,6,25,26,28,31,32	2,4,5,7,8,27,29,30	7	P00731,P06760,P25809,P62804,P70549,Q4G075,Q62975
4470	1,3,6,25,26,29,30,31	2,4,5,7,8,27,28,32	4	P01946,P22282,P25809,Q4G075
4471	1,3,6,25,26,29,30,32	2,4,5,7,8,27,28,31	3	P50116,P82471,Q4G075
4472	1,3,6,25,26,29,31,32	2,4,5,7,8,27,28,30	5	P19218,P25809,P62804,P70549,Q4G075
4473	1,3,6,25,26,30,31,32	2,4,5,7,8,27,28,29	8	P00507,P06760,P22273,P25809,P47967,P62804,Q00715,Q4G075
4474	1,3,6,25,27,28,29,30	2,4,5,7,8,26,31,32	2	P05539,P82471
4475	1,3,6,25,27,28,29,31	2,4,5,7,8,26,30,32	3	P05539,P20762,P70549
4476	1,3,6,25,27,28,29,32	2,4,5,7,8,26,30,31	6	P00758,P05539,P20762,P82471,Q5RJR2,Q62761;Q62762;Q62763
4477	1,3,6,25,27,28,30,31	2,4,5,7,8,26,29,32	1	P06760

4478	1,3,6,25,27,28,30,32	2,4,5,7,8,26,29,31	3	P06760,P82471,Q9QX74
4479	1,3,6,25,27,28,31,32	2,4,5,7,8,26,29,30	6	P06760,P20762,P22006,P62804,Q5RJR2,Q9QX74
4480	1,3,6,25,27,29,30,31	2,4,5,7,8,26,28,32	2	O54728,P20762
4481	1,3,6,25,27,29,30,32	2,4,5,7,8,26,28,31	3	P20762,P82471,Q5RLM2
4482	1,3,6,25,27,29,31,32	2,4,5,7,8,26,28,30	2	P20762,P62804
4483	1,3,6,25,27,30,31,32	2,4,5,7,8,26,28,29	8	P06760,P20646,P20762,P62804,Q00715,Q6P6R2,Q9QX74,Q9WTT6
4484	1,3,6,25,28,29,30,31	2,4,5,7,8,26,27,32	4	P20762,P25809,P50116,Q4G075
4485	1,3,6,25,28,29,30,32	2,4,5,7,8,26,27,31	6	P20762,P50116,P82471,Q4G075,Q5BJY9,Q62975
4486	1,3,6,25,28,29,31,32	2,4,5,7,8,26,27,30	5	P20762,P50116,P62804,P70549,Q4G075
4487	1,3,6,25,28,30,31,32	2,4,5,7,8,26,27,29	11	P06760,P20762,P22006,P25809,P50116,P62804,Q00715,Q4G075,Q62975,Q9QX74,Q9WTT6
4488	1,3,6,25,29,30,31,32	2,4,5,7,8,26,27,28	10	P07150,P20762,P22273,P25809,P50116,P62804,Q4G075,Q5BJY9,Q5RLM2,Q9WTT6
4489	1,3,6,26,27,28,29,30	2,4,5,7,8,25,31,32	3	P09656,P82471,Q811M5
4490	1,3,6,26,27,28,29,31	2,4,5,7,8,25,30,32	3	P05539,P70549,Q01205
4491	1,3,6,26,27,28,29,32	2,4,5,7,8,25,30,31	4	P00758,P09656,P70549,P82471
4492	1,3,6,26,27,28,30,31	2,4,5,7,8,25,29,32	1	P06760
4493	1,3,6,26,27,28,30,32	2,4,5,7,8,25,29,31	4	P06760,P62804,P82471,Q8VD89
4494	1,3,6,26,27,28,31,32	2,4,5,7,8,25,29,30	3	P06760,P22006,P62804
4495	1,3,6,26,27,29,30,31	2,4,5,7,8,25,28,32	2	P39069,Q811M5
4496	1,3,6,26,27,29,30,32	2,4,5,7,8,25,28,31	2	P82471,Q811M5
4497	1,3,6,26,27,29,31,32	2,4,5,7,8,25,28,30	2	P62804,P70549

4498	1,3,6,26,27,30,31,32	2,4,5,7,8,25,28,29	5	P06760,P22006,P39069,P62804,Q00715
4499	1,3,6,26,28,29,30,31	2,4,5,7,8,25,27,32	3	P50116,Q4G075,Q6TMA8
4500	1,3,6,26,28,29,30,32	2,4,5,7,8,25,27,31	4	P50115,P50116,P82471,Q4G075
4501	1,3,6,26,28,29,31,32	2,4,5,7,8,25,27,30	3	P62804,P70549,Q4G075
4502	1,3,6,26,28,30,31,32	2,4,5,7,8,25,27,29	9	O89117,P06760,P22006,P25809,P50115,P50116,P62804,Q00715,Q4G075
4503	1,3,6,26,29,30,31,32	2,4,5,7,8,25,27,28	6	P25809,P36860,P50115,P50116,P62804,Q4G075
4504	1,3,6,27,28,29,30,31	2,4,5,7,8,25,26,32	0	
4505	1,3,6,27,28,29,30,32	2,4,5,7,8,25,26,31	3	P00758,P82471,Q9Z0V6
4506	1,3,6,27,28,29,31,32	2,4,5,7,8,25,26,30	2	P20762,P22006
4507	1,3,6,27,28,30,31,32	2,4,5,7,8,25,26,29	6	P06760,P22006,P62804,Q8VD89,Q9QX74,Q9WTT6
4508	1,3,6,27,29,30,31,32	2,4,5,7,8,25,26,28	3	P07150,P20762,Q9WTT6
4509	1,3,6,28,29,30,31,32	2,4,5,7,8,25,26,27	7	P20762,P22006,P50115,P50116,Q4G075,Q5BJY9,Q9WTT6
4510	1,3,7,8,25,26,27,28	2,4,5,6,29,30,31,32	4	O55145,P26342,P36860,Q9WTT6
4511	1,3,7,8,25,26,27,29	2,4,5,6,28,30,31,32	4	P22006,P70549,Q9QX74,Q9WTT6
4512	1,3,7,8,25,26,27,30	2,4,5,6,28,29,31,32	2	O70417,Q05511
4513	1,3,7,8,25,26,27,31	2,4,5,6,28,29,30,32	1	O55145
4514	1,3,7,8,25,26,27,32	2,4,5,6,28,29,30,31	1	P01681
4515	1,3,7,8,25,26,28,29	2,4,5,6,27,30,31,32	9	P01039,P20646,P70549,P80299,Q64093,Q6P6R2,Q9QX74,Q9WTT6,Q9Z0J6
4516	1,3,7,8,25,26,28,30	2,4,5,6,27,29,31,32	2	P01039,P80299
4517	1,3,7,8,25,26,28,31	2,4,5,6,27,29,30,32	6	O55145,P01039,P70549,Q63598,Q811M5,Q923V8

4518	1,3,7,8,25,26,28,32	2,4,5,6,27,29,30,31	2	P01039,Q923V8
4519	1,3,7,8,25,26,29,30	2,4,5,6,27,28,31,32	4	P01039,P22006,P80299,Q9Z0J6
4520	1,3,7,8,25,26,29,31	2,4,5,6,27,28,30,32	4	P01039,P70549,Q64093,Q9QX74
4521	1,3,7,8,25,26,29,32	2,4,5,6,27,28,30,31	1	P01039
4522	1,3,7,8,25,26,30,31	2,4,5,6,27,28,29,32	0	
4523	1,3,7,8,25,26,30,32	2,4,5,6,27,28,29,31	2	P01681,P35280
4524	1,3,7,8,25,26,31,32	2,4,5,6,27,28,29,30	3	P01681,P21588,Q923V8
4525	1,3,7,8,25,27,28,29	2,4,5,6,26,30,31,32	4	P36860,Q812E4,Q9EQS0,Q9Z2L0
4526	1,3,7,8,25,27,28,30	2,4,5,6,26,29,31,32	2	O70417,P36860
4527	1,3,7,8,25,27,28,31	2,4,5,6,26,29,30,32	2	O55145,P36860
4528	1,3,7,8,25,27,28,32	2,4,5,6,26,29,30,31	5	P01681,P36860,Q5RJR2,Q6AYR9,Q812E4
4529	1,3,7,8,25,27,29,30	2,4,5,6,26,28,31,32	3	P22006,Q6IFW6,Q6IG02
4530	1,3,7,8,25,27,29,31	2,4,5,6,26,28,30,32	0	
4531	1,3,7,8,25,27,29,32	2,4,5,6,26,28,30,31	1	P01681
4532	1,3,7,8,25,27,30,31	2,4,5,6,26,28,29,32	2	Q05511,Q6IFW6
4533	1,3,7,8,25,27,30,32	2,4,5,6,26,28,29,31	5	O70417,P01681,P35280,Q05511,Q6IFW6
4534	1,3,7,8,25,27,31,32	2,4,5,6,26,28,29,30	3	P01681,P21588,Q80WL1
4535	1,3,7,8,25,28,29,30	2,4,5,6,26,27,31,32	2	P01039,Q9Z0J6
4536	1,3,7,8,25,28,29,31	2,4,5,6,26,27,30,32	4	P01039,P21588,P70549,Q811M5
4537	1,3,7,8,25,28,29,32	2,4,5,6,26,27,30,31	4	P01039,P30120,Q812E4,Q9Z0J6

4538	1,3,7,8,25,28,30,31	2,4,5,6,26,27,29,32	2	P01039,Q9JJ50
4539	1,3,7,8,25,28,30,32	2,4,5,6,26,27,29,31	4	P01039,P01681,P35280,Q62975
4540	1,3,7,8,25,28,31,32	2,4,5,6,26,27,29,30	6	P01039,P01681,P21588,Q80WL1,Q811M5,Q923V8
4541	1,3,7,8,25,29,30,31	2,4,5,6,26,27,28,32	2	P01039,Q6IFW6
4542	1,3,7,8,25,29,30,32	2,4,5,6,26,27,28,31	3	P01039,P01681,P35280
4543	1,3,7,8,25,29,31,32	2,4,5,6,26,27,28,30	4	P01039,P01681,P21588,Q80WL1
4544	1,3,7,8,25,30,31,32	2,4,5,6,26,27,28,29	4	P01681,P02625,P21588,P35280
4545	1,3,7,8,26,27,28,29	2,4,5,6,25,30,31,32	8	P20646,P47967,P70549,Q64093,Q6P6R2,Q812E4,Q9QX74,Q9WTT6
4546	1,3,7,8,26,27,28,30	2,4,5,6,25,29,31,32	1	Q9WTT6
4547	1,3,7,8,26,27,28,31	2,4,5,6,25,29,30,32	3	O55145,Q63598,Q9WTT6
4548	1,3,7,8,26,27,28,32	2,4,5,6,25,29,30,31	2	Q6AYR9,Q9QWN8
4549	1,3,7,8,26,27,29,30	2,4,5,6,25,28,31,32	0	
4550	1,3,7,8,26,27,29,31	2,4,5,6,25,28,30,32	2	P70549,Q9QX74
4551	1,3,7,8,26,27,29,32	2,4,5,6,25,28,30,31	0	
4552	1,3,7,8,26,27,30,31	2,4,5,6,25,28,29,32	0	
4553	1,3,7,8,26,27,30,32	2,4,5,6,25,28,29,31	1	P35280
4554	1,3,7,8,26,27,31,32	2,4,5,6,25,28,29,30	0	
4555	1,3,7,8,26,28,29,30	2,4,5,6,25,27,31,32	2	P01039,P21674
4556	1,3,7,8,26,28,29,31	2,4,5,6,25,27,30,32	7	P01039,P21674,P70549,Q63424,Q64093,Q6P6R2,Q9QX74
4557	1,3,7,8,26,28,29,32	2,4,5,6,25,27,30,31	4	P01039,P30120,P70549,Q64093

4558	1,3,7,8,26,<br>28,30,31	2,4,5,6,25,<br>27,29,32	0	
4559	1,3,7,8,26,<br>28,30,32	2,4,5,6,25,<br>27,29,31	2	P01039,P35280
4560	1,3,7,8,26,<br>28,31,32	2,4,5,6,25,<br>27,29,30	3	P01039,Q811M5,Q923V8
4561	1,3,7,8,26,<br>29,30,31	2,4,5,6,25,<br>27,28,32	0	
4562	1,3,7,8,26,<br>29,30,32	2,4,5,6,25,<br>27,28,31	2	P01039,P35280
4563	1,3,7,8,26,<br>29,31,32	2,4,5,6,25,<br>27,28,30	2	P70549,Q64093
4564	1,3,7,8,26,<br>30,31,32	2,4,5,6,25,<br>27,28,29	1	P35280
4565	1,3,7,8,27,<br>28,29,30	2,4,5,6,25,<br>26,31,32	3	P00507,P47967,Q812E4
4566	1,3,7,8,27,<br>28,29,31	2,4,5,6,25,<br>26,30,32	4	D3ZUC6,P00507,Q812E4,Q9EQS0
4567	1,3,7,8,27,<br>28,29,32	2,4,5,6,25,<br>26,30,31	7	P08723,P47727,P47967,Q6AYR9,Q812E4,Q9EQS0,Q9Z2L0
4568	1,3,7,8,27,<br>28,30,31	2,4,5,6,25,<br>26,29,32	0	
4569	1,3,7,8,27,<br>28,30,32	2,4,5,6,25,<br>26,29,31	2	P35280,Q6AYR9
4570	1,3,7,8,27,<br>28,31,32	2,4,5,6,25,<br>26,29,30	1	P21588
4571	1,3,7,8,27,<br>29,30,31	2,4,5,6,25,<br>26,28,32	2	Q6IFW6,Q6IG02
4572	1,3,7,8,27,<br>29,30,32	2,4,5,6,25,<br>26,28,31	1	P35280
4573	1,3,7,8,27,<br>29,31,32	2,4,5,6,25,<br>26,28,30	1	P21588
4574	1,3,7,8,27,<br>30,31,32	2,4,5,6,25,<br>26,28,29	1	P35280
4575	1,3,7,8,28,<br>29,30,31	2,4,5,6,25,<br>26,27,32	2	P01039,P21674
4576	1,3,7,8,28,<br>29,30,32	2,4,5,6,25,<br>26,27,31	2	P01039,P35280
4577	1,3,7,8,28,<br>29,31,32	2,4,5,6,25,<br>26,27,30	4	P01039,P21588,P21674,Q811M5

4578	1,3,7,8,28,<br>30,31,32	2,4,5,6,25,<br>26,27,29	2	P21588,P35280
4579	1,3,7,8,29,<br>30,31,32	2,4,5,6,25,<br>26,27,28	2	P21588,P35280
4580	1,3,7,25,2<br>6,27,28,29	2,4,5,6,8,3<br>0,31,32	4	O54858,P20646,P70549,Q63532
4581	1,3,7,25,2<br>6,27,28,30	2,4,5,6,8,2<br>9,31,32	0	
4582	1,3,7,25,2<br>6,27,28,31	2,4,5,6,8,2<br>9,30,32	1	O55145
4583	1,3,7,25,2<br>6,27,28,32	2,4,5,6,8,2<br>9,30,31	1	Q5RJR2
4584	1,3,7,25,2<br>6,27,29,30	2,4,5,6,8,2<br>8,31,32	1	Q63532
4585	1,3,7,25,2<br>6,27,29,31	2,4,5,6,8,2<br>8,30,32	1	P70549
4586	1,3,7,25,2<br>6,27,29,32	2,4,5,6,8,2<br>8,30,31	2	O54858,Q63532
4587	1,3,7,25,2<br>6,27,30,31	2,4,5,6,8,2<br>8,29,32	0	
4588	1,3,7,25,2<br>6,27,30,32	2,4,5,6,8,2<br>8,29,31	0	
4589	1,3,7,25,2<br>6,27,31,32	2,4,5,6,8,2<br>8,29,30	0	
4590	1,3,7,25,2<br>6,28,29,30	2,4,5,6,8,2<br>7,31,32	2	P01039,Q63532
4591	1,3,7,25,2<br>6,28,29,31	2,4,5,6,8,2<br>7,30,32	5	P01039,P19218,P70549,Q01205,Q64093
4592	1,3,7,25,2<br>6,28,29,32	2,4,5,6,8,2<br>7,30,31	4	O54858,P01039,P70549,Q63532
4593	1,3,7,25,2<br>6,28,30,31	2,4,5,6,8,2<br>7,29,32	2	P00714,Q62975
4594	1,3,7,25,2<br>6,28,30,32	2,4,5,6,8,2<br>7,29,31	1	Q62975
4595	1,3,7,25,2<br>6,28,31,32	2,4,5,6,8,2<br>7,29,30	4	P00731,P19218,Q10758,Q62975
4596	1,3,7,25,2<br>6,29,30,31	2,4,5,6,8,2<br>7,28,32	3	P22282,Q9QWN8,Q9QZQ5
4597	1,3,7,25,2<br>6,29,30,32	2,4,5,6,8,2<br>7,28,31	1	Q63532

4598	1,3,7,25,26,29,31,32	2,4,5,6,8,27,28,30	2 P19218,P70549
4599	1,3,7,25,26,30,31,32	2,4,5,6,8,27,28,29	3 P22273,P25809,Q00715
4600	1,3,7,25,27,28,29,30	2,4,5,6,8,26,31,32	2 O54858,Q63532
4601	1,3,7,25,27,28,29,31	2,4,5,6,8,26,30,32	1 P20762
4602	1,3,7,25,27,28,29,32	2,4,5,6,8,26,30,31	9 O54858,P00758,P20762,Q5RJR2,Q62761;Q62762;Q62763,Q63532,Q6AY61,Q812E4,Q8CGS4
4603	1,3,7,25,27,28,30,31	2,4,5,6,8,26,29,32	0
4604	1,3,7,25,27,28,30,32	2,4,5,6,8,26,29,31	1 Q5RJR2
4605	1,3,7,25,27,28,31,32	2,4,5,6,8,26,29,30	2 P20762,Q5RJR2
4606	1,3,7,25,27,29,30,31	2,4,5,6,8,26,28,32	2 O54728,P08649
4607	1,3,7,25,27,29,30,32	2,4,5,6,8,26,28,31	1 Q63532
4608	1,3,7,25,27,29,31,32	2,4,5,6,8,26,28,30	1 P20762
4609	1,3,7,25,27,30,31,32	2,4,5,6,8,26,28,29	0
4610	1,3,7,25,28,29,30,31	2,4,5,6,8,26,27,32	4 P01039,P17559,P20762,Q9JJ50
4611	1,3,7,25,28,29,30,32	2,4,5,6,8,26,27,31	5 P01039,P17559,P20762,Q5BJY9,Q62975
4612	1,3,7,25,28,29,31,32	2,4,5,6,8,26,27,30	4 P01039,P17559,P20762,P21588
4613	1,3,7,25,28,30,31,32	2,4,5,6,8,26,27,29	5 P17559,P20762,P25809,Q62975,Q9JJ50
4614	1,3,7,25,29,30,31,32	2,4,5,6,8,26,27,28	5 P08649,P17559,P20762,P25809,Q5BJY9
4615	1,3,7,26,27,28,29,30	2,4,5,6,8,25,31,32	3 O54858,P47967,Q63532
4616	1,3,7,26,27,28,29,31	2,4,5,6,8,25,30,32	3 O54858,P70549,Q01205
4617	1,3,7,26,27,28,29,32	2,4,5,6,8,25,30,31	4 O54858,P00758,P47967,Q63532

4618	1,3,7,26,27,28,30,31	2,4,5,6,8,25,29,32	0	
4619	1,3,7,26,27,28,30,32	2,4,5,6,8,25,29,31	0	
4620	1,3,7,26,27,28,31,32	2,4,5,6,8,25,29,30	0	
4621	1,3,7,26,27,29,30,31	2,4,5,6,8,25,28,32	2	P08649,Q9QZQ5
4622	1,3,7,26,27,29,30,32	2,4,5,6,8,25,28,31	3	O54858,P36376,Q63532
4623	1,3,7,26,27,29,31,32	2,4,5,6,8,25,28,30	0	
4624	1,3,7,26,27,30,31,32	2,4,5,6,8,25,28,29	0	
4625	1,3,7,26,28,29,30,31	2,4,5,6,8,25,27,32	3	P01039,P17559,Q9QZQ5
4626	1,3,7,26,28,29,30,32	2,4,5,6,8,25,27,31	3	O54858,P01039,P17559
4627	1,3,7,26,28,29,31,32	2,4,5,6,8,25,27,30	3	P01039,P17559,P70549
4628	1,3,7,26,28,30,31,32	2,4,5,6,8,25,27,29	1	P17559
4629	1,3,7,26,29,30,31,32	2,4,5,6,8,25,27,28	2	P17559,Q9QZQ5
4630	1,3,7,27,28,29,30,31	2,4,5,6,8,25,26,32	3	O54858,P00507,P08649
4631	1,3,7,27,28,29,30,32	2,4,5,6,8,25,26,31	4	O54858,P00758,Q63150,Q63532
4632	1,3,7,27,28,29,31,32	2,4,5,6,8,25,26,30	3	O54858,P08649,P20762
4633	1,3,7,27,28,30,31,32	2,4,5,6,8,25,26,29	1	P08649
4634	1,3,7,27,29,30,31,32	2,4,5,6,8,25,26,28	1	P08649
4635	1,3,7,28,29,30,31,32	2,4,5,6,8,25,26,27	4	P08649,P17559,P20762,Q5BJY9
4636	1,3,8,25,26,27,28,29	2,4,5,6,7,30,31,32	6	P26342,P36860,P70549,P80299,Q6P6R2,Q9Z0J6
4637	1,3,8,25,26,27,28,30	2,4,5,6,7,29,31,32	4	P00714,P26342,P36860,P80299

4638	1,3,8,25,26,27,28,31	2,4,5,6,7,29,30,32	5	O55145,P00714,P26342,P36860,Q80WL1
4639	1,3,8,25,26,27,28,32	2,4,5,6,7,29,30,31	6	P26342,P36860,Q5RJR2,Q6AYR9,Q80WL1,Q9QWN8
4640	1,3,8,25,26,27,29,30	2,4,5,6,7,28,31,32	4	P22006,P80299,Q6IFW6,Q9Z0J6
4641	1,3,8,25,26,27,29,31	2,4,5,6,7,28,30,32	2	P70549,Q9Z0J6
4642	1,3,8,25,26,27,29,32	2,4,5,6,7,28,30,31	2	P00762,Q9Z0J6
4643	1,3,8,25,26,27,30,31	2,4,5,6,7,28,29,32	1	P54921
4644	1,3,8,25,26,27,30,32	2,4,5,6,7,28,29,31	1	P00762
4645	1,3,8,25,26,27,31,32	2,4,5,6,7,28,29,30	3	P00762,P01681,Q80WL1
4646	1,3,8,25,26,28,29,30	2,4,5,6,7,27,31,32	5	P00714,P01039,P50116,P80299,Q9Z0J6
4647	1,3,8,25,26,28,29,31	2,4,5,6,7,27,30,32	6	P00714,P01039,P50116,P70549,Q80WL1,Q9Z0J6
4648	1,3,8,25,26,28,29,32	2,4,5,6,7,27,30,31	6	P01039,P50116,P70549,P80299,Q80WL1,Q9Z0J6
4649	1,3,8,25,26,28,30,31	2,4,5,6,7,27,29,32	6	O08839,P00714,P01039,P50116,P80299,Q6TMA8
4650	1,3,8,25,26,28,30,32	2,4,5,6,7,27,29,31	4	O08839,P01039,P50116,P80299
4651	1,3,8,25,26,28,31,32	2,4,5,6,7,27,29,30	5	P01039,P21588,P50116,Q80WL1,Q923V8
4652	1,3,8,25,26,29,30,31	2,4,5,6,7,27,28,32	4	P01039,P11598,P50116,Q9Z0J6
4653	1,3,8,25,26,29,30,32	2,4,5,6,7,27,28,31	5	O88797,P01039,P50116,P80299,Q9Z0J6
4654	1,3,8,25,26,29,31,32	2,4,5,6,7,27,28,30	7	P01039,P19218,P21588,P50116,P70549,Q80WL1,Q9Z0J6
4655	1,3,8,25,26,30,31,32	2,4,5,6,7,27,28,29	6	P00507,P01681,P11598,P50116,Q00715,Q63617
4656	1,3,8,25,27,28,29,30	2,4,5,6,7,26,31,32	4	Q6IFU7,Q6IFW6,Q9WVH8,Q9Z0J6
4657	1,3,8,25,27,28,29,31	2,4,5,6,7,26,30,32	5	D3ZTX0,P70549,Q80WL1,Q9R168,Q9Z0J6

4658	1,3,8,25,27,28,29,32	2,4,5,6,7,26,30,31	6 P00758,P36860,Q6AYR9,Q80WL1,Q812E4,Q9Z0J6
4659	1,3,8,25,27,28,30,31	2,4,5,6,7,26,29,32	0
4660	1,3,8,25,27,28,30,32	2,4,5,6,7,26,29,31	2 P00762,P01681
4661	1,3,8,25,27,28,31,32	2,4,5,6,7,26,29,30	4 P00762,P01681,P21588,Q80WL1
4662	1,3,8,25,27,29,30,31	2,4,5,6,7,26,28,32	3 Q6IFW6,Q6IG02,Q9Z0J6
4663	1,3,8,25,27,29,30,32	2,4,5,6,7,26,28,31	3 P00762,Q6IFW6,Q9Z0J6
4664	1,3,8,25,27,29,31,32	2,4,5,6,7,26,28,30	5 P00762,P01681,P21588,Q80WL1,Q9Z0J6
4665	1,3,8,25,27,30,31,32	2,4,5,6,7,26,28,29	4 P00762,P01681,P02625,P21588
4666	1,3,8,25,28,29,30,31	2,4,5,6,7,26,27,32	4 P01039,P50116,Q9JJ50,Q9Z0J6
4667	1,3,8,25,28,29,30,32	2,4,5,6,7,26,27,31	3 P01039,P50116,Q9Z0J6
4668	1,3,8,25,28,29,31,32	2,4,5,6,7,26,27,30	5 P01039,P21588,P50116,Q80WL1,Q9Z0J6
4669	1,3,8,25,28,30,31,32	2,4,5,6,7,26,27,29	6 O08839,P01039,P01681,P21588,P50116,Q9JJ50
4670	1,3,8,25,29,30,31,32	2,4,5,6,7,26,27,28	6 P01039,P01681,P11598,P21588,P50116,Q9Z0J6
4671	1,3,8,26,27,28,29,30	2,4,5,6,7,25,31,32	0
4672	1,3,8,26,27,28,29,31	2,4,5,6,7,25,30,32	4 P26342,P70549,Q6P6R2,Q9R168
4673	1,3,8,26,27,28,29,32	2,4,5,6,7,25,30,31	5 P00758,P08010,Q6AYR9,Q812E4,Q9QWN8
4674	1,3,8,26,27,28,30,31	2,4,5,6,7,25,29,32	0
4675	1,3,8,26,27,28,30,32	2,4,5,6,7,25,29,31	1 Q9QWN8
4676	1,3,8,26,27,28,31,32	2,4,5,6,7,25,29,30	2 Q80WL1,Q9QWN8
4677	1,3,8,26,27,29,30,31	2,4,5,6,7,25,28,32	0

4678	1,3,8,26,27,29,30,32	2,4,5,6,7,25,28,31	0	
4679	1,3,8,26,27,29,31,32	2,4,5,6,7,25,28,30	2	P70549,Q80WL1
4680	1,3,8,26,27,30,31,32	2,4,5,6,7,25,28,29	1	P00762
4681	1,3,8,26,28,29,30,31	2,4,5,6,7,25,27,32	3	P01039,P50116,Q03191
4682	1,3,8,26,28,29,30,32	2,4,5,6,7,25,27,31	2	P01039,P50116
4683	1,3,8,26,28,29,31,32	2,4,5,6,7,25,27,30	4	P01039,P50116,P70549,Q80WL1
4684	1,3,8,26,28,30,31,32	2,4,5,6,7,25,27,29	4	O08839,O89117,P01039,P50116
4685	1,3,8,26,29,30,31,32	2,4,5,6,7,25,27,28	3	P01039,P50115,P50116
4686	1,3,8,27,28,29,30,31	2,4,5,6,7,25,26,32	1	Q9WVH8
4687	1,3,8,27,28,29,30,32	2,4,5,6,7,25,26,31	4	Q63150,Q6AYR9,Q812E4,Q9WVH8
4688	1,3,8,27,28,29,31,32	2,4,5,6,7,25,26,30	5	P21588,Q6AYR9,Q80WL1,Q812E4,Q9EQS0
4689	1,3,8,27,28,30,31,32	2,4,5,6,7,25,26,29	1	Q9WVH8
4690	1,3,8,27,29,30,31,32	2,4,5,6,7,25,26,28	1	P00762
4691	1,3,8,28,29,30,31,32	2,4,5,6,7,25,26,27	4	P01039,P21588,P50115,P50116
4692	1,3,25,26,27,28,29,30	2,4,5,6,7,8,31,32	7	O54858,P00714,P02454,P82471,Q63532,Q9QZQ5,Q9Z0J6
4693	1,3,25,26,27,28,29,31	2,4,5,6,7,8,30,32	8	O54858,P00714,P02454,P02631,P70549,Q01205,Q99041,Q9Z0J6
4694	1,3,25,26,27,28,29,32	2,4,5,6,7,8,30,31	8	O54858,P00758,P82471,Q5RJR2,Q63532,Q8CGS4,Q99041,Q9Z0J6
4695	1,3,25,26,27,28,30,31	2,4,5,6,7,8,29,32	5	P00714,P02631,P22273,P62804,Q99041

4696	1,3,25,26,27,28,30,32	2,4,5,6,7,8,29,31	5	P22273,P62804,P82471,Q5RJR2,Q63532
4697	1,3,25,26,27,28,31,32	2,4,5,6,7,8,29,30	5	P22273,P62804,Q5RJR2,Q80WL1,Q99041
4698	1,3,25,26,27,29,30,31	2,4,5,6,7,8,28,32	12	O54858,P01946,P02454,P02631,P07171,P11598,P22273,P22282,Q63617,Q99041,Q9QZQ5,Q9Z0J6
4699	1,3,25,26,27,29,30,32	2,4,5,6,7,8,28,31	8	O54858,P07171,P22273,P50115,P82471,Q63532,Q99041,Q9Z0J6
4700	1,3,25,26,27,29,31,32	2,4,5,6,7,8,28,30	8	O54858,P02631,P07171,P19218,P22273,P62804,P70549,Q99041
4701	1,3,25,26,27,30,31,32	2,4,5,6,7,8,28,29	13	P00762,P02631,P06760,P06761,P07171,P11598,P21674,P22273,P50115,P62804,Q00715,Q63617,Q99041
4702	1,3,25,26,28,29,30,31	2,4,5,6,7,8,27,32	15	O54858,P00714,P01039,P02454,P02631,P22273,P22282,P50115,P50116,Q08163,Q4G075,Q6TMA8,Q99041,Q9QZQ5,Q9Z0J6
4703	1,3,25,26,28,29,30,32	2,4,5,6,7,8,27,31	12	O54858,P01039,P22273,P50115,P50116,P82471,Q4G075,Q62975,Q63532,Q99041,Q9QZQ5,Q9Z0J6
4704	1,3,25,26,28,29,31,32	2,4,5,6,7,8,27,30	14	O54858,P00731,P01039,P19218,P22273,P50115,P50116,P62804,P70549,Q08163,Q4G075,Q80WL1,Q99041,Q9Z0J6
4705	1,3,25,26,28,30,31,32	2,4,5,6,7,8,27,29	13	O08839,P06760,P22273,P22282,P25809,P50115,P50116,P62804,Q00715,Q08163,Q4G075,Q62975,Q99041
4706	1,3,25,26,29,30,31,32	2,4,5,6,7,8,27,28	16	P02631,P06761,P11598,P19218,P22273,P22282,P25809,P50115,P50116,P62804,Q00715,Q4G075,Q63617,Q99041,Q9QZQ5,Q9Z0J6
4707	1,3,25,27,28,29,30,31	2,4,5,6,7,8,26,32	9	O54858,O55004,P02454,P02631,P20762,P22273,Q99041,Q9QZQ5,Q9Z0J6
4708	1,3,25,27,28,29,30,32	2,4,5,6,7,8,26,31	11	O54858,P00758,P20762,P20766,P22273,P50115,P82471,Q5RJR2,Q63532,Q9WVH8,Q9Z0J6

4709	1,3,25,27,28,29,31,32	2,4,5,6,7,8,26,30	8	O54858,P20762,P22273,P62804,Q5RJR2,Q80WL1,Q99041,Q9Z0J6
4710	1,3,25,27,28,30,31,32	2,4,5,6,7,8,26,29	9	P06760,P13852,P20762,P22273,P50115,P62804,Q5RJR2,Q99041,Q9QX74
4711	1,3,25,27,29,30,31,32	2,4,5,6,7,8,26,28	11	O54858,P00762,P02631,P07150,P11598,P20762,P22273,P50115,P62804,Q5RLM2,Q99041
4712	1,3,25,28,29,30,31,32	2,4,5,6,7,8,26,27	16	O54858,P01039,P07150,P17559,P20762,P21588,P22273,P22282,P25809,P50115,P50116,Q4G075,Q99041,Q9JJ50,Q9QZQ5,Q9Z0J6
4713	1,3,26,27,28,29,30,31	2,4,5,6,7,8,25,32	5	O54858,P02454,P02631,P50115,Q9QZQ5
4714	1,3,26,27,28,29,30,32	2,4,5,6,7,8,25,31	7	O54858,P00758,P02631,P50115,P82471,Q63532,Q9QZQ5
4715	1,3,26,27,28,29,31,32	2,4,5,6,7,8,25,30	5	O54858,P02631,P50115,P62804,P70549
4716	1,3,26,27,28,30,31,32	2,4,5,6,7,8,25,29	5	P02631,P06760,P22273,P50115,P62804
4717	1,3,26,27,29,30,31,32	2,4,5,6,7,8,25,28	8	O54858,P02454,P02631,P22273,P50115,P62804,Q99041,Q9QZQ5
4718	1,3,26,28,29,30,31,32	2,4,5,6,7,8,25,27	11	O54858,P01039,P02631,P17559,P22273,P50115,P50116,P62804,Q4G075,Q99041,Q9QZQ5
4719	1,3,27,28,29,30,31,32	2,4,5,6,7,8,25,26	9	O54858,P02631,P08649,P13852,P20762,P22273,P50115,Q9QZQ5,Q9WVH8
4720	1,4,5,6,7,8,25,26	2,3,27,28,29,30,31,32	6	P06911,P13432,P22273,P49134,Q99041,Q9WVK7
4721	1,4,5,6,7,8,25,27	2,3,26,28,29,30,31,32	8	P06911,P22273,P22282,P50115,P50116,Q03191,Q4G075,Q99041

4722	1,4,5,6,7,8,25,28	2,3,26,27,29,30,31,32	9	P06761,P06911,P11598,P22273,P22282,P49134,Q63617,Q99041,Q9QW07
4723	1,4,5,6,7,8,25,29	2,3,26,27,28,30,31,32	7	P06760,P06911,P13432,P22273,P48032,P49134,Q99041
4724	1,4,5,6,7,8,25,30	2,3,26,27,28,29,31,32	8	P06911,P19218,P49134,Q4FZU2,Q6IMF3,Q6P6Q2,Q99041,Q9WVK7
4725	1,4,5,6,7,8,25,31	2,3,26,27,28,29,30,32	5	O54858,P06911,P49134,Q99041,Q9WVK7
4726	1,4,5,6,7,8,25,32	2,3,26,27,28,29,30,31	5	P01681,P06911,P13432,Q99041,Q9WVK7
4727	1,4,5,6,7,8,26,27	2,3,25,28,29,30,31,32	11	P06761,P11598,P21588,P22273,P22282,P50115,P50116,Q03191,Q4G075,Q99041,Q9Z0J6
4728	1,4,5,6,7,8,26,28	2,3,25,27,29,30,31,32	14	D3ZUC6,P00762,P06761,P06911,P07150,P11598,P21674,P22273,P22282,P49134,Q63617,Q99041,Q9JI85,Q9QW07
4729	1,4,5,6,7,8,26,29	2,3,25,27,28,30,31,32	8	P06760,P11598,P21674,P22273,P49134,Q5RJR2,Q99041,Q9QX74
4730	1,4,5,6,7,8,26,30	2,3,25,27,28,29,31,32	4	P49134,Q5RJR2,Q6P6Q2,Q99041
4731	1,4,5,6,7,8,26,31	2,3,25,27,28,29,30,32	6	P49134,P57113,Q5RJR2,Q63532,Q99041,Q9Z0J6
4732	1,4,5,6,7,8,26,32	2,3,25,27,28,29,30,31	6	P11598,P13432,P18418,P22282,P49134,Q99041
4733	1,4,5,6,7,8,27,28	2,3,25,26,29,30,31,32	17	D3ZUC6,P06761,P06911,P11598,P19218,P22273,P22282,P22283,P50115,P50116,Q03191,Q4G075,Q63617,Q99041,Q9JI85,Q9QW07,Q9Z0J6
4734	1,4,5,6,7,8,27,29	2,3,25,26,28,30,31,32	13	P06760,P06761,P11598,P19218,P22273,P22282,P50115,P50116,Q03191,Q4G075,Q5I0D1,Q63617,Q99041

4735	1,4,5,6,7,8,27,30	2,3,25,26,28,29,31,32	11	P19218,P22273,P22282,P49744,P50116,Q03191,Q4FZU2,Q6IMF3,Q6P6Q2,Q99041,Q9Z0J6
4736	1,4,5,6,7,8,27,31	2,3,25,26,28,29,30,32	11	P06911,P11598,P19218,P22273,P22282,P23739,P50115,P50116,Q03191,Q99041,Q9Z0J6
4737	1,4,5,6,7,8,27,32	2,3,25,26,28,29,30,31	9	P00714,P11598,P19218,P22273,P22282,P50116,Q03191,Q99041,Q9Z0J6
4738	1,4,5,6,7,8,28,29	2,3,25,26,27,30,31,32	18	D3ZUC6,P00762,P06760,P06761,P06911,P11598,P19218,P21674,P22273,P22282,P22283,P49134,P97840,Q63493,Q63617,Q99041,Q9JI85,Q9QW07
4739	1,4,5,6,7,8,28,30	2,3,25,26,27,29,31,32	11	P06911,P07171,P11598,P19218,P22273,P22282,P49134,Q63617,Q6P6Q2,Q99041,Q9QW07
4740	1,4,5,6,7,8,28,31	2,3,25,26,27,29,30,32	14	D3ZUC6,P06761,P06911,P07171,P11598,P21674,P22273,P22282,P49134,P82471,Q63532,Q63617,Q99041,Q9Z0J6
4741	1,4,5,6,7,8,28,32	2,3,25,26,27,29,30,31	8	P06761,P11598,P22273,P22282,Q63617,Q99041,Q9JI85,Q9QW07
4742	1,4,5,6,7,8,29,30	2,3,25,26,27,28,31,32	7	P19218,P22273,P49134,Q5RJR2,Q6IMF3,Q6P6Q2,Q99041
4743	1,4,5,6,7,8,29,31	2,3,25,26,27,28,30,32	6	P06911,P21674,P49134,P82471,Q5RJR2,Q99041
4744	1,4,5,6,7,8,29,32	2,3,25,26,27,28,30,31	7	P00714,P11598,P13432,P22273,P22282,P49134,Q99041
4745	1,4,5,6,7,8,30,31	2,3,25,26,27,28,29,32	8	P19218,P49134,Q5RJR2,Q63532,Q6IMF3,Q6P6Q2,Q99041,Q9Z0J6
4746	1,4,5,6,7,8,30,32	2,3,25,26,27,28,29,31	5	P19218,P49134,Q6IMF3,Q6P6Q2,Q99041
4747	1,4,5,6,7,8,31,32	2,3,25,26,27,28,29,30	5	P11598,P82471,Q63532,Q99041,Q9Z0J6
4748	1,4,5,6,7,25,26,27	2,3,8,28,29,30,31,32	3	P21588,P50116,Q03191

4749	1,4,5,6,7,25,26,28	2,3,8,27,29,30,31,32	3	P00762,P11598,Q5PQL7
4750	1,4,5,6,7,25,26,29	2,3,8,27,28,30,31,32	0	
4751	1,4,5,6,7,25,26,30	2,3,8,27,28,29,31,32	4	P21588,Q5PQL7,Q9EQS0,Q9WVK7
4752	1,4,5,6,7,25,26,31	2,3,8,27,28,29,30,32	3	P25809,Q63150,Q9WVK7
4753	1,4,5,6,7,25,26,32	2,3,8,27,28,29,30,31	2	Q5PQL7,Q9WVK7
4754	1,4,5,6,7,25,27,28	2,3,8,26,29,30,31,32	3	P11598,P50116,Q03191
4755	1,4,5,6,7,25,27,29	2,3,8,26,28,30,31,32	3	O89117,P50116,Q03191
4756	1,4,5,6,7,25,27,30	2,3,8,26,28,29,31,32	3	P50116,Q03191,Q99376
4757	1,4,5,6,7,25,27,31	2,3,8,26,28,29,30,32	3	P50116,Q03191,Q9QYP1
4758	1,4,5,6,7,25,27,32	2,3,8,26,28,29,30,31	2	P50116,Q03191
4759	1,4,5,6,7,25,28,29	2,3,8,26,27,30,31,32	2	P00762,Q63617
4760	1,4,5,6,7,25,28,30	2,3,8,26,27,29,31,32	1	Q5PQL7
4761	1,4,5,6,7,25,28,31	2,3,8,26,27,29,30,32	0	
4762	1,4,5,6,7,25,28,32	2,3,8,26,27,29,30,31	3	P11598,Q5PQL7,Q62975
4763	1,4,5,6,7,25,29,30	2,3,8,26,27,28,31,32	1	Q9QWN8
4764	1,4,5,6,7,25,29,31	2,3,8,26,27,28,30,32	1	Q9QWN8
4765	1,4,5,6,7,25,29,32	2,3,8,26,27,28,30,31	0	
4766	1,4,5,6,7,25,30,31	2,3,8,26,27,28,29,32	3	P25809,Q9QWN8,Q9WVK7
4767	1,4,5,6,7,25,30,32	2,3,8,26,27,28,29,31	7	P25809,P26342,Q5BJY9,Q5PQL7,Q6P6R2,Q9R168,Q9WVK7
4768	1,4,5,6,7,25,31,32	2,3,8,26,27,28,29,30	3	P10719,P25809,Q9WVK7

4769	1,4,5,6,7,26,27,28	2,3,8,25,29,30,31,32	8	P02625,P11598,P21588,P50116,P97840,Q63617,Q99041,Q9Z0J6
4770	1,4,5,6,7,26,27,29	2,3,8,25,28,30,31,32	4	P02625,P11598,P21588,P50116
4771	1,4,5,6,7,26,27,30	2,3,8,25,28,29,31,32	4	P21588,P50116,Q03191,Q9Z0J6
4772	1,4,5,6,7,26,27,31	2,3,8,25,28,29,30,32	3	P50116,Q03191,Q9Z0J6
4773	1,4,5,6,7,26,27,32	2,3,8,25,28,29,30,31	4	P11598,P50116,Q03191,Q9Z0J6
4774	1,4,5,6,7,26,28,29	2,3,8,25,27,30,31,32	8	P00762,P02625,P11598,P21588,P21674,P47967,P97840,Q63617
4775	1,4,5,6,7,26,28,30	2,3,8,25,27,29,31,32	3	P00762,P21588,P97840
4776	1,4,5,6,7,26,28,31	2,3,8,25,27,29,30,32	3	P00762,P11598,Q9Z0J6
4777	1,4,5,6,7,26,28,32	2,3,8,25,27,29,30,31	2	P11598,Q9Z0J6
4778	1,4,5,6,7,26,29,30	2,3,8,25,27,28,31,32	5	P00762,P21588,P36376,P36860,P49134
4779	1,4,5,6,7,26,29,31	2,3,8,25,27,28,30,32	4	B1H234,P00762,P36860,P49134
4780	1,4,5,6,7,26,29,32	2,3,8,25,27,28,30,31	2	P36376,P36860
4781	1,4,5,6,7,26,30,31	2,3,8,25,27,28,29,32	5	P25809,P36860,Q5RJR2,Q9WVK7,Q9Z0J6
4782	1,4,5,6,7,26,30,32	2,3,8,25,27,28,29,31	5	P36376,P36860,Q8VD89,Q9WVK7,Q9Z0J6
4783	1,4,5,6,7,26,31,32	2,3,8,25,27,28,29,30	3	P36860,Q9WVK7,Q9Z0J6
4784	1,4,5,6,7,27,28,29	2,3,8,25,26,30,31,32	7	P02625,P11598,P22283,P47967,P50116,P97840,Q63617
4785	1,4,5,6,7,27,28,30	2,3,8,25,26,29,31,32	9	P11598,P19218,P21588,P50116,P97840,Q03191,Q8VD89,Q99041,Q9Z0J6
4786	1,4,5,6,7,27,28,31	2,3,8,25,26,29,30,32	6	P11598,P50116,Q03191,Q63617,Q68G31,Q9Z0J6
4787	1,4,5,6,7,27,28,32	2,3,8,25,26,29,30,31	7	P11598,P22283,P50116,Q03191,Q62761;Q62762;Q62763,Q63617,Q9Z0J6
4788	1,4,5,6,7,27,29,30	2,3,8,25,26,28,31,32	4	P19218,P21588,Q03191,Q923V8

4789	1,4,5,6,7,27,29,31	2,3,8,25,26,28,30,32	1 Q03191
4790	1,4,5,6,7,27,29,32	2,3,8,25,26,28,30,31	3 P00714,P11598,Q03191
4791	1,4,5,6,7,27,30,31	2,3,8,25,26,28,29,32	4 P19218,P36860,Q03191,Q9Z0J6
4792	1,4,5,6,7,27,30,32	2,3,8,25,26,28,29,31	5 P00714,P19218,Q03191,Q8VD89,Q9Z0J6
4793	1,4,5,6,7,27,31,32	2,3,8,25,26,28,29,30	5 P00714,P22006,P80299,Q03191,Q9Z0J6
4794	1,4,5,6,7,28,29,30	2,3,8,25,26,27,31,32	4 P00762,P19218,P97840,Q63617
4795	1,4,5,6,7,28,29,31	2,3,8,25,26,27,30,32	5 B1H234,P00762,P11598,P21674,Q63617
4796	1,4,5,6,7,28,29,32	2,3,8,25,26,27,30,31	3 P11598,P97840,Q63617
4797	1,4,5,6,7,28,30,31	2,3,8,25,26,27,29,32	2 P00762,Q9Z0J6
4798	1,4,5,6,7,28,30,32	2,3,8,25,26,27,29,31	5 P11598,P19218,Q5BJY9,Q8VD89,Q9Z0J6
4799	1,4,5,6,7,28,31,32	2,3,8,25,26,27,29,30	3 P11598,P22006,Q9Z0J6
4800	1,4,5,6,7,29,30,31	2,3,8,25,26,27,28,32	5 B1H234,P26342,P36860,Q5RJR2,Q9QWN8
4801	1,4,5,6,7,29,30,32	2,3,8,25,26,27,28,31	5 P00714,P26342,P36376,P36860,Q5BJY9
4802	1,4,5,6,7,29,31,32	2,3,8,25,26,27,28,30	2 P26342,P36860
4803	1,4,5,6,7,30,31,32	2,3,8,25,26,27,28,29	8 P22006,P25809,P26342,P36860,Q5BJY9,Q6P6R2,Q9WVK7,Q9Z0J6
4804	1,4,5,6,8,25,26,27	2,3,7,28,29,30,31,32	3 P13432,P15399,P54921
4805	1,4,5,6,8,25,26,28	2,3,7,27,29,30,31,32	4 P08649,P13432,P80299,Q9QW07
4806	1,4,5,6,8,25,26,29	2,3,7,27,28,30,31,32	2 P13432,P49134
4807	1,4,5,6,8,25,26,30	2,3,7,27,28,29,31,32	7 P35952,P49134,P54921,P80299,Q6AY61,Q6P6Q2,Q9WVK7
4808	1,4,5,6,8,25,26,31	2,3,7,27,28,29,30,32	4 P13432,P54921,Q63150,Q9WVK7

4809	1,4,5,6,8,25,26,32	2,3,7,27,28,29,30,31	3 P00507,P13432,Q9WVK7
4810	1,4,5,6,8,25,27,28	2,3,7,26,29,30,31,32	4 P06911,P20766,P22282,Q9QW07
4811	1,4,5,6,8,25,27,29	2,3,7,26,28,30,31,32	1 P13432
4812	1,4,5,6,8,25,27,30	2,3,7,26,28,29,31,32	4 P19218,Q4FZU2,Q6IMF3,Q6P6Q2
4813	1,4,5,6,8,25,27,31	2,3,7,26,28,29,30,32	3 P23739,P54921,Q03191
4814	1,4,5,6,8,25,27,32	2,3,7,26,28,29,30,31	3 P01681,P13432,Q03191
4815	1,4,5,6,8,25,28,29	2,3,7,26,27,30,31,32	3 P13432,P20766,Q9QW07
4816	1,4,5,6,8,25,28,30	2,3,7,26,27,29,31,32	2 P20766,Q6P6Q2
4817	1,4,5,6,8,25,28,31	2,3,7,26,27,29,30,32	1 P06911
4818	1,4,5,6,8,25,28,32	2,3,7,26,27,29,30,31	4 P01681,P13432,P20766,Q9QW07
4819	1,4,5,6,8,25,29,30	2,3,7,26,27,28,31,32	4 P20766,Q4FZU2,Q6IMF3,Q6P6Q2
4820	1,4,5,6,8,25,29,31	2,3,7,26,27,28,30,32	1 P13432
4821	1,4,5,6,8,25,29,32	2,3,7,26,27,28,30,31	2 P13432,P20766
4822	1,4,5,6,8,25,30,31	2,3,7,26,27,28,29,32	5 P54921,Q4FZU2,Q6IMF3,Q6P6Q2,Q9WVK7
4823	1,4,5,6,8,25,30,32	2,3,7,26,27,28,29,31	6 O88797,P01681,P13432,Q6IMF3,Q6P6Q2,Q9WVK7
4824	1,4,5,6,8,25,31,32	2,3,7,26,27,28,29,30	3 P01681,P13432,Q9WVK7
4825	1,4,5,6,8,26,27,28	2,3,7,25,29,30,31,32	4 P15399,P21588,P22282,Q9JI85
4826	1,4,5,6,8,26,27,29	2,3,7,25,28,30,31,32	0
4827	1,4,5,6,8,26,27,30	2,3,7,25,28,29,31,32	4 P15399,P21588,Q30KJ2,Q6P6Q2
4828	1,4,5,6,8,26,27,31	2,3,7,25,28,29,30,32	1 P54921

4829	1,4,5,6,8,26,27,32	2,3,7,25,28,29,30,31	3	P13432,P15399,P18418
4830	1,4,5,6,8,26,28,29	2,3,7,25,27,30,31,32	5	P20766,P21674,P49134,Q9JI85,Q9QW07
4831	1,4,5,6,8,26,28,30	2,3,7,25,27,29,31,32	2	P20766,Q8VD89
4832	1,4,5,6,8,26,28,31	2,3,7,25,27,29,30,32	0	
4833	1,4,5,6,8,26,28,32	2,3,7,25,27,29,30,31	4	O54728,P13432,P18418,Q9JI85
4834	1,4,5,6,8,26,29,30	2,3,7,25,27,28,31,32	4	P49134,Q5RJR2,Q6IMF3,Q6P6Q2
4835	1,4,5,6,8,26,29,31	2,3,7,25,27,28,30,32	2	P49134,Q5RJR2
4836	1,4,5,6,8,26,29,32	2,3,7,25,27,28,30,31	1	P13432
4837	1,4,5,6,8,26,30,31	2,3,7,25,27,28,29,32	5	P49134,P54921,Q5RJR2,Q6AY61,Q6P6Q2
4838	1,4,5,6,8,26,30,32	2,3,7,25,27,28,29,31	1	Q8VD89
4839	1,4,5,6,8,26,31,32	2,3,7,25,27,28,29,30	3	O54728,P13432,P18418
4840	1,4,5,6,8,27,28,29	2,3,7,25,26,30,31,32	5	P19218,P20766,P22282,Q812E4,Q9JI85
4841	1,4,5,6,8,27,28,30	2,3,7,25,26,29,31,32	6	P19218,P20766,P22282,Q10758,Q6P6Q2,Q8VD89
4842	1,4,5,6,8,27,28,31	2,3,7,25,26,29,30,32	3	P19218,P22282,P23739
4843	1,4,5,6,8,27,28,32	2,3,7,25,26,29,30,31	7	B0LT89,P19218,P20766,P22282,Q6AYR9,Q8VD89,Q9JI85
4844	1,4,5,6,8,27,29,30	2,3,7,25,26,28,31,32	9	P19218,P20766,Q10758,Q4FZU2,Q6IFU7,Q6IFU8,Q6IG02,Q6IMF3,Q6P6Q2
4845	1,4,5,6,8,27,29,31	2,3,7,25,26,28,30,32	0	
4846	1,4,5,6,8,27,29,32	2,3,7,25,26,28,30,31	2	P13432,P22282
4847	1,4,5,6,8,27,30,31	2,3,7,25,26,28,29,32	5	P19218,P23739,Q10758,Q6IMF3,Q6P6Q2
4848	1,4,5,6,8,27,30,32	2,3,7,25,26,28,29,31	5	P19218,Q03191,Q6IMF3,Q6P6Q2,Q8VD89

4849	1,4,5,6,8,27,31,32	2,3,7,25,26,28,29,30	2	P23739,Q03191
4850	1,4,5,6,8,28,29,30	2,3,7,25,26,27,31,32	5	P19218,P20766,P21674,Q6IMF3,Q6P6Q2
4851	1,4,5,6,8,28,29,31	2,3,7,25,26,27,30,32	1	P21674
4852	1,4,5,6,8,28,29,32	2,3,7,25,26,27,30,31	3	P20766,Q9JI85,Q9QW07
4853	1,4,5,6,8,28,30,31	2,3,7,25,26,27,29,32	3	P19218,Q6P6Q2,Q8VD89
4854	1,4,5,6,8,28,30,32	2,3,7,25,26,27,29,31	4	P19218,P20766,Q6P6Q2,Q8VD89
4855	1,4,5,6,8,28,31,32	2,3,7,25,26,27,29,30	2	P22006,Q63532
4856	1,4,5,6,8,29,30,31	2,3,7,25,26,27,28,32	4	P36860,Q5RJR2,Q6IMF3,Q6P6Q2
4857	1,4,5,6,8,29,30,32	2,3,7,25,26,27,28,31	4	P20766,P36860,Q6IMF3,Q6P6Q2
4858	1,4,5,6,8,29,31,32	2,3,7,25,26,27,28,30	2	P13432,P36860
4859	1,4,5,6,8,30,31,32	2,3,7,25,26,27,28,29	6	P20646,P36860,Q6IMF3,Q6P6Q2,Q8VD89,Q9WVK7
4860	1,4,5,6,25,26,27,28	2,3,7,8,29,30,31,32	3	P20766,P21588,P35280
4861	1,4,5,6,25,26,27,29	2,3,7,8,28,30,31,32	3	P20766,P21588,P35280
4862	1,4,5,6,25,26,27,30	2,3,7,8,28,29,31,32	1	P21588
4863	1,4,5,6,25,26,27,31	2,3,7,8,28,29,30,32	3	P35280,P54921,P62804
4864	1,4,5,6,25,26,27,32	2,3,7,8,28,29,30,31	2	P62804,Q03191
4865	1,4,5,6,25,26,28,29	2,3,7,8,27,30,31,32	2	P20766,P35280
4866	1,4,5,6,25,26,28,30	2,3,7,8,27,29,31,32	3	P20766,P21588,Q5PQL7
4867	1,4,5,6,25,26,28,31	2,3,7,8,27,29,30,32	2	P35280,P62804
4868	1,4,5,6,25,26,28,32	2,3,7,8,27,29,30,31	3	P20766,P62804,Q5PQL7

4869	1,4,5,6,25, 26,29,30	2,3,7,8,27, 28,31,32	2	P20766,P21588
4870	1,4,5,6,25, 26,29,31	2,3,7,8,27, 28,30,32	1	P35280
4871	1,4,5,6,25, 26,29,32	2,3,7,8,27, 28,30,31	1	P20766
4872	1,4,5,6,25, 26,30,31	2,3,7,8,27, 28,29,32	7	P25809,P54921,P62804,Q00715,Q812E4,Q9EQS0,Q9WVK7
4873	1,4,5,6,25, 26,30,32	2,3,7,8,27, 28,29,31	8	P00507,P20766,P25809,P62804,Q00715,Q5PQL7,Q9EQS0,Q9WVK7
4874	1,4,5,6,25, 26,31,32	2,3,7,8,27, 28,29,30	5	P00507,P25809,P62804,Q00715,Q9WVK7
4875	1,4,5,6,25, 27,28,29	2,3,7,8,26, 30,31,32	3	P20766,P35280,P97615
4876	1,4,5,6,25, 27,28,30	2,3,7,8,26, 29,31,32	1	P20766
4877	1,4,5,6,25, 27,28,31	2,3,7,8,26, 29,30,32	2	P23739,P35280
4878	1,4,5,6,25, 27,28,32	2,3,7,8,26, 29,30,31	3	P20766,Q03191,Q62761;Q62762;Q62763
4879	1,4,5,6,25, 27,29,30	2,3,7,8,26, 28,31,32	2	P20766,Q923V8
4880	1,4,5,6,25, 27,29,31	2,3,7,8,26, 28,30,32	1	P35280
4881	1,4,5,6,25, 27,29,32	2,3,7,8,26, 28,30,31	2	P20766,Q03191
4882	1,4,5,6,25, 27,30,31	2,3,7,8,26, 28,29,32	4	P23739,P54921,Q03191,Q9QYP1
4883	1,4,5,6,25, 27,30,32	2,3,7,8,26, 28,29,31	7	P20766,Q03191,Q62687,Q63424,Q64093,Q6P6R2,Q9QX74
4884	1,4,5,6,25, 27,31,32	2,3,7,8,26, 28,29,30	4	P23739,P62804,Q03191,Q9QYP1
4885	1,4,5,6,25, 28,29,30	2,3,7,8,26, 27,31,32	1	P20766
4886	1,4,5,6,25, 28,29,31	2,3,7,8,26, 27,30,32	1	P35280
4887	1,4,5,6,25, 28,29,32	2,3,7,8,26, 27,30,31	1	P20766
4888	1,4,5,6,25, 28,30,31	2,3,7,8,26, 27,29,32	1	P25809

4889	1,4,5,6,25,<br>28,30,32	2,3,7,8,26,<br>27,29,31	6	P20766,P25809,Q5PQL7,Q62975,Q8VD89,Q9QX74
4890	1,4,5,6,25,<br>28,31,32	2,3,7,8,26,<br>27,29,30	3	P22006,P25809,P62804
4891	1,4,5,6,25,<br>29,30,31	2,3,7,8,26,<br>27,28,32	3	P25809,P36860,Q9QWN8
4892	1,4,5,6,25,<br>29,30,32	2,3,7,8,26,<br>27,28,31	6	P20766,P25809,P36860,Q5BJY9,Q9WTT6,Q9WVK7
4893	1,4,5,6,25,<br>29,31,32	2,3,7,8,26,<br>27,28,30	5	P11883,P25809,P36860,P62804,Q9WTT6
4894	1,4,5,6,25,<br>30,31,32	2,3,7,8,26,<br>27,28,29	11	P25809,P36860,P47967,P62804,P97584,Q00715,Q5U2V4,Q6P6R2,Q9QX74,Q9WTT6,Q9WVK7
4895	1,4,5,6,26,<br>27,28,29	2,3,7,8,25,<br>30,31,32	4	P02625,P20766,P21588,P35280
4896	1,4,5,6,26,<br>27,28,30	2,3,7,8,25,<br>29,31,32	3	P20766,P21588,Q8VD89
4897	1,4,5,6,26,<br>27,28,31	2,3,7,8,25,<br>29,30,32	1	P35280
4898	1,4,5,6,26,<br>27,28,32	2,3,7,8,25,<br>29,30,31	2	P20766,Q8VD89
4899	1,4,5,6,26,<br>27,29,30	2,3,7,8,25,<br>28,31,32	4	P20766,P21588,Q811M5,Q923V8
4900	1,4,5,6,26,<br>27,29,31	2,3,7,8,25,<br>28,30,32	1	P35280
4901	1,4,5,6,26,<br>27,29,32	2,3,7,8,25,<br>28,30,31	1	P20766
4902	1,4,5,6,26,<br>27,30,31	2,3,7,8,25,<br>28,29,32	1	P36860
4903	1,4,5,6,26,<br>27,30,32	2,3,7,8,25,<br>28,29,31	3	P21588,P36860,Q8VD89
4904	1,4,5,6,26,<br>27,31,32	2,3,7,8,25,<br>28,29,30	4	P22006,P36860,P62804,Q03191
4905	1,4,5,6,26,<br>28,29,30	2,3,7,8,25,<br>27,31,32	2	P20766,P21588
4906	1,4,5,6,26,<br>28,29,31	2,3,7,8,25,<br>27,30,32	1	P35280
4907	1,4,5,6,26,<br>28,29,32	2,3,7,8,25,<br>27,30,31	1	P20766
4908	1,4,5,6,26,<br>28,30,31	2,3,7,8,25,<br>27,29,32	1	Q8VD89

4909	1,4,5,6,26,<br>28,30,32	2,3,7,8,25,<br>27,29,31	2	P20766,Q8VD89
4910	1,4,5,6,26,<br>28,31,32	2,3,7,8,25,<br>27,29,30	3	O54728,P22006,P62804
4911	1,4,5,6,26,<br>29,30,31	2,3,7,8,25,<br>27,28,32	3	B1H234,P36860,Q5RJR2
4912	1,4,5,6,26,<br>29,30,32	2,3,7,8,25,<br>27,28,31	3	P20766,P36376,P36860
4913	1,4,5,6,26,<br>29,31,32	2,3,7,8,25,<br>27,28,30	2	P36860,P62804
4914	1,4,5,6,26,<br>30,31,32	2,3,7,8,25,<br>27,28,29	10	P22006,P25809,P36860,P62804,Q00715,Q4G075,Q8VD89,Q9EQS0,Q9WTT6,Q9WVK7
4915	1,4,5,6,27,<br>28,29,30	2,3,7,8,25,<br>26,31,32	4	P19218,P20766,P21588,Q923V8
4916	1,4,5,6,27,<br>28,29,31	2,3,7,8,25,<br>26,30,32	1	P35280
4917	1,4,5,6,27,<br>28,29,32	2,3,7,8,25,<br>26,30,31	4	O35547,P20766,Q62761;Q62762;Q62763,Q6AYR9
4918	1,4,5,6,27,<br>28,30,31	2,3,7,8,25,<br>26,29,32	2	P19218,Q8VD89
4919	1,4,5,6,27,<br>28,30,32	2,3,7,8,25,<br>26,29,31	4	P19218,P20766,Q8VD89,Q9QX74
4920	1,4,5,6,27,<br>28,31,32	2,3,7,8,25,<br>26,29,30	5	P22006,P23739,P62804,Q03191,Q8VD89
4921	1,4,5,6,27,<br>29,30,31	2,3,7,8,25,<br>26,28,32	3	B1H234,P36860,Q923V8
4922	1,4,5,6,27,<br>29,30,32	2,3,7,8,25,<br>26,28,31	4	P20766,P36860,Q63598,Q923V8
4923	1,4,5,6,27,<br>29,31,32	2,3,7,8,25,<br>26,28,30	2	P36860,Q03191
4924	1,4,5,6,27,<br>30,31,32	2,3,7,8,25,<br>26,28,29	9	P22006,P23739,P36860,P62804,Q03191,Q64093,Q6P6R2,Q8VD89,Q9WTT6
4925	1,4,5,6,28,<br>29,30,31	2,3,7,8,25,<br>26,27,32	2	B1H234,P36860
4926	1,4,5,6,28,<br>29,30,32	2,3,7,8,25,<br>26,27,31	3	P20766,P36860,Q5BJY9
4927	1,4,5,6,28,<br>29,31,32	2,3,7,8,25,<br>26,27,30	1	P22006
4928	1,4,5,6,28,<br>30,31,32	2,3,7,8,25,<br>26,27,29	9	P22006,P25809,P36860,P62804,Q4G075,Q5BJY9,Q8VD89,Q9QX74,Q9WTT6

4929	1,4,5,6,29,30,31,32	2,3,7,8,25,26,27,28	6 B1H234,P25809,P26342,P36860,Q5BJY9,Q9WTT6
4930	1,4,5,7,8,25,26,27	2,3,6,28,29,30,31,32	3 Q03191,Q4G075,Q9WTT6
4931	1,4,5,7,8,25,26,28	2,3,6,27,29,30,31,32	2 P49134,Q9WTT6
4932	1,4,5,7,8,25,26,29	2,3,6,27,28,30,31,32	5 P06760,P22006,P48032,P49134,Q9QX74
4933	1,4,5,7,8,25,26,30	2,3,6,27,28,29,31,32	5 P49134,Q4FZU2,Q6IMF3,Q6P6Q2,Q9WVK7
4934	1,4,5,7,8,25,26,31	2,3,6,27,28,29,30,32	3 P49134,Q9WVK7,Q9Z0V6
4935	1,4,5,7,8,25,26,32	2,3,6,27,28,29,30,31	3 P01681,P13432,Q9WVK7
4936	1,4,5,7,8,25,27,28	2,3,6,26,29,30,31,32	3 P22282,Q03191,Q4G075
4937	1,4,5,7,8,25,27,29	2,3,6,26,28,30,31,32	6 O89117,P06760,P22006,Q03191,Q4G075,Q99MH3
4938	1,4,5,7,8,25,27,30	2,3,6,26,28,29,31,32	7 Q03191,Q4FZU2,Q4G075,Q6IFU8,Q6IFW6,Q6IMF3,Q6P6Q2
4939	1,4,5,7,8,25,27,31	2,3,6,26,28,29,30,32	4 P23739,Q03191,Q4G075,Q68G31
4940	1,4,5,7,8,25,27,32	2,3,6,26,28,29,30,31	4 P01681,P22282,Q01984,Q03191
4941	1,4,5,7,8,25,28,29	2,3,6,26,27,30,31,32	3 P06760,P49134,Q99MH3
4942	1,4,5,7,8,25,28,30	2,3,6,26,27,29,31,32	2 Q6IMF3,Q6P6Q2
4943	1,4,5,7,8,25,28,31	2,3,6,26,27,29,30,32	2 P82471,Q811M5
4944	1,4,5,7,8,25,28,32	2,3,6,26,27,29,30,31	2 P01681,Q811M5
4945	1,4,5,7,8,25,29,30	2,3,6,26,27,28,31,32	9 P06760,P22006,P48032,P49134,Q4FZU2,Q6IFU8,Q6IFW6,Q6IMF3,Q6P6Q2
4946	1,4,5,7,8,25,29,31	2,3,6,26,27,28,30,32	3 P06760,P49134,P82471
4947	1,4,5,7,8,25,29,32	2,3,6,26,27,28,30,31	3 P01681,P06760,P13432
4948	1,4,5,7,8,25,30,31	2,3,6,26,27,28,29,32	5 P49134,Q4FZU2,Q6IMF3,Q6P6Q2,Q9WVK7

4949	1,4,5,7,8,25,30,32	2,3,6,26,27,28,29,31	5	P01681,Q4FZU2,Q6IMF3,Q6P6Q2,Q9WVK7
4950	1,4,5,7,8,25,31,32	2,3,6,26,27,28,29,30	4	P01681,P82471,Q811M5,Q9WVK7
4951	1,4,5,7,8,26,27,28	2,3,6,25,29,30,31,32	7	D3ZUC6,P07150,P22282,P47967,P97840,Q4G075,Q9WTT6
4952	1,4,5,7,8,26,27,29	2,3,6,25,28,30,31,32	4	P06760,Q4G075,Q9QX74,Q9WTT6
4953	1,4,5,7,8,26,27,30	2,3,6,25,28,29,31,32	3	Q4FZU2,Q6IMF3,Q6P6Q2
4954	1,4,5,7,8,26,27,31	2,3,6,25,28,29,30,32	2	P23739,Q68G31
4955	1,4,5,7,8,26,27,32	2,3,6,25,28,29,30,31	2	P22282,Q03191
4956	1,4,5,7,8,26,28,29	2,3,6,25,27,30,31,32	10	D3ZUC6,P06760,P20646,P21674,P47967,P49134,P97840,Q6P6R2,Q9QX74,Q9WTT6
4957	1,4,5,7,8,26,28,30	2,3,6,25,27,29,31,32	3	P49134,P97840,Q6P6Q2
4958	1,4,5,7,8,26,28,31	2,3,6,25,27,29,30,32	3	P49134,P82471,Q5RLM2
4959	1,4,5,7,8,26,28,32	2,3,6,25,27,29,30,31	1	O54728
4960	1,4,5,7,8,26,29,30	2,3,6,25,27,28,31,32	6	P06760,P49134,Q5RJR2,Q6IMF3,Q6P6Q2,Q9QX74
4961	1,4,5,7,8,26,29,31	2,3,6,25,27,28,30,32	5	P06760,P49134,P82471,Q5RJR2,Q9QX74
4962	1,4,5,7,8,26,29,32	2,3,6,25,27,28,30,31	3	P06760,P49134,Q9QX74
4963	1,4,5,7,8,26,30,31	2,3,6,25,27,28,29,32	4	P49134,Q5RJR2,Q6IMF3,Q6P6Q2
4964	1,4,5,7,8,26,30,32	2,3,6,25,27,28,29,31	2	P49134,Q6P6Q2
4965	1,4,5,7,8,26,31,32	2,3,6,25,27,28,29,30	2	O54728,P82471
4966	1,4,5,7,8,27,28,29	2,3,6,25,26,30,31,32	14	D3ZUC6,P06760,P22273,P22282,P22283,P47967,P97840,Q4G075,Q5I0D1,Q63493,Q63617,Q812E4,Q99MH3,Q9Z2L0
4967	1,4,5,7,8,27,28,30	2,3,6,25,26,29,31,32	6	P19218,P22282,P97840,Q4G075,Q6IMF3,Q6P6Q2
4968	1,4,5,7,8,27,28,31	2,3,6,25,26,29,30,32	5	D3ZUC6,P22282,P23739,P82471,Q68G31

4969	1,4,5,7,8,27,28,32	2,3,6,25,26,29,30,31	6	P04905,P22282,P97840,Q03191,Q6AYR9,Q812E4
4970	1,4,5,7,8,27,29,30	2,3,6,25,26,28,31,32	9	P06760,P19218,Q4FZU2,Q4G075,Q6IFU8,Q6IFW6,Q6IG02,Q6IMF3,Q6P6Q2
4971	1,4,5,7,8,27,29,31	2,3,6,25,26,28,30,32	6	P06760,P82471,Q4G075,Q68G31,Q99MH3,Q9QX74
4972	1,4,5,7,8,27,29,32	2,3,6,25,26,28,30,31	4	P06760,P22282,Q03191,Q4G075
4973	1,4,5,7,8,27,30,31	2,3,6,25,26,28,29,32	6	P19218,P23739,Q4FZU2,Q6IFU8,Q6IMF3,Q6P6Q2
4974	1,4,5,7,8,27,30,32	2,3,6,25,26,28,29,31	5	P19218,Q03191,Q4FZU2,Q6IMF3,Q6P6Q2
4975	1,4,5,7,8,27,31,32	2,3,6,25,26,28,29,30	3	P23739,P82471,Q03191
4976	1,4,5,7,8,28,29,30	2,3,6,25,26,27,31,32	7	P06760,P19218,P21674,P49134,P97840,Q6IMF3,Q6P6Q2
4977	1,4,5,7,8,28,29,31	2,3,6,25,26,27,30,32	6	D3ZUC6,P06760,P21674,P49134,P82471,Q63493
4978	1,4,5,7,8,28,29,32	2,3,6,25,26,27,30,31	7	P06760,P08723,P21674,P30120,P97840,Q63493,Q812E4
4979	1,4,5,7,8,28,30,31	2,3,6,25,26,27,29,32	5	P35745,P49134,P82471,Q6IMF3,Q6P6Q2
4980	1,4,5,7,8,28,30,32	2,3,6,25,26,27,29,31	3	P19218,Q6IMF3,Q6P6Q2
4981	1,4,5,7,8,28,31,32	2,3,6,25,26,27,29,30	2	P82471,Q811M5
4982	1,4,5,7,8,29,30,31	2,3,6,25,26,27,28,32	7	P49134,P82471,Q4FZU2,Q5RJR2,Q6IMF3,Q6P6Q2,Q9QWN8
4983	1,4,5,7,8,29,30,32	2,3,6,25,26,27,28,31	3	Q4FZU2,Q6IMF3,Q6P6Q2
4984	1,4,5,7,8,29,31,32	2,3,6,25,26,27,28,30	1	P82471
4985	1,4,5,7,8,30,31,32	2,3,6,25,26,27,28,29	5	P82471,Q4FZU2,Q6IMF3,Q6P6Q2,Q9WVK7
4986	1,4,5,7,25,26,27,28	2,3,6,8,29,30,31,32	1	P20646
4987	1,4,5,7,25,26,27,29	2,3,6,8,28,30,31,32	1	O89117
4988	1,4,5,7,25,26,27,30	2,3,6,8,28,29,31,32	2	P21588,Q03191

4989	1,4,5,7,25,<br>26,27,31	2,3,6,8,28,<br>29,30,32	4 P23739,Q03191,Q68G31,Q9QYP1
4990	1,4,5,7,25,<br>26,27,32	2,3,6,8,28,<br>29,30,31	1 Q03191
4991	1,4,5,7,25,<br>26,28,29	2,3,6,8,27,<br>30,31,32	1 P20646
4992	1,4,5,7,25,<br>26,28,30	2,3,6,8,27,<br>29,31,32	0
4993	1,4,5,7,25,<br>26,28,31	2,3,6,8,27,<br>29,30,32	0
4994	1,4,5,7,25,<br>26,28,32	2,3,6,8,27,<br>29,30,31	0
4995	1,4,5,7,25,<br>26,29,30	2,3,6,8,27,<br>28,31,32	0
4996	1,4,5,7,25,<br>26,29,31	2,3,6,8,27,<br>28,30,32	1 Q6P6S4
4997	1,4,5,7,25,<br>26,29,32	2,3,6,8,27,<br>28,30,31	1 P36376
4998	1,4,5,7,25,<br>26,30,31	2,3,6,8,27,<br>28,29,32	2 P25809,Q9WVK7
4999	1,4,5,7,25,<br>26,30,32	2,3,6,8,27,<br>28,29,31	2 Q9R168,Q9WVK7
5000	1,4,5,7,25,<br>26,31,32	2,3,6,8,27,<br>28,29,30	1 Q9WVK7
5001	1,4,5,7,25,<br>27,28,29	2,3,6,8,26,<br>30,31,32	4 O89117,P97615,P97840,Q99MH3
5002	1,4,5,7,25,<br>27,28,30	2,3,6,8,26,<br>29,31,32	1 Q03191
5003	1,4,5,7,25,<br>27,28,31	2,3,6,8,26,<br>29,30,32	4 P23739,Q03191,Q68G31,Q99MH3
5004	1,4,5,7,25,<br>27,28,32	2,3,6,8,26,<br>29,30,31	3 Q03191,Q62761;Q62762;Q62763,Q99MH3
5005	1,4,5,7,25,<br>27,29,30	2,3,6,8,26,<br>28,31,32	3 O89117,Q03191,Q99MH3
5006	1,4,5,7,25,<br>27,29,31	2,3,6,8,26,<br>28,30,32	4 O89117,Q03191,Q68G31,Q99MH3
5007	1,4,5,7,25,<br>27,29,32	2,3,6,8,26,<br>28,30,31	3 O89117,Q03191,Q99MH3
5008	1,4,5,7,25,<br>27,30,31	2,3,6,8,26,<br>28,29,32	3 P23739,Q03191,Q9QYP1

5009	1,4,5,7,25, 27,30,32	2,3,6,8,26, 28,29,31	1 Q03191
5010	1,4,5,7,25, 27,31,32	2,3,6,8,26, 28,29,30	3 P23739,Q03191,Q9QYP1
5011	1,4,5,7,25, 28,29,30	2,3,6,8,26, 27,31,32	1 Q99MH3
5012	1,4,5,7,25, 28,29,31	2,3,6,8,26, 27,30,32	1 Q99MH3
5013	1,4,5,7,25, 28,29,32	2,3,6,8,26, 27,30,31	1 Q99MH3
5014	1,4,5,7,25, 28,30,31	2,3,6,8,26, 27,29,32	1 P25809
5015	1,4,5,7,25, 28,30,32	2,3,6,8,26, 27,29,31	3 Q5BJY9,Q62975,Q9R168
5016	1,4,5,7,25, 28,31,32	2,3,6,8,26, 27,29,30	1 Q811M5
5017	1,4,5,7,25, 29,30,31	2,3,6,8,26, 27,28,32	2 Q9QWN8,Q9WVK7
5018	1,4,5,7,25, 29,30,32	2,3,6,8,26, 27,28,31	3 Q5BJY9,Q9R168,Q9WVK7
5019	1,4,5,7,25, 29,31,32	2,3,6,8,26, 27,28,30	1 Q9WVK7
5020	1,4,5,7,25, 30,31,32	2,3,6,8,26, 27,28,29	5 P25809,P26342,Q6P6R2,Q9QYP1,Q9WVK7
5021	1,4,5,7,26, 27,28,29	2,3,6,8,25, 30,31,32	5 P02625,P20646,P21588,P47967,P97840
5022	1,4,5,7,26, 27,28,30	2,3,6,8,25, 29,31,32	2 P21588,P97840
5023	1,4,5,7,26, 27,28,31	2,3,6,8,25, 29,30,32	1 Q68G31
5024	1,4,5,7,26, 27,28,32	2,3,6,8,25, 29,30,31	3 P97840,Q03191,Q62761;Q62762;Q62763
5025	1,4,5,7,26, 27,29,30	2,3,6,8,25, 28,31,32	3 P21588,P36376,P97840
5026	1,4,5,7,26, 27,29,31	2,3,6,8,25, 28,30,32	1 Q68G31
5027	1,4,5,7,26, 27,29,32	2,3,6,8,25, 28,30,31	2 P36376,Q03191
5028	1,4,5,7,26, 27,30,31	2,3,6,8,25, 28,29,32	0

5029	1,4,5,7,26,<br>27,30,32	2,3,6,8,25,<br>28,29,31	2 P36376,Q03191
5030	1,4,5,7,26,<br>27,31,32	2,3,6,8,25,<br>28,29,30	3 P23739,Q03191,Q9Z0J6
5031	1,4,5,7,26,<br>28,29,30	2,3,6,8,25,<br>27,31,32	1 P97840
5032	1,4,5,7,26,<br>28,29,31	2,3,6,8,25,<br>27,30,32	3 P02625,P97840,Q68G31
5033	1,4,5,7,26,<br>28,29,32	2,3,6,8,25,<br>27,30,31	1 P97840
5034	1,4,5,7,26,<br>28,30,31	2,3,6,8,25,<br>27,29,32	0
5035	1,4,5,7,26,<br>28,30,32	2,3,6,8,25,<br>27,29,31	2 P97840,Q9R168
5036	1,4,5,7,26,<br>28,31,32	2,3,6,8,25,<br>27,29,30	1 O54728
5037	1,4,5,7,26,<br>29,30,31	2,3,6,8,25,<br>27,28,32	2 P36860,Q9QWN8
5038	1,4,5,7,26,<br>29,30,32	2,3,6,8,25,<br>27,28,31	3 P36376,P36860,Q9R168
5039	1,4,5,7,26,<br>29,31,32	2,3,6,8,25,<br>27,28,30	1 P36860
5040	1,4,5,7,26,<br>30,31,32	2,3,6,8,25,<br>27,28,29	3 P25809,P36860,Q9WVK7
5041	1,4,5,7,27,<br>28,29,30	2,3,6,8,25,<br>26,31,32	3 P47967,P97840,Q99MH3
5042	1,4,5,7,27,<br>28,29,31	2,3,6,8,25,<br>26,30,32	3 P97840,Q68G31,Q99MH3
5043	1,4,5,7,27,<br>28,29,32	2,3,6,8,25,<br>26,30,31	8 O35547,P47967,P97840,Q03191,Q62761;Q62762;Q62763,Q6AYR9,Q812E4,Q99MH3
5044	1,4,5,7,27,<br>28,30,31	2,3,6,8,25,<br>26,29,32	2 P08649,P23739
5045	1,4,5,7,27,<br>28,30,32	2,3,6,8,25,<br>26,29,31	3 P97840,Q03191,Q8VD89
5046	1,4,5,7,27,<br>28,31,32	2,3,6,8,25,<br>26,29,30	3 P23739,Q03191,Q62761;Q62762;Q62763
5047	1,4,5,7,27,<br>29,30,31	2,3,6,8,25,<br>26,28,32	2 P08649,Q99MH3
5048	1,4,5,7,27,<br>29,30,32	2,3,6,8,25,<br>26,28,31	3 P08649,P36376,Q03191

5049	1,4,5,7,27, 29,31,32	2,3,6,8,25, 26,28,30	4	P08649,P80299,Q03191,Q99MH3
5050	1,4,5,7,27, 30,31,32	2,3,6,8,25, 26,28,29	6	P08649,P23739,P36860,Q03191,Q9QYP1,Q9Z0J6
5051	1,4,5,7,28, 29,30,31	2,3,6,8,25, 26,27,32	1	P08649
5052	1,4,5,7,28, 29,30,32	2,3,6,8,25, 26,27,31	2	P97840,Q5BJY9
5053	1,4,5,7,28, 29,31,32	2,3,6,8,25, 26,27,30	0	
5054	1,4,5,7,28, 30,31,32	2,3,6,8,25, 26,27,29	1	Q5BJY9
5055	1,4,5,7,29, 30,31,32	2,3,6,8,25, 26,27,28	6	P08649,P17559,P26342,P36860,P63081,Q5BJY9
5056	1,4,5,8,25, 26,27,28	2,3,6,7,29, 30,31,32	3	P01835,P20766,P26342
5057	1,4,5,8,25, 26,27,29	2,3,6,7,28, 30,31,32	2	P06760,P22006
5058	1,4,5,8,25, 26,27,30	2,3,6,7,28, 29,31,32	4	P01835,Q4FZU2,Q6IMF3,Q6P6Q2
5059	1,4,5,8,25, 26,27,31	2,3,6,7,28, 29,30,32	3	P01835,P23739,P54921
5060	1,4,5,8,25, 26,27,32	2,3,6,7,28, 29,30,31	3	P01681,P01835,P13432
5061	1,4,5,8,25, 26,28,29	2,3,6,7,27, 30,31,32	3	P06760,P20766,P80299
5062	1,4,5,8,25, 26,28,30	2,3,6,7,27, 29,31,32	3	P20766,P80299,Q6P6Q2
5063	1,4,5,8,25, 26,28,31	2,3,6,7,27, 29,30,32	0	
5064	1,4,5,8,25, 26,28,32	2,3,6,7,27, 29,30,31	4	O54728,P01681,P13432,P20766
5065	1,4,5,8,25, 26,29,30	2,3,6,7,27, 28,31,32	6	P20766,P22006,P80299,Q4FZU2,Q6IMF3,Q6P6Q2
5066	1,4,5,8,25, 26,29,31	2,3,6,7,27, 28,30,32	0	
5067	1,4,5,8,25, 26,29,32	2,3,6,7,27, 28,30,31	3	P00507,P13432,P20766
5068	1,4,5,8,25, 26,30,31	2,3,6,7,27, 28,29,32	5	P54921,Q6AY61,Q6IMF3,Q6P6Q2,Q9WVK7

5069	1,4,5,8,25,26,30,32	2,3,6,7,27,28,29,31	6	O88797,P00507,P01681,P20766,Q6P6Q2,Q9WVK7
5070	1,4,5,8,25,26,31,32	2,3,6,7,27,28,29,30	5	O54728,P00507,P01681,P13432,Q9WVK7
5071	1,4,5,8,25,27,28,29	2,3,6,7,26,30,31,32	3	P20766,Q812E4,Q99MH3
5072	1,4,5,8,25,27,28,30	2,3,6,7,26,29,31,32	4	P01835,P20766,Q6IMF3,Q6P6Q2
5073	1,4,5,8,25,27,28,31	2,3,6,7,26,29,30,32	2	P01835,P23739
5074	1,4,5,8,25,27,28,32	2,3,6,7,26,29,30,31	5	P01681,P01835,P20766,Q6AYR9,Q9QWN8
5075	1,4,5,8,25,27,29,30	2,3,6,7,26,28,31,32	8	P20766,Q4FZU2,Q6IFU7,Q6IFU8,Q6IFW6,Q6IG02,Q6IMF3,Q6P6Q2
5076	1,4,5,8,25,27,29,31	2,3,6,7,26,28,30,32	2	P23739,Q99MH3
5077	1,4,5,8,25,27,29,32	2,3,6,7,26,28,30,31	3	P01681,P13432,P20766
5078	1,4,5,8,25,27,30,31	2,3,6,7,26,28,29,32	9	P01835,P23739,P54921,Q4FZU2,Q6IFU8,Q6IFW6,Q6IMF3,Q6P6Q2,Q9QYP1
5079	1,4,5,8,25,27,30,32	2,3,6,7,26,28,29,31	7	P01681,P01835,P20766,Q03191,Q4FZU2,Q6IMF3,Q6P6Q2
5080	1,4,5,8,25,27,31,32	2,3,6,7,26,28,29,30	4	P01681,P01835,P23739,Q03191
5081	1,4,5,8,25,28,29,30	2,3,6,7,26,27,31,32	3	P20766,Q6IMF3,Q6P6Q2
5082	1,4,5,8,25,28,29,31	2,3,6,7,26,27,30,32	0	
5083	1,4,5,8,25,28,29,32	2,3,6,7,26,27,30,31	2	P01681,P20766
5084	1,4,5,8,25,28,30,31	2,3,6,7,26,27,29,32	2	Q6IMF3,Q6P6Q2
5085	1,4,5,8,25,28,30,32	2,3,6,7,26,27,29,31	3	P01681,P20766,Q6P6Q2
5086	1,4,5,8,25,28,31,32	2,3,6,7,26,27,29,30	2	P01681,Q811M5
5087	1,4,5,8,25,29,30,31	2,3,6,7,26,27,28,32	4	Q4FZU2,Q6IFW6,Q6IMF3,Q6P6Q2
5088	1,4,5,8,25,29,30,32	2,3,6,7,26,27,28,31	7	O88797,P01681,P20766,Q4FZU2,Q62894,Q6IMF3,Q6P6Q2

5089	1,4,5,8,25,29,31,32	2,3,6,7,26,27,28,30	2	P01681,P13432
5090	1,4,5,8,25,30,31,32	2,3,6,7,26,27,28,29	5	P01681,Q4FZU2,Q6IMF3,Q6P6Q2,Q9WVK7
5091	1,4,5,8,26,27,28,29	2,3,6,7,25,30,31,32	3	P06760,P20766,Q6P6R2
5092	1,4,5,8,26,27,28,30	2,3,6,7,25,29,31,32	2	P20766,Q6P6Q2
5093	1,4,5,8,26,27,28,31	2,3,6,7,25,29,30,32	1	P23739
5094	1,4,5,8,26,27,28,32	2,3,6,7,25,29,30,31	4	O54728,P20766,Q6AYR9,Q9QWN8
5095	1,4,5,8,26,27,29,30	2,3,6,7,25,28,31,32	5	P20766,Q6IFU8,Q6IG02,Q6IMF3,Q6P6Q2
5096	1,4,5,8,26,27,29,31	2,3,6,7,25,28,30,32	0	
5097	1,4,5,8,26,27,29,32	2,3,6,7,25,28,30,31	1	P20766
5098	1,4,5,8,26,27,30,31	2,3,6,7,25,28,29,32	4	P23739,Q6AY61,Q6IMF3,Q6P6Q2
5099	1,4,5,8,26,27,30,32	2,3,6,7,25,28,29,31	2	Q6IMF3,Q6P6Q2
5100	1,4,5,8,26,27,31,32	2,3,6,7,25,28,29,30	2	O54728,P23739
5101	1,4,5,8,26,28,29,30	2,3,6,7,25,27,31,32	2	P20766,Q6P6Q2
5102	1,4,5,8,26,28,29,31	2,3,6,7,25,27,30,32	0	
5103	1,4,5,8,26,28,29,32	2,3,6,7,25,27,30,31	2	O54728,P20766
5104	1,4,5,8,26,28,30,31	2,3,6,7,25,27,29,32	1	Q6P6Q2
5105	1,4,5,8,26,28,30,32	2,3,6,7,25,27,29,31	3	O54728,P20766,Q8VD89
5106	1,4,5,8,26,28,31,32	2,3,6,7,25,27,29,30	1	O54728
5107	1,4,5,8,26,29,30,31	2,3,6,7,25,27,28,32	3	Q5RJR2,Q6IMF3,Q6P6Q2
5108	1,4,5,8,26,29,30,32	2,3,6,7,25,27,28,31	4	O88797,P20766,Q6IMF3,Q6P6Q2

5109	1,4,5,8,26,29,31,32	2,3,6,7,25,27,28,30	1	O54728
5110	1,4,5,8,26,30,31,32	2,3,6,7,25,27,28,29	4	O54728,P63081,Q6P6Q2,Q9WVK7
5111	1,4,5,8,27,28,29,30	2,3,6,7,25,26,31,32	5	P19218,P20766,Q6IG02,Q6IMF3,Q6P6Q2
5112	1,4,5,8,27,28,29,31	2,3,6,7,25,26,30,32	1	Q99MH3
5113	1,4,5,8,27,28,29,32	2,3,6,7,25,26,30,31	5	O35547,P04905,P20766,Q6AYR9,Q812E4
5114	1,4,5,8,27,28,30,31	2,3,6,7,25,26,29,32	4	P19218,P23739,Q6IMF3,Q6P6Q2
5115	1,4,5,8,27,28,30,32	2,3,6,7,25,26,29,31	7	B0LT89,P19218,P20766,Q6AYR9,Q6IMF3,Q6P6Q2,Q8VD89
5116	1,4,5,8,27,28,31,32	2,3,6,7,25,26,29,30	1	P23739
5117	1,4,5,8,27,29,30,31	2,3,6,7,25,26,28,32	7	P23739,Q4FZU2,Q6IFU8,Q6IFW6,Q6IG02,Q6IMF3,Q6P6Q2
5118	1,4,5,8,27,29,30,32	2,3,6,7,25,26,28,31	6	P20766,Q4FZU2,Q6IFU8,Q6IG02,Q6IMF3,Q6P6Q2
5119	1,4,5,8,27,29,31,32	2,3,6,7,25,26,28,30	1	P23739
5120	1,4,5,8,27,30,31,32	2,3,6,7,25,26,28,29	4	P23739,Q4FZU2,Q6IMF3,Q6P6Q2
5121	1,4,5,8,28,29,30,31	2,3,6,7,25,26,27,32	2	Q6IMF3,Q6P6Q2
5122	1,4,5,8,28,29,30,32	2,3,6,7,25,26,27,31	4	P20766,Q62894,Q6IMF3,Q6P6Q2
5123	1,4,5,8,28,29,31,32	2,3,6,7,25,26,27,30	0	
5124	1,4,5,8,28,30,31,32	2,3,6,7,25,26,27,29	2	Q6P6Q2,Q8VD89
5125	1,4,5,8,29,30,31,32	2,3,6,7,25,26,27,28	5	P36860,P63081,Q4FZU2,Q6IMF3,Q6P6Q2
5126	1,4,5,25,26,27,28,29	2,3,6,7,8,30,31,32	6	O89117,P02625,P20646,P20766,P35280,P97615
5127	1,4,5,25,26,27,28,30	2,3,6,7,8,29,31,32	2	P20766,P21588
5128	1,4,5,25,26,27,28,31	2,3,6,7,8,29,30,32	2	P23739,P35280

5129	1,4,5,25,26,27,28,32	2,3,6,7,8,29,30,31	3	P20766,P62804,Q9QWN8
5130	1,4,5,25,26,27,29,30	2,3,6,7,8,28,31,32	3	O89117,P20766,P21588
5131	1,4,5,25,26,27,29,31	2,3,6,7,8,28,30,32	2	O89117,P35280
5132	1,4,5,25,26,27,29,32	2,3,6,7,8,28,30,31	1	P20766
5133	1,4,5,25,26,27,30,31	2,3,6,7,8,28,29,32	4	P23739,P54921,P62804,Q9QYP1
5134	1,4,5,25,26,27,30,32	2,3,6,7,8,28,29,31	3	P20766,P21674,P62804
5135	1,4,5,25,26,27,31,32	2,3,6,7,8,28,29,30	4	P23739,P62804,Q03191,Q9QYP1
5136	1,4,5,25,26,28,29,30	2,3,6,7,8,27,31,32	2	P20766,Q9QZK9
5137	1,4,5,25,26,28,29,31	2,3,6,7,8,27,30,32	1	P35280
5138	1,4,5,25,26,28,29,32	2,3,6,7,8,27,30,31	2	P20766,Q62894
5139	1,4,5,25,26,28,30,31	2,3,6,7,8,27,29,32	1	P25809
5140	1,4,5,25,26,28,30,32	2,3,6,7,8,27,29,31	4	P20766,P50115,Q00715,Q62894
5141	1,4,5,25,26,28,31,32	2,3,6,7,8,27,29,30	3	O54728,P00731,P62804
5142	1,4,5,25,26,29,30,31	2,3,6,7,8,27,28,32	0	
5143	1,4,5,25,26,29,30,32	2,3,6,7,8,27,28,31	3	P20766,P50115,Q62894
5144	1,4,5,25,26,29,31,32	2,3,6,7,8,27,28,30	3	P00507,P19218,P62804
5145	1,4,5,25,26,30,31,32	2,3,6,7,8,27,28,29	8	P00507,P04636,P25809,P50115,P62804,Q00715,Q4G075,Q9WVK7
5146	1,4,5,25,27,28,29,30	2,3,6,7,8,26,31,32	3	O89117,P20766,Q99MH3
5147	1,4,5,25,27,28,29,31	2,3,6,7,8,26,30,32	4	O89117,P35280,P97615,Q99MH3
5148	1,4,5,25,27,28,29,32	2,3,6,7,8,26,30,31	5	O35547,P20766,Q62761;Q62762;Q62763,Q8CGS4,Q99MH3

5149	1,4,5,25,27,28,30,31	2,3,6,7,8,26,29,32	2	P23739,Q9QYP1
5150	1,4,5,25,27,28,30,32	2,3,6,7,8,26,29,31	1	P20766
5151	1,4,5,25,27,28,31,32	2,3,6,7,8,26,29,30	3	P23739,P62804,Q03191
5152	1,4,5,25,27,29,30,31	2,3,6,7,8,26,28,32	1	Q9QYP1
5153	1,4,5,25,27,29,30,32	2,3,6,7,8,26,28,31	2	P20766,Q63598
5154	1,4,5,25,27,29,31,32	2,3,6,7,8,26,28,30	3	P23739,P62804,Q99MH3
5155	1,4,5,25,27,30,31,32	2,3,6,7,8,26,28,29	7	P23739,P62804,Q00715,Q03191,Q6P6R2,Q9QYP1,Q9WTT6
5156	1,4,5,25,28,29,30,31	2,3,6,7,8,26,27,32	1	P50115
5157	1,4,5,25,28,29,30,32	2,3,6,7,8,26,27,31	4	P20766,P50115,Q5BJY9,Q62894
5158	1,4,5,25,28,29,31,32	2,3,6,7,8,26,27,30	1	P50115
5159	1,4,5,25,28,30,31,32	2,3,6,7,8,26,27,29	5	P25809,P50115,P62804,Q00715,Q9WTT6
5160	1,4,5,25,29,30,31,32	2,3,6,7,8,26,27,28	7	P07150,P25809,P36860,P50115,Q5BJY9,Q9WTT6,Q9WVK7
5161	1,4,5,26,27,28,29,30	2,3,6,7,8,25,31,32	2	P20766,P21588
5162	1,4,5,26,27,28,29,31	2,3,6,7,8,25,30,32	4	P02625,P02631,P35280,P97615
5163	1,4,5,26,27,28,29,32	2,3,6,7,8,25,30,31	2	P20766,Q62761;Q62762;Q62763
5164	1,4,5,26,27,28,30,31	2,3,6,7,8,25,29,32	0	
5165	1,4,5,26,27,28,30,32	2,3,6,7,8,25,29,31	2	P20766,Q8VD89
5166	1,4,5,26,27,28,31,32	2,3,6,7,8,25,29,30	3	O54728,P23739,P62804
5167	1,4,5,26,27,29,30,31	2,3,6,7,8,25,28,32	2	P02631,P36860
5168	1,4,5,26,27,29,30,32	2,3,6,7,8,25,28,31	2	P20766,P36376

5169	1,4,5,26,27,29,31,32	2,3,6,7,8,25,28,30	3	O70417,P02631,P62804
5170	1,4,5,26,27,30,31,32	2,3,6,7,8,25,28,29	5	P02631,P23739,P36860,P62804,Q00715
5171	1,4,5,26,28,29,30,31	2,3,6,7,8,25,27,32	1	P50115
5172	1,4,5,26,28,29,30,32	2,3,6,7,8,25,27,31	3	P20766,P50115,Q62894
5173	1,4,5,26,28,29,31,32	2,3,6,7,8,25,27,30	3	O54728,O70417,P50115
5174	1,4,5,26,28,30,31,32	2,3,6,7,8,25,27,29	6	O54728,P50115,P62804,Q00715,Q4G075,Q8VD89
5175	1,4,5,26,29,30,31,32	2,3,6,7,8,25,27,28	6	P02631,P36860,P50115,P62804,P63081,Q5QE79
5176	1,4,5,27,28,29,30,31	2,3,6,7,8,25,26,32	2	P08649,Q99MH3
5177	1,4,5,27,28,29,30,32	2,3,6,7,8,25,26,31	1	P20766
5178	1,4,5,27,28,29,31,32	2,3,6,7,8,25,26,30	4	O35547,P36374,Q62761;Q62762;Q62763,Q99MH3
5179	1,4,5,27,28,30,31,32	2,3,6,7,8,25,26,29	3	P23739,P62804,Q8VD89
5180	1,4,5,27,29,30,31,32	2,3,6,7,8,25,26,28	2	P08649,P36860
5181	1,4,5,28,29,30,31,32	2,3,6,7,8,25,26,27	6	P17559,P36860,P50115,P63081,Q5BJY9,Q62894
5182	1,4,6,7,8,25,26,27	2,3,5,28,29,30,31,32	10	P06761,P06911,P11598,P13432,P15399,P22273,P22282,Q4G075,Q99041,Q9WTT6
5183	1,4,6,7,8,25,26,28	2,3,5,27,29,30,31,32	11	P06761,P06911,P08649,P11598,P22273,P22282,Q63617,Q99041,Q9JI85,Q9QW07,Q9WTT6
5184	1,4,6,7,8,25,26,29	2,3,5,27,28,30,31,32	7	P06911,P13432,P22273,P48032,P49134,P70549,Q9QX74
5185	1,4,6,7,8,25,26,30	2,3,5,27,28,29,31,32	4	P06911,P49134,Q6AY61,Q99041
5186	1,4,6,7,8,25,26,31	2,3,5,27,28,29,30,32	5	P06911,P12020,P13432,P49134,P57113
5187	1,4,6,7,8,25,26,32	2,3,5,27,28,29,30,31	5	P06911,P11598,P13432,Q99041,Q9WVK7
5188	1,4,6,7,8,25,27,28	2,3,5,26,29,30,31,32	18	P06761,P06911,P0C0A9,P11598,P19629,P22273,P22282,P22283,P63081,Q4G075,Q5GRG2,Q5M8C6,Q63617,Q6AYR9,Q812E4,Q99041,Q9JI85,Q9QW07

5189	1,4,6,7,8,25,27,29	2,3,5,26,28,30,31,32	9	P06761,P06911,P11598,P22273,P22282,P22283,Q4G075,Q63617,Q99041
5190	1,4,6,7,8,25,27,30	2,3,5,26,28,29,31,32	6	O70417,P06911,P22273,P22282,Q99041,Q99376
5191	1,4,6,7,8,25,27,31	2,3,5,26,28,29,30,32	7	P06911,P12020,P22273,P22282,Q5GRG2,Q99041,Q9QYP1
5192	1,4,6,7,8,25,27,32	2,3,5,26,28,29,30,31	11	P00714,P06911,P0C0A9,P11598,P13432,P22273,P22282,P98166,Q01984,Q99041,Q9JHB9
5193	1,4,6,7,8,25,28,29	2,3,5,26,27,30,31,32	20	P06761,P06911,P0C0A9,P11598,P12020,P21674,P22273,P22282,P22283,P47727,P48032,Q5GRG2,Q5M8C6,Q63493,Q63617,Q812E4,Q99041,Q9JI85,Q9QW07,Q9Z2L0
5194	1,4,6,7,8,25,28,30	2,3,5,26,27,29,31,32	7	O70417,P06911,P11598,P22282,Q63617,Q99041,Q9QW07
5195	1,4,6,7,8,25,28,31	2,3,5,26,27,29,30,32	8	P06911,P11598,P12020,P22282,Q5GRG2,Q63617,Q99041,Q9QW07
5196	1,4,6,7,8,25,28,32	2,3,5,26,27,29,30,31	14	P06761,P06911,P07647,P08723,P0C0A9,P11598,P13432,P22282,P22283,Q5M8C6,Q63617,Q99041,Q9JI85,Q9QW07
5197	1,4,6,7,8,25,29,30	2,3,5,26,27,28,31,32	3	P06911,P48032,Q99041
5198	1,4,6,7,8,25,29,31	2,3,5,26,27,28,30,32	3	P06911,P12020,Q5GRG2
5199	1,4,6,7,8,25,29,32	2,3,5,26,27,28,30,31	4	P06911,P11598,P13432,P22282
5200	1,4,6,7,8,25,30,31	2,3,5,26,27,28,29,32	4	P06911,P12020,Q6AY61,Q9WVK7
5201	1,4,6,7,8,25,30,32	2,3,5,26,27,28,29,31	4	P06911,Q5PQL7,Q99041,Q9WVK7
5202	1,4,6,7,8,25,31,32	2,3,5,26,27,28,29,30	4	P06911,P12020,P13432,Q9WVK7
5203	1,4,6,7,8,26,27,28	2,3,5,25,29,30,31,32	15	P02782,P06761,P06911,P07150,P11598,P15399,P22273,P22282,P22283,Q63617,Q6AYR9,Q99041,Q9JI85,Q9QW07,Q9WTT6
5204	1,4,6,7,8,26,27,29	2,3,5,25,28,30,31,32	12	P06761,P06911,P11598,P22273,P22282,P22283,P70549,Q4G075,Q63617,Q99041,Q9QX74,Q9WTT6
5205	1,4,6,7,8,26,27,30	2,3,5,25,28,29,31,32	6	P11598,P15399,P22273,P22282,Q30KJ2,Q99041
5206	1,4,6,7,8,26,27,31	2,3,5,25,28,29,30,32	6	P06911,P11598,P22273,P22282,Q62894,Q99041
5207	1,4,6,7,8,26,27,32	2,3,5,25,28,29,30,31	8	P06761,P11598,P13432,P15399,P18418,P22273,P22282,Q99041
5208	1,4,6,7,8,26,28,29	2,3,5,25,27,30,31,32	18	P06761,P06911,P11598,P20646,P21674,P22273,P22282,P22283,P49134,P70549,Q63493,Q63617,Q6P6R2,Q99041,Q9JI85,Q9QW07,Q9QX74,Q9WTT6

5209	1,4,6,7,8,26,28,30	2,3,5,25,27,29,31,32	9	P06911,P11598,P22273,P22282,P49134,Q63617,Q99041,Q9JI85,Q9QW07
5210	1,4,6,7,8,26,28,31	2,3,5,25,27,29,30,32	12	P06761,P06911,P11598,P21674,P22273,P22282,P49134,P57113,Q63617,Q99041,Q9JI85,Q9QW07
5211	1,4,6,7,8,26,28,32	2,3,5,25,27,29,30,31	13	O54728,P06761,P06911,P08723,P11598,P18418,P22273,P22282,P22283,Q63617,Q99041,Q9JI85,Q9QW07
5212	1,4,6,7,8,26,29,30	2,3,5,25,27,28,31,32	3	P49134,Q99041,Q9QX74
5213	1,4,6,7,8,26,29,31	2,3,5,25,27,28,30,32	8	P06911,P11598,P21674,P49134,P70549,Q63474,Q99041,Q9QX74
5214	1,4,6,7,8,26,29,32	2,3,5,25,27,28,30,31	5	P11598,P13432,P22282,Q63617,Q99041
5215	1,4,6,7,8,26,30,31	2,3,5,25,27,28,29,32	4	P06911,P49134,Q6AY61,Q99041
5216	1,4,6,7,8,26,30,32	2,3,5,25,27,28,29,31	1	Q99041
5217	1,4,6,7,8,26,31,32	2,3,5,25,27,28,29,30	6	O54728,P11598,P13432,P22006,P57113,Q99041
5218	1,4,6,7,8,27,28,29	2,3,5,25,26,30,31,32	22	P02782,P06761,P06911,P08723,P0C0A9,P11598,P21674,P22273,P22282,P22283,P46462,P47727,Q4G075,Q5M8C6,Q63493,Q63617,Q6AYR9,Q812E4,Q99041,Q9JI85,Q9QW07,Q9Z2L0
5219	1,4,6,7,8,27,28,30	2,3,5,25,26,29,31,32	12	P06761,P06911,P11598,P19218,P22273,P22282,P22283,Q63617,Q8VD89,Q99041,Q9JI85,Q9QW07
5220	1,4,6,7,8,27,28,31	2,3,5,25,26,29,30,32	14	P06761,P06911,P11598,P12020,P22273,P22282,P22283,Q5GRG2,Q63493,Q63617,Q99041,Q9JI85,Q9QW07,Q9QZK9
5221	1,4,6,7,8,27,28,32	2,3,5,25,26,29,30,31	24	P02780,P02781,P04905,P06761,P06911,P07647,P08723,P0C0A9,P11598,P22273,P22282,P22283,P46462,Q5M8C6,Q62902,Q63493,Q63617,Q6AYR9,Q6RUV5,Q812E4,Q99041,Q9JI85,Q9QW07,Q9Z2L0
5222	1,4,6,7,8,27,29,30	2,3,5,25,26,28,31,32	8	P06911,P11598,P19218,P22273,P22282,Q4G075,Q63617,Q99041
5223	1,4,6,7,8,27,29,31	2,3,5,25,26,28,30,32	10	P06761,P06911,P11598,P22273,P22282,P82471,Q4G075,Q63493,Q63617,Q99041
5224	1,4,6,7,8,27,29,32	2,3,5,25,26,28,30,31	12	P00714,P06761,P06911,P11598,P22273,P22282,P22283,Q4G075,Q63493,Q63617,Q99041,Q9Z2L0
5225	1,4,6,7,8,27,30,31	2,3,5,25,26,28,29,32	4	P06911,P19218,P22282,Q99041
5226	1,4,6,7,8,27,30,32	2,3,5,25,26,28,29,31	6	P00714,P11598,P19218,P22282,Q8VD89,Q99041
5227	1,4,6,7,8,27,31,32	2,3,5,25,26,28,29,30	8	P00714,P06911,P11598,P22006,P22282,P82471,Q99041,Q9QZK9
5228	1,4,6,7,8,28,29,30	2,3,5,25,26,27,31,32	12	P06761,P06911,P11598,P21674,P22273,P22282,P22283,Q63493,Q63617,Q99041,Q9JI85,Q9QW07

5229	1,4,6,7,8,28,29,31	2,3,5,25,26,27,30,32	17	P06761,P06911,P11598,P12020,P21674,P22273,P22282,P22283,P70549,P82471,Q5GRG2,Q63474,Q63493,Q63617,Q99041,Q9JI85,Q9QW07
5230	1,4,6,7,8,28,29,32	2,3,5,25,26,27,30,31	23	P02780,P04905,P06761,P06911,P07647,P08723,P0C0A9,P11598,P21674,P22273,P22282,P22283,P30120,P47727,Q5M8C6,Q63493,Q63617,Q6AYR9,Q812E4,Q99041,Q9JI85,Q9QW07,Q9Z2L0
5231	1,4,6,7,8,28,30,31	2,3,5,25,26,27,29,32	7	P06911,P11598,P12020,P35745,P82471,Q63617,Q99041
5232	1,4,6,7,8,28,30,32	2,3,5,25,26,27,29,31	10	P02780,P06911,P08723,P11598,P22282,Q63617,Q8VD89,Q99041,Q9JI85,Q9QW07
5233	1,4,6,7,8,28,31,32	2,3,5,25,26,27,29,30	14	P06911,P08723,P11598,P12020,P22006,P22282,P82471,Q63493,Q63532,Q63617,Q99041,Q9JI85,Q9QW07,Q9QZK9
5234	1,4,6,7,8,29,30,31	2,3,5,25,26,27,28,32	7	P06911,P12020,P49134,P82471,Q5RJR2,Q63474,Q99041
5235	1,4,6,7,8,29,30,32	2,3,5,25,26,27,28,31	2	P00714,Q99041
5236	1,4,6,7,8,29,31,32	2,3,5,25,26,27,28,30	7	P00714,P06911,P11598,P82471,Q63474,Q63493,Q99041
5237	1,4,6,7,8,30,31,32	2,3,5,25,26,27,28,29	5	P00714,P22006,P26342,P82471,Q99041
5238	1,4,6,7,25,26,27,28	2,3,5,8,29,30,31,32	5	P11598,P22283,P63081,Q63617,Q9QW07
5239	1,4,6,7,25,26,27,29	2,3,5,8,28,30,31,32	2	P36376,P70549
5240	1,4,6,7,25,26,27,30	2,3,5,8,28,29,31,32	2	P21588,P36376
5241	1,4,6,7,25,26,27,31	2,3,5,8,28,29,30,32	1	Q9QYP1
5242	1,4,6,7,25,26,27,32	2,3,5,8,28,29,30,31	1	P36376
5243	1,4,6,7,25,26,28,29	2,3,5,8,27,30,31,32	5	P11598,P20646,P70549,Q63617,Q9QW07
5244	1,4,6,7,25,26,28,30	2,3,5,8,27,29,31,32	2	Q5PQL7,Q62975
5245	1,4,6,7,25,26,28,31	2,3,5,8,27,29,30,32	1	Q6IFU7
5246	1,4,6,7,25,26,28,32	2,3,5,8,27,29,30,31	7	P00731,P07647,P11598,Q5PQL7,Q62975,Q6AYR8,Q9QW07
5247	1,4,6,7,25,26,29,30	2,3,5,8,27,28,31,32	1	P36376
5248	1,4,6,7,25,26,29,31	2,3,5,8,27,28,30,32	1	P70549

5249	1,4,6,7,25,<br>26,29,32	2,3,5,8,27,<br>28,30,31	2	P36376,Q6AYR8
5250	1,4,6,7,25,<br>26,30,31	2,3,5,8,27,<br>28,29,32	3	P25809,Q9QYP1,Q9WVK7
5251	1,4,6,7,25,<br>26,30,32	2,3,5,8,27,<br>28,29,31	5	P25809,P36376,Q5PQL7,Q9R168,Q9WVK7
5252	1,4,6,7,25,<br>26,31,32	2,3,5,8,27,<br>28,29,30	4	P00731,P25809,Q6IFU7,Q9WVK7
5253	1,4,6,7,25,<br>27,28,29	2,3,5,8,26,<br>30,31,32	9	P05539,P0C0A9,P11598,P22283,Q63617,Q812E4,Q99MH3,Q9QW07,Q9Z2L0
5254	1,4,6,7,25,<br>27,28,30	2,3,5,8,26,<br>29,31,32	0	
5255	1,4,6,7,25,<br>27,28,31	2,3,5,8,26,<br>29,30,32	4	O88797,P11598,Q63617,Q9QYP1
5256	1,4,6,7,25,<br>27,28,32	2,3,5,8,26,<br>29,30,31	8	P07647,P0C0A9,P11598,P22283,Q62761;Q62762;Q62763,Q63617,Q6AYR9,Q9QW07
5257	1,4,6,7,25,<br>27,29,30	2,3,5,8,26,<br>28,31,32	1	P36376
5258	1,4,6,7,25,<br>27,29,31	2,3,5,8,26,<br>28,30,32	1	Q9QYP1
5259	1,4,6,7,25,<br>27,29,32	2,3,5,8,26,<br>28,30,31	3	P00714,P36376,Q6AYR8
5260	1,4,6,7,25,<br>27,30,31	2,3,5,8,26,<br>28,29,32	1	Q9QYP1
5261	1,4,6,7,25,<br>27,30,32	2,3,5,8,26,<br>28,29,31	2	P36376,Q99376
5262	1,4,6,7,25,<br>27,31,32	2,3,5,8,26,<br>28,29,30	1	Q9QYP1
5263	1,4,6,7,25,<br>28,29,30	2,3,5,8,26,<br>27,31,32	2	Q63617,Q9QW07
5264	1,4,6,7,25,<br>28,29,31	2,3,5,8,26,<br>27,30,32	2	P70549,Q63617
5265	1,4,6,7,25,<br>28,29,32	2,3,5,8,26,<br>27,30,31	9	P07647,P08723,P0C0A9,P11598,P22283,Q63617,Q6AYR8,Q9QW07,Q9Z2L0
5266	1,4,6,7,25,<br>28,30,31	2,3,5,8,26,<br>27,29,32	2	P25809,Q9JJ50
5267	1,4,6,7,25,<br>28,30,32	2,3,5,8,26,<br>27,29,31	3	Q5PQL7,Q62975,Q9R168
5268	1,4,6,7,25,<br>28,31,32	2,3,5,8,26,<br>27,29,30	4	P00731,P11598,P22006,P25809

5269	1,4,6,7,25,<br>29,30,31	2,3,5,8,26,<br>27,28,32	2	P25809,Q9QWN8
5270	1,4,6,7,25,<br>29,30,32	2,3,5,8,26,<br>27,28,31	3	P36376,Q5BJY9,Q9R168
5271	1,4,6,7,25,<br>29,31,32	2,3,5,8,26,<br>27,28,30	1	Q6AYR8
5272	1,4,6,7,25,<br>30,31,32	2,3,5,8,26,<br>27,28,29	7	P22006,P25809,P26342,P47967,Q6P6R2,Q9QYP1,Q9WVK7
5273	1,4,6,7,26,<br>27,28,29	2,3,5,8,25,<br>30,31,32	9	P02625,P11598,P20646,P21588,P22283,P36376,P70549,Q63617,Q9QW07
5274	1,4,6,7,26,<br>27,28,30	2,3,5,8,25,<br>29,31,32	6	P11598,P21588,P36376,Q63617,Q80WL1,Q8VD89
5275	1,4,6,7,26,<br>27,28,31	2,3,5,8,25,<br>29,30,32	4	O88797,P11598,P22283,Q63617
5276	1,4,6,7,26,<br>27,28,32	2,3,5,8,25,<br>29,30,31	7	P11598,P22283,P36376,Q62761;Q62762;Q62763,Q63617,Q6AYR9,Q8CJ52
5277	1,4,6,7,26,<br>27,29,30	2,3,5,8,25,<br>28,31,32	4	P21588,P36376,Q80WL1,Q811M5
5278	1,4,6,7,26,<br>27,29,31	2,3,5,8,25,<br>28,30,32	1	P70549
5279	1,4,6,7,26,<br>27,29,32	2,3,5,8,25,<br>28,30,31	3	P00714,P11598,P36376
5280	1,4,6,7,26,<br>27,30,31	2,3,5,8,25,<br>28,29,32	1	Q9QYP1
5281	1,4,6,7,26,<br>27,30,32	2,3,5,8,25,<br>28,29,31	2	P36376,Q8VD89
5282	1,4,6,7,26,<br>27,31,32	2,3,5,8,25,<br>28,29,30	2	P11598,P22006
5283	1,4,6,7,26,<br>28,29,30	2,3,5,8,25,<br>27,31,32	4	P11598,P36376,Q63617,Q80WL1
5284	1,4,6,7,26,<br>28,29,31	2,3,5,8,25,<br>27,30,32	5	P11598,P21674,P70549,Q63474,Q63617
5285	1,4,6,7,26,<br>28,29,32	2,3,5,8,25,<br>27,30,31	8	P07647,P11598,P22283,P36376,Q63617,Q6AYR8,Q8CJ52,Q9QW07
5286	1,4,6,7,26,<br>28,30,31	2,3,5,8,25,<br>27,29,32	0	
5287	1,4,6,7,26,<br>28,30,32	2,3,5,8,25,<br>27,29,31	4	P11598,P36376,Q8VD89,Q9R168
5288	1,4,6,7,26,<br>28,31,32	2,3,5,8,25,<br>27,29,30	5	O54728,P00731,P11598,P22006,Q6IFU7

5289	1,4,6,7,26,<br>29,30,31	2,3,5,8,25,<br>27,28,32	3	B1H234,P36860,Q63474
5290	1,4,6,7,26,<br>29,30,32	2,3,5,8,25,<br>27,28,31	3	P36376,P36860,Q9R168
5291	1,4,6,7,26,<br>29,31,32	2,3,5,8,25,<br>27,28,30	3	P36376,P36860,Q63474
5292	1,4,6,7,26,<br>30,31,32	2,3,5,8,25,<br>27,28,29	4	P22006,P25809,P36860,Q9WVK7
5293	1,4,6,7,27,<br>28,29,30	2,3,5,8,25,<br>26,31,32	5	P11598,P22283,P36376,Q63617,Q9Z2L0
5294	1,4,6,7,27,<br>28,29,31	2,3,5,8,25,<br>26,30,32	5	O88797,P11598,P22283,P80299,Q63617
5295	1,4,6,7,27,<br>28,29,32	2,3,5,8,25,<br>26,30,31	19	O08815,O35547,P00714,P00758,P07647,P08723,P0C0A9,P11598,P22283,P36374,P36376,P8155<br>6,Q62761;Q62762;Q62763,Q63617,Q6AYR9,Q812E4,Q8CGS4,Q9QW07,Q9Z2L0
5296	1,4,6,7,27,<br>28,30,31	2,3,5,8,25,<br>26,29,32	3	P11598,Q63617,Q8VD89
5297	1,4,6,7,27,<br>28,30,32	2,3,5,8,25,<br>26,29,31	6	P11598,P22283,P36376,Q63617,Q6AYR9,Q8VD89
5298	1,4,6,7,27,<br>28,31,32	2,3,5,8,25,<br>26,29,30	8	O88797,P11598,P22006,P22283,P36374,P80299,Q62761;Q62762;Q62763,Q63617
5299	1,4,6,7,27,<br>29,30,31	2,3,5,8,25,<br>26,28,32	2	B1H234,P80299
5300	1,4,6,7,27,<br>29,30,32	2,3,5,8,25,<br>26,28,31	2	P00714,P36376
5301	1,4,6,7,27,<br>29,31,32	2,3,5,8,25,<br>26,28,30	3	P00714,P11598,P80299
5302	1,4,6,7,27,<br>30,31,32	2,3,5,8,25,<br>26,28,29	5	P00714,P22006,P80299,Q8VD89,Q9QYP1
5303	1,4,6,7,28,<br>29,30,31	2,3,5,8,25,<br>26,27,32	3	B1H234,Q63474,Q63617
5304	1,4,6,7,28,<br>29,30,32	2,3,5,8,25,<br>26,27,31	4	P11598,P36376,Q5BJY9,Q63617
5305	1,4,6,7,28,<br>29,31,32	2,3,5,8,25,<br>26,27,30	6	P11598,P22006,P22283,P80299,Q63474,Q63617
5306	1,4,6,7,28,<br>30,31,32	2,3,5,8,25,<br>26,27,29	3	P22006,P25809,Q8VD89
5307	1,4,6,7,29,<br>30,31,32	2,3,5,8,25,<br>26,27,28	7	B1H234,P00714,P26342,P36860,P80299,Q5BJY9,Q63474
5308	1,4,6,8,25,<br>26,27,28	2,3,5,7,29,<br>30,31,32	7	P06911,P08649,P15399,P22282,P26342,Q9JI85,Q9QW07

5309	1,4,6,8,25,<br>26,27,29	2,3,5,7,28,<br>30,31,32	3	P13432,P15399,P70549
5310	1,4,6,8,25,<br>26,27,30	2,3,5,7,28,<br>29,31,32	4	P15399,P54921,Q30KJ2,Q6AY61
5311	1,4,6,8,25,<br>26,27,31	2,3,5,7,28,<br>29,30,32	3	P15399,P47967,P54921
5312	1,4,6,8,25,<br>26,27,32	2,3,5,7,28,<br>29,30,31	3	P13432,P15399,P54921
5313	1,4,6,8,25,<br>26,28,29	2,3,5,7,27,<br>30,31,32	8	P06911,P08649,P13432,P20766,P70549,Q6P6R2,Q9JI85,Q9QW07
5314	1,4,6,8,25,<br>26,28,30	2,3,5,7,27,<br>29,31,32	4	P08649,P80299,Q6AY61,Q9QW07
5315	1,4,6,8,25,<br>26,28,31	2,3,5,7,27,<br>29,30,32	3	P06911,P08649,P70549
5316	1,4,6,8,25,<br>26,28,32	2,3,5,7,27,<br>29,30,31	5	O54728,P08649,P13432,Q9JI85,Q9QW07
5317	1,4,6,8,25,<br>26,29,30	2,3,5,7,27,<br>28,31,32	1	Q6AY61
5318	1,4,6,8,25,<br>26,29,31	2,3,5,7,27,<br>28,30,32	2	P13432,P70549
5319	1,4,6,8,25,<br>26,29,32	2,3,5,7,27,<br>28,30,31	2	P00507,P13432
5320	1,4,6,8,25,<br>26,30,31	2,3,5,7,27,<br>28,29,32	4	P47967,P54921,Q6AY61,Q9WVK7
5321	1,4,6,8,25,<br>26,30,32	2,3,5,7,27,<br>28,29,31	6	P00507,P13432,P47967,P54921,Q6AY61,Q9WVK7
5322	1,4,6,8,25,<br>26,31,32	2,3,5,7,27,<br>28,29,30	6	O54728,P00507,P13432,P47967,P54921,Q9WVK7
5323	1,4,6,8,25,<br>27,28,29	2,3,5,7,26,<br>30,31,32	10	P06911,P0C0A9,P20766,P22282,P47727,Q6AYR9,Q812E4,Q9JI85,Q9QW07,Q9Z2L0
5324	1,4,6,8,25,<br>27,28,30	2,3,5,7,26,<br>29,31,32	4	P06911,P15399,P20766,Q9QW07
5325	1,4,6,8,25,<br>27,28,31	2,3,5,7,26,<br>29,30,32	3	P06911,P19629,Q5GRG2
5326	1,4,6,8,25,<br>27,28,32	2,3,5,7,26,<br>29,30,31	13	P06911,P08723,P0C0A9,P13432,P15399,P19629,P20766,P22282,Q6AYR9,Q812E4,Q9JI85,Q9Q<br>W07,Q9QWN8
5327	1,4,6,8,25,<br>27,29,30	2,3,5,7,26,<br>28,31,32	0	
5328	1,4,6,8,25,<br>27,29,31	2,3,5,7,26,<br>28,30,32	1	P06911

5329	1,4,6,8,25, 27,29,32	2,3,5,7,26, 28,30,31	1	P13432
5330	1,4,6,8,25, 27,30,31	2,3,5,7,26, 28,29,32	5	P06911,P47967,P54921,Q6AY61,Q9QYP1
5331	1,4,6,8,25, 27,30,32	2,3,5,7,26, 28,29,31	2	P15399,P54921
5332	1,4,6,8,25, 27,31,32	2,3,5,7,26, 28,29,30	4	P13432,P47967,P54921,Q9QYP1
5333	1,4,6,8,25, 28,29,30	2,3,5,7,26, 27,31,32	3	P06911,P20766,Q9QW07
5334	1,4,6,8,25, 28,29,31	2,3,5,7,26, 27,30,32	5	P06911,P12020,P70549,Q5GRG2,Q9QW07
5335	1,4,6,8,25, 28,29,32	2,3,5,7,26, 27,30,31	9	P07647,P08723,P0C0A9,P13432,P20766,P30120,Q812E4,Q9JI85,Q9QW07
5336	1,4,6,8,25, 28,30,31	2,3,5,7,26, 27,29,32	3	P06911,Q6AY61,Q9JJ50
5337	1,4,6,8,25, 28,30,32	2,3,5,7,26, 27,29,31	3	P20766,Q5PQL7,Q9QW07
5338	1,4,6,8,25, 28,31,32	2,3,5,7,26, 27,29,30	3	P06911,P13432,P22006
5339	1,4,6,8,25, 29,30,31	2,3,5,7,26, 27,28,32	2	P47967,Q6AY61
5340	1,4,6,8,25, 29,30,32	2,3,5,7,26, 27,28,31	3	P13432,P20766,Q5RLM2
5341	1,4,6,8,25, 29,31,32	2,3,5,7,26, 27,28,30	2	P13432,P47967
5342	1,4,6,8,25, 30,31,32	2,3,5,7,26, 27,28,29	4	P47967,P54921,Q6AY61,Q9WVK7
5343	1,4,6,8,26, 27,28,29	2,3,5,7,25, 30,31,32	9	P02782,P15399,P22282,P70549,Q6AYR9,Q6P6R2,Q812E4,Q9JI85,Q9QW07
5344	1,4,6,8,26, 27,28,30	2,3,5,7,25, 29,31,32	4	P15399,P22282,Q6AY61,Q8VD89
5345	1,4,6,8,26, 27,28,31	2,3,5,7,25, 29,30,32	3	P15399,P22282,Q9JI85
5346	1,4,6,8,26, 27,28,32	2,3,5,7,25, 29,30,31	10	O54728,P08723,P15399,P18418,P22282,Q6AYR9,Q8VD89,Q9JI85,Q9QW07,Q9QWN8
5347	1,4,6,8,26, 27,29,30	2,3,5,7,25, 28,31,32	3	P15399,Q6AY61,Q811M5
5348	1,4,6,8,26, 27,29,31	2,3,5,7,25, 28,30,32	3	P15399,P70549,Q63474

5349	1,4,6,8,26,<br>27,29,32	2,3,5,7,25,<br>28,30,31	3	P13432,P15399,P22282
5350	1,4,6,8,26,<br>27,30,31	2,3,5,7,25,<br>28,29,32	3	P15399,P54921,Q6AY61
5351	1,4,6,8,26,<br>27,30,32	2,3,5,7,25,<br>28,29,31	3	P15399,P54921,Q8VD89
5352	1,4,6,8,26,<br>27,31,32	2,3,5,7,25,<br>28,29,30	4	O54728,P15399,P22006,P54921
5353	1,4,6,8,26,<br>28,29,30	2,3,5,7,25,<br>27,31,32	2	P20766,Q9QW07
5354	1,4,6,8,26,<br>28,29,31	2,3,5,7,25,<br>27,30,32	4	P21674,P70549,Q63474,Q9JI85
5355	1,4,6,8,26,<br>28,29,32	2,3,5,7,25,<br>27,30,31	7	O54728,P08723,P13432,P20766,P30120,Q9JI85,Q9QW07
5356	1,4,6,8,26,<br>28,30,31	2,3,5,7,25,<br>27,29,32	2	Q6AY61,Q8VD89
5357	1,4,6,8,26,<br>28,30,32	2,3,5,7,25,<br>27,29,31	5	O54728,P20766,Q8VD89,Q9JI85,Q9QW07
5358	1,4,6,8,26,<br>28,31,32	2,3,5,7,25,<br>27,29,30	3	O54728,P22006,Q9JI85
5359	1,4,6,8,26,<br>29,30,31	2,3,5,7,25,<br>27,28,32	3	Q5RJR2,Q63474,Q6AY61
5360	1,4,6,8,26,<br>29,30,32	2,3,5,7,25,<br>27,28,31	0	
5361	1,4,6,8,26,<br>29,31,32	2,3,5,7,25,<br>27,28,30	4	O54728,P13432,P70549,Q63474
5362	1,4,6,8,26,<br>30,31,32	2,3,5,7,25,<br>27,28,29	6	O54728,P00507,P22006,P54921,Q6AY61,Q8VD89
5363	1,4,6,8,27,<br>28,29,30	2,3,5,7,25,<br>26,31,32	7	P19218,P20766,P22282,Q6AYR9,Q812E4,Q9JI85,Q9QW07
5364	1,4,6,8,27,<br>28,29,31	2,3,5,7,25,<br>26,30,32	7	P22282,P36374,Q63474,Q63493,Q6AYR9,Q812E4,Q9JI85
5365	1,4,6,8,27,<br>28,29,32	2,3,5,7,25,<br>26,30,31	20	O08815,O35547,P00758,P04905,P07647,P08723,P0C0A9,P20766,P22282,P22283,P30120,P3637<br>4,P47727,Q63493,Q6AYR9,Q812E4,Q8CFN2,Q9JI85,Q9QW07,Q9Z2L0
5366	1,4,6,8,27,<br>28,30,31	2,3,5,7,25,<br>26,29,32	2	P19218,Q8VD89
5367	1,4,6,8,27,<br>28,30,32	2,3,5,7,25,<br>26,29,31	10	B0LT89,P15399,P19218,P20766,P22282,Q6AYR9,Q6P6S4,Q6RUV5,Q8VD89,Q9JI85
5368	1,4,6,8,27,<br>28,31,32	2,3,5,7,25,<br>26,29,30	9	B0LT89,P08723,P22006,P22282,P36374,Q6AYR9,Q8VD89,Q9JI85,Q9QZK9

5369	1,4,6,8,27,<br>29,30,31	2,3,5,7,25,<br>26,28,32	1	Q63474
5370	1,4,6,8,27,<br>29,30,32	2,3,5,7,25,<br>26,28,31	2	P00714,P20766
5371	1,4,6,8,27,<br>29,31,32	2,3,5,7,25,<br>26,28,30	2	P00714,Q63474
5372	1,4,6,8,27,<br>30,31,32	2,3,5,7,25,<br>26,28,29	3	P22006,P54921,Q8VD89
5373	1,4,6,8,28,<br>29,30,31	2,3,5,7,25,<br>26,27,32	1	Q63474
5374	1,4,6,8,28,<br>29,30,32	2,3,5,7,25,<br>26,27,31	5	P08723,P20766,P30120,Q9JI85,Q9QW07
5375	1,4,6,8,28,<br>29,31,32	2,3,5,7,25,<br>26,27,30	6	P08723,P22006,P30120,Q63474,Q63493,Q9JI85
5376	1,4,6,8,28,<br>30,31,32	2,3,5,7,25,<br>26,27,29	2	P22006,Q8VD89
5377	1,4,6,8,29,<br>30,31,32	2,3,5,7,25,<br>26,27,28	2	P36860,Q63474
5378	1,4,6,25,2<br>6,27,28,29	2,3,5,7,8,3<br>0,31,32	3	P20766,P35280,P70549
5379	1,4,6,25,2<br>6,27,28,30	2,3,5,7,8,2<br>9,31,32	2	P20766,P21588
5380	1,4,6,25,2<br>6,27,28,31	2,3,5,7,8,2<br>9,30,32	2	P35280,P62804
5381	1,4,6,25,2<br>6,27,28,32	2,3,5,7,8,2<br>9,30,31	4	P20766,P62804,Q6AYR9,Q9QWN8
5382	1,4,6,25,2<br>6,27,29,30	2,3,5,7,8,2<br>8,31,32	4	P20766,P21588,P36376,Q811M5
5383	1,4,6,25,2<br>6,27,29,31	2,3,5,7,8,2<br>8,30,32	2	P35280,P70549
5384	1,4,6,25,2<br>6,27,29,32	2,3,5,7,8,2<br>8,30,31	3	P20766,P36376,Q6AYR8
5385	1,4,6,25,2<br>6,27,30,31	2,3,5,7,8,2<br>8,29,32	4	P47967,P54921,P62804,Q9QYP1
5386	1,4,6,25,2<br>6,27,30,32	2,3,5,7,8,2<br>8,29,31	5	D3ZUC6,P06760,P36376,P54921,P62804
5387	1,4,6,25,2<br>6,27,31,32	2,3,5,7,8,2<br>8,29,30	5	P22006,P47967,P54921,P62804,Q9QYP1
5388	1,4,6,25,2<br>6,28,29,30	2,3,5,7,8,2<br>7,31,32	1	P20766

5389	1,4,6,25,26,28,29,31	2,3,5,7,8,27,30,32	2	P35280,P70549
5390	1,4,6,25,26,28,29,32	2,3,5,7,8,27,30,31	3	P00731,P20766,Q6AYR8
5391	1,4,6,25,26,28,30,31	2,3,5,7,8,27,29,32	1	P25809
5392	1,4,6,25,26,28,30,32	2,3,5,7,8,27,29,31	7	P00731,P06760,P20766,P25809,P62804,Q5PQL7,Q62975
5393	1,4,6,25,26,28,31,32	2,3,5,7,8,27,29,30	5	O54728,P00731,P22006,P25809,P62804
5394	1,4,6,25,26,29,30,31	2,3,5,7,8,27,28,32	2	P25809,P47967
5395	1,4,6,25,26,29,30,32	2,3,5,7,8,27,28,31	5	D3ZUC6,P00507,P20766,P36376,Q6AYR8
5396	1,4,6,25,26,29,31,32	2,3,5,7,8,27,28,30	7	P00507,P00731,P19218,P47967,P62804,P70549,Q6AYR8
5397	1,4,6,25,26,30,31,32	2,3,5,7,8,27,28,29	12	D3ZUC6,P00507,P06760,P22006,P25809,P47967,P54921,P62804,Q00715,Q4G075,Q9QYP1,Q9WVK7
5398	1,4,6,25,27,28,29,30	2,3,5,7,8,26,31,32	1	P20766
5399	1,4,6,25,27,28,29,31	2,3,5,7,8,26,30,32	2	P35280,P36374
5400	1,4,6,25,27,28,29,32	2,3,5,7,8,26,30,31	13	O08815,O35547,P00758,P07647,P0C0A9,P20766,P36374,Q62761;Q62762;Q62763,Q6AYR8,Q6AYR9,Q812E4,Q8CGS4,Q9Z2L0
5401	1,4,6,25,27,28,30,31	2,3,5,7,8,26,29,32	1	Q9QYP1
5402	1,4,6,25,27,28,30,32	2,3,5,7,8,26,29,31	4	P06760,P20766,Q8VD89,Q9QX74
5403	1,4,6,25,27,28,31,32	2,3,5,7,8,26,29,30	5	P22006,P36374,P62804,Q9QYP1,Q9WUW8
5404	1,4,6,25,27,29,30,31	2,3,5,7,8,26,28,32	1	Q9QYP1
5405	1,4,6,25,27,29,30,32	2,3,5,7,8,26,28,31	3	P20766,P36376,Q5RLM2
5406	1,4,6,25,27,29,31,32	2,3,5,7,8,26,28,30	2	P62804,Q9QYP1
5407	1,4,6,25,27,30,31,32	2,3,5,7,8,26,28,29	10	D3ZUC6,P06760,P22006,P47967,P54921,P62804,Q6P6R2,Q9QX74,Q9QYP1,Q9WTT6
5408	1,4,6,25,28,29,30,31	2,3,5,7,8,26,27,32	2	P25809,Q9JJ50

5409	1,4,6,25,28,29,30,32	2,3,5,7,8,26,27,31	1	P20766
5410	1,4,6,25,28,29,31,32	2,3,5,7,8,26,27,30	2	P00731,Q6AYR8
5411	1,4,6,25,28,30,31,32	2,3,5,7,8,26,27,29	11	P06760,P22006,P25809,P47967,P62804,Q00715,Q4G075,Q62975,Q9JJ50,Q9QX74,Q9WTT6
5412	1,4,6,25,29,30,31,32	2,3,5,7,8,26,27,28	6	D3ZUC6,P25809,P36860,P47967,Q5RLM2,Q9WTT6
5413	1,4,6,26,27,28,29,30	2,3,5,7,8,25,31,32	3	P20766,P21588,Q811M5
5414	1,4,6,26,27,28,29,31	2,3,5,7,8,25,30,32	4	P35280,P36374,P70549,Q63474
5415	1,4,6,26,27,28,29,32	2,3,5,7,8,25,30,31	7	O35547,P00758,P20766,P36374,P36376,Q6AYR9,Q8CGS4
5416	1,4,6,26,27,28,30,31	2,3,5,7,8,25,29,32	1	Q8VD89
5417	1,4,6,26,27,28,30,32	2,3,5,7,8,25,29,31	2	P20766,Q8VD89
5418	1,4,6,26,27,28,31,32	2,3,5,7,8,25,29,30	5	O54728,P22006,P36374,P62804,Q8VD89
5419	1,4,6,26,27,29,30,31	2,3,5,7,8,25,28,32	2	P36860,Q811M5
5420	1,4,6,26,27,29,30,32	2,3,5,7,8,25,28,31	3	P20766,P36376,Q811M5
5421	1,4,6,26,27,29,31,32	2,3,5,7,8,25,28,30	3	P22006,P62804,Q63474
5422	1,4,6,26,27,30,31,32	2,3,5,7,8,25,28,29	7	P06760,P22006,P36860,P54921,P62804,Q8VD89,Q9QYP1
5423	1,4,6,26,28,29,30,31	2,3,5,7,8,25,27,32	1	Q63474
5424	1,4,6,26,28,29,30,32	2,3,5,7,8,25,27,31	3	P20766,P36376,P50115
5425	1,4,6,26,28,29,31,32	2,3,5,7,8,25,27,30	5	O54728,P00731,P22006,P70549,Q63474
5426	1,4,6,26,28,30,31,32	2,3,5,7,8,25,27,29	9	O54728,P06760,P22006,P25809,P50115,P62804,Q00715,Q4G075,Q8VD89
5427	1,4,6,26,29,30,31,32	2,3,5,7,8,25,27,28	5	P22006,P36860,P50115,P62804,Q63474
5428	1,4,6,27,28,29,30,31	2,3,5,7,8,25,26,32	1	Q63474

5429	1,4,6,27,28,29,30,32	2,3,5,7,8,25,26,31	4	O35547,P00758,P20766,Q6AYR9
5430	1,4,6,27,28,29,31,32	2,3,5,7,8,25,26,30	7	O08815,O35547,P22006,P36374,P80299,Q63474,Q6AYR9
5431	1,4,6,27,28,30,31,32	2,3,5,7,8,25,26,29	5	P06760,P22006,P62804,Q8VD89,Q9QX74
5432	1,4,6,27,29,30,31,32	2,3,5,7,8,25,26,28	5	P00714,P22006,P36860,P80299,Q9WTT6
5433	1,4,6,28,29,30,31,32	2,3,5,7,8,25,26,27	5	P22006,P36860,P50115,Q63474,Q9WTT6
5434	1,4,7,8,25,26,27,28	2,3,5,6,29,30,31,32	11	P02782,P07150,P0C0A9,P20646,P22282,P26342,P36860,Q4G075,Q9QW07,Q9WTT6,Q9Z1F2
5435	1,4,7,8,25,26,27,29	2,3,5,6,28,30,31,32	4	P20646,Q4G075,Q9QX74,Q9WTT6
5436	1,4,7,8,25,26,27,30	2,3,5,6,28,29,31,32	1	Q9Z1F2
5437	1,4,7,8,25,26,27,31	2,3,5,6,28,29,30,32	4	Q5I0J9,Q9QYP1,Q9Z0V6,Q9Z1F2
5438	1,4,7,8,25,26,27,32	2,3,5,6,28,29,30,31	2	P13432,Q9JHB9
5439	1,4,7,8,25,26,28,29	2,3,5,6,27,30,31,32	8	P01039,P20646,Q5I0J9,Q6P6R2,Q9QW07,Q9QX74,Q9WTT6,Q9Z2L0
5440	1,4,7,8,25,26,28,30	2,3,5,6,27,29,31,32	1	Q9Z1F2
5441	1,4,7,8,25,26,28,31	2,3,5,6,27,29,30,32	4	Q5I0J9,Q63598,Q9Z0V6,Q9Z1F2
5442	1,4,7,8,25,26,28,32	2,3,5,6,27,29,30,31	5	O54728,P01039,P08723,Q9QW07,Q9Z1F2
5443	1,4,7,8,25,26,29,30	2,3,5,6,27,28,31,32	1	Q9QX74
5444	1,4,7,8,25,26,29,31	2,3,5,6,27,28,30,32	3	P70549,Q5I0J9,Q9QX74
5445	1,4,7,8,25,26,29,32	2,3,5,6,27,28,30,31	1	P13432
5446	1,4,7,8,25,26,30,31	2,3,5,6,27,28,29,32	3	Q6AY61,Q9WVK7,Q9Z1F2
5447	1,4,7,8,25,26,30,32	2,3,5,6,27,28,29,31	1	Q9WVK7
5448	1,4,7,8,25,26,31,32	2,3,5,6,27,28,29,30	4	O54728,P13432,Q5I0J9,Q9WVK7

5449	1,4,7,8,25,27,28,29	2,3,5,6,26,30,31,32	15	P0C0A9,P10758,P20646,P22282,P22283,P47727,Q4G075,Q63493,Q63617,Q6AYR9,Q812E4,Q99MH3,Q9QW07,Q9WTT6,Q9Z2L0
5450	1,4,7,8,25,27,28,30	2,3,5,6,26,29,31,32	3	P22282,Q4G075,Q9Z1F2
5451	1,4,7,8,25,27,28,31	2,3,5,6,26,29,30,32	5	P06911,P0C0A9,P22282,P82471,Q99MH3
5452	1,4,7,8,25,27,28,32	2,3,5,6,26,29,30,31	10	P02781,P04905,P08723,P0C0A9,P22282,Q6AYR9,Q812E4,Q9JHB9,Q9QW07,Q9Z2L0
5453	1,4,7,8,25,27,29,30	2,3,5,6,26,28,31,32	3	Q4G075,Q6P6Q2,Q99MH3
5454	1,4,7,8,25,27,29,31	2,3,5,6,26,28,30,32	4	P10758,P82471,Q4G075,Q99MH3
5455	1,4,7,8,25,27,29,32	2,3,5,6,26,28,30,31	5	P0C0A9,P22282,Q4G075,Q99MH3,Q9Z2L0
5456	1,4,7,8,25,27,30,31	2,3,5,6,26,28,29,32	1	Q9QYP1
5457	1,4,7,8,25,27,30,32	2,3,5,6,26,28,29,31	1	Q9JHB9
5458	1,4,7,8,25,27,31,32	2,3,5,6,26,28,29,30	3	P01681,P82471,Q9QYP1
5459	1,4,7,8,25,28,29,30	2,3,5,6,26,27,31,32	3	P01039,Q9QW07,Q9Z2L0
5460	1,4,7,8,25,28,29,31	2,3,5,6,26,27,30,32	6	P01039,P82471,Q5I0J9,Q63493,Q99MH3,Q9JJ50
5461	1,4,7,8,25,28,29,32	2,3,5,6,26,27,30,31	11	P01039,P08723,P0C0A9,P24368,P30120,P47727,Q63493,Q812E4,Q99MH3,Q9QW07,Q9Z2L0
5462	1,4,7,8,25,28,30,31	2,3,5,6,26,27,29,32	3	P82471,Q9JJ50,Q9Z1F2
5463	1,4,7,8,25,28,30,32	2,3,5,6,26,27,29,31	1	P08723
5464	1,4,7,8,25,28,31,32	2,3,5,6,26,27,29,30	5	P08723,P21588,P82471,Q811M5,Q9JJ50
5465	1,4,7,8,25,29,30,31	2,3,5,6,26,27,28,32	1	P82471
5466	1,4,7,8,25,29,30,32	2,3,5,6,26,27,28,31	0	
5467	1,4,7,8,25,29,31,32	2,3,5,6,26,27,28,30	2	P21588,P82471
5468	1,4,7,8,25,30,31,32	2,3,5,6,26,27,28,29	3	P82471,Q9QYP1,Q9WVK7

5469	1,4,7,8,26, 27,28,29	2,3,5,6,25, 30,31,32	16	P02782,P07150,P20646,P22273,P22282,P22283,P47727,Q4G075,Q63617,Q6AYR9,Q6P6R2,Q812E4,Q9QW07,Q9QX74,Q9WTT6,Q9Z2L0
5470	1,4,7,8,26, 27,28,30	2,3,5,6,25, 29,31,32	4	P07150,P22282,Q9WTT6,Q9Z1F2
5471	1,4,7,8,26, 27,28,31	2,3,5,6,25, 29,30,32	6	P02782,P07150,P22282,P97523,Q9WTT6,Q9Z1F2
5472	1,4,7,8,26, 27,28,32	2,3,5,6,25, 29,30,31	10	O54728,P02782,P07150,P08723,P22282,Q6AYR9,Q812E4,Q9QWN8,Q9WTT6,Q9Z1F2
5473	1,4,7,8,26, 27,29,30	2,3,5,6,25, 28,31,32	2	Q4G075,Q9QX74
5474	1,4,7,8,26, 27,29,31	2,3,5,6,25, 28,30,32	3	Q4G075,Q9QX74,Q9WTT6
5475	1,4,7,8,26, 27,29,32	2,3,5,6,25, 28,30,31	3	P22282,Q4G075,Q9QX74
5476	1,4,7,8,26, 27,30,31	2,3,5,6,25, 28,29,32	2	Q6AY61,Q9Z1F2
5477	1,4,7,8,26, 27,30,32	2,3,5,6,25, 28,29,31	0	
5478	1,4,7,8,26, 27,31,32	2,3,5,6,25, 28,29,30	2	O54728,P82471
5479	1,4,7,8,26, 28,29,30	2,3,5,6,25, 27,31,32	5	P01039,P20646,P21674,Q6P6R2,Q9QX74
5480	1,4,7,8,26, 28,29,31	2,3,5,6,25, 27,30,32	9	P01039,P20646,P21674,P70549,P82471,Q63474,Q6P6R2,Q9QX74,Q9WTT6
5481	1,4,7,8,26, 28,29,32	2,3,5,6,25, 27,30,31	10	O54728,P01039,P08723,P21674,P24368,P30120,Q63493,Q812E4,Q9QW07,Q9Z2L0
5482	1,4,7,8,26, 28,30,31	2,3,5,6,25, 27,29,32	2	P82471,Q9Z1F2
5483	1,4,7,8,26, 28,30,32	2,3,5,6,25, 27,29,31	2	O54728,Q9Z1F2
5484	1,4,7,8,26, 28,31,32	2,3,5,6,25, 27,29,30	4	O54728,P08723,P82471,Q9Z1F2
5485	1,4,7,8,26, 29,30,31	2,3,5,6,25, 27,28,32	4	P49134,P82471,Q63474,Q9QX74
5486	1,4,7,8,26, 29,30,32	2,3,5,6,25, 27,28,31	0	
5487	1,4,7,8,26, 29,31,32	2,3,5,6,25, 27,28,30	4	O54728,P82471,Q63474,Q9QX74
5488	1,4,7,8,26, 30,31,32	2,3,5,6,25, 27,28,29	2	O54728,P82471

5489	1,4,7,8,27,28,29,30	2,3,5,6,25,26,31,32	8	P22282,P47727,Q4G075,Q63617,Q6AYR9,Q812E4,Q99MH3,Q9Z2L0
5490	1,4,7,8,27,28,29,31	2,3,5,6,25,26,30,32	13	P22282,P22283,P36374,P47727,P82471,P97523,Q4G075,Q63493,Q63617,Q6AYR9,Q812E4,Q99MH3,Q9Z2L0
5491	1,4,7,8,27,28,29,32	2,3,5,6,25,26,30,31	23	O08815,O35547,P00758,P02780,P02781,P04905,P08723,P0C0A9,P18298,P22282,P22283,P24368,P30120,P36374,P47727,Q4G075,Q63493,Q63617,Q6AYR9,Q812E4,Q99MH3,Q9QW07,Q9Z2L0
5492	1,4,7,8,27,28,30,31	2,3,5,6,25,26,29,32	2	P82471,Q9Z1F2
5493	1,4,7,8,27,28,30,32	2,3,5,6,25,26,29,31	7	P08723,P18298,P22282,Q6AYR9,Q812E4,Q8VD89,Q9Z2L0
5494	1,4,7,8,27,28,31,32	2,3,5,6,25,26,29,30	6	P08723,P22282,P36374,P82471,Q6AYR9,Q812E4
5495	1,4,7,8,27,29,30,31	2,3,5,6,25,26,28,32	2	P82471,Q4G075
5496	1,4,7,8,27,29,30,32	2,3,5,6,25,26,28,31	2	P00714,Q4G075
5497	1,4,7,8,27,29,31,32	2,3,5,6,25,26,28,30	4	P00714,P80299,P82471,Q99MH3
5498	1,4,7,8,27,30,31,32	2,3,5,6,25,26,28,29	2	P82471,Q9QYP1
5499	1,4,7,8,28,29,30,31	2,3,5,6,25,26,27,32	5	P01039,P21674,P82471,Q63474,Q9JJ50
5500	1,4,7,8,28,29,30,32	2,3,5,6,25,26,27,31	6	P01039,P02780,P08723,P30120,Q812E4,Q9Z2L0
5501	1,4,7,8,28,29,31,32	2,3,5,6,25,26,27,30	8	P01039,P08723,P21674,P30120,P82471,Q63474,Q63493,Q812E4
5502	1,4,7,8,28,30,31,32	2,3,5,6,25,26,27,29	2	P82471,Q9JJ50
5503	1,4,7,8,29,30,31,32	2,3,5,6,25,26,27,28	3	B0BNN3,P82471,Q63474
5504	1,4,7,25,26,27,28,29	2,3,5,6,8,30,31,32	7	O89117,P10758,P20646,Q6P6R2,Q8CGS4,Q99MH3,Q9Z2L0
5505	1,4,7,25,26,27,28,30	2,3,5,6,8,29,31,32	2	P20646,Q9Z1F2
5506	1,4,7,25,26,27,28,31	2,3,5,6,8,29,30,32	4	O88797,P20646,Q5I0J9,Q9QYP1
5507	1,4,7,25,26,27,28,32	2,3,5,6,8,29,30,31	4	P0C0A9,Q5RJR2,Q62761;Q62762;Q62763,Q6AYR9
5508	1,4,7,25,26,27,29,30	2,3,5,6,8,28,31,32	1	P36376

5509	1,4,7,25,26,27,29,31	2,3,5,6,8,28,30,32	3	P10758,Q5I0J9,Q9QYP1
5510	1,4,7,25,26,27,29,32	2,3,5,6,8,28,30,31	3	P10758,P36376,Q6AYR8
5511	1,4,7,25,26,27,30,31	2,3,5,6,8,28,29,32	1	Q9QYP1
5512	1,4,7,25,26,27,30,32	2,3,5,6,8,28,29,31	2	P36376,Q9R168
5513	1,4,7,25,26,27,31,32	2,3,5,6,8,28,29,30	1	Q9QYP1
5514	1,4,7,25,26,28,29,30	2,3,5,6,8,27,31,32	3	P01039,P20646,P36376
5515	1,4,7,25,26,28,29,31	2,3,5,6,8,27,30,32	3	P20646,P70549,Q5I0J9
5516	1,4,7,25,26,28,29,32	2,3,5,6,8,27,30,31	5	P00731,P01039,P20646,P36376,Q6AYR8
5517	1,4,7,25,26,28,30,31	2,3,5,6,8,27,29,32	2	Q9JJ50,Q9Z1F2
5518	1,4,7,25,26,28,30,32	2,3,5,6,8,27,29,31	4	P00731,P36376,Q62975,Q9R168
5519	1,4,7,25,26,28,31,32	2,3,5,6,8,27,29,30	4	O54728,P00731,Q5I0J9,Q6IFU7
5520	1,4,7,25,26,29,30,31	2,3,5,6,8,27,28,32	0	
5521	1,4,7,25,26,29,30,32	2,3,5,6,8,27,28,31	2	P36376,Q9R168
5522	1,4,7,25,26,29,31,32	2,3,5,6,8,27,28,30	3	P19218,Q5I0J9,Q6AYR8
5523	1,4,7,25,26,30,31,32	2,3,5,6,8,27,28,29	4	P25809,Q00715,Q9QYP1,Q9WVK7
5524	1,4,7,25,27,28,29,30	2,3,5,6,8,26,31,32	2	Q99MH3,Q9Z2L0
5525	1,4,7,25,27,28,29,31	2,3,5,6,8,26,30,32	3	P10758,P36374,Q99MH3
5526	1,4,7,25,27,28,29,32	2,3,5,6,8,26,30,31	16	O08815,O35547,P00758,P08723,P0C0A9,P10758,P22283,P36374,Q5RJR2,Q62761;Q62762;Q62763,Q6AYR8,Q6AYR9,Q812E4,Q8CGS4,Q99MH3,Q9Z2L0
5527	1,4,7,25,27,28,30,31	2,3,5,6,8,26,29,32	2	Q99MH3,Q9QYP1
5528	1,4,7,25,27,28,30,32	2,3,5,6,8,26,29,31	1	Q9R168

5529	1,4,7,25,27,28,31,32	2,3,5,6,8,26,29,30	4	P36374,Q62761;Q62762;Q62763,Q99MH3,Q9QYP1
5530	1,4,7,25,27,29,30,31	2,3,5,6,8,26,28,32	2	Q99MH3,Q9QYP1
5531	1,4,7,25,27,29,30,32	2,3,5,6,8,26,28,31	3	P36376,Q99MH3,Q9R168
5532	1,4,7,25,27,29,31,32	2,3,5,6,8,26,28,30	4	P10758,P80299,Q99MH3,Q9QYP1
5533	1,4,7,25,27,30,31,32	2,3,5,6,8,26,28,29	1	Q9QYP1
5534	1,4,7,25,28,29,30,31	2,3,5,6,8,26,27,32	2	Q99MH3,Q9JJ50
5535	1,4,7,25,28,29,30,32	2,3,5,6,8,26,27,31	4	P36376,Q62975,Q99MH3,Q9R168
5536	1,4,7,25,28,29,31,32	2,3,5,6,8,26,27,30	2	Q99MH3,Q9JJ50
5537	1,4,7,25,28,30,31,32	2,3,5,6,8,26,27,29	4	P25809,Q62975,Q9JJ50,Q9QYP1
5538	1,4,7,25,29,30,31,32	2,3,5,6,8,26,27,28	3	P25809,Q9QYP1,Q9WVK7
5539	1,4,7,26,27,28,29,30	2,3,5,6,8,25,31,32	2	P20646,P36376
5540	1,4,7,26,27,28,29,31	2,3,5,6,8,25,30,32	4	O88797,P10758,P20646,P36374
5541	1,4,7,26,27,28,29,32	2,3,5,6,8,25,30,31	12	O35547,P00758,P10758,P20646,P22283,P36374,P36376,Q62761;Q62762;Q62763,Q6AYR9,Q812E4,Q8CGS4,Q9Z2L0
5542	1,4,7,26,27,28,30,31	2,3,5,6,8,25,29,32	1	Q9Z1F2
5543	1,4,7,26,27,28,30,32	2,3,5,6,8,25,29,31	3	P36376,Q8VD89,Q9R168
5544	1,4,7,26,27,28,31,32	2,3,5,6,8,25,29,30	4	O54728,O88797,P22006,P36374
5545	1,4,7,26,27,29,30,31	2,3,5,6,8,25,28,32	1	P36376
5546	1,4,7,26,27,29,30,32	2,3,5,6,8,25,28,31	1	P36376
5547	1,4,7,26,27,29,31,32	2,3,5,6,8,25,28,30	3	P10758,P36376,P80299
5548	1,4,7,26,27,30,31,32	2,3,5,6,8,25,28,29	2	P36376,Q9QYP1

5549	1,4,7,26,28,29,30,31	2,3,5,6,8,25,27,32	1	Q63474
5550	1,4,7,26,28,29,30,32	2,3,5,6,8,25,27,31	2	P36376,Q9R168
5551	1,4,7,26,28,29,31,32	2,3,5,6,8,25,27,30	3	O54728,Q63474,Q6IFU7
5552	1,4,7,26,28,30,31,32	2,3,5,6,8,25,27,29	2	O54728,P22006
5553	1,4,7,26,29,30,31,32	2,3,5,6,8,25,27,28	3	P36376,P36860,Q63474
5554	1,4,7,27,28,29,30,31	2,3,5,6,8,25,26,32	1	Q99MH3
5555	1,4,7,27,28,29,30,32	2,3,5,6,8,25,26,31	8	O35547,P00758,P36376,Q62761;Q62762;Q62763,Q6AYR9,Q812E4,Q99MH3,Q9Z2L0
5556	1,4,7,27,28,29,31,32	2,3,5,6,8,25,26,30	10	O08815,O35547,P10758,P36374,P80299,Q62761;Q62762;Q62763,Q6AYR9,Q812E4,Q8CGS4,Q99MH3
5557	1,4,7,27,28,30,31,32	2,3,5,6,8,25,26,29	4	P22006,P80299,Q8VD89,Q9QYP1
5558	1,4,7,27,29,30,31,32	2,3,5,6,8,25,26,28	6	B0BNN3,P00714,P08649,P80299,Q99MH3,Q9QYP1
5559	1,4,7,28,29,30,31,32	2,3,5,6,8,25,26,27	4	P17559,Q5BJY9,Q63474,Q9JJ50
5560	1,4,8,25,26,27,28,29	2,3,5,6,7,30,31,32	8	P20646,P20766,P26342,P47727,Q6AYR9,Q6P6R2,Q812E4,Q9Z2L0
5561	1,4,8,25,26,27,28,30	2,3,5,6,7,29,31,32	1	P20766
5562	1,4,8,25,26,27,28,31	2,3,5,6,7,29,30,32	2	P01835,P26342
5563	1,4,8,25,26,27,28,32	2,3,5,6,7,29,30,31	5	O54728,P20766,P36374,Q6AYR9,Q9QWN8
5564	1,4,8,25,26,27,29,30	2,3,5,6,7,28,31,32	1	P20766
5565	1,4,8,25,26,27,29,31	2,3,5,6,7,28,30,32	0	
5566	1,4,8,25,26,27,29,32	2,3,5,6,7,28,30,31	1	P13432
5567	1,4,8,25,26,27,30,31	2,3,5,6,7,28,29,32	4	P47967,P54921,Q6AY61,Q9QYP1
5568	1,4,8,25,26,27,30,32	2,3,5,6,7,28,29,31	2	P00762,P54921

5569	1,4,8,25,26,27,31,32	2,3,5,6,7,28,29,30	5	O54728,P00762,P47967,P54921,Q9QYP1
5570	1,4,8,25,26,28,29,30	2,3,5,6,7,27,31,32	4	P01039,P20766,P50116,Q62714
5571	1,4,8,25,26,28,29,31	2,3,5,6,7,27,30,32	4	P01039,P50116,P70549,Q5I0J9
5572	1,4,8,25,26,28,29,32	2,3,5,6,7,27,30,31	7	O54728,P01039,P08723,P13432,P20766,P30120,P50116
5573	1,4,8,25,26,28,30,31	2,3,5,6,7,27,29,32	5	O08839,P50116,Q07523,Q6AY61,Q9JJ50
5574	1,4,8,25,26,28,30,32	2,3,5,6,7,27,29,31	4	O54728,P01039,P20766,P50116
5575	1,4,8,25,26,28,31,32	2,3,5,6,7,27,29,30	3	O54728,P01039,P50116
5576	1,4,8,25,26,29,30,31	2,3,5,6,7,27,28,32	3	P47967,P50116,Q6AY61
5577	1,4,8,25,26,29,30,32	2,3,5,6,7,27,28,31	4	P00507,P01039,P20766,P50116
5578	1,4,8,25,26,29,31,32	2,3,5,6,7,27,28,30	7	O54728,P00507,P01039,P13432,P19218,P47967,P50116
5579	1,4,8,25,26,30,31,32	2,3,5,6,7,27,28,29	8	O54728,P00507,P47967,P50116,P54921,Q00715,Q6AY61,Q9WVK7
5580	1,4,8,25,27,28,29,30	2,3,5,6,7,26,31,32	6	P20766,Q62714,Q6AYR9,Q812E4,Q99MH3,Q9Z2L0
5581	1,4,8,25,27,28,29,31	2,3,5,6,7,26,30,32	4	D3ZTX0,P36374,Q812E4,Q99MH3
5582	1,4,8,25,27,28,29,32	2,3,5,6,7,26,30,31	14	O08815,O35547,P00758,P04905,P08723,P0C0A9,P20766,P30120,P36374,P47727,Q6AYR9,Q812E4,Q99MH3,Q9Z2L0
5583	1,4,8,25,27,28,30,31	2,3,5,6,7,26,29,32	1	Q9QYP1
5584	1,4,8,25,27,28,30,32	2,3,5,6,7,26,29,31	2	P20766,Q6AYR9
5585	1,4,8,25,27,28,31,32	2,3,5,6,7,26,29,30	2	P36374,Q6AYR9
5586	1,4,8,25,27,29,30,31	2,3,5,6,7,26,28,32	1	Q9QYP1
5587	1,4,8,25,27,29,30,32	2,3,5,6,7,26,28,31	3	P00762,P20766,Q5RLM2
5588	1,4,8,25,27,29,31,32	2,3,5,6,7,26,28,30	1	P00762

5589	1,4,8,25,27,30,31,32	2,3,5,6,7,26,28,29	4	P00762,P47967,P54921,Q9QYP1
5590	1,4,8,25,28,29,30,31	2,3,5,6,7,26,27,32	3	P01039,P50116,Q9JJ50
5591	1,4,8,25,28,29,30,32	2,3,5,6,7,26,27,31	5	P01039,P20766,P30120,P50116,Q62714
5592	1,4,8,25,28,29,31,32	2,3,5,6,7,26,27,30	6	P01039,P21588,P30120,P36374,P50116,Q9JJ50
5593	1,4,8,25,28,30,31,32	2,3,5,6,7,26,27,29	4	O08839,P47967,P50116,Q9JJ50
5594	1,4,8,25,29,30,31,32	2,3,5,6,7,26,27,28	3	P21588,P47967,P50116
5595	1,4,8,26,27,28,29,30	2,3,5,6,7,25,31,32	3	P20766,Q6AYR9,Q6P6R2
5596	1,4,8,26,27,28,29,31	2,3,5,6,7,25,30,32	4	P36374,P97523,Q63474,Q6P6R2
5597	1,4,8,26,27,28,29,32	2,3,5,6,7,25,30,31	17	O08815,O35547,O54728,P00758,P04905,P08010,P08723,P20766,P30120,P36374,P47727,Q6AYR9,Q6P6R2,Q812E4,Q8CFN2,Q9QWN8,Q9Z2L0
5598	1,4,8,26,27,28,30,31	2,3,5,6,7,25,29,32	1	Q6AY61
5599	1,4,8,26,27,28,30,32	2,3,5,6,7,25,29,31	6	B0LT89,O54728,P20766,Q6AYR9,Q8VD89,Q9QWN8
5600	1,4,8,26,27,28,31,32	2,3,5,6,7,25,29,30	4	O54728,P36374,Q6AYR9,Q9QWN8
5601	1,4,8,26,27,29,30,31	2,3,5,6,7,25,28,32	2	Q63474,Q6AY61
5602	1,4,8,26,27,29,30,32	2,3,5,6,7,25,28,31	1	P20766
5603	1,4,8,26,27,29,31,32	2,3,5,6,7,25,28,30	2	O54728,Q63474
5604	1,4,8,26,27,30,31,32	2,3,5,6,7,25,28,29	4	O54728,P54921,Q6AY61,Q9QYP1
5605	1,4,8,26,28,29,30,31	2,3,5,6,7,25,27,32	3	P01039,P50116,Q63474
5606	1,4,8,26,28,29,30,32	2,3,5,6,7,25,27,31	5	O54728,P01039,P20766,P30120,P50116
5607	1,4,8,26,28,29,31,32	2,3,5,6,7,25,27,30	5	O54728,P01039,P30120,P50116,Q63474
5608	1,4,8,26,28,30,31,32	2,3,5,6,7,25,27,29	3	O08839,O54728,P50116

5609	1,4,8,26,29,30,31,32	2,3,5,6,7,25,27,28	5	O54728,P50115,P50116,P63081,Q63474
5610	1,4,8,27,28,29,30,31	2,3,5,6,7,25,26,32	1	Q63474
5611	1,4,8,27,28,29,30,32	2,3,5,6,7,25,26,31	11	O35547,P00758,P08723,P18298,P20766,P36374,P60905,Q63150,Q6AYR9,Q812E4,Q9Z2L0
5612	1,4,8,27,28,29,31,32	2,3,5,6,7,25,26,30	9	O08815,O35547,P08723,P30120,P36374,P47727,Q63474,Q6AYR9,Q812E4
5613	1,4,8,27,28,30,31,32	2,3,5,6,7,25,26,29	4	B0LT89,P36374,Q6AYR9,Q8VD89
5614	1,4,8,27,29,30,31,32	2,3,5,6,7,25,26,28	2	B0BNN3,Q63474
5615	1,4,8,28,29,30,31,32	2,3,5,6,7,25,26,27	5	P01039,P50115,P50116,Q63474,Q9JJ50
5616	1,4,25,26,27,28,29,30	2,3,5,6,7,8,31,32	2	P20766,Q62714
5617	1,4,25,26,27,28,29,31	2,3,5,6,7,8,30,32	4	P10758,P20646,P35280,P36374
5618	1,4,25,26,27,28,29,32	2,3,5,6,7,8,30,31	8	O35547,P00758,P20766,P36374,Q5RJR2,Q6AYR8,Q6AYR9,Q8CGS4
5619	1,4,25,26,27,28,30,31	2,3,5,6,7,8,29,32	1	Q9QYP1
5620	1,4,25,26,27,28,30,32	2,3,5,6,7,8,29,31	2	O70594,P20766
5621	1,4,25,26,27,28,31,32	2,3,5,6,7,8,29,30	5	O54728,P36374,P62804,Q5RJR2,Q9QYP1
5622	1,4,25,26,27,29,30,31	2,3,5,6,7,8,28,32	1	Q9QYP1
5623	1,4,25,26,27,29,30,32	2,3,5,6,7,8,28,31	5	D3ZUC6,P20766,P36376,P50115,Q63532
5624	1,4,25,26,27,29,31,32	2,3,5,6,7,8,28,30	3	P19218,P62804,Q9QYP1

5625	1,4,25,26,27,30,31,32	2,3,5,6,7,8,28,29	8	D3ZUC6,P21674,P47967,P50115,P54921,P62804,Q00715,Q9QYP1
5626	1,4,25,26,28,29,30,31	2,3,5,6,7,8,27,32	6	P01039,P50115,P50116,Q62714,Q9JJ50,Q9QZQ5
5627	1,4,25,26,28,29,30,32	2,3,5,6,7,8,27,31	7	P00731,P01039,P20766,P36376,P50115,P50116,Q62714
5628	1,4,25,26,28,29,31,32	2,3,5,6,7,8,27,30	8	O54728,P00731,P01039,P19218,P36374,P50115,P50116,P62804
5629	1,4,25,26,28,30,31,32	2,3,5,6,7,8,27,29	11	O08839,O54728,P00731,P25809,P47967,P50115,P50116,P62804,Q00715,Q4G075,Q9JJ50
5630	1,4,25,26,29,30,31,32	2,3,5,6,7,8,27,28	9	D3ZUC6,P00507,P19218,P25809,P47967,P50115,P50116,P62804,Q00715
5631	1,4,25,27,28,29,30,31	2,3,5,6,7,8,26,32	4	P50115,Q62714,Q99MH3,Q9QYP1
5632	1,4,25,27,28,29,30,32	2,3,5,6,7,8,26,31	7	P00758,P20766,P36374,P50115,Q62714,Q6AYR9,Q99MH3
5633	1,4,25,27,28,29,31,32	2,3,5,6,7,8,26,30	8	O08815,O35547,P10758,P36374,P50115,Q6AYR9,Q8CGS4,Q99MH3
5634	1,4,25,27,28,30,31,32	2,3,5,6,7,8,26,29	7	O70594,P06760,P36374,P50115,P62804,Q9QX74,Q9QYP1
5635	1,4,25,27,29,30,31,32	2,3,5,6,7,8,26,28	5	D3ZUC6,P47967,P50115,Q5RLM2,Q9QYP1
5636	1,4,25,28,29,30,31,32	2,3,5,6,7,8,26,27	3	P50115,P50116,Q9JJ50
5637	1,4,26,27,28,29,30,31	2,3,5,6,7,8,25,32	2	P50115,Q63474

5638	1,4,26,27,28,29,30,32	2,3,5,6,7,8,25,31	5	P00758,P20766,P36376,P50115,Q6AYR9
5639	1,4,26,27,28,29,31,32	2,3,5,6,7,8,25,30	7	O35547,O54728,P36374,P50115,Q63474,Q6AYR9,Q8CGS4
5640	1,4,26,27,28,30,31,32	2,3,5,6,7,8,25,29	7	O54728,O70594,P22006,P36374,P50115,P62804,Q8VD89
5641	1,4,26,27,29,30,31,32	2,3,5,6,7,8,25,28	3	P02631,P36860,P50115
5642	1,4,26,28,29,30,31,32	2,3,5,6,7,8,25,27	4	O54728,P50115,P50116,Q63474
5643	1,4,27,28,29,30,31,32	2,3,5,6,7,8,25,26	3	P36374,P50115,Q63474
5644	1,5,6,7,8,25,26,27	2,3,4,28,29,30,31,32	4	P13432,P50115,P50116,Q9WTT6
5645	1,5,6,7,8,25,26,28	2,3,4,27,29,30,31,32	6	P00762,P13432,P35745,P80299,Q9QW07,Q9WTT6
5646	1,5,6,7,8,25,26,29	2,3,4,27,28,30,31,32	4	P06760,P13432,P49134,Q9QX74
5647	1,5,6,7,8,25,26,30	2,3,4,27,28,29,31,32	4	P35745,P49134,P80299,Q9WVK7
5648	1,5,6,7,8,25,26,31	2,3,4,27,28,29,30,32	5	P13432,P35745,P49134,P57113,Q9WVK7
5649	1,5,6,7,8,25,26,32	2,3,4,27,28,29,30,31	3	P13432,Q5PQL7,Q9WVK7
5650	1,5,6,7,8,25,27,28	2,3,4,26,29,30,31,32	5	P06911,P50115,P50116,P63081,Q9QW07
5651	1,5,6,7,8,25,27,29	2,3,4,26,28,30,31,32	3	P06760,P50115,Q4G075
5652	1,5,6,7,8,25,27,30	2,3,4,26,28,29,31,32	2	P19218,Q6IG05
5653	1,5,6,7,8,25,27,31	2,3,4,26,28,29,30,32	2	P23739,P50115
5654	1,5,6,7,8,25,27,32	2,3,4,26,28,29,30,31	2	P13432,Q03191

5655	1,5,6,7,8,25,28,29	2,3,4,26,27,30,31,32	5	P00762,P06760,P06911,P21674,Q9QW07
5656	1,5,6,7,8,25,28,30	2,3,4,26,27,29,31,32	3	P35745,Q5PQL7,Q9QW07
5657	1,5,6,7,8,25,28,31	2,3,4,26,27,29,30,32	2	P06911,P35745
5658	1,5,6,7,8,25,28,32	2,3,4,26,27,29,30,31	3	P13432,Q5PQL7,Q9QW07
5659	1,5,6,7,8,25,29,30	2,3,4,26,27,28,31,32	1	P35745
5660	1,5,6,7,8,25,29,31	2,3,4,26,27,28,30,32	1	P06760
5661	1,5,6,7,8,25,29,32	2,3,4,26,27,28,30,31	1	P13432
5662	1,5,6,7,8,25,30,31	2,3,4,26,27,28,29,32	2	P35745,Q9WVK7
5663	1,5,6,7,8,25,30,32	2,3,4,26,27,28,29,31	3	P35745,Q5PQL7,Q9WVK7
5664	1,5,6,7,8,25,31,32	2,3,4,26,27,28,29,30	2	P13432,Q9WVK7
5665	1,5,6,7,8,26,27,28	2,3,4,25,29,30,31,32	6	D3ZUC6,P07150,P29315,P50116,Q9QW07,Q9WTT6
5666	1,5,6,7,8,26,27,29	2,3,4,25,28,30,31,32	7	P06760,P29315,P50116,Q4G075,Q9JJ50,Q9QX74,Q9WTT6
5667	1,5,6,7,8,26,27,30	2,3,4,25,28,29,31,32	4	P19218,P29315,P35745,P50116
5668	1,5,6,7,8,26,27,31	2,3,4,25,28,29,30,32	2	P50116,Q62714
5669	1,5,6,7,8,26,27,32	2,3,4,25,28,29,30,31	3	P13432,P29315,P50116
5670	1,5,6,7,8,26,28,29	2,3,4,25,27,30,31,32	12	D3ZUC6,P00762,P06760,P21674,P29315,P35745,P49134,Q6P6R2,Q9QW07,Q9QX74,Q9QYP1,Q9WTT6
5671	1,5,6,7,8,26,28,30	2,3,4,25,27,29,31,32	4	P00762,P29315,P35745,P49134
5672	1,5,6,7,8,26,28,31	2,3,4,25,27,29,30,32	4	P00762,P35745,P49134,P57113
5673	1,5,6,7,8,26,28,32	2,3,4,25,27,29,30,31	4	P13432,P29315,P35745,Q9QW07
5674	1,5,6,7,8,26,29,30	2,3,4,25,27,28,31,32	6	P00762,P06760,P29315,P35745,P49134,Q9QX74

5675	1,5,6,7,8,26,29,31	2,3,4,25,27,28,30,32	5	P00762,P06760,P35745,P49134,Q9QX74
5676	1,5,6,7,8,26,29,32	2,3,4,25,27,28,30,31	4	P06760,P13432,P29315,Q9QX74
5677	1,5,6,7,8,26,30,31	2,3,4,25,27,28,29,32	4	P35745,P49134,Q5RJR2,Q9WVK7
5678	1,5,6,7,8,26,30,32	2,3,4,25,27,28,29,31	3	P29315,P35745,Q9WVK7
5679	1,5,6,7,8,26,31,32	2,3,4,25,27,28,29,30	5	P13432,P35745,P57113,Q62714,Q9WVK7
5680	1,5,6,7,8,27,28,29	2,3,4,25,26,30,31,32	13	D3ZUC6,P06760,P06761,P19218,P21674,P22283,P29315,P47967,Q4G075,Q63493,Q63617,Q812E4,Q9QW07
5681	1,5,6,7,8,27,28,30	2,3,4,25,26,29,31,32	3	P19218,P29315,P35745
5682	1,5,6,7,8,27,28,31	2,3,4,25,26,29,30,32	3	D3ZUC6,P19218,P35745
5683	1,5,6,7,8,27,28,32	2,3,4,25,26,29,30,31	8	P19218,P22282,P22283,P29315,Q6AYR9,Q812E4,Q9JI85,Q9QW07
5684	1,5,6,7,8,27,29,30	2,3,4,25,26,28,31,32	3	P19218,P29315,Q6IMF3
5685	1,5,6,7,8,27,29,31	2,3,4,25,26,28,30,32	1	P06760
5686	1,5,6,7,8,27,29,32	2,3,4,25,26,28,30,31	1	P29315
5687	1,5,6,7,8,27,30,31	2,3,4,25,26,28,29,32	3	P19218,P35745,Q6IG05
5688	1,5,6,7,8,27,30,32	2,3,4,25,26,28,29,31	2	P19218,P29315
5689	1,5,6,7,8,27,31,32	2,3,4,25,26,28,29,30	2	P23739,Q62714
5690	1,5,6,7,8,28,29,30	2,3,4,25,26,27,31,32	6	P00762,P19218,P21674,P29315,P35745,Q9QW07
5691	1,5,6,7,8,28,29,31	2,3,4,25,26,27,30,32	6	D3ZUC6,P00762,P06760,P21674,P35745,Q63493
5692	1,5,6,7,8,28,29,32	2,3,4,25,26,27,30,31	8	P21674,P29315,P30120,Q63493,Q63617,Q812E4,Q9QW07,Q9QYP1
5693	1,5,6,7,8,28,30,31	2,3,4,25,26,27,29,32	3	P00762,P19218,P35745
5694	1,5,6,7,8,28,30,32	2,3,4,25,26,27,29,31	3	P19218,P29315,P35745

5695	1,5,6,7,8,28,31,32	2,3,4,25,26,27,29,30	1	P35745
5696	1,5,6,7,8,29,30,31	2,3,4,25,26,27,28,32	5	P35745,P36860,P49134,Q5RJR2,Q9QWN8
5697	1,5,6,7,8,29,30,32	2,3,4,25,26,27,28,31	3	P29315,P35745,P36860
5698	1,5,6,7,8,29,31,32	2,3,4,25,26,27,28,30	1	P36860
5699	1,5,6,7,8,30,31,32	2,3,4,25,26,27,28,29	3	P35745,P36860,Q9WVK7
5700	1,5,6,7,25,26,27,28	2,3,4,8,29,30,31,32	1	P63081
5701	1,5,6,7,25,26,27,29	2,3,4,8,28,30,31,32	1	P05539
5702	1,5,6,7,25,26,27,30	2,3,4,8,28,29,31,32	1	P21588
5703	1,5,6,7,25,26,27,31	2,3,4,8,28,29,30,32	0	
5704	1,5,6,7,25,26,27,32	2,3,4,8,28,29,30,31	1	Q03191
5705	1,5,6,7,25,26,28,29	2,3,4,8,27,30,31,32	3	P00762,P02625,P05539
5706	1,5,6,7,25,26,28,30	2,3,4,8,27,29,31,32	2	P00762,Q5PQL7
5707	1,5,6,7,25,26,28,31	2,3,4,8,27,29,30,32	1	P00762
5708	1,5,6,7,25,26,28,32	2,3,4,8,27,29,30,31	2	Q5PQL7,Q6IG02
5709	1,5,6,7,25,26,29,30	2,3,4,8,27,28,31,32	3	P00762,P36376,Q9WVK7
5710	1,5,6,7,25,26,29,31	2,3,4,8,27,28,30,32	2	P00762,Q9WVK7
5711	1,5,6,7,25,26,29,32	2,3,4,8,27,28,30,31	2	P36376,Q9WVK7
5712	1,5,6,7,25,26,30,31	2,3,4,8,27,28,29,32	2	P25809,Q9WVK7
5713	1,5,6,7,25,26,30,32	2,3,4,8,27,28,29,31	4	P36376,Q5PQL7,Q9R168,Q9WVK7
5714	1,5,6,7,25,26,31,32	2,3,4,8,27,28,29,30	2	P25809,Q9WVK7

5715	1,5,6,7,25, 27,28,29	2,3,4,8,26, 30,31,32	4 P05539,P63081,P97615,Q9WUC4
5716	1,5,6,7,25, 27,28,30	2,3,4,8,26, 29,31,32	0
5717	1,5,6,7,25, 27,28,31	2,3,4,8,26, 29,30,32	0
5718	1,5,6,7,25, 27,28,32	2,3,4,8,26, 29,30,31	2 Q03191,Q62761;Q62762;Q62763
5719	1,5,6,7,25, 27,29,30	2,3,4,8,26, 28,31,32	2 O54728,P05539
5720	1,5,6,7,25, 27,29,31	2,3,4,8,26, 28,30,32	2 O54728,P05539
5721	1,5,6,7,25, 27,29,32	2,3,4,8,26, 28,30,31	2 P05539,Q03191
5722	1,5,6,7,25, 27,30,31	2,3,4,8,26, 28,29,32	1 O54728
5723	1,5,6,7,25, 27,30,32	2,3,4,8,26, 28,29,31	1 Q03191
5724	1,5,6,7,25, 27,31,32	2,3,4,8,26, 28,29,30	2 P23739,Q03191
5725	1,5,6,7,25, 28,29,30	2,3,4,8,26, 27,31,32	3 P00762,P05539,Q5BJY9
5726	1,5,6,7,25, 28,29,31	2,3,4,8,26, 27,30,32	2 P00762,P05539
5727	1,5,6,7,25, 28,29,32	2,3,4,8,26, 27,30,31	2 P05539,Q5BJY9
5728	1,5,6,7,25, 28,30,31	2,3,4,8,26, 27,29,32	4 P00762,P25809,P35745,Q5BJY9
5729	1,5,6,7,25, 28,30,32	2,3,4,8,26, 27,29,31	3 Q5BJY9,Q5PQL7,Q62975
5730	1,5,6,7,25, 28,31,32	2,3,4,8,26, 27,29,30	1 Q5BJY9
5731	1,5,6,7,25, 29,30,31	2,3,4,8,26, 27,28,32	5 O54728,P36860,Q5BJY9,Q9QWN8,Q9WVK7
5732	1,5,6,7,25, 29,30,32	2,3,4,8,26, 27,28,31	4 P36376,P36860,Q5BJY9,Q9WVK7
5733	1,5,6,7,25, 29,31,32	2,3,4,8,26, 27,28,30	3 P36860,Q5BJY9,Q9WVK7
5734	1,5,6,7,25, 30,31,32	2,3,4,8,26, 27,28,29	5 P25809,P36860,Q5BJY9,Q6P6R2,Q9WVK7

5735	1,5,6,7,26,<br>27,28,29	2,3,4,8,25,<br>30,31,32	6	P00762,P02625,P05539,P21588,P29315,P47967
5736	1,5,6,7,26,<br>27,28,30	2,3,4,8,25,<br>29,31,32	3	P00762,P21588,P29315
5737	1,5,6,7,26,<br>27,28,31	2,3,4,8,25,<br>29,30,32	2	O88797,P00762
5738	1,5,6,7,26,<br>27,28,32	2,3,4,8,25,<br>29,30,31	1	P29315
5739	1,5,6,7,26,<br>27,29,30	2,3,4,8,25,<br>28,31,32	5	P21588,P29315,P36376,Q811M5,Q923V8
5740	1,5,6,7,26,<br>27,29,31	2,3,4,8,25,<br>28,30,32	2	P02625,P05539
5741	1,5,6,7,26,<br>27,29,32	2,3,4,8,25,<br>28,30,31	3	P29315,P36376,Q9JJ50
5742	1,5,6,7,26,<br>27,30,31	2,3,4,8,25,<br>28,29,32	1	P36860
5743	1,5,6,7,26,<br>27,30,32	2,3,4,8,25,<br>28,29,31	3	P29315,P36376,P36860
5744	1,5,6,7,26,<br>27,31,32	2,3,4,8,25,<br>28,29,30	2	P36860,Q62714
5745	1,5,6,7,26,<br>28,29,30	2,3,4,8,25,<br>27,31,32	4	P00762,P01835,P29315,P35745
5746	1,5,6,7,26,<br>28,29,31	2,3,4,8,25,<br>27,30,32	6	P00762,P01835,P02625,P05539,P70549,Q01205
5747	1,5,6,7,26,<br>28,29,32	2,3,4,8,25,<br>27,30,31	4	P01835,P02625,P29315,P36376
5748	1,5,6,7,26,<br>28,30,31	2,3,4,8,25,<br>27,29,32	2	P00762,P35745
5749	1,5,6,7,26,<br>28,30,32	2,3,4,8,25,<br>27,29,31	2	P29315,Q5PQL7
5750	1,5,6,7,26,<br>28,31,32	2,3,4,8,25,<br>27,29,30	2	P22006,Q6IG02
5751	1,5,6,7,26,<br>29,30,31	2,3,4,8,25,<br>27,28,32	5	P00762,P01835,P35745,P36860,Q9QWN8
5752	1,5,6,7,26,<br>29,30,32	2,3,4,8,25,<br>27,28,31	5	P01835,P29315,P36376,P36860,Q5BJY9
5753	1,5,6,7,26,<br>29,31,32	2,3,4,8,25,<br>27,28,30	3	O70417,P01835,P36860
5754	1,5,6,7,26,<br>30,31,32	2,3,4,8,25,<br>27,28,29	3	P25809,P36860,Q9WVK7

5755	1,5,6,7,27,<br>28,29,30	2,3,4,8,25,<br>26,31,32	4	P00762,P05539,P19218,P29315
5756	1,5,6,7,27,<br>28,29,31	2,3,4,8,25,<br>26,30,32	3	P00762,P02625,P05539
5757	1,5,6,7,27,<br>28,29,32	2,3,4,8,25,<br>26,30,31	7	O35547,P05539,P22283,P29315,Q62761;Q62762;Q62763,Q6AY61,Q812E4
5758	1,5,6,7,27,<br>28,30,31	2,3,4,8,25,<br>26,29,32	2	P19218,P35745
5759	1,5,6,7,27,<br>28,30,32	2,3,4,8,25,<br>26,29,31	3	P19218,P29315,Q8VD89
5760	1,5,6,7,27,<br>28,31,32	2,3,4,8,25,<br>26,29,30	1	P22006
5761	1,5,6,7,27,<br>29,30,31	2,3,4,8,25,<br>26,28,32	2	O54728,P36860
5762	1,5,6,7,27,<br>29,30,32	2,3,4,8,25,<br>26,28,31	3	P29315,P36376,P36860
5763	1,5,6,7,27,<br>29,31,32	2,3,4,8,25,<br>26,28,30	1	P36860
5764	1,5,6,7,27,<br>30,31,32	2,3,4,8,25,<br>26,28,29	1	P36860
5765	1,5,6,7,28,<br>29,30,31	2,3,4,8,25,<br>26,27,32	6	P00762,P01835,P05539,P35745,P36860,Q5BJY9
5766	1,5,6,7,28,<br>29,30,32	2,3,4,8,25,<br>26,27,31	4	P01835,P29315,P36860,Q5BJY9
5767	1,5,6,7,28,<br>29,31,32	2,3,4,8,25,<br>26,27,30	4	O70417,P01835,P05539,Q5BJY9
5768	1,5,6,7,28,<br>30,31,32	2,3,4,8,25,<br>26,27,29	4	P22006,P35745,P36860,Q5BJY9
5769	1,5,6,7,29,<br>30,31,32	2,3,4,8,25,<br>26,27,28	5	P01835,P26342,P36860,Q5BJY9,Q9WVK7
5770	1,5,6,8,25,<br>26,27,28	2,3,4,7,29,<br>30,31,32	2	P26342,P80299
5771	1,5,6,8,25,<br>26,27,29	2,3,4,7,28,<br>30,31,32	2	P13432,P80299
5772	1,5,6,8,25,<br>26,27,30	2,3,4,7,28,<br>29,31,32	1	P80299
5773	1,5,6,8,25,<br>26,27,31	2,3,4,7,28,<br>29,30,32	3	P13432,P23739,P54921
5774	1,5,6,8,25,<br>26,27,32	2,3,4,7,28,<br>29,30,31	1	P13432

5775	1,5,6,8,25,26,28,29	2,3,4,7,27,30,31,32	4 P13432,P20766,P80299,Q9QW07
5776	1,5,6,8,25,26,28,30	2,3,4,7,27,29,31,32	3 P35745,P80299,Q5PQL7
5777	1,5,6,8,25,26,28,31	2,3,4,7,27,29,30,32	3 P00714,P13432,P80299
5778	1,5,6,8,25,26,28,32	2,3,4,7,27,29,30,31	3 P13432,P80299,Q5PQL7
5779	1,5,6,8,25,26,29,30	2,3,4,7,27,28,31,32	1 P80299
5780	1,5,6,8,25,26,29,31	2,3,4,7,27,28,30,32	2 P13432,P70549
5781	1,5,6,8,25,26,29,32	2,3,4,7,27,28,30,31	2 P00507,P13432
5782	1,5,6,8,25,26,30,31	2,3,4,7,27,28,29,32	4 P35745,P54921,P80299,Q9WVK7
5783	1,5,6,8,25,26,30,32	2,3,4,7,27,28,29,31	5 P00507,P13432,P80299,Q5PQL7,Q9WVK7
5784	1,5,6,8,25,26,31,32	2,3,4,7,27,28,29,30	4 P00507,P13432,Q80WL1,Q9WVK7
5785	1,5,6,8,25,27,28,29	2,3,4,7,26,30,31,32	3 P20766,Q812E4,Q9QW07
5786	1,5,6,8,25,27,28,30	2,3,4,7,26,29,31,32	3 P19218,P20766,P80299
5787	1,5,6,8,25,27,28,31	2,3,4,7,26,29,30,32	1 P23739
5788	1,5,6,8,25,27,28,32	2,3,4,7,26,29,30,31	3 P13432,Q6AYR9,Q9QWN8
5789	1,5,6,8,25,27,29,30	2,3,4,7,26,28,31,32	1 Q6IMF3
5790	1,5,6,8,25,27,29,31	2,3,4,7,26,28,30,32	0
5791	1,5,6,8,25,27,29,32	2,3,4,7,26,28,30,31	1 P13432
5792	1,5,6,8,25,27,30,31	2,3,4,7,26,28,29,32	3 P23739,P54921,Q6IG05
5793	1,5,6,8,25,27,30,32	2,3,4,7,26,28,29,31	1 Q63424
5794	1,5,6,8,25,27,31,32	2,3,4,7,26,28,29,30	3 P13432,P23739,Q80WL1

5795	1,5,6,8,25,<br>28,29,30	2,3,4,7,26,<br>27,31,32	2	P20766,P80299
5796	1,5,6,8,25,<br>28,29,31	2,3,4,7,26,<br>27,30,32	0	
5797	1,5,6,8,25,<br>28,29,32	2,3,4,7,26,<br>27,30,31	3	P13432,P20766,Q9QW07
5798	1,5,6,8,25,<br>28,30,31	2,3,4,7,26,<br>27,29,32	1	P35745
5799	1,5,6,8,25,<br>28,30,32	2,3,4,7,26,<br>27,29,31	2	P20766,Q5PQL7
5800	1,5,6,8,25,<br>28,31,32	2,3,4,7,26,<br>27,29,30	2	P13432,Q80WL1
5801	1,5,6,8,25,<br>29,30,31	2,3,4,7,26,<br>27,28,32	0	
5802	1,5,6,8,25,<br>29,30,32	2,3,4,7,26,<br>27,28,31	3	P13432,P20766,Q9WVK7
5803	1,5,6,8,25,<br>29,31,32	2,3,4,7,26,<br>27,28,30	2	P13432,Q80WL1
5804	1,5,6,8,25,<br>30,31,32	2,3,4,7,26,<br>27,28,29	3	P20646,P47967,Q9WVK7
5805	1,5,6,8,26,<br>27,28,29	2,3,4,7,25,<br>30,31,32	3	P26342,P29315,Q6P6R2
5806	1,5,6,8,26,<br>27,28,30	2,3,4,7,25,<br>29,31,32	2	P29315,P80299
5807	1,5,6,8,26,<br>27,28,31	2,3,4,7,25,<br>29,30,32	0	
5808	1,5,6,8,26,<br>27,28,32	2,3,4,7,25,<br>29,30,31	4	B0LT89,P29315,Q6AYR9,Q9QWN8
5809	1,5,6,8,26,<br>27,29,30	2,3,4,7,25,<br>28,31,32	2	P29315,Q811M5
5810	1,5,6,8,26,<br>27,29,31	2,3,4,7,25,<br>28,30,32	0	
5811	1,5,6,8,26,<br>27,29,32	2,3,4,7,25,<br>28,30,31	2	P13432,P29315
5812	1,5,6,8,26,<br>27,30,31	2,3,4,7,25,<br>28,29,32	0	
5813	1,5,6,8,26,<br>27,30,32	2,3,4,7,25,<br>28,29,31	1	P29315
5814	1,5,6,8,26,<br>27,31,32	2,3,4,7,25,<br>28,29,30	2	P13432,P23739

5815	1,5,6,8,26,<br>28,29,30	2,3,4,7,25,<br>27,31,32	5	P20766,P29315,P35745,P80299,Q9QYP1
5816	1,5,6,8,26,<br>28,29,31	2,3,4,7,25,<br>27,30,32	2	P00762,P70549
5817	1,5,6,8,26,<br>28,29,32	2,3,4,7,25,<br>27,30,31	4	P13432,P20766,P29315,Q9QYP1
5818	1,5,6,8,26,<br>28,30,31	2,3,4,7,25,<br>27,29,32	1	P35745
5819	1,5,6,8,26,<br>28,30,32	2,3,4,7,25,<br>27,29,31	3	P29315,P35745,Q8VD89
5820	1,5,6,8,26,<br>28,31,32	2,3,4,7,25,<br>27,29,30	0	
5821	1,5,6,8,26,<br>29,30,31	2,3,4,7,25,<br>27,28,32	3	P35745,P36860,Q5RJR2
5822	1,5,6,8,26,<br>29,30,32	2,3,4,7,25,<br>27,28,31	2	P29315,P36860
5823	1,5,6,8,26,<br>29,31,32	2,3,4,7,25,<br>27,28,30	2	P13432,P36860
5824	1,5,6,8,26,<br>30,31,32	2,3,4,7,25,<br>27,28,29	5	O89117,P00507,P35745,P36860,Q9WVK7
5825	1,5,6,8,27,<br>28,29,30	2,3,4,7,25,<br>26,31,32	3	P19218,P20766,P29315
5826	1,5,6,8,27,<br>28,29,31	2,3,4,7,25,<br>26,30,32	1	Q9R168
5827	1,5,6,8,27,<br>28,29,32	2,3,4,7,25,<br>26,30,31	5	O35547,P20766,P29315,Q6AYR9,Q812E4
5828	1,5,6,8,27,<br>28,30,31	2,3,4,7,25,<br>26,29,32	2	P19218,P35745
5829	1,5,6,8,27,<br>28,30,32	2,3,4,7,25,<br>26,29,31	6	B0LT89,P19218,P20766,P29315,Q5I0J9,Q8VD89
5830	1,5,6,8,27,<br>28,31,32	2,3,4,7,25,<br>26,29,30	4	B0LT89,P22006,P23739,Q80WL1
5831	1,5,6,8,27,<br>29,30,31	2,3,4,7,25,<br>26,28,32	1	Q6IMF3
5832	1,5,6,8,27,<br>29,30,32	2,3,4,7,25,<br>26,28,31	3	P19218,P29315,Q5I0J9
5833	1,5,6,8,27,<br>29,31,32	2,3,4,7,25,<br>26,28,30	0	
5834	1,5,6,8,27,<br>30,31,32	2,3,4,7,25,<br>26,28,29	2	P20646,P23739

5835	1,5,6,8,28,<br>29,30,31	2,3,4,7,25,<br>26,27,32	1	P35745
5836	1,5,6,8,28,<br>29,30,32	2,3,4,7,25,<br>26,27,31	2	P20766,P29315
5837	1,5,6,8,28,<br>29,31,32	2,3,4,7,25,<br>26,27,30	1	Q80WL1
5838	1,5,6,8,28,<br>30,31,32	2,3,4,7,25,<br>26,27,29	4	O89117,P22006,P35745,Q8VD89
5839	1,5,6,8,29,<br>30,31,32	2,3,4,7,25,<br>26,27,28	2	P20646,P36860
5840	1,5,6,25,2<br>6,27,28,29	2,3,4,7,8,3<br>0,31,32	6	P02625,P05539,P20766,P35280,P82471,P97615
5841	1,5,6,25,2<br>6,27,28,30	2,3,4,7,8,2<br>9,31,32	4	P20766,P21588,P80299,P82471
5842	1,5,6,25,2<br>6,27,28,31	2,3,4,7,8,2<br>9,30,32	2	P35280,P62804
5843	1,5,6,25,2<br>6,27,28,32	2,3,4,7,8,2<br>9,30,31	3	P20766,P62804,P82471
5844	1,5,6,25,2<br>6,27,29,30	2,3,4,7,8,2<br>8,31,32	4	P20766,P82471,Q811M5,Q923V8
5845	1,5,6,25,2<br>6,27,29,31	2,3,4,7,8,2<br>8,30,32	3	O70417,P05539,P35280
5846	1,5,6,25,2<br>6,27,29,32	2,3,4,7,8,2<br>8,30,31	2	P20766,P82471
5847	1,5,6,25,2<br>6,27,30,31	2,3,4,7,8,2<br>8,29,32	2	P54921,P62804
5848	1,5,6,25,2<br>6,27,30,32	2,3,4,7,8,2<br>8,29,31	2	P62804,P82471
5849	1,5,6,25,2<br>6,27,31,32	2,3,4,7,8,2<br>8,29,30	1	P62804
5850	1,5,6,25,2<br>6,28,29,30	2,3,4,7,8,2<br>7,31,32	3	P20766,P80299,P82471
5851	1,5,6,25,2<br>6,28,29,31	2,3,4,7,8,2<br>7,30,32	6	O70417,P00762,P05539,P35280,P70549,Q01205
5852	1,5,6,25,2<br>6,28,29,32	2,3,4,7,8,2<br>7,30,31	2	P20766,P82471
5853	1,5,6,25,2<br>6,28,30,31	2,3,4,7,8,2<br>7,29,32	4	P00714,P25809,Q4G075,Q6TMA8
5854	1,5,6,25,2<br>6,28,30,32	2,3,4,7,8,2<br>7,29,31	6	P20766,P62804,P82471,Q4G075,Q5PQL7,Q62975

5855	1,5,6,25,26,28,31,32	2,3,4,7,8,27,29,30	5	O70417,P00731,P62804,Q4G075,Q6IG02
5856	1,5,6,25,26,29,30,31	2,3,4,7,8,27,28,32	3	A0JPJ7,P36860,Q9WVK7
5857	1,5,6,25,26,29,30,32	2,3,4,7,8,27,28,31	6	P00507,P20766,P36860,P82471,Q5BJY9,Q9WVK7
5858	1,5,6,25,26,29,31,32	2,3,4,7,8,27,28,30	5	O70417,P00507,P36860,P62804,Q9WVK7
5859	1,5,6,25,26,30,31,32	2,3,4,7,8,27,28,29	10	P00507,P22273,P25809,P36860,P47967,P62804,Q00715,Q4G075,Q9EQS0,Q9WVK7
5860	1,5,6,25,27,28,29,30	2,3,4,7,8,26,31,32	3	P05539,P20766,P82471
5861	1,5,6,25,27,28,29,31	2,3,4,7,8,26,30,32	5	P05539,P20761,P20762,P35280,P97615
5862	1,5,6,25,27,28,29,32	2,3,4,7,8,26,30,31	5	O35547,P05539,P20766,P82471,Q62761;Q62762;Q62763
5863	1,5,6,25,27,28,30,31	2,3,4,7,8,26,29,32	1	P20761
5864	1,5,6,25,27,28,30,32	2,3,4,7,8,26,29,31	4	P20766,P82471,Q63424,Q9QX74
5865	1,5,6,25,27,28,31,32	2,3,4,7,8,26,29,30	3	P20761,P23739,P62804
5866	1,5,6,25,27,29,30,31	2,3,4,7,8,26,28,32	1	O54728
5867	1,5,6,25,27,29,30,32	2,3,4,7,8,26,28,31	2	P20766,P82471
5868	1,5,6,25,27,29,31,32	2,3,4,7,8,26,28,30	3	O70417,P20762,P62804
5869	1,5,6,25,27,30,31,32	2,3,4,7,8,26,28,29	7	P20646,P23739,P62804,Q63424,Q6P6R2,Q9QX74,Q9WTT6
5870	1,5,6,25,28,29,30,31	2,3,4,7,8,26,27,32	4	P05539,P20761,P20762,Q5BJY9
5871	1,5,6,25,28,29,30,32	2,3,4,7,8,26,27,31	3	P20762,P20766,Q5BJY9
5872	1,5,6,25,28,29,31,32	2,3,4,7,8,26,27,30	5	O70417,P05539,P20761,P20762,Q5BJY9
5873	1,5,6,25,28,30,31,32	2,3,4,7,8,26,27,29	10	P20761,P20762,P25809,P62804,Q00715,Q4G075,Q5BJY9,Q62975,Q9QX74,Q9WTT6
5874	1,5,6,25,29,30,31,32	2,3,4,7,8,26,27,28	9	P07150,P20762,P25809,P36860,P62804,Q4G075,Q5BJY9,Q9WTT6,Q9WVK7

5875	1,5,6,26,27,28,29,30	2,3,4,7,8,25,31,32	6	P05539,P20766,P21588,P29315,P82471,Q811M5
5876	1,5,6,26,27,28,29,31	2,3,4,7,8,25,30,32	4	O70417,P02625,P05539,P35280
5877	1,5,6,26,27,28,29,32	2,3,4,7,8,25,30,31	7	O35547,O70417,P02625,P05539,P20766,P29315,P82471
5878	1,5,6,26,27,28,30,31	2,3,4,7,8,25,29,32	0	
5879	1,5,6,26,27,28,30,32	2,3,4,7,8,25,29,31	4	P20766,P29315,P82471,Q8VD89
5880	1,5,6,26,27,28,31,32	2,3,4,7,8,25,29,30	3	O70417,P22006,P62804
5881	1,5,6,26,27,29,30,31	2,3,4,7,8,25,28,32	2	P36860,Q811M5
5882	1,5,6,26,27,29,30,32	2,3,4,7,8,25,28,31	6	P20766,P29315,P36376,P36860,P82471,Q811M5
5883	1,5,6,26,27,29,31,32	2,3,4,7,8,25,28,30	3	O70417,P36860,P62804
5884	1,5,6,26,27,30,31,32	2,3,4,7,8,25,28,29	3	P36860,P62804,Q8VD89
5885	1,5,6,26,28,29,30,31	2,3,4,7,8,25,27,32	3	O70417,P00762,P36860
5886	1,5,6,26,28,29,30,32	2,3,4,7,8,25,27,31	5	P20766,P29315,P36860,P82471,Q5BJY9
5887	1,5,6,26,28,29,31,32	2,3,4,7,8,25,27,30	2	O70417,P36860
5888	1,5,6,26,28,30,31,32	2,3,4,7,8,25,27,29	6	P22006,P36860,P62804,Q00715,Q4G075,Q8VD89
5889	1,5,6,26,29,30,31,32	2,3,4,7,8,25,27,28	5	O70417,P36860,P62804,Q4G075,Q5BJY9
5890	1,5,6,27,28,29,30,31	2,3,4,7,8,25,26,32	1	P05539
5891	1,5,6,27,28,29,30,32	2,3,4,7,8,25,26,31	3	P20766,P29315,Q5I0J9
5892	1,5,6,27,28,29,31,32	2,3,4,7,8,25,26,30	3	O35547,O70417,P05539
5893	1,5,6,27,28,30,31,32	2,3,4,7,8,25,26,29	3	P22006,P62804,Q8VD89
5894	1,5,6,27,29,30,31,32	2,3,4,7,8,25,26,28	1	P36860

5895	1,5,6,28,29,30,31,32	2,3,4,7,8,25,26,27	7	O70417,P20761,P20762,P36860,Q4G075,Q5BJY9,Q9WTT6
5896	1,5,7,8,25,26,27,28	2,3,4,6,29,30,31,32	4	O55145,P26342,P80299,Q9WTT6
5897	1,5,7,8,25,26,27,29	2,3,4,6,28,30,31,32	5	P06760,P22006,Q4G075,Q9QX74,Q9WTT6
5898	1,5,7,8,25,26,27,30	2,3,4,6,28,29,31,32	1	P80299
5899	1,5,7,8,25,26,27,31	2,3,4,6,28,29,30,32	4	O55145,P23739,Q9WTT6,Q9Z0V6
5900	1,5,7,8,25,26,27,32	2,3,4,6,28,29,30,31	1	P13432
5901	1,5,7,8,25,26,28,29	2,3,4,6,27,30,31,32	6	P06760,P22006,P80299,Q6P6R2,Q9QX74,Q9WTT6
5902	1,5,7,8,25,26,28,30	2,3,4,6,27,29,31,32	2	P35745,P80299
5903	1,5,7,8,25,26,28,31	2,3,4,6,27,29,30,32	5	O55145,P00714,P35745,Q9WTT6,Q9Z0V6
5904	1,5,7,8,25,26,28,32	2,3,4,6,27,29,30,31	1	P13432
5905	1,5,7,8,25,26,29,30	2,3,4,6,27,28,31,32	5	P06760,P22006,P35745,P80299,Q9QX74
5906	1,5,7,8,25,26,29,31	2,3,4,6,27,28,30,32	2	P06760,Q9QX74
5907	1,5,7,8,25,26,29,32	2,3,4,6,27,28,30,31	3	P06760,P13432,Q9QX74
5908	1,5,7,8,25,26,30,31	2,3,4,6,27,28,29,32	2	P35745,Q9WVK7
5909	1,5,7,8,25,26,30,32	2,3,4,6,27,28,29,31	1	Q9WVK7
5910	1,5,7,8,25,26,31,32	2,3,4,6,27,28,29,30	3	P13432,Q80WL1,Q9WVK7
5911	1,5,7,8,25,27,28,29	2,3,4,6,26,30,31,32	7	P06760,P22006,Q4G075,Q812E4,Q99MH3,Q9WTT6,Q9WUC4
5912	1,5,7,8,25,27,28,30	2,3,4,6,26,29,31,32	0	
5913	1,5,7,8,25,27,28,31	2,3,4,6,26,29,30,32	2	O55145,P23739
5914	1,5,7,8,25,27,28,32	2,3,4,6,26,29,30,31	2	Q6AYR9,Q812E4

5915	1,5,7,8,25,<br>27,29,30	2,3,4,6,26,<br>28,31,32	5 P06760,P22006,Q4G075,Q6IMF3,Q6P6Q2
5916	1,5,7,8,25,<br>27,29,31	2,3,4,6,26,<br>28,30,32	2 P06760,Q4G075
5917	1,5,7,8,25,<br>27,29,32	2,3,4,6,26,<br>28,30,31	1 P06760
5918	1,5,7,8,25,<br>27,30,31	2,3,4,6,26,<br>28,29,32	3 P23739,Q6IG05,Q6IMF3
5919	1,5,7,8,25,<br>27,30,32	2,3,4,6,26,<br>28,29,31	0
5920	1,5,7,8,25,<br>27,31,32	2,3,4,6,26,<br>28,29,30	2 P23739,Q80WL1
5921	1,5,7,8,25,<br>28,29,30	2,3,4,6,26,<br>27,31,32	2 P06760,P35745
5922	1,5,7,8,25,<br>28,29,31	2,3,4,6,26,<br>27,30,32	2 P06760,P35745
5923	1,5,7,8,25,<br>28,29,32	2,3,4,6,26,<br>27,30,31	3 P06760,P30120,Q812E4
5924	1,5,7,8,25,<br>28,30,31	2,3,4,6,26,<br>27,29,32	1 P35745
5925	1,5,7,8,25,<br>28,30,32	2,3,4,6,26,<br>27,29,31	1 P35745
5926	1,5,7,8,25,<br>28,31,32	2,3,4,6,26,<br>27,29,30	3 P21588,Q80WL1,Q811M5
5927	1,5,7,8,25,<br>29,30,31	2,3,4,6,26,<br>27,28,32	3 P35745,Q6IMF3,Q9WVK7
5928	1,5,7,8,25,<br>29,30,32	2,3,4,6,26,<br>27,28,31	2 Q6IMF3,Q9WVK7
5929	1,5,7,8,25,<br>29,31,32	2,3,4,6,26,<br>27,28,30	3 P21588,Q80WL1,Q9WVK7
5930	1,5,7,8,25,<br>30,31,32	2,3,4,6,26,<br>27,28,29	3 P09656,P35745,Q9WVK7
5931	1,5,7,8,26,<br>27,28,29	2,3,4,6,25,<br>30,31,32	8 P06760,P29315,P47967,Q4G075,Q6P6R2,Q812E4,Q9QX74,Q9WTT6
5932	1,5,7,8,26,<br>27,28,30	2,3,4,6,25,<br>29,31,32	3 P29315,P35745,Q9WTT6
5933	1,5,7,8,26,<br>27,28,31	2,3,4,6,25,<br>29,30,32	3 O55145,P35745,Q9WTT6
5934	1,5,7,8,26,<br>27,28,32	2,3,4,6,25,<br>29,30,31	4 P29315,Q6AYR9,Q9QWN8,Q9WTT6

5935	1,5,7,8,26, 27,29,30	2,3,4,6,25, 28,31,32	4	P06760,P29315,Q9QX74,Q9WTT6
5936	1,5,7,8,26, 27,29,31	2,3,4,6,25, 28,30,32	3	P06760,Q9QX74,Q9WTT6
5937	1,5,7,8,26, 27,29,32	2,3,4,6,25, 28,30,31	3	P06760,P29315,Q9QX74
5938	1,5,7,8,26, 27,30,31	2,3,4,6,25, 28,29,32	2	P23739,P35745
5939	1,5,7,8,26, 27,30,32	2,3,4,6,25, 28,29,31	1	P29315
5940	1,5,7,8,26, 27,31,32	2,3,4,6,25, 28,29,30	1	P23739
5941	1,5,7,8,26, 28,29,30	2,3,4,6,25, 27,31,32	6	P06760,P21674,P29315,P35745,Q9QX74,Q9WTT6
5942	1,5,7,8,26, 28,29,31	2,3,4,6,25, 27,30,32	6	P06760,P21674,P35745,Q6P6R2,Q9QX74,Q9WTT6
5943	1,5,7,8,26, 28,29,32	2,3,4,6,25, 27,30,31	5	P06760,P29315,P30120,Q9QX74,Q9QYP1
5944	1,5,7,8,26, 28,30,31	2,3,4,6,25, 27,29,32	1	P35745
5945	1,5,7,8,26, 28,30,32	2,3,4,6,25, 27,29,31	2	P29315,P35745
5946	1,5,7,8,26, 28,31,32	2,3,4,6,25, 27,29,30	1	P35745
5947	1,5,7,8,26, 29,30,31	2,3,4,6,25, 27,28,32	4	P06760,P35745,P49134,Q9QX74
5948	1,5,7,8,26, 29,30,32	2,3,4,6,25, 27,28,31	2	P29315,P35745
5949	1,5,7,8,26, 29,31,32	2,3,4,6,25, 27,28,30	2	P06760,Q9QX74
5950	1,5,7,8,26, 30,31,32	2,3,4,6,25, 27,28,29	3	B2RYW9,P35745,Q9WVK7
5951	1,5,7,8,27, 28,29,30	2,3,4,6,25, 26,31,32	8	P06760,P19218,P29315,P35745,P47967,Q4G075,Q6IMF3,Q812E4
5952	1,5,7,8,27, 28,29,31	2,3,4,6,25, 26,30,32	4	D3ZUC6,P06760,Q06000,Q812E4
5953	1,5,7,8,27, 28,29,32	2,3,4,6,25, 26,30,31	10	O35547,P06760,P08010,P29315,P30120,P47727,P47967,Q6AYR9,Q812E4,Q9Z2L0
5954	1,5,7,8,27, 28,30,31	2,3,4,6,25, 26,29,32	3	P19218,P23739,P35745

5955	1,5,7,8,27,28,30,32	2,3,4,6,25,26,29,31	3	P19218,P29315,P35745
5956	1,5,7,8,27,28,31,32	2,3,4,6,25,26,29,30	2	P23739,Q80WL1
5957	1,5,7,8,27,29,30,31	2,3,4,6,25,26,28,32	6	P06760,P08649,P35745,Q06000,Q6IMF3,Q6P6Q2
5958	1,5,7,8,27,29,30,32	2,3,4,6,25,26,28,31	3	P29315,Q6IMF3,Q6P6Q2
5959	1,5,7,8,27,29,31,32	2,3,4,6,25,26,28,30	0	
5960	1,5,7,8,27,30,31,32	2,3,4,6,25,26,28,29	4	P08649,P23739,P35745,Q6IMF3
5961	1,5,7,8,28,29,30,31	2,3,4,6,25,26,27,32	3	P21674,P35745,Q06000
5962	1,5,7,8,28,29,30,32	2,3,4,6,25,26,27,31	2	P29315,P35745
5963	1,5,7,8,28,29,31,32	2,3,4,6,25,26,27,30	2	P21588,P35745
5964	1,5,7,8,28,30,31,32	2,3,4,6,25,26,27,29	1	P35745
5965	1,5,7,8,29,30,31,32	2,3,4,6,25,26,27,28	4	P08649,P35745,P36860,Q6IMF3
5966	1,5,7,25,26,27,28,29	2,3,4,6,8,30,31,32	5	P02625,P20646,P97615,Q63532,Q9WUC4
5967	1,5,7,25,26,27,28,30	2,3,4,6,8,29,31,32	0	
5968	1,5,7,25,26,27,28,31	2,3,4,6,8,29,30,32	1	O55145
5969	1,5,7,25,26,27,28,32	2,3,4,6,8,29,30,31	1	Q5RJR2
5970	1,5,7,25,26,27,29,30	2,3,4,6,8,28,31,32	3	P22006,P36376,Q63532
5971	1,5,7,25,26,27,29,31	2,3,4,6,8,28,30,32	0	
5972	1,5,7,25,26,27,29,32	2,3,4,6,8,28,30,31	2	P36376,Q63532
5973	1,5,7,25,26,27,30,31	2,3,4,6,8,28,29,32	0	
5974	1,5,7,25,26,27,30,32	2,3,4,6,8,28,29,31	3	P36376,Q63532,Q9R168

5975	1,5,7,25,26,27,31,32	2,3,4,6,8,28,29,30	1 P23739
5976	1,5,7,25,26,28,29,30	2,3,4,6,8,27,31,32	1 Q63532
5977	1,5,7,25,26,28,29,31	2,3,4,6,8,27,30,32	1 Q01205
5978	1,5,7,25,26,28,29,32	2,3,4,6,8,27,30,31	1 Q63532
5979	1,5,7,25,26,28,30,31	2,3,4,6,8,27,29,32	2 P00714,P35745
5980	1,5,7,25,26,28,30,32	2,3,4,6,8,27,29,31	3 Q5PQL7,Q62975,Q9R168
5981	1,5,7,25,26,28,31,32	2,3,4,6,8,27,29,30	0
5982	1,5,7,25,26,29,30,31	2,3,4,6,8,27,28,32	2 Q9QWN8,Q9WVK7
5983	1,5,7,25,26,29,30,32	2,3,4,6,8,27,28,31	5 P36376,Q5BJY9,Q63532,Q9R168,Q9WVK7
5984	1,5,7,25,26,29,31,32	2,3,4,6,8,27,28,30	3 O70417,P19218,Q9WVK7
5985	1,5,7,25,26,30,31,32	2,3,4,6,8,27,28,29	3 P25809,Q00715,Q9WVK7
5986	1,5,7,25,27,28,29,30	2,3,4,6,8,26,31,32	3 Q05702,Q63532,Q99MH3
5987	1,5,7,25,27,28,29,31	2,3,4,6,8,26,30,32	2 P97615,Q99MH3
5988	1,5,7,25,27,28,29,32	2,3,4,6,8,26,30,31	7 Q05702,Q62761;Q62762;Q62763,Q63532,Q6AY61,Q812E4,Q99MH3,Q9WUC4
5989	1,5,7,25,27,28,30,31	2,3,4,6,8,26,29,32	1 P23739
5990	1,5,7,25,27,28,30,32	2,3,4,6,8,26,29,31	1 Q05702
5991	1,5,7,25,27,28,31,32	2,3,4,6,8,26,29,30	1 P23739
5992	1,5,7,25,27,29,30,31	2,3,4,6,8,26,28,32	2 O54728,P08649
5993	1,5,7,25,27,29,30,32	2,3,4,6,8,26,28,31	3 P08649,P36376,Q63532
5994	1,5,7,25,27,29,31,32	2,3,4,6,8,26,28,30	1 P08649

5995	1,5,7,25,27,30,31,32	2,3,4,6,8,26,28,29	2 P08649,P23739
5996	1,5,7,25,28,29,30,31	2,3,4,6,8,26,27,32	4 P08649,P17559,P35745,Q5BJY9
5997	1,5,7,25,28,29,30,32	2,3,4,6,8,26,27,31	4 P17559,Q5BJY9,Q63532,Q9R168
5998	1,5,7,25,28,29,31,32	2,3,4,6,8,26,27,30	3 P17559,P20762,Q5BJY9
5999	1,5,7,25,28,30,31,32	2,3,4,6,8,26,27,29	4 P17559,P25809,Q5BJY9,Q62975
6000	1,5,7,25,29,30,31,32	2,3,4,6,8,26,27,28	6 P08649,P17559,P20762,P36860,Q5BJY9,Q9WVK7
6001	1,5,7,26,27,28,29,30	2,3,4,6,8,25,31,32	5 P29315,P36376,P47967,Q05702,Q63532
6002	1,5,7,26,27,28,29,31	2,3,4,6,8,25,30,32	3 O70417,P02625,P97615
6003	1,5,7,26,27,28,29,32	2,3,4,6,8,25,30,31	7 P02625,P29315,P36376,P47967,Q62761;Q62762;Q62763,Q63532,Q812E4
6004	1,5,7,26,27,28,30,31	2,3,4,6,8,25,29,32	0
6005	1,5,7,26,27,28,30,32	2,3,4,6,8,25,29,31	2 P29315,P36376
6006	1,5,7,26,27,28,31,32	2,3,4,6,8,25,29,30	0
6007	1,5,7,26,27,29,30,31	2,3,4,6,8,25,28,32	3 P02631,P08649,P36860
6008	1,5,7,26,27,29,30,32	2,3,4,6,8,25,28,31	4 P08649,P29315,P36376,Q63532
6009	1,5,7,26,27,29,31,32	2,3,4,6,8,25,28,30	2 O70417,P08649
6010	1,5,7,26,27,30,31,32	2,3,4,6,8,25,28,29	2 P08649,P36860
6011	1,5,7,26,28,29,30,31	2,3,4,6,8,25,27,32	3 P00762,P35745,P98166
6012	1,5,7,26,28,29,30,32	2,3,4,6,8,25,27,31	5 P17559,P29315,P36376,Q5BJY9,Q63532
6013	1,5,7,26,28,29,31,32	2,3,4,6,8,25,27,30	2 O70417,P17559
6014	1,5,7,26,28,30,31,32	2,3,4,6,8,25,27,29	1 P35745

6015	1,5,7,26,29,30,31,32	2,3,4,6,8,25,27,28	6	O70417,P08649,P17559,P36860,Q5BJY9,Q9WVK7
6016	1,5,7,27,28,29,30,31	2,3,4,6,8,25,26,32	2	P08649,Q06000
6017	1,5,7,27,28,29,30,32	2,3,4,6,8,25,26,31	5	P08649,P29315,P36376,Q05702,Q63532
6018	1,5,7,27,28,29,31,32	2,3,4,6,8,25,26,30	4	O70417,P08649,Q62761;Q62762;Q62763,Q6AY61
6019	1,5,7,27,28,30,31,32	2,3,4,6,8,25,26,29	2	P08649,P23739
6020	1,5,7,27,29,30,31,32	2,3,4,6,8,25,26,28	3	P08649,P36860,Q99PP0
6021	1,5,7,28,29,30,31,32	2,3,4,6,8,25,26,27	5	P08649,P17559,P35745,P36860,Q5BJY9
6022	1,5,8,25,26,27,28,29	2,3,4,6,7,30,31,32	7	P00714,P06760,P20766,P26342,P80299,P97615,Q6P6R2
6023	1,5,8,25,26,27,28,30	2,3,4,6,7,29,31,32	4	P00714,P20766,P26342,P80299
6024	1,5,8,25,26,27,28,31	2,3,4,6,7,29,30,32	5	O55145,P00714,P23739,P26342,Q80WL1
6025	1,5,8,25,26,27,28,32	2,3,4,6,7,29,30,31	5	P20766,P26342,P80299,Q80WL1,Q9QWN8
6026	1,5,8,25,26,27,29,30	2,3,4,6,7,28,31,32	3	P20766,P22006,P80299
6027	1,5,8,25,26,27,29,31	2,3,4,6,7,28,30,32	1	Q80WL1
6028	1,5,8,25,26,27,29,32	2,3,4,6,7,28,30,31	2	P13432,Q80WL1
6029	1,5,8,25,26,27,30,31	2,3,4,6,7,28,29,32	1	P23739
6030	1,5,8,25,26,27,30,32	2,3,4,6,7,28,29,31	1	P80299
6031	1,5,8,25,26,27,31,32	2,3,4,6,7,28,29,30	3	P13432,P23739,Q80WL1
6032	1,5,8,25,26,28,29,30	2,3,4,6,7,27,31,32	3	P00714,P20766,P80299
6033	1,5,8,25,26,28,29,31	2,3,4,6,7,27,30,32	2	P00714,Q80WL1
6034	1,5,8,25,26,28,29,32	2,3,4,6,7,27,30,31	4	P13432,P20766,P80299,Q80WL1

6035	1,5,8,25,26,28,30,31	2,3,4,6,7,27,29,32	3	P00714,P35745,P80299
6036	1,5,8,25,26,28,30,32	2,3,4,6,7,27,29,31	2	P20766,P80299
6037	1,5,8,25,26,28,31,32	2,3,4,6,7,27,29,30	1	Q80WL1
6038	1,5,8,25,26,29,30,31	2,3,4,6,7,27,28,32	1	P11598
6039	1,5,8,25,26,29,30,32	2,3,4,6,7,27,28,31	3	P00507,P20766,P80299
6040	1,5,8,25,26,29,31,32	2,3,4,6,7,27,28,30	3	P00507,P13432,Q80WL1
6041	1,5,8,25,26,30,31,32	2,3,4,6,7,27,28,29	4	P00507,Q00715,Q63617,Q9WVK7
6042	1,5,8,25,27,28,29,30	2,3,4,6,7,26,31,32	2	P20766,Q9WVH8
6043	1,5,8,25,27,28,29,31	2,3,4,6,7,26,30,32	2	P97615,Q80WL1
6044	1,5,8,25,27,28,29,32	2,3,4,6,7,26,30,31	4	P20766,Q6AYR9,Q80WL1,Q812E4
6045	1,5,8,25,27,28,30,31	2,3,4,6,7,26,29,32	1	P23739
6046	1,5,8,25,27,28,30,32	2,3,4,6,7,26,29,31	1	P20766
6047	1,5,8,25,27,28,31,32	2,3,4,6,7,26,29,30	2	P23739,Q80WL1
6048	1,5,8,25,27,29,30,31	2,3,4,6,7,26,28,32	2	P23739,Q6IMF3
6049	1,5,8,25,27,29,30,32	2,3,4,6,7,26,28,31	2	P20766,Q6IMF3
6050	1,5,8,25,27,29,31,32	2,3,4,6,7,26,28,30	2	P23739,Q80WL1
6051	1,5,8,25,27,30,31,32	2,3,4,6,7,26,28,29	1	P23739
6052	1,5,8,25,28,29,30,31	2,3,4,6,7,26,27,32	1	P35745
6053	1,5,8,25,28,29,30,32	2,3,4,6,7,26,27,31	2	P20766,Q62894
6054	1,5,8,25,28,29,31,32	2,3,4,6,7,26,27,30	2	P21588,Q80WL1

6055	1,5,8,25,28,30,31,32	2,3,4,6,7,26,27,29	2	P21588,Q80WL1
6056	1,5,8,25,29,30,31,32	2,3,4,6,7,26,27,28	2	P21588,Q9WVK7
6057	1,5,8,26,27,28,29,30	2,3,4,6,7,25,31,32	2	P20766,P29315
6058	1,5,8,26,27,28,29,31	2,3,4,6,7,25,30,32	2	P26342,Q6P6R2
6059	1,5,8,26,27,28,29,32	2,3,4,6,7,25,30,31	6	P08010,P20766,P29315,Q6AYR9,Q812E4,Q9QWN8
6060	1,5,8,26,27,28,30,31	2,3,4,6,7,25,29,32	2	P23739,P35745
6061	1,5,8,26,27,28,30,32	2,3,4,6,7,25,29,31	4	B0LT89,P20766,P29315,Q9QWN8
6062	1,5,8,26,27,28,31,32	2,3,4,6,7,25,29,30	3	P23739,Q80WL1,Q9QWN8
6063	1,5,8,26,27,29,30,31	2,3,4,6,7,25,28,32	0	
6064	1,5,8,26,27,29,30,32	2,3,4,6,7,25,28,31	2	P20766,P29315
6065	1,5,8,26,27,29,31,32	2,3,4,6,7,25,28,30	1	Q80WL1
6066	1,5,8,26,27,30,31,32	2,3,4,6,7,25,28,29	1	P23739
6067	1,5,8,26,28,29,30,31	2,3,4,6,7,25,27,32	1	P35745
6068	1,5,8,26,28,29,30,32	2,3,4,6,7,25,27,31	3	P20766,P29315,Q62894
6069	1,5,8,26,28,29,31,32	2,3,4,6,7,25,27,30	1	Q80WL1
6070	1,5,8,26,28,30,31,32	2,3,4,6,7,25,27,29	1	P35745
6071	1,5,8,26,29,30,31,32	2,3,4,6,7,25,27,28	3	P36860,P63081,Q5QE79
6072	1,5,8,27,28,29,30,31	2,3,4,6,7,25,26,32	1	Q9WVH8
6073	1,5,8,27,28,29,30,32	2,3,4,6,7,25,26,31	6	P18298,P20766,P29315,Q6AYR9,Q812E4,Q9WVH8
6074	1,5,8,27,28,29,31,32	2,3,4,6,7,25,26,30	3	Q6AYR9,Q80WL1,Q812E4

6075	1,5,8,27,28,30,31,32	2,3,4,6,7,25,26,29	3	B0LT89,P23739,Q9WVH8
6076	1,5,8,27,29,30,31,32	2,3,4,6,7,25,26,28	3	P08649,P23739,Q6IMF3
6077	1,5,8,28,29,30,31,32	2,3,4,6,7,25,26,27	1	P35745
6078	1,5,25,26,27,28,29,30	2,3,4,6,7,8,31,32	6	O55004,P00714,P20766,P82471,P97615,Q63532
6079	1,5,25,26,27,28,29,31	2,3,4,6,7,8,30,32	6	O70417,P00714,P02631,P35280,P97615,Q99041
6080	1,5,25,26,27,28,29,32	2,3,4,6,7,8,30,31	6	O70417,P20766,P82471,P97615,Q5RJR2,Q63532
6081	1,5,25,26,27,28,30,31	2,3,4,6,7,8,29,32	5	O55004,P00714,P02631,P20761,P42854
6082	1,5,25,26,27,28,30,32	2,3,4,6,7,8,29,31	4	O70594,P20766,P82471,Q63532
6083	1,5,25,26,27,28,31,32	2,3,4,6,7,8,29,30	4	O70417,P23739,P62804,Q80WL1
6084	1,5,25,26,27,29,30,31	2,3,4,6,7,8,28,32	7	P02631,P11598,P22273,Q63617,Q99041,Q99PP0,Q9QZQ5
6085	1,5,25,26,27,29,30,32	2,3,4,6,7,8,28,31	7	P12020,P20766,P22273,P36376,P82471,Q63532,Q99PP0
6086	1,5,25,26,27,29,31,32	2,3,4,6,7,8,28,30	6	O70417,P02631,P22273,P62804,Q80WL1,Q99041
6087	1,5,25,26,27,30,31,32	2,3,4,6,7,8,28,29	10	P02631,P11598,P21674,P22273,P23739,P42854,P62804,Q00715,Q63617,Q99041
6088	1,5,25,26,28,29,30,31	2,3,4,6,7,8,27,32	10	O70417,P00714,P02631,P20761,P22273,P22282,P50115,P98166,Q99041,Q9QZQ5

6089	1,5,25,26,28,29,30,32	2,3,4,6,7,8,27,31	5	P20766,P22273,P50115,Q62894,Q63532
6090	1,5,25,26,28,29,31,32	2,3,4,6,7,8,27,30	7	O70417,P19218,P22273,P50115,P62804,Q80WL1,Q99041
6091	1,5,25,26,28,30,31,32	2,3,4,6,7,8,27,29	8	P20761,P22273,P25809,P50115,P62804,Q00715,Q4G075,Q99041
6092	1,5,25,26,29,30,31,32	2,3,4,6,7,8,27,28	16	O70417,P00507,P02631,P06761,P11598,P22273,P22282,P36860,P50115,P62804,Q00715,Q4G075,Q63617,Q99041,Q9QZQ5,Q9WVK7
6093	1,5,25,27,28,29,30,31	2,3,4,6,7,8,26,32	6	O55004,P02631,P20761,P20762,P97615,Q99PP0
6094	1,5,25,27,28,29,30,32	2,3,4,6,7,8,26,31	5	P20762,P20766,Q05702,Q63532,Q99PP0
6095	1,5,25,27,28,29,31,32	2,3,4,6,7,8,26,30	6	O70417,P20761,P20762,P97615,Q80WL1,Q99041
6096	1,5,25,27,28,30,31,32	2,3,4,6,7,8,26,29	7	O70594,P20761,P20762,P22273,P23739,P42854,P62804
6097	1,5,25,27,29,30,31,32	2,3,4,6,7,8,26,28	8	P02631,P07150,P08649,P11598,P20762,P22273,Q99041,Q99PP0
6098	1,5,25,28,29,30,31,32	2,3,4,6,7,8,26,27	10	O70417,P17559,P20761,P20762,P22273,P50115,P50116,Q5BJY9,Q62894,Q99041
6099	1,5,26,27,28,29,30,31	2,3,4,6,7,8,25,32	5	O55004,O70417,P02631,Q99PP0,Q9QZQ5
6100	1,5,26,27,28,29,30,32	2,3,4,6,7,8,25,31	8	O70417,P02631,P12020,P20766,P29315,P36376,Q63532,Q99PP0
6101	1,5,26,27,28,29,31,32	2,3,4,6,7,8,25,30	2	O70417,P02631

6102	1,5,26,27,28,30,31,32	2,3,4,6,7,8,25,29	6	O70417,P02631,P22273,P23739,P42854,P62804
6103	1,5,26,27,29,30,31,32	2,3,4,6,7,8,25,28	7	O70417,P02631,P08649,P22273,P36860,P62804,Q99PP0
6104	1,5,26,28,29,30,31,32	2,3,4,6,7,8,25,27	10	O70417,P02631,P17559,P22273,P36860,P50115,Q4G075,Q5BJY9,Q5QE79,Q9QZQ5
6105	1,5,27,28,29,30,31,32	2,3,4,6,7,8,25,26	8	O70417,P02631,P08649,P20761,P20762,P50115,Q99PP0,Q9WVH8
6106	1,6,7,8,25,26,27,28	2,3,4,5,29,30,31,32	10	P06911,P07150,P19629,P22283,P26342,P63081,P70549,Q63598,Q9QW07,Q9WTT6
6107	1,6,7,8,25,26,27,29	2,3,4,5,28,30,31,32	4	P13432,P70549,Q9QX74,Q9WTT6
6108	1,6,7,8,25,26,27,30	2,3,4,5,28,29,31,32	0	
6109	1,6,7,8,25,26,27,31	2,3,4,5,28,29,30,32	4	P06911,P19629,P70549,Q62894
6110	1,6,7,8,25,26,27,32	2,3,4,5,28,29,30,31	3	P13432,P19629,Q9JHB9
6111	1,6,7,8,25,26,28,29	2,3,4,5,27,30,31,32	7	P06911,P13432,P70549,Q6P6R2,Q9QW07,Q9QX74,Q9WTT6
6112	1,6,7,8,25,26,28,30	2,3,4,5,27,29,31,32	5	P06911,P35745,P80299,Q5PQL7,Q9QW07
6113	1,6,7,8,25,26,28,31	2,3,4,5,27,29,30,32	7	P06911,P19629,P35745,P57113,P70549,Q63598,Q9QW07
6114	1,6,7,8,25,26,28,32	2,3,4,5,27,29,30,31	5	P08723,P13432,P19629,Q5PQL7,Q9QW07
6115	1,6,7,8,25,26,29,30	2,3,4,5,27,28,31,32	0	
6116	1,6,7,8,25,26,29,31	2,3,4,5,27,28,30,32	3	P13432,P70549,Q9QX74
6117	1,6,7,8,25,26,29,32	2,3,4,5,27,28,30,31	2	P13432,P70549
6118	1,6,7,8,25,26,30,31	2,3,4,5,27,28,29,32	2	P35745,Q9WVK7
6119	1,6,7,8,25,26,30,32	2,3,4,5,27,28,29,31	3	P13432,Q5PQL7,Q9WVK7

6120	1,6,7,8,25,26,31,32	2,3,4,5,27,28,29,30	3	P13432,P57113,Q9WVK7
6121	1,6,7,8,25,27,28,29	2,3,4,5,26,30,31,32	19	P05539,P06761,P06911,P0C0A9,P19629,P22283,P47727,P63081,P70549,Q4G075,Q5GRG2,Q63617,Q6AYR9,Q812E4,Q9JI85,Q9QW07,Q9WTT6,Q9WUC4,Q9Z2L0
6122	1,6,7,8,25,27,28,30	2,3,4,5,26,29,31,32	3	P06911,P19629,Q9QW07
6123	1,6,7,8,25,27,28,31	2,3,4,5,26,29,30,32	4	P06911,P19629,Q5GRG2,Q9QW07
6124	1,6,7,8,25,27,28,32	2,3,4,5,26,29,30,31	10	P06911,P08723,P0C0A9,P19629,P22283,Q6AYR9,Q812E4,Q9JHB9,Q9JI85,Q9QW07
6125	1,6,7,8,25,27,29,30	2,3,4,5,26,28,31,32	0	
6126	1,6,7,8,25,27,29,31	2,3,4,5,26,28,30,32	2	P06911,P70549
6127	1,6,7,8,25,27,29,32	2,3,4,5,26,28,30,31	1	P13432
6128	1,6,7,8,25,27,30,31	2,3,4,5,26,28,29,32	1	P06911
6129	1,6,7,8,25,27,30,32	2,3,4,5,26,28,29,31	1	Q9JHB9
6130	1,6,7,8,25,27,31,32	2,3,4,5,26,28,29,30	2	P13432,P19629
6131	1,6,7,8,25,28,29,30	2,3,4,5,26,27,31,32	3	P06911,P35745,Q9QW07
6132	1,6,7,8,25,28,29,31	2,3,4,5,26,27,30,32	7	P06911,P12020,P21674,P35745,P70549,Q5GRG2,Q9QW07
6133	1,6,7,8,25,28,29,32	2,3,4,5,26,27,30,31	12	P06911,P08723,P13432,P22283,P24368,P30120,P47727,Q63617,Q812E4,Q9JI85,Q9QW07,Q9Z2L0
6134	1,6,7,8,25,28,30,31	2,3,4,5,26,27,29,32	4	P06911,P35745,Q5GRG2,Q9QW07
6135	1,6,7,8,25,28,30,32	2,3,4,5,26,27,29,31	4	P06911,P35745,Q5PQL7,Q9QW07
6136	1,6,7,8,25,28,31,32	2,3,4,5,26,27,29,30	6	P06911,P08723,P19629,P21588,Q5GRG2,Q9QW07
6137	1,6,7,8,25,29,30,31	2,3,4,5,26,27,28,32	2	P06911,P35745
6138	1,6,7,8,25,29,30,32	2,3,4,5,26,27,28,31	0	
6139	1,6,7,8,25,29,31,32	2,3,4,5,26,27,28,30	3	P13432,P21588,P70549

6140	1,6,7,8,25,30,31,32	2,3,4,5,26,27,28,29	3	P35745,P47967,Q9WVK7
6141	1,6,7,8,26,27,28,29	2,3,4,5,25,30,31,32	16	P02782,P06761,P21674,P22283,P29315,P47727,P70549,Q63617,Q6AYR9,Q6P6R2,Q812E4,Q9JI85,Q9QW07,Q9QX74,Q9WTT6,Q9Z2L0
6142	1,6,7,8,26,27,28,30	2,3,4,5,25,29,31,32	5	P07150,P29315,P35745,Q9QW07,Q9WTT6
6143	1,6,7,8,26,27,28,31	2,3,4,5,25,29,30,32	6	iRT-Kit_WR_fusion,P07150,P70549,Q63598,Q9QW07,Q9WTT6
6144	1,6,7,8,26,27,28,32	2,3,4,5,25,29,30,31	11	P07150,P08723,P22282,P22283,P29315,Q63617,Q6AYR9,Q812E4,Q9JI85,Q9QW07,Q9QWN8
6145	1,6,7,8,26,27,29,30	2,3,4,5,25,28,31,32	3	P29315,Q811M5,Q9QX74
6146	1,6,7,8,26,27,29,31	2,3,4,5,25,28,30,32	3	P70549,Q63474,Q9QX74
6147	1,6,7,8,26,27,29,32	2,3,4,5,25,28,30,31	3	P13432,P29315,P70549
6148	1,6,7,8,26,27,30,31	2,3,4,5,25,28,29,32	1	P35745
6149	1,6,7,8,26,27,30,32	2,3,4,5,25,28,29,31	1	P29315
6150	1,6,7,8,26,27,31,32	2,3,4,5,25,28,29,30	0	
6151	1,6,7,8,26,28,29,30	2,3,4,5,25,27,31,32	4	P21674,P29315,P35745,Q9QW07
6152	1,6,7,8,26,28,29,31	2,3,4,5,25,27,30,32	8	P21674,P35745,P70549,Q63474,Q63617,Q6P6R2,Q9QW07,Q9QX74
6153	1,6,7,8,26,28,29,32	2,3,4,5,25,27,30,31	10	P08723,P21674,P22283,P29315,P30120,P70549,Q63617,Q812E4,Q9JI85,Q9QW07
6154	1,6,7,8,26,28,30,31	2,3,4,5,25,27,29,32	1	P35745
6155	1,6,7,8,26,28,30,32	2,3,4,5,25,27,29,31	4	O89117,P29315,P35745,Q9QW07
6156	1,6,7,8,26,28,31,32	2,3,4,5,25,27,29,30	8	O89117,P08723,P22006,P35745,P57113,P70549,Q6IG02,Q9QW07
6157	1,6,7,8,26,29,30,31	2,3,4,5,25,27,28,32	3	P35745,Q63474,Q9QX74
6158	1,6,7,8,26,29,30,32	2,3,4,5,25,27,28,31	2	P29315,P35745
6159	1,6,7,8,26,29,31,32	2,3,4,5,25,27,28,30	4	P13432,P57113,P70549,Q63474

6160	1,6,7,8,26,30,31,32	2,3,4,5,25,27,28,29	5	B2RYW9,O89117,P35745,P57113,Q9WVK7
6161	1,6,7,8,27,28,29,30	2,3,4,5,25,26,31,32	11	P19218,P21674,P22283,P29315,P35745,P47727,Q63617,Q6AYR9,Q812E4,Q9QW07,Q9Z2L0
6162	1,6,7,8,27,28,29,31	2,3,4,5,25,26,30,32	12	P05539,P21674,P22283,P47727,P70549,P97523,Q5GRG2,Q63474,Q63493,Q63617,Q812E4,Q9QW07
6163	1,6,7,8,27,28,29,32	2,3,4,5,25,26,30,31	19	O35547,P00758,P04905,P06761,P08723,P18298,P22282,P22283,P29315,P30120,P47727,Q63493,Q63617,Q6AYR9,Q812E4,Q8CFN2,Q9JI85,Q9QW07,Q9Z2L0
6164	1,6,7,8,27,28,30,31	2,3,4,5,25,26,29,32	2	P19218,P35745
6165	1,6,7,8,27,28,30,32	2,3,4,5,25,26,29,31	9	B0LT89,P08723,P19218,P29315,Q6AYR9,Q6P6S4,Q812E4,Q8VD89,Q9QW07
6166	1,6,7,8,27,28,31,32	2,3,4,5,25,26,29,30	9	P08723,P22006,P22283,Q63617,Q6AYR9,Q812E4,Q9JI85,Q9QW07,Q9QZK9
6167	1,6,7,8,27,29,30,31	2,3,4,5,25,26,28,32	2	P35745,Q63474
6168	1,6,7,8,27,29,30,32	2,3,4,5,25,26,28,31	1	P29315
6169	1,6,7,8,27,29,31,32	2,3,4,5,25,26,28,30	1	Q63474
6170	1,6,7,8,27,30,31,32	2,3,4,5,25,26,28,29	2	P22006,Q9QZK9
6171	1,6,7,8,28,29,30,31	2,3,4,5,25,26,27,32	4	P21674,P35745,Q63474,Q9QW07
6172	1,6,7,8,28,29,30,32	2,3,4,5,25,26,27,31	8	P08723,P21674,P29315,P30120,P35745,Q63617,Q812E4,Q9QW07
6173	1,6,7,8,28,29,31,32	2,3,4,5,25,26,27,30	11	P08723,P21588,P21674,P30120,P35745,P70549,Q63474,Q63493,Q63617,Q812E4,Q9QW07
6174	1,6,7,8,28,30,31,32	2,3,4,5,25,26,27,29	4	O89117,P22006,P35745,Q9QZK9
6175	1,6,7,8,29,30,31,32	2,3,4,5,25,26,27,28	3	P35745,P36860,Q63474
6176	1,6,7,25,26,27,28,29	2,3,4,5,8,30,31,32	7	P05539,P22283,P36376,P63081,P70549,Q9QW07,Q9WUC4
6177	1,6,7,25,26,27,28,30	2,3,4,5,8,29,31,32	0	
6178	1,6,7,25,26,27,28,31	2,3,4,5,8,29,30,32	4	O88797,P70549,Q63598,Q6IFU7
6179	1,6,7,25,26,27,28,32	2,3,4,5,8,29,30,31	3	P36376,Q5RJR2,Q6IG02

6180	1,6,7,25,26,27,29,30	2,3,4,5,8,28,31,32	3	P05539,P36376,Q811M5
6181	1,6,7,25,26,27,29,31	2,3,4,5,8,28,30,32	2	P05539,P70549
6182	1,6,7,25,26,27,29,32	2,3,4,5,8,28,30,31	4	P05539,P36376,P70549,Q6AYR8
6183	1,6,7,25,26,27,30,31	2,3,4,5,8,28,29,32	0	
6184	1,6,7,25,26,27,30,32	2,3,4,5,8,28,29,31	2	P36376,Q9R168
6185	1,6,7,25,26,27,31,32	2,3,4,5,8,28,29,30	1	Q6IFU7
6186	1,6,7,25,26,28,29,30	2,3,4,5,8,27,31,32	2	P05539,P36376
6187	1,6,7,25,26,28,29,31	2,3,4,5,8,27,30,32	4	P05539,P70549,Q01205,Q6IFU7
6188	1,6,7,25,26,28,29,32	2,3,4,5,8,27,30,31	6	P00731,P05539,P36376,P70549,Q6AYR8,Q9QW07
6189	1,6,7,25,26,28,30,31	2,3,4,5,8,27,29,32	1	P35745
6190	1,6,7,25,26,28,30,32	2,3,4,5,8,27,29,31	5	P00731,P36376,Q5PQL7,Q62975,Q9R168
6191	1,6,7,25,26,28,31,32	2,3,4,5,8,27,29,30	7	P00731,P22006,P70549,Q10758,Q6IFU7,Q6IFU8,Q6IG02
6192	1,6,7,25,26,29,30,31	2,3,4,5,8,27,28,32	2	P01835,Q9WVK7
6193	1,6,7,25,26,29,30,32	2,3,4,5,8,27,28,31	4	P01835,P36376,Q9R168,Q9WVK7
6194	1,6,7,25,26,29,31,32	2,3,4,5,8,27,28,30	5	P19218,P70549,Q10758,Q6IFU7,Q9WVK7
6195	1,6,7,25,26,30,31,32	2,3,4,5,8,27,28,29	6	B2RYW9,P08721,P25809,P47967,Q00715,Q9WVK7
6196	1,6,7,25,27,28,29,30	2,3,4,5,8,26,31,32	1	P05539
6197	1,6,7,25,27,28,29,31	2,3,4,5,8,26,30,32	3	O88797,P05539,P70549
6198	1,6,7,25,27,28,29,32	2,3,4,5,8,26,30,31	15	O35547,P00758,P05539,P0C0A9,P22283,P36376,Q5RJR2,Q62761;Q62762;Q62763,Q6AYR8,Q6AYR9,Q812E4,Q8CGS4,Q9QW07,Q9WUC4,Q9Z2L0
6199	1,6,7,25,27,28,30,31	2,3,4,5,8,26,29,32	0	

6200	1,6,7,25,27,28,30,32	2,3,4,5,8,26,29,31	1	Q5RJR2
6201	1,6,7,25,27,28,31,32	2,3,4,5,8,26,29,30	4	O88797,P19629,P22006,Q5RJR2
6202	1,6,7,25,27,29,30,31	2,3,4,5,8,26,28,32	2	O54728,P05539
6203	1,6,7,25,27,29,30,32	2,3,4,5,8,26,28,31	3	P05539,P36376,Q5RLM2
6204	1,6,7,25,27,29,31,32	2,3,4,5,8,26,28,30	2	P05539,P20762
6205	1,6,7,25,27,30,31,32	2,3,4,5,8,26,28,29	0	
6206	1,6,7,25,28,29,30,31	2,3,4,5,8,26,27,32	2	P05539,Q5BJY9
6207	1,6,7,25,28,29,30,32	2,3,4,5,8,26,27,31	6	P01835,P05539,P36376,Q5BJY9,Q62975,Q9R168
6208	1,6,7,25,28,29,31,32	2,3,4,5,8,26,27,30	4	P05539,P20762,P70549,Q5BJY9
6209	1,6,7,25,28,30,31,32	2,3,4,5,8,26,27,29	4	P22006,P25809,Q5BJY9,Q62975
6210	1,6,7,25,29,30,31,32	2,3,4,5,8,26,27,28	6	P01835,P20762,P25809,P36860,Q5BJY9,Q9WVK7
6211	1,6,7,26,27,28,29,30	2,3,4,5,8,25,31,32	5	P01835,P05539,P29315,P36376,Q811M5
6212	1,6,7,26,27,28,29,31	2,3,4,5,8,25,30,32	6	O88797,P01835,P02625,P05539,P70549,Q63474
6213	1,6,7,26,27,28,29,32	2,3,4,5,8,25,30,31	10	O35547,P00758,P01835,P05539,P22283,P29315,P36376,P70549,Q6AYR9,Q812E4
6214	1,6,7,26,27,28,30,31	2,3,4,5,8,25,29,32	0	
6215	1,6,7,26,27,28,30,32	2,3,4,5,8,25,29,31	3	P29315,P36376,Q8VD89
6216	1,6,7,26,27,28,31,32	2,3,4,5,8,25,29,30	4	O88797,P22006,Q6IFU7,Q6IG02
6217	1,6,7,26,27,29,30,31	2,3,4,5,8,25,28,32	5	P01835,P05539,P36376,P36860,Q811M5
6218	1,6,7,26,27,29,30,32	2,3,4,5,8,25,28,31	4	P01835,P29315,P36376,Q811M5
6219	1,6,7,26,27,29,31,32	2,3,4,5,8,25,28,30	5	P01835,P05539,P36376,P70549,Q63474

6220	1,6,7,26,27,30,31,32	2,3,4,5,8,25,28,29	3 P22006,P36376,P36860
6221	1,6,7,26,28,29,30,31	2,3,4,5,8,25,27,32	6 P00762,P01835,P05539,P35745,Q63474,Q6DGG1
6222	1,6,7,26,28,29,30,32	2,3,4,5,8,25,27,31	4 P01835,P29315,P36376,Q9R168
6223	1,6,7,26,28,29,31,32	2,3,4,5,8,25,27,30	8 P01835,P05539,P22006,P70549,Q63474,Q6IFU7,Q6IFU8,Q6IG02
6224	1,6,7,26,28,30,31,32	2,3,4,5,8,25,27,29	4 O89117,P01835,P22006,P35745
6225	1,6,7,26,29,30,31,32	2,3,4,5,8,25,27,28	4 P01835,P36376,P36860,Q63474
6226	1,6,7,27,28,29,30,31	2,3,4,5,8,25,26,32	3 P01835,P05539,Q63474
6227	1,6,7,27,28,29,30,32	2,3,4,5,8,25,26,31	9 O35547,P00758,P01835,P05539,P22283,P29315,P36376,Q6AYR9,Q812E4
6228	1,6,7,27,28,29,31,32	2,3,4,5,8,25,26,30	9 O35547,O88797,P01835,P05539,P22006,P22283,Q63474,Q6AYR9,Q812E4
6229	1,6,7,27,28,30,31,32	2,3,4,5,8,25,26,29	2 P22006,Q8VD89
6230	1,6,7,27,29,30,31,32	2,3,4,5,8,25,26,28	3 P01835,P36376,P36860
6231	1,6,7,28,29,30,31,32	2,3,4,5,8,25,26,27	5 P01835,P22006,P36860,Q5BJY9,Q63474
6232	1,6,8,25,26,27,28,29	2,3,4,5,7,30,31,32	7 P26342,P47727,P70549,Q6AYR9,Q6P6R2,Q812E4,Q9QW07
6233	1,6,8,25,26,27,28,30	2,3,4,5,7,29,31,32	4 P15399,P26342,P80299,Q9QW07
6234	1,6,8,25,26,27,28,31	2,3,4,5,7,29,30,32	3 P19629,P26342,P70549
6235	1,6,8,25,26,27,28,32	2,3,4,5,7,29,30,31	6 P13432,P15399,P19629,Q6AYR9,Q9QW07,Q9QWN8
6236	1,6,8,25,26,27,29,30	2,3,4,5,7,28,31,32	1 Q811M5
6237	1,6,8,25,26,27,29,31	2,3,4,5,7,28,30,32	2 P13432,P70549
6238	1,6,8,25,26,27,29,32	2,3,4,5,7,28,30,31	2 P13432,P70549
6239	1,6,8,25,26,27,30,31	2,3,4,5,7,28,29,32	4 P47967,P54921,P97840,Q6AY61

6240	1,6,8,25,2 6,27,30,32	2,3,4,5,7,2 8,29,31	3	P13432,P15399,P54921
6241	1,6,8,25,2 6,27,31,32	2,3,4,5,7,2 8,29,30	4	P13432,P47967,P54921,P97840
6242	1,6,8,25,2 6,28,29,30	2,3,4,5,7,2 7,31,32	3	P20766,P80299,Q9QW07
6243	1,6,8,25,2 6,28,29,31	2,3,4,5,7,2 7,30,32	2	P70549,Q63474
6244	1,6,8,25,2 6,28,29,32	2,3,4,5,7,2 7,30,31	5	P13432,P20766,P30120,P70549,Q9QW07
6245	1,6,8,25,2 6,28,30,31	2,3,4,5,7,2 7,29,32	1	P35745
6246	1,6,8,25,2 6,28,30,32	2,3,4,5,7,2 7,29,31	4	P80299,Q5PQL7,Q68G31,Q9QW07
6247	1,6,8,25,2 6,28,31,32	2,3,4,5,7,2 7,29,30	4	P00731,P13432,P47967,P70549
6248	1,6,8,25,2 6,29,30,31	2,3,4,5,7,2 7,28,32	2	P47967,P97840
6249	1,6,8,25,2 6,29,30,32	2,3,4,5,7,2 7,28,31	3	P00507,P13432,Q68G31
6250	1,6,8,25,2 6,29,31,32	2,3,4,5,7,2 7,28,30	5	P00507,P13432,P47967,P70549,P97840
6251	1,6,8,25,2 6,30,31,32	2,3,4,5,7,2 7,28,29	9	P00507,P13432,P47967,P54921,P70712,P97840,Q00715,Q5I0D1,Q9WVK7
6252	1,6,8,25,2 7,28,29,30	2,3,4,5,7,2 6,31,32	3	P20766,Q812E4,Q9QW07
6253	1,6,8,25,2 7,28,29,31	2,3,4,5,7,2 6,30,32	5	P05539,P19629,P70549,Q5GRG2,Q812E4
6254	1,6,8,25,2 7,28,29,32	2,3,4,5,7,2 6,30,31	14	O35547,P00758,P08723,P13432,P19629,P20766,P30120,P47727,Q6AYR9,Q812E4,Q8CFN2,Q9JI 85,Q9QW07,Q9Z2L0
6255	1,6,8,25,2 7,28,30,31	2,3,4,5,7,2 6,29,32	1	P19629
6256	1,6,8,25,2 7,28,30,32	2,3,4,5,7,2 6,29,31	5	B0LT89,P19629,P20766,Q6AYR9,Q9QW07
6257	1,6,8,25,2 7,28,31,32	2,3,4,5,7,2 6,29,30	4	P19629,Q6AYR9,Q80WL1,Q9WUW8
6258	1,6,8,25,2 7,29,30,31	2,3,4,5,7,2 6,28,32	1	P97840
6259	1,6,8,25,2 7,29,30,32	2,3,4,5,7,2 6,28,31	1	Q5RLM2

6260	1,6,8,25,27,29,31,32	2,3,4,5,7,26,28,30	3	P13432,P97840,Q80WL1
6261	1,6,8,25,27,30,31,32	2,3,4,5,7,26,28,29	3	P47967,P54921,P97840
6262	1,6,8,25,28,29,30,31	2,3,4,5,7,26,27,32	1	P35745
6263	1,6,8,25,28,29,30,32	2,3,4,5,7,26,27,31	3	P20766,Q68G31,Q9QW07
6264	1,6,8,25,28,29,31,32	2,3,4,5,7,26,27,30	6	P13432,P21588,P30120,P70549,Q63474,Q80WL1
6265	1,6,8,25,28,30,31,32	2,3,4,5,7,26,27,29	3	P21588,P22006,P47967
6266	1,6,8,25,29,30,31,32	2,3,4,5,7,26,27,28	4	P21588,P47967,P97840,Q5RLM2
6267	1,6,8,26,27,28,29,30	2,3,4,5,7,25,31,32	5	P15399,P29315,Q6P6R2,Q811M5,Q9QW07
6268	1,6,8,26,27,28,29,31	2,3,4,5,7,25,30,32	3	P70549,Q63474,Q6P6R2
6269	1,6,8,26,27,28,29,32	2,3,4,5,7,25,30,31	15	O35547,P00758,P08010,P08723,P29315,P30120,P47727,P70549,Q6AYR9,Q6P6R2,Q812E4,Q8CFN2,Q9JI85,Q9QW07,Q9QWN8
6270	1,6,8,26,27,28,30,31	2,3,4,5,7,25,29,32	1	P15399
6271	1,6,8,26,27,28,30,32	2,3,4,5,7,25,29,31	7	B0LT89,P15399,P29315,Q6AYR9,Q6P6S4,Q8VD89,Q9QWN8
6272	1,6,8,26,27,28,31,32	2,3,4,5,7,25,29,30	5	B0LT89,P15399,P22006,Q6AYR9,Q9QWN8
6273	1,6,8,26,27,29,30,31	2,3,4,5,7,25,28,32	2	Q63474,Q811M5
6274	1,6,8,26,27,29,30,32	2,3,4,5,7,25,28,31	2	P29315,Q811M5
6275	1,6,8,26,27,29,31,32	2,3,4,5,7,25,28,30	2	P70549,Q63474
6276	1,6,8,26,27,30,31,32	2,3,4,5,7,25,28,29	3	O89117,P22006,P54921
6277	1,6,8,26,28,29,30,31	2,3,4,5,7,25,27,32	2	P35745,Q63474
6278	1,6,8,26,28,29,30,32	2,3,4,5,7,25,27,31	5	P20766,P29315,Q63474,Q68G31,Q9QW07
6279	1,6,8,26,28,29,31,32	2,3,4,5,7,25,27,30	3	P30120,P70549,Q63474

6280	1,6,8,26,28,30,31,32	2,3,4,5,7,25,27,29	5	O89117,P22006,P35745,Q63474,Q8VD89
6281	1,6,8,26,29,30,31,32	2,3,4,5,7,25,27,28	2	P36860,Q63474
6282	1,6,8,27,28,29,30,31	2,3,4,5,7,25,26,32	1	Q63474
6283	1,6,8,27,28,29,30,32	2,3,4,5,7,25,26,31	11	B0LT89,O35547,P00758,P18298,P20766,P29315,Q6AYR9,Q6P6S4,Q812E4,Q8CFN2,Q9QW07
6284	1,6,8,27,28,29,31,32	2,3,4,5,7,25,26,30	7	O35547,P08723,P36374,P47727,Q63474,Q6AYR9,Q812E4
6285	1,6,8,27,28,30,31,32	2,3,4,5,7,25,26,29	5	B0LT89,O89117,P22006,Q6AYR9,Q8VD89
6286	1,6,8,27,29,30,31,32	2,3,4,5,7,25,26,28	1	Q63474
6287	1,6,8,28,29,30,31,32	2,3,4,5,7,25,26,27	3	P22006,P35745,Q63474
6288	1,6,25,26,27,28,29,30	2,3,4,5,7,8,31,32	4	P05539,P20766,P82471,Q811M5
6289	1,6,25,26,27,28,29,31	2,3,4,5,7,8,30,32	3	P05539,P35280,P70549
6290	1,6,25,26,27,28,29,32	2,3,4,5,7,8,30,31	11	O35547,P00758,P05539,P20766,P36376,P70549,P82471,Q5RJR2,Q6AYR8,Q6AYR9,Q8CGS4
6291	1,6,25,26,27,28,30,31	2,3,4,5,7,8,29,32	0	
6292	1,6,25,26,27,28,30,32	2,3,4,5,7,8,29,31	5	O70594,P20766,P62804,P82471,Q5RJR2
6293	1,6,25,26,27,28,31,32	2,3,4,5,7,8,29,30	5	P22006,P62804,Q5RJR2,Q6IG02,Q9WUW8
6294	1,6,25,26,27,29,30,31	2,3,4,5,7,8,28,32	2	P05539,Q811M5
6295	1,6,25,26,27,29,30,32	2,3,4,5,7,8,28,31	7	D3ZUC6,P00758,P20766,P36376,P82471,Q5RLM2,Q811M5

6296	1,6,25,26,27,29,31,32	2,3,4,5,7,8,28,30	3	P05539,P62804,P70549
6297	1,6,25,26,27,30,31,32	2,3,4,5,7,8,28,29	7	D3ZUC6,P06760,P47967,P54921,P62804,P97840,Q00715
6298	1,6,25,26,28,29,30,31	2,3,4,5,7,8,27,32	2	P05539,Q6TMA8
6299	1,6,25,26,28,29,30,32	2,3,4,5,7,8,27,31	6	P00731,P20766,P36376,P50115,P82471,Q68G31
6300	1,6,25,26,28,29,31,32	2,3,4,5,7,8,27,30	9	O70417,P00731,P05539,P19218,P62804,P70549,Q4G075,Q6IFU7,Q6IFU8
6301	1,6,25,26,28,30,31,32	2,3,4,5,7,8,27,29	11	P00731,P06760,P22006,P25809,P47967,P50115,P62804,Q00715,Q4G075,Q5I0D1,Q62975
6302	1,6,25,26,29,30,31,32	2,3,4,5,7,8,27,28	14	A0JPJ7,D3ZUC6,P00507,P25809,P36860,P47967,P50115,P50116,P62804,P97840,Q00715,Q4G075,Q5I0D1,Q9WVK7
6303	1,6,25,27,28,29,30,31	2,3,4,5,7,8,26,32	2	P05539,P20762
6304	1,6,25,27,28,29,30,32	2,3,4,5,7,8,26,31	7	O35547,P00758,P05539,P20762,P20766,P82471,Q5RLM2
6305	1,6,25,27,28,29,31,32	2,3,4,5,7,8,26,30	6	O35547,P00758,P05539,P20762,P36374,Q9WUW8
6306	1,6,25,27,28,30,31,32	2,3,4,5,7,8,26,29	6	O70594,P06760,P20762,P22006,P62804,Q9QX74
6307	1,6,25,27,29,30,31,32	2,3,4,5,7,8,26,28	5	D3ZUC6,P20762,P47967,P97840,Q5RLM2
6308	1,6,25,28,29,30,31,32	2,3,4,5,7,8,26,27	7	P20762,P25809,P50115,P50116,Q4G075,Q5BJY9,Q5RLM2

6309	1,6,26,27,28,29,30,31	2,3,4,5,7,8,25,32	3	P05539,Q63474,Q811M5
6310	1,6,26,27,28,29,30,32	2,3,4,5,7,8,25,31	6	P00758,P20766,P29315,P36376,P82471,Q811M5
6311	1,6,26,27,28,29,31,32	2,3,4,5,7,8,25,30	8	O35547,O70417,P00758,P05539,P22006,P36374,P70549,Q63474
6312	1,6,26,27,28,30,31,32	2,3,4,5,7,8,25,29	4	P06760,P22006,P62804,Q8VD89
6313	1,6,26,27,29,30,31,32	2,3,4,5,7,8,25,28	5	P36376,P36860,P62804,Q63474,Q811M5
6314	1,6,26,28,29,30,31,32	2,3,4,5,7,8,25,27	7	P01835,P22006,P36860,P50115,P50116,Q4G075,Q63474
6315	1,6,27,28,29,30,31,32	2,3,4,5,7,8,25,26	5	O35547,P05539,P20762,P22006,Q63474
6316	1,7,8,25,26,27,28,29	2,3,4,5,6,30,31,32	13	P20646,P26342,P47727,P70549,Q4G075,Q6AYR9,Q6P6R2,Q812E4,Q9QW07,Q9QX74,Q9WTT6,Q9WUC4,Q9Z2L0
6317	1,7,8,25,26,27,28,30	2,3,4,5,6,29,31,32	2	O55145,Q9WTT6
6318	1,7,8,25,26,27,28,31	2,3,4,5,6,29,30,32	6	iRT-Kit_WR_fusion,O55145,P26342,Q63598,Q9WTT6,Q9Z0V6
6319	1,7,8,25,26,27,28,32	2,3,4,5,6,29,30,31	7	P08723,Q5RJR2,Q6AYR9,Q812E4,Q9JHB9,Q9QWN8,Q9WTT6
6320	1,7,8,25,26,27,29,30	2,3,4,5,6,28,31,32	2	Q9QX74,Q9WTT6
6321	1,7,8,25,26,27,29,31	2,3,4,5,6,28,30,32	3	P70549,Q9QX74,Q9WTT6
6322	1,7,8,25,26,27,29,32	2,3,4,5,6,28,30,31	2	P13432,Q9WTT6
6323	1,7,8,25,26,27,30,31	2,3,4,5,6,28,29,32	1	O55145
6324	1,7,8,25,26,27,30,32	2,3,4,5,6,28,29,31	1	Q9JHB9
6325	1,7,8,25,26,27,31,32	2,3,4,5,6,28,29,30	0	

6326	1,7,8,25,26,28,29,30	2,3,4,5,6,27,31,32	4	P01039,P35745,Q9QX74,Q9WTT6
6327	1,7,8,25,26,28,29,31	2,3,4,5,6,27,30,32	5	P01039,P70549,Q6P6R2,Q9QX74,Q9WTT6
6328	1,7,8,25,26,28,29,32	2,3,4,5,6,27,30,31	10	P01039,P08723,P13432,P24368,P30120,P47727,P70549,Q812E4,Q9QW07,Q9WTT6
6329	1,7,8,25,26,28,30,31	2,3,4,5,6,27,29,32	2	O08839,P35745
6330	1,7,8,25,26,28,30,32	2,3,4,5,6,27,29,31	2	P01039,P35745
6331	1,7,8,25,26,28,31,32	2,3,4,5,6,27,29,30	1	P21588
6332	1,7,8,25,26,29,30,31	2,3,4,5,6,27,28,32	2	P35745,Q9QX74
6333	1,7,8,25,26,29,30,32	2,3,4,5,6,27,28,31	0	
6334	1,7,8,25,26,29,31,32	2,3,4,5,6,27,28,30	4	P13432,P19218,P21588,P70549
6335	1,7,8,25,26,30,31,32	2,3,4,5,6,27,28,29	3	B2RYW9,P35745,Q9WVK7
6336	1,7,8,25,27,28,29,30	2,3,4,5,6,26,31,32	3	P47727,Q812E4,Q9Z2L0
6337	1,7,8,25,27,28,29,31	2,3,4,5,6,26,30,32	3	P47727,Q812E4,Q99MH3
6338	1,7,8,25,27,28,29,32	2,3,4,5,6,26,30,31	14	O35547,P00758,P08010,P08723,P0C0A9,P24368,P30120,P47727,Q5RJR2,Q6AYR9,Q812E4,Q9QW07,Q9WUC4,Q9Z2L0
6339	1,7,8,25,27,28,30,31	2,3,4,5,6,26,29,32	1	P35745
6340	1,7,8,25,27,28,30,32	2,3,4,5,6,26,29,31	3	Q6AYR9,Q812E4,Q9JHB9
6341	1,7,8,25,27,28,31,32	2,3,4,5,6,26,29,30	5	P08723,P21588,Q6AYR9,Q80WL1,Q812E4
6342	1,7,8,25,27,29,30,31	2,3,4,5,6,26,28,32	0	
6343	1,7,8,25,27,29,30,32	2,3,4,5,6,26,28,31	0	
6344	1,7,8,25,27,29,31,32	2,3,4,5,6,26,28,30	2	P21588,Q80WL1
6345	1,7,8,25,27,30,31,32	2,3,4,5,6,26,28,29	1	P21588

6346	1,7,8,25,28,29,30,31	2,3,4,5,6,26,27,32	3	P01039,P35745,Q9JJ50
6347	1,7,8,25,28,29,30,32	2,3,4,5,6,26,27,31	5	P01039,P08723,P30120,Q812E4,Q9Z2L0
6348	1,7,8,25,28,29,31,32	2,3,4,5,6,26,27,30	6	P01039,P08723,P21588,P30120,Q80WL1,Q812E4
6349	1,7,8,25,28,30,31,32	2,3,4,5,6,26,27,29	3	P21588,P35745,Q9JJ50
6350	1,7,8,25,29,30,31,32	2,3,4,5,6,26,27,28	2	P21588,Q9WVK7
6351	1,7,8,26,27,28,29,30	2,3,4,5,6,25,31,32	6	P29315,Q4KLZ6,Q6P6R2,Q812E4,Q9QX74,Q9WTT6
6352	1,7,8,26,27,28,29,31	2,3,4,5,6,25,30,32	11	iRT-Kit_WR_fusion,P20646,P70549,P97523,Q4KLZ6,Q63474,Q6P6R2,Q812E4,Q9ES87,Q9QX74,Q9WTT6
6353	1,7,8,26,27,28,29,32	2,3,4,5,6,25,30,31	17	O35547,P00758,P08010,P08723,P24368,P29315,P30120,P47727,Q4KLZ6,Q6AYR9,Q6P6R2,Q812E4,Q8CFN2,Q9QWN8,Q9QX74,Q9WTT6,Q9Z2L0
6354	1,7,8,26,27,28,30,31	2,3,4,5,6,25,29,32	5	iRT-Kit_WR_fusion,O55145,P35745,Q4KLZ6,Q9WTT6
6355	1,7,8,26,27,28,30,32	2,3,4,5,6,25,29,31	5	P29315,Q4KLZ6,Q6AYR9,Q812E4,Q9QWN8
6356	1,7,8,26,27,28,31,32	2,3,4,5,6,25,29,30	4	P08723,Q4KLZ6,Q6AYR9,Q9QWN8
6357	1,7,8,26,27,29,30,31	2,3,4,5,6,25,28,32	3	Q4KLZ6,Q63474,Q9QX74
6358	1,7,8,26,27,29,30,32	2,3,4,5,6,25,28,31	2	P29315,Q4KLZ6
6359	1,7,8,26,27,29,31,32	2,3,4,5,6,25,28,30	3	Q4KLZ6,Q63474,Q9QX74
6360	1,7,8,26,27,30,31,32	2,3,4,5,6,25,28,29	1	Q4KLZ6
6361	1,7,8,26,28,29,30,31	2,3,4,5,6,25,27,32	6	P01039,P21674,P35745,Q4KLZ6,Q63474,Q9QX74
6362	1,7,8,26,28,29,30,32	2,3,4,5,6,25,27,31	7	P01039,P08723,P29315,P30120,P35745,Q4KLZ6,Q812E4
6363	1,7,8,26,28,29,31,32	2,3,4,5,6,25,27,30	7	P01039,P08723,P30120,P70549,Q4KLZ6,Q63474,Q9QX74
6364	1,7,8,26,28,30,31,32	2,3,4,5,6,25,27,29	3	O08839,P35745,Q4KLZ6
6365	1,7,8,26,29,30,31,32	2,3,4,5,6,25,27,28	3	P35745,Q4KLZ6,Q63474

6366	1,7,8,27,28,29,30,31	2,3,4,5,6,25,26,32	5	P35745,Q06000,Q4KLZ6,Q63474,Q812E4
6367	1,7,8,27,28,29,30,32	2,3,4,5,6,25,26,31	11	P00758,P08723,P18298,P29315,P30120,P47727,Q4KLZ6,Q63150,Q6AYR9,Q812E4,Q9Z2L0
6368	1,7,8,27,28,29,31,32	2,3,4,5,6,25,26,30	13	O08815,O35547,P08723,P21588,P30120,P36374,P47727,Q06000,Q4KLZ6,Q63474,Q63493,Q6AYR9,Q812E4
6369	1,7,8,27,28,30,31,32	2,3,4,5,6,25,26,29	4	B0LT89,P35745,Q4KLZ6,Q6AYR9
6370	1,7,8,27,29,30,31,32	2,3,4,5,6,25,26,28	2	Q4KLZ6,Q63474
6371	1,7,8,28,29,30,31,32	2,3,4,5,6,25,26,27	7	P01039,P08723,P17559,P21588,P35745,Q4KLZ6,Q63474
6372	1,7,25,26,27,28,29,30	2,3,4,5,6,8,31,32	5	P20646,P36376,Q05702,Q63532,Q9QZQ5
6373	1,7,25,26,27,28,29,31	2,3,4,5,6,8,30,32	4	O88797,P20646,P70549,P97615
6374	1,7,25,26,27,28,29,32	2,3,4,5,6,8,30,31	10	P00758,P36376,Q05702,Q5RJR2,Q63532,Q6AYR9,Q812E4,Q8CGS4,Q9WUC4,Q9Z2L0
6375	1,7,25,26,27,28,30,31	2,3,4,5,6,8,29,32	3	O55145,P42854,Q9QZQ5
6376	1,7,25,26,27,28,30,32	2,3,4,5,6,8,29,31	5	P36376,Q05702,Q5RJR2,Q63532,Q9R168
6377	1,7,25,26,27,28,31,32	2,3,4,5,6,8,29,30	5	O88797,P42854,Q5RJR2,Q6IFU7,Q6IG02
6378	1,7,25,26,27,29,30,31	2,3,4,5,6,8,28,32	1	Q9QZQ5
6379	1,7,25,26,27,29,30,32	2,3,4,5,6,8,28,31	6	P00758,P36376,Q63532,Q99PP0,Q9QZQ5,Q9R168
6380	1,7,25,26,27,29,31,32	2,3,4,5,6,8,28,30	2	P19218,P36376

6381	1,7,25,26,27,30,31,32	2,3,4,5,6,8,28,29	2	D3ZUC6,P42854
6382	1,7,25,26,28,29,30,31	2,3,4,5,6,8,27,32	1	Q9QZQ5
6383	1,7,25,26,28,29,30,32	2,3,4,5,6,8,27,31	6	P01039,P17559,P36376,Q63532,Q9QZQ5,Q9R168
6384	1,7,25,26,28,29,31,32	2,3,4,5,6,8,27,30	6	P00731,P01039,P19218,P70549,Q10758,Q6IFU7
6385	1,7,25,26,28,30,31,32	2,3,4,5,6,8,27,29	5	O08839,P00731,P42854,Q62975,Q9QZQ5
6386	1,7,25,26,29,30,31,32	2,3,4,5,6,8,27,28	6	D3ZUC6,P17559,P19218,P36376,Q9QZQ5,Q9WVK7
6387	1,7,25,27,28,29,30,31	2,3,4,5,6,8,26,32	4	P20762,Q64240,Q99MH3,Q9QZQ5
6388	1,7,25,27,28,29,30,32	2,3,4,5,6,8,26,31	9	P00758,P36376,Q05702,Q5RJR2,Q63532,Q6AYR9,Q812E4,Q99PP0,Q9Z2L0
6389	1,7,25,27,28,29,31,32	2,3,4,5,6,8,26,30	7	P00758,P20762,P36374,Q5RJR2,Q812E4,Q8CGS4,Q99MH3
6390	1,7,25,27,28,30,31,32	2,3,4,5,6,8,26,29	2	P42854,Q5RJR2
6391	1,7,25,27,29,30,31,32	2,3,4,5,6,8,26,28	4	P08649,P20762,Q99PP0,Q9QZQ5
6392	1,7,25,28,29,30,31,32	2,3,4,5,6,8,26,27	5	P17559,P20762,Q5BJY9,Q9JJ50,Q9QZQ5
6393	1,7,26,27,28,29,30,31	2,3,4,5,6,8,25,32	3	Q4KLZ6,Q63474,Q9QZQ5

6394	1,7,26,27,28,29,30,32	2,3,4,5,6,8,25,31	10	P00758,P29315,P36376,Q05702,Q4KLZ6,Q63532,Q6AYR9,Q812E4,Q99PP0,Q9QZQ5
6395	1,7,26,27,28,29,31,32	2,3,4,5,6,8,25,30	5	O88797,P36374,P70549,Q4KLZ6,Q63474
6396	1,7,26,27,28,30,31,32	2,3,4,5,6,8,25,29	2	P42854,Q4KLZ6
6397	1,7,26,27,29,30,31,32	2,3,4,5,6,8,25,28	6	P08649,P36376,P36860,Q4KLZ6,Q99PP0,Q9QZQ5
6398	1,7,26,28,29,30,31,32	2,3,4,5,6,8,25,27	6	P01835,P17559,P36376,Q4KLZ6,Q63474,Q9QZQ5
6399	1,7,27,28,29,30,31,32	2,3,4,5,6,8,25,26	6	P08649,Q4KLZ6,Q63474,Q64240,Q99PP0,Q9QZQ5
6400	1,8,25,26,27,28,29,30	2,3,4,5,6,7,31,32	4	P20766,P26342,P80299,Q6P6R2
6401	1,8,25,26,27,28,29,31	2,3,4,5,6,7,30,32	3	P26342,P70549,Q6P6R2
6402	1,8,25,26,27,28,29,32	2,3,4,5,6,7,30,31	12	P00758,P08010,P20766,P26342,P30120,P47727,Q5RJR2,Q6AYR9,Q6P6R2,Q812E4,Q8CFN2,Q9QWN8
6403	1,8,25,26,27,28,30,31	2,3,4,5,6,7,29,32	3	O55145,P26342,P42854
6404	1,8,25,26,27,28,30,32	2,3,4,5,6,7,29,31	3	P20766,Q6AYR9,Q9QWN8
6405	1,8,25,26,27,28,31,32	2,3,4,5,6,7,29,30	4	Q5RJR2,Q6AYR9,Q80WL1,Q9QWN8
6406	1,8,25,26,27,29,30,31	2,3,4,5,6,7,28,32	0	

6407	1,8,25,26,27,29,30,32	2,3,4,5,6,7,28,31	1	P20766
6408	1,8,25,26,27,29,31,32	2,3,4,5,6,7,28,30	2	P13432,Q80WL1
6409	1,8,25,26,27,30,31,32	2,3,4,5,6,7,28,29	4	P42854,P47967,P54921,P97840
6410	1,8,25,26,28,29,30,31	2,3,4,5,6,7,27,32	5	O08839,P00714,P01039,P50116,Q5I0D7
6411	1,8,25,26,28,29,30,32	2,3,4,5,6,7,27,31	4	P01039,P20766,P30120,P50116
6412	1,8,25,26,28,29,31,32	2,3,4,5,6,7,27,30	8	P01039,P19218,P21588,P30120,P50116,P70549,Q63474,Q80WL1
6413	1,8,25,26,28,30,31,32	2,3,4,5,6,7,27,29	6	O08839,P01039,P42854,P47967,P50116,Q9JJ50
6414	1,8,25,26,29,30,31,32	2,3,4,5,6,7,27,28	4	P00507,P47967,P50115,P50116
6415	1,8,25,27,28,29,30,31	2,3,4,5,6,7,26,32	0	
6416	1,8,25,27,28,29,30,32	2,3,4,5,6,7,26,31	8	P00758,P20766,P47727,Q63150,Q6AYR9,Q812E4,Q9WVH8,Q9Z2L0
6417	1,8,25,27,28,29,31,32	2,3,4,5,6,7,26,30	8	O35547,P21588,P30120,P36374,P47727,Q6AYR9,Q80WL1,Q812E4
6418	1,8,25,27,28,30,31,32	2,3,4,5,6,7,26,29	2	P21588,P42854
6419	1,8,25,27,29,30,31,32	2,3,4,5,6,7,26,28	4	P21588,P47967,Q5RLM2,Q99PP0

6420	1,8,25,28,29,30,31,32	2,3,4,5,6,7,26,27	6	O08839,P01039,P21588,P50115,P50116,Q9JJ50
6421	1,8,26,27,28,29,30,31	2,3,4,5,6,7,25,32	2	Q4KLZ6,Q63474
6422	1,8,26,27,28,29,30,32	2,3,4,5,6,7,25,31	10	P00758,P20766,P29315,Q4KLZ6,Q6AYR9,Q812E4,Q8CFN2,Q99PP0,Q9QWN8,Q9WVH8
6423	1,8,26,27,28,29,31,32	2,3,4,5,6,7,25,30	9	O35547,P30120,P36374,P70549,Q4KLZ6,Q63474,Q6AYR9,Q812E4,Q9QWN8
6424	1,8,26,27,28,30,31,32	2,3,4,5,6,7,25,29	4	B0LT89,P42854,Q4KLZ6,Q9QWN8
6425	1,8,26,27,29,30,31,32	2,3,4,5,6,7,25,28	3	Q4KLZ6,Q63474,Q99PP0
6426	1,8,26,28,29,30,31,32	2,3,4,5,6,7,25,27	7	O08839,P01039,P50115,P50116,Q4KLZ6,Q5I0D7,Q63474
6427	1,8,27,28,29,30,31,32	2,3,4,5,6,7,25,26	7	Q4KLZ6,Q63150,Q63474,Q6AYR9,Q812E4,Q99PP0,Q9WVH8
6428	1,25,26,27,28,29,30,31	2,3,4,5,6,7,8,32	9	P02454,P02631,P42854,P50115,P52590,Q64240,Q99041,Q99PP0,Q9QZQ5
6429	1,25,26,27,28,29,30,32	2,3,4,5,6,7,8,31	11	P00758,P13852,P20766,P36376,P50115,P82471,Q05702,Q5RJR2,Q63532,Q99PP0,Q9QZQ5
6430	1,25,26,27,28,29,31,32	2,3,4,5,6,7,8,30	13	O70417,P00758,P13852,P19218,P36374,P50115,P62804,P70549,Q5RJR2,Q8CGS4,Q99041,Q99PP0,Q9QZQ5
6431	1,25,26,27,28,30,31,32	2,3,4,5,6,7,8,29	13	D3ZUC6,O70594,P13852,P22273,P42854,P50115,P52590,P62804,Q00715,Q5RJR2,Q99041,Q99PP0,Q9QZQ5
6432	1,25,26,27,29,30,31,32	2,3,4,5,6,7,8,28	11	D3ZUC6,P02631,P13852,P22273,P47967,P50115,P52590,P62804,Q99041,Q99PP0,Q9QZQ5

6433	1,25,26,28,29,30,31,32	2,3,4,5,6,7,8,27	14	D3ZUC6,O08839,P00731,P01039,P17559,P19218,P22273,P50115,P50116,P62804,Q4G075,Q99041,Q99PP0,Q9QZQ5
6434	1,25,27,28,29,30,31,32	2,3,4,5,6,7,8,26	12	P00758,P13852,P20761,P20762,P22273,P50115,Q5RJR2,Q5RLM2,Q64240,Q99041,Q99PP0,Q9QZQ5
6435	1,26,27,28,29,30,31,32	2,3,4,5,6,7,8,25	12	O70417,P00758,P02454,P02631,P13852,P22273,P50115,Q4KLZ6,Q63474,Q64240,Q99PP0,Q9QZQ5

Day 28

Random allocation	Group1	Group2	Numbers of differential urinary proteins	Differential protein ID
1	1,2,3,4,5,6,7,8	33,34,35,36,37,38,39,40	36	B5DFC9,O35276,O35568,O54858,O88339;Q4V882,P02454,P07897,P0C0K7,P17559,P19218,P20762,P23593,P26453,P33436,P35444,P36374,P36376,P42854,P48199,P49744,P52590,P80202,P85971,Q05702,Q09030,Q4AEF8,Q63149,Q6DGG1,Q8CG08,Q8K4G9,Q91XN4,Q99PP0,Q9JJ50,Q9QW30,Q9QZQ5,Q9Z1F2
2	1,2,3,4,5,6,7,33	8,34,35,36,37,38,39,40	19	O35276,O54800;Q5DWV2,P08721,P16636,P35444,P35745,P36374,P42854,P52590,P85971,Q09030,Q4AEF8,Q5I0D7,Q63475,Q6AYQ8,Q91XN4,Q99PP0,Q9QW30,Q9QZQ5
3	1,2,3,4,5,6,7,34	8,33,35,36,37,38,39,40	20	O35276,P33436,P36374,P42854,P48199,P52590,P60905,Q09030,Q4AEF8,Q4TU93,Q5I0D7,Q68G31,Q6AY61,Q8K4G9,Q91XN4,Q99PP0,Q9JJ50,Q9QW30,Q9QWN8,Q9Z1F2
4	1,2,3,4,5,6,7,35	8,33,34,36,37,38,39,40	18	P19132,P19218,P23593,P36374,P42854,P52590,P85971,P97840,Q09030,Q4AEF8,Q4TU93,Q5I0D7,Q5PPH0,Q63149,Q63474,Q8K4G9,Q91XN4,Q9QWN8
5	1,2,3,4,5,6,7,36	8,33,34,35,37,38,39,40	18	O35276,P09656,P19218,P36374,P42854,P47967,P52590,P97840,Q09030,Q4AEF8,Q5I0D1,Q5I0D7,Q8K4G9,Q91XN4,Q923V8,Q99PP0,Q9JJ50,Q9QWN8
6	1,2,3,4,5,6,7,37	8,33,34,35,36,38,39,40	18	P01835,P11598,P19132,P19218,P23593,P33436,P42854,P47967,P52590,P82471,P97840,Q00657,Q09030,Q4AEF8,Q6AY61,Q8K4G9,Q91XN4,Q9JJ50
7	1,2,3,4,5,6,7,38	8,33,34,35,36,37,39,40	17	O35276,P13432,P19218,P24368,P36374,P42854,P50116,P52590,P60905,P84039,Q09030,Q4AEF8,Q6P6R2,Q8K4G9,Q91XN4,Q923V8,Q9QWN8
8	1,2,3,4,5,6,7,39	8,33,34,35,36,37,38,40	12	P23593,P36374,P42854,P52590,P63029,Q09030,Q4AEF8,Q6AYQ8,Q8K4G9,Q91XN4,Q9QX79,Q9Z1F2
9	1,2,3,4,5,6,7,40	8,33,34,35,36,37,38,39	9	P36374,P42854,P52590,P82471,Q09030,Q4AEF8,Q6P6R2,Q91XN4,Q9QWN8

10	1,2,3,4,5,6,8,33	7,34,35,36,37,38,39,40	26	O35276,O54800;Q5DWV2,O54858,P02454,P16636,P17559,P23593,P35745,P36374,P36376,P42854,P52590,P80202,P85971,Q05702,Q09030,Q4AEF8,Q5I0D7,Q63475,Q6IG05,Q8CG08,Q91XN4,Q99MH3,Q99PP0,Q9QW30,Q9QZQ5
11	1,2,3,4,5,6,8,34	7,33,35,36,37,38,39,40	26	O35276,O54858,P17559,P20762,P33436,P36374,P36376,P42854,P48199,P52590,P80202,P85971,P97580,Q05702,Q09030,Q30KJ2,Q4AEF8,Q5I0D7,Q63475,Q6IG05,Q8CG08,Q8K4G9,Q91XN4,Q99PP0,Q9JJ50,Q9QZQ5
12	1,2,3,4,5,6,8,35	7,33,34,36,37,38,39,40	23	O35276,O54858,P19218,P23593,P30919,P36374,P36376,P42854,P48199,P52590,P80202,P82471,P85971,Q05702,Q09030,Q4AEF8,Q5PPH0,Q63149,Q64093,Q6DGG1,Q8CG08,Q8K4G9,Q91XN4
13	1,2,3,4,5,6,8,36	7,33,34,35,37,38,39,40	18	O35276,P09656,P19218,P36374,P36376,P42854,P48199,P52590,P80202,P82471,P85971,Q05702,Q09030,Q4AEF8,Q8K4G9,Q91XN4,Q99PP0,Q9QZQ5
14	1,2,3,4,5,6,8,37	7,33,34,35,36,38,39,40	16	P19218,P23593,P33436,P36376,P42854,P52590,P82471,P85971,Q00657,Q05702,Q09030,Q4AEF8,Q6DGG1,Q8K4G9,Q91XN4,Q9JJ50
15	1,2,3,4,5,6,8,38	7,33,34,35,36,37,39,40	16	O35276,O88339;Q4V882,P19218,P36374,P36376,P42854,P52590,P82471,P85971,Q05702,Q09030,Q4AEF8,Q6DGG1,Q6IG05,Q6P6Q2,Q91XN4
16	1,2,3,4,5,6,8,39	7,33,34,35,36,37,38,40	16	P20762,P23593,P30919,P36374,P36376,P42854,P52590,P80202,P82471,P85971,Q05702,Q09030,Q4AEF8,Q8K4G9,Q91XN4,Q9R168
17	1,2,3,4,5,6,8,40	7,33,34,35,36,37,38,39	18	B5DFC9,O35276,O35568,O54858,P23593,P25031,P36374,P36376,P42854,P52590,P80202,P82471,P85971,Q05702,Q09030,Q4AEF8,Q8CG08,Q91XN4
18	1,2,3,4,5,6,33,34	7,8,35,36,37,38,39,40	13	O54800;Q5DWV2,P33436,P35745,P42854,P52590,Q09030,Q4AEF8,Q5I0D7,Q63475,Q91XN4,Q99MH3,Q99PP0,Q9Z2L0
19	1,2,3,4,5,6,33,35	7,8,34,36,37,38,39,40	15	O54800;Q5DWV2,P35745,P42854,P52590,P62804,Q09030,Q4AEF8,Q5I0D7,Q5PPH0,Q63149,Q63475,Q64093,Q812E4,Q91XN4,Q9Z2L0
20	1,2,3,4,5,6,33,36	7,8,34,35,37,38,39,40	16	O54800;Q5DWV2,P09656,P11883,P16636,P35745,P42854,P52590,P97584,Q09030,Q4AEF8,Q5I0D7,Q91XN4,Q923V8,Q99MH3,Q99PP0,Q9QZQ5
21	1,2,3,4,5,6,33,37	7,8,34,35,36,38,39,40	9	D3ZTX0,O54800;Q5DWV2,P35745,P42854,P52590,Q00657,Q09030,Q4AEF8,Q91XN4
22	1,2,3,4,5,6,33,38	7,8,34,35,36,37,39,40	11	O54800;Q5DWV2,P35745,P42854,P52590,Q09030,Q4AEF8,Q63475,Q6P6R2,Q812E4,Q91XN4,Q923V8

23	1,2,3,4,5,6,33,39	7,8,34,35,36,37,38,40	11	O54800;Q5DWV2,P23593,P35745,P42854,P52590,Q09030,Q4AEF8,Q6AYQ8,Q91XN4,Q99MH3,Q9Z2L0
24	1,2,3,4,5,6,33,40	7,8,34,35,36,37,38,39	9	O54800;Q5DWV2,P35745,P42854,P52590,Q09030,Q4AEF8,Q63475,Q6P6R2,Q91XN4
25	1,2,3,4,5,6,34,35	7,8,33,36,37,38,39,40	13	P36374,P42854,P52590,Q09030,Q4AEF8,Q4TU93,Q5I0D7,Q64093,Q812E4,Q8K4G9,Q91XN4,Q9QWN8,Q9Z2L0
26	1,2,3,4,5,6,34,36	7,8,33,35,37,38,39,40	12	O55145,P11883,P42854,P52590,P97584,Q09030,Q4KLZ6,Q5I0D7,Q8K4G9,Q91XN4,Q9JJ50,Q9QWN8
27	1,2,3,4,5,6,34,37	7,8,33,35,36,38,39,40	9	P33436,P52590,Q4AEF8,Q4KLZ6,Q4TU93,Q5I0D7,Q8K4G9,Q91XN4,Q9JJ50
28	1,2,3,4,5,6,34,38	7,8,33,35,36,37,39,40	14	P01946,P02091,P33436,P36374,P42854,P52590,P60905,Q4KLZ6,Q4TU93,Q6P6R2,Q812E4,Q8K4G9,Q91XN4,Q9QWN8
29	1,2,3,4,5,6,34,39	7,8,33,35,36,37,38,40	8	P08723,P30919,P36374,P42854,P52590,Q4AEF8,Q8K4G9,Q91XN4
30	1,2,3,4,5,6,34,40	7,8,33,35,36,37,38,39	7	P33436,P36374,P42854,P52590,P97580,Q4KLZ6,Q91XN4
31	1,2,3,4,5,6,35,36	7,8,33,34,37,38,39,40	15	P09656,P19132,P19218,P21674,P42854,P52590,P53792,Q09030,Q4AEF8,Q4TU93,Q5I0D7,Q64093,Q8K4G9,Q91XN4,Q9QWN8
32	1,2,3,4,5,6,35,37	7,8,33,34,36,38,39,40	11	P19132,P19218,P30919,P52590,P82471,Q00657,Q09030,Q4AEF8,Q4TU93,Q64093,Q9QX74
33	1,2,3,4,5,6,35,38	7,8,33,34,36,37,39,40	19	P08723,P19218,P19814,P21674,P24368,P30120,P30919,P36374,P47727,P52590,Q09030,Q4AEF8,Q4TU93,Q63149,Q64093,Q6P6R2,Q812E4,Q9QWN8,Q9QX74
34	1,2,3,4,5,6,35,39	7,8,33,34,36,37,38,40	16	P08723,P09456,P23593,P24368,P30120,P30919,P36374,P47727,P52590,P62898,Q09030,Q4AEF8,Q4TU93,Q5PPH0,Q63149,Q8K4G9
35	1,2,3,4,5,6,35,40	7,8,33,34,36,37,38,39	14	P08723,P36374,P42854,P52590,P53792,P82471,Q09030,Q4AEF8,Q4TU93,Q63149,Q64093,Q6P6R2,Q91XN4,Q9QX74

36	1,2,3,4,5,6,36,37	7,8,33,34,35,38,39,40	15	P09656,P11883,P19132,P47967,P52590,P53792,P55091,P82471,P97840,Q00657,Q09030,Q4AEF8,Q8K4G9,Q91XN4,Q9JJ50
37	1,2,3,4,5,6,36,38	7,8,33,34,35,37,39,40	11	P09656,P19218,P21674,P24368,P52590,Q09030,Q4KLZ6,Q91XN4,Q923V8,Q9QWN8,Q9QX74
38	1,2,3,4,5,6,36,39	7,8,33,34,35,37,38,40	7	P09656,P42854,P52590,Q09030,Q4AEF8,Q8K4G9,Q91XN4
39	1,2,3,4,5,6,36,40	7,8,33,34,35,37,38,39	8	P42854,P52590,P53792,P82471,Q09030,Q91XN4,Q9QWN8,Q9QX74
40	1,2,3,4,5,6,37,38	7,8,33,34,35,36,39,40	8	P19218,P52590,P82471,Q00657,Q4AEF8,Q4KLZ6,Q4TU93,Q9QX74
41	1,2,3,4,5,6,37,39	7,8,33,34,35,36,38,40	8	P23593,P30919,P52590,P63029,P82471,Q4AEF8,Q8K4G9,Q9QX74
42	1,2,3,4,5,6,37,40	7,8,33,34,35,36,38,39	9	P19132,P33436,P52590,P82471,Q4AEF8,Q4KLZ6,Q91XN4,Q9JJ50,Q9QX74
43	1,2,3,4,5,6,38,39	7,8,33,34,35,36,37,40	10	P08723,P24368,P30120,P30919,P36374,P52590,Q4AEF8,Q6P6R2,Q9ES87,Q9QX74
44	1,2,3,4,5,6,38,40	7,8,33,34,35,36,37,39	9	P08723,P36374,P52590,P82471,Q4KLZ6,Q6P6R2,Q91XN4,Q9ES87,Q9QX74
45	1,2,3,4,5,6,39,40	7,8,33,34,35,36,37,38	11	P08723,P30919,P36374,P42854,P52590,P54921,P82471,Q4AEF8,Q6P6R2,Q91XN4,Q9QX74
46	1,2,3,4,5,7,8,33	6,34,35,36,37,38,39,40	10	B1H234,P23593,P51740,P70549,P85971,Q09030,Q4AEF8,Q63475,Q6AYQ8,Q91XN4
47	1,2,3,4,5,7,8,34	6,33,35,36,37,38,39,40	15	B1H234,O35276,P20762,P36374,P47967,P51740,P70549,Q30KJ2,Q4AEF8,Q66H69,Q68G31,Q6IFW6,Q8K4G9,Q91XN4,Q9Z1F2
48	1,2,3,4,5,7,8,35	6,33,34,36,37,38,39,40	13	P19218,P23593,P36374,P36376,P36860,P47967,P51740,P85971,P97840,Q4AEF8,Q63149,Q6DGG1,Q9Z1F2

49	1,2,3,4,5,7,8,36	6,33,34,35,37,38,39,40	12	B1H234,P19218,P36374,P47967,P51740,P97840,Q4AEF8,Q5I0D1,Q6DGG1,Q8K4G9,Q91XN4,Q9Z1F2
50	1,2,3,4,5,7,8,37	6,33,34,35,36,38,39,40	15	B1H234,P19218,P22006,P23593,P47967,P51740,P63029,P97840,Q00657,Q4AEF8,Q5I0D1,Q66H69,Q6AYQ8,Q6DGG1,Q6IFW6
51	1,2,3,4,5,7,8,38	6,33,34,35,36,37,39,40	15	B1H234,O35276,O88339;Q4V882,P19218,P22006,P35280,P36374,P36376,P70549,Q4AEF8,Q6DGG1,Q6IFU8,Q6IFW6,Q6IG05,Q6P6Q2
52	1,2,3,4,5,7,8,39	6,33,34,35,36,37,38,40	10	P20762,P23593,P36374,P36376,P63029,P70549,Q4AEF8,Q6AYQ8,Q8K4G9,Q9Z1F2
53	1,2,3,4,5,7,8,40	6,33,34,35,36,37,38,39	4	P36374,P36376,Q4AEF8,Q91XN4
54	1,2,3,4,5,7,33,34	6,8,35,36,37,38,39,40	13	P08721,P19629,P19939,P35745,P47967,P51740,P70549,P80299,Q5I0D7,Q63475,Q6AYQ8,Q6P6S4,Q9WUW8
55	1,2,3,4,5,7,33,35	6,8,34,36,37,38,39,40	8	P12020,P19629,P47967,P51740,P97840,Q4AEF8,Q5I0D7,Q63474
56	1,2,3,4,5,7,33,36	6,8,34,35,37,38,39,40	7	P01681,P08721,P47967,P51740,P97840,Q09030,Q5I0D7
57	1,2,3,4,5,7,33,37	6,8,34,35,36,38,39,40	5	P47967,P51740,P97840,Q6AY61,Q6AYQ8
58	1,2,3,4,5,7,33,38	6,8,34,35,36,37,39,40	3	P13432,P70549,Q6AYQ8
59	1,2,3,4,5,7,33,39	6,8,34,35,36,37,38,40	2	Q5RKI1,Q6AYQ8
60	1,2,3,4,5,7,33,40	6,8,34,35,36,37,38,39	1	Q6AYQ8
61	1,2,3,4,5,7,34,35	6,8,33,36,37,38,39,40	10	P19629,P36374,P47967,P51740,P70549,P97840,Q5I0D7,Q63474,Q9QWN8,Q9Z1F2

62	1,2,3,4,5,7,34,36	6,8,33,35,37,38,39,40	9	P47967,P51740,P55091,P97840,Q5I0D7,Q68G31,Q8K4G9,Q9QWN8,Q9Z1F2
63	1,2,3,4,5,7,34,37	6,8,33,35,36,38,39,40	6	P47967,P51740,P97840,Q6AY61,Q6AYQ8,Q9JJ50
64	1,2,3,4,5,7,34,38	6,8,33,35,36,37,39,40	10	P01946,P02091,P36374,P60905,P70549,P80299,Q4KLZ6,Q6AY61,Q6P6S4,Q9QWN8
65	1,2,3,4,5,7,34,39	6,8,33,35,36,37,38,40	9	P36374,P60905,P63029,P70549,Q68G31,Q6AY61,Q6AYQ8,Q8K4G9,Q9Z1F2
66	1,2,3,4,5,7,34,40	6,8,33,35,36,37,38,39	5	P36374,P70549,Q68G31,Q6AY61,Q9WUW8
67	1,2,3,4,5,7,35,36	6,8,33,34,37,38,39,40	9	P19132,P19218,P21674,P47967,P51740,P97840,Q5I0D1,Q5I0D7,Q9QWN8
68	1,2,3,4,5,7,35,37	6,8,33,34,36,38,39,40	5	P19132,P47967,P51740,P97840,Q5I0D1
69	1,2,3,4,5,7,35,38	6,8,33,34,36,37,39,40	7	P13432,P19218,P24368,P36374,P70549,Q6P6R2,Q9QWN8
70	1,2,3,4,5,7,35,39	6,8,33,34,36,37,38,40	5	P12020,P30120,P36374,Q63474,Q9Z1F2
71	1,2,3,4,5,7,35,40	6,8,33,34,36,37,38,39	4	P02793;Q7TP54,P36374,Q6P6R2,Q9QWN8
72	1,2,3,4,5,7,36,37	6,8,33,34,35,38,39,40	6	P19132,P47967,P51740,P55091,P97840,Q5I0D1
73	1,2,3,4,5,7,36,38	6,8,33,34,35,37,39,40	6	P13432,P19218,P47967,P97840,Q923V8,Q9QWN8
74	1,2,3,4,5,7,36,39	6,8,33,34,35,37,38,40	5	P63029,P97840,Q8K4G9,Q9QWN8,Q9Z1F2

75	1,2,3,4,5,7,36,40	6,8,33,34,35,37,38,39	2	P97840,Q9QWN8
76	1,2,3,4,5,7,37,38	6,8,33,34,35,36,39,40	4	P22006,P47967,P97840,Q6AY61
77	1,2,3,4,5,7,37,39	6,8,33,34,35,36,38,40	3	P63029,Q6AY61,Q6AYQ8
78	1,2,3,4,5,7,37,40	6,8,33,34,35,36,38,39	2	P19132,Q6AY61
79	1,2,3,4,5,7,38,39	6,8,33,34,35,36,37,40	7	P02770,P13432,P24368,P36374,P60905,P70549,Q63556
80	1,2,3,4,5,7,38,40	6,8,33,34,35,36,37,39	5	P36374,Q6AY61,Q6P6R2,Q9QWN8,Q9QX74
81	1,2,3,4,5,7,39,40	6,8,33,34,35,36,37,38	3	O89117,P36374,Q6AYQ8
82	1,2,3,4,5,8,33,34	6,7,35,36,37,38,39,40	7	G3V686,P51740,P70549,Q30KJ2,Q5I0D7,Q63475,Q6IG05
83	1,2,3,4,5,8,33,35	6,7,34,36,37,38,39,40	7	P01681,P19629,P36376,P51740,Q09030,Q63475,Q6DGG1
84	1,2,3,4,5,8,33,36	6,7,34,35,37,38,39,40	5	O54728,P01681,P51740,Q09030,Q63475
85	1,2,3,4,5,8,33,37	6,7,34,35,36,38,39,40	5	P51740,Q00657,Q63475,Q6AYQ8,Q6IFW6
86	1,2,3,4,5,8,33,38	6,7,34,35,36,37,39,40	7	P36376,P70549,Q63475,Q6IFU7,Q6IFW6,Q6IG05,Q6P6Q2
87	1,2,3,4,5,8,33,39	6,7,34,35,36,37,38,40	5	P01681,P23593,P36376,Q63475,Q6AYQ8

88	1,2,3,4,5,8,33,40	6,7,34,35,36,37,38,39	4	P00731,P01681,P36376,Q63475
89	1,2,3,4,5,8,34,35	6,7,33,36,37,38,39,40	9	P19629,P36374,P36376,P36860,P51740,P70549,P97580,Q30KJ2,Q5I0D7
90	1,2,3,4,5,8,34,36	6,7,33,35,37,38,39,40	8	G3V686,P51740,P55091,P97615,Q30KJ2,Q5I0D7,Q8K4G9,Q9Z1F2
91	1,2,3,4,5,8,34,37	6,7,33,35,36,38,39,40	6	P51740,Q30KJ2,Q6IFW6,Q6IG02,Q6IG05,Q6IMF3
92	1,2,3,4,5,8,34,38	6,7,33,35,36,37,39,40	15	B1H234,P01946,P02091,P36374,P36376,P70549,P97580,Q30KJ2,Q4KLZ6,Q6IFU7,Q6IFU8,Q6IFW6,Q6IG05,Q6IMF3,Q6P6Q2
93	1,2,3,4,5,8,34,39	6,7,33,35,36,37,38,40	9	P20762,P36376,P70549,P97580,Q30KJ2,Q6IFW6,Q8K4G9,Q9Z0V6,Q9Z1F2
94	1,2,3,4,5,8,34,40	6,7,33,35,36,37,38,39	5	P15399,P36374,P36376,P97580,Q30KJ2
95	1,2,3,4,5,8,35,36	6,7,33,34,37,38,39,40	5	P19218,P21674,P36376,P51740,Q6DGG1
96	1,2,3,4,5,8,35,37	6,7,33,34,36,38,39,40	7	F1M3L7,P19218,P36860,P51740,P82471,Q00657,Q6DGG1
97	1,2,3,4,5,8,35,38	6,7,33,34,36,37,39,40	6	P19218,P24368,P36374,P36376,Q6DGG1,Q6IG05
98	1,2,3,4,5,8,35,39	6,7,33,34,36,37,38,40	5	P20762,P23593,P36374,P36376,P36860
99	1,2,3,4,5,8,35,40	6,7,33,34,36,37,38,39	4	P36374,P36376,P82471,Q6DGG1
100	1,2,3,4,5,8,36,37	6,7,33,34,35,38,39,40	7	F1M3L7,P47967,P51740,P55091,P97840,Q00657,Q6DGG1

101	1,2,3,4,5,8,36,38	6,7,33,34,35,37,39,40	2	P19218,Q6IG05
102	1,2,3,4,5,8,36,39	6,7,33,34,35,37,38,40	5	P20762,P36376,Q8K4G9,Q9R168,Q9Z1F2
103	1,2,3,4,5,8,36,40	6,7,33,34,35,37,38,39	2	P36376,P82471
104	1,2,3,4,5,8,37,38	6,7,33,34,35,36,39,40	10	P19218,P22006,Q4FZU2,Q6DGG1,Q6IFU8,Q6IFW6,Q6IG02,Q6IG05,Q6IMF3,Q6P6Q2
105	1,2,3,4,5,8,37,39	6,7,33,34,35,36,38,40	8	P20762,P23593,P36376,P63029,P82471,Q6AYQ8,Q6IFW6,Q6IG02
106	1,2,3,4,5,8,37,40	6,7,33,34,35,36,38,39	3	P22006,P82471,Q6IFW6
107	1,2,3,4,5,8,38,39	6,7,33,34,35,36,37,40	7	P20762,P36374,P36376,P70549,Q6IFW6,Q6P6Q2,Q6PCU2
108	1,2,3,4,5,8,38,40	6,7,33,34,35,36,37,39	8	P22006,P36374,P36376,P82471,Q6IFW6,Q6IG05,Q6P6Q2,Q6P6R2
109	1,2,3,4,5,8,39,40	6,7,33,34,35,36,37,38	5	P20762,P23593,P36376,P82471,Q9R168
110	1,2,3,4,5,33,34,35	6,7,8,36,37,38,39,40	10	P06911,P12020,P19629,P35745,P51740,Q5GRG2,Q5I0D7,Q63475,Q812E4,Q9Z2L0
111	1,2,3,4,5,33,34,36	6,7,8,35,37,38,39,40	12	G3V686,P08721,P19939,P35745,P51740,P97584,P98089,Q5I0D7,Q5QE79,Q62635,Q63475,Q99041
112	1,2,3,4,5,33,34,37	6,7,8,35,36,38,39,40	5	P35745,P51740,P98089,Q5I0D7,Q6AYQ8
113	1,2,3,4,5,33,34,38	6,7,8,35,36,37,39,40	8	P01946,P02091,P35745,P70549,P80299,Q4KLZ6,Q63475,Q812E4
114	1,2,3,4,5,33,34,39	6,7,8,35,36,37,38,40	3	P35745,Q6AYQ8,Q99041
115	1,2,3,4,5,33,34,40	6,7,8,35,36,37,38,39	2	P35745,Q63475
116	1,2,3,4,5,33,35,36	6,7,8,34,37,38,39,40	7	P01681,P21674,P35745,P49134,P51740,P97840,Q5I0D7

117	1,2,3,4,5,33,35,37	6,7,8,34,36,38,39,40	2	F1M3L7,P51740
118	1,2,3,4,5,33,35,38	6,7,8,34,36,37,39,40	5	P12020,P13432,P19629,Q6P6R2,Q812E4
119	1,2,3,4,5,33,35,39	6,7,8,34,36,37,38,40	2	P12020,P62804
120	1,2,3,4,5,33,35,40	6,7,8,34,36,37,38,39	4	P01681,P12020,P35745,Q6P6R2
121	1,2,3,4,5,33,36,37	6,7,8,34,35,38,39,40	6	P47967,P51740,P55091,P97840,P98089,Q05695
122	1,2,3,4,5,33,36,38	6,7,8,34,35,37,39,40	3	P01681,P35745,Q923V8
123	1,2,3,4,5,33,36,39	6,7,8,34,35,37,38,40	2	P01681,Q99041
124	1,2,3,4,5,33,36,40	6,7,8,34,35,37,38,39	2	P01681,P35745
125	1,2,3,4,5,33,37,38	6,7,8,34,35,36,39,40	0	
126	1,2,3,4,5,33,37,39	6,7,8,34,35,36,38,40	1	Q6AYQ8
127	1,2,3,4,5,33,37,40	6,7,8,34,35,36,38,39	0	
128	1,2,3,4,5,33,38,39	6,7,8,34,35,36,37,40	1	Q9QW07
129	1,2,3,4,5,33,38,40	6,7,8,34,35,36,37,39	3	P01039,P35745,Q6P6R2
130	1,2,3,4,5,33,39,40	6,7,8,34,35,36,37,38	2	P35745,Q6AYQ8
131	1,2,3,4,5,34,35,36	6,7,8,33,37,38,39,40	5	P21674,P51740,P97840,Q5I0D7,Q9QWN8
132	1,2,3,4,5,34,35,37	6,7,8,33,36,38,39,40	2	P50115,P51740
133	1,2,3,4,5,34,35,38	6,7,8,33,36,37,39,40	8	P01946,P08723,P21674,P70549,Q4KLZ6,Q4TU93,Q812E4,Q9QWN8
134	1,2,3,4,5,34,35,39	6,7,8,33,36,37,38,40	4	P08723,P09456,P12020,P30120
135	1,2,3,4,5,34,35,40	6,7,8,33,36,37,38,39	0	
136	1,2,3,4,5,34,36,37	6,7,8,33,35,38,39,40	8	P47967,P51740,P55091,P97840,P98089,Q05695,Q4KLZ6,Q62635

137	1,2,3,4,5,34,36,38	6,7,8,33,35,37,39,40	5	P01946,P02091,P21674,Q4KLZ6,Q9QWN8
138	1,2,3,4,5,34,36,39	6,7,8,33,35,37,38,40	2	Q8K4G9,Q9Z1F2
139	1,2,3,4,5,34,36,40	6,7,8,33,35,37,38,39	1	Q4KLZ6
140	1,2,3,4,5,34,37,38	6,7,8,33,35,36,39,40	5	P01946,P02091,Q4KLZ6,Q6AY61,Q6IG02
141	1,2,3,4,5,34,37,39	6,7,8,33,35,36,38,40	1	Q6AYQ8
142	1,2,3,4,5,34,37,40	6,7,8,33,35,36,38,39	1	Q4KLZ6
143	1,2,3,4,5,34,38,39	6,7,8,33,35,36,37,40	7	P01946,P02091,P08723,P60905,P70549,Q4KLZ6,Q9QW07
144	1,2,3,4,5,34,38,40	6,7,8,33,35,36,37,39	6	P01946,P08723,P70549,P97580,Q4KLZ6,Q6P6R2
145	1,2,3,4,5,34,39,40	6,7,8,33,35,36,37,38	1	P08723
146	1,2,3,4,5,35,36,37	6,7,8,33,34,38,39,40	7	F1M3L7,P19132,P21674,P47967,P51740,P55091,P97840
147	1,2,3,4,5,35,36,38	6,7,8,33,34,37,39,40	5	P19218,P21674,P24368,Q9QW07,Q9QWN8
148	1,2,3,4,5,35,36,39	6,7,8,33,34,37,38,40	4	P23739,P30120,Q62714,Q9QW07
149	1,2,3,4,5,35,36,40	6,7,8,33,34,37,38,39	3	P02793;Q7TP54,P21674,P53792
150	1,2,3,4,5,35,37,38	6,7,8,33,34,36,39,40	2	P19218,Q9QX74
151	1,2,3,4,5,35,37,39	6,7,8,33,34,36,38,40	0	
152	1,2,3,4,5,35,37,40	6,7,8,33,34,36,38,39	4	P19132,P50115,P82471,Q9QX74
153	1,2,3,4,5,35,38,39	6,7,8,33,34,36,37,40	10	P08723,P09456,P13432,P24368,P30120,P47727,P62898,Q6P6R2,Q9QW07,Q9QX74
154	1,2,3,4,5,35,38,40	6,7,8,33,34,36,37,39	3	P08723,Q6P6R2,Q9QX74
155	1,2,3,4,5,35,39,40	6,7,8,33,34,36,37,38	4	P00774,P08723,Q6P6R2,Q9QX74
156	1,2,3,4,5,36,37,38	6,7,8,33,34,35,39,40	3	P97840,Q4KLZ6,Q9QX74

157	1,2,3,4,5,36,37,39	6,7,8,33,34,35,38,40	0	
158	1,2,3,4,5,36,37,40	6,7,8,33,34,35,38,39	3	P82471,Q4KLZ6,Q9QX74
159	1,2,3,4,5,36,38,39	6,7,8,33,34,35,37,40	3	P24368,Q9QW07,Q9QX74
160	1,2,3,4,5,36,38,40	6,7,8,33,34,35,37,39	3	Q4KLZ6,Q9QWN8,Q9QX74
161	1,2,3,4,5,36,39,40	6,7,8,33,34,35,37,38	1	Q9QX74
162	1,2,3,4,5,37,38,39	6,7,8,33,34,35,36,40	1	Q9QX74
163	1,2,3,4,5,37,38,40	6,7,8,33,34,35,36,39	3	P82471,Q4KLZ6,Q9QX74
164	1,2,3,4,5,37,39,40	6,7,8,33,34,35,36,38	3	O89117,P82471,Q9QX74
165	1,2,3,4,5,38,39,40	6,7,8,33,34,35,36,37	4	O89117,P08723,Q6P6R2,Q9QX74
166	1,2,3,4,6,7,8,33	5,34,35,36,37,38,39,40	21	G3V686,O54800;Q5DWV2,O54858,P02454,P02631,P0C0K7,P11598,P23593,P42854,P80202,P85971,Q05702,Q09030,Q4AEF8,Q4V885,Q63475,Q6AYQ8,Q91XN4,Q99PP0,Q9QW30,Q9QZQ5
167	1,2,3,4,6,7,8,34	5,33,35,36,37,38,39,40	24	G3V686,O54800;Q5DWV2,O54858,P02631,P0C0K7,P11598,P20762,P20766,P33436,P36374,P42854,P80202,P85971,P97580,P97615,Q05702,Q09030,Q30KJ2,Q4AEF8,Q63475,Q91XN4,Q99PP0,Q9QW30,Q9Z1F2
168	1,2,3,4,6,7,8,35	5,33,34,36,37,38,39,40	15	O54858,P19132,P19218,P23593,P36374,P36860,P42854,P80202,P85971,Q09030,Q4AEF8,Q5PPH0,Q63149,Q91XN4,Q9Z1F2
169	1,2,3,4,6,7,8,36	5,33,34,35,37,38,39,40	16	P09656,P0C0K7,P11598,P19218,P42854,P80202,P85971,P97615,Q05702,Q09030,Q4AEF8,Q63617,Q91XN4,Q99PP0,Q9QZQ5,Q9Z1F2
170	1,2,3,4,6,7,8,37	5,33,34,35,36,38,39,40	14	P02631,P0C0A9,P0C0K7,P11598,P19132,P22273,P23593,P85971,Q05702,Q09030,Q4AEF8,Q63617,Q91XN4,Q99041
171	1,2,3,4,6,7,8,38	5,33,34,35,36,37,39,40	11	O70417,O88339;Q4V882,P19218,P35280,P36374,P42854,P85971,Q05702,Q09030,Q4AEF8,Q91XN4
172	1,2,3,4,6,7,8,39	5,33,34,35,36,37,38,40	16	P20762,P20766,P23593,P36374,P36376,P42854,P63029,P80202,P85971,Q05702,Q09030,Q4AEF8,Q6AYQ8,Q91XN4,Q99PP0,Q9Z1F2

173	1,2,3,4,6,7,8,40	5,33,34,35,36,37,38,39	13	O54858,P02454,P02631,P23593,P36374,P42854,P80202,P85971,Q05702,Q09030,Q4AEF8,Q91XN4,Q99PP0
174	1,2,3,4,6,7,33,34	5,8,35,36,37,38,39,40	16	G3V686,O54800;Q5DWV2,P08721,P35745,P42854,P80299,Q09030,Q4AEF8,Q4V885,Q5I0D7,Q63475,Q6AYQ8,Q8K1G0,Q91XN4,Q99PP0,Q9QW30
175	1,2,3,4,6,7,33,35	5,8,34,36,37,38,39,40	10	O54800;Q5DWV2,P08721,P42854,Q09030,Q4AEF8,Q5I0D7,Q5PPH0,Q63149,Q63475,Q99PP0
176	1,2,3,4,6,7,33,36	5,8,34,35,37,38,39,40	11	O54800;Q5DWV2,P08721,P09656,P35745,P42854,P97615,Q09030,Q4AEF8,Q5I0D7,Q91XN4,Q99PP0
177	1,2,3,4,6,7,33,37	5,8,34,35,36,38,39,40	6	O54800;Q5DWV2,P11598,Q09030,Q4AEF8,Q6AYQ8,Q91XN4
178	1,2,3,4,6,7,33,38	5,8,34,35,36,37,39,40	6	O54800;Q5DWV2,Q09030,Q4AEF8,Q63475,Q91XN4,Q99PP0
179	1,2,3,4,6,7,33,39	5,8,34,35,36,37,38,40	7	O54800;Q5DWV2,P42854,Q09030,Q4AEF8,Q5RKI1,Q6AYQ8,Q99PP0
180	1,2,3,4,6,7,33,40	5,8,34,35,36,37,38,39	10	O54800;Q5DWV2,P06760,P35745,P42854,Q09030,Q4AEF8,Q63475,Q6AYQ8,Q91XN4,Q99PP0
181	1,2,3,4,6,7,34,35	5,8,33,36,37,38,39,40	8	O54800;Q5DWV2,P36374,P42854,Q4AEF8,Q5I0D7,Q80WL1,Q9QWN8,Q9Z1F2
182	1,2,3,4,6,7,34,36	5,8,33,35,37,38,39,40	11	G3V686,P11598,P42854,P80299,P97615,Q5I0D7,Q80WL1,Q91XN4,Q99PP0,Q9QWN8,Q9Z1F2
183	1,2,3,4,6,7,34,37	5,8,33,35,36,38,39,40	7	O54800;Q5DWV2,P01835,P11598,P19132,P33436,Q6AYQ8,Q80WL1
184	1,2,3,4,6,7,34,38	5,8,33,35,36,37,39,40	8	O54800;Q5DWV2,P01946,P02091,P36374,P80299,Q4KLZ6,Q80WL1,Q9QWN8
185	1,2,3,4,6,7,34,39	5,8,33,35,36,37,38,40	6	P20766,Q4AEF8,Q6AYQ8,Q80WL1,Q9QX79,Q9Z1F2

186	1,2,3,4,6,7,34,40	5,8,33,35,36,37,38,39	4	P42854,P97580,Q80WL1,Q91XN4
187	1,2,3,4,6,7,35,36	5,8,33,34,37,38,39,40	7	P02793;Q7TP54,P09656,P19132,P19218,Q4AEF8,Q9QWN8,Q9Z1F2
188	1,2,3,4,6,7,35,37	5,8,33,34,36,38,39,40	3	P19132,Q4AEF8,Q5PPH0
189	1,2,3,4,6,7,35,38	5,8,33,34,36,37,39,40	7	P06760,P13432,P19218,P36374,Q4AEF8,Q63149,Q9QWN8
190	1,2,3,4,6,7,35,39	5,8,33,34,36,37,38,40	7	P06760,P23593,P36374,Q4AEF8,Q5PPH0,Q63149,Q9Z1F2
191	1,2,3,4,6,7,35,40	5,8,33,34,36,37,38,39	6	P02793;Q7TP54,P06760,P19132,P36374,Q4AEF8,Q63149
192	1,2,3,4,6,7,36,37	5,8,33,34,35,38,39,40	9	P01835,P09656,P11598,P19132,P47967,P97840,Q4AEF8,Q63617,Q8CJD3
193	1,2,3,4,6,7,36,38	5,8,33,34,35,37,39,40	3	P09656,P19218,Q9QWN8
194	1,2,3,4,6,7,36,39	5,8,33,34,35,37,38,40	3	P09656,Q4AEF8,Q9Z1F2
195	1,2,3,4,6,7,36,40	5,8,33,34,35,37,38,39	7	P02793;Q7TP54,P06760,P09656,P19132,P42854,Q91XN4,Q9QWN8
196	1,2,3,4,6,7,37,38	5,8,33,34,35,36,39,40	4	P01835,P23739,Q4AEF8,Q9QX74
197	1,2,3,4,6,7,37,39	5,8,33,34,35,36,38,40	4	P01835,P63029,Q4AEF8,Q6AYQ8
198	1,2,3,4,6,7,37,40	5,8,33,34,35,36,38,39	6	P01835,P11598,P19132,P82471,Q4AEF8,Q9QX74

199	1,2,3,4,6,7,38,39	5,8,33,34,35,36,37,40	6	P02770,P06760,P13432,P36374,Q4AEF8,Q9QX79
200	1,2,3,4,6,7,38,40	5,8,33,34,35,36,37,39	4	P06760,P36374,Q6P6R2,Q9QX74
201	1,2,3,4,6,7,39,40	5,8,33,34,35,36,37,38	3	P06760,Q4AEF8,Q6AYQ8
202	1,2,3,4,6,8,33,34	5,7,35,36,37,38,39,40	21	G3V686,O54800;Q5DWV2,O55004,P08649,P35745,P42854,P80202,P85971,P97580,P97615,Q05702,Q09030,Q30KJ2,Q4V885,Q5I0D7,Q63475,Q64240,Q91XN4,Q99MH3,Q99PP0,Q9QZQ5
203	1,2,3,4,6,8,33,35	5,7,34,36,37,38,39,40	13	O54800;Q5DWV2,P23593,P42854,P80202,P85971,Q05702,Q09030,Q4V885,Q5PPH0,Q63149,Q63475,Q91XN4,Q99PP0
204	1,2,3,4,6,8,33,36	5,7,34,35,37,38,39,40	18	G3V686,O54728,O54800;Q5DWV2,P01681,P08649,P09656,P42854,P80202,P85971,P97615,Q05702,Q09030,Q4V885,Q63475,Q91XN4,Q99MH3,Q99PP0,Q9QZQ5
205	1,2,3,4,6,8,33,37	5,7,34,35,36,38,39,40	8	O54800;Q5DWV2,P23593,P85971,Q05702,Q09030,Q63475,Q91XN4,Q99PP0
206	1,2,3,4,6,8,33,38	5,7,34,35,36,37,39,40	11	O54800;Q5DWV2,O70417,P42854,P80202,P85971,Q05702,Q09030,Q63475,Q6IG05,Q91XN4,Q99PP0
207	1,2,3,4,6,8,33,39	5,7,34,35,36,37,38,40	14	O54800;Q5DWV2,P23593,P36376,P42854,P80202,P85971,Q05702,Q09030,Q63475,Q63514,Q6AYQ8,Q91XN4,Q99MH3,Q99PP0
208	1,2,3,4,6,8,33,40	5,7,34,35,36,37,38,39	9	O54800;Q5DWV2,P42854,P80202,P85971,Q05702,Q09030,Q63475,Q91XN4,Q99PP0
209	1,2,3,4,6,8,34,35	5,7,33,36,37,38,39,40	8	P36374,P36860,P42854,P85971,P97580,Q05702,Q30KJ2,Q5PPH0
210	1,2,3,4,6,8,34,36	5,7,33,35,37,38,39,40	12	G3V686,P08649,P42854,P97580,P97615,Q05702,Q09030,Q30KJ2,Q5I0D7,Q91XN4,Q99PP0,Q9Z1F2
211	1,2,3,4,6,8,34,37	5,7,33,35,36,38,39,40	5	P33436,P97580,Q05702,Q30KJ2,Q91XN4

212	1,2,3,4,6,8,34,38	5,7,33,35,36,37,39,40	12	O54800;Q5DWV2,P01946,P02091,P02625,P15399,P36374,P97580,Q05702,Q30KJ2,Q4KLZ6,Q6IG05,Q91XN4
213	1,2,3,4,6,8,34,39	5,7,33,35,36,37,38,40	9	P15399,P20762,P36376,P42854,P97580,Q05702,Q30KJ2,Q63514,Q9Z1F2
214	1,2,3,4,6,8,34,40	5,7,33,35,36,37,38,39	7	P15399,P42854,P80202,P97580,Q05702,Q30KJ2,Q91XN4
215	1,2,3,4,6,8,35,36	5,7,33,34,37,38,39,40	7	P09656,P19132,P19218,P42854,P85971,Q09030,Q5PPH0
216	1,2,3,4,6,8,35,37	5,7,33,34,36,38,39,40	7	P19132,P19218,P23593,P36860,P82471,P85971,Q5PPH0
217	1,2,3,4,6,8,35,38	5,7,33,34,36,37,39,40	5	O70417,P19218,P36374,P85971,Q63149
218	1,2,3,4,6,8,35,39	5,7,33,34,36,37,38,40	10	P20762,P23593,P36374,P36376,P36860,P54921,P85971,Q5PPH0,Q63149,Q9Z1F2
219	1,2,3,4,6,8,35,40	5,7,33,34,36,37,38,39	10	P02793;Q7TP54,P06760,P23593,P36374,P42854,P54921,P82471,P85971,Q5PPH0,Q63149
220	1,2,3,4,6,8,36,37	5,7,33,34,35,38,39,40	7	P09656,P19132,P82471,Q05702,Q09030,Q91XN4,Q9JI85
221	1,2,3,4,6,8,36,38	5,7,33,34,35,37,39,40	5	O70417,P09656,P19218,Q09030,Q91XN4
222	1,2,3,4,6,8,36,39	5,7,33,34,35,37,38,40	9	P08649,P09656,P36376,P85971,P97615,Q05702,Q09030,Q63514,Q9Z1F2
223	1,2,3,4,6,8,36,40	5,7,33,34,35,37,38,39	7	P09656,P42854,P82471,P97615,Q05702,Q09030,Q91XN4
224	1,2,3,4,6,8,37,38	5,7,33,34,35,36,39,40	2	P19218,Q05702

225	1,2,3,4,6,8,37,39	5,7,33,34,35,36,38,40	4	P23593,P36376,P82471,Q05702
226	1,2,3,4,6,8,37,40	5,7,33,34,35,36,38,39	3	P19132,P82471,Q05702
227	1,2,3,4,6,8,38,39	5,7,33,34,35,36,37,40	3	P20762,P36374,P36376
228	1,2,3,4,6,8,38,40	5,7,33,34,35,36,37,39	5	P06760,P36374,P82471,P97580,Q91XN4
229	1,2,3,4,6,8,39,40	5,7,33,34,35,36,37,38	8	P06760,P20762,P23593,P36376,P54921,P82471,P85971,Q9R168
230	1,2,3,4,6,33,34,35	5,7,8,36,37,38,39,40	12	D3ZUC6,O54800;Q5DWV2,P08721,P35745,P42854,P80020,P80299,Q09030,Q5I0D7,Q5PPH0,Q63475,Q8K1G0
231	1,2,3,4,6,33,34,36	5,7,8,35,37,38,39,40	21	D3ZUC6,G3V686,O54800;Q5DWV2,O55145,P08494,P08649,P08721,P09656,P11883,P35745,P42854,P80299,P97584,P97615,Q09030,Q5I0D7,Q63475,Q8K1G0,Q91XN4,Q99MH3,Q99PP0
232	1,2,3,4,6,33,34,37	5,7,8,35,36,38,39,40	8	O54800;Q5DWV2,O55145,P35745,P80020,Q5I0D7,Q63475,Q6AYQ8,Q8K1G0
233	1,2,3,4,6,33,34,38	5,7,8,35,36,37,39,40	8	O54800;Q5DWV2,P01946,P02091,P35745,P80299,Q4KLZ6,Q63475,Q8K1G0
234	1,2,3,4,6,33,34,39	5,7,8,35,36,37,38,40	8	O54800;Q5DWV2,P35745,P80299,Q63475,Q63514,Q6AYQ8,Q8K1G0,Q99MH3
235	1,2,3,4,6,33,34,40	5,7,8,35,36,37,38,39	7	O54800;Q5DWV2,P35745,P42854,P97580,Q63475,Q8K1G0,Q91XN4
236	1,2,3,4,6,33,35,36	5,7,8,34,37,38,39,40	10	O54800;Q5DWV2,P06760,P08721,P09656,P11762,P35745,P49134,Q09030,Q5I0D7,Q5PPH0
237	1,2,3,4,6,33,35,37	5,7,8,34,36,38,39,40	2	O54800;Q5DWV2,Q5PPH0
238	1,2,3,4,6,33,35,38	5,7,8,34,36,37,39,40	4	O54800;Q5DWV2,P06760,P49134,Q5PPH0
239	1,2,3,4,6,33,35,39	5,7,8,34,36,37,38,40	5	O54800;Q5DWV2,P06760,P49134,P62804,Q5PPH0
240	1,2,3,4,6,33,35,40	5,7,8,34,36,37,38,39	4	O54800;Q5DWV2,P06760,P35745,Q5PPH0
241	1,2,3,4,6,33,36,37	5,7,8,34,35,38,39,40	5	O54800;Q5DWV2,P09656,P11883,Q09030,Q9Z0V6
242	1,2,3,4,6,33,36,38	5,7,8,34,35,37,39,40	7	O54800;Q5DWV2,P06760,P09656,P35745,Q09030,Q923V8,Q9Z0V6

243	1,2,3,4,6,33,36,39	5,7,8,34,35,37,38,40	7	O54800;Q5DWV2,P06760,P09656,P35745,Q09030,Q63514,Q99MH3
244	1,2,3,4,6,33,36,40	5,7,8,34,35,37,38,39	10	O54800;Q5DWV2,P01681,P06760,P09656,P35745,P42854,Q09030,Q91XN4,Q9WTT6,Q9Z0V6
245	1,2,3,4,6,33,37,38	5,7,8,34,35,36,39,40	1	O54800;Q5DWV2
246	1,2,3,4,6,33,37,39	5,7,8,34,35,36,38,40	2	O54800;Q5DWV2,Q6AYQ8
247	1,2,3,4,6,33,37,40	5,7,8,34,35,36,38,39	3	O54800;Q5DWV2,P06760,P35745
248	1,2,3,4,6,33,38,39	5,7,8,34,35,36,37,40	2	O54800;Q5DWV2,P06760
249	1,2,3,4,6,33,38,40	5,7,8,34,35,36,37,39	6	O54800;Q5DWV2,P01039,P06760,P35745,Q6P6R2,Q9QX74
250	1,2,3,4,6,33,39,40	5,7,8,34,35,36,37,38	4	O54800;Q5DWV2,P06760,P35745,Q6AYQ8
251	1,2,3,4,6,34,35,36	5,7,8,33,37,38,39,40	3	P09656,Q5I0D7,Q9QWN8
252	1,2,3,4,6,34,35,37	5,7,8,33,36,38,39,40	2	P19132,P39069
253	1,2,3,4,6,34,35,38	5,7,8,33,36,37,39,40	7	O54800;Q5DWV2,P01946,P02091,P80299,P97580,Q4KLZ6,Q9QWN8
254	1,2,3,4,6,34,35,39	5,7,8,33,36,37,38,40	0	
255	1,2,3,4,6,34,35,40	5,7,8,33,36,37,38,39	3	P06760,P15399,P97580
256	1,2,3,4,6,34,36,37	5,7,8,33,35,38,39,40	4	O55145,P09656,P11883,Q4KLZ6
257	1,2,3,4,6,34,36,38	5,7,8,33,35,37,39,40	6	P01946,P02091,P09656,P80299,Q4KLZ6,Q9QWN8
258	1,2,3,4,6,34,36,39	5,7,8,33,35,37,38,40	2	P97615,Q9Z1F2
259	1,2,3,4,6,34,36,40	5,7,8,33,35,37,38,39	4	P06760,P97580,P97615,Q4KLZ6
260	1,2,3,4,6,34,37,38	5,7,8,33,35,36,39,40	3	P01946,P02091,Q4KLZ6
261	1,2,3,4,6,34,37,39	5,7,8,33,35,36,38,40	0	
262	1,2,3,4,6,34,37,40	5,7,8,33,35,36,38,39	3	P33436,P97580,Q4KLZ6

263	1,2,3,4,6,34,38,39	5,7,8,33,35,36,37,40	4	P01946,P02091,P97580,Q4KLZ6
264	1,2,3,4,6,34,38,40	5,7,8,33,35,36,37,39	6	P01946,P02091,P06760,P15399,P97580,Q4KLZ6
265	1,2,3,4,6,34,39,40	5,7,8,33,35,36,37,38	3	P06760,P15399,P97580
266	1,2,3,4,6,35,36,37	5,7,8,33,34,38,39,40	3	P06760,P09656,P19132
267	1,2,3,4,6,35,36,38	5,7,8,33,34,37,39,40	6	P06760,P09656,P19218,P21674,Q9QWN8,Q9QX74
268	1,2,3,4,6,35,36,39	5,7,8,33,34,37,38,40	3	P06760,P09656,Q62714
269	1,2,3,4,6,35,36,40	5,7,8,33,34,37,38,39	7	P02793;Q7TP54,P06760,P09656,P19132,Q9EQS0,Q9QX74,Q9Z0V6
270	1,2,3,4,6,35,37,38	5,7,8,33,34,36,39,40	2	P06760,Q9QX74
271	1,2,3,4,6,35,37,39	5,7,8,33,34,36,38,40	2	P06760,Q9QX74
272	1,2,3,4,6,35,37,40	5,7,8,33,34,36,38,39	5	P02793;Q7TP54,P06760,P19132,P82471,Q9QX74
273	1,2,3,4,6,35,38,39	5,7,8,33,34,36,37,40	3	P06760,Q6P6R2,Q9QX74
274	1,2,3,4,6,35,38,40	5,7,8,33,34,36,37,39	4	P02793;Q7TP54,P06760,Q6P6R2,Q9QX74
275	1,2,3,4,6,35,39,40	5,7,8,33,34,36,37,38	5	P00774,P06760,P54921,P82471,Q9QX74
276	1,2,3,4,6,36,37,38	5,7,8,33,34,35,39,40	4	P09656,Q4KLZ6,Q9QX74,Q9Z0V6
277	1,2,3,4,6,36,37,39	5,7,8,33,34,35,38,40	3	P06760,P09656,Q9QX74
278	1,2,3,4,6,36,37,40	5,7,8,33,34,35,38,39	7	P06760,P09656,P19132,P82471,Q4KLZ6,Q9QX74,Q9Z0V6
279	1,2,3,4,6,36,38,39	5,7,8,33,34,35,37,40	3	P06760,P09656,Q9QX74
280	1,2,3,4,6,36,38,40	5,7,8,33,34,35,37,39	5	P06760,P09656,Q4KLZ6,Q9QX74,Q9Z0V6
281	1,2,3,4,6,36,39,40	5,7,8,33,34,35,37,38	3	P06760,P54921,Q9QX74
282	1,2,3,4,6,37,38,39	5,7,8,33,34,35,36,40	2	P06760,Q9QX74

283	1,2,3,4,6,37,38,40	5,7,8,33,34,35,36,39	5	P06760,P23739,P82471,Q4KLZ6,Q9QX74
284	1,2,3,4,6,37,39,40	5,7,8,33,34,35,36,38	3	P06760,P82471,Q9QX74
285	1,2,3,4,6,38,39,40	5,7,8,33,34,35,36,37	6	P06760,P51740,P54921,P55091,Q6P6R2,Q9QX74
286	1,2,3,4,7,8,33,34	5,6,35,36,37,38,39,40	8	G3V686,P51740,P80299,P97615,Q30KJ2,Q4V885,Q63475,Q6AYQ8
287	1,2,3,4,7,8,33,35	5,6,34,36,37,38,39,40	4	P36860,P51740,Q5PPH0,Q63475
288	1,2,3,4,7,8,33,36	5,6,34,35,37,38,39,40	6	G3V686,O54728,P01681,P51740,P97615,Q63475
289	1,2,3,4,7,8,33,37	5,6,34,35,36,38,39,40	3	P51740,Q63475,Q6AYQ8
290	1,2,3,4,7,8,33,38	5,6,34,35,36,37,39,40	4	O70417,P35280,Q63475,Q6AYQ8
291	1,2,3,4,7,8,33,39	5,6,34,35,36,37,38,40	8	D3ZTE0,P04916,P20762,P36376,Q5RKI1,Q63475,Q63514,Q6AYQ8
292	1,2,3,4,7,8,33,40	5,6,34,35,36,37,38,39	3	P01681,Q63475,Q6AYQ8
293	1,2,3,4,7,8,34,35	5,6,33,36,37,38,39,40	7	P20766,P36860,P51740,P97580,Q30KJ2,Q66H69,Q9Z1F2
294	1,2,3,4,7,8,34,36	5,6,33,35,37,38,39,40	7	G3V686,P20766,P51740,P97615,Q30KJ2,Q66H69,Q9Z1F2
295	1,2,3,4,7,8,34,37	5,6,33,35,36,38,39,40	5	P36860,P51740,Q30KJ2,Q66H69,Q6AYQ8
296	1,2,3,4,7,8,34,38	5,6,33,35,36,37,39,40	5	B1H234,O70417,P35280,P97580,Q30KJ2

297	1,2,3,4,7,8,34,39	5,6,33,35,36,37,38,40	11	G3V686,P20762,P20766,P36376,P36860,P97580,Q30KJ2,Q63618,Q6AYQ8,Q9Z0V6,Q9Z1F2
298	1,2,3,4,7,8,34,40	5,6,33,35,36,37,38,39	5	P15399,P20766,P97580,Q30KJ2,Q66H69
299	1,2,3,4,7,8,35,36	5,6,33,34,37,38,39,40	6	P19218,P36860,P51740,P97840,Q66H69,Q9Z1F2
300	1,2,3,4,7,8,35,37	5,6,33,34,36,38,39,40	4	P19132,P36860,P51740,Q66H69
301	1,2,3,4,7,8,35,38	5,6,33,34,36,37,39,40	5	O70417,P19218,P35280,P36374,P36860
302	1,2,3,4,7,8,35,39	5,6,33,34,36,37,38,40	5	P20762,P23593,P36376,P36860,Q9Z1F2
303	1,2,3,4,7,8,35,40	5,6,33,34,36,37,38,39	5	P00774,P02793;Q7TP54,P36374,P36860,Q66H69
304	1,2,3,4,7,8,36,37	5,6,33,34,35,38,39,40	6	P19132,P47967,P51740,P55091,P97840,Q66H69
305	1,2,3,4,7,8,36,38	5,6,33,34,35,37,39,40	3	O70417,P19218,P35280
306	1,2,3,4,7,8,36,39	5,6,33,34,35,37,38,40	5	P20762,P36376,P63029,P97615,Q9Z1F2
307	1,2,3,4,7,8,36,40	5,6,33,34,35,37,38,39	2	P97615,Q66H69
308	1,2,3,4,7,8,37,38	5,6,33,34,35,36,39,40	4	P22006,P35280,Q6IFW6,Q9EQS0
309	1,2,3,4,7,8,37,39	5,6,33,34,35,36,38,40	4	P20762,P23593,P63029,Q6AYQ8

310	1,2,3,4,7,8,37,40	5,6,33,34,35,36,38,39	2	Q66H69,Q6AYQ8
311	1,2,3,4,7,8,38,39	5,6,33,34,35,36,37,40	10	D4A5U3,P02770,P11883,P20762,P35280,P36376,Q63556,Q63618,Q6AYQ8,Q9EQS0
312	1,2,3,4,7,8,38,40	5,6,33,34,35,36,37,39	2	O70417,P35280
313	1,2,3,4,7,8,39,40	5,6,33,34,35,36,37,38	4	P10758,P20762,P36376,Q6AYQ8
314	1,2,3,4,7,33,34,35	5,6,8,36,37,38,39,40	7	D3ZUC6,P08721,P51740,P80299,Q5I0D7,Q63475,Q8K1G0
315	1,2,3,4,7,33,34,36	5,6,8,35,37,38,39,40	9	G3V686,P08721,P51740,P80299,P97615,Q5I0D7,Q63475,Q6AYQ8,Q8K1G0
316	1,2,3,4,7,33,34,37	5,6,8,35,36,38,39,40	4	P51740,P80299,Q6AYQ8,Q8K1G0
317	1,2,3,4,7,33,34,38	5,6,8,35,36,37,39,40	4	P80299,Q63475,Q6AYQ8,Q8K1G0
318	1,2,3,4,7,33,34,39	5,6,8,35,36,37,38,40	6	G3V686,P80299,Q5RKI1,Q64093,Q6AYQ8,Q8K1G0
319	1,2,3,4,7,33,34,40	5,6,8,35,36,37,38,39	4	P80299,Q63475,Q6AYQ8,Q8K1G0
320	1,2,3,4,7,33,35,36	5,6,8,34,37,38,39,40	4	P08721,P49134,P51740,P97840
321	1,2,3,4,7,33,35,37	5,6,8,34,36,38,39,40	1	P51740
322	1,2,3,4,7,33,35,38	5,6,8,34,36,37,39,40	1	P13432
323	1,2,3,4,7,33,35,39	5,6,8,34,36,37,38,40	2	Q5RKI1,Q6AYQ8
324	1,2,3,4,7,33,35,40	5,6,8,34,36,37,38,39	0	
325	1,2,3,4,7,33,36,37	5,6,8,34,35,38,39,40	4	P47967,P51740,P97840,Q6AYQ8
326	1,2,3,4,7,33,36,38	5,6,8,34,35,37,39,40	0	
327	1,2,3,4,7,33,36,39	5,6,8,34,35,37,38,40	2	Q5RKI1,Q6AYQ8

328	1,2,3,4,7,33,36,40	5,6,8,34,35,37,38,39	1 P01681
329	1,2,3,4,7,33,37,38	5,6,8,34,35,36,39,40	1 Q6AYQ8
330	1,2,3,4,7,33,37,39	5,6,8,34,35,36,38,40	2 Q5RKI1,Q6AYQ8
331	1,2,3,4,7,33,37,40	5,6,8,34,35,36,38,39	1 Q6AYQ8
332	1,2,3,4,7,33,38,39	5,6,8,34,35,36,37,40	6 P02770,P13432,Q5RKI1,Q63556,Q64093,Q6AYQ8
333	1,2,3,4,7,33,38,40	5,6,8,34,35,36,37,39	2 Q6AYQ8,Q9QYP1
334	1,2,3,4,7,33,39,40	5,6,8,34,35,36,37,38	2 Q5RKI1,Q6AYQ8
335	1,2,3,4,7,34,35,36	5,6,8,33,37,38,39,40	6 P25031,P51740,P97840,Q5I0D7,Q9QWN8,Q9Z1F2
336	1,2,3,4,7,34,35,37	5,6,8,33,36,38,39,40	3 P19132,P25031,P51740
337	1,2,3,4,7,34,35,38	5,6,8,33,36,37,39,40	2 P80299,Q9QWN8
338	1,2,3,4,7,34,35,39	5,6,8,33,36,37,38,40	2 P20766,Q9Z1F2
339	1,2,3,4,7,34,35,40	5,6,8,33,36,37,38,39	1 P10758
340	1,2,3,4,7,34,36,37	5,6,8,33,35,38,39,40	5 P25031,P47967,P51740,P55091,P97840
341	1,2,3,4,7,34,36,38	5,6,8,33,35,37,39,40	4 P02091,P80299,Q4KLZ6,Q9QWN8
342	1,2,3,4,7,34,36,39	5,6,8,33,35,37,38,40	3 P20766,P25031,Q9Z1F2
343	1,2,3,4,7,34,36,40	5,6,8,33,35,37,38,39	0
344	1,2,3,4,7,34,37,38	5,6,8,33,35,36,39,40	2 Q4KLZ6,Q6AY61
345	1,2,3,4,7,34,37,39	5,6,8,33,35,36,38,40	2 P25031,Q6AYQ8
346	1,2,3,4,7,34,37,40	5,6,8,33,35,36,38,39	1 Q6AYQ8
347	1,2,3,4,7,34,38,39	5,6,8,33,35,36,37,40	8 P02770,P20766,P50503,P80299,Q63556,Q63618,Q64093,Q6AYQ8

348	1,2,3,4,7,34,38,40	5,6,8,33,35,36,37,39	4 P10758,P80299,P97580,Q4KLZ6
349	1,2,3,4,7,34,39,40	5,6,8,33,35,36,37,38	3 P10758,P20766,Q6AYQ8
350	1,2,3,4,7,35,36,37	5,6,8,33,34,38,39,40	6 P02793;Q7TP54,P19132,P25031,P47967,P51740,P97840
351	1,2,3,4,7,35,36,38	5,6,8,33,34,37,39,40	3 P13432,P21674,Q9QWN8
352	1,2,3,4,7,35,36,39	5,6,8,33,34,37,38,40	3 P25031,Q62714,Q9Z1F2
353	1,2,3,4,7,35,36,40	5,6,8,33,34,37,38,39	3 P02793;Q7TP54,P19132,Q9QWN8
354	1,2,3,4,7,35,37,38	5,6,8,33,34,36,39,40	0
355	1,2,3,4,7,35,37,39	5,6,8,33,34,36,38,40	1 P25031
356	1,2,3,4,7,35,37,40	5,6,8,33,34,36,38,39	2 P02793;Q7TP54,P19132
357	1,2,3,4,7,35,38,39	5,6,8,33,34,36,37,40	3 P02770,P10758,P13432
358	1,2,3,4,7,35,38,40	5,6,8,33,34,36,37,39	5 P00774,P02793;Q7TP54,P06760,P10758,Q6P6R2
359	1,2,3,4,7,35,39,40	5,6,8,33,34,36,37,38	3 P00774,P06760,P10758
360	1,2,3,4,7,36,37,38	5,6,8,33,34,35,39,40	1 P97840
361	1,2,3,4,7,36,37,39	5,6,8,33,34,35,38,40	3 P25031,P63029,Q6AYQ8
362	1,2,3,4,7,36,37,40	5,6,8,33,34,35,38,39	2 P02793;Q7TP54,P19132
363	1,2,3,4,7,36,38,39	5,6,8,33,34,35,37,40	1 P13432
364	1,2,3,4,7,36,38,40	5,6,8,33,34,35,37,39	1 Q9QWN8
365	1,2,3,4,7,36,39,40	5,6,8,33,34,35,37,38	1 P10758
366	1,2,3,4,7,37,38,39	5,6,8,33,34,35,36,40	2 P02770,Q6AYQ8
367	1,2,3,4,7,37,38,40	5,6,8,33,34,35,36,39	1 Q9QX74

368	1,2,3,4,7,37,39,40	5,6,8,33,34,35,36,38	1	Q6AYQ8
369	1,2,3,4,7,38,39,40	5,6,8,33,34,35,36,37	6	O89117,P02770,P06760,P10758,P55091,Q9QX74
370	1,2,3,4,8,33,34,35	5,6,7,36,37,38,39,40	8	D3ZUC6,G3V686,P00762,P36860,P51740,P97580,Q30KJ2,Q63475
371	1,2,3,4,8,33,34,36	5,6,7,35,37,38,39,40	11	G3V686,O54728,P00762,P01681,P02625,P51740,P97615,Q30KJ2,Q5QE79,Q63475,Q99MH3
372	1,2,3,4,8,33,34,37	5,6,7,35,36,38,39,40	4	P51740,Q30KJ2,Q63475,Q6AYQ8
373	1,2,3,4,8,33,34,38	5,6,7,35,36,37,39,40	9	G3V686,P00762,P02091,P02625,P80299,P97580,Q30KJ2,Q63475,Q6IG05
374	1,2,3,4,8,33,34,39	5,6,7,35,36,37,38,40	11	G3V686,P00762,P02651,P20762,P36376,P97580,Q30KJ2,Q63475,Q63514,Q6AYQ8,Q99MH3
375	1,2,3,4,8,33,34,40	5,6,7,35,36,37,38,39	6	P00762,P01681,P15399,P97580,Q30KJ2,Q63475
376	1,2,3,4,8,33,35,36	5,6,7,34,37,38,39,40	4	G3V686,O54728,P01681,P51740
377	1,2,3,4,8,33,35,37	5,6,7,34,36,38,39,40	3	P36860,P50116,P51740
378	1,2,3,4,8,33,35,38	5,6,7,34,36,37,39,40	2	O70417,Q63475
379	1,2,3,4,8,33,35,39	5,6,7,34,36,37,38,40	3	P36376,Q5PPH0,Q63514
380	1,2,3,4,8,33,35,40	5,6,7,34,36,37,38,39	2	P01681,Q63475
381	1,2,3,4,8,33,36,37	5,6,7,34,35,38,39,40	3	O54728,P01681,P51740
382	1,2,3,4,8,33,36,38	5,6,7,34,35,37,39,40	3	O54728,P01681,Q63475
383	1,2,3,4,8,33,36,39	5,6,7,34,35,37,38,40	5	G3V686,O54728,P01681,P36376,Q63514
384	1,2,3,4,8,33,36,40	5,6,7,34,35,37,38,39	3	O54728,P01681,Q63475
385	1,2,3,4,8,33,37,38	5,6,7,34,35,36,39,40	1	Q6IG05
386	1,2,3,4,8,33,37,39	5,6,7,34,35,36,38,40	1	Q6AYQ8
387	1,2,3,4,8,33,37,40	5,6,7,34,35,36,38,39	1	Q6AYQ8

388	1,2,3,4,8,33,38,39	5,6,7,34,35,36,37,40	3	P36376,Q63514,Q6AYQ8
389	1,2,3,4,8,33,38,40	5,6,7,34,35,36,37,39	2	P01681,Q63475
390	1,2,3,4,8,33,39,40	5,6,7,34,35,36,37,38	3	P01681,P36376,Q6AYQ8
391	1,2,3,4,8,34,35,36	5,6,7,33,37,38,39,40	7	G3V686,P36860,P50116,P51740,P97580,Q30KJ2,Q9Z1F2
392	1,2,3,4,8,34,35,37	5,6,7,33,36,38,39,40	5	P36860,P50116,P51740,P97580,Q30KJ2
393	1,2,3,4,8,34,35,38	5,6,7,33,36,37,39,40	3	P36860,P97580,Q30KJ2
394	1,2,3,4,8,34,35,39	5,6,7,33,36,37,38,40	7	P20762,P36376,P36860,P50116,P97580,Q30KJ2,Q9Z1F2
395	1,2,3,4,8,34,35,40	5,6,7,33,36,37,38,39	5	P00774,P15399,P36860,P97580,Q30KJ2
396	1,2,3,4,8,34,36,37	5,6,7,33,35,38,39,40	3	P50116,P51740,P55091
397	1,2,3,4,8,34,36,38	5,6,7,33,35,37,39,40	6	G3V686,P02091,P02625,P97580,Q30KJ2,Q4KLZ6
398	1,2,3,4,8,34,36,39	5,6,7,33,35,37,38,40	8	G3V686,P20762,P36376,P97580,P97615,Q30KJ2,Q63514,Q9Z1F2
399	1,2,3,4,8,34,36,40	5,6,7,33,35,37,38,39	4	P15399,P97580,P97615,Q30KJ2
400	1,2,3,4,8,34,37,38	5,6,7,33,35,36,39,40	7	P97580,Q30KJ2,Q4KLZ6,Q6IFU8,Q6IFW6,Q6IG02,Q6IG05
401	1,2,3,4,8,34,37,39	5,6,7,33,35,36,38,40	5	P20762,P50116,P97580,Q30KJ2,Q6AYQ8
402	1,2,3,4,8,34,37,40	5,6,7,33,35,36,38,39	4	P15399,P97580,Q30KJ2,Q923V8
403	1,2,3,4,8,34,38,39	5,6,7,33,35,36,37,40	8	D4A5U3,P02091,P20762,P36376,P50503,P97580,Q30KJ2,Q63618
404	1,2,3,4,8,34,38,40	5,6,7,33,35,36,37,39	4	P15399,P97580,Q30KJ2,Q4KLZ6
405	1,2,3,4,8,34,39,40	5,6,7,33,35,36,37,38	5	P15399,P20762,P36376,P97580,Q30KJ2
406	1,2,3,4,8,35,36,37	5,6,7,33,34,38,39,40	3	P19132,P50116,P51740
407	1,2,3,4,8,35,36,38	5,6,7,33,34,37,39,40	2	P19218,P21674

408	1,2,3,4,8,35,36,39	5,6,7,33,34,37,38,40	4	P36376,P50116,Q62714,Q9Z1F2
409	1,2,3,4,8,35,36,40	5,6,7,33,34,37,38,39	2	P02793;Q7TP54,P50116
410	1,2,3,4,8,35,37,38	5,6,7,33,34,36,39,40	1	P36860
411	1,2,3,4,8,35,37,39	5,6,7,33,34,36,38,40	3	P36376,P36860,P50116
412	1,2,3,4,8,35,37,40	5,6,7,33,34,36,38,39	4	P19132,P36860,P50116,P82471
413	1,2,3,4,8,35,38,39	5,6,7,33,34,36,37,40	1	P36376
414	1,2,3,4,8,35,38,40	5,6,7,33,34,36,37,39	3	P00774,P98089,Q62635
415	1,2,3,4,8,35,39,40	5,6,7,33,34,36,37,38	5	P00774,P36376,P36860,P50116,P54921
416	1,2,3,4,8,36,37,38	5,6,7,33,34,35,39,40	0	
417	1,2,3,4,8,36,37,39	5,6,7,33,34,35,38,40	1	P50116
418	1,2,3,4,8,36,37,40	5,6,7,33,34,35,38,39	2	P50116,P82471
419	1,2,3,4,8,36,38,39	5,6,7,33,34,35,37,40	2	P36376,Q5BJY9
420	1,2,3,4,8,36,38,40	5,6,7,33,34,35,37,39	0	
421	1,2,3,4,8,36,39,40	5,6,7,33,34,35,37,38	3	P36376,P54921,Q5BJY9
422	1,2,3,4,8,37,38,39	5,6,7,33,34,35,36,40	4	P36376,Q63618,Q6IFW6,Q6IG02
423	1,2,3,4,8,37,38,40	5,6,7,33,34,35,36,39	1	Q6IFW6
424	1,2,3,4,8,37,39,40	5,6,7,33,34,35,36,38	4	P36376,P50116,P82471,Q6AYQ8
425	1,2,3,4,8,38,39,40	5,6,7,33,34,35,36,37	7	D4A5U3,P36376,P54921,P55091,Q5BJY9,Q62635,Q8CIZ5
426	1,2,3,4,33,34,35,36	5,6,7,8,37,38,39,40	10	D3ZUC6,G3V686,P08721,P25031,P35745,P51740,Q5I0D7,Q5QE79,Q6PCU2,Q8K1G0
427	1,2,3,4,33,34,35,37	5,6,7,8,36,38,39,40	8	B0BNN3,D3ZUC6,P25031,P50115,P50116,P51740,P80020,Q8K1G0

428	1,2,3,4,33,<br>34,35,38	5,6,7,8,36,<br>37,39,40	4	D3ZUC6,P80299,Q63475,Q8K1G0
429	1,2,3,4,33,<br>34,35,39	5,6,7,8,36,<br>37,38,40	2	D3ZUC6,Q8K1G0
430	1,2,3,4,33,<br>34,35,40	5,6,7,8,36,<br>37,38,39	5	D3ZUC6,P35745,P50115,Q63475,Q8K1G0
431	1,2,3,4,33,<br>34,36,37	5,6,7,8,35,<br>38,39,40	5	D3ZUC6,P25031,P51740,P98089,Q5QE79
432	1,2,3,4,33,<br>34,36,38	5,6,7,8,35,<br>37,39,40	7	D3ZUC6,G3V686,P02091,P35745,P80299,Q4KLZ6,Q99041
433	1,2,3,4,33,<br>34,36,39	5,6,7,8,35,<br>37,38,40	5	D3ZUC6,G3V686,Q63514,Q99041,Q99MH3
434	1,2,3,4,33,<br>34,36,40	5,6,7,8,35,<br>37,38,39	3	D3ZUC6,P01681,P35745
435	1,2,3,4,33,<br>34,37,38	5,6,7,8,35,<br>36,39,40	3	B0BNN3,P80299,Q4KLZ6
436	1,2,3,4,33,<br>34,37,39	5,6,7,8,35,<br>36,38,40	1	Q6AYQ8
437	1,2,3,4,33,<br>34,37,40	5,6,7,8,35,<br>36,38,39	2	P50115,Q6AYQ8
438	1,2,3,4,33,<br>34,38,39	5,6,7,8,35,<br>36,37,40	3	P80299,Q6AYQ8,Q99041
439	1,2,3,4,33,<br>34,38,40	5,6,7,8,35,<br>36,37,39	6	P35745,P80299,P97580,Q4KLZ6,Q63475,Q9QYP1
440	1,2,3,4,33,<br>34,39,40	5,6,7,8,35,<br>36,37,38	3	P19218,P35745,Q6AYQ8
441	1,2,3,4,33,<br>35,36,37	5,6,7,8,34,<br>38,39,40	4	P25031,P50115,P50116,P51740
442	1,2,3,4,33,<br>35,36,38	5,6,7,8,34,<br>37,39,40	3	P06760,P21674,P49134
443	1,2,3,4,33,<br>35,36,39	5,6,7,8,34,<br>37,38,40	4	P01681,P06760,P49134,Q62714
444	1,2,3,4,33,<br>35,36,40	5,6,7,8,34,<br>37,38,39	4	P01681,P06760,P49134,Q9Z0V6
445	1,2,3,4,33,<br>35,37,38	5,6,7,8,34,<br>36,39,40	1	B0BNN3
446	1,2,3,4,33,<br>35,37,39	5,6,7,8,34,<br>36,38,40	1	P50116
447	1,2,3,4,33,<br>35,37,40	5,6,7,8,34,<br>36,38,39	3	P06760,P50115,P50116

448	1,2,3,4,33, 35,38,39	5,6,7,8,34, 36,37,40	2	P06760,P49134
449	1,2,3,4,33, 35,38,40	5,6,7,8,34, 36,37,39	3	P06760,Q6P6R2,Q9QYP1
450	1,2,3,4,33, 35,39,40	5,6,7,8,34, 36,37,38	1	P06760
451	1,2,3,4,33, 36,37,38	5,6,7,8,34, 35,39,40	0	
452	1,2,3,4,33, 36,37,39	5,6,7,8,34, 35,38,40	2	P50116,Q6AYQ8
453	1,2,3,4,33, 36,37,40	5,6,7,8,34, 35,38,39	3	P01681,Q9WTT6,Q9Z0V6
454	1,2,3,4,33, 36,38,39	5,6,7,8,34, 35,37,40	2	P06760,Q99041
455	1,2,3,4,33, 36,38,40	5,6,7,8,34, 35,37,39	3	P01681,P06760,Q9Z0V6
456	1,2,3,4,33, 36,39,40	5,6,7,8,34, 35,37,38	2	P01681,P06760
457	1,2,3,4,33, 37,38,39	5,6,7,8,34, 35,36,40	2	Q6AYQ8,Q9EQV9
458	1,2,3,4,33, 37,38,40	5,6,7,8,34, 35,36,39	2	Q9QX74,Q9Z1F2
459	1,2,3,4,33, 37,39,40	5,6,7,8,34, 35,36,38	2	P06760,Q6AYQ8
460	1,2,3,4,33, 38,39,40	5,6,7,8,34, 35,36,37	3	P06760,P55091,Q9QX74
461	1,2,3,4,34, 35,36,37	5,6,7,8,33, 38,39,40	5	P19132,P25031,P50115,P50116,P51740
462	1,2,3,4,34, 35,36,38	5,6,7,8,33, 37,39,40	3	P21674,Q4KLZ6,Q9QWN8
463	1,2,3,4,34, 35,36,39	5,6,7,8,33, 37,38,40	6	P25031,P50115,P50116,P50137,Q62714,Q9Z1F2
464	1,2,3,4,34, 35,36,40	5,6,7,8,33, 37,38,39	2	P50115,P50116
465	1,2,3,4,34, 35,37,38	5,6,7,8,33, 36,39,40	2	B0BNN3,Q4KLZ6
466	1,2,3,4,34, 35,37,39	5,6,7,8,33, 36,38,40	3	P25031,P50115,P50116
467	1,2,3,4,34, 35,37,40	5,6,7,8,33, 36,38,39	2	P50115,P50116

468	1,2,3,4,34, 35,38,39	5,6,7,8,33, 36,37,40	0	
469	1,2,3,4,34, 35,38,40	5,6,7,8,33, 36,37,39	4	P00774,P10758,P97580,Q4KLZ6
470	1,2,3,4,34, 35,39,40	5,6,7,8,33, 36,37,38	6	P00774,P06760,P10758,P50115,P50116,P97580
471	1,2,3,4,34, 36,37,38	5,6,7,8,33, 35,39,40	4	P02091,P25031,P29315,Q4KLZ6
472	1,2,3,4,34, 36,37,39	5,6,7,8,33, 35,38,40	3	P25031,P50115,P50116
473	1,2,3,4,34, 36,37,40	5,6,7,8,33, 35,38,39	3	P50115,P50116,Q4KLZ6
474	1,2,3,4,34, 36,38,39	5,6,7,8,33, 35,37,40	3	P01946,P02091,Q4KLZ6
475	1,2,3,4,34, 36,38,40	5,6,7,8,33, 35,37,39	2	P97580,Q4KLZ6
476	1,2,3,4,34, 36,39,40	5,6,7,8,33, 35,37,38	0	
477	1,2,3,4,34, 37,38,39	5,6,7,8,33, 35,36,40	1	Q4KLZ6
478	1,2,3,4,34, 37,38,40	5,6,7,8,33, 35,36,39	2	P97580,Q4KLZ6
479	1,2,3,4,34, 37,39,40	5,6,7,8,33, 35,36,38	3	P50115,P50116,Q6AYQ8
480	1,2,3,4,34, 38,39,40	5,6,7,8,33, 35,36,37	4	P10758,P55091,P97580,Q4KLZ6
481	1,2,3,4,35, 36,37,38	5,6,7,8,33, 34,39,40	2	P25031,Q9QX74
482	1,2,3,4,35, 36,37,39	5,6,7,8,33, 34,38,40	4	P25031,P50115,P50116,Q62714
483	1,2,3,4,35, 36,37,40	5,6,7,8,33, 34,38,39	7	P02793;Q7TP54,P06760,P19132,P50115,P50116,Q9QX74,Q9Z0V6
484	1,2,3,4,35, 36,38,39	5,6,7,8,33, 34,37,40	3	P06760,Q62714,Q9QW07
485	1,2,3,4,35, 36,38,40	5,6,7,8,33, 34,37,39	4	P02793;Q7TP54,P06760,Q9QX74,Q9Z0V6
486	1,2,3,4,35, 36,39,40	5,6,7,8,33, 34,37,38	3	P06760,P50115,P50116
487	1,2,3,4,35, 37,38,39	5,6,7,8,33, 34,36,40	1	Q9QX74

488	1,2,3,4,35,37,38,40	5,6,7,8,33,34,36,39	2	P06760,Q9QX74
489	1,2,3,4,35,37,39,40	5,6,7,8,33,34,36,38	5	P00774,P06760,P50115,P50116,Q9QX74
490	1,2,3,4,35,38,39,40	5,6,7,8,33,34,36,37	8	P00774,P06760,P10758,P55091,P98089,Q62635,Q6P6R2,Q9QX74
491	1,2,3,4,36,37,38,39	5,6,7,8,33,34,35,40	1	Q9QX74
492	1,2,3,4,36,37,38,40	5,6,7,8,33,34,35,39	4	P06760,Q4KLZ6,Q9QX74,Q9Z0V6
493	1,2,3,4,36,37,39,40	5,6,7,8,33,34,35,38	4	P06760,P50115,P50116,Q9QX74
494	1,2,3,4,36,38,39,40	5,6,7,8,33,34,35,37	2	P06760,Q9QX74
495	1,2,3,4,37,38,39,40	5,6,7,8,33,34,35,36	3	P06760,P19223,Q9QX74
496	1,2,3,5,6,7,8,33	4,34,35,36,37,38,39,40	18	D3ZTX0,O54800;Q5DWV2,O54858,P16636,P36374,P42854,P52590,P80202,P85971,Q05702,Q09030,Q4AEF8,Q6IG05,Q91XN4,Q99MH3,Q99PP0,Q9QW30,Q9QZQ5
497	1,2,3,5,6,7,8,34	4,33,35,36,37,38,39,40	14	O54858,P20766,P36374,P48199,P52590,P80202,Q05702,Q09030,Q4AEF8,Q6IG05,Q8K4G9,Q91XN4,Q9JJ50,Q9QW30
498	1,2,3,5,6,7,8,35	4,33,34,36,37,38,39,40	11	O54858,P19218,P23593,P36374,P52590,P80202,P85971,Q09030,Q4AEF8,Q63149,Q91XN4
499	1,2,3,5,6,7,8,36	4,33,34,35,37,38,39,40	14	B0BNN3,O54858,P19218,P36374,P47967,P52590,P80202,P85971,Q09030,Q4AEF8,Q8K4G9,Q91XN4,Q9JJ50,Q9QZQ5
500	1,2,3,5,6,7,8,37	4,33,34,35,36,38,39,40	14	B5DFC9,P01835,P19218,P47967,P52590,P80202,P82471,Q09030,Q4AEF8,Q6AY61,Q6AYR8,Q6IG05,Q91XN4,Q9JJ50
501	1,2,3,5,6,7,8,38	4,33,34,35,36,37,39,40	14	O54858,O88339;Q4V882,P19218,P35280,P36374,P50115,P50116,P52590,P80202,P85971,Q09030,Q4AEF8,Q6IG05,Q91XN4
502	1,2,3,5,6,7,8,39	4,33,34,35,36,37,38,40	14	B0BNN3,O54858,P00714,P20766,P23593,P36374,P36376,P80202,P85971,Q09030,Q4AEF8,Q8K4G9,Q91XN4,Q9Z1F2
503	1,2,3,5,6,7,8,40	4,33,34,35,36,37,38,39	12	B0BNN3,B5DFC9,O54858,P36374,P52590,P80202,P82471,P85971,Q09030,Q4AEF8,Q91XN4,Q9JJ50

504	1,2,3,5,6,7,33,34	4,8,35,36,37,38,39,40	10	O54800;Q5DWV2,P08721,P52590,P60905,Q09030,Q5I0D7,Q6AY61,Q91XN4,Q99MH3,Q9Z2L0
505	1,2,3,5,6,7,33,35	4,8,34,36,37,38,39,40	8	O54800;Q5DWV2,P08721,P36374,P52590,Q09030,Q4AEF8,Q5I0D7,Q91XN4
506	1,2,3,5,6,7,33,36	4,8,34,35,37,38,39,40	11	O54800;Q5DWV2,P08721,P16636,P47967,P52590,P98089,Q09030,Q5I0D7,Q91XN4,Q923V8,Q99MH3
507	1,2,3,5,6,7,33,37	4,8,34,35,36,38,39,40	10	D3ZTX0,O54800;Q5DWV2,P47967,P52590,P98089,Q09030,Q6AY61,Q8CIZ5,Q91XN4,Q9JJ50
508	1,2,3,5,6,7,33,38	4,8,34,35,36,37,39,40	9	O54800;Q5DWV2,P36374,P50116,P52590,P60905,Q09030,Q6AY61,Q91XN4,Q923V8
509	1,2,3,5,6,7,33,39	4,8,34,35,36,37,38,40	10	D3ZTX0,O54800;Q5DWV2,P00714,P52590,Q09030,Q5RKI1,Q6AY61,Q6AYQ8,Q91XN4,Q99MH3
510	1,2,3,5,6,7,33,40	4,8,34,35,36,37,38,39	6	O54800;Q5DWV2,P52590,Q09030,Q62714,Q6AY61,Q91XN4
511	1,2,3,5,6,7,34,35	4,8,33,36,37,38,39,40	5	P36374,P52590,Q5I0D7,Q91XN4,Q9QWN8
512	1,2,3,5,6,7,34,36	4,8,33,35,37,38,39,40	11	B0BNN3,P47967,P52590,P98089,Q09030,Q5I0D7,Q62635,Q8K4G9,Q91XN4,Q9JJ50,Q9QWN8
513	1,2,3,5,6,7,34,37	4,8,33,35,36,38,39,40	8	P01835,P20767,P47967,P52590,P98089,Q6AY61,Q91XN4,Q9JJ50
514	1,2,3,5,6,7,34,38	4,8,33,35,36,37,39,40	11	O54800;Q5DWV2,P01835,P01946,P02091,P36374,P50116,P52590,P60905,Q6AY61,Q91XN4,Q9QWN8
515	1,2,3,5,6,7,34,39	4,8,33,35,36,37,38,40	8	P01835,P20766,P36374,P60905,Q6AY61,Q8K4G9,Q91XN4,Q9QX79
516	1,2,3,5,6,7,34,40	4,8,33,35,36,37,38,39	7	P01835,P20766,P36374,P52590,Q6AY61,Q91XN4,Q9JJ50

517	1,2,3,5,6,7,35,36	4,8,33,34,37,38,39,40	12	B0BNN3,P00762,P19218,P36374,P47967,P52590,P53792,Q09030,Q5I0D7,Q91XN4,Q9JJS8,Q9QWN8
518	1,2,3,5,6,7,35,37	4,8,33,34,36,38,39,40	7	P00762,P01835,P19132,P19218,P47967,P52590,Q8CIZ5
519	1,2,3,5,6,7,35,38	4,8,33,34,36,37,39,40	8	P13432,P19218,P19814,P24368,P36374,Q62812,Q6P6R2,Q9QWN8
520	1,2,3,5,6,7,35,39	4,8,33,34,36,37,38,40	5	P00762,P08723,P24368,P30120,P36374
521	1,2,3,5,6,7,35,40	4,8,33,34,36,37,38,39	7	P00762,P36374,P52590,P82471,Q6P6R2,Q91XN4,Q9QWN8
522	1,2,3,5,6,7,36,37	4,8,33,34,35,38,39,40	15	P00762,P01835,P09656,P19132,P47967,P52590,P55091,P97840,P98089,Q07523,Q09030,Q5I0D1,Q91XN4,Q9JJ50,Q9JJS8
523	1,2,3,5,6,7,36,38	4,8,33,34,35,37,39,40	9	P01835,P19218,P36374,P47967,P52590,Q09030,Q91XN4,Q923V8,Q9QWN8
524	1,2,3,5,6,7,36,39	4,8,33,34,35,37,38,40	8	B0BNN3,P00714,P00762,P52590,Q09030,Q8K4G9,Q91XN4,Q9QWN8
525	1,2,3,5,6,7,36,40	4,8,33,34,35,37,38,39	11	B0BNN3,P00762,P01835,P36374,P52590,P53792,P82471,Q09030,Q91XN4,Q9JJ50,Q9QWN8
526	1,2,3,5,6,7,37,38	4,8,33,34,35,36,39,40	6	P00762,P01835,P19218,P20767,Q6AY61,Q9JJ50
527	1,2,3,5,6,7,37,39	4,8,33,34,35,36,38,40	3	P00762,P01835,Q6AY61
528	1,2,3,5,6,7,37,40	4,8,33,34,35,36,38,39	9	P00762,P01835,P20767,P52590,P82471,Q62714,Q6AY61,Q91XN4,Q9JJ50
529	1,2,3,5,6,7,38,39	4,8,33,34,35,36,37,40	10	P00762,P01835,P02770,P08723,P13432,P24368,P36374,P60905,Q63556,Q6AY61

530	1,2,3,5,6,7,38,40	4,8,33,34,35,36,37,39	11	P01835,P36374,P50116,P52590,Q62714,Q62812,Q6AY61,Q6P6R2,Q91XN4,Q9QWN8,Q9QX74
531	1,2,3,5,6,7,39,40	4,8,33,34,35,36,37,38	8	B0BNN3,P00762,P01835,P36374,P52590,P82471,Q6AY61,Q91XN4
532	1,2,3,5,6,8,33,34	4,7,35,36,37,38,39,40	14	O54800;Q5DWV2,O54858,O89117,P52590,P80202,Q05702,Q09030,Q5I0D7,Q6IG05,Q91XN4,Q99MH3,Q99PP0,Q9QZQ5,Q9Z2L0
533	1,2,3,5,6,8,33,35	4,7,34,36,37,38,39,40	9	O54800;Q5DWV2,P36374,P52590,P80202,P85971,Q09030,Q6IG05,Q91XN4,Q99MH3
534	1,2,3,5,6,8,33,36	4,7,34,35,37,38,39,40	12	O54800;Q5DWV2,P00714,P08649,P16636,P52590,P80202,Q09030,Q6IG05,Q91XN4,Q99MH3,Q99PP0,Q9QZQ5
535	1,2,3,5,6,8,33,37	4,7,34,35,36,38,39,40	10	D3ZTX0,O54800;Q5DWV2,P52590,P80202,P82471,Q09030,Q6IG05,Q91XN4,Q99MH3,Q9JJ50
536	1,2,3,5,6,8,33,38	4,7,34,35,36,37,39,40	8	O54800;Q5DWV2,P52590,P80202,Q09030,Q6IFU7,Q6IG05,Q91XN4,Q99MH3
537	1,2,3,5,6,8,33,39	4,7,34,35,36,37,38,40	8	O54800;Q5DWV2,P00714,P36376,P52590,P80202,Q09030,Q91XN4,Q99MH3
538	1,2,3,5,6,8,33,40	4,7,34,35,36,37,38,39	10	O54800;Q5DWV2,P00731,P25031,P52590,P80202,P82471,Q09030,Q6IG05,Q91XN4,Q99MH3
539	1,2,3,5,6,8,34,35	4,7,33,36,37,38,39,40	9	P36374,P52590,P80202,P97580,Q09030,Q30KJ2,Q5I0D7,Q6IG05,Q91XN4
540	1,2,3,5,6,8,34,36	4,7,33,35,37,38,39,40	12	P00714,P08649,P52590,P80202,P97615,Q09030,Q5I0D7,Q6IG05,Q8K4G9,Q91XN4,Q99MH3,Q9JJ50
541	1,2,3,5,6,8,34,37	4,7,33,35,36,38,39,40	6	P52590,Q09030,Q6AYR8,Q6IG05,Q91XN4,Q9JJ50
542	1,2,3,5,6,8,34,38	4,7,33,35,36,37,39,40	8	P01946,P02091,P36374,P97580,Q09030,Q30KJ2,Q6IG05,Q91XN4

543	1,2,3,5,6,8,34,39	4,7,33,35,36,37,38,40	10	P00714,P36374,P36376,P80202,P97580,Q09030,Q30KJ2,Q8K4G9,Q91XN4,Q99MH3
544	1,2,3,5,6,8,34,40	4,7,33,35,36,37,38,39	10	P15399,P36374,P52590,P80202,P97580,Q09030,Q30KJ2,Q6IG05,Q91XN4,Q9JJ50
545	1,2,3,5,6,8,35,36	4,7,33,34,37,38,39,40	8	P19218,P36374,P52590,P53792,P82471,Q09030,Q64093,Q91XN4
546	1,2,3,5,6,8,35,37	4,7,33,34,36,38,39,40	6	F1M3L7,P19218,P52590,P82471,Q09030,Q64093
547	1,2,3,5,6,8,35,38	4,7,33,34,36,37,39,40	9	P19218,P19814,P24368,P36374,P36376,P82471,Q09030,Q5I0J9,Q6IG05
548	1,2,3,5,6,8,35,39	4,7,33,34,36,37,38,40	5	P23593,P36374,P36376,P82471,Q09030
549	1,2,3,5,6,8,35,40	4,7,33,34,36,37,38,39	8	P36374,P36376,P52590,P80202,P82471,Q09030,Q64093,Q91XN4
550	1,2,3,5,6,8,36,37	4,7,33,34,35,38,39,40	9	F1M3L7,P09656,P19218,P52590,P55091,P82471,Q09030,Q91XN4,Q9JJ50
551	1,2,3,5,6,8,36,38	4,7,33,34,35,37,39,40	6	P19218,P36374,P52590,Q09030,Q6IG05,Q91XN4
552	1,2,3,5,6,8,36,39	4,7,33,34,35,37,38,40	10	B0BNN3,P00714,P36376,P52590,P82471,Q09030,Q8K4G9,Q91XN4,Q99MH3,Q9R168
553	1,2,3,5,6,8,36,40	4,7,33,34,35,37,38,39	8	B0BNN3,P52590,P53792,P80202,P82471,Q09030,Q91XN4,Q9JJ50
554	1,2,3,5,6,8,37,38	4,7,33,34,35,36,39,40	4	P19218,P82471,Q6IG05,Q91XN4
555	1,2,3,5,6,8,37,39	4,7,33,34,35,36,38,40	4	P00714,P36376,P82471,Q9R168

556	1,2,3,5,6,8,37,40	4,7,33,34,35,36,38,39	5	P52590,P82471,Q09030,Q91XN4,Q9JJ50
557	1,2,3,5,6,8,38,39	4,7,33,34,35,36,37,40	5	P24368,P36374,P36376,Q09030,Q6IG05
558	1,2,3,5,6,8,38,40	4,7,33,34,35,36,37,39	9	P25031,P36374,P36376,P52590,P82471,Q09030,Q6IG05,Q6P6R2,Q91XN4
559	1,2,3,5,6,8,39,40	4,7,33,34,35,36,37,38	10	B0BNN3,P25031,P36374,P36376,P52590,P80202,P82471,Q09030,Q91XN4,Q9R168
560	1,2,3,5,6,33,34,35	4,7,8,36,37,38,39,40	10	O54800;Q5DWV2,O89117,P08721,P52590,Q09030,Q5I0D7,Q812E4,Q91XN4,Q99MH3,Q9Z2L0
561	1,2,3,5,6,33,34,36	4,7,8,35,37,38,39,40	13	O54800;Q5DWV2,P08721,P52590,P97584,P98089,Q09030,Q5I0D7,Q5QE79,Q62635,Q91XN4,Q99041,Q99MH3,Q9Z2L0
562	1,2,3,5,6,33,34,37	4,7,8,35,36,38,39,40	10	O54800;Q5DWV2,P52590,P98089,Q09030,Q5I0D7,Q62635,Q6AY61,Q91XN4,Q99MH3,Q9JJ50
563	1,2,3,5,6,33,34,38	4,7,8,35,36,37,39,40	11	O54800;Q5DWV2,P01946,P02091,P52590,P60905,Q09030,Q6AY61,Q812E4,Q91XN4,Q99MH3,Q9Z2L0
564	1,2,3,5,6,33,34,39	4,7,8,35,36,37,38,40	7	O54800;Q5DWV2,P00714,P52590,Q09030,Q99041,Q99MH3,Q9Z2L0
565	1,2,3,5,6,33,34,40	4,7,8,35,36,37,38,39	8	O54800;Q5DWV2,P01039,P52590,Q09030,Q6AY61,Q91XN4,Q99MH3,Q9Z2L0
566	1,2,3,5,6,33,35,36	4,7,8,34,37,38,39,40	11	D4A5U3,P08721,P49134,P52590,P53792,Q09030,Q5I0D7,Q5RKI7,Q91XN4,Q99MH3,Q9Z0V6
567	1,2,3,5,6,33,35,37	4,7,8,34,36,38,39,40	5	F1M3L7,P52590,Q09030,Q64093,Q8CIZ5
568	1,2,3,5,6,33,35,38	4,7,8,34,36,37,39,40	9	O54800;Q5DWV2,P22282,P49134,P52590,Q09030,Q5I0J9,Q6P6R2,Q812E4,Q9Z2L0
569	1,2,3,5,6,33,35,39	4,7,8,34,36,37,38,40	5	P30120,P62804,Q09030,Q99MH3,Q9Z2L0
570	1,2,3,5,6,33,35,40	4,7,8,34,36,37,38,39	6	P22282,P52590,P53792,Q09030,Q6P6R2,Q9EQS0
571	1,2,3,5,6,33,36,37	4,7,8,34,35,38,39,40	14	D3ZTX0,D4A5U3,P09656,P11883,P52590,P98089,Q05695,Q09030,Q62635,Q8CIZ5,Q91XN4,Q99MH3,Q9JJ50,Q9Z0V6
572	1,2,3,5,6,33,36,38	4,7,8,34,35,37,39,40	8	O54800;Q5DWV2,P22282,P52590,Q09030,Q91XN4,Q923V8,Q99MH3,Q9Z0V6
573	1,2,3,5,6,33,36,39	4,7,8,34,35,37,38,40	5	P00714,P52590,Q09030,Q99041,Q99MH3

574	1,2,3,5,6,33,36,40	4,7,8,34,35,37,38,39	7	P52590,P53792,Q09030,Q91XN4,Q99MH3,Q9WTT6,Q9Z0V6
575	1,2,3,5,6,33,37,38	4,7,8,34,35,36,39,40	4	O54800;Q5DWV2,P52590,Q09030,Q6AY61
576	1,2,3,5,6,33,37,39	4,7,8,34,35,36,38,40	4	D3ZTX0,Q09030,Q6AY61,Q99MH3
577	1,2,3,5,6,33,37,40	4,7,8,34,35,36,38,39	6	P52590,P82471,Q09030,Q62714,Q6AY61,Q9Z0V6
578	1,2,3,5,6,33,38,39	4,7,8,34,35,36,37,40	5	O54800;Q5DWV2,P22282,Q09030,Q99041,Q99MH3
579	1,2,3,5,6,33,38,40	4,7,8,34,35,36,37,39	10	O54800;Q5DWV2,P01039,P22282,P52590,Q09030,Q62714,Q6P6R2,Q91XN4,Q9QX74,Q9Z0V6
580	1,2,3,5,6,33,39,40	4,7,8,34,35,36,37,38	4	P01039,P52590,Q09030,Q99MH3
581	1,2,3,5,6,34,35,36	4,7,8,33,37,38,39,40	5	P21674,P52590,P53792,Q5I0D7,Q9QWN8
582	1,2,3,5,6,34,35,37	4,7,8,33,36,38,39,40	0	
583	1,2,3,5,6,34,35,38	4,7,8,33,36,37,39,40	12	P01946,P02091,P02782,P08723,P0C0A9,P30120,P36374,Q4TU93,Q5I0J9,Q812E4,Q9QWN8,Q9Z2L0
584	1,2,3,5,6,34,35,39	4,7,8,33,36,37,38,40	4	P08723,P30120,P36374,Q9Z2L0
585	1,2,3,5,6,34,35,40	4,7,8,33,36,37,38,39	3	P08723,P36374,P52590
586	1,2,3,5,6,34,36,37	4,7,8,33,35,38,39,40	8	P09656,P47967,P52590,P55091,P98089,Q05695,Q62635,Q9JJ50
587	1,2,3,5,6,34,36,38	4,7,8,33,35,37,39,40	5	P01946,P02091,P52590,Q91XN4,Q9QWN8
588	1,2,3,5,6,34,36,39	4,7,8,33,35,37,38,40	6	B0BNN3,P00714,P52590,Q8K4G9,Q99041,Q99MH3
589	1,2,3,5,6,34,36,40	4,7,8,33,35,37,38,39	4	P52590,P53792,Q91XN4,Q9JJ50
590	1,2,3,5,6,34,37,38	4,7,8,33,35,36,39,40	6	P01835,P01946,P02091,Q4KLZ6,Q6AY61,Q9JJ50
591	1,2,3,5,6,34,37,39	4,7,8,33,35,36,38,40	2	P01835,Q6AY61
592	1,2,3,5,6,34,37,40	4,7,8,33,35,36,38,39	6	P01835,P20767,P52590,Q62714,Q6AY61,Q9JJ50
593	1,2,3,5,6,34,38,39	4,7,8,33,35,36,37,40	7	P01946,P02091,P08723,P0C0A9,P36374,P60905,Q6AY61

594	1,2,3,5,6,34,38,40	4,7,8,33,35,36,37,39	9	P01946,P02091,P08723,P0C0A9,P36374,P52590,P97580,Q6AY61,Q6P6R2
595	1,2,3,5,6,34,39,40	4,7,8,33,35,36,37,38	3	P08723,P52590,Q6AY61
596	1,2,3,5,6,35,36,37	4,7,8,33,34,38,39,40	12	D4A5U3,F1M3L7,P09656,P19132,P19218,P25236,P52590,P53792,P82471,Q64093,Q8CIZ5,Q9Z0V6
597	1,2,3,5,6,35,36,38	4,7,8,33,34,37,39,40	12	P0C0A9,P19218,P19814,P21674,P22282,P24368,P52590,P53792,Q5I0J9,Q923V8,Q9QWN8,Q9Z0V6
598	1,2,3,5,6,35,36,39	4,7,8,33,34,37,38,40	5	B0BNN3,P24368,P30120,P52590,P53792
599	1,2,3,5,6,35,36,40	4,7,8,33,34,37,38,39	8	P02793;Q7TP54,P20646,P52590,P53792,P82471,Q64093,Q9EQS0,Q9Z0V6
600	1,2,3,5,6,35,37,38	4,7,8,33,34,36,39,40	6	P19218,P19814,P20646,P82471,Q5I0J9,Q9QX74
601	1,2,3,5,6,35,37,39	4,7,8,33,34,36,38,40	3	P20646,P30919,P82471
602	1,2,3,5,6,35,37,40	4,7,8,33,34,36,38,39	8	P19132,P20646,P52590,P53792,P82471,Q64093,Q9QX74,Q9Z0V6
603	1,2,3,5,6,35,38,39	4,7,8,33,34,36,37,40	12	P08723,P0C0A9,P19814,P20646,P22282,P24368,P30120,P36374,P47727,P62898,Q6P6R2,Q9QX74
604	1,2,3,5,6,35,38,40	4,7,8,33,34,36,37,39	12	P08723,P0C0A9,P19814,P20646,P22282,P36374,P52590,P53792,P82471,Q5I0J9,Q6P6R2,Q9QX74
605	1,2,3,5,6,35,39,40	4,7,8,33,34,36,37,38	7	P08723,P0C0A9,P20646,P36374,P82471,Q6P6R2,Q9QX74
606	1,2,3,5,6,36,37,38	4,7,8,33,34,35,39,40	8	P01835,P09656,P19218,P52590,P82471,Q9JJ50,Q9QX74,Q9Z0V6
607	1,2,3,5,6,36,37,39	4,7,8,33,34,35,38,40	4	P00714,P09656,P52590,P82471
608	1,2,3,5,6,36,37,40	4,7,8,33,34,35,38,39	8	P01835,P09656,P52590,P53792,P82471,Q9JJ50,Q9QX74,Q9Z0V6
609	1,2,3,5,6,36,38,39	4,7,8,33,34,35,37,40	6	P00714,P0C0A9,P22282,P24368,Q923V8,Q9QX74
610	1,2,3,5,6,36,38,40	4,7,8,33,34,35,37,39	8	P0C0A9,P22282,P52590,P53792,P82471,Q9QWN8,Q9QX74,Q9Z0V6
611	1,2,3,5,6,36,39,40	4,7,8,33,34,35,37,38	4	B0BNN3,P52590,P82471,Q9QX74
612	1,2,3,5,6,37,38,39	4,7,8,33,34,35,36,40	4	P01835,P82471,Q6AY61,Q9QX74
613	1,2,3,5,6,37,38,40	4,7,8,33,34,35,36,39	9	P01835,P20646,P20767,P52590,P82471,Q62714,Q6AY61,Q9QX74,Q9Z0V6

614	1,2,3,5,6,37,39,40	4,7,8,33,34,35,36,38	6	P00762,P01835,P52590,P82471,Q6AY61,Q9QX74
615	1,2,3,5,6,38,39,40	4,7,8,33,34,35,36,37	10	P08723,P0C0A9,P20646,P22282,P36374,P51740,P82471,Q6AY61,Q6P6R2,Q9QX74
616	1,2,3,5,7,8,33,34	4,6,35,36,37,38,39,40	4	B1H234,P51740,Q6IG05,Q99MH3
617	1,2,3,5,7,8,33,35	4,6,34,36,37,38,39,40	1	P51740
618	1,2,3,5,7,8,33,36	4,6,34,35,37,38,39,40	7	B1H234,P00714,P01681,P47967,P51740,Q09030,Q99MH3
619	1,2,3,5,7,8,33,37	4,6,34,35,36,38,39,40	6	B1H234,P47967,P51740,Q6AYQ8,Q6AYR8,Q6IG05
620	1,2,3,5,7,8,33,38	4,6,34,35,36,37,39,40	3	B1H234,P35280,Q6IG05
621	1,2,3,5,7,8,33,39	4,6,34,35,36,37,38,40	4	P00714,P36376,Q5RKI1,Q6AYQ8
622	1,2,3,5,7,8,33,40	4,6,34,35,36,37,38,39	1	P00731
623	1,2,3,5,7,8,34,35	4,6,33,36,37,38,39,40	7	B1H234,P20766,P36374,P36860,P47967,P51740,Q9Z1F2
624	1,2,3,5,7,8,34,36	4,6,33,35,37,38,39,40	11	B1H234,P00714,P20766,P47967,P51740,P55091,P97615,Q66H69,Q6IG05,Q9JJ50,Q9Z1F2
625	1,2,3,5,7,8,34,37	4,6,33,35,36,38,39,40	10	B1H234,P06760,P47967,P51740,P55091,Q66H69,Q6AY61,Q6AYR8,Q6IG05,Q9JJ50
626	1,2,3,5,7,8,34,38	4,6,33,35,36,37,39,40	9	B1H234,P01946,P02091,P20766,P35280,P36374,P60905,Q6AY61,Q6IG05
627	1,2,3,5,7,8,34,39	4,6,33,35,36,37,38,40	7	P00714,P20762,P20766,P36374,P36376,Q9Z0V6,Q9Z1F2

628	1,2,3,5,7,8,34,40	4,6,33,35,36,37,38,39	4	P20766,P36374,P97580,Q9WUW8
629	1,2,3,5,7,8,35,36	4,6,33,34,37,38,39,40	6	P19218,P36374,P47967,P51740,P97840,Q5I0D1
630	1,2,3,5,7,8,35,37	4,6,33,34,36,38,39,40	7	F1M3L7,P19218,P36860,P47967,P51740,Q66H69,Q6AYR8
631	1,2,3,5,7,8,35,38	4,6,33,34,36,37,39,40	5	P19218,P19814,P35280,P36374,Q6IG05
632	1,2,3,5,7,8,35,39	4,6,33,34,36,37,38,40	4	P36374,P36376,P36860,Q9Z1F2
633	1,2,3,5,7,8,35,40	4,6,33,34,36,37,38,39	1	P36374
634	1,2,3,5,7,8,36,37	4,6,33,34,35,38,39,40	8	P47967,P51740,P55091,P97840,Q5I0D1,Q66H69,Q6AYR8,Q9JJ50
635	1,2,3,5,7,8,36,38	4,6,33,34,35,37,39,40	6	B1H234,P19218,P35280,P36374,P47967,Q6IG05
636	1,2,3,5,7,8,36,39	4,6,33,34,35,37,38,40	4	B0BNN3,P00714,P36376,Q9Z1F2
637	1,2,3,5,7,8,36,40	4,6,33,34,35,37,38,39	0	
638	1,2,3,5,7,8,37,38	4,6,33,34,35,36,39,40	7	B1H234,P19218,P22006,P35280,Q6AY61,Q6IFW6,Q6IG05
639	1,2,3,5,7,8,37,39	4,6,33,34,35,36,38,40	5	P00714,P36376,P63029,Q6AY61,Q6AYQ8
640	1,2,3,5,7,8,37,40	4,6,33,34,35,36,38,39	6	D3ZUC6,P22006,P82471,Q66H69,Q6AY61,Q6AYR8

641	1,2,3,5,7,8,38,39	4,6,33,34,35,36,37,40	7	P02770,P11883,P35280,P36374,P36376,Q63556,Q6IG05
642	1,2,3,5,7,8,38,40	4,6,33,34,35,36,37,39	4	P22006,P35280,P36374,Q6IG05
643	1,2,3,5,7,8,39,40	4,6,33,34,35,36,37,38	2	P36374,P36376
644	1,2,3,5,7,33,34,35	4,6,8,36,37,38,39,40	8	P06911,P08721,P12020,P19629,P47967,P51740,Q5GRG2,Q5I0D7
645	1,2,3,5,7,33,34,36	4,6,8,35,37,38,39,40	9	P08721,P47967,P51740,P98089,Q5I0D7,Q62635,Q99041,Q99MH3,Q9WUW8
646	1,2,3,5,7,33,34,37	4,6,8,35,36,38,39,40	8	P47967,P51740,P54921,P98089,Q62635,Q6AY61,Q6AYQ8,Q9WUW8
647	1,2,3,5,7,33,34,38	4,6,8,35,36,37,39,40	6	P01946,P02091,P60905,P80299,Q6AY61,Q9WUW8
648	1,2,3,5,7,33,34,39	4,6,8,35,36,37,38,40	8	P00714,P20766,P60905,Q5RKI1,Q6AY61,Q6AYQ8,Q99041,Q99MH3
649	1,2,3,5,7,33,34,40	4,6,8,35,36,37,38,39	2	Q6AY61,Q9WUW8
650	1,2,3,5,7,33,35,36	4,6,8,34,37,38,39,40	5	P08721,P47967,P49134,P51740,P97840
651	1,2,3,5,7,33,35,37	4,6,8,34,36,38,39,40	2	P47967,P51740
652	1,2,3,5,7,33,35,38	4,6,8,34,36,37,39,40	3	P12020,P13432,P22282
653	1,2,3,5,7,33,35,39	4,6,8,34,36,37,38,40	2	P12020,Q5RKI1
654	1,2,3,5,7,33,35,40	4,6,8,34,36,37,38,39	0	
655	1,2,3,5,7,33,36,37	4,6,8,34,35,38,39,40	6	P47967,P51740,P55091,P97840,P98089,Q62635
656	1,2,3,5,7,33,36,38	4,6,8,34,35,37,39,40	2	P47967,Q923V8
657	1,2,3,5,7,33,36,39	4,6,8,34,35,37,38,40	4	P00714,Q5RKI1,Q99041,Q99MH3
658	1,2,3,5,7,33,36,40	4,6,8,34,35,37,38,39	1	P01681
659	1,2,3,5,7,33,37,38	4,6,8,34,35,36,39,40	2	P47967,Q6AY61

660	1,2,3,5,7,33,37,39	4,6,8,34,35,36,38,40	4	P00714,Q5RKI1,Q6AY61,Q6AYQ8
661	1,2,3,5,7,33,37,40	4,6,8,34,35,36,38,39	2	Q62714,Q6AY61
662	1,2,3,5,7,33,38,39	4,6,8,34,35,36,37,40	7	P02770,P13432,P60905,Q5RKI1,Q63556,Q6AY61,Q99041
663	1,2,3,5,7,33,38,40	4,6,8,34,35,36,37,39	6	P01039,P22282,Q62714,Q6AY61,Q6P6R2,Q9WUW8
664	1,2,3,5,7,33,39,40	4,6,8,34,35,36,37,38	3	Q5RKI1,Q6AY61,Q6AYQ8
665	1,2,3,5,7,34,35,36	4,6,8,33,37,38,39,40	5	P47967,P51740,P97840,Q5I0D7,Q9QWN8
666	1,2,3,5,7,34,35,37	4,6,8,33,36,38,39,40	3	P47967,P51740,Q6AY61
667	1,2,3,5,7,34,35,38	4,6,8,33,36,37,39,40	4	P01946,P02091,P36374,Q9QWN8
668	1,2,3,5,7,34,35,39	4,6,8,33,36,37,38,40	3	P08723,P12020,P20766
669	1,2,3,5,7,34,35,40	4,6,8,33,36,37,38,39	1	P36374
670	1,2,3,5,7,34,36,37	4,6,8,33,35,38,39,40	9	P47967,P51740,P55091,P97840,P98089,Q05695,Q5I0D1,Q62635,Q9JJ50
671	1,2,3,5,7,34,36,38	4,6,8,33,35,37,39,40	6	P01946,P02091,P47967,Q5RLM2,Q9QWN8,Q9WUW8
672	1,2,3,5,7,34,36,39	4,6,8,33,35,37,38,40	4	P00714,P20766,Q99041,Q9Z1F2
673	1,2,3,5,7,34,36,40	4,6,8,33,35,37,38,39	2	Q9QWN8,Q9WUW8
674	1,2,3,5,7,34,37,38	4,6,8,33,35,36,39,40	8	P01835,P01946,P02091,P20767,P47967,P54921,Q6AY61,Q9WUW8
675	1,2,3,5,7,34,37,39	4,6,8,33,35,36,38,40	3	P01835,Q6AY61,Q6AYQ8
676	1,2,3,5,7,34,37,40	4,6,8,33,35,36,38,39	6	P01835,P20767,Q62714,Q6AY61,Q9JJ50,Q9WUW8
677	1,2,3,5,7,34,38,39	4,6,8,33,35,36,37,40	8	P01946,P02091,P02770,P08723,P20766,P60905,Q63556,Q6AY61
678	1,2,3,5,7,34,38,40	4,6,8,33,35,36,37,39	6	P01946,P02091,Q5RLM2,Q6AY61,Q9QWN8,Q9WUW8
679	1,2,3,5,7,34,39,40	4,6,8,33,35,36,37,38	2	P20766,Q6AY61

680	1,2,3,5,7,35,36,37	4,6,8,33,34,38,39,40	9	F1M3L7,P19132,P25031,P47967,P51740,P55091,P97840,Q5I0D1,Q9JJS8
681	1,2,3,5,7,35,36,38	4,6,8,33,34,37,39,40	6	P13432,P19218,P19814,P21674,P47967,Q9QWN8
682	1,2,3,5,7,35,36,39	4,6,8,33,34,37,38,40	2	P23739,Q9QWN8
683	1,2,3,5,7,35,36,40	4,6,8,33,34,37,38,39	3	P02793;Q7TP54,P53792,Q9QWN8
684	1,2,3,5,7,35,37,38	4,6,8,33,34,36,39,40	6	P08494,P08649,P19218,P19814,P47967,Q6AY61
685	1,2,3,5,7,35,37,39	4,6,8,33,34,36,38,40	1	P08649
686	1,2,3,5,7,35,37,40	4,6,8,33,34,36,38,39	3	P08649,P19132,P20767
687	1,2,3,5,7,35,38,39	4,6,8,33,34,36,37,40	10	P02770,P08649,P08723,P13432,P19814,P24368,P30120,P36374,Q63556,Q9QW07
688	1,2,3,5,7,35,38,40	4,6,8,33,34,36,37,39	7	P08649,P19814,P22282,P36374,Q62812,Q6P6R2,Q9QWN8
689	1,2,3,5,7,35,39,40	4,6,8,33,34,36,37,38	3	P08649,P08723,P36374
690	1,2,3,5,7,36,37,38	4,6,8,33,34,35,39,40	3	P01835,P47967,P97840
691	1,2,3,5,7,36,37,39	4,6,8,33,34,35,38,40	2	P00714,P47967
692	1,2,3,5,7,36,37,40	4,6,8,33,34,35,38,39	3	P01835,P47967,Q9JJ50
693	1,2,3,5,7,36,38,39	4,6,8,33,34,35,37,40	3	P00714,Q9QW07,Q9QWN8
694	1,2,3,5,7,36,38,40	4,6,8,33,34,35,37,39	3	P22282,Q5RLM2,Q9QWN8
695	1,2,3,5,7,36,39,40	4,6,8,33,34,35,37,38	1	B0BNN3
696	1,2,3,5,7,37,38,39	4,6,8,33,34,35,36,40	5	P01835,P02770,P08494,P08649,Q6AY61
697	1,2,3,5,7,37,38,40	4,6,8,33,34,35,36,39	8	P01835,P08494,P08649,P20767,P22006,Q62714,Q6AY61,Q9QX74
698	1,2,3,5,7,37,39,40	4,6,8,33,34,35,36,38	4	P00762,P01835,P08649,Q6AY61
699	1,2,3,5,7,38,39,40	4,6,8,33,34,35,36,37	9	O55004,P02770,P08649,P08723,P22282,Q63556,Q6AY61,Q6P6R2,Q9QX74

700	1,2,3,5,8,33,34,35	4,6,7,36,37,38,39,40	4	O89117,P51740,Q6IG05,Q99MH3
701	1,2,3,5,8,33,34,36	4,6,7,35,37,38,39,40	9	P00714,P01681,P51740,P98089,Q5I0D7,Q5QE79,Q6IG05,Q99041,Q99MH3
702	1,2,3,5,8,33,34,37	4,6,7,35,36,38,39,40	4	P51740,P98089,Q6AYR8,Q6IG05
703	1,2,3,5,8,33,34,38	4,6,7,35,36,37,39,40	6	B1H234,P01946,P02091,Q6IFU7,Q6IG05,Q99041
704	1,2,3,5,8,33,34,39	4,6,7,35,36,37,38,40	7	P00714,P02651,P02793;Q7TP54,P36376,Q6IFU7,Q99041,Q99MH3
705	1,2,3,5,8,33,34,40	4,6,7,35,36,37,38,39	4	P00731,P97580,Q6IFU7,Q6IG05
706	1,2,3,5,8,33,35,36	4,6,7,34,37,38,39,40	4	P00714,P01681,P51740,Q99MH3
707	1,2,3,5,8,33,35,37	4,6,7,34,36,38,39,40	2	F1M3L7,P51740
708	1,2,3,5,8,33,35,38	4,6,7,34,36,37,39,40	1	Q6IG05
709	1,2,3,5,8,33,35,39	4,6,7,34,36,37,38,40	3	P00714,P11598,P36376
710	1,2,3,5,8,33,35,40	4,6,7,34,36,37,38,39	1	P01681
711	1,2,3,5,8,33,36,37	4,6,7,34,35,38,39,40	8	F1M3L7,P00714,P47967,P51740,P55091,P98089,Q05695,Q6IG05
712	1,2,3,5,8,33,36,38	4,6,7,34,35,37,39,40	5	P00714,P01681,Q6IFU7,Q6IG05,Q99041
713	1,2,3,5,8,33,36,39	4,6,7,34,35,37,38,40	5	P00714,P01681,P36376,Q99041,Q99MH3
714	1,2,3,5,8,33,36,40	4,6,7,34,35,37,38,39	1	P01681
715	1,2,3,5,8,33,37,38	4,6,7,34,35,36,39,40	2	P22006,Q6IG05
716	1,2,3,5,8,33,37,39	4,6,7,34,35,36,38,40	3	P00714,P36376,Q6AYQ8
717	1,2,3,5,8,33,37,40	4,6,7,34,35,36,38,39	0	
718	1,2,3,5,8,33,38,39	4,6,7,34,35,36,37,40	6	P00714,P19132,P36376,Q6IFU7,Q6IG05,Q99041
719	1,2,3,5,8,33,38,40	4,6,7,34,35,36,37,39	2	Q6IFU7,Q6IG05

720	1,2,3,5,8,33,39,40	4,6,7,34,35,36,37,38	2	P01681,P36376
721	1,2,3,5,8,34,35,36	4,6,7,33,37,38,39,40	1	P51740
722	1,2,3,5,8,34,35,37	4,6,7,33,36,38,39,40	4	F1M3L7,P36860,P51740,Q6IG05
723	1,2,3,5,8,34,35,38	4,6,7,33,36,37,39,40	5	P01946,P02091,P36374,P97580,Q6IG05
724	1,2,3,5,8,34,35,39	4,6,7,33,36,37,38,40	3	P00714,P36376,P36860
725	1,2,3,5,8,34,35,40	4,6,7,33,36,37,38,39	2	P97580,Q30KJ2
726	1,2,3,5,8,34,36,37	4,6,7,33,35,38,39,40	9	P00714,P47967,P51740,P55091,P98089,Q05695,Q6AYR8,Q6IG05,Q9JJ50
727	1,2,3,5,8,34,36,38	4,6,7,33,35,37,39,40	3	P01946,P02091,Q6IG05
728	1,2,3,5,8,34,36,39	4,6,7,33,35,37,38,40	4	P00714,P36376,Q99041,Q9Z1F2
729	1,2,3,5,8,34,36,40	4,6,7,33,35,37,38,39	1	P97580
730	1,2,3,5,8,34,37,38	4,6,7,33,35,36,39,40	6	P01946,P02091,Q6IFU8,Q6IFW6,Q6IG05,Q6IMF3
731	1,2,3,5,8,34,37,39	4,6,7,33,35,36,38,40	2	P00714,P36376
732	1,2,3,5,8,34,37,40	4,6,7,33,35,36,38,39	3	P97580,Q6AYR8,Q6IG05
733	1,2,3,5,8,34,38,39	4,6,7,33,35,36,37,40	7	P00714,P01946,P02091,P36376,P97580,Q6IFU7,Q6IG05
734	1,2,3,5,8,34,38,40	4,6,7,33,35,36,37,39	7	P01946,P02091,P15399,P97580,Q30KJ2,Q6IFU7,Q6IG05
735	1,2,3,5,8,34,39,40	4,6,7,33,35,36,37,38	3	P36376,P97580,Q30KJ2
736	1,2,3,5,8,35,36,37	4,6,7,33,34,38,39,40	4	F1M3L7,P47967,P51740,P55091
737	1,2,3,5,8,35,36,38	4,6,7,33,34,37,39,40	4	P19218,P19814,P21674,Q6IG05
738	1,2,3,5,8,35,36,39	4,6,7,33,34,37,38,40	3	P00714,P23739,P36376
739	1,2,3,5,8,35,36,40	4,6,7,33,34,37,38,39	1	P82471

740	1,2,3,5,8,35,37,38	4,6,7,33,34,36,39,40	4	F1M3L7,P19218,P19814,Q6IG05
741	1,2,3,5,8,35,37,39	4,6,7,33,34,36,38,40	3	F1M3L7,P36376,P80299
742	1,2,3,5,8,35,37,40	4,6,7,33,34,36,38,39	3	F1M3L7,P80299,P82471
743	1,2,3,5,8,35,38,39	4,6,7,33,34,36,37,40	6	P08723,P0C0A9,P11598,P24368,P36374,P36376
744	1,2,3,5,8,35,38,40	4,6,7,33,34,36,37,39	5	P0C0A9,P11598,P19814,P36374,Q6IG05
745	1,2,3,5,8,35,39,40	4,6,7,33,34,36,37,38	3	P11598,P36376,P82471
746	1,2,3,5,8,36,37,38	4,6,7,33,34,35,39,40	3	F1M3L7,P19218,Q6IG05
747	1,2,3,5,8,36,37,39	4,6,7,33,34,35,38,40	3	P00714,P36376,P80299
748	1,2,3,5,8,36,37,40	4,6,7,33,34,35,38,39	3	F1M3L7,P80299,P82471
749	1,2,3,5,8,36,38,39	4,6,7,33,34,35,37,40	3	P00714,P36376,Q6IG05
750	1,2,3,5,8,36,38,40	4,6,7,33,34,35,37,39	1	Q6IG05
751	1,2,3,5,8,36,39,40	4,6,7,33,34,35,37,38	4	B0BNN3,P36376,P82471,Q9R168
752	1,2,3,5,8,37,38,39	4,6,7,33,34,35,36,40	4	P08494,P36376,Q6IFW6,Q6IG05
753	1,2,3,5,8,37,38,40	4,6,7,33,34,35,36,39	4	P08494,P22006,P82471,Q6IG05
754	1,2,3,5,8,37,39,40	4,6,7,33,34,35,36,38	3	P36376,P80299,P82471
755	1,2,3,5,8,38,39,40	4,6,7,33,34,35,36,37	5	P11598,P36376,Q63617,Q6IFU7,Q6PCU2
756	1,2,3,5,33,34,35,36	4,6,7,8,37,38,39,40	10	D3ZUC6,P08721,P12020,P22273,P22282,P51740,Q5I0D7,Q5QE79,Q99041,Q99MH3
757	1,2,3,5,33,34,35,37	4,6,7,8,36,38,39,40	4	P12020,P22273,P51740,Q5QE79
758	1,2,3,5,33,34,35,38	4,6,7,8,36,37,39,40	14	P01946,P02091,P06911,P0C0A9,P11598,P12020,P22273,P22282,P46462,Q5M8C6,Q63617,Q812E4,Q99041,Q9Z2L0
759	1,2,3,5,33,34,35,39	4,6,7,8,36,37,38,40	12	P00714,P06911,P0C0A9,P11598,P12020,P22273,P22282,Q5GRG2,Q63617,Q99041,Q99MH3,Q9Z2L0

760	1,2,3,5,33,34,35,40	4,6,7,8,36,37,38,39	8	P06911,P0C0A9,P12020,P22273,P22282,Q5M8C6,Q63617,Q99041
761	1,2,3,5,33,34,36,37	4,6,7,8,35,38,39,40	11	P22273,P47967,P51740,P54921,P55091,P98089,Q05695,Q5QE79,Q62635,Q99041,Q99MH3
762	1,2,3,5,33,34,36,38	4,6,7,8,35,37,39,40	6	P01946,P02091,P22273,P22282,Q63617,Q99041
763	1,2,3,5,33,34,36,39	4,6,7,8,35,37,38,40	6	P00714,P22273,P22282,Q63617,Q99041,Q99MH3
764	1,2,3,5,33,34,36,40	4,6,7,8,35,37,38,39	6	P22273,P22282,Q5QE79,Q63617,Q99041,Q99MH3
765	1,2,3,5,33,34,37,38	4,6,7,8,35,36,39,40	5	P01946,P02091,P54921,Q6AY61,Q99041
766	1,2,3,5,33,34,37,39	4,6,7,8,35,36,38,40	7	O70417,P00714,P02793;Q7TP54,Q6AY61,Q6AYQ8,Q99041,Q99376
767	1,2,3,5,33,34,37,40	4,6,7,8,35,36,38,39	2	Q62714,Q6AY61
768	1,2,3,5,33,34,38,39	4,6,7,8,35,36,37,40	13	P00714,P01946,P02091,P02793;Q7TP54,P0C0A9,P19132,P22273,P22282,P60905,Q63617,Q6AY61,Q99041,Q9QW07
769	1,2,3,5,33,34,38,40	4,6,7,8,35,36,37,39	10	P01039,P01946,P02091,P0C0A9,P22273,P22282,Q63617,Q6AY61,Q99041,Q9WUW8
770	1,2,3,5,33,34,39,40	4,6,7,8,35,36,37,38	7	P19218,P22273,P22282,Q63617,Q6AY61,Q99041,Q99MH3
771	1,2,3,5,33,35,36,37	4,6,7,8,34,38,39,40	6	D4A5U3,F1M3L7,P22273,P47967,P51740,Q5QE79
772	1,2,3,5,33,35,36,38	4,6,7,8,34,37,39,40	9	P0C0A9,P11598,P21674,P22273,P22282,P49134,Q63617,Q99041,Q9QW07
773	1,2,3,5,33,35,36,39	4,6,7,8,34,37,38,40	11	P00714,P0C0A9,P11598,P12020,P22273,P22282,P23739,P49134,Q63617,Q99041,Q99MH3
774	1,2,3,5,33,35,36,40	4,6,7,8,34,37,38,39	10	P01681,P0C0A9,P22273,P22282,P49134,P53792,Q63617,Q99041,Q9EQS0,Q9Z0V6
775	1,2,3,5,33,35,37,38	4,6,7,8,34,36,39,40	1	P22282
776	1,2,3,5,33,35,37,39	4,6,7,8,34,36,38,40	2	P12020,Q99041
777	1,2,3,5,33,35,37,40	4,6,7,8,34,36,38,39	1	F1M3L7
778	1,2,3,5,33,35,38,39	4,6,7,8,34,36,37,40	12	P06761,P0C0A9,P11598,P12020,P22273,P22282,P30120,P46462,P49134,Q63617,Q99041,Q9QW07
779	1,2,3,5,33,35,38,40	4,6,7,8,34,36,37,39	10	P06761,P0C0A9,P11598,P22273,P22282,P22283,P46462,P63029,Q63617,Q6P6R2

780	1,2,3,5,33, 35,39,40	4,6,7,8,34, 36,37,38	8	P06761,P0C0A9,P11598,P12020,P22273,P22282,Q63617,Q99041
781	1,2,3,5,33, 36,37,38	4,6,7,8,34, 35,39,40	3	P22282,Q99041,Q9Z0V6
782	1,2,3,5,33, 36,37,39	4,6,7,8,34, 35,38,40	2	P00714,Q99041
783	1,2,3,5,33, 36,37,40	4,6,7,8,34, 35,38,39	2	Q9WTT6,Q9Z0V6
784	1,2,3,5,33, 36,38,39	4,6,7,8,34, 35,37,40	8	P00714,P0C0A9,P22273,P22282,Q63617,Q923V8,Q99041,Q9QW07
785	1,2,3,5,33, 36,38,40	4,6,7,8,34, 35,37,39	6	P0C0A9,P22273,P22282,Q63617,Q99041,Q9Z0V6
786	1,2,3,5,33, 36,39,40	4,6,7,8,34, 35,37,38	5	P01681,P22273,P22282,Q63617,Q99041
787	1,2,3,5,33, 37,38,39	4,6,7,8,34, 35,36,40	3	P22282,Q6AY61,Q99041
788	1,2,3,5,33, 37,38,40	4,6,7,8,34, 35,36,39	6	P22282,Q62714,Q6AY61,Q9QX74,Q9Z0V6,Q9Z1F2
789	1,2,3,5,33, 37,39,40	4,6,7,8,34, 35,36,38	3	O70417,Q6AY61,Q99041
790	1,2,3,5,33, 38,39,40	4,6,7,8,34, 35,36,37	11	P01039,P06761,P0C0A9,P11598,P19132,P22273,P22282,P55091,Q63617,Q6P6R2,Q99041
791	1,2,3,5,34, 35,36,37	4,6,7,8,33, 38,39,40	7	F1M3L7,P22273,P25031,P47967,P50115,P51740,P55091
792	1,2,3,5,34, 35,36,38	4,6,7,8,33, 37,39,40	10	P01946,P02091,P0C0A9,P21674,P22273,P22282,Q63617,Q99041,Q9QW07,Q9QWN8
793	1,2,3,5,34, 35,36,39	4,6,7,8,33, 37,38,40	8	P00714,P0C0A9,P22273,P22282,P23739,Q63617,Q99041,Q9QW07
794	1,2,3,5,34, 35,36,40	4,6,7,8,33, 37,38,39	4	P0C0A9,P22273,P22282,Q63617
795	1,2,3,5,34, 35,37,38	4,6,7,8,33, 36,39,40	3	P01946,P02091,P22273
796	1,2,3,5,34, 35,37,39	4,6,7,8,33, 36,38,40	2	P22273,P50115
797	1,2,3,5,34, 35,37,40	4,6,7,8,33, 36,38,39	2	P22273,P50115
798	1,2,3,5,34, 35,38,39	4,6,7,8,33, 36,37,40	15	O08815,P01946,P02091,P06761,P08723,P0C0A9,P11598,P12020,P22273,P22282,P30120,P4646 2,Q63617,Q99041,Q9QW07
799	1,2,3,5,34, 35,38,40	4,6,7,8,33, 36,37,39	11	P01946,P02091,P06761,P08723,P0C0A9,P11598,P22273,P22282,P46462,Q63617,Q6P6R2

800	1,2,3,5,34, 35,39,40	4,6,7,8,33, 36,37,38	7	P06761,P08723,P0C0A9,P11598,P22273,P22282,Q63617
801	1,2,3,5,34, 36,37,38	4,6,7,8,33, 35,39,40	4	P01946,P02091,P22273,Q4KLZ6
802	1,2,3,5,34, 36,37,39	4,6,7,8,33, 35,38,40	4	O70417,P00714,P22273,Q99041
803	1,2,3,5,34, 36,37,40	4,6,7,8,33, 35,38,39	2	P22273,Q9JJ50
804	1,2,3,5,34, 36,38,39	4,6,7,8,33, 35,37,40	9	P00714,P01946,P02091,P0C0A9,P22273,P22282,Q63617,Q99041,Q9QW07
805	1,2,3,5,34, 36,38,40	4,6,7,8,33, 35,37,39	7	P01946,P02091,P0C0A9,P22273,P22282,Q63617,Q99041
806	1,2,3,5,34, 36,39,40	4,6,7,8,33, 35,37,38	5	P0C0A9,P22273,P22282,Q63617,Q99041
807	1,2,3,5,34, 37,38,39	4,6,7,8,33, 35,36,40	4	P01946,P02091,Q6AY61,Q99041
808	1,2,3,5,34, 37,38,40	4,6,7,8,33, 35,36,39	6	P01946,P02091,P20767,Q4KLZ6,Q62714,Q6AY61
809	1,2,3,5,34, 37,39,40	4,6,7,8,33, 35,36,38	2	O70417,Q6AY61
810	1,2,3,5,34, 38,39,40	4,6,7,8,33, 35,36,37	11	P01946,P02091,P06761,P08723,P0C0A9,P11598,P22273,P22282,Q63617,Q6AY61,Q99041
811	1,2,3,5,35, 36,37,38	4,6,7,8,33, 34,39,40	4	F1M3L7,P21674,P22273,P22282
812	1,2,3,5,35, 36,37,39	4,6,7,8,33, 34,38,40	2	F1M3L7,P22273
813	1,2,3,5,35, 36,37,40	4,6,7,8,33, 34,38,39	8	F1M3L7,P19132,P22273,P50115,P53792,P82471,Q4G075,Q9Z0V6
814	1,2,3,5,35, 36,38,39	4,6,7,8,33, 34,37,40	11	P06761,P0C0A9,P11598,P21674,P22273,P22282,P24368,P30120,Q63617,Q99041,Q9QW07
815	1,2,3,5,35, 36,38,40	4,6,7,8,33, 34,37,39	12	P06761,P0C0A9,P11598,P19814,P21674,P22273,P22282,P53792,Q63617,Q9QWN8,Q9QX74,Q9 Z0V6
816	1,2,3,5,35, 36,39,40	4,6,7,8,33, 34,37,38	7	P06761,P0C0A9,P11598,P22273,P22282,P23739,Q63617
817	1,2,3,5,35, 37,38,39	4,6,7,8,33, 34,36,40	5	P08494,P08649,P11598,P22282,Q9QX74
818	1,2,3,5,35, 37,38,40	4,6,7,8,33, 34,36,39	5	P08494,P08649,P22282,P82471,Q9QX74
819	1,2,3,5,35, 37,39,40	4,6,7,8,33, 34,36,38	7	O70417,P07150,P08649,P11598,P50115,P82471,Q9QX74

820	1,2,3,5,35,38,39,40	4,6,7,8,33,34,36,37	16	O55004,P06761,P08649,P08723,P0C0A9,P11598,P22273,P22282,P22283,P46462,P55091,P98089,Q62635,Q63617,Q6P6R2,Q9QX74
821	1,2,3,5,36,37,38,39	4,6,7,8,33,34,35,40	2	Q99041,Q9QX74
822	1,2,3,5,36,37,38,40	4,6,7,8,33,34,35,39	4	P22282,P82471,Q9QX74,Q9Z0V6
823	1,2,3,5,36,37,39,40	4,6,7,8,33,34,35,38	3	O70417,P82471,Q9QX74
824	1,2,3,5,36,38,39,40	4,6,7,8,33,34,35,37	8	P06761,P0C0A9,P11598,P22273,P22282,Q63617,Q99041,Q9QX74
825	1,2,3,5,37,38,39,40	4,6,7,8,33,34,35,36	6	P08494,P08649,P22282,P82471,Q6AY61,Q9QX74
826	1,2,3,6,7,8,33,34	4,5,35,36,37,38,39,40	11	iRT-Kit_WR_fusion,O54800;Q5DWV2,O89117,P02631,P20766,P80202,P97615,Q09030,Q91XN4,Q99MH3,Q99PP0
827	1,2,3,6,7,8,33,35	4,5,34,36,37,38,39,40	7	O54800;Q5DWV2,O89117,P80202,P85971,Q09030,Q5PPH0,Q91XN4
828	1,2,3,6,7,8,33,36	4,5,34,35,37,38,39,40	9	O54800;Q5DWV2,P08649,P80202,P97615,Q09030,Q91XN4,Q99MH3,Q99PP0,Q9QZQ5
829	1,2,3,6,7,8,33,37	4,5,34,35,36,38,39,40	4	O54800;Q5DWV2,P80202,Q09030,Q91XN4
830	1,2,3,6,7,8,33,38	4,5,34,35,36,37,39,40	8	iRT-Kit_WR_fusion,O54800;Q5DWV2,P35280,P50115,P80202,Q09030,Q6IG05,Q91XN4
831	1,2,3,6,7,8,33,39	4,5,34,35,36,37,38,40	8	O54800;Q5DWV2,P20766,P80202,Q09030,Q5RKI1,Q6AYQ8,Q91XN4,Q99MH3
832	1,2,3,6,7,8,33,40	4,5,34,35,36,37,38,39	5	O54800;Q5DWV2,P02631,P80202,Q09030,Q91XN4
833	1,2,3,6,7,8,34,35	4,5,33,36,37,38,39,40	9	O54800;Q5DWV2,P20766,P36374,P36860,P80202,P97580,Q30KJ2,Q91XN4,Q9Z1F2
834	1,2,3,6,7,8,34,36	4,5,33,35,37,38,39,40	8	iRT-Kit_WR_fusion,P08649,P20766,P80202,P97615,Q09030,Q91XN4,Q9Z1F2

835	1,2,3,6,7,8,34,37	4,5,33,35,36,38,39,40	3	P01835,Q66H69,Q91XN4
836	1,2,3,6,7,8,34,38	4,5,33,35,36,37,39,40	12	iRT-Kit_WR_fusion,O54800;Q5DWV2,P01946,P02091,P20766,P35280,P36374,P97580,Q30KJ2,Q62812,Q6IG05,Q91XN4
837	1,2,3,6,7,8,34,39	4,5,33,35,36,37,38,40	9	P02651,P20766,P36374,P80202,P97580,Q30KJ2,Q62894,Q9Z0V6,Q9Z1F2
838	1,2,3,6,7,8,34,40	4,5,33,35,36,37,38,39	7	P15399,P20766,P36374,P80202,P97580,Q30KJ2,Q91XN4
839	1,2,3,6,7,8,35,36	4,5,33,34,37,38,39,40	6	P19218,P85971,Q09030,Q91XN4,Q9JJS8,Q9Z1F2
840	1,2,3,6,7,8,35,37	4,5,33,34,36,38,39,40	3	P19132,P19218,P36860
841	1,2,3,6,7,8,35,38	4,5,33,34,36,37,39,40	4	P19218,P35280,P36374,Q62812
842	1,2,3,6,7,8,35,39	4,5,33,34,36,37,38,40	6	P20766,P36374,P36376,P36860,P85971,Q9Z1F2
843	1,2,3,6,7,8,35,40	4,5,33,34,36,37,38,39	3	P36374,P80202,P85971
844	1,2,3,6,7,8,36,37	4,5,33,34,35,38,39,40	5	P01835,P09656,P19132,Q91XN4,Q9JI85
845	1,2,3,6,7,8,36,38	4,5,33,34,35,37,39,40	3	P19218,P35280,Q91XN4
846	1,2,3,6,7,8,36,39	4,5,33,34,35,37,38,40	4	B0BNN3,P20766,P97615,Q9Z1F2
847	1,2,3,6,7,8,36,40	4,5,33,34,35,37,38,39	3	P80202,P97615,Q91XN4

848	1,2,3,6,7,8,37,38	4,5,33,34,35,36,39,40	4	P01835,P19218,P35280,Q6IG05
849	1,2,3,6,7,8,37,39	4,5,33,34,35,36,38,40	1	P01835
850	1,2,3,6,7,8,37,40	4,5,33,34,35,36,38,39	4	P01835,P82471,Q5GRG2,Q9QW07
851	1,2,3,6,7,8,38,39	4,5,33,34,35,36,37,40	5	P02770,P20766,P35280,P36374,P36376
852	1,2,3,6,7,8,38,40	4,5,33,34,35,36,37,39	5	P35280,P36374,P57113,Q62812,Q91XN4
853	1,2,3,6,7,8,39,40	4,5,33,34,35,36,37,38	4	P20766,P36374,P36376,P80202
854	1,2,3,6,7,33,34,35	4,5,8,36,37,38,39,40	6	D3ZUC6,O54800;Q5DWV2,O89117,P08721,Q5I0D7,Q99MH3
855	1,2,3,6,7,33,34,36	4,5,8,35,37,38,39,40	11	O54800;Q5DWV2,O89117,P08649,P08721,P80299,P97615,P98089,Q09030,Q5I0D7,Q91XN4,Q99MH3
856	1,2,3,6,7,33,34,37	4,5,8,35,36,38,39,40	5	O54800;Q5DWV2,P98089,Q6AY61,Q6AYQ8,Q99MH3
857	1,2,3,6,7,33,34,38	4,5,8,35,36,37,39,40	5	O54800;Q5DWV2,P01946,P02091,P80299,Q6AY61
858	1,2,3,6,7,33,34,39	4,5,8,35,36,37,38,40	7	O54800;Q5DWV2,P02651,P20766,Q5RKI1,Q62894,Q6AYQ8,Q99MH3
859	1,2,3,6,7,33,34,40	4,5,8,35,36,37,38,39	5	O54800;Q5DWV2,P20766,Q6AY61,Q91XN4,Q99MH3
860	1,2,3,6,7,33,35,36	4,5,8,34,37,38,39,40	6	O54800;Q5DWV2,P08721,P09656,P49134,Q09030,Q99MH3
861	1,2,3,6,7,33,35,37	4,5,8,34,36,38,39,40	1	O54800;Q5DWV2
862	1,2,3,6,7,33,35,38	4,5,8,34,36,37,39,40	2	O54800;Q5DWV2,Q62812
863	1,2,3,6,7,33,35,39	4,5,8,34,36,37,38,40	3	O54800;Q5DWV2,Q5RKI1,Q99MH3
864	1,2,3,6,7,33,35,40	4,5,8,34,36,37,38,39	2	O54800;Q5DWV2,P06760

865	1,2,3,6,7,33,36,37	4,5,8,34,35,38,39,40	4	O54800;Q5DWV2,P09656,P98089,Q09030
866	1,2,3,6,7,33,36,38	4,5,8,34,35,37,39,40	3	O54800;Q5DWV2,P09656,Q09030
867	1,2,3,6,7,33,36,39	4,5,8,34,35,37,38,40	4	O54800;Q5DWV2,Q09030,Q5RKI1,Q99MH3
868	1,2,3,6,7,33,36,40	4,5,8,34,35,37,38,39	3	O54800;Q5DWV2,Q09030,Q91XN4
869	1,2,3,6,7,33,37,38	4,5,8,34,35,36,39,40	4	O54800;Q5DWV2,P01835,P23739,Q6AY61
870	1,2,3,6,7,33,37,39	4,5,8,34,35,36,38,40	4	O54800;Q5DWV2,Q5RKI1,Q6AY61,Q6AYQ8
871	1,2,3,6,7,33,37,40	4,5,8,34,35,36,38,39	4	O54800;Q5DWV2,P01835,Q62714,Q6AY61
872	1,2,3,6,7,33,38,39	4,5,8,34,35,36,37,40	3	O54800;Q5DWV2,P02770,Q5RKI1
873	1,2,3,6,7,33,38,40	4,5,8,34,35,36,37,39	6	O54800;Q5DWV2,P01039,P06760,P23739,Q62714,Q62812
874	1,2,3,6,7,33,39,40	4,5,8,34,35,36,37,38	4	O54800;Q5DWV2,P06760,Q5RKI1,Q6AYQ8
875	1,2,3,6,7,34,35,36	4,5,8,33,37,38,39,40	3	P20766,Q5I0D7,Q9QWN8
876	1,2,3,6,7,34,35,37	4,5,8,33,36,38,39,40	3	P01835,P19132,P20760
877	1,2,3,6,7,34,35,38	4,5,8,33,36,37,39,40	7	O54800;Q5DWV2,P01946,P02091,P20766,P36374,Q62812,Q9QWN8
878	1,2,3,6,7,34,35,39	4,5,8,33,36,37,38,40	2	P20766,Q9Z1F2
879	1,2,3,6,7,34,35,40	4,5,8,33,36,37,38,39	1	P20766
880	1,2,3,6,7,34,36,37	4,5,8,33,35,38,39,40	5	P01835,P09656,P47967,P55091,P98089
881	1,2,3,6,7,34,36,38	4,5,8,33,35,37,39,40	7	O54800;Q5DWV2,P01835,P01946,P02091,P20766,Q62812,Q9QWN8
882	1,2,3,6,7,34,36,39	4,5,8,33,35,37,38,40	4	P20766,P97615,Q99MH3,Q9Z1F2
883	1,2,3,6,7,34,36,40	4,5,8,33,35,37,38,39	2	P01835,P20766
884	1,2,3,6,7,34,37,38	4,5,8,33,35,36,39,40	7	O54800;Q5DWV2,P01835,P01946,P02091,P20767,P23739,Q6AY61

885	1,2,3,6,7,34,37,39	4,5,8,33,35,36,38,40	3	P01835,P20766,Q6AY61
886	1,2,3,6,7,34,37,40	4,5,8,33,35,36,38,39	3	P01835,P20767,Q6AY61
887	1,2,3,6,7,34,38,39	4,5,8,33,35,36,37,40	9	O54800;Q5DWV2,P01835,P01946,P02091,P02770,P20766,P60905,Q6AY61,Q9QX79
888	1,2,3,6,7,34,38,40	4,5,8,33,35,36,37,39	9	O54800;Q5DWV2,P01835,P01946,P02091,P20766,P23739,P97580,Q62812,Q6AY61
889	1,2,3,6,7,34,39,40	4,5,8,33,35,36,37,38	3	P01835,P20766,Q6AY61
890	1,2,3,6,7,35,36,37	4,5,8,33,34,38,39,40	3	P09656,P19132,Q9JJS8
891	1,2,3,6,7,35,36,38	4,5,8,33,34,37,39,40	4	P09656,P19218,Q62812,Q9QWN8
892	1,2,3,6,7,35,36,39	4,5,8,33,34,37,38,40	0	
893	1,2,3,6,7,35,36,40	4,5,8,33,34,37,38,39	4	P02793;Q7TP54,P06760,P19132,Q9QWN8
894	1,2,3,6,7,35,37,38	4,5,8,33,34,36,39,40	3	P01835,P19218,Q62812
895	1,2,3,6,7,35,37,39	4,5,8,33,34,36,38,40	1	P01835
896	1,2,3,6,7,35,37,40	4,5,8,33,34,36,38,39	3	P01835,P19132,P20760
897	1,2,3,6,7,35,38,39	4,5,8,33,34,36,37,40	3	P06760,P13432,P36374
898	1,2,3,6,7,35,38,40	4,5,8,33,34,36,37,39	5	P06760,P36374,Q62812,Q6P6R2,Q9QX74
899	1,2,3,6,7,35,39,40	4,5,8,33,34,36,37,38	1	P06760
900	1,2,3,6,7,36,37,38	4,5,8,33,34,35,39,40	2	P01835,P09656
901	1,2,3,6,7,36,37,39	4,5,8,33,34,35,38,40	2	P01835,P09656
902	1,2,3,6,7,36,37,40	4,5,8,33,34,35,38,39	3	P01835,P09656,P19132
903	1,2,3,6,7,36,38,39	4,5,8,33,34,35,37,40	1	P01835
904	1,2,3,6,7,36,38,40	4,5,8,33,34,35,37,39	4	P01835,P06760,Q62812,Q9QWN8

905	1,2,3,6,7,36,39,40	4,5,8,33,34,35,37,38	3	B0BNN3,P01835,P06760
906	1,2,3,6,7,37,38,39	4,5,8,33,34,35,36,40	3	P01835,P02770,Q6AY61
907	1,2,3,6,7,37,38,40	4,5,8,33,34,35,36,39	7	P01835,P20767,P23739,Q62714,Q62812,Q6AY61,Q9QX74
908	1,2,3,6,7,37,39,40	4,5,8,33,34,35,36,38	4	P00762,P01835,Q6AY61,Q9QX74
909	1,2,3,6,7,38,39,40	4,5,8,33,34,35,36,37	6	P01835,P02770,P06760,P51740,P55091,Q9QX74
910	1,2,3,6,8,33,34,35	4,5,7,36,37,38,39,40	8	D3ZUC6,O54800;Q5DWV2,O89117,P80202,P97580,Q09030,Q30KJ2,Q99MH3
911	1,2,3,6,8,33,34,36	4,5,7,35,37,38,39,40	9	O54800;Q5DWV2,O89117,P02625,P08649,P80202,P97615,Q09030,Q91XN4,Q99MH3
912	1,2,3,6,8,33,34,37	4,5,7,35,36,38,39,40	3	O54800;Q5DWV2,O89117,Q99MH3
913	1,2,3,6,8,33,34,38	4,5,7,35,36,37,39,40	9	O54800;Q5DWV2,O89117,P01946,P02091,P02625,P97580,Q30KJ2,Q6IG05,Q99MH3
914	1,2,3,6,8,33,34,39	4,5,7,35,36,37,38,40	8	O54800;Q5DWV2,P02651,P08649,P80202,P97580,Q30KJ2,Q63532,Q99MH3
915	1,2,3,6,8,33,34,40	4,5,7,35,36,37,38,39	9	O54800;Q5DWV2,O89117,P15399,P80202,P97580,Q09030,Q30KJ2,Q91XN4,Q99MH3
916	1,2,3,6,8,33,35,36	4,5,7,34,37,38,39,40	7	O54800;Q5DWV2,O89117,P08649,P09656,P49134,Q09030,Q99MH3
917	1,2,3,6,8,33,35,37	4,5,7,34,36,38,39,40	3	O54800;Q5DWV2,O89117,Q09030
918	1,2,3,6,8,33,35,38	4,5,7,34,36,37,39,40	5	O54800;Q5DWV2,O89117,Q09030,Q68G31,Q6IG05
919	1,2,3,6,8,33,35,39	4,5,7,34,36,37,38,40	4	O54800;Q5DWV2,P36376,Q09030,Q99MH3
920	1,2,3,6,8,33,35,40	4,5,7,34,36,37,38,39	3	O54800;Q5DWV2,P80202,Q09030
921	1,2,3,6,8,33,36,37	4,5,7,34,35,38,39,40	5	O54800;Q5DWV2,P08649,P09656,Q09030,Q99MH3
922	1,2,3,6,8,33,36,38	4,5,7,34,35,37,39,40	6	O54800;Q5DWV2,P08649,P09656,Q09030,Q6IG05,Q99MH3
923	1,2,3,6,8,33,36,39	4,5,7,34,35,37,38,40	6	O54800;Q5DWV2,P00714,P08649,P80202,Q09030,Q99MH3
924	1,2,3,6,8,33,36,40	4,5,7,34,35,37,38,39	7	O54800;Q5DWV2,P01681,P08649,P80202,Q09030,Q91XN4,Q99MH3

925	1,2,3,6,8,33,37,38	4,5,7,34,35,36,39,40	3	O54800;Q5DWV2,Q68G31,Q6IG05
926	1,2,3,6,8,33,37,39	4,5,7,34,35,36,38,40	2	O54800;Q5DWV2,Q99MH3
927	1,2,3,6,8,33,37,40	4,5,7,34,35,36,38,39	3	O54800;Q5DWV2,P82471,Q9QW07
928	1,2,3,6,8,33,38,39	4,5,7,34,35,36,37,40	3	O54800;Q5DWV2,P36376,Q99MH3
929	1,2,3,6,8,33,38,40	4,5,7,34,35,36,37,39	5	O54800;Q5DWV2,P01039,P25031,P80202,Q6IG05
930	1,2,3,6,8,33,39,40	4,5,7,34,35,36,37,38	4	O54800;Q5DWV2,P36376,P80202,Q99MH3
931	1,2,3,6,8,34,35,36	4,5,7,33,37,38,39,40	3	P08649,Q30KJ2,Q99MH3
932	1,2,3,6,8,34,35,37	4,5,7,33,36,38,39,40	2	P36860,Q30KJ2
933	1,2,3,6,8,34,35,38	4,5,7,33,36,37,39,40	5	P01946,P02091,P97580,Q30KJ2,Q6IG05
934	1,2,3,6,8,34,35,39	4,5,7,33,36,37,38,40	4	P36376,P36860,P97580,Q30KJ2
935	1,2,3,6,8,34,35,40	4,5,7,33,36,37,38,39	3	P15399,P97580,Q30KJ2
936	1,2,3,6,8,34,36,37	4,5,7,33,35,38,39,40	4	P08649,P09656,P55091,Q9JI85
937	1,2,3,6,8,34,36,38	4,5,7,33,35,37,39,40	7	P01946,P02091,P02625,P08649,P97580,Q30KJ2,Q6IG05
938	1,2,3,6,8,34,36,39	4,5,7,33,35,37,38,40	5	P08649,P97580,P97615,Q99MH3,Q9Z1F2
939	1,2,3,6,8,34,36,40	4,5,7,33,35,37,38,39	6	P08649,P15399,P97580,P97615,Q30KJ2,Q91XN4
940	1,2,3,6,8,34,37,38	4,5,7,33,35,36,39,40	5	P01946,P02091,P97580,Q30KJ2,Q6IG05
941	1,2,3,6,8,34,37,39	4,5,7,33,35,36,38,40	1	P97580
942	1,2,3,6,8,34,37,40	4,5,7,33,35,36,38,39	3	P15399,P97580,Q30KJ2
943	1,2,3,6,8,34,38,39	4,5,7,33,35,36,37,40	6	P01946,P02091,P15399,P36376,P97580,Q30KJ2
944	1,2,3,6,8,34,38,40	4,5,7,33,35,36,37,39	6	P01946,P02091,P15399,P97580,Q30KJ2,Q6IG05

945	1,2,3,6,8,34,39,40	4,5,7,33,35,36,37,38	5 P15399,P36376,P97580,P97840,Q30KJ2
946	1,2,3,6,8,35,36,37	4,5,7,33,34,38,39,40	2 P09656,P19132
947	1,2,3,6,8,35,36,38	4,5,7,33,34,37,39,40	2 P09656,P19218
948	1,2,3,6,8,35,36,39	4,5,7,33,34,37,38,40	1 P36376
949	1,2,3,6,8,35,36,40	4,5,7,33,34,37,38,39	2 P02793;Q7TP54,P82471
950	1,2,3,6,8,35,37,38	4,5,7,33,34,36,39,40	2 P19218,Q6IG05
951	1,2,3,6,8,35,37,39	4,5,7,33,34,36,38,40	2 P36376,P82471
952	1,2,3,6,8,35,37,40	4,5,7,33,34,36,38,39	2 P19132,P82471
953	1,2,3,6,8,35,38,39	4,5,7,33,34,36,37,40	1 P36376
954	1,2,3,6,8,35,38,40	4,5,7,33,34,36,37,39	4 P06760,P36374,P82471,Q62635
955	1,2,3,6,8,35,39,40	4,5,7,33,34,36,37,38	4 P06760,P36376,P54921,P82471
956	1,2,3,6,8,36,37,38	4,5,7,33,34,35,39,40	2 P09656,Q6IG05
957	1,2,3,6,8,36,37,39	4,5,7,33,34,35,38,40	1 P09656
958	1,2,3,6,8,36,37,40	4,5,7,33,34,35,38,39	4 P09656,P19629,P82471,Q5GRG2
959	1,2,3,6,8,36,38,39	4,5,7,33,34,35,37,40	1 P36376
960	1,2,3,6,8,36,38,40	4,5,7,33,34,35,37,39	0
961	1,2,3,6,8,36,39,40	4,5,7,33,34,35,37,38	5 B0BNN3,P36376,P54921,P82471,Q9R168
962	1,2,3,6,8,37,38,39	4,5,7,33,34,35,36,40	0
963	1,2,3,6,8,37,38,40	4,5,7,33,34,35,36,39	2 P82471,Q5GRG2
964	1,2,3,6,8,37,39,40	4,5,7,33,34,35,36,38	2 P36376,P82471

965	1,2,3,6,8,38,39,40	4,5,7,33,34,35,36,37	7	P06760,P36376,P51740,P54921,P55091,P97840,Q62635
966	1,2,3,6,33,34,35,36	4,5,7,8,37,38,39,40	13	D3ZUC6,O54800;Q5DWV2,O89117,P08649,P08721,P22273,P39069,P49134,Q498R7,Q5I0D7,Q5QE79,Q6PCU2,Q99MH3
967	1,2,3,6,33,34,35,37	4,5,7,8,36,38,39,40	5	D3ZUC6,O54800;Q5DWV2,O89117,P39069,Q99MH3
968	1,2,3,6,33,34,35,38	4,5,7,8,36,37,39,40	7	D3ZUC6,O54800;Q5DWV2,O89117,P01946,P02091,P63029,P80299
969	1,2,3,6,33,34,35,39	4,5,7,8,36,37,38,40	3	D3ZUC6,O54800;Q5DWV2,Q99MH3
970	1,2,3,6,33,34,35,40	4,5,7,8,36,37,38,39	6	D3ZUC6,O54800;Q5DWV2,O89117,P06760,Q06000,Q99MH3
971	1,2,3,6,33,34,36,37	4,5,7,8,35,38,39,40	8	O54800;Q5DWV2,O89117,P08649,P09656,P98089,Q05695,Q62635,Q99MH3
[truncated: 512,523 more chars]
