# Supplementary material for: Dynamic Changes of Urine Proteome in Rat Models Inoculated with Two Different Hepatoma Cell Lines
Source: J Oncol. 2021 Jan 7;2021:8895330. doi: 10.1155/2021/8895330 (PMC7810548; doi:10.1155/2021/8895330)
Supplement: Supplementary Materials — Supplementary Figure 1. Functional analysis of differentially expressed proteins at days 5, 7, 14, and 28 in two models. (a) Cell component for the CBRH-7919 model. (b) Molecular function for the CBRH-7919 model. (c) Cell component for the RH-35 model. (d) Molecular function for the RH-35 model. Supplementary Table 1. All urinary proteins identified in the CBRH-7919 model. Supplementary Table 2. All urinary proteins identified in the RH-35 model. Supplementary Table 3. The details of 6435 random allocations in the CBRH-7919 model. Supplementary Table 4. The occurrence of the protein in 6435 random allocations in the CBRH-7919 model. Supplementary Table 5. The details of 12155 random allocations in the RH-35 model. Supplementary Table 6. The occurrence of the protein in 12155 random allocations in the RH-35 model. [file 8895330.f1.zip › 8895330.f1/Supplementary Table 4.pdf]

Title：Dynamic changes of urine proteome in rat models inoculated with two different hepatoma cell lines. Full author: Yameng Zhang, Yufei Gao, Jing Wei & Youhe Gao*.

Table S4 In CBRH-7919 model, the occurance of the protein in 6435 random allocations.

Day 5		Day 7		Day 14		Day 28	
Protein ID	Occurance	Protein ID	Occurance	Protein ID	Occurance	Protein ID	Occurance
Q5RKI1	485	P35280	495	P29315	400	P35280	495
P02091	479	Q4G075	396	P82471	386	Q5RKI1	495
P20760	364	Q9R168	371	P20766	375	P51740	492
P06760	355	P23739	371	Q03191	371	P02091	443
P00774	349	Q811M5	369	Q99041	370	P55091	390
P22283	345	iRT-Kit_WI	367	P62804	365	P19218	360
P00762	342	Q9WTT6	361	Q4G075	353	P52590	357
P07647	341	P25809	349	P52590	349	P49134	355
P02782	340	P52590	346	P06760	347	P97580	354
P02780	339	P06760	344	P22273	343	P36860	345
P97840	339	P30919	343	Q9WTT6	340	Q62635	343
P52590	338	P36860	341	P47967	340	P97840	337
Q9JI85	338	P00762	340	Q68G31	339	P00762	335
P47967	338	P01835	340	P80299	336	Q6AY61	335
Q5GRG2	336	Q6P6R2	339	P22006	329	Q68G31	334
Q9WUW8	335	O70417	337	O70417	326	D3ZUC6	333
P30120	335	P22006	337	Q9QX74	325	Q9QX74	333
Q8CIZ5	334	P47967	336	P00762	324	Q99041	332
P01835	333	Q5GRG2	333	P19218	323	P01681	330
Q9JHB9	332	Q6IMF3	331	P36860	318	P47967	326
P06761	330	Q9QX74	329	P21588	316	P01039	326
O70417	330	P80299	328	P50116	315	P80299	326
P80299	325	P19223	328	P97840	311	P50116	321
P01681	324	P20766	328	P01835	309	Q9EQS0	321
P35280	322	P10758	327	P25809	303	Q4KLZ6	320
O70594	321	P50116	326	P01681	302	P36376	319
P11598	321	Q99041	324	P22282	301	Q9Z1F2	315
P36860	319	Q6IG02	321	Q6IG02	297	P19132	315
P46844	319	P06911	316	Q6IMF3	290	P01835	312
P20766	316	P70549	308	Q6P6Q2	290	O70417	310
P50116	313	P19629	307	P70549	282	P15399	310
Q63493	312	Q6IFW6	304	P11598	281	P06760	308
Q99041	307	Q6P6Q2	304	P97580	278	P36374	307
P02781	304	P97840	297	Q812E4	278	P01946	305
iRT-Kit_WI	303	P11598	297	Q6IFW6	271	Q30KJ2	301
P08723	303	P22282	297	Q63617	268	P70549	298
Q6IFW6	299	P23593	296	P06911	257	P22273	296
P19629	297	Q68G31	296	P50115	251	Q64093	296
P97580	297	Q63617	295	Q6AYR9	249	P35745	290
Q62714	295	P01946	291	Q30KJ2	246	P20766	289
Q68G31	295	P02783	289	P02783	245	Q99MH3	287
P20646	294	Q99MH3	289	P01039	240	P09656	285
Q99MH3	291	Q9Z2L0	287	Q99MH3	239	P11598	285
P02783	289	D4A5U3	282	Q63532	238	Q9QWN8	280
P50280	287	Q4KLZ6	281	Q6IG05	238	Q91XN4	275
P22282	285	O89117	281	O54728	237	P98089	273
P70549	283	P01681	278	P26342	235	P25809	270
P06911	281	P97580	274	P00714	235	P13432	270
Q63617	279	P22273	274	Q9QW07	235	Q9Z0V6	270
Q63618	278	P36374	273	Q6IFU7	229	P20762	268
Q6P6Q2	275	Q03191	271	D3ZUC6	225	P08721	263
Q66H69	273	Q9Z0J6	267	Q9R168	225	P08649	258
P18418	271	P42854	264	Q6P6R2	224	Q6AYQ8	255
P57113	270	Q4FZU2	260	Q9QYP1	211	Q6IG05	242
P08937	267	O70594	259	P15399	210	Q09030	240
P02631	263	P02780	257	Q5RJR2	209	Q5I0D7	240
Q5M8C6	263	P02091	253	P36376	203	Q9R168	235
P25031	263	P12020	252	Q9QWN8	200	Q63617	230
Q03191	259	Q05702	250	Q80WL1	190	P21674	228
Q9Z1F2	258	Q30KJ2	248	Q4KLZ6	189	Q9QW07	226
P29315	257	P50115	246	P08649	183	P82471	220
Q6IMF3	256	Q63474	246	O54800;Q	182	P25031	216
Q6AY61	253	Q6AY61	241	Q811M5	182	O54858	198
Q6TMA8	252	P09656	240	P13432	178	P08723	190
Q4FZU2	251	Q00715	240	P02631	177	P63029	186
P48508	250	Q09030	235	P23739	176	P0C0A9	185
Q6IG02	248	Q812E4	232	P49134	171	P19814	184
P50115	244	P54921	231	Q9WVK7	171	Q923V8	184

Q30KJ2	243	Q9EQS0	218	Q63474	169	P22282	184
P55091	242	P01039	215	P21674	166	O89117	176
P22273	241	P08649	213	Q9JI85	166	P00774	176
Q812E4	239	P00507	208	P35280	165	Q4AEF8	171
Q4KLZ6	233	P62804	204	P35745	164	P50115	167
Q811M5	233	Q9JI85	204	O88797	156	P19629	167
P46462	233	P13676	202	Q09030	154	O54800;Q!	166
P0C0A9	231	Q66H69	202	P20646	154	P12020	165
Q4G075	230	P15399	198	Q9JJ50	152	Q62714	165
B0BNN3	230	P63029	191	Q4FZU2	147	O54728	165
P36374	229	P00714	190	Q9Z0J6	138	P11883	161
Q9R0T3	228	P20761	185	P54921	133	Q80WL1	157
D3ZUC6	228	O54800;Q!	184	Q8VD89	131	P08494	153
P0DMW0;F	228	Q9QW07	183	Q66H69	131	P62804	153
P20761	227	P50280	182	Q62975	131	P42854	148
D4A5U3	219	Q6IG05	179	P20762	131	Q63474	148
P20762	219	P05539	177	P05539	128	P14173	147
Q5RLM2	218	P22283	174	Q6AY61	128	P23739	145
Q8CJ52	218	Q9QYP1	174	Q5I0J9	127	Q5GRG2	144
Q9QW07	217	P47727	171	Q10758	127	P02793;Q7	142
P01946	216	P18418	170	O54858	120	P97829	135
P35745	216	P02631	170	Q5BJY9	117	P54921	134
P08649	214	Q5I0D1	169	O89117	116	F1M3L7	133
P19468	214	Q63532	168	Q00715	113	Q99PP0	131
Q9QX74	212	O54728	165	P12020	111	D3ZTX0	130
Q63598	207	P36376	162	P00731	111	B0BNN3	127
Q6Q0N1	206	P08723	162	Q6IFU8	102	Q9Z0J6	126
P12020	206	Q62714	160	P36374	100	P02625	122
Q9R168	204	D3ZUC6	159	P07150	98	Q9JJ50	119
P09456	204	B1H234	157	Q62761;Q(	98	Q63751	107
B1H234	202	O54858	157	P08723	96	P02631	106
P10758	201	P20646	157	Q9EQS0	95	Q6P6R2	105
Q6IFU8	201	P13432	154	P22283	92	P10758	102
Q5QE79	200	Q91ZS3	152	Q5GRG2	92	Q66H69	101
P54921	194	Q9JHB9	144	Q9Z2L0	91	Q9QZQ5	100
Q6IG05	192	B0LT89	138	P30120	88	G3V686	98
P15399	190	P30120	137	P06761	88	Q8CIZ5	97
P98089	189	P06761	136	P00507	87	P06911	97
Q9WTW7	189	Q9JJ50	133	P47727	86	Q6PCU2	95
O54728	189	Q9WVK7	132	Q62714	84	D4A5U3	95
O55004	186	P55091	131	P09656	83	Q6IFW6	94
P00714	176	P19218	127	Q9QZQ5	73	P30120	94
Q64093	175	P21674	117	Q5RLM2	72	Q63475	94
P21674	173	Q10743	115	Q9Z1F2	70	Q05702	93
Q63424	171	P49134	113	Q5PQL7	70	Q5QE79	93
P53790	168	Q6IFU7	111	P00758	69	P02770	92
P17988	167	P11762	110	P02625	67	Q812E4	92
P36376	167	Q5PQL7	106	P63081	66	P80202	91
Q6IFU7	165	P20762	105	O35547	65	iRT-Kit_WF	91
Q63270	163	Q9Z1F2	105	Q06000	64	P60905	88
Q63751	159	P16636	102	P57113	61	P00714	81
Q00715	149	P02625	100	P0C0A9	60	Q6P6Q2	81
O35077	146	P35745	99	P97615	59	P23593	77
B0LT89	144	Q63598	97	Q63598	59	Q9JI85	77
Q62761;Q(	144	Q8CJD3	94	P19223	57	O35276	76
Q6P6S4	143	Q6IFU8	90	P14173	56	P48199	75
P08721	142	Q10758	83	O55145	55	Q8K4G9	75
P07150	141	Q9QZQ5	81	P17559	53	P85971	70
P19223	139	Q63493	80	P42854	51	P26772	66
Q05175	139	Q5QE79	80	P19629	47	P24368	64
Q62635	139	P17559	78	Q05511	47	Q6AYR9	64
Q8R431	136	P08937	76	Q63493	45	P16636	62
Q923S2	136	P0C0A9	76	Q62894	45	P07174	61
O70377	131	Q80WL1	75	B1H234	43	Q5I0D1	61
P22006	129	Q9R0T3	75	G3V686	42	P80020	60
Q3ZAV1	124	P11883	74	Q99PP0	41	O55004	53
Q5I0E9	122	P31044	73	Q05702	38	Q6IG02	53
P31430	119	P25031	70	Q8CFN2	38	P97615	50
Q9Z0V6	118	P20760	69	Q9WVH8	38	Q9QW30	49
Q9Z2L0	117	P02782	68	Q9QZK9	37	Q5BJY9	47
Q03248	115	Q5I0J9	68	Q8CGS4	35	P19223	47
Q71MB6	115	P19132	66	P10758	35	Q9Z2L0	46
P19132	115	O35547	63	Q6AYR8	35	P29315	45
Q9Z0W7	114	P04762	58	Q923V8	34	Q4G075	44

P31044	113	O55145	57	Q5I0D1	32	Q03191	43
Q9Z0J6	113	Q5M8C6	53	Q9WUW8	30	P30919	42
Q5M7T9	111	P09456	53	P0DMW0;P	27	P06761	41
Q6MG61	111	P46462	52	O70594	25	Q9QYP1	41
P30904	110	Q6AYR9	51	O55004	25	B1H234	39
P19814	110	P46844	46	Q9Z0V6	24	P20767	39
P60905	110	P60905	44	P02454	23	P05539	38
Q10758	110	P36375	41	Q6TMA8	22	P02782	36
Q6P6R2	107	Q6AYQ8	40	Q63150	22	Q5I0J9	35
P18297	105	P04355	40	Q6P6S4	21	Q63618	34
P23593	105	Q6AYC4	36	Q64093	21	Q9WUC4	34
P04905	103	P07150	35	P97584	20	P17475	34
Q5I0D1	103	Q64268	35	Q01205	20	P02454	33
P07151	98	Q99PP0	35	B0LT89	19	Q6AYR8	33
O70257	97	Q91XN4	34	O08839	18	Q63149	32
P62804	96	O35077	34	P20761	17	Q6IFU7	32
O88797	94	P33436	32	P18298	16	P53792	32
Q80W57	93	P27590	32	Q5QE79	16	D3ZTE0	31
P40241	93	P24368	32	P98166	15	Q62812	31
P11883	89	G3V686	32	P05964	15	P22006	30
O35547	89	O08815	31	P97523	15	Q63556	30
O88339;Q4	88	P55159	29	iRT-Kit_WR	14	Q8K1G0	30
P13432	87	P97697	29	B0BNN3	14	Q5PPH0	30
Q05702	87	Q78P75	29	Q5M8C6	14	Q811M5	29
P25809	84	P07647	29	P50280	14	P02651	29
Q62902	80	P04905	28	B2RYW9	13	Q6DGG1	28
Q80WL1	80	P26772	28	P07171	13	P48032	28
P38918	78	Q3ZAV1	27	Q62902	12	P70502	28
Q9WUW9	77	P02781	27	P02780	12	Q5EGZ1	28
A2RUW1	76	Q62946	26	P04905	12	O35568	27
Q498D9	75	P97615	25	P07647	12	O55145	27
P18757	72	Q62761;Q	25	O08815	12	Q9WTT6	27
P23928	72	P80202	24	Q63424	12	O08815	26
P51907	71	Q62894	24	P84039	11	Q63493	26
Q64602	70	Q9WUW8	23	Q64240	10	Q6IMF3	24
Q9EQS0	70	Q06000	23	Q9WUC4	10	Q920G2	24
Q9JJ40	68	Q6TMA8	23	Q9JHB9	10	Q8VD89	24
P02761	68	Q5RKI1	23	P48032	10	O35547	23
Q5I0D7	65	Q64335	22	P18418	10	P22283	23
D3ZHA0	63	B0BNN3	20	Q9QX79	9	Q63514	22
Q5I0J9	61	P19939	19	P17046	9	Q00715	21
Q6B345	61	P19814	19	P08010	9	Q4FZU2	21
P10247	60	P02454	18	P80202	8	P20646	20
Q6AYQ8	60	P35952	18	P13852	8	Q4TU93	20
P09527	60	Q9QZK9	18	P35952	8	B5DFC9	19
Q09030	60	P05964	17	P02782	8	P19939	19
P49134	58	Q9Z0V6	17	P0DP29;P	7	P12368	19
D3ZTX0	58	P14173	17	P33436	7	Q99376	18
Q09326	58	P05369	17	Q08163	7	P47727	18
O35763	57	P97675	16	P24368	7	Q9WUW8	17
P24368	57	P0DP29;P	16	Q07523	7	Q9JHB9	16
P70545	56	Q4FZU4	14	Q99376	7	Q9QX79	15
O08557	55	Q6P9T8	14	P15999	6	Q00657	15
Q6AY41	55	Q8CJ52	14	P14562	6	P07379	15
Q63355	55	P39069	13	P04636	6	P17559	14
Q78P75	55	P55281	13	P06866	6	P33436	14
Q66HG3	52	Q8K1G0	12	Q01984	6	P97584	14
Q63317	51	Q6RUV5	12	Q68FX1	5	Q6IFU8	13
Q6PCU2	51	Q6Q7Y5	12	P01946	5	P46462	13
P08010	51	Q5BK81	11	O70257	5	Q05511	13
Q63474	49	P68511	11	Q5I0D7	5	Q63532	12
P02454	49	O55004	11	Q6RUV5	5	P39069	11
P05539	48	P02761	10	D3ZTX0	5	Q8CJ52	11
O88267	47	Q64093	10	Q4V885	4	P09456	11
P17559	46	P82471	10	P49744	4	Q05695	11
P05369	46	P07335	10	Q8CJD3	4	P46720	11
P63029	45	P23785	9	P11762	4	Q8CG08	10
P29975	44	F1M3L7	9	A0JPJ7	3	Q5M8C6	10
P48037	44	P18757	8	P02781	3	P18418	10
P28570	44	Q4AEF8	7	P46462	3	P04916	10
Q9QYP1	44	Q64240	7	P20759	3	Q4V885	10
P19218	44	P07151	7	Q9JJS8	3	P50280	10
P01039	44	P14046	7	P81556	2	Q9JJS8	9
Q6AYR9	42	O88797	7	Q91ZS3	2	Q6P6S4	9

P23739	40	Q9WVH8	7	O54861	2	Q9QZ76	8
Q4G063	39	P06866	7	P51792;P5	2	P07171	8
Q6Q7Y5	39	Q5RLM2	6	Q5M872	2	P11030	8
Q8CFN2	39	P38918	6	Q8CJ52	2	P0C0K7	7
Q62946	38	Q9WTW7	6	Q6DGG1	2	P00758	7
Q8CJD3	38	P07174	6	P39069	2	P06866	7
P11762	37	P17046	6	P09527	2	Q05175	7
P63081	36	P13265	6	P01836	1	Q9EQV9	7
Q9QYU4	36	P28648	6	Q91XN4	1	Q06000	7
Q5PQL7	36	Q63751	6	O35276	1	Q6AY41	7
P34901	35	P69897	6	P46844	1	Q91ZS3	7
Q9R1T5	33	Q5BJY9	6	Q9QZ76	1	Q6AYC4	7
Q9QZK9	33	P46720	5	Q64268	1	P25236	7
P63095	30	P34901	5	P08721	1	P00731	7
P01836	29	Q4V885	5	P70712	1	P50503	7
Q3T1J9	29	P16228	5	Q6P777	1	P50137	7
Q9JLJ3	29	A0JPJ7	5	P11442	1	O88339;Q	6
P14668	29	P40241	5	P60905	1	Q62894	6
P02625	27	P01836	4	P10247	1	P26453	5
P70709	27	Q5I0E9	4	Q62687	1	Q5RLM2	5
Q62812	25	P13852	4	P31044	1	Q5PQL7	5
Q9JJ19	25	P15978	4	P11883	1	P49744	4
P36970	25	P18297	4	Q5U2V4	1	P62898	4
Q68FT5	25	P98089	4	Q9ES87	1	P07861	4
Q64335	24	P70619	3	P10719	1	P35444	3
P05371	23	P38438	3	Q4AEF8	1	P57113	3
P35952	23	P10719	3	A2RUW1	0	Q9ES87	3
O89117	23	Q9WU74	3	A4KWA5;A	0	Q64119	3
P46720	22	Q62902	3	B0BNA5	0	Q8CJD3	3
P68035;P6	22	Q5M872	3	B0BND0	0	P08937	3
B2RYW9	22	Q5PPH0	3	B0BNE5	0	P35952	3
Q06000	22	O54861	3	B5DEN9	0	P07647	3
P07171	21	P01041	3	B5DFC9	0	P70712	3
O08815	20	P00774	3	D3Z8L7	0	Q64240	3
Q5BJY9	20	Q63467	3	D3ZHA0	0	P13852	3
Q923V8	19	Q9WUC4	3	D3ZTD8	0	Q5RKI7	3
Q9WVH8	19	O70257	3	D3ZTE0	0	P07897	2
P82471	19	Q5U2V4	3	D3ZTV3	0	Q498R7	2
P61206;P8	19	Q6RY07	3	D3ZUK3	0	Q78P75	2
P27590	16	P08721	3	D3ZW55	0	Q7TP52	2
P12368	16	P53790	2	D4A1J4	0	P20760	2
Q8K1G0	16	O08557	2	D4A1R8	0	P04355	2
P04904	15	P29315	2	D4A5U3	0	Q9WVH8	2
Q91ZS3	15	O88917	2	D4AE59	0	P04276	2
Q99MZ8	15	P00731	2	E9PT87;P2	0	Q4FZU6	2
P97523	15	Q9QZK8	2	F1LM93	0	P84039	1
P52847	14	P20759	2	F1M3L7	0	Q62997	1
Q8R5M3	14	Q9JJS8	2	G3V7W1	0	P17046	1
Q9WTT6	14	P34080	2	H1UBN0;Q	0	Q07523	1
Q923M1	14	Q07523	2	O08557	0	P07150	1
P60711;P6	13	D3ZHA0	1	O08628	0	P12346	1
Q6AYS7	13	P48508	1	O08651	0	P0DMW0;	1
Q6Q0N0	13	Q63556	1	O09175	0	P10719	1
Q9QZK8	13	P48037	1	O35077	0	Q62740	1
O54800;Q	13	Q05175	1	O35112	0	Q8R431	1
Q5U2Q3	12	Q80WY6	1	O35142	0	Q10758	1
Q99MA2	12	P17988	1	O35217	0	P11762	1
P63322	12	Q6DGG1	1	O35244	0	P69897	1
Q920G2	11	P61589	1	O35264	0	B2RYW9	1
Q8K3P7	11	P12368	1	O35331	0	P01015	1
Q3MIE4	11	Q9Z0W7	1	O35509	0	Q63467	1
Q62753	11	Q63317	1	O35568	0	A0JPJ7	0
P10536	11	Q8R431	1	O35760	0	A2RUW1	0
Q62687	11	Q9QX79	1	O35763	0	A4KWA5;A	0
P19112	10	Q63424	1	O35952	0	B0BNA5	0
P26772	10	Q63618	1	O35956	0	B0BND0	0
O54861	10	Q1WIM3	1	O54715	0	B0BNE5	0
P07943	9	P08753	1	O54975	0	B0LT89	0
O88917	9	P08934	1	O55006	0	B5DEN9	0
Q5U2V4	8	Q01984	1	O55096	0	D3Z8L7	0
Q3T1J1	8	O54715	1	O70215	0	D3ZHA0	0
Q498R7	8	P35053	1	O70244	0	D3ZTD8	0
P97605	8	Q01205	1	O70352	0	D3ZTV3	0
Q63357	8	Q63475	1	O70377	0	D3ZUK3	0

P09606	7	Q9QZ76	1	O70489	0	D3ZW55	0
Q8CGS4	7	P10247	1	O70513	0	D4A1J4	0
P15999	7	P07379	1	O70535	0	D4A1R8	0
P01041	7	A2RUW1	0	O70540	0	D4AE59	0
Q99376	7	A4KWA5;A	0	O88204	0	E9PT87;P2	0
Q4FZU6	7	B0BNA5	0	O88267	0	F1LM93	0
Q64319	7	B0BND0	0	O88339;Q4	0	G3V7W1	0
P97608	7	B0BNE5	0	O88600	0	H1UBN0;Q	0
P38438	6	B2RYW9	0	O88766	0	O08557	0
P16636	6	B5DEN9	0	O88767	0	O08628	0
Q5M872	6	B5DFC9	0	O88775	0	O08651	0
Q6RUV5	6	D3Z8L7	0	O88917	0	O08839	0
P20673	5	D3ZTD8	0	O88989	0	O09175	0
P04916	5	D3ZTE0	0	P00502	0	O35077	0
P97584	5	D3ZTV3	0	P00689	0	O35112	0
Q08463	5	D3ZTX0	0	P00774	0	O35142	0
P02793;Q7	5	D3ZUK3	0	P00786	0	O35217	0
Q6AYE5	5	D3ZW55	0	P00787	0	O35244	0
Q64268	5	D4A1J4	0	P00884	0	O35264	0
P10760	5	D4A1R8	0	P01015	0	O35331	0
P46413	5	D4AE59	0	P01026	0	O35509	0
P51792;P5	5	E9PT87;P2	0	P01041	0	O35760	0
P24268	4	F1LM93	0	P01048	0	O35763	0
Q6AY33	4	G3V7W1	0	P01830	0	O35952	0
Q7M0E3	4	H1UBN0;Q	0	P02091	0	O35956	0
P10719	4	O08628	0	P02650	0	O54715	0
P01015	4	O08651	0	P02651	0	O54861	0
Q6GMN2	4	O08839	0	P02680	0	O54975	0
P97697	4	O09175	0	P02696	0	O55006	0
Q5RKI0	3	O35112	0	P02761	0	O55096	0
Q6AYT0	3	O35142	0	P02764	0	O70215	0
Q1WIM3	3	O35217	0	P02767	0	O70244	0
Q9QWN8	3	O35244	0	P02770	0	O70257	0
P14046	3	O35264	0	P02793;Q7	0	O70352	0
P62630	3	O35276	0	P03994	0	O70377	0
Q8VI04	3	O35331	0	P04041	0	O70489	0
Q4QQT4	3	O35509	0	P04073	0	O70513	0
B0BNA5	2	O35568	0	P04218	0	O70535	0
Q91Y81	2	O35760	0	P04276	0	O70540	0
P70619	2	O35763	0	P04355	0	O70594	0
P37996	2	O35952	0	P04639	0	O88204	0
O55145	2	O35956	0	P04642	0	O88267	0
O35956	2	O54975	0	P04762	0	O88600	0
P18421	2	O55006	0	P04764	0	O88766	0
P81556	2	O55096	0	P04785	0	O88767	0
P35053	2	O70215	0	P04797	0	O88775	0
P18427	2	O70244	0	P04897	0	O88797	0
Q9QYP2	2	O70352	0	P04903	0	O88917	0
P15978	2	O70377	0	P04904	0	O88989	0
O35244	1	O70489	0	P04906	0	P00502	0
P00884	1	O70513	0	P04916	0	P00507	0
P41562	1	O70535	0	P04937	0	P00689	0
P38652	1	O70540	0	P05065	0	P00786	0
P45592	1	O88204	0	P05197	0	P00787	0
P22734	1	O88267	0	P05369	0	P00884	0
Q5U1Y4	1	O88339;Q4	0	P05371	0	P01026	0
P48032	1	O88600	0	P05544	0	P01041	0
Q10743	1	O88766	0	P05545	0	P01048	0
Q9JJ50	1	O88767	0	P05712	0	P01830	0
Q7TQ94	1	O88775	0	P06214	0	P01836	0
Q6P6T4	1	O88989	0	P06399	0	P02650	0
P43427	1	P00502	0	P06685	0	P02680	0
P08753	1	P00689	0	P07151	0	P02696	0
P06399	1	P00758	0	P07154	0	P02761	0
Q5U316	1	P00786	0	P07174	0	P02764	0
P33436	1	P00787	0	P07314	0	P02767	0
P06866	1	P00884	0	P07335	0	P02780	0
Q63199	1	P01015	0	P07340	0	P02781	0
A0JPJ7	0	P01026	0	P07379	0	P02783	0
A4KWA5;A	0	P01048	0	P07483	0	P03994	0
B0BND0	0	P01830	0	P07522	0	P04041	0
B0BNE5	0	P02650	0	P07632	0	P04073	0
B5DEN9	0	P02651	0	P07861	0	P04218	0
B5DFC9	0	P02680	0	P07897	0	P04636	0

D3Z8L7	0
D3ZTD8	0
D3ZTE0	0
D3ZTV3	0
D3ZUK3	0
D3ZW55	0
D4A1J4	0
D4A1R8	0
D4AE59	0
E9PT87;P2	0
F1LM93	0
F1M3L7	0
G3V686	0
G3V7W1	0
H1UBN0;Ç	0
O08628	0
O08651	0
O08839	0
O09175	0
O35112	0
O35142	0
O35217	0
O35264	0
O35276	0
O35331	0
O35509	0
O35568	0
O35760	0
O35952	0
O54715	0
O54858	0
O54975	0
O55006	0
O55096	0
O70215	0
O70244	0
O70352	0
O70489	0
O70513	0
O70535	0
O70540	0
O88204	0
O88600	0
O88766	0
O88767	0
O88775	0
O88989	0
P00502	0
P00507	0
P00689	0
P00731	0
P00758	0
P00786	0
P00787	0
P01026	0
P01048	0
P01830	0
P02650	0
P02651	0
P02680	0
P02696	0
P02764	0
P02767	0
P02770	0
P03994	0
P04041	0
P04073	0
P04218	0
P04276	0
P04355	0
P04636	0
P04639	0
P04642	0
P04762	0
P02696	0
P02764	0
P02767	0
P02770	0
P02793;Q7	0
P03994	0
P04041	0
P04073	0
P04218	0
P04276	0
P04636	0
P04639	0
P04642	0
P04764	0
P04785	0
P04797	0
P04897	0
P04903	0
P04904	0
P04906	0
P04916	0
P04937	0
P05065	0
P05197	0
P05371	0
P05544	0
P05545	0
P05712	0
P06214	0
P06399	0
P06685	0
P07154	0
P07171	0
P07314	0
P07340	0
P07483	0
P07522	0
P07632	0
P07861	0
P07897	0
P07943	0
P08010	0
P08289	0
P08290	0
P08460	0
P08494	0
P08592	0
P08644;P2	0
P08650	0
P08932	0
P09006	0
P09034	0
P09527	0
P09606	0
P0C0K7	0
P0CG51;P6	0
P0DMW0;P	0
P10111	0
P10252	0
P10536	0
P10760	0
P10824	0
P10959	0
P10960	0
P11030	0
P11232	0
P11348	0
P11442	0
P11980	0
P12346	0
P13221	0
P13596	0
P13635	0
P14408	0
P07943	0
P08289	0
P08290	0
P08460	0
P08494	0
P08592	0
P08644;P2	0
P08650	0
P08753	0
P08932	0
P08934	0
P08937	0
P09006	0
P09034	0
P09456	0
P09606	0
P0C0K7	0
P0CG51;P6	0
P10111	0
P10252	0
P10536	0
P10760	0
P10824	0
P10959	0
P10960	0
P11030	0
P11232	0
P11348	0
P11980	0
P12346	0
P12368	0
P13221	0
P13265	0
P13596	0
P13635	0
P13676	0
P14046	0
P14408	0
P14480	0
P14630	0
P14668	0
P14669	0
P14740	0
P14841	0
P14925	0
P14942	0
P15083	0
P15087	0
P15390	0
P15473	0
P15684	0
P15800	0
P15943	0
P15978	0
P16086	0
P16228	0
P16290	0
P16310	0
P16391	0
P16446	0
P16573	0
P16617	0
P16636	0
P17164	0
P17475	0
P17988	0
P18292	0
P18297	0
P18421	0
P18427	0
P18757	0
P19112	0
P19132	0
P19468	0
P04639	0
P04642	0
P04762	0
P04764	0
P04785	0
P04797	0
P04897	0
P04903	0
P04904	0
P04905	0
P04906	0
P04937	0
P05065	0
P05197	0
P05369	0
P05371	0
P05544	0
P05545	0
P05712	0
P05964	0
P06214	0
P06399	0
P06685	0
P07151	0
P07154	0
P07314	0
P07335	0
P07340	0
P07483	0
P07522	0
P07632	0
P07943	0
P08010	0
P08289	0
P08290	0
P08460	0
P08592	0
P08644;P2	0
P08650	0
P08753	0
P08932	0
P08934	0
P09006	0
P09034	0
P09527	0
P09606	0
P0CG51;P6	0
P0DP29;P0	0
P10111	0
P10247	0
P10252	0
P10536	0
P10760	0
P10824	0
P10959	0
P10960	0
P11232	0
P11348	0
P11442	0
P11980	0
P13221	0
P13265	0
P13596	0
P13635	0
P13676	0
P14046	0
P14408	0
P14480	0
P14562	0
P14630	0
P14668	0
P14669	0
P14740	0
P14841	0

P04764	0	P14480	0	P19804	0	P14925	0
P04785	0	P14562	0	P19814	0	P14942	0
P04797	0	P14630	0	P19939	0	P15083	0
P04897	0	P14668	0	P20059	0	P15087	0
P04903	0	P14669	0	P20611	0	P15390	0
P04906	0	P14740	0	P20673	0	P15473	0
P04937	0	P14841	0	P20760	0	P15684	0
P05065	0	P14925	0	P20767	0	P15800	0
P05197	0	P14942	0	P20786	0	P15943	0
P05544	0	P15083	0	P20961	0	P15978	0
P05545	0	P15087	0	P21581	0	P15999	0
P05712	0	P15390	0	P21670	0	P16086	0
P05964	0	P15473	0	P21704	0	P16228	0
P06214	0	P15684	0	P21708;P2	0	P16290	0
P06685	0	P15800	0	P22057	0	P16310	0
P07154	0	P15943	0	P22734	0	P16391	0
P07174	0	P15999	0	P22985	0	P16446	0
P07314	0	P16086	0	P23377	0	P16573	0
P07335	0	P16290	0	P23593	0	P16617	0
P07340	0	P16310	0	P23680	0	P17164	0
P07379	0	P16391	0	P23764	0	P17988	0
P07483	0	P16446	0	P23785	0	P18292	0
P07522	0	P16573	0	P23928	0	P18297	0
P07632	0	P16617	0	P24090	0	P18298	0
P07861	0	P17164	0	P24268	0	P18421	0
P07897	0	P17475	0	P24594	0	P18427	0
P08289	0	P18292	0	P25031	0	P18757	0
P08290	0	P18298	0	P25093	0	P19112	0
P08460	0	P18421	0	P25113	0	P19468	0
P08494	0	P18427	0	P25236	0	P19804	0
P08592	0	P19112	0	P26051	0	P20059	0
P08644;P2	0	P19468	0	P26453	0	P20611	0
P08650	0	P19804	0	P26644	0	P20673	0
P08932	0	P20059	0	P26772	0	P20759	0
P08934	0	P20611	0	P27139	0	P20761	0
P09006	0	P20673	0	P27274	0	P20786	0
P09034	0	P20767	0	P27590	0	P20961	0
P09656	0	P20786	0	P27605	0	P21581	0
P0C0K7	0	P20961	0	P27653	0	P21588	0
P0CG51;P6	0	P21581	0	P27867	0	P21670	0
P0DP29;P0	0	P21588	0	P28037	0	P21704	0
P10111	0	P21670	0	P28075	0	P21708;P2	0
P10252	0	P21704	0	P28480	0	P22057	0
P10824	0	P21708;P2	0	P28494	0	P22734	0
P10959	0	P22057	0	P28570	0	P22985	0
P10960	0	P22734	0	P28648	0	P23377	0
P11030	0	P22985	0	P28826	0	P23680	0
P11232	0	P23377	0	P29288	0	P23764	0
P11348	0	P23680	0	P29534	0	P23785	0
P11442	0	P23764	0	P29598	0	P23928	0
P11980	0	P23928	0	P29975	0	P24090	0
P12346	0	P24090	0	P30121	0	P24268	0
P13221	0	P24268	0	P30152	0	P24594	0
P13265	0	P24594	0	P30713	0	P25093	0
P13596	0	P25093	0	P30836	0	P25113	0
P13635	0	P25113	0	P30904	0	P26051	0
P13676	0	P25236	0	P30919	0	P26342	0
P13852	0	P26051	0	P31211	0	P26644	0
P14173	0	P26342	0	P31430	0	P27139	0
P14408	0	P26453	0	P31977	0	P27274	0
P14480	0	P26644	0	P32038	0	P27590	0
P14562	0	P27139	0	P32755	0	P27605	0
P14630	0	P27274	0	P34058	0	P27653	0
P14669	0	P27605	0	P34080	0	P27867	0
P14740	0	P27653	0	P34158	0	P28037	0
P14841	0	P27867	0	P34900	0	P28075	0
P14925	0	P28037	0	P34901	0	P28480	0
P14942	0	P28075	0	P35053	0	P28494	0
P15083	0	P28480	0	P35213	0	P28570	0
P15087	0	P28494	0	P35444	0	P28648	0
P15390	0	P28570	0	P35446	0	P28826	0
P15473	0	P28826	0	P35704	0	P29288	0
P15684	0	P29288	0	P35859	0	P29534	0
P15800	0	P29534	0	P36373	0	P29598	0

P15943	0	P29598	0	P36375	0	P29975	0
P16086	0	P29975	0	P36953	0	P30121	0
P16228	0	P30121	0	P36970	0	P30152	0
P16290	0	P30152	0	P36972	0	P30713	0
P16310	0	P30713	0	P37996	0	P30836	0
P16391	0	P30836	0	P38438	0	P30904	0
P16446	0	P30904	0	P38444	0	P31044	0
P16573	0	P31211	0	P38652	0	P31211	0
P16617	0	P31430	0	P38659	0	P31430	0
P17046	0	P31977	0	P38918	0	P31977	0
P17164	0	P32038	0	P40241	0	P32038	0
P17475	0	P32755	0	P41498	0	P32755	0
P18292	0	P34058	0	P41562	0	P34058	0
P18298	0	P34158	0	P41740	0	P34080	0
P19804	0	P34900	0	P42123	0	P34158	0
P19939	0	P35213	0	P43303	0	P34900	0
P20059	0	P35444	0	P43427	0	P34901	0
P20611	0	P35446	0	P45479	0	P35053	0
P20759	0	P35704	0	P45592	0	P35213	0
P20767	0	P35859	0	P46413	0	P35446	0
P20786	0	P36373	0	P46720	0	P35704	0
P20961	0	P36953	0	P46953	0	P35859	0
P21581	0	P36970	0	P47820	0	P36373	0
P21588	0	P36972	0	P47853	0	P36375	0
P21670	0	P37996	0	P48037	0	P36953	0
P21704	0	P38444	0	P48199	0	P36970	0
P21708;P2	0	P38652	0	P48284	0	P36972	0
P22057	0	P38659	0	P48500	0	P37996	0
P22985	0	P41498	0	P48508	0	P38438	0
P23377	0	P41562	0	P49002	0	P38444	0
P23680	0	P41740	0	P50123	0	P38652	0
P23764	0	P42123	0	P50137	0	P38659	0
P23785	0	P43303	0	P50398	0	P38918	0
P24090	0	P43427	0	P50399	0	P40241	0
P24594	0	P45479	0	P50430	0	P41498	0
P25093	0	P45592	0	P50503	0	P41562	0
P25113	0	P46413	0	P50609	0	P41740	0
P25236	0	P46953	0	P51635	0	P42123	0
P26051	0	P47820	0	P51647	0	P43303	0
P26342	0	P47853	0	P51740	0	P43427	0
P26453	0	P48032	0	P51886	0	P45479	0
P26644	0	P48199	0	P51907	0	P45592	0
P27139	0	P48284	0	P52759	0	P46413	0
P27274	0	P48500	0	P52796	0	P46844	0
P27605	0	P49002	0	P52847	0	P46953	0
P27653	0	P49744	0	P53369	0	P47820	0
P27867	0	P50123	0	P53790	0	P47853	0
P28037	0	P50137	0	P53792	0	P48037	0
P28075	0	P50398	0	P53812	0	P48284	0
P28480	0	P50399	0	P53813	0	P48500	0
P28494	0	P50430	0	P54311	0	P48508	0
P28648	0	P50503	0	P54313	0	P49002	0
P28826	0	P50609	0	P55018	0	P50123	0
P29288	0	P51635	0	P55053	0	P50398	0
P29534	0	P51647	0	P55091	0	P50399	0
P29598	0	P51740	0	P55146	0	P50430	0
P30121	0	P51792;P5	0	P55159	0	P50609	0
P30152	0	P51886	0	P55260	0	P51635	0
P30713	0	P51907	0	P55281	0	P51647	0
P30836	0	P52759	0	P55314	0	P51792;P5	0
P30919	0	P52796	0	P57097	0	P51886	0
P31211	0	P52847	0	P59647	0	P51907	0
P31977	0	P53369	0	P60711;P6	0	P52759	0
P32038	0	P53792	0	P60901	0	P52796	0
P32755	0	P53812	0	P61107	0	P52847	0
P34058	0	P53813	0	P61206;P8	0	P53369	0
P34080	0	P54311	0	P61459	0	P53790	0
P34158	0	P54313	0	P61589	0	P53812	0
P34900	0	P55018	0	P61943	0	P53813	0
P35213	0	P55053	0	P61972	0	P54311	0
P35444	0	P55146	0	P61983	0	P54313	0
P35446	0	P55260	0	P62260	0	P55018	0
P35704	0	P55314	0	P62630	0	P55053	0
P35859	0	P57097	0	P62749	0	P55146	0

P36373	0	P57113	0	P62775	0	P55159	0
P36375	0	P59647	0	P62815	0	P55260	0
P36953	0	P60711;P6	0	P62828;Q8	0	P55281	0
P36972	0	P60901	0	P62836	0	P55314	0
P38444	0	P61107	0	P62898	0	P57097	0
P38659	0	P61206;P8	0	P62959	0	P59647	0
P39069	0	P61459	0	P62963	0	P60711;P6	0
P41498	0	P61943	0	P63018	0	P60901	0
P41740	0	P61972	0	P63029	0	P61107	0
P42123	0	P61983	0	P63095	0	P61206;P8	0
P42854	0	P62260	0	P63102	0	P61459	0
P43303	0	P62630	0	P63322	0	P61589	0
P45479	0	P62749	0	P68035;P6	0	P61943	0
P46953	0	P62775	0	P68255	0	P61972	0
P47727	0	P62815	0	P68370	0	P61983	0
P47820	0	P62828;Q8	0	P68511	0	P62260	0
P47853	0	P62836	0	P69897	0	P62630	0
P48199	0	P62898	0	P70470	0	P62749	0
P48284	0	P62959	0	P70490	0	P62775	0
P48500	0	P62963	0	P70502	0	P62815	0
P49002	0	P63018	0	P70545	0	P62828;Q8	0
P49744	0	P63081	0	P70619	0	P62836	0
P50123	0	P63095	0	P70709	0	P62959	0
P50137	0	P63102	0	P80020	0	P62963	0
P50398	0	P63322	0	P80067	0	P63018	0
P50399	0	P68035;P6	0	P80201	0	P63081	0
P50430	0	P68255	0	P80204	0	P63095	0
P50503	0	P68370	0	P80254	0	P63102	0
P50609	0	P70470	0	P81827	0	P63322	0
P51635	0	P70490	0	P81828	0	P68035;P6	0
P51647	0	P70502	0	P82252	0	P68255	0
P51740	0	P70545	0	P82450	0	P68370	0
P51886	0	P70709	0	P82995	0	P68511	0
P52759	0	P70712	0	P83121	0	P70470	0
P52796	0	P80020	0	P85968	0	P70490	0
P53369	0	P80067	0	P85971	0	P70545	0
P53792	0	P80201	0	P85973	0	P70619	0
P53812	0	P80204	0	P97532	0	P70709	0
P53813	0	P80254	0	P97546	0	P80067	0
P54311	0	P81556	0	P97553	0	P80201	0
P54313	0	P81827	0	P97574	0	P80204	0
P55018	0	P81828	0	P97603	0	P80254	0
P55053	0	P82252	0	P97605	0	P81556	0
P55146	0	P82450	0	P97608	0	P81827	0
P55159	0	P82995	0	P97675	0	P81828	0
P55260	0	P83121	0	P97697	0	P82252	0
P55281	0	P84039	0	P97710	0	P82450	0
P55314	0	P85968	0	P97829	0	P82995	0
P57097	0	P85971	0	P98089	0	P83121	0
P59647	0	P85973	0	P98158	0	P85968	0
P60901	0	P97523	0	Q00238	0	P85973	0
P61107	0	P97532	0	Q00495	0	P97523	0
P61459	0	P97546	0	Q00657	0	P97532	0
P61589	0	P97553	0	Q00918	0	P97546	0
P61943	0	P97574	0	Q01177	0	P97553	0
P61972	0	P97584	0	Q01460	0	P97574	0
P61983	0	P97603	0	Q02445	0	P97603	0
P62260	0	P97605	0	Q02765	0	P97605	0
P62749	0	P97608	0	Q02974	0	P97608	0
P62775	0	P97710	0	Q03248	0	P97675	0
P62815	0	P97829	0	Q03336	0	P97697	0
P62828;Q8	0	P98158	0	Q03626	0	P97710	0
P62836	0	P98166	0	Q04589	0	P98158	0
P62898	0	Q00238	0	Q04807	0	P98166	0
P62959	0	Q00495	0	Q05030	0	Q00238	0
P62963	0	Q00657	0	Q05175	0	Q00495	0
P63018	0	Q00918	0	Q05695	0	Q00918	0
P63102	0	Q01177	0	Q05820	0	Q01177	0
P68255	0	Q01460	0	Q05982	0	Q01205	0
P68370	0	Q02445	0	Q06496	0	Q01460	0
P68511	0	Q02765	0	Q06880	0	Q01984	0
P69897	0	Q02974	0	Q07116	0	Q02445	0
P70470	0	Q03248	0	Q07936	0	Q02765	0
P70490	0	Q03336	0	Q08406	0	Q02974	0

P70502	0	Q03626	0	Q08415	0	Q03248	0
P70712	0	Q04589	0	Q08420	0	Q03336	0
P80020	0	Q04807	0	Q08463	0	Q03626	0
P80067	0	Q05030	0	Q08464	0	Q04589	0
P80201	0	Q05511	0	Q08849	0	Q04807	0
P80202	0	Q05695	0	Q09326	0	Q05030	0
P80204	0	Q05820	0	Q0PMD2	0	Q05820	0
P80254	0	Q05982	0	Q10743	0	Q05982	0
P81827	0	Q06496	0	Q1WIM1	0	Q06496	0
P81828	0	Q06880	0	Q1WIM3	0	Q06880	0
P82252	0	Q07116	0	Q32KJ6	0	Q07116	0
P82450	0	Q07936	0	Q32PY2	0	Q07936	0
P82995	0	Q08163	0	Q3KRC4	0	Q08163	0
P83121	0	Q08406	0	Q3KRD8	0	Q08406	0
P84039	0	Q08415	0	Q3MIE4	0	Q08415	0
P85968	0	Q08420	0	Q3MIF4	0	Q08420	0
P85971	0	Q08463	0	Q3T1J1	0	Q08463	0
P85973	0	Q08464	0	Q3T1J9	0	Q08464	0
P97532	0	Q08849	0	Q3T1K5	0	Q08849	0
P97546	0	Q09326	0	Q3ZAV1	0	Q09326	0
P97553	0	Q0PMD2	0	Q497B0	0	Q0PMD2	0
P97574	0	Q1WIM1	0	Q498D9	0	Q10743	0
P97603	0	Q32KJ6	0	Q498R7	0	Q1WIM1	0
P97615	0	Q32PY2	0	Q498S8	0	Q1WIM3	0
P97675	0	Q3KRC4	0	Q499T2	0	Q32KJ6	0
P97710	0	Q3KRD8	0	Q4FZU4	0	Q32PY2	0
P97829	0	Q3MIE4	0	Q4FZU6	0	Q3KRC4	0
P98158	0	Q3MIF4	0	Q4FZV0	0	Q3KRD8	0
P98166	0	Q3T1J1	0	Q4G063	0	Q3MIE4	0
Q00238	0	Q3T1J9	0	Q4KM73	0	Q3MIF4	0
Q00495	0	Q3T1K5	0	Q4QQT4	0	Q3T1J1	0
Q00657	0	Q497B0	0	Q4QQV8	0	Q3T1J9	0
Q00918	0	Q498D9	0	Q4QQW8	0	Q3T1K5	0
Q01177	0	Q498R7	0	Q4TU93	0	Q3ZAV1	0
Q01205	0	Q498S8	0	Q4V8I1	0	Q497B0	0
Q01460	0	Q499T2	0	Q4V8K5	0	Q498D9	0
Q01984	0	Q4FZU6	0	Q561R9	0	Q498S8	0
Q02445	0	Q4FZV0	0	Q562C9	0	Q499T2	0
Q02765	0	Q4G063	0	Q568Z6	0	Q4FZU4	0
Q02974	0	Q4KM73	0	Q56A20	0	Q4FZV0	0
Q03336	0	Q4QQT4	0	Q5BJP3	0	Q4G063	0
Q03626	0	Q4QQV8	0	Q5BK81	0	Q4KM73	0
Q04589	0	Q4QQW8	0	Q5EGZ1	0	Q4QQT4	0
Q04807	0	Q4TU93	0	Q5FVF9	0	Q4QQV8	0
Q05030	0	Q4V8I1	0	Q5FVH2	0	Q4QQW8	0
Q05511	0	Q4V8K5	0	Q5FVI6	0	Q4V8I1	0
Q05695	0	Q561R9	0	Q5FVR0	0	Q4V8K5	0
Q05820	0	Q562C9	0	Q5FVR3	0	Q561R9	0
Q05982	0	Q568Z6	0	Q5HZW5	0	Q562C9	0
Q06496	0	Q56A20	0	Q5HZW7	0	Q568Z6	0
Q06880	0	Q5BJP3	0	Q5I0D5	0	Q56A20	0
Q07116	0	Q5EGZ1	0	Q5I0E9	0	Q5BJP3	0
Q07523	0	Q5FVF9	0	Q5I0M2	0	Q5BK81	0
Q07936	0	Q5FVH2	0	Q5M7T9	0	Q5FVF9	0
Q08163	0	Q5FVI6	0	Q5M819	0	Q5FVH2	0
Q08406	0	Q5FVR0	0	Q5M843	0	Q5FVI6	0
Q08415	0	Q5FVR3	0	Q5M871	0	Q5FVR0	0
Q08420	0	Q5HZW5	0	Q5M876	0	Q5FVR3	0
Q08464	0	Q5HZW7	0	Q5PPH0	0	Q5HZW5	0
Q08849	0	Q5I0D5	0	Q5RJL6	0	Q5HZW7	0
Q0PMD2	0	Q5I0D7	0	Q5RKH6	0	Q5I0D5	0
Q1WIM1	0	Q5I0M2	0	Q5RKI0	0	Q5I0E9	0
Q32KJ6	0	Q5M7T9	0	Q5RKI1	0	Q5I0M2	0
Q32PY2	0	Q5M819	0	Q5RKI7	0	Q5M7T9	0
Q3KRC4	0	Q5M843	0	Q5U1Y4	0	Q5M819	0
Q3KRD8	0	Q5M871	0	Q5U2P2	0	Q5M843	0
Q3MIF4	0	Q5M876	0	Q5U2Q3	0	Q5M871	0
Q3T1K5	0	Q5RJL6	0	Q5U300	0	Q5M872	0
Q497B0	0	Q5RJR2	0	Q5U316	0	Q5M876	0
Q498S8	0	Q5RKH6	0	Q5U367	0	Q5RJL6	0
Q499T2	0	Q5RKI0	0	Q5XFX0	0	Q5RJR2	0
Q4AEF8	0	Q5RKI7	0	Q5XI43	0	Q5RKH6	0
Q4FZU4	0	Q5U1Y4	0	Q5XI73	0	Q5RKI0	0
Q4FZV0	0	Q5U2P2	0	Q5XI89	0	Q5U1Y4	0

Q4KM73	0	Q5U2Q3	0	Q5XID1	0	Q5U2P2	0
Q4QQV8	0	Q5U300	0	Q5XIE8	0	Q5U2Q3	0
Q4QQW8	0	Q5U316	0	Q5XIL0	0	Q5U2V4	0
Q4TU93	0	Q5U367	0	Q5ZQU0	0	Q5U300	0
Q4V885	0	Q5XFX0	0	Q62632	0	Q5U316	0
Q4V8I1	0	Q5XI43	0	Q62635	0	Q5U367	0
Q4V8K5	0	Q5XI73	0	Q62638	0	Q5XFX0	0
Q561R9	0	Q5XI89	0	Q62718	0	Q5XI43	0
Q562C9	0	Q5XID1	0	Q62740	0	Q5XI73	0
Q568Z6	0	Q5XIE8	0	Q62745	0	Q5XI89	0
Q56A20	0	Q5XIL0	0	Q62753	0	Q5XID1	0
Q5BJP3	0	Q5ZQU0	0	Q62786	0	Q5XIE8	0
Q5BK81	0	Q62632	0	Q62795	0	Q5XIL0	0
Q5EGZ1	0	Q62635	0	Q62812	0	Q5ZQU0	0
Q5FVF9	0	Q62638	0	Q62867	0	Q62632	0
Q5FVH2	0	Q62687	0	Q62930	0	Q62638	0
Q5FVI6	0	Q62718	0	Q62946	0	Q62687	0
Q5FVR0	0	Q62740	0	Q62997	0	Q62718	0
Q5FVR3	0	Q62745	0	Q63041	0	Q62745	0
Q5HZW5	0	Q62753	0	Q63060	0	Q62753	0
Q5HZW7	0	Q62786	0	Q63072	0	Q62761;Q	0
Q5I0D5	0	Q62795	0	Q63081	0	Q62786	0
Q5I0M2	0	Q62812	0	Q63083	0	Q62795	0
Q5M819	0	Q62867	0	Q63135	0	Q62867	0
Q5M843	0	Q62930	0	Q63149	0	Q62902	0
Q5M871	0	Q62975	0	Q63199	0	Q62930	0
Q5M876	0	Q62997	0	Q63228	0	Q62946	0
Q5PPH0	0	Q63041	0	Q63257	0	Q62975	0
Q5RJL6	0	Q63060	0	Q63270	0	Q63041	0
Q5RJR2	0	Q63072	0	Q63317	0	Q63060	0
Q5RKH6	0	Q63081	0	Q63355	0	Q63072	0
Q5RKI7	0	Q63083	0	Q63357	0	Q63081	0
Q5U2P2	0	Q63135	0	Q63416	0	Q63083	0
Q5U300	0	Q63149	0	Q63467	0	Q63135	0
Q5U367	0	Q63150	0	Q63475	0	Q63150	0
Q5XFX0	0	Q63199	0	Q63514	0	Q63199	0
Q5XI43	0	Q63228	0	Q63515	0	Q63228	0
Q5XI73	0	Q63257	0	Q63530	0	Q63257	0
Q5XI89	0	Q63270	0	Q63556	0	Q63270	0
Q5XID1	0	Q63355	0	Q63618	0	Q63317	0
Q5XIE8	0	Q63357	0	Q63621	0	Q63355	0
Q5XIL0	0	Q63416	0	Q63678	0	Q63357	0
Q5ZQU0	0	Q63514	0	Q63691	0	Q63416	0
Q62632	0	Q63515	0	Q63716	0	Q63424	0
Q62638	0	Q63530	0	Q63751	0	Q63515	0
Q62718	0	Q63621	0	Q63772	0	Q63530	0
Q62740	0	Q63678	0	Q63797	0	Q63598	0
Q62745	0	Q63691	0	Q64057	0	Q63621	0
Q62786	0	Q63716	0	Q64119	0	Q63678	0
Q62795	0	Q63772	0	Q641X3	0	Q63691	0
Q62867	0	Q63797	0	Q641Z6	0	Q63716	0
Q62894	0	Q64057	0	Q641Z7	0	Q63772	0
Q62930	0	Q64119	0	Q641Z8	0	Q63797	0
Q62975	0	Q641X3	0	Q64230	0	Q64057	0
Q62997	0	Q641Z6	0	Q642A7	0	Q641X3	0
Q63041	0	Q641Z7	0	Q64319	0	Q641Z6	0
Q63060	0	Q641Z8	0	Q64335	0	Q641Z7	0
Q63072	0	Q64230	0	Q64361	0	Q641Z8	0
Q63081	0	Q642A7	0	Q64537	0	Q64230	0
Q63083	0	Q64319	0	Q64573	0	Q64268	0
Q63135	0	Q64361	0	Q64602	0	Q642A7	0
Q63149	0	Q64537	0	Q64604	0	Q64319	0
Q63150	0	Q64573	0	Q64605	0	Q64335	0
Q63228	0	Q64602	0	Q64611	0	Q64361	0
Q63257	0	Q64604	0	Q64640	0	Q64537	0
Q63416	0	Q64605	0	Q66H12	0	Q64573	0
Q63467	0	Q64611	0	Q66H94	0	Q64602	0
Q63475	0	Q64640	0	Q66HG3	0	Q64604	0
Q63514	0	Q66H12	0	Q66HG4	0	Q64605	0
Q63515	0	Q66H94	0	Q675A5	0	Q64611	0
Q63530	0	Q66HG3	0	Q68FP1	0	Q64640	0
Q63532	0	Q66HG4	0	Q68FQ0	0	Q66H12	0
Q63556	0	Q675A5	0	Q68FQ2	0	Q66H94	0
Q63621	0	Q68FP1	0	Q68FS4	0	Q66HG3	0

Q63678	0	Q68FQ0	0	Q68FT5	0	Q66HG4	0
Q63691	0	Q68FQ2	0	Q6AXR4	0	Q675A5	0
Q63716	0	Q68FS4	0	Q6AXS4	0	Q68FP1	0
Q63772	0	Q68FT5	0	Q6AY33	0	Q68FQ0	0
Q63797	0	Q68FX1	0	Q6AY41	0	Q68FQ2	0
Q64057	0	Q6AXR4	0	Q6AYC4	0	Q68FS4	0
Q64119	0	Q6AXS4	0	Q6AYD4	0	Q68FT5	0
Q641X3	0	Q6AY33	0	Q6AYE5	0	Q68FX1	0
Q641Z6	0	Q6AY41	0	Q6AYH6	0	Q6AXR4	0
Q641Z7	0	Q6AYD4	0	Q6AYP5	0	Q6AXS4	0
Q641Z8	0	Q6AYE5	0	Q6AYQ8	0	Q6AY33	0
Q64230	0	Q6AYH6	0	Q6AYR5	0	Q6AYD4	0
Q64240	0	Q6AYP5	0	Q6AYR6	0	Q6AYE5	0
Q642A7	0	Q6AYR5	0	Q6AYS4	0	Q6AYH6	0
Q64361	0	Q6AYR6	0	Q6AYS7	0	Q6AYP5	0
Q64537	0	Q6AYR8	0	Q6AYT0	0	Q6AYR5	0
Q64573	0	Q6AYS4	0	Q6AYT8	0	Q6AYR6	0
Q64604	0	Q6AYS7	0	Q6B345	0	Q6AYS4	0
Q64605	0	Q6AYT0	0	Q6BEA2	0	Q6AYS7	0
Q64611	0	Q6AYT8	0	Q6GMN2	0	Q6AYT0	0
Q64640	0	Q6B345	0	Q6I7R3	0	Q6AYT8	0
Q66H12	0	Q6BEA2	0	Q6IE52	0	Q6B345	0
Q66H94	0	Q6GMN2	0	Q6IE64	0	Q6BEA2	0
Q66HG4	0	Q6I7R3	0	Q6IRE4	0	Q6GMN2	0
Q675A5	0	Q6IE52	0	Q6IRK9	0	Q6I7R3	0
Q68FP1	0	Q6IE64	0	Q6IUU3	0	Q6IE52	0
Q68FQ0	0	Q6IRE4	0	Q6MG61	0	Q6IE64	0
Q68FQ2	0	Q6IRK9	0	Q6MG71	0	Q6IRE4	0
Q68FS4	0	Q6IUU3	0	Q6MGD0	0	Q6IRK9	0
Q68FX1	0	Q6MG61	0	Q6NX65	0	Q6IUU3	0
Q6AXR4	0	Q6MG71	0	Q6NYB7	0	Q6MG61	0
Q6AXS4	0	Q6MGD0	0	Q6P6S9	0	Q6MG71	0
Q6AYC4	0	Q6NX65	0	Q6P6T1	0	Q6MGD0	0
Q6AYD4	0	Q6NYB7	0	Q6P6T4	0	Q6NX65	0
Q6AYH6	0	Q6P6S4	0	Q6P6V0	0	Q6NYB7	0
Q6AYP5	0	Q6P6S9	0	Q6P734	0	Q6P6S9	0
Q6AYR5	0	Q6P6T1	0	Q6P767	0	Q6P6T1	0
Q6AYR6	0	Q6P6T4	0	Q6P7A9	0	Q6P6T4	0
Q6AYR8	0	Q6P6V0	0	Q6P7Q4	0	Q6P6V0	0
Q6AYS4	0	Q6P734	0	Q6P7S1	0	Q6P734	0
Q6AYT8	0	Q6P767	0	Q6P9T8	0	Q6P767	0
Q6BEA2	0	Q6P777	0	Q6PCU2	0	Q6P777	0
Q6DGG1	0	Q6P7A9	0	Q6PEC4	0	Q6P7A9	0
Q6I7R3	0	Q6P7Q4	0	Q6Q0N0	0	Q6P7Q4	0
Q6IE52	0	Q6P7S1	0	Q6Q0N1	0	Q6P7S1	0
Q6IE64	0	Q6PCU2	0	Q6Q7Y5	0	Q6P9T8	0
Q6IRE4	0	Q6PEC4	0	Q6RY07	0	Q6PEC4	0
Q6IRK9	0	Q6Q0N0	0	Q6TUD4	0	Q6Q0N0	0
Q6IUU3	0	Q6Q0N1	0	Q6X936	0	Q6Q0N1	0
Q6MG71	0	Q6TUD4	0	Q6XQN1	0	Q6Q7Y5	0
Q6MGD0	0	Q6X936	0	Q711G3	0	Q6RUV5	0
Q6NX65	0	Q6XQN1	0	Q71MB6	0	Q6RY07	0
Q6NYB7	0	Q711G3	0	Q76HN1	0	Q6TMA8	0
Q6P6S9	0	Q71MB6	0	Q78P75	0	Q6TUD4	0
Q6P6T1	0	Q76HN1	0	Q793F9	0	Q6X936	0
Q6P6V0	0	Q793F9	0	Q794F9	0	Q6XQN1	0
Q6P734	0	Q794F9	0	Q7M0E3	0	Q711G3	0
Q6P767	0	Q7M0E3	0	Q7TP52	0	Q71MB6	0
Q6P777	0	Q7TP52	0	Q7TPB4	0	Q76HN1	0
Q6P7A9	0	Q7TPB4	0	Q7TQ94	0	Q793F9	0
Q6P7Q4	0	Q7TQ94	0	Q80W57	0	Q794F9	0
Q6P7S1	0	Q80W57	0	Q80WD0	0	Q7M0E3	0
Q6P9T8	0	Q80WD0	0	Q80WD1	0	Q7TPB4	0
Q6PEC4	0	Q80WD1	0	Q80WF4	0	Q7TQ94	0
Q6RY07	0	Q80WF4	0	Q80WY6	0	Q80W57	0
Q6TUD4	0	Q80YN4	0	Q80YN4	0	Q80WD0	0
Q6X936	0	Q810F4	0	Q810F4	0	Q80WD1	0
Q6XQN1	0	Q811A3	0	Q811A3	0	Q80WF4	0
Q711G3	0	Q811X6	0	Q811X6	0	Q80WY6	0
Q76HN1	0	Q812E9	0	Q812E9	0	Q80YN4	0
Q793F9	0	Q8CFN2	0	Q8CG08	0	Q810F4	0
Q794F9	0	Q8CG08	0	Q8CG45	0	Q811A3	0
Q7TP52	0	Q8CG45	0	Q8CGU6	0	Q811X6	0
Q7TPB4	0	Q8CGS4	0	Q8CHN3	0	Q812E9	0

Q80WD0	0	Q8CGU6	0	Q8CHN8	0	Q8CFN2	0
Q80WD1	0	Q8CHN3	0	Q8CIZ5	0	Q8CG45	0
Q80WF4	0	Q8CHN8	0	Q8JZQ0	0	Q8CGS4	0
Q80WY6	0	Q8CIZ5	0	Q8K1G0	0	Q8CGU6	0
Q80YN4	0	Q8JZQ0	0	Q8K3P7	0	Q8CHN3	0
Q810F4	0	Q8K3P7	0	Q8K3V3	0	Q8CHN8	0
Q811A3	0	Q8K3V3	0	Q8K4G9	0	Q8JZQ0	0
Q811X6	0	Q8K4G9	0	Q8K4Y7	0	Q8K3P7	0
Q812E9	0	Q8K4Y7	0	Q8N7M5	0	Q8K3V3	0
Q8CG08	0	Q8N7M5	0	Q8R431	0	Q8K4Y7	0
Q8CG45	0	Q8R491	0	Q8R491	0	Q8N7M5	0
Q8CGU6	0	Q8R4C0	0	Q8R4C0	0	Q8R491	0
Q8CHN3	0	Q8R4E1	0	Q8R4E1	0	Q8R4C0	0
Q8CHN8	0	Q8R5M3	0	Q8R5M3	0	Q8R4E1	0
Q8JZQ0	0	Q8R5M5	0	Q8R5M5	0	Q8R5M3	0
Q8K3V3	0	Q8VBX1	0	Q8VBX1	0	Q8R5M5	0
Q8K4G9	0	Q8VD89	0	Q8VI04	0	Q8VBX1	0
Q8K4Y7	0	Q8VI04	0	Q8VIF7	0	Q8VI04	0
Q8N7M5	0	Q8VIF7	0	Q91XT9	0	Q8VIF7	0
Q8R491	0	Q91XT9	0	Q91Y81	0	Q91XT9	0
Q8R4C0	0	Q91Y81	0	Q920A6	0	Q91Y81	0
Q8R4E1	0	Q920A6	0	Q920G2	0	Q920A6	0
Q8R5M5	0	Q920G2	0	Q920J4	0	Q920J4	0
Q8VBX1	0	Q920J4	0	Q920P0	0	Q920P0	0
Q8VD89	0	Q920P0	0	Q923M1	0	Q923M1	0
Q8VIF7	0	Q923M1	0	Q923S2	0	Q923S2	0
Q91XN4	0	Q923S2	0	Q924B5	0	Q924B5	0
Q91XT9	0	Q923V8	0	Q99068	0	Q99068	0
Q920A6	0	Q924B5	0	Q99J86	0	Q99J86	0
Q920J4	0	Q99068	0	Q99M75	0	Q99M75	0
Q920P0	0	Q99376	0	Q99MA2	0	Q99MA2	0
Q924B5	0	Q99J86	0	Q99MF4	0	Q99MF4	0
Q99068	0	Q99M75	0	Q99MZ8	0	Q99MZ8	0
Q99J86	0	Q99MA2	0	Q99PD4	0	Q99PD4	0
Q99M75	0	Q99MF4	0	Q99PS8	0	Q99PS8	0
Q99MF4	0	Q99MZ8	0	Q99PW3	0	Q99PW3	0
Q99PD4	0	Q99PD4	0	Q99PW7	0	Q99PW7	0
Q99PP0	0	Q99PS8	0	Q9EPB1	0	Q9EPB1	0
Q99PS8	0	Q99PW3	0	Q9EPF2	0	Q9EPF2	0
Q99PW3	0	Q99PW7	0	Q9EQT1	0	Q9EQT1	0
Q99PW7	0	Q9EPB1	0	Q9EQV6	0	Q9EQV6	0
Q9EPB1	0	Q9EPF2	0	Q9EQV9	0	Q9EQX9	0
Q9EPF2	0	Q9EQT1	0	Q9EQX9	0	Q9ESG3	0
Q9EQT1	0	Q9EQV6	0	Q9ESG3	0	Q9ESS6	0
Q9EQV6	0	Q9EQV9	0	Q9ESS6	0	Q9ESW0	0
Q9EQV9	0	Q9EQX9	0	Q9ESW0	0	Q9ET32	0
Q9EQX9	0	Q9ES87	0	Q9ET32	0	Q9JHW1	0
Q9ES87	0	Q9ESG3	0	Q9JHW1	0	Q9JHY1	0
Q9ESG3	0	Q9ESS6	0	Q9JHY1	0	Q9JI92	0
Q9ESS6	0	Q9ESW0	0	Q9JI92	0	Q9JIK1	0
Q9ESW0	0	Q9ET32	0	Q9JIK1	0	Q9JJ19	0
Q9ET32	0	Q9JHW1	0	Q9JJ19	0	Q9JJ22	0
Q9JHW1	0	Q9JHY1	0	Q9JJ22	0	Q9JJ40	0
Q9JHY1	0	Q9JI92	0	Q9JJ40	0	Q9JLJ3	0
Q9JI92	0	Q9JIK1	0	Q9JLJ3	0	Q9JLS4	0
Q9JIK1	0	Q9JJ19	0	Q9JLS4	0	Q9JLZ1	0
Q9JJ22	0	Q9JJ22	0	Q9JLZ1	0	Q9QUL6	0
Q9JJS8	0	Q9JJ40	0	Q9QUL6	0	Q9QWJ9	0
Q9JLS4	0	Q9JLJ3	0	Q9QW30	0	Q9QXN4	0
Q9JLZ1	0	Q9JLS4	0	Q9QWJ9	0	Q9QXQ0	0
Q9QUL6	0	Q9JLZ1	0	Q9QXN4	0	Q9QY17	0
Q9QW30	0	Q9QUL6	0	Q9QXQ0	0	Q9QYP2	0
Q9QWJ9	0	Q9QW30	0	Q9QY17	0	Q9QYU4	0
Q9QX79	0	Q9QWJ9	0	Q9QYP2	0	Q9QZA2	0
Q9QXN4	0	Q9QWN8	0	Q9QYU4	0	Q9QZA6	0
Q9QXQ0	0	Q9QXN4	0	Q9QZA2	0	Q9QZH0	0
Q9QY17	0	Q9QXQ0	0	Q9QZA6	0	Q9QZK8	0
Q9QZ76	0	Q9QY17	0	Q9QZH0	0	Q9QZK9	0
Q9QZA2	0	Q9QYP2	0	Q9QZK8	0	Q9R044	0
Q9QZA6	0	Q9QYU4	0	Q9R044	0	Q9R063	0
Q9QZH0	0	Q9QZA2	0	Q9R063	0	Q9R066	0
Q9QZQ5	0	Q9QZA6	0	Q9R066	0	Q9R0D6	0
Q9R044	0	Q9QZH0	0	Q9R0D6	0	Q9R0J8	0
Q9R063	0	Q9R044	0	Q9R0J8	0	Q9R0T3	0

Q9R066	0	Q9R063	0	Q9R0T3	0	Q9R0T4	0
Q9R0D6	0	Q9R066	0	Q9R0T4	0	Q9R1T3	0
Q9R0J8	0	Q9R0D6	0	Q9R1T3	0	Q9R1T5	0
Q9R0T4	0	Q9R0J8	0	Q9R1T5	0	Q9WTQ2	0
Q9R1T3	0	Q9R0T4	0	Q9WTQ2	0	Q9WTW7	0
Q9WTQ2	0	Q9R1T3	0	Q9WTW7	0	Q9WU49	0
Q9WU49	0	Q9R1T5	0	Q9WU49	0	Q9WU74	0
Q9WU74	0	Q9WTQ2	0	Q9WU74	0	Q9WUK5	0
Q9WUC4	0	Q9WU49	0	Q9WUK5	0	Q9WUW3	0
Q9WUK5	0	Q9WUK5	0	Q9WUW3	0	Q9WUW9	0
Q9WUW3	0	Q9WUW3	0	Q9WUW9	0	Q9WVK7	0
Q9WVK7	0	Q9WUW9	0	Q9Z0J8	0	Q9Z0J8	0
Q9Z0J8	0	Q9Z0J8	0	Q9Z0T0	0	Q9Z0T0	0
Q9Z0T0	0	Q9Z0T0	0	Q9Z0W7	0	Q9Z0W7	0
Q9Z1Y3	0	Q9Z1Y3	0	Q9Z1Y3	0	Q9Z1Y3	0
Q9Z2Y9	0	Q9Z2Y9	0	Q9Z2Y9	0	Q9Z2Y9	0
Q9Z339	0	Q9Z339	0	Q9Z339	0	Q9Z339	0
