# Supplementary material for: Dynamic Changes of Urine Proteome in Rat Models Inoculated with Two Different Hepatoma Cell Lines
Source: J Oncol. 2021 Jan 7;2021:8895330. doi: 10.1155/2021/8895330 (PMC7810548; doi:10.1155/2021/8895330)
Supplement: Supplementary Materials — Supplementary Figure 1. Functional analysis of differentially expressed proteins at days 5, 7, 14, and 28 in two models. (a) Cell component for the CBRH-7919 model. (b) Molecular function for the CBRH-7919 model. (c) Cell component for the RH-35 model. (d) Molecular function for the RH-35 model. Supplementary Table 1. All urinary proteins identified in the CBRH-7919 model. Supplementary Table 2. All urinary proteins identified in the RH-35 model. Supplementary Table 3. The details of 6435 random allocations in the CBRH-7919 model. Supplementary Table 4. The occurrence of the protein in 6435 random allocations in the CBRH-7919 model. Supplementary Table 5. The details of 12155 random allocations in the RH-35 model. Supplementary Table 6. The occurrence of the protein in 12155 random allocations in the RH-35 model. [file 8895330.f1.zip › 8895330.f1/Supplementary Table 5.pdf]

Title： Dynamic changes of urine proteome in rat models inoculated with two different hepatoma cell lines. Full author: Yameng Zhang, Yufei Gao, Jing Wei & Youhe Gao*.

Table S5 The details of 12155 random allocations in RH-35 model.

Day 5

Random allocation	Group1	Group2	Numbers of differential urinary proteins	Differential protein ID
1	1,2,3,4,5,6,7,8	9,10,11,12,13,14,15,16,17	64	D3ZHA0,O54858,O55004,O55006,O88267,P01041,P01681,P02631,P02761,P02764,P05371,P06761,P07171,P07647,P08649,P08723,P08932,P08937,P10536,P15399,P16086,P18418,P19629,P20646,P20760,P20761,P20762,P22282,P22283,P23593,P28648,P29975,P31044,P34901,P50280,P52590,P61206;P84079;P84082,P62815,P68370,P83748,P97571,P97580,P97615,Q08463,Q30KJ2,Q5GRG2,Q5M8C6,Q63317,Q63475,Q63617,Q63751,Q64361,Q6AYS7,Q6TMA8,Q8CJ52,Q8K1G0,Q8VD89,Q99041,Q9EQS0,Q9JI85,Q9QX79,Q9R0T3,Q9WTT6,Q9Z0V6
2	1,2,3,4,5,6,7,9	8,10,11,12,13,14,15,16,17	29	O55006,O88267,P01681,P02631,P05371,P08937,P15399,P16086,P18418,P20646,P20760,P20761,P20762,P23593,P28648,P34901,P52590,P61206;P84079;P84082,P83748,P97580,Q30KJ2,Q498R7,Q63317,Q63475,Q63751,Q63942,Q64361,Q9EQS0,Q9Z0V6
3	1,2,3,4,5,6,7,10	8,9,11,12,13,14,15,16,17	33	O54858,O55006,O88267,P01681,P02631,P05371,P08937,P12020,P15399,P16086,P18418,P20646,P20760,P20761,P20762,P28648,P34901,P52590,P61206;P84079;P84082,P68370,P83748,P97571,P97580,P97615,Q30KJ2,Q4FZU2,Q63317,Q63475,Q63751,Q64361,Q7TP52,Q9EQS0,Q9Z0V6
4	1,2,3,4,5,6,7,11	8,9,10,12,13,14,15,16,17	36	O54858,O55004,O88267,P01681,P01836,P02631,P05371,P08937,P15399,P16086,P18418,P20646,P20760,P20761,P20762,P28648,P34901,P52590,P55314,P61206;P84079;P84082,P68370,P83748,P97580,P97615,Q30KJ2,Q498R7,Q4FZU2,Q63317,Q63475,Q63751,Q64361,Q8VD89,Q9EQS0,Q9ESW0,Q9JI85,Q9Z0V6
5	1,2,3,4,5,6,7,12	8,9,10,11,13,14,15,16,17	44	O54858,O55004,O88267,P01041,P01681,P01836,P02631,P05371,P07647,P08723,P08937,P15399,P16086,P18418,P20760,P20761,P20762,P28648,P30120,P34901,P52590,P61206;P84079;P84082,P62815,P83748,P97580,P97615,Q10758,Q30KJ2,Q498R7,Q4FZU2,Q5M8C6,Q63317,Q63475,Q63751,Q64361,Q6AYS7,Q6IFW6,Q6IG02,Q6IMF3,Q6P6Q2,Q9EQS0,Q9ESW0,Q9JI85,Q9Z0V6
6	1,2,3,4,5,6,7,13	8,9,10,11,12,14,15,16,17	31	O88267,P01681,P01836,P02631,P05371,P05539,P06911,P07647,P08937,P12020,P15399,P16086,P18418,P20760,P20761,P20762,P28648,P34901,P52590,P61206;P84079;P84082,P62815,P83748,P97580,Q30KJ2,Q5GRG2,Q63317,Q63751,Q64361,Q8VD89,Q9JI85,Q9Z0V6
7	1,2,3,4,5,6,7,14	8,9,10,11,12,13,15,16,17	28	O88267,P01681,P02631,P05371,P08937,P12020,P15399,P16086,P18418,P20760,P20761,P20762,P28648,P34901,P52590,P61206;P84079;P84082,P83748,P97580,P97615,Q30KJ2,Q63317,Q63475,Q63532,Q63751,Q64361,Q6AYS7,Q9EQS0,Q9Z0V6
8	1,2,3,4,5,6,7,15	8,9,10,11,12,13,14,16,17	27	O55004,O55006,O88267,P01681,P01836,P02631,P05371,P08937,P15399,P16086,P18418,P20760,P20761,P20762,P28648,P34901,P52590,P61206;P84079;P84082,P83748,P97580,P97615,Q30KJ2,Q63317,Q63475,Q63751,Q64361,Q9Z0V6
9	1,2,3,4,5,6,7,16	8,9,10,11,12,13,14,15,17	26	G3V686,O88267,P01681,P01836,P02631,P05371,P08937,P15399,P16086,P18418,P20760,P20761,P20762,P23593,P28648,P34901,P52590,P61206;P84079;P84082,P83748,P97580,Q30KJ2,Q63317,Q63751,Q64361,Q8VD89,Q9Z0V6
10	1,2,3,4,5,6,7,17	8,9,10,11,12,13,14,15,16	36	O54858,O55004,O88267,P01041,P01681,P01946,P02091,P02631,P05371,P08937,P09605;P25809,P10536,P15399,P16086,P18418,P19223,P20646,P20760,P20761,P20762,P23593,P28648,P34901,P52590,P55091,P61206;P84079;P84082,P62815,P83748,P97580,P97615,Q30KJ2,Q63317,Q63751,Q64361,Q80WY6,Q9Z0V6
11	1,2,3,4,5,6,8,9	7,10,11,12,13,14,15,16,17	25	D3ZHA0,O55006,O88267,P01681,P02793;Q7TP54,P07647,P08649,P08937,P18418,P20646,P20761,P20762,P23593,P28648,P34901,Q5M8C6,Q63317,Q63751,Q63942,Q64361,Q6TMA8,Q9EQS0,Q9QX79,Q9WTT6,Q9Z0V6

12	1,2,3,4,5,6,8,10	7,9,11,12,13,14,15,16,17	27	D3ZHA0,O54858,O55006,O88267,P01681,P02761,P07647,P08649,P08937,P18418,P20760,P20761,P20762,P23593,P28648,P34901,P97615,Q08463,Q4FZU2,Q5M8C6,Q63317,Q63751,Q64361,Q7TP52,Q9EQS0,Q9WTT6,Q9Z0V6
13	1,2,3,4,5,6,8,11	7,9,10,12,13,14,15,16,17	38	D3ZHA0,O54715,O54858,O88267,P01681,P02761,P02793;Q7TP54,P04041,P07150,P07171,P07647,P08649,P08937,P14668,P14669,P18418,P20760,P20761,P20762,P28648,P34901,P50280,P55314,Q30KJ2,Q4FZU2,Q5I0D1,Q5M8C6,Q5XI22,Q63317,Q63751,Q64361,Q6AYS4,Q6IFW6,Q6P6Q2,Q6TMA8,Q9EQS0,Q9ESW0,Q9Z0V6
14	1,2,3,4,5,6,8,12	7,9,10,11,13,14,15,16,17	43	D3ZHA0,O54858,O88267,P01681,P02780,P02782,P07647,P08723,P08937,P16636,P18418,P20760,P20761,P20762,P23593,P28648,P30120,P34901,P35053,P50280,Q08463,Q10758,Q4FZU2,Q5M8C6,Q63317,Q63475,Q63751,Q64361,Q6AYS7,Q6IFU8,Q6IFW6,Q6IG02,Q6IMF3,Q6P6Q2,Q6TMA8,Q8CJ52,Q8K1G0,Q9EQS0,Q9ESW0,Q9JHB9,Q9JJS8,Q9WTT6,Q9Z0V6
15	1,2,3,4,5,6,8,13	7,9,10,11,12,14,15,16,17	26	D3ZHA0,O88267,P01681,P02764,P06911,P07647,P08937,P10536,P18418,P20760,P20761,P20762,P23593,P25236,P28648,P29975,P34901,Q5GRG2,Q5M8C6,Q63317,Q63751,Q64361,Q6PCU2,Q6TMA8,Q9WTT6,Q9Z0V6
16	1,2,3,4,5,6,8,14	7,9,10,11,12,13,15,16,17	25	D3ZHA0,O88267,P01681,P02793;Q7TP54,P07647,P08649,P08937,P10536,P18418,P20760,P20761,P20762,P28648,P29288,P34901,Q5M8C6,Q63317,Q63751,Q64361,Q6AYS7,Q6TMA8,Q923S2,Q9EQS0,Q9WTT6,Q9Z0V6
17	1,2,3,4,5,6,8,15	7,9,10,11,12,13,14,16,17	24	B0BNN3,D3ZHA0,O55006,P01681,P02631,P02761,P08649,P08937,P10536,P18418,P20760,P20761,P20762,P23593,P28648,P34901,Q5M8C6,Q62894,Q63317,Q63751,Q64361,Q6TMA8,Q9WTT6,Q9Z0V6
18	1,2,3,4,5,6,8,16	7,9,10,11,12,13,14,15,17	18	D3ZHA0,O88267,P01681,P08649,P08937,P14668,P20760,P20762,P23593,P28648,P34901,Q5M8C6,Q63317,Q63751,Q64361,Q6PCU2,Q9WTT6,Q9Z0V6
19	1,2,3,4,5,6,8,17	7,9,10,11,12,13,14,15,16	41	B0BNN3,D3ZHA0,O54858,O88267,P01681,P01946,P02761,P02770,P02793;Q7TP54,P06760,P07647,P08649,P08937,P10536,P16636,P17475,P18418,P19223,P20646,P20760,P20761,P20762,P23593,P23928,P28648,P34901,P50115,P52590,P62815,P97580,Q08463,Q30KJ2,Q4G075,Q5M8C6,Q63317,Q63751,Q64361,Q6PCU2,Q9QX79,Q9WTT6,Q9Z0V6
20	1,2,3,4,5,6,9,10	7,8,11,12,13,14,15,16,17	11	O55006,P01681,P08649,P14173,P23593,P28648,P34901,Q4FZU2,Q63942,Q64361,Q7TP52
21	1,2,3,4,5,6,9,11	7,8,10,12,13,14,15,16,17	9	P01681,P08649,P20762,P28648,P34901,P55314,Q30KJ2,Q4FZU2,Q63942
22	1,2,3,4,5,6,9,12	7,8,10,11,13,14,15,16,17	16	P01681,P01835,P20762,P23593,P34901,Q10758,Q498R7,Q4FZU2,Q5M8C6,Q64361,Q6IFU8,Q6IFW6,Q6IG02,Q6IMF3,Q6P6Q2,Q9JJS8
23	1,2,3,4,5,6,9,13	7,8,10,11,12,14,15,16,17	5	P01681,P23593,P34901,Q63317,Q63942
24	1,2,3,4,5,6,9,14	7,8,10,11,12,13,15,16,17	5	P01681,P20762,P34901,Q63317,Q63942
25	1,2,3,4,5,6,9,15	7,8,10,11,12,13,14,16,17	8	O54728,O55006,P01681,P20762,P23593,P34901,Q63317,Q63942
26	1,2,3,4,5,6,9,16	7,8,10,11,12,13,14,15,17	4	P01681,P23593,P34901,Q63942

27	1,2,3,4,5,6,9,17	7,8,10,11,12,13,14,15,16	10	P01681,P20760,P20761,P23593,P34901,P52590,Q30KJ2,Q63751,Q63942,Q9Z0V6
28	1,2,3,4,5,6,10,11	7,8,9,12,13,14,15,16,17	15	O54858,P01681,P08649,P15399,P20762,P28648,P34901,P55314,P97580,Q30KJ2,Q4FZU2,Q64361,Q6IFW6,Q6P6Q2,Q7TP52
29	1,2,3,4,5,6,10,12	7,8,9,11,13,14,15,16,17	16	P01681,P08937,P28648,P34901,Q08463,Q10758,Q4FZU2,Q5M8C6,Q64361,Q6IFU7,Q6IFU8,Q6IFW6,Q6IG02,Q6IMF3,Q6P6Q2,Q9JJS8
30	1,2,3,4,5,6,10,13	7,8,9,11,12,14,15,16,17	5	P01681,P28648,P34901,Q63317,Q64361
31	1,2,3,4,5,6,10,14	7,8,9,11,12,13,15,16,17	8	P01681,P08649,P20762,P28648,P34901,P97615,Q4FZU2,Q64361
32	1,2,3,4,5,6,10,15	7,8,9,11,12,13,14,16,17	8	P01681,P08649,P20760,P28648,P34901,Q4FZU2,Q64361,Q7TP52
33	1,2,3,4,5,6,10,16	7,8,9,11,12,13,14,15,17	9	P01681,P08649,P08721,P23593,P28648,P34901,Q4FZU2,Q62997,Q7TP52
34	1,2,3,4,5,6,10,17	7,8,9,11,12,13,14,15,16	19	O54858,P01681,P08649,P08937,P20760,P20761,P23593,P28648,P34901,P52590,P97580,Q08463,Q30KJ2,Q4FZU2,Q63355,Q63751,Q64361,Q7TP52,Q9Z0V6
35	1,2,3,4,5,6,11,12	7,8,9,10,13,14,15,16,17	25	P01681,P01836,P07647,P08937,P15399,P20762,P28648,P30120,P34901,P55314,Q10758,Q30KJ2,Q498R7,Q4FZU2,Q5M8C6,Q63751,Q64361,Q6IFU7,Q6IFU8,Q6IFW6,Q6IG02,Q6IMF3,Q6P6Q2,Q9ESW0,Q9JHB9
36	1,2,3,4,5,6,11,13	7,8,9,10,12,14,15,16,17	9	P01681,P05539,P20762,P28648,P34901,P55314,Q30KJ2,Q4FZU2,Q63317
37	1,2,3,4,5,6,11,14	7,8,9,10,12,13,15,16,17	10	P01681,P02793;Q7TP54,P15399,P20762,P28648,P34901,P35434,P55314,Q30KJ2,Q4FZU2
38	1,2,3,4,5,6,11,15	7,8,9,10,12,13,14,16,17	9	B0BNN3,P01681,P20762,P28648,P34901,P55314,Q30KJ2,Q4FZU2,Q5I0D1
39	1,2,3,4,5,6,11,16	7,8,9,10,12,13,14,15,17	8	G3V686,P01681,P14668,P28648,P34901,P55314,Q30KJ2,Q4FZU2
40	1,2,3,4,5,6,11,17	7,8,9,10,12,13,14,15,16	17	O54858,P01681,P01946,P08649,P08937,P15399,P20760,P20761,P20762,P28648,P34901,P55314,P97580,Q30KJ2,Q4FZU2,Q63751,Q9Z0V6
41	1,2,3,4,5,6,12,13	7,8,9,10,11,14,15,16,17	17	O88267,P01681,P01836,P07647,P08937,P25236,P30120,P34901,P82995,Q10758,Q4FZU2,Q5M8C6,Q63317,Q6IFW6,Q6IMF3,Q9JHB9,Q9JJS8

42	1,2,3,4,5,6,12,14	7,8,9,10,11,13,15,16,17	18	O88267,P01681,P01835,P20762,P25236,P34901,Q10758,Q4FZU2,Q5M8C6,Q63317,Q64361,Q6IFU8,Q6IFW6,Q6IG02,Q6IMF3,Q6P6Q2,Q9JJS8,Q9Z0V6
43	1,2,3,4,5,6,12,15	7,8,9,10,11,13,14,16,17	10	B0BNN3,P01681,P01836,P20762,P34901,Q10758,Q4FZU2,Q64361,Q6IFW6,Q6IMF3
44	1,2,3,4,5,6,12,16	7,8,9,10,11,13,14,15,17	14	G3V686,O88267,P01681,P01836,P23593,P34901,Q10758,Q4FZU2,Q6IFU8,Q6IFW6,Q6IG02,Q6IMF3,Q6P6Q2,Q9JJS8
45	1,2,3,4,5,6,12,17	7,8,9,10,11,13,14,15,16	33	B0BNN3,O54858,O88267,P01681,P01835,P01946,P08937,P13432,P16636,P19223,P20760,P20761,P20762,P23593,P34901,P55091,P62815,P97580,P98089,Q08463,Q10758,Q30KJ2,Q4FZU2,Q5M8C6,Q62635,Q63751,Q64361,Q6IFU8,Q6IFW6,Q6IG02,Q6IMF3,Q9JJS8,Q9Z0V6
46	1,2,3,4,5,6,13,14	7,8,9,10,11,12,15,16,17	6	P01681,P06911,P20762,P25236,P34901,Q63317
47	1,2,3,4,5,6,13,15	7,8,9,10,11,12,14,16,17	5	P01681,P20760,P34901,P82995,Q63317
48	1,2,3,4,5,6,13,16	7,8,9,10,11,12,14,15,17	4	G3V686,P01681,P23593,P34901
49	1,2,3,4,5,6,13,17	7,8,9,10,11,12,14,15,16	15	O88267,P01681,P01946,P08937,P20760,P20761,P23593,P34901,P62815,Q30KJ2,Q63317,Q63355,Q63751,Q6PCU2,Q9Z0V6
50	1,2,3,4,5,6,14,15	7,8,9,10,11,12,13,16,17	7	B0BNN3,P01681,P10536,P20760,P20762,P34901,Q63317
51	1,2,3,4,5,6,14,16	7,8,9,10,11,12,13,15,17	4	P01681,P20762,P34901,Q6P767
52	1,2,3,4,5,6,14,17	7,8,9,10,11,12,13,15,16	14	O88267,P01681,P01946,P02793;Q7TP54,P10536,P20760,P20761,P20762,P34901,P97580,Q30KJ2,Q63751,Q6PCU2,Q9Z0V6
53	1,2,3,4,5,6,15,16	7,8,9,10,11,12,13,14,17	3	P01681,P23593,P34901
54	1,2,3,4,5,6,15,17	7,8,9,10,11,12,13,14,16	11	B0BNN3,P01681,P10536,P20760,P20761,P20762,P23593,P34901,Q30KJ2,Q63751,Q9Z0V6
55	1,2,3,4,5,6,16,17	7,8,9,10,11,12,13,14,15	11	O88267,P01681,P20760,P20761,P23593,P34901,P97580,Q30KJ2,Q63751,Q6PCU2,Q9Z0V6
56	1,2,3,4,5,7,8,9	6,10,11,12,13,14,15,16,17	20	D3ZHA0,O55006,O88267,P02761,P05371,P08937,P18418,P20762,P28648,P31044,P34901,P36970,P52590,P97571,Q63317,Q63475,Q63617,Q9EQS0,Q9QX79,Q9R0T3

57	1,2,3,4,5,7,8,10	6,9,11,12,13,14,15,16,17	28	D3ZHA0,O54858,O55006,O88267,P02761,P05371,P07171,P08649,P08937,P16391,P18418,P20762,P28648,P31044,P34901,P36970,P46844,P52590,P61206;P84079;P84082,P68370,P97571,P97615,Q63317,Q63475,Q64361,Q9EQS0,Q9QX79,Q9R0T3
58	1,2,3,4,5,7,8,11	6,9,10,12,13,14,15,16,17	33	D3ZHA0,O54715,O54858,O55004,O88267,P01836,P02761,P05371,P07171,P08649,P08937,P10715,P14669,P18418,P19814,P20762,P28648,P31044,P34901,P52590,P55314,P61206;P84079;P84082,P68370,Q63317,Q63475,Q63751,Q64194,Q6AYS4,Q8VD89,Q9EQS0,Q9ESW0,Q9QX79,Q9R0T3
59	1,2,3,4,5,7,8,12	6,9,10,11,13,14,15,16,17	41	D3ZHA0,O54858,O55004,O88267,P01041,P01681,P01836,P02761,P05371,P06761,P08937,P10715,P18418,P19223,P20762,P28648,P31044,P34901,P36970,P52590,P83748,P97571,Q498R7,Q5M8C6,Q62894,Q63317,Q63475,Q63751,Q64361,Q68FS4,Q6AYS7,Q6IFW6,Q6IG02,Q80WL1,Q8CJ52,Q8K1G0,Q99041,Q9EQS0,Q9ESW0,Q9QX79,Q9R0T3
60	1,2,3,4,5,7,8,13	6,9,10,11,12,14,15,16,17	19	D3ZHA0,O88267,P01836,P05371,P08937,P18418,P20762,P28648,P31044,P34901,P36970,P52590,P97571,Q5GRG2,Q63317,Q63751,Q9EQS0,Q9QX79,Q9R0T3
61	1,2,3,4,5,7,8,14	6,9,10,11,12,13,15,16,17	23	D3ZHA0,O88267,P02761,P05371,P07171,P08937,P10536,P16086,P18418,P20762,P28648,P31044,P34901,P36970,P52590,P97571,P97615,Q63317,Q63475,Q6AYS7,Q9EQS0,Q9ESW0,Q9QX79
62	1,2,3,4,5,7,8,15	6,9,10,11,12,13,14,16,17	26	D3ZHA0,O54728,O88267,P01041,P01836,P02761,P05371,P07171,P08937,P10536,P18418,P20762,P28648,P31044,P34901,P36970,P52590,P97571,P97615,Q62894,Q63317,Q63475,Q63751,Q9EQS0,Q9ESW0,Q9QX79
63	1,2,3,4,5,7,8,16	6,9,10,11,12,13,14,15,17	21	B4F795,D3ZHA0,O88267,P01836,P05371,P08721,P08937,P18418,P20762,P28648,P31044,P34901,P52590,P97571,Q62894,Q63317,Q63475,Q8VD89,Q9EQS0,Q9QX79,Q9R0T3
64	1,2,3,4,5,7,8,17	6,9,10,11,12,13,14,15,16	41	D3ZHA0,O54858,O88267,P01041,P01681,P01946,P02091,P02761,P02770,P05371,P08937,P10536,P12346,P17475,P18418,P19223,P20760,P20761,P20762,P23928,P28648,P31044,P34901,P36970,P46844,P50115,P52590,P55091,P97571,Q4G075,Q62894,Q63317,Q63751,Q64361,Q6PCU2,Q99041,Q9EQS0,Q9ESW0,Q9QX79,Q9QZA6,Q9R0T3
65	1,2,3,4,5,7,9,10	6,8,11,12,13,14,15,16,17	7	O55006,P18418,P28648,P34901,P52590,P97584,Q63475
66	1,2,3,4,5,7,9,11	6,8,10,12,13,14,15,16,17	10	P01836,P05371,P08289,P28648,P34901,P52590,P55314,P61206;P84079;P84082,Q63475,Q9ESW0
67	1,2,3,4,5,7,9,12	6,8,10,11,13,14,15,16,17	8	P01836,P18418,P34901,P52590,Q498R7,Q63475,Q9EQS0,Q9ESW0
68	1,2,3,4,5,7,9,13	6,8,10,11,12,14,15,16,17	5	P01836,P05539,P34901,P52590,Q63317
69	1,2,3,4,5,7,9,14	6,8,10,11,12,13,15,16,17	4	P18418,P34901,P52590,Q63317
70	1,2,3,4,5,7,9,15	6,8,10,11,12,13,14,16,17	8	O54728,P01836,P34901,P47727,P52590,P97584,Q63317,Q63475
71	1,2,3,4,5,7,9,16	6,8,10,11,12,13,14,15,17	8	O54728,P01836,P08010,P23593,P34901,P47727,P52590,Q9QZK9

72	1,2,3,4,5,7,9,17	6,8,10,11,12,13,14,15,16	7	P01946,P02091,P19223,P23593,P34901,P47727,P52590
73	1,2,3,4,5,7,10,11	6,8,9,12,13,14,15,16,17	14	P01836,P05371,P05964,P18418,P28648,P34901,P52590,P55314,P61206;P84079;P84082,Q5FVI6,Q63475,Q7TP52,Q8VD89,Q9ESW0
74	1,2,3,4,5,7,10,12	6,8,9,11,13,14,15,16,17	16	O35783,P01041,P01836,P18418,P28648,P34901,P52590,Q10758,Q498R7,Q4FZU2,Q63475,Q6IFW6,Q6IG02,Q6IMF3,Q9EQS0,Q9ESW0
75	1,2,3,4,5,7,10,13	6,8,9,11,12,14,15,16,17	6	P01836,P18418,P28648,P34901,P52590,Q63317
76	1,2,3,4,5,7,10,14	6,8,9,11,12,13,15,16,17	8	P18418,P28648,P34901,P36970,P52590,P97615,Q63317,Q63475
77	1,2,3,4,5,7,10,15	6,8,9,11,12,13,14,16,17	8	O54728,P01836,P18418,P28648,P34901,P52590,P97615,Q63475
78	1,2,3,4,5,7,10,16	6,8,9,11,12,13,14,15,17	8	P01836,P08721,P18418,P28648,P34901,P52590,P61206;P84079;P84082,Q62997
79	1,2,3,4,5,7,10,17	6,8,9,11,12,13,14,15,16	10	O54858,P01946,P02091,P18418,P19223,P28648,P34901,P46844,P52590,P97615
80	1,2,3,4,5,7,11,12	6,8,9,10,13,14,15,16,17	23	P01681,P01836,P05371,P05964,P08937,P18418,P28648,P34901,P52590,P55314,P61206;P84079;P84082,Q498R7,Q4FZU2,Q63475,Q63751,Q6IFU8,Q6IFW6,Q6IG02,Q6IMF3,Q6P6Q2,Q9EQS0,Q9ESW0,Q9R1T1
81	1,2,3,4,5,7,11,13	6,8,9,10,12,14,15,16,17	11	P01836,P05371,P05539,P28648,P34901,P52590,P55314,P61206;P84079;P84082,Q63317,Q8VD89,Q9ESW0
82	1,2,3,4,5,7,11,14	6,8,9,10,12,13,15,16,17	12	P01836,P05371,P18418,P20762,P28648,P34901,P52590,P55314,P61206;P84079;P84082,Q63317,Q63475,Q9ESW0
83	1,2,3,4,5,7,11,15	6,8,9,10,12,13,14,16,17	12	O54728,P01836,P05371,P28648,P34901,P52590,P55314,P61206;P84079;P84082,Q5I0D1,Q63475,Q8VD89,Q9ESW0
84	1,2,3,4,5,7,11,16	6,8,9,10,12,13,14,15,17	10	P01836,P05371,P14408,P28648,P34901,P52590,P55314,P61206;P84079;P84082,Q8VD89,Q9ESW0
85	1,2,3,4,5,7,11,17	6,8,9,10,12,13,14,15,16	14	O54858,P01681,P01836,P01946,P02091,P05371,P08937,P28648,P34901,P52590,P55314,P61206;P84079;P84082,Q63751,Q9ESW0
86	1,2,3,4,5,7,12,13	6,8,9,10,11,14,15,16,17	11	O88267,P01836,P08937,P18418,P34901,P36970,P52590,P83748,Q498R7,Q63317,Q9ESW0

87	1,2,3,4,5,7,12,14	6,8,9,10,11,13,15,16,17	12	O88267,P01836,P11762,P18418,P34901,P36970,P52590,Q498R7,Q63317,Q63475,Q9EQS0,Q9ESW0
88	1,2,3,4,5,7,12,15	6,8,9,10,11,13,14,16,17	12	P01041,P01836,P11762,P18418,P34901,P52590,P83748,Q498R7,Q4FZU2,Q63317,Q63475,Q9ESW0
89	1,2,3,4,5,7,12,16	6,8,9,10,11,13,14,15,17	10	O88267,P01836,P08721,P34901,P52590,P83748,Q498R7,Q63475,Q6IFW6,Q9ESW0
90	1,2,3,4,5,7,12,17	6,8,9,10,11,13,14,15,16	17	O88267,P01041,P01681,P01836,P01946,P02091,P08937,P18418,P19223,P34901,P52590,P55091,P98089,Q10758,Q498R7,Q63751,Q9ESW0
91	1,2,3,4,5,7,13,14	6,8,9,10,11,12,15,16,17	6	O88267,P01836,P34901,P36970,P52590,Q63317
92	1,2,3,4,5,7,13,15	6,8,9,10,11,12,14,16,17	7	O54728,P01836,P05539,P34901,P52590,Q09030,Q63317
93	1,2,3,4,5,7,13,16	6,8,9,10,11,12,14,15,17	7	O88267,P01836,P08010,P34901,P52590,Q63317,Q8VD89
94	1,2,3,4,5,7,13,17	6,8,9,10,11,12,14,15,16	11	O88267,P01836,P01946,P02091,P08937,P34901,P52590,P55091,Q63317,Q63751,Q6PCU2
95	1,2,3,4,5,7,14,15	6,8,9,10,11,12,13,16,17	8	O54728,P01836,P11762,P34901,P52590,Q63317,Q63475,Q6IE51
96	1,2,3,4,5,7,14,16	6,8,9,10,11,12,13,15,17	7	O88267,P01836,P05371,P08721,P34901,P52590,Q63532
97	1,2,3,4,5,7,14,17	6,8,9,10,11,12,13,15,16	8	O88267,P01946,P02091,P10536,P34901,P52590,Q63317,Q6PCU2
98	1,2,3,4,5,7,15,16	6,8,9,10,11,12,13,14,17	5	O54728,P01836,P34901,P47727,P52590
99	1,2,3,4,5,7,15,17	6,8,9,10,11,12,13,14,16	11	P01041,P01836,P01946,P02091,P10536,P34901,P47727,P52590,Q63317,Q63751,Q9ESW0
100	1,2,3,4,5,7,16,17	6,8,9,10,11,12,13,14,15	9	O88267,P01836,P01946,P02091,P23593,P34901,P47727,P52590,Q6PCU2
101	1,2,3,4,5,8,9,10	6,7,11,12,13,14,15,16,17	8	D3ZHA0,O55006,P02761,P08649,P23593,P28648,P34901,Q63942

102	1,2,3,4,5,8,9,11	6,7,10,12,13,14,15,16,17	9	P02761,P08649,P14668,P28648,P34901,P55314,Q63942,Q6AYS4,Q6TMA8
103	1,2,3,4,5,8,9,12	6,7,10,11,13,14,15,16,17	12	D3ZHA0,P01681,P23593,P34901,P36970,Q5M8C6,Q62894,Q6IFW6,Q6IG02,Q6TMA8,Q9EQS0,Q9JJS8
104	1,2,3,4,5,8,9,13	6,7,10,11,12,14,15,16,17	6	P20766,P34901,P36970,Q63317,Q63942,Q6TMA8
105	1,2,3,4,5,8,9,14	6,7,10,11,12,13,15,16,17	7	D3ZHA0,P34901,P36970,Q63317,Q63942,Q9QX79,Q9QZK9
106	1,2,3,4,5,8,9,15	6,7,10,11,12,13,14,16,17	11	D3ZHA0,O54728,P02761,P23593,P34901,Q5QE79,Q62894,Q63317,Q63942,Q9QX79,Q9QZK9
107	1,2,3,4,5,8,9,16	6,7,10,11,12,13,14,15,17	5	P23593,P34901,Q62894,Q63942,Q9QZK9
108	1,2,3,4,5,8,9,17	6,7,10,11,12,13,14,15,16	11	P01681,P01946,P02761,P08649,P17475,P19223,P23593,P34901,P52590,Q63942,Q9QX79
109	1,2,3,4,5,8,10,11	6,7,9,12,13,14,15,16,17	12	D3ZHA0,O54858,P02761,P08649,P28648,P34901,P55314,Q5BJY9,Q63598,Q64194,Q6AYS4,Q9ESW0
110	1,2,3,4,5,8,10,12	6,7,9,11,13,14,15,16,17	19	D3ZHA0,P01681,P28648,P34901,P35053,P36970,Q10758,Q4FZU2,Q5M8C6,Q63475,Q64361,Q6IFW6,Q6IG02,Q6IMF3,Q6P6Q2,Q80WL1,Q9EQS0,Q9ESW0,Q9JJS8
111	1,2,3,4,5,8,10,13	6,7,9,11,12,14,15,16,17	5	D3ZHA0,P28648,P34901,P36970,Q63317
112	1,2,3,4,5,8,10,14	6,7,9,11,12,13,15,16,17	7	D3ZHA0,P02761,P08649,P28648,P29288,P34901,P36970
113	1,2,3,4,5,8,10,15	6,7,9,11,12,13,14,16,17	5	D3ZHA0,P02761,P08649,P28648,P34901
114	1,2,3,4,5,8,10,16	6,7,9,11,12,13,14,15,17	6	D3ZHA0,P08649,P08721,P28648,P34901,Q62997
115	1,2,3,4,5,8,10,17	6,7,9,11,12,13,14,15,16	14	D3ZHA0,O54858,P01681,P01946,P02761,P08649,P10959,P17475,P19223,P28648,P34901,P46844,P52590,Q08463
116	1,2,3,4,5,8,11,12	6,7,9,10,13,14,15,16,17	18	P01681,P01836,P28648,P34901,P35053,P55314,Q4FZU2,Q5M8C6,Q63475,Q6AYS4,Q6IFU8,Q6IFW6,Q6IG02,Q6IMF3,Q6P6Q2,Q80WL1,Q9EQS0,Q9ESW0

117	1,2,3,4,5,8,11,13	6,7,9,10,12,14,15,16,17	6	P28648,P34901,P55314,Q63317,Q6AYS4,Q6TMA8
118	1,2,3,4,5,8,11,14	6,7,9,10,12,13,15,16,17	15	P01681,P02793;Q7TP54,P08649,P14668,P19218,P20762,P28648,P34901,P55314,P97840,Q5I0D1,Q64194,Q6AYS4,Q9EQS0,Q9ESW0
119	1,2,3,4,5,8,11,15	6,7,9,10,12,13,14,16,17	13	O54728,P02761,P08649,P14668,P34901,P52303,P55314,P97840,Q5I0D1,Q62894,Q64194,Q6AYS4,Q9ESW0
120	1,2,3,4,5,8,11,16	6,7,9,10,12,13,14,15,17	8	P01681,P08649,P14668,P34901,P55314,Q64194,Q6AYS4,Q9ESW0
121	1,2,3,4,5,8,11,17	6,7,9,10,12,13,14,15,16	15	O54858,P01681,P01946,P02761,P08649,P17475,P19223,P28648,P34901,P50115,P55314,P97840,Q63751,Q6AYS4,Q9ESW0
122	1,2,3,4,5,8,12,13	6,7,9,10,11,14,15,16,17	13	O88267,P01681,P01836,P13265,P34901,P35053,P36970,Q5M8C6,Q62894,Q63317,Q6IG02,Q6TMA8,Q80WL1
123	1,2,3,4,5,8,12,14	6,7,9,10,11,13,15,16,17	14	D3ZHA0,O88267,P01681,P29288,P34901,P35053,P36970,Q5M8C6,Q63317,Q6IFW6,Q6IG02,Q9EQS0,Q9ESW0,Q9JJS8
124	1,2,3,4,5,8,12,15	6,7,9,10,11,13,14,16,17	14	B0BNN3,D3ZHA0,P01836,P02761,P34901,P35053,P36970,P83121,Q4FZU2,Q5M8C6,Q62894,Q63317,Q6IG02,Q9ESW0
125	1,2,3,4,5,8,12,16	6,7,9,10,11,13,14,15,17	12	O88267,P01681,P01836,P08721,P34901,P83121,Q4FZU2,Q5M8C6,Q62894,Q6IFW6,Q6IG02,Q9JJS8
126	1,2,3,4,5,8,12,17	6,7,9,10,11,13,14,15,16	26	B0BNN3,O54858,O88267,P01681,P01946,P02761,P16636,P17475,P19223,P34901,P35053,P55091,P83121,P98089,Q10758,Q4G075,Q5M8C6,Q62894,Q63751,Q6IFW6,Q6IG02,Q6PCU2,Q80WL1,Q9ESW0,Q9JJS8,Q9QZA6
127	1,2,3,4,5,8,13,14	6,7,9,10,11,12,15,16,17	7	P13265,P34901,P36970,Q63317,Q64093,Q6PCU2,Q6TMA8
128	1,2,3,4,5,8,13,15	6,7,9,10,11,12,14,16,17	5	P34901,P36970,Q62894,Q63317,Q6TMA8
129	1,2,3,4,5,8,13,16	6,7,9,10,11,12,14,15,17	4	P34901,Q62894,Q63317,Q6PCU2
130	1,2,3,4,5,8,13,17	6,7,9,10,11,12,14,15,16	9	O88267,P01681,P01946,P10536,P17475,P34901,P36970,Q63317,Q6PCU2
131	1,2,3,4,5,8,14,15	6,7,9,10,11,12,13,16,17	8	B0BNN3,O54728,P02761,P10536,P34901,P36970,P97840,Q63317

132	1,2,3,4,5,8,14,16	6,7,9,10,11,12,13,15,17	7	P08721,P29288,P34901,P36970,P97574,Q6PCU2,Q9QZK9
133	1,2,3,4,5,8,14,17	6,7,9,10,11,12,13,15,16	13	O88267,P01681,P01946,P02761,P02793;Q7TP54,P10536,P17475,P34901,P36970,P97840,Q63317,Q6PCU2,Q9QW07
134	1,2,3,4,5,8,15,16	6,7,9,10,11,12,13,14,17	6	O54728,P34901,P47727,P83121,Q62894,Q9QZK9
135	1,2,3,4,5,8,15,17	6,7,9,10,11,12,13,14,16	10	B0BNN3,P02761,P10536,P17475,P23593,P34901,P83121,P97840,Q62894,Q6PCU2
136	1,2,3,4,5,8,16,17	6,7,9,10,11,12,13,14,15	8	P01681,P01946,P17475,P23593,P23928,P34901,Q62894,Q6PCU2
137	1,2,3,4,5,9,10,11	6,7,8,12,13,14,15,16,17	6	P08649,P14173,P28648,P34901,P55314,Q63661
138	1,2,3,4,5,9,10,12	6,7,8,11,13,14,15,16,17	7	P34901,Q10758,Q4FZU2,Q6IFW6,Q6IG02,Q6IMF3,Q9JJS8
139	1,2,3,4,5,9,10,13	6,7,8,11,12,14,15,16,17	1	P34901
140	1,2,3,4,5,9,10,14	6,7,8,11,12,13,15,16,17	2	P34901,Q9QZK9
141	1,2,3,4,5,9,10,15	6,7,8,11,12,13,14,16,17	4	O54728,P34901,Q62902,Q9QZK9
142	1,2,3,4,5,9,10,16	6,7,8,11,12,13,14,15,17	5	P11598,P23593,P34901,Q62997,Q9QZK9
143	1,2,3,4,5,9,10,17	6,7,8,11,12,13,14,15,16	4	P23593,P34901,P52590,Q62902
144	1,2,3,4,5,9,11,12	6,7,8,10,13,14,15,16,17	11	P01681,P01836,P34901,P55314,Q4FZU2,Q6IFU8,Q6IFW6,Q6IG02,Q6IMF3,Q6P6Q2,Q9ESW0
145	1,2,3,4,5,9,11,13	6,7,8,10,12,14,15,16,17	3	P05539,P34901,P55314
146	1,2,3,4,5,9,11,14	6,7,8,10,12,13,15,16,17	3	P34901,P55314,Q9QZK9

147	1,2,3,4,5,9,11,15	6,7,8,10,12,13,14,16,17	4	O54728,P34901,P55314,Q9QZK9
148	1,2,3,4,5,9,11,16	6,7,8,10,12,13,14,15,17	4	O54728,P34901,P55314,Q9QZK9
149	1,2,3,4,5,9,11,17	6,7,8,10,12,13,14,15,16	5	P01681,P01946,P34901,P52590,P55314
150	1,2,3,4,5,9,12,13	6,7,8,10,11,14,15,16,17	2	P01836,P34901
151	1,2,3,4,5,9,12,14	6,7,8,10,11,13,15,16,17	2	P34901,Q9JJS8
152	1,2,3,4,5,9,12,15	6,7,8,10,11,13,14,16,17	3	O54728,P01836,P34901
153	1,2,3,4,5,9,12,16	6,7,8,10,11,13,14,15,17	5	P01836,P23593,P34901,Q6IFW6,Q9JJS8
154	1,2,3,4,5,9,12,17	6,7,8,10,11,13,14,15,16	5	P01681,P19223,P23593,P34901,P52590
155	1,2,3,4,5,9,13,14	6,7,8,10,11,12,15,16,17	2	P34901,Q63317
156	1,2,3,4,5,9,13,15	6,7,8,10,11,12,14,16,17	2	O54728,P34901
157	1,2,3,4,5,9,13,16	6,7,8,10,11,12,14,15,17	4	O54728,P08010,P34901,Q9QZK9
158	1,2,3,4,5,9,13,17	6,7,8,10,11,12,14,15,16	4	P01946,P23593,P34901,P52590
159	1,2,3,4,5,9,14,15	6,7,8,10,11,12,13,16,17	3	O54728,P34901,Q9QZK9
160	1,2,3,4,5,9,14,16	6,7,8,10,11,12,13,15,17	4	O54728,P34901,Q63942,Q9QZK9
161	1,2,3,4,5,9,14,17	6,7,8,10,11,12,13,15,16	2	P34901,P52590

162	1,2,3,4,5,9,15,16	6,7,8,10,11,12,13,14,17	8	O54728,P11598,P23593,P34901,P47727,Q62902,Q63942,Q9QZK9
163	1,2,3,4,5,9,15,17	6,7,8,10,11,12,13,14,16	6	O54728,P23593,P34901,P47727,P52590,Q62902
164	1,2,3,4,5,9,16,17	6,7,8,10,11,12,13,14,15	4	P23593,P34901,P47727,P52590
165	1,2,3,4,5,10,11,12	6,7,8,9,13,14,15,16,17	17	P01681,P01836,P05964,P28648,P34901,P55314,Q10758,Q4FZU2,Q62636,Q6IFU7,Q6IFU8,Q6IFW6,Q6IG02,Q6IMF3,Q6P6Q2,Q9ESW0,Q9JJS8
166	1,2,3,4,5,10,11,13	6,7,8,9,12,14,15,16,17	3	P28648,P34901,P55314
167	1,2,3,4,5,10,11,14	6,7,8,9,12,13,15,16,17	5	P08649,P28648,P34901,P55314,Q64194
168	1,2,3,4,5,10,11,15	6,7,8,9,12,13,14,16,17	5	O54728,P08649,P34901,P55314,Q64194
169	1,2,3,4,5,10,11,16	6,7,8,9,12,13,14,15,17	4	P08649,P28648,P34901,P55314
170	1,2,3,4,5,10,11,17	6,7,8,9,12,13,14,15,16	7	P01681,P01946,P08649,P28648,P34901,P55314,Q7TP52
171	1,2,3,4,5,10,12,13	6,7,8,9,11,14,15,16,17	6	P01836,P34901,Q10758,Q4FZU2,Q6IFW6,Q9JJS8
172	1,2,3,4,5,10,12,14	6,7,8,9,11,13,15,16,17	7	P34901,Q10758,Q4FZU2,Q6IFW6,Q6IG02,Q6IMF3,Q9JJS8
173	1,2,3,4,5,10,12,15	6,7,8,9,11,13,14,16,17	6	P01836,P34901,Q10758,Q4FZU2,Q6IFW6,Q9JJS8
174	1,2,3,4,5,10,12,16	6,7,8,9,11,13,14,15,17	11	P01836,P08721,P34901,Q10758,Q4FZU2,Q62997,Q6IFW6,Q6IG02,Q6IMF3,Q6P6Q2,Q9JJS8
175	1,2,3,4,5,10,12,17	6,7,8,9,11,13,14,15,16	10	P01681,P01946,P19223,P34901,Q10758,Q4FZU2,Q6IFW6,Q6IG02,Q6IMF3,Q9JJS8
176	1,2,3,4,5,10,13,14	6,7,8,9,11,12,15,16,17	2	P34901,P36970

177	1,2,3,4,5,10,13,15	6,7,8,9,11,12,14,16,17	1	P34901
178	1,2,3,4,5,10,13,16	6,7,8,9,11,12,14,15,17	2	P34901,Q62997
179	1,2,3,4,5,10,13,17	6,7,8,9,11,12,14,15,16	3	P01946,P34901,Q63355
180	1,2,3,4,5,10,14,15	6,7,8,9,11,12,13,16,17	2	O54728,P34901
181	1,2,3,4,5,10,14,16	6,7,8,9,11,12,13,15,17	4	P08721,P34901,Q62997,Q9QZK9
182	1,2,3,4,5,10,14,17	6,7,8,9,11,12,13,15,16	2	P01946,P34901
183	1,2,3,4,5,10,15,16	6,7,8,9,11,12,13,14,17	8	O54728,P02782,P08721,P34901,P47727,Q62902,Q62997,Q9QZK9
184	1,2,3,4,5,10,15,17	6,7,8,9,11,12,13,14,16	2	P34901,Q62902
185	1,2,3,4,5,10,16,17	6,7,8,9,11,12,13,14,15	5	P08721,P23593,P34901,Q62902,Q62997
186	1,2,3,4,5,11,12,13	6,7,8,9,10,14,15,16,17	10	P01681,P01836,P34901,P55314,Q4FZU2,Q6IFU8,Q6IFW6,Q6IG02,Q6P6Q2,Q9ESW0
187	1,2,3,4,5,11,12,14	6,7,8,9,10,13,15,16,17	11	P01681,P01836,P34901,P55314,Q4FZU2,Q6IFU8,Q6IFW6,Q6IG02,Q6IMF3,Q6P6Q2,Q9ESW0
188	1,2,3,4,5,11,12,15	6,7,8,9,10,13,14,16,17	11	P01681,P01836,P34901,P51907,P55314,Q4FZU2,Q6IFU8,Q6IFW6,Q6IG02,Q6P6Q2,Q9ESW0
189	1,2,3,4,5,11,12,16	6,7,8,9,10,13,14,15,17	12	P01681,P01836,P34901,P55314,Q4FZU2,Q6IFU8,Q6IFW6,Q6IG02,Q6IMF3,Q6P6Q2,Q9ESW0,Q9JJS8
190	1,2,3,4,5,11,12,17	6,7,8,9,10,13,14,15,16	14	P01681,P01836,P01946,P19223,P34901,P55314,Q10758,Q4FZU2,Q6IFU8,Q6IFW6,Q6IG02,Q6IMF3,Q6P6Q2,Q9ESW0
191	1,2,3,4,5,11,13,14	6,7,8,9,10,12,15,16,17	2	P34901,P55314

192	1,2,3,4,5,11,13,15	6,7,8,9,10,12,14,16,17	4	O54728,P01836,P34901,P55314
193	1,2,3,4,5,11,13,16	6,7,8,9,10,12,14,15,17	3	P01836,P34901,P55314
194	1,2,3,4,5,11,13,17	6,7,8,9,10,12,14,15,16	4	P01681,P01946,P34901,P55314
195	1,2,3,4,5,11,14,15	6,7,8,9,10,12,13,16,17	7	O54728,P19218,P34901,P55314,P97840,Q5I0D1,Q64194
196	1,2,3,4,5,11,14,16	6,7,8,9,10,12,13,15,17	3	P34901,P55314,Q9QZK9
197	1,2,3,4,5,11,14,17	6,7,8,9,10,12,13,15,16	7	P01681,P01946,P19218,P34901,P55314,P97840,Q9ESW0
198	1,2,3,4,5,11,15,16	6,7,8,9,10,12,13,14,17	8	O54728,P01836,P14408,P34901,P55314,Q5I0D1,Q6AYR8,Q9QZK9
199	1,2,3,4,5,11,15,17	6,7,8,9,10,12,13,14,16	5	B0BNN3,P34901,P55314,P97840,Q9ESW0
200	1,2,3,4,5,11,16,17	6,7,8,9,10,12,13,14,15	4	P01681,P01946,P34901,P55314
201	1,2,3,4,5,12,13,14	6,7,8,9,10,11,15,16,17	4	P01836,P34901,P36970,Q63317
202	1,2,3,4,5,12,13,15	6,7,8,9,10,11,14,16,17	3	P01836,P34901,P83121
203	1,2,3,4,5,12,13,16	6,7,8,9,10,11,14,15,17	3	P01836,P34901,Q9JJS8
204	1,2,3,4,5,12,13,17	6,7,8,9,10,11,14,15,16	5	P01681,P01836,P01946,P19223,P34901
205	1,2,3,4,5,12,14,15	6,7,8,9,10,11,13,16,17	5	A2RUV9,B0BNN3,P01836,P34901,Q4FZU2
206	1,2,3,4,5,12,14,16	6,7,8,9,10,11,13,15,17	4	P01836,P34901,Q6IFW6,Q9JJS8

207	1,2,3,4,5,12,14,17	6,7,8,9,10,11,13,15,16	8	P01681,P01946,P19223,P31430,P34901,P98089,Q10758,Q9JJS8
208	1,2,3,4,5,12,15,16	6,7,8,9,10,11,13,14,17	6	P01836,P34901,P47727,P83121,Q4FZU2,Q9JJS8
209	1,2,3,4,5,12,15,17	6,7,8,9,10,11,13,14,16	7	B0BNN3,P01681,P01836,P19223,P34901,P83121,Q9ESW0
210	1,2,3,4,5,12,16,17	6,7,8,9,10,11,13,14,15	9	P01681,P01836,P01946,P19223,P23593,P34901,P83121,Q10758,Q9JJS8
211	1,2,3,4,5,13,14,15	6,7,8,9,10,11,12,16,17	3	O54728,P34901,Q63317
212	1,2,3,4,5,13,14,16	6,7,8,9,10,11,12,15,17	1	P34901
213	1,2,3,4,5,13,14,17	6,7,8,9,10,11,12,15,16	4	P01946,P34901,Q6PCU2,Q9QW07
214	1,2,3,4,5,13,15,16	6,7,8,9,10,11,12,14,17	4	O54728,P01836,P34901,P47727
215	1,2,3,4,5,13,15,17	6,7,8,9,10,11,12,14,16	2	P01946,P34901
216	1,2,3,4,5,13,16,17	6,7,8,9,10,11,12,14,15	4	P01946,P08010,P34901,Q6PCU2
217	1,2,3,4,5,14,15,16	6,7,8,9,10,11,12,13,17	4	O54728,P34901,P60905,Q9QZK9
218	1,2,3,4,5,14,15,17	6,7,8,9,10,11,12,13,16	6	B0BNN3,P10536,P34901,P47967,P97840,Q9QW07
219	1,2,3,4,5,14,16,17	6,7,8,9,10,11,12,13,15	4	P01946,P34901,Q6PCU2,Q9QW07
220	1,2,3,4,5,15,16,17	6,7,8,9,10,11,12,13,14	6	P23593,P34901,P47727,P83121,Q62902,Q6PCU2
221	1,2,3,4,6,7,8,9	5,10,11,12,13,14,15,16,17	34	D3ZHA0,O55004,O55006,O88267,P01041,P02631,P02761,P05371,P06761,P07647,P08723,P15399,P16086,P18418,P19629,P20761,P20762,P22282,P22283,P31044,P68370,P97580,Q06496,Q30KJ2,Q63317,Q63617,Q63751,Q64361,Q6TMA8,Q9EQS0,Q9JI85,Q9QX79,Q9R0T3,Q9Z0V6

222	1,2,3,4,6,7,8,10	5,9,11,12,13,14,15,16,17	41	D3ZHA0,O55004,O55006,O88267,P01041,P02631,P02761,P05371,P06761,P07647,P08723,P08937,P12020,P15399,P16086,P18418,P19629,P20761,P20762,P22282,P22283,P31044,P35280,P46844,P50280,P68370,P97571,P97580,P97615,Q30KJ2,Q5BJY9,Q5GRG2,Q63317,Q63475,Q63617,Q63751,Q64361,Q9EQS0,Q9JI85,Q9R0T3,Q9Z0V6
223	1,2,3,4,6,7,8,11	5,9,10,12,13,14,15,16,17	52	D3ZHA0,O54715,O55004,O88267,P01041,P01681,P01836,P02631,P02761,P05371,P05964,P06761,P07150,P07171,P07647,P08723,P08937,P10715,P14669,P15399,P16086,P18418,P19629,P20761,P20762,P22282,P22283,P31044,P35280,P50280,P55314,P61206;P84079;P84082,P68370,P97580,Q30KJ2,Q5GRG2,Q5XI22,Q63317,Q63475,Q63493,Q63617,Q63751,Q64361,Q6TMA8,Q8CJ52,Q8VD89,Q99041,Q9EQS0,Q9JI85,Q9QX79,Q9R0T3,Q9Z0V6
224	1,2,3,4,6,7,8,12	5,9,10,11,13,14,15,16,17	66	D3ZHA0,O08557,O55004,O88267,P01041,P01681,P01836,P02631,P02761,P02780,P02781,P02782,P05371,P06761,P07647,P08723,P08937,P0C0A9,P10715,P11598,P13432,P15399,P16086,P18418,P19223,P19629,P20761,P20762,P22282,P22283,P25236,P29975,P30120,P31044,P35280,P40241,P50280,P62815,P68370,P83748,P97580,Q06496,Q30KJ2,Q498R7,Q5GRG2,Q5M8C6,Q63317,Q63475,Q63493,Q63617,Q63751,Q64361,Q6AYR9,Q6AYS7,Q6IFW6,Q6TMA8,Q80WL1,Q8CJ52,Q8K1G0,Q923M1,Q99041,Q9EQS0,Q9JHB9,Q9JI85,Q9R0T3,Q9Z0V6
225	1,2,3,4,6,7,8,13	5,9,10,11,12,14,15,16,17	48	D3ZHA0,O08557,O55004,O88267,P01041,P01836,P02631,P02761,P02780,P05371,P06761,P06911,P07647,P08723,P08937,P11598,P12020,P15399,P16086,P18418,P19629,P20761,P20762,P20766,P22282,P22283,P25236,P29975,P31044,P50280,P62815,P68370,P70545,P97580,Q06496,Q30KJ2,Q5GRG2,Q62902,Q63317,Q63617,Q63751,Q64361,Q6TMA8,Q8CJ52,Q9EQS0,Q9JI85,Q9R0T3,Q9Z0V6
226	1,2,3,4,6,7,8,14	5,9,10,11,12,13,15,16,17	53	D3ZHA0,O08557,O55004,O88267,P01041,P02631,P02761,P05371,P06761,P06911,P07171,P07647,P08723,P10536,P11598,P12020,P15399,P16086,P18418,P19629,P20761,P20762,P22282,P22283,P25236,P29975,P31044,P35280,P36970,P50280,P62815,P68370,P97580,P97615,Q30KJ2,Q5GRG2,Q63317,Q63475,Q63532,Q63617,Q63751,Q64361,Q6AYS7,Q6TMA8,Q8CJ52,Q923M1,Q923S2,Q9EQS0,Q9JI85,Q9QX79,Q9R0T3,Q9WTT6,Q9Z0V6
227	1,2,3,4,6,7,8,15	5,9,10,11,12,13,14,16,17	46	B0BNN3,D3ZHA0,O08557,O55004,O55006,O88267,P01041,P01836,P02631,P02761,P05371,P06761,P07171,P07647,P08723,P10536,P15399,P16086,P18418,P19629,P20761,P20762,P22282,P31044,P35280,P50280,P62815,P68370,P97571,P97580,P97615,Q30KJ2,Q5GRG2,Q5QE79,Q63317,Q63475,Q63617,Q63751,Q64361,Q6AYS7,Q6TMA8,Q9EQS0,Q9JI85,Q9QX79,Q9R0T3,Q9Z0V6
228	1,2,3,4,6,7,8,16	5,9,10,11,12,13,14,15,17	32	D3ZHA0,O55004,O88267,P01041,P01836,P02631,P05371,P06761,P07647,P08721,P08723,P15399,P16086,P18418,P19629,P20761,P20762,P22282,P31044,P68370,P97580,Q30KJ2,Q63617,Q63751,Q64361,Q6AYS7,Q8CJ52,Q8VD89,Q9EQS0,Q9JI85,Q9R0T3,Q9Z0V6
229	1,2,3,4,6,7,8,17	5,9,10,11,12,13,14,15,16	61	B0BNN3,D3ZHA0,O54858,O55004,O88267,P01041,P01681,P01946,P02091,P02631,P02761,P02770,P05371,P06761,P07647,P08723,P08937,P09605;P25809,P10536,P11598,P12346,P15399,P16086,P17475,P18418,P19223,P19629,P20760,P20761,P20762,P22282,P22283,P29975,P31044,P46844,P50115,P50280,P55091,P62815,P68370,P97571,P97580,P97615,Q30KJ2,Q4G075,Q63317,Q63617,Q63751,Q64361,Q6AYS7,Q6P6R2,Q6PCU2,Q99041,Q9EPB1,Q9EQS0,Q9EQV9,Q9JI85,Q9QX79,Q9QZA6,Q9R0T3,Q9Z0V6
230	1,2,3,4,6,7,9,10	5,8,11,12,13,14,15,16,17	11	O55006,P02631,P05371,P15399,P16086,P18418,P97580,Q30KJ2,Q5FVI6,Q91ZS3,Q9EQS0
231	1,2,3,4,6,7,9,11	5,8,10,12,13,14,15,16,17	15	O55004,P01836,P02631,P05371,P15399,P16086,P18418,P20762,P55314,P97580,Q30KJ2,Q498R7,Q91ZS3,Q9EQS0,Q9JI85
232	1,2,3,4,6,7,9,12	5,8,10,11,13,14,15,16,17	23	O35783,O55004,P01041,P01836,P02631,P05371,P07647,P08723,P15399,P16086,P18418,P19223,P20762,P25236,P30120,P34901,P83748,P97580,Q06496,Q30KJ2,Q498R7,Q9EQS0,Q9JI85
233	1,2,3,4,6,7,9,13	5,8,10,11,12,14,15,16,17	14	P01836,P02631,P05371,P06911,P12020,P15399,P16086,P18418,P25236,P97580,Q30KJ2,Q5GRG2,Q63317,Q9JI85
234	1,2,3,4,6,7,9,14	5,8,10,11,12,13,15,16,17	13	P02631,P05371,P12020,P15399,P16086,P18418,P20762,P25236,P34901,P97580,Q30KJ2,Q63532,Q9EQS0

235	1,2,3,4,6,7,9,15	5,8,10,11,12,13,14,16,17	12	O55006,P01041,P01836,P02631,P05371,P15399,P16086,P18418,P20762,P97580,Q30KJ2,Q5QE79
236	1,2,3,4,6,7,9,16	5,8,10,11,12,13,14,15,17	8	P01836,P02631,P05371,P15399,P16086,P18418,P97580,Q30KJ2
237	1,2,3,4,6,7,9,17	5,8,10,11,12,13,14,15,16	18	O55004,P01041,P01946,P02091,P02631,P02770,P05371,P09605;P25809,P15399,P16086,P18418,P19223,P20761,P23593,P52590,P97580,Q30KJ2,Q63751
238	1,2,3,4,6,7,10,11	5,8,9,12,13,14,15,16,17	25	O55004,P01836,P02631,P05371,P05964,P12020,P15399,P16086,P18418,P20762,P34901,P35280,P55314,P61206;P84079;P84082,P68370,P97580,P97615,Q30KJ2,Q5BJY9,Q5FVI6,Q63475,Q7TP52,Q8VD89,Q9EQS0,Q9JI85
239	1,2,3,4,6,7,10,12	5,8,9,11,13,14,15,16,17	34	O35783,O55004,P01041,P01836,P02631,P05371,P05964,P07647,P08723,P12020,P15399,P16086,P18418,P19223,P25236,P30120,P34901,P35280,P47967,P83748,P97580,P97615,P97840,Q10758,Q30KJ2,Q498R7,Q4FZU2,Q63475,Q64361,Q6IFW6,Q6IMF3,Q9EQS0,Q9JI85,Q9JJS8
240	1,2,3,4,6,7,10,13	5,8,9,11,12,14,15,16,17	14	P01836,P02631,P05371,P06911,P12020,P15399,P16086,P18418,P25236,P97580,Q30KJ2,Q5GRG2,Q63317,Q9JI85
241	1,2,3,4,6,7,10,14	5,8,9,11,12,13,15,16,17	16	P02631,P05371,P12020,P15399,P16086,P18418,P20762,P25236,P34901,P35280,P97580,P97615,Q30KJ2,Q5GRG2,Q63532,Q9EQS0
242	1,2,3,4,6,7,10,15	5,8,9,11,12,13,14,16,17	13	P01041,P01836,P02631,P05371,P15399,P16086,P18418,P20761,P35280,P97580,P97615,Q30KJ2,Q5FVI6
243	1,2,3,4,6,7,10,16	5,8,9,11,12,13,14,15,17	14	P01836,P02631,P05371,P08721,P15399,P16086,P18418,P34901,P47967,P97580,Q30KJ2,Q5FVI6,Q62997,Q63532
244	1,2,3,4,6,7,10,17	5,8,9,11,12,13,14,15,16	21	O55004,P01041,P01946,P02091,P02631,P02770,P05371,P09605;P25809,P12020,P15399,P16086,P17475,P18418,P19223,P20761,P46844,P97580,P97615,Q30KJ2,Q63751,Q7TP52
245	1,2,3,4,6,7,11,12	5,8,9,10,13,14,15,16,17	35	O35783,O54715,O55004,P01041,P01681,P01836,P02631,P05371,P05964,P07647,P08723,P15399,P16086,P18418,P20762,P25236,P30120,P34901,P35280,P55314,P61206;P84079;P84082,P97580,Q30KJ2,Q498R7,Q4FZU2,Q63475,Q63493,Q63751,Q6IFU8,Q6IFW6,Q6IMF3,Q6P6Q2,Q9EQS0,Q9JHB9,Q9JI85
246	1,2,3,4,6,7,11,13	5,8,9,10,12,14,15,16,17	21	P01836,P02631,P05371,P05539,P06911,P07647,P12020,P15399,P16086,P18418,P20762,P25236,P55314,P97580,Q30KJ2,Q5GRG2,Q5XI22,Q63317,Q63751,Q8VD89,Q9JI85
247	1,2,3,4,6,7,11,14	5,8,9,10,12,13,15,16,17	17	P01836,P02631,P05371,P12020,P15399,P16086,P18418,P20762,P25236,P34901,P35280,P55314,P97580,Q30KJ2,Q63532,Q9EQS0,Q9JI85
248	1,2,3,4,6,7,11,15	5,8,9,10,12,13,14,16,17	19	O55004,P01041,P01836,P02625,P02631,P05371,P15399,P16086,P18418,P20761,P20762,P22006,P35280,P55314,P97580,Q30KJ2,Q63751,Q8VD89,Q9JI85
249	1,2,3,4,6,7,11,16	5,8,9,10,12,13,14,15,17	15	G3V686,P01836,P02631,P05371,P15399,P16086,P18418,P55053,P55314,P61206;P84079;P84082,P97580,Q30KJ2,Q63532,Q8VD89,Q9JI85

250	1,2,3,4,6,7,11,17	5,8,9,10,12,13,14,15,16	24	O55004,P01041,P01681,P01836,P01946,P02091,P02631,P02770,P05371,P05964,P09605;P25809,P15399,P16086,P17475,P18418,P19223,P20761,P20762,P55091,P55314,P97580,Q30KJ2,Q63751,Q9JI85
251	1,2,3,4,6,7,12,13	5,8,9,10,11,14,15,16,17	31	O88267,P01041,P01836,P02631,P05371,P06911,P07647,P08723,P12020,P15399,P16086,P18418,P20762,P22282,P25236,P29975,P30120,P62815,P83748,P97580,Q30KJ2,Q498R7,Q5GRG2,Q62902,Q63317,Q63751,Q6AYR9,Q9EQS0,Q9JHB9,Q9JI85,Q9R0T3
252	1,2,3,4,6,7,12,14	5,8,9,10,11,13,15,16,17	27	A2RUV9,O88267,P01041,P01836,P02631,P05371,P07647,P08723,P12020,P15399,P16086,P18418,P20761,P20762,P25236,P29975,P30120,P34901,P83748,P97580,Q30KJ2,Q498R7,Q63475,Q63532,Q923M1,Q9EQS0,Q9JI85
253	1,2,3,4,6,7,12,15	5,8,9,10,11,13,14,16,17	25	B0BNN3,O55004,P01041,P01836,P02625,P02631,P05371,P08723,P15399,P16086,P18418,P20761,P20762,P25236,P34901,P35280,P83748,P97580,Q30KJ2,Q498R7,Q4FZU2,Q63475,Q63751,Q9EQS0,Q9JI85
254	1,2,3,4,6,7,12,16	5,8,9,10,11,13,14,15,17	23	G3V686,O55004,O88267,P01041,P01836,P02631,P05371,P08723,P15399,P16086,P18418,P25236,P34901,P47967,P83748,P97580,Q30KJ2,Q498R7,Q4FZU2,Q63532,Q9EQS0,Q9JI85,Q9JJS8
255	1,2,3,4,6,7,12,17	5,8,9,10,11,13,14,15,16	38	B0BNN3,O55004,O88267,P01041,P01681,P01836,P01946,P02091,P02631,P02770,P05371,P07647,P08723,P08937,P09605;P25809,P13432,P15399,P16086,P17475,P18418,P19223,P20761,P20762,P22282,P34901,P55091,P62815,P83748,P97580,P98089,Q10758,Q30KJ2,Q498R7,Q62635,Q63751,Q9EQS0,Q9JI85,Q9QZK9
256	1,2,3,4,6,7,13,14	5,8,9,10,11,12,15,16,17	18	O88267,P01836,P02631,P05371,P06911,P12020,P15399,P16086,P18418,P20762,P25236,P29975,P97580,Q30KJ2,Q5GRG2,Q63317,Q63532,Q9JI85
257	1,2,3,4,6,7,13,15	5,8,9,10,11,12,14,16,17	15	P01836,P02631,P05371,P06911,P12020,P15399,P16086,P18418,P20761,P20762,P25236,P97580,Q30KJ2,Q5GRG2,Q63317
258	1,2,3,4,6,7,13,16	5,8,9,10,11,12,14,15,17	15	G3V686,O88267,P01836,P02631,P05371,P06911,P12020,P15399,P16086,P18418,P25236,P97580,Q30KJ2,Q5GRG2,Q8VD89
259	1,2,3,4,6,7,13,17	5,8,9,10,11,12,14,15,16	27	O88267,P01836,P01946,P02091,P02631,P02770,P05371,P06911,P09605;P25809,P12020,P15399,P16086,P17475,P18418,P19223,P20761,P22282,P25236,P29975,P55091,P62815,P97580,Q30KJ2,Q5GRG2,Q63317,Q63751,Q9JI85
260	1,2,3,4,6,7,14,15	5,8,9,10,11,12,13,16,17	21	B0BNN3,P01041,P01836,P02631,P02803,P05371,P12020,P15399,P16086,P18418,P20761,P20762,P25236,P34901,P35280,P97580,Q30KJ2,Q5GRG2,Q63317,Q63532,Q6IE51
261	1,2,3,4,6,7,14,16	5,8,9,10,11,12,13,15,17	14	G3V686,P01836,P02631,P05371,P12020,P15399,P16086,P18418,P20762,P25236,P34901,P97580,Q30KJ2,Q63532
262	1,2,3,4,6,7,14,17	5,8,9,10,11,12,13,15,16	22	O88267,P01041,P01946,P02091,P02631,P05371,P09605;P25809,P12020,P15399,P16086,P17475,P18418,P19223,P20761,P20762,P34901,P55091,P62815,P97580,Q30KJ2,Q63532,Q63751
263	1,2,3,4,6,7,15,16	5,8,9,10,11,12,13,14,17	9	P01041,P01836,P02631,P05371,P16086,P18418,P97580,Q30KJ2,Q63532
264	1,2,3,4,6,7,15,17	5,8,9,10,11,12,13,14,16	22	B0BNN3,O55004,P01041,P01836,P01946,P02091,P02631,P02761,P05371,P09605;P25809,P15399,P16086,P17475,P18418,P19223,P20760,P20761,P20762,P62815,P97580,Q30KJ2,Q63751

265	1,2,3,4,6,7,16,17	5,8,9,10,11,12,13,14,15	18	O88267,P01041,P01836,P01946,P02091,P02631,P05371,P09605;P25809,P14480,P15399,P16086,P18418,P19223,P20761,P97580,Q30KJ2,Q63751,Q6PCU2
266	1,2,3,4,6,8,9,10	5,7,11,12,13,14,15,16,17	9	D3ZHA0,O55006,P02761,P08649,P16636,Q63942,Q64361,Q6TMA8,Q9EQS0
267	1,2,3,4,6,8,9,11	5,7,10,12,13,14,15,16,17	15	D3ZHA0,O54715,P01681,P02761,P07647,P08649,P14669,P16636,P20762,P55314,Q30KJ2,Q5XI22,Q63942,Q6TMA8,Q9EQS0
268	1,2,3,4,6,8,9,12	5,7,10,11,13,14,15,16,17	20	D3ZHA0,P01681,P02761,P02780,P02782,P07647,P08723,P0C0A9,P16636,P19223,P20762,P25236,P30120,Q06496,Q5M8C6,Q64361,Q6TMA8,Q9EQS0,Q9JJS8,Q9Z0V6
269	1,2,3,4,6,8,9,13	5,7,10,11,12,14,15,16,17	9	D3ZHA0,P06911,P07647,P20766,P25236,Q06496,Q63317,Q63942,Q6TMA8
270	1,2,3,4,6,8,9,14	5,7,10,11,12,13,15,16,17	8	D3ZHA0,P02761,P20762,P25236,Q63942,Q6TMA8,Q9EQS0,Q9Z0V6
271	1,2,3,4,6,8,9,15	5,7,10,11,12,13,14,16,17	9	B0BNN3,D3ZHA0,O55006,P02761,P20762,P23593,Q5QE79,Q63942,Q6TMA8
272	1,2,3,4,6,8,9,16	5,7,10,11,12,13,14,15,17	4	D3ZHA0,P23593,Q63942,Q6TMA8
273	1,2,3,4,6,8,9,17	5,7,10,11,12,13,14,15,16	17	B0BNN3,D3ZHA0,P01681,P02761,P02770,P16636,P17475,P19223,P20761,P23593,Q06496,Q30KJ2,Q4G075,Q63355,Q63942,Q6P6R2,Q9Z0V6
274	1,2,3,4,6,8,10,11	5,7,9,12,13,14,15,16,17	23	D3ZHA0,O54715,P01681,P02761,P07150,P07647,P08649,P14669,P15399,P16636,P20762,P35280,P50280,P55314,P97580,Q30KJ2,Q5BJY9,Q5XI22,Q63598,Q64361,Q6TMA8,Q7TP52,Q9EQS0
275	1,2,3,4,6,8,10,12	5,7,9,11,13,14,15,16,17	32	B0BNN3,D3ZHA0,P01681,P02761,P02782,P07647,P08723,P0C0A9,P16636,P18418,P19223,P25236,P30120,P35280,P35281,P50280,Q08463,Q10758,Q4FZU2,Q5M8C6,Q64361,Q6AYR9,Q6IFW6,Q6IG02,Q6IMF3,Q6P6Q2,Q6TMA8,Q80WL1,Q8K1G0,Q9EQS0,Q9JJS8,Q9Z0V6
276	1,2,3,4,6,8,10,13	5,7,9,11,12,14,15,16,17	11	D3ZHA0,P02761,P06911,P07647,P12020,P16636,P25236,Q5GRG2,Q63355,Q64361,Q6TMA8
277	1,2,3,4,6,8,10,14	5,7,9,11,12,13,15,16,17	11	D3ZHA0,P02761,P12020,P20762,P25236,P35280,Q64361,Q6TMA8,Q9EQS0,Q9WTT6,Q9Z0V6
278	1,2,3,4,6,8,10,15	5,7,9,11,12,13,14,16,17	6	B0BNN3,D3ZHA0,P02761,P35280,Q64361,Q9Z0V6
279	1,2,3,4,6,8,10,16	5,7,9,11,12,13,14,15,17	4	D3ZHA0,P08721,P16636,Q64361

280	1,2,3,4,6,8,10,17	5,7,9,11,12,13,14,15,16	20	B0BNN3,D3ZHA0,P01681,P02761,P02770,P08649,P12346,P16636,P17475,P19223,P20761,P46844,P97580,Q08463,Q30KJ2,Q4G075,Q63355,Q64361,Q6P6R2,Q9Z0V6
281	1,2,3,4,6,8,11,12	5,7,9,10,13,14,15,16,17	44	B0BNN3,D3ZHA0,O35547,O54715,O55004,P01681,P01836,P02761,P02780,P02781,P02782,P04905,P07647,P08723,P0C0A9,P14669,P15399,P16636,P20762,P25236,P30120,P35280,P40241,P50280,P55314,Q30KJ2,Q4FZU2,Q5M8C6,Q5XI22,Q63751,Q64361,Q6AYR9,Q6IFU8,Q6IFW6,Q6IG02,Q6IMF3,Q6P6Q2,Q6TMA8,Q80WL1,Q8CJ52,Q9EQS0,Q9JHB9,Q9JI85,Q9Z0V6
282	1,2,3,4,6,8,11,13	5,7,9,10,12,14,15,16,17	18	O54715,P01681,P02782,P06911,P07647,P08723,P14669,P16636,P20762,P20766,P25236,P50280,P55314,Q30KJ2,Q5GRG2,Q5XI22,Q6TMA8,Q9JI85
283	1,2,3,4,6,8,11,14	5,7,9,10,12,13,15,16,17	18	D3ZHA0,O54715,P01681,P02761,P07647,P14669,P15399,P19218,P20762,P25236,P35280,P35434,P50280,P55314,Q30KJ2,Q6TMA8,Q9EQS0,Q9Z0V6
284	1,2,3,4,6,8,11,15	5,7,9,10,12,13,14,16,17	14	B0BNN3,D3ZHA0,O54715,P02625,P02761,P14669,P20762,P35280,P50280,P55314,Q30KJ2,Q5QE79,Q5XI22,Q6TMA8
285	1,2,3,4,6,8,11,16	5,7,9,10,12,13,14,15,17	10	O54715,P01681,P07150,P14668,P14669,P16636,P55314,Q30KJ2,Q5XI22,Q6TMA8
286	1,2,3,4,6,8,11,17	5,7,9,10,12,13,14,15,16	23	B0BNN3,O54715,P01681,P02761,P02770,P07150,P07647,P08649,P14669,P15399,P16636,P17475,P19223,P20761,P20762,P50280,P55314,P97580,Q30KJ2,Q4G075,Q5XI22,Q63751,Q9Z0V6
287	1,2,3,4,6,8,12,13	5,7,9,10,11,14,15,16,17	34	D3ZHA0,O08557,O88267,P01681,P01836,P02780,P02781,P02782,P06761,P06911,P07647,P08723,P0C0A9,P16636,P20762,P22282,P25236,P29975,P30120,P50280,Q06496,Q5GRG2,Q5M8C6,Q63317,Q64093,Q64361,Q6AYR9,Q6TMA8,Q80WL1,Q8CJ52,Q9EQS0,Q9JHB9,Q9JI85,Q9JJS8
288	1,2,3,4,6,8,12,14	5,7,9,10,11,13,15,16,17	32	B0BNN3,D3ZHA0,O08557,O88267,P01681,P02780,P02782,P07647,P08723,P20762,P25236,P29975,P30120,P34901,P40241,P50280,Q10758,Q4FZU2,Q5M8C6,Q64093,Q64361,Q6AYR9,Q6AYS7,Q6IFW6,Q6Q0N1,Q6TMA8,Q923M1,Q9EQS0,Q9JHB9,Q9JJS8,Q9WTT6,Q9Z0V6
289	1,2,3,4,6,8,12,15	5,7,9,10,11,13,14,16,17	22	B0BNN3,D3ZHA0,O08557,P01681,P01836,P02761,P02782,P07647,P08723,P20761,P20762,P25236,P30120,P35280,P50280,Q4FZU2,Q5M8C6,Q5QE79,Q64361,Q6TMA8,Q9EQS0,Q9Z0V6
290	1,2,3,4,6,8,12,16	5,7,9,10,11,13,14,15,17	17	D3ZHA0,P01681,P01836,P02782,P07647,P08723,P16636,P25236,P30120,Q4FZU2,Q5M8C6,Q64361,Q6IFW6,Q6IMF3,Q9EQS0,Q9JJS8,Q9Z0V6
291	1,2,3,4,6,8,12,17	5,7,9,10,11,13,14,15,16	41	B0BNN3,D3ZHA0,O55004,O88267,P01681,P02761,P02770,P02782,P07647,P08723,P13432,P16636,P17475,P19223,P20761,P20762,P22282,P29975,P30120,P50280,P55091,P62815,P98089,Q06496,Q08463,Q10758,Q30KJ2,Q4G075,Q5M8C6,Q62635,Q63355,Q63751,Q64361,Q6AYR9,Q80WL1,Q8K1G0,Q9EQS0,Q9EQV9,Q9JJS8,Q9QZA6,Q9Z0V6
292	1,2,3,4,6,8,13,14	5,7,9,10,11,12,15,16,17	16	D3ZHA0,O08557,P06911,P07647,P12020,P20762,P20766,P25236,P29975,P36970,P50280,Q5GRG2,Q63317,Q64093,Q6TMA8,Q923M1
293	1,2,3,4,6,8,13,15	5,7,9,10,11,12,14,16,17	12	B0BNN3,D3ZHA0,O08557,P02761,P06911,P20762,P25236,Q5GRG2,Q5QE79,Q63317,Q64093,Q6TMA8
294	1,2,3,4,6,8,13,16	5,7,9,10,11,12,14,15,17	4	P06911,P25236,Q6PCU2,Q6TMA8

295	1,2,3,4,6,8,13,17	5,7,9,10,11,12,14,15,16	29	B0BNN3,O88267,P01681,P02761,P02770,P06911,P07647,P10536,P16636,P17475,P19223,P20761,P20766,P22282,P25236,P29975,P50280,P51907,P62815,Q06496,Q30KJ2,Q4G075,Q63317,Q63355,Q63751,Q64093,Q6PCU2,Q6TMA8,Q9Z0V6
296	1,2,3,4,6,8,14,15	5,7,9,10,11,12,13,16,17	13	B0BNN3,D3ZHA0,O08557,P02761,P10536,P20761,P20762,P25236,P35280,Q63317,Q64093,Q6TMA8,Q9Z0V6
297	1,2,3,4,6,8,14,16	5,7,9,10,11,12,13,15,17	7	D3ZHA0,P20762,P25236,P97574,Q6PCU2,Q6Q0N1,Q9Z0V6
298	1,2,3,4,6,8,14,17	5,7,9,10,11,12,13,15,16	21	B0BNN3,P01681,P02761,P02770,P02793;Q7TP54,P10536,P17475,P19223,P20761,P20762,P25236,P29975,P62815,P97580,Q30KJ2,Q4G075,Q64093,Q6PCU2,Q923M1,Q9WTT6,Q9Z0V6
299	1,2,3,4,6,8,15,16	5,7,9,10,11,12,13,14,17	4	B0BNN3,D3ZHA0,P02761,Q5QE79
300	1,2,3,4,6,8,15,17	5,7,9,10,11,12,13,14,16	19	B0BNN3,D3ZHA0,P01681,P02761,P02770,P10536,P17475,P19223,P20761,P20762,P23593,P62815,Q30KJ2,Q4G075,Q5QE79,Q63751,Q6PCU2,Q9EQV9,Q9Z0V6
301	1,2,3,4,6,8,16,17	5,7,9,10,11,12,13,14,15	14	B0BNN3,P01681,P02761,P02770,P16636,P17475,P19223,P20761,P23593,Q30KJ2,Q4G075,Q6P6R2,Q6PCU2,Q9Z0V6
302	1,2,3,4,6,9,10,11	5,7,8,12,13,14,15,16,17	8	P14173,P15399,P34901,P55314,Q30KJ2,Q63661,Q7TP52,Q91ZS3
303	1,2,3,4,6,9,10,12	5,7,8,11,13,14,15,16,17	8	P14173,P16636,P34901,Q10758,Q4FZU2,Q6IFW6,Q6IMF3,Q9JJS8
304	1,2,3,4,6,9,10,13	5,7,8,11,12,14,15,16,17	3	P14173,P25236,Q63355
305	1,2,3,4,6,9,10,14	5,7,8,11,12,13,15,16,17	3	P14173,P25236,P34901
306	1,2,3,4,6,9,10,15	5,7,8,11,12,13,14,16,17	0	
307	1,2,3,4,6,9,10,16	5,7,8,11,12,13,14,15,17	2	P14173,P23593
308	1,2,3,4,6,9,10,17	5,7,8,11,12,13,14,15,16	6	P14173,P16636,P23593,P97580,Q30KJ2,Q63355
309	1,2,3,4,6,9,11,12	5,7,8,10,13,14,15,16,17	14	P01681,P01835,P01836,P15399,P16636,P34901,P55314,Q498R7,Q4FZU2,Q6IFU7,Q6IFU8,Q6IFW6,Q6IMF3,Q6P6Q2

310	1,2,3,4,6,9,11,13	5,7,8,10,12,14,15,16,17	2	P25236,P55314
311	1,2,3,4,6,9,11,14	5,7,8,10,12,13,15,16,17	5	P15399,P25236,P34901,P55314,Q30KJ2
312	1,2,3,4,6,9,11,15	5,7,8,10,12,13,14,16,17	3	P02625,P55314,Q5QE79
313	1,2,3,4,6,9,11,16	5,7,8,10,12,13,14,15,17	1	P55314
314	1,2,3,4,6,9,11,17	5,7,8,10,12,13,14,15,16	7	P01681,P09605;P25809,P15399,P16636,P55314,P97580,Q30KJ2
315	1,2,3,4,6,9,12,13	5,7,8,10,11,14,15,16,17	3	P01836,P25236,Q06496
316	1,2,3,4,6,9,12,14	5,7,8,10,11,13,15,16,17	4	P01835,P25236,P34901,Q9JJS8
317	1,2,3,4,6,9,12,15	5,7,8,10,11,13,14,16,17	7	B0BNN3,P01836,P02625,P25236,P34901,Q4FZU2,Q5QE79
318	1,2,3,4,6,9,12,16	5,7,8,10,11,13,14,15,17	5	P01836,P23593,P25236,P34901,Q9JJS8
319	1,2,3,4,6,9,12,17	5,7,8,10,11,13,14,15,16	15	B0BNN3,P01681,P01835,P09605;P25809,P13432,P16636,P19223,P23593,P34901,P98089,Q06496,Q30KJ2,Q62635,Q63355,Q9JJS8
320	1,2,3,4,6,9,13,14	5,7,8,10,11,12,15,16,17	2	P06911,P25236
321	1,2,3,4,6,9,13,15	5,7,8,10,11,12,14,16,17	2	P25236,Q5QE79
322	1,2,3,4,6,9,13,16	5,7,8,10,11,12,14,15,17	1	P25236
323	1,2,3,4,6,9,13,17	5,7,8,10,11,12,14,15,16	3	P23593,Q30KJ2,Q63355
324	1,2,3,4,6,9,14,15	5,7,8,10,11,12,13,16,17	4	B0BNN3,P25236,P34901,Q5QE79

325	1,2,3,4,6,9,14,16	5,7,8,10,11,12,13,15,17	3	P25236,P34901,Q63942
326	1,2,3,4,6,9,14,17	5,7,8,10,11,12,13,15,16	3	P34901,Q30KJ2,Q5HZV9
327	1,2,3,4,6,9,15,16	5,7,8,10,11,12,13,14,17	3	P23593,Q5QE79,Q63942
328	1,2,3,4,6,9,15,17	5,7,8,10,11,12,13,14,16	4	B0BNN3,P20761,P23593,Q5QE79
329	1,2,3,4,6,9,16,17	5,7,8,10,11,12,13,14,15	3	P09605;P25809,P23593,Q30KJ2
330	1,2,3,4,6,10,11,12	5,7,8,9,13,14,15,16,17	23	P01681,P01836,P05964,P06757,P08723,P0C0A9,P15399,P16636,P30120,P34901,P35280,P55314,Q10758,Q30KJ2,Q4FZU2,Q6IFU7,Q6IFU8,Q6IFW6,Q6IG02,Q6IMF3,Q6P6Q2,Q7TP52,Q9JJS8
331	1,2,3,4,6,10,11,13	5,7,8,9,12,14,15,16,17	4	P15399,P25236,P55314,Q30KJ2
332	1,2,3,4,6,10,11,14	5,7,8,9,12,13,15,16,17	8	P15399,P25236,P34901,P35280,P55314,P97580,Q30KJ2,Q7TP52
333	1,2,3,4,6,10,11,15	5,7,8,9,12,13,14,16,17	8	B0BNN3,P15399,P34901,P35280,P55314,Q30KJ2,Q4FZU6,Q7TP52
334	1,2,3,4,6,10,11,16	5,7,8,9,12,13,14,15,17	5	P15399,P34901,P55314,Q30KJ2,Q7TP52
335	1,2,3,4,6,10,11,17	5,7,8,9,12,13,14,15,16	10	P01681,P05964,P15399,P16636,P17475,P34901,P55314,P97580,Q30KJ2,Q7TP52
336	1,2,3,4,6,10,12,13	5,7,8,9,11,14,15,16,17	12	P01836,P08723,P16636,P25236,P34901,P70709,Q10758,Q4FZU2,Q63355,Q6AYR9,Q6IMF3,Q9JJS8
337	1,2,3,4,6,10,12,14	5,7,8,9,11,13,15,16,17	8	P01681,P25236,P34901,Q10758,Q4FZU2,Q6IFW6,Q6IMF3,Q9JJS8
338	1,2,3,4,6,10,12,15	5,7,8,9,11,13,14,16,17	8	B0BNN3,P01836,P34901,P35280,Q10758,Q4FZU2,Q6IMF3,Q9JJS8
339	1,2,3,4,6,10,12,16	5,7,8,9,11,13,14,15,17	10	P01836,P08721,P16636,P34901,P47967,Q10758,Q4FZU2,Q6IFW6,Q6IMF3,Q9JJS8

340	1,2,3,4,6,10,12,17	5,7,8,9,11,13,14,15,16	16	B0BNN3,P01681,P16636,P17475,P19223,P34901,P97580,P98089,Q10758,Q30KJ2,Q4FZU2,Q62635,Q63355,Q6IFW6,Q6IMF3,Q9JJS8
341	1,2,3,4,6,10,13,14	5,7,8,9,11,12,15,16,17	4	P06911,P12020,P25236,Q5GRG2
342	1,2,3,4,6,10,13,15	5,7,8,9,11,12,14,16,17	2	P25236,Q4FZU6
343	1,2,3,4,6,10,13,16	5,7,8,9,11,12,14,15,17	2	P25236,Q62997
344	1,2,3,4,6,10,13,17	5,7,8,9,11,12,14,15,16	5	P16636,P17475,P97580,Q30KJ2,Q63355
345	1,2,3,4,6,10,14,15	5,7,8,9,11,12,13,16,17	4	B0BNN3,P25236,P34901,P35280
346	1,2,3,4,6,10,14,16	5,7,8,9,11,12,13,15,17	3	P08721,P25236,P34901
347	1,2,3,4,6,10,14,17	5,7,8,9,11,12,13,15,16	5	P15399,P17475,P34901,P97580,Q30KJ2
348	1,2,3,4,6,10,15,16	5,7,8,9,11,12,13,14,17	1	Q62997
349	1,2,3,4,6,10,15,17	5,7,8,9,11,12,13,14,16	6	B0BNN3,P02761,P17475,P20761,P97580,Q30KJ2
350	1,2,3,4,6,10,16,17	5,7,8,9,11,12,13,14,15	7	P16636,P23593,P97580,Q30KJ2,Q62997,Q63355,Q7TP52
351	1,2,3,4,6,11,12,13	5,7,8,9,10,14,15,16,17	18	P01681,P01836,P07647,P08723,P0C0A9,P15399,P16636,P25236,P30120,P34901,P55314,Q4FZU2,Q5XI22,Q6IFU8,Q6IFW6,Q6IMF3,Q6P6Q2,Q9JHB9
352	1,2,3,4,6,11,12,14	5,7,8,9,10,13,15,16,17	18	P01681,P01836,P04905,P08723,P15399,P25236,P30120,P34901,P55314,Q30KJ2,Q4FZU2,Q6IFU7,Q6IFU8,Q6IFW6,Q6IMF3,Q6P6Q2,Q9EQS0,Q9JHB9
353	1,2,3,4,6,11,12,15	5,7,8,9,10,13,14,16,17	13	B0BNN3,P01681,P01836,P02625,P34901,P35280,P55314,Q4FZU2,Q6IFU7,Q6IFU8,Q6IFW6,Q6IMF3,Q6P6Q2
354	1,2,3,4,6,11,12,16	5,7,8,9,10,13,14,15,17	13	G3V686,P01681,P01836,P16636,P34901,P55314,Q4FZU2,Q6IFU7,Q6IFU8,Q6IFW6,Q6IMF3,Q6P6Q2,Q9JJS8

355	1,2,3,4,6,11,12,17	5,7,8,9,10,13,14,15,16	23	B0BNN3,P01681,P01836,P05964,P09605;P25809,P13432,P15399,P16636,P17475,P19223,P34901,P55314,P97580,P98089,Q10758,Q30KJ2,Q4FZU2,Q62635,Q63751,Q6IFU8,Q6IFW6,Q6IMF3,Q6P6Q2
356	1,2,3,4,6,11,13,14	5,7,8,9,10,12,15,16,17	7	P06911,P15399,P25236,P47727,P55314,Q30KJ2,Q64093
357	1,2,3,4,6,11,13,15	5,7,8,9,10,12,14,16,17	5	P01836,P02625,P25236,P55314,Q5XI22
358	1,2,3,4,6,11,13,16	5,7,8,9,10,12,14,15,17	4	G3V686,P01836,P25236,P55314
359	1,2,3,4,6,11,13,17	5,7,8,9,10,12,14,15,16	6	P01681,P15399,P16636,P55314,P97580,Q30KJ2
360	1,2,3,4,6,11,14,15	5,7,8,9,10,12,13,16,17	9	B0BNN3,P02625,P19218,P25236,P34901,P35280,P35434,P55314,Q30KJ2
361	1,2,3,4,6,11,14,16	5,7,8,9,10,12,13,15,17	6	G3V686,P15399,P25236,P34901,P55314,Q30KJ2
362	1,2,3,4,6,11,14,17	5,7,8,9,10,12,13,15,16	7	P01681,P15399,P19218,P34901,P55314,P97580,Q30KJ2
363	1,2,3,4,6,11,15,16	5,7,8,9,10,12,13,14,17	2	P01836,P55314
364	1,2,3,4,6,11,15,17	5,7,8,9,10,12,13,14,16	9	B0BNN3,P01681,P02761,P15399,P17475,P20761,P55314,P97580,Q30KJ2
365	1,2,3,4,6,11,16,17	5,7,8,9,10,12,13,14,15	7	P01681,P09605;P25809,P15399,P16636,P55314,P97580,Q30KJ2
366	1,2,3,4,6,12,13,14	5,7,8,9,10,11,15,16,17	10	P01836,P06911,P08723,P25236,P29975,P30120,P34901,Q64093,Q6AYR9,Q9JJS8
367	1,2,3,4,6,12,13,15	5,7,8,9,10,11,14,16,17	5	B0BNN3,P01836,P02625,P25236,P82995
368	1,2,3,4,6,12,13,16	5,7,8,9,10,11,14,15,17	4	G3V686,P01836,P25236,Q9JJS8
369	1,2,3,4,6,12,13,17	5,7,8,9,10,11,14,15,16	15	B0BNN3,P01681,P01836,P13432,P16636,P17475,P19223,P20761,P25236,P55091,P62815,P98089,Q62635,Q63355,Q9QZK9

370	1,2,3,4,6,12,14,15	5,7,8,9,10,11,13,16,17	7	A2RUV9,B0BNN3,P01836,P02625,P25236,P34901,Q4FZU2
371	1,2,3,4,6,12,14,16	5,7,8,9,10,11,13,15,17	11	G3V686,P01681,P01836,P25236,P34901,Q10758,Q4FZU2,Q63532,Q6IFW6,Q6IMF3,Q9JJS8
372	1,2,3,4,6,12,14,17	5,7,8,9,10,11,13,15,16	15	B0BNN3,P01681,P01835,P13432,P19223,P20761,P25236,P31430,P34901,P98089,Q10758,Q30KJ2,Q62635,Q923M1,Q9JJS8
373	1,2,3,4,6,12,15,16	5,7,8,9,10,11,13,14,17	6	B0BNN3,P01836,P25236,P34901,Q4FZU2,Q9JJS8
374	1,2,3,4,6,12,15,17	5,7,8,9,10,11,13,14,16	10	B0BNN3,P01681,P01836,P17475,P19223,P20761,P34901,P98089,Q4FZU2,Q62635
375	1,2,3,4,6,12,16,17	5,7,8,9,10,11,13,14,15	15	B0BNN3,P01681,P01836,P09605;P25809,P13432,P14480,P16636,P19223,P23593,P34901,P98089,Q10758,Q30KJ2,Q62635,Q9JJS8
376	1,2,3,4,6,13,14,15	5,7,8,9,10,11,12,16,17	6	B0BNN3,P06911,P25236,Q5GRG2,Q63317,Q64093
377	1,2,3,4,6,13,14,16	5,7,8,9,10,11,12,15,17	4	G3V686,P06911,P25236,Q64093
378	1,2,3,4,6,13,14,17	5,7,8,9,10,11,12,15,16	6	P06911,P25236,Q30KJ2,Q64093,Q6PCU2,Q9QW07
379	1,2,3,4,6,13,15,16	5,7,8,9,10,11,12,14,17	2	P01836,P25236
380	1,2,3,4,6,13,15,17	5,7,8,9,10,11,12,14,16	4	B0BNN3,P17475,P20761,P25236
381	1,2,3,4,6,13,16,17	5,7,8,9,10,11,12,14,15	4	P14480,Q30KJ2,Q63355,Q6PCU2
382	1,2,3,4,6,14,15,16	5,7,8,9,10,11,12,13,17	3	B0BNN3,P25236,P34901
383	1,2,3,4,6,14,15,17	5,7,8,9,10,11,12,13,16	8	B0BNN3,P02803,P10536,P17475,P20761,P34901,Q30KJ2,Q5HZV9
384	1,2,3,4,6,14,16,17	5,7,8,9,10,11,12,13,15	4	P34901,Q30KJ2,Q5HZV9,Q6PCU2

385	1,2,3,4,6,15,16,17	5,7,8,9,10,11,12,13,14	3	B0BNN3,P20761,P23593
386	1,2,3,4,7,8,9,10	5,6,11,12,13,14,15,16,17	13	D3ZHA0,O55006,P01041,P02761,P18418,P31044,P36970,P97571,Q63475,Q80WL1,Q9EQS0,Q9QX79,Q9R0T3
387	1,2,3,4,7,8,9,11	5,6,10,12,13,14,15,16,17	15	D3ZHA0,O54715,P01836,P02761,P05371,P10715,P18418,P31044,Q5XI22,Q63475,Q63617,Q6TMA8,Q9EQS0,Q9QX79,Q9R0T3
388	1,2,3,4,7,8,9,12	5,6,10,11,13,14,15,16,17	16	D3ZHA0,O55004,P01041,P01836,P02761,P10715,P18418,P19223,P31044,P36970,Q498R7,Q63475,Q80WL1,Q8CJ52,Q9EQS0,Q9R0T3
389	1,2,3,4,7,8,9,13	5,6,10,11,12,14,15,16,17	11	D3ZHA0,P01836,P18418,P20766,P31044,P36970,Q63317,Q63617,Q6TMA8,Q9EQS0,Q9R0T3
390	1,2,3,4,7,8,9,14	5,6,10,11,12,13,15,16,17	9	D3ZHA0,P02761,P18418,P31044,P36970,Q63317,Q63617,Q9EQS0,Q9QX79
391	1,2,3,4,7,8,9,15	5,6,10,11,12,13,14,16,17	10	D3ZHA0,P01041,P01836,P02761,P18418,P31044,Q5QE79,Q63317,Q9EQS0,Q9QX79
392	1,2,3,4,7,8,9,16	5,6,10,11,12,13,14,15,17	8	B4F795,D3ZHA0,P01836,P18418,P31044,Q63617,Q9EQS0,Q9R0T3
393	1,2,3,4,7,8,9,17	5,6,10,11,12,13,14,15,16	16	D3ZHA0,P01041,P01946,P02091,P02761,P02770,P17475,P18418,P19223,P50115,P52590,P55091,Q63617,Q9EQS0,Q9QX79,Q9R0T3
394	1,2,3,4,7,8,10,11	5,6,9,12,13,14,15,16,17	20	D3ZHA0,O54715,P00714,P01041,P01836,P02761,P05371,P05964,P14669,P18418,P31044,P35280,P55314,P68370,Q5BJY9,Q5XI22,Q63475,Q80WL1,Q9EQS0,Q9R0T3
395	1,2,3,4,7,8,10,12	5,6,9,11,13,14,15,16,17	20	D3ZHA0,O35783,P01041,P01836,P02761,P05964,P08723,P10715,P18418,P19223,P31044,P35280,P36970,P46844,Q63475,Q80WL1,Q8CJ52,Q8K1G0,Q9EQS0,Q9R0T3
396	1,2,3,4,7,8,10,13	5,6,9,11,12,14,15,16,17	15	D3ZHA0,P01835,P01836,P02761,P06911,P18418,P20766,P31044,P36970,P46844,Q5GRG2,Q63317,Q80WL1,Q9EQS0,Q9R0T3
397	1,2,3,4,7,8,10,14	5,6,9,11,12,13,15,16,17	11	D3ZHA0,P01041,P02761,P16086,P18418,P31044,P35280,P36970,P97615,Q63475,Q9EQS0
398	1,2,3,4,7,8,10,15	5,6,9,11,12,13,14,16,17	13	D3ZHA0,P01041,P01835,P01836,P02761,P18418,P31044,P35280,P36970,P97571,P97615,Q63475,Q9EQS0
399	1,2,3,4,7,8,10,16	5,6,9,11,12,13,14,15,17	11	D3ZHA0,P01041,P01835,P01836,P02761,P08721,P18418,P31044,Q62997,Q9EQS0,Q9R0T3

400	1,2,3,4,7,8,10,17	5,6,9,11,12,13,14,15,16	18	D3ZHA0,P01041,P01946,P02091,P02761,P02770,P12346,P17475,P18418,P19223,P20761,P31044,P46844,P55091,P97571,Q80WL1,Q9EQS0,Q9R0T3
401	1,2,3,4,7,8,11,12	5,6,9,10,13,14,15,16,17	27	D3ZHA0,O54715,O55004,P01041,P01836,P02761,P05371,P05964,P07647,P08723,P10715,P14669,P18418,P19223,P31044,P35280,Q498R7,Q5XI22,Q63475,Q63493,Q80WL1,Q8CJ52,Q9EQS0,Q9ESW0,Q9JI85,Q9R0T3,Q9R1T1
402	1,2,3,4,7,8,11,13	5,6,9,10,12,14,15,16,17	16	P01836,P02761,P16086,P18418,P20766,P31044,P36970,Q5GRG2,Q5XI22,Q63317,Q6TMA8,Q80WL1,Q8VD89,Q9EQS0,Q9JI85,Q9R0T3
403	1,2,3,4,7,8,11,14	5,6,9,10,12,13,15,16,17	15	P01041,P01836,P02761,P05371,P16086,P18418,P19218,P19629,P20762,P31044,P35280,P36970,Q63475,Q63617,Q9EQS0
404	1,2,3,4,7,8,11,15	5,6,9,10,12,13,14,16,17	15	D3ZHA0,O55004,P01041,P01836,P02761,P05371,P10715,P14669,P18418,P22006,P31044,P35280,Q5XI22,Q63475,Q9EQS0
405	1,2,3,4,7,8,11,16	5,6,9,10,12,13,14,15,17	13	B4F795,P01041,P01836,P02761,P05371,P10715,P16086,P18418,P31044,P55314,Q8VD89,Q9EQS0,Q9R0T3
406	1,2,3,4,7,8,11,17	5,6,9,10,12,13,14,15,16	24	O55004,P01041,P01836,P01946,P02091,P02761,P02770,P05371,P10715,P12346,P14669,P16086,P17475,P18418,P19223,P20761,P31044,P50115,P55091,Q63751,Q80WL1,Q9EQS0,Q9QZA6,Q9R0T3
407	1,2,3,4,7,8,12,13	5,6,9,10,11,14,15,16,17	25	D3ZHA0,O08557,O88267,P01041,P01836,P06761,P07647,P08723,P10715,P18418,P19223,P20766,P22283,P29975,P31044,P36374,P36970,Q5GRG2,Q63317,Q6TMA8,Q80WL1,Q8CJ52,Q9EQS0,Q9JI85,Q9R0T3
408	1,2,3,4,7,8,12,14	5,6,9,10,11,13,15,16,17	25	A2RUV9,D3ZHA0,O08557,O88267,P01041,P01836,P02761,P08723,P10715,P18418,P19223,P19629,P20762,P29975,P31044,P34901,P36970,Q63317,Q63475,Q6AYS7,Q80WL1,Q8CJ52,Q923M1,Q9EQS0,Q9R0T3
409	1,2,3,4,7,8,12,15	5,6,9,10,11,13,14,16,17	16	B0BNN3,D3ZHA0,O08557,O55004,P01041,P01836,P02761,P10715,P18418,P19223,P35280,P36970,Q62894,Q63475,Q80WL1,Q9EQS0
410	1,2,3,4,7,8,12,16	5,6,9,10,11,13,14,15,17	13	D3ZHA0,O88267,P01041,P01836,P08721,P10715,P18418,P19223,P36970,Q80WL1,Q8CJ52,Q9EQS0,Q9R0T3
411	1,2,3,4,7,8,12,17	5,6,9,10,11,13,14,15,16	31	D3ZHA0,O55004,O88267,P01041,P01836,P01946,P02091,P02761,P02770,P10715,P12346,P17475,P18418,P19223,P20761,P31430,P36970,P46844,P50115,P55091,P63029,P98089,Q4G075,Q63751,Q80WL1,Q8CJ52,Q99041,Q9EQS0,Q9EQV9,Q9QZA6,Q9R0T3
412	1,2,3,4,7,8,13,14	5,6,9,10,11,12,15,16,17	13	P01836,P06911,P16086,P18418,P20766,P29975,P31044,P36970,Q5GRG2,Q63317,Q6TMA8,Q9EQS0,Q9R0T3
413	1,2,3,4,7,8,13,15	5,6,9,10,11,12,14,16,17	12	D3ZHA0,P01041,P01835,P01836,P02761,P18418,P20766,P31044,P36970,Q09030,Q5GRG2,Q63317
414	1,2,3,4,7,8,13,16	5,6,9,10,11,12,14,15,17	7	P01835,P01836,P18418,P20766,P31044,P36970,Q9R0T3

415	1,2,3,4,7,8,13,17	5,6,9,10,11,12,14,15,16	23	O88267,P01041,P01836,P01946,P02091,P02761,P02770,P17475,P18418,P19223,P20761,P20766,P29975,P31044,P36970,P46844,P55091,P63029,Q63317,Q63751,Q6PCU2,Q80WL1,Q9R0T3
416	1,2,3,4,7,8,14,15	5,6,9,10,11,12,13,16,17	13	D3ZHA0,P01041,P01836,P02761,P18418,P19629,P20762,P31044,P35280,P36970,Q63317,Q9EQS0,Q9QX79
417	1,2,3,4,7,8,14,16	5,6,9,10,11,12,13,15,17	13	D3ZHA0,P01041,P01836,P08721,P16086,P18418,P31044,P36970,P97574,Q566E6,Q63532,Q9EQS0,Q9R0T3
418	1,2,3,4,7,8,14,17	5,6,9,10,11,12,13,15,16	20	O88267,P01041,P01946,P02091,P02761,P02770,P10536,P12346,P16086,P17475,P18418,P19223,P20761,P36970,P55091,Q6PCU2,Q923M1,Q9EQS0,Q9QX79,Q9QZA6
419	1,2,3,4,7,8,15,16	5,6,9,10,11,12,13,14,17	10	B4F795,D3ZHA0,P01041,P01835,P01836,P02761,P08721,P18418,P31044,Q62894
420	1,2,3,4,7,8,15,17	5,6,9,10,11,12,13,14,16	18	B0BNN3,P01041,P01836,P01946,P02091,P02761,P02770,P10536,P12346,P17475,P18418,P19223,P20761,P55091,Q62894,Q9EQV9,Q9QX79,Q9QZA6
421	1,2,3,4,7,8,16,17	5,6,9,10,11,12,13,14,15	15	O88267,P01041,P01836,P01946,P02091,P02761,P02770,P08721,P17475,P18418,P19223,P55091,P63029,Q6PCU2,Q9R0T3
422	1,2,3,4,7,9,10,11	5,6,8,12,13,14,15,16,17	5	P01836,P05964,P55314,Q5FVI6,Q63661
423	1,2,3,4,7,9,10,12	5,6,8,11,13,14,15,16,17	8	O35783,P01041,P01836,P34901,P97840,Q498R7,Q63475,Q9EQS0
424	1,2,3,4,7,9,10,13	5,6,8,11,12,14,15,16,17	1	P01836
425	1,2,3,4,7,9,10,14	5,6,8,11,12,13,15,16,17	1	P34901
426	1,2,3,4,7,9,10,15	5,6,8,11,12,13,14,16,17	1	P01836
427	1,2,3,4,7,9,10,16	5,6,8,11,12,13,14,15,17	2	P01836,P08721
428	1,2,3,4,7,9,10,17	5,6,8,11,12,13,14,15,16	4	P01946,P02091,P19223,P52590
429	1,2,3,4,7,9,11,12	5,6,8,10,13,14,15,16,17	7	O35783,P01836,P05964,P34901,Q498R7,Q63475,Q9EQS0

430	1,2,3,4,7,9,11,13	5,6,8,10,12,14,15,16,17	2	P01836,P05539
431	1,2,3,4,7,9,11,14	5,6,8,10,12,13,15,16,17	2	P01836,P34901
432	1,2,3,4,7,9,11,15	5,6,8,10,12,13,14,16,17	3	O54728,P01836,P22006
433	1,2,3,4,7,9,11,16	5,6,8,10,12,13,14,15,17	2	P01836,P55314
434	1,2,3,4,7,9,11,17	5,6,8,10,12,13,14,15,16	4	P01836,P01946,P02091,P19223
435	1,2,3,4,7,9,12,13	5,6,8,10,11,14,15,16,17	2	P01836,Q498R7
436	1,2,3,4,7,9,12,14	5,6,8,10,11,13,15,16,17	7	A2RUV9,P01041,P01836,P34901,P36970,Q498R7,Q9EQS0
437	1,2,3,4,7,9,12,15	5,6,8,10,11,13,14,16,17	4	P01041,P01836,P34901,Q498R7
438	1,2,3,4,7,9,12,16	5,6,8,10,11,13,14,15,17	3	P01041,P01836,P34901
439	1,2,3,4,7,9,12,17	5,6,8,10,11,13,14,15,16	10	P01041,P01836,P01946,P02091,P09605;P25809,P19223,P34901,P55091,P98089,Q498R7
440	1,2,3,4,7,9,13,14	5,6,8,10,11,12,15,16,17	3	P01836,P06760,P36970
441	1,2,3,4,7,9,13,15	5,6,8,10,11,12,14,16,17	3	O54728,P01836,P06760
442	1,2,3,4,7,9,13,16	5,6,8,10,11,12,14,15,17	3	P01836,P06760,P08010
443	1,2,3,4,7,9,13,17	5,6,8,10,11,12,14,15,16	4	P01836,P01946,P02091,P19223
444	1,2,3,4,7,9,14,15	5,6,8,10,11,12,13,16,17	5	O54728,P01836,P06760,P34901,Q9QZK9

445	1,2,3,4,7,9,14,16	5,6,8,10,11,12,13,15,17	6	P01836,P06760,P08010,P34901,Q63532,Q9QZK9
446	1,2,3,4,7,9,14,17	5,6,8,10,11,12,13,15,16	4	P01946,P02091,P19223,P34901
447	1,2,3,4,7,9,15,16	5,6,8,10,11,12,13,14,17	5	O54728,P01836,P06760,P47727,Q9QZK9
448	1,2,3,4,7,9,15,17	5,6,8,10,11,12,13,14,16	6	P01041,P01836,P01946,P02091,P19223,P47727
449	1,2,3,4,7,9,16,17	5,6,8,10,11,12,13,14,15	6	P01836,P01946,P02091,P08010,P19223,P47727
450	1,2,3,4,7,10,11,12	5,6,8,9,13,14,15,16,17	14	O35783,P01041,P01836,P05964,P06757,P34901,P35280,P47967,P55314,Q498R7,Q5FVI6,Q62636,Q63475,Q9EQS0
451	1,2,3,4,7,10,11,13	5,6,8,9,12,14,15,16,17	3	P01836,P05964,P55314
452	1,2,3,4,7,10,11,14	5,6,8,9,12,13,15,16,17	7	P01836,P05964,P34901,P35280,P55314,Q63532,Q9EQS0
453	1,2,3,4,7,10,11,15	5,6,8,9,12,13,14,16,17	6	P01836,P02761,P22006,P35280,P55314,Q5FVI6
454	1,2,3,4,7,10,11,16	5,6,8,9,12,13,14,15,17	5	P01836,P08721,P55314,Q5FVI6,Q8VD89
455	1,2,3,4,7,10,11,17	5,6,8,9,12,13,14,15,16	7	P01041,P01836,P01946,P02091,P05964,P19223,P55314
456	1,2,3,4,7,10,12,13	5,6,8,9,11,14,15,16,17	3	O35783,P01836,P47967
457	1,2,3,4,7,10,12,14	5,6,8,9,11,13,15,16,17	9	A2RUV9,O35783,P01041,P01836,P34901,P36970,Q63475,Q63532,Q9EQS0
458	1,2,3,4,7,10,12,15	5,6,8,9,11,13,14,16,17	5	P01041,P01836,P34901,P35280,Q63475
459	1,2,3,4,7,10,12,16	5,6,8,9,11,13,14,15,17	8	O35783,P01041,P01836,P08721,P34901,P47967,P97840,Q9JJS8

460	1,2,3,4,7,10,12,17	5,6,8,9,11,13,14,15,16	11	O35783,P01041,P01836,P01946,P02091,P05964,P19223,P34901,P55091,P98089,Q10758
461	1,2,3,4,7,10,13,14	5,6,8,9,11,12,15,16,17	5	P01836,P06911,P12020,P36970,Q5GRG2
462	1,2,3,4,7,10,13,15	5,6,8,9,11,12,14,16,17	3	P01835,P01836,Q09030
463	1,2,3,4,7,10,13,16	5,6,8,9,11,12,14,15,17	3	P01835,P01836,Q62997
464	1,2,3,4,7,10,13,17	5,6,8,9,11,12,14,15,16	3	P01836,P01946,P02091
465	1,2,3,4,7,10,14,15	5,6,8,9,11,12,13,16,17	5	A2RUV9,P01041,P01836,P34901,P35280
466	1,2,3,4,7,10,14,16	5,6,8,9,11,12,13,15,17	5	P01836,P08721,P34901,Q62997,Q63532
467	1,2,3,4,7,10,14,17	5,6,8,9,11,12,13,15,16	4	P01041,P01946,P02091,P34901
468	1,2,3,4,7,10,15,16	5,6,8,9,11,12,13,14,17	5	P01041,P01835,P01836,P08721,Q62997
469	1,2,3,4,7,10,15,17	5,6,8,9,11,12,13,14,16	6	P01041,P01836,P01946,P02091,P02761,P17475
470	1,2,3,4,7,10,16,17	5,6,8,9,11,12,13,14,15	6	P01041,P01836,P01946,P02091,P08721,Q62997
471	1,2,3,4,7,11,12,13	5,6,8,9,10,14,15,16,17	5	P01836,P05964,P36374,Q498R7,Q9JI85
472	1,2,3,4,7,11,12,14	5,6,8,9,10,13,15,16,17	9	P01041,P01836,P05964,P34901,Q498R7,Q63475,Q63532,Q9EQS0,Q9ESW0
473	1,2,3,4,7,11,12,15	5,6,8,9,10,13,14,16,17	9	P01041,P01836,P22006,P34901,P35280,P51907,Q498R7,Q63475,Q9ESW0
474	1,2,3,4,7,11,12,16	5,6,8,9,10,13,14,15,17	6	P01041,P01836,P34901,P55314,Q498R7,Q9EQS0

475	1,2,3,4,7,11,12,17	5,6,8,9,10,13,14,15,16	12	P01041,P01836,P01946,P02091,P05964,P19223,P34901,P55091,P98089,Q498R7,Q6AYQ8,Q9ESW0
476	1,2,3,4,7,11,13,14	5,6,8,9,10,12,15,16,17	2	P01836,Q5GRG2
477	1,2,3,4,7,11,13,15	5,6,8,9,10,12,14,16,17	4	P01836,P05539,P22006,Q09030
478	1,2,3,4,7,11,13,16	5,6,8,9,10,12,14,15,17	3	P01836,P55314,Q8VD89
479	1,2,3,4,7,11,13,17	5,6,8,9,10,12,14,15,16	3	P01836,P01946,P02091
480	1,2,3,4,7,11,14,15	5,6,8,9,10,12,13,16,17	5	P01836,P19218,P22006,P34901,P35280
481	1,2,3,4,7,11,14,16	5,6,8,9,10,12,13,15,17	4	P01836,P34901,P55314,Q63532
482	1,2,3,4,7,11,14,17	5,6,8,9,10,12,13,15,16	5	P01836,P01946,P02091,P19218,P34901
483	1,2,3,4,7,11,15,16	5,6,8,9,10,12,13,14,17	3	P01836,P55314,Q8VD89
484	1,2,3,4,7,11,15,17	5,6,8,9,10,12,13,14,16	6	P01041,P01836,P01946,P02091,P02761,P22006
485	1,2,3,4,7,11,16,17	5,6,8,9,10,12,13,14,15	4	P01836,P01946,P02091,P55314
486	1,2,3,4,7,12,13,14	5,6,8,9,10,11,15,16,17	7	A2RUV9,P01836,P06911,P25236,P34901,P36970,Q5GRG2
487	1,2,3,4,7,12,13,15	5,6,8,9,10,11,14,16,17	3	P01041,P01836,Q09030
488	1,2,3,4,7,12,13,16	5,6,8,9,10,11,14,15,17	1	P01836
489	1,2,3,4,7,12,13,17	5,6,8,9,10,11,14,15,16	8	P01041,P01836,P01946,P02091,P19223,P36374,P55091,P98089

490	1,2,3,4,7,12,14,15	5,6,8,9,10,11,13,16,17	6	A2RUV9,P01041,P01836,P34901,P36970,Q63532
491	1,2,3,4,7,12,14,16	5,6,8,9,10,11,13,15,17	6	P01041,P01836,P08721,P34901,Q63532,Q9EQS0
492	1,2,3,4,7,12,14,17	5,6,8,9,10,11,13,15,16	10	A2RUV9,P01041,P01836,P01946,P02091,P19223,P31430,P34901,P55091,P98089
493	1,2,3,4,7,12,15,16	5,6,8,9,10,11,13,14,17	3	P01041,P01836,P34901
494	1,2,3,4,7,12,15,17	5,6,8,9,10,11,13,14,16	10	B0BNN3,P01041,P01836,P01946,P02091,P06866,P19223,P34901,P55091,P98089
495	1,2,3,4,7,12,16,17	5,6,8,9,10,11,13,14,15	9	P01041,P01836,P01946,P02091,P06866,P19223,P34901,P55091,P98089
496	1,2,3,4,7,13,14,15	5,6,8,9,10,11,12,16,17	7	A2RUV9,P01836,P06760,P36970,Q09030,Q5GRG2,Q63317
497	1,2,3,4,7,13,14,16	5,6,8,9,10,11,12,15,17	5	P01836,P06760,P08010,Q566E6,Q63532
498	1,2,3,4,7,13,14,17	5,6,8,9,10,11,12,15,16	5	P01836,P01946,P02091,Q6PCU2,Q9QW07
499	1,2,3,4,7,13,15,16	5,6,8,9,10,11,12,14,17	4	P01835,P01836,P06760,Q09030
500	1,2,3,4,7,13,15,17	5,6,8,9,10,11,12,14,16	4	P01041,P01836,P01946,P02091
501	1,2,3,4,7,13,16,17	5,6,8,9,10,11,12,14,15	5	P01836,P01946,P02091,P08010,Q6PCU2
502	1,2,3,4,7,14,15,16	5,6,8,9,10,11,12,13,17	5	P01836,P06760,P34901,Q63532,Q9QZK9
503	1,2,3,4,7,14,15,17	5,6,8,9,10,11,12,13,16	5	B0BNN3,P01041,P01836,P01946,P02091
504	1,2,3,4,7,14,16,17	5,6,8,9,10,11,12,13,15	6	P01836,P01946,P02091,P34901,Q63532,Q6PCU2

505	1,2,3,4,7,15,16,17	5,6,8,9,10,11,12,13,14	5	P01041,P01836,P01946,P02091,P47727
506	1,2,3,4,8,9,10,11	5,6,7,12,13,14,15,16,17	3	P02761,P08649,Q5XI22
507	1,2,3,4,8,9,10,12	5,6,7,11,13,14,15,16,17	7	D3ZHA0,P02761,P16636,P19223,Q80WL1,Q9EQS0,Q9JJS8
508	1,2,3,4,8,9,10,13	5,6,7,11,12,14,15,16,17	4	D3ZHA0,P20766,P36970,Q6TMA8
509	1,2,3,4,8,9,10,14	5,6,7,11,12,13,15,16,17	3	D3ZHA0,P02761,P36970
510	1,2,3,4,8,9,10,15	5,6,7,11,12,13,14,16,17	3	D3ZHA0,P02761,Q5QE79
511	1,2,3,4,8,9,10,16	5,6,7,11,12,13,14,15,17	3	D3ZHA0,P08721,Q9WUW8
512	1,2,3,4,8,9,10,17	5,6,7,11,12,13,14,15,16	6	P02761,P02770,P16636,P17475,P19223,Q63355
513	1,2,3,4,8,9,11,12	5,6,7,10,13,14,15,16,17	7	P01836,P02761,P16636,Q5XI22,Q6TMA8,Q80WL1,Q9EQS0
514	1,2,3,4,8,9,11,13	5,6,7,10,12,14,15,16,17	3	P20766,Q5XI22,Q6TMA8
515	1,2,3,4,8,9,11,14	5,6,7,10,12,13,15,16,17	4	P02761,P19218,Q6TMA8,Q9EQS0
516	1,2,3,4,8,9,11,15	5,6,7,10,12,13,14,16,17	4	P02761,Q5QE79,Q5XI22,Q6TMA8
517	1,2,3,4,8,9,11,16	5,6,7,10,12,13,14,15,17	3	B4F795,P55314,Q9QZK9
518	1,2,3,4,8,9,11,17	5,6,7,10,12,13,14,15,16	5	P02761,P02770,P16636,P17475,P19223
519	1,2,3,4,8,9,12,13	5,6,7,10,11,14,15,16,17	5	P01836,P20766,P36970,Q6TMA8,Q80WL1

520	1,2,3,4,8,9,12,14	5,6,7,10,11,13,15,16,17	6	P34901,P36970,Q6TMA8,Q80WL1,Q9EQS0,Q9JJS8
521	1,2,3,4,8,9,12,15	5,6,7,10,11,13,14,16,17	7	B0BNN3,D3ZHA0,P01836,P02761,Q5QE79,Q62894,Q6TMA8
522	1,2,3,4,8,9,12,16	5,6,7,10,11,13,14,15,17	3	P01836,Q80WL1,Q9JJS8
523	1,2,3,4,8,9,12,17	5,6,7,10,11,13,14,15,16	8	P02761,P02770,P16636,P17475,P19223,P55091,P98089,Q80WL1
524	1,2,3,4,8,9,13,14	5,6,7,10,11,12,15,16,17	3	P20766,P36970,Q6TMA8
525	1,2,3,4,8,9,13,15	5,6,7,10,11,12,14,16,17	4	P02761,P20766,Q5QE79,Q6TMA8
526	1,2,3,4,8,9,13,16	5,6,7,10,11,12,14,15,17	1	P20766
527	1,2,3,4,8,9,13,17	5,6,7,10,11,12,14,15,16	6	P02761,P02770,P17475,P19223,P20766,Q63355
528	1,2,3,4,8,9,14,15	5,6,7,10,11,12,13,16,17	4	P02761,P36970,Q5QE79,Q9QZK9
529	1,2,3,4,8,9,14,16	5,6,7,10,11,12,13,15,17	2	P97574,Q9QZK9
530	1,2,3,4,8,9,14,17	5,6,7,10,11,12,13,15,16	3	P02761,P17475,P19223
531	1,2,3,4,8,9,15,16	5,6,7,10,11,12,13,14,17	7	B4F795,O54728,P02761,P47727,Q5QE79,Q62894,Q9QZK9
532	1,2,3,4,8,9,15,17	5,6,7,10,11,12,13,14,16	7	B0BNN3,P02761,P02770,P17475,P19223,Q5QE79,Q68FV8
533	1,2,3,4,8,9,16,17	5,6,7,10,11,12,13,14,15	4	P17475,P19223,P23593,Q68FV8
534	1,2,3,4,8,10,11,12	5,6,7,9,13,14,15,16,17	16	D3ZHA0,P01836,P02761,P05964,P16636,P35280,P53792,Q5XI22,Q63598,Q6IFU8,Q6IFW6,Q6IG02,Q6P6Q2,Q80WL1,Q9EQS0,Q9JJS8

535	1,2,3,4,8,10,11,13	5,6,7,9,12,14,15,16,17	5	P02761,P20766,Q5XI22,Q63598,Q6TMA8
536	1,2,3,4,8,10,11,14	5,6,7,9,12,13,15,16,17	6	P02761,P19218,P35280,Q63598,Q64194,Q9EQS0
537	1,2,3,4,8,10,11,15	5,6,7,9,12,13,14,16,17	4	P02761,P35280,Q5XI22,Q64194
538	1,2,3,4,8,10,11,16	5,6,7,9,12,13,14,15,17	4	P08649,P08721,P53792,P55314
539	1,2,3,4,8,10,11,17	5,6,7,9,12,13,14,15,16	7	P02761,P02770,P08649,P16636,P17475,P19223,Q63598
540	1,2,3,4,8,10,12,13	5,6,7,9,11,14,15,16,17	6	D3ZHA0,P01836,P16636,P36970,Q80WL1,Q9JJS8
541	1,2,3,4,8,10,12,14	5,6,7,9,11,13,15,16,17	7	D3ZHA0,P02761,P34901,P36970,Q80WL1,Q9EQS0,Q9JJS8
542	1,2,3,4,8,10,12,15	5,6,7,9,11,13,14,16,17	6	B0BNN3,D3ZHA0,P01836,P02761,P35280,P36970
543	1,2,3,4,8,10,12,16	5,6,7,9,11,13,14,15,17	7	D3ZHA0,P01836,P08721,P16636,Q80WL1,Q9JJS8,Q9WUW8
544	1,2,3,4,8,10,12,17	5,6,7,9,11,13,14,15,16	14	B0BNN3,P02761,P02770,P16636,P17475,P19223,P55091,P98089,Q10758,Q4G075,Q63355,Q80WL1,Q9EQV9,Q9JJS8
545	1,2,3,4,8,10,13,14	5,6,7,9,11,12,15,16,17	2	P20766,P36970
546	1,2,3,4,8,10,13,15	5,6,7,9,11,12,14,16,17	3	P01835,P02761,P36970
547	1,2,3,4,8,10,13,16	5,6,7,9,11,12,14,15,17	2	P01835,Q62997
548	1,2,3,4,8,10,13,17	5,6,7,9,11,12,14,15,16	8	P02761,P02770,P16636,P17475,P19223,P20766,P46844,Q63355
549	1,2,3,4,8,10,14,15	5,6,7,9,11,12,13,16,17	5	B0BNN3,D3ZHA0,P02761,P35280,P36970

550	1,2,3,4,8,10,14,16	5,6,7,9,11,12,13,15,17	4	P08721,P36970,P97574,Q62997
551	1,2,3,4,8,10,14,17	5,6,7,9,11,12,13,15,16	4	P02761,P17475,P19223,P36970
552	1,2,3,4,8,10,15,16	5,6,7,9,11,12,13,14,17	4	P01835,P02761,P08721,Q62997
553	1,2,3,4,8,10,15,17	5,6,7,9,11,12,13,14,16	7	B0BNN3,P02761,P02770,P12346,P17475,P19223,Q9EQV9
554	1,2,3,4,8,10,16,17	5,6,7,9,11,12,13,14,15	6	P02761,P08721,P16636,P17475,P19223,Q62997
555	1,2,3,4,8,11,12,13	5,6,7,9,10,14,15,16,17	7	P01836,P16636,P36374,Q5XI22,Q6TMA8,Q80WL1,Q9EQS0
556	1,2,3,4,8,11,12,14	5,6,7,9,10,13,15,16,17	8	P01836,P02761,P19218,P34901,P36970,Q6TMA8,Q80WL1,Q9EQS0
557	1,2,3,4,8,11,12,15	5,6,7,9,10,13,14,16,17	6	B0BNN3,P01836,P02761,P35280,Q5XI22,Q9EQS0
558	1,2,3,4,8,11,12,16	5,6,7,9,10,13,14,15,17	7	P01836,P16636,P55314,Q6P6Q2,Q80WL1,Q9EQS0,Q9JJS8
559	1,2,3,4,8,11,12,17	5,6,7,9,10,13,14,15,16	14	B0BNN3,P01836,P02761,P02770,P16636,P17475,P19223,P55091,P98089,Q4G075,Q6AYQ8,Q80WL1,Q9EQS0,Q9QZA6
560	1,2,3,4,8,11,13,14	5,6,7,9,10,12,15,16,17	5	P19218,P20766,P36970,Q64093,Q6TMA8
561	1,2,3,4,8,11,13,15	5,6,7,9,10,12,14,16,17	5	P01836,P02761,P20766,Q5XI22,Q6TMA8
562	1,2,3,4,8,11,13,16	5,6,7,9,10,12,14,15,17	4	P01836,P20766,P55314,Q5XI22
563	1,2,3,4,8,11,13,17	5,6,7,9,10,12,14,15,16	6	P02761,P02770,P16636,P17475,P20766,Q5XI22
564	1,2,3,4,8,11,14,15	5,6,7,9,10,12,13,16,17	6	B0BNN3,P02761,P19218,P35280,P97840,Q64194

565	1,2,3,4,8,11,14,16	5,6,7,9,10,12,13,15,17	3	P19218,P55314,P97574
566	1,2,3,4,8,11,14,17	5,6,7,9,10,12,13,15,16	4	P02761,P17475,P19218,P97840
567	1,2,3,4,8,11,15,16	5,6,7,9,10,12,13,14,17	4	B4F795,P01836,P02761,P55314
568	1,2,3,4,8,11,15,17	5,6,7,9,10,12,13,14,16	4	B0BNN3,P02761,P17475,P97840
569	1,2,3,4,8,11,16,17	5,6,7,9,10,12,13,14,15	4	P02761,P16636,P17475,P55314
570	1,2,3,4,8,12,13,14	5,6,7,9,10,11,15,16,17	12	A2RUV9,O08557,P01836,P25236,P29975,P36970,Q64093,Q6TMA8,Q80WL1,Q923M1,Q9EQS0,Q9QUL6
571	1,2,3,4,8,12,13,15	5,6,7,9,10,11,14,16,17	8	B0BNN3,P01836,P02761,P36970,Q62894,Q64093,Q6TMA8,Q9QUL6
572	1,2,3,4,8,12,13,16	5,6,7,9,10,11,14,15,17	5	P01836,P36970,Q80WL1,Q9JJS8,Q9QUL6
573	1,2,3,4,8,12,13,17	5,6,7,9,10,11,14,15,16	12	P01836,P16636,P17475,P19223,P20766,P36970,P55091,P98089,Q62635,Q63355,Q6PCU2,Q80WL1
574	1,2,3,4,8,12,14,15	5,6,7,9,10,11,13,16,17	10	A2RUV9,B0BNN3,O08557,P01836,P02761,P34901,P36970,Q64093,Q923M1,Q9EQS0
575	1,2,3,4,8,12,14,16	5,6,7,9,10,11,13,15,17	9	P01836,P08721,P34901,P36970,P97574,Q566E6,Q80WL1,Q9EQS0,Q9JJS8
576	1,2,3,4,8,12,14,17	5,6,7,9,10,11,13,15,16	15	B0BNN3,P02761,P17475,P19223,P31430,P34901,P36970,P55091,P98089,Q6PCU2,Q80WL1,Q923M1,Q9EQS0,Q9JJS8,Q9QZA6
577	1,2,3,4,8,12,15,16	5,6,7,9,10,11,13,14,17	6	B0BNN3,P01836,P83121,Q62894,Q9JJS8,Q9QUL6
578	1,2,3,4,8,12,15,17	5,6,7,9,10,11,13,14,16	12	B0BNN3,P01836,P02761,P06866,P17475,P19223,P55091,P83121,P98089,Q62894,Q9EQV9,Q9QZA6
579	1,2,3,4,8,12,16,17	5,6,7,9,10,11,13,14,15	10	P01836,P16636,P17475,P19223,P55091,P83121,P98089,Q6PCU2,Q80WL1,Q9JJS8

580	1,2,3,4,8,13,14,15	5,6,7,9,10,11,12,16,17	6	P02761,P20766,P36970,Q63317,Q64093,Q6TMA8
581	1,2,3,4,8,13,14,16	5,6,7,9,10,11,12,15,17	5	P36970,P97574,Q566E6,Q64093,Q6PCU2
582	1,2,3,4,8,13,14,17	5,6,7,9,10,11,12,15,16	6	P17475,P20766,P36970,Q64093,Q6PCU2,Q9QW07
583	1,2,3,4,8,13,15,16	5,6,7,9,10,11,12,14,17	4	P01835,P01836,Q62894,Q9QUL6
584	1,2,3,4,8,13,15,17	5,6,7,9,10,11,12,14,16	6	B0BNN3,P02761,P17475,P20766,Q64093,Q6PCU2
585	1,2,3,4,8,13,16,17	5,6,7,9,10,11,12,14,15	3	P17475,P20766,Q6PCU2
586	1,2,3,4,8,14,15,16	5,6,7,9,10,11,12,13,17	2	P97574,Q9QZK9
587	1,2,3,4,8,14,15,17	5,6,7,9,10,11,12,13,16	7	B0BNN3,P02761,P10536,P17475,P36970,P97840,Q6PCU2
588	1,2,3,4,8,14,16,17	5,6,7,9,10,11,12,13,15	3	P17475,Q566E6,Q6PCU2
589	1,2,3,4,8,15,16,17	5,6,7,9,10,11,12,13,14	7	B0BNN3,P02761,P17475,P47727,P83121,Q62894,Q6PCU2
590	1,2,3,4,9,10,11,12	5,6,7,8,13,14,15,16,17	7	P01836,P05964,P14173,P34901,Q62636,Q63661,Q6IFU8
591	1,2,3,4,9,10,11,13	5,6,7,8,12,14,15,16,17	3	P12785,P14173,Q63661
592	1,2,3,4,9,10,11,14	5,6,7,8,12,13,15,16,17	4	P14173,P19218,P34901,Q63661
593	1,2,3,4,9,10,11,15	5,6,7,8,12,13,14,16,17	2	Q561R9,Q63661
594	1,2,3,4,9,10,11,16	5,6,7,8,12,13,14,15,17	6	P12785,P14173,P55314,Q63661,Q9QZK9,Q9WUW8

595	1,2,3,4,9,10,11,17	5,6,7,8,12,13,14,15,16	2	P12785,P16636
596	1,2,3,4,9,10,12,13	5,6,7,8,11,14,15,16,17	2	P01836,Q9JJS8
597	1,2,3,4,9,10,12,14	5,6,7,8,11,13,15,16,17	2	P34901,Q9JJS8
598	1,2,3,4,9,10,12,15	5,6,7,8,11,13,14,16,17	3	P01836,P10758,P34901
599	1,2,3,4,9,10,12,16	5,6,7,8,11,13,14,15,17	4	P01836,P34901,Q9JJS8,Q9WUW8
600	1,2,3,4,9,10,12,17	5,6,7,8,11,13,14,15,16	6	P06866,P16636,P19223,P34901,Q63355,Q9JJS8
601	1,2,3,4,9,10,13,14	5,6,7,8,11,12,15,16,17	0	
602	1,2,3,4,9,10,13,15	5,6,7,8,11,12,14,16,17	0	
603	1,2,3,4,9,10,13,16	5,6,7,8,11,12,14,15,17	0	
604	1,2,3,4,9,10,13,17	5,6,7,8,11,12,14,15,16	1	Q63355
605	1,2,3,4,9,10,14,15	5,6,7,8,11,12,13,16,17	2	P34901,Q9QZK9
606	1,2,3,4,9,10,14,16	5,6,7,8,11,12,13,15,17	2	P34901,Q9QZK9
607	1,2,3,4,9,10,14,17	5,6,7,8,11,12,13,15,16	1	P34901
608	1,2,3,4,9,10,15,16	5,6,7,8,11,12,13,14,17	4	O54728,P47727,Q62902,Q9QZK9
609	1,2,3,4,9,10,15,17	5,6,7,8,11,12,13,14,16	1	Q62902

610	1,2,3,4,9,10,16,17	5,6,7,8,11,12,13,14,15	1	Q62997
611	1,2,3,4,9,11,12,13	5,6,7,8,10,14,15,16,17	1	P01836
612	1,2,3,4,9,11,12,14	5,6,7,8,10,13,15,16,17	3	P01836,P34901,Q6IFU8
613	1,2,3,4,9,11,12,15	5,6,7,8,10,13,14,16,17	3	P01836,P34901,P51907
614	1,2,3,4,9,11,12,16	5,6,7,8,10,13,14,15,17	3	P01836,P34901,P55314
615	1,2,3,4,9,11,12,17	5,6,7,8,10,13,14,15,16	5	P01836,P16636,P19223,P34901,P98089
616	1,2,3,4,9,11,13,14	5,6,7,8,10,12,15,16,17	1	P06760
617	1,2,3,4,9,11,13,15	5,6,7,8,10,12,14,16,17	2	P01836,P06760
618	1,2,3,4,9,11,13,16	5,6,7,8,10,12,14,15,17	3	P01836,P06760,P55314
619	1,2,3,4,9,11,13,17	5,6,7,8,10,12,14,15,16	0	
620	1,2,3,4,9,11,14,15	5,6,7,8,10,12,13,16,17	5	O54728,P06760,P19218,P34901,Q9QZK9
621	1,2,3,4,9,11,14,16	5,6,7,8,10,12,13,15,17	6	P06760,P19218,P34901,P55314,Q99MH3,Q9QZK9
622	1,2,3,4,9,11,14,17	5,6,7,8,10,12,13,15,16	2	P19218,P34901
623	1,2,3,4,9,11,15,16	5,6,7,8,10,12,13,14,17	5	O54728,P01836,P06760,P55314,Q9QZK9
624	1,2,3,4,9,11,15,17	5,6,7,8,10,12,13,14,16	0	

625	1,2,3,4,9,11,16,17	5,6,7,8,10,12,13,14,15	2	P55314,Q498D9
626	1,2,3,4,9,12,13,14	5,6,7,8,10,11,15,16,17	5	A2RUV9,P01836,P25236,P34901,P36970
627	1,2,3,4,9,12,13,15	5,6,7,8,10,11,14,16,17	2	P01836,P10758
628	1,2,3,4,9,12,13,16	5,6,7,8,10,11,14,15,17	2	P01836,Q9JJS8
629	1,2,3,4,9,12,13,17	5,6,7,8,10,11,14,15,16	3	P01836,P19223,Q63355
630	1,2,3,4,9,12,14,15	5,6,7,8,10,11,13,16,17	4	A2RUV9,P01836,P10758,P34901
631	1,2,3,4,9,12,14,16	5,6,7,8,10,11,13,15,17	3	P01836,P34901,Q9JJS8
632	1,2,3,4,9,12,14,17	5,6,7,8,10,11,13,15,16	4	P19223,P31430,P34901,P98089
633	1,2,3,4,9,12,15,16	5,6,7,8,10,11,13,14,17	4	P01836,P06760,P34901,P47727
634	1,2,3,4,9,12,15,17	5,6,7,8,10,11,13,14,16	6	B0BNN3,P01836,P06866,P19223,P34901,P98089
635	1,2,3,4,9,12,16,17	5,6,7,8,10,11,13,14,15	6	P01836,P06866,P19223,P34901,P98089,Q9JJS8
636	1,2,3,4,9,13,14,15	5,6,7,8,10,11,12,16,17	2	P06760,Q9QZK9
637	1,2,3,4,9,13,14,16	5,6,7,8,10,11,12,15,17	3	P06760,P08010,Q9QZK9
638	1,2,3,4,9,13,14,17	5,6,7,8,10,11,12,15,16	1	Q9QW07
639	1,2,3,4,9,13,15,16	5,6,7,8,10,11,12,14,17	4	O54728,P01836,P06760,Q9QZK9

640	1,2,3,4,9,13,15,17	5,6,7,8,10,11,12,14,16	0	
641	1,2,3,4,9,13,16,17	5,6,7,8,10,11,12,14,15	1	P08010
642	1,2,3,4,9,14,15,16	5,6,7,8,10,11,12,13,17	4	O54728,P06760,P60905,Q9QZK9
643	1,2,3,4,9,14,15,17	5,6,7,8,10,11,12,13,16	1	B0BNN3
644	1,2,3,4,9,14,16,17	5,6,7,8,10,11,12,13,15	0	
645	1,2,3,4,9,15,16,17	5,6,7,8,10,11,12,13,14	3	P47727,Q62902,Q68FV8
646	1,2,3,4,10,11,12,13	5,6,7,8,9,14,15,16,17	5	P01836,P05964,P16636,P34901,Q62636
647	1,2,3,4,10,11,12,14	5,6,7,8,9,13,15,16,17	6	P01836,P05964,P34901,P53792,Q6IFU8,Q9JJS8
648	1,2,3,4,10,11,12,15	5,6,7,8,9,13,14,16,17	6	P01836,P34901,P35280,P51907,P53792,Q4FZU2
649	1,2,3,4,10,11,12,16	5,6,7,8,9,13,14,15,17	10	P01836,P06757,P34901,P53792,P55314,Q4FZU2,Q6IFU8,Q6P6Q2,Q9JJS8,Q9WUW8
650	1,2,3,4,10,11,12,17	5,6,7,8,9,13,14,15,16	10	P01836,P05964,P06757,P06866,P16636,P19223,P34901,P53792,Q10758,Q6IFU8
651	1,2,3,4,10,11,13,14	5,6,7,8,9,12,15,16,17	0	
652	1,2,3,4,10,11,13,15	5,6,7,8,9,12,14,16,17	1	P01836
653	1,2,3,4,10,11,13,16	5,6,7,8,9,12,14,15,17	2	P01836,P55314
654	1,2,3,4,10,11,13,17	5,6,7,8,9,12,14,15,16	1	P16636
655	1,2,3,4,10,11,14,15	5,6,7,8,9,12,13,16,17	3	P19218,P34901,P35280
656	1,2,3,4,10,11,14,16	5,6,7,8,9,12,13,15,17	3	P34901,P53792,P55314
657	1,2,3,4,10,11,14,17	5,6,7,8,9,12,13,15,16	2	P19218,P34901
658	1,2,3,4,10,11,15,16	5,6,7,8,9,12,13,14,17	4	P01836,P51907,P53792,P55314
659	1,2,3,4,10,11,15,17	5,6,7,8,9,12,13,14,16	2	B0BNN3,P02761

660	1,2,3,4,10, 11,16,17	5,6,7,8,9,1 2,13,14,15	1	P55314
661	1,2,3,4,10, 12,13,14	5,6,7,8,9,1 1,15,16,17	6	A2RUV9,P01836,P25236,P34901,P36970,Q9JJS8
662	1,2,3,4,10, 12,13,15	5,6,7,8,9,1 1,14,16,17	1	P01836
663	1,2,3,4,10, 12,13,16	5,6,7,8,9,1 1,14,15,17	2	P01836,Q9JJS8
664	1,2,3,4,10, 12,13,17	5,6,7,8,9,1 1,14,15,16	4	P01836,P16636,P19223,Q63355
665	1,2,3,4,10, 12,14,15	5,6,7,8,9,1 1,13,16,17	5	A2RUV9,B0BNN3,P01836,P34901,Q9JJS8
666	1,2,3,4,10, 12,14,16	5,6,7,8,9,1 1,13,15,17	4	P01836,P08721,P34901,Q9JJS8
667	1,2,3,4,10, 12,14,17	5,6,7,8,9,1 1,13,15,16	6	P19223,P31430,P34901,P98089,Q10758,Q9JJS8
668	1,2,3,4,10, 12,15,16	5,6,7,8,9,1 1,13,14,17	4	P01836,P08721,P34901,Q9JJS8
669	1,2,3,4,10, 12,15,17	5,6,7,8,9,1 1,13,14,16	6	B0BNN3,P01836,P06866,P19223,P34901,P98089
670	1,2,3,4,10, 12,16,17	5,6,7,8,9,1 1,13,14,15	8	P01836,P06866,P08721,P16636,P19223,P34901,Q10758,Q9JJS8
671	1,2,3,4,10, 13,14,15	5,6,7,8,9,1 1,12,16,17	1	P50115
672	1,2,3,4,10, 13,14,16	5,6,7,8,9,1 1,12,15,17	2	P50115,Q62997
673	1,2,3,4,10, 13,14,17	5,6,7,8,9,1 1,12,15,16	1	Q9QW07
674	1,2,3,4,10, 13,15,16	5,6,7,8,9,1 1,12,14,17	4	P01835,P01836,P50115,Q62997
675	1,2,3,4,10, 13,15,17	5,6,7,8,9,1 1,12,14,16	0	
676	1,2,3,4,10, 13,16,17	5,6,7,8,9,1 1,12,14,15	2	Q62997,Q63355
677	1,2,3,4,10, 14,15,16	5,6,7,8,9,1 1,12,13,17	5	P08721,P34901,P50115,Q62997,Q9QZK9
678	1,2,3,4,10, 14,15,17	5,6,7,8,9,1 1,12,13,16	2	B0BNN3,P34901
679	1,2,3,4,10, 14,16,17	5,6,7,8,9,1 1,12,13,15	2	P34901,Q62997
680	1,2,3,4,10, 15,16,17	5,6,7,8,9,1 1,12,13,14	3	P47727,Q62902,Q62997
681	1,2,3,4,11, 12,13,14	5,6,7,8,9,1 0,15,16,17	4	P01836,P25236,P34901,Q64093
682	1,2,3,4,11, 12,13,15	5,6,7,8,9,1 0,14,16,17	1	P01836

683	1,2,3,4,11,<br>12,13,16	5,6,7,8,9,1<br>0,14,15,17	2	P01836,P55314
684	1,2,3,4,11,<br>12,13,17	5,6,7,8,9,1<br>0,14,15,16	4	P01836,P16636,P19223,P36374
685	1,2,3,4,11,<br>12,14,15	5,6,7,8,9,1<br>0,13,16,17	5	A2RUV9,B0BNN3,P01836,P34901,P51907
686	1,2,3,4,11,<br>12,14,16	5,6,7,8,9,1<br>0,13,15,17	4	P01836,P34901,P55314,Q9JJS8
687	1,2,3,4,11,<br>12,14,17	5,6,7,8,9,1<br>0,13,15,16	7	P01836,P19218,P19223,P31430,P34901,P98089,Q6AYQ8
688	1,2,3,4,11,<br>12,15,16	5,6,7,8,9,1<br>0,13,14,17	4	P01836,P34901,P51907,P55314
689	1,2,3,4,11,<br>12,15,17	5,6,7,8,9,1<br>0,13,14,16	6	B0BNN3,P01836,P06866,P19223,P34901,P98089
690	1,2,3,4,11,<br>12,16,17	5,6,7,8,9,1<br>0,13,14,15	7	P01836,P06866,P16636,P19223,P34901,P55314,P98089
691	1,2,3,4,11,<br>13,14,15	5,6,7,8,9,1<br>0,12,16,17	3	P01836,P06760,Q64093
692	1,2,3,4,11,<br>13,14,16	5,6,7,8,9,1<br>0,12,15,17	3	P01836,P06760,P55314
693	1,2,3,4,11,<br>13,14,17	5,6,7,8,9,1<br>0,12,15,16	3	P19218,Q64093,Q9QW07
694	1,2,3,4,11,<br>13,15,16	5,6,7,8,9,1<br>0,12,14,17	3	P01836,P06760,P55314
695	1,2,3,4,11,<br>13,15,17	5,6,7,8,9,1<br>0,12,14,16	1	P01836
696	1,2,3,4,11,<br>13,16,17	5,6,7,8,9,1<br>0,12,14,15	2	P01836,P55314
697	1,2,3,4,11,<br>14,15,16	5,6,7,8,9,1<br>0,12,13,17	6	P01836,P06760,P19218,P34901,P55314,Q9QZK9
698	1,2,3,4,11,<br>14,15,17	5,6,7,8,9,1<br>0,12,13,16	4	B0BNN3,P19218,P34901,P97840
699	1,2,3,4,11,<br>14,16,17	5,6,7,8,9,1<br>0,12,13,15	3	P19218,P34901,P55314
700	1,2,3,4,11,<br>15,16,17	5,6,7,8,9,1<br>0,12,13,14	2	P01836,P55314
701	1,2,3,4,12,<br>13,14,15	5,6,7,8,9,1<br>0,11,16,17	8	A2RUV9,P01836,P10758,P25236,P34901,P36970,Q64093,Q9QUL6
702	1,2,3,4,12,<br>13,14,16	5,6,7,8,9,1<br>0,11,15,17	6	P01836,P25236,P34901,Q566E6,Q9JJS8,Q9QUL6
703	1,2,3,4,12,<br>13,14,17	5,6,7,8,9,1<br>0,11,15,16	7	A2RUV9,P01836,P31430,P34901,P98089,Q64093,Q9QW07
704	1,2,3,4,12,<br>13,15,16	5,6,7,8,9,1<br>0,11,14,17	3	P01836,P83121,Q9QUL6
705	1,2,3,4,12,<br>13,15,17	5,6,7,8,9,1<br>0,11,14,16	3	B0BNN3,P01836,P06866

706	1,2,3,4,12,13,16,17	5,6,7,8,9,10,11,14,15	2	P01836,P06866
707	1,2,3,4,12,14,15,16	5,6,7,8,9,10,11,13,17	5	A2RUV9,D3ZUC6,P01836,P34901,Q9JJS8
708	1,2,3,4,12,14,15,17	5,6,7,8,9,10,11,13,16	7	A2RUV9,B0BNN3,P01836,P06866,P31430,P34901,P98089
709	1,2,3,4,12,14,16,17	5,6,7,8,9,10,11,13,15	6	P01836,P06866,P31430,P34901,P98089,Q9JJS8
710	1,2,3,4,12,15,16,17	5,6,7,8,9,10,11,13,14	7	B0BNN3,P01836,P06866,P34901,P47727,P83121,P98089
711	1,2,3,4,13,14,15,16	5,6,7,8,9,10,11,12,17	7	P01836,P06760,P50115,P60905,Q566E6,Q64093,Q9QZK9
712	1,2,3,4,13,14,15,17	5,6,7,8,9,10,11,12,16	3	B0BNN3,Q64093,Q9QW07
713	1,2,3,4,13,14,16,17	5,6,7,8,9,10,11,12,15	4	P08010,Q566E6,Q6PCU2,Q9QW07
714	1,2,3,4,13,15,16,17	5,6,7,8,9,10,11,12,14	3	P01836,P47727,Q6PCU2
715	1,2,3,4,14,15,16,17	5,6,7,8,9,10,11,12,13	2	B0BNN3,P60905
716	1,2,3,5,6,7,8,9	4,10,11,12,13,14,15,16,17	21	O55006,O88267,P02631,P02764,P05371,P08932,P08937,P10715,P16086,P19629,P20762,P23593,P27213,P31044,P52590,P53792,P68370,P83748,Q63942,Q9QX79,Q9Z1F2
717	1,2,3,5,6,7,8,10	4,9,11,12,13,14,15,16,17	23	D3ZHA0,O54858,O55006,O88267,P02631,P05371,P08649,P08937,P12020,P16086,P19629,P27213,P28648,P31044,P52590,P68370,P83748,P97571,P97615,Q30KJ2,Q63751,Q64361,Q6AYS7
718	1,2,3,5,6,7,8,11	4,9,10,12,13,14,15,16,17	32	O54715,O54858,O88267,P02631,P04041,P05371,P07150,P08649,P08932,P08937,P10715,P14669,P15399,P16086,P19629,P20762,P27213,P28648,P31044,P52590,P55314,P68370,P97580,P97615,Q30KJ2,Q63493,Q63751,Q6IRE4,Q9EQS0,Q9JI85,Q9QUL6,Q9Z1F2
719	1,2,3,5,6,7,8,12	4,9,10,11,13,14,15,16,17	31	O88267,P02631,P02782,P05371,P06761,P08723,P08937,P10715,P13432,P16086,P19629,P20762,P22283,P27213,P30120,P31044,P38918,P52590,P68370,P83748,P97615,Q561R9,Q5M8C6,Q63751,Q64361,Q6AYS7,Q8CJ52,Q9EQS0,Q9JHB9,Q9JI85,Q9Z1F2
720	1,2,3,5,6,7,8,13	4,9,10,11,12,14,15,16,17	26	O88267,P02631,P02764,P05371,P06761,P06911,P08753,P08932,P08937,P12020,P16086,P19629,P20762,P29975,P31044,P51907,P52590,P53792,P68370,P70545,P83748,Q5GRG2,Q63317,Q63751,Q6AYS7,Q9Z1F2
721	1,2,3,5,6,7,8,14	4,9,10,11,12,13,15,16,17	22	O88267,P02631,P05371,P08937,P10536,P12020,P16086,P19629,P20762,P27213,P31044,P38918,P52590,P68370,P83748,P97615,Q30KJ2,Q561R9,Q5GRG2,Q63317,Q6AYS7,Q9Z1F2
722	1,2,3,5,6,7,8,15	4,9,10,11,12,13,14,16,17	20	O55006,O88267,P02631,P05371,P08932,P08937,P10536,P10715,P16086,P19629,P20762,P27213,P31044,P52590,P68370,P83748,P97615,Q63317,Q63751,Q6AYS7
723	1,2,3,5,6,7,8,16	4,9,10,11,12,13,14,15,17	17	O88267,P02631,P05371,P08937,P10715,P10758,P16086,P19629,P27213,P31044,P52590,P68370,P83748,Q561R9,Q63751,Q6AYS7,Q6IRE4

724	1,2,3,5,6,7,8,17	4,9,10,11,12,13,14,15,16	36	O54858,O88267,P01946,P02091,P02631,P02770,P05371,P06760,P08937,P10536,P10715,P10758,P12346,P16086,P17475,P19629,P20762,P23593,P23928,P31044,P50115,P51907,P52590,P53792,P55091,P68370,P83748,P97580,P97615,Q30KJ2,Q4G075,Q561R9,Q63751,Q6AYS7,Q6PCU2,Q9Z1F2
725	1,2,3,5,6,7,9,10	4,8,11,12,13,14,15,16,17	9	O55006,P02631,P05371,P16086,P27213,P52590,P83748,Q63942,Q9QUL6
726	1,2,3,5,6,7,9,11	4,8,10,12,13,14,15,16,17	12	P02631,P05371,P08937,P15399,P16086,P27213,P52590,P55314,Q30KJ2,Q6IRE4,Q91ZS3,Q9QUL6
727	1,2,3,5,6,7,9,12	4,8,10,11,13,14,15,16,17	10	O88267,P02631,P05371,P08937,P16086,P27213,P52590,P83748,Q498R7,Q9Z1F2
728	1,2,3,5,6,7,9,13	4,8,10,11,12,14,15,16,17	9	P02631,P08937,P16086,P52590,P53792,P83748,Q63942,Q6IRE4,Q9Z1F2
729	1,2,3,5,6,7,9,14	4,8,10,11,12,13,15,16,17	10	P02631,P05371,P16086,P27213,P52590,P53792,P83748,Q63942,Q6IRE4,Q9QUL6
730	1,2,3,5,6,7,9,15	4,8,10,11,12,13,14,16,17	9	O55006,P02631,P05371,P16086,P27213,P52590,P53792,P83748,Q63942
731	1,2,3,5,6,7,9,16	4,8,10,11,12,13,14,15,17	9	P02631,P05371,P16086,P23593,P27213,P52590,P83748,Q63942,Q6IRE4
732	1,2,3,5,6,7,9,17	4,8,10,11,12,13,14,15,16	15	O88267,P01946,P02091,P02631,P05371,P08937,P09605;P25809,P16086,P23593,P52590,P53792,P97580,Q30KJ2,Q561R9,Q9QUL6
733	1,2,3,5,6,7,10,11	4,8,9,12,13,14,15,16,17	15	P02631,P05371,P08937,P15399,P16086,P27213,P28648,P52590,P55314,P61206;P84079;P84082,P68370,P97580,P97615,Q30KJ2,Q9QUL6
734	1,2,3,5,6,7,10,12	4,8,9,11,13,14,15,16,17	13	P05371,P08937,P16086,P27213,P34901,P52590,P83748,P97615,P97840,Q10758,Q4FZU2,Q6IFW6,Q6IMF3
735	1,2,3,5,6,7,10,13	4,8,9,11,12,14,15,16,17	7	P08937,P12020,P16086,P52590,P83748,P97615,Q5GRG2
736	1,2,3,5,6,7,10,14	4,8,9,11,12,13,15,16,17	10	P02631,P05371,P08937,P12020,P16086,P27213,P52590,P83748,P97615,Q63532
737	1,2,3,5,6,7,10,15	4,8,9,11,12,13,14,16,17	8	P02631,P05371,P08937,P16086,P27213,P52590,P83748,P97615
738	1,2,3,5,6,7,10,16	4,8,9,11,12,13,14,15,17	7	P05371,P16086,P27213,P52590,P83748,P97615,Q6IRE4

739	1,2,3,5,6,7,10,17	4,8,9,11,12,13,14,15,16	13	P01946,P02091,P08937,P15399,P16086,P52590,P83748,P97580,P97615,Q30KJ2,Q561R9,Q63751,Q9QUL6
740	1,2,3,5,6,7,11,12	4,8,9,10,13,14,15,16,17	23	O88267,P02631,P05371,P08937,P15399,P16086,P17046,P27213,P52590,P55314,P83748,P97580,Q30KJ2,Q498R7,Q4FZU2,Q63751,Q6IFU8,Q6IFW6,Q6IMF3,Q6IRE4,Q6P6Q2,Q9JHB9,Q9JI85
741	1,2,3,5,6,7,11,13	4,8,9,10,12,14,15,16,17	11	P01048,P02631,P05371,P08937,P15399,P16086,P17046,P55314,Q30KJ2,Q6IRE4,Q9Z1F2
742	1,2,3,5,6,7,11,14	4,8,9,10,12,13,15,16,17	11	P02631,P05371,P08937,P15399,P16086,P27213,P55314,P97580,Q30KJ2,Q6IRE4,Q9QUL6
743	1,2,3,5,6,7,11,15	4,8,9,10,12,13,14,16,17	12	P02631,P05371,P08937,P15399,P16086,P27213,P52590,P55314,P83748,Q30KJ2,Q5I0D1,Q6IRE4
744	1,2,3,5,6,7,11,16	4,8,9,10,12,13,14,15,17	14	P02631,P05371,P08937,P10758,P15399,P16086,P17046,P27213,P55314,P61206;P84079;P84082,P97580,Q30KJ2,Q6IRE4,Q8VD89
745	1,2,3,5,6,7,11,17	4,8,9,10,12,13,14,15,16	14	P01946,P02091,P02631,P05371,P08937,P10758,P15399,P16086,P52590,P55314,P97580,Q30KJ2,Q63751,Q9QUL6
746	1,2,3,5,6,7,12,13	4,8,9,10,11,14,15,16,17	8	O88267,P08937,P16086,P17046,P83748,Q561R9,Q9JHB9,Q9Z1F2
747	1,2,3,5,6,7,12,14	4,8,9,10,11,13,15,16,17	13	O88267,P02631,P05371,P08937,P16086,P19629,P27213,P34901,P83748,Q561R9,Q63532,Q6AYS7,Q9Z1F2
748	1,2,3,5,6,7,12,15	4,8,9,10,11,13,14,16,17	7	P02631,P05371,P08937,P16086,P27213,P52590,P83748
749	1,2,3,5,6,7,12,16	4,8,9,10,11,13,14,15,17	9	O88267,P02631,P05371,P08937,P16086,P17046,P83748,Q561R9,Q6IRE4
750	1,2,3,5,6,7,12,17	4,8,9,10,11,13,14,15,16	20	O88267,P01681,P01946,P02091,P02631,P05371,P08937,P09605;P25809,P13432,P16086,P52590,P55091,P83748,P97580,Q10758,Q30KJ2,Q561R9,Q62635,Q63751,Q9Z1F2
751	1,2,3,5,6,7,13,14	4,8,9,10,11,12,15,16,17	11	O88267,P02631,P06911,P08937,P12020,P16086,P53792,P83748,Q561R9,Q5GRG2,Q9Z1F2
752	1,2,3,5,6,7,13,15	4,8,9,10,11,12,14,16,17	6	P02631,P08937,P16086,P53792,P83748,Q5GRG2
753	1,2,3,5,6,7,13,16	4,8,9,10,11,12,14,15,17	8	O88267,P05371,P08937,P16086,P17046,P83748,Q561R9,Q6IRE4

754	1,2,3,5,6,7,13,17	4,8,9,10,11,12,14,15,16	14	O88267,P01946,P02091,P08937,P16086,P51907,P52590,P53792,P83748,P97580,Q30KJ2,Q561R9,Q63751,Q9Z1F2
755	1,2,3,5,6,7,14,15	4,8,9,10,11,12,13,16,17	7	P02631,P02803,P05371,P16086,P27213,P53792,P83748
756	1,2,3,5,6,7,14,16	4,8,9,10,11,12,13,15,17	8	O88267,P02631,P05371,P16086,P83748,Q561R9,Q63532,Q6IRE4
757	1,2,3,5,6,7,14,17	4,8,9,10,11,12,13,15,16	13	O88267,P01946,P02091,P02631,P05371,P08937,P10536,P16086,P52590,P53792,P97580,Q30KJ2,Q561R9
758	1,2,3,5,6,7,15,16	4,8,9,10,11,12,13,14,17	6	P02631,P02803,P05371,P16086,P83748,Q6IRE4
759	1,2,3,5,6,7,15,17	4,8,9,10,11,12,13,14,16	15	P01946,P02091,P02631,P02803,P05371,P08937,P10536,P16086,P52590,P53792,P83748,P97580,Q30KJ2,Q561R9,Q63751
760	1,2,3,5,6,7,16,17	4,8,9,10,11,12,13,14,15	15	O88267,P01946,P02091,P02631,P05371,P08937,P10758,P16086,P17046,P23593,P52590,P83748,P97580,Q30KJ2,Q561R9
761	1,2,3,5,6,8,9,10	4,7,11,12,13,14,15,16,17	6	O55006,P08649,P23593,P27213,Q63942,Q9QUL6
762	1,2,3,5,6,8,9,11	4,7,10,12,13,14,15,16,17	8	P08649,P08932,P14669,P27213,P55314,Q63942,Q9QUL6,Q9Z1F2
763	1,2,3,5,6,8,9,12	4,7,10,11,13,14,15,16,17	7	P08937,P23593,P27213,Q5M8C6,Q63942,Q6IG02,Q9Z1F2
764	1,2,3,5,6,8,9,13	4,7,10,11,12,14,15,16,17	6	P08753,P23593,P51907,P53792,Q63942,Q9Z1F2
765	1,2,3,5,6,8,9,14	4,7,10,11,12,13,15,16,17	4	P02793;Q7TP54,P27213,Q63942,Q9Z1F2
766	1,2,3,5,6,8,9,15	4,7,10,11,12,13,14,16,17	5	O55006,P23593,P27213,Q5QE79,Q63942
767	1,2,3,5,6,8,9,16	4,7,10,11,12,13,14,15,17	4	P23593,P27213,Q63942,Q6IRE4
768	1,2,3,5,6,8,9,17	4,7,10,11,12,13,14,15,16	10	P06760,P08649,P23593,P50115,P51907,P52590,P53792,Q561R9,Q63942,Q9Z1F2

769	1,2,3,5,6,8,10,11	4,7,9,12,13,14,15,16,17	6	P08649,P14669,P27213,P28648,P55314,Q9QUL6
770	1,2,3,5,6,8,10,12	4,7,9,11,13,14,15,16,17	12	P08937,P27213,Q08463,Q10758,Q4FZU2,Q5M8C6,Q64361,Q6IFW6,Q6IG02,Q6IMF3,Q6P6Q2,Q9Z1F2
771	1,2,3,5,6,8,10,13	4,7,9,11,12,14,15,16,17	3	P08753,P51907,Q9Z1F2
772	1,2,3,5,6,8,10,14	4,7,9,11,12,13,15,16,17	4	P08649,P27213,P97615,Q9Z1F2
773	1,2,3,5,6,8,10,15	4,7,9,11,12,13,14,16,17	3	P08649,P27213,Q6IFV1
774	1,2,3,5,6,8,10,16	4,7,9,11,12,13,14,15,17	1	P08649
775	1,2,3,5,6,8,10,17	4,7,9,11,12,13,14,15,16	11	O54858,P01946,P06760,P08649,P08937,P17475,P23593,P50115,P51907,Q08463,Q561R9
776	1,2,3,5,6,8,11,12	4,7,9,10,13,14,15,16,17	19	O35547,O54715,P01681,P02782,P08937,P14669,P27213,P30120,P55314,P62804,Q4FZU2,Q5M8C6,Q6IFU8,Q6IFW6,Q6IG02,Q6IMF3,Q6P6Q2,Q9JHB9,Q9Z1F2
777	1,2,3,5,6,8,11,13	4,7,9,10,12,14,15,16,17	4	P08937,P14669,P55314,Q9Z1F2
778	1,2,3,5,6,8,11,14	4,7,9,10,12,13,15,16,17	7	P02793;Q7TP54,P08649,P14669,P27213,P55314,Q5I0D1,Q9Z1F2
779	1,2,3,5,6,8,11,15	4,7,9,10,12,13,14,16,17	5	P08649,P14669,P27213,P55314,Q5I0D1
780	1,2,3,5,6,8,11,16	4,7,9,10,12,13,14,15,17	4	P08649,P14668,P55314,Q6IRE4
781	1,2,3,5,6,8,11,17	4,7,9,10,12,13,14,15,16	12	O54858,P01681,P01946,P06760,P08649,P08937,P14669,P17475,P50115,P55314,Q63751,Q9QUL6
782	1,2,3,5,6,8,12,13	4,7,9,10,11,14,15,16,17	10	O88267,P02782,P08937,P30120,P51907,P82995,Q561R9,Q5M8C6,Q9JHB9,Q9Z1F2
783	1,2,3,5,6,8,12,14	4,7,9,10,11,13,15,16,17	10	O88267,P02782,P02793;Q7TP54,P08937,Q561R9,Q5M8C6,Q6AYS7,Q6IFW6,Q6IG02,Q9Z1F2

784	1,2,3,5,6,8,12,15	4,7,9,10,11,13,14,16,17	5	P08937,P83121,Q5M8C6,Q6IFV1,Q9Z1F2
785	1,2,3,5,6,8,12,16	4,7,9,10,11,13,14,15,17	7	O88267,P08937,Q561R9,Q5M8C6,Q6IFW6,Q6IMF3,Q9Z1F2
786	1,2,3,5,6,8,12,17	4,7,9,10,11,13,14,15,16	22	O88267,P01681,P01946,P06760,P08937,P13432,P16636,P17475,P23593,P50115,P51907,P55091,Q08463,Q10758,Q4G075,Q561R9,Q5M8C6,Q62635,Q63751,Q6IFV1,Q6IG02,Q9Z1F2
787	1,2,3,5,6,8,13,14	4,7,9,10,11,12,15,16,17	9	O88267,P06911,P08753,P51907,Q561R9,Q5FVI6,Q5GRG2,Q64093,Q9Z1F2
788	1,2,3,5,6,8,13,15	4,7,9,10,11,12,14,16,17	4	P08753,P82995,Q6IFV1,Q9Z1F2
789	1,2,3,5,6,8,13,16	4,7,9,10,11,12,14,15,17	4	P08753,P51907,Q6PCU2,Q9Z1F2
790	1,2,3,5,6,8,13,17	4,7,9,10,11,12,14,15,16	14	O88267,P01946,P06760,P08753,P08937,P10536,P23593,P51907,P53792,Q561R9,Q5FVI6,Q6IFV1,Q6PCU2,Q9Z1F2
791	1,2,3,5,6,8,14,15	4,7,9,10,11,12,13,16,17	3	P08753,P10536,Q9Z1F2
792	1,2,3,5,6,8,14,16	4,7,9,10,11,12,13,15,17	3	P08753,Q561R9,Q9Z1F2
793	1,2,3,5,6,8,14,17	4,7,9,10,11,12,13,15,16	13	O88267,P01946,P02793;Q7TP54,P06760,P08753,P08937,P10536,P50115,P51907,Q561R9,Q5FVI6,Q6PCU2,Q9Z1F2
794	1,2,3,5,6,8,15,16	4,7,9,10,11,12,13,14,17	1	P23593
795	1,2,3,5,6,8,15,17	4,7,9,10,11,12,13,14,16	8	P06760,P08753,P08937,P10536,P17475,P23593,Q561R9,Q6IFV1
796	1,2,3,5,6,8,16,17	4,7,9,10,11,12,13,14,15	10	O88267,P01946,P06760,P08753,P10758,P23593,P42854,P51907,Q561R9,Q6PCU2
797	1,2,3,5,6,9,10,11	4,7,8,12,13,14,15,16,17	7	P08649,P14173,P27213,P55314,Q63942,Q91ZS3,Q9QUL6
798	1,2,3,5,6,9,10,12	4,7,8,11,13,14,15,16,17	5	P27213,Q10758,Q63942,Q6IFW6,Q6IMF3

799	1,2,3,5,6,9,10,13	4,7,8,11,12,14,15,16,17	1	Q63942
800	1,2,3,5,6,9,10,14	4,7,8,11,12,13,15,16,17	3	P27213,Q63942,Q9QUL6
801	1,2,3,5,6,9,10,15	4,7,8,11,12,13,14,16,17	3	P27213,Q63942,Q9QUL6
802	1,2,3,5,6,9,10,16	4,7,8,11,12,13,14,15,17	5	P23593,P27213,Q63942,Q6IRE4,Q9QUL6
803	1,2,3,5,6,9,10,17	4,7,8,11,12,13,14,15,16	4	P23593,P52590,Q63942,Q9QUL6
804	1,2,3,5,6,9,11,12	4,7,8,10,13,14,15,16,17	9	P27213,P55314,Q4FZU2,Q6IFU7,Q6IFU8,Q6IFW6,Q6IG02,Q6IMF3,Q6P6Q2
805	1,2,3,5,6,9,11,13	4,7,8,10,12,14,15,16,17	3	P55314,Q63942,Q6IRE4
806	1,2,3,5,6,9,11,14	4,7,8,10,12,13,15,16,17	5	P27213,P55314,Q63942,Q6IRE4,Q9QUL6
807	1,2,3,5,6,9,11,15	4,7,8,10,12,13,14,16,17	5	P27213,P55314,Q63942,Q6IRE4,Q9QUL6
808	1,2,3,5,6,9,11,16	4,7,8,10,12,13,14,15,17	5	P27213,P55314,Q63942,Q6IRE4,Q9QUL6
809	1,2,3,5,6,9,11,17	4,7,8,10,12,13,14,15,16	3	P55314,Q63942,Q9QUL6
810	1,2,3,5,6,9,12,13	4,7,8,10,11,14,15,16,17	2	P00714,Q9Z1F2
811	1,2,3,5,6,9,12,14	4,7,8,10,11,13,15,16,17	5	P01835,P34901,Q561R9,Q63942,Q9Z1F2
812	1,2,3,5,6,9,12,15	4,7,8,10,11,13,14,16,17	2	P27213,Q63942
813	1,2,3,5,6,9,12,16	4,7,8,10,11,13,14,15,17	3	P23593,Q63942,Q6IRE4

814	1,2,3,5,6,9,12,17	4,7,8,10,11,13,14,15,16	5	P00714,P01681,P01835,P23593,Q561R9
815	1,2,3,5,6,9,13,14	4,7,8,10,11,12,15,16,17	4	P00714,P53792,Q63942,Q9Z1F2
816	1,2,3,5,6,9,13,15	4,7,8,10,11,12,14,16,17	3	P00714,P53792,Q63942
817	1,2,3,5,6,9,13,16	4,7,8,10,11,12,14,15,17	4	P00714,P23593,Q63942,Q6IRE4
818	1,2,3,5,6,9,13,17	4,7,8,10,11,12,14,15,16	9	P00714,P01946,P08753,P23593,P51907,P53792,Q561R9,Q63942,Q9Z1F2
819	1,2,3,5,6,9,14,15	4,7,8,10,11,12,13,16,17	4	P00714,P27213,P53792,Q63942
820	1,2,3,5,6,9,14,16	4,7,8,10,11,12,13,15,17	3	Q561R9,Q63942,Q6IRE4
821	1,2,3,5,6,9,14,17	4,7,8,10,11,12,13,15,16	5	P00714,P53792,Q561R9,Q63942,Q9QUL6
822	1,2,3,5,6,9,15,16	4,7,8,10,11,12,13,14,17	5	O54728,P00714,P23593,Q63942,Q6IRE4
823	1,2,3,5,6,9,15,17	4,7,8,10,11,12,13,14,16	4	P00714,P23593,P53792,Q63942
824	1,2,3,5,6,9,16,17	4,7,8,10,11,12,13,14,15	4	P00714,P23593,Q561R9,Q63942
825	1,2,3,5,6,10,11,12	4,7,8,9,13,14,15,16,17	11	P27213,P34901,P55314,Q10758,Q4FZU2,Q6IFU7,Q6IFU8,Q6IFW6,Q6IG02,Q6IMF3,Q6P6Q2
826	1,2,3,5,6,10,11,13	4,7,8,9,12,14,15,16,17	1	P55314
827	1,2,3,5,6,10,11,14	4,7,8,9,12,13,15,16,17	5	P08649,P27213,P34901,P55314,Q9QUL6
828	1,2,3,5,6,10,11,15	4,7,8,9,12,13,14,16,17	5	P08649,P27213,P55314,Q4FZU6,Q9QUL6

829	1,2,3,5,6,10,11,16	4,7,8,9,12,13,14,15,17	5	P08649,P27213,P55314,Q6IRE4,Q9QUL6
830	1,2,3,5,6,10,11,17	4,7,8,9,12,13,14,15,16	4	P01946,P08649,P55314,Q9QUL6
831	1,2,3,5,6,10,12,13	4,7,8,9,11,14,15,16,17	3	Q10758,Q6IMF3,Q9Z1F2
832	1,2,3,5,6,10,12,14	4,7,8,9,11,13,15,16,17	6	P34901,Q10758,Q4FZU2,Q561R9,Q6IFW6,Q6IMF3
833	1,2,3,5,6,10,12,15	4,7,8,9,11,13,14,16,17	4	P27213,Q10758,Q4FZU2,Q6IMF3
834	1,2,3,5,6,10,12,16	4,7,8,9,11,13,14,15,17	6	Q10758,Q4FZU2,Q6IFU7,Q6IFW6,Q6IMF3,Q6P6Q2
835	1,2,3,5,6,10,12,17	4,7,8,9,11,13,14,15,16	5	P01681,Q10758,Q561R9,Q6IMF3,Q99MH3
836	1,2,3,5,6,10,13,14	4,7,8,9,11,12,15,16,17	1	Q9Z1F2
837	1,2,3,5,6,10,13,15	4,7,8,9,11,12,14,16,17	1	P82995
838	1,2,3,5,6,10,13,16	4,7,8,9,11,12,14,15,17	0	
839	1,2,3,5,6,10,13,17	4,7,8,9,11,12,14,15,16	5	P01946,P08753,P51907,Q561R9,Q99MH3
840	1,2,3,5,6,10,14,15	4,7,8,9,11,12,13,16,17	1	P27213
841	1,2,3,5,6,10,14,16	4,7,8,9,11,12,13,15,17	0	
842	1,2,3,5,6,10,14,17	4,7,8,9,11,12,13,15,16	1	Q561R9
843	1,2,3,5,6,10,15,16	4,7,8,9,11,12,13,14,17	0	

844	1,2,3,5,6,10,15,17	4,7,8,9,11,12,13,14,16	0	
845	1,2,3,5,6,10,16,17	4,7,8,9,11,12,13,14,15	3	P10758,P23593,Q561R9
846	1,2,3,5,6,11,12,13	4,7,8,9,10,14,15,16,17	8	P55314,Q4FZU2,Q6IFU8,Q6IFW6,Q6IMF3,Q6P6Q2,Q9JHB9,Q9Z1F2
847	1,2,3,5,6,11,12,14	4,7,8,9,10,13,15,16,17	11	P01681,P34901,P55314,Q4FZU2,Q6IFU7,Q6IFU8,Q6IFW6,Q6IG02,Q6IMF3,Q6P6Q2,Q9Z1F2
848	1,2,3,5,6,11,12,15	4,7,8,9,10,13,14,16,17	8	P27213,P55314,Q4FZU2,Q6IFU7,Q6IFU8,Q6IFW6,Q6IMF3,Q6P6Q2
849	1,2,3,5,6,11,12,16	4,7,8,9,10,13,14,15,17	9	P17046,P55314,Q4FZU2,Q6IFU7,Q6IFU8,Q6IFW6,Q6IMF3,Q6IRE4,Q6P6Q2
850	1,2,3,5,6,11,12,17	4,7,8,9,10,13,14,15,16	11	P01681,P01946,P55314,Q10758,Q4FZU2,Q561R9,Q6IFU8,Q6IFW6,Q6IG02,Q6IMF3,Q6P6Q2
851	1,2,3,5,6,11,13,14	4,7,8,9,10,12,15,16,17	2	P55314,Q9Z1F2
852	1,2,3,5,6,11,13,15	4,7,8,9,10,12,14,16,17	1	P55314
853	1,2,3,5,6,11,13,16	4,7,8,9,10,12,14,15,17	3	P17046,P55314,Q6IRE4
854	1,2,3,5,6,11,13,17	4,7,8,9,10,12,14,15,16	2	P01946,P55314
855	1,2,3,5,6,11,14,15	4,7,8,9,10,12,13,16,17	3	P27213,P55314,Q5I0D1
856	1,2,3,5,6,11,14,16	4,7,8,9,10,12,13,15,17	2	P55314,Q6IRE4
857	1,2,3,5,6,11,14,17	4,7,8,9,10,12,13,15,16	4	P01946,P55314,Q561R9,Q9QUL6
858	1,2,3,5,6,11,15,16	4,7,8,9,10,12,13,14,17	3	P55314,Q5I0D1,Q6IRE4

859	1,2,3,5,6,11,15,17	4,7,8,9,10,12,13,14,16	1	P55314
860	1,2,3,5,6,11,16,17	4,7,8,9,10,12,13,14,15	4	P01946,P10758,P17046,P55314
861	1,2,3,5,6,12,13,14	4,7,8,9,10,11,15,16,17	3	P00714,Q561R9,Q9Z1F2
862	1,2,3,5,6,12,13,15	4,7,8,9,10,11,14,16,17	3	P00714,P82995,Q9Z1F2
863	1,2,3,5,6,12,13,16	4,7,8,9,10,11,14,15,17	4	P00714,P17046,Q561R9,Q9Z1F2
864	1,2,3,5,6,12,13,17	4,7,8,9,10,11,14,15,16	8	P00714,P01681,P01946,P51907,Q561R9,Q62635,Q99MH3,Q9Z1F2
865	1,2,3,5,6,12,14,15	4,7,8,9,10,11,13,16,17	2	P00714,P34901
866	1,2,3,5,6,12,14,16	4,7,8,9,10,11,13,15,17	3	P34901,Q561R9,Q6IMF3
867	1,2,3,5,6,12,14,17	4,7,8,9,10,11,13,15,16	8	P00714,P01681,P01946,P31430,P34901,Q10758,Q561R9,Q9Z1F2
868	1,2,3,5,6,12,15,16	4,7,8,9,10,11,13,14,17	2	P00714,P83121
869	1,2,3,5,6,12,15,17	4,7,8,9,10,11,13,14,16	3	P00714,P83121,Q561R9
870	1,2,3,5,6,12,16,17	4,7,8,9,10,11,13,14,15	7	P00714,P01681,P01946,P17046,P23593,Q10758,Q561R9
871	1,2,3,5,6,13,14,15	4,7,8,9,10,11,12,16,17	5	P00714,P08753,P53792,Q64093,Q9Z1F2
872	1,2,3,5,6,13,14,16	4,7,8,9,10,11,12,15,17	5	P00714,P08753,Q561R9,Q6IRE4,Q9Z1F2
873	1,2,3,5,6,13,14,17	4,7,8,9,10,11,12,15,16	10	P00714,P01946,P08753,P51907,P53792,Q561R9,Q5FVI6,Q6PCU2,Q9QW07,Q9Z1F2

874	1,2,3,5,6,13,15,16	4,7,8,9,10,11,12,14,17	3	P00714,P08753,P82995
875	1,2,3,5,6,13,15,17	4,7,8,9,10,11,12,14,16	5	P00714,P08753,P53792,Q561R9,Q6IFV1
876	1,2,3,5,6,13,16,17	4,7,8,9,10,11,12,14,15	8	P00714,P01946,P08753,P10758,P23593,P51907,Q561R9,Q6PCU2
877	1,2,3,5,6,14,15,16	4,7,8,9,10,11,12,13,17	4	P00714,P02803,P36374,Q6IRE4
878	1,2,3,5,6,14,15,17	4,7,8,9,10,11,12,13,16	5	P00714,P02803,P10536,P47967,Q561R9
879	1,2,3,5,6,14,16,17	4,7,8,9,10,11,12,13,15	6	P00714,P01946,P10758,Q561R9,Q6PCU2,Q9QW07
880	1,2,3,5,6,15,16,17	4,7,8,9,10,11,12,13,14	4	P00714,P02803,P23593,Q561R9
881	1,2,3,5,7,8,9,10	4,6,11,12,13,14,15,16,17	5	O55006,P27213,P31044,P52590,Q63942
882	1,2,3,5,7,8,9,11	4,6,10,12,13,14,15,16,17	6	P10715,P27213,P52590,P55314,Q6IRE4,Q9QUL6
883	1,2,3,5,7,8,9,12	4,6,10,11,13,14,15,16,17	5	P10715,P27213,P52590,Q9EQS0,Q9Z1F2
884	1,2,3,5,7,8,9,13	4,6,10,11,12,14,15,16,17	4	P20766,P52590,P53792,Q9Z1F2
885	1,2,3,5,7,8,9,14	4,6,10,11,12,13,15,16,17	4	P27213,P52590,Q63942,Q9QX79
886	1,2,3,5,7,8,9,15	4,6,10,11,12,13,14,16,17	6	O54728,P10715,P27213,P52590,Q5QE79,Q63942
887	1,2,3,5,7,8,9,16	4,6,10,11,12,13,14,15,17	5	P10715,P27213,P52590,Q63942,Q6IRE4
888	1,2,3,5,7,8,9,17	4,6,10,11,12,13,14,15,16	7	P01946,P02091,P10715,P50115,P52590,P53792,Q9QX79

889	1,2,3,5,7,8,10,11	4,6,9,12,13,14,15,16,17	9	P08649,P10715,P16391,P27213,P28648,P31044,P52590,P55314,P68370
890	1,2,3,5,7,8,10,12	4,6,9,11,13,14,15,16,17	7	P00731,P08937,P10715,P16391,P27213,P52590,Q9EQS0
891	1,2,3,5,7,8,10,13	4,6,9,11,12,14,15,16,17	3	P01835,P31044,P52590
892	1,2,3,5,7,8,10,14	4,6,9,11,12,13,15,16,17	5	P27213,P31044,P36970,P52590,P97615
893	1,2,3,5,7,8,10,15	4,6,9,11,12,13,14,16,17	6	P00731,P01835,P27213,P31044,P52590,P97615
894	1,2,3,5,7,8,10,16	4,6,9,11,12,13,14,15,17	4	P01835,P08721,P27213,P52590
895	1,2,3,5,7,8,10,17	4,6,9,11,12,13,14,15,16	8	P01946,P02091,P08937,P12346,P50115,P52590,P97571,P97615
896	1,2,3,5,7,8,11,12	4,6,9,10,13,14,15,16,17	8	P08937,P10715,P27213,P52590,P55314,Q6IG02,Q8CJ52,Q9EQS0
897	1,2,3,5,7,8,11,13	4,6,9,10,12,14,15,16,17	2	P10715,P55314
898	1,2,3,5,7,8,11,14	4,6,9,10,12,13,15,16,17	5	P10715,P19629,P27213,P52590,P55314
899	1,2,3,5,7,8,11,15	4,6,9,10,12,13,14,16,17	5	P10715,P27213,P52590,P55314,Q5I0D1
900	1,2,3,5,7,8,11,16	4,6,9,10,12,13,14,15,17	5	B4F795,P10715,P27213,P55314,Q6IRE4
901	1,2,3,5,7,8,11,17	4,6,9,10,12,13,14,15,16	8	P01946,P02091,P08937,P10715,P50115,P52590,P55314,Q63751
902	1,2,3,5,7,8,12,13	4,6,9,10,11,14,15,16,17	6	O88267,P08937,P10715,P36970,Q8CJ52,Q9Z1F2
903	1,2,3,5,7,8,12,14	4,6,9,10,11,13,15,16,17	9	O88267,P08937,P10715,P19629,P36970,P52590,Q6AYS7,Q9EQS0,Q9Z1F2

904	1,2,3,5,7,8,12,15	4,6,9,10,11,13,14,16,17	5	P08937,P10715,P27213,P52590,P83121
905	1,2,3,5,7,8,12,16	4,6,9,10,11,13,14,15,17	3	O88267,P10715,P83121
906	1,2,3,5,7,8,12,17	4,6,9,10,11,13,14,15,16	14	O88267,P01946,P02091,P08937,P10715,P19223,P23928,P50115,P52590,P55091,P83121,Q561R9,Q63751,Q9QZA6
907	1,2,3,5,7,8,13,14	4,6,9,10,11,12,15,16,17	8	O88267,P00762,P08753,P36970,Q08849,Q5GRG2,Q63317,Q9Z1F2
908	1,2,3,5,7,8,13,15	4,6,9,10,11,12,14,16,17	6	P00762,P01835,P08753,P52590,Q09030,Q63317
909	1,2,3,5,7,8,13,16	4,6,9,10,11,12,14,15,17	4	O88267,P00762,P01835,P08753
910	1,2,3,5,7,8,13,17	4,6,9,10,11,12,14,15,16	13	O88267,P01946,P02091,P08753,P08937,P23928,P50115,P51907,P52590,P53792,P55091,Q6PCU2,Q9Z1F2
911	1,2,3,5,7,8,14,15	4,6,9,10,11,12,13,16,17	5	P10536,P19629,P27213,P52590,Q08849
912	1,2,3,5,7,8,14,16	4,6,9,10,11,12,13,15,17	2	O88267,Q6IRE4
913	1,2,3,5,7,8,14,17	4,6,9,10,11,12,13,15,16	9	O88267,P01946,P02091,P10536,P19629,P50115,P52590,Q561R9,Q6PCU2
914	1,2,3,5,7,8,15,16	4,6,9,10,11,12,13,14,17	5	B4F795,P00762,P01835,P10715,P83121
915	1,2,3,5,7,8,15,17	4,6,9,10,11,12,13,14,16	7	P01946,P02091,P10536,P12346,P50115,P52590,P83121
916	1,2,3,5,7,8,16,17	4,6,9,10,11,12,13,14,15	10	O88267,P01946,P02091,P10715,P10758,P23928,P50115,P50116,P52590,Q6PCU2
917	1,2,3,5,7,9,10,11	4,6,8,12,13,14,15,16,17	5	P27213,P52590,P55314,Q6IRE4,Q9QUL6
918	1,2,3,5,7,9,10,12	4,6,8,11,13,14,15,16,17	3	P27213,P52590,P97840

919	1,2,3,5,7,9,10,13	4,6,8,11,12,14,15,16,17	1	P52590
920	1,2,3,5,7,9,10,14	4,6,8,11,12,13,15,16,17	3	P27213,P52590,Q9QUL6
921	1,2,3,5,7,9,10,15	4,6,8,11,12,13,14,16,17	3	O54728,P27213,P52590
922	1,2,3,5,7,9,10,16	4,6,8,11,12,13,14,15,17	3	P27213,P52590,Q6IRE4
923	1,2,3,5,7,9,10,17	4,6,8,11,12,13,14,15,16	4	P01946,P02091,P52590,Q9QUL6
924	1,2,3,5,7,9,11,12	4,6,8,10,13,14,15,16,17	5	P27213,P52590,P55314,Q498R7,Q6IRE4
925	1,2,3,5,7,9,11,13	4,6,8,10,12,14,15,16,17	3	P52590,P55314,Q6IRE4
926	1,2,3,5,7,9,11,14	4,6,8,10,12,13,15,16,17	5	P27213,P52590,P55314,Q6IRE4,Q9QUL6
927	1,2,3,5,7,9,11,15	4,6,8,10,12,13,14,16,17	6	O54728,P27213,P52590,P55314,Q6IRE4,Q9QUL6
928	1,2,3,5,7,9,11,16	4,6,8,10,12,13,14,15,17	5	P27213,P52590,P55314,Q6IRE4,Q9QUL6
929	1,2,3,5,7,9,11,17	4,6,8,10,12,13,14,15,16	5	P01946,P02091,P52590,P55314,Q9QUL6
930	1,2,3,5,7,9,12,13	4,6,8,10,11,14,15,16,17	2	P52590,Q9Z1F2
931	1,2,3,5,7,9,12,14	4,6,8,10,11,13,15,16,17	3	P27213,P34901,P52590
932	1,2,3,5,7,9,12,15	4,6,8,10,11,13,14,16,17	2	P27213,P52590
933	1,2,3,5,7,9,12,16	4,6,8,10,11,13,14,15,17	3	P17046,P52590,Q6IRE4

934	1,2,3,5,7,9,12,17	4,6,8,10,11,13,14,15,16	4	P01946,P02091,P52590,P55091
935	1,2,3,5,7,9,13,14	4,6,8,10,11,12,15,16,17	3	P52590,P53792,Q6IRE4
936	1,2,3,5,7,9,13,15	4,6,8,10,11,12,14,16,17	3	O54728,P52590,P53792
937	1,2,3,5,7,9,13,16	4,6,8,10,11,12,14,15,17	3	P08010,P52590,Q6IRE4
938	1,2,3,5,7,9,13,17	4,6,8,10,11,12,14,15,16	4	P01946,P02091,P52590,P53792
939	1,2,3,5,7,9,14,15	4,6,8,10,11,12,13,16,17	7	O54728,P0C0A9,P16636,P27213,P52590,P53792,Q9QZK9
940	1,2,3,5,7,9,14,16	4,6,8,10,11,12,13,15,17	4	P0C0A9,P52590,Q6IRE4,Q9QZK9
941	1,2,3,5,7,9,14,17	4,6,8,10,11,12,13,15,16	4	P01946,P02091,P52590,P53792
942	1,2,3,5,7,9,15,16	4,6,8,10,11,12,13,14,17	8	O54728,P02782,P0C0A9,P47727,P52590,Q6AYR9,Q6IRE4,Q9QZK9
943	1,2,3,5,7,9,15,17	4,6,8,10,11,12,13,14,16	7	O54728,P01946,P02091,P0C0A9,P47727,P52590,P53792
944	1,2,3,5,7,9,16,17	4,6,8,10,11,12,13,14,15	5	P01946,P02091,P0C0A9,P47727,P52590
945	1,2,3,5,7,10,11,12	4,6,8,9,13,14,15,16,17	5	P27213,P34901,P52590,P55314,Q6P6Q2
946	1,2,3,5,7,10,11,13	4,6,8,9,12,14,15,16,17	2	P52590,P55314
947	1,2,3,5,7,10,11,14	4,6,8,9,12,13,15,16,17	4	P27213,P52590,P55314,Q9QUL6
948	1,2,3,5,7,10,11,15	4,6,8,9,12,13,14,16,17	3	P27213,P52590,P55314

949	1,2,3,5,7,10,11,16	4,6,8,9,12,13,14,15,17	4	P17046,P27213,P55314,Q6IRE4
950	1,2,3,5,7,10,11,17	4,6,8,9,12,13,14,15,16	5	P01946,P02091,P52590,P55314,Q9QUL6
951	1,2,3,5,7,10,12,13	4,6,8,9,11,14,15,16,17	1	P52590
952	1,2,3,5,7,10,12,14	4,6,8,9,11,13,15,16,17	2	P34901,P52590
953	1,2,3,5,7,10,12,15	4,6,8,9,11,13,14,16,17	3	P00731,P27213,P52590
954	1,2,3,5,7,10,12,16	4,6,8,9,11,13,14,15,17	3	P08721,P17046,P97840
955	1,2,3,5,7,10,12,17	4,6,8,9,11,13,14,15,16	5	P01946,P02091,P52590,P55091,Q10758
956	1,2,3,5,7,10,13,14	4,6,8,9,11,12,15,16,17	0	
957	1,2,3,5,7,10,13,15	4,6,8,9,11,12,14,16,17	3	P01835,P52590,Q09030
958	1,2,3,5,7,10,13,16	4,6,8,9,11,12,14,15,17	1	P01835
959	1,2,3,5,7,10,13,17	4,6,8,9,11,12,14,15,16	3	P01946,P02091,P52590
960	1,2,3,5,7,10,14,15	4,6,8,9,11,12,13,16,17	2	P27213,P52590
961	1,2,3,5,7,10,14,16	4,6,8,9,11,12,13,15,17	3	P08721,Q63532,Q6IRE4
962	1,2,3,5,7,10,14,17	4,6,8,9,11,12,13,15,16	3	P01946,P02091,P52590
963	1,2,3,5,7,10,15,16	4,6,8,9,11,12,13,14,17	4	P01835,P02782,P0C0A9,P52590

964	1,2,3,5,7,10,15,17	4,6,8,9,11,12,13,14,16	4	P01946,P02091,P0C0A9,P52590
965	1,2,3,5,7,10,16,17	4,6,8,9,11,12,13,14,15	5	P01946,P02091,P0C0A9,P10758,P52590
966	1,2,3,5,7,11,12,13	4,6,8,9,10,14,15,16,17	2	P17046,P55314
967	1,2,3,5,7,11,12,14	4,6,8,9,10,13,15,16,17	4	P17046,P27213,P34901,P55314
968	1,2,3,5,7,11,12,15	4,6,8,9,10,13,14,16,17	3	P17046,P27213,P55314
969	1,2,3,5,7,11,12,16	4,6,8,9,10,13,14,15,17	3	P17046,P55314,Q6IRE4
970	1,2,3,5,7,11,12,17	4,6,8,9,10,13,14,15,16	6	P01946,P02091,P17046,P52590,P55091,P55314
971	1,2,3,5,7,11,13,14	4,6,8,9,10,12,15,16,17	2	P55314,Q6IRE4
972	1,2,3,5,7,11,13,15	4,6,8,9,10,12,14,16,17	2	P55314,Q09030
973	1,2,3,5,7,11,13,16	4,6,8,9,10,12,14,15,17	3	P17046,P55314,Q6IRE4
974	1,2,3,5,7,11,13,17	4,6,8,9,10,12,14,15,16	5	P01946,P02091,P17046,P52590,P55314
975	1,2,3,5,7,11,14,15	4,6,8,9,10,12,13,16,17	4	P16636,P27213,P55314,Q6IRE4
976	1,2,3,5,7,11,14,16	4,6,8,9,10,12,13,15,17	4	P17046,P55314,Q63532,Q6IRE4
977	1,2,3,5,7,11,14,17	4,6,8,9,10,12,13,15,16	5	P01946,P02091,P52590,P55314,Q9QUL6
978	1,2,3,5,7,11,15,16	4,6,8,9,10,12,13,14,17	4	O54728,P17046,P55314,Q6IRE4

979	1,2,3,5,7,11,15,17	4,6,8,9,10,12,13,14,16	4	P01946,P02091,P52590,P55314
980	1,2,3,5,7,11,16,17	4,6,8,9,10,12,13,14,15	7	P01946,P02091,P10758,P17046,P52590,P55314,Q6IRE4
981	1,2,3,5,7,12,13,14	4,6,8,9,10,11,15,16,17	2	A2RUV9,Q9Z1F2
982	1,2,3,5,7,12,13,15	4,6,8,9,10,11,14,16,17	2	P83121,Q09030
983	1,2,3,5,7,12,13,16	4,6,8,9,10,11,14,15,17	2	P17046,Q6IRE4
984	1,2,3,5,7,12,13,17	4,6,8,9,10,11,14,15,16	6	P01946,P02091,P17046,P52590,P55091,Q561R9
985	1,2,3,5,7,12,14,15	4,6,8,9,10,11,13,16,17	3	A2RUV9,P34901,Q8CJD3
986	1,2,3,5,7,12,14,16	4,6,8,9,10,11,13,15,17	4	P17046,P34901,Q63532,Q6IRE4
987	1,2,3,5,7,12,14,17	4,6,8,9,10,11,13,15,16	7	P01946,P02091,P31430,P34901,P52590,P55091,Q561R9
988	1,2,3,5,7,12,15,16	4,6,8,9,10,11,13,14,17	2	P17046,P83121
989	1,2,3,5,7,12,15,17	4,6,8,9,10,11,13,14,16	5	P01946,P02091,P52590,P55091,P83121
990	1,2,3,5,7,12,16,17	4,6,8,9,10,11,13,14,15	7	P01946,P02091,P17046,P52590,P55091,P83121,Q561R9
991	1,2,3,5,7,13,14,15	4,6,8,9,10,11,12,16,17	4	P00762,P16636,P53792,Q09030
992	1,2,3,5,7,13,14,16	4,6,8,9,10,11,12,15,17	2	P00762,Q6IRE4
993	1,2,3,5,7,13,14,17	4,6,8,9,10,11,12,15,16	9	P00714,P01946,P02091,P08753,P52590,P53792,Q561R9,Q6PCU2,Q9QW07

994	1,2,3,5,7,13,15,16	4,6,8,9,10,11,12,14,17	6	O54728,P00762,P01835,P0C0A9,Q09030,Q6IRE4
995	1,2,3,5,7,13,15,17	4,6,8,9,10,11,12,14,16	7	P00714,P01946,P02091,P0C0A9,P52590,P53792,Q09030
996	1,2,3,5,7,13,16,17	4,6,8,9,10,11,12,14,15	9	P00714,P01946,P02091,P0C0A9,P10758,P17046,P52590,Q561R9,Q6PCU2
997	1,2,3,5,7,14,15,16	4,6,8,9,10,11,12,13,17	6	O54728,P0C0A9,P16636,Q63532,Q6IRE4,Q9QZK9
998	1,2,3,5,7,14,15,17	4,6,8,9,10,11,12,13,16	5	P01946,P02091,P02803,P0C0A9,P52590
999	1,2,3,5,7,14,16,17	4,6,8,9,10,11,12,13,15	8	P01946,P02091,P0C0A9,P10758,P52590,Q561R9,Q6PCU2,Q9QW07
1000	1,2,3,5,7,15,16,17	4,6,8,9,10,11,12,13,14	8	P01946,P02091,P02780,P02782,P0C0A9,P47727,P52590,P83121
1001	1,2,3,5,8,9,10,11	4,6,7,12,13,14,15,16,17	5	P08649,P27213,P55314,Q63942,Q9QUL6
1002	1,2,3,5,8,9,10,12	4,6,7,11,13,14,15,16,17	2	P27213,Q6IG02
1003	1,2,3,5,8,9,10,13	4,6,7,11,12,14,15,16,17	1	Q63942
1004	1,2,3,5,8,9,10,14	4,6,7,11,12,13,15,16,17	2	P27213,Q63942
1005	1,2,3,5,8,9,10,15	4,6,7,11,12,13,14,16,17	3	O54728,P27213,Q63942
1006	1,2,3,5,8,9,10,16	4,6,7,11,12,13,14,15,17	2	Q63942,Q9QZK9
1007	1,2,3,5,8,9,10,17	4,6,7,11,12,13,14,15,16	3	P08649,P52590,Q63942
1008	1,2,3,5,8,9,11,12	4,6,7,10,13,14,15,16,17	2	P27213,Q6IG02

1009	1,2,3,5,8,9,11,13	4,6,7,10,12,14,15,16,17	2	P20766,Q63942
1010	1,2,3,5,8,9,11,14	4,6,7,10,12,13,15,16,17	2	P27213,Q63942
1011	1,2,3,5,8,9,11,15	4,6,7,10,12,13,14,16,17	4	O54728,P27213,Q5QE79,Q63942
1012	1,2,3,5,8,9,11,16	4,6,7,10,12,13,14,15,17	5	P27213,P55314,Q63942,Q6IRE4,Q9QZK9
1013	1,2,3,5,8,9,11,17	4,6,7,10,12,13,14,15,16	3	P01946,P08649,Q9QUL6
1014	1,2,3,5,8,9,12,13	4,6,7,10,11,14,15,16,17	2	Q5FVI6,Q9Z1F2
1015	1,2,3,5,8,9,12,14	4,6,7,10,11,13,15,16,17	3	Q5FVI6,Q63942,Q9Z1F2
1016	1,2,3,5,8,9,12,15	4,6,7,10,11,13,14,16,17	4	P27213,P83121,Q5QE79,Q63942
1017	1,2,3,5,8,9,12,16	4,6,7,10,11,13,14,15,17	2	P83121,Q63942
1018	1,2,3,5,8,9,12,17	4,6,7,10,11,13,14,15,16	2	P01946,Q5FVI6
1019	1,2,3,5,8,9,13,14	4,6,7,10,11,12,15,16,17	5	P08753,P20766,Q5FVI6,Q63942,Q9Z1F2
1020	1,2,3,5,8,9,13,15	4,6,7,10,11,12,14,16,17	5	O54728,P08753,Q5FVI6,Q5QE79,Q63942
1021	1,2,3,5,8,9,13,16	4,6,7,10,11,12,14,15,17	2	P08753,Q63942
1022	1,2,3,5,8,9,13,17	4,6,7,10,11,12,14,15,16	8	P01946,P08753,P20766,P51907,P53792,Q5FVI6,Q63942,Q9Z1F2
1023	1,2,3,5,8,9,14,15	4,6,7,10,11,12,13,16,17	6	O54728,P27213,Q5FVI6,Q5QE79,Q63942,Q9QZK9

1024	1,2,3,5,8,9,14,16	4,6,7,10,11,12,13,15,17	2	Q63942,Q9QZK9
1025	1,2,3,5,8,9,14,17	4,6,7,10,11,12,13,15,16	5	P01946,P08753,P47967,Q5FVI6,Q63942
1026	1,2,3,5,8,9,15,16	4,6,7,10,11,12,13,14,17	6	O54728,P47727,P83121,Q5QE79,Q63942,Q9QZK9
1027	1,2,3,5,8,9,15,17	4,6,7,10,11,12,13,14,16	5	P47727,P47967,Q5FVI6,Q5QE79,Q63942
1028	1,2,3,5,8,9,16,17	4,6,7,10,11,12,13,14,15	3	P23593,P47727,Q63942
1029	1,2,3,5,8,10,11,12	4,6,7,9,13,14,15,16,17	7	P00731,P27213,P55314,P62804,Q6IFU8,Q6IG02,Q6P6Q2
1030	1,2,3,5,8,10,11,13	4,6,7,9,12,14,15,16,17	1	P55314
1031	1,2,3,5,8,10,11,14	4,6,7,9,12,13,15,16,17	3	P08649,P27213,P55314
1032	1,2,3,5,8,10,11,15	4,6,7,9,12,13,14,16,17	4	P00731,P08649,P27213,P55314
1033	1,2,3,5,8,10,11,16	4,6,7,9,12,13,14,15,17	2	P08649,P55314
1034	1,2,3,5,8,10,11,17	4,6,7,9,12,13,14,15,16	3	P01946,P08649,P55314
1035	1,2,3,5,8,10,12,13	4,6,7,9,11,14,15,16,17	2	P83121,Q9Z1F2
1036	1,2,3,5,8,10,12,14	4,6,7,9,11,13,15,16,17	1	Q6IG02
1037	1,2,3,5,8,10,12,15	4,6,7,9,11,13,14,16,17	2	P00731,P83121
1038	1,2,3,5,8,10,12,16	4,6,7,9,11,13,14,15,17	3	P08721,P83121,Q6IG02

1039	1,2,3,5,8,10,12,17	4,6,7,9,11,13,14,15,16	4	P01946,P83121,Q10758,Q6IG02
1040	1,2,3,5,8,10,13,14	4,6,7,9,11,12,15,16,17	3	P08753,P09605;P25809,P36970
1041	1,2,3,5,8,10,13,15	4,6,7,9,11,12,14,16,17	4	P01835,P08753,P09605;P25809,P83121
1042	1,2,3,5,8,10,13,16	4,6,7,9,11,12,14,15,17	3	P01835,P08753,P83121
1043	1,2,3,5,8,10,13,17	4,6,7,9,11,12,14,15,16	4	P01946,P08753,P51907,P83121
1044	1,2,3,5,8,10,14,15	4,6,7,9,11,12,13,16,17	1	P09605;P25809
1045	1,2,3,5,8,10,14,16	4,6,7,9,11,12,13,15,17	1	P08721
1046	1,2,3,5,8,10,14,17	4,6,7,9,11,12,13,15,16	1	P01946
1047	1,2,3,5,8,10,15,16	4,6,7,9,11,12,13,14,17	2	P01835,P83121
1048	1,2,3,5,8,10,15,17	4,6,7,9,11,12,13,14,16	1	P83121
1049	1,2,3,5,8,10,16,17	4,6,7,9,11,12,13,14,15	2	P01946,P83121
1050	1,2,3,5,8,11,12,13	4,6,7,9,10,14,15,16,17	1	Q9Z1F2
1051	1,2,3,5,8,11,12,14	4,6,7,9,10,13,15,16,17	4	P62804,Q6IFU8,Q6IG02,Q6P6Q2
1052	1,2,3,5,8,11,12,15	4,6,7,9,10,13,14,16,17	2	P83121,Q5I0D1
1053	1,2,3,5,8,11,12,16	4,6,7,9,10,13,14,15,17	3	P55314,Q6IG02,Q6P6Q2

1054	1,2,3,5,8,11,12,17	4,6,7,9,10,13,14,15,16	2	P01946,Q6IG02
1055	1,2,3,5,8,11,13,14	4,6,7,9,10,12,15,16,17	1	Q9Z1F2
1056	1,2,3,5,8,11,13,15	4,6,7,9,10,12,14,16,17	0	
1057	1,2,3,5,8,11,13,16	4,6,7,9,10,12,14,15,17	2	P55314,Q6IRE4
1058	1,2,3,5,8,11,13,17	4,6,7,9,10,12,14,15,16	1	P01946
1059	1,2,3,5,8,11,14,15	4,6,7,9,10,12,13,16,17	3	P97840,Q5I0D1,Q64194
1060	1,2,3,5,8,11,14,16	4,6,7,9,10,12,13,15,17	3	P55314,Q6IRE4,Q9QZK9
1061	1,2,3,5,8,11,14,17	4,6,7,9,10,12,13,15,16	2	P01946,P97840
1062	1,2,3,5,8,11,15,16	4,6,7,9,10,12,13,14,17	3	P55314,Q5I0D1,Q9QZK9
1063	1,2,3,5,8,11,15,17	4,6,7,9,10,12,13,14,16	2	P01946,P97840
1064	1,2,3,5,8,11,16,17	4,6,7,9,10,12,13,14,15	2	P01946,P55314
1065	1,2,3,5,8,12,13,14	4,6,7,9,10,11,15,16,17	4	P36970,Q5FVI6,Q64093,Q9Z1F2
1066	1,2,3,5,8,12,13,15	4,6,7,9,10,11,14,16,17	3	P83121,Q5FVI6,Q9Z1F2
1067	1,2,3,5,8,12,13,16	4,6,7,9,10,11,14,15,17	2	P83121,Q9Z1F2
1068	1,2,3,5,8,12,13,17	4,6,7,9,10,11,14,15,16	9	P01946,P08753,P51907,P55091,P83121,Q561R9,Q5FVI6,Q6PCU2,Q9Z1F2

1069	1,2,3,5,8,12,14,15	4,6,7,9,10,11,13,16,17	2	P83121,Q5FVI6
1070	1,2,3,5,8,12,14,16	4,6,7,9,10,11,13,15,17	1	P83121
1071	1,2,3,5,8,12,14,17	4,6,7,9,10,11,13,15,16	6	P01946,P31430,P83121,Q561R9,Q5FVI6,Q9Z1F2
1072	1,2,3,5,8,12,15,16	4,6,7,9,10,11,13,14,17	1	P83121
1073	1,2,3,5,8,12,15,17	4,6,7,9,10,11,13,14,16	2	P83121,Q5FVI6
1074	1,2,3,5,8,12,16,17	4,6,7,9,10,11,13,14,15	5	P01946,P83121,Q561R9,Q5XFX0,Q6PCU2
1075	1,2,3,5,8,13,14,15	4,6,7,9,10,11,12,16,17	7	P06757,P08753,P09605;P25809,Q08849,Q5FVI6,Q64093,Q9Z1F2
1076	1,2,3,5,8,13,14,16	4,6,7,9,10,11,12,15,17	4	P08753,Q5FVI6,Q6PCU2,Q9Z1F2
1077	1,2,3,5,8,13,14,17	4,6,7,9,10,11,12,15,16	11	P01946,P08753,P10536,P51907,Q08849,Q561R9,Q5FVI6,Q64093,Q6PCU2,Q9QW07,Q9Z1F2
1078	1,2,3,5,8,13,15,16	4,6,7,9,10,11,12,14,17	3	P01835,P08753,P83121
1079	1,2,3,5,8,13,15,17	4,6,7,9,10,11,12,14,16	7	P00714,P01946,P08753,P10536,P83121,Q5FVI6,Q6PCU2
1080	1,2,3,5,8,13,16,17	4,6,7,9,10,11,12,14,15	6	P01946,P08753,P51907,P83121,Q5FVI6,Q6PCU2
1081	1,2,3,5,8,14,15,16	4,6,7,9,10,11,12,13,17	4	P08753,P36374,P83121,Q9QZK9
1082	1,2,3,5,8,14,15,17	4,6,7,9,10,11,12,13,16	8	P08753,P10536,P47967,P83121,P97840,Q08849,Q5FVI6,Q6PCU2
1083	1,2,3,5,8,14,16,17	4,6,7,9,10,11,12,13,15	6	P01946,P08753,Q561R9,Q5FVI6,Q6PCU2,Q9QW07

1084	1,2,3,5,8,15,16,17	4,6,7,9,10,11,12,13,14	6	P08753,P0C0A9,P47727,P83121,Q5XFX0,Q6PCU2
1085	1,2,3,5,9,10,11,12	4,6,7,8,13,14,15,16,17	7	P11598,P27213,P55314,Q63661,Q6IFU8,Q6IG02,Q6P6Q2
1086	1,2,3,5,9,10,11,13	4,6,7,8,12,14,15,16,17	5	P11598,P55314,Q4G075,Q63661,Q99041
1087	1,2,3,5,9,10,11,14	4,6,7,8,12,13,15,16,17	7	P11598,P27213,P55314,Q4G075,Q63661,Q99041,Q9QUL6
1088	1,2,3,5,9,10,11,15	4,6,7,8,12,13,14,16,17	11	O54728,P06761,P11598,P22282,P27213,P55314,Q62902,Q63661,Q99041,Q9QUL6,Q9QZK9
1089	1,2,3,5,9,10,11,16	4,6,7,8,12,13,14,15,17	10	P06761,P11598,P22282,P27213,P55314,Q63661,Q6IRE4,Q99041,Q9QUL6,Q9QZK9
1090	1,2,3,5,9,10,11,17	4,6,7,8,12,13,14,15,16	4	P11598,P55314,Q62902,Q9QUL6
1091	1,2,3,5,9,10,12,13	4,6,7,8,11,14,15,16,17	1	P07150
1092	1,2,3,5,9,10,12,14	4,6,7,8,11,13,15,16,17	1	P34901
1093	1,2,3,5,9,10,12,15	4,6,7,8,11,13,14,16,17	3	P07150,P11598,P27213
1094	1,2,3,5,9,10,12,16	4,6,7,8,11,13,14,15,17	2	P11598,Q9WUW8
1095	1,2,3,5,9,10,12,17	4,6,7,8,11,13,14,15,16	0	
1096	1,2,3,5,9,10,13,14	4,6,7,8,11,12,15,16,17	2	Q4G075,Q99041
1097	1,2,3,5,9,10,13,15	4,6,7,8,11,12,14,16,17	5	O54728,P07150,P11598,Q4G075,Q99041
1098	1,2,3,5,9,10,13,16	4,6,7,8,11,12,14,15,17	3	P11598,Q4G075,Q99041

1099	1,2,3,5,9,10,13,17	4,6,7,8,11,12,14,15,16	1	P01946
1100	1,2,3,5,9,10,14,15	4,6,7,8,11,12,13,16,17	11	O54728,P0C0A9,P11598,P22282,P22283,P27213,Q4G075,Q62902,Q63942,Q99041,Q9QZK9
1101	1,2,3,5,9,10,14,16	4,6,7,8,11,12,13,15,17	8	P06761,P11598,P22282,P22283,Q4G075,Q63942,Q99041,Q9QZK9
1102	1,2,3,5,9,10,14,17	4,6,7,8,11,12,13,15,16	3	Q62902,Q99041,Q9QUL6
1103	1,2,3,5,9,10,15,16	4,6,7,8,11,12,13,14,17	19	O54728,P02780,P02782,P06761,P07647,P0C0A9,P11598,P22282,P22283,P30120,P36374,P47727,Q4G075,Q62902,Q63942,Q6AYR9,Q99041,Q9JHB9,Q9QZK9
1104	1,2,3,5,9,10,15,17	4,6,7,8,11,12,13,14,16	9	P02780,P06761,P0C0A9,P11598,P22282,P22283,P47727,Q62902,Q8CJ52
1105	1,2,3,5,9,10,16,17	4,6,7,8,11,12,13,14,15	8	P02780,P06761,P0C0A9,P11598,P22282,P22283,P47727,Q62902
1106	1,2,3,5,9,11,12,13	4,6,7,8,10,14,15,16,17	0	
1107	1,2,3,5,9,11,12,14	4,6,7,8,10,13,15,16,17	3	P27213,P34901,Q6IFU8
1108	1,2,3,5,9,11,12,15	4,6,7,8,10,13,14,16,17	3	P11598,P12020,P27213
1109	1,2,3,5,9,11,12,16	4,6,7,8,10,13,14,15,17	4	P11598,P12020,P55314,Q6IRE4
1110	1,2,3,5,9,11,12,17	4,6,7,8,10,13,14,15,16	2	P01946,P12020
1111	1,2,3,5,9,11,13,14	4,6,7,8,10,12,15,16,17	3	Q4G075,Q6IRE4,Q99041
1112	1,2,3,5,9,11,13,15	4,6,7,8,10,12,14,16,17	4	O54728,P11598,Q4G075,Q99041
1113	1,2,3,5,9,11,13,16	4,6,7,8,10,12,14,15,17	6	P11598,P55314,Q4G075,Q6IRE4,Q99041,Q9QZK9

1114	1,2,3,5,9,11,13,17	4,6,7,8,10,12,14,15,16	1	P01946
1115	1,2,3,5,9,11,14,15	4,6,7,8,10,12,13,16,17	6	O54728,P11598,P27213,Q4G075,Q99041,Q9QZK9
1116	1,2,3,5,9,11,14,16	4,6,7,8,10,12,13,15,17	8	P06761,P11598,P55314,Q4G075,Q6IRE4,Q99041,Q99MH3,Q9QZK9
1117	1,2,3,5,9,11,14,17	4,6,7,8,10,12,13,15,16	2	P01946,Q9QUL6
1118	1,2,3,5,9,11,15,16	4,6,7,8,10,12,13,14,17	15	O54728,P02782,P06761,P06911,P11598,P12020,P22282,P22283,P55314,Q4G075,Q62902,Q6AYR9,Q6IRE4,Q99041,Q9QZK9
1119	1,2,3,5,9,11,15,17	4,6,7,8,10,12,13,14,16	5	P06911,P11598,P12020,Q62902,Q9QUL6
1120	1,2,3,5,9,11,16,17	4,6,7,8,10,12,13,14,15	7	P01946,P06761,P11598,P12020,P55314,Q6IRE4,Q9QUL6
1121	1,2,3,5,9,12,13,14	4,6,7,8,10,11,15,16,17	4	P00714,P07150,Q5FVI6,Q9Z1F2
1122	1,2,3,5,9,12,13,15	4,6,7,8,10,11,14,16,17	2	P00714,P07150
1123	1,2,3,5,9,12,13,16	4,6,7,8,10,11,14,15,17	4	P00714,P07150,P11598,P22006
1124	1,2,3,5,9,12,13,17	4,6,7,8,10,11,14,15,16	4	P00714,P01946,P35280,Q5FVI6
1125	1,2,3,5,9,12,14,15	4,6,7,8,10,11,13,16,17	2	P07150,P34901
1126	1,2,3,5,9,12,14,16	4,6,7,8,10,11,13,15,17	3	P11598,P34901,Q6IRE4
1127	1,2,3,5,9,12,14,17	4,6,7,8,10,11,13,15,16	4	P00714,P34901,Q561R9,Q5FVI6
1128	1,2,3,5,9,12,15,16	4,6,7,8,10,11,13,14,17	9	O54728,P00714,P07150,P11598,P12020,P22282,P22283,P47727,P83121

1129	1,2,3,5,9,12,15,17	4,6,7,8,10,11,13,14,16	5	P00714,P12020,P47727,P83121,Q5FVI6
1130	1,2,3,5,9,12,16,17	4,6,7,8,10,11,13,14,15	6	P00714,P11598,P12020,P22006,P35280,P47727
1131	1,2,3,5,9,13,14,15	4,6,7,8,10,11,12,16,17	10	O54728,P00714,P07150,P0C0A9,P53792,P60905,Q4G075,Q5FVI6,Q99041,Q9QZK9
1132	1,2,3,5,9,13,14,16	4,6,7,8,10,11,12,15,17	10	P00714,P07150,P11598,P14669,Q4G075,Q5FVI6,Q63942,Q6IRE4,Q99041,Q9QZK9
1133	1,2,3,5,9,13,14,17	4,6,7,8,10,11,12,15,16	7	P00714,P01946,P08753,P35280,P53792,Q5FVI6,Q9QW07
1134	1,2,3,5,9,13,15,16	4,6,7,8,10,11,12,14,17	14	O54728,P00714,P07150,P0C0A9,P11598,P22282,P22283,P47727,P60905,Q4G075,Q63942,Q6IRE4,Q99041,Q9QZK9
1135	1,2,3,5,9,13,15,17	4,6,7,8,10,11,12,14,16	6	P00714,P08753,P0C0A9,P47727,P53792,Q5FVI6
1136	1,2,3,5,9,13,16,17	4,6,7,8,10,11,12,14,15	9	P00714,P01946,P08753,P0C0A9,P11598,P22006,P35280,P47727,Q5FVI6
1137	1,2,3,5,9,14,15,16	4,6,7,8,10,11,12,13,17	22	O54728,P00714,P02780,P02781,P02782,P06761,P07150,P07647,P0C0A9,P11598,P22282,P22283,P36374,P60905,Q4G075,Q62902,Q63942,Q6AYR9,Q6IRE4,Q99041,Q9JHB9,Q9QZK9
1138	1,2,3,5,9,14,15,17	4,6,7,8,10,11,12,13,16	9	P00714,P02780,P0C0A9,P22283,P47967,P60905,Q5FVI6,Q62902,Q8CJ52
1139	1,2,3,5,9,14,16,17	4,6,7,8,10,11,12,13,15	5	P00714,P0C0A9,P11598,P22283,Q5FVI6
1140	1,2,3,5,9,15,16,17	4,6,7,8,10,11,12,13,14	23	O54728,P00714,P02780,P02781,P02782,P04905,P06761,P06911,P07647,P0C0A9,P11598,P12020,P22282,P22283,P23593,P24368,P30120,P47727,P60905,Q62902,Q6AYR9,Q8CJ52,Q9JHB9
1141	1,2,3,5,10,11,12,13	4,6,7,8,9,14,15,16,17	1	P55314
1142	1,2,3,5,10,11,12,14	4,6,7,8,9,13,15,16,17	5	P34901,P55314,Q6IFU8,Q6IG02,Q6P6Q2
1143	1,2,3,5,10,11,12,15	4,6,7,8,9,13,14,16,17	4	P00731,P27213,P55314,Q63617
1144	1,2,3,5,10,11,12,16	4,6,7,8,9,13,14,15,17	5	P11598,P55314,Q6IFU8,Q6IG02,Q6P6Q2
1145	1,2,3,5,10,11,12,17	4,6,7,8,9,13,14,15,16	5	P01946,P55314,Q10758,Q6IFU8,Q6IG02

1146	1,2,3,5,10,<br>11,13,14	4,6,7,8,9,1<br>2,15,16,17	3	P09605;P25809,P55314,Q99041
1147	1,2,3,5,10,<br>11,13,15	4,6,7,8,9,1<br>2,14,16,17	2	P55314,Q99041
1148	1,2,3,5,10,<br>11,13,16	4,6,7,8,9,1<br>2,14,15,17	4	P11598,P55314,Q6IRE4,Q99041
1149	1,2,3,5,10,<br>11,13,17	4,6,7,8,9,1<br>2,14,15,16	2	P01946,P55314
1150	1,2,3,5,10,<br>11,14,15	4,6,7,8,9,1<br>2,13,16,17	7	P09605;P25809,P11598,P22282,P27213,P55314,Q62902,Q99041
1151	1,2,3,5,10,<br>11,14,16	4,6,7,8,9,1<br>2,13,15,17	7	P06761,P11598,P22282,P55314,Q6IRE4,Q99041,Q9QZK9
1152	1,2,3,5,10,<br>11,14,17	4,6,7,8,9,1<br>2,13,15,16	3	P01946,P55314,Q9QUL6
1153	1,2,3,5,10,<br>11,15,16	4,6,7,8,9,1<br>2,13,14,17	11	P02780,P02782,P06761,P11598,P22282,P22283,P55314,Q62902,Q6AYR9,Q99041,Q9QZK9
1154	1,2,3,5,10,<br>11,15,17	4,6,7,8,9,1<br>2,13,14,16	2	P55314,Q62902
1155	1,2,3,5,10,<br>11,16,17	4,6,7,8,9,1<br>2,13,14,15	5	P01946,P06761,P11598,P55314,Q62902
1156	1,2,3,5,10,<br>12,13,14	4,6,7,8,9,1<br>1,15,16,17	0	
1157	1,2,3,5,10,<br>12,13,15	4,6,7,8,9,1<br>1,14,16,17	2	P83121,Q63617
1158	1,2,3,5,10,<br>12,13,16	4,6,7,8,9,1<br>1,14,15,17	1	P83121
1159	1,2,3,5,10,<br>12,13,17	4,6,7,8,9,1<br>1,14,15,16	2	P01946,P83121
1160	1,2,3,5,10,<br>12,14,15	4,6,7,8,9,1<br>1,13,16,17	2	P34901,Q63617
1161	1,2,3,5,10,<br>12,14,16	4,6,7,8,9,1<br>1,13,15,17	2	P11598,P34901
1162	1,2,3,5,10,<br>12,14,17	4,6,7,8,9,1<br>1,13,15,16	5	P01946,P31430,P34901,Q10758,Q561R9
1163	1,2,3,5,10,<br>12,15,16	4,6,7,8,9,1<br>1,13,14,17	6	P11598,P22282,P22283,P83121,Q62902,Q63617
1164	1,2,3,5,10,<br>12,15,17	4,6,7,8,9,1<br>1,13,14,16	3	P83121,Q62902,Q63617
1165	1,2,3,5,10,<br>12,16,17	4,6,7,8,9,1<br>1,13,14,15	4	P01946,P83121,Q10758,Q63617
1166	1,2,3,5,10,<br>13,14,15	4,6,7,8,9,1<br>1,12,16,17	2	P09605;P25809,Q99041
1167	1,2,3,5,10,<br>13,14,16	4,6,7,8,9,1<br>1,12,15,17	3	P09605;P25809,Q4G075,Q99041
1168	1,2,3,5,10,<br>13,14,17	4,6,7,8,9,1<br>1,12,15,16	3	P01946,P08753,Q9QW07

1169	1,2,3,5,10,13,15,16	4,6,7,8,9,11,12,14,17	9	P01835,P02780,P0C0A9,P11598,P22282,P22283,P83121,Q63617,Q99041
1170	1,2,3,5,10,13,15,17	4,6,7,8,9,11,12,14,16	4	P08753,P0C0A9,P83121,Q63617
1171	1,2,3,5,10,13,16,17	4,6,7,8,9,11,12,14,15	3	P01946,P0C0A9,P83121
1172	1,2,3,5,10,14,15,16	4,6,7,8,9,11,12,13,17	14	P02780,P02782,P06761,P07647,P09605;P25809,P0C0A9,P11598,P22282,P22283,P36374,Q62902,Q99041,Q9JHB9,Q9QZK9
1173	1,2,3,5,10,14,15,17	4,6,7,8,9,11,12,13,16	4	P02780,P0C0A9,P22283,Q62902
1174	1,2,3,5,10,14,16,17	4,6,7,8,9,11,12,13,15	5	P02780,P0C0A9,P11598,P22283,Q62902
1175	1,2,3,5,10,15,16,17	4,6,7,8,9,11,12,13,14	17	P02780,P02781,P02782,P06761,P07647,P0C0A9,P11598,P22282,P22283,P24368,P30120,P47727,P83121,Q62902,Q63617,Q6AYR9,Q9JHB9
1176	1,2,3,5,11,12,13,14	4,6,7,8,9,10,15,16,17	0	
1177	1,2,3,5,11,12,13,15	4,6,7,8,9,10,14,16,17	1	Q6P6R2
1178	1,2,3,5,11,12,13,16	4,6,7,8,9,10,14,15,17	3	P17046,P55314,Q6IRE4
1179	1,2,3,5,11,12,13,17	4,6,7,8,9,10,14,15,16	2	P01946,P17046
1180	1,2,3,5,11,12,14,15	4,6,7,8,9,10,13,16,17	1	P34901
1181	1,2,3,5,11,12,14,16	4,6,7,8,9,10,13,15,17	6	P11598,P17046,P34901,P55314,Q6IFU8,Q6IRE4
1182	1,2,3,5,11,12,14,17	4,6,7,8,9,10,13,15,16	3	P01946,P31430,P34901
1183	1,2,3,5,11,12,15,16	4,6,7,8,9,10,13,14,17	5	P11598,P12020,P17046,P55314,P83121
1184	1,2,3,5,11,12,15,17	4,6,7,8,9,10,13,14,16	3	P01946,P12020,P83121
1185	1,2,3,5,11,12,16,17	4,6,7,8,9,10,13,14,15	4	P01946,P12020,P17046,P55314
1186	1,2,3,5,11,13,14,15	4,6,7,8,9,10,12,16,17	2	Q6P6R2,Q99041
1187	1,2,3,5,11,13,14,16	4,6,7,8,9,10,12,15,17	3	P55314,Q6IRE4,Q99041
1188	1,2,3,5,11,13,14,17	4,6,7,8,9,10,12,15,16	3	P01946,Q5FVI6,Q9QW07
1189	1,2,3,5,11,13,15,16	4,6,7,8,9,10,12,14,17	5	O54728,P11598,P55314,Q6IRE4,Q99041
1190	1,2,3,5,11,13,15,17	4,6,7,8,9,10,12,14,16	2	P00714,P01946
1191	1,2,3,5,11,13,16,17	4,6,7,8,9,10,12,14,15	4	P00714,P01946,P17046,P55314

1192	1,2,3,5,11,14,15,16	4,6,7,8,9,10,12,13,17	11	O54728,P06761,P0C0A9,P11598,P22282,P22283,P55314,Q6AYR9,Q6IRE4,Q99041,Q9QZK9
1193	1,2,3,5,11,14,15,17	4,6,7,8,9,10,12,13,16	2	P47967,P97840
1194	1,2,3,5,11,14,16,17	4,6,7,8,9,10,12,13,15	3	P01946,P11598,P55314
1195	1,2,3,5,11,15,16,17	4,6,7,8,9,10,12,13,14	12	P02780,P02782,P06761,P06911,P0C0A9,P11598,P12020,P22282,P22283,P55314,Q62902,Q6AYR9
1196	1,2,3,5,12,13,14,15	4,6,7,8,9,10,11,16,17	5	A2RUV9,P00714,P07150,P83121,Q5FVI6
1197	1,2,3,5,12,13,14,16	4,6,7,8,9,10,11,15,17	2	P00714,Q5FVI6
1198	1,2,3,5,12,13,14,17	4,6,7,8,9,10,11,15,16	7	P00714,P01946,P31430,Q561R9,Q5FVI6,Q9QW07,Q9Z1F2
1199	1,2,3,5,12,13,15,16	4,6,7,8,9,10,11,14,17	3	P00714,P83121,Q63617
1200	1,2,3,5,12,13,15,17	4,6,7,8,9,10,11,14,16	5	P00714,P01946,P83121,Q5FVI6,Q63617
1201	1,2,3,5,12,13,16,17	4,6,7,8,9,10,11,14,15	8	P00714,P01946,P17046,P22006,P35280,P83121,Q561R9,Q5FVI6
1202	1,2,3,5,12,14,15,16	4,6,7,8,9,10,11,13,17	6	P00714,P11598,P22282,P22283,P34901,P83121
1203	1,2,3,5,12,14,15,17	4,6,7,8,9,10,11,13,16	3	P00714,P83121,Q5FVI6
1204	1,2,3,5,12,14,16,17	4,6,7,8,9,10,11,13,15	8	P00714,P01946,P31430,P34901,P83121,Q561R9,Q5FVI6,Q9QW07
1205	1,2,3,5,12,15,16,17	4,6,7,8,9,10,11,13,14	6	P00714,P12020,P47727,P83121,Q5XFX0,Q63617
1206	1,2,3,5,13,14,15,16	4,6,7,8,9,10,11,12,17	10	O54728,P00714,P08753,P0C0A9,P22282,P22283,P60905,Q4G075,Q99041,Q9QZK9
1207	1,2,3,5,13,14,15,17	4,6,7,8,9,10,11,12,16	9	P00714,P01946,P08753,P0C0A9,P47967,P60905,P63095,Q5FVI6,Q9QW07
1208	1,2,3,5,13,14,16,17	4,6,7,8,9,10,11,12,15	9	P00714,P01946,P08753,P0C0A9,P35280,Q561R9,Q5FVI6,Q6PCU2,Q9QW07
1209	1,2,3,5,13,15,16,17	4,6,7,8,9,10,11,12,14	9	P00714,P01946,P02780,P08753,P0C0A9,P22283,P47727,P60905,P83121
1210	1,2,3,5,14,15,16,17	4,6,7,8,9,10,11,12,13	23	P00714,P02780,P02781,P02782,P02803,P06761,P07647,P0C0A9,P11598,P22282,P22283,P24368,P30120,P36374,P47727,P60905,P83121,Q5FVI6,Q62902,Q6AYR9,Q8CFN2,Q9JHB9,Q9QW07
1211	1,2,3,6,7,8,9,10	4,5,11,12,13,14,15,16,17	10	O55006,P02631,P16086,P19629,P27213,P31044,P35280,P68370,Q63942,Q91ZS3
1212	1,2,3,6,7,8,9,11	4,5,10,12,13,14,15,16,17	16	O54715,P02631,P05371,P10715,P14669,P15399,P16086,P19629,P27213,P31044,P35280,P68370,Q30KJ2,Q63493,Q91ZS3,Q9JI85

1213	1,2,3,6,7,8,9,12	4,5,10,11,13,14,15,16,17	14	P02631,P02782,P08723,P10715,P13432,P16086,P19629,P27213,P30120,P31044,P68370,Q9EQS0,Q9JI85,Q9Z1F2
1214	1,2,3,6,7,8,9,13	4,5,10,11,12,14,15,16,17	13	P02631,P06911,P12020,P16086,P19629,P20766,P29975,P31044,P51907,P53792,P68370,Q5GRG2,Q9Z1F2
1215	1,2,3,6,7,8,9,14	4,5,10,11,12,13,15,16,17	10	P02631,P05371,P12020,P16086,P19629,P27213,P31044,P68370,Q63942,Q9Z1F2
1216	1,2,3,6,7,8,9,15	4,5,10,11,12,13,14,16,17	11	O55006,P02631,P10715,P16086,P19629,P27213,P31044,P35280,P68370,Q5QE79,Q63942
1217	1,2,3,6,7,8,9,16	4,5,10,11,12,13,14,15,17	8	P02631,P05371,P10715,P16086,P19629,P31044,P68370,Q63942
1218	1,2,3,6,7,8,9,17	4,5,10,11,12,13,14,15,16	17	P01946,P02091,P02631,P02770,P09605;P25809,P10715,P12346,P16086,P17475,P19629,P31044,P50115,P51907,P55091,P97580,Q30KJ2,Q6P6R2
1219	1,2,3,6,7,8,10,11	4,5,9,12,13,14,15,16,17	20	O54715,P02631,P05371,P10715,P12020,P14669,P15399,P16086,P19629,P27213,P31044,P35280,P55314,P68370,P97580,P97615,Q30KJ2,Q63493,Q9EQS0,Q9JI85
1220	1,2,3,6,7,8,10,12	4,5,9,11,13,14,15,16,17	19	O35783,P01041,P02631,P02782,P08723,P10715,P12020,P16086,P19629,P27213,P30120,P31044,P35280,P68370,P97615,P97840,Q8CJ52,Q9EQS0,Q9JI85
1221	1,2,3,6,7,8,10,13	4,5,9,11,12,14,15,16,17	10	P02631,P06911,P12020,P16086,P19629,P31044,P51907,P68370,Q5GRG2,Q9Z1F2
1222	1,2,3,6,7,8,10,14	4,5,9,11,12,13,15,16,17	12	P02631,P12020,P16086,P19629,P27213,P31044,P35280,P68370,P97615,Q30KJ2,Q5GRG2,Q9EQS0
1223	1,2,3,6,7,8,10,15	4,5,9,11,12,13,14,16,17	8	P02631,P16086,P19629,P27213,P31044,P35280,P68370,P97615
1224	1,2,3,6,7,8,10,16	4,5,9,11,12,13,14,15,17	7	P02631,P05371,P08721,P16086,P19629,P31044,P68370
1225	1,2,3,6,7,8,10,17	4,5,9,11,12,13,14,15,16	19	P01946,P02091,P02631,P02770,P12020,P12346,P15399,P16086,P17475,P19629,P31044,P50115,P51907,P55091,P68370,P97580,P97615,Q30KJ2,Q6P6R2
1226	1,2,3,6,7,8,11,12	4,5,9,10,13,14,15,16,17	30	O54715,P02631,P02781,P02782,P05371,P07647,P08723,P10715,P13432,P14669,P15399,P16086,P19629,P22282,P22283,P27213,P30120,P31044,P35280,P68370,P97580,Q30KJ2,Q498R7,Q63493,Q63751,Q8CJ52,Q9EQS0,Q9JHB9,Q9JI85,Q9Z1F2
1227	1,2,3,6,7,8,11,13	4,5,9,10,12,14,15,16,17	21	P02631,P05371,P06911,P10715,P12020,P14669,P15399,P16086,P19629,P20766,P22282,P22283,P31044,P68370,Q30KJ2,Q5GRG2,Q5XI22,Q62902,Q63493,Q9JI85,Q9Z1F2

1228	1,2,3,6,7,8,11,14	4,5,9,10,12,13,15,16,17	19	P02631,P05371,P10715,P12020,P14669,P15399,P16086,P19629,P27213,P31044,P35280,P47727,P68370,P97580,Q30KJ2,Q5GRG2,Q63493,Q9EQS0,Q9JI85
1229	1,2,3,6,7,8,11,15	4,5,9,10,12,13,14,16,17	13	P02631,P05371,P10715,P14669,P15399,P16086,P19629,P27213,P31044,P35280,P68370,Q30KJ2,Q9JI85
1230	1,2,3,6,7,8,11,16	4,5,9,10,12,13,14,15,17	14	O54715,P02631,P05371,P10715,P14669,P15399,P16086,P19629,P31044,P55314,P68370,P97580,Q30KJ2,Q9JI85
1231	1,2,3,6,7,8,11,17	4,5,9,10,12,13,14,15,16	22	P01946,P02091,P02631,P02770,P05371,P07150,P10715,P12346,P14669,P15399,P16086,P17475,P19629,P31044,P50115,P55091,P68370,P97580,Q30KJ2,Q63493,Q63751,Q9JI85
1232	1,2,3,6,7,8,12,13	4,5,9,10,11,14,15,16,17	28	O08557,O88267,P02631,P02782,P06761,P06911,P07647,P08723,P08937,P10715,P12020,P13432,P16086,P19629,P22282,P22283,P29975,P30120,P31044,P68370,P70545,Q5GRG2,Q62902,Q6AYR9,Q8CJ52,Q9JHB9,Q9JI85,Q9Z1F2
1233	1,2,3,6,7,8,12,14	4,5,9,10,11,13,15,16,17	24	O08557,O88267,P02631,P02782,P05371,P08723,P10715,P12020,P13432,P16086,P19629,P29975,P30120,P31044,P68370,Q5GRG2,Q63532,Q6AYS7,Q8CJ52,Q923M1,Q9EQS0,Q9JHB9,Q9JI85,Q9Z1F2
1234	1,2,3,6,7,8,12,15	4,5,9,10,11,13,14,16,17	11	O08557,P01041,P02631,P08723,P10715,P16086,P19629,P31044,P35280,P68370,Q9JI85
1235	1,2,3,6,7,8,12,16	4,5,9,10,11,13,14,15,17	13	O88267,P01041,P02631,P05371,P08723,P10715,P16086,P19629,P31044,P68370,Q8CJ52,Q9EQS0,Q9JI85
1236	1,2,3,6,7,8,12,17	4,5,9,10,11,13,14,15,16	36	O88267,P01041,P01946,P02091,P02631,P02770,P02782,P08723,P08937,P09605;P25809,P10715,P12346,P13432,P16086,P17475,P19223,P19629,P22282,P22283,P29975,P30120,P31044,P50115,P55091,P68370,P97580,P98089,Q30KJ2,Q4G075,Q561R9,Q62635,Q63751,Q8CJ52,Q9JI85,Q9QZK9,Q9Z1F2
1237	1,2,3,6,7,8,13,14	4,5,9,10,11,12,15,16,17	15	O08557,O88267,P02631,P06911,P12020,P16086,P19629,P20766,P29975,P31044,P51907,P68370,Q5GRG2,Q62902,Q9Z1F2
1238	1,2,3,6,7,8,13,15	4,5,9,10,11,12,14,16,17	10	P02631,P06911,P12020,P15999,P16086,P19629,P31044,P68370,Q5GRG2,Q9Z1F2
1239	1,2,3,6,7,8,13,16	4,5,9,10,11,12,14,15,17	11	O88267,P02631,P06911,P12020,P16086,P19629,P31044,P51907,P68370,Q5GRG2,Q9Z1F2
1240	1,2,3,6,7,8,13,17	4,5,9,10,11,12,14,15,16	26	O88267,P01946,P02091,P02631,P02770,P06911,P12020,P12346,P16086,P17475,P19629,P20766,P22282,P29975,P31044,P50115,P51907,P55091,P68370,P70545,P97580,Q30KJ2,Q5GRG2,Q63751,Q6PCU2,Q9Z1F2
1241	1,2,3,6,7,8,14,15	4,5,9,10,11,12,13,16,17	10	O08557,P02631,P05371,P12020,P16086,P19629,P31044,P35280,P68370,Q5GRG2
1242	1,2,3,6,7,8,14,16	4,5,9,10,11,12,13,15,17	8	P02631,P05371,P12020,P16086,P19629,P31044,P68370,Q63532

1243	1,2,3,6,7,8,14,17	4,5,9,10,11,12,13,15,16	23	O88267,P01946,P02091,P02631,P02770,P10536,P12020,P12346,P15399,P16086,P17475,P19629,P29975,P31044,P50115,P51907,P55091,P68370,P97580,Q30KJ2,Q561R9,Q6PCU2,Q9Z1F2
1244	1,2,3,6,7,8,15,16	4,5,9,10,11,12,13,14,17	7	P02631,P05371,P10715,P16086,P19629,P31044,P68370
1245	1,2,3,6,7,8,15,17	4,5,9,10,11,12,13,14,16	17	P01041,P01946,P02091,P02631,P02770,P10536,P10715,P12346,P16086,P17475,P19629,P31044,P50115,P55091,P97580,Q30KJ2,Q63751
1246	1,2,3,6,7,8,16,17	4,5,9,10,11,12,13,14,15	20	O88267,P01946,P02091,P02631,P02770,P05371,P09605;P25809,P10715,P10758,P12346,P16086,P17475,P19629,P31044,P50115,P51907,P97580,Q30KJ2,Q6P6R2,Q6PCU2
1247	1,2,3,6,7,9,10,11	4,5,8,12,13,14,15,16,17	9	P15399,P16086,P27213,P35280,P55314,P97580,Q30KJ2,Q91ZS3,Q9QUL6
1248	1,2,3,6,7,9,10,12	4,5,8,11,13,14,15,16,17	6	O35783,P16086,P27213,P47967,P97840,Q91ZS3
1249	1,2,3,6,7,9,10,13	4,5,8,11,12,14,15,16,17	4	P12020,P16086,P97840,Q91ZS3
1250	1,2,3,6,7,9,10,14	4,5,8,11,12,13,15,16,17	5	P02631,P12020,P16086,P27213,Q91ZS3
1251	1,2,3,6,7,9,10,15	4,5,8,11,12,13,14,16,17	6	P02631,P16086,P27213,P35280,P97840,Q91ZS3
1252	1,2,3,6,7,9,10,16	4,5,8,11,12,13,14,15,17	3	P16086,P97840,Q91ZS3
1253	1,2,3,6,7,9,10,17	4,5,8,11,12,13,14,15,16	8	P01946,P02091,P15399,P16086,P97580,Q30KJ2,Q91ZS3,Q9QUL6
1254	1,2,3,6,7,9,11,12	4,5,8,10,13,14,15,16,17	8	P02631,P15399,P16086,P27213,Q30KJ2,Q498R7,Q91ZS3,Q9JI85
1255	1,2,3,6,7,9,11,13	4,5,8,10,12,14,15,16,17	5	P02631,P15399,P16086,Q30KJ2,Q91ZS3
1256	1,2,3,6,7,9,11,14	4,5,8,10,12,13,15,16,17	8	P02631,P05371,P15399,P16086,P27213,Q30KJ2,Q91ZS3,Q9QUL6
1257	1,2,3,6,7,9,11,15	4,5,8,10,12,13,14,16,17	7	P02631,P15399,P16086,P27213,P35280,Q30KJ2,Q91ZS3

1258	1,2,3,6,7,9,11,16	4,5,8,10,12,13,14,15,17	7	P02631,P05371,P15399,P16086,P55314,Q30KJ2,Q91ZS3
1259	1,2,3,6,7,9,11,17	4,5,8,10,12,13,14,15,16	10	P01946,P02091,P02631,P09605;P25809,P15399,P16086,P97580,Q30KJ2,Q91ZS3,Q9QUL6
1260	1,2,3,6,7,9,12,13	4,5,8,10,11,14,15,16,17	3	P16086,P97840,Q9Z1F2
1261	1,2,3,6,7,9,12,14	4,5,8,10,11,13,15,16,17	3	P02631,P16086,Q63532
1262	1,2,3,6,7,9,12,15	4,5,8,10,11,13,14,16,17	2	P02631,P16086
1263	1,2,3,6,7,9,12,16	4,5,8,10,11,13,14,15,17	3	P16086,P97840,Q6AYS4
1264	1,2,3,6,7,9,12,17	4,5,8,10,11,13,14,15,16	8	P01946,P02091,P09605;P25809,P13432,P16086,P55091,P97580,Q30KJ2
1265	1,2,3,6,7,9,13,14	4,5,8,10,11,12,15,16,17	7	P02631,P06911,P12020,P16086,P53792,Q5GRG2,Q9Z1F2
1266	1,2,3,6,7,9,13,15	4,5,8,10,11,12,14,16,17	4	P02631,P16086,P53792,Q6AYS4
1267	1,2,3,6,7,9,13,16	4,5,8,10,11,12,14,15,17	2	P16086,Q6AYS4
1268	1,2,3,6,7,9,13,17	4,5,8,10,11,12,14,15,16	7	P01946,P02091,P09605;P25809,P16086,P51907,P53792,Q30KJ2
1269	1,2,3,6,7,9,14,15	4,5,8,10,11,12,13,16,17	3	P02631,P16086,Q6AYS4
1270	1,2,3,6,7,9,14,16	4,5,8,10,11,12,13,15,17	4	P02631,P16086,Q63532,Q6AYS4
1271	1,2,3,6,7,9,14,17	4,5,8,10,11,12,13,15,16	8	P01946,P02091,P02631,P15399,P16086,P53792,P97580,Q30KJ2
1272	1,2,3,6,7,9,15,16	4,5,8,10,11,12,13,14,17	3	P02631,P16086,Q6AYS4

1273	1,2,3,6,7,9,15,17	4,5,8,10,11,12,13,14,16	6	P01946,P02091,P02631,P16086,P53792,Q30KJ2
1274	1,2,3,6,7,9,16,17	4,5,8,10,11,12,13,14,15	6	P01946,P02091,P09605;P25809,P16086,P97580,Q30KJ2
1275	1,2,3,6,7,10,11,12	4,5,8,9,13,14,15,16,17	13	O35783,P05964,P15399,P16086,P27213,P35280,P47967,P55314,P97580,P97840,Q30KJ2,Q91ZS3,Q9JI85
1276	1,2,3,6,7,10,11,13	4,5,8,9,12,14,15,16,17	8	P12020,P15399,P16086,P55314,P97580,Q30KJ2,Q5GRG2,Q91ZS3
1277	1,2,3,6,7,10,11,14	4,5,8,9,12,13,15,16,17	11	P02631,P12020,P15399,P16086,P27213,P35280,P47727,P55314,P97580,Q30KJ2,Q63532
1278	1,2,3,6,7,10,11,15	4,5,8,9,12,13,14,16,17	9	P02631,P15399,P16086,P27213,P35280,P55314,P97580,Q30KJ2,Q4FZU6
1279	1,2,3,6,7,10,11,16	4,5,8,9,12,13,14,15,17	7	P05371,P15399,P16086,P47967,P55314,P97580,Q30KJ2
1280	1,2,3,6,7,10,11,17	4,5,8,9,12,13,14,15,16	8	P01946,P02091,P15399,P16086,P55314,P97580,Q30KJ2,Q9QUL6
1281	1,2,3,6,7,10,12,13	4,5,8,9,11,14,15,16,17	7	O35783,P06911,P12020,P16086,P47967,P97840,Q5GRG2
1282	1,2,3,6,7,10,12,14	4,5,8,9,11,13,15,16,17	6	A2RUV9,P12020,P15399,P16086,P97840,Q63532
1283	1,2,3,6,7,10,12,15	4,5,8,9,11,13,14,16,17	4	P02631,P16086,P35280,P97840
1284	1,2,3,6,7,10,12,16	4,5,8,9,11,13,14,15,17	4	P16086,P47967,P97840,Q63532
1285	1,2,3,6,7,10,12,17	4,5,8,9,11,13,14,15,16	14	P01041,P01946,P02091,P13432,P15399,P16086,P55091,P97580,P97840,Q10758,Q30KJ2,Q62635,Q99MH3,Q9QZK9
1286	1,2,3,6,7,10,13,14	4,5,8,9,11,12,15,16,17	5	P06911,P12020,P16086,Q5GRG2,Q63532
1287	1,2,3,6,7,10,13,15	4,5,8,9,11,12,14,16,17	4	P12020,P16086,Q4FZU6,Q5GRG2

1288	1,2,3,6,7,10,13,16	4,5,8,9,11,12,14,15,17	5	P12020,P16086,P47967,P97840,Q6AYS4
1289	1,2,3,6,7,10,13,17	4,5,8,9,11,12,14,15,16	8	P01946,P02091,P12020,P15399,P16086,P51907,P97580,Q30KJ2
1290	1,2,3,6,7,10,14,15	4,5,8,9,11,12,13,16,17	6	P02631,P02803,P12020,P16086,P35280,Q63532
1291	1,2,3,6,7,10,14,16	4,5,8,9,11,12,13,15,17	4	P12020,P16086,P97840,Q63532
1292	1,2,3,6,7,10,14,17	4,5,8,9,11,12,13,15,16	7	P01946,P02091,P12020,P15399,P16086,P97580,Q30KJ2
1293	1,2,3,6,7,10,15,16	4,5,8,9,11,12,13,14,17	3	P16086,P97840,Q6AYS4
1294	1,2,3,6,7,10,15,17	4,5,8,9,11,12,13,14,16	6	P01946,P02091,P02803,P16086,P97580,Q30KJ2
1295	1,2,3,6,7,10,16,17	4,5,8,9,11,12,13,14,15	6	P01946,P02091,P10758,P16086,P97580,Q30KJ2
1296	1,2,3,6,7,11,12,13	4,5,8,9,10,14,15,16,17	7	P15399,P16086,P30120,Q30KJ2,Q62902,Q9JHB9,Q9JI85
1297	1,2,3,6,7,11,12,14	4,5,8,9,10,13,15,16,17	10	P02631,P05371,P15399,P16086,P19629,P47727,Q30KJ2,Q63532,Q9JHB9,Q9JI85
1298	1,2,3,6,7,11,12,15	4,5,8,9,10,13,14,16,17	6	P02631,P15399,P16086,P35280,Q30KJ2,Q9JI85
1299	1,2,3,6,7,11,12,16	4,5,8,9,10,13,14,15,17	6	P05371,P15399,P16086,P17046,P55314,Q30KJ2
1300	1,2,3,6,7,11,12,17	4,5,8,9,10,13,14,15,16	14	P01946,P02091,P02631,P09605;P25809,P13432,P15399,P16086,P55091,P97580,Q30KJ2,Q62635,Q63751,Q9JI85,Q9QZK9
1301	1,2,3,6,7,11,13,14	4,5,8,9,10,12,15,16,17	9	P02631,P06911,P12020,P15399,P16086,P47727,Q30KJ2,Q5GRG2,Q63532
1302	1,2,3,6,7,11,13,15	4,5,8,9,10,12,14,16,17	5	P02631,P15399,P16086,Q30KJ2,Q5GRG2

1303	1,2,3,6,7,11,13,16	4,5,8,9,10,12,14,15,17	4	P15399,P16086,P55314,Q30KJ2
1304	1,2,3,6,7,11,13,17	4,5,8,9,10,12,14,15,16	6	P01946,P02091,P15399,P16086,P97580,Q30KJ2
1305	1,2,3,6,7,11,14,15	4,5,8,9,10,12,13,16,17	8	P02631,P05371,P15399,P16086,P19629,P35280,Q30KJ2,Q63532
1306	1,2,3,6,7,11,14,16	4,5,8,9,10,12,13,15,17	7	P02631,P05371,P15399,P16086,P55314,Q30KJ2,Q63532
1307	1,2,3,6,7,11,14,17	4,5,8,9,10,12,13,15,16	8	P01946,P02091,P02631,P15399,P16086,P19629,P97580,Q30KJ2
1308	1,2,3,6,7,11,15,16	4,5,8,9,10,12,13,14,17	5	P02631,P05371,P16086,P55314,Q30KJ2
1309	1,2,3,6,7,11,15,17	4,5,8,9,10,12,13,14,16	7	P01946,P02091,P02631,P15399,P16086,P97580,Q30KJ2
1310	1,2,3,6,7,11,16,17	4,5,8,9,10,12,13,14,15	10	P01946,P02091,P05371,P09605;P25809,P10758,P15399,P16086,P55314,P97580,Q30KJ2
1311	1,2,3,6,7,12,13,14	4,5,8,9,10,11,15,16,17	8	A2RUV9,P06911,P12020,P16086,P29975,Q5GRG2,Q63532,Q9Z1F2
1312	1,2,3,6,7,12,13,15	4,5,8,9,10,11,14,16,17	4	P02631,P16086,Q5GRG2,Q6AYS4
1313	1,2,3,6,7,12,13,16	4,5,8,9,10,11,14,15,17	3	P16086,P97840,Q6AYS4
1314	1,2,3,6,7,12,13,17	4,5,8,9,10,11,14,15,16	11	P01946,P02091,P09605;P25809,P13432,P16086,P55091,Q30KJ2,Q561R9,Q62635,Q9QZK9,Q9Z1F2
1315	1,2,3,6,7,12,14,15	4,5,8,9,10,11,13,16,17	6	A2RUV9,P02631,P16086,P19629,Q63532,Q6AYS4
1316	1,2,3,6,7,12,14,16	4,5,8,9,10,11,13,15,17	4	P02631,P16086,Q63532,Q6AYS4
1317	1,2,3,6,7,12,14,17	4,5,8,9,10,11,13,15,16	14	P01946,P02091,P02631,P13432,P15399,P16086,P19629,P31430,P55091,P97580,P98089,Q30KJ2,Q561R9,Q62635

1318	1,2,3,6,7,12,15,16	4,5,8,9,10,11,13,14,17	3	P02631,P16086,Q6AYS4
1319	1,2,3,6,7,12,15,17	4,5,8,9,10,11,13,14,16	9	P01041,P01946,P02091,P02631,P13432,P16086,P55091,Q30KJ2,Q62635
1320	1,2,3,6,7,12,16,17	4,5,8,9,10,11,13,14,15	9	P01946,P02091,P09605;P25809,P13432,P16086,P55091,P97580,Q30KJ2,Q561R9
1321	1,2,3,6,7,13,14,15	4,5,8,9,10,11,12,16,17	7	P02631,P06911,P12020,P16086,Q09030,Q5GRG2,Q6AYS4
1322	1,2,3,6,7,13,14,16	4,5,8,9,10,11,12,15,17	6	P06911,P12020,P16086,Q5GRG2,Q63532,Q6AYS4
1323	1,2,3,6,7,13,14,17	4,5,8,9,10,11,12,15,16	13	P01946,P02091,P06911,P12020,P16086,P51907,P53792,P97580,Q30KJ2,Q561R9,Q5GRG2,Q9QW07,Q9Z1F2
1324	1,2,3,6,7,13,15,16	4,5,8,9,10,11,12,14,17	2	P16086,Q6AYS4
1325	1,2,3,6,7,13,15,17	4,5,8,9,10,11,12,14,16	7	P00714,P01946,P02091,P02631,P16086,P53792,Q30KJ2
1326	1,2,3,6,7,13,16,17	4,5,8,9,10,11,12,14,15	7	P01946,P02091,P10758,P16086,P51907,Q30KJ2,Q561R9
1327	1,2,3,6,7,14,15,16	4,5,8,9,10,11,12,13,17	5	P02631,P02803,P16086,Q63532,Q6AYS4
1328	1,2,3,6,7,14,15,17	4,5,8,9,10,11,12,13,16	8	P01946,P02091,P02631,P02803,P16086,P19629,P97580,Q30KJ2
1329	1,2,3,6,7,14,16,17	4,5,8,9,10,11,12,13,15	8	P01946,P02091,P10758,P16086,P97580,Q30KJ2,Q561R9,Q63532
1330	1,2,3,6,7,15,16,17	4,5,8,9,10,11,12,13,14	8	P01946,P02091,P02631,P02803,P16086,P97580,Q30KJ2,Q6AYS4
1331	1,2,3,6,8,9,10,11	4,5,7,12,13,14,15,16,17	7	P08649,P27213,P35280,P55314,Q63942,Q91ZS3,Q9QUL6
1332	1,2,3,6,8,9,10,12	4,5,7,11,13,14,15,16,17	3	P02782,P16636,Q91ZS3

1333	1,2,3,6,8,9,10,13	4,5,7,11,12,14,15,16,17	4	P20766,P51907,Q63942,Q91ZS3
1334	1,2,3,6,8,9,10,14	4,5,7,11,12,13,15,16,17	2	P14408,Q63942
1335	1,2,3,6,8,9,10,15	4,5,7,11,12,13,14,16,17	5	P27213,P35280,Q4FZU6,Q5QE79,Q63942
1336	1,2,3,6,8,9,10,16	4,5,7,11,12,13,14,15,17	1	Q63942
1337	1,2,3,6,8,9,10,17	4,5,7,11,12,13,14,15,16	7	P02770,P16636,P17475,P51907,Q63355,Q63942,Q6P6R2
1338	1,2,3,6,8,9,11,12	4,5,7,10,13,14,15,16,17	8	P02782,P08723,P16636,P27213,P30120,Q6IFU8,Q91ZS3,Q9JHB9
1339	1,2,3,6,8,9,11,13	4,5,7,10,12,14,15,16,17	4	P20766,Q63942,Q91ZS3,Q9Z1F2
1340	1,2,3,6,8,9,11,14	4,5,7,10,12,13,15,16,17	2	P27213,Q63942
1341	1,2,3,6,8,9,11,15	4,5,7,10,12,13,14,16,17	4	P27213,P35280,Q5QE79,Q63942
1342	1,2,3,6,8,9,11,16	4,5,7,10,12,13,14,15,17	2	P55314,Q63942
1343	1,2,3,6,8,9,11,17	4,5,7,10,12,13,14,15,16	2	P16636,P17475
1344	1,2,3,6,8,9,12,13	4,5,7,10,11,14,15,16,17	7	P02782,P08723,P20766,P30120,Q06496,Q9JHB9,Q9Z1F2
1345	1,2,3,6,8,9,12,14	4,5,7,10,11,13,15,16,17	4	P02782,P08723,P14408,Q9Z1F2
1346	1,2,3,6,8,9,12,15	4,5,7,10,11,13,14,16,17	1	Q5QE79
1347	1,2,3,6,8,9,12,16	4,5,7,10,11,13,14,15,17	1	Q63942

1348	1,2,3,6,8,9,12,17	4,5,7,10,11,13,14,15,16	7	P02782,P13432,P16636,P17475,P98089,Q62635,Q9Z1F2
1349	1,2,3,6,8,9,13,14	4,5,7,10,11,12,15,16,17	6	P06911,P20766,P51907,Q62687,Q63942,Q9Z1F2
1350	1,2,3,6,8,9,13,15	4,5,7,10,11,12,14,16,17	4	P15999,P20766,Q5QE79,Q63942
1351	1,2,3,6,8,9,13,16	4,5,7,10,11,12,14,15,17	4	P20766,P51907,Q63942,Q9Z1F2
1352	1,2,3,6,8,9,13,17	4,5,7,10,11,12,14,15,16	6	P17475,P20766,P51907,Q63355,Q63942,Q9Z1F2
1353	1,2,3,6,8,9,14,15	4,5,7,10,11,12,13,16,17	2	Q5QE79,Q63942
1354	1,2,3,6,8,9,14,16	4,5,7,10,11,12,13,15,17	1	Q63942
1355	1,2,3,6,8,9,14,17	4,5,7,10,11,12,13,15,16	2	P51907,Q63942
1356	1,2,3,6,8,9,15,16	4,5,7,10,11,12,13,14,17	2	Q5QE79,Q63942
1357	1,2,3,6,8,9,15,17	4,5,7,10,11,12,13,14,16	3	P17475,Q5QE79,Q63942
1358	1,2,3,6,8,9,16,17	4,5,7,10,11,12,13,14,15	4	P23593,P51907,Q63942,Q6P6R2
1359	1,2,3,6,8,10,11,12	4,5,7,9,13,14,15,16,17	14	O35547,P02782,P08723,P16636,P30120,P35280,P55314,P62804,Q00715,Q6IFU8,Q6IG02,Q6IMF3,Q6P6Q2,Q9JHB9
1360	1,2,3,6,8,10,11,13	4,5,7,9,12,14,15,16,17	3	P47727,P55314,Q4FZU6
1361	1,2,3,6,8,10,11,14	4,5,7,9,12,13,15,16,17	3	P35280,P47727,P55314
1362	1,2,3,6,8,10,11,15	4,5,7,9,12,13,14,16,17	4	P27213,P35280,P55314,Q4FZU6

1363	1,2,3,6,8,10,11,16	4,5,7,9,12,13,14,15,17	2	P08649,P55314
1364	1,2,3,6,8,10,11,17	4,5,7,9,12,13,14,15,16	6	P08649,P12346,P14669,P16636,P17475,P55314
1365	1,2,3,6,8,10,12,13	4,5,7,9,11,14,15,16,17	7	P02782,P08723,P16636,P30120,Q8K4G9,Q9JHB9,Q9Z1F2
1366	1,2,3,6,8,10,12,14	4,5,7,9,11,13,15,16,17	4	P02782,P08723,Q10758,Q9Z1F2
1367	1,2,3,6,8,10,12,15	4,5,7,9,11,13,14,16,17	2	P35280,Q4FZU6
1368	1,2,3,6,8,10,12,16	4,5,7,9,11,13,14,15,17	2	P05539,P16636
1369	1,2,3,6,8,10,12,17	4,5,7,9,11,13,14,15,16	6	P02782,P13432,P16636,P17475,Q10758,Q62635
1370	1,2,3,6,8,10,13,14	4,5,7,9,11,12,15,16,17	6	P06911,P12020,P51907,Q5GRG2,Q64093,Q9Z1F2
1371	1,2,3,6,8,10,13,15	4,5,7,9,11,12,14,16,17	4	P15999,Q4FZU6,Q5GRG2,Q6IFV1
1372	1,2,3,6,8,10,13,16	4,5,7,9,11,12,14,15,17	1	P51907
1373	1,2,3,6,8,10,13,17	4,5,7,9,11,12,14,15,16	5	P16636,P17475,P51907,Q63355,Q6IFV1
1374	1,2,3,6,8,10,14,15	4,5,7,9,11,12,13,16,17	1	P35280
1375	1,2,3,6,8,10,14,16	4,5,7,9,11,12,13,15,17	1	P05539
1376	1,2,3,6,8,10,14,17	4,5,7,9,11,12,13,15,16	2	P17475,P51907
1377	1,2,3,6,8,10,15,16	4,5,7,9,11,12,13,14,17	0	

1378	1,2,3,6,8,10,15,17	4,5,7,9,11,12,13,14,16	4	P12346,P17475,Q4FZU6,Q6IFV1
1379	1,2,3,6,8,10,16,17	4,5,7,9,11,12,13,14,15	5	P05539,P16636,P17475,P51907,Q6P6R2
1380	1,2,3,6,8,11,12,13	4,5,7,9,10,14,15,16,17	9	P02781,P02782,P08723,P16636,P30120,P47727,Q5XI22,Q9JHB9,Q9Z1F2
1381	1,2,3,6,8,11,12,14	4,5,7,9,10,13,15,16,17	10	O35547,P02782,P08723,P30120,P47727,P62804,Q00715,Q6IFU8,Q9JHB9,Q9Z1F2
1382	1,2,3,6,8,11,12,15	4,5,7,9,10,13,14,16,17	3	O35547,P02782,P35280
1383	1,2,3,6,8,11,12,16	4,5,7,9,10,13,14,15,17	6	O35547,P02782,P16636,P55314,Q6IFU8,Q6P6Q2
1384	1,2,3,6,8,11,12,17	4,5,7,9,10,13,14,15,16	11	O35547,P02782,P08723,P13432,P16636,P17475,P55091,P62804,Q00715,Q62635,Q9JHB9
1385	1,2,3,6,8,11,13,14	4,5,7,9,10,12,15,16,17	6	P06911,P20766,P47727,Q5GRG2,Q64093,Q9Z1F2
1386	1,2,3,6,8,11,13,15	4,5,7,9,10,12,14,16,17	2	P15999,Q64093
1387	1,2,3,6,8,11,13,16	4,5,7,9,10,12,14,15,17	1	P55314
1388	1,2,3,6,8,11,13,17	4,5,7,9,10,12,14,15,16	4	P16636,P17475,P20766,Q9Z1F2
1389	1,2,3,6,8,11,14,15	4,5,7,9,10,12,13,16,17	3	P35280,P35434,Q64093
1390	1,2,3,6,8,11,14,16	4,5,7,9,10,12,13,15,17	1	P55314
1391	1,2,3,6,8,11,14,17	4,5,7,9,10,12,13,15,16	1	P17475
1392	1,2,3,6,8,11,15,16	4,5,7,9,10,12,13,14,17	1	P55314

1393	1,2,3,6,8,11,15,17	4,5,7,9,10,12,13,14,16	1	P17475
1394	1,2,3,6,8,11,16,17	4,5,7,9,10,12,13,14,15	3	P16636,P17475,P55314
1395	1,2,3,6,8,12,13,14	4,5,7,9,10,11,15,16,17	14	O08557,P02782,P06911,P08723,P29975,P30120,P46720,P51907,Q5GRG2,Q62687,Q63424,Q64093,Q9JHB9,Q9Z1F2
1396	1,2,3,6,8,12,13,15	4,5,7,9,10,11,14,16,17	6	O08557,P02782,P15999,Q64093,Q6IFV1,Q9Z1F2
1397	1,2,3,6,8,12,13,16	4,5,7,9,10,11,14,15,17	2	P02782,Q9Z1F2
1398	1,2,3,6,8,12,13,17	4,5,7,9,10,11,14,15,16	15	P02782,P13432,P16636,P17475,P29975,P51907,P55091,Q561R9,Q62635,Q63355,Q64093,Q6IFV1,Q9JHB9,Q9QZK9,Q9Z1F2
1399	1,2,3,6,8,12,14,15	4,5,7,9,10,11,13,16,17	3	O08557,Q64093,Q9Z1F2
1400	1,2,3,6,8,12,14,16	4,5,7,9,10,11,13,15,17	1	Q9Z1F2
1401	1,2,3,6,8,12,14,17	4,5,7,9,10,11,13,15,16	12	P02782,P13432,P17475,P31430,P51907,P55091,P98089,Q561R9,Q5FVI6,Q62635,Q64093,Q9Z1F2
1402	1,2,3,6,8,12,15,16	4,5,7,9,10,11,13,14,17	1	P83121
1403	1,2,3,6,8,12,15,17	4,5,7,9,10,11,13,14,16	6	P13432,P17475,P83121,P98089,Q62635,Q6IFV1
1404	1,2,3,6,8,12,16,17	4,5,7,9,10,11,13,14,15	7	P13432,P16636,P17475,P98089,Q561R9,Q5XFX0,Q62635
1405	1,2,3,6,8,13,14,15	4,5,7,9,10,11,12,16,17	8	O08557,P06911,P15999,Q5GRG2,Q62687,Q63424,Q64093,Q9Z1F2
1406	1,2,3,6,8,13,14,16	4,5,7,9,10,11,12,15,17	4	P06911,P51907,Q64093,Q9Z1F2
1407	1,2,3,6,8,13,14,17	4,5,7,9,10,11,12,15,16	15	P06911,P08753,P17475,P20766,P29975,P51907,P53790,Q561R9,Q5FVI6,Q62687,Q63424,Q64093,Q6PCU2,Q9QW07,Q9Z1F2

1408	1,2,3,6,8,13,15,16	4,5,7,9,10,11,12,14,17	1	P15999
1409	1,2,3,6,8,13,15,17	4,5,7,9,10,11,12,14,16	6	P08753,P15999,P17475,P51907,Q64093,Q6IFV1
1410	1,2,3,6,8,13,16,17	4,5,7,9,10,11,12,14,15	3	P17475,P51907,Q6PCU2
1411	1,2,3,6,8,14,15,16	4,5,7,9,10,11,12,13,17	0	
1412	1,2,3,6,8,14,15,17	4,5,7,9,10,11,12,13,16	3	P10536,P17475,Q64093
1413	1,2,3,6,8,14,16,17	4,5,7,9,10,11,12,13,15	3	P51907,Q561R9,Q6PCU2
1414	1,2,3,6,8,15,16,17	4,5,7,9,10,11,12,13,14	3	P17475,Q5XFX0,Q6PCU2
1415	1,2,3,6,9,10,11,12	4,5,7,8,13,14,15,16,17	8	P14173,P27213,P55314,Q4FZU6,Q63661,Q6IFU7,Q6IFU8,Q91ZS3
1416	1,2,3,6,9,10,11,13	4,5,7,8,12,14,15,16,17	4	P14173,P55314,Q4FZU6,Q91ZS3
1417	1,2,3,6,9,10,11,14	4,5,7,8,12,13,15,16,17	7	P14173,P27213,P55314,Q63661,Q91ZS3,Q99041,Q9QUL6
1418	1,2,3,6,9,10,11,15	4,5,7,8,12,13,14,16,17	6	P27213,P35280,P55314,Q4FZU6,Q63661,Q91ZS3
1419	1,2,3,6,9,10,11,16	4,5,7,8,12,13,14,15,17	3	P14173,P55314,Q91ZS3
1420	1,2,3,6,9,10,11,17	4,5,7,8,12,13,14,15,16	5	P16636,P55314,Q4FZU6,Q91ZS3,Q9QUL6
1421	1,2,3,6,9,10,12,13	4,5,7,8,11,14,15,16,17	3	P97840,Q6AYS4,Q91ZS3
1422	1,2,3,6,9,10,12,14	4,5,7,8,11,13,15,16,17	0	

1423	1,2,3,6,9,10,12,15	4,5,7,8,11,13,14,16,17	2	Q4FZU6,Q6AYS4
1424	1,2,3,6,9,10,12,16	4,5,7,8,11,13,14,15,17	3	P97840,Q6AYS4,Q9WUW8
1425	1,2,3,6,9,10,12,17	4,5,7,8,11,13,14,15,16	4	P07171,P16636,Q10758,Q63355
1426	1,2,3,6,9,10,13,14	4,5,7,8,11,12,15,16,17	2	Q6AYS4,Q99041
1427	1,2,3,6,9,10,13,15	4,5,7,8,11,12,14,16,17	3	Q4FZU6,Q6AYS4,Q99041
1428	1,2,3,6,9,10,13,16	4,5,7,8,11,12,14,15,17	2	Q6AYS4,Q99041
1429	1,2,3,6,9,10,13,17	4,5,7,8,11,12,14,15,16	2	P51907,Q63355
1430	1,2,3,6,9,10,14,15	4,5,7,8,11,12,13,16,17	3	Q4FZU6,Q6AYS4,Q99041
1431	1,2,3,6,9,10,14,16	4,5,7,8,11,12,13,15,17	3	Q63942,Q6AYS4,Q99041
1432	1,2,3,6,9,10,14,17	4,5,7,8,11,12,13,15,16	0	
1433	1,2,3,6,9,10,15,16	4,5,7,8,11,12,13,14,17	4	Q4FZU6,Q63942,Q6AYS4,Q99041
1434	1,2,3,6,9,10,15,17	4,5,7,8,11,12,13,14,16	1	Q4FZU6
1435	1,2,3,6,9,10,16,17	4,5,7,8,11,12,13,14,15	1	Q6AYS4
1436	1,2,3,6,9,11,12,13	4,5,7,8,10,14,15,16,17	2	Q91ZS3,Q9JHB9
1437	1,2,3,6,9,11,12,14	4,5,7,8,10,13,15,16,17	2	Q6IFU8,Q91ZS3

1438	1,2,3,6,9,11,12,15	4,5,7,8,10,13,14,16,17	0	
1439	1,2,3,6,9,11,12,16	4,5,7,8,10,13,14,15,17	4	P55314,Q6IFU7,Q6IFU8,Q91ZS3
1440	1,2,3,6,9,11,12,17	4,5,7,8,10,13,14,15,16	1	P16636
1441	1,2,3,6,9,11,13,14	4,5,7,8,10,12,15,16,17	1	Q91ZS3
1442	1,2,3,6,9,11,13,15	4,5,7,8,10,12,14,16,17	1	Q4FZU6
1443	1,2,3,6,9,11,13,16	4,5,7,8,10,12,14,15,17	1	P55314
1444	1,2,3,6,9,11,13,17	4,5,7,8,10,12,14,15,16	0	
1445	1,2,3,6,9,11,14,15	4,5,7,8,10,12,13,16,17	0	
1446	1,2,3,6,9,11,14,16	4,5,7,8,10,12,13,15,17	2	P55314,Q99MH3
1447	1,2,3,6,9,11,14,17	4,5,7,8,10,12,13,15,16	1	Q9QUL6
1448	1,2,3,6,9,11,15,16	4,5,7,8,10,12,13,14,17	1	P55314
1449	1,2,3,6,9,11,15,17	4,5,7,8,10,12,13,14,16	0	
1450	1,2,3,6,9,11,16,17	4,5,7,8,10,12,13,14,15	1	P55314
1451	1,2,3,6,9,12,13,14	4,5,7,8,10,11,15,16,17	3	P00714,Q6AYS4,Q9Z1F2
1452	1,2,3,6,9,12,13,15	4,5,7,8,10,11,14,16,17	2	P00714,Q6AYS4

1453	1,2,3,6,9,12,13,16	4,5,7,8,10,11,14,15,17	2	P00714,Q6AYS4
1454	1,2,3,6,9,12,13,17	4,5,7,8,10,11,14,15,16	6	P00714,P07171,Q62635,Q63355,Q6AYS4,Q9Z1F2
1455	1,2,3,6,9,12,14,15	4,5,7,8,10,11,13,16,17	1	Q6AYS4
1456	1,2,3,6,9,12,14,16	4,5,7,8,10,11,13,15,17	1	Q6AYS4
1457	1,2,3,6,9,12,14,17	4,5,7,8,10,11,13,15,16	3	P00714,P01835,Q561R9
1458	1,2,3,6,9,12,15,16	4,5,7,8,10,11,13,14,17	1	Q6AYS4
1459	1,2,3,6,9,12,15,17	4,5,7,8,10,11,13,14,16	2	P00714,Q6AYS4
1460	1,2,3,6,9,12,16,17	4,5,7,8,10,11,13,14,15	3	P00714,P07171,Q6AYS4
1461	1,2,3,6,9,13,14,15	4,5,7,8,10,11,12,16,17	4	P00714,P16391,Q6AYS4,Q99041
1462	1,2,3,6,9,13,14,16	4,5,7,8,10,11,12,15,17	3	P00714,Q6AYS4,Q99041
1463	1,2,3,6,9,13,14,17	4,5,7,8,10,11,12,15,16	6	P00714,P51907,P53792,Q5FVI6,Q6AYS4,Q9QW07
1464	1,2,3,6,9,13,15,16	4,5,7,8,10,11,12,14,17	2	P00714,Q6AYS4
1465	1,2,3,6,9,13,15,17	4,5,7,8,10,11,12,14,16	3	P00714,P53792,Q6AYS4
1466	1,2,3,6,9,13,16,17	4,5,7,8,10,11,12,14,15	4	P00714,P07171,P51907,Q6AYS4
1467	1,2,3,6,9,14,15,16	4,5,7,8,10,11,12,13,17	5	P00714,Q63942,Q6AYS4,Q99041,Q9QZK9

1468	1,2,3,6,9,14,15,17	4,5,7,8,10,11,12,13,16	2	P00714,Q6AYS4
1469	1,2,3,6,9,14,16,17	4,5,7,8,10,11,12,13,15	2	P00714,Q6AYS4
1470	1,2,3,6,9,15,16,17	4,5,7,8,10,11,12,13,14	3	P00714,P23593,Q6AYS4
1471	1,2,3,6,10,11,12,13	4,5,7,8,9,14,15,16,17	4	P47727,P55314,Q4FZU6,Q9JHB9
1472	1,2,3,6,10,11,12,14	4,5,7,8,9,13,15,16,17	5	P47727,P55314,P62804,Q6IFU7,Q6IFU8
1473	1,2,3,6,10,11,12,15	4,5,7,8,9,13,14,16,17	4	P35280,P55314,Q4FZU6,Q6IFU7
1474	1,2,3,6,10,11,12,16	4,5,7,8,9,13,14,15,17	6	P47967,P55314,Q6IFU7,Q6IFU8,Q6IMF3,Q6P6Q2
1475	1,2,3,6,10,11,12,17	4,5,7,8,9,13,14,15,16	5	P06757,P16636,P55314,Q10758,Q6IFU8
1476	1,2,3,6,10,11,13,14	4,5,7,8,9,12,15,16,17	3	P47727,P55314,Q4FZU6
1477	1,2,3,6,10,11,13,15	4,5,7,8,9,12,14,16,17	2	P55314,Q4FZU6
1478	1,2,3,6,10,11,13,16	4,5,7,8,9,12,14,15,17	2	P55314,Q4FZU6
1479	1,2,3,6,10,11,13,17	4,5,7,8,9,12,14,15,16	3	P16636,P55314,Q4FZU6
1480	1,2,3,6,10,11,14,15	4,5,7,8,9,12,13,16,17	3	P35280,P55314,Q4FZU6
1481	1,2,3,6,10,11,14,16	4,5,7,8,9,12,13,15,17	1	P55314
1482	1,2,3,6,10,11,14,17	4,5,7,8,9,12,13,15,16	2	P15399,P55314
1483	1,2,3,6,10,11,15,16	4,5,7,8,9,12,13,14,17	2	P55314,Q4FZU6
1484	1,2,3,6,10,11,15,17	4,5,7,8,9,12,13,14,16	2	P55314,Q4FZU6
1485	1,2,3,6,10,11,16,17	4,5,7,8,9,12,13,14,15	1	P55314
1486	1,2,3,6,10,12,13,14	4,5,7,8,9,11,15,16,17	2	Q05175,Q6AYS4
1487	1,2,3,6,10,12,13,15	4,5,7,8,9,11,14,16,17	2	Q4FZU6,Q6AYS4
1488	1,2,3,6,10,12,13,16	4,5,7,8,9,11,14,15,17	2	P97840,Q6AYS4

1489	1,2,3,6,10,<br>12,13,17	4,5,7,8,9,1<br>1,14,15,16	8	P07171,P16636,Q05175,Q10758,Q62635,Q63355,Q99MH3,Q9QZK9
1490	1,2,3,6,10,<br>12,14,15	4,5,7,8,9,1<br>1,13,16,17	3	A2RUV9,Q4FZU6,Q6AYS4
1491	1,2,3,6,10,<br>12,14,16	4,5,7,8,9,1<br>1,13,15,17	3	P97840,Q10758,Q6AYS4
1492	1,2,3,6,10,<br>12,14,17	4,5,7,8,9,1<br>1,13,15,16	2	Q10758,Q561R9
1493	1,2,3,6,10,<br>12,15,16	4,5,7,8,9,1<br>1,13,14,17	3	P97840,Q4FZU6,Q6AYS4
1494	1,2,3,6,10,<br>12,15,17	4,5,7,8,9,1<br>1,13,14,16	3	P06866,Q4FZU6,Q6AYS4
1495	1,2,3,6,10,<br>12,16,17	4,5,7,8,9,1<br>1,13,14,15	4	P07171,P16636,Q10758,Q6AYS4
1496	1,2,3,6,10,<br>13,14,15	4,5,7,8,9,1<br>1,12,16,17	3	Q4FZU6,Q6AYS4,Q99041
1497	1,2,3,6,10,<br>13,14,16	4,5,7,8,9,1<br>1,12,15,17	2	Q6AYS4,Q99041
1498	1,2,3,6,10,<br>13,14,17	4,5,7,8,9,1<br>1,12,15,16	2	P51907,Q9QW07
1499	1,2,3,6,10,<br>13,15,16	4,5,7,8,9,1<br>1,12,14,17	2	Q4FZU6,Q6AYS4
1500	1,2,3,6,10,<br>13,15,17	4,5,7,8,9,1<br>1,12,14,16	3	Q4FZU6,Q6AYS4,Q6IFV1
1501	1,2,3,6,10,<br>13,16,17	4,5,7,8,9,1<br>1,12,14,15	2	P51907,Q6AYS4
1502	1,2,3,6,10,<br>14,15,16	4,5,7,8,9,1<br>1,12,13,17	4	P02803,P36374,Q6AYS4,Q99041
1503	1,2,3,6,10,<br>14,15,17	4,5,7,8,9,1<br>1,12,13,16	3	P02803,Q4FZU6,Q6AYS4
1504	1,2,3,6,10,<br>14,16,17	4,5,7,8,9,1<br>1,12,13,15	1	Q6AYS4
1505	1,2,3,6,10,<br>15,16,17	4,5,7,8,9,1<br>1,12,13,14	3	P02803,Q4FZU6,Q6AYS4
1506	1,2,3,6,11,<br>12,13,14	4,5,7,8,9,1<br>0,15,16,17	4	P47727,Q64093,Q9JHB9,Q9Z1F2
1507	1,2,3,6,11,<br>12,13,15	4,5,7,8,9,1<br>0,14,16,17	0	
1508	1,2,3,6,11,<br>12,13,16	4,5,7,8,9,1<br>0,14,15,17	1	P55314
1509	1,2,3,6,11,<br>12,13,17	4,5,7,8,9,1<br>0,14,15,16	4	P16636,Q62635,Q9JHB9,Q9QZK9
1510	1,2,3,6,11,<br>12,14,15	4,5,7,8,9,1<br>0,13,16,17	0	
1511	1,2,3,6,11,<br>12,14,16	4,5,7,8,9,1<br>0,13,15,17	2	P55314,Q6IFU8

1512	1,2,3,6,11,12,14,17	4,5,7,8,9,10,13,15,16	2	Q62635,Q6IFU8
1513	1,2,3,6,11,12,15,16	4,5,7,8,9,10,13,14,17	1	P55314
1514	1,2,3,6,11,12,15,17	4,5,7,8,9,10,13,14,16	0	
1515	1,2,3,6,11,12,16,17	4,5,7,8,9,10,13,14,15	2	P16636,P55314
1516	1,2,3,6,11,13,14,15	4,5,7,8,9,10,12,16,17	1	Q64093
1517	1,2,3,6,11,13,14,16	4,5,7,8,9,10,12,15,17	1	P55314
1518	1,2,3,6,11,13,14,17	4,5,7,8,9,10,12,15,16	2	Q64093,Q9QW07
1519	1,2,3,6,11,13,15,16	4,5,7,8,9,10,12,14,17	1	P55314
1520	1,2,3,6,11,13,15,17	4,5,7,8,9,10,12,14,16	1	P00714
1521	1,2,3,6,11,13,16,17	4,5,7,8,9,10,12,14,15	1	P55314
1522	1,2,3,6,11,14,15,16	4,5,7,8,9,10,12,13,17	1	P55314
1523	1,2,3,6,11,14,15,17	4,5,7,8,9,10,12,13,16	0	
1524	1,2,3,6,11,14,16,17	4,5,7,8,9,10,12,13,15	1	P55314
1525	1,2,3,6,11,15,16,17	4,5,7,8,9,10,12,13,14	1	P55314
1526	1,2,3,6,12,13,14,15	4,5,7,8,9,10,11,16,17	5	A2RUV9,P00714,Q64093,Q6AYS4,Q9Z1F2
1527	1,2,3,6,12,13,14,16	4,5,7,8,9,10,11,15,17	4	P00714,Q64093,Q6AYS4,Q9Z1F2
1528	1,2,3,6,12,13,14,17	4,5,7,8,9,10,11,15,16	9	P00714,P51907,Q561R9,Q5FVI6,Q62635,Q64093,Q6AYS4,Q9QW07,Q9Z1F2
1529	1,2,3,6,12,13,15,16	4,5,7,8,9,10,11,14,17	2	P00714,Q6AYS4
1530	1,2,3,6,12,13,15,17	4,5,7,8,9,10,11,14,16	4	P00714,Q62635,Q6AYS4,Q6IFV1
1531	1,2,3,6,12,13,16,17	4,5,7,8,9,10,11,14,15	5	P00714,P07171,Q561R9,Q62635,Q6AYS4
1532	1,2,3,6,12,14,15,16	4,5,7,8,9,10,11,13,17	2	P00714,Q6AYS4
1533	1,2,3,6,12,14,15,17	4,5,7,8,9,10,11,13,16	2	P00714,Q6AYS4
1534	1,2,3,6,12,14,16,17	4,5,7,8,9,10,11,13,15	3	P00714,Q561R9,Q6AYS4

1535	1,2,3,6,12,15,16,17	4,5,7,8,9,10,11,13,14	5	P00714,P06866,P83121,Q5XFX0,Q6AYS4
1536	1,2,3,6,13,14,15,16	4,5,7,8,9,10,11,12,17	4	P00714,Q64093,Q6AYS4,Q99041
1537	1,2,3,6,13,14,15,17	4,5,7,8,9,10,11,12,16	4	P00714,Q64093,Q6AYS4,Q9QW07
1538	1,2,3,6,13,14,16,17	4,5,7,8,9,10,11,12,15	6	P00714,P51907,Q561R9,Q6AYS4,Q6PCU2,Q9QW07
1539	1,2,3,6,13,15,16,17	4,5,7,8,9,10,11,12,14	2	P00714,Q6AYS4
1540	1,2,3,6,14,15,16,17	4,5,7,8,9,10,11,12,13	3	P00714,P02803,Q6AYS4
1541	1,2,3,7,8,9,10,11	4,5,6,12,13,14,15,16,17	5	P10715,P27213,P31044,P35280,Q91ZS3
1542	1,2,3,7,8,9,10,12	4,5,6,11,13,14,15,16,17	4	P10715,P27213,P97840,Q9EQS0
1543	1,2,3,7,8,9,10,13	4,5,6,11,12,14,15,16,17	3	P01835,P20766,P31044
1544	1,2,3,7,8,9,10,14	4,5,6,11,12,13,15,16,17	2	P27213,P31044
1545	1,2,3,7,8,9,10,15	4,5,6,11,12,13,14,16,17	4	P01835,P27213,P35280,Q5QE79
1546	1,2,3,7,8,9,10,16	4,5,6,11,12,13,14,15,17	1	P01835
1547	1,2,3,7,8,9,10,17	4,5,6,11,12,13,14,15,16	4	P01946,P02091,P02770,P12346
1548	1,2,3,7,8,9,11,12	4,5,6,10,13,14,15,16,17	3	P10715,P27213,Q9EQS0
1549	1,2,3,7,8,9,11,13	4,5,6,10,12,14,15,16,17	2	P10715,P20766
1550	1,2,3,7,8,9,11,14	4,5,6,10,12,13,15,16,17	3	P10715,P19629,P27213
1551	1,2,3,7,8,9,11,15	4,5,6,10,12,13,14,16,17	4	P10715,P27213,P35280,Q5QE79

1552	1,2,3,7,8,9,11,16	4,5,6,10,12,13,14,15,17	2	B4F795,P10715
1553	1,2,3,7,8,9,11,17	4,5,6,10,12,13,14,15,16	5	P01946,P02091,P02770,P10715,P50115
1554	1,2,3,7,8,9,12,13	4,5,6,10,11,14,15,16,17	3	P10715,P20766,Q9Z1F2
1555	1,2,3,7,8,9,12,14	4,5,6,10,11,13,15,16,17	4	P10715,P19629,P36970,Q9EQS0
1556	1,2,3,7,8,9,12,15	4,5,6,10,11,13,14,16,17	2	P10715,Q5QE79
1557	1,2,3,7,8,9,12,16	4,5,6,10,11,13,14,15,17	1	P10715
1558	1,2,3,7,8,9,12,17	4,5,6,10,11,13,14,15,16	6	P01946,P02091,P02770,P10715,P19223,P55091
1559	1,2,3,7,8,9,13,14	4,5,6,10,11,12,15,16,17	4	P20766,P36970,Q08849,Q9Z1F2
1560	1,2,3,7,8,9,13,15	4,5,6,10,11,12,14,16,17	3	P01835,P20766,Q5QE79
1561	1,2,3,7,8,9,13,16	4,5,6,10,11,12,14,15,17	2	P01835,P20766
1562	1,2,3,7,8,9,13,17	4,5,6,10,11,12,14,15,16	6	P01946,P02091,P02770,P20766,P51907,Q6IFU7
1563	1,2,3,7,8,9,14,15	4,5,6,10,11,12,13,16,17	3	P19629,Q08849,Q5QE79
1564	1,2,3,7,8,9,14,16	4,5,6,10,11,12,13,15,17	2	Q08849,Q9QZK9
1565	1,2,3,7,8,9,14,17	4,5,6,10,11,12,13,15,16	4	P01946,P02091,P19629,Q08849
1566	1,2,3,7,8,9,15,16	4,5,6,10,11,12,13,14,17	5	B4F795,P01835,P10715,Q5QE79,Q9QZK9

1567	1,2,3,7,8,9,15,17	4,5,6,10,11,12,13,14,16	3	P01946,P02091,Q5QE79
1568	1,2,3,7,8,9,16,17	4,5,6,10,11,12,13,14,15	3	P01946,P02091,P10715
1569	1,2,3,7,8,10,11,12	4,5,6,9,13,14,15,16,17	7	O35783,P00731,P05964,P10715,P27213,P35280,Q9EQS0
1570	1,2,3,7,8,10,11,13	4,5,6,9,12,14,15,16,17	3	P01835,P20766,P31044
1571	1,2,3,7,8,10,11,14	4,5,6,9,12,13,15,16,17	3	P27213,P31044,P35280
1572	1,2,3,7,8,10,11,15	4,5,6,9,12,13,14,16,17	5	P00731,P01835,P27213,P31044,P35280
1573	1,2,3,7,8,10,11,16	4,5,6,9,12,13,14,15,17	3	P01835,P10715,P55314
1574	1,2,3,7,8,10,11,17	4,5,6,9,12,13,14,15,16	6	P01946,P02091,P02770,P10715,P12346,P17475
1575	1,2,3,7,8,10,12,13	4,5,6,9,11,14,15,16,17	0	
1576	1,2,3,7,8,10,12,14	4,5,6,9,11,13,15,16,17	4	A2RUV9,P19629,P36970,Q9EQS0
1577	1,2,3,7,8,10,12,15	4,5,6,9,11,13,14,16,17	4	P00731,P01041,P10715,P35280
1578	1,2,3,7,8,10,12,16	4,5,6,9,11,13,14,15,17	4	P08721,P10715,P47967,P97840
1579	1,2,3,7,8,10,12,17	4,5,6,9,11,13,14,15,16	9	P01041,P01946,P02091,P02770,P10715,P12346,P17475,P19223,P55091
1580	1,2,3,7,8,10,13,14	4,5,6,9,11,12,15,16,17	8	P01835,P06911,P12020,P20766,P31044,P36970,Q08849,Q5GRG2
1581	1,2,3,7,8,10,13,15	4,5,6,9,11,12,14,16,17	4	P01835,P15999,Q09030,Q5GRG2

1582	1,2,3,7,8,10,13,16	4,5,6,9,11,12,14,15,17	1	P01835
1583	1,2,3,7,8,10,13,17	4,5,6,9,11,12,14,15,16	8	P01835,P01946,P02091,P02770,P12346,P17475,P20766,P51907
1584	1,2,3,7,8,10,14,15	4,5,6,9,11,12,13,16,17	4	P01835,P19629,P35280,Q08849
1585	1,2,3,7,8,10,14,16	4,5,6,9,11,12,13,15,17	3	P01835,P08721,Q63532
1586	1,2,3,7,8,10,14,17	4,5,6,9,11,12,13,15,16	5	P01946,P02091,P12346,P17475,Q08849
1587	1,2,3,7,8,10,15,16	4,5,6,9,11,12,13,14,17	2	P01835,P08721
1588	1,2,3,7,8,10,15,17	4,5,6,9,11,12,13,14,16	5	P01835,P01946,P02091,P12346,P17475
1589	1,2,3,7,8,10,16,17	4,5,6,9,11,12,13,14,15	5	P01835,P01946,P02091,P08721,P12346
1590	1,2,3,7,8,11,12,13	4,5,6,9,10,14,15,16,17	2	P10715,Q8CJ52
1591	1,2,3,7,8,11,12,14	4,5,6,9,10,13,15,16,17	3	P10715,P19629,Q9EQS0
1592	1,2,3,7,8,11,12,15	4,5,6,9,10,13,14,16,17	4	P01041,P10715,P19629,P35280
1593	1,2,3,7,8,11,12,16	4,5,6,9,10,13,14,15,17	1	P10715
1594	1,2,3,7,8,11,12,17	4,5,6,9,10,13,14,15,16	10	P01041,P01946,P02091,P02770,P10715,P17475,P19223,P50115,P55091,Q9QZA6
1595	1,2,3,7,8,11,13,14	4,5,6,9,10,12,15,16,17	4	P19629,P20766,Q08849,Q5GRG2
1596	1,2,3,7,8,11,13,15	4,5,6,9,10,12,14,16,17	3	P01835,P20766,Q09030

1597	1,2,3,7,8,11,13,16	4,5,6,9,10,12,14,15,17	2	P01835,P10715
1598	1,2,3,7,8,11,13,17	4,5,6,9,10,12,14,15,16	4	P01946,P02091,P10715,P20766
1599	1,2,3,7,8,11,14,15	4,5,6,9,10,12,13,16,17	3	P19629,P35280,Q08849
1600	1,2,3,7,8,11,14,16	4,5,6,9,10,12,13,15,17	2	P19629,Q63532
1601	1,2,3,7,8,11,14,17	4,5,6,9,10,12,13,15,16	3	P01946,P02091,P19629
1602	1,2,3,7,8,11,15,16	4,5,6,9,10,12,13,14,17	3	B4F795,P01835,P10715
1603	1,2,3,7,8,11,15,17	4,5,6,9,10,12,13,14,16	5	P01946,P02091,P10715,P12346,P17475
1604	1,2,3,7,8,11,16,17	4,5,6,9,10,12,13,14,15	3	P01946,P02091,P10715
1605	1,2,3,7,8,12,13,14	4,5,6,9,10,11,15,16,17	7	A2RUV9,P06911,P19629,P29975,P36970,Q5GRG2,Q9Z1F2
1606	1,2,3,7,8,12,13,15	4,5,6,9,10,11,14,16,17	4	P10715,P15999,P83121,Q09030
1607	1,2,3,7,8,12,13,16	4,5,6,9,10,11,14,15,17	2	P10715,P63029
1608	1,2,3,7,8,12,13,17	4,5,6,9,10,11,14,15,16	9	P01946,P02091,P10715,P17475,P20766,P55091,P63029,Q62635,Q9Z1F2
1609	1,2,3,7,8,12,14,15	4,5,6,9,10,11,13,16,17	5	A2RUV9,P01041,P10715,P19629,P36970
1610	1,2,3,7,8,12,14,16	4,5,6,9,10,11,13,15,17	3	P10715,P19629,Q63532
1611	1,2,3,7,8,12,14,17	4,5,6,9,10,11,13,15,16	8	P01041,P01946,P02091,P10715,P17475,P19629,P31430,P55091

1612	1,2,3,7,8,12,15,16	4,5,6,9,10,11,13,14,17	3	P01041,P10715,P83121
1613	1,2,3,7,8,12,15,17	4,5,6,9,10,11,13,14,16	10	P01041,P01946,P02091,P10715,P12346,P17475,P19629,P55091,P83121,Q9QZA6
1614	1,2,3,7,8,12,16,17	4,5,6,9,10,11,13,14,15	7	P01041,P01946,P02091,P10715,P55091,P63029,P83121
1615	1,2,3,7,8,13,14,15	4,5,6,9,10,11,12,16,17	8	A2RUV9,P01835,P15999,P19629,P36970,Q08849,Q09030,Q5GRG2
1616	1,2,3,7,8,13,14,16	4,5,6,9,10,11,12,15,17	3	P01835,Q08849,Q566E6
1617	1,2,3,7,8,13,14,17	4,5,6,9,10,11,12,15,16	9	P01946,P02091,P19629,P20766,P51907,Q08849,Q6IFU7,Q6PCU2,Q9QW07
1618	1,2,3,7,8,13,15,16	4,5,6,9,10,11,12,14,17	3	P01835,Q08849,Q09030
1619	1,2,3,7,8,13,15,17	4,5,6,9,10,11,12,14,16	8	P01835,P01946,P02091,P15999,P17475,P20766,Q08849,Q6IFU7
1620	1,2,3,7,8,13,16,17	4,5,6,9,10,11,12,14,15	7	P01835,P01946,P02091,P20766,P51907,P63029,Q6PCU2
1621	1,2,3,7,8,14,15,16	4,5,6,9,10,11,12,13,17	4	P01835,P19629,Q08849,Q63532
1622	1,2,3,7,8,14,15,17	4,5,6,9,10,11,12,13,16	8	P01946,P02091,P10536,P12346,P17475,P19629,Q08849,Q6IFU7
1623	1,2,3,7,8,14,16,17	4,5,6,9,10,11,12,13,15	5	P01946,P02091,P19629,Q08849,Q6PCU2
1624	1,2,3,7,8,15,16,17	4,5,6,9,10,11,12,13,14	5	P01835,P01946,P02091,P0C0A9,P83121
1625	1,2,3,7,9,10,11,12	4,5,6,8,13,14,15,16,17	5	O35783,P27213,P97840,Q63661,Q91ZS3
1626	1,2,3,7,9,10,11,13	4,5,6,8,12,14,15,16,17	3	P06760,Q63661,Q91ZS3

1627	1,2,3,7,9,10,11,14	4,5,6,8,12,13,15,16,17	5	P06760,P27213,Q63661,Q91ZS3,Q9QUL6
1628	1,2,3,7,9,10,11,15	4,5,6,8,12,13,14,16,17	4	P06760,P27213,P35280,Q63661
1629	1,2,3,7,9,10,11,16	4,5,6,8,12,13,14,15,17	3	P06760,P55314,Q91ZS3
1630	1,2,3,7,9,10,11,17	4,5,6,8,12,13,14,15,16	3	P01946,P02091,Q9QUL6
1631	1,2,3,7,9,10,12,13	4,5,6,8,11,14,15,16,17	3	P06760,P14668,P97840
1632	1,2,3,7,9,10,12,14	4,5,6,8,11,13,15,16,17	4	A2RUV9,P06760,P14668,P97840
1633	1,2,3,7,9,10,12,15	4,5,6,8,11,13,14,16,17	3	P06760,P14668,P97840
1634	1,2,3,7,9,10,12,16	4,5,6,8,11,13,14,15,17	3	P06760,P47967,P97840
1635	1,2,3,7,9,10,12,17	4,5,6,8,11,13,14,15,16	4	P01946,P02091,P14668,P97840
1636	1,2,3,7,9,10,13,14	4,5,6,8,11,12,15,16,17	3	P06760,P14668,Q4G075
1637	1,2,3,7,9,10,13,15	4,5,6,8,11,12,14,16,17	4	P01835,P06760,P14668,Q4G075
1638	1,2,3,7,9,10,13,16	4,5,6,8,11,12,14,15,17	4	P01835,P06760,P97840,Q4G075
1639	1,2,3,7,9,10,13,17	4,5,6,8,11,12,14,15,16	3	P01946,P02091,P14668
1640	1,2,3,7,9,10,14,15	4,5,6,8,11,12,13,16,17	3	P06760,P14668,Q4G075
1641	1,2,3,7,9,10,14,16	4,5,6,8,11,12,13,15,17	4	P06760,Q4G075,Q63532,Q9QZK9

1642	1,2,3,7,9,10,14,17	4,5,6,8,11,12,13,15,16	3	P01946,P02091,P14668
1643	1,2,3,7,9,10,15,16	4,5,6,8,11,12,13,14,17	6	P01835,P02780,P02782,P06760,P0C0A9,Q9QZK9
1644	1,2,3,7,9,10,15,17	4,5,6,8,11,12,13,14,16	4	P01946,P02091,P0C0A9,P14668
1645	1,2,3,7,9,10,16,17	4,5,6,8,11,12,13,14,15	4	P01946,P02091,P0C0A9,P14668
1646	1,2,3,7,9,11,12,13	4,5,6,8,10,14,15,16,17	1	P06760
1647	1,2,3,7,9,11,12,14	4,5,6,8,10,13,15,16,17	1	P06760
1648	1,2,3,7,9,11,12,15	4,5,6,8,10,13,14,16,17	1	P06760
1649	1,2,3,7,9,11,12,16	4,5,6,8,10,13,14,15,17	1	P06760
1650	1,2,3,7,9,11,12,17	4,5,6,8,10,13,14,15,16	2	P01946,P02091
1651	1,2,3,7,9,11,13,14	4,5,6,8,10,12,15,16,17	2	P06760,Q4G075
1652	1,2,3,7,9,11,13,15	4,5,6,8,10,12,14,16,17	1	P06760
1653	1,2,3,7,9,11,13,16	4,5,6,8,10,12,14,15,17	2	P06760,Q4G075
1654	1,2,3,7,9,11,13,17	4,5,6,8,10,12,14,15,16	2	P01946,P02091
1655	1,2,3,7,9,11,14,15	4,5,6,8,10,12,13,16,17	2	P06760,Q4G075
1656	1,2,3,7,9,11,14,16	4,5,6,8,10,12,13,15,17	5	P06760,Q4G075,Q63532,Q99MH3,Q9QZK9

1657	1,2,3,7,9,11,14,17	4,5,6,8,10,12,13,15,16	2	P01946,P02091
1658	1,2,3,7,9,11,15,16	4,5,6,8,10,12,13,14,17	2	P06760,Q9QZK9
1659	1,2,3,7,9,11,15,17	4,5,6,8,10,12,13,14,16	2	P01946,P02091
1660	1,2,3,7,9,11,16,17	4,5,6,8,10,12,13,14,15	3	P01946,P02091,Q498D9
1661	1,2,3,7,9,12,13,14	4,5,6,8,10,11,15,16,17	3	A2RUV9,P06760,P14668
1662	1,2,3,7,9,12,13,15	4,5,6,8,10,11,14,16,17	2	P06760,P14668
1663	1,2,3,7,9,12,13,16	4,5,6,8,10,11,14,15,17	1	P06760
1664	1,2,3,7,9,12,13,17	4,5,6,8,10,11,14,15,16	4	P01946,P02091,P14668,P55091
1665	1,2,3,7,9,12,14,15	4,5,6,8,10,11,13,16,17	3	A2RUV9,P06760,P14668
1666	1,2,3,7,9,12,14,16	4,5,6,8,10,11,13,15,17	2	P06760,Q63532
1667	1,2,3,7,9,12,14,17	4,5,6,8,10,11,13,15,16	5	A2RUV9,P01946,P02091,P14668,P31430
1668	1,2,3,7,9,12,15,16	4,5,6,8,10,11,13,14,17	2	P06760,Q6AYS4
1669	1,2,3,7,9,12,15,17	4,5,6,8,10,11,13,14,16	3	P01946,P02091,P14668
1670	1,2,3,7,9,12,16,17	4,5,6,8,10,11,13,14,15	3	P01946,P02091,P14668
1671	1,2,3,7,9,13,14,15	4,5,6,8,10,11,12,16,17	5	A2RUV9,P06760,P14668,Q08849,Q4G075

1672	1,2,3,7,9,13,14,16	4,5,6,8,10,11,12,15,17	3	P06760,P08010,Q4G075
1673	1,2,3,7,9,13,14,17	4,5,6,8,10,11,12,15,16	5	P01946,P02091,P14668,P53792,Q9QW07
1674	1,2,3,7,9,13,15,16	4,5,6,8,10,11,12,14,17	5	P01835,P06760,P0C0A9,Q4G075,Q6AYS4
1675	1,2,3,7,9,13,15,17	4,5,6,8,10,11,12,14,16	6	P01946,P02091,P0C0A9,P14668,P53792,Q6IFU7
1676	1,2,3,7,9,13,16,17	4,5,6,8,10,11,12,14,15	5	P01946,P02091,P08010,P0C0A9,P14668
1677	1,2,3,7,9,14,15,16	4,5,6,8,10,11,12,13,17	7	P06760,P0C0A9,P16636,Q4G075,Q63532,Q6AYS4,Q9QZK9
1678	1,2,3,7,9,14,15,17	4,5,6,8,10,11,12,13,16	4	P01946,P02091,P0C0A9,P14668
1679	1,2,3,7,9,14,16,17	4,5,6,8,10,11,12,13,15	3	P01946,P02091,P0C0A9
1680	1,2,3,7,9,15,16,17	4,5,6,8,10,11,12,13,14	6	P01946,P02091,P02780,P02781,P0C0A9,P47727
1681	1,2,3,7,10,11,12,13	4,5,6,8,9,14,15,16,17	1	O35783
1682	1,2,3,7,10,11,12,14	4,5,6,8,9,13,15,16,17	1	O35783
1683	1,2,3,7,10,11,12,15	4,5,6,8,9,13,14,16,17	2	P00731,P35280
1684	1,2,3,7,10,11,12,16	4,5,6,8,9,13,14,15,17	5	P06757,P12368,P47967,P55314,P97840
1685	1,2,3,7,10,11,12,17	4,5,6,8,9,13,14,15,16	4	P01946,P02091,P05964,P06757
1686	1,2,3,7,10,11,13,14	4,5,6,8,9,12,15,16,17	1	P06760
1687	1,2,3,7,10,11,13,15	4,5,6,8,9,12,14,16,17	3	P01835,P06760,Q09030
1688	1,2,3,7,10,11,13,16	4,5,6,8,9,12,14,15,17	3	P01835,P06760,P55314
1689	1,2,3,7,10,11,13,17	4,5,6,8,9,12,14,15,16	2	P01946,P02091

1690	1,2,3,7,10,<br>11,14,15	4,5,6,8,9,1<br>2,13,16,17	2	P06760,P35280
1691	1,2,3,7,10,<br>11,14,16	4,5,6,8,9,1<br>2,13,15,17	3	P06760,P55314,Q63532
1692	1,2,3,7,10,<br>11,14,17	4,5,6,8,9,1<br>2,13,15,16	3	P01946,P02091,Q8R431
1693	1,2,3,7,10,<br>11,15,16	4,5,6,8,9,1<br>2,13,14,17	3	P01835,P06760,P55314
1694	1,2,3,7,10,<br>11,15,17	4,5,6,8,9,1<br>2,13,14,16	2	P01946,P02091
1695	1,2,3,7,10,<br>11,16,17	4,5,6,8,9,1<br>2,13,14,15	3	P01946,P02091,P55314
1696	1,2,3,7,10,<br>12,13,14	4,5,6,8,9,1<br>1,15,16,17	3	A2RUV9,P06760,P14668
1697	1,2,3,7,10,<br>12,13,15	4,5,6,8,9,1<br>1,14,16,17	3	P06760,P14668,Q09030
1698	1,2,3,7,10,<br>12,13,16	4,5,6,8,9,1<br>1,14,15,17	4	P06760,P14668,P47967,P97840
1699	1,2,3,7,10,<br>12,13,17	4,5,6,8,9,1<br>1,14,15,16	4	P01946,P02091,P14668,P55091
1700	1,2,3,7,10,<br>12,14,15	4,5,6,8,9,1<br>1,13,16,17	3	A2RUV9,P06760,P14668
1701	1,2,3,7,10,<br>12,14,16	4,5,6,8,9,1<br>1,13,15,17	4	P06760,P08721,P97840,Q63532
1702	1,2,3,7,10,<br>12,14,17	4,5,6,8,9,1<br>1,13,15,16	5	A2RUV9,P01946,P02091,P14668,P31430
1703	1,2,3,7,10,<br>12,15,16	4,5,6,8,9,1<br>1,13,14,17	2	P06760,P97840
1704	1,2,3,7,10,<br>12,15,17	4,5,6,8,9,1<br>1,13,14,16	5	P01041,P01946,P02091,P06866,P14668
1705	1,2,3,7,10,<br>12,16,17	4,5,6,8,9,1<br>1,13,14,15	5	P01946,P02091,P06866,P14668,P97840
1706	1,2,3,7,10,<br>13,14,15	4,5,6,8,9,1<br>1,12,16,17	5	A2RUV9,P01835,P06760,P14668,Q09030
1707	1,2,3,7,10,<br>13,14,16	4,5,6,8,9,1<br>1,12,15,17	3	P01835,P06760,Q63532
1708	1,2,3,7,10,<br>13,14,17	4,5,6,8,9,1<br>1,12,15,16	4	P01946,P02091,P14668,Q9QW07
1709	1,2,3,7,10,<br>13,15,16	4,5,6,8,9,1<br>1,12,14,17	5	P01835,P06760,P0C0A9,Q09030,Q6AYS4
1710	1,2,3,7,10,<br>13,15,17	4,5,6,8,9,1<br>1,12,14,16	5	P01835,P01946,P02091,P0C0A9,P14668
1711	1,2,3,7,10,<br>13,16,17	4,5,6,8,9,1<br>1,12,14,15	4	P01835,P01946,P02091,P14668
1712	1,2,3,7,10,<br>14,15,16	4,5,6,8,9,1<br>1,12,13,17	4	P01835,P06760,P0C0A9,Q63532

1713	1,2,3,7,10,<br>14,15,17	4,5,6,8,9,1<br>1,12,13,16	4	P01946,P02091,P0C0A9,P14668
1714	1,2,3,7,10,<br>14,16,17	4,5,6,8,9,1<br>1,12,13,15	4	P01946,P02091,P0C0A9,P14668
1715	1,2,3,7,10,<br>15,16,17	4,5,6,8,9,1<br>1,12,13,14	7	P01835,P01946,P02091,P02780,P02782,P0C0A9,P14668
1716	1,2,3,7,11,<br>12,13,14	4,5,6,8,9,1<br>0,15,16,17	2	A2RUV9,P06760
1717	1,2,3,7,11,<br>12,13,15	4,5,6,8,9,1<br>0,14,16,17	3	P01836,P06760,Q09030
1718	1,2,3,7,11,<br>12,13,16	4,5,6,8,9,1<br>0,14,15,17	3	P01836,P06760,P17046
1719	1,2,3,7,11,<br>12,13,17	4,5,6,8,9,1<br>0,14,15,16	3	P01946,P02091,P55091
1720	1,2,3,7,11,<br>12,14,15	4,5,6,8,9,1<br>0,13,16,17	2	A2RUV9,P06760
1721	1,2,3,7,11,<br>12,14,16	4,5,6,8,9,1<br>0,13,15,17	2	P06760,Q63532
1722	1,2,3,7,11,<br>12,14,17	4,5,6,8,9,1<br>0,13,15,16	3	P01946,P02091,P31430
1723	1,2,3,7,11,<br>12,15,16	4,5,6,8,9,1<br>0,13,14,17	2	P01836,P06760
1724	1,2,3,7,11,<br>12,15,17	4,5,6,8,9,1<br>0,13,14,16	3	P01946,P02091,P06866
1725	1,2,3,7,11,<br>12,16,17	4,5,6,8,9,1<br>0,13,14,15	3	P01946,P02091,P17046
1726	1,2,3,7,11,<br>13,14,15	4,5,6,8,9,1<br>0,12,16,17	2	P06760,Q09030
1727	1,2,3,7,11,<br>13,14,16	4,5,6,8,9,1<br>0,12,15,17	2	P06760,Q63532
1728	1,2,3,7,11,<br>13,14,17	4,5,6,8,9,1<br>0,12,15,16	3	P01946,P02091,Q9QW07
1729	1,2,3,7,11,<br>13,15,16	4,5,6,8,9,1<br>0,12,14,17	3	P01835,P06760,Q09030
1730	1,2,3,7,11,<br>13,15,17	4,5,6,8,9,1<br>0,12,14,16	2	P01946,P02091
1731	1,2,3,7,11,<br>13,16,17	4,5,6,8,9,1<br>0,12,14,15	2	P01946,P02091
1732	1,2,3,7,11,<br>14,15,16	4,5,6,8,9,1<br>0,12,13,17	3	P06760,Q63532,Q9QZK9
1733	1,2,3,7,11,<br>14,15,17	4,5,6,8,9,1<br>0,12,13,16	2	P01946,P02091
1734	1,2,3,7,11,<br>14,16,17	4,5,6,8,9,1<br>0,12,13,15	2	P01946,P02091
1735	1,2,3,7,11,<br>15,16,17	4,5,6,8,9,1<br>0,12,13,14	3	P01946,P02091,P0C0A9

1736	1,2,3,7,12,13,14,15	4,5,6,8,9,10,11,16,17	4	A2RUV9,P06760,P14668,Q09030
1737	1,2,3,7,12,13,14,16	4,5,6,8,9,10,11,15,17	5	A2RUV9,P06760,Q566E6,Q63532,Q6AYS4
1738	1,2,3,7,12,13,14,17	4,5,6,8,9,10,11,15,16	7	A2RUV9,P01946,P02091,P14668,P31430,P55091,Q9QW07
1739	1,2,3,7,12,13,15,16	4,5,6,8,9,10,11,14,17	5	P01836,P06760,P83121,Q09030,Q6AYS4
1740	1,2,3,7,12,13,15,17	4,5,6,8,9,10,11,14,16	5	P00714,P01946,P02091,P14668,P55091
1741	1,2,3,7,12,13,16,17	4,5,6,8,9,10,11,14,15	3	P01946,P02091,P14668
1742	1,2,3,7,12,14,15,16	4,5,6,8,9,10,11,13,17	4	A2RUV9,P06760,Q63532,Q6AYS4
1743	1,2,3,7,12,14,15,17	4,5,6,8,9,10,11,13,16	5	A2RUV9,P01946,P02091,P14668,P31430
1744	1,2,3,7,12,14,16,17	4,5,6,8,9,10,11,13,15	5	P01946,P02091,P14668,P31430,Q63532
1745	1,2,3,7,12,15,16,17	4,5,6,8,9,10,11,13,14	5	P01946,P02091,P06866,P14668,P83121
1746	1,2,3,7,13,14,15,16	4,5,6,8,9,10,11,12,17	8	P01835,P06760,P0C0A9,P16636,Q08849,Q09030,Q63532,Q6AYS4
1747	1,2,3,7,13,14,15,17	4,5,6,8,9,10,11,12,16	10	A2RUV9,P00714,P01946,P02091,P0C0A9,P14668,Q08849,Q09030,Q6IFU7,Q9QW07
1748	1,2,3,7,13,14,16,17	4,5,6,8,9,10,11,12,15	8	P00714,P01946,P02091,P0C0A9,P14668,Q08849,Q6PCU2,Q9QW07
1749	1,2,3,7,13,15,16,17	4,5,6,8,9,10,11,12,14	7	P00714,P01835,P01946,P02091,P0C0A9,P14668,Q09030
1750	1,2,3,7,14,15,16,17	4,5,6,8,9,10,11,12,13	6	P01946,P02091,P02780,P02803,P0C0A9,Q08849
1751	1,2,3,8,9,10,11,12	4,5,6,7,13,14,15,16,17	2	P27213,Q63661
1752	1,2,3,8,9,10,11,13	4,5,6,7,12,14,15,16,17	2	P20766,P35467
1753	1,2,3,8,9,10,11,14	4,5,6,7,12,13,15,16,17	1	P27213
1754	1,2,3,8,9,10,11,15	4,5,6,7,12,13,14,16,17	3	P27213,P35280,Q5QE79
1755	1,2,3,8,9,10,11,16	4,5,6,7,12,13,14,15,17	1	Q9WUW8

1756	1,2,3,8,9,10,11,17	4,5,6,7,12,13,14,15,16	0	
1757	1,2,3,8,9,10,12,13	4,5,6,7,11,14,15,16,17	1	P20766
1758	1,2,3,8,9,10,12,14	4,5,6,7,11,13,15,16,17	0	
1759	1,2,3,8,9,10,12,15	4,5,6,7,11,13,14,16,17	1	Q5QE79
1760	1,2,3,8,9,10,12,16	4,5,6,7,11,13,14,15,17	2	P05539,Q9WUW8
1761	1,2,3,8,9,10,12,17	4,5,6,7,11,13,14,15,16	1	P16636
1762	1,2,3,8,9,10,13,14	4,5,6,7,11,12,15,16,17	1	P20766
1763	1,2,3,8,9,10,13,15	4,5,6,7,11,12,14,16,17	4	P01835,P15999,P20766,Q5QE79
1764	1,2,3,8,9,10,13,16	4,5,6,7,11,12,14,15,17	2	P01835,P20766
1765	1,2,3,8,9,10,13,17	4,5,6,7,11,12,14,15,16	2	P20766,P51907
1766	1,2,3,8,9,10,14,15	4,5,6,7,11,12,13,16,17	2	Q5QE79,Q9QZK9
1767	1,2,3,8,9,10,14,16	4,5,6,7,11,12,13,15,17	3	P05539,Q63942,Q9QZK9
1768	1,2,3,8,9,10,14,17	4,5,6,7,11,12,13,15,16	0	
1769	1,2,3,8,9,10,15,16	4,5,6,7,11,12,13,14,17	4	P01835,Q5QE79,Q63942,Q9QZK9
1770	1,2,3,8,9,10,15,17	4,5,6,7,11,12,13,14,16	1	Q5QE79

1771	1,2,3,8,9,10,16,17	4,5,6,7,11,12,13,14,15	2	P05539,P25031
1772	1,2,3,8,9,11,12,13	4,5,6,7,10,14,15,16,17	1	P20766
1773	1,2,3,8,9,11,12,14	4,5,6,7,10,13,15,16,17	0	
1774	1,2,3,8,9,11,12,15	4,5,6,7,10,13,14,16,17	1	Q5QE79
1775	1,2,3,8,9,11,12,16	4,5,6,7,10,13,14,15,17	0	
1776	1,2,3,8,9,11,12,17	4,5,6,7,10,13,14,15,16	1	P16636
1777	1,2,3,8,9,11,13,14	4,5,6,7,10,12,15,16,17	1	P20766
1778	1,2,3,8,9,11,13,15	4,5,6,7,10,12,14,16,17	2	P20766,Q5QE79
1779	1,2,3,8,9,11,13,16	4,5,6,7,10,12,14,15,17	1	P20766
1780	1,2,3,8,9,11,13,17	4,5,6,7,10,12,14,15,16	1	P20766
1781	1,2,3,8,9,11,14,15	4,5,6,7,10,12,13,16,17	3	P19218,Q5QE79,Q9QZK9
1782	1,2,3,8,9,11,14,16	4,5,6,7,10,12,13,15,17	2	Q99MH3,Q9QZK9
1783	1,2,3,8,9,11,14,17	4,5,6,7,10,12,13,15,16	1	P19218
1784	1,2,3,8,9,11,15,16	4,5,6,7,10,12,13,14,17	3	B4F795,Q5QE79,Q9QZK9
1785	1,2,3,8,9,11,15,17	4,5,6,7,10,12,13,14,16	1	Q5QE79

1786	1,2,3,8,9,11,16,17	4,5,6,7,10,12,13,14,15	1	Q498D9
1787	1,2,3,8,9,12,13,14	4,5,6,7,10,11,15,16,17	4	P20766,P36970,Q5FVI6,Q9Z1F2
1788	1,2,3,8,9,12,13,15	4,5,6,7,10,11,14,16,17	4	P15999,P20766,P61206;P84079;P84082,Q5QE79
1789	1,2,3,8,9,12,13,16	4,5,6,7,10,11,14,15,17	0	
1790	1,2,3,8,9,12,13,17	4,5,6,7,10,11,14,15,16	2	P20766,Q5FVI6
1791	1,2,3,8,9,12,14,15	4,5,6,7,10,11,13,16,17	4	A2RUV9,P61206;P84079;P84082,Q5FVI6,Q5QE79
1792	1,2,3,8,9,12,14,16	4,5,6,7,10,11,13,15,17	0	
1793	1,2,3,8,9,12,14,17	4,5,6,7,10,11,13,15,16	3	P31430,Q5FVI6,Q8VD89
1794	1,2,3,8,9,12,15,16	4,5,6,7,10,11,13,14,17	2	P83121,Q5QE79
1795	1,2,3,8,9,12,15,17	4,5,6,7,10,11,13,14,16	4	P61206;P84079;P84082,P83121,Q5FVI6,Q5QE79
1796	1,2,3,8,9,12,16,17	4,5,6,7,10,11,13,14,15	0	
1797	1,2,3,8,9,13,14,15	4,5,6,7,10,11,12,16,17	7	P06757,P15999,P20766,P61206;P84079;P84082,Q08849,Q5FVI6,Q5QE79
1798	1,2,3,8,9,13,14,16	4,5,6,7,10,11,12,15,17	4	P20766,Q08849,Q5FVI6,Q9QZK9
1799	1,2,3,8,9,13,14,17	4,5,6,7,10,11,12,15,16	6	P20766,P51907,P61206;P84079;P84082,Q08849,Q5FVI6,Q9QW07
1800	1,2,3,8,9,13,15,16	4,5,6,7,10,11,12,14,17	4	P01835,P20766,Q5QE79,Q9QZK9

1801	1,2,3,8,9,13,15,17	4,5,6,7,10,11,12,14,16	5	P15999,P20766,P61206;P84079;P84082,Q5FVI6,Q5QE79
1802	1,2,3,8,9,13,16,17	4,5,6,7,10,11,12,14,15	3	P20766,P51907,Q5FVI6
1803	1,2,3,8,9,14,15,16	4,5,6,7,10,11,12,13,17	4	Q08849,Q5QE79,Q63942,Q9QZK9
1804	1,2,3,8,9,14,15,17	4,5,6,7,10,11,12,13,16	4	P61206;P84079;P84082,Q08849,Q5FVI6,Q5QE79
1805	1,2,3,8,9,14,16,17	4,5,6,7,10,11,12,13,15	2	Q08849,Q5FVI6
1806	1,2,3,8,9,15,16,17	4,5,6,7,10,11,12,13,14	4	P0C0A9,P47727,Q5QE79,Q68FV8
1807	1,2,3,8,10,11,12,13	4,5,6,7,9,14,15,16,17	0	
1808	1,2,3,8,10,11,12,14	4,5,6,7,9,13,15,16,17	2	P62804,Q00715
1809	1,2,3,8,10,11,12,15	4,5,6,7,9,13,14,16,17	3	P00731,P35280,P62804
1810	1,2,3,8,10,11,12,16	4,5,6,7,9,13,14,15,17	1	Q9WUW8
1811	1,2,3,8,10,11,12,17	4,5,6,7,9,13,14,15,16	4	P16636,P17475,P62804,Q00715
1812	1,2,3,8,10,11,13,14	4,5,6,7,9,12,15,16,17	1	P20766
1813	1,2,3,8,10,11,13,15	4,5,6,7,9,12,14,16,17	2	P01835,P15999
1814	1,2,3,8,10,11,13,16	4,5,6,7,9,12,14,15,17	1	P01835
1815	1,2,3,8,10,11,13,17	4,5,6,7,9,12,14,15,16	2	P16636,P20766
1816	1,2,3,8,10,11,14,15	4,5,6,7,9,12,13,16,17	2	P09605;P25809,P35280
1817	1,2,3,8,10,11,14,16	4,5,6,7,9,12,13,15,17	0	
1818	1,2,3,8,10,11,14,17	4,5,6,7,9,12,13,15,16	1	P19218
1819	1,2,3,8,10,11,15,16	4,5,6,7,9,12,13,14,17	1	P01835
1820	1,2,3,8,10,11,15,17	4,5,6,7,9,12,13,14,16	1	P17475

1821	1,2,3,8,10,<br>11,16,17	4,5,6,7,9,1<br>2,13,14,15	1	P05539
1822	1,2,3,8,10,<br>12,13,14	4,5,6,7,9,1<br>1,15,16,17	2	P36970,Q8K4G9
1823	1,2,3,8,10,<br>12,13,15	4,5,6,7,9,1<br>1,14,16,17	2	P15999,P83121
1824	1,2,3,8,10,<br>12,13,16	4,5,6,7,9,1<br>1,14,15,17	1	P83121
1825	1,2,3,8,10,<br>12,13,17	4,5,6,7,9,1<br>1,14,15,16	4	P16636,P17475,P83121,Q8K4G9
1826	1,2,3,8,10,<br>12,14,15	4,5,6,7,9,1<br>1,13,16,17	1	A2RUV9
1827	1,2,3,8,10,<br>12,14,16	4,5,6,7,9,1<br>1,13,15,17	2	P05539,P08721
1828	1,2,3,8,10,<br>12,14,17	4,5,6,7,9,1<br>1,13,15,16	2	P05539,P31430
1829	1,2,3,8,10,<br>12,15,16	4,5,6,7,9,1<br>1,13,14,17	2	P05539,P83121
1830	1,2,3,8,10,<br>12,15,17	4,5,6,7,9,1<br>1,13,14,16	4	P05539,P06866,P17475,P83121
1831	1,2,3,8,10,<br>12,16,17	4,5,6,7,9,1<br>1,13,14,15	4	P05539,P06866,P16636,P83121
1832	1,2,3,8,10,<br>13,14,15	4,5,6,7,9,1<br>1,12,16,17	5	P01835,P09605;P25809,P15999,Q08849,Q64093
1833	1,2,3,8,10,<br>13,14,16	4,5,6,7,9,1<br>1,12,15,17	2	P01835,Q08849
1834	1,2,3,8,10,<br>13,14,17	4,5,6,7,9,1<br>1,12,15,16	4	P20766,P51907,Q08849,Q9QW07
1835	1,2,3,8,10,<br>13,15,16	4,5,6,7,9,1<br>1,12,14,17	3	P01835,P15999,P83121
1836	1,2,3,8,10,<br>13,15,17	4,5,6,7,9,1<br>1,12,14,16	4	P01835,P15999,P17475,P83121
1837	1,2,3,8,10,<br>13,16,17	4,5,6,7,9,1<br>1,12,14,15	3	P01835,P51907,P83121
1838	1,2,3,8,10,<br>14,15,16	4,5,6,7,9,1<br>1,12,13,17	5	P01835,P05539,P09605;P25809,Q08849,Q9QZK9
1839	1,2,3,8,10,<br>14,15,17	4,5,6,7,9,1<br>1,12,13,16	2	P17475,Q08849
1840	1,2,3,8,10,<br>14,16,17	4,5,6,7,9,1<br>1,12,13,15	1	P05539
1841	1,2,3,8,10,<br>15,16,17	4,5,6,7,9,1<br>1,12,13,14	4	P01835,P05539,P0C0A9,P83121
1842	1,2,3,8,11,<br>12,13,14	4,5,6,7,9,1<br>0,15,16,17	2	Q64093,Q9Z1F2
1843	1,2,3,8,11,<br>12,13,15	4,5,6,7,9,1<br>0,14,16,17	1	P15999

1844	1,2,3,8,11,<br>12,13,16	4,5,6,7,9,1<br>0,14,15,17	0	
1845	1,2,3,8,11,<br>12,13,17	4,5,6,7,9,1<br>0,14,15,16	2	P16636,P20766
1846	1,2,3,8,11,<br>12,14,15	4,5,6,7,9,1<br>0,13,16,17	0	
1847	1,2,3,8,11,<br>12,14,16	4,5,6,7,9,1<br>0,13,15,17	0	
1848	1,2,3,8,11,<br>12,14,17	4,5,6,7,9,1<br>0,13,15,16	3	P31430,P62804,Q00715
1849	1,2,3,8,11,<br>12,15,16	4,5,6,7,9,1<br>0,13,14,17	1	P83121
1850	1,2,3,8,11,<br>12,15,17	4,5,6,7,9,1<br>0,13,14,16	2	P17475,P83121
1851	1,2,3,8,11,<br>12,16,17	4,5,6,7,9,1<br>0,13,14,15	1	P16636
1852	1,2,3,8,11,<br>13,14,15	4,5,6,7,9,1<br>0,12,16,17	3	P15999,Q08849,Q64093
1853	1,2,3,8,11,<br>13,14,16	4,5,6,7,9,1<br>0,12,15,17	0	
1854	1,2,3,8,11,<br>13,14,17	4,5,6,7,9,1<br>0,12,15,16	5	P20766,Q08849,Q5FVI6,Q64093,Q9QW07
1855	1,2,3,8,11,<br>13,15,16	4,5,6,7,9,1<br>0,12,14,17	1	P01835
1856	1,2,3,8,11,<br>13,15,17	4,5,6,7,9,1<br>0,12,14,16	3	P15999,P17475,P20766
1857	1,2,3,8,11,<br>13,16,17	4,5,6,7,9,1<br>0,12,14,15	1	P20766
1858	1,2,3,8,11,<br>14,15,16	4,5,6,7,9,1<br>0,12,13,17	1	Q9QZK9
1859	1,2,3,8,11,<br>14,15,17	4,5,6,7,9,1<br>0,12,13,16	3	P19218,P97840,Q08849
1860	1,2,3,8,11,<br>14,16,17	4,5,6,7,9,1<br>0,12,13,15	0	
1861	1,2,3,8,11,<br>15,16,17	4,5,6,7,9,1<br>0,12,13,14	0	
1862	1,2,3,8,12,<br>13,14,15	4,5,6,7,9,1<br>0,11,16,17	6	A2RUV9,P15999,P36970,P61206;P84079;P84082,Q5FVI6,Q64093
1863	1,2,3,8,12,<br>13,14,16	4,5,6,7,9,1<br>0,11,15,17	3	Q566E6,Q5FVI6,Q64093
1864	1,2,3,8,12,<br>13,14,17	4,5,6,7,9,1<br>0,11,15,16	10	P31430,P51907,P61206;P84079;P84082,Q5FVI6,Q62635,Q64093,Q6PCU2,Q80W57,Q9QW07,Q9Z1F2
1865	1,2,3,8,12,<br>13,15,16	4,5,6,7,9,1<br>0,11,14,17	2	P15999,P83121
1866	1,2,3,8,12,<br>13,15,17	4,5,6,7,9,1<br>0,11,14,16	7	P15999,P17475,P61206;P84079;P84082,P83121,Q5FVI6,Q5XFX0,Q64093

1867	1,2,3,8,12,<br>13,16,17	4,5,6,7,9,1<br>0,11,14,15	5	P63029,P83121,Q5FVI6,Q5XFX0,Q6PCU2
1868	1,2,3,8,12,<br>14,15,16	4,5,6,7,9,1<br>0,11,13,17	1	P83121
1869	1,2,3,8,12,<br>14,15,17	4,5,6,7,9,1<br>0,11,13,16	6	A2RUV9,P17475,P31430,P61206;P84079;P84082,P83121,Q5FVI6
1870	1,2,3,8,12,<br>14,16,17	4,5,6,7,9,1<br>0,11,13,15	5	P05539,P31430,P83121,Q5FVI6,Q6PCU2
1871	1,2,3,8,12,<br>15,16,17	4,5,6,7,9,1<br>0,11,13,14	4	P05539,P06866,P83121,Q5XFX0
1872	1,2,3,8,13,<br>14,15,16	4,5,6,7,9,1<br>0,11,12,17	6	P01835,P15999,Q08849,Q566E6,Q64093,Q9QZK9
1873	1,2,3,8,13,<br>14,15,17	4,5,6,7,9,1<br>0,11,12,16	11	P05712,P08753,P15999,P20766,P61206;P84079;P84082,Q08849,Q5FVI6,Q62687,Q64093,Q6PCU2,Q9QW07
1874	1,2,3,8,13,<br>14,16,17	4,5,6,7,9,1<br>0,11,12,15	8	P08753,P51907,Q08849,Q566E6,Q5FVI6,Q64093,Q6PCU2,Q9QW07
1875	1,2,3,8,13,<br>15,16,17	4,5,6,7,9,1<br>0,11,12,14	7	P00714,P01835,P15999,P83121,Q08849,Q5XFX0,Q6PCU2
1876	1,2,3,8,14,<br>15,16,17	4,5,6,7,9,1<br>0,11,12,13	5	P0C0A9,P83121,Q08849,Q5FVI6,Q6PCU2
1877	1,2,3,9,10,<br>11,12,13	4,5,6,7,8,1<br>4,15,16,17	4	P06760,P20760,Q63661,Q91ZS3
1878	1,2,3,9,10,<br>11,12,14	4,5,6,7,8,1<br>3,15,16,17	4	P06760,P20760,Q63661,Q99041
1879	1,2,3,9,10,<br>11,12,15	4,5,6,7,8,1<br>3,14,16,17	3	P06760,Q63661,Q99041
1880	1,2,3,9,10,<br>11,12,16	4,5,6,7,8,1<br>3,14,15,17	7	P06760,P06761,P07171,P10536,P20760,Q63661,Q9WUW8
1881	1,2,3,9,10,<br>11,12,17	4,5,6,7,8,1<br>3,14,15,16	3	P07171,P16636,Q63661
1882	1,2,3,9,10,<br>11,13,14	4,5,6,7,8,1<br>2,15,16,17	5	P06760,P20760,Q4G075,Q63661,Q99041
1883	1,2,3,9,10,<br>11,13,15	4,5,6,7,8,1<br>2,14,16,17	5	P06760,P06761,Q4G075,Q63661,Q99041
1884	1,2,3,9,10,<br>11,13,16	4,5,6,7,8,1<br>2,14,15,17	6	P06760,P06761,P20760,Q4G075,Q63661,Q99041
1885	1,2,3,9,10,<br>11,13,17	4,5,6,7,8,1<br>2,14,15,16	2	P07171,Q99041
1886	1,2,3,9,10,<br>11,14,15	4,5,6,7,8,1<br>2,13,16,17	9	P06760,P06761,P22282,P22283,P23928,Q4G075,Q63661,Q99041,Q9QZK9
1887	1,2,3,9,10,<br>11,14,16	4,5,6,7,8,1<br>2,13,15,17	11	P06760,P06761,P20760,P22282,P22283,Q4G075,Q63661,Q99041,Q99MH3,Q9QZK9,Q9WUW8
1888	1,2,3,9,10,<br>11,14,17	4,5,6,7,8,1<br>2,13,15,16	5	P06761,Q63661,Q8R431,Q99041,Q9QUL6
1889	1,2,3,9,10,<br>11,15,16	4,5,6,7,8,1<br>2,13,14,17	11	P02780,P06760,P06761,P11598,P22282,P22283,Q4G075,Q62902,Q63661,Q99041,Q9QZK9

1890	1,2,3,9,10,<br>11,15,17	4,5,6,7,8,1<br>2,13,14,16	3	P06761,Q62902,Q99041
1891	1,2,3,9,10,<br>11,16,17	4,5,6,7,8,1<br>2,13,14,15	3	P06761,P07171,Q99041
1892	1,2,3,9,10,<br>12,13,14	4,5,6,7,8,1<br>1,15,16,17	7	P06760,P07150,P07171,P14668,P20760,Q4G075,Q99041
1893	1,2,3,9,10,<br>12,13,15	4,5,6,7,8,1<br>1,14,16,17	6	P06760,P07150,P07171,P14668,Q6AYS4,Q99041
1894	1,2,3,9,10,<br>12,13,16	4,5,6,7,8,1<br>1,14,15,17	8	P06760,P07150,P07171,P14669,P20760,P97840,Q6AYS4,Q99041
1895	1,2,3,9,10,<br>12,13,17	4,5,6,7,8,1<br>1,14,15,16	3	P07150,P07171,P14668
1896	1,2,3,9,10,<br>12,14,15	4,5,6,7,8,1<br>1,13,16,17	5	A2RUV9,P06760,P07150,P14668,Q99041
1897	1,2,3,9,10,<br>12,14,16	4,5,6,7,8,1<br>1,13,15,17	8	P06760,P07150,P07171,P14669,P20760,Q6AYS4,Q99041,Q9WUW8
1898	1,2,3,9,10,<br>12,14,17	4,5,6,7,8,1<br>1,13,15,16	3	P07171,P14668,Q8VD89
1899	1,2,3,9,10,<br>12,15,16	4,5,6,7,8,1<br>1,13,14,17	10	P06760,P06761,P07150,P07171,P14669,P22282,P22283,Q6AYS4,Q99041,Q9WUW8
1900	1,2,3,9,10,<br>12,15,17	4,5,6,7,8,1<br>1,13,14,16	4	P06866,P07150,P07171,P14668
1901	1,2,3,9,10,<br>12,16,17	4,5,6,7,8,1<br>1,13,14,15	4	P05539,P06866,P07171,P14668
1902	1,2,3,9,10,<br>13,14,15	4,5,6,7,8,1<br>1,12,16,17	10	P06760,P07150,P14668,P20646,P50115,Q07936,Q4G075,Q6AYS4,Q99041,Q9QZK9
1903	1,2,3,9,10,<br>13,14,16	4,5,6,7,8,1<br>1,12,15,17	9	P06760,P07150,P14669,P20760,P50115,Q4G075,Q6AYS4,Q99041,Q9QZK9
1904	1,2,3,9,10,<br>13,14,17	4,5,6,7,8,1<br>1,12,15,16	5	P07150,P07171,P14668,Q99041,Q9QW07
1905	1,2,3,9,10,<br>13,15,16	4,5,6,7,8,1<br>1,12,14,17	14	P01835,P02780,P06760,P06761,P07150,P0C0A9,P14669,P22282,P22283,P50115,Q4G075,Q6AYS4,Q99041,Q9QZK9
1906	1,2,3,9,10,<br>13,15,17	4,5,6,7,8,1<br>1,12,14,16	7	P07150,P07171,P0C0A9,P14668,Q07936,Q8CJ52,Q99041
1907	1,2,3,9,10,<br>13,16,17	4,5,6,7,8,1<br>1,12,14,15	6	P06761,P07171,P0C0A9,P14668,P14669,Q99041
1908	1,2,3,9,10,<br>14,15,16	4,5,6,7,8,1<br>1,12,13,17	20	P02780,P02781,P06760,P06761,P07150,P07647,P0C0A9,P14669,P22282,P22283,P30120,P36374,P50115,Q4G075,Q62902,Q6AYS4,Q8CJ<br>52,Q99041,Q9JHB9,Q9QZK9
1909	1,2,3,9,10,<br>14,15,17	4,5,6,7,8,1<br>1,12,13,16	11	P02780,P06761,P07150,P0C0A9,P14668,P22282,P22283,Q62902,Q8CJ52,Q99041,Q9JHB9
1910	1,2,3,9,10,<br>14,16,17	4,5,6,7,8,1<br>1,12,13,15	9	P02780,P06761,P07171,P0C0A9,P14669,P22282,P22283,Q99041,Q9JHB9
1911	1,2,3,9,10,<br>15,16,17	4,5,6,7,8,1<br>1,12,13,14	16	P02780,P02781,P02782,P06761,P07171,P07647,P0C0A9,P22282,P22283,P30120,P46462,P47727,Q62902,Q8CJ52,Q99041,Q9JHB9
1912	1,2,3,9,11,<br>12,13,14	4,5,6,7,8,1<br>0,15,16,17	3	P06760,P20760,Q99041

1913	1,2,3,9,11,12,13,15	4,5,6,7,8,10,14,16,17	1	P06760
1914	1,2,3,9,11,12,13,16	4,5,6,7,8,10,14,15,17	3	P06760,P07171,P20760
1915	1,2,3,9,11,12,13,17	4,5,6,7,8,10,14,15,16	2	P07171,P82471
1916	1,2,3,9,11,12,14,15	4,5,6,7,8,10,13,16,17	2	P06760,Q99041
1917	1,2,3,9,11,12,14,16	4,5,6,7,8,10,13,15,17	4	P06760,P20760,Q99041,Q99MH3
1918	1,2,3,9,11,12,14,17	4,5,6,7,8,10,13,15,16	0	
1919	1,2,3,9,11,12,15,16	4,5,6,7,8,10,13,14,17	4	P06760,P06761,P12020,P22282
1920	1,2,3,9,11,12,15,17	4,5,6,7,8,10,13,14,16	2	P06866,P12020
1921	1,2,3,9,11,12,16,17	4,5,6,7,8,10,13,14,15	3	P06866,P07171,P12020
1922	1,2,3,9,11,13,14,15	4,5,6,7,8,10,12,16,17	6	P06760,P20646,P82471,Q4G075,Q99041,Q9QZK9
1923	1,2,3,9,11,13,14,16	4,5,6,7,8,10,12,15,17	7	P06760,P20760,Q4G075,Q7TPB1,Q99041,Q99MH3,Q9QZK9
1924	1,2,3,9,11,13,14,17	4,5,6,7,8,10,12,15,16	3	P82471,Q99041,Q9QW07
1925	1,2,3,9,11,13,15,16	4,5,6,7,8,10,12,14,17	7	P06760,P06761,P22282,P22283,Q4G075,Q99041,Q9QZK9
1926	1,2,3,9,11,13,15,17	4,5,6,7,8,10,12,14,16	1	P82471
1927	1,2,3,9,11,13,16,17	4,5,6,7,8,10,12,14,15	1	P07171
1928	1,2,3,9,11,14,15,16	4,5,6,7,8,10,12,13,17	10	P02780,P06760,P06761,P22282,P22283,Q4G075,Q6AYR9,Q99041,Q99MH3,Q9QZK9
1929	1,2,3,9,11,14,15,17	4,5,6,7,8,10,12,13,16	2	P06761,Q99041
1930	1,2,3,9,11,14,16,17	4,5,6,7,8,10,12,13,15	2	P06761,Q99041
1931	1,2,3,9,11,15,16,17	4,5,6,7,8,10,12,13,14	11	P02780,P06761,P06911,P0C0A9,P12020,P22282,P22283,Q498D9,Q62902,Q6AYR9,Q99041
1932	1,2,3,9,12,13,14,15	4,5,6,7,8,10,11,16,17	13	A2RUV9,P00714,P06760,P07150,P14668,P14669,P20646,P61206;P84079;P84082,P82471,Q4G075,Q5FVI6,Q6AYS4,Q99041
1933	1,2,3,9,12,13,14,16	4,5,6,7,8,10,11,15,17	10	P00714,P06760,P07150,P07171,P14669,P20760,P82471,Q4G075,Q6AYS4,Q99041
1934	1,2,3,9,12,13,14,17	4,5,6,7,8,10,11,15,16	9	P00714,P07150,P07171,P14668,P61206;P84079;P84082,P82471,Q5FVI6,Q8VD89,Q9QW07
1935	1,2,3,9,12,13,15,16	4,5,6,7,8,10,11,14,17	9	P00714,P06760,P07150,P07171,P14669,P82471,Q4G075,Q6AYS4,Q99041

1936	1,2,3,9,12,13,15,17	4,5,6,7,8,10,11,14,16	7	P00714,P07150,P07171,P14668,P61206;P84079;P84082,P82471,Q5FVI6
1937	1,2,3,9,12,13,16,17	4,5,6,7,8,10,11,14,15	7	P00714,P07150,P07171,P14668,P14669,P82471,Q6AYS4
1938	1,2,3,9,12,14,15,16	4,5,6,7,8,10,11,13,17	9	P06760,P06761,P07150,P14669,P22282,P22283,Q4G075,Q6AYS4,Q99041
1939	1,2,3,9,12,14,15,17	4,5,6,7,8,10,11,13,16	8	A2RUV9,P00714,P07150,P14668,P61206;P84079;P84082,P82471,Q5FVI6,Q8VD89
1940	1,2,3,9,12,14,16,17	4,5,6,7,8,10,11,13,15	3	P00714,P07171,P14669
1941	1,2,3,9,12,15,16,17	4,5,6,7,8,10,11,13,14	12	P00714,P06761,P06866,P06911,P07150,P07171,P12020,P14669,P22283,P47727,P83121,Q6AYS4
1942	1,2,3,9,13,14,15,16	4,5,6,7,8,10,11,12,17	16	P00714,P06760,P06761,P07150,P0C0A9,P14669,P20646,P22282,P22283,P50115,P60905,P82471,Q4G075,Q6AYS4,Q99041,Q9QZK9
1943	1,2,3,9,13,14,15,17	4,5,6,7,8,10,11,12,16	14	P00714,P07150,P0C0A9,P14668,P48199,P60905,P61206;P84079;P84082,P82471,Q07936,Q5FVI6,Q6AYS4,Q8CJ52,Q99041,Q9QW07
1944	1,2,3,9,13,14,16,17	4,5,6,7,8,10,11,12,15	11	P00714,P07150,P07171,P0C0A9,P14668,P14669,P82471,Q5FVI6,Q6AYS4,Q99041,Q9QW07
1945	1,2,3,9,13,15,16,17	4,5,6,7,8,10,11,12,14	18	P00714,P02780,P02781,P06761,P07150,P07171,P0C0A9,P14668,P14669,P22283,P30120,P47727,P60905,P61206;P84079;P84082,P82471,Q6AYS4,Q8CJ52,Q99041
1946	1,2,3,9,14,15,16,17	4,5,6,7,8,10,11,12,13	20	P00714,P02780,P02781,P06761,P07150,P07647,P0C0A9,P14669,P22282,P22283,P30120,P47727,P60905,P61206;P84079;P84082,P82471,Q62902,Q6AYS4,Q8CJ52,Q99041,Q9JHB9
1947	1,2,3,10,11,12,13,14	4,5,6,7,8,9,15,16,17	2	P06760,Q99041
1948	1,2,3,10,11,12,13,15	4,5,6,7,8,9,14,16,17	2	P06760,Q63617
1949	1,2,3,10,11,12,13,16	4,5,6,7,8,9,14,15,17	3	P06760,P07171,P20760
1950	1,2,3,10,11,12,13,17	4,5,6,7,8,9,14,15,16	2	P07171,P16636
1951	1,2,3,10,11,12,14,15	4,5,6,7,8,9,13,16,17	2	P06760,Q99041
1952	1,2,3,10,11,12,14,16	4,5,6,7,8,9,13,15,17	4	P06760,P20760,Q99041,Q9WUW8
1953	1,2,3,10,11,12,14,17	4,5,6,7,8,9,13,15,16	0	
1954	1,2,3,10,11,12,15,16	4,5,6,7,8,9,13,14,17	4	P06760,P06761,P22282,Q63617
1955	1,2,3,10,11,12,15,17	4,5,6,7,8,9,13,14,16	2	P06866,Q63617
1956	1,2,3,10,11,12,16,17	4,5,6,7,8,9,13,14,15	3	P06757,P06866,P07171
1957	1,2,3,10,11,13,14,15	4,5,6,7,8,9,12,16,17	6	P06760,P09605;P25809,P20646,P50115,Q4G075,Q99041
1958	1,2,3,10,11,13,14,16	4,5,6,7,8,9,12,15,17	6	P06760,P20646,P50115,Q4G075,Q7TPB1,Q99041

1959	1,2,3,10,11,13,14,17	4,5,6,7,8,9,12,15,16	2	Q99041,Q9QW07
1960	1,2,3,10,11,13,15,16	4,5,6,7,8,9,12,14,17	8	P01835,P06760,P06761,P22282,P22283,P50115,Q4G075,Q99041
1961	1,2,3,10,11,13,15,17	4,5,6,7,8,9,12,14,16	0	
1962	1,2,3,10,11,13,16,17	4,5,6,7,8,9,12,14,15	1	P07171
1963	1,2,3,10,11,14,15,16	4,5,6,7,8,9,12,13,17	9	P02780,P06760,P06761,P22282,P22283,P50115,Q4G075,Q99041,Q9QZK9
1964	1,2,3,10,11,14,15,17	4,5,6,7,8,9,12,13,16	2	P06761,Q99041
1965	1,2,3,10,11,14,16,17	4,5,6,7,8,9,12,13,15	2	P06761,Q99041
1966	1,2,3,10,11,15,16,17	4,5,6,7,8,9,12,13,14	6	P02780,P06761,P0C0A9,P22282,P22283,Q62902
1967	1,2,3,10,12,13,14,15	4,5,6,7,8,9,11,16,17	9	A2RUV9,P06760,P07150,P14668,P20646,P50115,Q63617,Q6AYS4,Q99041
1968	1,2,3,10,12,13,14,16	4,5,6,7,8,9,11,15,17	8	P06760,P07150,P07171,P14669,P20646,P50115,Q6AYS4,Q99041
1969	1,2,3,10,12,13,14,17	4,5,6,7,8,9,11,15,16	4	P07171,P14668,Q05175,Q9QW07
1970	1,2,3,10,12,13,15,16	4,5,6,7,8,9,11,14,17	9	P06760,P07150,P07171,P14669,P50115,P83121,Q63617,Q6AYS4,Q99041
1971	1,2,3,10,12,13,15,17	4,5,6,7,8,9,11,14,16	5	P06866,P07171,P14668,P83121,Q63617
1972	1,2,3,10,12,13,16,17	4,5,6,7,8,9,11,14,15	5	P06866,P07171,P14668,P14669,P83121
1973	1,2,3,10,12,14,15,16	4,5,6,7,8,9,11,13,17	9	P06760,P07150,P14669,P22282,P22283,P50115,Q63617,Q6AYS4,Q99041
1974	1,2,3,10,12,14,15,17	4,5,6,7,8,9,11,13,16	4	A2RUV9,P06866,P14668,Q63617
1975	1,2,3,10,12,14,16,17	4,5,6,7,8,9,11,13,15	4	P05539,P06866,P07171,P14668
1976	1,2,3,10,12,15,16,17	4,5,6,7,8,9,11,13,14	10	P05539,P06761,P06866,P07171,P14668,P22282,P22283,P83121,Q63617,Q6AYS4
1977	1,2,3,10,13,14,15,16	4,5,6,7,8,9,11,12,17	16	P01835,P02780,P06760,P06761,P07150,P09605;P25809,P0C0A9,P14669,P20646,P22282,P22283,P50115,Q4G075,Q6AYS4,Q99041,Q9QZK9
1978	1,2,3,10,13,14,15,17	4,5,6,7,8,9,11,12,16	5	P0C0A9,P14668,P61206;P84079;P84082,Q99041,Q9QW07
1979	1,2,3,10,13,14,16,17	4,5,6,7,8,9,11,12,15	6	P07171,P0C0A9,P14668,P14669,Q99041,Q9QW07
1980	1,2,3,10,13,15,16,17	4,5,6,7,8,9,11,12,14	14	P01835,P02780,P06761,P07171,P0C0A9,P14668,P14669,P22282,P22283,P30120,P83121,Q63617,Q6AYS4,Q99041
1981	1,2,3,10,14,15,16,17	4,5,6,7,8,9,11,12,13	14	P02780,P02781,P05539,P06761,P07647,P0C0A9,P22282,P22283,P30120,Q62902,Q6AYS4,Q8CJ52,Q99041,Q9JHB9

1982	1,2,3,11,12,13,14,15	4,5,6,7,8,9,10,16,17	4	A2RUV9,P06760,P20646,Q64093
1983	1,2,3,11,12,13,14,16	4,5,6,7,8,9,10,15,17	3	P06760,P20646,P20760
1984	1,2,3,11,12,13,14,17	4,5,6,7,8,9,10,15,16	2	Q64093,Q9QW07
1985	1,2,3,11,12,13,15,16	4,5,6,7,8,9,10,14,17	1	P06760
1986	1,2,3,11,12,13,15,17	4,5,6,7,8,9,10,14,16	2	P06866,P82471
1987	1,2,3,11,12,13,16,17	4,5,6,7,8,9,10,14,15	1	P07171
1988	1,2,3,11,12,14,15,16	4,5,6,7,8,9,10,13,17	2	P06760,Q99041
1989	1,2,3,11,12,14,15,17	4,5,6,7,8,9,10,13,16	1	P06866
1990	1,2,3,11,12,14,16,17	4,5,6,7,8,9,10,13,15	0	
1991	1,2,3,11,12,15,16,17	4,5,6,7,8,9,10,13,14	4	P06761,P06866,P12020,P83121
1992	1,2,3,11,13,14,15,16	4,5,6,7,8,9,10,12,17	8	P06760,P06761,P20646,P22283,P50115,Q4G075,Q99041,Q9QZK9
1993	1,2,3,11,13,14,15,17	4,5,6,7,8,9,10,12,16	5	P00714,P82471,Q64093,Q99041,Q9QW07
1994	1,2,3,11,13,14,16,17	4,5,6,7,8,9,10,12,15	2	Q99041,Q9QW07
1995	1,2,3,11,13,15,16,17	4,5,6,7,8,9,10,12,14	3	P00714,P06761,P0C0A9
1996	1,2,3,11,14,15,16,17	4,5,6,7,8,9,10,12,13	6	P02780,P06761,P0C0A9,P22282,P22283,Q99041
1997	1,2,3,12,13,14,15,16	4,5,6,7,8,9,10,11,17	11	A2RUV9,P00714,P06760,P07150,P14669,P20646,P50115,P83121,Q64093,Q6AYS4,Q99041
1998	1,2,3,12,13,14,15,17	4,5,6,7,8,9,10,11,16	10	A2RUV9,P00714,P07150,P14668,P61206;P84079;P84082,P82471,Q5FVI6,Q64093,Q6AYS4,Q9QW07
1999	1,2,3,12,13,14,16,17	4,5,6,7,8,9,10,11,15	8	P00714,P07171,P14668,P14669,P82471,Q5FVI6,Q6AYS4,Q9QW07
2000	1,2,3,12,13,15,16,17	4,5,6,7,8,9,10,11,14	11	P00714,P06866,P07171,P14668,P14669,P61206;P84079;P84082,P82471,P83121,Q5XFX0,Q63617,Q6AYS4
2001	1,2,3,12,14,15,16,17	4,5,6,7,8,9,10,11,13	7	P00714,P06866,P14669,P22283,P61206;P84079;P84082,P83121,Q6AYS4
2002	1,2,3,13,14,15,16,17	4,5,6,7,8,9,10,11,12	17	P00714,P02780,P02781,P0C0A9,P14668,P14669,P20646,P22283,P48199,P60905,P61206;P84079;P84082,P82471,Q08849,Q5FVI6,Q6AYS4,Q99041,Q9QW07
2003	1,2,4,5,6,7,8,9	3,10,11,12,13,14,15,16,17	36	D3ZHA0,O55004,O55006,O88267,P02631,P02764,P05371,P06761,P07171,P07647,P18418,P19629,P20646,P20760,P20762,P22282,P22283,P28648,P31044,P50280,P52590,P61206;P84079;P84082,P68370,P83748,P97571,Q30KJ2,Q63617,Q63942,Q64361,Q8CJ52,Q99041,Q9EQS0,Q9JI85,Q9R0T3,Q9WTT6,Q9Z0V6

2004	1,2,4,5,6,7,8,10	3,9,11,12,13,14,15,16,17	47	D3ZHA0,O54858,O55004,O55006,O88267,P02631,P02764,P05371,P06761,P07171,P07647,P08649,P08723,P12020,P15399,P18418,P19629,P20646,P20760,P20762,P22282,P22283,P28648,P31044,P50280,P52590,P55018,P61206;P84079;P84082,P68370,P82471,P83748,P97571,P97580,Q30KJ2,Q5BJY9,Q5GRG2,Q63475,Q63617,Q64361,Q7TP52,Q8CJ52,Q99041,Q9EQS0,Q9JI85,Q9R0T3,Q9WTT6,Q9Z0V6
2005	1,2,4,5,6,7,8,11	3,9,10,12,13,14,15,16,17	51	D3ZHA0,O54715,O54858,O55004,O88267,P02631,P02764,P02780,P05371,P06761,P07150,P07171,P07647,P08649,P08723,P10715,P14668,P14669,P15399,P18418,P19629,P20646,P20760,P20762,P22282,P22283,P28648,P31044,P50280,P52590,P61206;P84079;P84082,P68370,P83748,P97580,Q07936,Q30KJ2,Q5BJY9,Q5GRG2,Q63475,Q63493,Q63617,Q64361,Q6AYS4,Q8CJ52,Q8VD89,Q99041,Q9EQS0,Q9JHB9,Q9JI85,Q9R0T3,Q9Z0V6
2006	1,2,4,5,6,7,8,12	3,9,10,11,13,14,15,16,17	59	D3ZHA0,F1LM93,O54858,O55004,O88267,P02631,P02780,P02781,P02782,P05371,P06761,P07171,P07647,P08723,P0C0A9,P10715,P13432,P18418,P19223,P19629,P20646,P20760,P20761,P20762,P22282,P22283,P24368,P28648,P30120,P31044,P50280,P52590,P61206;P84079;P84082,P62815,P68370,P83748,P97571,Q10758,Q30KJ2,Q5GRG2,Q5M8C6,Q63475,Q63493,Q63617,Q64361,Q6AYR9,Q6AYS7,Q6IFW6,Q6IMF3,Q80WL1,Q8CJ52,Q99041,Q9EQS0,Q9JHB9,Q9JI85,Q9QZK8,Q9R0T3,Q9WTT6,Q9Z0V6
2007	1,2,4,5,6,7,8,13	3,9,10,11,12,14,15,16,17	40	D3ZHA0,O55004,O88267,P02631,P02764,P02780,P05371,P06761,P06911,P07171,P07647,P08723,P12020,P18418,P19629,P20760,P20762,P22282,P22283,P25236,P28648,P31044,P50280,P52590,P61206;P84079;P84082,P68370,P83748,P97571,Q30KJ2,Q5GRG2,Q62902,Q63617,Q64361,Q8CJ52,Q99041,Q9JHB9,Q9JI85,Q9R0T3,Q9WTT6,Q9Z0V6
2008	1,2,4,5,6,7,8,14	3,9,10,11,12,13,15,16,17	41	D3ZHA0,O55004,O88267,P02631,P05371,P06761,P06911,P07171,P07647,P08723,P10536,P12020,P15399,P18418,P19629,P20760,P20762,P22282,P22283,P25236,P28648,P31044,P50280,P52590,P61206;P84079;P84082,P68370,P83748,P97571,P97580,Q30KJ2,Q5GRG2,Q63532,Q63617,Q64361,Q6AYS7,Q8CJ52,Q9EQS0,Q9JI85,Q9R0T3,Q9WTT6,Q9Z0V6
2009	1,2,4,5,6,7,8,15	3,9,10,11,12,13,14,16,17	37	D3ZHA0,O55004,O55006,O88267,P02631,P02761,P05371,P06761,P07171,P07647,P10536,P18418,P19629,P20646,P20760,P20761,P20762,P22282,P22283,P28648,P31044,P50280,P52590,P61206;P84079;P84082,P68370,P83748,P97571,Q30KJ2,Q5GRG2,Q63617,Q64361,Q8CJ52,Q99041,Q9JI85,Q9R0T3,Q9WTT6,Q9Z0V6
2010	1,2,4,5,6,7,8,16	3,9,10,11,12,13,14,15,17	33	D3ZHA0,O55004,O88267,P02631,P05371,P06761,P07171,P07647,P18418,P19629,P20646,P20760,P20762,P22282,P22283,P28648,P31044,P50280,P52590,P61206;P84079;P84082,P68370,P83748,P97571,Q30KJ2,Q63617,Q64361,Q8CJ52,Q8VD89,Q99041,Q9JI85,Q9R0T3,Q9WTT6,Q9Z0V6
2011	1,2,4,5,6,7,8,17	3,9,10,11,12,13,14,15,16	49	D3ZHA0,O54858,O55004,O88267,P02631,P02761,P05371,P06760,P06761,P07171,P07647,P09605;P25809,P10536,P15399,P18418,P19223,P19629,P20646,P20760,P20761,P20762,P22282,P22283,P23928,P28648,P31044,P50115,P50280,P52590,P55091,P61206;P84079;P84082,P62815,P68370,P83748,P97571,P97580,Q30KJ2,Q4G075,Q63617,Q64361,Q6PCU2,Q8CJ52,Q99041,Q9EPB1,Q9JI85,Q9QZA6,Q9R0T3,Q9WTT6,Q9Z0V6
2012	1,2,4,5,6,7,9,10	3,8,11,12,13,14,15,16,17	16	O55006,P02631,P05371,P15399,P18418,P20646,P20760,P20762,P28648,P52590,P61206;P84079;P84082,P83748,P97580,Q30KJ2,Q63942,Q7TP52
2013	1,2,4,5,6,7,9,11	3,8,10,12,13,14,15,16,17	15	O55004,P02631,P05371,P15399,P18418,P20646,P20762,P28648,P52590,P61206;P84079;P84082,P68370,P83748,P97580,Q30KJ2,Q9JI85
2014	1,2,4,5,6,7,9,12	3,8,10,11,13,14,15,16,17	16	O55004,O88267,P02631,P05371,P07647,P08723,P13432,P18418,P20762,P30120,P52590,P61206;P84079;P84082,P83748,Q30KJ2,Q9JHB9,Q9JI85
2015	1,2,4,5,6,7,9,13	3,8,10,11,12,14,15,16,17	13	P02631,P05539,P06911,P07647,P12020,P18418,P20762,P52590,P61206;P84079;P84082,P83748,Q30KJ2,Q5GRG2,Q9JI85
2016	1,2,4,5,6,7,9,14	3,8,10,11,12,13,15,16,17	11	P02631,P05371,P18418,P20760,P20762,P52590,P61206;P84079;P84082,P83748,Q30KJ2,Q63532,Q63942
2017	1,2,4,5,6,7,9,15	3,8,10,11,12,13,14,16,17	10	O55006,P02631,P05371,P18418,P20760,P20762,P52590,P83748,Q30KJ2,Q63942

2018	1,2,4,5,6,7,9,16	3,8,10,11,12,13,14,15,17	9	P02631,P05371,P18418,P23593,P52590,P61206;P84079;P84082,P83748,Q30KJ2,Q63942
2019	1,2,4,5,6,7,9,17	3,8,10,11,12,13,14,15,16	18	O55004,O88267,P02631,P05371,P09605;P25809,P15399,P18418,P19223,P20646,P20760,P20761,P20762,P23593,P52590,P61206;P84079;P84082,P83748,P97580,Q30KJ2
2020	1,2,4,5,6,7,10,11	3,8,9,12,13,14,15,16,17	21	O54858,P02631,P05371,P05964,P15399,P18418,P20760,P20762,P28648,P52590,P55018,P61206;P84079;P84082,P68370,P83748,P97580,Q30KJ2,Q5BJY9,Q5FVI6,Q7TP52,Q8VD89,Q9JI85
2021	1,2,4,5,6,7,10,12	3,8,9,11,13,14,15,16,17	32	F1LM93,O55004,O88267,P02631,P04218,P05371,P07647,P08723,P12020,P15399,P17559,P18418,P20760,P20762,P28648,P30120,P52590,P61206;P84079;P84082,P68370,P83748,P97580,P97840,Q10758,Q30KJ2,Q63475,Q64361,Q6IFU7,Q6IFW6,Q6IMF3,Q7TP52,Q9JHB9,Q9JI85
2022	1,2,4,5,6,7,10,13	3,8,9,11,12,14,15,16,17	18	P02631,P06911,P07647,P12020,P15399,P18418,P20760,P20762,P28648,P52590,P61206;P84079;P84082,P68370,P83748,P97580,Q30KJ2,Q5GRG2,Q7TP52,Q9JI85
2023	1,2,4,5,6,7,10,14	3,8,9,11,12,13,15,16,17	18	P02631,P05371,P12020,P15399,P18418,P20760,P20762,P28648,P52590,P55018,P61206;P84079;P84082,P68370,P83748,P97580,Q30KJ2,Q5GRG2,Q63532,Q7TP52
2024	1,2,4,5,6,7,10,15	3,8,9,11,12,13,14,16,17	14	O55006,P02631,P05371,P18418,P20760,P20762,P28648,P52590,P55018,P61206;P84079;P84082,P83748,P97580,Q30KJ2,Q7TP52
2025	1,2,4,5,6,7,10,16	3,8,9,11,12,13,14,15,17	17	P02631,P05371,P08721,P18418,P20760,P28648,P52590,P55018,P61206;P84079;P84082,P83748,P97580,P97840,Q30KJ2,Q5FVI6,Q62997,Q63532,Q7TP52
2026	1,2,4,5,6,7,10,17	3,8,9,11,12,13,14,15,16	18	O54858,O88267,P02631,P05371,P15399,P18418,P20646,P20760,P20761,P20762,P28648,P52590,P61206;P84079;P84082,P83748,P97580,Q30KJ2,Q64361,Q7TP52
2027	1,2,4,5,6,7,11,12	3,8,9,10,13,14,15,16,17	39	O55004,O88267,P02631,P04905,P05371,P07647,P08723,P0C0A9,P13432,P15399,P18418,P20760,P20762,P22282,P28648,P30120,P36374,P50280,P52590,P61206;P84079;P84082,P68370,P83748,P97580,Q10758,Q30KJ2,Q498R7,Q63357,Q63475,Q63493,Q6IFU7,Q6IFU8,Q6IFW6,Q6IMF3,Q6P6Q2,Q8CJ52,Q9EQS0,Q9ESW0,Q9JHB9,Q9JI85
2028	1,2,4,5,6,7,11,13	3,8,9,10,12,14,15,16,17	21	P02631,P05371,P05539,P06911,P07647,P12020,P15399,P18418,P20760,P20762,P22282,P28648,P61206;P84079;P84082,P68370,P83748,P97580,Q30KJ2,Q5GRG2,Q8VD89,Q9JHB9,Q9JI85
2029	1,2,4,5,6,7,11,14	3,8,9,10,12,13,15,16,17	16	P02631,P05371,P12020,P15399,P18418,P20760,P20762,P28648,P52590,P61206;P84079;P84082,P68370,P83748,P97580,Q30KJ2,Q63532,Q9JI85
2030	1,2,4,5,6,7,11,15	3,8,9,10,12,13,14,16,17	17	O55004,P02631,P05371,P14408,P15399,P18418,P20760,P20762,P28648,P52590,P61206;P84079;P84082,P68370,P83748,P97580,Q30KJ2,Q8VD89,Q9JI85
2031	1,2,4,5,6,7,11,16	3,8,9,10,12,13,14,15,17	15	G3V686,P02631,P05371,P14408,P15399,P18418,P20762,P28648,P61206;P84079;P84082,P68370,P83748,P97580,Q30KJ2,Q8VD89,Q9JI85
2032	1,2,4,5,6,7,11,17	3,8,9,10,12,13,14,15,16	22	O54858,O55004,O88267,P02631,P05371,P09605;P25809,P15399,P18418,P20646,P20760,P20761,P20762,P28648,P52590,P55091,P61206;P84079;P84082,P83748,P97580,Q30KJ2,Q63493,Q7TP52,Q9JI85

2033	1,2,4,5,6,7,12,13	3,8,9,10,11,14,15,16,17	22	O88267,P02631,P05539,P06911,P07647,P08723,P12020,P18418,P20760,P20762,P22282,P25236,P30120,P36374,P61206;P84079;P84082,P83748,Q30KJ2,Q5GRG2,Q6AYR9,Q8CJ52,Q9JHB9,Q9JI85
2034	1,2,4,5,6,7,12,14	3,8,9,10,11,13,15,16,17	23	O88267,P02631,P05371,P07647,P08723,P12020,P13432,P15399,P18418,P20760,P20762,P25236,P30120,P35467,P52590,P61206;P84079;P84082,P83748,Q10758,Q30KJ2,Q63532,Q9EQS0,Q9JHB9,Q9JI85
2035	1,2,4,5,6,7,12,15	3,8,9,10,11,13,14,16,17	14	O55004,O88267,P02631,P05371,P07647,P18418,P20760,P20762,P35467,P52590,P61206;P84079;P84082,P83748,Q30KJ2,Q9JI85
2036	1,2,4,5,6,7,12,16	3,8,9,10,11,13,14,15,17	17	G3V686,O88267,P02631,P05371,P07647,P18418,P20760,P20762,P30120,P35467,P61206;P84079;P84082,P83748,Q10758,Q30KJ2,Q63532,Q6IMF3,Q9JI85
2037	1,2,4,5,6,7,12,17	3,8,9,10,11,13,14,15,16	34	O54858,O55004,O88267,P02631,P05371,P07647,P09605;P25809,P13432,P15399,P18418,P19223,P20646,P20760,P20761,P20762,P22282,P30120,P52590,P55091,P61206;P84079;P84082,P62815,P83748,P97580,P98089,Q10758,Q30KJ2,Q4G075,Q62635,Q64361,Q99041,Q9JHB9,Q9JI85,Q9QZK9,Q9Z0V6
2038	1,2,4,5,6,7,13,14	3,8,9,10,11,12,15,16,17	17	O88267,P02631,P05371,P05539,P06911,P07647,P12020,P18418,P20760,P20762,P25236,P61206;P84079;P84082,P83748,Q30KJ2,Q5GRG2,Q63532,Q9JI85
2039	1,2,4,5,6,7,13,15	3,8,9,10,11,12,14,16,17	12	P02631,P05539,P06911,P12020,P18418,P20760,P20762,P25236,P35467,P83748,Q30KJ2,Q5GRG2
2040	1,2,4,5,6,7,13,16	3,8,9,10,11,12,14,15,17	13	G3V686,O88267,P02631,P05371,P06911,P12020,P18418,P20760,P61206;P84079;P84082,P83748,Q30KJ2,Q5GRG2,Q8VD89
2041	1,2,4,5,6,7,13,17	3,8,9,10,11,12,14,15,16	21	O88267,P02631,P05539,P06911,P07647,P09605;P25809,P12020,P15399,P18418,P20760,P20761,P20762,P22282,P52590,P55091,P62815,P83748,P97580,Q30KJ2,Q5GRG2,Q9JI85
2042	1,2,4,5,6,7,14,15	3,8,9,10,11,12,13,16,17	13	P02631,P05371,P16636,P18418,P20760,P20762,P35467,P52590,P83748,Q30KJ2,Q5GRG2,Q63532,Q6IE51
2043	1,2,4,5,6,7,14,16	3,8,9,10,11,12,13,15,17	12	G3V686,O88267,P02631,P05371,P18418,P20760,P20762,P35467,P61206;P84079;P84082,P83748,Q30KJ2,Q63532
2044	1,2,4,5,6,7,14,17	3,8,9,10,11,12,13,15,16	17	O88267,P02631,P05371,P10536,P15399,P18418,P20760,P20761,P20762,P35467,P52590,P55091,P62815,P83748,P97580,Q30KJ2,Q9Z0V6
2045	1,2,4,5,6,7,15,16	3,8,9,10,11,12,13,14,17	9	P02631,P05371,P18418,P20760,P20762,P35467,P61206;P84079;P84082,P83748,Q30KJ2
2046	1,2,4,5,6,7,15,17	3,8,9,10,11,12,13,14,16	14	O55004,P02631,P05371,P10536,P15399,P18418,P20760,P20761,P20762,P35467,P52590,P83748,P97580,Q30KJ2
2047	1,2,4,5,6,7,16,17	3,8,9,10,11,12,13,14,15	16	O88267,P02631,P05371,P09605;P25809,P15399,P18418,P20646,P20760,P23593,P23928,P35467,P52590,P61206;P84079;P84082,P83748,P97580,Q30KJ2

2048	1,2,4,5,6,8,9,10	3,7,11,12,13,14,15,16,17	13	D3ZHA0,O55006,P07647,P08649,P18418,P20762,P23593,P28648,Q5M8C6,Q63942,Q64361,Q9WTT6,Q9Z0V6
2049	1,2,4,5,6,8,9,11	3,7,10,12,13,14,15,16,17	12	A2RUV9,D3ZHA0,O54715,P07647,P08649,P14668,P14669,P20762,P28648,Q5M8C6,Q63942,Q9Z0V6
2050	1,2,4,5,6,8,9,12	3,7,10,11,13,14,15,16,17	18	D3ZHA0,P02780,P02782,P07647,P08723,P0C0A9,P13432,P20762,P23593,P30120,Q5M8C6,Q63942,Q64361,Q6IFW6,Q6IMF3,Q9JHB9,Q9WTT6,Q9Z0V6
2051	1,2,4,5,6,8,9,13	3,7,10,11,12,14,15,16,17	10	D3ZHA0,P06911,P07647,P20762,P25236,Q5M8C6,Q63942,Q6TMA8,Q9WTT6,Q9Z0V6
2052	1,2,4,5,6,8,9,14	3,7,10,11,12,13,15,16,17	7	D3ZHA0,P02793;Q7TP54,P20762,P25236,Q63942,Q9WTT6,Q9Z0V6
2053	1,2,4,5,6,8,9,15	3,7,10,11,12,13,14,16,17	9	D3ZHA0,O55006,P20760,P20762,P23593,Q5QE79,Q63942,Q9WTT6,Q9Z0V6
2054	1,2,4,5,6,8,9,16	3,7,10,11,12,13,14,15,17	7	A2RUV9,D3ZHA0,P14668,P23593,Q63942,Q9WTT6,Q9Z0V6
2055	1,2,4,5,6,8,9,17	3,7,10,11,12,13,14,15,16	15	A2RUV9,P06760,P19223,P20646,P20760,P20762,P23593,P50115,P52590,Q4G075,Q5M8C6,Q63942,Q64361,Q9WTT6,Q9Z0V6
2056	1,2,4,5,6,8,10,11	3,7,9,12,13,14,15,16,17	22	A2RUV9,D3ZHA0,O54715,O54858,P07150,P07647,P08649,P14668,P14669,P18418,P20762,P28648,P50280,P55018,P68370,Q5BJY9,Q5M8C6,Q63598,Q64361,Q6AYS4,Q7TP52,Q9Z0V6
2057	1,2,4,5,6,8,10,12	3,7,9,11,13,14,15,16,17	29	D3ZHA0,F1LM93,P02780,P02782,P06760,P07647,P08723,P0C0A9,P18418,P20762,P28648,P30120,P35281,P50280,P55018,Q10758,Q5M8C6,Q63598,Q64361,Q6AYR9,Q6IFU7,Q6IFW6,Q6IMF3,Q6P6Q2,Q8CJ52,Q9EQS0,Q9JHB9,Q9WTT6,Q9Z0V6
2058	1,2,4,5,6,8,10,13	3,7,9,11,12,14,15,16,17	14	D3ZHA0,P06911,P07647,P12020,P18418,P20762,P28648,P50280,P55018,Q5GRG2,Q5M8C6,Q64361,Q9WTT6,Q9Z0V6
2059	1,2,4,5,6,8,10,14	3,7,9,11,12,13,15,16,17	11	D3ZHA0,P02793;Q7TP54,P07647,P08649,P18418,P20762,P28648,P55018,Q64361,Q9WTT6,Q9Z0V6
2060	1,2,4,5,6,8,10,15	3,7,9,11,12,13,14,16,17	10	D3ZHA0,P02761,P08649,P18418,P20760,P20762,P55018,Q64361,Q9WTT6,Q9Z0V6
2061	1,2,4,5,6,8,10,16	3,7,9,11,12,13,14,15,17	11	A2RUV9,D3ZHA0,P08649,P08721,P18418,P55018,Q62997,Q64361,Q7TP52,Q9WTT6,Q9Z0V6
2062	1,2,4,5,6,8,10,17	3,7,9,11,12,13,14,15,16	21	D3ZHA0,O54858,P02761,P06760,P07647,P08649,P18418,P19223,P20646,P20760,P20762,P23593,P28648,Q08463,Q4G075,Q5M8C6,Q64361,Q6P6R2,Q7TP52,Q9WTT6,Q9Z0V6

2063	1,2,4,5,6,8,11,12	3,7,9,10,13,14,15,16,17	39	D3ZHA0,O35547,O54715,O55004,P02780,P02781,P02782,P04905,P07150,P07647,P08723,P0C0A9,P13432,P14668,P14669,P20762,P22282,P24368,P28648,P30120,P35053,P50280,P62804,Q10758,Q5M8C6,Q63357,Q64361,Q6AYR9,Q6IFU7,Q6IFU8,Q6IFW6,Q6IMF3,Q6P6Q2,Q8CJ52,Q9EQS0,Q9ESW0,Q9JHB9,Q9JI85,Q9Z0V6
2064	1,2,4,5,6,8,11,13	3,7,9,10,12,14,15,16,17	17	P02780,P06911,P07150,P07647,P14668,P14669,P20762,P22282,P25236,P28648,P50280,Q5GRG2,Q5M8C6,Q6TMA8,Q9JHB9,Q9JI85,Q9Z0V6
2065	1,2,4,5,6,8,11,14	3,7,9,10,12,13,15,16,17	12	P02793;Q7TP54,P07150,P07171,P07647,P08649,P14668,P14669,P20762,P47727,P50280,Q5M8C6,Q9Z0V6
2066	1,2,4,5,6,8,11,15	3,7,9,10,12,13,14,16,17	12	P02761,P07150,P07171,P08649,P14668,P14669,P20760,P20762,P50280,Q5I0D1,Q64361,Q9Z0V6
2067	1,2,4,5,6,8,11,16	3,7,9,10,12,13,14,15,17	8	A2RUV9,O54715,P07150,P08649,P14668,P14669,P20762,Q9Z0V6
2068	1,2,4,5,6,8,11,17	3,7,9,10,12,13,14,15,16	21	A2RUV9,O54858,P02761,P06760,P07150,P07647,P08649,P14668,P14669,P20646,P20760,P20762,P28648,P50115,P50280,Q30KJ2,Q4G075,Q5M8C6,Q64361,Q6AYS4,Q9Z0V6
2069	1,2,4,5,6,8,12,13	3,7,9,10,11,14,15,16,17	27	D3ZHA0,O88267,P02780,P02781,P02782,P06761,P06911,P07647,P08723,P0C0A9,P20762,P22282,P22283,P25236,P30120,P36374,P50280,Q5GRG2,Q5M8C6,Q64361,Q6AYR9,Q6TMA8,Q8CJ52,Q9JHB9,Q9JI85,Q9WTT6,Q9Z0V6
2070	1,2,4,5,6,8,12,14	3,7,9,10,11,13,15,16,17	24	D3ZHA0,O88267,P02780,P02782,P02793;Q7TP54,P06761,P07647,P08723,P0C0A9,P20762,P25236,P30120,P50280,Q10758,Q5M8C6,Q64361,Q6AYR9,Q6IFW6,Q6IMF3,Q8CJ52,Q9EQS0,Q9JHB9,Q9WTT6,Q9Z0V6
2071	1,2,4,5,6,8,12,15	3,7,9,10,11,13,14,16,17	14	D3ZHA0,P07647,P08723,P20760,P20762,P30120,P35053,P50280,P83748,Q5M8C6,Q64361,Q9JHB9,Q9WTT6,Q9Z0V6
2072	1,2,4,5,6,8,12,16	3,7,9,10,11,13,14,15,17	16	D3ZHA0,O88267,P07647,P08723,P20762,P30120,Q10758,Q5M8C6,Q64361,Q6IFU7,Q6IFW6,Q6IMF3,Q6P6Q2,Q8CJ52,Q9WTT6,Q9Z0V6
2073	1,2,4,5,6,8,12,17	3,7,9,10,11,13,14,15,16	37	D3ZHA0,O54858,O88267,P01681,P02780,P02782,P06760,P07647,P08723,P10536,P13432,P19223,P20646,P20760,P20761,P20762,P22282,P23593,P23928,P30120,P50115,P50280,P55091,P62815,P98089,Q10758,Q4G075,Q5M8C6,Q62635,Q64361,Q6AYR9,Q6IMF3,Q8CJ52,Q9JHB9,Q9QZA6,Q9WTT6,Q9Z0V6
2074	1,2,4,5,6,8,13,14	3,7,9,10,11,12,15,16,17	12	O88267,P02793;Q7TP54,P06911,P07647,P12020,P20762,P25236,P50280,Q5GRG2,Q5M8C6,Q9WTT6,Q9Z0V6
2075	1,2,4,5,6,8,13,15	3,7,9,10,11,12,14,16,17	10	P06911,P07647,P20760,P20762,P25236,P50280,Q5GRG2,Q6IFV1,Q9WTT6,Q9Z0V6
2076	1,2,4,5,6,8,13,16	3,7,9,10,11,12,14,15,17	7	O88267,P06911,P07647,P25236,Q6PCU2,Q9WTT6,Q9Z0V6
2077	1,2,4,5,6,8,13,17	3,7,9,10,11,12,14,15,16	18	O88267,P06760,P06911,P07647,P10536,P20760,P20762,P22282,P23928,P50280,P62815,Q4G075,Q5M8C6,Q63355,Q6IFV1,Q6PCU2,Q9WTT6,Q9Z0V6

2078	1,2,4,5,6,8,14,15	3,7,9,10,11,12,13,16,17	9	D3ZHA0,P02793;Q7TP54,P07171,P10536,P20760,P20762,P25236,Q9WTT6,Q9Z0V6
2079	1,2,4,5,6,8,14,16	3,7,9,10,11,12,13,15,17	8	O88267,P02793;Q7TP54,P14668,P20762,P25236,P97574,Q9WTT6,Q9Z0V6
2080	1,2,4,5,6,8,14,17	3,7,9,10,11,12,13,15,16	14	O88267,P02793;Q7TP54,P06760,P10536,P20760,P20761,P20762,P50280,Q4G075,Q5M8C6,Q64361,Q6PCU2,Q9WTT6,Q9Z0V6
2081	1,2,4,5,6,8,15,16	3,7,9,10,11,12,13,14,17	4	P14668,P20760,P20762,Q9Z0V6
2082	1,2,4,5,6,8,15,17	3,7,9,10,11,12,13,14,16	14	B0BNN3,P02761,P06760,P10536,P20760,P20761,P20762,P23593,Q4G075,Q64361,Q6IFV1,Q9QZA6,Q9WTT6,Q9Z0V6
2083	1,2,4,5,6,8,16,17	3,7,9,10,11,12,13,14,15	11	A2RUV9,O88267,P06760,P20760,P20762,P23593,P23928,Q4G075,Q6PCU2,Q9WTT6,Q9Z0V6
2084	1,2,4,5,6,9,10,11	3,7,8,12,13,14,15,16,17	6	A2RUV9,P08649,P28648,Q63942,Q6IFU7,Q7TP52
2085	1,2,4,5,6,9,10,12	3,7,8,11,13,14,15,16,17	4	Q10758,Q6IFU7,Q6IFW6,Q6IMF3
2086	1,2,4,5,6,9,10,13	3,7,8,11,12,14,15,16,17	1	Q63942
2087	1,2,4,5,6,9,10,14	3,7,8,11,12,13,15,16,17	1	Q63942
2088	1,2,4,5,6,9,10,15	3,7,8,11,12,13,14,16,17	2	Q63942,Q7TP52
2089	1,2,4,5,6,9,10,16	3,7,8,11,12,13,14,15,17	4	A2RUV9,P23593,Q63942,Q7TP52
2090	1,2,4,5,6,9,10,17	3,7,8,11,12,13,14,15,16	5	P23593,P52590,Q63942,Q64335,Q7TP52
2091	1,2,4,5,6,9,11,12	3,7,8,10,13,14,15,16,17	9	P0C0A9,P30120,Q08849,Q6IFU7,Q6IFU8,Q6IFW6,Q6IMF3,Q6P6Q2,Q9JHB9
2092	1,2,4,5,6,9,11,13	3,7,8,10,12,14,15,16,17	2	P05539,Q63942

2093	1,2,4,5,6,9,11,14	3,7,8,10,12,13,15,16,17	2	P20762,Q63942
2094	1,2,4,5,6,9,11,15	3,7,8,10,12,13,14,16,17	1	Q63942
2095	1,2,4,5,6,9,11,16	3,7,8,10,12,13,14,15,17	3	A2RUV9,P14668,Q63942
2096	1,2,4,5,6,9,11,17	3,7,8,10,12,13,14,15,16	2	A2RUV9,Q63942
2097	1,2,4,5,6,9,12,13	3,7,8,10,11,14,15,16,17	5	P02803,P07647,P25236,P30120,Q9JHB9
2098	1,2,4,5,6,9,12,14	3,7,8,10,11,13,15,16,17	5	P01835,P25236,Q10758,Q63942,Q6IMF3
2099	1,2,4,5,6,9,12,15	3,7,8,10,11,13,14,16,17	1	Q63942
2100	1,2,4,5,6,9,12,16	3,7,8,10,11,13,14,15,17	4	P23593,Q63942,Q6IFU7,Q6IMF3
2101	1,2,4,5,6,9,12,17	3,7,8,10,11,13,14,15,16	7	P01835,P13432,P19223,P23593,P98089,Q10758,Q62635
2102	1,2,4,5,6,9,13,14	3,7,8,10,11,12,15,16,17	3	P06911,P25236,Q63942
2103	1,2,4,5,6,9,13,15	3,7,8,10,11,12,14,16,17	1	Q63942
2104	1,2,4,5,6,9,13,16	3,7,8,10,11,12,14,15,17	1	Q63942
2105	1,2,4,5,6,9,13,17	3,7,8,10,11,12,14,15,16	3	P23593,Q63355,Q63942
2106	1,2,4,5,6,9,14,15	3,7,8,10,11,12,13,16,17	1	Q63942
2107	1,2,4,5,6,9,14,16	3,7,8,10,11,12,13,15,17	1	Q63942

2108	1,2,4,5,6,9,14,17	3,7,8,10,11,12,13,15,16	1	Q63942
2109	1,2,4,5,6,9,15,16	3,7,8,10,11,12,13,14,17	2	P23593,Q63942
2110	1,2,4,5,6,9,15,17	3,7,8,10,11,12,13,14,16	3	P20760,P23593,Q63942
2111	1,2,4,5,6,9,16,17	3,7,8,10,11,12,13,14,15	3	A2RUV9,P23593,Q63942
2112	1,2,4,5,6,10,11,12	3,7,8,9,13,14,15,16,17	17	O35547,P06757,P07647,P0C0A9,P28648,P30120,Q08849,Q10758,Q63357,Q63598,Q6IFU7,Q6IFU8,Q6IFW6,Q6IMF3,Q6P6Q2,Q7TP52,Q9JHB9
2113	1,2,4,5,6,10,11,13	3,7,8,9,12,14,15,16,17	2	P28648,Q7TP52
2114	1,2,4,5,6,10,11,14	3,7,8,9,12,13,15,16,17	3	P08649,P55018,Q7TP52
2115	1,2,4,5,6,10,11,15	3,7,8,9,12,13,14,16,17	3	P08649,P55018,Q7TP52
2116	1,2,4,5,6,10,11,16	3,7,8,9,12,13,14,15,17	6	A2RUV9,P08649,P55018,P55314,Q6IFU7,Q7TP52
2117	1,2,4,5,6,10,11,17	3,7,8,9,12,13,14,15,16	8	A2RUV9,O54858,P08649,P15399,P20760,P28648,P97580,Q7TP52
2118	1,2,4,5,6,10,12,13	3,7,8,9,11,14,15,16,17	9	P07647,P25236,P30120,P70709,Q10758,Q6AYR9,Q6IFU7,Q6IMF3,Q9JHB9
2119	1,2,4,5,6,10,12,14	3,7,8,9,11,13,15,16,17	5	P25236,Q10758,Q6IFU7,Q6IFW6,Q6IMF3
2120	1,2,4,5,6,10,12,15	3,7,8,9,11,13,14,16,17	4	Q10758,Q6IFU7,Q6IFW6,Q6IMF3
2121	1,2,4,5,6,10,12,16	3,7,8,9,11,13,14,15,17	6	Q10758,Q6IFU7,Q6IFW6,Q6IMF3,Q6P6Q2,Q7TP52
2122	1,2,4,5,6,10,12,17	3,7,8,9,11,13,14,15,16	8	P19223,P20760,P98089,Q10758,Q62635,Q6IFW6,Q6IMF3,Q7TP52

2123	1,2,4,5,6,10,13,14	3,7,8,9,11,12,15,16,17	4	P06911,P12020,P25236,Q5GRG2
2124	1,2,4,5,6,10,13,15	3,7,8,9,11,12,14,16,17	0	
2125	1,2,4,5,6,10,13,16	3,7,8,9,11,12,14,15,17	2	Q62997,Q7TP52
2126	1,2,4,5,6,10,13,17	3,7,8,9,11,12,14,15,16	3	P20760,Q63355,Q7TP52
2127	1,2,4,5,6,10,14,15	3,7,8,9,11,12,13,16,17	1	P55018
2128	1,2,4,5,6,10,14,16	3,7,8,9,11,12,13,15,17	3	P55018,Q62997,Q7TP52
2129	1,2,4,5,6,10,14,17	3,7,8,9,11,12,13,15,16	2	P20760,Q7TP52
2130	1,2,4,5,6,10,15,16	3,7,8,9,11,12,13,14,17	3	P55018,Q62997,Q7TP52
2131	1,2,4,5,6,10,15,17	3,7,8,9,11,12,13,14,16	2	P20760,Q7TP52
2132	1,2,4,5,6,10,16,17	3,7,8,9,11,12,13,14,15	5	A2RUV9,P20760,P23593,Q62997,Q7TP52
2133	1,2,4,5,6,11,12,13	3,7,8,9,10,14,15,16,17	12	P04905,P07647,P0C0A9,P25236,P30120,P36374,Q6IFU7,Q6IFU8,Q6IFW6,Q6IMF3,Q6P6Q2,Q9JHB9
2134	1,2,4,5,6,11,12,14	3,7,8,9,10,13,15,16,17	14	O35547,P04905,P0C0A9,P20762,P25236,P30120,P47727,Q10758,Q6IFU7,Q6IFU8,Q6IFW6,Q6IMF3,Q6P6Q2,Q9JHB9
2135	1,2,4,5,6,11,12,15	3,7,8,9,10,13,14,16,17	7	P51907,Q6IFU7,Q6IFU8,Q6IFW6,Q6IMF3,Q6P6Q2,Q9JHB9
2136	1,2,4,5,6,11,12,16	3,7,8,9,10,13,14,15,17	8	G3V686,O35547,Q10758,Q6IFU7,Q6IFU8,Q6IFW6,Q6IMF3,Q6P6Q2
2137	1,2,4,5,6,11,12,17	3,7,8,9,10,13,14,15,16	16	O35547,P01681,P07647,P13432,P15399,P19223,P30120,P98089,Q10758,Q62635,Q6IFU7,Q6IFU8,Q6IFW6,Q6IMF3,Q6P6Q2,Q9JHB9

2138	1,2,4,5,6,11,13,14	3,7,8,9,10,12,15,16,17	5	P05539,P06911,P20762,P25236,P47727
2139	1,2,4,5,6,11,13,15	3,7,8,9,10,12,14,16,17	1	P05539
2140	1,2,4,5,6,11,13,16	3,7,8,9,10,12,14,15,17	2	A2RUV9,G3V686
2141	1,2,4,5,6,11,13,17	3,7,8,9,10,12,14,15,16	1	P20760
2142	1,2,4,5,6,11,14,15	3,7,8,9,10,12,13,16,17	1	P20762
2143	1,2,4,5,6,11,14,16	3,7,8,9,10,12,13,15,17	1	P14668
2144	1,2,4,5,6,11,14,17	3,7,8,9,10,12,13,15,16	3	P15399,P20760,P20762
2145	1,2,4,5,6,11,15,16	3,7,8,9,10,12,13,14,17	2	P14408,P14668
2146	1,2,4,5,6,11,15,17	3,7,8,9,10,12,13,14,16	1	P20760
2147	1,2,4,5,6,11,16,17	3,7,8,9,10,12,13,14,15	1	A2RUV9
2148	1,2,4,5,6,12,13,14	3,7,8,9,10,11,15,16,17	7	P06911,P07647,P25236,P30120,Q10758,Q6AYR9,Q9JHB9
2149	1,2,4,5,6,12,13,15	3,7,8,9,10,11,14,16,17	2	P25236,P83748
2150	1,2,4,5,6,12,13,16	3,7,8,9,10,11,14,15,17	3	G3V686,P25236,Q6IFU7
2151	1,2,4,5,6,12,13,17	3,7,8,9,10,11,14,15,16	11	P07647,P13432,P20760,P25236,P36374,P55091,P98089,Q10758,Q62635,Q63355,Q9JHB9
2152	1,2,4,5,6,12,14,15	3,7,8,9,10,11,13,16,17	3	P20762,P25236,P35467

2153	1,2,4,5,6,12,14,16	3,7,8,9,10,11,13,15,17	7	G3V686,P25236,P35467,Q10758,Q6IFU7,Q6IFW6,Q6IMF3
2154	1,2,4,5,6,12,14,17	3,7,8,9,10,11,13,15,16	8	P01835,P13432,P20760,P25236,P31430,P98089,Q10758,Q62635
2155	1,2,4,5,6,12,15,16	3,7,8,9,10,11,13,14,17	3	P35467,P83748,Q6IFU7
2156	1,2,4,5,6,12,15,17	3,7,8,9,10,11,13,14,16	6	B0BNN3,P13432,P20760,P98089,Q10758,Q62635
2157	1,2,4,5,6,12,16,17	3,7,8,9,10,11,13,14,15	6	P13432,P23593,P98089,Q10758,Q62635,Q6IMF3
2158	1,2,4,5,6,13,14,15	3,7,8,9,10,11,12,16,17	4	P06911,P25236,P35467,Q5GRG2
2159	1,2,4,5,6,13,14,16	3,7,8,9,10,11,12,15,17	3	G3V686,P06911,P25236
2160	1,2,4,5,6,13,14,17	3,7,8,9,10,11,12,15,16	4	P06911,P20760,P25236,Q6PCU2
2161	1,2,4,5,6,13,15,16	3,7,8,9,10,11,12,14,17	1	P35467
2162	1,2,4,5,6,13,15,17	3,7,8,9,10,11,12,14,16	2	P20760,Q6IFV1
2163	1,2,4,5,6,13,16,17	3,7,8,9,10,11,12,14,15	2	P20760,Q6PCU2
2164	1,2,4,5,6,14,15,16	3,7,8,9,10,11,12,13,17	1	P35467
2165	1,2,4,5,6,14,15,17	3,7,8,9,10,11,12,13,16	3	P10536,P20760,P35467
2166	1,2,4,5,6,14,16,17	3,7,8,9,10,11,12,13,15	3	P20760,P35467,Q6PCU2
2167	1,2,4,5,6,15,16,17	3,7,8,9,10,11,12,13,14	3	P20760,P23593,P35467

2168	1,2,4,5,7,8,9,10	3,6,11,12,13,14,15,16,17	10	D3ZHA0,O55006,P18418,P28648,P31044,P52590,P68370,P97571,Q63475,Q63942
2169	1,2,4,5,7,8,9,11	3,6,10,12,13,14,15,16,17	14	P02803,P07171,P10715,P14669,P18418,P20762,P28648,P31044,P52590,P61206;P84079;P84082,P68370,Q63617,Q6AYS4,Q9EQS0
2170	1,2,4,5,7,8,9,12	3,6,10,11,13,14,15,16,17	14	D3ZHA0,O88267,P02803,P10715,P18418,P19223,P31044,P52590,P83748,Q63475,Q80WL1,Q8CJ52,Q9EQS0,Q9R0T3
2171	1,2,4,5,7,8,9,13	3,6,10,11,12,14,15,16,17	7	D3ZHA0,P02803,P18418,P31044,P52590,Q63617,Q9R0T3
2172	1,2,4,5,7,8,9,14	3,6,10,11,12,13,15,16,17	9	D3ZHA0,P07171,P18418,P20762,P31044,P52590,Q63617,Q63942,Q9EQS0
2173	1,2,4,5,7,8,9,15	3,6,10,11,12,13,14,16,17	8	D3ZHA0,P02761,P07171,P18418,P31044,P52590,Q5QE79,Q63942
2174	1,2,4,5,7,8,9,16	3,6,10,11,12,13,14,15,17	7	D3ZHA0,P18418,P31044,P52590,Q63617,Q63942,Q9R0T3
2175	1,2,4,5,7,8,9,17	3,6,10,11,12,13,14,15,16	8	O88267,P18418,P19223,P50115,P52590,P55091,Q63617,Q9QZA6
2176	1,2,4,5,7,8,10,11	3,6,9,12,13,14,15,16,17	22	D3ZHA0,O54858,P00714,P02761,P07171,P08649,P14669,P16391,P18418,P28648,P31044,P52590,P55018,P61206;P84079;P84082,P68370,Q5BJY9,Q63475,Q63598,Q6AYS4,Q8CJ52,Q9EQS0,Q9R0T3
2177	1,2,4,5,7,8,10,12	3,6,9,11,13,14,15,16,17	17	D3ZHA0,P10715,P16391,P18418,P19223,P28648,P31044,P52590,P68370,P83748,P97571,Q10758,Q63475,Q80WL1,Q8CJ52,Q9EQS0,Q9R0T3
2178	1,2,4,5,7,8,10,13	3,6,9,11,12,14,15,16,17	10	D3ZHA0,P01835,P18418,P28648,P31044,P52590,P68370,P97571,Q5GRG2,Q9R0T3
2179	1,2,4,5,7,8,10,14	3,6,9,11,12,13,15,16,17	13	D3ZHA0,P07171,P18418,P20762,P28648,P31044,P36970,P52590,P55018,P68370,P97571,Q63475,Q9EQS0
2180	1,2,4,5,7,8,10,15	3,6,9,11,12,13,14,16,17	12	D3ZHA0,P01835,P02761,P07171,P18418,P28648,P31044,P52590,P55018,P68370,P97571,Q63475
2181	1,2,4,5,7,8,10,16	3,6,9,11,12,13,14,15,17	13	D3ZHA0,P01835,P08721,P18418,P28648,P31044,P52590,P55018,P61206;P84079;P84082,P68370,P97571,Q62997,Q9R0T3
2182	1,2,4,5,7,8,10,17	3,6,9,11,12,13,14,15,16	16	D3ZHA0,O54858,P02761,P16391,P18418,P19223,P28648,P31044,P46844,P50115,P52590,P55091,P97571,Q4G075,Q9QZA6,Q9R0T3

2183	1,2,4,5,7,8,11,12	3,6,9,10,13,14,15,16,17	32	D3ZHA0,O54715,O55004,P02780,P02781,P02803,P07171,P07647,P10715,P14669,P18418,P20762,P22283,P28648,P30120,P31044,P36374,P52590,P61206;P84079;P84082,P68370,Q63475,Q63493,Q68FS4,Q6AYS4,Q6P6Q2,Q80WL1,Q8CJ52,Q99041,Q9EQS0,Q9ESW0,Q9JI85,Q9R0T3
2184	1,2,4,5,7,8,11,13	3,6,9,10,12,14,15,16,17	17	P02803,P05539,P07171,P14669,P18418,P28648,P31044,P52590,P61206;P84079;P84082,P68370,Q5GRG2,Q63617,Q6AYS4,Q8CJ52,Q8VD89,Q9JI85,Q9R0T3
2185	1,2,4,5,7,8,11,14	3,6,9,10,12,13,15,16,17	16	P05371,P07171,P14669,P18418,P19814,P20762,P28648,P31044,P52590,P61206;P84079;P84082,P68370,Q63617,Q6AYS4,Q8CJ52,Q9EQS0,Q9ESW0
2186	1,2,4,5,7,8,11,15	3,6,9,10,12,13,14,16,17	14	P02761,P07171,P10715,P14408,P14669,P18418,P20762,P22006,P31044,P52590,P68370,Q63475,Q6AYS4,Q9ESW0
2187	1,2,4,5,7,8,11,16	3,6,9,10,12,13,14,15,17	14	P05371,P07171,P10715,P14408,P14668,P18418,P31044,P61206;P84079;P84082,P68370,Q63617,Q6AYS4,Q8CJ52,Q8VD89,Q9R0T3
2188	1,2,4,5,7,8,11,17	3,6,9,10,12,13,14,15,16	22	O54858,O55004,P02761,P07171,P10715,P14669,P18418,P19223,P20762,P28648,P31044,P50115,P52590,P55091,P61206;P84079;P84082,Q4G075,Q63617,Q6AYS4,Q99041,Q9ESW0,Q9QZA6,Q9R0T3
2189	1,2,4,5,7,8,12,13	3,6,9,10,11,14,15,16,17	22	D3ZHA0,O88267,P02780,P02803,P06761,P07647,P10715,P18418,P22282,P22283,P30120,P31044,P36374,P36970,P52590,P83748,Q5GRG2,Q80WL1,Q8CJ52,Q9EQS0,Q9JI85,Q9R0T3
2190	1,2,4,5,7,8,12,14	3,6,9,10,11,13,15,16,17	18	D3ZHA0,O88267,P06761,P07171,P10715,P18418,P19629,P20762,P31044,P36970,P52590,P83748,Q63475,Q68FS4,Q80WL1,Q8CJ52,Q9EQS0,Q9R0T3
2191	1,2,4,5,7,8,12,15	3,6,9,10,11,13,14,16,17	13	D3ZHA0,O55004,P07171,P10715,P18418,P20762,P52590,P83748,Q63475,Q8CJ52,Q9EQS0,Q9ESW0,Q9QZA6
2192	1,2,4,5,7,8,12,16	3,6,9,10,11,13,14,15,17	10	D3ZHA0,O88267,P08721,P10715,P18418,P83748,Q80WL1,Q8CJ52,Q9EQS0,Q9R0T3
2193	1,2,4,5,7,8,12,17	3,6,9,10,11,13,14,15,16	23	O54858,O55004,O88267,P06761,P10715,P18418,P19223,P23928,P36374,P50115,P52590,P55091,P83748,P98089,Q4G075,Q62635,Q80WL1,Q8CJ52,Q99041,Q9EQS0,Q9ESW0,Q9QZA6,Q9R0T3
2194	1,2,4,5,7,8,13,14	3,6,9,10,11,12,15,16,17	10	O88267,P06761,P06911,P07171,P18418,P20762,P31044,P36970,Q5GRG2,Q8CJ52
2195	1,2,4,5,7,8,13,15	3,6,9,10,11,12,14,16,17	7	P01835,P07171,P18418,P31044,P52590,Q09030,Q5GRG2
2196	1,2,4,5,7,8,13,16	3,6,9,10,11,12,14,15,17	6	O88267,P01835,P18418,P31044,Q8CJ52,Q9R0T3
2197	1,2,4,5,7,8,13,17	3,6,9,10,11,12,14,15,16	11	O88267,P06761,P10536,P18418,P19223,P23928,P50115,P52590,P55091,Q6PCU2,Q9R0T3

2198	1,2,4,5,7,8,14,15	3,6,9,10,11,12,13,16,17	8	D3ZHA0,P07171,P10536,P16636,P18418,P20762,P31044,P52590
2199	1,2,4,5,7,8,14,16	3,6,9,10,11,12,13,15,17	7	O88267,P07171,P08721,P18418,P31044,P97574,Q63532
2200	1,2,4,5,7,8,14,17	3,6,9,10,11,12,13,15,16	11	O88267,P07171,P10536,P18418,P19223,P20762,P50115,P52590,P55091,Q6PCU2,Q9QZA6
2201	1,2,4,5,7,8,15,16	3,6,9,10,11,12,13,14,17	5	P01835,P07171,P18418,P31044,P52590
2202	1,2,4,5,7,8,15,17	3,6,9,10,11,12,13,14,16	10	P02761,P07171,P10536,P18418,P19223,P20760,P50115,P52590,P55091,Q9QZA6
2203	1,2,4,5,7,8,16,17	3,6,9,10,11,12,13,14,15	11	O88267,P18418,P19223,P23928,P50115,P50116,P52590,P55091,Q6PCU2,Q9QZA6,Q9R0T3
2204	1,2,4,5,7,9,10,11	3,6,8,12,13,14,15,16,17	6	P18418,P28648,P52590,P61206;P84079;P84082,Q64093,Q7TP52
2205	1,2,4,5,7,9,10,12	3,6,8,11,13,14,15,16,17	4	P18418,P52590,P97840,Q63475
2206	1,2,4,5,7,9,10,13	3,6,8,11,12,14,15,16,17	1	P52590
2207	1,2,4,5,7,9,10,14	3,6,8,11,12,13,15,16,17	2	P18418,P52590
2208	1,2,4,5,7,9,10,15	3,6,8,11,12,13,14,16,17	1	P52590
2209	1,2,4,5,7,9,10,16	3,6,8,11,12,13,14,15,17	4	P52590,P61206;P84079;P84082,Q62997,Q64093
2210	1,2,4,5,7,9,10,17	3,6,8,11,12,13,14,15,16	2	P52590,Q64093
2211	1,2,4,5,7,9,11,12	3,6,8,10,13,14,15,16,17	5	P02803,P51907,P52590,P61206;P84079;P84082,Q9ESW0
2212	1,2,4,5,7,9,11,13	3,6,8,10,12,14,15,16,17	4	P02803,P05539,P52590,P61206;P84079;P84082

2213	1,2,4,5,7,9,11,14	3,6,8,10,12,13,15,16,17	3	P52590,P61206;P84079;P84082,Q99MH3
2214	1,2,4,5,7,9,11,15	3,6,8,10,12,13,14,16,17	3	P14408,P51907,P52590
2215	1,2,4,5,7,9,11,16	3,6,8,10,12,13,14,15,17	3	P14408,P52590,P61206;P84079;P84082
2216	1,2,4,5,7,9,11,17	3,6,8,10,12,13,14,15,16	1	P52590
2217	1,2,4,5,7,9,12,13	3,6,8,10,11,14,15,16,17	5	P02803,P05539,P36374,P52590,P83748
2218	1,2,4,5,7,9,12,14	3,6,8,10,11,13,15,16,17	1	P52590
2219	1,2,4,5,7,9,12,15	3,6,8,10,11,13,14,16,17	2	P52590,P83748
2220	1,2,4,5,7,9,12,16	3,6,8,10,11,13,14,15,17	2	P52590,P83748
2221	1,2,4,5,7,9,12,17	3,6,8,10,11,13,14,15,16	4	P19223,P52590,P55091,P98089
2222	1,2,4,5,7,9,13,14	3,6,8,10,11,12,15,16,17	2	P05539,P52590
2223	1,2,4,5,7,9,13,15	3,6,8,10,11,12,14,16,17	2	P05539,P52590
2224	1,2,4,5,7,9,13,16	3,6,8,10,11,12,14,15,17	1	P52590
2225	1,2,4,5,7,9,13,17	3,6,8,10,11,12,14,15,16	2	P05539,P52590
2226	1,2,4,5,7,9,14,15	3,6,8,10,11,12,13,16,17	3	P16636,P52590,Q9QZK9
2227	1,2,4,5,7,9,14,16	3,6,8,10,11,12,13,15,17	4	P52590,Q63532,Q99MH3,Q9QZK9

2228	1,2,4,5,7,9,14,17	3,6,8,10,11,12,13,15,16	1	P52590
2229	1,2,4,5,7,9,15,16	3,6,8,10,11,12,13,14,17	3	P47727,P52590,Q9QZK9
2230	1,2,4,5,7,9,15,17	3,6,8,10,11,12,13,14,16	2	P47727,P52590
2231	1,2,4,5,7,9,16,17	3,6,8,10,11,12,13,14,15	2	P47727,P52590
2232	1,2,4,5,7,10,11,12	3,6,8,9,13,14,15,16,17	11	P05964,P06757,P18418,P28648,P51907,P52590,P61206;P84079;P84082,Q62636,Q63475,Q6P6Q2,Q9ESW0
2233	1,2,4,5,7,10,11,13	3,6,8,9,12,14,15,16,17	4	P05539,P28648,P52590,P61206;P84079;P84082
2234	1,2,4,5,7,10,11,14	3,6,8,9,12,13,15,16,17	4	P18418,P28648,P52590,P61206;P84079;P84082
2235	1,2,4,5,7,10,11,15	3,6,8,9,12,13,14,16,17	7	P14408,P22006,P28648,P51907,P52590,P61206;P84079;P84082,Q5FVI6
2236	1,2,4,5,7,10,11,16	3,6,8,9,12,13,14,15,17	8	P14408,P28648,P51907,P52590,P61206;P84079;P84082,Q5FVI6,Q7TP52,Q8VD89
2237	1,2,4,5,7,10,11,17	3,6,8,9,12,13,14,15,16	5	P14408,P28648,P52590,P61206;P84079;P84082,Q7TP52
2238	1,2,4,5,7,10,12,13	3,6,8,9,11,14,15,16,17	4	P18418,P36374,P52590,P83748
2239	1,2,4,5,7,10,12,14	3,6,8,9,11,13,15,16,17	5	P18418,P52590,Q10758,Q63475,Q63532
2240	1,2,4,5,7,10,12,15	3,6,8,9,11,13,14,16,17	4	P18418,P52590,P83748,Q63475
2241	1,2,4,5,7,10,12,16	3,6,8,9,11,13,14,15,17	7	P08721,P18418,P52590,P61206;P84079;P84082,P83748,P97840,Q10758
2242	1,2,4,5,7,10,12,17	3,6,8,9,11,13,14,15,16	6	P18418,P19223,P52590,P55091,P98089,Q10758

2243	1,2,4,5,7,10,13,14	3,6,8,9,11,12,15,16,17	3	P12020,P18418,Q5GRG2
2244	1,2,4,5,7,10,13,15	3,6,8,9,11,12,14,16,17	3	P01835,P52590,Q09030
2245	1,2,4,5,7,10,13,16	3,6,8,9,11,12,14,15,17	2	P01835,Q62997
2246	1,2,4,5,7,10,13,17	3,6,8,9,11,12,14,15,16	1	P52590
2247	1,2,4,5,7,10,14,15	3,6,8,9,11,12,13,16,17	4	P16636,P18418,P35467,P52590
2248	1,2,4,5,7,10,14,16	3,6,8,9,11,12,13,15,17	3	P08721,Q62997,Q63532
2249	1,2,4,5,7,10,14,17	3,6,8,9,11,12,13,15,16	2	P18418,P52590
2250	1,2,4,5,7,10,15,16	3,6,8,9,11,12,13,14,17	6	P01835,P08721,P14408,P35467,P52590,Q62997
2251	1,2,4,5,7,10,15,17	3,6,8,9,11,12,13,14,16	1	P52590
2252	1,2,4,5,7,10,16,17	3,6,8,9,11,12,13,14,15	3	P08721,P52590,Q62997
2253	1,2,4,5,7,11,12,13	3,6,8,9,10,14,15,16,17	8	P01836,P02803,P05539,P14408,P36374,P61206;P84079;P84082,Q9ESW0,Q9JI85
2254	1,2,4,5,7,11,12,14	3,6,8,9,10,13,15,16,17	3	P51907,P61206;P84079;P84082,Q9ESW0
2255	1,2,4,5,7,11,12,15	3,6,8,9,10,13,14,16,17	4	P01836,P14408,P51907,Q9ESW0
2256	1,2,4,5,7,11,12,16	3,6,8,9,10,13,14,15,17	5	P01836,P14408,P51907,P61206;P84079;P84082,Q9ESW0
2257	1,2,4,5,7,11,12,17	3,6,8,9,10,13,14,15,16	7	P14408,P19223,P36374,P52590,P55091,P98089,Q9ESW0

2258	1,2,4,5,7,11,13,14	3,6,8,9,10,12,15,16,17	2	P05539,Q5GRG2
2259	1,2,4,5,7,11,13,15	3,6,8,9,10,12,14,16,17	3	P05539,P14408,Q09030
2260	1,2,4,5,7,11,13,16	3,6,8,9,10,12,14,15,17	3	P14408,P61206;P84079;P84082,Q8VD89
2261	1,2,4,5,7,11,13,17	3,6,8,9,10,12,14,15,16	3	P05539,P14408,P52590
2262	1,2,4,5,7,11,14,15	3,6,8,9,10,12,13,16,17	4	P14408,P16636,P22006,P51907
2263	1,2,4,5,7,11,14,16	3,6,8,9,10,12,13,15,17	4	P14408,P61206;P84079;P84082,Q63532,Q99MH3
2264	1,2,4,5,7,11,14,17	3,6,8,9,10,12,13,15,16	1	P52590
2265	1,2,4,5,7,11,15,16	3,6,8,9,10,12,13,14,17	4	P14408,P51907,P61206;P84079;P84082,Q8VD89
2266	1,2,4,5,7,11,15,17	3,6,8,9,10,12,13,14,16	2	P14408,P52590
2267	1,2,4,5,7,11,16,17	3,6,8,9,10,12,13,14,15	3	P14408,P52590,P61206;P84079;P84082
2268	1,2,4,5,7,12,13,14	3,6,8,9,10,11,15,16,17	1	P83748
2269	1,2,4,5,7,12,13,15	3,6,8,9,10,11,14,16,17	4	P01836,P05539,P83748,Q09030
2270	1,2,4,5,7,12,13,16	3,6,8,9,10,11,14,15,17	2	P01836,P83748
2271	1,2,4,5,7,12,13,17	3,6,8,9,10,11,14,15,16	6	O88267,P19223,P36374,P52590,P55091,P98089
2272	1,2,4,5,7,12,14,15	3,6,8,9,10,11,13,16,17	3	P16636,P35467,P83748

2273	1,2,4,5,7,12,14,16	3,6,8,9,10,11,13,15,17	3	P35467,P83748,Q63532
2274	1,2,4,5,7,12,14,17	3,6,8,9,10,11,13,15,16	6	O88267,P19223,P31430,P52590,P55091,P98089
2275	1,2,4,5,7,12,15,16	3,6,8,9,10,11,13,14,17	4	P01836,P14408,P35467,P83748
2276	1,2,4,5,7,12,15,17	3,6,8,9,10,11,13,14,16	5	P19223,P52590,P55091,P83748,P98089
2277	1,2,4,5,7,12,16,17	3,6,8,9,10,11,13,14,15	5	P19223,P52590,P55091,P83748,P98089
2278	1,2,4,5,7,13,14,15	3,6,8,9,10,11,12,16,17	5	P05539,P16636,P35467,Q09030,Q5GRG2
2279	1,2,4,5,7,13,14,16	3,6,8,9,10,11,12,15,17	2	P35467,Q63532
2280	1,2,4,5,7,13,14,17	3,6,8,9,10,11,12,15,16	2	P52590,Q6PCU2
2281	1,2,4,5,7,13,15,16	3,6,8,9,10,11,12,14,17	4	P01835,P14408,P35467,Q09030
2282	1,2,4,5,7,13,15,17	3,6,8,9,10,11,12,14,16	1	P52590
2283	1,2,4,5,7,13,16,17	3,6,8,9,10,11,12,14,15	2	P52590,Q6PCU2
2284	1,2,4,5,7,14,15,16	3,6,8,9,10,11,12,13,17	4	P16636,P35467,Q63532,Q9QZK9
2285	1,2,4,5,7,14,15,17	3,6,8,9,10,11,12,13,16	4	P10536,P16636,P35467,P52590
2286	1,2,4,5,7,14,16,17	3,6,8,9,10,11,12,13,15	3	P35467,P52590,Q6PCU2
2287	1,2,4,5,7,15,16,17	3,6,8,9,10,11,12,13,14	4	P14408,P35467,P47727,P52590

2288	1,2,4,5,8,9,10,11	3,6,7,12,13,14,15,16,17	6	A2RUV9,P02803,P08649,Q63598,Q63942,Q6AYS4
2289	1,2,4,5,8,9,10,12	3,6,7,11,13,14,15,16,17	3	D3ZHA0,P02803,Q80WL1
2290	1,2,4,5,8,9,10,13	3,6,7,11,12,14,15,16,17	2	P02803,Q63942
2291	1,2,4,5,8,9,10,14	3,6,7,11,12,13,15,16,17	1	Q63942
2292	1,2,4,5,8,9,10,15	3,6,7,11,12,13,14,16,17	3	D3ZHA0,P02761,Q63942
2293	1,2,4,5,8,9,10,16	3,6,7,11,12,13,14,15,17	4	A2RUV9,Q62997,Q63942,Q9QZK9
2294	1,2,4,5,8,9,10,17	3,6,7,11,12,13,14,15,16	4	P08649,P19223,P52590,Q63942
2295	1,2,4,5,8,9,11,12	3,6,7,10,13,14,15,16,17	5	P02803,Q5M8C6,Q6AYS4,Q6P6Q2,Q80WL1
2296	1,2,4,5,8,9,11,13	3,6,7,10,12,14,15,16,17	2	P02803,Q6AYS4
2297	1,2,4,5,8,9,11,14	3,6,7,10,12,13,15,16,17	3	Q63942,Q6AYS4,Q99MH3
2298	1,2,4,5,8,9,11,15	3,6,7,10,12,13,14,16,17	3	P02761,Q5QE79,Q63942
2299	1,2,4,5,8,9,11,16	3,6,7,10,12,13,14,15,17	5	A2RUV9,P02803,P14668,Q63942,Q9QZK9
2300	1,2,4,5,8,9,11,17	3,6,7,10,12,13,14,15,16	4	A2RUV9,P02803,P08649,Q6AYS4
2301	1,2,4,5,8,9,12,13	3,6,7,10,11,14,15,16,17	4	P02803,P36374,Q5M8C6,Q80WL1
2302	1,2,4,5,8,9,12,14	3,6,7,10,11,13,15,16,17	1	P02803

2303	1,2,4,5,8,9,12,15	3,6,7,10,11,13,14,16,17	1	Q5QE79
2304	1,2,4,5,8,9,12,16	3,6,7,10,11,13,14,15,17	2	P02803,Q63942
2305	1,2,4,5,8,9,12,17	3,6,7,10,11,13,14,15,16	7	P02803,P19223,P55091,P98089,Q5M8C6,Q80WL1,Q9QZA6
2306	1,2,4,5,8,9,13,14	3,6,7,10,11,12,15,16,17	2	P02803,Q63942
2307	1,2,4,5,8,9,13,15	3,6,7,10,11,12,14,16,17	2	Q5QE79,Q63942
2308	1,2,4,5,8,9,13,16	3,6,7,10,11,12,14,15,17	2	P02803,Q63942
2309	1,2,4,5,8,9,13,17	3,6,7,10,11,12,14,15,16	1	P02803
2310	1,2,4,5,8,9,14,15	3,6,7,10,11,12,13,16,17	3	Q5QE79,Q63942,Q9QZK9
2311	1,2,4,5,8,9,14,16	3,6,7,10,11,12,13,15,17	4	P97574,Q63942,Q99MH3,Q9QZK9
2312	1,2,4,5,8,9,14,17	3,6,7,10,11,12,13,15,16	1	Q63942
2313	1,2,4,5,8,9,15,16	3,6,7,10,11,12,13,14,17	3	Q5QE79,Q63942,Q9QZK9
2314	1,2,4,5,8,9,15,17	3,6,7,10,11,12,13,14,16	4	P02761,Q5QE79,Q63942,Q68FV8
2315	1,2,4,5,8,9,16,17	3,6,7,10,11,12,13,14,15	4	A2RUV9,P23593,Q63942,Q68FV8
2316	1,2,4,5,8,10,11,12	3,6,7,9,13,14,15,16,17	11	P02803,P62804,Q10758,Q63598,Q6AYS4,Q6IFU7,Q6IFU8,Q6IFW6,Q6IMF3,Q6P6Q2,Q80WL1
2317	1,2,4,5,8,10,11,13	3,6,7,9,12,14,15,16,17	3	P02803,Q63598,Q6AYS4

2318	1,2,4,5,8,10,11,14	3,6,7,9,12,13,15,16,17	3	P08649,Q63598,Q6AYS4
2319	1,2,4,5,8,10,11,15	3,6,7,9,12,13,14,16,17	4	P02761,P08649,Q63598,Q6AYS4
2320	1,2,4,5,8,10,11,16	3,6,7,9,12,13,14,15,17	5	A2RUV9,P08649,P14668,P55018,Q63598
2321	1,2,4,5,8,10,11,17	3,6,7,9,12,13,14,15,16	6	A2RUV9,O54858,P02761,P08649,Q63598,Q6AYS4
2322	1,2,4,5,8,10,12,13	3,6,7,9,11,14,15,16,17	3	P02803,Q5M8C6,Q80WL1
2323	1,2,4,5,8,10,12,14	3,6,7,9,11,13,15,16,17	2	D3ZHA0,Q10758
2324	1,2,4,5,8,10,12,15	3,6,7,9,11,13,14,16,17	1	D3ZHA0
2325	1,2,4,5,8,10,12,16	3,6,7,9,11,13,14,15,17	2	P08721,Q10758
2326	1,2,4,5,8,10,12,17	3,6,7,9,11,13,14,15,16	7	P19223,P55091,P98089,Q10758,Q4G075,Q80WL1,Q9QZA6
2327	1,2,4,5,8,10,13,14	3,6,7,9,11,12,15,16,17	1	P36970
2328	1,2,4,5,8,10,13,15	3,6,7,9,11,12,14,16,17	1	P01835
2329	1,2,4,5,8,10,13,16	3,6,7,9,11,12,14,15,17	2	P01835,Q62997
2330	1,2,4,5,8,10,13,17	3,6,7,9,11,12,14,15,16	0	
2331	1,2,4,5,8,10,14,15	3,6,7,9,11,12,13,16,17	1	P55018
2332	1,2,4,5,8,10,14,16	3,6,7,9,11,12,13,15,17	4	P08721,P55018,P97574,Q62997

2333	1,2,4,5,8,10,14,17	3,6,7,9,11,12,13,15,16	0	
2334	1,2,4,5,8,10,15,16	3,6,7,9,11,12,13,14,17	4	P01835,P08721,P55018,Q62997
2335	1,2,4,5,8,10,15,17	3,6,7,9,11,12,13,14,16	1	P02761
2336	1,2,4,5,8,10,16,17	3,6,7,9,11,12,13,14,15	3	A2RUV9,P08721,Q62997
2337	1,2,4,5,8,11,12,13	3,6,7,9,10,14,15,16,17	8	P02780,P02803,P35053,P36374,Q5M8C6,Q80WL1,Q8CJ52,Q9JHB9
2338	1,2,4,5,8,11,12,14	3,6,7,9,10,13,15,16,17	6	P35053,P62804,Q6AYS4,Q6IFU8,Q6P6Q2,Q9ESW0
2339	1,2,4,5,8,11,12,15	3,6,7,9,10,13,14,16,17	3	P35053,P51907,Q9ESW0
2340	1,2,4,5,8,11,12,16	3,6,7,9,10,13,14,15,17	5	P02803,P14668,Q6IFU7,Q6P6Q2,Q8CJ52
2341	1,2,4,5,8,11,12,17	3,6,7,9,10,13,14,15,16	12	P02803,P19223,P36374,P55091,P98089,Q4G075,Q5M8C6,Q6AYS4,Q6P6Q2,Q80WL1,Q9ESW0,Q9QZA6
2342	1,2,4,5,8,11,13,14	3,6,7,9,10,12,15,16,17	0	
2343	1,2,4,5,8,11,13,15	3,6,7,9,10,12,14,16,17	0	
2344	1,2,4,5,8,11,13,16	3,6,7,9,10,12,14,15,17	2	A2RUV9,P02803
2345	1,2,4,5,8,11,13,17	3,6,7,9,10,12,14,15,16	2	P02803,Q6AYS4
2346	1,2,4,5,8,11,14,15	3,6,7,9,10,12,13,16,17	4	P07171,P97840,Q5I0D1,Q64194
2347	1,2,4,5,8,11,14,16	3,6,7,9,10,12,13,15,17	3	P14668,P97574,Q99MH3

2348	1,2,4,5,8,11,14,17	3,6,7,9,10,12,13,15,16	2	P97840,Q6AYS4
2349	1,2,4,5,8,11,15,16	3,6,7,9,10,12,13,14,17	2	P14408,P14668
2350	1,2,4,5,8,11,15,17	3,6,7,9,10,12,13,14,16	4	P02761,P97840,Q6AYS4,Q9QZA6
2351	1,2,4,5,8,11,16,17	3,6,7,9,10,12,13,14,15	2	A2RUV9,Q6AYS4
2352	1,2,4,5,8,12,13,14	3,6,7,9,10,11,15,16,17	3	P02803,P25236,P36970
2353	1,2,4,5,8,12,13,15	3,6,7,9,10,11,14,16,17	1	P35053
2354	1,2,4,5,8,12,13,16	3,6,7,9,10,11,14,15,17	1	P02803
2355	1,2,4,5,8,12,13,17	3,6,7,9,10,11,14,15,16	9	P02803,P19223,P36374,P55091,P98089,Q5M8C6,Q62635,Q6PCU2,Q80WL1
2356	1,2,4,5,8,12,14,15	3,6,7,9,10,11,13,16,17	1	P35053
2357	1,2,4,5,8,12,14,16	3,6,7,9,10,11,13,15,17	0	
2358	1,2,4,5,8,12,14,17	3,6,7,9,10,11,13,15,16	6	P19223,P31430,P55091,P98089,Q6PCU2,Q9QZA6
2359	1,2,4,5,8,12,15,16	3,6,7,9,10,11,13,14,17	0	
2360	1,2,4,5,8,12,15,17	3,6,7,9,10,11,13,14,16	6	P02761,P19223,P55091,P98089,Q4G075,Q9QZA6
2361	1,2,4,5,8,12,16,17	3,6,7,9,10,11,13,14,15	7	P19223,P23928,P55091,P98089,Q4G075,Q6PCU2,Q9QZA6
2362	1,2,4,5,8,13,14,15	3,6,7,9,10,11,12,16,17	0	

2363	1,2,4,5,8,13,14,16	3,6,7,9,10,11,12,15,17	1	Q6PCU2
2364	1,2,4,5,8,13,14,17	3,6,7,9,10,11,12,15,16	2	P10536,Q6PCU2
2365	1,2,4,5,8,13,15,16	3,6,7,9,10,11,12,14,17	1	P01835
2366	1,2,4,5,8,13,15,17	3,6,7,9,10,11,12,14,16	2	P10536,Q6PCU2
2367	1,2,4,5,8,13,16,17	3,6,7,9,10,11,12,14,15	2	P23928,Q6PCU2
2368	1,2,4,5,8,14,15,16	3,6,7,9,10,11,12,13,17	2	P97574,Q9QZK9
2369	1,2,4,5,8,14,15,17	3,6,7,9,10,11,12,13,16	6	P02761,P10536,P47967,P97840,Q6PCU2,Q9QZA6
2370	1,2,4,5,8,14,16,17	3,6,7,9,10,11,12,13,15	1	Q6PCU2
2371	1,2,4,5,8,15,16,17	3,6,7,9,10,11,12,13,14	2	P47727,Q6PCU2
2372	1,2,4,5,9,10,11,12	3,6,7,8,13,14,15,16,17	7	P02803,P51907,Q62636,Q63661,Q6IFU7,Q6IFU8,Q6P6Q2
2373	1,2,4,5,9,10,11,13	3,6,7,8,12,14,15,16,17	1	P02803
2374	1,2,4,5,9,10,11,14	3,6,7,8,12,13,15,16,17	1	Q63661
2375	1,2,4,5,9,10,11,15	3,6,7,8,12,13,14,16,17	3	P51907,Q561R9,Q63661
2376	1,2,4,5,9,10,11,16	3,6,7,8,12,13,14,15,17	4	A2RUV9,P51907,Q9QZK9,Q9WUW8
2377	1,2,4,5,9,10,11,17	3,6,7,8,12,13,14,15,16	1	Q7TP52

2378	1,2,4,5,9,10,12,13	3,6,7,8,11,14,15,16,17	1	P02803
2379	1,2,4,5,9,10,12,14	3,6,7,8,11,13,15,16,17	0	
2380	1,2,4,5,9,10,12,15	3,6,7,8,11,13,14,16,17	0	
2381	1,2,4,5,9,10,12,16	3,6,7,8,11,13,14,15,17	1	Q9WUW8
2382	1,2,4,5,9,10,12,17	3,6,7,8,11,13,14,15,16	2	P19223,Q10758
2383	1,2,4,5,9,10,13,14	3,6,7,8,11,12,15,16,17	0	
2384	1,2,4,5,9,10,13,15	3,6,7,8,11,12,14,16,17	0	
2385	1,2,4,5,9,10,13,16	3,6,7,8,11,12,14,15,17	1	Q62997
2386	1,2,4,5,9,10,13,17	3,6,7,8,11,12,14,15,16	0	
2387	1,2,4,5,9,10,14,15	3,6,7,8,11,12,13,16,17	1	Q9QZK9
2388	1,2,4,5,9,10,14,16	3,6,7,8,11,12,13,15,17	2	Q63942,Q9QZK9
2389	1,2,4,5,9,10,14,17	3,6,7,8,11,12,13,15,16	0	
2390	1,2,4,5,9,10,15,16	3,6,7,8,11,12,13,14,17	5	P02782,Q62902,Q62997,Q63942,Q9QZK9
2391	1,2,4,5,9,10,15,17	3,6,7,8,11,12,13,14,16	1	Q62902
2392	1,2,4,5,9,10,16,17	3,6,7,8,11,12,13,14,15	2	Q62902,Q62997

2393	1,2,4,5,9,11,12,13	3,6,7,8,10,14,15,16,17	2	P02803,P36374
2394	1,2,4,5,9,11,12,14	3,6,7,8,10,13,15,16,17	3	P02803,P51907,Q6IFU8
2395	1,2,4,5,9,11,12,15	3,6,7,8,10,13,14,16,17	1	P51907
2396	1,2,4,5,9,11,12,16	3,6,7,8,10,13,14,15,17	3	P02803,P51907,Q6IFU7
2397	1,2,4,5,9,11,12,17	3,6,7,8,10,13,14,15,16	2	P02803,P19223
2398	1,2,4,5,9,11,13,14	3,6,7,8,10,12,15,16,17	2	P02803,Q99MH3
2399	1,2,4,5,9,11,13,15	3,6,7,8,10,12,14,16,17	1	P05539
2400	1,2,4,5,9,11,13,16	3,6,7,8,10,12,14,15,17	2	A2RUV9,P02803
2401	1,2,4,5,9,11,13,17	3,6,7,8,10,12,14,15,16	1	P02803
2402	1,2,4,5,9,11,14,15	3,6,7,8,10,12,13,16,17	4	P51907,Q03191,Q99MH3,Q9QZK9
2403	1,2,4,5,9,11,14,16	3,6,7,8,10,12,13,15,17	2	Q99MH3,Q9QZK9
2404	1,2,4,5,9,11,14,17	3,6,7,8,10,12,13,15,16	0	
2405	1,2,4,5,9,11,15,16	3,6,7,8,10,12,13,14,17	3	P14408,P51907,Q9QZK9
2406	1,2,4,5,9,11,15,17	3,6,7,8,10,12,13,14,16	0	
2407	1,2,4,5,9,11,16,17	3,6,7,8,10,12,13,14,15	1	A2RUV9

2408	1,2,4,5,9,12,13,14	3,6,7,8,10,11,15,16,17	1	P02803
2409	1,2,4,5,9,12,13,15	3,6,7,8,10,11,14,16,17	0	
2410	1,2,4,5,9,12,13,16	3,6,7,8,10,11,14,15,17	1	P02803
2411	1,2,4,5,9,12,13,17	3,6,7,8,10,11,14,15,16	2	P02803,P35280
2412	1,2,4,5,9,12,14,15	3,6,7,8,10,11,13,16,17	0	
2413	1,2,4,5,9,12,14,16	3,6,7,8,10,11,13,15,17	0	
2414	1,2,4,5,9,12,14,17	3,6,7,8,10,11,13,15,16	1	P31430
2415	1,2,4,5,9,12,15,16	3,6,7,8,10,11,13,14,17	0	
2416	1,2,4,5,9,12,15,17	3,6,7,8,10,11,13,14,16	1	P06866
2417	1,2,4,5,9,12,16,17	3,6,7,8,10,11,13,14,15	0	
2418	1,2,4,5,9,13,14,15	3,6,7,8,10,11,12,16,17	1	Q9QZK9
2419	1,2,4,5,9,13,14,16	3,6,7,8,10,11,12,15,17	2	Q99MH3,Q9QZK9
2420	1,2,4,5,9,13,14,17	3,6,7,8,10,11,12,15,16	0	
2421	1,2,4,5,9,13,15,16	3,6,7,8,10,11,12,14,17	1	Q9QZK9
2422	1,2,4,5,9,13,15,17	3,6,7,8,10,11,12,14,16	0	

2423	1,2,4,5,9,13,16,17	3,6,7,8,10,11,12,14,15	1	P35280
2424	1,2,4,5,9,14,15,16	3,6,7,8,10,11,12,13,17	3	Q63942,Q99MH3,Q9QZK9
2425	1,2,4,5,9,14,15,17	3,6,7,8,10,11,12,13,16	1	P47967
2426	1,2,4,5,9,14,16,17	3,6,7,8,10,11,12,13,15	0	
2427	1,2,4,5,9,15,16,17	3,6,7,8,10,11,12,13,14	3	P47727,Q62902,Q68FV8
2428	1,2,4,5,10,11,12,13	3,6,7,8,9,14,15,16,17	5	P02803,P36374,Q62636,Q6IFU7,Q6P6Q2
2429	1,2,4,5,10,11,12,14	3,6,7,8,9,13,15,16,17	5	P51907,Q10758,Q6IFU7,Q6IFU8,Q6P6Q2
2430	1,2,4,5,10,11,12,15	3,6,7,8,9,13,14,16,17	4	P51907,Q62636,Q6IFU7,Q6P6Q2
2431	1,2,4,5,10,11,12,16	3,6,7,8,9,13,14,15,17	11	P06757,P14408,P51907,P53792,Q10758,Q6IFU7,Q6IFU8,Q6IFW6,Q6IMF3,Q6P6Q2,Q9WUW8
2432	1,2,4,5,10,11,12,17	3,6,7,8,9,13,14,15,16	7	P06757,P06866,P19223,Q10758,Q62636,Q6IFU8,Q6P6Q2
2433	1,2,4,5,10,11,13,14	3,6,7,8,9,12,15,16,17	0	
2434	1,2,4,5,10,11,13,15	3,6,7,8,9,12,14,16,17	0	
2435	1,2,4,5,10,11,13,16	3,6,7,8,9,12,14,15,17	1	P14408
2436	1,2,4,5,10,11,13,17	3,6,7,8,9,12,14,15,16	0	
2437	1,2,4,5,10,11,14,15	3,6,7,8,9,12,13,16,17	1	P51907
2438	1,2,4,5,10,11,14,16	3,6,7,8,9,12,13,15,17	0	
2439	1,2,4,5,10,11,14,17	3,6,7,8,9,12,13,15,16	0	
2440	1,2,4,5,10,11,15,16	3,6,7,8,9,12,13,14,17	3	P14408,P51907,Q9QZK9
2441	1,2,4,5,10,11,15,17	3,6,7,8,9,12,13,14,16	2	Q62902,Q7TP52
2442	1,2,4,5,10,11,16,17	3,6,7,8,9,12,13,14,15	3	A2RUV9,P14408,Q7TP52

2443	1,2,4,5,10,<br>12,13,14	3,6,7,8,9,1<br>1,15,16,17	0	
2444	1,2,4,5,10,<br>12,13,15	3,6,7,8,9,1<br>1,14,16,17	0	
2445	1,2,4,5,10,<br>12,13,16	3,6,7,8,9,1<br>1,14,15,17	1	Q62997
2446	1,2,4,5,10,<br>12,13,17	3,6,7,8,9,1<br>1,14,15,16	1	Q10758
2447	1,2,4,5,10,<br>12,14,15	3,6,7,8,9,1<br>1,13,16,17	0	
2448	1,2,4,5,10,<br>12,14,16	3,6,7,8,9,1<br>1,13,15,17	2	P08721,Q10758
2449	1,2,4,5,10,<br>12,14,17	3,6,7,8,9,1<br>1,13,15,16	2	P31430,Q10758
2450	1,2,4,5,10,<br>12,15,16	3,6,7,8,9,1<br>1,13,14,17	1	Q62997
2451	1,2,4,5,10,<br>12,15,17	3,6,7,8,9,1<br>1,13,14,16	2	P06866,Q10758
2452	1,2,4,5,10,<br>12,16,17	3,6,7,8,9,1<br>1,13,14,15	3	P06866,Q10758,Q62997
2453	1,2,4,5,10,<br>13,14,15	3,6,7,8,9,1<br>1,12,16,17	0	
2454	1,2,4,5,10,<br>13,14,16	3,6,7,8,9,1<br>1,12,15,17	1	Q62997
2455	1,2,4,5,10,<br>13,14,17	3,6,7,8,9,1<br>1,12,15,16	0	
2456	1,2,4,5,10,<br>13,15,16	3,6,7,8,9,1<br>1,12,14,17	2	P01835,Q62997
2457	1,2,4,5,10,<br>13,15,17	3,6,7,8,9,1<br>1,12,14,16	0	
2458	1,2,4,5,10,<br>13,16,17	3,6,7,8,9,1<br>1,12,14,15	1	Q62997
2459	1,2,4,5,10,<br>14,15,16	3,6,7,8,9,1<br>1,12,13,17	2	Q62997,Q9QZK9
2460	1,2,4,5,10,<br>14,15,17	3,6,7,8,9,1<br>1,12,13,16	1	Q62902
2461	1,2,4,5,10,<br>14,16,17	3,6,7,8,9,1<br>1,12,13,15	1	Q62997
2462	1,2,4,5,10,<br>15,16,17	3,6,7,8,9,1<br>1,12,13,14	4	P02782,P47727,Q62902,Q62997
2463	1,2,4,5,11,<br>12,13,14	3,6,7,8,9,1<br>0,15,16,17	1	P02803
2464	1,2,4,5,11,<br>12,13,15	3,6,7,8,9,1<br>0,14,16,17	1	P51907
2465	1,2,4,5,11,<br>12,13,16	3,6,7,8,9,1<br>0,14,15,17	3	P02803,P14408,Q6IFU7

2466	1,2,4,5,11,12,13,17	3,6,7,8,9,10,14,15,16	2	P02803,P36374
2467	1,2,4,5,11,12,14,15	3,6,7,8,9,10,13,16,17	1	P51907
2468	1,2,4,5,11,12,14,16	3,6,7,8,9,10,13,15,17	3	P51907,Q6IFU7,Q6IFU8
2469	1,2,4,5,11,12,14,17	3,6,7,8,9,10,13,15,16	2	P31430,Q6AYQ8
2470	1,2,4,5,11,12,15,16	3,6,7,8,9,10,13,14,17	3	P14408,P51907,Q6IFU7
2471	1,2,4,5,11,12,15,17	3,6,7,8,9,10,13,14,16	3	P06866,P51907,Q9ESW0
2472	1,2,4,5,11,12,16,17	3,6,7,8,9,10,13,14,15	2	P06866,P14408
2473	1,2,4,5,11,13,14,15	3,6,7,8,9,10,12,16,17	0	
2474	1,2,4,5,11,13,14,16	3,6,7,8,9,10,12,15,17	1	P14408
2475	1,2,4,5,11,13,14,17	3,6,7,8,9,10,12,15,16	0	
2476	1,2,4,5,11,13,15,16	3,6,7,8,9,10,12,14,17	1	P14408
2477	1,2,4,5,11,13,15,17	3,6,7,8,9,10,12,14,16	0	
2478	1,2,4,5,11,13,16,17	3,6,7,8,9,10,12,14,15	2	A2RUV9,P14408
2479	1,2,4,5,11,14,15,16	3,6,7,8,9,10,12,13,17	4	P14408,P51907,Q99MH3,Q9QZK9
2480	1,2,4,5,11,14,15,17	3,6,7,8,9,10,12,13,16	2	P47967,P97840
2481	1,2,4,5,11,14,16,17	3,6,7,8,9,10,12,13,15	1	P14408
2482	1,2,4,5,11,15,16,17	3,6,7,8,9,10,12,13,14	1	P14408
2483	1,2,4,5,12,13,14,15	3,6,7,8,9,10,11,16,17	0	
2484	1,2,4,5,12,13,14,16	3,6,7,8,9,10,11,15,17	0	
2485	1,2,4,5,12,13,14,17	3,6,7,8,9,10,11,15,16	1	P31430
2486	1,2,4,5,12,13,15,16	3,6,7,8,9,10,11,14,17	0	
2487	1,2,4,5,12,13,15,17	3,6,7,8,9,10,11,14,16	0	
2488	1,2,4,5,12,13,16,17	3,6,7,8,9,10,11,14,15	1	P35280

2489	1,2,4,5,12,14,15,16	3,6,7,8,9,10,11,13,17	1	P35467
2490	1,2,4,5,12,14,15,17	3,6,7,8,9,10,11,13,16	2	P31430,P98089
2491	1,2,4,5,12,14,16,17	3,6,7,8,9,10,11,13,15	1	P31430
2492	1,2,4,5,12,15,16,17	3,6,7,8,9,10,11,13,14	2	P06866,P47727
2493	1,2,4,5,13,14,15,16	3,6,7,8,9,10,11,12,17	2	P35467,Q9QZK9
2494	1,2,4,5,13,14,15,17	3,6,7,8,9,10,11,12,16	1	P47967
2495	1,2,4,5,13,14,16,17	3,6,7,8,9,10,11,12,15	1	Q6PCU2
2496	1,2,4,5,13,15,16,17	3,6,7,8,9,10,11,12,14	0	
2497	1,2,4,5,14,15,16,17	3,6,7,8,9,10,11,12,13	1	P35467
2498	1,2,4,6,7,8,9,10	3,5,11,12,13,14,15,16,17	27	D3ZHA0,O55004,O55006,P02631,P02761,P06761,P07647,P08723,P12020,P15399,P18418,P20762,P22282,P22283,P31044,P35280,P50280,P68370,P97571,P97580,Q30KJ2,Q5GRG2,Q63617,Q6P6R2,Q9EQS0,Q9JI85,Q9R0T3
2499	1,2,4,6,7,8,9,11	3,5,10,12,13,14,15,16,17	34	D3ZHA0,O54715,O55004,P02631,P02761,P02780,P05371,P06761,P07150,P07171,P07647,P08723,P09456,P10715,P14669,P15399,P18418,P19629,P20762,P22282,P22283,P31044,P35280,P50280,P68370,P97580,Q30KJ2,Q63493,Q63617,Q8CJ52,Q9EQS0,Q9JHB9,Q9JI85,Q9R0T3
2500	1,2,4,6,7,8,9,12	3,5,10,11,13,14,15,16,17	40	D3ZHA0,O55004,O88267,P02631,P02780,P02781,P02782,P05371,P06761,P07647,P08723,P09456,P0C0A9,P10715,P11598,P13432,P18418,P19223,P19629,P20762,P22282,P22283,P24368,P25236,P30120,P31044,P36374,P50280,P68370,P83748,Q30KJ2,Q63617,Q6AYR9,Q80WL1,Q8CJ52,Q99041,Q9EQS0,Q9JHB9,Q9JI85,Q9R0T3
2501	1,2,4,6,7,8,9,13	3,5,10,11,12,14,15,16,17	28	D3ZHA0,O55004,P02631,P02780,P06761,P06911,P07647,P08723,P09456,P11598,P12020,P18418,P19629,P20762,P22282,P22283,P25236,P31044,P50280,P68370,Q30KJ2,Q5GRG2,Q62902,Q63617,Q6TMA8,Q8CJ52,Q9JI85,Q9R0T3
2502	1,2,4,6,7,8,9,14	3,5,10,11,12,13,15,16,17	28	D3ZHA0,O55004,P02631,P05371,P06761,P06911,P07647,P08723,P11598,P12020,P15399,P18418,P19629,P20762,P22282,P22283,P25236,P31044,P50280,P68370,Q30KJ2,Q5GRG2,Q63617,Q63942,Q9EQS0,Q9JI85,Q9R0T3,Q9WTT6
2503	1,2,4,6,7,8,9,15	3,5,10,11,12,13,14,16,17	23	D3ZHA0,O55004,O55006,P02631,P02761,P05371,P06761,P07647,P18418,P19629,P20762,P22282,P31044,P35280,P50280,P68370,Q30KJ2,Q5GRG2,Q5QE79,Q63617,Q63942,Q9JI85,Q9R0T3
2504	1,2,4,6,7,8,9,16	3,5,10,11,12,13,14,15,17	18	D3ZHA0,O55004,P02631,P05371,P06761,P07647,P18418,P19629,P20762,P22282,P31044,P68370,Q30KJ2,Q63617,Q63942,Q6P6R2,Q9JI85,Q9R0T3
2505	1,2,4,6,7,8,9,17	3,5,10,11,12,13,14,15,16	31	D3ZHA0,O55004,P02631,P02761,P06761,P07647,P08723,P09605;P25809,P11598,P15399,P18418,P19223,P19629,P20761,P20762,P22282,P22283,P31044,P50115,P50280,P55091,P68370,P97580,Q30KJ2,Q4G075,Q63617,Q6P6R2,Q99041,Q9JI85,Q9QZA6,Q9R0T3
2506	1,2,4,6,7,8,10,11	3,5,9,12,13,14,15,16,17	46	D3ZHA0,O54715,O55004,P00714,P02631,P02761,P02780,P05371,P05964,P06761,P07150,P07171,P07647,P08723,P11598,P12020,P14669,P15399,P18418,P19629,P20762,P22282,P22283,P24368,P31044,P35280,P47727,P50280,P61206;P84079;P84082,P68370,P97580,Q30KJ2,Q5BJY9,Q5FVI6,Q5GRG2,Q63493,Q63598,Q63617,Q64361,Q6AYL6,Q7TP52,Q8CJ52,Q9EQS0,Q9JHB9,Q9JI85,Q9R0T3

2507	1,2,4,6,7,8,10,12	3,5,9,11,13,14,15,16,17	55	D3ZHA0,F1LM93,O55004,P01041,P02631,P02761,P02780,P02781,P02782,P05964,P06761,P07647,P08723,P09456,P0C0A9,P10715,P11598,P12020,P13432,P15399,P18418,P19223,P19629,P20762,P22282,P22283,P24368,P25236,P30120,P31044,P35280,P35281,P36374,P47967,P50280,P60905,P68370,P83748,P97580,P97840,Q10758,Q30KJ2,Q5GRG2,Q63475,Q63493,Q63617,Q64361,Q6AYR9,Q80WL1,Q8CJ52,Q99041,Q9EQS0,Q9JHB9,Q9JI85,Q9R0T3
2508	1,2,4,6,7,8,10,13	3,5,9,11,12,14,15,16,17	26	D3ZHA0,P02631,P02780,P06761,P06911,P07647,P08723,P11598,P12020,P15399,P18418,P20762,P22282,P22283,P25236,P31044,P50280,P68370,P97580,Q30KJ2,Q5GRG2,Q62902,Q63617,Q8CJ52,Q9JI85,Q9R0T3
2509	1,2,4,6,7,8,10,14	3,5,9,11,12,13,15,16,17	33	D3ZHA0,P02631,P02761,P05371,P06761,P06911,P07171,P07647,P08723,P11598,P12020,P15399,P18418,P19629,P20762,P22282,P22283,P25236,P31044,P35280,P50280,P68370,P97580,Q30KJ2,Q5BJY9,Q5GRG2,Q63532,Q63617,Q8CJ52,Q9EQS0,Q9JI85,Q9R0T3,Q9WTT6
2510	1,2,4,6,7,8,10,15	3,5,9,11,12,13,14,16,17	29	D3ZHA0,O55004,P02631,P02761,P06761,P07171,P07647,P08723,P11598,P12020,P15399,P18418,P19629,P20762,P22006,P22282,P22283,P31044,P35280,P50280,P68370,P97571,P97580,Q30KJ2,Q5GRG2,Q63617,Q64361,Q9JI85,Q9R0T3
2511	1,2,4,6,7,8,10,16	3,5,9,11,12,13,14,15,17	26	D3ZHA0,O55004,P02631,P05371,P06761,P07647,P08721,P08723,P12020,P15399,P18418,P22282,P22283,P31044,P35280,P47967,P68370,P97580,Q30KJ2,Q5FVI6,Q5GRG2,Q63617,Q6P6R2,Q8CJ52,Q9JI85,Q9R0T3
2512	1,2,4,6,7,8,10,17	3,5,9,11,12,13,14,15,16	37	D3ZHA0,O55004,P02631,P02761,P06761,P07647,P08723,P11598,P12020,P15399,P18418,P19223,P19629,P20761,P20762,P22282,P22283,P31044,P46844,P50115,P50280,P55091,P68370,P97571,P97580,Q30KJ2,Q4G075,Q5GRG2,Q63617,Q64361,Q6P6R2,Q7TP52,Q99041,Q9JI85,Q9QZA6,Q9R0T3,Q9Z0V6
2513	1,2,4,6,7,8,11,12	3,5,9,10,13,14,15,16,17	59	D3ZHA0,O35547,O54715,O55004,P02631,P02780,P02781,P02782,P04905,P05371,P05964,P06761,P07150,P07647,P08723,P09456,P0C0A9,P10715,P11598,P13432,P14669,P15399,P18418,P19223,P19629,P20762,P22282,P22283,P24368,P25236,P30120,P31044,P35280,P36374,P40241,P46462,P47727,P50280,P60905,P68370,P97580,Q30KJ2,Q5GRG2,Q5M8C6,Q62902,Q63357,Q63493,Q63617,Q64361,Q6AYL6,Q6AYR9,Q6P6Q2,Q80WL1,Q8CJ52,Q99041,Q9EQS0,Q9JHB9,Q9JI85,Q9R0T3
2514	1,2,4,6,7,8,11,13	3,5,9,10,12,14,15,16,17	42	D3ZHA0,O54715,O55004,P02631,P02780,P02781,P02782,P05371,P06761,P06911,P07150,P07171,P07647,P08723,P09456,P11598,P12020,P14669,P15399,P18418,P19629,P20762,P22282,P22283,P24368,P25236,P31044,P47727,P50280,P68370,P97580,Q30KJ2,Q5GRG2,Q62902,Q63493,Q63617,Q8CJ52,Q8VD89,Q9EQS0,Q9JHB9,Q9JI85,Q9R0T3
2515	1,2,4,6,7,8,11,14	3,5,9,10,12,13,15,16,17	40	D3ZHA0,O54715,O55004,P02631,P02761,P02780,P05371,P06761,P06911,P07150,P07171,P07647,P08723,P11598,P12020,P14669,P15399,P16086,P18418,P19629,P20762,P22282,P22283,P25236,P31044,P35280,P47727,P50280,P68370,P97580,Q30KJ2,Q5GRG2,Q63493,Q63532,Q63617,Q8CJ52,Q9EQS0,Q9JHB9,Q9JI85,Q9R0T3
2516	1,2,4,6,7,8,11,15	3,5,9,10,12,13,14,16,17	34	D3ZHA0,O54715,O55004,P02631,P02761,P05371,P06761,P07150,P07171,P07647,P08723,P10715,P11598,P14669,P15399,P18418,P19629,P20762,P22006,P22282,P22283,P31044,P35280,P50280,P68370,P97580,Q30KJ2,Q5GRG2,Q63493,Q63617,Q6P9T8,Q8CJ52,Q9JI85,Q9R0T3
2517	1,2,4,6,7,8,11,16	3,5,9,10,12,13,14,15,17	34	D3ZHA0,O54715,O55004,P02631,P05371,P06761,P07150,P07171,P07647,P08723,P10715,P14668,P14669,P15399,P18418,P19629,P20762,P22282,P22283,P31044,P35280,P50280,P61206;P84079;P84082,P68370,P97580,Q30KJ2,Q63493,Q63617,Q6AYL6,Q8CJ52,Q8VD89,Q9EQS0,Q9JI85,Q9R0T3
2518	1,2,4,6,7,8,11,17	3,5,9,10,12,13,14,15,16	38	O54715,O55004,P02631,P02761,P05371,P06761,P07150,P07647,P08723,P09605;P25809,P10715,P11598,P14669,P15399,P18418,P19223,P19629,P20761,P20762,P22282,P22283,P31044,P50115,P50280,P55091,P68370,P97580,Q30KJ2,Q4G075,Q63493,Q63617,Q6P6R2,Q8CJ52,Q99041,Q9JI85,Q9QZA6,Q9R0T3,Q9Z0V6
2519	1,2,4,6,7,8,12,13	3,5,9,10,11,14,15,16,17	49	D3ZHA0,O08557,O55004,O88267,P02631,P02780,P02781,P02782,P06761,P06911,P07647,P08723,P09456,P0C0A9,P10715,P11598,P12020,P13432,P18418,P19223,P19629,P20762,P22282,P22283,P24368,P25236,P29975,P30120,P31044,P36374,P50280,P62815,P68370,P83748,Q30KJ2,Q5GRG2,Q5M8C6,Q62635,Q62902,Q63493,Q63617,Q6AYR9,Q80WL1,Q8CJ52,Q99041,Q9EQS0,Q9JHB9,Q9JI85,Q9R0T3
2520	1,2,4,6,7,8,12,14	3,5,9,10,11,13,15,16,17	52	D3ZHA0,O08557,O55004,O88267,P02631,P02780,P02781,P02782,P05371,P06761,P06911,P07647,P08723,P0C0A9,P10715,P11598,P12020,P13432,P15399,P18418,P19223,P19629,P20762,P22282,P22283,P24368,P25236,P29975,P30120,P31044,P35280,P40241,P50280,P68370,P83748,Q30KJ2,Q5GRG2,Q62902,Q63493,Q63532,Q63617,Q6AYR9,Q6AYS7,Q80WL1,Q8CJ52,Q923M1,Q99041,Q9EQS0,Q9JHB9,Q9JI85,Q9R0T3,Q9WTT6

2521	1,2,4,6,7,8,12,15	3,5,9,10,11,13,14,16,17	40	D3ZHA0,O08557,O55004,P01015,P01041,P02631,P02761,P02780,P02782,P05371,P06761,P07647,P08723,P10715,P11598,P13432,P18418,P19223,P19629,P20762,P22282,P22283,P25236,P30120,P31044,P35280,P50280,P68370,P83748,Q30KJ2,Q5GRG2,Q63617,Q64361,Q6AYR9,Q8CJ52,Q99041,Q9EQS0,Q9JHB9,Q9JI85,Q9R0T3
2522	1,2,4,6,7,8,12,16	3,5,9,10,11,13,14,15,17	37	D3ZHA0,O55004,O88267,P02631,P02780,P02782,P05371,P06761,P07647,P08723,P0C0A9,P10715,P11598,P13432,P18418,P19223,P19629,P20762,P22282,P22283,P25236,P30120,P31044,P50280,P68370,P83748,Q30KJ2,Q63532,Q63617,Q6AYR9,Q80WL1,Q8CJ52,Q99041,Q9EQS0,Q9JHB9,Q9JI85,Q9R0T3
2523	1,2,4,6,7,8,12,17	3,5,9,10,11,13,14,15,16	57	D3ZHA0,O55004,O88267,P01041,P02631,P02761,P02780,P02782,P06761,P07647,P08723,P09605;P25809,P0C0A9,P10715,P11598,P13432,P15399,P18418,P19223,P19629,P20761,P20762,P22282,P22283,P25236,P30120,P31044,P36374,P50115,P50280,P55091,P62815,P63029,P68370,P83748,P97580,P98089,Q30KJ2,Q4G075,Q5M8C6,Q62635,Q63493,Q63617,Q64361,Q6AYR9,Q6P6R2,Q80WL1,Q8CJ52,Q99041,Q9EQS0,Q9EQV9,Q9JHB9,Q9JI85,Q9QZA6,Q9QZK9,Q9R0T3,Q9Z0V6
2524	1,2,4,6,7,8,13,14	3,5,9,10,11,12,15,16,17	32	D3ZHA0,O08557,O88267,P02631,P02780,P02782,P06761,P06911,P07647,P08723,P11598,P12020,P15399,P18418,P19629,P20762,P22282,P22283,P25236,P29975,P31044,P50280,P68370,Q30KJ2,Q5GRG2,Q62902,Q63532,Q63617,Q8CJ52,Q9JHB9,Q9JI85,Q9R0T3
2525	1,2,4,6,7,8,13,15	3,5,9,10,11,12,14,16,17	26	D3ZHA0,O08557,O55004,P02631,P06761,P06911,P07647,P08723,P11598,P12020,P18418,P19629,P20762,P22282,P22283,P25236,P31044,P50280,P68370,P83748,Q30KJ2,Q5GRG2,Q63617,Q8CJ52,Q9JI85,Q9R0T3
2526	1,2,4,6,7,8,13,16	3,5,9,10,11,12,14,15,17	24	D3ZHA0,O88267,P02631,P06761,P06911,P07647,P08723,P11598,P12020,P18418,P19629,P22282,P22283,P25236,P31044,P50280,P68370,Q30KJ2,Q5GRG2,Q62902,Q63617,Q8CJ52,Q9JI85,Q9R0T3
2527	1,2,4,6,7,8,13,17	3,5,9,10,11,12,14,15,16	40	O55004,O88267,P02631,P02780,P02782,P06761,P06911,P07647,P08723,P09605;P25809,P11598,P12020,P15399,P18418,P19223,P19629,P20761,P20762,P22282,P22283,P25236,P31044,P50115,P50280,P55091,P62815,P63029,P68370,P70545,P97580,Q30KJ2,Q4G075,Q5GRG2,Q62902,Q63617,Q6PCU2,Q8CJ52,Q99041,Q9JI85,Q9R0T3
2528	1,2,4,6,7,8,14,15	3,5,9,10,11,12,13,16,17	30	D3ZHA0,O08557,O55004,P02631,P02761,P05371,P06761,P06911,P07171,P07647,P08723,P11598,P12020,P18418,P19629,P20762,P22006,P22282,P22283,P25236,P31044,P35280,P50280,P68370,Q30KJ2,Q5GRG2,Q63532,Q63617,Q9JI85,Q9R0T3
2529	1,2,4,6,7,8,14,16	3,5,9,10,11,12,13,15,17	28	D3ZHA0,O88267,P02631,P05371,P06761,P06911,P07647,P08723,P11598,P12020,P18418,P19629,P20762,P22282,P22283,P25236,P31044,P50280,P68370,P97574,Q30KJ2,Q5GRG2,Q63532,Q63617,Q8CJ52,Q9EQS0,Q9JI85,Q9R0T3
2530	1,2,4,6,7,8,14,17	3,5,9,10,11,12,13,15,16	43	D3ZHA0,O55004,O88267,P02631,P02761,P05371,P06761,P06911,P07647,P08723,P09605;P25809,P10536,P11598,P12020,P15399,P18418,P19223,P19629,P20761,P20762,P22282,P22283,P25236,P31044,P50115,P50280,P55091,P62815,P68370,P97580,Q30KJ2,Q4G075,Q5GRG2,Q63617,Q6P6R2,Q6PCU2,Q8CJ52,Q99041,Q9JI85,Q9QZA6,Q9R0T3,Q9WTT6,Q9Z0V6
2531	1,2,4,6,7,8,15,16	3,5,9,10,11,12,13,14,17	17	D3ZHA0,O55004,P02631,P05371,P06761,P18418,P19629,P20762,P31044,P35280,P68370,P83748,Q30KJ2,Q5GRG2,Q63617,Q9JI85,Q9R0T3
2532	1,2,4,6,7,8,15,17	3,5,9,10,11,12,13,14,16	37	B0BNN3,D3ZHA0,O55004,P01041,P02631,P02761,P06761,P07647,P09605;P25809,P10536,P11598,P15399,P18418,P19223,P19629,P20760,P20761,P20762,P22282,P22283,P31044,P50115,P50280,P55091,P62815,P68370,P97580,Q30KJ2,Q4G075,Q5GRG2,Q63617,Q99041,Q9EQV9,Q9JI85,Q9QZA6,Q9R0T3,Q9Z0V6
2533	1,2,4,6,7,8,16,17	3,5,9,10,11,12,13,14,15	33	O55004,O88267,P02631,P05371,P06761,P07647,P09605;P25809,P11598,P15399,P18418,P19223,P19629,P20761,P20762,P22282,P22283,P23928,P31044,P50115,P50280,P55091,P63029,P68370,P97580,Q30KJ2,Q4G075,Q63617,Q6P6R2,Q6PCU2,Q8CJ52,Q99041,Q9JI85,Q9R0T3
2534	1,2,4,6,7,9,10,11	3,5,8,12,13,14,15,16,17	14	O54861,P02631,P05964,P15399,P18418,P35280,P61206;P84079;P84082,P83121,P97580,Q30KJ2,Q5FVI6,Q7TP52,Q91ZS3,Q9JI85
2535	1,2,4,6,7,9,10,12	3,5,8,11,13,14,15,16,17	15	O55004,P07647,P08723,P09456,P15399,P18418,P25236,P30120,P47967,P83748,P97840,Q30KJ2,Q6AYR9,Q91ZS3,Q9JI85

2536	1,2,4,6,7,9,10,13	3,5,8,11,12,14,15,16,17	9	P06911,P12020,P15399,P18418,P25236,P97840,Q30KJ2,Q5GRG2,Q9JI85
2537	1,2,4,6,7,9,10,14	3,5,8,11,12,13,15,16,17	9	P02631,P12020,P15399,P18418,P25236,P97580,Q30KJ2,Q5GRG2,Q63532
2538	1,2,4,6,7,9,10,15	3,5,8,11,12,13,14,16,17	5	P02631,P18418,P35280,P97840,Q30KJ2
2539	1,2,4,6,7,9,10,16	3,5,8,11,12,13,14,15,17	5	P18418,P47967,P97840,Q30KJ2,Q5FVI6
2540	1,2,4,6,7,9,10,17	3,5,8,11,12,13,14,15,16	8	P09605;P25809,P15399,P18418,P19223,P97580,Q30KJ2,Q6P6R2,Q7TP52
2541	1,2,4,6,7,9,11,12	3,5,8,10,13,14,15,16,17	24	O54861,O55004,P02631,P04905,P05371,P07647,P08723,P09456,P0C0A9,P13432,P15399,P18418,P25236,P30120,P36374,P97580,Q30KJ2,Q63357,Q6AYL6,Q6IFU7,Q91ZS3,Q9EQS0,Q9JHB9,Q9JI85
2542	1,2,4,6,7,9,11,13	3,5,8,10,12,14,15,16,17	12	P02631,P05539,P06911,P07647,P09456,P15399,P25236,P97580,Q30KJ2,Q5GRG2,Q91ZS3,Q9JI85
2543	1,2,4,6,7,9,11,14	3,5,8,10,12,13,15,16,17	11	P02631,P05371,P15399,P18418,P20762,P25236,P83121,P97580,Q30KJ2,Q63532,Q9JI85
2544	1,2,4,6,7,9,11,15	3,5,8,10,12,13,14,16,17	9	O55004,P02631,P05371,P15399,P22006,P35280,P97580,Q30KJ2,Q9JI85
2545	1,2,4,6,7,9,11,16	3,5,8,10,12,13,14,15,17	7	P02631,P05371,P15399,P61206;P84079;P84082,P97580,Q30KJ2,Q9JI85
2546	1,2,4,6,7,9,11,17	3,5,8,10,12,13,14,15,16	9	O54861,O55004,P02631,P09605;P25809,P15399,P19223,P97580,Q30KJ2,Q9JI85
2547	1,2,4,6,7,9,12,13	3,5,8,10,11,14,15,16,17	19	P02631,P06911,P07647,P08723,P09456,P12020,P18418,P22282,P25236,P30120,P36374,P83748,P97840,Q30KJ2,Q5GRG2,Q62902,Q6AYR9,Q9JHB9,Q9JI85
2548	1,2,4,6,7,9,12,14	3,5,8,10,11,13,15,16,17	16	P02631,P07647,P08723,P13432,P15399,P18418,P20762,P25236,P30120,P83748,Q30KJ2,Q63532,Q6AYR9,Q9EQS0,Q9JHB9,Q9JI85
2549	1,2,4,6,7,9,12,15	3,5,8,10,11,13,14,16,17	8	O55004,P02631,P08723,P18418,P25236,P83748,Q30KJ2,Q9JI85
2550	1,2,4,6,7,9,12,16	3,5,8,10,11,13,14,15,17	10	G3V686,P02631,P08723,P25236,P47967,P83748,P97840,Q30KJ2,Q63532,Q9JI85

2551	1,2,4,6,7,9,12,17	3,5,8,10,11,13,14,15,16	18	O55004,P02631,P07647,P08723,P09605;P25809,P13432,P15399,P18418,P19223,P36374,P55091,P83748,P97580,P98089,Q30KJ2,Q62635,Q6AYR9,Q9JI85
2552	1,2,4,6,7,9,13,14	3,5,8,10,11,12,15,16,17	9	P02631,P06911,P12020,P18418,P25236,Q30KJ2,Q5GRG2,Q63532,Q9JI85
2553	1,2,4,6,7,9,13,15	3,5,8,10,11,12,14,16,17	7	P02631,P05539,P06911,P25236,Q30KJ2,Q5GRG2,Q9JI85
2554	1,2,4,6,7,9,13,16	3,5,8,10,11,12,14,15,17	5	G3V686,P06911,P25236,Q30KJ2,Q5GRG2
2555	1,2,4,6,7,9,13,17	3,5,8,10,11,12,14,15,16	8	P06911,P09605;P25809,P15399,P25236,P97580,Q30KJ2,Q5GRG2,Q9JI85
2556	1,2,4,6,7,9,14,15	3,5,8,10,11,12,13,16,17	5	P02631,P25236,Q30KJ2,Q63532,Q6IE51
2557	1,2,4,6,7,9,14,16	3,5,8,10,11,12,13,15,17	4	P02631,P25236,Q30KJ2,Q63532
2558	1,2,4,6,7,9,14,17	3,5,8,10,11,12,13,15,16	6	P02631,P09605;P25809,P15399,P25236,P97580,Q30KJ2
2559	1,2,4,6,7,9,15,16	3,5,8,10,11,12,13,14,17	2	P02631,Q30KJ2
2560	1,2,4,6,7,9,15,17	3,5,8,10,11,12,13,14,16	6	O55004,P02631,P09605;P25809,P15399,P97580,Q30KJ2
2561	1,2,4,6,7,9,16,17	3,5,8,10,11,12,13,14,15	7	P02631,P09605;P25809,P14480,P15399,P97580,Q30KJ2,Q6P6R2
2562	1,2,4,6,7,10,11,12	3,5,8,9,13,14,15,16,17	37	O54861,O55004,P02631,P04905,P05964,P06757,P07647,P08723,P09456,P0C0A9,P15399,P18418,P24368,P30120,P35280,P36374,P47727,P47967,P50280,P61206;P84079;P84082,P68370,P97580,P97840,Q10758,Q30KJ2,Q5FVI6,Q63357,Q63493,Q6AYL6,Q6AYR9,Q6IFU7,Q6IMF3,Q6P6Q2,Q7TP52,Q9EQS0,Q9JHB9,Q9JI85
2563	1,2,4,6,7,10,11,13	3,5,8,9,12,14,15,16,17	14	P06911,P07647,P12020,P15399,P18418,P25236,P47727,P61206;P84079;P84082,P97580,Q30KJ2,Q5FVI6,Q5GRG2,Q7TP52,Q9JI85
2564	1,2,4,6,7,10,11,14	3,5,8,9,12,13,15,16,17	20	P02631,P05371,P05964,P12020,P15399,P18418,P20762,P25236,P35280,P47727,P61206;P84079;P84082,P83121,P97580,Q30KJ2,Q5BJY9,Q5FVI6,Q5GRG2,Q63532,Q7TP52,Q9JI85
2565	1,2,4,6,7,10,11,15	3,5,8,9,12,13,14,16,17	13	O54861,O55004,P02631,P15399,P18418,P22006,P35280,P61206;P84079;P84082,P97580,Q30KJ2,Q5FVI6,Q7TP52,Q9JI85

2566	1,2,4,6,7,10,11,16	3,5,8,9,12,13,14,15,17	14	P02631,P05371,P15399,P18418,P35280,P47967,P61206;P84079;P84082,P97580,Q30KJ2,Q5FVI6,Q63532,Q7TP52,Q8VD89,Q9JI85
2567	1,2,4,6,7,10,11,17	3,5,8,9,12,13,14,15,16	14	O54861,O55004,P02631,P05964,P09605;P25809,P15399,P18418,P19223,P61206;P84079;P84082,P97580,Q30KJ2,Q5FVI6,Q7TP52,Q9JI85
2568	1,2,4,6,7,10,12,13	3,5,8,9,11,14,15,16,17	21	P06911,P07647,P08723,P09456,P0C0A9,P12020,P15399,P18418,P22282,P25236,P30120,P36374,P47967,P70709,P83748,P97840,Q30KJ2,Q5GRG2,Q6AYR9,Q9JHB9,Q9JI85
2569	1,2,4,6,7,10,12,14	3,5,8,9,11,13,15,16,17	20	P02631,P07647,P08723,P12020,P15399,P18418,P25236,P30120,P35280,P83748,P97580,P97840,Q10758,Q30KJ2,Q5GRG2,Q63532,Q6AYR9,Q9EQS0,Q9JHB9,Q9JI85
2570	1,2,4,6,7,10,12,15	3,5,8,9,11,13,14,16,17	11	O55004,P02631,P08723,P15399,P18418,P25236,P35280,P83748,P97840,Q30KJ2,Q9JI85
2571	1,2,4,6,7,10,12,16	3,5,8,9,11,13,14,15,17	13	P08721,P08723,P15399,P18418,P25236,P47967,P83748,P97840,Q10758,Q30KJ2,Q5FVI6,Q63532,Q9JI85
2572	1,2,4,6,7,10,12,17	3,5,8,9,11,13,14,15,16	23	O55004,P05964,P07647,P08723,P09605;P25809,P13432,P14480,P15399,P18418,P19223,P30120,P36374,P55091,P83748,P97580,P97840,P98089,Q10758,Q30KJ2,Q62635,Q6AYR9,Q9JI85,Q9QZK9
2573	1,2,4,6,7,10,13,14	3,5,8,9,11,12,15,16,17	9	P06911,P12020,P15399,P18418,P25236,Q30KJ2,Q5GRG2,Q63532,Q9JI85
2574	1,2,4,6,7,10,13,15	3,5,8,9,11,12,14,16,17	7	P02631,P06911,P12020,P18418,P25236,Q30KJ2,Q5GRG2
2575	1,2,4,6,7,10,13,16	3,5,8,9,11,12,14,15,17	9	P06911,P12020,P18418,P25236,P47967,P97840,Q30KJ2,Q5GRG2,Q63532
2576	1,2,4,6,7,10,13,17	3,5,8,9,11,12,14,15,16	9	P06911,P12020,P14480,P15399,P18418,P97580,Q30KJ2,Q5GRG2,Q9JI85
2577	1,2,4,6,7,10,14,15	3,5,8,9,11,12,13,16,17	11	P02631,P12020,P15399,P18418,P22006,P25236,P35280,P35467,Q30KJ2,Q5GRG2,Q63532
2578	1,2,4,6,7,10,14,16	3,5,8,9,11,12,13,15,17	11	P02631,P08721,P12020,P15399,P18418,P25236,P97580,P97840,Q30KJ2,Q5GRG2,Q63532
2579	1,2,4,6,7,10,14,17	3,5,8,9,11,12,13,15,16	7	P02631,P12020,P15399,P18418,P97580,Q30KJ2,Q63532
2580	1,2,4,6,7,10,15,16	3,5,8,9,11,12,13,14,17	8	P02631,P18418,P35280,P35467,P97840,Q30KJ2,Q5FVI6,Q63532

2581	1,2,4,6,7,10,15,17	3,5,8,9,11,12,13,14,16	6	P02631,P15399,P18418,P97580,Q30KJ2,Q7TP52
2582	1,2,4,6,7,10,16,17	3,5,8,9,11,12,13,14,15	9	P09605;P25809,P14480,P15399,P18418,P97580,Q30KJ2,Q62997,Q6P6R2,Q7TP52
2583	1,2,4,6,7,11,12,13	3,5,8,9,10,14,15,16,17	33	O55004,P01836,P02631,P02780,P02781,P04905,P05539,P06911,P07647,P08723,P09456,P0C0A9,P12020,P15399,P18418,P22282,P22283,P24368,P25236,P30120,P36374,P47727,P50280,P97580,Q30KJ2,Q5GRG2,Q62902,Q63357,Q63493,Q6AYR9,Q8CJ52,Q9JHB9,Q9JI85
2584	1,2,4,6,7,11,12,14	3,5,8,9,10,13,15,16,17	29	O55004,P02631,P04905,P05371,P07647,P08723,P09456,P0C0A9,P12020,P13432,P15399,P18418,P20762,P25236,P30120,P35280,P36374,P47727,P50280,P97580,Q30KJ2,Q63493,Q63532,Q6AYL6,Q6AYR9,Q8CJ52,Q9EQS0,Q9JHB9,Q9JI85
2585	1,2,4,6,7,11,12,15	3,5,8,9,10,13,14,16,17	22	O54861,O55004,P01015,P01836,P02631,P05371,P07647,P08723,P15399,P18418,P20762,P22006,P25236,P30120,P35280,P50280,P51907,P83748,P97580,Q30KJ2,Q9JHB9,Q9JI85
2586	1,2,4,6,7,11,12,16	3,5,8,9,10,13,14,15,17	21	G3V686,O55004,P01836,P02631,P05371,P07647,P08723,P15399,P18418,P25236,P30120,P47967,P61206;P84079;P84082,P97580,Q30KJ2,Q63532,Q6AYL6,Q6IFU7,Q8CJ52,Q9JHB9,Q9JI85
2587	1,2,4,6,7,11,12,17	3,5,8,9,10,13,14,15,16	29	O54861,O55004,P01015,P02631,P05964,P07647,P08723,P09605;P25809,P0C0A9,P13432,P15399,P18418,P19223,P22282,P30120,P36374,P50280,P55091,P97580,P98089,Q30KJ2,Q62635,Q63493,Q6AYQ8,Q6AYR9,Q99041,Q9JHB9,Q9JI85,Q9QZK9
2588	1,2,4,6,7,11,13,14	3,5,8,9,10,12,15,16,17	18	P02631,P05539,P06911,P07647,P12020,P15399,P18418,P20762,P25236,P47727,P50280,P97580,Q30KJ2,Q5GRG2,Q62902,Q63532,Q9JHB9,Q9JI85
2589	1,2,4,6,7,11,13,15	3,5,8,9,10,12,14,16,17	11	P01836,P02631,P05539,P06911,P15399,P22006,P25236,P97580,Q30KJ2,Q5GRG2,Q9JI85
2590	1,2,4,6,7,11,13,16	3,5,8,9,10,12,14,15,17	13	G3V686,P01836,P02631,P06911,P15399,P25236,P61206;P84079;P84082,P97580,Q30KJ2,Q5GRG2,Q63532,Q8VD89,Q9JI85
2591	1,2,4,6,7,11,13,17	3,5,8,9,10,12,14,15,16	13	P02631,P05539,P06911,P07647,P09605;P25809,P15399,P22282,P25236,P50280,P97580,Q30KJ2,Q5GRG2,Q9JI85
2592	1,2,4,6,7,11,14,15	3,5,8,9,10,12,13,16,17	13	P02631,P05371,P15399,P18418,P20762,P22006,P25236,P35280,P97580,Q30KJ2,Q5GRG2,Q63532,Q9JI85
2593	1,2,4,6,7,11,14,16	3,5,8,9,10,12,13,15,17	10	G3V686,P02631,P05371,P15399,P25236,P61206;P84079;P84082,P97580,Q30KJ2,Q63532,Q9JI85
2594	1,2,4,6,7,11,14,17	3,5,8,9,10,12,13,15,16	11	P02631,P05371,P09605;P25809,P15399,P18418,P20762,P97580,Q30KJ2,Q63493,Q63532,Q9JI85
2595	1,2,4,6,7,11,15,16	3,5,8,9,10,12,13,14,17	10	P01836,P02631,P05371,P15399,P35280,P97580,Q30KJ2,Q63532,Q8VD89,Q9JI85

2596	1,2,4,6,7,11,15,17	3,5,8,9,10,12,13,14,16	9	O55004,P01015,P02631,P09605;P25809,P15399,P22006,P97580,Q30KJ2,Q9JI85
2597	1,2,4,6,7,11,16,17	3,5,8,9,10,12,13,14,15	9	O55004,P02631,P05371,P09605;P25809,P14480,P15399,P97580,Q30KJ2,Q9JI85
2598	1,2,4,6,7,12,13,14	3,5,8,9,10,11,15,16,17	21	P02631,P06911,P07647,P08723,P09456,P12020,P15399,P18418,P22282,P25236,P30120,P36374,P50280,P83748,Q30KJ2,Q5GRG2,Q62902,Q63532,Q6AYR9,Q9JHB9,Q9JI85
2599	1,2,4,6,7,12,13,15	3,5,8,9,10,11,14,16,17	15	P01836,P02631,P06911,P07647,P08723,P12020,P18418,P25236,P30120,P83748,Q30KJ2,Q5GRG2,Q6AYR9,Q9JHB9,Q9JI85
2600	1,2,4,6,7,12,13,16	3,5,8,9,10,11,14,15,17	19	G3V686,P01836,P06911,P07647,P08723,P12020,P25236,P30120,P47967,P83748,P97840,Q30KJ2,Q5GRG2,Q62902,Q63532,Q6AYR9,Q9JHB9,Q9JI85,Q9R0T3
2601	1,2,4,6,7,12,13,17	3,5,8,9,10,11,14,15,16	31	O55004,O88267,P06911,P07647,P08723,P09605;P25809,P12020,P13432,P14480,P15399,P18418,P19223,P22282,P22283,P25236,P30120,P36374,P50280,P55091,P62815,P80254,P83748,P97580,P98089,Q30KJ2,Q5GRG2,Q62635,Q6AYR9,Q9JHB9,Q9JI85,Q9QZK9
2602	1,2,4,6,7,12,14,15	3,5,8,9,10,11,13,16,17	13	P02631,P08723,P15399,P18418,P20762,P25236,P35280,P35467,P83748,Q30KJ2,Q5GRG2,Q63532,Q9JI85
2603	1,2,4,6,7,12,14,16	3,5,8,9,10,11,13,15,17	13	D3ZUC6,G3V686,P02631,P08723,P15399,P18418,P25236,P30120,P35467,P83748,Q30KJ2,Q63532,Q9JI85
2604	1,2,4,6,7,12,14,17	3,5,8,9,10,11,13,15,16	28	O55004,O88267,P02631,P07647,P08723,P09605;P25809,P12020,P13432,P15399,P18418,P19223,P20762,P22282,P25236,P30120,P31430,P55091,P62815,P80254,P83748,P97580,P98089,Q30KJ2,Q62635,Q63532,Q6AYQ8,Q6AYR9,Q9JI85
2605	1,2,4,6,7,12,15,16	3,5,8,9,10,11,13,14,17	8	P01836,P02631,P25236,P35467,P83748,Q30KJ2,Q63532,Q9JI85
2606	1,2,4,6,7,12,15,17	3,5,8,9,10,11,13,14,16	19	O55004,P01015,P01041,P02631,P06866,P09605;P25809,P13432,P15399,P18418,P19223,P20761,P55091,P62815,P83748,P97580,P98089,Q30KJ2,Q62635,Q9JI85
2607	1,2,4,6,7,12,16,17	3,5,8,9,10,11,13,14,15	15	O55004,P09605;P25809,P13432,P14480,P15399,P16573,P19223,P55091,P80254,P83748,P97580,P98089,Q30KJ2,Q62635,Q9JI85
2608	1,2,4,6,7,13,14,15	3,5,8,9,10,11,12,16,17	10	P02631,P06911,P12020,P25236,P35467,Q30KJ2,Q5GRG2,Q63532,Q6IE51,Q9JI85
2609	1,2,4,6,7,13,14,16	3,5,8,9,10,11,12,15,17	9	G3V686,P02631,P06911,P12020,P25236,P35467,Q30KJ2,Q5GRG2,Q63532
2610	1,2,4,6,7,13,14,17	3,5,8,9,10,11,12,15,16	10	P02631,P06911,P12020,P15399,P22282,P25236,P97580,Q30KJ2,Q5GRG2,Q9JI85

2611	1,2,4,6,7,13,15,16	3,5,8,9,10,11,12,14,17	7	P02631,P06911,P25236,P35467,P83748,Q30KJ2,Q5GRG2
2612	1,2,4,6,7,13,15,17	3,5,8,9,10,11,12,14,16	7	P02631,P06911,P15399,P25236,P97580,Q30KJ2,Q5GRG2
2613	1,2,4,6,7,13,16,17	3,5,8,9,10,11,12,14,15	9	P06911,P09605;P25809,P14480,P15399,P25236,P97580,Q30KJ2,Q5GRG2,Q6PCU2
2614	1,2,4,6,7,14,15,16	3,5,8,9,10,11,12,13,17	6	P02631,P25236,P35467,Q30KJ2,Q63532,Q6IE51
2615	1,2,4,6,7,14,15,17	3,5,8,9,10,11,12,13,16	10	P02631,P15399,P20761,P20762,P25236,P35467,P97580,Q30KJ2,Q63532,Q6IE51
2616	1,2,4,6,7,14,16,17	3,5,8,9,10,11,12,13,15	9	P02631,P09605;P25809,P14480,P15399,P25236,P35467,P97580,Q30KJ2,Q63532
2617	1,2,4,6,7,15,16,17	3,5,8,9,10,11,12,13,14	5	P02631,P09605;P25809,P35467,P97580,Q30KJ2
2618	1,2,4,6,8,9,10,11	3,5,7,12,13,14,15,16,17	12	D3ZHA0,O54715,P02761,P07647,P08649,P14669,P35280,Q63598,Q63942,Q64335,Q7TP52,Q91ZS3
2619	1,2,4,6,8,9,10,12	3,5,7,11,13,14,15,16,17	15	D3ZHA0,P02780,P02782,P02803,P07647,P08723,P0C0A9,P25236,P30120,Q10758,Q5M8C6,Q6AYR9,Q80WL1,Q9EQS0,Q9JHB9
2620	1,2,4,6,8,9,10,13	3,5,7,11,12,14,15,16,17	8	D3ZHA0,P02803,P06911,P07647,P25236,Q5GRG2,Q63942,Q8CJD3
2621	1,2,4,6,8,9,10,14	3,5,7,11,12,13,15,16,17	5	D3ZHA0,P17046,P25236,Q63942,Q9WTT6
2622	1,2,4,6,8,9,10,15	3,5,7,11,12,13,14,16,17	6	D3ZHA0,P02761,P17046,P35280,Q5QE79,Q63942
2623	1,2,4,6,8,9,10,16	3,5,7,11,12,13,14,15,17	3	D3ZHA0,Q63942,Q6P6R2
2624	1,2,4,6,8,9,10,17	3,5,7,11,12,13,14,15,16	8	D3ZHA0,P02761,P17046,P19223,Q63355,Q63942,Q64335,Q6P6R2
2625	1,2,4,6,8,9,11,12	3,5,7,10,13,14,15,16,17	30	O35547,O54715,O55004,P02780,P02781,P02782,P02803,P04905,P07647,P08723,P0C0A9,P13432,P14669,P24368,P25236,P30120,P36374,P50280,Q5M8C6,Q63357,Q6AYR9,Q6IFU7,Q6IFU8,Q6P6Q2,Q6TMA8,Q80WL1,Q8CJ52,Q9EQS0,Q9JHB9,Q9JI85

2626	1,2,4,6,8,9,11,13	3,5,7,10,12,14,15,16,17	12	P02780,P02782,P02803,P06911,P07647,P14669,P25236,P50280,Q6TMA8,Q8CJD3,Q9JHB9,Q9JI85
2627	1,2,4,6,8,9,11,14	3,5,7,10,12,13,15,16,17	6	P07647,P14669,P20762,P25236,P47727,Q63942
2628	1,2,4,6,8,9,11,15	3,5,7,10,12,13,14,16,17	5	P02761,P14669,P35280,Q5QE79,Q63942
2629	1,2,4,6,8,9,11,16	3,5,7,10,12,13,14,15,17	4	A2RUV9,P14668,P14669,Q63942
2630	1,2,4,6,8,9,11,17	3,5,7,10,12,13,14,15,16	6	A2RUV9,P02761,P07647,P14669,P19223,Q6P6R2
2631	1,2,4,6,8,9,12,13	3,5,7,10,11,14,15,16,17	18	P02780,P02782,P02803,P06911,P07647,P08723,P0C0A9,P22282,P25236,P30120,P36374,P50280,Q5M8C6,Q6AYR9,Q6TMA8,Q80WL1,Q9JHB9,Q9JI85
2632	1,2,4,6,8,9,12,14	3,5,7,10,11,13,15,16,17	15	D3ZHA0,P02780,P02782,P07647,P08723,P0C0A9,P13432,P20762,P25236,P30120,Q5M8C6,Q6AYR9,Q9EQS0,Q9JHB9,Q9WTT6
2633	1,2,4,6,8,9,12,15	3,5,7,10,11,13,14,16,17	8	D3ZHA0,P02761,P02782,P07647,P08723,P25236,P30120,Q5QE79
2634	1,2,4,6,8,9,12,16	3,5,7,10,11,13,14,15,17	6	P02782,P07647,P08723,P25236,P30120,Q63942
2635	1,2,4,6,8,9,12,17	3,5,7,10,11,13,14,15,16	20	P02782,P07647,P08723,P0C0A9,P13432,P16636,P19223,P30120,P50280,P55091,P98089,Q4G075,Q5M8C6,Q62635,Q63355,Q6AYR9,Q6P6R2,Q80WL1,Q9JHB9,Q9QZA6
2636	1,2,4,6,8,9,13,14	3,5,7,10,11,12,15,16,17	7	P02782,P06911,P07647,P25236,Q5GRG2,Q63942,Q6TMA8
2637	1,2,4,6,8,9,13,15	3,5,7,10,11,12,14,16,17	5	P25236,Q5GRG2,Q5QE79,Q63942,Q6TMA8
2638	1,2,4,6,8,9,13,16	3,5,7,10,11,12,14,15,17	3	P25236,Q63942,Q8CJD3
2639	1,2,4,6,8,9,13,17	3,5,7,10,11,12,14,15,16	6	P07647,P19223,P22282,P25236,Q63355,Q8CJD3
2640	1,2,4,6,8,9,14,15	3,5,7,10,11,12,13,16,17	5	P02761,P17046,P25236,Q5QE79,Q63942

2641	1,2,4,6,8,9,14,16	3,5,7,10,11,12,13,15,17	3	P25236,P97574,Q63942
2642	1,2,4,6,8,9,14,17	3,5,7,10,11,12,13,15,16	5	P19223,P25236,Q63942,Q6P6R2,Q9WTT6
2643	1,2,4,6,8,9,15,16	3,5,7,10,11,12,13,14,17	2	Q5QE79,Q63942
2644	1,2,4,6,8,9,15,17	3,5,7,10,11,12,13,14,16	5	B0BNN3,P02761,P19223,Q5QE79,Q63942
2645	1,2,4,6,8,9,16,17	3,5,7,10,11,12,13,14,15	5	A2RUV9,P19223,P23593,Q63942,Q6P6R2
2646	1,2,4,6,8,10,11,12	3,5,7,9,13,14,15,16,17	40	D3ZHA0,O35547,O54715,P02780,P02781,P02782,P04905,P05964,P07647,P08723,P0C0A9,P14669,P15399,P16636,P24368,P25236,P30120,P35280,P35281,P36374,P47727,P50280,P53792,P60905,P62804,Q00715,Q10758,Q5M8C6,Q63357,Q63598,Q6AYR9,Q6IFU7,Q6IFU8,Q6IMF3,Q6P6Q2,Q80WL1,Q8CJ52,Q9EQS0,Q9JHB9,Q9JI85
2647	1,2,4,6,8,10,11,13	3,5,7,9,12,14,15,16,17	12	P06911,P07647,P08723,P14669,P25236,P47727,P50280,Q5GRG2,Q63598,Q8CJD3,Q9JHB9,Q9JI85
2648	1,2,4,6,8,10,11,14	3,5,7,9,12,13,15,16,17	11	P02761,P07647,P14669,P15399,P20762,P25236,P35280,P47727,P50280,Q5BJY9,Q63598
2649	1,2,4,6,8,10,11,15	3,5,7,9,12,13,14,16,17	7	D3ZHA0,P02761,P14669,P35280,P50280,Q63598,Q7TP52
2650	1,2,4,6,8,10,11,16	3,5,7,9,12,13,14,15,17	7	A2RUV9,O54715,P07150,P14669,P35280,Q63598,Q7TP52
2651	1,2,4,6,8,10,11,17	3,5,7,9,12,13,14,15,16	16	P02761,P07150,P07647,P08649,P14669,P15399,P16636,P19223,P50280,P97580,Q30KJ2,Q4G075,Q63598,Q64335,Q6P6R2,Q7TP52
2652	1,2,4,6,8,10,12,13	3,5,7,9,11,14,15,16,17	25	D3ZHA0,P02780,P02781,P02782,P02803,P06911,P07647,P08723,P0C0A9,P12020,P22282,P22283,P25236,P30120,P36374,P50280,P70709,Q10758,Q5GRG2,Q5M8C6,Q6AYR9,Q80WL1,Q8CJ52,Q9JHB9,Q9JI85
2653	1,2,4,6,8,10,12,14	3,5,7,9,11,13,15,16,17	15	D3ZHA0,P02780,P02782,P07647,P08723,P0C0A9,P25236,P30120,P35280,P50280,Q10758,Q6AYR9,Q9EQS0,Q9JHB9,Q9WTT6
2654	1,2,4,6,8,10,12,15	3,5,7,9,11,13,14,16,17	13	D3ZHA0,P01015,P02761,P02782,P07647,P08723,P0C0A9,P25236,P30120,P35280,P35281,P50280,Q6AYR9
2655	1,2,4,6,8,10,12,16	3,5,7,9,11,13,14,15,17	13	D3ZHA0,P02782,P07647,P08721,P08723,P0C0A9,P25236,P30120,P35281,P47967,Q10758,Q6AYR9,Q6IFU7

2656	1,2,4,6,8,10,12,17	3,5,7,9,11,13,14,15,16	27	D3ZHA0,D4A5U3,P02761,P02782,P07647,P08723,P0C0A9,P13432,P16636,P19223,P30120,P50280,P55091,P98089,Q10758,Q4G075,Q5M8C6,Q62635,Q63355,Q64361,Q6AYR9,Q6P6R2,Q80WL1,Q9EQV9,Q9JHB9,Q9QZA6,Q9QZK9
2657	1,2,4,6,8,10,13,14	3,5,7,9,11,12,15,16,17	6	P06911,P07647,P12020,P25236,P50280,Q5GRG2
2658	1,2,4,6,8,10,13,15	3,5,7,9,11,12,14,16,17	7	D3ZHA0,P06911,P15999,P25236,P50280,Q5GRG2,Q6IFV1
2659	1,2,4,6,8,10,13,16	3,5,7,9,11,12,14,15,17	4	P06911,P25236,Q5GRG2,Q8CJD3
2660	1,2,4,6,8,10,13,17	3,5,7,9,11,12,14,15,16	9	P06911,P07647,P16636,P25236,P50280,Q4G075,Q5GRG2,Q63355,Q6IFV1
2661	1,2,4,6,8,10,14,15	3,5,7,9,11,12,13,16,17	6	D3ZHA0,P02761,P17046,P25236,P35280,Q5GRG2
2662	1,2,4,6,8,10,14,16	3,5,7,9,11,12,13,15,17	3	P08721,P25236,P97574
2663	1,2,4,6,8,10,14,17	3,5,7,9,11,12,13,15,16	6	P02761,P25236,P50280,Q4G075,Q6P6R2,Q9WTT6
2664	1,2,4,6,8,10,15,16	3,5,7,9,11,12,13,14,17	2	D3ZHA0,P35280
2665	1,2,4,6,8,10,15,17	3,5,7,9,11,12,13,14,16	5	B0BNN3,P02761,Q4G075,Q6IFV1,Q9EQV9
2666	1,2,4,6,8,10,16,17	3,5,7,9,11,12,13,14,15	3	Q4G075,Q62997,Q6P6R2
2667	1,2,4,6,8,11,12,13	3,5,7,9,10,14,15,16,17	30	O35547,P02780,P02781,P02782,P02803,P04905,P06761,P06911,P07647,P08723,P0C0A9,P14669,P22282,P22283,P24368,P25236,P30120,P36374,P47727,P50280,Q5GRG2,Q5M8C6,Q62902,Q63357,Q6AYR9,Q6TMA8,Q80WL1,Q8CJ52,Q9JHB9,Q9JI85
2668	1,2,4,6,8,11,12,14	3,5,7,9,10,13,15,16,17	28	O35547,P02780,P02781,P02782,P04905,P07647,P08723,P0C0A9,P14669,P20762,P24368,P25236,P30120,P35280,P40241,P47727,P50280,P62804,Q00715,Q5M8C6,Q6AYR9,Q6IFU7,Q6IFU8,Q6P6Q2,Q8CJ52,Q9EQS0,Q9JHB9,Q9JI85
2669	1,2,4,6,8,11,12,15	3,5,7,9,10,13,14,16,17	21	B0BNN3,O35547,O54715,O55004,P01015,P02761,P02782,P07647,P08723,P0C0A9,P14669,P20762,P25236,P30120,P35280,P50280,Q00715,Q6IFU7,Q8CJ52,Q9JHB9,Q9JI85
2670	1,2,4,6,8,11,12,16	3,5,7,9,10,13,14,15,17	16	O35547,O54715,P02782,P07647,P08723,P0C0A9,P14669,P25236,P30120,P50280,Q6IFU7,Q6IFU8,Q6P6Q2,Q8CJ52,Q9JHB9,Q9JI85

2671	1,2,4,6,8,11,12,17	3,5,7,9,10,13,14,15,16	39	O35547,O54715,O55004,P01015,P02761,P02780,P02781,P02782,P04905,P07150,P07647,P08723,P0C0A9,P13432,P14669,P15399,P16636,P19223,P22282,P22283,P30120,P36374,P50280,P55091,P62804,P98089,Q00715,Q4G075,Q5M8C6,Q62635,Q63493,Q6AYQ8,Q6AYR9,Q80WL1,Q8CJ52,Q9JHB9,Q9JI85,Q9QZA6,Q9QZK9
2672	1,2,4,6,8,11,13,14	3,5,7,9,10,12,15,16,17	15	P02780,P02782,P06911,P07647,P08723,P14669,P20762,P22282,P25236,P47727,P50280,Q5GRG2,Q6TMA8,Q9JHB9,Q9JI85
2673	1,2,4,6,8,11,13,15	3,5,7,9,10,12,14,16,17	6	P07647,P14669,P25236,P50280,Q5GRG2,Q6TMA8
2674	1,2,4,6,8,11,13,16	3,5,7,9,10,12,14,15,17	6	P06911,P07647,P14669,P25236,P50280,Q8CJD3
2675	1,2,4,6,8,11,13,17	3,5,7,9,10,12,14,15,16	12	P02782,P06911,P07150,P07647,P14669,P22282,P25236,P50280,Q4G075,Q8CJD3,Q9JHB9,Q9JI85
2676	1,2,4,6,8,11,14,15	3,5,7,9,10,12,13,16,17	6	P02761,P14669,P20762,P25236,P35280,P50280
2677	1,2,4,6,8,11,14,16	3,5,7,9,10,12,13,15,17	4	P14668,P14669,P25236,P97574
2678	1,2,4,6,8,11,14,17	3,5,7,9,10,12,13,15,16	10	P02761,P07150,P07647,P14669,P15399,P20762,P25236,P50280,Q30KJ2,Q4G075
2679	1,2,4,6,8,11,15,16	3,5,7,9,10,12,13,14,17	3	P14668,P14669,P35280
2680	1,2,4,6,8,11,15,17	3,5,7,9,10,12,13,14,16	8	B0BNN3,P01015,P02761,P07150,P14669,P50280,Q4G075,Q9QZA6
2681	1,2,4,6,8,11,16,17	3,5,7,9,10,12,13,14,15	5	A2RUV9,P07150,P14669,Q4G075,Q6P6R2
2682	1,2,4,6,8,12,13,14	3,5,7,9,10,11,15,16,17	26	O08557,P02780,P02782,P06761,P06911,P07647,P08723,P0C0A9,P12020,P22282,P22283,P25236,P29975,P30120,P36374,P50280,Q5GRG2,Q5M8C6,Q62902,Q64093,Q6AYR9,Q6TMA8,Q8CJ52,Q923M1,Q9JHB9,Q9JI85
2683	1,2,4,6,8,12,13,15	3,5,7,9,10,11,14,16,17	14	O08557,P02782,P06911,P07647,P08723,P0C0A9,P15999,P25236,P30120,P50280,Q5GRG2,Q6AYR9,Q6IFV1,Q9JHB9
2684	1,2,4,6,8,12,13,16	3,5,7,9,10,11,14,15,17	12	P02780,P02782,P06911,P07647,P08723,P0C0A9,P25236,P30120,P50280,Q6AYR9,Q8CJ52,Q9JHB9
2685	1,2,4,6,8,12,13,17	3,5,7,9,10,11,14,15,16	29	P02780,P02782,P06761,P06911,P07647,P08723,P0C0A9,P13432,P16636,P19223,P22282,P22283,P25236,P30120,P36374,P50280,P55091,P62815,P98089,Q4G075,Q5M8C6,Q62635,Q63355,Q6AYR9,Q6IFV1,Q80WL1,Q8CJ52,Q9JHB9,Q9QZK9

2686	1,2,4,6,8,12,14,15	3,5,7,9,10,11,13,16,17	11	B0BNN3,O08557,P02782,P07647,P08723,P20762,P25236,P30120,P35280,P50280,Q6AYR9
2687	1,2,4,6,8,12,14,16	3,5,7,9,10,11,13,15,17	7	P02782,P07647,P08723,P25236,P30120,Q6AYR9,Q8CJ52
2688	1,2,4,6,8,12,14,17	3,5,7,9,10,11,13,15,16	24	P02782,P07647,P08723,P0C0A9,P13432,P19223,P20762,P22282,P25236,P30120,P31430,P50280,P55091,P98089,Q10758,Q4G075,Q5M8C6,Q62635,Q6AYQ8,Q6AYR9,Q923M1,Q9JHB9,Q9QZA6,Q9WTT6
2689	1,2,4,6,8,12,15,16	3,5,7,9,10,11,13,14,17	2	P08723,P25236
2690	1,2,4,6,8,12,15,17	3,5,7,9,10,11,13,14,16	17	B0BNN3,P01015,P02761,P02782,P06866,P07647,P08723,P13432,P19223,P50280,P55091,P98089,Q4G075,Q62635,Q6IFV1,Q9EQV9,Q9QZA6
2691	1,2,4,6,8,12,16,17	3,5,7,9,10,11,13,14,15	14	P02782,P07647,P08723,P13432,P14480,P16636,P19223,P30120,P55091,P98089,Q4G075,Q62635,Q6AYR9,Q6P6R2
2692	1,2,4,6,8,13,14,15	3,5,7,9,10,11,12,16,17	7	O08557,P06911,P15999,P25236,P50280,Q5GRG2,Q64093
2693	1,2,4,6,8,13,14,16	3,5,7,9,10,11,12,15,17	4	P06911,P25236,Q5GRG2,Q6PCU2
2694	1,2,4,6,8,13,14,17	3,5,7,9,10,11,12,15,16	8	P02782,P06911,P07647,P22282,P25236,P50280,Q5GRG2,Q6PCU2
2695	1,2,4,6,8,13,15,16	3,5,7,9,10,11,12,14,17	2	P25236,Q5GRG2
2696	1,2,4,6,8,13,15,17	3,5,7,9,10,11,12,14,16	8	B0BNN3,P02761,P15999,P25236,P50280,Q4G075,Q5GRG2,Q6IFV1
2697	1,2,4,6,8,13,16,17	3,5,7,9,10,11,12,14,15	4	P25236,Q63355,Q6PCU2,Q8CJD3
2698	1,2,4,6,8,14,15,16	3,5,7,9,10,11,12,13,17	2	P25236,P97574
2699	1,2,4,6,8,14,15,17	3,5,7,9,10,11,12,13,16	6	B0BNN3,P02761,P10536,P20762,P25236,P50280
2700	1,2,4,6,8,14,16,17	3,5,7,9,10,11,12,13,15	3	P25236,Q6P6R2,Q6PCU2

2701	1,2,4,6,8,15,16,17	3,5,7,9,10,11,12,13,14	4	B0BNN3,P02761,Q4G075,Q6PCU2
2702	1,2,4,6,9,10,11,12	3,5,7,8,13,14,15,16,17	13	P02803,P06757,P0C0A9,P30120,Q63357,Q63661,Q6IFU7,Q6IFU8,Q6IMF3,Q6P6Q2,Q7TP52,Q91ZS3,Q9JHB9
2703	1,2,4,6,9,10,11,13	3,5,7,8,12,14,15,16,17	4	P02803,P25236,Q8CJD3,Q91ZS3
2704	1,2,4,6,9,10,11,14	3,5,7,8,12,13,15,16,17	7	P15399,P25236,P47727,P83121,Q63661,Q7TP52,Q91ZS3
2705	1,2,4,6,9,10,11,15	3,5,7,8,12,13,14,16,17	3	P35280,Q561R9,Q7TP52
2706	1,2,4,6,9,10,11,16	3,5,7,8,12,13,14,15,17	4	A2RUV9,Q7TP52,Q91ZS3,Q9WUW8
2707	1,2,4,6,9,10,11,17	3,5,7,8,12,13,14,15,16	3	P15399,Q64335,Q7TP52
2708	1,2,4,6,9,10,12,13	3,5,7,8,11,14,15,16,17	8	P02803,P25236,P30120,P36374,P70709,P97840,Q6AYR9,Q9JHB9
2709	1,2,4,6,9,10,12,14	3,5,7,8,11,13,15,16,17	2	P25236,Q10758
2710	1,2,4,6,9,10,12,15	3,5,7,8,11,13,14,16,17	0	
2711	1,2,4,6,9,10,12,16	3,5,7,8,11,13,14,15,17	5	P47967,P97840,Q10758,Q6IFU7,Q9WUW8
2712	1,2,4,6,9,10,12,17	3,5,7,8,11,13,14,15,16	7	P13432,P16636,P19223,P98089,Q10758,Q62635,Q63355
2713	1,2,4,6,9,10,13,14	3,5,7,8,11,12,15,16,17	4	P06911,P12020,P25236,Q5GRG2
2714	1,2,4,6,9,10,13,15	3,5,7,8,11,12,14,16,17	1	P25236
2715	1,2,4,6,9,10,13,16	3,5,7,8,11,12,14,15,17	2	P25236,Q8CJD3

2716	1,2,4,6,9,10,13,17	3,5,7,8,11,12,14,15,16	2	Q63355,Q8CJD3
2717	1,2,4,6,9,10,14,15	3,5,7,8,11,12,13,16,17	2	P17046,P25236
2718	1,2,4,6,9,10,14,16	3,5,7,8,11,12,13,15,17	2	P25236,Q63942
2719	1,2,4,6,9,10,14,17	3,5,7,8,11,12,13,15,16	0	
2720	1,2,4,6,9,10,15,16	3,5,7,8,11,12,13,14,17	1	Q63942
2721	1,2,4,6,9,10,15,17	3,5,7,8,11,12,13,14,16	0	
2722	1,2,4,6,9,10,16,17	3,5,7,8,11,12,13,14,15	3	P14480,Q6P6R2,Q7TP52
2723	1,2,4,6,9,11,12,13	3,5,7,8,10,14,15,16,17	11	P02803,P04905,P07647,P0C0A9,P25236,P30120,P36374,Q6AYR9,Q6IFU7,Q9JHB9,Q9JI85
2724	1,2,4,6,9,11,12,14	3,5,7,8,10,13,15,16,17	8	P04905,P0C0A9,P25236,P30120,P47727,Q6IFU7,Q6IFU8,Q9JHB9
2725	1,2,4,6,9,11,12,15	3,5,7,8,10,13,14,16,17	3	P25236,P51907,Q6IFU7
2726	1,2,4,6,9,11,12,16	3,5,7,8,10,13,14,15,17	2	Q6IFU7,Q6IFU8
2727	1,2,4,6,9,11,12,17	3,5,7,8,10,13,14,15,16	10	P09605;P25809,P13432,P15399,P19223,P36374,P98089,Q62635,Q6AYQ8,Q6IFU7,Q9JHB9
2728	1,2,4,6,9,11,13,14	3,5,7,8,10,12,15,16,17	4	P06911,P25236,P47727,P83121
2729	1,2,4,6,9,11,13,15	3,5,7,8,10,12,14,16,17	1	P25236
2730	1,2,4,6,9,11,13,16	3,5,7,8,10,12,14,15,17	2	P25236,Q8CJD3

2731	1,2,4,6,9,11,13,17	3,5,7,8,10,12,14,15,16	1	Q8CJD3
2732	1,2,4,6,9,11,14,15	3,5,7,8,10,12,13,16,17	1	P25236
2733	1,2,4,6,9,11,14,16	3,5,7,8,10,12,13,15,17	2	P25236,Q99MH3
2734	1,2,4,6,9,11,14,17	3,5,7,8,10,12,13,15,16	1	P15399
2735	1,2,4,6,9,11,15,16	3,5,7,8,10,12,13,14,17	0	
2736	1,2,4,6,9,11,15,17	3,5,7,8,10,12,13,14,16	0	
2737	1,2,4,6,9,11,16,17	3,5,7,8,10,12,13,14,15	1	A2RUV9
2738	1,2,4,6,9,12,13,14	3,5,7,8,10,11,15,16,17	5	P06911,P25236,P30120,Q6AYR9,Q9JHB9
2739	1,2,4,6,9,12,13,15	3,5,7,8,10,11,14,16,17	1	P25236
2740	1,2,4,6,9,12,13,16	3,5,7,8,10,11,14,15,17	2	G3V686,P25236
2741	1,2,4,6,9,12,13,17	3,5,7,8,10,11,14,15,16	9	P13432,P19223,P25236,P36374,P98089,Q62635,Q63355,Q6AYR9,Q9JHB9
2742	1,2,4,6,9,12,14,15	3,5,7,8,10,11,13,16,17	1	P25236
2743	1,2,4,6,9,12,14,16	3,5,7,8,10,11,13,15,17	1	P25236
2744	1,2,4,6,9,12,14,17	3,5,7,8,10,11,13,15,16	9	P01835,P13432,P19223,P25236,P31430,P98089,Q5HZV9,Q62635,Q6AYQ8
2745	1,2,4,6,9,12,15,16	3,5,7,8,10,11,13,14,17	1	P25236

2746	1,2,4,6,9,12,15,17	3,5,7,8,10,11,13,14,16	6	B0BNN3,P06866,P13432,P19223,P98089,Q62635
2747	1,2,4,6,9,12,16,17	3,5,7,8,10,11,13,14,15	8	P06866,P09605;P25809,P13432,P14480,P16573,P19223,P98089,Q62635
2748	1,2,4,6,9,13,14,15	3,5,7,8,10,11,12,16,17	2	P16391,P25236
2749	1,2,4,6,9,13,14,16	3,5,7,8,10,11,12,15,17	1	P25236
2750	1,2,4,6,9,13,14,17	3,5,7,8,10,11,12,15,16	1	P25236
2751	1,2,4,6,9,13,15,16	3,5,7,8,10,11,12,14,17	1	P25236
2752	1,2,4,6,9,13,15,17	3,5,7,8,10,11,12,14,16	1	P25236
2753	1,2,4,6,9,13,16,17	3,5,7,8,10,11,12,14,15	3	P14480,Q63355,Q8CJD3
2754	1,2,4,6,9,14,15,16	3,5,7,8,10,11,12,13,17	3	P25236,Q63942,Q9QZK9
2755	1,2,4,6,9,14,15,17	3,5,7,8,10,11,12,13,16	1	Q5HZV9
2756	1,2,4,6,9,14,16,17	3,5,7,8,10,11,12,13,15	1	Q5HZV9
2757	1,2,4,6,9,15,16,17	3,5,7,8,10,11,12,13,14	0	
2758	1,2,4,6,10,11,12,13	3,5,7,8,9,14,15,16,17	13	P02803,P04905,P07647,P08723,P0C0A9,P25236,P30120,P36374,P47727,P70709,Q6AYR9,Q6IFU7,Q9JHB9
2759	1,2,4,6,10,11,12,14	3,5,7,8,9,13,15,16,17	15	P04905,P08723,P0C0A9,P15399,P25236,P30120,P35280,P47727,P62804,Q10758,Q6IFU7,Q6IFU8,Q6IMF3,Q6P6Q2,Q9JHB9
2760	1,2,4,6,10,11,12,15	3,5,7,8,9,13,14,16,17	4	P01015,P35280,P51907,Q6IFU7
2761	1,2,4,6,10,11,12,16	3,5,7,8,9,13,14,15,17	10	P06757,P47967,P53792,Q10758,Q6IFU7,Q6IFU8,Q6IMF3,Q6P6Q2,Q7TP52,Q9WUW8
2762	1,2,4,6,10,11,12,17	3,5,7,8,9,13,14,15,16	17	O08815,P05964,P06757,P06866,P0C0A9,P13432,P15399,P16636,P19223,P36374,P98089,Q10758,Q62635,Q6IFU7,Q6IFU8,Q7TP52,Q9JHB9

2763	1,2,4,6,10,<br>11,13,14	3,5,7,8,9,1<br>2,15,16,17	5	P06911,P12020,P25236,P47727,Q5GRG2
2764	1,2,4,6,10,<br>11,13,15	3,5,7,8,9,1<br>2,14,16,17	1	P25236
2765	1,2,4,6,10,<br>11,13,16	3,5,7,8,9,1<br>2,14,15,17	3	P25236,Q7TP52,Q8CJD3
2766	1,2,4,6,10,<br>11,13,17	3,5,7,8,9,1<br>2,14,15,16	3	P15399,Q7TP52,Q8CJD3
2767	1,2,4,6,10,<br>11,14,15	3,5,7,8,9,1<br>2,13,16,17	2	P25236,P35280
2768	1,2,4,6,10,<br>11,14,16	3,5,7,8,9,1<br>2,13,15,17	2	P25236,Q7TP52
2769	1,2,4,6,10,<br>11,14,17	3,5,7,8,9,1<br>2,13,15,16	4	P15399,P97580,Q30KJ2,Q7TP52
2770	1,2,4,6,10,<br>11,15,16	3,5,7,8,9,1<br>2,13,14,17	3	P35280,P51907,Q7TP52
2771	1,2,4,6,10,<br>11,15,17	3,5,7,8,9,1<br>2,13,14,16	2	P15399,Q7TP52
2772	1,2,4,6,10,<br>11,16,17	3,5,7,8,9,1<br>2,13,14,15	4	A2RUV9,P14480,P15399,Q7TP52
2773	1,2,4,6,10,<br>12,13,14	3,5,7,8,9,1<br>1,15,16,17	10	P06911,P08723,P12020,P25236,P30120,P70709,Q10758,Q5GRG2,Q6AYR9,Q9JHB9
2774	1,2,4,6,10,<br>12,13,15	3,5,7,8,9,1<br>1,14,16,17	2	P25236,P70709
2775	1,2,4,6,10,<br>12,13,16	3,5,7,8,9,1<br>1,14,15,17	6	P25236,P47967,P70709,P97840,Q10758,Q6IFU7
2776	1,2,4,6,10,<br>12,13,17	3,5,7,8,9,1<br>1,14,15,16	13	P14480,P16636,P19223,P25236,P36374,P70709,P98089,Q10758,Q62635,Q63355,Q6AYR9,Q9JHB9,Q9QZK9
2777	1,2,4,6,10,<br>12,14,15	3,5,7,8,9,1<br>1,13,16,17	3	P25236,P35280,Q10758
2778	1,2,4,6,10,<br>12,14,16	3,5,7,8,9,1<br>1,13,15,17	5	P25236,P97840,Q10758,Q63532,Q6IFU7
2779	1,2,4,6,10,<br>12,14,17	3,5,7,8,9,1<br>1,13,15,16	7	P19223,P25236,P31430,P98089,Q10758,Q62635,Q6AYR9
2780	1,2,4,6,10,<br>12,15,16	3,5,7,8,9,1<br>1,13,14,17	3	P25236,P97840,Q6IFU7
2781	1,2,4,6,10,<br>12,15,17	3,5,7,8,9,1<br>1,13,14,16	7	B0BNN3,P01015,P06866,P19223,P98089,Q10758,Q62635
2782	1,2,4,6,10,<br>12,16,17	3,5,7,8,9,1<br>1,13,14,15	6	P06866,P14480,P19223,P98089,Q10758,Q62635
2783	1,2,4,6,10,<br>13,14,15	3,5,7,8,9,1<br>1,12,16,17	4	P06911,P12020,P25236,Q5GRG2
2784	1,2,4,6,10,<br>13,14,16	3,5,7,8,9,1<br>1,12,15,17	4	P06911,P12020,P25236,Q5GRG2
2785	1,2,4,6,10,<br>13,14,17	3,5,7,8,9,1<br>1,12,15,16	4	P06911,P12020,P25236,Q5GRG2

2786	1,2,4,6,10,13,15,16	3,5,7,8,9,11,12,14,17	2	P25236,Q62997
2787	1,2,4,6,10,13,15,17	3,5,7,8,9,11,12,14,16	1	Q6IFV1
2788	1,2,4,6,10,13,16,17	3,5,7,8,9,11,12,14,15	4	P14480,Q62997,Q63355,Q8CJD3
2789	1,2,4,6,10,14,15,16	3,5,7,8,9,11,12,13,17	1	P25236
2790	1,2,4,6,10,14,15,17	3,5,7,8,9,11,12,13,16	0	
2791	1,2,4,6,10,14,16,17	3,5,7,8,9,11,12,13,15	1	P14480
2792	1,2,4,6,10,15,16,17	3,5,7,8,9,11,12,13,14	1	Q62997
2793	1,2,4,6,11,12,13,14	3,5,7,8,9,10,15,16,17	13	P04905,P06911,P07647,P08723,P0C0A9,P25236,P30120,P36374,P47727,Q6AYR9,Q6IFU7,Q9JHB9,Q9JI85
2794	1,2,4,6,11,12,13,15	3,5,7,8,9,10,14,16,17	4	P25236,P30120,Q6IFU7,Q9JHB9
2795	1,2,4,6,11,12,13,16	3,5,7,8,9,10,14,15,17	6	G3V686,P0C0A9,P25236,P30120,Q6IFU7,Q9JHB9
2796	1,2,4,6,11,12,13,17	3,5,7,8,9,10,14,15,16	12	P07647,P0C0A9,P13432,P15399,P25236,P30120,P36374,P98089,Q62635,Q6AYR9,Q9JHB9,Q9QZK9
2797	1,2,4,6,11,12,14,15	3,5,7,8,9,10,13,16,17	4	P25236,P35280,P51907,Q6IFU7
2798	1,2,4,6,11,12,14,16	3,5,7,8,9,10,13,15,17	5	G3V686,P25236,Q63532,Q6IFU7,Q6IFU8
2799	1,2,4,6,11,12,14,17	3,5,7,8,9,10,13,15,16	13	P13432,P15399,P25236,P30120,P31430,P47727,P98089,Q00715,Q10758,Q62635,Q6AYQ8,Q6IFU8,Q9JHB9
2800	1,2,4,6,11,12,15,16	3,5,7,8,9,10,13,14,17	3	P25236,P51907,Q6IFU7
2801	1,2,4,6,11,12,15,17	3,5,7,8,9,10,13,14,16	8	B0BNN3,P01015,P06866,P13432,P19223,P98089,Q62635,Q6AYQ8
2802	1,2,4,6,11,12,16,17	3,5,7,8,9,10,13,14,15	6	P06866,P13432,P14480,P98089,Q62635,Q6IFU7
2803	1,2,4,6,11,13,14,15	3,5,7,8,9,10,12,16,17	3	P06911,P25236,Q5GRG2
2804	1,2,4,6,11,13,14,16	3,5,7,8,9,10,12,15,17	3	G3V686,P06911,P25236
2805	1,2,4,6,11,13,14,17	3,5,7,8,9,10,12,15,16	4	P06911,P15399,P25236,P47727
2806	1,2,4,6,11,13,15,16	3,5,7,8,9,10,12,14,17	1	P25236
2807	1,2,4,6,11,13,15,17	3,5,7,8,9,10,12,14,16	0	
2808	1,2,4,6,11,13,16,17	3,5,7,8,9,10,12,14,15	2	P14480,Q8CJD3

2809	1,2,4,6,11,14,15,16	3,5,7,8,9,10,12,13,17	1	P25236
2810	1,2,4,6,11,14,15,17	3,5,7,8,9,10,12,13,16	0	
2811	1,2,4,6,11,14,16,17	3,5,7,8,9,10,12,13,15	0	
2812	1,2,4,6,11,15,16,17	3,5,7,8,9,10,12,13,14	0	
2813	1,2,4,6,12,13,14,15	3,5,7,8,9,10,11,16,17	3	P06911,P25236,Q5GRG2
2814	1,2,4,6,12,13,14,16	3,5,7,8,9,10,11,15,17	3	G3V686,P06911,P25236
2815	1,2,4,6,12,13,14,17	3,5,7,8,9,10,11,15,16	10	P06911,P13432,P25236,P30120,P31430,P36374,P98089,Q62635,Q6AYR9,Q9JHB9
2816	1,2,4,6,12,13,15,16	3,5,7,8,9,10,11,14,17	1	P25236
2817	1,2,4,6,12,13,15,17	3,5,7,8,9,10,11,14,16	8	B0BNN3,P06866,P13432,P25236,P55016,P98089,Q62635,Q6IFV1
2818	1,2,4,6,12,13,16,17	3,5,7,8,9,10,11,14,15	8	P13432,P14480,P16573,P25236,P27213,P98089,Q62635,Q63355
2819	1,2,4,6,12,14,15,16	3,5,7,8,9,10,11,13,17	4	D3ZUC6,P25236,P35467,Q63532
2820	1,2,4,6,12,14,15,17	3,5,7,8,9,10,11,13,16	9	B0BNN3,P06866,P13432,P25236,P31430,P98089,Q5HZV9,Q62635,Q6AYQ8
2821	1,2,4,6,12,14,16,17	3,5,7,8,9,10,11,13,15	8	P13432,P14480,P25236,P31430,P98089,Q10758,Q5HZV9,Q62635
2822	1,2,4,6,12,15,16,17	3,5,7,8,9,10,11,13,14	4	P06866,P16573,P98089,Q62635
2823	1,2,4,6,13,14,15,16	3,5,7,8,9,10,11,12,17	2	P25236,P35467
2824	1,2,4,6,13,14,15,17	3,5,7,8,9,10,11,12,16	1	P25236
2825	1,2,4,6,13,14,16,17	3,5,7,8,9,10,11,12,15	4	P14480,P25236,P27213,Q6PCU2
2826	1,2,4,6,13,15,16,17	3,5,7,8,9,10,11,12,14	2	P25236,P27213
2827	1,2,4,6,14,15,16,17	3,5,7,8,9,10,11,12,13	2	P35467,Q5HZV9
2828	1,2,4,7,8,9,10,11	3,5,6,12,13,14,15,16,17	13	D3ZHA0,P00714,P02761,P02803,P18418,P30919,P31044,P35280,Q561R9,Q63617,Q80WL1,Q9EQS0,Q9R0T3
2829	1,2,4,7,8,9,10,12	3,5,6,11,13,14,15,16,17	12	D3ZHA0,P02803,P10715,P18418,P19223,P31044,P36374,P97840,Q80WL1,Q8CJ52,Q9EQS0,Q9R0T3

2830	1,2,4,7,8,9,10,13	3,5,6,11,12,14,15,16,17	7	D3ZHA0,P02803,P18418,P31044,Q5GRG2,Q80WL1,Q9R0T3
2831	1,2,4,7,8,9,10,14	3,5,6,11,12,13,15,16,17	5	D3ZHA0,P18418,P31044,Q63617,Q9EQS0
2832	1,2,4,7,8,9,10,15	3,5,6,11,12,13,14,16,17	7	D3ZHA0,P02761,P18418,P31044,P35280,Q561R9,Q5QE79
2833	1,2,4,7,8,9,10,16	3,5,6,11,12,13,14,15,17	5	D3ZHA0,P08721,P18418,P31044,Q9R0T3
2834	1,2,4,7,8,9,10,17	3,5,6,11,12,13,14,15,16	7	P02761,P18418,P19223,P31044,P52590,P55091,Q80WL1
2835	1,2,4,7,8,9,11,12	3,5,6,10,13,14,15,16,17	16	O55004,P02780,P02803,P08723,P0C0A9,P10715,P18418,P19223,P31044,P36374,Q63617,Q80WL1,Q8CJ52,Q9EQS0,Q9JI85,Q9R0T3
2836	1,2,4,7,8,9,11,13	3,5,6,10,12,14,15,16,17	10	P02803,P18418,P20766,P31044,Q561R9,Q5GRG2,Q63617,Q80WL1,Q9JI85,Q9R0T3
2837	1,2,4,7,8,9,11,14	3,5,6,10,12,13,15,16,17	6	P18418,P31044,Q63617,Q99MH3,Q9EQS0,Q9JI85
2838	1,2,4,7,8,9,11,15	3,5,6,10,12,13,14,16,17	9	P02761,P10715,P18418,P22006,P31044,P35280,Q561R9,Q5QE79,Q63617
2839	1,2,4,7,8,9,11,16	3,5,6,10,12,13,14,15,17	5	P10715,P31044,Q63617,Q99MH3,Q9R0T3
2840	1,2,4,7,8,9,11,17	3,5,6,10,12,13,14,15,16	9	O55004,P02761,P10715,P18418,P19223,P55091,Q63617,Q80WL1,Q9QZA6
2841	1,2,4,7,8,9,12,13	3,5,6,10,11,14,15,16,17	17	P02780,P02803,P06761,P07647,P10715,P18418,P19223,P22283,P31044,P36374,Q5GRG2,Q6AYR9,Q80WL1,Q8CJ52,Q9EQS0,Q9JI85,Q9R0T3
2842	1,2,4,7,8,9,12,14	3,5,6,10,11,13,15,16,17	8	P10715,P18418,P19223,P36970,Q63617,Q80WL1,Q8CJ52,Q9EQS0
2843	1,2,4,7,8,9,12,15	3,5,6,10,11,13,14,16,17	8	D3ZHA0,P02761,P10715,P18418,P19223,Q5QE79,Q80WL1,Q9EQS0
2844	1,2,4,7,8,9,12,16	3,5,6,10,11,13,14,15,17	8	P02803,P10715,P18418,P19223,Q80WL1,Q8CJ52,Q9EQS0,Q9R0T3

2845	1,2,4,7,8,9,12,17	3,5,6,10,11,13,14,15,16	14	O55004,P02761,P10715,P18418,P19223,P36374,P55091,P98089,Q80WL1,Q8CJ52,Q99041,Q9EQS0,Q9QZA6,Q9R0T3
2846	1,2,4,7,8,9,13,14	3,5,6,10,11,12,15,16,17	6	P06911,P18418,P31044,P36970,Q5GRG2,Q63617
2847	1,2,4,7,8,9,13,15	3,5,6,10,11,12,14,16,17	3	P31044,Q5GRG2,Q5QE79
2848	1,2,4,7,8,9,13,16	3,5,6,10,11,12,14,15,17	4	P02803,P31044,Q63617,Q9R0T3
2849	1,2,4,7,8,9,13,17	3,5,6,10,11,12,14,15,16	6	P19223,P20766,P55091,Q63617,Q80WL1,Q9R0T3
2850	1,2,4,7,8,9,14,15	3,5,6,10,11,12,13,16,17	4	P02761,P18418,P31044,Q5QE79
2851	1,2,4,7,8,9,14,16	3,5,6,10,11,12,13,15,17	6	P31044,P97574,Q63532,Q63617,Q99MH3,Q9QZK9
2852	1,2,4,7,8,9,14,17	3,5,6,10,11,12,13,15,16	5	P02761,P18418,P19223,P55091,Q63617
2853	1,2,4,7,8,9,15,16	3,5,6,10,11,12,13,14,17	2	Q5QE79,Q9QZK9
2854	1,2,4,7,8,9,15,17	3,5,6,10,11,12,13,14,16	5	P02761,P19223,P55091,Q5QE79,Q9QZA6
2855	1,2,4,7,8,9,16,17	3,5,6,10,11,12,13,14,15	3	P19223,Q63617,Q9R0T3
2856	1,2,4,7,8,10,11,12	3,5,6,9,13,14,15,16,17	30	D3ZHA0,O54715,O55004,P00714,P02761,P02781,P02803,P05964,P08723,P0C0A9,P10715,P18418,P19223,P24368,P31044,P35280,P36374,P53792,P68370,Q63357,Q63475,Q63493,Q63598,Q64057,Q6AYL6,Q80WL1,Q8CJ52,Q9EQS0,Q9JI85,Q9R0T3
2857	1,2,4,7,8,10,11,13	3,5,6,9,12,14,15,16,17	12	P00714,P01835,P02803,P18418,P31044,P68370,Q561R9,Q5GRG2,Q80WL1,Q8CJ52,Q9JI85,Q9R0T3
2858	1,2,4,7,8,10,11,14	3,5,6,9,12,13,15,16,17	9	P00714,P02761,P18418,P31044,P35280,P68370,Q5BJY9,Q63598,Q9EQS0
2859	1,2,4,7,8,10,11,15	3,5,6,9,12,13,14,16,17	8	P00714,P01835,P02761,P18418,P22006,P31044,P35280,Q561R9

2860	1,2,4,7,8,10,11,16	3,5,6,9,12,13,14,15,17	9	P00714,P01835,P08721,P18418,P31044,P35280,Q561R9,Q5FVI6,Q9R0T3
2861	1,2,4,7,8,10,11,17	3,5,6,9,12,13,14,15,16	12	P00714,P02761,P05964,P14669,P18418,P19223,P31044,P55091,Q63598,Q80WL1,Q9QZA6,Q9R0T3
2862	1,2,4,7,8,10,12,13	3,5,6,9,11,14,15,16,17	17	D3ZHA0,P02803,P06761,P07647,P08723,P11598,P18418,P22283,P31044,P36374,Q5GRG2,Q6AYR9,Q80WL1,Q8CJ52,Q9EQS0,Q9JI85,Q9R0T3
2863	1,2,4,7,8,10,12,14	3,5,6,9,11,13,15,16,17	12	D3ZHA0,P08723,P18418,P31044,P35280,P36970,Q63532,Q6AYR9,Q80WL1,Q8CJ52,Q9EQS0,Q9R0T3
2864	1,2,4,7,8,10,12,15	3,5,6,9,11,13,14,16,17	11	D3ZHA0,P01041,P02761,P10715,P18418,P19223,P31044,P35280,Q80WL1,Q8CJ52,Q9EQS0
2865	1,2,4,7,8,10,12,16	3,5,6,9,11,13,14,15,17	11	D3ZHA0,P08721,P10715,P18418,P31044,P47967,P97840,Q80WL1,Q8CJ52,Q9EQS0,Q9R0T3
2866	1,2,4,7,8,10,12,17	3,5,6,9,11,13,14,15,16	21	D3ZHA0,P01041,P02761,P06866,P10715,P18418,P19223,P31044,P36374,P55091,P98089,Q4G075,Q62635,Q6AYR9,Q80WL1,Q8CJ52,Q99041,Q9EQS0,Q9EQV9,Q9QZA6,Q9R0T3
2867	1,2,4,7,8,10,13,14	3,5,6,9,11,12,15,16,17	8	P01835,P06911,P12020,P18418,P31044,P36970,Q5GRG2,Q9R0T3
2868	1,2,4,7,8,10,13,15	3,5,6,9,11,12,14,16,17	6	D3ZHA0,P01835,P02761,P18418,P31044,Q5GRG2
2869	1,2,4,7,8,10,13,16	3,5,6,9,11,12,14,15,17	6	P01835,P18418,P31044,Q5GRG2,Q62997,Q9R0T3
2870	1,2,4,7,8,10,13,17	3,5,6,9,11,12,14,15,16	11	P01835,P02761,P11598,P18418,P19223,P31044,P46844,P55091,Q5GRG2,Q80WL1,Q9R0T3
2871	1,2,4,7,8,10,14,15	3,5,6,9,11,12,13,16,17	8	D3ZHA0,P01835,P02761,P18418,P22006,P31044,P35280,Q5GRG2
2872	1,2,4,7,8,10,14,16	3,5,6,9,11,12,13,15,17	6	P01835,P08721,P18418,P31044,P97574,Q63532
2873	1,2,4,7,8,10,14,17	3,5,6,9,11,12,13,15,16	5	P02761,P18418,P19223,P31044,P55091
2874	1,2,4,7,8,10,15,16	3,5,6,9,11,12,13,14,17	7	D3ZHA0,P01835,P02761,P08721,P18418,P31044,P35280

2875	1,2,4,7,8,10,15,17	3,5,6,9,11,12,13,14,16	8	P01835,P02761,P18418,P19223,P31044,P55091,Q9EQV9,Q9QZA6
2876	1,2,4,7,8,10,16,17	3,5,6,9,11,12,13,14,15	7	P01835,P08721,P18418,P19223,P31044,Q62997,Q9R0T3
2877	1,2,4,7,8,11,12,13	3,5,6,9,10,14,15,16,17	27	P01836,P02780,P02781,P02803,P06761,P07647,P08723,P0C0A9,P10715,P11598,P18418,P22282,P22283,P24368,P30120,P31044,P36374,Q5GRG2,Q62902,Q63493,Q6AYR9,Q80WL1,Q8CJ52,Q9EQS0,Q9JHB9,Q9JI85,Q9R0T3
2878	1,2,4,7,8,11,12,14	3,5,6,9,10,13,15,16,17	17	P02780,P08723,P0C0A9,P10715,P18418,P19629,P24368,P30120,P31044,P35280,P36374,Q63493,Q80WL1,Q8CJ52,Q9EQS0,Q9JI85,Q9R0T3
2879	1,2,4,7,8,11,12,15	3,5,6,9,10,13,14,16,17	15	O55004,P01015,P01041,P01836,P02761,P10715,P18418,P22006,P35280,P51907,Q561R9,Q80WL1,Q8CJ52,Q9EQS0,Q9JI85
2880	1,2,4,7,8,11,12,16	3,5,6,9,10,13,14,15,17	8	P01836,P10715,P18418,Q80WL1,Q8CJ52,Q9EQS0,Q9JI85,Q9R0T3
2881	1,2,4,7,8,11,12,17	3,5,6,9,10,13,14,15,16	25	O55004,P01015,P01041,P02761,P05964,P10715,P14669,P18418,P19223,P22283,P36374,P50115,P55091,P98089,Q4G075,Q62635,Q63493,Q6AYQ8,Q80WL1,Q8CJ52,Q99041,Q9EQS0,Q9JI85,Q9QZA6,Q9R0T3
2882	1,2,4,7,8,11,13,14	3,5,6,9,10,12,15,16,17	7	P06911,P18418,P31044,Q5GRG2,Q63617,Q8CJ52,Q9JI85
2883	1,2,4,7,8,11,13,15	3,5,6,9,10,12,14,16,17	8	P01835,P01836,P02761,P22006,P31044,Q561R9,Q5GRG2,Q9JI85
2884	1,2,4,7,8,11,13,16	3,5,6,9,10,12,14,15,17	8	P01835,P01836,P31044,Q5GRG2,Q63617,Q8CJ52,Q8VD89,Q9R0T3
2885	1,2,4,7,8,11,13,17	3,5,6,9,10,12,14,15,16	8	P11598,P19223,P55091,Q5GRG2,Q63617,Q80WL1,Q9JI85,Q9R0T3
2886	1,2,4,7,8,11,14,15	3,5,6,9,10,12,13,16,17	7	P02761,P07171,P18418,P19629,P22006,P31044,P35280
2887	1,2,4,7,8,11,14,16	3,5,6,9,10,12,13,15,17	6	P18418,P31044,P97574,Q63532,Q63617,Q99MH3
2888	1,2,4,7,8,11,14,17	3,5,6,9,10,12,13,15,16	6	P02761,P18418,P19223,P55091,Q63617,Q9QZA6
2889	1,2,4,7,8,11,15,16	3,5,6,9,10,12,13,14,17	7	P01835,P01836,P10715,P22006,P31044,P35280,Q561R9

2890	1,2,4,7,8,11,15,17	3,5,6,9,10,12,13,14,16	9	O55004,P01015,P02761,P10715,P14669,P19223,P22006,P55091,Q9QZA6
2891	1,2,4,7,8,11,16,17	3,5,6,9,10,12,13,14,15	6	P10715,P19223,P55091,Q63617,Q9QZA6,Q9R0T3
2892	1,2,4,7,8,12,13,14	3,5,6,9,10,11,15,16,17	22	O08557,P02780,P06761,P06911,P07647,P08723,P11598,P18418,P22283,P25236,P30120,P31044,P36374,P36970,Q5GRG2,Q62902,Q6AYR9,Q80WL1,Q8CJ52,Q9EQS0,Q9JI85,Q9R0T3
2893	1,2,4,7,8,12,13,15	3,5,6,9,10,11,14,16,17	9	P01836,P10715,P11598,P18418,Q5GRG2,Q80WL1,Q8CJ52,Q9JI85,Q9R0T3
2894	1,2,4,7,8,12,13,16	3,5,6,9,10,11,14,15,17	9	P01836,P02803,P10715,P18418,P63029,Q5GRG2,Q80WL1,Q8CJ52,Q9R0T3
2895	1,2,4,7,8,12,13,17	3,5,6,9,10,11,14,15,16	21	P06761,P07647,P10715,P11598,P18418,P19223,P22282,P22283,P36374,P55091,P63029,P98089,Q4G075,Q62635,Q6AYR9,Q80WL1,Q8CJ52,Q99041,Q9JI85,Q9QZA6,Q9R0T3
2896	1,2,4,7,8,12,14,15	3,5,6,9,10,11,13,16,17	11	D3ZUC6,O08557,P01041,P02761,P18418,P19629,P35280,P36970,Q80WL1,Q8CJ52,Q9EQS0
2897	1,2,4,7,8,12,14,16	3,5,6,9,10,11,13,15,17	10	D3ZUC6,P08721,P10715,P18418,P97574,Q63532,Q80WL1,Q8CJ52,Q9EQS0,Q9R0T3
2898	1,2,4,7,8,12,14,17	3,5,6,9,10,11,13,15,16	18	P01041,P11598,P18418,P19223,P19629,P31430,P36374,P55091,P98089,Q4G075,Q62635,Q6AYQ8,Q80WL1,Q8CJ52,Q99041,Q9EQS0,Q9QZA6,Q9R0T3
2899	1,2,4,7,8,12,15,16	3,5,6,9,10,11,13,14,17	5	P01041,P01836,P10715,Q80WL1,Q8CJ52
2900	1,2,4,7,8,12,15,17	3,5,6,9,10,11,13,14,16	16	O55004,P01015,P01041,P02761,P06866,P10715,P18418,P19223,P55091,P98089,Q4G075,Q62635,Q80WL1,Q99041,Q9EQV9,Q9QZA6
2901	1,2,4,7,8,12,16,17	3,5,6,9,10,11,13,14,15	13	P01041,P06866,P10715,P19223,P55091,P63029,P98089,Q4G075,Q80WL1,Q8CJ52,Q99041,Q9QZA6,Q9R0T3
2902	1,2,4,7,8,13,14,15	3,5,6,9,10,11,12,16,17	8	P01835,P06911,P18418,P25236,P31044,P36970,Q09030,Q5GRG2
2903	1,2,4,7,8,13,14,16	3,5,6,9,10,11,12,15,17	8	P01835,P06911,P31044,P97574,Q5GRG2,Q63532,Q63617,Q9R0T3
2904	1,2,4,7,8,13,14,17	3,5,6,9,10,11,12,15,16	7	P06911,P11598,P18418,P19223,P55091,Q5GRG2,Q6PCU2

2905	1,2,4,7,8,13,15,16	3,5,6,9,10,11,12,14,17	4	P01835,P31044,Q09030,Q5GRG2
2906	1,2,4,7,8,13,15,17	3,5,6,9,10,11,12,14,16	7	P01835,P02761,P11598,P19223,P55091,Q5GRG2,Q9QZA6
2907	1,2,4,7,8,13,16,17	3,5,6,9,10,11,12,14,15	6	P01835,P19223,P55091,P63029,Q6PCU2,Q9R0T3
2908	1,2,4,7,8,14,15,16	3,5,6,9,10,11,12,13,17	4	P01835,P31044,P97574,Q63532
2909	1,2,4,7,8,14,15,17	3,5,6,9,10,11,12,13,16	6	P02761,P10536,P19223,P19629,P55091,Q9QZA6
2910	1,2,4,7,8,14,16,17	3,5,6,9,10,11,12,13,15	3	P55091,P97574,Q6PCU2
2911	1,2,4,7,8,15,16,17	3,5,6,9,10,11,12,13,14	4	P01835,P02761,P19223,Q9QZA6
2912	1,2,4,7,9,10,11,12	3,5,6,8,13,14,15,16,17	13	O54861,P02803,P05964,P06757,P08753,P36374,P47967,P51907,P97840,Q561R9,Q5FVI6,Q62636,Q63661
2913	1,2,4,7,9,10,11,13	3,5,6,8,12,14,15,16,17	2	P02803,Q561R9
2914	1,2,4,7,9,10,11,14	3,5,6,8,12,13,15,16,17	3	Q561R9,Q63661,Q99MH3
2915	1,2,4,7,9,10,11,15	3,5,6,8,12,13,14,16,17	7	O70594,P22006,P35280,P51907,Q561R9,Q5FVI6,Q63661
2916	1,2,4,7,9,10,11,16	3,5,6,8,12,13,14,15,17	7	O70594,P47967,P51907,Q561R9,Q5FVI6,Q64093,Q9WUW8
2917	1,2,4,7,9,10,11,17	3,5,6,8,12,13,14,15,16	1	P19223
2918	1,2,4,7,9,10,12,13	3,5,6,8,11,14,15,16,17	4	P02803,P36374,P47967,P97840
2919	1,2,4,7,9,10,12,14	3,5,6,8,11,13,15,16,17	2	P97840,Q63532

2920	1,2,4,7,9,10,12,15	3,5,6,8,11,13,14,16,17	1	P97840
2921	1,2,4,7,9,10,12,16	3,5,6,8,11,13,14,15,17	3	P47967,P97840,Q9WUW8
2922	1,2,4,7,9,10,12,17	3,5,6,8,11,13,14,15,16	6	P06866,P19223,P36374,P55091,P97840,P98089
2923	1,2,4,7,9,10,13,14	3,5,6,8,11,12,15,16,17	1	Q5GRG2
2924	1,2,4,7,9,10,13,15	3,5,6,8,11,12,14,16,17	1	Q561R9
2925	1,2,4,7,9,10,13,16	3,5,6,8,11,12,14,15,17	2	P47967,P97840
2926	1,2,4,7,9,10,13,17	3,5,6,8,11,12,14,15,16	0	
2927	1,2,4,7,9,10,14,15	3,5,6,8,11,12,13,16,17	0	
2928	1,2,4,7,9,10,14,16	3,5,6,8,11,12,13,15,17	3	Q63532,Q99MH3,Q9QZK9
2929	1,2,4,7,9,10,14,17	3,5,6,8,11,12,13,15,16	0	
2930	1,2,4,7,9,10,15,16	3,5,6,8,11,12,13,14,17	2	Q561R9,Q9QZK9
2931	1,2,4,7,9,10,15,17	3,5,6,8,11,12,13,14,16	0	
2932	1,2,4,7,9,10,16,17	3,5,6,8,11,12,13,14,15	0	
2933	1,2,4,7,9,11,12,13	3,5,6,8,10,14,15,16,17	4	P01836,P02803,P36374,Q9JI85
2934	1,2,4,7,9,11,12,14	3,5,6,8,10,13,15,16,17	3	D3ZUC6,P51907,Q99MH3

2935	1,2,4,7,9,11,12,15	3,5,6,8,10,13,14,16,17	4	P01836,P22006,P51907,Q561R9
2936	1,2,4,7,9,11,12,16	3,5,6,8,10,13,14,15,17	3	P01836,P02803,P51907
2937	1,2,4,7,9,11,12,17	3,5,6,8,10,13,14,15,16	6	P06866,P19223,P36374,P55091,P98089,Q6AYQ8
2938	1,2,4,7,9,11,13,14	3,5,6,8,10,12,15,16,17	2	P05539,Q99MH3
2939	1,2,4,7,9,11,13,15	3,5,6,8,10,12,14,16,17	4	P01836,P05539,P22006,Q561R9
2940	1,2,4,7,9,11,13,16	3,5,6,8,10,12,14,15,17	2	P01836,P02803
2941	1,2,4,7,9,11,13,17	3,5,6,8,10,12,14,15,16	0	
2942	1,2,4,7,9,11,14,15	3,5,6,8,10,12,13,16,17	5	P06760,P22006,P51907,Q561R9,Q99MH3
2943	1,2,4,7,9,11,14,16	3,5,6,8,10,12,13,15,17	4	P06760,Q63532,Q99MH3,Q9QZK9
2944	1,2,4,7,9,11,14,17	3,5,6,8,10,12,13,15,16	0	
2945	1,2,4,7,9,11,15,16	3,5,6,8,10,12,13,14,17	6	P01836,P06760,P51907,Q561R9,Q99MH3,Q9QZK9
2946	1,2,4,7,9,11,15,17	3,5,6,8,10,12,13,14,16	1	P22006
2947	1,2,4,7,9,11,16,17	3,5,6,8,10,12,13,14,15	0	
2948	1,2,4,7,9,12,13,14	3,5,6,8,10,11,15,16,17	4	D3ZUC6,P02803,P25236,P36374
2949	1,2,4,7,9,12,13,15	3,5,6,8,10,11,14,16,17	1	P01836

2950	1,2,4,7,9,12,13,16	3,5,6,8,10,11,14,15,17	2	P01836,P02803
2951	1,2,4,7,9,12,13,17	3,5,6,8,10,11,14,15,16	5	P02803,P19223,P36374,P55091,P98089
2952	1,2,4,7,9,12,14,15	3,5,6,8,10,11,13,16,17	1	D3ZUC6
2953	1,2,4,7,9,12,14,16	3,5,6,8,10,11,13,15,17	3	D3ZUC6,Q63532,Q99MH3
2954	1,2,4,7,9,12,14,17	3,5,6,8,10,11,13,15,16	6	D3ZUC6,P19223,P31430,P55091,P98089,Q6AYQ8
2955	1,2,4,7,9,12,15,16	3,5,6,8,10,11,13,14,17	1	P01836
2956	1,2,4,7,9,12,15,17	3,5,6,8,10,11,13,14,16	4	P06866,P19223,P55091,P98089
2957	1,2,4,7,9,12,16,17	3,5,6,8,10,11,13,14,15	4	P06866,P19223,P55091,P98089
2958	1,2,4,7,9,13,14,15	3,5,6,8,10,11,12,16,17	3	P06760,P16636,Q5GRG2
2959	1,2,4,7,9,13,14,16	3,5,6,8,10,11,12,15,17	3	P06760,Q63532,Q99MH3
2960	1,2,4,7,9,13,14,17	3,5,6,8,10,11,12,15,16	0	
2961	1,2,4,7,9,13,15,16	3,5,6,8,10,11,12,14,17	1	P06760
2962	1,2,4,7,9,13,15,17	3,5,6,8,10,11,12,14,16	0	
2963	1,2,4,7,9,13,16,17	3,5,6,8,10,11,12,14,15	0	
2964	1,2,4,7,9,14,15,16	3,5,6,8,10,11,12,13,17	6	D3ZUC6,P06760,P16636,Q63532,Q99MH3,Q9QZK9

2965	1,2,4,7,9,14,15,17	3,5,6,8,10,11,12,13,16	0	
2966	1,2,4,7,9,14,16,17	3,5,6,8,10,11,12,13,15	0	
2967	1,2,4,7,9,15,16,17	3,5,6,8,10,11,12,13,14	1	P47727
2968	1,2,4,7,10,11,12,13	3,5,6,8,9,14,15,16,17	7	P01836,P02803,P05964,P36374,P47967,Q62636,Q9JI85
2969	1,2,4,7,10,11,12,14	3,5,6,8,9,13,15,16,17	4	P05964,P35280,P51907,Q63532
2970	1,2,4,7,10,11,12,15	3,5,6,8,9,13,14,16,17	8	P01836,P05964,P22006,P35280,P51907,Q561R9,Q5FVI6,Q62636
2971	1,2,4,7,10,11,12,16	3,5,6,8,9,13,14,15,17	9	P01836,P05964,P06757,P47967,P51907,P53792,P97840,Q5FVI6,Q9WUW8
2972	1,2,4,7,10,11,12,17	3,5,6,8,9,13,14,15,16	9	O54861,P05964,P06757,P06866,P19223,P36374,P55091,P98089,Q62636
2973	1,2,4,7,10,11,13,14	3,5,6,8,9,12,15,16,17	1	Q5GRG2
2974	1,2,4,7,10,11,13,15	3,5,6,8,9,12,14,16,17	4	P01835,P01836,P22006,Q561R9
2975	1,2,4,7,10,11,13,16	3,5,6,8,9,12,14,15,17	4	P01835,P01836,P47967,Q5FVI6
2976	1,2,4,7,10,11,13,17	3,5,6,8,9,12,14,15,16	0	
2977	1,2,4,7,10,11,14,15	3,5,6,8,9,12,13,16,17	5	P22006,P35280,P51907,Q561R9,Q63532
2978	1,2,4,7,10,11,14,16	3,5,6,8,9,12,13,15,17	3	P51907,Q5FVI6,Q63532
2979	1,2,4,7,10,11,14,17	3,5,6,8,9,12,13,15,16	0	
2980	1,2,4,7,10,11,15,16	3,5,6,8,9,12,13,14,17	8	P01835,P01836,P22006,P35280,P51907,Q561R9,Q5FVI6,Q9Z1F2
2981	1,2,4,7,10,11,15,17	3,5,6,8,9,12,13,14,16	1	P22006
2982	1,2,4,7,10,11,16,17	3,5,6,8,9,12,13,14,15	1	Q5FVI6
2983	1,2,4,7,10,12,13,14	3,5,6,8,9,11,15,16,17	5	P12020,P25236,P36374,Q5GRG2,Q63532
2984	1,2,4,7,10,12,13,15	3,5,6,8,9,11,14,16,17	1	P01836
2985	1,2,4,7,10,12,13,16	3,5,6,8,9,11,14,15,17	3	P01836,P47967,P97840

2986	1,2,4,7,10,12,13,17	3,5,6,8,9,11,14,15,16	5	P06866,P19223,P36374,P55091,P98089
2987	1,2,4,7,10,12,14,15	3,5,6,8,9,11,13,16,17	3	D3ZUC6,P35280,Q63532
2988	1,2,4,7,10,12,14,16	3,5,6,8,9,11,13,15,17	5	D3ZUC6,P08721,P47967,P97840,Q63532
2989	1,2,4,7,10,12,14,17	3,5,6,8,9,11,13,15,16	5	P06866,P19223,P31430,P55091,P98089
2990	1,2,4,7,10,12,15,16	3,5,6,8,9,11,13,14,17	5	P01836,P08721,P47967,P51907,P97840
2991	1,2,4,7,10,12,15,17	3,5,6,8,9,11,13,14,16	5	P01041,P06866,P19223,P55091,P98089
2992	1,2,4,7,10,12,16,17	3,5,6,8,9,11,13,14,15	7	P06866,P08721,P14480,P19223,P55091,P97840,P98089
2993	1,2,4,7,10,13,14,15	3,5,6,8,9,11,12,16,17	3	P01835,Q09030,Q5GRG2
2994	1,2,4,7,10,13,14,16	3,5,6,8,9,11,12,15,17	3	P01835,Q5GRG2,Q63532
2995	1,2,4,7,10,13,14,17	3,5,6,8,9,11,12,15,16	1	Q5GRG2
2996	1,2,4,7,10,13,15,16	3,5,6,8,9,11,12,14,17	4	P01835,Q09030,Q62997,Q99PP0
2997	1,2,4,7,10,13,15,17	3,5,6,8,9,11,12,14,16	1	P01835
2998	1,2,4,7,10,13,16,17	3,5,6,8,9,11,12,14,15	3	P01835,P14480,Q62997
2999	1,2,4,7,10,14,15,16	3,5,6,8,9,11,12,13,17	4	P01835,P08721,P35467,Q63532
3000	1,2,4,7,10,14,15,17	3,5,6,8,9,11,12,13,16	0	
3001	1,2,4,7,10,14,16,17	3,5,6,8,9,11,12,13,15	2	Q62997,Q63532
3002	1,2,4,7,10,15,16,17	3,5,6,8,9,11,12,13,14	2	P01835,Q62997
3003	1,2,4,7,11,12,13,14	3,5,6,8,9,10,15,16,17	7	D3ZUC6,P01836,P25236,P36374,Q5GRG2,Q63532,Q9JI85
3004	1,2,4,7,11,12,13,15	3,5,6,8,9,10,14,16,17	4	P01836,P22006,P36374,P51907
3005	1,2,4,7,11,12,13,16	3,5,6,8,9,10,14,15,17	5	G3V686,P01836,P02803,P36374,P47967
3006	1,2,4,7,11,12,13,17	3,5,6,8,9,10,14,15,16	5	P01836,P19223,P36374,P55091,P98089
3007	1,2,4,7,11,12,14,15	3,5,6,8,9,10,13,16,17	6	D3ZUC6,P01836,P22006,P35280,P51907,Q63532
3008	1,2,4,7,11,12,14,16	3,5,6,8,9,10,13,15,17	4	D3ZUC6,P01836,P51907,Q63532

3009	1,2,4,7,11,<br>12,14,17	3,5,6,8,9,1<br>0,13,15,16	7	D3ZUC6,P19223,P31430,P36374,P55091,P98089,Q6AYQ8
3010	1,2,4,7,11,<br>12,15,16	3,5,6,8,9,1<br>0,13,14,17	3	P01836,P14408,P51907
3011	1,2,4,7,11,<br>12,15,17	3,5,6,8,9,1<br>0,13,14,16	9	P01015,P01836,P06866,P19223,P22006,P51907,P55091,P98089,Q6AYQ8
3012	1,2,4,7,11,<br>12,16,17	3,5,6,8,9,1<br>0,13,14,15	5	P01836,P06866,P19223,P55091,P98089
3013	1,2,4,7,11,<br>13,14,15	3,5,6,8,9,1<br>0,12,16,17	4	P01836,P22006,Q09030,Q5GRG2
3014	1,2,4,7,11,<br>13,14,16	3,5,6,8,9,1<br>0,12,15,17	3	P01836,Q63532,Q99MH3
3015	1,2,4,7,11,<br>13,14,17	3,5,6,8,9,1<br>0,12,15,16	0	
3016	1,2,4,7,11,<br>13,15,16	3,5,6,8,9,1<br>0,12,14,17	6	P01835,P01836,P14408,Q09030,Q561R9,Q99PP0
3017	1,2,4,7,11,<br>13,15,17	3,5,6,8,9,1<br>0,12,14,16	1	P22006
3018	1,2,4,7,11,<br>13,16,17	3,5,6,8,9,1<br>0,12,14,15	0	
3019	1,2,4,7,11,<br>14,15,16	3,5,6,8,9,1<br>0,12,13,17	7	D3ZUC6,P01836,P16636,P22006,P51907,Q63532,Q99MH3
3020	1,2,4,7,11,<br>14,15,17	3,5,6,8,9,1<br>0,12,13,16	1	P22006
3021	1,2,4,7,11,<br>14,16,17	3,5,6,8,9,1<br>0,12,13,15	2	D3ZUC6,Q63532
3022	1,2,4,7,11,<br>15,16,17	3,5,6,8,9,1<br>0,12,13,14	1	P14408
3023	1,2,4,7,12,<br>13,14,15	3,5,6,8,9,1<br>0,11,16,17	6	D3ZUC6,P01836,P25236,Q09030,Q5GRG2,Q63532
3024	1,2,4,7,12,<br>13,14,16	3,5,6,8,9,1<br>0,11,15,17	4	D3ZUC6,P01836,P25236,Q63532
3025	1,2,4,7,12,<br>13,14,17	3,5,6,8,9,1<br>0,11,15,16	7	D3ZUC6,P19223,P25236,P31430,P36374,P55091,P98089
3026	1,2,4,7,12,<br>13,15,16	3,5,6,8,9,1<br>0,11,14,17	2	P01836,Q09030
3027	1,2,4,7,12,<br>13,15,17	3,5,6,8,9,1<br>0,11,14,16	5	P01836,P06866,P19223,P55091,P98089
3028	1,2,4,7,12,<br>13,16,17	3,5,6,8,9,1<br>0,11,14,15	7	P01836,P06866,P14480,P19223,P27213,P55091,P98089
3029	1,2,4,7,12,<br>14,15,16	3,5,6,8,9,1<br>0,11,13,17	4	D3ZUC6,P01836,P35467,Q63532
3030	1,2,4,7,12,<br>14,15,17	3,5,6,8,9,1<br>0,11,13,16	8	D3ZUC6,P06866,P19223,P31430,P35467,P55091,P98089,Q6AYQ8
3031	1,2,4,7,12,<br>14,16,17	3,5,6,8,9,1<br>0,11,13,15	8	D3ZUC6,P06866,P14480,P19223,P31430,P55091,P98089,Q63532

3032	1,2,4,7,12,15,16,17	3,5,6,8,9,10,11,13,14	6	P01836,P06866,P19223,P35467,P55091,P98089
3033	1,2,4,7,13,14,15,16	3,5,6,8,9,10,11,12,17	8	D3ZUC6,P01835,P06760,P16636,P35467,Q09030,Q5GRG2,Q63532
3034	1,2,4,7,13,14,15,17	3,5,6,8,9,10,11,12,16	1	Q5GRG2
3035	1,2,4,7,13,14,16,17	3,5,6,8,9,10,11,12,15	4	P14480,P27213,Q63532,Q6PCU2
3036	1,2,4,7,13,15,16,17	3,5,6,8,9,10,11,12,14	1	P01835
3037	1,2,4,7,14,15,16,17	3,5,6,8,9,10,11,12,13	3	D3ZUC6,P35467,Q63532
3038	1,2,4,8,9,10,11,12	3,5,6,7,13,14,15,16,17	8	P00714,P02803,P0C0A9,P36374,P53792,Q561R9,Q63598,Q80WL1
3039	1,2,4,8,9,10,11,13	3,5,6,7,12,14,15,16,17	3	P02803,Q561R9,Q8CJD3
3040	1,2,4,8,9,10,11,14	3,5,6,7,12,13,15,16,17	3	P02803,Q561R9,Q99MH3
3041	1,2,4,8,9,10,11,15	3,5,6,7,12,13,14,16,17	4	P02761,P35280,Q561R9,Q5QE79
3042	1,2,4,8,9,10,11,16	3,5,6,7,12,13,14,15,17	4	A2RUV9,P02803,Q561R9,Q9WUW8
3043	1,2,4,8,9,10,11,17	3,5,6,7,12,13,14,15,16	3	P02761,P02803,P19223
3044	1,2,4,8,9,10,12,13	3,5,6,7,11,14,15,16,17	3	P02803,P36374,Q80WL1
3045	1,2,4,8,9,10,12,14	3,5,6,7,11,13,15,16,17	2	P02803,Q80WL1
3046	1,2,4,8,9,10,12,15	3,5,6,7,11,13,14,16,17	4	P02761,P10758,Q561R9,Q5QE79
3047	1,2,4,8,9,10,12,16	3,5,6,7,11,13,14,15,17	3	P02803,Q80WL1,Q9WUW8
3048	1,2,4,8,9,10,12,17	3,5,6,7,11,13,14,15,16	6	P02761,P02803,P06866,P19223,P98089,Q80WL1

3049	1,2,4,8,9,10,13,14	3,5,6,7,11,12,15,16,17	2	P02803,P17046
3050	1,2,4,8,9,10,13,15	3,5,6,7,11,12,14,16,17	3	P17046,Q561R9,Q5QE79
3051	1,2,4,8,9,10,13,16	3,5,6,7,11,12,14,15,17	2	P02803,Q8CJD3
3052	1,2,4,8,9,10,13,17	3,5,6,7,11,12,14,15,16	2	P02803,Q63355
3053	1,2,4,8,9,10,14,15	3,5,6,7,11,12,13,16,17	4	P02761,P17046,Q5QE79,Q9QZK9
3054	1,2,4,8,9,10,14,16	3,5,6,7,11,12,13,15,17	4	P97574,Q99MH3,Q9QZK9,Q9WUW8
3055	1,2,4,8,9,10,14,17	3,5,6,7,11,12,13,15,16	2	P02761,P17046
3056	1,2,4,8,9,10,15,16	3,5,6,7,11,12,13,14,17	3	Q561R9,Q5QE79,Q9QZK9
3057	1,2,4,8,9,10,15,17	3,5,6,7,11,12,13,14,16	4	P02761,P17046,P80201,Q5QE79
3058	1,2,4,8,9,10,16,17	3,5,6,7,11,12,13,14,15	1	P25031
3059	1,2,4,8,9,11,12,13	3,5,6,7,10,14,15,16,17	4	P02803,P0C0A9,P36374,Q80WL1
3060	1,2,4,8,9,11,12,14	3,5,6,7,10,13,15,16,17	3	P02803,Q80WL1,Q99MH3
3061	1,2,4,8,9,11,12,15	3,5,6,7,10,13,14,16,17	5	P02761,P10758,P51907,Q561R9,Q5QE79
3062	1,2,4,8,9,11,12,16	3,5,6,7,10,13,14,15,17	2	P02803,Q80WL1
3063	1,2,4,8,9,11,12,17	3,5,6,7,10,13,14,15,16	8	P02803,P19223,P36374,P55091,P98089,Q6AYQ8,Q80WL1,Q9QZA6

3064	1,2,4,8,9,11,13,14	3,5,6,7,10,12,15,16,17	2	P02803,Q99MH3
3065	1,2,4,8,9,11,13,15	3,5,6,7,10,12,14,16,17	2	Q561R9,Q5QE79
3066	1,2,4,8,9,11,13,16	3,5,6,7,10,12,14,15,17	3	P02803,Q561R9,Q8CJD3
3067	1,2,4,8,9,11,13,17	3,5,6,7,10,12,14,15,16	2	P02803,P20766
3068	1,2,4,8,9,11,14,15	3,5,6,7,10,12,13,16,17	6	P02761,P19218,Q561R9,Q5QE79,Q99MH3,Q9QZK9
3069	1,2,4,8,9,11,14,16	3,5,6,7,10,12,13,15,17	3	P97574,Q99MH3,Q9QZK9
3070	1,2,4,8,9,11,14,17	3,5,6,7,10,12,13,15,16	2	P02761,P19218
3071	1,2,4,8,9,11,15,16	3,5,6,7,10,12,13,14,17	4	Q561R9,Q5QE79,Q99MH3,Q9QZK9
3072	1,2,4,8,9,11,15,17	3,5,6,7,10,12,13,14,16	3	P02761,Q5QE79,Q68FV8
3073	1,2,4,8,9,11,16,17	3,5,6,7,10,12,13,14,15	3	A2RUV9,Q498D9,Q68FV8
3074	1,2,4,8,9,12,13,14	3,5,6,7,10,11,15,16,17	4	P02803,P25236,P36970,Q80WL1
3075	1,2,4,8,9,12,13,15	3,5,6,7,10,11,14,16,17	3	P02803,P10758,Q5QE79
3076	1,2,4,8,9,12,13,16	3,5,6,7,10,11,14,15,17	2	P02803,Q80WL1
3077	1,2,4,8,9,12,13,17	3,5,6,7,10,11,14,15,16	8	P02803,P19223,P36374,P55091,P98089,Q62635,Q63355,Q80WL1
3078	1,2,4,8,9,12,14,15	3,5,6,7,10,11,13,16,17	2	P10758,Q5QE79

3079	1,2,4,8,9,12,14,16	3,5,6,7,10,11,13,15,17	2	Q80WL1,Q99MH3
3080	1,2,4,8,9,12,14,17	3,5,6,7,10,11,13,15,16	6	P19223,P31430,P55091,P98089,Q6AYQ8,Q80WL1
3081	1,2,4,8,9,12,15,16	3,5,6,7,10,11,13,14,17	1	Q5QE79
3082	1,2,4,8,9,12,15,17	3,5,6,7,10,11,13,14,16	7	P02761,P06866,P19223,P98089,Q5QE79,Q68FV8,Q9QZA6
3083	1,2,4,8,9,12,16,17	3,5,6,7,10,11,13,14,15	4	P06866,P19223,P98089,Q80WL1
3084	1,2,4,8,9,13,14,15	3,5,6,7,10,11,12,16,17	2	P17046,Q5QE79
3085	1,2,4,8,9,13,14,16	3,5,6,7,10,11,12,15,17	2	Q99MH3,Q9QZK9
3086	1,2,4,8,9,13,14,17	3,5,6,7,10,11,12,15,16	0	
3087	1,2,4,8,9,13,15,16	3,5,6,7,10,11,12,14,17	2	Q5QE79,Q9QZK9
3088	1,2,4,8,9,13,15,17	3,5,6,7,10,11,12,14,16	2	P02761,Q5QE79
3089	1,2,4,8,9,13,16,17	3,5,6,7,10,11,12,14,15	1	Q8CJD3
3090	1,2,4,8,9,14,15,16	3,5,6,7,10,11,12,13,17	5	P97574,Q5QE79,Q63942,Q99MH3,Q9QZK9
3091	1,2,4,8,9,14,15,17	3,5,6,7,10,11,12,13,16	3	P02761,P17046,Q5QE79
3092	1,2,4,8,9,14,16,17	3,5,6,7,10,11,12,13,15	0	
3093	1,2,4,8,9,15,16,17	3,5,6,7,10,11,12,13,14	3	P47727,Q5QE79,Q68FV8

3094	1,2,4,8,10,<br>11,12,13	3,5,6,7,9,1<br>4,15,16,17	8	P02803,P0C0A9,P36374,P53792,Q561R9,Q63598,Q6AYR9,Q80WL1
3095	1,2,4,8,10,<br>11,12,14	3,5,6,7,9,1<br>3,15,16,17	9	P02803,P0C0A9,P35280,P47727,P53792,P62804,Q00715,Q63598,Q80WL1
3096	1,2,4,8,10,<br>11,12,15	3,5,6,7,9,1<br>3,14,16,17	6	P01015,P02761,P35280,P51907,P53792,Q561R9
3097	1,2,4,8,10,<br>11,12,16	3,5,6,7,9,1<br>3,14,15,17	7	P02803,P53792,Q63598,Q6IFU7,Q6P6Q2,Q80WL1,Q9WUW8
3098	1,2,4,8,10,<br>11,12,17	3,5,6,7,9,1<br>3,14,15,16	16	P02761,P02803,P05964,P06757,P06866,P16636,P19223,P36374,P53792,P55091,P62804,P98089,Q00715,Q63598,Q80WL1,Q9QZA6
3099	1,2,4,8,10,<br>11,13,14	3,5,6,7,9,1<br>2,15,16,17	3	P02803,P47727,Q63598
3100	1,2,4,8,10,<br>11,13,15	3,5,6,7,9,1<br>2,14,16,17	3	P01835,P02761,Q561R9
3101	1,2,4,8,10,<br>11,13,16	3,5,6,7,9,1<br>2,14,15,17	4	P01835,P02803,Q561R9,Q8CJD3
3102	1,2,4,8,10,<br>11,13,17	3,5,6,7,9,1<br>2,14,15,16	2	P02803,Q63598
3103	1,2,4,8,10,<br>11,14,15	3,5,6,7,9,1<br>2,13,16,17	4	P02761,P19218,P35280,Q561R9
3104	1,2,4,8,10,<br>11,14,16	3,5,6,7,9,1<br>2,13,15,17	2	P53792,P97574
3105	1,2,4,8,10,<br>11,14,17	3,5,6,7,9,1<br>2,13,15,16	3	P02761,P19218,Q63598
3106	1,2,4,8,10,<br>11,15,16	3,5,6,7,9,1<br>2,13,14,17	5	P01835,P35280,P51907,P53792,Q561R9
3107	1,2,4,8,10,<br>11,15,17	3,5,6,7,9,1<br>2,13,14,16	2	P02761,Q561R9
3108	1,2,4,8,10,<br>11,16,17	3,5,6,7,9,1<br>2,13,14,15	2	A2RUV9,P53792
3109	1,2,4,8,10,<br>12,13,14	3,5,6,7,9,1<br>1,15,16,17	5	P02803,P25236,P36970,Q6AYR9,Q80WL1
3110	1,2,4,8,10,<br>12,13,15	3,5,6,7,9,1<br>1,14,16,17	2	P10758,P15999
3111	1,2,4,8,10,<br>12,13,16	3,5,6,7,9,1<br>1,14,15,17	2	P02803,Q80WL1
3112	1,2,4,8,10,<br>12,13,17	3,5,6,7,9,1<br>1,14,15,16	10	P02803,P16636,P19223,P36374,P55091,P98089,Q62635,Q63355,Q6AYR9,Q80WL1
3113	1,2,4,8,10,<br>12,14,15	3,5,6,7,9,1<br>1,13,16,17	2	P02761,P35280
3114	1,2,4,8,10,<br>12,14,16	3,5,6,7,9,1<br>1,13,15,17	4	P08721,P97574,Q80WL1,Q9WUW8
3115	1,2,4,8,10,<br>12,14,17	3,5,6,7,9,1<br>1,13,15,16	8	P06866,P19223,P31430,P55091,P98089,Q10758,Q62635,Q80WL1
3116	1,2,4,8,10,<br>12,15,16	3,5,6,7,9,1<br>1,13,14,17	1	P08721

3117	1,2,4,8,10,<br>12,15,17	3,5,6,7,9,1<br>1,13,14,16	7	P01015,P02761,P06866,P19223,P98089,Q9EQV9,Q9QZA6
3118	1,2,4,8,10,<br>12,16,17	3,5,6,7,9,1<br>1,13,14,15	5	P06866,P08721,P19223,P98089,Q80WL1
3119	1,2,4,8,10,<br>13,14,15	3,5,6,7,9,1<br>1,12,16,17	4	P01835,P15999,P17046,Q5GRG2
3120	1,2,4,8,10,<br>13,14,16	3,5,6,7,9,1<br>1,12,15,17	1	P01835
3121	1,2,4,8,10,<br>13,14,17	3,5,6,7,9,1<br>1,12,15,16	0	
3122	1,2,4,8,10,<br>13,15,16	3,5,6,7,9,1<br>1,12,14,17	2	P01835,Q62997
3123	1,2,4,8,10,<br>13,15,17	3,5,6,7,9,1<br>1,12,14,16	3	P01835,P02761,P15999
3124	1,2,4,8,10,<br>13,16,17	3,5,6,7,9,1<br>1,12,14,15	3	P01835,Q62997,Q8CJD3
3125	1,2,4,8,10,<br>14,15,16	3,5,6,7,9,1<br>1,12,13,17	4	P01835,P08721,P97574,Q9QZK9
3126	1,2,4,8,10,<br>14,15,17	3,5,6,7,9,1<br>1,12,13,16	2	P02761,P17046
3127	1,2,4,8,10,<br>14,16,17	3,5,6,7,9,1<br>1,12,13,15	1	Q62997
3128	1,2,4,8,10,<br>15,16,17	3,5,6,7,9,1<br>1,12,13,14	3	P01835,P02761,Q62997
3129	1,2,4,8,11,<br>12,13,14	3,5,6,7,9,1<br>0,15,16,17	8	P02803,P0C0A9,P25236,P36374,P47727,Q6AYR9,Q80WL1,Q9JHB9
3130	1,2,4,8,11,<br>12,13,15	3,5,6,7,9,1<br>0,14,16,17	1	Q561R9
3131	1,2,4,8,11,<br>12,13,16	3,5,6,7,9,1<br>0,14,15,17	3	P02803,Q80WL1,Q8CJ52
3132	1,2,4,8,11,<br>12,13,17	3,5,6,7,9,1<br>0,14,15,16	7	P02803,P19223,P36374,P55091,P98089,Q62635,Q80WL1
3133	1,2,4,8,11,<br>12,14,15	3,5,6,7,9,1<br>0,13,16,17	5	D3ZUC6,P02761,P35280,P51907,Q00715
3134	1,2,4,8,11,<br>12,14,16	3,5,6,7,9,1<br>0,13,15,17	3	D3ZUC6,Q80WL1,Q99MH3
3135	1,2,4,8,11,<br>12,14,17	3,5,6,7,9,1<br>0,13,15,16	11	P19223,P31430,P36374,P55091,P62804,P98089,Q00715,Q62635,Q6AYQ8,Q80WL1,Q9QZA6
3136	1,2,4,8,11,<br>12,15,16	3,5,6,7,9,1<br>0,13,14,17	2	P51907,Q561R9
3137	1,2,4,8,11,<br>12,15,17	3,5,6,7,9,1<br>0,13,14,16	10	B0BNN3,P01015,P02761,P06866,P19223,P55091,P98089,Q00715,Q6AYQ8,Q9QZA6
3138	1,2,4,8,11,<br>12,16,17	3,5,6,7,9,1<br>0,13,14,15	5	P06866,P19223,P98089,Q80WL1,Q9QZA6
3139	1,2,4,8,11,<br>13,14,15	3,5,6,7,9,1<br>0,12,16,17	0	

3140	1,2,4,8,11,<br>13,14,16	3,5,6,7,9,1<br>0,12,15,17	1	Q99MH3
3141	1,2,4,8,11,<br>13,14,17	3,5,6,7,9,1<br>0,12,15,16	0	
3142	1,2,4,8,11,<br>13,15,16	3,5,6,7,9,1<br>0,12,14,17	2	P01835,Q561R9
3143	1,2,4,8,11,<br>13,15,17	3,5,6,7,9,1<br>0,12,14,16	1	P02761
3144	1,2,4,8,11,<br>13,16,17	3,5,6,7,9,1<br>0,12,14,15	1	Q8CJD3
3145	1,2,4,8,11,<br>14,15,16	3,5,6,7,9,1<br>0,12,13,17	3	P97574,Q99MH3,Q9QZK9
3146	1,2,4,8,11,<br>14,15,17	3,5,6,7,9,1<br>0,12,13,16	3	P02761,P19218,P97840
3147	1,2,4,8,11,<br>14,16,17	3,5,6,7,9,1<br>0,12,13,15	1	P19218
3148	1,2,4,8,11,<br>15,16,17	3,5,6,7,9,1<br>0,12,13,14	1	P02761
3149	1,2,4,8,12,<br>13,14,15	3,5,6,7,9,1<br>0,11,16,17	7	O08557,P10758,P15999,P25236,P36970,Q64093,Q9QUL6
3150	1,2,4,8,12,<br>13,14,16	3,5,6,7,9,1<br>0,11,15,17	3	P25236,Q80WL1,Q9QUL6
3151	1,2,4,8,12,<br>13,14,17	3,5,6,7,9,1<br>0,11,15,16	10	P19223,P25236,P31430,P36374,P55091,P98089,Q62635,Q6AYR9,Q6PCU2,Q80WL1
3152	1,2,4,8,12,<br>13,15,16	3,5,6,7,9,1<br>0,11,14,17	1	Q9QUL6
3153	1,2,4,8,12,<br>13,15,17	3,5,6,7,9,1<br>0,11,14,16	7	P06866,P15999,P19223,P55091,P98089,Q62635,Q9QZA6
3154	1,2,4,8,12,<br>13,16,17	3,5,6,7,9,1<br>0,11,14,15	9	P06866,P19223,P27213,P55091,P63029,P98089,Q62635,Q6PCU2,Q80WL1
3155	1,2,4,8,12,<br>14,15,16	3,5,6,7,9,1<br>0,11,13,17	2	D3ZUC6,P97574
3156	1,2,4,8,12,<br>14,15,17	3,5,6,7,9,1<br>0,11,13,16	9	B0BNN3,P02761,P06866,P19223,P31430,P55091,P98089,Q6AYQ8,Q9QZA6
3157	1,2,4,8,12,<br>14,16,17	3,5,6,7,9,1<br>0,11,13,15	7	D3ZUC6,P06866,P19223,P31430,P98089,Q6PCU2,Q80WL1
3158	1,2,4,8,12,<br>15,16,17	3,5,6,7,9,1<br>0,11,13,14	4	P06866,P19223,P98089,Q9QZA6
3159	1,2,4,8,13,<br>14,15,16	3,5,6,7,9,1<br>0,11,12,17	2	P01835,P97574
3160	1,2,4,8,13,<br>14,15,17	3,5,6,7,9,1<br>0,11,12,16	2	P15999,Q6PCU2
3161	1,2,4,8,13,<br>14,16,17	3,5,6,7,9,1<br>0,11,12,15	2	P27213,Q6PCU2
3162	1,2,4,8,13,<br>15,16,17	3,5,6,7,9,1<br>0,11,12,14	3	P01835,P27213,Q6PCU2

3163	1,2,4,8,14,<br>15,16,17	3,5,6,7,9,1<br>0,11,12,13	1	Q6PCU2
3164	1,2,4,9,10,<br>11,12,13	3,5,6,7,8,1<br>4,15,16,17	5	P02803,P36374,Q561R9,Q62636,Q63661
3165	1,2,4,9,10,<br>11,12,14	3,5,6,7,8,1<br>3,15,16,17	3	P02803,P51907,Q63661
3166	1,2,4,9,10,<br>11,12,15	3,5,6,7,8,1<br>3,14,16,17	5	P10758,P51907,Q561R9,Q62636,Q63661
3167	1,2,4,9,10,<br>11,12,16	3,5,6,7,8,1<br>3,14,15,17	10	P02803,P06757,P10536,P47967,P51907,P53792,Q561R9,Q63661,Q6IFU7,Q9WUW8
3168	1,2,4,9,10,<br>11,12,17	3,5,6,7,8,1<br>3,14,15,16	6	P02803,P06757,P06866,P19223,Q62636,Q63661
3169	1,2,4,9,10,<br>11,13,14	3,5,6,7,8,1<br>2,15,16,17	4	P02803,P50115,Q561R9,Q63661
3170	1,2,4,9,10,<br>11,13,15	3,5,6,7,8,1<br>2,14,16,17	3	P50115,Q561R9,Q63661
3171	1,2,4,9,10,<br>11,13,16	3,5,6,7,8,1<br>2,14,15,17	5	P02803,P50115,Q561R9,Q63661,Q8CJD3
3172	1,2,4,9,10,<br>11,13,17	3,5,6,7,8,1<br>2,14,15,16	1	P02803
3173	1,2,4,9,10,<br>11,14,15	3,5,6,7,8,1<br>2,13,16,17	7	P08937,P50115,P51907,Q561R9,Q63661,Q99MH3,Q9QZK9
3174	1,2,4,9,10,<br>11,14,16	3,5,6,7,8,1<br>2,13,15,17	8	P08937,P50115,P51907,Q561R9,Q63661,Q99MH3,Q9QZK9,Q9WUW8
3175	1,2,4,9,10,<br>11,14,17	3,5,6,7,8,1<br>2,13,15,16	0	
3176	1,2,4,9,10,<br>11,15,16	3,5,6,7,8,1<br>2,13,14,17	9	O70594,P08937,P50115,P51907,Q561R9,Q63661,Q9QZK9,Q9WUW8,Q9Z1F2
3177	1,2,4,9,10,<br>11,15,17	3,5,6,7,8,1<br>2,13,14,16	1	Q561R9
3178	1,2,4,9,10,<br>11,16,17	3,5,6,7,8,1<br>2,13,14,15	1	A2RUV9
3179	1,2,4,9,10,<br>12,13,14	3,5,6,7,8,1<br>1,15,16,17	4	P02803,P10758,P25236,P50115
3180	1,2,4,9,10,<br>12,13,15	3,5,6,7,8,1<br>1,14,16,17	3	P10758,P50115,Q561R9
3181	1,2,4,9,10,<br>12,13,16	3,5,6,7,8,1<br>1,14,15,17	5	P02803,P47967,P50115,P97840,Q9WUW8
3182	1,2,4,9,10,<br>12,13,17	3,5,6,7,8,1<br>1,14,15,16	5	P02803,P06866,P19223,P36374,Q63355
3183	1,2,4,9,10,<br>12,14,15	3,5,6,7,8,1<br>1,13,16,17	2	P10758,P50115
3184	1,2,4,9,10,<br>12,14,16	3,5,6,7,8,1<br>1,13,15,17	2	P50115,Q9WUW8
3185	1,2,4,9,10,<br>12,14,17	3,5,6,7,8,1<br>1,13,15,16	4	P06866,P19223,P31430,P98089

3186	1,2,4,9,10,<br>12,15,16	3,5,6,7,8,1<br>1,13,14,17	3	P50115,P51907,Q9WUW8
3187	1,2,4,9,10,<br>12,15,17	3,5,6,7,8,1<br>1,13,14,16	2	P06866,P19223
3188	1,2,4,9,10,<br>12,16,17	3,5,6,7,8,1<br>1,13,14,15	2	P06866,P19223
3189	1,2,4,9,10,<br>13,14,15	3,5,6,7,8,1<br>1,12,16,17	3	P08937,P17046,P50115
3190	1,2,4,9,10,<br>13,14,16	3,5,6,7,8,1<br>1,12,15,17	3	P08937,P50115,Q9QZK9
3191	1,2,4,9,10,<br>13,14,17	3,5,6,7,8,1<br>1,12,15,16	0	
3192	1,2,4,9,10,<br>13,15,16	3,5,6,7,8,1<br>1,12,14,17	4	P08937,P50115,Q561R9,Q9QZK9
3193	1,2,4,9,10,<br>13,15,17	3,5,6,7,8,1<br>1,12,14,16	0	
3194	1,2,4,9,10,<br>13,16,17	3,5,6,7,8,1<br>1,12,14,15	1	Q8CJD3
3195	1,2,4,9,10,<br>14,15,16	3,5,6,7,8,1<br>1,12,13,17	4	P08937,P50115,Q99MH3,Q9QZK9
3196	1,2,4,9,10,<br>14,15,17	3,5,6,7,8,1<br>1,12,13,16	1	P17046
3197	1,2,4,9,10,<br>14,16,17	3,5,6,7,8,1<br>1,12,13,15	0	
3198	1,2,4,9,10,<br>15,16,17	3,5,6,7,8,1<br>1,12,13,14	3	P47727,Q62902,Q9Z1F2
3199	1,2,4,9,11,<br>12,13,14	3,5,6,7,8,1<br>0,15,16,17	4	D3ZUC6,P02803,P25236,P36374
3200	1,2,4,9,11,<br>12,13,15	3,5,6,7,8,1<br>0,14,16,17	4	P02803,P10758,P51907,Q561R9
3201	1,2,4,9,11,<br>12,13,16	3,5,6,7,8,1<br>0,14,15,17	1	P02803
3202	1,2,4,9,11,<br>12,13,17	3,5,6,7,8,1<br>0,14,15,16	2	P02803,P36374
3203	1,2,4,9,11,<br>12,14,15	3,5,6,7,8,1<br>0,13,16,17	5	D3ZUC6,P10758,P51907,Q561R9,Q99MH3
3204	1,2,4,9,11,<br>12,14,16	3,5,6,7,8,1<br>0,13,15,17	3	D3ZUC6,P51907,Q99MH3
3205	1,2,4,9,11,<br>12,14,17	3,5,6,7,8,1<br>0,13,15,16	5	D3ZUC6,P06866,P31430,P98089,Q6AYQ8
3206	1,2,4,9,11,<br>12,15,16	3,5,6,7,8,1<br>0,13,14,17	2	P51907,Q561R9
3207	1,2,4,9,11,<br>12,15,17	3,5,6,7,8,1<br>0,13,14,16	4	P06866,P19223,P51907,Q6AYQ8
3208	1,2,4,9,11,<br>12,16,17	3,5,6,7,8,1<br>0,13,14,15	2	P02803,P06866

3209	1,2,4,9,11,<br>13,14,15	3,5,6,7,8,1<br>0,12,16,17	4	P06760,P50115,Q561R9,Q99MH3
3210	1,2,4,9,11,<br>13,14,16	3,5,6,7,8,1<br>0,12,15,17	6	P06760,P08937,P50115,Q7TPB1,Q99MH3,Q9QZK9
3211	1,2,4,9,11,<br>13,14,17	3,5,6,7,8,1<br>0,12,15,16	0	
3212	1,2,4,9,11,<br>13,15,16	3,5,6,7,8,1<br>0,12,14,17	5	P06760,P08937,P50115,Q561R9,Q9QZK9
3213	1,2,4,9,11,<br>13,15,17	3,5,6,7,8,1<br>0,12,14,16	0	
3214	1,2,4,9,11,<br>13,16,17	3,5,6,7,8,1<br>0,12,14,15	1	Q8CJD3
3215	1,2,4,9,11,<br>14,15,16	3,5,6,7,8,1<br>0,12,13,17	7	P06760,P08937,P50115,P51907,Q561R9,Q99MH3,Q9QZK9
3216	1,2,4,9,11,<br>14,15,17	3,5,6,7,8,1<br>0,12,13,16	0	
3217	1,2,4,9,11,<br>14,16,17	3,5,6,7,8,1<br>0,12,13,15	1	Q99MH3
3218	1,2,4,9,11,<br>15,16,17	3,5,6,7,8,1<br>0,12,13,14	2	Q498D9,Q68FV8
3219	1,2,4,9,12,<br>13,14,15	3,5,6,7,8,1<br>0,11,16,17	5	D3ZUC6,P06760,P10758,P25236,P50115
3220	1,2,4,9,12,<br>13,14,16	3,5,6,7,8,1<br>0,11,15,17	5	D3ZUC6,P02803,P06760,P25236,P50115
3221	1,2,4,9,12,<br>13,14,17	3,5,6,7,8,1<br>0,11,15,16	2	D3ZUC6,P31430
3222	1,2,4,9,12,<br>13,15,16	3,5,6,7,8,1<br>0,11,14,17	2	P06760,P50115
3223	1,2,4,9,12,<br>13,15,17	3,5,6,7,8,1<br>0,11,14,16	2	P06866,P82471
3224	1,2,4,9,12,<br>13,16,17	3,5,6,7,8,1<br>0,11,14,15	3	P02803,P06866,P27213
3225	1,2,4,9,12,<br>14,15,16	3,5,6,7,8,1<br>0,11,13,17	5	D3ZUC6,P06760,P08937,P50115,Q99MH3
3226	1,2,4,9,12,<br>14,15,17	3,5,6,7,8,1<br>0,11,13,16	5	D3ZUC6,P06866,P31430,P98089,Q6AYQ8
3227	1,2,4,9,12,<br>14,16,17	3,5,6,7,8,1<br>0,11,13,15	4	D3ZUC6,P06866,P31430,P98089
3228	1,2,4,9,12,<br>15,16,17	3,5,6,7,8,1<br>0,11,13,14	2	P06866,P98089
3229	1,2,4,9,13,<br>14,15,16	3,5,6,7,8,1<br>0,11,12,17	5	P06760,P08937,P50115,Q99MH3,Q9QZK9
3230	1,2,4,9,13,<br>14,15,17	3,5,6,7,8,1<br>0,11,12,16	1	P82471
3231	1,2,4,9,13,<br>14,16,17	3,5,6,7,8,1<br>0,11,12,15	1	P27213

3232	1,2,4,9,13,15,16,17	3,5,6,7,8,10,11,12,14	1	P27213
3233	1,2,4,9,14,15,16,17	3,5,6,7,8,10,11,12,13	1	P08937
3234	1,2,4,10,11,12,13,14	3,5,6,7,8,9,15,16,17	4	P02803,P25236,P36374,P47727
3235	1,2,4,10,11,12,13,15	3,5,6,7,8,9,14,16,17	2	P51907,Q561R9
3236	1,2,4,10,11,12,13,16	3,5,6,7,8,9,14,15,17	4	P02803,P47967,P53792,Q6IFU7
3237	1,2,4,10,11,12,13,17	3,5,6,7,8,9,14,15,16	4	P02803,P06757,P06866,P36374
3238	1,2,4,10,11,12,14,15	3,5,6,7,8,9,13,16,17	4	P35280,P51907,P53792,Q561R9
3239	1,2,4,10,11,12,14,16	3,5,6,7,8,9,13,15,17	5	D3ZUC6,P51907,P53792,Q6IFU7,Q9WUW8
3240	1,2,4,10,11,12,14,17	3,5,6,7,8,9,13,15,16	4	P06866,P31430,P98089,Q6AYQ8
3241	1,2,4,10,11,12,15,16	3,5,6,7,8,9,13,14,17	5	P51907,P53792,Q561R9,Q6IFU7,Q9WUW8
3242	1,2,4,10,11,12,15,17	3,5,6,7,8,9,13,14,16	5	P01015,P06757,P06866,P51907,P53792
3243	1,2,4,10,11,12,16,17	3,5,6,7,8,9,13,14,15	3	P06757,P06866,P53792
3244	1,2,4,10,11,13,14,15	3,5,6,7,8,9,12,16,17	2	P50115,Q561R9
3245	1,2,4,10,11,13,14,16	3,5,6,7,8,9,12,15,17	2	P50115,Q7TPB1
3246	1,2,4,10,11,13,14,17	3,5,6,7,8,9,12,15,16	0	
3247	1,2,4,10,11,13,15,16	3,5,6,7,8,9,12,14,17	4	P01835,P50115,P51907,Q561R9
3248	1,2,4,10,11,13,15,17	3,5,6,7,8,9,12,14,16	0	
3249	1,2,4,10,11,13,16,17	3,5,6,7,8,9,12,14,15	1	Q8CJD3
3250	1,2,4,10,11,14,15,16	3,5,6,7,8,9,12,13,17	7	P08937,P50115,P51907,P53792,Q561R9,Q99MH3,Q9QZK9
3251	1,2,4,10,11,14,15,17	3,5,6,7,8,9,12,13,16	0	
3252	1,2,4,10,11,14,16,17	3,5,6,7,8,9,12,13,15	0	
3253	1,2,4,10,11,15,16,17	3,5,6,7,8,9,12,13,14	2	P51907,Q9Z1F2
3254	1,2,4,10,12,13,14,15	3,5,6,7,8,9,11,16,17	3	P10758,P25236,P50115

3255	1,2,4,10,12,13,14,16	3,5,6,7,8,9,11,15,17	2	P25236,P50115
3256	1,2,4,10,12,13,14,17	3,5,6,7,8,9,11,15,16	2	P06866,P31430
3257	1,2,4,10,12,13,15,16	3,5,6,7,8,9,11,14,17	1	P50115
3258	1,2,4,10,12,13,15,17	3,5,6,7,8,9,11,14,16	1	P06866
3259	1,2,4,10,12,13,16,17	3,5,6,7,8,9,11,14,15	3	P06866,P14480,P27213
3260	1,2,4,10,12,14,15,16	3,5,6,7,8,9,11,13,17	3	D3ZUC6,P50115,P51907
3261	1,2,4,10,12,14,15,17	3,5,6,7,8,9,11,13,16	3	P06866,P31430,P98089
3262	1,2,4,10,12,14,16,17	3,5,6,7,8,9,11,13,15	6	D3ZUC6,P06866,P14480,P31430,P98089,Q10758
3263	1,2,4,10,12,15,16,17	3,5,6,7,8,9,11,13,14	1	P06866
3264	1,2,4,10,13,14,15,16	3,5,6,7,8,9,11,12,17	5	P01835,P08937,P50115,Q62997,Q9QZK9
3265	1,2,4,10,13,14,15,17	3,5,6,7,8,9,11,12,16	0	
3266	1,2,4,10,13,14,16,17	3,5,6,7,8,9,11,12,15	2	P27213,Q62997
3267	1,2,4,10,13,15,16,17	3,5,6,7,8,9,11,12,14	3	P01835,P27213,Q62997
3268	1,2,4,10,14,15,16,17	3,5,6,7,8,9,11,12,13	1	Q62997
3269	1,2,4,11,12,13,14,15	3,5,6,7,8,9,10,16,17	4	D3ZUC6,P10758,P25236,P51907
3270	1,2,4,11,12,13,14,16	3,5,6,7,8,9,10,15,17	2	D3ZUC6,P25236
3271	1,2,4,11,12,13,14,17	3,5,6,7,8,9,10,15,16	4	D3ZUC6,P31430,P36374,Q6AYQ8
3272	1,2,4,11,12,13,15,16	3,5,6,7,8,9,10,14,17	2	P01836,P51907
3273	1,2,4,11,12,13,15,17	3,5,6,7,8,9,10,14,16	1	P06866
3274	1,2,4,11,12,13,16,17	3,5,6,7,8,9,10,14,15	3	P02803,P06866,P27213
3275	1,2,4,11,12,14,15,16	3,5,6,7,8,9,10,13,17	4	D3ZUC6,O89117,P51907,Q99MH3
3276	1,2,4,11,12,14,15,17	3,5,6,7,8,9,10,13,16	6	D3ZUC6,P06866,P31430,P51907,P98089,Q6AYQ8
3277	1,2,4,11,12,14,16,17	3,5,6,7,8,9,10,13,15	5	D3ZUC6,P06866,P31430,P98089,Q6AYQ8

3278	1,2,4,11,12,15,16,17	3,5,6,7,8,9,10,13,14	2	P06866,P51907
3279	1,2,4,11,13,14,15,16	3,5,6,7,8,9,10,12,17	4	P06760,P50115,Q99MH3,Q9QZK9
3280	1,2,4,11,13,14,15,17	3,5,6,7,8,9,10,12,16	0	
3281	1,2,4,11,13,14,16,17	3,5,6,7,8,9,10,12,15	1	P27213
3282	1,2,4,11,13,15,16,17	3,5,6,7,8,9,10,12,14	1	P27213
3283	1,2,4,11,14,15,16,17	3,5,6,7,8,9,10,12,13	1	D3ZUC6
3284	1,2,4,12,13,14,15,16	3,5,6,7,8,9,10,11,17	5	D3ZUC6,P06760,P25236,P50115,Q9QUL6
3285	1,2,4,12,13,14,15,17	3,5,6,7,8,9,10,11,16	6	D3ZUC6,P06866,P25236,P27213,P31430,P98089
3286	1,2,4,12,13,14,16,17	3,5,6,7,8,9,10,11,15	6	D3ZUC6,P06866,P14480,P25236,P27213,P31430
3287	1,2,4,12,13,15,16,17	3,5,6,7,8,9,10,11,14	2	P06866,P27213
3288	1,2,4,12,14,15,16,17	3,5,6,7,8,9,10,11,13	5	D3ZUC6,P06866,P31430,P35467,P98089
3289	1,2,4,13,14,15,16,17	3,5,6,7,8,9,10,11,12	1	P27213
3290	1,2,5,6,7,8,9,10	3,4,11,12,13,14,15,16,17	10	O55006,P02631,P19629,P27213,P31044,P52590,P68370,P83748,Q63942,Q9QUL6
3291	1,2,5,6,7,8,9,11	3,4,10,12,13,14,15,16,17	15	O54715,P02631,P05371,P08932,P10715,P14669,P19629,P27213,P31044,P52590,P68370,Q63493,Q63942,Q9JI85,Q9QUL6
3292	1,2,5,6,7,8,9,12	3,4,10,11,13,14,15,16,17	19	O88267,P02631,P02782,P08723,P10715,P13432,P19629,P22283,P27213,P30120,P31044,P52590,P68370,P83748,Q63942,Q8CJ52,Q9JHB9,Q9JI85,Q9Z1F2
3293	1,2,5,6,7,8,9,13	3,4,10,11,12,14,15,16,17	13	P02631,P02764,P06911,P12020,P19629,P31044,P52590,P53792,P68370,P83748,Q5GRG2,Q63942,Q9Z1F2
3294	1,2,5,6,7,8,9,14	3,4,10,11,12,13,15,16,17	11	P02631,P19629,P27213,P31044,P52590,P53792,P68370,P83748,Q63942,Q9QUL6,Q9Z1F2
3295	1,2,5,6,7,8,9,15	3,4,10,11,12,13,14,16,17	11	O55006,P02631,P10715,P19629,P27213,P31044,P52590,P53792,P68370,P83748,Q63942
3296	1,2,5,6,7,8,9,16	3,4,10,11,12,13,14,15,17	8	P02631,P10715,P19629,P31044,P52590,P68370,P83748,Q63942

3297	1,2,5,6,7,8,9,17	3,4,10,11,12,13,14,15,16	14	O88267,P02631,P06760,P10715,P19629,P31044,P50115,P52590,P53792,P55091,P68370,P83748,Q63942,Q9QUL6
3298	1,2,5,6,7,8,10,11	3,4,9,12,13,14,15,16,17	18	O54715,P02631,P05371,P07150,P08649,P10715,P14669,P15399,P19629,P27213,P28648,P31044,P52590,P61206;P84079;P84082,P68370,Q63493,Q9JI85,Q9QUL6
3299	1,2,5,6,7,8,10,12	3,4,9,11,13,14,15,16,17	20	F1LM93,O88267,P02631,P02782,P08723,P10715,P12020,P19629,P22283,P27213,P30120,P31044,P52590,P68370,P83748,P97840,Q10758,Q8CJ52,Q9JHB9,Q9JI85
3300	1,2,5,6,7,8,10,13	3,4,9,11,12,14,15,16,17	10	P02764,P06911,P12020,P19629,P31044,P52590,P68370,P83748,Q5GRG2,Q9Z1F2
3301	1,2,5,6,7,8,10,14	3,4,9,11,12,13,15,16,17	10	P02631,P12020,P19629,P27213,P31044,P52590,P55018,P68370,P83748,Q5GRG2
3302	1,2,5,6,7,8,10,15	3,4,9,11,12,13,14,16,17	9	O55006,P02631,P19629,P27213,P31044,P52590,P55018,P68370,P83748
3303	1,2,5,6,7,8,10,16	3,4,9,11,12,13,14,15,17	6	P19629,P31044,P52590,P55018,P68370,P83748
3304	1,2,5,6,7,8,10,17	3,4,9,11,12,13,14,15,16	13	O54858,O88267,P02631,P06760,P19629,P31044,P50115,P52590,P55091,P68370,P83748,P97571,Q9QUL6
3305	1,2,5,6,7,8,11,12	3,4,9,10,13,14,15,16,17	30	O35547,O54715,O88267,P02631,P02781,P02782,P05371,P06761,P07647,P08723,P10715,P13432,P14669,P19629,P20762,P22282,P22283,P27213,P30120,P31044,P62804,P68370,P83748,Q63357,Q63493,Q6P6Q2,Q8CJ52,Q9JHB9,Q9JI85,Q9Z1F2
3306	1,2,5,6,7,8,11,13	3,4,9,10,12,14,15,16,17	21	P01048,P02631,P02764,P05371,P06911,P10715,P12020,P14669,P19629,P22282,P22283,P31044,P68370,P83748,Q5GRG2,Q62902,Q63493,Q8CJ52,Q9JHB9,Q9JI85,Q9Z1F2
3307	1,2,5,6,7,8,11,14	3,4,9,10,12,13,15,16,17	18	P02631,P05371,P07171,P10715,P12020,P14669,P15399,P19629,P20762,P27213,P31044,P47727,P68370,Q5GRG2,Q63493,Q8CJ52,Q9JI85,Q9QUL6
3308	1,2,5,6,7,8,11,15	3,4,9,10,12,13,14,16,17	11	P02631,P05371,P07171,P10715,P14669,P19629,P27213,P31044,P68370,P83748,Q9JI85
3309	1,2,5,6,7,8,11,16	3,4,9,10,12,13,14,15,17	15	A2RUV9,O54715,P02631,P05371,P07150,P10715,P14669,P19629,P31044,P61206;P84079;P84082,P68370,P83748,Q63493,Q8CJ52,Q9JI85
3310	1,2,5,6,7,8,11,17	3,4,9,10,12,13,14,15,16	20	O54858,O88267,P02631,P05371,P06760,P07150,P10715,P14669,P15399,P19629,P31044,P50115,P52590,P55091,P68370,P97580,Q30KJ2,Q63493,Q9JI85,Q9QUL6
3311	1,2,5,6,7,8,12,13	3,4,9,10,11,14,15,16,17	25	O88267,P02631,P02781,P02782,P06761,P06911,P07647,P08723,P10715,P12020,P13432,P19629,P22282,P22283,P30120,P31044,P68370,P83748,Q5GRG2,Q62902,Q6AYR9,Q8CJ52,Q9JHB9,Q9JI85,Q9Z1F2

3312	1,2,5,6,7,8,12,14	3,4,9,10,11,13,15,16,17	23	O88267,P02631,P02782,P06761,P08723,P10715,P12020,P13432,P19629,P20762,P22282,P22283,P30120,P31044,P68370,P83748,Q5GRG2,Q63493,Q6AYS7,Q8CJ52,Q9JHB9,Q9JI85,Q9Z1F2
3313	1,2,5,6,7,8,12,15	3,4,9,10,11,13,14,16,17	13	O88267,P02631,P08723,P10715,P13432,P19629,P30120,P31044,P68370,P83748,Q8CJ52,Q9JHB9,Q9JI85
3314	1,2,5,6,7,8,12,16	3,4,9,10,11,13,14,15,17	14	O88267,P02631,P08723,P10715,P13432,P19629,P30120,P31044,P68370,P83748,Q8CJ52,Q9JHB9,Q9JI85,Q9Z1F2
3315	1,2,5,6,7,8,12,17	3,4,9,10,11,13,14,15,16	32	F1LM93,O88267,P02631,P02782,P06760,P06761,P08723,P10715,P13432,P19629,P22282,P22283,P23928,P30120,P31044,P50115,P52590,P55091,P68370,P83748,P98089,Q4G075,Q561R9,Q62635,Q63493,Q8CJ52,Q99041,Q9JHB9,Q9JI85,Q9QZA6,Q9QZK9,Q9Z1F2
3316	1,2,5,6,7,8,13,14	3,4,9,10,11,12,15,16,17	15	O88267,P02631,P06761,P06911,P12020,P19629,P22282,P31044,P53792,P68370,P83748,Q5GRG2,Q5RLM2,Q62902,Q9Z1F2
3317	1,2,5,6,7,8,13,15	3,4,9,10,11,12,14,16,17	11	P02631,P06911,P12020,P19629,P31044,P53792,P68370,P83748,Q5GRG2,Q6IFV1,Q9Z1F2
3318	1,2,5,6,7,8,13,16	3,4,9,10,11,12,14,15,17	10	O88267,P02631,P06911,P12020,P19629,P31044,P68370,P83748,Q5GRG2,Q9Z1F2
3319	1,2,5,6,7,8,13,17	3,4,9,10,11,12,14,15,16	23	O88267,P02631,P06760,P06761,P06911,P12020,P19629,P22282,P22283,P23928,P31044,P50115,P51907,P52590,P53792,P55091,P68370,P70545,P83748,Q5GRG2,Q6IFV1,Q6PCU2,Q9Z1F2
3320	1,2,5,6,7,8,14,15	3,4,9,10,11,12,13,16,17	10	P02631,P07171,P10536,P16636,P19629,P20762,P31044,P68370,P83748,Q5GRG2
3321	1,2,5,6,7,8,14,16	3,4,9,10,11,12,13,15,17	7	O88267,P02631,P19629,P31044,P68370,P83748,Q63532
3322	1,2,5,6,7,8,14,17	3,4,9,10,11,12,13,15,16	17	O88267,P02631,P06760,P10536,P12020,P19629,P20762,P31044,P50115,P52590,P53792,P55091,P68370,P83748,Q561R9,Q6PCU2,Q9Z1F2
3323	1,2,5,6,7,8,15,16	3,4,9,10,11,12,13,14,17	6	P02631,P10715,P19629,P31044,P68370,P83748
3324	1,2,5,6,7,8,15,17	3,4,9,10,11,12,13,14,16	13	P02631,P06760,P10536,P10715,P19629,P31044,P50115,P52590,P53792,P55091,P68370,P83748,Q6IFV1
3325	1,2,5,6,7,8,16,17	3,4,9,10,11,12,13,14,15	14	O88267,P02631,P06760,P10715,P10758,P19629,P23928,P31044,P50115,P50116,P52590,P68370,P83748,Q6PCU2
3326	1,2,5,6,7,9,10,11	3,4,8,12,13,14,15,16,17	7	P15399,P27213,P52590,P61206;P84079;P84082,Q63942,Q91ZS3,Q9QUL6

3327	1,2,5,6,7,9,10,12	3,4,8,11,13,14,15,16,17	5	P27213,P52590,P83748,P97840,Q9QUL6
3328	1,2,5,6,7,9,10,13	3,4,8,11,12,14,15,16,17	6	P12020,P52590,P83748,P97840,Q63942,Q9QUL6
3329	1,2,5,6,7,9,10,14	3,4,8,11,12,13,15,16,17	6	P12020,P27213,P52590,P83748,Q63942,Q9QUL6
3330	1,2,5,6,7,9,10,15	3,4,8,11,12,13,14,16,17	6	P27213,P52590,P83748,P97840,Q63942,Q9QUL6
3331	1,2,5,6,7,9,10,16	3,4,8,11,12,13,14,15,17	6	P52590,P83748,P97840,Q63942,Q64093,Q9QUL6
3332	1,2,5,6,7,9,10,17	3,4,8,11,12,13,14,15,16	4	P52590,P83748,Q63942,Q9QUL6
3333	1,2,5,6,7,9,11,12	3,4,8,10,13,14,15,16,17	9	P27213,P30120,P52590,P83748,Q6IFU7,Q91ZS3,Q9JHB9,Q9JI85,Q9QUL6
3334	1,2,5,6,7,9,11,13	3,4,8,10,12,14,15,16,17	2	Q91ZS3,Q9QUL6
3335	1,2,5,6,7,9,11,14	3,4,8,10,12,13,15,16,17	5	P02631,P27213,P52590,Q63942,Q9QUL6
3336	1,2,5,6,7,9,11,15	3,4,8,10,12,13,14,16,17	6	P02631,P27213,P52590,P83748,Q63942,Q9QUL6
3337	1,2,5,6,7,9,11,16	3,4,8,10,12,13,14,15,17	5	A2RUV9,P05712,P61206;P84079;P84082,Q63942,Q9QUL6
3338	1,2,5,6,7,9,11,17	3,4,8,10,12,13,14,15,16	3	P15399,P52590,Q9QUL6
3339	1,2,5,6,7,9,12,13	3,4,8,10,11,14,15,16,17	5	P19218,P83748,P97840,Q9JHB9,Q9Z1F2
3340	1,2,5,6,7,9,12,14	3,4,8,10,11,13,15,16,17	4	P02631,P52590,P83748,Q9Z1F2
3341	1,2,5,6,7,9,12,15	3,4,8,10,11,13,14,16,17	3	P02631,P52590,P83748

3342	1,2,5,6,7,9,12,16	3,4,8,10,11,13,14,15,17	4	P19218,P83748,P97840,Q63942
3343	1,2,5,6,7,9,12,17	3,4,8,10,11,13,14,15,16	5	P13432,P52590,P55091,P83748,Q9QUL6
3344	1,2,5,6,7,9,13,14	3,4,8,10,11,12,15,16,17	7	P06911,P12020,P53792,P83748,Q5GRG2,Q63942,Q9Z1F2
3345	1,2,5,6,7,9,13,15	3,4,8,10,11,12,14,16,17	3	P53792,P83748,Q63942
3346	1,2,5,6,7,9,13,16	3,4,8,10,11,12,14,15,17	5	B0BNN3,P19218,P53792,P83748,Q63942
3347	1,2,5,6,7,9,13,17	3,4,8,10,11,12,14,15,16	4	P52590,P53792,P83748,Q9QUL6
3348	1,2,5,6,7,9,14,15	3,4,8,10,11,12,13,16,17	8	P02631,P16636,P35467,P52590,P53792,P83748,Q63942,Q9QUL6
3349	1,2,5,6,7,9,14,16	3,4,8,10,11,12,13,15,17	4	P83748,Q63532,Q63942,Q9QUL6
3350	1,2,5,6,7,9,14,17	3,4,8,10,11,12,13,15,16	4	P52590,P53792,Q63942,Q9QUL6
3351	1,2,5,6,7,9,15,16	3,4,8,10,11,12,13,14,17	4	P02631,P35467,P83748,Q63942
3352	1,2,5,6,7,9,15,17	3,4,8,10,11,12,13,14,16	6	P02631,P52590,P53792,P83748,Q63942,Q9QUL6
3353	1,2,5,6,7,9,16,17	3,4,8,10,11,12,13,14,15	5	P10758,P52590,P83748,Q63942,Q9QUL6
3354	1,2,5,6,7,10,11,12	3,4,8,9,13,14,15,16,17	15	P15399,P27213,P30120,P61206;P84079;P84082,P68370,P83748,P97840,Q10758,Q63357,Q6IFU7,Q6IMF3,Q6P6Q2,Q9JHB9,Q9JI85,Q9QUL6
3355	1,2,5,6,7,10,11,13	3,4,8,9,12,14,15,16,17	6	P12020,P15399,P61206;P84079;P84082,P68370,Q5GRG2,Q9QUL6
3356	1,2,5,6,7,10,11,14	3,4,8,9,12,13,15,16,17	8	P12020,P15399,P27213,P47727,P61206;P84079;P84082,P68370,Q63532,Q9QUL6

3357	1,2,5,6,7,10,11,15	3,4,8,9,12,13,14,16,17	4	P02631,P27213,P83748,Q9QUL6
3358	1,2,5,6,7,10,11,16	3,4,8,9,12,13,14,15,17	4	A2RUV9,P05712,P61206;P84079;P84082,Q9QUL6
3359	1,2,5,6,7,10,11,17	3,4,8,9,12,13,14,15,16	5	P15399,P52590,P97580,Q30KJ2,Q9QUL6
3360	1,2,5,6,7,10,12,13	3,4,8,9,11,14,15,16,17	6	P12020,P19218,P83748,P97840,Q5GRG2,Q9JHB9
3361	1,2,5,6,7,10,12,14	3,4,8,9,11,13,15,16,17	5	P12020,P83748,P97840,Q10758,Q63532
3362	1,2,5,6,7,10,12,15	3,4,8,9,11,13,14,16,17	2	P83748,P97840
3363	1,2,5,6,7,10,12,16	3,4,8,9,11,13,14,15,17	5	P19218,P47967,P83748,P97840,Q10758
3364	1,2,5,6,7,10,12,17	3,4,8,9,11,13,14,15,16	8	P13432,P52590,P55091,P83748,P97840,Q10758,Q62635,Q9QZK9
3365	1,2,5,6,7,10,13,14	3,4,8,9,11,12,15,16,17	4	P06911,P12020,P83748,Q5GRG2
3366	1,2,5,6,7,10,13,15	3,4,8,9,11,12,14,16,17	3	P12020,P83748,Q5GRG2
3367	1,2,5,6,7,10,13,16	3,4,8,9,11,12,14,15,17	4	P12020,P19218,P83748,P97840
3368	1,2,5,6,7,10,13,17	3,4,8,9,11,12,14,15,16	3	P12020,P52590,P83748
3369	1,2,5,6,7,10,14,15	3,4,8,9,11,12,13,16,17	6	P02631,P12020,P16636,P35467,P83748,Q63532
3370	1,2,5,6,7,10,14,16	3,4,8,9,11,12,13,15,17	5	P12020,P35467,P83748,P97840,Q63532
3371	1,2,5,6,7,10,14,17	3,4,8,9,11,12,13,15,16	4	P12020,P52590,P83748,Q9QUL6

3372	1,2,5,6,7,10,15,16	3,4,8,9,11,12,13,14,17	3	P35467,P83748,P97840
3373	1,2,5,6,7,10,15,17	3,4,8,9,11,12,13,14,16	3	P52590,P83748,Q9QUL6
3374	1,2,5,6,7,10,16,17	3,4,8,9,11,12,13,14,15	4	P10758,P52590,P83748,Q9QUL6
3375	1,2,5,6,7,11,12,13	3,4,8,9,10,14,15,16,17	5	P30120,P83748,Q9JHB9,Q9JI85,Q9Z1F2
3376	1,2,5,6,7,11,12,14	3,4,8,9,10,13,15,16,17	11	P02631,P15399,P19629,P30120,P47727,P83748,Q63493,Q63532,Q6IFU7,Q9JHB9,Q9JI85
3377	1,2,5,6,7,11,12,15	3,4,8,9,10,13,14,16,17	4	P02631,P83748,Q9JHB9,Q9JI85
3378	1,2,5,6,7,11,12,16	3,4,8,9,10,13,14,15,17	4	P17046,P61206;P84079;P84082,P83748,Q6IFU7
3379	1,2,5,6,7,11,12,17	3,4,8,9,10,13,14,15,16	11	P13432,P15399,P52590,P55091,P83748,Q62635,Q63493,Q9JHB9,Q9JI85,Q9QUL6,Q9QZK9
3380	1,2,5,6,7,11,13,14	3,4,8,9,10,12,15,16,17	4	P06911,P12020,P47727,Q5GRG2
3381	1,2,5,6,7,11,13,15	3,4,8,9,10,12,14,16,17	3	P02631,P83748,Q5GRG2
3382	1,2,5,6,7,11,13,16	3,4,8,9,10,12,14,15,17	1	P83748
3383	1,2,5,6,7,11,13,17	3,4,8,9,10,12,14,15,16	3	P15399,P52590,Q9QUL6
3384	1,2,5,6,7,11,14,15	3,4,8,9,10,12,13,16,17	4	P02631,P16636,P83748,Q9QUL6
3385	1,2,5,6,7,11,14,16	3,4,8,9,10,12,13,15,17	3	P02631,Q63532,Q9QUL6
3386	1,2,5,6,7,11,14,17	3,4,8,9,10,12,13,15,16	6	P02631,P15399,P52590,P97580,Q30KJ2,Q9QUL6

3387	1,2,5,6,7,11,15,16	3,4,8,9,10,12,13,14,17	2	P02631,P83748
3388	1,2,5,6,7,11,15,17	3,4,8,9,10,12,13,14,16	4	P02631,P15399,P52590,Q9QUL6
3389	1,2,5,6,7,11,16,17	3,4,8,9,10,12,13,14,15	6	A2RUV9,P10758,P15399,P52590,P97580,Q9QUL6
3390	1,2,5,6,7,12,13,14	3,4,8,9,10,11,15,16,17	9	O88267,P06911,P12020,P30120,P83748,Q5GRG2,Q63532,Q9JHB9,Q9Z1F2
3391	1,2,5,6,7,12,13,15	3,4,8,9,10,11,14,16,17	4	P19218,P35467,P83748,Q5GRG2
3392	1,2,5,6,7,12,13,16	3,4,8,9,10,11,14,15,17	3	P19218,P83748,P97840
3393	1,2,5,6,7,12,13,17	3,4,8,9,10,11,14,15,16	10	O88267,P13432,P52590,P55091,P83748,Q561R9,Q62635,Q9JHB9,Q9QZK9,Q9Z1F2
3394	1,2,5,6,7,12,14,15	3,4,8,9,10,11,13,16,17	6	P02631,P16636,P19629,P35467,P83748,Q63532
3395	1,2,5,6,7,12,14,16	3,4,8,9,10,11,13,15,17	3	P35467,P83748,Q63532
3396	1,2,5,6,7,12,14,17	3,4,8,9,10,11,13,15,16	9	O88267,P13432,P19629,P35467,P52590,P55091,P83748,Q561R9,Q62635
3397	1,2,5,6,7,12,15,16	3,4,8,9,10,11,13,14,17	2	P35467,P83748
3398	1,2,5,6,7,12,15,17	3,4,8,9,10,11,13,14,16	6	P13432,P35467,P52590,P55091,P83748,Q62635
3399	1,2,5,6,7,12,16,17	3,4,8,9,10,11,13,14,15	7	O88267,P13432,P35467,P52590,P55091,P83748,Q561R9
3400	1,2,5,6,7,13,14,15	3,4,8,9,10,11,12,16,17	8	P02631,P06911,P12020,P16636,P35467,P53792,P83748,Q5GRG2
3401	1,2,5,6,7,13,14,16	3,4,8,9,10,11,12,15,17	6	P06911,P12020,P35467,P83748,Q5GRG2,Q63532

3402	1,2,5,6,7,13,14,17	3,4,8,9,10,11,12,15,16	8	P06911,P12020,P52590,P53792,P83748,Q561R9,Q5GRG2,Q9Z1F2
3403	1,2,5,6,7,13,15,16	3,4,8,9,10,11,12,14,17	2	P35467,P83748
3404	1,2,5,6,7,13,15,17	3,4,8,9,10,11,12,14,16	7	P00714,P35467,P52590,P53792,P83748,Q6IFV1,Q9WUW8
3405	1,2,5,6,7,13,16,17	3,4,8,9,10,11,12,14,15	4	P10758,P52590,P83748,Q561R9
3406	1,2,5,6,7,14,15,16	3,4,8,9,10,11,12,13,17	5	P02631,P16636,P35467,P83748,Q63532
3407	1,2,5,6,7,14,15,17	3,4,8,9,10,11,12,13,16	6	P02631,P16636,P35467,P52590,P53792,P83748
3408	1,2,5,6,7,14,16,17	3,4,8,9,10,11,12,13,15	6	P10758,P35467,P52590,P83748,Q561R9,Q63532
3409	1,2,5,6,7,15,16,17	3,4,8,9,10,11,12,13,14	4	P10758,P35467,P52590,P83748
3410	1,2,5,6,8,9,10,11	3,4,7,12,13,14,15,16,17	7	A2RUV9,P08649,P14669,P27213,Q63942,Q91ZS3,Q9QUL6
3411	1,2,5,6,8,9,10,12	3,4,7,11,13,14,15,16,17	2	P27213,Q63942
3412	1,2,5,6,8,9,10,13	3,4,7,11,12,14,15,16,17	2	Q63942,Q9Z1F2
3413	1,2,5,6,8,9,10,14	3,4,7,11,12,13,15,16,17	3	P27213,Q63942,Q9QUL6
3414	1,2,5,6,8,9,10,15	3,4,7,11,12,13,14,16,17	3	P27213,Q63942,Q9QUL6
3415	1,2,5,6,8,9,10,16	3,4,7,11,12,13,14,15,17	3	A2RUV9,Q63942,Q9QUL6
3416	1,2,5,6,8,9,10,17	3,4,7,11,12,13,14,15,16	5	A2RUV9,P06760,P08649,Q63942,Q9QUL6

3417	1,2,5,6,8,9,11,12	3,4,7,10,13,14,15,16,17	12	O35547,P02782,P02803,P27213,P30120,Q5M8C6,Q63942,Q6IFU7,Q6IFU8,Q6P6Q2,Q9JHB9,Q9Z1F2
3418	1,2,5,6,8,9,11,13	3,4,7,10,12,14,15,16,17	4	A2RUV9,P02803,Q63942,Q9Z1F2
3419	1,2,5,6,8,9,11,14	3,4,7,10,12,13,15,16,17	3	P27213,Q63942,Q9QUL6
3420	1,2,5,6,8,9,11,15	3,4,7,10,12,13,14,16,17	3	P27213,Q63942,Q9QUL6
3421	1,2,5,6,8,9,11,16	3,4,7,10,12,13,14,15,17	3	A2RUV9,Q63942,Q9QUL6
3422	1,2,5,6,8,9,11,17	3,4,7,10,12,13,14,15,16	6	A2RUV9,P06760,P08649,P14669,Q63942,Q9QUL6
3423	1,2,5,6,8,9,12,13	3,4,7,10,11,14,15,16,17	7	P02782,P02803,P30120,Q5M8C6,Q63942,Q9JHB9,Q9Z1F2
3424	1,2,5,6,8,9,12,14	3,4,7,10,11,13,15,16,17	4	P02782,P30120,Q63942,Q9Z1F2
3425	1,2,5,6,8,9,12,15	3,4,7,10,11,13,14,16,17	1	Q63942
3426	1,2,5,6,8,9,12,16	3,4,7,10,11,13,14,15,17	1	Q63942
3427	1,2,5,6,8,9,12,17	3,4,7,10,11,13,14,15,16	7	P02782,P06760,P13432,Q5M8C6,Q62635,Q63942,Q9Z1F2
3428	1,2,5,6,8,9,13,14	3,4,7,10,11,12,15,16,17	5	P06911,P53792,Q5FVI6,Q63942,Q9Z1F2
3429	1,2,5,6,8,9,13,15	3,4,7,10,11,12,14,16,17	4	P53792,Q63942,Q6IFV1,Q9Z1F2
3430	1,2,5,6,8,9,13,16	3,4,7,10,11,12,14,15,17	3	A2RUV9,Q63942,Q9Z1F2
3431	1,2,5,6,8,9,13,17	3,4,7,10,11,12,14,15,16	8	P06760,P08753,P51907,P53792,Q5FVI6,Q63942,Q6IFV1,Q9Z1F2

3432	1,2,5,6,8,9,14,15	3,4,7,10,11,12,13,16,17	1	Q63942
3433	1,2,5,6,8,9,14,16	3,4,7,10,11,12,13,15,17	1	Q63942
3434	1,2,5,6,8,9,14,17	3,4,7,10,11,12,13,15,16	6	P06760,P53792,Q5FVI6,Q63942,Q9QUL6,Q9Z1F2
3435	1,2,5,6,8,9,15,16	3,4,7,10,11,12,13,14,17	2	Q5QE79,Q63942
3436	1,2,5,6,8,9,15,17	3,4,7,10,11,12,13,14,16	4	P06760,P53792,Q63942,Q6IFV1
3437	1,2,5,6,8,9,16,17	3,4,7,10,11,12,13,14,15	4	A2RUV9,P06760,P23593,Q63942
3438	1,2,5,6,8,10,11,12	3,4,7,9,13,14,15,16,17	15	F1LM93,O35547,P02782,P14669,P27213,P30120,P47727,P62804,Q10758,Q63357,Q6IFU7,Q6IFU8,Q6IMF3,Q6P6Q2,Q9JHB9
3439	1,2,5,6,8,10,11,13	3,4,7,9,12,14,15,16,17	3	A2RUV9,P14669,P47727
3440	1,2,5,6,8,10,11,14	3,4,7,9,12,13,15,16,17	6	P08649,P14669,P27213,P47727,P62804,Q9QUL6
3441	1,2,5,6,8,10,11,15	3,4,7,9,12,13,14,16,17	4	P08649,P14669,P27213,Q9QUL6
3442	1,2,5,6,8,10,11,16	3,4,7,9,12,13,14,15,17	3	A2RUV9,P08649,Q9QUL6
3443	1,2,5,6,8,10,11,17	3,4,7,9,12,13,14,15,16	5	A2RUV9,P06760,P08649,P14669,Q9QUL6
3444	1,2,5,6,8,10,12,13	3,4,7,9,11,14,15,16,17	6	P02782,P30120,Q5M8C6,Q6IFV1,Q9JHB9,Q9Z1F2
3445	1,2,5,6,8,10,12,14	3,4,7,9,11,13,15,16,17	5	P02782,P30120,P62804,Q10758,Q9Z1F2
3446	1,2,5,6,8,10,12,15	3,4,7,9,11,13,14,16,17	1	Q6IFV1

3447	1,2,5,6,8,10,12,16	3,4,7,9,11,13,14,15,17	2	Q10758,Q6IFU7
3448	1,2,5,6,8,10,12,17	3,4,7,9,11,13,14,15,16	7	F1LM93,P06760,P13432,Q10758,Q4G075,Q62635,Q6IFV1
3449	1,2,5,6,8,10,13,14	3,4,7,9,11,12,15,16,17	4	P06911,P12020,Q5GRG2,Q9Z1F2
3450	1,2,5,6,8,10,13,15	3,4,7,9,11,12,14,16,17	1	Q6IFV1
3451	1,2,5,6,8,10,13,16	3,4,7,9,11,12,14,15,17	1	A2RUV9
3452	1,2,5,6,8,10,13,17	3,4,7,9,11,12,14,15,16	3	P06760,P51907,Q6IFV1
3453	1,2,5,6,8,10,14,15	3,4,7,9,11,12,13,16,17	0	
3454	1,2,5,6,8,10,14,16	3,4,7,9,11,12,13,15,17	0	
3455	1,2,5,6,8,10,14,17	3,4,7,9,11,12,13,15,16	1	P06760
3456	1,2,5,6,8,10,15,16	3,4,7,9,11,12,13,14,17	0	
3457	1,2,5,6,8,10,15,17	3,4,7,9,11,12,13,14,16	2	P06760,Q6IFV1
3458	1,2,5,6,8,10,16,17	3,4,7,9,11,12,13,14,15	5	A2RUV9,P02625,P06760,P10758,P42854
3459	1,2,5,6,8,11,12,13	3,4,7,9,10,14,15,16,17	12	O35547,P02781,P02782,P02803,P07647,P30120,P47727,Q5M8C6,Q6IFU7,Q8CJ52,Q9JHB9,Q9Z1F2
3460	1,2,5,6,8,11,12,14	3,4,7,9,10,13,15,16,17	11	O35547,P02782,P30120,P47727,P62804,Q00715,Q6IFU7,Q6IFU8,Q6P6Q2,Q9JHB9,Q9Z1F2
3461	1,2,5,6,8,11,12,15	3,4,7,9,10,13,14,16,17	5	O35547,P14669,P62804,Q6IFU7,Q9JHB9

3462	1,2,5,6,8,11,12,16	3,4,7,9,10,13,14,15,17	6	O35547,P30120,Q6IFU7,Q6IFU8,Q6P6Q2,Q9JHB9
3463	1,2,5,6,8,11,12,17	3,4,7,9,10,13,14,15,16	15	O35547,P02782,P06760,P13432,P14669,P30120,P50115,P55091,P62804,Q00715,Q4G075,Q5M8C6,Q62635,Q6P6Q2,Q9JHB9
3464	1,2,5,6,8,11,13,14	3,4,7,9,10,12,15,16,17	4	P06911,P47727,Q5GRG2,Q9Z1F2
3465	1,2,5,6,8,11,13,15	3,4,7,9,10,12,14,16,17	1	Q6IFV1
3466	1,2,5,6,8,11,13,16	3,4,7,9,10,12,14,15,17	1	A2RUV9
3467	1,2,5,6,8,11,13,17	3,4,7,9,10,12,14,15,16	5	A2RUV9,P06760,P14669,Q6IFV1,Q9Z1F2
3468	1,2,5,6,8,11,14,15	3,4,7,9,10,12,13,16,17	1	Q5I0D1
3469	1,2,5,6,8,11,14,16	3,4,7,9,10,12,13,15,17	0	
3470	1,2,5,6,8,11,14,17	3,4,7,9,10,12,13,15,16	4	P02793;Q7TP54,P06760,P14669,Q9QUL6
3471	1,2,5,6,8,11,15,16	3,4,7,9,10,12,13,14,17	1	A2RUV9
3472	1,2,5,6,8,11,15,17	3,4,7,9,10,12,13,14,16	3	P06760,P14669,Q6IFV1
3473	1,2,5,6,8,11,16,17	3,4,7,9,10,12,13,14,15	3	A2RUV9,P06760,P10758
3474	1,2,5,6,8,12,13,14	3,4,7,9,10,11,15,16,17	7	P02782,P06911,P30120,Q5FVI6,Q5GRG2,Q9JHB9,Q9Z1F2
3475	1,2,5,6,8,12,13,15	3,4,7,9,10,11,14,16,17	4	P02782,Q6IFV1,Q9JHB9,Q9Z1F2
3476	1,2,5,6,8,12,13,16	3,4,7,9,10,11,14,15,17	3	P02782,Q9JHB9,Q9Z1F2

3477	1,2,5,6,8,12,13,17	3,4,7,9,10,11,14,15,16	14	P02782,P06760,P13432,P30120,P51907,P55091,Q561R9,Q5FVI6,Q5M8C6,Q62635,Q6IFV1,Q9JHB9,Q9QZK9,Q9Z1F2
3478	1,2,5,6,8,12,14,15	3,4,7,9,10,11,13,16,17	1	Q9Z1F2
3479	1,2,5,6,8,12,14,16	3,4,7,9,10,11,13,15,17	1	Q9Z1F2
3480	1,2,5,6,8,12,14,17	3,4,7,9,10,11,13,15,16	10	P02782,P06760,P13432,P31430,P55091,Q10758,Q561R9,Q5FVI6,Q62635,Q9Z1F2
3481	1,2,5,6,8,12,15,16	3,4,7,9,10,11,13,14,17	0	
3482	1,2,5,6,8,12,15,17	3,4,7,9,10,11,13,14,16	4	P06760,P13432,Q62635,Q6IFV1
3483	1,2,5,6,8,12,16,17	3,4,7,9,10,11,13,14,15	4	P06760,P13432,Q561R9,Q62635
3484	1,2,5,6,8,13,14,15	3,4,7,9,10,11,12,16,17	6	P06757,P06911,P08753,Q5FVI6,Q5GRG2,Q9Z1F2
3485	1,2,5,6,8,13,14,16	3,4,7,9,10,11,12,15,17	3	P06911,P08753,Q9Z1F2
3486	1,2,5,6,8,13,14,17	3,4,7,9,10,11,12,15,16	10	P06760,P06911,P08753,P10536,P51907,P53792,Q561R9,Q5FVI6,Q6PCU2,Q9Z1F2
3487	1,2,5,6,8,13,15,16	3,4,7,9,10,11,12,14,17	2	P08753,Q6IFV1
3488	1,2,5,6,8,13,15,17	3,4,7,9,10,11,12,14,16	7	P06760,P08753,P10536,P53792,Q5FVI6,Q6IFV1,Q9Z1F2
3489	1,2,5,6,8,13,16,17	3,4,7,9,10,11,12,14,15	7	A2RUV9,P06760,P08753,P51907,Q6IFV1,Q6PCU2,Q9Z1F2
3490	1,2,5,6,8,14,15,16	3,4,7,9,10,11,12,13,17	0	
3491	1,2,5,6,8,14,15,17	3,4,7,9,10,11,12,13,16	4	P06760,P10536,P47967,Q5FVI6

3492	1,2,5,6,8,14,16,17	3,4,7,9,10,11,12,13,15	4	P06760,P42854,Q561R9,Q6PCU2
3493	1,2,5,6,8,15,16,17	3,4,7,9,10,11,12,13,14	2	P06760,Q6IFV1
3494	1,2,5,6,9,10,11,12	3,4,7,8,13,14,15,16,17	8	P27213,Q566E6,Q6IFU7,Q6IFU8,Q6IMF3,Q6P6Q2,Q91ZS3,Q9QUL6
3495	1,2,5,6,9,10,11,13	3,4,7,8,12,14,15,16,17	3	Q63942,Q91ZS3,Q9QUL6
3496	1,2,5,6,9,10,11,14	3,4,7,8,12,13,15,16,17	3	P27213,Q63942,Q9QUL6
3497	1,2,5,6,9,10,11,15	3,4,7,8,12,13,14,16,17	4	P27213,Q566E6,Q63942,Q9QUL6
3498	1,2,5,6,9,10,11,16	3,4,7,8,12,13,14,15,17	3	A2RUV9,Q63942,Q9QUL6
3499	1,2,5,6,9,10,11,17	3,4,7,8,12,13,14,15,16	4	A2RUV9,Q566E6,Q63942,Q9QUL6
3500	1,2,5,6,9,10,12,13	3,4,7,8,11,14,15,16,17	1	P97840
3501	1,2,5,6,9,10,12,14	3,4,7,8,11,13,15,16,17	2	Q10758,Q63942
3502	1,2,5,6,9,10,12,15	3,4,7,8,11,13,14,16,17	1	Q63942
3503	1,2,5,6,9,10,12,16	3,4,7,8,11,13,14,15,17	3	P97840,Q63942,Q6IFU7
3504	1,2,5,6,9,10,12,17	3,4,7,8,11,13,14,15,16	2	Q10758,Q9QUL6
3505	1,2,5,6,9,10,13,14	3,4,7,8,11,12,15,16,17	1	Q63942
3506	1,2,5,6,9,10,13,15	3,4,7,8,11,12,14,16,17	1	Q63942

3507	1,2,5,6,9,10,13,16	3,4,7,8,11,12,14,15,17	1	Q63942
3508	1,2,5,6,9,10,13,17	3,4,7,8,11,12,14,15,16	2	Q63942,Q9QUL6
3509	1,2,5,6,9,10,14,15	3,4,7,8,11,12,13,16,17	2	Q63942,Q9QUL6
3510	1,2,5,6,9,10,14,16	3,4,7,8,11,12,13,15,17	2	Q63942,Q9QUL6
3511	1,2,5,6,9,10,14,17	3,4,7,8,11,12,13,15,16	2	Q63942,Q9QUL6
3512	1,2,5,6,9,10,15,16	3,4,7,8,11,12,13,14,17	1	Q63942
3513	1,2,5,6,9,10,15,17	3,4,7,8,11,12,13,14,16	2	Q63942,Q9QUL6
3514	1,2,5,6,9,10,16,17	3,4,7,8,11,12,13,14,15	4	A2RUV9,P23593,Q63942,Q9QUL6
3515	1,2,5,6,9,11,12,13	3,4,7,8,10,14,15,16,17	4	P02803,Q6IFU7,Q9JHB9,Q9Z1F2
3516	1,2,5,6,9,11,12,14	3,4,7,8,10,13,15,16,17	3	Q6IFU7,Q6IFU8,Q9QUL6
3517	1,2,5,6,9,11,12,15	3,4,7,8,10,13,14,16,17	1	Q6IFU7
3518	1,2,5,6,9,11,12,16	3,4,7,8,10,13,14,15,17	3	Q63942,Q6IFU7,Q6IFU8
3519	1,2,5,6,9,11,12,17	3,4,7,8,10,13,14,15,16	4	P13432,Q6IFU7,Q6IFU8,Q9QUL6
3520	1,2,5,6,9,11,13,14	3,4,7,8,10,12,15,16,17	2	Q63942,Q9QUL6
3521	1,2,5,6,9,11,13,15	3,4,7,8,10,12,14,16,17	1	Q63942

3522	1,2,5,6,9,11,13,16	3,4,7,8,10,12,14,15,17	2	A2RUV9,Q63942
3523	1,2,5,6,9,11,13,17	3,4,7,8,10,12,14,15,16	3	A2RUV9,Q63942,Q9QUL6
3524	1,2,5,6,9,11,14,15	3,4,7,8,10,12,13,16,17	2	Q63942,Q9QUL6
3525	1,2,5,6,9,11,14,16	3,4,7,8,10,12,13,15,17	3	Q63942,Q99MH3,Q9QUL6
3526	1,2,5,6,9,11,14,17	3,4,7,8,10,12,13,15,16	2	Q63942,Q9QUL6
3527	1,2,5,6,9,11,15,16	3,4,7,8,10,12,13,14,17	3	A2RUV9,Q63942,Q9QUL6
3528	1,2,5,6,9,11,15,17	3,4,7,8,10,12,13,14,16	2	Q63942,Q9QUL6
3529	1,2,5,6,9,11,16,17	3,4,7,8,10,12,13,14,15	3	A2RUV9,Q63942,Q9QUL6
3530	1,2,5,6,9,12,13,14	3,4,7,8,10,11,15,16,17	3	P00714,Q63942,Q9Z1F2
3531	1,2,5,6,9,12,13,15	3,4,7,8,10,11,14,16,17	2	P00714,Q63942
3532	1,2,5,6,9,12,13,16	3,4,7,8,10,11,14,15,17	3	P00714,P19218,Q63942
3533	1,2,5,6,9,12,13,17	3,4,7,8,10,11,14,15,16	4	P00714,P35280,Q62635,Q9Z1F2
3534	1,2,5,6,9,12,14,15	3,4,7,8,10,11,13,16,17	1	Q63942
3535	1,2,5,6,9,12,14,16	3,4,7,8,10,11,13,15,17	1	Q63942
3536	1,2,5,6,9,12,14,17	3,4,7,8,10,11,13,15,16	4	P13432,Q561R9,Q5FVI6,Q8VD89

3537	1,2,5,6,9,12,15,16	3,4,7,8,10,11,13,14,17	1	Q63942
3538	1,2,5,6,9,12,15,17	3,4,7,8,10,11,13,14,16	1	P00714
3539	1,2,5,6,9,12,16,17	3,4,7,8,10,11,13,14,15	2	P23593,Q63942
3540	1,2,5,6,9,13,14,15	3,4,7,8,10,11,12,16,17	3	P00714,P53792,Q63942
3541	1,2,5,6,9,13,14,16	3,4,7,8,10,11,12,15,17	2	P00714,Q63942
3542	1,2,5,6,9,13,14,17	3,4,7,8,10,11,12,15,16	5	P00714,P53792,Q5FVI6,Q63942,Q9Z1F2
3543	1,2,5,6,9,13,15,16	3,4,7,8,10,11,12,14,17	2	P00714,Q63942
3544	1,2,5,6,9,13,15,17	3,4,7,8,10,11,12,14,16	4	P00714,P53792,Q63942,Q6IFV1
3545	1,2,5,6,9,13,16,17	3,4,7,8,10,11,12,14,15	4	A2RUV9,P00714,P35280,Q63942
3546	1,2,5,6,9,14,15,16	3,4,7,8,10,11,12,13,17	3	P00714,Q63942,Q9QZK9
3547	1,2,5,6,9,14,15,17	3,4,7,8,10,11,12,13,16	5	P00714,P47967,P53792,Q63942,Q9QUL6
3548	1,2,5,6,9,14,16,17	3,4,7,8,10,11,12,13,15	3	P00714,Q63942,Q9QUL6
3549	1,2,5,6,9,15,16,17	3,4,7,8,10,11,12,13,14	3	P00714,P23593,Q63942
3550	1,2,5,6,10,11,12,13	3,4,7,8,9,14,15,16,17	4	P47727,Q6IFU7,Q6P6Q2,Q9JHB9
3551	1,2,5,6,10,11,12,14	3,4,7,8,9,13,15,16,17	8	O35547,P47727,P62804,Q10758,Q6IFU7,Q6IFU8,Q6IMF3,Q6P6Q2
3552	1,2,5,6,10,11,12,15	3,4,7,8,9,13,14,16,17	2	Q6IFU7,Q6P6Q2

3553	1,2,5,6,10,<br>11,12,16	3,4,7,8,9,1<br>3,14,15,17	6	O35547,Q10758,Q6IFU7,Q6IFU8,Q6IMF3,Q6P6Q2
3554	1,2,5,6,10,<br>11,12,17	3,4,7,8,9,1<br>3,14,15,16	8	O35547,P06757,P62804,Q10758,Q6IFU7,Q6IFU8,Q6P6Q2,Q9QUL6
3555	1,2,5,6,10,<br>11,13,14	3,4,7,8,9,1<br>2,15,16,17	1	P47727
3556	1,2,5,6,10,<br>11,13,15	3,4,7,8,9,1<br>2,14,16,17	0	
3557	1,2,5,6,10,<br>11,13,16	3,4,7,8,9,1<br>2,14,15,17	1	A2RUV9
3558	1,2,5,6,10,<br>11,13,17	3,4,7,8,9,1<br>2,14,15,16	1	Q9QUL6
3559	1,2,5,6,10,<br>11,14,15	3,4,7,8,9,1<br>2,13,16,17	1	Q9QUL6
3560	1,2,5,6,10,<br>11,14,16	3,4,7,8,9,1<br>2,13,15,17	1	Q9QUL6
3561	1,2,5,6,10,<br>11,14,17	3,4,7,8,9,1<br>2,13,15,16	1	Q9QUL6
3562	1,2,5,6,10,<br>11,15,16	3,4,7,8,9,1<br>2,13,14,17	2	A2RUV9,Q9QUL6
3563	1,2,5,6,10,<br>11,15,17	3,4,7,8,9,1<br>2,13,14,16	1	Q9QUL6
3564	1,2,5,6,10,<br>11,16,17	3,4,7,8,9,1<br>2,13,14,15	3	A2RUV9,P10758,Q9QUL6
3565	1,2,5,6,10,<br>12,13,14	3,4,7,8,9,1<br>1,15,16,17	2	Q10758,Q9Z1F2
3566	1,2,5,6,10,<br>12,13,15	3,4,7,8,9,1<br>1,14,16,17	1	Q6IFV1
3567	1,2,5,6,10,<br>12,13,16	3,4,7,8,9,1<br>1,14,15,17	3	P19218,P97840,Q6IFU7
3568	1,2,5,6,10,<br>12,13,17	3,4,7,8,9,1<br>1,14,15,16	4	Q10758,Q62635,Q6IFV1,Q9QZK9
3569	1,2,5,6,10,<br>12,14,15	3,4,7,8,9,1<br>1,13,16,17	1	Q10758
3570	1,2,5,6,10,<br>12,14,16	3,4,7,8,9,1<br>1,13,15,17	3	P97840,Q10758,Q6IFU7
3571	1,2,5,6,10,<br>12,14,17	3,4,7,8,9,1<br>1,13,15,16	3	Q10758,Q561R9,Q62635
3572	1,2,5,6,10,<br>12,15,16	3,4,7,8,9,1<br>1,13,14,17	2	P97840,Q6IFU7
3573	1,2,5,6,10,<br>12,15,17	3,4,7,8,9,1<br>1,13,14,16	2	Q10758,Q6IFV1
3574	1,2,5,6,10,<br>12,16,17	3,4,7,8,9,1<br>1,13,14,15	1	Q10758
3575	1,2,5,6,10,<br>13,14,15	3,4,7,8,9,1<br>1,12,16,17	0	

3576	1,2,5,6,10,<br>13,14,16	3,4,7,8,9,1<br>1,12,15,17	0	
3577	1,2,5,6,10,<br>13,14,17	3,4,7,8,9,1<br>1,12,15,16	0	
3578	1,2,5,6,10,<br>13,15,16	3,4,7,8,9,1<br>1,12,14,17	0	
3579	1,2,5,6,10,<br>13,15,17	3,4,7,8,9,1<br>1,12,14,16	1	Q6IFV1
3580	1,2,5,6,10,<br>13,16,17	3,4,7,8,9,1<br>1,12,14,15	0	
3581	1,2,5,6,10,<br>14,15,16	3,4,7,8,9,1<br>1,12,13,17	1	P35467
3582	1,2,5,6,10,<br>14,15,17	3,4,7,8,9,1<br>1,12,13,16	0	
3583	1,2,5,6,10,<br>14,16,17	3,4,7,8,9,1<br>1,12,13,15	0	
3584	1,2,5,6,10,<br>15,16,17	3,4,7,8,9,1<br>1,12,13,14	0	
3585	1,2,5,6,11,<br>12,13,14	3,4,7,8,9,1<br>0,15,16,17	5	P30120,P47727,Q6IFU7,Q9JHB9,Q9Z1F2
3586	1,2,5,6,11,<br>12,13,15	3,4,7,8,9,1<br>0,14,16,17	2	Q6IFU7,Q9JHB9
3587	1,2,5,6,11,<br>12,13,16	3,4,7,8,9,1<br>0,14,15,17	2	Q6IFU7,Q9JHB9
3588	1,2,5,6,11,<br>12,13,17	3,4,7,8,9,1<br>0,14,15,16	3	Q62635,Q9JHB9,Q9QZK9
3589	1,2,5,6,11,<br>12,14,15	3,4,7,8,9,1<br>0,13,16,17	1	Q6IFU7
3590	1,2,5,6,11,<br>12,14,16	3,4,7,8,9,1<br>0,13,15,17	2	Q6IFU7,Q6IFU8
3591	1,2,5,6,11,<br>12,14,17	3,4,7,8,9,1<br>0,13,15,16	4	P13432,P62804,Q62635,Q6IFU8
3592	1,2,5,6,11,<br>12,15,16	3,4,7,8,9,1<br>0,13,14,17	1	Q6IFU7
3593	1,2,5,6,11,<br>12,15,17	3,4,7,8,9,1<br>0,13,14,16	0	
3594	1,2,5,6,11,<br>12,16,17	3,4,7,8,9,1<br>0,13,14,15	1	Q6IFU7
3595	1,2,5,6,11,<br>13,14,15	3,4,7,8,9,1<br>0,12,16,17	0	
3596	1,2,5,6,11,<br>13,14,16	3,4,7,8,9,1<br>0,12,15,17	0	
3597	1,2,5,6,11,<br>13,14,17	3,4,7,8,9,1<br>0,12,15,16	0	
3598	1,2,5,6,11,<br>13,15,16	3,4,7,8,9,1<br>0,12,14,17	0	

3599	1,2,5,6,11,13,15,17	3,4,7,8,9,10,12,14,16	1	Q6IFV1
3600	1,2,5,6,11,13,16,17	3,4,7,8,9,10,12,14,15	1	A2RUV9
3601	1,2,5,6,11,14,15,16	3,4,7,8,9,10,12,13,17	0	
3602	1,2,5,6,11,14,15,17	3,4,7,8,9,10,12,13,16	1	Q9QUL6
3603	1,2,5,6,11,14,16,17	3,4,7,8,9,10,12,13,15	1	Q9QUL6
3604	1,2,5,6,11,15,16,17	3,4,7,8,9,10,12,13,14	1	A2RUV9
3605	1,2,5,6,12,13,14,15	3,4,7,8,9,10,11,16,17	2	P00714,Q9Z1F2
3606	1,2,5,6,12,13,14,16	3,4,7,8,9,10,11,15,17	2	P00714,Q9Z1F2
3607	1,2,5,6,12,13,14,17	3,4,7,8,9,10,11,15,16	5	P00714,Q561R9,Q5FVI6,Q62635,Q9Z1F2
3608	1,2,5,6,12,13,15,16	3,4,7,8,9,10,11,14,17	1	P00714
3609	1,2,5,6,12,13,15,17	3,4,7,8,9,10,11,14,16	3	P00714,Q62635,Q6IFV1
3610	1,2,5,6,12,13,16,17	3,4,7,8,9,10,11,14,15	4	P00714,P35280,Q561R9,Q62635
3611	1,2,5,6,12,14,15,16	3,4,7,8,9,10,11,13,17	2	P00714,P35467
3612	1,2,5,6,12,14,15,17	3,4,7,8,9,10,11,13,16	3	P00714,P35467,Q62635
3613	1,2,5,6,12,14,16,17	3,4,7,8,9,10,11,13,15	4	P00714,P35467,Q10758,Q561R9
3614	1,2,5,6,12,15,16,17	3,4,7,8,9,10,11,13,14	2	P00714,P35467
3615	1,2,5,6,13,14,15,16	3,4,7,8,9,10,11,12,17	2	P00714,P35467
3616	1,2,5,6,13,14,15,17	3,4,7,8,9,10,11,12,16	5	P00714,P08753,P35467,P53792,Q5FVI6
3617	1,2,5,6,13,14,16,17	3,4,7,8,9,10,11,12,15	4	P00714,P08753,Q561R9,Q6PCU2
3618	1,2,5,6,13,15,16,17	3,4,7,8,9,10,11,12,14	1	P00714
3619	1,2,5,6,14,15,16,17	3,4,7,8,9,10,11,12,13	2	P00714,P35467
3620	1,2,5,7,8,9,10,11	3,4,6,12,13,14,15,16,17	6	P10715,P27213,P31044,P52590,P68370,Q9QUL6

3621	1,2,5,7,8,9,10,12	3,4,6,11,13,14,15,16,17	5	P02803,P10715,P27213,P52590,P97840
3622	1,2,5,7,8,9,10,13	3,4,6,11,12,14,15,16,17	4	P01835,P02803,P31044,P52590
3623	1,2,5,7,8,9,10,14	3,4,6,11,12,13,15,16,17	5	P27213,P31044,P52590,Q63942,Q9QUL6
3624	1,2,5,7,8,9,10,15	3,4,6,11,12,13,14,16,17	4	P01835,P27213,P52590,Q63942
3625	1,2,5,7,8,9,10,16	3,4,6,11,12,13,14,15,17	6	B0BNN3,P01835,P02625,P52590,Q63942,Q64093
3626	1,2,5,7,8,9,10,17	3,4,6,11,12,13,14,15,16	3	P02625,P52590,Q9QUL6
3627	1,2,5,7,8,9,11,12	3,4,6,10,13,14,15,16,17	5	P02803,P10715,P27213,P52590,Q8CJ52
3628	1,2,5,7,8,9,11,13	3,4,6,10,12,14,15,16,17	3	P02803,P10715,P52590
3629	1,2,5,7,8,9,11,14	3,4,6,10,12,13,15,16,17	6	P10715,P27213,P52590,Q63942,Q99MH3,Q9QUL6
3630	1,2,5,7,8,9,11,15	3,4,6,10,12,13,14,16,17	5	P10715,P27213,P52590,Q63942,Q9QUL6
3631	1,2,5,7,8,9,11,16	3,4,6,10,12,13,14,15,17	4	A2RUV9,P10715,Q63942,Q9QUL6
3632	1,2,5,7,8,9,11,17	3,4,6,10,12,13,14,15,16	4	P10715,P50115,P52590,Q9QUL6
3633	1,2,5,7,8,9,12,13	3,4,6,10,11,14,15,16,17	4	P02803,P10715,P52590,Q9Z1F2
3634	1,2,5,7,8,9,12,14	3,4,6,10,11,13,15,16,17	3	P10715,P19629,P52590
3635	1,2,5,7,8,9,12,15	3,4,6,10,11,13,14,16,17	2	P10715,P52590

3636	1,2,5,7,8,9,12,16	3,4,6,10,11,13,14,15,17	1	P10715
3637	1,2,5,7,8,9,12,17	3,4,6,10,11,13,14,15,16	5	P10715,P50115,P52590,P55091,Q9QZA6
3638	1,2,5,7,8,9,13,14	3,4,6,10,11,12,15,16,17	4	P00762,P53792,Q63942,Q9Z1F2
3639	1,2,5,7,8,9,13,15	3,4,6,10,11,12,14,16,17	5	P00762,P01835,P52590,P53792,Q63942
3640	1,2,5,7,8,9,13,16	3,4,6,10,11,12,14,15,17	4	B0BNN3,P00762,P01835,Q63942
3641	1,2,5,7,8,9,13,17	3,4,6,10,11,12,14,15,16	3	P00762,P52590,P53792
3642	1,2,5,7,8,9,14,15	3,4,6,10,11,12,13,16,17	7	P00762,P16636,P19629,P52590,Q03191,Q63942,Q9QZK9
3643	1,2,5,7,8,9,14,16	3,4,6,10,11,12,13,15,17	5	P00762,P02625,Q63942,Q99MH3,Q9QZK9
3644	1,2,5,7,8,9,14,17	3,4,6,10,11,12,13,15,16	3	P00762,P52590,P53792
3645	1,2,5,7,8,9,15,16	3,4,6,10,11,12,13,14,17	6	P00762,P01835,P10715,P52590,Q63942,Q9QZK9
3646	1,2,5,7,8,9,15,17	3,4,6,10,11,12,13,14,16	3	P00762,P52590,P53792
3647	1,2,5,7,8,9,16,17	3,4,6,10,11,12,13,14,15	5	P00762,P02625,P10715,P52590,Q63942
3648	1,2,5,7,8,10,11,12	3,4,6,9,13,14,15,16,17	6	P10715,P16391,P27213,P62804,P68370,Q8CJ52
3649	1,2,5,7,8,10,11,13	3,4,6,9,12,14,15,16,17	4	P01835,P31044,P68370,Q5HZV9
3650	1,2,5,7,8,10,11,14	3,4,6,9,12,13,15,16,17	4	P27213,P31044,P68370,Q9QUL6

3651	1,2,5,7,8,10,11,15	3,4,6,9,12,13,14,16,17	2	P01835,P27213
3652	1,2,5,7,8,10,11,16	3,4,6,9,12,13,14,15,17	4	A2RUV9,P01835,P02625,P10715
3653	1,2,5,7,8,10,11,17	3,4,6,9,12,13,14,15,16	5	P10715,P16391,P50115,P52590,Q9QUL6
3654	1,2,5,7,8,10,12,13	3,4,6,9,11,14,15,16,17	2	P02803,Q8CJ52
3655	1,2,5,7,8,10,12,14	3,4,6,9,11,13,15,16,17	0	
3656	1,2,5,7,8,10,12,15	3,4,6,9,11,13,14,16,17	2	P00731,P10715
3657	1,2,5,7,8,10,12,16	3,4,6,9,11,13,14,15,17	4	P02625,P08721,P10715,P97840
3658	1,2,5,7,8,10,12,17	3,4,6,9,11,13,14,15,16	6	P10715,P16391,P50115,P52590,P55091,Q9QZA6
3659	1,2,5,7,8,10,13,14	3,4,6,9,11,12,15,16,17	5	P00762,P01835,P12020,P31044,Q5GRG2
3660	1,2,5,7,8,10,13,15	3,4,6,9,11,12,14,16,17	3	P00762,P01835,Q5GRG2
3661	1,2,5,7,8,10,13,16	3,4,6,9,11,12,14,15,17	4	B0BNN3,P00762,P01835,P02625
3662	1,2,5,7,8,10,13,17	3,4,6,9,11,12,14,15,16	3	P01835,P02625,P52590
3663	1,2,5,7,8,10,14,15	3,4,6,9,11,12,13,16,17	3	P00762,P01835,P16636
3664	1,2,5,7,8,10,14,16	3,4,6,9,11,12,13,15,17	4	P00762,P01835,P02625,P08721
3665	1,2,5,7,8,10,14,17	3,4,6,9,11,12,13,15,16	2	P02625,P52590

3666	1,2,5,7,8,10,15,16	3,4,6,9,11,12,13,14,17	2	P00762,P01835
3667	1,2,5,7,8,10,15,17	3,4,6,9,11,12,13,14,16	2	P01835,P52590
3668	1,2,5,7,8,10,16,17	3,4,6,9,11,12,13,14,15	4	P01835,P02625,P10758,P52590
3669	1,2,5,7,8,11,12,13	3,4,6,9,10,14,15,16,17	5	P02803,P10715,Q8CJ52,Q9JHB9,Q9Z1F2
3670	1,2,5,7,8,11,12,14	3,4,6,9,10,13,15,16,17	5	P10715,P19629,P62804,Q63493,Q8CJ52
3671	1,2,5,7,8,11,12,15	3,4,6,9,10,13,14,16,17	2	P10715,Q8CJ52
3672	1,2,5,7,8,11,12,16	3,4,6,9,10,13,14,15,17	2	P10715,Q8CJ52
3673	1,2,5,7,8,11,12,17	3,4,6,9,10,13,14,15,16	7	P10715,P50115,P52590,P55091,Q63493,Q8CJ52,Q9QZA6
3674	1,2,5,7,8,11,13,14	3,4,6,9,10,12,15,16,17	1	Q5GRG2
3675	1,2,5,7,8,11,13,15	3,4,6,9,10,12,14,16,17	2	P00762,P01835
3676	1,2,5,7,8,11,13,16	3,4,6,9,10,12,14,15,17	2	P01835,P10715
3677	1,2,5,7,8,11,13,17	3,4,6,9,10,12,14,15,16	3	P10715,P50115,P52590
3678	1,2,5,7,8,11,14,15	3,4,6,9,10,12,13,16,17	4	P07171,P16636,P19629,Q03191
3679	1,2,5,7,8,11,14,16	3,4,6,9,10,12,13,15,17	1	Q99MH3
3680	1,2,5,7,8,11,14,17	3,4,6,9,10,12,13,15,16	4	P19629,P50115,P52590,Q9QUL6

3681	1,2,5,7,8,11,15,16	3,4,6,9,10,12,13,14,17	2	P01835,P10715
3682	1,2,5,7,8,11,15,17	3,4,6,9,10,12,13,14,16	4	P10715,P50115,P52590,Q9QZA6
3683	1,2,5,7,8,11,16,17	3,4,6,9,10,12,13,14,15	6	A2RUV9,P10715,P10758,P50115,P50116,P52590
3684	1,2,5,7,8,12,13,14	3,4,6,9,10,11,15,16,17	5	P00762,P19629,Q5GRG2,Q8CJ52,Q9Z1F2
3685	1,2,5,7,8,12,13,15	3,4,6,9,10,11,14,16,17	2	P00762,P10715
3686	1,2,5,7,8,12,13,16	3,4,6,9,10,11,14,15,17	3	P00762,P10715,Q8CJ52
3687	1,2,5,7,8,12,13,17	3,4,6,9,10,11,14,15,16	9	O88267,P10715,P23928,P50115,P52590,P55091,Q62635,Q8CJ52,Q9Z1F2
3688	1,2,5,7,8,12,14,15	3,4,6,9,10,11,13,16,17	5	P00762,P10715,P16636,P19629,Q03191
3689	1,2,5,7,8,12,14,16	3,4,6,9,10,11,13,15,17	4	P10715,P19629,Q63532,Q8CJ52
3690	1,2,5,7,8,12,14,17	3,4,6,9,10,11,13,15,16	8	O88267,P10715,P19629,P31430,P50115,P52590,P55091,Q9QZA6
3691	1,2,5,7,8,12,15,16	3,4,6,9,10,11,13,14,17	3	P00762,P10715,P83121
3692	1,2,5,7,8,12,15,17	3,4,6,9,10,11,13,14,16	5	P10715,P50115,P52590,P55091,Q9QZA6
3693	1,2,5,7,8,12,16,17	3,4,6,9,10,11,13,14,15	9	P02625,P10715,P23928,P50115,P50116,P52590,P55091,P63029,Q9QZA6
3694	1,2,5,7,8,13,14,15	3,4,6,9,10,11,12,16,17	7	P00762,P01835,P06757,P16636,Q03191,Q09030,Q5GRG2
3695	1,2,5,7,8,13,14,16	3,4,6,9,10,11,12,15,17	3	P00762,P01835,P02625

3696	1,2,5,7,8,13,14,17	3,4,6,9,10,11,12,15,16	6	P00762,P08753,P52590,P53792,Q6PCU2,Q9Z1F2
3697	1,2,5,7,8,13,15,16	3,4,6,9,10,11,12,14,17	3	P00762,P01835,Q09030
3698	1,2,5,7,8,13,15,17	3,4,6,9,10,11,12,14,16	4	P00762,P01835,P52590,P53792
3699	1,2,5,7,8,13,16,17	3,4,6,9,10,11,12,14,15	6	P00762,P01835,P02625,P23928,P52590,Q6PCU2
3700	1,2,5,7,8,14,15,16	3,4,6,9,10,11,12,13,17	5	P00762,P01835,P16636,P19629,Q9QZK9
3701	1,2,5,7,8,14,15,17	3,4,6,9,10,11,12,13,16	5	P00762,P10536,P16636,P19629,P52590
3702	1,2,5,7,8,14,16,17	3,4,6,9,10,11,12,13,15	4	P00762,P02625,P52590,Q6PCU2
3703	1,2,5,7,8,15,16,17	3,4,6,9,10,11,12,13,14	3	P00762,P01835,P52590
3704	1,2,5,7,9,10,11,12	3,4,6,8,13,14,15,16,17	6	P02803,P27213,P52590,P97840,Q64093,Q9QUL6
3705	1,2,5,7,9,10,11,13	3,4,6,8,12,14,15,16,17	4	B0BNN3,P02803,P52590,Q9QUL6
3706	1,2,5,7,9,10,11,14	3,4,6,8,12,13,15,16,17	4	B0BNN3,P27213,P52590,Q9QUL6
3707	1,2,5,7,9,10,11,15	3,4,6,8,12,13,14,16,17	3	P27213,P52590,Q9QUL6
3708	1,2,5,7,9,10,11,16	3,4,6,8,12,13,14,15,17	5	B0BNN3,P52590,Q64093,Q80W57,Q9QUL6
3709	1,2,5,7,9,10,11,17	3,4,6,8,12,13,14,15,16	3	P52590,Q64093,Q9QUL6
3710	1,2,5,7,9,10,12,13	3,4,6,8,11,14,15,16,17	4	B0BNN3,P02803,P52590,P97840

3711	1,2,5,7,9,10,12,14	3,4,6,8,11,13,15,16,17	2	P52590,P97840
3712	1,2,5,7,9,10,12,15	3,4,6,8,11,13,14,16,17	2	P52590,P97840
3713	1,2,5,7,9,10,12,16	3,4,6,8,11,13,14,15,17	4	B0BNN3,P52590,P97840,Q64093
3714	1,2,5,7,9,10,12,17	3,4,6,8,11,13,14,15,16	4	P52590,P97840,Q64093,Q9QUL6
3715	1,2,5,7,9,10,13,14	3,4,6,8,11,12,15,16,17	2	B0BNN3,Q4G075
3716	1,2,5,7,9,10,13,15	3,4,6,8,11,12,14,16,17	2	P01835,P52590
3717	1,2,5,7,9,10,13,16	3,4,6,8,11,12,14,15,17	4	B0BNN3,P01835,P02625,P97840
3718	1,2,5,7,9,10,13,17	3,4,6,8,11,12,14,15,16	4	P02625,P14668,P52590,Q9QUL6
3719	1,2,5,7,9,10,14,15	3,4,6,8,11,12,13,16,17	5	P16636,P52590,Q03191,Q9QUL6,Q9QZK9
3720	1,2,5,7,9,10,14,16	3,4,6,8,11,12,13,15,17	4	B0BNN3,P02625,Q9QUL6,Q9QZK9
3721	1,2,5,7,9,10,14,17	3,4,6,8,11,12,13,15,16	3	P02625,P52590,Q9QUL6
3722	1,2,5,7,9,10,15,16	3,4,6,8,11,12,13,14,17	5	P01835,P02782,P52590,Q64093,Q9QZK9
3723	1,2,5,7,9,10,15,17	3,4,6,8,11,12,13,14,16	2	P52590,Q9QUL6
3724	1,2,5,7,9,10,16,17	3,4,6,8,11,12,13,14,15	4	P02625,P52590,Q64093,Q9QUL6
3725	1,2,5,7,9,11,12,13	3,4,6,8,10,14,15,16,17	1	P02803

3726	1,2,5,7,9,11,12,14	3,4,6,8,10,13,15,16,17	0	
3727	1,2,5,7,9,11,12,15	3,4,6,8,10,13,14,16,17	1	P51907
3728	1,2,5,7,9,11,12,16	3,4,6,8,10,13,14,15,17	0	
3729	1,2,5,7,9,11,12,17	3,4,6,8,10,13,14,15,16	2	P52590,Q9QUL6
3730	1,2,5,7,9,11,13,14	3,4,6,8,10,12,15,16,17	1	B0BNN3
3731	1,2,5,7,9,11,13,15	3,4,6,8,10,12,14,16,17	0	
3732	1,2,5,7,9,11,13,16	3,4,6,8,10,12,14,15,17	1	B0BNN3
3733	1,2,5,7,9,11,13,17	3,4,6,8,10,12,14,15,16	2	P52590,Q9QUL6
3734	1,2,5,7,9,11,14,15	3,4,6,8,10,12,13,16,17	5	P16636,Q03191,Q99MH3,Q9QUL6,Q9QZK9
3735	1,2,5,7,9,11,14,16	3,4,6,8,10,12,13,15,17	4	B0BNN3,Q99MH3,Q9QUL6,Q9QZK9
3736	1,2,5,7,9,11,14,17	3,4,6,8,10,12,13,15,16	2	P52590,Q9QUL6
3737	1,2,5,7,9,11,15,16	3,4,6,8,10,12,13,14,17	3	P16636,Q9QUL6,Q9QZK9
3738	1,2,5,7,9,11,15,17	3,4,6,8,10,12,13,14,16	2	P52590,Q9QUL6
3739	1,2,5,7,9,11,16,17	3,4,6,8,10,12,13,14,15	3	A2RUV9,P52590,Q9QUL6
3740	1,2,5,7,9,12,13,14	3,4,6,8,10,11,15,16,17	0	

3741	1,2,5,7,9,12,13,15	3,4,6,8,10,11,14,16,17	0	
3742	1,2,5,7,9,12,13,16	3,4,6,8,10,11,14,15,17	2	B0BNN3,P19218
3743	1,2,5,7,9,12,13,17	3,4,6,8,10,11,14,15,16	4	P14668,P35280,P52590,P55091
3744	1,2,5,7,9,12,14,15	3,4,6,8,10,11,13,16,17	2	P16636,Q03191
3745	1,2,5,7,9,12,14,16	3,4,6,8,10,11,13,15,17	1	Q63532
3746	1,2,5,7,9,12,14,17	3,4,6,8,10,11,13,15,16	1	P52590
3747	1,2,5,7,9,12,15,16	3,4,6,8,10,11,13,14,17	0	
3748	1,2,5,7,9,12,15,17	3,4,6,8,10,11,13,14,16	1	P52590
3749	1,2,5,7,9,12,16,17	3,4,6,8,10,11,13,14,15	1	P52590
3750	1,2,5,7,9,13,14,15	3,4,6,8,10,11,12,16,17	5	P00762,P16636,P53792,Q03191,Q4G075
3751	1,2,5,7,9,13,14,16	3,4,6,8,10,11,12,15,17	4	B0BNN3,P00762,Q4G075,Q9QZK9
3752	1,2,5,7,9,13,14,17	3,4,6,8,10,11,12,15,16	4	P00762,P14668,P52590,P53792
3753	1,2,5,7,9,13,15,16	3,4,6,8,10,11,12,14,17	5	P00762,P01835,P16636,Q4G075,Q9QZK9
3754	1,2,5,7,9,13,15,17	3,4,6,8,10,11,12,14,16	4	P00762,P14668,P52590,P53792
3755	1,2,5,7,9,13,16,17	3,4,6,8,10,11,12,14,15	5	B0BNN3,P00762,P02625,P35280,P52590

3756	1,2,5,7,9,14,15,16	3,4,6,8,10,11,12,13,17	8	P00762,P0C0A9,P16636,P35467,Q03191,Q4G075,Q99MH3,Q9QZK9
3757	1,2,5,7,9,14,15,17	3,4,6,8,10,11,12,13,16	5	P00762,P0C0A9,P16636,P52590,P53792
3758	1,2,5,7,9,14,16,17	3,4,6,8,10,11,12,13,15	3	P00762,P02625,P52590
3759	1,2,5,7,9,15,16,17	3,4,6,8,10,11,12,13,14	5	P00762,P02782,P0C0A9,P47727,P52590
3760	1,2,5,7,10,11,12,13	3,4,6,8,9,14,15,16,17	1	P02803
3761	1,2,5,7,10,11,12,14	3,4,6,8,9,13,15,16,17	1	O70417
3762	1,2,5,7,10,11,12,15	3,4,6,8,9,13,14,16,17	1	P51907
3763	1,2,5,7,10,11,12,16	3,4,6,8,9,13,14,15,17	2	P12368,P97840
3764	1,2,5,7,10,11,12,17	3,4,6,8,9,13,14,15,16	3	P06757,P52590,Q9QUL6
3765	1,2,5,7,10,11,13,14	3,4,6,8,9,12,15,16,17	2	B0BNN3,O70417
3766	1,2,5,7,10,11,13,15	3,4,6,8,9,12,14,16,17	1	P01835
3767	1,2,5,7,10,11,13,16	3,4,6,8,9,12,14,15,17	2	B0BNN3,P01835
3768	1,2,5,7,10,11,13,17	3,4,6,8,9,12,14,15,16	2	P52590,Q9QUL6
3769	1,2,5,7,10,11,14,15	3,4,6,8,9,12,13,16,17	4	O70417,P16636,Q03191,Q9QUL6
3770	1,2,5,7,10,11,14,16	3,4,6,8,9,12,13,15,17	3	B0BNN3,Q63532,Q9QUL6
3771	1,2,5,7,10,11,14,17	3,4,6,8,9,12,13,15,16	2	P52590,Q9QUL6
3772	1,2,5,7,10,11,15,16	3,4,6,8,9,12,13,14,17	2	P01835,Q80W57
3773	1,2,5,7,10,11,15,17	3,4,6,8,9,12,13,14,16	2	P52590,Q9QUL6
3774	1,2,5,7,10,11,16,17	3,4,6,8,9,12,13,14,15	4	P02625,P10758,P52590,Q9QUL6
3775	1,2,5,7,10,12,13,14	3,4,6,8,9,11,15,16,17	0	
3776	1,2,5,7,10,12,13,15	3,4,6,8,9,11,14,16,17	1	P97840

3777	1,2,5,7,10,<br>12,13,16	3,4,6,8,9,1<br>1,14,15,17	3	B0BNN3,P19218,P97840
3778	1,2,5,7,10,<br>12,13,17	3,4,6,8,9,1<br>1,14,15,16	3	P14668,P52590,P55091
3779	1,2,5,7,10,<br>12,14,15	3,4,6,8,9,1<br>1,13,16,17	1	P16636
3780	1,2,5,7,10,<br>12,14,16	3,4,6,8,9,1<br>1,13,15,17	3	P02625,P97840,Q63532
3781	1,2,5,7,10,<br>12,14,17	3,4,6,8,9,1<br>1,13,15,16	1	P52590
3782	1,2,5,7,10,<br>12,15,16	3,4,6,8,9,1<br>1,13,14,17	1	P97840
3783	1,2,5,7,10,<br>12,15,17	3,4,6,8,9,1<br>1,13,14,16	2	P06866,P52590
3784	1,2,5,7,10,<br>12,16,17	3,4,6,8,9,1<br>1,13,14,15	3	P02625,P52590,P97840
3785	1,2,5,7,10,<br>13,14,15	3,4,6,8,9,1<br>1,12,16,17	4	P00762,P01835,P16636,Q09030
3786	1,2,5,7,10,<br>13,14,16	3,4,6,8,9,1<br>1,12,15,17	5	B0BNN3,P00762,P01835,P02625,Q63532
3787	1,2,5,7,10,<br>13,14,17	3,4,6,8,9,1<br>1,12,15,16	3	P02625,P14668,P52590
3788	1,2,5,7,10,<br>13,15,16	3,4,6,8,9,1<br>1,12,14,17	3	P00762,P01835,Q09030
3789	1,2,5,7,10,<br>13,15,17	3,4,6,8,9,1<br>1,12,14,16	3	P01835,P14668,P52590
3790	1,2,5,7,10,<br>13,16,17	3,4,6,8,9,1<br>1,12,14,15	3	P01835,P02625,P52590
3791	1,2,5,7,10,<br>14,15,16	3,4,6,8,9,1<br>1,12,13,17	7	P00762,P01835,P02782,P16636,P35467,Q63532,Q9QZK9
3792	1,2,5,7,10,<br>14,15,17	3,4,6,8,9,1<br>1,12,13,16	3	P16636,P35467,P52590
3793	1,2,5,7,10,<br>14,16,17	3,4,6,8,9,1<br>1,12,13,15	2	P02625,P52590
3794	1,2,5,7,10,<br>15,16,17	3,4,6,8,9,1<br>1,12,13,14	4	P01835,P02782,P0C0A9,P52590
3795	1,2,5,7,11,<br>12,13,14	3,4,6,8,9,1<br>0,15,16,17	1	O70417
3796	1,2,5,7,11,<br>12,13,15	3,4,6,8,9,1<br>0,14,16,17	0	
3797	1,2,5,7,11,<br>12,13,16	3,4,6,8,9,1<br>0,14,15,17	1	P17046
3798	1,2,5,7,11,<br>12,13,17	3,4,6,8,9,1<br>0,14,15,16	1	P55091
3799	1,2,5,7,11,<br>12,14,15	3,4,6,8,9,1<br>0,13,16,17	2	P16636,Q03191

3800	1,2,5,7,11,<br>12,14,16	3,4,6,8,9,1<br>0,13,15,17	1	Q63532
3801	1,2,5,7,11,<br>12,14,17	3,4,6,8,9,1<br>0,13,15,16	0	
3802	1,2,5,7,11,<br>12,15,16	3,4,6,8,9,1<br>0,13,14,17	1	P51907
3803	1,2,5,7,11,<br>12,15,17	3,4,6,8,9,1<br>0,13,14,16	1	P52590
3804	1,2,5,7,11,<br>12,16,17	3,4,6,8,9,1<br>0,13,14,15	1	P17046
3805	1,2,5,7,11,<br>13,14,15	3,4,6,8,9,1<br>0,12,16,17	4	O70417,P16636,Q03191,Q09030
3806	1,2,5,7,11,<br>13,14,16	3,4,6,8,9,1<br>0,12,15,17	2	B0BNN3,Q7TPB1
3807	1,2,5,7,11,<br>13,14,17	3,4,6,8,9,1<br>0,12,15,16	0	
3808	1,2,5,7,11,<br>13,15,16	3,4,6,8,9,1<br>0,12,14,17	3	P01835,P16636,Q09030
3809	1,2,5,7,11,<br>13,15,17	3,4,6,8,9,1<br>0,12,14,16	0	
3810	1,2,5,7,11,<br>13,16,17	3,4,6,8,9,1<br>0,12,14,15	0	
3811	1,2,5,7,11,<br>14,15,16	3,4,6,8,9,1<br>0,12,13,17	5	P16636,Q03191,Q63532,Q99MH3,Q9QZK9
3812	1,2,5,7,11,<br>14,15,17	3,4,6,8,9,1<br>0,12,13,16	2	P16636,P52590
3813	1,2,5,7,11,<br>14,16,17	3,4,6,8,9,1<br>0,12,13,15	1	P10758
3814	1,2,5,7,11,<br>15,16,17	3,4,6,8,9,1<br>0,12,13,14	0	
3815	1,2,5,7,12,<br>13,14,15	3,4,6,8,9,1<br>0,11,16,17	5	P00762,P16636,P35467,Q03191,Q09030
3816	1,2,5,7,12,<br>13,14,16	3,4,6,8,9,1<br>0,11,15,17	2	P00762,Q63532
3817	1,2,5,7,12,<br>13,14,17	3,4,6,8,9,1<br>0,11,15,16	2	P14668,P55091
3818	1,2,5,7,12,<br>13,15,16	3,4,6,8,9,1<br>0,11,14,17	3	P00762,P35467,Q09030
3819	1,2,5,7,12,<br>13,15,17	3,4,6,8,9,1<br>0,11,14,16	2	P14668,P55091
3820	1,2,5,7,12,<br>13,16,17	3,4,6,8,9,1<br>0,11,14,15	1	P35280
3821	1,2,5,7,12,<br>14,15,16	3,4,6,8,9,1<br>0,11,13,17	3	P16636,P35467,Q63532
3822	1,2,5,7,12,<br>14,15,17	3,4,6,8,9,1<br>0,11,13,16	3	P16636,P35467,P52590

3823	1,2,5,7,12,14,16,17	3,4,6,8,9,10,11,13,15	1	P35467
3824	1,2,5,7,12,15,16,17	3,4,6,8,9,10,11,13,14	2	P06866,P35467
3825	1,2,5,7,13,14,15,16	3,4,6,8,9,10,11,12,17	5	P00762,P01835,P16636,P35467,Q09030
3826	1,2,5,7,13,14,15,17	3,4,6,8,9,10,11,12,16	6	P00714,P00762,P14668,P16636,P35467,P53792
3827	1,2,5,7,13,14,16,17	3,4,6,8,9,10,11,12,15	4	P00762,P02625,P35467,Q6PCU2
3828	1,2,5,7,13,15,16,17	3,4,6,8,9,10,11,12,14	5	P00714,P00762,P01835,P0C0A9,P35467
3829	1,2,5,7,14,15,16,17	3,4,6,8,9,10,11,12,13	4	P00762,P0C0A9,P16636,P35467
3830	1,2,5,8,9,10,11,12	3,4,6,7,13,14,15,16,17	3	P02803,P27213,P62804
3831	1,2,5,8,9,10,11,13	3,4,6,7,12,14,15,16,17	2	P02803,Q63942
3832	1,2,5,8,9,10,11,14	3,4,6,7,12,13,15,16,17	3	P27213,Q63942,Q9QUL6
3833	1,2,5,8,9,10,11,15	3,4,6,7,12,13,14,16,17	3	P27213,Q63942,Q9QUL6
3834	1,2,5,8,9,10,11,16	3,4,6,7,12,13,14,15,17	5	A2RUV9,Q63942,Q9QUL6,Q9QZK9,Q9WUW8
3835	1,2,5,8,9,10,11,17	3,4,6,7,12,13,14,15,16	4	A2RUV9,P08649,Q63942,Q9QUL6
3836	1,2,5,8,9,10,12,13	3,4,6,7,11,14,15,16,17	1	P02803
3837	1,2,5,8,9,10,12,14	3,4,6,7,11,13,15,16,17	1	Q63942
3838	1,2,5,8,9,10,12,15	3,4,6,7,11,13,14,16,17	1	Q63942
3839	1,2,5,8,9,10,12,16	3,4,6,7,11,13,14,15,17	4	P02625,P02803,Q63942,Q9WUW8

3840	1,2,5,8,9,10,12,17	3,4,6,7,11,13,14,15,16	1	P02803
3841	1,2,5,8,9,10,13,14	3,4,6,7,11,12,15,16,17	2	P09605;P25809,Q63942
3842	1,2,5,8,9,10,13,15	3,4,6,7,11,12,14,16,17	3	P01835,P09605;P25809,Q63942
3843	1,2,5,8,9,10,13,16	3,4,6,7,11,12,14,15,17	4	P01835,P02625,P02803,Q63942
3844	1,2,5,8,9,10,13,17	3,4,6,7,11,12,14,15,16	2	P02625,Q63942
3845	1,2,5,8,9,10,14,15	3,4,6,7,11,12,13,16,17	3	P09605;P25809,Q63942,Q9QZK9
3846	1,2,5,8,9,10,14,16	3,4,6,7,11,12,13,15,17	3	P02625,Q63942,Q9QZK9
3847	1,2,5,8,9,10,14,17	3,4,6,7,11,12,13,15,16	3	P02625,Q63942,Q9QUL6
3848	1,2,5,8,9,10,15,16	3,4,6,7,11,12,13,14,17	3	P01835,Q63942,Q9QZK9
3849	1,2,5,8,9,10,15,17	3,4,6,7,11,12,13,14,16	1	Q63942
3850	1,2,5,8,9,10,16,17	3,4,6,7,11,12,13,14,15	4	A2RUV9,P02625,P25031,Q63942
3851	1,2,5,8,9,11,12,13	3,4,6,7,10,14,15,16,17	1	P02803
3852	1,2,5,8,9,11,12,14	3,4,6,7,10,13,15,16,17	1	P02803
3853	1,2,5,8,9,11,12,15	3,4,6,7,10,13,14,16,17	0	
3854	1,2,5,8,9,11,12,16	3,4,6,7,10,13,14,15,17	2	P02803,Q63942

3855	1,2,5,8,9,11,12,17	3,4,6,7,10,13,14,15,16	1	P02803
3856	1,2,5,8,9,11,13,14	3,4,6,7,10,12,15,16,17	2	P02803,Q63942
3857	1,2,5,8,9,11,13,15	3,4,6,7,10,12,14,16,17	1	Q63942
3858	1,2,5,8,9,11,13,16	3,4,6,7,10,12,14,15,17	3	A2RUV9,P02803,Q63942
3859	1,2,5,8,9,11,13,17	3,4,6,7,10,12,14,15,16	3	A2RUV9,P02803,Q5FVI6
3860	1,2,5,8,9,11,14,15	3,4,6,7,10,12,13,16,17	4	Q03191,Q63942,Q99MH3,Q9QZK9
3861	1,2,5,8,9,11,14,16	3,4,6,7,10,12,13,15,17	3	Q63942,Q99MH3,Q9QZK9
3862	1,2,5,8,9,11,14,17	3,4,6,7,10,12,13,15,16	3	Q5FVI6,Q63942,Q9QUL6
3863	1,2,5,8,9,11,15,16	3,4,6,7,10,12,13,14,17	3	A2RUV9,Q63942,Q9QZK9
3864	1,2,5,8,9,11,15,17	3,4,6,7,10,12,13,14,16	2	Q63942,Q9QUL6
3865	1,2,5,8,9,11,16,17	3,4,6,7,10,12,13,14,15	3	A2RUV9,Q63942,Q9QUL6
3866	1,2,5,8,9,12,13,14	3,4,6,7,10,11,15,16,17	3	P02803,Q5FVI6,Q9Z1F2
3867	1,2,5,8,9,12,13,15	3,4,6,7,10,11,14,16,17	1	Q5FVI6
3868	1,2,5,8,9,12,13,16	3,4,6,7,10,11,14,15,17	3	P02803,Q5FVI6,Q63942
3869	1,2,5,8,9,12,13,17	3,4,6,7,10,11,14,15,16	4	P02803,P35280,Q5FVI6,Q9Z1F2

3870	1,2,5,8,9,12,14,15	3,4,6,7,10,11,13,16,17	3	Q03191,Q5FVI6,Q63942
3871	1,2,5,8,9,12,14,16	3,4,6,7,10,11,13,15,17	2	Q5FVI6,Q63942
3872	1,2,5,8,9,12,14,17	3,4,6,7,10,11,13,15,16	2	Q5FVI6,Q8VD89
3873	1,2,5,8,9,12,15,16	3,4,6,7,10,11,13,14,17	1	Q63942
3874	1,2,5,8,9,12,15,17	3,4,6,7,10,11,13,14,16	1	Q5FVI6
3875	1,2,5,8,9,12,16,17	3,4,6,7,10,11,13,14,15	2	Q5FVI6,Q63942
3876	1,2,5,8,9,13,14,15	3,4,6,7,10,11,12,16,17	7	P00762,P06757,P09605;P25809,Q03191,Q5FVI6,Q63942,Q9QZK9
3877	1,2,5,8,9,13,14,16	3,4,6,7,10,11,12,15,17	4	P00762,Q5FVI6,Q63942,Q9QZK9
3878	1,2,5,8,9,13,14,17	3,4,6,7,10,11,12,15,16	4	P08753,P53792,Q5FVI6,Q63942
3879	1,2,5,8,9,13,15,16	3,4,6,7,10,11,12,14,17	4	P00762,P01835,Q63942,Q9QZK9
3880	1,2,5,8,9,13,15,17	3,4,6,7,10,11,12,14,16	4	P08753,P53792,Q5FVI6,Q63942
3881	1,2,5,8,9,13,16,17	3,4,6,7,10,11,12,14,15	6	A2RUV9,P02625,P08753,P35280,Q5FVI6,Q63942
3882	1,2,5,8,9,14,15,16	3,4,6,7,10,11,12,13,17	5	P00762,Q03191,Q63942,Q99MH3,Q9QZK9
3883	1,2,5,8,9,14,15,17	3,4,6,7,10,11,12,13,16	3	P47967,Q5FVI6,Q63942
3884	1,2,5,8,9,14,16,17	3,4,6,7,10,11,12,13,15	3	P02625,Q5FVI6,Q63942

3885	1,2,5,8,9,15,16,17	3,4,6,7,10,11,12,13,14	2	P47727,Q63942
3886	1,2,5,8,10,11,12,13	3,4,6,7,9,14,15,16,17	1	P02803
3887	1,2,5,8,10,11,12,14	3,4,6,7,9,13,15,16,17	1	P62804
3888	1,2,5,8,10,11,12,15	3,4,6,7,9,13,14,16,17	2	P00731,P62804
3889	1,2,5,8,10,11,12,16	3,4,6,7,9,13,14,15,17	4	P02803,Q6IFU7,Q6P6Q2,Q9WUW8
3890	1,2,5,8,10,11,12,17	3,4,6,7,9,13,14,15,16	1	P62804
3891	1,2,5,8,10,11,13,14	3,4,6,7,9,12,15,16,17	1	P09605;P25809
3892	1,2,5,8,10,11,13,15	3,4,6,7,9,12,14,16,17	2	P01835,P09605;P25809
3893	1,2,5,8,10,11,13,16	3,4,6,7,9,12,14,15,17	2	A2RUV9,P01835
3894	1,2,5,8,10,11,13,17	3,4,6,7,9,12,14,15,16	0	
3895	1,2,5,8,10,11,14,15	3,4,6,7,9,12,13,16,17	1	P09605;P25809
3896	1,2,5,8,10,11,14,16	3,4,6,7,9,12,13,15,17	2	P02625,P09605;P25809
3897	1,2,5,8,10,11,14,17	3,4,6,7,9,12,13,15,16	1	Q9QUL6
3898	1,2,5,8,10,11,15,16	3,4,6,7,9,12,13,14,17	2	P01835,P09605;P25809
3899	1,2,5,8,10,11,15,17	3,4,6,7,9,12,13,14,16	0	
3900	1,2,5,8,10,11,16,17	3,4,6,7,9,12,13,14,15	3	A2RUV9,P02625,P08649
3901	1,2,5,8,10,12,13,14	3,4,6,7,9,11,15,16,17	2	P09605;P25809,Q9Z1F2
3902	1,2,5,8,10,12,13,15	3,4,6,7,9,11,14,16,17	3	P09605;P25809,P83121,Q6IFV1
3903	1,2,5,8,10,12,13,16	3,4,6,7,9,11,14,15,17	2	P02803,P83121
3904	1,2,5,8,10,12,13,17	3,4,6,7,9,11,14,15,16	3	P02803,P83121,Q6IFV1
3905	1,2,5,8,10,12,14,15	3,4,6,7,9,11,13,16,17	1	P09605;P25809
3906	1,2,5,8,10,12,14,16	3,4,6,7,9,11,13,15,17	1	P02625

3907	1,2,5,8,10,<br>12,14,17	3,4,6,7,9,1<br>1,13,15,16	2	P31430,Q10758
3908	1,2,5,8,10,<br>12,15,16	3,4,6,7,9,1<br>1,13,14,17	1	P83121
3909	1,2,5,8,10,<br>12,15,17	3,4,6,7,9,1<br>1,13,14,16	2	P83121,Q6IFV1
3910	1,2,5,8,10,<br>12,16,17	3,4,6,7,9,1<br>1,13,14,15	3	P02625,P05539,P83121
3911	1,2,5,8,10,<br>13,14,15	3,4,6,7,9,1<br>1,12,16,17	2	P01835,P09605;P25809
3912	1,2,5,8,10,<br>13,14,16	3,4,6,7,9,1<br>1,12,15,17	3	P01835,P02625,P09605;P25809
3913	1,2,5,8,10,<br>13,14,17	3,4,6,7,9,1<br>1,12,15,16	2	P02625,P08753
3914	1,2,5,8,10,<br>13,15,16	3,4,6,7,9,1<br>1,12,14,17	3	P01835,P09605;P25809,P83121
3915	1,2,5,8,10,<br>13,15,17	3,4,6,7,9,1<br>1,12,14,16	3	P01835,P83121,Q6IFV1
3916	1,2,5,8,10,<br>13,16,17	3,4,6,7,9,1<br>1,12,14,15	3	P01835,P02625,P83121
3917	1,2,5,8,10,<br>14,15,16	3,4,6,7,9,1<br>1,12,13,17	3	P01835,P09605;P25809,Q9QZK9
3918	1,2,5,8,10,<br>14,15,17	3,4,6,7,9,1<br>1,12,13,16	0	
3919	1,2,5,8,10,<br>14,16,17	3,4,6,7,9,1<br>1,12,13,15	2	P02625,P05539
3920	1,2,5,8,10,<br>15,16,17	3,4,6,7,9,1<br>1,12,13,14	2	P01835,P83121
3921	1,2,5,8,11,<br>12,13,14	3,4,6,7,9,1<br>0,15,16,17	3	P02803,Q5FVI6,Q9Z1F2
3922	1,2,5,8,11,<br>12,13,15	3,4,6,7,9,1<br>0,14,16,17	0	
3923	1,2,5,8,11,<br>12,13,16	3,4,6,7,9,1<br>0,14,15,17	1	P02803
3924	1,2,5,8,11,<br>12,13,17	3,4,6,7,9,1<br>0,14,15,16	3	P02803,Q5FVI6,Q62635
3925	1,2,5,8,11,<br>12,14,15	3,4,6,7,9,1<br>0,13,16,17	2	P62804,Q03191
3926	1,2,5,8,11,<br>12,14,16	3,4,6,7,9,1<br>0,13,15,17	0	
3927	1,2,5,8,11,<br>12,14,17	3,4,6,7,9,1<br>0,13,15,16	4	P31430,P62804,Q00715,Q5FVI6
3928	1,2,5,8,11,<br>12,15,16	3,4,6,7,9,1<br>0,13,14,17	0	
3929	1,2,5,8,11,<br>12,15,17	3,4,6,7,9,1<br>0,13,14,16	1	Q9QZA6

3930	1,2,5,8,11,<br>12,16,17	3,4,6,7,9,1<br>0,13,14,15	0	
3931	1,2,5,8,11,<br>13,14,15	3,4,6,7,9,1<br>0,12,16,17	2	P09605;P25809,Q03191
3932	1,2,5,8,11,<br>13,14,16	3,4,6,7,9,1<br>0,12,15,17	0	
3933	1,2,5,8,11,<br>13,14,17	3,4,6,7,9,1<br>0,12,15,16	1	Q5FVI6
3934	1,2,5,8,11,<br>13,15,16	3,4,6,7,9,1<br>0,12,14,17	1	P01835
3935	1,2,5,8,11,<br>13,15,17	3,4,6,7,9,1<br>0,12,14,16	0	
3936	1,2,5,8,11,<br>13,16,17	3,4,6,7,9,1<br>0,12,14,15	1	A2RUV9
3937	1,2,5,8,11,<br>14,15,16	3,4,6,7,9,1<br>0,12,13,17	4	P09605;P25809,Q03191,Q99MH3,Q9QZK9
3938	1,2,5,8,11,<br>14,15,17	3,4,6,7,9,1<br>0,12,13,16	2	P47967,P97840
3939	1,2,5,8,11,<br>14,16,17	3,4,6,7,9,1<br>0,12,13,15	0	
3940	1,2,5,8,11,<br>15,16,17	3,4,6,7,9,1<br>0,12,13,14	2	A2RUV9,P05369
3941	1,2,5,8,12,<br>13,14,15	3,4,6,7,9,1<br>0,11,16,17	4	P09605;P25809,Q03191,Q5FVI6,Q9Z1F2
3942	1,2,5,8,12,<br>13,14,16	3,4,6,7,9,1<br>0,11,15,17	2	Q5FVI6,Q9Z1F2
3943	1,2,5,8,12,<br>13,14,17	3,4,6,7,9,1<br>0,11,15,16	4	P31430,Q5FVI6,Q62635,Q9Z1F2
3944	1,2,5,8,12,<br>13,15,16	3,4,6,7,9,1<br>0,11,14,17	1	P83121
3945	1,2,5,8,12,<br>13,15,17	3,4,6,7,9,1<br>0,11,14,16	3	P83121,Q5FVI6,Q6IFV1
3946	1,2,5,8,12,<br>13,16,17	3,4,6,7,9,1<br>0,11,14,15	4	P35280,P83121,Q5FVI6,Q6PCU2
3947	1,2,5,8,12,<br>14,15,16	3,4,6,7,9,1<br>0,11,13,17	0	
3948	1,2,5,8,12,<br>14,15,17	3,4,6,7,9,1<br>0,11,13,16	1	Q5FVI6
3949	1,2,5,8,12,<br>14,16,17	3,4,6,7,9,1<br>0,11,13,15	3	P31430,Q5FVI6,Q6PCU2
3950	1,2,5,8,12,<br>15,16,17	3,4,6,7,9,1<br>0,11,13,14	4	P06866,P83121,Q5FVI6,Q5XFX0
3951	1,2,5,8,13,<br>14,15,16	3,4,6,7,9,1<br>0,11,12,17	6	P00762,P01835,P08753,P09605;P25809,Q5FVI6,Q9QZK9
3952	1,2,5,8,13,<br>14,15,17	3,4,6,7,9,1<br>0,11,12,16	5	P08753,P10536,P47967,Q5FVI6,Q6PCU2

3953	1,2,5,8,13,<br>14,16,17	3,4,6,7,9,1<br>0,11,12,15	4	P02625,P08753,Q5FVI6,Q6PCU2
3954	1,2,5,8,13,<br>15,16,17	3,4,6,7,9,1<br>0,11,12,14	6	P00762,P01835,P08753,P83121,Q5FVI6,Q6PCU2
3955	1,2,5,8,14,<br>15,16,17	3,4,6,7,9,1<br>0,11,12,13	2	Q5FVI6,Q6PCU2
3956	1,2,5,9,10,<br>11,12,13	3,4,6,7,8,1<br>4,15,16,17	3	P02803,P20761,Q63661
3957	1,2,5,9,10,<br>11,12,14	3,4,6,7,8,1<br>3,15,16,17	3	P20761,Q63661,Q9QUL6
3958	1,2,5,9,10,<br>11,12,15	3,4,6,7,8,1<br>3,14,16,17	2	P51907,Q63661
3959	1,2,5,9,10,<br>11,12,16	3,4,6,7,8,1<br>3,14,15,17	7	P02803,P11598,P12368,P20761,Q63661,Q6IFU7,Q9WUW8
3960	1,2,5,9,10,<br>11,12,17	3,4,6,7,8,1<br>3,14,15,16	2	P02803,Q9QUL6
3961	1,2,5,9,10,<br>11,13,14	3,4,6,7,8,1<br>2,15,16,17	8	B0BNN3,O70417,P09605;P25809,P20761,Q4G075,Q63661,Q99041,Q9QUL6
3962	1,2,5,9,10,<br>11,13,15	3,4,6,7,8,1<br>2,14,16,17	4	P09605;P25809,P20761,Q4G075,Q99041
3963	1,2,5,9,10,<br>11,13,16	3,4,6,7,8,1<br>2,14,15,17	8	A2RUV9,B0BNN3,P02803,P11598,P20761,P62815,Q4G075,Q99041
3964	1,2,5,9,10,<br>11,13,17	3,4,6,7,8,1<br>2,14,15,16	2	P02803,Q9QUL6
3965	1,2,5,9,10,<br>11,14,15	3,4,6,7,8,1<br>2,13,16,17	9	P06761,P09605;P25809,P22282,Q03191,Q4G075,Q63661,Q99041,Q9QUL6,Q9QZK9
3966	1,2,5,9,10,<br>11,14,16	3,4,6,7,8,1<br>2,13,15,17	12	B0BNN3,P06761,P11598,P20761,P22282,P62815,Q4G075,Q63661,Q99041,Q99MH3,Q9QUL6,Q9QZK9
3967	1,2,5,9,10,<br>11,14,17	3,4,6,7,8,1<br>2,13,15,16	1	Q9QUL6
3968	1,2,5,9,10,<br>11,15,16	3,4,6,7,8,1<br>2,13,14,17	13	P02782,P06761,P06911,P11598,P20761,P22282,P22283,P62815,Q4G075,Q62902,Q99041,Q9QUL6,Q9QZK9
3969	1,2,5,9,10,<br>11,15,17	3,4,6,7,8,1<br>2,13,14,16	4	P06761,P06911,Q62902,Q9QUL6
3970	1,2,5,9,10,<br>11,16,17	3,4,6,7,8,1<br>2,13,14,15	8	A2RUV9,O08557,P02625,P06761,P06911,P11598,Q62902,Q9QUL6
3971	1,2,5,9,10,<br>12,13,14	3,4,6,7,8,1<br>1,15,16,17	4	P02803,P07150,P20761,Q99041
3972	1,2,5,9,10,<br>12,13,15	3,4,6,7,8,1<br>1,14,16,17	1	P07150
3973	1,2,5,9,10,<br>12,13,16	3,4,6,7,8,1<br>1,14,15,17	6	B0BNN3,P02803,P07150,P14669,P20761,P97840
3974	1,2,5,9,10,<br>12,13,17	3,4,6,7,8,1<br>1,14,15,16	3	P02803,P14668,P35280
3975	1,2,5,9,10,<br>12,14,15	3,4,6,7,8,1<br>1,13,16,17	4	P07150,P09605;P25809,Q03191,Q99041

3976	1,2,5,9,10,<br>12,14,16	3,4,6,7,8,1<br>1,13,15,17	4	P11598,P20761,Q99041,Q9WUW8
3977	1,2,5,9,10,<br>12,14,17	3,4,6,7,8,1<br>1,13,15,16	1	Q8VD89
3978	1,2,5,9,10,<br>12,15,16	3,4,6,7,8,1<br>1,13,14,17	5	P07150,P11598,P20761,P22282,P22283
3979	1,2,5,9,10,<br>12,15,17	3,4,6,7,8,1<br>1,13,14,16	2	P06866,Q62902
3980	1,2,5,9,10,<br>12,16,17	3,4,6,7,8,1<br>1,13,14,15	1	P02625
3981	1,2,5,9,10,<br>13,14,15	3,4,6,7,8,1<br>1,12,16,17	7	P07150,P09605;P25809,P50115,Q4G075,Q63751,Q99041,Q9QZK9
3982	1,2,5,9,10,<br>13,14,16	3,4,6,7,8,1<br>1,12,15,17	12	B0BNN3,P02625,P07150,P09605;P25809,P14669,P20761,P50115,Q4G075,Q63751,Q63942,Q99041,Q9QZK9
3983	1,2,5,9,10,<br>13,14,17	3,4,6,7,8,1<br>1,12,15,16	2	P14668,Q99041
3984	1,2,5,9,10,<br>13,15,16	3,4,6,7,8,1<br>1,12,14,17	13	P01835,P07150,P09605;P25809,P11598,P14669,P20761,P22282,P22283,P50115,Q4G075,Q63942,Q99041,Q9QZK9
3985	1,2,5,9,10,<br>13,15,17	3,4,6,7,8,1<br>1,12,14,16	2	P14668,Q99041
3986	1,2,5,9,10,<br>13,16,17	3,4,6,7,8,1<br>1,12,14,15	2	P02625,P35280
3987	1,2,5,9,10,<br>14,15,16	3,4,6,7,8,1<br>1,12,13,17	18	P02780,P02782,P06761,P07150,P07647,P09605;P25809,P0C0A9,P11598,P20761,P22282,P22283,P50115,Q4G075,Q62902,Q63751,Q639<br>42,Q99041,Q9QZK9
3988	1,2,5,9,10,<br>14,15,17	3,4,6,7,8,1<br>1,12,13,16	5	P22282,P22283,Q62902,Q99041,Q9QUL6
3989	1,2,5,9,10,<br>14,16,17	3,4,6,7,8,1<br>1,12,13,15	8	P02625,P06761,P11598,P22282,P22283,Q62902,Q99041,Q9QUL6
3990	1,2,5,9,10,<br>15,16,17	3,4,6,7,8,1<br>1,12,13,14	15	P02780,P02781,P02782,P06761,P06911,P07647,P0C0A9,P11598,P22282,P22283,P30120,P47727,Q62902,Q99041,Q9JHB9
3991	1,2,5,9,11,<br>12,13,14	3,4,6,7,8,1<br>0,15,16,17	2	P02803,P20761
3992	1,2,5,9,11,<br>12,13,15	3,4,6,7,8,1<br>0,14,16,17	0	
3993	1,2,5,9,11,<br>12,13,16	3,4,6,7,8,1<br>0,14,15,17	2	P02803,P20761
3994	1,2,5,9,11,<br>12,13,17	3,4,6,7,8,1<br>0,14,15,16	2	P02803,P35280
3995	1,2,5,9,11,<br>12,14,15	3,4,6,7,8,1<br>0,13,16,17	1	Q03191
3996	1,2,5,9,11,<br>12,14,16	3,4,6,7,8,1<br>0,13,15,17	2	P20761,Q99MH3
3997	1,2,5,9,11,<br>12,14,17	3,4,6,7,8,1<br>0,13,15,16	0	
3998	1,2,5,9,11,<br>12,15,16	3,4,6,7,8,1<br>0,13,14,17	4	P06911,P11598,P12020,P51907

3999	1,2,5,9,11,12,15,17	3,4,6,7,8,10,13,14,16	2	P06911,P12020
4000	1,2,5,9,11,12,16,17	3,4,6,7,8,10,13,14,15	2	P06911,P12020
4001	1,2,5,9,11,13,14,15	3,4,6,7,8,10,12,16,17	4	Q03191,Q4G075,Q99041,Q9QZK9
4002	1,2,5,9,11,13,14,16	3,4,6,7,8,10,12,15,17	7	B0BNN3,P20761,Q4G075,Q7TPB1,Q99041,Q99MH3,Q9QZK9
4003	1,2,5,9,11,13,14,17	3,4,6,7,8,10,12,15,16	1	Q5FVI6
4004	1,2,5,9,11,13,15,16	3,4,6,7,8,10,12,14,17	5	P11598,P20761,Q4G075,Q99041,Q9QZK9
4005	1,2,5,9,11,13,15,17	3,4,6,7,8,10,12,14,16	0	
4006	1,2,5,9,11,13,16,17	3,4,6,7,8,10,12,14,15	2	A2RUV9,P35280
4007	1,2,5,9,11,14,15,16	3,4,6,7,8,10,12,13,17	11	P06761,P11598,P20761,P22282,P22283,Q03191,Q4G075,Q63942,Q99041,Q99MH3,Q9QZK9
4008	1,2,5,9,11,14,15,17	3,4,6,7,8,10,12,13,16	3	P47967,Q9QUL6,Q9R0T3
4009	1,2,5,9,11,14,16,17	3,4,6,7,8,10,12,13,15	2	Q99MH3,Q9QUL6
4010	1,2,5,9,11,15,16,17	3,4,6,7,8,10,12,13,14	11	A2RUV9,P02780,P06761,P06911,P11598,P12020,P22282,P22283,Q62902,Q6AYR9,Q9QUL6
4011	1,2,5,9,12,13,14,15	3,4,6,7,8,10,11,16,17	5	P00714,P07150,Q03191,Q5FVI6,Q99041
4012	1,2,5,9,12,13,14,16	3,4,6,7,8,10,11,15,17	7	P00714,P07150,P14669,P20761,Q4G075,Q5FVI6,Q99041
4013	1,2,5,9,12,13,14,17	3,4,6,7,8,10,11,15,16	5	P00714,P14668,P35280,Q5FVI6,Q8VD89
4014	1,2,5,9,12,13,15,16	3,4,6,7,8,10,11,14,17	3	P00714,P07150,P14669
4015	1,2,5,9,12,13,15,17	3,4,6,7,8,10,11,14,16	5	P00714,P07150,P14668,P35280,Q5FVI6
4016	1,2,5,9,12,13,16,17	3,4,6,7,8,10,11,14,15	5	P00714,P07171,P22006,P35280,Q5FVI6
4017	1,2,5,9,12,14,15,16	3,4,6,7,8,10,11,13,17	7	P07150,P11598,P14669,P22282,P22283,Q03191,Q99041
4018	1,2,5,9,12,14,15,17	3,4,6,7,8,10,11,13,16	3	P00714,Q5FVI6,Q8VD89
4019	1,2,5,9,12,14,16,17	3,4,6,7,8,10,11,13,15	3	P00714,P35280,Q5FVI6
4020	1,2,5,9,12,15,16,17	3,4,6,7,8,10,11,13,14	5	P00714,P06866,P06911,P12020,P47727
4021	1,2,5,9,13,14,15,16	3,4,6,7,8,10,11,12,17	16	P00714,P00762,P07150,P0C0A9,P14669,P20761,P22282,P22283,P50115,Q03191,Q4G075,Q5FVI6,Q63751,Q63942,Q99041,Q9QZK9

4022	1,2,5,9,13,<br>14,15,17	3,4,6,7,8,1<br>0,11,12,16	6	P00714,P07150,P47967,P53792,Q5FVI6,Q99041
4023	1,2,5,9,13,<br>14,16,17	3,4,6,7,8,1<br>0,11,12,15	6	P00714,P02625,P14669,P35280,Q5FVI6,Q99041
4024	1,2,5,9,13,<br>15,16,17	3,4,6,7,8,1<br>0,11,12,14	6	P00714,P0C0A9,P22283,P35280,P47727,Q5FVI6
4025	1,2,5,9,14,<br>15,16,17	3,4,6,7,8,1<br>0,11,12,13	15	P00714,P02780,P02781,P06761,P07647,P0C0A9,P11598,P22282,P22283,P30120,Q5FVI6,Q62902,Q63942,Q99041,Q9JHB9
4026	1,2,5,10,1<br>1,12,13,14	3,4,6,7,8,9<br>,15,16,17	3	O70417,P09605;P25809,P20761
4027	1,2,5,10,1<br>1,12,13,15	3,4,6,7,8,9<br>,14,16,17	1	P09605;P25809
4028	1,2,5,10,1<br>1,12,13,16	3,4,6,7,8,9<br>,14,15,17	3	P02803,P20761,Q6IFU7
4029	1,2,5,10,1<br>1,12,13,17	3,4,6,7,8,9<br>,14,15,16	1	P02803
4030	1,2,5,10,1<br>1,12,14,15	3,4,6,7,8,9<br>,13,16,17	4	O70417,P09605;P25809,P51907,Q03191
4031	1,2,5,10,1<br>1,12,14,16	3,4,6,7,8,9<br>,13,15,17	2	P20761,Q6IFU7
4032	1,2,5,10,1<br>1,12,14,17	3,4,6,7,8,9<br>,13,15,16	1	P62804
4033	1,2,5,10,1<br>1,12,15,16	3,4,6,7,8,9<br>,13,14,17	4	P11598,P22282,P51907,Q6IFU7
4034	1,2,5,10,1<br>1,12,15,17	3,4,6,7,8,9<br>,13,14,16	1	P06866
4035	1,2,5,10,1<br>1,12,16,17	3,4,6,7,8,9<br>,13,14,15	3	P05369,P06757,P06866
4036	1,2,5,10,1<br>1,13,14,15	3,4,6,7,8,9<br>,12,16,17	4	O70417,P09605;P25809,Q4G075,Q99041
4037	1,2,5,10,1<br>1,13,14,16	3,4,6,7,8,9<br>,12,15,17	7	B0BNN3,O70417,P09605;P25809,P20761,Q4G075,Q7TPB1,Q99041
4038	1,2,5,10,1<br>1,13,14,17	3,4,6,7,8,9<br>,12,15,16	1	O70417
4039	1,2,5,10,1<br>1,13,15,16	3,4,6,7,8,9<br>,12,14,17	5	P01835,P09605;P25809,P20761,P22282,Q99041
4040	1,2,5,10,1<br>1,13,15,17	3,4,6,7,8,9<br>,12,14,16	0	
4041	1,2,5,10,1<br>1,13,16,17	3,4,6,7,8,9<br>,12,14,15	2	A2RUV9,P02625
4042	1,2,5,10,1<br>1,14,15,16	3,4,6,7,8,9<br>,12,13,17	8	P06761,P09605;P25809,P11598,P20761,P22282,P22283,Q99041,Q9QZK9
4043	1,2,5,10,1<br>1,14,15,17	3,4,6,7,8,9<br>,12,13,16	2	Q62902,Q9QUL6
4044	1,2,5,10,1<br>1,14,16,17	3,4,6,7,8,9<br>,12,13,15	2	P02625,Q9QUL6

4045	1,2,5,10,11,15,16,17 3,4,6,7,8,9,12,13,14	9	P02780,P02782,P05369,P06761,P06911,P11598,P22282,P22283,Q62902
4046	1,2,5,10,12,13,14,15 3,4,6,7,8,9,11,16,17	2	P07150,P09605;P25809
4047	1,2,5,10,12,13,14,16 3,4,6,7,8,9,11,15,17	2	P09605;P25809,P20761
4048	1,2,5,10,12,13,14,17 3,4,6,7,8,9,11,15,16	1	P14668
4049	1,2,5,10,12,13,15,16 3,4,6,7,8,9,11,14,17	3	P09605;P25809,P83121,Q63617
4050	1,2,5,10,12,13,15,17 3,4,6,7,8,9,11,14,16	3	P14668,P83121,Q63617
4051	1,2,5,10,12,13,16,17 3,4,6,7,8,9,11,14,15	3	P02625,P35280,P83121
4052	1,2,5,10,12,14,15,16 3,4,6,7,8,9,11,13,17	5	P09605;P25809,P22282,P22283,Q63617,Q99041
4053	1,2,5,10,12,14,15,17 3,4,6,7,8,9,11,13,16	1	Q63617
4054	1,2,5,10,12,14,16,17 3,4,6,7,8,9,11,13,15	1	P02625
4055	1,2,5,10,12,15,16,17 3,4,6,7,8,9,11,13,14	4	P06866,P83121,Q62902,Q63617
4056	1,2,5,10,13,14,15,16 3,4,6,7,8,9,11,12,17	10	P01835,P09605;P25809,P19223,P20761,P22282,P22283,P50115,Q4G075,Q99041,Q9QZK9
4057	1,2,5,10,13,14,15,17 3,4,6,7,8,9,11,12,16	2	P14668,Q99041
4058	1,2,5,10,13,14,16,17 3,4,6,7,8,9,11,12,15	2	P02625,Q99041
4059	1,2,5,10,13,15,16,17 3,4,6,7,8,9,11,12,14	5	P01835,P02780,P0C0A9,P22283,P83121
4060	1,2,5,10,14,15,16,17 3,4,6,7,8,9,11,12,13	11	P02780,P02781,P02782,P06761,P07647,P0C0A9,P22282,P22283,P30120,Q62902,Q99041
4061	1,2,5,11,12,13,14,15 3,4,6,7,8,9,10,16,17	3	O70417,Q03191,Q6P6R2
4062	1,2,5,11,12,13,14,16 3,4,6,7,8,9,10,15,17	1	P20761
4063	1,2,5,11,12,13,14,17 3,4,6,7,8,9,10,15,16	1	Q5FVI6
4064	1,2,5,11,12,13,15,16 3,4,6,7,8,9,10,14,17	0	
4065	1,2,5,11,12,13,15,17 3,4,6,7,8,9,10,14,16	0	
4066	1,2,5,11,12,13,16,17 3,4,6,7,8,9,10,14,15	1	P35280
4067	1,2,5,11,12,14,15,16 3,4,6,7,8,9,10,13,17	2	P51907,Q03191

4068	1,2,5,11,12,14,15,17	3,4,6,7,8,9,10,13,16	0	
4069	1,2,5,11,12,14,16,17	3,4,6,7,8,9,10,13,15	0	
4070	1,2,5,11,12,15,16,17	3,4,6,7,8,9,10,13,14	4	P05369,P06866,P06911,P12020
4071	1,2,5,11,13,14,15,16	3,4,6,7,8,9,10,12,17	6	P09605;P25809,P19223,Q03191,Q4G075,Q99041,Q9QZK9
4072	1,2,5,11,13,14,15,17	3,4,6,7,8,9,10,12,16	3	P00714,P47967,Q5FVI6
4073	1,2,5,11,13,14,16,17	3,4,6,7,8,9,10,12,15	0	
4074	1,2,5,11,13,15,16,17	3,4,6,7,8,9,10,12,14	1	P00714
4075	1,2,5,11,14,15,16,17	3,4,6,7,8,9,10,12,13	4	P02780,P06761,P22282,P22283
4076	1,2,5,12,13,14,15,16	3,4,6,7,8,9,10,11,17	5	P00714,P07150,P14669,P35467,Q5FVI6
4077	1,2,5,12,13,14,15,17	3,4,6,7,8,9,10,11,16	3	P00714,P14668,Q5FVI6
4078	1,2,5,12,13,14,16,17	3,4,6,7,8,9,10,11,15	3	P00714,P35280,Q5FVI6
4079	1,2,5,12,13,15,16,17	3,4,6,7,8,9,10,11,14	5	P00714,P06866,P35280,P83121,Q5FVI6
4080	1,2,5,12,14,15,16,17	3,4,6,7,8,9,10,11,13	4	P00714,P06866,P35467,Q5FVI6
4081	1,2,5,13,14,15,16,17	3,4,6,7,8,9,10,11,12	7	P00714,P08753,P0C0A9,P22283,P23785,P35467,Q5FVI6
4082	1,2,6,7,8,9,10,11	3,4,5,12,13,14,15,16,17	14	P02631,P10715,P14669,P15399,P19629,P27213,P31044,P35280,P68370,Q63493,Q6AYL6,Q91ZS3,Q9JI85,Q9QUL6
4083	1,2,6,7,8,9,10,12	3,4,5,11,13,14,15,16,17	12	P02782,P08723,P10715,P19629,P30120,P31044,P68370,P97840,Q8CJ52,Q91ZS3,Q9JHB9,Q9JI85
4084	1,2,6,7,8,9,10,13	3,4,5,11,12,14,15,16,17	7	P06911,P12020,P31044,P68370,P97840,Q5GRG2,Q91ZS3
4085	1,2,6,7,8,9,10,14	3,4,5,11,12,13,15,16,17	7	P02631,P12020,P19629,P31044,P68370,Q5GRG2,Q63942
4086	1,2,6,7,8,9,10,15	3,4,5,11,12,13,14,16,17	7	P02631,P19629,P31044,P35280,P68370,P97840,Q63942
4087	1,2,6,7,8,9,10,16	3,4,5,11,12,13,14,15,17	6	P19629,P31044,P68370,P97840,Q63942,Q6P6R2

4088	1,2,6,7,8,9,10,17	3,4,5,11,12,13,14,15,16	5	P19629,P31044,P68370,Q6P6R2,Q9QUL6
4089	1,2,6,7,8,9,11,12	3,4,5,10,13,14,15,16,17	22	O54715,P02631,P02781,P02782,P07647,P08723,P09456,P10715,P13432,P14669,P19629,P22283,P30120,P31044,P68370,Q63357,Q63493,Q6AYL6,Q8CJ52,Q91ZS3,Q9JHB9,Q9JI85
4090	1,2,6,7,8,9,11,13	3,4,5,10,12,14,15,16,17	13	P02631,P06911,P10715,P14669,P19629,P22283,P31044,P68370,Q5GRG2,Q62902,Q91ZS3,Q9JHB9,Q9JI85
4091	1,2,6,7,8,9,11,14	3,4,5,10,12,13,15,16,17	12	P02631,P10715,P14669,P15399,P19629,P31044,P47727,P68370,Q63493,Q91ZS3,Q9JI85,Q9QUL6
4092	1,2,6,7,8,9,11,15	3,4,5,10,12,13,14,16,17	10	P02631,P10715,P14669,P19629,P27213,P31044,P35280,P68370,Q5QE79,Q9JI85
4093	1,2,6,7,8,9,11,16	3,4,5,10,12,13,14,15,17	9	A2RUV9,P02631,P10715,P19629,P31044,P68370,Q63942,Q91ZS3,Q9JI85
4094	1,2,6,7,8,9,11,17	3,4,5,10,12,13,14,15,16	12	P02631,P10715,P14669,P15399,P19629,P31044,P50115,P68370,Q30KJ2,Q63493,Q9JI85,Q9QUL6
4095	1,2,6,7,8,9,12,13	3,4,5,10,11,14,15,16,17	24	P02782,P02803,P06761,P06911,P07647,P08723,P09456,P10715,P12020,P13432,P19629,P22282,P22283,P30120,P31044,P68370,P97840,Q5GRG2,Q62902,Q6AYR9,Q8CJ52,Q9JHB9,Q9JI85,Q9Z1F2
4096	1,2,6,7,8,9,12,14	3,4,5,10,11,13,15,16,17	13	P02631,P02782,P08723,P10715,P13432,P19629,P30120,P31044,P68370,Q8CJ52,Q9JHB9,Q9JI85,Q9Z1F2
4097	1,2,6,7,8,9,12,15	3,4,5,10,11,13,14,16,17	9	P02631,P08723,P10715,P13432,P19629,P31044,P68370,Q5QE79,Q9JI85
4098	1,2,6,7,8,9,12,16	3,4,5,10,11,13,14,15,17	9	P08723,P10715,P13432,P19629,P31044,P68370,P97840,Q8CJ52,Q9JI85
4099	1,2,6,7,8,9,12,17	3,4,5,10,11,13,14,15,16	18	P02782,P08723,P09605;P25809,P10715,P13432,P19223,P19629,P22282,P22283,P30120,P31044,P50115,P55091,P98089,Q62635,Q6P6R2,Q9JHB9,Q9JI85
4100	1,2,6,7,8,9,13,14	3,4,5,10,11,12,15,16,17	12	P02631,P06911,P12020,P19629,P31044,P53792,P68370,Q5GRG2,Q62902,Q63942,Q9JI85,Q9Z1F2
4101	1,2,6,7,8,9,13,15	3,4,5,10,11,12,14,16,17	10	P02631,P06911,P15999,P19629,P31044,P53792,P68370,Q5GRG2,Q5QE79,Q63942
4102	1,2,6,7,8,9,13,16	3,4,5,10,11,12,14,15,17	6	P06911,P19629,P31044,P68370,Q5GRG2,Q63942

4103	1,2,6,7,8,9,13,17	3,4,5,10,11,12,14,15,16	8	P06911,P19629,P22282,P31044,P51907,P53792,Q5GRG2,Q9Z1F2
4104	1,2,6,7,8,9,14,15	3,4,5,10,11,12,13,16,17	6	P02631,P19629,P31044,P68370,Q5GRG2,Q63942
4105	1,2,6,7,8,9,14,16	3,4,5,10,11,12,13,15,17	6	P02631,P19629,P31044,P68370,Q63532,Q63942
4106	1,2,6,7,8,9,14,17	3,4,5,10,11,12,13,15,16	5	P02631,P19629,P31044,P53792,Q6P6R2
4107	1,2,6,7,8,9,15,16	3,4,5,10,11,12,13,14,17	6	P02631,P10715,P19629,P31044,Q5QE79,Q63942
4108	1,2,6,7,8,9,15,17	3,4,5,10,11,12,13,14,16	6	P02631,P10715,P19629,P31044,P53792,Q5QE79
4109	1,2,6,7,8,9,16,17	3,4,5,10,11,12,13,14,15	5	P10715,P19629,P31044,Q63942,Q6P6R2
4110	1,2,6,7,8,10,11,12	3,4,5,9,13,14,15,16,17	31	O35547,O54715,P02781,P02782,P05964,P07647,P08723,P0C0A9,P10715,P12020,P14669,P15399,P19629,P22283,P24368,P30120,P31044,P35280,P47727,P47967,P60905,P62804,P68370,P97840,Q00715,Q63357,Q63493,Q6AYL6,Q8CJ52,Q9JHB9,Q9JI85
4111	1,2,6,7,8,10,11,13	3,4,5,9,12,14,15,16,17	15	P01835,P06911,P12020,P14669,P15399,P19629,P22283,P31044,P47727,P68370,Q5GRG2,Q62902,Q63493,Q9JHB9,Q9JI85
4112	1,2,6,7,8,10,11,14	3,4,5,9,12,13,15,16,17	14	P02631,P12020,P14669,P15399,P19629,P31044,P35280,P47727,P68370,Q5GRG2,Q63493,Q63532,Q6AYL6,Q9JI85
4113	1,2,6,7,8,10,11,15	3,4,5,9,12,13,14,16,17	11	P01835,P02631,P10715,P14669,P15399,P19629,P22006,P31044,P35280,P68370,Q9JI85
4114	1,2,6,7,8,10,11,16	3,4,5,9,12,13,14,15,17	11	P01835,P10715,P14669,P15399,P19629,P31044,P35280,P47967,P68370,Q6AYL6,Q9JI85
4115	1,2,6,7,8,10,11,17	3,4,5,9,12,13,14,15,16	14	P07150,P10715,P14669,P15399,P19629,P31044,P50115,P68370,P97580,Q30KJ2,Q63493,Q6P6R2,Q9JI85,Q9QUL6
4116	1,2,6,7,8,10,12,13	3,4,5,9,11,14,15,16,17	23	P02782,P06761,P06911,P07647,P08723,P09456,P11598,P12020,P19629,P22282,P22283,P30120,P31044,P47967,P68370,P97840,Q5GRG2,Q62902,Q6AYR9,Q8CJ52,Q9JHB9,Q9JI85,Q9Z1F2
4117	1,2,6,7,8,10,12,14	3,4,5,9,11,13,15,16,17	16	P02782,P08723,P12020,P19629,P22283,P30120,P31044,P35280,P68370,P97840,Q5GRG2,Q63532,Q6AYR9,Q8CJ52,Q9JHB9,Q9JI85

4118	1,2,6,7,8,10,12,15	3,4,5,9,11,13,14,16,17	9	P02631,P08723,P10715,P19629,P31044,P35280,P68370,P97840,Q9JI85
4119	1,2,6,7,8,10,12,16	3,4,5,9,11,13,14,15,17	10	P08723,P10715,P19629,P30120,P31044,P47967,P68370,P97840,Q8CJ52,Q9JI85
4120	1,2,6,7,8,10,12,17	3,4,5,9,11,13,14,15,16	25	O54728,P02782,P08723,P10715,P11598,P13432,P19223,P19629,P22282,P22283,P30120,P31044,P50115,P55091,P68370,P97840,P98089,Q4G075,Q62635,Q6AYR9,Q6P6R2,Q8CJ52,Q9JHB9,Q9JI85,Q9QZK9
4121	1,2,6,7,8,10,13,14	3,4,5,9,11,12,15,16,17	8	P06911,P11598,P12020,P19629,P31044,P68370,Q5GRG2,Q62902
4122	1,2,6,7,8,10,13,15	3,4,5,9,11,12,14,16,17	9	P01835,P06911,P12020,P15999,P19629,P31044,P68370,Q5GRG2,Q6IFV1
4123	1,2,6,7,8,10,13,16	3,4,5,9,11,12,14,15,17	7	P01835,P06911,P12020,P31044,P68370,P97840,Q5GRG2
4124	1,2,6,7,8,10,13,17	3,4,5,9,11,12,14,15,16	11	P06911,P11598,P12020,P19629,P22282,P22283,P31044,P51907,P68370,Q5GRG2,Q6IFV1
4125	1,2,6,7,8,10,14,15	3,4,5,9,11,12,13,16,17	8	P02631,P12020,P19629,P31044,P35280,P68370,Q5GRG2,Q63532
4126	1,2,6,7,8,10,14,16	3,4,5,9,11,12,13,15,17	7	P12020,P19629,P31044,P68370,P97840,Q5GRG2,Q63532
4127	1,2,6,7,8,10,14,17	3,4,5,9,11,12,13,15,16	6	P12020,P19629,P31044,P68370,Q5GRG2,Q6P6R2
4128	1,2,6,7,8,10,15,16	3,4,5,9,11,12,13,14,17	7	P01835,P02631,P19629,P31044,P35280,P68370,P97840
4129	1,2,6,7,8,10,15,17	3,4,5,9,11,12,13,14,16	5	P02631,P19629,P31044,P68370,Q6IFV1
4130	1,2,6,7,8,10,16,17	3,4,5,9,11,12,13,14,15	5	P10758,P19629,P31044,P68370,Q6P6R2
4131	1,2,6,7,8,11,12,13	3,4,5,9,10,14,15,16,17	33	P02780,P02781,P02782,P04905,P06761,P06911,P07647,P08723,P09456,P0C0A9,P10715,P11598,P12020,P13432,P14669,P19629,P22282,P22283,P24368,P30120,P31044,P36374,P47727,P68370,Q5GRG2,Q62902,Q63357,Q63493,Q6AYR9,Q8CJ52,Q9JHB9,Q9JI85,Q9Z1F2
4132	1,2,6,7,8,11,12,14	3,4,5,9,10,13,15,16,17	30	P02631,P02781,P02782,P04905,P07647,P08723,P10715,P12020,P13432,P14669,P15399,P19629,P22282,P22283,P30120,P31044,P35280,P47727,P62804,P68370,Q00715,Q5GRG2,Q62902,Q63493,Q63532,Q6AYL6,Q8CJ52,Q9EQS0,Q9JHB9,Q9JI85

4133	1,2,6,7,8,11,12,15	3,4,5,9,10,13,14,16,17	16	P02631,P02782,P08723,P10715,P13432,P14669,P19629,P30120,P31044,P35280,P68370,Q63493,Q6AYL6,Q8CJ52,Q9JHB9,Q9JI85
4134	1,2,6,7,8,11,12,16	3,4,5,9,10,13,14,15,17	15	O54715,P02631,P02782,P08723,P10715,P13432,P19629,P30120,P31044,P68370,Q63493,Q6AYL6,Q8CJ52,Q9JHB9,Q9JI85
4135	1,2,6,7,8,11,12,17	3,4,5,9,10,13,14,15,16	31	O54728,P02631,P02782,P07647,P08723,P10715,P11598,P13432,P14669,P15399,P19629,P22282,P22283,P30120,P31044,P50115,P55091,P62804,P68370,P98089,Q00715,Q30KJ2,Q4G075,Q62635,Q63493,Q6AYL6,Q8CJ52,Q9JHB9,Q9JI85,Q9QZA6,Q9QZK9
4136	1,2,6,7,8,11,13,14	3,4,5,9,10,12,15,16,17	20	P02631,P02782,P06911,P11598,P12020,P14669,P15399,P19629,P22282,P22283,P31044,P47727,P68370,Q5GRG2,Q62902,Q63493,Q8CJ52,Q9JHB9,Q9JI85,Q9Z1F2
4137	1,2,6,7,8,11,13,15	3,4,5,9,10,12,14,16,17	11	P01835,P02631,P06911,P10715,P14669,P15999,P19629,P31044,P68370,Q5GRG2,Q9JI85
4138	1,2,6,7,8,11,13,16	3,4,5,9,10,12,14,15,17	10	P01835,P06911,P10715,P19629,P31044,P68370,Q5GRG2,Q62902,Q8CJ52,Q9JI85
4139	1,2,6,7,8,11,13,17	3,4,5,9,10,12,14,15,16	19	P06911,P10715,P11598,P12020,P14669,P15399,P19629,P22282,P22283,P31044,P50115,P55091,P68370,Q30KJ2,Q5GRG2,Q62902,Q63493,Q9JHB9,Q9JI85
4140	1,2,6,7,8,11,14,15	3,4,5,9,10,12,13,16,17	10	P02631,P14669,P19629,P22006,P31044,P35280,P47727,P68370,Q5GRG2,Q9JI85
4141	1,2,6,7,8,11,14,16	3,4,5,9,10,12,13,15,17	9	P02631,P10715,P19629,P31044,P47727,P68370,Q63493,Q63532,Q9JI85
4142	1,2,6,7,8,11,14,17	3,4,5,9,10,12,13,15,16	12	P02631,P14669,P15399,P19629,P31044,P47727,P50115,P68370,P97580,Q30KJ2,Q63493,Q9JI85
4143	1,2,6,7,8,11,15,16	3,4,5,9,10,12,13,14,17	7	P01835,P02631,P10715,P19629,P31044,P35280,P68370
4144	1,2,6,7,8,11,15,17	3,4,5,9,10,12,13,14,16	10	P02631,P10715,P14669,P15399,P19629,P31044,P50115,P68370,Q30KJ2,Q9JI85
4145	1,2,6,7,8,11,16,17	3,4,5,9,10,12,13,14,15	13	A2RUV9,P02631,P07150,P10715,P10758,P14669,P15399,P19629,P31044,P50115,P68370,Q30KJ2,Q63493
4146	1,2,6,7,8,12,13,14	3,4,5,9,10,11,15,16,17	23	O08557,P02782,P06761,P06911,P07647,P08723,P11598,P12020,P13432,P19629,P22282,P22283,P29975,P30120,P31044,P68370,Q5GRG2,Q62902,Q6AYR9,Q8CJ52,Q9JHB9,Q9JI85,Q9Z1F2
4147	1,2,6,7,8,12,13,15	3,4,5,9,10,11,14,16,17	20	O08557,P02631,P02782,P06911,P08723,P10715,P11598,P12020,P15999,P19629,P30120,P31044,P68370,Q5GRG2,Q62902,Q6IFV1,Q8CJ52,Q9JHB9,Q9JI85,Q9Z1F2

4148	1,2,6,7,8,12,13,16	3,4,5,9,10,11,14,15,17	17	P02782,P06911,P08723,P10715,P12020,P13432,P19629,P30120,P31044,P68370,P97840,Q5GRG2,Q62902,Q8CJ52,Q9JHB9,Q9JI85,Q9Z1F2
4149	1,2,6,7,8,12,13,17	3,4,5,9,10,11,14,15,16	33	O54728,O88267,P02782,P06761,P06911,P07647,P08723,P10715,P11598,P12020,P13432,P19629,P22282,P22283,P30120,P31044,P50115,P55091,P63029,P68370,P70545,P98089,Q4G075,Q5GRG2,Q62635,Q62902,Q6AYR9,Q6IFV1,Q8CJ52,Q9JHB9,Q9JI85,Q9QZK9,Q9Z1F2
4150	1,2,6,7,8,12,14,15	3,4,5,9,10,11,13,16,17	15	O08557,P02631,P02782,P08723,P10715,P13432,P19629,P30120,P31044,P35280,P68370,Q5GRG2,Q63532,Q8CJ52,Q9JI85
4151	1,2,6,7,8,12,14,16	3,4,5,9,10,11,13,15,17	13	P02631,P02782,P08723,P10715,P12020,P13432,P19629,P30120,P31044,P68370,Q63532,Q8CJ52,Q9JI85
4152	1,2,6,7,8,12,14,17	3,4,5,9,10,11,13,15,16	27	O54728,O88267,P02631,P02782,P08723,P10715,P11598,P12020,P13432,P19629,P22282,P22283,P30120,P31044,P31430,P50115,P55091,P68370,P98089,Q62635,Q63493,Q6AYR9,Q8CJ52,Q9JHB9,Q9JI85,Q9QZA6,Q9Z1F2
4153	1,2,6,7,8,12,15,16	3,4,5,9,10,11,13,14,17	6	P02631,P10715,P19629,P31044,P68370,Q8CJ52
4154	1,2,6,7,8,12,15,17	3,4,5,9,10,11,13,14,16	15	P02631,P08723,P10715,P11598,P13432,P19629,P31044,P50115,P55091,P98089,Q4G075,Q62635,Q6IFV1,Q9JI85,Q9QZA6
4155	1,2,6,7,8,12,16,17	3,4,5,9,10,11,13,14,15	13	P08723,P10715,P13432,P19629,P31044,P50115,P55091,P63029,P98089,Q4G075,Q62635,Q6P6R2,Q8CJ52
4156	1,2,6,7,8,13,14,15	3,4,5,9,10,11,12,16,17	10	O08557,P02631,P06911,P11598,P12020,P15999,P19629,P31044,P68370,Q5GRG2
4157	1,2,6,7,8,13,14,16	3,4,5,9,10,11,12,15,17	8	P06911,P12020,P19629,P31044,P68370,Q5GRG2,Q62902,Q63532
4158	1,2,6,7,8,13,14,17	3,4,5,9,10,11,12,15,16	14	P06911,P11598,P12020,P19629,P22282,P22283,P31044,P51907,P55091,P68370,Q5GRG2,Q62902,Q6PCU2,Q9Z1F2
4159	1,2,6,7,8,13,15,16	3,4,5,9,10,11,12,14,17	8	P01835,P02631,P06911,P15999,P19629,P31044,P68370,Q5GRG2
4160	1,2,6,7,8,13,15,17	3,4,5,9,10,11,12,14,16	8	P02631,P06911,P11598,P15999,P19629,P31044,Q5GRG2,Q6IFV1
4161	1,2,6,7,8,13,16,17	3,4,5,9,10,11,12,14,15	7	P06911,P19629,P31044,P51907,P63029,Q5GRG2,Q6PCU2
4162	1,2,6,7,8,14,15,16	3,4,5,9,10,11,12,13,17	6	P02631,P19629,P31044,P68370,Q5GRG2,Q63532

4163	1,2,6,7,8,14,15,17	3,4,5,9,10,11,12,13,16	5	P02631,P10536,P19629,P31044,Q5GRG2
4164	1,2,6,7,8,14,16,17	3,4,5,9,10,11,12,13,15	6	P02631,P19629,P31044,Q63532,Q6P6R2,Q6PCU2
4165	1,2,6,7,8,15,16,17	3,4,5,9,10,11,12,13,14	3	P02631,P19629,P31044
4166	1,2,6,7,9,10,11,12	3,4,5,8,13,14,15,16,17	11	O54861,P15399,P47967,P97840,Q63357,Q6AYL6,Q6IFU7,Q91ZS3,Q9JHB9,Q9JI85,Q9QUL6
4167	1,2,6,7,9,10,11,13	3,4,5,8,12,14,15,16,17	5	P12020,P15399,P97840,Q91ZS3,Q9QUL6
4168	1,2,6,7,9,10,11,14	3,4,5,8,12,13,15,16,17	7	P12020,P15399,P47727,P83121,Q63532,Q91ZS3,Q9QUL6
4169	1,2,6,7,9,10,11,15	3,4,5,8,12,13,14,16,17	6	O54861,P27213,P35280,P97840,Q91ZS3,Q9QUL6
4170	1,2,6,7,9,10,11,16	3,4,5,8,12,13,14,15,17	4	P47967,P97840,Q91ZS3,Q9QUL6
4171	1,2,6,7,9,10,11,17	3,4,5,8,12,13,14,15,16	5	O54861,P15399,P97580,Q91ZS3,Q9QUL6
4172	1,2,6,7,9,10,12,13	3,4,5,8,11,14,15,16,17	7	P09456,P12020,P19218,P47967,P97840,Q91ZS3,Q9JHB9
4173	1,2,6,7,9,10,12,14	3,4,5,8,11,13,15,16,17	4	P12020,P97840,Q63532,Q91ZS3
4174	1,2,6,7,9,10,12,15	3,4,5,8,11,13,14,16,17	3	P97840,Q6AYS4,Q91ZS3
4175	1,2,6,7,9,10,12,16	3,4,5,8,11,13,14,15,17	4	P47967,P97840,Q6AYS4,Q91ZS3
4176	1,2,6,7,9,10,12,17	3,4,5,8,11,13,14,15,16	3	P13432,P14668,P97840
4177	1,2,6,7,9,10,13,14	3,4,5,8,11,12,15,16,17	5	P06911,P12020,P97840,Q5GRG2,Q91ZS3

4178	1,2,6,7,9,10,13,15	3,4,5,8,11,12,14,16,17	4	P12020,P97840,Q5GRG2,Q6AYS4
4179	1,2,6,7,9,10,13,16	3,4,5,8,11,12,14,15,17	5	P12020,P47967,P97840,Q6AYS4,Q91ZS3
4180	1,2,6,7,9,10,13,17	3,4,5,8,11,12,14,15,16	3	P12020,P14668,P97840
4181	1,2,6,7,9,10,14,15	3,4,5,8,11,12,13,16,17	3	P97840,Q63532,Q6AYS4
4182	1,2,6,7,9,10,14,16	3,4,5,8,11,12,13,15,17	3	P97840,Q63532,Q6AYS4
4183	1,2,6,7,9,10,14,17	3,4,5,8,11,12,13,15,16	1	Q9QUL6
4184	1,2,6,7,9,10,15,16	3,4,5,8,11,12,13,14,17	2	P97840,Q6AYS4
4185	1,2,6,7,9,10,15,17	3,4,5,8,11,12,13,14,16	2	P97840,Q9QUL6
4186	1,2,6,7,9,10,16,17	3,4,5,8,11,12,13,14,15	3	P97840,Q6P6R2,Q9QUL6
4187	1,2,6,7,9,11,12,13	3,4,5,8,10,14,15,16,17	8	P02803,P09456,P30120,P97840,Q62902,Q91ZS3,Q9JHB9,Q9JI85
4188	1,2,6,7,9,11,12,14	3,4,5,8,10,13,15,16,17	9	P15399,P19629,P30120,P47727,Q63532,Q6AYL6,Q91ZS3,Q9JHB9,Q9JI85
4189	1,2,6,7,9,11,12,15	3,4,5,8,10,13,14,16,17	4	O54861,P02631,Q91ZS3,Q9JI85
4190	1,2,6,7,9,11,12,16	3,4,5,8,10,13,14,15,17	6	P12368,P47967,P97840,Q6AYL6,Q6IFU7,Q91ZS3
4191	1,2,6,7,9,11,12,17	3,4,5,8,10,13,14,15,16	9	O54861,P09605;P25809,P13432,P15399,Q62635,Q91ZS3,Q9JHB9,Q9JI85,Q9QUL6
4192	1,2,6,7,9,11,13,14	3,4,5,8,10,12,15,16,17	5	P06911,P12020,P47727,Q5GRG2,Q91ZS3

4193	1,2,6,7,9,11,13,15	3,4,5,8,10,12,14,16,17	1	Q91ZS3
4194	1,2,6,7,9,11,13,16	3,4,5,8,10,12,14,15,17	1	Q91ZS3
4195	1,2,6,7,9,11,13,17	3,4,5,8,10,12,14,15,16	3	P15399,Q91ZS3,Q9QUL6
4196	1,2,6,7,9,11,14,15	3,4,5,8,10,12,13,16,17	2	P02631,Q9QUL6
4197	1,2,6,7,9,11,14,16	3,4,5,8,10,12,13,15,17	3	Q63532,Q99MH3,Q9QUL6
4198	1,2,6,7,9,11,14,17	3,4,5,8,10,12,13,15,16	3	P15399,Q30KJ2,Q9QUL6
4199	1,2,6,7,9,11,15,16	3,4,5,8,10,12,13,14,17	0	
4200	1,2,6,7,9,11,15,17	3,4,5,8,10,12,13,14,16	3	P02631,P15399,Q9QUL6
4201	1,2,6,7,9,11,16,17	3,4,5,8,10,12,13,14,15	3	P09605;P25809,P15399,Q9QUL6
4202	1,2,6,7,9,12,13,14	3,4,5,8,10,11,15,16,17	7	P06911,P12020,P97840,Q5GRG2,Q6AYS4,Q9JHB9,Q9Z1F2
4203	1,2,6,7,9,12,13,15	3,4,5,8,10,11,14,16,17	2	P97840,Q6AYS4
4204	1,2,6,7,9,12,13,16	3,4,5,8,10,11,14,15,17	4	P19218,P47967,P97840,Q6AYS4
4205	1,2,6,7,9,12,13,17	3,4,5,8,10,11,14,15,16	8	P09605;P25809,P13432,P14668,P55091,P97840,Q62635,Q64194,Q9JHB9
4206	1,2,6,7,9,12,14,15	3,4,5,8,10,11,13,16,17	4	P02631,P19629,Q63532,Q6AYS4
4207	1,2,6,7,9,12,14,16	3,4,5,8,10,11,13,15,17	3	P97840,Q63532,Q6AYS4

4208	1,2,6,7,9,12,14,17	3,4,5,8,10,11,13,15,16	3	P13432,P98089,Q62635
4209	1,2,6,7,9,12,15,16	3,4,5,8,10,11,13,14,17	2	P97840,Q6AYS4
4210	1,2,6,7,9,12,15,17	3,4,5,8,10,11,13,14,16	1	P13432
4211	1,2,6,7,9,12,16,17	3,4,5,8,10,11,13,14,15	4	P09605;P25809,P13432,P97840,Q6AYS4
4212	1,2,6,7,9,13,14,15	3,4,5,8,10,11,12,16,17	6	P06911,P12020,P16636,P53792,Q5GRG2,Q6AYS4
4213	1,2,6,7,9,13,14,16	3,4,5,8,10,11,12,15,17	5	P06911,P12020,Q5GRG2,Q63532,Q6AYS4
4214	1,2,6,7,9,13,14,17	3,4,5,8,10,11,12,15,16	5	P06911,P12020,P14668,P53792,Q5GRG2
4215	1,2,6,7,9,13,15,16	3,4,5,8,10,11,12,14,17	1	Q6AYS4
4216	1,2,6,7,9,13,15,17	3,4,5,8,10,11,12,14,16	3	P14668,P53792,Q6AYS4
4217	1,2,6,7,9,13,16,17	3,4,5,8,10,11,12,14,15	1	Q6AYS4
4218	1,2,6,7,9,14,15,16	3,4,5,8,10,11,12,13,17	5	P02631,P16636,P35467,Q63532,Q6AYS4
4219	1,2,6,7,9,14,15,17	3,4,5,8,10,11,12,13,16	2	P02631,P53792
4220	1,2,6,7,9,14,16,17	3,4,5,8,10,11,12,13,15	2	Q63532,Q6AYS4
4221	1,2,6,7,9,15,16,17	3,4,5,8,10,11,12,13,14	1	Q6AYS4
4222	1,2,6,7,10,11,12,13	3,4,5,8,9,14,15,16,17	11	P09456,P12020,P15399,P30120,P47727,P47967,P97840,Q5GRG2,Q91ZS3,Q9JHB9,Q9JI85
4223	1,2,6,7,10,11,12,14	3,4,5,8,9,13,15,16,17	12	P12020,P15399,P30120,P35280,P47727,P62804,P97840,Q63532,Q6AYL6,Q6IFU7,Q9JHB9,Q9JI85

4224	1,2,6,7,10,<br>11,12,15	3,4,5,8,9,1<br>3,14,16,17	5	O54861,P15399,P35280,P97840,Q9JI85
4225	1,2,6,7,10,<br>11,12,16	3,4,5,8,9,1<br>3,14,15,17	9	O54861,P06757,P12368,P15399,P47967,P97840,Q63532,Q6AYL6,Q6IFU7
4226	1,2,6,7,10,<br>11,12,17	3,4,5,8,9,1<br>3,14,15,16	14	O54728,O54861,P05964,P06757,P13432,P15399,P55091,P97580,P97840,Q62635,Q63493,Q9JHB9,Q9JI85,Q9QZK9
4227	1,2,6,7,10,<br>11,13,14	3,4,5,8,9,1<br>2,15,16,17	6	P06911,P12020,P15399,P47727,Q5GRG2,Q63532
4228	1,2,6,7,10,<br>11,13,15	3,4,5,8,9,1<br>2,14,16,17	3	P01835,P12020,Q5GRG2
4229	1,2,6,7,10,<br>11,13,16	3,4,5,8,9,1<br>2,14,15,17	4	P01835,P12020,P47967,P97840
4230	1,2,6,7,10,<br>11,13,17	3,4,5,8,9,1<br>2,14,15,16	3	P12020,P15399,P97580
4231	1,2,6,7,10,<br>11,14,15	3,4,5,8,9,1<br>2,13,16,17	5	P02631,P12020,P15399,P35280,Q63532
4232	1,2,6,7,10,<br>11,14,16	3,4,5,8,9,1<br>2,13,15,17	4	P12020,P15399,P97840,Q63532
4233	1,2,6,7,10,<br>11,14,17	3,4,5,8,9,1<br>2,13,15,16	6	P12020,P15399,P47727,P97580,Q30KJ2,Q9QUL6
4234	1,2,6,7,10,<br>11,15,16	3,4,5,8,9,1<br>2,13,14,17	3	P01835,P35280,P97840
4235	1,2,6,7,10,<br>11,15,17	3,4,5,8,9,1<br>2,13,14,16	4	O54861,P15399,P97580,Q9QUL6
4236	1,2,6,7,10,<br>11,16,17	3,4,5,8,9,1<br>2,13,14,15	4	P10758,P15399,P97580,Q9QUL6
4237	1,2,6,7,10,<br>12,13,14	3,4,5,8,9,1<br>1,15,16,17	8	P06911,P12020,P30120,P97840,Q5GRG2,Q63532,Q6AYS4,Q9JHB9
4238	1,2,6,7,10,<br>12,13,15	3,4,5,8,9,1<br>1,14,16,17	4	P12020,P97840,Q5GRG2,Q6AYS4
4239	1,2,6,7,10,<br>12,13,16	3,4,5,8,9,1<br>1,14,15,17	6	P12020,P19218,P47967,P97840,Q63532,Q6AYS4
4240	1,2,6,7,10,<br>12,13,17	3,4,5,8,9,1<br>1,14,15,16	9	O54728,P12020,P13432,P14668,P55091,P97840,Q62635,Q9JHB9,Q9QZK9
4241	1,2,6,7,10,<br>12,14,15	3,4,5,8,9,1<br>1,13,16,17	5	P12020,P35280,P97840,Q63532,Q6AYS4
4242	1,2,6,7,10,<br>12,14,16	3,4,5,8,9,1<br>1,13,15,17	5	P12020,P47967,P97840,Q63532,Q6AYS4
4243	1,2,6,7,10,<br>12,14,17	3,4,5,8,9,1<br>1,13,15,16	9	O54728,P12020,P13432,P14668,P15399,P55091,P97840,Q62635,Q63532
4244	1,2,6,7,10,<br>12,15,16	3,4,5,8,9,1<br>1,13,14,17	4	P47967,P97840,Q63532,Q6AYS4
4245	1,2,6,7,10,<br>12,15,17	3,4,5,8,9,1<br>1,13,14,16	5	P06866,P13432,P14668,P97840,Q62635
4246	1,2,6,7,10,<br>12,16,17	3,4,5,8,9,1<br>1,13,14,15	3	P06866,P13432,P97840

4247	1,2,6,7,10,<br>13,14,15	3,4,5,8,9,1<br>1,12,16,17	5	P06911,P12020,Q5GRG2,Q63532,Q6AYS4
4248	1,2,6,7,10,<br>13,14,16	3,4,5,8,9,1<br>1,12,15,17	6	P06911,P12020,P97840,Q5GRG2,Q63532,Q6AYS4
4249	1,2,6,7,10,<br>13,14,17	3,4,5,8,9,1<br>1,12,15,16	4	P06911,P12020,P14668,Q5GRG2
4250	1,2,6,7,10,<br>13,15,16	3,4,5,8,9,1<br>1,12,14,17	5	P01835,P12020,P97840,Q5GRG2,Q6AYS4
4251	1,2,6,7,10,<br>13,15,17	3,4,5,8,9,1<br>1,12,14,16	4	P12020,P14668,Q5GRG2,Q6IFV1
4252	1,2,6,7,10,<br>13,16,17	3,4,5,8,9,1<br>1,12,14,15	3	P12020,P97840,Q6AYS4
4253	1,2,6,7,10,<br>14,15,16	3,4,5,8,9,1<br>1,12,13,17	4	P35467,P97840,Q63532,Q6AYS4
4254	1,2,6,7,10,<br>14,15,17	3,4,5,8,9,1<br>1,12,13,16	1	P35467
4255	1,2,6,7,10,<br>14,16,17	3,4,5,8,9,1<br>1,12,13,15	3	P12020,P97840,Q63532
4256	1,2,6,7,10,<br>15,16,17	3,4,5,8,9,1<br>1,12,13,14	2	P97840,Q6AYS4
4257	1,2,6,7,11,<br>12,13,14	3,4,5,8,9,1<br>0,15,16,17	10	P06911,P12020,P15399,P30120,P47727,Q5GRG2,Q62902,Q63532,Q9JHB9,Q9JI85
4258	1,2,6,7,11,<br>12,13,15	3,4,5,8,9,1<br>0,14,16,17	3	Q5GRG2,Q9JHB9,Q9JI85
4259	1,2,6,7,11,<br>12,13,16	3,4,5,8,9,1<br>0,14,15,17	5	P47967,P97840,Q62902,Q9JHB9,Q9JI85
4260	1,2,6,7,11,<br>12,13,17	3,4,5,8,9,1<br>0,14,15,16	9	P13432,P15399,P30120,P36374,P55091,Q62635,Q9JHB9,Q9JI85,Q9QZK9
4261	1,2,6,7,11,<br>12,14,15	3,4,5,8,9,1<br>0,13,16,17	7	P02631,P19629,P25031,P35280,P47727,Q63532,Q9JI85
4262	1,2,6,7,11,<br>12,14,16	3,4,5,8,9,1<br>0,13,15,17	5	P15399,P19629,P47727,Q63532,Q6AYL6
4263	1,2,6,7,11,<br>12,14,17	3,4,5,8,9,1<br>0,13,15,16	9	P13432,P15399,P19629,P47727,P55091,Q62635,Q63493,Q9JHB9,Q9JI85
4264	1,2,6,7,11,<br>12,15,16	3,4,5,8,9,1<br>0,13,14,17	0	
4265	1,2,6,7,11,<br>12,15,17	3,4,5,8,9,1<br>0,13,14,16	5	O54861,P06866,P13432,P15399,Q62635
4266	1,2,6,7,11,<br>12,16,17	3,4,5,8,9,1<br>0,13,14,15	4	P09605;P25809,P13432,P15399,Q62635
4267	1,2,6,7,11,<br>13,14,15	3,4,5,8,9,1<br>0,12,16,17	6	P02631,P06911,P12020,P47727,Q5GRG2,Q63532
4268	1,2,6,7,11,<br>13,14,16	3,4,5,8,9,1<br>0,12,15,17	5	P06911,P12020,P47727,Q5GRG2,Q63532
4269	1,2,6,7,11,<br>13,14,17	3,4,5,8,9,1<br>0,12,15,16	5	P06911,P12020,P15399,P47727,Q5GRG2

4270	1,2,6,7,11,<br>13,15,16	3,4,5,8,9,1<br>0,12,14,17	1	P01835
4271	1,2,6,7,11,<br>13,15,17	3,4,5,8,9,1<br>0,12,14,16	1	P15399
4272	1,2,6,7,11,<br>13,16,17	3,4,5,8,9,1<br>0,12,14,15	1	P15399
4273	1,2,6,7,11,<br>14,15,16	3,4,5,8,9,1<br>0,12,13,17	3	P02631,P16636,Q63532
4274	1,2,6,7,11,<br>14,15,17	3,4,5,8,9,1<br>0,12,13,16	3	P02631,P15399,P19629
4275	1,2,6,7,11,<br>14,16,17	3,4,5,8,9,1<br>0,12,13,15	4	P10758,P15399,Q30KJ2,Q63532
4276	1,2,6,7,11,<br>15,16,17	3,4,5,8,9,1<br>0,12,13,14	0	
4277	1,2,6,7,12,<br>13,14,15	3,4,5,8,9,1<br>0,11,16,17	7	P06911,P12020,P19629,P35467,Q5GRG2,Q63532,Q6AYS4
4278	1,2,6,7,12,<br>13,14,16	3,4,5,8,9,1<br>0,11,15,17	6	P06911,P12020,P97840,Q5GRG2,Q63532,Q6AYS4
4279	1,2,6,7,12,<br>13,14,17	3,4,5,8,9,1<br>0,11,15,16	11	P06911,P12020,P13432,P14668,P55091,P80254,Q5GRG2,Q62635,Q9JHB9,Q9QZK9,Q9Z1F2
4280	1,2,6,7,12,<br>13,15,16	3,4,5,8,9,1<br>0,11,14,17	3	P35467,P97840,Q6AYS4
4281	1,2,6,7,12,<br>13,15,17	3,4,5,8,9,1<br>0,11,14,16	8	P13432,P14668,P55016,P55091,Q62635,Q6AYS4,Q6IFV1,Q9QZK9
4282	1,2,6,7,12,<br>13,16,17	3,4,5,8,9,1<br>0,11,14,15	6	P13432,P55091,P80254,P97840,Q62635,Q6AYS4
4283	1,2,6,7,12,<br>14,15,16	3,4,5,8,9,1<br>0,11,13,17	4	P19629,P35467,Q63532,Q6AYS4
4284	1,2,6,7,12,<br>14,15,17	3,4,5,8,9,1<br>0,11,13,16	7	P13432,P19629,P35467,P55091,P98089,Q62635,Q6AYS4
4285	1,2,6,7,12,<br>14,16,17	3,4,5,8,9,1<br>0,11,13,15	6	P13432,P35467,P98089,Q62635,Q63532,Q6AYS4
4286	1,2,6,7,12,<br>15,16,17	3,4,5,8,9,1<br>0,11,13,14	4	P06866,P13432,P35467,Q6AYS4
4287	1,2,6,7,13,<br>14,15,16	3,4,5,8,9,1<br>0,11,12,17	7	P06911,P12020,P16636,P35467,Q5GRG2,Q63532,Q6AYS4
4288	1,2,6,7,13,<br>14,15,17	3,4,5,8,9,1<br>0,11,12,16	7	P06911,P12020,P14668,P35467,P53792,Q5GRG2,Q6AYS4
4289	1,2,6,7,13,<br>14,16,17	3,4,5,8,9,1<br>0,11,12,15	5	P06911,P12020,Q5GRG2,Q63532,Q6AYS4
4290	1,2,6,7,13,<br>15,16,17	3,4,5,8,9,1<br>0,11,12,14	3	P00714,P35467,Q6AYS4
4291	1,2,6,7,14,<br>15,16,17	3,4,5,8,9,1<br>0,11,12,13	3	P35467,Q63532,Q6AYS4

4292	1,2,6,8,9,10,11,12	3,4,5,7,13,14,15,16,17	12	O35547,P02782,P02803,P08723,P0C0A9,P30120,P47727,P62804,Q63357,Q6IFU7,Q91ZS3,Q9JHB9
4293	1,2,6,8,9,10,11,13	3,4,5,7,12,14,15,16,17	5	P02803,P47727,Q63942,Q8CJD3,Q91ZS3
4294	1,2,6,8,9,10,11,14	3,4,5,7,12,13,15,16,17	4	P47727,Q63942,Q91ZS3,Q9QUL6
4295	1,2,6,8,9,10,11,15	3,4,5,7,12,13,14,16,17	5	P35280,Q4FZU6,Q63942,Q91ZS3,Q9QUL6
4296	1,2,6,8,9,10,11,16	3,4,5,7,12,13,14,15,17	4	A2RUV9,Q63942,Q91ZS3,Q9WUW8
4297	1,2,6,8,9,10,11,17	3,4,5,7,12,13,14,15,16	4	A2RUV9,Q6P6R2,Q91ZS3,Q9QUL6
4298	1,2,6,8,9,10,12,13	3,4,5,7,11,14,15,16,17	8	P02782,P02803,P08723,P30120,P97840,Q8K4G9,Q91ZS3,Q9JHB9
4299	1,2,6,8,9,10,12,14	3,4,5,7,11,13,15,16,17	3	P02782,P08723,Q63942
4300	1,2,6,8,9,10,12,15	3,4,5,7,11,13,14,16,17	1	Q63942
4301	1,2,6,8,9,10,12,16	3,4,5,7,11,13,14,15,17	3	P97840,Q63942,Q9WUW8
4302	1,2,6,8,9,10,12,17	3,4,5,7,11,13,14,15,16	4	P02782,P13432,Q62635,Q6P6R2
4303	1,2,6,8,9,10,13,14	3,4,5,7,11,12,15,16,17	5	P06911,P12020,Q5GRG2,Q63942,Q8K4G9
4304	1,2,6,8,9,10,13,15	3,4,5,7,11,12,14,16,17	3	P15999,Q63942,Q6IFV1
4305	1,2,6,8,9,10,13,16	3,4,5,7,11,12,14,15,17	2	Q63942,Q8CJD3
4306	1,2,6,8,9,10,13,17	3,4,5,7,11,12,14,15,16	5	P51907,Q63355,Q63942,Q6IFV1,Q8CJD3

4307	1,2,6,8,9,10,14,15	3,4,5,7,11,12,13,16,17	2	P17046,Q63942
4308	1,2,6,8,9,10,14,16	3,4,5,7,11,12,13,15,17	1	Q63942
4309	1,2,6,8,9,10,14,17	3,4,5,7,11,12,13,15,16	2	Q63942,Q6P6R2
4310	1,2,6,8,9,10,15,16	3,4,5,7,11,12,13,14,17	2	Q5QE79,Q63942
4311	1,2,6,8,9,10,15,17	3,4,5,7,11,12,13,14,16	2	Q63942,Q6IFV1
4312	1,2,6,8,9,10,16,17	3,4,5,7,11,12,13,14,15	3	A2RUV9,Q63942,Q6P6R2
4313	1,2,6,8,9,11,12,13	3,4,5,7,10,14,15,16,17	9	P02782,P02803,P08723,P0C0A9,P30120,P47727,Q91ZS3,Q9JHB9,Q9Z1F2
4314	1,2,6,8,9,11,12,14	3,4,5,7,10,13,15,16,17	7	P02782,P08723,P30120,P47727,P62804,Q00715,Q9JHB9
4315	1,2,6,8,9,11,12,15	3,4,5,7,10,13,14,16,17	3	P02782,Q5QE79,Q9JHB9
4316	1,2,6,8,9,11,12,16	3,4,5,7,10,13,14,15,17	3	P02782,Q6IFU7,Q9JHB9
4317	1,2,6,8,9,11,12,17	3,4,5,7,10,13,14,15,16	7	P02782,P13432,P30120,P62804,Q00715,Q62635,Q9JHB9
4318	1,2,6,8,9,11,13,14	3,4,5,7,10,12,15,16,17	5	P02782,P06911,P47727,Q63942,Q9Z1F2
4319	1,2,6,8,9,11,13,15	3,4,5,7,10,12,14,16,17	2	Q5QE79,Q63942
4320	1,2,6,8,9,11,13,16	3,4,5,7,10,12,14,15,17	3	A2RUV9,Q63942,Q8CJD3
4321	1,2,6,8,9,11,13,17	3,4,5,7,10,12,14,15,16	1	Q8CJD3

4322	1,2,6,8,9,11,14,15	3,4,5,7,10,12,13,16,17	2	Q5QE79,Q63942
4323	1,2,6,8,9,11,14,16	3,4,5,7,10,12,13,15,17	2	Q63942,Q99MH3
4324	1,2,6,8,9,11,14,17	3,4,5,7,10,12,13,15,16	2	Q63942,Q9QUL6
4325	1,2,6,8,9,11,15,16	3,4,5,7,10,12,13,14,17	2	Q5QE79,Q63942
4326	1,2,6,8,9,11,15,17	3,4,5,7,10,12,13,14,16	2	Q5QE79,Q63942
4327	1,2,6,8,9,11,16,17	3,4,5,7,10,12,13,14,15	3	A2RUV9,Q63942,Q6P6R2
4328	1,2,6,8,9,12,13,14	3,4,5,7,10,11,15,16,17	8	P02782,P06911,P08723,P30120,Q5FVI6,Q8K4G9,Q9JHB9,Q9Z1F2
4329	1,2,6,8,9,12,13,15	3,4,5,7,10,11,14,16,17	6	P02782,P15999,Q5QE79,Q6IFV1,Q9JHB9,Q9Z1F2
4330	1,2,6,8,9,12,13,16	3,4,5,7,10,11,14,15,17	4	P02782,P02803,Q63942,Q9Z1F2
4331	1,2,6,8,9,12,13,17	3,4,5,7,10,11,14,15,16	7	P02782,P13432,Q5FVI6,Q62635,Q6IFV1,Q9JHB9,Q9Z1F2
4332	1,2,6,8,9,12,14,15	3,4,5,7,10,11,13,16,17	2	P02782,Q63942
4333	1,2,6,8,9,12,14,16	3,4,5,7,10,11,13,15,17	2	P02782,Q63942
4334	1,2,6,8,9,12,14,17	3,4,5,7,10,11,13,15,16	7	P02782,P13432,P98089,Q5FVI6,Q62635,Q8VD89,Q9Z1F2
4335	1,2,6,8,9,12,15,16	3,4,5,7,10,11,13,14,17	2	Q5QE79,Q63942
4336	1,2,6,8,9,12,15,17	3,4,5,7,10,11,13,14,16	4	P13432,Q5QE79,Q62635,Q6IFV1

4337	1,2,6,8,9,12,16,17	3,4,5,7,10,11,13,14,15	3	P13432,Q62635,Q6P6R2
4338	1,2,6,8,9,13,14,15	3,4,5,7,10,11,12,16,17	4	P15999,Q5GRG2,Q5QE79,Q63942
4339	1,2,6,8,9,13,14,16	3,4,5,7,10,11,12,15,17	1	Q63942
4340	1,2,6,8,9,13,14,17	3,4,5,7,10,11,12,15,16	5	P51907,P53792,Q5FVI6,Q63942,Q9Z1F2
4341	1,2,6,8,9,13,15,16	3,4,5,7,10,11,12,14,17	2	Q5QE79,Q63942
4342	1,2,6,8,9,13,15,17	3,4,5,7,10,11,12,14,16	5	P15999,P53792,Q5QE79,Q63942,Q6IFV1
4343	1,2,6,8,9,13,16,17	3,4,5,7,10,11,12,14,15	3	P51907,Q63942,Q8CJD3
4344	1,2,6,8,9,14,15,16	3,4,5,7,10,11,12,13,17	3	Q5QE79,Q63942,Q9QZK9
4345	1,2,6,8,9,14,15,17	3,4,5,7,10,11,12,13,16	2	Q5QE79,Q63942
4346	1,2,6,8,9,14,16,17	3,4,5,7,10,11,12,13,15	2	Q63942,Q6P6R2
4347	1,2,6,8,9,15,16,17	3,4,5,7,10,11,12,13,14	2	Q5QE79,Q63942
4348	1,2,6,8,10,11,12,13	3,4,5,7,9,14,15,16,17	13	O35547,P02781,P02782,P02803,P08723,P0C0A9,P30120,P47727,P62804,Q6AYR9,Q6IFU7,Q8K4G9,Q9JHB9
4349	1,2,6,8,10,11,12,14	3,4,5,7,9,13,15,16,17	11	O35547,P02782,P08723,P0C0A9,P30120,P35280,P47727,P62804,Q00715,Q6IFU7,Q9JHB9
4350	1,2,6,8,10,11,12,15	3,4,5,7,9,13,14,16,17	7	O35547,P08010,P35280,P62804,Q00715,Q6IFU7,Q9JHB9
4351	1,2,6,8,10,11,12,16	3,4,5,7,9,13,14,15,17	6	O35547,P62804,Q6IFU7,Q6P6Q2,Q9JHB9,Q9WUW8
4352	1,2,6,8,10,11,12,17	3,4,5,7,9,13,14,15,16	13	O35547,O54728,P02782,P08723,P13432,P16636,P30120,P47727,P62804,Q00715,Q62635,Q9JHB9,Q9QZK9
4353	1,2,6,8,10,11,13,14	3,4,5,7,9,12,15,16,17	5	P06911,P12020,P47727,Q5GRG2,Q8K4G9

4354	1,2,6,8,10,<br>11,13,15	3,4,5,7,9,1<br>2,14,16,17	5	P01835,P15999,Q4FZU6,Q5GRG2,Q6IFV1
4355	1,2,6,8,10,<br>11,13,16	3,4,5,7,9,1<br>2,14,15,17	3	A2RUV9,P01835,Q8CJD3
4356	1,2,6,8,10,<br>11,13,17	3,4,5,7,9,1<br>2,14,15,16	3	P47727,Q6IFV1,Q8CJD3
4357	1,2,6,8,10,<br>11,14,15	3,4,5,7,9,1<br>2,13,16,17	2	P35280,P47727
4358	1,2,6,8,10,<br>11,14,16	3,4,5,7,9,1<br>2,13,15,17	1	P47727
4359	1,2,6,8,10,<br>11,14,17	3,4,5,7,9,1<br>2,13,15,16	3	P47727,P62804,Q00715
4360	1,2,6,8,10,<br>11,15,16	3,4,5,7,9,1<br>2,13,14,17	2	P01835,P35280
4361	1,2,6,8,10,<br>11,15,17	3,4,5,7,9,1<br>2,13,14,16	1	Q6IFV1
4362	1,2,6,8,10,<br>11,16,17	3,4,5,7,9,1<br>2,13,14,15	2	A2RUV9,Q6P6R2
4363	1,2,6,8,10,<br>12,13,14	3,4,5,7,9,1<br>1,15,16,17	11	P02782,P06911,P08723,P12020,P30120,P47727,Q5GRG2,Q6AYR9,Q8K4G9,Q9JHB9,Q9Z1F2
4364	1,2,6,8,10,<br>12,13,15	3,4,5,7,9,1<br>1,14,16,17	5	P02782,P15999,Q5GRG2,Q6IFV1,Q9JHB9
4365	1,2,6,8,10,<br>12,13,16	3,4,5,7,9,1<br>1,14,15,17	3	P02782,P97840,Q8K4G9
4366	1,2,6,8,10,<br>12,13,17	3,4,5,7,9,1<br>1,14,15,16	12	O54728,P02782,P13432,P16636,P30120,Q62635,Q63355,Q6AYR9,Q6IFV1,Q8K4G9,Q9JHB9,Q9QZK9
4367	1,2,6,8,10,<br>12,14,15	3,4,5,7,9,1<br>1,13,16,17	1	P35280
4368	1,2,6,8,10,<br>12,14,16	3,4,5,7,9,1<br>1,13,15,17	3	P05539,P97840,Q8K4G9
4369	1,2,6,8,10,<br>12,14,17	3,4,5,7,9,1<br>1,13,15,16	8	O54728,P02782,P13432,P62804,P98089,Q10758,Q62635,Q8K4G9
4370	1,2,6,8,10,<br>12,15,16	3,4,5,7,9,1<br>1,13,14,17	2	P05539,P97840
4371	1,2,6,8,10,<br>12,15,17	3,4,5,7,9,1<br>1,13,14,16	3	P06866,Q62635,Q6IFV1
4372	1,2,6,8,10,<br>12,16,17	3,4,5,7,9,1<br>1,13,14,15	7	O54728,P05539,P13432,Q10758,Q62635,Q6P6R2,Q8K4G9
4373	1,2,6,8,10,<br>13,14,15	3,4,5,7,9,1<br>1,12,16,17	4	P06911,P12020,P15999,Q5GRG2
4374	1,2,6,8,10,<br>13,14,16	3,4,5,7,9,1<br>1,12,15,17	4	P06911,P12020,Q5GRG2,Q8K4G9
4375	1,2,6,8,10,<br>13,14,17	3,4,5,7,9,1<br>1,12,15,16	5	P06911,P12020,P51907,Q5GRG2,Q8K4G9
4376	1,2,6,8,10,<br>13,15,16	3,4,5,7,9,1<br>1,12,14,17	2	P01835,P15999

4377	1,2,6,8,10,<br>13,15,17	3,4,5,7,9,1<br>1,12,14,16	2	P15999,Q6IFV1
4378	1,2,6,8,10,<br>13,16,17	3,4,5,7,9,1<br>1,12,14,15	3	P51907,Q8CJD3,Q8K4G9
4379	1,2,6,8,10,<br>14,15,16	3,4,5,7,9,1<br>1,12,13,17	0	
4380	1,2,6,8,10,<br>14,15,17	3,4,5,7,9,1<br>1,12,13,16	0	
4381	1,2,6,8,10,<br>14,16,17	3,4,5,7,9,1<br>1,12,13,15	2	P05539,Q6P6R2
4382	1,2,6,8,10,<br>15,16,17	3,4,5,7,9,1<br>1,12,13,14	2	P05539,Q6IFV1
4383	1,2,6,8,11,<br>12,13,14	3,4,5,7,9,1<br>0,15,16,17	16	O35547,P02782,P04905,P06911,P08723,P0C0A9,P30120,P47727,P62804,Q00715,Q5GRG2,Q62902,Q6AYR9,Q8K4G9,Q9JHB9,Q9Z1F2
4384	1,2,6,8,11,<br>12,13,15	3,4,5,7,9,1<br>0,14,16,17	5	P02782,P15999,P30120,Q6IFV1,Q9JHB9
4385	1,2,6,8,11,<br>12,13,16	3,4,5,7,9,1<br>0,14,15,17	3	P02782,P30120,Q9JHB9
4386	1,2,6,8,11,<br>12,13,17	3,4,5,7,9,1<br>0,14,15,16	13	O35547,O54728,P02782,P08723,P13432,P30120,P47727,Q00715,Q62635,Q6IFV1,Q9JHB9,Q9QZK9,Q9Z1F2
4387	1,2,6,8,11,<br>12,14,15	3,4,5,7,9,1<br>0,13,16,17	7	O35547,P02782,P35280,P47727,P62804,Q00715,Q9JHB9
4388	1,2,6,8,11,<br>12,14,16	3,4,5,7,9,1<br>0,13,15,17	7	O35547,P02782,P30120,P47727,P62804,Q6IFU7,Q9JHB9
4389	1,2,6,8,11,<br>12,14,17	3,4,5,7,9,1<br>0,13,15,16	12	O35547,O54728,P02782,P08723,P13432,P30120,P47727,P62804,P98089,Q00715,Q62635,Q9JHB9
4390	1,2,6,8,11,<br>12,15,16	3,4,5,7,9,1<br>0,13,14,17	2	O35547,Q6IFU7
4391	1,2,6,8,11,<br>12,15,17	3,4,5,7,9,1<br>0,13,14,16	7	O35547,P02782,P13432,P62804,Q00715,Q62635,Q6IFV1
4392	1,2,6,8,11,<br>12,16,17	3,4,5,7,9,1<br>0,13,14,15	4	O35547,P02782,P13432,Q62635
4393	1,2,6,8,11,<br>13,14,15	3,4,5,7,9,1<br>0,12,16,17	5	P06911,P15999,P47727,Q5GRG2,Q64093
4394	1,2,6,8,11,<br>13,14,16	3,4,5,7,9,1<br>0,12,15,17	2	P06911,P47727
4395	1,2,6,8,11,<br>13,14,17	3,4,5,7,9,1<br>0,12,15,16	3	P02782,P06911,P47727
4396	1,2,6,8,11,<br>13,15,16	3,4,5,7,9,1<br>0,12,14,17	1	P01835
4397	1,2,6,8,11,<br>13,15,17	3,4,5,7,9,1<br>0,12,14,16	2	P15999,Q6IFV1
4398	1,2,6,8,11,<br>13,16,17	3,4,5,7,9,1<br>0,12,14,15	2	A2RUV9,Q8CJD3
4399	1,2,6,8,11,<br>14,15,16	3,4,5,7,9,1<br>0,12,13,17	0	

4400	1,2,6,8,11,14,15,17	3,4,5,7,9,10,12,13,16	0	
4401	1,2,6,8,11,14,16,17	3,4,5,7,9,10,12,13,15	0	
4402	1,2,6,8,11,15,16,17	3,4,5,7,9,10,12,13,14	0	
4403	1,2,6,8,12,13,14,15	3,4,5,7,9,10,11,16,17	8	O08557,P02782,P06911,P15999,Q5GRG2,Q64093,Q9JHB9,Q9Z1F2
4404	1,2,6,8,12,13,14,16	3,4,5,7,9,10,11,15,17	6	P02782,P06911,P30120,Q8K4G9,Q9JHB9,Q9Z1F2
4405	1,2,6,8,12,13,14,17	3,4,5,7,9,10,11,15,16	12	O54728,P02782,P06911,P13432,P30120,P51907,Q5FVI6,Q62635,Q6AYR9,Q8K4G9,Q9JHB9,Q9Z1F2
4406	1,2,6,8,12,13,15,16	3,4,5,7,9,10,11,14,17	1	P15999
4407	1,2,6,8,12,13,15,17	3,4,5,7,9,10,11,14,16	6	P02782,P13432,P15999,P55016,Q62635,Q6IFV1
4408	1,2,6,8,12,13,16,17	3,4,5,7,9,10,11,14,15	4	P02782,P13432,Q62635,Q8K4G9
4409	1,2,6,8,12,14,15,16	3,4,5,7,9,10,11,13,17	0	
4410	1,2,6,8,12,14,15,17	3,4,5,7,9,10,11,13,16	5	P02782,P13432,P98089,Q5FVI6,Q62635
4411	1,2,6,8,12,14,16,17	3,4,5,7,9,10,11,13,15	4	P02782,P13432,P98089,Q62635
4412	1,2,6,8,12,15,16,17	3,4,5,7,9,10,11,13,14	5	P06866,P13432,Q5XFX0,Q62635,Q6IFV1
4413	1,2,6,8,13,14,15,16	3,4,5,7,9,10,11,12,17	2	P15999,Q5GRG2
4414	1,2,6,8,13,14,15,17	3,4,5,7,9,10,11,12,16	4	P15999,Q5FVI6,Q5GRG2,Q64093
4415	1,2,6,8,13,14,16,17	3,4,5,7,9,10,11,12,15	4	P51907,Q5FVI6,Q6PCU2,Q8K4G9
4416	1,2,6,8,13,15,16,17	3,4,5,7,9,10,11,12,14	3	P15999,Q6IFV1,Q6PCU2
4417	1,2,6,8,14,15,16,17	3,4,5,7,9,10,11,12,13	0	
4418	1,2,6,9,10,11,12,13	3,4,5,7,8,14,15,16,17	7	P02803,P47727,P97840,Q63661,Q6IFU7,Q91ZS3,Q9JHB9
4419	1,2,6,9,10,11,12,14	3,4,5,7,8,13,15,16,17	4	P47727,Q63661,Q6IFU7,Q91ZS3
4420	1,2,6,9,10,11,12,15	3,4,5,7,8,13,14,16,17	4	Q4FZU6,Q63661,Q6IFU7,Q91ZS3
4421	1,2,6,9,10,11,12,16	3,4,5,7,8,13,14,15,17	8	P10536,P12368,P47967,P97840,Q63661,Q6IFU7,Q91ZS3,Q9WUW8
4422	1,2,6,9,10,11,12,17	3,4,5,7,8,13,14,15,16	4	P06757,Q6IFU7,Q91ZS3,Q9QUL6

4423	1,2,6,9,10,11,13,14	3,4,5,7,8,12,15,16,17	3	P47727,Q91ZS3,Q99041
4424	1,2,6,9,10,11,13,15	3,4,5,7,8,12,14,16,17	2	Q4FZU6,Q91ZS3
4425	1,2,6,9,10,11,13,16	3,4,5,7,8,12,14,15,17	2	Q8CJD3,Q91ZS3
4426	1,2,6,9,10,11,13,17	3,4,5,7,8,12,14,15,16	3	Q8CJD3,Q91ZS3,Q9QUL6
4427	1,2,6,9,10,11,14,15	3,4,5,7,8,12,13,16,17	5	Q4FZU6,Q63661,Q91ZS3,Q99041,Q9QUL6
4428	1,2,6,9,10,11,14,16	3,4,5,7,8,12,13,15,17	4	Q91ZS3,Q99041,Q99MH3,Q9QUL6
4429	1,2,6,9,10,11,14,17	3,4,5,7,8,12,13,15,16	2	Q91ZS3,Q9QUL6
4430	1,2,6,9,10,11,15,16	3,4,5,7,8,12,13,14,17	3	Q4FZU6,Q91ZS3,Q99041
4431	1,2,6,9,10,11,15,17	3,4,5,7,8,12,13,14,16	2	Q4FZU6,Q9QUL6
4432	1,2,6,9,10,11,16,17	3,4,5,7,8,12,13,14,15	4	A2RUV9,P36970,Q91ZS3,Q9QUL6
4433	1,2,6,9,10,12,13,14	3,4,5,7,8,11,15,16,17	2	P97840,Q6AYS4
4434	1,2,6,9,10,12,13,15	3,4,5,7,8,11,14,16,17	3	P97840,Q4FZU6,Q6AYS4
4435	1,2,6,9,10,12,13,16	3,4,5,7,8,11,14,15,17	4	P07171,P47967,P97840,Q6AYS4
4436	1,2,6,9,10,12,13,17	3,4,5,7,8,11,14,15,16	6	P07171,P14668,P97840,Q62635,Q63355,Q6AYS4
4437	1,2,6,9,10,12,14,15	3,4,5,7,8,11,13,16,17	2	P97840,Q6AYS4
4438	1,2,6,9,10,12,14,16	3,4,5,7,8,11,13,15,17	3	P97840,Q6AYS4,Q9WUW8
4439	1,2,6,9,10,12,14,17	3,4,5,7,8,11,13,15,16	1	Q8VD89
4440	1,2,6,9,10,12,15,16	3,4,5,7,8,11,13,14,17	2	P97840,Q6AYS4
4441	1,2,6,9,10,12,15,17	3,4,5,7,8,11,13,14,16	2	P06866,Q6AYS4
4442	1,2,6,9,10,12,16,17	3,4,5,7,8,11,13,14,15	4	P06866,P07171,P97840,Q6AYS4
4443	1,2,6,9,10,13,14,15	3,4,5,7,8,11,12,16,17	4	P50115,Q6AYS4,Q99041,Q9ESW0
4444	1,2,6,9,10,13,14,16	3,4,5,7,8,11,12,15,17	5	P50115,Q63942,Q6AYS4,Q99041,Q9ESW0
4445	1,2,6,9,10,13,14,17	3,4,5,7,8,11,12,15,16	2	P14668,Q6AYS4

4446	1,2,6,9,10,<br>13,15,16	3,4,5,7,8,1<br>1,12,14,17	6	P50115,P97840,Q63942,Q6AYS4,Q99041,Q9ESW0
4447	1,2,6,9,10,<br>13,15,17	3,4,5,7,8,1<br>1,12,14,16	4	P14668,Q4FZU6,Q6AYS4,Q6IFV1
4448	1,2,6,9,10,<br>13,16,17	3,4,5,7,8,1<br>1,12,14,15	4	P07171,Q6AYS4,Q8CJD3,Q9ESW0
4449	1,2,6,9,10,<br>14,15,16	3,4,5,7,8,1<br>1,12,13,17	5	P50115,Q63942,Q6AYS4,Q99041,Q9QZK9
4450	1,2,6,9,10,<br>14,15,17	3,4,5,7,8,1<br>1,12,13,16	1	Q6AYS4
4451	1,2,6,9,10,<br>14,16,17	3,4,5,7,8,1<br>1,12,13,15	1	Q6AYS4
4452	1,2,6,9,10,<br>15,16,17	3,4,5,7,8,1<br>1,12,13,14	1	Q6AYS4
4453	1,2,6,9,11,<br>12,13,14	3,4,5,7,8,1<br>0,15,16,17	2	P47727,Q9JHB9
4454	1,2,6,9,11,<br>12,13,15	3,4,5,7,8,1<br>0,14,16,17	0	
4455	1,2,6,9,11,<br>12,13,16	3,4,5,7,8,1<br>0,14,15,17	2	P02803,Q6IFU7
4456	1,2,6,9,11,<br>12,13,17	3,4,5,7,8,1<br>0,14,15,16	2	Q62635,Q9JHB9
4457	1,2,6,9,11,<br>12,14,15	3,4,5,7,8,1<br>0,13,16,17	0	
4458	1,2,6,9,11,<br>12,14,16	3,4,5,7,8,1<br>0,13,15,17	2	Q6IFU7,Q99MH3
4459	1,2,6,9,11,<br>12,14,17	3,4,5,7,8,1<br>0,13,15,16	1	P13432
4460	1,2,6,9,11,<br>12,15,16	3,4,5,7,8,1<br>0,13,14,17	1	Q6IFU7
4461	1,2,6,9,11,<br>12,15,17	3,4,5,7,8,1<br>0,13,14,16	1	P06866
4462	1,2,6,9,11,<br>12,16,17	3,4,5,7,8,1<br>0,13,14,15	0	
4463	1,2,6,9,11,<br>13,14,15	3,4,5,7,8,1<br>0,12,16,17	1	Q99041
4464	1,2,6,9,11,<br>13,14,16	3,4,5,7,8,1<br>0,12,15,17	3	Q7TPB1,Q99041,Q99MH3
4465	1,2,6,9,11,<br>13,14,17	3,4,5,7,8,1<br>0,12,15,16	0	
4466	1,2,6,9,11,<br>13,15,16	3,4,5,7,8,1<br>0,12,14,17	0	
4467	1,2,6,9,11,<br>13,15,17	3,4,5,7,8,1<br>0,12,14,16	0	
4468	1,2,6,9,11,<br>13,16,17	3,4,5,7,8,1<br>0,12,14,15	2	A2RUV9,Q8CJD3

4469	1,2,6,9,11,<br>14,15,16	3,4,5,7,8,1<br>0,12,13,17	4	Q63942,Q99041,Q99MH3,Q9QZK9
4470	1,2,6,9,11,<br>14,15,17	3,4,5,7,8,1<br>0,12,13,16	1	Q9QUL6
4471	1,2,6,9,11,<br>14,16,17	3,4,5,7,8,1<br>0,12,13,15	1	Q9QUL6
4472	1,2,6,9,11,<br>15,16,17	3,4,5,7,8,1<br>0,12,13,14	1	P36970
4473	1,2,6,9,12,<br>13,14,15	3,4,5,7,8,1<br>0,11,16,17	2	P00714,Q6AYS4
4474	1,2,6,9,12,<br>13,14,16	3,4,5,7,8,1<br>0,11,15,17	2	P00714,Q6AYS4
4475	1,2,6,9,12,<br>13,14,17	3,4,5,7,8,1<br>0,11,15,16	6	P00714,P14668,Q5FVI6,Q62635,Q6AYS4,Q8VD89
4476	1,2,6,9,12,<br>13,15,16	3,4,5,7,8,1<br>0,11,14,17	2	P00714,Q6AYS4
4477	1,2,6,9,12,<br>13,15,17	3,4,5,7,8,1<br>0,11,14,16	5	P00714,P14668,Q62635,Q6AYS4,Q6IFV1
4478	1,2,6,9,12,<br>13,16,17	3,4,5,7,8,1<br>0,11,14,15	5	P00714,P07171,Q62635,Q64194,Q6AYS4
4479	1,2,6,9,12,<br>14,15,16	3,4,5,7,8,1<br>0,11,13,17	1	Q6AYS4
4480	1,2,6,9,12,<br>14,15,17	3,4,5,7,8,1<br>0,11,13,16	3	P00714,Q6AYS4,Q8VD89
4481	1,2,6,9,12,<br>14,16,17	3,4,5,7,8,1<br>0,11,13,15	1	Q6AYS4
4482	1,2,6,9,12,<br>15,16,17	3,4,5,7,8,1<br>0,11,13,14	3	P00714,P06866,Q6AYS4
4483	1,2,6,9,13,<br>14,15,16	3,4,5,7,8,1<br>0,11,12,17	7	P00714,P16391,P50115,Q63942,Q6AYS4,Q99041,Q9ESW0
4484	1,2,6,9,13,<br>14,15,17	3,4,5,7,8,1<br>0,11,12,16	6	P00714,P14668,P16391,P53792,Q5FVI6,Q6AYS4
4485	1,2,6,9,13,<br>14,16,17	3,4,5,7,8,1<br>0,11,12,15	3	P00714,Q6AYS4,Q9ESW0
4486	1,2,6,9,13,<br>15,16,17	3,4,5,7,8,1<br>0,11,12,14	2	P00714,Q6AYS4
4487	1,2,6,9,14,<br>15,16,17	3,4,5,7,8,1<br>0,11,12,13	3	P00714,Q63942,Q6AYS4
4488	1,2,6,10,1<br>1,12,13,14	3,4,5,7,8,9<br>,15,16,17	3	P47727,Q6IFU7,Q9JHB9
4489	1,2,6,10,1<br>1,12,13,15	3,4,5,7,8,9<br>,14,16,17	3	Q4FZU6,Q6IFU7,Q9JHB9
4490	1,2,6,10,1<br>1,12,13,16	3,4,5,7,8,9<br>,14,15,17	3	P47967,P97840,Q6IFU7
4491	1,2,6,10,1<br>1,12,13,17	3,4,5,7,8,9<br>,14,15,16	5	O54728,P47727,Q62635,Q9JHB9,Q9QZK9

4492	1,2,6,10,11,12,14,15	3,4,5,7,8,9,13,16,17	4	P35280,P47727,P62804,Q6IFU7
4493	1,2,6,10,11,12,14,16	3,4,5,7,8,9,13,15,17	3	P47727,P97840,Q6IFU7
4494	1,2,6,10,11,12,14,17	3,4,5,7,8,9,13,15,16	6	O54728,P47727,P62804,Q00715,Q10758,Q62635
4495	1,2,6,10,11,12,15,16	3,4,5,7,8,9,13,14,17	2	P97840,Q6IFU7
4496	1,2,6,10,11,12,15,17	3,4,5,7,8,9,13,14,16	2	P06866,Q4FZU6
4497	1,2,6,10,11,12,16,17	3,4,5,7,8,9,13,14,15	3	P06757,P06866,Q6IFU7
4498	1,2,6,10,11,13,14,15	3,4,5,7,8,9,12,16,17	3	P47727,Q4FZU6,Q99041
4499	1,2,6,10,11,13,14,16	3,4,5,7,8,9,12,15,17	3	P47727,Q7TPB1,Q99041
4500	1,2,6,10,11,13,14,17	3,4,5,7,8,9,12,15,16	1	P47727
4501	1,2,6,10,11,13,15,16	3,4,5,7,8,9,12,14,17	2	P01835,Q4FZU6
4502	1,2,6,10,11,13,15,17	3,4,5,7,8,9,12,14,16	1	Q4FZU6
4503	1,2,6,10,11,13,16,17	3,4,5,7,8,9,12,14,15	1	Q8CJD3
4504	1,2,6,10,11,14,15,16	3,4,5,7,8,9,12,13,17	1	Q99041
4505	1,2,6,10,11,14,15,17	3,4,5,7,8,9,12,13,16	0	
4506	1,2,6,10,11,14,16,17	3,4,5,7,8,9,12,13,15	0	
4507	1,2,6,10,11,15,16,17	3,4,5,7,8,9,12,13,14	0	
4508	1,2,6,10,12,13,14,15	3,4,5,7,8,9,11,16,17	1	Q6AYS4
4509	1,2,6,10,12,13,14,16	3,4,5,7,8,9,11,15,17	2	P97840,Q6AYS4
4510	1,2,6,10,12,13,14,17	3,4,5,7,8,9,11,15,16	6	O54728,P14668,Q05175,Q62635,Q6AYS4,Q8K4G9
4511	1,2,6,10,12,13,15,16	3,4,5,7,8,9,11,14,17	2	P97840,Q6AYS4
4512	1,2,6,10,12,13,15,17	3,4,5,7,8,9,11,14,16	6	P06866,P14668,P55016,Q62635,Q6AYS4,Q6IFV1
4513	1,2,6,10,12,13,16,17	3,4,5,7,8,9,11,14,15	4	P07171,P97840,Q62635,Q6AYS4
4514	1,2,6,10,12,14,15,16	3,4,5,7,8,9,11,13,17	2	P97840,Q6AYS4

4515	1,2,6,10,1 2,14,15,17	3,4,5,7,8,9 ,11,13,16	3	P06866,Q62635,Q6AYS4
4516	1,2,6,10,1 2,14,16,17	3,4,5,7,8,9 ,11,13,15	4	P05539,P97840,Q10758,Q6AYS4
4517	1,2,6,10,1 2,15,16,17	3,4,5,7,8,9 ,11,13,14	3	P06866,P97840,Q6AYS4
4518	1,2,6,10,1 3,14,15,16	3,4,5,7,8,9 ,11,12,17	3	P50115,Q6AYS4,Q99041
4519	1,2,6,10,1 3,14,15,17	3,4,5,7,8,9 ,11,12,16	2	P14668,Q6AYS4
4520	1,2,6,10,1 3,14,16,17	3,4,5,7,8,9 ,11,12,15	1	Q6AYS4
4521	1,2,6,10,1 3,15,16,17	3,4,5,7,8,9 ,11,12,14	1	Q6AYS4
4522	1,2,6,10,1 4,15,16,17	3,4,5,7,8,9 ,11,12,13	1	Q6AYS4
4523	1,2,6,11,1 2,13,14,15	3,4,5,7,8,9 ,10,16,17	2	P47727,Q9JHB9
4524	1,2,6,11,1 2,13,14,16	3,4,5,7,8,9 ,10,15,17	3	P47727,Q6IFU7,Q9JHB9
4525	1,2,6,11,1 2,13,14,17	3,4,5,7,8,9 ,10,15,16	3	P47727,Q62635,Q9JHB9
4526	1,2,6,11,1 2,13,15,16	3,4,5,7,8,9 ,10,14,17	2	Q6AYS4,Q6IFU7
4527	1,2,6,11,1 2,13,15,17	3,4,5,7,8,9 ,10,14,16	2	Q62635,Q6IFV1
4528	1,2,6,11,1 2,13,16,17	3,4,5,7,8,9 ,10,14,15	1	Q62635
4529	1,2,6,11,1 2,14,15,16	3,4,5,7,8,9 ,10,13,17	1	Q6IFU7
4530	1,2,6,11,1 2,14,15,17	3,4,5,7,8,9 ,10,13,16	2	Q00715,Q62635
4531	1,2,6,11,1 2,14,16,17	3,4,5,7,8,9 ,10,13,15	0	
4532	1,2,6,11,1 2,15,16,17	3,4,5,7,8,9 ,10,13,14	1	P06866
4533	1,2,6,11,1 3,14,15,16	3,4,5,7,8,9 ,10,12,17	1	Q6AYS4
4534	1,2,6,11,1 3,14,15,17	3,4,5,7,8,9 ,10,12,16	0	
4535	1,2,6,11,1 3,14,16,17	3,4,5,7,8,9 ,10,12,15	0	
4536	1,2,6,11,1 3,15,16,17	3,4,5,7,8,9 ,10,12,14	0	
4537	1,2,6,11,1 4,15,16,17	3,4,5,7,8,9 ,10,12,13	0	

4538	1,2,6,12,13,14,15,16	3,4,5,7,8,9,10,11,17	3	P00714,P35467,Q6AYS4
4539	1,2,6,12,13,14,15,17	3,4,5,7,8,9,10,11,16	5	P00714,P14668,Q5FVI6,Q62635,Q6AYS4
4540	1,2,6,12,13,14,16,17	3,4,5,7,8,9,10,11,15	3	P00714,Q62635,Q6AYS4
4541	1,2,6,12,13,15,16,17	3,4,5,7,8,9,10,11,14	4	P00714,P06866,Q62635,Q6AYS4
4542	1,2,6,12,14,15,16,17	3,4,5,7,8,9,10,11,13	4	P00714,P06866,P35467,Q6AYS4
4543	1,2,6,13,14,15,16,17	3,4,5,7,8,9,10,11,12	3	P00714,P35467,Q6AYS4
4544	1,2,7,8,9,10,11,12	3,4,5,6,13,14,15,16,17	5	P02803,P10715,P97840,Q6AYL6,Q91ZS3
4545	1,2,7,8,9,10,11,13	3,4,5,6,12,14,15,16,17	4	P01835,P02803,P31044,Q91ZS3
4546	1,2,7,8,9,10,11,14	3,4,5,6,12,13,15,16,17	1	Q9QUL6
4547	1,2,7,8,9,10,11,15	3,4,5,6,12,13,14,16,17	3	P01835,P10715,P35280
4548	1,2,7,8,9,10,11,16	3,4,5,6,12,13,14,15,17	3	P01835,P10715,Q9WUW8
4549	1,2,7,8,9,10,11,17	3,4,5,6,12,13,14,15,16	2	P10715,Q9QUL6
4550	1,2,7,8,9,10,12,13	3,4,5,6,11,14,15,16,17	2	P02803,P97840
4551	1,2,7,8,9,10,12,14	3,4,5,6,11,13,15,16,17	1	P97840
4552	1,2,7,8,9,10,12,15	3,4,5,6,11,13,14,16,17	2	P10715,P97840
4553	1,2,7,8,9,10,12,16	3,4,5,6,11,13,14,15,17	4	P10715,P47967,P97840,Q9WUW8
4554	1,2,7,8,9,10,12,17	3,4,5,6,11,13,14,15,16	4	P10715,P19223,P55091,P97840

4555	1,2,7,8,9,10,13,14	3,4,5,6,11,12,15,16,17	2	P01835,Q5GRG2
4556	1,2,7,8,9,10,13,15	3,4,5,6,11,12,14,16,17	2	P01835,P15999
4557	1,2,7,8,9,10,13,16	3,4,5,6,11,12,14,15,17	4	B0BNN3,P01835,P02625,P97840
4558	1,2,7,8,9,10,13,17	3,4,5,6,11,12,14,15,16	3	P01835,P14668,Q4FZU2
4559	1,2,7,8,9,10,14,15	3,4,5,6,11,12,13,16,17	1	P01835
4560	1,2,7,8,9,10,14,16	3,4,5,6,11,12,13,15,17	2	P01835,P02625
4561	1,2,7,8,9,10,14,17	3,4,5,6,11,12,13,15,16	0	
4562	1,2,7,8,9,10,15,16	3,4,5,6,11,12,13,14,17	1	P01835
4563	1,2,7,8,9,10,15,17	3,4,5,6,11,12,13,14,16	1	P01835
4564	1,2,7,8,9,10,16,17	3,4,5,6,11,12,13,14,15	2	P01835,P02625
4565	1,2,7,8,9,11,12,13	3,4,5,6,10,14,15,16,17	4	P02803,P10715,P36374,Q8CJ52
4566	1,2,7,8,9,11,12,14	3,4,5,6,10,13,15,16,17	3	P02803,P10715,P19629
4567	1,2,7,8,9,11,12,15	3,4,5,6,10,13,14,16,17	1	P10715
4568	1,2,7,8,9,11,12,16	3,4,5,6,10,13,14,15,17	2	P02803,P10715
4569	1,2,7,8,9,11,12,17	3,4,5,6,10,13,14,15,16	4	P02803,P10715,P55091,Q9QZA6

4570	1,2,7,8,9,11,13,14	3,4,5,6,10,12,15,16,17	2	P02803,Q99MH3
4571	1,2,7,8,9,11,13,15	3,4,5,6,10,12,14,16,17	1	P01835
4572	1,2,7,8,9,11,13,16	3,4,5,6,10,12,14,15,17	4	B0BNN3,P01835,P02803,P10715
4573	1,2,7,8,9,11,13,17	3,4,5,6,10,12,14,15,16	3	P02803,P10715,P20766
4574	1,2,7,8,9,11,14,15	3,4,5,6,10,12,13,16,17	3	P16636,P19629,Q99MH3
4575	1,2,7,8,9,11,14,16	3,4,5,6,10,12,13,15,17	1	Q99MH3
4576	1,2,7,8,9,11,14,17	3,4,5,6,10,12,13,15,16	1	P19629
4577	1,2,7,8,9,11,15,16	3,4,5,6,10,12,13,14,17	3	P01835,P10715,Q99MH3
4578	1,2,7,8,9,11,15,17	3,4,5,6,10,12,13,14,16	1	P10715
4579	1,2,7,8,9,11,16,17	3,4,5,6,10,12,13,14,15	1	P10715
4580	1,2,7,8,9,12,13,14	3,4,5,6,10,11,15,16,17	3	P02803,P19629,Q9Z1F2
4581	1,2,7,8,9,12,13,15	3,4,5,6,10,11,14,16,17	1	P10715
4582	1,2,7,8,9,12,13,16	3,4,5,6,10,11,14,15,17	2	P02803,P10715
4583	1,2,7,8,9,12,13,17	3,4,5,6,10,11,14,15,16	5	P02803,P10715,P14668,P55091,Q62635
4584	1,2,7,8,9,12,14,15	3,4,5,6,10,11,13,16,17	2	P10715,P19629

4585	1,2,7,8,9,12,14,16	3,4,5,6,10,11,13,15,17	3	P10715,P19629,Q63532
4586	1,2,7,8,9,12,14,17	3,4,5,6,10,11,13,15,16	4	P10715,P19629,P31430,P55091
4587	1,2,7,8,9,12,15,16	3,4,5,6,10,11,13,14,17	1	P10715
4588	1,2,7,8,9,12,15,17	3,4,5,6,10,11,13,14,16	3	P10715,P55091,Q9QZA6
4589	1,2,7,8,9,12,16,17	3,4,5,6,10,11,13,14,15	2	P10715,P55091
4590	1,2,7,8,9,13,14,15	3,4,5,6,10,11,12,16,17	7	P00762,P01835,P15999,P16636,Q4FZU2,Q5GRG2,Q6IFW6
4591	1,2,7,8,9,13,14,16	3,4,5,6,10,11,12,15,17	4	P00762,P01835,Q4FZU2,Q99MH3
4592	1,2,7,8,9,13,14,17	3,4,5,6,10,11,12,15,16	3	P53792,Q4FZU2,Q6IFW6
4593	1,2,7,8,9,13,15,16	3,4,5,6,10,11,12,14,17	4	P00762,P01835,Q4FZU2,Q6IFW6
4594	1,2,7,8,9,13,15,17	3,4,5,6,10,11,12,14,16	4	P01835,P53792,Q4FZU2,Q6IFW6
4595	1,2,7,8,9,13,16,17	3,4,5,6,10,11,12,14,15	2	P01835,Q4FZU2
4596	1,2,7,8,9,14,15,16	3,4,5,6,10,11,12,13,17	4	P01835,P16636,Q99MH3,Q9QZK9
4597	1,2,7,8,9,14,15,17	3,4,5,6,10,11,12,13,16	3	P19629,Q4FZU2,Q6IFW6
4598	1,2,7,8,9,14,16,17	3,4,5,6,10,11,12,13,15	1	Q4FZU2
4599	1,2,7,8,9,15,16,17	3,4,5,6,10,11,12,13,14	2	P01835,Q4FZU2

4600	1,2,7,8,10, 11,12,13	3,4,5,6,9,1 4,15,16,17	6	P01835,P02803,P10715,P36374,P47727,Q8CJ52
4601	1,2,7,8,10, 11,12,14	3,4,5,6,9,1 3,15,16,17	8	P10715,P19629,P35280,P47727,P62804,Q00715,Q6AYL6,Q8CJ52
4602	1,2,7,8,10, 11,12,15	3,4,5,6,9,1 3,14,16,17	4	P00731,P01835,P10715,P35280
4603	1,2,7,8,10, 11,12,16	3,4,5,6,9,1 3,14,15,17	7	P01835,P10715,P47967,P97840,Q6AYL6,Q8CJ52,Q9WUW8
4604	1,2,7,8,10, 11,12,17	3,4,5,6,9,1 3,14,15,16	5	P10715,P55091,P62804,Q00715,Q9QZA6
4605	1,2,7,8,10, 11,13,14	3,4,5,6,9,1 2,15,16,17	4	P01835,P31044,P47727,Q5GRG2
4606	1,2,7,8,10, 11,13,15	3,4,5,6,9,1 2,14,16,17	3	P01835,P15999,Q5GRG2
4607	1,2,7,8,10, 11,13,16	3,4,5,6,9,1 2,14,15,17	1	P01835
4608	1,2,7,8,10, 11,13,17	3,4,5,6,9,1 2,14,15,16	1	P01835
4609	1,2,7,8,10, 11,14,15	3,4,5,6,9,1 2,13,16,17	4	P01835,P19629,P22006,P35280
4610	1,2,7,8,10, 11,14,16	3,4,5,6,9,1 2,13,15,17	2	P01835,Q63532
4611	1,2,7,8,10, 11,14,17	3,4,5,6,9,1 2,13,15,16	0	
4612	1,2,7,8,10, 11,15,16	3,4,5,6,9,1 2,13,14,17	2	P01835,P35280
4613	1,2,7,8,10, 11,15,17	3,4,5,6,9,1 2,13,14,16	1	P01835
4614	1,2,7,8,10, 11,16,17	3,4,5,6,9,1 2,13,14,15	2	P01835,P02625
4615	1,2,7,8,10, 12,13,14	3,4,5,6,9,1 1,15,16,17	3	P12020,Q5GRG2,Q8K4G9
4616	1,2,7,8,10, 12,13,15	3,4,5,6,9,1 1,14,16,17	3	P01835,P15999,Q5GRG2
4617	1,2,7,8,10, 12,13,16	3,4,5,6,9,1 1,14,15,17	3	P01835,P47967,P97840
4618	1,2,7,8,10, 12,13,17	3,4,5,6,9,1 1,14,15,16	3	P14668,P55091,Q62635
4619	1,2,7,8,10, 12,14,15	3,4,5,6,9,1 1,13,16,17	2	P19629,P35280
4620	1,2,7,8,10, 12,14,16	3,4,5,6,9,1 1,13,15,17	3	P08721,P97840,Q63532
4621	1,2,7,8,10, 12,14,17	3,4,5,6,9,1 1,13,15,16	3	P19629,P31430,P55091
4622	1,2,7,8,10, 12,15,16	3,4,5,6,9,1 1,13,14,17	4	P01835,P08721,P10715,P97840

4623	1,2,7,8,10,<br>12,15,17	3,4,5,6,9,1<br>1,13,14,16	4	P06866,P10715,P55091,Q9QZA6
4624	1,2,7,8,10,<br>12,16,17	3,4,5,6,9,1<br>1,13,14,15	5	P02625,P06866,P10715,P55091,P97840
4625	1,2,7,8,10,<br>13,14,15	3,4,5,6,9,1<br>1,12,16,17	3	P01835,P15999,Q5GRG2
4626	1,2,7,8,10,<br>13,14,16	3,4,5,6,9,1<br>1,12,15,17	4	P01835,P02625,Q5GRG2,Q63532
4627	1,2,7,8,10,<br>13,14,17	3,4,5,6,9,1<br>1,12,15,16	3	P01835,P14668,Q5GRG2
4628	1,2,7,8,10,<br>13,15,16	3,4,5,6,9,1<br>1,12,14,17	2	P01835,P15999
4629	1,2,7,8,10,<br>13,15,17	3,4,5,6,9,1<br>1,12,14,16	3	P01835,P14668,P15999
4630	1,2,7,8,10,<br>13,16,17	3,4,5,6,9,1<br>1,12,14,15	2	P01835,P02625
4631	1,2,7,8,10,<br>14,15,16	3,4,5,6,9,1<br>1,12,13,17	2	P01835,Q63532
4632	1,2,7,8,10,<br>14,15,17	3,4,5,6,9,1<br>1,12,13,16	1	P01835
4633	1,2,7,8,10,<br>14,16,17	3,4,5,6,9,1<br>1,12,13,15	2	P01835,P02625
4634	1,2,7,8,10,<br>15,16,17	3,4,5,6,9,1<br>1,12,13,14	1	P01835
4635	1,2,7,8,11,<br>12,13,14	3,4,5,6,9,1<br>0,15,16,17	8	P02803,P10715,P19629,P47727,Q5GRG2,Q62902,Q8CJ52,Q9JHB9
4636	1,2,7,8,11,<br>12,13,15	3,4,5,6,9,1<br>0,14,16,17	4	P01835,P10715,P15999,Q8CJ52
4637	1,2,7,8,11,<br>12,13,16	3,4,5,6,9,1<br>0,14,15,17	4	P01835,P02803,P10715,Q8CJ52
4638	1,2,7,8,11,<br>12,13,17	3,4,5,6,9,1<br>0,14,15,16	6	P02803,P10715,P36374,P55091,Q62635,Q8CJ52
4639	1,2,7,8,11,<br>12,14,15	3,4,5,6,9,1<br>0,13,16,17	3	P10715,P19629,P35280
4640	1,2,7,8,11,<br>12,14,16	3,4,5,6,9,1<br>0,13,15,17	4	P10715,P19629,Q63532,Q8CJ52
4641	1,2,7,8,11,<br>12,14,17	3,4,5,6,9,1<br>0,13,15,16	8	P10715,P19629,P31430,P55091,P62804,Q00715,Q63493,Q9QZA6
4642	1,2,7,8,11,<br>12,15,16	3,4,5,6,9,1<br>0,13,14,17	2	P01835,P10715
4643	1,2,7,8,11,<br>12,15,17	3,4,5,6,9,1<br>0,13,14,16	5	P06866,P10715,P19629,P55091,Q9QZA6
4644	1,2,7,8,11,<br>12,16,17	3,4,5,6,9,1<br>0,13,14,15	4	P06866,P10715,P55091,Q9QZA6
4645	1,2,7,8,11,<br>13,14,15	3,4,5,6,9,1<br>0,12,16,17	5	P01835,P15999,P19629,Q09030,Q5GRG2

4646	1,2,7,8,11,13,14,16	3,4,5,6,9,10,12,15,17	3	P01835,Q7TPB1,Q99MH3
4647	1,2,7,8,11,13,14,17	3,4,5,6,9,10,12,15,16	1	P19629
4648	1,2,7,8,11,13,15,16	3,4,5,6,9,10,12,14,17	1	P01835
4649	1,2,7,8,11,13,15,17	3,4,5,6,9,10,12,14,16	2	P01835,P15999
4650	1,2,7,8,11,13,16,17	3,4,5,6,9,10,12,14,15	1	P01835
4651	1,2,7,8,11,14,15,16	3,4,5,6,9,10,12,13,17	5	P01835,P16636,P19629,Q63532,Q99MH3
4652	1,2,7,8,11,14,15,17	3,4,5,6,9,10,12,13,16	1	P19629
4653	1,2,7,8,11,14,16,17	3,4,5,6,9,10,12,13,15	1	P19629
4654	1,2,7,8,11,15,16,17	3,4,5,6,9,10,12,13,14	2	P01835,P10715
4655	1,2,7,8,12,13,14,15	3,4,5,6,9,10,11,16,17	3	P15999,P19629,Q5GRG2
4656	1,2,7,8,12,13,14,16	3,4,5,6,9,10,11,15,17	3	P19629,Q63532,Q8CJ52
4657	1,2,7,8,12,13,14,17	3,4,5,6,9,10,11,15,16	5	P14668,P19629,P31430,P55091,Q62635
4658	1,2,7,8,12,13,15,16	3,4,5,6,9,10,11,14,17	2	P01835,P10715
4659	1,2,7,8,12,13,15,17	3,4,5,6,9,10,11,14,16	5	P10715,P14668,P15999,P55091,Q62635
4660	1,2,7,8,12,13,16,17	3,4,5,6,9,10,11,14,15	4	P10715,P55091,P63029,Q62635
4661	1,2,7,8,12,14,15,16	3,4,5,6,9,10,11,13,17	3	P10715,P19629,Q63532
4662	1,2,7,8,12,14,15,17	3,4,5,6,9,10,11,13,16	5	P10715,P19629,P31430,P55091,Q9QZA6
4663	1,2,7,8,12,14,16,17	3,4,5,6,9,10,11,13,15	4	P10715,P19629,P31430,P55091
4664	1,2,7,8,12,15,16,17	3,4,5,6,9,10,11,13,14	4	P06866,P10715,P55091,Q9QZA6
4665	1,2,7,8,13,14,15,16	3,4,5,6,9,10,11,12,17	8	P00762,P01835,P15999,P16636,Q08849,Q09030,Q4FZU2,Q5GRG2
4666	1,2,7,8,13,14,15,17	3,4,5,6,9,10,11,12,16	7	P01835,P15999,P19629,Q08849,Q4FZU2,Q5GRG2,Q6IFW6
4667	1,2,7,8,13,14,16,17	3,4,5,6,9,10,11,12,15	4	P01835,Q08849,Q4FZU2,Q6PCU2
4668	1,2,7,8,13,15,16,17	3,4,5,6,9,10,11,12,14	4	P00762,P01835,Q4FZU2,Q6IFW6

4669	1,2,7,8,14,15,16,17	3,4,5,6,9,10,11,12,13	3	P01835,P19629,Q08849
4670	1,2,7,9,10,11,12,13	3,4,5,6,8,14,15,16,17	7	P02803,P06760,P10536,P47967,P97840,Q63661,Q91ZS3
4671	1,2,7,9,10,11,12,14	3,4,5,6,8,13,15,16,17	4	P06760,P97840,Q63661,Q91ZS3
4672	1,2,7,9,10,11,12,15	3,4,5,6,8,13,14,16,17	4	P06760,P51907,P97840,Q63661
4673	1,2,7,9,10,11,12,16	3,4,5,6,8,13,14,15,17	9	P06760,P10536,P12368,P47967,P97840,Q63661,Q64093,Q91ZS3,Q9WUW8
4674	1,2,7,9,10,11,12,17	3,4,5,6,8,13,14,15,16	5	O54861,P06757,P06866,P97840,Q9QUL6
4675	1,2,7,9,10,11,13,14	3,4,5,6,8,12,15,16,17	4	B0BNN3,P06760,Q4G075,Q91ZS3
4676	1,2,7,9,10,11,13,15	3,4,5,6,8,12,14,16,17	4	P01835,P06760,Q4G075,Q9Z0V6
4677	1,2,7,9,10,11,13,16	3,4,5,6,8,12,14,15,17	8	B0BNN3,P01835,P02793;Q7TP54,P06760,P10536,P97840,Q4G075,Q9Z0V6
4678	1,2,7,9,10,11,13,17	3,4,5,6,8,12,14,15,16	2	P14668,Q9QUL6
4679	1,2,7,9,10,11,14,15	3,4,5,6,8,12,13,16,17	4	P06760,P16636,Q4G075,Q63661
4680	1,2,7,9,10,11,14,16	3,4,5,6,8,12,13,15,17	5	B0BNN3,P06760,Q4G075,Q63532,Q99MH3
4681	1,2,7,9,10,11,14,17	3,4,5,6,8,12,13,15,16	2	P82995,Q9QUL6
4682	1,2,7,9,10,11,15,16	3,4,5,6,8,12,13,14,17	5	P01835,P06760,P51907,Q4G075,Q9QZK9
4683	1,2,7,9,10,11,15,17	3,4,5,6,8,12,13,14,16	1	Q9QUL6
4684	1,2,7,9,10,11,16,17	3,4,5,6,8,12,13,14,15	2	Q64093,Q9QUL6
4685	1,2,7,9,10,12,13,14	3,4,5,6,8,11,15,16,17	3	P06760,P14668,P97840
4686	1,2,7,9,10,12,13,15	3,4,5,6,8,11,14,16,17	4	P02793;Q7TP54,P06760,P14668,P97840
4687	1,2,7,9,10,12,13,16	3,4,5,6,8,11,14,15,17	7	B0BNN3,P02793;Q7TP54,P02803,P06760,P14668,P47967,P97840
4688	1,2,7,9,10,12,13,17	3,4,5,6,8,11,14,15,16	2	P14668,P97840
4689	1,2,7,9,10,12,14,15	3,4,5,6,8,11,13,16,17	3	P06760,P14668,P97840
4690	1,2,7,9,10,12,14,16	3,4,5,6,8,11,13,15,17	4	P06760,P14668,P97840,Q63532
4691	1,2,7,9,10,12,14,17	3,4,5,6,8,11,13,15,16	2	P14668,P97840

4692	1,2,7,9,10,<br>12,15,16	3,4,5,6,8,1<br>1,13,14,17	5	P02793;Q7TP54,P06760,P14668,P97840,Q6AYS4
4693	1,2,7,9,10,<br>12,15,17	3,4,5,6,8,1<br>1,13,14,16	3	P06866,P14668,P97840
4694	1,2,7,9,10,<br>12,16,17	3,4,5,6,8,1<br>1,13,14,15	3	P06866,P14668,P97840
4695	1,2,7,9,10,<br>13,14,15	3,4,5,6,8,1<br>1,12,16,17	6	P01835,P06760,P14668,P16636,Q4G075,Q6IFW6
4696	1,2,7,9,10,<br>13,14,16	3,4,5,6,8,1<br>1,12,15,17	8	B0BNN3,P01835,P06760,P14668,P97840,Q4G075,Q63532,Q63751
4697	1,2,7,9,10,<br>13,14,17	3,4,5,6,8,1<br>1,12,15,16	2	P14668,Q4FZU2
4698	1,2,7,9,10,<br>13,15,16	3,4,5,6,8,1<br>1,12,14,17	8	P01835,P02793;Q7TP54,P06760,P14668,P97840,Q4G075,Q6AYS4,Q9Z0V6
4699	1,2,7,9,10,<br>13,15,17	3,4,5,6,8,1<br>1,12,14,16	3	P01835,P14668,Q6IFW6
4700	1,2,7,9,10,<br>13,16,17	3,4,5,6,8,1<br>1,12,14,15	5	P01835,P02625,P14668,P97840,Q4FZU2
4701	1,2,7,9,10,<br>14,15,16	3,4,5,6,8,1<br>1,12,13,17	8	P01835,P06760,P16636,Q4G075,Q63532,Q63751,Q6AYS4,Q9QZK9
4702	1,2,7,9,10,<br>14,15,17	3,4,5,6,8,1<br>1,12,13,16	1	P14668
4703	1,2,7,9,10,<br>14,16,17	3,4,5,6,8,1<br>1,12,13,15	2	P02625,P14668
4704	1,2,7,9,10,<br>15,16,17	3,4,5,6,8,1<br>1,12,13,14	4	P01835,P02780,P0C0A9,P14668
4705	1,2,7,9,11,<br>12,13,14	3,4,5,6,8,1<br>0,15,16,17	2	P02803,P06760
4706	1,2,7,9,11,<br>12,13,15	3,4,5,6,8,1<br>0,14,16,17	1	P06760
4707	1,2,7,9,11,<br>12,13,16	3,4,5,6,8,1<br>0,14,15,17	4	B0BNN3,P02803,P06760,Q9Z0V6
4708	1,2,7,9,11,<br>12,13,17	3,4,5,6,8,1<br>0,14,15,16	2	P02803,P14668
4709	1,2,7,9,11,<br>12,14,15	3,4,5,6,8,1<br>0,13,16,17	4	D3ZUC6,P06760,P51907,Q99MH3
4710	1,2,7,9,11,<br>12,14,16	3,4,5,6,8,1<br>0,13,15,17	4	D3ZUC6,P06760,Q63532,Q99MH3
4711	1,2,7,9,11,<br>12,14,17	3,4,5,6,8,1<br>0,13,15,16	1	D3ZUC6
4712	1,2,7,9,11,<br>12,15,16	3,4,5,6,8,1<br>0,13,14,17	2	P06760,P51907
4713	1,2,7,9,11,<br>12,15,17	3,4,5,6,8,1<br>0,13,14,16	1	P06866
4714	1,2,7,9,11,<br>12,16,17	3,4,5,6,8,1<br>0,13,14,15	1	P06866

4715	1,2,7,9,11,13,14,15	3,4,5,6,8,10,12,16,17	4	P06760,P16636,Q4G075,Q99MH3
4716	1,2,7,9,11,13,14,16	3,4,5,6,8,10,12,15,17	6	B0BNN3,P06760,Q4G075,Q7TPB1,Q99MH3,Q9Z0V6
4717	1,2,7,9,11,13,14,17	3,4,5,6,8,10,12,15,16	1	P14668
4718	1,2,7,9,11,13,15,16	3,4,5,6,8,10,12,14,17	4	P01835,P06760,Q4G075,Q9Z0V6
4719	1,2,7,9,11,13,15,17	3,4,5,6,8,10,12,14,16	1	P14668
4720	1,2,7,9,11,13,16,17	3,4,5,6,8,10,12,14,15	0	
4721	1,2,7,9,11,14,15,16	3,4,5,6,8,10,12,13,17	6	P06760,P16636,Q4G075,Q63532,Q99MH3,Q9QZK9
4722	1,2,7,9,11,14,15,17	3,4,5,6,8,10,12,13,16	0	
4723	1,2,7,9,11,14,16,17	3,4,5,6,8,10,12,13,15	2	P82995,Q99MH3
4724	1,2,7,9,11,15,16,17	3,4,5,6,8,10,12,13,14	0	
4725	1,2,7,9,12,13,14,15	3,4,5,6,8,10,11,16,17	7	D3ZUC6,P06760,P07150,P08649,P14668,P16636,Q6AYS4
4726	1,2,7,9,12,13,14,16	3,4,5,6,8,10,11,15,17	6	D3ZUC6,P06760,P08649,P14668,Q63532,Q6AYS4
4727	1,2,7,9,12,13,14,17	3,4,5,6,8,10,11,15,16	1	P14668
4728	1,2,7,9,12,13,15,16	3,4,5,6,8,10,11,14,17	6	P02793;Q7TP54,P06760,P08649,P14668,P97840,Q6AYS4
4729	1,2,7,9,12,13,15,17	3,4,5,6,8,10,11,14,16	1	P14668
4730	1,2,7,9,12,13,16,17	3,4,5,6,8,10,11,14,15	1	P14668
4731	1,2,7,9,12,14,15,16	3,4,5,6,8,10,11,13,17	6	D3ZUC6,P06760,P16636,Q63532,Q6AYS4,Q99MH3
4732	1,2,7,9,12,14,15,17	3,4,5,6,8,10,11,13,16	2	D3ZUC6,P14668
4733	1,2,7,9,12,14,16,17	3,4,5,6,8,10,11,13,15	2	D3ZUC6,P14668
4734	1,2,7,9,12,15,16,17	3,4,5,6,8,10,11,13,14	3	P06866,P14668,Q6AYS4
4735	1,2,7,9,13,14,15,16	3,4,5,6,8,10,11,12,17	13	P00762,P01835,P06760,P14668,P16636,Q4FZU2,Q4G075,Q6AYS4,Q6IFW6,Q6IG02,Q99MH3,Q9QZK9,Q9Z0V6
4736	1,2,7,9,13,14,15,17	3,4,5,6,8,10,11,12,16	8	P14668,P16636,P53792,Q4FZU2,Q6IFW6,Q6IG02,Q6IMF3,Q6P6Q2
4737	1,2,7,9,13,14,16,17	3,4,5,6,8,10,11,12,15	3	P14668,Q4FZU2,Q6IFW6

4738	1,2,7,9,13,<br>15,16,17	3,4,5,6,8,1<br>0,11,12,14	7	P01835,P0C0A9,P14668,Q4FZU2,Q6AYS4,Q6IFW6,Q6IG02
4739	1,2,7,9,14,<br>15,16,17	3,4,5,6,8,1<br>0,11,12,13	7	P02781,P0C0A9,P14668,P16636,Q4FZU2,Q6AYS4,Q6IFW6
4740	1,2,7,10,1<br>1,12,13,14	3,4,5,6,8,9<br>,15,16,17	2	P23593,P47727
4741	1,2,7,10,1<br>1,12,13,15	3,4,5,6,8,9<br>,14,16,17	2	P01835,P02793;Q7TP54
4742	1,2,7,10,1<br>1,12,13,16	3,4,5,6,8,9<br>,14,15,17	5	P01835,P02793;Q7TP54,P12368,P47967,P97840
4743	1,2,7,10,1<br>1,12,13,17	3,4,5,6,8,9<br>,14,15,16	1	P14668
4744	1,2,7,10,1<br>1,12,14,15	3,4,5,6,8,9<br>,13,16,17	4	P06760,P23593,P35280,P51907
4745	1,2,7,10,1<br>1,12,14,16	3,4,5,6,8,9<br>,13,15,17	4	P06760,P47967,P97840,Q63532
4746	1,2,7,10,1<br>1,12,14,17	3,4,5,6,8,9<br>,13,15,16	0	
4747	1,2,7,10,1<br>1,12,15,16	3,4,5,6,8,9<br>,13,14,17	6	P01835,P02793;Q7TP54,P06760,P47967,P51907,P97840
4748	1,2,7,10,1<br>1,12,15,17	3,4,5,6,8,9<br>,13,14,16	1	P06866
4749	1,2,7,10,1<br>1,12,16,17	3,4,5,6,8,9<br>,13,14,15	3	P06757,P06866,P97840
4750	1,2,7,10,1<br>1,13,14,15	3,4,5,6,8,9<br>,12,16,17	5	P01835,P06760,P16636,P23593,Q09030
4751	1,2,7,10,1<br>1,13,14,16	3,4,5,6,8,9<br>,12,15,17	5	B0BNN3,P01835,P06760,Q63532,Q7TPB1
4752	1,2,7,10,1<br>1,13,14,17	3,4,5,6,8,9<br>,12,15,16	1	P14668
4753	1,2,7,10,1<br>1,13,15,16	3,4,5,6,8,9<br>,12,14,17	4	P01835,P02793;Q7TP54,P06760,Q9Z0V6
4754	1,2,7,10,1<br>1,13,15,17	3,4,5,6,8,9<br>,12,14,16	2	P01835,P14668
4755	1,2,7,10,1<br>1,13,16,17	3,4,5,6,8,9<br>,12,14,15	1	P01835
4756	1,2,7,10,1<br>1,14,15,16	3,4,5,6,8,9<br>,12,13,17	5	P01835,P06760,P16636,P51907,Q63532
4757	1,2,7,10,1<br>1,14,15,17	3,4,5,6,8,9<br>,12,13,16	0	
4758	1,2,7,10,1<br>1,14,16,17	3,4,5,6,8,9<br>,12,13,15	1	Q63532
4759	1,2,7,10,1<br>1,15,16,17	3,4,5,6,8,9<br>,12,13,14	1	P01835
4760	1,2,7,10,1<br>2,13,14,15	3,4,5,6,8,9<br>,11,16,17	3	P06760,P14668,P97840

4761	1,2,7,10,12,13,14,16 3,4,5,6,8,9,11,15,17	6	P06760,P14668,P47967,P97840,Q63532,Q6AYS4
4762	1,2,7,10,12,13,14,17 3,4,5,6,8,9,11,15,16	1	P14668
4763	1,2,7,10,12,13,15,16 3,4,5,6,8,9,11,14,17	7	P01835,P02793;Q7TP54,P06760,P14668,P47967,P97840,Q6AYS4
4764	1,2,7,10,12,13,15,17 3,4,5,6,8,9,11,14,16	3	P02793;Q7TP54,P06866,P14668
4765	1,2,7,10,12,13,16,17 3,4,5,6,8,9,11,14,15	4	P02793;Q7TP54,P06866,P14668,P97840
4766	1,2,7,10,12,14,15,16 3,4,5,6,8,9,11,13,17	6	P06760,P14668,P35467,P97840,Q63532,Q6AYS4
4767	1,2,7,10,12,14,15,17 3,4,5,6,8,9,11,13,16	2	P06866,P14668
4768	1,2,7,10,12,14,16,17 3,4,5,6,8,9,11,13,15	4	P06866,P14668,P97840,Q63532
4769	1,2,7,10,12,15,16,17 3,4,5,6,8,9,11,13,14	4	P02793;Q7TP54,P06866,P14668,P97840
4770	1,2,7,10,13,14,15,16 3,4,5,6,8,9,11,12,17	8	P01835,P06760,P14668,P16636,Q09030,Q4G075,Q63532,Q6AYS4
4771	1,2,7,10,13,14,15,17 3,4,5,6,8,9,11,12,16	3	P01835,P14668,Q6IFW6
4772	1,2,7,10,13,14,16,17 3,4,5,6,8,9,11,12,15	3	P01835,P02625,P14668
4773	1,2,7,10,13,15,16,17 3,4,5,6,8,9,11,12,14	4	P01835,P02793;Q7TP54,P0C0A9,P14668
4774	1,2,7,10,14,15,16,17 3,4,5,6,8,9,11,12,13	6	P01835,P02780,P0C0A9,P14668,P35467,Q63532
4775	1,2,7,11,12,13,14,15 3,4,5,6,8,9,10,16,17	5	D3ZUC6,P06760,P23593,P25031,Q09030
4776	1,2,7,11,12,13,14,16 3,4,5,6,8,9,10,15,17	4	D3ZUC6,P06760,Q63532,Q7TPB1
4777	1,2,7,11,12,13,14,17 3,4,5,6,8,9,10,15,16	2	D3ZUC6,P14668
4778	1,2,7,11,12,13,15,16 3,4,5,6,8,9,10,14,17	3	P01835,P02793;Q7TP54,P06760
4779	1,2,7,11,12,13,15,17 3,4,5,6,8,9,10,14,16	2	P06866,P14668
4780	1,2,7,11,12,13,16,17 3,4,5,6,8,9,10,14,15	1	P06866
4781	1,2,7,11,12,14,15,16 3,4,5,6,8,9,10,13,17	5	D3ZUC6,P06760,P16636,P51907,Q63532
4782	1,2,7,11,12,14,15,17 3,4,5,6,8,9,10,13,16	2	D3ZUC6,P06866
4783	1,2,7,11,12,14,16,17 3,4,5,6,8,9,10,13,15	3	D3ZUC6,P06866,Q63532

4784	1,2,7,11,12,15,16,17	3,4,5,6,8,9,10,13,14	1	P06866
4785	1,2,7,11,13,14,15,16	3,4,5,6,8,9,10,12,17	7	P01835,P06760,P16636,Q09030,Q4G075,Q63532,Q99MH3
4786	1,2,7,11,13,14,15,17	3,4,5,6,8,9,10,12,16	1	P14668
4787	1,2,7,11,13,14,16,17	3,4,5,6,8,9,10,12,15	0	
4788	1,2,7,11,13,15,16,17	3,4,5,6,8,9,10,12,14	1	P01835
4789	1,2,7,11,14,15,16,17	3,4,5,6,8,9,10,12,13	1	P16636
4790	1,2,7,12,13,14,15,16	3,4,5,6,8,9,10,11,17	9	D3ZUC6,P06760,P08649,P14668,P16636,P35467,Q09030,Q63532,Q6AYS4
4791	1,2,7,12,13,14,15,17	3,4,5,6,8,9,10,11,16	3	D3ZUC6,P08649,P14668
4792	1,2,7,12,13,14,16,17	3,4,5,6,8,9,10,11,15	3	D3ZUC6,P14668,Q6AYS4
4793	1,2,7,12,13,15,16,17	3,4,5,6,8,9,10,11,14	4	P02793;Q7TP54,P06866,P14668,Q6AYS4
4794	1,2,7,12,14,15,16,17	3,4,5,6,8,9,10,11,13	6	D3ZUC6,P06866,P14668,P35467,Q63532,Q6AYS4
4795	1,2,7,13,14,15,16,17	3,4,5,6,8,9,10,11,12	10	P00714,P01835,P0C0A9,P14668,P16636,P35467,Q4FZU2,Q6AYS4,Q6IFW6,Q6IG02
4796	1,2,8,9,10,11,12,13	3,4,5,6,7,14,15,16,17	1	P02803
4797	1,2,8,9,10,11,12,14	3,4,5,6,7,13,15,16,17	2	P02803,P62804
4798	1,2,8,9,10,11,12,15	3,4,5,6,7,13,14,16,17	0	
4799	1,2,8,9,10,11,12,16	3,4,5,6,7,13,14,15,17	3	P02803,P20760,Q9WUW8
4800	1,2,8,9,10,11,12,17	3,4,5,6,7,13,14,15,16	2	P02803,P62804
4801	1,2,8,9,10,11,13,14	3,4,5,6,7,12,15,16,17	1	P02803
4802	1,2,8,9,10,11,13,15	3,4,5,6,7,12,14,16,17	1	P01835
4803	1,2,8,9,10,11,13,16	3,4,5,6,7,12,14,15,17	3	P01835,P02803,Q8CJD3
4804	1,2,8,9,10,11,13,17	3,4,5,6,7,12,14,15,16	1	P02803
4805	1,2,8,9,10,11,14,15	3,4,5,6,7,12,13,16,17	3	P09605;P25809,Q99MH3,Q9QZK9
4806	1,2,8,9,10,11,14,16	3,4,5,6,7,12,13,15,17	4	P08937,Q99MH3,Q9QZK9,Q9WUW8

4807	1,2,8,9,10,<br>11,14,17	3,4,5,6,7,1<br>2,13,15,16	1	Q9QUL6
4808	1,2,8,9,10,<br>11,15,16	3,4,5,6,7,1<br>2,13,14,17	3	P01835,Q9QZK9,Q9WUW8
4809	1,2,8,9,10,<br>11,15,17	3,4,5,6,7,1<br>2,13,14,16	0	
4810	1,2,8,9,10,<br>11,16,17	3,4,5,6,7,1<br>2,13,14,15	1	A2RUV9
4811	1,2,8,9,10,<br>12,13,14	3,4,5,6,7,1<br>1,15,16,17	2	P02803,Q8K4G9
4812	1,2,8,9,10,<br>12,13,15	3,4,5,6,7,1<br>1,14,16,17	1	P15999
4813	1,2,8,9,10,<br>12,13,16	3,4,5,6,7,1<br>1,14,15,17	2	P02803,P97840
4814	1,2,8,9,10,<br>12,13,17	3,4,5,6,7,1<br>1,14,15,16	3	P02803,P14668,Q8K4G9
4815	1,2,8,9,10,<br>12,14,15	3,4,5,6,7,1<br>1,13,16,17	0	
4816	1,2,8,9,10,<br>12,14,16	3,4,5,6,7,1<br>1,13,15,17	3	P05539,Q63751,Q9WUW8
4817	1,2,8,9,10,<br>12,14,17	3,4,5,6,7,1<br>1,13,15,16	3	P05539,Q8K4G9,Q8VD89
4818	1,2,8,9,10,<br>12,15,16	3,4,5,6,7,1<br>1,13,14,17	2	P05539,Q9WUW8
4819	1,2,8,9,10,<br>12,15,17	3,4,5,6,7,1<br>1,13,14,16	1	P06866
4820	1,2,8,9,10,<br>12,16,17	3,4,5,6,7,1<br>1,13,14,15	2	P05539,P06866
4821	1,2,8,9,10,<br>13,14,15	3,4,5,6,7,1<br>1,12,16,17	4	P01835,P09605;P25809,P15999,Q63751
4822	1,2,8,9,10,<br>13,14,16	3,4,5,6,7,1<br>1,12,15,17	3	P01835,Q63751,Q9QZK9
4823	1,2,8,9,10,<br>13,14,17	3,4,5,6,7,1<br>1,12,15,16	2	Q4FZU2,Q8K4G9
4824	1,2,8,9,10,<br>13,15,16	3,4,5,6,7,1<br>1,12,14,17	3	P01835,Q63751,Q9QZK9
4825	1,2,8,9,10,<br>13,15,17	3,4,5,6,7,1<br>1,12,14,16	2	P01835,P15999
4826	1,2,8,9,10,<br>13,16,17	3,4,5,6,7,1<br>1,12,14,15	3	P01835,P02625,Q8CJD3
4827	1,2,8,9,10,<br>14,15,16	3,4,5,6,7,1<br>1,12,13,17	9	P01835,P05539,P08937,P09605;P25809,Q63751,Q63942,Q99041,Q99MH3,Q9QZK9
4828	1,2,8,9,10,<br>14,15,17	3,4,5,6,7,1<br>1,12,13,16	1	P17046
4829	1,2,8,9,10,<br>14,16,17	3,4,5,6,7,1<br>1,12,13,15	2	P02625,P05539

4830	1,2,8,9,10,<br>15,16,17	3,4,5,6,7,1<br>1,12,13,14	2	P01835,P05539
4831	1,2,8,9,11,<br>12,13,14	3,4,5,6,7,1<br>0,15,16,17	1	P02803
4832	1,2,8,9,11,<br>12,13,15	3,4,5,6,7,1<br>0,14,16,17	1	P02803
4833	1,2,8,9,11,<br>12,13,16	3,4,5,6,7,1<br>0,14,15,17	2	P02803,P20760
4834	1,2,8,9,11,<br>12,13,17	3,4,5,6,7,1<br>0,14,15,16	1	P02803
4835	1,2,8,9,11,<br>12,14,15	3,4,5,6,7,1<br>0,13,16,17	1	Q99MH3
4836	1,2,8,9,11,<br>12,14,16	3,4,5,6,7,1<br>0,13,15,17	3	P02803,P20760,Q99MH3
4837	1,2,8,9,11,<br>12,14,17	3,4,5,6,7,1<br>0,13,15,16	2	P62804,Q00715
4838	1,2,8,9,11,<br>12,15,16	3,4,5,6,7,1<br>0,13,14,17	0	
4839	1,2,8,9,11,<br>12,15,17	3,4,5,6,7,1<br>0,13,14,16	1	P06866
4840	1,2,8,9,11,<br>12,16,17	3,4,5,6,7,1<br>0,13,14,15	2	P02803,P06866
4841	1,2,8,9,11,<br>13,14,15	3,4,5,6,7,1<br>0,12,16,17	1	Q99MH3
4842	1,2,8,9,11,<br>13,14,16	3,4,5,6,7,1<br>0,12,15,17	4	P02803,Q7TPB1,Q99MH3,Q9QZK9
4843	1,2,8,9,11,<br>13,14,17	3,4,5,6,7,1<br>0,12,15,16	1	Q5FVI6
4844	1,2,8,9,11,<br>13,15,16	3,4,5,6,7,1<br>0,12,14,17	2	P01835,Q9QZK9
4845	1,2,8,9,11,<br>13,15,17	3,4,5,6,7,1<br>0,12,14,16	0	
4846	1,2,8,9,11,<br>13,16,17	3,4,5,6,7,1<br>0,12,14,15	3	A2RUV9,P02803,Q8CJD3
4847	1,2,8,9,11,<br>14,15,16	3,4,5,6,7,1<br>0,12,13,17	3	P08937,Q99MH3,Q9QZK9
4848	1,2,8,9,11,<br>14,15,17	3,4,5,6,7,1<br>0,12,13,16	0	
4849	1,2,8,9,11,<br>14,16,17	3,4,5,6,7,1<br>0,12,13,15	1	Q99MH3
4850	1,2,8,9,11,<br>15,16,17	3,4,5,6,7,1<br>0,12,13,14	1	Q498D9
4851	1,2,8,9,12,<br>13,14,15	3,4,5,6,7,1<br>0,11,16,17	3	P15999,P61206;P84079;P84082,Q5FVI6
4852	1,2,8,9,12,<br>13,14,16	3,4,5,6,7,1<br>0,11,15,17	2	P02803,Q5FVI6

4853	1,2,8,9,12,<br>13,14,17	3,4,5,6,7,1<br>0,11,15,16	5	P02803,P61206;P84079;P84082,Q5FVI6,Q8K4G9,Q8VD89
4854	1,2,8,9,12,<br>13,15,16	3,4,5,6,7,1<br>0,11,14,17	0	
4855	1,2,8,9,12,<br>13,15,17	3,4,5,6,7,1<br>0,11,14,16	3	P15999,P61206;P84079;P84082,Q5FVI6
4856	1,2,8,9,12,<br>13,16,17	3,4,5,6,7,1<br>0,11,14,15	2	P02803,Q5FVI6
4857	1,2,8,9,12,<br>14,15,16	3,4,5,6,7,1<br>0,11,13,17	1	Q99MH3
4858	1,2,8,9,12,<br>14,15,17	3,4,5,6,7,1<br>0,11,13,16	3	P61206;P84079;P84082,Q5FVI6,Q8VD89
4859	1,2,8,9,12,<br>14,16,17	3,4,5,6,7,1<br>0,11,13,15	3	P05539,Q5FVI6,Q8VD89
4860	1,2,8,9,12,<br>15,16,17	3,4,5,6,7,1<br>0,11,13,14	1	P06866
4861	1,2,8,9,13,<br>14,15,16	3,4,5,6,7,1<br>0,11,12,17	8	P01835,P08937,Q4FZU2,Q5FVI6,Q63751,Q63942,Q99MH3,Q9QZK9
4862	1,2,8,9,13,<br>14,15,17	3,4,5,6,7,1<br>0,11,12,16	5	P15999,P61206;P84079;P84082,Q4FZU2,Q5FVI6,Q6IFW6
4863	1,2,8,9,13,<br>14,16,17	3,4,5,6,7,1<br>0,11,12,15	2	Q4FZU2,Q5FVI6
4864	1,2,8,9,13,<br>15,16,17	3,4,5,6,7,1<br>0,11,12,14	5	P01835,P61206;P84079;P84082,Q4FZU2,Q5FVI6,Q6IFW6
4865	1,2,8,9,14,<br>15,16,17	3,4,5,6,7,1<br>0,11,12,13	3	P61206;P84079;P84082,Q4FZU2,Q5FVI6
4866	1,2,8,10,1<br>1,12,13,14	3,4,5,6,7,9<br>,15,16,17	4	P02803,P47727,P62804,Q8K4G9
4867	1,2,8,10,1<br>1,12,13,15	3,4,5,6,7,9<br>,14,16,17	2	P01835,P15999
4868	1,2,8,10,1<br>1,12,13,16	3,4,5,6,7,9<br>,14,15,17	2	P01835,P02803
4869	1,2,8,10,1<br>1,12,13,17	3,4,5,6,7,9<br>,14,15,16	4	P02803,P62804,Q62635,Q8K4G9
4870	1,2,8,10,1<br>1,12,14,15	3,4,5,6,7,9<br>,13,16,17	4	P09605;P25809,P35280,P62804,Q00715
4871	1,2,8,10,1<br>1,12,14,16	3,4,5,6,7,9<br>,13,15,17	3	P05539,P62804,Q9WUW8
4872	1,2,8,10,1<br>1,12,14,17	3,4,5,6,7,9<br>,13,15,16	2	P62804,Q00715
4873	1,2,8,10,1<br>1,12,15,16	3,4,5,6,7,9<br>,13,14,17	2	P01835,Q9WUW8
4874	1,2,8,10,1<br>1,12,15,17	3,4,5,6,7,9<br>,13,14,16	3	P06866,P62804,Q00715
4875	1,2,8,10,1<br>1,12,16,17	3,4,5,6,7,9<br>,13,14,15	2	P05539,P06866

4876	1,2,8,10,11,13,14,15	3,4,5,6,7,9,12,16,17	3	P01835,P09605;P25809,P15999
4877	1,2,8,10,11,13,14,16	3,4,5,6,7,9,12,15,17	3	P01835,P09605;P25809,Q7TPB1
4878	1,2,8,10,11,13,14,17	3,4,5,6,7,9,12,15,16	1	Q8K4G9
4879	1,2,8,10,11,13,15,16	3,4,5,6,7,9,12,14,17	2	P01835,P09605;P25809
4880	1,2,8,10,11,13,15,17	3,4,5,6,7,9,12,14,16	2	P01835,P15999
4881	1,2,8,10,11,13,16,17	3,4,5,6,7,9,12,14,15	2	P01835,Q8CJD3
4882	1,2,8,10,11,14,15,16	3,4,5,6,7,9,12,13,17	3	P01835,P09605;P25809,Q9QZK9
4883	1,2,8,10,11,14,15,17	3,4,5,6,7,9,12,13,16	0	
4884	1,2,8,10,11,14,16,17	3,4,5,6,7,9,12,13,15	1	P05539
4885	1,2,8,10,11,15,16,17	3,4,5,6,7,9,12,13,14	1	P01835
4886	1,2,8,10,12,13,14,15	3,4,5,6,7,9,11,16,17	3	P09605;P25809,P15999,Q8K4G9
4887	1,2,8,10,12,13,14,16	3,4,5,6,7,9,11,15,17	1	Q8K4G9
4888	1,2,8,10,12,13,14,17	3,4,5,6,7,9,11,15,16	3	P14668,Q62635,Q8K4G9
4889	1,2,8,10,12,13,15,16	3,4,5,6,7,9,11,14,17	3	P01835,P15999,P83121
4890	1,2,8,10,12,13,15,17	3,4,5,6,7,9,11,14,16	6	P06866,P14668,P15999,P83121,Q6IFV1,Q8K4G9
4891	1,2,8,10,12,13,16,17	3,4,5,6,7,9,11,14,15	3	P06866,P83121,Q8K4G9
4892	1,2,8,10,12,14,15,16	3,4,5,6,7,9,11,13,17	2	P05539,P09605;P25809
4893	1,2,8,10,12,14,15,17	3,4,5,6,7,9,11,13,16	2	P05539,P06866
4894	1,2,8,10,12,14,16,17	3,4,5,6,7,9,11,13,15	4	P02625,P05539,P06866,Q8K4G9
4895	1,2,8,10,12,15,16,17	3,4,5,6,7,9,11,13,14	3	P05539,P06866,P83121
4896	1,2,8,10,13,14,15,16	3,4,5,6,7,9,11,12,17	4	P01835,P09605;P25809,P15999,Q63751
4897	1,2,8,10,13,14,15,17	3,4,5,6,7,9,11,12,16	3	P01835,P15999,P61206;P84079;P84082
4898	1,2,8,10,13,14,16,17	3,4,5,6,7,9,11,12,15	3	P01835,P02625,Q8K4G9

4899	1,2,8,10,13,15,16,17	3,4,5,6,7,9,11,12,14	3	P01835,P15999,P83121
4900	1,2,8,10,14,15,16,17	3,4,5,6,7,9,11,12,13	2	P01835,P05539
4901	1,2,8,11,12,13,14,15	3,4,5,6,7,9,10,16,17	1	P15999
4902	1,2,8,11,12,13,14,16	3,4,5,6,7,9,10,15,17	1	P02803
4903	1,2,8,11,12,13,14,17	3,4,5,6,7,9,10,15,16	4	Q00715,Q5FVI6,Q62635,Q8K4G9
4904	1,2,8,11,12,13,15,16	3,4,5,6,7,9,10,14,17	1	P01835
4905	1,2,8,11,12,13,15,17	3,4,5,6,7,9,10,14,16	3	P06866,P15999,Q62635
4906	1,2,8,11,12,13,16,17	3,4,5,6,7,9,10,14,15	1	P02803
4907	1,2,8,11,12,14,15,16	3,4,5,6,7,9,10,13,17	0	
4908	1,2,8,11,12,14,15,17	3,4,5,6,7,9,10,13,16	3	P06866,P62804,Q00715
4909	1,2,8,11,12,14,16,17	3,4,5,6,7,9,10,13,15	0	
4910	1,2,8,11,12,15,16,17	3,4,5,6,7,9,10,13,14	1	P06866
4911	1,2,8,11,13,14,15,16	3,4,5,6,7,9,10,12,17	3	P01835,P15999,Q99MH3
4912	1,2,8,11,13,14,15,17	3,4,5,6,7,9,10,12,16	2	P15999,Q5FVI6
4913	1,2,8,11,13,14,16,17	3,4,5,6,7,9,10,12,15	0	
4914	1,2,8,11,13,15,16,17	3,4,5,6,7,9,10,12,14	1	P01835
4915	1,2,8,11,14,15,16,17	3,4,5,6,7,9,10,12,13	0	
4916	1,2,8,12,13,14,15,16	3,4,5,6,7,9,10,11,17	2	P15999,Q5FVI6
4917	1,2,8,12,13,14,15,17	3,4,5,6,7,9,10,11,16	4	P15999,P61206;P84079;P84082,Q5FVI6,Q62635
4918	1,2,8,12,13,14,16,17	3,4,5,6,7,9,10,11,15	3	Q5FVI6,Q6PCU2,Q8K4G9
4919	1,2,8,12,13,15,16,17	3,4,5,6,7,9,10,11,14	6	P06866,P15999,P61206;P84079;P84082,P83121,Q5FVI6,Q5XFX0
4920	1,2,8,12,14,15,16,17	3,4,5,6,7,9,10,11,13	4	P05539,P06866,P61206;P84079;P84082,Q5FVI6
4921	1,2,8,13,14,15,16,17	3,4,5,6,7,9,10,11,12	8	P01835,P15999,P61206;P84079;P84082,Q08849,Q4FZU2,Q5FVI6,Q6IFW6,Q6PCU2

4922	1,2,9,10,11,12,13,14	3,4,5,6,7,8,15,16,17	11	P02803,P06760,P08937,P20760,P20761,P50115,Q4G075,Q63661,Q63751,Q99041,Q9Z0V6
4923	1,2,9,10,11,12,13,15	3,4,5,6,7,8,14,16,17	8	P06760,P08937,P20760,P20761,P50115,Q63661,Q99041,Q9Z0V6
4924	1,2,9,10,11,12,13,16	3,4,5,6,7,8,14,15,17	14	P02803,P06760,P07171,P08937,P10536,P20760,P20761,P50115,P97840,Q63661,Q63751,Q99041,Q9WUW8,Q9Z0V6
4925	1,2,9,10,11,12,13,17	3,4,5,6,7,8,14,15,16	3	P02803,P07171,P14668
4926	1,2,9,10,11,12,14,15	3,4,5,6,7,8,13,16,17	11	P06760,P08937,P20760,P20761,P23928,P50115,P51907,Q63661,Q63751,Q99041,Q9Z0V6
4927	1,2,9,10,11,12,14,16	3,4,5,6,7,8,13,15,17	14	P06760,P08937,P10536,P20760,P20761,P50115,P62815,Q4G075,Q63661,Q63751,Q99041,Q99MH3,Q9WUW8,Q9Z0V6
4928	1,2,9,10,11,12,14,17	3,4,5,6,7,8,13,15,16	3	P06866,Q63661,Q8VD89
4929	1,2,9,10,11,12,15,16	3,4,5,6,7,8,13,14,17	15	P06760,P06761,P08937,P10536,P20760,P20761,P22282,P50115,P51907,P62815,Q63661,Q63751,Q99041,Q9WUW8,Q9Z0V6
4930	1,2,9,10,11,12,15,17	3,4,5,6,7,8,13,14,16	2	P06866,Q63661
4931	1,2,9,10,11,12,16,17	3,4,5,6,7,8,13,14,15	4	P06757,P06761,P06866,P07171
4932	1,2,9,10,11,13,14,15	3,4,5,6,7,8,12,16,17	12	P06760,P08937,P09605;P25809,P20646,P20761,P23928,P50115,Q4G075,Q63661,Q63751,Q99041,Q9Z0V6
4933	1,2,9,10,11,13,14,16	3,4,5,6,7,8,12,15,17	18	B0BNN3,P06760,P06761,P08937,P10536,P20646,P20760,P20761,P50115,P62815,Q4G075,Q63661,Q63751,Q7TPB1,Q99041,Q99MH3,Q9QZK9,Q9Z0V6
4934	1,2,9,10,11,13,14,17	3,4,5,6,7,8,12,15,16	3	P08937,P14668,Q99041
4935	1,2,9,10,11,13,15,16	3,4,5,6,7,8,12,14,17	17	P01835,P06760,P06761,P08937,P10536,P20760,P20761,P22282,P22283,P50115,P62815,Q4G075,Q63661,Q63751,Q99041,Q9QZK9,Q9Z0V6
4936	1,2,9,10,11,13,15,17	3,4,5,6,7,8,12,14,16	4	P08937,P14668,Q99041,Q9Z0V6
4937	1,2,9,10,11,13,16,17	3,4,5,6,7,8,12,14,15	6	P06761,P07171,P08937,Q8CJD3,Q99041,Q9Z0V6
4938	1,2,9,10,11,14,15,16	3,4,5,6,7,8,12,13,17	19	P02780,P06760,P06761,P07647,P08937,P20760,P20761,P22282,P22283,P50115,P51907,P62815,Q4G075,Q63661,Q63751,Q99041,Q99MH3,Q9QZK9,Q9Z0V6
4939	1,2,9,10,11,14,15,17	3,4,5,6,7,8,12,13,16	4	P06761,P08937,Q99041,Q9QUL6
4940	1,2,9,10,11,14,16,17	3,4,5,6,7,8,12,13,15	4	P06761,P08937,Q99041,Q99MH3
4941	1,2,9,10,11,15,16,17	3,4,5,6,7,8,12,13,14	8	P02780,P06761,P06911,P08937,P22282,P22283,Q62902,Q99041
4942	1,2,9,10,12,13,14,15	3,4,5,6,7,8,11,16,17	14	P06760,P07150,P08937,P09605;P25809,P14668,P14669,P20646,P20761,P50115,Q4G075,Q63751,Q6AYS4,Q99041,Q9Z0V6
4943	1,2,9,10,12,13,14,16	3,4,5,6,7,8,11,15,17	16	P06760,P07150,P07171,P08937,P14668,P14669,P20646,P20760,P20761,P50115,P97840,Q4G075,Q63751,Q6AYS4,Q99041,Q9Z0V6
4944	1,2,9,10,12,13,14,17	3,4,5,6,7,8,11,15,16	4	P07150,P07171,P14668,Q8VD89

4945	1,2,9,10,1 2,13,15,16	3,4,5,6,7,8 ,11,14,17	16	P02793;Q7TP54,P06760,P07150,P07171,P08937,P14668,P14669,P20760,P20761,P50115,P97840,Q4G075,Q63751,Q6AYS4,Q99041,Q9Z0V6
4946	1,2,9,10,1 2,13,15,17	3,4,5,6,7,8 ,11,14,16	5	P06866,P07150,P07171,P14668,P61206;P84079;P84082
4947	1,2,9,10,1 2,13,16,17	3,4,5,6,7,8 ,11,14,15	7	P06866,P07150,P07171,P14668,P14669,P97840,Q6AYS4
4948	1,2,9,10,1 2,14,15,16	3,4,5,6,7,8 ,11,13,17	16	P06760,P06761,P07150,P08937,P14669,P20760,P20761,P22282,P22283,P50115,Q4G075,Q63751,Q6AYS4,Q99041,Q9WUW8,Q9Z0V6
4949	1,2,9,10,1 2,14,15,17	3,4,5,6,7,8 ,11,13,16	7	P06866,P07150,P07171,P08937,P14668,P61206;P84079;P84082,Q8VD89
4950	1,2,9,10,1 2,14,16,17	3,4,5,6,7,8 ,11,13,15	9	P05539,P06866,P07171,P08937,P14668,P14669,Q63751,Q6AYS4,Q8VD89
4951	1,2,9,10,1 2,15,16,17	3,4,5,6,7,8 ,11,13,14	11	P06761,P06866,P06911,P07150,P07171,P08937,P14668,P14669,P22282,P22283,Q6AYS4
4952	1,2,9,10,1 3,14,15,16	3,4,5,6,7,8 ,11,12,17	20	P01835,P06760,P06761,P07150,P08937,P09605;P25809,P14668,P14669,P20646,P20761,P22282,P22283,P50115,Q4G075,Q63751,Q6AYS4,Q99041,Q9ESW0,Q9QZK9,Q9Z0V6
4953	1,2,9,10,1 3,14,15,17	3,4,5,6,7,8 ,11,12,16	8	P07150,P08937,P14668,P61206;P84079;P84082,Q63751,Q6AYS4,Q6IFW6,Q99041
4954	1,2,9,10,1 3,14,16,17	3,4,5,6,7,8 ,11,12,15	10	P02625,P07150,P07171,P08937,P14668,P14669,Q63751,Q6AYS4,Q99041,Q9ESW0
4955	1,2,9,10,1 3,15,16,17	3,4,5,6,7,8 ,11,12,14	16	P01835,P02780,P02781,P06761,P07150,P07171,P08937,P0C0A9,P14668,P14669,P22282,P22283,Q63751,Q6AYS4,Q99041,Q9Z0V6
4956	1,2,9,10,1 4,15,16,17	3,4,5,6,7,8 ,11,12,13	18	P02780,P02781,P06761,P07150,P07647,P08937,P0C0A9,P14668,P14669,P22282,P22283,P30120,Q62902,Q63751,Q6AYS4,Q8CJ52,Q99041,Q9JHB9
4957	1,2,9,11,1 2,13,14,15	3,4,5,6,7,8 ,10,16,17	11	P06760,P08937,P20646,P20760,P20761,P50115,P82471,Q4G075,Q63751,Q99041,Q9Z0V6
4958	1,2,9,11,1 2,13,14,16	3,4,5,6,7,8 ,10,15,17	14	D3ZUC6,P02803,P06760,P08937,P20646,P20760,P20761,P50115,Q4G075,Q63751,Q7TPB1,Q99041,Q99MH3,Q9Z0V6
4959	1,2,9,11,1 2,13,14,17	3,4,5,6,7,8 ,10,15,16	4	P02803,P14668,P82471,Q5FVI6
4960	1,2,9,11,1 2,13,15,16	3,4,5,6,7,8 ,10,14,17	8	P06760,P08937,P20760,P20761,P50115,Q4G075,Q99041,Q9Z0V6
4961	1,2,9,11,1 2,13,15,17	3,4,5,6,7,8 ,10,14,16	3	P06866,P14668,P82471
4962	1,2,9,11,1 2,13,16,17	3,4,5,6,7,8 ,10,14,15	5	P02803,P06866,P07171,P82471,Q9Z0V6
4963	1,2,9,11,1 2,14,15,16	3,4,5,6,7,8 ,10,13,17	14	D3ZUC6,P06760,P06761,P08937,P20760,P20761,P22282,P50115,P51907,Q4G075,Q63751,Q99041,Q99MH3,Q9Z0V6
4964	1,2,9,11,1 2,14,15,17	3,4,5,6,7,8 ,10,13,16	4	D3ZUC6,P06866,P82471,Q8VD89
4965	1,2,9,11,1 2,14,16,17	3,4,5,6,7,8 ,10,13,15	5	D3ZUC6,P06866,P07171,P08937,Q99MH3
4966	1,2,9,11,1 2,15,16,17	3,4,5,6,7,8 ,10,13,14	6	P06761,P06866,P06911,P07171,P08937,P12020
4967	1,2,9,11,1 3,14,15,16	3,4,5,6,7,8 ,10,12,17	16	P06760,P06761,P08937,P20646,P20760,P20761,P22282,P22283,P50115,Q4G075,Q63751,Q7TPB1,Q99041,Q99MH3,Q9QZK9,Q9Z0V6

4968	1,2,9,11,13,14,15,17	3,4,5,6,7,8,10,12,16	3	P08937,P82471,Q99041
4969	1,2,9,11,13,14,16,17	3,4,5,6,7,8,10,12,15	6	D3ZHA0,P08937,P82471,Q99041,Q99MH3,Q9Z0V6
4970	1,2,9,11,13,15,16,17	3,4,5,6,7,8,10,12,14	6	D3ZHA0,P06761,P08937,P82471,Q99041,Q9Z0V6
4971	1,2,9,11,14,15,16,17	3,4,5,6,7,8,10,12,13	8	P02780,P06761,P07647,P08937,P22282,P22283,Q99041,Q99MH3
4972	1,2,9,12,13,14,15,16	3,4,5,6,7,8,10,11,17	19	D3ZUC6,P00714,P04041,P06760,P07150,P08937,P14668,P14669,P20646,P20760,P20761,P50115,P61206;P84079;P84082,P82471,Q4G075,Q63751,Q6AYS4,Q99041,Q9Z0V6
4973	1,2,9,12,13,14,15,17	3,4,5,6,7,8,10,11,16	10	P00714,P07150,P07171,P14668,P14669,P61206;P84079;P84082,P82471,Q5FVI6,Q6AYS4,Q8VD89
4974	1,2,9,12,13,14,16,17	3,4,5,6,7,8,10,11,15	11	P00714,P07150,P07171,P08937,P14668,P14669,P61206;P84079;P84082,P82471,Q5FVI6,Q6AYS4,Q8VD89
4975	1,2,9,12,13,15,16,17	3,4,5,6,7,8,10,11,14	10	P00714,P06866,P07150,P07171,P14668,P14669,P61206;P84079;P84082,P82471,Q5BJY9,Q6AYS4
4976	1,2,9,12,14,15,16,17	3,4,5,6,7,8,10,11,13	13	D3ZUC6,P00714,P06866,P07150,P07171,P08937,P14668,P14669,P22283,P61206;P84079;P84082,P82471,Q6AYS4,Q8VD89
4977	1,2,9,13,14,15,16,17	3,4,5,6,7,8,10,11,12	22	P00714,P02780,P02781,P04041,P06761,P07150,P08937,P0C0A9,P14668,P14669,P20646,P22283,P61206;P84079;P84082,P82471,Q4FZU2,Q5FVI6,Q63751,Q6AYS4,Q6IFW6,Q6IG02,Q99041,Q9Z0V6
4978	1,2,10,11,12,13,14,15	3,4,5,6,7,8,9,16,17	8	P06760,P09605;P25809,P20646,P20761,P23593,P50115,Q99041,Q9Z0V6
4979	1,2,10,11,12,13,14,16	3,4,5,6,7,8,9,15,17	10	P06760,P08937,P20646,P20760,P20761,P50115,Q63751,Q7TPB1,Q99041,Q9Z0V6
4980	1,2,10,11,12,13,14,17	3,4,5,6,7,8,9,15,16	1	P14668
4981	1,2,10,11,12,13,15,16	3,4,5,6,7,8,9,14,17	7	P01835,P06760,P08937,P20761,P50115,Q99041,Q9Z0V6
4982	1,2,10,11,12,13,15,17	3,4,5,6,7,8,9,14,16	2	P06866,P14668
4983	1,2,10,11,12,13,16,17	3,4,5,6,7,8,9,14,15	2	P06866,P07171
4984	1,2,10,11,12,14,15,16	3,4,5,6,7,8,9,13,17	10	P06760,P06761,P08937,P20761,P22282,P50115,P51907,Q63751,Q99041,Q9Z0V6
4985	1,2,10,11,12,14,15,17	3,4,5,6,7,8,9,13,16	1	P06866

4986	1,2,10,11,12,14,16,17	3,4,5,6,7,8,9,13,15	1	P06866
4987	1,2,10,11,12,15,16,17	3,4,5,6,7,8,9,13,14	2	P06761,P06866
4988	1,2,10,11,13,14,15,16	3,4,5,6,7,8,9,12,17	15	P01835,P06760,P06761,P08937,P09605;P25809,P19223,P20646,P20761,P22282,P50115,Q4G075,Q63751,Q7TPB1,Q99041,Q9Z0V6
4989	1,2,10,11,13,14,15,17	3,4,5,6,7,8,9,12,16	2	P14668,Q99041
4990	1,2,10,11,13,14,16,17	3,4,5,6,7,8,9,12,15	3	P08937,Q7TPB1,Q99041
4991	1,2,10,11,13,15,16,17	3,4,5,6,7,8,9,12,14	5	P01835,P06761,P08937,Q99041,Q9Z0V6
4992	1,2,10,11,14,15,16,17	3,4,5,6,7,8,9,12,13	7	P02780,P06761,P07647,P08937,P22282,P22283,Q99041
4993	1,2,10,12,13,14,15,16	3,4,5,6,7,8,9,11,17	13	P06760,P07150,P08937,P09605;P25809,P14668,P14669,P20646,P20761,P50115,Q63751,Q6AYS4,Q99041,Q9Z0V6
4994	1,2,10,12,13,14,15,17	3,4,5,6,7,8,9,11,16	5	P06866,P07150,P14668,P61206;P84079;P84082,Q6AYS4
4995	1,2,10,12,13,14,16,17	3,4,5,6,7,8,9,11,15	5	P06866,P07171,P14668,P14669,Q6AYS4
4996	1,2,10,12,13,15,16,17	3,4,5,6,7,8,9,11,14	7	P06866,P07171,P14668,P14669,P83121,Q63617,Q6AYS4
4997	1,2,10,12,14,15,16,17	3,4,5,6,7,8,9,11,13	8	P05539,P06866,P07171,P08937,P14668,P14669,Q63617,Q6AYS4
4998	1,2,10,13,14,15,16,17	3,4,5,6,7,8,9,11,12	12	P01835,P02780,P06761,P08937,P0C0A9,P14668,P14669,P20646,P22283,Q63751,Q6AYS4,Q99041
4999	1,2,11,12,13,14,15,16	3,4,5,6,7,8,9,10,17	8	D3ZUC6,P06760,P08937,P20646,P20761,P50115,Q99041,Q9Z0V6
5000	1,2,11,12,13,14,15,17	3,4,5,6,7,8,9,10,16	4	D3ZUC6,P06866,P14668,P82471

5001	1,2,11,12,13,14,16,17	3,4,5,6,7,8,9,10,15	5	D3ZHA0,D3ZUC6,O55006,P06866,P07171
5002	1,2,11,12,13,15,16,17	3,4,5,6,7,8,9,10,14	2	P06866,P82471
5003	1,2,11,12,14,15,16,17	3,4,5,6,7,8,9,10,13	2	D3ZUC6,P06866
5004	1,2,11,13,14,15,16,17	3,4,5,6,7,8,9,10,12	6	D3ZHA0,P00714,P08937,P20646,P82471,Q99041
5005	1,2,12,13,14,15,16,17	3,4,5,6,7,8,9,10,11	13	D3ZUC6,P00714,P04041,P06866,P07150,P07171,P14668,P14669,P20646,P61206;P84079;P84082,P82471,Q5FVI6,Q6AYS4
5006	1,3,4,5,6,7,8,9	2,10,11,12,13,14,15,16,17	29	D3ZHA0,O55006,O88267,P01681,P06761,P06866,P07171,P07647,P08937,P16086,P18418,P20646,P20761,P22282,P22283,P23593,P31044,P52590,P61206;P84079;P84082,P97571,Q63617,Q63751,Q64361,Q99041,Q9JI85,Q9QX79,Q9R0T3,Q9WTT6,Q9Z0V6
5007	1,3,4,5,6,7,8,10	2,9,11,12,13,14,15,16,17	39	D3ZHA0,O55006,P01681,P04041,P06761,P07171,P07647,P08649,P08723,P08937,P12020,P16086,P18418,P20646,P20760,P20761,P22282,P22283,P28648,P31044,P61206;P84079;P84082,P68370,P82471,P97571,P97615,Q4FZU2,Q5BJY9,Q5FVI6,Q63617,Q63751,Q64361,Q8CJ52,Q8VD89,Q99041,Q99MH3,Q9QX79,Q9R0T3,Q9WTT6,Q9Z0V6
5008	1,3,4,5,6,7,8,11	2,9,10,12,13,14,15,16,17	44	D3ZHA0,O88267,P01681,P04041,P05371,P06761,P07150,P07171,P07647,P08649,P08723,P08937,P10715,P14668,P14669,P15399,P16086,P18418,P19629,P20646,P20761,P22282,P22283,P28648,P31044,P55314,P61206;P84079;P84082,P68370,Q30KJ2,Q4FZU2,Q5I0D1,Q63493,Q63617,Q63751,Q64361,Q6AYS4,Q8CJ52,Q8VD89,Q99041,Q9ESW0,Q9JI85,Q9QX79,Q9R0T3,Q9Z0V6
5009	1,3,4,5,6,7,8,12	2,9,10,11,13,14,15,16,17	50	D3ZHA0,F1LM93,O55006,O88267,P01681,P02780,P02781,P02782,P06761,P07171,P07647,P08723,P08937,P0C0A9,P10715,P13432,P16086,P18418,P19629,P20646,P20761,P22282,P22283,P30120,P31044,P61206;P84079;P84082,P62815,P83748,Q10758,Q4FZU2,Q5M8C6,Q63493,Q63617,Q63751,Q64361,Q6AYS7,Q6IFW6,Q6IG02,Q6IMF3,Q6P6Q2,Q8CJ52,Q99041,Q99MH3,Q9ESW0,Q9JHB9,Q9JI85,Q9QX79,Q9R0T3,Q9WTT6,Q9Z0V6
5010	1,3,4,5,6,7,8,13	2,9,10,11,12,14,15,16,17	32	D3ZHA0,O88267,P01681,P02764,P05539,P06761,P06866,P06911,P07171,P07647,P08723,P08937,P12020,P16086,P18418,P20760,P20761,P22282,P22283,P31044,Q5GRG2,Q62902,Q63617,Q63751,Q8CJ52,Q8VD89,Q99041,Q99MH3,Q9JI85,Q9QX79,Q9R0T3,Q9Z0V6
5011	1,3,4,5,6,7,8,14	2,9,10,11,12,13,15,16,17	35	D3ZHA0,O88267,P01681,P02793;Q7TP54,P05371,P06761,P06866,P06911,P07171,P07647,P08723,P08937,P10536,P12020,P16086,P18418,P19629,P20760,P20761,P22282,P22283,P31044,Q63617,Q63618,Q63751,Q64361,Q6AYS7,Q8CJ52,Q923S2,Q99041,Q9JI85,Q9QX79,Q9R0T3,Q9WTT6,Q9Z0V6
5012	1,3,4,5,6,7,8,15	2,9,10,11,12,13,14,16,17	26	B0BNN3,D3ZHA0,O54728,O55006,P01681,P02631,P06761,P07171,P08937,P10536,P16086,P18418,P19629,P20760,P20761,P22282,P22283,P31044,P97571,Q63617,Q63751,Q64361,Q8VD89,Q99041,Q9QX79,Q9Z0V6
5013	1,3,4,5,6,7,8,16	2,9,10,11,12,13,14,15,17	26	D3ZHA0,O88267,P01681,P05371,P06761,P07171,P08937,P14668,P16086,P18418,P20646,P20761,P22282,P22283,P23593,P31044,P61206;P84079;P84082,Q63617,Q63751,Q6IRE4,Q8CJ52,Q8VD89,Q99041,Q9QX79,Q9R0T3,Q9Z0V6
5014	1,3,4,5,6,7,8,17	2,9,10,11,12,13,14,15,16	39	D3ZHA0,O54858,O88267,P01681,P01946,P02091,P06760,P06761,P07171,P07647,P08937,P10536,P16086,P18418,P20646,P20760,P20761,P22282,P22283,P23593,P23928,P31044,P50115,P52590,P62815,P62898,P97571,Q30KJ2,Q4G075,Q63617,Q63751,Q64361,Q99041,Q99MH3,Q9EPB1,Q9QX79,Q9R0T3,Q9WTT6,Q9Z0V6
5015	1,3,4,5,6,7,9,10	2,8,11,12,13,14,15,16,17	8	O55006,P01681,P08937,P18418,P23593,P52590,P61206;P84079;P84082,Q5FVI6

5016	1,3,4,5,6,7,9,11	2,8,10,12,13,14,15,16,17	10	P01681,P05539,P08937,P15399,P18418,P55314,P61206;P84079;P84082,Q4FZU2,Q63751,Q6IRE4
5017	1,3,4,5,6,7,9,12	2,8,10,11,13,14,15,16,17	13	O55006,P01681,P01835,P08937,P13432,P18418,P23593,P61206;P84079;P84082,Q4FZU2,Q63751,Q6IFW6,Q6IG02,Q6IMF3
5018	1,3,4,5,6,7,9,13	2,8,10,11,12,14,15,16,17	7	P01681,P05539,P06866,P08937,P18418,P62804,Q6IRE4
5019	1,3,4,5,6,7,9,14	2,8,10,11,12,13,15,16,17	5	P01681,P06866,P08937,P18418,Q6IRE4
5020	1,3,4,5,6,7,9,15	2,8,10,11,12,13,14,16,17	7	O54728,O55006,P01681,P08937,P18418,P23593,P62804
5021	1,3,4,5,6,7,9,16	2,8,10,11,12,13,14,15,17	8	O54728,P01681,P18418,P23593,P61206;P84079;P84082,P62804,Q00715,Q6IRE4
5022	1,3,4,5,6,7,9,17	2,8,10,11,12,13,14,15,16	12	P01681,P01946,P02091,P08937,P09605;P25809,P18418,P20646,P20760,P20761,P23593,P52590,Q63751
5023	1,3,4,5,6,7,10,11	2,8,9,12,13,14,15,16,17	15	P01681,P08937,P15399,P18418,P28648,P55314,P61206;P84079;P84082,Q4FZU2,Q5BJY9,Q5FVI6,Q63751,Q6IFW6,Q6P6Q2,Q8VD89,Q9ESW0
5024	1,3,4,5,6,7,10,12	2,8,9,11,13,14,15,16,17	17	F1LM93,P01681,P08723,P08937,P18418,P61206;P84079;P84082,P83748,Q10758,Q4FZU2,Q5FVI6,Q63751,Q6IFW6,Q6IG02,Q6IMF3,Q6P6Q2,Q99MH3,Q9ESW0
5025	1,3,4,5,6,7,10,13	2,8,9,11,12,14,15,16,17	12	P01681,P05539,P06911,P08937,P12020,P18418,P61206;P84079;P84082,Q5FVI6,Q5GRG2,Q63751,Q8VD89,Q99MH3
5026	1,3,4,5,6,7,10,14	2,8,9,11,12,13,15,16,17	5	P01681,P08937,P12020,P18418,P61206;P84079;P84082
5027	1,3,4,5,6,7,10,15	2,8,9,11,12,13,14,16,17	10	O54728,O55006,P01681,P08937,P18418,P61206;P84079;P84082,Q4FZU2,Q5FVI6,Q8VD89,Q99MH3
5028	1,3,4,5,6,7,10,16	2,8,9,11,12,13,14,15,17	9	P01681,P08721,P08937,P18418,P61206;P84079;P84082,Q4FZU2,Q5FVI6,Q6IRE4,Q8VD89
5029	1,3,4,5,6,7,10,17	2,8,9,11,12,13,14,15,16	14	P01681,P01946,P02091,P08937,P18418,P20646,P20760,P20761,P23593,P52590,P61206;P84079;P84082,P97580,Q63751,Q99MH3
5030	1,3,4,5,6,7,11,12	2,8,9,10,13,14,15,16,17	23	P01681,P04905,P07647,P08723,P08937,P15399,P17046,P18418,P30120,P55314,P61206;P84079;P84082,Q4FZU2,Q63493,Q63751,Q6IFU8,Q6IFW6,Q6IG02,Q6IMF3,Q6P6Q2,Q99041,Q9ESW0,Q9JHB9,Q9JI85

5031	1,3,4,5,6,7,11,13	2,8,9,10,12,14,15,16,17	13	P01681,P05539,P06911,P08937,P16086,P18418,P55314,P61206;P84079;P84082,Q4FZU2,Q63751,Q6IRE4,Q8VD89,Q9JI85
5032	1,3,4,5,6,7,11,14	2,8,9,10,12,13,15,16,17	12	P01681,P05371,P08937,P15399,P16086,P18418,P55314,P61206;P84079;P84082,Q4FZU2,Q63751,Q6IRE4,Q9ESW0
5033	1,3,4,5,6,7,11,15	2,8,9,10,12,13,14,16,17	14	O54728,P01681,P05539,P08937,P18418,P22006,P55314,P61206;P84079;P84082,Q4FZU2,Q5I0D1,Q63751,Q6IRE4,Q8VD89,Q9ESW0
5034	1,3,4,5,6,7,11,16	2,8,9,10,12,13,14,15,17	13	P01681,P05371,P08937,P14668,P17046,P18418,P55314,P61206;P84079;P84082,Q4FZU2,Q5FVI6,Q63751,Q6IRE4,Q8VD89
5035	1,3,4,5,6,7,11,17	2,8,9,10,12,13,14,15,16	18	P01681,P01946,P02091,P08937,P09605;P25809,P15399,P18418,P20646,P20760,P20761,P52590,P55314,P61206;P84079;P84082,P97580,Q30KJ2,Q4FZU2,Q63751,Q9ESW0
5036	1,3,4,5,6,7,12,13	2,8,9,10,11,14,15,16,17	20	O88267,P01681,P05539,P06911,P07647,P08723,P08937,P18418,P22282,P30120,P36374,P83748,Q4FZU2,Q63751,Q6IFW6,Q6IG02,Q6IMF3,Q99MH3,Q9JHB9,Q9JI85
5037	1,3,4,5,6,7,12,14	2,8,9,10,11,13,15,16,17	15	O88267,P01681,P01835,P08723,P08937,P18418,P30120,P83748,Q10758,Q4FZU2,Q63751,Q6IFW6,Q6IG02,Q6IMF3,Q9ESW0
5038	1,3,4,5,6,7,12,15	2,8,9,10,11,13,14,16,17	11	P01681,P08937,P18418,P83748,Q4FZU2,Q63751,Q6IFW6,Q6IG02,Q6IMF3,Q99MH3,Q9ESW0
5039	1,3,4,5,6,7,12,16	2,8,9,10,11,13,14,15,17	15	O88267,P01681,P08937,P17046,P18418,P61206;P84079;P84082,P83748,Q10758,Q4FZU2,Q63751,Q6IFW6,Q6IG02,Q6IMF3,Q6IRE4,Q6P6Q2
5040	1,3,4,5,6,7,12,17	2,8,9,10,11,13,14,15,16	25	O88267,P01681,P01835,P01946,P02091,P08937,P09605;P25809,P13432,P18418,P20760,P20761,P22282,P23593,P52590,P62815,Q10758,Q4FZU2,Q63751,Q6IFW6,Q6IG02,Q6IMF3,Q99041,Q99MH3,Q9ESW0,Q9QZK9
5041	1,3,4,5,6,7,13,14	2,8,9,10,11,12,15,16,17	9	P01681,P05539,P06866,P06911,P08937,P12020,P16086,P18418,Q5GRG2
5042	1,3,4,5,6,7,13,15	2,8,9,10,11,12,14,16,17	9	O54728,P01681,P05539,P08937,P18418,P62804,Q63751,Q8VD89,Q99MH3
5043	1,3,4,5,6,7,13,16	2,8,9,10,11,12,14,15,17	10	P01681,P05539,P08937,P17046,P61206;P84079;P84082,P62804,Q00715,Q63751,Q6IRE4,Q8VD89
5044	1,3,4,5,6,7,13,17	2,8,9,10,11,12,14,15,16	14	O88267,P01681,P01946,P02091,P05539,P08937,P18418,P20760,P20761,P22282,P52590,P62815,Q63751,Q99MH3
5045	1,3,4,5,6,7,14,15	2,8,9,10,11,12,13,16,17	5	O54728,P01681,P02803,P08937,P18418

5046	1,3,4,5,6,7,14,16	2,8,9,10,11,12,13,15,17	5	P01681,P08937,P18418,P61206;P84079;P84082,Q6IRE4
5047	1,3,4,5,6,7,14,17	2,8,9,10,11,12,13,15,16	13	O88267,P01681,P01946,P02091,P08937,P10536,P18418,P20760,P20761,P52590,Q30KJ2,Q63751,Q9QW07
5048	1,3,4,5,6,7,15,16	2,8,9,10,11,12,13,14,17	8	O54728,P01681,P02803,P08937,P61206;P84079;P84082,P62804,Q6IRE4,Q8VD89
5049	1,3,4,5,6,7,15,17	2,8,9,10,11,12,13,14,16	14	B0BNN3,P01681,P01946,P02091,P02803,P08937,P10536,P18418,P20760,P20761,P23593,P52590,Q63751,Q99MH3
5050	1,3,4,5,6,7,16,17	2,8,9,10,11,12,13,14,15	12	O88267,P01681,P01946,P02091,P08937,P09605;P25809,P20760,P20761,P23593,P52590,P61206;P84079;P84082,Q63751
5051	1,3,4,5,6,8,9,10	2,7,11,12,13,14,15,16,17	9	D3ZHA0,D3ZUC6,O55006,P01681,P08649,P23593,Q5M8C6,Q9WTT6,Q9Z0V6
5052	1,3,4,5,6,8,9,11	2,7,10,12,13,14,15,16,17	12	P01681,P02793;Q7TP54,P04041,P08649,P14668,P14669,P55314,Q4FZU2,Q5I0D1,Q5M8C6,Q63751,Q9Z0V6
5053	1,3,4,5,6,8,9,12	2,7,10,11,13,14,15,16,17	18	D3ZHA0,P01681,P01835,P02782,P07647,P08723,P08937,P23593,P30120,Q4FZU2,Q5M8C6,Q63751,Q6IFW6,Q6IG02,Q6IMF3,Q6P6Q2,Q9WTT6,Q9Z0V6
5054	1,3,4,5,6,8,9,13	2,7,10,11,12,14,15,16,17	5	P01681,P06866,P23593,Q5M8C6,Q9Z0V6
5055	1,3,4,5,6,8,9,14	2,7,10,11,12,13,15,16,17	5	P01681,P02793;Q7TP54,P06866,Q9WTT6,Q9Z0V6
5056	1,3,4,5,6,8,9,15	2,7,10,11,12,13,14,16,17	7	B0BNN3,O54728,O55006,P01681,P23593,Q5QE79,Q9Z0V6
5057	1,3,4,5,6,8,9,16	2,7,10,11,12,13,14,15,17	5	P01681,P14668,P23593,Q6IRE4,Q9Z0V6
5058	1,3,4,5,6,8,9,17	2,7,10,11,12,13,14,15,16	13	P01681,P02793;Q7TP54,P06760,P08649,P08937,P16636,P20761,P23593,Q4G075,Q5M8C6,Q63751,Q9WTT6,Q9Z0V6
5059	1,3,4,5,6,8,10,11	2,7,9,12,13,14,15,16,17	18	P01681,P04041,P07150,P08649,P08937,P14668,P14669,P16636,P55314,Q4FZU2,Q5BJY9,Q5I0D1,Q5M8C6,Q63751,Q64361,Q6IFW6,Q6P6Q2,Q9Z0V6
5060	1,3,4,5,6,8,10,12	2,7,9,11,13,14,15,16,17	24	D3ZHA0,F1LM93,P01681,P02781,P07647,P08723,P08937,P0C0A9,P16636,P30120,P35281,Q08463,Q10758,Q4FZU2,Q5M8C6,Q63751,Q64361,Q6IFW6,Q6IG02,Q6IMF3,Q6P6Q2,Q99MH3,Q9WTT6,Q9Z0V6

5061	1,3,4,5,6,8,10,13	2,7,9,11,12,14,15,16,17	8	D3ZUC6,P01681,P06911,P08937,P82995,Q5M8C6,Q99MH3,Q9Z0V6
5062	1,3,4,5,6,8,10,14	2,7,9,11,12,13,15,16,17	7	D3ZHA0,P01681,P02793;Q7TP54,P08649,Q4FZU2,Q9WTT6,Q9Z0V6
5063	1,3,4,5,6,8,10,15	2,7,9,11,12,13,14,16,17	9	B0BNN3,D3ZHA0,O55006,P01681,P08649,Q4FZU2,Q64361,Q99MH3,Q9Z0V6
5064	1,3,4,5,6,8,10,16	2,7,9,11,12,13,14,15,17	7	P01681,P08649,P08721,P14668,P23593,Q4FZU2,Q9Z0V6
5065	1,3,4,5,6,8,10,17	2,7,9,11,12,13,14,15,16	18	O54858,P01681,P06760,P08649,P08937,P16636,P20760,P20761,P23593,Q08463,Q4FZU2,Q4G075,Q5M8C6,Q63751,Q64361,Q99MH3,Q9WTT6,Q9Z0V6
5066	1,3,4,5,6,8,11,12	2,7,9,10,13,14,15,16,17	33	F1LM93,O35547,P01681,P02780,P02781,P02782,P04041,P04905,P07150,P07647,P08723,P08937,P0C0A9,P14668,P14669,P16636,P30120,P55314,Q10758,Q4FZU2,Q5I0D1,Q5M8C6,Q63751,Q6IFU7,Q6IFU8,Q6IFW6,Q6IG02,Q6IMF3,Q6P6Q2,Q8CJ52,Q9ESW0,Q9JHB9,Q9Z0V6
5067	1,3,4,5,6,8,11,13	2,7,9,10,12,14,15,16,17	11	P01681,P07647,P08937,P14668,P14669,P55314,Q4FZU2,Q5M8C6,Q63751,Q8VD89,Q9Z0V6
5068	1,3,4,5,6,8,11,14	2,7,9,10,12,13,15,16,17	16	P01681,P02793;Q7TP54,P07150,P07171,P08649,P08937,P14668,P14669,P35434,P55314,P97840,Q4FZU2,Q5I0D1,Q63751,Q6P6Q2,Q9Z0V6
5069	1,3,4,5,6,8,11,15	2,7,9,10,12,13,14,16,17	15	B0BNN3,O54728,P01681,P07171,P08649,P08937,P14668,P14669,P55314,P97840,Q4FZU2,Q5I0D1,Q63751,Q9ESW0,Q9Z0V6
5070	1,3,4,5,6,8,11,16	2,7,9,10,12,13,14,15,17	14	P01681,P07150,P08649,P14668,P14669,P55314,Q4FZU2,Q5I0D1,Q63751,Q6IFW6,Q6IRE4,Q6P6Q2,Q8VD89,Q9Z0V6
5071	1,3,4,5,6,8,11,17	2,7,9,10,12,13,14,15,16	23	O54858,P01681,P02793;Q7TP54,P04041,P06760,P07150,P08649,P08937,P14668,P14669,P16636,P20761,P50115,P55314,P97840,Q4FZU2,Q4G075,Q5I0D1,Q5M8C6,Q63751,Q6AYS4,Q9ESW0,Q9Z0V6
5072	1,3,4,5,6,8,12,13	2,7,9,10,11,14,15,16,17	25	P01681,P02780,P02781,P02782,P06761,P06911,P07647,P08723,P08937,P0C0A9,P22282,P22283,P30120,P82995,Q4FZU2,Q5M8C6,Q63751,Q6IFW6,Q6IG02,Q6IMF3,Q6P6Q2,Q8CJ52,Q99MH3,Q9JHB9,Q9Z0V6
5073	1,3,4,5,6,8,12,14	2,7,9,10,11,13,15,16,17	17	P01681,P01835,P02782,P02793;Q7TP54,P07647,P08723,P08937,P30120,Q10758,Q4FZU2,Q5M8C6,Q6IFW6,Q6IG02,Q6IMF3,Q6P6Q2,Q9WTT6,Q9Z0V6
5074	1,3,4,5,6,8,12,15	2,7,9,10,11,13,14,16,17	16	B0BNN3,P01681,P08723,P08937,Q4FZU2,Q5M8C6,Q62894,Q63751,Q64361,Q6IFW6,Q6IG02,Q6IMF3,Q6P6Q2,Q99MH3,Q9ESW0,Q9Z0V6
5075	1,3,4,5,6,8,12,16	2,7,9,10,11,13,14,15,17	14	P01681,P08723,P08937,P14668,P23593,Q10758,Q4FZU2,Q5M8C6,Q63751,Q6IFW6,Q6IG02,Q6IMF3,Q6P6Q2,Q9Z0V6

5076	1,3,4,5,6,8,12,17	2,7,9,10,11,13,14,15,16	37	B0BNN3,F1LM93,O88267,P01681,P01835,P02782,P06760,P07647,P08723,P08937,P10536,P13432,P16636,P19223,P20761,P22282,P23593,P23928,P30120,P62815,Q08463,Q10758,Q4FZU2,Q4G075,Q5M8C6,Q63751,Q64361,Q6IFW6,Q6IG02,Q6IMF3,Q6P6Q2,Q99041,Q99MH3,Q9ESW0,Q9QZK9,Q9WTT6,Q9Z0V6
5077	1,3,4,5,6,8,13,14	2,7,9,10,11,12,15,16,17	10	P01681,P02793;Q7TP54,P06866,P06911,P08937,P10536,P51907,Q64093,Q9WTT6,Q9Z0V6
5078	1,3,4,5,6,8,13,15	2,7,9,10,11,12,14,16,17	6	B0BNN3,P01681,P08937,P10536,P82995,Q9Z0V6
5079	1,3,4,5,6,8,13,16	2,7,9,10,11,12,14,15,17	5	P01681,P14668,P23593,P82995,Q9Z0V6
5080	1,3,4,5,6,8,13,17	2,7,9,10,11,12,14,15,16	16	P01681,P06760,P08937,P10536,P16636,P20761,P22282,P23593,P23928,P51907,Q4G075,Q5M8C6,Q63751,Q6PCU2,Q99MH3,Q9Z0V6
5081	1,3,4,5,6,8,14,15	2,7,9,10,11,12,13,16,17	7	B0BNN3,O54728,P01681,P02793;Q7TP54,P10536,P97840,Q9Z0V6
5082	1,3,4,5,6,8,14,16	2,7,9,10,11,12,13,15,17	4	P01681,P02793;Q7TP54,P14668,Q9Z0V6
5083	1,3,4,5,6,8,14,17	2,7,9,10,11,12,13,15,16	15	B0BNN3,P01681,P02793;Q7TP54,P06760,P08937,P10536,P20760,P20761,P51907,P97840,Q4G075,Q63751,Q9QW07,Q9WTT6,Q9Z0V6
5084	1,3,4,5,6,8,15,16	2,7,9,10,11,12,13,14,17	7	B0BNN3,O54728,P01681,P14668,P23593,Q62894,Q9Z0V6
5085	1,3,4,5,6,8,15,17	2,7,9,10,11,12,13,14,16	13	B0BNN3,P01681,P06760,P08937,P10536,P20760,P20761,P23593,P97840,Q4G075,Q63751,Q99MH3,Q9Z0V6
5086	1,3,4,5,6,8,16,17	2,7,9,10,11,12,13,14,15	11	P01681,P06760,P08937,P20761,P23593,P23928,P42854,Q4G075,Q63751,Q6PCU2,Q9Z0V6
5087	1,3,4,5,6,9,10,11	2,7,8,12,13,14,15,16,17	5	P01681,P08649,P55314,Q4FZU2,Q6P6Q2
5088	1,3,4,5,6,9,10,12	2,7,8,11,13,14,15,16,17	9	P01681,P01835,P23593,Q10758,Q4FZU2,Q6IFW6,Q6IG02,Q6IMF3,Q6P6Q2
5089	1,3,4,5,6,9,10,13	2,7,8,11,12,14,15,16,17	1	P01681
5090	1,3,4,5,6,9,10,14	2,7,8,11,12,13,15,16,17	1	P01681

5091	1,3,4,5,6,9,10,15	2,7,8,11,12,13,14,16,17	5	O54728,O55006,P01681,P23593,Q4FZU2
5092	1,3,4,5,6,9,10,16	2,7,8,11,12,13,14,15,17	3	P01681,P23593,Q4FZU2
5093	1,3,4,5,6,9,10,17	2,7,8,11,12,13,14,15,16	3	P01681,P23593,Q99MH3
5094	1,3,4,5,6,9,11,12	2,7,8,10,13,14,15,16,17	11	P01681,P01835,P55314,Q4FZU2,Q6IFU7,Q6IFU8,Q6IFW6,Q6IG02,Q6IMF3,Q6P6Q2,Q9ESW0
5095	1,3,4,5,6,9,11,13	2,7,8,10,12,14,15,16,17	5	P01681,P05539,P55314,Q4FZU2,Q6IRE4
5096	1,3,4,5,6,9,11,14	2,7,8,10,12,13,15,16,17	5	P01681,P01835,P55314,Q4FZU2,Q6IRE4
5097	1,3,4,5,6,9,11,15	2,7,8,10,12,13,14,16,17	6	O54728,P01681,P55314,Q4FZU2,Q5I0D1,Q6IRE4
5098	1,3,4,5,6,9,11,16	2,7,8,10,12,13,14,15,17	6	O54728,P01681,P14668,P55314,Q4FZU2,Q6IRE4
5099	1,3,4,5,6,9,11,17	2,7,8,10,12,13,14,15,16	6	P01681,P01835,P23593,P55314,Q4FZU2,Q63751
5100	1,3,4,5,6,9,12,13	2,7,8,10,11,14,15,16,17	7	P01681,P01835,P82995,Q4FZU2,Q6IFW6,Q6IG02,Q6IMF3
5101	1,3,4,5,6,9,12,14	2,7,8,10,11,13,15,16,17	6	P01681,P01835,Q4FZU2,Q6IFW6,Q6IG02,Q6IMF3
5102	1,3,4,5,6,9,12,15	2,7,8,10,11,13,14,16,17	9	B0BNN3,O54728,P01681,P01835,P23593,Q4FZU2,Q6IFW6,Q6IG02,Q6IMF3
5103	1,3,4,5,6,9,12,16	2,7,8,10,11,13,14,15,17	9	P01681,P01835,P23593,Q4FZU2,Q6IFW6,Q6IG02,Q6IMF3,Q6IRE4,Q6P6Q2
5104	1,3,4,5,6,9,12,17	2,7,8,10,11,13,14,15,16	11	P01681,P01835,P13432,P16636,P23593,Q10758,Q4FZU2,Q6IFW6,Q6IG02,Q6IMF3,Q99MH3
5105	1,3,4,5,6,9,13,14	2,7,8,10,11,12,15,16,17	2	P01681,P06866

5106	1,3,4,5,6,9,13,15	2,7,8,10,11,12,14,16,17	6	O54728,P01681,P05539,P23593,P62804,P82995
5107	1,3,4,5,6,9,13,16	2,7,8,10,11,12,14,15,17	6	O54728,P01681,P23593,P62804,Q00715,Q6IRE4
5108	1,3,4,5,6,9,13,17	2,7,8,10,11,12,14,15,16	2	P01681,P23593
5109	1,3,4,5,6,9,14,15	2,7,8,10,11,12,13,16,17	2	O54728,P01681
5110	1,3,4,5,6,9,14,16	2,7,8,10,11,12,13,15,17	3	O54728,P01681,Q6IRE4
5111	1,3,4,5,6,9,14,17	2,7,8,10,11,12,13,15,16	2	P01681,P01835
5112	1,3,4,5,6,9,15,16	2,7,8,10,11,12,13,14,17	6	O54728,P01681,P23593,P62804,Q00715,Q6IRE4
5113	1,3,4,5,6,9,15,17	2,7,8,10,11,12,13,14,16	4	B0BNN3,O54728,P01681,P23593
5114	1,3,4,5,6,9,16,17	2,7,8,10,11,12,13,14,15	3	P01681,P23593,P62804
5115	1,3,4,5,6,10,11,12	2,7,8,9,13,14,15,16,17	13	O35547,P01681,P16636,P55314,Q10758,Q4FZU2,Q6IFU7,Q6IFU8,Q6IFW6,Q6IG02,Q6IMF3,Q6P6Q2,Q9ESW0
5116	1,3,4,5,6,10,11,13	2,7,8,9,12,14,15,16,17	3	P01681,P55314,Q4FZU2
5117	1,3,4,5,6,10,11,14	2,7,8,9,12,13,15,16,17	6	P01681,P08649,P55314,Q4FZU2,Q6IFW6,Q6P6Q2
5118	1,3,4,5,6,10,11,15	2,7,8,9,12,13,14,16,17	6	O54728,P01681,P08649,P55314,Q4FZU2,Q5I0D1
5119	1,3,4,5,6,10,11,16	2,7,8,9,12,13,14,15,17	10	P01681,P08649,P14668,P55314,Q4FZU2,Q5FVI6,Q6IFW6,Q6IRE4,Q6P6Q2,Q8VD89
5120	1,3,4,5,6,10,11,17	2,7,8,9,12,13,14,15,16	7	P01681,P08649,P16636,P55314,Q4FZU2,Q63751,Q99MH3

5121	1,3,4,5,6,10,12,13	2,7,8,9,11,14,15,16,17	10	P01681,P82995,Q10758,Q498D9,Q4FZU2,Q6IFW6,Q6IG02,Q6IMF3,Q6P6Q2,Q99MH3
5122	1,3,4,5,6,10,12,14	2,7,8,9,11,13,15,16,17	10	P01681,P01835,P34901,Q10758,Q498D9,Q4FZU2,Q6IFW6,Q6IG02,Q6IMF3,Q6P6Q2
5123	1,3,4,5,6,10,12,15	2,7,8,9,11,13,14,16,17	10	B0BNN3,P01681,P20766,Q10758,Q4FZU2,Q6IFW6,Q6IG02,Q6IMF3,Q6P6Q2,Q99MH3
5124	1,3,4,5,6,10,12,16	2,7,8,9,11,13,14,15,17	10	P01681,P08721,P20766,Q10758,Q4FZU2,Q6IFU7,Q6IFW6,Q6IG02,Q6IMF3,Q6P6Q2
5125	1,3,4,5,6,10,12,17	2,7,8,9,11,13,14,15,16	14	P01681,P01835,P08937,P16636,P23593,Q10758,Q4FZU2,Q63751,Q6IFW6,Q6IG02,Q6IMF3,Q6P6Q2,Q99MH3,Q9QZK9
5126	1,3,4,5,6,10,13,14	2,7,8,9,11,12,15,16,17	3	P01681,P06911,Q498D9
5127	1,3,4,5,6,10,13,15	2,7,8,9,11,12,14,16,17	4	P01681,P82995,Q4FZU2,Q99MH3
5128	1,3,4,5,6,10,13,16	2,7,8,9,11,12,14,15,17	1	P01681
5129	1,3,4,5,6,10,13,17	2,7,8,9,11,12,14,15,16	2	P01681,Q99MH3
5130	1,3,4,5,6,10,14,15	2,7,8,9,11,12,13,16,17	2	P01681,Q4FZU2
5131	1,3,4,5,6,10,14,16	2,7,8,9,11,12,13,15,17	2	P01681,Q4FZU2
5132	1,3,4,5,6,10,14,17	2,7,8,9,11,12,13,15,16	1	P01681
5133	1,3,4,5,6,10,15,16	2,7,8,9,11,12,13,14,17	5	O54728,P01681,P20766,P23593,Q4FZU2
5134	1,3,4,5,6,10,15,17	2,7,8,9,11,12,13,14,16	5	B0BNN3,P01681,P23593,Q4FZU2,Q99MH3
5135	1,3,4,5,6,10,16,17	2,7,8,9,11,12,13,14,15	4	P01681,P23593,Q4FZU2,Q99MH3

5136	1,3,4,5,6,11,12,13	2,7,8,9,10,14,15,16,17	15	P01681,P04905,P05539,P0C0A9,P30120,P36374,P55314,Q4FZU2,Q6IFU7,Q6IFU8,Q6IFW6,Q6IG02,Q6IMF3,Q6P6Q2,Q9JHB9
5137	1,3,4,5,6,11,12,14	2,7,8,9,10,13,15,16,17	14	P01681,P01835,P04905,P34901,P55314,Q10758,Q4FZU2,Q6IFU7,Q6IFU8,Q6IFW6,Q6IG02,Q6IMF3,Q6P6Q2,Q9ESW0
5138	1,3,4,5,6,11,12,15	2,7,8,9,10,13,14,16,17	13	B0BNN3,P01681,P02625,P55314,Q4FZU2,Q5I0D1,Q6IFU7,Q6IFU8,Q6IFW6,Q6IG02,Q6IMF3,Q6P6Q2,Q9ESW0
5139	1,3,4,5,6,11,12,16	2,7,8,9,10,13,14,15,17	14	P01681,P14668,P17046,P55314,Q10758,Q4FZU2,Q6IFU7,Q6IFU8,Q6IFW6,Q6IG02,Q6IMF3,Q6IRE4,Q6P6Q2,Q9ESW0
5140	1,3,4,5,6,11,12,17	2,7,8,9,10,13,14,15,16	17	P01681,P01835,P08937,P13432,P16636,P55314,Q10758,Q4FZU2,Q63751,Q6IFU8,Q6IFW6,Q6IG02,Q6IMF3,Q6P6Q2,Q99MH3,Q9ESW0,Q9QZK9
5141	1,3,4,5,6,11,13,14	2,7,8,9,10,12,15,16,17	4	P01681,P05539,P55314,Q4FZU2
5142	1,3,4,5,6,11,13,15	2,7,8,9,10,12,14,16,17	6	O54728,P01681,P05539,P55314,P82995,Q4FZU2
5143	1,3,4,5,6,11,13,16	2,7,8,9,10,12,14,15,17	5	P01681,P55314,Q4FZU2,Q6IRE4,Q8VD89
5144	1,3,4,5,6,11,13,17	2,7,8,9,10,12,14,15,16	6	P01681,P05539,P55314,Q4FZU2,Q63751,Q99MH3
5145	1,3,4,5,6,11,14,15	2,7,8,9,10,12,13,16,17	6	O54728,P01681,P55314,P97840,Q4FZU2,Q5I0D1
5146	1,3,4,5,6,11,14,16	2,7,8,9,10,12,13,15,17	6	P01681,P14668,P55314,Q4FZU2,Q5I0D1,Q6IRE4
5147	1,3,4,5,6,11,14,17	2,7,8,9,10,12,13,15,16	7	P01681,P01835,P02793;Q7TP54,P55314,P97840,Q4FZU2,Q63751
5148	1,3,4,5,6,11,15,16	2,7,8,9,10,12,13,14,17	8	O54728,P01681,P14668,P55314,Q4FZU2,Q5I0D1,Q6IRE4,Q8VD89
5149	1,3,4,5,6,11,15,17	2,7,8,9,10,12,13,14,16	7	B0BNN3,P01681,P55314,P97840,Q4FZU2,Q5I0D1,Q63751
5150	1,3,4,5,6,11,16,17	2,7,8,9,10,12,13,14,15	4	P01681,P55314,Q4FZU2,Q63751

5151	1,3,4,5,6,12,13,14	2,7,8,9,10,11,15,16,17	9	P01681,P01835,P06911,P82995,Q498D9,Q4FZU2,Q6IFW6,Q6IG02,Q6IMF3
5152	1,3,4,5,6,12,13,15	2,7,8,9,10,11,14,16,17	8	B0BNN3,P01681,P82995,Q4FZU2,Q6IFW6,Q6IG02,Q6IMF3,Q99MH3
5153	1,3,4,5,6,12,13,16	2,7,8,9,10,11,14,15,17	8	P01681,P17046,P82995,Q4FZU2,Q6IFW6,Q6IG02,Q6IMF3,Q6P6Q2
5154	1,3,4,5,6,12,13,17	2,7,8,9,10,11,14,15,16	13	P01681,P01835,P08937,P13432,P16636,Q10758,Q4FZU2,Q63751,Q6IFW6,Q6IG02,Q6IMF3,Q99MH3,Q9QZK9
5155	1,3,4,5,6,12,14,15	2,7,8,9,10,11,13,16,17	7	B0BNN3,P01681,P01835,Q4FZU2,Q6IFW6,Q6IG02,Q6IMF3
5156	1,3,4,5,6,12,14,16	2,7,8,9,10,11,13,15,17	8	P01681,P01835,Q10758,Q4FZU2,Q6IFW6,Q6IG02,Q6IMF3,Q6P6Q2
5157	1,3,4,5,6,12,14,17	2,7,8,9,10,11,13,15,16	10	B0BNN3,P01681,P01835,P13432,Q10758,Q4FZU2,Q6IFW6,Q6IG02,Q6IMF3,Q9QW07
5158	1,3,4,5,6,12,15,16	2,7,8,9,10,11,13,14,17	7	P01681,P20766,P23593,Q4FZU2,Q6IFW6,Q6IG02,Q6IMF3
5159	1,3,4,5,6,12,15,17	2,7,8,9,10,11,13,14,16	12	B0BNN3,P01681,P01835,P13432,P23593,Q10758,Q4FZU2,Q63751,Q6IFW6,Q6IG02,Q6IMF3,Q99MH3
5160	1,3,4,5,6,12,16,17	2,7,8,9,10,11,13,14,15	12	P01681,P01835,P13432,P23593,Q10758,Q4FZU2,Q63751,Q6IFW6,Q6IG02,Q6IMF3,Q6P6Q2,Q99MH3
5161	1,3,4,5,6,13,14,15	2,7,8,9,10,11,12,16,17	4	O54728,P01681,P82995,Q64093
5162	1,3,4,5,6,13,14,16	2,7,8,9,10,11,12,15,17	2	P01681,Q6IRE4
5163	1,3,4,5,6,13,14,17	2,7,8,9,10,11,12,15,16	3	P01681,P51907,Q9QW07
5164	1,3,4,5,6,13,15,16	2,7,8,9,10,11,12,14,17	5	O54728,P01681,P62804,P82995,Q00715
5165	1,3,4,5,6,13,15,17	2,7,8,9,10,11,12,14,16	4	B0BNN3,P01681,P23593,Q99MH3

5166	1,3,4,5,6,13,16,17	2,7,8,9,10,11,12,14,15	4	P01681,P23593,P62804,Q99MH3
5167	1,3,4,5,6,14,15,16	2,7,8,9,10,11,12,13,17	3	O54728,P01681,P02803
5168	1,3,4,5,6,14,15,17	2,7,8,9,10,11,12,13,16	7	B0BNN3,P01681,P02803,P10536,P47967,P97840,Q9QW07
5169	1,3,4,5,6,14,16,17	2,7,8,9,10,11,12,13,15	2	P01681,Q9QW07
5170	1,3,4,5,6,15,16,17	2,7,8,9,10,11,12,13,14	4	B0BNN3,P01681,P02803,P23593
5171	1,3,4,5,7,8,9,10	2,6,11,12,13,14,15,16,17	7	D3ZHA0,O54728,O55006,P18418,P52590,P97571,Q9QX79
5172	1,3,4,5,7,8,9,11	2,6,10,12,13,14,15,16,17	9	O54728,P07171,P10715,P18418,Q63617,Q6AYS4,Q6IRE4,Q9ESW0,Q9QX79
5173	1,3,4,5,7,8,9,12	2,6,10,11,13,14,15,16,17	10	P01681,P08937,P10715,P18418,Q4FZU6,Q6IG02,Q8CJ52,Q99041,Q9ESW0,Q9QX79
5174	1,3,4,5,7,8,9,13	2,6,10,11,12,14,15,16,17	6	O54728,P05539,P18418,Q4FZU6,Q63617,Q9QX79
5175	1,3,4,5,7,8,9,14	2,6,10,11,12,13,15,16,17	5	O54728,P18418,Q4FZU6,Q63617,Q9QX79
5176	1,3,4,5,7,8,9,15	2,6,10,11,12,13,14,16,17	5	O54728,O55006,P18418,Q62894,Q9QX79
5177	1,3,4,5,7,8,9,16	2,6,10,11,12,13,14,15,17	8	B4F795,O54728,P18418,P62804,Q4FZU6,Q63617,Q6IRE4,Q9QX79
5178	1,3,4,5,7,8,9,17	2,6,10,11,12,13,14,15,16	13	P01681,P01946,P02091,P08937,P18418,P23593,P50115,P52590,Q4FZU6,Q63617,Q63751,Q99041,Q9QX79
5179	1,3,4,5,7,8,10,11	2,6,9,12,13,14,15,16,17	15	P00714,P04041,P07171,P08649,P08937,P16391,P18418,P55314,P61206;P84079;P84082,Q5BJY9,Q5FVI6,Q6AYS4,Q8VD89,Q9ESW0,Q9QX79
5180	1,3,4,5,7,8,10,12	2,6,9,11,13,14,15,16,17	15	D3ZHA0,F1LM93,P01681,P08937,P18418,Q10758,Q4FZU2,Q6IFW6,Q6IG02,Q6IMF3,Q6P6Q2,Q8CJ52,Q99041,Q99MH3,Q9ESW0

5181	1,3,4,5,7,8,10,13	2,6,9,11,12,14,15,16,17	3	P08937,P18418,Q8VD89
5182	1,3,4,5,7,8,10,14	2,6,9,11,12,13,15,16,17	5	D3ZHA0,P07171,P18418,P36970,Q9QX79
5183	1,3,4,5,7,8,10,15	2,6,9,11,12,13,14,16,17	7	D3ZHA0,O54728,P07171,P18418,P97571,Q8VD89,Q9QX79
5184	1,3,4,5,7,8,10,16	2,6,9,11,12,13,14,15,17	3	P08721,P18418,Q8VD89
5185	1,3,4,5,7,8,10,17	2,6,9,11,12,13,14,15,16	14	P01681,P01946,P02091,P08937,P12346,P18418,P46844,P50115,P52590,P97571,Q63751,Q99041,Q99MH3,Q9QX79
5186	1,3,4,5,7,8,11,12	2,6,9,10,13,14,15,16,17	20	P01681,P02781,P08937,P10715,P18418,P22283,P36374,P61206;P84079;P84082,Q4FZU2,Q4FZU6,Q63493,Q63751,Q6AYS4,Q6IFW6,Q6IG02,Q6IMF3,Q6P6Q2,Q8CJ52,Q99041,Q9ESW0
5187	1,3,4,5,7,8,11,13	2,6,9,10,12,14,15,16,17	10	P05539,P07171,P08937,P18418,Q63617,Q63751,Q6AYS4,Q8CJ52,Q8VD89,Q9ESW0
5188	1,3,4,5,7,8,11,14	2,6,9,10,12,13,15,16,17	11	P01681,P07171,P18418,P97840,Q4FZU6,Q5I0D1,Q63617,Q64194,Q6AYS4,Q9ESW0,Q9QX79
5189	1,3,4,5,7,8,11,15	2,6,9,10,12,13,14,16,17	12	O54728,P07171,P10715,P18418,P22006,Q5I0D1,Q63751,Q64194,Q6AYS4,Q8VD89,Q9ESW0,Q9QX79
5190	1,3,4,5,7,8,11,16	2,6,9,10,12,13,14,15,17	13	B4F795,P07171,P10715,P14668,P18418,P55314,P61206;P84079;P84082,Q4FZU6,Q63617,Q6AYS4,Q6IRE4,Q8VD89,Q9ESW0
5191	1,3,4,5,7,8,11,17	2,6,9,10,12,13,14,15,16	18	P01681,P01946,P02091,P04041,P08937,P10715,P14669,P18418,P50115,P52590,P97840,Q4FZU6,Q63617,Q63751,Q6AYS4,Q99041,Q9ESW0,Q9QX79
5192	1,3,4,5,7,8,12,13	2,6,9,10,11,14,15,16,17	14	O88267,P01681,P06761,P08937,P18418,P22283,P36374,P36970,Q4FZU6,Q63751,Q6IG02,Q8CJ52,Q99041,Q9ESW0
5193	1,3,4,5,7,8,12,14	2,6,9,10,11,13,15,16,17	14	P01681,P06761,P08937,P18418,P36970,Q4FZU6,Q68FS4,Q6IFW6,Q6IG02,Q8CJ52,Q8CJD3,Q99041,Q9ESW0,Q9QX79
5194	1,3,4,5,7,8,12,15	2,6,9,10,11,13,14,16,17	11	O54728,P08937,P10715,P18418,Q62894,Q63751,Q6IG02,Q8CJ52,Q99041,Q9ESW0,Q9QX79
5195	1,3,4,5,7,8,12,16	2,6,9,10,11,13,14,15,17	12	P01681,P08721,P08937,P10715,P18418,Q4FZU6,Q62894,Q6IFW6,Q6IG02,Q8CJ52,Q99041,Q9ESW0

5196	1,3,4,5,7,8,12,17	2,6,9,10,11,13,14,15,16	22	O88267,P01681,P01946,P02091,P08937,P10715,P18418,P19223,P23928,P50115,P52590,P55091,Q4FZU6,Q4G075,Q63751,Q6IG02,Q8CJ52,Q99041,Q99MH3,Q9ESW0,Q9QX79,Q9QZA6
5197	1,3,4,5,7,8,13,14	2,6,9,10,11,12,15,16,17	7	P06866,P06911,P18418,P36970,Q4FZU6,Q5GRG2,Q9QX79
5198	1,3,4,5,7,8,13,15	2,6,9,10,11,12,14,16,17	6	O54728,P07171,P18418,Q62894,Q8VD89,Q9QX79
5199	1,3,4,5,7,8,13,16	2,6,9,10,11,12,14,15,17	4	P18418,P62804,Q4FZU6,Q8VD89
5200	1,3,4,5,7,8,13,17	2,6,9,10,11,12,14,15,16	13	P01681,P01946,P02091,P08937,P10536,P18418,P23928,P52590,Q4FZU6,Q63751,Q99041,Q99MH3,Q9QX79
5201	1,3,4,5,7,8,14,15	2,6,9,10,11,12,13,16,17	6	O54728,P07171,P10536,P18418,P36970,Q9QX79
5202	1,3,4,5,7,8,14,16	2,6,9,10,11,12,13,15,17	6	B4F795,P08721,P18418,Q4FZU6,Q6IRE4,Q9QX79
5203	1,3,4,5,7,8,14,17	2,6,9,10,11,12,13,15,16	13	P01681,P01946,P02091,P08937,P10536,P18418,P52590,P97840,Q4FZU6,Q63751,Q9ESW0,Q9QW07,Q9QX79
5204	1,3,4,5,7,8,15,16	2,6,9,10,11,12,13,14,17	7	B4F795,O54728,P08721,P18418,Q62894,Q8VD89,Q9QX79
5205	1,3,4,5,7,8,15,17	2,6,9,10,11,12,13,14,16	13	B0BNN3,O54728,P01946,P02091,P08937,P10536,P18418,P52590,Q62894,Q63751,Q99041,Q9ESW0,Q9QX79
5206	1,3,4,5,7,8,16,17	2,6,9,10,11,12,13,14,15	12	B4F795,P01681,P01946,P02091,P08937,P18418,P23928,P52590,Q4FZU6,Q63751,Q99041,Q9QX79
5207	1,3,4,5,7,9,10,11	2,6,8,12,13,14,15,16,17	7	O54728,P52590,P55314,P61206;P84079;P84082,Q5FVI6,Q62635,Q6IRE4
5208	1,3,4,5,7,9,10,12	2,6,8,11,13,14,15,16,17	4	P52590,Q4FZU2,Q6IFW6,Q6IG02
5209	1,3,4,5,7,9,10,13	2,6,8,11,12,14,15,16,17	4	O54728,P62804,Q00715,Q62635
5210	1,3,4,5,7,9,10,14	2,6,8,11,12,13,15,16,17	2	O54728,Q62635

5211	1,3,4,5,7,9,10,15	2,6,8,11,12,13,14,16,17	3	O54728,P52590,Q62635
5212	1,3,4,5,7,9,10,16	2,6,8,11,12,13,14,15,17	7	O54728,P08721,P62804,Q00715,Q5FVI6,Q62635,Q6IRE4
5213	1,3,4,5,7,9,10,17	2,6,8,11,12,13,14,15,16	3	P01946,P02091,P52590
5214	1,3,4,5,7,9,11,12	2,6,8,10,13,14,15,16,17	8	O54728,P01681,Q4FZU2,Q6IFW6,Q6IG02,Q6IRE4,Q6P6Q2,Q9ESW0
5215	1,3,4,5,7,9,11,13	2,6,8,10,12,14,15,16,17	4	O54728,P05539,Q62635,Q6IRE4
5216	1,3,4,5,7,9,11,14	2,6,8,10,12,13,15,16,17	3	O54728,Q62635,Q6IRE4
5217	1,3,4,5,7,9,11,15	2,6,8,10,12,13,14,16,17	5	O54728,Q62635,Q6IRE4,Q8K4G9,Q9ESW0
5218	1,3,4,5,7,9,11,16	2,6,8,10,12,13,14,15,17	8	B4F795,O54728,P15999,P55314,P61206;P84079;P84082,Q62635,Q6IRE4,Q8K4G9
5219	1,3,4,5,7,9,11,17	2,6,8,10,12,13,14,15,16	6	O54728,P01681,P01946,P02091,P52590,Q9ESW0
5220	1,3,4,5,7,9,12,13	2,6,8,10,11,14,15,16,17	1	P05539
5221	1,3,4,5,7,9,12,14	2,6,8,10,11,13,15,16,17	2	Q4FZU6,Q6IG02
5222	1,3,4,5,7,9,12,15	2,6,8,10,11,13,14,16,17	2	O54728,Q9ESW0
5223	1,3,4,5,7,9,12,16	2,6,8,10,11,13,14,15,17	4	O54728,Q4FZU6,Q6IG02,Q6IRE4
5224	1,3,4,5,7,9,12,17	2,6,8,10,11,13,14,15,16	7	P01681,P01946,P02091,P52590,Q4FZU6,Q6IG02,Q9ESW0
5225	1,3,4,5,7,9,13,14	2,6,8,10,11,12,15,16,17	4	O54728,P05539,P62804,Q4FZU6

5226	1,3,4,5,7,9,13,15	2,6,8,10,11,12,14,16,17	5	O54728,P05539,P62804,Q00715,Q62635
5227	1,3,4,5,7,9,13,16	2,6,8,10,11,12,14,15,17	6	O54728,P62804,Q00715,Q4FZU6,Q62635,Q6IRE4
5228	1,3,4,5,7,9,13,17	2,6,8,10,11,12,14,15,16	6	P01946,P02091,P05539,P52590,P62804,Q4FZU6
5229	1,3,4,5,7,9,14,15	2,6,8,10,11,12,13,16,17	3	O54728,P62804,Q62635
5230	1,3,4,5,7,9,14,16	2,6,8,10,11,12,13,15,17	7	O54728,P62804,Q00715,Q4FZU6,Q62635,Q6IRE4,Q9QZK9
5231	1,3,4,5,7,9,14,17	2,6,8,10,11,12,13,15,16	5	O54728,P01946,P02091,P52590,Q4FZU6
5232	1,3,4,5,7,9,15,16	2,6,8,10,11,12,13,14,17	10	B4F795,O54728,P02782,P47727,P62804,Q00715,Q62635,Q6IRE4,Q8K4G9,Q9QZK9
5233	1,3,4,5,7,9,15,17	2,6,8,10,11,12,13,14,16	5	O54728,P02091,P47727,P52590,P62804
5234	1,3,4,5,7,9,16,17	2,6,8,10,11,12,13,14,15	9	O54728,P01946,P02091,P23593,P47727,P52590,P62804,Q00715,Q4FZU6
5235	1,3,4,5,7,10,11,12	2,6,8,9,13,14,15,16,17	11	P01681,P06757,P55314,P61206;P84079;P84082,Q4FZU2,Q5FVI6,Q6IFW6,Q6IG02,Q6IMF3,Q6P6Q2,Q9ESW0
5236	1,3,4,5,7,10,11,13	2,6,8,9,12,14,15,16,17	5	P05539,P55314,P61206;P84079;P84082,Q5FVI6,Q8VD89
5237	1,3,4,5,7,10,11,14	2,6,8,9,12,13,15,16,17	5	P55314,P61206;P84079;P84082,Q5FVI6,Q62635,Q9ESW0
5238	1,3,4,5,7,10,11,15	2,6,8,9,12,13,14,16,17	8	O54728,P22006,P55314,P61206;P84079;P84082,Q5FVI6,Q62635,Q8VD89,Q9ESW0
5239	1,3,4,5,7,10,11,16	2,6,8,9,12,13,14,15,17	7	P08721,P55314,P61206;P84079;P84082,Q5FVI6,Q62635,Q6IRE4,Q8VD89
5240	1,3,4,5,7,10,11,17	2,6,8,9,12,13,14,15,16	8	P01681,P01946,P02091,P52590,P55314,P61206;P84079;P84082,Q5FVI6,Q9ESW0

5241	1,3,4,5,7,10,12,13	2,6,8,9,11,14,15,16,17	3	Q4FZU2,Q6IG02,Q99MH3
5242	1,3,4,5,7,10,12,14	2,6,8,9,11,13,15,16,17	5	Q10758,Q4FZU2,Q6IFW6,Q6IG02,Q9ESW0
5243	1,3,4,5,7,10,12,15	2,6,8,9,11,13,14,16,17	5	O54728,Q4FZU2,Q6IG02,Q99MH3,Q9ESW0
5244	1,3,4,5,7,10,12,16	2,6,8,9,11,13,14,15,17	6	P08721,Q4FZU2,Q5FVI6,Q6IFW6,Q6IG02,Q6IMF3
5245	1,3,4,5,7,10,12,17	2,6,8,9,11,13,14,15,16	10	P01681,P01946,P02091,P52590,Q10758,Q4FZU2,Q6IFW6,Q6IG02,Q99MH3,Q9ESW0
5246	1,3,4,5,7,10,13,14	2,6,8,9,11,12,15,16,17	0	
5247	1,3,4,5,7,10,13,15	2,6,8,9,11,12,14,16,17	3	O54728,P62804,Q8VD89
5248	1,3,4,5,7,10,13,16	2,6,8,9,11,12,14,15,17	4	P62804,Q00715,Q62635,Q8VD89
5249	1,3,4,5,7,10,13,17	2,6,8,9,11,12,14,15,16	4	P01946,P02091,P52590,Q99MH3
5250	1,3,4,5,7,10,14,15	2,6,8,9,11,12,13,16,17	2	O54728,Q62635
5251	1,3,4,5,7,10,14,16	2,6,8,9,11,12,13,15,17	2	P08721,Q62635
5252	1,3,4,5,7,10,14,17	2,6,8,9,11,12,13,15,16	4	P01946,P02091,P18427,P52590
5253	1,3,4,5,7,10,15,16	2,6,8,9,11,12,13,14,17	8	O54728,P02782,P08721,P62804,Q00715,Q5FVI6,Q62635,Q8VD89
5254	1,3,4,5,7,10,15,17	2,6,8,9,11,12,13,14,16	4	O54728,P02091,P52590,Q99MH3
5255	1,3,4,5,7,10,16,17	2,6,8,9,11,12,13,14,15	4	P01946,P02091,P08721,P52590

5256	1,3,4,5,7,11,12,13	2,6,8,9,10,14,15,16,17	9	P01681,P05539,P17046,P36374,Q4FZU2,Q6IFW6,Q6IG02,Q6P6Q2,Q9ESW0
5257	1,3,4,5,7,11,12,14	2,6,8,9,10,13,15,16,17	7	P01681,Q4FZU2,Q4FZU6,Q6IFW6,Q6IG02,Q6P6Q2,Q9ESW0
5258	1,3,4,5,7,11,12,15	2,6,8,9,10,13,14,16,17	7	O54728,Q4FZU2,Q5I0D1,Q6IFW6,Q6IG02,Q6P6Q2,Q9ESW0
5259	1,3,4,5,7,11,12,16	2,6,8,9,10,13,14,15,17	12	P01681,P17046,P55314,P61206;P84079;P84082,Q4FZU2,Q4FZU6,Q6IFW6,Q6IG02,Q6IMF3,Q6IRE4,Q6P6Q2,Q9ESW0
5260	1,3,4,5,7,11,12,17	2,6,8,9,10,13,14,15,16	11	P01681,P01946,P02091,P08937,P17046,Q4FZU2,Q63751,Q6IFW6,Q6IG02,Q6P6Q2,Q9ESW0
5261	1,3,4,5,7,11,13,14	2,6,8,9,10,12,15,16,17	3	P05539,Q6IRE4,Q6P6R2
5262	1,3,4,5,7,11,13,15	2,6,8,9,10,12,14,16,17	5	O54728,P05539,Q6P6R2,Q8VD89,Q9ESW0
5263	1,3,4,5,7,11,13,16	2,6,8,9,10,12,14,15,17	7	O54728,P05539,P17046,P55314,P61206;P84079;P84082,Q6IRE4,Q8VD89
5264	1,3,4,5,7,11,13,17	2,6,8,9,10,12,14,15,16	6	P01681,P01946,P02091,P05539,Q63751,Q9ESW0
5265	1,3,4,5,7,11,14,15	2,6,8,9,10,12,13,16,17	7	O54728,P22006,Q5I0D1,Q62635,Q6IRE4,Q6P6R2,Q9ESW0
5266	1,3,4,5,7,11,14,16	2,6,8,9,10,12,13,15,17	7	O54728,P55314,P61206;P84079;P84082,Q4FZU6,Q62635,Q6IRE4,Q9ESW0
5267	1,3,4,5,7,11,14,17	2,6,8,9,10,12,13,15,16	6	P01681,P01946,P02091,P97840,Q4FZU6,Q9ESW0
5268	1,3,4,5,7,11,15,16	2,6,8,9,10,12,13,14,17	11	B4F795,O54728,P14408,P17046,P55314,Q5I0D1,Q62635,Q6IRE4,Q8K4G9,Q8VD89,Q9ESW0
5269	1,3,4,5,7,11,15,17	2,6,8,9,10,12,13,14,16	5	O54728,P01946,P02091,Q63751,Q9ESW0
5270	1,3,4,5,7,11,16,17	2,6,8,9,10,12,13,14,15	8	P01681,P01946,P02091,P17046,P55314,Q4FZU6,Q6IRE4,Q9ESW0

5271	1,3,4,5,7,12,13,14	2,6,8,9,10,11,15,16,17	2	A2RUV9,Q4FZU6
5272	1,3,4,5,7,12,13,15	2,6,8,9,10,11,14,16,17	4	O54728,P05539,Q6P6R2,Q9ESW0
5273	1,3,4,5,7,12,13,16	2,6,8,9,10,11,14,15,17	2	P17046,Q4FZU6
5274	1,3,4,5,7,12,13,17	2,6,8,9,10,11,14,15,16	9	P01681,P01946,P02091,P08937,P36374,Q4FZU6,Q63751,Q99MH3,Q9ESW0
5275	1,3,4,5,7,12,14,15	2,6,8,9,10,11,13,16,17	4	A2RUV9,O54728,Q8CJD3,Q9ESW0
5276	1,3,4,5,7,12,14,16	2,6,8,9,10,11,13,15,17	6	P08721,P17046,Q4FZU6,Q6IG02,Q6IRE4,Q9ESW0
5277	1,3,4,5,7,12,14,17	2,6,8,9,10,11,13,15,16	9	P01681,P01946,P02091,P31430,Q4FZU6,Q6IG02,Q8CJD3,Q9ESW0,Q9QW07
5278	1,3,4,5,7,12,15,16	2,6,8,9,10,11,13,14,17	3	O54728,P17046,Q9ESW0
5279	1,3,4,5,7,12,15,17	2,6,8,9,10,11,13,14,16	5	P01681,P01946,P02091,Q99MH3,Q9ESW0
5280	1,3,4,5,7,12,16,17	2,6,8,9,10,11,13,14,15	7	P01681,P01946,P02091,P17046,Q4FZU6,Q6IG02,Q9ESW0
5281	1,3,4,5,7,13,14,15	2,6,8,9,10,11,12,16,17	5	O54728,P05539,P62804,Q09030,Q6P6R2
5282	1,3,4,5,7,13,14,16	2,6,8,9,10,11,12,15,17	5	O54728,P62804,Q00715,Q4FZU6,Q6IRE4
5283	1,3,4,5,7,13,14,17	2,6,8,9,10,11,12,15,16	4	P01946,P02091,Q4FZU6,Q9QW07
5284	1,3,4,5,7,13,15,16	2,6,8,9,10,11,12,14,17	7	O54728,P62804,Q00715,Q09030,Q62635,Q6IRE4,Q8VD89
5285	1,3,4,5,7,13,15,17	2,6,8,9,10,11,12,14,16	7	O54728,P01946,P02091,P05539,P62804,Q99MH3,Q9WUW8

5286	1,3,4,5,7,13,16,17	2,6,8,9,10,11,12,14,15	6	P01946,P02091,P17046,P62804,Q00715,Q4FZU6
5287	1,3,4,5,7,14,15,16	2,6,8,9,10,11,12,13,17	4	O54728,P62804,Q62635,Q6IRE4
5288	1,3,4,5,7,14,15,17	2,6,8,9,10,11,12,13,16	7	O54728,P01946,P02091,P02803,P10536,Q8CJD3,Q9QW07
5289	1,3,4,5,7,14,16,17	2,6,8,9,10,11,12,13,15	4	P01946,P02091,Q4FZU6,Q9QW07
5290	1,3,4,5,7,15,16,17	2,6,8,9,10,11,12,13,14	6	O54728,P01946,P02091,P02782,P47727,P62804
5291	1,3,4,5,8,9,10,11	2,6,7,12,13,14,15,16,17	3	O54728,P08649,Q6AYS4
5292	1,3,4,5,8,9,10,12	2,6,7,11,13,14,15,16,17	6	P01681,Q4FZU2,Q6IFW6,Q6IG02,Q6IMF3,Q6P6Q2
5293	1,3,4,5,8,9,10,13	2,6,7,11,12,14,15,16,17	1	O54728
5294	1,3,4,5,8,9,10,14	2,6,7,11,12,13,15,16,17	1	O54728
5295	1,3,4,5,8,9,10,15	2,6,7,11,12,13,14,16,17	2	O54728,Q62635
5296	1,3,4,5,8,9,10,16	2,6,7,11,12,13,14,15,17	4	O54728,P08721,P23593,Q62635
5297	1,3,4,5,8,9,10,17	2,6,7,11,12,13,14,15,16	5	P01681,P08649,P23593,P25031,Q63532
5298	1,3,4,5,8,9,11,12	2,6,7,10,13,14,15,16,17	9	P01681,Q4FZU2,Q6AYS4,Q6IFU8,Q6IFW6,Q6IG02,Q6IMF3,Q6P6Q2,Q9ESW0
5299	1,3,4,5,8,9,11,13	2,6,7,10,12,14,15,16,17	3	O54728,P97840,Q6AYS4
5300	1,3,4,5,8,9,11,14	2,6,7,10,12,13,15,16,17	5	O54728,P01681,P14668,P97840,Q6AYS4

5301	1,3,4,5,8,9,11,15	2,6,7,10,12,13,14,16,17	5	O54728,P14480,P14668,P97840,Q5I0D1
5302	1,3,4,5,8,9,11,16	2,6,7,10,12,13,14,15,17	6	B4F795,O54728,P14668,P55314,Q62635,Q6IRE4
5303	1,3,4,5,8,9,11,17	2,6,7,10,12,13,14,15,16	5	P01681,P08649,P97840,Q63532,Q6AYS4
5304	1,3,4,5,8,9,12,13	2,6,7,10,11,14,15,16,17	3	P01681,Q5M8C6,Q6IG02
5305	1,3,4,5,8,9,12,14	2,6,7,10,11,13,15,16,17	4	P01681,Q4FZU6,Q6IFW6,Q6IG02
5306	1,3,4,5,8,9,12,15	2,6,7,10,11,13,14,16,17	3	O54728,Q62894,Q6IG02
5307	1,3,4,5,8,9,12,16	2,6,7,10,11,13,14,15,17	6	P01681,P23593,Q4FZU6,Q62894,Q6IFW6,Q6IG02
5308	1,3,4,5,8,9,12,17	2,6,7,10,11,13,14,15,16	7	P01681,P16636,P19223,P23593,Q5M8C6,Q63532,Q6IG02
5309	1,3,4,5,8,9,13,14	2,6,7,10,11,12,15,16,17	3	O54728,P06866,P97840
5310	1,3,4,5,8,9,13,15	2,6,7,10,11,12,14,16,17	3	O54728,P97840,Q62894
5311	1,3,4,5,8,9,13,16	2,6,7,10,11,12,14,15,17	3	O54728,P62804,Q00715
5312	1,3,4,5,8,9,13,17	2,6,7,10,11,12,14,15,16	4	P01681,P23593,P97840,Q63532
5313	1,3,4,5,8,9,14,15	2,6,7,10,11,12,13,16,17	4	O54728,P47967,P97840,Q9QZK9
5314	1,3,4,5,8,9,14,16	2,6,7,10,11,12,13,15,17	4	O54728,Q4FZU6,Q62635,Q9QZK9
5315	1,3,4,5,8,9,14,17	2,6,7,10,11,12,13,15,16	5	P01681,P02793;Q7TP54,P47967,P97840,Q4FZU6

5316	1,3,4,5,8,9,15,16	2,6,7,10,11,12,13,14,17	8	B4F795,O54728,P23593,P47727,Q5QE79,Q62635,Q62894,Q9QZK9
5317	1,3,4,5,8,9,15,17	2,6,7,10,11,12,13,14,16	9	B0BNN3,O54728,P23593,P47727,P47967,P97840,Q5QE79,Q62894,Q63532
5318	1,3,4,5,8,9,16,17	2,6,7,10,11,12,13,14,15	5	P01681,P23593,P25031,P47727,Q4FZU6
5319	1,3,4,5,8,10,11,12	2,6,7,9,13,14,15,16,17	11	P01681,P16636,Q10758,Q4FZU2,Q6AYS4,Q6IFU8,Q6IFW6,Q6IG02,Q6IMF3,Q6P6Q2,Q9ESW0
5320	1,3,4,5,8,10,11,13	2,6,7,9,12,14,15,16,17	1	Q6AYS4
5321	1,3,4,5,8,10,11,14	2,6,7,9,12,13,15,16,17	4	P01681,P08649,Q64194,Q6AYS4
5322	1,3,4,5,8,10,11,15	2,6,7,9,12,13,14,16,17	6	O54728,P08649,Q5I0D1,Q64194,Q6AYS4,Q9ESW0
5323	1,3,4,5,8,10,11,16	2,6,7,9,12,13,14,15,17	4	P08649,P08721,P14668,P55314
5324	1,3,4,5,8,10,11,17	2,6,7,9,12,13,14,15,16	5	P01681,P08649,P16636,Q6AYS4,Q9ESW0
5325	1,3,4,5,8,10,12,13	2,6,7,9,11,14,15,16,17	5	P01681,Q4FZU2,Q6IFW6,Q6IG02,Q99MH3
5326	1,3,4,5,8,10,12,14	2,6,7,9,11,13,15,16,17	8	P01681,P36970,Q10758,Q4FZU2,Q6IFW6,Q6IG02,Q6IMF3,Q6P6Q2
5327	1,3,4,5,8,10,12,15	2,6,7,9,11,13,14,16,17	5	B0BNN3,Q4FZU2,Q6IFW6,Q6IG02,Q99MH3
5328	1,3,4,5,8,10,12,16	2,6,7,9,11,13,14,15,17	8	P01681,P08721,Q10758,Q4FZU2,Q6IFW6,Q6IG02,Q6IMF3,Q6P6Q2
5329	1,3,4,5,8,10,12,17	2,6,7,9,11,13,14,15,16	9	P01681,P16636,Q10758,Q4FZU2,Q4G075,Q6IFW6,Q6IG02,Q99MH3,Q9ESW0
5330	1,3,4,5,8,10,13,14	2,6,7,9,11,12,15,16,17	1	P36970

5331	1,3,4,5,8,10,13,15	2,6,7,9,11,12,14,16,17	1	O54728
5332	1,3,4,5,8,10,13,16	2,6,7,9,11,12,14,15,17	0	
5333	1,3,4,5,8,10,13,17	2,6,7,9,11,12,14,15,16	2	P01681,Q99MH3
5334	1,3,4,5,8,10,14,15	2,6,7,9,11,12,13,16,17	2	O54728,P09605;P25809
5335	1,3,4,5,8,10,14,16	2,6,7,9,11,12,13,15,17	1	P08721
5336	1,3,4,5,8,10,14,17	2,6,7,9,11,12,13,15,16	1	P01681
5337	1,3,4,5,8,10,15,16	2,6,7,9,11,12,13,14,17	3	O54728,P08721,Q62635
5338	1,3,4,5,8,10,15,17	2,6,7,9,11,12,13,14,16	2	B0BNN3,Q99MH3
5339	1,3,4,5,8,10,16,17	2,6,7,9,11,12,13,14,15	4	P01681,P08721,P23593,P25031
5340	1,3,4,5,8,11,12,13	2,6,7,9,10,14,15,16,17	9	P01681,P02781,P36374,Q4FZU2,Q5M8C6,Q6IFW6,Q6IG02,Q6P6Q2,Q9ESW0
5341	1,3,4,5,8,11,12,14	2,6,7,9,10,13,15,16,17	11	P01681,P97840,Q4FZU2,Q5I0D1,Q64194,Q6IFU8,Q6IFW6,Q6IG02,Q6IMF3,Q6P6Q2,Q9ESW0
5342	1,3,4,5,8,11,12,15	2,6,7,9,10,13,14,16,17	10	B0BNN3,O54728,P01681,P35053,Q4FZU2,Q5I0D1,Q6IFW6,Q6IG02,Q6P6Q2,Q9ESW0
5343	1,3,4,5,8,11,12,16	2,6,7,9,10,13,14,15,17	10	P01681,P14668,P55314,Q4FZU2,Q6IFU8,Q6IFW6,Q6IG02,Q6IMF3,Q6P6Q2,Q9ESW0
5344	1,3,4,5,8,11,12,17	2,6,7,9,10,13,14,15,16	13	P01681,P16636,P97840,Q4FZU2,Q4G075,Q5M8C6,Q63751,Q6AYS4,Q6IFW6,Q6IG02,Q6IMF3,Q6P6Q2,Q9ESW0
5345	1,3,4,5,8,11,13,14	2,6,7,9,10,12,15,16,17	1	P97840

5346	1,3,4,5,8,11,13,15	2,6,7,9,10,12,14,16,17	3	O54728,P97840,Q5I0D1
5347	1,3,4,5,8,11,13,16	2,6,7,9,10,12,14,15,17	5	P14668,P55314,P97840,Q6IRE4,Q8VD89
5348	1,3,4,5,8,11,13,17	2,6,7,9,10,12,14,15,16	4	P01681,P97840,Q63532,Q6AYS4
5349	1,3,4,5,8,11,14,15	2,6,7,9,10,12,13,16,17	6	O54728,P14668,P97840,Q5I0D1,Q64194,Q9ESW0
5350	1,3,4,5,8,11,14,16	2,6,7,9,10,12,13,15,17	7	P01681,P14668,P55314,P97840,Q5I0D1,Q64194,Q6IRE4
5351	1,3,4,5,8,11,14,17	2,6,7,9,10,12,13,15,16	6	P01681,P02793;Q7TP54,P47967,P97840,Q6AYS4,Q9ESW0
5352	1,3,4,5,8,11,15,16	2,6,7,9,10,12,13,14,17	9	B4F795,O54728,P14668,P55314,P97840,Q5I0D1,Q62894,Q64194,Q8VD89
5353	1,3,4,5,8,11,15,17	2,6,7,9,10,12,13,14,16	9	B0BNN3,O54728,P01681,P47967,P97840,Q5I0D1,Q63532,Q6AYS4,Q9ESW0
5354	1,3,4,5,8,11,16,17	2,6,7,9,10,12,13,14,15	5	P01681,P14668,P55314,P97840,Q9ESW0
5355	1,3,4,5,8,12,13,14	2,6,7,9,10,11,15,16,17	5	P01681,P36970,Q64093,Q6IG02,Q9QUL6
5356	1,3,4,5,8,12,13,15	2,6,7,9,10,11,14,16,17	4	O54728,Q62894,Q6IG02,Q9QUL6
5357	1,3,4,5,8,12,13,16	2,6,7,9,10,11,14,15,17	3	P01681,Q6IG02,Q9QUL6
5358	1,3,4,5,8,12,13,17	2,6,7,9,10,11,14,15,16	6	P01681,P16636,P36374,Q5M8C6,Q6IG02,Q99MH3
5359	1,3,4,5,8,12,14,15	2,6,7,9,10,11,13,16,17	6	B0BNN3,O54728,P01681,P36970,Q6IG02,Q9ESW0
5360	1,3,4,5,8,12,14,16	2,6,7,9,10,11,13,15,17	5	P01681,P08721,Q4FZU6,Q6IFW6,Q6IG02

5361	1,3,4,5,8,12,14,17	2,6,7,9,10,11,13,15,16	8	P01681,P10536,P31430,P97840,Q4FZU6,Q6IG02,Q9ESW0,Q9QW07
5362	1,3,4,5,8,12,15,16	2,6,7,9,10,11,13,14,17	5	O54728,Q4FZU2,Q62894,Q6IG02,Q9QUL6
5363	1,3,4,5,8,12,15,17	2,6,7,9,10,11,13,14,16	8	B0BNN3,P01681,P10536,P97840,Q62894,Q6IG02,Q99MH3,Q9ESW0
5364	1,3,4,5,8,12,16,17	2,6,7,9,10,11,13,14,15	7	P01681,P23593,P23928,Q4FZU6,Q62894,Q6IFW6,Q6IG02
5365	1,3,4,5,8,13,14,15	2,6,7,9,10,11,12,16,17	4	O54728,P36970,P97840,Q64093
5366	1,3,4,5,8,13,14,16	2,6,7,9,10,11,12,15,17	1	Q4FZU6
5367	1,3,4,5,8,13,14,17	2,6,7,9,10,11,12,15,16	7	P01681,P10536,P47967,P51907,P97840,Q6PCU2,Q9QW07
5368	1,3,4,5,8,13,15,16	2,6,7,9,10,11,12,14,17	4	O54728,P62804,Q62894,Q9QUL6
5369	1,3,4,5,8,13,15,17	2,6,7,9,10,11,12,14,16	8	B0BNN3,O54728,P10536,P47967,P97840,Q62894,Q63532,Q99MH3
5370	1,3,4,5,8,13,16,17	2,6,7,9,10,11,12,14,15	4	P01681,P23928,P97840,Q6PCU2
5371	1,3,4,5,8,14,15,16	2,6,7,9,10,11,12,13,17	3	O54728,P97840,Q9QZK9
5372	1,3,4,5,8,14,15,17	2,6,7,9,10,11,12,13,16	6	B0BNN3,O54728,P10536,P47967,P97840,Q9QW07
5373	1,3,4,5,8,14,16,17	2,6,7,9,10,11,12,13,15	5	P01681,P97840,Q4FZU6,Q6PCU2,Q9QW07
5374	1,3,4,5,8,15,16,17	2,6,7,9,10,11,12,13,14	6	B0BNN3,O54728,P23593,P47727,P97840,Q62894
5375	1,3,4,5,9,10,11,12	2,6,7,8,13,14,15,16,17	7	P01681,Q4FZU2,Q6IFU8,Q6IFW6,Q6IG02,Q6IMF3,Q6P6Q2

5376	1,3,4,5,9,10,11,13	2,6,7,8,12,14,15,16,17	3	O54728,P12785,Q62635
5377	1,3,4,5,9,10,11,14	2,6,7,8,12,13,15,16,17	2	O54728,Q62635
5378	1,3,4,5,9,10,11,15	2,6,7,8,12,13,14,16,17	2	O54728,Q62635
5379	1,3,4,5,9,10,11,16	2,6,7,8,12,13,14,15,17	5	O54728,P11598,P55314,Q62635,Q6IRE4
5380	1,3,4,5,9,10,11,17	2,6,7,8,12,13,14,15,16	3	P01681,P12785,P18427
5381	1,3,4,5,9,10,12,13	2,6,7,8,11,14,15,16,17	1	Q6IG02
5382	1,3,4,5,9,10,12,14	2,6,7,8,11,13,15,16,17	3	Q4FZU2,Q6IFW6,Q6IG02
5383	1,3,4,5,9,10,12,15	2,6,7,8,11,13,14,16,17	4	O54728,P10758,Q4FZU2,Q6IG02
5384	1,3,4,5,9,10,12,16	2,6,7,8,11,13,14,15,17	4	Q4FZU2,Q6IFW6,Q6IG02,Q6IMF3
5385	1,3,4,5,9,10,12,17	2,6,7,8,11,13,14,15,16	4	P01681,Q10758,Q4FZU2,Q6IG02
5386	1,3,4,5,9,10,13,14	2,6,7,8,11,12,15,16,17	2	O54728,Q62635
5387	1,3,4,5,9,10,13,15	2,6,7,8,11,12,14,16,17	3	O54728,P62804,Q62635
5388	1,3,4,5,9,10,13,16	2,6,7,8,11,12,14,15,17	4	O54728,P62804,Q00715,Q62635
5389	1,3,4,5,9,10,13,17	2,6,7,8,11,12,14,15,16	0	
5390	1,3,4,5,9,10,14,15	2,6,7,8,11,12,13,16,17	4	O54728,Q62635,Q62902,Q9QZK9

5391	1,3,4,5,9,10,14,16	2,6,7,8,11,12,13,15,17	3	O54728,Q62635,Q9QZK9
5392	1,3,4,5,9,10,14,17	2,6,7,8,11,12,13,15,16	1	P18427
5393	1,3,4,5,9,10,15,16	2,6,7,8,11,12,13,14,17	10	O54728,P02782,P11598,P47727,P62804,Q00715,Q62635,Q62902,Q9JHB9,Q9QZK9
5394	1,3,4,5,9,10,15,17	2,6,7,8,11,12,13,14,16	3	O54728,P47727,Q62902
5395	1,3,4,5,9,10,16,17	2,6,7,8,11,12,13,14,15	3	P23593,P47727,Q62902
5396	1,3,4,5,9,11,12,13	2,6,7,8,10,14,15,16,17	6	P01681,P05539,Q4FZU2,Q6IFW6,Q6IG02,Q6P6Q2
5397	1,3,4,5,9,11,12,14	2,6,7,8,10,13,15,16,17	9	P01681,P01835,Q4FZU2,Q6IFU8,Q6IFW6,Q6IG02,Q6IMF3,Q6P6Q2,Q9ESW0
5398	1,3,4,5,9,11,12,15	2,6,7,8,10,13,14,16,17	7	O54728,P12020,Q4FZU2,Q6IFW6,Q6IG02,Q6P6Q2,Q9ESW0
5399	1,3,4,5,9,11,12,16	2,6,7,8,10,13,14,15,17	12	O54728,P01681,P12020,P15999,P55314,Q4FZU2,Q6IFU8,Q6IFW6,Q6IG02,Q6IMF3,Q6IRE4,Q6P6Q2
5400	1,3,4,5,9,11,12,17	2,6,7,8,10,13,14,15,16	8	P01681,P01835,P12020,Q4FZU2,Q6IFW6,Q6IG02,Q6P6Q2,Q9ESW0
5401	1,3,4,5,9,11,13,14	2,6,7,8,10,12,15,16,17	5	O54728,P05539,P97840,Q62635,Q6IRE4
5402	1,3,4,5,9,11,13,15	2,6,7,8,10,12,14,16,17	5	O54728,P05539,P97840,Q62635,Q6P6R2
5403	1,3,4,5,9,11,13,16	2,6,7,8,10,12,14,15,17	4	O54728,P55314,Q62635,Q6IRE4
5404	1,3,4,5,9,11,13,17	2,6,7,8,10,12,14,15,16	4	P01681,P05539,P97840,Q63532
5405	1,3,4,5,9,11,14,15	2,6,7,8,10,12,13,16,17	4	O54728,P97840,Q62635,Q9QZK9

5406	1,3,4,5,9,11,14,16	2,6,7,8,10,12,13,15,17	5	O54728,P55314,Q62635,Q6IRE4,Q9QZK9
5407	1,3,4,5,9,11,14,17	2,6,7,8,10,12,13,15,16	2	P01681,P97840
5408	1,3,4,5,9,11,15,16	2,6,7,8,10,12,13,14,17	7	O54728,P11598,P12020,P55314,Q62635,Q6IRE4,Q9QZK9
5409	1,3,4,5,9,11,15,17	2,6,7,8,10,12,13,14,16	4	O54728,P12020,P97840,Q63532
5410	1,3,4,5,9,11,16,17	2,6,7,8,10,12,13,14,15	5	O54728,P01681,P12020,P15999,P55314
5411	1,3,4,5,9,12,13,14	2,6,7,8,10,11,15,16,17	1	Q6IG02
5412	1,3,4,5,9,12,13,15	2,6,7,8,10,11,14,16,17	3	O54728,P10758,Q6P6R2
5413	1,3,4,5,9,12,13,16	2,6,7,8,10,11,14,15,17	2	O54728,P62804
5414	1,3,4,5,9,12,13,17	2,6,7,8,10,11,14,15,16	2	P01681,P35280
5415	1,3,4,5,9,12,14,15	2,6,7,8,10,11,13,16,17	3	O54728,P10758,Q6IG02
5416	1,3,4,5,9,12,14,16	2,6,7,8,10,11,13,15,17	3	O54728,Q4FZU6,Q6IG02
5417	1,3,4,5,9,12,14,17	2,6,7,8,10,11,13,15,16	3	P01681,P01835,Q6IG02
5418	1,3,4,5,9,12,15,16	2,6,7,8,10,11,13,14,17	4	O54728,P12020,P47727,Q6IG02
5419	1,3,4,5,9,12,15,17	2,6,7,8,10,11,13,14,16	6	B0BNN3,O54728,P01681,P12020,P47727,Q6IG02
5420	1,3,4,5,9,12,16,17	2,6,7,8,10,11,13,14,15	6	P01681,P12020,P23593,P35280,P47727,Q6IG02

5421	1,3,4,5,9,13,14,15	2,6,7,8,10,11,12,16,17	5	O54728,P60905,P62804,Q62635,Q6P6R2
5422	1,3,4,5,9,13,14,16	2,6,7,8,10,11,12,15,17	6	O54728,P62804,Q00715,Q62635,Q6IRE4,Q9QZK9
5423	1,3,4,5,9,13,14,17	2,6,7,8,10,11,12,15,16	3	P35280,P97840,Q9QW07
5424	1,3,4,5,9,13,15,16	2,6,7,8,10,11,12,14,17	8	O54728,P47727,P60905,P62804,Q00715,Q62635,Q6IRE4,Q9QZK9
5425	1,3,4,5,9,13,15,17	2,6,7,8,10,11,12,14,16	5	O54728,P47727,P62804,P97840,Q63532
5426	1,3,4,5,9,13,16,17	2,6,7,8,10,11,12,14,15	6	O54728,P23593,P35280,P47727,P62804,Q00715
5427	1,3,4,5,9,14,15,16	2,6,7,8,10,11,12,13,17	9	O54728,P02782,P60905,P62804,Q00715,Q62635,Q6IRE4,Q9JHB9,Q9QZK9
5428	1,3,4,5,9,14,15,17	2,6,7,8,10,11,12,13,16	4	O54728,P47967,P60905,P97840
5429	1,3,4,5,9,14,16,17	2,6,7,8,10,11,12,13,15	4	O54728,P62804,Q4FZU6,Q9QW07
5430	1,3,4,5,9,15,16,17	2,6,7,8,10,11,12,13,14	10	O54728,P02782,P12020,P23593,P47727,P60905,P62804,Q00715,Q62902,Q9JHB9
5431	1,3,4,5,10,11,12,13	2,6,7,8,9,14,15,16,17	6	P01681,Q4FZU2,Q6IFW6,Q6IG02,Q6IMF3,Q6P6Q2
5432	1,3,4,5,10,11,12,14	2,6,7,8,9,13,15,16,17	10	P01681,P34901,Q10758,Q4FZU2,Q6IFU8,Q6IFW6,Q6IG02,Q6IMF3,Q6P6Q2,Q9ESW0
5433	1,3,4,5,10,11,12,15	2,6,7,8,9,13,14,16,17	8	O54728,P51907,Q4FZU2,Q6IFW6,Q6IG02,Q6IMF3,Q6P6Q2,Q9ESW0
5434	1,3,4,5,10,11,12,16	2,6,7,8,9,13,14,15,17	13	P01681,P06757,P53792,P55314,Q10758,Q4FZU2,Q6IFU7,Q6IFU8,Q6IFW6,Q6IG02,Q6IMF3,Q6P6Q2,Q9ESW0
5435	1,3,4,5,10,11,12,17	2,6,7,8,9,13,14,15,16	12	P01681,P06757,P16636,Q10758,Q4FZU2,Q6IFU8,Q6IFW6,Q6IG02,Q6IMF3,Q6P6Q2,Q99MH3,Q9ESW0
5436	1,3,4,5,10,11,13,14	2,6,7,8,9,12,15,16,17	0	
5437	1,3,4,5,10,11,13,15	2,6,7,8,9,12,14,16,17	3	O54728,Q62635,Q6P6R2
5438	1,3,4,5,10,11,13,16	2,6,7,8,9,12,14,15,17	2	P55314,Q62635

5439	1,3,4,5,10,<br>11,13,17	2,6,7,8,9,1<br>2,14,15,16	1	P01681
5440	1,3,4,5,10,<br>11,14,15	2,6,7,8,9,1<br>2,13,16,17	3	O54728,Q62635,Q64194
5441	1,3,4,5,10,<br>11,14,16	2,6,7,8,9,1<br>2,13,15,17	3	P55314,Q62635,Q6IRE4
5442	1,3,4,5,10,<br>11,14,17	2,6,7,8,9,1<br>2,13,15,16	2	P01681,P18427
5443	1,3,4,5,10,<br>11,15,16	2,6,7,8,9,1<br>2,13,14,17	4	O54728,P02782,P55314,Q62635
5444	1,3,4,5,10,<br>11,15,17	2,6,7,8,9,1<br>2,13,14,16	2	O54728,Q62902
5445	1,3,4,5,10,<br>11,16,17	2,6,7,8,9,1<br>2,13,14,15	2	P01681,P55314
5446	1,3,4,5,10,<br>12,13,14	2,6,7,8,9,1<br>1,15,16,17	2	Q4FZU2,Q6IG02
5447	1,3,4,5,10,<br>12,13,15	2,6,7,8,9,1<br>1,14,16,17	3	Q4FZU2,Q6IG02,Q99MH3
5448	1,3,4,5,10,<br>12,13,16	2,6,7,8,9,1<br>1,14,15,17	3	Q4FZU2,Q6IFW6,Q6IG02
5449	1,3,4,5,10,<br>12,13,17	2,6,7,8,9,1<br>1,14,15,16	4	P01681,Q10758,Q6IG02,Q99MH3
5450	1,3,4,5,10,<br>12,14,15	2,6,7,8,9,1<br>1,13,16,17	4	A2RUV9,O54728,Q4FZU2,Q6IG02
5451	1,3,4,5,10,<br>12,14,16	2,6,7,8,9,1<br>1,13,15,17	6	P08721,Q10758,Q4FZU2,Q6IFW6,Q6IG02,Q6IMF3
5452	1,3,4,5,10,<br>12,14,17	2,6,7,8,9,1<br>1,13,15,16	5	P01681,Q10758,Q4FZU2,Q6IFW6,Q6IG02
5453	1,3,4,5,10,<br>12,15,16	2,6,7,8,9,1<br>1,13,14,17	6	O54728,P08721,P20766,Q4FZU2,Q6IFW6,Q6IG02
5454	1,3,4,5,10,<br>12,15,17	2,6,7,8,9,1<br>1,13,14,16	6	B0BNN3,P01681,Q10758,Q4FZU2,Q6IG02,Q99MH3
5455	1,3,4,5,10,<br>12,16,17	2,6,7,8,9,1<br>1,13,14,15	6	P01681,Q10758,Q4FZU2,Q6IFW6,Q6IG02,Q99MH3
5456	1,3,4,5,10,<br>13,14,15	2,6,7,8,9,1<br>1,12,16,17	4	O54728,P09605;P25809,Q62635,Q6P6R2
5457	1,3,4,5,10,<br>13,14,16	2,6,7,8,9,1<br>1,12,15,17	1	Q62635
5458	1,3,4,5,10,<br>13,14,17	2,6,7,8,9,1<br>1,12,15,16	1	Q9QW07
5459	1,3,4,5,10,<br>13,15,16	2,6,7,8,9,1<br>1,12,14,17	4	O54728,P62804,Q00715,Q62635
5460	1,3,4,5,10,<br>13,15,17	2,6,7,8,9,1<br>1,12,14,16	1	Q99MH3
5461	1,3,4,5,10,<br>13,16,17	2,6,7,8,9,1<br>1,12,14,15	2	P62804,Q62997

5462	1,3,4,5,10,14,15,16 2,6,7,8,9,11,12,13,17	7	O54728,P02782,P08721,Q62635,Q62902,Q9JHB9,Q9QZK9
5463	1,3,4,5,10,14,15,17 2,6,7,8,9,11,12,13,16	2	O54728,Q62902
5464	1,3,4,5,10,14,16,17 2,6,7,8,9,11,12,13,15	1	Q9QW07
5465	1,3,4,5,10,15,16,17 2,6,7,8,9,11,12,13,14	6	O54728,P02782,P47727,Q62902,Q62997,Q9JHB9
5466	1,3,4,5,11,12,13,14 2,6,7,8,9,10,15,16,17	7	P01681,Q4FZU2,Q6IFW6,Q6IG02,Q6P6Q2,Q6P6R2,Q9ESW0
5467	1,3,4,5,11,12,13,15 2,6,7,8,9,10,14,16,17	6	O54728,Q4FZU2,Q6IFW6,Q6IG02,Q6P6R2,Q9ESW0
5468	1,3,4,5,11,12,13,16 2,6,7,8,9,10,14,15,17	8	P01681,P17046,P55314,Q4FZU2,Q6IFW6,Q6IG02,Q6IRE4,Q6P6Q2
5469	1,3,4,5,11,12,13,17 2,6,7,8,9,10,14,15,16	6	P01681,P36374,Q4FZU2,Q6IFW6,Q6IG02,Q9ESW0
5470	1,3,4,5,11,12,14,15 2,6,7,8,9,10,13,16,17	9	O54728,P01681,Q4FZU2,Q5I0D1,Q6IFW6,Q6IG02,Q6P6Q2,Q6P6R2,Q9ESW0
5471	1,3,4,5,11,12,14,16 2,6,7,8,9,10,13,15,17	10	P01681,P55314,Q4FZU2,Q6IFU8,Q6IFW6,Q6IG02,Q6IMF3,Q6IRE4,Q6P6Q2,Q9ESW0
5472	1,3,4,5,11,12,14,17 2,6,7,8,9,10,13,15,16	9	P01681,P01835,P97840,Q4FZU2,Q6IFU8,Q6IFW6,Q6IG02,Q6P6Q2,Q9ESW0
5473	1,3,4,5,11,12,15,16 2,6,7,8,9,10,13,14,17	10	O54728,P12020,P51907,P55314,Q4FZU2,Q5I0D1,Q6IFW6,Q6IG02,Q6P6Q2,Q9ESW0
5474	1,3,4,5,11,12,15,17 2,6,7,8,9,10,13,14,16	8	B0BNN3,P01681,P12020,P97840,Q4FZU2,Q6IFW6,Q6IG02,Q9ESW0
5475	1,3,4,5,11,12,16,17 2,6,7,8,9,10,13,14,15	10	P01681,P12020,P17046,P55314,Q4FZU2,Q6IFW6,Q6IG02,Q6IMF3,Q6P6Q2,Q9ESW0
5476	1,3,4,5,11,13,14,15 2,6,7,8,9,10,12,16,17	4	O54728,P05539,P97840,Q6P6R2
5477	1,3,4,5,11,13,14,16 2,6,7,8,9,10,12,15,17	4	O54728,P55314,Q62635,Q6IRE4
5478	1,3,4,5,11,13,14,17 2,6,7,8,9,10,12,15,16	3	P01681,P97840,Q9QW07
5479	1,3,4,5,11,13,15,16 2,6,7,8,9,10,12,14,17	5	O54728,P55314,Q62635,Q6IRE4,Q6P6R2
5480	1,3,4,5,11,13,15,17 2,6,7,8,9,10,12,14,16	2	O54728,P97840
5481	1,3,4,5,11,13,16,17 2,6,7,8,9,10,12,14,15	3	P01681,P55314,P97840
5482	1,3,4,5,11,14,15,16 2,6,7,8,9,10,12,13,17	8	O54728,P55314,P97840,Q5I0D1,Q62635,Q64194,Q6IRE4,Q9QZK9
5483	1,3,4,5,11,14,15,17 2,6,7,8,9,10,12,13,16	4	O54728,P01681,P47967,P97840
5484	1,3,4,5,11,14,16,17 2,6,7,8,9,10,12,13,15	4	P01681,P55314,P97840,Q9QW07

5485	1,3,4,5,11,15,16,17	2,6,7,8,9,10,12,13,14	4	O54728,P12020,P55314,P97840
5486	1,3,4,5,12,13,14,15	2,6,7,8,9,10,11,16,17	5	A2RUV9,O54728,P10758,Q6P6R2,Q9QUL6
5487	1,3,4,5,12,13,14,16	2,6,7,8,9,10,11,15,17	1	Q9QUL6
5488	1,3,4,5,12,13,14,17	2,6,7,8,9,10,11,15,16	2	P01681,Q9QW07
5489	1,3,4,5,12,13,15,16	2,6,7,8,9,10,11,14,17	3	O54728,Q6P6R2,Q9QUL6
5490	1,3,4,5,12,13,15,17	2,6,7,8,9,10,11,14,16	3	B0BNN3,P01681,Q99MH3
5491	1,3,4,5,12,13,16,17	2,6,7,8,9,10,11,14,15	3	P01681,P17046,P35280
5492	1,3,4,5,12,14,15,16	2,6,7,8,9,10,11,13,17	3	O54728,P20766,Q4FZU2
5493	1,3,4,5,12,14,15,17	2,6,7,8,9,10,11,13,16	5	B0BNN3,P01681,Q6IG02,Q8CJD3,Q9QW07
5494	1,3,4,5,12,14,16,17	2,6,7,8,9,10,11,13,15	4	P01681,Q4FZU6,Q6IG02,Q9QW07
5495	1,3,4,5,12,15,16,17	2,6,7,8,9,10,11,13,14	4	B0BNN3,P01681,P12020,P47727
5496	1,3,4,5,13,14,15,16	2,6,7,8,9,10,11,12,17	6	O54728,P60905,P62804,Q00715,Q62635,Q6P6R2
5497	1,3,4,5,13,14,15,17	2,6,7,8,9,10,11,12,16	5	O54728,P47967,P60905,P97840,Q9QW07
5498	1,3,4,5,13,14,16,17	2,6,7,8,9,10,11,12,15	4	P35280,P62804,Q6PCU2,Q9QW07
5499	1,3,4,5,13,15,16,17	2,6,7,8,9,10,11,12,14	5	O54728,P47727,P60905,P62804,Q00715
5500	1,3,4,5,14,15,16,17	2,6,7,8,9,10,11,12,13	7	O54728,P02803,P47727,P60905,P97840,Q9JHB9,Q9QW07
5501	1,3,4,6,7,8,9,10	2,5,11,12,13,14,15,16,17	14	D3ZHA0,O55006,P06761,P08723,P16086,P18418,P22282,P22283,P31044,P35280,Q5FVI6,Q63617,Q9JI85,Q9R0T3
5502	1,3,4,6,7,8,9,11	2,5,10,12,13,14,15,16,17	19	P06761,P07150,P07647,P08723,P10715,P14669,P15399,P16086,P18418,P22282,P22283,P31044,P35280,Q63493,Q63617,Q63751,Q99041,Q9JI85,Q9R0T3
5503	1,3,4,6,7,8,9,12	2,5,10,11,13,14,15,16,17	28	D3ZHA0,O55006,P01681,P02780,P02781,P02782,P06761,P07647,P08723,P0C0A9,P10715,P13432,P16086,P18418,P19629,P22282,P22283,P30120,P31044,Q63617,Q63751,Q6AYR9,Q6IG02,Q8CJ52,Q99041,Q9JHB9,Q9JI85,Q9R0T3
5504	1,3,4,6,7,8,9,13	2,5,10,11,12,14,15,16,17	16	P06761,P06911,P07647,P08723,P11598,P12020,P16086,P18418,P22282,P22283,P31044,Q5GRG2,Q62902,Q63617,Q9JI85,Q9R0T3

5505	1,3,4,6,7,8,9,14	2,5,10,11,12,13,15,16,17	12	D3ZHA0,P06761,P06911,P08723,P16086,P18418,P19629,P22282,P22283,P31044,Q63617,Q9JI85
5506	1,3,4,6,7,8,9,15	2,5,10,11,12,13,14,16,17	11	D3ZHA0,O55006,P06761,P16086,P18418,P22006,P22282,P31044,P35280,Q5QE79,Q63617
5507	1,3,4,6,7,8,9,16	2,5,10,11,12,13,14,15,17	7	P06761,P16086,P18418,P22282,P31044,Q63617,Q9R0T3
5508	1,3,4,6,7,8,9,17	2,5,10,11,12,13,14,15,16	19	P01681,P02091,P02770,P06761,P09605;P25809,P16086,P18418,P19223,P20761,P22282,P22283,P23593,P31044,P50115,Q4G075,Q63617,Q63751,Q99041,Q9R0T3
5509	1,3,4,6,7,8,10,11	2,5,9,12,13,14,15,16,17	27	D3ZHA0,P00714,P01681,P06761,P07150,P07647,P08723,P14669,P15399,P16086,P18418,P22282,P22283,P31044,P35280,P61206;P84079;P84082,Q30KJ2,Q5BJY9,Q5FVI6,Q63493,Q63617,Q63751,Q8CJ52,Q8VD89,Q99041,Q9JI85,Q9R0T3
5510	1,3,4,6,7,8,10,12	2,5,9,11,13,14,15,16,17	39	D3ZHA0,F1LM93,P01681,P02780,P02781,P02782,P06761,P07647,P08723,P08937,P0C0A9,P11598,P12020,P13432,P16086,P18418,P22282,P22283,P30120,P31044,P35280,P35281,Q10758,Q4FZU2,Q5FVI6,Q63617,Q63751,Q64361,Q6AYR9,Q6IFW6,Q6IG02,Q6IMF3,Q812E4,Q8CJ52,Q99041,Q99MH3,Q9JHB9,Q9JI85,Q9R0T3
5511	1,3,4,6,7,8,10,13	2,5,9,11,12,14,15,16,17	20	D3ZHA0,P06761,P06911,P07647,P08723,P11598,P12020,P16086,P18418,P22282,P22283,P31044,Q5GRG2,Q62902,Q63617,Q8CJ52,Q8VD89,Q99MH3,Q9JI85,Q9R0T3
5512	1,3,4,6,7,8,10,14	2,5,9,11,12,13,15,16,17	17	D3ZHA0,P06761,P06911,P08723,P11598,P12020,P16086,P18418,P19629,P22282,P22283,P31044,P35280,Q5GRG2,Q63617,Q9JI85,Q9R0T3
5513	1,3,4,6,7,8,10,15	2,5,9,11,12,13,14,16,17	15	B0BNN3,D3ZHA0,O55006,P06761,P08723,P16086,P18418,P22006,P22282,P22283,P31044,P35280,Q5FVI6,Q63617,Q8VD89
5514	1,3,4,6,7,8,10,16	2,5,9,11,12,13,14,15,17	13	D3ZHA0,P06761,P08721,P08723,P16086,P18418,P22282,P22283,P31044,Q5FVI6,Q63617,Q8VD89,Q9R0T3
5515	1,3,4,6,7,8,10,17	2,5,9,11,12,13,14,15,16	26	D3ZHA0,P01681,P02091,P02770,P06761,P08723,P08937,P11598,P12346,P15399,P16086,P17475,P18418,P20761,P22282,P22283,P31044,P46844,P97580,Q30KJ2,Q4G075,Q63617,Q63751,Q99041,Q99MH3,Q9R0T3
5516	1,3,4,6,7,8,11,12	2,5,9,10,13,14,15,16,17	47	O55004,P01681,P02780,P02781,P02782,P04905,P06761,P07150,P07647,P08723,P08937,P0C0A9,P10715,P11598,P13432,P14669,P15399,P16086,P18418,P19629,P22282,P22283,P24368,P30120,P31044,P35280,P36374,P40241,P46462,Q4FZU2,Q62902,Q63357,Q63493,Q63617,Q63751,Q6AYR9,Q6IFW6,Q6IG02,Q6IMF3,Q6P6Q2,Q812E4,Q8CJ52,Q99041,Q9ESW0,Q9JHB9,Q9JI85,Q9R0T3
5517	1,3,4,6,7,8,11,13	2,5,9,10,12,14,15,16,17	29	P02780,P02781,P05539,P06761,P06911,P07150,P07647,P08723,P11598,P12020,P14669,P15399,P16086,P18418,P22282,P22283,P31044,Q5GRG2,Q5XI22,Q62902,Q63493,Q63617,Q63751,Q8CJ52,Q8VD89,Q99041,Q9JHB9,Q9JI85,Q9R0T3
5518	1,3,4,6,7,8,11,14	2,5,9,10,12,13,15,16,17	27	P01681,P06761,P06911,P07150,P07171,P07647,P08723,P11598,P12020,P14669,P15399,P16086,P18418,P19629,P22282,P22283,P31044,P35280,P35434,P47727,Q30KJ2,Q63493,Q63617,Q63751,Q8CJ52,Q9JI85,Q9R0T3
5519	1,3,4,6,7,8,11,15	2,5,9,10,12,13,14,16,17	21	B0BNN3,P02625,P06761,P07150,P07171,P08723,P10715,P14669,P16086,P18418,P19629,P22006,P22282,P22283,P31044,P35280,Q63617,Q63751,Q8VD89,Q99041,Q9JI85

5520	1,3,4,6,7,8,11,16	2,5,9,10,12,13,14,15,17	23	P01681,P06761,P07150,P08723,P10715,P14668,P14669,P16086,P18418,P22282,P22283,P31044,P55314,P61206;P84079;P84082,Q5FVI6,Q63493,Q63617,Q63751,Q8CJ52,Q8VD89,Q99041,Q9JI85,Q9R0T3
5521	1,3,4,6,7,8,11,17	2,5,9,10,12,13,14,15,16	32	P01681,P02091,P02770,P06761,P07150,P07647,P08723,P08937,P09605;P25809,P10715,P11598,P12346,P14669,P15399,P16086,P18418,P19629,P20761,P22282,P22283,P31044,P50115,P97580,Q30KJ2,Q4G075,Q63493,Q63617,Q63751,Q8CJ52,Q99041,Q9JI85,Q9R0T3
5522	1,3,4,6,7,8,12,13	2,5,9,10,11,14,15,16,17	38	D3ZHA0,O08557,P01681,P02780,P02781,P02782,P06761,P06911,P07647,P08723,P08937,P09456,P0C0A9,P11598,P12020,P13432,P16086,P18418,P19629,P22282,P22283,P29975,P30120,P31044,P36374,P62815,Q5GRG2,Q62902,Q63617,Q63751,Q6AYR9,Q8CJ52,Q99041,Q99MH3,Q9JHB9,Q9JI85,Q9JJ50,Q9R0T3
5523	1,3,4,6,7,8,12,14	2,5,9,10,11,13,15,16,17	37	D3ZHA0,O08557,P01681,P02780,P02781,P02782,P06761,P06911,P07647,P08723,P0C0A9,P11598,P12020,P13432,P16086,P18418,P19629,P22282,P22283,P29975,P30120,P31044,P40241,Q62902,Q63493,Q63617,Q63618,Q63751,Q6AYR9,Q6AYS7,Q6IG02,Q8CJ52,Q923M1,Q99041,Q9JHB9,Q9JI85,Q9R0T3
5524	1,3,4,6,7,8,12,15	2,5,9,10,11,13,14,16,17	27	B0BNN3,D3ZHA0,O08557,P06761,P07647,P08723,P10715,P11598,P13432,P16086,P18418,P19629,P20761,P22006,P22282,P22283,P30120,P31044,P35280,Q4FZU2,Q63617,Q63751,Q812E4,Q8CJ52,Q99041,Q9JI85,Q9R0T3
5525	1,3,4,6,7,8,12,16	2,5,9,10,11,13,14,15,17	23	D3ZHA0,P01681,P06761,P07647,P08721,P08723,P10715,P13432,P16086,P18418,P19629,P22282,P22283,P30120,P31044,Q63617,Q63751,Q6IFW6,Q6IG02,Q8CJ52,Q99041,Q9JI85,Q9R0T3
5526	1,3,4,6,7,8,12,17	2,5,9,10,11,13,14,15,16	42	B0BNN3,O88267,P01681,P02091,P02770,P02780,P02782,P06761,P07647,P08723,P08937,P09605;P25809,P10715,P11598,P13432,P16086,P17475,P18418,P19223,P19629,P20761,P22282,P22283,P30120,P36374,P50115,P55091,P62815,Q4G075,Q63493,Q63617,Q63751,Q6AYR9,Q6IG02,Q8CJ52,Q99041,Q99MH3,Q9JI85,Q9QZA6,Q9QZK9,Q9R0T3,Q9Z0V6
5527	1,3,4,6,7,8,13,14	2,5,9,10,11,12,15,16,17	20	O08557,P06761,P06911,P07647,P08723,P11598,P12020,P16086,P18418,P19629,P22282,P22283,P29975,P31044,Q5GRG2,Q62902,Q63617,Q8CJ52,Q9JI85,Q9R0T3
5528	1,3,4,6,7,8,13,15	2,5,9,10,11,12,14,16,17	18	O08557,P06761,P06911,P07647,P08723,P11598,P12020,P16086,P18418,P22282,P22283,P31044,Q5GRG2,Q63617,Q63751,Q8VD89,Q9JI85,Q9R0T3
5529	1,3,4,6,7,8,13,16	2,5,9,10,11,12,14,15,17	17	P06761,P06911,P07647,P08723,P12020,P16086,P18418,P22282,P22283,P31044,Q5GRG2,Q62902,Q63617,Q8CJ52,Q8VD89,Q9JI85,Q9R0T3
5530	1,3,4,6,7,8,13,17	2,5,9,10,11,12,14,15,16	28	P01681,P02091,P02770,P06761,P06911,P07647,P08723,P08937,P11598,P12020,P16086,P18418,P20761,P22282,P22283,P29975,P31044,P51907,P62815,Q4G075,Q5GRG2,Q62902,Q63617,Q63751,Q99041,Q99MH3,Q9JI85,Q9R0T3
5531	1,3,4,6,7,8,14,15	2,5,9,10,11,12,13,16,17	19	B0BNN3,O08557,P06761,P07171,P08723,P10536,P11598,P16086,P18418,P19629,P20761,P22006,P22282,P22283,P31044,P35280,Q5GRG2,Q63617,Q63618
5532	1,3,4,6,7,8,14,16	2,5,9,10,11,12,13,15,17	11	P06761,P06911,P08723,P16086,P18418,P19629,P22282,P22283,P31044,Q63617,Q9R0T3
5533	1,3,4,6,7,8,14,17	2,5,9,10,11,12,13,15,16	23	P01681,P02091,P02770,P06761,P08723,P10536,P11598,P12020,P16086,P18418,P19629,P20761,P22282,P22283,P31044,P62815,Q30KJ2,Q4G075,Q63617,Q63751,Q99041,Q9R0T3,Q9Z0V6
5534	1,3,4,6,7,8,15,16	2,5,9,10,11,12,13,14,17	5	P16086,P18418,P31044,Q63617,Q8VD89

5535	1,3,4,6,7,8,15,17	2,5,9,10,11,12,13,14,16	21	B0BNN3,P02091,P02770,P06761,P10536,P11598,P12346,P16086,P17475,P18418,P19629,P20761,P22282,P22283,P31044,P62815,Q4G075,Q63617,Q63751,Q99041,Q99MH3
5536	1,3,4,6,7,8,16,17	2,5,9,10,11,12,13,14,15	17	P01681,P02091,P02770,P06761,P09605;P25809,P16086,P18418,P20761,P22282,P22283,P23928,P31044,Q4G075,Q63617,Q63751,Q99041,Q9R0T3
5537	1,3,4,6,7,9,10,11	2,5,8,12,13,14,15,16,17	5	P15399,P35280,P61206;P84079;P84082,P83121,Q5FVI6
5538	1,3,4,6,7,9,10,12	2,5,8,11,13,14,15,16,17	6	P08723,P18418,P97840,Q4FZU2,Q5FVI6,Q6IG02
5539	1,3,4,6,7,9,10,13	2,5,8,11,12,14,15,16,17	3	P06911,P12020,Q5FVI6
5540	1,3,4,6,7,9,10,14	2,5,8,11,12,13,15,16,17	2	P12020,Q5FVI6
5541	1,3,4,6,7,9,10,15	2,5,8,11,12,13,14,16,17	3	O55006,P35280,Q5FVI6
5542	1,3,4,6,7,9,10,16	2,5,8,11,12,13,14,15,17	2	P62804,Q5FVI6
5543	1,3,4,6,7,9,10,17	2,5,8,11,12,13,14,15,16	3	P02091,P09605;P25809,Q5FVI6
5544	1,3,4,6,7,9,11,12	2,5,8,10,13,14,15,16,17	16	P01681,P01835,P04905,P08723,P0C0A9,P13432,P15399,P30120,P36374,Q4FZU2,Q6IFW6,Q6IG02,Q6IMF3,Q6P6Q2,Q9JHB9,Q9JI85
5545	1,3,4,6,7,9,11,13	2,5,8,10,12,14,15,16,17	4	P05539,P15399,P16086,Q9JI85
5546	1,3,4,6,7,9,11,14	2,5,8,10,12,13,15,16,17	3	P15399,P16086,P83121
5547	1,3,4,6,7,9,11,15	2,5,8,10,12,13,14,16,17	5	O54728,P02625,P22006,P35280,Q5FVI6
5548	1,3,4,6,7,9,11,16	2,5,8,10,12,13,14,15,17	2	P55314,Q5FVI6
5549	1,3,4,6,7,9,11,17	2,5,8,10,12,13,14,15,16	7	P01681,P02091,P09605;P25809,P15399,P97580,Q30KJ2,Q63751

5550	1,3,4,6,7,9,12,13	2,5,8,10,11,14,15,16,17	10	P05539,P06911,P07647,P08723,P09456,P22282,P30120,P36374,Q62902,Q9JI85
5551	1,3,4,6,7,9,12,14	2,5,8,10,11,13,15,16,17	2	P01835,P08723
5552	1,3,4,6,7,9,12,15	2,5,8,10,11,13,14,16,17	1	P02625
5553	1,3,4,6,7,9,12,16	2,5,8,10,11,13,14,15,17	0	
5554	1,3,4,6,7,9,12,17	2,5,8,10,11,13,14,15,16	9	P01681,P01835,P02091,P08723,P09605;P25809,P13432,P19223,P20761,Q63751
5555	1,3,4,6,7,9,13,14	2,5,8,10,11,12,15,16,17	5	P05539,P06911,P12020,P16086,Q5GRG2
5556	1,3,4,6,7,9,13,15	2,5,8,10,11,12,14,16,17	3	O54728,P05539,P62804
5557	1,3,4,6,7,9,13,16	2,5,8,10,11,12,14,15,17	2	P62804,Q00715
5558	1,3,4,6,7,9,13,17	2,5,8,10,11,12,14,15,16	3	P02091,P09605;P25809,P62804
5559	1,3,4,6,7,9,14,15	2,5,8,10,11,12,13,16,17	1	O54728
5560	1,3,4,6,7,9,14,16	2,5,8,10,11,12,13,15,17	1	P62804
5561	1,3,4,6,7,9,14,17	2,5,8,10,11,12,13,15,16	2	P02091,P09605;P25809
5562	1,3,4,6,7,9,15,16	2,5,8,10,11,12,13,14,17	2	O54728,P62804
5563	1,3,4,6,7,9,15,17	2,5,8,10,11,12,13,14,16	4	B0BNN3,P02091,P09605;P25809,P20761
5564	1,3,4,6,7,9,16,17	2,5,8,10,11,12,13,14,15	4	P02091,P09605;P25809,P23593,P62804

5565	1,3,4,6,7,10,11,12	2,5,8,9,13,14,15,16,17	24	O35783,P01681,P04905,P06757,P08723,P0C0A9,P15399,P18418,P30120,P35280,P36374,P61206;P84079;P84082,Q10758,Q4FZU2,Q5FVI6,Q63357,Q6IFU8,Q6IFW6,Q6IG02,Q6IMF3,Q6P6Q2,Q812E4,Q9JHB9,Q9JI85
5566	1,3,4,6,7,10,11,13	2,5,8,9,12,14,15,16,17	8	P06911,P12020,P15399,P16086,Q5FVI6,Q5GRG2,Q8VD89,Q9JI85
5567	1,3,4,6,7,10,11,14	2,5,8,9,12,13,15,16,17	8	P12020,P15399,P16086,P18418,P35280,P47727,Q30KJ2,Q5FVI6
5568	1,3,4,6,7,10,11,15	2,5,8,9,12,13,14,16,17	5	P15399,P22006,P35280,Q5FVI6,Q8VD89
5569	1,3,4,6,7,10,11,16	2,5,8,9,12,13,14,15,17	5	P15399,P55314,P61206;P84079;P84082,Q5FVI6,Q8VD89
5570	1,3,4,6,7,10,11,17	2,5,8,9,12,13,14,15,16	8	O08815,P01681,P02091,P15399,P97580,Q30KJ2,Q5FVI6,Q63751
5571	1,3,4,6,7,10,12,13	2,5,8,9,11,14,15,16,17	14	P06911,P07647,P08723,P12020,P18418,P22282,P30120,P36374,Q4FZU2,Q5FVI6,Q5GRG2,Q6AYR9,Q99MH3,Q9JI85
5572	1,3,4,6,7,10,12,14	2,5,8,9,11,13,15,16,17	9	A2RUV9,P08723,P12020,P18418,Q10758,Q4FZU2,Q5FVI6,Q6IFW6,Q6IMF3
5573	1,3,4,6,7,10,12,15	2,5,8,9,11,13,14,16,17	6	B0BNN3,P08723,P35280,Q4FZU2,Q5FVI6,Q99MH3
5574	1,3,4,6,7,10,12,16	2,5,8,9,11,13,14,15,17	9	P08721,P08723,P47967,P97840,Q10758,Q4FZU2,Q5FVI6,Q6IFW6,Q6IMF3
5575	1,3,4,6,7,10,12,17	2,5,8,9,11,13,14,15,16	16	O08815,P01681,P02091,P08723,P09605;P25809,P13432,P15399,P18418,P19223,P20761,Q10758,Q4FZU2,Q5FVI6,Q63751,Q99MH3,Q9QZK9
5576	1,3,4,6,7,10,13,14	2,5,8,9,11,12,15,16,17	4	P06911,P12020,P16086,Q5GRG2
5577	1,3,4,6,7,10,13,15	2,5,8,9,11,12,14,16,17	6	P06911,P12020,Q5FVI6,Q5GRG2,Q8VD89,Q99MH3
5578	1,3,4,6,7,10,13,16	2,5,8,9,11,12,14,15,17	5	P06911,P12020,P62804,Q5FVI6,Q8VD89
5579	1,3,4,6,7,10,13,17	2,5,8,9,11,12,14,15,16	4	P02091,P06911,P12020,Q99MH3

5580	1,3,4,6,7,10,14,15	2,5,8,9,11,12,13,16,17	5	P02803,P12020,P22006,P35280,Q5FVI6
5581	1,3,4,6,7,10,14,16	2,5,8,9,11,12,13,15,17	3	P08721,P12020,Q5FVI6
5582	1,3,4,6,7,10,14,17	2,5,8,9,11,12,13,15,16	4	P02091,P12020,P15399,Q30KJ2
5583	1,3,4,6,7,10,15,16	2,5,8,9,11,12,13,14,17	3	P08721,Q5FVI6,Q8VD89
5584	1,3,4,6,7,10,15,17	2,5,8,9,11,12,13,14,16	6	B0BNN3,P02091,P02803,P20761,Q5FVI6,Q99MH3
5585	1,3,4,6,7,10,16,17	2,5,8,9,11,12,13,14,15	2	P02091,Q5FVI6
5586	1,3,4,6,7,11,12,13	2,5,8,9,10,14,15,16,17	22	P01681,P01836,P02781,P04905,P05539,P06911,P07647,P08723,P09456,P0C0A9,P15399,P22282,P22283,P30120,P36374,Q4FZU2,Q62902,Q63751,Q6AYR9,Q6IFW6,Q9JHB9,Q9JI85
5587	1,3,4,6,7,11,12,14	2,5,8,9,10,13,15,16,17	16	P01681,P04905,P08723,P0C0A9,P15399,P16086,P30120,P47727,Q4FZU2,Q63493,Q6IFW6,Q6IG02,Q6IMF3,Q6P6Q2,Q9JHB9,Q9JI85
5588	1,3,4,6,7,11,12,15	2,5,8,9,10,13,14,16,17	11	B0BNN3,P01681,P01836,P02625,P08723,P15399,P22006,P35280,Q4FZU2,Q6IFW6,Q9JI85
5589	1,3,4,6,7,11,12,16	2,5,8,9,10,13,14,15,17	12	P01681,P01836,P08723,P15399,P55314,Q4FZU2,Q5FVI6,Q6IFW6,Q6IG02,Q6IMF3,Q6P6Q2,Q9JI85
5590	1,3,4,6,7,11,12,17	2,5,8,9,10,13,14,15,16	22	P01681,P02091,P08723,P09605;P25809,P13432,P15399,P19223,P20761,P22282,P30120,P36374,P97580,Q30KJ2,Q4FZU2,Q63493,Q63751,Q6IFW6,Q6IG02,Q6IMF3,Q99041,Q9JI85,Q9QZK9
5591	1,3,4,6,7,11,13,14	2,5,8,9,10,12,15,16,17	9	P05539,P06911,P12020,P15399,P16086,P47727,Q5GRG2,Q62902,Q9JI85
5592	1,3,4,6,7,11,13,15	2,5,8,9,10,12,14,16,17	7	P02625,P05539,P16086,P22006,Q5GRG2,Q8VD89,Q9JI85
5593	1,3,4,6,7,11,13,16	2,5,8,9,10,12,14,15,17	3	P16086,P55314,Q8VD89
5594	1,3,4,6,7,11,13,17	2,5,8,9,10,12,14,15,16	11	P01681,P02091,P05539,P09605;P25809,P15399,P16086,P22282,P97580,Q30KJ2,Q63751,Q9JI85

5595	1,3,4,6,7,11,14,15	2,5,8,9,10,12,13,16,17	5	P02625,P15399,P16086,P22006,P35280
5596	1,3,4,6,7,11,14,16	2,5,8,9,10,12,13,15,17	3	P15399,P16086,P55314
5597	1,3,4,6,7,11,14,17	2,5,8,9,10,12,13,15,16	8	P01681,P02091,P15399,P16086,P20761,P97580,Q30KJ2,Q63751
5598	1,3,4,6,7,11,15,16	2,5,8,9,10,12,13,14,17	4	P22006,P55314,Q5FVI6,Q8VD89
5599	1,3,4,6,7,11,15,17	2,5,8,9,10,12,13,14,16	9	B0BNN3,P02091,P09605;P25809,P15399,P20761,P22006,P97580,Q30KJ2,Q63751
5600	1,3,4,6,7,11,16,17	2,5,8,9,10,12,13,14,15	8	P01681,P02091,P09605;P25809,P15399,P55314,P97580,Q30KJ2,Q63751
5601	1,3,4,6,7,12,13,14	2,5,8,9,10,11,15,16,17	13	A2RUV9,P06911,P07647,P08723,P12020,P16086,P22282,P30120,Q5GRG2,Q62902,Q6AYR9,Q9JHB9,Q9JI85
5602	1,3,4,6,7,12,13,15	2,5,8,9,10,11,14,16,17	5	P02625,P06911,P08723,Q5GRG2,Q99MH3
5603	1,3,4,6,7,12,13,16	2,5,8,9,10,11,14,15,17	3	P06911,P08723,Q62902
5604	1,3,4,6,7,12,13,17	2,5,8,9,10,11,14,15,16	17	P01681,P02091,P06911,P07647,P08723,P09605;P25809,P13432,P20761,P22282,P36374,P62815,Q63751,Q6AYR9,Q99041,Q99MH3,Q9JI85,Q9QZK9
5605	1,3,4,6,7,12,14,15	2,5,8,9,10,11,13,16,17	4	A2RUV9,B0BNN3,P02625,P08723
5606	1,3,4,6,7,12,14,16	2,5,8,9,10,11,13,15,17	1	P08723
5607	1,3,4,6,7,12,14,17	2,5,8,9,10,11,13,15,16	12	P01681,P01835,P02091,P08723,P09605;P25809,P13432,P15399,P20761,P31430,P62815,Q63751,Q9QZK9
5608	1,3,4,6,7,12,15,16	2,5,8,9,10,11,13,14,17	1	Q4FZU2
5609	1,3,4,6,7,12,15,17	2,5,8,9,10,11,13,14,16	11	B0BNN3,P01681,P02091,P07151,P09605;P25809,P13432,P20761,Q63751,Q99041,Q99MH3,Q9QZK9

5610	1,3,4,6,7,12,16,17	2,5,8,9,10,11,13,14,15	6	P01681,P02091,P09605;P25809,P13432,Q63751,Q99041
5611	1,3,4,6,7,13,14,15	2,5,8,9,10,11,12,16,17	5	A2RUV9,P06911,P12020,P16086,Q5GRG2
5612	1,3,4,6,7,13,14,16	2,5,8,9,10,11,12,15,17	5	P06911,P12020,P16086,P62804,Q5GRG2
5613	1,3,4,6,7,13,14,17	2,5,8,9,10,11,12,15,16	8	P02091,P06911,P12020,P16086,P20761,P22282,Q5GRG2,Q9QW07
5614	1,3,4,6,7,13,15,16	2,5,8,9,10,11,12,14,17	2	P62804,Q8VD89
5615	1,3,4,6,7,13,15,17	2,5,8,9,10,11,12,14,16	5	B0BNN3,P02091,P20761,Q63751,Q99MH3
5616	1,3,4,6,7,13,16,17	2,5,8,9,10,11,12,14,15	3	P02091,P09605;P25809,P62804
5617	1,3,4,6,7,14,15,16	2,5,8,9,10,11,12,13,17	1	P02803
5618	1,3,4,6,7,14,15,17	2,5,8,9,10,11,12,13,16	4	B0BNN3,P02091,P02803,P20761
5619	1,3,4,6,7,14,16,17	2,5,8,9,10,11,12,13,15	3	P02091,P09605;P25809,P16086
5620	1,3,4,6,7,15,16,17	2,5,8,9,10,11,12,13,14	6	B0BNN3,P02091,P02803,P09605;P25809,P20761,P62804
5621	1,3,4,6,8,9,10,11	2,5,7,12,13,14,15,16,17	6	P01681,P08649,P14669,P16636,P35280,P35467
5622	1,3,4,6,8,9,10,12	2,5,7,11,13,14,15,16,17	14	D3ZHA0,P01681,P02782,P07647,P08723,P0C0A9,P16636,P30120,P35281,Q4FZU2,Q5M8C6,Q6IFW6,Q6IG02,Q6IMF3
5623	1,3,4,6,8,9,10,13	2,5,7,11,12,14,15,16,17	2	P06911,P16636
5624	1,3,4,6,8,9,10,14	2,5,7,11,12,13,15,16,17	0	

5625	1,3,4,6,8,9,10,15	2,5,7,11,12,13,14,16,17	3	B0BNN3,P35280,Q5QE79
5626	1,3,4,6,8,9,10,16	2,5,7,11,12,13,14,15,17	2	P16636,P23593
5627	1,3,4,6,8,9,10,17	2,5,7,11,12,13,14,15,16	3	P01681,P16636,P23593
5628	1,3,4,6,8,9,11,12	2,5,7,10,13,14,15,16,17	20	P01681,P01835,P02780,P02781,P02782,P04905,P07647,P08723,P0C0A9,P14669,P16636,P30120,Q4FZU2,Q5M8C6,Q6IFU8,Q6IFW6,Q6IG02,Q6IMF3,Q6P6Q2,Q9JHB9
5629	1,3,4,6,8,9,11,13	2,5,7,10,12,14,15,16,17	3	P07647,P14669,P16636
5630	1,3,4,6,8,9,11,14	2,5,7,10,12,13,15,16,17	2	P01681,P14669
5631	1,3,4,6,8,9,11,15	2,5,7,10,12,13,14,16,17	6	B0BNN3,P02625,P14668,P14669,P35280,Q5QE79
5632	1,3,4,6,8,9,11,16	2,5,7,10,12,13,14,15,17	2	P01681,P14668
5633	1,3,4,6,8,9,11,17	2,5,7,10,12,13,14,15,16	3	P01681,P14669,P16636
5634	1,3,4,6,8,9,12,13	2,5,7,10,11,14,15,16,17	15	P01681,P02780,P02782,P07647,P08723,P0C0A9,P16636,P22282,P30120,P36374,Q06496,Q5M8C6,Q6AYR9,Q6IG02,Q9JHB9
5635	1,3,4,6,8,9,12,14	2,5,7,10,11,13,15,16,17	7	P01681,P01835,P02782,P08723,P0C0A9,P30120,Q6IG02
5636	1,3,4,6,8,9,12,15	2,5,7,10,11,13,14,16,17	5	B0BNN3,P02625,P08723,Q5QE79,Q6IG02
5637	1,3,4,6,8,9,12,16	2,5,7,10,11,13,14,15,17	5	P01681,P08723,P16636,P23593,Q6IG02
5638	1,3,4,6,8,9,12,17	2,5,7,10,11,13,14,15,16	12	B0BNN3,P01681,P01835,P02782,P08723,P13432,P16636,P19223,P23593,Q4G075,Q5M8C6,Q6IG02
5639	1,3,4,6,8,9,13,14	2,5,7,10,11,12,15,16,17	1	P06911

5640	1,3,4,6,8,9,13,15	2,5,7,10,11,12,14,16,17	1	Q5QE79
5641	1,3,4,6,8,9,13,16	2,5,7,10,11,12,14,15,17	0	
5642	1,3,4,6,8,9,13,17	2,5,7,10,11,12,14,15,16	4	P01681,P16636,P23593,P51907
5643	1,3,4,6,8,9,14,15	2,5,7,10,11,12,13,16,17	2	B0BNN3,Q5QE79
5644	1,3,4,6,8,9,14,16	2,5,7,10,11,12,13,15,17	0	
5645	1,3,4,6,8,9,14,17	2,5,7,10,11,12,13,15,16	1	P01681
5646	1,3,4,6,8,9,15,16	2,5,7,10,11,12,13,14,17	3	O54728,P23593,Q5QE79
5647	1,3,4,6,8,9,15,17	2,5,7,10,11,12,13,14,16	4	B0BNN3,P20761,P23593,Q5QE79
5648	1,3,4,6,8,9,16,17	2,5,7,10,11,12,13,14,15	4	O70417,P01681,P16636,P23593
5649	1,3,4,6,8,10,11,12	2,5,7,9,13,14,15,16,17	28	O35547,P01681,P02780,P02781,P02782,P04905,P07647,P08723,P0C0A9,P14669,P16636,P30120,P35280,P35281,P47727,Q10758,Q4FZU2,Q5M8C6,Q63357,Q6IFU7,Q6IFU8,Q6IFW6,Q6IG02,Q6IMF3,Q6P6Q2,Q812E4,Q8CJ52,Q9JHB9
5650	1,3,4,6,8,10,11,13	2,5,7,9,12,14,15,16,17	6	P01681,P06911,P07647,P14669,P16636,P47727
5651	1,3,4,6,8,10,11,14	2,5,7,9,12,13,15,16,17	5	P01681,P14669,P35280,P47727,Q5BJY9
5652	1,3,4,6,8,10,11,15	2,5,7,9,12,13,14,16,17	4	B0BNN3,P14669,P35280,P35281
5653	1,3,4,6,8,10,11,16	2,5,7,9,12,13,14,15,17	7	P01681,P14668,P14669,P16636,P55314,Q5FVI6,Q8VD89
5654	1,3,4,6,8,10,11,17	2,5,7,9,12,13,14,15,16	6	P01681,P07150,P08649,P14669,P16636,Q4G075

5655	1,3,4,6,8,10,12,13	2,5,7,9,11,14,15,16,17	21	P01681,P02780,P02781,P02782,P06911,P07647,P08723,P0C0A9,P16636,P22282,P22283,P30120,P35281,P36374,Q4FZU2,Q5M8C6,Q6AYR9,Q6IFW6,Q6IG02,Q99MH3,Q9JHB9
5656	1,3,4,6,8,10,12,14	2,5,7,9,11,13,15,16,17	13	P01681,P02782,P07647,P08723,P0C0A9,P30120,Q10758,Q4FZU2,Q6AYR9,Q6IFW6,Q6IG02,Q6IMF3,Q6P6Q2
5657	1,3,4,6,8,10,12,15	2,5,7,9,11,13,14,16,17	12	B0BNN3,D3ZHA0,P01681,P08723,P35280,P35281,Q4FZU2,Q6IFW6,Q6IG02,Q6IMF3,Q812E4,Q99MH3
5658	1,3,4,6,8,10,12,16	2,5,7,9,11,13,14,15,17	11	P01681,P08721,P08723,P16636,P35281,Q10758,Q4FZU2,Q6IFW6,Q6IG02,Q6IMF3,Q6P6Q2
5659	1,3,4,6,8,10,12,17	2,5,7,9,11,13,14,15,16	20	B0BNN3,F1LM93,P01681,P02782,P07647,P08723,P13432,P16636,P17475,P19223,Q10758,Q4FZU2,Q4G075,Q5M8C6,Q6AYR9,Q6IFW6,Q6IG02,Q6IMF3,Q99MH3,Q9QZK9
5660	1,3,4,6,8,10,13,14	2,5,7,9,11,12,15,16,17	4	P06911,P12020,Q5GRG2,Q64093
5661	1,3,4,6,8,10,13,15	2,5,7,9,11,12,14,16,17	3	B0BNN3,P82995,Q99MH3
5662	1,3,4,6,8,10,13,16	2,5,7,9,11,12,14,15,17	2	P06911,P16636
5663	1,3,4,6,8,10,13,17	2,5,7,9,11,12,14,15,16	7	P01681,P06911,P16636,P51907,Q4G075,Q63355,Q99MH3
5664	1,3,4,6,8,10,14,15	2,5,7,9,11,12,13,16,17	2	B0BNN3,P35280
5665	1,3,4,6,8,10,14,16	2,5,7,9,11,12,13,15,17	1	P08721
5666	1,3,4,6,8,10,14,17	2,5,7,9,11,12,13,15,16	2	B0BNN3,P01681
5667	1,3,4,6,8,10,15,16	2,5,7,9,11,12,13,14,17	2	B0BNN3,P08721
5668	1,3,4,6,8,10,15,17	2,5,7,9,11,12,13,14,16	5	B0BNN3,P17475,P20761,Q4G075,Q99MH3
5669	1,3,4,6,8,10,16,17	2,5,7,9,11,12,13,14,15	5	P01681,P16636,P23593,P25031,Q4G075

5670	1,3,4,6,8,11,12,13	2,5,7,9,10,14,15,16,17	30	O35547,P01681,P02780,P02781,P02782,P04905,P06911,P07647,P08723,P0C0A9,P14669,P16636,P22282,P22283,P30120,P36374,P40241,P47727,Q4FZU2,Q5M8C6,Q5XI22,Q62902,Q6AYR9,Q6IFW6,Q6IG02,Q6IMF3,Q6P6Q2,Q8CJ52,Q9JHB9,Q9JI85
5671	1,3,4,6,8,11,12,14	2,5,7,9,10,13,15,16,17	23	B0BNN3,O35547,P01681,P02780,P02781,P02782,P04905,P07647,P08723,P0C0A9,P14669,P30120,P40241,P47727,Q4FZU2,Q5M8C6,Q6IFU8,Q6IFW6,Q6IG02,Q6IMF3,Q6P6Q2,Q8CJ52,Q9JHB9
5672	1,3,4,6,8,11,12,15	2,5,7,9,10,13,14,16,17	16	B0BNN3,O35547,P01681,P02625,P02782,P08723,P0C0A9,P14669,P30120,P35280,Q4FZU2,Q6IFW6,Q6IG02,Q6IMF3,Q6P6Q2,Q812E4
5673	1,3,4,6,8,11,12,16	2,5,7,9,10,13,14,15,17	16	O35547,P01681,P02782,P08723,P0C0A9,P14668,P16636,P30120,Q4FZU2,Q6IFU7,Q6IFU8,Q6IFW6,Q6IG02,Q6IMF3,Q6P6Q2,Q8CJ52
5674	1,3,4,6,8,11,12,17	2,5,7,9,10,13,14,15,16	29	B0BNN3,O35547,P01681,P02781,P02782,P04905,P07150,P07647,P08723,P0C0A9,P13432,P14669,P16636,P19223,P22282,P22283,P30120,P36374,Q4FZU2,Q4G075,Q5M8C6,Q63751,Q6IFU8,Q6IFW6,Q6IG02,Q6IMF3,Q6P6Q2,Q9JHB9,Q9QZK9
5675	1,3,4,6,8,11,13,14	2,5,7,9,10,12,15,16,17	8	P01681,P02782,P06911,P07647,P14669,P47727,Q5GRG2,Q64093
5676	1,3,4,6,8,11,13,15	2,5,7,9,10,12,14,16,17	5	B0BNN3,P02625,P14669,Q5XI22,Q64093
5677	1,3,4,6,8,11,13,16	2,5,7,9,10,12,14,15,17	4	P01681,P14668,P16636,Q8VD89
5678	1,3,4,6,8,11,13,17	2,5,7,9,10,12,14,15,16	7	P01681,P07647,P14669,P16636,P22282,Q4G075,Q63751
5679	1,3,4,6,8,11,14,15	2,5,7,9,10,12,13,16,17	6	B0BNN3,P02625,P14669,P35280,P35434,P97840
5680	1,3,4,6,8,11,14,16	2,5,7,9,10,12,13,15,17	2	P01681,P14668
5681	1,3,4,6,8,11,14,17	2,5,7,9,10,12,13,15,16	6	B0BNN3,P01681,P07150,P14669,P97840,Q4G075
5682	1,3,4,6,8,11,15,16	2,5,7,9,10,12,13,14,17	3	B0BNN3,P14668,Q8VD89
5683	1,3,4,6,8,11,15,17	2,5,7,9,10,12,13,14,16	8	B0BNN3,P01681,P14669,P17475,P20761,P97840,Q4G075,Q63751
5684	1,3,4,6,8,11,16,17	2,5,7,9,10,12,13,14,15	6	P01681,P07150,P14668,P14669,P16636,Q4G075

5685	1,3,4,6,8,12,13,14	2,5,7,9,10,11,15,16,17	23	O08557,P01681,P02780,P02781,P02782,P06761,P06911,P07647,P08723,P0C0A9,P22282,P22283,P29975,P30120,Q5GRG2,Q5M8C6,Q62902,Q64093,Q6AYR9,Q80W57,Q923M1,Q9JHB9,Q9QUL6
5686	1,3,4,6,8,12,13,15	2,5,7,9,10,11,14,16,17	11	B0BNN3,O08557,P02625,P02782,P07647,P08723,P30120,P82995,Q64093,Q99MH3,Q9QUL6
5687	1,3,4,6,8,12,13,16	2,5,7,9,10,11,14,15,17	7	P01681,P02782,P07647,P08723,P16636,P30120,Q9QUL6
5688	1,3,4,6,8,12,13,17	2,5,7,9,10,11,14,15,16	23	B0BNN3,P01681,P02780,P02782,P07647,P08723,P13432,P16636,P22282,P22283,P30120,P36374,P51907,P62815,Q4G075,Q5M8C6,Q62635,Q63751,Q6AYR9,Q80W57,Q99MH3,Q9JHB9,Q9QZK9
5689	1,3,4,6,8,12,14,15	2,5,7,9,10,11,13,16,17	9	B0BNN3,O08557,P01681,P02625,P02782,P08723,Q4FZU2,Q64093,Q6IG02
5690	1,3,4,6,8,12,14,16	2,5,7,9,10,11,13,15,17	6	P01681,P02782,P08723,Q4FZU2,Q6IFW6,Q6IG02
5691	1,3,4,6,8,12,14,17	2,5,7,9,10,11,13,15,16	17	B0BNN3,P01681,P01835,P02782,P08723,P13432,P16636,P20761,P22282,P30120,P31430,Q10758,Q4G075,Q5M8C6,Q6IG02,Q923M1,Q9QYU4
5692	1,3,4,6,8,12,15,16	2,5,7,9,10,11,13,14,17	5	B0BNN3,P01681,P08723,Q4FZU2,Q9QUL6
5693	1,3,4,6,8,12,15,17	2,5,7,9,10,11,13,14,16	10	B0BNN3,P01681,P08723,P13432,P17475,P20761,Q4G075,Q63751,Q6IG02,Q99MH3
5694	1,3,4,6,8,12,16,17	2,5,7,9,10,11,13,14,15	8	B0BNN3,P01681,P08723,P13432,P16636,P23593,Q4G075,Q6IG02
5695	1,3,4,6,8,13,14,15	2,5,7,9,10,11,12,16,17	6	B0BNN3,O08557,P06911,Q5GRG2,Q62687,Q64093
5696	1,3,4,6,8,13,14,16	2,5,7,9,10,11,12,15,17	2	P06911,Q64093
5697	1,3,4,6,8,13,14,17	2,5,7,9,10,11,12,15,16	10	P01681,P06911,P10536,P22282,P51907,Q62687,Q64093,Q6PCU2,Q80W57,Q9QW07
5698	1,3,4,6,8,13,15,16	2,5,7,9,10,11,12,14,17	2	P82995,Q9QUL6
5699	1,3,4,6,8,13,15,17	2,5,7,9,10,11,12,14,16	5	B0BNN3,P10536,P20761,Q64093,Q99MH3

5700	1,3,4,6,8,13,16,17	2,5,7,9,10,11,12,14,15	4	P01681,P16636,P51907,Q6PCU2
5701	1,3,4,6,8,14,15,16	2,5,7,9,10,11,12,13,17	1	B0BNN3
5702	1,3,4,6,8,14,15,17	2,5,7,9,10,11,12,13,16	6	B0BNN3,P01681,P10536,P20761,P97840,Q64093
5703	1,3,4,6,8,14,16,17	2,5,7,9,10,11,12,13,15	2	P01681,Q6PCU2
5704	1,3,4,6,8,15,16,17	2,5,7,9,10,11,12,13,14	3	B0BNN3,O70417,P23593
5705	1,3,4,6,9,10,11,12	2,5,7,8,13,14,15,16,17	13	P00762,P01681,P01835,P06757,P0C0A9,P16636,Q4FZU2,Q6IFU7,Q6IFU8,Q6IFW6,Q6IG02,Q6IMF3,Q6P6Q2
5706	1,3,4,6,9,10,11,13	2,5,7,8,12,14,15,16,17	1	P12785
5707	1,3,4,6,9,10,11,14	2,5,7,8,12,13,15,16,17	1	P83121
5708	1,3,4,6,9,10,11,15	2,5,7,8,12,13,14,16,17	2	P35280,Q5FVI6
5709	1,3,4,6,9,10,11,16	2,5,7,8,12,13,14,15,17	2	P55314,Q5FVI6
5710	1,3,4,6,9,10,11,17	2,5,7,8,12,13,14,15,16	3	P01681,P12785,P16636
5711	1,3,4,6,9,10,12,13	2,5,7,8,11,14,15,16,17	2	P16636,Q4FZU2
5712	1,3,4,6,9,10,12,14	2,5,7,8,11,13,15,16,17	6	P01835,Q10758,Q4FZU2,Q6IFW6,Q6IG02,Q6IMF3
5713	1,3,4,6,9,10,12,15	2,5,7,8,11,13,14,16,17	2	B0BNN3,Q4FZU2
5714	1,3,4,6,9,10,12,16	2,5,7,8,11,13,14,15,17	5	P16636,Q4FZU2,Q6IFW6,Q6IG02,Q6IMF3

5715	1,3,4,6,9,10,12,17	2,5,7,8,11,13,14,15,16	8	O08815,P01681,P01835,P16636,Q10758,Q4FZU2,Q6IG02,Q99MH3
5716	1,3,4,6,9,10,13,14	2,5,7,8,11,12,15,16,17	1	P06911
5717	1,3,4,6,9,10,13,15	2,5,7,8,11,12,14,16,17	0	
5718	1,3,4,6,9,10,13,16	2,5,7,8,11,12,14,15,17	1	P62804
5719	1,3,4,6,9,10,13,17	2,5,7,8,11,12,14,15,16	3	P16636,Q63355,Q99MH3
5720	1,3,4,6,9,10,14,15	2,5,7,8,11,12,13,16,17	0	
5721	1,3,4,6,9,10,14,16	2,5,7,8,11,12,13,15,17	0	
5722	1,3,4,6,9,10,14,17	2,5,7,8,11,12,13,15,16	0	
5723	1,3,4,6,9,10,15,16	2,5,7,8,11,12,13,14,17	1	O54728
5724	1,3,4,6,9,10,15,17	2,5,7,8,11,12,13,14,16	1	B0BNN3
5725	1,3,4,6,9,10,16,17	2,5,7,8,11,12,13,14,15	2	P16636,P23593
5726	1,3,4,6,9,11,12,13	2,5,7,8,10,14,15,16,17	11	P01681,P01835,P04905,P0C0A9,P16636,P30120,P36374,Q4FZU2,Q6IFW6,Q6IG02,Q9JHB9
5727	1,3,4,6,9,11,12,14	2,5,7,8,10,13,15,16,17	10	P01681,P01835,P04905,P0C0A9,Q4FZU2,Q6IFU8,Q6IFW6,Q6IG02,Q6IMF3,Q6P6Q2
5728	1,3,4,6,9,11,12,15	2,5,7,8,10,13,14,16,17	7	B0BNN3,P01681,P01835,P02625,Q4FZU2,Q6IFW6,Q6IG02
5729	1,3,4,6,9,11,12,16	2,5,7,8,10,13,14,15,17	10	P01681,P01835,P16636,Q4FZU2,Q6IFU7,Q6IFU8,Q6IFW6,Q6IG02,Q6IMF3,Q6P6Q2

5730	1,3,4,6,9,11,12,17	2,5,7,8,10,13,14,15,16	9	P01681,P01835,P13432,P16636,Q4FZU2,Q6IFU8,Q6IFW6,Q6IG02,Q6IMF3
5731	1,3,4,6,9,11,13,14	2,5,7,8,10,12,15,16,17	0	
5732	1,3,4,6,9,11,13,15	2,5,7,8,10,12,14,16,17	2	P02625,P05539
5733	1,3,4,6,9,11,13,16	2,5,7,8,10,12,14,15,17	0	
5734	1,3,4,6,9,11,13,17	2,5,7,8,10,12,14,15,16	2	P01681,P16636
5735	1,3,4,6,9,11,14,15	2,5,7,8,10,12,13,16,17	2	O54728,P02625
5736	1,3,4,6,9,11,14,16	2,5,7,8,10,12,13,15,17	0	
5737	1,3,4,6,9,11,14,17	2,5,7,8,10,12,13,15,16	2	P01681,P01835
5738	1,3,4,6,9,11,15,16	2,5,7,8,10,12,13,14,17	1	O54728
5739	1,3,4,6,9,11,15,17	2,5,7,8,10,12,13,14,16	2	B0BNN3,P02625
5740	1,3,4,6,9,11,16,17	2,5,7,8,10,12,13,14,15	2	P01681,P16636
5741	1,3,4,6,9,12,13,14	2,5,7,8,10,11,15,16,17	1	P01835
5742	1,3,4,6,9,12,13,15	2,5,7,8,10,11,14,16,17	2	P02625,P10758
5743	1,3,4,6,9,12,13,16	2,5,7,8,10,11,14,15,17	0	
5744	1,3,4,6,9,12,13,17	2,5,7,8,10,11,14,15,16	6	P01681,P01835,P13432,P16636,P36374,Q99MH3

5745	1,3,4,6,9,12,14,15	2,5,7,8,10,11,13,16,17	5	A2RUV9,B0BNN3,P01835,P02625,P10758
5746	1,3,4,6,9,12,14,16	2,5,7,8,10,11,13,15,17	1	P01835
5747	1,3,4,6,9,12,14,17	2,5,7,8,10,11,13,15,16	3	P01681,P01835,P13432
5748	1,3,4,6,9,12,15,16	2,5,7,8,10,11,13,14,17	0	
5749	1,3,4,6,9,12,15,17	2,5,7,8,10,11,13,14,16	4	B0BNN3,P01681,P01835,P13432
5750	1,3,4,6,9,12,16,17	2,5,7,8,10,11,13,14,15	5	P01681,P01835,P13432,P16636,P23593
5751	1,3,4,6,9,13,14,15	2,5,7,8,10,11,12,16,17	1	P16391
5752	1,3,4,6,9,13,14,16	2,5,7,8,10,11,12,15,17	1	P62804
5753	1,3,4,6,9,13,14,17	2,5,7,8,10,11,12,15,16	1	Q9QW07
5754	1,3,4,6,9,13,15,16	2,5,7,8,10,11,12,14,17	3	O54728,P62804,Q00715
5755	1,3,4,6,9,13,15,17	2,5,7,8,10,11,12,14,16	2	B0BNN3,P62804
5756	1,3,4,6,9,13,16,17	2,5,7,8,10,11,12,14,15	2	P23593,P62804
5757	1,3,4,6,9,14,15,16	2,5,7,8,10,11,12,13,17	2	O54728,P62804
5758	1,3,4,6,9,14,15,17	2,5,7,8,10,11,12,13,16	2	B0BNN3,Q5HZV9
5759	1,3,4,6,9,14,16,17	2,5,7,8,10,11,12,13,15	0	

5760	1,3,4,6,9,15,16,17	2,5,7,8,10,11,12,13,14	3	B0BNN3,P23593,P62804
5761	1,3,4,6,10,11,12,13	2,5,7,8,9,14,15,16,17	15	P01681,P04905,P08723,P0C0A9,P16636,P30120,P36374,P47727,Q4FZU2,Q6IFU7,Q6IFW6,Q6IG02,Q6IMF3,Q6P6Q2,Q9JHB9
5762	1,3,4,6,10,11,12,14	2,5,7,8,9,13,15,16,17	13	P01681,P04905,P08723,P0C0A9,P47727,Q10758,Q4FZU2,Q6IFU7,Q6IFU8,Q6IFW6,Q6IG02,Q6IMF3,Q6P6Q2
5763	1,3,4,6,10,11,12,15	2,5,7,8,9,13,14,16,17	11	B0BNN3,P01681,P02625,P35280,Q4FZU2,Q5FVI6,Q6IFU7,Q6IFW6,Q6IG02,Q6IMF3,Q6P6Q2
5764	1,3,4,6,10,11,12,16	2,5,7,8,9,13,14,15,17	14	P01681,P06757,P16636,P53792,P55314,Q10758,Q4FZU2,Q5FVI6,Q6IFU7,Q6IFU8,Q6IFW6,Q6IG02,Q6IMF3,Q6P6Q2
5765	1,3,4,6,10,11,12,17	2,5,7,8,9,13,14,15,16	14	O08815,P00762,P01681,P06757,P16636,Q10758,Q4FZU2,Q6IFU8,Q6IFW6,Q6IG02,Q6IMF3,Q6P6Q2,Q99MH3,Q9QZK9
5766	1,3,4,6,10,11,13,14	2,5,7,8,9,12,15,16,17	2	P06911,P47727
5767	1,3,4,6,10,11,13,15	2,5,7,8,9,12,14,16,17	0	
5768	1,3,4,6,10,11,13,16	2,5,7,8,9,12,14,15,17	2	P55314,Q5FVI6
5769	1,3,4,6,10,11,13,17	2,5,7,8,9,12,14,15,16	3	P01681,P16636,Q99MH3
5770	1,3,4,6,10,11,14,15	2,5,7,8,9,12,13,16,17	1	P35280
5771	1,3,4,6,10,11,14,16	2,5,7,8,9,12,13,15,17	1	P55314
5772	1,3,4,6,10,11,14,17	2,5,7,8,9,12,13,15,16	1	P01681
5773	1,3,4,6,10,11,15,16	2,5,7,8,9,12,13,14,17	2	P55314,Q5FVI6
5774	1,3,4,6,10,11,15,17	2,5,7,8,9,12,13,14,16	2	B0BNN3,P01681
5775	1,3,4,6,10,11,16,17	2,5,7,8,9,12,13,14,15	3	P01681,P16636,P55314
5776	1,3,4,6,10,12,13,14	2,5,7,8,9,11,15,16,17	3	P06911,Q10758,Q4FZU2
5777	1,3,4,6,10,12,13,15	2,5,7,8,9,11,14,16,17	4	B0BNN3,P82995,Q4FZU2,Q99MH3
5778	1,3,4,6,10,12,13,16	2,5,7,8,9,11,14,15,17	2	P16636,Q4FZU2
5779	1,3,4,6,10,12,13,17	2,5,7,8,9,11,14,15,16	8	P01681,P16636,P36374,Q10758,Q4FZU2,Q63355,Q99MH3,Q9QZK9
5780	1,3,4,6,10,12,14,15	2,5,7,8,9,11,13,16,17	3	A2RUV9,B0BNN3,Q4FZU2
5781	1,3,4,6,10,12,14,16	2,5,7,8,9,11,13,15,17	5	Q10758,Q4FZU2,Q6IFW6,Q6IG02,Q6IMF3

5782	1,3,4,6,10,<br>12,14,17	2,5,7,8,9,1<br>1,13,15,16	8	B0BNN3,P01681,P01835,Q10758,Q4FZU2,Q6IG02,Q6IMF3,Q99MH3
5783	1,3,4,6,10,<br>12,15,16	2,5,7,8,9,1<br>1,13,14,17	3	B0BNN3,Q4FZU2,Q6IMF3
5784	1,3,4,6,10,<br>12,15,17	2,5,7,8,9,1<br>1,13,14,16	5	B0BNN3,P01681,Q10758,Q4FZU2,Q99MH3
5785	1,3,4,6,10,<br>12,16,17	2,5,7,8,9,1<br>1,13,14,15	8	P01681,P16636,Q10758,Q4FZU2,Q6IFW6,Q6IG02,Q6IMF3,Q99MH3
5786	1,3,4,6,10,<br>13,14,15	2,5,7,8,9,1<br>1,12,16,17	2	P06911,P50115
5787	1,3,4,6,10,<br>13,14,16	2,5,7,8,9,1<br>1,12,15,17	2	P06911,P50115
5788	1,3,4,6,10,<br>13,14,17	2,5,7,8,9,1<br>1,12,15,16	2	P06911,Q9QW07
5789	1,3,4,6,10,<br>13,15,16	2,5,7,8,9,1<br>1,12,14,17	2	P50115,P62804
5790	1,3,4,6,10,<br>13,15,17	2,5,7,8,9,1<br>1,12,14,16	2	B0BNN3,Q99MH3
5791	1,3,4,6,10,<br>13,16,17	2,5,7,8,9,1<br>1,12,14,15	2	P16636,Q99MH3
5792	1,3,4,6,10,<br>14,15,16	2,5,7,8,9,1<br>1,12,13,17	2	P02803,P50115
5793	1,3,4,6,10,<br>14,15,17	2,5,7,8,9,1<br>1,12,13,16	2	B0BNN3,P02803
5794	1,3,4,6,10,<br>14,16,17	2,5,7,8,9,1<br>1,12,13,15	0	
5795	1,3,4,6,10,<br>15,16,17	2,5,7,8,9,1<br>1,12,13,14	2	B0BNN3,P02803
5796	1,3,4,6,11,<br>12,13,14	2,5,7,8,9,1<br>0,15,16,17	11	P01681,P04905,P06911,P08723,P0C0A9,P30120,P47727,Q4FZU2,Q64093,Q6IFW6,Q9JHB9
5797	1,3,4,6,11,<br>12,13,15	2,5,7,8,9,1<br>0,14,16,17	5	B0BNN3,P01681,P02625,Q4FZU2,Q6IFW6
5798	1,3,4,6,11,<br>12,13,16	2,5,7,8,9,1<br>0,14,15,17	6	P01681,P16636,Q4FZU2,Q6IFW6,Q6IMF3,Q6P6Q2
5799	1,3,4,6,11,<br>12,13,17	2,5,7,8,9,1<br>0,14,15,16	9	P01681,P13432,P16636,P36374,Q4FZU2,Q6IFW6,Q99MH3,Q9JHB9,Q9QZK9
5800	1,3,4,6,11,<br>12,14,15	2,5,7,8,9,1<br>0,13,16,17	6	B0BNN3,P01681,P02625,Q4FZU2,Q6IFW6,Q6IG02
5801	1,3,4,6,11,<br>12,14,16	2,5,7,8,9,1<br>0,13,15,17	8	P01681,Q4FZU2,Q6IFU7,Q6IFU8,Q6IFW6,Q6IG02,Q6IMF3,Q6P6Q2
5802	1,3,4,6,11,<br>12,14,17	2,5,7,8,9,1<br>0,13,15,16	10	B0BNN3,P01681,P01835,P13432,Q4FZU2,Q6IFU8,Q6IFW6,Q6IG02,Q6IMF3,Q6P6Q2
5803	1,3,4,6,11,<br>12,15,16	2,5,7,8,9,1<br>0,13,14,17	8	B0BNN3,P01681,P02625,Q4FZU2,Q6IFU7,Q6IFW6,Q6IMF3,Q6P6Q2
5804	1,3,4,6,11,<br>12,15,17	2,5,7,8,9,1<br>0,13,14,16	7	B0BNN3,P01681,P02625,P13432,Q4FZU2,Q6IFW6,Q6IG02

5805	1,3,4,6,11,12,16,17	2,5,7,8,9,10,13,14,15	9	P01681,P13432,P16636,Q4FZU2,Q6IFU8,Q6IFW6,Q6IG02,Q6IMF3,Q6P6Q2
5806	1,3,4,6,11,13,14,15	2,5,7,8,9,10,12,16,17	2	P02625,Q64093
5807	1,3,4,6,11,13,14,16	2,5,7,8,9,10,12,15,17	0	
5808	1,3,4,6,11,13,14,17	2,5,7,8,9,10,12,15,16	3	P01681,Q64093,Q9QW07
5809	1,3,4,6,11,13,15,16	2,5,7,8,9,10,12,14,17	0	
5810	1,3,4,6,11,13,15,17	2,5,7,8,9,10,12,14,16	2	B0BNN3,P02625
5811	1,3,4,6,11,13,16,17	2,5,7,8,9,10,12,14,15	2	P01681,P16636
5812	1,3,4,6,11,14,15,16	2,5,7,8,9,10,12,13,17	0	
5813	1,3,4,6,11,14,15,17	2,5,7,8,9,10,12,13,16	3	B0BNN3,P01681,P97840
5814	1,3,4,6,11,14,16,17	2,5,7,8,9,10,12,13,15	1	P01681
5815	1,3,4,6,11,15,16,17	2,5,7,8,9,10,12,13,14	2	B0BNN3,P01681
5816	1,3,4,6,12,13,14,15	2,5,7,8,9,10,11,16,17	5	A2RUV9,B0BNN3,P02625,Q64093,Q9QUL6
5817	1,3,4,6,12,13,14,16	2,5,7,8,9,10,11,15,17	1	Q9QUL6
5818	1,3,4,6,12,13,14,17	2,5,7,8,9,10,11,15,16	6	P01681,P01835,P13432,Q64093,Q9QW07,Q9QZK9
5819	1,3,4,6,12,13,15,16	2,5,7,8,9,10,11,14,17	2	P82995,Q9QUL6
5820	1,3,4,6,12,13,15,17	2,5,7,8,9,10,11,14,16	4	B0BNN3,P01681,Q99MH3,Q9QZK9
5821	1,3,4,6,12,13,16,17	2,5,7,8,9,10,11,14,15	5	P01681,P13432,P16636,P27213,Q99MH3
5822	1,3,4,6,12,14,15,16	2,5,7,8,9,10,11,13,17	2	B0BNN3,Q4FZU2
5823	1,3,4,6,12,14,15,17	2,5,7,8,9,10,11,13,16	5	A2RUV9,B0BNN3,P01681,P01835,P13432
5824	1,3,4,6,12,14,16,17	2,5,7,8,9,10,11,13,15	4	P01681,P01835,P13432,Q10758
5825	1,3,4,6,12,15,16,17	2,5,7,8,9,10,11,13,14	3	B0BNN3,P01681,Q4FZU2
5826	1,3,4,6,13,14,15,16	2,5,7,8,9,10,11,12,17	4	P16391,P50115,P62804,Q64093
5827	1,3,4,6,13,14,15,17	2,5,7,8,9,10,11,12,16	3	B0BNN3,Q64093,Q9QW07

5828	1,3,4,6,13,14,16,17	2,5,7,8,9,10,11,12,15	2	P27213,Q9QW07
5829	1,3,4,6,13,15,16,17	2,5,7,8,9,10,11,12,14	2	B0BNN3,P62804
5830	1,3,4,6,14,15,16,17	2,5,7,8,9,10,11,12,13	2	B0BNN3,P02803
5831	1,3,4,7,8,9,10,11	2,5,6,12,13,14,15,16,17	6	P00714,P18418,P35280,Q561R9,Q5FVI6,Q63617
5832	1,3,4,7,8,9,10,12	2,5,6,11,13,14,15,16,17	4	D3ZHA0,P00714,P18418,Q6IG02
5833	1,3,4,7,8,9,10,13	2,5,6,11,12,14,15,16,17	0	
5834	1,3,4,7,8,9,10,14	2,5,6,11,12,13,15,16,17	2	P18418,Q63617
5835	1,3,4,7,8,9,10,15	2,5,6,11,12,13,14,16,17	3	O54728,P22006,P35280
5836	1,3,4,7,8,9,10,16	2,5,6,11,12,13,14,15,17	2	P08721,Q5FVI6
5837	1,3,4,7,8,9,10,17	2,5,6,11,12,13,14,15,16	2	P02091,P02770
5838	1,3,4,7,8,9,11,12	2,5,6,10,13,14,15,16,17	5	P10715,P36374,Q63617,Q6IG02,Q8CJ52
5839	1,3,4,7,8,9,11,13	2,5,6,10,12,14,15,16,17	1	Q63617
5840	1,3,4,7,8,9,11,14	2,5,6,10,12,13,15,16,17	1	Q63617
5841	1,3,4,7,8,9,11,15	2,5,6,10,12,13,14,16,17	7	O54728,P10715,P22006,P35280,Q561R9,Q5QE79,Q63617
5842	1,3,4,7,8,9,11,16	2,5,6,10,12,13,14,15,17	3	B4F795,P10715,Q63617
5843	1,3,4,7,8,9,11,17	2,5,6,10,12,13,14,15,16	3	P02091,P10715,Q63617

5844	1,3,4,7,8,9,12,13	2,5,6,10,11,14,15,16,17	3	P22283,P36374,Q8CJ52
5845	1,3,4,7,8,9,12,14	2,5,6,10,11,13,15,16,17	3	A2RUV9,P36970,Q63617
5846	1,3,4,7,8,9,12,15	2,5,6,10,11,13,14,16,17	2	P10715,Q5QE79
5847	1,3,4,7,8,9,12,16	2,5,6,10,11,13,14,15,17	2	P10715,Q4FZU6
5848	1,3,4,7,8,9,12,17	2,5,6,10,11,13,14,15,16	5	P02091,P10715,P19223,P36374,Q99041
5849	1,3,4,7,8,9,13,14	2,5,6,10,11,12,15,16,17	3	P06911,P36970,Q63617
5850	1,3,4,7,8,9,13,15	2,5,6,10,11,12,14,16,17	3	O54728,P62804,Q5QE79
5851	1,3,4,7,8,9,13,16	2,5,6,10,11,12,14,15,17	3	B4F795,P62804,Q63617
5852	1,3,4,7,8,9,13,17	2,5,6,10,11,12,14,15,16	2	P02091,Q63617
5853	1,3,4,7,8,9,14,15	2,5,6,10,11,12,13,16,17	3	O54728,P22006,Q5QE79
5854	1,3,4,7,8,9,14,16	2,5,6,10,11,12,13,15,17	3	B4F795,Q4FZU6,Q63617
5855	1,3,4,7,8,9,14,17	2,5,6,10,11,12,13,15,16	2	P02091,Q63617
5856	1,3,4,7,8,9,15,16	2,5,6,10,11,12,13,14,17	4	B4F795,O54728,P62804,Q5QE79
5857	1,3,4,7,8,9,15,17	2,5,6,10,11,12,13,14,16	2	P02091,Q5QE79
5858	1,3,4,7,8,9,16,17	2,5,6,10,11,12,13,14,15	4	B4F795,P02091,Q4FZU6,Q63617

5859	1,3,4,7,8,10,11,12	2,5,6,9,13,14,15,16,17	13	P00714,P02781,P08723,P10715,P18418,P35280,P36374,Q5FVI6,Q63357,Q6IG02,Q6P6Q2,Q8CJ52,Q9ESW0
5860	1,3,4,7,8,10,11,13	2,5,6,9,12,14,15,16,17	3	P00714,Q5FVI6,Q8VD89
5861	1,3,4,7,8,10,11,14	2,5,6,9,12,13,15,16,17	5	P00714,P18418,P35280,Q5BJY9,Q5FVI6
5862	1,3,4,7,8,10,11,15	2,5,6,9,12,13,14,16,17	6	P00714,P22006,P35280,Q561R9,Q5FVI6,Q8VD89
5863	1,3,4,7,8,10,11,16	2,5,6,9,12,13,14,15,17	4	P00714,P08721,Q5FVI6,Q8VD89
5864	1,3,4,7,8,10,11,17	2,5,6,9,12,13,14,15,16	4	P00714,P02091,P12346,Q5FVI6
5865	1,3,4,7,8,10,12,13	2,5,6,9,11,14,15,16,17	9	P08723,P18418,P22283,P36374,P36970,Q8CJ52,Q99MH3,Q9QUL6,Q9R0T3
5866	1,3,4,7,8,10,12,14	2,5,6,9,11,13,15,16,17	6	A2RUV9,P08723,P18418,P36970,Q6IG02,Q8CJ52
5867	1,3,4,7,8,10,12,15	2,5,6,9,11,13,14,16,17	3	P18418,P22006,P35280
5868	1,3,4,7,8,10,12,16	2,5,6,9,11,13,14,15,17	5	P08721,P47967,Q5FVI6,Q6IG02,Q8CJ52
5869	1,3,4,7,8,10,12,17	2,5,6,9,11,13,14,15,16	10	P02091,P12346,P18418,P19223,P36374,P46844,Q4G075,Q6IG02,Q99041,Q99MH3
5870	1,3,4,7,8,10,13,14	2,5,6,9,11,12,15,16,17	5	P06911,P12020,P18418,P36970,Q5GRG2
5871	1,3,4,7,8,10,13,15	2,5,6,9,11,12,14,16,17	2	P01835,P22006
5872	1,3,4,7,8,10,13,16	2,5,6,9,11,12,14,15,17	3	P01835,P08721,Q8VD89
5873	1,3,4,7,8,10,13,17	2,5,6,9,11,12,14,15,16	3	P02091,P46844,Q99MH3

5874	1,3,4,7,8,10,14,15	2,5,6,9,11,12,13,16,17	2	P22006,P35280
5875	1,3,4,7,8,10,14,16	2,5,6,9,11,12,13,15,17	1	P08721
5876	1,3,4,7,8,10,14,17	2,5,6,9,11,12,13,15,16	2	P02091,P12346
5877	1,3,4,7,8,10,15,16	2,5,6,9,11,12,13,14,17	4	P01835,P08721,Q5FVI6,Q8VD89
5878	1,3,4,7,8,10,15,17	2,5,6,9,11,12,13,14,16	6	B0BNN3,P02091,P02761,P12346,P22006,Q99MH3
5879	1,3,4,7,8,10,16,17	2,5,6,9,11,12,13,14,15	3	P02091,P08721,P25031
5880	1,3,4,7,8,11,12,13	2,5,6,9,10,14,15,16,17	13	P01836,P02780,P02781,P08723,P10715,P22282,P22283,P36374,Q62902,Q6IG02,Q8CJ52,Q99041,Q9JI85
5881	1,3,4,7,8,11,12,14	2,5,6,9,10,13,15,16,17	6	P08723,P10715,Q63493,Q6IG02,Q8CJ52,Q9ESW0
5882	1,3,4,7,8,11,12,15	2,5,6,9,10,13,14,16,17	7	P01836,P10715,P22006,P35280,Q6IG02,Q8CJ52,Q9ESW0
5883	1,3,4,7,8,11,12,16	2,5,6,9,10,13,14,15,17	6	P01836,P10715,Q6IG02,Q6P6Q2,Q8CJ52,Q9ESW0
5884	1,3,4,7,8,11,12,17	2,5,6,9,10,13,14,15,16	13	P02091,P10715,P19223,P22283,P36374,Q4G075,Q63493,Q63751,Q6IG02,Q8CJ52,Q99041,Q9ESW0,Q9QZA6
5885	1,3,4,7,8,11,13,14	2,5,6,9,10,12,15,16,17	3	P06911,Q5GRG2,Q63617
5886	1,3,4,7,8,11,13,15	2,5,6,9,10,12,14,16,17	2	P22006,Q8VD89
5887	1,3,4,7,8,11,13,16	2,5,6,9,10,12,14,15,17	3	B4F795,Q63617,Q8VD89
5888	1,3,4,7,8,11,13,17	2,5,6,9,10,12,14,15,16	3	P01946,P02091,Q63617

5889	1,3,4,7,8,11,14,15	2,5,6,9,10,12,13,16,17	2	P22006,P35280
5890	1,3,4,7,8,11,14,16	2,5,6,9,10,12,13,15,17	2	B4F795,Q63617
5891	1,3,4,7,8,11,14,17	2,5,6,9,10,12,13,15,16	3	P02091,P97840,Q63617
5892	1,3,4,7,8,11,15,16	2,5,6,9,10,12,13,14,17	4	B4F795,O54728,P22006,Q8VD89
5893	1,3,4,7,8,11,15,17	2,5,6,9,10,12,13,14,16	4	B0BNN3,P02091,P02761,P22006
5894	1,3,4,7,8,11,16,17	2,5,6,9,10,12,13,14,15	4	B4F795,P02091,P10715,Q63617
5895	1,3,4,7,8,12,13,14	2,5,6,9,10,11,15,16,17	12	A2RUV9,O08557,P06761,P06911,P08723,P22283,P36374,P36970,Q5GRG2,Q62902,Q8CJ52,Q9QUL6
5896	1,3,4,7,8,12,13,15	2,5,6,9,10,11,14,16,17	3	P36970,Q8CJ52,Q9QUL6
5897	1,3,4,7,8,12,13,16	2,5,6,9,10,11,14,15,17	3	Q8CJ52,Q9QUL6,Q9R0T3
5898	1,3,4,7,8,12,13,17	2,5,6,9,10,11,14,15,16	15	P01946,P02091,P06761,P11598,P19223,P22282,P22283,P36374,P55091,Q4G075,Q8CJ52,Q99041,Q99MH3,Q9QUL6,Q9QZK9
5899	1,3,4,7,8,12,14,15	2,5,6,9,10,11,13,16,17	6	A2RUV9,B0BNN3,O08557,P22006,P36970,Q9QUL6
5900	1,3,4,7,8,12,14,16	2,5,6,9,10,11,13,15,17	4	P08721,Q4FZU6,Q8CJ52,Q9QUL6
5901	1,3,4,7,8,12,14,17	2,5,6,9,10,11,13,15,16	10	A2RUV9,P02091,P19223,P31430,P36970,Q4FZU6,Q4G075,Q8CJ52,Q99041,Q9QZA6
5902	1,3,4,7,8,12,15,16	2,5,6,9,10,11,13,14,17	4	P08721,P10715,Q62894,Q9QUL6
5903	1,3,4,7,8,12,15,17	2,5,6,9,10,11,13,14,16	8	B0BNN3,P02091,P10715,P19223,Q4G075,Q99041,Q99MH3,Q9QZA6

5904	1,3,4,7,8,12,16,17	2,5,6,9,10,11,13,14,15	7	P02091,P10715,P19223,Q4FZU6,Q4G075,Q8CJ52,Q99041
5905	1,3,4,7,8,13,14,15	2,5,6,9,10,11,12,16,17	6	A2RUV9,P06911,P22006,P36970,Q5GRG2,Q9QUL6
5906	1,3,4,7,8,13,14,16	2,5,6,9,10,11,12,15,17	2	P06911,Q9QUL6
5907	1,3,4,7,8,13,14,17	2,5,6,9,10,11,12,15,16	6	P01946,P02091,P06911,P36970,Q6IFU7,Q9QW07
5908	1,3,4,7,8,13,15,16	2,5,6,9,10,11,12,14,17	6	B4F795,O54728,P01835,P62804,Q8VD89,Q9QUL6
5909	1,3,4,7,8,13,15,17	2,5,6,9,10,11,12,14,16	2	P02091,Q6IFU7
5910	1,3,4,7,8,13,16,17	2,5,6,9,10,11,12,14,15	3	P02091,P62804,Q6PCU2
5911	1,3,4,7,8,14,15,16	2,5,6,9,10,11,12,13,17	4	B4F795,O54728,P08721,P97574
5912	1,3,4,7,8,14,15,17	2,5,6,9,10,11,12,13,16	4	B0BNN3,P02091,P10536,P22006
5913	1,3,4,7,8,14,16,17	2,5,6,9,10,11,12,13,15	2	P02091,Q4FZU6
5914	1,3,4,7,8,15,16,17	2,5,6,9,10,11,12,13,14	2	B4F795,P02091
5915	1,3,4,7,9,10,11,12	2,5,6,8,13,14,15,16,17	5	P06757,P08753,P09527,Q5FVI6,Q6IG02
5916	1,3,4,7,9,10,11,13	2,5,6,8,12,14,15,16,17	3	P12785,Q5FVI6,Q62635
5917	1,3,4,7,9,10,11,14	2,5,6,8,12,13,15,16,17	2	Q5FVI6,Q62635
5918	1,3,4,7,9,10,11,15	2,5,6,8,12,13,14,16,17	6	O54728,P22006,P35280,Q561R9,Q5FVI6,Q62635

5919	1,3,4,7,9,10,11,16	2,5,6,8,12,13,14,15,17	2	Q5FVI6,Q62635
5920	1,3,4,7,9,10,11,17	2,5,6,8,12,13,14,15,16	3	P02091,P12785,Q5FVI6
5921	1,3,4,7,9,10,12,13	2,5,6,8,11,14,15,16,17	1	P36374
5922	1,3,4,7,9,10,12,14	2,5,6,8,11,13,15,16,17	1	A2RUV9
5923	1,3,4,7,9,10,12,15	2,5,6,8,11,13,14,16,17	1	Q5FVI6
5924	1,3,4,7,9,10,12,16	2,5,6,8,11,13,14,15,17	3	P08721,P47967,Q5FVI6
5925	1,3,4,7,9,10,12,17	2,5,6,8,11,13,14,15,16	2	P02091,P19223
5926	1,3,4,7,9,10,13,14	2,5,6,8,11,12,15,16,17	1	P06760
5927	1,3,4,7,9,10,13,15	2,5,6,8,11,12,14,16,17	5	O54728,P06760,P62804,Q5U2V4,Q62635
5928	1,3,4,7,9,10,13,16	2,5,6,8,11,12,14,15,17	4	P06760,P62804,Q00715,Q62635
5929	1,3,4,7,9,10,13,17	2,5,6,8,11,12,14,15,16	2	P02091,P62804
5930	1,3,4,7,9,10,14,15	2,5,6,8,11,12,13,16,17	4	O54728,P06760,P22006,Q62635
5931	1,3,4,7,9,10,14,16	2,5,6,8,11,12,13,15,17	4	P06760,P08721,P62804,Q62635
5932	1,3,4,7,9,10,14,17	2,5,6,8,11,12,13,15,16	1	P02091
5933	1,3,4,7,9,10,15,16	2,5,6,8,11,12,13,14,17	8	O54728,P02782,P06760,P62804,Q00715,Q5FVI6,Q62635,Q9Z1F2

5934	1,3,4,7,9,10,15,17	2,5,6,8,11,12,13,14,16	1	P02091
5935	1,3,4,7,9,10,16,17	2,5,6,8,11,12,13,14,15	2	P02091,P62804
5936	1,3,4,7,9,11,12,13	2,5,6,8,10,14,15,16,17	3	P01836,P05539,P36374
5937	1,3,4,7,9,11,12,14	2,5,6,8,10,13,15,16,17	0	
5938	1,3,4,7,9,11,12,15	2,5,6,8,10,13,14,16,17	4	O54728,P01836,P22006,P51907
5939	1,3,4,7,9,11,12,16	2,5,6,8,10,13,14,15,17	1	P01836
5940	1,3,4,7,9,11,12,17	2,5,6,8,10,13,14,15,16	3	P02091,P19223,P36374
5941	1,3,4,7,9,11,13,14	2,5,6,8,10,12,15,16,17	2	P05539,P06760
5942	1,3,4,7,9,11,13,15	2,5,6,8,10,12,14,16,17	6	O54728,P05539,P06760,P22006,Q561R9,Q62635
5943	1,3,4,7,9,11,13,16	2,5,6,8,10,12,14,15,17	3	P06760,P62804,Q62635
5944	1,3,4,7,9,11,13,17	2,5,6,8,10,12,14,15,16	2	P02091,P05539
5945	1,3,4,7,9,11,14,15	2,5,6,8,10,12,13,16,17	4	O54728,P06760,P22006,Q62635
5946	1,3,4,7,9,11,14,16	2,5,6,8,10,12,13,15,17	2	P06760,Q62635
5947	1,3,4,7,9,11,14,17	2,5,6,8,10,12,13,15,16	1	P02091
5948	1,3,4,7,9,11,15,16	2,5,6,8,10,12,13,14,17	5	B4F795,O54728,P06760,P22006,Q62635

5949	1,3,4,7,9,11,15,17	2,5,6,8,10,12,13,14,16	3	O54728,P02091,P22006
5950	1,3,4,7,9,11,16,17	2,5,6,8,10,12,13,14,15	1	P02091
5951	1,3,4,7,9,12,13,14	2,5,6,8,10,11,15,16,17	2	A2RUV9,P06760
5952	1,3,4,7,9,12,13,15	2,5,6,8,10,11,14,16,17	3	O54728,P06760,P10758
5953	1,3,4,7,9,12,13,16	2,5,6,8,10,11,14,15,17	2	P06760,P62804
5954	1,3,4,7,9,12,13,17	2,5,6,8,10,11,14,15,16	2	P02091,P36374
5955	1,3,4,7,9,12,14,15	2,5,6,8,10,11,13,16,17	4	A2RUV9,O54728,P06760,P10758
5956	1,3,4,7,9,12,14,16	2,5,6,8,10,11,13,15,17	2	P06760,Q4FZU6
5957	1,3,4,7,9,12,14,17	2,5,6,8,10,11,13,15,16	2	A2RUV9,P02091
5958	1,3,4,7,9,12,15,16	2,5,6,8,10,11,13,14,17	3	O54728,P06760,P62804
5959	1,3,4,7,9,12,15,17	2,5,6,8,10,11,13,14,16	1	P02091
5960	1,3,4,7,9,12,16,17	2,5,6,8,10,11,13,14,15	1	P02091
5961	1,3,4,7,9,13,14,15	2,5,6,8,10,11,12,16,17	4	A2RUV9,O54728,P06760,P62804
5962	1,3,4,7,9,13,14,16	2,5,6,8,10,11,12,15,17	4	P06760,P62804,Q00715,Q62635
5963	1,3,4,7,9,13,14,17	2,5,6,8,10,11,12,15,16	3	P02091,P62804,Q9QW07

5964	1,3,4,7,9,13,15,16	2,5,6,8,10,11,12,14,17	5	O54728,P06760,P62804,Q00715,Q62635
5965	1,3,4,7,9,13,15,17	2,5,6,8,10,11,12,14,16	3	O54728,P02091,P62804
5966	1,3,4,7,9,13,16,17	2,5,6,8,10,11,12,14,15	3	P02091,P62804,Q00715
5967	1,3,4,7,9,14,15,16	2,5,6,8,10,11,12,13,17	5	O54728,P06760,P62804,Q62635,Q9QZK9
5968	1,3,4,7,9,14,15,17	2,5,6,8,10,11,12,13,16	2	O54728,P02091
5969	1,3,4,7,9,14,16,17	2,5,6,8,10,11,12,13,15	3	P02091,P62804,Q4FZU6
5970	1,3,4,7,9,15,16,17	2,5,6,8,10,11,12,13,14	4	O54728,P02091,P47727,P62804
5971	1,3,4,7,10,11,12,13	2,5,6,8,9,14,15,16,17	4	P01836,P36374,Q5FVI6,Q63942
5972	1,3,4,7,10,11,12,14	2,5,6,8,9,13,15,16,17	2	Q5FVI6,Q6IG02
5973	1,3,4,7,10,11,12,15	2,5,6,8,9,13,14,16,17	5	P01836,P22006,P35280,P51907,Q5FVI6
5974	1,3,4,7,10,11,12,16	2,5,6,8,9,13,14,15,17	8	P01836,P06757,P08721,P47967,P53792,Q5FVI6,Q6IG02,Q6P6Q2
5975	1,3,4,7,10,11,12,17	2,5,6,8,9,13,14,15,16	7	O08815,P02091,P06757,P36374,Q5FVI6,Q63942,Q6IG02
5976	1,3,4,7,10,11,13,14	2,5,6,8,9,12,15,16,17	0	
5977	1,3,4,7,10,11,13,15	2,5,6,8,9,12,14,16,17	4	P22006,Q561R9,Q5FVI6,Q8VD89
5978	1,3,4,7,10,11,13,16	2,5,6,8,9,12,14,15,17	2	Q5FVI6,Q8VD89
5979	1,3,4,7,10,11,13,17	2,5,6,8,9,12,14,15,16	1	P02091
5980	1,3,4,7,10,11,14,15	2,5,6,8,9,12,13,16,17	4	P22006,P35280,Q5FVI6,Q62635
5981	1,3,4,7,10,11,14,16	2,5,6,8,9,12,13,15,17	2	Q5FVI6,Q62635
5982	1,3,4,7,10,11,14,17	2,5,6,8,9,12,13,15,16	1	P02091

5983	1,3,4,7,10,<br>11,15,16	2,5,6,8,9,1<br>2,13,14,17	7	O54728,P22006,Q561R9,Q5FVI6,Q62635,Q8VD89,Q9Z1F2
5984	1,3,4,7,10,<br>11,15,17	2,5,6,8,9,1<br>2,13,14,16	5	O35077,P02091,P22006,Q5FVI6,Q9Z1F2
5985	1,3,4,7,10,<br>11,16,17	2,5,6,8,9,1<br>2,13,14,15	3	P02091,Q5FVI6,Q9Z1F2
5986	1,3,4,7,10,<br>12,13,14	2,5,6,8,9,1<br>1,15,16,17	1	A2RUV9
5987	1,3,4,7,10,<br>12,13,15	2,5,6,8,9,1<br>1,14,16,17	2	Q99MH3,Q9QUL6
5988	1,3,4,7,10,<br>12,13,16	2,5,6,8,9,1<br>1,14,15,17	2	P47967,Q9QUL6
5989	1,3,4,7,10,<br>12,13,17	2,5,6,8,9,1<br>1,14,15,16	5	P02091,P36374,Q63942,Q99MH3,Q9QZK9
5990	1,3,4,7,10,<br>12,14,15	2,5,6,8,9,1<br>1,13,16,17	2	A2RUV9,P22006
5991	1,3,4,7,10,<br>12,14,16	2,5,6,8,9,1<br>1,13,15,17	1	P08721
5992	1,3,4,7,10,<br>12,14,17	2,5,6,8,9,1<br>1,13,15,16	3	A2RUV9,P02091,P31430
5993	1,3,4,7,10,<br>12,15,16	2,5,6,8,9,1<br>1,13,14,17	2	P08721,Q5FVI6
5994	1,3,4,7,10,<br>12,15,17	2,5,6,8,9,1<br>1,13,14,16	3	B0BNN3,P02091,Q99MH3
5995	1,3,4,7,10,<br>12,16,17	2,5,6,8,9,1<br>1,13,14,15	2	P02091,P08721
5996	1,3,4,7,10,<br>13,14,15	2,5,6,8,9,1<br>1,12,16,17	3	A2RUV9,P06760,P22006
5997	1,3,4,7,10,<br>13,14,16	2,5,6,8,9,1<br>1,12,15,17	2	P06760,P62804
5998	1,3,4,7,10,<br>13,14,17	2,5,6,8,9,1<br>1,12,15,16	2	P02091,Q9QW07
5999	1,3,4,7,10,<br>13,15,16	2,5,6,8,9,1<br>1,12,14,17	7	O54728,P01835,P06760,P62804,Q5FVI6,Q62635,Q8VD89
6000	1,3,4,7,10,<br>13,15,17	2,5,6,8,9,1<br>1,12,14,16	2	P02091,Q99MH3
6001	1,3,4,7,10,<br>13,16,17	2,5,6,8,9,1<br>1,12,14,15	2	P02091,P62804
6002	1,3,4,7,10,<br>14,15,16	2,5,6,8,9,1<br>1,12,13,17	5	O54728,P06760,P08721,Q5FVI6,Q62635
6003	1,3,4,7,10,<br>14,15,17	2,5,6,8,9,1<br>1,12,13,16	2	P02091,P22006
6004	1,3,4,7,10,<br>14,16,17	2,5,6,8,9,1<br>1,12,13,15	2	P02091,P08721
6005	1,3,4,7,10,<br>15,16,17	2,5,6,8,9,1<br>1,12,13,14	5	P02091,P02782,P62804,Q5FVI6,Q9Z1F2

6006	1,3,4,7,11,12,13,14	2,5,6,8,9,10,15,16,17	3	A2RUV9,P01836,P36374
6007	1,3,4,7,11,12,13,15	2,5,6,8,9,10,14,16,17	4	P01836,P05539,P22006,Q6P6R2
6008	1,3,4,7,11,12,13,16	2,5,6,8,9,10,14,15,17	2	P01836,P17046
6009	1,3,4,7,11,12,13,17	2,5,6,8,9,10,14,15,16	5	P01946,P02091,P36374,Q63942,Q9QZK9
6010	1,3,4,7,11,12,14,15	2,5,6,8,9,10,13,16,17	3	A2RUV9,P01836,P22006
6011	1,3,4,7,11,12,14,16	2,5,6,8,9,10,13,15,17	1	P01836
6012	1,3,4,7,11,12,14,17	2,5,6,8,9,10,13,15,16	3	P02091,Q63942,Q9ESW0
6013	1,3,4,7,11,12,15,16	2,5,6,8,9,10,13,14,17	3	P01836,P22006,P51907
6014	1,3,4,7,11,12,15,17	2,5,6,8,9,10,13,14,16	5	B0BNN3,P02091,P22006,Q63942,Q9ESW0
6015	1,3,4,7,11,12,16,17	2,5,6,8,9,10,13,14,15	1	P02091
6016	1,3,4,7,11,13,14,15	2,5,6,8,9,10,12,16,17	6	O54728,P05539,P06760,P22006,Q09030,Q6P6R2
6017	1,3,4,7,11,13,14,16	2,5,6,8,9,10,12,15,17	1	P06760
6018	1,3,4,7,11,13,14,17	2,5,6,8,9,10,12,15,16	3	P01946,P02091,Q9QW07
6019	1,3,4,7,11,13,15,16	2,5,6,8,9,10,12,14,17	5	O54728,P01836,P06760,P62804,Q8VD89
6020	1,3,4,7,11,13,15,17	2,5,6,8,9,10,12,14,16	3	P02091,P05539,P22006
6021	1,3,4,7,11,13,16,17	2,5,6,8,9,10,12,14,15	2	P01946,P02091
6022	1,3,4,7,11,14,15,16	2,5,6,8,9,10,12,13,17	5	B4F795,O54728,P06760,P22006,Q62635
6023	1,3,4,7,11,14,15,17	2,5,6,8,9,10,12,13,16	2	P02091,P22006
6024	1,3,4,7,11,14,16,17	2,5,6,8,9,10,12,13,15	1	P02091
6025	1,3,4,7,11,15,16,17	2,5,6,8,9,10,12,13,14	1	P02091
6026	1,3,4,7,12,13,14,15	2,5,6,8,9,10,11,16,17	3	A2RUV9,P06760,Q9QUL6
6027	1,3,4,7,12,13,14,16	2,5,6,8,9,10,11,15,17	3	A2RUV9,P06760,Q9QUL6
6028	1,3,4,7,12,13,14,17	2,5,6,8,9,10,11,15,16	5	A2RUV9,P02091,P31430,P36374,Q9QW07

6029	1,3,4,7,12,13,15,16	2,5,6,8,9,10,11,14,17	4	P01836,P06760,P62804,Q9QUL6
6030	1,3,4,7,12,13,15,17	2,5,6,8,9,10,11,14,16	3	P02091,Q99MH3,Q9QUL6
6031	1,3,4,7,12,13,16,17	2,5,6,8,9,10,11,14,15	2	P02091,Q9QUL6
6032	1,3,4,7,12,14,15,16	2,5,6,8,9,10,11,13,17	3	A2RUV9,P06760,Q9QUL6
6033	1,3,4,7,12,14,15,17	2,5,6,8,9,10,11,13,16	3	A2RUV9,B0BNN3,P02091
6034	1,3,4,7,12,14,16,17	2,5,6,8,9,10,11,13,15	3	P02091,P31430,Q4FZU6
6035	1,3,4,7,12,15,16,17	2,5,6,8,9,10,11,13,14	1	P02091
6036	1,3,4,7,13,14,15,16	2,5,6,8,9,10,11,12,17	5	O54728,P06760,P62804,Q09030,Q9QUL6
6037	1,3,4,7,13,14,15,17	2,5,6,8,9,10,11,12,16	3	A2RUV9,P02091,Q9QW07
6038	1,3,4,7,13,14,16,17	2,5,6,8,9,10,11,12,15	3	P02091,P62804,Q9QW07
6039	1,3,4,7,13,15,16,17	2,5,6,8,9,10,11,12,14	2	P02091,P62804
6040	1,3,4,7,14,15,16,17	2,5,6,8,9,10,11,12,13	3	P02091,P02803,P62804
6041	1,3,4,8,9,10,11,12	2,5,6,7,13,14,15,16,17	5	P00714,P16636,P35467,Q6IG02,Q6P6Q2
6042	1,3,4,8,9,10,11,13	2,5,6,7,12,14,15,16,17	3	P35467,P83748,Q561R9
6043	1,3,4,8,9,10,11,14	2,5,6,7,12,13,15,16,17	1	P83748
6044	1,3,4,8,9,10,11,15	2,5,6,7,12,13,14,16,17	3	O54728,P35280,Q561R9
6045	1,3,4,8,9,10,11,16	2,5,6,7,12,13,14,15,17	3	P83748,Q62635,Q9WUW8
6046	1,3,4,8,9,10,11,17	2,5,6,7,12,13,14,15,16	3	P16636,P35467,P83748
6047	1,3,4,8,9,10,12,13	2,5,6,7,11,14,15,16,17	3	P10758,P16636,Q6IG02

6048	1,3,4,8,9,10,12,14	2,5,6,7,11,13,15,16,17	2	P10758,Q6IG02
6049	1,3,4,8,9,10,12,15	2,5,6,7,11,13,14,16,17	2	P10758,Q6IG02
6050	1,3,4,8,9,10,12,16	2,5,6,7,11,13,14,15,17	4	P08721,P16636,Q6IG02,Q9WUW8
6051	1,3,4,8,9,10,12,17	2,5,6,7,11,13,14,15,16	3	P16636,P19223,Q6IG02
6052	1,3,4,8,9,10,13,14	2,5,6,7,11,12,15,16,17	0	
6053	1,3,4,8,9,10,13,15	2,5,6,7,11,12,14,16,17	1	O54728
6054	1,3,4,8,9,10,13,16	2,5,6,7,11,12,14,15,17	0	
6055	1,3,4,8,9,10,13,17	2,5,6,7,11,12,14,15,16	2	P16636,Q63532
6056	1,3,4,8,9,10,14,15	2,5,6,7,11,12,13,16,17	1	O54728
6057	1,3,4,8,9,10,14,16	2,5,6,7,11,12,13,15,17	2	P08721,Q62635
6058	1,3,4,8,9,10,14,17	2,5,6,7,11,12,13,15,16	0	
6059	1,3,4,8,9,10,15,16	2,5,6,7,11,12,13,14,17	4	O54728,Q5QE79,Q62635,Q9QZK9
6060	1,3,4,8,9,10,15,17	2,5,6,7,11,12,13,14,16	2	B0BNN3,Q5QE79
6061	1,3,4,8,9,10,16,17	2,5,6,7,11,12,13,14,15	2	P16636,P25031
6062	1,3,4,8,9,11,12,13	2,5,6,7,10,14,15,16,17	5	P10758,P16636,P35467,P36374,Q6IG02

6063	1,3,4,8,9,11,12,14	2,5,6,7,10,13,15,16,17	2	P10758,Q6IG02
6064	1,3,4,8,9,11,12,15	2,5,6,7,10,13,14,16,17	3	P10758,Q5QE79,Q6IG02
6065	1,3,4,8,9,11,12,16	2,5,6,7,10,13,14,15,17	1	Q6IG02
6066	1,3,4,8,9,11,12,17	2,5,6,7,10,13,14,15,16	4	P16636,P19223,P36374,Q6IG02
6067	1,3,4,8,9,11,13,14	2,5,6,7,10,12,15,16,17	2	P83748,P97840
6068	1,3,4,8,9,11,13,15	2,5,6,7,10,12,14,16,17	4	O54728,P97840,Q561R9,Q5QE79
6069	1,3,4,8,9,11,13,16	2,5,6,7,10,12,14,15,17	2	B4F795,P83748
6070	1,3,4,8,9,11,13,17	2,5,6,7,10,12,14,15,16	5	P16636,P35467,P83748,P97840,Q63532
6071	1,3,4,8,9,11,14,15	2,5,6,7,10,12,13,16,17	3	O54728,P97840,Q5QE79
6072	1,3,4,8,9,11,14,16	2,5,6,7,10,12,13,15,17	3	B4F795,P83748,Q9QZK9
6073	1,3,4,8,9,11,14,17	2,5,6,7,10,12,13,15,16	3	P19218,P83748,P97840
6074	1,3,4,8,9,11,15,16	2,5,6,7,10,12,13,14,17	6	B4F795,O54728,Q561R9,Q5QE79,Q62635,Q9QZK9
6075	1,3,4,8,9,11,15,17	2,5,6,7,10,12,13,14,16	5	B0BNN3,P83748,P97840,Q5QE79,Q63532
6076	1,3,4,8,9,11,16,17	2,5,6,7,10,12,13,14,15	3	B4F795,P16636,P83748
6077	1,3,4,8,9,12,13,14	2,5,6,7,10,11,15,16,17	3	P10758,P36970,Q9QUL6

6078	1,3,4,8,9,12,13,15	2,5,6,7,10,11,14,16,17	3	P10758,Q5QE79,Q9QUL6
6079	1,3,4,8,9,12,13,16	2,5,6,7,10,11,14,15,17	1	Q9QUL6
6080	1,3,4,8,9,12,13,17	2,5,6,7,10,11,14,15,16	2	P16636,P36374
6081	1,3,4,8,9,12,14,15	2,5,6,7,10,11,13,16,17	4	A2RUV9,B0BNN3,P10758,Q5QE79
6082	1,3,4,8,9,12,14,16	2,5,6,7,10,11,13,15,17	0	
6083	1,3,4,8,9,12,14,17	2,5,6,7,10,11,13,15,16	1	P31430
6084	1,3,4,8,9,12,15,16	2,5,6,7,10,11,13,14,17	4	O54728,Q5QE79,Q62894,Q9QUL6
6085	1,3,4,8,9,12,15,17	2,5,6,7,10,11,13,14,16	2	B0BNN3,Q5QE79
6086	1,3,4,8,9,12,16,17	2,5,6,7,10,11,13,14,15	1	P16636
6087	1,3,4,8,9,13,14,15	2,5,6,7,10,11,12,16,17	2	O54728,Q5QE79
6088	1,3,4,8,9,13,14,16	2,5,6,7,10,11,12,15,17	0	
6089	1,3,4,8,9,13,14,17	2,5,6,7,10,11,12,15,16	2	P97840,Q9QW07
6090	1,3,4,8,9,13,15,16	2,5,6,7,10,11,12,14,17	4	O54728,P62804,Q5QE79,Q9QUL6
6091	1,3,4,8,9,13,15,17	2,5,6,7,10,11,12,14,16	3	P97840,Q5QE79,Q63532
6092	1,3,4,8,9,13,16,17	2,5,6,7,10,11,12,14,15	1	P62804

6093	1,3,4,8,9,14,15,16	2,5,6,7,10,11,12,13,17	5	B4F795,O54728,Q5QE79,Q62635,Q9QZK9
6094	1,3,4,8,9,14,15,17	2,5,6,7,10,11,12,13,16	4	B0BNN3,P47967,P97840,Q5QE79
6095	1,3,4,8,9,14,16,17	2,5,6,7,10,11,12,13,15	0	
6096	1,3,4,8,9,15,16,17	2,5,6,7,10,11,12,13,14	4	B4F795,O54728,P47727,Q5QE79
6097	1,3,4,8,10,11,12,13	2,5,6,7,9,14,15,16,17	4	P16636,P36374,Q6IG02,Q6P6Q2
6098	1,3,4,8,10,11,12,14	2,5,6,7,9,13,15,16,17	3	P53792,Q6IG02,Q6P6Q2
6099	1,3,4,8,10,11,12,15	2,5,6,7,9,13,14,16,17	8	B0BNN3,P10758,P35280,P53792,Q4FZU2,Q561R9,Q6IG02,Q6P6Q2
6100	1,3,4,8,10,11,12,16	2,5,6,7,9,13,14,15,17	7	P08721,P16636,P53792,Q6IFW6,Q6IG02,Q6P6Q2,Q9WUW8
6101	1,3,4,8,10,11,12,17	2,5,6,7,9,13,14,15,16	7	P06757,P16636,P19223,P36374,P53792,Q6IG02,Q6P6Q2
6102	1,3,4,8,10,11,13,14	2,5,6,7,9,12,15,16,17	1	P83748
6103	1,3,4,8,10,11,13,15	2,5,6,7,9,12,14,16,17	1	Q561R9
6104	1,3,4,8,10,11,13,16	2,5,6,7,9,12,14,15,17	0	
6105	1,3,4,8,10,11,13,17	2,5,6,7,9,12,14,15,16	2	P16636,P83748
6106	1,3,4,8,10,11,14,15	2,5,6,7,9,12,13,16,17	2	P35280,Q64194
6107	1,3,4,8,10,11,14,16	2,5,6,7,9,12,13,15,17	3	P08721,P53792,P83748
6108	1,3,4,8,10,11,14,17	2,5,6,7,9,12,13,15,16	2	P19218,P83748
6109	1,3,4,8,10,11,15,16	2,5,6,7,9,12,13,14,17	2	P53792,Q561R9
6110	1,3,4,8,10,11,15,17	2,5,6,7,9,12,13,14,16	3	B0BNN3,P02761,P83748
6111	1,3,4,8,10,11,16,17	2,5,6,7,9,12,13,14,15	2	P16636,P83748
6112	1,3,4,8,10,12,13,14	2,5,6,7,9,11,15,16,17	2	P36970,Q9QUL6
6113	1,3,4,8,10,12,13,15	2,5,6,7,9,11,14,16,17	3	P10758,Q99MH3,Q9QUL6

6114	1,3,4,8,10,<br>12,13,16	2,5,6,7,9,1<br>1,14,15,17	2	P16636,Q9QUL6
6115	1,3,4,8,10,<br>12,13,17	2,5,6,7,9,1<br>1,14,15,16	4	P16636,P36374,Q99MH3,Q9QUL6
6116	1,3,4,8,10,<br>12,14,15	2,5,6,7,9,1<br>1,13,16,17	5	A2RUV9,B0BNN3,P10758,Q6IG02,Q9QUL6
6117	1,3,4,8,10,<br>12,14,16	2,5,6,7,9,1<br>1,13,15,17	3	P08721,Q6IG02,Q9QUL6
6118	1,3,4,8,10,<br>12,14,17	2,5,6,7,9,1<br>1,13,15,16	2	P31430,Q6IG02
6119	1,3,4,8,10,<br>12,15,16	2,5,6,7,9,1<br>1,13,14,17	2	P08721,Q9QUL6
6120	1,3,4,8,10,<br>12,15,17	2,5,6,7,9,1<br>1,13,14,16	3	B0BNN3,Q6IG02,Q99MH3
6121	1,3,4,8,10,<br>12,16,17	2,5,6,7,9,1<br>1,13,14,15	3	P08721,P16636,Q6IG02
6122	1,3,4,8,10,<br>13,14,15	2,5,6,7,9,1<br>1,12,16,17	3	P09605;P25809,Q64093,Q9QUL6
6123	1,3,4,8,10,<br>13,14,16	2,5,6,7,9,1<br>1,12,15,17	1	Q9QUL6
6124	1,3,4,8,10,<br>13,14,17	2,5,6,7,9,1<br>1,12,15,16	1	Q9QW07
6125	1,3,4,8,10,<br>13,15,16	2,5,6,7,9,1<br>1,12,14,17	2	P01835,Q9QUL6
6126	1,3,4,8,10,<br>13,15,17	2,5,6,7,9,1<br>1,12,14,16	2	B0BNN3,Q99MH3
6127	1,3,4,8,10,<br>13,16,17	2,5,6,7,9,1<br>1,12,14,15	2	P16636,P25031
6128	1,3,4,8,10,<br>14,15,16	2,5,6,7,9,1<br>1,12,13,17	1	P08721
6129	1,3,4,8,10,<br>14,15,17	2,5,6,7,9,1<br>1,12,13,16	1	B0BNN3
6130	1,3,4,8,10,<br>14,16,17	2,5,6,7,9,1<br>1,12,13,15	1	P08721
6131	1,3,4,8,10,<br>15,16,17	2,5,6,7,9,1<br>1,12,13,14	1	B0BNN3
6132	1,3,4,8,11,<br>12,13,14	2,5,6,7,9,1<br>0,15,16,17	4	P36374,Q64093,Q6IG02,Q9QUL6
6133	1,3,4,8,11,<br>12,13,15	2,5,6,7,9,1<br>0,14,16,17	2	P10758,Q9QUL6
6134	1,3,4,8,11,<br>12,13,16	2,5,6,7,9,1<br>0,14,15,17	3	P16636,Q6IG02,Q9QUL6
6135	1,3,4,8,11,<br>12,13,17	2,5,6,7,9,1<br>0,14,15,16	3	P16636,P36374,Q6IG02
6136	1,3,4,8,11,<br>12,14,15	2,5,6,7,9,1<br>0,13,16,17	3	B0BNN3,P10758,Q6IG02

6137	1,3,4,8,11,<br>12,14,16	2,5,6,7,9,1<br>0,13,15,17	2	Q6IG02,Q6P6Q2
6138	1,3,4,8,11,<br>12,14,17	2,5,6,7,9,1<br>0,13,15,16	4	B0BNN3,P31430,P97840,Q6IG02
6139	1,3,4,8,11,<br>12,15,16	2,5,6,7,9,1<br>0,13,14,17	2	Q6IG02,Q9QUL6
6140	1,3,4,8,11,<br>12,15,17	2,5,6,7,9,1<br>0,13,14,16	3	B0BNN3,P97840,Q6IG02
6141	1,3,4,8,11,<br>12,16,17	2,5,6,7,9,1<br>0,13,14,15	2	P16636,Q6IG02
6142	1,3,4,8,11,<br>13,14,15	2,5,6,7,9,1<br>0,12,16,17	2	P97840,Q64093
6143	1,3,4,8,11,<br>13,14,16	2,5,6,7,9,1<br>0,12,15,17	0	
6144	1,3,4,8,11,<br>13,14,17	2,5,6,7,9,1<br>0,12,15,16	4	P83748,P97840,Q64093,Q9QW07
6145	1,3,4,8,11,<br>13,15,16	2,5,6,7,9,1<br>0,12,14,17	2	B4F795,Q9QUL6
6146	1,3,4,8,11,<br>13,15,17	2,5,6,7,9,1<br>0,12,14,16	2	B0BNN3,P97840
6147	1,3,4,8,11,<br>13,16,17	2,5,6,7,9,1<br>0,12,14,15	3	P16636,P83748,P97840
6148	1,3,4,8,11,<br>14,15,16	2,5,6,7,9,1<br>0,12,13,17	3	B4F795,O54728,P97840
6149	1,3,4,8,11,<br>14,15,17	2,5,6,7,9,1<br>0,12,13,16	4	B0BNN3,P19218,P83748,P97840
6150	1,3,4,8,11,<br>14,16,17	2,5,6,7,9,1<br>0,12,13,15	2	P83748,P97840
6151	1,3,4,8,11,<br>15,16,17	2,5,6,7,9,1<br>0,12,13,14	3	B0BNN3,B4F795,P97840
6152	1,3,4,8,12,<br>13,14,15	2,5,6,7,9,1<br>0,11,16,17	6	A2RUV9,B0BNN3,P10758,P36970,Q64093,Q9QUL6
6153	1,3,4,8,12,<br>13,14,16	2,5,6,7,9,1<br>0,11,15,17	2	Q64093,Q9QUL6
6154	1,3,4,8,12,<br>13,14,17	2,5,6,7,9,1<br>0,11,15,16	6	P31430,P36970,Q64093,Q80W57,Q9QUL6,Q9QW07
6155	1,3,4,8,12,<br>13,15,16	2,5,6,7,9,1<br>0,11,14,17	1	Q9QUL6
6156	1,3,4,8,12,<br>13,15,17	2,5,6,7,9,1<br>0,11,14,16	3	B0BNN3,Q99MH3,Q9QUL6
6157	1,3,4,8,12,<br>13,16,17	2,5,6,7,9,1<br>0,11,14,15	3	P16636,P27213,Q9QUL6
6158	1,3,4,8,12,<br>14,15,16	2,5,6,7,9,1<br>0,11,13,17	2	B0BNN3,Q9QUL6
6159	1,3,4,8,12,<br>14,15,17	2,5,6,7,9,1<br>0,11,13,16	4	A2RUV9,B0BNN3,P31430,Q9QUL6

6160	1,3,4,8,12, 14,16,17	2,5,6,7,9,1 0,11,13,15	2	P31430,Q9QUL6
6161	1,3,4,8,12, 15,16,17	2,5,6,7,9,1 0,11,13,14	4	B0BNN3,Q5XFX0,Q62894,Q9QUL6
6162	1,3,4,8,13, 14,15,16	2,5,6,7,9,1 0,11,12,17	2	Q64093,Q9QUL6
6163	1,3,4,8,13, 14,15,17	2,5,6,7,9,1 0,11,12,16	6	B0BNN3,P05712,P97840,Q64093,Q9QUL6,Q9QW07
6164	1,3,4,8,13, 14,16,17	2,5,6,7,9,1 0,11,12,15	5	P27213,Q64093,Q6PCU2,Q9QUL6,Q9QW07
6165	1,3,4,8,13, 15,16,17	2,5,6,7,9,1 0,11,12,14	2	P27213,Q9QUL6
6166	1,3,4,8,14, 15,16,17	2,5,6,7,9,1 0,11,12,13	2	B0BNN3,P97840
6167	1,3,4,9,10, 11,12,13	2,5,6,7,8,1 4,15,16,17	3	P10758,P36374,Q6IG02
6168	1,3,4,9,10, 11,12,14	2,5,6,7,8,1 3,15,16,17	2	P10758,Q6IG02
6169	1,3,4,9,10, 11,12,15	2,5,6,7,8,1 3,14,16,17	4	P10758,P51907,Q561R9,Q6IG02
6170	1,3,4,9,10, 11,12,16	2,5,6,7,8,1 3,14,15,17	6	P06757,P10536,P53792,Q6IG02,Q6P6Q2,Q9WUW8
6171	1,3,4,9,10, 11,12,17	2,5,6,7,8,1 3,14,15,16	3	P06757,P16636,Q6IG02
6172	1,3,4,9,10, 11,13,14	2,5,6,7,8,1 2,15,16,17	4	P12785,P50115,P83748,Q62635
6173	1,3,4,9,10, 11,13,15	2,5,6,7,8,1 2,14,16,17	5	O54728,P12785,P50115,Q561R9,Q62635
6174	1,3,4,9,10, 11,13,16	2,5,6,7,8,1 2,14,15,17	4	P12785,P50115,P83748,Q62635
6175	1,3,4,9,10, 11,13,17	2,5,6,7,8,1 2,14,15,16	2	P12785,P83748
6176	1,3,4,9,10, 11,14,15	2,5,6,7,8,1 2,13,16,17	6	O54728,P50115,P55091,P83748,Q561R9,Q62635
6177	1,3,4,9,10, 11,14,16	2,5,6,7,8,1 2,13,15,17	6	P12785,P50115,P55091,P83748,Q62635,Q9QZK9
6178	1,3,4,9,10, 11,14,17	2,5,6,7,8,1 2,13,15,16	2	P12785,P83748
6179	1,3,4,9,10, 11,15,16	2,5,6,7,8,1 2,13,14,17	8	O54728,P06760,P50115,P55091,Q561R9,Q62635,Q9QZK9,Q9Z1F2
6180	1,3,4,9,10, 11,15,17	2,5,6,7,8,1 2,13,14,16	2	P83748,Q9Z1F2
6181	1,3,4,9,10, 11,16,17	2,5,6,7,8,1 2,13,14,15	3	P12785,P83748,Q9Z1F2
6182	1,3,4,9,10, 12,13,14	2,5,6,7,8,1 1,15,16,17	3	A2RUV9,P10758,P50115

6183	1,3,4,9,10,<br>12,13,15	2,5,6,7,8,1<br>1,14,16,17	2	P10758,P50115
6184	1,3,4,9,10,<br>12,13,16	2,5,6,7,8,1<br>1,14,15,17	1	P50115
6185	1,3,4,9,10,<br>12,13,17	2,5,6,7,8,1<br>1,14,15,16	1	P16636
6186	1,3,4,9,10,<br>12,14,15	2,5,6,7,8,1<br>1,13,16,17	3	A2RUV9,P10758,P50115
6187	1,3,4,9,10,<br>12,14,16	2,5,6,7,8,1<br>1,13,15,17	2	P50115,Q9WUW8
6188	1,3,4,9,10,<br>12,14,17	2,5,6,7,8,1<br>1,13,15,16	0	
6189	1,3,4,9,10,<br>12,15,16	2,5,6,7,8,1<br>1,13,14,17	2	O54728,P50115
6190	1,3,4,9,10,<br>12,15,17	2,5,6,7,8,1<br>1,13,14,16	1	B0BNN3
6191	1,3,4,9,10,<br>12,16,17	2,5,6,7,8,1<br>1,13,14,15	1	P16636
6192	1,3,4,9,10,<br>13,14,15	2,5,6,7,8,1<br>1,12,16,17	4	O54728,P06760,P50115,Q62635
6193	1,3,4,9,10,<br>13,14,16	2,5,6,7,8,1<br>1,12,15,17	4	P06760,P50115,P62804,Q62635
6194	1,3,4,9,10,<br>13,14,17	2,5,6,7,8,1<br>1,12,15,16	2	P83748,Q9QW07
6195	1,3,4,9,10,<br>13,15,16	2,5,6,7,8,1<br>1,12,14,17	7	O54728,P06760,P50115,P55091,P62804,Q00715,Q62635
6196	1,3,4,9,10,<br>13,15,17	2,5,6,7,8,1<br>1,12,14,16	0	
6197	1,3,4,9,10,<br>13,16,17	2,5,6,7,8,1<br>1,12,14,15	1	P62804
6198	1,3,4,9,10,<br>14,15,16	2,5,6,7,8,1<br>1,12,13,17	7	O54728,P06760,P50115,P55091,Q62635,Q9JHB9,Q9QZK9
6199	1,3,4,9,10,<br>14,15,17	2,5,6,7,8,1<br>1,12,13,16	0	
6200	1,3,4,9,10,<br>14,16,17	2,5,6,7,8,1<br>1,12,13,15	1	Q9JHB9
6201	1,3,4,9,10,<br>15,16,17	2,5,6,7,8,1<br>1,12,13,14	7	O54728,P02782,P47727,P62804,Q62902,Q9JHB9,Q9Z1F2
6202	1,3,4,9,11,<br>12,13,14	2,5,6,7,8,1<br>0,15,16,17	1	P10758
6203	1,3,4,9,11,<br>12,13,15	2,5,6,7,8,1<br>0,14,16,17	4	O54728,P02625,P06760,P10758
6204	1,3,4,9,11,<br>12,13,16	2,5,6,7,8,1<br>0,14,15,17	1	P06760
6205	1,3,4,9,11,<br>12,13,17	2,5,6,7,8,1<br>0,14,15,16	2	P16636,P36374

6206	1,3,4,9,11,12,14,15	2,5,6,7,8,10,13,16,17	3	O54728,P06760,P10758
6207	1,3,4,9,11,12,14,16	2,5,6,7,8,10,13,15,17	1	P06760
6208	1,3,4,9,11,12,14,17	2,5,6,7,8,10,13,15,16	1	P01835
6209	1,3,4,9,11,12,15,16	2,5,6,7,8,10,13,14,17	4	O54728,P06760,P12020,P51907
6210	1,3,4,9,11,12,15,17	2,5,6,7,8,10,13,14,16	2	B0BNN3,P12020
6211	1,3,4,9,11,12,16,17	2,5,6,7,8,10,13,14,15	2	P12020,P16636
6212	1,3,4,9,11,13,14,15	2,5,6,7,8,10,12,16,17	6	O54728,P06760,P50115,P83748,Q62635,Q6P6R2
6213	1,3,4,9,11,13,14,16	2,5,6,7,8,10,12,15,17	4	P06760,P50115,P83748,Q62635
6214	1,3,4,9,11,13,14,17	2,5,6,7,8,10,12,15,16	3	P83748,P97840,Q9QW07
6215	1,3,4,9,11,13,15,16	2,5,6,7,8,10,12,14,17	6	O54728,P06760,P50115,P62804,Q561R9,Q62635
6216	1,3,4,9,11,13,15,17	2,5,6,7,8,10,12,14,16	3	O54728,P83748,P97840
6217	1,3,4,9,11,13,16,17	2,5,6,7,8,10,12,14,15	1	P83748
6218	1,3,4,9,11,14,15,16	2,5,6,7,8,10,12,13,17	6	O54728,P06760,P50115,P55091,Q62635,Q9QZK9
6219	1,3,4,9,11,14,15,17	2,5,6,7,8,10,12,13,16	3	O54728,P83748,P97840
6220	1,3,4,9,11,14,16,17	2,5,6,7,8,10,12,13,15	1	P83748
6221	1,3,4,9,11,15,16,17	2,5,6,7,8,10,12,13,14	4	O54728,P12020,P83748,Q9Z1F2
6222	1,3,4,9,12,13,14,15	2,5,6,7,8,10,11,16,17	6	A2RUV9,P06760,P10758,P50115,P82471,Q9QUL6
6223	1,3,4,9,12,13,14,16	2,5,6,7,8,10,11,15,17	3	P06760,P50115,Q9QUL6
6224	1,3,4,9,12,13,14,17	2,5,6,7,8,10,11,15,16	2	P82471,Q9QW07
6225	1,3,4,9,12,13,15,16	2,5,6,7,8,10,11,14,17	5	O54728,P06760,P50115,P62804,Q9QUL6
6226	1,3,4,9,12,13,15,17	2,5,6,7,8,10,11,14,16	1	P82471
6227	1,3,4,9,12,13,16,17	2,5,6,7,8,10,11,14,15	2	P07171,P27213
6228	1,3,4,9,12,14,15,16	2,5,6,7,8,10,11,13,17	3	O54728,P06760,P50115

6229	1,3,4,9,12,<br>14,15,17	2,5,6,7,8,1<br>0,11,13,16	2	A2RUV9,B0BNN3
6230	1,3,4,9,12,<br>14,16,17	2,5,6,7,8,1<br>0,11,13,15	0	
6231	1,3,4,9,12,<br>15,16,17	2,5,6,7,8,1<br>0,11,13,14	3	P06866,P12020,P47727
6232	1,3,4,9,13,<br>14,15,16	2,5,6,7,8,1<br>0,11,12,17	8	O54728,P06760,P50115,P60905,P62804,Q00715,Q62635,Q9QZK9
6233	1,3,4,9,13,<br>14,15,17	2,5,6,7,8,1<br>0,11,12,16	6	O54728,P48199,P60905,P62804,P82471,Q9QW07
6234	1,3,4,9,13,<br>14,16,17	2,5,6,7,8,1<br>0,11,12,15	3	P27213,P62804,Q9QW07
6235	1,3,4,9,13,<br>15,16,17	2,5,6,7,8,1<br>0,11,12,14	6	O54728,P47727,P60905,P62804,P82471,Q00715
6236	1,3,4,9,14,<br>15,16,17	2,5,6,7,8,1<br>0,11,12,13	4	O54728,P60905,P62804,Q9JHB9
6237	1,3,4,10,1<br>1,12,13,14	2,5,6,7,8,9<br>,15,16,17	1	P50115
6238	1,3,4,10,1<br>1,12,13,15	2,5,6,7,8,9<br>,14,16,17	2	P10758,P50115
6239	1,3,4,10,1<br>1,12,13,16	2,5,6,7,8,9<br>,14,15,17	2	P50115,P53792
6240	1,3,4,10,1<br>1,12,13,17	2,5,6,7,8,9<br>,14,15,16	4	P06757,P16636,P36374,Q99MH3
6241	1,3,4,10,1<br>1,12,14,15	2,5,6,7,8,9<br>,13,16,17	4	P10758,P50115,P51907,Q6IG02
6242	1,3,4,10,1<br>1,12,14,16	2,5,6,7,8,9<br>,13,15,17	4	P50115,P53792,Q6IG02,Q6P6Q2
6243	1,3,4,10,1<br>1,12,14,17	2,5,6,7,8,9<br>,13,15,16	1	Q6IG02
6244	1,3,4,10,1<br>1,12,15,16	2,5,6,7,8,9<br>,13,14,17	5	P50115,P51907,P53792,Q4FZU2,Q9Z1F2
6245	1,3,4,10,1<br>1,12,15,17	2,5,6,7,8,9<br>,13,14,16	3	B0BNN3,P06866,Q6IG02
6246	1,3,4,10,1<br>1,12,16,17	2,5,6,7,8,9<br>,13,14,15	5	P06757,P06866,P16636,P53792,Q6IG02
6247	1,3,4,10,1<br>1,13,14,15	2,5,6,7,8,9<br>,12,16,17	1	P50115
6248	1,3,4,10,1<br>1,13,14,16	2,5,6,7,8,9<br>,12,15,17	3	P50115,P83748,Q62635
6249	1,3,4,10,1<br>1,13,14,17	2,5,6,7,8,9<br>,12,15,16	2	P83748,Q9QW07
6250	1,3,4,10,1<br>1,13,15,16	2,5,6,7,8,9<br>,12,14,17	3	P50115,Q561R9,Q62635
6251	1,3,4,10,1<br>1,13,15,17	2,5,6,7,8,9<br>,12,14,16	1	P83748

6252	1,3,4,10,1 2,5,6,7,8,9 1,13,16,17 ,12,14,15	2	P12785,P83748
6253	1,3,4,10,1 2,5,6,7,8,9 1,14,15,16 ,12,13,17	5	O54728,P50115,P55091,Q62635,Q9Z1F2
6254	1,3,4,10,1 2,5,6,7,8,9 1,14,15,17 ,12,13,16	1	P83748
6255	1,3,4,10,1 2,5,6,7,8,9 1,14,16,17 ,12,13,15	1	P83748
6256	1,3,4,10,1 2,5,6,7,8,9 1,15,16,17 ,12,13,14	2	P83748,Q9Z1F2
6257	1,3,4,10,1 2,5,6,7,8,9 2,13,14,15 ,11,16,17	4	A2RUV9,P10758,P50115,Q9QUL6
6258	1,3,4,10,1 2,5,6,7,8,9 2,13,14,16 ,11,15,17	2	P50115,Q9QUL6
6259	1,3,4,10,1 2,5,6,7,8,9 2,13,14,17 ,11,15,16	2	A2RUV9,Q9QW07
6260	1,3,4,10,1 2,5,6,7,8,9 2,13,15,16 ,11,14,17	2	P50115,Q9QUL6
6261	1,3,4,10,1 2,5,6,7,8,9 2,13,15,17 ,11,14,16	3	B0BNN3,Q99MH3,Q9QUL6
6262	1,3,4,10,1 2,5,6,7,8,9 2,13,16,17 ,11,14,15	4	P16636,P27213,Q99MH3,Q9QUL6
6263	1,3,4,10,1 2,5,6,7,8,9 2,14,15,16 ,11,13,17	3	P08721,P50115,Q9QUL6
6264	1,3,4,10,1 2,5,6,7,8,9 2,14,15,17 ,11,13,16	2	A2RUV9,B0BNN3
6265	1,3,4,10,1 2,5,6,7,8,9 2,14,16,17 ,11,13,15	0	
6266	1,3,4,10,1 2,5,6,7,8,9 2,15,16,17 ,11,13,14	2	B0BNN3,P06866
6267	1,3,4,10,1 2,5,6,7,8,9 3,14,15,16 ,11,12,17	5	P06760,P50115,P62804,Q62635,Q9QUL6
6268	1,3,4,10,1 2,5,6,7,8,9 3,14,15,17 ,11,12,16	1	Q9QW07
6269	1,3,4,10,1 2,5,6,7,8,9 3,14,16,17 ,11,12,15	2	P27213,Q9QW07
6270	1,3,4,10,1 2,5,6,7,8,9 3,15,16,17 ,11,12,14	2	P27213,P62804
6271	1,3,4,10,1 2,5,6,7,8,9 4,15,16,17 ,11,12,13	1	Q9JHB9
6272	1,3,4,11,1 2,5,6,7,8,9 2,13,14,15 ,10,16,17	7	A2RUV9,P02625,P10758,P50115,Q64093,Q6P6R2,Q9QUL6
6273	1,3,4,11,1 2,5,6,7,8,9 2,13,14,16 ,10,15,17	2	P50115,Q9QUL6
6274	1,3,4,11,1 2,5,6,7,8,9 2,13,14,17 ,10,15,16	2	P36374,Q9QW07

6275	1,3,4,11,12,13,15,16	2,5,6,7,8,9,10,14,17	4	P01836,P06760,P50115,Q9QUL6
6276	1,3,4,11,12,13,15,17	2,5,6,7,8,9,10,14,16	1	B0BNN3
6277	1,3,4,11,12,13,16,17	2,5,6,7,8,9,10,14,15	2	P16636,P27213
6278	1,3,4,11,12,14,15,16	2,5,6,7,8,9,10,13,17	3	P06760,P50115,P51907
6279	1,3,4,11,12,14,15,17	2,5,6,7,8,9,10,13,16	1	B0BNN3
6280	1,3,4,11,12,14,16,17	2,5,6,7,8,9,10,13,15	0	
6281	1,3,4,11,12,15,16,17	2,5,6,7,8,9,10,13,14	3	B0BNN3,P06866,P12020
6282	1,3,4,11,13,14,15,16	2,5,6,7,8,9,10,12,17	5	O54728,P06760,P50115,Q62635,Q6P6R2
6283	1,3,4,11,13,14,15,17	2,5,6,7,8,9,10,12,16	4	P83748,P97840,Q64093,Q9QW07
6284	1,3,4,11,13,14,16,17	2,5,6,7,8,9,10,12,15	3	P27213,P83748,Q9QW07
6285	1,3,4,11,13,15,16,17	2,5,6,7,8,9,10,12,14	0	
6286	1,3,4,11,14,15,16,17	2,5,6,7,8,9,10,12,13	2	P83748,P97840
6287	1,3,4,12,13,14,15,16	2,5,6,7,8,9,10,11,17	4	A2RUV9,P06760,P50115,Q9QUL6
6288	1,3,4,12,13,14,15,17	2,5,6,7,8,9,10,11,16	7	A2RUV9,B0BNN3,P27213,P82471,Q64093,Q9QUL6,Q9QW07
6289	1,3,4,12,13,14,16,17	2,5,6,7,8,9,10,11,15	3	P27213,Q9QUL6,Q9QW07
6290	1,3,4,12,13,15,16,17	2,5,6,7,8,9,10,11,14	2	P27213,Q9QUL6
6291	1,3,4,12,14,15,16,17	2,5,6,7,8,9,10,11,13	2	B0BNN3,Q9QUL6
6292	1,3,4,13,14,15,16,17	2,5,6,7,8,9,10,11,12	6	P27213,P48199,P60905,P62804,Q9QUL6,Q9QW07
6293	1,3,5,6,7,8,9,10	2,4,11,12,13,14,15,16,17	7	D3ZUC6,O55006,P06866,P08937,P27213,P31044,Q6IRE4
6294	1,3,5,6,7,8,9,11	2,4,10,12,13,14,15,16,17	12	P01048,P04041,P06866,P08932,P08937,P10715,P14669,P19629,P27213,P31044,Q6IRE4,Q9QUL6
6295	1,3,5,6,7,8,9,12	2,4,10,11,13,14,15,16,17	12	O55006,P08723,P08937,P10715,P13432,P19629,P27213,P30120,Q6IG02,Q6IRE4,Q8CJ52,Q9Z1F2

6296	1,3,5,6,7,8,9,13	2,4,10,11,12,14,15,16,17	8	P01048,P06866,P08937,P31044,P51907,P53792,Q6IRE4,Q9Z1F2
6297	1,3,5,6,7,8,9,14	2,4,10,11,12,13,15,16,17	8	P06866,P08937,P19629,P27213,P51907,P53792,Q6IRE4,Q9Z1F2
6298	1,3,5,6,7,8,9,15	2,4,10,11,12,13,14,16,17	7	O54728,O55006,P08937,P19629,P27213,P53792,Q6IRE4
6299	1,3,5,6,7,8,9,16	2,4,10,11,12,13,14,15,17	4	P06866,P08937,P23593,Q6IRE4
6300	1,3,5,6,7,8,9,17	2,4,10,11,12,13,14,15,16	12	P01946,P02091,P06760,P08937,P19629,P23593,P50115,P51907,P52590,P53792,Q63751,Q6IRE4
6301	1,3,5,6,7,8,10,11	2,4,9,12,13,14,15,16,17	14	D3ZUC6,P04041,P07150,P08649,P08937,P14669,P27213,P31044,P55314,P61206;P84079;P84082,P68370,Q63493,Q6IRE4,Q8VD89
6302	1,3,5,6,7,8,10,12	2,4,9,11,13,14,15,16,17	17	F1LM93,P08723,P08937,P10715,P19629,P22283,P27213,P30120,P31044,Q10758,Q6IFW6,Q6IG02,Q6IMF3,Q6P6Q2,Q8CJ52,Q99MH3,Q9ET32
6303	1,3,5,6,7,8,10,13	2,4,9,11,12,14,15,16,17	10	D3ZUC6,P06866,P06911,P08937,P12020,P31044,P51907,Q5GRG2,Q6IRE4,Q99MH3
6304	1,3,5,6,7,8,10,14	2,4,9,11,12,13,15,16,17	7	P06866,P08937,P12020,P19629,P27213,P31044,Q6IRE4
6305	1,3,5,6,7,8,10,15	2,4,9,11,12,13,14,16,17	6	O55006,P08937,P19629,P27213,P31044,Q99MH3
6306	1,3,5,6,7,8,10,16	2,4,9,11,12,13,14,15,17	5	P08721,P08937,P31044,Q6IRE4,Q8VD89
6307	1,3,5,6,7,8,10,17	2,4,9,11,12,13,14,15,16	12	D3ZUC6,P01946,P02091,P06760,P08937,P12346,P31044,P50115,P51907,P52590,Q63751,Q99MH3
6308	1,3,5,6,7,8,11,12	2,4,9,10,13,14,15,16,17	30	F1LM93,O35547,P01681,P02781,P02782,P04041,P08723,P08937,P10715,P13432,P14669,P17046,P19629,P22282,P22283,P24388,P27213,P30120,Q4FZU2,Q63493,Q63751,Q6IFW6,Q6IG02,Q6IMF3,Q6IRE4,Q6P6Q2,Q8CJ52,Q9ET32,Q9JHB9,Q9JI85
6309	1,3,5,6,7,8,11,13	2,4,9,10,12,14,15,16,17	17	P01048,P06866,P06911,P08932,P08937,P14669,P16086,P19629,P22282,P22283,P31044,Q62902,Q63493,Q63751,Q6IRE4,Q8VD89,Q9Z1F2
6310	1,3,5,6,7,8,11,14	2,4,9,10,12,13,15,16,17	10	P06866,P08937,P14669,P16086,P19629,P27213,P31044,Q5I0D1,Q63493,Q6IRE4

6311	1,3,5,6,7,8,11,15	2,4,9,10,12,13,14,16,17	9	P08937,P10715,P14669,P19629,P27213,Q5I0D1,Q63751,Q6IRE4,Q8VD89
6312	1,3,5,6,7,8,11,16	2,4,9,10,12,13,14,15,17	7	P08937,P10715,P17046,P19629,P55314,Q6IRE4,Q8VD89
6313	1,3,5,6,7,8,11,17	2,4,9,10,12,13,14,15,16	14	P01681,P01946,P02091,P04041,P06760,P07150,P08937,P10715,P14669,P19629,P50115,Q63493,Q63751,Q6IRE4
6314	1,3,5,6,7,8,12,13	2,4,9,10,11,14,15,16,17	23	O88267,P02781,P02782,P06761,P06911,P08723,P08937,P10715,P13432,P19218,P19629,P22282,P22283,P30120,P51907,Q62902,Q63751,Q6IG02,Q6IRE4,Q8CJ52,Q99MH3,Q9JHB9,Q9Z1F2
6315	1,3,5,6,7,8,12,14	2,4,9,10,11,13,15,16,17	17	O88267,P02782,P06761,P08723,P08937,P10715,P13432,P19629,P22283,P30120,Q561R9,Q6AYS7,Q6IG02,Q6IRE4,Q8CJ52,Q9ET32,Q9Z1F2
6316	1,3,5,6,7,8,12,15	2,4,9,10,11,13,14,16,17	7	P08937,P10715,P19629,Q63751,Q6IG02,Q6IRE4,Q99MH3
6317	1,3,5,6,7,8,12,16	2,4,9,10,11,13,14,15,17	10	O88267,P08937,P10715,P13432,P17046,P19629,Q6IG02,Q6IRE4,Q8CJ52,Q9ET32
6318	1,3,5,6,7,8,12,17	2,4,9,10,11,13,14,15,16	25	F1LM93,O88267,P01681,P01946,P02091,P06760,P08723,P08937,P10715,P13432,P19629,P22282,P22283,P23928,P50115,P51907,Q4G075,Q561R9,Q63751,Q6IG02,Q8CJ52,Q99041,Q99MH3,Q9QZK9,Q9Z1F2
6319	1,3,5,6,7,8,13,14	2,4,9,10,11,12,15,16,17	13	P06866,P06911,P08937,P12020,P16086,P19629,P31044,P51907,P53792,Q498D9,Q5GRG2,Q6IRE4,Q9Z1F2
6320	1,3,5,6,7,8,13,15	2,4,9,10,11,12,14,16,17	8	P06866,P08937,P19629,P51907,P53792,Q5GRG2,Q6IRE4,Q99MH3
6321	1,3,5,6,7,8,13,16	2,4,9,10,11,12,14,15,17	6	P06866,P08937,P17046,P51907,Q6IRE4,Q8VD89
6322	1,3,5,6,7,8,13,17	2,4,9,10,11,12,14,15,16	18	O88267,P01946,P02091,P06760,P08937,P10536,P19629,P22282,P22283,P23928,P50115,P51907,P53792,Q561R9,Q63751,Q99MH3,Q9WUW8,Q9Z1F2
6323	1,3,5,6,7,8,14,15	2,4,9,10,11,12,13,16,17	6	P02803,P08937,P10536,P19629,P53792,Q6IRE4
6324	1,3,5,6,7,8,14,16	2,4,9,10,11,12,13,15,17	5	P06866,P08937,P19629,Q561R9,Q6IRE4
6325	1,3,5,6,7,8,14,17	2,4,9,10,11,12,13,15,16	12	O88267,P01946,P02091,P06760,P08937,P10536,P19629,P50115,P51907,P53792,Q561R9,Q63751

6326	1,3,5,6,7,8,15,16	2,4,9,10,11,12,13,14,17	4	P08937,P19629,Q6IRE4,Q8VD89
6327	1,3,5,6,7,8,15,17	2,4,9,10,11,12,13,14,16	11	P01946,P02091,P02803,P06760,P08937,P10536,P19629,P50115,P53792,Q63751,Q99MH3
6328	1,3,5,6,7,8,16,17	2,4,9,10,11,12,13,14,15	15	O88267,P01946,P02091,P06760,P08937,P10758,P17046,P19629,P23593,P23928,P50115,P51907,Q561R9,Q63751,Q6IRE4
6329	1,3,5,6,7,9,10,11	2,4,8,12,13,14,15,16,17	4	P27213,P55314,Q6IRE4,Q9QUL6
6330	1,3,5,6,7,9,10,12	2,4,8,11,13,14,15,16,17	5	P19218,P27213,P97840,Q6IG02,Q6IRE4
6331	1,3,5,6,7,9,10,13	2,4,8,11,12,14,15,16,17	4	P06866,P19218,Q5U2V4,Q6IRE4
6332	1,3,5,6,7,9,10,14	2,4,8,11,12,13,15,16,17	4	P06866,P27213,Q6IRE4,Q9QUL6
6333	1,3,5,6,7,9,10,15	2,4,8,11,12,13,14,16,17	5	O54728,O55006,P27213,Q6IRE4,Q80WL1
6334	1,3,5,6,7,9,10,16	2,4,8,11,12,13,14,15,17	3	P27213,Q00715,Q6IRE4
6335	1,3,5,6,7,9,10,17	2,4,8,11,12,13,14,15,16	6	P01946,P02091,P52590,Q6IRE4,Q99MH3,Q9QUL6
6336	1,3,5,6,7,9,11,12	2,4,8,10,13,14,15,16,17	9	P08937,P17046,P27213,Q4FZU2,Q6IFW6,Q6IG02,Q6IMF3,Q6IRE4,Q6P6Q2
6337	1,3,5,6,7,9,11,13	2,4,8,10,12,14,15,16,17	4	P01048,P06866,P17046,Q6IRE4
6338	1,3,5,6,7,9,11,14	2,4,8,10,12,13,15,16,17	4	P06866,P27213,Q6IRE4,Q9QUL6
6339	1,3,5,6,7,9,11,15	2,4,8,10,12,13,14,16,17	5	O54728,P27213,Q6IRE4,Q80WL1,Q9QUL6
6340	1,3,5,6,7,9,11,16	2,4,8,10,12,13,14,15,17	5	P17046,P27213,P55314,Q6IRE4,Q9QUL6

6341	1,3,5,6,7,9,11,17	2,4,8,10,12,13,14,15,16	6	P01946,P02091,P08937,P52590,Q6IRE4,Q9QUL6
6342	1,3,5,6,7,9,12,13	2,4,8,10,11,14,15,16,17	6	P08937,P17046,P19218,P53792,Q6IRE4,Q9Z1F2
6343	1,3,5,6,7,9,12,14	2,4,8,10,11,13,15,16,17	2	P01835,Q6IRE4
6344	1,3,5,6,7,9,12,15	2,4,8,10,11,13,14,16,17	2	P27213,Q6IRE4
6345	1,3,5,6,7,9,12,16	2,4,8,10,11,13,14,15,17	3	P17046,P19218,Q6IRE4
6346	1,3,5,6,7,9,12,17	2,4,8,10,11,13,14,15,16	10	P01835,P01946,P02091,P08937,P13432,P52590,P53792,Q561R9,Q6IRE4,Q99MH3
6347	1,3,5,6,7,9,13,14	2,4,8,10,11,12,15,16,17	5	P06866,P06911,P51907,P53792,Q6IRE4
6348	1,3,5,6,7,9,13,15	2,4,8,10,11,12,14,16,17	6	O54728,P53792,P62804,Q5U2V4,Q6IRE4,Q80WL1
6349	1,3,5,6,7,9,13,16	2,4,8,10,11,12,14,15,17	7	P06866,P17046,P19218,P53792,P62804,Q00715,Q6IRE4
6350	1,3,5,6,7,9,13,17	2,4,8,10,11,12,14,15,16	9	P01946,P02091,P08937,P51907,P52590,P53792,Q561R9,Q6IRE4,Q99MH3
6351	1,3,5,6,7,9,14,15	2,4,8,10,11,12,13,16,17	5	O54728,P27213,P53792,Q6IRE4,Q80WL1
6352	1,3,5,6,7,9,14,16	2,4,8,10,11,12,13,15,17	3	P53792,Q6IRE4,Q80WL1
6353	1,3,5,6,7,9,14,17	2,4,8,10,11,12,13,15,16	7	P01946,P02091,P51907,P52590,P53792,Q561R9,Q6IRE4
6354	1,3,5,6,7,9,15,16	2,4,8,10,11,12,13,14,17	5	O54728,P62804,Q00715,Q6IRE4,Q80WL1
6355	1,3,5,6,7,9,15,17	2,4,8,10,11,12,13,14,16	7	O54728,P02091,P23593,P52590,P53792,Q6IRE4,Q80WL1

6356	1,3,5,6,7,9,16,17	2,4,8,10,11,12,13,14,15	9	P01946,P02091,P17046,P23593,P52590,P53792,P62804,Q561R9,Q6IRE4
6357	1,3,5,6,7,10,11,12	2,4,8,9,13,14,15,16,17	14	F1LM93,P08937,P17046,P27213,P55314,Q10758,Q4FZU2,Q6IFU8,Q6IFW6,Q6IG02,Q6IMF3,Q6IRE4,Q6P6Q2,Q99MH3
6358	1,3,5,6,7,10,11,13	2,4,8,9,12,14,15,16,17	5	P08937,P17046,P55314,Q6IRE4,Q8VD89
6359	1,3,5,6,7,10,11,14	2,4,8,9,12,13,15,16,17	4	P27213,P55314,Q6IRE4,Q9QUL6
6360	1,3,5,6,7,10,11,15	2,4,8,9,12,13,14,16,17	5	P27213,P55314,Q6IRE4,Q80WL1,Q8VD89
6361	1,3,5,6,7,10,11,16	2,4,8,9,12,13,14,15,17	6	P17046,P27213,P55314,P61206;P84079;P84082,Q6IRE4,Q8VD89
6362	1,3,5,6,7,10,11,17	2,4,8,9,12,13,14,15,16	7	P01946,P02091,P08937,P55314,Q6IRE4,Q99MH3,Q9QUL6
6363	1,3,5,6,7,10,12,13	2,4,8,9,11,14,15,16,17	7	P08937,P17046,P19218,Q498D9,Q6IG02,Q6IRE4,Q99MH3
6364	1,3,5,6,7,10,12,14	2,4,8,9,11,13,15,16,17	7	P08937,Q10758,Q498D9,Q6IG02,Q6IMF3,Q6IRE4,Q9ET32
6365	1,3,5,6,7,10,12,15	2,4,8,9,11,13,14,16,17	5	P08937,P20766,Q4FZU2,Q6IG02,Q99MH3
6366	1,3,5,6,7,10,12,16	2,4,8,9,11,13,14,15,17	10	P17046,P19218,P20766,P97840,Q10758,Q6IFW6,Q6IG02,Q6IMF3,Q6IRE4,Q99MH3
6367	1,3,5,6,7,10,12,17	2,4,8,9,11,13,14,15,16	10	F1LM93,P01946,P02091,P08937,P13432,Q10758,Q561R9,Q6IG02,Q99MH3,Q9QZK9
6368	1,3,5,6,7,10,13,14	2,4,8,9,11,12,15,16,17	5	P06866,P06911,P12020,Q498D9,Q6IRE4
6369	1,3,5,6,7,10,13,15	2,4,8,9,11,12,14,16,17	3	Q6IRE4,Q80WL1,Q99MH3
6370	1,3,5,6,7,10,13,16	2,4,8,9,11,12,14,15,17	5	P17046,P19218,Q6AYQ8,Q6IRE4,Q8VD89

6371	1,3,5,6,7,10,13,17	2,4,8,9,11,12,14,15,16	6	P01946,P02091,P08937,P51907,Q561R9,Q99MH3
6372	1,3,5,6,7,10,14,15	2,4,8,9,11,12,13,16,17	3	P02803,Q6IRE4,Q80WL1
6373	1,3,5,6,7,10,14,16	2,4,8,9,11,12,13,15,17	2	Q6IRE4,Q80WL1
6374	1,3,5,6,7,10,14,17	2,4,8,9,11,12,13,15,16	6	P01946,P02091,P08689,P08937,P18427,Q561R9
6375	1,3,5,6,7,10,15,16	2,4,8,9,11,12,13,14,17	4	P02803,P20766,Q6IRE4,Q80WL1
6376	1,3,5,6,7,10,15,17	2,4,8,9,11,12,13,14,16	5	P02091,P02803,P08937,Q80WL1,Q99MH3
6377	1,3,5,6,7,10,16,17	2,4,8,9,11,12,13,14,15	7	P01946,P02091,P10758,P17046,Q561R9,Q6IRE4,Q99MH3
6378	1,3,5,6,7,11,12,13	2,4,8,9,10,14,15,16,17	9	P08937,P17046,P30120,Q4FZU2,Q6IFW6,Q6IG02,Q6IRE4,Q6P6Q2,Q9JHB9
6379	1,3,5,6,7,11,12,14	2,4,8,9,10,13,15,16,17	8	P08937,P17046,Q4FZU2,Q6IFW6,Q6IG02,Q6IMF3,Q6IRE4,Q6P6Q2
6380	1,3,5,6,7,11,12,15	2,4,8,9,10,13,14,16,17	8	P08937,P17046,P27213,Q4FZU2,Q6IFW6,Q6IG02,Q6IRE4,Q6P6Q2
6381	1,3,5,6,7,11,12,16	2,4,8,9,10,13,14,15,17	9	P08937,P17046,P55314,Q4FZU2,Q6IFW6,Q6IG02,Q6IMF3,Q6IRE4,Q6P6Q2
6382	1,3,5,6,7,11,12,17	2,4,8,9,10,13,14,15,16	15	P01681,P01946,P02091,P08937,P13432,P17046,Q4FZU2,Q63751,Q6IFW6,Q6IG02,Q6IMF3,Q6IRE4,Q6P6Q2,Q99MH3,Q9QZK9
6383	1,3,5,6,7,11,13,14	2,4,8,9,10,12,15,16,17	4	P06866,P06911,P17046,Q6IRE4
6384	1,3,5,6,7,11,13,15	2,4,8,9,10,12,14,16,17	4	P17046,Q6IRE4,Q80WL1,Q8VD89
6385	1,3,5,6,7,11,13,16	2,4,8,9,10,12,14,15,17	4	P17046,P55314,Q6IRE4,Q8VD89

6386	1,3,5,6,7,11,13,17	2,4,8,9,10,12,14,15,16	7	P01946,P02091,P08937,P17046,Q63751,Q6IRE4,Q99MH3
6387	1,3,5,6,7,11,14,15	2,4,8,9,10,12,13,16,17	5	P02803,P27213,Q5I0D1,Q6IRE4,Q80WL1
6388	1,3,5,6,7,11,14,16	2,4,8,9,10,12,13,15,17	4	P17046,P55314,Q6IRE4,Q80WL1
6389	1,3,5,6,7,11,14,17	2,4,8,9,10,12,13,15,16	6	P01946,P02091,P08937,P17046,Q561R9,Q6IRE4
6390	1,3,5,6,7,11,15,16	2,4,8,9,10,12,13,14,17	7	O54728,P17046,P55314,Q5I0D1,Q6IRE4,Q80WL1,Q8VD89
6391	1,3,5,6,7,11,15,17	2,4,8,9,10,12,13,14,16	7	P01946,P02091,P08937,P17046,Q63751,Q6IRE4,Q80WL1
6392	1,3,5,6,7,11,16,17	2,4,8,9,10,12,13,14,15	7	P01946,P02091,P08937,P10758,P17046,P55314,Q6IRE4
6393	1,3,5,6,7,12,13,14	2,4,8,9,10,11,15,16,17	7	P06911,P08937,P17046,Q498D9,Q561R9,Q6IRE4,Q9Z1F2
6394	1,3,5,6,7,12,13,15	2,4,8,9,10,11,14,16,17	5	P08937,P17046,P19218,Q6IRE4,Q99MH3
6395	1,3,5,6,7,12,13,16	2,4,8,9,10,11,14,15,17	5	P08937,P17046,P19218,Q561R9,Q6IRE4
6396	1,3,5,6,7,12,13,17	2,4,8,9,10,11,14,15,16	12	P01946,P02091,P08937,P13432,P17046,P19218,P51907,Q561R9,Q63751,Q99MH3,Q9QZK9,Q9Z1F2
6397	1,3,5,6,7,12,14,15	2,4,8,9,10,11,13,16,17	3	A2RUV9,Q6IRE4,Q8CJD3
6398	1,3,5,6,7,12,14,16	2,4,8,9,10,11,13,15,17	5	P17046,P20766,Q561R9,Q6IRE4,Q9ET32
6399	1,3,5,6,7,12,14,17	2,4,8,9,10,11,13,15,16	7	P01681,P01946,P02091,P08937,P13432,P17046,Q561R9
6400	1,3,5,6,7,12,15,16	2,4,8,9,10,11,13,14,17	3	P17046,P20766,Q6IRE4

6401	1,3,5,6,7,12,15,17	2,4,8,9,10,11,13,14,16	8	P01946,P02091,P08937,P13432,P17046,Q561R9,Q63751,Q99MH3
6402	1,3,5,6,7,12,16,17	2,4,8,9,10,11,13,14,15	8	P01946,P02091,P08937,P13432,P17046,Q561R9,Q6IRE4,Q99MH3
6403	1,3,5,6,7,13,14,15	2,4,8,9,10,11,12,16,17	3	P53792,Q6IRE4,Q80WL1
6404	1,3,5,6,7,13,14,16	2,4,8,9,10,11,12,15,17	6	P06866,P06911,P17046,Q561R9,Q6IRE4,Q80WL1
6405	1,3,5,6,7,13,14,17	2,4,8,9,10,11,12,15,16	9	P01946,P02091,P08937,P51907,P53792,Q561R9,Q6IRE4,Q9QW07,Q9WUW8
6406	1,3,5,6,7,13,15,16	2,4,8,9,10,11,12,14,17	7	O54728,P17046,P62804,Q00715,Q6IRE4,Q80WL1,Q8VD89
6407	1,3,5,6,7,13,15,17	2,4,8,9,10,11,12,14,16	8	P01946,P02091,P08937,P51907,P53792,Q80WL1,Q99MH3,Q9WUW8
6408	1,3,5,6,7,13,16,17	2,4,8,9,10,11,12,14,15	11	P01946,P02091,P08937,P10758,P17046,P51907,P53792,P62804,Q561R9,Q6IRE4,Q99MH3
6409	1,3,5,6,7,14,15,16	2,4,8,9,10,11,12,13,17	4	O54728,P02803,Q6IRE4,Q80WL1
6410	1,3,5,6,7,14,15,17	2,4,8,9,10,11,12,13,16	8	P01946,P02091,P02803,P10536,P53792,Q561R9,Q6IRE4,Q80WL1
6411	1,3,5,6,7,14,16,17	2,4,8,9,10,11,12,13,15	9	P01946,P02091,P02803,P10758,P17046,Q561R9,Q6IRE4,Q80WL1,Q9QW07
6412	1,3,5,6,7,15,16,17	2,4,8,9,10,11,12,13,14	6	P02091,P02803,P17046,Q561R9,Q6IRE4,Q80WL1
6413	1,3,5,6,8,9,10,11	2,4,7,12,13,14,15,16,17	6	D3ZUC6,P04041,P08649,P27213,Q6IRE4,Q9QUL6
6414	1,3,5,6,8,9,10,12	2,4,7,11,13,14,15,16,17	6	F1LM93,P27213,Q6IFW6,Q6IG02,Q6IMF3,Q6P6Q2
6415	1,3,5,6,8,9,10,13	2,4,7,11,12,14,15,16,17	3	D3ZUC6,P06866,P51907

6416	1,3,5,6,8,9,10,14	2,4,7,11,12,13,15,16,17	3	P06866,P27213,Q6IRE4
6417	1,3,5,6,8,9,10,15	2,4,7,11,12,13,14,16,17	4	D3ZUC6,O54728,O55006,P27213
6418	1,3,5,6,8,9,10,16	2,4,7,11,12,13,14,15,17	4	D3ZUC6,P23593,Q63942,Q6IRE4
6419	1,3,5,6,8,9,10,17	2,4,7,11,12,13,14,15,16	6	D3ZUC6,P06760,P08649,P23593,P51907,Q99MH3
6420	1,3,5,6,8,9,11,12	2,4,7,10,13,14,15,16,17	11	O35547,P01681,P27213,Q4FZU2,Q5M8C6,Q6IFU8,Q6IFW6,Q6IG02,Q6IMF3,Q6IRE4,Q6P6Q2
6421	1,3,5,6,8,9,11,13	2,4,7,10,12,14,15,16,17	4	P01048,P06866,Q6IRE4,Q9Z1F2
6422	1,3,5,6,8,9,11,14	2,4,7,10,12,13,15,16,17	4	P02793;Q7TP54,P06866,P27213,Q6IRE4
6423	1,3,5,6,8,9,11,15	2,4,7,10,12,13,14,16,17	5	O54728,P14480,P27213,Q5I0D1,Q6IRE4
6424	1,3,5,6,8,9,11,16	2,4,7,10,12,13,14,15,17	3	P14668,P55314,Q6IRE4
6425	1,3,5,6,8,9,11,17	2,4,7,10,12,13,14,15,16	6	P01681,P06760,P08649,Q63532,Q6IRE4,Q9QUL6
6426	1,3,5,6,8,9,12,13	2,4,7,10,11,14,15,16,17	7	P02782,P51907,P82995,Q5M8C6,Q6IG02,Q6IRE4,Q9Z1F2
6427	1,3,5,6,8,9,12,14	2,4,7,10,11,13,15,16,17	4	P01835,Q6IG02,Q6IRE4,Q9Z1F2
6428	1,3,5,6,8,9,12,15	2,4,7,10,11,13,14,16,17	1	Q6IG02
6429	1,3,5,6,8,9,12,16	2,4,7,10,11,13,14,15,17	3	P23593,Q6IG02,Q6IRE4
6430	1,3,5,6,8,9,12,17	2,4,7,10,11,13,14,15,16	12	P01681,P01835,P06760,P13432,P16636,P23593,P51907,Q561R9,Q5M8C6,Q6IG02,Q99MH3,Q9Z1F2

6431	1,3,5,6,8,9,13,14	2,4,7,10,11,12,15,16,17	6	P06866,P08753,P51907,P53792,Q6IRE4,Q9Z1F2
6432	1,3,5,6,8,9,13,15	2,4,7,10,11,12,14,16,17	5	O54728,P51907,P53792,P82995,Q6IRE4
6433	1,3,5,6,8,9,13,16	2,4,7,10,11,12,14,15,17	4	P06866,P23593,P51907,Q6IRE4
6434	1,3,5,6,8,9,13,17	2,4,7,10,11,12,14,15,16	7	P06760,P08753,P23593,P51907,P53792,Q63532,Q9Z1F2
6435	1,3,5,6,8,9,14,15	2,4,7,10,11,12,13,16,17	5	O54728,P14480,P53792,Q63942,Q6IRE4
6436	1,3,5,6,8,9,14,16	2,4,7,10,11,12,13,15,17	3	P06866,Q63942,Q6IRE4
6437	1,3,5,6,8,9,14,17	2,4,7,10,11,12,13,15,16	6	P02793;Q7TP54,P06760,P47967,P51907,P53792,Q561R9
6438	1,3,5,6,8,9,15,16	2,4,7,10,11,12,13,14,17	4	O54728,P23593,Q63942,Q6IRE4
6439	1,3,5,6,8,9,15,17	2,4,7,10,11,12,13,14,16	6	P06760,P23593,P47967,P51907,P53792,Q63532
6440	1,3,5,6,8,9,16,17	2,4,7,10,11,12,13,14,15	6	P06760,P23593,P42854,P51907,Q561R9,Q6IRE4
6441	1,3,5,6,8,10,11,12	2,4,7,9,13,14,15,16,17	14	F1LM93,O35547,P01681,P02781,P16636,P27213,Q10758,Q4FZU2,Q6IFU7,Q6IFU8,Q6IFW6,Q6IG02,Q6IMF3,Q6P6Q2
6442	1,3,5,6,8,10,11,13	2,4,7,9,12,14,15,16,17	2	D3ZUC6,Q6IRE4
6443	1,3,5,6,8,10,11,14	2,4,7,9,12,13,15,16,17	4	P08649,P27213,P47727,Q6IRE4
6444	1,3,5,6,8,10,11,15	2,4,7,9,12,13,14,16,17	5	D3ZUC6,P08649,P27213,Q5I0D1,Q6IRE4
6445	1,3,5,6,8,10,11,16	2,4,7,9,12,13,14,15,17	3	P08649,P55314,Q6IRE4

6446	1,3,5,6,8,10,11,17	2,4,7,9,12,13,14,15,16	7	D3ZUC6,P01681,P04041,P06760,P08649,P16636,Q99MH3
6447	1,3,5,6,8,10,12,13	2,4,7,9,11,14,15,16,17	6	P02782,P82995,Q498D9,Q6IG02,Q99MH3,Q9Z1F2
6448	1,3,5,6,8,10,12,14	2,4,7,9,11,13,15,16,17	6	Q10758,Q498D9,Q6IFW6,Q6IG02,Q6IMF3,Q6P6Q2
6449	1,3,5,6,8,10,12,15	2,4,7,9,11,13,14,16,17	5	Q4FZU2,Q6IFV1,Q6IG02,Q6IMF3,Q99MH3
6450	1,3,5,6,8,10,12,16	2,4,7,9,11,13,14,15,17	7	Q10758,Q4FZU2,Q6IFW6,Q6IG02,Q6IMF3,Q6IRE4,Q6P6Q2
6451	1,3,5,6,8,10,12,17	2,4,7,9,11,13,14,15,16	15	F1LM93,P01681,P06760,P08937,P13432,P16636,Q08463,Q10758,Q561R9,Q6IFV1,Q6IFW6,Q6IG02,Q6IMF3,Q99MH3,Q9QZK9
6452	1,3,5,6,8,10,13,14	2,4,7,9,11,12,15,16,17	5	P06866,P06911,P51907,Q498D9,Q9Z1F2
6453	1,3,5,6,8,10,13,15	2,4,7,9,11,12,14,16,17	4	D3ZUC6,P82995,Q6IFV1,Q99MH3
6454	1,3,5,6,8,10,13,16	2,4,7,9,11,12,14,15,17	4	D3ZUC6,P51907,Q6AYQ8,Q6IRE4
6455	1,3,5,6,8,10,13,17	2,4,7,9,11,12,14,15,16	6	D3ZUC6,P06760,P08753,P51907,Q6IFV1,Q99MH3
6456	1,3,5,6,8,10,14,15	2,4,7,9,11,12,13,16,17	0	
6457	1,3,5,6,8,10,14,16	2,4,7,9,11,12,13,15,17	1	Q6IRE4
6458	1,3,5,6,8,10,14,17	2,4,7,9,11,12,13,15,16	3	P06760,P51907,Q561R9
6459	1,3,5,6,8,10,15,16	2,4,7,9,11,12,13,14,17	1	Q6IRE4
6460	1,3,5,6,8,10,15,17	2,4,7,9,11,12,13,14,16	4	D3ZUC6,P06760,Q6IFV1,Q99MH3

6461	1,3,5,6,8,10,16,17	2,4,7,9,11,12,13,14,15	6	P06760,P23593,P25031,P42854,P51907,Q99MH3
6462	1,3,5,6,8,11,12,13	2,4,7,9,10,14,15,16,17	15	O35547,P01681,P02781,P02782,P08937,P30120,Q4FZU2,Q5M8C6,Q6IFW6,Q6IG02,Q6IMF3,Q6IRE4,Q6P6Q2,Q9JHB9,Q9Z1F2
6463	1,3,5,6,8,11,12,14	2,4,7,9,10,13,15,16,17	14	O35547,P01681,P02782,P30120,P47727,Q4FZU2,Q5I0D1,Q6IFU8,Q6IFW6,Q6IG02,Q6IMF3,Q6IRE4,Q6P6Q2,Q9Z1F2
6464	1,3,5,6,8,11,12,15	2,4,7,9,10,13,14,16,17	9	O35547,P01681,Q4FZU2,Q5I0D1,Q6IFW6,Q6IG02,Q6IMF3,Q6IRE4,Q6P6Q2
6465	1,3,5,6,8,11,12,16	2,4,7,9,10,13,14,15,17	12	O35547,P01681,P17046,P55314,Q4FZU2,Q6IFU7,Q6IFU8,Q6IFW6,Q6IG02,Q6IMF3,Q6IRE4,Q6P6Q2
6466	1,3,5,6,8,11,12,17	2,4,7,9,10,13,14,15,16	18	O35547,P01681,P02782,P06760,P08937,P13432,P16636,Q4FZU2,Q4G075,Q5M8C6,Q63751,Q6IFU8,Q6IFW6,Q6IG02,Q6IMF3,Q6P6Q2,Q99MH3,Q9QZK9
6467	1,3,5,6,8,11,13,14	2,4,7,9,10,12,15,16,17	5	P06866,P47727,Q64093,Q6IRE4,Q9Z1F2
6468	1,3,5,6,8,11,13,15	2,4,7,9,10,12,14,16,17	3	P82995,Q5I0D1,Q6IRE4
6469	1,3,5,6,8,11,13,16	2,4,7,9,10,12,14,15,17	3	P17046,P55314,Q6IRE4
6470	1,3,5,6,8,11,13,17	2,4,7,9,10,12,14,15,16	5	P01681,P06760,P08937,P51907,Q99MH3
6471	1,3,5,6,8,11,14,15	2,4,7,9,10,12,13,16,17	4	P14480,P97840,Q5I0D1,Q6IRE4
6472	1,3,5,6,8,11,14,16	2,4,7,9,10,12,13,15,17	4	P14668,P55314,Q5I0D1,Q6IRE4
6473	1,3,5,6,8,11,14,17	2,4,7,9,10,12,13,15,16	5	P01681,P02793;Q7TP54,P06760,P47967,P97840
6474	1,3,5,6,8,11,15,16	2,4,7,9,10,12,13,14,17	4	P14668,P55314,Q5I0D1,Q6IRE4
6475	1,3,5,6,8,11,15,17	2,4,7,9,10,12,13,14,16	4	P06760,P47967,P97840,Q5I0D1

6476	1,3,5,6,8,11,16,17	2,4,7,9,10,12,13,14,15	4	P01681,P06760,P55314,Q6IRE4
6477	1,3,5,6,8,12,13,14	2,4,7,9,10,11,15,16,17	9	P02782,P06911,P30120,P51907,Q498D9,Q561R9,Q64093,Q6IG02,Q9Z1F2
6478	1,3,5,6,8,12,13,15	2,4,7,9,10,11,14,16,17	5	P82995,Q6IFV1,Q6IG02,Q99MH3,Q9Z1F2
6479	1,3,5,6,8,12,13,16	2,4,7,9,10,11,14,15,17	6	P17046,P51907,P82995,Q6IG02,Q6IRE4,Q9Z1F2
6480	1,3,5,6,8,12,13,17	2,4,7,9,10,11,14,15,16	14	P01681,P02782,P06760,P08937,P13432,P16636,P51907,Q561R9,Q5M8C6,Q6IFV1,Q6IG02,Q99MH3,Q9QZK9,Q9Z1F2
6481	1,3,5,6,8,12,14,15	2,4,7,9,10,11,13,16,17	1	Q6IG02
6482	1,3,5,6,8,12,14,16	2,4,7,9,10,11,13,15,17	4	Q561R9,Q6IFW6,Q6IG02,Q6IRE4
6483	1,3,5,6,8,12,14,17	2,4,7,9,10,11,13,15,16	12	O70594,P01681,P02793;Q7TP54,P06760,P08937,P13432,P51907,Q10758,Q561R9,Q6IG02,Q99PP0,Q9Z1F2
6484	1,3,5,6,8,12,15,16	2,4,7,9,10,11,13,14,17	2	Q6IG02,Q6IRE4
6485	1,3,5,6,8,12,15,17	2,4,7,9,10,11,13,14,16	7	P01681,P06760,P08937,P13432,Q6IFV1,Q6IG02,Q99MH3
6486	1,3,5,6,8,12,16,17	2,4,7,9,10,11,13,14,15	9	P01681,P06760,P13432,P23593,P51907,Q561R9,Q5XFX0,Q6IG02,Q99MH3
6487	1,3,5,6,8,13,14,15	2,4,7,9,10,11,12,16,17	8	P06757,P06866,P08753,P51907,P53792,P82995,Q64093,Q9Z1F2
6488	1,3,5,6,8,13,14,16	2,4,7,9,10,11,12,15,17	6	P06866,P08753,P51907,Q561R9,Q6IRE4,Q9Z1F2
6489	1,3,5,6,8,13,14,17	2,4,7,9,10,11,12,15,16	11	O70594,P06760,P08753,P10536,P47967,P51907,P53792,Q561R9,Q64093,Q9QW07,Q9Z1F2
6490	1,3,5,6,8,13,15,16	2,4,7,9,10,11,12,14,17	3	P08753,P82995,Q6IRE4

6491	1,3,5,6,8,13,15,17	2,4,7,9,10,11,12,14,16	8	P06760,P08753,P10536,P47967,P51907,P53792,Q6IFV1,Q99MH3
6492	1,3,5,6,8,13,16,17	2,4,7,9,10,11,12,14,15	9	P06760,P08753,P23593,P42854,P51907,Q561R9,Q6IRE4,Q6PCU2,Q99MH3
6493	1,3,5,6,8,14,15,16	2,4,7,9,10,11,12,13,17	2	P02803,Q6IRE4
6494	1,3,5,6,8,14,15,17	2,4,7,9,10,11,12,13,16	9	P02793;Q7TP54,P02803,P06760,P10536,P47967,P51907,P97840,Q561R9,Q9QW07
6495	1,3,5,6,8,14,16,17	2,4,7,9,10,11,12,13,15	6	P06760,P42854,P51907,Q561R9,Q6IRE4,Q9QW07
6496	1,3,5,6,8,15,16,17	2,4,7,9,10,11,12,13,14	3	P06760,P23593,Q5XFX0
6497	1,3,5,6,9,10,11,12	2,4,7,8,13,14,15,16,17	9	P27213,Q4FZU2,Q6IFU7,Q6IFU8,Q6IFW6,Q6IG02,Q6IMF3,Q6IRE4,Q6P6Q2
6498	1,3,5,6,9,10,11,13	2,4,7,8,12,14,15,16,17	2	Q6IRE4,Q80WL1
6499	1,3,5,6,9,10,11,14	2,4,7,8,12,13,15,16,17	5	P27213,P55091,Q6IRE4,Q80WL1,Q9QUL6
6500	1,3,5,6,9,10,11,15	2,4,7,8,12,13,14,16,17	6	O54728,P27213,P55091,Q6IRE4,Q80WL1,Q9QUL6
6501	1,3,5,6,9,10,11,16	2,4,7,8,12,13,14,15,17	6	P55091,P55314,Q62635,Q6IRE4,Q80WL1,Q9QUL6
6502	1,3,5,6,9,10,11,17	2,4,7,8,12,13,14,15,16	4	P18427,Q6IRE4,Q80WL1,Q9QUL6
6503	1,3,5,6,9,10,12,13	2,4,7,8,11,14,15,16,17	4	P19218,Q6IG02,Q6IRE4,Q99MH3
6504	1,3,5,6,9,10,12,14	2,4,7,8,11,13,15,16,17	5	P01835,Q10758,Q6IG02,Q6IMF3,Q6IRE4
6505	1,3,5,6,9,10,12,15	2,4,7,8,11,13,14,16,17	1	Q6IG02

6506	1,3,5,6,9,10,12,16	2,4,7,8,11,13,14,15,17	4	Q6IFW6,Q6IG02,Q6IMF3,Q6IRE4
6507	1,3,5,6,9,10,12,17	2,4,7,8,11,13,14,15,16	5	P01835,P23593,Q10758,Q6IG02,Q99MH3
6508	1,3,5,6,9,10,13,14	2,4,7,8,11,12,15,16,17	4	P06866,P19223,Q6IRE4,Q80WL1
6509	1,3,5,6,9,10,13,15	2,4,7,8,11,12,14,16,17	4	O54728,Q5U2V4,Q6IRE4,Q80WL1
6510	1,3,5,6,9,10,13,16	2,4,7,8,11,12,14,15,17	7	P19223,P55091,Q00715,Q6AYQ8,Q6IRE4,Q80WL1,Q9QZA6
6511	1,3,5,6,9,10,13,17	2,4,7,8,11,12,14,15,16	3	P51907,Q80WL1,Q99MH3
6512	1,3,5,6,9,10,14,15	2,4,7,8,11,12,13,16,17	4	O54728,P55091,Q6IRE4,Q80WL1
6513	1,3,5,6,9,10,14,16	2,4,7,8,11,12,13,15,17	4	P19223,P55091,Q6IRE4,Q80WL1
6514	1,3,5,6,9,10,14,17	2,4,7,8,11,12,13,15,16	4	P08689,P18427,Q80WL1,Q9QUL6
6515	1,3,5,6,9,10,15,16	2,4,7,8,11,12,13,14,17	5	O54728,P55091,Q62635,Q6IRE4,Q80WL1
6516	1,3,5,6,9,10,15,17	2,4,7,8,11,12,13,14,16	3	P23593,Q80WL1,Q99MH3
6517	1,3,5,6,9,10,16,17	2,4,7,8,11,12,13,14,15	3	P23593,Q6IRE4,Q80WL1
6518	1,3,5,6,9,11,12,13	2,4,7,8,10,14,15,16,17	4	Q6IFW6,Q6IG02,Q6IRE4,Q6P6Q2
6519	1,3,5,6,9,11,12,14	2,4,7,8,10,13,15,16,17	8	P01835,Q4FZU2,Q6IFU8,Q6IFW6,Q6IG02,Q6IMF3,Q6IRE4,Q6P6Q2
6520	1,3,5,6,9,11,12,15	2,4,7,8,10,13,14,16,17	5	P27213,Q4FZU2,Q6IFW6,Q6IG02,Q6IRE4

6521	1,3,5,6,9,11,12,16	2,4,7,8,10,13,14,15,17	10	P17046,P55314,Q4FZU2,Q6IFU7,Q6IFU8,Q6IFW6,Q6IG02,Q6IMF3,Q6IRE4,Q6P6Q2
6522	1,3,5,6,9,11,12,17	2,4,7,8,10,13,14,15,16	10	P01681,P01835,P13432,Q4FZU2,Q6IFU8,Q6IFW6,Q6IG02,Q6IMF3,Q6IRE4,Q6P6Q2
6523	1,3,5,6,9,11,13,14	2,4,7,8,10,12,15,16,17	4	P06866,P19223,Q6IRE4,Q80WL1
6524	1,3,5,6,9,11,13,15	2,4,7,8,10,12,14,16,17	4	O54728,P19223,Q6IRE4,Q80WL1
6525	1,3,5,6,9,11,13,16	2,4,7,8,10,12,14,15,17	6	P17046,P19223,P55091,P55314,Q6IRE4,Q80WL1
6526	1,3,5,6,9,11,13,17	2,4,7,8,10,12,14,15,16	2	Q6IRE4,Q80WL1
6527	1,3,5,6,9,11,14,15	2,4,7,8,10,12,13,16,17	7	O54728,P14480,P19223,P27213,P55091,Q6IRE4,Q80WL1
6528	1,3,5,6,9,11,14,16	2,4,7,8,10,12,13,15,17	5	P19223,P55091,P55314,Q6IRE4,Q80WL1
6529	1,3,5,6,9,11,14,17	2,4,7,8,10,12,13,15,16	5	P01835,P04218,Q6IRE4,Q80WL1,Q9QUL6
6530	1,3,5,6,9,11,15,16	2,4,7,8,10,12,13,14,17	7	O54728,P19223,P55091,P55314,Q62635,Q6IRE4,Q80WL1
6531	1,3,5,6,9,11,15,17	2,4,7,8,10,12,13,14,16	3	Q6IRE4,Q80WL1,Q9QUL6
6532	1,3,5,6,9,11,16,17	2,4,7,8,10,12,13,14,15	5	P04218,P55314,Q6IRE4,Q80WL1,Q9QUL6
6533	1,3,5,6,9,12,13,14	2,4,7,8,10,11,15,16,17	3	P01835,Q6IRE4,Q9Z1F2
6534	1,3,5,6,9,12,13,15	2,4,7,8,10,11,14,16,17	3	P00714,P82995,Q6IRE4
6535	1,3,5,6,9,12,13,16	2,4,7,8,10,11,14,15,17	4	P02761,P17046,P19218,Q6IRE4

6536	1,3,5,6,9,12,13,17	2,4,7,8,10,11,14,15,16	7	P00714,P01835,P35280,P51907,P53792,Q561R9,Q99MH3
6537	1,3,5,6,9,12,14,15	2,4,7,8,10,11,13,16,17	3	P01835,Q6IRE4,Q80WL1
6538	1,3,5,6,9,12,14,16	2,4,7,8,10,11,13,15,17	2	P01835,Q6IRE4
6539	1,3,5,6,9,12,14,17	2,4,7,8,10,11,13,15,16	4	P01681,P01835,Q561R9,Q99PP0
6540	1,3,5,6,9,12,15,16	2,4,7,8,10,11,13,14,17	3	O54728,Q6IRE4,Q80WL1
6541	1,3,5,6,9,12,15,17	2,4,7,8,10,11,13,14,16	4	P00714,P01835,P23593,Q99MH3
6542	1,3,5,6,9,12,16,17	2,4,7,8,10,11,13,14,15	5	P01835,P23593,P35280,Q561R9,Q6IRE4
6543	1,3,5,6,9,13,14,15	2,4,7,8,10,11,12,16,17	6	O54728,P00714,P19223,P53792,Q6IRE4,Q80WL1
6544	1,3,5,6,9,13,14,16	2,4,7,8,10,11,12,15,17	10	P00714,P01015,P02761,P06866,P19223,P53792,P62804,Q00715,Q6IRE4,Q80WL1
6545	1,3,5,6,9,13,14,17	2,4,7,8,10,11,12,15,16	8	P00714,P35280,P51907,P53792,Q561R9,Q6IRE4,Q80WL1,Q9QW07
6546	1,3,5,6,9,13,15,16	2,4,7,8,10,11,12,14,17	8	O54728,P00714,P19223,P55091,P62804,Q00715,Q6IRE4,Q80WL1
6547	1,3,5,6,9,13,15,17	2,4,7,8,10,11,12,14,16	4	P00714,P51907,P53792,Q80WL1
6548	1,3,5,6,9,13,16,17	2,4,7,8,10,11,12,14,15	10	P00714,P23593,P35280,P51907,P53792,P62804,Q00715,Q561R9,Q6IRE4,Q80WL1
6549	1,3,5,6,9,14,15,16	2,4,7,8,10,11,12,13,17	6	O54728,P19223,P55091,Q62635,Q6IRE4,Q80WL1
6550	1,3,5,6,9,14,15,17	2,4,7,8,10,11,12,13,16	5	P00714,P02803,P47967,P53792,Q80WL1

6551	1,3,5,6,9,14,16,17	2,4,7,8,10,11,12,13,15	4	P00714,Q561R9,Q6IRE4,Q80WL1
6552	1,3,5,6,9,15,16,17	2,4,7,8,10,11,12,13,14	6	O54728,P00714,P23593,P47727,Q6IRE4,Q80WL1
6553	1,3,5,6,10,11,12,13	2,4,7,8,9,14,15,16,17	8	Q4FZU2,Q6IFU7,Q6IFW6,Q6IG02,Q6IMF3,Q6IRE4,Q6P6Q2,Q99MH3
6554	1,3,5,6,10,11,12,14	2,4,7,8,9,13,15,16,17	10	P47727,Q10758,Q4FZU2,Q6IFU7,Q6IFU8,Q6IFW6,Q6IG02,Q6IMF3,Q6IRE4,Q6P6Q2
6555	1,3,5,6,10,11,12,15	2,4,7,8,9,13,14,16,17	9	P20766,Q4FZU2,Q6IFU7,Q6IFU8,Q6IFW6,Q6IG02,Q6IMF3,Q6IRE4,Q6P6Q2
6556	1,3,5,6,10,11,12,16	2,4,7,8,9,13,14,15,17	12	P17046,P20766,P55314,Q10758,Q4FZU2,Q6IFU7,Q6IFU8,Q6IFW6,Q6IG02,Q6IMF3,Q6IRE4,Q6P6Q2
6557	1,3,5,6,10,11,12,17	2,4,7,8,9,13,14,15,16	12	O08815,P01681,P16636,Q10758,Q4FZU2,Q6IFU8,Q6IFW6,Q6IG02,Q6IMF3,Q6P6Q2,Q99MH3,Q9QZK9
6558	1,3,5,6,10,11,13,14	2,4,7,8,9,12,15,16,17	4	P19223,P47727,Q6IRE4,Q80WL1
6559	1,3,5,6,10,11,13,15	2,4,7,8,9,12,14,16,17	3	P19223,Q6IRE4,Q80WL1
6560	1,3,5,6,10,11,13,16	2,4,7,8,9,12,14,15,17	5	P17046,P19223,P55314,Q6IRE4,Q80WL1
6561	1,3,5,6,10,11,13,17	2,4,7,8,9,12,14,15,16	1	Q99MH3
6562	1,3,5,6,10,11,14,15	2,4,7,8,9,12,13,16,17	4	P19223,P55091,Q6IRE4,Q80WL1
6563	1,3,5,6,10,11,14,16	2,4,7,8,9,12,13,15,17	5	P19223,P55091,P55314,Q6IRE4,Q80WL1
6564	1,3,5,6,10,11,14,17	2,4,7,8,9,12,13,15,16	4	P08689,P18427,Q80WL1,Q9QUL6
6565	1,3,5,6,10,11,15,16	2,4,7,8,9,12,13,14,17	6	P19223,P20766,P55091,P55314,Q6IRE4,Q80WL1
6566	1,3,5,6,10,11,15,17	2,4,7,8,9,12,13,14,16	2	Q80WL1,Q99MH3
6567	1,3,5,6,10,11,16,17	2,4,7,8,9,12,13,14,15	3	P55314,Q6IRE4,Q80WL1
6568	1,3,5,6,10,12,13,14	2,4,7,8,9,11,15,16,17	4	Q05175,Q10758,Q498D9,Q6IG02
6569	1,3,5,6,10,12,13,15	2,4,7,8,9,11,14,16,17	3	P82995,Q498D9,Q99MH3
6570	1,3,5,6,10,12,13,16	2,4,7,8,9,11,14,15,17	6	P17046,P19218,P82995,Q6IG02,Q6IRE4,Q99MH3
6571	1,3,5,6,10,12,13,17	2,4,7,8,9,11,14,15,16	6	Q05175,Q10758,Q561R9,Q6IG02,Q99MH3,Q9QZK9
6572	1,3,5,6,10,12,14,15	2,4,7,8,9,11,13,16,17	4	P20766,Q498D9,Q4FZU2,Q6IG02

6573	1,3,5,6,10,12,14,16	2,4,7,8,9,11,13,15,17	7	P20766,Q10758,Q4FZU2,Q6IFW6,Q6IG02,Q6IMF3,Q6IRE4
6574	1,3,5,6,10,12,14,17	2,4,7,8,9,11,13,15,16	7	P01681,P08689,Q10758,Q561R9,Q6IG02,Q6IMF3,Q99MH3
6575	1,3,5,6,10,12,15,16	2,4,7,8,9,11,13,14,17	6	P20766,Q4FZU2,Q6IG02,Q6IMF3,Q6IRE4,Q80WL1
6576	1,3,5,6,10,12,15,17	2,4,7,8,9,11,13,14,16	5	P20766,Q10758,Q4FZU2,Q6IG02,Q99MH3
6577	1,3,5,6,10,12,16,17	2,4,7,8,9,11,13,14,15	6	P20766,Q10758,Q561R9,Q6IG02,Q6IMF3,Q99MH3
6578	1,3,5,6,10,13,14,15	2,4,7,8,9,11,12,16,17	3	P19223,Q498D9,Q80WL1
6579	1,3,5,6,10,13,14,16	2,4,7,8,9,11,12,15,17	4	P19223,Q6IRE4,Q80WL1,Q9QZA6
6580	1,3,5,6,10,13,14,17	2,4,7,8,9,11,12,15,16	5	P51907,Q561R9,Q80WL1,Q99MH3,Q9QW07
6581	1,3,5,6,10,13,15,16	2,4,7,8,9,11,12,14,17	6	P19223,P55091,P82995,Q6AYQ8,Q6IRE4,Q80WL1
6582	1,3,5,6,10,13,15,17	2,4,7,8,9,11,12,14,16	3	Q6IFV1,Q80WL1,Q99MH3
6583	1,3,5,6,10,13,16,17	2,4,7,8,9,11,12,14,15	4	P51907,Q561R9,Q80WL1,Q99MH3
6584	1,3,5,6,10,14,15,16	2,4,7,8,9,11,12,13,17	6	P02803,P19223,P20766,P55091,Q6IRE4,Q80WL1
6585	1,3,5,6,10,14,15,17	2,4,7,8,9,11,12,13,16	2	P02803,Q80WL1
6586	1,3,5,6,10,14,16,17	2,4,7,8,9,11,12,13,15	3	Q561R9,Q6IRE4,Q80WL1
6587	1,3,5,6,10,15,16,17	2,4,7,8,9,11,12,13,14	4	P02803,P20766,Q80WL1,Q99MH3
6588	1,3,5,6,11,12,13,14	2,4,7,8,9,10,15,16,17	8	P17046,P47727,Q4FZU2,Q6IFW6,Q6IG02,Q6IRE4,Q6P6Q2,Q9Z1F2
6589	1,3,5,6,11,12,13,15	2,4,7,8,9,10,14,16,17	6	P17046,P82995,Q4FZU2,Q6IFW6,Q6IG02,Q6IRE4
6590	1,3,5,6,11,12,13,16	2,4,7,8,9,10,14,15,17	9	P17046,P55314,Q4FZU2,Q6IFU7,Q6IFW6,Q6IG02,Q6IMF3,Q6IRE4,Q6P6Q2
6591	1,3,5,6,11,12,13,17	2,4,7,8,9,10,14,15,16	7	P01681,P17046,Q4FZU2,Q6IFW6,Q6IG02,Q99MH3,Q9QZK9
6592	1,3,5,6,11,12,14,15	2,4,7,8,9,10,13,16,17	7	Q4FZU2,Q5I0D1,Q6IFW6,Q6IG02,Q6IRE4,Q6P6Q2,Q80WL1
6593	1,3,5,6,11,12,14,16	2,4,7,8,9,10,13,15,17	11	P17046,P20766,P55314,Q4FZU2,Q6IFU7,Q6IFU8,Q6IFW6,Q6IG02,Q6IMF3,Q6IRE4,Q6P6Q2
6594	1,3,5,6,11,12,14,17	2,4,7,8,9,10,13,15,16	10	P01681,P01835,Q4FZU2,Q561R9,Q6IFU8,Q6IFW6,Q6IG02,Q6IMF3,Q6IRE4,Q6P6Q2
6595	1,3,5,6,11,12,15,16	2,4,7,8,9,10,13,14,17	12	P17046,P20766,P55314,Q4FZU2,Q5I0D1,Q6IFU7,Q6IFW6,Q6IG02,Q6IMF3,Q6IRE4,Q6P6Q2,Q80WL1

6596	1,3,5,6,11,12,15,17	2,4,7,8,9,10,13,14,16	5	P01681,Q4FZU2,Q6IFW6,Q6IG02,Q99MH3
6597	1,3,5,6,11,12,16,17	2,4,7,8,9,10,13,14,15	10	P01681,P17046,P55314,Q4FZU2,Q6IFU8,Q6IFW6,Q6IG02,Q6IMF3,Q6IRE4,Q6P6Q2
6598	1,3,5,6,11,13,14,15	2,4,7,8,9,10,12,16,17	3	P19223,Q6IRE4,Q80WL1
6599	1,3,5,6,11,13,14,16	2,4,7,8,9,10,12,15,17	5	P17046,P19223,P55314,Q6IRE4,Q80WL1
6600	1,3,5,6,11,13,14,17	2,4,7,8,9,10,12,15,16	3	Q6IRE4,Q80WL1,Q9QW07
6601	1,3,5,6,11,13,15,16	2,4,7,8,9,10,12,14,17	7	O54728,P17046,P19223,P55091,P55314,Q6IRE4,Q80WL1
6602	1,3,5,6,11,13,15,17	2,4,7,8,9,10,12,14,16	3	Q6IRE4,Q80WL1,Q99MH3
6603	1,3,5,6,11,13,16,17	2,4,7,8,9,10,12,14,15	4	P17046,P55314,Q6IRE4,Q80WL1
6604	1,3,5,6,11,14,15,16	2,4,7,8,9,10,12,13,17	8	O54728,P02803,P19223,P55091,P55314,Q5I0D1,Q6IRE4,Q80WL1
6605	1,3,5,6,11,14,15,17	2,4,7,8,9,10,12,13,16	5	P02803,P47967,P97840,Q6IRE4,Q80WL1
6606	1,3,5,6,11,14,16,17	2,4,7,8,9,10,12,13,15	5	P17046,P55314,Q561R9,Q6IRE4,Q80WL1
6607	1,3,5,6,11,15,16,17	2,4,7,8,9,10,12,13,14	4	P17046,P55314,Q6IRE4,Q80WL1
6608	1,3,5,6,12,13,14,15	2,4,7,8,9,10,11,16,17	6	P00714,P82995,Q498D9,Q64093,Q6IRE4,Q80WL1
6609	1,3,5,6,12,13,14,16	2,4,7,8,9,10,11,15,17	5	P00714,P02761,P17046,Q561R9,Q6IRE4
6610	1,3,5,6,12,13,14,17	2,4,7,8,9,10,11,15,16	7	P00714,P01681,P51907,Q561R9,Q99MH3,Q9QW07,Q9Z1F2
6611	1,3,5,6,12,13,15,16	2,4,7,8,9,10,11,14,17	6	P00714,P17046,P19218,P82995,Q6IRE4,Q80WL1
6612	1,3,5,6,12,13,15,17	2,4,7,8,9,10,11,14,16	4	P00714,Q561R9,Q6IFV1,Q99MH3
6613	1,3,5,6,12,13,16,17	2,4,7,8,9,10,11,14,15	7	P00714,P17046,P35280,P51907,Q561R9,Q6IRE4,Q99MH3
6614	1,3,5,6,12,14,15,16	2,4,7,8,9,10,11,13,17	3	P20766,Q6IRE4,Q80WL1
6615	1,3,5,6,12,14,15,17	2,4,7,8,9,10,11,13,16	4	P00714,P02803,Q561R9,Q9QW07
6616	1,3,5,6,12,14,16,17	2,4,7,8,9,10,11,13,15	7	P00714,P01681,P17046,Q10758,Q561R9,Q6IRE4,Q9QW07
6617	1,3,5,6,12,15,16,17	2,4,7,8,9,10,11,13,14	7	P00714,P17046,P20766,Q561R9,Q5XFX0,Q80WL1,Q99MH3
6618	1,3,5,6,13,14,15,16	2,4,7,8,9,10,11,12,17	5	O54728,P00714,P19223,Q6IRE4,Q80WL1

6619	1,3,5,6,13,14,15,17	2,4,7,8,9,10,11,12,16	10	P00714,P02803,P08753,P47967,P51907,P53792,Q561R9,Q80WL1,Q9QW07,Q9WUW8
6620	1,3,5,6,13,14,16,17	2,4,7,8,9,10,11,12,15	8	P00714,P08753,P35280,P51907,Q561R9,Q6IRE4,Q80WL1,Q9QW07
6621	1,3,5,6,13,15,16,17	2,4,7,8,9,10,11,12,14	7	P00714,P08753,P62804,Q6IRE4,Q80WL1,Q99MH3,Q9EQS0
6622	1,3,5,6,14,15,16,17	2,4,7,8,9,10,11,12,13	6	P00714,P02803,Q561R9,Q6IRE4,Q80WL1,Q9QW07
6623	1,3,5,7,8,9,10,11	2,4,6,12,13,14,15,16,17	6	O54728,P14480,P25236,P27213,Q62635,Q6IRE4
6624	1,3,5,7,8,9,10,12	2,4,6,11,13,14,15,16,17	3	P10715,P27213,Q6IG02
6625	1,3,5,7,8,9,10,13	2,4,6,11,12,14,15,16,17	1	Q6IRE4
6626	1,3,5,7,8,9,10,14	2,4,6,11,12,13,15,16,17	4	O54728,P27213,Q62635,Q6IRE4
6627	1,3,5,7,8,9,10,15	2,4,6,11,12,13,14,16,17	6	O54728,P14480,P25236,P27213,Q62635,Q6IRE4
6628	1,3,5,7,8,9,10,16	2,4,6,11,12,13,14,15,17	4	O54728,P25236,Q62635,Q6IRE4
6629	1,3,5,7,8,9,10,17	2,4,6,11,12,13,14,15,16	5	P01946,P02091,P18427,P25236,P52590
6630	1,3,5,7,8,9,11,12	2,4,6,10,13,14,15,16,17	4	P10715,P27213,Q6IG02,Q6IRE4
6631	1,3,5,7,8,9,11,13	2,4,6,10,12,14,15,16,17	5	O54728,P01048,P06866,P14480,Q6IRE4
6632	1,3,5,7,8,9,11,14	2,4,6,10,12,13,15,16,17	6	O54728,P06866,P14480,P27213,Q62635,Q6IRE4
6633	1,3,5,7,8,9,11,15	2,4,6,10,12,13,14,16,17	7	O54728,P10715,P14480,P25236,P27213,Q62635,Q6IRE4
6634	1,3,5,7,8,9,11,16	2,4,6,10,12,13,14,15,17	7	B4F795,O54728,P10715,P25236,P27213,Q62635,Q6IRE4

6635	1,3,5,7,8,9,11,17	2,4,6,10,12,13,14,15,16	7	P01946,P02091,P10715,P25236,P50115,P52590,Q6IRE4
6636	1,3,5,7,8,9,12,13	2,4,6,10,11,14,15,16,17	3	P10715,Q6IRE4,Q9Z1F2
6637	1,3,5,7,8,9,12,14	2,4,6,10,11,13,15,16,17	4	P10715,Q4FZU6,Q6IG02,Q6IRE4
6638	1,3,5,7,8,9,12,15	2,4,6,10,11,13,14,16,17	4	O54728,P10715,P14480,Q6IRE4
6639	1,3,5,7,8,9,12,16	2,4,6,10,11,13,14,15,17	3	P10715,Q4FZU6,Q6IRE4
6640	1,3,5,7,8,9,12,17	2,4,6,10,11,13,14,15,16	6	P01946,P02091,P10715,P50115,P52590,Q4FZU6
6641	1,3,5,7,8,9,13,14	2,4,6,10,11,12,15,16,17	5	O54728,P06866,P51907,P53792,Q6IRE4
6642	1,3,5,7,8,9,13,15	2,4,6,10,11,12,14,16,17	4	O54728,P14480,P53792,Q6IRE4
6643	1,3,5,7,8,9,13,16	2,4,6,10,11,12,14,15,17	4	O54728,P62804,Q00715,Q6IRE4
6644	1,3,5,7,8,9,13,17	2,4,6,10,11,12,14,15,16	5	P01946,P02091,P51907,P52590,P53792
6645	1,3,5,7,8,9,14,15	2,4,6,10,11,12,13,16,17	5	O54728,P14480,P53792,Q62635,Q6IRE4
6646	1,3,5,7,8,9,14,16	2,4,6,10,11,12,13,15,17	5	B4F795,O54728,Q4FZU6,Q62635,Q6IRE4
6647	1,3,5,7,8,9,14,17	2,4,6,10,11,12,13,15,16	6	P01946,P02091,P51907,P52590,P53792,Q4FZU6
6648	1,3,5,7,8,9,15,16	2,4,6,10,11,12,13,14,17	7	B4F795,O54728,P25236,P47727,P62804,Q62635,Q6IRE4
6649	1,3,5,7,8,9,15,17	2,4,6,10,11,12,13,14,16	7	O54728,P01946,P02091,P25236,P47727,P52590,P53792

6650	1,3,5,7,8,9,16,17	2,4,6,10,11,12,13,14,15	7	P01946,P02091,P25236,P47727,P52590,Q4FZU6,Q6IRE4
6651	1,3,5,7,8,10,11,12	2,4,6,9,13,14,15,16,17	10	F1LM93,P00731,P10715,P16391,P24388,P27213,Q6IG02,Q6IRE4,Q6P6Q2,Q8CJ52
6652	1,3,5,7,8,10,11,13	2,4,6,9,12,14,15,16,17	3	Q5HZV9,Q6IRE4,Q8VD89
6653	1,3,5,7,8,10,11,14	2,4,6,9,12,13,15,16,17	2	P27213,Q6IRE4
6654	1,3,5,7,8,10,11,15	2,4,6,9,12,13,14,16,17	7	O54728,P00731,P14480,P25236,P27213,Q62635,Q6IRE4
6655	1,3,5,7,8,10,11,16	2,4,6,9,12,13,14,15,17	5	P25236,P55314,Q62635,Q6IRE4,Q8VD89
6656	1,3,5,7,8,10,11,17	2,4,6,9,12,13,14,15,16	4	P01946,P02091,P18427,P25236
6657	1,3,5,7,8,10,12,13	2,4,6,9,11,14,15,16,17	2	Q6IG02,Q99MH3
6658	1,3,5,7,8,10,12,14	2,4,6,9,11,13,15,16,17	2	Q6IG02,Q9ET32
6659	1,3,5,7,8,10,12,15	2,4,6,9,11,13,14,16,17	4	P00731,P10715,Q6IG02,Q99MH3
6660	1,3,5,7,8,10,12,16	2,4,6,9,11,13,14,15,17	4	P08721,P10715,Q6IG02,Q6IRE4
6661	1,3,5,7,8,10,12,17	2,4,6,9,11,13,14,15,16	7	F1LM93,P01946,P02091,P08937,P10715,Q6IG02,Q99MH3
6662	1,3,5,7,8,10,13,14	2,4,6,9,11,12,15,16,17	1	P06866
6663	1,3,5,7,8,10,13,15	2,4,6,9,11,12,14,16,17	2	O54728,P01835
6664	1,3,5,7,8,10,13,16	2,4,6,9,11,12,14,15,17	3	P01835,Q6AYQ8,Q6IRE4

6665	1,3,5,7,8,10,13,17	2,4,6,9,11,12,14,15,16	4	P01946,P02091,P51907,Q99MH3
6666	1,3,5,7,8,10,14,15	2,4,6,9,11,12,13,16,17	2	O54728,P14480
6667	1,3,5,7,8,10,14,16	2,4,6,9,11,12,13,15,17	3	P08721,Q62635,Q6IRE4
6668	1,3,5,7,8,10,14,17	2,4,6,9,11,12,13,15,16	3	P01946,P02091,P18427
6669	1,3,5,7,8,10,15,16	2,4,6,9,11,12,13,14,17	6	O54728,P01835,P08721,P25236,Q62635,Q6IRE4
6670	1,3,5,7,8,10,15,17	2,4,6,9,11,12,13,14,16	4	P01946,P02091,P25236,Q99MH3
6671	1,3,5,7,8,10,16,17	2,4,6,9,11,12,13,14,15	4	P01946,P02091,P25031,P25236
6672	1,3,5,7,8,11,12,13	2,4,6,9,10,14,15,16,17	5	P10715,P17046,Q6IG02,Q6IRE4,Q8CJ52
6673	1,3,5,7,8,11,12,14	2,4,6,9,10,13,15,16,17	5	P10715,Q6IG02,Q6IRE4,Q8CJ52,Q9ET32
6674	1,3,5,7,8,11,12,15	2,4,6,9,10,13,14,16,17	5	P10715,P14480,Q5I0D1,Q6IG02,Q6IRE4
6675	1,3,5,7,8,11,12,16	2,4,6,9,10,13,14,15,17	7	P10715,P17046,P24388,Q6IG02,Q6IRE4,Q6P6Q2,Q8CJ52
6676	1,3,5,7,8,11,12,17	2,4,6,9,10,13,14,15,16	8	P01946,P02091,P08937,P10715,P17046,P50115,Q63751,Q6IG02
6677	1,3,5,7,8,11,13,14	2,4,6,9,10,12,15,16,17	2	P06866,Q6IRE4
6678	1,3,5,7,8,11,13,15	2,4,6,9,10,12,14,16,17	4	O54728,P14480,Q6IRE4,Q8VD89
6679	1,3,5,7,8,11,13,16	2,4,6,9,10,12,14,15,17	3	P17046,Q6IRE4,Q8VD89

6680	1,3,5,7,8,11,13,17	2,4,6,9,10,12,14,15,16	3	P01946,P02091,Q6IRE4
6681	1,3,5,7,8,11,14,15	2,4,6,9,10,12,13,16,17	5	O54728,P14480,Q5I0D1,Q63355,Q6IRE4
6682	1,3,5,7,8,11,14,16	2,4,6,9,10,12,13,15,17	3	B4F795,P17046,Q6IRE4
6683	1,3,5,7,8,11,14,17	2,4,6,9,10,12,13,15,16	4	P01946,P02091,P97840,Q6IRE4
6684	1,3,5,7,8,11,15,16	2,4,6,9,10,12,13,14,17	9	B4F795,O54728,P10715,P17046,P25236,Q5I0D1,Q62635,Q6IRE4,Q8VD89
6685	1,3,5,7,8,11,15,17	2,4,6,9,10,12,13,14,16	4	P01946,P02091,P10715,P25236
6686	1,3,5,7,8,11,16,17	2,4,6,9,10,12,13,14,15	7	B4F795,P01946,P02091,P10715,P17046,P25236,Q6IRE4
6687	1,3,5,7,8,12,13,14	2,4,6,9,10,11,15,16,17	2	Q6IRE4,Q9Z1F2
6688	1,3,5,7,8,12,13,15	2,4,6,9,10,11,14,16,17	1	P10715
6689	1,3,5,7,8,12,13,16	2,4,6,9,10,11,14,15,17	4	P10715,P17046,Q4FZU6,Q6IRE4
6690	1,3,5,7,8,12,13,17	2,4,6,9,10,11,14,15,16	7	P01946,P02091,P08937,P10715,P17046,P51907,Q99MH3
6691	1,3,5,7,8,12,14,15	2,4,6,9,10,11,13,16,17	4	A2RUV9,P10715,P19629,Q8CJD3
6692	1,3,5,7,8,12,14,16	2,4,6,9,10,11,13,15,17	5	P10715,P17046,Q4FZU6,Q6IRE4,Q9ET32
6693	1,3,5,7,8,12,14,17	2,4,6,9,10,11,13,15,16	6	P01946,P02091,P10715,Q4FZU6,Q561R9,Q8CJD3
6694	1,3,5,7,8,12,15,16	2,4,6,9,10,11,13,14,17	4	P10715,P17046,P83121,Q6IRE4

6695	1,3,5,7,8,12,15,17	2,4,6,9,10,11,13,14,16	4	P01946,P02091,P10715,Q99MH3
6696	1,3,5,7,8,12,16,17	2,4,6,9,10,11,13,14,15	6	P01946,P02091,P10715,P17046,P23928,Q4FZU6
6697	1,3,5,7,8,13,14,15	2,4,6,9,10,11,12,16,17	7	O54728,P00762,P06757,P53792,Q08849,Q09030,Q6IRE4
6698	1,3,5,7,8,13,14,16	2,4,6,9,10,11,12,15,17	4	P00762,Q08849,Q4FZU6,Q6IRE4
6699	1,3,5,7,8,13,14,17	2,4,6,9,10,11,12,15,16	7	P01946,P02091,P08753,P51907,P53792,Q08849,Q9QW07
6700	1,3,5,7,8,13,15,16	2,4,6,9,10,11,12,14,17	5	O54728,P00762,P01835,P62804,Q6IRE4
6701	1,3,5,7,8,13,15,17	2,4,6,9,10,11,12,14,16	7	P01946,P02091,P51907,P53792,Q6IFU7,Q99MH3,Q9WUW8
6702	1,3,5,7,8,13,16,17	2,4,6,9,10,11,12,14,15	7	P01946,P02091,P17046,P23928,P51907,Q4FZU6,Q6IRE4
6703	1,3,5,7,8,14,15,16	2,4,6,9,10,11,12,13,17	5	B4F795,O54728,Q08849,Q62635,Q6IRE4
6704	1,3,5,7,8,14,15,17	2,4,6,9,10,11,12,13,16	7	P01946,P02091,P10536,P47967,Q08849,Q8CJD3,Q9QW07
6705	1,3,5,7,8,14,16,17	2,4,6,9,10,11,12,13,15	6	P01946,P02091,Q4FZU6,Q561R9,Q6IRE4,Q9QW07
6706	1,3,5,7,8,15,16,17	2,4,6,9,10,11,12,13,14	7	O54728,P01946,P02091,P0C0A9,P25236,P47727,Q6IRE4
6707	1,3,5,7,9,10,11,12	2,4,6,8,13,14,15,16,17	3	P27213,Q6IG02,Q6IRE4
6708	1,3,5,7,9,10,11,13	2,4,6,8,12,14,15,16,17	4	O54728,P98089,Q62635,Q6IRE4
6709	1,3,5,7,9,10,11,14	2,4,6,8,12,13,15,16,17	6	O54728,P18427,P27213,Q62635,Q6IRE4,Q9QUL6

6710	1,3,5,7,9,10,11,15	2,4,6,8,12,13,14,16,17	6	O54728,P14480,P25236,P27213,Q62635,Q6IRE4
6711	1,3,5,7,9,10,11,16	2,4,6,8,12,13,14,15,17	7	O54728,P25236,P27213,P55314,P98089,Q62635,Q6IRE4
6712	1,3,5,7,9,10,11,17	2,4,6,8,12,13,14,15,16	8	P01946,P02091,P08689,P18427,P25236,P52590,Q6IRE4,Q9QUL6
6713	1,3,5,7,9,10,12,13	2,4,6,8,11,14,15,16,17	3	P19218,Q5U2V4,Q6IRE4
6714	1,3,5,7,9,10,12,14	2,4,6,8,11,13,15,16,17	1	Q6IRE4
6715	1,3,5,7,9,10,12,15	2,4,6,8,11,13,14,16,17	3	O54728,P27213,Q6IRE4
6716	1,3,5,7,9,10,12,16	2,4,6,8,11,13,14,15,17	1	Q6IRE4
6717	1,3,5,7,9,10,12,17	2,4,6,8,11,13,14,15,16	6	P01946,P02091,P08689,P25236,P52590,Q99MH3
6718	1,3,5,7,9,10,13,14	2,4,6,8,11,12,15,16,17	5	O54728,P98089,Q4G075,Q62635,Q6IRE4
6719	1,3,5,7,9,10,13,15	2,4,6,8,11,12,14,16,17	7	O54728,P62804,P98089,Q00715,Q5U2V4,Q62635,Q6IRE4
6720	1,3,5,7,9,10,13,16	2,4,6,8,11,12,14,15,17	9	O54728,P01015,P62804,P98089,Q00715,Q4G075,Q62635,Q6AYQ8,Q6IRE4
6721	1,3,5,7,9,10,13,17	2,4,6,8,11,12,14,15,16	3	P01946,P02091,P52590
6722	1,3,5,7,9,10,14,15	2,4,6,8,11,12,13,16,17	7	O54728,P16636,P27213,Q4G075,Q62635,Q6IRE4,Q80WL1
6723	1,3,5,7,9,10,14,16	2,4,6,8,11,12,13,15,17	6	O54728,P01015,Q00715,Q4G075,Q62635,Q6IRE4
6724	1,3,5,7,9,10,14,17	2,4,6,8,11,12,13,15,16	5	P01946,P02091,P08689,P18427,P52590

6725	1,3,5,7,9,10,15,16	2,4,6,8,11,12,13,14,17	13	O54728,P02780,P02782,P0C0A9,P25236,P47727,P62804,Q00715,Q62635,Q6AYR9,Q6IRE4,Q80WL1,Q9JHB9
6726	1,3,5,7,9,10,15,17	2,4,6,8,11,12,13,14,16	8	O54728,P02091,P02782,P0C0A9,P18427,P25236,P47727,P52590
6727	1,3,5,7,9,10,16,17	2,4,6,8,11,12,13,14,15	9	P01946,P02091,P02782,P0C0A9,P25236,P47727,P52590,Q00715,Q6IRE4
6728	1,3,5,7,9,11,12,13	2,4,6,8,10,14,15,16,17	2	P17046,Q6IRE4
6729	1,3,5,7,9,11,12,14	2,4,6,8,10,13,15,16,17	2	Q6IG02,Q6IRE4
6730	1,3,5,7,9,11,12,15	2,4,6,8,10,13,14,16,17	4	O54728,P14480,P27213,Q6IRE4
6731	1,3,5,7,9,11,12,16	2,4,6,8,10,13,14,15,17	2	P17046,Q6IRE4
6732	1,3,5,7,9,11,12,17	2,4,6,8,10,13,14,15,16	5	P01946,P02091,P17046,P25236,Q6IRE4
6733	1,3,5,7,9,11,13,14	2,4,6,8,10,12,15,16,17	6	O54728,P06866,Q4G075,Q62635,Q6IRE4,Q6P6R2
6734	1,3,5,7,9,11,13,15	2,4,6,8,10,12,14,16,17	6	O54728,P14480,P98089,Q62635,Q6IRE4,Q6P6R2
6735	1,3,5,7,9,11,13,16	2,4,6,8,10,12,14,15,17	6	O54728,P17046,P62804,P98089,Q62635,Q6IRE4
6736	1,3,5,7,9,11,13,17	2,4,6,8,10,12,14,15,16	4	P01946,P02091,P17046,Q6IRE4
6737	1,3,5,7,9,11,14,15	2,4,6,8,10,12,13,16,17	9	O54728,P14480,P16636,P27213,Q62635,Q63355,Q6IRE4,Q6P6R2,Q80WL1
6738	1,3,5,7,9,11,14,16	2,4,6,8,10,12,13,15,17	5	O54728,P17046,Q4G075,Q62635,Q6IRE4
6739	1,3,5,7,9,11,14,17	2,4,6,8,10,12,13,15,16	4	P01946,P02091,Q6IRE4,Q9QUL6

6740	1,3,5,7,9,11,15,16	2,4,6,8,10,12,13,14,17	8	O54728,P02782,P17046,P25236,Q62635,Q6AYR9,Q6IRE4,Q80WL1
6741	1,3,5,7,9,11,15,17	2,4,6,8,10,12,13,14,16	5	O54728,P01946,P02091,P25236,Q6IRE4
6742	1,3,5,7,9,11,16,17	2,4,6,8,10,12,13,14,15	5	P01946,P02091,P17046,P25236,Q6IRE4
6743	1,3,5,7,9,12,13,14	2,4,6,8,10,11,15,16,17	2	A2RUV9,Q6IRE4
6744	1,3,5,7,9,12,13,15	2,4,6,8,10,11,14,16,17	4	O54728,Q5U2V4,Q6IRE4,Q6P6R2
6745	1,3,5,7,9,12,13,16	2,4,6,8,10,11,14,15,17	6	P02761,P17046,P19218,P62804,Q00715,Q6IRE4
6746	1,3,5,7,9,12,13,17	2,4,6,8,10,11,14,15,16	5	P01946,P02091,P17046,P35280,P53792
6747	1,3,5,7,9,12,14,15	2,4,6,8,10,11,13,16,17	4	A2RUV9,O54728,Q6IRE4,Q8CJD3
6748	1,3,5,7,9,12,14,16	2,4,6,8,10,11,13,15,17	3	P17046,Q4FZU6,Q6IRE4
6749	1,3,5,7,9,12,14,17	2,4,6,8,10,11,13,15,16	4	P01946,P02091,Q6IRE4,Q8CJD3
6750	1,3,5,7,9,12,15,16	2,4,6,8,10,11,13,14,17	4	O54728,P17046,P47727,Q6IRE4
6751	1,3,5,7,9,12,15,17	2,4,6,8,10,11,13,14,16	3	O54728,P02091,P47727
6752	1,3,5,7,9,12,16,17	2,4,6,8,10,11,13,14,15	7	P01946,P02091,P17046,P35280,P47727,Q4FZU6,Q6IRE4
6753	1,3,5,7,9,13,14,15	2,4,6,8,10,11,12,16,17	9	O54728,P16636,P53792,P62804,Q4G075,Q62635,Q6IRE4,Q6P6R2,Q80WL1
6754	1,3,5,7,9,13,14,16	2,4,6,8,10,11,12,15,17	8	O54728,P01015,P53792,P62804,Q00715,Q4G075,Q62635,Q6IRE4

6755	1,3,5,7,9,13,14,17	2,4,6,8,10,11,12,15,16	7	P01946,P02091,P35280,P51907,P53792,Q6IRE4,Q9QW07
6756	1,3,5,7,9,13,15,16	2,4,6,8,10,11,12,14,17	11	O54728,P0C0A9,P47727,P62804,P98089,Q00715,Q4G075,Q62635,Q63598,Q6IRE4,Q80WL1
6757	1,3,5,7,9,13,15,17	2,4,6,8,10,11,12,14,16	8	O54728,P01946,P02091,P0C0A9,P47727,P53792,P62804,Q6IRE4
6758	1,3,5,7,9,13,16,17	2,4,6,8,10,11,12,14,15	10	P01946,P02091,P0C0A9,P17046,P35280,P47727,P53792,P62804,Q00715,Q6IRE4
6759	1,3,5,7,9,14,15,16	2,4,6,8,10,11,12,13,17	13	O54728,P02782,P0C0A9,P16636,P62804,Q00715,Q4G075,Q62635,Q6AYR9,Q6IRE4,Q80WL1,Q9JHB9,Q9QZK9
6760	1,3,5,7,9,14,15,17	2,4,6,8,10,11,12,13,16	8	O54728,P02091,P0C0A9,P47967,P53792,Q6IRE4,Q80WL1,Q8CJD3
6761	1,3,5,7,9,14,16,17	2,4,6,8,10,11,12,13,15	6	P01946,P02091,P0C0A9,P62804,Q4FZU6,Q6IRE4
6762	1,3,5,7,9,15,16,17	2,4,6,8,10,11,12,13,14	16	O54728,P02091,P02780,P02782,P0C0A9,P24368,P25236,P30120,P47727,P62804,Q00715,Q63598,Q6AYR9,Q6IRE4,Q80WL1,Q9JHB9
6763	1,3,5,7,10,11,12,13	2,4,6,8,9,14,15,16,17	3	P17046,Q6IG02,Q6IRE4
6764	1,3,5,7,10,11,12,14	2,4,6,8,9,13,15,16,17	2	Q6IG02,Q6IRE4
6765	1,3,5,7,10,11,12,15	2,4,6,8,9,13,14,16,17	4	P00731,P27213,Q6IG02,Q6IRE4
6766	1,3,5,7,10,11,12,16	2,4,6,8,9,13,14,15,17	6	P12368,P17046,P55314,Q6IG02,Q6IRE4,Q6P6Q2
6767	1,3,5,7,10,11,12,17	2,4,6,8,9,13,14,15,16	8	P01946,P02091,P06757,P08689,P17046,P25236,Q6IG02,Q99MH3
6768	1,3,5,7,10,11,13,14	2,4,6,8,9,12,15,16,17	4	P98089,Q62635,Q6IRE4,Q6P6R2
6769	1,3,5,7,10,11,13,15	2,4,6,8,9,12,14,16,17	5	O54728,P98089,Q62635,Q6IRE4,Q6P6R2
6770	1,3,5,7,10,11,13,16	2,4,6,8,9,12,14,15,17	6	P17046,P55314,P98089,Q62635,Q6IRE4,Q8VD89
6771	1,3,5,7,10,11,13,17	2,4,6,8,9,12,14,15,16	4	P01946,P02091,P17046,Q6IRE4
6772	1,3,5,7,10,11,14,15	2,4,6,8,9,12,13,16,17	4	O54728,P27213,Q62635,Q6IRE4
6773	1,3,5,7,10,11,14,16	2,4,6,8,9,12,13,15,17	4	P17046,P55314,Q62635,Q6IRE4

6774	1,3,5,7,10,<br>11,14,17	2,4,6,8,9,1<br>2,13,15,16	5	P01946,P02091,P08689,P18427,Q6IRE4
6775	1,3,5,7,10,<br>11,15,16	2,4,6,8,9,1<br>2,13,14,17	11	O54728,P02782,P17046,P25236,P32755,P55314,Q62635,Q6AYR9,Q6IRE4,Q80WL1,Q8VD89
6776	1,3,5,7,10,<br>11,15,17	2,4,6,8,9,1<br>2,13,14,16	4	P01946,P02091,P18427,P25236
6777	1,3,5,7,10,<br>11,16,17	2,4,6,8,9,1<br>2,13,14,15	6	P01946,P02091,P17046,P25236,P55314,Q6IRE4
6778	1,3,5,7,10,<br>12,13,14	2,4,6,8,9,1<br>1,15,16,17	3	A2RUV9,Q498D9,Q5QE79
6779	1,3,5,7,10,<br>12,13,15	2,4,6,8,9,1<br>1,14,16,17	2	Q6P6R2,Q99MH3
6780	1,3,5,7,10,<br>12,13,16	2,4,6,8,9,1<br>1,14,15,17	3	P17046,P19218,Q6IRE4
6781	1,3,5,7,10,<br>12,13,17	2,4,6,8,9,1<br>1,14,15,16	5	P01946,P02091,P14668,P17046,Q99MH3
6782	1,3,5,7,10,<br>12,14,15	2,4,6,8,9,1<br>1,13,16,17	2	A2RUV9,Q8CJD3
6783	1,3,5,7,10,<br>12,14,16	2,4,6,8,9,1<br>1,13,15,17	3	P08721,P17046,Q6IRE4
6784	1,3,5,7,10,<br>12,14,17	2,4,6,8,9,1<br>1,13,15,16	4	P01946,P02091,P08689,Q8CJD3
6785	1,3,5,7,10,<br>12,15,16	2,4,6,8,9,1<br>1,13,14,17	3	P17046,P20766,Q6IRE4
6786	1,3,5,7,10,<br>12,15,17	2,4,6,8,9,1<br>1,13,14,16	4	P01946,P02091,P25236,Q99MH3
6787	1,3,5,7,10,<br>12,16,17	2,4,6,8,9,1<br>1,13,14,15	6	P01946,P02091,P17046,P25236,Q6TMA8,Q99MH3
6788	1,3,5,7,10,<br>13,14,15	2,4,6,8,9,1<br>1,12,16,17	5	O54728,P16636,Q62635,Q6IRE4,Q6P6R2
6789	1,3,5,7,10,<br>13,14,16	2,4,6,8,9,1<br>1,12,15,17	6	P01015,P19223,P98089,Q00715,Q62635,Q6IRE4
6790	1,3,5,7,10,<br>13,14,17	2,4,6,8,9,1<br>1,12,15,16	3	P01946,P02091,Q9QW07
6791	1,3,5,7,10,<br>13,15,16	2,4,6,8,9,1<br>1,12,14,17	11	O54728,P01835,P02782,P0C0A9,P62804,P98089,Q00715,Q62635,Q6AYQ8,Q6IRE4,Q80WL1
6792	1,3,5,7,10,<br>13,15,17	2,4,6,8,9,1<br>1,12,14,16	4	P01946,P02091,P0C0A9,Q99MH3
6793	1,3,5,7,10,<br>13,16,17	2,4,6,8,9,1<br>1,12,14,15	6	P01946,P02091,P0C0A9,P17046,P62804,Q6IRE4
6794	1,3,5,7,10,<br>14,15,16	2,4,6,8,9,1<br>1,12,13,17	11	O54728,P02780,P02782,P02803,P0C0A9,P16636,P19223,Q62635,Q6IRE4,Q80WL1,Q9JHB9
6795	1,3,5,7,10,<br>14,15,17	2,4,6,8,9,1<br>1,12,13,16	7	P01946,P02091,P02803,P08689,P0C0A9,P18427,Q8CJD3
6796	1,3,5,7,10,<br>14,16,17	2,4,6,8,9,1<br>1,12,13,15	5	P01946,P02091,P0C0A9,Q6IRE4,Q6TMA8

6797	1,3,5,7,10,<br>15,16,17	2,4,6,8,9,1<br>1,12,13,14	11	P02091,P02780,P02782,P0C0A9,P25236,P30120,P47727,Q6IRE4,Q6TMA8,Q80WL1,Q9JHB9
6798	1,3,5,7,11,<br>12,13,14	2,4,6,8,9,1<br>0,15,16,17	3	P17046,Q6IRE4,Q6P6R2
6799	1,3,5,7,11,<br>12,13,15	2,4,6,8,9,1<br>0,14,16,17	3	P17046,Q6IRE4,Q6P6R2
6800	1,3,5,7,11,<br>12,13,16	2,4,6,8,9,1<br>0,14,15,17	2	P17046,Q6IRE4
6801	1,3,5,7,11,<br>12,13,17	2,4,6,8,9,1<br>0,14,15,16	4	P01946,P02091,P17046,Q6IRE4
6802	1,3,5,7,11,<br>12,14,15	2,4,6,8,9,1<br>0,13,16,17	5	P17046,Q63355,Q6IRE4,Q6P6R2,Q8CJD3
6803	1,3,5,7,11,<br>12,14,16	2,4,6,8,9,1<br>0,13,15,17	2	P17046,Q6IRE4
6804	1,3,5,7,11,<br>12,14,17	2,4,6,8,9,1<br>0,13,15,16	5	P01946,P02091,P17046,Q6IRE4,Q8CJD3
6805	1,3,5,7,11,<br>12,15,16	2,4,6,8,9,1<br>0,13,14,17	3	O54728,P17046,Q6IRE4
6806	1,3,5,7,11,<br>12,15,17	2,4,6,8,9,1<br>0,13,14,16	3	P01946,P02091,P17046
6807	1,3,5,7,11,<br>12,16,17	2,4,6,8,9,1<br>0,13,14,15	5	P01946,P02091,P17046,P25236,Q6IRE4
6808	1,3,5,7,11,<br>13,14,15	2,4,6,8,9,1<br>0,12,16,17	8	O54728,P16636,P19223,Q09030,Q62635,Q6IRE4,Q6P6R2,Q80WL1
6809	1,3,5,7,11,<br>13,14,16	2,4,6,8,9,1<br>0,12,15,17	5	P17046,P19223,Q62635,Q6IRE4,Q6P6R2
6810	1,3,5,7,11,<br>13,14,17	2,4,6,8,9,1<br>0,12,15,16	5	P01946,P02091,P17046,Q6IRE4,Q9QW07
6811	1,3,5,7,11,<br>13,15,16	2,4,6,8,9,1<br>0,12,14,17	10	O54728,P17046,P19223,P98089,Q09030,Q62635,Q6IRE4,Q6P6R2,Q80WL1,Q8VD89
6812	1,3,5,7,11,<br>13,15,17	2,4,6,8,9,1<br>0,12,14,16	4	P01946,P02091,P17046,Q6IRE4
6813	1,3,5,7,11,<br>13,16,17	2,4,6,8,9,1<br>0,12,14,15	4	P01946,P02091,P17046,Q6IRE4
6814	1,3,5,7,11,<br>14,15,16	2,4,6,8,9,1<br>0,12,13,17	10	O54728,P16636,P17046,P19223,Q62635,Q63355,Q6AYR9,Q6IRE4,Q6P6R2,Q80WL1
6815	1,3,5,7,11,<br>14,15,17	2,4,6,8,9,1<br>0,12,13,16	6	O54728,P01946,P02091,Q6IRE4,Q80WL1,Q8CJD3
6816	1,3,5,7,11,<br>14,16,17	2,4,6,8,9,1<br>0,12,13,15	4	P01946,P02091,P17046,Q6IRE4
6817	1,3,5,7,11,<br>15,16,17	2,4,6,8,9,1<br>0,12,13,14	10	O54728,P01946,P02091,P02782,P0C0A9,P17046,P25236,Q6AYR9,Q6IRE4,Q80WL1
6818	1,3,5,7,12,<br>13,14,15	2,4,6,8,9,1<br>0,11,16,17	3	A2RUV9,Q6IRE4,Q6P6R2
6819	1,3,5,7,12,<br>13,14,16	2,4,6,8,9,1<br>0,11,15,17	3	P02761,P17046,Q6IRE4

6820	1,3,5,7,12,13,14,17	2,4,6,8,9,10,11,15,16	5	P01946,P02091,P17046,Q561R9,Q9QW07
6821	1,3,5,7,12,13,15,16	2,4,6,8,9,10,11,14,17	5	O54728,P17046,P19218,Q6IRE4,Q6P6R2
6822	1,3,5,7,12,13,15,17	2,4,6,8,9,10,11,14,16	4	P01946,P02091,P17046,Q99MH3
6823	1,3,5,7,12,13,16,17	2,4,6,8,9,10,11,14,15	5	P01946,P02091,P17046,P35280,Q6IRE4
6824	1,3,5,7,12,14,15,16	2,4,6,8,9,10,11,13,17	5	O54728,P17046,P20766,Q6IRE4,Q8CJD3
6825	1,3,5,7,12,14,15,17	2,4,6,8,9,10,11,13,16	4	A2RUV9,P01946,P02091,Q8CJD3
6826	1,3,5,7,12,14,16,17	2,4,6,8,9,10,11,13,15	7	P01946,P02091,P17046,Q4FZU6,Q561R9,Q6IRE4,Q9QW07
6827	1,3,5,7,12,15,16,17	2,4,6,8,9,10,11,13,14	5	P01946,P02091,P17046,P47727,Q6IRE4
6828	1,3,5,7,13,14,15,16	2,4,6,8,9,10,11,12,17	13	O54728,P00762,P0C0A9,P16636,P17046,P19223,P62804,Q00715,Q09030,Q62635,Q6IRE4,Q6P6R2,Q80WL1
6829	1,3,5,7,13,14,15,17	2,4,6,8,9,10,11,12,16	6	P01946,P02091,P0C0A9,P53792,Q9QW07,Q9WUW8
6830	1,3,5,7,13,14,16,17	2,4,6,8,9,10,11,12,15	9	P01946,P02091,P0C0A9,P17046,P35280,P62804,Q561R9,Q6IRE4,Q9QW07
6831	1,3,5,7,13,15,16,17	2,4,6,8,9,10,11,12,14	11	O54728,P00714,P01946,P02091,P0C0A9,P17046,P47727,P62804,Q00715,Q6IRE4,Q80WL1
6832	1,3,5,7,14,15,16,17	2,4,6,8,9,10,11,12,13	11	O54728,P01946,P02091,P02780,P02782,P02803,P0C0A9,Q6IRE4,Q80WL1,Q9JHB9,Q9QW07
6833	1,3,5,8,9,10,11,12	2,4,6,7,13,14,15,16,17	5	P27213,P35467,Q6IG02,Q6IRE4,Q6P6Q2
6834	1,3,5,8,9,10,11,13	2,4,6,7,12,14,15,16,17	6	P09605;P25809,P14480,P35467,P98089,Q63532,Q6IRE4
6835	1,3,5,8,9,10,11,14	2,4,6,7,12,13,15,16,17	6	P09605;P25809,P14480,P18427,P27213,Q62635,Q6IRE4
6836	1,3,5,8,9,10,11,15	2,4,6,7,12,13,14,16,17	8	O54728,P09605;P25809,P14480,P25236,P27213,Q62635,Q63532,Q6IRE4
6837	1,3,5,8,9,10,11,16	2,4,6,7,12,13,14,15,17	4	O54728,P25236,Q62635,Q6IRE4
6838	1,3,5,8,9,10,11,17	2,4,6,7,12,13,14,15,16	5	P08649,P18427,P25236,P35467,Q63532

6839	1,3,5,8,9,10,12,13	2,4,6,7,11,14,15,16,17	1	Q6IG02
6840	1,3,5,8,9,10,12,14	2,4,6,7,11,13,15,16,17	2	P09605;P25809,Q6IG02
6841	1,3,5,8,9,10,12,15	2,4,6,7,11,13,14,16,17	4	O54728,P09605;P25809,P14480,Q6IG02
6842	1,3,5,8,9,10,12,16	2,4,6,7,11,13,14,15,17	2	Q6IG02,Q6IRE4
6843	1,3,5,8,9,10,12,17	2,4,6,7,11,13,14,15,16	3	P25236,Q63532,Q6IG02
6844	1,3,5,8,9,10,13,14	2,4,6,7,11,12,15,16,17	1	P09605;P25809
6845	1,3,5,8,9,10,13,15	2,4,6,7,11,12,14,16,17	5	O54728,P09605;P25809,P14480,P98089,Q63532
6846	1,3,5,8,9,10,13,16	2,4,6,7,11,12,14,15,17	6	O54728,P09605;P25809,P98089,Q62635,Q6AYQ8,Q6IRE4
6847	1,3,5,8,9,10,13,17	2,4,6,7,11,12,14,15,16	2	P51907,Q63532
6848	1,3,5,8,9,10,14,15	2,4,6,7,11,12,13,16,17	4	O54728,P09605;P25809,P14480,Q62635
6849	1,3,5,8,9,10,14,16	2,4,6,7,11,12,13,15,17	5	O54728,P09605;P25809,Q62635,Q6IRE4,Q9QZK9
6850	1,3,5,8,9,10,14,17	2,4,6,7,11,12,13,15,16	1	P18427
6851	1,3,5,8,9,10,15,16	2,4,6,7,11,12,13,14,17	6	O54728,P09605;P25809,P25236,Q62635,Q6IRE4,Q9QZK9
6852	1,3,5,8,9,10,15,17	2,4,6,7,11,12,13,14,16	5	G3V686,O54728,P18427,P25236,Q63532
6853	1,3,5,8,9,10,16,17	2,4,6,7,11,12,13,14,15	2	P25031,P25236

6854	1,3,5,8,9,11,12,13	2,4,6,7,10,14,15,16,17	3	Q63532,Q6IG02,Q6IRE4
6855	1,3,5,8,9,11,12,14	2,4,6,7,10,13,15,16,17	3	P14480,Q6IG02,Q6IRE4
6856	1,3,5,8,9,11,12,15	2,4,6,7,10,13,14,16,17	5	O54728,P12020,P14480,Q6IG02,Q6IRE4
6857	1,3,5,8,9,11,12,16	2,4,6,7,10,13,14,15,17	4	P12020,Q6IG02,Q6IRE4,Q6P6Q2
6858	1,3,5,8,9,11,12,17	2,4,6,7,10,13,14,15,16	3	P12020,Q63532,Q6IG02
6859	1,3,5,8,9,11,13,14	2,4,6,7,10,12,15,16,17	6	O54728,P06866,P09605;P25809,P14480,P97840,Q6IRE4
6860	1,3,5,8,9,11,13,15	2,4,6,7,10,12,14,16,17	5	O54728,P14480,P97840,Q63532,Q6IRE4
6861	1,3,5,8,9,11,13,16	2,4,6,7,10,12,14,15,17	3	O54728,P98089,Q6IRE4
6862	1,3,5,8,9,11,13,17	2,4,6,7,10,12,14,15,16	3	P04218,P97840,Q63532
6863	1,3,5,8,9,11,14,15	2,4,6,7,10,12,13,16,17	10	O54728,P04218,P09605;P25809,P14480,P47967,P97840,Q5I0D1,Q62635,Q6IRE4,Q9QZK9
6864	1,3,5,8,9,11,14,16	2,4,6,7,10,12,13,15,17	5	O54728,P04218,Q62635,Q6IRE4,Q9QZK9
6865	1,3,5,8,9,11,14,17	2,4,6,7,10,12,13,15,16	4	P04218,P47967,P97840,Q6IRE4
6866	1,3,5,8,9,11,15,16	2,4,6,7,10,12,13,14,17	10	B4F795,O54728,P04218,P12020,P14480,P25236,Q62635,Q6AYR9,Q6IRE4,Q9QZK9
6867	1,3,5,8,9,11,15,17	2,4,6,7,10,12,13,14,16	7	O54728,P04218,P12020,P25236,P47967,P97840,Q63532
6868	1,3,5,8,9,11,16,17	2,4,6,7,10,12,13,14,15	4	P04218,P12020,P25236,Q6IRE4

6869	1,3,5,8,9,12,13,14	2,4,6,7,10,11,15,16,17	2	Q5FVI6,Q9Z1F2
6870	1,3,5,8,9,12,13,15	2,4,6,7,10,11,14,16,17	3	O54728,P14480,Q63532
6871	1,3,5,8,9,12,13,16	2,4,6,7,10,11,14,15,17	1	Q6IRE4
6872	1,3,5,8,9,12,13,17	2,4,6,7,10,11,14,15,16	4	P35280,P51907,Q5FVI6,Q63532
6873	1,3,5,8,9,12,14,15	2,4,6,7,10,11,13,16,17	2	O54728,P14480
6874	1,3,5,8,9,12,14,16	2,4,6,7,10,11,13,15,17	2	Q6IG02,Q6IRE4
6875	1,3,5,8,9,12,14,17	2,4,6,7,10,11,13,15,16	3	Q5FVI6,Q6IG02,Q99PP0
6876	1,3,5,8,9,12,15,16	2,4,6,7,10,11,13,14,17	3	O54728,P12020,Q6IRE4
6877	1,3,5,8,9,12,15,17	2,4,6,7,10,11,13,14,16	2	P12020,Q63532
6878	1,3,5,8,9,12,16,17	2,4,6,7,10,11,13,14,15	3	P12020,P35280,Q6IG02
6879	1,3,5,8,9,13,14,15	2,4,6,7,10,11,12,16,17	8	O54728,P06757,P08753,P09605;P25809,P14480,P47967,P53792,Q6IRE4
6880	1,3,5,8,9,13,14,16	2,4,6,7,10,11,12,15,17	3	O54728,P01015,Q6IRE4
6881	1,3,5,8,9,13,14,17	2,4,6,7,10,11,12,15,16	8	P08753,P35280,P47967,P51907,P53792,P97840,Q5FVI6,Q9QW07
6882	1,3,5,8,9,13,15,16	2,4,6,7,10,11,12,14,17	5	O54728,P62804,Q00715,Q62635,Q6IRE4
6883	1,3,5,8,9,13,15,17	2,4,6,7,10,11,12,14,16	8	O54728,P08753,P47967,P51907,P53792,P97840,Q5FVI6,Q63532

6884	1,3,5,8,9,13,16,17	2,4,6,7,10,11,12,14,15	4	P08753,P35280,P51907,Q6IRE4
6885	1,3,5,8,9,14,15,16	2,4,6,7,10,11,12,13,17	6	O54728,P09605;P25809,P14480,Q62635,Q6IRE4,Q9QZK9
6886	1,3,5,8,9,14,15,17	2,4,6,7,10,11,12,13,16	6	O54728,P04218,P47967,P53792,P97840,Q5FVI6
6887	1,3,5,8,9,14,16,17	2,4,6,7,10,11,12,13,15	2	P04218,Q6IRE4
6888	1,3,5,8,9,15,16,17	2,4,6,7,10,11,12,13,14	9	O54728,P04218,P0C0A9,P12020,P23593,P25236,P47727,Q6IRE4,Q9JHB9
6889	1,3,5,8,10,11,12,13	2,4,6,7,9,14,15,16,17	3	P09605;P25809,Q6IG02,Q6P6Q2
6890	1,3,5,8,10,11,12,14	2,4,6,7,9,13,15,16,17	3	P09605;P25809,Q6IG02,Q6P6Q2
6891	1,3,5,8,10,11,12,15	2,4,6,7,9,13,14,16,17	5	P00731,P09605;P25809,P14480,Q6IG02,Q6P6Q2
6892	1,3,5,8,10,11,12,16	2,4,6,7,9,13,14,15,17	3	Q6IG02,Q6IRE4,Q6P6Q2
6893	1,3,5,8,10,11,12,17	2,4,6,7,9,13,14,15,16	5	P16636,P25236,Q6IG02,Q6P6Q2,Q99MH3
6894	1,3,5,8,10,11,13,14	2,4,6,7,9,12,15,16,17	2	P09605;P25809,Q6IRE4
6895	1,3,5,8,10,11,13,15	2,4,6,7,9,12,14,16,17	4	O54728,P09605;P25809,P14480,P98089
6896	1,3,5,8,10,11,13,16	2,4,6,7,9,12,14,15,17	3	P09605;P25809,P98089,Q6IRE4
6897	1,3,5,8,10,11,13,17	2,4,6,7,9,12,14,15,16	1	Q63532
6898	1,3,5,8,10,11,14,15	2,4,6,7,9,12,13,16,17	4	O54728,P09605;P25809,P14480,Q62635
6899	1,3,5,8,10,11,14,16	2,4,6,7,9,12,13,15,17	3	P09605;P25809,Q62635,Q6IRE4
6900	1,3,5,8,10,11,14,17	2,4,6,7,9,12,13,15,16	1	P18427
6901	1,3,5,8,10,11,15,16	2,4,6,7,9,12,13,14,17	6	O54728,P09605;P25809,P25236,P32755,Q62635,Q6IRE4
6902	1,3,5,8,10,11,15,17	2,4,6,7,9,12,13,14,16	4	G3V686,P18427,P25236,Q63532
6903	1,3,5,8,10,11,16,17	2,4,6,7,9,12,13,14,15	2	P08649,P25236

6904	1,3,5,8,10,<br>12,13,14	2,4,6,7,9,1<br>1,15,16,17	2	P09605;P25809,Q6IG02
6905	1,3,5,8,10,<br>12,13,15	2,4,6,7,9,1<br>1,14,16,17	4	P09605;P25809,P83121,Q6IG02,Q99MH3
6906	1,3,5,8,10,<br>12,13,16	2,4,6,7,9,1<br>1,14,15,17	3	P09605;P25809,P83121,Q6IG02
6907	1,3,5,8,10,<br>12,13,17	2,4,6,7,9,1<br>1,14,15,16	4	P83121,Q63532,Q6IG02,Q99MH3
6908	1,3,5,8,10,<br>12,14,15	2,4,6,7,9,1<br>1,13,16,17	2	P09605;P25809,Q6IG02
6909	1,3,5,8,10,<br>12,14,16	2,4,6,7,9,1<br>1,13,15,17	3	P08721,P09605;P25809,Q6IG02
6910	1,3,5,8,10,<br>12,14,17	2,4,6,7,9,1<br>1,13,15,16	2	Q6IG02,Q99PP0
6911	1,3,5,8,10,<br>12,15,16	2,4,6,7,9,1<br>1,13,14,17	3	P09605;P25809,P83121,Q6IG02
6912	1,3,5,8,10,<br>12,15,17	2,4,6,7,9,1<br>1,13,14,16	4	P83121,Q63532,Q6IG02,Q99MH3
6913	1,3,5,8,10,<br>12,16,17	2,4,6,7,9,1<br>1,13,14,15	3	P83121,Q6IG02,Q99MH3
6914	1,3,5,8,10,<br>13,14,15	2,4,6,7,9,1<br>1,12,16,17	3	O54728,P09605;P25809,P14480
6915	1,3,5,8,10,<br>13,14,16	2,4,6,7,9,1<br>1,12,15,17	2	P09605;P25809,Q6IRE4
6916	1,3,5,8,10,<br>13,14,17	2,4,6,7,9,1<br>1,12,15,16	3	P08753,P51907,Q9QW07
6917	1,3,5,8,10,<br>13,15,16	2,4,6,7,9,1<br>1,12,14,17	6	O54728,P01835,P09605;P25809,P83121,P98089,Q6AYQ8
6918	1,3,5,8,10,<br>13,15,17	2,4,6,7,9,1<br>1,12,14,16	4	P08753,P83121,Q63532,Q99MH3
6919	1,3,5,8,10,<br>13,16,17	2,4,6,7,9,1<br>1,12,14,15	4	P08753,P25031,P51907,P83121
6920	1,3,5,8,10,<br>14,15,16	2,4,6,7,9,1<br>1,12,13,17	5	O54728,P09605;P25809,Q62635,Q6IRE4,Q9QZK9
6921	1,3,5,8,10,<br>14,15,17	2,4,6,7,9,1<br>1,12,13,16	3	G3V686,P18427,P47967
6922	1,3,5,8,10,<br>14,16,17	2,4,6,7,9,1<br>1,12,13,15	1	Q9QW07
6923	1,3,5,8,10,<br>15,16,17	2,4,6,7,9,1<br>1,12,13,14	4	P0C0A9,P25236,P83121,Q9JHB9
6924	1,3,5,8,11,<br>12,13,14	2,4,6,7,9,1<br>0,15,16,17	3	P09605;P25809,Q6IG02,Q6IRE4
6925	1,3,5,8,11,<br>12,13,15	2,4,6,7,9,1<br>0,14,16,17	3	P14480,Q6IG02,Q6P6R2
6926	1,3,5,8,11,<br>12,13,16	2,4,6,7,9,1<br>0,14,15,17	3	P17046,Q6IG02,Q6IRE4

6927	1,3,5,8,11,<br>12,13,17	2,4,6,7,9,1<br>0,14,15,16	2	Q63532,Q6IG02
6928	1,3,5,8,11,<br>12,14,15	2,4,6,7,9,1<br>0,13,16,17	5	P09605;P25809,P14480,Q5I0D1,Q6IG02,Q6IRE4
6929	1,3,5,8,11,<br>12,14,16	2,4,6,7,9,1<br>0,13,15,17	3	Q6IG02,Q6IRE4,Q6P6Q2
6930	1,3,5,8,11,<br>12,14,17	2,4,6,7,9,1<br>0,13,15,16	3	P97840,Q6IG02,Q99PP0
6931	1,3,5,8,11,<br>12,15,16	2,4,6,7,9,1<br>0,13,14,17	4	P12020,Q5I0D1,Q6IG02,Q6IRE4
6932	1,3,5,8,11,<br>12,15,17	2,4,6,7,9,1<br>0,13,14,16	4	P12020,P97840,Q63532,Q6IG02
6933	1,3,5,8,11,<br>12,16,17	2,4,6,7,9,1<br>0,13,14,15	5	P12020,P17046,Q6IG02,Q6IRE4,Q6P6Q2
6934	1,3,5,8,11,<br>13,14,15	2,4,6,7,9,1<br>0,12,16,17	8	O54728,P09605;P25809,P14480,P19223,P47967,P97840,Q6IRE4,Q6P6R2
6935	1,3,5,8,11,<br>13,14,16	2,4,6,7,9,1<br>0,12,15,17	3	P09605;P25809,P19223,Q6IRE4
6936	1,3,5,8,11,<br>13,14,17	2,4,6,7,9,1<br>0,12,15,16	3	P47967,P97840,Q9QW07
6937	1,3,5,8,11,<br>13,15,16	2,4,6,7,9,1<br>0,12,14,17	4	O54728,P09605;P25809,P19223,Q6IRE4
6938	1,3,5,8,11,<br>13,15,17	2,4,6,7,9,1<br>0,12,14,16	3	P47967,P97840,Q63532
6939	1,3,5,8,11,<br>13,16,17	2,4,6,7,9,1<br>0,12,14,15	3	P17046,P97840,Q6IRE4
6940	1,3,5,8,11,<br>14,15,16	2,4,6,7,9,1<br>0,12,13,17	9	O54728,P09605;P25809,P14480,P19223,P97840,Q5I0D1,Q62635,Q6IRE4,Q9QZK9
6941	1,3,5,8,11,<br>14,15,17	2,4,6,7,9,1<br>0,12,13,16	4	P04218,P47967,P97840,Q5I0D1
6942	1,3,5,8,11,<br>14,16,17	2,4,6,7,9,1<br>0,12,13,15	4	P04218,P97840,Q6IRE4,Q9QW07
6943	1,3,5,8,11,<br>15,16,17	2,4,6,7,9,1<br>0,12,13,14	5	P04218,P12020,P25236,P97840,Q6IRE4
6944	1,3,5,8,12,<br>13,14,15	2,4,6,7,9,1<br>0,11,16,17	4	P09605;P25809,Q5FVI6,Q64093,Q6P6R2
6945	1,3,5,8,12,<br>13,14,16	2,4,6,7,9,1<br>0,11,15,17	1	Q6IRE4
6946	1,3,5,8,12,<br>13,14,17	2,4,6,7,9,1<br>0,11,15,16	6	O70594,P51907,Q561R9,Q5FVI6,Q9QW07,Q9Z1F2
6947	1,3,5,8,12,<br>13,15,16	2,4,6,7,9,1<br>0,11,14,17	2	P83121,Q6IRE4
6948	1,3,5,8,12,<br>13,15,17	2,4,6,7,9,1<br>0,11,14,16	5	P83121,Q5FVI6,Q5XFX0,Q63532,Q99MH3
6949	1,3,5,8,12,<br>13,16,17	2,4,6,7,9,1<br>0,11,14,15	5	P17046,P35280,P51907,P83121,Q5XFX0

6950	1,3,5,8,12,14,15,16	2,4,6,7,9,10,11,13,17	2	P09605;P25809,Q6IRE4
6951	1,3,5,8,12,14,15,17	2,4,6,7,9,10,11,13,16	4	P47967,Q5FVI6,Q8CJD3,Q9QW07
6952	1,3,5,8,12,14,16,17	2,4,6,7,9,10,11,13,15	3	Q561R9,Q6IG02,Q9QW07
6953	1,3,5,8,12,15,16,17	2,4,6,7,9,10,11,13,14	3	P12020,P83121,Q5XFX0
6954	1,3,5,8,13,14,15,16	2,4,6,7,9,10,11,12,17	6	O54728,P08753,P09605;P25809,P19223,Q08849,Q6IRE4
6955	1,3,5,8,13,14,15,17	2,4,6,7,9,10,11,12,16	9	P08753,P10536,P47967,P51907,P97840,Q08849,Q5FVI6,Q64093,Q9QW07
6956	1,3,5,8,13,14,16,17	2,4,6,7,9,10,11,12,15	6	P08753,P35280,P51907,Q5FVI6,Q6PCU2,Q9QW07
6957	1,3,5,8,13,15,16,17	2,4,6,7,9,10,11,12,14	4	P08753,P0C0A9,P83121,Q5XFX0
6958	1,3,5,8,14,15,16,17	2,4,6,7,9,10,11,12,13	4	P0C0A9,P47967,P97840,Q9QW07
6959	1,3,5,9,10,11,12,13	2,4,6,7,8,14,15,16,17	2	Q6IG02,Q6IRE4
6960	1,3,5,9,10,11,12,14	2,4,6,7,8,13,15,16,17	2	Q6IG02,Q6IRE4
6961	1,3,5,9,10,11,12,15	2,4,6,7,8,13,14,16,17	6	O54728,P06911,P11598,P14480,Q6IG02,Q6IRE4
6962	1,3,5,9,10,11,12,16	2,4,6,7,8,13,14,15,17	9	P06911,P11598,P12368,P20761,Q5GRG2,Q6IG02,Q6IRE4,Q6P6Q2,Q9WUW8
6963	1,3,5,9,10,11,12,17	2,4,6,7,8,13,14,15,16	5	P06911,P08689,P25236,Q5GRG2,Q6IG02
6964	1,3,5,9,10,11,13,14	2,4,6,7,8,12,15,16,17	10	O54728,P09605;P25809,P19223,P20761,P55091,P98089,Q4G075,Q62635,Q6IRE4,Q99041
6965	1,3,5,9,10,11,13,15	2,4,6,7,8,12,14,16,17	14	O54728,P09605;P25809,P11598,P14480,P19223,P55091,P98089,Q4G075,Q62635,Q6IRE4,Q6P6R2,Q80WL1,Q99041,Q9R0T3
6966	1,3,5,9,10,11,13,16	2,4,6,7,8,12,14,15,17	12	O54728,P11598,P19223,P20761,P20762,P55091,P62815,P98089,Q4G075,Q62635,Q6IRE4,Q99041
6967	1,3,5,9,10,11,13,17	2,4,6,7,8,12,14,15,16	2	Q63532,Q920P0
6968	1,3,5,9,10,11,14,15	2,4,6,7,8,12,13,16,17	19	O54728,P06761,P09605;P25809,P11598,P14480,P18427,P19223,P22282,P55091,P63029,P98089,Q4G075,Q62635,Q62902,Q6IRE4,Q80WL1,Q99041,Q9QZK9,Q9R0T3
6969	1,3,5,9,10,11,14,16	2,4,6,7,8,12,13,15,17	15	O54728,P06761,P11598,P19223,P20761,P22282,P55091,P62815,P98089,Q4G075,Q62635,Q6IRE4,Q80WL1,Q99041,Q9QZK9
6970	1,3,5,9,10,11,14,17	2,4,6,7,8,12,13,15,16	6	P08689,P18427,P25236,Q6IRE4,Q9QUL6,Q9R0T3
6971	1,3,5,9,10,11,15,16	2,4,6,7,8,12,13,14,17	25	O54728,P02780,P02782,P06761,P06911,P07647,P11598,P12020,P19223,P22282,P22283,P25236,P50280,P55091,P62815,P98089,Q4G075,Q62635,Q62902,Q6AYR9,Q6IRE4,Q80WL1,Q99041,Q9QZK9,Q9R0T3
6972	1,3,5,9,10,11,15,17	2,4,6,7,8,12,13,14,16	12	O54728,P06761,P06911,P11598,P12020,P18427,P25236,Q62902,Q63532,Q6AYR9,Q80WL1,Q9R0T3

6973	1,3,5,9,10,<br>11,16,17	2,4,6,7,8,1<br>2,13,14,15	10	O08557,P06761,P06911,P11598,P12020,P25236,Q5GRG2,Q62902,Q6AYR9,Q6IRE4
6974	1,3,5,9,10,<br>12,13,14	2,4,6,7,8,1<br>1,15,16,17	3	P07150,P09605;P25809,P14669
6975	1,3,5,9,10,<br>12,13,15	2,4,6,7,8,1<br>1,14,16,17	4	O54728,P07150,P09605;P25809,Q6P6R2
6976	1,3,5,9,10,<br>12,13,16	2,4,6,7,8,1<br>1,14,15,17	9	P02631,P02761,P07150,P07171,P11598,P14669,P20761,P20762,Q6IRE4
6977	1,3,5,9,10,<br>12,13,17	2,4,6,7,8,1<br>1,14,15,16	5	P02631,P07171,P35280,Q63532,Q99MH3
6978	1,3,5,9,10,<br>12,14,15	2,4,6,7,8,1<br>1,13,16,17	5	O54728,P07150,P09605;P25809,P11598,Q9R0T3
6979	1,3,5,9,10,<br>12,14,16	2,4,6,7,8,1<br>1,13,15,17	4	P07150,P11598,P14669,Q6IRE4
6980	1,3,5,9,10,<br>12,14,17	2,4,6,7,8,1<br>1,13,15,16	1	P08689
6981	1,3,5,9,10,<br>12,15,16	2,4,6,7,8,1<br>1,13,14,17	12	O54728,P06761,P06911,P07150,P11598,P12020,P14669,P22282,P22283,P50280,Q62902,Q6IRE4
6982	1,3,5,9,10,<br>12,15,17	2,4,6,7,8,1<br>1,13,14,16	4	P06911,P25236,Q62902,Q63532
6983	1,3,5,9,10,<br>12,16,17	2,4,6,7,8,1<br>1,13,14,15	9	P02631,P06911,P07171,P11598,P14669,P20762,P25236,P35280,Q5GRG2
6984	1,3,5,9,10,<br>13,14,15	2,4,6,7,8,1<br>1,12,16,17	17	O54728,O55004,P07150,P09605;P25809,P14669,P19223,P50115,P55091,P98089,Q07936,Q4G075,Q62635,Q6IRE4,Q6P6R2,Q80WL1,Q9<br>9041,Q9R0T3
6985	1,3,5,9,10,<br>13,14,16	2,4,6,7,8,1<br>1,12,15,17	20	O54728,O55004,P01015,P07150,P09605;P25809,P11598,P14669,P19223,P20761,P50115,P50280,P55091,P98089,Q00715,Q4G075,Q626<br>35,Q6IRE4,Q80WL1,Q99041,Q9QZA6
6986	1,3,5,9,10,<br>13,14,17	2,4,6,7,8,1<br>1,12,15,16	2	P35280,Q9QW07
6987	1,3,5,9,10,<br>13,15,16	2,4,6,7,8,1<br>1,12,14,17	32	O54728,O55004,P02780,P02782,P06761,P07150,P07647,P09605;P25809,P0C0A9,P11598,P14669,P19223,P20762,P22282,P22283,P3012<br>0,P50115,P50280,P55091,P62804,P98089,Q00715,Q4G075,Q62635,Q63493,Q6AYQ8,Q6AYR9,Q6IRE4,Q80WL1,Q99041,Q9EQS0,Q9QZ<br>K9
6988	1,3,5,9,10,<br>13,15,17	2,4,6,7,8,1<br>1,12,14,16	6	O54728,P0C0A9,Q63532,Q80WL1,Q9EQS0,Q9R0T3
6989	1,3,5,9,10,<br>13,16,17	2,4,6,7,8,1<br>1,12,14,15	12	P02631,P07171,P0C0A9,P11598,P14669,P20762,P35280,P50280,P62804,Q00715,Q6IRE4,Q9EQS0
6990	1,3,5,9,10,<br>14,15,16	2,4,6,7,8,1<br>1,12,13,17	30	O54728,O55004,P02780,P02782,P06761,P07150,P07647,P09605;P25809,P0C0A9,P11598,P14669,P19223,P22282,P22283,P30120,P5011<br>5,P50280,P55091,P60905,P98089,Q4G075,Q62635,Q62902,Q6AYR9,Q6IRE4,Q80WL1,Q99041,Q9JHB9,Q9QZK9,Q9R0T3
6991	1,3,5,9,10,<br>14,15,17	2,4,6,7,8,1<br>1,12,13,16	18	O54728,P02780,P02782,P06761,P07647,P08689,P0C0A9,P11598,P18427,P22282,P22283,P24368,P30120,Q62902,Q80WL1,Q8CJ52,Q9J<br>HB9,Q9R0T3
6992	1,3,5,9,10,<br>14,16,17	2,4,6,7,8,1<br>1,12,13,15	15	P02780,P02782,P06761,P07647,P0C0A9,P11598,P14669,P22282,P22283,P30120,P50280,Q62902,Q6IRE4,Q80WL1,Q9JHB9
6993	1,3,5,9,10,<br>15,16,17	2,4,6,7,8,1<br>1,12,13,14	28	O54728,P02780,P02781,P02782,P06761,P06911,P07647,P0C0A9,P11598,P12020,P14669,P20762,P22282,P22283,P24368,P25236,P3012<br>0,P47727,P50280,Q5GRG2,Q62902,Q6AYR9,Q80WL1,Q8CJ52,Q9EQS0,Q9JHB9,Q9JI85,Q9R0T3
6994	1,3,5,9,11,<br>12,13,14	2,4,6,7,8,1<br>0,15,16,17	2	Q6IRE4,Q6P6R2

6995	1,3,5,9,11,12,13,15	2,4,6,7,8,10,14,16,17	4	O54728,P14480,Q6IRE4,Q6P6R2
6996	1,3,5,9,11,12,13,16	2,4,6,7,8,10,14,15,17	4	P02761,P11598,P17046,Q6IRE4
6997	1,3,5,9,11,12,13,17	2,4,6,7,8,10,14,15,16	3	P35280,Q63532,Q6IRE4
6998	1,3,5,9,11,12,14,15	2,4,6,7,8,10,13,16,17	5	O54728,P14480,Q6IRE4,Q6P6R2,Q9R0T3
6999	1,3,5,9,11,12,14,16	2,4,6,7,8,10,13,15,17	3	P11598,Q6IG02,Q6IRE4
7000	1,3,5,9,11,12,14,17	2,4,6,7,8,10,13,15,16	3	P01835,Q6IG02,Q6IRE4
7001	1,3,5,9,11,12,15,16	2,4,6,7,8,10,13,14,17	9	O54728,P06761,P06911,P11598,P12020,P17046,P22282,Q5GRG2,Q6IRE4
7002	1,3,5,9,11,12,15,17	2,4,6,7,8,10,13,14,16	6	P06911,P12020,P25236,Q5GRG2,Q63532,Q9R0T3
7003	1,3,5,9,11,12,16,17	2,4,6,7,8,10,13,14,15	9	P06911,P11598,P12020,P17046,P25236,P35280,Q5GRG2,Q6IG02,Q6IRE4
7004	1,3,5,9,11,13,14,15	2,4,6,7,8,10,12,16,17	13	O54728,P09605;P25809,P14480,P19223,P55091,P98089,Q4G075,Q62635,Q6IRE4,Q6P6R2,Q80WL1,Q99041,Q9R0T3
7005	1,3,5,9,11,13,14,16	2,4,6,7,8,10,12,15,17	17	O54728,O55004,P01015,P02761,P04218,P11598,P19223,P20761,P55091,P98089,Q4G075,Q62635,Q6IRE4,Q6P6R2,Q80WL1,Q99041,Q9QZK9
7006	1,3,5,9,11,13,14,17	2,4,6,7,8,10,12,15,16	7	D3ZHA0,P04218,P35280,P47967,P97840,Q6IRE4,Q9QW07
7007	1,3,5,9,11,13,15,16	2,4,6,7,8,10,12,14,17	17	O54728,P06761,P11598,P12020,P19223,P22282,P55091,P98089,Q4G075,Q62635,Q6AYR9,Q6IRE4,Q6P6R2,Q80WL1,Q99041,Q9QZK9,Q9R0T3
7008	1,3,5,9,11,13,15,17	2,4,6,7,8,10,12,14,16	9	O54728,P04218,P47967,P97840,Q63532,Q6IRE4,Q80WL1,Q9EQS0,Q9R0T3
7009	1,3,5,9,11,13,16,17	2,4,6,7,8,10,12,14,15	6	D3ZHA0,P04218,P11598,P17046,P35280,Q6IRE4
7010	1,3,5,9,11,14,15,16	2,4,6,7,8,10,12,13,17	21	O54728,P02780,P02782,P04218,P06761,P07647,P11598,P12020,P19223,P22282,P22283,P55091,P98089,Q4G075,Q62635,Q6AYR9,Q6IRE4,Q80WL1,Q99041,Q9QZK9,Q9R0T3
7011	1,3,5,9,11,14,15,17	2,4,6,7,8,10,12,13,16	10	O54728,P04218,P06761,P12020,P47967,P97840,Q6AYR9,Q6IRE4,Q80WL1,Q9R0T3
7012	1,3,5,9,11,14,16,17	2,4,6,7,8,10,12,13,15	8	D3ZHA0,P04218,P06761,P11598,P12020,Q6AYR9,Q6IRE4,Q80WL1
7013	1,3,5,9,11,15,16,17	2,4,6,7,8,10,12,13,14	22	O54728,P02780,P02782,P04218,P06761,P06911,P07647,P0C0A9,P11598,P12020,P22282,P22283,P24368,P25236,P30120,Q5GRG2,Q62902,Q6AYR9,Q6IRE4,Q80WL1,Q9JHB9,Q9R0T3
7014	1,3,5,9,12,13,14,15	2,4,6,7,8,10,11,16,17	10	A2RUV9,O54728,P00714,P07150,P09605;P25809,P14669,Q4G075,Q6IRE4,Q6P6R2,Q9R0T3
7015	1,3,5,9,12,13,14,16	2,4,6,7,8,10,11,15,17	6	P00714,P02761,P07150,P14669,Q4G075,Q6IRE4
7016	1,3,5,9,12,13,14,17	2,4,6,7,8,10,11,15,16	6	P00714,P07150,P14669,P35280,Q5FVI6,Q9QW07
7017	1,3,5,9,12,13,15,16	2,4,6,7,8,10,11,14,17	8	O54728,P00714,P07150,P11598,P14669,P62804,Q6IRE4,Q6P6R2

7018	1,3,5,9,12,13,15,17	2,4,6,7,8,10,11,14,16	5	P00714,P07150,P35280,Q5BJY9,Q63532
7019	1,3,5,9,12,13,16,17	2,4,6,7,8,10,11,14,15	9	P00714,P02631,P07171,P14669,P17046,P20762,P35280,Q5BJY9,Q6IRE4
7020	1,3,5,9,12,14,15,16	2,4,6,7,8,10,11,13,17	9	O54728,P07150,P11598,P14669,P22282,P22283,Q4G075,Q6IRE4,Q9R0T3
7021	1,3,5,9,12,14,15,17	2,4,6,7,8,10,11,13,16	5	P00714,P07150,P47967,Q8CJD3,Q9R0T3
7022	1,3,5,9,12,14,16,17	2,4,6,7,8,10,11,13,15	3	P14669,P35280,Q6IRE4
7023	1,3,5,9,12,15,16,17	2,4,6,7,8,10,11,13,14	11	O54728,P00714,P06911,P11598,P12020,P14669,P25236,P35280,P47727,Q5GRG2,Q6IRE4
7024	1,3,5,9,13,14,15,16	2,4,6,7,8,10,11,12,17	29	O54728,O55004,P00714,P02780,P07150,P07647,P09605;P25809,P0C0A9,P11598,P14669,P19223,P22282,P22283,P50115,P50280,P55091,P60905,P62804,P98089,Q00715,Q4G075,Q62635,Q6AYR9,Q6IRE4,Q6P6R2,Q80WL1,Q99041,Q9QZK9,Q9R0T3
7025	1,3,5,9,13,14,15,17	2,4,6,7,8,10,11,12,16	16	O54728,P00714,P07150,P0C0A9,P14669,P35280,P47967,P53792,P60905,P82471,Q07936,Q5FVI6,Q80WL1,Q9EQS0,Q9QW07,Q9R0T3
7026	1,3,5,9,13,14,16,17	2,4,6,7,8,10,11,12,15	13	D3ZHA0,O55004,P00714,P01015,P0C0A9,P14669,P35280,P60905,P62804,Q00715,Q6IRE4,Q80WL1,Q9QW07
7027	1,3,5,9,13,15,16,17	2,4,6,7,8,10,11,12,14	24	O54728,P00714,P02780,P07647,P0C0A9,P11598,P12020,P14669,P20762,P22283,P24368,P30120,P35280,P47727,P50280,P60905,P62804,Q00715,Q6AYR9,Q6IRE4,Q80WL1,Q9EQS0,Q9JHB9,Q9R0T3
7028	1,3,5,9,14,15,16,17	2,4,6,7,8,10,11,12,13	29	O54728,P00714,P02780,P02781,P02782,P04218,P06761,P07647,P0C0A9,P11598,P12020,P14669,P22282,P22283,P24368,P30120,P47727,P47967,P50280,P60905,P62804,Q62902,Q6AYR9,Q6IRE4,Q80WL1,Q8CJ52,Q9JHB9,Q9QW07,Q9R0T3
7029	1,3,5,10,11,12,13,14	2,4,6,7,8,9,15,16,17	4	P09605;P25809,Q6IG02,Q6IRE4,Q6P6R2
7030	1,3,5,10,11,12,13,15	2,4,6,7,8,9,14,16,17	3	P09605;P25809,Q6IG02,Q6P6R2
7031	1,3,5,10,11,12,13,16	2,4,6,7,8,9,14,15,17	5	P02631,P17046,Q6IG02,Q6IRE4,Q6P6Q2
7032	1,3,5,10,11,12,13,17	2,4,6,7,8,9,14,15,16	3	P02631,Q6IG02,Q99MH3
7033	1,3,5,10,11,12,14,15	2,4,6,7,8,9,13,16,17	4	P09605;P25809,Q6IG02,Q6P6R2,Q9R0T3
7034	1,3,5,10,11,12,14,16	2,4,6,7,8,9,13,15,17	5	P11598,P17046,Q6IG02,Q6IRE4,Q6P6Q2
7035	1,3,5,10,11,12,14,17	2,4,6,7,8,9,13,15,16	2	P08689,Q6IG02
7036	1,3,5,10,11,12,15,16	2,4,6,7,8,9,13,14,17	7	P06911,P11598,P17046,P20766,P22282,Q6IG02,Q6IRE4
7037	1,3,5,10,11,12,15,17	2,4,6,7,8,9,13,14,16	4	P06911,P25236,Q6IG02,Q99MH3
7038	1,3,5,10,11,12,16,17	2,4,6,7,8,9,13,14,15	8	P06757,P06911,P17046,P25236,Q5GRG2,Q6IG02,Q6IRE4,Q6P6Q2
7039	1,3,5,10,11,13,14,15	2,4,6,7,8,9,12,16,17	12	O54728,P09605;P25809,P19223,P55091,P98089,Q4G075,Q62635,Q6IRE4,Q6P6R2,Q80WL1,Q99041,Q9R0T3
7040	1,3,5,10,11,13,14,16	2,4,6,7,8,9,12,15,17	11	O55004,P09605;P25809,P19223,P20761,P50115,P55091,P98089,Q4G075,Q62635,Q6IRE4,Q99041

7041	1,3,5,10,1<br>1,13,14,17	2,4,6,7,8,9<br>,12,15,16	2	P18427,Q9QW07
7042	1,3,5,10,1<br>1,13,15,16	2,4,6,7,8,9<br>,12,14,17	15	O54728,P06761,P09605;P25809,P11598,P19223,P22282,P50115,P55091,P98089,Q4G075,Q62635,Q6IRE4,Q6P6R2,Q80WL1,Q99041
7043	1,3,5,10,1<br>1,13,15,17	2,4,6,7,8,9<br>,12,14,16	2	Q63532,Q80WL1
7044	1,3,5,10,1<br>1,13,16,17	2,4,6,7,8,9<br>,12,14,15	4	P02631,P17046,P20762,Q6IRE4
7045	1,3,5,10,1<br>1,14,15,16	2,4,6,7,8,9<br>,12,13,17	22	O54728,P02780,P02782,P06761,P07647,P09605;P25809,P11598,P19223,P22282,P22283,P50115,P55091,P98089,Q4G075,Q62635,Q629<br>02,Q6AYR9,Q6IRE4,Q80WL1,Q99041,Q9QZK9,Q9R0T3
7046	1,3,5,10,1<br>1,14,15,17	2,4,6,7,8,9<br>,12,13,16	5	P18427,P25236,Q62902,Q80WL1,Q9R0T3
7047	1,3,5,10,1<br>1,14,16,17	2,4,6,7,8,9<br>,12,13,15	5	P06761,P11598,P18427,P25236,Q6IRE4
7048	1,3,5,10,1<br>1,15,16,17	2,4,6,7,8,9<br>,12,13,14	17	P02780,P02782,P06761,P06911,P07647,P0C0A9,P11598,P12020,P22282,P22283,P25236,P30120,Q62902,Q6AYR9,Q6IRE4,Q80WL1,Q9J<br>HB9
7049	1,3,5,10,1<br>2,13,14,15	2,4,6,7,8,9<br>,11,16,17	5	A2RUV9,P07150,P09605;P25809,P50115,Q6P6R2
7050	1,3,5,10,1<br>2,13,14,16	2,4,6,7,8,9<br>,11,15,17	7	O55004,P02631,P02761,P09605;P25809,P14669,P50115,Q6IRE4
7051	1,3,5,10,1<br>2,13,14,17	2,4,6,7,8,9<br>,11,15,16	3	P02631,Q99MH3,Q9QW07
7052	1,3,5,10,1<br>2,13,15,16	2,4,6,7,8,9<br>,11,14,17	9	P07150,P09605;P25809,P14669,P20762,P50115,P83121,Q63617,Q6IRE4,Q6P6R2
7053	1,3,5,10,1<br>2,13,15,17	2,4,6,7,8,9<br>,11,14,16	3	P83121,Q63617,Q99MH3
7054	1,3,5,10,1<br>2,13,16,17	2,4,6,7,8,9<br>,11,14,15	8	P02631,P07171,P14669,P17046,P20762,P35280,P83121,Q99MH3
7055	1,3,5,10,1<br>2,14,15,16	2,4,6,7,8,9<br>,11,13,17	9	P09605;P25809,P11598,P14669,P20766,P22282,P22283,P50115,Q63617,Q6IRE4
7056	1,3,5,10,1<br>2,14,15,17	2,4,6,7,8,9<br>,11,13,16	4	P08689,Q63617,Q8CJD3,Q9R0T3
7057	1,3,5,10,1<br>2,14,16,17	2,4,6,7,8,9<br>,11,13,15	1	P14669
7058	1,3,5,10,1<br>2,15,16,17	2,4,6,7,8,9<br>,11,13,14	11	P06911,P11598,P14669,P20766,P22282,P25236,P83121,Q62902,Q63617,Q6TMA8,Q99MH3
7059	1,3,5,10,1<br>3,14,15,16	2,4,6,7,8,9<br>,11,12,17	23	O54728,O55004,P02780,P07150,P07647,P09605;P25809,P0C0A9,P11598,P14669,P19223,P22282,P22283,P50115,P55091,P60905,P9808<br>9,Q4G075,Q62635,Q6IRE4,Q6P6R2,Q80WL1,Q99041,Q9R0T3
7060	1,3,5,10,1<br>3,14,15,17	2,4,6,7,8,9<br>,11,12,16	5	P0C0A9,Q80WL1,Q9EQS0,Q9QW07,Q9R0T3
7061	1,3,5,10,1<br>3,14,16,17	2,4,6,7,8,9<br>,11,12,15	6	O55004,P02631,P0C0A9,P14669,P35280,Q9QW07
7062	1,3,5,10,1<br>3,15,16,17	2,4,6,7,8,9<br>,11,12,14	14	P02780,P02782,P07647,P0C0A9,P14669,P20762,P22283,P24368,P30120,P62804,P83121,Q80WL1,Q9EQS0,Q9JHB9
7063	1,3,5,10,1<br>4,15,16,17	2,4,6,7,8,9<br>,11,12,13	22	P02780,P02781,P02782,P02803,P06761,P07647,P0C0A9,P11598,P14669,P22282,P22283,P24368,P30120,P60905,Q62902,Q6AYR9,Q6TM<br>A8,Q80WL1,Q9JHB9,Q9JI85,Q9QW07,Q9R0T3

7064	1,3,5,11,12,13,14,15	2,4,6,7,8,9,10,16,17	3	P09605;P25809,Q6IRE4,Q6P6R2
7065	1,3,5,11,12,13,14,16	2,4,6,7,8,9,10,15,17	4	P02761,P17046,Q6IRE4,Q6P6R2
7066	1,3,5,11,12,13,14,17	2,4,6,7,8,9,10,15,16	2	P17046,Q9QW07
7067	1,3,5,11,12,13,15,16	2,4,6,7,8,9,10,14,17	4	O54728,P17046,Q6IRE4,Q6P6R2
7068	1,3,5,11,12,13,15,17	2,4,6,7,8,9,10,14,16	2	P17046,Q6P6R2
7069	1,3,5,11,12,13,16,17	2,4,6,7,8,9,10,14,15	4	D3ZHA0,P17046,P35280,Q6IRE4
7070	1,3,5,11,12,14,15,16	2,4,6,7,8,9,10,13,17	7	O54728,P11598,P17046,P20766,Q6IRE4,Q6P6R2,Q9R0T3
7071	1,3,5,11,12,14,15,17	2,4,6,7,8,9,10,13,16	3	P47967,Q8CJD3,Q9R0T3
7072	1,3,5,11,12,14,16,17	2,4,6,7,8,9,10,13,15	3	P17046,Q6IG02,Q6IRE4
7073	1,3,5,11,12,15,16,17	2,4,6,7,8,9,10,13,14	6	P06911,P12020,P17046,P25236,Q5GRG2,Q6IRE4
7074	1,3,5,11,13,14,15,16	2,4,6,7,8,9,10,12,17	15	D3ZHA0,O54728,P09605;P25809,P19223,P50115,P55091,P60905,P98089,Q4G075,Q62635,Q6IRE4,Q6P6R2,Q80WL1,Q99041,Q9R0T3
7075	1,3,5,11,13,14,15,17	2,4,6,7,8,9,10,12,16	7	D3ZHA0,P47967,P97840,Q6P6R2,Q80WL1,Q9QW07,Q9R0T3
7076	1,3,5,11,13,14,16,17	2,4,6,7,8,9,10,12,15	5	D3ZHA0,P17046,P35280,Q6IRE4,Q9QW07
7077	1,3,5,11,13,15,16,17	2,4,6,7,8,9,10,12,14	9	D3ZHA0,O54728,P00714,P0C0A9,P17046,Q6AYR9,Q6IRE4,Q80WL1,Q9EQS0
7078	1,3,5,11,14,15,16,17	2,4,6,7,8,9,10,12,13	20	D3ZHA0,O54728,P02780,P02782,P04218,P06761,P07647,P0C0A9,P11598,P12020,P22282,P22283,P47967,P97840,Q62902,Q6AYR9,Q6IRE4,Q80WL1,Q9QW07,Q9R0T3
7079	1,3,5,12,13,14,15,16	2,4,6,7,8,9,10,11,17	9	O54728,P00714,P07150,P09605;P25809,P14669,P19223,P50115,Q6IRE4,Q6P6R2
7080	1,3,5,12,13,14,15,17	2,4,6,7,8,9,10,11,16	6	A2RUV9,P00714,P47967,Q5FVI6,Q6P6R2,Q9QW07
7081	1,3,5,12,13,14,16,17	2,4,6,7,8,9,10,11,15	6	P00714,P14669,P17046,P35280,Q561R9,Q9QW07
7082	1,3,5,12,13,15,16,17	2,4,6,7,8,9,10,11,14	6	P00714,P14669,P17046,P35280,P83121,Q5XFX0
7083	1,3,5,12,14,15,16,17	2,4,6,7,8,9,10,11,13	4	P00714,P14669,Q9QW07,Q9R0T3
7084	1,3,5,13,14,15,16,17	2,4,6,7,8,9,10,11,12	23	D3ZHA0,O54728,O55004,P00714,P02780,P07647,P08753,P0C0A9,P14669,P22283,P24368,P30120,P35280,P47967,P60905,P62804,Q6IRE4,Q6RUV5,Q80WL1,Q9EQS0,Q9JHB9,Q9QW07,Q9R0T3
7085	1,3,6,7,8,9,10,11	2,4,5,12,13,14,15,16,17	3	P27213,P31044,P35280

7086	1,3,6,7,8,9,10,12	2,4,5,11,13,14,15,16,17	5	P08723,P10715,P19629,P31044,P97840
7087	1,3,6,7,8,9,10,13	2,4,5,11,12,14,15,16,17	5	P06911,P12020,P31044,P51907,Q5GRG2
7088	1,3,6,7,8,9,10,14	2,4,5,11,12,13,15,16,17	3	P12020,P19629,P31044
7089	1,3,6,7,8,9,10,15	2,4,5,11,12,13,14,16,17	4	O55006,P27213,P31044,P35280
7090	1,3,6,7,8,9,10,16	2,4,5,11,12,13,14,15,17	1	P31044
7091	1,3,6,7,8,9,10,17	2,4,5,11,12,13,14,15,16	2	P02091,P51907
7092	1,3,6,7,8,9,11,12	2,4,5,10,13,14,15,16,17	15	P02781,P02782,P08723,P10715,P13432,P19629,P22283,P27213,P30120,Q62997,Q63493,Q6IG02,Q8CJ52,Q9JHB9,Q9JI85
7093	1,3,6,7,8,9,11,13	2,4,5,10,12,14,15,16,17	4	P16086,P22283,Q62902,Q9JI85
7094	1,3,6,7,8,9,11,14	2,4,5,10,12,13,15,16,17	3	P16086,P19629,P27213
7095	1,3,6,7,8,9,11,15	2,4,5,10,12,13,14,16,17	4	P10715,P19629,P27213,P35280
7096	1,3,6,7,8,9,11,16	2,4,5,10,12,13,14,15,17	2	P10715,P19629
7097	1,3,6,7,8,9,11,17	2,4,5,10,12,13,14,15,16	4	P02091,P10715,P19629,P50115
7098	1,3,6,7,8,9,12,13	2,4,5,10,11,14,15,16,17	14	P02782,P06911,P08723,P10715,P13432,P19629,P22282,P22283,P30120,P51907,Q62902,Q8CJ52,Q9JHB9,Q9Z1F2
7099	1,3,6,7,8,9,12,14	2,4,5,10,11,13,15,16,17	6	P02782,P08723,P10715,P13432,P19629,P30120
7100	1,3,6,7,8,9,12,15	2,4,5,10,11,13,14,16,17	4	P08723,P10715,P13432,P19629

7101	1,3,6,7,8,9,12,16	2,4,5,10,11,13,14,15,17	4	P08723,P10715,P13432,P19629
7102	1,3,6,7,8,9,12,17	2,4,5,10,11,13,14,15,16	10	P02091,P02782,P08723,P10715,P13432,P19629,P22283,P50115,P51907,Q9QZK9
7103	1,3,6,7,8,9,13,14	2,4,5,10,11,12,15,16,17	10	P06866,P06911,P12020,P16086,P19629,P51907,P53792,Q5GRG2,Q62902,Q9Z1F2
7104	1,3,6,7,8,9,13,15	2,4,5,10,11,12,14,16,17	2	P19629,P53792
7105	1,3,6,7,8,9,13,16	2,4,5,10,11,12,14,15,17	1	P51907
7106	1,3,6,7,8,9,13,17	2,4,5,10,11,12,14,15,16	4	P02091,P22282,P51907,P53792
7107	1,3,6,7,8,9,14,15	2,4,5,10,11,12,13,16,17	2	P19629,P53792
7108	1,3,6,7,8,9,14,16	2,4,5,10,11,12,13,15,17	1	P19629
7109	1,3,6,7,8,9,14,17	2,4,5,10,11,12,13,15,16	4	P02091,P19629,P51907,P53792
7110	1,3,6,7,8,9,15,16	2,4,5,10,11,12,13,14,17	2	P19629,Q5QE79
7111	1,3,6,7,8,9,15,17	2,4,5,10,11,12,13,14,16	4	P02091,P19629,P53792,Q5QE79
7112	1,3,6,7,8,9,16,17	2,4,5,10,11,12,13,14,15	2	P02091,P51907
7113	1,3,6,7,8,10,11,12	2,4,5,9,13,14,15,16,17	20	F1LM93,P02781,P02782,P08723,P10715,P14669,P19629,P22283,P30120,P31044,P35280,P47727,Q63357,Q63493,Q6AYL6,Q6IG02,Q6P6Q2,Q8CJ52,Q9JHB9,Q9JI85
7114	1,3,6,7,8,10,11,13	2,4,5,9,12,14,15,16,17	9	P06911,P12020,P16086,P22283,P31044,P47727,Q5GRG2,Q62902,Q8VD89
7115	1,3,6,7,8,10,11,14	2,4,5,9,12,13,15,16,17	7	P12020,P16086,P19629,P31044,P35280,P47727,Q63493

7116	1,3,6,7,8,10,11,15	2,4,5,9,12,13,14,16,17	6	P14669,P19629,P22006,P27213,P31044,P35280
7117	1,3,6,7,8,10,11,16	2,4,5,9,12,13,14,15,17	2	P31044,Q8VD89
7118	1,3,6,7,8,10,11,17	2,4,5,9,12,13,14,15,16	8	P02091,P07150,P12346,P14669,P15399,P19629,Q63493,Q99MH3
7119	1,3,6,7,8,10,12,13	2,4,5,9,11,14,15,16,17	15	P02781,P02782,P06911,P08723,P12020,P19629,P22282,P22283,P30120,P31044,Q5GRG2,Q62902,Q8CJ52,Q99MH3,Q9JHB9
7120	1,3,6,7,8,10,12,14	2,4,5,9,11,13,15,16,17	10	P02782,P08723,P12020,P19629,P22283,P30120,P31044,P35280,Q8CJ52,Q9ET32
7121	1,3,6,7,8,10,12,15	2,4,5,9,11,13,14,16,17	5	P08723,P10715,P19629,P35280,Q99MH3
7122	1,3,6,7,8,10,12,16	2,4,5,9,11,13,14,15,17	7	P08721,P08723,P10715,P19629,P47967,P97840,Q8CJ52
7123	1,3,6,7,8,10,12,17	2,4,5,9,11,13,14,15,16	11	F1LM93,P02091,P08723,P10715,P12346,P13432,P19629,P22283,Q4G075,Q99MH3,Q9QZK9
7124	1,3,6,7,8,10,13,14	2,4,5,9,11,12,15,16,17	7	P06911,P12020,P16086,P19629,P31044,P51907,Q5GRG2
7125	1,3,6,7,8,10,13,15	2,4,5,9,11,12,14,16,17	6	P06911,P12020,P15999,P31044,Q5GRG2,Q99MH3
7126	1,3,6,7,8,10,13,16	2,4,5,9,11,12,14,15,17	5	P06911,P12020,P31044,P51907,Q5GRG2
7127	1,3,6,7,8,10,13,17	2,4,5,9,11,12,14,15,16	6	P02091,P06911,P12020,P22283,P51907,Q99MH3
7128	1,3,6,7,8,10,14,15	2,4,5,9,11,12,13,16,17	4	P12020,P19629,P31044,P35280
7129	1,3,6,7,8,10,14,16	2,4,5,9,11,12,13,15,17	3	P12020,P19629,P31044
7130	1,3,6,7,8,10,14,17	2,4,5,9,11,12,13,15,16	6	P02091,P08689,P12020,P12346,P19629,P51907

7131	1,3,6,7,8,10,15,16	2,4,5,9,11,12,13,14,17	1	P31044
7132	1,3,6,7,8,10,15,17	2,4,5,9,11,12,13,14,16	4	P02091,P12346,P19629,Q99MH3
7133	1,3,6,7,8,10,16,17	2,4,5,9,11,12,13,14,15	3	P02091,P51907,Q99MH3
7134	1,3,6,7,8,11,12,13	2,4,5,9,10,14,15,16,17	19	P02781,P02782,P04905,P06761,P06911,P08723,P10715,P13432,P19629,P22282,P22283,P30120,P47727,Q62902,Q63493,Q8CJ52,Q9JHB9,Q9JI85,Q9Z1F2
7135	1,3,6,7,8,11,12,14	2,4,5,9,10,13,15,16,17	17	P02781,P02782,P04905,P08723,P10715,P13432,P16086,P19629,P22282,P22283,P30120,P35280,P47727,Q63493,Q8CJ52,Q9JHB9,Q9JI85
7136	1,3,6,7,8,11,12,15	2,4,5,9,10,13,14,16,17	8	P08723,P10715,P19629,P22006,P35280,Q63493,Q8CJ52,Q9JI85
7137	1,3,6,7,8,11,12,16	2,4,5,9,10,13,14,15,17	9	P08723,P10715,P13432,P17046,P19629,P30120,Q63493,Q8CJ52,Q9JI85
7138	1,3,6,7,8,11,12,17	2,4,5,9,10,13,14,15,16	20	P02091,P02782,P08723,P10715,P13432,P14669,P19629,P22282,P22283,P30120,P50115,Q4G075,Q63493,Q63751,Q8CJ52,Q99041,Q99MH3,Q9JHB9,Q9JI85,Q9QZK9
7139	1,3,6,7,8,11,13,14	2,4,5,9,10,12,15,16,17	12	P06866,P06911,P12020,P16086,P19629,P22282,P22283,P47727,Q5GRG2,Q62902,Q63493,Q9JI85
7140	1,3,6,7,8,11,13,15	2,4,5,9,10,12,14,16,17	4	P16086,P19629,Q5GRG2,Q8VD89
7141	1,3,6,7,8,11,13,16	2,4,5,9,10,12,14,15,17	3	P16086,Q62902,Q8VD89
7142	1,3,6,7,8,11,13,17	2,4,5,9,10,12,14,15,16	10	P01946,P02091,P16086,P19629,P22282,P22283,P51907,Q62902,Q63493,Q63751
7143	1,3,6,7,8,11,14,15	2,4,5,9,10,12,13,16,17	4	P16086,P19629,P22006,P35280
7144	1,3,6,7,8,11,14,16	2,4,5,9,10,12,13,15,17	2	P16086,P19629
7145	1,3,6,7,8,11,14,17	2,4,5,9,10,12,13,15,16	5	P02091,P14669,P16086,P19629,Q63493

7146	1,3,6,7,8,11,15,16	2,4,5,9,10,12,13,14,17	3	P10715,P19629,Q8VD89
7147	1,3,6,7,8,11,15,17	2,4,5,9,10,12,13,14,16	5	P02091,P10715,P14669,P19629,Q63751
7148	1,3,6,7,8,11,16,17	2,4,5,9,10,12,13,14,15	3	P02091,P10715,P19629
7149	1,3,6,7,8,12,13,14	2,4,5,9,10,11,15,16,17	21	A2RUV9,O08557,P02782,P06761,P06911,P08723,P11598,P12020,P13432,P16086,P19629,P22282,P22283,P29975,P30120,P51907,Q5GRG2,Q62902,Q8CJ52,Q9JHB9,Q9Z1F2
7150	1,3,6,7,8,12,13,15	2,4,5,9,10,11,14,16,17	9	P02782,P06911,P08723,P10715,P15999,P19629,Q5GRG2,Q62902,Q99MH3
7151	1,3,6,7,8,12,13,16	2,4,5,9,10,11,14,15,17	7	P02782,P06911,P08723,P10715,P19629,Q62902,Q8CJ52
7152	1,3,6,7,8,12,13,17	2,4,5,9,10,11,14,15,16	21	P02091,P02782,P06761,P06911,P08723,P10715,P11598,P13432,P19629,P22282,P22283,P30120,P51907,Q62902,Q63751,Q8CJ52,Q99041,Q99MH3,Q9JHB9,Q9QZK9,Q9Z1F2
7153	1,3,6,7,8,12,14,15	2,4,5,9,10,11,13,16,17	6	A2RUV9,O08557,P08723,P10715,P19629,P35280
7154	1,3,6,7,8,12,14,16	2,4,5,9,10,11,13,15,17	5	P08723,P10715,P19629,Q8CJ52,Q9ET32
7155	1,3,6,7,8,12,14,17	2,4,5,9,10,11,13,15,16	12	P02091,P02782,P08723,P10715,P13432,P19629,P22282,P22283,P51907,Q561R9,Q9QYU4,Q9QZK9
7156	1,3,6,7,8,12,15,16	2,4,5,9,10,11,13,14,17	2	P10715,P19629
7157	1,3,6,7,8,12,15,17	2,4,5,9,10,11,13,14,16	8	P02091,P08723,P10715,P13432,P19629,Q4G075,Q99MH3,Q9QZK9
7158	1,3,6,7,8,12,16,17	2,4,5,9,10,11,13,14,15	7	P02091,P08723,P10715,P13432,P19629,Q99MH3,Q9QZK9
7159	1,3,6,7,8,13,14,15	2,4,5,9,10,11,12,16,17	7	P06911,P12020,P15999,P16086,P19629,Q5GRG2,Q62687
7160	1,3,6,7,8,13,14,16	2,4,5,9,10,11,12,15,17	7	P06911,P12020,P16086,P19629,P51907,Q5GRG2,Q62902

7161	1,3,6,7,8,13,14,17	2,4,5,9,10,11,12,15,16	16	P02091,P06911,P11598,P12020,P16086,P19629,P22282,P22283,P51907,P53790,P53792,Q5GRG2,Q62687,Q62902,Q9QW07,Q9Z1F2
7162	1,3,6,7,8,13,15,16	2,4,5,9,10,11,12,14,17	1	P19629
7163	1,3,6,7,8,13,15,17	2,4,5,9,10,11,12,14,16	7	P02091,P15999,P19629,P51907,P53792,Q99MH3,Q9WUW8
7164	1,3,6,7,8,13,16,17	2,4,5,9,10,11,12,14,15	2	P02091,P51907
7165	1,3,6,7,8,14,15,16	2,4,5,9,10,11,12,13,17	2	P02803,P19629
7166	1,3,6,7,8,14,15,17	2,4,5,9,10,11,12,13,16	4	P02091,P02803,P10536,P19629
7167	1,3,6,7,8,14,16,17	2,4,5,9,10,11,12,13,15	5	P02091,P16086,P19629,P51907,Q561R9
7168	1,3,6,7,8,15,16,17	2,4,5,9,10,11,12,13,14	3	P02091,P02803,P19629
7169	1,3,6,7,9,10,11,12	2,4,5,8,13,14,15,16,17	3	P09527,P27213,P97840
7170	1,3,6,7,9,10,11,13	2,4,5,8,12,14,15,16,17	0	
7171	1,3,6,7,9,10,11,14	2,4,5,8,12,13,15,16,17	2	P27213,P83121
7172	1,3,6,7,9,10,11,15	2,4,5,8,12,13,14,16,17	2	P27213,P35280
7173	1,3,6,7,9,10,11,16	2,4,5,8,12,13,14,15,17	0	
7174	1,3,6,7,9,10,11,17	2,4,5,8,12,13,14,15,16	3	P02091,P08689,Q9QUL6
7175	1,3,6,7,9,10,12,13	2,4,5,8,11,14,15,16,17	2	P19218,P97840

7176	1,3,6,7,9,10,12,14	2,4,5,8,11,13,15,16,17	1	P97840
7177	1,3,6,7,9,10,12,15	2,4,5,8,11,13,14,16,17	2	P97840,Q6AYS4
7178	1,3,6,7,9,10,12,16	2,4,5,8,11,13,14,15,17	3	P47967,P97840,Q6AYS4
7179	1,3,6,7,9,10,12,17	2,4,5,8,11,13,14,15,16	6	P02091,P08689,P13432,P97840,Q99MH3,Q9QZK9
7180	1,3,6,7,9,10,13,14	2,4,5,8,11,12,15,16,17	2	P06911,P12020
7181	1,3,6,7,9,10,13,15	2,4,5,8,11,12,14,16,17	2	Q5U2V4,Q6AYS4
7182	1,3,6,7,9,10,13,16	2,4,5,8,11,12,14,15,17	4	P62804,P97840,Q00715,Q6AYS4
7183	1,3,6,7,9,10,13,17	2,4,5,8,11,12,14,15,16	3	P02091,P51907,Q99MH3
7184	1,3,6,7,9,10,14,15	2,4,5,8,11,12,13,16,17	2	Q6AYS4,Q80WL1
7185	1,3,6,7,9,10,14,16	2,4,5,8,11,12,13,15,17	1	Q6AYS4
7186	1,3,6,7,9,10,14,17	2,4,5,8,11,12,13,15,16	2	P02091,P08689
7187	1,3,6,7,9,10,15,16	2,4,5,8,11,12,13,14,17	2	Q6AYS4,Q80WL1
7188	1,3,6,7,9,10,15,17	2,4,5,8,11,12,13,14,16	2	P02091,P08689
7189	1,3,6,7,9,10,16,17	2,4,5,8,11,12,13,14,15	1	P02091
7190	1,3,6,7,9,11,12,13	2,4,5,8,10,14,15,16,17	1	Q9JHB9

7191	1,3,6,7,9,11,12,14	2,4,5,8,10,13,15,16,17	1	Q62997
7192	1,3,6,7,9,11,12,15	2,4,5,8,10,13,14,16,17	0	
7193	1,3,6,7,9,11,12,16	2,4,5,8,10,13,14,15,17	2	P12368,P17046
7194	1,3,6,7,9,11,12,17	2,4,5,8,10,13,14,15,16	5	P02091,P08689,P09605;P25809,P13432,Q9QZK9
7195	1,3,6,7,9,11,13,14	2,4,5,8,10,12,15,16,17	0	
7196	1,3,6,7,9,11,13,15	2,4,5,8,10,12,14,16,17	0	
7197	1,3,6,7,9,11,13,16	2,4,5,8,10,12,14,15,17	0	
7198	1,3,6,7,9,11,13,17	2,4,5,8,10,12,14,15,16	1	P02091
7199	1,3,6,7,9,11,14,15	2,4,5,8,10,12,13,16,17	1	Q80WL1
7200	1,3,6,7,9,11,14,16	2,4,5,8,10,12,13,15,17	0	
7201	1,3,6,7,9,11,14,17	2,4,5,8,10,12,13,15,16	2	P02091,P08689
7202	1,3,6,7,9,11,15,16	2,4,5,8,10,12,13,14,17	1	Q80WL1
7203	1,3,6,7,9,11,15,17	2,4,5,8,10,12,13,14,16	1	P02091
7204	1,3,6,7,9,11,16,17	2,4,5,8,10,12,13,14,15	1	P02091
7205	1,3,6,7,9,12,13,14	2,4,5,8,10,11,15,16,17	3	A2RUV9,P06911,Q6AYS4

7206	1,3,6,7,9,12,13,15	2,4,5,8,10,11,14,16,17	1	Q6AYS4
7207	1,3,6,7,9,12,13,16	2,4,5,8,10,11,14,15,17	2	P19218,Q6AYS4
7208	1,3,6,7,9,12,13,17	2,4,5,8,10,11,14,15,16	5	P02091,P13432,P51907,Q99MH3,Q9QZK9
7209	1,3,6,7,9,12,14,15	2,4,5,8,10,11,13,16,17	2	A2RUV9,Q6AYS4
7210	1,3,6,7,9,12,14,16	2,4,5,8,10,11,13,15,17	1	Q6AYS4
7211	1,3,6,7,9,12,14,17	2,4,5,8,10,11,13,15,16	4	P01835,P02091,P08689,P13432
7212	1,3,6,7,9,12,15,16	2,4,5,8,10,11,13,14,17	1	Q6AYS4
7213	1,3,6,7,9,12,15,17	2,4,5,8,10,11,13,14,16	3	P02091,P13432,Q6AYS4
7214	1,3,6,7,9,12,16,17	2,4,5,8,10,11,13,14,15	4	P02091,P09605;P25809,P13432,Q6AYS4
7215	1,3,6,7,9,13,14,15	2,4,5,8,10,11,12,16,17	3	P53792,Q6AYS4,Q80WL1
7216	1,3,6,7,9,13,14,16	2,4,5,8,10,11,12,15,17	2	P62804,Q6AYS4
7217	1,3,6,7,9,13,14,17	2,4,5,8,10,11,12,15,16	4	P02091,P51907,P53792,Q9QW07
7218	1,3,6,7,9,13,15,16	2,4,5,8,10,11,12,14,17	5	P62804,Q00715,Q63598,Q6AYS4,Q80WL1
7219	1,3,6,7,9,13,15,17	2,4,5,8,10,11,12,14,16	4	P02091,P53792,P62804,Q6AYS4
7220	1,3,6,7,9,13,16,17	2,4,5,8,10,11,12,14,15	5	P02091,P51907,P53792,P62804,Q6AYS4

7221	1,3,6,7,9,14,15,16	2,4,5,8,10,11,12,13,17	4	P02803,P62804,Q6AYS4,Q80WL1
7222	1,3,6,7,9,14,15,17	2,4,5,8,10,11,12,13,16	6	P02091,P02803,P08689,P53792,Q6AYS4,Q80WL1
7223	1,3,6,7,9,14,16,17	2,4,5,8,10,11,12,13,15	2	P02091,Q6AYS4
7224	1,3,6,7,9,15,16,17	2,4,5,8,10,11,12,13,14	6	P02091,P02803,P62804,Q63598,Q6AYS4,Q80WL1
7225	1,3,6,7,10,11,12,13	2,4,5,8,9,14,15,16,17	3	P47727,Q99MH3,Q9JHB9
7226	1,3,6,7,10,11,12,14	2,4,5,8,9,13,15,16,17	2	P35280,P47727
7227	1,3,6,7,10,11,12,15	2,4,5,8,9,13,14,16,17	1	P35280
7228	1,3,6,7,10,11,12,16	2,4,5,8,9,13,14,15,17	6	P06757,P12368,P17046,P47967,P97840,Q6P6Q2
7229	1,3,6,7,10,11,12,17	2,4,5,8,9,13,14,15,16	8	O08815,P02091,P06757,P08689,P13432,P15399,Q99MH3,Q9QZK9
7230	1,3,6,7,10,11,13,14	2,4,5,8,9,12,15,16,17	4	P06911,P12020,P47727,Q5GRG2
7231	1,3,6,7,10,11,13,15	2,4,5,8,9,12,14,16,17	0	
7232	1,3,6,7,10,11,13,16	2,4,5,8,9,12,14,15,17	1	Q8VD89
7233	1,3,6,7,10,11,13,17	2,4,5,8,9,12,14,15,16	2	P02091,Q99MH3
7234	1,3,6,7,10,11,14,15	2,4,5,8,9,12,13,16,17	3	P22006,P35280,Q80WL1
7235	1,3,6,7,10,11,14,16	2,4,5,8,9,12,13,15,17	0	
7236	1,3,6,7,10,11,14,17	2,4,5,8,9,12,13,15,16	3	P02091,P08689,P15399
7237	1,3,6,7,10,11,15,16	2,4,5,8,9,12,13,14,17	2	Q80WL1,Q8VD89
7238	1,3,6,7,10,11,15,17	2,4,5,8,9,12,13,14,16	3	P02091,P08689,Q99MH3
7239	1,3,6,7,10,11,16,17	2,4,5,8,9,12,13,14,15	1	P02091
7240	1,3,6,7,10,12,13,14	2,4,5,8,9,11,15,16,17	7	A2RUV9,P06911,P12020,Q05175,Q498D9,Q5GRG2,Q6AYS4
7241	1,3,6,7,10,12,13,15	2,4,5,8,9,11,14,16,17	3	Q05175,Q6AYS4,Q99MH3

7242	1,3,6,7,10,<br>12,13,16	2,4,5,8,9,1<br>1,14,15,17	5	P19218,P47967,P97840,Q6AYS4,Q99MH3
7243	1,3,6,7,10,<br>12,13,17	2,4,5,8,9,1<br>1,14,15,16	6	P02091,P13432,P14668,Q05175,Q99MH3,Q9QZK9
7244	1,3,6,7,10,<br>12,14,15	2,4,5,8,9,1<br>1,13,16,17	3	A2RUV9,P35280,Q6AYS4
7245	1,3,6,7,10,<br>12,14,16	2,4,5,8,9,1<br>1,13,15,17	2	P97840,Q6AYS4
7246	1,3,6,7,10,<br>12,14,17	2,4,5,8,9,1<br>1,13,15,16	5	P02091,P08689,P13432,Q99MH3,Q9QZK9
7247	1,3,6,7,10,<br>12,15,16	2,4,5,8,9,1<br>1,13,14,17	2	P97840,Q6AYS4
7248	1,3,6,7,10,<br>12,15,17	2,4,5,8,9,1<br>1,13,14,16	4	P02091,P08689,Q99MH3,Q9QZK9
7249	1,3,6,7,10,<br>12,16,17	2,4,5,8,9,1<br>1,13,14,15	5	P02091,P13432,P97840,Q99MH3,Q9QZK9
7250	1,3,6,7,10,<br>13,14,15	2,4,5,8,9,1<br>1,12,16,17	4	P06911,P12020,Q5GRG2,Q6AYS4
7251	1,3,6,7,10,<br>13,14,16	2,4,5,8,9,1<br>1,12,15,17	3	P06911,P12020,Q6AYS4
7252	1,3,6,7,10,<br>13,14,17	2,4,5,8,9,1<br>1,12,15,16	6	P02091,P06911,P12020,P51907,Q99MH3,Q9QW07
7253	1,3,6,7,10,<br>13,15,16	2,4,5,8,9,1<br>1,12,14,17	3	P62804,Q6AYS4,Q80WL1
7254	1,3,6,7,10,<br>13,15,17	2,4,5,8,9,1<br>1,12,14,16	2	P02091,Q99MH3
7255	1,3,6,7,10,<br>13,16,17	2,4,5,8,9,1<br>1,12,14,15	4	P02091,P51907,Q6AYS4,Q99MH3
7256	1,3,6,7,10,<br>14,15,16	2,4,5,8,9,1<br>1,12,13,17	3	P02803,Q6AYS4,Q80WL1
7257	1,3,6,7,10,<br>14,15,17	2,4,5,8,9,1<br>1,12,13,16	3	P02091,P02803,P08689
7258	1,3,6,7,10,<br>14,16,17	2,4,5,8,9,1<br>1,12,13,15	2	P02091,Q6AYS4
7259	1,3,6,7,10,<br>15,16,17	2,4,5,8,9,1<br>1,12,13,14	5	P02091,P02803,Q6AYS4,Q80WL1,Q99MH3
7260	1,3,6,7,11,<br>12,13,14	2,4,5,8,9,1<br>0,15,16,17	5	P06911,P30120,P47727,Q62902,Q9JHB9
7261	1,3,6,7,11,<br>12,13,15	2,4,5,8,9,1<br>0,14,16,17	0	
7262	1,3,6,7,11,<br>12,13,16	2,4,5,8,9,1<br>0,14,15,17	1	P17046
7263	1,3,6,7,11,<br>12,13,17	2,4,5,8,9,1<br>0,14,15,16	7	P01946,P02091,P13432,P17046,Q99MH3,Q9JHB9,Q9QZK9
7264	1,3,6,7,11,<br>12,14,15	2,4,5,8,9,1<br>0,13,16,17	2	A2RUV9,P35280

7265	1,3,6,7,11,12,14,16	2,4,5,8,9,10,13,15,17	1	P17046
7266	1,3,6,7,11,12,14,17	2,4,5,8,9,10,13,15,16	4	P02091,P08689,P13432,Q9QZK9
7267	1,3,6,7,11,12,15,16	2,4,5,8,9,10,13,14,17	1	P17046
7268	1,3,6,7,11,12,15,17	2,4,5,8,9,10,13,14,16	3	P02091,P13432,Q9QZK9
7269	1,3,6,7,11,12,16,17	2,4,5,8,9,10,13,14,15	3	P02091,P13432,P17046
7270	1,3,6,7,11,13,14,15	2,4,5,8,9,10,12,16,17	1	Q80WL1
7271	1,3,6,7,11,13,14,16	2,4,5,8,9,10,12,15,17	0	
7272	1,3,6,7,11,13,14,17	2,4,5,8,9,10,12,15,16	3	P01946,P02091,Q9QW07
7273	1,3,6,7,11,13,15,16	2,4,5,8,9,10,12,14,17	2	Q80WL1,Q8VD89
7274	1,3,6,7,11,13,15,17	2,4,5,8,9,10,12,14,16	1	P02091
7275	1,3,6,7,11,13,16,17	2,4,5,8,9,10,12,14,15	2	P02091,P17046
7276	1,3,6,7,11,14,15,16	2,4,5,8,9,10,12,13,17	2	P02803,Q80WL1
7277	1,3,6,7,11,14,15,17	2,4,5,8,9,10,12,13,16	3	P02091,P02803,Q80WL1
7278	1,3,6,7,11,14,16,17	2,4,5,8,9,10,12,13,15	1	P02091
7279	1,3,6,7,11,15,16,17	2,4,5,8,9,10,12,13,14	3	P02091,P02803,Q80WL1
7280	1,3,6,7,12,13,14,15	2,4,5,8,9,10,11,16,17	3	A2RUV9,P06911,Q6AYS4
7281	1,3,6,7,12,13,14,16	2,4,5,8,9,10,11,15,17	2	P06911,Q6AYS4
7282	1,3,6,7,12,13,14,17	2,4,5,8,9,10,11,15,16	9	A2RUV9,P02091,P06911,P13432,P51907,Q561R9,Q6AYS4,Q9QW07,Q9QZK9
7283	1,3,6,7,12,13,15,16	2,4,5,8,9,10,11,14,17	2	P19218,Q6AYS4
7284	1,3,6,7,12,13,15,17	2,4,5,8,9,10,11,14,16	5	P02091,P13432,Q6AYS4,Q99MH3,Q9QZK9
7285	1,3,6,7,12,13,16,17	2,4,5,8,9,10,11,14,15	6	P02091,P13432,P17046,Q6AYS4,Q99MH3,Q9QZK9
7286	1,3,6,7,12,14,15,16	2,4,5,8,9,10,11,13,17	3	A2RUV9,P02803,Q6AYS4
7287	1,3,6,7,12,14,15,17	2,4,5,8,9,10,11,13,16	6	A2RUV9,P02091,P02803,P08689,P13432,Q6AYS4

7288	1,3,6,7,12,14,16,17	2,4,5,8,9,10,11,13,15	4	P02091,P13432,Q561R9,Q6AYS4
7289	1,3,6,7,12,15,16,17	2,4,5,8,9,10,11,13,14	4	P02091,P02803,P13432,Q6AYS4
7290	1,3,6,7,13,14,15,16	2,4,5,8,9,10,11,12,17	5	P02803,P19223,P62804,Q6AYS4,Q80WL1
7291	1,3,6,7,13,14,15,17	2,4,5,8,9,10,11,12,16	7	P02091,P02803,P53792,Q6AYS4,Q80WL1,Q9QW07,Q9WUW8
7292	1,3,6,7,13,14,16,17	2,4,5,8,9,10,11,12,15	5	P02091,P51907,Q561R9,Q6AYS4,Q9QW07
7293	1,3,6,7,13,15,16,17	2,4,5,8,9,10,11,12,14	5	P02091,P02803,P62804,Q6AYS4,Q80WL1
7294	1,3,6,7,14,15,16,17	2,4,5,8,9,10,11,12,13	4	P02091,P02803,Q6AYS4,Q80WL1
7295	1,3,6,8,9,10,11,12	2,4,5,7,13,14,15,16,17	6	P02782,P08723,P16636,P35467,Q6IG02,Q6P6Q2
7296	1,3,6,8,9,10,11,13	2,4,5,7,12,14,15,16,17	1	P35467
7297	1,3,6,8,9,10,11,14	2,4,5,7,12,13,15,16,17	1	P47727
7298	1,3,6,8,9,10,11,15	2,4,5,7,12,13,14,16,17	2	P27213,P35280
7299	1,3,6,8,9,10,11,16	2,4,5,7,12,13,14,15,17	0	
7300	1,3,6,8,9,10,11,17	2,4,5,7,12,13,14,15,16	3	P16636,P35467,Q09030
7301	1,3,6,8,9,10,12,13	2,4,5,7,11,14,15,16,17	3	P02782,P14408,P16636
7302	1,3,6,8,9,10,12,14	2,4,5,7,11,13,15,16,17	2	P02782,P14408
7303	1,3,6,8,9,10,12,15	2,4,5,7,11,13,14,16,17	0	
7304	1,3,6,8,9,10,12,16	2,4,5,7,11,13,14,15,17	1	P16636

7305	1,3,6,8,9,10,12,17	2,4,5,7,11,13,14,15,16	5	P13432,P14408,P16636,Q09030,Q99MH3
7306	1,3,6,8,9,10,13,14	2,4,5,7,11,12,15,16,17	3	P06911,P14408,P51907
7307	1,3,6,8,9,10,13,15	2,4,5,7,11,12,14,16,17	1	P15999
7308	1,3,6,8,9,10,13,16	2,4,5,7,11,12,14,15,17	1	P51907
7309	1,3,6,8,9,10,13,17	2,4,5,7,11,12,14,15,16	4	P14408,P16636,P51907,Q99MH3
7310	1,3,6,8,9,10,14,15	2,4,5,7,11,12,13,16,17	1	P14408
7311	1,3,6,8,9,10,14,16	2,4,5,7,11,12,13,15,17	0	
7312	1,3,6,8,9,10,14,17	2,4,5,7,11,12,13,15,16	3	P08689,P14408,P51907
7313	1,3,6,8,9,10,15,16	2,4,5,7,11,12,13,14,17	0	
7314	1,3,6,8,9,10,15,17	2,4,5,7,11,12,13,14,16	0	
7315	1,3,6,8,9,10,16,17	2,4,5,7,11,12,13,14,15	3	P16636,P25031,P51907
7316	1,3,6,8,9,11,12,13	2,4,5,7,10,14,15,16,17	5	P02782,P08723,P16636,P30120,Q9JHB9
7317	1,3,6,8,9,11,12,14	2,4,5,7,10,13,15,16,17	5	P02782,P08723,P47727,Q62997,Q6IG02
7318	1,3,6,8,9,11,12,15	2,4,5,7,10,13,14,16,17	0	
7319	1,3,6,8,9,11,12,16	2,4,5,7,10,13,14,15,17	1	Q6IG02

7320	1,3,6,8,9,11,12,17	2,4,5,7,10,13,14,15,16	6	P02782,P04218,P13432,P16636,Q09030,Q6IG02
7321	1,3,6,8,9,11,13,14	2,4,5,7,10,12,15,16,17	2	P04218,P47727
7322	1,3,6,8,9,11,13,15	2,4,5,7,10,12,14,16,17	0	
7323	1,3,6,8,9,11,13,16	2,4,5,7,10,12,14,15,17	1	P04218
7324	1,3,6,8,9,11,13,17	2,4,5,7,10,12,14,15,16	3	P04218,P16636,P51907
7325	1,3,6,8,9,11,14,15	2,4,5,7,10,12,13,16,17	1	P04218
7326	1,3,6,8,9,11,14,16	2,4,5,7,10,12,13,15,17	1	P04218
7327	1,3,6,8,9,11,14,17	2,4,5,7,10,12,13,15,16	1	P04218
7328	1,3,6,8,9,11,15,16	2,4,5,7,10,12,13,14,17	2	P04218,Q5QE79
7329	1,3,6,8,9,11,15,17	2,4,5,7,10,12,13,14,16	2	P04218,Q5QE79
7330	1,3,6,8,9,11,16,17	2,4,5,7,10,12,13,14,15	2	P04218,P16636
7331	1,3,6,8,9,12,13,14	2,4,5,7,10,11,15,16,17	5	P02782,P14408,P51907,Q62687,Q9Z1F2
7332	1,3,6,8,9,12,13,15	2,4,5,7,10,11,14,16,17	1	P02782
7333	1,3,6,8,9,12,13,16	2,4,5,7,10,11,14,15,17	1	P02782
7334	1,3,6,8,9,12,13,17	2,4,5,7,10,11,14,15,16	8	P02782,P13432,P14408,P16636,P51907,Q99MH3,Q9QZK9,Q9Z1F2

7335	1,3,6,8,9,12,14,15	2,4,5,7,10,11,13,16,17	1	P14408
7336	1,3,6,8,9,12,14,16	2,4,5,7,10,11,13,15,17	0	
7337	1,3,6,8,9,12,14,17	2,4,5,7,10,11,13,15,16	5	P01835,P02782,P13432,P14408,P51907
7338	1,3,6,8,9,12,15,16	2,4,5,7,10,11,13,14,17	1	Q5QE79
7339	1,3,6,8,9,12,15,17	2,4,5,7,10,11,13,14,16	1	P13432
7340	1,3,6,8,9,12,16,17	2,4,5,7,10,11,13,14,15	2	P13432,P16636
7341	1,3,6,8,9,13,14,15	2,4,5,7,10,11,12,16,17	6	P06757,P14408,P15999,P51907,P53792,Q62687
7342	1,3,6,8,9,13,14,16	2,4,5,7,10,11,12,15,17	1	P51907
7343	1,3,6,8,9,13,14,17	2,4,5,7,10,11,12,15,16	6	P04218,P14408,P51907,P53792,Q62687,Q9QW07
7344	1,3,6,8,9,13,15,16	2,4,5,7,10,11,12,14,17	1	Q5QE79
7345	1,3,6,8,9,13,15,17	2,4,5,7,10,11,12,14,16	3	P51907,P53792,Q5QE79
7346	1,3,6,8,9,13,16,17	2,4,5,7,10,11,12,14,15	2	P04218,P51907
7347	1,3,6,8,9,14,15,16	2,4,5,7,10,11,12,13,17	2	P04218,Q5QE79
7348	1,3,6,8,9,14,15,17	2,4,5,7,10,11,12,13,16	1	P04218
7349	1,3,6,8,9,14,16,17	2,4,5,7,10,11,12,13,15	2	P04218,P51907

7350	1,3,6,8,9,15,16,17	2,4,5,7,10,11,12,13,14	2	P04218,Q5QE79
7351	1,3,6,8,10,11,12,13	2,4,5,7,9,14,15,16,17	10	O35547,P02781,P02782,P08723,P16636,P30120,P47727,Q6IG02,Q6P6Q2,Q9JHB9
7352	1,3,6,8,10,11,12,14	2,4,5,7,9,13,15,16,17	7	O35547,P02782,P08723,P35280,P47727,Q6IG02,Q6P6Q2
7353	1,3,6,8,10,11,12,15	2,4,5,7,9,13,14,16,17	4	O35547,P35280,Q6IG02,Q6P6Q2
7354	1,3,6,8,10,11,12,16	2,4,5,7,9,13,14,15,17	6	O35547,P16636,Q6IFU7,Q6IG02,Q6IMF3,Q6P6Q2
7355	1,3,6,8,10,11,12,17	2,4,5,7,9,13,14,15,16	9	O08815,O35547,P02782,P13432,P16636,Q6IG02,Q6P6Q2,Q99MH3,Q9QZK9
7356	1,3,6,8,10,11,13,14	2,4,5,7,9,12,15,16,17	2	P06911,P47727
7357	1,3,6,8,10,11,13,15	2,4,5,7,9,12,14,16,17	1	P15999
7358	1,3,6,8,10,11,13,16	2,4,5,7,9,12,14,15,17	0	
7359	1,3,6,8,10,11,13,17	2,4,5,7,9,12,14,15,16	2	P16636,Q99MH3
7360	1,3,6,8,10,11,14,15	2,4,5,7,9,12,13,16,17	1	P35280
7361	1,3,6,8,10,11,14,16	2,4,5,7,9,12,13,15,17	0	
7362	1,3,6,8,10,11,14,17	2,4,5,7,9,12,13,15,16	1	P47727
7363	1,3,6,8,10,11,15,16	2,4,5,7,9,12,13,14,17	0	
7364	1,3,6,8,10,11,15,17	2,4,5,7,9,12,13,14,16	0	
7365	1,3,6,8,10,11,16,17	2,4,5,7,9,12,13,14,15	1	P16636
7366	1,3,6,8,10,12,13,14	2,4,5,7,9,11,15,16,17	8	P02782,P06911,P08723,P14408,Q05175,Q498D9,Q64093,Q9Z1F2
7367	1,3,6,8,10,12,13,15	2,4,5,7,9,11,14,16,17	5	P15999,P82995,Q05175,Q6IFV1,Q99MH3
7368	1,3,6,8,10,12,13,16	2,4,5,7,9,11,14,15,17	1	P16636
7369	1,3,6,8,10,12,13,17	2,4,5,7,9,11,14,15,16	8	P02782,P13432,P16636,P51907,Q05175,Q6IFV1,Q99MH3,Q9QZK9
7370	1,3,6,8,10,12,14,15	2,4,5,7,9,11,13,16,17	1	P35280
7371	1,3,6,8,10,12,14,16	2,4,5,7,9,11,13,15,17	1	P05539

7372	1,3,6,8,10,12,14,17	2,4,5,7,9,11,13,15,16	6	P02782,P08689,P13432,P14408,Q10758,Q99MH3
7373	1,3,6,8,10,12,15,16	2,4,5,7,9,11,13,14,17	0	
7374	1,3,6,8,10,12,15,17	2,4,5,7,9,11,13,14,16	2	Q6IFV1,Q99MH3
7375	1,3,6,8,10,12,16,17	2,4,5,7,9,11,13,14,15	3	P05539,P16636,Q99MH3
7376	1,3,6,8,10,13,14,15	2,4,5,7,9,11,12,16,17	3	P06911,P15999,Q64093
7377	1,3,6,8,10,13,14,16	2,4,5,7,9,11,12,15,17	2	P06911,P51907
7378	1,3,6,8,10,13,14,17	2,4,5,7,9,11,12,15,16	4	P06911,P14408,P51907,Q9QW07
7379	1,3,6,8,10,13,15,16	2,4,5,7,9,11,12,14,17	1	P15999
7380	1,3,6,8,10,13,15,17	2,4,5,7,9,11,12,14,16	4	P15999,P51907,Q6IFV1,Q99MH3
7381	1,3,6,8,10,13,16,17	2,4,5,7,9,11,12,14,15	3	P16636,P51907,Q99MH3
7382	1,3,6,8,10,14,15,16	2,4,5,7,9,11,12,13,17	0	
7383	1,3,6,8,10,14,15,17	2,4,5,7,9,11,12,13,16	1	P02803
7384	1,3,6,8,10,14,16,17	2,4,5,7,9,11,12,13,15	2	P05539,P51907
7385	1,3,6,8,10,15,16,17	2,4,5,7,9,11,12,13,14	0	
7386	1,3,6,8,11,12,13,14	2,4,5,7,9,10,15,16,17	11	P02781,P02782,P04905,P06911,P08723,P30120,P47727,Q62902,Q64093,Q9JHB9,Q9Z1F2
7387	1,3,6,8,11,12,13,15	2,4,5,7,9,10,14,16,17	4	P02782,P15999,Q64093,Q9JHB9
7388	1,3,6,8,11,12,13,16	2,4,5,7,9,10,14,15,17	3	P02782,P16636,Q9JHB9
7389	1,3,6,8,11,12,13,17	2,4,5,7,9,10,14,15,16	7	P02782,P13432,P16636,P30120,Q99MH3,Q9JHB9,Q9QZK9
7390	1,3,6,8,11,12,14,15	2,4,5,7,9,10,13,16,17	2	P35280,Q64093
7391	1,3,6,8,11,12,14,16	2,4,5,7,9,10,13,15,17	3	P02782,Q6IG02,Q6P6Q2
7392	1,3,6,8,11,12,14,17	2,4,5,7,9,10,13,15,16	4	P02782,P13432,P47727,Q6IG02
7393	1,3,6,8,11,12,15,16	2,4,5,7,9,10,13,14,17	0	
7394	1,3,6,8,11,12,15,17	2,4,5,7,9,10,13,14,16	1	P13432

7395	1,3,6,8,11,<br>12,16,17	2,4,5,7,9,1<br>0,13,14,15	3	P13432,P16636,Q6IG02
7396	1,3,6,8,11,<br>13,14,15	2,4,5,7,9,1<br>0,12,16,17	2	P15999,Q64093
7397	1,3,6,8,11,<br>13,14,16	2,4,5,7,9,1<br>0,12,15,17	1	Q64093
7398	1,3,6,8,11,<br>13,14,17	2,4,5,7,9,1<br>0,12,15,16	5	P04218,P47727,P51907,Q64093,Q9QW07
7399	1,3,6,8,11,<br>13,15,16	2,4,5,7,9,1<br>0,12,14,17	0	
7400	1,3,6,8,11,<br>13,15,17	2,4,5,7,9,1<br>0,12,14,16	1	P15999
7401	1,3,6,8,11,<br>13,16,17	2,4,5,7,9,1<br>0,12,14,15	1	P16636
7402	1,3,6,8,11,<br>14,15,16	2,4,5,7,9,1<br>0,12,13,17	0	
7403	1,3,6,8,11,<br>14,15,17	2,4,5,7,9,1<br>0,12,13,16	2	P04218,P97840
7404	1,3,6,8,11,<br>14,16,17	2,4,5,7,9,1<br>0,12,13,15	1	P04218
7405	1,3,6,8,11,<br>15,16,17	2,4,5,7,9,1<br>0,12,13,14	1	P04218
7406	1,3,6,8,12,<br>13,14,15	2,4,5,7,9,1<br>0,11,16,17	8	A2RUV9,O08557,P02782,P15999,Q62687,Q63424,Q64093,Q9Z1F2
7407	1,3,6,8,12,<br>13,14,16	2,4,5,7,9,1<br>0,11,15,17	4	P02782,P51907,Q64093,Q9Z1F2
7408	1,3,6,8,12,<br>13,14,17	2,4,5,7,9,1<br>0,11,15,16	14	O70594,P02782,P13432,P14408,P46720,P51907,Q561R9,Q62687,Q63424,Q64093,Q80W57,Q9QW07,Q9QZK9,Q9Z1F2
7409	1,3,6,8,12,<br>13,15,16	2,4,5,7,9,1<br>0,11,14,17	1	P82995
7410	1,3,6,8,12,<br>13,15,17	2,4,5,7,9,1<br>0,11,14,16	9	P02782,P13432,P15999,P51907,Q5XFX0,Q64093,Q6IFV1,Q99MH3,Q9QZK9
7411	1,3,6,8,12,<br>13,16,17	2,4,5,7,9,1<br>0,11,14,15	6	P02782,P13432,P16636,P51907,Q5XFX0,Q99MH3
7412	1,3,6,8,12,<br>14,15,16	2,4,5,7,9,1<br>0,11,13,17	0	
7413	1,3,6,8,12,<br>14,15,17	2,4,5,7,9,1<br>0,11,13,16	2	P13432,Q64093
7414	1,3,6,8,12,<br>14,16,17	2,4,5,7,9,1<br>0,11,13,15	3	P13432,P51907,Q561R9
7415	1,3,6,8,12,<br>15,16,17	2,4,5,7,9,1<br>0,11,13,14	2	P13432,Q5XFX0
7416	1,3,6,8,13,<br>14,15,16	2,4,5,7,9,1<br>0,11,12,17	2	P15999,Q64093
7417	1,3,6,8,13,<br>14,15,17	2,4,5,7,9,1<br>0,11,12,16	6	P15999,P51907,Q62687,Q63424,Q64093,Q9QW07

7418	1,3,6,8,13,14,16,17	2,4,5,7,9,10,11,12,15	4	P51907,Q64093,Q6PCU2,Q9QW07
7419	1,3,6,8,13,15,16,17	2,4,5,7,9,10,11,12,14	2	P51907,Q5XFX0
7420	1,3,6,8,14,15,16,17	2,4,5,7,9,10,11,12,13	1	P02803
7421	1,3,6,9,10,11,12,13	2,4,5,7,8,14,15,16,17	0	
7422	1,3,6,9,10,11,12,14	2,4,5,7,8,13,15,16,17	1	P47727
7423	1,3,6,9,10,11,12,15	2,4,5,7,8,13,14,16,17	1	Q6PCU2
7424	1,3,6,9,10,11,12,16	2,4,5,7,8,13,14,15,17	5	P10536,P12368,Q6IFU7,Q6P6Q2,Q9WUW8
7425	1,3,6,9,10,11,12,17	2,4,5,7,8,13,14,15,16	5	O08815,P06757,P08689,P16636,Q09030
7426	1,3,6,9,10,11,13,14	2,4,5,7,8,12,15,16,17	2	P47727,P55091
7427	1,3,6,9,10,11,13,15	2,4,5,7,8,12,14,16,17	2	P55091,Q80WL1
7428	1,3,6,9,10,11,13,16	2,4,5,7,8,12,14,15,17	2	P55091,Q9QZA6
7429	1,3,6,9,10,11,13,17	2,4,5,7,8,12,14,15,16	0	
7430	1,3,6,9,10,11,14,15	2,4,5,7,8,12,13,16,17	3	P55091,P63029,Q80WL1
7431	1,3,6,9,10,11,14,16	2,4,5,7,8,12,13,15,17	2	P55091,Q80WL1
7432	1,3,6,9,10,11,14,17	2,4,5,7,8,12,13,15,16	1	P08689
7433	1,3,6,9,10,11,15,16	2,4,5,7,8,12,13,14,17	3	P55091,Q62635,Q80WL1
7434	1,3,6,9,10,11,15,17	2,4,5,7,8,12,13,14,16	2	P08689,Q80WL1
7435	1,3,6,9,10,11,16,17	2,4,5,7,8,12,13,14,15	0	
7436	1,3,6,9,10,12,13,14	2,4,5,7,8,11,15,16,17	3	P14408,Q05175,Q6AYS4
7437	1,3,6,9,10,12,13,15	2,4,5,7,8,11,14,16,17	1	Q6AYS4
7438	1,3,6,9,10,12,13,16	2,4,5,7,8,11,14,15,17	3	P07171,P97840,Q6AYS4
7439	1,3,6,9,10,12,13,17	2,4,5,7,8,11,14,15,16	4	P07171,P16636,Q6AYS4,Q99MH3
7440	1,3,6,9,10,12,14,15	2,4,5,7,8,11,13,16,17	1	Q6AYS4

7441	1,3,6,9,10,12,14,16	2,4,5,7,8,11,13,15,17	1	Q6AYS4
7442	1,3,6,9,10,12,14,17	2,4,5,7,8,11,13,15,16	4	P01835,P07171,P08689,Q6AYS4
7443	1,3,6,9,10,12,15,16	2,4,5,7,8,11,13,14,17	1	Q6AYS4
7444	1,3,6,9,10,12,15,17	2,4,5,7,8,11,13,14,16	4	P07171,P08689,Q6AYS4,Q99MH3
7445	1,3,6,9,10,12,16,17	2,4,5,7,8,11,13,14,15	3	P07171,P16636,Q6AYS4
7446	1,3,6,9,10,13,14,15	2,4,5,7,8,11,12,16,17	6	P19223,P50115,P55091,Q6AYS4,Q80WL1,Q99041
7447	1,3,6,9,10,13,14,16	2,4,5,7,8,11,12,15,17	7	P19223,P50115,P55091,Q6AYS4,Q80WL1,Q99041,Q9QZA6
7448	1,3,6,9,10,13,14,17	2,4,5,7,8,11,12,15,16	3	P51907,Q6AYS4,Q9QW07
7449	1,3,6,9,10,13,15,16	2,4,5,7,8,11,12,14,17	7	P19223,P50115,P55091,P62804,Q6AYS4,Q80WL1,Q9QZA6
7450	1,3,6,9,10,13,15,17	2,4,5,7,8,11,12,14,16	3	Q6AYS4,Q80WL1,Q99MH3
7451	1,3,6,9,10,13,16,17	2,4,5,7,8,11,12,14,15	3	P07171,P51907,Q6AYS4
7452	1,3,6,9,10,14,15,16	2,4,5,7,8,11,12,13,17	6	P19223,P50115,P55091,Q6AYS4,Q80WL1,Q99041
7453	1,3,6,9,10,14,15,17	2,4,5,7,8,11,12,13,16	3	P08689,Q6AYS4,Q80WL1
7454	1,3,6,9,10,14,16,17	2,4,5,7,8,11,12,13,15	2	Q6AYS4,Q80WL1
7455	1,3,6,9,10,15,16,17	2,4,5,7,8,11,12,13,14	2	Q6AYS4,Q80WL1
7456	1,3,6,9,11,12,13,14	2,4,5,7,8,10,15,16,17	1	P47727
7457	1,3,6,9,11,12,13,15	2,4,5,7,8,10,14,16,17	0	
7458	1,3,6,9,11,12,13,16	2,4,5,7,8,10,14,15,17	0	
7459	1,3,6,9,11,12,13,17	2,4,5,7,8,10,14,15,16	1	P16636
7460	1,3,6,9,11,12,14,15	2,4,5,7,8,10,13,16,17	0	
7461	1,3,6,9,11,12,14,16	2,4,5,7,8,10,13,15,17	0	
7462	1,3,6,9,11,12,14,17	2,4,5,7,8,10,13,15,16	3	P01835,P04218,P08689
7463	1,3,6,9,11,12,15,16	2,4,5,7,8,10,13,14,17	0	

7464	1,3,6,9,11,<br>12,15,17	2,4,5,7,8,1<br>0,13,14,16	0	
7465	1,3,6,9,11,<br>12,16,17	2,4,5,7,8,1<br>0,13,14,15	3	P04218,P07171,P16636
7466	1,3,6,9,11,<br>13,14,15	2,4,5,7,8,1<br>0,12,16,17	4	P16391,P19223,P55091,Q80WL1
7467	1,3,6,9,11,<br>13,14,16	2,4,5,7,8,1<br>0,12,15,17	5	P04218,P19223,P55091,Q80WL1,Q9QZA6
7468	1,3,6,9,11,<br>13,14,17	2,4,5,7,8,1<br>0,12,15,16	2	P04218,Q9QW07
7469	1,3,6,9,11,<br>13,15,16	2,4,5,7,8,1<br>0,12,14,17	3	P19223,P55091,Q80WL1
7470	1,3,6,9,11,<br>13,15,17	2,4,5,7,8,1<br>0,12,14,16	2	P04218,Q80WL1
7471	1,3,6,9,11,<br>13,16,17	2,4,5,7,8,1<br>0,12,14,15	1	P04218
7472	1,3,6,9,11,<br>14,15,16	2,4,5,7,8,1<br>0,12,13,17	4	P04218,P19223,P55091,Q80WL1
7473	1,3,6,9,11,<br>14,15,17	2,4,5,7,8,1<br>0,12,13,16	2	P04218,Q80WL1
7474	1,3,6,9,11,<br>14,16,17	2,4,5,7,8,1<br>0,12,13,15	2	P04218,Q80WL1
7475	1,3,6,9,11,<br>15,16,17	2,4,5,7,8,1<br>0,12,13,14	2	P04218,Q80WL1
7476	1,3,6,9,12,<br>13,14,15	2,4,5,7,8,1<br>0,11,16,17	4	A2RUV9,P00714,P16391,Q6AYS4
7477	1,3,6,9,12,<br>13,14,16	2,4,5,7,8,1<br>0,11,15,17	2	P02761,Q6AYS4
7478	1,3,6,9,12,<br>13,14,17	2,4,5,7,8,1<br>0,11,15,16	6	P00714,P01835,P07171,P51907,Q6AYS4,Q9QW07
7479	1,3,6,9,12,<br>13,15,16	2,4,5,7,8,1<br>0,11,14,17	2	P00714,Q6AYS4
7480	1,3,6,9,12,<br>13,15,17	2,4,5,7,8,1<br>0,11,14,16	4	P00714,P07171,Q6AYS4,Q99MH3
7481	1,3,6,9,12,<br>13,16,17	2,4,5,7,8,1<br>0,11,14,15	3	P00714,P07171,Q6AYS4
7482	1,3,6,9,12,<br>14,15,16	2,4,5,7,8,1<br>0,11,13,17	1	Q6AYS4
7483	1,3,6,9,12,<br>14,15,17	2,4,5,7,8,1<br>0,11,13,16	3	P01835,P08689,Q6AYS4
7484	1,3,6,9,12,<br>14,16,17	2,4,5,7,8,1<br>0,11,13,15	3	P01835,P07171,Q6AYS4
7485	1,3,6,9,12,<br>15,16,17	2,4,5,7,8,1<br>0,11,13,14	3	P00714,P07171,Q6AYS4
7486	1,3,6,9,13,<br>14,15,16	2,4,5,7,8,1<br>0,11,12,17	10	P00714,P16391,P19223,P50115,P55091,P62804,Q4G075,Q6AYS4,Q80WL1,Q99041

7487	1,3,6,9,13,<br>14,15,17	2,4,5,7,8,1<br>0,11,12,16	7	P00714,P16391,P53792,P61206;P84079;P84082,Q6AYS4,Q80WL1,Q9QW07
7488	1,3,6,9,13,<br>14,16,17	2,4,5,7,8,1<br>0,11,12,15	7	P00714,P04218,P07171,P51907,Q6AYS4,Q80WL1,Q9QW07
7489	1,3,6,9,13,<br>15,16,17	2,4,5,7,8,1<br>0,11,12,14	6	P00714,P62804,Q63598,Q6AYS4,Q80WL1,Q9EQS0
7490	1,3,6,9,14,<br>15,16,17	2,4,5,7,8,1<br>0,11,12,13	5	P00714,P02803,P04218,Q6AYS4,Q80WL1
7491	1,3,6,10,1<br>1,12,13,14	2,4,5,7,8,9<br>,15,16,17	1	P47727
7492	1,3,6,10,1<br>1,12,13,15	2,4,5,7,8,9<br>,14,16,17	0	
7493	1,3,6,10,1<br>1,12,13,16	2,4,5,7,8,9<br>,14,15,17	1	Q6IFU7
7494	1,3,6,10,1<br>1,12,13,17	2,4,5,7,8,9<br>,14,15,16	4	O08815,P16636,Q99MH3,Q9QZK9
7495	1,3,6,10,1<br>1,12,14,15	2,4,5,7,8,9<br>,13,16,17	1	P35280
7496	1,3,6,10,1<br>1,12,14,16	2,4,5,7,8,9<br>,13,15,17	2	Q6IFU7,Q6P6Q2
7497	1,3,6,10,1<br>1,12,14,17	2,4,5,7,8,9<br>,13,15,16	4	O08815,P08689,P47727,Q10758
7498	1,3,6,10,1<br>1,12,15,16	2,4,5,7,8,9<br>,13,14,17	1	Q6IFU7
7499	1,3,6,10,1<br>1,12,15,17	2,4,5,7,8,9<br>,13,14,16	2	P08689,Q99MH3
7500	1,3,6,10,1<br>1,12,16,17	2,4,5,7,8,9<br>,13,14,15	2	P06757,P16636
7501	1,3,6,10,1<br>1,13,14,15	2,4,5,7,8,9<br>,12,16,17	4	P19223,P50115,P55091,Q80WL1
7502	1,3,6,10,1<br>1,13,14,16	2,4,5,7,8,9<br>,12,15,17	5	P19223,P50115,P55091,Q80WL1,Q9QZA6
7503	1,3,6,10,1<br>1,13,14,17	2,4,5,7,8,9<br>,12,15,16	2	P47727,Q9QW07
7504	1,3,6,10,1<br>1,13,15,16	2,4,5,7,8,9<br>,12,14,17	4	P19223,P50115,P55091,Q80WL1
7505	1,3,6,10,1<br>1,13,15,17	2,4,5,7,8,9<br>,12,14,16	2	Q80WL1,Q99MH3
7506	1,3,6,10,1<br>1,13,16,17	2,4,5,7,8,9<br>,12,14,15	0	
7507	1,3,6,10,1<br>1,14,15,16	2,4,5,7,8,9<br>,12,13,17	4	P19223,P50115,P55091,Q80WL1
7508	1,3,6,10,1<br>1,14,15,17	2,4,5,7,8,9<br>,12,13,16	2	P08689,Q80WL1
7509	1,3,6,10,1<br>1,14,16,17	2,4,5,7,8,9<br>,12,13,15	0	

7510	1,3,6,10,1 1,15,16,17	2,4,5,7,8,9 ,12,13,14	1	Q80WL1
7511	1,3,6,10,1 2,13,14,15	2,4,5,7,8,9 ,11,16,17	4	A2RUV9,P50115,Q05175,Q6AYS4
7512	1,3,6,10,1 2,13,14,16	2,4,5,7,8,9 ,11,15,17	3	P50115,Q05175,Q6AYS4
7513	1,3,6,10,1 2,13,14,17	2,4,5,7,8,9 ,11,15,16	6	P07171,Q05175,Q6AYS4,Q99MH3,Q9QW07,Q9QZK9
7514	1,3,6,10,1 2,13,15,16	2,4,5,7,8,9 ,11,14,17	2	P50115,Q6AYS4
7515	1,3,6,10,1 2,13,15,17	2,4,5,7,8,9 ,11,14,16	6	P07171,Q05175,Q6AYS4,Q6IFV1,Q99MH3,Q9QZK9
7516	1,3,6,10,1 2,13,16,17	2,4,5,7,8,9 ,11,14,15	5	P07171,P16636,Q05175,Q6AYS4,Q99MH3
7517	1,3,6,10,1 2,14,15,16	2,4,5,7,8,9 ,11,13,17	3	P20766,P50115,Q6AYS4
7518	1,3,6,10,1 2,14,15,17	2,4,5,7,8,9 ,11,13,16	3	P08689,Q6AYS4,Q99MH3
7519	1,3,6,10,1 2,14,16,17	2,4,5,7,8,9 ,11,13,15	3	P07171,Q10758,Q6AYS4
7520	1,3,6,10,1 2,15,16,17	2,4,5,7,8,9 ,11,13,14	3	P07171,Q6AYS4,Q99MH3
7521	1,3,6,10,1 3,14,15,16	2,4,5,7,8,9 ,11,12,17	6	P19223,P50115,P55091,Q6AYS4,Q80WL1,Q99041
7522	1,3,6,10,1 3,14,15,17	2,4,5,7,8,9 ,11,12,16	3	Q6AYS4,Q80WL1,Q9QW07
7523	1,3,6,10,1 3,14,16,17	2,4,5,7,8,9 ,11,12,15	3	P51907,Q6AYS4,Q9QW07
7524	1,3,6,10,1 3,15,16,17	2,4,5,7,8,9 ,11,12,14	4	Q6AYS4,Q80WL1,Q99MH3,Q9EQS0
7525	1,3,6,10,1 4,15,16,17	2,4,5,7,8,9 ,11,12,13	3	P02803,Q6AYS4,Q80WL1
7526	1,3,6,11,1 2,13,14,15	2,4,5,7,8,9 ,10,16,17	2	P47727,Q64093
7527	1,3,6,11,1 2,13,14,16	2,4,5,7,8,9 ,10,15,17	1	P47727
7528	1,3,6,11,1 2,13,14,17	2,4,5,7,8,9 ,10,15,16	4	P47727,Q64093,Q9QW07,Q9QZK9
7529	1,3,6,11,1 2,13,15,16	2,4,5,7,8,9 ,10,14,17	1	Q6AYS4
7530	1,3,6,11,1 2,13,15,17	2,4,5,7,8,9 ,10,14,16	2	Q99MH3,Q9QZK9
7531	1,3,6,11,1 2,13,16,17	2,4,5,7,8,9 ,10,14,15	3	P07171,P16636,P17046
7532	1,3,6,11,1 2,14,15,16	2,4,5,7,8,9 ,10,13,17	1	Q6AYS4

7533	1,3,6,11,12,14,15,17	2,4,5,7,8,9,10,13,16	0	
7534	1,3,6,11,12,14,16,17	2,4,5,7,8,9,10,13,15	0	
7535	1,3,6,11,12,15,16,17	2,4,5,7,8,9,10,13,14	0	
7536	1,3,6,11,13,14,15,16	2,4,5,7,8,9,10,12,17	5	P19223,P50115,P55091,Q6AYS4,Q80WL1
7537	1,3,6,11,13,14,15,17	2,4,5,7,8,9,10,12,16	3	Q64093,Q80WL1,Q9QW07
7538	1,3,6,11,13,14,16,17	2,4,5,7,8,9,10,12,15	4	D3ZHA0,P04218,Q80WL1,Q9QW07
7539	1,3,6,11,13,15,16,17	2,4,5,7,8,9,10,12,14	2	Q80WL1,Q9EQS0
7540	1,3,6,11,14,15,16,17	2,4,5,7,8,9,10,12,13	3	P02803,P04218,Q80WL1
7541	1,3,6,12,13,14,15,16	2,4,5,7,8,9,10,11,17	4	P00714,P50115,Q64093,Q6AYS4
7542	1,3,6,12,13,14,15,17	2,4,5,7,8,9,10,11,16	5	A2RUV9,P00714,Q64093,Q6AYS4,Q9QW07
7543	1,3,6,12,13,14,16,17	2,4,5,7,8,9,10,11,15	6	P00714,P07171,P51907,Q561R9,Q6AYS4,Q9QW07
7544	1,3,6,12,13,15,16,17	2,4,5,7,8,9,10,11,14	5	P00714,P07171,Q5XFX0,Q6AYS4,Q99MH3
7545	1,3,6,12,14,15,16,17	2,4,5,7,8,9,10,11,13	3	P00714,P02803,Q6AYS4
7546	1,3,6,13,14,15,16,17	2,4,5,7,8,9,10,11,12	8	P00714,P02803,P16391,Q64093,Q6AYS4,Q80WL1,Q9EQS0,Q9QW07
7547	1,3,7,8,9,10,11,12	2,4,5,6,13,14,15,16,17	2	P10715,Q6IG02
7548	1,3,7,8,9,10,11,13	2,4,5,6,12,14,15,16,17	1	P35467
7549	1,3,7,8,9,10,11,14	2,4,5,6,12,13,15,16,17	1	Q62635
7550	1,3,7,8,9,10,11,15	2,4,5,6,12,13,14,16,17	6	O54728,P14480,P22006,P27213,P35280,Q62635
7551	1,3,7,8,9,10,11,16	2,4,5,6,12,13,14,15,17	1	Q62635
7552	1,3,7,8,9,10,11,17	2,4,5,6,12,13,14,15,16	2	P02091,P25236

7553	1,3,7,8,9,10,12,13	2,4,5,6,11,14,15,16,17	0	
7554	1,3,7,8,9,10,12,14	2,4,5,6,11,13,15,16,17	0	
7555	1,3,7,8,9,10,12,15	2,4,5,6,11,13,14,16,17	1	P10715
7556	1,3,7,8,9,10,12,16	2,4,5,6,11,13,14,15,17	1	P10715
7557	1,3,7,8,9,10,12,17	2,4,5,6,11,13,14,15,16	3	P02091,P08689,P10715
7558	1,3,7,8,9,10,13,14	2,4,5,6,11,12,15,16,17	0	
7559	1,3,7,8,9,10,13,15	2,4,5,6,11,12,14,16,17	2	O54728,P01835
7560	1,3,7,8,9,10,13,16	2,4,5,6,11,12,14,15,17	1	P01835
7561	1,3,7,8,9,10,13,17	2,4,5,6,11,12,14,15,16	2	P02091,P51907
7562	1,3,7,8,9,10,14,15	2,4,5,6,11,12,13,16,17	2	O54728,Q62635
7563	1,3,7,8,9,10,14,16	2,4,5,6,11,12,13,15,17	1	Q62635
7564	1,3,7,8,9,10,14,17	2,4,5,6,11,12,13,15,16	2	P02091,P08689
7565	1,3,7,8,9,10,15,16	2,4,5,6,11,12,13,14,17	3	O54728,P01835,Q62635
7566	1,3,7,8,9,10,15,17	2,4,5,6,11,12,13,14,16	3	G3V686,P02091,P25236
7567	1,3,7,8,9,10,16,17	2,4,5,6,11,12,13,14,15	3	P02091,P25031,P25236

7568	1,3,7,8,9,11,12,13	2,4,5,6,10,14,15,16,17	1	P10715
7569	1,3,7,8,9,11,12,14	2,4,5,6,10,13,15,16,17	1	P10715
7570	1,3,7,8,9,11,12,15	2,4,5,6,10,13,14,16,17	1	P10715
7571	1,3,7,8,9,11,12,16	2,4,5,6,10,13,14,15,17	1	P10715
7572	1,3,7,8,9,11,12,17	2,4,5,6,10,13,14,15,16	2	P02091,P10715
7573	1,3,7,8,9,11,13,14	2,4,5,6,10,12,15,16,17	0	
7574	1,3,7,8,9,11,13,15	2,4,5,6,10,12,14,16,17	1	O54728
7575	1,3,7,8,9,11,13,16	2,4,5,6,10,12,14,15,17	1	B4F795
7576	1,3,7,8,9,11,13,17	2,4,5,6,10,12,14,15,16	1	P02091
7577	1,3,7,8,9,11,14,15	2,4,5,6,10,12,13,16,17	4	O54728,P14480,P22006,Q62635
7578	1,3,7,8,9,11,14,16	2,4,5,6,10,12,13,15,17	3	B4F795,P04218,Q62635
7579	1,3,7,8,9,11,14,17	2,4,5,6,10,12,13,15,16	2	P02091,P04218
7580	1,3,7,8,9,11,15,16	2,4,5,6,10,12,13,14,17	4	B4F795,O54728,P10715,Q62635
7581	1,3,7,8,9,11,15,17	2,4,5,6,10,12,13,14,16	4	P02091,P04218,P10715,P25236
7582	1,3,7,8,9,11,16,17	2,4,5,6,10,12,13,14,15	5	B4F795,P02091,P04218,P10715,P25236

7583	1,3,7,8,9,12,13,14	2,4,5,6,10,11,15,16,17	1	A2RUV9
7584	1,3,7,8,9,12,13,15	2,4,5,6,10,11,14,16,17	1	P10715
7585	1,3,7,8,9,12,13,16	2,4,5,6,10,11,14,15,17	1	P10715
7586	1,3,7,8,9,12,13,17	2,4,5,6,10,11,14,15,16	3	P02091,P10715,P51907
7587	1,3,7,8,9,12,14,15	2,4,5,6,10,11,13,16,17	2	A2RUV9,P10715
7588	1,3,7,8,9,12,14,16	2,4,5,6,10,11,13,15,17	1	P10715
7589	1,3,7,8,9,12,14,17	2,4,5,6,10,11,13,15,16	2	P02091,P10715
7590	1,3,7,8,9,12,15,16	2,4,5,6,10,11,13,14,17	1	P10715
7591	1,3,7,8,9,12,15,17	2,4,5,6,10,11,13,14,16	2	P02091,P10715
7592	1,3,7,8,9,12,16,17	2,4,5,6,10,11,13,14,15	2	P02091,P10715
7593	1,3,7,8,9,13,14,15	2,4,5,6,10,11,12,16,17	5	O54728,P06757,P53792,Q08849,Q6IFU7
7594	1,3,7,8,9,13,14,16	2,4,5,6,10,11,12,15,17	1	Q08849
7595	1,3,7,8,9,13,14,17	2,4,5,6,10,11,12,15,16	6	P02091,P51907,P53792,Q08849,Q6IFU7,Q9QW07
7596	1,3,7,8,9,13,15,16	2,4,5,6,10,11,12,14,17	4	B4F795,O54728,P01835,P62804
7597	1,3,7,8,9,13,15,17	2,4,5,6,10,11,12,14,16	3	P02091,P53792,Q6IFU7

7598	1,3,7,8,9,13,16,17	2,4,5,6,10,11,12,14,15	4	P02091,P51907,P62804,Q6IFU7
7599	1,3,7,8,9,14,15,16	2,4,5,6,10,11,12,13,17	5	B4F795,O54728,Q08849,Q62635,Q9QZK9
7600	1,3,7,8,9,14,15,17	2,4,5,6,10,11,12,13,16	3	P02091,Q08849,Q6IFU7
7601	1,3,7,8,9,14,16,17	2,4,5,6,10,11,12,13,15	3	P02091,P04218,Q08849
7602	1,3,7,8,9,15,16,17	2,4,5,6,10,11,12,13,14	3	B4F795,P02091,P47727
7603	1,3,7,8,10,11,12,13	2,4,5,6,9,14,15,16,17	3	P10715,Q5HZV9,Q8CJ52
7604	1,3,7,8,10,11,12,14	2,4,5,6,9,13,15,16,17	3	P10715,P35280,Q6IG02
7605	1,3,7,8,10,11,12,15	2,4,5,6,9,13,14,16,17	4	P00731,P10715,P22006,P35280
7606	1,3,7,8,10,11,12,16	2,4,5,6,9,13,14,15,17	1	P10715
7607	1,3,7,8,10,11,12,17	2,4,5,6,9,13,14,15,16	4	P02091,P08689,P10715,Q9QZK9
7608	1,3,7,8,10,11,13,14	2,4,5,6,9,12,15,16,17	1	P47727
7609	1,3,7,8,10,11,13,15	2,4,5,6,9,12,14,16,17	2	P01835,P22006
7610	1,3,7,8,10,11,13,16	2,4,5,6,9,12,14,15,17	2	P01835,Q8VD89
7611	1,3,7,8,10,11,13,17	2,4,5,6,9,12,14,15,16	2	P01946,P02091
7612	1,3,7,8,10,11,14,15	2,4,5,6,9,12,13,16,17	2	P22006,P35280
7613	1,3,7,8,10,11,14,16	2,4,5,6,9,12,13,15,17	0	
7614	1,3,7,8,10,11,14,17	2,4,5,6,9,12,13,15,16	2	P02091,P08689
7615	1,3,7,8,10,11,15,16	2,4,5,6,9,12,13,14,17	2	P01835,Q62635
7616	1,3,7,8,10,11,15,17	2,4,5,6,9,12,13,14,16	4	P02091,P12346,P22006,P25236
7617	1,3,7,8,10,11,16,17	2,4,5,6,9,12,13,14,15	2	P02091,P25236

7618	1,3,7,8,10,12,13,14	2,4,5,6,9,11,15,16,17	1	A2RUV9
7619	1,3,7,8,10,12,13,15	2,4,5,6,9,11,14,16,17	2	P15999,Q99MH3
7620	1,3,7,8,10,12,13,16	2,4,5,6,9,11,14,15,17	0	
7621	1,3,7,8,10,12,13,17	2,4,5,6,9,11,14,15,16	3	P02091,Q99MH3,Q9QZK9
7622	1,3,7,8,10,12,14,15	2,4,5,6,9,11,13,16,17	2	A2RUV9,P35280
7623	1,3,7,8,10,12,14,16	2,4,5,6,9,11,13,15,17	1	P08721
7624	1,3,7,8,10,12,14,17	2,4,5,6,9,11,13,15,16	2	P02091,P08689
7625	1,3,7,8,10,12,15,16	2,4,5,6,9,11,13,14,17	2	P08721,P10715
7626	1,3,7,8,10,12,15,17	2,4,5,6,9,11,13,14,16	3	P02091,P10715,Q99MH3
7627	1,3,7,8,10,12,16,17	2,4,5,6,9,11,13,14,15	3	P02091,P08721,P10715
7628	1,3,7,8,10,13,14,15	2,4,5,6,9,11,12,16,17	4	A2RUV9,P01835,P15999,Q08849
7629	1,3,7,8,10,13,14,16	2,4,5,6,9,11,12,15,17	2	P01835,Q08849
7630	1,3,7,8,10,13,14,17	2,4,5,6,9,11,12,15,16	4	P02091,P51907,Q08849,Q9QW07
7631	1,3,7,8,10,13,15,16	2,4,5,6,9,11,12,14,17	1	P01835
7632	1,3,7,8,10,13,15,17	2,4,5,6,9,11,12,14,16	5	P01835,P02091,P15999,Q6IFU7,Q99MH3
7633	1,3,7,8,10,13,16,17	2,4,5,6,9,11,12,14,15	3	P01835,P02091,P51907
7634	1,3,7,8,10,14,15,16	2,4,5,6,9,11,12,13,17	4	P01835,P08721,Q08849,Q62635
7635	1,3,7,8,10,14,15,17	2,4,5,6,9,11,12,13,16	2	P02091,Q08849
7636	1,3,7,8,10,14,16,17	2,4,5,6,9,11,12,13,15	2	P02091,Q08849
7637	1,3,7,8,10,15,16,17	2,4,5,6,9,11,12,13,14	3	P01835,P02091,P25236
7638	1,3,7,8,11,12,13,14	2,4,5,6,9,10,15,16,17	4	P10715,P47727,Q62902,Q8CJ52
7639	1,3,7,8,11,12,13,15	2,4,5,6,9,10,14,16,17	1	P10715
7640	1,3,7,8,11,12,13,16	2,4,5,6,9,10,14,15,17	3	P10715,P17046,Q8CJ52

7641	1,3,7,8,11,12,13,17	2,4,5,6,9,10,14,15,16	5	P01946,P02091,P10715,P36374,Q9QZK9
7642	1,3,7,8,11,12,14,15	2,4,5,6,9,10,13,16,17	5	A2RUV9,P10715,P19629,P22006,P35280
7643	1,3,7,8,11,12,14,16	2,4,5,6,9,10,13,15,17	1	P10715
7644	1,3,7,8,11,12,14,17	2,4,5,6,9,10,13,15,16	4	P01946,P02091,P10715,P19629
7645	1,3,7,8,11,12,15,16	2,4,5,6,9,10,13,14,17	1	P10715
7646	1,3,7,8,11,12,15,17	2,4,5,6,9,10,13,14,16	2	P02091,P10715
7647	1,3,7,8,11,12,16,17	2,4,5,6,9,10,13,14,15	3	P02091,P10715,P17046
7648	1,3,7,8,11,13,14,15	2,4,5,6,9,10,12,16,17	2	P22006,Q08849
7649	1,3,7,8,11,13,14,16	2,4,5,6,9,10,12,15,17	1	Q08849
7650	1,3,7,8,11,13,14,17	2,4,5,6,9,10,12,15,16	4	P01946,P02091,Q08849,Q9QW07
7651	1,3,7,8,11,13,15,16	2,4,5,6,9,10,12,14,17	3	B4F795,P01835,Q8VD89
7652	1,3,7,8,11,13,15,17	2,4,5,6,9,10,12,14,16	1	P02091
7653	1,3,7,8,11,13,16,17	2,4,5,6,9,10,12,14,15	2	P01946,P02091
7654	1,3,7,8,11,14,15,16	2,4,5,6,9,10,12,13,17	3	B4F795,Q08849,Q63355
7655	1,3,7,8,11,14,15,17	2,4,5,6,9,10,12,13,16	3	P02091,P22006,Q08849
7656	1,3,7,8,11,14,16,17	2,4,5,6,9,10,12,13,15	2	P02091,P04218
7657	1,3,7,8,11,15,16,17	2,4,5,6,9,10,12,13,14	4	B4F795,P02091,P10715,P25236
7658	1,3,7,8,12,13,14,15	2,4,5,6,9,10,11,16,17	3	A2RUV9,P15999,Q08849
7659	1,3,7,8,12,13,14,16	2,4,5,6,9,10,11,15,17	0	
7660	1,3,7,8,12,13,14,17	2,4,5,6,9,10,11,15,16	5	A2RUV9,P01946,P02091,P51907,Q9QW07
7661	1,3,7,8,12,13,15,16	2,4,5,6,9,10,11,14,17	1	P10715
7662	1,3,7,8,12,13,15,17	2,4,5,6,9,10,11,14,16	4	P02091,P10715,P15999,Q99MH3
7663	1,3,7,8,12,13,16,17	2,4,5,6,9,10,11,14,15	5	P01946,P02091,P10715,P63029,Q5XFX0

7664	1,3,7,8,12,<br>14,15,16	2,4,5,6,9,1<br>0,11,13,17	1	A2RUV9
7665	1,3,7,8,12,<br>14,15,17	2,4,5,6,9,1<br>0,11,13,16	3	A2RUV9,P02091,P19629
7666	1,3,7,8,12,<br>14,16,17	2,4,5,6,9,1<br>0,11,13,15	1	P02091
7667	1,3,7,8,12,<br>15,16,17	2,4,5,6,9,1<br>0,11,13,14	3	P02091,P10715,Q5XFX0
7668	1,3,7,8,13,<br>14,15,16	2,4,5,6,9,1<br>0,11,12,17	3	P01835,Q08849,Q09030
7669	1,3,7,8,13,<br>14,15,17	2,4,5,6,9,1<br>0,11,12,16	5	P02091,P15999,Q08849,Q6IFU7,Q9QW07
7670	1,3,7,8,13,<br>14,16,17	2,4,5,6,9,1<br>0,11,12,15	5	P02091,P51907,Q08849,Q6IFU7,Q9QW07
7671	1,3,7,8,13,<br>15,16,17	2,4,5,6,9,1<br>0,11,12,14	5	P01835,P02091,Q08849,Q5XFX0,Q6IFU7
7672	1,3,7,8,14,<br>15,16,17	2,4,5,6,9,1<br>0,11,12,13	3	P02091,P02803,Q08849
7673	1,3,7,9,10,<br>11,12,13	2,4,5,6,8,1<br>4,15,16,17	3	P06760,P20760,Q9Z0V6
7674	1,3,7,9,10,<br>11,12,14	2,4,5,6,8,1<br>3,15,16,17	3	P06760,P08689,P20760
7675	1,3,7,9,10,<br>11,12,15	2,4,5,6,8,1<br>3,14,16,17	2	P06760,Q6PCU2
7676	1,3,7,9,10,<br>11,12,16	2,4,5,6,8,1<br>3,14,15,17	7	P06760,P10536,P12368,P20760,P47967,Q9WUW8,Q9Z0V6
7677	1,3,7,9,10,<br>11,12,17	2,4,5,6,8,1<br>3,14,15,16	3	P02091,P06757,P08689
7678	1,3,7,9,10,<br>11,13,14	2,4,5,6,8,1<br>2,15,16,17	5	P06760,P20760,Q4G075,Q62635,Q9Z0V6
7679	1,3,7,9,10,<br>11,13,15	2,4,5,6,8,1<br>2,14,16,17	5	O54728,P06760,Q4G075,Q62635,Q9Z0V6
7680	1,3,7,9,10,<br>11,13,16	2,4,5,6,8,1<br>2,14,15,17	7	P06760,P10536,P20760,P98089,Q4G075,Q62635,Q9Z0V6
7681	1,3,7,9,10,<br>11,13,17	2,4,5,6,8,1<br>2,14,15,16	1	P02091
7682	1,3,7,9,10,<br>11,14,15	2,4,5,6,8,1<br>2,13,16,17	5	O54728,P06760,P22006,Q4G075,Q62635
7683	1,3,7,9,10,<br>11,14,16	2,4,5,6,8,1<br>2,13,15,17	5	P06760,P20760,Q4G075,Q62635,Q9Z0V6
7684	1,3,7,9,10,<br>11,14,17	2,4,5,6,8,1<br>2,13,15,16	3	P02091,P08689,Q8R431
7685	1,3,7,9,10,<br>11,15,16	2,4,5,6,8,1<br>2,13,14,17	7	O54728,P02782,P06760,Q4G075,Q62635,Q9WTT6,Q9Z0V6
7686	1,3,7,9,10,<br>11,15,17	2,4,5,6,8,1<br>2,13,14,16	4	O35077,P02091,P08689,P25236

7687	1,3,7,9,10,<br>11,16,17	2,4,5,6,8,1<br>2,13,14,15	2	P02091,P25236
7688	1,3,7,9,10,<br>12,13,14	2,4,5,6,8,1<br>1,15,16,17	4	A2RUV9,P06760,P14668,P20760
7689	1,3,7,9,10,<br>12,13,15	2,4,5,6,8,1<br>1,14,16,17	5	P06760,P07150,P14668,Q5U2V4,Q9Z0V6
7690	1,3,7,9,10,<br>12,13,16	2,4,5,6,8,1<br>1,14,15,17	9	P02793;Q7TP54,P06760,P14668,P14669,P20760,P20762,P47967,P97840,Q9Z0V6
7691	1,3,7,9,10,<br>12,13,17	2,4,5,6,8,1<br>1,14,15,16	3	P02091,P14668,Q99MH3
7692	1,3,7,9,10,<br>12,14,15	2,4,5,6,8,1<br>1,13,16,17	3	A2RUV9,P06760,P14668
7693	1,3,7,9,10,<br>12,14,16	2,4,5,6,8,1<br>1,13,15,17	3	P06760,P14669,P20760
7694	1,3,7,9,10,<br>12,14,17	2,4,5,6,8,1<br>1,13,15,16	4	A2RUV9,P02091,P08689,P14668
7695	1,3,7,9,10,<br>12,15,16	2,4,5,6,8,1<br>1,13,14,17	3	P06760,P97840,Q6AYS4
7696	1,3,7,9,10,<br>12,15,17	2,4,5,6,8,1<br>1,13,14,16	3	P02091,P08689,P14668
7697	1,3,7,9,10,<br>12,16,17	2,4,5,6,8,1<br>1,13,14,15	4	P02091,P07171,P14668,P20762
7698	1,3,7,9,10,<br>13,14,15	2,4,5,6,8,1<br>1,12,16,17	8	A2RUV9,O54728,P06760,P14668,Q07936,Q4G075,Q62635,Q9Z0V6
7699	1,3,7,9,10,<br>13,14,16	2,4,5,6,8,1<br>1,12,15,17	9	P06760,P14669,P20760,P62804,Q00715,Q4G075,Q62635,Q6AYS4,Q9Z0V6
7700	1,3,7,9,10,<br>13,14,17	2,4,5,6,8,1<br>1,12,15,16	3	P02091,P14668,Q9QW07
7701	1,3,7,9,10,<br>13,15,16	2,4,5,6,8,1<br>1,12,14,17	14	O54728,P01835,P02793;Q7TP54,P06760,P0C0A9,P14669,P20762,P62804,Q00715,Q4G075,Q5U2V4,Q62635,Q6AYS4,Q9Z0V6
7702	1,3,7,9,10,<br>13,15,17	2,4,5,6,8,1<br>1,12,14,16	3	P02091,P14668,P62804
7703	1,3,7,9,10,<br>13,16,17	2,4,5,6,8,1<br>1,12,14,15	5	P02091,P14668,P20762,P62804,Q00715
7704	1,3,7,9,10,<br>14,15,16	2,4,5,6,8,1<br>1,12,13,17	12	O54728,P02780,P02782,P06760,P07647,P0C0A9,P14669,Q4G075,Q62635,Q6AYS4,Q9JHB9,Q9QZK9
7705	1,3,7,9,10,<br>14,15,17	2,4,5,6,8,1<br>1,12,13,16	4	P02091,P08689,P0C0A9,P14668
7706	1,3,7,9,10,<br>14,16,17	2,4,5,6,8,1<br>1,12,13,15	4	P02091,P08689,P0C0A9,P14668
7707	1,3,7,9,10,<br>15,16,17	2,4,5,6,8,1<br>1,12,13,14	12	P02091,P02780,P02782,P07647,P0C0A9,P14668,P20762,P25236,P30120,P47727,P62804,Q9JHB9
7708	1,3,7,9,11,<br>12,13,14	2,4,5,6,8,1<br>0,15,16,17	3	P06760,P20760,Q9Z0V6
7709	1,3,7,9,11,<br>12,13,15	2,4,5,6,8,1<br>0,14,16,17	3	P06760,Q6P6R2,Q9Z0V6

7710	1,3,7,9,11,<br>12,13,16	2,4,5,6,8,1<br>0,14,15,17	4	P06760,P17046,P20760,Q9Z0V6
7711	1,3,7,9,11,<br>12,13,17	2,4,5,6,8,1<br>0,14,15,16	1	P02091
7712	1,3,7,9,11,<br>12,14,15	2,4,5,6,8,1<br>0,13,16,17	2	A2RUV9,P06760
7713	1,3,7,9,11,<br>12,14,16	2,4,5,6,8,1<br>0,13,15,17	2	P06760,P20760
7714	1,3,7,9,11,<br>12,14,17	2,4,5,6,8,1<br>0,13,15,16	2	P02091,P08689
7715	1,3,7,9,11,<br>12,15,16	2,4,5,6,8,1<br>0,13,14,17	3	P06760,Q9WTT6,Q9Z0V6
7716	1,3,7,9,11,<br>12,15,17	2,4,5,6,8,1<br>0,13,14,16	1	P02091
7717	1,3,7,9,11,<br>12,16,17	2,4,5,6,8,1<br>0,13,14,15	2	P02091,P17046
7718	1,3,7,9,11,<br>13,14,15	2,4,5,6,8,1<br>0,12,16,17	7	D4A5U3,O54728,P06760,Q4G075,Q62635,Q6P6R2,Q9Z0V6
7719	1,3,7,9,11,<br>13,14,16	2,4,5,6,8,1<br>0,12,15,17	5	P06760,P20760,Q4G075,Q62635,Q9Z0V6
7720	1,3,7,9,11,<br>13,14,17	2,4,5,6,8,1<br>0,12,15,16	3	P02091,P04218,Q9QW07
7721	1,3,7,9,11,<br>13,15,16	2,4,5,6,8,1<br>0,12,14,17	7	O54728,P06760,P62804,Q4G075,Q62635,Q9WTT6,Q9Z0V6
7722	1,3,7,9,11,<br>13,15,17	2,4,5,6,8,1<br>0,12,14,16	1	P02091
7723	1,3,7,9,11,<br>13,16,17	2,4,5,6,8,1<br>0,12,14,15	2	P02091,P04218
7724	1,3,7,9,11,<br>14,15,16	2,4,5,6,8,1<br>0,12,13,17	7	O54728,P06760,Q4G075,Q62635,Q63355,Q9QZK9,Q9Z0V6
7725	1,3,7,9,11,<br>14,15,17	2,4,5,6,8,1<br>0,12,13,16	3	P02091,P04218,P08689
7726	1,3,7,9,11,<br>14,16,17	2,4,5,6,8,1<br>0,12,13,15	2	P02091,P04218
7727	1,3,7,9,11,<br>15,16,17	2,4,5,6,8,1<br>0,12,13,14	5	O54728,P02091,P04218,P0C0A9,P25236
7728	1,3,7,9,12,<br>13,14,15	2,4,5,6,8,1<br>0,11,16,17	6	A2RUV9,P06760,P07150,P08649,P14668,Q6AYS4
7729	1,3,7,9,12,<br>13,14,16	2,4,5,6,8,1<br>0,11,15,17	6	P06760,P08649,P14669,P20760,Q6AYS4,Q9Z0V6
7730	1,3,7,9,12,<br>13,14,17	2,4,5,6,8,1<br>0,11,15,16	4	A2RUV9,P02091,P14668,Q9QW07
7731	1,3,7,9,12,<br>13,15,16	2,4,5,6,8,1<br>0,11,14,17	8	P06760,P07150,P08649,P14669,P62804,Q63598,Q6AYS4,Q9Z0V6
7732	1,3,7,9,12,<br>13,15,17	2,4,5,6,8,1<br>0,11,14,16	3	P02091,P14668,Q63598

7733	1,3,7,9,12,13,16,17	2,4,5,6,8,10,11,14,15	4	P02091,P07171,P14668,P14669
7734	1,3,7,9,12,14,15,16	2,4,5,6,8,10,11,13,17	5	A2RUV9,P06760,P14669,Q63598,Q6AYS4
7735	1,3,7,9,12,14,15,17	2,4,5,6,8,10,11,13,16	4	A2RUV9,P02091,P08689,P14668
7736	1,3,7,9,12,14,16,17	2,4,5,6,8,10,11,13,15	3	P02091,P14668,P14669
7737	1,3,7,9,12,15,16,17	2,4,5,6,8,10,11,13,14	5	P02091,P14668,P47727,Q63598,Q6AYS4
7738	1,3,7,9,13,14,15,16	2,4,5,6,8,10,11,12,17	14	O54728,P06760,P07150,P0C0A9,P14669,P16636,P19223,P62804,Q00715,Q4G075,Q62635,Q63598,Q6AYS4,Q9Z0V6
7739	1,3,7,9,13,14,15,17	2,4,5,6,8,10,11,12,16	9	P02091,P0C0A9,P14668,P53792,P62804,Q08849,Q63598,Q6IFU7,Q9QW07
7740	1,3,7,9,13,14,16,17	2,4,5,6,8,10,11,12,15	6	P02091,P14668,P14669,P62804,Q63598,Q9QW07
7741	1,3,7,9,13,15,16,17	2,4,5,6,8,10,11,12,14	10	P02091,P0C0A9,P14668,P14669,P20762,P47727,P62804,Q00715,Q63598,Q6AYS4
7742	1,3,7,9,14,15,16,17	2,4,5,6,8,10,11,12,13	12	O54728,P02091,P02780,P02781,P07647,P0C0A9,P14669,P30120,P62804,Q63598,Q6AYS4,Q9JHB9
7743	1,3,7,10,11,12,13,14	2,4,5,6,8,9,15,16,17	4	A2RUV9,P06760,P20760,P47727
7744	1,3,7,10,11,12,13,15	2,4,5,6,8,9,14,16,17	1	P06760
7745	1,3,7,10,11,12,13,16	2,4,5,6,8,9,14,15,17	6	P06760,P12368,P17046,P20760,P47967,Q9Z0V6
7746	1,3,7,10,11,12,13,17	2,4,5,6,8,9,14,15,16	5	P01946,P02091,Q63942,Q99MH3,Q9QZK9
7747	1,3,7,10,11,12,14,15	2,4,5,6,8,9,13,16,17	4	A2RUV9,P06760,P22006,P35280
7748	1,3,7,10,11,12,14,16	2,4,5,6,8,9,13,15,17	2	P06760,P20760
7749	1,3,7,10,11,12,14,17	2,4,5,6,8,9,13,15,16	2	P02091,P08689
7750	1,3,7,10,11,12,15,16	2,4,5,6,8,9,13,14,17	1	P06760
7751	1,3,7,10,11,12,15,17	2,4,5,6,8,9,13,14,16	2	P02091,P08689
7752	1,3,7,10,11,12,16,17	2,4,5,6,8,9,13,14,15	3	P02091,P06757,P17046
7753	1,3,7,10,11,13,14,15	2,4,5,6,8,9,12,16,17	5	P06760,P22006,Q62635,Q6P6R2,Q9Z0V6
7754	1,3,7,10,11,13,14,16	2,4,5,6,8,9,12,15,17	6	P06760,P20760,Q4G075,Q62635,Q7TPB1,Q9Z0V6
7755	1,3,7,10,11,13,14,17	2,4,5,6,8,9,12,15,16	2	P02091,Q9QW07

7756	1,3,7,10,1<br>1,13,15,16	2,4,5,6,8,9<br>,12,14,17	5	P01835,P06760,Q62635,Q9WTT6,Q9Z0V6
7757	1,3,7,10,1<br>1,13,15,17	2,4,5,6,8,9<br>,12,14,16	1	P02091
7758	1,3,7,10,1<br>1,13,16,17	2,4,5,6,8,9<br>,12,14,15	1	P02091
7759	1,3,7,10,1<br>1,14,15,16	2,4,5,6,8,9<br>,12,13,17	2	P06760,Q62635
7760	1,3,7,10,1<br>1,14,15,17	2,4,5,6,8,9<br>,12,13,16	3	P02091,P08689,P22006
7761	1,3,7,10,1<br>1,14,16,17	2,4,5,6,8,9<br>,12,13,15	2	P02091,P08689
7762	1,3,7,10,1<br>1,15,16,17	2,4,5,6,8,9<br>,12,13,14	6	O35077,P02091,P02780,P02782,P25236,Q6TMA8
7763	1,3,7,10,1<br>2,13,14,15	2,4,5,6,8,9<br>,11,16,17	4	A2RUV9,P06760,P14668,Q6AYS4
7764	1,3,7,10,1<br>2,13,14,16	2,4,5,6,8,9<br>,11,15,17	6	A2RUV9,P06760,P14668,P14669,P20760,Q6AYS4
7765	1,3,7,10,1<br>2,13,14,17	2,4,5,6,8,9<br>,11,15,16	5	A2RUV9,P02091,P14668,Q05175,Q9QW07
7766	1,3,7,10,1<br>2,13,15,16	2,4,5,6,8,9<br>,11,14,17	6	P02793;Q7TP54,P06760,P14668,P14669,Q6AYS4,Q9Z0V6
7767	1,3,7,10,1<br>2,13,15,17	2,4,5,6,8,9<br>,11,14,16	3	P02091,P14668,Q99MH3
7768	1,3,7,10,1<br>2,13,16,17	2,4,5,6,8,9<br>,11,14,15	5	P02091,P07171,P14668,P20762,Q99MH3
7769	1,3,7,10,1<br>2,14,15,16	2,4,5,6,8,9<br>,11,13,17	3	A2RUV9,P06760,Q6AYS4
7770	1,3,7,10,1<br>2,14,15,17	2,4,5,6,8,9<br>,11,13,16	4	A2RUV9,P02091,P08689,P14668
7771	1,3,7,10,1<br>2,14,16,17	2,4,5,6,8,9<br>,11,13,15	4	P02091,P08689,P14668,Q6TMA8
7772	1,3,7,10,1<br>2,15,16,17	2,4,5,6,8,9<br>,11,13,14	4	P02091,P06866,P14668,Q6TMA8
7773	1,3,7,10,1<br>3,14,15,16	2,4,5,6,8,9<br>,11,12,17	11	P01835,P06760,P0C0A9,P14669,P19223,P50115,P62804,Q4G075,Q62635,Q6AYS4,Q9Z0V6
7774	1,3,7,10,1<br>3,14,15,17	2,4,5,6,8,9<br>,11,12,16	4	A2RUV9,P02091,P14668,Q9QW07
7775	1,3,7,10,1<br>3,14,16,17	2,4,5,6,8,9<br>,11,12,15	3	P02091,P14668,Q9QW07
7776	1,3,7,10,1<br>3,15,16,17	2,4,5,6,8,9<br>,11,12,14	7	P01835,P02091,P0C0A9,P14668,P20762,P62804,Q6AYS4
7777	1,3,7,10,1<br>4,15,16,17	2,4,5,6,8,9<br>,11,12,13	10	P02091,P02780,P02782,P02803,P07647,P0C0A9,P14668,P30120,Q6TMA8,Q9JHB9
7778	1,3,7,11,1<br>2,13,14,15	2,4,5,6,8,9<br>,10,16,17	3	A2RUV9,P06760,Q6P6R2

7779	1,3,7,11,12,13,14,16	2,4,5,6,8,9,10,15,17	3	P06760,P17046,P20760
7780	1,3,7,11,12,13,14,17	2,4,5,6,8,9,10,15,16	4	P01946,P02091,Q63942,Q9QW07
7781	1,3,7,11,12,13,15,16	2,4,5,6,8,9,10,14,17	5	P06760,P17046,Q6P6R2,Q9WTT6,Q9Z0V6
7782	1,3,7,11,12,13,15,17	2,4,5,6,8,9,10,14,16	2	P02091,Q63942
7783	1,3,7,11,12,13,16,17	2,4,5,6,8,9,10,14,15	3	P01946,P02091,P17046
7784	1,3,7,11,12,14,15,16	2,4,5,6,8,9,10,13,17	2	P06760,Q63355
7785	1,3,7,11,12,14,15,17	2,4,5,6,8,9,10,13,16	3	A2RUV9,P02091,P08689
7786	1,3,7,11,12,14,16,17	2,4,5,6,8,9,10,13,15	2	P02091,P17046
7787	1,3,7,11,12,15,16,17	2,4,5,6,8,9,10,13,14	2	P02091,P17046
7788	1,3,7,11,13,14,15,16	2,4,5,6,8,9,10,12,17	8	P06760,P19223,Q09030,Q4G075,Q62635,Q6P6R2,Q9WTT6,Q9Z0V6
7789	1,3,7,11,13,14,15,17	2,4,5,6,8,9,10,12,16	2	P02091,Q9QW07
7790	1,3,7,11,13,14,16,17	2,4,5,6,8,9,10,12,15	4	D3ZHA0,P01946,P02091,Q9QW07
7791	1,3,7,11,13,15,16,17	2,4,5,6,8,9,10,12,14	2	P02091,Q9WTT6
7792	1,3,7,11,14,15,16,17	2,4,5,6,8,9,10,12,13	3	P02091,P02803,P0C0A9
7793	1,3,7,12,13,14,15,16	2,4,5,6,8,9,10,11,17	7	A2RUV9,P06760,P08649,P14669,P20646,Q09030,Q6AYS4
7794	1,3,7,12,13,14,15,17	2,4,5,6,8,9,10,11,16	4	A2RUV9,P02091,P14668,Q9QW07
7795	1,3,7,12,13,14,16,17	2,4,5,6,8,9,10,11,15	5	P02091,P14668,P14669,Q6AYS4,Q9QW07
7796	1,3,7,12,13,15,16,17	2,4,5,6,8,9,10,11,14	5	P02091,P14668,Q5XFX0,Q63598,Q6AYS4
7797	1,3,7,12,14,15,16,17	2,4,5,6,8,9,10,11,13	4	A2RUV9,P02091,P14668,Q6AYS4
7798	1,3,7,13,14,15,16,17	2,4,5,6,8,9,10,11,12	10	P00714,P02091,P0C0A9,P14668,P14669,P62804,Q08849,Q63598,Q6AYS4,Q9QW07
7799	1,3,8,9,10,11,12,13	2,4,5,6,7,14,15,16,17	3	P20760,P35467,P83748
7800	1,3,8,9,10,11,12,14	2,4,5,6,7,13,15,16,17	3	P20760,P83748,Q6IG02
7801	1,3,8,9,10,11,12,15	2,4,5,6,7,13,14,16,17	2	P20760,P83748

7802	1,3,8,9,10,<br>11,12,16	2,4,5,6,7,1<br>3,14,15,17	5	P20760,P35467,P83748,Q6IG02,Q9WUW8
7803	1,3,8,9,10,<br>11,12,17	2,4,5,6,7,1<br>3,14,15,16	5	P16636,P35467,P83748,Q63532,Q6IG02
7804	1,3,8,9,10,<br>11,13,14	2,4,5,6,7,1<br>2,15,16,17	4	P09605;P25809,P20760,P35467,P83748
7805	1,3,8,9,10,<br>11,13,15	2,4,5,6,7,1<br>2,14,16,17	6	O54728,P09605;P25809,P14480,P20760,P35467,P83748
7806	1,3,8,9,10,<br>11,13,16	2,4,5,6,7,1<br>2,14,15,17	3	P20760,P35467,P83748
7807	1,3,8,9,10,<br>11,13,17	2,4,5,6,7,1<br>2,14,15,16	3	P35467,P83748,Q63532
7808	1,3,8,9,10,<br>11,14,15	2,4,5,6,7,1<br>2,13,16,17	6	O54728,P09605;P25809,P14480,P20760,P83748,Q62635
7809	1,3,8,9,10,<br>11,14,16	2,4,5,6,7,1<br>2,13,15,17	4	P20760,P55091,P83748,Q62635
7810	1,3,8,9,10,<br>11,14,17	2,4,5,6,7,1<br>2,13,15,16	2	P08689,P83748
7811	1,3,8,9,10,<br>11,15,16	2,4,5,6,7,1<br>2,13,14,17	5	O54728,P20760,P55091,P83748,Q62635
7812	1,3,8,9,10,<br>11,15,17	2,4,5,6,7,1<br>2,13,14,16	4	G3V686,P25236,P83748,Q63532
7813	1,3,8,9,10,<br>11,16,17	2,4,5,6,7,1<br>2,13,14,15	2	P25236,P83748
7814	1,3,8,9,10,<br>12,13,14	2,4,5,6,7,1<br>1,15,16,17	3	P09605;P25809,P14408,P20760
7815	1,3,8,9,10,<br>12,13,15	2,4,5,6,7,1<br>1,14,16,17	1	P20760
7816	1,3,8,9,10,<br>12,13,16	2,4,5,6,7,1<br>1,14,15,17	2	P20760,P20762
7817	1,3,8,9,10,<br>12,13,17	2,4,5,6,7,1<br>1,14,15,16	3	P16636,P83748,Q63532
7818	1,3,8,9,10,<br>12,14,15	2,4,5,6,7,1<br>1,13,16,17	2	P09605;P25809,P20760
7819	1,3,8,9,10,<br>12,14,16	2,4,5,6,7,1<br>1,13,15,17	2	P05539,P20760
7820	1,3,8,9,10,<br>12,14,17	2,4,5,6,7,1<br>1,13,15,16	3	P08689,P14408,P83748
7821	1,3,8,9,10,<br>12,15,16	2,4,5,6,7,1<br>1,13,14,17	1	P20760
7822	1,3,8,9,10,<br>12,15,17	2,4,5,6,7,1<br>1,13,14,16	3	G3V686,P61206;P84079;P84082,Q63532
7823	1,3,8,9,10,<br>12,16,17	2,4,5,6,7,1<br>1,13,14,15	4	P05539,P16636,P20762,P83748
7824	1,3,8,9,10,<br>13,14,15	2,4,5,6,7,1<br>1,12,16,17	7	O54728,P09605;P25809,P15999,P20760,P61206;P84079;P84082,P83748,Q08849

7825	1,3,8,9,10,<br>13,14,16	2,4,5,6,7,1<br>1,12,15,17	3	P09605;P25809,P20760,P83748
7826	1,3,8,9,10,<br>13,14,17	2,4,5,6,7,1<br>1,12,15,16	5	P14408,P51907,P61206;P84079;P84082,P83748,Q9QW07
7827	1,3,8,9,10,<br>13,15,16	2,4,5,6,7,1<br>1,12,14,17	7	O54728,P01835,P09605;P25809,P20760,P20762,P83748,Q62635
7828	1,3,8,9,10,<br>13,15,17	2,4,5,6,7,1<br>1,12,14,16	4	G3V686,P61206;P84079;P84082,P83748,Q63532
7829	1,3,8,9,10,<br>13,16,17	2,4,5,6,7,1<br>1,12,14,15	4	P20762,P25031,P51907,P83748
7830	1,3,8,9,10,<br>14,15,16	2,4,5,6,7,1<br>1,12,13,17	8	O54728,P09605;P25809,P20760,P55091,P83748,Q62635,Q9JHB9,Q9QZK9
7831	1,3,8,9,10,<br>14,15,17	2,4,5,6,7,1<br>1,12,13,16	3	G3V686,P61206;P84079;P84082,P83748
7832	1,3,8,9,10,<br>14,16,17	2,4,5,6,7,1<br>1,12,13,15	2	P05539,P83748
7833	1,3,8,9,10,<br>15,16,17	2,4,5,6,7,1<br>1,12,13,14	5	P20762,P25031,P25236,P83748,Q9JHB9
7834	1,3,8,9,11,<br>12,13,14	2,4,5,6,7,1<br>0,15,16,17	3	P04218,P20760,P83748
7835	1,3,8,9,11,<br>12,13,15	2,4,5,6,7,1<br>0,14,16,17	2	P20760,P83748
7836	1,3,8,9,11,<br>12,13,16	2,4,5,6,7,1<br>0,14,15,17	3	P04218,P20760,P83748
7837	1,3,8,9,11,<br>12,13,17	2,4,5,6,7,1<br>0,14,15,16	5	P04218,P16636,P35467,P83748,Q63532
7838	1,3,8,9,11,<br>12,14,15	2,4,5,6,7,1<br>0,13,16,17	4	P04218,P14480,P20760,P83748
7839	1,3,8,9,11,<br>12,14,16	2,4,5,6,7,1<br>0,13,15,17	3	P04218,P20760,P83748
7840	1,3,8,9,11,<br>12,14,17	2,4,5,6,7,1<br>0,13,15,16	2	P04218,P83748
7841	1,3,8,9,11,<br>12,15,16	2,4,5,6,7,1<br>0,13,14,17	4	P04218,P12020,P20760,P83748
7842	1,3,8,9,11,<br>12,15,17	2,4,5,6,7,1<br>0,13,14,16	4	P04218,P12020,P83748,Q63532
7843	1,3,8,9,11,<br>12,16,17	2,4,5,6,7,1<br>0,13,14,15	5	P04218,P12020,P16636,P36376,P83748
7844	1,3,8,9,11,<br>13,14,15	2,4,5,6,7,1<br>0,12,16,17	6	O54728,P04218,P09605;P25809,P14480,P20760,P83748
7845	1,3,8,9,11,<br>13,14,16	2,4,5,6,7,1<br>0,12,15,17	3	P04218,P20760,P83748
7846	1,3,8,9,11,<br>13,14,17	2,4,5,6,7,1<br>0,12,15,16	4	P04218,P83748,P97840,Q9QW07
7847	1,3,8,9,11,<br>13,15,16	2,4,5,6,7,1<br>0,12,14,17	4	O54728,P04218,P20760,P83748

7848	1,3,8,9,11,13,15,17	2,4,5,6,7,10,12,14,16	4	P04218,P83748,P97840,Q63532
7849	1,3,8,9,11,13,16,17	2,4,5,6,7,10,12,14,15	2	P04218,P83748
7850	1,3,8,9,11,14,15,16	2,4,5,6,7,10,12,13,17	8	B4F795,O54728,P04218,P20760,P55091,P83748,Q62635,Q9QZK9
7851	1,3,8,9,11,14,15,17	2,4,5,6,7,10,12,13,16	4	P04218,P47967,P83748,P97840
7852	1,3,8,9,11,14,16,17	2,4,5,6,7,10,12,13,15	2	P04218,P83748
7853	1,3,8,9,11,15,16,17	2,4,5,6,7,10,12,13,14	5	B4F795,P04218,P12020,P25236,P83748
7854	1,3,8,9,12,13,14,15	2,4,5,6,7,10,11,16,17	4	A2RUV9,P10758,P20760,P61206;P84079;P84082
7855	1,3,8,9,12,13,14,16	2,4,5,6,7,10,11,15,17	1	P20760
7856	1,3,8,9,12,13,14,17	2,4,5,6,7,10,11,15,16	6	P04218,P51907,P61206;P84079;P84082,P83748,Q5FVI6,Q9QW07
7857	1,3,8,9,12,13,15,16	2,4,5,6,7,10,11,14,17	2	P20760,P61206;P84079;P84082
7858	1,3,8,9,12,13,15,17	2,4,5,6,7,10,11,14,16	2	P61206;P84079;P84082,Q63532
7859	1,3,8,9,12,13,16,17	2,4,5,6,7,10,11,14,15	1	P61206;P84079;P84082
7860	1,3,8,9,12,14,15,16	2,4,5,6,7,10,11,13,17	2	P20760,P61206;P84079;P84082
7861	1,3,8,9,12,14,15,17	2,4,5,6,7,10,11,13,16	2	P04218,P61206;P84079;P84082
7862	1,3,8,9,12,14,16,17	2,4,5,6,7,10,11,13,15	3	P04218,P61206;P84079;P84082,P83748
7863	1,3,8,9,12,15,16,17	2,4,5,6,7,10,11,13,14	3	P04218,P12020,P61206;P84079;P84082
7864	1,3,8,9,13,14,15,16	2,4,5,6,7,10,11,12,17	9	O54728,P04218,P09605;P25809,P20646,P20760,P61206;P84079;P84082,P83748,Q08849,Q9QZK9
7865	1,3,8,9,13,14,15,17	2,4,5,6,7,10,11,12,16	10	P04218,P47967,P51907,P61206;P84079;P84082,P83748,Q08849,Q5FVI6,Q62687,Q6IFU7,Q9QW07
7866	1,3,8,9,13,14,16,17	2,4,5,6,7,10,11,12,15	7	P04218,P51907,P61206;P84079;P84082,P83748,Q08849,Q5FVI6,Q9QW07
7867	1,3,8,9,13,15,16,17	2,4,5,6,7,10,11,12,14	4	P04218,P61206;P84079;P84082,P62804,P83748
7868	1,3,8,9,14,15,16,17	2,4,5,6,7,10,11,12,13	6	P04218,P0C0A9,P61206;P84079;P84082,P83748,Q08849,Q9JHB9
7869	1,3,8,10,11,12,13,14	2,4,5,6,7,9,15,16,17	4	P09605;P25809,P20760,P47727,P83748
7870	1,3,8,10,11,12,13,15	2,4,5,6,7,9,14,16,17	4	P09605;P25809,P15999,P20760,P83748

7871	1,3,8,10,1 1,12,13,16	2,4,5,6,7,9 ,14,15,17	2	P20760,P83748
7872	1,3,8,10,1 1,12,13,17	2,4,5,6,7,9 ,14,15,16	3	P16636,P83748,Q99MH3
7873	1,3,8,10,1 1,12,14,15	2,4,5,6,7,9 ,13,16,17	3	P09605;P25809,P35280,P83748
7874	1,3,8,10,1 1,12,14,16	2,4,5,6,7,9 ,13,15,17	3	P20760,P83748,Q6IG02
7875	1,3,8,10,1 1,12,14,17	2,4,5,6,7,9 ,13,15,16	3	P08689,P83748,Q6IG02
7876	1,3,8,10,1 1,12,15,16	2,4,5,6,7,9 ,13,14,17	1	P20760
7877	1,3,8,10,1 1,12,15,17	2,4,5,6,7,9 ,13,14,16	1	P83748
7878	1,3,8,10,1 1,12,16,17	2,4,5,6,7,9 ,13,14,15	3	P16636,P83748,Q6IG02
7879	1,3,8,10,1 1,13,14,15	2,4,5,6,7,9 ,12,16,17	3	P09605;P25809,P15999,P83748
7880	1,3,8,10,1 1,13,14,16	2,4,5,6,7,9 ,12,15,17	4	P09605;P25809,P20760,P83748,Q7TPB1
7881	1,3,8,10,1 1,13,14,17	2,4,5,6,7,9 ,12,15,16	2	P83748,Q9QW07
7882	1,3,8,10,1 1,13,15,16	2,4,5,6,7,9 ,12,14,17	4	P01835,P09605;P25809,P20760,P83748
7883	1,3,8,10,1 1,13,15,17	2,4,5,6,7,9 ,12,14,16	3	P15999,P83748,Q63532
7884	1,3,8,10,1 1,13,16,17	2,4,5,6,7,9 ,12,14,15	1	P83748
7885	1,3,8,10,1 1,14,15,16	2,4,5,6,7,9 ,12,13,17	5	P09605;P25809,P20760,P55091,P83748,Q62635
7886	1,3,8,10,1 1,14,15,17	2,4,5,6,7,9 ,12,13,16	2	G3V686,P83748
7887	1,3,8,10,1 1,14,16,17	2,4,5,6,7,9 ,12,13,15	1	P83748
7888	1,3,8,10,1 1,15,16,17	2,4,5,6,7,9 ,12,13,14	2	P25236,P83748
7889	1,3,8,10,1 2,13,14,15	2,4,5,6,7,9 ,11,16,17	5	A2RUV9,P09605;P25809,P15999,P61206;P84079;P84082,Q64093
7890	1,3,8,10,1 2,13,14,16	2,4,5,6,7,9 ,11,15,17	2	P09605;P25809,P20760
7891	1,3,8,10,1 2,13,14,17	2,4,5,6,7,9 ,11,15,16	4	P61206;P84079;P84082,P83748,Q05175,Q9QW07
7892	1,3,8,10,1 2,13,15,16	2,4,5,6,7,9 ,11,14,17	2	P09605;P25809,P83121
7893	1,3,8,10,1 2,13,15,17	2,4,5,6,7,9 ,11,14,16	4	P15999,P61206;P84079;P84082,P83121,Q99MH3

7894	1,3,8,10,12,13,16,17	2,4,5,6,7,9,11,14,15	5	P02631,P16636,P20762,P83121,Q99MH3
7895	1,3,8,10,12,14,15,16	2,4,5,6,7,9,11,13,17	2	P05539,P09605;P25809
7896	1,3,8,10,12,14,15,17	2,4,5,6,7,9,11,13,16	1	P61206;P84079;P84082
7897	1,3,8,10,12,14,16,17	2,4,5,6,7,9,11,13,15	2	P05539,P83748
7898	1,3,8,10,12,15,16,17	2,4,5,6,7,9,11,13,14	2	P05539,P83121
7899	1,3,8,10,13,14,15,16	2,4,5,6,7,9,11,12,17	7	P01835,P09605;P25809,P15999,P19223,P20646,P83748,Q08849
7900	1,3,8,10,13,14,15,17	2,4,5,6,7,9,11,12,16	5	P15999,P61206;P84079;P84082,P83748,Q08849,Q9QW07
7901	1,3,8,10,13,14,16,17	2,4,5,6,7,9,11,12,15	4	P51907,P83748,Q08849,Q9QW07
7902	1,3,8,10,13,15,16,17	2,4,5,6,7,9,11,12,14	4	P01835,P20762,P83121,P83748
7903	1,3,8,10,14,15,16,17	2,4,5,6,7,9,11,12,13	4	P05539,P83748,Q08849,Q9JHB9
7904	1,3,8,11,12,13,14,15	2,4,5,6,7,9,10,16,17	6	P09605;P25809,P15999,P20760,P83748,Q64093,Q6P6R2
7905	1,3,8,11,12,13,14,16	2,4,5,6,7,9,10,15,17	2	P20760,P83748
7906	1,3,8,11,12,13,14,17	2,4,5,6,7,9,10,15,16	3	P83748,Q64093,Q9QW07
7907	1,3,8,11,12,13,15,16	2,4,5,6,7,9,10,14,17	1	P20760
7908	1,3,8,11,12,13,15,17	2,4,5,6,7,9,10,14,16	2	P83748,Q63532
7909	1,3,8,11,12,13,16,17	2,4,5,6,7,9,10,14,15	3	P16636,P36376,P83748
7910	1,3,8,11,12,14,15,16	2,4,5,6,7,9,10,13,17	1	P20760
7911	1,3,8,11,12,14,15,17	2,4,5,6,7,9,10,13,16	2	P04218,P83748
7912	1,3,8,11,12,14,16,17	2,4,5,6,7,9,10,13,15	2	P04218,P83748
7913	1,3,8,11,12,15,16,17	2,4,5,6,7,9,10,13,14	3	P04218,P12020,P83748
7914	1,3,8,11,13,14,15,16	2,4,5,6,7,9,10,12,17	8	P04218,P09605;P25809,P19223,P20646,P20760,P83748,Q08849,Q64093
7915	1,3,8,11,13,14,15,17	2,4,5,6,7,9,10,12,16	9	P04218,P15999,P47967,P61206;P84079;P84082,P83748,P97840,Q08849,Q64093,Q9QW07
7916	1,3,8,11,13,14,16,17	2,4,5,6,7,9,10,12,15	3	P04218,P83748,Q9QW07

7917	1,3,8,11,1<br>3,15,16,17	2,4,5,6,7,9<br>,10,12,14	2	P04218,P83748
7918	1,3,8,11,1<br>4,15,16,17	2,4,5,6,7,9<br>,10,12,13	3	P04218,P83748,P97840
7919	1,3,8,12,1<br>3,14,15,16	2,4,5,6,7,9<br>,10,11,17	4	P09605;P25809,P20646,P61206;P84079;P84082,Q64093
7920	1,3,8,12,1<br>3,14,15,17	2,4,5,6,7,9<br>,10,11,16	8	A2RUV9,P05712,P15999,P61206;P84079;P84082,Q5FVI6,Q62687,Q64093,Q9QW07
7921	1,3,8,12,1<br>3,14,16,17	2,4,5,6,7,9<br>,10,11,15	5	P51907,P61206;P84079;P84082,Q5FVI6,Q64093,Q9QW07
7922	1,3,8,12,1<br>3,15,16,17	2,4,5,6,7,9<br>,10,11,14	3	P61206;P84079;P84082,P83121,Q5XFX0
7923	1,3,8,12,1<br>4,15,16,17	2,4,5,6,7,9<br>,10,11,13	1	P61206;P84079;P84082
7924	1,3,8,13,1<br>4,15,16,17	2,4,5,6,7,9<br>,10,11,12	7	P04218,P48199,P61206;P84079;P84082,P83748,Q08849,Q64093,Q9QW07
7925	1,3,9,10,1<br>1,12,13,14	2,4,5,6,7,8<br>,15,16,17	10	P06760,P20646,P20760,P20761,P20762,P50115,P83748,Q4G075,Q99041,Q9Z0V6
7926	1,3,9,10,1<br>1,12,13,15	2,4,5,6,7,8<br>,14,16,17	9	O88267,P06760,P20760,P20761,P20762,P50115,P83748,Q4G075,Q9Z0V6
7927	1,3,9,10,1<br>1,12,13,16	2,4,5,6,7,8<br>,14,15,17	11	P02631,P06760,P07171,P10536,P20760,P20761,P20762,P50115,P83748,Q4G075,Q9Z0V6
7928	1,3,9,10,1<br>1,12,13,17	2,4,5,6,7,8<br>,14,15,16	6	P02631,P07171,P20760,P20762,P83748,Q9Z0V6
7929	1,3,9,10,1<br>1,12,14,15	2,4,5,6,7,8<br>,13,16,17	10	O88267,P06760,P20760,P23928,P50115,P83748,Q4G075,Q99041,Q9R0T3,Q9Z0V6
7930	1,3,9,10,1<br>1,12,14,16	2,4,5,6,7,8<br>,13,15,17	14	P06760,P06761,P07171,P10536,P20760,P20761,P20762,P50115,P62815,P83748,Q4G075,Q99041,Q9WUW8,Q9Z0V6
7931	1,3,9,10,1<br>1,12,14,17	2,4,5,6,7,8<br>,13,15,16	4	P07171,P08689,P20760,P83748
7932	1,3,9,10,1<br>1,12,15,16	2,4,5,6,7,8<br>,13,14,17	17	O88267,P06760,P06761,P06911,P07171,P10536,P12020,P20760,P20761,P20762,P22282,P50115,P62815,P83748,Q9WTT6,Q9WUW8,Q9<br>Z0V6
7933	1,3,9,10,1<br>1,12,15,17	2,4,5,6,7,8<br>,13,14,16	7	P06761,P06911,P07171,P08689,P12020,P20762,P83748
7934	1,3,9,10,1<br>1,12,16,17	2,4,5,6,7,8<br>,13,14,15	11	P02631,P06757,P06761,P06911,P07171,P12020,P20760,P20762,P83748,Q5GRG2,Q9Z0V6
7935	1,3,9,10,1<br>1,13,14,15	2,4,5,6,7,8<br>,12,16,17	23	O54728,O88267,P06760,P06761,P09605;P25809,P19223,P20646,P20760,P20761,P20762,P23928,P50115,P50116,P55091,P83748,P9808<br>9,Q4G075,Q62635,Q6P6R2,Q80WL1,Q99041,Q9R0T3,Q9Z0V6
7936	1,3,9,10,1<br>1,13,14,16	2,4,5,6,7,8<br>,12,15,17	20	O55004,P02631,P06760,P06761,P19223,P20646,P20760,P20761,P20762,P50115,P55091,P62815,P83748,P98089,Q4G075,Q62635,Q7TP<br>B1,Q99041,Q9QZA6,Q9Z0V6
7937	1,3,9,10,1<br>1,13,14,17	2,4,5,6,7,8<br>,12,15,16	6	P07171,P20760,P20762,P83748,Q99041,Q9Z0V6
7938	1,3,9,10,1<br>1,13,15,16	2,4,5,6,7,8<br>,12,14,17	22	O54728,O88267,P06760,P06761,P07647,P19223,P20760,P20761,P20762,P22282,P22283,P50115,P55091,P62815,P83748,P98089,Q4G07<br>5,Q62635,Q80WL1,Q99041,Q9WTT6,Q9Z0V6
7939	1,3,9,10,1<br>1,13,15,17	2,4,5,6,7,8<br>,12,14,16	9	O88267,P06761,P07171,P20762,P83748,Q63532,Q9EQS0,Q9R0T3,Q9Z0V6

7940	1,3,9,10,11,13,16,17	2,4,5,6,7,8,12,14,15	8	D3ZHA0,P02631,P06761,P07171,P20760,P20762,P83748,Q9Z0V6
7941	1,3,9,10,11,14,15,16	2,4,5,6,7,8,12,13,17	29	O54728,O88267,P02780,P06760,P06761,P07647,P09605;P25809,P11598,P19223,P20760,P20761,P20762,P22282,P22283,P50115,P55091,P62815,P70545,P83748,P98089,Q4G075,Q62635,Q6AYR9,Q80WL1,Q99041,Q9JHB9,Q9QZK9,Q9R0T3,Q9Z0V6
7942	1,3,9,10,11,14,15,17	2,4,5,6,7,8,12,13,16	11	O88267,P02780,P06761,P07647,P08689,P22282,P83748,Q80WL1,Q8CJ52,Q99041,Q9R0T3
7943	1,3,9,10,11,14,16,17	2,4,5,6,7,8,12,13,15	11	P06761,P07171,P07647,P08689,P20760,P20762,P22282,P83748,Q8R431,Q99041,Q9Z0V6
7944	1,3,9,10,11,15,16,17	2,4,5,6,7,8,12,13,14	23	O88267,P02780,P02782,P06761,P06911,P07171,P07647,P12020,P20762,P22282,P22283,P25236,P30120,P83748,Q5GRG2,Q62902,Q6AYR9,Q80WL1,Q8CJ52,Q9JHB9,Q9R0T3,Q9WTT6,Q9Z0V6
7945	1,3,9,10,12,13,14,15	2,4,5,6,7,8,11,16,17	20	A2RUV9,P06760,P07150,P07171,P09605;P25809,P10758,P14668,P14669,P20646,P20760,P20762,P50115,P50116,P61206;P84079;P84082,Q07936,Q4G075,Q6AYS4,Q99041,Q9R0T3,Q9Z0V6
7946	1,3,9,10,12,13,14,16	2,4,5,6,7,8,11,15,17	15	O55004,P02631,P06760,P07150,P07171,P14669,P20646,P20760,P20761,P20762,P50115,Q4G075,Q6AYS4,Q99041,Q9Z0V6
7947	1,3,9,10,12,13,14,17	2,4,5,6,7,8,11,15,16	11	P02631,P07150,P07171,P14668,P14669,P20760,P20762,P61206;P84079;P84082,P83748,Q07936,Q9QW07
7948	1,3,9,10,12,13,15,16	2,4,5,6,7,8,11,14,17	14	P02631,P06760,P07150,P07171,P14669,P20760,P20761,P20762,P50115,Q07936,Q4G075,Q6AYS4,Q99041,Q9Z0V6
7949	1,3,9,10,12,13,15,17	2,4,5,6,7,8,11,14,16	10	P02631,P07150,P07171,P14668,P14669,P20762,P61206;P84079;P84082,Q07936,Q6AYS4,Q9Z0V6
7950	1,3,9,10,12,13,16,17	2,4,5,6,7,8,11,14,15	10	P02631,P07150,P07171,P14668,P14669,P20760,P20762,P83748,Q6AYS4,Q9Z0V6
7951	1,3,9,10,12,14,15,16	2,4,5,6,7,8,11,13,17	17	P06760,P06761,P07150,P07171,P07647,P14669,P20760,P20761,P20762,P22282,P22283,P50115,Q4G075,Q6AYS4,Q99041,Q9R0T3,Q9Z0V6
7952	1,3,9,10,12,14,15,17	2,4,5,6,7,8,11,13,16	11	A2RUV9,P07150,P07171,P08689,P14668,P14669,P20762,P61206;P84079;P84082,P83748,Q07936,Q9R0T3
7953	1,3,9,10,12,14,16,17	2,4,5,6,7,8,11,13,15	10	P02631,P07150,P07171,P08689,P14668,P14669,P20760,P20762,P83748,Q6AYS4
7954	1,3,9,10,12,15,16,17	2,4,5,6,7,8,11,13,14	16	P02631,P02780,P06761,P06866,P06911,P07150,P07171,P07647,P12020,P14668,P14669,P20762,P22282,P22283,Q5GRG2,Q6AYS4
7955	1,3,9,10,13,14,15,16	2,4,5,6,7,8,11,12,17	37	O54728,O55004,P02780,P06760,P06761,P07150,P07647,P09605;P25809,P0C0A9,P14669,P19223,P20646,P20760,P20761,P20762,P22282,P22283,P30120,P50115,P50280,P55091,P60905,P62804,P83748,P98089,Q07936,Q4G075,Q62635,Q63493,Q6AYS4,Q80WL1,Q99041,Q9JHB9,Q9QZA6,Q9QZK9,Q9R0T3,Q9Z0V6
7956	1,3,9,10,13,14,15,17	2,4,5,6,7,8,11,12,16	18	P07150,P07171,P07647,P0C0A9,P14668,P14669,P20646,P20762,P61206;P84079;P84082,P83748,Q07936,Q6AYS4,Q8CJ52,Q99041,Q9EQS0,Q9QW07,Q9R0T3,Q9Z0V6
7957	1,3,9,10,13,14,16,17	2,4,5,6,7,8,11,12,15	16	O55004,P02631,P06761,P07150,P07171,P07647,P14668,P14669,P20760,P20762,P83748,Q07936,Q6AYS4,Q99041,Q9QW07,Q9Z0V6
7958	1,3,9,10,13,15,16,17	2,4,5,6,7,8,11,12,14	27	P02631,P02780,P06761,P07150,P07171,P07647,P0C0A9,P14668,P14669,P20762,P22282,P22283,P24368,P30120,P50280,P61206;P84079;P84082,P62804,P83748,Q07936,Q63493,Q6AYS4,Q80WL1,Q8CJ52,Q99041,Q9EQS0,Q9JHB9,Q9Z0V6
7959	1,3,9,10,14,15,16,17	2,4,5,6,7,8,11,12,13	26	P02780,P02781,P02782,P06761,P07150,P07171,P07647,P0C0A9,P14669,P20762,P22282,P22283,P24368,P30120,P50280,P60905,P61206;P84079;P84082,P83748,Q62902,Q6AYR9,Q6AYS4,Q80WL1,Q8CJ52,Q99041,Q9JHB9,Q9R0T3
7960	1,3,9,11,12,13,14,15	2,4,5,6,7,8,10,16,17	12	A2RUV9,P06760,P20646,P20760,P50115,P82471,P83748,Q4G075,Q6P6R2,Q99041,Q9R0T3,Q9Z0V6
7961	1,3,9,11,12,13,14,16	2,4,5,6,7,8,10,15,17	12	D3ZHA0,P04218,P06760,P07171,P20646,P20760,P20761,P20762,P50115,P83748,Q4G075,Q9Z0V6

7962	1,3,9,11,12,13,14,17	2,4,5,6,7,8,10,15,16	8	D3ZHA0,P04218,P07171,P20760,P82471,P83748,Q9QW07,Q9Z0V6
7963	1,3,9,11,12,13,15,16	2,4,5,6,7,8,10,14,17	11	P06760,P07171,P20760,P20761,P20762,P50115,P82471,P83748,Q4G075,Q9WTT6,Q9Z0V6
7964	1,3,9,11,12,13,15,17	2,4,5,6,7,8,10,14,16	8	D3ZHA0,P04218,P07171,P20762,P82471,P83748,Q63532,Q9Z0V6
7965	1,3,9,11,12,13,16,17	2,4,5,6,7,8,10,14,15	9	D3ZHA0,P02631,P04218,P07171,P20760,P20762,P82471,P83748,Q9Z0V6
7966	1,3,9,11,12,14,15,16	2,4,5,6,7,8,10,13,17	13	P04218,P06760,P06761,P12020,P20760,P20761,P22282,P50115,P83748,Q4G075,Q99041,Q9R0T3,Q9Z0V6
7967	1,3,9,11,12,14,15,17	2,4,5,6,7,8,10,13,16	6	P04218,P08689,P12020,P82471,P83748,Q9R0T3
7968	1,3,9,11,12,14,16,17	2,4,5,6,7,8,10,13,15	7	D3ZHA0,P04218,P07171,P12020,P20760,P20762,P83748
7969	1,3,9,11,12,15,16,17	2,4,5,6,7,8,10,13,14	12	D3ZHA0,P04218,P06761,P06911,P07171,P12020,P20760,P20762,P83748,Q5GRG2,Q9WTT6,Q9Z0V6
7970	1,3,9,11,13,14,15,16	2,4,5,6,7,8,10,12,17	28	D3ZHA0,O54728,P04218,P06760,P06761,P07647,P19223,P20646,P20760,P20761,P20762,P22282,P22283,P50115,P55091,P82471,P83748,P98089,Q4G075,Q62635,Q64361,Q6P6R2,Q80WL1,Q99041,Q9QZK9,Q9R0T3,Q9WTT6,Q9Z0V6
7971	1,3,9,11,13,14,15,17	2,4,5,6,7,8,10,12,16	11	D3ZHA0,P04218,P20646,P61206;P84079;P84082,P82471,P83748,Q80WL1,Q99041,Q9QW07,Q9R0T3,Q9Z0V6
7972	1,3,9,11,13,14,16,17	2,4,5,6,7,8,10,12,15	12	D3ZHA0,P04218,P06761,P07171,P20760,P20762,P82471,P83748,Q64361,Q99041,Q9QW07,Q9Z0V6
7973	1,3,9,11,13,15,16,17	2,4,5,6,7,8,10,12,14	14	D3ZHA0,P02780,P04218,P06761,P07171,P07647,P12020,P20762,P82471,P83748,Q80WL1,Q9EQS0,Q9WTT6,Q9Z0V6
7974	1,3,9,11,14,15,16,17	2,4,5,6,7,8,10,12,13	19	D3ZHA0,P02780,P04218,P06761,P07647,P0C0A9,P12020,P20762,P22282,P22283,P30120,P82471,P83748,Q6AYR9,Q80WL1,Q99041,Q9JHB9,Q9R0T3,Q9Z0V6
7975	1,3,9,12,13,14,15,16	2,4,5,6,7,8,10,11,17	20	A2RUV9,P00714,P06760,P07150,P07171,P14669,P20646,P20760,P20761,P20762,P50115,P61206;P84079;P84082,P82471,Q07936,Q4G075,Q63598,Q6AYS4,Q99041,Q9R0T3,Q9Z0V6
7976	1,3,9,12,13,14,15,17	2,4,5,6,7,8,10,11,16	16	A2RUV9,P00714,P07150,P07171,P14668,P14669,P20646,P20762,P61206;P84079;P84082,P82471,Q07936,Q5BJY9,Q5FVI6,Q6AYS4,Q9QW07,Q9R0T3
7977	1,3,9,12,13,14,16,17	2,4,5,6,7,8,10,11,15	18	D3ZHA0,P00714,P02631,P04218,P07150,P07171,P14668,P14669,P20646,P20760,P20762,P61206;P84079;P84082,P82471,P83748,Q5BJY9,Q6AYS4,Q9QW07,Q9Z0V6
7978	1,3,9,12,13,15,16,17	2,4,5,6,7,8,10,11,14	14	D3ZHA0,P00714,P07150,P07171,P14668,P14669,P20762,P61206;P84079;P84082,P82471,Q5BJY9,Q63598,Q6AYS4,Q9EQS0,Q9Z0V6
7979	1,3,9,12,14,15,16,17	2,4,5,6,7,8,10,11,13	16	P00714,P04218,P06761,P07150,P07171,P07647,P12020,P14669,P20762,P22282,P22283,P61206;P84079;P84082,P82471,Q63598,Q6AYS4,Q9R0T3
7980	1,3,9,13,14,15,16,17	2,4,5,6,7,8,10,11,12	36	D3ZHA0,O55004,P00714,P02780,P02781,P04218,P06761,P07150,P07171,P07647,P0C0A9,P14668,P14669,P20646,P20762,P22283,P24368,P30120,P48199,P60905,P61206;P84079;P84082,P62804,P82471,P83748,Q07936,Q63598,Q64361,Q6AYS4,Q80WL1,Q8CJ52,Q99041,Q9EQS0,Q9JHB9,Q9QW07,Q9R0T3,Q9Z0V6
7981	1,3,10,11,12,13,14,15	2,4,5,6,7,8,9,16,17	9	A2RUV9,P06760,P09605;P25809,P20646,P20760,P50115,P83748,Q6P6R2,Q9Z0V6
7982	1,3,10,11,12,13,14,16	2,4,5,6,7,8,9,15,17	11	P02631,P06760,P07171,P20646,P20760,P20761,P20762,P50115,P83748,Q7TPB1,Q9Z0V6

7983	1,3,10,11,12,13,14,17	2,4,5,6,7,8,9,15,16	5	P02631,P07171,P20760,P83748,Q9QW07
7984	1,3,10,11,12,13,15,16	2,4,5,6,7,8,9,14,17	11	P02631,P06760,P07171,P20646,P20760,P20761,P20762,P50115,P83748,Q9WTT6,Q9Z0V6
7985	1,3,10,11,12,13,15,17	2,4,5,6,7,8,9,14,16	5	P07171,P20762,P83748,Q99MH3,Q9Z0V6
7986	1,3,10,11,12,13,16,17	2,4,5,6,7,8,9,14,15	6	P02631,P07171,P20760,P20762,P83748,Q9Z0V6
7987	1,3,10,11,12,14,15,16	2,4,5,6,7,8,9,13,17	10	P06760,P06761,P09605;P25809,P20760,P20761,P20762,P22282,P50115,P83748,Q9Z0V6
7988	1,3,10,11,12,14,15,17	2,4,5,6,7,8,9,13,16	3	P08689,P83748,Q9R0T3
7989	1,3,10,11,12,14,16,17	2,4,5,6,7,8,9,13,15	6	P02631,P07171,P08689,P20760,P20762,P83748
7990	1,3,10,11,12,15,16,17	2,4,5,6,7,8,9,13,14	7	P06761,P06866,P06911,P07171,P12020,P20762,P83748
7991	1,3,10,11,13,14,15,16	2,4,5,6,7,8,9,12,17	23	O55004,P06760,P06761,P07647,P09605;P25809,P19223,P20646,P20760,P20761,P20762,P22282,P50115,P55091,P83748,P98089,Q4G075,Q62635,Q6P6R2,Q80WL1,Q99041,Q9R0T3,Q9WTT6,Q9Z0V6
7992	1,3,10,11,13,14,15,17	2,4,5,6,7,8,9,12,16	8	D3ZHA0,P20646,P20762,P83748,Q99041,Q9QW07,Q9R0T3,Q9Z0V6
7993	1,3,10,11,13,14,16,17	2,4,5,6,7,8,9,12,15	10	D3ZHA0,P02631,P07171,P20646,P20760,P20762,P83748,Q99041,Q9QW07,Q9Z0V6
7994	1,3,10,11,13,15,16,17	2,4,5,6,7,8,9,12,14	11	D3ZHA0,P02631,P02780,P06761,P07171,P07647,P20762,P83748,Q9EQS0,Q9WTT6,Q9Z0V6
7995	1,3,10,11,14,15,16,17	2,4,5,6,7,8,9,12,13	15	D3ZHA0,P02780,P06761,P07647,P0C0A9,P20762,P22282,P22283,P30120,P83748,Q62902,Q80WL1,Q99041,Q9JHB9,Q9R0T3
7996	1,3,10,12,13,14,15,16	2,4,5,6,7,8,9,11,17	16	A2RUV9,O55004,P06760,P07150,P07171,P09605;P25809,P14669,P20646,P20760,P20761,P20762,P50115,Q4G075,Q6AYS4,Q99041,Q9Z0V6
7997	1,3,10,12,13,14,15,17	2,4,5,6,7,8,9,11,16	12	A2RUV9,P07150,P07171,P14668,P14669,P20646,P20762,P61206;P84079;P84082,Q05175,Q07936,Q6AYS4,Q9QW07

7998	1,3,10,12,13,14,16,17	2,4,5,6,7,8,9,11,15	10	P02631,P07171,P14668,P14669,P20646,P20760,P20762,P83748,Q6AYS4,Q9QW07
7999	1,3,10,12,13,15,16,17	2,4,5,6,7,8,9,11,14	11	P02631,P07150,P07171,P14668,P14669,P20762,P83121,Q63617,Q6AYS4,Q99MH3,Q9Z0V6
8000	1,3,10,12,14,15,16,17	2,4,5,6,7,8,9,11,13	10	P02780,P06761,P07171,P07647,P14668,P14669,P20762,P22282,Q63617,Q6AYS4
8001	1,3,10,13,14,15,16,17	2,4,5,6,7,8,9,11,12	27	D3ZHA0,O55004,P02631,P02780,P06761,P07150,P07171,P07647,P0C0A9,P14668,P14669,P20646,P20762,P22282,P22283,P30120,P60905,P61206;P84079;P84082,P83748,Q6AYS4,Q80WL1,Q99041,Q9EQS0,Q9JHB9,Q9QW07,Q9R0T3,Q9Z0V6
8002	1,3,11,12,13,14,15,16	2,4,5,6,7,8,9,10,17	12	D3ZHA0,P06760,P19223,P20646,P20760,P20762,P50115,P52590,P83748,Q6P6R2,Q9WTT6,Q9Z0V6
8003	1,3,11,12,13,14,15,17	2,4,5,6,7,8,9,10,16	9	A2RUV9,D3ZHA0,P20646,P61206;P84079;P84082,P82471,P83748,Q64093,Q6P6R2,Q9QW07
8004	1,3,11,12,13,14,16,17	2,4,5,6,7,8,9,10,15	10	D3ZHA0,O55006,P07171,P20646,P20760,P20762,P82471,P83748,Q9QW07,Q9Z0V6
8005	1,3,11,12,13,15,16,17	2,4,5,6,7,8,9,10,14	7	D3ZHA0,P07171,P20762,P82471,P83748,Q9WTT6,Q9Z0V6
8006	1,3,11,12,14,15,16,17	2,4,5,6,7,8,9,10,13	6	D3ZHA0,P04218,P06761,P12020,P83748,Q9R0T3
8007	1,3,11,13,14,15,16,17	2,4,5,6,7,8,9,10,12	15	D3ZHA0,P02780,P04218,P06761,P07647,P20646,P20762,P82471,P83748,Q80WL1,Q9EQS0,Q9QW07,Q9R0T3,Q9WTT6,Q9Z0V6
8008	1,3,12,13,14,15,16,17	2,4,5,6,7,8,9,10,11	14	A2RUV9,D3ZHA0,P00714,P07150,P07171,P14668,P14669,P20646,P20762,P48199,P61206;P84079;P84082,P82471,Q6AYS4,Q9QW07
8009	1,4,5,6,7,8,9,10	2,3,11,12,13,14,15,16,17	19	D3ZHA0,O55006,P00714,P06761,P07171,P07647,P18418,P20646,P22282,P22283,P31044,P52590,P61206;P84079;P84082,P97571,Q63617,Q99041,Q9JI85,Q9R0T3,Q9WTT6
8010	1,4,5,6,7,8,9,11	2,3,10,12,13,14,15,16,17	24	P02780,P04041,P06761,P07150,P07171,P07647,P10715,P14668,P14669,P18418,P20646,P22282,P22283,P31044,P61206;P84079;P84082,P68370,P83121,Q63493,Q63617,Q6AYS4,Q8CJ52,Q99041,Q9JI85,Q9R0T3
8011	1,4,5,6,7,8,9,12	2,3,10,11,13,14,15,16,17	31	D3ZHA0,F1LM93,O55006,P02780,P02781,P02782,P06761,P07647,P08723,P0C0A9,P10715,P13432,P18418,P20646,P22282,P22283,P30120,P31044,P36374,P61206;P84079;P84082,P83748,Q5M8C6,Q63617,Q6AYR9,Q8CJ52,Q99041,Q9JHB9,Q9JI85,Q9R0T3,Q9WTT6,Q9Z0V6
8012	1,4,5,6,7,8,9,13	2,3,10,11,12,14,15,16,17	19	P02764,P02780,P05539,P06761,P06866,P06911,P07171,P07647,P18418,P22282,P22283,P31044,Q5GRG2,Q62902,Q63617,Q8CJ52,Q99041,Q9JI85,Q9R0T3

8013	1,4,5,6,7,8,9,14	2,3,10,11,12,13,15,16,17	13	P06761,P06866,P07171,P07647,P18418,P19629,P22282,P22283,P31044,Q63617,Q8CJ52,Q9JI85,Q9WTT6
8014	1,4,5,6,7,8,9,15	2,3,10,11,12,13,14,16,17	9	O55006,P06761,P07171,P18418,P22282,P22283,P31044,Q63617,Q99041
8015	1,4,5,6,7,8,9,16	2,3,10,11,12,13,14,15,17	13	P06761,P07171,P14668,P18418,P20646,P22282,P22283,P31044,P61206;P84079;P84082,Q63617,Q8CJ52,Q99041,Q9R0T3
8016	1,4,5,6,7,8,9,17	2,3,10,11,12,13,14,15,16	18	P06760,P06761,P07647,P18418,P20646,P20760,P22282,P22283,P23593,P31044,P50115,P52590,Q4G075,Q63617,Q99041,Q9R0T3,Q9WTT6,Q9Z0V6
8017	1,4,5,6,7,8,10,11	2,3,9,12,13,14,15,16,17	31	D3ZHA0,P00714,P02781,P04041,P06761,P07150,P07171,P07647,P08649,P08723,P14668,P14669,P18418,P20646,P22282,P22283,P31044,P55018,P61206;P84079;P84082,P68370,P82471,Q5BJY9,Q5FVI6,Q63493,Q63617,Q6AYS4,Q8CJ52,Q8VD89,Q99041,Q9JI85,Q9R0T3
8018	1,4,5,6,7,8,10,12	2,3,9,11,13,14,15,16,17	41	D3ZHA0,F1LM93,P02780,P02781,P02782,P06761,P07647,P08723,P0C0A9,P12020,P13432,P18418,P20646,P22282,P22283,P30120,P31044,P35281,P36374,P61206;P84079;P84082,P68370,P82471,P83748,Q10758,Q5M8C6,Q63493,Q63617,Q64361,Q6AYR9,Q6IFW6,Q6IG02,Q6IMF3,Q6P6Q2,Q8CJ52,Q99041,Q99MH3,Q9JHB9,Q9JI85,Q9R0T3,Q9WTT6,Q9Z0V6
8019	1,4,5,6,7,8,10,13	2,3,9,11,12,14,15,16,17	22	D3ZHA0,P02780,P06761,P06911,P07171,P07647,P08723,P12020,P18418,P22282,P22283,P31044,P61206;P84079;P84082,P68370,Q498D9,Q5GRG2,Q63617,Q8CJ52,Q8VD89,Q99041,Q9JI85,Q9R0T3
8020	1,4,5,6,7,8,10,14	2,3,9,11,12,13,15,16,17	24	D3ZHA0,P06761,P06911,P07171,P07647,P08723,P12020,P18418,P19629,P22282,P22283,P31044,P55018,P61206;P84079;P84082,P68370,Q498D9,Q5BJY9,Q5GRG2,Q63617,Q8CJ52,Q9JI85,Q9R0T3,Q9WTT6,Q9Z0V6
8021	1,4,5,6,7,8,10,15	2,3,9,11,12,13,14,16,17	17	D3ZHA0,O55006,P06761,P07171,P18418,P20760,P22282,P22283,P31044,P55018,P61206;P84079;P84082,P97571,Q5FVI6,Q63617,Q8VD89,Q99041,Q9Z0V6
8022	1,4,5,6,7,8,10,16	2,3,9,11,12,13,14,15,17	18	D3ZHA0,P06761,P07171,P08721,P18418,P20646,P22282,P22283,P31044,P55018,P61206;P84079;P84082,P82471,Q5FVI6,Q63617,Q8CJ52,Q8VD89,Q99041,Q9R0T3
8023	1,4,5,6,7,8,10,17	2,3,9,11,12,13,14,15,16	23	P06760,P06761,P07647,P18418,P20646,P20760,P20761,P22282,P22283,P23928,P31044,P50115,P52590,P61206;P84079;P84082,P97571,Q4G075,Q63617,Q8CJ52,Q99041,Q99MH3,Q9R0T3,Q9WTT6,Q9Z0V6
8024	1,4,5,6,7,8,11,12	2,3,9,10,13,14,15,16,17	46	F1LM93,O35547,P02780,P02781,P02782,P04041,P04905,P06761,P07150,P07171,P07647,P08723,P0C0A9,P10715,P13432,P14668,P14669,P18418,P19629,P20646,P22282,P22283,P24368,P30120,P31044,P36374,P46462,P61206;P84079;P84082,P68370,Q5M8C6,Q63357,Q63493,Q63617,Q6AYR9,Q6IFU7,Q6IFW6,Q6IG02,Q6IMF3,Q6P6Q2,Q8CJ52,Q99041,Q9ESW0,Q9JHB9,Q9JI85,Q9R0T3,Q9Z0V6
8025	1,4,5,6,7,8,11,13	2,3,9,10,12,14,15,16,17	29	P02780,P02781,P05539,P06761,P06911,P07150,P07171,P07647,P08723,P12020,P14668,P14669,P18418,P22282,P22283,P31044,P46462,P61206;P84079;P84082,P68370,Q5GRG2,Q62902,Q63493,Q63617,Q8CJ52,Q8VD89,Q99041,Q9JHB9,Q9JI85,Q9R0T3
8026	1,4,5,6,7,8,11,14	2,3,9,10,12,13,15,16,17	23	P02780,P06761,P07150,P07171,P07647,P08723,P14668,P14669,P18418,P19629,P22282,P22283,P31044,P46462,P47727,P61206;P84079;P84082,P68370,Q63493,Q63617,Q8CJ52,Q99041,Q9JI85,Q9Z0V6
8027	1,4,5,6,7,8,11,15	2,3,9,10,12,13,14,16,17	22	P06761,P07150,P07171,P07647,P10715,P14668,P14669,P18418,P19629,P20760,P22006,P22282,P22283,P31044,P61206;P84079;P84082,Q5I0D1,Q63493,Q63617,Q8CJ52,Q8VD89,Q99041,Q9JI85

8028	1,4,5,6,7,8,11,16	2,3,9,10,12,13,14,15,17	21	A2RUV9,P06761,P07150,P07171,P07647,P10715,P14668,P14669,P18418,P20646,P22282,P22283,P31044,P61206;P84079;P84082,Q63493,Q63617,Q8CJ52,Q8VD89,Q99041,Q9JI85,Q9R0T3
8029	1,4,5,6,7,8,11,17	2,3,9,10,12,13,14,15,16	28	P04041,P06760,P06761,P07150,P07171,P07647,P10715,P14668,P14669,P15399,P18418,P20646,P20760,P20761,P22282,P22283,P31044,P50115,P61206;P84079;P84082,Q4G075,Q63493,Q63617,Q6AYS4,Q8CJ52,Q99041,Q9JI85,Q9R0T3,Q9Z0V6
8030	1,4,5,6,7,8,12,13	2,3,9,10,11,14,15,16,17	33	F1LM93,O88267,P02780,P02781,P02782,P06761,P06911,P07647,P08723,P09456,P0C0A9,P11598,P12020,P13432,P18418,P22282,P22283,P30120,P31044,P36374,P46462,P83748,Q498D9,Q5GRG2,Q5M8C6,Q62902,Q63617,Q6AYR9,Q8CJ52,Q99041,Q9JHB9,Q9JI85,Q9R0T3
8031	1,4,5,6,7,8,12,14	2,3,9,10,11,13,15,16,17	35	F1LM93,O88267,P02780,P02781,P02782,P06761,P06911,P07171,P07647,P08723,P0C0A9,P11598,P12020,P13432,P18418,P19629,P22282,P22283,P30120,P31044,P36374,P83748,Q498D9,Q5M8C6,Q63493,Q63617,Q68FS4,Q6AYR9,Q8CJ52,Q99041,Q9JHB9,Q9JI85,Q9R0T3,Q9WTT6,Q9Z0V6
8032	1,4,5,6,7,8,12,15	2,3,9,10,11,13,14,16,17	24	D3ZHA0,F1LM93,P02780,P06761,P07171,P07647,P08723,P10715,P13432,P18418,P19629,P20760,P22282,P22283,P30120,P31044,P83748,Q63617,Q8CJ52,Q99041,Q9JHB9,Q9JI85,Q9R0T3,Q9Z0V6
8033	1,4,5,6,7,8,12,16	2,3,9,10,11,13,14,15,17	25	F1LM93,O88267,P02780,P06761,P07647,P08723,P0C0A9,P10715,P13432,P18418,P22282,P22283,P30120,P31044,P61206;P84079;P84082,P83748,Q63617,Q6IFW6,Q6IMF3,Q8CJ52,Q99041,Q9JHB9,Q9JI85,Q9R0T3,Q9Z0V6
8034	1,4,5,6,7,8,12,17	2,3,9,10,11,13,14,15,16	44	F1LM93,O88267,P02780,P02781,P02782,P06760,P06761,P07647,P08723,P0C0A9,P10715,P11598,P13432,P18418,P19223,P19629,P20646,P20760,P20761,P22282,P22283,P23928,P30120,P36374,P50115,P55091,P62815,P83748,Q10758,Q4G075,Q5M8C6,Q63493,Q63617,Q6AYR9,Q8CJ52,Q99041,Q99MH3,Q9JHB9,Q9JI85,Q9QZA6,Q9QZK9,Q9R0T3,Q9WTT6,Q9Z0V6
8035	1,4,5,6,7,8,13,14	2,3,9,10,11,12,15,16,17	25	O88267,P02780,P05539,P06761,P06866,P06911,P07171,P07647,P08723,P11598,P12020,P18418,P19629,P22282,P22283,P31044,Q498D9,Q5GRG2,Q62902,Q63617,Q8CJ52,Q99041,Q9JI85,Q9R0T3,Q9WTT6
8036	1,4,5,6,7,8,13,15	2,3,9,10,11,12,14,16,17	16	P05539,P06761,P06911,P07171,P07647,P18418,P20760,P22282,P22283,P31044,Q5GRG2,Q63617,Q8CJ52,Q8VD89,Q99041,Q9JI85
8037	1,4,5,6,7,8,13,16	2,3,9,10,11,12,14,15,17	19	O88267,P06761,P06911,P07171,P07647,P12020,P18418,P22282,P22283,P31044,P61206;P84079;P84082,Q5GRG2,Q62902,Q63617,Q8CJ52,Q8VD89,Q99041,Q9JI85,Q9R0T3
8038	1,4,5,6,7,8,13,17	2,3,9,10,11,12,14,15,16	26	O88267,P06760,P06761,P06911,P07647,P10536,P11598,P12020,P18418,P20760,P20761,P22282,P22283,P23928,P31044,P50115,P62815,Q4G075,Q5GRG2,Q63617,Q8CJ52,Q99041,Q99MH3,Q9JI85,Q9R0T3,Q9Z0V6
8039	1,4,5,6,7,8,14,15	2,3,9,10,11,12,13,16,17	13	P06761,P07171,P10536,P18418,P19629,P20760,P22282,P22283,P31044,Q5GRG2,Q63617,Q99041,Q9Z0V6
8040	1,4,5,6,7,8,14,16	2,3,9,10,11,12,13,15,17	13	P06761,P07171,P18418,P19629,P22282,P22283,P31044,P61206;P84079;P84082,Q63617,Q8CJ52,Q99041,Q9R0T3,Q9Z0V6
8041	1,4,5,6,7,8,14,17	2,3,9,10,11,12,13,15,16	22	O88267,P02793;Q7TP54,P06760,P06761,P07171,P07647,P10536,P11598,P18418,P19629,P20760,P20761,P22282,P22283,P31044,P50115,Q4G075,Q63617,Q8CJ52,Q99041,Q9WTT6,Q9Z0V6
8042	1,4,5,6,7,8,15,16	2,3,9,10,11,12,13,14,17	11	P06761,P07171,P14668,P18418,P20760,P31044,P61206;P84079;P84082,Q63617,Q8CJ52,Q8VD89,Q99041

8043	1,4,5,6,7,8,15,17	2,3,9,10,11,12,13,14,16	18	P06760,P06761,P07171,P10536,P18418,P19629,P20646,P20760,P20761,P22282,P22283,P31044,P50115,Q4G075,Q63617,Q99041,Q9QZA6,Q9Z0V6
8044	1,4,5,6,7,8,16,17	2,3,9,10,11,12,13,14,15	18	O88267,P06760,P06761,P18418,P20646,P20760,P22282,P22283,P23928,P31044,P50115,P61206;P84079;P84082,Q4G075,Q63617,Q8CJ52,Q99041,Q9R0T3,Q9Z0V6
8045	1,4,5,6,7,9,10,11	2,3,8,12,13,14,15,16,17	6	P18418,P61206;P84079;P84082,P83121,Q566E6,Q5FVI6,Q64093
8046	1,4,5,6,7,9,10,12	2,3,8,11,13,14,15,16,17	11	F1LM93,P08723,P18418,P20766,P36374,P61206;P84079;P84082,P83748,P97840,Q10758,Q5FVI6,Q6IMF3
8047	1,4,5,6,7,9,10,13	2,3,8,11,12,14,15,16,17	4	P05539,P12020,P18418,P61206;P84079;P84082
8048	1,4,5,6,7,9,10,14	2,3,8,11,12,13,15,16,17	4	P12020,P18418,P61206;P84079;P84082,P83121
8049	1,4,5,6,7,9,10,15	2,3,8,11,12,13,14,16,17	5	O55006,P18418,P20766,P61206;P84079;P84082,Q5FVI6
8050	1,4,5,6,7,9,10,16	2,3,8,11,12,13,14,15,17	5	P18418,P20766,P61206;P84079;P84082,Q5FVI6,Q64093
8051	1,4,5,6,7,9,10,17	2,3,8,11,12,13,14,15,16	4	P18418,P20646,P52590,P61206;P84079;P84082
8052	1,4,5,6,7,9,11,12	2,3,8,10,13,14,15,16,17	16	P04905,P07647,P08723,P09456,P0C0A9,P13432,P30120,P36374,P61206;P84079;P84082,Q63357,Q6IFU7,Q6IFW6,Q6IMF3,Q6P6Q2,Q9JHB9,Q9JI85
8053	1,4,5,6,7,9,11,13	2,3,8,10,12,14,15,16,17	4	P05539,P61206;P84079;P84082,P83121,Q9JI85
8054	1,4,5,6,7,9,11,14	2,3,8,10,12,13,15,16,17	3	P05539,P61206;P84079;P84082,P83121
8055	1,4,5,6,7,9,11,15	2,3,8,10,12,13,14,16,17	3	P05539,P22006,P61206;P84079;P84082
8056	1,4,5,6,7,9,11,16	2,3,8,10,12,13,14,15,17	3	A2RUV9,P15999,P61206;P84079;P84082
8057	1,4,5,6,7,9,11,17	2,3,8,10,12,13,14,15,16	6	P09605;P25809,P15399,P20646,P52590,P61206;P84079;P84082,P83121

8058	1,4,5,6,7,9,12,13	2,3,8,10,11,14,15,16,17	12	P05539,P07647,P08723,P09456,P13432,P19218,P22282,P30120,P36374,P83748,Q9JHB9,Q9JI85
8059	1,4,5,6,7,9,12,14	2,3,8,10,11,13,15,16,17	6	P01835,P08723,P13432,P18418,P30120,P83748
8060	1,4,5,6,7,9,12,15	2,3,8,10,11,13,14,16,17	2	P20766,P83748
8061	1,4,5,6,7,9,12,16	2,3,8,10,11,13,14,15,17	5	P13432,P19218,P20766,P61206;P84079;P84082,P83748
8062	1,4,5,6,7,9,12,17	2,3,8,10,11,13,14,15,16	6	P01835,P09605;P25809,P13432,P36374,P52590,Q99041
8063	1,4,5,6,7,9,13,14	2,3,8,10,11,12,15,16,17	5	P05539,P06911,P12020,P83121,Q5GRG2
8064	1,4,5,6,7,9,13,15	2,3,8,10,11,12,14,16,17	2	P05539,P62804
8065	1,4,5,6,7,9,13,16	2,3,8,10,11,12,14,15,17	5	P05539,P19218,P61206;P84079;P84082,P62804,Q00715
8066	1,4,5,6,7,9,13,17	2,3,8,10,11,12,14,15,16	3	P05539,P22282,P52590
8067	1,4,5,6,7,9,14,15	2,3,8,10,11,12,13,16,17	1	P16636
8068	1,4,5,6,7,9,14,16	2,3,8,10,11,12,13,15,17	1	P61206;P84079;P84082
8069	1,4,5,6,7,9,14,17	2,3,8,10,11,12,13,15,16	1	P52590
8070	1,4,5,6,7,9,15,16	2,3,8,10,11,12,13,14,17	3	P20766,P62804,Q00715
8071	1,4,5,6,7,9,15,17	2,3,8,10,11,12,13,14,16	2	P20760,P52590
8072	1,4,5,6,7,9,16,17	2,3,8,10,11,12,13,14,15	4	P09605;P25809,P23593,P52590,P62804

8073	1,4,5,6,7,10,11,12	2,3,8,9,13,14,15,16,17	26	F1LM93,P02781,P04905,P06757,P07647,P08723,P0C0A9,P15399,P18418,P20766,P30120,P36374,P61206;P84079;P84082,Q10758,Q5FVI6,Q63357,Q63493,Q6IFU7,Q6IFU8,Q6IFW6,Q6IG02,Q6IMF3,Q6P6Q2,Q8CJ52,Q9JHB9,Q9JI85
8074	1,4,5,6,7,10,11,13	2,3,8,9,12,14,15,16,17	9	P05539,P06911,P12020,P18418,P61206;P84079;P84082,Q5FVI6,Q5GRG2,Q8VD89,Q9JI85
8075	1,4,5,6,7,10,11,14	2,3,8,9,12,13,15,16,17	10	P12020,P15399,P18418,P20766,P47727,P55018,P61206;P84079;P84082,P83121,Q5BJY9,Q5FVI6
8076	1,4,5,6,7,10,11,15	2,3,8,9,12,13,14,16,17	7	P18418,P20766,P22006,P61206;P84079;P84082,Q566E6,Q5FVI6,Q8VD89
8077	1,4,5,6,7,10,11,16	2,3,8,9,12,13,14,15,17	7	A2RUV9,P18418,P20766,P55018,P61206;P84079;P84082,Q5FVI6,Q8VD89
8078	1,4,5,6,7,10,11,17	2,3,8,9,12,13,14,15,16	6	P15399,P18418,P20766,P61206;P84079;P84082,Q566E6,Q5FVI6
8079	1,4,5,6,7,10,12,13	2,3,8,9,11,14,15,16,17	20	F1LM93,P06911,P07647,P08723,P0C0A9,P12020,P18418,P19218,P22282,P30120,P36374,P61206;P84079;P84082,P83748,Q10758,Q498D9,Q5GRG2,Q6AYR9,Q99MH3,Q9JHB9,Q9JI85
8080	1,4,5,6,7,10,12,14	2,3,8,9,11,13,15,16,17	12	F1LM93,P08723,P12020,P18418,P20766,P30120,P61206;P84079;P84082,P83748,Q10758,Q498D9,Q6IFW6,Q6IMF3
8081	1,4,5,6,7,10,12,15	2,3,8,9,11,13,14,16,17	9	F1LM93,P18418,P20766,P61206;P84079;P84082,P83748,Q10758,Q5FVI6,Q6IMF3,Q99MH3
8082	1,4,5,6,7,10,12,16	2,3,8,9,11,13,14,15,17	13	F1LM93,P08721,P18418,P19218,P20766,P61206;P84079;P84082,P83748,P97840,Q10758,Q5FVI6,Q6IFU7,Q6IFW6,Q6IMF3
8083	1,4,5,6,7,10,12,17	2,3,8,9,11,13,14,15,16	12	F1LM93,P13432,P18418,P20760,P20766,P36374,P61206;P84079;P84082,Q10758,Q6IMF3,Q99041,Q99MH3,Q9QZK9
8084	1,4,5,6,7,10,13,14	2,3,8,9,11,12,15,16,17	6	P06911,P12020,P18418,P61206;P84079;P84082,Q498D9,Q5GRG2
8085	1,4,5,6,7,10,13,15	2,3,8,9,11,12,14,16,17	7	P05539,P12020,P18418,Q498D9,Q5FVI6,Q5GRG2,Q8VD89
8086	1,4,5,6,7,10,13,16	2,3,8,9,11,12,14,15,17	6	P12020,P18418,P19218,P61206;P84079;P84082,Q5FVI6,Q8VD89
8087	1,4,5,6,7,10,13,17	2,3,8,9,11,12,14,15,16	4	P12020,P18418,P20760,Q99MH3

8088	1,4,5,6,7,10,14,15	2,3,8,9,11,12,13,16,17	5	P12020,P18418,P20766,P55018,Q498D9
8089	1,4,5,6,7,10,14,16	2,3,8,9,11,12,13,15,17	6	P12020,P18418,P20766,P55018,P61206;P84079;P84082,Q5FVI6
8090	1,4,5,6,7,10,14,17	2,3,8,9,11,12,13,15,16	4	P12020,P18418,P20760,P20766
8091	1,4,5,6,7,10,15,16	2,3,8,9,11,12,13,14,17	5	P20766,P55018,P61206;P84079;P84082,Q5FVI6,Q8VD89
8092	1,4,5,6,7,10,15,17	2,3,8,9,11,12,13,14,16	4	P18418,P20760,P20766,Q99MH3
8093	1,4,5,6,7,10,16,17	2,3,8,9,11,12,13,14,15	4	P18418,P20766,P61206;P84079;P84082,Q5FVI6
8094	1,4,5,6,7,11,12,13	2,3,8,9,10,14,15,16,17	26	P02780,P02781,P04905,P05539,P07647,P08723,P09456,P0C0A9,P13432,P14046,P22282,P22283,P30120,P36374,P46462,P61206;P84079;P84082,Q62902,Q63493,Q6AYR9,Q6IFU7,Q6IFW6,Q6IMF3,Q6P6Q2,Q8CJ52,Q9JHB9,Q9JI85
8095	1,4,5,6,7,11,12,14	2,3,8,9,10,13,15,16,17	21	P04905,P07647,P08723,P0C0A9,P13432,P14046,P18418,P20766,P30120,P36374,P47727,P61206;P84079;P84082,Q63493,Q6IFU7,Q6IFW6,Q6IMF3,Q6P6Q2,Q8CJ52,Q9ESW0,Q9JHB9,Q9JI85
8096	1,4,5,6,7,11,12,15	2,3,8,9,10,13,14,16,17	11	P20766,P22006,P30120,P61206;P84079;P84082,Q6IFU7,Q6IFW6,Q6IMF3,Q6P6Q2,Q9ESW0,Q9JHB9,Q9JI85
8097	1,4,5,6,7,11,12,16	2,3,8,9,10,13,14,15,17	12	P14408,P17046,P20766,P30120,P61206;P84079;P84082,Q6IFU7,Q6IFW6,Q6IMF3,Q6P6Q2,Q8CJ52,Q9JHB9,Q9JI85
8098	1,4,5,6,7,11,12,17	2,3,8,9,10,13,14,15,16	24	F1LM93,P01681,P07647,P08723,P09605;P25809,P0C0A9,P13432,P15399,P20766,P22282,P22283,P30120,P36374,P61206;P84079;P84082,Q10758,Q63493,Q6IFW6,Q6IMF3,Q6P6Q2,Q99041,Q9ESW0,Q9JHB9,Q9JI85,Q9QZK9
8099	1,4,5,6,7,11,13,14	2,3,8,9,10,12,15,16,17	9	P05539,P06911,P12020,P22282,P47727,P61206;P84079;P84082,P83121,Q5GRG2,Q9JI85
8100	1,4,5,6,7,11,13,15	2,3,8,9,10,12,14,16,17	6	P05539,P22006,P61206;P84079;P84082,Q5GRG2,Q8VD89,Q9JI85
8101	1,4,5,6,7,11,13,16	2,3,8,9,10,12,14,15,17	4	P05539,P14408,P61206;P84079;P84082,Q8VD89
8102	1,4,5,6,7,11,13,17	2,3,8,9,10,12,14,15,16	4	P05539,P22282,P61206;P84079;P84082,Q9JI85

8103	1,4,5,6,7,11,14,15	2,3,8,9,10,12,13,16,17	3	P20766,P22006,P61206;P84079;P84082
8104	1,4,5,6,7,11,14,16	2,3,8,9,10,12,13,15,17	2	P20766,P61206;P84079;P84082
8105	1,4,5,6,7,11,14,17	2,3,8,9,10,12,13,15,16	2	P15399,P61206;P84079;P84082
8106	1,4,5,6,7,11,15,16	2,3,8,9,10,12,13,14,17	5	P14408,P20766,P61206;P84079;P84082,Q5FVI6,Q8VD89
8107	1,4,5,6,7,11,15,17	2,3,8,9,10,12,13,14,16	4	P20760,P20766,P22006,P61206;P84079;P84082
8108	1,4,5,6,7,11,16,17	2,3,8,9,10,12,13,14,15	4	A2RUV9,P14408,P20766,P61206;P84079;P84082
8109	1,4,5,6,7,12,13,14	2,3,8,9,10,11,15,16,17	16	P05539,P06911,P07647,P08723,P12020,P22282,P30120,P36374,P83748,Q498D9,Q5GRG2,Q62902,Q6AYR9,Q8CJ52,Q9JHB9,Q9JI85
8110	1,4,5,6,7,12,13,15	2,3,8,9,10,11,14,16,17	7	P05539,P19218,P30120,P83748,Q498D9,Q5GRG2,Q9JHB9
8111	1,4,5,6,7,12,13,16	2,3,8,9,10,11,14,15,17	5	P19218,P30120,P61206;P84079;P84082,P83748,Q8CJ52
8112	1,4,5,6,7,12,13,17	2,3,8,9,10,11,14,15,16	15	P05539,P07647,P13432,P19218,P22282,P22283,P30120,P36374,P83748,Q6AYR9,Q99041,Q99MH3,Q9JHB9,Q9JI85,Q9QZK9
8113	1,4,5,6,7,12,14,15	2,3,8,9,10,11,13,16,17	4	P20766,P35467,P83748,Q498D9
8114	1,4,5,6,7,12,14,16	2,3,8,9,10,11,13,15,17	3	P20766,P61206;P84079;P84082,P83748
8115	1,4,5,6,7,12,14,17	2,3,8,9,10,11,13,15,16	8	P01835,P13432,P20760,P20766,P22282,Q10758,Q99041,Q9QZK9
8116	1,4,5,6,7,12,15,16	2,3,8,9,10,11,13,14,17	3	P20766,P35467,P83748
8117	1,4,5,6,7,12,15,17	2,3,8,9,10,11,13,14,16	7	P13432,P20760,P20766,P83748,Q99041,Q99MH3,Q9QZK9

8118	1,4,5,6,7,12,16,17	2,3,8,9,10,11,13,14,15	6	P09605;P25809,P13432,P20766,P83748,Q10758,Q99041
8119	1,4,5,6,7,13,14,15	2,3,8,9,10,11,12,16,17	6	P05539,P06911,P12020,P16636,Q498D9,Q5GRG2
8120	1,4,5,6,7,13,14,16	2,3,8,9,10,11,12,15,17	4	P06911,P12020,P62804,Q5GRG2
8121	1,4,5,6,7,13,14,17	2,3,8,9,10,11,12,15,16	7	P05539,P06911,P12020,P20760,P22282,Q5GRG2,Q9WUW8
8122	1,4,5,6,7,13,15,16	2,3,8,9,10,11,12,14,17	4	P05539,P62804,Q00715,Q8VD89
8123	1,4,5,6,7,13,15,17	2,3,8,9,10,11,12,14,16	4	P05539,P20760,Q99MH3,Q9WUW8
8124	1,4,5,6,7,13,16,17	2,3,8,9,10,11,12,14,15	1	P62804
8125	1,4,5,6,7,14,15,16	2,3,8,9,10,11,12,13,17	3	P16636,P20766,P35467
8126	1,4,5,6,7,14,15,17	2,3,8,9,10,11,12,13,16	4	P10536,P20760,P20766,P35467
8127	1,4,5,6,7,14,16,17	2,3,8,9,10,11,12,13,15	2	P20766,P35467
8128	1,4,5,6,7,15,16,17	2,3,8,9,10,11,12,13,14	3	P20760,P20766,P35467
8129	1,4,5,6,8,9,10,11	2,3,7,12,13,14,15,16,17	6	A2RUV9,P00714,P08649,P14668,P14669,Q566E6
8130	1,4,5,6,8,9,10,12	2,3,7,11,13,14,15,16,17	12	F1LM93,P07647,P08723,P0C0A9,P30120,Q10758,Q5M8C6,Q6IFW6,Q6IG02,Q6IMF3,Q6P6Q2,Q9WTT6
8131	1,4,5,6,8,9,10,13	2,3,7,11,12,14,15,16,17	0	
8132	1,4,5,6,8,9,10,14	2,3,7,11,12,13,15,16,17	1	Q9WTT6

8133	1,4,5,6,8,9,10,15	2,3,7,11,12,13,14,16,17	1	O55006
8134	1,4,5,6,8,9,10,16	2,3,7,11,12,13,14,15,17	4	A2RUV9,P14668,P23593,Q63942
8135	1,4,5,6,8,9,10,17	2,3,7,11,12,13,14,15,16	6	A2RUV9,P06760,P08649,P23593,Q09030,Q9WTT6
8136	1,4,5,6,8,9,11,12	2,3,7,10,13,14,15,16,17	24	O35547,P02780,P02781,P02782,P02803,P04905,P07647,P08723,P0C0A9,P13432,P14668,P14669,P30120,P36374,Q5M8C6,Q63357,Q6IFU7,Q6IFU8,Q6IFW6,Q6IG02,Q6IMF3,Q6P6Q2,Q8CJ52,Q9JHB9
8137	1,4,5,6,8,9,11,13	2,3,7,10,12,14,15,16,17	6	A2RUV9,P02803,P05539,P07647,P14668,P14669
8138	1,4,5,6,8,9,11,14	2,3,7,10,12,13,15,16,17	4	P02793;Q7TP54,P14668,P14669,P83121
8139	1,4,5,6,8,9,11,15	2,3,7,10,12,13,14,16,17	3	P14668,P14669,Q5I0D1
8140	1,4,5,6,8,9,11,16	2,3,7,10,12,13,14,15,17	3	A2RUV9,P14668,P14669
8141	1,4,5,6,8,9,11,17	2,3,7,10,12,13,14,15,16	7	A2RUV9,P06760,P07150,P08649,P14668,P14669,Q09030
8142	1,4,5,6,8,9,12,13	2,3,7,10,11,14,15,16,17	17	P02780,P02781,P02782,P02803,P06761,P07647,P08723,P0C0A9,P13432,P22282,P22283,P30120,P36374,Q5M8C6,Q6AYR9,Q8CJ52,Q9JHB9
8143	1,4,5,6,8,9,12,14	2,3,7,10,11,13,15,16,17	11	P01835,P02780,P02782,P02793;Q7TP54,P07647,P08723,P0C0A9,P13432,P30120,Q5M8C6,Q9WTT6
8144	1,4,5,6,8,9,12,15	2,3,7,10,11,13,14,16,17	1	Q5M8C6
8145	1,4,5,6,8,9,12,16	2,3,7,10,11,13,14,15,17	6	P14668,P23593,P30120,Q5M8C6,Q6IFW6,Q6IMF3
8146	1,4,5,6,8,9,12,17	2,3,7,10,11,13,14,15,16	16	F1LM93,P01835,P02782,P06760,P07647,P08723,P0C0A9,P13432,P23593,P30120,P36374,Q09030,Q4G075,Q5M8C6,Q9WTT6,Q9Z0V6
8147	1,4,5,6,8,9,13,14	2,3,7,10,11,12,15,16,17	3	P06866,P06911,Q9WTT6

8148	1,4,5,6,8,9,13,15	2,3,7,10,11,12,14,16,17	0	
8149	1,4,5,6,8,9,13,16	2,3,7,10,11,12,14,15,17	1	A2RUV9
8150	1,4,5,6,8,9,13,17	2,3,7,10,11,12,14,15,16	3	P06760,P22282,P23593
8151	1,4,5,6,8,9,14,15	2,3,7,10,11,12,13,16,17	1	P02793;Q7TP54
8152	1,4,5,6,8,9,14,16	2,3,7,10,11,12,13,15,17	3	P02793;Q7TP54,P14668,Q63942
8153	1,4,5,6,8,9,14,17	2,3,7,10,11,12,13,15,16	3	P02793;Q7TP54,P06760,Q9WTT6
8154	1,4,5,6,8,9,15,16	2,3,7,10,11,12,13,14,17	3	P14668,P23593,Q63942
8155	1,4,5,6,8,9,15,17	2,3,7,10,11,12,13,14,16	2	P06760,P23593
8156	1,4,5,6,8,9,16,17	2,3,7,10,11,12,13,14,15	3	A2RUV9,P06760,P23593
8157	1,4,5,6,8,10,11,12	2,3,7,9,13,14,15,16,17	30	F1LM93,O35547,P00714,P02780,P02781,P02782,P04905,P06760,P07150,P07647,P08723,P0C0A9,P14668,P14669,P22283,P30120,P35281,P36374,P47727,Q10758,Q5M8C6,Q63357,Q6IFU7,Q6IFU8,Q6IFW6,Q6IG02,Q6IMF3,Q6P6Q2,Q8CJ52,Q9JHB9
8158	1,4,5,6,8,10,11,13	2,3,7,9,12,14,15,16,17	4	P07647,P14668,P14669,P47727
8159	1,4,5,6,8,10,11,14	2,3,7,9,12,13,15,16,17	8	P02793;Q7TP54,P07150,P08649,P14668,P14669,P47727,P55018,Q5BJY9
8160	1,4,5,6,8,10,11,15	2,3,7,9,12,13,14,16,17	6	P08649,P14668,P14669,P35281,P55018,Q5I0D1
8161	1,4,5,6,8,10,11,16	2,3,7,9,12,13,14,15,17	7	A2RUV9,P07150,P08649,P14668,P14669,P55018,Q6P6Q2
8162	1,4,5,6,8,10,11,17	2,3,7,9,12,13,14,15,16	9	A2RUV9,P04041,P06760,P07150,P08649,P14668,P14669,Q4G075,Q6AYS4

8163	1,4,5,6,8,10,12,13	2,3,7,9,11,14,15,16,17	23	F1LM93,P02780,P02781,P02782,P06761,P06911,P07647,P08723,P0C0A9,P22282,P22283,P30120,P36374,Q10758,Q498D9,Q5M8C6,Q6AYR9,Q6IFW6,Q6IMF3,Q6P6Q2,Q8CJ52,Q99MH3,Q9JHB9
8164	1,4,5,6,8,10,12,14	2,3,7,9,11,13,15,16,17	17	F1LM93,P02780,P02782,P07647,P08723,P0C0A9,P30120,Q10758,Q498D9,Q5M8C6,Q6AYR9,Q6IFW6,Q6IG02,Q6IMF3,Q6P6Q2,Q8CJ52,Q9WTT6
8165	1,4,5,6,8,10,12,15	2,3,7,9,11,13,14,16,17	10	F1LM93,P08723,P35281,Q10758,Q5M8C6,Q6IFW6,Q6IG02,Q6IMF3,Q6P6Q2,Q99MH3
8166	1,4,5,6,8,10,12,16	2,3,7,9,11,13,14,15,17	13	F1LM93,P08721,P08723,P0C0A9,P30120,P35281,Q10758,Q6IFU7,Q6IFW6,Q6IG02,Q6IMF3,Q6P6Q2,Q8CJ52
8167	1,4,5,6,8,10,12,17	2,3,7,9,11,13,14,15,16	22	F1LM93,P01681,P06760,P07647,P08723,P0C0A9,P13432,P16636,P30120,P36374,Q10758,Q4G075,Q5M8C6,Q6AYR9,Q6IFW6,Q6IG02,Q6IMF3,Q6P6Q2,Q99MH3,Q9QZK9,Q9WTT6,Q9Z0V6
8168	1,4,5,6,8,10,13,14	2,3,7,9,11,12,15,16,17	5	P06911,P12020,Q498D9,Q5GRG2,Q9WTT6
8169	1,4,5,6,8,10,13,15	2,3,7,9,11,12,14,16,17	1	Q6IFV1
8170	1,4,5,6,8,10,13,16	2,3,7,9,11,12,14,15,17	0	
8171	1,4,5,6,8,10,13,17	2,3,7,9,11,12,14,15,16	4	P06760,Q4G075,Q6IFV1,Q99MH3
8172	1,4,5,6,8,10,14,15	2,3,7,9,11,12,13,16,17	1	P55018
8173	1,4,5,6,8,10,14,16	2,3,7,9,11,12,13,15,17	1	P55018
8174	1,4,5,6,8,10,14,17	2,3,7,9,11,12,13,15,16	5	P02793;Q7TP54,P06760,Q4G075,Q9WTT6,Q9Z0V6
8175	1,4,5,6,8,10,15,16	2,3,7,9,11,12,13,14,17	1	P55018
8176	1,4,5,6,8,10,15,17	2,3,7,9,11,12,13,14,16	5	P06760,P20760,Q4G075,Q6IFV1,Q99MH3
8177	1,4,5,6,8,10,16,17	2,3,7,9,11,12,13,14,15	6	A2RUV9,P06760,P23593,P25031,P42854,Q4G075

8178	1,4,5,6,8,11,12,13	2,3,7,9,10,14,15,16,17	27	O35547,P02780,P02781,P02782,P02803,P04905,P06761,P07647,P08723,P0C0A9,P14668,P14669,P22282,P22283,P30120,P36374,P47727,Q5M8C6,Q6AYR9,Q6IFU7,Q6IFW6,Q6IG02,Q6IMF3,Q6P6Q2,Q8CJ52,Q9JHB9,Q9JI85
8179	1,4,5,6,8,11,12,14	2,3,7,9,10,13,15,16,17	26	O35547,P02780,P02781,P02782,P02793;Q7TP54,P04905,P07647,P08723,P0C0A9,P13432,P14668,P14669,P22282,P22283,P30120,P36374,P47727,Q5M8C6,Q6IFU7,Q6IFU8,Q6IFW6,Q6IG02,Q6IMF3,Q6P6Q2,Q8CJ52,Q9JHB9
8180	1,4,5,6,8,11,12,15	2,3,7,9,10,13,14,16,17	17	O35547,P02781,P07647,P08723,P0C0A9,P14668,P14669,P30120,Q5I0D1,Q5M8C6,Q6IFU7,Q6IFW6,Q6IG02,Q6IMF3,Q6P6Q2,Q8CJ52,Q9JHB9
8181	1,4,5,6,8,11,12,16	2,3,7,9,10,13,14,15,17	18	O35547,P02781,P07150,P07647,P08723,P0C0A9,P14668,P14669,P30120,Q5M8C6,Q6IFU7,Q6IFU8,Q6IFW6,Q6IG02,Q6IMF3,Q6P6Q2,Q8CJ52,Q9JHB9
8182	1,4,5,6,8,11,12,17	2,3,7,9,10,13,14,15,16	34	F1LM93,O35547,P01681,P02780,P02781,P02782,P04905,P06760,P07150,P07647,P08723,P0C0A9,P13432,P14668,P14669,P22282,P22283,P30120,P36374,Q10758,Q4G075,Q5M8C6,Q6IFU7,Q6IFU8,Q6IFW6,Q6IG02,Q6IMF3,Q6P6Q2,Q8CJ52,Q99041,Q9ESW0,Q9JHB9,Q9QZK9,Q9Z0V6
8183	1,4,5,6,8,11,13,14	2,3,7,9,10,12,15,16,17	9	P02780,P02793;Q7TP54,P06911,P07647,P14668,P14669,P22282,P47727,Q5GRG2
8184	1,4,5,6,8,11,13,15	2,3,7,9,10,12,14,16,17	3	P05539,P14668,P14669
8185	1,4,5,6,8,11,13,16	2,3,7,9,10,12,14,15,17	4	A2RUV9,P14668,P14669,Q8VD89
8186	1,4,5,6,8,11,13,17	2,3,7,9,10,12,14,15,16	8	A2RUV9,P06760,P07150,P07647,P14669,P22282,P22283,Q4G075
8187	1,4,5,6,8,11,14,15	2,3,7,9,10,12,13,16,17	6	P02793;Q7TP54,P07171,P14668,P14669,P97840,Q5I0D1
8188	1,4,5,6,8,11,14,16	2,3,7,9,10,12,13,15,17	4	P02793;Q7TP54,P07150,P14668,P14669
8189	1,4,5,6,8,11,14,17	2,3,7,9,10,12,13,15,16	8	P02793;Q7TP54,P06760,P07150,P14668,P14669,P97840,Q4G075,Q9Z0V6
8190	1,4,5,6,8,11,15,16	2,3,7,9,10,12,13,14,17	4	A2RUV9,P14668,P14669,Q5I0D1
8191	1,4,5,6,8,11,15,17	2,3,7,9,10,12,13,14,16	7	P06760,P07150,P14668,P14669,P97840,Q4G075,Q5I0D1
8192	1,4,5,6,8,11,16,17	2,3,7,9,10,12,13,14,15	6	A2RUV9,P06760,P07150,P14668,P14669,Q4G075

8193	1,4,5,6,8,12,13,14	2,3,7,9,10,11,15,16,17	19	P02780,P02781,P02782,P06761,P06911,P07647,P08723,P0C0A9,P22282,P22283,P30120,P36374,Q498D9,Q5GRG2,Q5M8C6,Q6AYR9,Q8CJ52,Q9JHB9,Q9WTT6
8194	1,4,5,6,8,12,13,15	2,3,7,9,10,11,14,16,17	10	P02782,P06761,P07647,P08723,P0C0A9,P30120,Q5M8C6,Q6IFV1,Q8CJ52,Q9JHB9
8195	1,4,5,6,8,12,13,16	2,3,7,9,10,11,14,15,17	8	P02782,P07647,P08723,P0C0A9,P30120,Q5M8C6,Q8CJ52,Q9JHB9
8196	1,4,5,6,8,12,13,17	2,3,7,9,10,11,14,15,16	23	P02780,P02782,P06760,P06761,P07647,P08723,P0C0A9,P13432,P22282,P22283,P23928,P30120,P36374,Q4G075,Q5M8C6,Q62635,Q6AYR9,Q6IFV1,Q8CJ52,Q99041,Q99MH3,Q9JHB9,Q9QZK9
8197	1,4,5,6,8,12,14,15	2,3,7,9,10,11,13,16,17	2	P08723,P30120
8198	1,4,5,6,8,12,14,16	2,3,7,9,10,11,13,15,17	5	P08723,P30120,Q6IFW6,Q6IMF3,Q8CJ52
8199	1,4,5,6,8,12,14,17	2,3,7,9,10,11,13,15,16	17	P01681,P01835,P02782,P02793;Q7TP54,P06760,P07647,P08723,P0C0A9,P10536,P13432,P22282,P30120,Q10758,Q4G075,Q5M8C6,Q9WTT6,Q9Z0V6
8200	1,4,5,6,8,12,15,16	2,3,7,9,10,11,13,14,17	1	P14668
8201	1,4,5,6,8,12,15,17	2,3,7,9,10,11,13,14,16	12	B0BNN3,P06760,P10536,P13432,P20760,Q4G075,Q5M8C6,Q6IFV1,Q99041,Q99MH3,Q9QZA6,Q9Z0V6
8202	1,4,5,6,8,12,16,17	2,3,7,9,10,11,13,14,15	12	F1LM93,P01681,P06760,P13432,P23593,P23928,Q10758,Q4G075,Q5M8C6,Q6IMF3,Q99041,Q9Z0V6
8203	1,4,5,6,8,13,14,15	2,3,7,9,10,11,12,16,17	4	P06911,P10536,Q498D9,Q5GRG2
8204	1,4,5,6,8,13,14,16	2,3,7,9,10,11,12,15,17	1	P06911
8205	1,4,5,6,8,13,14,17	2,3,7,9,10,11,12,15,16	7	P02793;Q7TP54,P06760,P06911,P10536,P22282,P51907,Q9WTT6
8206	1,4,5,6,8,13,15,16	2,3,7,9,10,11,12,14,17	0	
8207	1,4,5,6,8,13,15,17	2,3,7,9,10,11,12,14,16	6	P06760,P10536,P20760,Q4G075,Q6IFV1,Q99MH3

8208	1,4,5,6,8,13,16,17	2,3,7,9,10,11,12,14,15	5	A2RUV9,P06760,P23928,Q4G075,Q6PCU2
8209	1,4,5,6,8,14,15,16	2,3,7,9,10,11,12,13,17	1	P14668
8210	1,4,5,6,8,14,15,17	2,3,7,9,10,11,12,13,16	9	B0BNN3,P02793;Q7TP54,P06760,P10536,P20760,P47967,P97840,Q4G075,Q9Z0V6
8211	1,4,5,6,8,14,16,17	2,3,7,9,10,11,12,13,15	3	P02793;Q7TP54,P06760,Q6PCU2
8212	1,4,5,6,8,15,16,17	2,3,7,9,10,11,12,13,14	3	P06760,P23593,Q4G075
8213	1,4,5,6,9,10,11,12	2,3,7,8,13,14,15,16,17	12	P0C0A9,P20766,Q08849,Q10758,Q566E6,Q63357,Q6IFU7,Q6IFU8,Q6IFW6,Q6IG02,Q6IMF3,Q6P6Q2
8214	1,4,5,6,9,10,11,13	2,3,7,8,12,14,15,16,17	2	P05539,Q566E6
8215	1,4,5,6,9,10,11,14	2,3,7,8,12,13,15,16,17	1	P83121
8216	1,4,5,6,9,10,11,15	2,3,7,8,12,13,14,16,17	2	P20766,Q566E6
8217	1,4,5,6,9,10,11,16	2,3,7,8,12,13,14,15,17	4	A2RUV9,P20766,Q5FVI6,Q6IFU7
8218	1,4,5,6,9,10,11,17	2,3,7,8,12,13,14,15,16	3	A2RUV9,Q09030,Q566E6
8219	1,4,5,6,9,10,12,13	2,3,7,8,11,14,15,16,17	2	P19218,P36374
8220	1,4,5,6,9,10,12,14	2,3,7,8,11,13,15,16,17	6	P01835,P20766,Q10758,Q6IFU7,Q6IFW6,Q6IMF3
8221	1,4,5,6,9,10,12,15	2,3,7,8,11,13,14,16,17	2	P20766,Q6IMF3
8222	1,4,5,6,9,10,12,16	2,3,7,8,11,13,14,15,17	5	P20766,Q10758,Q6IFU7,Q6IFW6,Q6IMF3

8223	1,4,5,6,9,10,12,17	2,3,7,8,11,13,14,15,16	6	P01835,P13432,P20766,Q09030,Q10758,Q6IMF3
8224	1,4,5,6,9,10,13,14	2,3,7,8,11,12,15,16,17	0	
8225	1,4,5,6,9,10,13,15	2,3,7,8,11,12,14,16,17	0	
8226	1,4,5,6,9,10,13,16	2,3,7,8,11,12,14,15,17	2	P62804,Q00715
8227	1,4,5,6,9,10,13,17	2,3,7,8,11,12,14,15,16	0	
8228	1,4,5,6,9,10,14,15	2,3,7,8,11,12,13,16,17	1	P20766
8229	1,4,5,6,9,10,14,16	2,3,7,8,11,12,13,15,17	1	P20766
8230	1,4,5,6,9,10,14,17	2,3,7,8,11,12,13,15,16	0	
8231	1,4,5,6,9,10,15,16	2,3,7,8,11,12,13,14,17	1	P20766
8232	1,4,5,6,9,10,15,17	2,3,7,8,11,12,13,14,16	1	P20766
8233	1,4,5,6,9,10,16,17	2,3,7,8,11,12,13,14,15	3	A2RUV9,P20766,P23593
8234	1,4,5,6,9,11,12,13	2,3,7,8,10,14,15,16,17	11	P02803,P04905,P05539,P0C0A9,P30120,P36374,Q6IFU7,Q6IFW6,Q6IMF3,Q6P6Q2,Q9JHB9
8235	1,4,5,6,9,11,12,14	2,3,7,8,10,13,15,16,17	9	P01835,P04905,P0C0A9,P30120,Q6IFU7,Q6IFU8,Q6IFW6,Q6IMF3,Q6P6Q2
8236	1,4,5,6,9,11,12,15	2,3,7,8,10,13,14,16,17	5	P20766,Q6IFU7,Q6IFW6,Q6IMF3,Q6P6Q2
8237	1,4,5,6,9,11,12,16	2,3,7,8,10,13,14,15,17	7	P20766,Q08849,Q6IFU7,Q6IFU8,Q6IFW6,Q6IMF3,Q6P6Q2

8238	1,4,5,6,9,11,12,17	2,3,7,8,10,13,14,15,16	10	P01681,P01835,P13432,P36374,Q09030,Q6IFU7,Q6IFU8,Q6IFW6,Q6IMF3,Q6P6Q2
8239	1,4,5,6,9,11,13,14	2,3,7,8,10,12,15,16,17	2	P05539,P83121
8240	1,4,5,6,9,11,13,15	2,3,7,8,10,12,14,16,17	1	P05539
8241	1,4,5,6,9,11,13,16	2,3,7,8,10,12,14,15,17	2	A2RUV9,P05539
8242	1,4,5,6,9,11,13,17	2,3,7,8,10,12,14,15,16	1	P05539
8243	1,4,5,6,9,11,14,15	2,3,7,8,10,12,13,16,17	0	
8244	1,4,5,6,9,11,14,16	2,3,7,8,10,12,13,15,17	2	P14668,P83121
8245	1,4,5,6,9,11,14,17	2,3,7,8,10,12,13,15,16	2	P01835,P83121
8246	1,4,5,6,9,11,15,16	2,3,7,8,10,12,13,14,17	3	A2RUV9,P14668,P20766
8247	1,4,5,6,9,11,15,17	2,3,7,8,10,12,13,14,16	0	
8248	1,4,5,6,9,11,16,17	2,3,7,8,10,12,13,14,15	1	A2RUV9
8249	1,4,5,6,9,12,13,14	2,3,7,8,10,11,15,16,17	1	P01835
8250	1,4,5,6,9,12,13,15	2,3,7,8,10,11,14,16,17	1	P05539
8251	1,4,5,6,9,12,13,16	2,3,7,8,10,11,14,15,17	1	P19218
8252	1,4,5,6,9,12,13,17	2,3,7,8,10,11,14,15,16	4	P01835,P13432,P35280,P36374

8253	1,4,5,6,9,12,14,15	2,3,7,8,10,11,13,16,17	2	P01835,P20766
8254	1,4,5,6,9,12,14,16	2,3,7,8,10,11,13,15,17	2	P01835,P20766
8255	1,4,5,6,9,12,14,17	2,3,7,8,10,11,13,15,16	3	P01835,P13432,Q10758
8256	1,4,5,6,9,12,15,16	2,3,7,8,10,11,13,14,17	1	P20766
8257	1,4,5,6,9,12,15,17	2,3,7,8,10,11,13,14,16	3	P01835,P13432,P20766
8258	1,4,5,6,9,12,16,17	2,3,7,8,10,11,13,14,15	4	P01835,P13432,P20766,P23593
8259	1,4,5,6,9,13,14,15	2,3,7,8,10,11,12,16,17	1	P05539
8260	1,4,5,6,9,13,14,16	2,3,7,8,10,11,12,15,17	1	P62804
8261	1,4,5,6,9,13,14,17	2,3,7,8,10,11,12,15,16	0	
8262	1,4,5,6,9,13,15,16	2,3,7,8,10,11,12,14,17	2	P62804,Q00715
8263	1,4,5,6,9,13,15,17	2,3,7,8,10,11,12,14,16	0	
8264	1,4,5,6,9,13,16,17	2,3,7,8,10,11,12,14,15	3	P23593,P35280,P62804
8265	1,4,5,6,9,14,15,16	2,3,7,8,10,11,12,13,17	1	P20766
8266	1,4,5,6,9,14,15,17	2,3,7,8,10,11,12,13,16	1	P47967
8267	1,4,5,6,9,14,16,17	2,3,7,8,10,11,12,13,15	0	

8268	1,4,5,6,9,15,16,17	2,3,7,8,10,11,12,13,14	3	P20766,P23593,P62804
8269	1,4,5,6,10,11,12,13	2,3,7,8,9,14,15,16,17	12	O35547,P04905,P0C0A9,P30120,P36374,P47727,Q10758,Q6IFU7,Q6IFW6,Q6IMF3,Q6P6Q2,Q9JHB9
8270	1,4,5,6,10,11,12,14	2,3,7,8,9,13,15,16,17	13	O35547,P04905,P0C0A9,P20766,P30120,P47727,Q10758,Q6IFU7,Q6IFU8,Q6IFW6,Q6IG02,Q6IMF3,Q6P6Q2
8271	1,4,5,6,10,11,12,15	2,3,7,8,9,13,14,16,17	11	O35547,P20766,P51907,Q10758,Q566E6,Q6IFU7,Q6IFU8,Q6IFW6,Q6IG02,Q6IMF3,Q6P6Q2
8272	1,4,5,6,10,11,12,16	2,3,7,8,9,13,14,15,17	12	O35547,P06757,P20766,Q08849,Q10758,Q5FVI6,Q6IFU7,Q6IFU8,Q6IFW6,Q6IG02,Q6IMF3,Q6P6Q2
8273	1,4,5,6,10,11,12,17	2,3,7,8,9,13,14,15,16	20	F1LM93,O08815,O35547,P01681,P06757,P0C0A9,P13432,P20766,P36374,Q08849,Q10758,Q566E6,Q6IFU7,Q6IFU8,Q6IFW6,Q6IG02,Q6IMF3,Q6P6Q2,Q99MH3,Q9QZK9
8274	1,4,5,6,10,11,13,14	2,3,7,8,9,12,15,16,17	1	P47727
8275	1,4,5,6,10,11,13,15	2,3,7,8,9,12,14,16,17	0	
8276	1,4,5,6,10,11,13,16	2,3,7,8,9,12,14,15,17	1	A2RUV9
8277	1,4,5,6,10,11,13,17	2,3,7,8,9,12,14,15,16	0	
8278	1,4,5,6,10,11,14,15	2,3,7,8,9,12,13,16,17	1	P20766
8279	1,4,5,6,10,11,14,16	2,3,7,8,9,12,13,15,17	2	P20766,Q6IFU7
8280	1,4,5,6,10,11,14,17	2,3,7,8,9,12,13,15,16	1	P20766
8281	1,4,5,6,10,11,15,16	2,3,7,8,9,12,13,14,17	2	P20766,Q5FVI6
8282	1,4,5,6,10,11,15,17	2,3,7,8,9,12,13,14,16	2	P20766,Q566E6
8283	1,4,5,6,10,11,16,17	2,3,7,8,9,12,13,14,15	2	A2RUV9,P20766
8284	1,4,5,6,10,12,13,14	2,3,7,8,9,11,15,16,17	5	P06911,P30120,Q10758,Q498D9,Q6IMF3
8285	1,4,5,6,10,12,13,15	2,3,7,8,9,11,14,16,17	2	Q498D9,Q99MH3
8286	1,4,5,6,10,12,13,16	2,3,7,8,9,11,14,15,17	4	P19218,Q10758,Q6IFU7,Q6IMF3
8287	1,4,5,6,10,12,13,17	2,3,7,8,9,11,14,15,16	4	P36374,Q10758,Q99MH3,Q9QZK9
8288	1,4,5,6,10,12,14,15	2,3,7,8,9,11,13,16,17	4	P20766,Q10758,Q498D9,Q6IMF3
8289	1,4,5,6,10,12,14,16	2,3,7,8,9,11,13,15,17	6	P20766,Q10758,Q6IFU7,Q6IFW6,Q6IMF3,Q6P6Q2

8290	1,4,5,6,10, 12,14,17	2,3,7,8,9,1 1,13,15,16	6	P01835,P13432,P20766,Q10758,Q6IFW6,Q6IMF3
8291	1,4,5,6,10, 12,15,16	2,3,7,8,9,1 1,13,14,17	5	P20766,Q10758,Q6IFU7,Q6IFW6,Q6IMF3
8292	1,4,5,6,10, 12,15,17	2,3,7,8,9,1 1,13,14,16	3	P20766,Q10758,Q99MH3
8293	1,4,5,6,10, 12,16,17	2,3,7,8,9,1 1,13,14,15	6	P20766,Q10758,Q6IFU7,Q6IFW6,Q6IMF3,Q99MH3
8294	1,4,5,6,10, 13,14,15	2,3,7,8,9,1 1,12,16,17	1	Q498D9
8295	1,4,5,6,10, 13,14,16	2,3,7,8,9,1 1,12,15,17	0	
8296	1,4,5,6,10, 13,14,17	2,3,7,8,9,1 1,12,15,16	0	
8297	1,4,5,6,10, 13,15,16	2,3,7,8,9,1 1,12,14,17	0	
8298	1,4,5,6,10, 13,15,17	2,3,7,8,9,1 1,12,14,16	2	Q6IFV1,Q99MH3
8299	1,4,5,6,10, 13,16,17	2,3,7,8,9,1 1,12,14,15	0	
8300	1,4,5,6,10, 14,15,16	2,3,7,8,9,1 1,12,13,17	2	P20766,P55018
8301	1,4,5,6,10, 14,15,17	2,3,7,8,9,1 1,12,13,16	1	P20766
8302	1,4,5,6,10, 14,16,17	2,3,7,8,9,1 1,12,13,15	1	P20766
8303	1,4,5,6,10, 15,16,17	2,3,7,8,9,1 1,12,13,14	1	P20766
8304	1,4,5,6,11, 12,13,14	2,3,7,8,9,1 0,15,16,17	11	P04905,P05539,P0C0A9,P30120,P36374,P47727,Q6IFU7,Q6IFW6,Q6IMF3,Q6P6Q2,Q9JHB9
8305	1,4,5,6,11, 12,13,15	2,3,7,8,9,1 0,14,16,17	4	P05539,Q6IFU7,Q6IFW6,Q9JHB9
8306	1,4,5,6,11, 12,13,16	2,3,7,8,9,1 0,14,15,17	6	P30120,Q6IFU7,Q6IFW6,Q6IMF3,Q6P6Q2,Q9JHB9
8307	1,4,5,6,11, 12,13,17	2,3,7,8,9,1 0,14,15,16	10	P01681,P0C0A9,P13432,P30120,P36374,Q6IFW6,Q6IMF3,Q6P6Q2,Q9JHB9,Q9QZK9
8308	1,4,5,6,11, 12,14,15	2,3,7,8,9,1 0,13,16,17	5	P20766,Q6IFU7,Q6IFW6,Q6IMF3,Q6P6Q2
8309	1,4,5,6,11, 12,14,16	2,3,7,8,9,1 0,13,15,17	6	P20766,Q6IFU7,Q6IFU8,Q6IFW6,Q6IMF3,Q6P6Q2
8310	1,4,5,6,11, 12,14,17	2,3,7,8,9,1 0,13,15,16	11	P01681,P01835,P04905,P13432,P20766,Q10758,Q6IFU7,Q6IFU8,Q6IFW6,Q6IMF3,Q6P6Q2
8311	1,4,5,6,11, 12,15,16	2,3,7,8,9,1 0,13,14,17	6	P20766,P51907,Q6IFU7,Q6IFW6,Q6IMF3,Q6P6Q2
8312	1,4,5,6,11, 12,15,17	2,3,7,8,9,1 0,13,14,16	6	P13432,P20766,Q6IFU7,Q6IFW6,Q6IMF3,Q6P6Q2

8313	1,4,5,6,11,12,16,17	2,3,7,8,9,10,13,14,15	9	P01681,P13432,P20766,Q10758,Q6IFU7,Q6IFU8,Q6IFW6,Q6IMF3,Q6P6Q2
8314	1,4,5,6,11,13,14,15	2,3,7,8,9,10,12,16,17	1	P05539
8315	1,4,5,6,11,13,14,16	2,3,7,8,9,10,12,15,17	0	
8316	1,4,5,6,11,13,14,17	2,3,7,8,9,10,12,15,16	1	P05539
8317	1,4,5,6,11,13,15,16	2,3,7,8,9,10,12,14,17	0	
8318	1,4,5,6,11,13,15,17	2,3,7,8,9,10,12,14,16	1	P05539
8319	1,4,5,6,11,13,16,17	2,3,7,8,9,10,12,14,15	1	A2RUV9
8320	1,4,5,6,11,14,15,16	2,3,7,8,9,10,12,13,17	2	P14668,P20766
8321	1,4,5,6,11,14,15,17	2,3,7,8,9,10,12,13,16	3	P20766,P47967,P97840
8322	1,4,5,6,11,14,16,17	2,3,7,8,9,10,12,13,15	1	P20766
8323	1,4,5,6,11,15,16,17	2,3,7,8,9,10,12,13,14	2	A2RUV9,P20766
8324	1,4,5,6,12,13,14,15	2,3,7,8,9,10,11,16,17	1	Q498D9
8325	1,4,5,6,12,13,14,16	2,3,7,8,9,10,11,15,17	0	
8326	1,4,5,6,12,13,14,17	2,3,7,8,9,10,11,15,16	4	P01835,P13432,P36374,Q10758
8327	1,4,5,6,12,13,15,16	2,3,7,8,9,10,11,14,17	0	
8328	1,4,5,6,12,13,15,17	2,3,7,8,9,10,11,14,16	3	P13432,Q6IFV1,Q99MH3
8329	1,4,5,6,12,13,16,17	2,3,7,8,9,10,11,14,15	3	P13432,P27213,P35280
8330	1,4,5,6,12,14,15,16	2,3,7,8,9,10,11,13,17	2	P20766,P35467
8331	1,4,5,6,12,14,15,17	2,3,7,8,9,10,11,13,16	4	B0BNN3,P01835,P13432,P20766
8332	1,4,5,6,12,14,16,17	2,3,7,8,9,10,11,13,15	4	P01835,P13432,P20766,Q10758
8333	1,4,5,6,12,15,16,17	2,3,7,8,9,10,11,13,14	2	P13432,P20766
8334	1,4,5,6,13,14,15,16	2,3,7,8,9,10,11,12,17	0	
8335	1,4,5,6,13,14,15,17	2,3,7,8,9,10,11,12,16	1	P47967

8336	1,4,5,6,13,14,16,17	2,3,7,8,9,10,11,12,15	0	
8337	1,4,5,6,13,15,16,17	2,3,7,8,9,10,11,12,14	1	P62804
8338	1,4,5,6,14,15,16,17	2,3,7,8,9,10,11,12,13	2	P20766,P35467
8339	1,4,5,7,8,9,10,11	2,3,6,12,13,14,15,16,17	8	P00714,P07171,P18418,P61206;P84079;P84082,Q561R9,Q5FVI6,Q63617,Q6AYS4
8340	1,4,5,7,8,9,10,12	2,3,6,11,13,14,15,16,17	7	F1LM93,P00714,P02803,P18418,P36374,Q4FZU6,Q8CJ52
8341	1,4,5,7,8,9,10,13	2,3,6,11,12,14,15,16,17	3	P02803,P18418,Q63617
8342	1,4,5,7,8,9,10,14	2,3,6,11,12,13,15,16,17	3	P18418,Q4FZU6,Q63617
8343	1,4,5,7,8,9,10,15	2,3,6,11,12,13,14,16,17	1	P18418
8344	1,4,5,7,8,9,10,16	2,3,6,11,12,13,14,15,17	5	P08721,P18418,Q4FZU6,Q63617,Q64093
8345	1,4,5,7,8,9,10,17	2,3,6,11,12,13,14,15,16	4	P18418,P52590,Q4FZU6,Q63617
8346	1,4,5,7,8,9,11,12	2,3,6,10,13,14,15,16,17	14	P02780,P02781,P02803,P0C0A9,P10715,P18418,P22283,P36374,Q4FZU6,Q63617,Q6AYS4,Q8CJ52,Q99041,Q9ESW0
8347	1,4,5,7,8,9,11,13	2,3,6,10,12,14,15,16,17	6	P02803,P05539,Q4FZU6,Q63617,Q6AYS4,Q8CJ52
8348	1,4,5,7,8,9,11,14	2,3,6,10,12,13,15,16,17	4	P07171,Q4FZU6,Q63617,Q6AYS4
8349	1,4,5,7,8,9,11,15	2,3,6,10,12,13,14,16,17	6	P07171,P10715,P22006,Q561R9,Q63617,Q6AYS4
8350	1,4,5,7,8,9,11,16	2,3,6,10,12,13,14,15,17	6	A2RUV9,P10715,P14668,P61206;P84079;P84082,Q4FZU6,Q63617
8351	1,4,5,7,8,9,11,17	2,3,6,10,12,13,14,15,16	6	P10715,P52590,Q4FZU6,Q63617,Q6AYS4,Q99041

8352	1,4,5,7,8,9,12,13	2,3,6,10,11,14,15,16,17	8	P02780,P02803,P06761,P22283,P36374,Q4FZU6,Q63617,Q8CJ52
8353	1,4,5,7,8,9,12,14	2,3,6,10,11,13,15,16,17	4	P18418,Q4FZU6,Q63617,Q8CJ52
8354	1,4,5,7,8,9,12,15	2,3,6,10,11,13,14,16,17	2	P10715,Q4FZU6
8355	1,4,5,7,8,9,12,16	2,3,6,10,11,13,14,15,17	4	P10715,Q4FZU6,Q63617,Q8CJ52
8356	1,4,5,7,8,9,12,17	2,3,6,10,11,13,14,15,16	9	P10715,P19223,P36374,P52590,Q4FZU6,Q63617,Q8CJ52,Q99041,Q9QZA6
8357	1,4,5,7,8,9,13,14	2,3,6,10,11,12,15,16,17	2	Q4FZU6,Q63617
8358	1,4,5,7,8,9,13,15	2,3,6,10,11,12,14,16,17	2	P05539,Q63617
8359	1,4,5,7,8,9,13,16	2,3,6,10,11,12,14,15,17	4	P62804,Q00715,Q4FZU6,Q63617
8360	1,4,5,7,8,9,13,17	2,3,6,10,11,12,14,15,16	3	P52590,Q4FZU6,Q63617
8361	1,4,5,7,8,9,14,15	2,3,6,10,11,12,13,16,17	4	P07171,P16636,Q4FZU6,Q63617
8362	1,4,5,7,8,9,14,16	2,3,6,10,11,12,13,15,17	2	Q4FZU6,Q63617
8363	1,4,5,7,8,9,14,17	2,3,6,10,11,12,13,15,16	3	P52590,Q4FZU6,Q63617
8364	1,4,5,7,8,9,15,16	2,3,6,10,11,12,13,14,17	3	O54728,P62804,Q4FZU6
8365	1,4,5,7,8,9,15,17	2,3,6,10,11,12,13,14,16	2	P52590,Q4FZU6
8366	1,4,5,7,8,9,16,17	2,3,6,10,11,12,13,14,15	3	P52590,Q4FZU6,Q63617

8367	1,4,5,7,8,10,11,12	2,3,6,9,13,14,15,16,17	20	F1LM93,P00714,P02781,P02803,P0C0A9,P10715,P16391,P18418,P22283,P36374,P61206;P84079;P84082,Q5FVI6,Q63357,Q63493,Q6AYS4,Q6IG02,Q6P6Q2,Q8CJ52,Q99041,Q9ESW0
8368	1,4,5,7,8,10,11,13	2,3,6,9,12,14,15,16,17	7	P00714,P07171,P18418,P61206;P84079;P84082,Q6AYS4,Q8CJ52,Q8VD89
8369	1,4,5,7,8,10,11,14	2,3,6,9,12,13,15,16,17	8	P00714,P07171,P18418,P61206;P84079;P84082,Q5BJY9,Q63617,Q6AYS4,Q8CJ52
8370	1,4,5,7,8,10,11,15	2,3,6,9,12,13,14,16,17	9	P00714,P07171,P18418,P22006,P61206;P84079;P84082,Q561R9,Q5FVI6,Q6AYS4,Q8VD89
8371	1,4,5,7,8,10,11,16	2,3,6,9,12,13,14,15,17	9	P00714,P08721,P18418,P55018,P61206;P84079;P84082,Q5FVI6,Q6AYS4,Q8CJ52,Q8VD89
8372	1,4,5,7,8,10,11,17	2,3,6,9,12,13,14,15,16	6	P00714,P16391,P18418,P61206;P84079;P84082,Q6AYS4,Q99041
8373	1,4,5,7,8,10,12,13	2,3,6,9,11,14,15,16,17	7	F1LM93,P02803,P06761,P18418,P22283,P36374,Q8CJ52
8374	1,4,5,7,8,10,12,14	2,3,6,9,11,13,15,16,17	4	F1LM93,P18418,Q4FZU6,Q8CJ52
8375	1,4,5,7,8,10,12,15	2,3,6,9,11,13,14,16,17	3	F1LM93,P18418,Q8CJ52
8376	1,4,5,7,8,10,12,16	2,3,6,9,11,13,14,15,17	5	F1LM93,P08721,P18418,Q4FZU6,Q8CJ52
8377	1,4,5,7,8,10,12,17	2,3,6,9,11,13,14,15,16	11	F1LM93,P18418,P19223,P36374,Q10758,Q4FZU6,Q4G075,Q8CJ52,Q99041,Q99MH3,Q9QZA6
8378	1,4,5,7,8,10,13,14	2,3,6,9,11,12,15,16,17	4	P06911,P12020,P18418,Q5GRG2
8379	1,4,5,7,8,10,13,15	2,3,6,9,11,12,14,16,17	2	P01835,P18418
8380	1,4,5,7,8,10,13,16	2,3,6,9,11,12,14,15,17	4	P01835,P18418,Q4FZU6,Q8VD89
8381	1,4,5,7,8,10,13,17	2,3,6,9,11,12,14,15,16	2	P18418,Q99MH3

8382	1,4,5,7,8,10,14,15	2,3,6,9,11,12,13,16,17	4	P07171,P18418,P22006,P55018
8383	1,4,5,7,8,10,14,16	2,3,6,9,11,12,13,15,17	4	P08721,P18418,P55018,Q4FZU6
8384	1,4,5,7,8,10,14,17	2,3,6,9,11,12,13,15,16	2	P18418,Q4FZU6
8385	1,4,5,7,8,10,15,16	2,3,6,9,11,12,13,14,17	5	P01835,P08721,P18418,P55018,Q8VD89
8386	1,4,5,7,8,10,15,17	2,3,6,9,11,12,13,14,16	2	P18418,P52590
8387	1,4,5,7,8,10,16,17	2,3,6,9,11,12,13,14,15	4	P08721,P18418,P23928,Q4FZU6
8388	1,4,5,7,8,11,12,13	2,3,6,9,10,14,15,16,17	20	P02780,P02781,P02803,P05539,P06761,P07647,P0C0A9,P10715,P18418,P22282,P22283,P30120,P36374,Q4FZU6,Q6AYS4,Q8CJ52,Q99041,Q9ESW0,Q9JHB9,Q9JI85
8389	1,4,5,7,8,11,12,14	2,3,6,9,10,13,15,16,17	16	P02780,P02781,P07171,P0C0A9,P10715,P18418,P22283,P36374,Q4FZU6,Q63493,Q63617,Q68FS4,Q6AYS4,Q8CJ52,Q99041,Q9ESW0
8390	1,4,5,7,8,11,12,15	2,3,6,9,10,13,14,16,17	6	P07171,P10715,P22006,Q8CJ52,Q99041,Q9ESW0
8391	1,4,5,7,8,11,12,16	2,3,6,9,10,13,14,15,17	7	P10715,P61206;P84079;P84082,Q4FZU6,Q6P6Q2,Q8CJ52,Q99041,Q9ESW0
8392	1,4,5,7,8,11,12,17	2,3,6,9,10,13,14,15,16	15	F1LM93,P10715,P18418,P19223,P22283,P36374,P50115,Q4FZU6,Q4G075,Q63493,Q6AYS4,Q8CJ52,Q99041,Q9ESW0,Q9QZA6
8393	1,4,5,7,8,11,13,14	2,3,6,9,10,12,15,16,17	8	P05539,P06911,P07171,Q4FZU6,Q5GRG2,Q63617,Q6AYS4,Q8CJ52
8394	1,4,5,7,8,11,13,15	2,3,6,9,10,12,14,16,17	4	P05539,P07171,P22006,Q8VD89
8395	1,4,5,7,8,11,13,16	2,3,6,9,10,12,14,15,17	5	P61206;P84079;P84082,Q4FZU6,Q63617,Q8CJ52,Q8VD89
8396	1,4,5,7,8,11,13,17	2,3,6,9,10,12,14,15,16	8	P05539,P22283,P36374,Q4FZU6,Q63617,Q6AYS4,Q8CJ52,Q99041

8397	1,4,5,7,8,11,14,15	2,3,6,9,10,12,13,16,17	4	P07171,P22006,Q4FZU6,Q5I0D1
8398	1,4,5,7,8,11,14,16	2,3,6,9,10,12,13,15,17	6	P07171,P14668,P61206;P84079;P84082,Q4FZU6,Q63617,Q8CJ52
8399	1,4,5,7,8,11,14,17	2,3,6,9,10,12,13,15,16	6	P07171,P97840,Q4FZU6,Q63617,Q6AYS4,Q99041
8400	1,4,5,7,8,11,15,16	2,3,6,9,10,12,13,14,17	5	P07171,P14408,P14668,Q4FZU6,Q8VD89
8401	1,4,5,7,8,11,15,17	2,3,6,9,10,12,13,14,16	6	P07171,P22006,Q6AYS4,Q99041,Q9ESW0,Q9QZA6
8402	1,4,5,7,8,11,16,17	2,3,6,9,10,12,13,14,15	6	A2RUV9,P10715,Q4FZU6,Q63617,Q6AYS4,Q99041
8403	1,4,5,7,8,12,13,14	2,3,6,9,10,11,15,16,17	10	P02780,P06761,P06911,P18418,P22282,P22283,P36374,Q4FZU6,Q5GRG2,Q8CJ52
8404	1,4,5,7,8,12,13,15	2,3,6,9,10,11,14,16,17	5	P06761,P36374,Q4FZU6,Q8CJ52,Q99041
8405	1,4,5,7,8,12,13,16	2,3,6,9,10,11,14,15,17	4	P36374,Q4FZU6,Q8CJ52,Q99041
8406	1,4,5,7,8,12,13,17	2,3,6,9,10,11,14,15,16	12	P06761,P22282,P22283,P23928,P36374,P55091,Q4FZU6,Q4G075,Q8CJ52,Q99041,Q99MH3,Q9QZK9
8407	1,4,5,7,8,12,14,15	2,3,6,9,10,11,13,16,17	4	P07171,P18418,Q4FZU6,Q8CJ52
8408	1,4,5,7,8,12,14,16	2,3,6,9,10,11,13,15,17	4	P08721,Q4FZU6,Q68FS4,Q8CJ52
8409	1,4,5,7,8,12,14,17	2,3,6,9,10,11,13,15,16	9	P06761,P18418,P31430,P36374,Q4FZU6,Q4G075,Q8CJ52,Q99041,Q9QZA6
8410	1,4,5,7,8,12,15,16	2,3,6,9,10,11,13,14,17	4	P10715,Q4FZU6,Q8CJ52,Q99041
8411	1,4,5,7,8,12,15,17	2,3,6,9,10,11,13,14,16	7	P10715,Q4FZU6,Q4G075,Q8CJ52,Q99041,Q9ESW0,Q9QZA6

8412	1,4,5,7,8,12,16,17	2,3,6,9,10,11,13,14,15	7	P10715,P23928,Q4FZU6,Q4G075,Q8CJ52,Q99041,Q9QZA6
8413	1,4,5,7,8,13,14,15	2,3,6,9,10,11,12,16,17	4	P07171,P16636,Q4FZU6,Q5GRG2
8414	1,4,5,7,8,13,14,16	2,3,6,9,10,11,12,15,17	2	Q4FZU6,Q63617
8415	1,4,5,7,8,13,14,17	2,3,6,9,10,11,12,15,16	3	P10536,Q4FZU6,Q63617
8416	1,4,5,7,8,13,15,16	2,3,6,9,10,11,12,14,17	4	P01835,P62804,Q4FZU6,Q8VD89
8417	1,4,5,7,8,13,15,17	2,3,6,9,10,11,12,14,16	2	P10536,Q4FZU6
8418	1,4,5,7,8,13,16,17	2,3,6,9,10,11,12,14,15	3	P23928,Q4FZU6,Q99041
8419	1,4,5,7,8,14,15,16	2,3,6,9,10,11,12,13,17	3	P07171,P16636,Q4FZU6
8420	1,4,5,7,8,14,15,17	2,3,6,9,10,11,12,13,16	4	P07171,P10536,Q4FZU6,Q9QZA6
8421	1,4,5,7,8,14,16,17	2,3,6,9,10,11,12,13,15	3	P23928,Q4FZU6,Q63617
8422	1,4,5,7,8,15,16,17	2,3,6,9,10,11,12,13,14	1	Q4FZU6
8423	1,4,5,7,9,10,11,12	2,3,6,8,13,14,15,16,17	7	P02803,P36374,P51907,P61206;P84079;P84082,Q5FVI6,Q62636,Q64093
8424	1,4,5,7,9,10,11,13	2,3,6,8,12,14,15,16,17	4	P02803,P05539,P61206;P84079;P84082,Q5FVI6
8425	1,4,5,7,9,10,11,14	2,3,6,8,12,13,15,16,17	3	P61206;P84079;P84082,P83121,Q62635
8426	1,4,5,7,9,10,11,15	2,3,6,8,12,13,14,16,17	6	P22006,P51907,Q561R9,Q5FVI6,Q62635,Q64093

8427	1,4,5,7,9,10,11,16	2,3,6,8,12,13,14,15,17	7	P15999,P61206;P84079;P84082,Q5FVI6,Q62635,Q63424,Q64093,Q80W57
8428	1,4,5,7,9,10,11,17	2,3,6,8,12,13,14,15,16	2	P52590,Q64093
8429	1,4,5,7,9,10,12,13	2,3,6,8,11,14,15,16,17	3	P02803,P19218,P36374
8430	1,4,5,7,9,10,12,14	2,3,6,8,11,13,15,16,17	0	
8431	1,4,5,7,9,10,12,15	2,3,6,8,11,13,14,16,17	1	P20766
8432	1,4,5,7,9,10,12,16	2,3,6,8,11,13,14,15,17	3	P20766,Q4FZU6,Q64093
8433	1,4,5,7,9,10,12,17	2,3,6,8,11,13,14,15,16	3	P36374,P52590,Q64093
8434	1,4,5,7,9,10,13,14	2,3,6,8,11,12,15,16,17	0	
8435	1,4,5,7,9,10,13,15	2,3,6,8,11,12,14,16,17	3	P05539,P62804,Q62635
8436	1,4,5,7,9,10,13,16	2,3,6,8,11,12,14,15,17	3	P62804,Q00715,Q62635
8437	1,4,5,7,9,10,13,17	2,3,6,8,11,12,14,15,16	1	P52590
8438	1,4,5,7,9,10,14,15	2,3,6,8,11,12,13,16,17	2	P16636,Q62635
8439	1,4,5,7,9,10,14,16	2,3,6,8,11,12,13,15,17	2	Q4FZU6,Q62635
8440	1,4,5,7,9,10,14,17	2,3,6,8,11,12,13,15,16	1	P52590
8441	1,4,5,7,9,10,15,16	2,3,6,8,11,12,13,14,17	8	O54728,P02782,P20766,P62804,Q00715,Q5FVI6,Q62635,Q64093

8442	1,4,5,7,9,10,15,17	2,3,6,8,11,12,13,14,16	1	P52590
8443	1,4,5,7,9,10,16,17	2,3,6,8,11,12,13,14,15	4	P52590,P62804,Q4FZU6,Q64093
8444	1,4,5,7,9,11,12,13	2,3,6,8,10,14,15,16,17	4	P02803,P05539,P36374,Q4FZU6
8445	1,4,5,7,9,11,12,14	2,3,6,8,10,13,15,16,17	2	P36374,Q4FZU6
8446	1,4,5,7,9,11,12,15	2,3,6,8,10,13,14,16,17	2	P51907,Q9ESW0
8447	1,4,5,7,9,11,12,16	2,3,6,8,10,13,14,15,17	6	P14408,P15999,P20766,P51907,P61206;P84079;P84082,Q4FZU6
8448	1,4,5,7,9,11,12,17	2,3,6,8,10,13,14,15,16	3	P36374,Q4FZU6,Q9ESW0
8449	1,4,5,7,9,11,13,14	2,3,6,8,10,12,15,16,17	3	P05539,P83121,Q4FZU6
8450	1,4,5,7,9,11,13,15	2,3,6,8,10,12,14,16,17	4	O54728,P05539,Q561R9,Q6P6R2
8451	1,4,5,7,9,11,13,16	2,3,6,8,10,12,14,15,17	5	P05539,P14408,P62804,Q4FZU6,Q62635
8452	1,4,5,7,9,11,13,17	2,3,6,8,10,12,14,15,16	2	P05539,Q4FZU6
8453	1,4,5,7,9,11,14,15	2,3,6,8,10,12,13,16,17	5	O54728,P16636,P22006,P51907,Q62635
8454	1,4,5,7,9,11,14,16	2,3,6,8,10,12,13,15,17	2	Q4FZU6,Q62635
8455	1,4,5,7,9,11,14,17	2,3,6,8,10,12,13,15,16	1	Q4FZU6
8456	1,4,5,7,9,11,15,16	2,3,6,8,10,12,13,14,17	5	O54728,P14408,P51907,Q62635,Q8K4G9

8457	1,4,5,7,9,11,15,17	2,3,6,8,10,12,13,14,16	0	
8458	1,4,5,7,9,11,16,17	2,3,6,8,10,12,13,14,15	4	P14408,P15999,Q4FZU6,Q64093
8459	1,4,5,7,9,12,13,14	2,3,6,8,10,11,15,16,17	3	P05539,P36374,Q4FZU6
8460	1,4,5,7,9,12,13,15	2,3,6,8,10,11,14,16,17	1	P05539
8461	1,4,5,7,9,12,13,16	2,3,6,8,10,11,14,15,17	3	P19218,P62804,Q4FZU6
8462	1,4,5,7,9,12,13,17	2,3,6,8,10,11,14,15,16	4	P05539,P35280,P36374,Q4FZU6
8463	1,4,5,7,9,12,14,15	2,3,6,8,10,11,13,16,17	3	P16636,P20766,Q4FZU6
8464	1,4,5,7,9,12,14,16	2,3,6,8,10,11,13,15,17	2	P20766,Q4FZU6
8465	1,4,5,7,9,12,14,17	2,3,6,8,10,11,13,15,16	1	Q4FZU6
8466	1,4,5,7,9,12,15,16	2,3,6,8,10,11,13,14,17	2	P20766,Q4FZU6
8467	1,4,5,7,9,12,15,17	2,3,6,8,10,11,13,14,16	1	Q4FZU6
8468	1,4,5,7,9,12,16,17	2,3,6,8,10,11,13,14,15	1	Q4FZU6
8469	1,4,5,7,9,13,14,15	2,3,6,8,10,11,12,16,17	4	O54728,P05539,P16636,P62804
8470	1,4,5,7,9,13,14,16	2,3,6,8,10,11,12,15,17	4	P62804,Q00715,Q4FZU6,Q62635
8471	1,4,5,7,9,13,14,17	2,3,6,8,10,11,12,15,16	2	P05539,Q4FZU6

8472	1,4,5,7,9,13,15,16	2,3,6,8,10,11,12,14,17	5	O54728,P05539,P62804,Q00715,Q62635
8473	1,4,5,7,9,13,15,17	2,3,6,8,10,11,12,14,16	2	P05539,P62804
8474	1,4,5,7,9,13,16,17	2,3,6,8,10,11,12,14,15	4	P35280,P62804,Q00715,Q4FZU6
8475	1,4,5,7,9,14,15,16	2,3,6,8,10,11,12,13,17	7	O54728,P16636,P62804,Q00715,Q4FZU6,Q62635,Q9QZK9
8476	1,4,5,7,9,14,15,17	2,3,6,8,10,11,12,13,16	2	P16636,Q4FZU6
8477	1,4,5,7,9,14,16,17	2,3,6,8,10,11,12,13,15	2	P62804,Q4FZU6
8478	1,4,5,7,9,15,16,17	2,3,6,8,10,11,12,13,14	4	P47727,P62804,Q00715,Q4FZU6
8479	1,4,5,7,10,11,12,13	2,3,6,8,9,14,15,16,17	6	P02803,P05539,P36374,P61206;P84079;P84082,Q5FVI6,Q5QE79
8480	1,4,5,7,10,11,12,14	2,3,6,8,9,13,15,16,17	7	P20766,P36374,P51907,P61206;P84079;P84082,Q5QE79,Q6P6Q2,Q9ESW0
8481	1,4,5,7,10,11,12,15	2,3,6,8,9,13,14,16,17	5	P20766,P22006,P51907,Q5FVI6,Q9ESW0
8482	1,4,5,7,10,11,12,16	2,3,6,8,9,13,14,15,17	9	P06757,P14408,P20766,P51907,P61206;P84079;P84082,Q5FVI6,Q63424,Q6IFU7,Q6P6Q2
8483	1,4,5,7,10,11,12,17	2,3,6,8,9,13,14,15,16	5	P06757,P20766,P36374,Q10758,Q9ESW0
8484	1,4,5,7,10,11,13,14	2,3,6,8,9,12,15,16,17	2	P05539,Q5QE79
8485	1,4,5,7,10,11,13,15	2,3,6,8,9,12,14,16,17	5	P05539,P22006,Q561R9,Q5FVI6,Q8VD89
8486	1,4,5,7,10,11,13,16	2,3,6,8,9,12,14,15,17	4	P14408,P61206;P84079;P84082,Q5FVI6,Q8VD89
8487	1,4,5,7,10,11,13,17	2,3,6,8,9,12,14,15,16	1	P05539
8488	1,4,5,7,10,11,14,15	2,3,6,8,9,12,13,16,17	6	P16636,P20766,P22006,P51907,Q5FVI6,Q62635
8489	1,4,5,7,10,11,14,16	2,3,6,8,9,12,13,15,17	5	P14408,P20766,P61206;P84079;P84082,Q5FVI6,Q62635
8490	1,4,5,7,10,11,14,17	2,3,6,8,9,12,13,15,16	0	

8491	1,4,5,7,10,<br>11,15,16	2,3,6,8,9,1<br>2,13,14,17	9	P14408,P20766,P51907,P61206;P84079;P84082,Q5FVI6,Q62635,Q63424,Q8VD89,Q9Z1F2
8492	1,4,5,7,10,<br>11,15,17	2,3,6,8,9,1<br>2,13,14,16	4	P14408,P20766,P22006,Q5FVI6
8493	1,4,5,7,10,<br>11,16,17	2,3,6,8,9,1<br>2,13,14,15	4	P14408,P20766,P61206;P84079;P84082,Q5FVI6
8494	1,4,5,7,10,<br>12,13,14	2,3,6,8,9,1<br>1,15,16,17	3	P36374,Q498D9,Q5QE79
8495	1,4,5,7,10,<br>12,13,15	2,3,6,8,9,1<br>1,14,16,17	0	
8496	1,4,5,7,10,<br>12,13,16	2,3,6,8,9,1<br>1,14,15,17	1	P19218
8497	1,4,5,7,10,<br>12,13,17	2,3,6,8,9,1<br>1,14,15,16	3	P36374,Q99MH3,Q9QZK9
8498	1,4,5,7,10,<br>12,14,15	2,3,6,8,9,1<br>1,13,16,17	1	P20766
8499	1,4,5,7,10,<br>12,14,16	2,3,6,8,9,1<br>1,13,15,17	3	P08721,P20766,Q4FZU6
8500	1,4,5,7,10,<br>12,14,17	2,3,6,8,9,1<br>1,13,15,16	3	P20766,Q10758,Q4FZU6
8501	1,4,5,7,10,<br>12,15,16	2,3,6,8,9,1<br>1,13,14,17	3	P08721,P20766,Q5FVI6
8502	1,4,5,7,10,<br>12,15,17	2,3,6,8,9,1<br>1,13,14,16	2	P20766,Q99MH3
8503	1,4,5,7,10,<br>12,16,17	2,3,6,8,9,1<br>1,13,14,15	4	P08721,P20766,Q10758,Q4FZU6
8504	1,4,5,7,10,<br>13,14,15	2,3,6,8,9,1<br>1,12,16,17	1	P16636
8505	1,4,5,7,10,<br>13,14,16	2,3,6,8,9,1<br>1,12,15,17	0	
8506	1,4,5,7,10,<br>13,14,17	2,3,6,8,9,1<br>1,12,15,16	0	
8507	1,4,5,7,10,<br>13,15,16	2,3,6,8,9,1<br>1,12,14,17	4	P01835,P62804,Q00715,Q8VD89
8508	1,4,5,7,10,<br>13,15,17	2,3,6,8,9,1<br>1,12,14,16	1	Q99MH3
8509	1,4,5,7,10,<br>13,16,17	2,3,6,8,9,1<br>1,12,14,15	1	P62804
8510	1,4,5,7,10,<br>14,15,16	2,3,6,8,9,1<br>1,12,13,17	4	P08721,P16636,P20766,Q62635
8511	1,4,5,7,10,<br>14,15,17	2,3,6,8,9,1<br>1,12,13,16	1	P20766
8512	1,4,5,7,10,<br>14,16,17	2,3,6,8,9,1<br>1,12,13,15	2	P20766,Q4FZU6
8513	1,4,5,7,10,<br>15,16,17	2,3,6,8,9,1<br>1,12,13,14	3	P02782,P20766,Q9Z1F2

8514	1,4,5,7,11,12,13,14	2,3,6,8,9,10,15,16,17	5	P05539,P14046,P36374,Q4FZU6,Q6P6R2
8515	1,4,5,7,11,12,13,15	2,3,6,8,9,10,14,16,17	4	P05539,P14408,P36374,Q6P6R2
8516	1,4,5,7,11,12,13,16	2,3,6,8,9,10,14,15,17	5	P05539,P14408,P17046,P36374,Q4FZU6
8517	1,4,5,7,11,12,13,17	2,3,6,8,9,10,14,15,16	4	P05539,P36374,Q4FZU6,Q9ESW0
8518	1,4,5,7,11,12,14,15	2,3,6,8,9,10,13,16,17	5	P20766,P22006,P51907,Q6P6R2,Q9ESW0
8519	1,4,5,7,11,12,14,16	2,3,6,8,9,10,13,15,17	4	P14408,P20766,P51907,Q4FZU6
8520	1,4,5,7,11,12,14,17	2,3,6,8,9,10,13,15,16	4	P20766,P36374,Q4FZU6,Q9ESW0
8521	1,4,5,7,11,12,15,16	2,3,6,8,9,10,13,14,17	4	P14408,P20766,P51907,Q9ESW0
8522	1,4,5,7,11,12,15,17	2,3,6,8,9,10,13,14,16	3	P14408,P20766,Q9ESW0
8523	1,4,5,7,11,12,16,17	2,3,6,8,9,10,13,14,15	5	P14408,P17046,P20766,Q4FZU6,Q9ESW0
8524	1,4,5,7,11,13,14,15	2,3,6,8,9,10,12,16,17	4	P05539,P16636,P22006,Q6P6R2
8525	1,4,5,7,11,13,14,16	2,3,6,8,9,10,12,15,17	3	P05539,P14408,Q4FZU6
8526	1,4,5,7,11,13,14,17	2,3,6,8,9,10,12,15,16	2	P05539,Q4FZU6
8527	1,4,5,7,11,13,15,16	2,3,6,8,9,10,12,14,17	4	P05539,P14408,Q6P6R2,Q8VD89
8528	1,4,5,7,11,13,15,17	2,3,6,8,9,10,12,14,16	2	P05539,P14408
8529	1,4,5,7,11,13,16,17	2,3,6,8,9,10,12,14,15	2	P14408,Q4FZU6
8530	1,4,5,7,11,14,15,16	2,3,6,8,9,10,12,13,17	7	P14408,P16636,P20766,P51907,Q4FZU6,Q62635,Q63355
8531	1,4,5,7,11,14,15,17	2,3,6,8,9,10,12,13,16	1	P22006
8532	1,4,5,7,11,14,16,17	2,3,6,8,9,10,12,13,15	3	P14408,P20766,Q4FZU6
8533	1,4,5,7,11,15,16,17	2,3,6,8,9,10,12,13,14	2	P14408,P20766
8534	1,4,5,7,12,13,14,15	2,3,6,8,9,10,11,16,17	4	P05539,P16636,Q4FZU6,Q6P6R2
8535	1,4,5,7,12,13,14,16	2,3,6,8,9,10,11,15,17	1	Q4FZU6
8536	1,4,5,7,12,13,14,17	2,3,6,8,9,10,11,15,16	2	P36374,Q4FZU6

8537	1,4,5,7,12,13,15,16	2,3,6,8,9,10,11,14,17	1	Q4FZU6
8538	1,4,5,7,12,13,15,17	2,3,6,8,9,10,11,14,16	3	P05539,P36374,Q99MH3
8539	1,4,5,7,12,13,16,17	2,3,6,8,9,10,11,14,15	3	P35280,P36374,Q4FZU6
8540	1,4,5,7,12,14,15,16	2,3,6,8,9,10,11,13,17	4	P16636,P20766,P35467,Q4FZU6
8541	1,4,5,7,12,14,15,17	2,3,6,8,9,10,11,13,16	2	P20766,Q4FZU6
8542	1,4,5,7,12,14,16,17	2,3,6,8,9,10,11,13,15	2	P20766,Q4FZU6
8543	1,4,5,7,12,15,16,17	2,3,6,8,9,10,11,13,14	2	P20766,Q4FZU6
8544	1,4,5,7,13,14,15,16	2,3,6,8,9,10,11,12,17	5	P16636,P35467,P62804,Q00715,Q4FZU6
8545	1,4,5,7,13,14,15,17	2,3,6,8,9,10,11,12,16	5	P05539,P11883,P16636,Q4FZU6,Q9WUW8
8546	1,4,5,7,13,14,16,17	2,3,6,8,9,10,11,12,15	2	P62804,Q4FZU6
8547	1,4,5,7,13,15,16,17	2,3,6,8,9,10,11,12,14	4	P11883,P62804,Q00715,Q4FZU6
8548	1,4,5,7,14,15,16,17	2,3,6,8,9,10,11,12,13	4	P16636,P20766,P35467,Q4FZU6
8549	1,4,5,8,9,10,11,12	2,3,6,7,13,14,15,16,17	7	P00714,P02803,P0C0A9,P36374,Q6AYS4,Q6IG02,Q6P6Q2
8550	1,4,5,8,9,10,11,13	2,3,6,7,12,14,15,16,17	3	P02803,Q561R9,Q6AYS4
8551	1,4,5,8,9,10,11,14	2,3,6,7,12,13,15,16,17	2	P00714,Q6AYS4
8552	1,4,5,8,9,10,11,15	2,3,6,7,12,13,14,16,17	1	Q561R9
8553	1,4,5,8,9,10,11,16	2,3,6,7,12,13,14,15,17	4	A2RUV9,P02803,P14668,Q62635
8554	1,4,5,8,9,10,11,17	2,3,6,7,12,13,14,15,16	4	A2RUV9,P08649,Q63532,Q6AYS4
8555	1,4,5,8,9,10,12,13	2,3,6,7,11,14,15,16,17	2	P02803,P36374

8556	1,4,5,8,9,10,12,14	2,3,6,7,11,13,15,16,17	0	
8557	1,4,5,8,9,10,12,15	2,3,6,7,11,13,14,16,17	1	P10758
8558	1,4,5,8,9,10,12,16	2,3,6,7,11,13,14,15,17	2	P02803,Q9WUW8
8559	1,4,5,8,9,10,12,17	2,3,6,7,11,13,14,15,16	2	P02803,P36374
8560	1,4,5,8,9,10,13,14	2,3,6,7,11,12,15,16,17	1	P09605;P25809
8561	1,4,5,8,9,10,13,15	2,3,6,7,11,12,14,16,17	0	
8562	1,4,5,8,9,10,13,16	2,3,6,7,11,12,14,15,17	1	P02803
8563	1,4,5,8,9,10,13,17	2,3,6,7,11,12,14,15,16	2	P02803,Q63532
8564	1,4,5,8,9,10,14,15	2,3,6,7,11,12,13,16,17	1	P09605;P25809
8565	1,4,5,8,9,10,14,16	2,3,6,7,11,12,13,15,17	1	Q9QZK9
8566	1,4,5,8,9,10,14,17	2,3,6,7,11,12,13,15,16	0	
8567	1,4,5,8,9,10,15,16	2,3,6,7,11,12,13,14,17	2	Q62635,Q9QZK9
8568	1,4,5,8,9,10,15,17	2,3,6,7,11,12,13,14,16	1	Q63532
8569	1,4,5,8,9,10,16,17	2,3,6,7,11,12,13,14,15	2	A2RUV9,P25031
8570	1,4,5,8,9,11,12,13	2,3,6,7,10,14,15,16,17	3	P02803,P0C0A9,P36374

8571	1,4,5,8,9,11,12,14	2,3,6,7,10,13,15,16,17	2	P02803,Q4FZU6
8572	1,4,5,8,9,11,12,15	2,3,6,7,10,13,14,16,17	1	P10758
8573	1,4,5,8,9,11,12,16	2,3,6,7,10,13,14,15,17	4	P02803,P14668,Q4FZU6,Q6P6Q2
8574	1,4,5,8,9,11,12,17	2,3,6,7,10,13,14,15,16	4	P02803,P36374,Q4FZU6,Q6AYS4
8575	1,4,5,8,9,11,13,14	2,3,6,7,10,12,15,16,17	2	P02803,P97840
8576	1,4,5,8,9,11,13,15	2,3,6,7,10,12,14,16,17	2	P97840,Q561R9
8577	1,4,5,8,9,11,13,16	2,3,6,7,10,12,14,15,17	2	A2RUV9,P02803
8578	1,4,5,8,9,11,13,17	2,3,6,7,10,12,14,15,16	4	P02803,P97840,Q63532,Q6AYS4
8579	1,4,5,8,9,11,14,15	2,3,6,7,10,12,13,16,17	1	P97840
8580	1,4,5,8,9,11,14,16	2,3,6,7,10,12,13,15,17	3	P14668,Q4FZU6,Q9QZK9
8581	1,4,5,8,9,11,14,17	2,3,6,7,10,12,13,15,16	4	P47967,P97840,Q4FZU6,Q6AYS4
8582	1,4,5,8,9,11,15,16	2,3,6,7,10,12,13,14,17	6	A2RUV9,O54728,P14668,Q561R9,Q62635,Q9QZK9
8583	1,4,5,8,9,11,15,17	2,3,6,7,10,12,13,14,16	3	P47967,P97840,Q63532
8584	1,4,5,8,9,11,16,17	2,3,6,7,10,12,13,14,15	2	A2RUV9,Q4FZU6
8585	1,4,5,8,9,12,13,14	2,3,6,7,10,11,15,16,17	3	P02803,P36374,Q4FZU6

8586	1,4,5,8,9,12,13,15	2,3,6,7,10,11,14,16,17	1	P10758
8587	1,4,5,8,9,12,13,16	2,3,6,7,10,11,14,15,17	2	P02803,Q4FZU6
8588	1,4,5,8,9,12,13,17	2,3,6,7,10,11,14,15,16	5	P02803,P35280,P36374,Q4FZU6,Q63532
8589	1,4,5,8,9,12,14,15	2,3,6,7,10,11,13,16,17	1	P10758
8590	1,4,5,8,9,12,14,16	2,3,6,7,10,11,13,15,17	1	Q4FZU6
8591	1,4,5,8,9,12,14,17	2,3,6,7,10,11,13,15,16	1	Q4FZU6
8592	1,4,5,8,9,12,15,16	2,3,6,7,10,11,13,14,17	0	
8593	1,4,5,8,9,12,15,17	2,3,6,7,10,11,13,14,16	1	Q63532
8594	1,4,5,8,9,12,16,17	2,3,6,7,10,11,13,14,15	1	Q4FZU6
8595	1,4,5,8,9,13,14,15	2,3,6,7,10,11,12,16,17	0	
8596	1,4,5,8,9,13,14,16	2,3,6,7,10,11,12,15,17	1	Q4FZU6
8597	1,4,5,8,9,13,14,17	2,3,6,7,10,11,12,15,16	3	P47967,P97840,Q4FZU6
8598	1,4,5,8,9,13,15,16	2,3,6,7,10,11,12,14,17	2	O54728,P62804
8599	1,4,5,8,9,13,15,17	2,3,6,7,10,11,12,14,16	3	P47967,P97840,Q63532
8600	1,4,5,8,9,13,16,17	2,3,6,7,10,11,12,14,15	2	P35280,Q4FZU6

8601	1,4,5,8,9,14,15,16	2,3,6,7,10,11,12,13,17	3	O54728,Q4FZU6,Q9QZK9
8602	1,4,5,8,9,14,15,17	2,3,6,7,10,11,12,13,16	2	P47967,P97840
8603	1,4,5,8,9,14,16,17	2,3,6,7,10,11,12,13,15	1	Q4FZU6
8604	1,4,5,8,9,15,16,17	2,3,6,7,10,11,12,13,14	1	P47727
8605	1,4,5,8,10,11,12,13	2,3,6,7,9,14,15,16,17	7	P02781,P02803,P0C0A9,P36374,Q6IG02,Q6P6Q2,Q8CJ52
8606	1,4,5,8,10,11,12,14	2,3,6,7,9,13,15,16,17	5	P00714,P0C0A9,Q6IFW6,Q6IG02,Q6P6Q2
8607	1,4,5,8,10,11,12,15	2,3,6,7,9,13,14,16,17	4	P51907,Q561R9,Q6IG02,Q6P6Q2
8608	1,4,5,8,10,11,12,16	2,3,6,7,9,13,14,15,17	7	P02803,P53792,Q6IFU7,Q6IFW6,Q6IG02,Q6IMF3,Q6P6Q2
8609	1,4,5,8,10,11,12,17	2,3,6,7,9,13,14,15,16	7	F1LM93,P36374,Q10758,Q4G075,Q6AYS4,Q6IG02,Q6P6Q2
8610	1,4,5,8,10,11,13,14	2,3,6,7,9,12,15,16,17	1	P09605;P25809
8611	1,4,5,8,10,11,13,15	2,3,6,7,9,12,14,16,17	2	P09605;P25809,Q561R9
8612	1,4,5,8,10,11,13,16	2,3,6,7,9,12,14,15,17	2	A2RUV9,P02803
8613	1,4,5,8,10,11,13,17	2,3,6,7,9,12,14,15,16	1	Q6AYS4
8614	1,4,5,8,10,11,14,15	2,3,6,7,9,12,13,16,17	1	P09605;P25809
8615	1,4,5,8,10,11,14,16	2,3,6,7,9,12,13,15,17	1	P14668
8616	1,4,5,8,10,11,14,17	2,3,6,7,9,12,13,15,16	1	Q6AYS4
8617	1,4,5,8,10,11,15,16	2,3,6,7,9,12,13,14,17	2	P14668,Q561R9
8618	1,4,5,8,10,11,15,17	2,3,6,7,9,12,13,14,16	1	Q6AYS4
8619	1,4,5,8,10,11,16,17	2,3,6,7,9,12,13,14,15	1	A2RUV9
8620	1,4,5,8,10,12,13,14	2,3,6,7,9,11,15,16,17	3	P09605;P25809,P36374,Q498D9
8621	1,4,5,8,10,12,13,15	2,3,6,7,9,11,14,16,17	1	P10758

8622	1,4,5,8,10,<br>12,13,16	2,3,6,7,9,1<br>1,14,15,17	1	P02803
8623	1,4,5,8,10,<br>12,13,17	2,3,6,7,9,1<br>1,14,15,16	3	P02803,P36374,Q99MH3
8624	1,4,5,8,10,<br>12,14,15	2,3,6,7,9,1<br>1,13,16,17	1	P09605;P25809
8625	1,4,5,8,10,<br>12,14,16	2,3,6,7,9,1<br>1,13,15,17	1	P08721
8626	1,4,5,8,10,<br>12,14,17	2,3,6,7,9,1<br>1,13,15,16	1	Q10758
8627	1,4,5,8,10,<br>12,15,16	2,3,6,7,9,1<br>1,13,14,17	1	P08721
8628	1,4,5,8,10,<br>12,15,17	2,3,6,7,9,1<br>1,13,14,16	1	Q99MH3
8629	1,4,5,8,10,<br>12,16,17	2,3,6,7,9,1<br>1,13,14,15	2	P08721,Q10758
8630	1,4,5,8,10,<br>13,14,15	2,3,6,7,9,1<br>1,12,16,17	1	P09605;P25809
8631	1,4,5,8,10,<br>13,14,16	2,3,6,7,9,1<br>1,12,15,17	1	P09605;P25809
8632	1,4,5,8,10,<br>13,14,17	2,3,6,7,9,1<br>1,12,15,16	0	
8633	1,4,5,8,10,<br>13,15,16	2,3,6,7,9,1<br>1,12,14,17	2	P01835,P09605;P25809
8634	1,4,5,8,10,<br>13,15,17	2,3,6,7,9,1<br>1,12,14,16	0	
8635	1,4,5,8,10,<br>13,16,17	2,3,6,7,9,1<br>1,12,14,15	1	P25031
8636	1,4,5,8,10,<br>14,15,16	2,3,6,7,9,1<br>1,12,13,17	3	P08721,P09605;P25809,P55018
8637	1,4,5,8,10,<br>14,15,17	2,3,6,7,9,1<br>1,12,13,16	0	
8638	1,4,5,8,10,<br>14,16,17	2,3,6,7,9,1<br>1,12,13,15	0	
8639	1,4,5,8,10,<br>15,16,17	2,3,6,7,9,1<br>1,12,13,14	0	
8640	1,4,5,8,11,<br>12,13,14	2,3,6,7,9,1<br>0,15,16,17	5	P02803,P0C0A9,P36374,Q4FZU6,Q8CJ52
8641	1,4,5,8,11,<br>12,13,15	2,3,6,7,9,1<br>0,14,16,17	1	P36374
8642	1,4,5,8,11,<br>12,13,16	2,3,6,7,9,1<br>0,14,15,17	5	P02803,P36374,Q4FZU6,Q6P6Q2,Q8CJ52
8643	1,4,5,8,11,<br>12,13,17	2,3,6,7,9,1<br>0,14,15,16	2	P02803,P36374
8644	1,4,5,8,11,<br>12,14,15	2,3,6,7,9,1<br>0,13,16,17	2	Q5I0D1,Q9ESW0

8645	1,4,5,8,11,<br>12,14,16	2,3,6,7,9,1<br>0,13,15,17	3	P14668,Q4FZU6,Q6P6Q2
8646	1,4,5,8,11,<br>12,14,17	2,3,6,7,9,1<br>0,13,15,16	5	P36374,P97840,Q4FZU6,Q6AYS4,Q9ESW0
8647	1,4,5,8,11,<br>12,15,16	2,3,6,7,9,1<br>0,13,14,17	3	P14668,P51907,Q6P6Q2
8648	1,4,5,8,11,<br>12,15,17	2,3,6,7,9,1<br>0,13,14,16	2	Q9ESW0,Q9QZA6
8649	1,4,5,8,11,<br>12,16,17	2,3,6,7,9,1<br>0,13,14,15	2	Q4FZU6,Q6P6Q2
8650	1,4,5,8,11,<br>13,14,15	2,3,6,7,9,1<br>0,12,16,17	3	P09605;P25809,P97840,Q6P6R2
8651	1,4,5,8,11,<br>13,14,16	2,3,6,7,9,1<br>0,12,15,17	1	Q4FZU6
8652	1,4,5,8,11,<br>13,14,17	2,3,6,7,9,1<br>0,12,15,16	2	P97840,Q4FZU6
8653	1,4,5,8,11,<br>13,15,16	2,3,6,7,9,1<br>0,12,14,17	0	
8654	1,4,5,8,11,<br>13,15,17	2,3,6,7,9,1<br>0,12,14,16	2	P97840,Q63532
8655	1,4,5,8,11,<br>13,16,17	2,3,6,7,9,1<br>0,12,14,15	3	A2RUV9,P97840,Q4FZU6
8656	1,4,5,8,11,<br>14,15,16	2,3,6,7,9,1<br>0,12,13,17	3	P14668,P97840,Q5I0D1
8657	1,4,5,8,11,<br>14,15,17	2,3,6,7,9,1<br>0,12,13,16	2	P47967,P97840
8658	1,4,5,8,11,<br>14,16,17	2,3,6,7,9,1<br>0,12,13,15	2	P97840,Q4FZU6
8659	1,4,5,8,11,<br>15,16,17	2,3,6,7,9,1<br>0,12,13,14	2	A2RUV9,P97840
8660	1,4,5,8,12,<br>13,14,15	2,3,6,7,9,1<br>0,11,16,17	2	P10758,Q9QUL6
8661	1,4,5,8,12,<br>13,14,16	2,3,6,7,9,1<br>0,11,15,17	2	Q4FZU6,Q9QUL6
8662	1,4,5,8,12,<br>13,14,17	2,3,6,7,9,1<br>0,11,15,16	2	P36374,Q4FZU6
8663	1,4,5,8,12,<br>13,15,16	2,3,6,7,9,1<br>0,11,14,17	1	Q9QUL6
8664	1,4,5,8,12,<br>13,15,17	2,3,6,7,9,1<br>0,11,14,16	0	
8665	1,4,5,8,12,<br>13,16,17	2,3,6,7,9,1<br>0,11,14,15	4	P23928,P27213,P35280,Q4FZU6
8666	1,4,5,8,12,<br>14,15,16	2,3,6,7,9,1<br>0,11,13,17	1	Q4FZU6
8667	1,4,5,8,12,<br>14,15,17	2,3,6,7,9,1<br>0,11,13,16	1	Q9QZA6

8668	1,4,5,8,12,<br>14,16,17	2,3,6,7,9,1<br>0,11,13,15	1	Q4FZU6
8669	1,4,5,8,12,<br>15,16,17	2,3,6,7,9,1<br>0,11,13,14	0	
8670	1,4,5,8,13,<br>14,15,16	2,3,6,7,9,1<br>0,11,12,17	1	P09605;P25809
8671	1,4,5,8,13,<br>14,15,17	2,3,6,7,9,1<br>0,11,12,16	3	P10536,P47967,P97840
8672	1,4,5,8,13,<br>14,16,17	2,3,6,7,9,1<br>0,11,12,15	3	P27213,Q4FZU6,Q6PCU2
8673	1,4,5,8,13,<br>15,16,17	2,3,6,7,9,1<br>0,11,12,14	0	
8674	1,4,5,8,14,<br>15,16,17	2,3,6,7,9,1<br>0,11,12,13	3	P47967,P97840,Q4FZU6
8675	1,4,5,9,10,<br>11,12,13	2,3,6,7,8,1<br>4,15,16,17	2	P02803,P36374
8676	1,4,5,9,10,<br>11,12,14	2,3,6,7,8,1<br>3,15,16,17	2	P51907,Q6P6Q2
8677	1,4,5,9,10,<br>11,12,15	2,3,6,7,8,1<br>3,14,16,17	4	P10758,P20766,P51907,Q561R9
8678	1,4,5,9,10,<br>11,12,16	2,3,6,7,8,1<br>3,14,15,17	7	P02803,P06757,P20766,P51907,Q6IFU7,Q6P6Q2,Q9WUW8
8679	1,4,5,9,10,<br>11,12,17	2,3,6,7,8,1<br>3,14,15,16	3	P02803,P06757,P36374
8680	1,4,5,9,10,<br>11,13,14	2,3,6,7,8,1<br>2,15,16,17	1	Q62635
8681	1,4,5,9,10,<br>11,13,15	2,3,6,7,8,1<br>2,14,16,17	2	Q561R9,Q62635
8682	1,4,5,9,10,<br>11,13,16	2,3,6,7,8,1<br>2,14,15,17	3	A2RUV9,P02803,Q62635
8683	1,4,5,9,10,<br>11,13,17	2,3,6,7,8,1<br>2,14,15,16	1	P02803
8684	1,4,5,9,10,<br>11,14,15	2,3,6,7,8,1<br>2,13,16,17	5	P51907,P55091,P63029,Q561R9,Q62635
8685	1,4,5,9,10,<br>11,14,16	2,3,6,7,8,1<br>2,13,15,17	3	P55091,Q62635,Q9QZK9
8686	1,4,5,9,10,<br>11,14,17	2,3,6,7,8,1<br>2,13,15,16	0	
8687	1,4,5,9,10,<br>11,15,16	2,3,6,7,8,1<br>2,13,14,17	10	O54728,P02782,P20766,P50115,P51907,P55091,Q561R9,Q62635,Q9QZK9,Q9Z1F2
8688	1,4,5,9,10,<br>11,15,17	2,3,6,7,8,1<br>2,13,14,16	2	Q566E6,Q62902
8689	1,4,5,9,10,<br>11,16,17	2,3,6,7,8,1<br>2,13,14,15	1	A2RUV9
8690	1,4,5,9,10,<br>12,13,14	2,3,6,7,8,1<br>1,15,16,17	1	P02803

8691	1,4,5,9,10,<br>12,13,15	2,3,6,7,8,1<br>1,14,16,17	3	P10758,P16086,P50115
8692	1,4,5,9,10,<br>12,13,16	2,3,6,7,8,1<br>1,14,15,17	3	P02803,P16086,P50115
8693	1,4,5,9,10,<br>12,13,17	2,3,6,7,8,1<br>1,14,15,16	4	P02803,P16086,P35280,P36374
8694	1,4,5,9,10,<br>12,14,15	2,3,6,7,8,1<br>1,13,16,17	3	P10758,P20766,P50115
8695	1,4,5,9,10,<br>12,14,16	2,3,6,7,8,1<br>1,13,15,17	2	P20766,P50115
8696	1,4,5,9,10,<br>12,14,17	2,3,6,7,8,1<br>1,13,15,16	1	Q10758
8697	1,4,5,9,10,<br>12,15,16	2,3,6,7,8,1<br>1,13,14,17	3	P16086,P20766,P50115
8698	1,4,5,9,10,<br>12,15,17	2,3,6,7,8,1<br>1,13,14,16	2	P16086,P20766
8699	1,4,5,9,10,<br>12,16,17	2,3,6,7,8,1<br>1,13,14,15	2	P16086,P20766
8700	1,4,5,9,10,<br>13,14,15	2,3,6,7,8,1<br>1,12,16,17	3	P09605;P25809,P50115,Q62635
8701	1,4,5,9,10,<br>13,14,16	2,3,6,7,8,1<br>1,12,15,17	4	P50115,P62804,Q00715,Q62635
8702	1,4,5,9,10,<br>13,14,17	2,3,6,7,8,1<br>1,12,15,16	0	
8703	1,4,5,9,10,<br>13,15,16	2,3,6,7,8,1<br>1,12,14,17	5	P50115,P55091,P62804,Q00715,Q62635
8704	1,4,5,9,10,<br>13,15,17	2,3,6,7,8,1<br>1,12,14,16	0	
8705	1,4,5,9,10,<br>13,16,17	2,3,6,7,8,1<br>1,12,14,15	3	P35280,P62804,Q00715
8706	1,4,5,9,10,<br>14,15,16	2,3,6,7,8,1<br>1,12,13,17	7	O54728,P02782,P20766,P50115,P55091,Q62635,Q9QZK9
8707	1,4,5,9,10,<br>14,15,17	2,3,6,7,8,1<br>1,12,13,16	1	Q62902
8708	1,4,5,9,10,<br>14,16,17	2,3,6,7,8,1<br>1,12,13,15	0	
8709	1,4,5,9,10,<br>15,16,17	2,3,6,7,8,1<br>1,12,13,14	7	P02782,P16086,P20766,P47727,Q62902,Q9JHB9,Q9Z1F2
8710	1,4,5,9,11,<br>12,13,14	2,3,6,7,8,1<br>0,15,16,17	3	P02803,P36374,Q6P6R2
8711	1,4,5,9,11,<br>12,13,15	2,3,6,7,8,1<br>0,14,16,17	3	P05539,P10758,Q6P6R2
8712	1,4,5,9,11,<br>12,13,16	2,3,6,7,8,1<br>0,14,15,17	1	P02803
8713	1,4,5,9,11,<br>12,13,17	2,3,6,7,8,1<br>0,14,15,16	3	P02803,P35280,P36374

8714	1,4,5,9,11,<br>12,14,15	2,3,6,7,8,1<br>0,13,16,17	3	P10758,P20766,P51907
8715	1,4,5,9,11,<br>12,14,16	2,3,6,7,8,1<br>0,13,15,17	3	P20766,P51907,Q4FZU6
8716	1,4,5,9,11,<br>12,14,17	2,3,6,7,8,1<br>0,13,15,16	2	P01835,Q4FZU6
8717	1,4,5,9,11,<br>12,15,16	2,3,6,7,8,1<br>0,13,14,17	3	P12020,P20766,P51907
8718	1,4,5,9,11,<br>12,15,17	2,3,6,7,8,1<br>0,13,14,16	1	P12020
8719	1,4,5,9,11,<br>12,16,17	2,3,6,7,8,1<br>0,13,14,15	2	P12020,Q4FZU6
8720	1,4,5,9,11,<br>13,14,15	2,3,6,7,8,1<br>0,12,16,17	4	O54728,P05539,Q62635,Q6P6R2
8721	1,4,5,9,11,<br>13,14,16	2,3,6,7,8,1<br>0,12,15,17	1	Q62635
8722	1,4,5,9,11,<br>13,14,17	2,3,6,7,8,1<br>0,12,15,16	1	P97840
8723	1,4,5,9,11,<br>13,15,16	2,3,6,7,8,1<br>0,12,14,17	3	O54728,P50115,Q62635
8724	1,4,5,9,11,<br>13,15,17	2,3,6,7,8,1<br>0,12,14,16	3	P05539,P97840,Q63532
8725	1,4,5,9,11,<br>13,16,17	2,3,6,7,8,1<br>0,12,14,15	2	A2RUV9,P35280
8726	1,4,5,9,11,<br>14,15,16	2,3,6,7,8,1<br>0,12,13,17	7	O54728,P20766,P50115,P51907,P55091,Q62635,Q9QZK9
8727	1,4,5,9,11,<br>14,15,17	2,3,6,7,8,1<br>0,12,13,16	2	P47967,P97840
8728	1,4,5,9,11,<br>14,16,17	2,3,6,7,8,1<br>0,12,13,15	1	Q4FZU6
8729	1,4,5,9,11,<br>15,16,17	2,3,6,7,8,1<br>0,12,13,14	2	P06911,P12020
8730	1,4,5,9,12,<br>13,14,15	2,3,6,7,8,1<br>0,11,16,17	3	P10758,P50115,Q6P6R2
8731	1,4,5,9,12,<br>13,14,16	2,3,6,7,8,1<br>0,11,15,17	2	P50115,Q4FZU6
8732	1,4,5,9,12,<br>13,14,17	2,3,6,7,8,1<br>0,11,15,16	3	P35280,P36374,Q4FZU6
8733	1,4,5,9,12,<br>13,15,16	2,3,6,7,8,1<br>0,11,14,17	3	P16086,P50115,P62804
8734	1,4,5,9,12,<br>13,15,17	2,3,6,7,8,1<br>0,11,14,16	2	P16086,P35280
8735	1,4,5,9,12,<br>13,16,17	2,3,6,7,8,1<br>0,11,14,15	2	P35280,Q4FZU6
8736	1,4,5,9,12,<br>14,15,16	2,3,6,7,8,1<br>0,11,13,17	2	P20766,P50115

8737	1,4,5,9,12,<br>14,15,17	2,3,6,7,8,1<br>0,11,13,16	0	
8738	1,4,5,9,12,<br>14,16,17	2,3,6,7,8,1<br>0,11,13,15	3	P20766,P35280,Q4FZU6
8739	1,4,5,9,12,<br>15,16,17	2,3,6,7,8,1<br>0,11,13,14	4	P12020,P16086,P20766,P47727
8740	1,4,5,9,13,<br>14,15,16	2,3,6,7,8,1<br>0,11,12,17	7	O54728,P11883,P50115,P62804,Q00715,Q62635,Q9QZK9
8741	1,4,5,9,13,<br>14,15,17	2,3,6,7,8,1<br>0,11,12,16	2	P11883,P47967
8742	1,4,5,9,13,<br>14,16,17	2,3,6,7,8,1<br>0,11,12,15	3	P35280,P62804,Q4FZU6
8743	1,4,5,9,13,<br>15,16,17	2,3,6,7,8,1<br>0,11,12,14	6	P11883,P35280,P47727,P62804,Q00715,Q9EQS0
8744	1,4,5,9,14,<br>15,16,17	2,3,6,7,8,1<br>0,11,12,13	2	P11883,P62804
8745	1,4,5,10,1<br>1,12,13,14	2,3,6,7,8,9<br>,15,16,17	2	P36374,Q5QE79
8746	1,4,5,10,1<br>1,12,13,15	2,3,6,7,8,9<br>,14,16,17	2	P51907,Q6P6R2
8747	1,4,5,10,1<br>1,12,13,16	2,3,6,7,8,9<br>,14,15,17	3	P02803,Q6IFU7,Q6P6Q2
8748	1,4,5,10,1<br>1,12,13,17	2,3,6,7,8,9<br>,14,15,16	2	P02803,P36374
8749	1,4,5,10,1<br>1,12,14,15	2,3,6,7,8,9<br>,13,16,17	2	P20766,P51907
8750	1,4,5,10,1<br>1,12,14,16	2,3,6,7,8,9<br>,13,15,17	5	P20766,P51907,P53792,Q6IFU7,Q6P6Q2
8751	1,4,5,10,1<br>1,12,14,17	2,3,6,7,8,9<br>,13,15,16	2	P20766,Q10758
8752	1,4,5,10,1<br>1,12,15,16	2,3,6,7,8,9<br>,13,14,17	6	P14408,P20766,P51907,P53792,Q6IFU7,Q6P6Q2
8753	1,4,5,10,1<br>1,12,15,17	2,3,6,7,8,9<br>,13,14,16	2	P20766,P51907
8754	1,4,5,10,1<br>1,12,16,17	2,3,6,7,8,9<br>,13,14,15	5	P06757,P20766,Q10758,Q6IFU7,Q6P6Q2
8755	1,4,5,10,1<br>1,13,14,15	2,3,6,7,8,9<br>,12,16,17	3	P09605;P25809,P50115,Q6P6R2
8756	1,4,5,10,1<br>1,13,14,16	2,3,6,7,8,9<br>,12,15,17	1	P50115
8757	1,4,5,10,1<br>1,13,14,17	2,3,6,7,8,9<br>,12,15,16	0	
8758	1,4,5,10,1<br>1,13,15,16	2,3,6,7,8,9<br>,12,14,17	4	P14408,P50115,Q561R9,Q62635
8759	1,4,5,10,1<br>1,13,15,17	2,3,6,7,8,9<br>,12,14,16	0	

8760	1,4,5,10,1<br>1,13,16,17	2,3,6,7,8,9<br>,12,14,15	1	A2RUV9
8761	1,4,5,10,1<br>1,14,15,16	2,3,6,7,8,9<br>,12,13,17	8	P09605;P25809,P20766,P50115,P51907,P55091,Q62635,Q9QZK9,Q9Z1F2
8762	1,4,5,10,1<br>1,14,15,17	2,3,6,7,8,9<br>,12,13,16	1	P20766
8763	1,4,5,10,1<br>1,14,16,17	2,3,6,7,8,9<br>,12,13,15	1	P20766
8764	1,4,5,10,1<br>1,15,16,17	2,3,6,7,8,9<br>,12,13,14	6	P02782,P05369,P14408,P20766,Q62902,Q9Z1F2
8765	1,4,5,10,1<br>2,13,14,15	2,3,6,7,8,9<br>,11,16,17	5	P09605;P25809,P10758,P50115,Q498D9,Q6P6R2
8766	1,4,5,10,1<br>2,13,14,16	2,3,6,7,8,9<br>,11,15,17	1	P50115
8767	1,4,5,10,1<br>2,13,14,17	2,3,6,7,8,9<br>,11,15,16	1	P36374
8768	1,4,5,10,1<br>2,13,15,16	2,3,6,7,8,9<br>,11,14,17	2	P16086,P50115
8769	1,4,5,10,1<br>2,13,15,17	2,3,6,7,8,9<br>,11,14,16	2	P16086,Q99MH3
8770	1,4,5,10,1<br>2,13,16,17	2,3,6,7,8,9<br>,11,14,15	3	P02631,P27213,P35280
8771	1,4,5,10,1<br>2,14,15,16	2,3,6,7,8,9<br>,11,13,17	2	P20766,P50115
8772	1,4,5,10,1<br>2,14,15,17	2,3,6,7,8,9<br>,11,13,16	1	P20766
8773	1,4,5,10,1<br>2,14,16,17	2,3,6,7,8,9<br>,11,13,15	2	P20766,Q10758
8774	1,4,5,10,1<br>2,15,16,17	2,3,6,7,8,9<br>,11,13,14	3	P06866,P16086,P20766
8775	1,4,5,10,1<br>3,14,15,16	2,3,6,7,8,9<br>,11,12,17	3	P09605;P25809,P50115,Q62635
8776	1,4,5,10,1<br>3,14,15,17	2,3,6,7,8,9<br>,11,12,16	0	
8777	1,4,5,10,1<br>3,14,16,17	2,3,6,7,8,9<br>,11,12,15	1	P27213
8778	1,4,5,10,1<br>3,15,16,17	2,3,6,7,8,9<br>,11,12,14	2	P62804,Q9EQS0
8779	1,4,5,10,1<br>4,15,16,17	2,3,6,7,8,9<br>,11,12,13	3	P02782,P20766,Q62902
8780	1,4,5,11,1<br>2,13,14,15	2,3,6,7,8,9<br>,10,16,17	2	P10758,Q6P6R2
8781	1,4,5,11,1<br>2,13,14,16	2,3,6,7,8,9<br>,10,15,17	2	Q4FZU6,Q6P6R2
8782	1,4,5,11,1<br>2,13,14,17	2,3,6,7,8,9<br>,10,15,16	1	P36374

8783	1,4,5,11,12,13,15,16	2,3,6,7,8,9,10,14,17	3	P14408,P51907,Q6P6R2
8784	1,4,5,11,12,13,15,17	2,3,6,7,8,9,10,14,16	1	P36374
8785	1,4,5,11,12,13,16,17	2,3,6,7,8,9,10,14,15	2	P35280,P36374
8786	1,4,5,11,12,14,15,16	2,3,6,7,8,9,10,13,17	2	P20766,P51907
8787	1,4,5,11,12,14,15,17	2,3,6,7,8,9,10,13,16	1	P20766
8788	1,4,5,11,12,14,16,17	2,3,6,7,8,9,10,13,15	2	P20766,Q4FZU6
8789	1,4,5,11,12,15,16,17	2,3,6,7,8,9,10,13,14	6	P05369,P06866,P12020,P14408,P20766,P51907
8790	1,4,5,11,13,14,15,16	2,3,6,7,8,9,10,12,17	2	P50115,Q6P6R2
8791	1,4,5,11,13,14,15,17	2,3,6,7,8,9,10,12,16	2	P47967,P97840
8792	1,4,5,11,13,14,16,17	2,3,6,7,8,9,10,12,15	1	Q4FZU6
8793	1,4,5,11,13,15,16,17	2,3,6,7,8,9,10,12,14	1	P14408
8794	1,4,5,11,14,15,16,17	2,3,6,7,8,9,10,12,13	3	P14408,P20766,P97840
8795	1,4,5,12,13,14,15,16	2,3,6,7,8,9,10,11,17	3	P50115,Q6P6R2,Q9QUL6
8796	1,4,5,12,13,14,15,17	2,3,6,7,8,9,10,11,16	2	P11883,P27213
8797	1,4,5,12,13,14,16,17	2,3,6,7,8,9,10,11,15	3	P27213,P35280,Q4FZU6
8798	1,4,5,12,13,15,16,17	2,3,6,7,8,9,10,11,14	3	P11883,P27213,P35280
8799	1,4,5,12,14,15,16,17	2,3,6,7,8,9,10,11,13	1	P20766
8800	1,4,5,13,14,15,16,17	2,3,6,7,8,9,10,11,12	3	P11883,P27213,P62804
8801	1,4,6,7,8,9,10,11	2,3,5,12,13,14,15,16,17	24	P00714,P06761,P07150,P07647,P08723,P09456,P0C0A9,P14669,P15399,P18418,P22282,P22283,P31044,P35280,P61206;P84079;P84082,P83121,Q561R9,Q5FVI6,Q63493,Q63617,Q6AYL6,Q8CJ52,Q9JI85,Q9R0T3
8802	1,4,6,7,8,9,10,12	2,3,5,11,13,14,15,16,17	29	D3ZHA0,F1LM93,P00714,P02780,P02781,P02782,P06761,P07647,P08723,P09456,P0C0A9,P11598,P13432,P18418,P22282,P22283,P30120,P31044,P35281,P36374,P97840,Q5FVI6,Q63617,Q6AYR9,Q8CJ52,Q99041,Q9JHB9,Q9JI85,Q9R0T3
8803	1,4,6,7,8,9,10,13	2,3,5,11,12,14,15,16,17	16	P06761,P06911,P07647,P08723,P09456,P11598,P12020,P18418,P22282,P22283,P31044,Q5GRG2,Q62902,Q63617,Q9JI85,Q9R0T3

8804	1,4,6,7,8,9,10,14	2,3,5,11,12,13,15,16,17	14	P00714,P06761,P08723,P11598,P12020,P18418,P19629,P22282,P22283,P31044,P83121,Q5GRG2,Q63617,Q9JI85
8805	1,4,6,7,8,9,10,15	2,3,5,11,12,13,14,16,17	10	D3ZHA0,O55006,P06761,P18418,P22006,P22283,P31044,P35280,Q5FVI6,Q63617
8806	1,4,6,7,8,9,10,16	2,3,5,11,12,13,14,15,17	7	P00714,P18418,P22283,P31044,Q5FVI6,Q63617,Q9R0T3
8807	1,4,6,7,8,9,10,17	2,3,5,11,12,13,14,15,16	11	P06761,P08723,P11598,P18418,P22282,P22283,P31044,Q4G075,Q63617,Q99041,Q9R0T3
8808	1,4,6,7,8,9,11,12	2,3,5,10,13,14,15,16,17	35	P00714,P02780,P02781,P02782,P04905,P06761,P07150,P07647,P08723,P09456,P0C0A9,P10715,P11598,P13432,P14669,P18418,P19629,P22282,P22283,P24368,P30120,P31044,P36374,P46462,Q62902,Q63357,Q63493,Q63617,Q6AYL6,Q6AYR9,Q8CJ52,Q99041,Q9JHB9,Q9JI85,Q9R0T3
8809	1,4,6,7,8,9,11,13	2,3,5,10,12,14,15,16,17	26	P02780,P02781,P02782,P05539,P06761,P06911,P07647,P08723,P09456,P0C0A9,P11598,P14669,P18418,P22282,P22283,P31044,P36374,P83121,Q5GRG2,Q62902,Q63493,Q63617,Q8CJ52,Q9JHB9,Q9JI85,Q9R0T3
8810	1,4,6,7,8,9,11,14	2,3,5,10,12,13,15,16,17	19	P02780,P06761,P07150,P07171,P07647,P08723,P11598,P14669,P18418,P19629,P22282,P22283,P31044,P47727,P83121,Q63493,Q63617,Q8CJ52,Q9JI85
8811	1,4,6,7,8,9,11,15	2,3,5,10,12,13,14,16,17	13	P06761,P07171,P10715,P14669,P18418,P22006,P22282,P22283,P31044,P35280,Q561R9,Q63617,Q9JI85
8812	1,4,6,7,8,9,11,16	2,3,5,10,12,13,14,15,17	15	P06761,P07150,P10715,P14668,P14669,P18418,P22282,P22283,P31044,P83121,Q63617,Q6AYL6,Q8CJ52,Q9JI85,Q9R0T3
8813	1,4,6,7,8,9,11,17	2,3,5,10,12,13,14,15,16	20	P06761,P07150,P07647,P08723,P09605;P25809,P10715,P11598,P14669,P15399,P18418,P22282,P22283,P31044,P83121,Q4G075,Q63493,Q63617,Q99041,Q9JI85,Q9R0T3
8814	1,4,6,7,8,9,12,13	2,3,5,10,11,14,15,16,17	28	P02780,P02781,P02782,P02803,P06761,P06911,P07647,P08723,P09456,P0C0A9,P11598,P12020,P13432,P18418,P22282,P22283,P30120,P31044,P36374,Q5GRG2,Q62902,Q63617,Q6AYR9,Q8CJ52,Q99041,Q9JHB9,Q9JI85,Q9R0T3
8815	1,4,6,7,8,9,12,14	2,3,5,10,11,13,15,16,17	25	P02780,P02781,P02782,P06761,P07647,P08723,P09456,P0C0A9,P11598,P13432,P18418,P19629,P22282,P22283,P30120,P31044,P36374,Q62902,Q63617,Q6AYR9,Q8CJ52,Q99041,Q9JHB9,Q9JI85,Q9R0T3
8816	1,4,6,7,8,9,12,15	2,3,5,10,11,13,14,16,17	19	P02782,P06761,P07647,P08723,P0C0A9,P10715,P11598,P13432,P18418,P19629,P22006,P22282,P22283,P30120,P31044,Q63617,Q8CJ52,Q99041,Q9JI85
8817	1,4,6,7,8,9,12,16	2,3,5,10,11,13,14,15,17	18	P02780,P02782,P06761,P07647,P08723,P0C0A9,P10715,P13432,P18418,P22282,P22283,P30120,P31044,Q63617,Q8CJ52,Q99041,Q9JI85,Q9R0T3
8818	1,4,6,7,8,9,12,17	2,3,5,10,11,13,14,15,16	27	P02780,P02782,P06761,P07647,P08723,P09605;P25809,P0C0A9,P10715,P11598,P13432,P18418,P19223,P19629,P22282,P22283,P30120,P36374,Q4G075,Q63617,Q6AYR9,Q8CJ52,Q99041,Q9JHB9,Q9JI85,Q9QZA6,Q9QZK9,Q9R0T3

8819	1,4,6,7,8,9,13,14	2,3,5,10,11,12,15,16,17	18	P02780,P06761,P06911,P07647,P08723,P11598,P12020,P18418,P19629,P22282,P22283,P31044,P83121,Q5GRG2,Q62902,Q63617,Q8CJ52,Q9JI85
8820	1,4,6,7,8,9,13,15	2,3,5,10,11,12,14,16,17	11	P06761,P06911,P11598,P18418,P22006,P22282,P22283,P31044,Q5GRG2,Q63617,Q9JI85
8821	1,4,6,7,8,9,13,16	2,3,5,10,11,12,14,15,17	13	P06761,P06911,P11598,P18418,P22282,P22283,P31044,Q5GRG2,Q62902,Q63617,Q8CJ52,Q9JI85,Q9R0T3
8822	1,4,6,7,8,9,13,17	2,3,5,10,11,12,14,15,16	15	P06761,P06911,P07647,P08723,P11598,P18418,P22282,P22283,P31044,Q5GRG2,Q62902,Q63617,Q99041,Q9JI85,Q9R0T3
8823	1,4,6,7,8,9,14,15	2,3,5,10,11,12,13,16,17	10	P06761,P11598,P18418,P19629,P22006,P22282,P22283,P31044,Q5GRG2,Q63617
8824	1,4,6,7,8,9,14,16	2,3,5,10,11,12,13,15,17	7	P06761,P18418,P19629,P22282,P22283,P31044,Q63617
8825	1,4,6,7,8,9,14,17	2,3,5,10,11,12,13,15,16	11	P06761,P08723,P11598,P18418,P19629,P22282,P22283,P31044,Q63617,Q99041,Q9JI85
8826	1,4,6,7,8,9,15,16	2,3,5,10,11,12,13,14,17	3	P18418,P31044,Q63617
8827	1,4,6,7,8,9,15,17	2,3,5,10,11,12,13,14,16	9	P06761,P11598,P18418,P22006,P22282,P22283,P31044,Q63617,Q99041
8828	1,4,6,7,8,9,16,17	2,3,5,10,11,12,13,14,15	8	P06761,P09605;P25809,P18418,P22282,P22283,Q63617,Q99041,Q9R0T3
8829	1,4,6,7,8,10,11,12	2,3,5,9,13,14,15,16,17	45	F1LM93,O35547,P00714,P02780,P02781,P02782,P04905,P06761,P07150,P07647,P08723,P09456,P0C0A9,P10715,P11598,P13432,P14669,P15399,P18418,P22282,P22283,P24368,P30120,P31044,P35280,P35281,P36374,P46462,P47727,P60905,P61206;P84079;P84082,Q5FVI6,Q63357,Q63493,Q63617,Q6AYL6,Q6AYR9,Q6IFU7,Q6P6Q2,Q812E4,Q8CJ52,Q99041,Q9JHB9,Q9JI85,Q9R0T3
8830	1,4,6,7,8,10,11,13	2,3,5,9,12,14,15,16,17	29	P00714,P02780,P02781,P06761,P06911,P07150,P07647,P08723,P09456,P0C0A9,P11598,P12020,P14669,P18418,P22282,P22283,P31044,P36374,P47727,Q5FVI6,Q5GRG2,Q62902,Q63493,Q63617,Q8CJ52,Q8VD89,Q9JHB9,Q9JI85,Q9R0T3
8831	1,4,6,7,8,10,11,14	2,3,5,9,12,13,15,16,17	28	P00714,P06761,P06911,P07150,P07171,P07647,P08723,P11598,P12020,P14669,P15399,P18418,P19629,P22282,P22283,P31044,P35280,P47727,P83121,Q5BJY9,Q5FVI6,Q5GRG2,Q63493,Q63617,Q6AYL6,Q8CJ52,Q9JI85,Q9R0T3
8832	1,4,6,7,8,10,11,15	2,3,5,9,12,13,14,16,17	21	P00714,P06761,P07150,P07171,P08723,P11598,P14669,P18418,P22006,P22282,P22283,P31044,P35280,P35281,Q561R9,Q5BJY9,Q5FVI6,Q63617,Q8CJ52,Q8VD89,Q9JI85
8833	1,4,6,7,8,10,11,16	2,3,5,9,12,13,14,15,17	20	P00714,P06761,P07150,P08723,P14669,P18418,P22282,P22283,P31044,P35280,P61206;P84079;P84082,Q5BJY9,Q5FVI6,Q63493,Q63617,Q6AYL6,Q8CJ52,Q8VD89,Q9JI85,Q9R0T3

8834	1,4,6,7,8,10,11,17	2,3,5,9,12,13,14,15,16	20	P00714,P06761,P07150,P07647,P08723,P11598,P14669,P15399,P18418,P22282,P22283,P31044,Q4G075,Q5FVI6,Q63493,Q63617,Q8CJ52,Q99041,Q9JI85,Q9R0T3
8835	1,4,6,7,8,10,12,13	2,3,5,9,11,14,15,16,17	32	F1LM93,P02780,P02781,P02782,P04905,P06761,P06911,P07647,P08723,P09456,P0C0A9,P11598,P12020,P13432,P18418,P22282,P22283,P24368,P30120,P31044,P35281,P36374,Q5GRG2,Q62902,Q63617,Q6AYR9,Q8CJ52,Q99041,Q99MH3,Q9JHB9,Q9JI85,Q9R0T3
8836	1,4,6,7,8,10,12,14	2,3,5,9,11,13,15,16,17	33	D3ZHA0,F1LM93,P00714,P02780,P02781,P02782,P04905,P06761,P06911,P07647,P08723,P0C0A9,P11598,P12020,P13432,P18418,P19629,P22282,P22283,P24368,P30120,P31044,P35280,P36374,Q5GRG2,Q63493,Q63617,Q6AYR9,Q8CJ52,Q99041,Q9JHB9,Q9JI85,Q9R0T3
8837	1,4,6,7,8,10,12,15	2,3,5,9,11,13,14,16,17	24	D3ZHA0,F1LM93,P06761,P07647,P08723,P0C0A9,P11598,P13432,P18418,P19629,P22006,P22282,P22283,P30120,P31044,P35280,P35281,Q5FVI6,Q63617,Q6AYR9,Q8CJ52,Q99041,Q9JI85,Q9R0T3
8838	1,4,6,7,8,10,12,16	2,3,5,9,11,13,14,15,17	23	F1LM93,P06761,P07647,P08721,P08723,P0C0A9,P11598,P13432,P18418,P22282,P22283,P30120,P31044,P35281,P47967,P97840,Q5FVI6,Q63617,Q6AYR9,Q8CJ52,Q99041,Q9JI85,Q9R0T3
8839	1,4,6,7,8,10,12,17	2,3,5,9,11,13,14,15,16	30	F1LM93,P02780,P02781,P02782,P06761,P07647,P08723,P0C0A9,P11598,P13432,P18418,P19223,P22282,P22283,P30120,P31044,P36374,Q10758,Q4G075,Q63493,Q63617,Q6AYR9,Q8CJ52,Q99041,Q99MH3,Q9JHB9,Q9JI85,Q9QZA6,Q9QZK9,Q9R0T3
8840	1,4,6,7,8,10,13,14	2,3,5,9,11,12,15,16,17	17	P06761,P06911,P07647,P08723,P11598,P12020,P18418,P19629,P22282,P22283,P31044,Q5GRG2,Q62902,Q63617,Q8CJ52,Q9JI85,Q9R0T3
8841	1,4,6,7,8,10,13,15	2,3,5,9,11,12,14,16,17	14	P06761,P06911,P07647,P08723,P11598,P12020,P18418,P22006,P22282,P22283,P31044,Q5GRG2,Q63617,Q9JI85
8842	1,4,6,7,8,10,13,16	2,3,5,9,11,12,14,15,17	18	P06761,P06911,P07647,P08723,P11598,P12020,P18418,P22282,P22283,P31044,Q5FVI6,Q5GRG2,Q62902,Q63617,Q8CJ52,Q8VD89,Q9JI85,Q9R0T3
8843	1,4,6,7,8,10,13,17	2,3,5,9,11,12,14,15,16	18	P06761,P06911,P07647,P08723,P11598,P12020,P18418,P22282,P22283,P31044,Q4G075,Q5GRG2,Q63617,Q8CJ52,Q99041,Q99MH3,Q9JI85,Q9R0T3
8844	1,4,6,7,8,10,14,15	2,3,5,9,11,12,13,16,17	14	P06761,P07171,P08723,P11598,P12020,P18418,P19629,P22006,P22282,P22283,P31044,P35280,Q5GRG2,Q63617
8845	1,4,6,7,8,10,14,16	2,3,5,9,11,12,13,15,17	14	P06761,P08721,P08723,P12020,P18418,P19629,P22282,P22283,P31044,Q5FVI6,Q5GRG2,Q63617,Q8CJ52,Q9R0T3
8846	1,4,6,7,8,10,14,17	2,3,5,9,11,12,13,15,16	14	P06761,P08723,P11598,P12020,P18418,P19629,P22282,P22283,P31044,Q4G075,Q5GRG2,Q63617,Q99041,Q9R0T3
8847	1,4,6,7,8,10,15,16	2,3,5,9,11,12,13,14,17	7	P08721,P18418,P31044,P35280,Q5FVI6,Q63617,Q8VD89
8848	1,4,6,7,8,10,15,17	2,3,5,9,11,12,13,14,16	11	P06761,P11598,P18418,P22006,P22282,P22283,P31044,Q4G075,Q63617,Q99041,Q99MH3

8849	1,4,6,7,8,10,16,17	2,3,5,9,11,12,13,14,15	11	P06761,P11598,P18418,P22282,P22283,P31044,Q4G075,Q5FVI6,Q63617,Q99041,Q9R0T3
8850	1,4,6,7,8,11,12,13	2,3,5,9,10,14,15,16,17	37	P02780,P02781,P02782,P04905,P06761,P06911,P07150,P07647,P08723,P09456,P0C0A9,P10715,P11598,P12020,P13432,P14669,P18418,P19629,P22282,P22283,P24368,P30120,P31044,P36374,P46462,P47727,Q5GRG2,Q62902,Q63357,Q63493,Q63617,Q6AYR9,Q8CJ52,Q99041,Q9JHB9,Q9JI85,Q9R0T3
8851	1,4,6,7,8,11,12,14	2,3,5,9,10,13,15,16,17	40	P02780,P02781,P02782,P04905,P06761,P07150,P07647,P08723,P09456,P0C0A9,P10715,P11598,P12020,P13432,P14669,P15399,P18418,P19629,P22282,P22283,P24368,P30120,P31044,P35280,P36374,P40241,P46462,P47727,Q5GRG2,Q62902,Q63357,Q63493,Q63617,Q6AYL6,Q6AYR9,Q8CJ52,Q99041,Q9JHB9,Q9JI85,Q9R0T3
8852	1,4,6,7,8,11,12,15	2,3,5,9,10,13,14,16,17	32	P02780,P02781,P02782,P04905,P06761,P07150,P07647,P08723,P0C0A9,P10715,P11598,P13432,P14669,P18418,P19629,P22006,P22282,P22283,P30120,P31044,P35280,P36374,Q63357,Q63493,Q63617,Q6AYL6,Q6AYR9,Q8CJ52,Q99041,Q9JHB9,Q9JI85,Q9R0T3
8853	1,4,6,7,8,11,12,16	2,3,5,9,10,13,14,15,17	33	P02780,P02781,P02782,P04905,P06761,P07150,P07647,P08723,P0C0A9,P10715,P11598,P13432,P14669,P18418,P19629,P22282,P22283,P24368,P30120,P31044,P36374,Q62902,Q63357,Q63493,Q63617,Q6AYL6,Q6AYR9,Q6P6Q2,Q8CJ52,Q99041,Q9JHB9,Q9JI85,Q9R0T3
8854	1,4,6,7,8,11,12,17	2,3,5,9,10,13,14,15,16	39	F1LM93,O55004,P02780,P02781,P02782,P04905,P06761,P07150,P07647,P08723,P09605;P25809,P0C0A9,P10715,P11598,P13432,P14669,P15399,P18418,P19223,P19629,P22282,P22283,P30120,P36374,P46462,P50115,Q4G075,Q63357,Q63493,Q63617,Q6AYL6,Q6AYR9,Q8CJ52,Q99041,Q9JHB9,Q9JI85,Q9QZA6,Q9QZK9,Q9R0T3
8855	1,4,6,7,8,11,13,14	2,3,5,9,10,12,15,16,17	30	P02780,P02781,P02782,P04905,P06761,P06911,P07150,P07171,P07647,P08723,P0C0A9,P11598,P12020,P14669,P18418,P19629,P22282,P22283,P31044,P46462,P47727,P83121,Q5GRG2,Q62902,Q63493,Q63617,Q8CJ52,Q9JHB9,Q9JI85,Q9R0T3
8856	1,4,6,7,8,11,13,15	2,3,5,9,10,12,14,16,17	21	P05539,P06761,P06911,P07150,P07171,P07647,P08723,P11598,P14669,P18418,P22006,P22282,P22283,P31044,Q5GRG2,Q62902,Q63617,Q8CJ52,Q8VD89,Q99041,Q9JI85
8857	1,4,6,7,8,11,13,16	2,3,5,9,10,12,14,15,17	19	P06761,P06911,P07150,P07647,P08723,P11598,P14669,P18418,P22282,P22283,P31044,Q5GRG2,Q62902,Q63617,Q8CJ52,Q8VD89,Q99041,Q9JI85,Q9R0T3
8858	1,4,6,7,8,11,13,17	2,3,5,9,10,12,14,15,16	25	P02780,P02782,P06761,P06911,P07150,P07647,P08723,P11598,P14669,P15399,P18418,P22282,P22283,P31044,P36374,Q4G075,Q5GRG2,Q62902,Q63493,Q63617,Q8CJ52,Q99041,Q9JHB9,Q9JI85,Q9R0T3
8859	1,4,6,7,8,11,14,15	2,3,5,9,10,12,13,16,17	19	P06761,P07150,P07171,P08723,P11598,P14669,P18418,P19629,P22006,P22282,P22283,P31044,P35280,P47727,Q5GRG2,Q63493,Q63617,Q8CJ52,Q9JI85
8860	1,4,6,7,8,11,14,16	2,3,5,9,10,12,13,15,17	17	P06761,P07150,P08723,P11598,P14668,P14669,P18418,P19629,P22282,P22283,P31044,P47727,Q63493,Q63617,Q8CJ52,Q9JI85,Q9R0T3
8861	1,4,6,7,8,11,14,17	2,3,5,9,10,12,13,15,16	19	P06761,P07150,P07647,P08723,P11598,P14669,P15399,P18418,P19629,P22282,P22283,P31044,P47727,Q4G075,Q63493,Q63617,Q8CJ52,Q99041,Q9JI85
8862	1,4,6,7,8,11,15,16	2,3,5,9,10,12,13,14,17	14	P07150,P07171,P10715,P14668,P14669,P18418,P19629,P22006,P31044,P35280,Q63617,Q8CJ52,Q8VD89,Q9JI85
8863	1,4,6,7,8,11,15,17	2,3,5,9,10,12,13,14,16	16	P06761,P07150,P11598,P14669,P18418,P19629,P22006,P22282,P22283,P31044,Q4G075,Q63493,Q63617,Q99041,Q9JI85,Q9QZA6

8864	1,4,6,7,8,11,16,17	2,3,5,9,10,12,13,14,15	17	P06761,P07150,P09605;P25809,P10715,P11598,P14669,P18418,P22282,P22283,P31044,Q4G075,Q63493,Q63617,Q8CJ52,Q99041,Q9JI85,Q9R0T3
8865	1,4,6,7,8,12,13,14	2,3,5,9,10,11,15,16,17	33	O08557,P02780,P02781,P02782,P04905,P06761,P06911,P07647,P08723,P09456,P0C0A9,P11598,P12020,P13432,P18418,P19629,P22282,P22283,P24368,P30120,P31044,P36374,P46462,Q498D9,Q5GRG2,Q62902,Q63617,Q6AYR9,Q8CJ52,Q99041,Q9JHB9,Q9JI85,Q9R0T3
8866	1,4,6,7,8,12,13,15	2,3,5,9,10,11,14,16,17	29	O08557,P02780,P02781,P02782,P06761,P06911,P07647,P08723,P0C0A9,P11598,P12020,P13432,P18418,P19629,P22006,P22282,P22283,P30120,P31044,P36374,Q5GRG2,Q62902,Q63617,Q6AYR9,Q8CJ52,Q99041,Q9JHB9,Q9JI85,Q9R0T3
8867	1,4,6,7,8,12,13,16	2,3,5,9,10,11,14,15,17	26	P02780,P02781,P02782,P06761,P06911,P07647,P08723,P0C0A9,P11598,P12020,P13432,P18418,P22282,P22283,P30120,P31044,P36374,Q5GRG2,Q62902,Q63617,Q6AYR9,Q8CJ52,Q99041,Q9JHB9,Q9JI85,Q9R0T3
8868	1,4,6,7,8,12,13,17	2,3,5,9,10,11,14,15,16	34	P02780,P02781,P02782,P06761,P06911,P07647,P08723,P0C0A9,P11598,P12020,P13432,P18418,P19223,P19629,P22282,P22283,P30120,P36374,P55091,P62815,Q4G075,Q5GRG2,Q62635,Q62902,Q63493,Q63617,Q6AYR9,Q8CJ52,Q99041,Q99MH3,Q9JHB9,Q9JI85,Q9QZK9,Q9R0T3
8869	1,4,6,7,8,12,14,15	2,3,5,9,10,11,13,16,17	23	O08557,P02780,P02782,P06761,P07647,P08723,P0C0A9,P11598,P13432,P18418,P19629,P22006,P22282,P22283,P30120,P31044,P35280,Q5GRG2,Q63617,Q6AYR9,Q8CJ52,Q99041,Q9JI85
8870	1,4,6,7,8,12,14,16	2,3,5,9,10,11,13,15,17	21	P02780,P02782,P06761,P07647,P08723,P0C0A9,P11598,P13432,P18418,P19629,P22282,P22283,P30120,P31044,Q62902,Q63617,Q6AYR9,Q8CJ52,Q99041,Q9JI85,Q9R0T3
8871	1,4,6,7,8,12,14,17	2,3,5,9,10,11,13,15,16	30	P02780,P02782,P06761,P07647,P08723,P0C0A9,P11598,P13432,P18418,P19223,P19629,P22282,P22283,P30120,P31430,P36374,P55091,P62815,Q4G075,Q62635,Q63493,Q63617,Q6AYR9,Q8CJ52,Q99041,Q9JHB9,Q9JI85,Q9QZA6,Q9QZK9,Q9R0T3
8872	1,4,6,7,8,12,15,16	2,3,5,9,10,11,13,14,17	15	P06761,P08723,P10715,P11598,P13432,P18418,P19629,P22282,P22283,P30120,Q63617,Q8CJ52,Q99041,Q9JI85,Q9R0T3
8873	1,4,6,7,8,12,15,17	2,3,5,9,10,11,13,14,16	27	B0BNN3,P02782,P06761,P07647,P08723,P10715,P11598,P13432,P18418,P19223,P19629,P20761,P22006,P22282,P22283,P30120,P36374,Q4G075,Q63617,Q6AYR9,Q8CJ52,Q99041,Q99MH3,Q9JI85,Q9QZA6,Q9QZK9,Q9R0T3
8874	1,4,6,7,8,12,16,17	2,3,5,9,10,11,13,14,15	24	P02782,P06761,P07647,P08723,P09605;P25809,P10715,P11598,P13432,P18418,P19223,P19629,P22282,P22283,P23928,P30120,P36374,Q4G075,Q63617,Q6AYR9,Q8CJ52,Q99041,Q9JI85,Q9QZK9,Q9R0T3
8875	1,4,6,7,8,13,14,15	2,3,5,9,10,11,12,16,17	16	O08557,P06761,P06911,P08723,P11598,P12020,P18418,P19629,P22006,P22282,P22283,P31044,Q5GRG2,Q62902,Q63617,Q9JI85
8876	1,4,6,7,8,13,14,16	2,3,5,9,10,11,12,15,17	16	P06761,P06911,P08723,P11598,P12020,P18418,P19629,P22282,P22283,P31044,Q5GRG2,Q62902,Q63617,Q8CJ52,Q9JI85,Q9R0T3
8877	1,4,6,7,8,13,14,17	2,3,5,9,10,11,12,15,16	19	P06761,P06911,P07647,P08723,P11598,P12020,P18418,P19629,P22282,P22283,P31044,Q4G075,Q5GRG2,Q62902,Q63617,Q8CJ52,Q99041,Q9JI85,Q9R0T3
8878	1,4,6,7,8,13,15,16	2,3,5,9,10,11,12,14,17	12	P06761,P06911,P11598,P18418,P22282,P22283,P31044,Q5GRG2,Q63617,Q8CJ52,Q8VD89,Q9R0T3

8879	1,4,6,7,8,13,15,17	2,3,5,9,10,11,12,14,16	12	P06761,P06911,P11598,P18418,P22282,P22283,P31044,Q4G075,Q5GRG2,Q63617,Q99041,Q9JI85
8880	1,4,6,7,8,13,16,17	2,3,5,9,10,11,12,14,15	15	P06761,P06911,P11598,P18418,P22282,P22283,P23928,P31044,Q4G075,Q5GRG2,Q62902,Q63617,Q8CJ52,Q99041,Q9R0T3
8881	1,4,6,7,8,14,15,16	2,3,5,9,10,11,12,13,17	6	P06761,P18418,P19629,P31044,Q5GRG2,Q63617
8882	1,4,6,7,8,14,15,17	2,3,5,9,10,11,12,13,16	12	P06761,P10536,P11598,P18418,P19629,P22006,P22282,P22283,P31044,Q4G075,Q63617,Q99041
8883	1,4,6,7,8,14,16,17	2,3,5,9,10,11,12,13,15	10	P06761,P11598,P18418,P19629,P22282,P22283,Q4G075,Q63617,Q99041,Q9R0T3
8884	1,4,6,7,8,15,16,17	2,3,5,9,10,11,12,13,14	6	P06761,P11598,P22282,Q4G075,Q63617,Q99041
8885	1,4,6,7,9,10,11,12	2,3,5,8,13,14,15,16,17	19	P00714,P04905,P06757,P08723,P08753,P09456,P0C0A9,P15399,P30120,P36374,P47967,P83121,P97840,Q5FVI6,Q63357,Q6AYL6,Q6IFU7,Q9JHB9,Q9JI85
8886	1,4,6,7,9,10,11,13	2,3,5,8,12,14,15,16,17	6	P05539,P09456,P12020,P83121,Q5FVI6,Q9JI85
8887	1,4,6,7,9,10,11,14	2,3,5,8,12,13,15,16,17	5	P15399,P47727,P83121,Q5FVI6,Q9JI85
8888	1,4,6,7,9,10,11,15	2,3,5,8,12,13,14,16,17	6	P22006,P35280,P83121,Q561R9,Q566E6,Q5FVI6
8889	1,4,6,7,9,10,11,16	2,3,5,8,12,13,14,15,17	3	P61206;P84079;P84082,P83121,Q5FVI6
8890	1,4,6,7,9,10,11,17	2,3,5,8,12,13,14,15,16	4	P15399,P83121,Q566E6,Q5FVI6
8891	1,4,6,7,9,10,12,13	2,3,5,8,11,14,15,16,17	14	P06911,P07647,P08723,P09456,P0C0A9,P12020,P19218,P30120,P36374,P97840,Q5FVI6,Q6AYR9,Q9JHB9,Q9JI85
8892	1,4,6,7,9,10,12,14	2,3,5,8,11,13,15,16,17	7	P08723,P0C0A9,P12020,P30120,P83121,P97840,Q6AYR9
8893	1,4,6,7,9,10,12,15	2,3,5,8,11,13,14,16,17	2	P97840,Q5FVI6

8894	1,4,6,7,9,10,12,16	2,3,5,8,11,13,14,15,17	3	P47967,P97840,Q5FVI6
8895	1,4,6,7,9,10,12,17	2,3,5,8,11,13,14,15,16	8	O08815,P08723,P09605;P25809,P13432,P19223,P36374,P97840,Q9QZK9
8896	1,4,6,7,9,10,13,14	2,3,5,8,11,12,15,16,17	4	P06911,P12020,P83121,Q5GRG2
8897	1,4,6,7,9,10,13,15	2,3,5,8,11,12,14,16,17	2	Q5FVI6,Q5GRG2
8898	1,4,6,7,9,10,13,16	2,3,5,8,11,12,14,15,17	4	P12020,P62804,P97840,Q5FVI6
8899	1,4,6,7,9,10,13,17	2,3,5,8,11,12,14,15,16	1	P12020
8900	1,4,6,7,9,10,14,15	2,3,5,8,11,12,13,16,17	3	P22006,P83121,Q5FVI6
8901	1,4,6,7,9,10,14,16	2,3,5,8,11,12,13,15,17	2	P83121,Q5FVI6
8902	1,4,6,7,9,10,14,17	2,3,5,8,11,12,13,15,16	1	P83121
8903	1,4,6,7,9,10,15,16	2,3,5,8,11,12,13,14,17	1	Q5FVI6
8904	1,4,6,7,9,10,15,17	2,3,5,8,11,12,13,14,16	1	Q5FVI6
8905	1,4,6,7,9,10,16,17	2,3,5,8,11,12,13,14,15	1	Q5FVI6
8906	1,4,6,7,9,11,12,13	2,3,5,8,10,14,15,16,17	19	P02780,P02803,P04905,P05539,P07647,P08723,P09456,P0C0A9,P13432,P22282,P22283,P30120,P36374,P83121,Q62902,Q63357,Q6AYR9,Q9JHB9,Q9JI85
8907	1,4,6,7,9,11,12,14	2,3,5,8,10,13,15,16,17	12	P04905,P08723,P09456,P0C0A9,P13432,P30120,P36374,P47727,P83121,Q6AYL6,Q9JHB9,Q9JI85
8908	1,4,6,7,9,11,12,15	2,3,5,8,10,13,14,16,17	5	P08723,P08753,P22006,P51907,Q9JI85

8909	1,4,6,7,9,11,12,16	2,3,5,8,10,13,14,15,17	9	P08723,P08753,P0C0A9,P13432,P30120,Q5FVI6,Q6AYL6,Q6IFU7,Q9JI85
8910	1,4,6,7,9,11,12,17	2,3,5,8,10,13,14,15,16	12	P08723,P09605;P25809,P0C0A9,P13432,P15399,P22282,P30120,P36374,Q99041,Q9JHB9,Q9JI85,Q9QZK9
8911	1,4,6,7,9,11,13,14	2,3,5,8,10,12,15,16,17	9	P05539,P06911,P09456,P12020,P47727,P83121,Q5GRG2,Q62902,Q9JI85
8912	1,4,6,7,9,11,13,15	2,3,5,8,10,12,14,16,17	5	P05539,P22006,P83121,Q561R9,Q9JI85
8913	1,4,6,7,9,11,13,16	2,3,5,8,10,12,14,15,17	2	P05539,P83121
8914	1,4,6,7,9,11,13,17	2,3,5,8,10,12,14,15,16	7	P05539,P09605;P25809,P15399,P22282,P36374,P83121,Q9JI85
8915	1,4,6,7,9,11,14,15	2,3,5,8,10,12,13,16,17	2	P22006,P83121
8916	1,4,6,7,9,11,14,16	2,3,5,8,10,12,13,15,17	1	P83121
8917	1,4,6,7,9,11,14,17	2,3,5,8,10,12,13,15,16	2	P15399,P83121
8918	1,4,6,7,9,11,15,16	2,3,5,8,10,12,13,14,17	2	P22006,Q5FVI6
8919	1,4,6,7,9,11,15,17	2,3,5,8,10,12,13,14,16	2	P09605;P25809,P22006
8920	1,4,6,7,9,11,16,17	2,3,5,8,10,12,13,14,15	2	P09605;P25809,P83121
8921	1,4,6,7,9,12,13,14	2,3,5,8,10,11,15,16,17	14	P06911,P08723,P09456,P0C0A9,P12020,P13432,P22282,P30120,P36374,Q5GRG2,Q62902,Q6AYR9,Q9JHB9,Q9JI85
8922	1,4,6,7,9,12,13,15	2,3,5,8,10,11,14,16,17	3	P05539,P36374,Q9JI85
8923	1,4,6,7,9,12,13,16	2,3,5,8,10,11,14,15,17	3	P19218,P36374,Q62902

8924	1,4,6,7,9,12,13,17	2,3,5,8,10,11,14,15,16	11	P07647,P08723,P09605;P25809,P13432,P22282,P30120,P36374,Q6AYR9,Q9JHB9,Q9JI85,Q9QZK9
8925	1,4,6,7,9,12,14,15	2,3,5,8,10,11,13,16,17	0	
8926	1,4,6,7,9,12,14,16	2,3,5,8,10,11,13,15,17	0	
8927	1,4,6,7,9,12,14,17	2,3,5,8,10,11,13,15,16	5	P01835,P08723,P09605;P25809,P13432,P36374
8928	1,4,6,7,9,12,15,16	2,3,5,8,10,11,13,14,17	1	P20766
8929	1,4,6,7,9,12,15,17	2,3,5,8,10,11,13,14,16	2	P09605;P25809,P13432
8930	1,4,6,7,9,12,16,17	2,3,5,8,10,11,13,14,15	2	P09605;P25809,P13432
8931	1,4,6,7,9,13,14,15	2,3,5,8,10,11,12,16,17	4	P05539,P06911,P12020,Q5GRG2
8932	1,4,6,7,9,13,14,16	2,3,5,8,10,11,12,15,17	5	P06911,P12020,P62804,P83121,Q5GRG2
8933	1,4,6,7,9,13,14,17	2,3,5,8,10,11,12,15,16	3	P06911,P12020,P83121
8934	1,4,6,7,9,13,15,16	2,3,5,8,10,11,12,14,17	2	P62804,Q00715
8935	1,4,6,7,9,13,15,17	2,3,5,8,10,11,12,14,16	2	P05539,P62804
8936	1,4,6,7,9,13,16,17	2,3,5,8,10,11,12,14,15	2	P09605;P25809,P62804
8937	1,4,6,7,9,14,15,16	2,3,5,8,10,11,12,13,17	1	P62804
8938	1,4,6,7,9,14,15,17	2,3,5,8,10,11,12,13,16	0	

8939	1,4,6,7,9,14,16,17	2,3,5,8,10,11,12,13,15	0	
8940	1,4,6,7,9,15,16,17	2,3,5,8,10,11,12,13,14	2	P09605;P25809,P62804
8941	1,4,6,7,10,11,12,13	2,3,5,8,9,14,15,16,17	24	P02781,P04905,P06911,P07647,P08723,P09456,P0C0A9,P12020,P15399,P22282,P22283,P30120,P36374,P47727,P47967,Q5FVI6,Q5GRG2,Q62902,Q63357,Q6AYR9,Q6IFU7,Q8CJ52,Q9JHB9,Q9JI85
8942	1,4,6,7,10,11,12,14	2,3,5,8,9,13,15,16,17	21	P04905,P08723,P08753,P09456,P0C0A9,P12020,P15399,P20766,P30120,P35280,P36374,P47727,P83121,Q5FVI6,Q63357,Q63493,Q6AYL6,Q6AYR9,Q6IFU7,Q9JHB9,Q9JI85
8943	1,4,6,7,10,11,12,15	2,3,5,8,9,13,14,16,17	11	P08723,P08753,P0C0A9,P20766,P22006,P35280,P51907,Q5FVI6,Q63357,Q6IFU7,Q9JI85
8944	1,4,6,7,10,11,12,16	2,3,5,8,9,13,14,15,17	16	P04905,P06757,P08723,P08753,P0C0A9,P20766,P30120,P47967,P61206;P84079;P84082,P97840,Q5FVI6,Q63357,Q6AYL6,Q6IFU7,Q6P6Q2,Q9JI85
8945	1,4,6,7,10,11,12,17	2,3,5,8,9,13,14,15,16	18	O08815,P04905,P06757,P08723,P0C0A9,P13432,P15399,P30120,P36374,Q10758,Q5FVI6,Q63357,Q63493,Q6AYR9,Q99041,Q9JHB9,Q9JI85,Q9QZK9
8946	1,4,6,7,10,11,13,14	2,3,5,8,9,12,15,16,17	8	P06911,P12020,P15399,P47727,P83121,Q5FVI6,Q5GRG2,Q9JI85
8947	1,4,6,7,10,11,13,15	2,3,5,8,9,12,14,16,17	8	P05539,P12020,P22006,Q561R9,Q5FVI6,Q5GRG2,Q8VD89,Q9JI85
8948	1,4,6,7,10,11,13,16	2,3,5,8,9,12,14,15,17	5	P12020,P47967,P61206;P84079;P84082,Q5FVI6,Q8VD89
8949	1,4,6,7,10,11,13,17	2,3,5,8,9,12,14,15,16	6	P12020,P15399,P22282,P36374,Q5FVI6,Q9JI85
8950	1,4,6,7,10,11,14,15	2,3,5,8,9,12,13,16,17	6	P15399,P22006,P35280,P47727,P83121,Q5FVI6
8951	1,4,6,7,10,11,14,16	2,3,5,8,9,12,13,15,17	6	P15399,P20766,P47727,P61206;P84079;P84082,P83121,Q5FVI6
8952	1,4,6,7,10,11,14,17	2,3,5,8,9,12,13,15,16	5	P12020,P15399,P47727,P83121,Q5FVI6
8953	1,4,6,7,10,11,15,16	2,3,5,8,9,12,13,14,17	6	P20766,P22006,P35280,Q561R9,Q5FVI6,Q8VD89
8954	1,4,6,7,10,11,15,17	2,3,5,8,9,12,13,14,16	3	P15399,P22006,Q5FVI6
8955	1,4,6,7,10,11,16,17	2,3,5,8,9,12,13,14,15	2	P15399,Q5FVI6
8956	1,4,6,7,10,12,13,14	2,3,5,8,9,11,15,16,17	16	P04905,P06911,P07647,P08723,P09456,P0C0A9,P12020,P22282,P30120,P36374,Q498D9,Q5GRG2,Q62902,Q6AYR9,Q9JHB9,Q9JI85
8957	1,4,6,7,10,12,13,15	2,3,5,8,9,11,14,16,17	8	P08723,P12020,P36374,P97840,Q5FVI6,Q5GRG2,Q6AYR9,Q99MH3
8958	1,4,6,7,10,12,13,16	2,3,5,8,9,11,14,15,17	9	P06911,P08723,P12020,P19218,P36374,P47967,P97840,Q5FVI6,Q6AYR9
8959	1,4,6,7,10,12,13,17	2,3,5,8,9,11,14,15,16	14	P06911,P07647,P08723,P12020,P13432,P22282,P22283,P30120,P36374,Q6AYR9,Q99MH3,Q9JHB9,Q9JI85,Q9QZK9
8960	1,4,6,7,10,12,14,15	2,3,5,8,9,11,13,16,17	6	P08723,P12020,P20766,P22006,P35280,Q5FVI6

8961	1,4,6,7,10,12,14,16	2,3,5,8,9,11,13,15,17	6	P08721,P08723,P12020,P20766,P97840,Q5FVI6
8962	1,4,6,7,10,12,14,17	2,3,5,8,9,11,13,15,16	8	P08723,P12020,P13432,P20766,P36374,Q10758,Q6AYR9,Q9QZK9
8963	1,4,6,7,10,12,15,16	2,3,5,8,9,11,13,14,17	3	P20766,P97840,Q5FVI6
8964	1,4,6,7,10,12,15,17	2,3,5,8,9,11,13,14,16	5	P13432,P20766,Q5FVI6,Q99MH3,Q9QZK9
8965	1,4,6,7,10,12,16,17	2,3,5,8,9,11,13,14,15	6	P13432,P20766,P97840,Q10758,Q5FVI6,Q9QZK9
8966	1,4,6,7,10,13,14,15	2,3,5,8,9,11,12,16,17	4	P06911,P12020,P22006,Q5GRG2
8967	1,4,6,7,10,13,14,16	2,3,5,8,9,11,12,15,17	4	P06911,P12020,Q5FVI6,Q5GRG2
8968	1,4,6,7,10,13,14,17	2,3,5,8,9,11,12,15,16	3	P06911,P12020,Q5GRG2
8969	1,4,6,7,10,13,15,16	2,3,5,8,9,11,12,14,17	4	P62804,Q5FVI6,Q5GRG2,Q8VD89
8970	1,4,6,7,10,13,15,17	2,3,5,8,9,11,12,14,16	3	P12020,Q5GRG2,Q99MH3
8971	1,4,6,7,10,13,16,17	2,3,5,8,9,11,12,14,15	2	P12020,Q5FVI6
8972	1,4,6,7,10,14,15,16	2,3,5,8,9,11,12,13,17	2	P20766,Q5FVI6
8973	1,4,6,7,10,14,15,17	2,3,5,8,9,11,12,13,16	1	P22006
8974	1,4,6,7,10,14,16,17	2,3,5,8,9,11,12,13,15	0	
8975	1,4,6,7,10,15,16,17	2,3,5,8,9,11,12,13,14	2	P20766,Q5FVI6
8976	1,4,6,7,11,12,13,14	2,3,5,8,9,10,15,16,17	22	P02780,P02781,P04905,P05539,P06911,P07647,P08723,P09456,P0C0A9,P12020,P22282,P22283,P30120,P36374,P47727,Q5GRG2,Q62902,Q63493,Q6AYR9,Q8CJ52,Q9JHB9,Q9JI85
8977	1,4,6,7,11,12,13,15	2,3,5,8,9,10,14,16,17	11	P04905,P05539,P08723,P0C0A9,P22006,P30120,P36374,Q5GRG2,Q62902,Q9JHB9,Q9JI85
8978	1,4,6,7,11,12,13,16	2,3,5,8,9,10,14,15,17	9	P04905,P08723,P0C0A9,P30120,P36374,Q62902,Q8CJ52,Q9JHB9,Q9JI85
8979	1,4,6,7,11,12,13,17	2,3,5,8,9,10,14,15,16	19	P04905,P05539,P07647,P08723,P09605;P25809,P0C0A9,P13432,P15399,P22282,P22283,P30120,P36374,Q62902,Q63493,Q6AYR9,Q99041,Q9JHB9,Q9JI85,Q9QZK9
8980	1,4,6,7,11,12,14,15	2,3,5,8,9,10,13,16,17	8	P04905,P08723,P20766,P22006,P30120,P35280,P47727,Q9JI85
8981	1,4,6,7,11,12,14,16	2,3,5,8,9,10,13,15,17	9	P04905,P08723,P0C0A9,P20766,P30120,P47727,Q6AYL6,Q6IFU7,Q9JI85
8982	1,4,6,7,11,12,14,17	2,3,5,8,9,10,13,15,16	14	P04905,P08723,P0C0A9,P13432,P15399,P22282,P30120,P36374,P47727,Q63493,Q6AYR9,Q9JHB9,Q9JI85,Q9QZK9
8983	1,4,6,7,11,12,15,16	2,3,5,8,9,10,13,14,17	6	P20766,P22006,P51907,Q5FVI6,Q6IFU7,Q9JI85

8984	1,4,6,7,11,<br>12,15,17	2,3,5,8,9,1<br>0,13,14,16	8	P09605;P25809,P13432,P15399,P22006,P36374,Q99041,Q9JI85,Q9QZK9
8985	1,4,6,7,11,<br>12,16,17	2,3,5,8,9,1<br>0,13,14,15	8	P09605;P25809,P13432,P15399,P20766,P36374,Q99041,Q9JI85,Q9QZK9
8986	1,4,6,7,11,<br>13,14,15	2,3,5,8,9,1<br>0,12,16,17	8	P05539,P06911,P12020,P22006,P47727,P83121,Q5GRG2,Q9JI85
8987	1,4,6,7,11,<br>13,14,16	2,3,5,8,9,1<br>0,12,15,17	6	P06911,P12020,P47727,P83121,Q5GRG2,Q62902
8988	1,4,6,7,11,<br>13,14,17	2,3,5,8,9,1<br>0,12,15,16	9	P05539,P06911,P12020,P15399,P22282,P47727,P83121,Q5GRG2,Q9JI85
8989	1,4,6,7,11,<br>13,15,16	2,3,5,8,9,1<br>0,12,14,17	3	P05539,P22006,Q8VD89
8990	1,4,6,7,11,<br>13,15,17	2,3,5,8,9,1<br>0,12,14,16	2	P05539,P22006
8991	1,4,6,7,11,<br>13,16,17	2,3,5,8,9,1<br>0,12,14,15	0	
8992	1,4,6,7,11,<br>14,15,16	2,3,5,8,9,1<br>0,12,13,17	2	P20766,P22006
8993	1,4,6,7,11,<br>14,15,17	2,3,5,8,9,1<br>0,12,13,16	2	P15399,P22006
8994	1,4,6,7,11,<br>14,16,17	2,3,5,8,9,1<br>0,12,13,15	1	P15399
8995	1,4,6,7,11,<br>15,16,17	2,3,5,8,9,1<br>0,12,13,14	1	P22006
8996	1,4,6,7,12,<br>13,14,15	2,3,5,8,9,1<br>0,11,16,17	6	P06911,P08723,P12020,P30120,Q5GRG2,Q9JI85
8997	1,4,6,7,12,<br>13,14,16	2,3,5,8,9,1<br>0,11,15,17	7	P06911,P08723,P12020,P30120,Q5GRG2,Q62902,Q6AYR9
8998	1,4,6,7,12,<br>13,14,17	2,3,5,8,9,1<br>0,11,15,16	14	P06911,P08723,P12020,P13432,P22282,P22283,P30120,P36374,Q5GRG2,Q62902,Q6AYR9,Q9JHB9,Q9JI85,Q9QZK9
8999	1,4,6,7,12,<br>13,15,16	2,3,5,8,9,1<br>0,11,14,17	1	P19218
9000	1,4,6,7,12,<br>13,15,17	2,3,5,8,9,1<br>0,11,14,16	5	P13432,P22282,P36374,Q99MH3,Q9QZK9
9001	1,4,6,7,12,<br>13,16,17	2,3,5,8,9,1<br>0,11,14,15	6	P09605;P25809,P13432,P22282,P27213,P36374,Q9QZK9
9002	1,4,6,7,12,<br>14,15,16	2,3,5,8,9,1<br>0,11,13,17	2	P20766,P35467
9003	1,4,6,7,12,<br>14,15,17	2,3,5,8,9,1<br>0,11,13,16	2	P13432,P20766
9004	1,4,6,7,12,<br>14,16,17	2,3,5,8,9,1<br>0,11,13,15	2	P13432,P20766
9005	1,4,6,7,12,<br>15,16,17	2,3,5,8,9,1<br>0,11,13,14	3	P09605;P25809,P13432,P20766
9006	1,4,6,7,13,<br>14,15,16	2,3,5,8,9,1<br>0,11,12,17	5	P06911,P12020,P35467,P62804,Q5GRG2

9007	1,4,6,7,13,14,15,17	2,3,5,8,9,10,11,12,16	4	P06911,P12020,Q5GRG2,Q9WUW8
9008	1,4,6,7,13,14,16,17	2,3,5,8,9,10,11,12,15	3	P06911,P12020,P27213
9009	1,4,6,7,13,15,16,17	2,3,5,8,9,10,11,12,14	2	P27213,P62804
9010	1,4,6,7,14,15,16,17	2,3,5,8,9,10,11,12,13	2	P20766,P35467
9011	1,4,6,8,9,10,11,12	2,3,5,7,13,14,15,16,17	23	O35547,P00714,P02780,P02781,P02782,P02803,P04905,P07647,P08723,P0C0A9,P14669,P16636,P30120,P35281,P36374,P47727,Q09030,Q63357,Q6AYR9,Q6IFU7,Q6IMF3,Q6P6Q2,Q9JHB9
9012	1,4,6,8,9,10,11,13	2,3,5,7,12,14,15,16,17	6	P02803,P0C0A9,P14669,P47727,Q561R9,Q8CJD3
9013	1,4,6,8,9,10,11,14	2,3,5,7,12,13,15,16,17	4	P00714,P14669,P47727,P83121
9014	1,4,6,8,9,10,11,15	2,3,5,7,12,13,14,16,17	4	P14669,P35280,P80201,Q561R9
9015	1,4,6,8,9,10,11,16	2,3,5,7,12,13,14,15,17	4	A2RUV9,P00714,P14668,Q9WUW8
9016	1,4,6,8,9,10,11,17	2,3,5,7,12,13,14,15,16	4	A2RUV9,P14669,P16636,Q09030
9017	1,4,6,8,9,10,12,13	2,3,5,7,11,14,15,16,17	13	P02780,P02781,P02782,P02803,P07647,P08723,P0C0A9,P22282,P22283,P30120,P36374,Q6AYR9,Q9JHB9
9018	1,4,6,8,9,10,12,14	2,3,5,7,11,13,15,16,17	5	P02782,P08723,P0C0A9,P30120,Q6AYR9
9019	1,4,6,8,9,10,12,15	2,3,5,7,11,13,14,16,17	5	P08723,P0C0A9,P10758,P35281,P80201
9020	1,4,6,8,9,10,12,16	2,3,5,7,11,13,14,15,17	3	P08723,P0C0A9,Q9WUW8
9021	1,4,6,8,9,10,12,17	2,3,5,7,11,13,14,15,16	13	P02782,P08644,P08723,P0C0A9,P13432,P16636,P19223,P30120,P36374,Q09030,Q10758,Q4G075,Q6AYR9
9022	1,4,6,8,9,10,13,14	2,3,5,7,11,12,15,16,17	3	P06911,P12020,Q5GRG2

9023	1,4,6,8,9,10,13,15	2,3,5,7,11,12,14,16,17	0	
9024	1,4,6,8,9,10,13,16	2,3,5,7,11,12,14,15,17	1	Q8CJD3
9025	1,4,6,8,9,10,13,17	2,3,5,7,11,12,14,15,16	0	
9026	1,4,6,8,9,10,14,15	2,3,5,7,11,12,13,16,17	1	P17046
9027	1,4,6,8,9,10,14,16	2,3,5,7,11,12,13,15,17	0	
9028	1,4,6,8,9,10,14,17	2,3,5,7,11,12,13,15,16	1	Q09030
9029	1,4,6,8,9,10,15,16	2,3,5,7,11,12,13,14,17	0	
9030	1,4,6,8,9,10,15,17	2,3,5,7,11,12,13,14,16	1	P80201
9031	1,4,6,8,9,10,16,17	2,3,5,7,11,12,13,14,15	4	A2RUV9,P25031,Q09030,Q6P6R2
9032	1,4,6,8,9,11,12,13	2,3,5,7,10,14,15,16,17	19	P02780,P02781,P02782,P02803,P04905,P07647,P08723,P0C0A9,P13432,P22282,P22283,P30120,P36374,P47727,Q62902,Q6AYR9,Q8CJ52,Q9JHB9,Q9JI85
9033	1,4,6,8,9,11,12,14	2,3,5,7,10,13,15,16,17	14	P02780,P02781,P02782,P04905,P07647,P08723,P0C0A9,P13432,P14669,P30120,P36374,P47727,Q6AYR9,Q9JHB9
9034	1,4,6,8,9,11,12,15	2,3,5,7,10,13,14,16,17	7	P02782,P08723,P0C0A9,P10758,P14669,P30120,Q9JHB9
9035	1,4,6,8,9,11,12,16	2,3,5,7,10,13,14,15,17	9	P02782,P08723,P0C0A9,P14668,P30120,Q6IFU7,Q6P6Q2,Q8CJ52,Q9JHB9
9036	1,4,6,8,9,11,12,17	2,3,5,7,10,13,14,15,16	19	P02780,P02781,P02782,P04905,P07647,P08723,P0C0A9,P13432,P14669,P16636,P19223,P22282,P22283,P30120,P36374,Q09030,Q4G075,Q6AYR9,Q9JHB9
9037	1,4,6,8,9,11,13,14	2,3,5,7,10,12,15,16,17	4	P02782,P06911,P47727,P83121

9038	1,4,6,8,9,11,13,15	2,3,5,7,10,12,14,16,17	1	Q561R9
9039	1,4,6,8,9,11,13,16	2,3,5,7,10,12,14,15,17	2	A2RUV9,Q8CJD3
9040	1,4,6,8,9,11,13,17	2,3,5,7,10,12,14,15,16	2	P14669,P22282
9041	1,4,6,8,9,11,14,15	2,3,5,7,10,12,13,16,17	1	P14669
9042	1,4,6,8,9,11,14,16	2,3,5,7,10,12,13,15,17	2	P14668,P83121
9043	1,4,6,8,9,11,14,17	2,3,5,7,10,12,13,15,16	2	P14669,P83121
9044	1,4,6,8,9,11,15,16	2,3,5,7,10,12,13,14,17	3	P14668,Q561R9,Q5QE79
9045	1,4,6,8,9,11,15,17	2,3,5,7,10,12,13,14,16	1	P14669
9046	1,4,6,8,9,11,16,17	2,3,5,7,10,12,13,14,15	2	A2RUV9,Q09030
9047	1,4,6,8,9,12,13,14	2,3,5,7,10,11,15,16,17	13	P02780,P02782,P06911,P07647,P08723,P0C0A9,P22282,P22283,P30120,P36374,Q62902,Q6AYR9,Q9JHB9
9048	1,4,6,8,9,12,13,15	2,3,5,7,10,11,14,16,17	9	P02782,P07647,P08723,P0C0A9,P10758,P30120,P36374,Q6AYR9,Q9JHB9
9049	1,4,6,8,9,12,13,16	2,3,5,7,10,11,14,15,17	9	P02782,P02803,P07647,P08723,P0C0A9,P30120,P36374,Q6AYR9,Q9JHB9
9050	1,4,6,8,9,12,13,17	2,3,5,7,10,11,14,15,16	16	P02780,P02782,P07647,P08723,P0C0A9,P13432,P16636,P22282,P22283,P30120,P36374,Q4G075,Q62635,Q6AYR9,Q9JHB9,Q9QZK9
9051	1,4,6,8,9,12,14,15	2,3,5,7,10,11,13,16,17	3	P02782,P08723,P10758
9052	1,4,6,8,9,12,14,16	2,3,5,7,10,11,13,15,17	4	P02782,P08723,P0C0A9,P30120

9053	1,4,6,8,9,12,14,17	2,3,5,7,10,11,13,15,16	9	P01835,P02782,P08723,P0C0A9,P13432,P30120,P36374,Q09030,Q6AYR9
9054	1,4,6,8,9,12,15,16	2,3,5,7,10,11,13,14,17	0	
9055	1,4,6,8,9,12,15,17	2,3,5,7,10,11,13,14,16	4	B0BNN3,P08723,P13432,Q4G075
9056	1,4,6,8,9,12,16,17	2,3,5,7,10,11,13,14,15	5	P08723,P13432,P16636,Q09030,Q4G075
9057	1,4,6,8,9,13,14,15	2,3,5,7,10,11,12,16,17	2	P06911,Q5GRG2
9058	1,4,6,8,9,13,14,16	2,3,5,7,10,11,12,15,17	1	P06911
9059	1,4,6,8,9,13,14,17	2,3,5,7,10,11,12,15,16	1	P06911
9060	1,4,6,8,9,13,15,16	2,3,5,7,10,11,12,14,17	0	
9061	1,4,6,8,9,13,15,17	2,3,5,7,10,11,12,14,16	0	
9062	1,4,6,8,9,13,16,17	2,3,5,7,10,11,12,14,15	0	
9063	1,4,6,8,9,14,15,16	2,3,5,7,10,11,12,13,17	0	
9064	1,4,6,8,9,14,15,17	2,3,5,7,10,11,12,13,16	0	
9065	1,4,6,8,9,14,16,17	2,3,5,7,10,11,12,13,15	0	
9066	1,4,6,8,9,15,16,17	2,3,5,7,10,11,12,13,14	1	Q5QE79
9067	1,4,6,8,10,11,12,13	2,3,5,7,9,14,15,16,17	26	O35547,P02780,P02781,P02782,P02803,P04905,P07647,P08723,P0C0A9,P14669,P16636,P22282,P22283,P24368,P30120,P35281,P36374,P47727,Q62975,Q63357,Q6AYR9,Q6IFU7,Q6P6Q2,Q8CJ52,Q9JHB9,Q9JI85
9068	1,4,6,8,10,11,12,14	2,3,5,7,9,13,15,16,17	23	O35547,P00714,P02780,P02781,P02782,P04905,P07647,P08723,P0C0A9,P14669,P24368,P30120,P35280,P35281,P36374,P47727,Q63357,Q6AYR9,Q6IFU7,Q6IMF3,Q6P6Q2,Q8CJ52,Q9JHB9

9069	1,4,6,8,10,11,12,15	2,3,5,7,9,13,14,16,17	15	O35547,P02782,P04905,P08010,P08723,P0C0A9,P14669,P30120,P35280,P35281,Q561R9,Q63357,Q6IFU7,Q6P6Q2,Q9JHB9
9070	1,4,6,8,10,11,12,16	2,3,5,7,9,13,14,15,17	17	O35547,P00714,P02782,P04905,P08723,P0C0A9,P16636,P30120,P35281,P53792,Q63357,Q6IFU7,Q6IMF3,Q6P6Q2,Q8CJ52,Q9JHB9,Q9WUW8
9071	1,4,6,8,10,11,12,17	2,3,5,7,9,13,14,15,16	28	F1LM93,O08815,O35547,P02780,P02781,P02782,P04905,P06757,P07150,P07647,P08723,P0C0A9,P13432,P14669,P16636,P22282,P22283,P30120,P36374,P47727,Q10758,Q4G075,Q63357,Q6AYR9,Q6P6Q2,Q8CJ52,Q9JHB9,Q9QZK9
9072	1,4,6,8,10,11,13,14	2,3,5,7,9,12,15,16,17	6	P06911,P0C0A9,P12020,P14669,P47727,Q5GRG2
9073	1,4,6,8,10,11,13,15	2,3,5,7,9,12,14,16,17	2	P14669,Q561R9
9074	1,4,6,8,10,11,13,16	2,3,5,7,9,12,14,15,17	1	Q8CJD3
9075	1,4,6,8,10,11,13,17	2,3,5,7,9,12,14,15,16	4	P14669,P16636,P22282,P47727
9076	1,4,6,8,10,11,14,15	2,3,5,7,9,12,13,16,17	3	P14669,P35280,P47727
9077	1,4,6,8,10,11,14,16	2,3,5,7,9,12,13,15,17	1	P47727
9078	1,4,6,8,10,11,14,17	2,3,5,7,9,12,13,15,16	3	P07150,P14669,P47727
9079	1,4,6,8,10,11,15,16	2,3,5,7,9,12,13,14,17	5	P14668,P35280,P35281,Q561R9,Q5FVI6
9080	1,4,6,8,10,11,15,17	2,3,5,7,9,12,13,14,16	2	P14669,Q4G075
9081	1,4,6,8,10,11,16,17	2,3,5,7,9,12,13,14,15	5	A2RUV9,P07150,P14669,P16636,Q4G075
9082	1,4,6,8,10,12,13,14	2,3,5,7,9,11,15,16,17	19	P02780,P02781,P02782,P04905,P06911,P07647,P08723,P0C0A9,P12020,P22282,P22283,P30120,P36374,P47727,Q498D9,Q5GRG2,Q6AYR9,Q8CJ52,Q9JHB9
9083	1,4,6,8,10,12,13,15	2,3,5,7,9,11,14,16,17	11	P02782,P07647,P08723,P0C0A9,P15999,P30120,P35281,Q6AYR9,Q6IFV1,Q99MH3,Q9JHB9
9084	1,4,6,8,10,12,13,16	2,3,5,7,9,11,14,15,17	8	P02782,P07647,P08723,P0C0A9,P30120,Q6AYR9,Q8CJ52,Q9JHB9
9085	1,4,6,8,10,12,13,17	2,3,5,7,9,11,14,15,16	19	P02780,P02782,P07647,P08723,P0C0A9,P13432,P16636,P22282,P22283,P30120,P36374,Q10758,Q4G075,Q62635,Q6AYR9,Q6IFV1,Q99MH3,Q9JHB9,Q9QZK9
9086	1,4,6,8,10,12,14,15	2,3,5,7,9,11,13,16,17	3	P08723,P0C0A9,P35280
9087	1,4,6,8,10,12,14,16	2,3,5,7,9,11,13,15,17	5	P08721,P08723,P0C0A9,P30120,Q10758
9088	1,4,6,8,10,12,14,17	2,3,5,7,9,11,13,15,16	10	P02782,P08723,P0C0A9,P13432,P30120,P36374,Q10758,Q4G075,Q6AYR9,Q9QZK9
9089	1,4,6,8,10,12,15,16	2,3,5,7,9,11,13,14,17	2	P08723,P35281
9090	1,4,6,8,10,12,15,17	2,3,5,7,9,11,13,14,16	7	B0BNN3,P08723,P13432,Q4G075,Q6IFV1,Q99MH3,Q9QZK9
9091	1,4,6,8,10,12,16,17	2,3,5,7,9,11,13,14,15	5	P08723,P13432,P16636,Q10758,Q4G075

9092	1,4,6,8,10,13,14,15	2,3,5,7,9,11,12,16,17	4	P06911,P12020,P15999,Q5GRG2
9093	1,4,6,8,10,13,14,16	2,3,5,7,9,11,12,15,17	3	P06911,P12020,Q5GRG2
9094	1,4,6,8,10,13,14,17	2,3,5,7,9,11,12,15,16	3	P06911,P12020,Q5GRG2
9095	1,4,6,8,10,13,15,16	2,3,5,7,9,11,12,14,17	0	
9096	1,4,6,8,10,13,15,17	2,3,5,7,9,11,12,14,16	3	P15999,Q6IFV1,Q99MH3
9097	1,4,6,8,10,13,16,17	2,3,5,7,9,11,12,14,15	0	
9098	1,4,6,8,10,14,15,16	2,3,5,7,9,11,12,13,17	0	
9099	1,4,6,8,10,14,15,17	2,3,5,7,9,11,12,13,16	1	B0BNN3
9100	1,4,6,8,10,14,16,17	2,3,5,7,9,11,12,13,15	0	
9101	1,4,6,8,10,15,16,17	2,3,5,7,9,11,12,13,14	0	
9102	1,4,6,8,11,12,13,14	2,3,5,7,9,10,15,16,17	21	O35547,P02780,P02781,P02782,P04905,P06911,P07647,P08723,P0C0A9,P22282,P22283,P24368,P30120,P36374,P47727,Q5GRG2,Q62902,Q6AYR9,Q8CJ52,Q9JHB9,Q9JI85
9103	1,4,6,8,11,12,13,15	2,3,5,7,9,10,14,16,17	16	O35547,P02780,P02781,P02782,P04905,P07647,P08723,P0C0A9,P14669,P30120,P36374,P47727,Q6AYR9,Q8CJ52,Q9JHB9,Q9JI85
9104	1,4,6,8,11,12,13,16	2,3,5,7,9,10,14,15,17	16	O35547,P02780,P02781,P02782,P04905,P07647,P08723,P0C0A9,P30120,P36374,P47727,Q62902,Q6AYR9,Q6IFU7,Q8CJ52,Q9JHB9
9105	1,4,6,8,11,12,13,17	2,3,5,7,9,10,14,15,16	24	O35547,P02780,P02781,P02782,P04905,P07647,P08723,P0C0A9,P13432,P14669,P16636,P22282,P22283,P30120,P36374,P47727,Q4G075,Q62635,Q62902,Q6AYR9,Q8CJ52,Q9JHB9,Q9JI85,Q9QZK9
9106	1,4,6,8,11,12,14,15	2,3,5,7,9,10,13,16,17	10	O35547,P02782,P04905,P08723,P0C0A9,P14669,P30120,P35280,P47727,Q9JHB9
9107	1,4,6,8,11,12,14,16	2,3,5,7,9,10,13,15,17	12	O35547,P02782,P04905,P08723,P0C0A9,P14668,P30120,P47727,Q6IFU7,Q6P6Q2,Q8CJ52,Q9JHB9
9108	1,4,6,8,11,12,14,17	2,3,5,7,9,10,13,15,16	19	O35547,P02780,P02781,P02782,P04905,P07647,P08723,P0C0A9,P13432,P14669,P22282,P22283,P30120,P36374,P47727,Q4G075,Q6AYR9,Q8CJ52,Q9JHB9
9109	1,4,6,8,11,12,15,16	2,3,5,7,9,10,13,14,17	5	O35547,P08723,P0C0A9,P14668,Q6IFU7
9110	1,4,6,8,11,12,15,17	2,3,5,7,9,10,13,14,16	11	B0BNN3,O35547,P02782,P08723,P0C0A9,P13432,P14669,P30120,P36374,Q4G075,Q9QZA6
9111	1,4,6,8,11,12,16,17	2,3,5,7,9,10,13,14,15	12	O35547,P02782,P07150,P08723,P0C0A9,P13432,P16636,P30120,P36374,Q4G075,Q6P6Q2,Q8CJ52
9112	1,4,6,8,11,13,14,15	2,3,5,7,9,10,12,16,17	3	P06911,P47727,Q5GRG2
9113	1,4,6,8,11,13,14,16	2,3,5,7,9,10,12,15,17	2	P06911,P47727
9114	1,4,6,8,11,13,14,17	2,3,5,7,9,10,12,15,16	5	P02782,P06911,P14669,P22282,P47727

9115	1,4,6,8,11,13,15,16	2,3,5,7,9,10,12,14,17	0	
9116	1,4,6,8,11,13,15,17	2,3,5,7,9,10,12,14,16	1	P14669
9117	1,4,6,8,11,13,16,17	2,3,5,7,9,10,12,14,15	1	A2RUV9
9118	1,4,6,8,11,14,15,16	2,3,5,7,9,10,12,13,17	1	P14668
9119	1,4,6,8,11,14,15,17	2,3,5,7,9,10,12,13,16	3	B0BNN3,P14669,P97840
9120	1,4,6,8,11,14,16,17	2,3,5,7,9,10,12,13,15	1	P07150
9121	1,4,6,8,11,15,16,17	2,3,5,7,9,10,12,13,14	0	
9122	1,4,6,8,12,13,14,15	2,3,5,7,9,10,11,16,17	14	O08557,P02782,P06911,P07647,P08723,P0C0A9,P10758,P15999,P30120,Q5GRG2,Q64093,Q6AYR9,Q9JHB9,Q9QUL6
9123	1,4,6,8,12,13,14,16	2,3,5,7,9,10,11,15,17	11	P02782,P06911,P07647,P08723,P0C0A9,P30120,Q62902,Q6AYR9,Q8CJ52,Q9JHB9,Q9QUL6
9124	1,4,6,8,12,13,14,17	2,3,5,7,9,10,11,15,16	18	P02780,P02782,P06761,P06911,P07647,P08723,P0C0A9,P13432,P22282,P22283,P27213,P30120,P36374,Q4G075,Q62635,Q6AYR9,Q9JHB9,Q9QZK9
9125	1,4,6,8,12,13,15,16	2,3,5,7,9,10,11,14,17	3	P02782,P08723,Q9QUL6
9126	1,4,6,8,12,13,15,17	2,3,5,7,9,10,11,14,16	14	B0BNN3,P02782,P07647,P08723,P13432,P22282,P30120,P36374,Q4G075,Q62635,Q6AYR9,Q6IFV1,Q99MH3,Q9QZK9
9127	1,4,6,8,12,13,16,17	2,3,5,7,9,10,11,14,15	13	P02782,P07647,P08723,P13432,P16636,P22282,P27213,P30120,P36374,Q4G075,Q62635,Q6AYR9,Q9QZK9
9128	1,4,6,8,12,14,15,16	2,3,5,7,9,10,11,13,17	1	P08723
9129	1,4,6,8,12,14,15,17	2,3,5,7,9,10,11,13,16	5	B0BNN3,P02782,P08723,P13432,Q4G075
9130	1,4,6,8,12,14,16,17	2,3,5,7,9,10,11,13,15	4	P02782,P08723,P13432,Q4G075
9131	1,4,6,8,12,15,16,17	2,3,5,7,9,10,11,13,14	3	B0BNN3,P13432,Q4G075
9132	1,4,6,8,13,14,15,16	2,3,5,7,9,10,11,12,17	2	P06911,Q5GRG2
9133	1,4,6,8,13,14,15,17	2,3,5,7,9,10,11,12,16	4	P06911,P15999,Q5GRG2,Q64093
9134	1,4,6,8,13,14,16,17	2,3,5,7,9,10,11,12,15	3	P06911,P27213,Q6PCU2
9135	1,4,6,8,13,15,16,17	2,3,5,7,9,10,11,12,14	1	P27213
9136	1,4,6,8,14,15,16,17	2,3,5,7,9,10,11,12,13	0	
9137	1,4,6,9,10,11,12,13	2,3,5,7,8,14,15,16,17	8	P02803,P04905,P0C0A9,P30120,P36374,P47727,Q6IFU7,Q9JHB9

9138	1,4,6,9,10,11,12,14	2,3,5,7,8,13,15,16,17	5	P04905,P0C0A9,P47727,P83121,Q6IFU7
9139	1,4,6,9,10,11,12,15	2,3,5,7,8,13,14,16,17	7	P08753,P0C0A9,P10758,P51907,Q561R9,Q566E6,Q6IFU7
9140	1,4,6,9,10,11,12,16	2,3,5,7,8,13,14,15,17	9	P06757,P08753,P0C0A9,P10536,Q5FVI6,Q6IFU7,Q6IMF3,Q6P6Q2,Q9WUW8
9141	1,4,6,9,10,11,12,17	2,3,5,7,8,13,14,15,16	9	O08815,P06757,P0C0A9,P13432,P16636,P36374,Q09030,Q566E6,Q6IFU7
9142	1,4,6,9,10,11,13,14	2,3,5,7,8,12,15,16,17	3	P47727,P50115,P83121
9143	1,4,6,9,10,11,13,15	2,3,5,7,8,12,14,16,17	2	P50115,Q561R9
9144	1,4,6,9,10,11,13,16	2,3,5,7,8,12,14,15,17	2	P50115,Q8CJD3
9145	1,4,6,9,10,11,13,17	2,3,5,7,8,12,14,15,16	0	
9146	1,4,6,9,10,11,14,15	2,3,5,7,8,12,13,16,17	3	P50115,P83121,Q561R9
9147	1,4,6,9,10,11,14,16	2,3,5,7,8,12,13,15,17	3	P50115,P55091,P83121
9148	1,4,6,9,10,11,14,17	2,3,5,7,8,12,13,15,16	1	P83121
9149	1,4,6,9,10,11,15,16	2,3,5,7,8,12,13,14,17	5	P50115,P55091,Q561R9,Q5FVI6,Q9Z1F2
9150	1,4,6,9,10,11,15,17	2,3,5,7,8,12,13,14,16	1	Q566E6
9151	1,4,6,9,10,11,16,17	2,3,5,7,8,12,13,14,15	2	A2RUV9,Q09030
9152	1,4,6,9,10,12,13,14	2,3,5,7,8,11,15,16,17	4	P0C0A9,P36374,P50115,Q6AYR9
9153	1,4,6,9,10,12,13,15	2,3,5,7,8,11,14,16,17	2	P10758,P50115
9154	1,4,6,9,10,12,13,16	2,3,5,7,8,11,14,15,17	2	P50115,P97840
9155	1,4,6,9,10,12,13,17	2,3,5,7,8,11,14,15,16	3	P36374,Q6AYR9,Q9QZK9
9156	1,4,6,9,10,12,14,15	2,3,5,7,8,11,13,16,17	2	P10758,P50115
9157	1,4,6,9,10,12,14,16	2,3,5,7,8,11,13,15,17	2	P20766,P50115
9158	1,4,6,9,10,12,14,17	2,3,5,7,8,11,13,15,16	4	P01835,P13432,Q09030,Q10758
9159	1,4,6,9,10,12,15,16	2,3,5,7,8,11,13,14,17	2	P20766,P50115
9160	1,4,6,9,10,12,15,17	2,3,5,7,8,11,13,14,16	0	

9161	1,4,6,9,10,<br>12,16,17	2,3,5,7,8,1<br>1,13,14,15	2	Q09030,Q10758
9162	1,4,6,9,10,<br>13,14,15	2,3,5,7,8,1<br>1,12,16,17	1	P50115
9163	1,4,6,9,10,<br>13,14,16	2,3,5,7,8,1<br>1,12,15,17	1	P50115
9164	1,4,6,9,10,<br>13,14,17	2,3,5,7,8,1<br>1,12,15,16	0	
9165	1,4,6,9,10,<br>13,15,16	2,3,5,7,8,1<br>1,12,14,17	2	P50115,P62804
9166	1,4,6,9,10,<br>13,15,17	2,3,5,7,8,1<br>1,12,14,16	0	
9167	1,4,6,9,10,<br>13,16,17	2,3,5,7,8,1<br>1,12,14,15	1	Q8CJD3
9168	1,4,6,9,10,<br>14,15,16	2,3,5,7,8,1<br>1,12,13,17	2	P50115,P55091
9169	1,4,6,9,10,<br>14,15,17	2,3,5,7,8,1<br>1,12,13,16	0	
9170	1,4,6,9,10,<br>14,16,17	2,3,5,7,8,1<br>1,12,13,15	0	
9171	1,4,6,9,10,<br>15,16,17	2,3,5,7,8,1<br>1,12,13,14	0	
9172	1,4,6,9,11,<br>12,13,14	2,3,5,7,8,1<br>0,15,16,17	7	P04905,P0C0A9,P30120,P36374,P47727,P83121,Q9JHB9
9173	1,4,6,9,11,<br>12,13,15	2,3,5,7,8,1<br>0,14,16,17	4	P0C0A9,P10758,P36374,Q9JHB9
9174	1,4,6,9,11,<br>12,13,16	2,3,5,7,8,1<br>0,14,15,17	5	P02803,P0C0A9,P36374,Q6IFU7,Q9JHB9
9175	1,4,6,9,11,<br>12,13,17	2,3,5,7,8,1<br>0,14,15,16	5	P02803,P0C0A9,P13432,P36374,Q9JHB9
9176	1,4,6,9,11,<br>12,14,15	2,3,5,7,8,1<br>0,13,16,17	1	P10758
9177	1,4,6,9,11,<br>12,14,16	2,3,5,7,8,1<br>0,13,15,17	1	Q6IFU7
9178	1,4,6,9,11,<br>12,14,17	2,3,5,7,8,1<br>0,13,15,16	5	P01835,P04905,P0C0A9,P13432,P36374
9179	1,4,6,9,11,<br>12,15,16	2,3,5,7,8,1<br>0,13,14,17	3	P20766,P51907,Q6IFU7
9180	1,4,6,9,11,<br>12,15,17	2,3,5,7,8,1<br>0,13,14,16	1	P13432
9181	1,4,6,9,11,<br>12,16,17	2,3,5,7,8,1<br>0,13,14,15	3	P13432,Q09030,Q6IFU7
9182	1,4,6,9,11,<br>13,14,15	2,3,5,7,8,1<br>0,12,16,17	3	P16391,P50115,P83121
9183	1,4,6,9,11,<br>13,14,16	2,3,5,7,8,1<br>0,12,15,17	2	P50115,P83121

9184	1,4,6,9,11,<br>13,14,17	2,3,5,7,8,1<br>0,12,15,16	1	P83121
9185	1,4,6,9,11,<br>13,15,16	2,3,5,7,8,1<br>0,12,14,17	2	P50115,Q561R9
9186	1,4,6,9,11,<br>13,15,17	2,3,5,7,8,1<br>0,12,14,16	0	
9187	1,4,6,9,11,<br>13,16,17	2,3,5,7,8,1<br>0,12,14,15	2	A2RUV9,Q8CJD3
9188	1,4,6,9,11,<br>14,15,16	2,3,5,7,8,1<br>0,12,13,17	2	P50115,P55091
9189	1,4,6,9,11,<br>14,15,17	2,3,5,7,8,1<br>0,12,13,16	0	
9190	1,4,6,9,11,<br>14,16,17	2,3,5,7,8,1<br>0,12,13,15	1	P83121
9191	1,4,6,9,11,<br>15,16,17	2,3,5,7,8,1<br>0,12,13,14	0	
9192	1,4,6,9,12,<br>13,14,15	2,3,5,7,8,1<br>0,11,16,17	3	P10758,P16391,P50115
9193	1,4,6,9,12,<br>13,14,16	2,3,5,7,8,1<br>0,11,15,17	1	P50115
9194	1,4,6,9,12,<br>13,14,17	2,3,5,7,8,1<br>0,11,15,16	3	P01835,P13432,P36374
9195	1,4,6,9,12,<br>13,15,16	2,3,5,7,8,1<br>0,11,14,17	1	P50115
9196	1,4,6,9,12,<br>13,15,17	2,3,5,7,8,1<br>0,11,14,16	1	P13432
9197	1,4,6,9,12,<br>13,16,17	2,3,5,7,8,1<br>0,11,14,15	2	P13432,P27213
9198	1,4,6,9,12,<br>14,15,16	2,3,5,7,8,1<br>0,11,13,17	2	P20766,P50115
9199	1,4,6,9,12,<br>14,15,17	2,3,5,7,8,1<br>0,11,13,16	3	P01835,P13432,Q5HZV9
9200	1,4,6,9,12,<br>14,16,17	2,3,5,7,8,1<br>0,11,13,15	3	P01835,P13432,Q5HZV9
9201	1,4,6,9,12,<br>15,16,17	2,3,5,7,8,1<br>0,11,13,14	1	P13432
9202	1,4,6,9,13,<br>14,15,16	2,3,5,7,8,1<br>0,11,12,17	3	P16391,P50115,P62804
9203	1,4,6,9,13,<br>14,15,17	2,3,5,7,8,1<br>0,11,12,16	1	P16391
9204	1,4,6,9,13,<br>14,16,17	2,3,5,7,8,1<br>0,11,12,15	2	P00731,P27213
9205	1,4,6,9,13,<br>15,16,17	2,3,5,7,8,1<br>0,11,12,14	2	P27213,P62804
9206	1,4,6,9,14,<br>15,16,17	2,3,5,7,8,1<br>0,11,12,13	1	Q5HZV9

9207	1,4,6,10,1<br>1,12,13,14	2,3,5,7,8,9<br>,15,16,17	10	P04905,P06911,P08723,P0C0A9,P30120,P36374,P47727,Q6AYR9,Q6IFU7,Q9JHB9
9208	1,4,6,10,1<br>1,12,13,15	2,3,5,7,8,9<br>,14,16,17	5	P0C0A9,P36374,P47727,Q6IFU7,Q9JHB9
9209	1,4,6,10,1<br>1,12,13,16	2,3,5,7,8,9<br>,14,15,17	7	P04905,P0C0A9,P36374,P47727,Q6IFU7,Q6P6Q2,Q9JHB9
9210	1,4,6,10,1<br>1,12,13,17	2,3,5,7,8,9<br>,14,15,16	12	O08815,P04905,P06757,P0C0A9,P16636,P30120,P36374,P47727,Q6AYR9,Q99MH3,Q9JHB9,Q9QZK9
9211	1,4,6,10,1<br>1,12,14,15	2,3,5,7,8,9<br>,13,16,17	7	P04905,P0C0A9,P20766,P35280,P47727,P51907,Q6IFU7
9212	1,4,6,10,1<br>1,12,14,16	2,3,5,7,8,9<br>,13,15,17	8	P04905,P0C0A9,P20766,P47727,P53792,Q6IFU7,Q6IMF3,Q6P6Q2
9213	1,4,6,10,1<br>1,12,14,17	2,3,5,7,8,9<br>,13,15,16	9	O08815,P04905,P0C0A9,P13432,P20766,P36374,P47727,Q10758,Q6IFU7
9214	1,4,6,10,1<br>1,12,15,16	2,3,5,7,8,9<br>,13,14,17	6	P20766,P51907,P53792,Q5FVI6,Q6IFU7,Q6P6Q2
9215	1,4,6,10,1<br>1,12,15,17	2,3,5,7,8,9<br>,13,14,16	4	P06757,P06866,P20766,Q6IFU7
9216	1,4,6,10,1<br>1,12,16,17	2,3,5,7,8,9<br>,13,14,15	8	O08815,P06757,P16636,P20766,P53792,Q10758,Q6IFU7,Q6P6Q2
9217	1,4,6,10,1<br>1,13,14,15	2,3,5,7,8,9<br>,12,16,17	2	P47727,P50115
9218	1,4,6,10,1<br>1,13,14,16	2,3,5,7,8,9<br>,12,15,17	2	P47727,P50115
9219	1,4,6,10,1<br>1,13,14,17	2,3,5,7,8,9<br>,12,15,16	1	P47727
9220	1,4,6,10,1<br>1,13,15,16	2,3,5,7,8,9<br>,12,14,17	3	P50115,Q561R9,Q5FVI6
9221	1,4,6,10,1<br>1,13,15,17	2,3,5,7,8,9<br>,12,14,16	0	
9222	1,4,6,10,1<br>1,13,16,17	2,3,5,7,8,9<br>,12,14,15	0	
9223	1,4,6,10,1<br>1,14,15,16	2,3,5,7,8,9<br>,12,13,17	2	P20766,P50115
9224	1,4,6,10,1<br>1,14,15,17	2,3,5,7,8,9<br>,12,13,16	0	
9225	1,4,6,10,1<br>1,14,16,17	2,3,5,7,8,9<br>,12,13,15	1	P20766
9226	1,4,6,10,1<br>1,15,16,17	2,3,5,7,8,9<br>,12,13,14	2	P20766,Q9Z1F2
9227	1,4,6,10,1<br>2,13,14,15	2,3,5,7,8,9<br>,11,16,17	3	P10758,P50115,Q498D9
9228	1,4,6,10,1<br>2,13,14,16	2,3,5,7,8,9<br>,11,15,17	1	P50115
9229	1,4,6,10,1<br>2,13,14,17	2,3,5,7,8,9<br>,11,15,16	5	P27213,P36374,Q10758,Q6AYR9,Q9QZK9

9230	1,4,6,10,12,13,15,16	2,3,5,7,8,9,11,14,17	1	P50115
9231	1,4,6,10,12,13,15,17	2,3,5,7,8,9,11,14,16	3	Q6IFV1,Q99MH3,Q9QZK9
9232	1,4,6,10,12,13,16,17	2,3,5,7,8,9,11,14,15	3	P27213,Q10758,Q99MH3
9233	1,4,6,10,12,14,15,16	2,3,5,7,8,9,11,13,17	2	P20766,P50115
9234	1,4,6,10,12,14,15,17	2,3,5,7,8,9,11,13,16	3	B0BNN3,P20766,Q10758
9235	1,4,6,10,12,14,16,17	2,3,5,7,8,9,11,13,15	2	P20766,Q10758
9236	1,4,6,10,12,15,16,17	2,3,5,7,8,9,11,13,14	2	P06866,P20766
9237	1,4,6,10,13,14,15,16	2,3,5,7,8,9,11,12,17	1	P50115
9238	1,4,6,10,13,14,15,17	2,3,5,7,8,9,11,12,16	0	
9239	1,4,6,10,13,14,16,17	2,3,5,7,8,9,11,12,15	1	P27213
9240	1,4,6,10,13,15,16,17	2,3,5,7,8,9,11,12,14	1	P27213
9241	1,4,6,10,14,15,16,17	2,3,5,7,8,9,11,12,13	1	P20766
9242	1,4,6,11,12,13,14,15	2,3,5,7,8,9,10,16,17	5	P04905,P0C0A9,P10758,P47727,Q9JHB9
9243	1,4,6,11,12,13,14,16	2,3,5,7,8,9,10,15,17	6	P04905,P0C0A9,P30120,P47727,Q6IFU7,Q9JHB9
9244	1,4,6,11,12,13,14,17	2,3,5,7,8,9,10,15,16	9	P04905,P0C0A9,P13432,P30120,P36374,P47727,Q6AYR9,Q9JHB9,Q9QZK9
9245	1,4,6,11,12,13,15,16	2,3,5,7,8,9,10,14,17	1	Q6IFU7
9246	1,4,6,11,12,13,15,17	2,3,5,7,8,9,10,14,16	3	P13432,P36374,Q9QZK9
9247	1,4,6,11,12,13,16,17	2,3,5,7,8,9,10,14,15	3	P13432,P27213,P36374
9248	1,4,6,11,12,14,15,16	2,3,5,7,8,9,10,13,17	3	P20766,P51907,Q6IFU7
9249	1,4,6,11,12,14,15,17	2,3,5,7,8,9,10,13,16	3	B0BNN3,P13432,P20766
9250	1,4,6,11,12,14,16,17	2,3,5,7,8,9,10,13,15	3	P13432,P20766,Q6IFU7
9251	1,4,6,11,12,15,16,17	2,3,5,7,8,9,10,13,14	4	P06866,P13432,P20766,Q6IFU7
9252	1,4,6,11,13,14,15,16	2,3,5,7,8,9,10,12,17	1	P50115

9253	1,4,6,11,13,14,15,17	2,3,5,7,8,9,10,12,16	0	
9254	1,4,6,11,13,14,16,17	2,3,5,7,8,9,10,12,15	1	P27213
9255	1,4,6,11,13,15,16,17	2,3,5,7,8,9,10,12,14	1	P27213
9256	1,4,6,11,14,15,16,17	2,3,5,7,8,9,10,12,13	1	P20766
9257	1,4,6,12,13,14,15,16	2,3,5,7,8,9,10,11,17	2	P50115,Q9QUL6
9258	1,4,6,12,13,14,15,17	2,3,5,7,8,9,10,11,16	2	P13432,P27213
9259	1,4,6,12,13,14,16,17	2,3,5,7,8,9,10,11,15	2	P13432,P27213
9260	1,4,6,12,13,15,16,17	2,3,5,7,8,9,10,11,14	1	P27213
9261	1,4,6,12,14,15,16,17	2,3,5,7,8,9,10,11,13	3	P13432,P20766,Q5HZV9
9262	1,4,6,13,14,15,16,17	2,3,5,7,8,9,10,11,12	2	P16391,P27213
9263	1,4,7,8,9,10,11,12	2,3,5,6,13,14,15,16,17	12	P00714,P02803,P08753,P0C0A9,P10715,P36374,Q561R9,Q5FVI6,Q63357,Q63617,Q6AYL6,Q8CJ52
9264	1,4,7,8,9,10,11,13	2,3,5,6,12,14,15,16,17	6	P00714,P02803,P36374,Q561R9,Q5FVI6,Q63617
9265	1,4,7,8,9,10,11,14	2,3,5,6,12,13,15,16,17	4	P00714,P83121,Q561R9,Q63617
9266	1,4,7,8,9,10,11,15	2,3,5,6,12,13,14,16,17	5	P00714,P22006,P35280,Q561R9,Q5FVI6
9267	1,4,7,8,9,10,11,16	2,3,5,6,12,13,14,15,17	4	P00714,Q561R9,Q5FVI6,Q63617
9268	1,4,7,8,9,10,11,17	2,3,5,6,12,13,14,15,16	3	P00714,Q561R9,Q63617
9269	1,4,7,8,9,10,12,13	2,3,5,6,11,14,15,16,17	5	P02803,P22283,P36374,Q6AYR9,Q8CJ52
9270	1,4,7,8,9,10,12,14	2,3,5,6,11,13,15,16,17	3	P00714,P36374,Q8CJ52

9271	1,4,7,8,9,10,12,15	2,3,5,6,11,13,14,16,17	3	P10758,P22006,Q561R9
9272	1,4,7,8,9,10,12,16	2,3,5,6,11,13,14,15,17	5	P00714,P08721,P47967,Q8CJ52,Q9WUW8
9273	1,4,7,8,9,10,12,17	2,3,5,6,11,13,14,15,16	3	P19223,P36374,Q99041
9274	1,4,7,8,9,10,13,14	2,3,5,6,11,12,15,16,17	3	P06911,Q5GRG2,Q63617
9275	1,4,7,8,9,10,13,15	2,3,5,6,11,12,14,16,17	2	P22006,Q561R9
9276	1,4,7,8,9,10,13,16	2,3,5,6,11,12,14,15,17	1	Q63617
9277	1,4,7,8,9,10,13,17	2,3,5,6,11,12,14,15,16	1	Q63617
9278	1,4,7,8,9,10,14,15	2,3,5,6,11,12,13,16,17	2	P22006,Q561R9
9279	1,4,7,8,9,10,14,16	2,3,5,6,11,12,13,15,17	2	P08721,Q63617
9280	1,4,7,8,9,10,14,17	2,3,5,6,11,12,13,15,16	1	Q63617
9281	1,4,7,8,9,10,15,16	2,3,5,6,11,12,13,14,17	2	Q561R9,Q5FVI6
9282	1,4,7,8,9,10,15,17	2,3,5,6,11,12,13,14,16	1	P22006
9283	1,4,7,8,9,10,16,17	2,3,5,6,11,12,13,14,15	2	P25031,Q63617
9284	1,4,7,8,9,11,12,13	2,3,5,6,10,14,15,16,17	13	P02780,P02781,P02803,P0C0A9,P10715,P22283,P30120,P36374,Q561R9,Q62902,Q63617,Q8CJ52,Q9JI85
9285	1,4,7,8,9,11,12,14	2,3,5,6,10,13,15,16,17	8	P02803,P0C0A9,P10715,P36374,Q4FZU6,Q63617,Q8CJ52,Q9JI85

9286	1,4,7,8,9,11,12,15	2,3,5,6,10,13,14,16,17	7	P10715,P10758,P22006,P36374,P51907,Q561R9,Q8CJ52
9287	1,4,7,8,9,11,12,16	2,3,5,6,10,13,14,15,17	7	P02803,P10715,P36374,Q4FZU6,Q561R9,Q63617,Q8CJ52
9288	1,4,7,8,9,11,12,17	2,3,5,6,10,13,14,15,16	10	P02803,P10715,P19223,P22283,P36374,Q4FZU6,Q63617,Q8CJ52,Q99041,Q9QZA6
9289	1,4,7,8,9,11,13,14	2,3,5,6,10,12,15,16,17	3	P02803,P83121,Q63617
9290	1,4,7,8,9,11,13,15	2,3,5,6,10,12,14,16,17	4	P05539,P22006,Q561R9,Q63617
9291	1,4,7,8,9,11,13,16	2,3,5,6,10,12,14,15,17	3	P02803,Q561R9,Q63617
9292	1,4,7,8,9,11,13,17	2,3,5,6,10,12,14,15,16	3	P02803,P36374,Q63617
9293	1,4,7,8,9,11,14,15	2,3,5,6,10,12,13,16,17	3	P22006,Q561R9,Q63617
9294	1,4,7,8,9,11,14,16	2,3,5,6,10,12,13,15,17	3	Q4FZU6,Q63617,Q99MH3
9295	1,4,7,8,9,11,14,17	2,3,5,6,10,12,13,15,16	2	Q4FZU6,Q63617
9296	1,4,7,8,9,11,15,16	2,3,5,6,10,12,13,14,17	4	B4F795,P22006,Q561R9,Q63617
9297	1,4,7,8,9,11,15,17	2,3,5,6,10,12,13,14,16	3	P22006,Q561R9,Q63617
9298	1,4,7,8,9,11,16,17	2,3,5,6,10,12,13,14,15	3	P10715,Q4FZU6,Q63617
9299	1,4,7,8,9,12,13,14	2,3,5,6,10,11,15,16,17	10	P02780,P02803,P06911,P22283,P36374,Q4FZU6,Q62902,Q63617,Q6AYR9,Q8CJ52
9300	1,4,7,8,9,12,13,15	2,3,5,6,10,11,14,16,17	2	P10758,P36374

9301	1,4,7,8,9,12,13,16	2,3,5,6,10,11,14,15,17	5	P02803,P36374,Q4FZU6,Q63617,Q8CJ52
9302	1,4,7,8,9,12,13,17	2,3,5,6,10,11,14,15,16	10	P02803,P11598,P19223,P22282,P22283,P36374,Q4FZU6,Q63617,Q8CJ52,Q99041
9303	1,4,7,8,9,12,14,15	2,3,5,6,10,11,13,16,17	2	P10758,P22006
9304	1,4,7,8,9,12,14,16	2,3,5,6,10,11,13,15,17	3	Q4FZU6,Q63617,Q8CJ52
9305	1,4,7,8,9,12,14,17	2,3,5,6,10,11,13,15,16	5	P19223,P36374,Q4FZU6,Q63617,Q99041
9306	1,4,7,8,9,12,15,16	2,3,5,6,10,11,13,14,17	1	P10715
9307	1,4,7,8,9,12,15,17	2,3,5,6,10,11,13,14,16	5	P10715,P19223,P36374,Q99041,Q9QZA6
9308	1,4,7,8,9,12,16,17	2,3,5,6,10,11,13,14,15	5	P10715,P19223,Q4FZU6,Q63617,Q99041
9309	1,4,7,8,9,13,14,15	2,3,5,6,10,11,12,16,17	3	P22006,Q5GRG2,Q63617
9310	1,4,7,8,9,13,14,16	2,3,5,6,10,11,12,15,17	2	Q4FZU6,Q63617
9311	1,4,7,8,9,13,14,17	2,3,5,6,10,11,12,15,16	2	Q4FZU6,Q63617
9312	1,4,7,8,9,13,15,16	2,3,5,6,10,11,12,14,17	3	P62804,Q561R9,Q63617
9313	1,4,7,8,9,13,15,17	2,3,5,6,10,11,12,14,16	1	Q63617
9314	1,4,7,8,9,13,16,17	2,3,5,6,10,11,12,14,15	3	P62804,Q4FZU6,Q63617
9315	1,4,7,8,9,14,15,16	2,3,5,6,10,11,12,13,17	2	Q63617,Q9QZK9

9316	1,4,7,8,9,14,15,17	2,3,5,6,10,11,12,13,16	2	P22006,Q63617
9317	1,4,7,8,9,14,16,17	2,3,5,6,10,11,12,13,15	2	Q4FZU6,Q63617
9318	1,4,7,8,9,15,16,17	2,3,5,6,10,11,12,13,14	0	
9319	1,4,7,8,10,11,12,13	2,3,5,6,9,14,15,16,17	16	P00714,P02780,P02781,P02803,P08723,P0C0A9,P22283,P30120,P36374,P47727,Q561R9,Q63357,Q6AYR9,Q8CJ52,Q9JHB9,Q9JI85
9320	1,4,7,8,10,11,12,14	2,3,5,6,9,13,15,16,17	12	P00714,P02781,P08723,P0C0A9,P22283,P35280,P36374,P47727,Q63357,Q63493,Q6AYL6,Q8CJ52
9321	1,4,7,8,10,11,12,15	2,3,5,6,9,13,14,16,17	10	P00714,P10715,P22006,P35280,P36374,P51907,Q561R9,Q5FVI6,Q63357,Q8CJ52
9322	1,4,7,8,10,11,12,16	2,3,5,6,9,13,14,15,17	12	P00714,P06757,P08721,P10715,P36374,P47967,P53792,Q561R9,Q5FVI6,Q63357,Q6AYL6,Q8CJ52
9323	1,4,7,8,10,11,12,17	2,3,5,6,9,13,14,15,16	13	P00714,P06757,P10715,P19223,P22283,P36374,Q4G075,Q63357,Q63493,Q8CJ52,Q99041,Q9QZA6,Q9QZK9
9324	1,4,7,8,10,11,13,14	2,3,5,6,9,12,15,16,17	7	P00714,P06911,P47727,Q561R9,Q5GRG2,Q63617,Q8CJ52
9325	1,4,7,8,10,11,13,15	2,3,5,6,9,12,14,16,17	7	P00714,P01835,P22006,Q561R9,Q5FVI6,Q5GRG2,Q8VD89
9326	1,4,7,8,10,11,13,16	2,3,5,6,9,12,14,15,17	6	P00714,P01835,Q561R9,Q5FVI6,Q8CJ52,Q8VD89
9327	1,4,7,8,10,11,13,17	2,3,5,6,9,12,14,15,16	3	P00714,P22283,P36374
9328	1,4,7,8,10,11,14,15	2,3,5,6,9,12,13,16,17	5	P00714,P22006,P35280,Q561R9,Q5FVI6
9329	1,4,7,8,10,11,14,16	2,3,5,6,9,12,13,15,17	4	P00714,P08721,Q5FVI6,Q63617
9330	1,4,7,8,10,11,14,17	2,3,5,6,9,12,13,15,16	2	P00714,Q63617
9331	1,4,7,8,10,11,15,16	2,3,5,6,9,12,13,14,17	8	P00714,P01835,P22006,P35280,Q561R9,Q5FVI6,Q8VD89,Q9Z1F2
9332	1,4,7,8,10,11,15,17	2,3,5,6,9,12,13,14,16	4	P00714,P22006,Q561R9,Q5FVI6
9333	1,4,7,8,10,11,16,17	2,3,5,6,9,12,13,14,15	2	P00714,Q5FVI6
9334	1,4,7,8,10,12,13,14	2,3,5,6,9,11,15,16,17	10	P06761,P06911,P08723,P11598,P12020,P22283,P36374,Q5GRG2,Q6AYR9,Q8CJ52
9335	1,4,7,8,10,12,13,15	2,3,5,6,9,11,14,16,17	4	P22006,P36374,Q8CJ52,Q9QUL6
9336	1,4,7,8,10,12,13,16	2,3,5,6,9,11,14,15,17	5	P36374,P47967,Q8CJ52,Q9QUL6,Q9R0T3

9337	1,4,7,8,10,<br>12,13,17	2,3,5,6,9,1<br>1,14,15,16	10	P11598,P19223,P22282,P22283,P36374,Q6AYR9,Q8CJ52,Q99041,Q99MH3,Q9QZK9
9338	1,4,7,8,10,<br>12,14,15	2,3,5,6,9,1<br>1,13,16,17	2	P22006,P35280
9339	1,4,7,8,10,<br>12,14,16	2,3,5,6,9,1<br>1,13,15,17	2	P08721,Q8CJ52
9340	1,4,7,8,10,<br>12,14,17	2,3,5,6,9,1<br>1,13,15,16	6	P11598,P19223,P31430,P36374,Q8CJ52,Q99041
9341	1,4,7,8,10,<br>12,15,16	2,3,5,6,9,1<br>1,13,14,17	3	P08721,Q5FVI6,Q8CJ52
9342	1,4,7,8,10,<br>12,15,17	2,3,5,6,9,1<br>1,13,14,16	5	P19223,P22006,Q4G075,Q99041,Q9QZA6
9343	1,4,7,8,10,<br>12,16,17	2,3,5,6,9,1<br>1,13,14,15	5	P08721,P19223,Q4G075,Q8CJ52,Q99041
9344	1,4,7,8,10,<br>13,14,15	2,3,5,6,9,1<br>1,12,16,17	4	P01835,P06911,P22006,Q5GRG2
9345	1,4,7,8,10,<br>13,14,16	2,3,5,6,9,1<br>1,12,15,17	3	P01835,P06911,Q5GRG2
9346	1,4,7,8,10,<br>13,14,17	2,3,5,6,9,1<br>1,12,15,16	3	P06911,P11598,Q5GRG2
9347	1,4,7,8,10,<br>13,15,16	2,3,5,6,9,1<br>1,12,14,17	2	P01835,Q561R9
9348	1,4,7,8,10,<br>13,15,17	2,3,5,6,9,1<br>1,12,14,16	2	P01835,P22006
9349	1,4,7,8,10,<br>13,16,17	2,3,5,6,9,1<br>1,12,14,15	1	P01835
9350	1,4,7,8,10,<br>14,15,16	2,3,5,6,9,1<br>1,12,13,17	3	P01835,P08721,P22006
9351	1,4,7,8,10,<br>14,15,17	2,3,5,6,9,1<br>1,12,13,16	1	P22006
9352	1,4,7,8,10,<br>14,16,17	2,3,5,6,9,1<br>1,12,13,15	1	P08721
9353	1,4,7,8,10,<br>15,16,17	2,3,5,6,9,1<br>1,12,13,14	1	P01835
9354	1,4,7,8,11,<br>12,13,14	2,3,5,6,9,1<br>0,15,16,17	21	P02780,P02781,P02803,P04905,P06761,P06911,P08723,P0C0A9,P11598,P22282,P22283,P30120,P36374,P47727,Q5GRG2,Q62902,Q636<br>17,Q6AYR9,Q8CJ52,Q9JHB9,Q9JI85
9355	1,4,7,8,11,<br>12,13,15	2,3,5,6,9,1<br>0,14,16,17	6	P10715,P22006,P36374,Q561R9,Q8CJ52,Q9JI85
9356	1,4,7,8,11,<br>12,13,16	2,3,5,6,9,1<br>0,14,15,17	7	P02803,P10715,P36374,Q4FZU6,Q62902,Q8CJ52,Q9R0T3
9357	1,4,7,8,11,<br>12,13,17	2,3,5,6,9,1<br>0,14,15,16	14	P02780,P02781,P02803,P10715,P11598,P19223,P22282,P22283,P36374,Q6AYR9,Q8CJ52,Q99041,Q9JI85,Q9QZK9
9358	1,4,7,8,11,<br>12,14,15	2,3,5,6,9,1<br>0,13,16,17	4	P10715,P22006,P35280,Q8CJ52
9359	1,4,7,8,11,<br>12,14,16	2,3,5,6,9,1<br>0,13,15,17	4	P10715,Q4FZU6,Q63617,Q8CJ52

9360	1,4,7,8,11,12,14,17	2,3,5,6,9,10,13,15,16	11	P10715,P11598,P19223,P22283,P36374,Q4FZU6,Q63493,Q63617,Q8CJ52,Q99041,Q9QZA6
9361	1,4,7,8,11,12,15,16	2,3,5,6,9,10,13,14,17	5	P10715,P22006,P51907,Q561R9,Q8CJ52
9362	1,4,7,8,11,12,15,17	2,3,5,6,9,10,13,14,16	8	P10715,P19223,P22006,P36374,Q4G075,Q8CJ52,Q99041,Q9QZA6
9363	1,4,7,8,11,12,16,17	2,3,5,6,9,10,13,14,15	8	P10715,P19223,P36374,Q4FZU6,Q4G075,Q8CJ52,Q99041,Q9QZA6
9364	1,4,7,8,11,13,14,15	2,3,5,6,9,10,12,16,17	4	P22006,Q561R9,Q5GRG2,Q63617
9365	1,4,7,8,11,13,14,16	2,3,5,6,9,10,12,15,17	3	Q4FZU6,Q63617,Q8CJ52
9366	1,4,7,8,11,13,14,17	2,3,5,6,9,10,12,15,16	4	P11598,P22283,Q4FZU6,Q63617
9367	1,4,7,8,11,13,15,16	2,3,5,6,9,10,12,14,17	4	P01835,P22006,Q561R9,Q8VD89
9368	1,4,7,8,11,13,15,17	2,3,5,6,9,10,12,14,16	1	P22006
9369	1,4,7,8,11,13,16,17	2,3,5,6,9,10,12,14,15	2	Q4FZU6,Q63617
9370	1,4,7,8,11,14,15,16	2,3,5,6,9,10,12,13,17	2	P22006,Q561R9
9371	1,4,7,8,11,14,15,17	2,3,5,6,9,10,12,13,16	1	P22006
9372	1,4,7,8,11,14,16,17	2,3,5,6,9,10,12,13,15	2	Q4FZU6,Q63617
9373	1,4,7,8,11,15,16,17	2,3,5,6,9,10,12,13,14	1	P22006
9374	1,4,7,8,12,13,14,15	2,3,5,6,9,10,11,16,17	7	P10758,P11598,P22006,P36374,Q5GRG2,Q8CJ52,Q9QUL6
9375	1,4,7,8,12,13,14,16	2,3,5,6,9,10,11,15,17	6	P06911,P36374,Q4FZU6,Q62902,Q8CJ52,Q9QUL6
9376	1,4,7,8,12,13,14,17	2,3,5,6,9,10,11,15,16	11	P06761,P06911,P11598,P22282,P22283,P31430,P36374,Q4FZU6,Q6AYR9,Q8CJ52,Q99041
9377	1,4,7,8,12,13,15,16	2,3,5,6,9,10,11,14,17	2	Q8CJ52,Q9QUL6
9378	1,4,7,8,12,13,15,17	2,3,5,6,9,10,11,14,16	6	P11598,P36374,Q8CJ52,Q99041,Q9QUL6,Q9QZA6
9379	1,4,7,8,12,13,16,17	2,3,5,6,9,10,11,14,15	8	P27213,P36374,P63029,Q4FZU6,Q8CJ52,Q99041,Q9QUL6,Q9R0T3
9380	1,4,7,8,12,14,15,16	2,3,5,6,9,10,11,13,17	3	Q4FZU6,Q8CJ52,Q9QUL6
9381	1,4,7,8,12,14,15,17	2,3,5,6,9,10,11,13,16	4	P11598,P22006,Q99041,Q9QZA6
9382	1,4,7,8,12,14,16,17	2,3,5,6,9,10,11,13,15	4	P31430,Q4FZU6,Q8CJ52,Q99041

9383	1,4,7,8,12,<br>15,16,17	2,3,5,6,9,1<br>0,11,13,14	4	P06866,P10715,Q99041,Q9QZA6
9384	1,4,7,8,13,<br>14,15,16	2,3,5,6,9,1<br>0,11,12,17	3	P01835,Q5GRG2,Q9QUL6
9385	1,4,7,8,13,<br>14,15,17	2,3,5,6,9,1<br>0,11,12,16	3	P11598,P22006,Q5GRG2
9386	1,4,7,8,13,<br>14,16,17	2,3,5,6,9,1<br>0,11,12,15	3	P27213,Q4FZU6,Q63617
9387	1,4,7,8,13,<br>15,16,17	2,3,5,6,9,1<br>0,11,12,14	2	P01835,P27213
9388	1,4,7,8,14,<br>15,16,17	2,3,5,6,9,1<br>0,11,12,13	1	Q4FZU6
9389	1,4,7,9,10,<br>11,12,13	2,3,5,6,8,1<br>4,15,16,17	4	P02803,P36374,Q561R9,Q5FVI6
9390	1,4,7,9,10,<br>11,12,14	2,3,5,6,8,1<br>3,15,16,17	7	P00714,P08753,P36374,P51907,P83121,Q561R9,Q5FVI6
9391	1,4,7,9,10,<br>11,12,15	2,3,5,6,8,1<br>3,14,16,17	7	P08753,P10758,P22006,P51907,Q561R9,Q5FVI6,Q6PCU2
9392	1,4,7,9,10,<br>11,12,16	2,3,5,6,8,1<br>3,14,15,17	10	P06757,P08753,P10536,P12368,P47967,P51907,Q561R9,Q5FVI6,Q64093,Q9WUW8
9393	1,4,7,9,10,<br>11,12,17	2,3,5,6,8,1<br>3,14,15,16	4	P06757,P08753,P36374,Q5FVI6
9394	1,4,7,9,10,<br>11,13,14	2,3,5,6,8,1<br>2,15,16,17	2	P83121,Q561R9
9395	1,4,7,9,10,<br>11,13,15	2,3,5,6,8,1<br>2,14,16,17	3	P22006,Q561R9,Q5FVI6
9396	1,4,7,9,10,<br>11,13,16	2,3,5,6,8,1<br>2,14,15,17	3	Q561R9,Q5FVI6,Q62635
9397	1,4,7,9,10,<br>11,13,17	2,3,5,6,8,1<br>2,14,15,16	0	
9398	1,4,7,9,10,<br>11,14,15	2,3,5,6,8,1<br>2,13,16,17	7	P22006,P51907,P83121,Q561R9,Q5FVI6,Q62635,Q9Z1F2
9399	1,4,7,9,10,<br>11,14,16	2,3,5,6,8,1<br>2,13,15,17	5	P83121,Q561R9,Q5FVI6,Q62635,Q9Z1F2
9400	1,4,7,9,10,<br>11,14,17	2,3,5,6,8,1<br>2,13,15,16	1	P83121
9401	1,4,7,9,10,<br>11,15,16	2,3,5,6,8,1<br>2,13,14,17	6	P22006,P51907,Q561R9,Q5FVI6,Q62635,Q9Z1F2
9402	1,4,7,9,10,<br>11,15,17	2,3,5,6,8,1<br>2,13,14,16	5	O35077,P22006,Q561R9,Q5FVI6,Q9Z1F2
9403	1,4,7,9,10,<br>11,16,17	2,3,5,6,8,1<br>2,13,14,15	3	Q5FVI6,Q64093,Q9Z1F2
9404	1,4,7,9,10,<br>12,13,14	2,3,5,6,8,1<br>1,15,16,17	1	P36374
9405	1,4,7,9,10,<br>12,13,15	2,3,5,6,8,1<br>1,14,16,17	3	P10758,P36374,Q561R9

9406	1,4,7,9,10,<br>12,13,16	2,3,5,6,8,1<br>1,14,15,17	4	P02803,P36374,P47967,P97840
9407	1,4,7,9,10,<br>12,13,17	2,3,5,6,8,1<br>1,14,15,16	1	P36374
9408	1,4,7,9,10,<br>12,14,15	2,3,5,6,8,1<br>1,13,16,17	2	P10758,P22006
9409	1,4,7,9,10,<br>12,14,16	2,3,5,6,8,1<br>1,13,15,17	0	
9410	1,4,7,9,10,<br>12,14,17	2,3,5,6,8,1<br>1,13,15,16	1	P36374
9411	1,4,7,9,10,<br>12,15,16	2,3,5,6,8,1<br>1,13,14,17	4	P97840,Q561R9,Q5FVI6,Q9Z1F2
9412	1,4,7,9,10,<br>12,15,17	2,3,5,6,8,1<br>1,13,14,16	1	P06866
9413	1,4,7,9,10,<br>12,16,17	2,3,5,6,8,1<br>1,13,14,15	1	Q64093
9414	1,4,7,9,10,<br>13,14,15	2,3,5,6,8,1<br>1,12,16,17	4	P06760,P22006,P50115,Q561R9
9415	1,4,7,9,10,<br>13,14,16	2,3,5,6,8,1<br>1,12,15,17	3	P06760,P50115,P62804
9416	1,4,7,9,10,<br>13,14,17	2,3,5,6,8,1<br>1,12,15,16	0	
9417	1,4,7,9,10,<br>13,15,16	2,3,5,6,8,1<br>1,12,14,17	6	P06760,P50115,P62804,Q00715,Q561R9,Q62635
9418	1,4,7,9,10,<br>13,15,17	2,3,5,6,8,1<br>1,12,14,16	1	P62804
9419	1,4,7,9,10,<br>13,16,17	2,3,5,6,8,1<br>1,12,14,15	1	P62804
9420	1,4,7,9,10,<br>14,15,16	2,3,5,6,8,1<br>1,12,13,17	7	P06760,P16636,P50115,Q561R9,Q62635,Q9QZK9,Q9Z1F2
9421	1,4,7,9,10,<br>14,15,17	2,3,5,6,8,1<br>1,12,13,16	1	P22006
9422	1,4,7,9,10,<br>14,16,17	2,3,5,6,8,1<br>1,12,13,15	0	
9423	1,4,7,9,10,<br>15,16,17	2,3,5,6,8,1<br>1,12,13,14	2	P62804,Q9Z1F2
9424	1,4,7,9,11,<br>12,13,14	2,3,5,6,8,1<br>0,15,16,17	3	P02803,P05539,P36374
9425	1,4,7,9,11,<br>12,13,15	2,3,5,6,8,1<br>0,14,16,17	6	P05539,P10758,P22006,P36374,P51907,Q561R9
9426	1,4,7,9,11,<br>12,13,16	2,3,5,6,8,1<br>0,14,15,17	2	P02803,P36374
9427	1,4,7,9,11,<br>12,13,17	2,3,5,6,8,1<br>0,14,15,16	2	P02803,P36374
9428	1,4,7,9,11,<br>12,14,15	2,3,5,6,8,1<br>0,13,16,17	4	P10758,P22006,P51907,Q561R9

9429	1,4,7,9,11,12,14,16	2,3,5,6,8,10,13,15,17	3	P08753,P51907,Q4FZU6
9430	1,4,7,9,11,12,14,17	2,3,5,6,8,10,13,15,16	2	P36374,Q4FZU6
9431	1,4,7,9,11,12,15,16	2,3,5,6,8,10,13,14,17	5	P06760,P08753,P22006,P51907,Q561R9
9432	1,4,7,9,11,12,15,17	2,3,5,6,8,10,13,14,16	3	P22006,P36374,P51907
9433	1,4,7,9,11,12,16,17	2,3,5,6,8,10,13,14,15	2	P36374,Q4FZU6
9434	1,4,7,9,11,13,14,15	2,3,5,6,8,10,12,16,17	5	P05539,P06760,P16636,P22006,Q561R9
9435	1,4,7,9,11,13,14,16	2,3,5,6,8,10,12,15,17	2	P06760,P83121
9436	1,4,7,9,11,13,14,17	2,3,5,6,8,10,12,15,16	2	P05539,P83121
9437	1,4,7,9,11,13,15,16	2,3,5,6,8,10,12,14,17	5	P06760,P22006,P62804,Q561R9,Q62635
9438	1,4,7,9,11,13,15,17	2,3,5,6,8,10,12,14,16	3	P05539,P22006,Q561R9
9439	1,4,7,9,11,13,16,17	2,3,5,6,8,10,12,14,15	0	
9440	1,4,7,9,11,14,15,16	2,3,5,6,8,10,12,13,17	8	P06760,P16636,P22006,P51907,Q561R9,Q62635,Q99MH3,Q9QZK9
9441	1,4,7,9,11,14,15,17	2,3,5,6,8,10,12,13,16	1	P22006
9442	1,4,7,9,11,14,16,17	2,3,5,6,8,10,12,13,15	1	Q4FZU6
9443	1,4,7,9,11,15,16,17	2,3,5,6,8,10,12,13,14	2	P22006,Q9Z1F2
9444	1,4,7,9,12,13,14,15	2,3,5,6,8,10,11,16,17	3	P06760,P08649,P10758
9445	1,4,7,9,12,13,14,16	2,3,5,6,8,10,11,15,17	3	P06760,P08649,Q4FZU6
9446	1,4,7,9,12,13,14,17	2,3,5,6,8,10,11,15,16	2	P36374,Q4FZU6
9447	1,4,7,9,12,13,15,16	2,3,5,6,8,10,11,14,17	3	P06760,P08649,P62804
9448	1,4,7,9,12,13,15,17	2,3,5,6,8,10,11,14,16	1	P36374
9449	1,4,7,9,12,13,16,17	2,3,5,6,8,10,11,14,15	3	P27213,P36374,Q4FZU6
9450	1,4,7,9,12,14,15,16	2,3,5,6,8,10,11,13,17	1	P06760
9451	1,4,7,9,12,14,15,17	2,3,5,6,8,10,11,13,16	0	

9452	1,4,7,9,12,<br>14,16,17	2,3,5,6,8,1<br>0,11,13,15	1	Q4FZU6
9453	1,4,7,9,12,<br>15,16,17	2,3,5,6,8,1<br>0,11,13,14	1	P06866
9454	1,4,7,9,13,<br>14,15,16	2,3,5,6,8,1<br>0,11,12,17	6	P06760,P11883,P16636,P50115,P62804,Q00715
9455	1,4,7,9,13,<br>14,15,17	2,3,5,6,8,1<br>0,11,12,16	2	P11883,P62804
9456	1,4,7,9,13,<br>14,16,17	2,3,5,6,8,1<br>0,11,12,15	3	P27213,P62804,Q4FZU6
9457	1,4,7,9,13,<br>15,16,17	2,3,5,6,8,1<br>0,11,12,14	4	P11883,P27213,P62804,Q00715
9458	1,4,7,9,14,<br>15,16,17	2,3,5,6,8,1<br>0,11,12,13	2	P11883,P62804
9459	1,4,7,10,1<br>1,12,13,14	2,3,5,6,8,9<br>,15,16,17	2	P36374,P47727
9460	1,4,7,10,1<br>1,12,13,15	2,3,5,6,8,9<br>,14,16,17	5	P22006,P36374,P51907,Q561R9,Q5FVI6
9461	1,4,7,10,1<br>1,12,13,16	2,3,5,6,8,9<br>,14,15,17	5	P06757,P36374,P47967,Q561R9,Q5FVI6
9462	1,4,7,10,1<br>1,12,13,17	2,3,5,6,8,9<br>,14,15,16	4	P06757,P36374,Q63942,Q9QZK9
9463	1,4,7,10,1<br>1,12,14,15	2,3,5,6,8,9<br>,13,16,17	7	P08753,P20766,P22006,P35280,P51907,Q561R9,Q5FVI6
9464	1,4,7,10,1<br>1,12,14,16	2,3,5,6,8,9<br>,13,15,17	5	P08753,P20766,P51907,P53792,Q5FVI6
9465	1,4,7,10,1<br>1,12,14,17	2,3,5,6,8,9<br>,13,15,16	1	P36374
9466	1,4,7,10,1<br>1,12,15,16	2,3,5,6,8,9<br>,13,14,17	8	P08753,P20766,P22006,P51907,P53792,Q561R9,Q5FVI6,Q9Z1F2
9467	1,4,7,10,1<br>1,12,15,17	2,3,5,6,8,9<br>,13,14,16	6	P06757,P06866,P22006,P36374,P51907,Q5FVI6
9468	1,4,7,10,1<br>1,12,16,17	2,3,5,6,8,9<br>,13,14,15	6	P06757,P06866,P20766,P36374,P53792,Q5FVI6
9469	1,4,7,10,1<br>1,13,14,15	2,3,5,6,8,9<br>,12,16,17	2	P22006,Q561R9
9470	1,4,7,10,1<br>1,13,14,16	2,3,5,6,8,9<br>,12,15,17	2	Q5FVI6,Q7TPB1
9471	1,4,7,10,1<br>1,13,14,17	2,3,5,6,8,9<br>,12,15,16	0	
9472	1,4,7,10,1<br>1,13,15,16	2,3,5,6,8,9<br>,12,14,17	6	P01835,P22006,Q561R9,Q5FVI6,Q8VD89,Q9Z1F2
9473	1,4,7,10,1<br>1,13,15,17	2,3,5,6,8,9<br>,12,14,16	2	P22006,Q561R9
9474	1,4,7,10,1<br>1,13,16,17	2,3,5,6,8,9<br>,12,14,15	1	Q5FVI6

9475	1,4,7,10,1 2,3,5,6,8,9 1,14,15,16 ,12,13,17	7	P20766,P22006,P51907,Q561R9,Q5FVI6,Q62635,Q9Z1F2
9476	1,4,7,10,1 2,3,5,6,8,9 1,14,15,17 ,12,13,16	2	P22006,Q9Z1F2
9477	1,4,7,10,1 2,3,5,6,8,9 1,14,16,17 ,12,13,15	2	Q5FVI6,Q9Z1F2
9478	1,4,7,10,1 2,3,5,6,8,9 1,15,16,17 ,12,13,14	5	O35077,P22006,Q561R9,Q5FVI6,Q9Z1F2
9479	1,4,7,10,1 2,3,5,6,8,9 2,13,14,15 ,11,16,17	2	P10758,P22006
9480	1,4,7,10,1 2,3,5,6,8,9 2,13,14,16 ,11,15,17	0	
9481	1,4,7,10,1 2,3,5,6,8,9 2,13,14,17 ,11,15,16	1	P36374
9482	1,4,7,10,1 2,3,5,6,8,9 2,13,15,16 ,11,14,17	2	P02793;Q7TP54,Q9QUL6
9483	1,4,7,10,1 2,3,5,6,8,9 2,13,15,17 ,11,14,16	2	P36374,Q99MH3
9484	1,4,7,10,1 2,3,5,6,8,9 2,13,16,17 ,11,14,15	2	P27213,P36374
9485	1,4,7,10,1 2,3,5,6,8,9 2,14,15,16 ,11,13,17	2	P08721,P20766
9486	1,4,7,10,1 2,3,5,6,8,9 2,14,15,17 ,11,13,16	3	P06866,P20766,P22006
9487	1,4,7,10,1 2,3,5,6,8,9 2,14,16,17 ,11,13,15	1	P20766
9488	1,4,7,10,1 2,3,5,6,8,9 2,15,16,17 ,11,13,14	3	P06866,P20766,Q9Z1F2
9489	1,4,7,10,1 2,3,5,6,8,9 3,14,15,16 ,11,12,17	4	P01835,P06760,P50115,P62804
9490	1,4,7,10,1 2,3,5,6,8,9 3,14,15,17 ,11,12,16	1	P22006
9491	1,4,7,10,1 2,3,5,6,8,9 3,14,16,17 ,11,12,15	1	P27213
9492	1,4,7,10,1 2,3,5,6,8,9 3,15,16,17 ,11,12,14	3	P01835,P27213,P62804
9493	1,4,7,10,1 2,3,5,6,8,9 4,15,16,17 ,11,12,13	2	P20766,Q9Z1F2
9494	1,4,7,11,1 2,3,5,6,8,9 2,13,14,15 ,10,16,17	5	P05539,P10758,P22006,P36374,Q6P6R2
9495	1,4,7,11,1 2,3,5,6,8,9 2,13,14,16 ,10,15,17	2	P36374,Q4FZU6
9496	1,4,7,11,1 2,3,5,6,8,9 2,13,14,17 ,10,15,16	2	P36374,Q63942
9497	1,4,7,11,1 2,3,5,6,8,9 2,13,15,16 ,10,14,17	3	P22006,P51907,Q561R9

9498	1,4,7,11,12,13,15,17	2,3,5,6,8,9,10,14,16	3	P22006,P36374,Q63942
9499	1,4,7,11,12,13,16,17	2,3,5,6,8,9,10,14,15	2	P27213,P36374
9500	1,4,7,11,12,14,15,16	2,3,5,6,8,9,10,13,17	3	P20766,P22006,P51907
9501	1,4,7,11,12,14,15,17	2,3,5,6,8,9,10,13,16	1	P22006
9502	1,4,7,11,12,14,16,17	2,3,5,6,8,9,10,13,15	1	Q4FZU6
9503	1,4,7,11,12,15,16,17	2,3,5,6,8,9,10,13,14	4	P06866,P20766,P22006,P51907
9504	1,4,7,11,13,14,15,16	2,3,5,6,8,9,10,12,17	3	P06760,P16636,P22006
9505	1,4,7,11,13,14,15,17	2,3,5,6,8,9,10,12,16	1	P22006
9506	1,4,7,11,13,14,16,17	2,3,5,6,8,9,10,12,15	2	P27213,Q4FZU6
9507	1,4,7,11,13,15,16,17	2,3,5,6,8,9,10,12,14	1	P27213
9508	1,4,7,11,14,15,16,17	2,3,5,6,8,9,10,12,13	2	P22006,Q9Z1F2
9509	1,4,7,12,13,14,15,16	2,3,5,6,8,9,10,11,17	4	P06760,P08649,P11883,Q9QUL6
9510	1,4,7,12,13,14,15,17	2,3,5,6,8,9,10,11,16	4	P08649,P11883,P27213,P36374
9511	1,4,7,12,13,14,16,17	2,3,5,6,8,9,10,11,15	4	P08649,P27213,P36374,Q4FZU6
9512	1,4,7,12,13,15,16,17	2,3,5,6,8,9,10,11,14	5	P06866,P08649,P11883,P27213,Q9QUL6
9513	1,4,7,12,14,15,16,17	2,3,5,6,8,9,10,11,13	3	P06866,P11883,P20766
9514	1,4,7,13,14,15,16,17	2,3,5,6,8,9,10,11,12	3	P11883,P27213,P62804
9515	1,4,8,9,10,11,12,13	2,3,5,6,7,14,15,16,17	5	P02803,P0C0A9,P10758,P36374,Q561R9
9516	1,4,8,9,10,11,12,14	2,3,5,6,7,13,15,16,17	6	P00714,P02803,P0C0A9,P10758,P36374,Q561R9
9517	1,4,8,9,10,11,12,15	2,3,5,6,7,13,14,16,17	4	P00714,P10758,P51907,Q561R9
9518	1,4,8,9,10,11,12,16	2,3,5,6,7,13,14,15,17	5	P00714,P02803,P53792,Q561R9,Q9WUW8
9519	1,4,8,9,10,11,12,17	2,3,5,6,7,13,14,15,16	4	P02803,P06757,P16636,P36374
9520	1,4,8,9,10,11,13,14	2,3,5,6,7,12,15,16,17	3	P02803,P83748,Q561R9

9521	1,4,8,9,10,<br>11,13,15	2,3,5,6,7,1<br>2,14,16,17	1	Q561R9
9522	1,4,8,9,10,<br>11,13,16	2,3,5,6,7,1<br>2,14,15,17	3	P02803,Q561R9,Q8CJD3
9523	1,4,8,9,10,<br>11,13,17	2,3,5,6,7,1<br>2,14,15,16	3	P02803,P83748,Q561R9
9524	1,4,8,9,10,<br>11,14,15	2,3,5,6,7,1<br>2,13,16,17	1	Q561R9
9525	1,4,8,9,10,<br>11,14,16	2,3,5,6,7,1<br>2,13,15,17	4	P00714,P83748,Q561R9,Q9WUW8
9526	1,4,8,9,10,<br>11,14,17	2,3,5,6,7,1<br>2,13,15,16	1	P83748
9527	1,4,8,9,10,<br>11,15,16	2,3,5,6,7,1<br>2,13,14,17	3	Q561R9,Q62635,Q9Z1F2
9528	1,4,8,9,10,<br>11,15,17	2,3,5,6,7,1<br>2,13,14,16	3	P80201,P83748,Q561R9
9529	1,4,8,9,10,<br>11,16,17	2,3,5,6,7,1<br>2,13,14,15	3	A2RUV9,P83748,Q561R9
9530	1,4,8,9,10,<br>12,13,14	2,3,5,6,7,1<br>1,15,16,17	3	P02803,P10758,P36374
9531	1,4,8,9,10,<br>12,13,15	2,3,5,6,7,1<br>1,14,16,17	2	P10758,Q561R9
9532	1,4,8,9,10,<br>12,13,16	2,3,5,6,7,1<br>1,14,15,17	1	P02803
9533	1,4,8,9,10,<br>12,13,17	2,3,5,6,7,1<br>1,14,15,16	2	P02803,P36374
9534	1,4,8,9,10,<br>12,14,15	2,3,5,6,7,1<br>1,13,16,17	2	P10758,Q561R9
9535	1,4,8,9,10,<br>12,14,16	2,3,5,6,7,1<br>1,13,15,17	1	Q9WUW8
9536	1,4,8,9,10,<br>12,14,17	2,3,5,6,7,1<br>1,13,15,16	0	
9537	1,4,8,9,10,<br>12,15,16	2,3,5,6,7,1<br>1,13,14,17	2	Q561R9,Q9WUW8
9538	1,4,8,9,10,<br>12,15,17	2,3,5,6,7,1<br>1,13,14,16	1	P80201
9539	1,4,8,9,10,<br>12,16,17	2,3,5,6,7,1<br>1,13,14,15	0	
9540	1,4,8,9,10,<br>13,14,15	2,3,5,6,7,1<br>1,12,16,17	3	P09605;P25809,P17046,Q561R9
9541	1,4,8,9,10,<br>13,14,16	2,3,5,6,7,1<br>1,12,15,17	0	
9542	1,4,8,9,10,<br>13,14,17	2,3,5,6,7,1<br>1,12,15,16	0	
9543	1,4,8,9,10,<br>13,15,16	2,3,5,6,7,1<br>1,12,14,17	1	Q561R9

9544	1,4,8,9,10,<br>13,15,17	2,3,5,6,7,1<br>1,12,14,16	0	
9545	1,4,8,9,10,<br>13,16,17	2,3,5,6,7,1<br>1,12,14,15	1	P25031
9546	1,4,8,9,10,<br>14,15,16	2,3,5,6,7,1<br>1,12,13,17	3	P08937,Q561R9,Q9QZK9
9547	1,4,8,9,10,<br>14,15,17	2,3,5,6,7,1<br>1,12,13,16	1	P17046
9548	1,4,8,9,10,<br>14,16,17	2,3,5,6,7,1<br>1,12,13,15	0	
9549	1,4,8,9,10,<br>15,16,17	2,3,5,6,7,1<br>1,12,13,14	1	P25031
9550	1,4,8,9,11,<br>12,13,14	2,3,5,6,7,1<br>0,15,16,17	5	P02803,P0C0A9,P10758,P36374,P47727
9551	1,4,8,9,11,<br>12,13,15	2,3,5,6,7,1<br>0,14,16,17	4	P02803,P10758,P36374,Q561R9
9552	1,4,8,9,11,<br>12,13,16	2,3,5,6,7,1<br>0,14,15,17	3	P02803,P36374,Q561R9
9553	1,4,8,9,11,<br>12,13,17	2,3,5,6,7,1<br>0,14,15,16	2	P02803,P36374
9554	1,4,8,9,11,<br>12,14,15	2,3,5,6,7,1<br>0,13,16,17	2	P10758,Q561R9
9555	1,4,8,9,11,<br>12,14,16	2,3,5,6,7,1<br>0,13,15,17	1	P02803
9556	1,4,8,9,11,<br>12,14,17	2,3,5,6,7,1<br>0,13,15,16	2	P02803,P36374
9557	1,4,8,9,11,<br>12,15,16	2,3,5,6,7,1<br>0,13,14,17	2	P51907,Q561R9
9558	1,4,8,9,11,<br>12,15,17	2,3,5,6,7,1<br>0,13,14,16	1	Q561R9
9559	1,4,8,9,11,<br>12,16,17	2,3,5,6,7,1<br>0,13,14,15	1	P02803
9560	1,4,8,9,11,<br>13,14,15	2,3,5,6,7,1<br>0,12,16,17	1	Q561R9
9561	1,4,8,9,11,<br>13,14,16	2,3,5,6,7,1<br>0,12,15,17	1	P02803
9562	1,4,8,9,11,<br>13,14,17	2,3,5,6,7,1<br>0,12,15,16	3	P02803,P83748,P97840
9563	1,4,8,9,11,<br>13,15,16	2,3,5,6,7,1<br>0,12,14,17	1	Q561R9
9564	1,4,8,9,11,<br>13,15,17	2,3,5,6,7,1<br>0,12,14,16	3	P97840,Q561R9,Q63532
9565	1,4,8,9,11,<br>13,16,17	2,3,5,6,7,1<br>0,12,14,15	2	P02803,P83748
9566	1,4,8,9,11,<br>14,15,16	2,3,5,6,7,1<br>0,12,13,17	3	Q561R9,Q99MH3,Q9QZK9

9567	1,4,8,9,11,<br>14,15,17	2,3,5,6,7,1<br>0,12,13,16	2	P83748,P97840
9568	1,4,8,9,11,<br>14,16,17	2,3,5,6,7,1<br>0,12,13,15	1	P83748
9569	1,4,8,9,11,<br>15,16,17	2,3,5,6,7,1<br>0,12,13,14	1	Q561R9
9570	1,4,8,9,12,<br>13,14,15	2,3,5,6,7,1<br>0,11,16,17	2	P10758,Q9QUL6
9571	1,4,8,9,12,<br>13,14,16	2,3,5,6,7,1<br>0,11,15,17	2	P02803,Q9QUL6
9572	1,4,8,9,12,<br>13,14,17	2,3,5,6,7,1<br>0,11,15,16	3	P02803,P27213,P36374
9573	1,4,8,9,12,<br>13,15,16	2,3,5,6,7,1<br>0,11,14,17	2	P10758,Q9QUL6
9574	1,4,8,9,12,<br>13,15,17	2,3,5,6,7,1<br>0,11,14,16	2	P10758,P36374
9575	1,4,8,9,12,<br>13,16,17	2,3,5,6,7,1<br>0,11,14,15	3	P02803,P27213,P36374
9576	1,4,8,9,12,<br>14,15,16	2,3,5,6,7,1<br>0,11,13,17	0	
9577	1,4,8,9,12,<br>14,15,17	2,3,5,6,7,1<br>0,11,13,16	0	
9578	1,4,8,9,12,<br>14,16,17	2,3,5,6,7,1<br>0,11,13,15	1	Q4FZU6
9579	1,4,8,9,12,<br>15,16,17	2,3,5,6,7,1<br>0,11,13,14	1	P06866
9580	1,4,8,9,13,<br>14,15,16	2,3,5,6,7,1<br>0,11,12,17	1	Q9QZK9
9581	1,4,8,9,13,<br>14,15,17	2,3,5,6,7,1<br>0,11,12,16	1	P11883
9582	1,4,8,9,13,<br>14,16,17	2,3,5,6,7,1<br>0,11,12,15	1	P27213
9583	1,4,8,9,13,<br>15,16,17	2,3,5,6,7,1<br>0,11,12,14	3	P11883,P27213,P62804
9584	1,4,8,9,14,<br>15,16,17	2,3,5,6,7,1<br>0,11,12,13	0	
9585	1,4,8,10,1<br>1,12,13,14	2,3,5,6,7,9<br>,15,16,17	6	P02803,P04905,P0C0A9,P10758,P36374,P47727
9586	1,4,8,10,1<br>1,12,13,15	2,3,5,6,7,9<br>,14,16,17	3	P10758,P36374,Q561R9
9587	1,4,8,10,1<br>1,12,13,16	2,3,5,6,7,9<br>,14,15,17	4	P02803,P36374,P53792,Q561R9
9588	1,4,8,10,1<br>1,12,13,17	2,3,5,6,7,9<br>,14,15,16	3	P02803,P16636,P36374
9589	1,4,8,10,1<br>1,12,14,15	2,3,5,6,7,9<br>,13,16,17	5	P10758,P35280,P51907,P53792,Q561R9

9590	1,4,8,10,1<br>1,12,14,16	2,3,5,6,7,9<br>,13,15,17	2	P53792,Q9WUW8
9591	1,4,8,10,1<br>1,12,14,17	2,3,5,6,7,9<br>,13,15,16	1	P36374
9592	1,4,8,10,1<br>1,12,15,16	2,3,5,6,7,9<br>,13,14,17	4	P51907,P53792,Q561R9,Q9WUW8
9593	1,4,8,10,1<br>1,12,15,17	2,3,5,6,7,9<br>,13,14,16	2	P06866,Q561R9
9594	1,4,8,10,1<br>1,12,16,17	2,3,5,6,7,9<br>,13,14,15	4	P06757,P06866,P16636,P53792
9595	1,4,8,10,1<br>1,13,14,15	2,3,5,6,7,9<br>,12,16,17	2	P09605;P25809,Q561R9
9596	1,4,8,10,1<br>1,13,14,16	2,3,5,6,7,9<br>,12,15,17	1	Q561R9
9597	1,4,8,10,1<br>1,13,14,17	2,3,5,6,7,9<br>,12,15,16	1	P83748
9598	1,4,8,10,1<br>1,13,15,16	2,3,5,6,7,9<br>,12,14,17	2	P01835,Q561R9
9599	1,4,8,10,1<br>1,13,15,17	2,3,5,6,7,9<br>,12,14,16	1	Q561R9
9600	1,4,8,10,1<br>1,13,16,17	2,3,5,6,7,9<br>,12,14,15	1	P83748
9601	1,4,8,10,1<br>1,14,15,16	2,3,5,6,7,9<br>,12,13,17	2	P53792,Q561R9
9602	1,4,8,10,1<br>1,14,15,17	2,3,5,6,7,9<br>,12,13,16	0	
9603	1,4,8,10,1<br>1,14,16,17	2,3,5,6,7,9<br>,12,13,15	1	P83748
9604	1,4,8,10,1<br>1,15,16,17	2,3,5,6,7,9<br>,12,13,14	2	Q561R9,Q9Z1F2
9605	1,4,8,10,1<br>2,13,14,15	2,3,5,6,7,9<br>,11,16,17	4	P09605;P25809,P10758,P15999,Q9QUL6
9606	1,4,8,10,1<br>2,13,14,16	2,3,5,6,7,9<br>,11,15,17	1	Q9QUL6
9607	1,4,8,10,1<br>2,13,14,17	2,3,5,6,7,9<br>,11,15,16	2	P27213,P36374
9608	1,4,8,10,1<br>2,13,15,16	2,3,5,6,7,9<br>,11,14,17	1	Q9QUL6
9609	1,4,8,10,1<br>2,13,15,17	2,3,5,6,7,9<br>,11,14,16	4	P27213,P36374,Q99MH3,Q9QUL6
9610	1,4,8,10,1<br>2,13,16,17	2,3,5,6,7,9<br>,11,14,15	3	P27213,P36374,Q9QUL6
9611	1,4,8,10,1<br>2,14,15,16	2,3,5,6,7,9<br>,11,13,17	2	P08721,Q9QUL6
9612	1,4,8,10,1<br>2,14,15,17	2,3,5,6,7,9<br>,11,13,16	0	

9613	1,4,8,10,12,14,16,17	2,3,5,6,7,9,11,13,15	0	
9614	1,4,8,10,12,15,16,17	2,3,5,6,7,9,11,13,14	1	P06866
9615	1,4,8,10,13,14,15,16	2,3,5,6,7,9,11,12,17	3	P01835,P09605;P25809,Q9QUL6
9616	1,4,8,10,13,14,15,17	2,3,5,6,7,9,11,12,16	1	P15999
9617	1,4,8,10,13,14,16,17	2,3,5,6,7,9,11,12,15	1	P27213
9618	1,4,8,10,13,15,16,17	2,3,5,6,7,9,11,12,14	2	P01835,P27213
9619	1,4,8,10,14,15,16,17	2,3,5,6,7,9,11,12,13	0	
9620	1,4,8,11,12,13,14,15	2,3,5,6,7,9,10,16,17	4	P10758,P36374,Q561R9,Q9QUL6
9621	1,4,8,11,12,13,14,16	2,3,5,6,7,9,10,15,17	3	P02803,P36374,Q9QUL6
9622	1,4,8,11,12,13,14,17	2,3,5,6,7,9,10,15,16	3	P02803,P27213,P36374
9623	1,4,8,11,12,13,15,16	2,3,5,6,7,9,10,14,17	2	Q561R9,Q9QUL6
9624	1,4,8,11,12,13,15,17	2,3,5,6,7,9,10,14,16	1	P36374
9625	1,4,8,11,12,13,16,17	2,3,5,6,7,9,10,14,15	3	P02803,P27213,P36374
9626	1,4,8,11,12,14,15,16	2,3,5,6,7,9,10,13,17	1	P51907
9627	1,4,8,11,12,14,15,17	2,3,5,6,7,9,10,13,16	0	
9628	1,4,8,11,12,14,16,17	2,3,5,6,7,9,10,13,15	0	
9629	1,4,8,11,12,15,16,17	2,3,5,6,7,9,10,13,14	1	P06866
9630	1,4,8,11,13,14,15,16	2,3,5,6,7,9,10,12,17	1	Q561R9
9631	1,4,8,11,13,14,15,17	2,3,5,6,7,9,10,12,16	1	P97840
9632	1,4,8,11,13,14,16,17	2,3,5,6,7,9,10,12,15	2	P27213,P83748
9633	1,4,8,11,13,15,16,17	2,3,5,6,7,9,10,12,14	1	P27213
9634	1,4,8,11,14,15,16,17	2,3,5,6,7,9,10,12,13	1	P97840
9635	1,4,8,12,13,14,15,16	2,3,5,6,7,9,10,11,17	2	P27213,Q9QUL6

9636	1,4,8,12,13,14,15,17	2,3,5,6,7,9,10,11,16	2	P27213,Q9QUL6
9637	1,4,8,12,13,14,16,17	2,3,5,6,7,9,10,11,15	3	P27213,Q4FZU6,Q9QUL6
9638	1,4,8,12,13,15,16,17	2,3,5,6,7,9,10,11,14	2	P27213,Q9QUL6
9639	1,4,8,12,14,15,16,17	2,3,5,6,7,9,10,11,13	3	P06866,P27213,Q9QUL6
9640	1,4,8,13,14,15,16,17	2,3,5,6,7,9,10,11,12	3	P11883,P27213,Q9QUL6
9641	1,4,9,10,11,12,13,14	2,3,5,6,7,8,15,16,17	6	P02803,P10758,P36374,P47727,P50115,Q561R9
9642	1,4,9,10,11,12,13,15	2,3,5,6,7,8,14,16,17	5	P10758,P36374,P50115,P51907,Q561R9
9643	1,4,9,10,11,12,13,16	2,3,5,6,7,8,14,15,17	7	P02631,P02803,P10536,P36374,P50115,Q561R9,Q9WUW8
9644	1,4,9,10,11,12,13,17	2,3,5,6,7,8,14,15,16	4	P02631,P02803,P06757,P36374
9645	1,4,9,10,11,12,14,15	2,3,5,6,7,8,13,16,17	5	P08937,P10758,P50115,P51907,Q561R9
9646	1,4,9,10,11,12,14,16	2,3,5,6,7,8,13,15,17	7	P08937,P10536,P50115,P51907,P53792,Q561R9,Q9WUW8
9647	1,4,9,10,11,12,14,17	2,3,5,6,7,8,13,15,16	1	P36374
9648	1,4,9,10,11,12,15,16	2,3,5,6,7,8,13,14,17	11	O88267,P08753,P08937,P10536,P20766,P50115,P51907,P53792,Q561R9,Q9WUW8,Q9Z1F2
9649	1,4,9,10,11,12,15,17	2,3,5,6,7,8,13,14,16	4	P06866,P51907,Q561R9,Q9Z1F2
9650	1,4,9,10,11,12,16,17	2,3,5,6,7,8,13,14,15	4	P06757,P06866,P53792,Q9Z1F2
9651	1,4,9,10,11,13,14,15	2,3,5,6,7,8,12,16,17	7	O88267,P08937,P50115,P50116,P55091,Q561R9,Q62635
9652	1,4,9,10,11,13,14,16	2,3,5,6,7,8,12,15,17	7	P08937,P50115,P55091,P83748,Q561R9,Q62635,Q7TPB1
9653	1,4,9,10,11,13,14,17	2,3,5,6,7,8,12,15,16	1	P83748
9654	1,4,9,10,11,13,15,16	2,3,5,6,7,8,12,14,17	8	O88267,P06760,P08937,P50115,P55091,Q561R9,Q62635,Q9Z1F2
9655	1,4,9,10,11,13,15,17	2,3,5,6,7,8,12,14,16	2	P83748,Q561R9
9656	1,4,9,10,11,13,16,17	2,3,5,6,7,8,12,14,15	2	P02631,P83748
9657	1,4,9,10,11,14,15,16	2,3,5,6,7,8,12,13,17	12	O88267,P06760,P08937,P50115,P51907,P55091,P70545,Q561R9,Q62635,Q63751,Q9QZK9,Q9Z1F2
9658	1,4,9,10,11,14,15,17	2,3,5,6,7,8,12,13,16	5	O88267,P08937,P83748,Q561R9,Q9Z1F2

9659	1,4,9,10,11,14,16,17 2,3,5,6,7,8,12,13,15	3	P08937,P83748,Q9Z1F2
9660	1,4,9,10,11,15,16,17 2,3,5,6,7,8,12,13,14	5	O88267,P08937,P83748,Q561R9,Q9Z1F2
9661	1,4,9,10,12,13,14,15 2,3,5,6,7,8,11,16,17	4	P08937,P10758,P50115,P50116
9662	1,4,9,10,12,13,14,16 2,3,5,6,7,8,11,15,17	4	P02631,P08937,P50115,Q63751
9663	1,4,9,10,12,13,14,17 2,3,5,6,7,8,11,15,16	1	P36374
9664	1,4,9,10,12,13,15,16 2,3,5,6,7,8,11,14,17	5	P08937,P10758,P16086,P50115,Q561R9
9665	1,4,9,10,12,13,15,17 2,3,5,6,7,8,11,14,16	2	P10758,P16086
9666	1,4,9,10,12,13,16,17 2,3,5,6,7,8,11,14,15	4	P02631,P02803,P07171,P27213
9667	1,4,9,10,12,14,15,16 2,3,5,6,7,8,11,13,17	5	P08937,P10758,P20766,P50115,Q63751
9668	1,4,9,10,12,14,15,17 2,3,5,6,7,8,11,13,16	2	P06866,P10758
9669	1,4,9,10,12,14,16,17 2,3,5,6,7,8,11,13,15	0	
9670	1,4,9,10,12,15,16,17 2,3,5,6,7,8,11,13,14	3	P06866,P16086,Q9Z1F2
9671	1,4,9,10,13,14,15,16 2,3,5,6,7,8,11,12,17	11	P06760,P08937,P11883,P50115,P55091,P62804,Q4G075,Q561R9,Q62635,Q63751,Q9QZK9
9672	1,4,9,10,13,14,15,17 2,3,5,6,7,8,11,12,16	2	P08937,P11883
9673	1,4,9,10,13,14,16,17 2,3,5,6,7,8,11,12,15	3	P02631,P08937,P27213
9674	1,4,9,10,13,15,16,17 2,3,5,6,7,8,11,12,14	6	P08937,P11883,P27213,P62804,Q9EQS0,Q9Z1F2
9675	1,4,9,10,14,15,16,17 2,3,5,6,7,8,11,12,13	4	P08937,P11883,Q9JHB9,Q9Z1F2
9676	1,4,9,11,12,13,14,15 2,3,5,6,7,8,10,16,17	5	P06760,P10758,P50115,Q561R9,Q6P6R2
9677	1,4,9,11,12,13,14,16 2,3,5,6,7,8,10,15,17	5	P02803,P06760,P08937,P50115,P97615
9678	1,4,9,11,12,13,14,17 2,3,5,6,7,8,10,15,16	2	P02803,P36374
9679	1,4,9,11,12,13,15,16 2,3,5,6,7,8,10,14,17	7	P06760,P08937,P10758,P50115,P51907,P97615,Q561R9
9680	1,4,9,11,12,13,15,17 2,3,5,6,7,8,10,14,16	2	P36374,P82471
9681	1,4,9,11,12,13,16,17 2,3,5,6,7,8,10,14,15	4	P02803,P27213,P36374,P97615

9682	1,4,9,11,12,14,15,16	2,3,5,6,7,8,10,13,17	5	P06760,P08937,P50115,P51907,Q561R9
9683	1,4,9,11,12,14,15,17	2,3,5,6,7,8,10,13,16	0	
9684	1,4,9,11,12,14,16,17	2,3,5,6,7,8,10,13,15	0	
9685	1,4,9,11,12,15,16,17	2,3,5,6,7,8,10,13,14	3	P06866,P12020,P51907
9686	1,4,9,11,13,14,15,16	2,3,5,6,7,8,10,12,17	8	P06760,P08937,P50115,P55091,Q4G075,Q561R9,Q62635,Q9QZK9
9687	1,4,9,11,13,14,15,17	2,3,5,6,7,8,10,12,16	2	P82471,P83748
9688	1,4,9,11,13,14,16,17	2,3,5,6,7,8,10,12,15	3	P08937,P27213,P83748
9689	1,4,9,11,13,15,16,17	2,3,5,6,7,8,10,12,14	3	P08937,P27213,Q561R9
9690	1,4,9,11,14,15,16,17	2,3,5,6,7,8,10,12,13	3	P08937,P83748,Q9Z1F2
9691	1,4,9,12,13,14,15,16	2,3,5,6,7,8,10,11,17	7	P06760,P08937,P10758,P11883,P14669,P50115,Q9QUL6
9692	1,4,9,12,13,14,15,17	2,3,5,6,7,8,10,11,16	4	P10758,P11883,P27213,P82471
9693	1,4,9,12,13,14,16,17	2,3,5,6,7,8,10,11,15	2	P27213,P82471
9694	1,4,9,12,13,15,16,17	2,3,5,6,7,8,10,11,14	5	P06866,P11883,P16086,P27213,P82471
9695	1,4,9,12,14,15,16,17	2,3,5,6,7,8,10,11,13	3	P06866,P08937,P11883
9696	1,4,9,13,14,15,16,17	2,3,5,6,7,8,10,11,12	5	P08937,P11883,P27213,P62804,P82471
9697	1,4,10,11,12,13,14,15	2,3,5,6,7,8,9,16,17	5	P10758,P50115,P51907,Q561R9,Q6P6R2
9698	1,4,10,11,12,13,14,16	2,3,5,6,7,8,9,15,17	2	P50115,P53792
9699	1,4,10,11,12,13,14,17	2,3,5,6,7,8,9,15,16	2	P27213,P36374
9700	1,4,10,11,12,13,15,16	2,3,5,6,7,8,9,14,17	4	P50115,P51907,P53792,Q561R9
9701	1,4,10,11,12,13,15,17	2,3,5,6,7,8,9,14,16	3	P06866,P36374,Q561R9

9702	1,4,10,11,12,13,16,17	2,3,5,6,7,8,9,14,15	7	P02631,P06757,P06866,P27213,P36374,P53792,P97605
9703	1,4,10,11,12,14,15,16	2,3,5,6,7,8,9,13,17	7	P08937,P20766,P50115,P51907,P53792,Q561R9,Q9Z1F2
9704	1,4,10,11,12,14,15,17	2,3,5,6,7,8,9,13,16	3	P06866,P20766,P51907
9705	1,4,10,11,12,14,16,17	2,3,5,6,7,8,9,13,15	4	P06757,P06866,P20766,P53792
9706	1,4,10,11,12,15,16,17	2,3,5,6,7,8,9,13,14	6	P06757,P06866,P20766,P51907,P53792,Q9Z1F2
9707	1,4,10,11,13,14,15,16	2,3,5,6,7,8,9,12,17	5	P08937,P09605;P25809,P50115,P55091,Q561R9
9708	1,4,10,11,13,14,15,17	2,3,5,6,7,8,9,12,16	1	P83748
9709	1,4,10,11,13,14,16,17	2,3,5,6,7,8,9,12,15	2	P27213,P83748
9710	1,4,10,11,13,15,16,17	2,3,5,6,7,8,9,12,14	3	P27213,Q561R9,Q9Z1F2
9711	1,4,10,11,14,15,16,17	2,3,5,6,7,8,9,12,13	3	P08937,P20766,Q9Z1F2
9712	1,4,10,12,13,14,15,16	2,3,5,6,7,8,9,11,17	4	P08937,P27213,P50115,Q9QUL6
9713	1,4,10,12,13,14,15,17	2,3,5,6,7,8,9,11,16	1	P27213
9714	1,4,10,12,13,14,16,17	2,3,5,6,7,8,9,11,15	3	P02631,P08932,P27213
9715	1,4,10,12,13,15,16,17	2,3,5,6,7,8,9,11,14	4	P02631,P06866,P27213,Q9QUL6
9716	1,4,10,12,14,15,16,17	2,3,5,6,7,8,9,11,13	3	P06866,P08932,P20766

9717	1,4,10,13,14,15,16,17	2,3,5,6,7,8,9,11,12	3	P08937,P11883,P27213
9718	1,4,11,12,13,14,15,16	2,3,5,6,7,8,9,10,17	6	P06760,P50115,P51907,P52590,Q6P6R2,Q9QUL6
9719	1,4,11,12,13,14,15,17	2,3,5,6,7,8,9,10,16	2	P27213,P36374
9720	1,4,11,12,13,14,16,17	2,3,5,6,7,8,9,10,15	3	O55006,P27213,P36374
9721	1,4,11,12,13,15,16,17	2,3,5,6,7,8,9,10,14	2	P06866,P27213
9722	1,4,11,12,14,15,16,17	2,3,5,6,7,8,9,10,13	3	P06866,P20766,P51907
9723	1,4,11,13,14,15,16,17	2,3,5,6,7,8,9,10,12	1	P27213
9724	1,4,12,13,14,15,16,17	2,3,5,6,7,8,9,10,11	5	P06866,P11883,P27213,P82471,Q9QUL6
9725	1,5,6,7,8,9,10,11	2,3,4,12,13,14,15,16,17	6	A2RUV9,P14669,P27213,P31044,Q566E6,Q9QUL6
9726	1,5,6,7,8,9,10,12	2,3,4,11,13,14,15,16,17	11	F1LM93,P08723,P10715,P13432,P19218,P22283,P27213,P30120,P31044,P97840,Q8CJ52
9727	1,5,6,7,8,9,10,13	2,3,4,11,12,14,15,16,17	5	P06866,P12020,P19218,P31044,Q5GRG2
9728	1,5,6,7,8,9,10,14	2,3,4,11,12,13,15,16,17	7	P01836,P06866,P12020,P19629,P27213,P31044,Q9QUL6
9729	1,5,6,7,8,9,10,15	2,3,4,11,12,13,14,16,17	3	O55006,P27213,P31044
9730	1,5,6,7,8,9,10,16	2,3,4,11,12,13,14,15,17	2	P19218,P31044
9731	1,5,6,7,8,9,10,17	2,3,4,11,12,13,14,15,16	4	P01836,P06760,P52590,Q9QUL6

9732	1,5,6,7,8,9,11,12	2,3,4,10,13,14,15,16,17	16	F1LM93,P02781,P02782,P08723,P10715,P13432,P14669,P19629,P22283,P27213,P30120,Q63357,Q63493,Q8CJ52,Q9JHB9,Q9JI85
9733	1,5,6,7,8,9,11,13	2,3,4,10,12,14,15,16,17	6	P01048,P06866,P22282,P22283,Q62902,Q9JI85
9734	1,5,6,7,8,9,11,14	2,3,4,10,12,13,15,16,17	5	P06866,P14669,P19629,P27213,Q9QUL6
9735	1,5,6,7,8,9,11,15	2,3,4,10,12,13,14,16,17	5	P10715,P14669,P19629,P27213,Q9QUL6
9736	1,5,6,7,8,9,11,16	2,3,4,10,12,13,14,15,17	3	A2RUV9,P10715,Q9QUL6
9737	1,5,6,7,8,9,11,17	2,3,4,10,12,13,14,15,16	7	A2RUV9,P06760,P10715,P14669,P19629,P50115,Q9QUL6
9738	1,5,6,7,8,9,12,13	2,3,4,10,11,14,15,16,17	14	P02782,P06761,P08723,P10715,P13432,P19218,P22282,P22283,P30120,P53792,Q62902,Q8CJ52,Q9JHB9,Q9Z1F2
9739	1,5,6,7,8,9,12,14	2,3,4,10,11,13,15,16,17	9	P02782,P08723,P10715,P13432,P19629,P22283,P30120,Q8CJ52,Q9Z1F2
9740	1,5,6,7,8,9,12,15	2,3,4,10,11,13,14,16,17	4	P10715,P13432,P19218,P19629
9741	1,5,6,7,8,9,12,16	2,3,4,10,11,13,14,15,17	5	P10715,P13432,P19218,P19629,Q8CJ52
9742	1,5,6,7,8,9,12,17	2,3,4,10,11,13,14,15,16	10	F1LM93,P06760,P10715,P13432,P19629,P22282,P22283,P50115,Q8CJ52,Q99041
9743	1,5,6,7,8,9,13,14	2,3,4,10,11,12,15,16,17	9	P06866,P06911,P12020,P19629,P51907,P53792,Q5GRG2,Q62902,Q9Z1F2
9744	1,5,6,7,8,9,13,15	2,3,4,10,11,12,14,16,17	2	P19218,P53792
9745	1,5,6,7,8,9,13,16	2,3,4,10,11,12,14,15,17	2	P19218,P53792
9746	1,5,6,7,8,9,13,17	2,3,4,10,11,12,14,15,16	5	P06760,P22282,P22283,P51907,P53792

9747	1,5,6,7,8,9,14,15	2,3,4,10,11,12,13,16,17	3	P16636,P19629,P53792
9748	1,5,6,7,8,9,14,16	2,3,4,10,11,12,13,15,17	1	P19629
9749	1,5,6,7,8,9,14,17	2,3,4,10,11,12,13,15,16	5	P01836,P06760,P19629,P51907,P53792
9750	1,5,6,7,8,9,15,16	2,3,4,10,11,12,13,14,17	0	
9751	1,5,6,7,8,9,15,17	2,3,4,10,11,12,13,14,16	3	P06760,P19629,P53792
9752	1,5,6,7,8,9,16,17	2,3,4,10,11,12,13,14,15	3	A2RUV9,P06760,P53792
9753	1,5,6,7,8,10,11,12	2,3,4,9,13,14,15,16,17	24	F1LM93,O35547,P02781,P02782,P08723,P0C0A9,P10715,P14669,P19629,P22283,P27213,P30120,P31044,P47727,P60905,P68370,Q63357,Q63493,Q6GMN2,Q6IFU7,Q6P6Q2,Q8CJ52,Q9JHB9,Q9JI85
9754	1,5,6,7,8,10,11,13	2,3,4,9,12,14,15,16,17	11	P06911,P12020,P14669,P22283,P31044,P47727,P68370,Q5GRG2,Q63493,Q8CJ52,Q8VD89
9755	1,5,6,7,8,10,11,14	2,3,4,9,12,13,15,16,17	8	P12020,P14669,P19629,P27213,P31044,P47727,Q63493,Q9QUL6
9756	1,5,6,7,8,10,11,15	2,3,4,9,12,13,14,16,17	5	P14669,P19629,P27213,P31044,Q566E6
9757	1,5,6,7,8,10,11,16	2,3,4,9,12,13,14,15,17	4	A2RUV9,P31044,P61206;P84079;P84082,Q8VD89
9758	1,5,6,7,8,10,11,17	2,3,4,9,12,13,14,15,16	9	A2RUV9,P06760,P07150,P14669,P22283,P50115,Q566E6,Q63493,Q9QUL6
9759	1,5,6,7,8,10,12,13	2,3,4,9,11,14,15,16,17	18	F1LM93,P02781,P02782,P06761,P06911,P08723,P12020,P19218,P22282,P22283,P30120,P31044,Q498D9,Q5GRG2,Q62902,Q8CJ52,Q99MH3,Q9JHB9
9760	1,5,6,7,8,10,12,14	2,3,4,9,11,13,15,16,17	11	F1LM93,P02782,P08723,P12020,P19629,P22283,P30120,P31044,Q498D9,Q8CJ52,Q9ET32
9761	1,5,6,7,8,10,12,15	2,3,4,9,11,13,14,16,17	6	F1LM93,P10715,P19218,P19629,Q8CJ52,Q99MH3

9762	1,5,6,7,8,10,12,16	2,3,4,9,11,13,14,15,17	5	F1LM93,P10715,P19218,P97840,Q8CJ52
9763	1,5,6,7,8,10,12,17	2,3,4,9,11,13,14,15,16	15	F1LM93,P06760,P08723,P10715,P13432,P19629,P22282,P22283,P50115,Q10758,Q4G075,Q8CJ52,Q99041,Q99MH3,Q9QZK9
9764	1,5,6,7,8,10,13,14	2,3,4,9,11,12,15,16,17	8	P06866,P06911,P12020,P19629,P22283,P31044,Q498D9,Q5GRG2
9765	1,5,6,7,8,10,13,15	2,3,4,9,11,12,14,16,17	6	P12020,P19218,P31044,Q498D9,Q5GRG2,Q6IFV1
9766	1,5,6,7,8,10,13,16	2,3,4,9,11,12,14,15,17	4	P12020,P19218,P31044,Q5GRG2
9767	1,5,6,7,8,10,13,17	2,3,4,9,11,12,14,15,16	8	P06760,P12020,P19218,P22282,P22283,P51907,Q6IFV1,Q99MH3
9768	1,5,6,7,8,10,14,15	2,3,4,9,11,12,13,16,17	4	P12020,P19629,P31044,Q498D9
9769	1,5,6,7,8,10,14,16	2,3,4,9,11,12,13,15,17	3	P12020,P19629,P31044
9770	1,5,6,7,8,10,14,17	2,3,4,9,11,12,13,15,16	4	P01836,P06760,P12020,P19629
9771	1,5,6,7,8,10,15,16	2,3,4,9,11,12,13,14,17	1	P31044
9772	1,5,6,7,8,10,15,17	2,3,4,9,11,12,13,14,16	3	P06760,Q6IFV1,Q99MH3
9773	1,5,6,7,8,10,16,17	2,3,4,9,11,12,13,14,15	4	A2RUV9,P02625,P06760,P10758
9774	1,5,6,7,8,11,12,13	2,3,4,9,10,14,15,16,17	26	F1LM93,P02780,P02781,P02782,P04905,P06761,P07647,P08723,P0C0A9,P10715,P13432,P14669,P19629,P22282,P22283,P30120,P36374,P46462,P47727,Q62902,Q63493,Q6GMN2,Q8CJ52,Q9JHB9,Q9JI85,Q9Z1F2
9775	1,5,6,7,8,11,12,14	2,3,4,9,10,13,15,16,17	20	F1LM93,O35547,P02781,P02782,P04905,P08723,P10715,P13432,P14669,P19629,P22282,P22283,P30120,P46462,P47727,Q63493,Q8CJ52,Q9ET32,Q9JHB9,Q9JI85
9776	1,5,6,7,8,11,12,15	2,3,4,9,10,13,14,16,17	12	F1LM93,O35547,P08723,P10715,P13432,P14669,P19629,P30120,Q63493,Q8CJ52,Q9JHB9,Q9JI85

9777	1,5,6,7,8,11,12,16	2,3,4,9,10,13,14,15,17	13	F1LM93,O35547,P08723,P10715,P13432,P17046,P19629,P30120,Q63493,Q6P6Q2,Q8CJ52,Q9JHB9,Q9JI85
9778	1,5,6,7,8,11,12,17	2,3,4,9,10,13,14,15,16	22	F1LM93,O35547,P02781,P02782,P06760,P07150,P08723,P10715,P13432,P14669,P19629,P22282,P22283,P30120,P50115,Q4G075,Q63493,Q8CJ52,Q99041,Q9JHB9,Q9JI85,Q9QZK9
9779	1,5,6,7,8,11,13,14	2,3,4,9,10,12,15,16,17	13	P06866,P06911,P12020,P19629,P22282,P22283,P47727,Q5GRG2,Q62902,Q63493,Q8CJ52,Q9JI85,Q9Z1F2
9780	1,5,6,7,8,11,13,15	2,3,4,9,10,12,14,16,17	5	P14669,P19629,Q5GRG2,Q8VD89,Q9JJS8
9781	1,5,6,7,8,11,13,16	2,3,4,9,10,12,14,15,17	4	A2RUV9,Q62902,Q8CJ52,Q8VD89
9782	1,5,6,7,8,11,13,17	2,3,4,9,10,12,14,15,16	7	P06760,P14669,P19629,P22282,P22283,P50115,Q63493
9783	1,5,6,7,8,11,14,15	2,3,4,9,10,12,13,16,17	4	P07171,P14669,P19629,Q5I0D1
9784	1,5,6,7,8,11,14,16	2,3,4,9,10,12,13,15,17	2	P19629,Q8CJ52
9785	1,5,6,7,8,11,14,17	2,3,4,9,10,12,13,15,16	7	P06760,P07150,P14669,P19629,P22283,P50115,Q63493
9786	1,5,6,7,8,11,15,16	2,3,4,9,10,12,13,14,17	3	P10715,P19629,Q8VD89
9787	1,5,6,7,8,11,15,17	2,3,4,9,10,12,13,14,16	6	P06760,P10715,P14669,P19629,P50115,Q9JJS8
9788	1,5,6,7,8,11,16,17	2,3,4,9,10,12,13,14,15	7	A2RUV9,P06760,P07150,P10715,P10758,P19629,P50115
9789	1,5,6,7,8,12,13,14	2,3,4,9,10,11,15,16,17	17	P02781,P02782,P06761,P06911,P08723,P12020,P13432,P19629,P22282,P22283,P30120,Q498D9,Q5GRG2,Q62902,Q8CJ52,Q9JHB9,Q9Z1F2
9790	1,5,6,7,8,12,13,15	2,3,4,9,10,11,14,16,17	14	P02782,P06761,P10715,P13432,P19218,P19629,P22283,P30120,Q498D9,Q5GRG2,Q62902,Q6IFV1,Q8CJ52,Q9JHB9
9791	1,5,6,7,8,12,13,16	2,3,4,9,10,11,14,15,17	11	P02782,P06761,P10715,P13432,P19218,P22283,P30120,Q62902,Q8CJ52,Q9JHB9,Q9Z1F2

9792	1,5,6,7,8,12,13,17	2,3,4,9,10,11,14,15,16	26	F1LM93,P02782,P06760,P06761,P08723,P10715,P13432,P19218,P19629,P22282,P22283,P23928,P30120,P36374,P50115,P51907,Q4G075,Q62635,Q62902,Q6IFV1,Q8CJ52,Q99041,Q99MH3,Q9JHB9,Q9QZK9,Q9Z1F2
9793	1,5,6,7,8,12,14,15	2,3,4,9,10,11,13,16,17	5	P10715,P13432,P19629,Q498D9,Q8CJ52
9794	1,5,6,7,8,12,14,16	2,3,4,9,10,11,13,15,17	5	P10715,P13432,P19629,Q8CJ52,Q9ET32
9795	1,5,6,7,8,12,14,17	2,3,4,9,10,11,13,15,16	17	F1LM93,P02782,P06760,P06761,P08723,P10715,P13432,P19629,P22282,P22283,P30120,P50115,Q4G075,Q561R9,Q8CJ52,Q99041,Q9QZK9
9796	1,5,6,7,8,12,15,16	2,3,4,9,10,11,13,14,17	4	P10715,P19218,P19629,Q8CJ52
9797	1,5,6,7,8,12,15,17	2,3,4,9,10,11,13,14,16	11	F1LM93,P06760,P10715,P13432,P19629,P50115,Q4G075,Q6IFV1,Q99041,Q99MH3,Q9QZK9
9798	1,5,6,7,8,12,16,17	2,3,4,9,10,11,13,14,15	11	F1LM93,P06760,P10715,P13432,P19218,P19629,P23928,P50115,Q4G075,Q8CJ52,Q99041
9799	1,5,6,7,8,13,14,15	2,3,4,9,10,11,12,16,17	7	P06911,P12020,P16636,P19629,P53792,Q498D9,Q5GRG2
9800	1,5,6,7,8,13,14,16	2,3,4,9,10,11,12,15,17	6	P06866,P06911,P12020,P19629,Q5GRG2,Q62902
9801	1,5,6,7,8,13,14,17	2,3,4,9,10,11,12,15,16	13	P06760,P06761,P06911,P10536,P12020,P19629,P22282,P22283,P51907,P53792,Q5GRG2,Q9WUW8,Q9Z1F2
9802	1,5,6,7,8,13,15,16	2,3,4,9,10,11,12,14,17	1	P19218
9803	1,5,6,7,8,13,15,17	2,3,4,9,10,11,12,14,16	6	P06760,P19629,P53792,Q6IFV1,Q99MH3,Q9WUW8
9804	1,5,6,7,8,13,16,17	2,3,4,9,10,11,12,14,15	5	P06760,P19218,P23928,P51907,Q63661
9805	1,5,6,7,8,14,15,16	2,3,4,9,10,11,12,13,17	2	P16636,P19629
9806	1,5,6,7,8,14,15,17	2,3,4,9,10,11,12,13,16	4	P06760,P10536,P19629,P53792

9807	1,5,6,7,8,14,16,17	2,3,4,9,10,11,12,13,15	4	P06760,P10758,P19629,Q561R9
9808	1,5,6,7,8,15,16,17	2,3,4,9,10,11,12,13,14	2	P06760,P19629
9809	1,5,6,7,9,10,11,12	2,3,4,8,13,14,15,16,17	9	P12368,P20766,P27213,P97840,Q566E6,Q63357,Q64093,Q6IFU7,Q9QUL6
9810	1,5,6,7,9,10,11,13	2,3,4,8,12,14,15,16,17	3	B0BNN3,Q566E6,Q9QUL6
9811	1,5,6,7,9,10,11,14	2,3,4,8,12,13,15,16,17	3	P27213,P83121,Q9QUL6
9812	1,5,6,7,9,10,11,15	2,3,4,8,12,13,14,16,17	5	P20766,P27213,Q566E6,Q80WL1,Q9QUL6
9813	1,5,6,7,9,10,11,16	2,3,4,8,12,13,14,15,17	7	A2RUV9,B0BNN3,P05712,P20766,P61206;P84079;P84082,Q64093,Q9QUL6
9814	1,5,6,7,9,10,11,17	2,3,4,8,12,13,14,15,16	3	Q566E6,Q64093,Q9QUL6
9815	1,5,6,7,9,10,12,13	2,3,4,8,11,14,15,16,17	2	P19218,P97840
9816	1,5,6,7,9,10,12,14	2,3,4,8,11,13,15,16,17	2	P20766,P97840
9817	1,5,6,7,9,10,12,15	2,3,4,8,11,13,14,16,17	3	P19218,P20766,P97840
9818	1,5,6,7,9,10,12,16	2,3,4,8,11,13,14,15,17	4	P19218,P20766,P97840,Q64093
9819	1,5,6,7,9,10,12,17	2,3,4,8,11,13,14,15,16	7	F1LM93,P13432,P19218,P20766,P97840,Q99MH3,Q9QUL6
9820	1,5,6,7,9,10,13,14	2,3,4,8,11,12,15,16,17	1	P12020
9821	1,5,6,7,9,10,13,15	2,3,4,8,11,12,14,16,17	3	P19218,Q5U2V4,Q80WL1

9822	1,5,6,7,9,10,13,16	2,3,4,8,11,12,14,15,17	6	B0BNN3,P02761,P19218,P62804,P97840,Q00715
9823	1,5,6,7,9,10,13,17	2,3,4,8,11,12,14,15,16	1	P19218
9824	1,5,6,7,9,10,14,15	2,3,4,8,11,12,13,16,17	3	P16636,P20766,Q80WL1
9825	1,5,6,7,9,10,14,16	2,3,4,8,11,12,13,15,17	2	P20766,Q80WL1
9826	1,5,6,7,9,10,14,17	2,3,4,8,11,12,13,15,16	2	P01836,Q9QUL6
9827	1,5,6,7,9,10,15,16	2,3,4,8,11,12,13,14,17	4	P19218,P20766,Q64093,Q80WL1
9828	1,5,6,7,9,10,15,17	2,3,4,8,11,12,13,14,16	3	P20766,Q80WL1,Q9QUL6
9829	1,5,6,7,9,10,16,17	2,3,4,8,11,12,13,14,15	5	P19218,P20766,Q64093,Q6TMA8,Q9QUL6
9830	1,5,6,7,9,11,12,13	2,3,4,8,10,14,15,16,17	2	P30120,Q9JHB9
9831	1,5,6,7,9,11,12,14	2,3,4,8,10,13,15,16,17	1	P20766
9832	1,5,6,7,9,11,12,15	2,3,4,8,10,13,14,16,17	1	P20766
9833	1,5,6,7,9,11,12,16	2,3,4,8,10,13,14,15,17	5	P05712,P12368,P17046,P20766,Q6IFU7
9834	1,5,6,7,9,11,12,17	2,3,4,8,10,13,14,15,16	2	P13432,Q9QUL6
9835	1,5,6,7,9,11,13,14	2,3,4,8,10,12,15,16,17	2	P06866,P83121
9836	1,5,6,7,9,11,13,15	2,3,4,8,10,12,14,16,17	2	Q80WL1,Q9JJS8

9837	1,5,6,7,9,11,13,16	2,3,4,8,10,12,14,15,17	2	B0BNN3,P02761
9838	1,5,6,7,9,11,13,17	2,3,4,8,10,12,14,15,16	3	P53792,Q9JJS8,Q9QUL6
9839	1,5,6,7,9,11,14,15	2,3,4,8,10,12,13,16,17	3	P16636,Q80WL1,Q9QUL6
9840	1,5,6,7,9,11,14,16	2,3,4,8,10,12,13,15,17	2	Q80WL1,Q9QUL6
9841	1,5,6,7,9,11,14,17	2,3,4,8,10,12,13,15,16	2	P83121,Q9QUL6
9842	1,5,6,7,9,11,15,16	2,3,4,8,10,12,13,14,17	3	P20766,P36970,Q80WL1
9843	1,5,6,7,9,11,15,17	2,3,4,8,10,12,13,14,16	4	Q566E6,Q80WL1,Q9JJS8,Q9QUL6
9844	1,5,6,7,9,11,16,17	2,3,4,8,10,12,13,14,15	3	A2RUV9,P36970,Q9QUL6
9845	1,5,6,7,9,12,13,14	2,3,4,8,10,11,15,16,17	3	P02761,P53792,Q9Z1F2
9846	1,5,6,7,9,12,13,15	2,3,4,8,10,11,14,16,17	2	P19218,P53792
9847	1,5,6,7,9,12,13,16	2,3,4,8,10,11,14,15,17	2	P02761,P19218
9848	1,5,6,7,9,12,13,17	2,3,4,8,10,11,14,15,16	4	P13432,P19218,P53792,Q9QZK9
9849	1,5,6,7,9,12,14,15	2,3,4,8,10,11,13,16,17	2	P16636,P20766
9850	1,5,6,7,9,12,14,16	2,3,4,8,10,11,13,15,17	2	P02761,P20766
9851	1,5,6,7,9,12,14,17	2,3,4,8,10,11,13,15,16	2	P13432,P53792

9852	1,5,6,7,9,12,15,16	2,3,4,8,10,11,13,14,17	2	P19218,P20766
9853	1,5,6,7,9,12,15,17	2,3,4,8,10,11,13,14,16	4	P13432,P19218,P20766,P53792
9854	1,5,6,7,9,12,16,17	2,3,4,8,10,11,13,14,15	3	P13432,P19218,P20766
9855	1,5,6,7,9,13,14,15	2,3,4,8,10,11,12,16,17	4	P16636,P19223,P53792,Q80WL1
9856	1,5,6,7,9,13,14,16	2,3,4,8,10,11,12,15,17	7	B0BNN3,P02761,P19223,P53792,P62804,Q00715,Q80WL1
9857	1,5,6,7,9,13,14,17	2,3,4,8,10,11,12,15,16	2	P51907,P53792
9858	1,5,6,7,9,13,15,16	2,3,4,8,10,11,12,14,17	6	P19218,P19223,P53792,P62804,Q00715,Q80WL1
9859	1,5,6,7,9,13,15,17	2,3,4,8,10,11,12,14,16	4	P19218,P53792,Q80WL1,Q9JJS8
9860	1,5,6,7,9,13,16,17	2,3,4,8,10,11,12,14,15	5	P19218,P35280,P53792,P62804,Q00715
9861	1,5,6,7,9,14,15,16	2,3,4,8,10,11,12,13,17	5	P16636,P19223,P20766,P53792,Q80WL1
9862	1,5,6,7,9,14,15,17	2,3,4,8,10,11,12,13,16	3	P16636,P53792,Q80WL1
9863	1,5,6,7,9,14,16,17	2,3,4,8,10,11,12,13,15	3	P53792,Q5XI22,Q80WL1
9864	1,5,6,7,9,15,16,17	2,3,4,8,10,11,12,13,14	4	P20766,P53792,P62804,Q80WL1
9865	1,5,6,7,10,11,12,13	2,3,4,8,9,14,15,16,17	6	P30120,P47727,Q5QE79,Q6GMN2,Q6IFU7,Q9JHB9
9866	1,5,6,7,10,11,12,14	2,3,4,8,9,13,15,16,17	5	P20766,P47727,Q5QE79,Q6IFU7,Q6P6Q2
9867	1,5,6,7,10,11,12,15	2,3,4,8,9,13,14,16,17	3	P20766,Q566E6,Q6IFU7

9868	1,5,6,7,10, 11,12,16	2,3,4,8,9,1 3,14,15,17	9	P05712,P12368,P17046,P20766,P61206;P84079;P84082,P97840,Q5QE79,Q6IFU7,Q6P6Q2
9869	1,5,6,7,10, 11,12,17	2,3,4,8,9,1 3,14,15,16	11	F1LM93,O08815,P06757,P13432,P20766,Q10758,Q566E6,Q5QE79,Q99MH3,Q9QUL6,Q9QZK9
9870	1,5,6,7,10, 11,13,14	2,3,4,8,9,1 2,15,16,17	5	P06911,P12020,P47727,Q5GRG2,Q5QE79
9871	1,5,6,7,10, 11,13,15	2,3,4,8,9,1 2,14,16,17	2	Q566E6,Q80WL1
9872	1,5,6,7,10, 11,13,16	2,3,4,8,9,1 2,14,15,17	3	B0BNN3,Q5QE79,Q8VD89
9873	1,5,6,7,10, 11,13,17	2,3,4,8,9,1 2,14,15,16	3	Q5QE79,Q99MH3,Q9QUL6
9874	1,5,6,7,10, 11,14,15	2,3,4,8,9,1 2,13,16,17	4	P16636,P20766,Q80WL1,Q9QUL6
9875	1,5,6,7,10, 11,14,16	2,3,4,8,9,1 2,13,15,17	2	P20766,Q5QE79
9876	1,5,6,7,10, 11,14,17	2,3,4,8,9,1 2,13,15,16	3	P20766,Q5QE79,Q9QUL6
9877	1,5,6,7,10, 11,15,16	2,3,4,8,9,1 2,13,14,17	3	P20766,Q80WL1,Q8VD89
9878	1,5,6,7,10, 11,15,17	2,3,4,8,9,1 2,13,14,16	4	P20766,Q566E6,Q80WL1,Q9QUL6
9879	1,5,6,7,10, 11,16,17	2,3,4,8,9,1 2,13,14,15	6	A2RUV9,P10758,P20766,P36970,Q6TMA8,Q9QUL6
9880	1,5,6,7,10, 12,13,14	2,3,4,8,9,1 1,15,16,17	5	P06911,P12020,Q498D9,Q5GRG2,Q5QE79
9881	1,5,6,7,10, 12,13,15	2,3,4,8,9,1 1,14,16,17	4	P19218,P97840,Q498D9,Q99MH3
9882	1,5,6,7,10, 12,13,16	2,3,4,8,9,1 1,14,15,17	4	P02761,P19218,P97840,Q5QE79
9883	1,5,6,7,10, 12,13,17	2,3,4,8,9,1 1,14,15,16	5	P13432,P19218,Q5QE79,Q99MH3,Q9QZK9
9884	1,5,6,7,10, 12,14,15	2,3,4,8,9,1 1,13,16,17	2	P20766,Q498D9
9885	1,5,6,7,10, 12,14,16	2,3,4,8,9,1 1,13,15,17	3	P20766,P97840,Q5QE79
9886	1,5,6,7,10, 12,14,17	2,3,4,8,9,1 1,13,15,16	5	P13432,P20766,Q10758,Q5QE79,Q9QZK9
9887	1,5,6,7,10, 12,15,16	2,3,4,8,9,1 1,13,14,17	3	P19218,P20766,P97840
9888	1,5,6,7,10, 12,15,17	2,3,4,8,9,1 1,13,14,16	6	F1LM93,P13432,P19218,P20766,Q99MH3,Q9QZK9
9889	1,5,6,7,10, 12,16,17	2,3,4,8,9,1 1,13,14,15	8	F1LM93,P13432,P19218,P20766,P97840,Q10758,Q6TMA8,Q99MH3
9890	1,5,6,7,10, 13,14,15	2,3,4,8,9,1 1,12,16,17	6	P12020,P16636,P19223,Q498D9,Q5GRG2,Q80WL1

9891	1,5,6,7,10,<br>13,14,16	2,3,4,8,9,1<br>1,12,15,17	4	P02761,P12020,P19223,Q5QE79
9892	1,5,6,7,10,<br>13,14,17	2,3,4,8,9,1<br>1,12,15,16	2	P12020,Q5QE79
9893	1,5,6,7,10,<br>13,15,16	2,3,4,8,9,1<br>1,12,14,17	3	P19218,P19223,Q80WL1
9894	1,5,6,7,10,<br>13,15,17	2,3,4,8,9,1<br>1,12,14,16	4	P19218,Q6IFV1,Q80WL1,Q99MH3
9895	1,5,6,7,10,<br>13,16,17	2,3,4,8,9,1<br>1,12,14,15	2	P19218,Q99MH3
9896	1,5,6,7,10,<br>14,15,16	2,3,4,8,9,1<br>1,12,13,17	5	P16636,P19223,P20766,P35467,Q80WL1
9897	1,5,6,7,10,<br>14,15,17	2,3,4,8,9,1<br>1,12,13,16	3	P20766,Q6TMA8,Q80WL1
9898	1,5,6,7,10,<br>14,16,17	2,3,4,8,9,1<br>1,12,13,15	4	P20766,Q5QE79,Q5XI22,Q6TMA8
9899	1,5,6,7,10,<br>15,16,17	2,3,4,8,9,1<br>1,12,13,14	3	P20766,Q6TMA8,Q80WL1
9900	1,5,6,7,11,<br>12,13,14	2,3,4,8,9,1<br>0,15,16,17	5	P30120,P47727,Q5QE79,Q62902,Q9JHB9
9901	1,5,6,7,11,<br>12,13,15	2,3,4,8,9,1<br>0,14,16,17	1	Q9JHB9
9902	1,5,6,7,11,<br>12,13,16	2,3,4,8,9,1<br>0,14,15,17	3	P02761,P17046,Q9JHB9
9903	1,5,6,7,11,<br>12,13,17	2,3,4,8,9,1<br>0,14,15,16	5	P13432,P17046,P36374,Q9JHB9,Q9QZK9
9904	1,5,6,7,11,<br>12,14,15	2,3,4,8,9,1<br>0,13,16,17	1	P20766
9905	1,5,6,7,11,<br>12,14,16	2,3,4,8,9,1<br>0,13,15,17	3	P17046,P20766,Q6IFU7
9906	1,5,6,7,11,<br>12,14,17	2,3,4,8,9,1<br>0,13,15,16	3	P13432,P20766,Q9QZK9
9907	1,5,6,7,11,<br>12,15,16	2,3,4,8,9,1<br>0,13,14,17	3	P17046,P20766,Q6IFU7
9908	1,5,6,7,11,<br>12,15,17	2,3,4,8,9,1<br>0,13,14,16	3	P13432,P20766,Q9QZK9
9909	1,5,6,7,11,<br>12,16,17	2,3,4,8,9,1<br>0,13,14,15	3	P13432,P17046,P20766
9910	1,5,6,7,11,<br>13,14,15	2,3,4,8,9,1<br>0,12,16,17	4	P16636,P19223,Q80WL1,Q9JJS8
9911	1,5,6,7,11,<br>13,14,16	2,3,4,8,9,1<br>0,12,15,17	4	P02761,P17046,P19223,Q80WL1
9912	1,5,6,7,11,<br>13,14,17	2,3,4,8,9,1<br>0,12,15,16	0	
9913	1,5,6,7,11,<br>13,15,16	2,3,4,8,9,1<br>0,12,14,17	4	P17046,P19223,Q80WL1,Q8VD89

9914	1,5,6,7,11,13,15,17	2,3,4,8,9,10,12,14,16	2	Q80WL1,Q9JJS8
9915	1,5,6,7,11,13,16,17	2,3,4,8,9,10,12,14,15	2	P10758,P17046
9916	1,5,6,7,11,14,15,16	2,3,4,8,9,10,12,13,17	5	P16636,P19223,P20766,Q63355,Q80WL1
9917	1,5,6,7,11,14,15,17	2,3,4,8,9,10,12,13,16	3	P20766,Q80WL1,Q9JJS8
9918	1,5,6,7,11,14,16,17	2,3,4,8,9,10,12,13,15	3	P10758,P20766,Q80WL1
9919	1,5,6,7,11,15,16,17	2,3,4,8,9,10,12,13,14	3	P20766,P36970,Q80WL1
9920	1,5,6,7,12,13,14,15	2,3,4,8,9,10,11,16,17	2	P16636,Q498D9
9921	1,5,6,7,12,13,14,16	2,3,4,8,9,10,11,15,17	2	P02761,P17046
9922	1,5,6,7,12,13,14,17	2,3,4,8,9,10,11,15,16	4	P13432,P53792,Q561R9,Q9QZK9
9923	1,5,6,7,12,13,15,16	2,3,4,8,9,10,11,14,17	2	P17046,P19218
9924	1,5,6,7,12,13,15,17	2,3,4,8,9,10,11,14,16	6	P13432,P19218,P53792,Q6IFV1,Q99MH3,Q9QZK9
9925	1,5,6,7,12,13,16,17	2,3,4,8,9,10,11,14,15	7	P02761,P13432,P17046,P19218,P35280,Q561R9,Q9QZK9
9926	1,5,6,7,12,14,15,16	2,3,4,8,9,10,11,13,17	3	P16636,P20766,P35467
9927	1,5,6,7,12,14,15,17	2,3,4,8,9,10,11,13,16	3	P13432,P20766,P35467
9928	1,5,6,7,12,14,16,17	2,3,4,8,9,10,11,13,15	3	P13432,P20766,Q561R9
9929	1,5,6,7,12,15,16,17	2,3,4,8,9,10,11,13,14	5	P13432,P19218,P20766,P35467,Q6TMA8
9930	1,5,6,7,13,14,15,16	2,3,4,8,9,10,11,12,17	5	P16636,P19223,P35467,P53792,Q80WL1
9931	1,5,6,7,13,14,15,17	2,3,4,8,9,10,11,12,16	4	P16636,P53792,Q80WL1,Q9WUW8
9932	1,5,6,7,13,14,16,17	2,3,4,8,9,10,11,12,15	3	P02761,P53792,Q561R9
9933	1,5,6,7,13,15,16,17	2,3,4,8,9,10,11,12,14	5	P19218,P53792,P62804,Q80WL1,Q9EQS0
9934	1,5,6,7,14,15,16,17	2,3,4,8,9,10,11,12,13	6	P16636,P20766,P35467,Q5XI22,Q6TMA8,Q80WL1
9935	1,5,6,8,9,10,11,12	2,3,4,7,13,14,15,16,17	10	F1LM93,O35547,P02803,P30120,Q566E6,Q6IFU7,Q6IG02,Q6IMF3,Q6P6Q2,Q9JHB9

9936	1,5,6,8,9,10,11,13	2,3,4,7,12,14,15,16,17	3	A2RUV9,P01836,Q566E6
9937	1,5,6,8,9,10,11,14	2,3,4,7,12,13,15,16,17	3	P01836,P47727,Q9QUL6
9938	1,5,6,8,9,10,11,15	2,3,4,7,12,13,14,16,17	4	P01836,P27213,Q566E6,Q9QUL6
9939	1,5,6,8,9,10,11,16	2,3,4,7,12,13,14,15,17	3	A2RUV9,P01836,Q9QUL6
9940	1,5,6,8,9,10,11,17	2,3,4,7,12,13,14,15,16	7	A2RUV9,P01836,P06760,P08649,Q09030,Q566E6,Q9QUL6
9941	1,5,6,8,9,10,12,13	2,3,4,7,11,14,15,16,17	4	P02782,P02803,P19218,Q9JHB9
9942	1,5,6,8,9,10,12,14	2,3,4,7,11,13,15,16,17	1	P01836
9943	1,5,6,8,9,10,12,15	2,3,4,7,11,13,14,16,17	0	
9944	1,5,6,8,9,10,12,16	2,3,4,7,11,13,14,15,17	1	P19218
9945	1,5,6,8,9,10,12,17	2,3,4,7,11,13,14,15,16	6	F1LM93,P01836,P06760,P13432,Q09030,Q10758
9946	1,5,6,8,9,10,13,14	2,3,4,7,11,12,15,16,17	2	P01836,P06866
9947	1,5,6,8,9,10,13,15	2,3,4,7,11,12,14,16,17	2	P01836,Q6IFV1
9948	1,5,6,8,9,10,13,16	2,3,4,7,11,12,14,15,17	4	A2RUV9,P01836,P19218,Q63942
9949	1,5,6,8,9,10,13,17	2,3,4,7,11,12,14,15,16	4	P01836,P06760,P51907,Q6IFV1
9950	1,5,6,8,9,10,14,15	2,3,4,7,11,12,13,16,17	2	P01836,Q63942

9951	1,5,6,8,9,10,14,16	2,3,4,7,11,12,13,15,17	2	P01836,Q63942
9952	1,5,6,8,9,10,14,17	2,3,4,7,11,12,13,15,16	4	P01836,P06760,Q09030,Q9QUL6
9953	1,5,6,8,9,10,15,16	2,3,4,7,11,12,13,14,17	2	P01836,Q63942
9954	1,5,6,8,9,10,15,17	2,3,4,7,11,12,13,14,16	3	P01836,P06760,Q6IFV1
9955	1,5,6,8,9,10,16,17	2,3,4,7,11,12,13,14,15	5	A2RUV9,P01836,P06760,P25031,Q09030
9956	1,5,6,8,9,11,12,13	2,3,4,7,10,14,15,16,17	7	P02781,P02782,P02803,P0C0A9,P30120,Q9JHB9,Q9Z1F2
9957	1,5,6,8,9,11,12,14	2,3,4,7,10,13,15,16,17	5	O35547,P02782,P30120,P47727,Q9JHB9
9958	1,5,6,8,9,11,12,15	2,3,4,7,10,13,14,16,17	1	O35547
9959	1,5,6,8,9,11,12,16	2,3,4,7,10,13,14,15,17	4	A2RUV9,O35547,Q6IFU7,Q6P6Q2
9960	1,5,6,8,9,11,12,17	2,3,4,7,10,13,14,15,16	5	O35547,P02782,P06760,P13432,Q09030
9961	1,5,6,8,9,11,13,14	2,3,4,7,10,12,15,16,17	3	P01836,P06866,P47727
9962	1,5,6,8,9,11,13,15	2,3,4,7,10,12,14,16,17	0	
9963	1,5,6,8,9,11,13,16	2,3,4,7,10,12,14,15,17	1	A2RUV9
9964	1,5,6,8,9,11,13,17	2,3,4,7,10,12,14,15,16	4	A2RUV9,P01836,P06760,Q63532
9965	1,5,6,8,9,11,14,15	2,3,4,7,10,12,13,16,17	2	P01836,P14480

9966	1,5,6,8,9,11,14,16	2,3,4,7,10,12,13,15,17	2	P01836,Q63942
9967	1,5,6,8,9,11,14,17	2,3,4,7,10,12,13,15,16	4	P01836,P04218,P06760,Q9QUL6
9968	1,5,6,8,9,11,15,16	2,3,4,7,10,12,13,14,17	2	A2RUV9,Q63942
9969	1,5,6,8,9,11,15,17	2,3,4,7,10,12,13,14,16	4	A2RUV9,P01836,P06760,Q9QUL6
9970	1,5,6,8,9,11,16,17	2,3,4,7,10,12,13,14,15	4	A2RUV9,P01836,P06760,Q9QUL6
9971	1,5,6,8,9,12,13,14	2,3,4,7,10,11,15,16,17	4	P02782,P30120,Q9JHB9,Q9Z1F2
9972	1,5,6,8,9,12,13,15	2,3,4,7,10,11,14,16,17	2	P19218,Q6IFV1
9973	1,5,6,8,9,12,13,16	2,3,4,7,10,11,14,15,17	1	P19218
9974	1,5,6,8,9,12,13,17	2,3,4,7,10,11,14,15,16	7	P02782,P06760,P13432,P19218,P51907,Q6IFV1,Q9Z1F2
9975	1,5,6,8,9,12,14,15	2,3,4,7,10,11,13,16,17	0	
9976	1,5,6,8,9,12,14,16	2,3,4,7,10,11,13,15,17	0	
9977	1,5,6,8,9,12,14,17	2,3,4,7,10,11,13,15,16	6	P01836,P02782,P06760,P13432,Q09030,Q99PP0
9978	1,5,6,8,9,12,15,16	2,3,4,7,10,11,13,14,17	0	
9979	1,5,6,8,9,12,15,17	2,3,4,7,10,11,13,14,16	3	P06760,P13432,Q6IFV1
9980	1,5,6,8,9,12,16,17	2,3,4,7,10,11,13,14,15	3	P06760,P13432,Q09030

9981	1,5,6,8,9,13,14,15	2,3,4,7,10,11,12,16,17	3	P01836,P06757,P53792
9982	1,5,6,8,9,13,14,16	2,3,4,7,10,11,12,15,17	2	P01836,Q63942
9983	1,5,6,8,9,13,14,17	2,3,4,7,10,11,12,15,16	6	P01836,P06760,P51907,P53792,Q5FVI6,Q9Z1F2
9984	1,5,6,8,9,13,15,16	2,3,4,7,10,11,12,14,17	1	Q63942
9985	1,5,6,8,9,13,15,17	2,3,4,7,10,11,12,14,16	6	P01836,P06760,P51907,P53792,Q63532,Q6IFV1
9986	1,5,6,8,9,13,16,17	2,3,4,7,10,11,12,14,15	6	A2RUV9,P01836,P06760,P35280,P51907,P53792
9987	1,5,6,8,9,14,15,16	2,3,4,7,10,11,12,13,17	3	P01836,Q63942,Q80WL1
9988	1,5,6,8,9,14,15,17	2,3,4,7,10,11,12,13,16	4	P01836,P06760,P47967,P53792
9989	1,5,6,8,9,14,16,17	2,3,4,7,10,11,12,13,15	3	P01836,P06760,Q63942
9990	1,5,6,8,9,15,16,17	2,3,4,7,10,11,12,13,14	4	A2RUV9,P01836,P23593,Q63942
9991	1,5,6,8,10,11,12,13	2,3,4,7,9,14,15,16,17	11	F1LM93,O35547,P02781,P02782,P02803,P0C0A9,P30120,P47727,Q6IFU7,Q6P6Q2,Q9JHB9
9992	1,5,6,8,10,11,12,14	2,3,4,7,9,13,15,16,17	12	F1LM93,O35547,P02782,P04905,P08010,P30120,P47727,Q6IFU7,Q6IG02,Q6IMF3,Q6P6Q2,Q9JHB9
9993	1,5,6,8,10,11,12,15	2,3,4,7,9,13,14,16,17	6	F1LM93,O35547,P08010,Q566E6,Q6IFU7,Q6P6Q2
9994	1,5,6,8,10,11,12,16	2,3,4,7,9,13,14,15,17	6	F1LM93,O35547,Q6IFU7,Q6IG02,Q6IMF3,Q6P6Q2
9995	1,5,6,8,10,11,12,17	2,3,4,7,9,13,14,15,16	12	F1LM93,O35547,P06760,P08010,P13432,Q10758,Q566E6,Q6IFU7,Q6IG02,Q6P6Q2,Q99MH3,Q9QZK9
9996	1,5,6,8,10,11,13,14	2,3,4,7,9,12,15,16,17	2	P01836,P47727
9997	1,5,6,8,10,11,13,15	2,3,4,7,9,12,14,16,17	1	Q6IFV1
9998	1,5,6,8,10,11,13,16	2,3,4,7,9,12,14,15,17	1	A2RUV9

9999	1,5,6,8,10,<br>11,13,17	2,3,4,7,9,1<br>2,14,15,16	4	A2RUV9,P01836,P06760,Q6IFV1
10000	1,5,6,8,10,<br>11,14,15	2,3,4,7,9,1<br>2,13,16,17	1	P01836
10001	1,5,6,8,10,<br>11,14,16	2,3,4,7,9,1<br>2,13,15,17	1	P01836
10002	1,5,6,8,10,<br>11,14,17	2,3,4,7,9,1<br>2,13,15,16	4	P01836,P06760,P47727,Q9QUL6
10003	1,5,6,8,10,<br>11,15,16	2,3,4,7,9,1<br>2,13,14,17	1	A2RUV9
10004	1,5,6,8,10,<br>11,15,17	2,3,4,7,9,1<br>2,13,14,16	4	P01836,P06760,Q566E6,Q6IFV1
10005	1,5,6,8,10,<br>11,16,17	2,3,4,7,9,1<br>2,13,14,15	3	A2RUV9,P01836,P06760
10006	1,5,6,8,10,<br>12,13,14	2,3,4,7,9,1<br>1,15,16,17	6	P02782,P06911,P30120,Q498D9,Q9JHB9,Q9Z1F2
10007	1,5,6,8,10,<br>12,13,15	2,3,4,7,9,1<br>1,14,16,17	4	P19218,Q498D9,Q6IFV1,Q99MH3
10008	1,5,6,8,10,<br>12,13,16	2,3,4,7,9,1<br>1,14,15,17	1	P19218
10009	1,5,6,8,10,<br>12,13,17	2,3,4,7,9,1<br>1,14,15,16	9	F1LM93,P02782,P06760,P13432,P19218,Q10758,Q6IFV1,Q99MH3,Q9QZK9
10010	1,5,6,8,10,<br>12,14,15	2,3,4,7,9,1<br>1,13,16,17	1	Q498D9
10011	1,5,6,8,10,<br>12,14,16	2,3,4,7,9,1<br>1,13,15,17	1	Q10758
10012	1,5,6,8,10,<br>12,14,17	2,3,4,7,9,1<br>1,13,15,16	6	F1LM93,P01836,P06760,P13432,Q10758,Q99PP0
10013	1,5,6,8,10,<br>12,15,16	2,3,4,7,9,1<br>1,13,14,17	1	P20766
10014	1,5,6,8,10,<br>12,15,17	2,3,4,7,9,1<br>1,13,14,16	5	F1LM93,P06760,P13432,Q6IFV1,Q99MH3
10015	1,5,6,8,10,<br>12,16,17	2,3,4,7,9,1<br>1,13,14,15	5	F1LM93,P06760,P13432,Q10758,Q99MH3
10016	1,5,6,8,10,<br>13,14,15	2,3,4,7,9,1<br>1,12,16,17	4	P01836,P09605;P25809,P19223,Q498D9
10017	1,5,6,8,10,<br>13,14,16	2,3,4,7,9,1<br>1,12,15,17	2	P01836,P19223
10018	1,5,6,8,10,<br>13,14,17	2,3,4,7,9,1<br>1,12,15,16	3	P01836,P06760,P51907
10019	1,5,6,8,10,<br>13,15,16	2,3,4,7,9,1<br>1,12,14,17	2	P19223,Q6IFV1
10020	1,5,6,8,10,<br>13,15,17	2,3,4,7,9,1<br>1,12,14,16	4	P01836,P06760,Q6IFV1,Q99MH3
10021	1,5,6,8,10,<br>13,16,17	2,3,4,7,9,1<br>1,12,14,15	5	A2RUV9,P01836,P06760,P51907,Q6IFV1

10022	1,5,6,8,10,<br>14,15,16	2,3,4,7,9,1<br>1,12,13,17	2	P01836,P19223
10023	1,5,6,8,10,<br>14,15,17	2,3,4,7,9,1<br>1,12,13,16	2	P01836,P06760
10024	1,5,6,8,10,<br>14,16,17	2,3,4,7,9,1<br>1,12,13,15	3	P01836,P06760,P42854
10025	1,5,6,8,10,<br>15,16,17	2,3,4,7,9,1<br>1,12,13,14	3	P01836,P06760,Q6IFV1
10026	1,5,6,8,11,<br>12,13,14	2,3,4,7,9,1<br>0,15,16,17	8	O35547,P02781,P02782,P04905,P30120,P47727,Q9JHB9,Q9Z1F2
10027	1,5,6,8,11,<br>12,13,15	2,3,4,7,9,1<br>0,14,16,17	5	O35547,P02782,P30120,Q6IFV1,Q9JHB9
10028	1,5,6,8,11,<br>12,13,16	2,3,4,7,9,1<br>0,14,15,17	7	O35547,P02782,P30120,Q6IFU7,Q6P6Q2,Q8CJ52,Q9JHB9
10029	1,5,6,8,11,<br>12,13,17	2,3,4,7,9,1<br>0,14,15,16	9	O35547,P02782,P06760,P13432,P30120,P36374,Q6IFV1,Q9JHB9,Q9QZK9
10030	1,5,6,8,11,<br>12,14,15	2,3,4,7,9,1<br>0,13,16,17	3	O35547,P47727,Q5I0D1
10031	1,5,6,8,11,<br>12,14,16	2,3,4,7,9,1<br>0,13,15,17	4	O35547,P47727,Q6IFU7,Q6P6Q2
10032	1,5,6,8,11,<br>12,14,17	2,3,4,7,9,1<br>0,13,15,16	8	O35547,P02782,P06760,P13432,P30120,P47727,Q99PP0,Q9JHB9
10033	1,5,6,8,11,<br>12,15,16	2,3,4,7,9,1<br>0,13,14,17	3	O35547,Q6IFU7,Q6P6Q2
10034	1,5,6,8,11,<br>12,15,17	2,3,4,7,9,1<br>0,13,14,16	4	O35547,P06760,P13432,Q6IFV1
10035	1,5,6,8,11,<br>12,16,17	2,3,4,7,9,1<br>0,13,14,15	5	A2RUV9,O35547,P06760,P13432,Q6P6Q2
10036	1,5,6,8,11,<br>13,14,15	2,3,4,7,9,1<br>0,12,16,17	2	P19223,P47727
10037	1,5,6,8,11,<br>13,14,16	2,3,4,7,9,1<br>0,12,15,17	2	P19223,P47727
10038	1,5,6,8,11,<br>13,14,17	2,3,4,7,9,1<br>0,12,15,16	3	P01836,P06760,P47727
10039	1,5,6,8,11,<br>13,15,16	2,3,4,7,9,1<br>0,12,14,17	1	P19223
10040	1,5,6,8,11,<br>13,15,17	2,3,4,7,9,1<br>0,12,14,16	2	P06760,Q6IFV1
10041	1,5,6,8,11,<br>13,16,17	2,3,4,7,9,1<br>0,12,14,15	2	A2RUV9,P06760
10042	1,5,6,8,11,<br>14,15,16	2,3,4,7,9,1<br>0,12,13,17	2	P19223,Q5I0D1
10043	1,5,6,8,11,<br>14,15,17	2,3,4,7,9,1<br>0,12,13,16	4	P01836,P06760,P47967,P97840
10044	1,5,6,8,11,<br>14,16,17	2,3,4,7,9,1<br>0,12,13,15	2	P01836,P06760

10045	1,5,6,8,11,<br>15,16,17	2,3,4,7,9,1<br>0,12,13,14	2	A2RUV9,P06760
10046	1,5,6,8,12,<br>13,14,15	2,3,4,7,9,1<br>0,11,16,17	3	P02782,Q498D9,Q9Z1F2
10047	1,5,6,8,12,<br>13,14,16	2,3,4,7,9,1<br>0,11,15,17	2	P02782,Q9Z1F2
10048	1,5,6,8,12,<br>13,14,17	2,3,4,7,9,1<br>0,11,15,16	8	P02782,P06760,P13432,P51907,Q561R9,Q5FVI6,Q9QZK9,Q9Z1F2
10049	1,5,6,8,12,<br>13,15,16	2,3,4,7,9,1<br>0,11,14,17	2	P19218,Q6IFV1
10050	1,5,6,8,12,<br>13,15,17	2,3,4,7,9,1<br>0,11,14,16	5	P06760,P13432,Q6IFV1,Q99MH3,Q9QZK9
10051	1,5,6,8,12,<br>13,16,17	2,3,4,7,9,1<br>0,11,14,15	7	P06760,P13432,P19218,P35280,P51907,Q5XFX0,Q6IFV1
10052	1,5,6,8,12,<br>14,15,16	2,3,4,7,9,1<br>0,11,13,17	0	
10053	1,5,6,8,12,<br>14,15,17	2,3,4,7,9,1<br>0,11,13,16	2	P06760,P13432
10054	1,5,6,8,12,<br>14,16,17	2,3,4,7,9,1<br>0,11,13,15	3	P06760,P13432,Q561R9
10055	1,5,6,8,12,<br>15,16,17	2,3,4,7,9,1<br>0,11,13,14	4	P06760,P13432,Q5XFX0,Q6IFV1
10056	1,5,6,8,13,<br>14,15,16	2,3,4,7,9,1<br>0,11,12,17	1	P19223
10057	1,5,6,8,13,<br>14,15,17	2,3,4,7,9,1<br>0,11,12,16	8	P01836,P06760,P08753,P10536,P47967,P51907,P53792,Q6IFV1
10058	1,5,6,8,13,<br>14,16,17	2,3,4,7,9,1<br>0,11,12,15	5	P01836,P06760,P08753,P51907,Q561R9
10059	1,5,6,8,13,<br>15,16,17	2,3,4,7,9,1<br>0,11,12,14	2	P06760,Q6IFV1
10060	1,5,6,8,14,<br>15,16,17	2,3,4,7,9,1<br>0,11,12,13	2	P01836,P06760
10061	1,5,6,9,10,<br>11,12,13	2,3,4,7,8,1<br>4,15,16,17	4	P02803,Q566E6,Q6IFU7,Q9JHB9
10062	1,5,6,9,10,<br>11,12,14	2,3,4,7,8,1<br>3,15,16,17	4	P20766,P47727,Q6IFU7,Q6P6Q2
10063	1,5,6,9,10,<br>11,12,15	2,3,4,7,8,1<br>3,14,16,17	4	P20766,Q566E6,Q6IFU7,Q6PCU2
10064	1,5,6,9,10,<br>11,12,16	2,3,4,7,8,1<br>3,14,15,17	6	P12368,P20766,P36970,Q6IFU7,Q6IMF3,Q6P6Q2
10065	1,5,6,9,10,<br>11,12,17	2,3,4,7,8,1<br>3,14,15,16	5	P20766,Q09030,Q566E6,Q6IFU7,Q9QUL6
10066	1,5,6,9,10,<br>11,13,14	2,3,4,7,8,1<br>2,15,16,17	6	P01041,P01836,P19223,P47727,P55091,Q80WL1
10067	1,5,6,9,10,<br>11,13,15	2,3,4,7,8,1<br>2,14,16,17	5	P19223,P31430,P55091,Q566E6,Q80WL1

10068	1,5,6,9,10,<br>11,13,16	2,3,4,7,8,1<br>2,14,15,17	9	A2RUV9,B0BNN3,P01041,P02761,P19223,P31430,P36970,P55091,Q80WL1
10069	1,5,6,9,10,<br>11,13,17	2,3,4,7,8,1<br>2,14,15,16	5	A2RUV9,P01836,Q566E6,Q920P0,Q9QUL6
10070	1,5,6,9,10,<br>11,14,15	2,3,4,7,8,1<br>2,13,16,17	7	P01836,P19223,P20766,P55091,P63029,Q80WL1,Q9QUL6
10071	1,5,6,9,10,<br>11,14,16	2,3,4,7,8,1<br>2,13,15,17	6	P01836,P19223,P20766,P55091,Q80WL1,Q9QUL6
10072	1,5,6,9,10,<br>11,14,17	2,3,4,7,8,1<br>2,13,15,16	3	P01836,Q80WL1,Q9QUL6
10073	1,5,6,9,10,<br>11,15,16	2,3,4,7,8,1<br>2,13,14,17	8	A2RUV9,P19223,P20766,P31430,P36970,P55091,Q80WL1,Q9QUL6
10074	1,5,6,9,10,<br>11,15,17	2,3,4,7,8,1<br>2,13,14,16	6	P01836,P20766,P36970,Q566E6,Q80WL1,Q9QUL6
10075	1,5,6,9,10,<br>11,16,17	2,3,4,7,8,1<br>2,13,14,15	6	A2RUV9,P01836,P20766,P36970,Q80WL1,Q9QUL6
10076	1,5,6,9,10,<br>12,13,14	2,3,4,7,8,1<br>1,15,16,17	1	P02761
10077	1,5,6,9,10,<br>12,13,15	2,3,4,7,8,1<br>1,14,16,17	1	P19218
10078	1,5,6,9,10,<br>12,13,16	2,3,4,7,8,1<br>1,14,15,17	3	P02761,P19218,P97840
10079	1,5,6,9,10,<br>12,13,17	2,3,4,7,8,1<br>1,14,15,16	2	P19218,Q99MH3
10080	1,5,6,9,10,<br>12,14,15	2,3,4,7,8,1<br>1,13,16,17	1	P20766
10081	1,5,6,9,10,<br>12,14,16	2,3,4,7,8,1<br>1,13,15,17	2	P02761,P20766
10082	1,5,6,9,10,<br>12,14,17	2,3,4,7,8,1<br>1,13,15,16	4	P01836,P20766,Q09030,Q10758
10083	1,5,6,9,10,<br>12,15,16	2,3,4,7,8,1<br>1,13,14,17	3	P19218,P20766,Q80WL1
10084	1,5,6,9,10,<br>12,15,17	2,3,4,7,8,1<br>1,13,14,16	1	P20766
10085	1,5,6,9,10,<br>12,16,17	2,3,4,7,8,1<br>1,13,14,15	4	P19218,P20766,Q09030,Q10758
10086	1,5,6,9,10,<br>13,14,15	2,3,4,7,8,1<br>1,12,16,17	4	P01836,P19223,P55091,Q80WL1
10087	1,5,6,9,10,<br>13,14,16	2,3,4,7,8,1<br>1,12,15,17	7	P01041,P01836,P02761,P19223,P55091,Q80WL1,Q9QZA6
10088	1,5,6,9,10,<br>13,14,17	2,3,4,7,8,1<br>1,12,15,16	2	P01836,Q80WL1
10089	1,5,6,9,10,<br>13,15,16	2,3,4,7,8,1<br>1,12,14,17	9	P19218,P19223,P31430,P50115,P55091,Q00715,Q6AYQ8,Q80WL1,Q9EQS0
10090	1,5,6,9,10,<br>13,15,17	2,3,4,7,8,1<br>1,12,14,16	4	P01836,Q6IFV1,Q80WL1,Q9EQS0

10091	1,5,6,9,10,<br>13,16,17	2,3,4,7,8,1<br>1,12,14,15	6	A2RUV9,P01836,P19218,P35280,Q80WL1,Q9EQS0
10092	1,5,6,9,10,<br>14,15,16	2,3,4,7,8,1<br>1,12,13,17	6	P01836,P19223,P20766,P50115,P55091,Q80WL1
10093	1,5,6,9,10,<br>14,15,17	2,3,4,7,8,1<br>1,12,13,16	3	P01836,P20766,Q80WL1
10094	1,5,6,9,10,<br>14,16,17	2,3,4,7,8,1<br>1,12,13,15	4	P01836,P20766,Q5XI22,Q80WL1
10095	1,5,6,9,10,<br>15,16,17	2,3,4,7,8,1<br>1,12,13,14	6	P01836,P20766,P36970,Q6TMA8,Q80WL1,Q9EQS0
10096	1,5,6,9,11,<br>12,13,14	2,3,4,7,8,1<br>0,15,16,17	3	P02761,P47727,Q9JHB9
10097	1,5,6,9,11,<br>12,13,15	2,3,4,7,8,1<br>0,14,16,17	0	
10098	1,5,6,9,11,<br>12,13,16	2,3,4,7,8,1<br>0,14,15,17	2	P02761,Q6IFU7
10099	1,5,6,9,11,<br>12,13,17	2,3,4,7,8,1<br>0,14,15,16	2	P13432,P97574
10100	1,5,6,9,11,<br>12,14,15	2,3,4,7,8,1<br>0,13,16,17	2	P20766,Q80WL1
10101	1,5,6,9,11,<br>12,14,16	2,3,4,7,8,1<br>0,13,15,17	3	P02761,P20766,Q6IFU7
10102	1,5,6,9,11,<br>12,14,17	2,3,4,7,8,1<br>0,13,15,16	2	P01835,P13432
10103	1,5,6,9,11,<br>12,15,16	2,3,4,7,8,1<br>0,13,14,17	4	P20766,P36970,Q6IFU7,Q80WL1
10104	1,5,6,9,11,<br>12,15,17	2,3,4,7,8,1<br>0,13,14,16	2	P20766,Q566E6
10105	1,5,6,9,11,<br>12,16,17	2,3,4,7,8,1<br>0,13,14,15	4	P13432,P20766,P36970,Q6IFU7
10106	1,5,6,9,11,<br>13,14,15	2,3,4,7,8,1<br>0,12,16,17	3	P19223,P55091,Q80WL1
10107	1,5,6,9,11,<br>13,14,16	2,3,4,7,8,1<br>0,12,15,17	5	P01041,P02761,P19223,P55091,Q80WL1
10108	1,5,6,9,11,<br>13,14,17	2,3,4,7,8,1<br>0,12,15,16	2	P01836,Q80WL1
10109	1,5,6,9,11,<br>13,15,16	2,3,4,7,8,1<br>0,12,14,17	6	P19223,P31430,P36970,P55091,Q80WL1,Q9EQS0
10110	1,5,6,9,11,<br>13,15,17	2,3,4,7,8,1<br>0,12,14,16	2	Q80WL1,Q9EQS0
10111	1,5,6,9,11,<br>13,16,17	2,3,4,7,8,1<br>0,12,14,15	5	A2RUV9,P35280,P36970,Q80WL1,Q9EQS0
10112	1,5,6,9,11,<br>14,15,16	2,3,4,7,8,1<br>0,12,13,17	4	P19223,P20766,P55091,Q80WL1
10113	1,5,6,9,11,<br>14,15,17	2,3,4,7,8,1<br>0,12,13,16	4	P01836,P47967,Q80WL1,Q9QUL6

10114	1,5,6,9,11,<br>14,16,17	2,3,4,7,8,1<br>0,12,13,15	5	P01836,P04218,P36970,Q80WL1,Q9QUL6
10115	1,5,6,9,11,<br>15,16,17	2,3,4,7,8,1<br>0,12,13,14	5	A2RUV9,P20766,P36970,Q80WL1,Q9EQS0
10116	1,5,6,9,12,<br>13,14,15	2,3,4,7,8,1<br>0,11,16,17	1	Q80WL1
10117	1,5,6,9,12,<br>13,14,16	2,3,4,7,8,1<br>0,11,15,17	1	P02761
10118	1,5,6,9,12,<br>13,14,17	2,3,4,7,8,1<br>0,11,15,16	3	P00714,P35280,P53792
10119	1,5,6,9,12,<br>13,15,16	2,3,4,7,8,1<br>0,11,14,17	4	P00714,P02761,P19218,Q80WL1
10120	1,5,6,9,12,<br>13,15,17	2,3,4,7,8,1<br>0,11,14,16	4	P00714,P19218,P53792,Q6IFV1
10121	1,5,6,9,12,<br>13,16,17	2,3,4,7,8,1<br>0,11,14,15	4	P00714,P02761,P19218,P35280
10122	1,5,6,9,12,<br>14,15,16	2,3,4,7,8,1<br>0,11,13,17	2	P20766,Q80WL1
10123	1,5,6,9,12,<br>14,15,17	2,3,4,7,8,1<br>0,11,13,16	2	P20766,Q80WL1
10124	1,5,6,9,12,<br>14,16,17	2,3,4,7,8,1<br>0,11,13,15	3	P20766,P35280,Q5XI22
10125	1,5,6,9,12,<br>15,16,17	2,3,4,7,8,1<br>0,11,13,14	3	P19218,P20766,Q80WL1
10126	1,5,6,9,13,<br>14,15,16	2,3,4,7,8,1<br>0,11,12,17	10	P00714,P02761,P16391,P19223,P50115,P53792,P55091,P62804,Q80WL1,Q9EQS0
10127	1,5,6,9,13,<br>14,15,17	2,3,4,7,8,1<br>0,11,12,16	6	P00714,P01836,P47967,P53792,Q80WL1,Q9EQS0
10128	1,5,6,9,13,<br>14,16,17	2,3,4,7,8,1<br>0,11,12,15	8	P00714,P01836,P02761,P35280,P53792,Q5XI22,Q80WL1,Q9EQS0
10129	1,5,6,9,13,<br>15,16,17	2,3,4,7,8,1<br>0,11,12,14	7	P00714,P19218,P35280,P53792,P62804,Q80WL1,Q9EQS0
10130	1,5,6,9,14,<br>15,16,17	2,3,4,7,8,1<br>0,11,12,13	6	P00714,P01836,P20766,Q5XI22,Q80WL1,Q9EQS0
10131	1,5,6,10,1<br>1,12,13,14	2,3,4,7,8,9<br>,15,16,17	4	P47727,Q5QE79,Q6IFU7,Q9JHB9
10132	1,5,6,10,1<br>1,12,13,15	2,3,4,7,8,9<br>,14,16,17	2	Q566E6,Q6IFU7
10133	1,5,6,10,1<br>1,12,13,16	2,3,4,7,8,9<br>,14,15,17	4	P02761,Q5QE79,Q6IFU7,Q6P6Q2
10134	1,5,6,10,1<br>1,12,13,17	2,3,4,7,8,9<br>,14,15,16	5	Q566E6,Q5QE79,Q6IFU7,Q99MH3,Q9QZK9
10135	1,5,6,10,1<br>1,12,14,15	2,3,4,7,8,9<br>,13,16,17	3	P20766,P47727,Q6IFU7
10136	1,5,6,10,1<br>1,12,14,16	2,3,4,7,8,9<br>,13,15,17	6	P20766,P47727,Q5QE79,Q6IFU7,Q6IMF3,Q6P6Q2

10137	1,5,6,10,1 1,12,14,17	2,3,4,7,8,9 ,13,15,16	5	P20766,P47727,Q10758,Q5QE79,Q6IFU7
10138	1,5,6,10,1 1,12,15,16	2,3,4,7,8,9 ,13,14,17	3	P20766,Q6IFU7,Q6P6Q2
10139	1,5,6,10,1 1,12,15,17	2,3,4,7,8,9 ,13,14,16	4	P20766,Q566E6,Q6IFU7,Q99MH3
10140	1,5,6,10,1 1,12,16,17	2,3,4,7,8,9 ,13,14,15	6	P06757,P20766,P36970,Q10758,Q6IFU7,Q6P6Q2
10141	1,5,6,10,1 1,13,14,15	2,3,4,7,8,9 ,12,16,17	4	P19223,P47727,P55091,Q80WL1
10142	1,5,6,10,1 1,13,14,16	2,3,4,7,8,9 ,12,15,17	7	P02761,P19223,P47727,P55091,Q5QE79,Q7TPB1,Q80WL1
10143	1,5,6,10,1 1,13,14,17	2,3,4,7,8,9 ,12,15,16	4	P01836,P47727,Q5QE79,Q80WL1
10144	1,5,6,10,1 1,13,15,16	2,3,4,7,8,9 ,12,14,17	5	P19223,P31430,P55091,Q80WL1,Q9EQS0
10145	1,5,6,10,1 1,13,15,17	2,3,4,7,8,9 ,12,14,16	4	Q566E6,Q6IFV1,Q80WL1,Q9EQS0
10146	1,5,6,10,1 1,13,16,17	2,3,4,7,8,9 ,12,14,15	4	A2RUV9,P36970,Q80WL1,Q9EQS0
10147	1,5,6,10,1 1,14,15,16	2,3,4,7,8,9 ,12,13,17	4	P19223,P20766,P55091,Q80WL1
10148	1,5,6,10,1 1,14,15,17	2,3,4,7,8,9 ,12,13,16	4	P01836,P20766,Q80WL1,Q9QUL6
10149	1,5,6,10,1 1,14,16,17	2,3,4,7,8,9 ,12,13,15	5	P01836,P20766,Q6TMA8,Q80WL1,Q9QUL6
10150	1,5,6,10,1 1,15,16,17	2,3,4,7,8,9 ,12,13,14	7	A2RUV9,P05369,P20766,P36970,Q6TMA8,Q80WL1,Q9EQS0
10151	1,5,6,10,1 2,13,14,15	2,3,4,7,8,9 ,11,16,17	1	Q498D9
10152	1,5,6,10,1 2,13,14,16	2,3,4,7,8,9 ,11,15,17	2	P02761,Q5QE79
10153	1,5,6,10,1 2,13,14,17	2,3,4,7,8,9 ,11,15,16	5	Q10758,Q498D9,Q5QE79,Q99MH3,Q9QZK9
10154	1,5,6,10,1 2,13,15,16	2,3,4,7,8,9 ,11,14,17	1	P19218
10155	1,5,6,10,1 2,13,15,17	2,3,4,7,8,9 ,11,14,16	4	P19218,Q6IFV1,Q99MH3,Q9QZK9
10156	1,5,6,10,1 2,13,16,17	2,3,4,7,8,9 ,11,14,15	5	P02761,P19218,P35280,Q10758,Q99MH3
10157	1,5,6,10,1 2,14,15,16	2,3,4,7,8,9 ,11,13,17	2	P20766,Q80WL1
10158	1,5,6,10,1 2,14,15,17	2,3,4,7,8,9 ,11,13,16	2	P20766,Q10758
10159	1,5,6,10,1 2,14,16,17	2,3,4,7,8,9 ,11,13,15	5	P20766,Q10758,Q5QE79,Q5XI22,Q6TMA8

10160	1,5,6,10,1<br>2,15,16,17	2,3,4,7,8,9<br>,11,13,14	4	P19218,P20766,Q6TMA8,Q99MH3
10161	1,5,6,10,1<br>3,14,15,16	2,3,4,7,8,9<br>,11,12,17	5	P19223,P50115,P55091,Q80WL1,Q9EQS0
10162	1,5,6,10,1<br>3,14,15,17	2,3,4,7,8,9<br>,11,12,16	3	P01836,Q80WL1,Q9EQS0
10163	1,5,6,10,1<br>3,14,16,17	2,3,4,7,8,9<br>,11,12,15	4	P01836,P02761,Q80WL1,Q9EQS0
10164	1,5,6,10,1<br>3,15,16,17	2,3,4,7,8,9<br>,11,12,14	3	P19218,Q80WL1,Q9EQS0
10165	1,5,6,10,1<br>4,15,16,17	2,3,4,7,8,9<br>,11,12,13	6	P01836,P20766,Q5XI22,Q6TMA8,Q80WL1,Q9EQS0
10166	1,5,6,11,1<br>2,13,14,15	2,3,4,7,8,9<br>,10,16,17	1	P47727
10167	1,5,6,11,1<br>2,13,14,16	2,3,4,7,8,9<br>,10,15,17	4	P02761,P19223,P47727,Q6IFU7
10168	1,5,6,11,1<br>2,13,14,17	2,3,4,7,8,9<br>,10,15,16	2	P47727,Q9QZK9
10169	1,5,6,11,1<br>2,13,15,16	2,3,4,7,8,9<br>,10,14,17	2	Q6IFU7,Q80WL1
10170	1,5,6,11,1<br>2,13,15,17	2,3,4,7,8,9<br>,10,14,16	1	Q6IFV1
10171	1,5,6,11,1<br>2,13,16,17	2,3,4,7,8,9<br>,10,14,15	3	P02761,P17046,P35280
10172	1,5,6,11,1<br>2,14,15,16	2,3,4,7,8,9<br>,10,13,17	3	P20766,Q6IFU7,Q80WL1
10173	1,5,6,11,1<br>2,14,15,17	2,3,4,7,8,9<br>,10,13,16	1	P20766
10174	1,5,6,11,1<br>2,14,16,17	2,3,4,7,8,9<br>,10,13,15	2	P20766,Q6IFU7
10175	1,5,6,11,1<br>2,15,16,17	2,3,4,7,8,9<br>,10,13,14	5	P05369,P20766,P36970,Q6IFU7,Q80WL1
10176	1,5,6,11,1<br>3,14,15,16	2,3,4,7,8,9<br>,10,12,17	4	P19223,P55091,Q80WL1,Q9EQS0
10177	1,5,6,11,1<br>3,14,15,17	2,3,4,7,8,9<br>,10,12,16	4	D3ZHA0,P47967,Q80WL1,Q9EQS0
10178	1,5,6,11,1<br>3,14,16,17	2,3,4,7,8,9<br>,10,12,15	4	D3ZHA0,P02761,Q80WL1,Q9EQS0
10179	1,5,6,11,1<br>3,15,16,17	2,3,4,7,8,9<br>,10,12,14	4	D3ZHA0,P36970,Q80WL1,Q9EQS0
10180	1,5,6,11,1<br>4,15,16,17	2,3,4,7,8,9<br>,10,12,13	3	P20766,Q80WL1,Q9EQS0
10181	1,5,6,12,1<br>3,14,15,16	2,3,4,7,8,9<br>,10,11,17	4	P00714,P02761,P19223,Q80WL1
10182	1,5,6,12,1<br>3,14,15,17	2,3,4,7,8,9<br>,10,11,16	1	P00714

10183	1,5,6,12,13,14,16,17	2,3,4,7,8,9,10,11,15	4	P00714,P02761,P35280,Q561R9
10184	1,5,6,12,13,15,16,17	2,3,4,7,8,9,10,11,14	4	P00714,P19218,P35280,Q9EQS0
10185	1,5,6,12,14,15,16,17	2,3,4,7,8,9,10,11,13	5	P00714,P20766,P35467,Q5XI22,Q80WL1
10186	1,5,6,13,14,15,16,17	2,3,4,7,8,9,10,11,12	3	P00714,Q80WL1,Q9EQS0
10187	1,5,7,8,9,10,11,12	2,3,4,6,13,14,15,16,17	3	P02803,P10715,P27213
10188	1,5,7,8,9,10,11,13	2,3,4,6,12,14,15,16,17	3	B0BNN3,P02803,Q5HZV9
10189	1,5,7,8,9,10,11,14	2,3,4,6,12,13,15,16,17	2	P27213,Q9QUL6
10190	1,5,7,8,9,10,11,15	2,3,4,6,12,13,14,16,17	4	P14480,P25236,P27213,Q62635
10191	1,5,7,8,9,10,11,16	2,3,4,6,12,13,14,15,17	5	A2RUV9,B0BNN3,P25236,Q62635,Q64093
10192	1,5,7,8,9,10,11,17	2,3,4,6,12,13,14,15,16	2	P25236,Q9QUL6
10193	1,5,7,8,9,10,12,13	2,3,4,6,11,14,15,16,17	2	P02803,P19218
10194	1,5,7,8,9,10,12,14	2,3,4,6,11,13,15,16,17	0	
10195	1,5,7,8,9,10,12,15	2,3,4,6,11,13,14,16,17	1	P10715
10196	1,5,7,8,9,10,12,16	2,3,4,6,11,13,14,15,17	4	P02625,P10715,P19218,Q64093
10197	1,5,7,8,9,10,12,17	2,3,4,6,11,13,14,15,16	3	F1LM93,P10715,P52590
10198	1,5,7,8,9,10,13,14	2,3,4,6,11,12,15,16,17	1	B0BNN3

10199	1,5,7,8,9,10,13,15	2,3,4,6,11,12,14,16,17	1	P01835
10200	1,5,7,8,9,10,13,16	2,3,4,6,11,12,14,15,17	4	B0BNN3,P01835,P02625,P19218
10201	1,5,7,8,9,10,13,17	2,3,4,6,11,12,14,15,16	1	P02625
10202	1,5,7,8,9,10,14,15	2,3,4,6,11,12,13,16,17	3	P14480,P16636,Q62635
10203	1,5,7,8,9,10,14,16	2,3,4,6,11,12,13,15,17	3	B0BNN3,P02625,Q62635
10204	1,5,7,8,9,10,14,17	2,3,4,6,11,12,13,15,16	2	P01836,P02625
10205	1,5,7,8,9,10,15,16	2,3,4,6,11,12,13,14,17	3	P01835,P25236,Q62635
10206	1,5,7,8,9,10,15,17	2,3,4,6,11,12,13,14,16	3	G3V686,P25236,P52590
10207	1,5,7,8,9,10,16,17	2,3,4,6,11,12,13,14,15	4	P02625,P25031,P25236,Q64093
10208	1,5,7,8,9,11,12,13	2,3,4,6,10,14,15,16,17	4	P02803,P10715,P36374,Q8CJ52
10209	1,5,7,8,9,11,12,14	2,3,4,6,10,13,15,16,17	2	P10715,Q4FZU6
10210	1,5,7,8,9,11,12,15	2,3,4,6,10,13,14,16,17	2	P10715,P14480
10211	1,5,7,8,9,11,12,16	2,3,4,6,10,13,14,15,17	3	P10715,Q4FZU6,Q8CJ52
10212	1,5,7,8,9,11,12,17	2,3,4,6,10,13,14,15,16	2	P10715,Q4FZU6
10213	1,5,7,8,9,11,13,14	2,3,4,6,10,12,15,16,17	2	B0BNN3,Q4FZU6

10214	1,5,7,8,9,11,13,15	2,3,4,6,10,12,14,16,17	2	P14480,Q9JJS8
10215	1,5,7,8,9,11,13,16	2,3,4,6,10,12,14,15,17	2	B0BNN3,Q4FZU6
10216	1,5,7,8,9,11,13,17	2,3,4,6,10,12,14,15,16	1	Q9JJS8
10217	1,5,7,8,9,11,14,15	2,3,4,6,10,12,13,16,17	4	P14480,P16636,Q63355,Q9JJS8
10218	1,5,7,8,9,11,14,16	2,3,4,6,10,12,13,15,17	3	B0BNN3,Q4FZU6,Q62635
10219	1,5,7,8,9,11,14,17	2,3,4,6,10,12,13,15,16	2	Q4FZU6,Q9QUL6
10220	1,5,7,8,9,11,15,16	2,3,4,6,10,12,13,14,17	3	P10715,P25236,Q62635
10221	1,5,7,8,9,11,15,17	2,3,4,6,10,12,13,14,16	3	P10715,P25236,Q9JJS8
10222	1,5,7,8,9,11,16,17	2,3,4,6,10,12,13,14,15	4	A2RUV9,P10715,P25236,Q4FZU6
10223	1,5,7,8,9,12,13,14	2,3,4,6,10,11,15,16,17	1	Q4FZU6
10224	1,5,7,8,9,12,13,15	2,3,4,6,10,11,14,16,17	2	P10715,P19218
10225	1,5,7,8,9,12,13,16	2,3,4,6,10,11,14,15,17	4	P02803,P10715,P19218,Q4FZU6
10226	1,5,7,8,9,12,13,17	2,3,4,6,10,11,14,15,16	4	P10715,P19218,P36374,Q4FZU6
10227	1,5,7,8,9,12,14,15	2,3,4,6,10,11,13,16,17	2	P10715,P16636
10228	1,5,7,8,9,12,14,16	2,3,4,6,10,11,13,15,17	2	P10715,Q4FZU6

10229	1,5,7,8,9,12,14,17	2,3,4,6,10,11,13,15,16	2	P10715,Q4FZU6
10230	1,5,7,8,9,12,15,16	2,3,4,6,10,11,13,14,17	1	P10715
10231	1,5,7,8,9,12,15,17	2,3,4,6,10,11,13,14,16	1	P10715
10232	1,5,7,8,9,12,16,17	2,3,4,6,10,11,13,14,15	2	P10715,Q4FZU6
10233	1,5,7,8,9,13,14,15	2,3,4,6,10,11,12,16,17	4	P00762,P06757,P16636,P53792
10234	1,5,7,8,9,13,14,16	2,3,4,6,10,11,12,15,17	3	B0BNN3,P00762,Q4FZU6
10235	1,5,7,8,9,13,14,17	2,3,4,6,10,11,12,15,16	4	P00762,P51907,P53792,Q4FZU6
10236	1,5,7,8,9,13,15,16	2,3,4,6,10,11,12,14,17	4	P00762,P01835,P62804,Q00715
10237	1,5,7,8,9,13,15,17	2,3,4,6,10,11,12,14,16	3	P00762,P53792,Q9JJS8
10238	1,5,7,8,9,13,16,17	2,3,4,6,10,11,12,14,15	5	P00762,P02625,P35280,P53792,Q4FZU6
10239	1,5,7,8,9,14,15,16	2,3,4,6,10,11,12,13,17	5	P00762,P16636,Q4FZU6,Q62635,Q9QZK9
10240	1,5,7,8,9,14,15,17	2,3,4,6,10,11,12,13,16	4	P00762,P16636,P47967,P53792
10241	1,5,7,8,9,14,16,17	2,3,4,6,10,11,12,13,15	3	P00762,P02625,Q4FZU6
10242	1,5,7,8,9,15,16,17	2,3,4,6,10,11,12,13,14	3	P00762,P25236,P47727
10243	1,5,7,8,10,11,12,13	2,3,4,6,9,14,15,16,17	5	P02803,P10715,P36374,Q5HZV9,Q8CJ52
10244	1,5,7,8,10,11,12,14	2,3,4,6,9,13,15,16,17	3	P10715,P47727,Q8CJ52

10245	1,5,7,8,10,11,12,15	2,3,4,6,9,13,14,16,17	2	P00731,P10715
10246	1,5,7,8,10,11,12,16	2,3,4,6,9,13,14,15,17	2	P10715,Q8CJ52
10247	1,5,7,8,10,11,12,17	2,3,4,6,9,13,14,15,16	3	F1LM93,P10715,P16391
10248	1,5,7,8,10,11,13,14	2,3,4,6,9,12,15,16,17	1	P47727
10249	1,5,7,8,10,11,13,15	2,3,4,6,9,12,14,16,17	3	P01835,Q5HZV9,Q9JJS8
10250	1,5,7,8,10,11,13,16	2,3,4,6,9,12,14,15,17	4	B0BNN3,P01835,Q5HZV9,Q8VD89
10251	1,5,7,8,10,11,13,17	2,3,4,6,9,12,14,15,16	0	
10252	1,5,7,8,10,11,14,15	2,3,4,6,9,12,13,16,17	4	P09605;P25809,P14480,P16636,Q63355
10253	1,5,7,8,10,11,14,16	2,3,4,6,9,12,13,15,17	1	P02625
10254	1,5,7,8,10,11,14,17	2,3,4,6,9,12,13,15,16	0	
10255	1,5,7,8,10,11,15,16	2,3,4,6,9,12,13,14,17	3	P01835,P25236,Q62635
10256	1,5,7,8,10,11,15,17	2,3,4,6,9,12,13,14,16	2	P25236,Q9JJS8
10257	1,5,7,8,10,11,16,17	2,3,4,6,9,12,13,14,15	3	A2RUV9,P02625,P25236
10258	1,5,7,8,10,12,13,14	2,3,4,6,9,11,15,16,17	2	Q498D9,Q8CJ52
10259	1,5,7,8,10,12,13,15	2,3,4,6,9,11,14,16,17	1	P19218
10260	1,5,7,8,10,12,13,16	2,3,4,6,9,11,14,15,17	3	P02625,P19218,Q8CJ52
10261	1,5,7,8,10,12,13,17	2,3,4,6,9,11,14,15,16	3	P19218,Q99MH3,Q9QZK9
10262	1,5,7,8,10,12,14,15	2,3,4,6,9,11,13,16,17	0	
10263	1,5,7,8,10,12,14,16	2,3,4,6,9,11,13,15,17	2	P02625,P08721
10264	1,5,7,8,10,12,14,17	2,3,4,6,9,11,13,15,16	0	
10265	1,5,7,8,10,12,15,16	2,3,4,6,9,11,13,14,17	1	P10715
10266	1,5,7,8,10,12,15,17	2,3,4,6,9,11,13,14,16	2	P10715,Q99MH3
10267	1,5,7,8,10,12,16,17	2,3,4,6,9,11,13,14,15	2	P02625,P10715

10268	1,5,7,8,10,<br>13,14,15	2,3,4,6,9,1<br>1,12,16,17	4	P00762,P01835,P09605;P25809,P16636
10269	1,5,7,8,10,<br>13,14,16	2,3,4,6,9,1<br>1,12,15,17	4	B0BNN3,P00762,P01835,P02625
10270	1,5,7,8,10,<br>13,14,17	2,3,4,6,9,1<br>1,12,15,16	1	P02625
10271	1,5,7,8,10,<br>13,15,16	2,3,4,6,9,1<br>1,12,14,17	2	P00762,P01835
10272	1,5,7,8,10,<br>13,15,17	2,3,4,6,9,1<br>1,12,14,16	2	P01835,Q99MH3
10273	1,5,7,8,10,<br>13,16,17	2,3,4,6,9,1<br>1,12,14,15	2	P01835,P02625
10274	1,5,7,8,10,<br>14,15,16	2,3,4,6,9,1<br>1,12,13,17	3	P01835,P09605;P25809,P16636
10275	1,5,7,8,10,<br>14,15,17	2,3,4,6,9,1<br>1,12,13,16	0	
10276	1,5,7,8,10,<br>14,16,17	2,3,4,6,9,1<br>1,12,13,15	1	P02625
10277	1,5,7,8,10,<br>15,16,17	2,3,4,6,9,1<br>1,12,13,14	2	P01835,P25236
10278	1,5,7,8,11,<br>12,13,14	2,3,4,6,9,1<br>0,15,16,17	4	P10715,P47727,Q4FZU6,Q8CJ52
10279	1,5,7,8,11,<br>12,13,15	2,3,4,6,9,1<br>0,14,16,17	2	P10715,Q8CJ52
10280	1,5,7,8,11,<br>12,13,16	2,3,4,6,9,1<br>0,14,15,17	4	P10715,P17046,Q4FZU6,Q8CJ52
10281	1,5,7,8,11,<br>12,13,17	2,3,4,6,9,1<br>0,14,15,16	5	P10715,P36374,Q4FZU6,Q8CJ52,Q9QZK9
10282	1,5,7,8,11,<br>12,14,15	2,3,4,6,9,1<br>0,13,16,17	3	P10715,P19629,Q63355
10283	1,5,7,8,11,<br>12,14,16	2,3,4,6,9,1<br>0,13,15,17	3	P10715,Q4FZU6,Q8CJ52
10284	1,5,7,8,11,<br>12,14,17	2,3,4,6,9,1<br>0,13,15,16	3	P10715,Q4FZU6,Q8CJ52
10285	1,5,7,8,11,<br>12,15,16	2,3,4,6,9,1<br>0,13,14,17	1	P10715
10286	1,5,7,8,11,<br>12,15,17	2,3,4,6,9,1<br>0,13,14,16	2	P10715,Q9QZA6
10287	1,5,7,8,11,<br>12,16,17	2,3,4,6,9,1<br>0,13,14,15	4	P10715,P17046,Q4FZU6,Q8CJ52
10288	1,5,7,8,11,<br>13,14,15	2,3,4,6,9,1<br>0,12,16,17	4	P16636,Q63355,Q6P6R2,Q9JJS8
10289	1,5,7,8,11,<br>13,14,16	2,3,4,6,9,1<br>0,12,15,17	2	B0BNN3,Q4FZU6
10290	1,5,7,8,11,<br>13,14,17	2,3,4,6,9,1<br>0,12,15,16	2	Q4FZU6,Q9JJS8

10291	1,5,7,8,11,13,15,16	2,3,4,6,9,10,12,14,17	3	P01835,Q8VD89,Q9JJS8
10292	1,5,7,8,11,13,15,17	2,3,4,6,9,10,12,14,16	2	P04073,Q9JJS8
10293	1,5,7,8,11,13,16,17	2,3,4,6,9,10,12,14,15	1	Q4FZU6
10294	1,5,7,8,11,14,15,16	2,3,4,6,9,10,12,13,17	2	P16636,Q63355
10295	1,5,7,8,11,14,15,17	2,3,4,6,9,10,12,13,16	3	P14173,P47967,Q9JJS8
10296	1,5,7,8,11,14,16,17	2,3,4,6,9,10,12,13,15	1	Q4FZU6
10297	1,5,7,8,11,15,16,17	2,3,4,6,9,10,12,13,14	3	P10715,P25236,Q9JJS8
10298	1,5,7,8,12,13,14,15	2,3,4,6,9,10,11,16,17	3	P00762,P14173,P16636
10299	1,5,7,8,12,13,14,16	2,3,4,6,9,10,11,15,17	3	P00762,Q4FZU6,Q8CJ52
10300	1,5,7,8,12,13,14,17	2,3,4,6,9,10,11,15,16	2	P14173,Q4FZU6
10301	1,5,7,8,12,13,15,16	2,3,4,6,9,10,11,14,17	4	P00762,P10715,P14173,P19218
10302	1,5,7,8,12,13,15,17	2,3,4,6,9,10,11,14,16	3	P10715,P14173,Q99MH3
10303	1,5,7,8,12,13,16,17	2,3,4,6,9,10,11,14,15	6	P10715,P14173,P19218,P23928,P35280,Q4FZU6
10304	1,5,7,8,12,14,15,16	2,3,4,6,9,10,11,13,17	3	P14173,P16636,Q4FZU6
10305	1,5,7,8,12,14,15,17	2,3,4,6,9,10,11,13,16	2	P14173,Q4FZU6
10306	1,5,7,8,12,14,16,17	2,3,4,6,9,10,11,13,15	2	P14173,Q4FZU6
10307	1,5,7,8,12,15,16,17	2,3,4,6,9,10,11,13,14	3	P10715,P14173,Q4FZU6
10308	1,5,7,8,13,14,15,16	2,3,4,6,9,10,11,12,17	4	P00762,P01835,P14173,P16636
10309	1,5,7,8,13,14,15,17	2,3,4,6,9,10,11,12,16	7	P00762,P14173,P16636,P47967,P53792,Q9JJS8,Q9WUW8
10310	1,5,7,8,13,14,16,17	2,3,4,6,9,10,11,12,15	4	P00762,P02625,P14173,Q4FZU6
10311	1,5,7,8,13,15,16,17	2,3,4,6,9,10,11,12,14	3	P00762,P01835,P14173
10312	1,5,7,8,14,15,16,17	2,3,4,6,9,10,11,12,13	4	P00762,P14173,P16636,Q4FZU6
10313	1,5,7,9,10,11,12,13	2,3,4,6,8,14,15,16,17	3	B0BNN3,P02803,P20761

10314	1,5,7,9,10,<br>11,12,14	2,3,4,6,8,1<br>3,15,16,17	1	P20761
10315	1,5,7,9,10,<br>11,12,15	2,3,4,6,8,1<br>3,14,16,17	5	P20766,P51907,Q566E6,Q64093,Q6PCU2
10316	1,5,7,9,10,<br>11,12,16	2,3,4,6,8,1<br>3,14,15,17	7	B0BNN3,P10536,P12368,P20761,P20766,Q63424,Q64093
10317	1,5,7,9,10,<br>11,12,17	2,3,4,6,8,1<br>3,14,15,16	5	P06757,P25236,Q566E6,Q64093,Q9QUL6
10318	1,5,7,9,10,<br>11,13,14	2,3,4,6,8,1<br>2,15,16,17	5	B0BNN3,P20761,P98089,Q4G075,Q62635
10319	1,5,7,9,10,<br>11,13,15	2,3,4,6,8,1<br>2,14,16,17	5	P20761,P98089,Q4G075,Q62635,Q9JJS8
10320	1,5,7,9,10,<br>11,13,16	2,3,4,6,8,1<br>2,14,15,17	5	B0BNN3,P20761,P98089,Q4G075,Q62635
10321	1,5,7,9,10,<br>11,13,17	2,3,4,6,8,1<br>2,14,15,16	2	B0BNN3,Q9QUL6
10322	1,5,7,9,10,<br>11,14,15	2,3,4,6,8,1<br>2,13,16,17	6	P16636,Q4G075,Q62635,Q63355,Q80WL1,Q9QUL6
10323	1,5,7,9,10,<br>11,14,16	2,3,4,6,8,1<br>2,13,15,17	6	B0BNN3,P20761,P62815,Q4G075,Q62635,Q9QUL6
10324	1,5,7,9,10,<br>11,14,17	2,3,4,6,8,1<br>2,13,15,16	2	Q6TMA8,Q9QUL6
10325	1,5,7,9,10,<br>11,15,16	2,3,4,6,8,1<br>2,13,14,17	12	P02782,P20761,P20766,P25236,P62815,P98089,Q62635,Q63424,Q64093,Q64319,Q80W57,Q80WL1
10326	1,5,7,9,10,<br>11,15,17	2,3,4,6,8,1<br>2,13,14,16	6	P25236,Q566E6,Q64093,Q6TMA8,Q9JJS8,Q9QUL6
10327	1,5,7,9,10,<br>11,16,17	2,3,4,6,8,1<br>2,13,14,15	8	A2RUV9,B0BNN3,O08557,P02625,P25236,Q64093,Q6TMA8,Q9QUL6
10328	1,5,7,9,10,<br>12,13,14	2,3,4,6,8,1<br>1,15,16,17	2	B0BNN3,P20761
10329	1,5,7,9,10,<br>12,13,15	2,3,4,6,8,1<br>1,14,16,17	1	P19218
10330	1,5,7,9,10,<br>12,13,16	2,3,4,6,8,1<br>1,14,15,17	5	B0BNN3,P02761,P19218,P20761,P97840
10331	1,5,7,9,10,<br>12,13,17	2,3,4,6,8,1<br>1,14,15,16	2	P14668,P19218
10332	1,5,7,9,10,<br>12,14,15	2,3,4,6,8,1<br>1,13,16,17	2	P16636,P20766
10333	1,5,7,9,10,<br>12,14,16	2,3,4,6,8,1<br>1,13,15,17	4	B0BNN3,P20761,P20766,Q5XI22
10334	1,5,7,9,10,<br>12,14,17	2,3,4,6,8,1<br>1,13,15,16	2	Q5XI22,Q6TMA8
10335	1,5,7,9,10,<br>12,15,16	2,3,4,6,8,1<br>1,13,14,17	4	P19218,P20766,P97840,Q64093
10336	1,5,7,9,10,<br>12,15,17	2,3,4,6,8,1<br>1,13,14,16	3	P20766,P25236,Q6TMA8

10337	1,5,7,9,10, 12,16,17	2,3,4,6,8,1 1,13,14,15	6	P02625,P19218,P20766,P25236,Q64093,Q6TMA8
10338	1,5,7,9,10, 13,14,15	2,3,4,6,8,1 1,12,16,17	6	P09605;P25809,P16636,P19223,P98089,Q4G075,Q62635
10339	1,5,7,9,10, 13,14,16	2,3,4,6,8,1 1,12,15,17	11	B0BNN3,P01015,P02625,P02761,P19223,P20761,P62804,P98089,Q00715,Q4G075,Q62635
10340	1,5,7,9,10, 13,14,17	2,3,4,6,8,1 1,12,15,16	1	P14668
10341	1,5,7,9,10, 13,15,16	2,3,4,6,8,1 1,12,14,17	12	B0BNN3,P01835,P16636,P19218,P19223,P20761,P62804,P98089,Q00715,Q4G075,Q62635,Q80WL1
10342	1,5,7,9,10, 13,15,17	2,3,4,6,8,1 1,12,14,16	3	P14668,Q9EQS0,Q9JJS8
10343	1,5,7,9,10, 13,16,17	2,3,4,6,8,1 1,12,14,15	7	B0BNN3,P02625,P19218,P35280,P62804,Q00715,Q6TMA8
10344	1,5,7,9,10, 14,15,16	2,3,4,6,8,1 1,12,13,17	11	P02782,P07647,P16636,P19223,P20766,Q4G075,Q5XI22,Q62635,Q6TMA8,Q80WL1,Q9QZK9
10345	1,5,7,9,10, 14,15,17	2,3,4,6,8,1 1,12,13,16	3	P16636,Q5XI22,Q6TMA8
10346	1,5,7,9,10, 14,16,17	2,3,4,6,8,1 1,12,13,15	3	P02625,Q5XI22,Q6TMA8
10347	1,5,7,9,10, 15,16,17	2,3,4,6,8,1 1,12,13,14	10	P02780,P02782,P0C0A9,P20766,P25236,P47727,Q64093,Q6TMA8,Q80WL1,Q9JHB9
10348	1,5,7,9,11, 12,13,14	2,3,4,6,8,1 0,15,16,17	3	B0BNN3,P20761,Q6P6R2
10349	1,5,7,9,11, 12,13,15	2,3,4,6,8,1 0,14,16,17	1	Q6P6R2
10350	1,5,7,9,11, 12,13,16	2,3,4,6,8,1 0,14,15,17	6	B0BNN3,P02761,P02803,P12368,P17046,P20761
10351	1,5,7,9,11, 12,13,17	2,3,4,6,8,1 0,14,15,16	2	P02803,P36374
10352	1,5,7,9,11, 12,14,15	2,3,4,6,8,1 0,13,16,17	4	P16636,P20766,Q63355,Q6P6R2
10353	1,5,7,9,11, 12,14,16	2,3,4,6,8,1 0,13,15,17	5	B0BNN3,P02761,P20761,P20766,Q4FZU6
10354	1,5,7,9,11, 12,14,17	2,3,4,6,8,1 0,13,15,16	1	Q4FZU6
10355	1,5,7,9,11, 12,15,16	2,3,4,6,8,1 0,13,14,17	2	P20766,P51907
10356	1,5,7,9,11, 12,15,17	2,3,4,6,8,1 0,13,14,16	0	
10357	1,5,7,9,11, 12,16,17	2,3,4,6,8,1 0,13,14,15	5	P17046,P25236,Q4FZU6,Q64093,Q6TMA8
10358	1,5,7,9,11, 13,14,15	2,3,4,6,8,1 0,12,16,17	8	D4A5U3,P16636,P19223,Q4G075,Q62635,Q6P6R2,Q80WL1,Q9JJS8
10359	1,5,7,9,11, 13,14,16	2,3,4,6,8,1 0,12,15,17	9	B0BNN3,P02761,P19223,P20761,P98089,Q4FZU6,Q4G075,Q62635,Q7TPB1

10360	1,5,7,9,11,<br>13,14,17	2,3,4,6,8,1<br>0,12,15,16	1	Q9JJS8
10361	1,5,7,9,11,<br>13,15,16	2,3,4,6,8,1<br>0,12,14,17	9	P16636,P19223,P20761,P98089,Q4G075,Q62635,Q6P6R2,Q80WL1,Q9JJS8
10362	1,5,7,9,11,<br>13,15,17	2,3,4,6,8,1<br>0,12,14,16	2	P04073,Q9JJS8
10363	1,5,7,9,11,<br>13,16,17	2,3,4,6,8,1<br>0,12,14,15	3	B0BNN3,D3ZHA0,P35280
10364	1,5,7,9,11,<br>14,15,16	2,3,4,6,8,1<br>0,12,13,17	9	D4A5U3,P16636,P19223,P20766,Q4G075,Q62635,Q63355,Q80WL1,Q9QZK9
10365	1,5,7,9,11,<br>14,15,17	2,3,4,6,8,1<br>0,12,13,16	4	P16636,Q80WL1,Q9JJS8,Q9QUL6
10366	1,5,7,9,11,<br>14,16,17	2,3,4,6,8,1<br>0,12,13,15	3	Q4FZU6,Q6TMA8,Q9QUL6
10367	1,5,7,9,11,<br>15,16,17	2,3,4,6,8,1<br>0,12,13,14	5	P02782,P25236,Q6TMA8,Q80WL1,Q9JJS8
10368	1,5,7,9,12,<br>13,14,15	2,3,4,6,8,1<br>0,11,16,17	2	P16636,Q6P6R2
10369	1,5,7,9,12,<br>13,14,16	2,3,4,6,8,1<br>0,11,15,17	5	B0BNN3,P02761,P14669,P20761,Q4FZU6
10370	1,5,7,9,12,<br>13,14,17	2,3,4,6,8,1<br>0,11,15,16	4	P14668,P35280,P53792,Q4FZU6
10371	1,5,7,9,12,<br>13,15,16	2,3,4,6,8,1<br>0,11,14,17	3	P02761,P19218,P62804
10372	1,5,7,9,12,<br>13,15,17	2,3,4,6,8,1<br>0,11,14,16	3	P14668,P19218,P53792
10373	1,5,7,9,12,<br>13,16,17	2,3,4,6,8,1<br>0,11,14,15	4	P02761,P19218,P35280,Q4FZU6
10374	1,5,7,9,12,<br>14,15,16	2,3,4,6,8,1<br>0,11,13,17	3	P16636,P20766,Q5XI22
10375	1,5,7,9,12,<br>14,15,17	2,3,4,6,8,1<br>0,11,13,16	1	P16636
10376	1,5,7,9,12,<br>14,16,17	2,3,4,6,8,1<br>0,11,13,15	5	P20766,P35280,Q4FZU6,Q5XI22,Q6TMA8
10377	1,5,7,9,12,<br>15,16,17	2,3,4,6,8,1<br>0,11,13,14	3	P20766,P47727,Q6TMA8
10378	1,5,7,9,13,<br>14,15,16	2,3,4,6,8,1<br>0,11,12,17	13	P00762,P02761,P11883,P14669,P16636,P19223,P53792,P62804,Q00715,Q4G075,Q62635,Q80WL1,Q9QZK9
10379	1,5,7,9,13,<br>14,15,17	2,3,4,6,8,1<br>0,11,12,16	7	P00762,P11883,P14668,P16636,P53792,Q80WL1,Q9JJS8
10380	1,5,7,9,13,<br>14,16,17	2,3,4,6,8,1<br>0,11,12,15	10	B0BNN3,P00762,P02625,P02761,P35280,P53792,P62804,Q00715,Q4FZU6,Q5XI22
10381	1,5,7,9,13,<br>15,16,17	2,3,4,6,8,1<br>0,11,12,14	14	P00762,P0C0A9,P11883,P19218,P35280,P47727,P53792,P62804,Q00715,Q63598,Q6TMA8,Q80WL1,Q9EQS0,Q9JJS8
10382	1,5,7,9,14,<br>15,16,17	2,3,4,6,8,1<br>0,11,12,13	10	P00762,P02782,P07647,P0C0A9,P11883,P16636,P62804,Q5XI22,Q6TMA8,Q80WL1

10383	1,5,7,10,11,12,13,14	2,3,4,6,8,9,15,16,17	3	P47727,Q5QE79,Q6P6R2
10384	1,5,7,10,11,12,13,15	2,3,4,6,8,9,14,16,17	1	Q6P6R2
10385	1,5,7,10,11,12,13,16	2,3,4,6,8,9,14,15,17	6	B0BNN3,P02761,P12368,P17046,P20761,Q5QE79
10386	1,5,7,10,11,12,13,17	2,3,4,6,8,9,14,15,16	3	P36374,Q5QE79,Q9QZK9
10387	1,5,7,10,11,12,14,15	2,3,4,6,8,9,13,16,17	5	P16636,P20766,P51907,Q63355,Q6P6R2
10388	1,5,7,10,11,12,14,16	2,3,4,6,8,9,13,15,17	3	P20761,P20766,Q5QE79
10389	1,5,7,10,11,12,14,17	2,3,4,6,8,9,13,15,16	3	P20766,Q5QE79,Q6TMA8
10390	1,5,7,10,11,12,15,16	2,3,4,6,8,9,13,14,17	4	P12368,P20766,P51907,Q6TMA8
10391	1,5,7,10,11,12,15,17	2,3,4,6,8,9,13,14,16	3	P20766,P25236,Q6TMA8
10392	1,5,7,10,11,12,16,17	2,3,4,6,8,9,13,14,15	8	P05369,P06757,P12368,P17046,P20766,P25236,Q5QE79,Q6TMA8
10393	1,5,7,10,11,13,14,15	2,3,4,6,8,9,12,16,17	7	P09605;P25809,P16636,P19223,P98089,Q62635,Q6P6R2,Q9JJS8
10394	1,5,7,10,11,13,14,16	2,3,4,6,8,9,12,15,17	8	B0BNN3,P02761,P19223,P20761,P98089,Q5QE79,Q62635,Q7TPB1
10395	1,5,7,10,11,13,14,17	2,3,4,6,8,9,12,15,16	1	Q5QE79
10396	1,5,7,10,11,13,15,16	2,3,4,6,8,9,12,14,17	5	P01835,P19223,P20761,P98089,Q62635
10397	1,5,7,10,11,13,15,17	2,3,4,6,8,9,12,14,16	2	Q6TMA8,Q9JJS8
10398	1,5,7,10,11,13,16,17	2,3,4,6,8,9,12,14,15	4	B0BNN3,P02625,Q5QE79,Q6TMA8
10399	1,5,7,10,11,14,15,16	2,3,4,6,8,9,12,13,17	8	P02782,P16636,P19223,P20766,Q62635,Q63355,Q6TMA8,Q80WL1
10400	1,5,7,10,11,14,15,17	2,3,4,6,8,9,12,13,16	3	P16636,P20766,Q6TMA8
10401	1,5,7,10,11,14,16,17	2,3,4,6,8,9,12,13,15	4	P02625,P20766,Q5QE79,Q6TMA8
10402	1,5,7,10,11,15,16,17	2,3,4,6,8,9,12,13,14	5	P02782,P05369,P20766,P25236,Q6TMA8
10403	1,5,7,10,12,13,14,15	2,3,4,6,8,9,11,16,17	4	P09605;P25809,P16636,Q498D9,Q6P6R2
10404	1,5,7,10,12,13,14,16	2,3,4,6,8,9,11,15,17	4	B0BNN3,P02761,P20761,Q5QE79
10405	1,5,7,10,12,13,14,17	2,3,4,6,8,9,11,15,16	2	P14668,Q5QE79

10406	1,5,7,10,12,13,15,16	2,3,4,6,8,9,11,14,17	1	P19218
10407	1,5,7,10,12,13,15,17	2,3,4,6,8,9,11,14,16	4	P14668,P19218,Q6TMA8,Q99MH3
10408	1,5,7,10,12,13,16,17	2,3,4,6,8,9,11,14,15	5	P02625,P19218,P35280,Q5QE79,Q6TMA8
10409	1,5,7,10,12,14,15,16	2,3,4,6,8,9,11,13,17	3	P16636,P20766,Q6TMA8
10410	1,5,7,10,12,14,15,17	2,3,4,6,8,9,11,13,16	2	P20766,Q6TMA8
10411	1,5,7,10,12,14,16,17	2,3,4,6,8,9,11,13,15	5	P02625,P20766,Q5QE79,Q5XI22,Q6TMA8
10412	1,5,7,10,12,15,16,17	2,3,4,6,8,9,11,13,14	3	P19218,P20766,Q6TMA8
10413	1,5,7,10,13,14,15,16	2,3,4,6,8,9,11,12,17	9	P00762,P01835,P09605;P25809,P16636,P19223,P98089,Q4G075,Q62635,Q80WL1
10414	1,5,7,10,13,14,15,17	2,3,4,6,8,9,11,12,16	3	P14668,P16636,Q6TMA8
10415	1,5,7,10,13,14,16,17	2,3,4,6,8,9,11,12,15	4	P02625,Q5QE79,Q5XI22,Q6TMA8
10416	1,5,7,10,13,15,16,17	2,3,4,6,8,9,11,12,14	6	P01835,P0C0A9,P19218,P62804,Q6TMA8,Q9EQS0
10417	1,5,7,10,14,15,16,17	2,3,4,6,8,9,11,12,13	9	P02780,P02782,P07647,P0C0A9,P16636,P20766,Q5XI22,Q6TMA8,Q80WL1
10418	1,5,7,11,12,13,14,15	2,3,4,6,8,9,10,16,17	3	P16636,Q63355,Q6P6R2
10419	1,5,7,11,12,13,14,16	2,3,4,6,8,9,10,15,17	7	B0BNN3,P02761,P17046,P20761,Q4FZU6,Q5QE79,Q6P6R2
10420	1,5,7,11,12,13,14,17	2,3,4,6,8,9,10,15,16	1	Q5QE79
10421	1,5,7,11,12,13,15,16	2,3,4,6,8,9,10,14,17	2	P17046,Q6P6R2
10422	1,5,7,11,12,13,15,17	2,3,4,6,8,9,10,14,16	0	
10423	1,5,7,11,12,13,16,17	2,3,4,6,8,9,10,14,15	4	P17046,P35280,Q4FZU6,Q5QE79
10424	1,5,7,11,12,14,15,16	2,3,4,6,8,9,10,13,17	4	P16636,P20766,Q63355,Q6P6R2
10425	1,5,7,11,12,14,15,17	2,3,4,6,8,9,10,13,16	2	P20766,Q6TMA8
10426	1,5,7,11,12,14,16,17	2,3,4,6,8,9,10,13,15	5	P17046,P20766,Q4FZU6,Q5QE79,Q6TMA8
10427	1,5,7,11,12,15,16,17	2,3,4,6,8,9,10,13,14	4	P05369,P17046,P20766,Q6TMA8
10428	1,5,7,11,13,14,15,16	2,3,4,6,8,9,10,12,17	8	D4A5U3,P16636,P19223,Q62635,Q63355,Q6P6R2,Q80WL1,Q9JJS8

10429	1,5,7,11,1<br>3,14,15,17	2,3,4,6,8,9<br>,10,12,16	4	D3ZHA0,P16636,Q6P6R2,Q9JJS8
10430	1,5,7,11,1<br>3,14,16,17	2,3,4,6,8,9<br>,10,12,15	5	D3ZHA0,P17046,Q4FZU6,Q5QE79,Q6TMA8
10431	1,5,7,11,1<br>3,15,16,17	2,3,4,6,8,9<br>,10,12,14	5	D3ZHA0,P17046,Q6TMA8,Q9EQS0,Q9JJS8
10432	1,5,7,11,1<br>4,15,16,17	2,3,4,6,8,9<br>,10,12,13	5	D3ZHA0,P16636,P20766,Q6TMA8,Q80WL1
10433	1,5,7,12,1<br>3,14,15,16	2,3,4,6,8,9<br>,10,11,17	4	P00762,P02761,P16636,Q6P6R2
10434	1,5,7,12,1<br>3,14,15,17	2,3,4,6,8,9<br>,10,11,16	4	P11883,P14173,P14668,P16636
10435	1,5,7,12,1<br>3,14,16,17	2,3,4,6,8,9<br>,10,11,15	7	P02761,P17046,P35280,Q4FZU6,Q5QE79,Q5XI22,Q6TMA8
10436	1,5,7,12,1<br>3,15,16,17	2,3,4,6,8,9<br>,10,11,14	6	P11883,P14173,P17046,P19218,P35280,Q6TMA8
10437	1,5,7,12,1<br>4,15,16,17	2,3,4,6,8,9<br>,10,11,13	7	P14173,P16636,P20766,P35467,Q4FZU6,Q5XI22,Q6TMA8
10438	1,5,7,13,1<br>4,15,16,17	2,3,4,6,8,9<br>,10,11,12	11	D3ZHA0,P00762,P0C0A9,P11883,P14173,P16636,P62804,Q5XI22,Q6TMA8,Q80WL1,Q9EQS0
10439	1,5,8,9,10,<br>11,12,13	2,3,4,6,7,1<br>4,15,16,17	3	P02803,P09605;P25809,P20761
10440	1,5,8,9,10,<br>11,12,14	2,3,4,6,7,1<br>3,15,16,17	3	P02803,P09605;P25809,P20761
10441	1,5,8,9,10,<br>11,12,15	2,3,4,6,7,1<br>3,14,16,17	2	P09605;P25809,P14480
10442	1,5,8,9,10,<br>11,12,16	2,3,4,6,7,1<br>3,14,15,17	3	P02803,P20761,Q9WUW8
10443	1,5,8,9,10,<br>11,12,17	2,3,4,6,7,1<br>3,14,15,16	3	P02803,P25236,Q63532
10444	1,5,8,9,10,<br>11,13,14	2,3,4,6,7,1<br>2,15,16,17	5	P01836,P02803,P09605;P25809,P20761,P98089
10445	1,5,8,9,10,<br>11,13,15	2,3,4,6,7,1<br>2,14,16,17	5	P09605;P25809,P14480,P20761,P98089,Q63532
10446	1,5,8,9,10,<br>11,13,16	2,3,4,6,7,1<br>2,14,15,17	7	A2RUV9,B0BNN3,P02803,P09605;P25809,P20761,P62815,P98089
10447	1,5,8,9,10,<br>11,13,17	2,3,4,6,7,1<br>2,14,15,16	6	A2RUV9,P01836,P02803,P04073,Q63532,Q920P0
10448	1,5,8,9,10,<br>11,14,15	2,3,4,6,7,1<br>2,13,16,17	5	P01836,P09605;P25809,P14480,P20761,Q62635
10449	1,5,8,9,10,<br>11,14,16	2,3,4,6,7,1<br>2,13,15,17	7	P01836,P09605;P25809,P20761,P55091,P62815,Q62635,Q9QZK9
10450	1,5,8,9,10,<br>11,14,17	2,3,4,6,7,1<br>2,13,15,16	2	P01836,Q9QUL6
10451	1,5,8,9,10,<br>11,15,16	2,3,4,6,7,1<br>2,13,14,17	8	A2RUV9,P09605;P25809,P20761,P25236,P55091,P62815,Q62635,Q9QZK9

10452	1,5,8,9,10, 11,15,17	2,3,4,6,7,1 2,13,14,16	5	G3V686,P01836,P25236,Q566E6,Q63532
10453	1,5,8,9,10, 11,16,17	2,3,4,6,7,1 2,13,14,15	4	A2RUV9,P01836,P02625,P25236
10454	1,5,8,9,10, 12,13,14	2,3,4,6,7,1 1,15,16,17	3	P02803,P09605;P25809,P20761
10455	1,5,8,9,10, 12,13,15	2,3,4,6,7,1 1,14,16,17	2	P09605;P25809,P16086
10456	1,5,8,9,10, 12,13,16	2,3,4,6,7,1 1,14,15,17	5	P02631,P02803,P09605;P25809,P19218,P20761
10457	1,5,8,9,10, 12,13,17	2,3,4,6,7,1 1,14,15,16	4	P02631,P02803,P16086,Q63532
10458	1,5,8,9,10, 12,14,15	2,3,4,6,7,1 1,13,16,17	1	P09605;P25809
10459	1,5,8,9,10, 12,14,16	2,3,4,6,7,1 1,13,15,17	2	P09605;P25809,P20761
10460	1,5,8,9,10, 12,14,17	2,3,4,6,7,1 1,13,15,16	2	P01836,Q99PP0
10461	1,5,8,9,10, 12,15,16	2,3,4,6,7,1 1,13,14,17	3	P09605;P25809,P16086,P20761
10462	1,5,8,9,10, 12,15,17	2,3,4,6,7,1 1,13,14,16	3	G3V686,P16086,Q63532
10463	1,5,8,9,10, 12,16,17	2,3,4,6,7,1 1,13,14,15	2	P02625,P16086
10464	1,5,8,9,10, 13,14,15	2,3,4,6,7,1 1,12,16,17	5	P01836,P09605;P25809,P14480,P19223,P20761
10465	1,5,8,9,10, 13,14,16	2,3,4,6,7,1 1,12,15,17	6	B0BNN3,P01836,P02625,P09605;P25809,P19223,P20761
10466	1,5,8,9,10, 13,14,17	2,3,4,6,7,1 1,12,15,16	2	P01836,P02625
10467	1,5,8,9,10, 13,15,16	2,3,4,6,7,1 1,12,14,17	5	P01835,P09605;P25809,P19223,P20761,P98089
10468	1,5,8,9,10, 13,15,17	2,3,4,6,7,1 1,12,14,16	3	G3V686,P01836,Q63532
10469	1,5,8,9,10, 13,16,17	2,3,4,6,7,1 1,12,14,15	6	A2RUV9,P01836,P02625,P02631,P25031,P35280
10470	1,5,8,9,10, 14,15,16	2,3,4,6,7,1 1,12,13,17	7	P01836,P09605;P25809,P19223,P20761,P55091,Q62635,Q9QZK9
10471	1,5,8,9,10, 14,15,17	2,3,4,6,7,1 1,12,13,16	3	G3V686,P01836,P47967
10472	1,5,8,9,10, 14,16,17	2,3,4,6,7,1 1,12,13,15	2	P01836,P02625
10473	1,5,8,9,10, 15,16,17	2,3,4,6,7,1 1,12,13,14	4	P01836,P16086,P25031,P25236
10474	1,5,8,9,11, 12,13,14	2,3,4,6,7,1 0,15,16,17	3	P02803,P09605;P25809,P20761

10475	1,5,8,9,11,<br>12,13,15	2,3,4,6,7,1<br>0,14,16,17	2	P14480,Q63532
10476	1,5,8,9,11,<br>12,13,16	2,3,4,6,7,1<br>0,14,15,17	2	P02803,P20761
10477	1,5,8,9,11,<br>12,13,17	2,3,4,6,7,1<br>0,14,15,16	4	P02803,P35280,P36374,Q63532
10478	1,5,8,9,11,<br>12,14,15	2,3,4,6,7,1<br>0,13,16,17	2	P09605;P25809,P14480
10479	1,5,8,9,11,<br>12,14,16	2,3,4,6,7,1<br>0,13,15,17	1	P20761
10480	1,5,8,9,11,<br>12,14,17	2,3,4,6,7,1<br>0,13,15,16	1	Q99PP0
10481	1,5,8,9,11,<br>12,15,16	2,3,4,6,7,1<br>0,13,14,17	2	P12020,P20761
10482	1,5,8,9,11,<br>12,15,17	2,3,4,6,7,1<br>0,13,14,16	2	P12020,Q63532
10483	1,5,8,9,11,<br>12,16,17	2,3,4,6,7,1<br>0,13,14,15	1	P12020
10484	1,5,8,9,11,<br>13,14,15	2,3,4,6,7,1<br>0,12,16,17	5	P09605;P25809,P14480,P19223,P20761,Q6P6R2
10485	1,5,8,9,11,<br>13,14,16	2,3,4,6,7,1<br>0,12,15,17	5	B0BNN3,P01041,P09605;P25809,P19223,P20761
10486	1,5,8,9,11,<br>13,14,17	2,3,4,6,7,1<br>0,12,15,16	4	P01836,P04218,P47967,P97840
10487	1,5,8,9,11,<br>13,15,16	2,3,4,6,7,1<br>0,12,14,17	4	P09605;P25809,P19223,P20761,P98089
10488	1,5,8,9,11,<br>13,15,17	2,3,4,6,7,1<br>0,12,14,16	6	P04073,P04218,P47967,P97840,Q63532,Q9JJS8
10489	1,5,8,9,11,<br>13,16,17	2,3,4,6,7,1<br>0,12,14,15	3	A2RUV9,P04218,P35280
10490	1,5,8,9,11,<br>14,15,16	2,3,4,6,7,1<br>0,12,13,17	7	P04218,P09605;P25809,P19223,P20761,P55091,Q62635,Q9QZK9
10491	1,5,8,9,11,<br>14,15,17	2,3,4,6,7,1<br>0,12,13,16	4	P01836,P04218,P47967,P97840
10492	1,5,8,9,11,<br>14,16,17	2,3,4,6,7,1<br>0,12,13,15	2	P01836,P04218
10493	1,5,8,9,11,<br>15,16,17	2,3,4,6,7,1<br>0,12,13,14	5	A2RUV9,P04218,P06911,P12020,P25236
10494	1,5,8,9,12,<br>13,14,15	2,3,4,6,7,1<br>0,11,16,17	2	P09605;P25809,Q5FVI6
10495	1,5,8,9,12,<br>13,14,16	2,3,4,6,7,1<br>0,11,15,17	3	P09605;P25809,P20761,Q4FZU6
10496	1,5,8,9,12,<br>13,14,17	2,3,4,6,7,1<br>0,11,15,16	2	P35280,Q5FVI6
10497	1,5,8,9,12,<br>13,15,16	2,3,4,6,7,1<br>0,11,14,17	2	P16086,P20761

10498	1,5,8,9,12,<br>13,15,17	2,3,4,6,7,1<br>0,11,14,16	3	P16086,Q5FVI6,Q63532
10499	1,5,8,9,12,<br>13,16,17	2,3,4,6,7,1<br>0,11,14,15	3	P02803,P35280,Q4FZU6
10500	1,5,8,9,12,<br>14,15,16	2,3,4,6,7,1<br>0,11,13,17	1	P09605;P25809
10501	1,5,8,9,12,<br>14,15,17	2,3,4,6,7,1<br>0,11,13,16	2	P47967,Q5FVI6
10502	1,5,8,9,12,<br>14,16,17	2,3,4,6,7,1<br>0,11,13,15	3	P35280,Q4FZU6,Q5FVI6
10503	1,5,8,9,12,<br>15,16,17	2,3,4,6,7,1<br>0,11,13,14	2	P12020,P16086
10504	1,5,8,9,13,<br>14,15,16	2,3,4,6,7,1<br>0,11,12,17	6	P00762,P05964,P09605;P25809,P19223,P20761,Q9QZK9
10505	1,5,8,9,13,<br>14,15,17	2,3,4,6,7,1<br>0,11,12,16	4	P01836,P47967,P53792,Q5FVI6
10506	1,5,8,9,13,<br>14,16,17	2,3,4,6,7,1<br>0,11,12,15	5	P01836,P02625,P35280,Q4FZU6,Q5FVI6
10507	1,5,8,9,13,<br>15,16,17	2,3,4,6,7,1<br>0,11,12,14	2	P35280,Q9EQS0
10508	1,5,8,9,14,<br>15,16,17	2,3,4,6,7,1<br>0,11,12,13	3	P01836,P04218,P47967
10509	1,5,8,10,1<br>1,12,13,14	2,3,4,6,7,9<br>,15,16,17	4	P02803,P09605;P25809,P20761,P47727
10510	1,5,8,10,1<br>1,12,13,15	2,3,4,6,7,9<br>,14,16,17	1	P09605;P25809
10511	1,5,8,10,1<br>1,12,13,16	2,3,4,6,7,9<br>,14,15,17	3	P02803,P09605;P25809,P20761
10512	1,5,8,10,1<br>1,12,13,17	2,3,4,6,7,9<br>,14,15,16	2	P02803,Q63532
10513	1,5,8,10,1<br>1,12,14,15	2,3,4,6,7,9<br>,13,16,17	1	P09605;P25809
10514	1,5,8,10,1<br>1,12,14,16	2,3,4,6,7,9<br>,13,15,17	2	P09605;P25809,P20761
10515	1,5,8,10,1<br>1,12,14,17	2,3,4,6,7,9<br>,13,15,16	0	
10516	1,5,8,10,1<br>1,12,15,16	2,3,4,6,7,9<br>,13,14,17	2	P09605;P25809,P20761
10517	1,5,8,10,1<br>1,12,15,17	2,3,4,6,7,9<br>,13,14,16	0	
10518	1,5,8,10,1<br>1,12,16,17	2,3,4,6,7,9<br>,13,14,15	2	P05369,P25236
10519	1,5,8,10,1<br>1,13,14,15	2,3,4,6,7,9<br>,12,16,17	3	P09605;P25809,P14480,P19223
10520	1,5,8,10,1<br>1,13,14,16	2,3,4,6,7,9<br>,12,15,17	5	P09605;P25809,P19223,P20761,P98089,Q7TPB1

10521	1,5,8,10,11,13,14,17	2,3,4,6,7,9,12,15,16	1	P01836
10522	1,5,8,10,11,13,15,16	2,3,4,6,7,9,12,14,17	5	P01835,P09605;P25809,P19223,P20761,P98089
10523	1,5,8,10,11,13,15,17	2,3,4,6,7,9,12,14,16	2	P04073,Q63532
10524	1,5,8,10,11,13,16,17	2,3,4,6,7,9,12,14,15	3	A2RUV9,P02625,P02631
10525	1,5,8,10,11,14,15,16	2,3,4,6,7,9,12,13,17	5	P09605;P25809,P19223,P20761,P55091,Q62635
10526	1,5,8,10,11,14,15,17	2,3,4,6,7,9,12,13,16	2	G3V686,P01836
10527	1,5,8,10,11,14,16,17	2,3,4,6,7,9,12,13,15	2	P01836,P02625
10528	1,5,8,10,11,15,16,17	2,3,4,6,7,9,12,13,14	3	A2RUV9,P05369,P25236
10529	1,5,8,10,12,13,14,15	2,3,4,6,7,9,11,16,17	2	P09605;P25809,Q498D9
10530	1,5,8,10,12,13,14,16	2,3,4,6,7,9,11,15,17	2	P09605;P25809,P20761
10531	1,5,8,10,12,13,14,17	2,3,4,6,7,9,11,15,16	0	
10532	1,5,8,10,12,13,15,16	2,3,4,6,7,9,11,14,17	3	P09605;P25809,P16086,P83121
10533	1,5,8,10,12,13,15,17	2,3,4,6,7,9,11,14,16	5	P16086,P83121,Q63532,Q6IFV1,Q99MH3
10534	1,5,8,10,12,13,16,17	2,3,4,6,7,9,11,14,15	4	P02625,P02631,P35280,P83121
10535	1,5,8,10,12,14,15,16	2,3,4,6,7,9,11,13,17	1	P09605;P25809
10536	1,5,8,10,12,14,15,17	2,3,4,6,7,9,11,13,16	0	
10537	1,5,8,10,12,14,16,17	2,3,4,6,7,9,11,13,15	1	P02625
10538	1,5,8,10,12,15,16,17	2,3,4,6,7,9,11,13,14	2	P16086,P83121
10539	1,5,8,10,13,14,15,16	2,3,4,6,7,9,11,12,17	5	P01835,P09605;P25809,P19223,P20761,Q498R7
10540	1,5,8,10,13,14,15,17	2,3,4,6,7,9,11,12,16	2	P01836,Q498R7
10541	1,5,8,10,13,14,16,17	2,3,4,6,7,9,11,12,15	3	P01836,P02625,Q498R7
10542	1,5,8,10,13,15,16,17	2,3,4,6,7,9,11,12,14	4	P01835,P83121,Q498R7,Q9EQS0
10543	1,5,8,10,14,15,16,17	2,3,4,6,7,9,11,12,13	2	P01836,Q498R7

10544	1,5,8,11,12,13,14,15	2,3,4,6,7,9,10,16,17	2	P09605;P25809,Q6P6R2
10545	1,5,8,11,12,13,14,16	2,3,4,6,7,9,10,15,17	2	P09605;P25809,P20761
10546	1,5,8,11,12,13,14,17	2,3,4,6,7,9,10,15,16	0	
10547	1,5,8,11,12,13,15,16	2,3,4,6,7,9,10,14,17	1	P09605;P25809
10548	1,5,8,11,12,13,15,17	2,3,4,6,7,9,10,14,16	1	Q63532
10549	1,5,8,11,12,13,16,17	2,3,4,6,7,9,10,14,15	1	P35280
10550	1,5,8,11,12,14,15,16	2,3,4,6,7,9,10,13,17	1	P09605;P25809
10551	1,5,8,11,12,14,15,17	2,3,4,6,7,9,10,13,16	1	P47967
10552	1,5,8,11,12,14,16,17	2,3,4,6,7,9,10,13,15	1	Q4FZU6
10553	1,5,8,11,12,15,16,17	2,3,4,6,7,9,10,13,14	2	P05369,P12020
10554	1,5,8,11,13,14,15,16	2,3,4,6,7,9,10,12,17	5	P02696,P09605;P25809,P19223,P20761,Q6P6R2
10555	1,5,8,11,13,14,15,17	2,3,4,6,7,9,10,12,16	2	P47967,P97840
10556	1,5,8,11,13,14,16,17	2,3,4,6,7,9,10,12,15	0	
10557	1,5,8,11,13,15,16,17	2,3,4,6,7,9,10,12,14	1	Q9EQS0
10558	1,5,8,11,14,15,16,17	2,3,4,6,7,9,10,12,13	3	P04218,P47967,P97840
10559	1,5,8,12,13,14,15,16	2,3,4,6,7,9,10,11,17	1	P09605;P25809
10560	1,5,8,12,13,14,15,17	2,3,4,6,7,9,10,11,16	2	P47967,Q5FVI6
10561	1,5,8,12,13,14,16,17	2,3,4,6,7,9,10,11,15	3	P35280,Q4FZU6,Q5FVI6
10562	1,5,8,12,13,15,16,17	2,3,4,6,7,9,10,11,14	3	P35280,P83121,Q5XFX0
10563	1,5,8,12,14,15,16,17	2,3,4,6,7,9,10,11,13	1	P14173
10564	1,5,8,13,14,15,16,17	2,3,4,6,7,9,10,11,12	7	P08753,P11883,P14173,P47967,Q498R7,Q5FVI6,Q9EQS0
10565	1,5,9,10,11,12,13,14	2,3,4,6,7,8,15,16,17	7	P02631,P02803,P09605;P25809,P16086,P20761,P50115,Q4G075
10566	1,5,9,10,11,12,13,15	2,3,4,6,7,8,14,16,17	7	P02631,P09605;P25809,P16086,P20761,P50115,Q6P6R2,Q9Z0V6

10567	1,5,9,10,11,12,13,16	2,3,4,6,7,8,14,15,17	12	B0BNN3,P02631,P02761,P02803,P10536,P12368,P16086,P20761,P20762,P50115,P62815,Q9Z0V6
10568	1,5,9,10,11,12,13,17	2,3,4,6,7,8,14,15,16	6	P02631,P02803,P16086,P20761,P20762,Q63532
10569	1,5,9,10,11,12,14,15	2,3,4,6,7,8,13,16,17	7	P09605;P25809,P16086,P20761,P20766,P50115,P51907,Q9R0T3
10570	1,5,9,10,11,12,14,16	2,3,4,6,7,8,13,15,17	10	P02631,P02761,P09605;P25809,P11598,P16086,P20761,P20766,P50115,P62815,Q9WUW8
10571	1,5,9,10,11,12,14,17	2,3,4,6,7,8,13,15,16	2	P02631,P16086
10572	1,5,9,10,11,12,15,16	2,3,4,6,7,8,13,14,17	14	P06761,P06911,P11598,P12020,P16086,P20761,P20762,P20766,P22282,P29975,P50115,P51907,P53790,P62815
10573	1,5,9,10,11,12,15,17	2,3,4,6,7,8,13,14,16	7	P06911,P12020,P16086,P20766,P25236,Q566E6,Q9R0T3
10574	1,5,9,10,11,12,16,17	2,3,4,6,7,8,13,14,15	12	O08557,P02631,P06757,P06911,P12020,P16086,P20761,P20762,P20766,P25236,Q5GRG2,Q6TMA8
10575	1,5,9,10,11,13,14,15	2,3,4,6,7,8,12,16,17	17	P01041,P09605;P25809,P19223,P20761,P50115,P55091,P63029,P98089,Q4G075,Q62635,Q63751,Q6P6R2,Q80WL1,Q99041,Q9EQS0,Q9R0T3,Q9Z0V6
10576	1,5,9,10,11,13,14,16	2,3,4,6,7,8,12,15,17	20	B0BNN3,D3ZHA0,O55004,P01041,P02631,P02761,P09605;P25809,P19223,P20761,P20762,P50115,P55091,P62815,P98089,Q4G075,Q62635,Q63751,Q7TPB1,Q99041,Q9Z0V6
10577	1,5,9,10,11,13,14,17	2,3,4,6,7,8,12,15,16	6	D3ZHA0,P01041,P01836,P02631,P20761,Q9EQS0
10578	1,5,9,10,11,13,15,16	2,3,4,6,7,8,12,14,17	26	O88267,P01041,P02631,P06761,P09605;P25809,P11598,P16086,P19223,P20761,P20762,P22282,P31430,P50115,P55091,P62815,P98089,Q4G075,Q62635,Q63751,Q80WL1,Q923M1,Q99041,Q9EQS0,Q9QYU4,Q9R0T3,Q9Z0V6
10579	1,5,9,10,11,13,15,17	2,3,4,6,7,8,12,14,16	10	D3ZHA0,P02631,P04073,P16086,P20762,Q63532,Q80WL1,Q920P0,Q9EQS0,Q9R0T3
10580	1,5,9,10,11,13,16,17	2,3,4,6,7,8,12,14,15	14	A2RUV9,B0BNN3,D3ZHA0,O08557,P01041,P02631,P16086,P20761,P20762,P35280,Q64611,Q920P0,Q923M1,Q9EQS0
10581	1,5,9,10,11,14,15,16	2,3,4,6,7,8,12,13,17	29	O88267,P02780,P02782,P06761,P07647,P09605;P25809,P11598,P16086,P19223,P20761,P20766,P22282,P22283,P29975,P50115,P55091,P62815,P70545,P98089,Q4G075,Q62635,Q62902,Q63751,Q6AYR9,Q80WL1,Q99041,Q9EQS0,Q9QZK9,Q9R0T3
10582	1,5,9,10,11,14,15,17	2,3,4,6,7,8,12,13,16	10	P01836,P06761,P07647,P16086,Q62902,Q6TMA8,Q80WL1,Q9EQS0,Q9QUL6,Q9R0T3
10583	1,5,9,10,11,14,16,17	2,3,4,6,7,8,12,13,15	16	D3ZHA0,O08557,P01836,P02625,P02631,P06761,P07647,P11598,P16086,P20761,P20762,P25236,P62815,Q6TMA8,Q9EQS0,Q9QUL6
10584	1,5,9,10,11,15,16,17	2,3,4,6,7,8,12,13,14	28	A2RUV9,D3ZHA0,O08557,P02780,P02782,P06761,P06911,P07647,P11598,P12020,P16086,P20761,P20762,P20766,P22282,P22283,P25236,P29975,P30120,P62815,Q5GRG2,Q62902,Q6AYR9,Q6TMA8,Q80WL1,Q923M1,Q9EQS0,Q9R0T3
10585	1,5,9,10,12,13,14,15	2,3,4,6,7,8,11,16,17	11	P07150,P09605;P25809,P14669,P16086,P20761,P50115,Q4G075,Q63751,Q6P6R2,Q99041,Q9R0T3
10586	1,5,9,10,12,13,14,16	2,3,4,6,7,8,11,15,17	13	B0BNN3,O55004,P02631,P02761,P07150,P09605;P25809,P14669,P16086,P20761,P20762,P50115,Q4G075,Q63751
10587	1,5,9,10,12,13,14,17	2,3,4,6,7,8,11,15,16	6	P02631,P07150,P14668,P14669,P16086,P35280
10588	1,5,9,10,12,13,15,16	2,3,4,6,7,8,11,14,17	14	P02631,P02761,P07150,P09605;P25809,P14669,P16086,P19218,P20761,P20762,P50115,Q4G075,Q63751,Q9EQS0,Q9Z0V6
10589	1,5,9,10,12,13,15,17	2,3,4,6,7,8,11,14,16	8	P02631,P07150,P14668,P14669,P16086,P20762,Q63532,Q9EQS0

10590	1,5,9,10,12,13,16,17	2,3,4,6,7,8,11,14,15	12	P02625,P02631,P02761,P07171,P14669,P16086,P19218,P20761,P20762,P35280,Q6TMA8,Q9EQS0
10591	1,5,9,10,12,14,15,16	2,3,4,6,7,8,11,13,17	16	P07150,P09605;P25809,P11598,P14669,P16086,P20761,P20762,P20766,P22282,P22283,P50115,Q4G075,Q5XI22,Q63751,Q99041,Q9R0T3
10592	1,5,9,10,12,14,15,17	2,3,4,6,7,8,11,13,16	6	P07150,P16086,P20766,Q5XI22,Q6TMA8,Q9R0T3
10593	1,5,9,10,12,14,16,17	2,3,4,6,7,8,11,13,15	10	P02625,P02631,P14669,P16086,P20761,P20762,P20766,P35280,Q5XI22,Q6TMA8
10594	1,5,9,10,12,15,16,17	2,3,4,6,7,8,11,13,14	14	P02631,P06761,P06911,P12020,P14669,P16086,P20762,P20766,P22282,P25236,Q5GRG2,Q62902,Q6TMA8,Q9EQS0
10595	1,5,9,10,13,14,15,16	2,3,4,6,7,8,11,12,17	30	O55004,P02631,P02761,P02780,P07150,P07647,P09605;P25809,P11883,P14669,P16086,P19223,P20761,P20762,P22282,P22283,P50115,P50280,P55091,P98089,Q00715,Q4G075,Q5XI22,Q62635,Q63751,Q80WL1,Q99041,Q9EQS0,Q9QZK9,Q9R0T3,Q9Z0V6
10596	1,5,9,10,13,14,15,17	2,3,4,6,7,8,11,12,16	12	O55004,P01836,P02631,P07150,P11883,P14668,P14669,P16086,Q80WL1,Q99041,Q9EQS0,Q9R0T3
10597	1,5,9,10,13,14,16,17	2,3,4,6,7,8,11,12,15	16	D3ZHA0,O55004,P01836,P02625,P02631,P02761,P14669,P16086,P20761,P20762,P35280,Q498R7,Q5XI22,Q6TMA8,Q99041,Q9EQS0
10598	1,5,9,10,13,15,16,17	2,3,4,6,7,8,11,12,14	23	D3ZHA0,P02631,P02780,P02782,P06761,P07647,P0C0A9,P11883,P14669,P16086,P20761,P20762,P22282,P22283,P24368,P30120,P35280,P62804,Q00715,Q6TMA8,Q80WL1,Q9EQS0,Q9R0T3
10599	1,5,9,10,14,15,16,17	2,3,4,6,7,8,11,12,13	28	P01836,P02780,P02781,P02782,P06761,P07647,P0C0A9,P11598,P11883,P14669,P16086,P20762,P20766,P22282,P22283,P24368,P30120,P50280,Q498R7,Q5XI22,Q62902,Q6AYR9,Q6TMA8,Q80WL1,Q99041,Q9EQS0,Q9JHB9,Q9R0T3
10600	1,5,9,11,12,13,14,15	2,3,4,6,7,8,10,16,17	8	D3ZHA0,P09605;P25809,P16086,P20761,P50115,Q4G075,Q6P6R2,Q9R0T3
10601	1,5,9,11,12,13,14,16	2,3,4,6,7,8,10,15,17	10	B0BNN3,D3ZHA0,P02631,P02761,P19223,P20761,P50115,Q4G075,Q6P6R2,Q9Z0V6
10602	1,5,9,11,12,13,14,17	2,3,4,6,7,8,10,15,16	2	D3ZHA0,P35280
10603	1,5,9,11,12,13,15,16	2,3,4,6,7,8,10,14,17	11	D3ZHA0,P02761,P12020,P16086,P20761,P20762,P50115,Q4G075,Q6P6R2,Q9EQS0,Q9Z0V6
10604	1,5,9,11,12,13,15,17	2,3,4,6,7,8,10,14,16	5	D3ZHA0,P12020,P16086,Q63532,Q9EQS0
10605	1,5,9,11,12,13,16,17	2,3,4,6,7,8,10,14,15	10	D3ZHA0,P02631,P02761,P02803,P16086,P20761,P20762,P35280,P97615,Q9EQS0
10606	1,5,9,11,12,14,15,16	2,3,4,6,7,8,10,13,17	11	D3ZHA0,P11598,P12020,P16086,P20761,P20766,P22282,P50115,Q4G075,Q6P6R2,Q9R0T3
10607	1,5,9,11,12,14,15,17	2,3,4,6,7,8,10,13,16	4	D3ZHA0,P12020,P16086,Q9R0T3
10608	1,5,9,11,12,14,16,17	2,3,4,6,7,8,10,13,15	6	D3ZHA0,P12020,P16086,P20761,P20766,P35280
10609	1,5,9,11,12,15,16,17	2,3,4,6,7,8,10,13,14	11	D3ZHA0,P05369,P06911,P12020,P16086,P20762,P20766,P25236,Q5GRG2,Q6TMA8,Q9EQS0
10610	1,5,9,11,13,14,15,16	2,3,4,6,7,8,10,12,17	22	D3ZHA0,D4A5U3,O55004,P01041,P02696,P02761,P09605;P25809,P19223,P20761,P50115,P55091,P98089,Q4G075,Q62635,Q63751,Q6P6R2,Q80WL1,Q99041,Q9EQS0,Q9QZK9,Q9R0T3,Q9Z0V6
10611	1,5,9,11,13,14,15,17	2,3,4,6,7,8,10,12,16	8	D3ZHA0,P04218,P47967,Q6P6R2,Q80WL1,Q9EQS0,Q9JJS8,Q9R0T3
10612	1,5,9,11,13,14,16,17	2,3,4,6,7,8,10,12,15	10	D3ZHA0,P01041,P02631,P02761,P04218,P20761,P20762,P35280,Q80WL1,Q9EQS0

10613	1,5,9,11,13,15,16,17	2,3,4,6,7,8,10,12,14	10	D3ZHA0,P04218,P06761,P12020,P16086,P20762,P35280,Q80WL1,Q9EQS0,Q9R0T3
10614	1,5,9,11,14,15,16,17	2,3,4,6,7,8,10,12,13	15	D3ZHA0,P02780,P04218,P06761,P07647,P11598,P12020,P16086,P22282,P22283,Q6AYR9,Q6TMA8,Q80WL1,Q9EQS0,Q9R0T3
10615	1,5,9,12,13,14,15,16	2,3,4,6,7,8,10,11,17	17	O55004,P00714,P02761,P07150,P09605;P25809,P11883,P14669,P16086,P19223,P20761,P20762,P50115,Q4G075,Q63751,Q6P6R2,Q9EQS0,Q9R0T3
10616	1,5,9,12,13,14,15,17	2,3,4,6,7,8,10,11,16	13	D3ZHA0,P00714,P07150,P11883,P14668,P14669,P16086,P35280,P82471,Q5BJY9,Q5FVI6,Q9EQS0,Q9R0T3
10617	1,5,9,12,13,14,16,17	2,3,4,6,7,8,10,11,15	14	D3ZHA0,P00714,P02631,P02761,P07150,P07171,P14669,P16086,P20762,P35280,Q5BJY9,Q5FVI6,Q5XI22,Q9EQS0
10618	1,5,9,12,13,15,16,17	2,3,4,6,7,8,10,11,14	13	D3ZHA0,P00714,P02631,P07150,P11883,P12020,P14669,P16086,P19218,P20762,P35280,Q5BJY9,Q9EQS0
10619	1,5,9,12,14,15,16,17	2,3,4,6,7,8,10,11,13	12	D3ZHA0,P07150,P11883,P12020,P14669,P16086,P20766,P35280,Q5XI22,Q6TMA8,Q9EQS0,Q9R0T3
10620	1,5,9,13,14,15,16,17	2,3,4,6,7,8,10,11,12	22	D3ZHA0,O55004,P00714,P02780,P05964,P07150,P07647,P0C0A9,P11883,P14669,P16086,P20762,P22283,P24368,P35280,P47967,P62804,Q5XI22,Q80WL1,Q99041,Q9EQS0,Q9R0T3
10621	1,5,10,11,12,13,14,15	2,3,4,6,7,8,9,16,17	7	P09605;P25809,P16086,P19223,P20761,P50115,Q6P6R2,Q9R0T3
10622	1,5,10,11,12,13,14,16	2,3,4,6,7,8,9,15,17	8	P02631,P02761,P09605;P25809,P19223,P20761,P50115,Q5QE79,Q7TPB1
10623	1,5,10,11,12,13,14,17	2,3,4,6,7,8,9,15,16	3	D3ZHA0,P02631,Q5QE79
10624	1,5,10,11,12,13,15,16	2,3,4,6,7,8,9,14,17	10	P02631,P09605;P25809,P16086,P19223,P20761,P20762,P50115,Q6P6R2,Q9EQS0,Q9Z0V6
10625	1,5,10,11,12,13,15,17	2,3,4,6,7,8,9,14,16	3	P02631,P16086,Q9EQS0
10626	1,5,10,11,12,13,16,17	2,3,4,6,7,8,9,14,15	9	D3ZHA0,P02631,P16086,P20761,P20762,P35280,Q5QE79,Q6TMA8,Q9EQS0
10627	1,5,10,11,12,14,15,16	2,3,4,6,7,8,9,13,17	9	P09605;P25809,P16086,P19223,P20761,P20766,P22282,P50115,P51907,Q9R0T3
10628	1,5,10,11,12,14,15,17	2,3,4,6,7,8,9,13,16	4	P16086,P20766,Q6TMA8,Q9R0T3
10629	1,5,10,11,12,14,16,17	2,3,4,6,7,8,9,13,15	6	D3ZHA0,P02631,P16086,P20766,Q5QE79,Q6TMA8
10630	1,5,10,11,12,15,16,17	2,3,4,6,7,8,9,13,14	11	P02631,P05369,P06866,P06911,P12020,P16086,P20762,P20766,P25236,Q6TMA8,Q9EQS0

10631	1,5,10,11,13,14,15,16	2,3,4,6,7,8,9,12,17	19	D3ZHA0,O55004,P09605;P25809,P19223,P20761,P22282,P50115,P55091,P98089,Q4G075,Q62635,Q63751,Q6P6R2,Q7TPB1,Q80WL1,Q99041,Q9EQS0,Q9R0T3,Q9Z0V6
10632	1,5,10,11,13,14,15,17	2,3,4,6,7,8,9,12,16	4	D3ZHA0,Q80WL1,Q9EQS0,Q9R0T3
10633	1,5,10,11,13,14,16,17	2,3,4,6,7,8,9,12,15	8	D3ZHA0,O55004,P02631,P20761,P20762,Q5QE79,Q6TMA8,Q9EQS0
10634	1,5,10,11,13,15,16,17	2,3,4,6,7,8,9,12,14	8	D3ZHA0,P02631,P06761,P16086,P20762,Q6TMA8,Q80WL1,Q9EQS0
10635	1,5,10,11,14,15,16,17	2,3,4,6,7,8,9,12,13	14	D3ZHA0,P02780,P02782,P06761,P07647,P16086,P20766,P22282,P22283,Q62902,Q6TMA8,Q80WL1,Q9EQS0,Q9R0T3
10636	1,5,10,12,13,14,15,16	2,3,4,6,7,8,9,11,17	14	O55004,P02631,P02761,P07150,P09605;P25809,P14669,P16086,P19223,P20761,P20762,P50115,Q63751,Q6P6R2,Q9EQS0
10637	1,5,10,12,13,14,15,17	2,3,4,6,7,8,9,11,16	5	P02631,P14668,P16086,Q9EQS0,Q9R0T3
10638	1,5,10,12,13,14,16,17	2,3,4,6,7,8,9,11,15	13	D3ZHA0,O55004,P02625,P02631,P02761,P14669,P16086,P20762,P35280,Q5QE79,Q5XI22,Q6TMA8,Q9EQS0
10639	1,5,10,12,13,15,16,17	2,3,4,6,7,8,9,11,14	10	P02631,P14669,P16086,P19218,P20762,P35280,P83121,Q63617,Q6TMA8,Q9EQS0
10640	1,5,10,12,14,15,16,17	2,3,4,6,7,8,9,11,13	9	P02631,P14669,P16086,P20762,P20766,Q5XI22,Q6TMA8,Q9EQS0,Q9R0T3
10641	1,5,10,13,14,15,16,17	2,3,4,6,7,8,9,11,12	16	D3ZHA0,O55004,P02631,P02780,P07647,P0C0A9,P11883,P14669,P16086,P20762,Q498R7,Q5XI22,Q6TMA8,Q80WL1,Q9EQS0,Q9R0T3
10642	1,5,11,12,13,14,15,16	2,3,4,6,7,8,9,10,17	8	D3ZHA0,P02761,P09605;P25809,P16086,P19223,P20761,P50115,Q6P6R2
10643	1,5,11,12,13,14,15,17	2,3,4,6,7,8,9,10,16	4	D3ZHA0,P16086,Q6P6R2,Q9R0T3
10644	1,5,11,12,13,14,16,17	2,3,4,6,7,8,9,10,15	6	D3ZHA0,O55006,P02631,P02761,P35280,Q5QE79
10645	1,5,11,12,13,15,16,17	2,3,4,6,7,8,9,10,14	7	D3ZHA0,P02631,P12020,P16086,P20762,P35280,Q9EQS0

10646	1,5,11,12,14,15,16,17	2,3,4,6,7,8,9,10,13	6	D3ZHA0,P12020,P16086,P20766,Q6TMA8,Q9R0T3
10647	1,5,11,13,14,15,16,17	2,3,4,6,7,8,9,10,12	6	D3ZHA0,P19223,Q6P6R2,Q80WL1,Q9EQS0,Q9R0T3
10648	1,5,12,13,14,15,16,17	2,3,4,6,7,8,9,10,11	10	D3ZHA0,P00714,P02631,P11883,P14669,P16086,P35280,Q5XI22,Q6TMA8,Q9EQS0
10649	1,6,7,8,9,10,11,12	2,3,4,5,13,14,15,16,17	20	F1LM93,P02781,P02782,P08723,P09456,P0C0A9,P10715,P13432,P19629,P22283,P30120,P47727,P60905,P97840,Q63357,Q63493,Q6AYL6,Q8CJ52,Q9JHB9,Q9JI85
10650	1,6,7,8,9,10,11,13	2,3,4,5,12,14,15,16,17	7	P12020,P22283,P31044,P47727,Q5GRG2,Q62902,Q9JI85
10651	1,6,7,8,9,10,11,14	2,3,4,5,12,13,15,16,17	6	P19629,P31044,P35280,P47727,P83121,Q6AYL6
10652	1,6,7,8,9,10,11,15	2,3,4,5,12,13,14,16,17	4	P22006,P31044,P35280,Q566E6
10653	1,6,7,8,9,10,11,16	2,3,4,5,12,13,14,15,17	4	A2RUV9,P31044,P63095,Q6AYL6
10654	1,6,7,8,9,10,11,17	2,3,4,5,12,13,14,15,16	1	Q9QUL6
10655	1,6,7,8,9,10,12,13	2,3,4,5,11,14,15,16,17	17	P02782,P06911,P08723,P09456,P12020,P19218,P22282,P22283,P30120,P36374,P63036,P97840,Q5GRG2,Q62902,Q6AYR9,Q8CJ52,Q9JHB9
10656	1,6,7,8,9,10,12,14	2,3,4,5,11,13,15,16,17	6	P02782,P08723,P12020,P19629,P30120,P97840
10657	1,6,7,8,9,10,12,15	2,3,4,5,11,13,14,16,17	4	P08723,P10715,P19629,P97840
10658	1,6,7,8,9,10,12,16	2,3,4,5,11,13,14,15,17	5	P08723,P10715,P19218,P47967,P97840
10659	1,6,7,8,9,10,12,17	2,3,4,5,11,13,14,15,16	8	F1LM93,P02782,P08723,P10715,P13432,P22283,P97840,Q9QZK9
10660	1,6,7,8,9,10,13,14	2,3,4,5,11,12,15,16,17	6	P06911,P12020,P19629,P31044,Q5GRG2,Q62902

10661	1,6,7,8,9,10,13,15	2,3,4,5,11,12,14,16,17	3	P31044,P34901,Q5GRG2
10662	1,6,7,8,9,10,13,16	2,3,4,5,11,12,14,15,17	5	P12020,P19218,P31044,P63036,P97840
10663	1,6,7,8,9,10,13,17	2,3,4,5,11,12,14,15,16	3	P12020,P22283,P51907
10664	1,6,7,8,9,10,14,15	2,3,4,5,11,12,13,16,17	3	P19629,P31044,P35280
10665	1,6,7,8,9,10,14,16	2,3,4,5,11,12,13,15,17	2	P19629,P31044
10666	1,6,7,8,9,10,14,17	2,3,4,5,11,12,13,15,16	2	P01836,P19629
10667	1,6,7,8,9,10,15,16	2,3,4,5,11,12,13,14,17	0	
10668	1,6,7,8,9,10,15,17	2,3,4,5,11,12,13,14,16	0	
10669	1,6,7,8,9,10,16,17	2,3,4,5,11,12,13,14,15	1	P25031
10670	1,6,7,8,9,11,12,13	2,3,4,5,10,14,15,16,17	22	P02780,P02781,P02782,P02803,P04905,P08723,P09456,P0C0A9,P10715,P13432,P19629,P22282,P22283,P30120,P36374,P47727,Q62902,Q62997,Q63493,Q8CJ52,Q9JHB9,Q9JI85
10671	1,6,7,8,9,11,12,14	2,3,4,5,10,13,15,16,17	18	P02781,P02782,P04905,P08723,P0C0A9,P10715,P13432,P19629,P22283,P30120,P47727,Q62902,Q62997,Q63493,Q6AYL6,Q8CJ52,Q9JHB9,Q9JI85
10672	1,6,7,8,9,11,12,15	2,3,4,5,10,13,14,16,17	11	P02782,P08723,P10715,P13432,P19629,P22006,P30120,Q62997,Q8CJ52,Q9JHB9,Q9JI85
10673	1,6,7,8,9,11,12,16	2,3,4,5,10,13,14,15,17	10	P02782,P08723,P10715,P13432,P19629,P30120,Q6AYL6,Q8CJ52,Q9JHB9,Q9JI85
10674	1,6,7,8,9,11,12,17	2,3,4,5,10,13,14,15,16	17	P02782,P08723,P10715,P13432,P19629,P22282,P22283,P30120,P36374,Q62997,Q63493,Q6AYL6,Q8CJ52,Q99068,Q9JHB9,Q9JI85,Q9QZK9
10675	1,6,7,8,9,11,13,14	2,3,4,5,10,12,15,16,17	9	P06911,P12020,P19629,P22283,P47727,P83121,Q5GRG2,Q62902,Q9JI85

10676	1,6,7,8,9,11,13,15	2,3,4,5,10,12,14,16,17	3	P19629,P34901,Q9JJS8
10677	1,6,7,8,9,11,13,16	2,3,4,5,10,12,14,15,17	1	Q62902
10678	1,6,7,8,9,11,13,17	2,3,4,5,10,12,14,15,16	5	P22282,P22283,P34901,Q62902,Q9JJS8
10679	1,6,7,8,9,11,14,15	2,3,4,5,10,12,13,16,17	3	P19629,P22006,P35280
10680	1,6,7,8,9,11,14,16	2,3,4,5,10,12,13,15,17	2	P19629,P83121
10681	1,6,7,8,9,11,14,17	2,3,4,5,10,12,13,15,16	2	P19629,P83121
10682	1,6,7,8,9,11,15,16	2,3,4,5,10,12,13,14,17	2	P10715,P19629
10683	1,6,7,8,9,11,15,17	2,3,4,5,10,12,13,14,16	3	P10715,P19629,Q9JJS8
10684	1,6,7,8,9,11,16,17	2,3,4,5,10,12,13,14,15	2	A2RUV9,P10715
10685	1,6,7,8,9,12,13,14	2,3,4,5,10,11,15,16,17	19	P02782,P06761,P06911,P08723,P09456,P11598,P12020,P13432,P19629,P22282,P22283,P30120,Q5GRG2,Q62902,Q6AYR9,Q8CJ52,Q9JHB9,Q9JI85,Q9Z1F2
10686	1,6,7,8,9,12,13,15	2,3,4,5,10,11,14,16,17	8	P02782,P08723,P10715,P13432,P19218,P19629,Q62902,Q9JHB9
10687	1,6,7,8,9,12,13,16	2,3,4,5,10,11,14,15,17	10	P02782,P08723,P10715,P13432,P19218,P30120,P63036,Q62902,Q8CJ52,Q9JHB9
10688	1,6,7,8,9,12,13,17	2,3,4,5,10,11,14,15,16	16	P02782,P08723,P10715,P11598,P13432,P19218,P19629,P22282,P22283,P30120,P36374,P51907,Q62902,Q8CJ52,Q9JHB9,Q9QZK9
10689	1,6,7,8,9,12,14,15	2,3,4,5,10,11,13,16,17	4	P08723,P10715,P13432,P19629
10690	1,6,7,8,9,12,14,16	2,3,4,5,10,11,13,15,17	5	P08723,P10715,P13432,P19629,Q8CJ52

10691	1,6,7,8,9,12,14,17	2,3,4,5,10,11,13,15,16	7	P02782,P08723,P10715,P13432,P19629,P22282,P22283
10692	1,6,7,8,9,12,15,16	2,3,4,5,10,11,13,14,17	3	P10715,P13432,P19629
10693	1,6,7,8,9,12,15,17	2,3,4,5,10,11,13,14,16	3	P10715,P13432,P19629
10694	1,6,7,8,9,12,16,17	2,3,4,5,10,11,13,14,15	3	P10715,P13432,P19629
10695	1,6,7,8,9,13,14,15	2,3,4,5,10,11,12,16,17	5	P06911,P12020,P19629,P53792,Q5GRG2
10696	1,6,7,8,9,13,14,16	2,3,4,5,10,11,12,15,17	5	P06911,P12020,P19629,Q5GRG2,Q62902
10697	1,6,7,8,9,13,14,17	2,3,4,5,10,11,12,15,16	10	P06911,P11598,P12020,P19629,P22282,P22283,P51907,P53792,Q5GRG2,Q62902
10698	1,6,7,8,9,13,15,16	2,3,4,5,10,11,12,14,17	1	P34901
10699	1,6,7,8,9,13,15,17	2,3,4,5,10,11,12,14,16	3	P34901,P53792,Q9JJS8
10700	1,6,7,8,9,13,16,17	2,3,4,5,10,11,12,14,15	2	P34901,P51907
10701	1,6,7,8,9,14,15,16	2,3,4,5,10,11,12,13,17	1	P19629
10702	1,6,7,8,9,14,15,17	2,3,4,5,10,11,12,13,16	2	P19629,P53792
10703	1,6,7,8,9,14,16,17	2,3,4,5,10,11,12,13,15	1	P19629
10704	1,6,7,8,9,15,16,17	2,3,4,5,10,11,12,13,14	1	P19629
10705	1,6,7,8,10,11,12,13	2,3,4,5,9,14,15,16,17	27	F1LM93,P02781,P02782,P04905,P06911,P08723,P09456,P0C0A9,P10715,P11598,P12020,P19629,P22282,P22283,P30120,P31044,P36374,P47727,P60905,Q5GRG2,Q62902,Q63357,Q63493,Q6AYR9,Q8CJ52,Q9JHB9,Q9JI85
10706	1,6,7,8,10,11,12,14	2,3,4,5,9,13,15,16,17	18	P02781,P02782,P04905,P08723,P0C0A9,P10715,P12020,P19629,P22283,P30120,P35280,P47727,Q63357,Q63493,Q6AYL6,Q8CJ52,Q9JHB9,Q9JI85

10707	1,6,7,8,10,11,12,15	2,3,4,5,9,13,14,16,17	15	F1LM93,P08010,P08723,P10715,P19629,P22006,P30120,P35280,P47727,Q63357,Q63493,Q6AYL6,Q8CJ52,Q9JHB9,Q9JI85
10708	1,6,7,8,10,11,12,16	2,3,4,5,9,13,14,15,17	14	F1LM93,P08723,P10715,P19629,P30120,P47727,P47967,P97840,Q63357,Q63493,Q6AYL6,Q8CJ52,Q9JHB9,Q9JI85
10709	1,6,7,8,10,11,12,17	2,3,4,5,9,13,14,15,16	23	F1LM93,O08815,P02781,P02782,P08723,P10715,P13432,P14669,P19629,P22282,P22283,P30120,P36374,P47727,P60905,Q4G075,Q63357,Q63493,Q6AYL6,Q8CJ52,Q9JHB9,Q9JI85,Q9QZK9
10710	1,6,7,8,10,11,13,14	2,3,4,5,9,12,15,16,17	10	P06911,P12020,P19629,P22283,P31044,P47727,Q5GRG2,Q62902,Q63493,Q9JI85
10711	1,6,7,8,10,11,13,15	2,3,4,5,9,12,14,16,17	7	P01835,P12020,P15999,P22006,P31044,P47727,Q5GRG2
10712	1,6,7,8,10,11,13,16	2,3,4,5,9,12,14,15,17	7	P01835,P12020,P31044,P47727,Q5GRG2,Q62902,Q8VD89
10713	1,6,7,8,10,11,13,17	2,3,4,5,9,12,14,15,16	7	P11598,P12020,P22282,P22283,P47727,Q5GRG2,Q63493
10714	1,6,7,8,10,11,14,15	2,3,4,5,9,12,13,16,17	5	P19629,P22006,P31044,P35280,P47727
10715	1,6,7,8,10,11,14,16	2,3,4,5,9,12,13,15,17	4	P19629,P31044,P47727,Q6AYL6
10716	1,6,7,8,10,11,14,17	2,3,4,5,9,12,13,15,16	4	P12020,P19629,P47727,Q63493
10717	1,6,7,8,10,11,15,16	2,3,4,5,9,12,13,14,17	2	P01835,P35280
10718	1,6,7,8,10,11,15,17	2,3,4,5,9,12,13,14,16	3	P14669,P19629,P22006
10719	1,6,7,8,10,11,16,17	2,3,4,5,9,12,13,14,15	1	A2RUV9
10720	1,6,7,8,10,12,13,14	2,3,4,5,9,11,15,16,17	17	P02782,P06761,P06911,P08723,P11598,P12020,P19629,P22282,P22283,P30120,P47727,Q498D9,Q5GRG2,Q62902,Q6AYR9,Q8CJ52,Q9JHB9
10721	1,6,7,8,10,12,13,15	2,3,4,5,9,11,14,16,17	17	P02782,P06911,P08723,P11598,P12020,P15999,P19218,P19629,P22283,P30120,P97840,Q5GRG2,Q62902,Q6IFV1,Q8CJ52,Q99MH3,Q9JHB9
10722	1,6,7,8,10,12,13,16	2,3,4,5,9,11,14,15,17	13	P02782,P06911,P08723,P12020,P19218,P22283,P30120,P47967,P97840,Q5GRG2,Q62902,Q8CJ52,Q9JHB9
10723	1,6,7,8,10,12,13,17	2,3,4,5,9,11,14,15,16	22	F1LM93,P02782,P06761,P06911,P08723,P11598,P12020,P13432,P19218,P22282,P22283,P30120,P36374,Q5GRG2,Q62635,Q62902,Q6AYR9,Q6IFV1,Q8CJ52,Q99MH3,Q9JHB9,Q9QZK9
10724	1,6,7,8,10,12,14,15	2,3,4,5,9,11,13,16,17	4	P08723,P12020,P19629,P35280
10725	1,6,7,8,10,12,14,16	2,3,4,5,9,11,13,15,17	5	P08723,P12020,P19629,P97840,Q8CJ52
10726	1,6,7,8,10,12,14,17	2,3,4,5,9,11,13,15,16	10	F1LM93,P02782,P08723,P11598,P12020,P13432,P19629,P22283,Q8CJ52,Q9QZK9
10727	1,6,7,8,10,12,15,16	2,3,4,5,9,11,13,14,17	3	P10715,P19629,P97840
10728	1,6,7,8,10,12,15,17	2,3,4,5,9,11,13,14,16	8	F1LM93,P08723,P10715,P13432,P19629,Q6IFV1,Q99MH3,Q9QZK9
10729	1,6,7,8,10,12,16,17	2,3,4,5,9,11,13,14,15	7	F1LM93,P08723,P10715,P13432,P97840,Q8CJ52,Q9QZK9

10730	1,6,7,8,10,13,14,15	2,3,4,5,9,11,12,16,17	6	P06911,P12020,P15999,P19629,P31044,Q5GRG2
10731	1,6,7,8,10,13,14,16	2,3,4,5,9,11,12,15,17	6	P06911,P12020,P19629,P31044,Q5GRG2,Q62902
10732	1,6,7,8,10,13,14,17	2,3,4,5,9,11,12,15,16	8	P06911,P11598,P12020,P19629,P22282,P22283,P51907,Q5GRG2
10733	1,6,7,8,10,13,15,16	2,3,4,5,9,11,12,14,17	3	P01835,P12020,Q5GRG2
10734	1,6,7,8,10,13,15,17	2,3,4,5,9,11,12,14,16	7	P11598,P12020,P15999,P34901,Q5GRG2,Q6IFV1,Q99MH3
10735	1,6,7,8,10,13,16,17	2,3,4,5,9,11,12,14,15	2	P12020,P51907
10736	1,6,7,8,10,14,15,16	2,3,4,5,9,11,12,13,17	1	P19629
10737	1,6,7,8,10,14,15,17	2,3,4,5,9,11,12,13,16	1	P19629
10738	1,6,7,8,10,14,16,17	2,3,4,5,9,11,12,13,15	1	P19629
10739	1,6,7,8,10,15,16,17	2,3,4,5,9,11,12,13,14	0	
10740	1,6,7,8,11,12,13,14	2,3,4,5,9,10,15,16,17	27	P02780,P02781,P02782,P04905,P06761,P06911,P08723,P09456,P0C0A9,P10715,P11598,P12020,P13432,P19629,P22282,P22283,P30120,P36374,P46462,P47727,Q5GRG2,Q62902,Q63493,Q6AYR9,Q8CJ52,Q9JHB9,Q9JI85
10741	1,6,7,8,11,12,13,15	2,3,4,5,9,10,14,16,17	17	P02781,P02782,P08723,P10715,P11598,P13432,P19629,P22006,P22282,P22283,P30120,P47727,Q5GRG2,Q62902,Q8CJ52,Q9JHB9,Q9JI85
10742	1,6,7,8,11,12,13,16	2,3,4,5,9,10,14,15,17	15	P02781,P02782,P08723,P10715,P13432,P19629,P22282,P22283,P30120,P47727,Q62902,Q63493,Q8CJ52,Q9JHB9,Q9JI85
10743	1,6,7,8,11,12,13,17	2,3,4,5,9,10,14,15,16	22	P02781,P02782,P06761,P08723,P10715,P11598,P13432,P19629,P22282,P22283,P30120,P36374,P47727,Q62635,Q62902,Q63493,Q6AYR9,Q8CJ52,Q99041,Q9JHB9,Q9JI85,Q9QZK9
10744	1,6,7,8,11,12,14,15	2,3,4,5,9,10,13,16,17	13	P02782,P08723,P10715,P13432,P19629,P22006,P30120,P35280,P47727,Q63493,Q8CJ52,Q9JHB9,Q9JI85
10745	1,6,7,8,11,12,14,16	2,3,4,5,9,10,13,15,17	13	P02782,P08723,P10715,P13432,P19629,P30120,P47727,Q62902,Q63493,Q6AYL6,Q8CJ52,Q9JHB9,Q9JI85
10746	1,6,7,8,11,12,14,17	2,3,4,5,9,10,13,15,16	15	P02782,P08723,P10715,P11598,P13432,P19629,P22282,P22283,P30120,P47727,Q63493,Q8CJ52,Q9JHB9,Q9JI85,Q9QZK9
10747	1,6,7,8,11,12,15,16	2,3,4,5,9,10,13,14,17	4	P10715,P19629,Q6AYL6,Q8CJ52
10748	1,6,7,8,11,12,15,17	2,3,4,5,9,10,13,14,16	12	P02782,P08723,P10715,P13432,P19629,P22006,P22283,Q63493,Q8CJ52,Q99041,Q9JI85,Q9QZK9
10749	1,6,7,8,11,12,16,17	2,3,4,5,9,10,13,14,15	11	P02782,P08723,P10715,P13432,P19629,P22283,Q63493,Q6AYL6,Q8CJ52,Q99041,Q9QZK9
10750	1,6,7,8,11,13,14,15	2,3,4,5,9,10,12,16,17	9	P06911,P12020,P15999,P19629,P22006,P47727,Q5GRG2,Q62902,Q9JJS8
10751	1,6,7,8,11,13,14,16	2,3,4,5,9,10,12,15,17	7	P06911,P12020,P19629,P47727,Q5GRG2,Q62902,Q8CJ52
10752	1,6,7,8,11,13,14,17	2,3,4,5,9,10,12,15,16	10	P06911,P11598,P12020,P19629,P22282,P22283,P47727,Q5GRG2,Q62902,Q63493

10753	1,6,7,8,11,13,15,16	2,3,4,5,9,10,12,14,17	5	P01835,P19629,P34901,Q5GRG2,Q8VD89
10754	1,6,7,8,11,13,15,17	2,3,4,5,9,10,12,14,16	4	P11598,P19629,P34901,Q9JJS8
10755	1,6,7,8,11,13,16,17	2,3,4,5,9,10,12,14,15	1	Q62902
10756	1,6,7,8,11,14,15,16	2,3,4,5,9,10,12,13,17	1	P19629
10757	1,6,7,8,11,14,15,17	2,3,4,5,9,10,12,13,16	3	P19629,P22006,Q9JJS8
10758	1,6,7,8,11,14,16,17	2,3,4,5,9,10,12,13,15	1	P19629
10759	1,6,7,8,11,15,16,17	2,3,4,5,9,10,12,13,14	2	P10715,P19629
10760	1,6,7,8,12,13,14,15	2,3,4,5,9,10,11,16,17	15	O08557,P02782,P06911,P08723,P11598,P12020,P15999,P19629,P22283,P30120,Q498D9,Q5GRG2,Q62902,Q8CJ52,Q9JHB9
10761	1,6,7,8,12,13,14,16	2,3,4,5,9,10,11,15,17	12	P02782,P06911,P08723,P12020,P13432,P19629,P22283,P30120,Q5GRG2,Q62902,Q8CJ52,Q9JHB9
10762	1,6,7,8,12,13,14,17	2,3,4,5,9,10,11,15,16	21	P02782,P06761,P06911,P08723,P11598,P12020,P13432,P19629,P22282,P22283,P30120,P36374,P51907,Q5GRG2,Q62635,Q62902,Q6AYR9,Q8CJ52,Q9JHB9,Q9QZK9,Q9Z1F2
10763	1,6,7,8,12,13,15,16	2,3,4,5,9,10,11,14,17	5	P10715,P19218,P19629,Q62902,Q8CJ52
10764	1,6,7,8,12,13,15,17	2,3,4,5,9,10,11,14,16	14	P02782,P08723,P10715,P11598,P13432,P15999,P19629,P22282,P22283,Q62635,Q62902,Q6IFV1,Q99MH3,Q9QZK9
10765	1,6,7,8,12,13,16,17	2,3,4,5,9,10,11,14,15	13	P02782,P08723,P10715,P11598,P13432,P19218,P19629,P22282,P22283,P63029,Q62902,Q8CJ52,Q9QZK9
10766	1,6,7,8,12,14,15,16	2,3,4,5,9,10,11,13,17	1	P19629
10767	1,6,7,8,12,14,15,17	2,3,4,5,9,10,11,13,16	4	P08723,P11598,P13432,P19629
10768	1,6,7,8,12,14,16,17	2,3,4,5,9,10,11,13,15	5	P08723,P10715,P13432,P19629,Q8CJ52
10769	1,6,7,8,12,15,16,17	2,3,4,5,9,10,11,13,14	4	P10715,P13432,P19629,Q5XFX0
10770	1,6,7,8,13,14,15,16	2,3,4,5,9,10,11,12,17	4	P06911,P12020,P19629,Q5GRG2
10771	1,6,7,8,13,14,15,17	2,3,4,5,9,10,11,12,16	8	P06911,P11598,P12020,P15999,P19629,P53792,Q5GRG2,Q9WUW8
10772	1,6,7,8,13,14,16,17	2,3,4,5,9,10,11,12,15	6	P06911,P12020,P19629,P51907,Q5GRG2,Q62902
10773	1,6,7,8,13,15,16,17	2,3,4,5,9,10,11,12,14	1	P34901
10774	1,6,7,8,14,15,16,17	2,3,4,5,9,10,11,12,13	1	P19629
10775	1,6,7,9,10,11,12,13	2,3,4,5,8,14,15,16,17	5	P09456,P36374,P47727,P97840,Q9JHB9

10776	1,6,7,9,10,<br>11,12,14	2,3,4,5,8,1<br>3,15,16,17	4	P47727,P83121,P97840,Q6AYL6
10777	1,6,7,9,10,<br>11,12,15	2,3,4,5,8,1<br>3,14,16,17	3	P97840,Q566E6,Q6PCU2
10778	1,6,7,9,10,<br>11,12,16	2,3,4,5,8,1<br>3,14,15,17	8	P06757,P10536,P12368,P20766,P47967,P97840,Q6AYL6,Q6IFU7
10779	1,6,7,9,10,<br>11,12,17	2,3,4,5,8,1<br>3,14,15,16	6	O08815,P06757,P13432,P97840,Q566E6,Q9QZK9
10780	1,6,7,9,10,<br>11,13,14	2,3,4,5,8,1<br>2,15,16,17	3	P12020,P47727,P83121
10781	1,6,7,9,10,<br>11,13,15	2,3,4,5,8,1<br>2,14,16,17	2	P31430,Q9JJS8
10782	1,6,7,9,10,<br>11,13,16	2,3,4,5,8,1<br>2,14,15,17	3	B0BNN3,P10536,P97840
10783	1,6,7,9,10,<br>11,13,17	2,3,4,5,8,1<br>2,14,15,16	0	
10784	1,6,7,9,10,<br>11,14,15	2,3,4,5,8,1<br>2,13,16,17	4	P22006,P35280,P83121,Q80WL1
10785	1,6,7,9,10,<br>11,14,16	2,3,4,5,8,1<br>2,13,15,17	1	P83121
10786	1,6,7,9,10,<br>11,14,17	2,3,4,5,8,1<br>2,13,15,16	2	P83121,Q9QUL6
10787	1,6,7,9,10,<br>11,15,16	2,3,4,5,8,1<br>2,13,14,17	3	P31430,P36970,Q80WL1
10788	1,6,7,9,10,<br>11,15,17	2,3,4,5,8,1<br>2,13,14,16	3	P36970,Q566E6,Q9QUL6
10789	1,6,7,9,10,<br>11,16,17	2,3,4,5,8,1<br>2,13,14,15	4	P35053,P36970,Q64093,Q9QUL6
10790	1,6,7,9,10,<br>12,13,14	2,3,4,5,8,1<br>1,15,16,17	4	P06911,P12020,P97840,Q6AYS4
10791	1,6,7,9,10,<br>12,13,15	2,3,4,5,8,1<br>1,14,16,17	3	P19218,P97840,Q6AYS4
10792	1,6,7,9,10,<br>12,13,16	2,3,4,5,8,1<br>1,14,15,17	5	P02761,P19218,P47967,P97840,Q6AYS4
10793	1,6,7,9,10,<br>12,13,17	2,3,4,5,8,1<br>1,14,15,16	8	P13432,P14668,P19218,P36374,P97840,Q64194,Q99MH3,Q9QZK9
10794	1,6,7,9,10,<br>12,14,15	2,3,4,5,8,1<br>1,13,16,17	3	P20766,P97840,Q6AYS4
10795	1,6,7,9,10,<br>12,14,16	2,3,4,5,8,1<br>1,13,15,17	3	P20766,P97840,Q6AYS4
10796	1,6,7,9,10,<br>12,14,17	2,3,4,5,8,1<br>1,13,15,16	3	P13432,P14668,P97840
10797	1,6,7,9,10,<br>12,15,16	2,3,4,5,8,1<br>1,13,14,17	4	P19218,P20766,P97840,Q6AYS4
10798	1,6,7,9,10,<br>12,15,17	2,3,4,5,8,1<br>1,13,14,16	4	P13432,P14668,P97840,Q6AYS4

10799	1,6,7,9,10,<br>12,16,17	2,3,4,5,8,1<br>1,13,14,15	4	P13432,P19218,P97840,Q6AYS4
10800	1,6,7,9,10,<br>13,14,15	2,3,4,5,8,1<br>1,12,16,17	4	P12020,Q5GRG2,Q6AYS4,Q80WL1
10801	1,6,7,9,10,<br>13,14,16	2,3,4,5,8,1<br>1,12,15,17	5	B0BNN3,P12020,P35053,P97840,Q6AYS4
10802	1,6,7,9,10,<br>13,14,17	2,3,4,5,8,1<br>1,12,15,16	2	P12020,P14668
10803	1,6,7,9,10,<br>13,15,16	2,3,4,5,8,1<br>1,12,14,17	5	P19218,P62804,P97840,Q6AYS4,Q80WL1
10804	1,6,7,9,10,<br>13,15,17	2,3,4,5,8,1<br>1,12,14,16	2	P14668,Q6AYS4
10805	1,6,7,9,10,<br>13,16,17	2,3,4,5,8,1<br>1,12,14,15	5	P19218,P35053,P97840,Q64194,Q6AYS4
10806	1,6,7,9,10,<br>14,15,16	2,3,4,5,8,1<br>1,12,13,17	3	P16636,Q6AYS4,Q80WL1
10807	1,6,7,9,10,<br>14,15,17	2,3,4,5,8,1<br>1,12,13,16	1	Q6AYS4
10808	1,6,7,9,10,<br>14,16,17	2,3,4,5,8,1<br>1,12,13,15	3	P35053,Q6AYS4,Q6TMA8
10809	1,6,7,9,10,<br>15,16,17	2,3,4,5,8,1<br>1,12,13,14	4	P35053,Q6AYS4,Q6TMA8,Q80WL1
10810	1,6,7,9,11,<br>12,13,14	2,3,4,5,8,1<br>0,15,16,17	7	P09456,P30120,P47727,P83121,Q62902,Q62997,Q9JHB9
10811	1,6,7,9,11,<br>12,13,15	2,3,4,5,8,1<br>0,14,16,17	2	Q62997,Q9JHB9
10812	1,6,7,9,11,<br>12,13,16	2,3,4,5,8,1<br>0,14,15,17	4	P02761,P12368,Q62902,Q9JHB9
10813	1,6,7,9,11,<br>12,13,17	2,3,4,5,8,1<br>0,14,15,16	4	P13432,P36374,Q9JHB9,Q9QZK9
10814	1,6,7,9,11,<br>12,14,15	2,3,4,5,8,1<br>0,13,16,17	1	Q62997
10815	1,6,7,9,11,<br>12,14,16	2,3,4,5,8,1<br>0,13,15,17	1	Q6AYL6
10816	1,6,7,9,11,<br>12,14,17	2,3,4,5,8,1<br>0,13,15,16	2	P13432,Q62997
10817	1,6,7,9,11,<br>12,15,16	2,3,4,5,8,1<br>0,13,14,17	2	P12368,P20766
10818	1,6,7,9,11,<br>12,15,17	2,3,4,5,8,1<br>0,13,14,16	1	P13432
10819	1,6,7,9,11,<br>12,16,17	2,3,4,5,8,1<br>0,13,14,15	3	P12368,P13432,P36970
10820	1,6,7,9,11,<br>13,14,15	2,3,4,5,8,1<br>0,12,16,17	4	P16636,P83121,Q80WL1,Q9JJS8
10821	1,6,7,9,11,<br>13,14,16	2,3,4,5,8,1<br>0,12,15,17	2	P02761,P83121

10822	1,6,7,9,11,13,14,17	2,3,4,5,8,10,12,15,16	2	P83121,Q9JJS8
10823	1,6,7,9,11,13,15,16	2,3,4,5,8,10,12,14,17	3	P31430,Q80WL1,Q9JJS8
10824	1,6,7,9,11,13,15,17	2,3,4,5,8,10,12,14,16	1	Q9JJS8
10825	1,6,7,9,11,13,16,17	2,3,4,5,8,10,12,14,15	1	P36970
10826	1,6,7,9,11,14,15,16	2,3,4,5,8,10,12,13,17	2	P16636,Q80WL1
10827	1,6,7,9,11,14,15,17	2,3,4,5,8,10,12,13,16	2	Q80WL1,Q9JJS8
10828	1,6,7,9,11,14,16,17	2,3,4,5,8,10,12,13,15	1	P83121
10829	1,6,7,9,11,15,16,17	2,3,4,5,8,10,12,13,14	2	P36970,Q80WL1
10830	1,6,7,9,12,13,14,15	2,3,4,5,8,10,11,16,17	2	P08649,Q6AYS4
10831	1,6,7,9,12,13,14,16	2,3,4,5,8,10,11,15,17	3	P02761,P08649,Q6AYS4
10832	1,6,7,9,12,13,14,17	2,3,4,5,8,10,11,15,16	5	P13432,P14668,P53792,Q64194,Q6AYS4
10833	1,6,7,9,12,13,15,16	2,3,4,5,8,10,11,14,17	4	P08649,P19218,P97840,Q6AYS4
10834	1,6,7,9,12,13,15,17	2,3,4,5,8,10,11,14,16	6	P13432,P14668,P19218,P53792,Q64194,Q6AYS4
10835	1,6,7,9,12,13,16,17	2,3,4,5,8,10,11,14,15	4	P13432,P19218,Q64194,Q6AYS4
10836	1,6,7,9,12,14,15,16	2,3,4,5,8,10,11,13,17	2	P20766,Q6AYS4
10837	1,6,7,9,12,14,15,17	2,3,4,5,8,10,11,13,16	2	P13432,Q6AYS4
10838	1,6,7,9,12,14,16,17	2,3,4,5,8,10,11,13,15	2	P13432,Q6AYS4
10839	1,6,7,9,12,15,16,17	2,3,4,5,8,10,11,13,14	4	P13432,P19218,P20766,Q6AYS4
10840	1,6,7,9,13,14,15,16	2,3,4,5,8,10,11,12,17	6	P16391,P16636,P19223,P62804,Q6AYS4,Q80WL1
10841	1,6,7,9,13,14,15,17	2,3,4,5,8,10,11,12,16	5	P14668,P53792,Q6AYS4,Q80WL1,Q9JJS8
10842	1,6,7,9,13,14,16,17	2,3,4,5,8,10,11,12,15	3	P35053,P53792,Q6AYS4
10843	1,6,7,9,13,15,16,17	2,3,4,5,8,10,11,12,14	7	P19218,P53792,P62804,Q63598,Q6AYS4,Q80WL1,Q9EQS0
10844	1,6,7,9,14,15,16,17	2,3,4,5,8,10,11,12,13	2	Q6AYS4,Q80WL1

10845	1,6,7,10,11,12,13,14	2,3,4,5,8,9,15,16,17	9	P04905,P06911,P12020,P30120,P47727,Q5GRG2,Q5QE79,Q62902,Q9JHB9
10846	1,6,7,10,11,12,13,15	2,3,4,5,8,9,14,16,17	2	P47727,Q9JHB9
10847	1,6,7,10,11,12,13,16	2,3,4,5,8,9,14,15,17	5	P12368,P47727,P47967,P97840,Q9JHB9
10848	1,6,7,10,11,12,13,17	2,3,4,5,8,9,14,15,16	7	O08815,P13432,P36374,P47727,Q99MH3,Q9JHB9,Q9QZK9
10849	1,6,7,10,11,12,14,15	2,3,4,5,8,9,13,16,17	4	P20766,P22006,P35280,P47727
10850	1,6,7,10,11,12,14,16	2,3,4,5,8,9,13,15,17	6	P12368,P20766,P47727,P97840,Q6AYL6,Q6IFU7
10851	1,6,7,10,11,12,14,17	2,3,4,5,8,9,13,15,16	5	O08815,P13432,P20766,P47727,Q9QZK9
10852	1,6,7,10,11,12,15,16	2,3,4,5,8,9,13,14,17	4	P12368,P20766,P97840,Q6IFU7
10853	1,6,7,10,11,12,15,17	2,3,4,5,8,9,13,14,16	3	P13432,P20766,Q9QZK9
10854	1,6,7,10,11,12,16,17	2,3,4,5,8,9,13,14,15	7	P06757,P12368,P13432,P20766,P97840,Q6TMA8,Q9QZK9
10855	1,6,7,10,11,13,14,15	2,3,4,5,8,9,12,16,17	5	P12020,P19223,P22006,P47727,Q5GRG2
10856	1,6,7,10,11,13,14,16	2,3,4,5,8,9,12,15,17	4	P12020,P19223,P47727,Q7TPB1
10857	1,6,7,10,11,13,14,17	2,3,4,5,8,9,12,15,16	2	P12020,P47727
10858	1,6,7,10,11,13,15,16	2,3,4,5,8,9,12,14,17	3	P01835,P19223,Q80WL1
10859	1,6,7,10,11,13,15,17	2,3,4,5,8,9,12,14,16	1	Q9JJS8
10860	1,6,7,10,11,13,16,17	2,3,4,5,8,9,12,14,15	0	
10861	1,6,7,10,11,14,15,16	2,3,4,5,8,9,12,13,17	3	P19223,P20766,Q80WL1
10862	1,6,7,10,11,14,15,17	2,3,4,5,8,9,12,13,16	1	P22006
10863	1,6,7,10,11,14,16,17	2,3,4,5,8,9,12,13,15	2	P20766,Q6TMA8
10864	1,6,7,10,11,15,16,17	2,3,4,5,8,9,12,13,14	3	P20766,P36970,Q6TMA8
10865	1,6,7,10,12,13,14,15	2,3,4,5,8,9,11,16,17	6	P06911,P12020,P97840,Q498D9,Q5GRG2,Q6AYS4
10866	1,6,7,10,12,13,14,16	2,3,4,5,8,9,11,15,17	5	P02761,P06911,P12020,P97840,Q6AYS4
10867	1,6,7,10,12,13,14,17	2,3,4,5,8,9,11,15,16	7	P06911,P12020,P13432,P14668,Q05175,Q6AYS4,Q9QZK9

10868	1,6,7,10,1<br>2,13,15,16	2,3,4,5,8,9<br>,11,14,17	3	P19218,P97840,Q6AYS4
10869	1,6,7,10,1<br>2,13,15,17	2,3,4,5,8,9<br>,11,14,16	6	P14668,P19218,Q6AYS4,Q6IFV1,Q99MH3,Q9QZK9
10870	1,6,7,10,1<br>2,13,16,17	2,3,4,5,8,9<br>,11,14,15	7	P13432,P19218,P97840,Q64194,Q6AYS4,Q99MH3,Q9QZK9
10871	1,6,7,10,1<br>2,14,15,16	2,3,4,5,8,9<br>,11,13,17	3	P20766,P97840,Q6AYS4
10872	1,6,7,10,1<br>2,14,15,17	2,3,4,5,8,9<br>,11,13,16	4	P13432,P14668,P20766,Q6AYS4
10873	1,6,7,10,1<br>2,14,16,17	2,3,4,5,8,9<br>,11,13,15	5	P13432,P20766,P97840,Q6AYS4,Q6TMA8
10874	1,6,7,10,1<br>2,15,16,17	2,3,4,5,8,9<br>,11,13,14	5	P19218,P20766,P97840,Q6AYS4,Q6TMA8
10875	1,6,7,10,1<br>3,14,15,16	2,3,4,5,8,9<br>,11,12,17	5	P12020,P19223,Q5GRG2,Q6AYS4,Q80WL1
10876	1,6,7,10,1<br>3,14,15,17	2,3,4,5,8,9<br>,11,12,16	4	P12020,P14668,Q5GRG2,Q6AYS4
10877	1,6,7,10,1<br>3,14,16,17	2,3,4,5,8,9<br>,11,12,15	2	P12020,Q6AYS4
10878	1,6,7,10,1<br>3,15,16,17	2,3,4,5,8,9<br>,11,12,14	4	P19218,Q6AYS4,Q6TMA8,Q9EQS0
10879	1,6,7,10,1<br>4,15,16,17	2,3,4,5,8,9<br>,11,12,13	4	P20766,Q6AYS4,Q6TMA8,Q80WL1
10880	1,6,7,11,1<br>2,13,14,15	2,3,4,5,8,9<br>,10,16,17	3	P47727,Q5GRG2,Q9JHB9
10881	1,6,7,11,1<br>2,13,14,16	2,3,4,5,8,9<br>,10,15,17	4	P02761,P47727,Q62902,Q9JHB9
10882	1,6,7,11,1<br>2,13,14,17	2,3,4,5,8,9<br>,10,15,16	6	P13432,P36374,P47727,Q62902,Q9JHB9,Q9QZK9
10883	1,6,7,11,1<br>2,13,15,16	2,3,4,5,8,9<br>,10,14,17	0	
10884	1,6,7,11,1<br>2,13,15,17	2,3,4,5,8,9<br>,10,14,16	2	P13432,Q9QZK9
10885	1,6,7,11,1<br>2,13,16,17	2,3,4,5,8,9<br>,10,14,15	3	P13432,P17046,Q9QZK9
10886	1,6,7,11,1<br>2,14,15,16	2,3,4,5,8,9<br>,10,13,17	1	P20766
10887	1,6,7,11,1<br>2,14,15,17	2,3,4,5,8,9<br>,10,13,16	2	P13432,P20766
10888	1,6,7,11,1<br>2,14,16,17	2,3,4,5,8,9<br>,10,13,15	2	P13432,P20766
10889	1,6,7,11,1<br>2,15,16,17	2,3,4,5,8,9<br>,10,13,14	2	P13432,P20766
10890	1,6,7,11,1<br>3,14,15,16	2,3,4,5,8,9<br>,10,12,17	3	P16636,P19223,Q80WL1

10891	1,6,7,11,13,14,15,17	2,3,4,5,8,9,10,12,16	1	Q9JJS8
10892	1,6,7,11,13,14,16,17	2,3,4,5,8,9,10,12,15	0	
10893	1,6,7,11,13,15,16,17	2,3,4,5,8,9,10,12,14	2	Q80WL1,Q9JJS8
10894	1,6,7,11,14,15,16,17	2,3,4,5,8,9,10,12,13	2	P20766,Q80WL1
10895	1,6,7,12,13,14,15,16	2,3,4,5,8,9,10,11,17	2	P08649,Q6AYS4
10896	1,6,7,12,13,14,15,17	2,3,4,5,8,9,10,11,16	5	P08649,P13432,P14668,Q6AYS4,Q9QZK9
10897	1,6,7,12,13,14,16,17	2,3,4,5,8,9,10,11,15	3	P08649,P13432,Q6AYS4
10898	1,6,7,12,13,15,16,17	2,3,4,5,8,9,10,11,14	4	P08649,P13432,P19218,Q6AYS4
10899	1,6,7,12,14,15,16,17	2,3,4,5,8,9,10,11,13	5	P13432,P20766,P35467,Q6AYS4,Q6TMA8
10900	1,6,7,13,14,15,16,17	2,3,4,5,8,9,10,11,12	2	Q6AYS4,Q80WL1
10901	1,6,8,9,10,11,12,13	2,3,4,5,7,14,15,16,17	9	P02781,P02782,P02803,P08723,P0C0A9,P30120,P36374,P47727,Q9JHB9
10902	1,6,8,9,10,11,12,14	2,3,4,5,7,13,15,16,17	6	P02782,P04905,P08723,P0C0A9,P30120,P47727
10903	1,6,8,9,10,11,12,15	2,3,4,5,7,13,14,16,17	2	P08010,Q566E6
10904	1,6,8,9,10,11,12,16	2,3,4,5,7,13,14,15,17	2	Q6IFU7,Q9WUW8
10905	1,6,8,9,10,11,12,17	2,3,4,5,7,13,14,15,16	6	P02782,P13432,P16636,Q09030,Q566E6,Q99068
10906	1,6,8,9,10,11,13,14	2,3,4,5,7,12,15,16,17	2	P01836,P47727
10907	1,6,8,9,10,11,13,15	2,3,4,5,7,12,14,16,17	0	
10908	1,6,8,9,10,11,13,16	2,3,4,5,7,12,14,15,17	3	A2RUV9,P11762,Q8CJD3
10909	1,6,8,9,10,11,13,17	2,3,4,5,7,12,14,15,16	1	P01836
10910	1,6,8,9,10,11,14,15	2,3,4,5,7,12,13,16,17	3	P01836,P35280,P47727
10911	1,6,8,9,10,11,14,16	2,3,4,5,7,12,13,15,17	2	P01836,P47727
10912	1,6,8,9,10,11,14,17	2,3,4,5,7,12,13,15,16	3	P01836,P47727,Q09030
10913	1,6,8,9,10,11,15,16	2,3,4,5,7,12,13,14,17	1	P55091

10914	1,6,8,9,10,<br>11,15,17	2,3,4,5,7,1<br>2,13,14,16	2	P01836,Q566E6
10915	1,6,8,9,10,<br>11,16,17	2,3,4,5,7,1<br>2,13,14,15	3	A2RUV9,P01836,Q09030
10916	1,6,8,9,10,<br>12,13,14	2,3,4,5,7,1<br>1,15,16,17	3	P02782,P14408,P30120
10917	1,6,8,9,10,<br>12,13,15	2,3,4,5,7,1<br>1,14,16,17	3	P02782,P15999,Q6IFV1
10918	1,6,8,9,10,<br>12,13,16	2,3,4,5,7,1<br>1,14,15,17	3	P02782,P19218,P97840
10919	1,6,8,9,10,<br>12,13,17	2,3,4,5,7,1<br>1,14,15,16	4	P02782,P13432,Q6IFV1,Q9QZK9
10920	1,6,8,9,10,<br>12,14,15	2,3,4,5,7,1<br>1,13,16,17	0	
10921	1,6,8,9,10,<br>12,14,16	2,3,4,5,7,1<br>1,13,15,17	0	
10922	1,6,8,9,10,<br>12,14,17	2,3,4,5,7,1<br>1,13,15,16	4	P01836,P02782,P13432,Q09030
10923	1,6,8,9,10,<br>12,15,16	2,3,4,5,7,1<br>1,13,14,17	0	
10924	1,6,8,9,10,<br>12,15,17	2,3,4,5,7,1<br>1,13,14,16	2	P13432,Q6IFV1
10925	1,6,8,9,10,<br>12,16,17	2,3,4,5,7,1<br>1,13,14,15	2	P13432,Q09030
10926	1,6,8,9,10,<br>13,14,15	2,3,4,5,7,1<br>1,12,16,17	2	P01836,P15999
10927	1,6,8,9,10,<br>13,14,16	2,3,4,5,7,1<br>1,12,15,17	2	P01836,Q9ESW0
10928	1,6,8,9,10,<br>13,14,17	2,3,4,5,7,1<br>1,12,15,16	2	P01836,P51907
10929	1,6,8,9,10,<br>13,15,16	2,3,4,5,7,1<br>1,12,14,17	2	P34901,Q9ESW0
10930	1,6,8,9,10,<br>13,15,17	2,3,4,5,7,1<br>1,12,14,16	4	P01836,P15999,P34901,Q6IFV1
10931	1,6,8,9,10,<br>13,16,17	2,3,4,5,7,1<br>1,12,14,15	6	P01836,P25031,P34901,P51907,Q8CJD3,Q9ESW0
10932	1,6,8,9,10,<br>14,15,16	2,3,4,5,7,1<br>1,12,13,17	1	P01836
10933	1,6,8,9,10,<br>14,15,17	2,3,4,5,7,1<br>1,12,13,16	1	P01836
10934	1,6,8,9,10,<br>14,16,17	2,3,4,5,7,1<br>1,12,13,15	2	P01836,Q09030
10935	1,6,8,9,10,<br>15,16,17	2,3,4,5,7,1<br>1,12,13,14	1	P01836
10936	1,6,8,9,11,<br>12,13,14	2,3,4,5,7,1<br>0,15,16,17	10	P02782,P02803,P04905,P08723,P0C0A9,P30120,P47727,Q62902,Q62997,Q9JHB9

10937	1,6,8,9,11,<br>12,13,15	2,3,4,5,7,1<br>0,14,16,17	3	P02782,Q62997,Q9JHB9
10938	1,6,8,9,11,<br>12,13,16	2,3,4,5,7,1<br>0,14,15,17	3	P02782,P02803,Q9JHB9
10939	1,6,8,9,11,<br>12,13,17	2,3,4,5,7,1<br>0,14,15,16	8	P02782,P02803,P13432,P30120,P36374,Q99068,Q9JHB9,Q9QZK9
10940	1,6,8,9,11,<br>12,14,15	2,3,4,5,7,1<br>0,13,16,17	3	P02782,P47727,Q62997
10941	1,6,8,9,11,<br>12,14,16	2,3,4,5,7,1<br>0,13,15,17	2	P02782,P47727
10942	1,6,8,9,11,<br>12,14,17	2,3,4,5,7,1<br>0,13,15,16	6	P02782,P04218,P13432,P47727,Q09030,Q62997
10943	1,6,8,9,11,<br>12,15,16	2,3,4,5,7,1<br>0,13,14,17	0	
10944	1,6,8,9,11,<br>12,15,17	2,3,4,5,7,1<br>0,13,14,16	2	P13432,Q99068
10945	1,6,8,9,11,<br>12,16,17	2,3,4,5,7,1<br>0,13,14,15	4	P04218,P13432,Q09030,Q99068
10946	1,6,8,9,11,<br>13,14,15	2,3,4,5,7,1<br>0,12,16,17	1	P47727
10947	1,6,8,9,11,<br>13,14,16	2,3,4,5,7,1<br>0,12,15,17	2	P04218,P47727
10948	1,6,8,9,11,<br>13,14,17	2,3,4,5,7,1<br>0,12,15,16	3	P01836,P04218,P47727
10949	1,6,8,9,11,<br>13,15,16	2,3,4,5,7,1<br>0,12,14,17	1	P34901
10950	1,6,8,9,11,<br>13,15,17	2,3,4,5,7,1<br>0,12,14,16	2	P04218,P34901
10951	1,6,8,9,11,<br>13,16,17	2,3,4,5,7,1<br>0,12,14,15	5	A2RUV9,P04218,P11762,P34901,Q8CJD3
10952	1,6,8,9,11,<br>14,15,16	2,3,4,5,7,1<br>0,12,13,17	1	P04218
10953	1,6,8,9,11,<br>14,15,17	2,3,4,5,7,1<br>0,12,13,16	2	P01836,P04218
10954	1,6,8,9,11,<br>14,16,17	2,3,4,5,7,1<br>0,12,13,15	2	P01836,P04218
10955	1,6,8,9,11,<br>15,16,17	2,3,4,5,7,1<br>0,12,13,14	2	A2RUV9,P04218
10956	1,6,8,9,12,<br>13,14,15	2,3,4,5,7,1<br>0,11,16,17	2	P02782,P15999
10957	1,6,8,9,12,<br>13,14,16	2,3,4,5,7,1<br>0,11,15,17	1	P02782
10958	1,6,8,9,12,<br>13,14,17	2,3,4,5,7,1<br>0,11,15,16	4	P02782,P13432,P51907,Q9Z1F2
10959	1,6,8,9,12,<br>13,15,16	2,3,4,5,7,1<br>0,11,14,17	1	P19218

10960	1,6,8,9,12,<br>13,15,17	2,3,4,5,7,1<br>0,11,14,16	4	P02782,P13432,P15999,Q6IFV1
10961	1,6,8,9,12,<br>13,16,17	2,3,4,5,7,1<br>0,11,14,15	2	P02782,P13432
10962	1,6,8,9,12,<br>14,15,16	2,3,4,5,7,1<br>0,11,13,17	0	
10963	1,6,8,9,12,<br>14,15,17	2,3,4,5,7,1<br>0,11,13,16	1	P13432
10964	1,6,8,9,12,<br>14,16,17	2,3,4,5,7,1<br>0,11,13,15	2	P13432,Q09030
10965	1,6,8,9,12,<br>15,16,17	2,3,4,5,7,1<br>0,11,13,14	1	P13432
10966	1,6,8,9,13,<br>14,15,16	2,3,4,5,7,1<br>0,11,12,17	1	P19223
10967	1,6,8,9,13,<br>14,15,17	2,3,4,5,7,1<br>0,11,12,16	4	P01836,P15999,P53792,P61206;P84079;P84082
10968	1,6,8,9,13,<br>14,16,17	2,3,4,5,7,1<br>0,11,12,15	3	P01836,P04218,P51907
10969	1,6,8,9,13,<br>15,16,17	2,3,4,5,7,1<br>0,11,12,14	1	P34901
10970	1,6,8,9,14,<br>15,16,17	2,3,4,5,7,1<br>0,11,12,13	2	P01836,P04218
10971	1,6,8,10,1<br>1,12,13,14	2,3,4,5,7,9<br>,15,16,17	10	O35547,P02781,P02782,P04905,P06911,P08723,P0C0A9,P30120,P47727,Q9JHB9
10972	1,6,8,10,1<br>1,12,13,15	2,3,4,5,7,9<br>,14,16,17	7	O35547,P02782,P08010,P15999,P47727,Q6IFV1,Q9JHB9
10973	1,6,8,10,1<br>1,12,13,16	2,3,4,5,7,9<br>,14,15,17	6	O35547,P02782,P30120,P47727,Q6IFU7,Q9JHB9
10974	1,6,8,10,1<br>1,12,13,17	2,3,4,5,7,9<br>,14,15,16	12	O35547,P02782,P13432,P16636,P30120,P36374,P47727,P97605,Q6IFV1,Q99MH3,Q9JHB9,Q9QZK9
10975	1,6,8,10,1<br>1,12,14,15	2,3,4,5,7,9<br>,13,16,17	4	O35547,P08010,P35280,P47727
10976	1,6,8,10,1<br>1,12,14,16	2,3,4,5,7,9<br>,13,15,17	3	O35547,P47727,Q6IFU7
10977	1,6,8,10,1<br>1,12,14,17	2,3,4,5,7,9<br>,13,15,16	6	O35547,P02782,P08010,P13432,P47727,Q9QZK9
10978	1,6,8,10,1<br>1,12,15,16	2,3,4,5,7,9<br>,13,14,17	2	O35547,Q6IFU7
10979	1,6,8,10,1<br>1,12,15,17	2,3,4,5,7,9<br>,13,14,16	5	O35547,P08010,P13432,Q6IFV1,Q9QZK9
10980	1,6,8,10,1<br>1,12,16,17	2,3,4,5,7,9<br>,13,14,15	5	O35547,P06757,P13432,P16636,P97605
10981	1,6,8,10,1<br>1,13,14,15	2,3,4,5,7,9<br>,12,16,17	2	P15999,P47727
10982	1,6,8,10,1<br>1,13,14,16	2,3,4,5,7,9<br>,12,15,17	3	P19223,P47727,Q7TPB1

10983	1,6,8,10,1<br>1,13,14,17	2,3,4,5,7,9<br>,12,15,16	2	P01836,P47727
10984	1,6,8,10,1<br>1,13,15,16	2,3,4,5,7,9<br>,12,14,17	1	P01835
10985	1,6,8,10,1<br>1,13,15,17	2,3,4,5,7,9<br>,12,14,16	2	P15999,Q6IFV1
10986	1,6,8,10,1<br>1,13,16,17	2,3,4,5,7,9<br>,12,14,15	2	A2RUV9,P11762
10987	1,6,8,10,1<br>1,14,15,16	2,3,4,5,7,9<br>,12,13,17	0	
10988	1,6,8,10,1<br>1,14,15,17	2,3,4,5,7,9<br>,12,13,16	1	P01836
10989	1,6,8,10,1<br>1,14,16,17	2,3,4,5,7,9<br>,12,13,15	1	P01836
10990	1,6,8,10,1<br>1,15,16,17	2,3,4,5,7,9<br>,12,13,14	0	
10991	1,6,8,10,1<br>2,13,14,15	2,3,4,5,7,9<br>,11,16,17	3	P02782,P15999,Q498D9
10992	1,6,8,10,1<br>2,13,14,16	2,3,4,5,7,9<br>,11,15,17	2	P02782,Q8K4G9
10993	1,6,8,10,1<br>2,13,14,17	2,3,4,5,7,9<br>,11,15,16	5	P02782,P13432,Q05175,Q8K4G9,Q9QZK9
10994	1,6,8,10,1<br>2,13,15,16	2,3,4,5,7,9<br>,11,14,17	3	P15999,P19218,Q6IFV1
10995	1,6,8,10,1<br>2,13,15,17	2,3,4,5,7,9<br>,11,14,16	5	P02782,P15999,Q6IFV1,Q99MH3,Q9QZK9
10996	1,6,8,10,1<br>2,13,16,17	2,3,4,5,7,9<br>,11,14,15	6	P02782,P13432,Q6IFV1,Q8K4G9,Q99MH3,Q9QZK9
10997	1,6,8,10,1<br>2,14,15,16	2,3,4,5,7,9<br>,11,13,17	0	
10998	1,6,8,10,1<br>2,14,15,17	2,3,4,5,7,9<br>,11,13,16	1	P13432
10999	1,6,8,10,1<br>2,14,16,17	2,3,4,5,7,9<br>,11,13,15	2	P05539,P13432
11000	1,6,8,10,1<br>2,15,16,17	2,3,4,5,7,9<br>,11,13,14	2	P05539,Q6IFV1
11001	1,6,8,10,1<br>3,14,15,16	2,3,4,5,7,9<br>,11,12,17	2	P15999,P19223
11002	1,6,8,10,1<br>3,14,15,17	2,3,4,5,7,9<br>,11,12,16	3	P01836,P15999,Q6IFV1
11003	1,6,8,10,1<br>3,14,16,17	2,3,4,5,7,9<br>,11,12,15	3	P01836,P51907,Q498R7
11004	1,6,8,10,1<br>3,15,16,17	2,3,4,5,7,9<br>,11,12,14	3	P15999,P34901,Q6IFV1
11005	1,6,8,10,1<br>4,15,16,17	2,3,4,5,7,9<br>,11,12,13	2	P01836,Q498R7

11006	1,6,8,11,12,13,14,15	2,3,4,5,7,9,10,16,17	6	P02782,P15999,P30120,P47727,P52590,Q9JHB9
11007	1,6,8,11,12,13,14,16	2,3,4,5,7,9,10,15,17	6	P02782,P30120,P47727,P52590,Q62902,Q9JHB9
11008	1,6,8,11,12,13,14,17	2,3,4,5,7,9,10,15,16	10	P02782,P04905,P08723,P13432,P30120,P36374,P47727,Q62635,Q9JHB9,Q9QZK9
11009	1,6,8,11,12,13,15,16	2,3,4,5,7,9,10,14,17	1	P52590
11010	1,6,8,11,12,13,15,17	2,3,4,5,7,9,10,14,16	6	P02782,P13432,P15999,Q6IFV1,Q9JHB9,Q9QZK9
11011	1,6,8,11,12,13,16,17	2,3,4,5,7,9,10,14,15	5	P02782,P13432,P36376,Q9JHB9,Q9QZK9
11012	1,6,8,11,12,14,15,16	2,3,4,5,7,9,10,13,17	1	P52590
11013	1,6,8,11,12,14,15,17	2,3,4,5,7,9,10,13,16	2	P02782,P13432
11014	1,6,8,11,12,14,16,17	2,3,4,5,7,9,10,13,15	4	O35547,P02782,P13432,P47727
11015	1,6,8,11,12,15,16,17	2,3,4,5,7,9,10,13,14	2	P13432,Q99068
11016	1,6,8,11,13,14,15,16	2,3,4,5,7,9,10,12,17	2	P19223,P52590
11017	1,6,8,11,13,14,15,17	2,3,4,5,7,9,10,12,16	2	P15999,P47727
11018	1,6,8,11,13,14,16,17	2,3,4,5,7,9,10,12,15	2	P04218,P47727
11019	1,6,8,11,13,15,16,17	2,3,4,5,7,9,10,12,14	1	P34901
11020	1,6,8,11,14,15,16,17	2,3,4,5,7,9,10,12,13	1	P04218
11021	1,6,8,12,13,14,15,16	2,3,4,5,7,9,10,11,17	2	P15999,P52590
11022	1,6,8,12,13,14,15,17	2,3,4,5,7,9,10,11,16	5	P02782,P13432,P15999,Q64093,Q6IFV1
11023	1,6,8,12,13,14,16,17	2,3,4,5,7,9,10,11,15	3	P02782,P13432,P51907
11024	1,6,8,12,13,15,16,17	2,3,4,5,7,9,10,11,14	4	P13432,P15999,Q5XFX0,Q6IFV1
11025	1,6,8,12,14,15,16,17	2,3,4,5,7,9,10,11,13	1	P13432
11026	1,6,8,13,14,15,16,17	2,3,4,5,7,9,10,11,12	1	P15999
11027	1,6,9,10,11,12,13,14	2,3,4,5,7,8,15,16,17	2	P47727,P50115
11028	1,6,9,10,11,12,13,15	2,3,4,5,7,8,14,16,17	3	P50115,Q566E6,Q6PCU2

11029	1,6,9,10,1<br>1,12,13,16	2,3,4,5,7,8<br>,14,15,17	6	P02761,P10536,P12368,P50115,P97840,Q6IFU7
11030	1,6,9,10,1<br>1,12,13,17	2,3,4,5,7,8<br>,14,15,16	3	P07171,P36374,Q9QZK9
11031	1,6,9,10,1<br>1,12,14,15	2,3,4,5,7,8<br>,13,16,17	4	P20766,P47727,P50115,Q6PCU2
11032	1,6,9,10,1<br>1,12,14,16	2,3,4,5,7,8<br>,13,15,17	6	P10536,P20766,P47727,P50115,Q6IFU7,Q9WUW8
11033	1,6,9,10,1<br>1,12,14,17	2,3,4,5,7,8<br>,13,15,16	2	P47727,Q09030
11034	1,6,9,10,1<br>1,12,15,16	2,3,4,5,7,8<br>,13,14,17	8	P10536,P12368,P20766,P36970,P50115,Q5M819,Q6IFU7,Q9WUW8
11035	1,6,9,10,1<br>1,12,15,17	2,3,4,5,7,8<br>,13,14,16	2	P36970,Q566E6
11036	1,6,9,10,1<br>1,12,16,17	2,3,4,5,7,8<br>,13,14,15	6	P06757,P07171,P12368,P36970,Q09030,Q6IFU7
11037	1,6,9,10,1<br>1,13,14,15	2,3,4,5,7,8<br>,12,16,17	9	O88267,P19223,P47727,P50115,P50116,P55091,Q4G075,Q80WL1,Q99041
11038	1,6,9,10,1<br>1,13,14,16	2,3,4,5,7,8<br>,12,15,17	10	P02761,P19223,P47727,P50115,P55091,Q4G075,Q7TPB1,Q80WL1,Q99041,Q9QZA6
11039	1,6,9,10,1<br>1,13,14,17	2,3,4,5,7,8<br>,12,15,16	2	P01836,P47727
11040	1,6,9,10,1<br>1,13,15,16	2,3,4,5,7,8<br>,12,14,17	9	O88267,P19223,P31430,P36970,P50115,P55091,Q6AYS4,Q80WL1,Q9EQS0
11041	1,6,9,10,1<br>1,13,15,17	2,3,4,5,7,8<br>,12,14,16	4	P36970,Q566E6,Q80WL1,Q9EQS0
11042	1,6,9,10,1<br>1,13,16,17	2,3,4,5,7,8<br>,12,14,15	6	A2RUV9,P11762,P36970,Q64611,Q8CJD3,Q9EQS0
11043	1,6,9,10,1<br>1,14,15,16	2,3,4,5,7,8<br>,12,13,17	8	O88267,P08937,P19223,P50115,P55091,Q5M819,Q80WL1,Q99041
11044	1,6,9,10,1<br>1,14,15,17	2,3,4,5,7,8<br>,12,13,16	2	P01836,Q80WL1
11045	1,6,9,10,1<br>1,14,16,17	2,3,4,5,7,8<br>,12,13,15	3	P01836,P36970,Q80WL1
11046	1,6,9,10,1<br>1,15,16,17	2,3,4,5,7,8<br>,12,13,14	4	P36970,Q5M819,Q80WL1,Q9EQS0
11047	1,6,9,10,1<br>2,13,14,15	2,3,4,5,7,8<br>,11,16,17	3	P50115,P50116,Q6AYS4
11048	1,6,9,10,1<br>2,13,14,16	2,3,4,5,7,8<br>,11,15,17	5	P02761,P07171,P50115,P97840,Q6AYS4
11049	1,6,9,10,1<br>2,13,14,17	2,3,4,5,7,8<br>,11,15,16	3	P07171,P14668,Q6AYS4
11050	1,6,9,10,1<br>2,13,15,16	2,3,4,5,7,8<br>,11,14,17	6	P07171,P19218,P20762,P50115,P97840,Q6AYS4
11051	1,6,9,10,1<br>2,13,15,17	2,3,4,5,7,8<br>,11,14,16	5	P07171,P14668,P19218,Q6AYS4,Q6IFV1

11052	1,6,9,10,1<br>2,13,16,17	2,3,4,5,7,8<br>,11,14,15	6	P07171,P19218,P20762,P97840,Q64194,Q6AYS4
11053	1,6,9,10,1<br>2,14,15,16	2,3,4,5,7,8<br>,11,13,17	3	P20766,P50115,Q6AYS4
11054	1,6,9,10,1<br>2,14,15,17	2,3,4,5,7,8<br>,11,13,16	1	Q6AYS4
11055	1,6,9,10,1<br>2,14,16,17	2,3,4,5,7,8<br>,11,13,15	4	P07171,P20766,Q09030,Q6AYS4
11056	1,6,9,10,1<br>2,15,16,17	2,3,4,5,7,8<br>,11,13,14	3	P07171,P20766,Q6AYS4
11057	1,6,9,10,1<br>3,14,15,16	2,3,4,5,7,8<br>,11,12,17	12	P08937,P16391,P19223,P50115,P55091,Q4G075,Q63751,Q6AYS4,Q80WL1,Q99041,Q9EQS0,Q9ESW0
11058	1,6,9,10,1<br>3,14,15,17	2,3,4,5,7,8<br>,11,12,16	6	P01836,P14668,Q6AYS4,Q80WL1,Q9EQS0,Q9ESW0
11059	1,6,9,10,1<br>3,14,16,17	2,3,4,5,7,8<br>,11,12,15	6	P01836,P07171,P35053,Q6AYS4,Q9EQS0,Q9ESW0
11060	1,6,9,10,1<br>3,15,16,17	2,3,4,5,7,8<br>,11,12,14	6	P07171,P20762,Q6AYS4,Q80WL1,Q9EQS0,Q9ESW0
11061	1,6,9,10,1<br>4,15,16,17	2,3,4,5,7,8<br>,11,12,13	5	P01836,Q6AYS4,Q6TMA8,Q80WL1,Q9EQS0
11062	1,6,9,11,1<br>2,13,14,15	2,3,4,5,7,8<br>,10,16,17	3	P47727,P50115,Q62997
11063	1,6,9,11,1<br>2,13,14,16	2,3,4,5,7,8<br>,10,15,17	4	P02761,P47727,P50115,Q6AYS4
11064	1,6,9,11,1<br>2,13,14,17	2,3,4,5,7,8<br>,10,15,16	2	D3ZHA0,P47727
11065	1,6,9,11,1<br>2,13,15,16	2,3,4,5,7,8<br>,10,14,17	2	P50115,Q6AYS4
11066	1,6,9,11,1<br>2,13,15,17	2,3,4,5,7,8<br>,10,14,16	0	
11067	1,6,9,11,1<br>2,13,16,17	2,3,4,5,7,8<br>,10,14,15	3	D3ZHA0,P07171,P36970
11068	1,6,9,11,1<br>2,14,15,16	2,3,4,5,7,8<br>,10,13,17	3	P20766,P50115,Q6AYS4
11069	1,6,9,11,1<br>2,14,15,17	2,3,4,5,7,8<br>,10,13,16	0	
11070	1,6,9,11,1<br>2,14,16,17	2,3,4,5,7,8<br>,10,13,15	2	D3ZHA0,P04218
11071	1,6,9,11,1<br>2,15,16,17	2,3,4,5,7,8<br>,10,13,14	2	P20766,P36970
11072	1,6,9,11,1<br>3,14,15,16	2,3,4,5,7,8<br>,10,12,17	9	D3ZHA0,P16391,P19223,P50115,P55091,Q4G075,Q6AYS4,Q80WL1,Q9EQS0
11073	1,6,9,11,1<br>3,14,15,17	2,3,4,5,7,8<br>,10,12,16	5	D3ZHA0,P04218,P16391,Q80WL1,Q9EQS0
11074	1,6,9,11,1<br>3,14,16,17	2,3,4,5,7,8<br>,10,12,15	5	D3ZHA0,P04218,Q63475,Q80WL1,Q9EQS0

11075	1,6,9,11,13,15,16,17	2,3,4,5,7,8,10,12,14	5	D3ZHA0,P04218,P36970,Q80WL1,Q9EQS0
11076	1,6,9,11,14,15,16,17	2,3,4,5,7,8,10,12,13	5	D3ZHA0,P04218,P36970,Q80WL1,Q9EQS0
11077	1,6,9,12,13,14,15,16	2,3,4,5,7,8,10,11,17	5	P00714,P02761,P16391,P50115,Q6AYS4
11078	1,6,9,12,13,14,15,17	2,3,4,5,7,8,10,11,16	5	P00714,P14668,P16391,P61206;P84079;P84082,Q6AYS4
11079	1,6,9,12,13,14,16,17	2,3,4,5,7,8,10,11,15	4	P00714,P02761,P07171,Q6AYS4
11080	1,6,9,12,13,15,16,17	2,3,4,5,7,8,10,11,14	7	P00714,P07171,P19218,Q5BJY9,Q64194,Q6AYS4,Q9EQS0
11081	1,6,9,12,14,15,16,17	2,3,4,5,7,8,10,11,13	2	P20766,Q6AYS4
11082	1,6,9,13,14,15,16,17	2,3,4,5,7,8,10,11,12	8	D3ZHA0,P00714,P16391,Q63475,Q6AYS4,Q80WL1,Q9EQS0,Q9ESW0
11083	1,6,10,11,12,13,14,15	2,3,4,5,7,8,9,16,17	3	P47727,P50115,P52590
11084	1,6,10,11,12,13,14,16	2,3,4,5,7,8,9,15,17	6	P02761,P47727,P50115,P52590,Q6IFU7,Q7TPB1
11085	1,6,10,11,12,13,14,17	2,3,4,5,7,8,9,15,16	2	P47727,Q9QZK9
11086	1,6,10,11,12,13,15,16	2,3,4,5,7,8,9,14,17	4	P50115,P52590,Q6AYS4,Q6IFU7
11087	1,6,10,11,12,13,15,17	2,3,4,5,7,8,9,14,16	4	P97605,Q6IFV1,Q99MH3,Q9QZK9
11088	1,6,10,11,12,13,16,17	2,3,4,5,7,8,9,14,15	3	P07171,P97605,Q9QZK9
11089	1,6,10,11,12,14,15,16	2,3,4,5,7,8,9,13,17	5	P20766,P50115,P52590,Q6AYS4,Q6IFU7
11090	1,6,10,11,12,14,15,17	2,3,4,5,7,8,9,13,16	1	P20766
11091	1,6,10,11,12,14,16,17	2,3,4,5,7,8,9,13,15	3	P20766,P97605,Q6IFU7
11092	1,6,10,11,12,15,16,17	2,3,4,5,7,8,9,13,14	6	P06866,P20766,P36970,P97605,Q6IFU7,Q6TMA8

11093	1,6,10,11,13,14,15,16	2,3,4,5,7,8,9,12,17	7	P19223,P50115,P52590,P55091,Q6AYS4,Q80WL1,Q9EQS0
11094	1,6,10,11,13,14,15,17	2,3,4,5,7,8,9,12,16	3	P47727,Q80WL1,Q9EQS0
11095	1,6,10,11,13,14,16,17	2,3,4,5,7,8,9,12,15	3	D3ZHA0,P47727,Q9EQS0
11096	1,6,10,11,13,15,16,17	2,3,4,5,7,8,9,12,14	4	D3ZHA0,P36970,Q80WL1,Q9EQS0
11097	1,6,10,11,14,15,16,17	2,3,4,5,7,8,9,12,13	5	P20766,P36970,Q6TMA8,Q80WL1,Q9EQS0
11098	1,6,10,12,13,14,15,16	2,3,4,5,7,8,9,11,17	3	P50115,P52590,Q6AYS4
11099	1,6,10,12,13,14,15,17	2,3,4,5,7,8,9,11,16	3	P14668,Q05175,Q6AYS4
11100	1,6,10,12,13,14,16,17	2,3,4,5,7,8,9,11,15	2	P07171,Q6AYS4
11101	1,6,10,12,13,15,16,17	2,3,4,5,7,8,9,11,14	5	P07171,P19218,Q6AYS4,Q99MH3,Q9EQS0
11102	1,6,10,12,14,15,16,17	2,3,4,5,7,8,9,11,13	3	P20766,Q6AYS4,Q6TMA8
11103	1,6,10,13,14,15,16,17	2,3,4,5,7,8,9,11,12	4	Q498R7,Q6AYS4,Q80WL1,Q9EQS0
11104	1,6,11,12,13,14,15,16	2,3,4,5,7,8,9,10,17	4	P19223,P50115,P52590,Q6AYS4
11105	1,6,11,12,13,14,15,17	2,3,4,5,7,8,9,10,16	2	D3ZHA0,P47727
11106	1,6,11,12,13,14,16,17	2,3,4,5,7,8,9,10,15	2	D3ZHA0,P47727
11107	1,6,11,12,13,15,16,17	2,3,4,5,7,8,9,10,14	4	D3ZHA0,P36970,Q6AYS4,Q9EQS0

11108	1,6,11,12,14,15,16,17	2,3,4,5,7,8,9,10,13	2	D3ZHA0,P20766
11109	1,6,11,13,14,15,16,17	2,3,4,5,7,8,9,10,12	4	D3ZHA0,Q6AYS4,Q80WL1,Q9EQS0
11110	1,6,12,13,14,15,16,17	2,3,4,5,7,8,9,10,11	4	D3ZHA0,P00714,Q6AYS4,Q9EQS0
11111	1,7,8,9,10,11,12,13	2,3,4,5,6,14,15,16,17	4	P02803,P10715,P20760,P36374
11112	1,7,8,9,10,11,12,14	2,3,4,5,6,13,15,16,17	4	P10715,P20760,P47727,Q6AYL6
11113	1,7,8,9,10,11,12,15	2,3,4,5,6,13,14,16,17	2	P10715,P22006
11114	1,7,8,9,10,11,12,16	2,3,4,5,6,13,14,15,17	6	P02803,P10715,P20760,P47967,Q6AYL6,Q9WUW8
11115	1,7,8,9,10,11,12,17	2,3,4,5,6,13,14,15,16	3	P02803,P10715,P36374
11116	1,7,8,9,10,11,13,14	2,3,4,5,6,12,15,16,17	1	P47727
11117	1,7,8,9,10,11,13,15	2,3,4,5,6,12,14,16,17	4	P01835,P22006,P34901,Q9JJS8
11118	1,7,8,9,10,11,13,16	2,3,4,5,6,12,14,15,17	4	B0BNN3,P01835,P02803,P20760
11119	1,7,8,9,10,11,13,17	2,3,4,5,6,12,14,15,16	2	P02803,Q9JJS8
11120	1,7,8,9,10,11,14,15	2,3,4,5,6,12,13,16,17	2	P22006,P35280
11121	1,7,8,9,10,11,14,16	2,3,4,5,6,12,13,15,17	3	B0BNN3,P20760,Q62635
11122	1,7,8,9,10,11,14,17	2,3,4,5,6,12,13,15,16	0	
11123	1,7,8,9,10,11,15,16	2,3,4,5,6,12,13,14,17	2	P01835,Q62635
11124	1,7,8,9,10,11,15,17	2,3,4,5,6,12,13,14,16	4	G3V686,P22006,P25236,Q9JJS8
11125	1,7,8,9,10,11,16,17	2,3,4,5,6,12,13,14,15	1	P25236
11126	1,7,8,9,10,12,13,14	2,3,4,5,6,11,15,16,17	1	P02803
11127	1,7,8,9,10,12,13,15	2,3,4,5,6,11,14,16,17	0	
11128	1,7,8,9,10,12,13,16	2,3,4,5,6,11,14,15,17	5	P02803,P19218,P20760,P47967,P97840

11129	1,7,8,9,10,12,13,17	2,3,4,5,6,11,14,15,16	3	P02803,P14668,P36374
11130	1,7,8,9,10,12,14,15	2,3,4,5,6,11,13,16,17	0	
11131	1,7,8,9,10,12,14,16	2,3,4,5,6,11,13,15,17	0	
11132	1,7,8,9,10,12,14,17	2,3,4,5,6,11,13,15,16	0	
11133	1,7,8,9,10,12,15,16	2,3,4,5,6,11,13,14,17	2	P10715,P97840
11134	1,7,8,9,10,12,15,17	2,3,4,5,6,11,13,14,16	2	G3V686,P10715
11135	1,7,8,9,10,12,16,17	2,3,4,5,6,11,13,14,15	1	P10715
11136	1,7,8,9,10,13,14,15	2,3,4,5,6,11,12,16,17	1	P01835
11137	1,7,8,9,10,13,14,16	2,3,4,5,6,11,12,15,17	2	B0BNN3,P01835
11138	1,7,8,9,10,13,14,17	2,3,4,5,6,11,12,15,16	2	P14668,Q4FZU2
11139	1,7,8,9,10,13,15,16	2,3,4,5,6,11,12,14,17	2	P01835,P34901
11140	1,7,8,9,10,13,15,17	2,3,4,5,6,11,12,14,16	7	G3V686,P01835,P14668,P34901,Q4FZU2,Q6IFW6,Q9JJS8
11141	1,7,8,9,10,13,16,17	2,3,4,5,6,11,12,14,15	4	P01835,P02625,P34901,Q4FZU2
11142	1,7,8,9,10,14,15,16	2,3,4,5,6,11,12,13,17	3	P01835,P16636,Q62635
11143	1,7,8,9,10,14,15,17	2,3,4,5,6,11,12,13,16	1	G3V686
11144	1,7,8,9,10,14,16,17	2,3,4,5,6,11,12,13,15	1	P02625
11145	1,7,8,9,10,15,16,17	2,3,4,5,6,11,12,13,14	2	P01835,P25236
11146	1,7,8,9,11,12,13,14	2,3,4,5,6,10,15,16,17	7	P02803,P10715,P20760,P36374,P47727,Q62902,Q62997
11147	1,7,8,9,11,12,13,15	2,3,4,5,6,10,14,16,17	1	P10715
11148	1,7,8,9,11,12,13,16	2,3,4,5,6,10,14,15,17	4	P02803,P10715,P20760,Q8CJ52
11149	1,7,8,9,11,12,13,17	2,3,4,5,6,10,14,15,16	3	P02803,P10715,P36374
11150	1,7,8,9,11,12,14,15	2,3,4,5,6,10,13,16,17	3	P10715,P22006,Q62997
11151	1,7,8,9,11,12,14,16	2,3,4,5,6,10,13,15,17	2	P10715,P20760

11152	1,7,8,9,11,12,14,17	2,3,4,5,6,10,13,15,16	1	P10715
11153	1,7,8,9,11,12,15,16	2,3,4,5,6,10,13,14,17	1	P10715
11154	1,7,8,9,11,12,15,17	2,3,4,5,6,10,13,14,16	1	P10715
11155	1,7,8,9,11,12,16,17	2,3,4,5,6,10,13,14,15	1	P10715
11156	1,7,8,9,11,13,14,15	2,3,4,5,6,10,12,16,17	3	P16636,P22006,Q9JJS8
11157	1,7,8,9,11,13,14,16	2,3,4,5,6,10,12,15,17	3	B0BNN3,P20760,Q7TPB1
11158	1,7,8,9,11,13,14,17	2,3,4,5,6,10,12,15,16	2	P04218,Q9JJS8
11159	1,7,8,9,11,13,15,16	2,3,4,5,6,10,12,14,17	3	P01835,P34901,Q9JJS8
11160	1,7,8,9,11,13,15,17	2,3,4,5,6,10,12,14,16	3	P04073,P34901,Q9JJS8
11161	1,7,8,9,11,13,16,17	2,3,4,5,6,10,12,14,15	2	P34901,Q9JJS8
11162	1,7,8,9,11,14,15,16	2,3,4,5,6,10,12,13,17	5	P16636,Q62635,Q63355,Q99MH3,Q9QZK9
11163	1,7,8,9,11,14,15,17	2,3,4,5,6,10,12,13,16	3	P04218,P22006,Q9JJS8
11164	1,7,8,9,11,14,16,17	2,3,4,5,6,10,12,13,15	1	P04218
11165	1,7,8,9,11,15,16,17	2,3,4,5,6,10,12,13,14	3	P04218,P10715,Q9JJS8
11166	1,7,8,9,12,13,14,15	2,3,4,5,6,10,11,16,17	0	
11167	1,7,8,9,12,13,14,16	2,3,4,5,6,10,11,15,17	2	P20760,Q4FZU6
11168	1,7,8,9,12,13,14,17	2,3,4,5,6,10,11,15,16	1	P14668
11169	1,7,8,9,12,13,15,16	2,3,4,5,6,10,11,14,17	2	P10715,P19218
11170	1,7,8,9,12,13,15,17	2,3,4,5,6,10,11,14,16	2	P10715,P14668
11171	1,7,8,9,12,13,16,17	2,3,4,5,6,10,11,14,15	1	P10715
11172	1,7,8,9,12,14,15,16	2,3,4,5,6,10,11,13,17	0	
11173	1,7,8,9,12,14,15,17	2,3,4,5,6,10,11,13,16	0	
11174	1,7,8,9,12,14,16,17	2,3,4,5,6,10,11,13,15	2	P10715,Q4FZU6

11175	1,7,8,9,12,15,16,17	2,3,4,5,6,10,11,13,14	1	P10715
11176	1,7,8,9,13,14,15,16	2,3,4,5,6,10,11,12,17	5	P00762,P01835,P16636,Q4FZU2,Q6IFW6
11177	1,7,8,9,13,14,15,17	2,3,4,5,6,10,11,12,16	7	P11883,P53792,Q4FZU2,Q6IFU7,Q6IFW6,Q6IMF3,Q9JJS8
11178	1,7,8,9,13,14,16,17	2,3,4,5,6,10,11,12,15	3	Q4FZU2,Q4FZU6,Q6IFW6
11179	1,7,8,9,13,15,16,17	2,3,4,5,6,10,11,12,14	8	P01835,P11883,P34901,P62804,Q4FZU2,Q6IFW6,Q6IMF3,Q9JJS8
11180	1,7,8,9,14,15,16,17	2,3,4,5,6,10,11,12,13	2	Q4FZU2,Q6IFW6
11181	1,7,8,10,11,12,13,14	2,3,4,5,6,9,15,16,17	4	P23593,P36374,P47727,Q8CJ52
11182	1,7,8,10,11,12,13,15	2,3,4,5,6,9,14,16,17	3	P01835,P10715,P22006
11183	1,7,8,10,11,12,13,16	2,3,4,5,6,9,14,15,17	5	P01835,P02803,P10715,P47967,Q8CJ52
11184	1,7,8,10,11,12,13,17	2,3,4,5,6,9,14,15,16	4	P02803,P10715,P36374,Q9QZK9
11185	1,7,8,10,11,12,14,15	2,3,4,5,6,9,13,16,17	3	P22006,P23593,P35280
11186	1,7,8,10,11,12,14,16	2,3,4,5,6,9,13,15,17	3	P10715,Q6AYL6,Q8CJ52
11187	1,7,8,10,11,12,14,17	2,3,4,5,6,9,13,15,16	1	P47727
11188	1,7,8,10,11,12,15,16	2,3,4,5,6,9,13,14,17	2	P01835,P10715
11189	1,7,8,10,11,12,15,17	2,3,4,5,6,9,13,14,16	2	P10715,P22006
11190	1,7,8,10,11,12,16,17	2,3,4,5,6,9,13,14,15	2	P06757,P10715
11191	1,7,8,10,11,13,14,15	2,3,4,5,6,9,12,16,17	6	P01835,P09605;P25809,P15999,P22006,P23593,Q9JJS8
11192	1,7,8,10,11,13,14,16	2,3,4,5,6,9,12,15,17	3	B0BNN3,P01835,Q7TPB1
11193	1,7,8,10,11,13,14,17	2,3,4,5,6,9,12,15,16	1	P47727
11194	1,7,8,10,11,13,15,16	2,3,4,5,6,9,12,14,17	1	P01835
11195	1,7,8,10,11,13,15,17	2,3,4,5,6,9,12,14,16	4	P01835,P22006,P34901,Q9JJS8
11196	1,7,8,10,11,13,16,17	2,3,4,5,6,9,12,14,15	1	P01835
11197	1,7,8,10,11,14,15,16	2,3,4,5,6,9,12,13,17	3	P01835,P22006,Q63355

11198	1,7,8,10,1<br>1,14,15,17	2,3,4,5,6,9<br>,12,13,16	1	P22006
11199	1,7,8,10,1<br>1,14,16,17	2,3,4,5,6,9<br>,12,13,15	0	
11200	1,7,8,10,1<br>1,15,16,17	2,3,4,5,6,9<br>,12,13,14	2	P01835,P25236
11201	1,7,8,10,1<br>2,13,14,15	2,3,4,5,6,9<br>,11,16,17	1	P15999
11202	1,7,8,10,1<br>2,13,14,16	2,3,4,5,6,9<br>,11,15,17	0	
11203	1,7,8,10,1<br>2,13,14,17	2,3,4,5,6,9<br>,11,15,16	1	P14668
11204	1,7,8,10,1<br>2,13,15,16	2,3,4,5,6,9<br>,11,14,17	2	P01835,P19218
11205	1,7,8,10,1<br>2,13,15,17	2,3,4,5,6,9<br>,11,14,16	3	P14668,P15999,Q99MH3
11206	1,7,8,10,1<br>2,13,16,17	2,3,4,5,6,9<br>,11,14,15	0	
11207	1,7,8,10,1<br>2,14,15,16	2,3,4,5,6,9<br>,11,13,17	0	
11208	1,7,8,10,1<br>2,14,15,17	2,3,4,5,6,9<br>,11,13,16	0	
11209	1,7,8,10,1<br>2,14,16,17	2,3,4,5,6,9<br>,11,13,15	1	P02625
11210	1,7,8,10,1<br>2,15,16,17	2,3,4,5,6,9<br>,11,13,14	1	P06866
11211	1,7,8,10,1<br>3,14,15,16	2,3,4,5,6,9<br>,11,12,17	3	P01835,P09605;P25809,P16636
11212	1,7,8,10,1<br>3,14,15,17	2,3,4,5,6,9<br>,11,12,16	3	P01835,P14668,P15999
11213	1,7,8,10,1<br>3,14,16,17	2,3,4,5,6,9<br>,11,12,15	2	P01835,P02625
11214	1,7,8,10,1<br>3,15,16,17	2,3,4,5,6,9<br>,11,12,14	2	P01835,P34901
11215	1,7,8,10,1<br>4,15,16,17	2,3,4,5,6,9<br>,11,12,13	1	P01835
11216	1,7,8,11,1<br>2,13,14,15	2,3,4,5,6,9<br>,10,16,17	2	P22006,P23593
11217	1,7,8,11,1<br>2,13,14,16	2,3,4,5,6,9<br>,10,15,17	2	Q62902,Q8CJ52
11218	1,7,8,11,1<br>2,13,14,17	2,3,4,5,6,9<br>,10,15,16	3	P36374,P47727,Q8CJ52
11219	1,7,8,11,1<br>2,13,15,16	2,3,4,5,6,9<br>,10,14,17	2	P01835,P10715
11220	1,7,8,11,1<br>2,13,15,17	2,3,4,5,6,9<br>,10,14,16	1	P10715

11221	1,7,8,11,12,13,16,17	2,3,4,5,6,9,10,14,15	3	P10715,P36376,Q8CJ52
11222	1,7,8,11,12,14,15,16	2,3,4,5,6,9,10,13,17	2	P10715,Q63355
11223	1,7,8,11,12,14,15,17	2,3,4,5,6,9,10,13,16	4	P10715,P14173,P19629,P22006
11224	1,7,8,11,12,14,16,17	2,3,4,5,6,9,10,13,15	2	P10715,Q4FZU6
11225	1,7,8,11,12,15,16,17	2,3,4,5,6,9,10,13,14	1	P10715
11226	1,7,8,11,13,14,15,16	2,3,4,5,6,9,10,12,17	4	P01835,P16636,Q63355,Q9JJS8
11227	1,7,8,11,13,14,15,17	2,3,4,5,6,9,10,12,16	3	P14173,P22006,Q9JJS8
11228	1,7,8,11,13,14,16,17	2,3,4,5,6,9,10,12,15	0	
11229	1,7,8,11,13,15,16,17	2,3,4,5,6,9,10,12,14	3	P01835,P34901,Q9JJS8
11230	1,7,8,11,14,15,16,17	2,3,4,5,6,9,10,12,13	1	P14173
11231	1,7,8,12,13,14,15,16	2,3,4,5,6,9,10,11,17	2	P08649,P14173
11232	1,7,8,12,13,14,15,17	2,3,4,5,6,9,10,11,16	3	P14173,P14668,P15999
11233	1,7,8,12,13,14,16,17	2,3,4,5,6,9,10,11,15	2	P14173,Q4FZU6
11234	1,7,8,12,13,15,16,17	2,3,4,5,6,9,10,11,14	2	P14173,Q5XFX0
11235	1,7,8,12,14,15,16,17	2,3,4,5,6,9,10,11,13	1	P14173
11236	1,7,8,13,14,15,16,17	2,3,4,5,6,9,10,11,12	6	P01835,P11883,P14173,Q08849,Q4FZU2,Q6IFW6
11237	1,7,9,10,11,12,13,14	2,3,4,5,6,8,15,16,17	8	B0BNN3,P02803,P06760,P20760,P20761,P23593,P47727,Q9Z0V6
11238	1,7,9,10,11,12,13,15	2,3,4,5,6,8,14,16,17	6	P02793;Q7TP54,P06760,P20760,P20761,Q6PCU2,Q9Z0V6
11239	1,7,9,10,11,12,13,16	2,3,4,5,6,8,14,15,17	12	B0BNN3,P02793;Q7TP54,P02803,P06760,P10536,P12368,P20760,P20761,P20762,P47967,P97840,Q9Z0V6
11240	1,7,9,10,11,12,13,17	2,3,4,5,6,8,14,15,16	5	P02803,P14668,P20762,P36374,Q9Z0V6
11241	1,7,9,10,11,12,14,15	2,3,4,5,6,8,13,16,17	7	P06760,P20760,P20761,P22006,P51907,Q6PCU2,Q9Z0V6
11242	1,7,9,10,11,12,14,16	2,3,4,5,6,8,13,15,17	8	B0BNN3,P06760,P10536,P12368,P20760,P20761,P62815,Q9Z0V6
11243	1,7,9,10,11,12,14,17	2,3,4,5,6,8,13,15,16	1	Q9Z0V6

11244	1,7,9,10,11,12,15,16	2,3,4,5,6,8,13,14,17	13	P02793;Q7TP54,P06760,P10536,P12368,P20760,P20761,P20762,P20766,P51907,P62815,P97840,Q6PCU2,Q9Z0V6
11245	1,7,9,10,11,12,15,17	2,3,4,5,6,8,13,14,16	2	Q6TMA8,Q9Z0V6
11246	1,7,9,10,11,12,16,17	2,3,4,5,6,8,13,14,15	7	P06757,P10536,P12368,P20762,Q64093,Q6TMA8,Q9Z0V6
11247	1,7,9,10,11,13,14,15	2,3,4,5,6,8,12,16,17	12	P06760,P16636,P19223,P20760,P20761,P22006,P23593,P50115,Q4G075,Q62635,Q9JJS8,Q9Z0V6
11248	1,7,9,10,11,13,14,16	2,3,4,5,6,8,12,15,17	14	B0BNN3,P06760,P10536,P19223,P20760,P20761,P20762,P50115,P62815,Q4G075,Q62635,Q63751,Q7TPB1,Q9Z0V6
11249	1,7,9,10,11,13,14,17	2,3,4,5,6,8,12,15,16	2	P14668,Q9Z0V6
11250	1,7,9,10,11,13,15,16	2,3,4,5,6,8,12,14,17	17	P01835,P02793;Q7TP54,P06760,P10536,P19223,P20760,P20761,P20762,P31430,P50115,P62815,P98089,Q4G075,Q62635,Q9JJS8,Q9WTT6,Q9Z0V6
11251	1,7,9,10,11,13,15,17	2,3,4,5,6,8,12,14,16	4	P14668,P20762,Q9JJS8,Q9Z0V6
11252	1,7,9,10,11,13,16,17	2,3,4,5,6,8,12,14,15	6	B0BNN3,P02793;Q7TP54,P20762,P35053,Q6TMA8,Q9Z0V6
11253	1,7,9,10,11,14,15,16	2,3,4,5,6,8,12,13,17	16	O88267,P06760,P08937,P16636,P19223,P20760,P20761,P50115,P55091,P62815,Q4G075,Q62635,Q63355,Q63751,Q9QZK9,Q9Z0V6
11254	1,7,9,10,11,14,15,17	2,3,4,5,6,8,12,13,16	4	P22006,Q6TMA8,Q9JJS8,Q9Z0V6
11255	1,7,9,10,11,14,16,17	2,3,4,5,6,8,12,13,15	3	P35053,Q6TMA8,Q9Z0V6
11256	1,7,9,10,11,15,16,17	2,3,4,5,6,8,12,13,14	6	O35077,P20762,P25236,Q64093,Q6TMA8,Q9Z0V6
11257	1,7,9,10,12,13,14,15	2,3,4,5,6,8,11,16,17	9	P06760,P07150,P14668,P20760,P20761,P50115,Q4G075,Q6AYS4,Q9Z0V6
11258	1,7,9,10,12,13,14,16	2,3,4,5,6,8,11,15,17	14	B0BNN3,P02761,P06760,P14668,P14669,P20760,P20761,P20762,P50115,P97840,Q4G075,Q63751,Q6AYS4,Q9Z0V6
11259	1,7,9,10,12,13,14,17	2,3,4,5,6,8,11,15,16	2	P14668,Q9Z0V6
11260	1,7,9,10,12,13,15,16	2,3,4,5,6,8,11,14,17	13	P02793;Q7TP54,P06760,P07150,P14668,P14669,P19218,P20760,P20761,P20762,P50115,P97840,Q6AYS4,Q9Z0V6
11261	1,7,9,10,12,13,15,17	2,3,4,5,6,8,11,14,16	4	P02793;Q7TP54,P14668,P20762,Q9Z0V6
11262	1,7,9,10,12,13,16,17	2,3,4,5,6,8,11,14,15	10	P02793;Q7TP54,P07171,P14668,P14669,P19218,P20762,P97840,Q64194,Q6TMA8,Q9Z0V6
11263	1,7,9,10,12,14,15,16	2,3,4,5,6,8,11,13,17	12	P06760,P14668,P14669,P16636,P20760,P20761,P20766,P50115,P97840,Q63751,Q6AYS4,Q9Z0V6
11264	1,7,9,10,12,14,15,17	2,3,4,5,6,8,11,13,16	3	P14668,Q6TMA8,Q9Z0V6
11265	1,7,9,10,12,14,16,17	2,3,4,5,6,8,11,13,15	5	P14668,P14669,P20762,Q6TMA8,Q9Z0V6
11266	1,7,9,10,12,15,16,17	2,3,4,5,6,8,11,13,14	9	P02793;Q7TP54,P06866,P14668,P14669,P20762,P97840,Q6AYS4,Q6TMA8,Q9Z0V6

11267	1,7,9,10,13,14,15,16	2,3,4,5,6,8,11,12,17	20	P01835,P06760,P07150,P08937,P11883,P14668,P14669,P16636,P19223,P20760,P20761,P20762,P50115,P62804,Q4G075,Q62635,Q63751,Q6AYS4,Q6IFW6,Q9Z0V6
11268	1,7,9,10,13,14,15,17	2,3,4,5,6,8,11,12,16	6	P11883,P14668,Q4FZU2,Q6IFW6,Q9JJS8,Q9Z0V6
11269	1,7,9,10,13,14,16,17	2,3,4,5,6,8,11,12,15	9	B0BNN3,P02625,P14668,P14669,P20762,P35053,Q4FZU2,Q6TMA8,Q9Z0V6
11270	1,7,9,10,13,15,16,17	2,3,4,5,6,8,11,12,14	12	P01835,P02793;Q7TP54,P11883,P14668,P14669,P20762,P62804,Q6AYS4,Q6IFW6,Q6TMA8,Q9EQS0,Q9Z0V6
11271	1,7,9,10,14,15,16,17	2,3,4,5,6,8,11,12,13	14	P02780,P02782,P07647,P0C0A9,P11883,P14668,P14669,P20762,P35053,Q5XI22,Q6AYS4,Q6TMA8,Q9JHB9,Q9Z0V6
11272	1,7,9,11,12,13,14,15	2,3,4,5,6,8,10,16,17	8	P06760,P08649,P16636,P20760,P20761,P23593,Q6P6R2,Q9Z0V6
11273	1,7,9,11,12,13,14,16	2,3,4,5,6,8,10,15,17	9	B0BNN3,P02761,P06760,P08649,P20760,P20761,Q4G075,Q7TPB1,Q9Z0V6
11274	1,7,9,11,12,13,14,17	2,3,4,5,6,8,10,15,16	4	D3ZHA0,P14668,P36374,Q9Z0V6
11275	1,7,9,11,12,13,15,16	2,3,4,5,6,8,10,14,17	8	P02793;Q7TP54,P06760,P08649,P20760,P20761,P20762,Q9WTT6,Q9Z0V6
11276	1,7,9,11,12,13,15,17	2,3,4,5,6,8,10,14,16	2	P14668,Q9Z0V6
11277	1,7,9,11,12,13,16,17	2,3,4,5,6,8,10,14,15	3	D3ZHA0,P20762,Q9Z0V6
11278	1,7,9,11,12,14,15,16	2,3,4,5,6,8,10,13,17	7	P06760,P16636,P20760,P20761,P51907,Q63355,Q9Z0V6
11279	1,7,9,11,12,14,15,17	2,3,4,5,6,8,10,13,16	1	Q9Z0V6
11280	1,7,9,11,12,14,16,17	2,3,4,5,6,8,10,13,15	3	D3ZHA0,Q6TMA8,Q9Z0V6
11281	1,7,9,11,12,15,16,17	2,3,4,5,6,8,10,13,14	4	P06866,P20762,Q6TMA8,Q9Z0V6
11282	1,7,9,11,13,14,15,16	2,3,4,5,6,8,10,12,17	15	D3ZHA0,D4A5U3,P06760,P08937,P16636,P19223,P20760,P20761,P50115,Q4G075,Q62635,Q64361,Q9JJS8,Q9WTT6,Q9Z0V6
11283	1,7,9,11,13,14,15,17	2,3,4,5,6,8,10,12,16	5	D3ZHA0,P14668,Q64361,Q9JJS8,Q9Z0V6
11284	1,7,9,11,13,14,16,17	2,3,4,5,6,8,10,12,15	4	D3ZHA0,P04218,Q64361,Q9Z0V6
11285	1,7,9,11,13,15,16,17	2,3,4,5,6,8,10,12,14	7	D3ZHA0,P20762,Q64361,Q9EQS0,Q9JJS8,Q9WTT6,Q9Z0V6
11286	1,7,9,11,14,15,16,17	2,3,4,5,6,8,10,12,13	7	D3ZHA0,P04218,P16636,Q64361,Q6TMA8,Q9JJS8,Q9Z0V6
11287	1,7,9,12,13,14,15,16	2,3,4,5,6,8,10,11,17	15	P06760,P07150,P08649,P11883,P14668,P14669,P16636,P20646,P20760,P20761,P50115,Q4G075,Q63598,Q6AYS4,Q9Z0V6
11288	1,7,9,12,13,14,15,17	2,3,4,5,6,8,10,11,16	5	P08649,P11883,P14668,Q6AYS4,Q9Z0V6
11289	1,7,9,12,13,14,16,17	2,3,4,5,6,8,10,11,15	8	D3ZHA0,P02761,P08649,P14668,P14669,P20762,Q6AYS4,Q9Z0V6

11290	1,7,9,12,13,15,16,17	2,3,4,5,6,8,10,11,14	11	P02793;Q7TP54,P08649,P11883,P14668,P14669,P19218,P20762,Q5BJY9,Q63598,Q6AYS4,Q9Z0V6
11291	1,7,9,12,14,15,16,17	2,3,4,5,6,8,10,11,13	7	P11883,P14668,P14669,Q63598,Q6AYS4,Q6TMA8,Q9Z0V6
11292	1,7,9,13,14,15,16,17	2,3,4,5,6,8,10,11,12	19	D3ZHA0,P0C0A9,P11883,P14668,P14669,P16636,P20762,P62804,Q4FZU2,Q63598,Q64361,Q6AYS4,Q6IFW6,Q6IMF3,Q6P6Q2,Q6TMA8,Q9EQS0,Q9JJS8,Q9Z0V6
11293	1,7,10,11,12,13,14,15	2,3,4,5,6,8,9,16,17	6	P06760,P20761,P22006,P23593,Q6P6R2,Q9Z0V6
11294	1,7,10,11,12,13,14,16	2,3,4,5,6,8,9,15,17	8	B0BNN3,P06760,P20760,P20761,P23593,Q5QE79,Q7TPB1,Q9Z0V6
11295	1,7,10,11,12,13,14,17	2,3,4,5,6,8,9,15,16	6	P14668,P23593,P36374,P47727,Q5QE79,Q9Z0V6
11296	1,7,10,11,12,13,15,16	2,3,4,5,6,8,9,14,17	9	P01835,P02793;Q7TP54,P06760,P20760,P20761,P20762,P50115,Q9WTT6,Q9Z0V6
11297	1,7,10,11,12,13,15,17	2,3,4,5,6,8,9,14,16	3	P02793;Q7TP54,P14668,Q9Z0V6
11298	1,7,10,11,12,13,16,17	2,3,4,5,6,8,9,14,15	6	P02793;Q7TP54,P06757,P20762,Q5QE79,Q6TMA8,Q9Z0V6
11299	1,7,10,11,12,14,15,16	2,3,4,5,6,8,9,13,17	9	P06760,P20760,P20761,P20766,P23593,P51907,Q63355,Q6TMA8,Q9Z0V6
11300	1,7,10,11,12,14,15,17	2,3,4,5,6,8,9,13,16	4	P20766,P22006,Q6TMA8,Q9Z0V6
11301	1,7,10,11,12,14,16,17	2,3,4,5,6,8,9,13,15	4	P20766,Q5QE79,Q6TMA8,Q9Z0V6
11302	1,7,10,11,12,15,16,17	2,3,4,5,6,8,9,13,14	7	P02793;Q7TP54,P06757,P06866,P20762,P20766,Q6TMA8,Q9Z0V6
11303	1,7,10,11,13,14,15,16	2,3,4,5,6,8,9,12,17	12	P01835,P06760,P16636,P19223,P20646,P20761,P23593,P50115,Q4G075,Q62635,Q7TPB1,Q9Z0V6
11304	1,7,10,11,13,14,15,17	2,3,4,5,6,8,9,12,16	5	P14668,P22006,P23593,Q9JJS8,Q9Z0V6
11305	1,7,10,11,13,14,16,17	2,3,4,5,6,8,9,12,15	5	D3ZHA0,P20762,Q5QE79,Q6TMA8,Q9Z0V6

11306	1,7,10,11,13,15,16,17	2,3,4,5,6,8,9,12,14	9	D3ZHA0,P01835,P02793;Q7TP54,P20762,Q6TMA8,Q9EQS0,Q9JJS8,Q9WTT6,Q9Z0V6
11307	1,7,10,11,14,15,16,17	2,3,4,5,6,8,9,12,13	3	P20766,Q6TMA8,Q9Z0V6
11308	1,7,10,12,13,14,15,16	2,3,4,5,6,8,9,11,17	10	P06760,P08649,P14668,P14669,P20646,P20761,P20762,P50115,Q6AYS4,Q9Z0V6
11309	1,7,10,12,13,14,15,17	2,3,4,5,6,8,9,11,16	4	P14668,Q6AYS4,Q6TMA8,Q9Z0V6
11310	1,7,10,12,13,14,16,17	2,3,4,5,6,8,9,11,15	7	P14668,P14669,P20762,Q5QE79,Q6AYS4,Q6TMA8,Q9Z0V6
11311	1,7,10,12,13,15,16,17	2,3,4,5,6,8,9,11,14	9	P02793;Q7TP54,P06866,P14668,P14669,P19218,P20762,Q6AYS4,Q6TMA8,Q9Z0V6
11312	1,7,10,12,14,15,16,17	2,3,4,5,6,8,9,11,13	6	P06866,P14668,P20766,Q6AYS4,Q6TMA8,Q9Z0V6
11313	1,7,10,13,14,15,16,17	2,3,4,5,6,8,9,11,12	10	P01835,P11883,P14668,P14669,P20762,Q6AYS4,Q6IFW6,Q6TMA8,Q9EQS0,Q9Z0V6
11314	1,7,11,12,13,14,15,16	2,3,4,5,6,8,9,10,17	12	D3ZHA0,P06760,P08649,P16636,P20646,P20760,P20761,P23593,P50115,Q63355,Q6P6R2,Q9Z0V6
11315	1,7,11,12,13,14,15,17	2,3,4,5,6,8,9,10,16	4	D3ZHA0,P14668,P23593,Q9Z0V6
11316	1,7,11,12,13,14,16,17	2,3,4,5,6,8,9,10,15	3	D3ZHA0,O55006,Q9Z0V6
11317	1,7,11,12,13,15,16,17	2,3,4,5,6,8,9,10,14	6	D3ZHA0,P02793;Q7TP54,P20762,Q6TMA8,Q9WTT6,Q9Z0V6
11318	1,7,11,12,14,15,16,17	2,3,4,5,6,8,9,10,13	5	D3ZHA0,P06866,P20766,Q6TMA8,Q9Z0V6
11319	1,7,11,13,14,15,16,17	2,3,4,5,6,8,9,10,12	7	D3ZHA0,P16636,Q64361,Q6TMA8,Q9EQS0,Q9JJS8,Q9Z0V6
11320	1,7,12,13,14,15,16,17	2,3,4,5,6,8,9,10,11	9	D3ZHA0,P08649,P11883,P14173,P14668,P14669,Q6AYS4,Q6TMA8,Q9Z0V6

11321	1,8,9,10,1<br>1,12,13,14	2,3,4,5,6,7<br>,15,16,17	6	P02803,P09605;P25809,P20760,P20761,P47727,P83748
11322	1,8,9,10,1<br>1,12,13,15	2,3,4,5,6,7<br>,14,16,17	5	P02803,P09605;P25809,P20760,P20761,P83748
11323	1,8,9,10,1<br>1,12,13,16	2,3,4,5,6,7<br>,14,15,17	10	P02631,P02803,P10536,P20760,P20761,P20762,P36376,P62815,P83748,Q9WUW8
11324	1,8,9,10,1<br>1,12,13,17	2,3,4,5,6,7<br>,14,15,16	8	P02631,P02803,P20760,P20762,P36374,P36376,P83748,Q63532
11325	1,8,9,10,1<br>1,12,14,15	2,3,4,5,6,7<br>,13,16,17	4	P09605;P25809,P20760,P20761,P83748
11326	1,8,9,10,1<br>1,12,14,16	2,3,4,5,6,7<br>,13,15,17	7	P08937,P20760,P20761,P62815,P83748,Q63751,Q9WUW8
11327	1,8,9,10,1<br>1,12,14,17	2,3,4,5,6,7<br>,13,15,16	2	P20760,P83748
11328	1,8,9,10,1<br>1,12,15,16	2,3,4,5,6,7<br>,13,14,17	6	O88267,P20760,P20761,P20762,P62815,Q9WUW8
11329	1,8,9,10,1<br>1,12,15,17	2,3,4,5,6,7<br>,13,14,16	2	G3V686,P83748
11330	1,8,9,10,1<br>1,12,16,17	2,3,4,5,6,7<br>,13,14,15	6	P02631,P02803,P20760,P20762,P36376,P83748
11331	1,8,9,10,1<br>1,13,14,15	2,3,4,5,6,7<br>,12,16,17	8	O88267,P08937,P09605;P25809,P20760,P20761,P55091,P83748,Q63751
11332	1,8,9,10,1<br>1,13,14,16	2,3,4,5,6,7<br>,12,15,17	11	P08937,P09605;P25809,P19223,P20760,P20761,P20762,P55091,P62815,P83748,Q63751,Q7TPB1
11333	1,8,9,10,1<br>1,13,14,17	2,3,4,5,6,7<br>,12,15,16	3	P01836,P20760,P83748
11334	1,8,9,10,1<br>1,13,15,16	2,3,4,5,6,7<br>,12,14,17	13	O88267,P01835,P08937,P09605;P25809,P20760,P20761,P20762,P34901,P55091,P62815,P83748,Q63751,Q9Z0V6
11335	1,8,9,10,1<br>1,13,15,17	2,3,4,5,6,7<br>,12,14,16	6	G3V686,P04073,P20762,P34901,P83748,Q63532
11336	1,8,9,10,1<br>1,13,16,17	2,3,4,5,6,7<br>,12,14,15	8	A2RUV9,P02631,P02803,P11762,P20760,P20762,P34901,P83748
11337	1,8,9,10,1<br>1,14,15,16	2,3,4,5,6,7<br>,12,13,17	11	O88267,P08937,P09605;P25809,P20760,P20761,P55091,P62815,P83748,Q62635,Q63751,Q9QZK9
11338	1,8,9,10,1<br>1,14,15,17	2,3,4,5,6,7<br>,12,13,16	4	G3V686,O88267,P01836,P83748
11339	1,8,9,10,1<br>1,14,16,17	2,3,4,5,6,7<br>,12,13,15	4	P01836,P08937,P20760,P83748
11340	1,8,9,10,1<br>1,15,16,17	2,3,4,5,6,7<br>,12,13,14	5	O88267,P08937,P20762,P25236,P83748
11341	1,8,9,10,1<br>2,13,14,15	2,3,4,5,6,7<br>,11,16,17	7	P09605;P25809,P10758,P15999,P20760,P20761,P61206;P84079;P84082,Q63751
11342	1,8,9,10,1<br>2,13,14,16	2,3,4,5,6,7<br>,11,15,17	7	P02631,P02803,P09605;P25809,P20760,P20761,P20762,Q63751
11343	1,8,9,10,1<br>2,13,14,17	2,3,4,5,6,7<br>,11,15,16	4	P02631,P14668,P61206;P84079;P84082,P83748

11344	1,8,9,10,12,13,15,16 2,3,4,5,6,7,11,14,17	7	P02631,P09605;P25809,P16086,P20760,P20761,P20762,Q63751
11345	1,8,9,10,12,13,15,17 2,3,4,5,6,7,11,14,16	8	G3V686,P02631,P14668,P15999,P16086,P20762,P61206;P84079;P84082,Q63532
11346	1,8,9,10,12,13,16,17 2,3,4,5,6,7,11,14,15	5	P02631,P02803,P20760,P20762,P36376
11347	1,8,9,10,12,14,15,16 2,3,4,5,6,7,11,13,17	5	P08937,P09605;P25809,P20760,P20761,Q63751
11348	1,8,9,10,12,14,15,17 2,3,4,5,6,7,11,13,16	3	G3V686,P61206;P84079;P84082,Q8VD89
11349	1,8,9,10,12,14,16,17 2,3,4,5,6,7,11,13,15	5	P02631,P05539,P20760,P20762,P83748
11350	1,8,9,10,12,15,16,17 2,3,4,5,6,7,11,13,14	4	P05539,P06866,P16086,P20762
11351	1,8,9,10,13,14,15,16 2,3,4,5,6,7,11,12,17	13	P01835,P08937,P09605;P25809,P19223,P20646,P20760,P20761,P20762,P55091,P83748,Q498R7,Q63751,Q9QZK9
11352	1,8,9,10,13,14,15,17 2,3,4,5,6,7,11,12,16	7	G3V686,P01836,P15999,P61206;P84079;P84082,P83748,Q498R7,Q6IFW6
11353	1,8,9,10,13,14,16,17 2,3,4,5,6,7,11,12,15	8	P01836,P02625,P02631,P08937,P20762,P83748,Q498R7,Q4FZU2
11354	1,8,9,10,13,15,16,17 2,3,4,5,6,7,11,12,14	9	P01835,P02631,P08937,P20762,P34901,P61206;P84079;P84082,P83748,Q498R7,Q9EQS0
11355	1,8,9,10,14,15,16,17 2,3,4,5,6,7,11,12,13	8	P01836,P05539,P08937,P20762,P61206;P84079;P84082,P83748,Q498R7,Q63751
11356	1,8,9,11,12,13,14,15 2,3,4,5,6,7,10,16,17	5	P09605;P25809,P10758,P20760,P20761,P83748
11357	1,8,9,11,12,13,14,16 2,3,4,5,6,7,10,15,17	8	P02803,P04218,P20760,P20761,P36376,P83748,P97615,Q7TPB1
11358	1,8,9,11,12,13,14,17 2,3,4,5,6,7,10,15,16	5	P02803,P04218,P20760,P36376,P83748
11359	1,8,9,11,12,13,15,16 2,3,4,5,6,7,10,14,17	5	P20760,P20761,P20762,P36376,P97615
11360	1,8,9,11,12,13,15,17 2,3,4,5,6,7,10,14,16	5	P04218,P36376,P61206;P84079;P84082,P83748,Q63532
11361	1,8,9,11,12,13,16,17 2,3,4,5,6,7,10,14,15	8	P02631,P02803,P04218,P20760,P20762,P36376,P83748,P97615
11362	1,8,9,11,12,14,15,16 2,3,4,5,6,7,10,13,17	4	P04218,P08937,P20760,P20761
11363	1,8,9,11,12,14,15,17 2,3,4,5,6,7,10,13,16	3	P04218,P61206;P84079;P84082,P83748
11364	1,8,9,11,12,14,16,17 2,3,4,5,6,7,10,13,15	4	P04218,P20760,P36376,P83748
11365	1,8,9,11,12,15,16,17 2,3,4,5,6,7,10,13,14	5	P04218,P12020,P20762,P36376,P83748
11366	1,8,9,11,13,14,15,16 2,3,4,5,6,7,10,12,17	12	P04218,P08937,P09605;P25809,P19223,P20646,P20760,P20761,P55091,P83748,Q63751,Q9QZK9,Q9Z0V6

11367	1,8,9,11,13,14,15,17	2,3,4,5,6,7,10,12,16	4	P04218,P61206;P84079;P84082,P83748,Q9JJS8
11368	1,8,9,11,13,14,16,17	2,3,4,5,6,7,10,12,15	3	P04218,P20760,P83748
11369	1,8,9,11,13,15,16,17	2,3,4,5,6,7,10,12,14	6	P04218,P20762,P34901,P36376,P83748,Q9EQS0
11370	1,8,9,11,14,15,16,17	2,3,4,5,6,7,10,12,13	3	P04218,P08937,P83748
11371	1,8,9,12,13,14,15,16	2,3,4,5,6,7,10,11,17	8	P08937,P09605;P25809,P20646,P20760,P20761,P28648,P61206;P84079;P84082,Q63751
11372	1,8,9,12,13,14,15,17	2,3,4,5,6,7,10,11,16	4	P11883,P15999,P61206;P84079;P84082,Q5FVI6
11373	1,8,9,12,13,14,16,17	2,3,4,5,6,7,10,11,15	7	P02631,P04218,P20760,P20762,P36376,P61206;P84079;P84082,Q5FVI6
11374	1,8,9,12,13,15,16,17	2,3,4,5,6,7,10,11,14	6	P11883,P16086,P20762,P36376,P61206;P84079;P84082,Q5BJY9
11375	1,8,9,12,14,15,16,17	2,3,4,5,6,7,10,11,13	3	P04218,P28648,P61206;P84079;P84082
11376	1,8,9,13,14,15,16,17	2,3,4,5,6,7,10,11,12	12	P04218,P08937,P11883,P20762,P28648,P61206;P84079;P84082,P83748,Q498R7,Q4FZU2,Q5FVI6,Q6IFW6,Q9EQS0
11377	1,8,10,11,12,13,14,15	2,3,4,5,6,7,9,16,17	7	P09605;P25809,P15999,P20646,P20760,P20761,P23593,P47727
11378	1,8,10,11,12,13,14,16	2,3,4,5,6,7,9,15,17	8	P02631,P09605;P25809,P20760,P20761,P47727,P52590,P83748,Q7TPB1
11379	1,8,10,11,12,13,14,17	2,3,4,5,6,7,9,15,16	4	P02631,P47727,P83748,P97605
11380	1,8,10,11,12,13,15,16	2,3,4,5,6,7,9,14,17	6	P01835,P09605;P25809,P20760,P20761,P20762,P52590
11381	1,8,10,11,12,13,15,17	2,3,4,5,6,7,9,14,16	3	P15999,P83748,P97605
11382	1,8,10,11,12,13,16,17	2,3,4,5,6,7,9,14,15	6	P02631,P02803,P20762,P36376,P83748,P97605
11383	1,8,10,11,12,14,15,16	2,3,4,5,6,7,9,13,17	4	P09605;P25809,P20760,P20761,P52590
11384	1,8,10,11,12,14,15,17	2,3,4,5,6,7,9,13,16	1	P83748

11385	1,8,10,11,12,14,16,17	2,3,4,5,6,7,9,13,15	3	P05539,P83748,P97605
11386	1,8,10,11,12,15,16,17	2,3,4,5,6,7,9,13,14	4	P06866,P20762,P83748,P97605
11387	1,8,10,11,13,14,15,16	2,3,4,5,6,7,9,12,17	13	P01835,P08937,P09605;P25809,P19223,P20646,P20760,P20761,P52590,P55091,P83748,Q498R7,Q63751,Q7TPB1
11388	1,8,10,11,13,14,15,17	2,3,4,5,6,7,9,12,16	3	P15999,P83748,Q498R7
11389	1,8,10,11,13,14,16,17	2,3,4,5,6,7,9,12,15	4	P02631,P20762,P83748,Q498R7
11390	1,8,10,11,13,15,16,17	2,3,4,5,6,7,9,12,14	7	P01835,P20762,P34901,P83748,P97605,Q498R7,Q9EQS0
11391	1,8,10,11,14,15,16,17	2,3,4,5,6,7,9,12,13	2	P83748,Q498R7
11392	1,8,10,12,13,14,15,16	2,3,4,5,6,7,9,11,17	8	P09605;P25809,P15999,P20646,P20760,P20761,P20762,P52590,Q63751
11393	1,8,10,12,13,14,15,17	2,3,4,5,6,7,9,11,16	3	P14668,P15999,P61206;P84079;P84082
11394	1,8,10,12,13,14,16,17	2,3,4,5,6,7,9,11,15	3	P02631,P20762,Q8K4G9
11395	1,8,10,12,13,15,16,17	2,3,4,5,6,7,9,11,14	5	P02631,P15999,P20762,P61206;P84079;P84082,P83121
11396	1,8,10,12,14,15,16,17	2,3,4,5,6,7,9,11,13	3	P05539,P06866,P61206;P84079;P84082
11397	1,8,10,13,14,15,16,17	2,3,4,5,6,7,9,11,12	8	P01835,P11883,P15999,P20762,P61206;P84079;P84082,P83748,Q498R7,Q9EQS0
11398	1,8,11,12,13,14,15,16	2,3,4,5,6,7,9,10,17	5	P09605;P25809,P20646,P20760,P20761,P52590
11399	1,8,11,12,13,14,15,17	2,3,4,5,6,7,9,10,16	3	P15999,P61206;P84079;P84082,P83748

11400	1,8,11,12,13,14,16,17	2,3,4,5,6,7,9,10,15	3	O55006,P36376,P83748
11401	1,8,11,12,13,15,16,17	2,3,4,5,6,7,9,10,14	3	P20762,P36376,P97605
11402	1,8,11,12,14,15,16,17	2,3,4,5,6,7,9,10,13	2	P04218,P83748
11403	1,8,11,13,14,15,16,17	2,3,4,5,6,7,9,10,12	3	P04218,P83748,Q498R7
11404	1,8,12,13,14,15,16,17	2,3,4,5,6,7,9,10,11	6	P11883,P14173,P15999,P28648,P61206;P84079;P84082,Q5FVI6
11405	1,9,10,11,12,13,14,15	2,3,4,5,6,7,8,16,17	21	O88267,P02631,P06760,P08937,P09605;P25809,P10758,P16086,P20646,P20760,P20761,P20762,P23928,P50115,P50116,P83748,Q4G075,Q63751,Q6P6R2,Q99041,Q9R0T3,Q9Z0V6
11406	1,9,10,11,12,13,14,16	2,3,4,5,6,7,8,15,17	23	B0BNN3,D3ZHA0,P02631,P02761,P02803,P06760,P07171,P08937,P09605;P25809,P10536,P20646,P20760,P20761,P20762,P50115,P62815,P83748,Q4G075,Q63751,Q64361,Q7TPB1,Q99041,Q9Z0V6
11407	1,9,10,11,12,13,14,17	2,3,4,5,6,7,8,15,16	11	D3ZHA0,P02631,P02803,P07171,P08937,P14668,P20760,P20761,P20762,P83748,Q9Z0V6
11408	1,9,10,11,12,13,15,16	2,3,4,5,6,7,8,14,17	23	D3ZHA0,O88267,P02631,P02793;Q7TP54,P06760,P07171,P08937,P10536,P16086,P20646,P20760,P20761,P20762,P50115,P55091,P62815,P83748,Q4G075,Q63751,Q99041,Q9EQS0,Q9WTT6,Q9Z0V6
11409	1,9,10,11,12,13,15,17	2,3,4,5,6,7,8,14,16	13	D3ZHA0,O88267,P02631,P07171,P08937,P14668,P16086,P20760,P20761,P20762,P83748,Q9EQS0,Q9Z0V6
11410	1,9,10,11,12,13,16,17	2,3,4,5,6,7,8,14,15	16	D3ZHA0,O55096,P02631,P02803,P07171,P08937,P10536,P16086,P20760,P20761,P20762,P83748,P97605,P97615,Q9EQS0,Q9Z0V6
11411	1,9,10,11,12,14,15,16	2,3,4,5,6,7,8,13,17	25	O88267,P06760,P06761,P08937,P09605;P25809,P10536,P16086,P20760,P20761,P20762,P20766,P22282,P50115,P51907,P55091,P62815,P70545,P83748,Q4G075,Q63751,Q99041,Q9EPB1,Q9R0T3,Q9WUW8,Q9Z0V6
11412	1,9,10,11,12,14,15,17	2,3,4,5,6,7,8,13,16	10	D3ZHA0,O88267,P08937,P16086,P20760,P20761,P20762,P83748,Q9R0T3,Q9Z0V6
11413	1,9,10,11,12,14,16,17	2,3,4,5,6,7,8,13,15	14	D3ZHA0,P02631,P06761,P07171,P08937,P16086,P20760,P20761,P20762,P62815,P83748,Q63751,Q6TMA8,Q9Z0V6
11414	1,9,10,11,12,15,16,17	2,3,4,5,6,7,8,13,14	20	D3ZHA0,O88267,P02631,P06761,P06866,P06911,P07171,P08937,P12020,P16086,P20760,P20761,P20762,P22282,P62815,P83748,P97605,Q6TMA8,Q9EQS0,Q9Z0V6

11415	1,9,10,11,13,14,15,16	2,3,4,5,6,7,8,12,17	35	D3ZHA0,O55004,O88267,P02631,P06760,P06761,P07647,P08937,P09605;P25809,P10536,P19223,P20646,P20760,P20761,P20762,P22282,P50115,P55091,P62815,P83748,P98089,Q4G075,Q62635,Q63751,Q64361,Q6AYS7,Q7TPB1,Q80WL1,Q99041,Q9EPB1,Q9EQS0,Q9QZK9,Q9R0T3,Q9WTT6,Q9Z0V6
11416	1,9,10,11,13,14,15,17	2,3,4,5,6,7,8,12,16	18	D3ZHA0,O88267,P02631,P06761,P08937,P14668,P20646,P20760,P20761,P20762,P83748,Q63751,Q64361,Q6AYS7,Q99041,Q9EQS0,Q9R0T3,Q9Z0V6
11417	1,9,10,11,13,14,16,17	2,3,4,5,6,7,8,12,15	21	D3ZHA0,O55004,O88267,P02631,P06761,P07171,P08937,P20646,P20760,P20761,P20762,P62815,P83748,Q63751,Q64361,Q64611,Q6AYS7,Q7TPB1,Q99041,Q9EQS0,Q9Z0V6
11418	1,9,10,11,13,15,16,17	2,3,4,5,6,7,8,12,14	24	D3ZHA0,O88267,P02631,P04636,P06761,P07171,P07647,P08937,P16086,P20760,P20761,P20762,P62815,P83748,Q63751,Q64361,Q6AYS7,Q6TMA8,Q80WL1,Q99041,Q9EQS0,Q9R0T3,Q9WTT6,Q9Z0V6
11419	1,9,10,11,14,15,16,17	2,3,4,5,6,7,8,12,13	25	D3ZHA0,O88267,P02631,P02780,P06761,P07647,P08937,P16086,P20760,P20761,P20762,P22282,P22283,P30120,P62815,P83748,Q498R7,Q63751,Q64361,Q6TMA8,Q80WL1,Q99041,Q9EQS0,Q9R0T3,Q9Z0V6
11420	1,9,10,12,13,14,15,16	2,3,4,5,6,7,8,11,17	28	O55004,O88267,P02631,P06760,P07150,P07171,P08937,P09605;P25809,P11883,P14668,P14669,P16086,P20646,P20760,P20761,P20762,P28648,P50115,P55091,P61206;P84079;P84082,Q4G075,Q63751,Q64361,Q6AYS4,Q99041,Q9EQS0,Q9R0T3,Q9Z0V6
11421	1,9,10,12,13,14,15,17	2,3,4,5,6,7,8,11,16	21	D3ZHA0,P02631,P07150,P07171,P08937,P11883,P14668,P14669,P16086,P20646,P20760,P20761,P20762,P61206;P84079;P84082,Q07936,Q63751,Q6AYS4,Q8VD89,Q9EQS0,Q9R0T3,Q9Z0V6
11422	1,9,10,12,13,14,16,17	2,3,4,5,6,7,8,11,15	19	D3ZHA0,O55004,P02631,P02761,P07150,P07171,P08937,P14668,P14669,P16086,P20646,P20760,P20761,P20762,P83748,Q63751,Q6AYS4,Q9EQS0,Q9Z0V6
11423	1,9,10,12,13,15,16,17	2,3,4,5,6,7,8,11,14	20	D3ZHA0,P02631,P02793;Q7TP54,P06866,P07150,P07171,P08937,P11883,P14668,P14669,P16086,P20760,P20761,P20762,P61206;P84079;P84082,Q63751,Q6AYS4,Q6TMA8,Q9EQS0,Q9Z0V6
11424	1,9,10,12,14,15,16,17	2,3,4,5,6,7,8,11,13	27	D3ZHA0,P02631,P06761,P06866,P07150,P07171,P07647,P08937,P11883,P14668,P14669,P16086,P20760,P20761,P20762,P20766,P22282,P22283,P28648,P61206;P84079;P84082,Q5XI22,Q63751,Q6AYS4,Q6TMA8,Q9EQS0,Q9R0T3,Q9Z0V6
11425	1,9,10,13,14,15,16,17	2,3,4,5,6,7,8,11,12	40	D3ZHA0,O55004,O88267,P02631,P02780,P02781,P06761,P07150,P07171,P07647,P08937,P0C0A9,P11883,P14668,P14669,P16086,P20646,P20760,P20761,P20762,P22282,P22283,P28648,P30120,P50115,P61206;P84079;P84082,P83748,Q07936,Q498R7,Q63751,Q64361,Q6AYS4,Q6IFW6,Q6TMA8,Q80WL1,Q99041,Q9EQS0,Q9ESW0,Q9R0T3,Q9Z0V6
11426	1,9,11,12,13,14,15,16	2,3,4,5,6,7,8,10,17	28	D3ZHA0,O88267,P06760,P08937,P16086,P19223,P20646,P20760,P20761,P20762,P28648,P46844,P50115,P52590,P62898,P82471,P83748,P97571,P97615,Q4G075,Q63751,Q64361,Q6P6R2,Q99041,Q9EPB1,Q9R0T3,Q9WTT6,Q9Z0V6
11427	1,9,11,12,13,14,15,17	2,3,4,5,6,7,8,10,16	17	D3ZHA0,P04218,P08937,P14668,P16086,P20646,P20760,P20761,P20762,P61206;P84079;P84082,P82471,P83748,P97571,P97615,Q64361,Q9R0T3,Q9Z0V6
11428	1,9,11,12,13,14,16,17	2,3,4,5,6,7,8,10,15	17	D3ZHA0,O55006,P02631,P02761,P04218,P07171,P08937,P20646,P20760,P20761,P20762,P82471,P83748,P97571,P97615,Q64361,Q9Z0V6
11429	1,9,11,12,13,15,16,17	2,3,4,5,6,7,8,10,14	21	D3ZHA0,P02631,P04218,P07171,P08937,P12020,P16086,P20760,P20761,P20762,P28648,P36376,P82471,P83748,P97571,P97615,Q5BJY9,Q64361,Q9EQS0,Q9WTT6,Q9Z0V6

11430	1,9,11,12,14,15,16,17	2,3,4,5,6,7,8,10,13	20	D3ZHA0,P04218,P06761,P06866,P07171,P08937,P12020,P16086,P20760,P20761,P20762,P28648,P82471,P83748,P97571,P97615,Q64361,Q6TMA8,Q9R0T3,Q9Z0V6
11431	1,9,11,13,14,15,16,17	2,3,4,5,6,7,8,10,12	25	D3ZHA0,O88267,P04218,P06761,P07647,P08937,P11883,P20646,P20760,P20761,P20762,P28648,P82471,P83748,P97571,P97615,Q63475,Q63751,Q64361,Q80WL1,Q99041,Q9EQS0,Q9R0T3,Q9WTT6,Q9Z0V6
11432	1,9,12,13,14,15,16,17	2,3,4,5,6,7,8,10,11	27	D3ZHA0,P00714,P02631,P07150,P07171,P08649,P08937,P11883,P14668,P14669,P16086,P20646,P20760,P20761,P20762,P28648,P61206;P84079;P84082,P82471,P97615,Q5BJY9,Q63598,Q63751,Q64361,Q6AYS4,Q9EQS0,Q9R0T3,Q9Z0V6
11433	1,10,11,12,13,14,15,16	2,3,4,5,6,7,8,9,17	23	D3ZHA0,O88267,P02631,P06760,P08937,P09605;P25809,P16086,P19223,P20646,P20760,P20761,P20762,P23593,P50115,P52590,Q4G075,Q63751,Q6P6R2,Q7TPB1,Q99041,Q9EPB1,Q9WTT6,Q9Z0V6
11434	1,10,11,12,13,14,15,17	2,3,4,5,6,7,8,9,16	10	D3ZHA0,P02631,P14668,P16086,P20646,P20761,P20762,P83748,P97605,Q9Z0V6
11435	1,10,11,12,13,14,16,17	2,3,4,5,6,7,8,9,15	14	D3ZHA0,O55006,P02631,P07171,P08937,P20646,P20760,P20761,P20762,P83748,P97605,Q5QE79,Q6TMA8,Q9Z0V6
11436	1,10,11,12,13,15,16,17	2,3,4,5,6,7,8,9,14	15	D3ZHA0,P02631,P06866,P07171,P08937,P16086,P20760,P20761,P20762,P83748,P97605,Q6TMA8,Q9EQS0,Q9WTT6,Q9Z0V6
11437	1,10,11,12,14,15,16,17	2,3,4,5,6,7,8,9,13	17	D3ZHA0,O88267,P02631,P02764,P06761,P06866,P08937,P16086,P20760,P20761,P20762,P20766,P83748,P97605,Q6TMA8,Q9R0T3,Q9Z0V6
11438	1,10,11,13,14,15,16,17	2,3,4,5,6,7,8,9,12	22	D3ZHA0,O55004,O55006,O88267,P02631,P06761,P07647,P08937,P20646,P20760,P20761,P20762,P83748,Q498R7,Q63751,Q64361,Q6TMA8,Q80WL1,Q99041,Q9EQS0,Q9R0T3,Q9Z0V6
11439	1,10,12,13,14,15,16,17	2,3,4,5,6,7,8,9,11	21	D3ZHA0,P02631,P06866,P07150,P07171,P08932,P08937,P11883,P14668,P14669,P16086,P20646,P20761,P20762,P28648,P61206;P84079;P84082,Q63751,Q6AYS4,Q6TMA8,Q9EQS0,Q9Z0V6
11440	1,11,12,13,14,15,16,17	2,3,4,5,6,7,8,9,10	17	D3ZHA0,O55006,P02631,P07171,P08937,P20646,P20760,P20761,P20762,P28648,P82471,P83748,P97571,P97615,Q64361,Q9EQS0,Q9Z0V6
11441	2,3,4,5,6,7,8,9	1,10,11,12,13,14,15,16,17	28	D3ZHA0,O55006,O88267,P02631,P02764,P06761,P06866,P07171,P08937,P16086,P20646,P20760,P20761,P20762,P28648,P50115,P52590,P83748,P97571,P97605,Q63617,Q63751,Q64361,Q6TMA8,Q9EQS0,Q9R0T3,Q9WTT6,Q9Z0V6
11442	2,3,4,5,6,7,8,10	1,9,11,12,13,14,15,16,17	30	D3ZHA0,O55006,O88267,P02631,P04218,P06761,P07171,P08649,P08937,P11883,P16086,P20646,P20760,P20761,P20762,P28648,P61206;P84079;P84082,P82471,P83748,P97571,P97615,Q63475,Q63751,Q64361,Q6AYS4,Q6TMA8,Q9EQS0,Q9R0T3,Q9WTT6,Q9Z0V6
11443	2,3,4,5,6,7,8,11	1,9,10,12,13,14,15,16,17	31	D3ZHA0,O55004,O88267,P02631,P06761,P07150,P07171,P07647,P08937,P11883,P14668,P14669,P16086,P20646,P20760,P20761,P20762,P22282,P28648,P50115,P61206;P84079;P84082,P83748,Q63617,Q63751,Q64361,Q6AYS4,Q6TMA8,Q99041,Q9EQS0,Q9R0T3,Q9Z0V6
11444	2,3,4,5,6,7,8,12	1,9,10,11,13,14,15,16,17	33	D3ZHA0,O55004,O88267,P02631,P02780,P06761,P07171,P07647,P08937,P16086,P19223,P20646,P20760,P20761,P20762,P22282,P22283,P30120,P50115,P62815,P83748,Q498R7,Q63475,Q63751,Q64361,Q6AYS7,Q6TMA8,Q80WL1,Q99041,Q9EQS0,Q9R0T3,Q9WTT6,Q9Z0V6

11445	2,3,4,5,6,7,8,13	1,9,10,11,12,14,15,16,17	28	D3ZHA0,O88267,P02631,P02764,P06761,P06866,P06911,P07171,P07647,P08937,P16086,P20760,P20761,P20762,P20766,P22282,P22283,P62815,P83748,Q63617,Q63751,Q64361,Q6TMA8,Q99041,Q9EQS0,Q9R0T3,Q9WTT6,Q9Z0V6
11446	2,3,4,5,6,7,8,14	1,9,10,11,12,13,15,16,17	28	D3ZHA0,O55096,O88267,P02631,P02793;Q7TP54,P06761,P06866,P07171,P08937,P10536,P16086,P20760,P20761,P20762,P62815,P83748,P97605,P97615,Q63617,Q63751,Q64361,Q6AYS7,Q6TMA8,Q923S2,Q9EQS0,Q9R0T3,Q9WTT6,Q9Z0V6
11447	2,3,4,5,6,7,8,15	1,9,10,11,12,13,14,16,17	26	D3ZHA0,O55004,O55006,O88267,P02631,P02761,P06761,P07171,P08937,P10536,P16086,P20646,P20760,P20761,P20762,P62815,P83748,P97571,P97605,P97615,Q63751,Q64361,Q6TMA8,Q9EQS0,Q9WTT6,Q9Z0V6
11448	2,3,4,5,6,7,8,16	1,9,10,11,12,13,14,15,17	21	D3ZHA0,O88267,P02631,P07171,P08937,P14668,P16086,P20646,P20760,P20761,P20762,P61206;P84079;P84082,P83748,Q63617,Q63751,Q64361,Q6TMA8,Q9EQS0,Q9R0T3,Q9WTT6,Q9Z0V6
11449	2,3,4,5,6,7,8,17	1,9,10,11,12,13,14,15,16	36	D3ZHA0,O54858,O55004,O88267,P02631,P02761,P06760,P06761,P07171,P08937,P09605;P25809,P10536,P16086,P19223,P20646,P20760,P20761,P20762,P22282,P50115,P52590,P55091,P62815,P83748,P97571,P97615,Q4G075,Q63617,Q63751,Q64361,Q6AYS4,Q99041,Q9EQS0,Q9R0T3,Q9WTT6,Q9Z0V6
11450	2,3,4,5,6,7,9,10	1,8,11,12,13,14,15,16,17	13	O55006,P02631,P04218,P16086,P20646,P20760,P20762,P28648,P34901,P36376,P52590,P61206;P84079;P84082,P83748
11451	2,3,4,5,6,7,9,11	1,8,10,12,13,14,15,16,17	10	P02631,P08937,P15999,P16086,P20646,P20760,P20762,P61206;P84079;P84082,P83748,Q63751
11452	2,3,4,5,6,7,9,12	1,8,10,11,13,14,15,16,17	12	P01835,P02631,P08937,P20646,P20760,P20761,P20762,P34901,P83748,Q498R7,Q9EQS0,Q9Z0V6
11453	2,3,4,5,6,7,9,13	1,8,10,11,12,14,15,16,17	9	P02631,P05539,P06866,P08937,P16086,P20760,P20762,P83748,P97605
11454	2,3,4,5,6,7,9,14	1,8,10,11,12,13,15,16,17	9	P02631,P16086,P20760,P20762,P34901,P36376,P83748,P97605,Q9Z0V6
11455	2,3,4,5,6,7,9,15	1,8,10,11,12,13,14,16,17	8	O55006,P02631,P20760,P20761,P20762,P36376,P83748,P97605
11456	2,3,4,5,6,7,9,16	1,8,10,11,12,13,14,15,17	7	P02631,P15999,P16086,P20760,P20762,P61206;P84079;P84082,P83748
11457	2,3,4,5,6,7,9,17	1,8,10,11,12,13,14,15,16	13	P02631,P08937,P09605;P25809,P16086,P20646,P20760,P20761,P20762,P23593,P52590,P83748,Q63751,Q9Z0V6
11458	2,3,4,5,6,7,10,11	1,8,9,12,13,14,15,16,17	16	P02631,P04218,P08937,P11883,P16086,P20760,P20762,P28648,P34901,P36376,P61206;P84079;P84082,P83748,P97615,Q5FVI6,Q63751,Q9Z0V6
11459	2,3,4,5,6,7,10,12	1,8,9,11,13,14,15,16,17	18	P04218,P08937,P17559,P20760,P20761,P20762,P28648,P34901,P61206;P84079;P84082,P83748,P97615,Q498R7,Q4FZU2,Q63475,Q63751,Q64361,Q9EQS0,Q9Z0V6

11460	2,3,4,5,6,7,10,13	1,8,9,11,12,14,15,16,17	10	P04218,P08937,P12020,P16086,P20760,P20762,P36376,P61206;P84079;P84082,P83748,P97615
11461	2,3,4,5,6,7,10,14	1,8,9,11,12,13,15,16,17	13	P02631,P04218,P08937,P12020,P16086,P20760,P20762,P34901,P36376,P61206;P84079;P84082,P83748,P97615,Q9Z0V6
11462	2,3,4,5,6,7,10,15	1,8,9,11,12,13,14,16,17	11	P02631,P02803,P04218,P08937,P20760,P20761,P20762,P36376,P83748,P97615,Q9Z0V6
11463	2,3,4,5,6,7,10,16	1,8,9,11,12,13,14,15,17	8	P04218,P16086,P20760,P34901,P36376,P61206;P84079;P84082,P83748,Q62997
11464	2,3,4,5,6,7,10,17	1,8,9,11,12,13,14,15,16	14	P04218,P08937,P09605;P25809,P16086,P20646,P20760,P20761,P20762,P36376,P52590,P83748,P97615,Q63751,Q9Z0V6
11465	2,3,4,5,6,7,11,12	1,8,9,10,13,14,15,16,17	18	P01836,P02631,P08937,P11883,P16086,P20760,P20761,P20762,P34901,P61206;P84079;P84082,P83748,Q498R7,Q4FZU2,Q63751,Q6IFW6,Q9EQS0,Q9ESW0,Q9Z0V6
11466	2,3,4,5,6,7,11,13	1,8,9,10,12,14,15,16,17	9	P02631,P05539,P08937,P16086,P20760,P20762,P61206;P84079;P84082,P83748,Q63751
11467	2,3,4,5,6,7,11,14	1,8,9,10,12,13,15,16,17	12	P02631,P08937,P16086,P20760,P20761,P20762,P34901,P36376,P61206;P84079;P84082,P83748,Q63751,Q9Z0V6
11468	2,3,4,5,6,7,11,15	1,8,9,10,12,13,14,16,17	9	P02631,P08937,P16086,P20760,P20761,P20762,P61206;P84079;P84082,P83748,Q63751
11469	2,3,4,5,6,7,11,16	1,8,9,10,12,13,14,15,17	12	G3V686,P02631,P08937,P14668,P15999,P16086,P20760,P20762,P34901,P61206;P84079;P84082,P83748,Q63751
11470	2,3,4,5,6,7,11,17	1,8,9,10,12,13,14,15,16	13	P02631,P08937,P09605;P25809,P16086,P20646,P20760,P20761,P20762,P52590,P61206;P84079;P84082,P83748,Q63751,Q9Z0V6
11471	2,3,4,5,6,7,12,13	1,8,9,10,11,14,15,16,17	13	O88267,P01836,P08937,P16086,P20760,P20761,P20762,P34901,P62815,P83748,Q498R7,Q63751,Q9Z0V6
11472	2,3,4,5,6,7,12,14	1,8,9,10,11,13,15,16,17	14	O88267,P01835,P02631,P08937,P11762,P16086,P20760,P20761,P20762,P34901,P83748,Q498R7,Q9EQS0,Q9Z0V6
11473	2,3,4,5,6,7,12,15	1,8,9,10,11,13,14,16,17	12	P01836,P02631,P08937,P11762,P20760,P20761,P20762,P34901,P83748,Q498R7,Q63751,Q9Z0V6
11474	2,3,4,5,6,7,12,16	1,8,9,10,11,13,14,15,17	13	G3V686,O88267,P01836,P02631,P08937,P16086,P20760,P20762,P34901,P61206;P84079;P84082,P83748,Q498R7,Q9Z0V6

11475	2,3,4,5,6,7,12,17	1,8,9,10,11,13,14,15,16	23	O88267,P01835,P02631,P08937,P09605;P25809,P16086,P19223,P20646,P20760,P20761,P20762,P34901,P52590,P55091,P62815,P83748,P98089,Q498R7,Q62635,Q63751,Q99041,Q9QZK9,Q9Z0V6
11476	2,3,4,5,6,7,13,14	1,8,9,10,11,12,15,16,17	13	O88267,P02631,P06866,P06911,P08937,P12020,P16086,P20760,P20761,P20762,P25236,P36376,P83748
11477	2,3,4,5,6,7,13,15	1,8,9,10,11,12,14,16,17	8	P02631,P08937,P16086,P20760,P20761,P20762,P36376,P83748
11478	2,3,4,5,6,7,13,16	1,8,9,10,11,12,14,15,17	6	G3V686,O88267,P08937,P16086,P20760,P83748
11479	2,3,4,5,6,7,13,17	1,8,9,10,11,12,14,15,16	12	O88267,P08937,P09605;P25809,P16086,P20760,P20761,P20762,P62815,P83748,Q63751,Q9WUW8,Q9Z0V6
11480	2,3,4,5,6,7,14,15	1,8,9,10,11,12,13,16,17	12	P02631,P02803,P11762,P16086,P20760,P20761,P20762,P34901,P36376,P83748,P97605,Q9Z0V6
11481	2,3,4,5,6,7,14,16	1,8,9,10,11,12,13,15,17	12	G3V686,O88267,P02631,P16086,P20760,P20762,P34901,P36376,P83748,Q63532,Q6P767,Q9Z0V6
11482	2,3,4,5,6,7,14,17	1,8,9,10,11,12,13,15,16	14	O88267,P02631,P08937,P09605;P25809,P16086,P20760,P20761,P20762,P34901,P36376,P62815,P83748,Q63751,Q9Z0V6
11483	2,3,4,5,6,7,15,16	1,8,9,10,11,12,13,14,17	8	P02631,P02803,P16086,P20760,P20761,P20762,P36376,P83748
11484	2,3,4,5,6,7,15,17	1,8,9,10,11,12,13,14,16	14	P02631,P02803,P08937,P09605;P25809,P16086,P20646,P20760,P20761,P20762,P52590,P62815,P83748,Q63751,Q9Z0V6
11485	2,3,4,5,6,7,16,17	1,8,9,10,11,12,13,14,15	13	O88267,P02631,P08937,P09605;P25809,P10758,P16086,P20646,P20760,P20761,P20762,P83748,Q63751,Q9Z0V6
11486	2,3,4,5,6,8,9,10	1,7,11,12,13,14,15,16,17	8	D3ZHA0,O55006,P08649,P20762,Q64361,Q6TMA8,Q9WTT6,Q9Z0V6
11487	2,3,4,5,6,8,9,11	1,7,10,12,13,14,15,16,17	9	D3ZHA0,P08649,P11883,P14668,P14669,P20762,Q6AYS4,Q6TMA8,Q9Z0V6
11488	2,3,4,5,6,8,9,12	1,7,10,11,13,14,15,16,17	11	D3ZHA0,P01835,P08937,P20762,Q5M8C6,Q64361,Q6TMA8,Q9EQS0,Q9JJS8,Q9WTT6,Q9Z0V6
11489	2,3,4,5,6,8,9,13	1,7,10,11,12,14,15,16,17	7	D3ZHA0,P06866,P20762,P20766,Q6TMA8,Q9WTT6,Q9Z0V6

11490	2,3,4,5,6,8,9,14	1,7,10,11,12,13,15,16,17	8	D3ZHA0,P02793;Q7TP54,P06866,P14668,P20762,Q6TMA8,Q9WTT6,Q9Z0V6
11491	2,3,4,5,6,8,9,15	1,7,10,11,12,13,14,16,17	11	D3ZHA0,O55006,P14668,P20760,P20761,P20762,P23593,Q5QE79,Q6TMA8,Q9WTT6,Q9Z0V6
11492	2,3,4,5,6,8,9,16	1,7,10,11,12,13,14,15,17	8	D3ZHA0,P14668,P20762,P23593,Q63942,Q6TMA8,Q9WTT6,Q9Z0V6
11493	2,3,4,5,6,8,9,17	1,7,10,11,12,13,14,15,16	12	P06760,P20646,P20760,P20761,P20762,P23593,P50115,Q4G075,Q63751,Q6TMA8,Q9WTT6,Q9Z0V6
11494	2,3,4,5,6,8,10,11	1,7,9,12,13,14,15,16,17	14	D3ZHA0,P07150,P08649,P11883,P14668,P14669,P20762,P28648,Q5BJY9,Q63598,Q64361,Q6AYS4,Q6TMA8,Q9Z0V6
11495	2,3,4,5,6,8,10,12	1,7,9,11,13,14,15,16,17	17	D3ZHA0,P04218,P08937,P11883,P16636,P20760,P20762,P34901,Q4FZU2,Q5M8C6,Q64361,Q6IFW6,Q6TMA8,Q9EQS0,Q9JJS8,Q9WTT6,Q9Z0V6
11496	2,3,4,5,6,8,10,13	1,7,9,11,12,14,15,16,17	8	D3ZHA0,P20760,P20762,P20766,Q64361,Q6TMA8,Q9WTT6,Q9Z0V6
11497	2,3,4,5,6,8,10,14	1,7,9,11,12,13,15,16,17	9	D3ZHA0,P02793;Q7TP54,P08649,P20760,P20762,Q64361,Q6TMA8,Q9WTT6,Q9Z0V6
11498	2,3,4,5,6,8,10,15	1,7,9,11,12,13,14,16,17	10	D3ZHA0,P02761,P08649,P20760,P20761,P20762,Q64361,Q6TMA8,Q9WTT6,Q9Z0V6
11499	2,3,4,5,6,8,10,16	1,7,9,11,12,13,14,15,17	7	D3ZHA0,P08649,P14668,Q62997,Q64361,Q9WTT6,Q9Z0V6
11500	2,3,4,5,6,8,10,17	1,7,9,11,12,13,14,15,16	16	D3ZHA0,P06760,P08649,P08937,P16636,P20646,P20760,P20761,P20762,P50115,Q4G075,Q63751,Q64361,Q6P6R2,Q9WTT6,Q9Z0V6
11501	2,3,4,5,6,8,11,12	1,7,9,10,13,14,15,16,17	23	D3ZHA0,P07150,P07647,P08937,P0C0A9,P11883,P14668,P14669,P20762,P34901,P35053,P62804,Q4FZU2,Q5M8C6,Q63751,Q64361,Q6AYS4,Q6IFU8,Q6IFW6,Q6IMF3,Q6TMA8,Q9EQS0,Q9Z0V6
11502	2,3,4,5,6,8,11,13	1,7,9,10,12,14,15,16,17	11	P08937,P11883,P14668,P14669,P20762,P20766,Q5XI22,Q63751,Q6AYS4,Q6TMA8,Q9Z0V6
11503	2,3,4,5,6,8,11,14	1,7,9,10,12,13,15,16,17	14	P02793;Q7TP54,P07150,P07171,P08649,P11883,P14668,P14669,P19218,P20762,Q64194,Q6AYS4,Q6TMA8,Q9EQS0,Q9Z0V6
11504	2,3,4,5,6,8,11,15	1,7,9,10,12,13,14,16,17	12	P02761,P07171,P08649,P14668,P14669,P20760,P20761,P20762,Q64194,Q6AYS4,Q6TMA8,Q9Z0V6

11505	2,3,4,5,6,8,11,16	1,7,9,10,12,13,14,15,17	9	P07150,P08649,P11883,P14668,P14669,P20762,Q6AYS4,Q6TMA8,Q9Z0V6
11506	2,3,4,5,6,8,11,17	1,7,9,10,12,13,14,15,16	17	P06760,P07150,P08649,P08937,P14668,P14669,P20646,P20760,P20761,P20762,P50115,P97840,Q4G075,Q63751,Q6AYS4,Q6TMA8,Q9Z0V6
11507	2,3,4,5,6,8,12,13	1,7,9,10,11,14,15,16,17	12	D3ZHA0,O88267,P02780,P02782,P07647,P08937,P20762,Q5M8C6,Q64361,Q6TMA8,Q9WTT6,Q9Z0V6
11508	2,3,4,5,6,8,12,14	1,7,9,10,11,13,15,16,17	15	D3ZHA0,O88267,P01835,P02793;Q7TP54,P08937,P20760,P20761,P20762,P34901,Q64361,Q6TMA8,Q9EQS0,Q9JJS8,Q9WTT6,Q9Z0V6
11509	2,3,4,5,6,8,12,15	1,7,9,10,11,13,14,16,17	12	B0BNN3,D3ZHA0,P08937,P20760,P20761,P20762,P35053,Q4FZU2,Q64361,Q6TMA8,Q9WTT6,Q9Z0V6
11510	2,3,4,5,6,8,12,16	1,7,9,10,11,13,14,15,17	9	D3ZHA0,O88267,P14668,P20762,Q4FZU2,Q6TMA8,Q9JJS8,Q9WTT6,Q9Z0V6
11511	2,3,4,5,6,8,12,17	1,7,9,10,11,13,14,15,16	23	B0BNN3,O88267,P01835,P06760,P08937,P16636,P19223,P20646,P20760,P20761,P20762,P50115,P55091,P62815,P98089,Q4G075,Q5M8C6,Q62635,Q63751,Q64361,Q9JJS8,Q9WTT6,Q9Z0V6
11512	2,3,4,5,6,8,13,14	1,7,9,10,11,12,15,16,17	11	P02793;Q7TP54,P06866,P06911,P20760,P20762,P20766,P25236,Q64093,Q6TMA8,Q9WTT6,Q9Z0V6
11513	2,3,4,5,6,8,13,15	1,7,9,10,11,12,14,16,17	6	P20760,P20761,P20762,Q6TMA8,Q9WTT6,Q9Z0V6
11514	2,3,4,5,6,8,13,16	1,7,9,10,11,12,14,15,17	4	P14668,Q6TMA8,Q9WTT6,Q9Z0V6
11515	2,3,4,5,6,8,13,17	1,7,9,10,11,12,14,15,16	18	O88267,P06760,P08937,P10536,P20760,P20761,P20762,P20766,P50115,P51907,P62815,Q4G075,Q63355,Q63751,Q6PCU2,Q6TMA8,Q9WTT6,Q9Z0V6
11516	2,3,4,5,6,8,14,15	1,7,9,10,11,12,13,16,17	13	B0BNN3,D3ZHA0,P02793;Q7TP54,P07171,P10536,P12368,P14668,P20760,P20761,P20762,Q6TMA8,Q9WTT6,Q9Z0V6
11517	2,3,4,5,6,8,14,16	1,7,9,10,11,12,13,15,17	6	P02793;Q7TP54,P14668,P20762,Q6TMA8,Q9WTT6,Q9Z0V6
11518	2,3,4,5,6,8,14,17	1,7,9,10,11,12,13,15,16	15	O88267,P02793;Q7TP54,P06760,P08937,P10536,P12368,P20760,P20761,P20762,P50115,P62815,Q4G075,Q63751,Q9WTT6,Q9Z0V6
11519	2,3,4,5,6,8,15,16	1,7,9,10,11,12,13,14,17	5	P14668,P20760,P20762,Q6TMA8,Q9Z0V6

11520	2,3,4,5,6,8,15,17	1,7,9,10,11,12,13,14,16	14	B0BNN3,P02761,P06760,P08937,P10536,P20760,P20761,P20762,P50115,P62815,Q4G075,Q63751,Q9WTT6,Q9Z0V6
11521	2,3,4,5,6,8,16,17	1,7,9,10,11,12,13,14,15	16	O88267,P06760,P14668,P20646,P20760,P20761,P20762,P23593,P50115,P50116,Q4G075,Q63751,Q6JE36,Q6PCU2,Q9WTT6,Q9Z0V6
11522	2,3,4,5,6,9,10,11	1,7,8,12,13,14,15,16,17	4	P08649,P11883,P14173,P34901
11523	2,3,4,5,6,9,10,12	1,7,8,11,13,14,15,16,17	5	P01835,P14173,P34901,Q4FZU2,Q9JJS8
11524	2,3,4,5,6,9,10,13	1,7,8,11,12,14,15,16,17	2	P10715,P14173
11525	2,3,4,5,6,9,10,14	1,7,8,11,12,13,15,16,17	3	P10715,P14173,P34901
11526	2,3,4,5,6,9,10,15	1,7,8,11,12,13,14,16,17	1	Q4FZU6
11527	2,3,4,5,6,9,10,16	1,7,8,11,12,13,14,15,17	3	P14173,P15999,P23593
11528	2,3,4,5,6,9,10,17	1,7,8,11,12,13,14,15,16	4	P14173,P20760,P23593,Q63355
11529	2,3,4,5,6,9,11,12	1,7,8,10,13,14,15,16,17	6	P01835,P15999,P34901,Q4FZU2,Q6IFU8,Q6IFW6
11530	2,3,4,5,6,9,11,13	1,7,8,10,12,14,15,16,17	1	Q6TMA8
11531	2,3,4,5,6,9,11,14	1,7,8,10,12,13,15,16,17	3	P01835,P20762,P34901
11532	2,3,4,5,6,9,11,15	1,7,8,10,12,13,14,16,17	1	P02625
11533	2,3,4,5,6,9,11,16	1,7,8,10,12,13,14,15,17	2	P14668,P15999
11534	2,3,4,5,6,9,11,17	1,7,8,10,12,13,14,15,16	3	P01835,P15999,P20760

11535	2,3,4,5,6,9,12,13	1,7,8,10,11,14,15,16,17	1	P01835
11536	2,3,4,5,6,9,12,14	1,7,8,10,11,13,15,16,17	3	P01835,P34901,Q9JJS8
11537	2,3,4,5,6,9,12,15	1,7,8,10,11,13,14,16,17	2	P01835,P34901
11538	2,3,4,5,6,9,12,16	1,7,8,10,11,13,14,15,17	5	P01835,P15999,P23593,P34901,Q9JJS8
11539	2,3,4,5,6,9,12,17	1,7,8,10,11,13,14,15,16	10	P01835,P09605;P25809,P19223,P20760,P20761,P23593,P34901,P98089,Q9JJS8,Q9Z0V6
11540	2,3,4,5,6,9,13,14	1,7,8,10,11,12,15,16,17	2	P06866,P10715
11541	2,3,4,5,6,9,13,15	1,7,8,10,11,12,14,16,17	0	
11542	2,3,4,5,6,9,13,16	1,7,8,10,11,12,14,15,17	0	
11543	2,3,4,5,6,9,13,17	1,7,8,10,11,12,14,15,16	3	P20760,P23593,Q63355
11544	2,3,4,5,6,9,14,15	1,7,8,10,11,12,13,16,17	1	P34901
11545	2,3,4,5,6,9,14,16	1,7,8,10,11,12,13,15,17	2	P15999,P34901
11546	2,3,4,5,6,9,14,17	1,7,8,10,11,12,13,15,16	6	P01835,P20760,P20761,P34901,Q5HZV9,Q9Z0V6
11547	2,3,4,5,6,9,15,16	1,7,8,10,11,12,13,14,17	3	P23593,P36374,P47727
11548	2,3,4,5,6,9,15,17	1,7,8,10,11,12,13,14,16	4	P20760,P20761,P23593,P47727
11549	2,3,4,5,6,9,16,17	1,7,8,10,11,12,13,14,15	4	P15999,P20760,P23593,P47727

11550	2,3,4,5,6,10,11,12	1,7,8,9,13,14,15,16,17	9	P11883,P34901,Q4FZU2,Q6IFU7,Q6IFU8,Q6IFW6,Q6IMF3,Q6P6Q2,Q9JJS8
11551	2,3,4,5,6,10,11,13	1,7,8,9,12,14,15,16,17	0	
11552	2,3,4,5,6,10,11,14	1,7,8,9,12,13,15,16,17	2	P20762,P34901
11553	2,3,4,5,6,10,11,15	1,7,8,9,12,13,14,16,17	2	P34901,Q4FZU6
11554	2,3,4,5,6,10,11,16	1,7,8,9,12,13,14,15,17	2	P14668,P34901
11555	2,3,4,5,6,10,11,17	1,7,8,9,12,13,14,15,16	3	P08649,P20760,P34901
11556	2,3,4,5,6,10,12,13	1,7,8,9,11,14,15,16,17	4	P04218,P34901,Q4FZU2,Q9JJS8
11557	2,3,4,5,6,10,12,14	1,7,8,9,11,13,15,16,17	5	P01835,P04218,P34901,Q4FZU2,Q9JJS8
11558	2,3,4,5,6,10,12,15	1,7,8,9,11,13,14,16,17	4	P04218,P34901,Q4FZU2,Q9JJS8
11559	2,3,4,5,6,10,12,16	1,7,8,9,11,13,14,15,17	5	P04218,P34901,Q4FZU2,Q6IFW6,Q9JJS8
11560	2,3,4,5,6,10,12,17	1,7,8,9,11,13,14,15,16	12	P01835,P16636,P19223,P20760,P20761,P34901,P98089,Q10758,Q4FZU2,Q62635,Q9JJS8,Q9Z0V6
11561	2,3,4,5,6,10,13,14	1,7,8,9,11,12,15,16,17	2	P10715,P34901
11562	2,3,4,5,6,10,13,15	1,7,8,9,11,12,14,16,17	3	P04218,P10715,Q4FZU6
11563	2,3,4,5,6,10,13,16	1,7,8,9,11,12,14,15,17	2	P10715,Q62997
11564	2,3,4,5,6,10,13,17	1,7,8,9,11,12,14,15,16	3	P10715,P20760,Q63355

11565	2,3,4,5,6,10,14,15	1,7,8,9,11,12,13,16,17	4	P02803,P10715,P34901,P36376
11566	2,3,4,5,6,10,14,16	1,7,8,9,11,12,13,15,17	3	P10715,P34901,Q62997
11567	2,3,4,5,6,10,14,17	1,7,8,9,11,12,13,15,16	5	P10715,P20760,P20761,P34901,Q9Z0V6
11568	2,3,4,5,6,10,15,16	1,7,8,9,11,12,13,14,17	3	P02803,P36374,Q62997
11569	2,3,4,5,6,10,15,17	1,7,8,9,11,12,13,14,16	5	B0BNN3,P02803,P20760,P20761,Q9Z0V6
11570	2,3,4,5,6,10,16,17	1,7,8,9,11,12,13,14,15	3	P20760,P23593,Q62997
11571	2,3,4,5,6,11,12,13	1,7,8,9,10,14,15,16,17	3	P34901,Q4FZU2,Q6IFW6
11572	2,3,4,5,6,11,12,14	1,7,8,9,10,13,15,16,17	6	P01835,P20762,P34901,Q4FZU2,Q6IFU8,Q6IFW6
11573	2,3,4,5,6,11,12,15	1,7,8,9,10,13,14,16,17	4	P02625,P34901,Q4FZU2,Q6IFW6
11574	2,3,4,5,6,11,12,16	1,7,8,9,10,13,14,15,17	8	G3V686,P14668,P34901,Q4FZU2,Q6IFU8,Q6IFW6,Q6IMF3,Q9JJS8
11575	2,3,4,5,6,11,12,17	1,7,8,9,10,13,14,15,16	12	P01835,P16636,P20760,P20761,P34901,P98089,Q4FZU2,Q62635,Q63751,Q6IFU8,Q6IFW6,Q9Z0V6
11576	2,3,4,5,6,11,13,14	1,7,8,9,10,12,15,16,17	1	P20762
11577	2,3,4,5,6,11,13,15	1,7,8,9,10,12,14,16,17	1	P02625
11578	2,3,4,5,6,11,13,16	1,7,8,9,10,12,14,15,17	2	G3V686,P14668
11579	2,3,4,5,6,11,13,17	1,7,8,9,10,12,14,15,16	1	P20760

11580	2,3,4,5,6,11,14,15	1,7,8,9,10,12,13,16,17	3	P19218,P20762,P34901
11581	2,3,4,5,6,11,14,16	1,7,8,9,10,12,13,15,17	3	P14668,P34901,Q6P767
11582	2,3,4,5,6,11,14,17	1,7,8,9,10,12,13,15,16	7	P01835,P19218,P20760,P20761,P20762,P34901,Q9Z0V6
11583	2,3,4,5,6,11,15,16	1,7,8,9,10,12,13,14,17	2	P14668,Q6AYR8
11584	2,3,4,5,6,11,15,17	1,7,8,9,10,12,13,14,16	3	B0BNN3,P20760,P20761
11585	2,3,4,5,6,11,16,17	1,7,8,9,10,12,13,14,15	2	P15999,P20760
11586	2,3,4,5,6,12,13,14	1,7,8,9,10,11,15,16,17	4	P01835,P25236,P34901,Q9JJS8
11587	2,3,4,5,6,12,13,15	1,7,8,9,10,11,14,16,17	1	P82995
11588	2,3,4,5,6,12,13,16	1,7,8,9,10,11,14,15,17	2	G3V686,Q9JJS8
11589	2,3,4,5,6,12,13,17	1,7,8,9,10,11,14,15,16	8	P01835,P20760,P20761,P62815,P98089,Q62635,Q63355,Q9QZK9
11590	2,3,4,5,6,12,14,15	1,7,8,9,10,11,13,16,17	4	B0BNN3,P01835,P20762,P34901
11591	2,3,4,5,6,12,14,16	1,7,8,9,10,11,13,15,17	4	G3V686,P01835,P34901,Q9JJS8
11592	2,3,4,5,6,12,14,17	1,7,8,9,10,11,13,15,16	10	P01835,P20760,P20761,P20762,P31430,P34901,P98089,Q62635,Q9JJS8,Q9Z0V6
11593	2,3,4,5,6,12,15,16	1,7,8,9,10,11,13,14,17	3	P34901,Q4FZU2,Q9JJS8
11594	2,3,4,5,6,12,15,17	1,7,8,9,10,11,13,14,16	8	B0BNN3,P01835,P20760,P20761,P34901,P98089,Q62635,Q9Z0V6

11595	2,3,4,5,6,12,16,17	1,7,8,9,10,11,13,14,15	8	P01835,P20760,P20761,P23593,P34901,P98089,Q9JJS8,Q9Z0V6
11596	2,3,4,5,6,13,14,15	1,7,8,9,10,11,12,16,17	2	P10715,P25236
11597	2,3,4,5,6,13,14,16	1,7,8,9,10,11,12,15,17	2	G3V686,P10715
11598	2,3,4,5,6,13,14,17	1,7,8,9,10,11,12,15,16	4	P10715,P20760,P20761,Q9QW07
11599	2,3,4,5,6,13,15,16	1,7,8,9,10,11,12,14,17	0	
11600	2,3,4,5,6,13,15,17	1,7,8,9,10,11,12,14,16	3	P20760,P20761,Q6AYL6
11601	2,3,4,5,6,13,16,17	1,7,8,9,10,11,12,14,15	1	P20760
11602	2,3,4,5,6,14,15,16	1,7,8,9,10,11,12,13,17	3	P02803,P34901,P36374
11603	2,3,4,5,6,14,15,17	1,7,8,9,10,11,12,13,16	9	B0BNN3,P02803,P10536,P20760,P20761,P34901,P47967,Q5HZV9,Q9Z0V6
11604	2,3,4,5,6,14,16,17	1,7,8,9,10,11,12,13,15	4	P20760,P20761,P34901,Q9Z0V6
11605	2,3,4,5,6,15,16,17	1,7,8,9,10,11,12,13,14	6	P02803,P20760,P20761,P23593,P36374,P47727
11606	2,3,4,5,7,8,9,10	1,6,11,12,13,14,15,16,17	6	D3ZHA0,O55006,P52590,P97571,Q6AYS4,Q9EQS0
11607	2,3,4,5,7,8,9,11	1,6,10,12,13,14,15,16,17	6	P07171,P14668,P20762,Q6AYS4,Q6TMA8,Q9EQS0
11608	2,3,4,5,7,8,9,12	1,6,10,11,13,14,15,16,17	7	D3ZHA0,P19223,Q498R7,Q6AYS4,Q6TMA8,Q80WL1,Q9EQS0
11609	2,3,4,5,7,8,9,13	1,6,10,11,12,14,15,16,17	4	P20766,P36970,Q6AYS4,Q6TMA8

11610	2,3,4,5,7,8,9,14	1,6,10,11,12,13,15,16,17	8	D3ZHA0,P07171,P20762,P36970,P97605,Q6AYS4,Q6TMA8,Q9EQS0
11611	2,3,4,5,7,8,9,15	1,6,10,11,12,13,14,16,17	7	D3ZHA0,P02761,P07171,P47727,P97605,Q5QE79,Q6TMA8
11612	2,3,4,5,7,8,9,16	1,6,10,11,12,13,14,15,17	2	D3ZHA0,P47727
11613	2,3,4,5,7,8,9,17	1,6,10,11,12,13,14,15,16	7	P19223,P20761,P47727,P50115,P52590,P55091,Q6AYS4
11614	2,3,4,5,7,8,10,11	1,6,9,12,13,14,15,16,17	14	D3ZHA0,P00714,P07171,P08649,P14669,P16391,P20762,P28648,P61206;P84079;P84082,Q5BJY9,Q63475,Q64194,Q6AYS4,Q9EQS0
11615	2,3,4,5,7,8,10,12	1,6,9,11,13,14,15,16,17	9	D3ZHA0,P04218,P08937,P16391,P36970,Q63475,Q6AYS4,Q80WL1,Q9EQS0
11616	2,3,4,5,7,8,10,13	1,6,9,11,12,14,15,16,17	6	D3ZHA0,P04218,P20766,P36970,Q6AYS4,Q80WL1
11617	2,3,4,5,7,8,10,14	1,6,9,11,12,13,15,16,17	7	D3ZHA0,P07171,P20762,P36970,P97615,Q6AYS4,Q9EQS0
11618	2,3,4,5,7,8,10,15	1,6,9,11,12,13,14,16,17	7	D3ZHA0,P02761,P04218,P07171,P97571,P97615,Q6AYS4
11619	2,3,4,5,7,8,10,16	1,6,9,11,12,13,14,15,17	2	D3ZHA0,Q62997
11620	2,3,4,5,7,8,10,17	1,6,9,11,12,13,14,15,16	15	D3ZHA0,P02761,P08937,P16391,P19223,P20760,P20761,P46844,P50115,P52590,P55091,P97571,P97615,Q6AYS4,Q80WL1
11621	2,3,4,5,7,8,11,12	1,6,9,10,13,14,15,16,17	14	D3ZHA0,P01836,P07171,P08937,P20762,Q498R7,Q63475,Q63751,Q6AYS4,Q6TMA8,Q80WL1,Q99041,Q9EQS0,Q9ESW0
11622	2,3,4,5,7,8,11,13	1,6,9,10,12,14,15,16,17	5	P07171,P20762,P20766,Q6AYS4,Q6TMA8
11623	2,3,4,5,7,8,11,14	1,6,9,10,12,13,15,16,17	8	P07171,P14668,P19218,P20762,Q64194,Q6AYS4,Q6TMA8,Q9EQS0
11624	2,3,4,5,7,8,11,15	1,6,9,10,12,13,14,16,17	6	P02761,P07171,P14668,P20762,Q64194,Q6AYS4

11625	2,3,4,5,7,8,11,16	1,6,9,10,12,13,14,15,17	5	P07171,P14668,P61206;P84079;P84082,Q6AYS4,Q9EQS0
11626	2,3,4,5,7,8,11,17	1,6,9,10,12,13,14,15,16	11	P02761,P08937,P14669,P19223,P20761,P20762,P50115,P52590,P55091,Q63751,Q6AYS4
11627	2,3,4,5,7,8,12,13	1,6,9,10,11,14,15,16,17	10	D3ZHA0,O88267,P01836,P08937,P20766,P36970,Q5M819,Q6TMA8,Q80WL1,Q9EQS0
11628	2,3,4,5,7,8,12,14	1,6,9,10,11,13,15,16,17	9	D3ZHA0,O88267,P20762,P34901,P36970,Q63475,Q6AYS4,Q80WL1,Q9EQS0
11629	2,3,4,5,7,8,12,15	1,6,9,10,11,13,14,16,17	10	D3ZHA0,P01836,P08937,P11762,P20762,P36970,Q63475,Q64611,Q80WL1,Q9EQS0
11630	2,3,4,5,7,8,12,16	1,6,9,10,11,13,14,15,17	5	D3ZHA0,O88267,P01836,Q80WL1,Q9EQS0
11631	2,3,4,5,7,8,12,17	1,6,9,10,11,13,14,15,16	16	O88267,P08937,P19223,P20761,P20762,P50115,P52590,P55091,P98089,Q4G075,Q63751,Q6AYS4,Q80WL1,Q99041,Q9EQS0,Q9QZA6
11632	2,3,4,5,7,8,13,14	1,6,9,10,11,12,15,16,17	7	O88267,P07171,P20762,P20766,P36970,Q5M819,Q6TMA8
11633	2,3,4,5,7,8,13,15	1,6,9,10,11,12,14,16,17	5	P07171,P20766,P36970,Q09030,Q6TMA8
11634	2,3,4,5,7,8,13,16	1,6,9,10,11,12,14,15,17	1	P20766
11635	2,3,4,5,7,8,13,17	1,6,9,10,11,12,14,15,16	11	O88267,P08937,P20761,P20766,P50115,P52590,P55091,Q5M819,Q63751,Q6AYS4,Q6IFU7
11636	2,3,4,5,7,8,14,15	1,6,9,10,11,12,13,16,17	5	D3ZHA0,P07171,P20762,P36970,P97605
11637	2,3,4,5,7,8,14,16	1,6,9,10,11,12,13,15,17	2	P36970,Q566E6
11638	2,3,4,5,7,8,14,17	1,6,9,10,11,12,13,15,16	12	O88267,P10536,P20760,P20761,P20762,P36970,P50115,P52590,P55091,Q5M819,Q6AYS4,Q6IFU7
11639	2,3,4,5,7,8,15,16	1,6,9,10,11,12,13,14,17	2	P07171,P47727

11640	2,3,4,5,7,8,15,17	1,6,9,10,11,12,13,14,16	11	P02761,P10536,P20760,P20761,P47727,P50115,P52590,P55091,Q63751,Q6AYS4,Q6IFU7
11641	2,3,4,5,7,8,16,17	1,6,9,10,11,12,13,14,15	7	O88267,P20761,P47727,P50115,P50116,P55091,Q6AYS4
11642	2,3,4,5,7,9,10,11	1,6,8,12,13,14,15,16,17	3	P15999,P34901,P61206;P84079;P84082
11643	2,3,4,5,7,9,10,12	1,6,8,11,13,14,15,16,17	2	P04218,P34901
11644	2,3,4,5,7,9,10,13	1,6,8,11,12,14,15,16,17	0	
11645	2,3,4,5,7,9,10,14	1,6,8,11,12,13,15,16,17	1	P34901
11646	2,3,4,5,7,9,10,15	1,6,8,11,12,13,14,16,17	2	P02782,P47727
11647	2,3,4,5,7,9,10,16	1,6,8,11,12,13,14,15,17	4	P02782,P15999,P47727,Q62997
11648	2,3,4,5,7,9,10,17	1,6,8,11,12,13,14,15,16	2	P47727,P52590
11649	2,3,4,5,7,9,11,12	1,6,8,10,13,14,15,16,17	5	P01836,P15999,P34901,Q498R7,Q9EQS0
11650	2,3,4,5,7,9,11,13	1,6,8,10,12,14,15,16,17	1	P05539
11651	2,3,4,5,7,9,11,14	1,6,8,10,12,13,15,16,17	4	P15999,P34901,Q6IFV1,Q99MH3
11652	2,3,4,5,7,9,11,15	1,6,8,10,12,13,14,16,17	1	Q8K4G9
11653	2,3,4,5,7,9,11,16	1,6,8,10,12,13,14,15,17	2	P15999,Q6IFV1
11654	2,3,4,5,7,9,11,17	1,6,8,10,12,13,14,15,16	2	P15999,P52590

11655	2,3,4,5,7,9,12,13	1,6,8,10,11,14,15,16,17	1	P01836
11656	2,3,4,5,7,9,12,14	1,6,8,10,11,13,15,16,17	1	P34901
11657	2,3,4,5,7,9,12,15	1,6,8,10,11,13,14,16,17	3	P01836,P34901,P47727
11658	2,3,4,5,7,9,12,16	1,6,8,10,11,13,14,15,17	4	P01836,P15999,P34901,P47727
11659	2,3,4,5,7,9,12,17	1,6,8,10,11,13,14,15,16	7	P15999,P19223,P34901,P47727,P52590,P55091,P98089
11660	2,3,4,5,7,9,13,14	1,6,8,10,11,12,15,16,17	1	P97605
11661	2,3,4,5,7,9,13,15	1,6,8,10,11,12,14,16,17	1	P47727
11662	2,3,4,5,7,9,13,16	1,6,8,10,11,12,14,15,17	2	P08010,P47727
11663	2,3,4,5,7,9,13,17	1,6,8,10,11,12,14,15,16	2	P47727,P52590
11664	2,3,4,5,7,9,14,15	1,6,8,10,11,12,13,16,17	4	P16636,P34901,P97605,Q9QZK9
11665	2,3,4,5,7,9,14,16	1,6,8,10,11,12,13,15,17	6	P08010,P15999,P34901,Q6IFV1,Q99MH3,Q9QZK9
11666	2,3,4,5,7,9,14,17	1,6,8,10,11,12,13,15,16	2	P34901,P52590
11667	2,3,4,5,7,9,15,16	1,6,8,10,11,12,13,14,17	4	P02782,P47727,Q9JHB9,Q9QZK9
11668	2,3,4,5,7,9,15,17	1,6,8,10,11,12,13,14,16	3	P02782,P47727,P52590
11669	2,3,4,5,7,9,16,17	1,6,8,10,11,12,13,14,15	5	P02782,P08010,P15999,P47727,P52590

11670	2,3,4,5,7,10,11,12	1,6,8,9,13,14,15,16,17	7	P01836,P04218,P34901,P61206;P84079;P84082,Q63475,Q9EQS0,Q9ESW0
11671	2,3,4,5,7,10,11,13	1,6,8,9,12,14,15,16,17	0	
11672	2,3,4,5,7,10,11,14	1,6,8,9,12,13,15,16,17	1	P34901
11673	2,3,4,5,7,10,11,15	1,6,8,9,12,13,14,16,17	2	P02782,P51907
11674	2,3,4,5,7,10,11,16	1,6,8,9,12,13,14,15,17	5	P02782,P15999,P34901,P61206;P84079;P84082,Q5FVI6
11675	2,3,4,5,7,10,11,17	1,6,8,9,12,13,14,15,16	1	P52590
11676	2,3,4,5,7,10,12,13	1,6,8,9,11,14,15,16,17	3	P01836,P04218,P34901
11677	2,3,4,5,7,10,12,14	1,6,8,9,11,13,15,16,17	4	A2RUV9,P04218,P34901,Q9EQS0
11678	2,3,4,5,7,10,12,15	1,6,8,9,11,13,14,16,17	3	P01836,P04218,P34901
11679	2,3,4,5,7,10,12,16	1,6,8,9,11,13,14,15,17	4	P01836,P04218,P34901,Q9JJS8
11680	2,3,4,5,7,10,12,17	1,6,8,9,11,13,14,15,16	6	P04218,P19223,P34901,P52590,P55091,P98089
11681	2,3,4,5,7,10,13,14	1,6,8,9,11,12,15,16,17	2	P04218,Q99068
11682	2,3,4,5,7,10,13,15	1,6,8,9,11,12,14,16,17	2	P04218,Q09030
11683	2,3,4,5,7,10,13,16	1,6,8,9,11,12,14,15,17	2	P04218,Q62997
11684	2,3,4,5,7,10,13,17	1,6,8,9,11,12,14,15,16	2	P04218,P52590

11685	2,3,4,5,7,10,14,15	1,6,8,9,11,12,13,16,17	6	P02782,P02803,P04218,P34901,P36376,Q99068
11686	2,3,4,5,7,10,14,16	1,6,8,9,11,12,13,15,17	5	P02782,P34901,Q62997,Q63532,Q99068
11687	2,3,4,5,7,10,14,17	1,6,8,9,11,12,13,15,16	2	P34901,P52590
11688	2,3,4,5,7,10,15,16	1,6,8,9,11,12,13,14,17	6	P02782,P04218,P13432,P47727,Q62997,Q9JHB9
11689	2,3,4,5,7,10,15,17	1,6,8,9,11,12,13,14,16	3	P02782,P47727,P52590
11690	2,3,4,5,7,10,16,17	1,6,8,9,11,12,13,14,15	4	P02782,P47727,P52590,Q62997
11691	2,3,4,5,7,11,12,13	1,6,8,9,10,14,15,16,17	3	P01836,P34901,Q498R7
11692	2,3,4,5,7,11,12,14	1,6,8,9,10,13,15,16,17	4	P01836,P34901,Q9EQS0,Q9ESW0
11693	2,3,4,5,7,11,12,15	1,6,8,9,10,13,14,16,17	5	P01836,P34901,P51907,Q498R7,Q9ESW0
11694	2,3,4,5,7,11,12,16	1,6,8,9,10,13,14,15,17	3	P01836,P15999,P34901
11695	2,3,4,5,7,11,12,17	1,6,8,9,10,13,14,15,16	7	P01836,P19223,P34901,P55091,P98089,Q63751,Q9ESW0
11696	2,3,4,5,7,11,13,14	1,6,8,9,10,12,15,16,17	0	
11697	2,3,4,5,7,11,13,15	1,6,8,9,10,12,14,16,17	3	P01836,P05539,Q09030
11698	2,3,4,5,7,11,13,16	1,6,8,9,10,12,14,15,17	1	P01836
11699	2,3,4,5,7,11,13,17	1,6,8,9,10,12,14,15,16	0	

11700	2,3,4,5,7,11,14,15	1,6,8,9,10,12,13,16,17	3	P19218,P34901,Q64194
11701	2,3,4,5,7,11,14,16	1,6,8,9,10,12,13,15,17	4	P15999,P34901,Q6IFV1,Q99MH3
11702	2,3,4,5,7,11,14,17	1,6,8,9,10,12,13,15,16	2	P19218,P34901
11703	2,3,4,5,7,11,15,16	1,6,8,9,10,12,13,14,17	4	P01836,P02782,P14408,Q6AYR8
11704	2,3,4,5,7,11,15,17	1,6,8,9,10,12,13,14,16	0	
11705	2,3,4,5,7,11,16,17	1,6,8,9,10,12,13,14,15	1	P15999
11706	2,3,4,5,7,12,13,14	1,6,8,9,10,11,15,16,17	4	A2RUV9,P01836,P34901,P36970
11707	2,3,4,5,7,12,13,15	1,6,8,9,10,11,14,16,17	2	P01836,Q09030
11708	2,3,4,5,7,12,13,16	1,6,8,9,10,11,14,15,17	1	P01836
11709	2,3,4,5,7,12,13,17	1,6,8,9,10,11,14,15,16	3	P01836,P19223,P55091
11710	2,3,4,5,7,12,14,15	1,6,8,9,10,11,13,16,17	5	A2RUV9,P01836,P11762,P34901,Q8CJD3
11711	2,3,4,5,7,12,14,16	1,6,8,9,10,11,13,15,17	3	P01836,P34901,Q63532
11712	2,3,4,5,7,12,14,17	1,6,8,9,10,11,13,15,16	6	P19223,P31430,P34901,P55091,P98089,Q8CJD3
11713	2,3,4,5,7,12,15,16	1,6,8,9,10,11,13,14,17	3	P01836,P34901,P47727
11714	2,3,4,5,7,12,15,17	1,6,8,9,10,11,13,14,16	6	P01836,P19223,P34901,P47727,P55091,P98089

11715	2,3,4,5,7,12,16,17	1,6,8,9,10,11,13,14,15	7	P01836,P15999,P19223,P34901,P47727,P55091,P98089
11716	2,3,4,5,7,13,14,15	1,6,8,9,10,11,12,16,17	3	P16636,Q09030,Q99068
11717	2,3,4,5,7,13,14,16	1,6,8,9,10,11,12,15,17	3	P08010,Q566E6,Q99068
11718	2,3,4,5,7,13,14,17	1,6,8,9,10,11,12,15,16	3	Q6IFU7,Q9QW07,Q9WUW8
11719	2,3,4,5,7,13,15,16	1,6,8,9,10,11,12,14,17	4	P01836,P02782,P47727,Q09030
11720	2,3,4,5,7,13,15,17	1,6,8,9,10,11,12,14,16	3	P47727,Q6IFU7,Q9WUW8
11721	2,3,4,5,7,13,16,17	1,6,8,9,10,11,12,14,15	2	P08010,P47727
11722	2,3,4,5,7,14,15,16	1,6,8,9,10,11,12,13,17	8	P02782,P02803,P16636,P34901,P36374,P47727,Q9JHB9,Q9QZK9
11723	2,3,4,5,7,14,15,17	1,6,8,9,10,11,12,13,16	2	P02803,P34901
11724	2,3,4,5,7,14,16,17	1,6,8,9,10,11,12,13,15	2	P08010,P34901
11725	2,3,4,5,7,15,16,17	1,6,8,9,10,11,12,13,14	7	P02782,P02803,P04905,P0C0A9,P30120,P47727,Q9JHB9
11726	2,3,4,5,8,9,10,11	1,6,7,12,13,14,15,16,17	4	P08649,P14668,Q6AYS4,Q6TMA8
11727	2,3,4,5,8,9,10,12	1,6,7,11,13,14,15,16,17	4	D3ZHA0,Q6TMA8,Q80WL1,Q9JJS8
11728	2,3,4,5,8,9,10,13	1,6,7,11,12,14,15,16,17	2	P20766,Q6TMA8
11729	2,3,4,5,8,9,10,14	1,6,7,11,12,13,15,16,17	0	

11730	2,3,4,5,8,9,10,15	1,6,7,11,12,13,14,16,17	2	D3ZHA0,Q5QE79
11731	2,3,4,5,8,9,10,16	1,6,7,11,12,13,14,15,17	2	Q62997,Q9QZK9
11732	2,3,4,5,8,9,10,17	1,6,7,11,12,13,14,15,16	2	P08649,Q6AYS4
11733	2,3,4,5,8,9,11,12	1,6,7,10,13,14,15,16,17	5	P14668,Q6AYS4,Q6TMA8,Q80WL1,Q9EQS0
11734	2,3,4,5,8,9,11,13	1,6,7,10,12,14,15,16,17	3	P20766,Q6AYS4,Q6TMA8
11735	2,3,4,5,8,9,11,14	1,6,7,10,12,13,15,16,17	7	P14668,P19218,P97840,Q64194,Q6AYS4,Q6TMA8,Q99MH3
11736	2,3,4,5,8,9,11,15	1,6,7,10,12,13,14,16,17	6	P14668,P97840,Q5QE79,Q64194,Q6AYS4,Q6TMA8
11737	2,3,4,5,8,9,11,16	1,6,7,10,12,13,14,15,17	4	P14668,Q6AYS4,Q6TMA8,Q9QZK9
11738	2,3,4,5,8,9,11,17	1,6,7,10,12,13,14,15,16	3	P50115,P97840,Q6AYS4
11739	2,3,4,5,8,9,12,13	1,6,7,10,11,14,15,16,17	3	P20766,Q6TMA8,Q80WL1
11740	2,3,4,5,8,9,12,14	1,6,7,10,11,13,15,16,17	6	P34901,P36970,Q6TMA8,Q80WL1,Q9EQS0,Q9JJS8
11741	2,3,4,5,8,9,12,15	1,6,7,10,11,13,14,16,17	2	Q5QE79,Q6TMA8
11742	2,3,4,5,8,9,12,16	1,6,7,10,11,13,14,15,17	2	Q80WL1,Q9JJS8
11743	2,3,4,5,8,9,12,17	1,6,7,10,11,13,14,15,16	6	P19223,P50115,P55091,P98089,Q6AYS4,Q80WL1
11744	2,3,4,5,8,9,13,14	1,6,7,10,11,12,15,16,17	3	P20766,P36970,Q6TMA8

11745	2,3,4,5,8,9,13,15	1,6,7,10,11,12,14,16,17	3	P20766,Q5QE79,Q6TMA8
11746	2,3,4,5,8,9,13,16	1,6,7,10,11,12,14,15,17	2	P20766,Q6TMA8
11747	2,3,4,5,8,9,13,17	1,6,7,10,11,12,14,15,16	1	P20766
11748	2,3,4,5,8,9,14,15	1,6,7,10,11,12,13,16,17	4	P97840,Q5QE79,Q6TMA8,Q9QZK9
11749	2,3,4,5,8,9,14,16	1,6,7,10,11,12,13,15,17	2	Q99MH3,Q9QZK9
11750	2,3,4,5,8,9,14,17	1,6,7,10,11,12,13,15,16	2	P97840,Q6AYS4
11751	2,3,4,5,8,9,15,16	1,6,7,10,11,12,13,14,17	4	P36374,P47727,Q5QE79,Q9QZK9
11752	2,3,4,5,8,9,15,17	1,6,7,10,11,12,13,14,16	4	P02761,P47727,P97840,Q5QE79
11753	2,3,4,5,8,9,16,17	1,6,7,10,11,12,13,14,15	2	P23593,P47727
11754	2,3,4,5,8,10,11,12	1,6,7,9,13,14,15,16,17	8	P34901,P35053,P53792,P62804,Q63598,Q6AYS4,Q80WL1,Q9EQS0
11755	2,3,4,5,8,10,11,13	1,6,7,9,12,14,15,16,17	3	P20766,Q6AYS4,Q6TMA8
11756	2,3,4,5,8,10,11,14	1,6,7,9,12,13,15,16,17	6	P08649,P14668,P19218,Q63598,Q64194,Q6AYS4
11757	2,3,4,5,8,10,11,15	1,6,7,9,12,13,14,16,17	5	P02761,P08649,P14668,Q64194,Q6AYS4
11758	2,3,4,5,8,10,11,16	1,6,7,9,12,13,14,15,17	5	P08649,P14668,P53792,Q64194,Q6AYS4
11759	2,3,4,5,8,10,11,17	1,6,7,9,12,13,14,15,16	3	P02761,P08649,Q6AYS4

11760	2,3,4,5,8,10,12,13	1,6,7,9,11,14,15,16,17	4	P36970,Q6TMA8,Q80WL1,Q9JJS8
11761	2,3,4,5,8,10,12,14	1,6,7,9,11,13,15,16,17	5	P34901,P36970,Q80WL1,Q9EQS0,Q9JJS8
11762	2,3,4,5,8,10,12,15	1,6,7,9,11,13,14,16,17	2	D3ZHA0,P35053
11763	2,3,4,5,8,10,12,16	1,6,7,9,11,13,14,15,17	2	Q80WL1,Q9JJS8
11764	2,3,4,5,8,10,12,17	1,6,7,9,11,13,14,15,16	8	P16636,P19223,P55091,P98089,Q4G075,Q6AYS4,Q80WL1,Q9JJS8
11765	2,3,4,5,8,10,13,14	1,6,7,9,11,12,15,16,17	3	P20766,P36970,Q6TMA8
11766	2,3,4,5,8,10,13,15	1,6,7,9,11,12,14,16,17	2	P13432,Q6TMA8
11767	2,3,4,5,8,10,13,16	1,6,7,9,11,12,14,15,17	1	Q62997
11768	2,3,4,5,8,10,13,17	1,6,7,9,11,12,14,15,16	2	P20766,Q6AYS4
11769	2,3,4,5,8,10,14,15	1,6,7,9,11,12,13,16,17	1	P36970
11770	2,3,4,5,8,10,14,16	1,6,7,9,11,12,13,15,17	1	Q62997
11771	2,3,4,5,8,10,14,17	1,6,7,9,11,12,13,15,16	1	Q6AYS4
11772	2,3,4,5,8,10,15,16	1,6,7,9,11,12,13,14,17	4	P13432,P36374,P47727,Q62997
11773	2,3,4,5,8,10,15,17	1,6,7,9,11,12,13,14,16	3	P02761,P47727,Q62902
11774	2,3,4,5,8,10,16,17	1,6,7,9,11,12,13,14,15	2	P47727,Q62997

11775	2,3,4,5,8,11,12,13	1,6,7,9,10,14,15,16,17	5	P20766,P35053,Q6AYS4,Q6TMA8,Q80WL1
11776	2,3,4,5,8,11,12,14	1,6,7,9,10,13,15,16,17	10	P14668,P19218,P34901,P35053,P62804,Q64194,Q6AYS4,Q6TMA8,Q80WL1,Q9EQS0
11777	2,3,4,5,8,11,12,15	1,6,7,9,10,13,14,16,17	6	P14668,P35053,Q64194,Q6AYS4,Q6TMA8,Q9ESW0
11778	2,3,4,5,8,11,12,16	1,6,7,9,10,13,14,15,17	3	P14668,Q6AYS4,Q80WL1
11779	2,3,4,5,8,11,12,17	1,6,7,9,10,13,14,15,16	10	P19223,P35053,P50115,P55091,P62804,P98089,Q4G075,Q6AYS4,Q80WL1,Q9ESW0
11780	2,3,4,5,8,11,13,14	1,6,7,9,10,12,15,16,17	6	P19218,P20766,P97840,Q64194,Q6AYS4,Q6TMA8
11781	2,3,4,5,8,11,13,15	1,6,7,9,10,12,14,16,17	4	P20766,P97840,Q6AYS4,Q6TMA8
11782	2,3,4,5,8,11,13,16	1,6,7,9,10,12,14,15,17	4	P14668,P20766,Q6AYS4,Q6TMA8
11783	2,3,4,5,8,11,13,17	1,6,7,9,10,12,14,15,16	3	P20766,P97840,Q6AYS4
11784	2,3,4,5,8,11,14,15	1,6,7,9,10,12,13,16,17	6	P14668,P19218,P97840,Q64194,Q6AYS4,Q6TMA8
11785	2,3,4,5,8,11,14,16	1,6,7,9,10,12,13,15,17	6	P14668,P19218,P97840,Q64194,Q6AYS4,Q99MH3
11786	2,3,4,5,8,11,14,17	1,6,7,9,10,12,13,15,16	3	P19218,P97840,Q6AYS4
11787	2,3,4,5,8,11,15,16	1,6,7,9,10,12,13,14,17	4	P14668,P97840,Q64194,Q6AYS4
11788	2,3,4,5,8,11,15,17	1,6,7,9,10,12,13,14,16	3	P02761,P97840,Q6AYS4
11789	2,3,4,5,8,11,16,17	1,6,7,9,10,12,13,14,15	3	P14668,P97840,Q6AYS4

11790	2,3,4,5,8,12,13,14	1,6,7,9,10,11,15,16,17	4	P20766,P36970,Q6TMA8,Q80WL1
11791	2,3,4,5,8,12,13,15	1,6,7,9,10,11,14,16,17	4	P35053,P36970,Q6TMA8,Q9QUL6
11792	2,3,4,5,8,12,13,16	1,6,7,9,10,11,14,15,17	4	Q6TMA8,Q80WL1,Q9JJS8,Q9QUL6
11793	2,3,4,5,8,12,13,17	1,6,7,9,10,11,14,15,16	6	P19223,P20766,P55091,P98089,Q62635,Q80WL1
11794	2,3,4,5,8,12,14,15	1,6,7,9,10,11,13,16,17	4	P34901,P35053,P36970,Q6TMA8
11795	2,3,4,5,8,12,14,16	1,6,7,9,10,11,13,15,17	3	P34901,Q566E6,Q9JJS8
11796	2,3,4,5,8,12,14,17	1,6,7,9,10,11,13,15,16	7	P19223,P31430,P34901,P36970,P55091,P98089,Q80WL1
11797	2,3,4,5,8,12,15,16	1,6,7,9,10,11,13,14,17	2	P47727,P83121
11798	2,3,4,5,8,12,15,17	1,6,7,9,10,11,13,14,16	8	B0BNN3,P02761,P19223,P35053,P47727,P55091,P83121,P98089
11799	2,3,4,5,8,12,16,17	1,6,7,9,10,11,13,14,15	5	P19223,P47727,P98089,Q80WL1,Q9JJS8
11800	2,3,4,5,8,13,14,15	1,6,7,9,10,11,12,16,17	8	P06757,P12368,P20766,P36970,P97840,Q64093,Q6AYL6,Q6TMA8
11801	2,3,4,5,8,13,14,16	1,6,7,9,10,11,12,15,17	3	P20766,P36970,Q566E6
11802	2,3,4,5,8,13,14,17	1,6,7,9,10,11,12,15,16	6	P12368,P20766,P36970,P97840,Q6PCU2,Q9QW07
11803	2,3,4,5,8,13,15,16	1,6,7,9,10,11,12,14,17	1	P47727
11804	2,3,4,5,8,13,15,17	1,6,7,9,10,11,12,14,16	4	P12368,P20766,P97840,Q6AYL6

11805	2,3,4,5,8,13,16,17	1,6,7,9,10,11,12,14,15	2	P20766,Q6PCU2
11806	2,3,4,5,8,14,15,16	1,6,7,9,10,11,12,13,17	2	P36374,Q9QZK9
11807	2,3,4,5,8,14,15,17	1,6,7,9,10,11,12,13,16	6	B0BNN3,P02761,P10536,P12368,P47967,P97840
11808	2,3,4,5,8,14,16,17	1,6,7,9,10,11,12,13,15	3	P97840,Q566E6,Q6PCU2
11809	2,3,4,5,8,15,16,17	1,6,7,9,10,11,12,13,14	4	P09456,P47727,P97840,Q9JHB9
11810	2,3,4,5,9,10,11,12	1,6,7,8,13,14,15,16,17	4	P14173,P15999,P19629,P34901
11811	2,3,4,5,9,10,11,13	1,6,7,8,12,14,15,16,17	4	P12785,P13432,P14173,P19629
11812	2,3,4,5,9,10,11,14	1,6,7,8,12,13,15,16,17	5	P13432,P14173,P19218,P34901,Q5XFX0
11813	2,3,4,5,9,10,11,15	1,6,7,8,12,13,14,16,17	7	P02782,P06911,P11598,P13432,P19629,Q62902,Q8CJ52
11814	2,3,4,5,9,10,11,16	1,6,7,8,12,13,14,15,17	10	P02782,P06911,P11598,P13432,P14173,P15999,P19629,Q5GRG2,Q62902,Q9QZK9
11815	2,3,4,5,9,10,11,17	1,6,7,8,12,13,14,15,16	5	P06911,P15999,P19629,Q5GRG2,Q62902
11816	2,3,4,5,9,10,12,13	1,6,7,8,11,14,15,16,17	1	P19629
11817	2,3,4,5,9,10,12,14	1,6,7,8,11,13,15,16,17	2	P34901,Q9JJS8
11818	2,3,4,5,9,10,12,15	1,6,7,8,11,13,14,16,17	3	P19629,P34901,Q62902
11819	2,3,4,5,9,10,12,16	1,6,7,8,11,13,14,15,17	6	P11598,P15999,P19629,P34901,Q5GRG2,Q9JJS8

11820	2,3,4,5,9,10,12,17	1,6,7,8,11,13,14,15,16	5	P19223,P19629,P34901,Q5GRG2,Q9JJS8
11821	2,3,4,5,9,10,13,14	1,6,7,8,11,12,15,16,17	3	P10715,P13432,P19629
11822	2,3,4,5,9,10,13,15	1,6,7,8,11,12,14,16,17	6	P10715,P13432,P19629,Q63493,Q6AYL6,Q8CJ52
11823	2,3,4,5,9,10,13,16	1,6,7,8,11,12,14,15,17	4	P10715,P13432,P19629,Q62997
11824	2,3,4,5,9,10,13,17	1,6,7,8,11,12,14,15,16	5	P10715,P19629,Q63355,Q6AYL6,Q8CJ52
11825	2,3,4,5,9,10,14,15	1,6,7,8,11,12,13,16,17	9	P02782,P10715,P13432,P22283,P34901,Q62902,Q8CJ52,Q9JHB9,Q9QZK9
11826	2,3,4,5,9,10,14,16	1,6,7,8,11,12,13,15,17	12	P02782,P10715,P11598,P13432,P19629,P22283,P34901,Q62902,Q62997,Q8CJ52,Q9JHB9,Q9QZK9
11827	2,3,4,5,9,10,14,17	1,6,7,8,11,12,13,15,16	5	P10715,P34901,Q62902,Q8CJ52,Q9JHB9
11828	2,3,4,5,9,10,15,16	1,6,7,8,11,12,13,14,17	20	P02781,P02782,P04905,P06911,P08723,P11598,P13432,P19629,P22282,P22283,P30120,P36374,P47727,Q62902,Q62997,Q63493,Q8CJ52,Q9JHB9,Q9JI85,Q9QZK9
11829	2,3,4,5,9,10,15,17	1,6,7,8,11,12,13,14,16	9	P02782,P06911,P08723,P19629,P30120,P47727,Q62902,Q8CJ52,Q9JHB9
11830	2,3,4,5,9,10,16,17	1,6,7,8,11,12,13,14,15	14	P02782,P06911,P08723,P11598,P15999,P19629,P30120,P47727,Q5GRG2,Q62902,Q62997,Q8CJ52,Q9JHB9,Q9JI85
11831	2,3,4,5,9,11,12,13	1,6,7,8,10,14,15,16,17	0	
11832	2,3,4,5,9,11,12,14	1,6,7,8,10,13,15,16,17	2	P01835,P34901
11833	2,3,4,5,9,11,12,15	1,6,7,8,10,13,14,16,17	4	P06911,P12020,P34901,P51907
11834	2,3,4,5,9,11,12,16	1,6,7,8,10,13,14,15,17	6	P06911,P11598,P12020,P15999,P34901,Q5GRG2

11835	2,3,4,5,9,11,12,17	1,6,7,8,10,13,14,15,16	6	P01835,P06911,P12020,P15999,P34901,Q5GRG2
11836	2,3,4,5,9,11,13,14	1,6,7,8,10,12,15,16,17	0	
11837	2,3,4,5,9,11,13,15	1,6,7,8,10,12,14,16,17	2	P13432,P19629
11838	2,3,4,5,9,11,13,16	1,6,7,8,10,12,14,15,17	2	P13432,P19629
11839	2,3,4,5,9,11,13,17	1,6,7,8,10,12,14,15,16	1	P19629
11840	2,3,4,5,9,11,14,15	1,6,7,8,10,12,13,16,17	6	P13432,P19218,P34901,P97840,Q99MH3,Q9QZK9
11841	2,3,4,5,9,11,14,16	1,6,7,8,10,12,13,15,17	8	P11598,P13432,P15999,P19218,P34901,Q6IFV1,Q99MH3,Q9QZK9
11842	2,3,4,5,9,11,14,17	1,6,7,8,10,12,13,15,16	3	P19218,P34901,P97840
11843	2,3,4,5,9,11,15,16	1,6,7,8,10,12,13,14,17	13	P02782,P06911,P08723,P11598,P12020,P13432,P19629,P22282,P22283,Q5GRG2,Q62902,Q6AYR9,Q9QZK9
11844	2,3,4,5,9,11,15,17	1,6,7,8,10,12,13,14,16	7	P06911,P08723,P12020,P97840,Q5GRG2,Q62902,Q8CJ52
11845	2,3,4,5,9,11,16,17	1,6,7,8,10,12,13,14,15	9	P06911,P08723,P11598,P12020,P15999,P19629,Q498D9,Q5GRG2,Q62902
11846	2,3,4,5,9,12,13,14	1,6,7,8,10,11,15,16,17	1	P34901
11847	2,3,4,5,9,12,13,15	1,6,7,8,10,11,14,16,17	0	
11848	2,3,4,5,9,12,13,16	1,6,7,8,10,11,14,15,17	1	P19629
11849	2,3,4,5,9,12,13,17	1,6,7,8,10,11,14,15,16	2	P19629,P35280

11850	2,3,4,5,9,12,14,15	1,6,7,8,10,11,13,16,17	1	P34901
11851	2,3,4,5,9,12,14,16	1,6,7,8,10,11,13,15,17	3	P15999,P34901,Q9JJS8
11852	2,3,4,5,9,12,14,17	1,6,7,8,10,11,13,15,16	4	P01835,P31430,P34901,Q8VD89
11853	2,3,4,5,9,12,15,16	1,6,7,8,10,11,13,14,17	7	P06911,P11598,P12020,P22283,P34901,P47727,Q5GRG2
11854	2,3,4,5,9,12,15,17	1,6,7,8,10,11,13,14,16	6	P06911,P12020,P34901,P47727,Q5GRG2,Q62902
11855	2,3,4,5,9,12,16,17	1,6,7,8,10,11,13,14,15	9	P06911,P12020,P15999,P19629,P34901,P35280,P47727,Q5GRG2,Q9JJS8
11856	2,3,4,5,9,13,14,15	1,6,7,8,10,11,12,16,17	6	F1LM93,P10715,P13432,Q6AYL6,Q8CJ52,Q9QZK9
11857	2,3,4,5,9,13,14,16	1,6,7,8,10,11,12,15,17	5	F1LM93,P08010,P10715,P13432,Q9QZK9
11858	2,3,4,5,9,13,14,17	1,6,7,8,10,11,12,15,16	3	P10715,P35280,Q6AYL6
11859	2,3,4,5,9,13,15,16	1,6,7,8,10,11,12,14,17	12	P02782,P08723,P13432,P19629,P22283,P30120,P47727,Q63493,Q6AYL6,Q8CJ52,Q9JHB9,Q9QZK9
11860	2,3,4,5,9,13,15,17	1,6,7,8,10,11,12,14,16	5	P08723,P19629,P47727,Q6AYL6,Q8CJ52
11861	2,3,4,5,9,13,16,17	1,6,7,8,10,11,12,14,15	7	P08010,P08723,P19629,P22006,P35280,P47727,Q6AYL6
11862	2,3,4,5,9,14,15,16	1,6,7,8,10,11,12,13,17	18	P02781,P02782,P08723,P11598,P13432,P22282,P22283,P30120,P34901,P36374,P47727,Q62902,Q63493,Q8CJ52,Q99MH3,Q9JHB9,Q9JI85,Q9QZK9
11863	2,3,4,5,9,14,15,17	1,6,7,8,10,11,12,13,16	10	P02782,P08723,P30120,P47727,P47967,P97840,Q62902,Q63357,Q8CJ52,Q9JHB9
11864	2,3,4,5,9,14,16,17	1,6,7,8,10,11,12,13,15	11	P02782,P08723,P15999,P30120,P34901,P47727,Q5GRG2,Q62902,Q8CJ52,Q9JHB9,Q9JI85

11865	2,3,4,5,9,15,16,17	1,6,7,8,10,11,12,13,14	20	P02781,P02782,P04905,P06911,P08723,P09456,P0C0A9,P11598,P12020,P19629,P22283,P30120,P36374,P47727,Q5GRG2,Q62902,Q63493,Q8CJ52,Q9JHB9,Q9JI85
11866	2,3,4,5,10,11,12,13	1,6,7,8,9,14,15,16,17	2	P19629,P34901
11867	2,3,4,5,10,11,12,14	1,6,7,8,9,13,15,16,17	2	P34901,P53792
11868	2,3,4,5,10,11,12,15	1,6,7,8,9,13,14,16,17	3	P34901,P51907,P53792
11869	2,3,4,5,10,11,12,16	1,6,7,8,9,13,14,15,17	4	P19629,P34901,P53792,Q9JJS8
11870	2,3,4,5,10,11,12,17	1,6,7,8,9,13,14,15,16	1	P34901
11871	2,3,4,5,10,11,13,14	1,6,7,8,9,12,15,16,17	3	P10715,P13432,P34901
11872	2,3,4,5,10,11,13,15	1,6,7,8,9,12,14,16,17	2	P13432,P19629
11873	2,3,4,5,10,11,13,16	1,6,7,8,9,12,14,15,17	2	P13432,P19629
11874	2,3,4,5,10,11,13,17	1,6,7,8,9,12,14,15,16	1	P19629
11875	2,3,4,5,10,11,14,15	1,6,7,8,9,12,13,16,17	5	P13432,P19218,P34901,Q62902,Q64194
11876	2,3,4,5,10,11,14,16	1,6,7,8,9,12,13,15,17	4	P13432,P19218,P34901,Q6IFV1
11877	2,3,4,5,10,11,14,17	1,6,7,8,9,12,13,15,16	2	P19218,P34901
11878	2,3,4,5,10,11,15,16	1,6,7,8,9,12,13,14,17	11	P02782,P06911,P08723,P11598,P13432,P19629,P22282,P22283,P51907,Q62902,Q9Z1F2
11879	2,3,4,5,10,11,15,17	1,6,7,8,9,12,13,14,16	4	P02782,P06911,P19629,Q62902
11880	2,3,4,5,10,11,16,17	1,6,7,8,9,12,13,14,15	5	P02782,P06911,P19629,Q5GRG2,Q62902
11881	2,3,4,5,10,12,13,14	1,6,7,8,9,11,15,16,17	2	P34901,Q9JJS8
11882	2,3,4,5,10,12,13,15	1,6,7,8,9,11,14,16,17	2	P04218,P19629
11883	2,3,4,5,10,12,13,16	1,6,7,8,9,11,14,15,17	4	P19629,P34901,Q62997,Q9JJS8
11884	2,3,4,5,10,12,13,17	1,6,7,8,9,11,14,15,16	1	P19629
11885	2,3,4,5,10,12,14,15	1,6,7,8,9,11,13,16,17	2	A2RUV9,P34901
11886	2,3,4,5,10,12,14,16	1,6,7,8,9,11,13,15,17	2	P34901,Q9JJS8

11887	2,3,4,5,10,12,14,17	1,6,7,8,9,11,13,15,16	3	P31430,P34901,Q9JJS8
11888	2,3,4,5,10,12,15,16	1,6,7,8,9,11,13,14,17	6	P19629,P34901,P47727,Q62902,Q62997,Q9JJS8
11889	2,3,4,5,10,12,15,17	1,6,7,8,9,11,13,14,16	3	P34901,P47727,Q62902
11890	2,3,4,5,10,12,16,17	1,6,7,8,9,11,13,14,15	6	P19629,P34901,P47727,Q5GRG2,Q62997,Q9JJS8
11891	2,3,4,5,10,13,14,15	1,6,7,8,9,11,12,16,17	4	P10715,P13432,Q6AYL6,Q99068
11892	2,3,4,5,10,13,14,16	1,6,7,8,9,11,12,15,17	5	P10715,P13432,P19629,Q62997,Q99068
11893	2,3,4,5,10,13,14,17	1,6,7,8,9,11,12,15,16	2	P10715,Q6AYL6
11894	2,3,4,5,10,13,15,16	1,6,7,8,9,11,12,14,17	10	P02782,P08723,P10715,P13432,P19629,P22283,P47727,Q62997,Q63493,Q9JHB9
11895	2,3,4,5,10,13,15,17	1,6,7,8,9,11,12,14,16	5	P10715,P19629,P47727,Q6AYL6,Q8CJ52
11896	2,3,4,5,10,13,16,17	1,6,7,8,9,11,12,14,15	4	P10715,P19629,P47727,Q62997
11897	2,3,4,5,10,14,15,16	1,6,7,8,9,11,12,13,17	16	P02782,P08723,P10715,P13432,P22282,P22283,P30120,P34901,P36374,Q62902,Q62997,Q63493,Q8CJ52,Q9JHB9,Q9JI85,Q9QZK9
11898	2,3,4,5,10,14,15,17	1,6,7,8,9,11,12,13,16	8	P02782,P02803,P10715,P30120,P34901,Q62902,Q8CJ52,Q9JHB9
11899	2,3,4,5,10,14,16,17	1,6,7,8,9,11,12,13,15	8	P02782,P10715,P30120,P34901,Q62902,Q62997,Q9JHB9,Q9JI85
11900	2,3,4,5,10,15,16,17	1,6,7,8,9,11,12,13,14	18	P02781,P02782,P04905,P06911,P08723,P09456,P0C0A9,P19629,P22282,P22283,P30120,P36374,P47727,Q62902,Q62997,Q8CJ52,Q9JHB9,Q9JI85
11901	2,3,4,5,11,12,13,14	1,6,7,8,9,10,15,16,17	1	P34901
11902	2,3,4,5,11,12,13,15	1,6,7,8,9,10,14,16,17	1	P01836
11903	2,3,4,5,11,12,13,16	1,6,7,8,9,10,14,15,17	1	P01836
11904	2,3,4,5,11,12,13,17	1,6,7,8,9,10,14,15,16	0	
11905	2,3,4,5,11,12,14,15	1,6,7,8,9,10,13,16,17	4	P19218,P34901,P51907,Q64194
11906	2,3,4,5,11,12,14,16	1,6,7,8,9,10,13,15,17	2	P34901,Q6IFV1
11907	2,3,4,5,11,12,14,17	1,6,7,8,9,10,13,15,16	5	P01835,P19218,P31430,P34901,Q6AYQ8
11908	2,3,4,5,11,12,15,16	1,6,7,8,9,10,13,14,17	5	P01836,P06911,P12020,P34901,P51907
11909	2,3,4,5,11,12,15,17	1,6,7,8,9,10,13,14,16	4	P06911,P12020,P34901,P63036

11910	2,3,4,5,11,12,16,17	1,6,7,8,9,10,13,14,15	5	P06911,P12020,P15999,P34901,Q5GRG2
11911	2,3,4,5,11,13,14,15	1,6,7,8,9,10,12,16,17	2	P13432,P97840
11912	2,3,4,5,11,13,14,16	1,6,7,8,9,10,12,15,17	1	P13432
11913	2,3,4,5,11,13,14,17	1,6,7,8,9,10,12,15,16	2	P19218,P97840
11914	2,3,4,5,11,13,15,16	1,6,7,8,9,10,12,14,17	4	P08723,P13432,P19629,Q6AYR8
11915	2,3,4,5,11,13,15,17	1,6,7,8,9,10,12,14,16	1	P97840
11916	2,3,4,5,11,13,16,17	1,6,7,8,9,10,12,14,15	1	P19629
11917	2,3,4,5,11,14,15,16	1,6,7,8,9,10,12,13,17	10	P02782,P08723,P13432,P19218,P22283,P34901,P36374,Q64194,Q99MH3,Q9QZK9
11918	2,3,4,5,11,14,15,17	1,6,7,8,9,10,12,13,16	6	P19218,P34901,P47967,P63036,P97840,Q62902
11919	2,3,4,5,11,14,16,17	1,6,7,8,9,10,12,13,15	3	P19218,P34901,P97840
11920	2,3,4,5,11,15,16,17	1,6,7,8,9,10,12,13,14	11	P02782,P06911,P08723,P09456,P12020,P22283,P30120,P97840,Q5GRG2,Q62902,Q9JHB9
11921	2,3,4,5,12,13,14,15	1,6,7,8,9,10,11,16,17	3	A2RUV9,P34901,Q9QUL6
11922	2,3,4,5,12,13,14,16	1,6,7,8,9,10,11,15,17	4	P34901,Q566E6,Q9JJS8,Q9QUL6
11923	2,3,4,5,12,13,14,17	1,6,7,8,9,10,11,15,16	3	P31430,P34901,Q9QW07
11924	2,3,4,5,12,13,15,16	1,6,7,8,9,10,11,14,17	3	P01836,P47727,Q9QUL6
11925	2,3,4,5,12,13,15,17	1,6,7,8,9,10,11,14,16	1	Q6AYL6
11926	2,3,4,5,12,13,16,17	1,6,7,8,9,10,11,14,15	3	P19629,P22006,P35280
11927	2,3,4,5,12,14,15,16	1,6,7,8,9,10,11,13,17	1	P34901
11928	2,3,4,5,12,14,15,17	1,6,7,8,9,10,11,13,16	4	B0BNN3,P31430,P34901,Q8CJD3
11929	2,3,4,5,12,14,16,17	1,6,7,8,9,10,11,13,15	3	P31430,P34901,Q9JJS8
11930	2,3,4,5,12,15,16,17	1,6,7,8,9,10,11,13,14	7	P06911,P12020,P34901,P47727,P83121,Q5GRG2,Q62902
11931	2,3,4,5,13,14,15,16	1,6,7,8,9,10,11,12,17	13	F1LM93,P08723,P10715,P13432,P22283,P36374,P60905,Q566E6,Q63357,Q63493,Q6AYL6,Q99068,Q9QZK9
11932	2,3,4,5,13,14,15,17	1,6,7,8,9,10,11,12,16	9	P10715,P12368,P47967,P63095,P97840,Q63357,Q6AYL6,Q8CJ52,Q9QW07

11933	2,3,4,5,13,14,16,17	1,6,7,8,9,10,11,12,15	8	P08010,P10715,P35280,Q566E6,Q63357,Q6AYL6,Q6PCU2,Q9QW07
11934	2,3,4,5,13,15,16,17	1,6,7,8,9,10,11,12,14	11	P02782,P04905,P08723,P0C0A9,P19629,P30120,P47727,Q63357,Q63493,Q6AYL6,Q9JHB9
11935	2,3,4,5,14,15,16,17	1,6,7,8,9,10,11,12,13	16	P02781,P02782,P02803,P04905,P08723,P09456,P0C0A9,P22283,P30120,P36374,P47727,Q62902,Q63357,Q8CJ52,Q9JHB9,Q9JI85
11936	2,3,4,6,7,8,9,10	1,5,11,12,13,14,15,16,17	13	D3ZHA0,O55006,P02631,P02761,P16086,P20761,P20762,Q63617,Q6P6R2,Q6TMA8,Q9EQS0,Q9R0T3,Q9Z0V6
11937	2,3,4,6,7,8,9,11	1,5,10,12,13,14,15,16,17	16	D3ZHA0,O55004,P02631,P02761,P07150,P11883,P14669,P16086,P20761,P20762,Q5XI22,Q63617,Q6TMA8,Q9EQS0,Q9R0T3,Q9Z0V6
11938	2,3,4,6,7,8,9,12	1,5,10,11,13,14,15,16,17	20	D3ZHA0,O55004,P02631,P02780,P06761,P07647,P16086,P19223,P20761,P20762,P22282,P22283,Q498R7,Q63617,Q6TMA8,Q80WL1,Q99041,Q9EQS0,Q9R0T3,Q9Z0V6
11939	2,3,4,6,7,8,9,13	1,5,10,11,12,14,15,16,17	13	D3ZHA0,P02631,P06761,P06911,P16086,P20761,P20762,P20766,P22282,Q63617,Q6TMA8,Q9EQS0,Q9R0T3
11940	2,3,4,6,7,8,9,14	1,5,10,11,12,13,15,16,17	9	D3ZHA0,P02631,P16086,P20761,P20762,Q63617,Q6TMA8,Q9EQS0,Q9Z0V6
11941	2,3,4,6,7,8,9,15	1,5,10,11,12,13,14,16,17	12	D3ZHA0,O55006,P02631,P02761,P16086,P20761,P20762,Q5QE79,Q63617,Q6TMA8,Q9EQS0,Q9Z0V6
11942	2,3,4,6,7,8,9,16	1,5,10,11,12,13,14,15,17	11	D3ZHA0,P02631,P16086,P20761,P20762,Q63617,Q6P6R2,Q6TMA8,Q9EQS0,Q9R0T3,Q9Z0V6
11943	2,3,4,6,7,8,9,17	1,5,10,11,12,13,14,15,16	20	D3ZHA0,O55004,P02631,P02761,P09605;P25809,P16086,P19223,P20761,P20762,P50115,P55091,Q4G075,Q63617,Q63751,Q6P6R2,Q6TMA8,Q99041,Q9EQS0,Q9R0T3,Q9Z0V6
11944	2,3,4,6,7,8,10,11	1,5,9,12,13,14,15,16,17	21	D3ZHA0,O55004,P00714,P02631,P02761,P07150,P11883,P14669,P16086,P20761,P20762,P35280,P82471,Q5BJY9,Q5FVI6,Q5XI22,Q63617,Q6TMA8,Q9EQS0,Q9R0T3,Q9Z0V6
11945	2,3,4,6,7,8,10,12	1,5,9,11,13,14,15,16,17	23	D3ZHA0,O55004,P01041,P02631,P04218,P06761,P07647,P08723,P16086,P19223,P20761,P20762,P22282,P22283,P35280,Q64361,Q6AYR9,Q6TMA8,Q80WL1,Q99041,Q9EQS0,Q9R0T3,Q9Z0V6
11946	2,3,4,6,7,8,10,13	1,5,9,11,12,14,15,16,17	17	D3ZHA0,P02631,P04218,P06761,P06911,P07647,P12020,P16086,P20761,P20762,P20766,P22282,Q5GRG2,Q6TMA8,Q9EQS0,Q9R0T3,Q9Z0V6
11947	2,3,4,6,7,8,10,14	1,5,9,11,12,13,15,16,17	13	D3ZHA0,P02631,P06761,P12020,P16086,P20761,P20762,P35280,P97615,Q6TMA8,Q9EQS0,Q9R0T3,Q9Z0V6
11948	2,3,4,6,7,8,10,15	1,5,9,11,12,13,14,16,17	11	D3ZHA0,P02631,P02761,P16086,P20761,P20762,P35280,P97615,Q6TMA8,Q9EQS0,Q9Z0V6

11949	2,3,4,6,7,8,10,16	1,5,9,11,12,13,14,15,17	9	D3ZHA0,P02631,P16086,P20761,P82471,Q6P6R2,Q9EQS0,Q9R0T3,Q9Z0V6
11950	2,3,4,6,7,8,10,17	1,5,9,11,12,13,14,15,16	21	D3ZHA0,O55004,P02631,P02761,P09605;P25809,P16086,P19223,P20760,P20761,P20762,P46844,P50115,P55091,P97615,Q4G075,Q63751,Q6P6R2,Q99041,Q9EQS0,Q9R0T3,Q9Z0V6
11951	2,3,4,6,7,8,11,12	1,5,9,10,13,14,15,16,17	38	D3ZHA0,O55004,P01836,P02631,P02780,P02781,P02782,P06761,P07150,P07647,P08723,P0C0A9,P11883,P14669,P16086,P19223,P20761,P20762,P22282,P22283,P24368,P30120,P35280,P50280,Q498R7,Q5XI22,Q63493,Q63617,Q63751,Q6TMA8,Q80WL1,Q8CJ52,Q99041,Q9EQS0,Q9JHB9,Q9JI85,Q9R0T3,Q9Z0V6
11952	2,3,4,6,7,8,11,13	1,5,9,10,12,14,15,16,17	21	P02631,P06761,P06911,P07150,P07647,P11598,P14669,P16086,P20761,P20762,P20766,P22282,P22283,P50280,Q5XI22,Q62902,Q63617,Q63751,Q6TMA8,Q9EQS0,Q9R0T3
11953	2,3,4,6,7,8,11,14	1,5,9,10,12,13,15,16,17	18	D3ZHA0,P02631,P06761,P07150,P07171,P11883,P14669,P16086,P19218,P20761,P20762,P22282,P35280,Q63617,Q6TMA8,Q9EQS0,Q9R0T3,Q9Z0V6
11954	2,3,4,6,7,8,11,15	1,5,9,10,12,13,14,16,17	18	D3ZHA0,O55004,P02625,P02631,P02761,P07150,P07171,P14669,P16086,P20761,P20762,P35280,Q5XI22,Q63617,Q63751,Q6TMA8,Q9EQS0,Q9Z0V6
11955	2,3,4,6,7,8,11,16	1,5,9,10,12,13,14,15,17	13	D3ZHA0,P02631,P07150,P14668,P14669,P16086,P20761,P20762,Q63617,Q6TMA8,Q9EQS0,Q9R0T3,Q9Z0V6
11956	2,3,4,6,7,8,11,17	1,5,9,10,12,13,14,15,16	23	O55004,P02631,P02761,P07150,P09605;P25809,P14669,P16086,P19223,P20761,P20762,P22282,P22283,P50115,P55091,Q4G075,Q5XI22,Q63617,Q63751,Q6P6R2,Q99041,Q9EQS0,Q9R0T3,Q9Z0V6
11957	2,3,4,6,7,8,12,13	1,5,9,10,11,14,15,16,17	35	D3ZHA0,O08557,O88267,P01836,P02631,P02780,P02782,P06761,P06911,P07647,P08723,P08937,P11598,P12020,P16086,P20761,P20762,P20766,P22282,P22283,P25236,P29975,P30120,P50280,P62815,Q62902,Q63751,Q6AYR9,Q6TMA8,Q80WL1,Q99041,Q9EQS0,Q9JHB9,Q9R0T3,Q9Z0V6
11958	2,3,4,6,7,8,12,14	1,5,9,10,11,13,15,16,17	31	D3ZHA0,O08557,O88267,P01041,P02631,P02780,P02782,P06761,P07647,P08723,P11598,P16086,P20761,P20762,P22282,P22283,P25236,P29975,P30120,P62815,Q63618,Q6AYR9,Q6AYS7,Q6TMA8,Q80WL1,Q920P0,Q923M1,Q99041,Q9EQS0,Q9R0T3,Q9Z0V6
11959	2,3,4,6,7,8,12,15	1,5,9,10,11,13,14,16,17	24	B0BNN3,D3ZHA0,O08557,O55004,P01041,P01836,P02625,P02631,P02761,P06761,P16086,P20761,P20762,P35280,P62815,P83748,Q63751,Q64611,Q6TMA8,Q80WL1,Q99041,Q9EQS0,Q9R0T3,Q9Z0V6
11960	2,3,4,6,7,8,12,16	1,5,9,10,11,13,14,15,17	16	D3ZHA0,O55004,O88267,P01041,P01836,P02631,P06761,P16086,P20761,P20762,Q6TMA8,Q80WL1,Q99041,Q9EQS0,Q9R0T3,Q9Z0V6
11961	2,3,4,6,7,8,12,17	1,5,9,10,11,13,14,15,16	32	D3ZHA0,O55004,O88267,P01041,P02631,P02761,P06761,P07647,P08937,P09605;P25809,P11598,P16086,P19223,P20760,P20761,P20762,P22282,P22283,P50115,P55091,P62815,P98089,Q4G075,Q62635,Q63751,Q6P6R2,Q80WL1,Q99041,Q9EQS0,Q9QZK9,Q9R0T3,Q9Z0V6
11962	2,3,4,6,7,8,13,14	1,5,9,10,11,12,15,16,17	23	D3ZHA0,O08557,P02631,P06761,P06911,P11598,P12020,P16086,P20761,P20762,P20766,P22282,P25236,P29975,P53790,P62815,Q5GRG2,Q62902,Q63617,Q6TMA8,Q9EQS0,Q9R0T3,Q9Z0V6
11963	2,3,4,6,7,8,13,15	1,5,9,10,11,12,14,16,17	12	D3ZHA0,P02631,P06761,P06911,P16086,P20761,P20762,P20766,P62815,Q5GRG2,Q6TMA8,Q9Z0V6

11964	2,3,4,6,7,8,13,16	1,5,9,10,11,12,14,15,17	11	D3ZHA0,P02631,P06761,P06911,P16086,P20761,P20762,P20766,Q63617,Q6TMA8,Q9R0T3
11965	2,3,4,6,7,8,13,17	1,5,9,10,11,12,14,15,16	27	O88267,P02631,P06761,P06911,P09605;P25809,P11598,P16086,P19223,P20760,P20761,P20762,P20766,P22282,P22283,P29975,P50115,P51907,P55091,P62815,P70545,Q4G075,Q63617,Q63751,Q6TMA8,Q99041,Q9R0T3,Q9Z0V6
11966	2,3,4,6,7,8,14,15	1,5,9,10,11,12,13,16,17	15	B0BNN3,D3ZHA0,O08557,P02631,P02761,P02803,P07171,P16086,P20761,P20762,P35280,Q6TMA8,Q923S2,Q9EQS0,Q9Z0V6
11967	2,3,4,6,7,8,14,16	1,5,9,10,11,12,13,15,17	13	D3ZHA0,P02631,P16086,P20761,P20762,P97574,Q566E6,Q63532,Q63617,Q6TMA8,Q9EQS0,Q9R0T3,Q9Z0V6
11968	2,3,4,6,7,8,14,17	1,5,9,10,11,12,13,15,16	25	O88267,P02631,P02761,P06761,P09605;P25809,P10536,P11598,P16086,P19223,P20760,P20761,P20762,P22282,P50115,P55091,P62815,Q4G075,Q63617,Q63751,Q6P6R2,Q923S2,Q99041,Q9EQS0,Q9R0T3,Q9Z0V6
11969	2,3,4,6,7,8,15,16	1,5,9,10,11,12,13,14,17	7	D3ZHA0,P02631,P02803,P16086,P20761,P20762,Q9Z0V6
11970	2,3,4,6,7,8,15,17	1,5,9,10,11,12,13,14,16	21	B0BNN3,D3ZHA0,O55004,P01041,P02631,P02761,P02803,P09605;P25809,P10536,P16086,P19223,P20760,P20761,P20762,P50115,P55091,P62815,Q4G075,Q63751,Q99041,Q9Z0V6
11971	2,3,4,6,7,8,16,17	1,5,9,10,11,12,13,14,15	16	O88267,P02631,P09605;P25809,P16086,P19223,P20761,P20762,P50115,P55091,Q4G075,Q63617,Q63751,Q6P6R2,Q99041,Q9R0T3,Q9Z0V6
11972	2,3,4,6,7,9,10,11	1,5,8,12,13,14,15,16,17	4	P11883,P14173,P16086,Q5FVI6
11973	2,3,4,6,7,9,10,12	1,5,8,11,13,14,15,16,17	5	P04218,P47967,P97840,Q498R7,Q9EQS0
11974	2,3,4,6,7,9,10,13	1,5,8,11,12,14,15,16,17	1	P16086
11975	2,3,4,6,7,9,10,14	1,5,8,11,12,13,15,16,17	2	P02631,P16086
11976	2,3,4,6,7,9,10,15	1,5,8,11,12,13,14,16,17	2	P02631,Q4FZU6
11977	2,3,4,6,7,9,10,16	1,5,8,11,12,13,14,15,17	3	P16086,P47967,Q5FVI6
11978	2,3,4,6,7,9,10,17	1,5,8,11,12,13,14,15,16	5	P09605;P25809,P16086,P19223,P20761,Q6P6R2

11979	2,3,4,6,7,9,11,12	1,5,8,10,13,14,15,16,17	6	P01835,P01836,P02631,P16086,Q498R7,Q9EQS0
11980	2,3,4,6,7,9,11,13	1,5,8,10,12,14,15,16,17	2	P02631,P16086
11981	2,3,4,6,7,9,11,14	1,5,8,10,12,13,15,16,17	4	P02631,P16086,P20762,P83121
11982	2,3,4,6,7,9,11,15	1,5,8,10,12,13,14,16,17	3	P02625,P02631,P16086
11983	2,3,4,6,7,9,11,16	1,5,8,10,12,13,14,15,17	2	P02631,P16086
11984	2,3,4,6,7,9,11,17	1,5,8,10,12,13,14,15,16	4	P02631,P09605;P25809,P16086,P20761
11985	2,3,4,6,7,9,12,13	1,5,8,10,11,14,15,16,17	4	P01836,P16086,P25236,Q498R7
11986	2,3,4,6,7,9,12,14	1,5,8,10,11,13,15,16,17	8	A2RUV9,P01835,P02631,P16086,P20762,P25236,Q498R7,Q9EQS0
11987	2,3,4,6,7,9,12,15	1,5,8,10,11,13,14,16,17	4	P01836,P02625,P02631,Q498R7
11988	2,3,4,6,7,9,12,16	1,5,8,10,11,13,14,15,17	1	P01836
11989	2,3,4,6,7,9,12,17	1,5,8,10,11,13,14,15,16	8	P01835,P09605;P25809,P19223,P20761,P55091,P98089,Q498R7,Q62635
11990	2,3,4,6,7,9,13,14	1,5,8,10,11,12,15,16,17	5	P02631,P06911,P12020,P16086,P25236
11991	2,3,4,6,7,9,13,15	1,5,8,10,11,12,14,16,17	2	P02631,P16086
11992	2,3,4,6,7,9,13,16	1,5,8,10,11,12,14,15,17	1	P16086
11993	2,3,4,6,7,9,13,17	1,5,8,10,11,12,14,15,16	4	P09605;P25809,P16086,P20761,P20766

11994	2,3,4,6,7,9,14,15	1,5,8,10,11,12,13,16,17	4	P02631,P02803,P16086,P20762
11995	2,3,4,6,7,9,14,16	1,5,8,10,11,12,13,15,17	3	P02631,P16086,Q63532
11996	2,3,4,6,7,9,14,17	1,5,8,10,11,12,13,15,16	4	P02631,P09605;P25809,P16086,P20761
11997	2,3,4,6,7,9,15,16	1,5,8,10,11,12,13,14,17	1	P02631
11998	2,3,4,6,7,9,15,17	1,5,8,10,11,12,13,14,16	3	P02631,P09605;P25809,P20761
11999	2,3,4,6,7,9,16,17	1,5,8,10,11,12,13,14,15	4	P09605;P25809,P16086,P20761,Q6P6R2
12000	2,3,4,6,7,10,11,12	1,5,8,9,13,14,15,16,17	10	P01836,P04218,P05964,P11883,P16086,P35280,P47967,Q498R7,Q5FVI6,Q9EQS0
12001	2,3,4,6,7,10,11,13	1,5,8,9,12,14,15,16,17	3	P12020,P16086,Q5FVI6
12002	2,3,4,6,7,10,11,14	1,5,8,9,12,13,15,16,17	6	P02631,P16086,P20762,P35280,Q5FVI6,Q63532
12003	2,3,4,6,7,10,11,15	1,5,8,9,12,13,14,16,17	5	P02631,P16086,P35280,Q4FZU6,Q5FVI6
12004	2,3,4,6,7,10,11,16	1,5,8,9,12,13,14,15,17	4	P16086,P47967,P61206;P84079;P84082,Q5FVI6
12005	2,3,4,6,7,10,11,17	1,5,8,9,12,13,14,15,16	4	P09605;P25809,P16086,P20761,Q5FVI6
12006	2,3,4,6,7,10,12,13	1,5,8,9,11,14,15,16,17	7	P01836,P04218,P12020,P16086,P25236,P47967,P97840
12007	2,3,4,6,7,10,12,14	1,5,8,9,11,13,15,16,17	8	A2RUV9,P04218,P12020,P16086,P25236,Q63532,Q9EQS0,Q9JJS8
12008	2,3,4,6,7,10,12,15	1,5,8,9,11,13,14,16,17	6	P01041,P01836,P02631,P04218,P35280,Q4FZU6

12009	2,3,4,6,7,10,12,16	1,5,8,9,11,13,14,15,17	8	P01836,P04073,P04218,P16086,P47967,P97840,Q5FVI6,Q9JJS8
12010	2,3,4,6,7,10,12,17	1,5,8,9,11,13,14,15,16	10	P01041,P02696,P04218,P09605;P25809,P19223,P20761,P55091,P98089,Q62635,Q9QZK9
12011	2,3,4,6,7,10,13,14	1,5,8,9,11,12,15,16,17	5	P04218,P06911,P12020,P16086,P25236
12012	2,3,4,6,7,10,13,15	1,5,8,9,11,12,14,16,17	3	P04218,P16086,Q4FZU6
12013	2,3,4,6,7,10,13,16	1,5,8,9,11,12,14,15,17	3	P04218,P16086,P47967
12014	2,3,4,6,7,10,13,17	1,5,8,9,11,12,14,15,16	4	P09605;P25809,P12020,P16086,P20761
12015	2,3,4,6,7,10,14,15	1,5,8,9,11,12,13,16,17	5	P02631,P02803,P16086,P35280,Q63532
12016	2,3,4,6,7,10,14,16	1,5,8,9,11,12,13,15,17	3	P04073,P16086,Q63532
12017	2,3,4,6,7,10,14,17	1,5,8,9,11,12,13,15,16	3	P09605;P25809,P16086,P20761
12018	2,3,4,6,7,10,15,16	1,5,8,9,11,12,13,14,17	3	P02803,P16086,Q5FVI6
12019	2,3,4,6,7,10,15,17	1,5,8,9,11,12,13,14,16	5	P02803,P09605;P25809,P16086,P20761,Q4FZU6
12020	2,3,4,6,7,10,16,17	1,5,8,9,11,12,13,14,15	6	P09605;P25809,P14480,P16086,P20761,Q62997,Q6P6R2
12021	2,3,4,6,7,11,12,13	1,5,8,9,10,14,15,16,17	6	P01836,P16086,P25236,Q498R7,Q5XI22,Q9JHB9
12022	2,3,4,6,7,11,12,14	1,5,8,9,10,13,15,16,17	8	P01836,P02631,P16086,P20762,P25236,Q498R7,Q63532,Q9EQS0
12023	2,3,4,6,7,11,12,15	1,5,8,9,10,13,14,16,17	8	O55004,P01836,P02625,P02631,P16086,P20762,P35280,Q498R7

12024	2,3,4,6,7,11,12,16	1,5,8,9,10,13,14,15,17	4	G3V686,P01836,P16086,Q498R7
12025	2,3,4,6,7,11,12,17	1,5,8,9,10,13,14,15,16	13	O55004,P01836,P02631,P09605;P25809,P16086,P19223,P20761,P55091,P98089,Q498R7,Q62635,Q63751,Q9QZK9
12026	2,3,4,6,7,11,13,14	1,5,8,9,10,12,15,16,17	6	P02631,P06911,P12020,P16086,P20762,P25236
12027	2,3,4,6,7,11,13,15	1,5,8,9,10,12,14,16,17	4	P01836,P02625,P02631,P16086
12028	2,3,4,6,7,11,13,16	1,5,8,9,10,12,14,15,17	3	G3V686,P01836,P16086
12029	2,3,4,6,7,11,13,17	1,5,8,9,10,12,14,15,16	4	P09605;P25809,P16086,P20761,Q63751
12030	2,3,4,6,7,11,14,15	1,5,8,9,10,12,13,16,17	7	P02625,P02631,P02803,P16086,P19218,P20762,P35280
12031	2,3,4,6,7,11,14,16	1,5,8,9,10,12,13,15,17	4	G3V686,P02631,P16086,Q63532
12032	2,3,4,6,7,11,14,17	1,5,8,9,10,12,13,15,16	6	P02631,P09605;P25809,P16086,P19218,P20761,P20762
12033	2,3,4,6,7,11,15,16	1,5,8,9,10,12,13,14,17	3	P01836,P02631,P16086
12034	2,3,4,6,7,11,15,17	1,5,8,9,10,12,13,14,16	6	P02631,P02803,P09605;P25809,P16086,P20761,Q63751
12035	2,3,4,6,7,11,16,17	1,5,8,9,10,12,13,14,15	3	P09605;P25809,P16086,P20761
12036	2,3,4,6,7,12,13,14	1,5,8,9,10,11,15,16,17	8	A2RUV9,P01836,P06911,P12020,P16086,P20762,P25236,Q63532
12037	2,3,4,6,7,12,13,15	1,5,8,9,10,11,14,16,17	6	P01836,P02625,P02631,P16086,P25236,P83748
12038	2,3,4,6,7,12,13,16	1,5,8,9,10,11,14,15,17	4	G3V686,P01836,P16086,P25236

12039	2,3,4,6,7,12,13,17	1,5,8,9,10,11,14,15,16	11	P01836,P09605;P25809,P16086,P19223,P20761,P55091,P62815,P98089,Q62635,Q63751,Q9QZK9
12040	2,3,4,6,7,12,14,15	1,5,8,9,10,11,13,16,17	10	A2RUV9,P01836,P02625,P02631,P02803,P16086,P20761,P20762,P25236,Q63532
12041	2,3,4,6,7,12,14,16	1,5,8,9,10,11,13,15,17	9	G3V686,P01836,P02631,P04073,P16086,P25236,Q63532,Q920P0,Q9JJS8
12042	2,3,4,6,7,12,14,17	1,5,8,9,10,11,13,15,16	13	A2RUV9,P01835,P02631,P09605;P25809,P16086,P19223,P20761,P20762,P31430,P55091,P62815,P98089,Q62635
12043	2,3,4,6,7,12,15,16	1,5,8,9,10,11,13,14,17	3	P01836,P02631,P83748
12044	2,3,4,6,7,12,15,17	1,5,8,9,10,11,13,14,16	12	B0BNN3,P01041,P01836,P02631,P09605;P25809,P19223,P20761,P55091,P62815,P98089,Q62635,Q63751
12045	2,3,4,6,7,12,16,17	1,5,8,9,10,11,13,14,15	9	P01836,P09605;P25809,P14480,P16086,P19223,P20761,P55091,P98089,Q62635
12046	2,3,4,6,7,13,14,15	1,5,8,9,10,11,12,16,17	7	A2RUV9,P02631,P02803,P06911,P12020,P16086,P25236
12047	2,3,4,6,7,13,14,16	1,5,8,9,10,11,12,15,17	7	G3V686,P06911,P12020,P16086,P25236,Q566E6,Q63532
12048	2,3,4,6,7,13,14,17	1,5,8,9,10,11,12,15,16	7	P06911,P09605;P25809,P12020,P16086,P20761,P25236,P62815
12049	2,3,4,6,7,13,15,16	1,5,8,9,10,11,12,14,17	3	P01836,P16086,Q99PP0
12050	2,3,4,6,7,13,15,17	1,5,8,9,10,11,12,14,16	6	P02631,P09605;P25809,P16086,P20761,P62815,Q9WUW8
12051	2,3,4,6,7,13,16,17	1,5,8,9,10,11,12,14,15	4	P09605;P25809,P14480,P16086,P20761
12052	2,3,4,6,7,14,15,16	1,5,8,9,10,11,12,13,17	4	P02631,P02803,P16086,Q63532
12053	2,3,4,6,7,14,15,17	1,5,8,9,10,11,12,13,16	8	B0BNN3,P02631,P02803,P09605;P25809,P16086,P20761,P20762,P62815

12054	2,3,4,6,7,14,16,17	1,5,8,9,10,11,12,13,15	6	P02803,P09605;P25809,P14480,P16086,P20761,Q63532
12055	2,3,4,6,7,15,16,17	1,5,8,9,10,11,12,13,14	5	P02631,P02803,P09605;P25809,P16086,P20761
12056	2,3,4,6,8,9,10,11	1,5,7,12,13,14,15,16,17	11	D3ZHA0,P00714,P02761,P08649,P11883,P14173,P14669,P16636,Q4FZU6,Q5XI22,Q6TMA8
12057	2,3,4,6,8,9,10,12	1,5,7,11,13,14,15,16,17	6	D3ZHA0,P16636,Q6TMA8,Q80WL1,Q9EQS0,Q9JJS8
12058	2,3,4,6,8,9,10,13	1,5,7,11,12,14,15,16,17	5	D3ZHA0,P14173,P17046,P20766,Q6TMA8
12059	2,3,4,6,8,9,10,14	1,5,7,11,12,13,15,16,17	3	D3ZHA0,P17046,Q6TMA8
12060	2,3,4,6,8,9,10,15	1,5,7,11,12,13,14,16,17	6	D3ZHA0,P02761,P17046,Q4FZU6,Q5QE79,Q6TMA8
12061	2,3,4,6,8,9,10,16	1,5,7,11,12,13,14,15,17	3	D3ZHA0,Q6P6R2,Q6TMA8
12062	2,3,4,6,8,9,10,17	1,5,7,11,12,13,14,15,16	9	D3ZHA0,P02761,P16636,P17046,P19223,P20761,Q63355,Q6P6R2,Q9Z0V6
12063	2,3,4,6,8,9,11,12	1,5,7,10,13,14,15,16,17	10	P01835,P02782,P0C0A9,P11883,P14669,P16636,Q5XI22,Q6TMA8,Q80WL1,Q9EQS0
12064	2,3,4,6,8,9,11,13	1,5,7,10,12,14,15,16,17	4	P14669,P20766,Q5XI22,Q6TMA8
12065	2,3,4,6,8,9,11,14	1,5,7,10,12,13,15,16,17	6	P14668,P14669,P19218,P20762,Q6TMA8,Q9EQS0
12066	2,3,4,6,8,9,11,15	1,5,7,10,12,13,14,16,17	7	P02625,P02761,P14668,P14669,Q5QE79,Q5XI22,Q6TMA8
12067	2,3,4,6,8,9,11,16	1,5,7,10,12,13,14,15,17	2	P14668,Q6TMA8
12068	2,3,4,6,8,9,11,17	1,5,7,10,12,13,14,15,16	10	P02761,P09605;P25809,P14669,P16636,P19223,P20761,P50115,Q6P6R2,Q6TMA8,Q9Z0V6

12069	2,3,4,6,8,9,12,13	1,5,7,10,11,14,15,16,17	8	P02782,P07647,P16636,P20766,P25236,Q06496,Q6TMA8,Q80WL1
12070	2,3,4,6,8,9,12,14	1,5,7,10,11,13,15,16,17	9	D3ZHA0,P01835,P02782,P20762,P25236,Q6TMA8,Q80WL1,Q9EQS0,Q9JJS8
12071	2,3,4,6,8,9,12,15	1,5,7,10,11,13,14,16,17	5	B0BNN3,D3ZHA0,P02625,Q5QE79,Q6TMA8
12072	2,3,4,6,8,9,12,16	1,5,7,10,11,13,14,15,17	3	Q6TMA8,Q80WL1,Q9JJS8
12073	2,3,4,6,8,9,12,17	1,5,7,10,11,13,14,15,16	15	P01835,P09605;P25809,P16636,P19223,P20761,P50115,P55091,P98089,Q4G075,Q62635,Q63355,Q6P6R2,Q6TMA8,Q80WL1,Q9Z0V6
12074	2,3,4,6,8,9,13,14	1,5,7,10,11,12,15,16,17	3	P20766,P25236,Q6TMA8
12075	2,3,4,6,8,9,13,15	1,5,7,10,11,12,14,16,17	3	P20766,Q5QE79,Q6TMA8
12076	2,3,4,6,8,9,13,16	1,5,7,10,11,12,14,15,17	2	P20766,Q6TMA8
12077	2,3,4,6,8,9,13,17	1,5,7,10,11,12,14,15,16	6	P16636,P20761,P20766,P51907,Q63355,Q6TMA8
12078	2,3,4,6,8,9,14,15	1,5,7,10,11,12,13,16,17	5	B0BNN3,P17046,P20762,Q5QE79,Q6TMA8
12079	2,3,4,6,8,9,14,16	1,5,7,10,11,12,13,15,17	1	Q6TMA8
12080	2,3,4,6,8,9,14,17	1,5,7,10,11,12,13,15,16	5	P17046,P20761,Q6P6R2,Q6TMA8,Q9Z0V6
12081	2,3,4,6,8,9,15,16	1,5,7,10,11,12,13,14,17	2	Q5QE79,Q6TMA8
12082	2,3,4,6,8,9,15,17	1,5,7,10,11,12,13,14,16	6	B0BNN3,P02761,P20761,Q5QE79,Q6TMA8,Q9Z0V6
12083	2,3,4,6,8,9,16,17	1,5,7,10,11,12,13,14,15	5	P09605;P25809,P16636,P20761,P23593,Q6P6R2

12084	2,3,4,6,8,10,11,12	1,5,7,9,13,14,15,16,17	18	D3ZHA0,P00714,P00762,P0C0A9,P11883,P14669,P16636,P35280,P53792,P62804,Q00715,Q4FZU6,Q5XI22,Q63598,Q6TMA8,Q80WL1,Q9EQS0,Q9JJS8
12085	2,3,4,6,8,10,11,13	1,5,7,9,12,14,15,16,17	7	P11883,P14669,P16636,P20766,Q4FZU6,Q5XI22,Q6TMA8
12086	2,3,4,6,8,10,11,14	1,5,7,9,12,13,15,16,17	9	P11883,P14669,P19218,P20762,P35280,P62804,Q63598,Q6TMA8,Q9EQS0
12087	2,3,4,6,8,10,11,15	1,5,7,9,12,13,14,16,17	7	D3ZHA0,P02761,P14669,P35280,Q4FZU6,Q5XI22,Q6TMA8
12088	2,3,4,6,8,10,11,16	1,5,7,9,12,13,14,15,17	6	P11883,P14668,P14669,P16636,P53792,Q6TMA8
12089	2,3,4,6,8,10,11,17	1,5,7,9,12,13,14,15,16	11	P02761,P07150,P08649,P11883,P14669,P16636,P20761,Q4FZU6,Q4G075,Q6P6R2,Q9Z0V6
12090	2,3,4,6,8,10,12,13	1,5,7,9,11,14,15,16,17	10	D3ZHA0,P02782,P07647,P16636,P20766,P25236,Q6AYR9,Q6TMA8,Q80WL1,Q9JJS8
12091	2,3,4,6,8,10,12,14	1,5,7,9,11,13,15,16,17	7	D3ZHA0,P25236,Q6TMA8,Q80WL1,Q9EQS0,Q9JJS8,Q9Z0V6
12092	2,3,4,6,8,10,12,15	1,5,7,9,11,13,14,16,17	7	B0BNN3,D3ZHA0,P02761,P35280,Q4FZU6,Q6TMA8,Q9JJS8
12093	2,3,4,6,8,10,12,16	1,5,7,9,11,13,14,15,17	4	D3ZHA0,P16636,Q80WL1,Q9JJS8
12094	2,3,4,6,8,10,12,17	1,5,7,9,11,13,14,15,16	17	B0BNN3,D3ZHA0,D4A5U3,P02761,P16636,P19223,P20761,P55091,P98089,Q4G075,Q62635,Q63355,Q6P6R2,Q80WL1,Q9JJS8,Q9QZK9,Q9Z0V6
12095	2,3,4,6,8,10,13,14	1,5,7,9,11,12,15,16,17	5	P06911,P12020,P20766,P25236,Q6TMA8
12096	2,3,4,6,8,10,13,15	1,5,7,9,11,12,14,16,17	4	D3ZHA0,P20766,Q4FZU6,Q6TMA8
12097	2,3,4,6,8,10,13,16	1,5,7,9,11,12,14,15,17	2	P20766,Q6TMA8
12098	2,3,4,6,8,10,13,17	1,5,7,9,11,12,14,15,16	7	P16636,P20761,P20766,Q4G075,Q63355,Q6TMA8,Q9Z0V6

12099	2,3,4,6,8,10,14,15	1,5,7,9,11,12,13,16,17	7	B0BNN3,D3ZHA0,P02761,P17046,P35280,Q4FZU6,Q6TMA8
12100	2,3,4,6,8,10,14,16	1,5,7,9,11,12,13,15,17	0	
12101	2,3,4,6,8,10,14,17	1,5,7,9,11,12,13,15,16	4	P02761,P20761,Q6P6R2,Q9Z0V6
12102	2,3,4,6,8,10,15,16	1,5,7,9,11,12,13,14,17	2	D3ZHA0,Q4FZU6
12103	2,3,4,6,8,10,15,17	1,5,7,9,11,12,13,14,16	6	B0BNN3,P02761,P20761,Q4FZU6,Q4G075,Q9Z0V6
12104	2,3,4,6,8,10,16,17	1,5,7,9,11,12,13,14,15	6	P16636,P20761,Q4G075,Q62997,Q6P6R2,Q9Z0V6
12105	2,3,4,6,8,11,12,13	1,5,7,9,10,14,15,16,17	14	P02780,P02782,P07647,P0C0A9,P14669,P16636,P20766,P25236,P30120,Q5XI22,Q6TMA8,Q80WL1,Q9EQS0,Q9JHB9
12106	2,3,4,6,8,11,12,14	1,5,7,9,10,13,15,16,17	13	P02782,P0C0A9,P11883,P14669,P19218,P20762,P25236,P30120,P62804,Q00715,Q6TMA8,Q80WL1,Q9EQS0
12107	2,3,4,6,8,11,12,15	1,5,7,9,10,13,14,16,17	10	B0BNN3,P02625,P02761,P14669,P20762,P35280,P62804,Q00715,Q5XI22,Q6TMA8
12108	2,3,4,6,8,11,12,16	1,5,7,9,10,13,14,15,17	7	P11883,P14668,P16636,Q6TMA8,Q80WL1,Q9EQS0,Q9JJS8
12109	2,3,4,6,8,11,12,17	1,5,7,9,10,13,14,15,16	23	B0BNN3,P00762,P02761,P02782,P07150,P09605;P25809,P14669,P16636,P19223,P20761,P50115,P55091,P62804,P98089,Q00715,Q4G075,Q62635,Q63751,Q6TMA8,Q80WL1,Q9EQS0,Q9QZK9,Q9Z0V6
12110	2,3,4,6,8,11,13,14	1,5,7,9,10,12,15,16,17	8	P06911,P14669,P19218,P20762,P20766,P25236,Q64093,Q6TMA8
12111	2,3,4,6,8,11,13,15	1,5,7,9,10,12,14,16,17	6	P02625,P14669,P20766,Q4FZU6,Q5XI22,Q6TMA8
12112	2,3,4,6,8,11,13,16	1,5,7,9,10,12,14,15,17	4	P14668,P20766,Q5XI22,Q6TMA8
12113	2,3,4,6,8,11,13,17	1,5,7,9,10,12,14,15,16	7	P14669,P16636,P20761,P20766,Q4G075,Q5XI22,Q6TMA8

12114	2,3,4,6,8,11,14,15	1,5,7,9,10,12,13,16,17	9	B0BNN3,P02625,P02761,P14668,P14669,P19218,P20762,P35280,Q6TMA8
12115	2,3,4,6,8,11,14,16	1,5,7,9,10,12,13,15,17	3	P14668,P19218,Q6TMA8
12116	2,3,4,6,8,11,14,17	1,5,7,9,10,12,13,15,16	9	P02761,P07150,P14669,P19218,P20761,P20762,Q4G075,Q6TMA8,Q9Z0V6
12117	2,3,4,6,8,11,15,16	1,5,7,9,10,12,13,14,17	2	P14668,Q6TMA8
12118	2,3,4,6,8,11,15,17	1,5,7,9,10,12,13,14,16	6	B0BNN3,P02761,P14669,P20761,Q4G075,Q9Z0V6
12119	2,3,4,6,8,11,16,17	1,5,7,9,10,12,13,14,15	8	P07150,P14668,P14669,P16636,P20761,Q4G075,Q6P6R2,Q9Z0V6
12120	2,3,4,6,8,12,13,14	1,5,7,9,10,11,15,16,17	15	O08557,P02782,P06911,P07647,P20762,P20766,P25236,P29975,Q64093,Q6AYR9,Q6TMA8,Q80WL1,Q923M1,Q9EQS0,Q9QUL6
12121	2,3,4,6,8,12,13,15	1,5,7,9,10,11,14,16,17	7	B0BNN3,P02625,P20766,P25236,Q64093,Q6TMA8,Q9QUL6
12122	2,3,4,6,8,12,13,16	1,5,7,9,10,11,14,15,17	7	P16636,P20766,P25236,Q6TMA8,Q80WL1,Q9JJS8,Q9QUL6
12123	2,3,4,6,8,12,13,17	1,5,7,9,10,11,14,15,16	18	P02782,P07647,P16636,P19223,P20761,P20766,P25236,P55091,P62815,P98089,Q4G075,Q62635,Q63355,Q6AYR9,Q6TMA8,Q80WL1,Q9QZK9,Q9Z0V6
12124	2,3,4,6,8,12,14,15	1,5,7,9,10,11,13,16,17	9	A2RUV9,B0BNN3,O08557,P02625,P20762,P25236,Q64093,Q6TMA8,Q9Z0V6
12125	2,3,4,6,8,12,14,16	1,5,7,9,10,11,13,15,17	6	P04073,P25236,Q566E6,Q6TMA8,Q9EQS0,Q9JJS8
12126	2,3,4,6,8,12,14,17	1,5,7,9,10,11,13,15,16	18	B0BNN3,P01835,P02782,P19223,P20761,P20762,P25236,P31430,P55091,P62815,P98089,Q4G075,Q62635,Q6TMA8,Q80WL1,Q923M1,Q9JJS8,Q9Z0V6
12127	2,3,4,6,8,12,15,16	1,5,7,9,10,11,13,14,17	4	B0BNN3,Q6TMA8,Q9JJS8,Q9QUL6
12128	2,3,4,6,8,12,15,17	1,5,7,9,10,11,13,14,16	10	B0BNN3,P02761,P19223,P20761,P55091,P62815,P98089,Q4G075,Q62635,Q9Z0V6

12129	2,3,4,6,8,12,16,17	1,5,7,9,10,11,13,14,15	12	P09605;P25809,P16636,P19223,P20761,P55091,P98089,Q4G075,Q62635,Q6P6R2,Q80WL1,Q9JJS8,Q9Z0V6
12130	2,3,4,6,8,13,14,15	1,5,7,9,10,11,12,16,17	7	B0BNN3,P06757,P12368,P20766,P25236,Q64093,Q6TMA8
12131	2,3,4,6,8,13,14,16	1,5,7,9,10,11,12,15,17	5	P20766,P25236,Q566E6,Q64093,Q6TMA8
12132	2,3,4,6,8,13,14,17	1,5,7,9,10,11,12,15,16	13	P12368,P20761,P20766,P25236,P51907,P53790,P62815,Q63424,Q64093,Q64319,Q6PCU2,Q6TMA8,Q9Z0V6
12133	2,3,4,6,8,13,15,16	1,5,7,9,10,11,12,14,17	3	P20766,Q6TMA8,Q9QUL6
12134	2,3,4,6,8,13,15,17	1,5,7,9,10,11,12,14,16	7	B0BNN3,P02761,P20761,P20766,P62815,Q6TMA8,Q9Z0V6
12135	2,3,4,6,8,13,16,17	1,5,7,9,10,11,12,14,15	6	P16636,P20761,P20766,P51907,Q63355,Q6PCU2
12136	2,3,4,6,8,14,15,16	1,5,7,9,10,11,12,13,17	4	B0BNN3,P02803,P36374,Q6TMA8
12137	2,3,4,6,8,14,15,17	1,5,7,9,10,11,12,13,16	8	B0BNN3,P02761,P02803,P10536,P12368,P20761,P20762,Q9Z0V6
12138	2,3,4,6,8,14,16,17	1,5,7,9,10,11,12,13,15	4	P20761,Q6P6R2,Q6PCU2,Q9Z0V6
12139	2,3,4,6,8,15,16,17	1,5,7,9,10,11,12,13,14	4	B0BNN3,P02803,P20761,Q9Z0V6
12140	2,3,4,6,9,10,11,12	1,5,7,8,13,14,15,16,17	6	P00762,P01835,P11883,P14173,P16636,Q4FZU6
12141	2,3,4,6,9,10,11,13	1,5,7,8,12,14,15,16,17	3	P12785,P14173,Q4FZU6
12142	2,3,4,6,9,10,11,14	1,5,7,8,12,13,15,16,17	3	P14173,P19218,Q4FZU6
12143	2,3,4,6,9,10,11,15	1,5,7,8,12,13,14,16,17	2	P14173,Q4FZU6

12144	2,3,4,6,9,10,11,16	1,5,7,8,12,13,14,15,17	2	P14173,Q4FZU6
12145	2,3,4,6,9,10,11,17	1,5,7,8,12,13,14,15,16	4	P00762,P14173,P16636,Q4FZU6
12146	2,3,4,6,9,10,12,13	1,5,7,8,11,14,15,16,17	5	P14173,P25236,Q4FZU6,Q63355,Q9JJS8
12147	2,3,4,6,9,10,12,14	1,5,7,8,11,13,15,16,17	4	P01835,P14173,P25236,Q9JJS8
12148	2,3,4,6,9,10,12,15	1,5,7,8,11,13,14,16,17	1	Q4FZU6
12149	2,3,4,6,9,10,12,16	1,5,7,8,11,13,14,15,17	2	P14173,Q9JJS8
12150	2,3,4,6,9,10,12,17	1,5,7,8,11,13,14,15,16	7	P00762,P01835,P16636,P19223,P98089,Q63355,Q9JJS8
12151	2,3,4,6,9,10,13,14	1,5,7,8,11,12,15,16,17	3	P10715,P14173,P25236
12152	2,3,4,6,9,10,13,15	1,5,7,8,11,12,14,16,17	2	P10715,Q4FZU6
12153	2,3,4,6,9,10,13,16	1,5,7,8,11,12,14,15,17	2	P10715,P14173
12154	2,3,4,6,9,10,13,17	1,5,7,8,11,12,14,15,16	4	P10715,P14173,Q4FZU6,Q63355
12155	2,3,4,6,9,10,14,15	1,5,7,8,11,12,13,16,17	4	P10715,P17046,Q4FZU6,Q8CJ52

Day 7

Random allocation	Group1	Group2	Numbers of differential urinary proteins	Differential protein ID
1	1,2,3,4,5,6,7,8	18,19,20,21,22,23,24,25,26	29	O35783,O55006,P00731,P01681,P01836,P02631,P02803,P05371,P08010,P10715,P16086,P18418,P20762,P50398,P52590,P61206;P84079;P84082,Q09030,Q30KJ2,Q4V885,Q63317,Q63475,Q63617,Q64057,Q6AYQ8,Q6IE51,Q7TP52,Q8N7M5,Q91ZS3,Q9ESW0

2	1,2,3,4,5,6,7,18	8,19,20,21,22,23,24,25,26	22	O35783,O55006,P00731,P01681,P01836,P02631,P05371,P05964,P16086,P18418,P20762,P25031,P34901,P52590,P61206;P84079;P84082,Q63317,Q63475,Q63942,Q6IE51,Q7TP52,Q91ZS3,Q9ESW0
3	1,2,3,4,5,6,7,19	8,18,20,21,22,23,24,25,26	19	O55006,P00731,P01681,P02631,P15399,P16086,P18418,P20762,P52590,P61206;P84079;P84082,P97580,Q09030,Q30KJ2,Q63317,Q63475,Q63532,Q6IE51,Q7TP52,Q8N7M5
4	1,2,3,4,5,6,7,20	8,18,19,21,22,23,24,25,26	19	O35783,O55006,P00731,P01681,P01836,P02631,P08010,P16086,P18418,P20762,P42854,P52590,P61206;P84079;P84082,Q63317,Q63475,Q6IE51,Q6IRE4,Q7TP52,Q91ZS3
5	1,2,3,4,5,6,7,21	8,18,19,20,22,23,24,25,26	24	O35783,O54800;Q5DWV2,O55006,P00731,P01681,P01836,P02631,P16086,P18418,P20646,P20762,P25031,P34901,P42854,P52590,P61206;P84079;P84082,Q4V885,Q63317,Q63475,Q6IE51,Q6IRE4,Q7TP52,Q91ZS3,Q9ESW0
6	1,2,3,4,5,6,7,22	8,18,19,20,21,23,24,25,26	14	P01681,P01836,P02631,P08010,P16086,P18418,P20762,P34901,P52590,Q63317,Q63475,Q6IE51,Q7TP52,Q9QW07
7	1,2,3,4,5,6,7,23	8,18,19,20,21,22,24,25,26	20	P01681,P02631,P05371,P15399,P16086,P18418,P20762,P28648,P34901,P36376,P52590,P55053,P55314,P97580,Q09030,Q30KJ2,Q63317,Q63475,Q6IE51,Q7TP52
8	1,2,3,4,5,6,7,24	8,18,19,20,21,22,23,25,26	15	O55006,P01681,P01836,P02631,P05371,P08010,P18418,P20762,P34901,P52590,P61206;P84079;P84082,Q63317,Q63475,Q6IE51,Q7TP52
9	1,2,3,4,5,6,7,25	8,18,19,20,21,22,23,24,26	17	O35783,P01681,P01836,P02631,P05371,P12020,P16086,P18418,P20762,P52590,P55314,P61206;P84079;P84082,Q5GRG2,Q63475,Q6IE51,Q7TP52,Q91ZS3
10	1,2,3,4,5,6,7,26	8,18,19,20,21,22,23,24,25	23	O35783,P01681,P01836,P01946,P02631,P05539,P08010,P18418,P20760,P20761,P20762,P36376,P52590,P61206;P84079;P84082,P83748,Q30KJ2,Q4FZU2,Q63475,Q63661,Q6AYQ8,Q6IE51,Q7TP52,Q9ESW0
11	1,2,3,4,5,6,8,18	7,19,20,21,22,23,24,25,26	15	O55006,P00731,P01681,P02803,P18418,P20762,P22006,P25031,P52590,Q63317,Q63475,Q63942,Q64361,Q7TP52,Q9ESW0
12	1,2,3,4,5,6,8,19	7,18,20,21,22,23,24,25,26	10	O55006,P00731,P01681,P02803,P20762,P22006,Q30KJ2,Q63317,Q63475,Q7TP52
13	1,2,3,4,5,6,8,20	7,18,19,21,22,23,24,25,26	7	O55006,P01681,P20762,P22006,Q63317,Q63475,Q7TP52
14	1,2,3,4,5,6,8,21	7,18,19,20,22,23,24,25,26	9	P00731,P01681,P02761,P20646,P20762,P52590,Q63317,Q63475,Q9Z0V6
15	1,2,3,4,5,6,8,22	7,18,19,20,21,23,24,25,26	7	P01681,P20762,P20766,P22006,Q63317,Q7TP52,Q9QW07
16	1,2,3,4,5,6,8,23	7,18,19,20,21,22,24,25,26	11	P01681,P20762,P22006,P28648,P34901,P55314,P97580,Q30KJ2,Q63317,Q63475,Q9Z0V6

17	1,2,3,4,5,6,8,24	7,18,19,20,21,22,23,25,26	9	D3ZHA0,O55006,P01681,P08010,P20762,P34901,Q63317,Q63475,Q7TP52
18	1,2,3,4,5,6,8,25	7,18,19,20,21,22,23,24,26	7	P01681,P20762,P55314,Q5GRG2,Q63475,Q7TP52,Q9Z0V6
19	1,2,3,4,5,6,8,26	7,18,19,20,21,22,23,24,25	12	P01681,P10959,P20760,P20761,P20762,P83748,Q4FZU2,Q5QE79,Q6AY33,Q6AYQ8,Q7TP52,Q9Z0V6
20	1,2,3,4,5,6,18,19	7,8,20,21,22,23,24,25,26	11	O55006,P00731,P01681,P02803,P14173,P22006,P25031,Q30KJ2,Q63661,Q63942,Q7TP52
21	1,2,3,4,5,6,18,20	7,8,19,21,22,23,24,25,26	8	P00731,P01681,P20762,P22006,P25031,Q4FZU2,Q7TP52,Q9ESW0
22	1,2,3,4,5,6,18,21	7,8,19,20,22,23,24,25,26	8	P00731,P01681,P01835,P25031,P34901,Q4FZU2,Q63661,Q9ESW0
23	1,2,3,4,5,6,18,22	7,8,19,20,21,23,24,25,26	5	P01681,P22006,P34901,Q7TP52,Q9QW07
24	1,2,3,4,5,6,18,23	7,8,19,20,21,22,24,25,26	15	P01681,P12785,P20762,P22006,P25031,P28648,P34901,P55314,P97580,Q4FZU2,Q5XI22,Q63661,Q63942,Q6IFW6,Q7TP52
25	1,2,3,4,5,6,18,24	7,8,19,20,21,22,23,25,26	4	P01681,P34901,Q63942,Q7TP52
26	1,2,3,4,5,6,18,25	7,8,19,20,21,22,23,24,26	7	P00731,P01681,P14173,P25031,P55314,P98089,Q7TP52
27	1,2,3,4,5,6,18,26	7,8,19,20,21,22,23,24,25	15	P00774,P01681,P20760,P20761,P20762,P25031,P34901,P83748,Q4FZU2,Q63355,Q63661,Q63942,Q6IFW6,Q7TP52,Q9ESW0
28	1,2,3,4,5,6,19,20	7,8,18,21,22,23,24,25,26	5	P00731,P01681,P22006,Q30KJ2,Q7TP52
29	1,2,3,4,5,6,19,21	7,8,18,20,22,23,24,25,26	4	P00731,P01681,P34901,Q30KJ2
30	1,2,3,4,5,6,19,22	7,8,18,20,21,23,24,25,26	5	P01681,P22006,Q30KJ2,Q7TP52,Q9QW07
31	1,2,3,4,5,6,19,23	7,8,18,20,21,22,24,25,26	9	P01681,P15399,P22006,P28648,P34901,P55314,P97580,Q30KJ2,Q7TP52

32	1,2,3,4,5,6,19,24	7,8,18,20,21,22,23,25,26	5	P01681,P34901,Q30KJ2,Q63532,Q7TP52
33	1,2,3,4,5,6,19,25	7,8,18,20,21,22,23,24,26	6	P00731,P01681,P14173,P55314,Q30KJ2,Q7TP52
34	1,2,3,4,5,6,19,26	7,8,18,20,21,22,23,24,25	8	P01681,P20760,P20761,P83748,Q30KJ2,Q4FZU2,Q63661,Q7TP52
35	1,2,3,4,5,6,20,21	7,8,18,19,22,23,24,25,26	8	P00731,P01681,P01835,P01836,P20762,P34901,Q6IRE4,Q7TPB1
36	1,2,3,4,5,6,20,22	7,8,18,19,21,23,24,25,26	6	P01681,P22006,P82995,Q62997,Q7TP52,Q9QW07
37	1,2,3,4,5,6,20,23	7,8,18,19,21,22,24,25,26	9	P01681,P20762,P22006,P28648,P34901,P55314,P82995,P97580,Q4FZU2
38	1,2,3,4,5,6,20,24	7,8,18,19,21,22,23,25,26	5	P01681,P08010,P34901,P82995,Q7TP52
39	1,2,3,4,5,6,20,25	7,8,18,19,21,22,23,24,26	4	P01681,P55314,Q7TP52,Q91ZS3
40	1,2,3,4,5,6,20,26	7,8,18,19,21,22,23,24,25	8	P01681,P01836,P05539,P20760,P20761,P20762,Q4FZU2,Q7TP52
41	1,2,3,4,5,6,21,22	7,8,18,19,20,23,24,25,26	4	P01681,P34901,Q7TP52,Q9QW07
42	1,2,3,4,5,6,21,23	7,8,18,19,20,22,24,25,26	10	P01681,P01835,P20762,P25236,P28648,P34901,P55314,P97580,Q4FZU2,Q6P777
43	1,2,3,4,5,6,21,24	7,8,18,19,20,22,23,25,26	2	P01681,P34901
44	1,2,3,4,5,6,21,25	7,8,18,19,20,22,23,24,26	8	P00731,P01681,P01835,P20646,P34901,P55314,Q6P777,Q91ZS3
45	1,2,3,4,5,6,21,26	7,8,18,19,20,22,23,24,25	13	P01681,P01835,P01836,P20760,P20761,P20762,P25236,P34901,P83748,Q4FZU2,Q63661,Q7TP52,Q9EQV9
46	1,2,3,4,5,6,22,23	7,8,18,19,20,21,24,25,26	12	G3V686,P01681,P22006,P28648,P34901,P36376,P55314,P82995,P97580,Q6P767,Q7TP52,Q9QW07

47	1,2,3,4,5,6,22,24	7,8,18,19,20,21,23,25,26	6	P01681,P08010,P34901,P82995,Q7TP52,Q9QW07
48	1,2,3,4,5,6,22,25	7,8,18,19,20,21,23,24,26	5	P01681,P06911,P55314,Q7TP52,Q9QW07
49	1,2,3,4,5,6,22,26	7,8,18,19,20,21,23,24,25	11	O54728,P01681,P05539,P20760,P20761,P20762,P34901,P83748,Q4FZU2,Q7TP52,Q9QW07
50	1,2,3,4,5,6,23,24	7,8,18,19,20,21,22,25,26	7	P01681,P28648,P34901,P55314,P82995,P97580,Q63751
51	1,2,3,4,5,6,23,25	7,8,18,19,20,21,22,24,26	8	P01681,P28648,P34901,P36376,P55314,P97580,Q63751,Q6P777
52	1,2,3,4,5,6,23,26	7,8,18,19,20,21,22,24,25	18	P01681,P01946,P20760,P20761,P20762,P28648,P34901,P36376,P55314,P97580,Q30KJ2,Q4FZU2,Q63661,Q6IFU8,Q6IFW6,Q6IMF3,Q6P6Q2,Q7TP52
53	1,2,3,4,5,6,24,25	7,8,18,19,20,21,22,23,26	4	P01681,P34901,P55314,Q7TP52
54	1,2,3,4,5,6,24,26	7,8,18,19,20,21,22,23,25	7	P01681,P08010,P20760,P20761,P34901,Q4FZU2,Q7TP52
55	1,2,3,4,5,6,25,26	7,8,18,19,20,21,22,23,24	6	P01681,P20760,P20761,P55314,Q4FZU2,Q7TP52
56	1,2,3,4,5,7,8,18	6,19,20,21,22,23,24,25,26	19	O35783,O55006,P00731,P01836,P02803,P10715,P18418,P20762,P25031,P42854,P50398,P52590,Q09030,Q4V885,Q63317,Q63475,Q64057,Q9ESW0,Q9QZA6
57	1,2,3,4,5,7,8,19	6,18,20,21,22,23,24,25,26	13	O55006,P00731,P02803,P18418,P52590,Q09030,Q4V885,Q63317,Q63475,Q63532,Q64057,Q6IE51,Q9ESW0
58	1,2,3,4,5,7,8,20	6,18,19,21,22,23,24,25,26	14	O55006,P01681,P01836,P08010,P10715,P18418,P20762,P42854,P52590,P97608,Q63317,Q63475,Q6IRE4,Q9ESW0
59	1,2,3,4,5,7,8,21	6,18,19,20,22,23,24,25,26	17	O35783,O54800;Q5DWV2,P00731,P01836,P02761,P02803,P18418,P20762,P42854,P52590,Q09030,Q4V885,Q63317,Q63475,Q64057,Q80WL1,Q9ESW0
60	1,2,3,4,5,7,8,22	6,18,19,20,21,23,24,25,26	11	P01836,P08010,P18418,P20766,P52590,P83121,Q63317,Q63475,Q7TP52,Q9ESW0,Q9QW07
61	1,2,3,4,5,7,8,23	6,18,19,20,21,22,24,25,26	13	P01836,P02803,P18418,P20762,P28648,P34901,P36970,P52590,Q09030,Q63317,Q63475,Q64057,Q9ESW0

62	1,2,3,4,5,7,8,24	6,18,19,20,21,22,23,25,26	11	O55006,P01836,P08010,P18418,P34901,P52590,P83121,Q09030,Q63317,Q63475,Q9ESW0
63	1,2,3,4,5,7,8,25	6,18,19,20,21,22,23,24,26	13	P01836,P02803,P10715,P18418,P19629,P52590,P55314,Q09030,Q5GRG2,Q63475,Q64057,Q6AYS4,Q9ESW0
64	1,2,3,4,5,7,8,26	6,18,19,20,21,22,23,24,25	21	O35783,P01041,P01681,P01836,P01946,P02770,P08010,P10959,P18418,P20760,P20761,P20762,P52590,P83748,Q09030,Q63475,Q64057,Q6AYQ8,Q9ESW0,Q9QX79,Q9R1T1
65	1,2,3,4,5,7,18,19	6,8,20,21,22,23,24,25,26	17	O89117,P00731,P01836,P02803,P05964,P07150,P18418,P25031,P52590,Q03191,Q09030,Q63475,Q63532,Q63661,Q6IE51,Q9ESW0,Q9WTT6
66	1,2,3,4,5,7,18,20	6,8,19,21,22,23,24,25,26	12	O35783,P00731,P01836,P07150,P18418,P25031,P42854,P52590,P61206;P84079;P84082,Q63475,Q6IE51,Q9ESW0
67	1,2,3,4,5,7,18,21	6,8,19,20,22,23,24,25,26	16	O35783,O89117,P00731,P01836,P05964,P18418,P25031,P34901,P42854,P52590,P61206;P84079;P84082,Q09030,Q63467,Q63475,Q63661,Q9ESW0
68	1,2,3,4,5,7,18,22	6,8,19,20,21,23,24,25,26	10	P01836,P07150,P18418,P25031,P34901,P52590,Q63475,Q7TP52,Q9ESW0,Q9QW07
69	1,2,3,4,5,7,18,23	6,8,19,20,21,22,24,25,26	16	P01836,P05964,P12785,P18418,P25031,P28648,P34901,P52590,Q09030,Q5XI22,Q62636,Q63475,Q63661,Q6IE51,Q99068,Q9ESW0
70	1,2,3,4,5,7,18,24	6,8,19,20,21,22,23,25,26	10	P01836,P08010,P18418,P25031,P34901,P52590,Q09030,Q63475,Q6IE51,Q9ESW0
71	1,2,3,4,5,7,18,25	6,8,19,20,21,22,23,24,26	13	O35783,P00731,P01836,P08721,P18418,P25031,P52590,P55314,P61206;P84079;P84082,Q09030,Q63475,Q6IE51,Q9ESW0
72	1,2,3,4,5,7,18,26	6,8,19,20,21,22,23,24,25	20	O35783,P01041,P01836,P01946,P16636,P18418,P25031,P52590,P61206;P84079;P84082,P83748,Q09030,Q4FZU2,Q63475,Q63661,Q63942,Q6AYQ8,Q6IE51,Q8VD89,Q9ESW0,Q9R1T1
73	1,2,3,4,5,7,19,20	6,8,18,21,22,23,24,25,26	8	P01836,P07150,P14669,P42854,P52590,Q63475,Q63532,Q6IE51
74	1,2,3,4,5,7,19,21	6,8,18,20,22,23,24,25,26	11	O89117,P00731,P01836,P34901,P42854,P52590,Q09030,Q63475,Q63532,Q6IE51,Q9ESW0
75	1,2,3,4,5,7,19,22	6,8,18,20,21,23,24,25,26	13	P01836,P07150,P08010,P14669,P18418,P52590,Q63317,Q63475,Q63532,Q6IE51,Q7TP52,Q9QW07,Q9WTT6
76	1,2,3,4,5,7,19,23	6,8,18,20,21,22,24,25,26	11	P15399,P28648,P34901,P52590,P97580,Q09030,Q30KJ2,Q63317,Q63475,Q63532,Q6IE51

77	1,2,3,4,5,7,19,24	6,8,18,20,21,22,23,25,26	12	P01836,P07150,P08010,P18418,P34901,P52590,Q09030,Q63317,Q63475,Q63532,Q6IE51,Q9WTT6
78	1,2,3,4,5,7,19,25	6,8,18,20,21,22,23,24,26	6	P52590,P55314,Q09030,Q63475,Q63532,Q6IE51
79	1,2,3,4,5,7,19,26	6,8,18,20,21,22,23,24,25	11	O54728,P01836,P01946,P05539,P52590,P83748,Q09030,Q63475,Q63532,Q63661,Q6IE51
80	1,2,3,4,5,7,20,21	6,8,18,19,22,23,24,25,26	9	O35783,P01681,P01836,P34901,P42854,P52590,Q63475,Q6IRE4,Q9ESW0
81	1,2,3,4,5,7,20,22	6,8,18,19,21,23,24,25,26	9	P01836,P07150,P08010,P14669,P52590,Q62997,Q63475,Q7TP52,Q9QW07
82	1,2,3,4,5,7,20,23	6,8,18,19,21,22,24,25,26	8	P01836,P14408,P28648,P34901,P52590,Q63475,Q6IE51,Q9ESW0
83	1,2,3,4,5,7,20,24	6,8,18,19,21,22,23,25,26	8	P01836,P07150,P08010,P14669,P34901,P52590,Q63475,Q6IRE4
84	1,2,3,4,5,7,20,25	6,8,18,19,21,22,23,24,26	4	P01836,P52590,P55314,Q63475
85	1,2,3,4,5,7,20,26	6,8,18,19,21,22,23,24,25	12	O35783,O54728,P01041,P01681,P01836,P01946,P05539,P08010,P52590,P83748,Q63475,Q9ESW0
86	1,2,3,4,5,7,21,22	6,8,18,19,20,23,24,25,26	7	P01836,P34901,P42854,P52590,Q63475,Q9ESW0,Q9QW07
87	1,2,3,4,5,7,21,23	6,8,18,19,20,22,24,25,26	9	P01836,P28648,P34901,P42854,P52590,Q09030,Q63475,Q6IE51,Q9ESW0
88	1,2,3,4,5,7,21,24	6,8,18,19,20,22,23,25,26	8	O89117,P01836,P34901,P42854,P52590,Q09030,Q63475,Q9ESW0
89	1,2,3,4,5,7,21,25	6,8,18,19,20,22,23,24,26	10	O35783,P01836,P34901,P42854,P52590,P55314,P61206;P84079;P84082,Q09030,Q63475,Q9ESW0
90	1,2,3,4,5,7,21,26	6,8,18,19,20,22,23,24,25	15	O35783,O54800;Q5DWV2,P01041,P01681,P01836,P01946,P34901,P42854,P52590,P83748,Q09030,Q63475,Q63661,Q9EQV9,Q9ESW0
91	1,2,3,4,5,7,22,23	6,8,18,19,20,21,24,25,26	8	P01836,P28648,P34901,P36376,P52590,Q63317,Q63475,Q9QW07

92	1,2,3,4,5,7,22,24	6,8,18,19,20,21,23,25,26	10	P01836,P07150,P08010,P14669,P34901,P52590,Q63317,Q63475,Q9QW07,Q9WTT6
93	1,2,3,4,5,7,22,25	6,8,18,19,20,21,23,24,26	6	P01836,P52590,P55314,Q63475,Q7TP52,Q9QW07
94	1,2,3,4,5,7,22,26	6,8,18,19,20,21,23,24,25	11	O54728,P01836,P01946,P05539,P08010,P34901,P52590,P55159,P83748,Q7TP52,Q9QW07
95	1,2,3,4,5,7,23,24	6,8,18,19,20,21,22,25,26	9	P01836,P28648,P34901,P52590,Q09030,Q63317,Q63475,Q6IE51,Q8K4G9
96	1,2,3,4,5,7,23,25	6,8,18,19,20,21,22,24,26	11	P01836,P05371,P28648,P34901,P36376,P52590,P55314,Q09030,Q63475,Q63751,Q6IE51
97	1,2,3,4,5,7,23,26	6,8,18,19,20,21,22,24,25	17	O54728,P01836,P01946,P02091,P05539,P28648,P34901,P36376,P52590,P83748,Q09030,Q4FZU2,Q63475,Q63661,Q6IE51,Q9ESW0,Q9R1T1
98	1,2,3,4,5,7,24,25	6,8,18,19,20,21,22,23,26	7	P01836,P08010,P34901,P52590,P55314,Q09030,Q63475
99	1,2,3,4,5,7,24,26	6,8,18,19,20,21,22,23,25	11	P01836,P01946,P02091,P08010,P20760,P34901,P52590,P83748,Q09030,Q63475,Q8CJD3
100	1,2,3,4,5,7,25,26	6,8,18,19,20,21,22,23,24	12	O35783,P01836,P01946,P02091,P05539,P52590,P55314,P61206;P84079;P84082,P83748,Q09030,Q63475,Q9R1T1
101	1,2,3,4,5,8,18,19	6,7,20,21,22,23,24,25,26	7	P00731,P02803,P22006,P83121,Q03191,Q63942,Q9ESW0
102	1,2,3,4,5,8,18,20	6,7,19,21,22,23,24,25,26	6	P01681,P22006,P83121,Q63475,Q9ESW0,Q9QZK9
103	1,2,3,4,5,8,18,21	6,7,19,20,22,23,24,25,26	11	O89117,P00731,P01681,P02761,P02803,P10758,P25031,P83121,Q63475,Q6AYS4,Q9ESW0
104	1,2,3,4,5,8,18,22	6,7,19,20,21,23,24,25,26	5	P20766,P22006,P83121,Q9ESW0,Q9QW07
105	1,2,3,4,5,8,18,23	6,7,19,20,21,22,24,25,26	11	P02803,P12785,P22006,P28648,P34901,Q5XI22,Q63475,Q63942,Q64194,Q6AY61,Q9ESW0
106	1,2,3,4,5,8,18,24	6,7,19,20,21,22,23,25,26	4	P34901,P83121,Q63475,Q9ESW0

107	1,2,3,4,5,8,18,25	6,7,19,20,21,22,23,24,26	4	P02803,P08721,Q6AYS4,Q9ESW0
108	1,2,3,4,5,8,18,26	6,7,19,20,21,22,23,24,25	6	P01681,P10959,Q4FZU2,Q63661,Q63942,Q9ESW0
109	1,2,3,4,5,8,19,20	6,7,18,21,22,23,24,25,26	4	P01681,P22006,P83121,Q62894
110	1,2,3,4,5,8,19,21	6,7,18,20,22,23,24,25,26	6	P00731,P01681,P02761,P02803,P35281,P83121
111	1,2,3,4,5,8,19,22	6,7,18,20,21,23,24,25,26	4	P20766,P22006,P83121,Q9QW07
112	1,2,3,4,5,8,19,23	6,7,18,20,21,22,24,25,26	6	P02803,P22006,P28648,P34901,P36970,Q63317
113	1,2,3,4,5,8,19,24	6,7,18,20,21,22,23,25,26	2	P34901,P83121
114	1,2,3,4,5,8,19,25	6,7,18,20,21,22,23,24,26	1	P02803
115	1,2,3,4,5,8,19,26	6,7,18,20,21,22,23,24,25	4	O54728,P01681,P10959,Q63661
116	1,2,3,4,5,8,20,21	6,7,18,19,22,23,24,25,26	6	P01681,P01836,P02761,P42854,Q63475,Q9ESW0
117	1,2,3,4,5,8,20,22	6,7,18,19,21,23,24,25,26	5	P01681,P22006,P83121,Q62997,Q9QW07
118	1,2,3,4,5,8,20,23	6,7,18,19,21,22,24,25,26	7	P01681,P22006,P28648,P34901,P97840,Q63475,Q9QZK9
119	1,2,3,4,5,8,20,24	6,7,18,19,21,22,23,25,26	6	P08010,P34901,P83121,P97840,Q63475,Q9QZK9
120	1,2,3,4,5,8,20,25	6,7,18,19,21,22,23,24,26	2	P01681,P55314
121	1,2,3,4,5,8,20,26	6,7,18,19,21,22,23,24,25	5	O54728,P01681,P01836,P10959,P97840

122	1,2,3,4,5,8,21,22	6,7,18,19,20,23,24,25,26	5	P01681,P34901,P83121,Q80WL1,Q9QW07
123	1,2,3,4,5,8,21,23	6,7,18,19,20,22,24,25,26	4	P01681,P34901,Q63475,Q9ESW0
124	1,2,3,4,5,8,21,24	6,7,18,19,20,22,23,25,26	4	P02761,P34901,P83121,Q63475
125	1,2,3,4,5,8,21,25	6,7,18,19,20,22,23,24,26	1	Q6AYS4
126	1,2,3,4,5,8,21,26	6,7,18,19,20,22,23,24,25	8	P01681,P01836,P02761,P10959,Q5QE79,Q63661,Q9EQV9,Q9ESW0
127	1,2,3,4,5,8,22,23	6,7,18,19,20,21,24,25,26	7	P20766,P22006,P28648,P34901,P36970,Q63317,Q9QW07
128	1,2,3,4,5,8,22,24	6,7,18,19,20,21,23,25,26	5	P08010,P34901,P83121,Q63317,Q9QW07
129	1,2,3,4,5,8,22,25	6,7,18,19,20,21,23,24,26	2	P20766,Q9QW07
130	1,2,3,4,5,8,22,26	6,7,18,19,20,21,23,24,25	7	O54728,P01681,P10959,P20766,P55159,Q5QE79,Q9QW07
131	1,2,3,4,5,8,23,24	6,7,18,19,20,21,22,25,26	7	P28648,P34901,P36970,P83121,P97840,Q63317,Q63475
132	1,2,3,4,5,8,23,25	6,7,18,19,20,21,22,24,26	3	P28648,P34901,P55314
133	1,2,3,4,5,8,23,26	6,7,18,19,20,21,22,24,25	15	O54728,P01681,P01946,P10959,P28648,P34901,P47967,P97840,Q4FZU2,Q5I0D1,Q5QE79,Q63661,Q6IFU8,Q6IFW6,Q6IG02
134	1,2,3,4,5,8,24,25	6,7,18,19,20,21,22,23,26	1	P34901
135	1,2,3,4,5,8,24,26	6,7,18,19,20,21,22,23,25	6	P08010,P10959,P34901,P47967,P97840,Q62902
136	1,2,3,4,5,8,25,26	6,7,18,19,20,21,22,23,24	3	P10959,P55314,Q62902

137	1,2,3,4,5,18,19,20	6,7,8,21,22,23,24,25,26	6	P00731,P07150,P22006,P83121,Q9QZK9,Q9WTT6
138	1,2,3,4,5,18,19,21	6,7,8,20,22,23,24,25,26	9	O89117,P00731,P02803,P05964,P10758,P25031,P34901,Q63661,Q9ESW0
139	1,2,3,4,5,18,19,22	6,7,8,20,21,23,24,25,26	6	P07150,P22006,P83121,Q6P6R2,Q9QW07,Q9WTT6
140	1,2,3,4,5,18,19,23	6,7,8,20,21,22,24,25,26	11	P02803,P05964,P12785,P22006,P28648,P34901,Q03191,Q63661,Q64194,Q99068,Q99MH3
141	1,2,3,4,5,18,19,24	6,7,8,20,21,22,23,25,26	4	P07150,P34901,P83121,Q9WTT6
142	1,2,3,4,5,18,19,25	6,7,8,20,21,22,23,24,26	3	P00731,P02803,P14173
143	1,2,3,4,5,18,19,26	6,7,8,20,21,22,23,24,25	4	Q03191,Q4FZU2,Q63661,Q63942
144	1,2,3,4,5,18,20,21	6,7,8,19,22,23,24,25,26	8	P00731,P01681,P01836,P25031,P34901,P42854,Q9ESW0,Q9QZK9
145	1,2,3,4,5,18,20,22	6,7,8,19,21,23,24,25,26	7	P07150,P22006,P83121,Q62997,Q9QW07,Q9QZK9,Q9WTT6
146	1,2,3,4,5,18,20,23	6,7,8,19,21,22,24,25,26	8	P12785,P22006,P28648,P34901,Q4FZU2,Q6IG02,Q9ESW0,Q9QZK9
147	1,2,3,4,5,18,20,24	6,7,8,19,21,22,23,25,26	4	P07150,P34901,P83121,Q9QZK9
148	1,2,3,4,5,18,20,25	6,7,8,19,21,22,23,24,26	0	
149	1,2,3,4,5,18,20,26	6,7,8,19,21,22,23,24,25	9	P01681,P01836,Q4FZU2,Q63355,Q63661,Q63942,Q6IG02,Q99041,Q9ESW0
150	1,2,3,4,5,18,21,22	6,7,8,19,20,23,24,25,26	4	P25031,P34901,Q9ESW0,Q9QW07
151	1,2,3,4,5,18,21,23	6,7,8,19,20,22,24,25,26	8	P12785,P25031,P28648,P34901,Q4FZU2,Q62636,Q63661,Q9ESW0

152	1,2,3,4,5,18,21,24	6,7,8,19,20,22,23,25,26	4	O89117,P25031,P34901,Q9ESW0
153	1,2,3,4,5,18,21,25	6,7,8,19,20,22,23,24,26	7	P00731,P08721,P25031,P31430,P34901,P98089,Q9ESW0
154	1,2,3,4,5,18,21,26	6,7,8,19,20,22,23,24,25	10	P00774,P01681,P01836,P25031,P34901,Q4FZU2,Q63661,Q63942,Q9EQV9,Q9ESW0
155	1,2,3,4,5,18,22,23	6,7,8,19,20,21,24,25,26	5	P12785,P22006,P28648,P34901,Q9QW07
156	1,2,3,4,5,18,22,24	6,7,8,19,20,21,23,25,26	6	P07150,P34901,P83121,Q9QW07,Q9QZK9,Q9WTT6
157	1,2,3,4,5,18,22,25	6,7,8,19,20,21,23,24,26	1	Q9QW07
158	1,2,3,4,5,18,22,26	6,7,8,19,20,21,23,24,25	11	O54728,P34901,P55159,P63036,Q4FZU2,Q63661,Q63942,Q6P6R2,Q7TP52,Q99041,Q9QW07
159	1,2,3,4,5,18,23,24	6,7,8,19,20,21,22,25,26	5	P12785,P28648,P34901,Q99068,Q9QZK9
160	1,2,3,4,5,18,23,25	6,7,8,19,20,21,22,24,26	6	P12785,P28648,P34901,P55314,Q99068,Q99MH3
161	1,2,3,4,5,18,23,26	6,7,8,19,20,21,22,24,25	18	P01946,P12785,P19939,P28648,P34901,P35467,Q4FZU2,Q62636,Q63661,Q63942,Q64194,Q6AY61,Q6IFU8,Q6IFW6,Q6IG02,Q6IMF3,Q6P6Q2,Q9ESW0
162	1,2,3,4,5,18,24,25	6,7,8,19,20,21,22,23,26	2	P34901,Q62902
163	1,2,3,4,5,18,24,26	6,7,8,19,20,21,22,23,25	6	P34901,Q4FZU2,Q62902,Q63661,Q63942,Q99041
164	1,2,3,4,5,18,25,26	6,7,8,19,20,21,22,23,24	7	P00774,P08721,P55314,Q4FZU2,Q62902,Q63661,Q63942
165	1,2,3,4,5,19,20,21	6,7,8,18,22,23,24,25,26	2	P01681,P01836
166	1,2,3,4,5,19,20,22	6,7,8,18,21,23,24,25,26	10	O54728,P07150,P14669,P22006,P55091,P83121,Q62997,Q6P6R2,Q9QW07,Q9WTT6

167	1,2,3,4,5,19,20,23	6,7,8,18,21,22,24,25,26	3	P22006,P34901,Q9QZK9
168	1,2,3,4,5,19,20,24	6,7,8,18,21,22,23,25,26	6	P07150,P14669,P34901,P83121,Q9QZK9,Q9WTT6
169	1,2,3,4,5,19,20,25	6,7,8,18,21,22,23,24,26	0	
170	1,2,3,4,5,19,20,26	6,7,8,18,21,22,23,24,25	4	O54728,P01681,P01836,P07150
171	1,2,3,4,5,19,21,22	6,7,8,18,20,23,24,25,26	2	P34901,Q9QW07
172	1,2,3,4,5,19,21,23	6,7,8,18,20,22,24,25,26	2	P34901,Q63661
173	1,2,3,4,5,19,21,24	6,7,8,18,20,22,23,25,26	2	O89117,P34901
174	1,2,3,4,5,19,21,25	6,7,8,18,20,22,23,24,26	0	
175	1,2,3,4,5,19,21,26	6,7,8,18,20,22,23,24,25	3	P01681,P01836,Q63661
176	1,2,3,4,5,19,22,23	6,7,8,18,20,21,24,25,26	4	O54728,P22006,P34901,Q9QW07
177	1,2,3,4,5,19,22,24	6,7,8,18,20,21,23,25,26	7	P07150,P08010,P14669,P34901,P83121,Q9QW07,Q9WTT6
178	1,2,3,4,5,19,22,25	6,7,8,18,20,21,23,24,26	1	Q9QW07
179	1,2,3,4,5,19,22,26	6,7,8,18,20,21,23,24,25	8	O54728,P07150,P55159,P63036,Q63661,Q6P6R2,Q7TP52,Q9QW07
180	1,2,3,4,5,19,23,24	6,7,8,18,20,21,22,25,26	1	P34901
181	1,2,3,4,5,19,23,25	6,7,8,18,20,21,22,24,26	2	P34901,P55314

182	1,2,3,4,5,19,23,26	6,7,8,18,20,21,22,24,25	7	O54728,P01946,P28648,P34901,Q4FZU2,Q63661,Q6IFU8
183	1,2,3,4,5,19,24,25	6,7,8,18,20,21,22,23,26	2	P34901,Q62902
184	1,2,3,4,5,19,24,26	6,7,8,18,20,21,22,23,25	4	O54728,P34901,Q62902,Q63661
185	1,2,3,4,5,19,25,26	6,7,8,18,20,21,22,23,24	3	P55314,Q62902,Q63661
186	1,2,3,4,5,20,21,22	6,7,8,18,19,23,24,25,26	6	P01681,P01836,P34901,Q62997,Q7TPB1,Q9QW07
187	1,2,3,4,5,20,21,23	6,7,8,18,19,22,24,25,26	3	P01681,P01836,P34901
188	1,2,3,4,5,20,21,24	6,7,8,18,19,22,23,25,26	3	P01681,P01836,P34901
189	1,2,3,4,5,20,21,25	6,7,8,18,19,22,23,24,26	2	P01681,P01836
190	1,2,3,4,5,20,21,26	6,7,8,18,19,22,23,24,25	5	P01681,P01836,P34901,Q4FZU2,Q63661
191	1,2,3,4,5,20,22,23	6,7,8,18,19,21,24,25,26	5	P22006,P34901,Q62997,Q9QW07,Q9QZK9
192	1,2,3,4,5,20,22,24	6,7,8,18,19,21,23,25,26	10	P01836,P07150,P08010,P14669,P34901,P83121,Q62997,Q9QW07,Q9QZK9,Q9WTT6
193	1,2,3,4,5,20,22,25	6,7,8,18,19,21,23,24,26	2	Q62997,Q9QW07
194	1,2,3,4,5,20,22,26	6,7,8,18,19,21,23,24,25	9	O54728,P01681,P01836,P05539,P07150,P55159,Q62997,Q99041,Q9QW07
195	1,2,3,4,5,20,23,24	6,7,8,18,19,21,22,25,26	4	P34901,P82995,P97840,Q9QZK9
196	1,2,3,4,5,20,23,25	6,7,8,18,19,21,22,24,26	2	P34901,P55314

197	1,2,3,4,5,20,23,26	6,7,8,18,19,21,22,24,25	11	O54728,P01681,P01836,P01946,P34901,P97840,Q4FZU2,Q6IFU8,Q6IFW6,Q6IG02,Q6IMF3
198	1,2,3,4,5,20,24,25	6,7,8,18,19,21,22,23,26	1	P34901
199	1,2,3,4,5,20,24,26	6,7,8,18,19,21,22,23,25	6	P01681,P01836,P08010,P34901,P97840,Q99041
200	1,2,3,4,5,20,25,26	6,7,8,18,19,21,22,23,24	3	P01681,P01836,P55314
201	1,2,3,4,5,21,22,23	6,7,8,18,19,20,24,25,26	2	P34901,Q9QW07
202	1,2,3,4,5,21,22,24	6,7,8,18,19,20,23,25,26	3	P01836,P34901,Q9QW07
203	1,2,3,4,5,21,22,25	6,7,8,18,19,20,23,24,26	2	P34901,Q9QW07
204	1,2,3,4,5,21,22,26	6,7,8,18,19,20,23,24,25	7	O54728,P01681,P01836,P34901,P63036,Q63661,Q9QW07
205	1,2,3,4,5,21,23,24	6,7,8,18,19,20,22,25,26	1	P34901
206	1,2,3,4,5,21,23,25	6,7,8,18,19,20,22,24,26	3	P34901,P55314,Q6P777
207	1,2,3,4,5,21,23,26	6,7,8,18,19,20,22,24,25	8	P01681,P01836,P01946,P34901,Q4FZU2,Q63661,Q6IFU8,Q6IMF3
208	1,2,3,4,5,21,24,25	6,7,8,18,19,20,22,23,26	1	P34901
209	1,2,3,4,5,21,24,26	6,7,8,18,19,20,22,23,25	7	P01681,P01836,P34901,P80020,Q63661,Q8CJD3,Q9EQV9
210	1,2,3,4,5,21,25,26	6,7,8,18,19,20,22,23,24	5	P01681,P01836,P34901,P55314,Q63661
211	1,2,3,4,5,22,23,24	6,7,8,18,19,20,21,25,26	3	P34901,P82471,Q9QW07

212	1,2,3,4,5,22,23,25	6,7,8,18,19,20,21,24,26	3	P34901,P55314,Q9QW07
213	1,2,3,4,5,22,23,26	6,7,8,18,19,20,21,24,25	9	O54728,P01946,P05539,P34901,P36376,P55159,Q4FZU2,Q63661,Q9QW07
214	1,2,3,4,5,22,24,25	6,7,8,18,19,20,21,23,26	2	P34901,Q9QW07
215	1,2,3,4,5,22,24,26	6,7,8,18,19,20,21,23,25	6	O54728,P01836,P08010,P34901,Q99041,Q9QW07
216	1,2,3,4,5,22,25,26	6,7,8,18,19,20,21,23,24	4	O54728,P05539,P55314,Q9QW07
217	1,2,3,4,5,23,24,25	6,7,8,18,19,20,21,22,26	4	P34901,P55314,Q62902,Q63751
218	1,2,3,4,5,23,24,26	6,7,8,18,19,20,21,22,25	10	O54728,P01946,P02091,P34901,P47967,P97840,Q4FZU2,Q62902,Q63661,Q6IFU8
219	1,2,3,4,5,23,25,26	6,7,8,18,19,20,21,22,24	10	P01946,P02091,P34901,P36376,P55314,Q4FZU2,Q62902,Q63661,Q6IFU8,Q6IFW6
220	1,2,3,4,5,24,25,26	6,7,8,18,19,20,21,22,23	6	P01946,P34901,P55314,P60905,Q62902,Q8CJD3
221	1,2,3,4,6,7,8,18	5,19,20,21,22,23,24,25,26	24	O35783,O54800;Q5DWV2,O55006,P00731,P01836,P02631,P02803,P05964,P10715,P16086,P18418,P20762,P25031,P35280,P42854,Q30KJ2,Q63475,Q63617,Q64057,Q6AYQ8,Q6IE51,Q7TP52,Q91ZS3,Q9R0T3
222	1,2,3,4,6,7,8,19	5,18,20,21,22,23,24,25,26	20	O55006,P00731,P01836,P02631,P02803,P15399,P16086,P18418,P20762,P35280,P97580,Q30KJ2,Q63317,Q63475,Q63532,Q63617,Q64057,Q6IE51,Q7TP52,Q9QZQ5
223	1,2,3,4,6,7,8,20	5,18,19,21,22,23,24,25,26	21	D3ZUK3,O35783,O55006,P00731,P01041,P01681,P01836,P02631,P10715,P16086,P18418,P20762,P35280,P42854,Q30KJ2,Q63317,Q63475,Q63617,Q64057,Q8K1G0,Q91ZS3
224	1,2,3,4,6,7,8,21	5,18,19,20,22,23,24,25,26	24	D4A1J4,O35783,O54800;Q5DWV2,P00731,P01681,P01836,P02631,P02761,P08723,P16086,P18418,P20762,P25236,P42854,Q30KJ2,Q4V885,Q63317,Q63475,Q63617,Q64057,Q80WL1,Q91ZS3,Q9JI85,Q9R0T3
225	1,2,3,4,6,7,8,22	5,18,19,20,21,23,24,25,26	15	D3ZUK3,D4A1J4,O54800;Q5DWV2,P01836,P02631,P16086,P18418,P20762,P20766,Q30KJ2,Q63317,Q63475,Q63617,Q7TP52,Q9R0T3
226	1,2,3,4,6,7,8,23	5,18,19,20,21,22,24,25,26	19	D3ZUK3,O35783,P01836,P02631,P05371,P15399,P16086,P18418,P20762,P34901,P50280,P97580,Q30KJ2,Q5XI22,Q63317,Q63475,Q63617,Q64057,Q6IE51

227	1,2,3,4,6,7,8,24	5,18,19,20,21,22,23,25,26	13	D3ZHA0,O54800;Q5DWV2,O55006,P01836,P02631,P08010,P16086,P18418,P20762,Q30KJ2,Q63317,Q63475,Q63617
228	1,2,3,4,6,7,8,25	5,18,19,20,21,22,23,24,26	24	O35783,O54800;Q5DWV2,P01836,P02631,P05371,P06911,P10715,P12020,P16086,P18418,P19629,P20762,P20766,P31044,P35280,P55314,P68370,Q30KJ2,Q5GRG2,Q63475,Q63617,Q64057,Q6AYQ8,Q91ZS3
229	1,2,3,4,6,7,8,26	5,18,19,20,21,22,23,24,25	27	O35783,O54800;Q5DWV2,P01041,P01681,P01836,P01946,P02631,P02770,P10959,P16086,P18418,P20760,P20761,P20762,P20766,P35280,P83748,Q30KJ2,Q5QE79,Q63475,Q63617,Q64057,Q6AY33,Q6AYQ8,Q7TP52,Q91ZS3,Q9R0T3
230	1,2,3,4,6,7,18,19	5,8,20,21,22,23,24,25,26	17	O55006,P00731,P01836,P02631,P05964,P06760,P15399,P16086,P18418,P25031,P35280,P97580,Q30KJ2,Q5FVI6,Q63532,Q6IE51,Q7TP52
231	1,2,3,4,6,7,18,20	5,8,19,21,22,23,24,25,26	16	O35783,P00731,P01041,P01836,P02631,P06760,P16086,P18418,P20762,P25031,P35280,P42854,Q63475,Q6IE51,Q7TP52,Q91ZS3
232	1,2,3,4,6,7,18,21	5,8,19,20,22,23,24,25,26	17	O35783,O54800;Q5DWV2,P00731,P01836,P02631,P05964,P16086,P18418,P25031,P25236,P42854,P98089,Q63475,Q63661,Q6IE51,Q91ZS3,Q9JI85
233	1,2,3,4,6,7,18,22	5,8,19,20,21,23,24,25,26	8	P01836,P02631,P06760,P16086,P18418,P25031,Q6IE51,Q7TP52
234	1,2,3,4,6,7,18,23	5,8,19,20,21,22,24,25,26	18	O35783,P01836,P02631,P05371,P05964,P12785,P15399,P16086,P18418,P20762,P25031,P34901,P55053,P97580,Q30KJ2,Q5XI22,Q63475,Q6IE51
235	1,2,3,4,6,7,18,24	5,8,19,20,21,22,23,25,26	10	O35783,P01836,P02631,P06760,P16086,P18418,P25031,Q63475,Q6IE51,Q7TP52
236	1,2,3,4,6,7,18,25	5,8,19,20,21,22,23,24,26	20	O35783,O54800;Q5DWV2,P00731,P01836,P02631,P05371,P05964,P12020,P16086,P18418,P25031,P35280,P55314,P61206;P84079;P84082,P98089,Q5GRG2,Q63475,Q6IE51,Q7TP52,Q91ZS3
237	1,2,3,4,6,7,18,26	5,8,19,20,21,22,23,24,25	26	O35783,O54800;Q5DWV2,P00774,P01041,P01836,P01946,P02631,P02770,P05964,P06760,P16086,P18418,P20761,P20762,P25031,P35280,P83748,Q30KJ2,Q4FZU2,Q63661,Q63942,Q6AYQ8,Q6IE51,Q7TP52,Q8VD89,Q91ZS3
238	1,2,3,4,6,7,19,20	5,8,18,21,22,23,24,25,26	16	P00731,P01836,P02631,P15399,P16086,P18418,P35280,P42854,P97580,Q30KJ2,Q5FVI6,Q63475,Q63532,Q6IE51,Q7TP52,Q91ZS3
239	1,2,3,4,6,7,19,21	5,8,18,20,22,23,24,25,26	17	O54800;Q5DWV2,P00731,P01836,P02631,P05964,P15399,P16086,P18418,P25236,P42854,P47967,P97580,Q30KJ2,Q63475,Q63532,Q6IE51,Q91ZS3
240	1,2,3,4,6,7,19,22	5,8,18,20,21,23,24,25,26	10	P01836,P02631,P15399,P16086,P18418,P97580,Q30KJ2,Q63532,Q6IE51,Q7TP52
241	1,2,3,4,6,7,19,23	5,8,18,20,21,22,24,25,26	13	P01836,P02631,P05964,P15399,P16086,P18418,P34901,P97580,Q30KJ2,Q5FVI6,Q63475,Q63532,Q6IE51

242	1,2,3,4,6,7,19,24	5,8,18,20,21,22,23,25,26	10	P01836,P02631,P15399,P16086,P18418,P97580,Q30KJ2,Q63475,Q63532,Q6IE51
243	1,2,3,4,6,7,19,25	5,8,18,20,21,22,23,24,26	21	P00731,P01836,P02631,P05371,P06911,P12020,P15399,P16086,P18418,P35280,P47967,P55314,P97580,P97840,Q30KJ2,Q5FVI6,Q5GRG2,Q63532,Q6IE51,Q7TP52,Q91ZS3
244	1,2,3,4,6,7,19,26	5,8,18,20,21,22,23,24,25	19	P01041,P01836,P01946,P02631,P02770,P06760,P15399,P16086,P18418,P20761,P35280,P83748,P97580,Q30KJ2,Q5FVI6,Q63532,Q63661,Q6IE51,Q7TP52
245	1,2,3,4,6,7,20,21	5,8,18,19,22,23,24,25,26	14	O35783,O54800;Q5DWV2,P00731,P01041,P01681,P01836,P02631,P16086,P18418,P20762,P25236,P42854,Q63475,Q91ZS3
246	1,2,3,4,6,7,20,22	5,8,18,19,21,23,24,25,26	7	P01836,P02631,P16086,P18418,Q62997,Q7TP52,Q91ZS3
247	1,2,3,4,6,7,20,23	5,8,18,19,21,22,24,25,26	14	O35783,P01836,P02631,P15399,P16086,P18418,P20762,P34901,P55053,P97580,Q30KJ2,Q63475,Q6IE51,Q91ZS3
248	1,2,3,4,6,7,20,24	5,8,18,19,21,22,23,25,26	8	P01836,P02631,P08010,P16086,P18418,Q63475,Q6IE51,Q91ZS3
249	1,2,3,4,6,7,20,25	5,8,18,19,21,22,23,24,26	13	O35783,P01836,P02631,P05371,P12020,P16086,P18418,P35280,P55314,Q5GRG2,Q63475,Q6IE51,Q91ZS3
250	1,2,3,4,6,7,20,26	5,8,18,19,21,22,23,24,25	20	O35783,P01041,P01681,P01836,P01946,P02631,P05539,P06760,P16086,P18418,P20761,P20762,P35280,P83748,Q30KJ2,Q5FVI6,Q6AYQ8,Q6IE51,Q7TP52,Q91ZS3
251	1,2,3,4,6,7,21,22	5,8,18,19,20,23,24,25,26	12	D4A1J4,O35783,O54800;Q5DWV2,P01836,P02631,P13432,P16086,P18418,P25236,P42854,Q7TP52,Q91ZS3
252	1,2,3,4,6,7,21,23	5,8,18,19,20,22,24,25,26	17	O35783,P01836,P02631,P05964,P15399,P16086,P18418,P20762,P25236,P34901,P42854,P55053,P97580,Q30KJ2,Q63475,Q6IE51,Q91ZS3
253	1,2,3,4,6,7,21,24	5,8,18,19,20,22,23,25,26	12	O35783,O54800;Q5DWV2,P01836,P02631,P13432,P16086,P18418,P25236,P42854,Q63475,Q6IE51,Q91ZS3
254	1,2,3,4,6,7,21,25	5,8,18,19,20,22,23,24,26	18	O35783,O54800;Q5DWV2,P00731,P01836,P02631,P05371,P09605;P25809,P12020,P16086,P18418,P19629,P25236,P42854,P47967,P55314,Q5GRG2,Q63475,Q91ZS3
255	1,2,3,4,6,7,21,26	5,8,18,19,20,22,23,24,25	20	O35783,O54800;Q5DWV2,P01041,P01681,P01836,P01946,P02631,P02770,P16086,P18418,P20761,P20762,P25236,P42854,P80020,P83748,Q30KJ2,Q63661,Q91ZS3,Q9EQV9
256	1,2,3,4,6,7,22,23	5,8,18,19,20,21,24,25,26	12	G3V686,P01836,P02631,P15399,P16086,P18418,P20762,P34901,P97580,Q30KJ2,Q6IE51,Q7TP52

257	1,2,3,4,6,7,22,24	5,8,18,19,20,21,23,25,26	7	P01836,P02631,P08010,P16086,P18418,Q6IE51,Q7TP52
258	1,2,3,4,6,7,22,25	5,8,18,19,20,21,23,24,26	13	G3V686,P01836,P02631,P05371,P06911,P12020,P16086,P18418,P19629,P55314,Q5GRG2,Q7TP52,Q91ZS3
259	1,2,3,4,6,7,22,26	5,8,18,19,20,21,23,24,25	16	O35783,O54800;Q5DWV2,P01041,P01836,P01946,P02631,P05539,P08010,P16086,P18418,P20761,P20762,P55159,P83748,Q30KJ2,Q7TP52
260	1,2,3,4,6,7,23,24	5,8,18,19,20,21,22,25,26	11	P01836,P02631,P15399,P16086,P18418,P34901,P97580,Q30KJ2,Q63475,Q63751,Q6IE51
261	1,2,3,4,6,7,23,25	5,8,18,19,20,21,22,24,26	21	G3V686,O35783,P01836,P02631,P05371,P06911,P12020,P15399,P16086,P18418,P19629,P34901,P50280,P55314,P97580,Q30KJ2,Q5GRG2,Q63475,Q63751,Q6IE51,Q91ZS3
262	1,2,3,4,6,7,23,26	5,8,18,19,20,21,22,24,25	20	O35783,P01836,P01946,P02091,P02631,P05539,P15399,P16086,P20761,P20762,P25236,P34901,P55053,P83748,P97580,Q30KJ2,Q4FZU2,Q63661,Q6AYQ8,Q6IE51
263	1,2,3,4,6,7,24,25	5,8,18,19,20,21,22,23,26	12	P01836,P02631,P05371,P12020,P16086,P18418,P19629,P55314,Q5GRG2,Q63475,Q6IE51,Q91ZS3
264	1,2,3,4,6,7,24,26	5,8,18,19,20,21,22,23,25	16	O35783,P01041,P01836,P01946,P02091,P02631,P02770,P08010,P18418,P20760,P20761,P83748,Q30KJ2,Q6AYQ8,Q6IE51,Q7TP52
265	1,2,3,4,6,7,25,26	5,8,18,19,20,21,22,23,24	23	O35783,O54800;Q5DWV2,P01041,P01836,P01946,P02091,P02631,P05371,P05539,P12020,P18418,P19629,P20761,P35280,P55314,P83748,Q30KJ2,Q5FVI6,Q5GRG2,Q6AYQ8,Q6IE51,Q7TP52,Q91ZS3
266	1,2,3,4,6,8,18,19	5,7,20,21,22,23,24,25,26	8	P00731,P02803,P05964,P22006,P35280,Q30KJ2,Q63355,Q63942
267	1,2,3,4,6,8,18,20	5,7,19,21,22,23,24,25,26	7	P00731,P01681,P20762,P22006,P35280,Q63355,Q91ZS3
268	1,2,3,4,6,8,18,21	5,7,19,20,22,23,24,25,26	13	O35547,P00731,P01681,P02761,P02803,P05964,P08723,P0C0A9,P10758,P25031,P25236,P98089,Q91ZS3
269	1,2,3,4,6,8,18,22	5,7,19,20,21,23,24,25,26	3	P20766,P22006,Q7TP52
270	1,2,3,4,6,8,18,23	5,7,19,20,21,22,24,25,26	9	P02803,P05964,P12785,P20762,P34901,P35467,P97580,Q5XI22,Q63942
271	1,2,3,4,6,8,18,24	5,7,19,20,21,22,23,25,26	0	

272	1,2,3,4,6,8,18,25	5,7,19,20,21,22,23,24,26	5	P00731,P02803,P35280,P98089,Q91ZS3
273	1,2,3,4,6,8,18,26	5,7,19,20,21,22,23,24,25	15	P00774,P01681,P02770,P10959,P20761,P20762,P35280,Q4FZU2,Q5QE79,Q63355,Q63661,Q63942,Q6AY33,Q6AYQ8,Q7TP52
274	1,2,3,4,6,8,19,20	5,7,18,21,22,23,24,25,26	7	P00731,P01681,P22006,P35280,Q30KJ2,Q63355,Q66H69
275	1,2,3,4,6,8,19,21	5,7,18,20,22,23,24,25,26	8	P00731,P01681,P02761,P02782,P0C0A9,P25236,P35281,Q30KJ2
276	1,2,3,4,6,8,19,22	5,7,18,20,21,23,24,25,26	3	P20766,Q30KJ2,Q7TP52
277	1,2,3,4,6,8,19,23	5,7,18,20,21,22,24,25,26	5	P15399,P50280,P97580,Q30KJ2,Q5XI22
278	1,2,3,4,6,8,19,24	5,7,18,20,21,22,23,25,26	1	Q30KJ2
279	1,2,3,4,6,8,19,25	5,7,18,20,21,22,23,24,26	5	P06911,P35280,Q30KJ2,Q5GRG2,Q9QUL6
280	1,2,3,4,6,8,19,26	5,7,18,20,21,22,23,24,25	8	P01681,P02770,P10959,P20761,P35280,Q30KJ2,Q5QE79,Q63355
281	1,2,3,4,6,8,20,21	5,7,18,19,22,23,24,25,26	11	P00731,P01681,P01836,P02761,P20762,P25236,P42854,P62804,Q00715,Q8K1G0,Q91ZS3
282	1,2,3,4,6,8,20,22	5,7,18,19,21,23,24,25,26	5	P01681,P20766,P22006,Q62997,Q7TP52
283	1,2,3,4,6,8,20,23	5,7,18,19,21,22,24,25,26	5	P01681,P20762,P22006,P97580,Q5XI22
284	1,2,3,4,6,8,20,24	5,7,18,19,21,22,23,25,26	1	P01681
285	1,2,3,4,6,8,20,25	5,7,18,19,21,22,23,24,26	4	P01681,P35280,Q5GRG2,Q91ZS3
286	1,2,3,4,6,8,20,26	5,7,18,19,21,22,23,24,25	13	P01681,P01836,P02770,P10959,P20761,P20762,P25236,P35280,P62804,Q00715,Q5QE79,Q63355,Q6AYQ8

287	1,2,3,4,6,8,21,22	5,7,18,19,20,23,24,25,26	7	P01681,P02761,P02782,P08723,P13432,P25236,Q80WL1
288	1,2,3,4,6,8,21,23	5,7,18,19,20,22,24,25,26	7	P01681,P02761,P20762,P25236,P34901,P97580,Q5XI22
289	1,2,3,4,6,8,21,24	5,7,18,19,20,22,23,25,26	5	D3ZHA0,P01836,P02761,P13432,P25236
290	1,2,3,4,6,8,21,25	5,7,18,19,20,22,23,24,26	7	P00731,P01681,P25236,Q6P6R2,Q91ZS3,Q9WTT6,Q9Z0V6
291	1,2,3,4,6,8,21,26	5,7,18,19,20,22,23,24,25	12	P01681,P01836,P02761,P02770,P10959,P20761,P20762,P25236,Q00715,Q5QE79,Q63661,Q9EQV9
292	1,2,3,4,6,8,22,23	5,7,18,19,20,21,24,25,26	6	P20762,P20766,P34901,P50280,P97580,Q5XI22
293	1,2,3,4,6,8,22,24	5,7,18,19,20,21,23,25,26	2	P20766,Q7TP52
294	1,2,3,4,6,8,22,25	5,7,18,19,20,21,23,24,26	5	P06911,P12020,P20766,Q5GRG2,Q7TP52
295	1,2,3,4,6,8,22,26	5,7,18,19,20,21,23,24,25	8	P01681,P02770,P10959,P20761,P20762,P20766,Q5QE79,Q7TP52
296	1,2,3,4,6,8,23,24	5,7,18,19,20,21,22,25,26	4	P34901,P97580,Q30KJ2,Q5XI22
297	1,2,3,4,6,8,23,25	5,7,18,19,20,21,22,24,26	12	P06911,P12020,P50280,P55314,P97580,Q30KJ2,Q5GRG2,Q5XI22,Q63751,Q6P6R2,Q9WTT6,Q9Z0V6
298	1,2,3,4,6,8,23,26	5,7,18,19,20,21,22,24,25	15	D4A5U3,P01681,P02770,P10959,P20761,P20762,P25236,P34901,P97580,Q30KJ2,Q4FZU2,Q5QE79,Q6AYQ8,Q6IFU8,Q6IFW6
299	1,2,3,4,6,8,24,25	5,7,18,19,20,21,22,23,26	1	Q5GRG2
300	1,2,3,4,6,8,24,26	5,7,18,19,20,21,22,23,25	7	D3ZHA0,P02770,P10959,P20760,P20761,Q5QE79,Q6AYQ8
301	1,2,3,4,6,8,25,26	5,7,18,19,20,21,22,23,24	10	P01681,P02770,P10959,P20761,P20766,P35280,Q5QE79,Q6AYQ8,Q91ZS3,Q9Z0V6

302	1,2,3,4,6,18,19,20	5,7,8,21,22,23,24,25,26	5	P00731,P22006,P35280,Q30KJ2,Q63355
303	1,2,3,4,6,18,19,21	5,7,8,20,22,23,24,25,26	9	O89117,P00731,P05964,P10758,P14173,P25031,P25236,Q30KJ2,Q63661
304	1,2,3,4,6,18,19,22	5,7,8,20,21,23,24,25,26	4	P02625,P22006,Q30KJ2,Q7TP52
305	1,2,3,4,6,18,19,23	5,7,8,20,21,22,24,25,26	10	P02625,P05964,P12785,P15399,P34901,P97580,Q30KJ2,Q5XI22,Q63661,Q8CJD3
306	1,2,3,4,6,18,19,24	5,7,8,20,21,22,23,25,26	2	Q30KJ2,Q63532
307	1,2,3,4,6,18,19,25	5,7,8,20,21,22,23,24,26	6	P00731,P14173,P35280,P98089,Q30KJ2,Q4FZU6
308	1,2,3,4,6,18,19,26	5,7,8,20,21,22,23,24,25	9	P05964,P06760,P35280,Q30KJ2,Q4FZU2,Q63355,Q63661,Q63942,Q7TP52
309	1,2,3,4,6,18,20,21	5,7,8,19,22,23,24,25,26	8	P00731,P01681,P01835,P01836,P25031,P25236,P42854,Q91ZS3
310	1,2,3,4,6,18,20,22	5,7,8,19,21,23,24,25,26	2	P22006,Q7TP52
311	1,2,3,4,6,18,20,23	5,7,8,19,21,22,24,25,26	5	P12785,P22006,P34901,Q4FZU2,Q5XI22
312	1,2,3,4,6,18,20,24	5,7,8,19,21,22,23,25,26	0	
313	1,2,3,4,6,18,20,25	5,7,8,19,21,22,23,24,26	3	P35280,P98089,Q91ZS3
314	1,2,3,4,6,18,20,26	5,7,8,19,21,22,23,24,25	7	P01681,P01836,P35280,Q4FZU2,Q63355,Q63661,Q63942
315	1,2,3,4,6,18,21,22	5,7,8,19,20,23,24,25,26	3	P01836,P25031,P25236
316	1,2,3,4,6,18,21,23	5,7,8,19,20,22,24,25,26	8	P05964,P12785,P25031,P25236,P34901,Q4FZU2,Q5XI22,Q63661

317	1,2,3,4,6,18,21,24	5,7,8,19,20,22,23,25,26	4	P01836,P13432,P25031,P25236
318	1,2,3,4,6,18,21,25	5,7,8,19,20,22,23,24,26	7	P00731,P00774,P25031,P25236,P31430,P98089,Q91ZS3
319	1,2,3,4,6,18,21,26	5,7,8,19,20,22,23,24,25	10	P00774,P01681,P01836,P20761,P25031,P25236,Q4FZU2,Q63661,Q63942,Q9EQV9
320	1,2,3,4,6,18,22,23	5,7,8,19,20,21,24,25,26	5	G3V686,P02625,P12785,P34901,Q5XI22
321	1,2,3,4,6,18,22,24	5,7,8,19,20,21,23,25,26	1	Q7TP52
322	1,2,3,4,6,18,22,25	5,7,8,19,20,21,23,24,26	3	P98089,Q4FZU6,Q7TP52
323	1,2,3,4,6,18,22,26	5,7,8,19,20,21,23,24,25	5	P20761,Q4FZU2,Q63661,Q63942,Q7TP52
324	1,2,3,4,6,18,23,24	5,7,8,19,20,21,22,25,26	3	P12785,P34901,Q5XI22
325	1,2,3,4,6,18,23,25	5,7,8,19,20,21,22,24,26	6	P00774,P12785,P34901,P55314,P98089,Q5XI22
326	1,2,3,4,6,18,23,26	5,7,8,19,20,21,22,24,25	17	P00774,P12785,P20761,P25236,P34901,P35467,Q4FZU2,Q5XI22,Q63355,Q63661,Q63942,Q6AY61,Q6IFU8,Q6IFW6,Q6IG02,Q6IMF3,Q6P6Q2
327	1,2,3,4,6,18,24,25	5,7,8,19,20,21,22,23,26	2	P98089,Q4FZU6
328	1,2,3,4,6,18,24,26	5,7,8,19,20,21,22,23,25	6	P00774,P01836,P20761,Q4FZU2,Q63661,Q63942
329	1,2,3,4,6,18,25,26	5,7,8,19,20,21,22,23,24	8	P00774,P35280,P55091,P98089,Q4FZU2,Q63661,Q63942,Q91ZS3
330	1,2,3,4,6,19,20,21	5,7,8,18,22,23,24,25,26	5	P00731,P01681,P01836,P25236,Q30KJ2
331	1,2,3,4,6,19,20,22	5,7,8,18,21,23,24,25,26	5	P07150,P22006,Q30KJ2,Q62997,Q7TP52

332	1,2,3,4,6,19,20,23	5,7,8,18,21,22,24,25,26	6	P01681,P15399,P22006,P82995,P97580,Q30KJ2
333	1,2,3,4,6,19,20,24	5,7,8,18,21,22,23,25,26	2	P82995,Q30KJ2
334	1,2,3,4,6,19,20,25	5,7,8,18,21,22,23,24,26	2	P35280,Q30KJ2
335	1,2,3,4,6,19,20,26	5,7,8,18,21,22,23,24,25	6	P01681,P01836,P25236,P35280,Q30KJ2,Q63355
336	1,2,3,4,6,19,21,22	5,7,8,18,20,23,24,25,26	2	P25236,Q30KJ2
337	1,2,3,4,6,19,21,23	5,7,8,18,20,22,24,25,26	5	P15399,P25236,P34901,P97580,Q30KJ2
338	1,2,3,4,6,19,21,24	5,7,8,18,20,22,23,25,26	2	P25236,Q30KJ2
339	1,2,3,4,6,19,21,25	5,7,8,18,20,22,23,24,26	7	P00731,P14173,P25236,P47967,P97840,Q30KJ2,Q91ZS3
340	1,2,3,4,6,19,21,26	5,7,8,18,20,22,23,24,25	7	P01681,P01836,P20761,P25236,Q30KJ2,Q63661,Q9EQV9
341	1,2,3,4,6,19,22,23	5,7,8,18,20,21,24,25,26	5	P02625,P15399,P34901,P97580,Q30KJ2
342	1,2,3,4,6,19,22,24	5,7,8,18,20,21,23,25,26	2	Q30KJ2,Q7TP52
343	1,2,3,4,6,19,22,25	5,7,8,18,20,21,23,24,26	4	P06911,Q30KJ2,Q4FZU6,Q7TP52
344	1,2,3,4,6,19,22,26	5,7,8,18,20,21,23,24,25	4	P20761,P55159,Q30KJ2,Q7TP52
345	1,2,3,4,6,19,23,24	5,7,8,18,20,21,22,25,26	6	P15399,P34901,P82995,P97580,Q30KJ2,Q63532
346	1,2,3,4,6,19,23,25	5,7,8,18,20,21,22,24,26	7	P15399,P34901,P50280,P55314,P97580,Q30KJ2,Q4FZU6

347	1,2,3,4,6,19,23,26	5,7,8,18,20,21,22,24,25	10	P01946,P15399,P20761,P25236,P34901,P97580,Q30KJ2,Q4FZU2,Q63661,Q6IFU8
348	1,2,3,4,6,19,24,25	5,7,8,18,20,21,22,23,26	2	Q30KJ2,Q4FZU6
349	1,2,3,4,6,19,24,26	5,7,8,18,20,21,22,23,25	2	P20761,Q30KJ2
350	1,2,3,4,6,19,25,26	5,7,8,18,20,21,22,23,24	3	P35280,Q30KJ2,Q63661
351	1,2,3,4,6,20,21,22	5,7,8,18,19,23,24,25,26	4	P01681,P01836,P25236,Q7TPB1
352	1,2,3,4,6,20,21,23	5,7,8,18,19,22,24,25,26	5	P01681,P01835,P01836,P25236,P34901
353	1,2,3,4,6,20,21,24	5,7,8,18,19,22,23,25,26	4	P01681,P01836,P13432,P25236
354	1,2,3,4,6,20,21,25	5,7,8,18,19,22,23,24,26	4	P01681,P01836,P25236,Q91ZS3
355	1,2,3,4,6,20,21,26	5,7,8,18,19,22,23,24,25	9	P01681,P01835,P01836,P20761,P25236,P80020,Q4FZU2,Q91ZS3,Q9EQV9
356	1,2,3,4,6,20,22,23	5,7,8,18,19,21,24,25,26	5	G3V686,P22006,P34901,P82995,Q62997
357	1,2,3,4,6,20,22,24	5,7,8,18,19,21,23,25,26	3	P01836,P82995,Q7TP52
358	1,2,3,4,6,20,22,25	5,7,8,18,19,21,23,24,26	2	P06911,Q7TP52
359	1,2,3,4,6,20,22,26	5,7,8,18,19,21,23,24,25	6	P01681,P01836,P20761,P55159,Q62997,Q7TP52
360	1,2,3,4,6,20,23,24	5,7,8,18,19,21,22,25,26	2	P34901,P82995
361	1,2,3,4,6,20,23,25	5,7,8,18,19,21,22,24,26	4	P34901,P55314,P70709,Q91ZS3

362	1,2,3,4,6,20,23,26	5,7,8,18,19,21,22,24,25	10	P01681,P01836,P20761,P20762,P25236,P34901,Q4FZU2,Q6IFU8,Q6IFW6,Q6IMF3
363	1,2,3,4,6,20,24,25	5,7,8,18,19,21,22,23,26	1	Q91ZS3
364	1,2,3,4,6,20,24,26	5,7,8,18,19,21,22,23,25	3	P01681,P01836,P20761
365	1,2,3,4,6,20,25,26	5,7,8,18,19,21,22,23,24	5	P01681,P01836,P25236,P35280,Q91ZS3
366	1,2,3,4,6,21,22,23	5,7,8,18,19,20,24,25,26	2	P25236,P34901
367	1,2,3,4,6,21,22,24	5,7,8,18,19,20,23,25,26	4	P01836,P13432,P25236,P34901
368	1,2,3,4,6,21,22,25	5,7,8,18,19,20,23,24,26	4	P06911,P09605;P25809,P25236,Q91ZS3
369	1,2,3,4,6,21,22,26	5,7,8,18,19,20,23,24,25	5	P01681,P01836,P20761,P25236,Q63661
370	1,2,3,4,6,21,23,24	5,7,8,18,19,20,22,25,26	3	P01836,P25236,P34901
371	1,2,3,4,6,21,23,25	5,7,8,18,19,20,22,24,26	7	P25236,P34901,P55314,P70709,Q63751,Q6P6R2,Q91ZS3
372	1,2,3,4,6,21,23,26	5,7,8,18,19,20,22,24,25	11	P01681,P01836,P01946,P20761,P25236,P34901,Q4FZU2,Q63661,Q6IFU8,Q6IMF3,Q9EQV9
373	1,2,3,4,6,21,24,25	5,7,8,18,19,20,22,23,26	4	P01836,P25236,Q4FZU6,Q91ZS3
374	1,2,3,4,6,21,24,26	5,7,8,18,19,20,22,23,25	6	P01681,P01836,P20761,P25236,P80020,Q9EQV9
375	1,2,3,4,6,21,25,26	5,7,8,18,19,20,22,23,24	7	P01681,P01836,P19223,P20761,P25236,Q63661,Q91ZS3
376	1,2,3,4,6,22,23,24	5,7,8,18,19,20,21,25,26	3	P34901,P82471,P82995

377	1,2,3,4,6,22,23,25	5,7,8,18,19,20,21,24,26	8	G3V686,P06911,P34901,P50280,P55314,P70709,Q4FZU6,Q5GRG2
378	1,2,3,4,6,22,23,26	5,7,8,18,19,20,21,24,25	5	P20761,P25236,P34901,Q4FZU2,Q7TP52
379	1,2,3,4,6,22,24,25	5,7,8,18,19,20,21,23,26	3	P06911,Q4FZU6,Q7TP52
380	1,2,3,4,6,22,24,26	5,7,8,18,19,20,21,23,25	4	P01836,P08010,P20761,Q7TP52
381	1,2,3,4,6,22,25,26	5,7,8,18,19,20,21,23,24	1	Q7TP52
382	1,2,3,4,6,23,24,25	5,7,8,18,19,20,21,22,26	6	P34901,P55314,P70709,P97580,Q4FZU6,Q63751
383	1,2,3,4,6,23,24,26	5,7,8,18,19,20,21,22,25	8	P01946,P20761,P25236,P27590,P34901,Q30KJ2,Q4FZU2,Q6IFU8
384	1,2,3,4,6,23,25,26	5,7,8,18,19,20,21,22,24	8	P01946,P25236,P34901,P55314,Q30KJ2,Q4FZU2,Q63661,Q6IFU8
385	1,2,3,4,6,24,25,26	5,7,8,18,19,20,21,22,23	2	P14480,P20761
386	1,2,3,4,7,8,18,19	5,6,20,21,22,23,24,25,26	13	P00731,P01836,P02803,P05964,P06760,P16086,P18418,P35280,P42854,Q09030,Q63475,Q63532,Q64057
387	1,2,3,4,7,8,18,20	5,6,19,21,22,23,24,25,26	11	O35783,P00731,P01041,P01836,P10715,P18418,P35280,P42854,Q63475,Q64057,Q9QZA6
388	1,2,3,4,7,8,18,21	5,6,19,20,22,23,24,25,26	17	O35783,O54800;Q5DWV2,O89117,P00731,P01041,P01836,P02761,P02803,P05964,P18418,P25031,P42854,Q63475,Q64057,Q80WL1,Q9ESW0,Q9QZA6
389	1,2,3,4,7,8,18,22	5,6,19,20,21,23,24,25,26	6	P01836,P18418,P20766,Q63475,Q64057,Q80WL1
390	1,2,3,4,7,8,18,23	5,6,19,20,21,22,24,25,26	11	P01836,P02803,P05964,P12785,P18418,P34901,Q09030,Q498D9,Q5XI22,Q63475,Q64057
391	1,2,3,4,7,8,18,24	5,6,19,20,21,22,23,25,26	4	P01836,P10715,P18418,Q63475

392	1,2,3,4,7,8,18,25	5,6,19,20,21,22,23,24,26	14	O35783,O54800;Q5DWV2,P01836,P02803,P08721,P10715,P18418,P19629,P35280,P98089,Q09030,Q63475,Q64057,Q9QZA6
393	1,2,3,4,7,8,18,26	5,6,19,20,21,22,23,24,25	24	O35783,O54800;Q5DWV2,P01041,P01836,P01946,P02770,P05964,P06760,P10959,P18418,P19223,P20766,P35280,P55091,P82995,Q5QE79,Q63475,Q63661,Q63942,Q64057,Q6AYQ8,Q8VD89,Q9ESW0,Q9R1T1
394	1,2,3,4,7,8,19,20	5,6,18,21,22,23,24,25,26	8	P01836,P18418,P35280,P36970,P42854,Q63475,Q63532,Q64057
395	1,2,3,4,7,8,19,21	5,6,18,20,22,23,24,25,26	15	O89117,P00731,P01836,P02761,P02803,P05964,P16086,P18418,P35281,P36970,P42854,Q63475,Q63532,Q64057,Q80WL1
396	1,2,3,4,7,8,19,22	5,6,18,20,21,23,24,25,26	7	P01836,P16086,P18418,P20766,P36970,Q63532,Q80WL1
397	1,2,3,4,7,8,19,23	5,6,18,20,21,22,24,25,26	15	P01836,P02803,P05964,P15399,P16086,P18418,P36970,P97580,Q09030,Q30KJ2,Q5XI22,Q63475,Q63532,Q64057,Q99041
398	1,2,3,4,7,8,19,24	5,6,18,20,21,22,23,25,26	5	P01836,P18418,P36970,Q63475,Q63532
399	1,2,3,4,7,8,19,25	5,6,18,20,21,22,23,24,26	15	P01836,P02803,P10715,P18418,P19629,P20766,P35280,P47967,Q09030,Q5GRG2,Q63475,Q63532,Q64057,Q6IFU7,Q9QUL6
400	1,2,3,4,7,8,19,26	5,6,18,20,21,22,23,24,25	13	P01041,P01836,P01946,P02770,P06760,P10959,P15978,P18418,P20766,P35280,Q63532,Q64057,Q6AYQ8
401	1,2,3,4,7,8,20,21	5,6,18,19,22,23,24,25,26	11	O35783,O54800;Q5DWV2,P01041,P01836,P02761,P18418,P42854,Q63475,Q64057,Q80WL1,Q8K1G0
402	1,2,3,4,7,8,20,22	5,6,18,19,21,23,24,25,26	6	P01836,P18418,P20766,P36970,Q62997,Q63475
403	1,2,3,4,7,8,20,23	5,6,18,19,21,22,24,25,26	7	P01836,P18418,P36970,Q5XI22,Q63475,Q63617,Q64057
404	1,2,3,4,7,8,20,24	5,6,18,19,21,22,23,25,26	8	P01836,P02761,P08010,P10715,P18418,P36970,Q63475,Q63617
405	1,2,3,4,7,8,20,25	5,6,18,19,21,22,23,24,26	9	O35783,P01836,P10715,P18418,P19629,P35280,Q5GRG2,Q63475,Q64057
406	1,2,3,4,7,8,20,26	5,6,18,19,21,22,23,24,25	13	O35783,P01041,P01836,P01946,P02770,P10959,P18418,P20761,P20762,P35280,Q5QE79,Q63475,Q6AYQ8

407	1,2,3,4,7,8,21,22	5,6,18,19,20,23,24,25,26	11	D4A1J4,O54800;Q5DWV2,P01836,P02761,P18418,P20766,P36970,P42854,Q63475,Q64057,Q80WL1
408	1,2,3,4,7,8,21,23	5,6,18,19,20,22,24,25,26	11	O35783,P01836,P02761,P18418,P34901,P36970,P42854,Q5XI22,Q63475,Q64057,Q80WL1
409	1,2,3,4,7,8,21,24	5,6,18,19,20,22,23,25,26	8	O54800;Q5DWV2,P01836,P02761,P18418,P36970,P42854,Q63475,Q80WL1
410	1,2,3,4,7,8,21,25	5,6,18,19,20,22,23,24,26	15	O35783,O54800;Q5DWV2,P01836,P02761,P08721,P10715,P18418,P19629,P42854,Q5GRG2,Q63475,Q64057,Q6IFU7,Q80WL1,Q91ZS3
411	1,2,3,4,7,8,21,26	5,6,18,19,20,22,23,24,25	19	O35783,O54800;Q5DWV2,P01041,P01836,P01946,P02761,P02770,P10959,P18418,P19223,P20761,P42854,Q5QE79,Q63475,Q63661,Q64057,Q6AYQ8,Q80WL1,Q9EQV9
412	1,2,3,4,7,8,22,23	5,6,18,19,20,21,24,25,26	9	D3ZUK3,P01836,P18418,P20766,P34901,P36970,Q63475,Q64057,Q80WL1
413	1,2,3,4,7,8,22,24	5,6,18,19,20,21,23,25,26	6	P01836,P08010,P18418,P20766,P36970,Q63475
414	1,2,3,4,7,8,22,25	5,6,18,19,20,21,23,24,26	9	P01836,P06911,P10715,P18418,P19629,P20766,Q5GRG2,Q80WL1,Q9QUL6
415	1,2,3,4,7,8,22,26	5,6,18,19,20,21,23,24,25	14	O54800;Q5DWV2,P01041,P01836,P01946,P02770,P08010,P10959,P18418,P20761,P20766,P55159,Q5QE79,Q6AYQ8,Q80WL1
416	1,2,3,4,7,8,23,24	5,6,18,19,20,21,22,25,26	5	P01836,P18418,P34901,P36970,Q63475
417	1,2,3,4,7,8,23,25	5,6,18,19,20,21,22,24,26	13	P01836,P10715,P18418,P19629,P20766,P36970,P55314,Q09030,Q5GRG2,Q63475,Q63751,Q64057,Q99041
418	1,2,3,4,7,8,23,26	5,6,18,19,20,21,22,24,25	16	O35783,P01836,P01946,P02091,P02770,P10959,P18418,P20761,P20762,P20766,Q5QE79,Q63475,Q64057,Q6AYQ8,Q6IE52,Q9R1T1
419	1,2,3,4,7,8,24,25	5,6,18,19,20,21,22,23,26	7	P01836,P10715,P18418,P19629,Q5GRG2,Q63475,Q9QUL6
420	1,2,3,4,7,8,24,26	5,6,18,19,20,21,22,23,25	13	D3ZHA0,P01041,P01836,P01946,P02091,P02770,P08010,P10959,P18418,P20761,Q5QE79,Q63475,Q6AYQ8
421	1,2,3,4,7,8,25,26	5,6,18,19,20,21,22,23,24	18	O35783,O54800;Q5DWV2,P01041,P01836,P01946,P02091,P02770,P08721,P10715,P10959,P18418,P19223,P19629,P20766,P35280,Q5QE79,Q64057,Q6AYQ8

422	1,2,3,4,7,18,19,20	5,6,8,21,22,23,24,25,26	10	P00731,P01836,P05964,P06760,P07150,P35280,P42854,Q63532,Q6IE51,Q9WTT6
423	1,2,3,4,7,18,19,21	5,6,8,20,22,23,24,25,26	12	D3ZUC6,O89117,P00731,P01836,P05964,P06760,P10758,P25031,P42854,Q63532,Q63661,Q6IE51
424	1,2,3,4,7,18,19,22	5,6,8,20,21,23,24,25,26	6	P01836,P05964,P06760,P07150,Q63532,Q9WTT6
425	1,2,3,4,7,18,19,23	5,6,8,20,21,22,24,25,26	17	P01836,P05964,P06760,P12785,P15399,P16086,P34901,P97580,Q03191,Q09030,Q30KJ2,Q498D9,Q5XI22,Q63532,Q63661,Q6IE51,Q99MH3
426	1,2,3,4,7,18,19,24	5,6,8,20,21,22,23,25,26	7	O89117,P01836,P05964,P06760,Q63532,Q6IE51,Q9WTT6
427	1,2,3,4,7,18,19,25	5,6,8,20,21,22,23,24,26	8	P00731,P01836,P05964,P35280,P47967,Q09030,Q63532,Q6IE51
428	1,2,3,4,7,18,19,26	5,6,8,20,21,22,23,24,25	10	P01041,P01836,P01946,P05964,P06760,P35280,Q63532,Q63661,Q6IE51,Q99PP0
429	1,2,3,4,7,18,20,21	5,6,8,19,22,23,24,25,26	10	O35783,O54800;Q5DWV2,P00731,P01041,P01836,P06760,P25031,P42854,Q63475,Q9ESW0
430	1,2,3,4,7,18,20,22	5,6,8,19,21,23,24,25,26	6	P01836,P06760,P07150,P42854,Q62997,Q9WTT6
431	1,2,3,4,7,18,20,23	5,6,8,19,21,22,24,25,26	7	A2RUV9,P01836,P06760,P12785,P34901,Q5XI22,Q63475
432	1,2,3,4,7,18,20,24	5,6,8,19,21,22,23,25,26	6	P01836,P06760,P07150,Q63475,Q9QZK9,Q9WTT6
433	1,2,3,4,7,18,20,25	5,6,8,19,21,22,23,24,26	3	O35783,P01836,P35280
434	1,2,3,4,7,18,20,26	5,6,8,19,21,22,23,24,25	7	O35783,P01041,P01836,P01946,P06760,P35280,Q63661
435	1,2,3,4,7,18,21,22	5,6,8,19,20,23,24,25,26	7	O35783,O54800;Q5DWV2,P01836,P06760,P25031,P42854,Q80WL1
436	1,2,3,4,7,18,21,23	5,6,8,19,20,22,24,25,26	14	D3ZUC6,O35783,P01836,P05964,P12785,P14668,P25031,P34901,P42854,Q5XI22,Q62636,Q63475,Q63661,Q64057

437	1,2,3,4,7,18,21,24	5,6,8,19,20,22,23,25,26	10	D3ZUC6,O35783,O54800;Q5DWV2,O89117,P01836,P05964,P06760,P25031,P42854,Q63475
438	1,2,3,4,7,18,21,25	5,6,8,19,20,22,23,24,26	12	O35783,O54800;Q5DWV2,P00731,P01836,P05964,P08721,P25031,P31430,P42854,P47967,P98089,Q91ZS3
439	1,2,3,4,7,18,21,26	5,6,8,19,20,22,23,24,25	19	D3ZUC6,O35783,O54800;Q5DWV2,O89117,P00774,P01041,P01836,P01946,P02770,P05964,P06760,P19223,P25031,P42854,P55091,P82995,Q63661,Q9EQV9,Q9ESW0
440	1,2,3,4,7,18,22,23	5,6,8,19,20,21,24,25,26	5	P01836,P06760,P12785,P34901,Q5XI22
441	1,2,3,4,7,18,22,24	5,6,8,19,20,21,23,25,26	5	P01836,P06760,P07150,P08010,Q9WTT6
442	1,2,3,4,7,18,22,25	5,6,8,19,20,21,23,24,26	1	P01836
443	1,2,3,4,7,18,22,26	5,6,8,19,20,21,23,24,25	8	O35783,P01041,P01836,P01946,P06760,P55159,Q63661,Q8VD89
444	1,2,3,4,7,18,23,24	5,6,8,19,20,21,22,25,26	9	A2RUV9,P01836,P06760,P12785,P34901,Q5XI22,Q63475,Q6IE51,Q99068
445	1,2,3,4,7,18,23,25	5,6,8,19,20,21,22,24,26	10	A2RUV9,P01836,P05964,P12785,P34901,P55314,Q09030,Q5XI22,Q99068,Q99MH3
446	1,2,3,4,7,18,23,26	5,6,8,19,20,21,22,24,25	21	A2RUV9,O35783,P01836,P01946,P02091,P05964,P06760,P12785,P34901,P35467,Q4FZU2,Q5XI22,Q62636,Q63661,Q6AY61,Q6IFU8,Q6IFW6,Q6IG02,Q6P6Q2,Q8VD89,Q9R1T1
447	1,2,3,4,7,18,24,25	5,6,8,19,20,21,22,23,26	2	P01836,P08721
448	1,2,3,4,7,18,24,26	5,6,8,19,20,21,22,23,25	10	O35783,P01041,P01836,P01946,P02091,P02770,P06760,P55091,Q63661,Q8VD89
449	1,2,3,4,7,18,25,26	5,6,8,19,20,21,22,23,24	13	O35783,P00774,P01041,P01836,P01946,P02091,P08721,P19223,P35280,P55091,P82995,Q63661,Q6AYQ8
450	1,2,3,4,7,19,20,21	5,6,8,18,22,23,24,25,26	4	P00731,P01836,P42854,Q63532
451	1,2,3,4,7,19,20,22	5,6,8,18,21,23,24,25,26	7	P01836,P06760,P07150,P14669,Q62997,Q63532,Q9WTT6

452	1,2,3,4,7,19,20,23	5,6,8,18,21,22,24,25,26	8	P01836,P06760,P15399,P16086,P97580,Q30KJ2,Q63532,Q6IE51
453	1,2,3,4,7,19,20,24	5,6,8,18,21,22,23,25,26	7	P01836,P06760,P07150,P14669,Q63532,Q6IE51,Q9WTT6
454	1,2,3,4,7,19,20,25	5,6,8,18,21,22,23,24,26	4	P01836,P35280,P47967,Q63532
455	1,2,3,4,7,19,20,26	5,6,8,18,21,22,23,24,25	7	P01041,P01836,P01946,P06760,P35280,Q5FVI6,Q63532
456	1,2,3,4,7,19,21,22	5,6,8,18,20,23,24,25,26	3	P01836,P42854,Q63532
457	1,2,3,4,7,19,21,23	5,6,8,18,20,22,24,25,26	12	D3ZUC6,P01836,P05964,P14668,P15399,P16086,P34901,P42854,P97580,Q30KJ2,Q63532,Q6IE51
458	1,2,3,4,7,19,21,24	5,6,8,18,20,22,23,25,26	6	D3ZUC6,O89117,P01836,P42854,Q63532,Q6IE51
459	1,2,3,4,7,19,21,25	5,6,8,18,20,22,23,24,26	5	P01836,P42854,P47967,P97840,Q63532
460	1,2,3,4,7,19,21,26	5,6,8,18,20,22,23,24,25	8	P01041,P01836,P01946,P06760,P42854,Q63532,Q63661,Q9EQV9
461	1,2,3,4,7,19,22,23	5,6,8,18,20,21,24,25,26	10	P01836,P06760,P15399,P16086,P34901,P97580,Q30KJ2,Q63532,Q6IE51,Q9WTT6
462	1,2,3,4,7,19,22,24	5,6,8,18,20,21,23,25,26	7	P01836,P06760,P07150,P08010,P14669,Q63532,Q9WTT6
463	1,2,3,4,7,19,22,25	5,6,8,18,20,21,23,24,26	3	P01836,P47967,Q63532
464	1,2,3,4,7,19,22,26	5,6,8,18,20,21,23,24,25	8	O54728,P01836,P01946,P05539,P06760,P55159,Q63532,Q9WTT6
465	1,2,3,4,7,19,23,24	5,6,8,18,20,21,22,25,26	8	P01836,P06760,P15399,P34901,P97580,Q30KJ2,Q63532,Q6IE51
466	1,2,3,4,7,19,23,25	5,6,8,18,20,21,22,24,26	8	P01836,P15399,P55314,P97580,Q09030,Q30KJ2,Q63532,Q6IE51

467	1,2,3,4,7,19,23,26	5,6,8,18,20,21,22,24,25	12	O54728,P01836,P01946,P02091,P06760,P15399,P34901,P97580,Q30KJ2,Q63532,Q63661,Q6IE51
468	1,2,3,4,7,19,24,25	5,6,8,18,20,21,22,23,26	3	P01836,Q63532,Q6IE51
469	1,2,3,4,7,19,24,26	5,6,8,18,20,21,22,23,25	7	P01836,P01946,P02091,P06760,Q63532,Q6IE51,Q9WTT6
470	1,2,3,4,7,19,25,26	5,6,8,18,20,21,22,23,24	7	P01836,P01946,P02091,P35280,Q5FVI6,Q63532,Q63661
471	1,2,3,4,7,20,21,22	5,6,8,18,19,23,24,25,26	2	P01836,P42854
472	1,2,3,4,7,20,21,23	5,6,8,18,19,22,24,25,26	5	O35783,P01836,P34901,P42854,Q63475
473	1,2,3,4,7,20,21,24	5,6,8,18,19,22,23,25,26	4	P01836,P02761,P42854,Q63475
474	1,2,3,4,7,20,21,25	5,6,8,18,19,22,23,24,26	5	O35783,P01836,P42854,P47967,Q91ZS3
475	1,2,3,4,7,20,21,26	5,6,8,18,19,22,23,24,25	9	O35783,O54800;Q5DWV2,P01041,P01836,P01946,P06760,P19223,P42854,Q9EQV9
476	1,2,3,4,7,20,22,23	5,6,8,18,19,21,24,25,26	4	P01836,P06760,P34901,Q62997
477	1,2,3,4,7,20,22,24	5,6,8,18,19,21,23,25,26	6	P01836,P06760,P07150,P08010,P14669,Q9WTT6
478	1,2,3,4,7,20,22,25	5,6,8,18,19,21,23,24,26	1	P01836
479	1,2,3,4,7,20,22,26	5,6,8,18,19,21,23,24,25	10	O54728,P01041,P01836,P01946,P05539,P06760,P07150,P08010,P55159,Q62997
480	1,2,3,4,7,20,23,24	5,6,8,18,19,21,22,25,26	4	A2RUV9,P01836,P34901,Q63475
481	1,2,3,4,7,20,23,25	5,6,8,18,19,21,22,24,26	4	A2RUV9,P01836,P34901,P55314

482	1,2,3,4,7,20,23,26	5,6,8,18,19,21,22,24,25	9	A2RUV9,O35783,P01041,P01836,P01946,P02091,P05539,P06760,P34901
483	1,2,3,4,7,20,24,25	5,6,8,18,19,21,22,23,26	1	P01836
484	1,2,3,4,7,20,24,26	5,6,8,18,19,21,22,23,25	6	P01041,P01836,P01946,P02091,P06760,P08010
485	1,2,3,4,7,20,25,26	5,6,8,18,19,21,22,23,24	8	O35783,P01041,P01836,P01946,P02091,P05539,P19223,P35280
486	1,2,3,4,7,21,22,23	5,6,8,18,19,20,24,25,26	3	P01836,P34901,P42854
487	1,2,3,4,7,21,22,24	5,6,8,18,19,20,23,25,26	1	P01836
488	1,2,3,4,7,21,22,25	5,6,8,18,19,20,23,24,26	4	O54800;Q5DWV2,P01836,P42854,P47967
489	1,2,3,4,7,21,22,26	5,6,8,18,19,20,23,24,25	10	O35783,O54800;Q5DWV2,P01041,P01836,P01946,P06760,P42854,Q63661,Q80WL1,Q9EQV9
490	1,2,3,4,7,21,23,24	5,6,8,18,19,20,22,25,26	4	D3ZUC6,P01836,P34901,Q63475
491	1,2,3,4,7,21,23,25	5,6,8,18,19,20,22,24,26	5	O35783,P01836,P34901,P42854,P55314
492	1,2,3,4,7,21,23,26	5,6,8,18,19,20,22,24,25	9	O35783,P01041,P01836,P01946,P02091,P34901,P42854,Q63661,Q9EQV9
493	1,2,3,4,7,21,24,25	5,6,8,18,19,20,22,23,26	3	O35783,P01836,P22006
494	1,2,3,4,7,21,24,26	5,6,8,18,19,20,22,23,25	9	O35783,O54800;Q5DWV2,P01041,P01836,P01946,P02091,P80020,Q63661,Q9EQV9
495	1,2,3,4,7,21,25,26	5,6,8,18,19,20,22,23,24	11	O35783,O54800;Q5DWV2,P01041,P01836,P01946,P02091,P08721,P19223,P22006,P42854,Q63661
496	1,2,3,4,7,22,23,24	5,6,8,18,19,20,21,25,26	3	P01836,P34901,P82471

497	1,2,3,4,7,22,23,25	5,6,8,18,19,20,21,24,26	5	P01836,P06911,P34901,P55314,Q5GRG2
498	1,2,3,4,7,22,23,26	5,6,8,18,19,20,21,24,25	8	O54728,P01836,P01946,P02091,P05539,P06760,P34901,P55159
499	1,2,3,4,7,22,24,25	5,6,8,18,19,20,21,23,26	1	P01836
500	1,2,3,4,7,22,24,26	5,6,8,18,19,20,21,23,25	5	P01836,P01946,P02091,P06760,P08010
501	1,2,3,4,7,22,25,26	5,6,8,18,19,20,21,23,24	4	P01836,P01946,P02091,P05539
502	1,2,3,4,7,23,24,25	5,6,8,18,19,20,21,22,26	4	P01836,P34901,P55314,Q63751
503	1,2,3,4,7,23,24,26	5,6,8,18,19,20,21,22,25	6	A2RUV9,P01836,P01946,P02091,P06760,P34901
504	1,2,3,4,7,23,25,26	5,6,8,18,19,20,21,22,24	7	O35783,P01836,P01946,P02091,P34901,P55314,Q63661
505	1,2,3,4,7,24,25,26	5,6,8,18,19,20,21,22,23	4	O35783,P01836,P01946,P02091
506	1,2,3,4,8,18,19,20	5,6,7,21,22,23,24,25,26	5	P00731,P22006,P35280,P83121,Q63355
507	1,2,3,4,8,18,19,21	5,6,7,20,22,23,24,25,26	9	O89117,P00731,P02761,P02803,P05964,P10758,P35281,Q64057,Q80WL1
508	1,2,3,4,8,18,19,22	5,6,7,20,21,23,24,25,26	3	P20766,P22006,P83121
509	1,2,3,4,8,18,19,23	5,6,7,20,21,22,24,25,26	10	P02803,P05964,P12785,P35467,Q03191,Q498D9,Q5XI22,Q64194,Q6AY61,Q99MH3
510	1,2,3,4,8,18,19,24	5,6,7,20,21,22,23,25,26	1	P83121
511	1,2,3,4,8,18,19,25	5,6,7,20,21,22,23,24,26	2	P02803,P35280

512	1,2,3,4,8,18,19,26	5,6,7,20,21,22,23,24,25	6	P02770,P10959,P35280,Q63355,Q63661,Q63942
513	1,2,3,4,8,18,20,21	5,6,7,19,22,23,24,25,26	5	P00731,P01836,P02761,P10758,P42854
514	1,2,3,4,8,18,20,22	5,6,7,19,21,23,24,25,26	4	P22006,P83121,Q62997,Q9QZK9
515	1,2,3,4,8,18,20,23	5,6,7,19,21,22,24,25,26	6	P12785,P22006,Q498D9,Q5XI22,Q6TMA8,Q9QZK9
516	1,2,3,4,8,18,20,24	5,6,7,19,21,22,23,25,26	2	P83121,Q9QZK9
517	1,2,3,4,8,18,20,25	5,6,7,19,21,22,23,24,26	1	P35280
518	1,2,3,4,8,18,20,26	5,6,7,19,21,22,23,24,25	6	P01836,P02770,P10959,P35280,Q5QE79,Q63355
519	1,2,3,4,8,18,21,22	5,6,7,19,20,23,24,25,26	4	P01836,P02761,P10758,Q80WL1
520	1,2,3,4,8,18,21,23	5,6,7,19,20,22,24,25,26	10	P02761,P02803,P05964,P10758,P12785,P34901,Q5XI22,Q64057,Q6AY61,Q80WL1
521	1,2,3,4,8,18,21,24	5,6,7,19,20,22,23,25,26	3	P01836,P02761,P10758
522	1,2,3,4,8,18,21,25	5,6,7,19,20,22,23,24,26	7	P00731,P02761,P02803,P08721,P31430,P98089,Q80WL1
523	1,2,3,4,8,18,21,26	5,6,7,19,20,22,23,24,25	12	P00774,P01836,P02761,P02770,P10758,P10959,P19223,Q5QE79,Q63661,Q63942,Q80WL1,Q9EQV9
524	1,2,3,4,8,18,22,23	5,6,7,19,20,21,24,25,26	3	P20766,P34901,Q5XI22
525	1,2,3,4,8,18,22,24	5,6,7,19,20,21,23,25,26	1	P83121
526	1,2,3,4,8,18,22,25	5,6,7,19,20,21,23,24,26	1	P20766

527	1,2,3,4,8,18,22,26	5,6,7,19,20,21,23,24,25	6	P02770,P10959,P20766,Q5QE79,Q63942,Q80WL1
528	1,2,3,4,8,18,23,24	5,6,7,19,20,21,22,25,26	3	P12785,P34901,Q5XI22
529	1,2,3,4,8,18,23,25	5,6,7,19,20,21,22,24,26	6	P00714,P02803,P12785,Q5XI22,Q63598,Q99MH3
530	1,2,3,4,8,18,23,26	5,6,7,19,20,21,22,24,25	18	P01946,P02770,P10959,P12785,P34901,P35467,Q4FZU2,Q5QE79,Q5XI22,Q63661,Q63942,Q64194,Q6AY61,Q6IFU8,Q6IFW6,Q6IG02,Q6IMF3,Q6P6Q2
531	1,2,3,4,8,18,24,25	5,6,7,19,20,21,22,23,26	1	P08721
532	1,2,3,4,8,18,24,26	5,6,7,19,20,21,22,23,25	5	P01836,P02770,P10959,Q5QE79,Q63942
533	1,2,3,4,8,18,25,26	5,6,7,19,20,21,22,23,24	9	P00774,P02770,P08721,P10959,P19223,P35280,P55091,Q5QE79,Q63661
534	1,2,3,4,8,19,20,21	5,6,7,18,22,23,24,25,26	4	P01836,P02761,P35281,P42854
535	1,2,3,4,8,19,20,22	5,6,7,18,21,23,24,25,26	5	P20766,P22006,P36970,P83121,Q62997
536	1,2,3,4,8,19,20,23	5,6,7,18,21,22,24,25,26	3	P22006,P36970,Q5XI22
537	1,2,3,4,8,19,20,24	5,6,7,18,21,22,23,25,26	2	P36970,P83121
538	1,2,3,4,8,19,20,25	5,6,7,18,21,22,23,24,26	2	P35280,Q9QUL6
539	1,2,3,4,8,19,20,26	5,6,7,18,21,22,23,24,25	5	P01836,P02770,P10959,P35280,Q63355
540	1,2,3,4,8,19,21,22	5,6,7,18,20,23,24,25,26	3	P02761,P36970,Q80WL1
541	1,2,3,4,8,19,21,23	5,6,7,18,20,22,24,25,26	5	P02761,P34901,P35281,P36970,Q5XI22

542	1,2,3,4,8,19,21,24	5,6,7,18,20,22,23,25,26	2	P02761,P36970
543	1,2,3,4,8,19,21,25	5,6,7,18,20,22,23,24,26	2	P47967,Q9QUL6
544	1,2,3,4,8,19,21,26	5,6,7,18,20,22,23,24,25	9	P01836,P02761,P02770,P10959,P35281,Q5QE79,Q63661,Q80WL1,Q9EQV9
545	1,2,3,4,8,19,22,23	5,6,7,18,20,21,24,25,26	2	P20766,P36970
546	1,2,3,4,8,19,22,24	5,6,7,18,20,21,23,25,26	4	P20766,P36970,P83121,Q9QUL6
547	1,2,3,4,8,19,22,25	5,6,7,18,20,21,23,24,26	2	P20766,Q9QUL6
548	1,2,3,4,8,19,22,26	5,6,7,18,20,21,23,24,25	5	O54728,P02770,P10959,P20766,P55159
549	1,2,3,4,8,19,23,24	5,6,7,18,20,21,22,25,26	2	P34901,P36970
550	1,2,3,4,8,19,23,25	5,6,7,18,20,21,22,24,26	2	P36970,Q9QUL6
551	1,2,3,4,8,19,23,26	5,6,7,18,20,21,22,24,25	5	P01946,P02770,P10959,Q63661,Q6AY61
552	1,2,3,4,8,19,24,25	5,6,7,18,20,21,22,23,26	1	Q9QUL6
553	1,2,3,4,8,19,24,26	5,6,7,18,20,21,22,23,25	2	P02770,P10959
554	1,2,3,4,8,19,25,26	5,6,7,18,20,21,22,23,24	1	P35280
555	1,2,3,4,8,20,21,22	5,6,7,18,19,23,24,25,26	4	P01836,P02761,Q7TPB1,Q80WL1
556	1,2,3,4,8,20,21,23	5,6,7,18,19,22,24,25,26	5	P01836,P02761,P34901,P36970,Q5XI22

557	1,2,3,4,8,20,21,24	5,6,7,18,19,22,23,25,26	3	P01836,P02761,Q5XFX0
558	1,2,3,4,8,20,21,25	5,6,7,18,19,22,23,24,26	2	P01836,P02761
559	1,2,3,4,8,20,21,26	5,6,7,18,19,22,23,24,25	8	P01836,P02761,P02770,P19223,P62804,Q00715,Q5QE79,Q9EQV9
560	1,2,3,4,8,20,22,23	5,6,7,18,19,21,24,25,26	4	P20766,P22006,P36970,Q62997
561	1,2,3,4,8,20,22,24	5,6,7,18,19,21,23,25,26	3	P01836,P36970,P83121
562	1,2,3,4,8,20,22,25	5,6,7,18,19,21,23,24,26	2	P20766,Q9QUL6
563	1,2,3,4,8,20,22,26	5,6,7,18,19,21,23,24,25	7	O54728,P01836,P10959,P20766,P55159,Q5QE79,Q62997
564	1,2,3,4,8,20,23,24	5,6,7,18,19,21,22,25,26	4	P34901,P36970,P97840,Q9QZK9
565	1,2,3,4,8,20,23,25	5,6,7,18,19,21,22,24,26	0	
566	1,2,3,4,8,20,23,26	5,6,7,18,19,21,22,24,25	7	P01836,P01946,P10959,P19218,P97840,Q5QE79,Q6IG02
567	1,2,3,4,8,20,24,25	5,6,7,18,19,21,22,23,26	1	Q9QUL6
568	1,2,3,4,8,20,24,26	5,6,7,18,19,21,22,23,25	6	P01836,P02761,P02770,P10959,P97840,Q5QE79
569	1,2,3,4,8,20,25,26	5,6,7,18,19,21,22,23,24	4	P01836,P19223,P35280,Q5QE79
570	1,2,3,4,8,21,22,23	5,6,7,18,19,20,24,25,26	3	P34901,P36970,Q80WL1
571	1,2,3,4,8,21,22,24	5,6,7,18,19,20,23,25,26	4	P01836,P02761,Q5XFX0,Q80WL1

572	1,2,3,4,8,21,22,25	5,6,7,18,19,20,23,24,26	2	P20766,Q80WL1
573	1,2,3,4,8,21,22,26	5,6,7,18,19,20,23,24,25	8	P01836,P02761,P02770,P20766,P63036,Q5QE79,Q80WL1,Q9EQV9
574	1,2,3,4,8,21,23,24	5,6,7,18,19,20,22,25,26	4	P01836,P02761,P34901,P36970
575	1,2,3,4,8,21,23,25	5,6,7,18,19,20,22,24,26	1	P34901
576	1,2,3,4,8,21,23,26	5,6,7,18,19,20,22,24,25	11	P01836,P01946,P02761,P02770,P10959,P34901,Q5QE79,Q63661,Q6AY61,Q80WL1,Q9EQV9
577	1,2,3,4,8,21,24,25	5,6,7,18,19,20,22,23,26	2	P01836,P02761
578	1,2,3,4,8,21,24,26	5,6,7,18,19,20,22,23,25	7	P01836,P02761,P02770,P10959,Q5QE79,Q68FV8,Q9EQV9
579	1,2,3,4,8,21,25,26	5,6,7,18,19,20,22,23,24	7	P01836,P02761,P08721,P19223,Q5QE79,Q80WL1,Q9EQV9
580	1,2,3,4,8,22,23,24	5,6,7,18,19,20,21,25,26	3	P20766,P34901,P36970
581	1,2,3,4,8,22,23,25	5,6,7,18,19,20,21,24,26	4	P06911,P20766,P34901,P36970
582	1,2,3,4,8,22,23,26	5,6,7,18,19,20,21,24,25	7	O54728,P01946,P10959,P20766,P34901,Q5QE79,Q6IE52
583	1,2,3,4,8,22,24,25	5,6,7,18,19,20,21,23,26	2	P20766,Q9QUL6
584	1,2,3,4,8,22,24,26	5,6,7,18,19,20,21,23,25	7	P01836,P02770,P10959,P20766,P36953,Q5QE79,Q64573
585	1,2,3,4,8,22,25,26	5,6,7,18,19,20,21,23,24	2	P20766,Q5QE79
586	1,2,3,4,8,23,24,25	5,6,7,18,19,20,21,22,26	3	P34901,P36970,Q63751

587	1,2,3,4,8,23,24,26	5,6,7,18,19,20,21,22,25	6	P01946,P02770,P10959,P34901,P97840,Q5QE79
588	1,2,3,4,8,23,25,26	5,6,7,18,19,20,21,22,24	4	P01946,P10959,P55314,Q5QE79
589	1,2,3,4,8,24,25,26	5,6,7,18,19,20,21,22,23	3	P10959,Q5QE79,Q68FV8
590	1,2,3,4,18,19,20,21	5,6,7,8,22,23,24,25,26	4	P00731,P01836,P10758,P42854
591	1,2,3,4,18,19,20,22	5,6,7,8,21,23,24,25,26	8	P06760,P07150,P14669,P22006,P83121,Q62997,Q9QZK9,Q9WTT6
592	1,2,3,4,18,19,20,23	5,6,7,8,21,22,24,25,26	6	P06760,P12785,P22006,Q498D9,Q5XI22,Q9QZK9
593	1,2,3,4,18,19,20,24	5,6,7,8,21,22,23,25,26	5	P06760,P07150,P83121,Q9QZK9,Q9WTT6
594	1,2,3,4,18,19,20,25	5,6,7,8,21,22,23,24,26	1	P35280
595	1,2,3,4,18,19,20,26	5,6,7,8,21,22,23,24,25	5	P01836,P06760,P35280,Q63355,Q63661
596	1,2,3,4,18,19,21,22	5,6,7,8,20,23,24,25,26	3	O89117,P10758,Q63661
597	1,2,3,4,18,19,21,23	5,6,7,8,20,22,24,25,26	10	D3ZUC6,O89117,P05964,P10758,P12785,P34901,Q03191,Q5XI22,Q63661,Q99MH3
598	1,2,3,4,18,19,21,24	5,6,7,8,20,22,23,25,26	4	D3ZUC6,O89117,P01836,P10758
599	1,2,3,4,18,19,21,25	5,6,7,8,20,22,23,24,26	6	P00731,P05964,P31430,P47967,P98089,Q63661
600	1,2,3,4,18,19,21,26	5,6,7,8,20,22,23,24,25	8	D3ZUC6,O89117,P01836,P05964,P06760,P10758,Q63661,Q9EQV9
601	1,2,3,4,18,19,22,23	5,6,7,8,20,21,24,25,26	6	P02625,P06760,P12785,P34901,Q5XI22,Q9WTT6

602	1,2,3,4,18,19,22,24	5,6,7,8,20,21,23,25,26	4	P06760,P07150,P83121,Q9WTT6
603	1,2,3,4,18,19,22,25	5,6,7,8,20,21,23,24,26	0	
604	1,2,3,4,18,19,22,26	5,6,7,8,20,21,23,24,25	4	P06760,P55159,Q63661,Q9WTT6
605	1,2,3,4,18,19,23,24	5,6,7,8,20,21,22,25,26	6	P06760,P12785,P34901,Q03191,Q5XI22,Q9WTT6
606	1,2,3,4,18,19,23,25	5,6,7,8,20,21,22,24,26	5	P05964,P12785,P34901,Q5XI22,Q99MH3
607	1,2,3,4,18,19,23,26	5,6,7,8,20,21,22,24,25	14	P01946,P05964,P06760,P12785,P34901,P35467,Q03191,Q4FZU2,Q63661,Q63942,Q64194,Q6AY61,Q6IFU8,Q6P6Q2
608	1,2,3,4,18,19,24,25	5,6,7,8,20,21,22,23,26	0	
609	1,2,3,4,18,19,24,26	5,6,7,8,20,21,22,23,25	3	P06760,Q63661,Q9WTT6
610	1,2,3,4,18,19,25,26	5,6,7,8,20,21,22,23,24	2	P35280,Q63661
611	1,2,3,4,18,20,21,22	5,6,7,8,19,23,24,25,26	2	P01836,P10758
612	1,2,3,4,18,20,21,23	5,6,7,8,19,22,24,25,26	6	P01836,P10758,P12785,P34901,Q5XI22,Q9QZK9
613	1,2,3,4,18,20,21,24	5,6,7,8,19,22,23,25,26	2	P01836,Q9QZK9
614	1,2,3,4,18,20,21,25	5,6,7,8,19,22,23,24,26	3	P01836,P31430,P98089
615	1,2,3,4,18,20,21,26	5,6,7,8,19,22,23,24,25	6	P01836,P06760,Q4FZU2,Q63661,Q99041,Q9EQV9
616	1,2,3,4,18,20,22,23	5,6,7,8,19,21,24,25,26	7	P06760,P12785,P22006,P34901,Q5XI22,Q62997,Q9QZK9

617	1,2,3,4,18,20,22,24	5,6,7,8,19,21,23,25,26	8	P01836,P06760,P07150,P14669,P83121,Q99041,Q9QZK9,Q9WTT6
618	1,2,3,4,18,20,22,25	5,6,7,8,19,21,23,24,26	0	
619	1,2,3,4,18,20,22,26	5,6,7,8,19,21,23,24,25	5	P01836,P06760,P55159,Q62997,Q99041
620	1,2,3,4,18,20,23,24	5,6,7,8,19,21,22,25,26	5	A2RUV9,P12785,P34901,Q5XI22,Q9QZK9
621	1,2,3,4,18,20,23,25	5,6,7,8,19,21,22,24,26	3	A2RUV9,P12785,P34901
622	1,2,3,4,18,20,23,26	5,6,7,8,19,21,22,24,25	16	A2RUV9,P01836,P01946,P06760,P12785,P19218,P34901,P35467,Q4FZU2,Q63661,Q6AY61,Q6IFU8,Q6IFW6,Q6IG02,Q6IMF3,Q6P6Q2
623	1,2,3,4,18,20,24,25	5,6,7,8,19,21,22,23,26	0	
624	1,2,3,4,18,20,24,26	5,6,7,8,19,21,22,23,25	4	P01836,P06760,Q99041,Q9QZK9
625	1,2,3,4,18,20,25,26	5,6,7,8,19,21,22,23,24	2	P01836,P35280
626	1,2,3,4,18,21,22,23	5,6,7,8,19,20,24,25,26	6	P01836,P10758,P12785,P34901,Q5XI22,Q63661
627	1,2,3,4,18,21,22,24	5,6,7,8,19,20,23,25,26	2	P01836,P34901
628	1,2,3,4,18,21,22,25	5,6,7,8,19,20,23,24,26	3	P01836,P31430,P98089
629	1,2,3,4,18,21,22,26	5,6,7,8,19,20,23,24,25	5	P01836,P06760,P63036,Q63661,Q99041
630	1,2,3,4,18,21,23,24	5,6,7,8,19,20,22,25,26	5	D3ZUC6,P01836,P12785,P34901,Q5XI22
631	1,2,3,4,18,21,23,25	5,6,7,8,19,20,22,24,26	7	P12785,P31430,P34901,P98089,Q62636,Q63661,Q99MH3

632	1,2,3,4,18,21,23,26	5,6,7,8,19,20,22,24,25	16	D3ZUC6,P00774,P01836,P01946,P12785,P34901,Q4FZU2,Q62636,Q63661,Q6AY61,Q6IFU8,Q6IFW6,Q6IG02,Q6IMF3,Q6P6Q2,Q9EQV9
633	1,2,3,4,18,21,24,25	5,6,7,8,19,20,22,23,26	4	P01836,P08721,P31430,P98089
634	1,2,3,4,18,21,24,26	5,6,7,8,19,20,22,23,25	5	D3ZUC6,P01836,Q63661,Q99041,Q9EQV9
635	1,2,3,4,18,21,25,26	5,6,7,8,19,20,22,23,24	9	P00774,P01836,P08721,P19223,P25031,P31430,P55091,P98089,Q63661
636	1,2,3,4,18,22,23,24	5,6,7,8,19,20,21,25,26	4	P12785,P34901,P82471,Q9QZK9
637	1,2,3,4,18,22,23,25	5,6,7,8,19,20,21,24,26	2	P12785,P34901
638	1,2,3,4,18,22,23,26	5,6,7,8,19,20,21,24,25	9	P01946,P06760,P12785,P34901,Q4FZU2,Q63661,Q6AY61,Q6IFU8,Q6P6Q2
639	1,2,3,4,18,22,24,25	5,6,7,8,19,20,21,23,26	0	
640	1,2,3,4,18,22,24,26	5,6,7,8,19,20,21,23,25	4	P01836,P06760,Q99041,Q9WTT6
641	1,2,3,4,18,22,25,26	5,6,7,8,19,20,21,23,24	2	P01836,Q63661
642	1,2,3,4,18,23,24,25	5,6,7,8,19,20,21,22,26	2	P12785,P34901
643	1,2,3,4,18,23,24,26	5,6,7,8,19,20,21,22,25	12	A2RUV9,P01836,P01946,P06760,P12785,P34901,Q4FZU2,Q63661,Q6IFU8,Q6IFW6,Q6IG02,Q6P6Q2
644	1,2,3,4,18,23,25,26	5,6,7,8,19,20,21,22,24	13	P00774,P01946,P12785,P34901,P55314,Q4FZU2,Q63661,Q6AY61,Q6IFU8,Q6IFW6,Q6IG02,Q6IMF3,Q6P6Q2
645	1,2,3,4,18,24,25,26	5,6,7,8,19,20,21,22,23	6	P00774,P01836,P08721,P55091,Q62902,Q63661
646	1,2,3,4,19,20,21,22	5,6,7,8,18,23,24,25,26	1	P01836

647	1,2,3,4,19,20,21,23	5,6,7,8,18,22,24,25,26	2	P01836,P34901
648	1,2,3,4,19,20,21,24	5,6,7,8,18,22,23,25,26	1	P01836
649	1,2,3,4,19,20,21,25	5,6,7,8,18,22,23,24,26	2	P01836,P47967
650	1,2,3,4,19,20,21,26	5,6,7,8,18,22,23,24,25	3	P01836,Q63661,Q9EQV9
651	1,2,3,4,19,20,22,23	5,6,7,8,18,21,24,25,26	5	P22006,P34901,P55091,Q62997,Q9WTT6
652	1,2,3,4,19,20,22,24	5,6,7,8,18,21,23,25,26	7	P01836,P06760,P07150,P14669,P83121,Q62997,Q9WTT6
653	1,2,3,4,19,20,22,25	5,6,7,8,18,21,23,24,26	1	Q62997
654	1,2,3,4,19,20,22,26	5,6,7,8,18,21,23,24,25	7	O54728,P01836,P06760,P07150,P55159,Q62997,Q9WTT6
655	1,2,3,4,19,20,23,24	5,6,7,8,18,21,22,25,26	2	P34901,Q9QZK9
656	1,2,3,4,19,20,23,25	5,6,7,8,18,21,22,24,26	0	
657	1,2,3,4,19,20,23,26	5,6,7,8,18,21,22,24,25	6	O54728,P01836,P01946,P06760,P19218,Q63661
658	1,2,3,4,19,20,24,25	5,6,7,8,18,21,22,23,26	0	
659	1,2,3,4,19,20,24,26	5,6,7,8,18,21,22,23,25	3	P01836,P06760,Q9WTT6
660	1,2,3,4,19,20,25,26	5,6,7,8,18,21,22,23,24	2	P01836,P35280
661	1,2,3,4,19,21,22,23	5,6,7,8,18,20,24,25,26	1	P34901

662	1,2,3,4,19,21,22,24	5,6,7,8,18,20,23,25,26	1	P01836
663	1,2,3,4,19,21,22,25	5,6,7,8,18,20,23,24,26	1	P47967
664	1,2,3,4,19,21,22,26	5,6,7,8,18,20,23,24,25	3	P01836,P63036,Q63661
665	1,2,3,4,19,21,23,24	5,6,7,8,18,20,22,25,26	2	D3ZUC6,P34901
666	1,2,3,4,19,21,23,25	5,6,7,8,18,20,22,24,26	1	P34901
667	1,2,3,4,19,21,23,26	5,6,7,8,18,20,22,24,25	5	D3ZUC6,P01836,P01946,P34901,Q63661
668	1,2,3,4,19,21,24,25	5,6,7,8,18,20,22,23,26	0	
669	1,2,3,4,19,21,24,26	5,6,7,8,18,20,22,23,25	4	D3ZUC6,P01836,Q63661,Q9EQV9
670	1,2,3,4,19,21,25,26	5,6,7,8,18,20,22,23,24	3	P01836,P19223,Q63661
671	1,2,3,4,19,22,23,24	5,6,7,8,18,20,21,25,26	3	P34901,P82471,Q9WTT6
672	1,2,3,4,19,22,23,25	5,6,7,8,18,20,21,24,26	2	P34901,P82471
673	1,2,3,4,19,22,23,26	5,6,7,8,18,20,21,24,25	7	O54728,P01946,P06760,P34901,P55159,P82471,Q63661
674	1,2,3,4,19,22,24,25	5,6,7,8,18,20,21,23,26	1	P82471
675	1,2,3,4,19,22,24,26	5,6,7,8,18,20,21,23,25	4	O54728,P01836,P06760,Q9WTT6
676	1,2,3,4,19,22,25,26	5,6,7,8,18,20,21,23,24	0	

677	1,2,3,4,19,23,24,25	5,6,7,8,18,20,21,22,26	2	P34901,P82471
678	1,2,3,4,19,23,24,26	5,6,7,8,18,20,21,22,25	5	P01946,P02091,P34901,P82471,Q63661
679	1,2,3,4,19,23,25,26	5,6,7,8,18,20,21,22,24	4	P01946,P02091,P55314,Q63661
680	1,2,3,4,19,24,25,26	5,6,7,8,18,20,21,22,23	2	P01946,P14480
681	1,2,3,4,20,21,22,23	5,6,7,8,18,19,24,25,26	2	P01836,P34901
682	1,2,3,4,20,21,22,24	5,6,7,8,18,19,23,25,26	2	P01836,Q5XFX0
683	1,2,3,4,20,21,22,25	5,6,7,8,18,19,23,24,26	1	P01836
684	1,2,3,4,20,21,22,26	5,6,7,8,18,19,23,24,25	2	P01836,Q99041
685	1,2,3,4,20,21,23,24	5,6,7,8,18,19,22,25,26	2	P01836,P34901
686	1,2,3,4,20,21,23,25	5,6,7,8,18,19,22,24,26	2	P01836,P34901
687	1,2,3,4,20,21,23,26	5,6,7,8,18,19,22,24,25	6	P01836,P01946,P34901,Q4FZU2,Q63661,Q6IFU8
688	1,2,3,4,20,21,24,25	5,6,7,8,18,19,22,23,26	1	P01836
689	1,2,3,4,20,21,24,26	5,6,7,8,18,19,22,23,25	5	P01015,P01836,P80020,Q99041,Q9EQV9
690	1,2,3,4,20,21,25,26	5,6,7,8,18,19,22,23,24	2	P01836,P19223
691	1,2,3,4,20,22,23,24	5,6,7,8,18,19,21,25,26	5	P01836,P34901,P82995,Q62997,Q9QZK9

692	1,2,3,4,20,22,23,25	5,6,7,8,18,19,21,24,26	2	P34901,Q62997
693	1,2,3,4,20,22,23,26	5,6,7,8,18,19,21,24,25	6	O54728,P01836,P01946,P34901,P55159,Q62997
694	1,2,3,4,20,22,24,25	5,6,7,8,18,19,21,23,26	1	P01836
695	1,2,3,4,20,22,24,26	5,6,7,8,18,19,21,23,25	7	P01836,P06760,P07150,P08010,P36953,Q62997,Q99041
696	1,2,3,4,20,22,25,26	5,6,7,8,18,19,21,23,24	2	P01836,Q62997
697	1,2,3,4,20,23,24,25	5,6,7,8,18,19,21,22,26	1	P34901
698	1,2,3,4,20,23,24,26	5,6,7,8,18,19,21,22,25	5	P01836,P01946,P19218,P34901,P97840
699	1,2,3,4,20,23,25,26	5,6,7,8,18,19,21,22,24	4	P01836,P01946,P19218,P55314
700	1,2,3,4,20,24,25,26	5,6,7,8,18,19,21,22,23	1	P01836
701	1,2,3,4,21,22,23,24	5,6,7,8,18,19,20,25,26	2	P01836,P34901
702	1,2,3,4,21,22,23,25	5,6,7,8,18,19,20,24,26	1	P34901
703	1,2,3,4,21,22,23,26	5,6,7,8,18,19,20,24,25	4	P01836,P01946,P34901,Q63661
704	1,2,3,4,21,22,24,25	5,6,7,8,18,19,20,23,26	1	P01836
705	1,2,3,4,21,22,24,26	5,6,7,8,18,19,20,23,25	4	P01836,P80020,Q99041,Q9EQV9
706	1,2,3,4,21,22,25,26	5,6,7,8,18,19,20,23,24	1	P01836

707	1,2,3,4,21,23,24,25	5,6,7,8,18,19,20,22,26	3	P01836,P34901,Q63751
708	1,2,3,4,21,23,24,26	5,6,7,8,18,19,20,22,25	5	P01836,P01946,P34901,Q63661,Q9EQV9
709	1,2,3,4,21,23,25,26	5,6,7,8,18,19,20,22,24	5	P01836,P01946,P34901,P55314,Q63661
710	1,2,3,4,21,24,25,26	5,6,7,8,18,19,20,22,23	3	P01836,Q68FV8,Q9EQV9
711	1,2,3,4,22,23,24,25	5,6,7,8,18,19,20,21,26	2	P34901,P82471
712	1,2,3,4,22,23,24,26	5,6,7,8,18,19,20,21,25	7	O54728,P00731,P01836,P01946,P34901,P36953,P82471
713	1,2,3,4,22,23,25,26	5,6,7,8,18,19,20,21,24	4	P01946,P34901,P55314,P82471
714	1,2,3,4,22,24,25,26	5,6,7,8,18,19,20,21,23	3	P01836,P36953,P82471
715	1,2,3,4,23,24,25,26	5,6,7,8,18,19,20,21,22	7	P01946,P02091,P34901,P55314,P82471,Q62902,Q7TPB1
716	1,2,3,5,6,7,8,18	4,19,20,21,22,23,24,25,26	13	O55006,P00731,P02631,P02803,P10715,P16086,P25031,P52590,Q63942,Q6IRE4,Q8N7M5,Q91ZS3,Q9ET32
717	1,2,3,5,6,7,8,19	4,18,20,21,22,23,24,25,26	11	O55006,P00731,P02631,P16086,P52590,P53792,P97580,Q30KJ2,Q63532,Q6IRE4,Q8N7M5
718	1,2,3,5,6,7,8,20	4,18,19,21,22,23,24,25,26	10	O55006,P02631,P10715,P16086,P20762,P27213,P52590,Q6IRE4,Q91ZS3,Q9ET32
719	1,2,3,5,6,7,8,21	4,18,19,20,22,23,24,25,26	10	O55006,P00731,P02631,P16086,P52590,P53792,Q6IRE4,Q8N7M5,Q91ZS3,Q9ET32
720	1,2,3,5,6,7,8,22	4,18,19,20,21,23,24,25,26	9	P02631,P16086,P20766,P27213,P52590,Q6IRE4,Q8N7M5,Q9ET32,Q9QW07
721	1,2,3,5,6,7,8,23	4,18,19,20,21,22,24,25,26	11	P02631,P16086,P20762,P28648,P51907,P52590,P97580,Q30KJ2,Q6IRE4,Q8N7M5,Q9ET32

722	1,2,3,5,6,7,8,24	4,18,19,20,21,22,23,25,26	8	O55006,P02631,P10715,P52590,P83121,Q6IRE4,Q8N7M5,Q9ET32
723	1,2,3,5,6,7,8,25	4,18,19,20,21,22,23,24,26	16	P02631,P05371,P10715,P12020,P16086,P19629,P36374,P52590,P53792,P55314,P68370,Q5GRG2,Q6IRE4,Q8N7M5,Q91ZS3,Q9ET32
724	1,2,3,5,6,7,8,26	4,18,19,20,21,22,23,24,25	9	P01946,P02631,P10715,P20762,P30904,P52590,P83748,Q6IRE4,Q91ZS3
725	1,2,3,5,6,7,18,19	4,8,20,21,22,23,24,25,26	11	O55006,P00731,P02631,P16086,P25031,P52590,P53792,Q30KJ2,Q63532,Q63942,Q6IE51
726	1,2,3,5,6,7,18,20	4,8,19,21,22,23,24,25,26	8	P00731,P02631,P16086,P25031,P27213,P52590,Q6IRE4,Q91ZS3
727	1,2,3,5,6,7,18,21	4,8,19,20,22,23,24,25,26	10	O35783,P00731,P02631,P16086,P25031,P42854,P52590,P53792,Q6IRE4,Q91ZS3
728	1,2,3,5,6,7,18,22	4,8,19,20,21,23,24,25,26	6	P02631,P16086,P25031,P27213,P52590,Q9QW07
729	1,2,3,5,6,7,18,23	4,8,19,20,21,22,24,25,26	10	P02631,P16086,P25031,P28648,P34901,P52590,P97580,Q63942,Q6IE51,Q6IRE4
730	1,2,3,5,6,7,18,24	4,8,19,20,21,22,23,25,26	7	O55006,P02631,P25031,P27213,P52590,Q6IE51,Q6IRE4
731	1,2,3,5,6,7,18,25	4,8,19,20,21,22,23,24,26	11	P00731,P02631,P05371,P10715,P19629,P25031,P52590,P55314,P61206;P84079;P84082,Q6IRE4,Q91ZS3
732	1,2,3,5,6,7,18,26	4,8,19,20,21,22,23,24,25	12	O35783,P01946,P02631,P16636,P25031,P30904,P52590,P83748,Q63661,Q63942,Q6IRE4,Q91ZS3
733	1,2,3,5,6,7,19,20	4,8,18,21,22,23,24,25,26	11	P00714,P02631,P07150,P16086,P27213,P52590,P53792,Q30KJ2,Q63532,Q6IE51,Q6IRE4
734	1,2,3,5,6,7,19,21	4,8,18,20,22,23,24,25,26	11	P00714,P00731,P02631,P16086,P52590,P53792,Q30KJ2,Q63532,Q6IE51,Q6IRE4,Q8N7M5
735	1,2,3,5,6,7,19,22	4,8,18,20,21,23,24,25,26	10	P00714,P02631,P16086,P17046,P27213,P52590,Q30KJ2,Q63532,Q8N7M5,Q9QW07
736	1,2,3,5,6,7,19,23	4,8,18,20,21,22,24,25,26	8	P02631,P15399,P16086,P52590,P97580,Q30KJ2,Q63532,Q6IE51

737	1,2,3,5,6,7,19,24	4,8,18,20,21,22,23,25,26	11	P00714,P02631,P16086,P30904,P52590,P53792,Q30KJ2,Q63532,Q6IE51,Q6IRE4,Q8N7M5
738	1,2,3,5,6,7,19,25	4,8,18,20,21,22,23,24,26	14	P02631,P12020,P16086,P17046,P52590,P53792,P55314,P97580,P97840,Q30KJ2,Q63532,Q6IE51,Q8N7M5,Q91ZS3
739	1,2,3,5,6,7,19,26	4,8,18,20,21,22,23,24,25	10	P01946,P02631,P16086,P30904,P52590,P53792,P83748,Q30KJ2,Q63532,Q6IE51
740	1,2,3,5,6,7,20,21	4,8,18,19,22,23,24,25,26	9	P00714,P00731,P02631,P16086,P42854,P52590,P53792,Q6IRE4,Q91ZS3
741	1,2,3,5,6,7,20,22	4,8,18,19,21,23,24,25,26	8	P00714,P02631,P07150,P16086,P27213,P52590,Q6IRE4,Q9QW07
742	1,2,3,5,6,7,20,23	4,8,18,19,21,22,24,25,26	6	P02631,P16086,P52590,P97580,Q6IRE4,Q91ZS3
743	1,2,3,5,6,7,20,24	4,8,18,19,21,22,23,25,26	6	P00714,P02631,P08010,P27213,P52590,Q6IRE4
744	1,2,3,5,6,7,20,25	4,8,18,19,21,22,23,24,26	7	P02631,P10715,P12020,P52590,P55314,Q6IRE4,Q91ZS3
745	1,2,3,5,6,7,20,26	4,8,18,19,21,22,23,24,25	7	P01681,P01946,P30904,P52590,P83748,Q6IRE4,Q91ZS3
746	1,2,3,5,6,7,21,22	4,8,18,19,20,23,24,25,26	6	P00714,P16086,P19218,P52590,Q6IRE4,Q9QW07
747	1,2,3,5,6,7,21,23	4,8,18,19,20,22,24,25,26	8	P02631,P16086,P34901,P52590,P97580,Q30KJ2,Q6IRE4,Q91ZS3
748	1,2,3,5,6,7,21,24	4,8,18,19,20,22,23,25,26	6	P00714,P02631,P13432,P52590,P53792,Q6IRE4
749	1,2,3,5,6,7,21,25	4,8,18,19,20,22,23,24,26	7	P02631,P19629,P52590,P53792,P55314,Q6IRE4,Q91ZS3
750	1,2,3,5,6,7,21,26	4,8,18,19,20,22,23,24,25	8	O35783,P01946,P30904,P52590,P53792,P83748,Q6IRE4,Q91ZS3
751	1,2,3,5,6,7,22,23	4,8,18,19,20,21,24,25,26	6	P02631,P16086,P34901,P52590,P97580,Q9QW07

752	1,2,3,5,6,7,22,24	4,8,18,19,20,21,23,25,26	8	P00714,P02631,P08010,P27213,P52590,Q6IRE4,Q8N7M5,Q9QW07
753	1,2,3,5,6,7,22,25	4,8,18,19,20,21,23,24,26	9	P06911,P12020,P17046,P19629,P52590,P55314,Q5GRG2,Q91ZS3,Q9QW07
754	1,2,3,5,6,7,22,26	4,8,18,19,20,21,23,24,25	5	O54728,P01946,P52590,P83748,Q9QW07
755	1,2,3,5,6,7,23,24	4,8,18,19,20,21,22,25,26	9	P02631,P16086,P34901,P36374,P52590,P97580,Q30KJ2,Q6IE51,Q6IRE4
756	1,2,3,5,6,7,23,25	4,8,18,19,20,21,22,24,26	15	P02631,P05371,P12020,P16086,P17046,P19629,P36374,P52590,P55314,P97580,Q30KJ2,Q5GRG2,Q63751,Q6IRE4,Q91ZS3
757	1,2,3,5,6,7,23,26	4,8,18,19,20,21,22,24,25	14	P01946,P02091,P02631,P16086,P28648,P34901,P36376,P52590,P55314,P83748,P97580,Q30KJ2,Q4FZU2,Q6IRE4
758	1,2,3,5,6,7,24,25	4,8,18,19,20,21,22,23,26	9	P02631,P12020,P19629,P36374,P52590,P55314,Q6IRE4,Q8N7M5,Q91ZS3
759	1,2,3,5,6,7,24,26	4,8,18,19,20,21,22,23,25	8	P01946,P02091,P08010,P30904,P52590,P83748,Q6IRE4,Q8CJD3
760	1,2,3,5,6,7,25,26	4,8,18,19,20,21,22,23,24	9	P01946,P02091,P19629,P30904,P52590,P55314,P83748,Q6IRE4,Q91ZS3
761	1,2,3,5,6,8,18,19	4,7,20,21,22,23,24,25,26	8	O55006,P00731,P02803,P22006,P53792,P83121,Q63942,Q9Z1F2
762	1,2,3,5,6,8,18,20	4,7,19,21,22,23,24,25,26	6	P00731,P22006,P27213,P83121,Q63942,Q6IRE4
763	1,2,3,5,6,8,18,21	4,7,19,20,22,23,24,25,26	4	O35547,P00731,P25031,Q63942
764	1,2,3,5,6,8,18,22	4,7,19,20,21,23,24,25,26	5	P22006,P27213,P83121,Q63942,Q9QW07
765	1,2,3,5,6,8,18,23	4,7,19,20,21,22,24,25,26	6	P22006,P28648,P51907,Q5XI22,Q63942,Q9Z1F2
766	1,2,3,5,6,8,18,24	4,7,19,20,21,22,23,25,26	3	O55006,P83121,Q63942

767	1,2,3,5,6,8,18,25	4,7,19,20,21,22,23,24,26	4	P55314,Q63942,Q91ZS3,Q9Z1F2
768	1,2,3,5,6,8,18,26	4,7,19,20,21,22,23,24,25	2	Q4FZU2,Q63942
769	1,2,3,5,6,8,19,20	4,7,18,21,22,23,24,25,26	4	P22006,P53792,P83121,Q6IRE4
770	1,2,3,5,6,8,19,21	4,7,18,20,22,23,24,25,26	3	P00731,P35281,P53792
771	1,2,3,5,6,8,19,22	4,7,18,20,21,23,24,25,26	5	P00714,P08753,P22006,P83121,Q9QW07
772	1,2,3,5,6,8,19,23	4,7,18,20,21,22,24,25,26	6	P15399,P22006,P51907,P97580,Q30KJ2,Q9Z1F2
773	1,2,3,5,6,8,19,24	4,7,18,20,21,22,23,25,26	1	P83121
774	1,2,3,5,6,8,19,25	4,7,18,20,21,22,23,24,26	4	P08753,P53792,P55314,Q9Z1F2
775	1,2,3,5,6,8,19,26	4,7,18,20,21,22,23,24,25	0	
776	1,2,3,5,6,8,20,21	4,7,18,19,22,23,24,25,26	5	P00714,P01681,P62804,Q6IRE4,Q91ZS3
777	1,2,3,5,6,8,20,22	4,7,18,19,21,23,24,25,26	8	P00714,P08753,P22006,P27213,P82995,P83121,Q6IRE4,Q9QW07
778	1,2,3,5,6,8,20,23	4,7,18,19,21,22,24,25,26	3	P22006,P82995,Q6IRE4
779	1,2,3,5,6,8,20,24	4,7,18,19,21,22,23,25,26	6	P00714,P22006,P27213,P82995,P83121,Q6IRE4
780	1,2,3,5,6,8,20,25	4,7,18,19,21,22,23,24,26	3	P55314,Q6IRE4,Q91ZS3
781	1,2,3,5,6,8,20,26	4,7,18,19,21,22,23,24,25	4	P01681,P22006,P62804,Q6IRE4

782	1,2,3,5,6,8,21,22	4,7,18,19,20,23,24,25,26	2	P00714,Q9QW07
783	1,2,3,5,6,8,21,23	4,7,18,19,20,22,24,25,26	3	P34901,P51907,Q9Z1F2
784	1,2,3,5,6,8,21,24	4,7,18,19,20,22,23,25,26	4	P00714,P13432,P83121,Q6IRE4
785	1,2,3,5,6,8,21,25	4,7,18,19,20,22,23,24,26	4	P06760,P55314,Q91ZS3,Q9WTT6
786	1,2,3,5,6,8,21,26	4,7,18,19,20,22,23,24,25	2	P01681,Q00715
787	1,2,3,5,6,8,22,23	4,7,18,19,20,21,24,25,26	5	P08753,P22006,P51907,Q9QW07,Q9Z1F2
788	1,2,3,5,6,8,22,24	4,7,18,19,20,21,23,25,26	5	P00714,P08753,P27213,P83121,Q9QW07
789	1,2,3,5,6,8,22,25	4,7,18,19,20,21,23,24,26	5	P06911,P08753,P20766,P55314,Q9QW07
790	1,2,3,5,6,8,22,26	4,7,18,19,20,21,23,24,25	3	P08753,P20766,Q9QW07
791	1,2,3,5,6,8,23,24	4,7,18,19,20,21,22,25,26	4	P34901,P36374,P51907,P82995
792	1,2,3,5,6,8,23,25	4,7,18,19,20,21,22,24,26	5	P08753,P36374,P51907,P55314,Q9Z1F2
793	1,2,3,5,6,8,23,26	4,7,18,19,20,21,22,24,25	5	P01946,P51907,Q4FZU2,Q5I0D1,Q6IFU8
794	1,2,3,5,6,8,24,25	4,7,18,19,20,21,22,23,26	3	P08753,P36374,P55314
795	1,2,3,5,6,8,24,26	4,7,18,19,20,21,22,23,25	0	
796	1,2,3,5,6,8,25,26	4,7,18,19,20,21,22,23,24	3	P08753,P55314,Q91ZS3

797	1,2,3,5,6,18,19,20	4,7,8,21,22,23,24,25,26	4	P00731,P22006,P83121,Q02253
798	1,2,3,5,6,18,19,21	4,7,8,20,22,23,24,25,26	4	P00731,P25031,P53792,Q63942
799	1,2,3,5,6,18,19,22	4,7,8,20,21,23,24,25,26	4	P22006,P83121,Q63942,Q9QW07
800	1,2,3,5,6,18,19,23	4,7,8,20,21,22,24,25,26	7	P15399,P22006,P97580,Q03191,Q30KJ2,Q63942,Q9Z1F2
801	1,2,3,5,6,18,19,24	4,7,8,20,21,22,23,25,26	2	P83121,Q63942
802	1,2,3,5,6,18,19,25	4,7,8,20,21,22,23,24,26	3	P00731,P14173,P55314
803	1,2,3,5,6,18,19,26	4,7,8,20,21,22,23,24,25	2	Q63661,Q63942
804	1,2,3,5,6,18,20,21	4,7,8,19,22,23,24,25,26	6	P00731,P01681,P01835,P25031,Q6IRE4,Q91ZS3
805	1,2,3,5,6,18,20,22	4,7,8,19,21,23,24,25,26	6	P07150,P22006,P27213,P83121,Q99041,Q9QW07
806	1,2,3,5,6,18,20,23	4,7,8,19,21,22,24,25,26	4	P22006,Q4FZU2,Q63942,Q6IRE4
807	1,2,3,5,6,18,20,24	4,7,8,19,21,22,23,25,26	5	P22006,P27213,P83121,Q6IRE4,Q99041
808	1,2,3,5,6,18,20,25	4,7,8,19,21,22,23,24,26	3	P55314,Q6IRE4,Q91ZS3
809	1,2,3,5,6,18,20,26	4,7,8,19,21,22,23,24,25	6	P01681,P22006,Q4FZU2,Q63942,Q6IRE4,Q99041
810	1,2,3,5,6,18,21,22	4,7,8,19,20,23,24,25,26	3	P00714,P25031,Q9QW07
811	1,2,3,5,6,18,21,23	4,7,8,19,20,22,24,25,26	3	P25031,P34901,Q63942

812	1,2,3,5,6,18,21,24	4,7,8,19,20,22,23,25,26	2	P00714,P25031
813	1,2,3,5,6,18,21,25	4,7,8,19,20,22,23,24,26	6	P00731,P25031,P31430,P55314,P98089,Q91ZS3
814	1,2,3,5,6,18,21,26	4,7,8,19,20,22,23,24,25	8	P00774,P25031,P83748,Q4FZU2,Q63661,Q63942,Q99041,Q9QUL6
815	1,2,3,5,6,18,22,23	4,7,8,19,20,21,24,25,26	4	P22006,P34901,Q63942,Q9QW07
816	1,2,3,5,6,18,22,24	4,7,8,19,20,21,23,25,26	5	P00714,P27213,P83121,Q99041,Q9QW07
817	1,2,3,5,6,18,22,25	4,7,8,19,20,21,23,24,26	2	P55314,Q9QW07
818	1,2,3,5,6,18,22,26	4,7,8,19,20,21,23,24,25	3	Q63942,Q99041,Q9QW07
819	1,2,3,5,6,18,23,24	4,7,8,19,20,21,22,25,26	2	P34901,Q63942
820	1,2,3,5,6,18,23,25	4,7,8,19,20,21,22,24,26	2	P55314,Q63942
821	1,2,3,5,6,18,23,26	4,7,8,19,20,21,22,24,25	13	P01946,P28648,P34901,Q4FZU2,Q63661,Q63942,Q6IFU7,Q6IFU8,Q6IFW6,Q6IG02,Q6IMF3,Q6P6Q2,Q9QUL6
822	1,2,3,5,6,18,24,25	4,7,8,19,20,21,22,23,26	1	P55314
823	1,2,3,5,6,18,24,26	4,7,8,19,20,21,22,23,25	2	Q63942,Q99041
824	1,2,3,5,6,18,25,26	4,7,8,19,20,21,22,23,24	6	P00774,P55314,Q4FZU2,Q63661,Q63942,Q91ZS3
825	1,2,3,5,6,19,20,21	4,7,8,18,22,23,24,25,26	5	P00714,P00731,P01681,P53792,Q6IRE4
826	1,2,3,5,6,19,20,22	4,7,8,18,21,23,24,25,26	9	P00714,P07150,P14669,P22006,P27213,P55091,P82995,P83121,Q9QW07

827	1,2,3,5,6,19,20,23	4,7,8,18,21,22,24,25,26	6	P15399,P22006,P55091,P82995,P97580,Q30KJ2
828	1,2,3,5,6,19,20,24	4,7,8,18,21,22,23,25,26	8	P00714,P07150,P22006,P27213,P82995,P83121,Q6IRE4,Q80WL1
829	1,2,3,5,6,19,20,25	4,7,8,18,21,22,23,24,26	1	P55314
830	1,2,3,5,6,19,20,26	4,7,8,18,21,22,23,24,25	3	P00714,P01681,P22006
831	1,2,3,5,6,19,21,22	4,7,8,18,20,23,24,25,26	2	P00714,Q9QW07
832	1,2,3,5,6,19,21,23	4,7,8,18,20,22,24,25,26	5	P00714,P15399,P34901,P97580,Q30KJ2
833	1,2,3,5,6,19,21,24	4,7,8,18,20,22,23,25,26	2	P00714,P53792
834	1,2,3,5,6,19,21,25	4,7,8,18,20,22,23,24,26	3	P53792,P55314,P97840
835	1,2,3,5,6,19,21,26	4,7,8,18,20,22,23,24,25	4	P00714,P01681,P53792,Q63661
836	1,2,3,5,6,19,22,23	4,7,8,18,20,21,24,25,26	9	P00714,P15399,P22006,P34901,P55091,P82995,P97580,Q30KJ2,Q9QW07
837	1,2,3,5,6,19,22,24	4,7,8,18,20,21,23,25,26	7	P00714,P07150,P14669,P27213,P82995,P83121,Q9QW07
838	1,2,3,5,6,19,22,25	4,7,8,18,20,21,23,24,26	4	P17046,P55314,Q4FZU6,Q9QW07
839	1,2,3,5,6,19,22,26	4,7,8,18,20,21,23,24,25	5	O54728,P00714,P55159,Q9QW07,Q9R044
840	1,2,3,5,6,19,23,24	4,7,8,18,20,21,22,25,26	6	P00714,P15399,P34901,P82995,P97580,Q30KJ2
841	1,2,3,5,6,19,23,25	4,7,8,18,20,21,22,24,26	5	P15399,P17046,P55314,P97580,Q30KJ2

842	1,2,3,5,6,19,23,26	4,7,8,18,20,21,22,24,25	6	P01946,P15399,P97580,Q30KJ2,Q63661,Q6IFU8
843	1,2,3,5,6,19,24,25	4,7,8,18,20,21,22,23,26	2	P55314,Q4FZU6
844	1,2,3,5,6,19,24,26	4,7,8,18,20,21,22,23,25	2	P00714,Q9R044
845	1,2,3,5,6,19,25,26	4,7,8,18,20,21,22,23,24	1	P55314
846	1,2,3,5,6,20,21,22	4,7,8,18,19,23,24,25,26	5	P00714,P01681,P27213,Q6IRE4,Q9QW07
847	1,2,3,5,6,20,21,23	4,7,8,18,19,22,24,25,26	5	P00714,P01681,P01835,P34901,Q6IRE4
848	1,2,3,5,6,20,21,24	4,7,8,18,19,22,23,25,26	3	P00714,Q5XFX0,Q6IRE4
849	1,2,3,5,6,20,21,25	4,7,8,18,19,22,23,24,26	3	P55314,Q6IRE4,Q91ZS3
850	1,2,3,5,6,20,21,26	4,7,8,18,19,22,23,24,25	5	P00714,P01681,P62804,Q6IRE4,Q99041
851	1,2,3,5,6,20,22,23	4,7,8,18,19,21,24,25,26	7	P00714,P22006,P27213,P34901,P55091,P82995,Q9QW07
852	1,2,3,5,6,20,22,24	4,7,8,18,19,21,23,25,26	10	P00714,P07150,P14669,P22006,P27213,P82995,P83121,Q6IRE4,Q99041,Q9QW07
853	1,2,3,5,6,20,22,25	4,7,8,18,19,21,23,24,26	2	P55314,Q9QW07
854	1,2,3,5,6,20,22,26	4,7,8,18,19,21,23,24,25	6	O54728,P00714,P01681,P22006,Q99041,Q9QW07
855	1,2,3,5,6,20,23,24	4,7,8,18,19,21,22,25,26	5	P00714,P34901,P82995,Q6IRE4,Q80WL1
856	1,2,3,5,6,20,23,25	4,7,8,18,19,21,22,24,26	4	P36374,P55314,P82995,Q6IRE4

857	1,2,3,5,6,20,23,26	4,7,8,18,19,21,22,24,25	8	P01681,P01946,Q4FZU2,Q6IFU8,Q6IFW6,Q6IG02,Q6IMF3,Q6IRE4
858	1,2,3,5,6,20,24,25	4,7,8,18,19,21,22,23,26	3	P55314,Q6IRE4,Q80WL1
859	1,2,3,5,6,20,24,26	4,7,8,18,19,21,22,23,25	3	P00714,Q6IRE4,Q99041
860	1,2,3,5,6,20,25,26	4,7,8,18,19,21,22,23,24	3	P55314,Q6IRE4,Q91ZS3
861	1,2,3,5,6,21,22,23	4,7,8,18,19,20,24,25,26	3	P00714,P34901,Q9QW07
862	1,2,3,5,6,21,22,24	4,7,8,18,19,20,23,25,26	4	P00714,P13432,Q5XFX0,Q9QW07
863	1,2,3,5,6,21,22,25	4,7,8,18,19,20,23,24,26	2	P55314,Q9QW07
864	1,2,3,5,6,21,22,26	4,7,8,18,19,20,23,24,25	3	P00714,Q99041,Q9QW07
865	1,2,3,5,6,21,23,24	4,7,8,18,19,20,22,25,26	2	P00714,P34901
866	1,2,3,5,6,21,23,25	4,7,8,18,19,20,22,24,26	4	P34901,P55314,Q6P6R2,Q91ZS3
867	1,2,3,5,6,21,23,26	4,7,8,18,19,20,22,24,25	8	P01681,P01946,P34901,Q4FZU2,Q63661,Q6IFU8,Q6IFV1,Q6IMF3
868	1,2,3,5,6,21,24,25	4,7,8,18,19,20,22,23,26	1	P55314
869	1,2,3,5,6,21,24,26	4,7,8,18,19,20,22,23,25	3	P00714,Q8CJD3,Q99041
870	1,2,3,5,6,21,25,26	4,7,8,18,19,20,22,23,24	2	P55314,Q91ZS3
871	1,2,3,5,6,22,23,24	4,7,8,18,19,20,21,25,26	4	P00714,P34901,P82995,Q9QW07

872	1,2,3,5,6,22,23,25	4,7,8,18,19,20,21,24,26	5	P06911,P34901,P55314,Q4FZU6,Q9QW07
873	1,2,3,5,6,22,23,26	4,7,8,18,19,20,21,24,25	4	O54728,P01946,P34901,Q9QW07
874	1,2,3,5,6,22,24,25	4,7,8,18,19,20,21,23,26	3	P55314,Q4FZU6,Q9QW07
875	1,2,3,5,6,22,24,26	4,7,8,18,19,20,21,23,25	3	P00714,Q99041,Q9QW07
876	1,2,3,5,6,22,25,26	4,7,8,18,19,20,21,23,24	2	P55314,Q9QW07
877	1,2,3,5,6,23,24,25	4,7,8,18,19,20,21,22,26	5	P34901,P36374,P55314,Q4FZU6,Q63751
878	1,2,3,5,6,23,24,26	4,7,8,18,19,20,21,22,25	5	P01946,P02091,P34901,P47967,Q6IFU8
879	1,2,3,5,6,23,25,26	4,7,8,18,19,20,21,22,24	5	P01946,P02091,P55314,Q4FZU2,Q6IFU8
880	1,2,3,5,6,24,25,26	4,7,8,18,19,20,21,22,23	4	P01946,P55314,Q62902,Q8CJD3
881	1,2,3,5,7,8,18,19	4,6,20,21,22,23,24,25,26	9	P00731,P02803,P52590,P53792,P83121,Q03191,Q09030,Q63532,Q63942
882	1,2,3,5,7,8,18,20	4,6,19,21,22,23,24,25,26	5	P10715,P27213,P52590,P83121,Q6IRE4
883	1,2,3,5,7,8,18,21	4,6,19,20,22,23,24,25,26	9	P00731,P02803,P25031,P42854,P52590,P83121,Q09030,Q63467,Q6IRE4
884	1,2,3,5,7,8,18,22	4,6,19,20,21,23,24,25,26	5	P20766,P27213,P52590,P83121,Q9QW07
885	1,2,3,5,7,8,18,23	4,6,19,20,21,22,24,25,26	7	P02803,P28648,P52590,Q03191,Q09030,Q5XI22,Q63942
886	1,2,3,5,7,8,18,24	4,6,19,20,21,22,23,25,26	4	P10715,P52590,P83121,Q09030

887	1,2,3,5,7,8,18,25	4,6,19,20,21,22,23,24,26	5	P02803,P10715,P19629,P52590,Q09030
888	1,2,3,5,7,8,18,26	4,6,19,20,21,22,23,24,25	6	P01946,P10715,P16636,P52590,Q63942,Q9R1T1
889	1,2,3,5,7,8,19,20	4,6,18,21,22,23,24,25,26	5	P27213,P52590,P53792,P83121,Q6IRE4
890	1,2,3,5,7,8,19,21	4,6,18,20,22,23,24,25,26	5	P00731,P52590,P53792,P83121,Q09030
891	1,2,3,5,7,8,19,22	4,6,18,20,21,23,24,25,26	5	P20766,P27213,P52590,P83121,Q9QW07
892	1,2,3,5,7,8,19,23	4,6,18,20,21,22,24,25,26	5	P52590,P97580,Q03191,Q09030,Q63532
893	1,2,3,5,7,8,19,24	4,6,18,20,21,22,23,25,26	4	P52590,P83121,Q09030,Q63532
894	1,2,3,5,7,8,19,25	4,6,18,20,21,22,23,24,26	6	P10715,P19629,P52590,P53792,Q09030,Q6IFU7
895	1,2,3,5,7,8,19,26	4,6,18,20,21,22,23,24,25	4	O54728,P01946,P52590,Q09030
896	1,2,3,5,7,8,20,21	4,6,18,19,22,23,24,25,26	3	P42854,P52590,Q6IRE4
897	1,2,3,5,7,8,20,22	4,6,18,19,21,23,24,25,26	5	P27213,P52590,P83121,Q6IRE4,Q9QW07
898	1,2,3,5,7,8,20,23	4,6,18,19,21,22,24,25,26	2	P52590,Q6IRE4
899	1,2,3,5,7,8,20,24	4,6,18,19,21,22,23,25,26	5	P10715,P27213,P52590,P83121,Q6IRE4
900	1,2,3,5,7,8,20,25	4,6,18,19,21,22,23,24,26	4	P10715,P19629,P52590,Q6IRE4
901	1,2,3,5,7,8,20,26	4,6,18,19,21,22,23,24,25	4	P01946,P10715,P52590,Q6IRE4

902	1,2,3,5,7,8,21,22	4,6,18,19,20,23,24,25,26	3	P52590,P83121,Q9QW07
903	1,2,3,5,7,8,21,23	4,6,18,19,20,22,24,25,26	4	P34901,P52590,Q09030,Q6IRE4
904	1,2,3,5,7,8,21,24	4,6,18,19,20,22,23,25,26	3	P52590,P83121,Q6IRE4
905	1,2,3,5,7,8,21,25	4,6,18,19,20,22,23,24,26	5	P10715,P19629,P52590,Q6IFU7,Q6IRE4
906	1,2,3,5,7,8,21,26	4,6,18,19,20,22,23,24,25	4	P01946,P52590,P83748,Q6IRE4
907	1,2,3,5,7,8,22,23	4,6,18,19,20,21,24,25,26	3	P20766,P52590,Q9QW07
908	1,2,3,5,7,8,22,24	4,6,18,19,20,21,23,25,26	5	P08010,P27213,P52590,P83121,Q9QW07
909	1,2,3,5,7,8,22,25	4,6,18,19,20,21,23,24,26	6	P10715,P19629,P20766,P52590,Q5GRG2,Q9QW07
910	1,2,3,5,7,8,22,26	4,6,18,19,20,21,23,24,25	6	O54728,P01946,P20766,P52590,P55159,Q9QW07
911	1,2,3,5,7,8,23,24	4,6,18,19,20,21,22,25,26	4	P34901,P52590,P83121,Q09030
912	1,2,3,5,7,8,23,25	4,6,18,19,20,21,22,24,26	6	P10715,P19629,P36374,P52590,P55314,Q09030
913	1,2,3,5,7,8,23,26	4,6,18,19,20,21,22,24,25	5	O54728,P01946,P02091,P52590,Q09030
914	1,2,3,5,7,8,24,25	4,6,18,19,20,21,22,23,26	3	P10715,P19629,P52590
915	1,2,3,5,7,8,24,26	4,6,18,19,20,21,22,23,25	5	P01946,P02091,P08010,P52590,Q8CJD3
916	1,2,3,5,7,8,25,26	4,6,18,19,20,21,22,23,24	5	P01946,P02091,P10715,P19629,P52590

917	1,2,3,5,7,18,19,20	4,6,8,21,22,23,24,25,26	6	P07150,P27213,P52590,P83121,Q6IRE4,Q9WTT6
918	1,2,3,5,7,18,19,21	4,6,8,20,22,23,24,25,26	7	P00731,P14668,P25031,P52590,P53792,Q03191,Q09030
919	1,2,3,5,7,18,19,22	4,6,8,20,21,23,24,25,26	8	P07150,P14669,P27213,P52590,P83121,Q6P6R2,Q9QW07,Q9WTT6
920	1,2,3,5,7,18,19,23	4,6,8,20,21,22,24,25,26	5	P52590,Q03191,Q09030,Q63532,Q6IE51
921	1,2,3,5,7,18,19,24	4,6,8,20,21,22,23,25,26	7	P07150,P52590,P83121,Q03191,Q09030,Q63532,Q9WTT6
922	1,2,3,5,7,18,19,25	4,6,8,20,21,22,23,24,26	3	P17046,P52590,Q09030
923	1,2,3,5,7,18,19,26	4,6,8,20,21,22,23,24,25	8	P01946,P06760,P16636,P52590,Q03191,Q63661,Q63942,Q9WTT6
924	1,2,3,5,7,18,20,21	4,6,8,19,22,23,24,25,26	5	P00731,P25031,P42854,P52590,Q6IRE4
925	1,2,3,5,7,18,20,22	4,6,8,19,21,23,24,25,26	9	P07150,P14669,P27213,P52590,P83121,Q6IRE4,Q6P6R2,Q9QW07,Q9WTT6
926	1,2,3,5,7,18,20,23	4,6,8,19,21,22,24,25,26	3	P52590,Q6IRE4,Q9QZK9
927	1,2,3,5,7,18,20,24	4,6,8,19,21,22,23,25,26	7	P07150,P27213,P52590,P83121,Q6IRE4,Q9QZK9,Q9WTT6
928	1,2,3,5,7,18,20,25	4,6,8,19,21,22,23,24,26	3	P10715,P52590,Q6IRE4
929	1,2,3,5,7,18,20,26	4,6,8,19,21,22,23,24,25	4	P01946,P06760,P52590,Q6IRE4
930	1,2,3,5,7,18,21,22	4,6,8,19,20,23,24,25,26	3	P25031,P52590,Q9QW07
931	1,2,3,5,7,18,21,23	4,6,8,19,20,22,24,25,26	7	P14668,P25031,P34901,P52590,Q09030,Q63467,Q6IRE4

932	1,2,3,5,7,18,21,24	4,6,8,19,20,22,23,25,26	3	P25031,P52590,Q6IRE4
933	1,2,3,5,7,18,21,25	4,6,8,19,20,22,23,24,26	4	P25031,P31430,P52590,Q6IRE4
934	1,2,3,5,7,18,21,26	4,6,8,19,20,22,23,24,25	7	O35783,P01946,P25031,P52590,P82995,P83748,Q63661
935	1,2,3,5,7,18,22,23	4,6,8,19,20,21,24,25,26	3	P34901,P52590,Q9QW07
936	1,2,3,5,7,18,22,24	4,6,8,19,20,21,23,25,26	7	P07150,P14669,P27213,P52590,P83121,Q9QW07,Q9WTT6
937	1,2,3,5,7,18,22,25	4,6,8,19,20,21,23,24,26	3	P17046,P52590,Q9QW07
938	1,2,3,5,7,18,22,26	4,6,8,19,20,21,23,24,25	8	O54728,P01946,P06760,P16636,P52590,P55159,Q6P6R2,Q9QW07
939	1,2,3,5,7,18,23,24	4,6,8,19,20,21,22,25,26	3	P34901,P52590,Q09030
940	1,2,3,5,7,18,23,25	4,6,8,19,20,21,22,24,26	4	P52590,P55314,Q09030,Q99MH3
941	1,2,3,5,7,18,23,26	4,6,8,19,20,21,22,24,25	12	P01946,P02091,P16636,P28648,P34901,P52590,Q07523,Q63661,Q63942,Q6IFU8,Q6IG02,Q6P6Q2
942	1,2,3,5,7,18,24,25	4,6,8,19,20,21,22,23,26	1	P52590
943	1,2,3,5,7,18,24,26	4,6,8,19,20,21,22,23,25	6	P01946,P02091,P16636,P52590,Q63942,Q8CJD3
944	1,2,3,5,7,18,25,26	4,6,8,19,20,21,22,23,24	6	P01946,P02091,P52590,P55091,P82995,Q63661
945	1,2,3,5,7,19,20,21	4,6,8,18,22,23,24,25,26	5	P00714,P42854,P52590,P53792,Q6IRE4
946	1,2,3,5,7,19,20,22	4,6,8,18,21,23,24,25,26	10	P00714,P00774,P07150,P14669,P27213,P52590,P83121,Q6P6R2,Q9QW07,Q9WTT6

947	1,2,3,5,7,19,20,23	4,6,8,18,21,22,24,25,26	2	P52590,Q6IRE4
948	1,2,3,5,7,19,20,24	4,6,8,18,21,22,23,25,26	8	P00714,P07150,P14669,P27213,P52590,P83121,Q6IRE4,Q9WTT6
949	1,2,3,5,7,19,20,25	4,6,8,18,21,22,23,24,26	3	P17046,P52590,Q6IRE4
950	1,2,3,5,7,19,20,26	4,6,8,18,21,22,23,24,25	5	O54728,P01946,P07150,P52590,Q6IRE4
951	1,2,3,5,7,19,21,22	4,6,8,18,20,23,24,25,26	4	P00714,P17046,P52590,Q9QW07
952	1,2,3,5,7,19,21,23	4,6,8,18,20,22,24,25,26	4	P14668,P34901,P52590,Q09030
953	1,2,3,5,7,19,21,24	4,6,8,18,20,22,23,25,26	3	P00714,P52590,P53792
954	1,2,3,5,7,19,21,25	4,6,8,18,20,22,23,24,26	5	P17046,P47967,P52590,P53792,P97840
955	1,2,3,5,7,19,21,26	4,6,8,18,20,22,23,24,25	5	P01946,P52590,P53792,P83748,Q63661
956	1,2,3,5,7,19,22,23	4,6,8,18,20,21,24,25,26	5	P17046,P52590,Q63532,Q9QW07,Q9WTT6
957	1,2,3,5,7,19,22,24	4,6,8,18,20,21,23,25,26	11	P00714,P07150,P14669,P17046,P27213,P52590,P83121,Q63532,Q6P6R2,Q9QW07,Q9WTT6
958	1,2,3,5,7,19,22,25	4,6,8,18,20,21,23,24,26	4	P08644,P17046,P52590,Q9QW07
959	1,2,3,5,7,19,22,26	4,6,8,18,20,21,23,24,25	8	O54728,P01946,P07150,P52590,P55159,Q6P6R2,Q9QW07,Q9WTT6
960	1,2,3,5,7,19,23,24	4,6,8,18,20,21,22,25,26	5	P34901,P52590,Q09030,Q63532,Q9WTT6
961	1,2,3,5,7,19,23,25	4,6,8,18,20,21,22,24,26	6	P17046,P52590,P55314,P97580,Q09030,Q63532

962	1,2,3,5,7,19,23,26	4,6,8,18,20,21,22,24,25	7	O54728,P01946,P02091,P52590,Q09030,Q63532,Q63661
963	1,2,3,5,7,19,24,25	4,6,8,18,20,21,22,23,26	3	P17046,P52590,Q63532
964	1,2,3,5,7,19,24,26	4,6,8,18,20,21,22,23,25	5	P01946,P02091,P52590,Q63532,Q9WTT6
965	1,2,3,5,7,19,25,26	4,6,8,18,20,21,22,23,24	5	P01946,P02091,P08644,P17046,P52590
966	1,2,3,5,7,20,21,22	4,6,8,18,19,23,24,25,26	5	P00714,P27213,P52590,Q6IRE4,Q9QW07
967	1,2,3,5,7,20,21,23	4,6,8,18,19,22,24,25,26	4	P14668,P34901,P52590,Q6IRE4
968	1,2,3,5,7,20,21,24	4,6,8,18,19,22,23,25,26	3	P00714,P52590,Q6IRE4
969	1,2,3,5,7,20,21,25	4,6,8,18,19,22,23,24,26	3	P52590,Q6IRE4,Q91ZS3
970	1,2,3,5,7,20,21,26	4,6,8,18,19,22,23,24,25	4	P01041,P01946,P52590,Q6IRE4
971	1,2,3,5,7,20,22,23	4,6,8,18,19,21,24,25,26	5	P27213,P34901,P52590,Q6IRE4,Q9QW07
972	1,2,3,5,7,20,22,24	4,6,8,18,19,21,23,25,26	10	P00714,P07150,P08010,P14669,P27213,P52590,P83121,Q6IRE4,Q9QW07,Q9WTT6
973	1,2,3,5,7,20,22,25	4,6,8,18,19,21,23,24,26	4	P17046,P52590,Q6IRE4,Q9QW07
974	1,2,3,5,7,20,22,26	4,6,8,18,19,21,23,24,25	9	O54728,P01946,P07150,P14669,P27213,P52590,P55159,Q6P6R2,Q9QW07
975	1,2,3,5,7,20,23,24	4,6,8,18,19,21,22,25,26	3	P34901,P52590,Q6IRE4
976	1,2,3,5,7,20,23,25	4,6,8,18,19,21,22,24,26	3	P52590,P55314,Q6IRE4

977	1,2,3,5,7,20,23,26	4,6,8,18,19,21,22,24,25	5	O54728,P01946,P02091,P52590,Q6IRE4
978	1,2,3,5,7,20,24,25	4,6,8,18,19,21,22,23,26	3	P0C0A9,P52590,Q6IRE4
979	1,2,3,5,7,20,24,26	4,6,8,18,19,21,22,23,25	7	P01946,P02091,P07150,P08010,P52590,Q6IRE4,Q8CJD3
980	1,2,3,5,7,20,25,26	4,6,8,18,19,21,22,23,24	4	P01946,P02091,P52590,Q6IRE4
981	1,2,3,5,7,21,22,23	4,6,8,18,19,20,24,25,26	4	P14668,P34901,P52590,Q9QW07
982	1,2,3,5,7,21,22,24	4,6,8,18,19,20,23,25,26	4	P00714,P52590,P83121,Q9QW07
983	1,2,3,5,7,21,22,25	4,6,8,18,19,20,23,24,26	3	P17046,P52590,Q9QW07
984	1,2,3,5,7,21,22,26	4,6,8,18,19,20,23,24,25	5	O54728,P01946,P52590,P83748,Q9QW07
985	1,2,3,5,7,21,23,24	4,6,8,18,19,20,22,25,26	4	P00507,P34901,P52590,Q6IRE4
986	1,2,3,5,7,21,23,25	4,6,8,18,19,20,22,24,26	4	P34901,P52590,P55314,Q6IRE4
987	1,2,3,5,7,21,23,26	4,6,8,18,19,20,22,24,25	5	P01946,P02091,P34901,P52590,Q63661
988	1,2,3,5,7,21,24,25	4,6,8,18,19,20,22,23,26	2	P52590,Q6IRE4
989	1,2,3,5,7,21,24,26	4,6,8,18,19,20,22,23,25	5	P01946,P02091,P52590,Q6IRE4,Q8CJD3
990	1,2,3,5,7,21,25,26	4,6,8,18,19,20,22,23,24	3	P01946,P02091,P52590
991	1,2,3,5,7,22,23,24	4,6,8,18,19,20,21,25,26	3	P34901,P52590,Q9QW07

992	1,2,3,5,7,22,23,25	4,6,8,18,19,20,21,24,26	5	P17046,P34901,P52590,P55314,Q9QW07
993	1,2,3,5,7,22,23,26	4,6,8,18,19,20,21,24,25	7	O54728,P01946,P02091,P34901,P52590,P55159,Q9QW07
994	1,2,3,5,7,22,24,25	4,6,8,18,19,20,21,23,26	5	P0C0A9,P17046,P52590,Q6AYR9,Q9QW07
995	1,2,3,5,7,22,24,26	4,6,8,18,19,20,21,23,25	9	O54728,P01946,P02091,P07150,P08010,P52590,Q6AYR9,Q8CJD3,Q9QW07
996	1,2,3,5,7,22,25,26	4,6,8,18,19,20,21,23,24	6	O54728,P01946,P02091,P17046,P52590,Q9QW07
997	1,2,3,5,7,23,24,25	4,6,8,18,19,20,21,22,26	7	P0C0A9,P17046,P34901,P36374,P52590,P55314,Q63751
998	1,2,3,5,7,23,24,26	4,6,8,18,19,20,21,22,25	4	P01946,P02091,P34901,P52590
999	1,2,3,5,7,23,25,26	4,6,8,18,19,20,21,22,24	4	P01946,P02091,P52590,P55314
1000	1,2,3,5,7,24,25,26	4,6,8,18,19,20,21,22,23	7	P01946,P02091,P0C0A9,P52590,Q62902,Q6AYR9,Q8CJD3
1001	1,2,3,5,8,18,19,20	4,6,7,21,22,23,24,25,26	4	P09605;P25809,P22006,P83121,Q9QZK9
1002	1,2,3,5,8,18,19,21	4,6,7,20,22,23,24,25,26	4	P00731,P02803,P83121,Q03191
1003	1,2,3,5,8,18,19,22	4,6,7,20,21,23,24,25,26	4	P09605;P25809,P22006,P83121,Q9QW07
1004	1,2,3,5,8,18,19,23	4,6,7,20,21,22,24,25,26	8	P02803,P09605;P25809,P22006,P35467,P83121,Q03191,Q63942,Q64194
1005	1,2,3,5,8,18,19,24	4,6,7,20,21,22,23,25,26	5	P05539,P09605;P25809,P83121,Q03191,Q63942
1006	1,2,3,5,8,18,19,25	4,6,7,20,21,22,23,24,26	3	P02803,P62815,P83121

1007	1,2,3,5,8,18,19,26	4,6,7,20,21,22,23,24,25	5	P09605;P25809,P83121,Q03191,Q63661,Q63942
1008	1,2,3,5,8,18,20,21	4,6,7,19,22,23,24,25,26	3	P62804,P83121,Q6IRE4
1009	1,2,3,5,8,18,20,22	4,6,7,19,21,23,24,25,26	6	P22006,P27213,P83121,Q99041,Q9QW07,Q9QZK9
1010	1,2,3,5,8,18,20,23	4,6,7,19,21,22,24,25,26	5	P09605;P25809,P22006,P83121,Q6IG02,Q9QZK9
1011	1,2,3,5,8,18,20,24	4,6,7,19,21,22,23,25,26	6	P09605;P25809,P22006,P27213,P83121,Q99041,Q9QZK9
1012	1,2,3,5,8,18,20,25	4,6,7,19,21,22,23,24,26	1	P83121
1013	1,2,3,5,8,18,20,26	4,6,7,19,21,22,23,24,25	5	P09605;P25809,P22006,P83121,Q63942,Q99041
1014	1,2,3,5,8,18,21,22	4,6,7,19,20,23,24,25,26	3	P83121,Q5FVI6,Q9QW07
1015	1,2,3,5,8,18,21,23	4,6,7,19,20,22,24,25,26	1	P34901
1016	1,2,3,5,8,18,21,24	4,6,7,19,20,22,23,25,26	3	P05539,P83121,Q5FVI6
1017	1,2,3,5,8,18,21,25	4,6,7,19,20,22,23,24,26	1	P31430
1018	1,2,3,5,8,18,21,26	4,6,7,19,20,22,23,24,25	2	Q63661,Q63942
1019	1,2,3,5,8,18,22,23	4,6,7,19,20,21,24,25,26	4	P22006,P83121,Q5FVI6,Q9QW07
1020	1,2,3,5,8,18,22,24	4,6,7,19,20,21,23,25,26	4	P27213,P83121,Q5FVI6,Q9QW07
1021	1,2,3,5,8,18,22,25	4,6,7,19,20,21,23,24,26	2	P83121,Q9QW07

1022	1,2,3,5,8,18,22,26	4,6,7,19,20,21,23,24,25	4	P83121,Q63942,Q99041,Q9QW07
1023	1,2,3,5,8,18,23,24	4,6,7,19,20,21,22,25,26	5	P09605;P25809,P34901,P83121,Q63942,Q9QZK9
1024	1,2,3,5,8,18,23,25	4,6,7,19,20,21,22,24,26	2	P55314,Q99MH3
1025	1,2,3,5,8,18,23,26	4,6,7,19,20,21,22,24,25	13	P01946,P09605;P25809,P35467,P47967,Q4FZU2,Q63661,Q63942,Q64194,Q6IFU8,Q6IFW6,Q6IG02,Q6IMF3,Q6P6Q2
1026	1,2,3,5,8,18,24,25	4,6,7,19,20,21,22,23,26	1	P83121
1027	1,2,3,5,8,18,24,26	4,6,7,19,20,21,22,23,25	5	P09605;P25809,P47967,P83121,Q63942,Q99041
1028	1,2,3,5,8,18,25,26	4,6,7,19,20,21,22,23,24	2	P55091,Q63942
1029	1,2,3,5,8,19,20,21	4,6,7,18,22,23,24,25,26	3	P00714,P62804,P83121
1030	1,2,3,5,8,19,20,22	4,6,7,18,21,23,24,25,26	6	P00714,P00774,P22006,P27213,P83121,Q9QW07
1031	1,2,3,5,8,19,20,23	4,6,7,18,21,22,24,25,26	3	P09605;P25809,P22006,P83121
1032	1,2,3,5,8,19,20,24	4,6,7,18,21,22,23,25,26	3	P00714,P09605;P25809,P83121
1033	1,2,3,5,8,19,20,25	4,6,7,18,21,22,23,24,26	1	P83121
1034	1,2,3,5,8,19,20,26	4,6,7,18,21,22,23,24,25	4	O54728,P09605;P25809,P22006,P83121
1035	1,2,3,5,8,19,21,22	4,6,7,18,20,23,24,25,26	3	P00714,P83121,Q9QW07
1036	1,2,3,5,8,19,21,23	4,6,7,18,20,22,24,25,26	0	

1037	1,2,3,5,8,19,21,24	4,6,7,18,20,22,23,25,26	2	P00714,P83121
1038	1,2,3,5,8,19,21,25	4,6,7,18,20,22,23,24,26	0	
1039	1,2,3,5,8,19,21,26	4,6,7,18,20,22,23,24,25	0	
1040	1,2,3,5,8,19,22,23	4,6,7,18,20,21,24,25,26	3	P22006,P83121,Q9QW07
1041	1,2,3,5,8,19,22,24	4,6,7,18,20,21,23,25,26	3	P00714,P83121,Q9QW07
1042	1,2,3,5,8,19,22,25	4,6,7,18,20,21,23,24,26	4	P08753,P20766,P83121,Q9QW07
1043	1,2,3,5,8,19,22,26	4,6,7,18,20,21,23,24,25	5	O54728,P20766,P55159,P83121,Q9QW07
1044	1,2,3,5,8,19,23,24	4,6,7,18,20,21,22,25,26	3	P09605;P25809,P34901,P83121
1045	1,2,3,5,8,19,23,25	4,6,7,18,20,21,22,24,26	1	P55314
1046	1,2,3,5,8,19,23,26	4,6,7,18,20,21,22,24,25	3	O54728,P01946,P09605;P25809
1047	1,2,3,5,8,19,24,25	4,6,7,18,20,21,22,23,26	1	P83121
1048	1,2,3,5,8,19,24,26	4,6,7,18,20,21,22,23,25	2	P09605;P25809,P83121
1049	1,2,3,5,8,19,25,26	4,6,7,18,20,21,22,23,24	0	
1050	1,2,3,5,8,20,21,22	4,6,7,18,19,23,24,25,26	5	P00714,P83121,Q5FVI6,Q5XFX0,Q9QW07
1051	1,2,3,5,8,20,21,23	4,6,7,18,19,22,24,25,26	1	Q6IRE4

1052	1,2,3,5,8,20,21,24	4,6,7,18,19,22,23,25,26	4	P00714,P83121,Q5XFX0,Q6IRE4
1053	1,2,3,5,8,20,21,25	4,6,7,18,19,22,23,24,26	1	Q6IRE4
1054	1,2,3,5,8,20,21,26	4,6,7,18,19,22,23,24,25	2	P62804,Q6IRE4
1055	1,2,3,5,8,20,22,23	4,6,7,18,19,21,24,25,26	4	P22006,P83121,Q9QW07,Q9QZK9
1056	1,2,3,5,8,20,22,24	4,6,7,18,19,21,23,25,26	7	P00714,P27213,P83121,Q5FVI6,Q5XFX0,Q9QW07,Q9QZK9
1057	1,2,3,5,8,20,22,25	4,6,7,18,19,21,23,24,26	2	P83121,Q9QW07
1058	1,2,3,5,8,20,22,26	4,6,7,18,19,21,23,24,25	5	O54728,P22006,P83121,Q99041,Q9QW07
1059	1,2,3,5,8,20,23,24	4,6,7,18,19,21,22,25,26	6	P09605;P25809,P34901,P47967,P83121,P97840,Q9QZK9
1060	1,2,3,5,8,20,23,25	4,6,7,18,19,21,22,24,26	1	P55314
1061	1,2,3,5,8,20,23,26	4,6,7,18,19,21,22,24,25	6	O54728,P01946,P09605;P25809,P47967,P97840,Q6IG02
1062	1,2,3,5,8,20,24,25	4,6,7,18,19,21,22,23,26	2	P08649,P83121
1063	1,2,3,5,8,20,24,26	4,6,7,18,19,21,22,23,25	4	P47967,P83121,P97840,Q99041
1064	1,2,3,5,8,20,25,26	4,6,7,18,19,21,22,23,24	0	
1065	1,2,3,5,8,21,22,23	4,6,7,18,19,20,24,25,26	3	P34901,Q5FVI6,Q9QW07
1066	1,2,3,5,8,21,22,24	4,6,7,18,19,20,23,25,26	5	P00714,P83121,Q5FVI6,Q5XFX0,Q9QW07

1067	1,2,3,5,8,21,22,25	4,6,7,18,19,20,23,24,26	2	Q5FVI6,Q9QW07
1068	1,2,3,5,8,21,22,26	4,6,7,18,19,20,23,24,25	1	Q9QW07
1069	1,2,3,5,8,21,23,24	4,6,7,18,19,20,22,25,26	3	P34901,P83121,Q5FVI6
1070	1,2,3,5,8,21,23,25	4,6,7,18,19,20,22,24,26	1	P55314
1071	1,2,3,5,8,21,23,26	4,6,7,18,19,20,22,24,25	1	P01946
1072	1,2,3,5,8,21,24,25	4,6,7,18,19,20,22,23,26	1	P83121
1073	1,2,3,5,8,21,24,26	4,6,7,18,19,20,22,23,25	2	P83121,Q8CJD3
1074	1,2,3,5,8,21,25,26	4,6,7,18,19,20,22,23,24	0	
1075	1,2,3,5,8,22,23,24	4,6,7,18,19,20,21,25,26	5	P34901,P47967,P83121,Q5FVI6,Q9QW07
1076	1,2,3,5,8,22,23,25	4,6,7,18,19,20,21,24,26	4	P08753,P20766,P55314,Q9QW07
1077	1,2,3,5,8,22,23,26	4,6,7,18,19,20,21,24,25	5	O54728,P01946,P20766,P47967,Q9QW07
1078	1,2,3,5,8,22,24,25	4,6,7,18,19,20,21,23,26	4	P08753,P83121,Q5FVI6,Q9QW07
1079	1,2,3,5,8,22,24,26	4,6,7,18,19,20,21,23,25	5	O54728,P47967,P83121,Q99041,Q9QW07
1080	1,2,3,5,8,22,25,26	4,6,7,18,19,20,21,23,24	3	P08753,P20766,Q9QW07
1081	1,2,3,5,8,23,24,25	4,6,7,18,19,20,21,22,26	3	P34901,P36374,P55314

1082	1,2,3,5,8,23,24,26	4,6,7,18,19,20,21,22,25	6	P01946,P02091,P09605;P25809,P34901,P47967,P97840
1083	1,2,3,5,8,23,25,26	4,6,7,18,19,20,21,22,24	3	P01946,P02091,P55314
1084	1,2,3,5,8,24,25,26	4,6,7,18,19,20,21,22,23	3	P01946,Q62902,Q8CJD3
1085	1,2,3,5,18,19,20,21	4,6,7,8,22,23,24,25,26	5	P00731,P06911,P83121,Q02253,Q63617
1086	1,2,3,5,18,19,20,22	4,6,7,8,21,23,24,25,26	10	P07150,P14669,P22006,P27213,P83121,Q6P6R2,Q99041,Q9QW07,Q9QZK9,Q9WTT6
1087	1,2,3,5,18,19,20,23	4,6,7,8,21,22,24,25,26	6	P09605;P25809,P22006,P83121,Q03191,Q9QZK9,Q9WTT6
1088	1,2,3,5,18,19,20,24	4,6,7,8,21,22,23,25,26	8	P06911,P07150,P14669,P22006,P83121,Q99041,Q9QZK9,Q9WTT6
1089	1,2,3,5,18,19,20,25	4,6,7,8,21,22,23,24,26	4	P22282,P62815,P83121,Q02253
1090	1,2,3,5,18,19,20,26	4,6,7,8,21,22,23,24,25	10	P06911,P07150,P09605;P25809,P22006,P83121,Q63617,Q63942,Q6P6R2,Q99041,Q9WTT6
1091	1,2,3,5,18,19,21,22	4,6,7,8,20,23,24,25,26	4	P00714,P83121,Q63617,Q9QW07
1092	1,2,3,5,18,19,21,23	4,6,7,8,20,22,24,25,26	5	P14668,P34901,Q03191,Q63617,Q63661
1093	1,2,3,5,18,19,21,24	4,6,7,8,20,22,23,25,26	2	P83121,Q63617
1094	1,2,3,5,18,19,21,25	4,6,7,8,20,22,23,24,26	2	P31430,Q63617
1095	1,2,3,5,18,19,21,26	4,6,7,8,20,22,23,24,25	5	P06911,Q63617,Q63661,Q63942,Q99041
1096	1,2,3,5,18,19,22,23	4,6,7,8,20,21,24,25,26	6	P22006,P34901,P83121,Q03191,Q9QW07,Q9WTT6

1097	1,2,3,5,18,19,22,24	4,6,7,8,20,21,23,25,26	6	P07150,P14669,P83121,Q6P6R2,Q99041,Q9WTT6
1098	1,2,3,5,18,19,22,25	4,6,7,8,20,21,23,24,26	3	P83121,Q63617,Q9QW07
1099	1,2,3,5,18,19,22,26	4,6,7,8,20,21,23,24,25	11	O54728,P07150,P55159,P83121,Q63617,Q63661,Q63942,Q6P6R2,Q99041,Q9QW07,Q9WTT6
1100	1,2,3,5,18,19,23,24	4,6,7,8,20,21,22,25,26	6	P09605;P25809,P34901,P83121,Q03191,Q9QZK9,Q9WTT6
1101	1,2,3,5,18,19,23,25	4,6,7,8,20,21,22,24,26	3	P55314,Q03191,Q99MH3
1102	1,2,3,5,18,19,23,26	4,6,7,8,20,21,22,24,25	11	P01946,P09605;P25809,P35467,Q03191,Q07523,Q63617,Q63661,Q63942,Q6IFU8,Q6IG02,Q6P6Q2
1103	1,2,3,5,18,19,24,25	4,6,7,8,20,21,22,23,26	3	P22282,P83121,Q63617
1104	1,2,3,5,18,19,24,26	4,6,7,8,20,21,22,23,25	9	P06911,P09605;P25809,P83121,Q03191,Q63617,Q63661,Q63942,Q99041,Q9WTT6
1105	1,2,3,5,18,19,25,26	4,6,7,8,20,21,22,23,24	3	Q63617,Q63661,Q63942
1106	1,2,3,5,18,20,21,22	4,6,7,8,19,23,24,25,26	5	P00714,P83121,Q99041,Q9QW07,Q9QZK9
1107	1,2,3,5,18,20,21,23	4,6,7,8,19,22,24,25,26	3	P34901,Q6IRE4,Q9QZK9
1108	1,2,3,5,18,20,21,24	4,6,7,8,19,22,23,25,26	7	P00714,P06911,P12020,P83121,Q6IRE4,Q99041,Q9QZK9
1109	1,2,3,5,18,20,21,25	4,6,7,8,19,22,23,24,26	2	P31430,Q6IRE4
1110	1,2,3,5,18,20,21,26	4,6,7,8,19,22,23,24,25	5	P06911,P12020,Q63617,Q63661,Q99041
1111	1,2,3,5,18,20,22,23	4,6,7,8,19,21,24,25,26	5	P22006,P34901,P83121,Q9QW07,Q9QZK9

1112	1,2,3,5,18,20,22,24	4,6,7,8,19,21,23,25,26	12	P00714,P07150,P14669,P22006,P27213,P83121,Q5FVI6,Q6P6R2,Q99041,Q9QW07,Q9QZK9,Q9WTT6
1113	1,2,3,5,18,20,22,25	4,6,7,8,19,21,23,24,26	3	P83121,Q99041,Q9QW07
1114	1,2,3,5,18,20,22,26	4,6,7,8,19,21,23,24,25	9	O54728,P07150,P22006,P55159,P83121,Q6P6R2,Q99041,Q9QW07,Q9QZK9
1115	1,2,3,5,18,20,23,24	4,6,7,8,19,21,22,25,26	3	P34901,P83121,Q9QZK9
1116	1,2,3,5,18,20,23,25	4,6,7,8,19,21,22,24,26	1	P55314
1117	1,2,3,5,18,20,23,26	4,6,7,8,19,21,22,24,25	12	P01946,P09605;P25809,P22006,Q4FZU2,Q63942,Q6IFU8,Q6IFW6,Q6IG02,Q6IMF3,Q6P6Q2,Q9JJ50,Q9QZK9
1118	1,2,3,5,18,20,24,25	4,6,7,8,19,21,22,23,26	3	P22282,P83121,Q99041
1119	1,2,3,5,18,20,24,26	4,6,7,8,19,21,22,23,25	7	P06911,P07150,P12020,P47967,P83121,Q99041,Q9QZK9
1120	1,2,3,5,18,20,25,26	4,6,7,8,19,21,22,23,24	1	Q99041
1121	1,2,3,5,18,21,22,23	4,6,7,8,19,20,24,25,26	2	P34901,Q9QW07
1122	1,2,3,5,18,21,22,24	4,6,7,8,19,20,23,25,26	6	P00714,P83121,Q5FVI6,Q63617,Q99041,Q9QW07
1123	1,2,3,5,18,21,22,25	4,6,7,8,19,20,23,24,26	3	P31430,Q63617,Q9QW07
1124	1,2,3,5,18,21,22,26	4,6,7,8,19,20,23,24,25	5	P63036,Q63617,Q63661,Q99041,Q9QW07
1125	1,2,3,5,18,21,23,24	4,6,7,8,19,20,22,25,26	1	P34901
1126	1,2,3,5,18,21,23,25	4,6,7,8,19,20,22,24,26	5	P31430,P34901,P55314,Q63617,Q99MH3

1127	1,2,3,5,18,21,23,26	4,6,7,8,19,20,22,24,25	11	P01946,P34901,Q4FZU2,Q63617,Q63661,Q63942,Q6IFU8,Q6IFW6,Q6IG02,Q6IMF3,Q6P6Q2
1128	1,2,3,5,18,21,24,25	4,6,7,8,19,20,22,23,26	2	P31430,Q63617
1129	1,2,3,5,18,21,24,26	4,6,7,8,19,20,22,23,25	6	P06911,P12020,Q63617,Q63661,Q8CJD3,Q99041
1130	1,2,3,5,18,21,25,26	4,6,7,8,19,20,22,23,24	7	P00774,P25031,P31430,P55091,Q63617,Q63661,Q99041
1131	1,2,3,5,18,22,23,24	4,6,7,8,19,20,21,25,26	6	P34901,P83121,Q4G075,Q5FVI6,Q9QZK9,Q9WTT6
1132	1,2,3,5,18,22,23,25	4,6,7,8,19,20,21,24,26	4	P01015,P34901,P55314,Q9QW07
1133	1,2,3,5,18,22,23,26	4,6,7,8,19,20,21,24,25	9	O54728,P01946,P34901,Q63661,Q63942,Q6IFU8,Q6IG02,Q6P6Q2,Q9QW07
1134	1,2,3,5,18,22,24,25	4,6,7,8,19,20,21,23,26	3	P83121,Q5FVI6,Q99041
1135	1,2,3,5,18,22,24,26	4,6,7,8,19,20,21,23,25	5	P83121,Q63617,Q6P6R2,Q99041,Q9WTT6
1136	1,2,3,5,18,22,25,26	4,6,7,8,19,20,21,23,24	3	Q63617,Q99041,Q9QW07
1137	1,2,3,5,18,23,24,25	4,6,7,8,19,20,21,22,26	2	P34901,P55314
1138	1,2,3,5,18,23,24,26	4,6,7,8,19,20,21,22,25	12	P01946,P02091,P09605;P25809,P34901,P47967,Q07523,Q63661,Q63942,Q6IFU8,Q6IFW6,Q6IG02,Q6P6Q2
1139	1,2,3,5,18,23,25,26	4,6,7,8,19,20,21,22,24	12	P01946,P02091,P55314,Q4FZU2,Q63617,Q63661,Q63942,Q6IFU8,Q6IFW6,Q6IG02,Q6IMF3,Q6P6Q2
1140	1,2,3,5,18,24,25,26	4,6,7,8,19,20,21,22,23	8	P01946,P22282,P55091,Q62902,Q63617,Q7TPB1,Q8CJD3,Q99041
1141	1,2,3,5,19,20,21,22	4,6,7,8,18,23,24,25,26	3	P00714,P83121,Q9QW07

1142	1,2,3,5,19,20,21,23	4,6,7,8,18,22,24,25,26	1	P00714
1143	1,2,3,5,19,20,21,24	4,6,7,8,18,22,23,25,26	3	P00714,P11598,P83121
1144	1,2,3,5,19,20,21,25	4,6,7,8,18,22,23,24,26	1	Q8VD89
1145	1,2,3,5,19,20,21,26	4,6,7,8,18,22,23,24,25	4	P00714,P06911,Q63617,Q99041
1146	1,2,3,5,19,20,22,23	4,6,7,8,18,21,24,25,26	13	O54728,P00714,P07150,P11598,P14669,P22006,P55091,P82995,P83121,Q62997,Q9QW07,Q9QZK9,Q9WTT6
1147	1,2,3,5,19,20,22,24	4,6,7,8,18,21,23,25,26	12	P00714,P00774,P07150,P11598,P14669,P27213,P82995,P83121,Q6P6R2,Q99041,Q9QZK9,Q9WTT6
1148	1,2,3,5,19,20,22,25	4,6,7,8,18,21,23,24,26	5	P07150,P11598,P14669,P17046,P83121
1149	1,2,3,5,19,20,22,26	4,6,7,8,18,21,23,24,25	12	O54728,P00714,P07150,P14669,P22006,P55159,P83121,Q62997,Q6P6R2,Q99041,Q9QW07,Q9WTT6
1150	1,2,3,5,19,20,23,24	4,6,7,8,18,21,22,25,26	7	P00714,P11598,P34901,P82995,P83121,Q9QZK9,Q9WTT6
1151	1,2,3,5,19,20,23,25	4,6,7,8,18,21,22,24,26	2	P11598,P55314
1152	1,2,3,5,19,20,23,26	4,6,7,8,18,21,22,24,25	3	O54728,P01946,P11598
1153	1,2,3,5,19,20,24,25	4,6,7,8,18,21,22,23,26	5	P0C0A9,P11598,P22282,P22283,P83121
1154	1,2,3,5,19,20,24,26	4,6,7,8,18,21,22,23,25	8	O54728,P00714,P07150,P11598,P14669,P83121,Q99041,Q9WTT6
1155	1,2,3,5,19,20,25,26	4,6,7,8,18,21,22,23,24	1	P11598
1156	1,2,3,5,19,21,22,23	4,6,7,8,18,20,24,25,26	4	P00714,P34901,Q63617,Q9QW07

1157	1,2,3,5,19,21,22,24	4,6,7,8,18,20,23,25,26	4	P00714,P83121,Q63617,Q9QW07
1158	1,2,3,5,19,21,22,25	4,6,7,8,18,20,23,24,26	3	P17046,Q63617,Q9QW07
1159	1,2,3,5,19,21,22,26	4,6,7,8,18,20,23,24,25	6	O54728,P00714,P63036,Q63617,Q99041,Q9QW07
1160	1,2,3,5,19,21,23,24	4,6,7,8,18,20,22,25,26	3	P00714,P11598,P34901
1161	1,2,3,5,19,21,23,25	4,6,7,8,18,20,22,24,26	2	P55314,Q63617
1162	1,2,3,5,19,21,23,26	4,6,7,8,18,20,22,24,25	4	P01946,P34901,Q63617,Q63661
1163	1,2,3,5,19,21,24,25	4,6,7,8,18,20,22,23,26	2	P11598,Q63617
1164	1,2,3,5,19,21,24,26	4,6,7,8,18,20,22,23,25	3	P00714,Q63617,Q8CJD3
1165	1,2,3,5,19,21,25,26	4,6,7,8,18,20,22,23,24	2	Q63617,Q63661
1166	1,2,3,5,19,22,23,24	4,6,7,8,18,20,21,25,26	7	P00714,P11598,P34901,P82471,P82995,P83121,Q9WTT6
1167	1,2,3,5,19,22,23,25	4,6,7,8,18,20,21,24,26	5	P11598,P17046,P55314,P82471,Q6AYR8
1168	1,2,3,5,19,22,23,26	4,6,7,8,18,20,21,24,25	5	O54728,P01946,P55159,P82471,Q63617
1169	1,2,3,5,19,22,24,25	4,6,7,8,18,20,21,23,26	7	P02780,P0C0A9,P11598,P14669,P17046,P22283,P83121
1170	1,2,3,5,19,22,24,26	4,6,7,8,18,20,21,23,25	12	O54728,P00714,P07150,P11598,P14669,P24368,P83121,Q63617,Q6P6R2,Q99041,Q9R044,Q9WTT6
1171	1,2,3,5,19,22,25,26	4,6,7,8,18,20,21,23,24	2	O54728,Q63617

1172	1,2,3,5,19,23,24,25	4,6,7,8,18,20,21,22,26	4	P11598,P22283,P34901,P55314
1173	1,2,3,5,19,23,24,26	4,6,7,8,18,20,21,22,25	7	O54728,P01946,P02091,P09605;P25809,P11598,P34901,Q63617
1174	1,2,3,5,19,23,25,26	4,6,7,8,18,20,21,22,24	6	P01946,P02091,P11598,P13432,P55314,Q63617
1175	1,2,3,5,19,24,25,26	4,6,7,8,18,20,21,22,23	6	P01946,P11598,P22283,Q62902,Q63617,Q8CJD3
1176	1,2,3,5,20,21,22,23	4,6,7,8,18,19,24,25,26	3	P00714,P34901,Q9QW07
1177	1,2,3,5,20,21,22,24	4,6,7,8,18,19,23,25,26	7	P00714,P27213,P83121,Q5FVI6,Q5XFX0,Q99041,Q9QW07
1178	1,2,3,5,20,21,22,25	4,6,7,8,18,19,23,24,26	1	Q9QW07
1179	1,2,3,5,20,21,22,26	4,6,7,8,18,19,23,24,25	5	O54728,P00714,Q5BJY9,Q99041,Q9QW07
1180	1,2,3,5,20,21,23,24	4,6,7,8,18,19,22,25,26	5	P00714,P11598,P34901,Q6IRE4,Q9QZK9
1181	1,2,3,5,20,21,23,25	4,6,7,8,18,19,22,24,26	3	P34901,P55314,Q6IRE4
1182	1,2,3,5,20,21,23,26	4,6,7,8,18,19,22,24,25	3	P01946,P34901,Q6IFU8
1183	1,2,3,5,20,21,24,25	4,6,7,8,18,19,22,23,26	3	P11598,Q5XFX0,Q6IRE4
1184	1,2,3,5,20,21,24,26	4,6,7,8,18,19,22,23,25	6	P00714,P06911,P12020,Q6IRE4,Q8CJD3,Q99041
1185	1,2,3,5,20,21,25,26	4,6,7,8,18,19,22,23,24	1	Q63617
1186	1,2,3,5,20,22,23,24	4,6,7,8,18,19,21,25,26	10	P00714,P0C0A9,P11598,P14669,P34901,P82995,P83121,Q4G075,Q5FVI6,Q9QZK9

1187	1,2,3,5,20,22,23,25	4,6,7,8,18,19,21,24,26	3	P0C0A9,P11598,P55314
1188	1,2,3,5,20,22,23,26	4,6,7,8,18,19,21,24,25	4	O54728,P01946,P47967,Q9QW07
1189	1,2,3,5,20,22,24,25	4,6,7,8,18,19,21,23,26	7	P0C0A9,P11598,P14669,P22282,P22283,P83121,Q99041
1190	1,2,3,5,20,22,24,26	4,6,7,8,18,19,21,23,25	10	O54728,P00714,P07150,P0C0A9,P11598,P14669,P47967,P83121,Q6P6R2,Q99041
1191	1,2,3,5,20,22,25,26	4,6,7,8,18,19,21,23,24	4	O54728,P0C0A9,Q99041,Q9QW07
1192	1,2,3,5,20,23,24,25	4,6,7,8,18,19,21,22,26	7	P0C0A9,P11598,P22282,P22283,P34901,P36374,P55314
1193	1,2,3,5,20,23,24,26	4,6,7,8,18,19,21,22,25	9	D4A1J4,P01946,P02091,P0C0A9,P11598,P34901,P47967,P97840,Q9QZK9
1194	1,2,3,5,20,23,25,26	4,6,7,8,18,19,21,22,24	5	P01946,P02091,P0C0A9,P11598,P55314
1195	1,2,3,5,20,24,25,26	4,6,7,8,18,19,21,22,23	9	P01946,P08723,P0C0A9,P11598,P22282,P22283,Q62902,Q8CJD3,Q99041
1196	1,2,3,5,21,22,23,24	4,6,7,8,18,19,20,25,26	6	P00714,P11598,P34901,P35280,Q5FVI6,Q9QW07
1197	1,2,3,5,21,22,23,25	4,6,7,8,18,19,20,24,26	4	P34901,P35280,P55314,Q9QW07
1198	1,2,3,5,21,22,23,26	4,6,7,8,18,19,20,24,25	5	O54728,P01946,P34901,Q63617,Q9QW07
1199	1,2,3,5,21,22,24,25	4,6,7,8,18,19,20,23,26	7	P11598,P35280,P35467,Q5FVI6,Q5XFX0,Q63617,Q9QW07
1200	1,2,3,5,21,22,24,26	4,6,7,8,18,19,20,23,25	7	P00714,P07171,Q5BJY9,Q63617,Q8CJD3,Q99041,Q9QW07
1201	1,2,3,5,21,22,25,26	4,6,7,8,18,19,20,23,24	2	Q63617,Q9QW07

1202	1,2,3,5,21,23,24,25	4,6,7,8,18,19,20,22,26	4	P11598,P34901,P35280,P55314
1203	1,2,3,5,21,23,24,26	4,6,7,8,18,19,20,22,25	4	P01946,P02091,P34901,Q63617
1204	1,2,3,5,21,23,25,26	4,6,7,8,18,19,20,22,24	6	P01946,P02091,P34901,P55314,Q63617,Q63661
1205	1,2,3,5,21,24,25,26	4,6,7,8,18,19,20,22,23	3	P01946,Q63617,Q8CJD3
1206	1,2,3,5,22,23,24,25	4,6,7,8,18,19,20,21,26	8	P0C0A9,P11598,P34901,P35280,P55314,P82471,Q5FVI6,Q6AYR9
1207	1,2,3,5,22,23,24,26	4,6,7,8,18,19,20,21,25	11	O54728,P00731,P01946,P02091,P11598,P34901,P36953,P47967,P82471,Q63556,Q6AYR9
1208	1,2,3,5,22,23,25,26	4,6,7,8,18,19,20,21,24	8	O54728,P01946,P02091,P11598,P34901,P55314,P82471,Q6AYR9
1209	1,2,3,5,22,24,25,26	4,6,7,8,18,19,20,21,23	11	P01946,P02780,P0C0A9,P11598,P24368,P36953,Q62902,Q63617,Q6AYR9,Q8CJD3,Q99041
1210	1,2,3,5,23,24,25,26	4,6,7,8,18,19,20,21,22	12	P01946,P02091,P08723,P0C0A9,P11598,P22283,P34901,P55314,P60905,Q62902,Q6AYR9,Q7TPB1
1211	1,2,3,6,7,8,18,19	4,5,20,21,22,23,24,25,26	11	O55006,P00731,P02631,P02803,P05964,P16086,P35280,Q30KJ2,Q63532,Q63942,Q91ZS3
1212	1,2,3,6,7,8,18,20	4,5,19,21,22,23,24,25,26	6	P00731,P02631,P10715,P16086,P35280,Q91ZS3
1213	1,2,3,6,7,8,18,21	4,5,19,20,22,23,24,25,26	10	D4A1J4,O35783,O54800;Q5DWV2,P00731,P02631,P16086,P25031,P42854,Q6AYL6,Q91ZS3
1214	1,2,3,6,7,8,18,22	4,5,19,20,21,23,24,25,26	5	D4A1J4,P02631,P16086,P20766,Q91ZS3
1215	1,2,3,6,7,8,18,23	4,5,19,20,21,22,24,25,26	8	P02631,P16086,P51907,P97580,Q30KJ2,Q5XI22,Q63942,Q91ZS3
1216	1,2,3,6,7,8,18,24	4,5,19,20,21,22,23,25,26	5	O55006,P02631,P10715,P16086,Q91ZS3

1217	1,2,3,6,7,8,18,25	4,5,19,20,21,22,23,24,26	12	O35783,P00731,P02631,P10715,P12020,P16086,P19629,P31044,P35280,P68370,Q5GRG2,Q91ZS3
1218	1,2,3,6,7,8,18,26	4,5,19,20,21,22,23,24,25	12	O35783,O54800;Q5DWV2,P01041,P01946,P02631,P02770,P10715,P16086,P35280,P83748,Q63942,Q91ZS3
1219	1,2,3,6,7,8,19,20	4,5,18,21,22,23,24,25,26	6	P02631,P16086,P35280,Q30KJ2,Q63532,Q91ZS3
1220	1,2,3,6,7,8,19,21	4,5,18,20,22,23,24,25,26	10	D4A1J4,P00731,P02631,P15399,P16086,P35281,P53792,Q30KJ2,Q63532,Q91ZS3
1221	1,2,3,6,7,8,19,22	4,5,18,20,21,23,24,25,26	6	D4A1J4,P02631,P16086,P20766,Q30KJ2,Q63532
1222	1,2,3,6,7,8,19,23	4,5,18,20,21,22,24,25,26	7	P02631,P15399,P16086,P51907,P97580,Q30KJ2,Q63532
1223	1,2,3,6,7,8,19,24	4,5,18,20,21,22,23,25,26	4	P02631,P16086,Q30KJ2,Q63532
1224	1,2,3,6,7,8,19,25	4,5,18,20,21,22,23,24,26	16	P02631,P06911,P10715,P12020,P16086,P19629,P31044,P35280,P47967,P68370,P97580,P97840,Q30KJ2,Q5GRG2,Q63532,Q91ZS3
1225	1,2,3,6,7,8,19,26	4,5,18,20,21,22,23,24,25	8	P01946,P02631,P02770,P16086,P35280,Q30KJ2,Q63532,Q91ZS3
1226	1,2,3,6,7,8,20,21	4,5,18,19,22,23,24,25,26	6	O35783,P02631,P16086,P42854,P62804,Q91ZS3
1227	1,2,3,6,7,8,20,22	4,5,18,19,21,23,24,25,26	6	P02631,P10715,P16086,P20766,P27213,Q91ZS3
1228	1,2,3,6,7,8,20,23	4,5,18,19,21,22,24,25,26	5	P02631,P16086,P97580,Q30KJ2,Q91ZS3
1229	1,2,3,6,7,8,20,24	4,5,18,19,21,22,23,25,26	4	P02631,P10715,P16086,Q91ZS3
1230	1,2,3,6,7,8,20,25	4,5,18,19,21,22,23,24,26	10	P02631,P10715,P12020,P16086,P19629,P31044,P35280,P68370,Q5GRG2,Q91ZS3
1231	1,2,3,6,7,8,20,26	4,5,18,19,21,22,23,24,25	10	O35783,P01041,P01946,P02631,P02770,P10715,P16086,P35280,P62804,Q91ZS3

1232	1,2,3,6,7,8,21,22	4,5,18,19,20,23,24,25,26	6	D4A1J4,O54800;Q5DWV2,P02631,P13432,P16086,Q91ZS3
1233	1,2,3,6,7,8,21,23	4,5,18,19,20,22,24,25,26	6	P02631,P15399,P16086,P97580,Q30KJ2,Q91ZS3
1234	1,2,3,6,7,8,21,24	4,5,18,19,20,22,23,25,26	4	P02631,P13432,P16086,Q91ZS3
1235	1,2,3,6,7,8,21,25	4,5,18,19,20,22,23,24,26	13	O35783,O54800;Q5DWV2,P02631,P10715,P12020,P16086,P19629,P47967,P68370,Q5GRG2,Q6P6R2,Q91ZS3,Q9WTT6
1236	1,2,3,6,7,8,21,26	4,5,18,19,20,22,23,24,25	11	O35783,O54800;Q5DWV2,P01041,P01946,P02631,P02770,P16086,P83748,Q00715,Q5QE79,Q91ZS3
1237	1,2,3,6,7,8,22,23	4,5,18,19,20,21,24,25,26	6	P02631,P16086,P20766,P51907,P97580,Q30KJ2
1238	1,2,3,6,7,8,22,24	4,5,18,19,20,21,23,25,26	3	P02631,P16086,P20766
1239	1,2,3,6,7,8,22,25	4,5,18,19,20,21,23,24,26	11	P02631,P06911,P10715,P12020,P16086,P19629,P20766,P31044,P68370,Q5GRG2,Q91ZS3
1240	1,2,3,6,7,8,22,26	4,5,18,19,20,21,23,24,25	6	P01946,P02631,P02770,P16086,P20766,Q91ZS3
1241	1,2,3,6,7,8,23,24	4,5,18,19,20,21,22,25,26	5	P02631,P16086,P51907,P97580,Q30KJ2
1242	1,2,3,6,7,8,23,25	4,5,18,19,20,21,22,24,26	18	P02631,P05371,P06911,P10715,P12020,P16086,P19629,P31044,P51907,P55314,P68370,P97580,Q30KJ2,Q5GRG2,Q63751,Q6P6R2,Q6P9T8,Q91ZS3
1243	1,2,3,6,7,8,23,26	4,5,18,19,20,21,22,24,25	10	P01946,P02091,P02631,P02770,P16086,P51907,P97580,Q30KJ2,Q5QE79,Q91ZS3
1244	1,2,3,6,7,8,24,25	4,5,18,19,20,21,22,23,26	8	P02631,P10715,P12020,P16086,P19629,P68370,Q5GRG2,Q91ZS3
1245	1,2,3,6,7,8,24,26	4,5,18,19,20,21,22,23,25	5	P01946,P02091,P02631,P02770,Q91ZS3
1246	1,2,3,6,7,8,25,26	4,5,18,19,20,21,22,23,24	14	O35783,P01946,P02091,P02631,P02770,P10715,P12020,P19629,P20766,P35280,P68370,Q5GRG2,Q6P9T8,Q91ZS3

1247	1,2,3,6,7,18,19,20	4,5,8,21,22,23,24,25,26	9	P00731,P02631,P06760,P16086,P35280,Q30KJ2,Q63532,Q6AYS4,Q91ZS3
1248	1,2,3,6,7,18,19,21	4,5,8,20,22,23,24,25,26	12	P00731,P02631,P05964,P14668,P15399,P16086,P25031,P53792,P97840,Q30KJ2,Q63532,Q91ZS3
1249	1,2,3,6,7,18,19,22	4,5,8,20,21,23,24,25,26	5	P02631,P06760,P16086,Q30KJ2,Q63532
1250	1,2,3,6,7,18,19,23	4,5,8,20,21,22,24,25,26	10	P02631,P05964,P15399,P16086,P97580,Q03191,Q30KJ2,Q5XI22,Q63532,Q6IE51
1251	1,2,3,6,7,18,19,24	4,5,8,20,21,22,23,25,26	7	P02631,P06760,P16086,Q30KJ2,Q63532,Q6AYS4,Q6IE51
1252	1,2,3,6,7,18,19,25	4,5,8,20,21,22,23,24,26	10	P00731,P02631,P12020,P16086,P35280,P47967,P97840,Q30KJ2,Q63532,Q91ZS3
1253	1,2,3,6,7,18,19,26	4,5,8,20,21,22,23,24,25	9	P01946,P02631,P06760,P16086,P35280,Q30KJ2,Q63532,Q63661,Q63942
1254	1,2,3,6,7,18,20,21	4,5,8,19,22,23,24,25,26	7	O35783,P00731,P02631,P16086,P25031,P42854,Q91ZS3
1255	1,2,3,6,7,18,20,22	4,5,8,19,21,23,24,25,26	5	P02631,P06760,P16086,P27213,Q91ZS3
1256	1,2,3,6,7,18,20,23	4,5,8,19,21,22,24,25,26	6	A2RUV9,P02631,P16086,P97580,Q5XI22,Q91ZS3
1257	1,2,3,6,7,18,20,24	4,5,8,19,21,22,23,25,26	4	P02631,P06760,P16086,Q91ZS3
1258	1,2,3,6,7,18,20,25	4,5,8,19,21,22,23,24,26	5	P02631,P10715,P16086,P35280,Q91ZS3
1259	1,2,3,6,7,18,20,26	4,5,8,19,21,22,23,24,25	6	O35783,P01041,P01946,P06760,P35280,Q91ZS3
1260	1,2,3,6,7,18,21,22	4,5,8,19,20,23,24,25,26	5	D4A1J4,P13432,P16086,P25031,Q91ZS3
1261	1,2,3,6,7,18,21,23	4,5,8,19,20,22,24,25,26	9	P02631,P05964,P14668,P15399,P16086,P25031,P97580,Q5XI22,Q91ZS3

1262	1,2,3,6,7,18,21,24	4,5,8,19,20,22,23,25,26	5	P02631,P13432,P16086,P25031,Q91ZS3
1263	1,2,3,6,7,18,21,25	4,5,8,19,20,22,23,24,26	12	O35783,O54800;Q5DWV2,P00731,P02631,P16086,P19629,P25031,P31430,P47967,P97840,P98089,Q91ZS3
1264	1,2,3,6,7,18,21,26	4,5,8,19,20,22,23,24,25	10	O35783,O54800;Q5DWV2,P00774,P01041,P01946,P16086,P25031,P83748,Q63661,Q91ZS3
1265	1,2,3,6,7,18,22,23	4,5,8,19,20,21,24,25,26	3	P02631,P16086,P97580
1266	1,2,3,6,7,18,22,24	4,5,8,19,20,21,23,25,26	3	P02631,P06760,P16086
1267	1,2,3,6,7,18,22,25	4,5,8,19,20,21,23,24,26	7	P06911,P12020,P16086,P19629,P97840,Q5GRG2,Q91ZS3
1268	1,2,3,6,7,18,22,26	4,5,8,19,20,21,23,24,25	4	P01946,P06760,P16086,P83748
1269	1,2,3,6,7,18,23,24	4,5,8,19,20,21,22,25,26	3	P02631,P16086,P97580
1270	1,2,3,6,7,18,23,25	4,5,8,19,20,21,22,24,26	9	P02631,P05371,P12020,P16086,P19629,P55314,P97580,Q5GRG2,Q91ZS3
1271	1,2,3,6,7,18,23,26	4,5,8,19,20,21,22,24,25	13	P01946,P02091,P02631,P16086,P55053,P97580,Q30KJ2,Q63661,Q63942,Q6IFU8,Q6IMF3,Q6P6Q2,Q91ZS3
1272	1,2,3,6,7,18,24,25	4,5,8,19,20,21,22,23,26	3	P02631,P19629,Q91ZS3
1273	1,2,3,6,7,18,24,26	4,5,8,19,20,21,22,23,25	5	P01946,P02091,P06760,Q63942,Q91ZS3
1274	1,2,3,6,7,18,25,26	4,5,8,19,20,21,22,23,24	8	O35783,P00774,P01946,P02091,P19629,P35280,P55091,Q91ZS3
1275	1,2,3,6,7,19,20,21	4,5,8,18,22,23,24,25,26	10	P00714,P00731,P02631,P15399,P16086,P42854,P53792,Q30KJ2,Q63532,Q91ZS3
1276	1,2,3,6,7,19,20,22	4,5,8,18,21,23,24,25,26	10	P00714,P02631,P06760,P07150,P14669,P16086,P27213,Q30KJ2,Q63532,Q6AYS4

1277	1,2,3,6,7,19,20,23	4,5,8,18,21,22,24,25,26	7	P02631,P15399,P16086,P97580,Q30KJ2,Q63532,Q6AYS4
1278	1,2,3,6,7,19,20,24	4,5,8,18,21,22,23,25,26	6	P00714,P02631,P16086,Q30KJ2,Q63532,Q6AYS4
1279	1,2,3,6,7,19,20,25	4,5,8,18,21,22,23,24,26	9	P02631,P12020,P16086,P35280,P47967,P97840,Q30KJ2,Q63532,Q91ZS3
1280	1,2,3,6,7,19,20,26	4,5,8,18,21,22,23,24,25	8	P01946,P06760,P16086,P35280,Q30KJ2,Q63532,Q6AYS4,Q91ZS3
1281	1,2,3,6,7,19,21,22	4,5,8,18,20,23,24,25,26	7	D4A1J4,P00714,P02631,P16086,P97840,Q30KJ2,Q63532
1282	1,2,3,6,7,19,21,23	4,5,8,18,20,22,24,25,26	7	P02631,P14668,P15399,P16086,P97580,Q30KJ2,Q63532
1283	1,2,3,6,7,19,21,24	4,5,8,18,20,22,23,25,26	6	P00714,P02631,P16086,P53792,Q30KJ2,Q63532
1284	1,2,3,6,7,19,21,25	4,5,8,18,20,22,23,24,26	12	P00731,P02631,P12020,P15399,P16086,P19629,P47967,P53792,P97840,Q30KJ2,Q63532,Q91ZS3
1285	1,2,3,6,7,19,21,26	4,5,8,18,20,22,23,24,25	8	P01946,P16086,P53792,P83748,Q30KJ2,Q63532,Q63661,Q91ZS3
1286	1,2,3,6,7,19,22,23	4,5,8,18,20,21,24,25,26	6	P02631,P15399,P16086,P97580,Q30KJ2,Q63532
1287	1,2,3,6,7,19,22,24	4,5,8,18,20,21,23,25,26	6	P00714,P02631,P16086,Q30KJ2,Q63532,Q6AYS4
1288	1,2,3,6,7,19,22,25	4,5,8,18,20,21,23,24,26	13	P02631,P06911,P12020,P16086,P17046,P19629,P47967,P97840,Q30KJ2,Q4FZU6,Q5GRG2,Q63532,Q91ZS3
1289	1,2,3,6,7,19,22,26	4,5,8,18,20,21,23,24,25	7	P01946,P06760,P16086,P55159,Q30KJ2,Q63532,Q6AYS4
1290	1,2,3,6,7,19,23,24	4,5,8,18,20,21,22,25,26	8	P02631,P15399,P16086,P97580,Q30KJ2,Q63532,Q6AYS4,Q6IE51
1291	1,2,3,6,7,19,23,25	4,5,8,18,20,21,22,24,26	14	P02631,P06911,P12020,P15399,P16086,P19629,P55314,P97580,P97840,Q30KJ2,Q4FZU6,Q5GRG2,Q63532,Q91ZS3

1292	1,2,3,6,7,19,23,26	4,5,8,18,20,21,22,24,25	9	P01946,P02091,P02631,P15399,P16086,P97580,Q30KJ2,Q63532,Q63661
1293	1,2,3,6,7,19,24,25	4,5,8,18,20,21,22,23,26	9	P02631,P12020,P16086,P19629,P97840,Q30KJ2,Q4FZU6,Q63532,Q91ZS3
1294	1,2,3,6,7,19,24,26	4,5,8,18,20,21,22,23,25	8	P01946,P02091,P02631,P06760,P16086,Q30KJ2,Q63532,Q6AYS4
1295	1,2,3,6,7,19,25,26	4,5,8,18,20,21,22,23,24	10	P01946,P02091,P12020,P16086,P19629,P35280,P97840,Q30KJ2,Q63532,Q91ZS3
1296	1,2,3,6,7,20,21,22	4,5,8,18,19,23,24,25,26	4	P00714,P13432,P16086,Q91ZS3
1297	1,2,3,6,7,20,21,23	4,5,8,18,19,22,24,25,26	6	P02631,P14668,P15399,P16086,P97580,Q91ZS3
1298	1,2,3,6,7,20,21,24	4,5,8,18,19,22,23,25,26	4	P00714,P13432,P16086,Q91ZS3
1299	1,2,3,6,7,20,21,25	4,5,8,18,19,22,23,24,26	6	O35783,P09605;P25809,P16086,P19629,P47967,Q91ZS3
1300	1,2,3,6,7,20,21,26	4,5,8,18,19,22,23,24,25	6	O35783,P01041,P01946,P16086,P83748,Q91ZS3
1301	1,2,3,6,7,20,22,23	4,5,8,18,19,21,24,25,26	3	P02631,P16086,P97580
1302	1,2,3,6,7,20,22,24	4,5,8,18,19,21,23,25,26	5	P00714,P14669,P16086,P27213,Q6AYS4
1303	1,2,3,6,7,20,22,25	4,5,8,18,19,21,23,24,26	6	P06911,P12020,P16086,P19629,Q5GRG2,Q91ZS3
1304	1,2,3,6,7,20,22,26	4,5,8,18,19,21,23,24,25	6	P01041,P01946,P06760,P55159,Q6AYS4,Q91ZS3
1305	1,2,3,6,7,20,23,24	4,5,8,18,19,21,22,25,26	4	P02631,P16086,P97580,Q6AYS4
1306	1,2,3,6,7,20,23,25	4,5,8,18,19,21,22,24,26	8	P02631,P12020,P16086,P19629,P55314,P97580,Q5GRG2,Q91ZS3

1307	1,2,3,6,7,20,23,26	4,5,8,18,19,21,22,24,25	6	P01946,P02091,P16086,P97580,Q30KJ2,Q91ZS3
1308	1,2,3,6,7,20,24,25	4,5,8,18,19,21,22,23,26	4	P10715,P12020,P19629,Q91ZS3
1309	1,2,3,6,7,20,24,26	4,5,8,18,19,21,22,23,25	6	P01041,P01946,P02091,P06760,Q6AYS4,Q91ZS3
1310	1,2,3,6,7,20,25,26	4,5,8,18,19,21,22,23,24	6	O35783,P01946,P02091,P12020,P35280,Q91ZS3
1311	1,2,3,6,7,21,22,23	4,5,8,18,19,20,24,25,26	3	P14668,P16086,P97580
1312	1,2,3,6,7,21,22,24	4,5,8,18,19,20,23,25,26	3	P00714,P13432,P16086
1313	1,2,3,6,7,21,22,25	4,5,8,18,19,20,23,24,26	9	P06911,P09605;P25809,P12020,P16086,P19629,P47967,P97840,Q5GRG2,Q91ZS3
1314	1,2,3,6,7,21,22,26	4,5,8,18,19,20,23,24,25	5	O54800;Q5DWV2,P01946,P16086,P83748,Q91ZS3
1315	1,2,3,6,7,21,23,24	4,5,8,18,19,20,22,25,26	3	P02631,P16086,P97580
1316	1,2,3,6,7,21,23,25	4,5,8,18,19,20,22,24,26	12	P02631,P12020,P14668,P15399,P16086,P19629,P55314,P97580,P97840,Q5GRG2,Q6P6R2,Q91ZS3
1317	1,2,3,6,7,21,23,26	4,5,8,18,19,20,22,24,25	8	O35783,P01946,P02091,P16086,P97580,Q30KJ2,Q63661,Q91ZS3
1318	1,2,3,6,7,21,24,25	4,5,8,18,19,20,22,23,26	4	P16086,P19629,P97840,Q91ZS3
1319	1,2,3,6,7,21,24,26	4,5,8,18,19,20,22,23,25	5	O35783,P01946,P02091,P83748,Q91ZS3
1320	1,2,3,6,7,21,25,26	4,5,8,18,19,20,22,23,24	8	O35783,O54800;Q5DWV2,P01946,P02091,P19629,P83748,Q91ZS3,Q9QZK9
1321	1,2,3,6,7,22,23,24	4,5,8,18,19,20,21,25,26	3	P02631,P16086,P97580

1322	1,2,3,6,7,22,23,25	4,5,8,18,19,20,21,24,26	9	P06911,P12020,P16086,P19629,P55314,P97580,Q4FZU6,Q5GRG2,Q91ZS3
1323	1,2,3,6,7,22,23,26	4,5,8,18,19,20,21,24,25	5	P01946,P02091,P16086,P97580,Q30KJ2
1324	1,2,3,6,7,22,24,25	4,5,8,18,19,20,21,23,26	6	P06911,P12020,P19629,Q4FZU6,Q5GRG2,Q91ZS3
1325	1,2,3,6,7,22,24,26	4,5,8,18,19,20,21,23,25	4	P01946,P02091,P06760,P08010
1326	1,2,3,6,7,22,25,26	4,5,8,18,19,20,21,23,24	7	P01946,P02091,P06911,P12020,P19629,Q5GRG2,Q91ZS3
1327	1,2,3,6,7,23,24,25	4,5,8,18,19,20,21,22,26	11	P02631,P12020,P16086,P19629,P36374,P55314,P97580,Q4FZU6,Q5GRG2,Q63751,Q91ZS3
1328	1,2,3,6,7,23,24,26	4,5,8,18,19,20,21,22,25	5	P01946,P02091,P16086,P97580,Q30KJ2
1329	1,2,3,6,7,23,25,26	4,5,8,18,19,20,21,22,24	10	P01946,P02091,P12020,P16086,P19629,P55314,P97580,Q30KJ2,Q5GRG2,Q91ZS3
1330	1,2,3,6,7,24,25,26	4,5,8,18,19,20,21,22,23	4	P01946,P02091,P19629,Q91ZS3
1331	1,2,3,6,8,18,19,20	4,5,7,21,22,23,24,25,26	5	P00731,P22006,P35280,P83121,Q66H69
1332	1,2,3,6,8,18,19,21	4,5,7,20,22,23,24,25,26	4	O35547,P00731,P05539,P35281
1333	1,2,3,6,8,18,19,22	4,5,7,20,21,23,24,25,26	2	P22006,P83121
1334	1,2,3,6,8,18,19,23	4,5,7,20,21,22,24,25,26	11	P15399,P22006,P35467,P51907,P97580,Q03191,Q30KJ2,Q5XI22,Q63942,Q8CJD3,Q9Z1F2
1335	1,2,3,6,8,18,19,24	4,5,7,20,21,22,23,25,26	3	P05539,P83121,Q63942
1336	1,2,3,6,8,18,19,25	4,5,7,20,21,22,23,24,26	6	P00731,P35280,Q4FZU6,Q62635,Q91ZS3,Q9Z1F2

1337	1,2,3,6,8,18,19,26	4,5,7,20,21,22,23,24,25	4	P02770,P35280,Q63355,Q63942
1338	1,2,3,6,8,18,20,21	4,5,7,19,22,23,24,25,26	4	O35547,P00731,P62804,Q91ZS3
1339	1,2,3,6,8,18,20,22	4,5,7,19,21,23,24,25,26	2	P22006,P83121
1340	1,2,3,6,8,18,20,23	4,5,7,19,21,22,24,25,26	2	P22006,Q5XI22
1341	1,2,3,6,8,18,20,24	4,5,7,19,21,22,23,25,26	1	P83121
1342	1,2,3,6,8,18,20,25	4,5,7,19,21,22,23,24,26	2	P35280,Q91ZS3
1343	1,2,3,6,8,18,20,26	4,5,7,19,21,22,23,24,25	6	P02770,P35280,P62804,Q63355,Q63942,Q91ZS3
1344	1,2,3,6,8,18,21,22	4,5,7,19,20,23,24,25,26	3	O35547,P02782,P13432
1345	1,2,3,6,8,18,21,23	4,5,7,19,20,22,24,25,26	2	O35547,Q5XI22
1346	1,2,3,6,8,18,21,24	4,5,7,19,20,22,23,25,26	2	P05539,P13432
1347	1,2,3,6,8,18,21,25	4,5,7,19,20,22,23,24,26	5	P00731,P31430,P98089,Q91ZS3,Q9WTT6
1348	1,2,3,6,8,18,21,26	4,5,7,19,20,22,23,24,25	7	P00774,P02770,Q00715,Q5QE79,Q63661,Q63942,Q91ZS3
1349	1,2,3,6,8,18,22,23	4,5,7,19,20,21,24,25,26	4	P22006,P51907,Q5XI22,Q9Z1F2
1350	1,2,3,6,8,18,22,24	4,5,7,19,20,21,23,25,26	1	P83121
1351	1,2,3,6,8,18,22,25	4,5,7,19,20,21,23,24,26	3	P20766,Q4FZU6,Q91ZS3

1352	1,2,3,6,8,18,22,26	4,5,7,19,20,21,23,24,25	4	P02770,P20766,Q5QE79,Q63942
1353	1,2,3,6,8,18,23,24	4,5,7,19,20,21,22,25,26	4	P05539,P51907,Q5XI22,Q63942
1354	1,2,3,6,8,18,23,25	4,5,7,19,20,21,22,24,26	5	P51907,P55314,Q5XI22,Q91ZS3,Q9Z1F2
1355	1,2,3,6,8,18,23,26	4,5,7,19,20,21,22,24,25	13	P01946,P02770,P35467,P51907,Q4FZU2,Q5QE79,Q5XI22,Q63942,Q6IFU8,Q6IFW6,Q6IG02,Q6IMF3,Q6P6Q2
1356	1,2,3,6,8,18,24,25	4,5,7,19,20,21,22,23,26	3	P05539,Q4FZU6,Q91ZS3
1357	1,2,3,6,8,18,24,26	4,5,7,19,20,21,22,23,25	2	P02770,Q63942
1358	1,2,3,6,8,18,25,26	4,5,7,19,20,21,22,23,24	6	P00774,P35280,P55091,Q5QE79,Q63942,Q91ZS3
1359	1,2,3,6,8,19,20,21	4,5,7,18,22,23,24,25,26	4	P00714,P35281,P62804,Q91ZS3
1360	1,2,3,6,8,19,20,22	4,5,7,18,21,23,24,25,26	3	P00714,P22006,P83121
1361	1,2,3,6,8,19,20,23	4,5,7,18,21,22,24,25,26	6	P15399,P22006,P82995,P97580,Q30KJ2,Q66H69
1362	1,2,3,6,8,19,20,24	4,5,7,18,21,22,23,25,26	4	P00714,P82995,P83121,Q66H69
1363	1,2,3,6,8,19,20,25	4,5,7,18,21,22,23,24,26	3	P35280,Q66H69,Q91ZS3
1364	1,2,3,6,8,19,20,26	4,5,7,18,21,22,23,24,25	3	P35280,P62804,Q66H69
1365	1,2,3,6,8,19,21,22	4,5,7,18,20,23,24,25,26	3	P00714,P02782,P35281
1366	1,2,3,6,8,19,21,23	4,5,7,18,20,22,24,25,26	5	P15399,P35281,P51907,P97580,Q30KJ2

1367	1,2,3,6,8,19,21,24	4,5,7,18,20,22,23,25,26	3	P00714,P05539,P35281
1368	1,2,3,6,8,19,21,25	4,5,7,18,20,22,23,24,26	4	P47967,P97840,Q4FZU6,Q91ZS3
1369	1,2,3,6,8,19,21,26	4,5,7,18,20,22,23,24,25	3	P02770,P35281,Q00715
1370	1,2,3,6,8,19,22,23	4,5,7,18,20,21,24,25,26	6	P15399,P20766,P22006,P51907,P97580,Q30KJ2
1371	1,2,3,6,8,19,22,24	4,5,7,18,20,21,23,25,26	2	P00714,P83121
1372	1,2,3,6,8,19,22,25	4,5,7,18,20,21,23,24,26	4	P06911,P20766,Q4FZU6,Q5GRG2
1373	1,2,3,6,8,19,22,26	4,5,7,18,20,21,23,24,25	1	P20766
1374	1,2,3,6,8,19,23,24	4,5,7,18,20,21,22,25,26	4	P15399,P51907,P97580,Q30KJ2
1375	1,2,3,6,8,19,23,25	4,5,7,18,20,21,22,24,26	8	P06911,P15399,P51907,P55314,P97580,Q30KJ2,Q4FZU6,Q9Z1F2
1376	1,2,3,6,8,19,23,26	4,5,7,18,20,21,22,24,25	5	P01946,P15399,P51907,P97580,Q30KJ2
1377	1,2,3,6,8,19,24,25	4,5,7,18,20,21,22,23,26	1	Q4FZU6
1378	1,2,3,6,8,19,24,26	4,5,7,18,20,21,22,23,25	1	P02770
1379	1,2,3,6,8,19,25,26	4,5,7,18,20,21,22,23,24	1	P35280
1380	1,2,3,6,8,20,21,22	4,5,7,18,19,23,24,25,26	6	P00714,P02782,P13432,P62804,Q5XFX0,Q91ZS3
1381	1,2,3,6,8,20,21,23	4,5,7,18,19,22,24,25,26	2	P62804,Q91ZS3

1382	1,2,3,6,8,20,21,24	4,5,7,18,19,22,23,25,26	4	P00714,P13432,Q5XFX0,Q91ZS3
1383	1,2,3,6,8,20,21,25	4,5,7,18,19,22,23,24,26	4	P62804,Q6P6R2,Q91ZS3,Q9WTT6
1384	1,2,3,6,8,20,21,26	4,5,7,18,19,22,23,24,25	4	P62804,Q00715,Q5QE79,Q91ZS3
1385	1,2,3,6,8,20,22,23	4,5,7,18,19,21,24,25,26	2	P22006,P82995
1386	1,2,3,6,8,20,22,24	4,5,7,18,19,21,23,25,26	5	P00714,P27213,P82995,P83121,Q5XFX0
1387	1,2,3,6,8,20,22,25	4,5,7,18,19,21,23,24,26	2	P06911,Q91ZS3
1388	1,2,3,6,8,20,22,26	4,5,7,18,19,21,23,24,25	2	P20766,Q5QE79
1389	1,2,3,6,8,20,23,24	4,5,7,18,19,21,22,25,26	1	P82995
1390	1,2,3,6,8,20,23,25	4,5,7,18,19,21,22,24,26	3	P55314,Q6P6R2,Q91ZS3
1391	1,2,3,6,8,20,23,26	4,5,7,18,19,21,22,24,25	2	P01946,Q5QE79
1392	1,2,3,6,8,20,24,25	4,5,7,18,19,21,22,23,26	1	Q91ZS3
1393	1,2,3,6,8,20,24,26	4,5,7,18,19,21,22,23,25	1	P02770
1394	1,2,3,6,8,20,25,26	4,5,7,18,19,21,22,23,24	2	P35280,Q91ZS3
1395	1,2,3,6,8,21,22,23	4,5,7,18,19,20,24,25,26	1	P02782
1396	1,2,3,6,8,21,22,24	4,5,7,18,19,20,23,25,26	5	P00714,P02782,P13432,P14408,Q5XFX0

1397	1,2,3,6,8,21,22,25	4,5,7,18,19,20,23,24,26	4	P06911,Q4FZU6,Q91ZS3,Q9WTT6
1398	1,2,3,6,8,21,22,26	4,5,7,18,19,20,23,24,25	2	Q00715,Q5QE79
1399	1,2,3,6,8,21,23,24	4,5,7,18,19,20,22,25,26	1	P05539
1400	1,2,3,6,8,21,23,25	4,5,7,18,19,20,22,24,26	4	P55314,Q6P6R2,Q91ZS3,Q9WTT6
1401	1,2,3,6,8,21,23,26	4,5,7,18,19,20,22,24,25	4	P01946,Q00715,Q5QE79,Q6IFV1
1402	1,2,3,6,8,21,24,25	4,5,7,18,19,20,22,23,26	6	P05539,Q4FZU6,Q5XFX0,Q6P6R2,Q91ZS3,Q9WTT6
1403	1,2,3,6,8,21,24,26	4,5,7,18,19,20,22,23,25	2	P02770,Q5QE79
1404	1,2,3,6,8,21,25,26	4,5,7,18,19,20,22,23,24	4	Q00715,Q5QE79,Q91ZS3,Q9WTT6
1405	1,2,3,6,8,22,23,24	4,5,7,18,19,20,21,25,26	2	P51907,P82995
1406	1,2,3,6,8,22,23,25	4,5,7,18,19,20,21,24,26	6	P06911,P20766,P51907,P55314,Q4FZU6,Q5GRG2
1407	1,2,3,6,8,22,23,26	4,5,7,18,19,20,21,24,25	2	P20766,Q5QE79
1408	1,2,3,6,8,22,24,25	4,5,7,18,19,20,21,23,26	2	P06911,Q4FZU6
1409	1,2,3,6,8,22,24,26	4,5,7,18,19,20,21,23,25	3	P02770,P20766,Q5QE79
1410	1,2,3,6,8,22,25,26	4,5,7,18,19,20,21,23,24	2	P20766,Q5QE79
1411	1,2,3,6,8,23,24,25	4,5,7,18,19,20,21,22,26	7	P19629,P36374,P51907,P55314,Q4FZU6,Q63751,Q6P6R2

1412	1,2,3,6,8,23,24,26	4,5,7,18,19,20,21,22,25	3	P01946,P51907,Q5QE79
1413	1,2,3,6,8,23,25,26	4,5,7,18,19,20,21,22,24	7	P01946,P51907,P55314,Q5QE79,Q6P6R2,Q91ZS3,Q9WTT6
1414	1,2,3,6,8,24,25,26	4,5,7,18,19,20,21,22,23	0	
1415	1,2,3,6,18,19,20,21	4,5,7,8,22,23,24,25,26	3	P00731,Q02253,Q91ZS3
1416	1,2,3,6,18,19,20,22	4,5,7,8,21,23,24,25,26	5	P06760,P07150,P22006,P83121,Q6AYS4
1417	1,2,3,6,18,19,20,23	4,5,7,8,21,22,24,25,26	5	P15399,P22006,P97580,Q30KJ2,Q6AYS4
1418	1,2,3,6,18,19,20,24	4,5,7,8,21,22,23,25,26	2	P83121,Q6AYS4
1419	1,2,3,6,18,19,20,25	4,5,7,8,21,22,23,24,26	3	P35280,Q02253,Q91ZS3
1420	1,2,3,6,18,19,20,26	4,5,7,8,21,22,23,24,25	6	P06760,P35280,Q63355,Q63942,Q6AYS4,Q99041
1421	1,2,3,6,18,19,21,22	4,5,7,8,20,23,24,25,26	0	
1422	1,2,3,6,18,19,21,23	4,5,7,8,20,22,24,25,26	6	P05964,P14668,P15399,P97580,Q30KJ2,Q63661
1423	1,2,3,6,18,19,21,24	4,5,7,8,20,22,23,25,26	1	P05539
1424	1,2,3,6,18,19,21,25	4,5,7,8,20,22,23,24,26	7	P00731,P31430,P47967,P97840,P98089,Q4FZU6,Q91ZS3
1425	1,2,3,6,18,19,21,26	4,5,7,8,20,22,23,24,25	2	Q63661,Q63942
1426	1,2,3,6,18,19,22,23	4,5,7,8,20,21,24,25,26	5	P15399,P22006,P97580,Q30KJ2,Q8CJD3

1427	1,2,3,6,18,<br>19,22,24	4,5,7,8,20,<br>21,23,25,2<br>6	2	P83121,Q6AYS4
1428	1,2,3,6,18,<br>19,22,25	4,5,7,8,20,<br>21,23,24,2<br>6	2	P97840,Q4FZU6
1429	1,2,3,6,18,<br>19,22,26	4,5,7,8,20,<br>21,23,24,2<br>5	4	P06760,Q63661,Q63942,Q99041
1430	1,2,3,6,18,<br>19,23,24	4,5,7,8,20,<br>21,22,25,2<br>6	4	P15399,P97580,Q30KJ2,Q6AYS4
1431	1,2,3,6,18,<br>19,23,25	4,5,7,8,20,<br>21,22,24,2<br>6	6	P15399,P55314,P97580,Q30KJ2,Q4FZU6,Q99MH3
1432	1,2,3,6,18,<br>19,23,26	4,5,7,8,20,<br>21,22,24,2<br>5	9	P01946,P15399,P35467,P97580,Q30KJ2,Q63661,Q63942,Q6IFU8,Q6P6Q2
1433	1,2,3,6,18,<br>19,24,25	4,5,7,8,20,<br>21,22,23,2<br>6	1	Q4FZU6
1434	1,2,3,6,18,<br>19,24,26	4,5,7,8,20,<br>21,22,23,2<br>5	3	P06760,Q63942,Q6AYS4
1435	1,2,3,6,18,<br>19,25,26	4,5,7,8,20,<br>21,22,23,2<br>4	4	P35280,Q4FZU6,Q63661,Q63942
1436	1,2,3,6,18,<br>20,21,22	4,5,7,8,19,<br>23,24,25,2<br>6	3	P00714,Q91ZS3,Q99041
1437	1,2,3,6,18,<br>20,21,23	4,5,7,8,19,<br>22,24,25,2<br>6	1	Q91ZS3
1438	1,2,3,6,18,<br>20,21,24	4,5,7,8,19,<br>22,23,25,2<br>6	4	P00714,P13432,Q91ZS3,Q99041
1439	1,2,3,6,18,<br>20,21,25	4,5,7,8,19,<br>22,23,24,2<br>6	3	P31430,P98089,Q91ZS3
1440	1,2,3,6,18,<br>20,21,26	4,5,7,8,19,<br>22,23,24,2<br>5	4	P25031,P62804,Q91ZS3,Q99041
1441	1,2,3,6,18,<br>20,22,23	4,5,7,8,19,<br>21,24,25,2<br>6	1	P22006

1442	1,2,3,6,18,20,22,24	4,5,7,8,19,21,23,25,26	5	P00714,P27213,P83121,Q6AYS4,Q99041
1443	1,2,3,6,18,20,22,25	4,5,7,8,19,21,23,24,26	1	Q91ZS3
1444	1,2,3,6,18,20,22,26	4,5,7,8,19,21,23,24,25	2	P06760,Q99041
1445	1,2,3,6,18,20,23,24	4,5,7,8,19,21,22,25,26	1	Q6AYS4
1446	1,2,3,6,18,20,23,25	4,5,7,8,19,21,22,24,26	2	P55314,Q91ZS3
1447	1,2,3,6,18,20,23,26	4,5,7,8,19,21,22,24,25	8	P01946,Q4FZU2,Q63942,Q6IFU8,Q6IFW6,Q6IG02,Q6IMF3,Q6P6Q2
1448	1,2,3,6,18,20,24,25	4,5,7,8,19,21,22,23,26	1	Q91ZS3
1449	1,2,3,6,18,20,24,26	4,5,7,8,19,21,22,23,25	3	P06760,Q6AYS4,Q99041
1450	1,2,3,6,18,20,25,26	4,5,7,8,19,21,22,23,24	3	P35280,Q91ZS3,Q99041
1451	1,2,3,6,18,21,22,23	4,5,7,8,19,20,24,25,26	0	
1452	1,2,3,6,18,21,22,24	4,5,7,8,19,20,23,25,26	3	P00714,P13432,Q99041
1453	1,2,3,6,18,21,22,25	4,5,7,8,19,20,23,24,26	5	P31430,P97840,P98089,Q4FZU6,Q91ZS3
1454	1,2,3,6,18,21,22,26	4,5,7,8,19,20,23,24,25	2	Q63661,Q99041
1455	1,2,3,6,18,21,23,24	4,5,7,8,19,20,22,25,26	2	P05539,P34901
1456	1,2,3,6,18,21,23,25	4,5,7,8,19,20,22,24,26	5	P31430,P55314,P98089,Q6P6R2,Q91ZS3

1457	1,2,3,6,18,21,23,26	4,5,7,8,19,20,22,24,25	9	P00774,P01946,Q4FZU2,Q63661,Q63942,Q6IFU8,Q6IFV1,Q6IMF3,Q6P6Q2
1458	1,2,3,6,18,21,24,25	4,5,7,8,19,20,22,23,26	5	P05539,P31430,P98089,Q4FZU6,Q91ZS3
1459	1,2,3,6,18,21,24,26	4,5,7,8,19,20,22,23,25	2	Q63661,Q99041
1460	1,2,3,6,18,21,25,26	4,5,7,8,19,20,22,23,24	7	P00774,P25031,P31430,P55091,P98089,Q63661,Q91ZS3
1461	1,2,3,6,18,22,23,24	4,5,7,8,19,20,21,25,26	0	
1462	1,2,3,6,18,22,23,25	4,5,7,8,19,20,21,24,26	2	P55314,Q4FZU6
1463	1,2,3,6,18,22,23,26	4,5,7,8,19,20,21,24,25	6	Q63661,Q63942,Q6IFU7,Q6IFU8,Q6IMF3,Q6P6Q2
1464	1,2,3,6,18,22,24,25	4,5,7,8,19,20,21,23,26	1	Q4FZU6
1465	1,2,3,6,18,22,24,26	4,5,7,8,19,20,21,23,25	2	P06760,Q99041
1466	1,2,3,6,18,22,25,26	4,5,7,8,19,20,21,23,24	2	Q4FZU6,Q99041
1467	1,2,3,6,18,23,24,25	4,5,7,8,19,20,21,22,26	2	P55314,Q4FZU6
1468	1,2,3,6,18,23,24,26	4,5,7,8,19,20,21,22,25	6	P01946,Q63942,Q6IFU7,Q6IFU8,Q6IMF3,Q6P6Q2
1469	1,2,3,6,18,23,25,26	4,5,7,8,19,20,21,22,24	10	P00774,P01946,P55314,Q4FZU2,Q63661,Q63942,Q6IFU8,Q6IMF3,Q6P6Q2,Q91ZS3
1470	1,2,3,6,18,24,25,26	4,5,7,8,19,20,21,22,23	5	P00774,P08494,P55091,Q4FZU6,Q7TPB1
1471	1,2,3,6,19,20,21,22	4,5,7,8,18,23,24,25,26	2	P00714,Q6AYS4

1472	1,2,3,6,19,20,21,23	4,5,7,8,18,22,24,25,26	5	P00714,P15399,P97580,Q30KJ2,Q6AYS4
1473	1,2,3,6,19,20,21,24	4,5,7,8,18,22,23,25,26	2	P00714,Q6AYS4
1474	1,2,3,6,19,20,21,25	4,5,7,8,18,22,23,24,26	4	P47967,P97840,Q8VD89,Q91ZS3
1475	1,2,3,6,19,20,21,26	4,5,7,8,18,22,23,24,25	3	P00714,P62804,Q6AYS4
1476	1,2,3,6,19,20,22,23	4,5,7,8,18,21,24,25,26	8	P00714,P15399,P22006,P55091,P82995,P97580,Q30KJ2,Q6AYS4
1477	1,2,3,6,19,20,22,24	4,5,7,8,18,21,23,25,26	6	P00714,P07150,P14669,P82995,P83121,Q6AYS4
1478	1,2,3,6,19,20,22,25	4,5,7,8,18,21,23,24,26	2	Q4FZU6,Q6AYS4
1479	1,2,3,6,19,20,22,26	4,5,7,8,18,21,23,24,25	3	P00714,P55159,Q6AYS4
1480	1,2,3,6,19,20,23,24	4,5,7,8,18,21,22,25,26	6	P00714,P15399,P82995,P97580,Q30KJ2,Q6AYS4
1481	1,2,3,6,19,20,23,25	4,5,7,8,18,21,22,24,26	5	P15399,P55314,P97580,Q30KJ2,Q6AYS4
1482	1,2,3,6,19,20,23,26	4,5,7,8,18,21,22,24,25	6	P01946,P15399,P97580,Q30KJ2,Q66H69,Q6AYS4
1483	1,2,3,6,19,20,24,25	4,5,7,8,18,21,22,23,26	2	Q4FZU6,Q6AYS4
1484	1,2,3,6,19,20,24,26	4,5,7,8,18,21,22,23,25	2	P00714,Q6AYS4
1485	1,2,3,6,19,20,25,26	4,5,7,8,18,21,22,23,24	3	P35280,Q6AYS4,Q91ZS3
1486	1,2,3,6,19,21,22,23	4,5,7,8,18,20,24,25,26	4	P00714,P15399,P97580,Q30KJ2

1487	1,2,3,6,19,21,22,24	4,5,7,8,18,20,23,25,26	3	P00714,P07171,Q6AYS4
1488	1,2,3,6,19,21,22,25	4,5,7,8,18,20,23,24,26	3	P47967,P97840,Q4FZU6
1489	1,2,3,6,19,21,22,26	4,5,7,8,18,20,23,24,25	2	P00714,P07171
1490	1,2,3,6,19,21,23,24	4,5,7,8,18,20,22,25,26	5	P00714,P15399,P97580,Q30KJ2,Q6AYS4
1491	1,2,3,6,19,21,23,25	4,5,7,8,18,20,22,24,26	7	P15399,P55314,P97580,P97840,Q30KJ2,Q4FZU6,Q6P6R2
1492	1,2,3,6,19,21,23,26	4,5,7,8,18,20,22,24,25	5	P01946,P15399,P97580,Q30KJ2,Q63661
1493	1,2,3,6,19,21,24,25	4,5,7,8,18,20,22,23,26	2	P97840,Q4FZU6
1494	1,2,3,6,19,21,24,26	4,5,7,8,18,20,22,23,25	3	P00714,P07171,Q6AYS4
1495	1,2,3,6,19,21,25,26	4,5,7,8,18,20,22,23,24	2	P97840,Q91ZS3
1496	1,2,3,6,19,22,23,24	4,5,7,8,18,20,21,25,26	7	P00714,P15399,P82471,P82995,P97580,Q30KJ2,Q6AYS4
1497	1,2,3,6,19,22,23,25	4,5,7,8,18,20,21,24,26	6	P06911,P15399,P55314,P97580,Q30KJ2,Q4FZU6
1498	1,2,3,6,19,22,23,26	4,5,7,8,18,20,21,24,25	5	P01946,P15399,P97580,Q30KJ2,Q6AYS4
1499	1,2,3,6,19,22,24,25	4,5,7,8,18,20,21,23,26	2	Q4FZU6,Q6AYS4
1500	1,2,3,6,19,22,24,26	4,5,7,8,18,20,21,23,25	4	P00714,P20646,Q6AYS4,Q9R044
1501	1,2,3,6,19,22,25,26	4,5,7,8,18,20,21,23,24	1	Q4FZU6

1502	1,2,3,6,19,23,24,25	4,5,7,8,18,20,21,22,26	5	P15399,P55314,P97580,Q30KJ2,Q4FZU6
1503	1,2,3,6,19,23,24,26	4,5,7,8,18,20,21,22,25	6	P01946,P02091,P15399,P97580,Q30KJ2,Q6AYS4
1504	1,2,3,6,19,23,25,26	4,5,7,8,18,20,21,22,24	7	P01946,P02091,P15399,P55314,P97580,Q30KJ2,Q4FZU6
1505	1,2,3,6,19,24,25,26	4,5,7,8,18,20,21,22,23	4	P01946,P14480,Q4FZU6,Q6AYS4
1506	1,2,3,6,20,21,22,23	4,5,7,8,18,19,24,25,26	2	P00714,P82995
1507	1,2,3,6,20,21,22,24	4,5,7,8,18,19,23,25,26	6	P00714,P13432,P82995,Q5XFX0,Q6AYS4,Q99041
1508	1,2,3,6,20,21,22,25	4,5,7,8,18,19,23,24,26	2	Q03191,Q91ZS3
1509	1,2,3,6,20,21,22,26	4,5,7,8,18,19,23,24,25	2	P00714,Q99041
1510	1,2,3,6,20,21,23,24	4,5,7,8,18,19,22,25,26	3	P00714,P82995,Q6AYS4
1511	1,2,3,6,20,21,23,25	4,5,7,8,18,19,22,24,26	3	P55314,Q6P6R2,Q91ZS3
1512	1,2,3,6,20,21,23,26	4,5,7,8,18,19,22,24,25	3	P01946,Q6IFU8,Q6IFV1
1513	1,2,3,6,20,21,24,25	4,5,7,8,18,19,22,23,26	2	Q5XFX0,Q91ZS3
1514	1,2,3,6,20,21,24,26	4,5,7,8,18,19,22,23,25	3	P00714,Q6AYS4,Q99041
1515	1,2,3,6,20,21,25,26	4,5,7,8,18,19,22,23,24	1	Q91ZS3
1516	1,2,3,6,20,22,23,24	4,5,7,8,18,19,21,25,26	3	P00714,P82995,Q6AYS4

1517	1,2,3,6,20,22,23,25	4,5,7,8,18,19,21,24,26	3	P06911,P55314,P82995
1518	1,2,3,6,20,22,23,26	4,5,7,8,18,19,21,24,25	1	Q6AYS4
1519	1,2,3,6,20,22,24,25	4,5,7,8,18,19,21,23,26	4	P82995,Q4FZU6,Q5XFX0,Q6AYS4
1520	1,2,3,6,20,22,24,26	4,5,7,8,18,19,21,23,25	5	P00714,P02803,Q6AYS4,Q99041,Q99MH3
1521	1,2,3,6,20,22,25,26	4,5,7,8,18,19,21,23,24	1	Q91ZS3
1522	1,2,3,6,20,23,24,25	4,5,7,8,18,19,21,22,26	3	P55314,P82995,Q4FZU6
1523	1,2,3,6,20,23,24,26	4,5,7,8,18,19,21,22,25	3	P01946,P02091,Q6AYS4
1524	1,2,3,6,20,23,25,26	4,5,7,8,18,19,21,22,24	3	P01946,P55314,Q91ZS3
1525	1,2,3,6,20,24,25,26	4,5,7,8,18,19,21,22,23	2	Q6AYS4,Q91ZS3
1526	1,2,3,6,21,22,23,24	4,5,7,8,18,19,20,25,26	3	P00714,P13432,P34901
1527	1,2,3,6,21,22,23,25	4,5,7,8,18,19,20,24,26	4	P06911,P55314,Q4FZU6,Q6P6R2
1528	1,2,3,6,21,22,23,26	4,5,7,8,18,19,20,24,25	1	P01946
1529	1,2,3,6,21,22,24,25	4,5,7,8,18,19,20,23,26	3	P35467,Q4FZU6,Q5XFX0
1530	1,2,3,6,21,22,24,26	4,5,7,8,18,19,20,23,25	4	P00714,P07171,P13432,Q99041
1531	1,2,3,6,21,22,25,26	4,5,7,8,18,19,20,23,24	3	P07171,Q4FZU6,Q91ZS3

1532	1,2,3,6,21,23,24,25	4,5,7,8,18,19,20,22,26	4	P55314,Q4FZU6,Q63751,Q6P6R2
1533	1,2,3,6,21,23,24,26	4,5,7,8,18,19,20,22,25	2	P01946,Q6IFV1
1534	1,2,3,6,21,23,25,26	4,5,7,8,18,19,20,22,24	4	P01946,P55314,Q6P6R2,Q91ZS3
1535	1,2,3,6,21,24,25,26	4,5,7,8,18,19,20,22,23	2	Q4FZU6,Q91ZS3
1536	1,2,3,6,22,23,24,25	4,5,7,8,18,19,20,21,26	5	P06911,P55314,P82471,P82995,Q4FZU6
1537	1,2,3,6,22,23,24,26	4,5,7,8,18,19,20,21,25	4	P00731,P01946,P82471,Q6AYS4
1538	1,2,3,6,22,23,25,26	4,5,7,8,18,19,20,21,24	3	P01946,P55314,Q4FZU6
1539	1,2,3,6,22,24,25,26	4,5,7,8,18,19,20,21,23	1	Q4FZU6
1540	1,2,3,6,23,24,25,26	4,5,7,8,18,19,20,21,22	5	P01946,P02091,P55314,Q4FZU6,Q7TPB1
1541	1,2,3,7,8,18,19,20	4,5,6,21,22,23,24,25,26	4	P06760,P35280,P83121,Q08849
1542	1,2,3,7,8,18,19,21	4,5,6,20,22,23,24,25,26	4	P00731,P02803,P05964,P42854
1543	1,2,3,7,8,18,19,22	4,5,6,20,21,23,24,25,26	4	P01835,P06760,P20766,P83121
1544	1,2,3,7,8,18,19,23	4,5,6,20,21,22,24,25,26	9	P02803,P05964,P16086,P35467,Q03191,Q498D9,Q5XI22,Q63532,Q99MH3
1545	1,2,3,7,8,18,19,24	4,5,6,20,21,22,23,25,26	4	P01835,P06760,P83121,Q63532
1546	1,2,3,7,8,18,19,25	4,5,6,20,21,22,23,24,26	5	P01835,P02803,P10715,P19629,P35280

1547	1,2,3,7,8,18,19,26	4,5,6,20,21,22,23,24,25	5	P01946,P02770,P06760,P35280,Q63942
1548	1,2,3,7,8,18,20,21	4,5,6,19,22,23,24,25,26	2	P42854,Q91ZS3
1549	1,2,3,7,8,18,20,22	4,5,6,19,21,23,24,25,26	5	P06760,P10715,P27213,P83121,Q08849
1550	1,2,3,7,8,18,20,23	4,5,6,19,21,22,24,25,26	4	A2RUV9,P10715,Q08849,Q5XI22
1551	1,2,3,7,8,18,20,24	4,5,6,19,21,22,23,25,26	3	P06760,P10715,P83121
1552	1,2,3,7,8,18,20,25	4,5,6,19,21,22,23,24,26	4	P10715,P19629,P35280,Q91ZS3
1553	1,2,3,7,8,18,20,26	4,5,6,19,21,22,23,24,25	7	P01041,P01946,P02770,P06760,P10715,P35280,Q08849
1554	1,2,3,7,8,18,21,22	4,5,6,19,20,23,24,25,26	1	D4A1J4
1555	1,2,3,7,8,18,21,23	4,5,6,19,20,22,24,25,26	2	P14668,Q5XI22
1556	1,2,3,7,8,18,21,24	4,5,6,19,20,22,23,25,26	1	P05539
1557	1,2,3,7,8,18,21,25	4,5,6,19,20,22,23,24,26	8	O35783,P08721,P10715,P19629,P25031,P31430,P98089,Q91ZS3
1558	1,2,3,7,8,18,21,26	4,5,6,19,20,22,23,24,25	9	O35783,O54800;Q5DWV2,P01041,P01946,P02770,P25031,P55091,P82995,Q63661
1559	1,2,3,7,8,18,22,23	4,5,6,19,20,21,24,25,26	2	P20766,Q5XI22
1560	1,2,3,7,8,18,22,24	4,5,6,19,20,21,23,25,26	3	P01835,P06760,P83121
1561	1,2,3,7,8,18,22,25	4,5,6,19,20,21,23,24,26	4	P01835,P10715,P19629,P20766

1562	1,2,3,7,8,18,22,26	4,5,6,19,20,21,23,24,25	5	P01041,P01946,P02770,P06760,P20766
1563	1,2,3,7,8,18,23,24	4,5,6,19,20,21,22,25,26	1	Q5XI22
1564	1,2,3,7,8,18,23,25	4,5,6,19,20,21,22,24,26	4	P10715,P19629,Q5XI22,Q99MH3
1565	1,2,3,7,8,18,23,26	4,5,6,19,20,21,22,24,25	7	A2RUV9,P01946,P02091,P02770,P35467,Q63942,Q6P6Q2
1566	1,2,3,7,8,18,24,25	4,5,6,19,20,21,22,23,26	3	P01835,P10715,P19629
1567	1,2,3,7,8,18,24,26	4,5,6,19,20,21,22,23,25	6	P01946,P02091,P02770,P06760,P10715,P55091
1568	1,2,3,7,8,18,25,26	4,5,6,19,20,21,22,23,24	10	O35783,P01946,P02091,P02770,P08721,P10715,P19629,P35280,P55091,P82995
1569	1,2,3,7,8,19,20,21	4,5,6,18,22,23,24,25,26	2	P42854,Q6IFU7
1570	1,2,3,7,8,19,20,22	4,5,6,18,21,23,24,25,26	3	P20766,P83121,Q08849
1571	1,2,3,7,8,19,20,23	4,5,6,18,21,22,24,25,26	2	P16086,Q08849
1572	1,2,3,7,8,19,20,24	4,5,6,18,21,22,23,25,26	1	P83121
1573	1,2,3,7,8,19,20,25	4,5,6,18,21,22,23,24,26	5	P10715,P19629,P35280,Q08849,Q6IFU7
1574	1,2,3,7,8,19,20,26	4,5,6,18,21,22,23,24,25	6	P01041,P01946,P02770,P06760,P35280,Q08849
1575	1,2,3,7,8,19,21,22	4,5,6,18,20,23,24,25,26	1	D4A1J4
1576	1,2,3,7,8,19,21,23	4,5,6,18,20,22,24,25,26	3	P14668,P15399,P16086

1577	1,2,3,7,8,19,21,24	4,5,6,18,20,22,23,25,26	0	
1578	1,2,3,7,8,19,21,25	4,5,6,18,20,22,23,24,26	5	P10715,P19629,P47967,P97840,Q6IFU7
1579	1,2,3,7,8,19,21,26	4,5,6,18,20,22,23,24,25	2	P01946,P02770
1580	1,2,3,7,8,19,22,23	4,5,6,18,20,21,24,25,26	4	P01835,P16086,P20766,Q63532
1581	1,2,3,7,8,19,22,24	4,5,6,18,20,21,23,25,26	6	P01835,P20646,P20766,P83121,Q63532,Q6IMF3
1582	1,2,3,7,8,19,22,25	4,5,6,18,20,21,23,24,26	6	P01835,P10715,P19629,P20766,Q5GRG2,Q6IFU7
1583	1,2,3,7,8,19,22,26	4,5,6,18,20,21,23,24,25	7	O54728,P01835,P01946,P02770,P06760,P20766,P55159
1584	1,2,3,7,8,19,23,24	4,5,6,18,20,21,22,25,26	2	P01835,Q63532
1585	1,2,3,7,8,19,23,25	4,5,6,18,20,21,22,24,26	4	P01835,P10715,P19629,P97580
1586	1,2,3,7,8,19,23,26	4,5,6,18,20,21,22,24,25	3	P01946,P02091,Q08849
1587	1,2,3,7,8,19,24,25	4,5,6,18,20,21,22,23,26	4	P01835,P10715,P19629,Q63532
1588	1,2,3,7,8,19,24,26	4,5,6,18,20,21,22,23,25	6	P01835,P01946,P02091,P02770,P06760,Q63532
1589	1,2,3,7,8,19,25,26	4,5,6,18,20,21,22,23,24	6	P01835,P01946,P02091,P10715,P19629,P35280
1590	1,2,3,7,8,20,21,22	4,5,6,18,19,23,24,25,26	1	P42854
1591	1,2,3,7,8,20,21,23	4,5,6,18,19,22,24,25,26	0	

1592	1,2,3,7,8,20,21,24	4,5,6,18,19,22,23,25,26	1	Q5XFX0
1593	1,2,3,7,8,20,21,25	4,5,6,18,19,22,23,24,26	4	P10715,P19629,Q6IFU7,Q91ZS3
1594	1,2,3,7,8,20,21,26	4,5,6,18,19,22,23,24,25	6	O35783,P01041,P01946,P02770,P62804,Q00715
1595	1,2,3,7,8,20,22,23	4,5,6,18,19,21,24,25,26	2	P20766,Q08849
1596	1,2,3,7,8,20,22,24	4,5,6,18,19,21,23,25,26	3	P27213,P83121,Q5XFX0
1597	1,2,3,7,8,20,22,25	4,5,6,18,19,21,23,24,26	5	P10715,P19629,P20766,Q08849,Q5GRG2
1598	1,2,3,7,8,20,22,26	4,5,6,18,19,21,23,24,25	6	P01041,P01946,P06760,P20766,P55159,Q08849
1599	1,2,3,7,8,20,23,24	4,5,6,18,19,21,22,25,26	1	Q08849
1600	1,2,3,7,8,20,23,25	4,5,6,18,19,21,22,24,26	3	P10715,P19629,Q08849
1601	1,2,3,7,8,20,23,26	4,5,6,18,19,21,22,24,25	3	P01946,P02091,Q08849
1602	1,2,3,7,8,20,24,25	4,5,6,18,19,21,22,23,26	2	P10715,P19629
1603	1,2,3,7,8,20,24,26	4,5,6,18,19,21,22,23,25	5	P01041,P01946,P02091,P02770,P10715
1604	1,2,3,7,8,20,25,26	4,5,6,18,19,21,22,23,24	8	P01041,P01946,P02091,P10715,P19629,P35280,Q08849,Q91ZS3
1605	1,2,3,7,8,21,22,23	4,5,6,18,19,20,24,25,26	0	
1606	1,2,3,7,8,21,22,24	4,5,6,18,19,20,23,25,26	2	P00714,Q5XFX0

1607	1,2,3,7,8,21,22,25	4,5,6,18,19,20,23,24,26	4	P10715,P19629,P20766,Q6IFU7
1608	1,2,3,7,8,21,22,26	4,5,6,18,19,20,23,24,25	5	O54800;Q5DWV2,P01041,P01946,P02770,P20766
1609	1,2,3,7,8,21,23,24	4,5,6,18,19,20,22,25,26	0	
1610	1,2,3,7,8,21,23,25	4,5,6,18,19,20,22,24,26	2	P10715,P19629
1611	1,2,3,7,8,21,23,26	4,5,6,18,19,20,22,24,25	3	P01946,P02091,P02770
1612	1,2,3,7,8,21,24,25	4,5,6,18,19,20,22,23,26	2	P10715,P19629
1613	1,2,3,7,8,21,24,26	4,5,6,18,19,20,22,23,25	4	P01041,P01946,P02091,P02770
1614	1,2,3,7,8,21,25,26	4,5,6,18,19,20,22,23,24	7	O35783,O54800;Q5DWV2,P01946,P02091,P10715,P19629,Q91ZS3
1615	1,2,3,7,8,22,23,24	4,5,6,18,19,20,21,25,26	2	P01835,P20766
1616	1,2,3,7,8,22,23,25	4,5,6,18,19,20,21,24,26	6	P01835,P06911,P10715,P19629,P20766,Q5GRG2
1617	1,2,3,7,8,22,23,26	4,5,6,18,19,20,21,24,25	4	O54728,P01946,P02091,P20766
1618	1,2,3,7,8,22,24,25	4,5,6,18,19,20,21,23,26	5	P01835,P10715,P19629,P20766,Q5GRG2
1619	1,2,3,7,8,22,24,26	4,5,6,18,19,20,21,23,25	5	P01835,P01946,P02091,P02770,P20766
1620	1,2,3,7,8,22,25,26	4,5,6,18,19,20,21,23,24	6	P01835,P01946,P02091,P10715,P19629,P20766
1621	1,2,3,7,8,23,24,25	4,5,6,18,19,20,21,22,26	4	P01835,P10715,P19629,Q63751

1622	1,2,3,7,8,23,24,26	4,5,6,18,19,20,21,22,25	3	P01946,P02091,P02770
1623	1,2,3,7,8,23,25,26	4,5,6,18,19,20,21,22,24	4	P01946,P02091,P10715,P19629
1624	1,2,3,7,8,24,25,26	4,5,6,18,19,20,21,22,23	5	P01835,P01946,P02091,P10715,P19629
1625	1,2,3,7,18,19,20,21	4,5,6,8,22,23,24,25,26	6	P00731,P06760,P14668,P25031,P42854,Q9Z0V6
1626	1,2,3,7,18,19,20,22	4,5,6,8,21,23,24,25,26	6	P06760,P07150,P14669,P83121,Q9WTT6,Q9Z0V6
1627	1,2,3,7,18,19,20,23	4,5,6,8,21,22,24,25,26	4	P06760,P14668,Q9WTT6,Q9Z0V6
1628	1,2,3,7,18,19,20,24	4,5,6,8,21,22,23,25,26	7	P06760,P07150,P14669,P83121,Q6AYS4,Q9WTT6,Q9Z0V6
1629	1,2,3,7,18,19,20,25	4,5,6,8,21,22,23,24,26	2	P35280,P47967
1630	1,2,3,7,18,19,20,26	4,5,6,8,21,22,23,24,25	7	P01041,P01946,P06760,P07150,P35280,Q9WTT6,Q9Z0V6
1631	1,2,3,7,18,19,21,22	4,5,6,8,20,23,24,25,26	3	P06760,P14668,P97840
1632	1,2,3,7,18,19,21,23	4,5,6,8,20,22,24,25,26	8	D3ZUC6,P05964,P14668,P15399,P16086,Q03191,Q63661,Q99MH3
1633	1,2,3,7,18,19,21,24	4,5,6,8,20,22,23,25,26	2	D3ZUC6,P06760
1634	1,2,3,7,18,19,21,25	4,5,6,8,20,22,23,24,26	7	P00731,P14668,P20760,P25031,P31430,P47967,P97840
1635	1,2,3,7,18,19,21,26	4,5,6,8,20,22,23,24,25	6	D3ZUC6,P01946,P06760,P25031,P82995,Q63661
1636	1,2,3,7,18,19,22,23	4,5,6,8,20,21,24,25,26	4	P06760,P14668,Q63532,Q9WTT6

1637	1,2,3,7,18,19,22,24	4,5,6,8,20,21,23,25,26	9	P01835,P06760,P07150,P14669,P20646,P83121,Q63532,Q9WTT6,Q9Z0V6
1638	1,2,3,7,18,19,22,25	4,5,6,8,20,21,23,24,26	3	P01835,P47967,P97840
1639	1,2,3,7,18,19,22,26	4,5,6,8,20,21,23,24,25	7	P01946,P06760,P07150,P55159,Q63661,Q9WTT6,Q9Z0V6
1640	1,2,3,7,18,19,23,24	4,5,6,8,20,21,22,25,26	4	P06760,Q03191,Q63532,Q9WTT6
1641	1,2,3,7,18,19,23,25	4,5,6,8,20,21,22,24,26	4	P14668,Q03191,Q63532,Q99MH3
1642	1,2,3,7,18,19,23,26	4,5,6,8,20,21,22,24,25	8	P01946,P02091,P06760,P35467,Q03191,Q07523,Q63532,Q63661
1643	1,2,3,7,18,19,24,25	4,5,6,8,20,21,22,23,26	2	P01835,Q63532
1644	1,2,3,7,18,19,24,26	4,5,6,8,20,21,22,23,25	5	P01946,P02091,P06760,Q63532,Q9WTT6
1645	1,2,3,7,18,19,25,26	4,5,6,8,20,21,22,23,24	6	P01946,P02091,P35280,P55091,P82995,Q63661
1646	1,2,3,7,18,20,21,22	4,5,6,8,19,23,24,25,26	2	P06760,P42854
1647	1,2,3,7,18,20,21,23	4,5,6,8,19,22,24,25,26	3	A2RUV9,P14668,P25031
1648	1,2,3,7,18,20,21,24	4,5,6,8,19,22,23,25,26	1	P06760
1649	1,2,3,7,18,20,21,25	4,5,6,8,19,22,23,24,26	4	O35783,P25031,P31430,Q91ZS3
1650	1,2,3,7,18,20,21,26	4,5,6,8,19,22,23,24,25	6	O35783,P01041,P01946,P06760,P25031,P42854
1651	1,2,3,7,18,20,22,23	4,5,6,8,19,21,24,25,26	3	A2RUV9,P06760,Q9WTT6

1652	1,2,3,7,18,20,22,24	4,5,6,8,19,21,23,25,26	9	P06760,P07150,P14669,P27213,P83121,Q99041,Q9QZK9,Q9WTT6,Q9Z0V6
1653	1,2,3,7,18,20,22,25	4,5,6,8,19,21,23,24,26	0	
1654	1,2,3,7,18,20,22,26	4,5,6,8,19,21,23,24,25	8	P01041,P01946,P06760,P07150,P55159,Q99041,Q9WTT6,Q9Z0V6
1655	1,2,3,7,18,20,23,24	4,5,6,8,19,21,22,25,26	3	A2RUV9,P06760,Q9QZK9
1656	1,2,3,7,18,20,23,25	4,5,6,8,19,21,22,24,26	1	A2RUV9
1657	1,2,3,7,18,20,23,26	4,5,6,8,19,21,22,24,25	7	A2RUV9,P01946,P02091,P06760,Q6IFU8,Q6IG02,Q6P6Q2
1658	1,2,3,7,18,20,24,25	4,5,6,8,19,21,22,23,26	1	P10715
1659	1,2,3,7,18,20,24,26	4,5,6,8,19,21,22,23,25	6	P01041,P01946,P02091,P06760,Q99041,Q9WTT6
1660	1,2,3,7,18,20,25,26	4,5,6,8,19,21,22,23,24	6	O35783,P01041,P01946,P02091,P35280,Q91ZS3
1661	1,2,3,7,18,21,22,23	4,5,6,8,19,20,24,25,26	1	P14668
1662	1,2,3,7,18,21,22,24	4,5,6,8,19,20,23,25,26	1	P06760
1663	1,2,3,7,18,21,22,25	4,5,6,8,19,20,23,24,26	3	P25031,P31430,P97840
1664	1,2,3,7,18,21,22,26	4,5,6,8,19,20,23,24,25	5	P01041,P01946,P06760,P25031,Q63661
1665	1,2,3,7,18,21,23,24	4,5,6,8,19,20,22,25,26	2	D3ZUC6,P14668
1666	1,2,3,7,18,21,23,25	4,5,6,8,19,20,22,24,26	4	P14668,P25031,P31430,Q99MH3

1667	1,2,3,7,18,21,23,26	4,5,6,8,19,20,22,24,25	10	A2RUV9,D3ZUC6,O35783,P01946,P02091,P14668,P25031,Q63661,Q6IFU8,Q6P6Q2
1668	1,2,3,7,18,21,24,25	4,5,6,8,19,20,22,23,26	2	P31430,P98089
1669	1,2,3,7,18,21,24,26	4,5,6,8,19,20,22,23,25	8	D3ZUC6,P01041,P01946,P02091,P06760,P25031,P55091,Q63661
1670	1,2,3,7,18,21,25,26	4,5,6,8,19,20,22,23,24	12	O35783,P00774,P01946,P02091,P08721,P25031,P31430,P55091,P82995,P98089,Q63661,Q91ZS3
1671	1,2,3,7,18,22,23,24	4,5,6,8,19,20,21,25,26	2	P06760,Q9WTT6
1672	1,2,3,7,18,22,23,25	4,5,6,8,19,20,21,24,26	0	
1673	1,2,3,7,18,22,23,26	4,5,6,8,19,20,21,24,25	5	A2RUV9,P01946,P02091,P06760,Q63661
1674	1,2,3,7,18,22,24,25	4,5,6,8,19,20,21,23,26	1	P01835
1675	1,2,3,7,18,22,24,26	4,5,6,8,19,20,21,23,25	5	P01946,P02091,P06760,Q99041,Q9WTT6
1676	1,2,3,7,18,22,25,26	4,5,6,8,19,20,21,23,24	3	P01946,P02091,P55091
1677	1,2,3,7,18,23,24,25	4,5,6,8,19,20,21,22,26	1	A2RUV9
1678	1,2,3,7,18,23,24,26	4,5,6,8,19,20,21,22,25	5	A2RUV9,P01946,P02091,P06760,Q07523
1679	1,2,3,7,18,23,25,26	4,5,6,8,19,20,21,22,24	5	A2RUV9,P01946,P02091,P55091,Q63661
1680	1,2,3,7,18,24,25,26	4,5,6,8,19,20,21,22,23	4	P01946,P02091,P55091,Q7TPB1
1681	1,2,3,7,19,20,21,22	4,5,6,8,18,23,24,25,26	3	P00714,P06760,Q9Z0V6

1682	1,2,3,7,19,20,21,23	4,5,6,8,18,22,24,25,26	3	P14668,P15399,P16086
1683	1,2,3,7,19,20,21,24	4,5,6,8,18,22,23,25,26	2	P00714,P06760
1684	1,2,3,7,19,20,21,25	4,5,6,8,18,22,23,24,26	2	P47967,P97840
1685	1,2,3,7,19,20,21,26	4,5,6,8,18,22,23,24,25	3	P01041,P01946,P06760
1686	1,2,3,7,19,20,22,23	4,5,6,8,18,21,24,25,26	5	P06760,P07150,P20646,Q08849,Q9WTT6
1687	1,2,3,7,19,20,22,24	4,5,6,8,18,21,23,25,26	12	P00714,P00774,P06760,P07150,P14669,P20646,P27213,P83121,Q6AYS4,Q6P6Q2,Q9WTT6,Q9Z0V6
1688	1,2,3,7,19,20,22,25	4,5,6,8,18,21,23,24,26	1	P14669
1689	1,2,3,7,19,20,22,26	4,5,6,8,18,21,23,24,25	10	O54728,P01946,P06760,P07150,P14669,P20646,P55159,Q6AYS4,Q9WTT6,Q9Z0V6
1690	1,2,3,7,19,20,23,24	4,5,6,8,18,21,22,25,26	4	P06760,Q63532,Q6AYS4,Q9WTT6
1691	1,2,3,7,19,20,23,25	4,5,6,8,18,21,22,24,26	1	Q08849
1692	1,2,3,7,19,20,23,26	4,5,6,8,18,21,22,24,25	4	P01946,P02091,P06760,Q08849
1693	1,2,3,7,19,20,24,25	4,5,6,8,18,21,22,23,26	0	
1694	1,2,3,7,19,20,24,26	4,5,6,8,18,21,22,23,25	7	P01946,P02091,P06760,P07150,P20646,Q6AYS4,Q9WTT6
1695	1,2,3,7,19,20,25,26	4,5,6,8,18,21,22,23,24	3	P01946,P02091,P35280
1696	1,2,3,7,19,21,22,23	4,5,6,8,18,20,24,25,26	2	P14668,P16086

1697	1,2,3,7,19,21,22,24	4,5,6,8,18,20,23,25,26	3	P00714,P06760,Q6IG02
1698	1,2,3,7,19,21,22,25	4,5,6,8,18,20,23,24,26	2	P47967,P97840
1699	1,2,3,7,19,21,22,26	4,5,6,8,18,20,23,24,25	2	P01946,P06760
1700	1,2,3,7,19,21,23,24	4,5,6,8,18,20,22,25,26	1	Q63532
1701	1,2,3,7,19,21,23,25	4,5,6,8,18,20,22,24,26	4	P14668,P15399,P47967,P97840
1702	1,2,3,7,19,21,23,26	4,5,6,8,18,20,22,24,25	4	P01946,P02091,P14668,Q63661
1703	1,2,3,7,19,21,24,25	4,5,6,8,18,20,22,23,26	2	P47967,P97840
1704	1,2,3,7,19,21,24,26	4,5,6,8,18,20,22,23,25	3	P01946,P02091,P06760
1705	1,2,3,7,19,21,25,26	4,5,6,8,18,20,22,23,24	4	P01946,P02091,P47967,P97840
1706	1,2,3,7,19,22,23,24	4,5,6,8,18,20,21,25,26	6	P01835,P06760,P20646,P82471,Q63532,Q9WTT6
1707	1,2,3,7,19,22,23,25	4,5,6,8,18,20,21,24,26	2	P01835,P17046
1708	1,2,3,7,19,22,23,26	4,5,6,8,18,20,21,24,25	7	O54728,P01946,P02091,P06760,P20646,P55159,Q9WTT6
1709	1,2,3,7,19,22,24,25	4,5,6,8,18,20,21,23,26	4	P01835,P17046,Q6IMF3,Q6P6Q2
1710	1,2,3,7,19,22,24,26	4,5,6,8,18,20,21,23,25	8	P01835,P01946,P02091,P06760,P07150,P20646,Q6AYS4,Q9WTT6
1711	1,2,3,7,19,22,25,26	4,5,6,8,18,20,21,23,24	3	P01835,P01946,P02091

1712	1,2,3,7,19,23,24,25	4,5,6,8,18,20,21,22,26	2	P01835,Q63532
1713	1,2,3,7,19,23,24,26	4,5,6,8,18,20,21,22,25	5	P01946,P02091,P06760,P20646,Q63532
1714	1,2,3,7,19,23,25,26	4,5,6,8,18,20,21,22,24	2	P01946,P02091
1715	1,2,3,7,19,24,25,26	4,5,6,8,18,20,21,22,23	4	P01835,P01946,P02091,Q7TPB1
1716	1,2,3,7,20,21,22,23	4,5,6,8,18,19,24,25,26	1	P14668
1717	1,2,3,7,20,21,22,24	4,5,6,8,18,19,23,25,26	2	P00714,Q5XFX0
1718	1,2,3,7,20,21,22,25	4,5,6,8,18,19,23,24,26	0	
1719	1,2,3,7,20,21,22,26	4,5,6,8,18,19,23,24,25	5	P00714,P01041,P01836,P01946,P06760
1720	1,2,3,7,20,21,23,24	4,5,6,8,18,19,22,25,26	0	
1721	1,2,3,7,20,21,23,25	4,5,6,8,18,19,22,24,26	2	P14668,Q91ZS3
1722	1,2,3,7,20,21,23,26	4,5,6,8,18,19,22,24,25	2	P01946,P02091
1723	1,2,3,7,20,21,24,25	4,5,6,8,18,19,22,23,26	0	
1724	1,2,3,7,20,21,24,26	4,5,6,8,18,19,22,23,25	5	P01041,P01836,P01946,P02091,P06760
1725	1,2,3,7,20,21,25,26	4,5,6,8,18,19,22,23,24	5	O35783,P01041,P01946,P02091,Q91ZS3
1726	1,2,3,7,20,22,23,24	4,5,6,8,18,19,21,25,26	3	P06760,P20646,Q9WTT6

1727	1,2,3,7,20,22,23,25	4,5,6,8,18,19,21,24,26	1	Q08849
1728	1,2,3,7,20,22,23,26	4,5,6,8,18,19,21,24,25	6	O54728,P01946,P02091,P06760,P55159,Q08849
1729	1,2,3,7,20,22,24,25	4,5,6,8,18,19,21,23,26	2	P0C0A9,P14669
1730	1,2,3,7,20,22,24,26	4,5,6,8,18,19,21,23,25	11	P00714,P01946,P02091,P02803,P06760,P07150,P08010,P14669,P20646,Q99041,Q9WTT6
1731	1,2,3,7,20,22,25,26	4,5,6,8,18,19,21,23,24	2	P01946,P02091
1732	1,2,3,7,20,23,24,25	4,5,6,8,18,19,21,22,26	2	A2RUV9,P0C0A9
1733	1,2,3,7,20,23,24,26	4,5,6,8,18,19,21,22,25	4	A2RUV9,P01946,P02091,P06760
1734	1,2,3,7,20,23,25,26	4,5,6,8,18,19,21,22,24	4	A2RUV9,P01946,P02091,Q08849
1735	1,2,3,7,20,24,25,26	4,5,6,8,18,19,21,22,23	3	P01946,P02091,P0C0A9
1736	1,2,3,7,21,22,23,24	4,5,6,8,18,19,20,25,26	2	P00714,P34901
1737	1,2,3,7,21,22,23,25	4,5,6,8,18,19,20,24,26	1	P14668
1738	1,2,3,7,21,22,23,26	4,5,6,8,18,19,20,24,25	2	P01946,P02091
1739	1,2,3,7,21,22,24,25	4,5,6,8,18,19,20,23,26	1	P35467
1740	1,2,3,7,21,22,24,26	4,5,6,8,18,19,20,23,25	4	P00714,P01946,P02091,P06760
1741	1,2,3,7,21,22,25,26	4,5,6,8,18,19,20,23,24	2	P01946,P02091

1742	1,2,3,7,21,23,24,25	4,5,6,8,18,19,20,22,26	1	P22006
1743	1,2,3,7,21,23,24,26	4,5,6,8,18,19,20,22,25	2	P01946,P02091
1744	1,2,3,7,21,23,25,26	4,5,6,8,18,19,20,22,24	3	P01946,P02091,P22006
1745	1,2,3,7,21,24,25,26	4,5,6,8,18,19,20,22,23	3	P01946,P02091,P22006
1746	1,2,3,7,22,23,24,25	4,5,6,8,18,19,20,21,26	3	P01835,P0C0A9,P82471
1747	1,2,3,7,22,23,24,26	4,5,6,8,18,19,20,21,25	6	P00731,P01946,P02091,P06760,P20646,P82471
1748	1,2,3,7,22,23,25,26	4,5,6,8,18,19,20,21,24	2	P01946,P02091
1749	1,2,3,7,22,24,25,26	4,5,6,8,18,19,20,21,23	4	P01835,P01946,P02091,P0C0A9
1750	1,2,3,7,23,24,25,26	4,5,6,8,18,19,20,21,22	3	P01946,P02091,Q7TPB1
1751	1,2,3,8,18,19,20,21	4,5,6,7,22,23,24,25,26	5	P05539,P20760,P35281,P62804,P83121
1752	1,2,3,8,18,19,20,22	4,5,6,7,21,23,24,25,26	3	P22006,P83121,Q08849
1753	1,2,3,8,18,19,20,23	4,5,6,7,21,22,24,25,26	6	P22006,P35467,P83121,Q08849,Q5XI22,Q9QZK9
1754	1,2,3,8,18,19,20,24	4,5,6,7,21,22,23,25,26	3	P05539,P83121,Q9QZK9
1755	1,2,3,8,18,19,20,25	4,5,6,7,21,22,23,24,26	4	P20760,P35280,P83121,Q08849
1756	1,2,3,8,18,19,20,26	4,5,6,7,21,22,23,24,25	6	P06760,P35280,P83121,Q08849,Q63355,Q63942

1757	1,2,3,8,18,19,21,22	4,5,6,7,20,23,24,25,26	1	P83121
1758	1,2,3,8,18,19,21,23	4,5,6,7,20,22,24,25,26	5	P05539,P20760,Q03191,Q5XI22,Q99MH3
1759	1,2,3,8,18,19,21,24	4,5,6,7,20,22,23,25,26	2	P05539,P83121
1760	1,2,3,8,18,19,21,25	4,5,6,7,20,22,23,24,26	5	P05539,P20760,P31430,P47967,P98089
1761	1,2,3,8,18,19,21,26	4,5,6,7,20,22,23,24,25	2	Q63661,Q63942
1762	1,2,3,8,18,19,22,23	4,5,6,7,20,21,24,25,26	4	P20646,P22006,P83121,Q5XI22
1763	1,2,3,8,18,19,22,24	4,5,6,7,20,21,23,25,26	4	P01835,P05539,P20646,P83121
1764	1,2,3,8,18,19,22,25	4,5,6,7,20,21,23,24,26	4	P01835,P20760,P20766,P83121
1765	1,2,3,8,18,19,22,26	4,5,6,7,20,21,23,24,25	5	P06760,P20646,P20766,P83121,Q63942
1766	1,2,3,8,18,19,23,24	4,5,6,7,20,21,22,25,26	5	P05539,P09605;P25809,P83121,Q03191,Q5XI22
1767	1,2,3,8,18,19,23,25	4,5,6,7,20,21,22,24,26	2	P20760,Q99MH3
1768	1,2,3,8,18,19,23,26	4,5,6,7,20,21,22,24,25	7	P01946,P09605;P25809,P35467,Q03191,Q63661,Q63942,Q6P6Q2
1769	1,2,3,8,18,19,24,25	4,5,6,7,20,21,22,23,26	3	P01835,P05539,P83121
1770	1,2,3,8,18,19,24,26	4,5,6,7,20,21,22,23,25	5	P02770,P06760,P09605;P25809,P83121,Q63942
1771	1,2,3,8,18,19,25,26	4,5,6,7,20,21,22,23,24	2	P35280,Q63942

1772	1,2,3,8,18,20,21,22	4,5,6,7,19,23,24,25,26	2	P83121,Q99041
1773	1,2,3,8,18,20,21,23	4,5,6,7,19,22,24,25,26	1	Q5XI22
1774	1,2,3,8,18,20,21,24	4,5,6,7,19,22,23,25,26	2	P05539,P83121
1775	1,2,3,8,18,20,21,25	4,5,6,7,19,22,23,24,26	2	P20760,P31430
1776	1,2,3,8,18,20,21,26	4,5,6,7,19,22,23,24,25	3	P62804,Q00715,Q99041
1777	1,2,3,8,18,20,22,23	4,5,6,7,19,21,24,25,26	4	P22006,P83121,Q08849,Q9QZK9
1778	1,2,3,8,18,20,22,24	4,5,6,7,19,21,23,25,26	4	P83121,Q5FVI6,Q99041,Q9QZK9
1779	1,2,3,8,18,20,22,25	4,5,6,7,19,21,23,24,26	3	P20760,P83121,Q08849
1780	1,2,3,8,18,20,22,26	4,5,6,7,19,21,23,24,25	3	P83121,Q08849,Q99041
1781	1,2,3,8,18,20,23,24	4,5,6,7,19,21,22,25,26	4	P05539,P83121,Q5XI22,Q9QZK9
1782	1,2,3,8,18,20,23,25	4,5,6,7,19,21,22,24,26	2	P20760,Q08849
1783	1,2,3,8,18,20,23,26	4,5,6,7,19,21,22,24,25	8	P01946,P19218,P35467,Q08849,Q63942,Q6IFU8,Q6IG02,Q6P6Q2
1784	1,2,3,8,18,20,24,25	4,5,6,7,19,21,22,23,26	2	P05539,P83121
1785	1,2,3,8,18,20,24,26	4,5,6,7,19,21,22,23,25	2	P83121,Q99041
1786	1,2,3,8,18,20,25,26	4,5,6,7,19,21,22,23,24	2	P35280,Q08849

1787	1,2,3,8,18,21,22,23	4,5,6,7,19,20,24,25,26	1	Q5XI22
1788	1,2,3,8,18,21,22,24	4,5,6,7,19,20,23,25,26	3	P05539,P83121,Q5FVI6
1789	1,2,3,8,18,21,22,25	4,5,6,7,19,20,23,24,26	2	P20760,P31430
1790	1,2,3,8,18,21,22,26	4,5,6,7,19,20,23,24,25	2	Q5QE79,Q99041
1791	1,2,3,8,18,21,23,24	4,5,6,7,19,20,22,25,26	2	P05539,Q5XI22
1792	1,2,3,8,18,21,23,25	4,5,6,7,19,20,22,24,26	5	P05539,P20760,P31430,P98089,Q99MH3
1793	1,2,3,8,18,21,23,26	4,5,6,7,19,20,22,24,25	8	P01946,P35467,Q5QE79,Q63661,Q63942,Q6IFU8,Q6IFV1,Q6P6Q2
1794	1,2,3,8,18,21,24,25	4,5,6,7,19,20,22,23,26	3	P05539,P31430,P98089
1795	1,2,3,8,18,21,24,26	4,5,6,7,19,20,22,23,25	2	P02770,Q99041
1796	1,2,3,8,18,21,25,26	4,5,6,7,19,20,22,23,24	5	P00774,P08721,P31430,P55091,P98089
1797	1,2,3,8,18,22,23,24	4,5,6,7,19,20,21,25,26	2	P83121,Q5FVI6
1798	1,2,3,8,18,22,23,25	4,5,6,7,19,20,21,24,26	2	P20760,P20766
1799	1,2,3,8,18,22,23,26	4,5,6,7,19,20,21,24,25	5	P01946,P20766,P35467,Q63942,Q6P6Q2
1800	1,2,3,8,18,22,24,25	4,5,6,7,19,20,21,23,26	3	P01835,P83121,Q5FVI6
1801	1,2,3,8,18,22,24,26	4,5,6,7,19,20,21,23,25	2	P83121,Q99041

1802	1,2,3,8,18,22,25,26	4,5,6,7,19,20,21,23,24	1	P20766
1803	1,2,3,8,18,23,24,25	4,5,6,7,19,20,21,22,26	1	P05539
1804	1,2,3,8,18,23,24,26	4,5,6,7,19,20,21,22,25	5	P01946,P09605;P25809,P35467,Q63942,Q6P6Q2
1805	1,2,3,8,18,23,25,26	4,5,6,7,19,20,21,22,24	6	P01946,P35467,P55091,Q63942,Q6IFU8,Q6P6Q2
1806	1,2,3,8,18,24,25,26	4,5,6,7,19,20,21,22,23	2	P55091,Q7TPB1
1807	1,2,3,8,19,20,21,22	4,5,6,7,18,23,24,25,26	2	P00714,P83121
1808	1,2,3,8,19,20,21,23	4,5,6,7,18,22,24,25,26	0	
1809	1,2,3,8,19,20,21,24	4,5,6,7,18,22,23,25,26	3	P00714,P05539,P83121
1810	1,2,3,8,19,20,21,25	4,5,6,7,18,22,23,24,26	2	P20760,Q8VD89
1811	1,2,3,8,19,20,21,26	4,5,6,7,18,22,23,24,25	3	P62804,Q00715,Q8K4G9
1812	1,2,3,8,19,20,22,23	4,5,6,7,18,21,24,25,26	5	P20646,P22006,P61206;P84079;P84082,P83121,Q08849
1813	1,2,3,8,19,20,22,24	4,5,6,7,18,21,23,25,26	5	P00714,P00774,P20646,P61206;P84079;P84082,P83121
1814	1,2,3,8,19,20,22,25	4,5,6,7,18,21,23,24,26	3	P20760,P83121,Q08849
1815	1,2,3,8,19,20,22,26	4,5,6,7,18,21,23,24,25	5	O54728,P20646,P55159,P83121,Q08849
1816	1,2,3,8,19,20,23,24	4,5,6,7,18,21,22,25,26	5	P20646,P61206;P84079;P84082,P83121,P83748,Q08849

1817	1,2,3,8,19,20,23,25	4,5,6,7,18,21,22,24,26	2	P20760,Q08849
1818	1,2,3,8,19,20,23,26	4,5,6,7,18,21,22,24,25	2	P01946,Q08849
1819	1,2,3,8,19,20,24,25	4,5,6,7,18,21,22,23,26	1	P83121
1820	1,2,3,8,19,20,24,26	4,5,6,7,18,21,22,23,25	2	P20646,P83121
1821	1,2,3,8,19,20,25,26	4,5,6,7,18,21,22,23,24	3	P35280,Q08849,Q8K4G9
1822	1,2,3,8,19,21,22,23	4,5,6,7,18,20,24,25,26	1	P61206;P84079;P84082
1823	1,2,3,8,19,21,22,24	4,5,6,7,18,20,23,25,26	4	P00714,P05539,P61206;P84079;P84082,P83121
1824	1,2,3,8,19,21,22,25	4,5,6,7,18,20,23,24,26	1	P20760
1825	1,2,3,8,19,21,22,26	4,5,6,7,18,20,23,24,25	0	
1826	1,2,3,8,19,21,23,24	4,5,6,7,18,20,22,25,26	2	P05539,P61206;P84079;P84082
1827	1,2,3,8,19,21,23,25	4,5,6,7,18,20,22,24,26	1	P20760
1828	1,2,3,8,19,21,23,26	4,5,6,7,18,20,22,24,25	2	P01946,Q63661
1829	1,2,3,8,19,21,24,25	4,5,6,7,18,20,22,23,26	1	P05539
1830	1,2,3,8,19,21,24,26	4,5,6,7,18,20,22,23,25	0	
1831	1,2,3,8,19,21,25,26	4,5,6,7,18,20,22,23,24	0	

1832	1,2,3,8,19,22,23,24	4,5,6,7,18,20,21,25,26	5	P01835,P20646,P61206;P84079;P84082,P82471,P83121
1833	1,2,3,8,19,22,23,25	4,5,6,7,18,20,21,24,26	4	P01835,P20760,P20766,Q08849
1834	1,2,3,8,19,22,23,26	4,5,6,7,18,20,21,24,25	5	O54728,P01946,P20646,P20766,Q08849
1835	1,2,3,8,19,22,24,25	4,5,6,7,18,20,21,23,26	2	P01835,P83121
1836	1,2,3,8,19,22,24,26	4,5,6,7,18,20,21,23,25	4	P01835,P20646,P36953,P83121
1837	1,2,3,8,19,22,25,26	4,5,6,7,18,20,21,23,24	2	P01835,P20766
1838	1,2,3,8,19,23,24,25	4,5,6,7,18,20,21,22,26	2	P01835,P83748
1839	1,2,3,8,19,23,24,26	4,5,6,7,18,20,21,22,25	4	P01946,P02091,P09605;P25809,P20646
1840	1,2,3,8,19,23,25,26	4,5,6,7,18,20,21,22,24	3	P01946,P02091,Q08849
1841	1,2,3,8,19,24,25,26	4,5,6,7,18,20,21,22,23	2	P01835,P01946
1842	1,2,3,8,20,21,22,23	4,5,6,7,18,19,24,25,26	0	
1843	1,2,3,8,20,21,22,24	4,5,6,7,18,19,23,25,26	4	P00714,P83121,Q5FVI6,Q5XFX0
1844	1,2,3,8,20,21,22,25	4,5,6,7,18,19,23,24,26	2	P20760,Q5XFX0
1845	1,2,3,8,20,21,22,26	4,5,6,7,18,19,23,24,25	1	Q99041
1846	1,2,3,8,20,21,23,24	4,5,6,7,18,19,22,25,26	1	P05539

1847	1,2,3,8,20,21,23,25	4,5,6,7,18,19,22,24,26	1	P20760
1848	1,2,3,8,20,21,23,26	4,5,6,7,18,19,22,24,25	3	P01946,P62804,Q5QE79
1849	1,2,3,8,20,21,24,25	4,5,6,7,18,19,22,23,26	2	P05539,Q5XFX0
1850	1,2,3,8,20,21,24,26	4,5,6,7,18,19,22,23,25	1	Q99041
1851	1,2,3,8,20,21,25,26	4,5,6,7,18,19,22,23,24	0	
1852	1,2,3,8,20,22,23,24	4,5,6,7,18,19,21,25,26	7	P20646,P61206;P84079;P84082,P82995,P83121,Q08849,Q5FVI6,Q9QZK9
1853	1,2,3,8,20,22,23,25	4,5,6,7,18,19,21,24,26	1	Q08849
1854	1,2,3,8,20,22,23,26	4,5,6,7,18,19,21,24,25	3	O54728,P01946,Q08849
1855	1,2,3,8,20,22,24,25	4,5,6,7,18,19,21,23,26	3	P83121,Q5FVI6,Q5XFX0
1856	1,2,3,8,20,22,24,26	4,5,6,7,18,19,21,23,25	5	P20646,P36953,P83121,Q64573,Q99041
1857	1,2,3,8,20,22,25,26	4,5,6,7,18,19,21,23,24	2	P20766,Q08849
1858	1,2,3,8,20,23,24,25	4,5,6,7,18,19,21,22,26	2	P83748,Q08849
1859	1,2,3,8,20,23,24,26	4,5,6,7,18,19,21,22,25	5	P01946,P19218,P47967,P97840,Q08849
1860	1,2,3,8,20,23,25,26	4,5,6,7,18,19,21,22,24	3	P01946,P19218,Q08849
1861	1,2,3,8,20,24,25,26	4,5,6,7,18,19,21,22,23	0	

1862	1,2,3,8,21,22,23,24	4,5,6,7,18,19,20,25,26	2	P61206;P84079;P84082,Q5FVI6
1863	1,2,3,8,21,22,23,25	4,5,6,7,18,19,20,24,26	1	P20760
1864	1,2,3,8,21,22,23,26	4,5,6,7,18,19,20,24,25	2	P01946,Q5QE79
1865	1,2,3,8,21,22,24,25	4,5,6,7,18,19,20,23,26	2	Q5FVI6,Q5XFX0
1866	1,2,3,8,21,22,24,26	4,5,6,7,18,19,20,23,25	1	Q99041
1867	1,2,3,8,21,22,25,26	4,5,6,7,18,19,20,23,24	2	P20766,Q5QE79
1868	1,2,3,8,21,23,24,25	4,5,6,7,18,19,20,22,26	1	P05539
1869	1,2,3,8,21,23,24,26	4,5,6,7,18,19,20,22,25	2	P01946,Q5QE79
1870	1,2,3,8,21,23,25,26	4,5,6,7,18,19,20,22,24	2	P01946,Q5QE79
1871	1,2,3,8,21,24,25,26	4,5,6,7,18,19,20,22,23	1	P01946
1872	1,2,3,8,22,23,24,25	4,5,6,7,18,19,20,21,26	3	P01835,P82471,Q5FVI6
1873	1,2,3,8,22,23,24,26	4,5,6,7,18,19,20,21,25	7	P00731,P01946,P20646,P36953,P47967,P82471,Q63556
1874	1,2,3,8,22,23,25,26	4,5,6,7,18,19,20,21,24	2	P01946,P20766
1875	1,2,3,8,22,24,25,26	4,5,6,7,18,19,20,21,23	3	P01835,P20766,P36953
1876	1,2,3,8,23,24,25,26	4,5,6,7,18,19,20,21,22	3	P01946,P02091,Q7TPB1

1877	1,2,3,18,19,20,21,22	4,5,6,7,8,23,24,25,26	7	P00714,P06760,P20760,P83121,Q02253,Q99041,Q9Z0V6
1878	1,2,3,18,19,20,21,23	4,5,6,7,8,22,24,25,26	2	P14668,P20760
1879	1,2,3,18,19,20,21,24	4,5,6,7,8,22,23,25,26	7	P00714,P05539,P06760,P06911,P83121,Q99041,Q9Z0V6
1880	1,2,3,18,19,20,21,25	4,5,6,7,8,22,23,24,26	4	P20760,P31430,P47967,Q02253
1881	1,2,3,18,19,20,21,26	4,5,6,7,8,22,23,24,25	5	P06760,P06911,Q63617,Q63661,Q99041
1882	1,2,3,18,19,20,22,23	4,5,6,7,8,21,24,25,26	8	P06760,P20646,P20760,P22006,P83121,Q9QZK9,Q9WTT6,Q9Z0V6
1883	1,2,3,18,19,20,22,24	4,5,6,7,8,21,23,25,26	12	P00714,P06760,P07150,P14669,P20646,P83121,Q6AYS4,Q6P6R2,Q99041,Q9QZK9,Q9WTT6,Q9Z0V6
1884	1,2,3,18,19,20,22,25	4,5,6,7,8,21,23,24,26	2	P20760,P83121
1885	1,2,3,18,19,20,22,26	4,5,6,7,8,21,23,24,25	9	P06760,P07150,P20646,P55159,P83121,Q6P6R2,Q99041,Q9WTT6,Q9Z0V6
1886	1,2,3,18,19,20,23,24	4,5,6,7,8,21,22,25,26	8	P06760,P20646,P83121,P83748,Q6AYS4,Q9QZK9,Q9WTT6,Q9Z0V6
1887	1,2,3,18,19,20,23,25	4,5,6,7,8,21,22,24,26	1	P20760
1888	1,2,3,18,19,20,23,26	4,5,6,7,8,21,22,24,25	7	P01946,P06760,P35467,Q63661,Q6IFU8,Q6IG02,Q6P6Q2
1889	1,2,3,18,19,20,24,25	4,5,6,7,8,21,22,23,26	3	P20760,P22282,P83121
1890	1,2,3,18,19,20,24,26	4,5,6,7,8,21,22,23,25	9	P06760,P06911,P07150,P20646,P83121,Q6AYS4,Q99041,Q9WTT6,Q9Z0V6
1891	1,2,3,18,19,20,25,26	4,5,6,7,8,21,22,23,24	1	P35280
1892	1,2,3,18,19,21,22,23	4,5,6,7,8,20,24,25,26	2	P14668,P20760
1893	1,2,3,18,19,21,22,24	4,5,6,7,8,20,23,25,26	7	P00714,P05539,P83121,Q63617,Q99041,Q9WTT6,Q9Z0V6
1894	1,2,3,18,19,21,22,25	4,5,6,7,8,20,23,24,26	5	P20760,P31430,P47967,P97840,Q63617
1895	1,2,3,18,19,21,22,26	4,5,6,7,8,20,23,24,25	4	P06760,Q63617,Q63661,Q99041
1896	1,2,3,18,19,21,23,24	4,5,6,7,8,20,22,25,26	3	D3ZUC6,P05539,Q03191
1897	1,2,3,18,19,21,23,25	4,5,6,7,8,20,22,24,26	5	P14668,P20760,P31430,Q63617,Q99MH3
1898	1,2,3,18,19,21,23,26	4,5,6,7,8,20,22,24,25	9	D3ZUC6,P01946,P35467,Q03191,Q07523,Q63617,Q63661,Q6IFU8,Q6P6Q2
1899	1,2,3,18,19,21,24,25	4,5,6,7,8,20,22,23,26	4	P05539,P20760,P31430,Q63617

1900	1,2,3,18,1<br>9,21,24,26	4,5,6,7,8,2<br>0,22,23,25	6	D3ZUC6,P06760,P06911,Q63617,Q63661,Q99041
1901	1,2,3,18,1<br>9,21,25,26	4,5,6,7,8,2<br>0,22,23,24	3	P31430,Q63617,Q63661
1902	1,2,3,18,1<br>9,22,23,24	4,5,6,7,8,2<br>0,21,25,26	5	P06760,P20646,P82471,P83121,Q9WTT6
1903	1,2,3,18,1<br>9,22,23,25	4,5,6,7,8,2<br>0,21,24,26	2	P20760,Q99MH3
1904	1,2,3,18,1<br>9,22,23,26	4,5,6,7,8,2<br>0,21,24,25	6	P01946,P06760,P20646,P35467,Q63661,Q9WTT6
1905	1,2,3,18,1<br>9,22,24,25	4,5,6,7,8,2<br>0,21,23,26	3	P01835,P20760,P83121
1906	1,2,3,18,1<br>9,22,24,26	4,5,6,7,8,2<br>0,21,23,25	9	P06760,P07150,P20646,P83121,Q63617,Q6P6R2,Q99041,Q9WTT6,Q9Z0V6
1907	1,2,3,18,1<br>9,22,25,26	4,5,6,7,8,2<br>0,21,23,24	1	Q63617
1908	1,2,3,18,1<br>9,23,24,25	4,5,6,7,8,2<br>0,21,22,26	2	P20760,Q99MH3
1909	1,2,3,18,1<br>9,23,24,26	4,5,6,7,8,2<br>0,21,22,25	10	P01946,P02091,P06760,P09605;P25809,P20646,P35467,Q03191,Q07523,Q63661,Q9WTT6
1910	1,2,3,18,1<br>9,23,25,26	4,5,6,7,8,2<br>0,21,22,24	6	P01946,P02091,P35467,Q63617,Q63661,Q99MH3
1911	1,2,3,18,1<br>9,24,25,26	4,5,6,7,8,2<br>0,21,22,23	3	P01946,Q63617,Q7TPB1
1912	1,2,3,18,2<br>0,21,22,23	4,5,6,7,8,1<br>9,24,25,26	2	P20760,Q9QZK9
1913	1,2,3,18,2<br>0,21,22,24	4,5,6,7,8,1<br>9,23,25,26	5	P00714,P83121,Q5FVI6,Q99041,Q9QZK9
1914	1,2,3,18,2<br>0,21,22,25	4,5,6,7,8,1<br>9,23,24,26	3	P20760,P31430,Q99041
1915	1,2,3,18,2<br>0,21,22,26	4,5,6,7,8,1<br>9,23,24,25	2	P06760,Q99041
1916	1,2,3,18,2<br>0,21,23,24	4,5,6,7,8,1<br>9,22,25,26	2	P05539,Q9QZK9
1917	1,2,3,18,2<br>0,21,23,25	4,5,6,7,8,1<br>9,22,24,26	2	P20760,P31430
1918	1,2,3,18,2<br>0,21,23,26	4,5,6,7,8,1<br>9,22,24,25	5	P01946,Q63661,Q6IFU8,Q6IG02,Q6P6Q2
1919	1,2,3,18,2<br>0,21,24,25	4,5,6,7,8,1<br>9,22,23,26	4	P05539,P20760,P31430,Q99041
1920	1,2,3,18,2<br>0,21,24,26	4,5,6,7,8,1<br>9,22,23,25	4	P06760,P06911,P12020,Q99041
1921	1,2,3,18,2<br>0,21,25,26	4,5,6,7,8,1<br>9,22,23,24	2	P31430,Q99041
1922	1,2,3,18,2<br>0,22,23,24	4,5,6,7,8,1<br>9,21,25,26	6	P06760,P20646,P83121,Q4G075,Q9QZK9,Q9WTT6

1923	1,2,3,18,20,22,23,25	4,5,6,7,8,19,21,24,26	1	P20760
1924	1,2,3,18,20,22,23,26	4,5,6,7,8,19,21,24,25	6	P01946,P06760,P20646,Q6IG02,Q6P6Q2,Q9QZK9
1925	1,2,3,18,20,22,24,25	4,5,6,7,8,19,21,23,26	3	P20760,P83121,Q99041
1926	1,2,3,18,20,22,24,26	4,5,6,7,8,19,21,23,25	8	P06760,P07150,P20646,P83121,Q99041,Q9QZK9,Q9WTT6,Q9Z0V6
1927	1,2,3,18,20,22,25,26	4,5,6,7,8,19,21,23,24	1	Q99041
1928	1,2,3,18,20,23,24,25	4,5,6,7,8,19,21,22,26	4	A2RUV9,P20760,P22282,P83748
1929	1,2,3,18,20,23,24,26	4,5,6,7,8,19,21,22,25	8	A2RUV9,P01946,P06760,P19218,Q6IFU8,Q6IG02,Q6P6Q2,Q9QZK9
1930	1,2,3,18,20,23,25,26	4,5,6,7,8,19,21,22,24	6	A2RUV9,P01946,P19218,Q6IFU8,Q6IG02,Q6P6Q2
1931	1,2,3,18,20,24,25,26	4,5,6,7,8,19,21,22,23	3	P22282,Q7TPB1,Q99041
1932	1,2,3,18,21,22,23,24	4,5,6,7,8,19,20,25,26	2	P34901,Q5FVI6
1933	1,2,3,18,21,22,23,25	4,5,6,7,8,19,20,24,26	2	P20760,P31430
1934	1,2,3,18,21,22,23,26	4,5,6,7,8,19,20,24,25	4	P01946,Q63617,Q63661,Q6P6Q2
1935	1,2,3,18,21,22,24,25	4,5,6,7,8,19,20,23,26	3	P20760,P31430,Q99041
1936	1,2,3,18,21,22,24,26	4,5,6,7,8,19,20,23,25	3	P06760,Q63617,Q99041
1937	1,2,3,18,21,22,25,26	4,5,6,7,8,19,20,23,24	3	P31430,Q63617,Q99041
1938	1,2,3,18,21,23,24,25	4,5,6,7,8,19,20,22,26	3	P05539,P20760,P31430
1939	1,2,3,18,21,23,24,26	4,5,6,7,8,19,20,22,25	6	D3ZUC6,P01946,Q63617,Q63661,Q6IFU8,Q6P6Q2
1940	1,2,3,18,21,23,25,26	4,5,6,7,8,19,20,22,24	8	P00774,P01946,P31430,P55091,Q63617,Q63661,Q6IFU8,Q6P6Q2
1941	1,2,3,18,21,24,25,26	4,5,6,7,8,19,20,22,23	6	P00774,P01946,P31430,P55091,Q63617,Q99041
1942	1,2,3,18,22,23,24,25	4,5,6,7,8,19,20,21,26	2	P20760,P82471
1943	1,2,3,18,22,23,24,26	4,5,6,7,8,19,20,21,25	5	P00731,P01946,P06760,P20646,P82471
1944	1,2,3,18,22,23,25,26	4,5,6,7,8,19,20,21,24	1	P01946
1945	1,2,3,18,22,24,25,26	4,5,6,7,8,19,20,21,23	2	Q7TPB1,Q99041

1946	1,2,3,18,23,24,25,26	4,5,6,7,8,19,20,21,22	5	P01946,P02091,P55091,Q6P6Q2,Q7TPB1
1947	1,2,3,19,20,21,22,23	4,5,6,7,8,18,24,25,26	3	P00714,P20760,P61206;P84079;P84082
1948	1,2,3,19,20,21,22,24	4,5,6,7,8,18,23,25,26	6	P00714,P61206;P84079;P84082,P83121,Q6AYS4,Q99041,Q9Z0V6
1949	1,2,3,19,20,21,22,25	4,5,6,7,8,18,23,24,26	2	P20760,Q8VD89
1950	1,2,3,19,20,21,22,26	4,5,6,7,8,18,23,24,25	4	O70377,P00714,P07171,Q99041
1951	1,2,3,19,20,21,23,24	4,5,6,7,8,18,22,25,26	3	P00714,P61206;P84079;P84082,Q6AYS4
1952	1,2,3,19,20,21,23,25	4,5,6,7,8,18,22,24,26	2	P20760,Q8VD89
1953	1,2,3,19,20,21,23,26	4,5,6,7,8,18,22,24,25	1	P01946
1954	1,2,3,19,20,21,24,25	4,5,6,7,8,18,22,23,26	2	P20760,Q8VD89
1955	1,2,3,19,20,21,24,26	4,5,6,7,8,18,22,23,25	4	P00714,P06911,Q6AYS4,Q99041
1956	1,2,3,19,20,21,25,26	4,5,6,7,8,18,22,23,24	1	Q63617
1957	1,2,3,19,20,22,23,24	4,5,6,7,8,18,21,25,26	11	P00714,P07150,P14669,P20646,P61206;P84079;P84082,P82995,P83121,P83748,Q6AYS4,Q9QZK9,Q9WTT6
1958	1,2,3,19,20,22,23,25	4,5,6,7,8,18,21,24,26	3	P20760,P83748,Q08849
1959	1,2,3,19,20,22,23,26	4,5,6,7,8,18,21,24,25	8	O54728,P01946,P06760,P20646,P55159,Q08849,Q6AYS4,Q9WTT6
1960	1,2,3,19,20,22,24,25	4,5,6,7,8,18,21,23,26	5	P0C0A9,P14669,P83121,P83748,Q6AYS4
1961	1,2,3,19,20,22,24,26	4,5,6,7,8,18,21,23,25	11	O54728,P00714,P06760,P07150,P14669,P20646,P83121,Q6AYS4,Q99041,Q9WTT6,Q9Z0V6
1962	1,2,3,19,20,22,25,26	4,5,6,7,8,18,21,23,24	0	
1963	1,2,3,19,20,23,24,25	4,5,6,7,8,18,21,22,26	3	P20760,P83748,Q6AYS4
1964	1,2,3,19,20,23,24,26	4,5,6,7,8,18,21,22,25	6	P01946,P02091,P06760,P19218,P20646,Q6AYS4
1965	1,2,3,19,20,23,25,26	4,5,6,7,8,18,21,22,24	4	P01946,P02091,P19218,Q08849
1966	1,2,3,19,20,24,25,26	4,5,6,7,8,18,21,22,23	3	P01946,P22283,Q6AYS4
1967	1,2,3,19,21,22,23,24	4,5,6,7,8,18,20,25,26	3	P00714,P61206;P84079;P84082,P82471
1968	1,2,3,19,21,22,23,25	4,5,6,7,8,18,20,24,26	1	P20760

1969	1,2,3,19,2<br>1,22,23,26	4,5,6,7,8,1<br>8,20,24,25	3	P01946,Q63617,Q63661
1970	1,2,3,19,2<br>1,22,24,25	4,5,6,7,8,1<br>8,20,23,26	2	P07171,P20760
1971	1,2,3,19,2<br>1,22,24,26	4,5,6,7,8,1<br>8,20,23,25	5	O70377,P00714,P07171,Q63617,Q99041
1972	1,2,3,19,2<br>1,22,25,26	4,5,6,7,8,1<br>8,20,23,24	2	P07171,Q63617
1973	1,2,3,19,2<br>1,23,24,25	4,5,6,7,8,1<br>8,20,22,26	1	P20760
1974	1,2,3,19,2<br>1,23,24,26	4,5,6,7,8,1<br>8,20,22,25	4	P01946,P02091,Q63617,Q63661
1975	1,2,3,19,2<br>1,23,25,26	4,5,6,7,8,1<br>8,20,22,24	4	P01946,P02091,Q63617,Q63661
1976	1,2,3,19,2<br>1,24,25,26	4,5,6,7,8,1<br>8,20,22,23	3	P01946,P07171,Q63617
1977	1,2,3,19,2<br>2,23,24,25	4,5,6,7,8,1<br>8,20,21,26	4	P01835,P20646,P82471,P83748
1978	1,2,3,19,2<br>2,23,24,26	4,5,6,7,8,1<br>8,20,21,25	12	O54728,P00731,P01946,P02091,P06760,P20646,P36953,P61206;P84079;P84082,P82471,Q63556,Q6AYS4,Q9WTT6
1979	1,2,3,19,2<br>2,23,25,26	4,5,6,7,8,1<br>8,20,21,24	3	P01946,P02091,P82471
1980	1,2,3,19,2<br>2,24,25,26	4,5,6,7,8,1<br>8,20,21,23	6	P01835,P01946,P20646,P36953,P82471,Q63617
1981	1,2,3,19,2<br>3,24,25,26	4,5,6,7,8,1<br>8,20,21,22	4	P01946,P02091,P82471,Q7TPB1
1982	1,2,3,20,2<br>1,22,23,24	4,5,6,7,8,1<br>8,19,25,26	3	P00714,P61206;P84079;P84082,Q5FVI6
1983	1,2,3,20,2<br>1,22,23,25	4,5,6,7,8,1<br>8,19,24,26	1	P20760
1984	1,2,3,20,2<br>1,22,23,26	4,5,6,7,8,1<br>8,19,24,25	1	P01946
1985	1,2,3,20,2<br>1,22,24,25	4,5,6,7,8,1<br>8,19,23,26	4	P20760,P35467,Q5XFX0,Q99041
1986	1,2,3,20,2<br>1,22,24,26	4,5,6,7,8,1<br>8,19,23,25	6	O70377,P00714,P02803,P07171,Q5BJY9,Q99041
1987	1,2,3,20,2<br>1,22,25,26	4,5,6,7,8,1<br>8,19,23,24	1	Q99041
1988	1,2,3,20,2<br>1,23,24,25	4,5,6,7,8,1<br>8,19,22,26	1	P20760
1989	1,2,3,20,2<br>1,23,24,26	4,5,6,7,8,1<br>8,19,22,25	1	P01946
1990	1,2,3,20,2<br>1,23,25,26	4,5,6,7,8,1<br>8,19,22,24	1	P01946
1991	1,2,3,20,2<br>1,24,25,26	4,5,6,7,8,1<br>8,19,22,23	2	P01946,Q99041

1992	1,2,3,20,22,23,24,25	4,5,6,7,8,18,19,21,26	2	P0C0A9,P83748
1993	1,2,3,20,22,23,24,26	4,5,6,7,8,18,19,21,25	6	P00731,P01946,P02803,P20646,P36953,Q6AYS4
1994	1,2,3,20,22,23,25,26	4,5,6,7,8,18,19,21,24	3	P01946,P0C0A9,Q08849
1995	1,2,3,20,22,24,25,26	4,5,6,7,8,18,19,21,23	5	P00731,P02803,P0C0A9,P36953,Q99041
1996	1,2,3,20,23,24,25,26	4,5,6,7,8,18,19,21,22	5	P01946,P02091,P0C0A9,P19218,Q7TPB1
1997	1,2,3,21,22,23,24,25	4,5,6,7,8,18,19,20,26	2	P82471,Q5FVI6
1998	1,2,3,21,22,23,24,26	4,5,6,7,8,18,19,20,25	6	P00714,P00731,P01946,P34901,P36953,P82471
1999	1,2,3,21,22,23,25,26	4,5,6,7,8,18,19,20,24	1	P01946
2000	1,2,3,21,22,24,25,26	4,5,6,7,8,18,19,20,23	4	P07171,Q5BJY9,Q63617,Q99041
2001	1,2,3,21,23,24,25,26	4,5,6,7,8,18,19,20,22	3	P01946,P02091,Q7TPB1
2002	1,2,3,22,23,24,25,26	4,5,6,7,8,18,19,20,21	7	P00731,P01946,P02091,P0C0A9,P36953,P82471,Q7TPB1
2003	1,2,4,5,6,7,8,18	3,19,20,21,22,23,24,25,26	22	O55006,P00731,P02631,P02803,P0DP29;P0DP30;P0DP31,P10715,P18418,P20762,P25031,P50398,P52590,P61206;P84079;P84082,Q63475,Q63617,Q63942,Q64057,Q6AYQ8,Q6IE51,Q7TP52,Q9ESW0,Q9QZA6,Q9R0T3
2004	1,2,4,5,6,7,8,19	3,18,20,21,22,23,24,25,26	17	O55006,P00731,P02631,P02803,P15399,P18418,P20762,P50398,P52590,P97580,Q30KJ2,Q63475,Q63532,Q63617,Q6IE51,Q7TP52,Q9QZQ5
2005	1,2,4,5,6,7,8,20	3,18,19,21,22,23,24,25,26	14	O55006,P00731,P02631,P02803,P10715,P18418,P20762,P50398,P52590,P61206;P84079;P84082,P68370,Q63475,Q63617,Q7TP52
2006	1,2,4,5,6,7,8,21	3,18,19,20,22,23,24,25,26	22	O55006,P00731,P02631,P02803,P0C0A9,P13432,P18418,P20646,P20762,P25236,P42854,P52590,P61206;P84079;P84082,P68370,P83748,Q63475,Q63617,Q64057,Q7TP52,Q8CJ52,Q9JI85,Q9R0T3
2007	1,2,4,5,6,7,8,22	3,18,19,20,21,23,24,25,26	10	O55006,O88267,P02631,P18418,P20762,P52590,P68370,Q63475,Q63617,Q7TP52
2008	1,2,4,5,6,7,8,23	3,18,19,20,21,22,24,25,26	17	P02631,P02803,P05371,P0DP29;P0DP30;P0DP31,P15399,P18418,P20762,P28648,P50280,P52590,P97580,Q30KJ2,Q63475,Q63617,Q6IE51,Q7TP52,Q99041
2009	1,2,4,5,6,7,8,24	3,18,19,20,21,22,23,25,26	10	D3ZHA0,O55006,O88267,P02631,P18418,P20762,P52590,Q63475,Q63617,Q7TP52
2010	1,2,4,5,6,7,8,25	3,18,19,20,21,22,23,24,26	21	P00731,P02631,P02803,P05371,P06911,P10715,P12020,P18418,P19629,P20762,P31044,P52590,P61206;P84079;P84082,P68370,Q5GRG2,Q63475,Q63617,Q6AYQ8,Q7TP52,Q91ZS3,Q9Z0V6

2011	1,2,4,5,6,7,8,26	3,18,19,20,21,22,23,24,25	16	P02631,P02803,P05539,P18418,P20760,P20761,P20762,P52590,P61206;P84079;P84082,P83748,Q1WIM3,Q63475,Q63617,Q6AY33,Q6AYQ8,Q7TP52
2012	1,2,4,5,6,7,18,19	3,8,20,21,22,23,24,25,26	18	O55006,P00731,P02631,P02803,P05964,P15399,P16636,P18418,P25031,P52590,P61206;P84079;P84082,P97580,Q03191,Q30KJ2,Q63532,Q63942,Q6IE51,Q7TP52
2013	1,2,4,5,6,7,18,20	3,8,19,21,22,23,24,25,26	10	P00731,P02631,P18418,P20762,P25031,P52590,P61206;P84079;P84082,Q63475,Q6IE51,Q7TP52
2014	1,2,4,5,6,7,18,21	3,8,19,20,22,23,24,25,26	21	O89117,P00731,P02631,P02803,P05964,P18418,P20646,P20762,P25031,P25236,P42854,P52590,P61206;P84079;P84082,P83748,Q08849,Q63467,Q63475,Q6IE51,Q7TP52,Q9ESW0,Q9JI85
2015	1,2,4,5,6,7,18,22	3,8,19,20,21,23,24,25,26	8	P02631,P18418,P20762,P25031,P52590,P61206;P84079;P84082,Q6IE51,Q7TP52
2016	1,2,4,5,6,7,18,23	3,8,19,20,21,22,24,25,26	17	P02631,P02803,P05964,P15399,P18418,P20762,P25031,P28648,P52590,P55053,P61206;P84079;P84082,P97580,Q30KJ2,Q63475,Q63942,Q6IE51,Q7TP52
2017	1,2,4,5,6,7,18,24	3,8,19,20,21,22,23,25,26	10	O55006,P02631,P16636,P18418,P25031,P52590,P61206;P84079;P84082,Q63475,Q6IE51,Q7TP52
2018	1,2,4,5,6,7,18,25	3,8,19,20,21,22,23,24,26	14	P00731,P02631,P02803,P05371,P12020,P18418,P25031,P52590,P61206;P84079;P84082,P98089,Q5GRG2,Q6IE51,Q7TP52,Q91ZS3
2019	1,2,4,5,6,7,18,26	3,8,19,20,21,22,23,24,25	20	P02631,P05539,P16636,P18418,P20760,P20761,P20762,P25031,P51886,P52590,P61206;P84079;P84082,P70502,P83748,Q63661,Q63942,Q6AY33,Q6AYQ8,Q6IE51,Q7TP52,Q8VD89
2020	1,2,4,5,6,7,19,20	3,8,18,21,22,23,24,25,26	11	P00731,P02631,P15399,P18418,P20762,P52590,P97580,Q30KJ2,Q63532,Q6IE51,Q7TP52
2021	1,2,4,5,6,7,19,21	3,8,18,20,22,23,24,25,26	15	P00731,P02631,P02803,P15399,P18418,P20762,P25236,P42854,P52590,P61206;P84079;P84082,P97580,Q30KJ2,Q63532,Q6IE51,Q7TP52
2022	1,2,4,5,6,7,19,22	3,8,18,20,21,23,24,25,26	8	P02631,P18418,P52590,P97580,Q30KJ2,Q63532,Q6IE51,Q7TP52
2023	1,2,4,5,6,7,19,23	3,8,18,20,21,22,24,25,26	12	P02631,P02803,P15399,P18418,P20762,P28648,P52590,P97580,Q30KJ2,Q63532,Q6IE51,Q7TP52
2024	1,2,4,5,6,7,19,24	3,8,18,20,21,22,23,25,26	10	O55006,P02631,P15399,P18418,P52590,P97580,Q30KJ2,Q63532,Q6IE51,Q7TP52
2025	1,2,4,5,6,7,19,25	3,8,18,20,21,22,23,24,26	15	P00731,P02631,P02803,P12020,P15399,P18418,P52590,P61206;P84079;P84082,P97580,P97840,Q30KJ2,Q5GRG2,Q63532,Q6IE51,Q7TP52

2026	1,2,4,5,6,7,19,26	3,8,18,20,21,22,23,24,25	15	P02631,P05539,P18418,P20760,P20761,P20762,P52590,P61206;P84079;P84082,P83748,P97580,Q30KJ2,Q5FVI6,Q63532,Q6IE51,Q7TP52
2027	1,2,4,5,6,7,20,21	3,8,18,19,22,23,24,25,26	13	P00731,P02631,P18418,P20646,P20762,P25236,P35467,P42854,P52590,P61206;P84079;P84082,Q63475,Q7TP52,Q91ZS3
2028	1,2,4,5,6,7,20,22	3,8,18,19,21,23,24,25,26	7	P02631,P18418,P20762,P35467,P52590,Q62997,Q7TP52
2029	1,2,4,5,6,7,20,23	3,8,18,19,21,22,24,25,26	12	P02631,P14408,P15399,P18418,P20762,P28648,P52590,P97580,Q30KJ2,Q63475,Q6IE51,Q7TP52
2030	1,2,4,5,6,7,20,24	3,8,18,19,21,22,23,25,26	9	P02631,P18418,P20762,P35467,P52590,P61206;P84079;P84082,Q63475,Q6IE51,Q7TP52
2031	1,2,4,5,6,7,20,25	3,8,18,19,21,22,23,24,26	11	P02631,P12020,P18418,P20762,P35467,P52590,P61206;P84079;P84082,P68370,Q5GRG2,Q7TP52,Q91ZS3
2032	1,2,4,5,6,7,20,26	3,8,18,19,21,22,23,24,25	12	P02631,P05539,P14408,P18418,P20760,P20761,P20762,P52590,P61206;P84079;P84082,P83748,Q6AYQ8,Q7TP52
2033	1,2,4,5,6,7,21,22	3,8,18,19,20,23,24,25,26	9	P02631,P13432,P18418,P19218,P20762,P35467,P52590,P83748,Q7TP52
2034	1,2,4,5,6,7,21,23	3,8,18,19,20,22,24,25,26	13	P02631,P02803,P15399,P18418,P20762,P25236,P28648,P52590,P61206;P84079;P84082,P97580,Q30KJ2,Q63475,Q6IE51
2035	1,2,4,5,6,7,21,24	3,8,18,19,20,22,23,25,26	10	P02631,P13432,P18418,P20762,P35467,P52590,P61206;P84079;P84082,Q08849,Q63475,Q6IE51
2036	1,2,4,5,6,7,21,25	3,8,18,19,20,22,23,24,26	16	P00731,P02631,P02803,P12020,P18418,P20646,P25236,P35467,P52590,P61206;P84079;P84082,P68370,P97840,Q5GRG2,Q63475,Q7TP52,Q91ZS3
2037	1,2,4,5,6,7,21,26	3,8,18,19,20,22,23,24,25	12	P02631,P05539,P18418,P20760,P20761,P20762,P25236,P52590,P61206;P84079;P84082,P83748,Q7TP52,Q9EQV9
2038	1,2,4,5,6,7,22,23	3,8,18,19,20,21,24,25,26	11	G3V686,P02631,P18418,P20762,P28648,P36376,P52590,P97580,Q30KJ2,Q6IE51,Q7TP52
2039	1,2,4,5,6,7,22,24	3,8,18,19,20,21,23,25,26	7	O88267,P02631,P18418,P35467,P52590,Q6IE51,Q7TP52
2040	1,2,4,5,6,7,22,25	3,8,18,19,20,21,23,24,26	11	P02631,P05371,P06911,P12020,P18418,P19629,P35467,P52590,P61206;P84079;P84082,Q5GRG2,Q7TP52

2041	1,2,4,5,6,7,22,26	3,8,18,19,20,21,23,24,25	9	P05539,P18418,P20760,P20761,P20762,P52590,P61206;P84079;P84082,P83748,Q7TP52
2042	1,2,4,5,6,7,23,24	3,8,18,19,20,21,22,25,26	12	P02631,P15399,P18418,P20762,P28648,P34901,P52590,P97580,Q30KJ2,Q63475,Q6IE51,Q7TP52
2043	1,2,4,5,6,7,23,25	3,8,18,19,20,21,22,24,26	20	P02631,P02803,P05371,P06911,P12020,P15399,P18418,P19629,P20762,P28648,P36376,P50280,P52590,P61206;P84079;P84082,P70709,P97580,Q30KJ2,Q5GRG2,Q6IE51,Q7TP52
2044	1,2,4,5,6,7,23,26	3,8,18,19,20,21,22,24,25	17	P02631,P05539,P18418,P20760,P20761,P20762,P28648,P36376,P52590,P55053,P61206;P84079;P84082,P83748,P97580,Q30KJ2,Q6AYQ8,Q6IE51,Q7TP52
2045	1,2,4,5,6,7,24,25	3,8,18,19,20,21,22,23,26	11	P02631,P05371,P12020,P18418,P19629,P35467,P52590,P61206;P84079;P84082,Q5GRG2,Q6IE51,Q7TP52
2046	1,2,4,5,6,7,24,26	3,8,18,19,20,21,22,23,25	11	P02631,P18418,P20760,P20761,P20762,P52590,P61206;P84079;P84082,P83748,Q6AYQ8,Q6IE51,Q7TP52
2047	1,2,4,5,6,7,25,26	3,8,18,19,20,21,22,23,24	14	P02631,P05539,P12020,P18418,P20760,P20762,P52590,P61206;P84079;P84082,P83748,Q5GRG2,Q6AY33,Q6AYQ8,Q7TP52,Q91ZS3
2048	1,2,4,5,6,8,18,19	3,7,20,21,22,23,24,25,26	8	O55006,P00731,P02803,P18418,P22006,Q03191,Q63942,Q7TP52
2049	1,2,4,5,6,8,18,20	3,7,19,21,22,23,24,25,26	6	P00731,P02803,P20762,P22006,Q63942,Q7TP52
2050	1,2,4,5,6,8,18,21	3,7,19,20,22,23,24,25,26	11	O35547,P00731,P02803,P0C0A9,P18418,P20646,P20762,P25031,P25236,Q63942,Q9QZA6
2051	1,2,4,5,6,8,18,22	3,7,19,20,21,23,24,25,26	5	P18418,P20762,P22006,Q63942,Q7TP52
2052	1,2,4,5,6,8,18,23	3,7,19,20,21,22,24,25,26	6	P02803,P20762,P22006,P28648,Q63598,Q63942
2053	1,2,4,5,6,8,18,24	3,7,19,20,21,22,23,25,26	4	O55006,P18418,Q63942,Q7TP52
2054	1,2,4,5,6,8,18,25	3,7,19,20,21,22,23,24,26	6	P00731,P02803,P18418,P98089,Q63942,Q7TP52
2055	1,2,4,5,6,8,18,26	3,7,19,20,21,22,23,24,25	8	P02803,P20760,P20762,P83748,Q63942,Q6AY33,Q6AYQ8,Q7TP52

2056	1,2,4,5,6,8,19,20	3,7,18,21,22,23,24,25,26	5	P00731,P02803,P20762,P22006,Q7TP52
2057	1,2,4,5,6,8,19,21	3,7,18,20,22,23,24,25,26	8	A2RUV9,O35547,P00731,P02803,P0C0A9,P20762,P25236,P35281
2058	1,2,4,5,6,8,19,22	3,7,18,20,21,23,24,25,26	3	A2RUV9,P22006,Q7TP52
2059	1,2,4,5,6,8,19,23	3,7,18,20,21,22,24,25,26	7	P02803,P15399,P20762,P28648,P50280,P97580,Q30KJ2
2060	1,2,4,5,6,8,19,24	3,7,18,20,21,22,23,25,26	2	A2RUV9,Q7TP52
2061	1,2,4,5,6,8,19,25	3,7,18,20,21,22,23,24,26	5	A2RUV9,P00731,P02803,Q5GRG2,Q7TP52
2062	1,2,4,5,6,8,19,26	3,7,18,20,21,22,23,24,25	5	P02803,P20760,P20762,P83748,Q7TP52
2063	1,2,4,5,6,8,20,21	3,7,18,19,22,23,24,25,26	5	P00731,P01681,P20762,P25236,P62804
2064	1,2,4,5,6,8,20,22	3,7,18,19,21,23,24,25,26	4	P20762,P22006,Q62997,Q7TP52
2065	1,2,4,5,6,8,20,23	3,7,18,19,21,22,24,25,26	2	P20762,P22006
2066	1,2,4,5,6,8,20,24	3,7,18,19,21,22,23,25,26	2	P20762,Q7TP52
2067	1,2,4,5,6,8,20,25	3,7,18,19,21,22,23,24,26	3	P20762,Q5GRG2,Q7TP52
2068	1,2,4,5,6,8,20,26	3,7,18,19,21,22,23,24,25	7	P01681,P20760,P20762,P62804,P83748,Q6AYQ8,Q7TP52
2069	1,2,4,5,6,8,21,22	3,7,18,19,20,23,24,25,26	7	A2RUV9,O35547,P13432,P20762,P25236,Q7TP52,Q80WL1
2070	1,2,4,5,6,8,21,23	3,7,18,19,20,22,24,25,26	6	P02803,P0C0A9,P20762,P25236,P50280,Q9Z0V6

2071	1,2,4,5,6,8,21,24	3,7,18,19,20,22,23,25,26	2	P13432,P20762
2072	1,2,4,5,6,8,21,25	3,7,18,19,20,22,23,24,26	8	P00731,P02803,P06760,P20646,P25236,Q6P6R2,Q9WTT6,Q9Z0V6
2073	1,2,4,5,6,8,21,26	3,7,18,19,20,22,23,24,25	9	P0C0A9,P20760,P20761,P20762,P25236,P83748,Q00715,Q9EQV9,Q9Z0V6
2074	1,2,4,5,6,8,22,23	3,7,18,19,20,21,24,25,26	3	P20762,P50280,Q7TP52
2075	1,2,4,5,6,8,22,24	3,7,18,19,20,21,23,25,26	1	Q7TP52
2076	1,2,4,5,6,8,22,25	3,7,18,19,20,21,23,24,26	4	P06911,Q5GRG2,Q7TP52,Q9Z0V6
2077	1,2,4,5,6,8,22,26	3,7,18,19,20,21,23,24,25	5	P20760,P20761,P20762,P83748,Q7TP52
2078	1,2,4,5,6,8,23,24	3,7,18,19,20,21,22,25,26	1	P20762
2079	1,2,4,5,6,8,23,25	3,7,18,19,20,21,22,24,26	11	P02803,P06760,P06911,P12020,P20762,P28648,P50280,Q5GRG2,Q6P6R2,Q9WTT6,Q9Z0V6
2080	1,2,4,5,6,8,23,26	3,7,18,19,20,21,22,24,25	13	D4A5U3,P02793;Q7TP54,P20760,P20761,P20762,P28648,Q6AYQ8,Q6IFU8,Q6IFW6,Q6IMF3,Q6P6Q2,Q7TP52,Q9Z0V6
2081	1,2,4,5,6,8,24,25	3,7,18,19,20,21,22,23,26	3	Q5GRG2,Q7TP52,Q9Z0V6
2082	1,2,4,5,6,8,24,26	3,7,18,19,20,21,22,23,25	5	P20760,P20761,P20762,Q6AYQ8,Q7TP52
2083	1,2,4,5,6,8,25,26	3,7,18,19,20,21,22,23,24	10	P02803,P20760,P20762,P83748,Q66HD0,Q6AY33,Q6AYQ8,Q7TP52,Q9WTT6,Q9Z0V6
2084	1,2,4,5,6,18,19,20	3,7,8,21,22,23,24,25,26	4	P00731,P02803,P22006,Q7TP52
2085	1,2,4,5,6,18,19,21	3,7,8,20,22,23,24,25,26	7	O35547,O89117,P00731,P02803,P25031,P25236,Q08849

2086	1,2,4,5,6,18,19,22	3,7,8,20,21,23,24,25,26	2	P22006,Q7TP52
2087	1,2,4,5,6,18,19,23	3,7,8,20,21,22,24,25,26	9	P02803,P15399,P22006,P28648,P97580,Q03191,Q30KJ2,Q63942,Q7TP52
2088	1,2,4,5,6,18,19,24	3,7,8,20,21,22,23,25,26	2	Q63942,Q7TP52
2089	1,2,4,5,6,18,19,25	3,7,8,20,21,22,23,24,26	4	P00731,P02803,P98089,Q7TP52
2090	1,2,4,5,6,18,19,26	3,7,8,20,21,22,23,24,25	7	P02803,P20760,P83748,Q63355,Q63661,Q63942,Q7TP52
2091	1,2,4,5,6,18,20,21	3,7,8,19,22,23,24,25,26	4	P00731,P01835,P25031,P25236
2092	1,2,4,5,6,18,20,22	3,7,8,19,21,23,24,25,26	3	P22006,Q62997,Q7TP52
2093	1,2,4,5,6,18,20,23	3,7,8,19,21,22,24,25,26	3	P22006,P28648,Q6IFU7
2094	1,2,4,5,6,18,20,24	3,7,8,19,21,22,23,25,26	1	Q7TP52
2095	1,2,4,5,6,18,20,25	3,7,8,19,21,22,23,24,26	2	P98089,Q7TP52
2096	1,2,4,5,6,18,20,26	3,7,8,19,21,22,23,24,25	6	P20760,P20762,P83748,Q63355,Q63942,Q7TP52
2097	1,2,4,5,6,18,21,22	3,7,8,19,20,23,24,25,26	5	O35547,P25031,P25236,Q08849,Q7TP52
2098	1,2,4,5,6,18,21,23	3,7,8,19,20,22,24,25,26	6	O35547,P02803,P25031,P25236,P28648,Q63942
2099	1,2,4,5,6,18,21,24	3,7,8,19,20,22,23,25,26	3	P13432,P25031,Q08849
2100	1,2,4,5,6,18,21,25	3,7,8,19,20,22,23,24,26	8	P00731,P02803,P20646,P25031,P25236,P31430,P98089,Q08849

2101	1,2,4,5,6,18,21,26	3,7,8,19,20,22,23,24,25	8	P20760,P25031,P25236,P83748,Q08849,Q63661,Q63942,Q7TP52
2102	1,2,4,5,6,18,22,23	3,7,8,19,20,21,24,25,26	5	P22006,P28648,Q63942,Q6IFU7,Q7TP52
2103	1,2,4,5,6,18,22,24	3,7,8,19,20,21,23,25,26	1	Q7TP52
2104	1,2,4,5,6,18,22,25	3,7,8,19,20,21,23,24,26	2	P98089,Q7TP52
2105	1,2,4,5,6,18,22,26	3,7,8,19,20,21,23,24,25	4	P20760,P83748,Q63942,Q7TP52
2106	1,2,4,5,6,18,23,24	3,7,8,19,20,21,22,25,26	4	P28648,P34901,Q63942,Q6IFU7
2107	1,2,4,5,6,18,23,25	3,7,8,19,20,21,22,24,26	4	P02803,P28648,P98089,Q99MH3
2108	1,2,4,5,6,18,23,26	3,7,8,19,20,21,22,24,25	12	P20760,P20762,P28648,Q63661,Q63942,Q6IFU7,Q6IFU8,Q6IFW6,Q6IG02,Q6IMF3,Q6P6Q2,Q7TP52
2109	1,2,4,5,6,18,24,25	3,7,8,19,20,21,22,23,26	2	P98089,Q7TP52
2110	1,2,4,5,6,18,24,26	3,7,8,19,20,21,22,23,25	4	P20760,P83748,Q63942,Q7TP52
2111	1,2,4,5,6,18,25,26	3,7,8,19,20,21,22,23,24	8	P00774,P20760,P55091,P83748,P98089,Q63942,Q6AY33,Q7TP52
2112	1,2,4,5,6,19,20,21	3,7,8,18,22,23,24,25,26	2	P00731,P25236
2113	1,2,4,5,6,19,20,22	3,7,8,18,21,23,24,25,26	3	P22006,Q62997,Q7TP52
2114	1,2,4,5,6,19,20,23	3,7,8,18,21,22,24,25,26	4	P15399,P22006,P97580,Q30KJ2
2115	1,2,4,5,6,19,20,24	3,7,8,18,21,22,23,25,26	1	Q7TP52

2116	1,2,4,5,6,19,20,25	3,7,8,18,21,22,23,24,26	1	Q7TP52
2117	1,2,4,5,6,19,20,26	3,7,8,18,21,22,23,24,25	3	P05539,P20760,Q7TP52
2118	1,2,4,5,6,19,21,22	3,7,8,18,20,23,24,25,26	4	A2RUV9,P25236,P35467,Q7TP52
2119	1,2,4,5,6,19,21,23	3,7,8,18,20,22,24,25,26	5	P02803,P15399,P25236,P97580,Q30KJ2
2120	1,2,4,5,6,19,21,24	3,7,8,18,20,22,23,25,26	3	A2RUV9,P35467,Q08849
2121	1,2,4,5,6,19,21,25	3,7,8,18,20,22,23,24,26	7	A2RUV9,P00731,P02803,P20646,P25236,P35467,P97840
2122	1,2,4,5,6,19,21,26	3,7,8,18,20,22,23,24,25	5	P20760,P25236,P83748,Q63661,Q7TP52
2123	1,2,4,5,6,19,22,23	3,7,8,18,20,21,24,25,26	4	P15399,P97580,Q30KJ2,Q7TP52
2124	1,2,4,5,6,19,22,24	3,7,8,18,20,21,23,25,26	3	A2RUV9,P35467,Q7TP52
2125	1,2,4,5,6,19,22,25	3,7,8,18,20,21,23,24,26	4	A2RUV9,P06911,P35467,Q7TP52
2126	1,2,4,5,6,19,22,26	3,7,8,18,20,21,23,24,25	4	P05539,P20760,P83748,Q7TP52
2127	1,2,4,5,6,19,23,24	3,7,8,18,20,21,22,25,26	3	P15399,P97580,Q30KJ2
2128	1,2,4,5,6,19,23,25	3,7,8,18,20,21,22,24,26	5	P02803,P15399,P50280,P97580,Q30KJ2
2129	1,2,4,5,6,19,23,26	3,7,8,18,20,21,22,24,25	7	P15399,P20760,P28648,P97580,Q30KJ2,Q6IFU8,Q7TP52
2130	1,2,4,5,6,19,24,25	3,7,8,18,20,21,22,23,26	2	P35467,Q7TP52

2131	1,2,4,5,6,19,24,26	3,7,8,18,20,21,22,23,25	2	P20760,Q7TP52
2132	1,2,4,5,6,19,25,26	3,7,8,18,20,21,22,23,24	2	P20760,Q7TP52
2133	1,2,4,5,6,20,21,22	3,7,8,18,19,23,24,25,26	4	P25236,P35467,Q7TP52,Q7TPB1
2134	1,2,4,5,6,20,21,23	3,7,8,18,19,22,24,25,26	3	P01835,P20762,P25236
2135	1,2,4,5,6,20,21,24	3,7,8,18,19,22,23,25,26	3	P13432,P25236,P35467
2136	1,2,4,5,6,20,21,25	3,7,8,18,19,22,23,24,26	4	P20646,P25236,P35467,Q91ZS3
2137	1,2,4,5,6,20,21,26	3,7,8,18,19,22,23,24,25	8	P01681,P01835,P20760,P20762,P25236,P62804,P80020,P83748
2138	1,2,4,5,6,20,22,23	3,7,8,18,19,21,24,25,26	3	P22006,Q62997,Q7TP52
2139	1,2,4,5,6,20,22,24	3,7,8,18,19,21,23,25,26	2	P35467,Q7TP52
2140	1,2,4,5,6,20,22,25	3,7,8,18,19,21,23,24,26	4	P35467,Q07523,Q62997,Q7TP52
2141	1,2,4,5,6,20,22,26	3,7,8,18,19,21,23,24,25	4	P05539,P20760,Q62997,Q7TP52
2142	1,2,4,5,6,20,23,24	3,7,8,18,19,21,22,25,26	1	P34901
2143	1,2,4,5,6,20,23,25	3,7,8,18,19,21,22,24,26	1	P70709
2144	1,2,4,5,6,20,23,26	3,7,8,18,19,21,22,24,25	8	P05539,P20760,P20762,P36376,Q6IFU8,Q6IFW6,Q6IMF3,Q7TP52
2145	1,2,4,5,6,20,24,25	3,7,8,18,19,21,22,23,26	2	P35467,Q7TP52

2146	1,2,4,5,6,20,24,26	3,7,8,18,19,21,22,23,25	2	P20760,Q7TP52
2147	1,2,4,5,6,20,25,26	3,7,8,18,19,21,22,23,24	3	P05539,P20760,Q7TP52
2148	1,2,4,5,6,21,22,23	3,7,8,18,19,20,24,25,26	1	P25236
2149	1,2,4,5,6,21,22,24	3,7,8,18,19,20,23,25,26	4	P13432,P35467,Q08849,Q7TP52
2150	1,2,4,5,6,21,22,25	3,7,8,18,19,20,23,24,26	3	P25236,P35467,Q7TP52
2151	1,2,4,5,6,21,22,26	3,7,8,18,19,20,23,24,25	4	P20760,P25236,P83748,Q7TP52
2152	1,2,4,5,6,21,23,24	3,7,8,18,19,20,22,25,26	2	P25236,P34901
2153	1,2,4,5,6,21,23,25	3,7,8,18,19,20,22,24,26	6	P06760,P20646,P25236,P50280,P70709,Q6P6R2
2154	1,2,4,5,6,21,23,26	3,7,8,18,19,20,22,24,25	8	P20760,P20762,P25236,P83748,Q63661,Q6IFU8,Q6IMF3,Q6P6Q2
2155	1,2,4,5,6,21,24,25	3,7,8,18,19,20,22,23,26	4	P20646,P25236,P35467,Q08849
2156	1,2,4,5,6,21,24,26	3,7,8,18,19,20,22,23,25	6	P20760,P25236,P80020,P83748,Q08849,Q9EQV9
2157	1,2,4,5,6,21,25,26	3,7,8,18,19,20,22,23,24	4	P20646,P20760,P25236,P83748
2158	1,2,4,5,6,22,23,24	3,7,8,18,19,20,21,25,26	3	P34901,Q6IFU7,Q7TP52
2159	1,2,4,5,6,22,23,25	3,7,8,18,19,20,21,24,26	6	G3V686,P06911,P50280,P70709,Q5GRG2,Q7TP52
2160	1,2,4,5,6,22,23,26	3,7,8,18,19,20,21,24,25	5	P05539,P20760,P36376,Q6IFU7,Q7TP52

2161	1,2,4,5,6,22,24,25	3,7,8,18,19,20,21,23,26	2	P35467,Q7TP52
2162	1,2,4,5,6,22,24,26	3,7,8,18,19,20,21,23,25	2	P20760,Q7TP52
2163	1,2,4,5,6,22,25,26	3,7,8,18,19,20,21,23,24	3	P05539,P20760,Q7TP52
2164	1,2,4,5,6,23,24,25	3,7,8,18,19,20,21,22,26	2	P34901,P70709
2165	1,2,4,5,6,23,24,26	3,7,8,18,19,20,21,22,25	8	P20760,P27590,P34901,P47967,Q6IFU7,Q6IFU8,Q6IMF3,Q7TP52
2166	1,2,4,5,6,23,25,26	3,7,8,18,19,20,21,22,24	6	P05539,P20760,P36376,Q6IFU8,Q6IMF3,Q7TP52
2167	1,2,4,5,6,24,25,26	3,7,8,18,19,20,21,22,23	2	P20760,Q7TP52
2168	1,2,4,5,7,8,18,19	3,6,20,21,22,23,24,25,26	12	P00731,P02803,P05964,P16636,P18418,P52590,Q03191,Q63475,Q63532,Q63942,Q64057,Q9QZA6
2169	1,2,4,5,7,8,18,20	3,6,19,21,22,23,24,25,26	8	P00731,P02803,P10715,P18418,P52590,Q63475,Q9ESW0,Q9QZA6
2170	1,2,4,5,7,8,18,21	3,6,19,20,22,23,24,25,26	15	O89117,P00731,P02803,P18418,P25031,P42854,P52590,P61206;P84079;P84082,Q63467,Q63475,Q64057,Q6AYS4,Q80WL1,Q9ESW0,Q9QZA6
2171	1,2,4,5,7,8,18,22	3,6,19,20,21,23,24,25,26	4	P18418,P52590,Q7TP52,Q80WL1
2172	1,2,4,5,7,8,18,23	3,6,19,20,21,22,24,25,26	12	P02803,P18418,P28648,P52590,Q03191,Q63467,Q63475,Q63942,Q64057,Q99MH3,Q9ESW0,Q9QZA6
2173	1,2,4,5,7,8,18,24	3,6,19,20,21,22,23,25,26	6	P10715,P16636,P18418,P52590,Q63475,Q9QZA6
2174	1,2,4,5,7,8,18,25	3,6,19,20,21,22,23,24,26	14	P00714,P00731,P02803,P08721,P10715,P18418,P19629,P52590,P61206;P84079;P84082,P98089,Q63475,Q64057,Q6AYS4,Q9QZA6
2175	1,2,4,5,7,8,18,26	3,6,19,20,21,22,23,24,25	17	P02803,P16636,P18418,P20762,P51886,P52590,P55091,P61206;P84079;P84082,P82995,P83748,Q63942,Q64057,Q6AY33,Q6AYQ8,Q8VD89,Q9ESW0,Q9QZA6

2176	1,2,4,5,7,8,19,20	3,6,18,21,22,23,24,25,26	5	P02803,P18418,P52590,Q63475,Q63532
2177	1,2,4,5,7,8,19,21	3,6,18,20,22,23,24,25,26	11	O89117,P00731,P02803,P18418,P35281,P42854,P52590,Q63475,Q63532,Q64057,Q80WL1
2178	1,2,4,5,7,8,19,22	3,6,18,20,21,23,24,25,26	4	P18418,P52590,Q63532,Q7TP52
2179	1,2,4,5,7,8,19,23	3,6,18,20,21,22,24,25,26	12	P02803,P15399,P18418,P28648,P52590,P97580,Q03191,Q30KJ2,Q63475,Q63532,Q64057,Q99041
2180	1,2,4,5,7,8,19,24	3,6,18,20,21,22,23,25,26	4	P18418,P52590,Q63475,Q63532
2181	1,2,4,5,7,8,19,25	3,6,18,20,21,22,23,24,26	8	P02803,P10715,P18418,P52590,P68370,P80204,Q5GRG2,Q63532
2182	1,2,4,5,7,8,19,26	3,6,18,20,21,22,23,24,25	6	P02803,P18418,P20760,P52590,P83748,Q63532
2183	1,2,4,5,7,8,20,21	3,6,18,19,22,23,24,25,26	8	P02761,P02803,P18418,P20762,P42854,P52590,Q63475,Q9QZA6
2184	1,2,4,5,7,8,20,22	3,6,18,19,21,23,24,25,26	5	P18418,P52590,Q62997,Q63475,Q63617
2185	1,2,4,5,7,8,20,23	3,6,18,19,21,22,24,25,26	8	P02803,P14408,P18418,P20762,P28648,P52590,Q63475,Q63617
2186	1,2,4,5,7,8,20,24	3,6,18,19,21,22,23,25,26	5	P10715,P18418,P52590,Q63475,Q63617
2187	1,2,4,5,7,8,20,25	3,6,18,19,21,22,23,24,26	7	P02803,P10715,P18418,P52590,P68370,Q5GRG2,Q63475
2188	1,2,4,5,7,8,20,26	3,6,18,19,21,22,23,24,25	8	P05539,P18418,P20760,P20762,P52590,P83748,Q63475,Q6AYQ8
2189	1,2,4,5,7,8,21,22	3,6,18,19,20,23,24,25,26	4	P18418,P52590,Q63475,Q80WL1
2190	1,2,4,5,7,8,21,23	3,6,18,19,20,22,24,25,26	7	P02803,P18418,P20762,P52590,Q63475,Q64057,Q80WL1

2191	1,2,4,5,7,8,21,24	3,6,18,19,20,22,23,25,26	4	P02761,P18418,P52590,Q63475
2192	1,2,4,5,7,8,21,25	3,6,18,19,20,22,23,24,26	13	P02803,P10715,P18418,P19629,P20646,P52590,P68370,Q5GRG2,Q63475,Q64057,Q6AYS4,Q80WL1,Q9QZA6
2193	1,2,4,5,7,8,21,26	3,6,18,19,20,22,23,24,25	11	P02803,P18418,P19223,P20762,P52590,P82995,P83748,Q63475,Q64057,Q80WL1,Q9EQV9
2194	1,2,4,5,7,8,22,23	3,6,18,19,20,21,24,25,26	4	P18418,P28648,P52590,Q63475
2195	1,2,4,5,7,8,22,24	3,6,18,19,20,21,23,25,26	3	P18418,P52590,Q63475
2196	1,2,4,5,7,8,22,25	3,6,18,19,20,21,23,24,26	9	P06911,P10715,P18418,P19629,P52590,P68370,P80204,Q5GRG2,Q6AYS4
2197	1,2,4,5,7,8,22,26	3,6,18,19,20,21,23,24,25	8	P05539,P18418,P20760,P52590,P83748,Q6AYQ8,Q7TP52,Q80WL1
2198	1,2,4,5,7,8,23,24	3,6,18,19,20,21,22,25,26	4	P18418,P28648,P52590,Q63475
2199	1,2,4,5,7,8,23,25	3,6,18,19,20,21,22,24,26	10	P02803,P10715,P18418,P19629,P28648,P52590,Q5GRG2,Q63475,Q6AYS4,Q99041
2200	1,2,4,5,7,8,23,26	3,6,18,19,20,21,22,24,25	9	P02803,P18418,P20760,P20762,P28648,P52590,P83748,Q63475,Q6AYQ8
2201	1,2,4,5,7,8,24,25	3,6,18,19,20,21,22,23,26	6	P10715,P18418,P19629,P52590,Q5GRG2,Q63475
2202	1,2,4,5,7,8,24,26	3,6,18,19,20,21,22,23,25	6	P18418,P20760,P52590,P83748,Q63475,Q6AYQ8
2203	1,2,4,5,7,8,25,26	3,6,18,19,20,21,22,23,24	10	P02803,P10715,P18418,P19629,P52590,P61206;P84079;P84082,P82995,P83748,Q6AY33,Q6AYQ8
2204	1,2,4,5,7,18,19,20	3,6,8,21,22,23,24,25,26	9	P00731,P02803,P07150,P14408,P18418,P52590,Q63532,Q6IE51,Q9WTT6
2205	1,2,4,5,7,18,19,21	3,6,8,20,22,23,24,25,26	13	D3ZUC6,O89117,P00731,P02803,P05964,P18418,P25031,P42854,P52590,Q03191,Q63467,Q63532,Q6IE51

2206	1,2,4,5,7,18,19,22	3,6,8,20,21,23,24,25,26	8	P07150,P16636,P18418,P52590,Q03191,Q63532,Q7TP52,Q9WTT6
2207	1,2,4,5,7,18,19,23	3,6,8,20,21,22,24,25,26	13	P02803,P05964,P14408,P15399,P16636,P18418,P28648,P52590,P97580,Q03191,Q63532,Q6IE51,Q99MH3
2208	1,2,4,5,7,18,19,24	3,6,8,20,21,22,23,25,26	8	O89117,P16636,P18418,P52590,Q03191,Q63532,Q6IE51,Q9WTT6
2209	1,2,4,5,7,18,19,25	3,6,8,20,21,22,23,24,26	8	P00731,P02803,P18418,P52590,P61206;P84079;P84082,Q03191,Q63532,Q6IE51
2210	1,2,4,5,7,18,19,26	3,6,8,20,21,22,23,24,25	11	P02803,P16636,P51886,P52590,P82995,P83748,Q03191,Q63532,Q63661,Q63942,Q6IE51
2211	1,2,4,5,7,18,20,21	3,6,8,19,22,23,24,25,26	7	P00731,P25031,P42854,P52590,P61206;P84079;P84082,Q63467,Q9ESW0
2212	1,2,4,5,7,18,20,22	3,6,8,19,21,23,24,25,26	4	P07150,P52590,Q62997,Q7TP52
2213	1,2,4,5,7,18,20,23	3,6,8,19,21,22,24,25,26	4	P14408,P28648,P52590,Q9QZK9
2214	1,2,4,5,7,18,20,24	3,6,8,19,21,22,23,25,26	3	P18418,P52590,Q9QZK9
2215	1,2,4,5,7,18,20,25	3,6,8,19,21,22,23,24,26	3	P14408,P52590,P61206;P84079;P84082
2216	1,2,4,5,7,18,20,26	3,6,8,19,21,22,23,24,25	7	P05539,P14408,P16636,P52590,P61206;P84079;P84082,P83748,Q9ESW0
2217	1,2,4,5,7,18,21,22	3,6,8,19,20,23,24,25,26	5	O89117,P18418,P25031,P42854,P52590
2218	1,2,4,5,7,18,21,23	3,6,8,19,20,22,24,25,26	12	D3ZUC6,O89117,P02803,P05964,P25031,P28648,P52590,Q03191,Q62636,Q63467,Q99MH3,Q9ESW0
2219	1,2,4,5,7,18,21,24	3,6,8,19,20,22,23,25,26	6	D3ZUC6,O89117,P18418,P25031,P52590,Q63475
2220	1,2,4,5,7,18,21,25	3,6,8,19,20,22,23,24,26	10	P00731,P02803,P08721,P18418,P20646,P25031,P31430,P52590,P61206;P84079;P84082,P98089

2221	1,2,4,5,7,18,21,26	3,6,8,19,20,22,23,24,25	14	D3ZUC6,O89117,P16636,P25031,P52590,P55091,P61206;P84079;P84082,P82995,P83748,Q62636,Q63661,Q71MB6,Q9EQV9,Q9ESW0
2222	1,2,4,5,7,18,22,23	3,6,8,19,20,21,24,25,26	2	P28648,P52590
2223	1,2,4,5,7,18,22,24	3,6,8,19,20,21,23,25,26	4	P16636,P18418,P52590,Q9WTT6
2224	1,2,4,5,7,18,22,25	3,6,8,19,20,21,23,24,26	4	P18418,P52590,P61206;P84079;P84082,Q7TP52
2225	1,2,4,5,7,18,22,26	3,6,8,19,20,21,23,24,25	7	O88797,P05539,P16636,P52590,P82995,P83748,Q7TP52
2226	1,2,4,5,7,18,23,24	3,6,8,19,20,21,22,25,26	6	P16636,P28648,P34901,P52590,Q03191,Q6IE51
2227	1,2,4,5,7,18,23,25	3,6,8,19,20,21,22,24,26	6	P02803,P14408,P28648,P52590,P61206;P84079;P84082,Q99MH3
2228	1,2,4,5,7,18,23,26	3,6,8,19,20,21,22,24,25	13	P14408,P16636,P28648,P52590,P83748,Q03191,Q62636,Q63661,Q63942,Q6IFU8,Q6IFW6,Q6IMF3,Q6P6Q2
2229	1,2,4,5,7,18,24,25	3,6,8,19,20,21,22,23,26	3	P18418,P52590,P61206;P84079;P84082
2230	1,2,4,5,7,18,24,26	3,6,8,19,20,21,22,23,25	7	P16636,P52590,P55091,P82995,P83748,Q63942,Q8VD89
2231	1,2,4,5,7,18,25,26	3,6,8,19,20,21,22,23,24	8	P02803,P08721,P16636,P52590,P55091,P61206;P84079;P84082,P82995,P83748
2232	1,2,4,5,7,19,20,21	3,6,8,18,22,23,24,25,26	3	P00731,P42854,P52590
2233	1,2,4,5,7,19,20,22	3,6,8,18,21,23,24,25,26	9	P00774,P07150,P14408,P14669,P52590,Q62997,Q63532,Q7TP52,Q9WTT6
2234	1,2,4,5,7,19,20,23	3,6,8,18,21,22,24,25,26	6	P14408,P15399,P52590,P97580,Q63532,Q6IE51
2235	1,2,4,5,7,19,20,24	3,6,8,18,21,22,23,25,26	6	P07150,P14408,P52590,Q63532,Q6IE51,Q9WTT6

2236	1,2,4,5,7,19,20,25	3,6,8,18,21,22,23,24,26	2	P14408,P52590
2237	1,2,4,5,7,19,20,26	3,6,8,18,21,22,23,24,25	4	P05539,P14408,P52590,P83748
2238	1,2,4,5,7,19,21,22	3,6,8,18,20,23,24,25,26	4	O89117,P35467,P52590,Q63532
2239	1,2,4,5,7,19,21,23	3,6,8,18,20,22,24,25,26	8	D3ZUC6,O89117,P02803,P15399,P52590,P97580,Q63532,Q6IE51
2240	1,2,4,5,7,19,21,24	3,6,8,18,20,22,23,25,26	6	D3ZUC6,O89117,P35467,P52590,Q63532,Q6IE51
2241	1,2,4,5,7,19,21,25	3,6,8,18,20,22,23,24,26	5	P02803,P35467,P47967,P52590,P97840
2242	1,2,4,5,7,19,21,26	3,6,8,18,20,22,23,24,25	4	O89117,P52590,P83748,Q63661
2243	1,2,4,5,7,19,22,23	3,6,8,18,20,21,24,25,26	4	P52590,P97580,Q63532,Q6IE51
2244	1,2,4,5,7,19,22,24	3,6,8,18,20,21,23,25,26	5	P07150,P35467,P52590,Q63532,Q9WTT6
2245	1,2,4,5,7,19,22,25	3,6,8,18,20,21,23,24,26	4	P35467,P52590,Q63532,Q7TP52
2246	1,2,4,5,7,19,22,26	3,6,8,18,20,21,23,24,25	5	P05539,P52590,P83748,Q63532,Q7TP52
2247	1,2,4,5,7,19,23,24	3,6,8,18,20,21,22,25,26	6	P15399,P52590,P97580,Q03191,Q63532,Q6IE51
2248	1,2,4,5,7,19,23,25	3,6,8,18,20,21,22,24,26	8	P02803,P14408,P15399,P52590,P97580,Q63532,Q6IE51,Q99041
2249	1,2,4,5,7,19,23,26	3,6,8,18,20,21,22,24,25	9	P05539,P14408,P28648,P52590,P97580,Q03191,Q30KJ2,Q63532,Q6IE51
2250	1,2,4,5,7,19,24,25	3,6,8,18,20,21,22,23,26	4	P35467,P52590,Q63532,Q6IE51

2251	1,2,4,5,7,19,24,26	3,6,8,18,20,21,22,23,25	6	P16636,P20760,P52590,P83748,Q63532,Q6IE51
2252	1,2,4,5,7,19,25,26	3,6,8,18,20,21,22,23,24	5	P05539,P14408,P52590,P83748,Q63532
2253	1,2,4,5,7,20,21,22	3,6,8,18,19,23,24,25,26	4	P01836,P35467,P42854,P52590
2254	1,2,4,5,7,20,21,23	3,6,8,18,19,22,24,25,26	4	P14408,P42854,P52590,Q63475
2255	1,2,4,5,7,20,21,24	3,6,8,18,19,22,23,25,26	4	P01836,P35467,P52590,Q63475
2256	1,2,4,5,7,20,21,25	3,6,8,18,19,22,23,24,26	4	P35467,P42854,P52590,P61206;P84079;P84082
2257	1,2,4,5,7,20,21,26	3,6,8,18,19,22,23,24,25	7	P01041,P01836,P05539,P14408,P42854,P52590,P83748
2258	1,2,4,5,7,20,22,23	3,6,8,18,19,21,24,25,26	3	P14408,P52590,Q62997
2259	1,2,4,5,7,20,22,24	3,6,8,18,19,21,23,25,26	6	P07150,P14669,P35467,P52590,Q62997,Q9WTT6
2260	1,2,4,5,7,20,22,25	3,6,8,18,19,21,23,24,26	4	P14408,P35467,P52590,Q62997
2261	1,2,4,5,7,20,22,26	3,6,8,18,19,21,23,24,25	6	P05539,P14408,P52590,P83748,Q62997,Q7TP52
2262	1,2,4,5,7,20,23,24	3,6,8,18,19,21,22,25,26	3	P14408,P52590,Q63475
2263	1,2,4,5,7,20,23,25	3,6,8,18,19,21,22,24,26	2	P14408,P52590
2264	1,2,4,5,7,20,23,26	3,6,8,18,19,21,22,24,25	4	P05539,P14408,P36376,P52590
2265	1,2,4,5,7,20,24,25	3,6,8,18,19,21,22,23,26	3	P14408,P35467,P52590

2266	1,2,4,5,7,20,24,26	3,6,8,18,19,21,22,23,25	4	P01836,P14408,P20760,P52590
2267	1,2,4,5,7,20,25,26	3,6,8,18,19,21,22,23,24	4	P05539,P14408,P52590,P61206;P84079;P84082
2268	1,2,4,5,7,21,22,23	3,6,8,18,19,20,24,25,26	1	P52590
2269	1,2,4,5,7,21,22,24	3,6,8,18,19,20,23,25,26	2	P35467,P52590
2270	1,2,4,5,7,21,22,25	3,6,8,18,19,20,23,24,26	2	P35467,P52590
2271	1,2,4,5,7,21,22,26	3,6,8,18,19,20,23,24,25	4	P01836,P05539,P52590,P83748
2272	1,2,4,5,7,21,23,24	3,6,8,18,19,20,22,25,26	4	D3ZUC6,P34901,P52590,Q63475
2273	1,2,4,5,7,21,23,25	3,6,8,18,19,20,22,24,26	2	P02803,P52590
2274	1,2,4,5,7,21,23,26	3,6,8,18,19,20,22,24,25	3	P52590,P83748,Q63661
2275	1,2,4,5,7,21,24,25	3,6,8,18,19,20,22,23,26	3	P22006,P35467,P52590
2276	1,2,4,5,7,21,24,26	3,6,8,18,19,20,22,23,25	6	P01836,P20760,P52590,P80020,P83748,Q9EQV9
2277	1,2,4,5,7,21,25,26	3,6,8,18,19,20,22,23,24	5	P19223,P52590,P61206;P84079;P84082,P82995,P83748
2278	1,2,4,5,7,22,23,24	3,6,8,18,19,20,21,25,26	2	P34901,P52590
2279	1,2,4,5,7,22,23,25	3,6,8,18,19,20,21,24,26	3	P06911,P52590,Q5GRG2
2280	1,2,4,5,7,22,23,26	3,6,8,18,19,20,21,24,25	4	P05539,P36376,P52590,P83748

2281	1,2,4,5,7,22,24,25	3,6,8,18,19,20,21,23,26	2	P35467,P52590
2282	1,2,4,5,7,22,24,26	3,6,8,18,19,20,21,23,25	6	P05539,P08010,P20760,P52590,P83748,Q7TP52
2283	1,2,4,5,7,22,25,26	3,6,8,18,19,20,21,23,24	4	P05539,P52590,P83748,Q7TP52
2284	1,2,4,5,7,23,24,25	3,6,8,18,19,20,21,22,26	1	P52590
2285	1,2,4,5,7,23,24,26	3,6,8,18,19,20,21,22,25	4	P01946,P20760,P34901,P52590
2286	1,2,4,5,7,23,25,26	3,6,8,18,19,20,21,22,24	5	P01946,P05539,P14408,P36376,P52590
2287	1,2,4,5,7,24,25,26	3,6,8,18,19,20,21,22,23	1	P52590
2288	1,2,4,5,8,18,19,20	3,6,7,21,22,23,24,25,26	5	P00731,P02803,P22006,P83121,Q9QZK9
2289	1,2,4,5,8,18,19,21	3,6,7,20,22,23,24,25,26	6	O89117,P00731,P02803,P10758,P35281,Q03191
2290	1,2,4,5,8,18,19,22	3,6,7,20,21,23,24,25,26	2	P22006,P83121
2291	1,2,4,5,8,18,19,23	3,6,7,20,21,22,24,25,26	9	P02803,P09605;P25809,P22006,P28648,Q03191,Q63598,Q63942,Q64194,Q99MH3
2292	1,2,4,5,8,18,19,24	3,6,7,20,21,22,23,25,26	3	P09605;P25809,P83121,Q03191
2293	1,2,4,5,8,18,19,25	3,6,7,20,21,22,23,24,26	3	P00731,P02803,Q03191
2294	1,2,4,5,8,18,19,26	3,6,7,20,21,22,23,24,25	4	P02803,P09605;P25809,Q03191,Q63942
2295	1,2,4,5,8,18,20,21	3,6,7,19,22,23,24,25,26	6	P00731,P02761,P02803,P62804,Q9ESW0,Q9QZA6

2296	1,2,4,5,8,18,20,22	3,6,7,19,21,23,24,25,26	4	P22006,P83121,Q62997,Q9QZK9
2297	1,2,4,5,8,18,20,23	3,6,7,19,21,22,24,25,26	4	P02803,P22006,P28648,Q9QZK9
2298	1,2,4,5,8,18,20,24	3,6,7,19,21,22,23,25,26	2	P83121,Q9QZK9
2299	1,2,4,5,8,18,20,25	3,6,7,19,21,22,23,24,26	1	P02803
2300	1,2,4,5,8,18,20,26	3,6,7,19,21,22,23,24,25	1	Q63942
2301	1,2,4,5,8,18,21,22	3,6,7,19,20,23,24,25,26	1	Q80WL1
2302	1,2,4,5,8,18,21,23	3,6,7,19,20,22,24,25,26	5	P02803,Q03191,Q62636,Q63598,Q99MH3
2303	1,2,4,5,8,18,21,24	3,6,7,19,20,22,23,25,26	1	O89117
2304	1,2,4,5,8,18,21,25	3,6,7,19,20,22,23,24,26	8	P00731,P02803,P08721,P20646,P31430,P98089,Q6AYS4,Q9QZA6
2305	1,2,4,5,8,18,21,26	3,6,7,19,20,22,23,24,25	7	P02803,Q63661,Q63942,Q80WL1,Q9EQV9,Q9ESW0,Q9QZA6
2306	1,2,4,5,8,18,22,23	3,6,7,19,20,21,24,25,26	1	P22006
2307	1,2,4,5,8,18,22,24	3,6,7,19,20,21,23,25,26	1	P83121
2308	1,2,4,5,8,18,22,25	3,6,7,19,20,21,23,24,26	1	Q6AYS4
2309	1,2,4,5,8,18,22,26	3,6,7,19,20,21,23,24,25	1	Q63942
2310	1,2,4,5,8,18,23,24	3,6,7,19,20,21,22,25,26	4	P09605;P25809,P28648,Q03191,Q63942

2311	1,2,4,5,8,18,23,25	3,6,7,19,20,21,22,24,26	6	P00714,P02803,P28648,Q63598,Q6AYS4,Q99MH3
2312	1,2,4,5,8,18,23,26	3,6,7,19,20,21,22,24,25	12	P02803,P09605;P25809,P28648,Q03191,Q63942,Q64194,Q6AY61,Q6IFU8,Q6IFW6,Q6IG02,Q6IMF3,Q6P6Q2
2313	1,2,4,5,8,18,24,25	3,6,7,19,20,21,22,23,26	0	
2314	1,2,4,5,8,18,24,26	3,6,7,19,20,21,22,23,25	1	Q63942
2315	1,2,4,5,8,18,25,26	3,6,7,19,20,21,22,23,24	6	P00714,P02803,P08721,P55091,Q63942,Q6AY33
2316	1,2,4,5,8,19,20,21	3,6,7,18,22,23,24,25,26	4	P02761,P02803,P35281,P62804
2317	1,2,4,5,8,19,20,22	3,6,7,18,21,23,24,25,26	4	P00774,P22006,P83121,Q62997
2318	1,2,4,5,8,19,20,23	3,6,7,18,21,22,24,25,26	2	P02803,P22006
2319	1,2,4,5,8,19,20,24	3,6,7,18,21,22,23,25,26	1	P83121
2320	1,2,4,5,8,19,20,25	3,6,7,18,21,22,23,24,26	1	P02803
2321	1,2,4,5,8,19,20,26	3,6,7,18,21,22,23,24,25	0	
2322	1,2,4,5,8,19,21,22	3,6,7,18,20,23,24,25,26	2	A2RUV9,Q80WL1
2323	1,2,4,5,8,19,21,23	3,6,7,18,20,22,24,25,26	2	P02803,P35281
2324	1,2,4,5,8,19,21,24	3,6,7,18,20,22,23,25,26	2	A2RUV9,O89117
2325	1,2,4,5,8,19,21,25	3,6,7,18,20,22,23,24,26	2	A2RUV9,P02803

2326	1,2,4,5,8,19,21,26	3,6,7,18,20,22,23,24,25	2	P02803,Q9EQV9
2327	1,2,4,5,8,19,22,23	3,6,7,18,20,21,24,25,26	0	
2328	1,2,4,5,8,19,22,24	3,6,7,18,20,21,23,25,26	2	A2RUV9,P83121
2329	1,2,4,5,8,19,22,25	3,6,7,18,20,21,23,24,26	1	A2RUV9
2330	1,2,4,5,8,19,22,26	3,6,7,18,20,21,23,24,25	0	
2331	1,2,4,5,8,19,23,24	3,6,7,18,20,21,22,25,26	2	P09605;P25809,Q03191
2332	1,2,4,5,8,19,23,25	3,6,7,18,20,21,22,24,26	1	P02803
2333	1,2,4,5,8,19,23,26	3,6,7,18,20,21,22,24,25	3	P02803,P09605;P25809,Q03191
2334	1,2,4,5,8,19,24,25	3,6,7,18,20,21,22,23,26	0	
2335	1,2,4,5,8,19,24,26	3,6,7,18,20,21,22,23,25	0	
2336	1,2,4,5,8,19,25,26	3,6,7,18,20,21,22,23,24	1	P02803
2337	1,2,4,5,8,20,21,22	3,6,7,18,19,23,24,25,26	0	
2338	1,2,4,5,8,20,21,23	3,6,7,18,19,22,24,25,26	0	
2339	1,2,4,5,8,20,21,24	3,6,7,18,19,22,23,25,26	1	P02761
2340	1,2,4,5,8,20,21,25	3,6,7,18,19,22,23,24,26	0	

2341	1,2,4,5,8,20,21,26	3,6,7,18,19,22,23,24,25	3	P02761,P62804,Q9EQV9
2342	1,2,4,5,8,20,22,23	3,6,7,18,19,21,24,25,26	2	P22006,Q62997
2343	1,2,4,5,8,20,22,24	3,6,7,18,19,21,23,25,26	3	P83121,Q62997,Q9QZK9
2344	1,2,4,5,8,20,22,25	3,6,7,18,19,21,23,24,26	1	Q62997
2345	1,2,4,5,8,20,22,26	3,6,7,18,19,21,23,24,25	1	Q62997
2346	1,2,4,5,8,20,23,24	3,6,7,18,19,21,22,25,26	2	P97840,Q9QZK9
2347	1,2,4,5,8,20,23,25	3,6,7,18,19,21,22,24,26	0	
2348	1,2,4,5,8,20,23,26	3,6,7,18,19,21,22,24,25	1	P97840
2349	1,2,4,5,8,20,24,25	3,6,7,18,19,21,22,23,26	0	
2350	1,2,4,5,8,20,24,26	3,6,7,18,19,21,22,23,25	2	P47967,P97840
2351	1,2,4,5,8,20,25,26	3,6,7,18,19,21,22,23,24	0	
2352	1,2,4,5,8,21,22,23	3,6,7,18,19,20,24,25,26	1	Q80WL1
2353	1,2,4,5,8,21,22,24	3,6,7,18,19,20,23,25,26	0	
2354	1,2,4,5,8,21,22,25	3,6,7,18,19,20,23,24,26	2	Q6AYS4,Q80WL1
2355	1,2,4,5,8,21,22,26	3,6,7,18,19,20,23,24,25	2	Q80WL1,Q9EQV9

2356	1,2,4,5,8,21,23,24	3,6,7,18,19,20,22,25,26	1	P34901
2357	1,2,4,5,8,21,23,25	3,6,7,18,19,20,22,24,26	3	P02803,P06760,Q6AYS4
2358	1,2,4,5,8,21,23,26	3,6,7,18,19,20,22,24,25	1	Q9EQV9
2359	1,2,4,5,8,21,24,25	3,6,7,18,19,20,22,23,26	0	
2360	1,2,4,5,8,21,24,26	3,6,7,18,19,20,22,23,25	2	P02761,Q9EQV9
2361	1,2,4,5,8,21,25,26	3,6,7,18,19,20,22,23,24	4	P02803,P19223,Q6AYS4,Q9EQV9
2362	1,2,4,5,8,22,23,24	3,6,7,18,19,20,21,25,26	0	
2363	1,2,4,5,8,22,23,25	3,6,7,18,19,20,21,24,26	0	
2364	1,2,4,5,8,22,23,26	3,6,7,18,19,20,21,24,25	0	
2365	1,2,4,5,8,22,24,25	3,6,7,18,19,20,21,23,26	0	
2366	1,2,4,5,8,22,24,26	3,6,7,18,19,20,21,23,25	0	
2367	1,2,4,5,8,22,25,26	3,6,7,18,19,20,21,23,24	0	
2368	1,2,4,5,8,23,24,25	3,6,7,18,19,20,21,22,26	0	
2369	1,2,4,5,8,23,24,26	3,6,7,18,19,20,21,22,25	2	P47967,P97840
2370	1,2,4,5,8,23,25,26	3,6,7,18,19,20,21,22,24	1	P02803

2371	1,2,4,5,8,24,25,26	3,6,7,18,19,20,21,22,23	1	Q68FV8
2372	1,2,4,5,18,19,20,21	3,6,7,8,22,23,24,25,26	3	P00731,P02803,Q63751
2373	1,2,4,5,18,19,20,22	3,6,7,8,21,23,24,25,26	7	P07150,P22006,Q62997,Q63751,Q6P6R2,Q9QZK9,Q9WTT6
2374	1,2,4,5,18,19,20,23	3,6,7,8,21,22,24,25,26	5	P02803,P14408,P22006,Q03191,Q9QZK9
2375	1,2,4,5,18,19,20,24	3,6,7,8,21,22,23,25,26	4	P07150,P83121,Q9QZK9,Q9WTT6
2376	1,2,4,5,18,19,20,25	3,6,7,8,21,22,23,24,26	2	P02803,P14408
2377	1,2,4,5,18,19,20,26	3,6,7,8,21,22,23,24,25	4	P14408,Q63355,Q63751,Q63942
2378	1,2,4,5,18,19,21,22	3,6,7,8,20,23,24,25,26	1	O89117
2379	1,2,4,5,18,19,21,23	3,6,7,8,20,22,24,25,26	7	D3ZUC6,O89117,P02803,P05369,P05964,Q03191,Q99MH3
2380	1,2,4,5,18,19,21,24	3,6,7,8,20,22,23,25,26	3	D3ZUC6,O89117,Q03191
2381	1,2,4,5,18,19,21,25	3,6,7,8,20,22,23,24,26	5	O89117,P00731,P02803,P31430,P98089
2382	1,2,4,5,18,19,21,26	3,6,7,8,20,22,23,24,25	7	D3ZUC6,O89117,P02803,Q03191,Q63661,Q63751,Q63942
2383	1,2,4,5,18,19,22,23	3,6,7,8,20,21,24,25,26	2	P22006,Q03191
2384	1,2,4,5,18,19,22,24	3,6,7,8,20,21,23,25,26	4	P07150,P83121,Q6P6R2,Q9WTT6
2385	1,2,4,5,18,19,22,25	3,6,7,8,20,21,23,24,26	0	

2386	1,2,4,5,18,19,22,26	3,6,7,8,20,21,23,24,25	6	Q63661,Q63751,Q63942,Q6P6R2,Q7TP52,Q9WTT6
2387	1,2,4,5,18,19,23,24	3,6,7,8,20,21,22,25,26	2	Q03191,Q99MH3
2388	1,2,4,5,18,19,23,25	3,6,7,8,20,21,22,24,26	3	P02803,Q03191,Q99MH3
2389	1,2,4,5,18,19,23,26	3,6,7,8,20,21,22,24,25	11	P02803,P09605;P25809,P28648,Q03191,Q5XFX0,Q63661,Q63942,Q6IFU8,Q6IFW6,Q6P6Q2,Q99MH3
2390	1,2,4,5,18,19,24,25	3,6,7,8,20,21,22,23,26	0	
2391	1,2,4,5,18,19,24,26	3,6,7,8,20,21,22,23,25	2	Q03191,Q63942
2392	1,2,4,5,18,19,25,26	3,6,7,8,20,21,22,23,24	3	P02803,Q63661,Q63942
2393	1,2,4,5,18,20,21,22	3,6,7,8,19,23,24,25,26	1	Q9QZK9
2394	1,2,4,5,18,20,21,23	3,6,7,8,19,22,24,25,26	1	Q9QZK9
2395	1,2,4,5,18,20,21,24	3,6,7,8,19,22,23,25,26	1	Q9QZK9
2396	1,2,4,5,18,20,21,25	3,6,7,8,19,22,23,24,26	2	P31430,P98089
2397	1,2,4,5,18,20,21,26	3,6,7,8,19,22,23,24,25	1	P25031
2398	1,2,4,5,18,20,22,23	3,6,7,8,19,21,24,25,26	3	P22006,Q62997,Q9QZK9
2399	1,2,4,5,18,20,22,24	3,6,7,8,19,21,23,25,26	5	P07150,P83121,Q99041,Q9QZK9,Q9WTT6
2400	1,2,4,5,18,20,22,25	3,6,7,8,19,21,23,24,26	1	Q62997

2401	1,2,4,5,18,20,22,26	3,6,7,8,19,21,23,24,25	3	Q62997,Q6P6R2,Q99041
2402	1,2,4,5,18,20,23,24	3,6,7,8,19,21,22,25,26	1	Q9QZK9
2403	1,2,4,5,18,20,23,25	3,6,7,8,19,21,22,24,26	2	P14408,Q99MH3
2404	1,2,4,5,18,20,23,26	3,6,7,8,19,21,22,24,25	9	P14408,Q63942,Q6IFU8,Q6IFW6,Q6IG02,Q6IMF3,Q6P6Q2,Q9JJ50,Q9QZK9
2405	1,2,4,5,18,20,24,25	3,6,7,8,19,21,22,23,26	0	
2406	1,2,4,5,18,20,24,26	3,6,7,8,19,21,22,23,25	2	Q99041,Q9QZK9
2407	1,2,4,5,18,20,25,26	3,6,7,8,19,21,22,23,24	1	P14408
2408	1,2,4,5,18,21,22,23	3,6,7,8,19,20,24,25,26	0	
2409	1,2,4,5,18,21,22,24	3,6,7,8,19,20,23,25,26	0	
2410	1,2,4,5,18,21,22,25	3,6,7,8,19,20,23,24,26	2	P31430,P98089
2411	1,2,4,5,18,21,22,26	3,6,7,8,19,20,23,24,25	1	Q63661
2412	1,2,4,5,18,21,23,24	3,6,7,8,19,20,22,25,26	3	D3ZUC6,O89117,P34901
2413	1,2,4,5,18,21,23,25	3,6,7,8,19,20,22,24,26	5	P02803,P31430,P98089,Q62636,Q99MH3
2414	1,2,4,5,18,21,23,26	3,6,7,8,19,20,22,24,25	8	D3ZUC6,Q62636,Q63661,Q63942,Q6IFU8,Q6IFW6,Q6IMF3,Q6P6Q2
2415	1,2,4,5,18,21,24,25	3,6,7,8,19,20,22,23,26	2	P31430,P98089

2416	1,2,4,5,18,21,24,26	3,6,7,8,19,20,22,23,25	4	D3ZUC6,O89117,Q63661,Q9EQV9
2417	1,2,4,5,18,21,25,26	3,6,7,8,19,20,22,23,24	6	P08721,P25031,P31430,P55091,P98089,Q63661
2418	1,2,4,5,18,22,23,24	3,6,7,8,19,20,21,25,26	3	P34901,Q6IFU7,Q9QZK9
2419	1,2,4,5,18,22,23,25	3,6,7,8,19,20,21,24,26	1	Q99MH3
2420	1,2,4,5,18,22,23,26	3,6,7,8,19,20,21,24,25	7	Q63661,Q63942,Q6IFU7,Q6IFU8,Q6IFW6,Q6IMF3,Q6P6Q2
2421	1,2,4,5,18,22,24,25	3,6,7,8,19,20,21,23,26	0	
2422	1,2,4,5,18,22,24,26	3,6,7,8,19,20,21,23,25	1	Q99041
2423	1,2,4,5,18,22,25,26	3,6,7,8,19,20,21,23,24	1	O55145
2424	1,2,4,5,18,23,24,25	3,6,7,8,19,20,21,22,26	2	P34901,Q99MH3
2425	1,2,4,5,18,23,24,26	3,6,7,8,19,20,21,22,25	8	P34901,P47967,Q63942,Q6IFU7,Q6IFU8,Q6IFW6,Q6IMF3,Q6P6Q2
2426	1,2,4,5,18,23,25,26	3,6,7,8,19,20,21,22,24	7	Q63661,Q63942,Q6IFU8,Q6IFW6,Q6IMF3,Q6P6Q2,Q99MH3
2427	1,2,4,5,18,24,25,26	3,6,7,8,19,20,21,22,23	3	P55091,Q62902,Q7TPB1
2428	1,2,4,5,19,20,21,22	3,6,7,8,18,23,24,25,26	2	P35467,Q62997
2429	1,2,4,5,19,20,21,23	3,6,7,8,18,22,24,25,26	0	
2430	1,2,4,5,19,20,21,24	3,6,7,8,18,22,23,25,26	1	P35467

2431	1,2,4,5,19,20,21,25	3,6,7,8,18,22,23,24,26	1	P35467
2432	1,2,4,5,19,20,21,26	3,6,7,8,18,22,23,24,25	1	Q63751
2433	1,2,4,5,19,20,22,23	3,6,7,8,18,21,24,25,26	5	P00774,P22006,P55091,Q62997,Q9QZK9
2434	1,2,4,5,19,20,22,24	3,6,7,8,18,21,23,25,26	8	P00774,P07150,P14669,P35467,P83121,Q62997,Q9QZK9,Q9WTT6
2435	1,2,4,5,19,20,22,25	3,6,7,8,18,21,23,24,26	2	P35467,Q62997
2436	1,2,4,5,19,20,22,26	3,6,7,8,18,21,23,24,25	6	O54728,P05539,P07150,Q62997,Q63751,Q6P6R2
2437	1,2,4,5,19,20,23,24	3,6,7,8,18,21,22,25,26	1	Q9QZK9
2438	1,2,4,5,19,20,23,25	3,6,7,8,18,21,22,24,26	1	P14408
2439	1,2,4,5,19,20,23,26	3,6,7,8,18,21,22,24,25	1	P14408
2440	1,2,4,5,19,20,24,25	3,6,7,8,18,21,22,23,26	1	P35467
2441	1,2,4,5,19,20,24,26	3,6,7,8,18,21,22,23,25	0	
2442	1,2,4,5,19,20,25,26	3,6,7,8,18,21,22,23,24	1	P14408
2443	1,2,4,5,19,21,22,23	3,6,7,8,18,20,24,25,26	0	
2444	1,2,4,5,19,21,22,24	3,6,7,8,18,20,23,25,26	2	A2RUV9,P35467
2445	1,2,4,5,19,21,22,25	3,6,7,8,18,20,23,24,26	2	A2RUV9,P35467

2446	1,2,4,5,19,21,22,26	3,6,7,8,18,20,23,24,25	0	
2447	1,2,4,5,19,21,23,24	3,6,7,8,18,20,22,25,26	2	D3ZUC6,P34901
2448	1,2,4,5,19,21,23,25	3,6,7,8,18,20,22,24,26	1	P02803
2449	1,2,4,5,19,21,23,26	3,6,7,8,18,20,22,24,25	1	Q63661
2450	1,2,4,5,19,21,24,25	3,6,7,8,18,20,22,23,26	1	P35467
2451	1,2,4,5,19,21,24,26	3,6,7,8,18,20,22,23,25	2	O89117,Q9EQV9
2452	1,2,4,5,19,21,25,26	3,6,7,8,18,20,22,23,24	0	
2453	1,2,4,5,19,22,23,24	3,6,7,8,18,20,21,25,26	3	P34901,P82471,Q9WTT6
2454	1,2,4,5,19,22,23,25	3,6,7,8,18,20,21,24,26	0	
2455	1,2,4,5,19,22,23,26	3,6,7,8,18,20,21,24,25	2	O54728,P05539
2456	1,2,4,5,19,22,24,25	3,6,7,8,18,20,21,23,26	1	P35467
2457	1,2,4,5,19,22,24,26	3,6,7,8,18,20,21,23,25	2	Q6P6R2,Q9WTT6
2458	1,2,4,5,19,22,25,26	3,6,7,8,18,20,21,23,24	1	P05539
2459	1,2,4,5,19,23,24,25	3,6,7,8,18,20,21,22,26	0	
2460	1,2,4,5,19,23,24,26	3,6,7,8,18,20,21,22,25	0	

2461	1,2,4,5,19,23,25,26	3,6,7,8,18,20,21,22,24	0	
2462	1,2,4,5,19,24,25,26	3,6,7,8,18,20,21,22,23	2	P14480,Q62902
2463	1,2,4,5,20,21,22,23	3,6,7,8,18,19,24,25,26	1	Q62997
2464	1,2,4,5,20,21,22,24	3,6,7,8,18,19,23,25,26	1	P35467
2465	1,2,4,5,20,21,22,25	3,6,7,8,18,19,23,24,26	1	P35467
2466	1,2,4,5,20,21,22,26	3,6,7,8,18,19,23,24,25	1	P51907
2467	1,2,4,5,20,21,23,24	3,6,7,8,18,19,22,25,26	2	P34901,Q9QZK9
2468	1,2,4,5,20,21,23,25	3,6,7,8,18,19,22,24,26	0	
2469	1,2,4,5,20,21,23,26	3,6,7,8,18,19,22,24,25	1	Q6IFU8
2470	1,2,4,5,20,21,24,25	3,6,7,8,18,19,22,23,26	1	P35467
2471	1,2,4,5,20,21,24,26	3,6,7,8,18,19,22,23,25	3	P01015,P80020,Q9EQV9
2472	1,2,4,5,20,21,25,26	3,6,7,8,18,19,22,23,24	0	
2473	1,2,4,5,20,22,23,24	3,6,7,8,18,19,21,25,26	3	P34901,Q62997,Q9QZK9
2474	1,2,4,5,20,22,23,25	3,6,7,8,18,19,21,24,26	1	Q62997
2475	1,2,4,5,20,22,23,26	3,6,7,8,18,19,21,24,25	2	P05539,Q62997

2476	1,2,4,5,20,22,24,25	3,6,7,8,18,19,21,23,26	1	P35467
2477	1,2,4,5,20,22,24,26	3,6,7,8,18,19,21,23,25	3	O70594,Q62997,Q99041
2478	1,2,4,5,20,22,25,26	3,6,7,8,18,19,21,23,24	2	P05539,Q62997
2479	1,2,4,5,20,23,24,25	3,6,7,8,18,19,21,22,26	0	
2480	1,2,4,5,20,23,24,26	3,6,7,8,18,19,21,22,25	3	D4A1J4,P47967,P97840
2481	1,2,4,5,20,23,25,26	3,6,7,8,18,19,21,22,24	2	P05539,P14408
2482	1,2,4,5,20,24,25,26	3,6,7,8,18,19,21,22,23	0	
2483	1,2,4,5,21,22,23,24	3,6,7,8,18,19,20,25,26	2	P34901,P35280
2484	1,2,4,5,21,22,23,25	3,6,7,8,18,19,20,24,26	0	
2485	1,2,4,5,21,22,23,26	3,6,7,8,18,19,20,24,25	0	
2486	1,2,4,5,21,22,24,25	3,6,7,8,18,19,20,23,26	1	P35467
2487	1,2,4,5,21,22,24,26	3,6,7,8,18,19,20,23,25	0	
2488	1,2,4,5,21,22,25,26	3,6,7,8,18,19,20,23,24	0	
2489	1,2,4,5,21,23,24,25	3,6,7,8,18,19,20,22,26	2	P34901,P35280
2490	1,2,4,5,21,23,24,26	3,6,7,8,18,19,20,22,25	1	P34901

2491	1,2,4,5,21,23,25,26	3,6,7,8,18,19,20,22,24	0	
2492	1,2,4,5,21,24,25,26	3,6,7,8,18,19,20,22,23	0	
2493	1,2,4,5,22,23,24,25	3,6,7,8,18,19,20,21,26	3	P34901,P35280,P82471
2494	1,2,4,5,22,23,24,26	3,6,7,8,18,19,20,21,25	4	P00731,P34901,P47967,P82471
2495	1,2,4,5,22,23,25,26	3,6,7,8,18,19,20,21,24	1	P05539
2496	1,2,4,5,22,24,25,26	3,6,7,8,18,19,20,21,23	0	
2497	1,2,4,5,23,24,25,26	3,6,7,8,18,19,20,21,22	2	Q62902,Q7TPB1
2498	1,2,4,6,7,8,18,19	3,5,20,21,22,23,24,25,26	22	O55006,P00731,P02631,P02803,P05964,P0C0A9,P11598,P15399,P18418,P31044,P35280,P97580,Q30KJ2,Q63532,Q63617,Q64057,Q6IE51,Q7TP52,Q8CJD3,Q9JI85,Q9QZQ5,Q9R0T3
2499	1,2,4,6,7,8,18,20	3,5,19,21,22,23,24,25,26	16	P00731,P02631,P02803,P10715,P11598,P18418,P20762,P31044,P35280,Q63617,Q64057,Q6AYR8,Q91ZS3,Q9JI85,Q9QZA6,Q9R0T3
2500	1,2,4,6,7,8,18,21	3,5,19,20,22,23,24,25,26	31	P00731,P02631,P02761,P02782,P02803,P05964,P07647,P08723,P0C0A9,P11598,P13432,P18418,P20762,P22282,P22283,P25031,P25236,P42854,P98089,Q63475,Q63617,Q64057,Q6AYL6,Q6AYR8,Q6AYR9,Q80WL1,Q8CJ52,Q91ZS3,Q9JI85,Q9QZA6,Q9R0T3
2501	1,2,4,6,7,8,18,22	3,5,19,20,21,23,24,25,26	11	P02631,P11598,P18418,P20762,P22282,P31044,Q63617,Q7TP52,Q80WL1,Q9JI85,Q9R0T3
2502	1,2,4,6,7,8,18,23	3,5,19,20,21,22,24,25,26	19	P02631,P02803,P05964,P11598,P15399,P18418,P20762,P31044,P50280,P97580,Q30KJ2,Q5XI22,Q63617,Q63942,Q64057,Q8CJD3,Q9JI85,Q9QZA6,Q9R0T3
2503	1,2,4,6,7,8,18,24	3,5,19,20,21,22,23,25,26	9	O55006,P02631,P10715,P18418,P20762,Q63475,Q63617,Q9JI85,Q9R0T3
2504	1,2,4,6,7,8,18,25	3,5,19,20,21,22,23,24,26	23	P00714,P00731,P02631,P02803,P05371,P06911,P10715,P11598,P12020,P18418,P19629,P31044,P35280,P61206;P84079;P84082,P68370,P98089,Q5GRG2,Q63617,Q64057,Q6AYQ8,Q91ZS3,Q9JI85,Q9QZA6
2505	1,2,4,6,7,8,18,26	3,5,19,20,21,22,23,24,25	23	P01041,P02631,P02803,P11598,P18418,P20761,P20762,P25236,P35280,P51886,P55091,P82995,P83748,Q63617,Q63942,Q64057,Q6AY33,Q6AYQ8,Q7TP52,Q8VD89,Q91ZS3,Q9QZA6,Q9R0T3

2506	1,2,4,6,7,8,19,20	3,5,18,21,22,23,24,25,26	13	P00731,P02631,P02803,P15399,P18418,P20762,P35280,P97580,Q30KJ2,Q63532,Q63617,Q9JI85,Q9QZQ5
2507	1,2,4,6,7,8,19,21	3,5,18,20,22,23,24,25,26	25	P00731,P02631,P02761,P02782,P02803,P07647,P08723,P0C0A9,P15399,P18418,P20762,P22282,P25236,P35281,P42854,P97580,Q30KJ2,Q63532,Q63617,Q64057,Q6AYL6,Q80WL1,Q8CJ52,Q9JI85,Q9R0T3
2508	1,2,4,6,7,8,19,22	3,5,18,20,21,23,24,25,26	14	P02631,P12020,P15399,P18418,P22282,P31044,P97580,Q30KJ2,Q63532,Q63617,Q7TP52,Q8CJD3,Q9JI85,Q9R0T3
2509	1,2,4,6,7,8,19,23	3,5,18,20,21,22,24,25,26	18	P02631,P02803,P12020,P15399,P16086,P18418,P20762,P25236,P31044,P50280,P97580,Q30KJ2,Q63532,Q63617,Q64057,Q6IE51,Q8CJD3,Q99041
2510	1,2,4,6,7,8,19,24	3,5,18,20,21,22,23,25,26	9	O55006,P02631,P15399,P18418,P97580,Q30KJ2,Q63532,Q63617,Q6IE51
2511	1,2,4,6,7,8,19,25	3,5,18,20,21,22,23,24,26	21	P00731,P02631,P02803,P06911,P10715,P12020,P15399,P18418,P19629,P31044,P35280,P47967,P68370,P97580,P97840,Q30KJ2,Q5GRG2,Q63532,Q63617,Q64057,Q91ZS3
2512	1,2,4,6,7,8,19,26	3,5,18,20,21,22,23,24,25	18	P02631,P02803,P15399,P18418,P20760,P20761,P20762,P25236,P35280,P83748,P97580,Q30KJ2,Q5FVI6,Q63532,Q63617,Q6AYQ8,Q7TP52,Q9R0T3
2513	1,2,4,6,7,8,20,21	3,5,18,19,22,23,24,25,26	24	O55004,P00731,P02631,P02761,P02782,P07647,P08723,P0C0A9,P13432,P18418,P20762,P25236,P42854,P62804,Q00715,Q63475,Q63617,Q64057,Q6AYL6,Q6AYR8,Q8CJ52,Q91ZS3,Q9JI85,Q9R0T3
2514	1,2,4,6,7,8,20,22	3,5,18,19,21,23,24,25,26	8	P02631,P18418,P20762,Q07523,Q63617,Q7TP52,Q9JI85,Q9R0T3
2515	1,2,4,6,7,8,20,23	3,5,18,19,21,22,24,25,26	12	P02631,P15399,P18418,P20762,P25236,P50280,P97580,Q30KJ2,Q63475,Q63617,Q8CJD3,Q9JI85
2516	1,2,4,6,7,8,20,24	3,5,18,19,21,22,23,25,26	6	P02631,P10715,P18418,P20762,Q63475,Q63617
2517	1,2,4,6,7,8,20,25	3,5,18,19,21,22,23,24,26	13	P02631,P06911,P10715,P12020,P18418,P19629,P20762,P31044,P35280,P68370,Q5GRG2,Q63617,Q91ZS3
2518	1,2,4,6,7,8,20,26	3,5,18,19,21,22,23,24,25	15	P01041,P02631,P18418,P20760,P20761,P20762,P25236,P35280,P62804,P83748,Q63617,Q6AYQ8,Q7TP52,Q91ZS3,Q9R0T3
2519	1,2,4,6,7,8,21,22	3,5,18,19,20,23,24,25,26	17	D4A1J4,P02631,P02782,P07647,P08723,P0C0A9,P13432,P18418,P20762,P22282,P25236,P42854,Q63617,Q80WL1,Q8CJ52,Q9JI85,Q9R0T3
2520	1,2,4,6,7,8,21,23	3,5,18,19,20,22,24,25,26	22	P02631,P02782,P02803,P08723,P0C0A9,P15399,P18418,P20762,P22282,P25236,P50280,P97580,Q30KJ2,Q63475,Q63617,Q64057,Q6AYL6,Q80WL1,Q8CJ52,Q99041,Q9JI85,Q9R0T3

2521	1,2,4,6,7,8,21,24	3,5,18,19,20,22,23,25,26	15	D3ZHA0,P02631,P02761,P02782,P08723,P13432,P18418,P20762,P25236,Q63475,Q63617,Q6AYL6,Q8CJ52,Q9JI85,Q9R0T3
2522	1,2,4,6,7,8,21,25	3,5,18,19,20,22,23,24,26	25	P00731,P02631,P02803,P06911,P08723,P09605;P25809,P10715,P12020,P18418,P19629,P20646,P25236,P31044,P47967,P68370,Q5GRG2,Q63617,Q64057,Q6AYL6,Q6P6R2,Q6P9T8,Q80WL1,Q91ZS3,Q9JI85,Q9WTT6
2523	1,2,4,6,7,8,21,26	3,5,18,19,20,22,23,24,25	27	O54800;Q5DWV2,P01041,P02631,P02761,P02782,P0C0A9,P18418,P19223,P20760,P20761,P20762,P22282,P25236,P42854,P83748,Q00715,Q5QE79,Q63617,Q64057,Q6AYQ8,Q6AYR8,Q80WL1,Q8CJ52,Q91ZS3,Q9EQV9,Q9R0T3,Q9WUF4
2524	1,2,4,6,7,8,22,23	3,5,18,19,20,21,24,25,26	13	P02631,P12020,P15399,P18418,P20762,P22282,P50280,P97580,Q30KJ2,Q63617,Q7TP52,Q8CJD3,Q9R0T3
2525	1,2,4,6,7,8,22,24	3,5,18,19,20,21,23,25,26	4	P02631,P18418,Q63617,Q7TP52
2526	1,2,4,6,7,8,22,25	3,5,18,19,20,21,23,24,26	13	P02631,P06911,P10715,P12020,P18418,P19629,P31044,P68370,Q07523,Q5GRG2,Q63617,Q7TP52,Q91ZS3
2527	1,2,4,6,7,8,22,26	3,5,18,19,20,21,23,24,25	13	P02631,P18418,P20760,P20761,P20762,P22282,P25236,P83748,Q63617,Q6AYQ8,Q7TP52,Q80WL1,Q9R0T3
2528	1,2,4,6,7,8,23,24	3,5,18,19,20,21,22,25,26	9	P02631,P15399,P18418,P20762,P50280,P97580,Q30KJ2,Q63475,Q63617
2529	1,2,4,6,7,8,23,25	3,5,18,19,20,21,22,24,26	25	P02631,P02803,P05371,P06911,P10715,P12020,P15399,P18418,P19629,P20762,P25236,P31044,P50280,P68370,P70709,P97580,Q30KJ2,Q5GRG2,Q63617,Q64057,Q6P6R2,Q6P9T8,Q91ZS3,Q99041,Q9WTT6
2530	1,2,4,6,7,8,23,26	3,5,18,19,20,21,22,24,25	15	P02631,P15399,P18418,P20760,P20761,P20762,P22282,P25236,P50280,P83748,P97580,Q30KJ2,Q63617,Q6AYQ8,Q9R0T3
2531	1,2,4,6,7,8,24,25	3,5,18,19,20,21,22,23,26	11	P02631,P06911,P10715,P12020,P18418,P19629,P31044,P68370,Q5GRG2,Q63617,Q91ZS3
2532	1,2,4,6,7,8,24,26	3,5,18,19,20,21,22,23,25	10	D3ZHA0,P02631,P18418,P20760,P20761,P20762,P83748,Q63617,Q6AYQ8,Q7TP52
2533	1,2,4,6,7,8,25,26	3,5,18,19,20,21,22,23,24	21	P02631,P06911,P10715,P12020,P18418,P19223,P19629,P20761,P20762,P25236,P31044,P35280,P68370,P83748,Q5GRG2,Q63617,Q6AY33,Q6AYQ8,Q6P9T8,Q7TP52,Q91ZS3
2534	1,2,4,6,7,18,19,20	3,5,8,21,22,23,24,25,26	11	P00731,P02631,P15399,P18418,P35280,P97580,Q30KJ2,Q63532,Q6IE51,Q7TP52,Q9JI85
2535	1,2,4,6,7,18,19,21	3,5,8,20,22,23,24,25,26	21	D3ZUC6,O89117,P00731,P02631,P02803,P05964,P08723,P0C0A9,P15399,P18418,P25031,P25236,P42854,P47967,P97580,P97840,Q30KJ2,Q63532,Q6AYL6,Q6IE51,Q9JI85

2536	1,2,4,6,7,18,19,22	3,5,8,20,21,23,24,25,26	10	P02631,P15399,P18418,P97580,Q30KJ2,Q63532,Q6IE51,Q7TP52,Q8CJD3,Q9JI85
2537	1,2,4,6,7,18,19,23	3,5,8,20,21,22,24,25,26	13	P02631,P02803,P05964,P15399,P18418,P97580,Q03191,Q30KJ2,Q5BJY9,Q63532,Q6IE51,Q8CJD3,Q99MH3
2538	1,2,4,6,7,18,19,24	3,5,8,20,21,22,23,25,26	9	P02631,P05964,P15399,P18418,P97580,Q30KJ2,Q63532,Q6IE51,Q7TP52
2539	1,2,4,6,7,18,19,25	3,5,8,20,21,22,23,24,26	19	P00731,P02631,P02803,P05964,P12020,P15399,P18418,P35280,P47967,P97580,P97840,P98089,Q30KJ2,Q5GRG2,Q62635,Q63532,Q6IE51,Q7TP52,Q91ZS3
2540	1,2,4,6,7,18,19,26	3,5,8,20,21,22,23,24,25	17	P02631,P05964,P06760,P15399,P18418,P25236,P35280,P83748,P97580,Q30KJ2,Q5FVI6,Q63532,Q63661,Q63942,Q6IE51,Q7TP52,Q99PP0
2541	1,2,4,6,7,18,20,21	3,5,8,19,22,23,24,25,26	14	O54861,P00731,P02631,P08723,P13432,P18418,P25031,P25236,P42854,Q6AYL6,Q6AYR8,Q6AYR9,Q91ZS3,Q9JI85
2542	1,2,4,6,7,18,20,22	3,5,8,19,21,23,24,25,26	4	P02631,P18418,Q7TP52,Q9JI85
2543	1,2,4,6,7,18,20,23	3,5,8,19,21,22,24,25,26	11	P02631,P15399,P18418,P20762,P25236,P55053,P97580,Q30KJ2,Q6IE51,Q8CJD3,Q9JI85
2544	1,2,4,6,7,18,20,24	3,5,8,19,21,22,23,25,26	2	P02631,P18418
2545	1,2,4,6,7,18,20,25	3,5,8,19,21,22,23,24,26	8	P02631,P12020,P18418,P35280,P61206;P84079;P84082,P98089,Q5GRG2,Q91ZS3
2546	1,2,4,6,7,18,20,26	3,5,8,19,21,22,23,24,25	10	P01041,P02631,P18418,P20762,P25236,P35280,P83748,Q6AYQ8,Q7TP52,Q91ZS3
2547	1,2,4,6,7,18,21,22	3,5,8,19,20,23,24,25,26	9	P02631,P08723,P13432,P18418,P25031,P25236,P42854,Q7TP52,Q9JI85
2548	1,2,4,6,7,18,21,23	3,5,8,19,20,22,24,25,26	14	D3ZUC6,P02631,P02803,P05964,P15399,P18418,P25031,P25236,P42854,P97580,Q30KJ2,Q6AYL6,Q6IE51,Q9JI85
2549	1,2,4,6,7,18,21,24	3,5,8,19,20,22,23,25,26	9	D3ZUC6,P02631,P13432,P18418,P25031,P25236,P98089,Q6AYL6,Q9JI85
2550	1,2,4,6,7,18,21,25	3,5,8,19,20,22,23,24,26	20	O54728,P00731,P02631,P02803,P05964,P09605;P25809,P12020,P18418,P25031,P25236,P31430,P42854,P47967,P61206;P84079;P84082,P97840,P98089,Q5GRG2,Q6AYL6,Q91ZS3,Q9JI85

2551	1,2,4,6,7,18,21,26	3,5,8,19,20,22,23,24,25	18	D3ZUC6,O35783,P01041,P02631,P05964,P18418,P19223,P25031,P25236,P42854,P55091,P61206;P84079;P84082,P82995,P83748,P98089,Q63661,Q91ZS3,Q9EQV9
2552	1,2,4,6,7,18,22,23	3,5,8,19,20,21,24,25,26	8	G3V686,P02631,P15399,P18418,P97580,Q30KJ2,Q7TP52,Q8CJD3
2553	1,2,4,6,7,18,22,24	3,5,8,19,20,21,23,25,26	3	P02631,P18418,Q7TP52
2554	1,2,4,6,7,18,22,25	3,5,8,19,20,21,23,24,26	9	P02631,P06911,P12020,P18418,P97840,P98089,Q5GRG2,Q7TP52,Q91ZS3
2555	1,2,4,6,7,18,22,26	3,5,8,19,20,21,23,24,25	3	P18418,P83748,Q7TP52
2556	1,2,4,6,7,18,23,24	3,5,8,19,20,21,22,25,26	6	P02631,P15399,P18418,P97580,Q30KJ2,Q6IE51
2557	1,2,4,6,7,18,23,25	3,5,8,19,20,21,22,24,26	18	P00714,P02631,P02803,P05371,P05964,P06911,P12020,P15399,P18418,P19629,P50280,P97580,P98089,Q30KJ2,Q5GRG2,Q6IE51,Q91ZS3,Q99MH3
2558	1,2,4,6,7,18,23,26	3,5,8,19,20,21,22,24,25	18	P02631,P05964,P15399,P18418,P20762,P25236,P55053,P83748,P97580,Q30KJ2,Q63661,Q63942,Q6AYQ8,Q6IE51,Q6IFU8,Q6IMF3,Q6P6Q2,Q8VD89
2559	1,2,4,6,7,18,24,25	3,5,8,19,20,21,22,23,26	7	P02631,P12020,P18418,P19629,P98089,Q5GRG2,Q91ZS3
2560	1,2,4,6,7,18,24,26	3,5,8,19,20,21,22,23,25	7	P02631,P18418,P55091,P83748,Q6AYQ8,Q7TP52,Q8VD89
2561	1,2,4,6,7,18,25,26	3,5,8,19,20,21,22,23,24	17	P00774,P02631,P12020,P18418,P25031,P25236,P35280,P55091,P61206;P84079;P84082,P82995,P83748,P98089,Q5GRG2,Q6AY33,Q6AYQ8,Q7TP52,Q91ZS3
2562	1,2,4,6,7,19,20,21	3,5,8,18,22,23,24,25,26	13	O54861,P00731,P02631,P15399,P18418,P25236,P42854,P47967,P97580,Q30KJ2,Q63532,Q6IE51,Q9JI85
2563	1,2,4,6,7,19,20,22	3,5,8,18,21,23,24,25,26	8	P02631,P15399,P18418,P97580,Q30KJ2,Q62997,Q63532,Q7TP52
2564	1,2,4,6,7,19,20,23	3,5,8,18,21,22,24,25,26	8	P02631,P15399,P25236,P97580,Q30KJ2,Q63532,Q6IE51,Q8CJD3
2565	1,2,4,6,7,19,20,24	3,5,8,18,21,22,23,25,26	7	P02631,P15399,P18418,P97580,Q30KJ2,Q63532,Q6IE51

2566	1,2,4,6,7,19,20,25	3,5,8,18,21,22,23,24,26	13	P02631,P12020,P15399,P18418,P35280,P47967,P97580,P97840,Q30KJ2,Q5GRG2,Q63532,Q6IE51,Q91ZS3
2567	1,2,4,6,7,19,20,26	3,5,8,18,21,22,23,24,25	12	P02631,P05539,P15399,P25236,P35280,P83748,P97580,Q30KJ2,Q5FVI6,Q63532,Q6IE51,Q7TP52
2568	1,2,4,6,7,19,21,22	3,5,8,18,20,23,24,25,26	15	D4A1J4,P02631,P08723,P15399,P18418,P25236,P35467,P47967,P97580,P97840,Q30KJ2,Q63532,Q6IG02,Q7TP52,Q9JI85
2569	1,2,4,6,7,19,21,23	3,5,8,18,20,22,24,25,26	15	D3ZUC6,P02631,P02803,P05964,P15399,P16086,P18418,P25236,P50280,P97580,Q30KJ2,Q63532,Q6IE51,Q8CJD3,Q9JI85
2570	1,2,4,6,7,19,21,24	3,5,8,18,20,22,23,25,26	12	D3ZUC6,P02631,P13432,P15399,P18418,P25236,P35467,P97580,Q30KJ2,Q63532,Q6IE51,Q6IG02
2571	1,2,4,6,7,19,21,25	3,5,8,18,20,22,23,24,26	18	P00731,P02631,P02803,P09605;P25809,P12020,P15399,P18418,P25236,P35467,P47967,P97580,P97840,Q30KJ2,Q5GRG2,Q63532,Q6IE51,Q8CIZ5,Q91ZS3
2572	1,2,4,6,7,19,21,26	3,5,8,18,20,22,23,24,25	12	D3ZUC6,P02631,P15399,P18418,P25236,P83748,P97580,Q30KJ2,Q5FVI6,Q63532,Q6IE51,Q9EQV9
2573	1,2,4,6,7,19,22,23	3,5,8,18,20,21,24,25,26	10	P02631,P15399,P18418,P50280,P97580,Q30KJ2,Q63532,Q6IE51,Q7TP52,Q8CJD3
2574	1,2,4,6,7,19,22,24	3,5,8,18,20,21,23,25,26	9	P02631,P18418,P35467,P97580,Q30KJ2,Q63532,Q6IE51,Q6IG02,Q7TP52
2575	1,2,4,6,7,19,22,25	3,5,8,18,20,21,23,24,26	14	P02631,P06911,P12020,P15399,P18418,P35467,P47967,P97580,P97840,Q30KJ2,Q5GRG2,Q63532,Q6IG02,Q7TP52
2576	1,2,4,6,7,19,22,26	3,5,8,18,20,21,23,24,25	10	P02631,P05539,P18418,P25236,P83748,P97580,Q30KJ2,Q5FVI6,Q63532,Q7TP52
2577	1,2,4,6,7,19,23,24	3,5,8,18,20,21,22,25,26	8	P02631,P15399,P18418,P50280,P97580,Q30KJ2,Q63532,Q6IE51
2578	1,2,4,6,7,19,23,25	3,5,8,18,20,21,22,24,26	17	P02631,P02803,P06911,P12020,P15399,P18418,P19629,P25236,P47967,P50280,P97580,P97840,Q30KJ2,Q5GRG2,Q63532,Q6IE51,Q99041
2579	1,2,4,6,7,19,23,26	3,5,8,18,20,21,22,24,25	9	P02631,P15399,P25236,P83748,P97580,Q30KJ2,Q5FVI6,Q63532,Q6IE51
2580	1,2,4,6,7,19,24,25	3,5,8,18,20,21,22,23,26	12	P02631,P06911,P12020,P15399,P18418,P35467,P97580,P97840,Q30KJ2,Q5GRG2,Q63532,Q6IE51

2581	1,2,4,6,7,19,24,26	3,5,8,18,20,21,22,23,25	11	P02631,P14480,P18418,P20760,P83748,P97580,Q30KJ2,Q5FVI6,Q63532,Q6IE51,Q7TP52
2582	1,2,4,6,7,19,25,26	3,5,8,18,20,21,22,23,24	17	P02631,P05539,P12020,P14480,P15399,P18418,P25236,P35280,P83748,P97580,P97840,Q30KJ2,Q5FVI6,Q5GRG2,Q63532,Q6IE51,Q7TP52
2583	1,2,4,6,7,20,21,22	3,5,8,18,19,23,24,25,26	10	O55004,P01836,P08723,P13432,P18418,P25236,P35467,P42854,Q07523,Q9JI85
2584	1,2,4,6,7,20,21,23	3,5,8,18,19,22,24,25,26	10	P02631,P15399,P20762,P25236,P42854,P97580,Q30KJ2,Q6AYL6,Q91ZS3,Q9JI85
2585	1,2,4,6,7,20,21,24	3,5,8,18,19,22,23,25,26	10	O54861,P01836,P02631,P13432,P18418,P25236,P35467,P42854,Q91ZS3,Q9JI85
2586	1,2,4,6,7,20,21,25	3,5,8,18,19,22,23,24,26	8	P09605;P25809,P12020,P25236,P35467,P42854,P47967,Q5GRG2,Q91ZS3
2587	1,2,4,6,7,20,21,26	3,5,8,18,19,22,23,24,25	14	O54861,O55004,P01015,P01041,P01836,P19223,P20761,P20762,P25236,P42854,P80020,P83748,Q91ZS3,Q9EQV9
2588	1,2,4,6,7,20,22,23	3,5,8,18,19,21,24,25,26	8	G3V686,P02631,P15399,P97580,Q30KJ2,Q62997,Q7TP52,Q8CJD3
2589	1,2,4,6,7,20,22,24	3,5,8,18,19,21,23,25,26	4	P02631,P18418,P35467,Q7TP52
2590	1,2,4,6,7,20,22,25	3,5,8,18,19,21,23,24,26	8	P06911,P12020,P18418,P35467,Q07523,Q5GRG2,Q7TP52,Q91ZS3
2591	1,2,4,6,7,20,22,26	3,5,8,18,19,21,23,24,25	6	P01041,P01836,P05539,P25236,P83748,Q7TP52
2592	1,2,4,6,7,20,23,24	3,5,8,18,19,21,22,25,26	5	P02631,P15399,P97580,Q30KJ2,Q6IE51
2593	1,2,4,6,7,20,23,25	3,5,8,18,19,21,22,24,26	11	P02631,P06911,P12020,P15399,P25236,P50280,P70709,P97580,Q30KJ2,Q5GRG2,Q91ZS3
2594	1,2,4,6,7,20,23,26	3,5,8,18,19,21,22,24,25	8	P02631,P05539,P15399,P20762,P25236,P55053,P97580,Q30KJ2
2595	1,2,4,6,7,20,24,25	3,5,8,18,19,21,22,23,26	6	P02631,P12020,P18418,P35467,Q5GRG2,Q91ZS3

2596	1,2,4,6,7,20,24,26	3,5,8,18,19,21,22,23,25	6	P01015,P01836,P20760,P20761,P83748,Q7TP52
2597	1,2,4,6,7,20,25,26	3,5,8,18,19,21,22,23,24	9	P05539,P12020,P25236,P35280,P83748,Q5GRG2,Q6AYQ8,Q7TP52,Q91ZS3
2598	1,2,4,6,7,21,22,23	3,5,8,18,19,20,24,25,26	9	G3V686,P02631,P13432,P15399,P25236,P50280,P97580,Q30KJ2,Q9JI85
2599	1,2,4,6,7,21,22,24	3,5,8,18,19,20,23,25,26	6	P02631,P13432,P18418,P25236,P35467,Q6IG02
2600	1,2,4,6,7,21,22,25	3,5,8,18,19,20,23,24,26	11	P06911,P09605;P25809,P12020,P18418,P19629,P25236,P35467,P47967,P97840,Q5GRG2,Q91ZS3
2601	1,2,4,6,7,21,22,26	3,5,8,18,19,20,23,24,25	7	P01836,P05539,P13432,P20761,P25236,P83748,Q7TP52
2602	1,2,4,6,7,21,23,24	3,5,8,18,19,20,22,25,26	8	D3ZUC6,P02631,P13432,P15399,P18418,P25236,P97580,Q30KJ2
2603	1,2,4,6,7,21,23,25	3,5,8,18,19,20,22,24,26	16	P02631,P06911,P09605;P25809,P12020,P15399,P19629,P25236,P50280,P70709,P83121,P97580,P97840,Q30KJ2,Q5GRG2,Q6P6R2,Q91ZS3
2604	1,2,4,6,7,21,23,26	3,5,8,18,19,20,22,24,25	11	D3ZUC6,P02631,P15399,P20761,P20762,P25236,P83121,P83748,P97580,Q30KJ2,Q9EQV9
2605	1,2,4,6,7,21,24,25	3,5,8,18,19,20,22,23,26	13	P02631,P09605;P25809,P12020,P13432,P18418,P19629,P22006,P25236,P35467,P97840,Q5GRG2,Q6P6R2,Q91ZS3
2606	1,2,4,6,7,21,24,26	3,5,8,18,19,20,22,23,25	9	D3ZUC6,P01836,P13432,P20760,P20761,P25236,P80020,P83748,Q9EQV9
2607	1,2,4,6,7,21,25,26	3,5,8,18,19,20,22,23,24	11	P09605;P25809,P12020,P19223,P19629,P22006,P25236,P83121,P83748,Q5GRG2,Q91ZS3,Q9QZK9
2608	1,2,4,6,7,22,23,24	3,5,8,18,19,20,21,25,26	5	G3V686,P02631,P18418,P97580,Q30KJ2
2609	1,2,4,6,7,22,23,25	3,5,8,18,19,20,21,24,26	12	G3V686,P02631,P06911,P12020,P15399,P19629,P50280,P70709,P97580,Q30KJ2,Q5GRG2,Q7TP52
2610	1,2,4,6,7,22,23,26	3,5,8,18,19,20,21,24,25	8	G3V686,P05539,P20761,P25236,P83748,P97580,Q30KJ2,Q7TP52

2611	1,2,4,6,7,22,24,25	3,5,8,18,19,20,21,23,26	9	P02631,P06911,P12020,P18418,P19629,P35467,Q5GRG2,Q6IG02,Q7TP52
2612	1,2,4,6,7,22,24,26	3,5,8,18,19,20,21,23,25	5	P18418,P20760,P20761,P83748,Q7TP52
2613	1,2,4,6,7,22,25,26	3,5,8,18,19,20,21,23,24	8	P05539,P06911,P12020,P19629,P25236,P83748,Q5GRG2,Q7TP52
2614	1,2,4,6,7,23,24,25	3,5,8,18,19,20,21,22,26	14	P02631,P05371,P06911,P12020,P15399,P18418,P19629,P50280,P70709,P97580,Q30KJ2,Q5GRG2,Q6IE51,Q6P6R2
2615	1,2,4,6,7,23,24,26	3,5,8,18,19,20,21,22,25	7	P02631,P20760,P20761,P25236,P97580,Q30KJ2,Q6IE51
2616	1,2,4,6,7,23,25,26	3,5,8,18,19,20,21,22,24	16	P01946,P02631,P05539,P06911,P12020,P15399,P19629,P25236,P50280,P83121,P83748,P97580,Q30KJ2,Q5GRG2,Q6AYQ8,Q91ZS3
2617	1,2,4,6,7,24,25,26	3,5,8,18,19,20,21,22,23	8	P12020,P14480,P18418,P19629,P83748,Q5GRG2,Q6AYQ8,Q7TP52
2618	1,2,4,6,8,18,19,20	3,5,7,21,22,23,24,25,26	7	P00731,P02803,P22006,P35280,P35281,Q63355,Q8CJD3
2619	1,2,4,6,8,18,19,21	3,5,7,20,22,23,24,25,26	16	O35547,P00731,P02761,P02782,P02803,P05964,P07647,P08723,P0C0A9,P10758,P25236,P35281,P98089,Q62635,Q6AYR9,Q8CJD3
2620	1,2,4,6,8,18,19,22	3,5,7,20,21,23,24,25,26	2	Q7TP52,Q8CJD3
2621	1,2,4,6,8,18,19,23	3,5,7,20,21,22,24,25,26	16	P02803,P05964,P0C0A9,P15399,P25236,P35281,P50280,P97580,Q03191,Q30KJ2,Q5BJY9,Q5XI22,Q63598,Q63942,Q8CJD3,Q99MH3
2622	1,2,4,6,8,18,19,24	3,5,7,20,21,22,23,25,26	0	
2623	1,2,4,6,8,18,19,25	3,5,7,20,21,22,23,24,26	5	P00731,P02803,P35280,P98089,Q62635
2624	1,2,4,6,8,18,19,26	3,5,7,20,21,22,23,24,25	7	P02803,P0C0A9,P25236,P35280,Q63355,Q63942,Q7TP52
2625	1,2,4,6,8,18,20,21	3,5,7,19,22,23,24,25,26	17	O35547,P00731,P02761,P02782,P02803,P07647,P08723,P0C0A9,P13432,P17046,P25236,P62804,Q00715,Q6AYR8,Q6AYR9,Q91ZS3,Q9QZA6

2626	1,2,4,6,8,18,20,22	3,5,7,19,21,23,24,25,26	3	P22006,Q7TP52,Q8CJD3
2627	1,2,4,6,8,18,20,23	3,5,7,19,21,22,24,25,26	5	P20762,P22006,P25236,Q5XI22,Q8CJD3
2628	1,2,4,6,8,18,20,24	3,5,7,19,21,22,23,25,26	0	
2629	1,2,4,6,8,18,20,25	3,5,7,19,21,22,23,24,26	4	P02803,P35280,P98089,Q91ZS3
2630	1,2,4,6,8,18,20,26	3,5,7,19,21,22,23,24,25	8	P17046,P20762,P25236,P35280,P62804,Q63355,Q63942,Q6AYQ8
2631	1,2,4,6,8,18,21,22	3,5,7,19,20,23,24,25,26	8	O35547,P02782,P07647,P08723,P0C0A9,P13432,P25236,Q80WL1
2632	1,2,4,6,8,18,21,23	3,5,7,19,20,22,24,25,26	12	O35547,P02782,P02803,P05964,P08644,P08723,P0C0A9,P25236,P98089,Q5XI22,Q63598,Q8CJD3
2633	1,2,4,6,8,18,21,24	3,5,7,19,20,22,23,25,26	8	O54728,P02761,P02782,P08723,P0C0A9,P13432,P25236,P98089
2634	1,2,4,6,8,18,21,25	3,5,7,19,20,22,23,24,26	11	O54728,P00731,P02803,P08723,P0C0A9,P25236,P31430,P98089,Q6P6R2,Q91ZS3,Q9WTT6
2635	1,2,4,6,8,18,21,26	3,5,7,19,20,22,23,24,25	15	P02761,P02782,P02803,P0C0A9,P17046,P19223,P25031,P25236,P98089,Q00715,Q5QE79,Q63942,Q6AYR8,Q80WL1,Q9EQV9
2636	1,2,4,6,8,18,22,23	3,5,7,19,20,21,24,25,26	3	P50280,Q5XI22,Q8CJD3
2637	1,2,4,6,8,18,22,24	3,5,7,19,20,21,23,25,26	1	Q7TP52
2638	1,2,4,6,8,18,22,25	3,5,7,19,20,21,23,24,26	4	P06911,P98089,Q5GRG2,Q7TP52
2639	1,2,4,6,8,18,22,26	3,5,7,19,20,21,23,24,25	3	P25236,Q63942,Q7TP52
2640	1,2,4,6,8,18,23,24	3,5,7,19,20,21,22,25,26	2	Q5XI22,Q63942

2641	1,2,4,6,8,18,23,25	3,5,7,19,20,21,22,24,26	12	P00714,P02803,P06911,P25236,P50280,P98089,Q5GRG2,Q5XI22,Q63598,Q6P6R2,Q8CJD3,Q99MH3
2642	1,2,4,6,8,18,23,26	3,5,7,19,20,21,22,24,25	11	P02803,P20762,P25236,Q5QE79,Q63942,Q6AYQ8,Q6IFU7,Q6IFU8,Q6IFW6,Q6IMF3,Q6P6Q2
2643	1,2,4,6,8,18,24,25	3,5,7,19,20,21,22,23,26	2	O54728,P98089
2644	1,2,4,6,8,18,24,26	3,5,7,19,20,21,22,23,25	3	P17046,Q63942,Q6AYQ8
2645	1,2,4,6,8,18,25,26	3,5,7,19,20,21,22,23,24	11	P00714,P00774,P02803,P19223,P25236,P35280,P55091,P98089,Q63942,Q6AY33,Q6AYQ8
2646	1,2,4,6,8,19,20,21	3,5,7,18,22,23,24,25,26	10	P00731,P02761,P02782,P08723,P0C0A9,P25236,P30120,P35281,P62804,Q00715
2647	1,2,4,6,8,19,20,22	3,5,7,18,21,23,24,25,26	4	P22006,Q62997,Q7TP52,Q8CJD3
2648	1,2,4,6,8,19,20,23	3,5,7,18,21,22,24,25,26	7	P15399,P25236,P35281,P50280,P97580,Q30KJ2,Q8CJD3
2649	1,2,4,6,8,19,20,24	3,5,7,18,21,22,23,25,26	0	
2650	1,2,4,6,8,19,20,25	3,5,7,18,21,22,23,24,26	2	P35280,Q5GRG2
2651	1,2,4,6,8,19,20,26	3,5,7,18,21,22,23,24,25	5	P25236,P35280,P62804,Q00715,Q63355
2652	1,2,4,6,8,19,21,22	3,5,7,18,20,23,24,25,26	9	A2RUV9,O35547,P02782,P07647,P08723,P0C0A9,P25236,P35281,Q80WL1
2653	1,2,4,6,8,19,21,23	3,5,7,18,20,22,24,25,26	11	P02782,P02803,P08723,P0C0A9,P15399,P25236,P35281,P50280,P97580,Q30KJ2,Q8CJD3
2654	1,2,4,6,8,19,21,24	3,5,7,18,20,22,23,25,26	6	P02761,P02782,P0C0A9,P13432,P25236,P35281
2655	1,2,4,6,8,19,21,25	3,5,7,18,20,22,23,24,26	12	P00731,P02803,P0C0A9,P25236,P35281,P47967,P97840,Q5GRG2,Q62635,Q6P6R2,Q8CIZ5,Q9WTT6

2656	1,2,4,6,8,19,21,26	3,5,7,18,20,22,23,24,25	7	P02761,P02782,P0C0A9,P25236,P35281,Q00715,Q9EQV9
2657	1,2,4,6,8,19,22,23	3,5,7,18,20,21,24,25,26	6	P15399,P25236,P50280,P97580,Q30KJ2,Q8CJD3
2658	1,2,4,6,8,19,22,24	3,5,7,18,20,21,23,25,26	1	Q7TP52
2659	1,2,4,6,8,19,22,25	3,5,7,18,20,21,23,24,26	4	P06911,P12020,Q5GRG2,Q7TP52
2660	1,2,4,6,8,19,22,26	3,5,7,18,20,21,23,24,25	2	P25236,Q7TP52
2661	1,2,4,6,8,19,23,24	3,5,7,18,20,21,22,25,26	4	P15399,P50280,P97580,Q30KJ2
2662	1,2,4,6,8,19,23,25	3,5,7,18,20,21,22,24,26	12	P02803,P06911,P12020,P15399,P25236,P50280,P97580,Q30KJ2,Q5GRG2,Q6P6R2,Q8CJD3,Q99041
2663	1,2,4,6,8,19,23,26	3,5,7,18,20,21,22,24,25	6	D4A5U3,P15399,P25236,P50280,P97580,Q30KJ2
2664	1,2,4,6,8,19,24,25	3,5,7,18,20,21,22,23,26	1	Q5GRG2
2665	1,2,4,6,8,19,24,26	3,5,7,18,20,21,22,23,25	1	P25236
2666	1,2,4,6,8,19,25,26	3,5,7,18,20,21,22,23,24	4	P02803,P25236,P35280,Q5GRG2
2667	1,2,4,6,8,20,21,22	3,5,7,18,19,23,24,25,26	8	P02761,P02782,P07647,P08723,P13432,P25236,P62804,Q07523
2668	1,2,4,6,8,20,21,23	3,5,7,18,19,22,24,25,26	7	P02761,P02782,P0C0A9,P20762,P25236,P62804,Q8CJD3
2669	1,2,4,6,8,20,21,24	3,5,7,18,19,22,23,25,26	4	P02761,P02782,P13432,P25236
2670	1,2,4,6,8,20,21,25	3,5,7,18,19,22,23,24,26	8	P25236,P30120,P47727,P62804,Q07523,Q6P6R2,Q91ZS3,Q9WTT6

2671	1,2,4,6,8,20,21,26	3,5,7,18,19,22,23,24,25	13	P01015,P02761,P02782,P0C0A9,P17046,P19223,P20762,P25236,P62804,Q00715,Q5QE79,Q6AYR8,Q9EQV9
2672	1,2,4,6,8,20,22,23	3,5,7,18,19,21,24,25,26	4	P25236,P50280,Q62997,Q8CJD3
2673	1,2,4,6,8,20,22,24	3,5,7,18,19,21,23,25,26	1	Q07523
2674	1,2,4,6,8,20,22,25	3,5,7,18,19,21,23,24,26	4	P06911,P12020,Q07523,Q5GRG2
2675	1,2,4,6,8,20,22,26	3,5,7,18,19,21,23,24,25	2	P25236,Q7TP52
2676	1,2,4,6,8,20,23,24	3,5,7,18,19,21,22,25,26	0	
2677	1,2,4,6,8,20,23,25	3,5,7,18,19,21,22,24,26	8	P06911,P12020,P25236,P50280,P70709,Q5GRG2,Q6P6R2,Q9WTT6
2678	1,2,4,6,8,20,23,26	3,5,7,18,19,21,22,24,25	3	P20762,P25236,Q5QE79
2679	1,2,4,6,8,20,24,25	3,5,7,18,19,21,22,23,26	1	Q5GRG2
2680	1,2,4,6,8,20,24,26	3,5,7,18,19,21,22,23,25	3	P01015,P17046,P25236
2681	1,2,4,6,8,20,25,26	3,5,7,18,19,21,22,23,24	5	P19223,P25236,P35280,Q6AYQ8,Q91ZS3
2682	1,2,4,6,8,21,22,23	3,5,7,18,19,20,24,25,26	8	P02782,P08723,P0C0A9,P13432,P25236,P50280,Q80WL1,Q8CJD3
2683	1,2,4,6,8,21,22,24	3,5,7,18,19,20,23,25,26	4	P02782,P08723,P13432,P25236
2684	1,2,4,6,8,21,22,25	3,5,7,18,19,20,23,24,26	8	P02782,P06911,P08723,P25236,Q07523,Q5GRG2,Q80WL1,Q9WTT6
2685	1,2,4,6,8,21,22,26	3,5,7,18,19,20,23,24,25	9	P02782,P0C0A9,P13432,P20761,P25236,Q00715,Q5QE79,Q80WL1,Q9EQV9

2686	1,2,4,6,8,21,23,24	3,5,7,18,19,20,22,25,26	4	P02782,P13432,P25236,P50280
2687	1,2,4,6,8,21,23,25	3,5,7,18,19,20,22,24,26	12	P02803,P06911,P07150,P12020,P14669,P25236,P50280,P70709,Q5GRG2,Q6P6R2,Q9WTT6,Q9Z0V6
2688	1,2,4,6,8,21,23,26	3,5,7,18,19,20,22,24,25	12	D4A5U3,P02782,P0C0A9,P14669,P20761,P20762,P25236,Q00715,Q5QE79,Q6IFV1,Q9EQV9,Q9WTT6
2689	1,2,4,6,8,21,24,25	3,5,7,18,19,20,22,23,26	5	O54728,P13432,P25236,Q6P6R2,Q9WTT6
2690	1,2,4,6,8,21,24,26	3,5,7,18,19,20,22,23,25	9	P01015,P02761,P02782,P13432,P20761,P25236,P80020,Q5QE79,Q9EQV9
2691	1,2,4,6,8,21,25,26	3,5,7,18,19,20,22,23,24	10	P19223,P25236,Q00715,Q5QE79,Q66HD0,Q6P6R2,Q91ZS3,Q9EQV9,Q9WTT6,Q9Z0V6
2692	1,2,4,6,8,22,23,24	3,5,7,18,19,20,21,25,26	1	P50280
2693	1,2,4,6,8,22,23,25	3,5,7,18,19,20,21,24,26	7	P06911,P12020,P25236,P50280,P70709,Q5GRG2,Q8CJD3
2694	1,2,4,6,8,22,23,26	3,5,7,18,19,20,21,24,25	4	D4A5U3,P25236,P50280,Q5QE79
2695	1,2,4,6,8,22,24,25	3,5,7,18,19,20,21,23,26	3	P06911,Q07523,Q5GRG2
2696	1,2,4,6,8,22,24,26	3,5,7,18,19,20,21,23,25	2	P20761,Q7TP52
2697	1,2,4,6,8,22,25,26	3,5,7,18,19,20,21,23,24	5	P06911,P25236,Q5GRG2,Q5QE79,Q7TP52
2698	1,2,4,6,8,23,24,25	3,5,7,18,19,20,21,22,26	6	P06911,P12020,P50280,P70709,Q5GRG2,Q6P6R2
2699	1,2,4,6,8,23,24,26	3,5,7,18,19,20,21,22,25	4	D4A5U3,P20761,P25236,P27590
2700	1,2,4,6,8,23,25,26	3,5,7,18,19,20,21,22,24	11	D4A5U3,P06911,P12020,P25236,P50280,Q5GRG2,Q5QE79,Q6AYQ8,Q6P6R2,Q9WTT6,Q9Z0V6

2701	1,2,4,6,8,24,25,26	3,5,7,18,19,20,21,22,23	3	P14480,P25236,Q6AYQ8
2702	1,2,4,6,18,19,20,21	3,5,7,8,22,23,24,25,26	4	P00731,P25236,P35281,Q6AYR9
2703	1,2,4,6,18,19,20,22	3,5,7,8,21,23,24,25,26	4	P22006,Q62997,Q7TP52,Q8CJD3
2704	1,2,4,6,18,19,20,23	3,5,7,8,21,22,24,25,26	6	P15399,P22006,P25236,P97580,Q30KJ2,Q8CJD3
2705	1,2,4,6,18,19,20,24	3,5,7,8,21,22,23,25,26	0	
2706	1,2,4,6,18,19,20,25	3,5,7,8,21,22,23,24,26	2	P35280,P98089
2707	1,2,4,6,18,19,20,26	3,5,7,8,21,22,23,24,25	5	P25236,P35280,Q63355,Q63751,Q63942
2708	1,2,4,6,18,19,21,22	3,5,7,8,20,23,24,25,26	5	D3ZUC6,O35547,P08723,P25236,Q8CJD3
2709	1,2,4,6,18,19,21,23	3,5,7,8,20,22,24,25,26	11	D3ZUC6,O35547,P02803,P05964,P0C0A9,P15399,P25236,P97580,Q30KJ2,Q8CJD3,Q99MH3
2710	1,2,4,6,18,19,21,24	3,5,7,8,20,22,23,25,26	4	D3ZUC6,O89117,P13432,P25236
2711	1,2,4,6,18,19,21,25	3,5,7,8,20,22,23,24,26	9	P00731,P02803,P25236,P31430,P47967,P97840,P98089,Q62635,Q8CIZ5
2712	1,2,4,6,18,19,21,26	3,5,7,8,20,22,23,24,25	8	D3ZUC6,P05964,P0C0A9,P25031,P25236,Q63661,Q63942,Q9EQV9
2713	1,2,4,6,18,19,22,23	3,5,7,8,20,21,24,25,26	4	P15399,P97580,Q30KJ2,Q8CJD3
2714	1,2,4,6,18,19,22,24	3,5,7,8,20,21,23,25,26	1	Q7TP52
2715	1,2,4,6,18,19,22,25	3,5,7,8,20,21,23,24,26	4	P06911,P97840,P98089,Q7TP52

2716	1,2,4,6,18,19,22,26	3,5,7,8,20,21,23,24,25	3	P25236,Q63942,Q7TP52
2717	1,2,4,6,18,19,23,24	3,5,7,8,20,21,22,25,26	4	P15399,P97580,Q30KJ2,Q6IFU7
2718	1,2,4,6,18,19,23,25	3,5,7,8,20,21,22,24,26	11	P02803,P15399,P25236,P50280,P97580,P98089,Q30KJ2,Q5BJY9,Q62635,Q8CJD3,Q99MH3
2719	1,2,4,6,18,19,23,26	3,5,7,8,20,21,22,24,25	11	P15399,P25236,P97580,Q03191,Q30KJ2,Q63661,Q63942,Q6IFU7,Q6IFU8,Q6IMF3,Q6P6Q2
2720	1,2,4,6,18,19,24,25	3,5,7,8,20,21,22,23,26	1	P98089
2721	1,2,4,6,18,19,24,26	3,5,7,8,20,21,22,23,25	2	P25236,Q63942
2722	1,2,4,6,18,19,25,26	3,5,7,8,20,21,22,23,24	4	P25236,P35280,P98089,Q63942
2723	1,2,4,6,18,20,21,22	3,5,7,8,19,23,24,25,26	3	P08723,P13432,P25236
2724	1,2,4,6,18,20,21,23	3,5,7,8,19,22,24,25,26	2	P25236,Q8CJD3
2725	1,2,4,6,18,20,21,24	3,5,7,8,19,22,23,25,26	2	P13432,P25236
2726	1,2,4,6,18,20,21,25	3,5,7,8,19,22,23,24,26	6	O54728,P25031,P25236,P31430,P98089,Q91ZS3
2727	1,2,4,6,18,20,21,26	3,5,7,8,19,22,23,24,25	5	P01015,P25031,P25236,P62804,Q9EQV9
2728	1,2,4,6,18,20,22,23	3,5,7,8,19,21,24,25,26	4	P22006,P25236,Q6IFU7,Q8CJD3
2729	1,2,4,6,18,20,22,24	3,5,7,8,19,21,23,25,26	1	Q7TP52
2730	1,2,4,6,18,20,22,25	3,5,7,8,19,21,23,24,26	2	P98089,Q7TP52

2731	1,2,4,6,18,20,22,26	3,5,7,8,19,21,23,24,25	2	P25236,Q7TP52
2732	1,2,4,6,18,20,23,24	3,5,7,8,19,21,22,25,26	1	Q6IFU7
2733	1,2,4,6,18,20,23,25	3,5,7,8,19,21,22,24,26	2	P25236,P98089
2734	1,2,4,6,18,20,23,26	3,5,7,8,19,21,22,24,25	9	P25236,Q63355,Q63942,Q6IFU7,Q6IFU8,Q6IFW6,Q6IG02,Q6IMF3,Q6P6Q2
2735	1,2,4,6,18,20,24,25	3,5,7,8,19,21,22,23,26	1	P98089
2736	1,2,4,6,18,20,24,26	3,5,7,8,19,21,22,23,25	2	P01015,P25236
2737	1,2,4,6,18,20,25,26	3,5,7,8,19,21,22,23,24	4	P25236,P35280,P98089,Q91ZS3
2738	1,2,4,6,18,21,22,23	3,5,7,8,19,20,24,25,26	2	P25236,Q8CJD3
2739	1,2,4,6,18,21,22,24	3,5,7,8,19,20,23,25,26	2	P13432,P25236
2740	1,2,4,6,18,21,22,25	3,5,7,8,19,20,23,24,26	4	P25236,P31430,P97840,P98089
2741	1,2,4,6,18,21,22,26	3,5,7,8,19,20,23,24,25	2	P25031,P25236
2742	1,2,4,6,18,21,23,24	3,5,7,8,19,20,22,25,26	4	D3ZUC6,P13432,P25236,Q6IFU7
2743	1,2,4,6,18,21,23,25	3,5,7,8,19,20,22,24,26	8	P02803,P25236,P31430,P50280,P70709,P98089,Q6P6R2,Q99MH3
2744	1,2,4,6,18,21,23,26	3,5,7,8,19,20,22,24,25	13	D3ZUC6,P25031,P25236,Q62636,Q63661,Q63942,Q6IFU7,Q6IFU8,Q6IFV1,Q6IFW6,Q6IMF3,Q6P6Q2,Q9EQV9
2745	1,2,4,6,18,21,24,25	3,5,7,8,19,20,22,23,26	4	O54728,P25236,P31430,P98089

2746	1,2,4,6,18,21,24,26	3,5,7,8,19,20,22,23,25	5	D3ZUC6,P13432,P25236,P80020,Q9EQV9
2747	1,2,4,6,18,21,25,26	3,5,7,8,19,20,22,23,24	9	P00774,P19223,P25031,P25236,P31430,P55091,P98089,Q63661,Q91ZS3
2748	1,2,4,6,18,22,23,24	3,5,7,8,19,20,21,25,26	1	Q6IFU7
2749	1,2,4,6,18,22,23,25	3,5,7,8,19,20,21,24,26	7	G3V686,P06911,P25236,P50280,P98089,Q5GRG2,Q8CJD3
2750	1,2,4,6,18,22,23,26	3,5,7,8,19,20,21,24,25	7	P25236,Q63942,Q6IFU7,Q6IFU8,Q6IMF3,Q6P6Q2,Q7TP52
2751	1,2,4,6,18,22,24,25	3,5,7,8,19,20,21,23,26	2	P98089,Q7TP52
2752	1,2,4,6,18,22,24,26	3,5,7,8,19,20,21,23,25	1	Q7TP52
2753	1,2,4,6,18,22,25,26	3,5,7,8,19,20,21,23,24	3	P25236,P98089,Q7TP52
2754	1,2,4,6,18,23,24,25	3,5,7,8,19,20,21,22,26	2	P98089,Q6IFU7
2755	1,2,4,6,18,23,24,26	3,5,7,8,19,20,21,22,25	8	P25236,P27590,Q63942,Q6IFU7,Q6IFU8,Q6IFW6,Q6IMF3,Q6P6Q2
2756	1,2,4,6,18,23,25,26	3,5,7,8,19,20,21,22,24	11	P00714,P00774,P25236,P55091,P98089,Q63942,Q6IFU7,Q6IFU8,Q6IFW6,Q6IMF3,Q6P6Q2
2757	1,2,4,6,18,24,25,26	3,5,7,8,19,20,21,22,23	5	P14480,P25236,P55091,P98089,Q7TPB1
2758	1,2,4,6,19,20,21,22	3,5,7,8,18,23,24,25,26	2	P25236,P35467
2759	1,2,4,6,19,20,21,23	3,5,7,8,18,22,24,25,26	5	P15399,P25236,P97580,Q30KJ2,Q8CJD3
2760	1,2,4,6,19,20,21,24	3,5,7,8,18,22,23,25,26	3	P13432,P25236,P35467

2761	1,2,4,6,19,20,21,25	3,5,7,8,18,22,23,24,26	4	P25236,P35467,P47967,P97840
2762	1,2,4,6,19,20,21,26	3,5,7,8,18,22,23,24,25	5	P01015,P25236,P62804,P80020,Q9EQV9
2763	1,2,4,6,19,20,22,23	3,5,7,8,18,21,24,25,26	6	P15399,P25236,P97580,Q30KJ2,Q62997,Q8CJD3
2764	1,2,4,6,19,20,22,24	3,5,7,8,18,21,23,25,26	2	P35467,Q7TP52
2765	1,2,4,6,19,20,22,25	3,5,7,8,18,21,23,24,26	4	P06911,P35467,Q62997,Q7TP52
2766	1,2,4,6,19,20,22,26	3,5,7,8,18,21,23,24,25	3	P25236,Q62997,Q7TP52
2767	1,2,4,6,19,20,23,24	3,5,7,8,18,21,22,25,26	3	P15399,P97580,Q30KJ2
2768	1,2,4,6,19,20,23,25	3,5,7,8,18,21,22,24,26	7	P15399,P25236,P50280,P70709,P97580,Q30KJ2,Q8CJD3
2769	1,2,4,6,19,20,23,26	3,5,7,8,18,21,22,24,25	4	P15399,P25236,P97580,Q30KJ2
2770	1,2,4,6,19,20,24,25	3,5,7,8,18,21,22,23,26	1	P35467
2771	1,2,4,6,19,20,24,26	3,5,7,8,18,21,22,23,25	2	P01015,P25236
2772	1,2,4,6,19,20,25,26	3,5,7,8,18,21,22,23,24	2	P25236,P35280
2773	1,2,4,6,19,21,22,23	3,5,7,8,18,20,24,25,26	6	P15399,P25236,P50280,P97580,Q30KJ2,Q8CJD3
2774	1,2,4,6,19,21,22,24	3,5,7,8,18,20,23,25,26	4	P13432,P25236,P35467,Q6IG02
2775	1,2,4,6,19,21,22,25	3,5,7,8,18,20,23,24,26	6	P06911,P25236,P35467,P47967,P97840,Q6IG02

2776	1,2,4,6,19,21,22,26	3,5,7,8,18,20,23,24,25	1	P25236
2777	1,2,4,6,19,21,23,24	3,5,7,8,18,20,22,25,26	5	D3ZUC6,P15399,P25236,P97580,Q30KJ2
2778	1,2,4,6,19,21,23,25	3,5,7,8,18,20,22,24,26	9	P15399,P25236,P47967,P50280,P70709,P97580,P97840,Q30KJ2,Q6P6R2
2779	1,2,4,6,19,21,23,26	3,5,7,8,18,20,22,24,25	6	D3ZUC6,P15399,P25236,P97580,Q30KJ2,Q63661
2780	1,2,4,6,19,21,24,25	3,5,7,8,18,20,22,23,26	4	P25236,P35467,P47967,P97840
2781	1,2,4,6,19,21,24,26	3,5,7,8,18,20,22,23,25	4	D3ZUC6,P25236,P80020,Q9EQV9
2782	1,2,4,6,19,21,25,26	3,5,7,8,18,20,22,23,24	2	P25236,P97840
2783	1,2,4,6,19,22,23,24	3,5,7,8,18,20,21,25,26	4	P15399,P50280,P97580,Q30KJ2
2784	1,2,4,6,19,22,23,25	3,5,7,8,18,20,21,24,26	11	P06911,P12020,P15399,P25236,P50280,P70709,P97580,Q30KJ2,Q4FZU6,Q5GRG2,Q8CJD3
2785	1,2,4,6,19,22,23,26	3,5,7,8,18,20,21,24,25	5	P15399,P25236,P97580,Q30KJ2,Q7TP52
2786	1,2,4,6,19,22,24,25	3,5,7,8,18,20,21,23,26	4	P06911,P35467,Q4FZU6,Q6IG02
2787	1,2,4,6,19,22,24,26	3,5,7,8,18,20,21,23,25	2	P14480,Q7TP52
2788	1,2,4,6,19,22,25,26	3,5,7,8,18,20,21,23,24	3	P06911,P25236,Q7TP52
2789	1,2,4,6,19,23,24,25	3,5,7,8,18,20,21,22,26	6	P15399,P50280,P70709,P97580,Q30KJ2,Q4FZU6
2790	1,2,4,6,19,23,24,26	3,5,7,8,18,20,21,22,25	6	P14480,P15399,P25236,P97580,Q30KJ2,Q6IFU7

2791	1,2,4,6,19,23,25,26	3,5,7,8,18,20,21,22,24	7	P14480,P15399,P25236,P27213,P50280,P97580,Q30KJ2
2792	1,2,4,6,19,24,25,26	3,5,7,8,18,20,21,22,23	2	P14480,P25236
2793	1,2,4,6,20,21,22,23	3,5,7,8,18,19,24,25,26	2	P25236,Q8CJD3
2794	1,2,4,6,20,21,22,24	3,5,7,8,18,19,23,25,26	4	P13432,P25236,P35467,Q07523
2795	1,2,4,6,20,21,22,25	3,5,7,8,18,19,23,24,26	5	P09605;P25809,P25236,P35467,P70709,Q07523
2796	1,2,4,6,20,21,22,26	3,5,7,8,18,19,23,24,25	3	P01015,P25236,P80020
2797	1,2,4,6,20,21,23,24	3,5,7,8,18,19,22,25,26	2	P13432,P25236
2798	1,2,4,6,20,21,23,25	3,5,7,8,18,19,22,24,26	4	P25236,P70709,Q6P6R2,Q91ZS3
2799	1,2,4,6,20,21,23,26	3,5,7,8,18,19,22,24,25	3	P01015,P25236,Q6IFU8
2800	1,2,4,6,20,21,24,25	3,5,7,8,18,19,22,23,26	3	P25236,P35467,P70709
2801	1,2,4,6,20,21,24,26	3,5,7,8,18,19,22,23,25	5	P01015,P13432,P25236,P80020,Q9EQV9
2802	1,2,4,6,20,21,25,26	3,5,7,8,18,19,22,23,24	3	P19223,P25236,Q91ZS3
2803	1,2,4,6,20,22,23,24	3,5,7,8,18,19,21,25,26	1	P82995
2804	1,2,4,6,20,22,23,25	3,5,7,8,18,19,21,24,26	6	G3V686,P06911,P25236,P50280,P70709,Q5GRG2
2805	1,2,4,6,20,22,23,26	3,5,7,8,18,19,21,24,25	3	P05539,P25236,Q62997

2806	1,2,4,6,20,22,24,25	3,5,7,8,18,19,21,23,26	2	P35467,Q07523
2807	1,2,4,6,20,22,24,26	3,5,7,8,18,19,21,23,25	4	O70594,P01015,P25236,Q7TP52
2808	1,2,4,6,20,22,25,26	3,5,7,8,18,19,21,23,24	3	P05539,P25236,Q7TP52
2809	1,2,4,6,20,23,24,25	3,5,7,8,18,19,21,22,26	2	P25236,P70709
2810	1,2,4,6,20,23,24,26	3,5,7,8,18,19,21,22,25	2	P25236,Q6IFU7
2811	1,2,4,6,20,23,25,26	3,5,7,8,18,19,21,22,24	1	P25236
2812	1,2,4,6,20,24,25,26	3,5,7,8,18,19,21,22,23	1	P25236
2813	1,2,4,6,21,22,23,24	3,5,7,8,18,19,20,25,26	2	P13432,P25236
2814	1,2,4,6,21,22,23,25	3,5,7,8,18,19,20,24,26	6	P06911,P25236,P50280,P70709,Q5GRG2,Q6P6R2
2815	1,2,4,6,21,22,23,26	3,5,7,8,18,19,20,24,25	1	P25236
2816	1,2,4,6,21,22,24,25	3,5,7,8,18,19,20,23,26	6	P09605;P25809,P13432,P25236,P35467,P70709,Q6IG02
2817	1,2,4,6,21,22,24,26	3,5,7,8,18,19,20,23,25	4	P13432,P25236,P80020,Q9EQV9
2818	1,2,4,6,21,22,25,26	3,5,7,8,18,19,20,23,24	1	P25236
2819	1,2,4,6,21,23,24,25	3,5,7,8,18,19,20,22,26	4	P25236,P50280,P70709,Q6P6R2
2820	1,2,4,6,21,23,24,26	3,5,7,8,18,19,20,22,25	6	D3ZUC6,P25236,P80020,Q6IFU7,Q6IFV1,Q9EQV9

2821	1,2,4,6,21,23,25,26	3,5,7,8,18,19,20,22,24	5	P25236,P27213,P70709,P83121,Q6P6R2
2822	1,2,4,6,21,24,25,26	3,5,7,8,18,19,20,22,23	4	P14480,P25236,P80020,Q9EQV9
2823	1,2,4,6,22,23,24,25	3,5,7,8,18,19,20,21,26	6	P06911,P50280,P70709,P82471,Q4FZU6,Q5GRG2
2824	1,2,4,6,22,23,24,26	3,5,7,8,18,19,20,21,25	3	P00731,P25236,Q6IFU7
2825	1,2,4,6,22,23,25,26	3,5,7,8,18,19,20,21,24	5	P06911,P25236,P50280,P70709,Q5GRG2
2826	1,2,4,6,22,24,25,26	3,5,7,8,18,19,20,21,23	2	P14480,Q7TP52
2827	1,2,4,6,23,24,25,26	3,5,7,8,18,19,20,21,22	4	P14480,P25236,P27590,Q7TPB1
2828	1,2,4,7,8,18,19,20	3,5,6,21,22,23,24,25,26	7	P00731,P02803,P18418,P35280,Q63532,Q64057,Q9QZA6
2829	1,2,4,7,8,18,19,21	3,5,6,20,22,23,24,25,26	14	D3ZUC6,O89117,P00731,P02761,P02803,P05964,P10758,P18418,P35281,P42854,Q63532,Q64057,Q80WL1,Q9QZA6
2830	1,2,4,7,8,18,19,22	3,5,6,20,21,23,24,25,26	5	P01835,P18418,Q63532,Q80WL1,Q8CJD3
2831	1,2,4,7,8,18,19,23	3,5,6,20,21,22,24,25,26	12	P02803,P05964,P15399,P18418,P97580,Q03191,Q5BJY9,Q5XI22,Q63532,Q64057,Q8CJD3,Q99MH3
2832	1,2,4,7,8,18,19,24	3,5,6,20,21,22,23,25,26	3	P01835,P18418,Q63532
2833	1,2,4,7,8,18,19,25	3,5,6,20,21,22,23,24,26	13	P00714,P00731,P02803,P05964,P10715,P18418,P31044,P35280,P47967,P98089,Q63532,Q64057,Q99MH3
2834	1,2,4,7,8,18,19,26	3,5,6,20,21,22,23,24,25	10	P02803,P05964,P15978,P18418,P35280,P82995,Q63532,Q63942,Q64057,Q6AYQ8
2835	1,2,4,7,8,18,20,21	3,5,6,19,22,23,24,25,26	12	P00731,P02761,P02803,P18418,P25031,P42854,Q64057,Q6AYL6,Q6AYR8,Q80WL1,Q9JI85,Q9QZA6

2836	1,2,4,7,8,18,20,22	3,5,6,19,21,23,24,25,26	2	P18418,Q63617
2837	1,2,4,7,8,18,20,23	3,5,6,19,21,22,24,25,26	7	P02803,P18418,Q5XI22,Q63617,Q64057,Q8CJD3,Q9QZA6
2838	1,2,4,7,8,18,20,24	3,5,6,19,21,22,23,25,26	4	P10715,P18418,Q63617,Q9QZA6
2839	1,2,4,7,8,18,20,25	3,5,6,19,21,22,23,24,26	8	P02803,P10715,P18418,P35280,P98089,Q63617,Q64057,Q9QZA6
2840	1,2,4,7,8,18,20,26	3,5,6,19,21,22,23,24,25	8	P01041,P10715,P18418,P19223,P35280,P82995,Q6AYQ8,Q9QZA6
2841	1,2,4,7,8,18,21,22	3,5,6,19,20,23,24,25,26	8	D3ZUC6,P18418,P42854,Q64057,Q80WL1,Q8CJ52,Q9QZA6,Q9R0T3
2842	1,2,4,7,8,18,21,23	3,5,6,19,20,22,24,25,26	12	D3ZUC6,P02803,P05964,P18418,P42854,Q5XI22,Q62636,Q64057,Q6AYL6,Q80WL1,Q99MH3,Q9QZA6
2843	1,2,4,7,8,18,21,24	3,5,6,19,20,22,23,25,26	6	D3ZUC6,O89117,P02761,P18418,Q80WL1,Q9QZA6
2844	1,2,4,7,8,18,21,25	3,5,6,19,20,22,23,24,26	15	P00731,P02803,P08721,P10715,P18418,P19629,P25031,P31430,P42854,P47967,P55091,P98089,Q64057,Q80WL1,Q9QZA6
2845	1,2,4,7,8,18,21,26	3,5,6,19,20,22,23,24,25	18	D3ZUC6,P01041,P02761,P02803,P05964,P18418,P19223,P25031,P42854,P55091,P82995,Q64057,Q6AYQ8,Q6AYR8,Q80WL1,Q9EQV9,Q9QZA6,Q9WUF4
2846	1,2,4,7,8,18,22,23	3,5,6,19,20,21,24,25,26	5	P18418,Q63617,Q64057,Q80WL1,Q8CJD3
2847	1,2,4,7,8,18,22,24	3,5,6,19,20,21,23,25,26	2	P01835,P18418
2848	1,2,4,7,8,18,22,25	3,5,6,19,20,21,23,24,26	7	P10715,P18418,P19629,P31044,P98089,Q5GRG2,Q80WL1
2849	1,2,4,7,8,18,22,26	3,5,6,19,20,21,23,24,25	5	P01041,P18418,P82995,Q6AYQ8,Q80WL1
2850	1,2,4,7,8,18,23,24	3,5,6,19,20,21,22,25,26	3	P18418,Q5XI22,Q63617

2851	1,2,4,7,8,18,23,25	3,5,6,19,20,21,22,24,26	12	P00714,P02803,P10715,P18418,P19629,P31044,P98089,Q5GRG2,Q63598,Q64057,Q99MH3,Q9QZA6
2852	1,2,4,7,8,18,23,26	3,5,6,19,20,21,22,24,25	9	P02803,P18418,P55091,P82995,Q63942,Q64057,Q6AYQ8,Q6P6Q2,Q9QZA6
2853	1,2,4,7,8,18,24,25	3,5,6,19,20,21,22,23,26	7	P08721,P10715,P18418,P19629,P55091,P98089,Q9QZA6
2854	1,2,4,7,8,18,24,26	3,5,6,19,20,21,22,23,25	4	P18418,P55091,P82995,Q6AYQ8
2855	1,2,4,7,8,18,25,26	3,5,6,19,20,21,22,23,24	15	P00714,P02803,P08721,P10715,P18418,P19223,P19629,P24594,P35280,P55091,P82995,P98089,Q64057,Q6AYQ8,Q9QZA6
2856	1,2,4,7,8,19,20,21	3,5,6,18,22,23,24,25,26	8	P00731,P02761,P02803,P18418,P35281,P42854,Q64057,Q80WL1
2857	1,2,4,7,8,19,20,22	3,5,6,18,21,23,24,25,26	5	P00774,P18418,Q62997,Q63532,Q63617
2858	1,2,4,7,8,19,20,23	3,5,6,18,21,22,24,25,26	7	P02803,P15399,P18418,P97580,Q63532,Q63617,Q8CJD3
2859	1,2,4,7,8,19,20,24	3,5,6,18,21,22,23,25,26	3	P18418,Q63532,Q63617
2860	1,2,4,7,8,19,20,25	3,5,6,18,21,22,23,24,26	7	P02803,P10715,P18418,P35280,P47967,Q5GRG2,Q63617
2861	1,2,4,7,8,19,20,26	3,5,6,18,21,22,23,24,25	4	P01041,P15978,P18418,P35280
2862	1,2,4,7,8,19,21,22	3,5,6,18,20,23,24,25,26	3	D4A1J4,P18418,Q80WL1
2863	1,2,4,7,8,19,21,23	3,5,6,18,20,22,24,25,26	11	D3ZUC6,P02803,P05964,P15399,P18418,P35281,P97580,Q63532,Q64057,Q80WL1,Q99041
2864	1,2,4,7,8,19,21,24	3,5,6,18,20,22,23,25,26	5	D3ZUC6,O89117,P02761,P18418,Q63532
2865	1,2,4,7,8,19,21,25	3,5,6,18,20,22,23,24,26	8	P02803,P18418,P19629,P47967,P97840,Q5GRG2,Q64057,Q80WL1

2866	1,2,4,7,8,19,21,26	3,5,6,18,20,22,23,24,25	12	D3ZUC6,P02761,P02803,P15978,P18418,P19223,P35281,P82995,Q64057,Q80WL1,Q9EQV9,Q9WUF4
2867	1,2,4,7,8,19,22,23	3,5,6,18,20,21,24,25,26	7	P01835,P15399,P18418,P97580,Q63532,Q80WL1,Q8CJD3
2868	1,2,4,7,8,19,22,24	3,5,6,18,20,21,23,25,26	5	P01835,P18418,Q4FZU2,Q63532,Q6IG02
2869	1,2,4,7,8,19,22,25	3,5,6,18,20,21,23,24,26	10	P01835,P06911,P12020,P18418,P19629,P31044,P47967,Q5GRG2,Q63532,Q80WL1
2870	1,2,4,7,8,19,22,26	3,5,6,18,20,21,23,24,25	4	P01835,P18418,Q63532,Q80WL1
2871	1,2,4,7,8,19,23,24	3,5,6,18,20,21,22,25,26	5	P01835,P15399,P18418,P97580,Q63532
2872	1,2,4,7,8,19,23,25	3,5,6,18,20,21,22,24,26	12	P02803,P06911,P12020,P15399,P18418,P19629,P31044,P97580,Q5GRG2,Q63532,Q64057,Q99041
2873	1,2,4,7,8,19,23,26	3,5,6,18,20,21,22,24,25	6	P02803,P15399,P97580,Q30KJ2,Q63532,Q64057
2874	1,2,4,7,8,19,24,25	3,5,6,18,20,21,22,23,26	6	P01835,P10715,P18418,P19629,Q5GRG2,Q63532
2875	1,2,4,7,8,19,24,26	3,5,6,18,20,21,22,23,25	3	P01835,P18418,Q63532
2876	1,2,4,7,8,19,25,26	3,5,6,18,20,21,22,23,24	10	P01835,P02803,P10715,P15978,P18418,P19223,P35280,P82995,Q5GRG2,Q63532
2877	1,2,4,7,8,20,21,22	3,5,6,18,19,23,24,25,26	7	P01836,P02761,P18418,P42854,Q07523,Q63617,Q80WL1
2878	1,2,4,7,8,20,21,23	3,5,6,18,19,22,24,25,26	4	P02761,P42854,Q63617,Q64057
2879	1,2,4,7,8,20,21,24	3,5,6,18,19,22,23,25,26	5	P01836,P02761,P18418,P42854,Q63617
2880	1,2,4,7,8,20,21,25	3,5,6,18,19,22,23,24,26	8	P10715,P18418,P19629,P42854,Q5GRG2,Q64057,Q80WL1,Q9QZA6

2881	1,2,4,7,8,20,21,26	3,5,6,18,19,22,23,24,25	13	O55004,P01015,P01041,P01836,P02761,P19223,P30836,P42854,P62804,Q00715,Q6AYR8,Q80WL1,Q9EQV9
2882	1,2,4,7,8,20,22,23	3,5,6,18,19,21,24,25,26	3	Q62997,Q63617,Q8CJD3
2883	1,2,4,7,8,20,22,24	3,5,6,18,19,21,23,25,26	2	P18418,Q63617
2884	1,2,4,7,8,20,22,25	3,5,6,18,19,21,23,24,26	7	P06911,P10715,P18418,P19629,Q07523,Q5GRG2,Q63617
2885	1,2,4,7,8,20,22,26	3,5,6,18,19,21,23,24,25	4	P01041,P01836,Q62997,Q63617
2886	1,2,4,7,8,20,23,24	3,5,6,18,19,21,22,25,26	2	P18418,Q63617
2887	1,2,4,7,8,20,23,25	3,5,6,18,19,21,22,24,26	4	P10715,P19629,Q5GRG2,Q63617
2888	1,2,4,7,8,20,23,26	3,5,6,18,19,21,22,24,25	2	Q63617,Q6AYQ8
2889	1,2,4,7,8,20,24,25	3,5,6,18,19,21,22,23,26	5	P10715,P18418,P19629,Q5GRG2,Q63617
2890	1,2,4,7,8,20,24,26	3,5,6,18,19,21,22,23,25	5	P01015,P01041,P01836,Q63617,Q6AYQ8
2891	1,2,4,7,8,20,25,26	3,5,6,18,19,21,22,23,24	5	P01041,P10715,P19223,P35280,Q6AYQ8
2892	1,2,4,7,8,21,22,23	3,5,6,18,19,20,24,25,26	3	P18418,Q64057,Q80WL1
2893	1,2,4,7,8,21,22,24	3,5,6,18,19,20,23,25,26	4	P02761,P18418,P35467,Q80WL1
2894	1,2,4,7,8,21,22,25	3,5,6,18,19,20,23,24,26	5	P06911,P18418,P19629,Q5GRG2,Q80WL1
2895	1,2,4,7,8,21,22,26	3,5,6,18,19,20,23,24,25	9	P01041,P01836,P18418,P19223,P30836,P82995,Q80WL1,Q8CJ52,Q9EQV9

2896	1,2,4,7,8,21,23,24	3,5,6,18,19,20,22,25,26	3	D3ZUC6,P02761,P18418
2897	1,2,4,7,8,21,23,25	3,5,6,18,19,20,22,24,26	7	P02803,P18418,P19629,Q5GRG2,Q64057,Q80WL1,Q99041
2898	1,2,4,7,8,21,23,26	3,5,6,18,19,20,22,24,25	5	D3ZUC6,P25236,Q64057,Q80WL1,Q9EQV9
2899	1,2,4,7,8,21,24,25	3,5,6,18,19,20,22,23,26	5	P10715,P18418,P19629,P22006,Q5GRG2
2900	1,2,4,7,8,21,24,26	3,5,6,18,19,20,22,23,25	8	D3ZUC6,P01041,P01836,P02761,P18418,P19223,Q80WL1,Q9EQV9
2901	1,2,4,7,8,21,25,26	3,5,6,18,19,20,22,23,24	10	P08721,P10715,P19223,P19629,P22006,P55091,P82995,Q64057,Q80WL1,Q9EQV9
2902	1,2,4,7,8,22,23,24	3,5,6,18,19,20,21,25,26	3	P01835,P18418,Q63617
2903	1,2,4,7,8,22,23,25	3,5,6,18,19,20,21,24,26	8	P06911,P10715,P12020,P18418,P19629,Q5GRG2,Q63617,Q80WL1
2904	1,2,4,7,8,22,23,26	3,5,6,18,19,20,21,24,25	1	Q80WL1
2905	1,2,4,7,8,22,24,25	3,5,6,18,19,20,21,23,26	6	P01835,P06911,P10715,P18418,P19629,Q5GRG2
2906	1,2,4,7,8,22,24,26	3,5,6,18,19,20,21,23,25	2	P01835,P18418
2907	1,2,4,7,8,22,25,26	3,5,6,18,19,20,21,23,24	10	P01835,P06911,P10715,P18418,P19223,P19629,P20766,Q5GRG2,Q6AYQ8,Q80WL1
2908	1,2,4,7,8,23,24,25	3,5,6,18,19,20,21,22,26	5	P10715,P18418,P19629,Q5GRG2,Q63617
2909	1,2,4,7,8,23,24,26	3,5,6,18,19,20,21,22,25	1	Q6AYQ8
2910	1,2,4,7,8,23,25,26	3,5,6,18,19,20,21,22,24	6	P00714,P01946,P10715,P19629,Q5GRG2,Q6AYQ8

2911	1,2,4,7,8,24,25,26	3,5,6,18,19,20,21,22,23	7	P01835,P10715,P18418,P19223,P19629,P55091,Q6AYQ8
2912	1,2,4,7,18,19,20,21	3,5,6,8,22,23,24,25,26	6	D3ZUC6,O89117,P00731,P05964,P25031,P42854
2913	1,2,4,7,18,19,20,22	3,5,6,8,21,23,24,25,26	5	P06760,P07150,Q62997,Q63532,Q9WTT6
2914	1,2,4,7,18,19,20,23	3,5,6,8,21,22,24,25,26	8	P05964,P06760,P15399,P97580,Q03191,Q63532,Q8CJD3,Q99MH3
2915	1,2,4,7,18,19,20,24	3,5,6,8,21,22,23,25,26	3	P06760,Q63532,Q9WTT6
2916	1,2,4,7,18,19,20,25	3,5,6,8,21,22,23,24,26	2	P35280,P47967
2917	1,2,4,7,18,19,20,26	3,5,6,8,21,22,23,24,25	6	P01041,P06760,P35280,P82995,Q63532,Q63751
2918	1,2,4,7,18,19,21,22	3,5,6,8,20,23,24,25,26	7	D3ZUC6,O89117,P05964,P42854,P97840,Q63532,Q80WL1
2919	1,2,4,7,18,19,21,23	3,5,6,8,20,22,24,25,26	11	D3ZUC6,O89117,P02803,P05964,P14668,P15399,P97580,Q03191,Q63532,Q8CJD3,Q99MH3
2920	1,2,4,7,18,19,21,24	3,5,6,8,20,22,23,25,26	4	D3ZUC6,O89117,P05964,Q63532
2921	1,2,4,7,18,19,21,25	3,5,6,8,20,22,23,24,26	12	D3ZUC6,O89117,P00731,P02803,P05964,P06757,P25031,P31430,P47967,P97840,P98089,Q99MH3
2922	1,2,4,7,18,19,21,26	3,5,6,8,20,22,23,24,25	9	D3ZUC6,O89117,P05964,P06760,P25031,P36374,P82995,Q63661,Q9EQV9
2923	1,2,4,7,18,19,22,23	3,5,6,8,20,21,24,25,26	9	P05964,P06760,P15399,P97580,Q03191,Q63532,Q8CJD3,Q99MH3,Q9WTT6
2924	1,2,4,7,18,19,22,24	3,5,6,8,20,21,23,25,26	4	P01835,P06760,Q63532,Q9WTT6
2925	1,2,4,7,18,19,22,25	3,5,6,8,20,21,23,24,26	3	P47967,P97840,Q63532

2926	1,2,4,7,18,19,22,26	3,5,6,8,20,21,23,24,25	4	P06760,P82995,Q63532,Q9WTT6
2927	1,2,4,7,18,19,23,24	3,5,6,8,20,21,22,25,26	9	D3ZUC6,P05964,P15399,P97580,Q03191,Q63532,Q6IE51,Q99MH3,Q9WTT6
2928	1,2,4,7,18,19,23,25	3,5,6,8,20,21,22,24,26	9	P00714,P02803,P05964,P15399,P97580,Q03191,Q5BJY9,Q63532,Q99MH3
2929	1,2,4,7,18,19,23,26	3,5,6,8,20,21,22,24,25	11	D3ZUC6,P05964,P06760,P15399,P82995,P97580,Q03191,Q5XFX0,Q63532,Q63661,Q99MH3
2930	1,2,4,7,18,19,24,25	3,5,6,8,20,21,22,23,26	1	Q63532
2931	1,2,4,7,18,19,24,26	3,5,6,8,20,21,22,23,25	5	D3ZUC6,P06760,P82995,Q63532,Q9WTT6
2932	1,2,4,7,18,19,25,26	3,5,6,8,20,21,22,23,24	6	P00714,P35280,P55091,P82995,Q63532,Q63661
2933	1,2,4,7,18,20,21,22	3,5,6,8,19,23,24,25,26	3	P01836,P25031,P42854
2934	1,2,4,7,18,20,21,23	3,5,6,8,19,22,24,25,26	4	D3ZUC6,P25031,P42854,Q62636
2935	1,2,4,7,18,20,21,24	3,5,6,8,19,22,23,25,26	3	D3ZUC6,P01836,P42854
2936	1,2,4,7,18,20,21,25	3,5,6,8,19,22,23,24,26	5	P25031,P31430,P42854,P47967,P98089
2937	1,2,4,7,18,20,21,26	3,5,6,8,19,22,23,24,25	8	D3ZUC6,P01041,P01836,P19223,P25031,P42854,P82995,Q9EQV9
2938	1,2,4,7,18,20,22,23	3,5,6,8,19,21,24,25,26	2	Q62997,Q8CJD3
2939	1,2,4,7,18,20,22,24	3,5,6,8,19,21,23,25,26	3	P06760,P07150,Q9WTT6
2940	1,2,4,7,18,20,22,25	3,5,6,8,19,21,23,24,26	0	

2941	1,2,4,7,18,20,22,26	3,5,6,8,19,21,23,24,25	4	O70594,P01041,P01836,P06760
2942	1,2,4,7,18,20,23,24	3,5,6,8,19,21,22,25,26	1	Q9QZK9
2943	1,2,4,7,18,20,23,25	3,5,6,8,19,21,22,24,26	1	Q99MH3
2944	1,2,4,7,18,20,23,26	3,5,6,8,19,21,22,24,25	3	P06760,Q6IFU8,Q6P6Q2
2945	1,2,4,7,18,20,24,25	3,5,6,8,19,21,22,23,26	0	
2946	1,2,4,7,18,20,24,26	3,5,6,8,19,21,22,23,25	4	O70594,P01041,P01836,P06760
2947	1,2,4,7,18,20,25,26	3,5,6,8,19,21,22,23,24	5	P01041,P19223,P35280,P55091,P82995
2948	1,2,4,7,18,21,22,23	3,5,6,8,19,20,24,25,26	2	D3ZUC6,Q80WL1
2949	1,2,4,7,18,21,22,24	3,5,6,8,19,20,23,25,26	3	D3ZUC6,O89117,P01836
2950	1,2,4,7,18,21,22,25	3,5,6,8,19,20,23,24,26	6	P25031,P31430,P47967,P97840,P98089,Q80WL1
2951	1,2,4,7,18,21,22,26	3,5,6,8,19,20,23,24,25	8	D3ZUC6,P01041,P01836,P25031,P36374,P82995,Q63661,Q80WL1
2952	1,2,4,7,18,21,23,24	3,5,6,8,19,20,22,25,26	2	D3ZUC6,O89117
2953	1,2,4,7,18,21,23,25	3,5,6,8,19,20,22,24,26	8	D3ZUC6,P02803,P05964,P25031,P31430,P98089,Q62636,Q99MH3
2954	1,2,4,7,18,21,23,26	3,5,6,8,19,20,22,24,25	10	D3ZUC6,P05964,P25031,P55091,P82995,Q62636,Q63661,Q6IFU8,Q6P6Q2,Q9EQV9
2955	1,2,4,7,18,21,24,25	3,5,6,8,19,20,22,23,26	7	D3ZUC6,P08721,P22006,P25031,P31430,P55091,P98089

2956	1,2,4,7,18,21,24,26	3,5,6,8,19,20,22,23,25	9	D3ZUC6,O89117,P01041,P01836,P25031,P55091,P82995,Q9EQV9,Q9JID2
2957	1,2,4,7,18,21,25,26	3,5,6,8,19,20,22,23,24	10	D3ZUC6,P08721,P19223,P22006,P25031,P31430,P55091,P82995,P98089,Q63661
2958	1,2,4,7,18,22,23,24	3,5,6,8,19,20,21,25,26	0	
2959	1,2,4,7,18,22,23,25	3,5,6,8,19,20,21,24,26	2	Q5GRG2,Q99MH3
2960	1,2,4,7,18,22,23,26	3,5,6,8,19,20,21,24,25	1	P06760
2961	1,2,4,7,18,22,24,25	3,5,6,8,19,20,21,23,26	0	
2962	1,2,4,7,18,22,24,26	3,5,6,8,19,20,21,23,25	2	O70594,P06760
2963	1,2,4,7,18,22,25,26	3,5,6,8,19,20,21,23,24	2	P55091,P82995
2964	1,2,4,7,18,23,24,25	3,5,6,8,19,20,21,22,26	1	Q99MH3
2965	1,2,4,7,18,23,24,26	3,5,6,8,19,20,21,22,25	3	D3ZUC6,P06760,P55091
2966	1,2,4,7,18,23,25,26	3,5,6,8,19,20,21,22,24	7	P00714,P01946,P24594,P55091,P82995,Q6P6Q2,Q99MH3
2967	1,2,4,7,18,24,25,26	3,5,6,8,19,20,21,22,23	4	P08721,P55091,P82995,Q7TPB1
2968	1,2,4,7,19,20,21,22	3,5,6,8,18,23,24,25,26	3	P01836,P35467,P42854
2969	1,2,4,7,19,20,21,23	3,5,6,8,18,22,24,25,26	4	D3ZUC6,P15399,P42854,P97580
2970	1,2,4,7,19,20,21,24	3,5,6,8,18,22,23,25,26	4	D3ZUC6,P01836,P35467,Q63532

2971	1,2,4,7,19,20,21,25	3,5,6,8,18,22,23,24,26	4	P35467,P42854,P47967,P97840
2972	1,2,4,7,19,20,21,26	3,5,6,8,18,22,23,24,25	7	D3ZUC6,P01015,P01041,P01836,P42854,Q63751,Q9EQV9
2973	1,2,4,7,19,20,22,23	3,5,6,8,18,21,24,25,26	7	P00774,P15399,P97580,Q62997,Q63532,Q8CJD3,Q9WTT6
2974	1,2,4,7,19,20,22,24	3,5,6,8,18,21,23,25,26	8	P00774,P06760,P07150,P14669,P35467,Q62997,Q63532,Q9WTT6
2975	1,2,4,7,19,20,22,25	3,5,6,8,18,21,23,24,26	3	P35467,P47967,Q62997
2976	1,2,4,7,19,20,22,26	3,5,6,8,18,21,23,24,25	6	P01836,P05539,P06760,Q62997,Q63751,Q9WTT6
2977	1,2,4,7,19,20,23,24	3,5,6,8,18,21,22,25,26	3	P15399,P97580,Q63532
2978	1,2,4,7,19,20,23,25	3,5,6,8,18,21,22,24,26	3	P15399,P97580,Q63532
2979	1,2,4,7,19,20,23,26	3,5,6,8,18,21,22,24,25	5	P05539,P06760,P15399,P97580,Q63532
2980	1,2,4,7,19,20,24,25	3,5,6,8,18,21,22,23,26	2	P35467,Q63532
2981	1,2,4,7,19,20,24,26	3,5,6,8,18,21,22,23,25	5	P01015,P01836,P06760,Q63532,Q9WTT6
2982	1,2,4,7,19,20,25,26	3,5,6,8,18,21,22,23,24	2	P05539,P35280
2983	1,2,4,7,19,21,22,23	3,5,6,8,18,20,24,25,26	4	D3ZUC6,P15399,P97580,Q63532
2984	1,2,4,7,19,21,22,24	3,5,6,8,18,20,23,25,26	4	D3ZUC6,P35467,Q63532,Q6IG02
2985	1,2,4,7,19,21,22,25	3,5,6,8,18,20,23,24,26	4	P35467,P47967,P97840,Q6IG02

2986	1,2,4,7,19,21,22,26	3,5,6,8,18,20,23,24,25	3	D3ZUC6,P01836,Q80WL1
2987	1,2,4,7,19,21,23,24	3,5,6,8,18,20,22,25,26	5	D3ZUC6,O89117,P15399,P97580,Q63532
2988	1,2,4,7,19,21,23,25	3,5,6,8,18,20,22,24,26	8	D3ZUC6,P02803,P15399,P47967,P97580,P97840,Q63532,Q99041
2989	1,2,4,7,19,21,23,26	3,5,6,8,18,20,22,24,25	6	D3ZUC6,P15399,P25236,P97580,Q63532,Q63661
2990	1,2,4,7,19,21,24,25	3,5,6,8,18,20,22,23,26	7	D3ZUC6,P22006,P35467,P47967,P97840,Q63532,Q6IG02
2991	1,2,4,7,19,21,24,26	3,5,6,8,18,20,22,23,25	6	D3ZUC6,O89117,P01836,P80020,Q63532,Q9EQV9
2992	1,2,4,7,19,21,25,26	3,5,6,8,18,20,22,23,24	6	D3ZUC6,P19223,P22006,P47967,P82995,P97840
2993	1,2,4,7,19,22,23,24	3,5,6,8,18,20,21,25,26	4	P01835,P97580,Q63532,Q9WTT6
2994	1,2,4,7,19,22,23,25	3,5,6,8,18,20,21,24,26	5	P06911,P15399,P97580,Q5GRG2,Q63532
2995	1,2,4,7,19,22,23,26	3,5,6,8,18,20,21,24,25	3	P05539,P97580,Q63532
2996	1,2,4,7,19,22,24,25	3,5,6,8,18,20,21,23,26	5	P01835,P35467,Q4FZU2,Q63532,Q6IG02
2997	1,2,4,7,19,22,24,26	3,5,6,8,18,20,21,23,25	4	P01835,P06760,Q63532,Q9WTT6
2998	1,2,4,7,19,22,25,26	3,5,6,8,18,20,21,23,24	2	P01835,P05539
2999	1,2,4,7,19,23,24,25	3,5,6,8,18,20,21,22,26	3	P15399,P97580,Q63532
3000	1,2,4,7,19,23,24,26	3,5,6,8,18,20,21,22,25	4	P01946,P14480,P97580,Q63532

3001	1,2,4,7,19,23,25,26	3,5,6,8,18,20,21,22,24	5	P01946,P14480,P15399,P97580,Q63532
3002	1,2,4,7,19,24,25,26	3,5,6,8,18,20,21,22,23	3	P01835,P14480,Q63532
3003	1,2,4,7,20,21,22,23	3,5,6,8,18,19,24,25,26	1	P01836
3004	1,2,4,7,20,21,22,24	3,5,6,8,18,19,23,25,26	3	P01836,P35467,P51907
3005	1,2,4,7,20,21,22,25	3,5,6,8,18,19,23,24,26	6	P01836,P09605;P25809,P35467,P47967,P51907,Q07523
3006	1,2,4,7,20,21,22,26	3,5,6,8,18,19,23,24,25	6	P01015,P01041,P01836,P05539,P30836,P51907
3007	1,2,4,7,20,21,23,24	3,5,6,8,18,19,22,25,26	2	D3ZUC6,P01836
3008	1,2,4,7,20,21,23,25	3,5,6,8,18,19,22,24,26	0	
3009	1,2,4,7,20,21,23,26	3,5,6,8,18,19,22,24,25	3	D3ZUC6,P01836,P25236
3010	1,2,4,7,20,21,24,25	3,5,6,8,18,19,22,23,26	4	P01836,P22006,P35467,P51907
3011	1,2,4,7,20,21,24,26	3,5,6,8,18,19,22,23,25	7	D3ZUC6,P01015,P01041,P01836,P51907,P80020,Q9EQV9
3012	1,2,4,7,20,21,25,26	3,5,6,8,18,19,22,23,24	5	P01041,P01836,P19223,P51907,P82995
3013	1,2,4,7,20,22,23,24	3,5,6,8,18,19,21,25,26	0	
3014	1,2,4,7,20,22,23,25	3,5,6,8,18,19,21,24,26	2	P06911,Q5GRG2
3015	1,2,4,7,20,22,23,26	3,5,6,8,18,19,21,24,25	3	P01836,P05539,Q62997

3016	1,2,4,7,20,<br>22,24,25	3,5,6,8,18,<br>19,21,23,2<br>6	1	P35467
3017	1,2,4,7,20,<br>22,24,26	3,5,6,8,18,<br>19,21,23,2<br>5	3	O70594,P01836,P06760
3018	1,2,4,7,20,<br>22,25,26	3,5,6,8,18,<br>19,21,23,2<br>4	2	P01836,P05539
3019	1,2,4,7,20,<br>23,24,25	3,5,6,8,18,<br>19,21,22,2<br>6	0	
3020	1,2,4,7,20,<br>23,24,26	3,5,6,8,18,<br>19,21,22,2<br>5	1	P01836
3021	1,2,4,7,20,<br>23,25,26	3,5,6,8,18,<br>19,21,22,2<br>4	2	P01946,P05539
3022	1,2,4,7,20,<br>24,25,26	3,5,6,8,18,<br>19,21,22,2<br>3	1	P01836
3023	1,2,4,7,21,<br>22,23,24	3,5,6,8,18,<br>19,20,25,2<br>6	1	D3ZUC6
3024	1,2,4,7,21,<br>22,23,25	3,5,6,8,18,<br>19,20,24,2<br>6	2	P06911,Q5GRG2
3025	1,2,4,7,21,<br>22,23,26	3,5,6,8,18,<br>19,20,24,2<br>5	3	D3ZUC6,P01836,Q80WL1
3026	1,2,4,7,21,<br>22,24,25	3,5,6,8,18,<br>19,20,23,2<br>6	3	P22006,P35467,Q6IG02
3027	1,2,4,7,21,<br>22,24,26	3,5,6,8,18,<br>19,20,23,2<br>5	4	D3ZUC6,P01836,P80020,Q9EQV9
3028	1,2,4,7,21,<br>22,25,26	3,5,6,8,18,<br>19,20,23,2<br>4	5	P01836,P19223,P22006,P82995,Q80WL1
3029	1,2,4,7,21,<br>23,24,25	3,5,6,8,18,<br>19,20,22,2<br>6	2	D3ZUC6,P22006
3030	1,2,4,7,21,<br>23,24,26	3,5,6,8,18,<br>19,20,22,2<br>5	5	D3ZUC6,P01836,P01946,P22006,Q9EQV9

3031	1,2,4,7,21,23,25,26	3,5,6,8,18,19,20,22,24	8	D3ZUC6,P01946,P19223,P22006,P25236,P27213,P82995,P83121
3032	1,2,4,7,21,24,25,26	3,5,6,8,18,19,20,22,23	7	D3ZUC6,P01836,P19223,P22006,P55091,P82995,Q9EQV9
3033	1,2,4,7,22,23,24,25	3,5,6,8,18,19,20,21,26	2	P06911,Q5GRG2
3034	1,2,4,7,22,23,24,26	3,5,6,8,18,19,20,21,25	1	P00731
3035	1,2,4,7,22,23,25,26	3,5,6,8,18,19,20,21,24	3	P01946,P05539,Q5GRG2
3036	1,2,4,7,22,24,25,26	3,5,6,8,18,19,20,21,23	2	O70594,P01835
3037	1,2,4,7,23,24,25,26	3,5,6,8,18,19,20,21,22	3	P01946,P14480,Q7TPB1
3038	1,2,4,8,18,19,20,21	3,5,6,7,22,23,24,25,26	7	P00731,P02761,P02803,P10758,P35281,P62804,Q63751
3039	1,2,4,8,18,19,20,22	3,5,6,7,21,23,24,25,26	4	P22006,Q62997,Q63751,Q8CJD3
3040	1,2,4,8,18,19,20,23	3,5,6,7,21,22,24,25,26	7	P02803,P22006,Q03191,Q5BJY9,Q5XI22,Q8CJD3,Q99MH3
3041	1,2,4,8,18,19,20,24	3,5,6,7,21,22,23,25,26	1	Q9QZK9
3042	1,2,4,8,18,19,20,25	3,5,6,7,21,22,23,24,26	2	P02803,P35280
3043	1,2,4,8,18,19,20,26	3,5,6,7,21,22,23,24,25	4	P35280,Q63355,Q63751,Q63942
3044	1,2,4,8,18,19,21,22	3,5,6,7,20,23,24,25,26	4	D3ZUC6,P10758,P35281,Q80WL1
3045	1,2,4,8,18,19,21,23	3,5,6,7,20,22,24,25,26	11	D3ZUC6,O89117,P02803,P05964,P10758,P35281,Q03191,Q5XI22,Q80WL1,Q8CJD3,Q99MH3

3046	1,2,4,8,18,19,21,24	3,5,6,7,20,22,23,25,26	5	D3ZUC6,O89117,P02761,P10758,P35281
3047	1,2,4,8,18,19,21,25	3,5,6,7,20,22,23,24,26	8	P00731,P02803,P31430,P35281,P47967,P98089,Q80WL1,Q99MH3
3048	1,2,4,8,18,19,21,26	3,5,6,7,20,22,23,24,25	12	D3ZUC6,O89117,P02761,P02803,P10758,P19223,P35281,Q63661,Q63751,Q63942,Q80WL1,Q9EQV9
3049	1,2,4,8,18,19,22,23	3,5,6,7,20,21,24,25,26	3	Q03191,Q8CJD3,Q99MH3
3050	1,2,4,8,18,19,22,24	3,5,6,7,20,21,23,25,26	2	P01835,P83121
3051	1,2,4,8,18,19,22,25	3,5,6,7,20,21,23,24,26	0	
3052	1,2,4,8,18,19,22,26	3,5,6,7,20,21,23,24,25	3	Q63751,Q63942,Q80WL1
3053	1,2,4,8,18,19,23,24	3,5,6,7,20,21,22,25,26	2	Q03191,Q99MH3
3054	1,2,4,8,18,19,23,25	3,5,6,7,20,21,22,24,26	7	P00714,P02803,Q03191,Q5BJY9,Q63598,Q8CJD3,Q99MH3
3055	1,2,4,8,18,19,23,26	3,5,6,7,20,21,22,24,25	8	P02803,P35467,Q03191,Q63661,Q63942,Q6AY61,Q6P6Q2,Q99MH3
3056	1,2,4,8,18,19,24,25	3,5,6,7,20,21,22,23,26	0	
3057	1,2,4,8,18,19,24,26	3,5,6,7,20,21,22,23,25	1	Q63942
3058	1,2,4,8,18,19,25,26	3,5,6,7,20,21,22,23,24	7	P00714,P02803,P19223,P35280,P55091,Q561R9,Q63942
3059	1,2,4,8,18,20,21,22	3,5,6,7,19,23,24,25,26	3	P02761,P10758,Q80WL1
3060	1,2,4,8,18,20,21,23	3,5,6,7,19,22,24,25,26	6	P02761,P02803,P10758,P17046,Q5XI22,Q6TMA8

3061	1,2,4,8,18,20,21,24	3,5,6,7,19,22,23,25,26	3	P02761,P10758,P17046
3062	1,2,4,8,18,20,21,25	3,5,6,7,19,22,23,24,26	4	P02803,P31430,P98089,Q9QZA6
3063	1,2,4,8,18,20,21,26	3,5,6,7,19,22,23,24,25	11	P01015,P02761,P10758,P17046,P19223,P62804,Q00715,Q6AYR8,Q80WL1,Q9EQV9,Q9QZA6
3064	1,2,4,8,18,20,22,23	3,5,6,7,19,21,24,25,26	4	P22006,Q62997,Q8CJD3,Q9QZK9
3065	1,2,4,8,18,20,22,24	3,5,6,7,19,21,23,25,26	2	P83121,Q9QZK9
3066	1,2,4,8,18,20,22,25	3,5,6,7,19,21,23,24,26	0	
3067	1,2,4,8,18,20,22,26	3,5,6,7,19,21,23,24,25	0	
3068	1,2,4,8,18,20,23,24	3,5,6,7,19,21,22,25,26	1	Q9QZK9
3069	1,2,4,8,18,20,23,25	3,5,6,7,19,21,22,24,26	3	P00714,P02803,Q99MH3
3070	1,2,4,8,18,20,23,26	3,5,6,7,19,21,22,24,25	6	P17046,P19218,Q63942,Q6IFU8,Q6IG02,Q6P6Q2
3071	1,2,4,8,18,20,24,25	3,5,6,7,19,21,22,23,26	0	
3072	1,2,4,8,18,20,24,26	3,5,6,7,19,21,22,23,25	2	P01015,P17046
3073	1,2,4,8,18,20,25,26	3,5,6,7,19,21,22,23,24	3	P19223,P35280,P55091
3074	1,2,4,8,18,21,22,23	3,5,6,7,19,20,24,25,26	4	D3ZUC6,P10758,Q80WL1,Q8CJD3
3075	1,2,4,8,18,21,22,24	3,5,6,7,19,20,23,25,26	4	D3ZUC6,P02761,P10758,Q80WL1

3076	1,2,4,8,18,21,22,25	3,5,6,7,19,20,23,24,26	3	P31430,P98089,Q80WL1
3077	1,2,4,8,18,21,22,26	3,5,6,7,19,20,23,24,25	5	D3ZUC6,P19223,P36374,Q80WL1,Q9EQV9
3078	1,2,4,8,18,21,23,24	3,5,6,7,19,20,22,25,26	3	D3ZUC6,P02761,P10758
3079	1,2,4,8,18,21,23,25	3,5,6,7,19,20,22,24,26	6	P02803,P31430,P98089,Q63598,Q80WL1,Q99MH3
3080	1,2,4,8,18,21,23,26	3,5,6,7,19,20,22,24,25	13	D3ZUC6,P02803,P17046,P25236,Q62636,Q63661,Q63942,Q6AY61,Q6IFU8,Q6IFV1,Q6P6Q2,Q80WL1,Q9EQV9
3081	1,2,4,8,18,21,24,25	3,5,6,7,19,20,22,23,26	4	O54728,P08721,P31430,P98089
3082	1,2,4,8,18,21,24,26	3,5,6,7,19,20,22,23,25	6	D3ZUC6,P02761,P17046,P55091,Q9EQV9,Q9JID2
3083	1,2,4,8,18,21,25,26	3,5,6,7,19,20,22,23,24	8	P02803,P08721,P19223,P31430,P55091,P98089,Q80WL1,Q9EQV9
3084	1,2,4,8,18,22,23,24	3,5,6,7,19,20,21,25,26	0	
3085	1,2,4,8,18,22,23,25	3,5,6,7,19,20,21,24,26	3	Q63598,Q8CJD3,Q99MH3
3086	1,2,4,8,18,22,23,26	3,5,6,7,19,20,21,24,25	3	Q63942,Q6P6Q2,Q80WL1
3087	1,2,4,8,18,22,24,25	3,5,6,7,19,20,21,23,26	0	
3088	1,2,4,8,18,22,24,26	3,5,6,7,19,20,21,23,25	0	
3089	1,2,4,8,18,22,25,26	3,5,6,7,19,20,21,23,24	2	P55091,Q80WL1
3090	1,2,4,8,18,23,24,25	3,5,6,7,19,20,21,22,26	2	Q63598,Q99MH3

3091	1,2,4,8,18,23,24,26	3,5,6,7,19,20,21,22,25	3	P17046,Q63942,Q6P6Q2
3092	1,2,4,8,18,23,25,26	3,5,6,7,19,20,21,22,24	8	P00714,P02803,P55091,Q63598,Q63942,Q6IFU8,Q6P6Q2,Q99MH3
3093	1,2,4,8,18,24,25,26	3,5,6,7,19,20,21,22,23	3	P08721,P55091,Q7TPB1
3094	1,2,4,8,19,20,21,22	3,5,6,7,18,23,24,25,26	4	P02761,P35281,Q63751,Q80WL1
3095	1,2,4,8,19,20,21,23	3,5,6,7,18,22,24,25,26	3	P02761,P35281,Q8CJD3
3096	1,2,4,8,19,20,21,24	3,5,6,7,18,22,23,25,26	2	P02761,P35281
3097	1,2,4,8,19,20,21,25	3,5,6,7,18,22,23,24,26	2	P35281,P47967
3098	1,2,4,8,19,20,21,26	3,5,6,7,18,22,23,24,25	8	P01015,P02761,P19223,P35281,P62804,Q00715,Q63751,Q9EQV9
3099	1,2,4,8,19,20,22,23	3,5,6,7,18,21,24,25,26	3	P00774,Q62997,Q8CJD3
3100	1,2,4,8,19,20,22,24	3,5,6,7,18,21,23,25,26	3	P00774,P83121,Q62997
3101	1,2,4,8,19,20,22,25	3,5,6,7,18,21,23,24,26	1	Q62997
3102	1,2,4,8,19,20,22,26	3,5,6,7,18,21,23,24,25	2	Q62997,Q63751
3103	1,2,4,8,19,20,23,24	3,5,6,7,18,21,22,25,26	0	
3104	1,2,4,8,19,20,23,25	3,5,6,7,18,21,22,24,26	0	
3105	1,2,4,8,19,20,23,26	3,5,6,7,18,21,22,24,25	1	P19218

3106	1,2,4,8,19,20,24,25	3,5,6,7,18,21,22,23,26	0	
3107	1,2,4,8,19,20,24,26	3,5,6,7,18,21,22,23,25	1	P01015
3108	1,2,4,8,19,20,25,26	3,5,6,7,18,21,22,23,24	1	P35280
3109	1,2,4,8,19,21,22,23	3,5,6,7,18,20,24,25,26	3	P35281,Q80WL1,Q8CJD3
3110	1,2,4,8,19,21,22,24	3,5,6,7,18,20,23,25,26	2	Q6IG02,Q80WL1
3111	1,2,4,8,19,21,22,25	3,5,6,7,18,20,23,24,26	2	P47967,Q80WL1
3112	1,2,4,8,19,21,22,26	3,5,6,7,18,20,23,24,25	3	Q63751,Q80WL1,Q9EQV9
3113	1,2,4,8,19,21,23,24	3,5,6,7,18,20,22,25,26	1	D3ZUC6
3114	1,2,4,8,19,21,23,25	3,5,6,7,18,20,22,24,26	2	P02803,P27213
3115	1,2,4,8,19,21,23,26	3,5,6,7,18,20,22,24,25	5	D3ZUC6,P25236,P35281,Q80WL1,Q9EQV9
3116	1,2,4,8,19,21,24,25	3,5,6,7,18,20,22,23,26	0	
3117	1,2,4,8,19,21,24,26	3,5,6,7,18,20,22,23,25	3	D3ZUC6,P02761,Q9EQV9
3118	1,2,4,8,19,21,25,26	3,5,6,7,18,20,22,23,24	5	P02803,P19223,P27213,Q80WL1,Q9EQV9
3119	1,2,4,8,19,22,23,24	3,5,6,7,18,20,21,25,26	1	P01835
3120	1,2,4,8,19,22,23,25	3,5,6,7,18,20,21,24,26	3	P06911,Q5GRG2,Q8CJD3

3121	1,2,4,8,19,22,23,26	3,5,6,7,18,20,21,24,25	0	
3122	1,2,4,8,19,22,24,25	3,5,6,7,18,20,21,23,26	2	P01835,Q6IG02
3123	1,2,4,8,19,22,24,26	3,5,6,7,18,20,21,23,25	1	P01835
3124	1,2,4,8,19,22,25,26	3,5,6,7,18,20,21,23,24	1	P01835
3125	1,2,4,8,19,23,24,25	3,5,6,7,18,20,21,22,26	0	
3126	1,2,4,8,19,23,24,26	3,5,6,7,18,20,21,22,25	0	
3127	1,2,4,8,19,23,25,26	3,5,6,7,18,20,21,22,24	2	P02803,P27213
3128	1,2,4,8,19,24,25,26	3,5,6,7,18,20,21,22,23	2	P01835,P14480
3129	1,2,4,8,20,21,22,23	3,5,6,7,18,19,24,25,26	1	Q80WL1
3130	1,2,4,8,20,21,22,24	3,5,6,7,18,19,23,25,26	2	P02761,Q5XFX0
3131	1,2,4,8,20,21,22,25	3,5,6,7,18,19,23,24,26	2	Q07523,Q80WL1
3132	1,2,4,8,20,21,22,26	3,5,6,7,18,19,23,24,25	5	P01015,P02761,Q00715,Q80WL1,Q9EQV9
3133	1,2,4,8,20,21,23,24	3,5,6,7,18,19,22,25,26	1	P02761
3134	1,2,4,8,20,21,23,25	3,5,6,7,18,19,22,24,26	0	
3135	1,2,4,8,20,21,23,26	3,5,6,7,18,19,22,24,25	4	P02761,P17046,P25236,Q9EQV9

3136	1,2,4,8,20,21,24,25	3,5,6,7,18,19,22,23,26	1	P02761
3137	1,2,4,8,20,21,24,26	3,5,6,7,18,19,22,23,25	4	P01015,P02761,P17046,Q9EQV9
3138	1,2,4,8,20,21,25,26	3,5,6,7,18,19,22,23,24	3	P19223,Q00715,Q9EQV9
3139	1,2,4,8,20,22,23,24	3,5,6,7,18,19,21,25,26	1	Q9QZK9
3140	1,2,4,8,20,22,23,25	3,5,6,7,18,19,21,24,26	0	
3141	1,2,4,8,20,22,23,26	3,5,6,7,18,19,21,24,25	1	Q62997
3142	1,2,4,8,20,22,24,25	3,5,6,7,18,19,21,23,26	1	Q07523
3143	1,2,4,8,20,22,24,26	3,5,6,7,18,19,21,23,25	1	P01015
3144	1,2,4,8,20,22,25,26	3,5,6,7,18,19,21,23,24	0	
3145	1,2,4,8,20,23,24,25	3,5,6,7,18,19,21,22,26	0	
3146	1,2,4,8,20,23,24,26	3,5,6,7,18,19,21,22,25	2	P19218,P97840
3147	1,2,4,8,20,23,25,26	3,5,6,7,18,19,21,22,24	1	P19218
3148	1,2,4,8,20,24,25,26	3,5,6,7,18,19,21,22,23	0	
3149	1,2,4,8,21,22,23,24	3,5,6,7,18,19,20,25,26	1	Q80WL1
3150	1,2,4,8,21,22,23,25	3,5,6,7,18,19,20,24,26	1	Q80WL1

3151	1,2,4,8,21,22,23,26	3,5,6,7,18,19,20,24,25	4	P25236,Q5QE79,Q80WL1,Q9EQV9
3152	1,2,4,8,21,22,24,25	3,5,6,7,18,19,20,23,26	2	P35467,Q80WL1
3153	1,2,4,8,21,22,24,26	3,5,6,7,18,19,20,23,25	3	P02761,Q80WL1,Q9EQV9
3154	1,2,4,8,21,22,25,26	3,5,6,7,18,19,20,23,24	2	P19223,Q80WL1
3155	1,2,4,8,21,23,24,25	3,5,6,7,18,19,20,22,26	1	Q5U2V4
3156	1,2,4,8,21,23,24,26	3,5,6,7,18,19,20,22,25	4	D3ZUC6,P02761,Q68FV8,Q9EQV9
3157	1,2,4,8,21,23,25,26	3,5,6,7,18,19,20,22,24	7	P14669,P19223,P25236,P27213,Q80WL1,Q9EQV9,Q9WTT6
3158	1,2,4,8,21,24,25,26	3,5,6,7,18,19,20,22,23	3	P19223,Q68FV8,Q9EQV9
3159	1,2,4,8,22,23,24,25	3,5,6,7,18,19,20,21,26	1	P06911
3160	1,2,4,8,22,23,24,26	3,5,6,7,18,19,20,21,25	1	P00731
3161	1,2,4,8,22,23,25,26	3,5,6,7,18,19,20,21,24	0	
3162	1,2,4,8,22,24,25,26	3,5,6,7,18,19,20,21,23	1	P01835
3163	1,2,4,8,23,24,25,26	3,5,6,7,18,19,20,21,22	2	Q68FV8,Q7TPB1
3164	1,2,4,18,19,20,21,22	3,5,6,7,8,23,24,25,26	4	D3ZUC6,P10758,P36374,Q63751
3165	1,2,4,18,19,20,21,23	3,5,6,7,8,22,24,25,26	4	D3ZUC6,P10758,Q8CJD3,Q99MH3
3166	1,2,4,18,19,20,21,24	3,5,6,7,8,22,23,25,26	2	D3ZUC6,P10758

3167	1,2,4,18,19,20,21,25	3,5,6,7,8,22,23,24,26	3	P31430,P47967,P98089
3168	1,2,4,18,19,20,21,26	3,5,6,7,8,22,23,24,25	7	D3ZUC6,P01015,P10758,P36374,Q63661,Q63751,Q9EQV9
3169	1,2,4,18,19,20,22,23	3,5,6,7,8,21,24,25,26	5	P22006,Q62997,Q8CJD3,Q9QZK9,Q9WTT6
3170	1,2,4,18,19,20,22,24	3,5,6,7,8,21,23,25,26	4	P06760,P07150,Q9QZK9,Q9WTT6
3171	1,2,4,18,19,20,22,25	3,5,6,7,8,21,23,24,26	1	Q62997
3172	1,2,4,18,19,20,22,26	3,5,6,7,8,21,23,24,25	5	P06760,P08937,Q62997,Q63751,Q9WTT6
3173	1,2,4,18,19,20,23,24	3,5,6,7,8,21,22,25,26	2	Q9QZK9,Q9WTT6
3174	1,2,4,18,19,20,23,25	3,5,6,7,8,21,22,24,26	2	Q8CJD3,Q99MH3
3175	1,2,4,18,19,20,23,26	3,5,6,7,8,21,22,24,25	3	P19218,Q6IFU8,Q6P6Q2
3176	1,2,4,18,19,20,24,25	3,5,6,7,8,21,22,23,26	0	
3177	1,2,4,18,19,20,24,26	3,5,6,7,8,21,22,23,25	3	P06760,Q63751,Q9WTT6
3178	1,2,4,18,19,20,25,26	3,5,6,7,8,21,22,23,24	1	P35280
3179	1,2,4,18,19,21,22,23	3,5,6,7,8,20,24,25,26	4	D3ZUC6,P10758,Q8CJD3,Q99MH3
3180	1,2,4,18,19,21,22,24	3,5,6,7,8,20,23,25,26	3	D3ZUC6,O89117,P10758
3181	1,2,4,18,19,21,22,25	3,5,6,7,8,20,23,24,26	5	D3ZUC6,P31430,P47967,P97840,P98089
3182	1,2,4,18,19,21,22,26	3,5,6,7,8,20,23,24,25	5	D3ZUC6,P10758,P36374,Q63661,Q63751
3183	1,2,4,18,19,21,23,24	3,5,6,7,8,20,22,25,26	5	D3ZUC6,O89117,P10758,Q03191,Q99MH3
3184	1,2,4,18,19,21,23,25	3,5,6,7,8,20,22,24,26	7	D3ZUC6,P02803,P05964,P27213,P31430,P98089,Q99MH3
3185	1,2,4,18,19,21,23,26	3,5,6,7,8,20,22,24,25	9	D3ZUC6,P05964,P10758,P25236,Q03191,Q63661,Q6IFU8,Q6P6Q2,Q99MH3
3186	1,2,4,18,19,21,24,25	3,5,6,7,8,20,22,23,26	3	D3ZUC6,P31430,P98089
3187	1,2,4,18,19,21,24,26	3,5,6,7,8,20,22,23,25	4	D3ZUC6,O89117,Q63661,Q9EQV9
3188	1,2,4,18,19,21,25,26	3,5,6,7,8,20,22,23,24	7	D3ZUC6,P19223,P27213,P31430,P55091,P98089,Q63661
3189	1,2,4,18,19,22,23,24	3,5,6,7,8,20,21,25,26	1	Q9WTT6

3190	1,2,4,18,1 9,22,23,25	3,5,6,7,8,2 0,21,24,26	2	Q8CJD3,Q99MH3
3191	1,2,4,18,1 9,22,23,26	3,5,6,7,8,2 0,21,24,25	1	Q63661
3192	1,2,4,18,1 9,22,24,25	3,5,6,7,8,2 0,21,23,26	0	
3193	1,2,4,18,1 9,22,24,26	3,5,6,7,8,2 0,21,23,25	2	P06760,Q9WTT6
3194	1,2,4,18,1 9,22,25,26	3,5,6,7,8,2 0,21,23,24	0	
3195	1,2,4,18,1 9,23,24,25	3,5,6,7,8,2 0,21,22,26	1	Q99MH3
3196	1,2,4,18,1 9,23,24,26	3,5,6,7,8,2 0,21,22,25	4	D3ZUC6,Q03191,Q63661,Q6IFU7
3197	1,2,4,18,1 9,23,25,26	3,5,6,7,8,2 0,21,22,24	6	P00714,P27213,Q03191,Q63661,Q6P6Q2,Q99MH3
3198	1,2,4,18,1 9,24,25,26	3,5,6,7,8,2 0,21,22,23	3	P14480,P55091,Q7TPB1
3199	1,2,4,18,2 0,21,22,23	3,5,6,7,8,1 9,24,25,26	2	P10758,Q8CJD3
3200	1,2,4,18,2 0,21,22,24	3,5,6,7,8,1 9,23,25,26	3	P51907,Q99041,Q9QZK9
3201	1,2,4,18,2 0,21,22,25	3,5,6,7,8,1 9,23,24,26	3	P31430,P51907,P98089
3202	1,2,4,18,2 0,21,22,26	3,5,6,7,8,1 9,23,24,25	4	P36374,P51907,Q63751,Q99041
3203	1,2,4,18,2 0,21,23,24	3,5,6,7,8,1 9,22,25,26	2	D3ZUC6,Q9QZK9
3204	1,2,4,18,2 0,21,23,25	3,5,6,7,8,1 9,22,24,26	3	P31430,P98089,Q99MH3
3205	1,2,4,18,2 0,21,23,26	3,5,6,7,8,1 9,22,24,25	7	D3ZUC6,P25236,Q62636,Q63661,Q6IFU8,Q6IMF3,Q6P6Q2
3206	1,2,4,18,2 0,21,24,25	3,5,6,7,8,1 9,22,23,26	3	P31430,P51907,P98089
3207	1,2,4,18,2 0,21,24,26	3,5,6,7,8,1 9,22,23,25	6	D3ZUC6,P01015,P51907,P80020,Q99041,Q9EQV9
3208	1,2,4,18,2 0,21,25,26	3,5,6,7,8,1 9,22,23,24	5	P19223,P31430,P51907,P55091,P98089
3209	1,2,4,18,2 0,22,23,24	3,5,6,7,8,1 9,21,25,26	2	P53792,Q9QZK9
3210	1,2,4,18,2 0,22,23,25	3,5,6,7,8,1 9,21,24,26	0	
3211	1,2,4,18,2 0,22,23,26	3,5,6,7,8,1 9,21,24,25	3	P53792,Q62997,Q6P6Q2
3212	1,2,4,18,2 0,22,24,25	3,5,6,7,8,1 9,21,23,26	0	

3213	1,2,4,18,20,22,24,26	3,5,6,7,8,19,21,23,25	5	O70594,P06760,Q99041,Q9QZK9,Q9WTT6
3214	1,2,4,18,20,22,25,26	3,5,6,7,8,19,21,23,24	0	
3215	1,2,4,18,20,23,24,25	3,5,6,7,8,19,21,22,26	0	
3216	1,2,4,18,20,23,24,26	3,5,6,7,8,19,21,22,25	5	P19218,Q6IFU7,Q6IFU8,Q6P6Q2,Q9QZK9
3217	1,2,4,18,20,23,25,26	3,5,6,7,8,19,21,22,24	3	P19218,Q6IFU8,Q6P6Q2
3218	1,2,4,18,20,24,25,26	3,5,6,7,8,19,21,22,23	1	P55091
3219	1,2,4,18,21,22,23,24	3,5,6,7,8,19,20,25,26	1	D3ZUC6
3220	1,2,4,18,21,22,23,25	3,5,6,7,8,19,20,24,26	3	P31430,P98089,Q99MH3
3221	1,2,4,18,21,22,23,26	3,5,6,7,8,19,20,24,25	4	D3ZUC6,Q63661,Q6IFU8,Q6P6Q2
3222	1,2,4,18,21,22,24,25	3,5,6,7,8,19,20,23,26	2	P31430,P98089
3223	1,2,4,18,21,22,24,26	3,5,6,7,8,19,20,23,25	4	D3ZUC6,Q99041,Q9EQV9,Q9JID2
3224	1,2,4,18,21,22,25,26	3,5,6,7,8,19,20,23,24	4	P19223,P31430,P55091,P98089
3225	1,2,4,18,21,23,24,25	3,5,6,7,8,19,20,22,26	4	D3ZUC6,P31430,P98089,Q99MH3
3226	1,2,4,18,21,23,24,26	3,5,6,7,8,19,20,22,25	6	D3ZUC6,Q63661,Q6IFU7,Q6IFU8,Q6P6Q2,Q9EQV9
3227	1,2,4,18,21,23,25,26	3,5,6,7,8,19,20,22,24	12	D3ZUC6,P00774,P25236,P27213,P31430,P55091,P98089,Q62636,Q63661,Q6IFU8,Q6P6Q2,Q99MH3
3228	1,2,4,18,21,24,25,26	3,5,6,7,8,19,20,22,23	6	D3ZUC6,P08721,P31430,P55091,P98089,Q9EQV9
3229	1,2,4,18,22,23,24,25	3,5,6,7,8,19,20,21,26	1	P53792
3230	1,2,4,18,22,23,24,26	3,5,6,7,8,19,20,21,25	3	P00731,P53792,Q6IFU7
3231	1,2,4,18,22,23,25,26	3,5,6,7,8,19,20,21,24	1	Q6P6Q2
3232	1,2,4,18,22,24,25,26	3,5,6,7,8,19,20,21,23	2	O70594,P55091
3233	1,2,4,18,23,24,25,26	3,5,6,7,8,19,20,21,22	3	P55091,Q6P6Q2,Q7TPB1
3234	1,2,4,19,20,21,22,23	3,5,6,7,8,18,24,25,26	1	Q8CJD3
3235	1,2,4,19,20,21,22,24	3,5,6,7,8,18,23,25,26	2	P35467,P51907

3236	1,2,4,19,20,21,22,25	3,5,6,7,8,18,23,24,26	3	P35467,P47967,P51907
3237	1,2,4,19,20,21,22,26	3,5,6,7,8,18,23,24,25	6	O70377,P01015,P08937,P36374,P51907,Q63751
3238	1,2,4,19,20,21,23,24	3,5,6,7,8,18,22,25,26	1	D3ZUC6
3239	1,2,4,19,20,21,23,25	3,5,6,7,8,18,22,24,26	0	
3240	1,2,4,19,20,21,23,26	3,5,6,7,8,18,22,24,25	2	D3ZUC6,P25236
3241	1,2,4,19,20,21,24,25	3,5,6,7,8,18,22,23,26	1	P35467
3242	1,2,4,19,20,21,24,26	3,5,6,7,8,18,22,23,25	5	D3ZUC6,P01015,P51907,P80020,Q9EQV9
3243	1,2,4,19,20,21,25,26	3,5,6,7,8,18,22,23,24	2	P19223,P51907
3244	1,2,4,19,20,22,23,24	3,5,6,7,8,18,21,25,26	4	P00774,Q62997,Q9QZK9,Q9WTT6
3245	1,2,4,19,20,22,23,25	3,5,6,7,8,18,21,24,26	2	Q62997,Q8CJD3
3246	1,2,4,19,20,22,23,26	3,5,6,7,8,18,21,24,25	1	Q62997
3247	1,2,4,19,20,22,24,25	3,5,6,7,8,18,21,23,26	1	P35467
3248	1,2,4,19,20,22,24,26	3,5,6,7,8,18,21,23,25	4	P01015,P06760,Q62997,Q9WTT6
3249	1,2,4,19,20,22,25,26	3,5,6,7,8,18,21,23,24	1	Q62997
3250	1,2,4,19,20,23,24,25	3,5,6,7,8,18,21,22,26	0	
3251	1,2,4,19,20,23,24,26	3,5,6,7,8,18,21,22,25	1	P19218
3252	1,2,4,19,20,23,25,26	3,5,6,7,8,18,21,22,24	1	P19218
3253	1,2,4,19,20,24,25,26	3,5,6,7,8,18,21,22,23	1	P14480
3254	1,2,4,19,21,22,23,24	3,5,6,7,8,18,20,25,26	1	D3ZUC6
3255	1,2,4,19,21,22,23,25	3,5,6,7,8,18,20,24,26	0	
3256	1,2,4,19,21,22,23,26	3,5,6,7,8,18,20,24,25	2	D3ZUC6,P25236
3257	1,2,4,19,21,22,24,25	3,5,6,7,8,18,20,23,26	2	P35467,Q6IG02
3258	1,2,4,19,21,22,24,26	3,5,6,7,8,18,20,23,25	2	D3ZUC6,O70377

3259	1,2,4,19,21,22,25,26	3,5,6,7,8,18,20,23,24	0	
3260	1,2,4,19,21,23,24,25	3,5,6,7,8,18,20,22,26	1	D3ZUC6
3261	1,2,4,19,21,23,24,26	3,5,6,7,8,18,20,22,25	2	D3ZUC6,Q9EQV9
3262	1,2,4,19,21,23,25,26	3,5,6,7,8,18,20,22,24	4	D3ZUC6,P25236,P27213,Q63661
3263	1,2,4,19,21,24,25,26	3,5,6,7,8,18,20,22,23	3	D3ZUC6,P14480,P27213
3264	1,2,4,19,22,23,24,25	3,5,6,7,8,18,20,21,26	1	P82471
3265	1,2,4,19,22,23,24,26	3,5,6,7,8,18,20,21,25	2	P00731,P82471
3266	1,2,4,19,22,23,25,26	3,5,6,7,8,18,20,21,24	1	P82471
3267	1,2,4,19,22,24,25,26	3,5,6,7,8,18,20,21,23	2	P01835,P14480
3268	1,2,4,19,23,24,25,26	3,5,6,7,8,18,20,21,22	3	P14480,P27213,Q7TPB1
3269	1,2,4,20,21,22,23,24	3,5,6,7,8,18,19,25,26	0	
3270	1,2,4,20,21,22,23,25	3,5,6,7,8,18,19,24,26	0	
3271	1,2,4,20,21,22,23,26	3,5,6,7,8,18,19,24,25	1	P25236
3272	1,2,4,20,21,22,24,25	3,5,6,7,8,18,19,23,26	3	P35467,P51907,Q07523
3273	1,2,4,20,21,22,24,26	3,5,6,7,8,18,19,23,25	7	O70377,O70594,P01015,P01836,P51907,P80020,Q99041
3274	1,2,4,20,21,22,25,26	3,5,6,7,8,18,19,23,24	2	P19223,P51907
3275	1,2,4,20,21,23,24,25	3,5,6,7,8,18,19,22,26	0	
3276	1,2,4,20,21,23,24,26	3,5,6,7,8,18,19,22,25	5	D3ZUC6,P01015,P25236,P80020,Q9EQV9
3277	1,2,4,20,21,23,25,26	3,5,6,7,8,18,19,22,24	1	P25236
3278	1,2,4,20,21,24,25,26	3,5,6,7,8,18,19,22,23	2	P01015,P51907
3279	1,2,4,20,22,23,24,25	3,5,6,7,8,18,19,21,26	1	P53792
3280	1,2,4,20,22,23,24,26	3,5,6,7,8,18,19,21,25	3	P00731,P53792,Q62997
3281	1,2,4,20,22,23,25,26	3,5,6,7,8,18,19,21,24	1	Q62997

3282	1,2,4,20,22,24,25,26	3,5,6,7,8,18,19,21,23	3	O70594,P00731,P51907
3283	1,2,4,20,23,24,25,26	3,5,6,7,8,18,19,21,22	2	P19218,Q7TPB1
3284	1,2,4,21,22,23,24,25	3,5,6,7,8,18,19,20,26	0	
3285	1,2,4,21,22,23,24,26	3,5,6,7,8,18,19,20,25	2	D3ZUC6,P00731
3286	1,2,4,21,22,23,25,26	3,5,6,7,8,18,19,20,24	2	P25236,P27213
3287	1,2,4,21,22,24,25,26	3,5,6,7,8,18,19,20,23	1	P35467
3288	1,2,4,21,23,24,25,26	3,5,6,7,8,18,19,20,22	4	D3ZUC6,P22006,P27213,Q7TPB1
3289	1,2,4,22,23,24,25,26	3,5,6,7,8,18,19,20,21	4	P00731,P53792,P82471,Q7TPB1
3290	1,2,5,6,7,8,18,19	3,4,20,21,22,23,24,25,26	10	O55006,P00731,P02631,P02803,P16636,P52590,P53792,Q03191,Q63532,Q63942
3291	1,2,5,6,7,8,18,20	3,4,19,21,22,23,24,25,26	7	P00731,P02631,P02803,P10715,P52590,Q63942,Q91ZS3
3292	1,2,5,6,7,8,18,21	3,4,19,20,22,23,24,25,26	11	O35547,P00731,P02631,P02803,P13432,P25031,P52590,P53792,Q63467,Q63942,Q91ZS3
3293	1,2,5,6,7,8,18,22	3,4,19,20,21,23,24,25,26	3	P02631,P52590,Q63942
3294	1,2,5,6,7,8,18,23	3,4,19,20,21,22,24,25,26	7	P02631,P02803,P0DP29;P0DP30;P0DP31,P28648,P52590,Q03191,Q63942
3295	1,2,5,6,7,8,18,24	3,4,19,20,21,22,23,25,26	6	O55006,P02631,P10715,P16636,P52590,Q63942
3296	1,2,5,6,7,8,18,25	3,4,19,20,21,22,23,24,26	13	P00731,P02631,P02803,P10715,P12020,P19629,P31044,P52590,P61206;P84079;P84082,P68370,Q5GRG2,Q63942,Q91ZS3
3297	1,2,5,6,7,8,18,26	3,4,19,20,21,22,23,24,25	8	P02631,P02803,P10715,P16636,P52590,P83748,Q63942,Q6AY33
3298	1,2,5,6,7,8,19,20	3,4,18,21,22,23,24,25,26	5	P02631,P12785,P52590,P53792,Q63532
3299	1,2,5,6,7,8,19,21	3,4,18,20,22,23,24,25,26	10	A2RUV9,P00731,P02631,P02803,P12785,P19218,P35281,P52590,P53792,Q63532

3300	1,2,5,6,7,8,19,22	3,4,18,20,21,23,24,25,26	6	A2RUV9,P02631,P12785,P52590,Q63532,Q8N7M5
3301	1,2,5,6,7,8,19,23	3,4,18,20,21,22,24,25,26	7	P02631,P02803,P15399,P52590,P97580,Q30KJ2,Q63532
3302	1,2,5,6,7,8,19,24	3,4,18,20,21,22,23,25,26	7	O55006,P02631,P12785,P52590,P53792,Q63532,Q8N7M5
3303	1,2,5,6,7,8,19,25	3,4,18,20,21,22,23,24,26	13	P02631,P02803,P10715,P12020,P12785,P19629,P31044,P52590,P53792,P68370,P97840,Q5GRG2,Q63532
3304	1,2,5,6,7,8,19,26	3,4,18,20,21,22,23,24,25	6	P02631,P52590,P53792,P83748,Q30KJ2,Q63532
3305	1,2,5,6,7,8,20,21	3,4,18,19,22,23,24,25,26	9	P00731,P02631,P12785,P13432,P52590,P53792,P62804,P68370,Q91ZS3
3306	1,2,5,6,7,8,20,22	3,4,18,19,21,23,24,25,26	5	P02631,P10715,P12785,P27213,P52590
3307	1,2,5,6,7,8,20,23	3,4,18,19,21,22,24,25,26	3	P02631,P20762,P52590
3308	1,2,5,6,7,8,20,24	3,4,18,19,21,22,23,25,26	4	P02631,P10715,P12785,P52590
3309	1,2,5,6,7,8,20,25	3,4,18,19,21,22,23,24,26	10	P02631,P10715,P12020,P12785,P19629,P31044,P52590,P68370,Q5GRG2,Q91ZS3
3310	1,2,5,6,7,8,20,26	3,4,18,19,21,22,23,24,25	6	P10715,P20762,P52590,P62804,P83748,Q91ZS3
3311	1,2,5,6,7,8,21,22	3,4,18,19,20,23,24,25,26	5	P02631,P12785,P13432,P19218,P52590
3312	1,2,5,6,7,8,21,23	3,4,18,19,20,22,24,25,26	3	P02631,P02803,P52590
3313	1,2,5,6,7,8,21,24	3,4,18,19,20,22,23,25,26	4	P02631,P12785,P13432,P52590
3314	1,2,5,6,7,8,21,25	3,4,18,19,20,22,23,24,26	14	P02631,P02803,P06760,P10715,P12020,P12785,P19629,P20646,P52590,P53792,P68370,Q5GRG2,Q91ZS3,Q9WTT6

3315	1,2,5,6,7,8,21,26	3,4,18,19,20,22,23,24,25	4	P52590,P53792,P83748,Q91ZS3
3316	1,2,5,6,7,8,22,23	3,4,18,19,20,21,24,25,26	2	P02631,P52590
3317	1,2,5,6,7,8,22,24	3,4,18,19,20,21,23,25,26	4	O88267,P02631,P12785,P52590
3318	1,2,5,6,7,8,22,25	3,4,18,19,20,21,23,24,26	10	P02631,P06911,P10715,P12020,P12785,P19629,P31044,P52590,P68370,Q5GRG2
3319	1,2,5,6,7,8,22,26	3,4,18,19,20,21,23,24,25	3	P12785,P52590,P83748
3320	1,2,5,6,7,8,23,24	3,4,18,19,20,21,22,25,26	2	P02631,P52590
3321	1,2,5,6,7,8,23,25	3,4,18,19,20,21,22,24,26	13	P02631,P02803,P05371,P06911,P10715,P12020,P19629,P31044,P52590,P68370,Q5GRG2,Q6P9T8,Q91ZS3
3322	1,2,5,6,7,8,23,26	3,4,18,19,20,21,22,24,25	4	P02631,P20762,P52590,P83748
3323	1,2,5,6,7,8,24,25	3,4,18,19,20,21,22,23,26	9	P02631,P10715,P12020,P12785,P19629,P52590,P68370,Q5GRG2,Q91ZS3
3324	1,2,5,6,7,8,24,26	3,4,18,19,20,21,22,23,25	2	P52590,P83748
3325	1,2,5,6,7,8,25,26	3,4,18,19,20,21,22,23,24	9	P10715,P12020,P19629,P52590,P68370,P83748,Q5GRG2,Q6P9T8,Q91ZS3
3326	1,2,5,6,7,18,19,20	3,4,8,21,22,23,24,25,26	5	P00731,P02631,P52590,P53792,Q63532
3327	1,2,5,6,7,18,19,21	3,4,8,20,22,23,24,25,26	11	P00731,P02631,P02803,P19218,P25031,P52590,P53792,P97840,Q03191,Q63532,Q9QUL6
3328	1,2,5,6,7,18,19,22	3,4,8,20,21,23,24,25,26	4	P02631,P16636,P52590,Q63532
3329	1,2,5,6,7,18,19,23	3,4,8,20,21,22,24,25,26	11	P02631,P02803,P15399,P16636,P52590,P97580,Q03191,Q30KJ2,Q63532,Q63942,Q6IE51

3330	1,2,5,6,7,18,19,24	3,4,8,20,21,22,23,25,26	6	P02631,P16636,P52590,Q03191,Q63532,Q6IE51
3331	1,2,5,6,7,18,19,25	3,4,8,20,21,22,23,24,26	8	P00731,P02631,P02803,P12020,P52590,P53792,P97840,Q63532
3332	1,2,5,6,7,18,19,26	3,4,8,20,21,22,23,24,25	8	P02631,P16636,P52590,P83748,Q03191,Q63532,Q63942,Q9QUL6
3333	1,2,5,6,7,18,20,21	3,4,8,19,22,23,24,25,26	5	P00731,P25031,P52590,Q91ZS3,Q9QUL6
3334	1,2,5,6,7,18,20,22	3,4,8,19,21,23,24,25,26	3	P27213,P52590,Q9QUL6
3335	1,2,5,6,7,18,20,23	3,4,8,19,21,22,24,25,26	3	P02631,P52590,Q9QUL6
3336	1,2,5,6,7,18,20,24	3,4,8,19,21,22,23,25,26	3	P27213,P52590,Q9QUL6
3337	1,2,5,6,7,18,20,25	3,4,8,19,21,22,23,24,26	4	P10715,P52590,P61206;P84079;P84082,Q91ZS3
3338	1,2,5,6,7,18,20,26	3,4,8,19,21,22,23,24,25	7	P16636,P25031,P52590,P83748,Q63942,Q91ZS3,Q9QUL6
3339	1,2,5,6,7,18,21,22	3,4,8,19,20,23,24,25,26	5	P13432,P19218,P25031,P52590,Q9QUL6
3340	1,2,5,6,7,18,21,23	3,4,8,19,20,22,24,25,26	6	P02631,P14668,P25031,P52590,Q63467,Q9QUL6
3341	1,2,5,6,7,18,21,24	3,4,8,19,20,22,23,25,26	4	P13432,P25031,P52590,Q9QUL6
3342	1,2,5,6,7,18,21,25	3,4,8,19,20,22,23,24,26	10	P00731,P02803,P19629,P25031,P31430,P52590,P61206;P84079;P84082,P97840,P98089,Q91ZS3
3343	1,2,5,6,7,18,21,26	3,4,8,19,20,22,23,24,25	8	P16636,P25031,P52590,P82995,P83748,Q63942,Q91ZS3,Q9QUL6
3344	1,2,5,6,7,18,22,23	3,4,8,19,20,21,24,25,26	3	P02631,P52590,Q9QUL6

3345	1,2,5,6,7,18,22,24	3,4,8,19,20,21,23,25,26	2	P16636,P52590
3346	1,2,5,6,7,18,22,25	3,4,8,19,20,21,23,24,26	5	P12020,P19629,P52590,P97840,Q5GRG2
3347	1,2,5,6,7,18,22,26	3,4,8,19,20,21,23,24,25	5	P16636,P52590,P83748,Q63942,Q9QUL6
3348	1,2,5,6,7,18,23,24	3,4,8,19,20,21,22,25,26	4	P02631,P16636,P52590,Q9QUL6
3349	1,2,5,6,7,18,23,25	3,4,8,19,20,21,22,24,26	8	P02631,P02803,P12020,P19629,P52590,Q5GRG2,Q91ZS3,Q99MH3
3350	1,2,5,6,7,18,23,26	3,4,8,19,20,21,22,24,25	9	P16636,P28648,P52590,P83748,Q63942,Q6IFU8,Q6IMF3,Q6P6Q2,Q9QUL6
3351	1,2,5,6,7,18,24,25	3,4,8,19,20,21,22,23,26	2	P19629,P52590
3352	1,2,5,6,7,18,24,26	3,4,8,19,20,21,22,23,25	5	P16636,P52590,P83748,Q63942,Q9QUL6
3353	1,2,5,6,7,18,25,26	3,4,8,19,20,21,22,23,24	10	P16636,P25031,P52590,P55091,P61206;P84079;P84082,P82995,P83748,Q63942,Q91ZS3,Q9QUL6
3354	1,2,5,6,7,19,20,21	3,4,8,18,22,23,24,25,26	6	P00714,P00731,P35467,P52590,P53792,Q63532
3355	1,2,5,6,7,19,20,22	3,4,8,18,21,23,24,25,26	7	P00714,P00774,P07150,P12785,P27213,P35467,Q63532
3356	1,2,5,6,7,19,20,23	3,4,8,18,21,22,24,25,26	5	P02631,P15399,P97580,Q30KJ2,Q63532
3357	1,2,5,6,7,19,20,24	3,4,8,18,21,22,23,25,26	4	P00714,P02631,P35467,Q63532
3358	1,2,5,6,7,19,20,25	3,4,8,18,21,22,23,24,26	6	P12020,P35467,P53792,P97840,Q63532,Q91ZS3
3359	1,2,5,6,7,19,20,26	3,4,8,18,21,22,23,24,25	2	P83748,Q63532

3360	1,2,5,6,7,19,21,22	3,4,8,18,20,23,24,25,26	8	A2RUV9,P00714,P12785,P19218,P35467,P52590,P97840,Q63532
3361	1,2,5,6,7,19,21,23	3,4,8,18,20,22,24,25,26	6	P02631,P15399,P52590,P97580,Q30KJ2,Q63532
3362	1,2,5,6,7,19,21,24	3,4,8,18,20,22,23,25,26	6	P00714,P02631,P35467,P52590,P53792,Q63532
3363	1,2,5,6,7,19,21,25	3,4,8,18,20,22,23,24,26	9	P00731,P12020,P35467,P47967,P52590,P53792,P97840,Q63532,Q91ZS3
3364	1,2,5,6,7,19,21,26	3,4,8,18,20,22,23,24,25	4	P52590,P53792,P83748,Q63532
3365	1,2,5,6,7,19,22,23	3,4,8,18,20,21,24,25,26	5	P02631,P15399,P97580,Q30KJ2,Q63532
3366	1,2,5,6,7,19,22,24	3,4,8,18,20,21,23,25,26	5	P00714,P02631,P12785,P35467,Q63532
3367	1,2,5,6,7,19,22,25	3,4,8,18,20,21,23,24,26	9	P06911,P12020,P12785,P17046,P19629,P35467,P97840,Q5GRG2,Q63532
3368	1,2,5,6,7,19,22,26	3,4,8,18,20,21,23,24,25	2	P83748,Q63532
3369	1,2,5,6,7,19,23,24	3,4,8,18,20,21,22,25,26	7	P02631,P15399,P52590,P97580,Q30KJ2,Q63532,Q6IE51
3370	1,2,5,6,7,19,23,25	3,4,8,18,20,21,22,24,26	10	P02631,P12020,P15399,P19629,P52590,P97580,P97840,Q30KJ2,Q5GRG2,Q63532
3371	1,2,5,6,7,19,23,26	3,4,8,18,20,21,22,24,25	6	P02631,P15399,P83748,P97580,Q30KJ2,Q63532
3372	1,2,5,6,7,19,24,25	3,4,8,18,20,21,22,23,26	7	P02631,P12020,P19629,P35467,P52590,P97840,Q63532
3373	1,2,5,6,7,19,24,26	3,4,8,18,20,21,22,23,25	3	P16636,P83748,Q63532
3374	1,2,5,6,7,19,25,26	3,4,8,18,20,21,22,23,24	5	P12020,P53792,P83748,P97840,Q63532

3375	1,2,5,6,7,20,21,22	3,4,8,18,19,23,24,25,26	5	P00714,P12785,P13432,P35467,P52590
3376	1,2,5,6,7,20,21,23	3,4,8,18,19,22,24,25,26	2	P52590,Q9QUL6
3377	1,2,5,6,7,20,21,24	3,4,8,18,19,22,23,25,26	4	P00714,P13432,P35467,P52590
3378	1,2,5,6,7,20,21,25	3,4,8,18,19,22,23,24,26	4	P35467,P52590,P97840,Q91ZS3
3379	1,2,5,6,7,20,21,26	3,4,8,18,19,22,23,24,25	5	P52590,P62804,P83748,Q91ZS3,Q9QUL6
3380	1,2,5,6,7,20,22,23	3,4,8,18,19,21,24,25,26	1	P52590
3381	1,2,5,6,7,20,22,24	3,4,8,18,19,21,23,25,26	4	P00714,P12785,P27213,P35467
3382	1,2,5,6,7,20,22,25	3,4,8,18,19,21,23,24,26	6	P12020,P12785,P35467,P52590,Q5GRG2,Q91ZS3
3383	1,2,5,6,7,20,22,26	3,4,8,18,19,21,23,24,25	2	P83748,Q9QUL6
3384	1,2,5,6,7,20,23,24	3,4,8,18,19,21,22,25,26	1	P52590
3385	1,2,5,6,7,20,23,25	3,4,8,18,19,21,22,24,26	5	P12020,P19629,P52590,Q5GRG2,Q91ZS3
3386	1,2,5,6,7,20,23,26	3,4,8,18,19,21,22,24,25	2	P83748,Q9QUL6
3387	1,2,5,6,7,20,24,25	3,4,8,18,19,21,22,23,26	6	P10715,P12785,P19629,P35467,P52590,Q91ZS3
3388	1,2,5,6,7,20,24,26	3,4,8,18,19,21,22,23,25	2	P83748,Q9QUL6
3389	1,2,5,6,7,20,25,26	3,4,8,18,19,21,22,23,24	3	P10758,P83748,Q91ZS3

3390	1,2,5,6,7,21,22,23	3,4,8,18,19,20,24,25,26	1	P52590
3391	1,2,5,6,7,21,22,24	3,4,8,18,19,20,23,25,26	6	P00714,P12785,P13432,P19218,P35467,P52590
3392	1,2,5,6,7,21,22,25	3,4,8,18,19,20,23,24,26	9	P12020,P12785,P19218,P19629,P35467,P52590,P97840,Q5GRG2,Q91ZS3
3393	1,2,5,6,7,21,22,26	3,4,8,18,19,20,23,24,25	3	P52590,P83748,Q9QUL6
3394	1,2,5,6,7,21,23,24	3,4,8,18,19,20,22,25,26	2	P13432,P52590
3395	1,2,5,6,7,21,23,25	3,4,8,18,19,20,22,24,26	7	P12020,P19629,P52590,P97840,Q5GRG2,Q6P6R2,Q91ZS3
3396	1,2,5,6,7,21,23,26	3,4,8,18,19,20,22,24,25	4	P52590,P83748,Q6IFV1,Q9QUL6
3397	1,2,5,6,7,21,24,25	3,4,8,18,19,20,22,23,26	5	P19629,P35467,P52590,P97840,Q91ZS3
3398	1,2,5,6,7,21,24,26	3,4,8,18,19,20,22,23,25	4	P13432,P52590,P83748,Q9QUL6
3399	1,2,5,6,7,21,25,26	3,4,8,18,19,20,22,23,24	4	P19629,P52590,P83748,Q91ZS3
3400	1,2,5,6,7,22,23,24	3,4,8,18,19,20,21,25,26	1	P52590
3401	1,2,5,6,7,22,23,25	3,4,8,18,19,20,21,24,26	5	P06911,P12020,P19629,P52590,Q5GRG2
3402	1,2,5,6,7,22,23,26	3,4,8,18,19,20,21,24,25	2	P83748,Q9QUL6
3403	1,2,5,6,7,22,24,25	3,4,8,18,19,20,21,23,26	7	P06911,P12020,P12785,P19629,P35467,P52590,Q5GRG2
3404	1,2,5,6,7,22,24,26	3,4,8,18,19,20,21,23,25	1	P83748

3405	1,2,5,6,7,22,25,26	3,4,8,18,19,20,21,23,24	5	P10758,P12020,P19629,P83748,Q5GRG2
3406	1,2,5,6,7,23,24,25	3,4,8,18,19,20,21,22,26	4	P12020,P19629,P52590,Q5GRG2
3407	1,2,5,6,7,23,24,26	3,4,8,18,19,20,21,22,25	4	P01946,P52590,P83748,Q9QUL6
3408	1,2,5,6,7,23,25,26	3,4,8,18,19,20,21,22,24	7	P01946,P10758,P12020,P19629,P52590,P83748,Q5GRG2
3409	1,2,5,6,7,24,25,26	3,4,8,18,19,20,21,22,23	4	P10758,P19629,P52590,P83748
3410	1,2,5,6,8,18,19,20	3,4,7,21,22,23,24,25,26	6	P00731,P02803,P22006,P62804,P83121,Q63942
3411	1,2,5,6,8,18,19,21	3,4,7,20,22,23,24,25,26	9	A2RUV9,O35547,P00731,P02803,P35281,P53792,Q03191,Q63942,Q9Z1F2
3412	1,2,5,6,8,18,19,22	3,4,7,20,21,23,24,25,26	5	A2RUV9,P22006,P83121,Q63942,Q9Z1F2
3413	1,2,5,6,8,18,19,23	3,4,7,20,21,22,24,25,26	6	P02803,P15399,P22006,Q03191,Q63942,Q9Z1F2
3414	1,2,5,6,8,18,19,24	3,4,7,20,21,22,23,25,26	3	P83121,Q03191,Q63942
3415	1,2,5,6,8,18,19,25	3,4,7,20,21,22,23,24,26	5	P00731,P02803,Q62635,Q63942,Q9Z1F2
3416	1,2,5,6,8,18,19,26	3,4,7,20,21,22,23,24,25	3	P02803,Q03191,Q63942
3417	1,2,5,6,8,18,20,21	3,4,7,19,22,23,24,25,26	4	O35547,P00731,P62804,Q63942
3418	1,2,5,6,8,18,20,22	3,4,7,19,21,23,24,25,26	4	P22006,P27213,P83121,Q63942
3419	1,2,5,6,8,18,20,23	3,4,7,19,21,22,24,25,26	3	P22006,Q63942,Q9Z1F2

3420	1,2,5,6,8,18,20,24	3,4,7,19,21,22,23,25,26	3	P22006,P83121,Q63942
3421	1,2,5,6,8,18,20,25	3,4,7,19,21,22,23,24,26	2	Q63942,Q91ZS3
3422	1,2,5,6,8,18,20,26	3,4,7,19,21,22,23,24,25	3	P22006,P62804,Q63942
3423	1,2,5,6,8,18,21,22	3,4,7,19,20,23,24,25,26	3	O35547,P13432,Q63942
3424	1,2,5,6,8,18,21,23	3,4,7,19,20,22,24,25,26	4	O35547,P02803,Q63942,Q9Z1F2
3425	1,2,5,6,8,18,21,24	3,4,7,19,20,22,23,25,26	3	P05539,P13432,Q63942
3426	1,2,5,6,8,18,21,25	3,4,7,19,20,22,23,24,26	9	O35547,P00731,P02803,P06760,P31430,P98089,Q63942,Q91ZS3,Q9WTT6
3427	1,2,5,6,8,18,21,26	3,4,7,19,20,22,23,24,25	4	P25031,P83748,Q63942,Q9QUL6
3428	1,2,5,6,8,18,22,23	3,4,7,19,20,21,24,25,26	3	P22006,Q63942,Q9Z1F2
3429	1,2,5,6,8,18,22,24	3,4,7,19,20,21,23,25,26	2	P83121,Q63942
3430	1,2,5,6,8,18,22,25	3,4,7,19,20,21,23,24,26	1	Q63942
3431	1,2,5,6,8,18,22,26	3,4,7,19,20,21,23,24,25	1	Q63942
3432	1,2,5,6,8,18,23,24	3,4,7,19,20,21,22,25,26	1	Q63942
3433	1,2,5,6,8,18,23,25	3,4,7,19,20,21,22,24,26	4	P02803,Q63942,Q99MH3,Q9Z1F2
3434	1,2,5,6,8,18,23,26	3,4,7,19,20,21,22,24,25	9	Q63942,Q6IFU7,Q6IFU8,Q6IFV1,Q6IFW6,Q6IG02,Q6IMF3,Q6P6Q2,Q9QUL6

3435	1,2,5,6,8,18,24,25	3,4,7,19,20,21,22,23,26	1	Q63942
3436	1,2,5,6,8,18,24,26	3,4,7,19,20,21,22,23,25	1	Q63942
3437	1,2,5,6,8,18,25,26	3,4,7,19,20,21,22,23,24	2	P55091,Q63942
3438	1,2,5,6,8,19,20,21	3,4,7,18,22,23,24,25,26	8	A2RUV9,O35547,P00714,P00731,P12785,P35281,P53792,P62804
3439	1,2,5,6,8,19,20,22	3,4,7,18,21,23,24,25,26	6	A2RUV9,P00714,P00774,P12785,P22006,P83121
3440	1,2,5,6,8,19,20,23	3,4,7,18,21,22,24,25,26	3	P15399,P22006,Q9Z1F2
3441	1,2,5,6,8,19,20,24	3,4,7,18,21,22,23,25,26	2	P12785,P83121
3442	1,2,5,6,8,19,20,25	3,4,7,18,21,22,23,24,26	1	P12785
3443	1,2,5,6,8,19,20,26	3,4,7,18,21,22,23,24,25	2	P22006,P62804
3444	1,2,5,6,8,19,21,22	3,4,7,18,20,23,24,25,26	6	A2RUV9,O35547,P00714,P02782,P12785,P19218
3445	1,2,5,6,8,19,21,23	3,4,7,18,20,22,24,25,26	5	O35547,P02803,P15399,P35281,Q9Z1F2
3446	1,2,5,6,8,19,21,24	3,4,7,18,20,22,23,25,26	4	A2RUV9,P00714,P12785,P53792
3447	1,2,5,6,8,19,21,25	3,4,7,18,20,22,23,24,26	7	A2RUV9,P02803,P06760,P12785,P53792,P97840,Q9WTT6
3448	1,2,5,6,8,19,21,26	3,4,7,18,20,22,23,24,25	4	A2RUV9,P35281,P53792,Q00715
3449	1,2,5,6,8,19,22,23	3,4,7,18,20,21,24,25,26	3	P15399,P22006,Q9Z1F2

3450	1,2,5,6,8,19,22,24	3,4,7,18,20,21,23,25,26	4	A2RUV9,P00714,P12785,P83121
3451	1,2,5,6,8,19,22,25	3,4,7,18,20,21,23,24,26	4	A2RUV9,P06911,P12785,Q5GRG2
3452	1,2,5,6,8,19,22,26	3,4,7,18,20,21,23,24,25	2	A2RUV9,P12785
3453	1,2,5,6,8,19,23,24	3,4,7,18,20,21,22,25,26	2	P15399,P97580
3454	1,2,5,6,8,19,23,25	3,4,7,18,20,21,22,24,26	6	P02803,P06760,P15399,P50280,P97580,Q9Z1F2
3455	1,2,5,6,8,19,23,26	3,4,7,18,20,21,22,24,25	1	P15399
3456	1,2,5,6,8,19,24,25	3,4,7,18,20,21,22,23,26	2	A2RUV9,P12785
3457	1,2,5,6,8,19,24,26	3,4,7,18,20,21,22,23,25	0	
3458	1,2,5,6,8,19,25,26	3,4,7,18,20,21,22,23,24	1	A2RUV9
3459	1,2,5,6,8,20,21,22	3,4,7,18,19,23,24,25,26	4	P00714,P12785,P13432,P62804
3460	1,2,5,6,8,20,21,23	3,4,7,18,19,22,24,25,26	1	P62804
3461	1,2,5,6,8,20,21,24	3,4,7,18,19,22,23,25,26	5	P00714,P12785,P13432,P62804,Q5XFX0
3462	1,2,5,6,8,20,21,25	3,4,7,18,19,22,23,24,26	5	P06760,P12785,P62804,Q91ZS3,Q9WTT6
3463	1,2,5,6,8,20,21,26	3,4,7,18,19,22,23,24,25	2	P62804,Q00715
3464	1,2,5,6,8,20,22,23	3,4,7,18,19,21,24,25,26	1	P22006

3465	1,2,5,6,8,20,22,24	3,4,7,18,19,21,23,25,26	5	P00714,P12785,P27213,P83121,Q5XFX0
3466	1,2,5,6,8,20,22,25	3,4,7,18,19,21,23,24,26	1	P12785
3467	1,2,5,6,8,20,22,26	3,4,7,18,19,21,23,24,25	2	P12785,P22006
3468	1,2,5,6,8,20,23,24	3,4,7,18,19,21,22,25,26	0	
3469	1,2,5,6,8,20,23,25	3,4,7,18,19,21,22,24,26	1	P06760
3470	1,2,5,6,8,20,23,26	3,4,7,18,19,21,22,24,25	2	P62804,Q6IFV1
3471	1,2,5,6,8,20,24,25	3,4,7,18,19,21,22,23,26	1	P12785
3472	1,2,5,6,8,20,24,26	3,4,7,18,19,21,22,23,25	0	
3473	1,2,5,6,8,20,25,26	3,4,7,18,19,21,22,23,24	1	Q91ZS3
3474	1,2,5,6,8,21,22,23	3,4,7,18,19,20,24,25,26	1	O35547
3475	1,2,5,6,8,21,22,24	3,4,7,18,19,20,23,25,26	6	A2RUV9,P00714,P12785,P13432,P35467,Q5XFX0
3476	1,2,5,6,8,21,22,25	3,4,7,18,19,20,23,24,26	5	A2RUV9,P06760,P12785,P35467,Q9WTT6
3477	1,2,5,6,8,21,22,26	3,4,7,18,19,20,23,24,25	2	P12785,P83748
3478	1,2,5,6,8,21,23,24	3,4,7,18,19,20,22,25,26	1	P13432
3479	1,2,5,6,8,21,23,25	3,4,7,18,19,20,22,24,26	5	P02803,P06760,Q6P6R2,Q9WTT6,Q9Z1F2

3480	1,2,5,6,8,21,23,26	3,4,7,18,19,20,22,24,25	1	Q6IFV1
3481	1,2,5,6,8,21,24,25	3,4,7,18,19,20,22,23,26	4	P06760,P12785,P35467,Q9WTT6
3482	1,2,5,6,8,21,24,26	3,4,7,18,19,20,22,23,25	1	P13432
3483	1,2,5,6,8,21,25,26	3,4,7,18,19,20,22,23,24	3	P06760,Q91ZS3,Q9WTT6
3484	1,2,5,6,8,22,23,24	3,4,7,18,19,20,21,25,26	0	
3485	1,2,5,6,8,22,23,25	3,4,7,18,19,20,21,24,26	4	P06760,P06911,Q5GRG2,Q9Z1F2
3486	1,2,5,6,8,22,23,26	3,4,7,18,19,20,21,24,25	0	
3487	1,2,5,6,8,22,24,25	3,4,7,18,19,20,21,23,26	3	P06911,P12785,P35467
3488	1,2,5,6,8,22,24,26	3,4,7,18,19,20,21,23,25	1	P12785
3489	1,2,5,6,8,22,25,26	3,4,7,18,19,20,21,23,24	1	P12785
3490	1,2,5,6,8,23,24,25	3,4,7,18,19,20,21,22,26	1	P06760
3491	1,2,5,6,8,23,24,26	3,4,7,18,19,20,21,22,25	2	P47967,Q6IFV1
3492	1,2,5,6,8,23,25,26	3,4,7,18,19,20,21,22,24	2	P06760,Q9WTT6
3493	1,2,5,6,8,24,25,26	3,4,7,18,19,20,21,22,23	0	
3494	1,2,5,6,18,19,20,21	3,4,7,8,22,23,24,25,26	3	O35547,P00731,Q9QUL6

3495	1,2,5,6,18,19,20,22	3,4,7,8,21,23,24,25,26	2	P22006,Q63751
3496	1,2,5,6,18,19,20,23	3,4,7,8,21,22,24,25,26	5	P15399,P22006,Q03191,Q63942,Q9QUL6
3497	1,2,5,6,18,19,20,24	3,4,7,8,21,22,23,25,26	1	P22006
3498	1,2,5,6,18,19,20,25	3,4,7,8,21,22,23,24,26	0	
3499	1,2,5,6,18,19,20,26	3,4,7,8,21,22,23,24,25	4	P22006,Q63751,Q63942,Q9QUL6
3500	1,2,5,6,18,19,21,22	3,4,7,8,20,23,24,25,26	2	A2RUV9,O35547
3501	1,2,5,6,18,19,21,23	3,4,7,8,20,22,24,25,26	7	O35547,P02803,P05369,P15399,Q03191,Q63942,Q9QUL6
3502	1,2,5,6,18,19,21,24	3,4,7,8,20,22,23,25,26	0	
3503	1,2,5,6,18,19,21,25	3,4,7,8,20,22,23,24,26	4	P00731,P02803,P97840,P98089
3504	1,2,5,6,18,19,21,26	3,4,7,8,20,22,23,24,25	4	P25031,Q63661,Q63942,Q9QUL6
3505	1,2,5,6,18,19,22,23	3,4,7,8,20,21,24,25,26	5	P15399,P22006,Q03191,Q63942,Q6IFU7
3506	1,2,5,6,18,19,22,24	3,4,7,8,20,21,23,25,26	1	P83121
3507	1,2,5,6,18,19,22,25	3,4,7,8,20,21,23,24,26	1	P97840
3508	1,2,5,6,18,19,22,26	3,4,7,8,20,21,23,24,25	3	Q63751,Q63942,Q9QUL6
3509	1,2,5,6,18,19,23,24	3,4,7,8,20,21,22,25,26	4	P15399,Q03191,Q63942,Q6IFU7

3510	1,2,5,6,18,19,23,25	3,4,7,8,20,21,22,24,26	6	P02803,P15399,Q03191,Q63942,Q99MH3,Q9Z1F2
3511	1,2,5,6,18,19,23,26	3,4,7,8,20,21,22,24,25	9	P15399,Q03191,Q63661,Q63942,Q6IFU7,Q6IFU8,Q6IMF3,Q6P6Q2,Q9QUL6
3512	1,2,5,6,18,19,24,25	3,4,7,8,20,21,22,23,26	0	
3513	1,2,5,6,18,19,24,26	3,4,7,8,20,21,22,23,25	2	Q63942,Q9QUL6
3514	1,2,5,6,18,19,25,26	3,4,7,8,20,21,22,23,24	1	Q63942
3515	1,2,5,6,18,20,21,22	3,4,7,8,19,23,24,25,26	4	O35547,P00714,Q99041,Q9QUL6
3516	1,2,5,6,18,20,21,23	3,4,7,8,19,22,24,25,26	1	Q9QUL6
3517	1,2,5,6,18,20,21,24	3,4,7,8,19,22,23,25,26	4	P00714,P13432,Q99041,Q9QUL6
3518	1,2,5,6,18,20,21,25	3,4,7,8,19,22,23,24,26	4	P25031,P31430,P98089,Q91ZS3
3519	1,2,5,6,18,20,21,26	3,4,7,8,19,22,23,24,25	5	P25031,P62804,Q63942,Q99041,Q9QUL6
3520	1,2,5,6,18,20,22,23	3,4,7,8,19,21,24,25,26	3	P22006,Q6IFU7,Q9QUL6
3521	1,2,5,6,18,20,22,24	3,4,7,8,19,21,23,25,26	5	P00714,P22006,P27213,Q99041,Q9QUL6
3522	1,2,5,6,18,20,22,25	3,4,7,8,19,21,23,24,26	0	
3523	1,2,5,6,18,20,22,26	3,4,7,8,19,21,23,24,25	4	P22006,Q63942,Q99041,Q9QUL6
3524	1,2,5,6,18,20,23,24	3,4,7,8,19,21,22,25,26	2	Q6IFU7,Q9QUL6

3525	1,2,5,6,18,20,23,25	3,4,7,8,19,21,22,24,26	0	
3526	1,2,5,6,18,20,23,26	3,4,7,8,19,21,22,24,25	9	Q63942,Q6IFU7,Q6IFU8,Q6IFV1,Q6IFW6,Q6IG02,Q6IMF3,Q6P6Q2,Q9QUL6
3527	1,2,5,6,18,20,24,25	3,4,7,8,19,21,22,23,26	0	
3528	1,2,5,6,18,20,24,26	3,4,7,8,19,21,22,23,25	3	Q63942,Q99041,Q9QUL6
3529	1,2,5,6,18,20,25,26	3,4,7,8,19,21,22,23,24	3	Q63942,Q91ZS3,Q9QUL6
3530	1,2,5,6,18,21,22,23	3,4,7,8,19,20,24,25,26	2	O35547,Q9QUL6
3531	1,2,5,6,18,21,22,24	3,4,7,8,19,20,23,25,26	4	P00714,P13432,Q99041,Q9QUL6
3532	1,2,5,6,18,21,22,25	3,4,7,8,19,20,23,24,26	3	P31430,P97840,P98089
3533	1,2,5,6,18,21,22,26	3,4,7,8,19,20,23,24,25	5	P25031,P83748,Q63942,Q99041,Q9QUL6
3534	1,2,5,6,18,21,23,24	3,4,7,8,19,20,22,25,26	2	Q6IFU7,Q9QUL6
3535	1,2,5,6,18,21,23,25	3,4,7,8,19,20,22,24,26	5	P31430,P98089,Q6P6R2,Q99MH3,Q9QUL6
3536	1,2,5,6,18,21,23,26	3,4,7,8,19,20,22,24,25	9	P25031,Q63661,Q63942,Q6IFU7,Q6IFU8,Q6IFV1,Q6IMF3,Q6P6Q2,Q9QUL6
3537	1,2,5,6,18,21,24,25	3,4,7,8,19,20,22,23,26	3	O54728,P31430,P98089
3538	1,2,5,6,18,21,24,26	3,4,7,8,19,20,22,23,25	3	Q63942,Q99041,Q9QUL6
3539	1,2,5,6,18,21,25,26	3,4,7,8,19,20,22,23,24	7	P25031,P31430,P55091,P98089,Q63942,Q91ZS3,Q9QUL6

3540	1,2,5,6,18,22,23,24	3,4,7,8,19,20,21,25,26	2	Q6IFU7,Q9QUL6
3541	1,2,5,6,18,22,23,25	3,4,7,8,19,20,21,24,26	0	
3542	1,2,5,6,18,22,23,26	3,4,7,8,19,20,21,24,25	6	Q63942,Q6IFU7,Q6IFU8,Q6IMF3,Q6P6Q2,Q9QUL6
3543	1,2,5,6,18,22,24,25	3,4,7,8,19,20,21,23,26	0	
3544	1,2,5,6,18,22,24,26	3,4,7,8,19,20,21,23,25	3	Q63942,Q99041,Q9QUL6
3545	1,2,5,6,18,22,25,26	3,4,7,8,19,20,21,23,24	2	O55145,Q63942
3546	1,2,5,6,18,23,24,25	3,4,7,8,19,20,21,22,26	1	Q6IFU7
3547	1,2,5,6,18,23,24,26	3,4,7,8,19,20,21,22,25	8	P47967,Q63942,Q6IFU7,Q6IFU8,Q6IFV1,Q6IMF3,Q6P6Q2,Q9QUL6
3548	1,2,5,6,18,23,25,26	3,4,7,8,19,20,21,22,24	7	Q63942,Q6IFU7,Q6IFU8,Q6IFW6,Q6IMF3,Q6P6Q2,Q9QUL6
3549	1,2,5,6,18,24,25,26	3,4,7,8,19,20,21,22,23	3	P55091,Q63942,Q7TPB1
3550	1,2,5,6,19,20,21,22	3,4,7,8,18,23,24,25,26	4	A2RUV9,P00714,P12785,P35467
3551	1,2,5,6,19,20,21,23	3,4,7,8,18,22,24,25,26	2	P00714,P15399
3552	1,2,5,6,19,20,21,24	3,4,7,8,18,22,23,25,26	2	P00714,P35467
3553	1,2,5,6,19,20,21,25	3,4,7,8,18,22,23,24,26	3	P35467,P97840,Q8VD89
3554	1,2,5,6,19,20,21,26	3,4,7,8,18,22,23,24,25	3	P00714,P62804,Q63751

3555	1,2,5,6,19,20,22,23	3,4,7,8,18,21,24,25,26	7	P00714,P00774,P15399,P22006,P31430,P55091,P82995
3556	1,2,5,6,19,20,22,24	3,4,7,8,18,21,23,25,26	8	P00714,P00774,P07150,P12785,P14669,P31430,P35467,P82995
3557	1,2,5,6,19,20,22,25	3,4,7,8,18,21,23,24,26	2	P12785,P35467
3558	1,2,5,6,19,20,22,26	3,4,7,8,18,21,23,24,25	3	P00714,P22006,Q63751
3559	1,2,5,6,19,20,23,24	3,4,7,8,18,21,22,25,26	5	P00714,P15399,P31430,P82995,Q80WL1
3560	1,2,5,6,19,20,23,25	3,4,7,8,18,21,22,24,26	1	P15399
3561	1,2,5,6,19,20,23,26	3,4,7,8,18,21,22,24,25	1	P15399
3562	1,2,5,6,19,20,24,25	3,4,7,8,18,21,22,23,26	2	P35467,Q80WL1
3563	1,2,5,6,19,20,24,26	3,4,7,8,18,21,22,23,25	1	P00714
3564	1,2,5,6,19,20,25,26	3,4,7,8,18,21,22,23,24	0	
3565	1,2,5,6,19,21,22,23	3,4,7,8,18,20,24,25,26	2	P00714,P15399
3566	1,2,5,6,19,21,22,24	3,4,7,8,18,20,23,25,26	5	A2RUV9,P00714,P12785,P13432,P35467
3567	1,2,5,6,19,21,22,25	3,4,7,8,18,20,23,24,26	4	A2RUV9,P12785,P35467,P97840
3568	1,2,5,6,19,21,22,26	3,4,7,8,18,20,23,24,25	2	A2RUV9,P00714
3569	1,2,5,6,19,21,23,24	3,4,7,8,18,20,22,25,26	2	P00714,P15399

3570	1,2,5,6,19,21,23,25	3,4,7,8,18,20,22,24,26	3	P15399,P97840,Q6P6R2
3571	1,2,5,6,19,21,23,26	3,4,7,8,18,20,22,24,25	2	P15399,Q6IFV1
3572	1,2,5,6,19,21,24,25	3,4,7,8,18,20,22,23,26	3	A2RUV9,P35467,P97840
3573	1,2,5,6,19,21,24,26	3,4,7,8,18,20,22,23,25	1	P00714
3574	1,2,5,6,19,21,25,26	3,4,7,8,18,20,22,23,24	1	P97840
3575	1,2,5,6,19,22,23,24	3,4,7,8,18,20,21,25,26	2	P00714,P31430
3576	1,2,5,6,19,22,23,25	3,4,7,8,18,20,21,24,26	2	P06911,P15399
3577	1,2,5,6,19,22,23,26	3,4,7,8,18,20,21,24,25	0	
3578	1,2,5,6,19,22,24,25	3,4,7,8,18,20,21,23,26	4	A2RUV9,P12785,P35467,Q4FZU6
3579	1,2,5,6,19,22,24,26	3,4,7,8,18,20,21,23,25	1	P00714
3580	1,2,5,6,19,22,25,26	3,4,7,8,18,20,21,23,24	0	
3581	1,2,5,6,19,23,24,25	3,4,7,8,18,20,21,22,26	1	P15399
3582	1,2,5,6,19,23,24,26	3,4,7,8,18,20,21,22,25	1	Q6IFU7
3583	1,2,5,6,19,23,25,26	3,4,7,8,18,20,21,22,24	1	P15399
3584	1,2,5,6,19,24,25,26	3,4,7,8,18,20,21,22,23	0	

3585	1,2,5,6,20,21,22,23	3,4,7,8,18,19,24,25,26	1	P00714
3586	1,2,5,6,20,21,22,24	3,4,7,8,18,19,23,25,26	6	P00714,P12785,P13432,P35467,Q5XFX0,Q99041
3587	1,2,5,6,20,21,22,25	3,4,7,8,18,19,23,24,26	2	P12785,P35467
3588	1,2,5,6,20,21,22,26	3,4,7,8,18,19,23,24,25	3	P00714,Q99041,Q9QUL6
3589	1,2,5,6,20,21,23,24	3,4,7,8,18,19,22,25,26	1	P00714
3590	1,2,5,6,20,21,23,25	3,4,7,8,18,19,22,24,26	2	P06760,Q6P6R2
3591	1,2,5,6,20,21,23,26	3,4,7,8,18,19,22,24,25	3	Q6IFU8,Q6IFV1,Q9QUL6
3592	1,2,5,6,20,21,24,25	3,4,7,8,18,19,22,23,26	1	P35467
3593	1,2,5,6,20,21,24,26	3,4,7,8,18,19,22,23,25	3	P00714,Q99041,Q9QUL6
3594	1,2,5,6,20,21,25,26	3,4,7,8,18,19,22,23,24	1	Q91ZS3
3595	1,2,5,6,20,22,23,24	3,4,7,8,18,19,21,25,26	2	P00714,P82995
3596	1,2,5,6,20,22,23,25	3,4,7,8,18,19,21,24,26	0	
3597	1,2,5,6,20,22,23,26	3,4,7,8,18,19,21,24,25	2	Q6IFU7,Q9QUL6
3598	1,2,5,6,20,22,24,25	3,4,7,8,18,19,21,23,26	2	P12785,P35467
3599	1,2,5,6,20,22,24,26	3,4,7,8,18,19,21,23,25	2	P00714,Q99041

3600	1,2,5,6,20,<br>22,25,26	3,4,7,8,18,<br>19,21,23,2<br>4	0	
3601	1,2,5,6,20,<br>23,24,25	3,4,7,8,18,<br>19,21,22,2<br>6	1	Q80WL1
3602	1,2,5,6,20,<br>23,24,26	3,4,7,8,18,<br>19,21,22,2<br>5	4	D4A1J4,P47967,Q6IFU7,Q9QUL6
3603	1,2,5,6,20,<br>23,25,26	3,4,7,8,18,<br>19,21,22,2<br>4	0	
3604	1,2,5,6,20,<br>24,25,26	3,4,7,8,18,<br>19,21,22,2<br>3	0	
3605	1,2,5,6,21,<br>22,23,24	3,4,7,8,18,<br>19,20,25,2<br>6	3	P00714,P13432,P35280
3606	1,2,5,6,21,<br>22,23,25	3,4,7,8,18,<br>19,20,24,2<br>6	1	P06760
3607	1,2,5,6,21,<br>22,23,26	3,4,7,8,18,<br>19,20,24,2<br>5	2	Q6IFV1,Q9QUL6
3608	1,2,5,6,21,<br>22,24,25	3,4,7,8,18,<br>19,20,23,2<br>6	3	P12785,P13432,P35467
3609	1,2,5,6,21,<br>22,24,26	3,4,7,8,18,<br>19,20,23,2<br>5	3	P00714,P13432,Q99041
3610	1,2,5,6,21,<br>22,25,26	3,4,7,8,18,<br>19,20,23,2<br>4	0	
3611	1,2,5,6,21,<br>23,24,25	3,4,7,8,18,<br>19,20,22,2<br>6	2	P06760,Q6P6R2
3612	1,2,5,6,21,<br>23,24,26	3,4,7,8,18,<br>19,20,22,2<br>5	3	Q6IFU7,Q6IFV1,Q9QUL6
3613	1,2,5,6,21,<br>23,25,26	3,4,7,8,18,<br>19,20,22,2<br>4	2	Q6IFV1,Q6P6R2
3614	1,2,5,6,21,<br>24,25,26	3,4,7,8,18,<br>19,20,22,2<br>3	0	

3615	1,2,5,6,22,23,24,25	3,4,7,8,18,19,20,21,26	1	P35280
3616	1,2,5,6,22,23,24,26	3,4,7,8,18,19,20,21,25	3	P00731,P47967,Q6IFU7
3617	1,2,5,6,22,23,25,26	3,4,7,8,18,19,20,21,24	0	
3618	1,2,5,6,22,24,25,26	3,4,7,8,18,19,20,21,23	1	Q5XI22
3619	1,2,5,6,23,24,25,26	3,4,7,8,18,19,20,21,22	2	Q6IFU7,Q7TPB1
3620	1,2,5,7,8,18,19,20	3,4,6,21,22,23,24,25,26	5	P00762,P02803,P52590,P83121,Q03191
3621	1,2,5,7,8,18,19,21	3,4,6,20,22,23,24,25,26	6	P00731,P02803,P52590,P53792,Q03191,Q63467
3622	1,2,5,7,8,18,19,22	3,4,6,20,21,23,24,25,26	6	P00762,P01835,P16636,P52590,P83121,Q03191
3623	1,2,5,7,8,18,19,23	3,4,6,20,21,22,24,25,26	6	P02803,P16636,P52590,Q03191,Q63942,Q99MH3
3624	1,2,5,7,8,18,19,24	3,4,6,20,21,22,23,25,26	7	P00762,P01835,P16636,P52590,P83121,Q03191,Q63532
3625	1,2,5,7,8,18,19,25	3,4,6,20,21,22,23,24,26	9	P00762,P01835,P02803,P10715,P16636,P19629,P52590,P62815,Q03191
3626	1,2,5,7,8,18,19,26	3,4,6,20,21,22,23,24,25	6	P02803,P16636,P52590,P82995,Q03191,Q63942
3627	1,2,5,7,8,18,20,21	3,4,6,19,22,23,24,25,26	7	P00731,P02803,P25031,P52590,P62804,Q63467,Q9QZA6
3628	1,2,5,7,8,18,20,22	3,4,6,19,21,23,24,25,26	5	P00762,P10715,P27213,P52590,P83121
3629	1,2,5,7,8,18,20,23	3,4,6,19,21,22,24,25,26	2	P10715,P52590

3630	1,2,5,7,8,18,20,24	3,4,6,19,21,22,23,25,26	4	P00762,P10715,P52590,P83121
3631	1,2,5,7,8,18,20,25	3,4,6,19,21,22,23,24,26	5	P00762,P02803,P10715,P19629,P52590
3632	1,2,5,7,8,18,20,26	3,4,6,19,21,22,23,24,25	5	P00762,P10715,P16636,P52590,Q63942
3633	1,2,5,7,8,18,21,22	3,4,6,19,20,23,24,25,26	1	P52590
3634	1,2,5,7,8,18,21,23	3,4,6,19,20,22,24,25,26	6	P02803,P05369,P52590,Q03191,Q63467,Q99MH3
3635	1,2,5,7,8,18,21,24	3,4,6,19,20,22,23,25,26	1	P52590
3636	1,2,5,7,8,18,21,25	3,4,6,19,20,22,23,24,26	7	P02803,P10715,P19629,P25031,P31430,P52590,P98089
3637	1,2,5,7,8,18,21,26	3,4,6,19,20,22,23,24,25	8	P02803,P16636,P25031,P52590,P55091,P82995,P83748,Q63942
3638	1,2,5,7,8,18,22,23	3,4,6,19,20,21,24,25,26	1	P52590
3639	1,2,5,7,8,18,22,24	3,4,6,19,20,21,23,25,26	5	P00762,P01835,P16636,P52590,P83121
3640	1,2,5,7,8,18,22,25	3,4,6,19,20,21,23,24,26	5	P00762,P01835,P10715,P19629,P52590
3641	1,2,5,7,8,18,22,26	3,4,6,19,20,21,23,24,25	5	P00762,P16636,P52590,P82995,Q63942
3642	1,2,5,7,8,18,23,24	3,4,6,19,20,21,22,25,26	3	P16636,P52590,Q03191
3643	1,2,5,7,8,18,23,25	3,4,6,19,20,21,22,24,26	5	P02803,P10715,P19629,P52590,Q99MH3
3644	1,2,5,7,8,18,23,26	3,4,6,19,20,21,22,24,25	7	P02803,P16636,P52590,P82995,Q03191,Q63942,Q6P6Q2

3645	1,2,5,7,8,18,24,25	3,4,6,19,20,21,22,23,26	5	P00762,P01835,P10715,P19629,P52590
3646	1,2,5,7,8,18,24,26	3,4,6,19,20,21,22,23,25	7	P00762,P10715,P16636,P52590,P55091,P82995,Q63942
3647	1,2,5,7,8,18,25,26	3,4,6,19,20,21,22,23,24	9	P00762,P02803,P10715,P16636,P19629,P52590,P55091,P82995,Q63942
3648	1,2,5,7,8,19,20,21	3,4,6,18,22,23,24,25,26	4	P12785,P52590,P53792,P62804
3649	1,2,5,7,8,19,20,22	3,4,6,18,21,23,24,25,26	6	P00762,P00774,P01835,P12785,P27213,P83121
3650	1,2,5,7,8,19,20,23	3,4,6,18,21,22,24,25,26	2	P00774,P52590
3651	1,2,5,7,8,19,20,24	3,4,6,18,21,22,23,25,26	6	P00762,P00774,P01835,P12785,P52590,P83121
3652	1,2,5,7,8,19,20,25	3,4,6,18,21,22,23,24,26	4	P00762,P10715,P12785,P52590
3653	1,2,5,7,8,19,20,26	3,4,6,18,21,22,23,24,25	2	P00762,Q63751
3654	1,2,5,7,8,19,21,22	3,4,6,18,20,23,24,25,26	2	P12785,P52590
3655	1,2,5,7,8,19,21,23	3,4,6,18,20,22,24,25,26	3	P02803,P52590,Q03191
3656	1,2,5,7,8,19,21,24	3,4,6,18,20,22,23,25,26	3	P12785,P52590,P53792
3657	1,2,5,7,8,19,21,25	3,4,6,18,20,22,23,24,26	7	P02803,P10715,P12785,P19629,P52590,P53792,P97840
3658	1,2,5,7,8,19,21,26	3,4,6,18,20,22,23,24,25	3	P52590,P53792,P83748
3659	1,2,5,7,8,19,22,23	3,4,6,18,20,21,24,25,26	3	P00774,P01835,P52590

3660	1,2,5,7,8,19,22,24	3,4,6,18,20,21,23,25,26	7	P00762,P00774,P01835,P12785,P52590,P83121,Q4FZU2
3661	1,2,5,7,8,19,22,25	3,4,6,18,20,21,23,24,26	8	P00762,P01835,P10715,P12785,P19629,P52590,Q4FZU2,Q5GRG2
3662	1,2,5,7,8,19,22,26	3,4,6,18,20,21,23,24,25	3	P00762,P01835,P12785
3663	1,2,5,7,8,19,23,24	3,4,6,18,20,21,22,25,26	4	P01835,P52590,Q03191,Q63532
3664	1,2,5,7,8,19,23,25	3,4,6,18,20,21,22,24,26	5	P01835,P02803,P10715,P19629,P52590
3665	1,2,5,7,8,19,23,26	3,4,6,18,20,21,22,24,25	2	P52590,Q03191
3666	1,2,5,7,8,19,24,25	3,4,6,18,20,21,22,23,26	7	P00762,P01835,P10715,P12785,P19629,P52590,Q4FZU2
3667	1,2,5,7,8,19,24,26	3,4,6,18,20,21,22,23,25	2	P01835,P16636
3668	1,2,5,7,8,19,25,26	3,4,6,18,20,21,22,23,24	6	P00762,P01835,P02803,P10715,P19629,P52590
3669	1,2,5,7,8,20,21,22	3,4,6,18,19,23,24,25,26	2	P12785,P52590
3670	1,2,5,7,8,20,21,23	3,4,6,18,19,22,24,25,26	1	P52590
3671	1,2,5,7,8,20,21,24	3,4,6,18,19,22,23,25,26	3	P02625,P12785,P52590
3672	1,2,5,7,8,20,21,25	3,4,6,18,19,22,23,24,26	6	P02625,P10715,P12785,P19629,P52590,P68370
3673	1,2,5,7,8,20,21,26	3,4,6,18,19,22,23,24,25	4	P02625,P52590,P62804,P83748
3674	1,2,5,7,8,20,22,23	3,4,6,18,19,21,24,25,26	2	P00774,P52590

3675	1,2,5,7,8,20,22,24	3,4,6,18,19,21,23,25,26	7	P00762,P00774,P01835,P12785,P27213,P52590,P83121
3676	1,2,5,7,8,20,22,25	3,4,6,18,19,21,23,24,26	7	P00762,P02625,P10715,P12785,P19629,P52590,Q5GRG2
3677	1,2,5,7,8,20,22,26	3,4,6,18,19,21,23,24,25	2	P00762,P12785
3678	1,2,5,7,8,20,23,24	3,4,6,18,19,21,22,25,26	1	P52590
3679	1,2,5,7,8,20,23,25	3,4,6,18,19,21,22,24,26	3	P10715,P19629,P52590
3680	1,2,5,7,8,20,23,26	3,4,6,18,19,21,22,24,25	1	P52590
3681	1,2,5,7,8,20,24,25	3,4,6,18,19,21,22,23,26	6	P00762,P02625,P10715,P12785,P19629,P52590
3682	1,2,5,7,8,20,24,26	3,4,6,18,19,21,22,23,25	4	P00762,P02625,P10715,P52590
3683	1,2,5,7,8,20,25,26	3,4,6,18,19,21,22,23,24	5	P00762,P02625,P10715,P19629,P52590
3684	1,2,5,7,8,21,22,23	3,4,6,18,19,20,24,25,26	1	P52590
3685	1,2,5,7,8,21,22,24	3,4,6,18,19,20,23,25,26	3	P12785,P35467,P52590
3686	1,2,5,7,8,21,22,25	3,4,6,18,19,20,23,24,26	5	P10715,P12785,P19629,P35467,P52590
3687	1,2,5,7,8,21,22,26	3,4,6,18,19,20,23,24,25	3	P12785,P52590,P83748
3688	1,2,5,7,8,21,23,24	3,4,6,18,19,20,22,25,26	1	P52590
3689	1,2,5,7,8,21,23,25	3,4,6,18,19,20,22,24,26	4	P02803,P10715,P19629,P52590

3690	1,2,5,7,8,21,23,26	3,4,6,18,19,20,22,24,25	1	P52590
3691	1,2,5,7,8,21,24,25	3,4,6,18,19,20,22,23,26	6	P02625,P10715,P12785,P19629,P35467,P52590
3692	1,2,5,7,8,21,24,26	3,4,6,18,19,20,22,23,25	1	P52590
3693	1,2,5,7,8,21,25,26	3,4,6,18,19,20,22,23,24	6	P02625,P10715,P19629,P52590,P82995,P83748
3694	1,2,5,7,8,22,23,24	3,4,6,18,19,20,21,25,26	2	P01835,P52590
3695	1,2,5,7,8,22,23,25	3,4,6,18,19,20,21,24,26	6	P01835,P06911,P10715,P19629,P52590,Q5GRG2
3696	1,2,5,7,8,22,23,26	3,4,6,18,19,20,21,24,25	1	P52590
3697	1,2,5,7,8,22,24,25	3,4,6,18,19,20,21,23,26	9	P00762,P01835,P10715,P12785,P19629,P35467,P52590,Q4FZU2,Q5GRG2
3698	1,2,5,7,8,22,24,26	3,4,6,18,19,20,21,23,25	4	P00762,P01835,P12785,P52590
3699	1,2,5,7,8,22,25,26	3,4,6,18,19,20,21,23,24	6	P00762,P01835,P10715,P12785,P19629,P52590
3700	1,2,5,7,8,23,24,25	3,4,6,18,19,20,21,22,26	4	P01835,P10715,P19629,P52590
3701	1,2,5,7,8,23,24,26	3,4,6,18,19,20,21,22,25	2	P01946,P52590
3702	1,2,5,7,8,23,25,26	3,4,6,18,19,20,21,22,24	4	P01946,P10715,P19629,P52590
3703	1,2,5,7,8,24,25,26	3,4,6,18,19,20,21,22,23	6	P00762,P01835,P02625,P10715,P19629,P52590
3704	1,2,5,7,18,19,20,21	3,4,6,8,22,23,24,25,26	3	P00731,P25031,Q63751

3705	1,2,5,7,18,19,20,22	3,4,6,8,21,23,24,25,26	8	P00762,P07150,P14669,P27213,Q63751,Q6P6R2,Q9WTT6,Q9Z0V6
3706	1,2,5,7,18,19,20,23	3,4,6,8,21,22,24,25,26	2	Q03191,Q9WTT6
3707	1,2,5,7,18,19,20,24	3,4,6,8,21,22,23,25,26	5	P00762,P07150,P16636,P83121,Q9WTT6
3708	1,2,5,7,18,19,20,25	3,4,6,8,21,22,23,24,26	1	P00762
3709	1,2,5,7,18,19,20,26	3,4,6,8,21,22,23,24,25	5	P16636,Q03191,Q63751,Q63942,Q9WTT6
3710	1,2,5,7,18,19,21,22	3,4,6,8,20,23,24,25,26	1	P97840
3711	1,2,5,7,18,19,21,23	3,4,6,8,20,22,24,25,26	8	D3ZUC6,P02803,P05369,P14668,P25031,Q03191,Q63467,Q99MH3
3712	1,2,5,7,18,19,21,24	3,4,6,8,20,22,23,25,26	3	D3ZUC6,P16636,Q03191
3713	1,2,5,7,18,19,21,25	3,4,6,8,20,22,23,24,26	7	P00731,P02803,P25031,P31430,P47967,P52590,P97840
3714	1,2,5,7,18,19,21,26	3,4,6,8,20,22,23,24,25	10	D3ZUC6,P16636,P25031,P82995,P83748,Q03191,Q63661,Q63751,Q63942,Q9QUL6
3715	1,2,5,7,18,19,22,23	3,4,6,8,20,21,24,25,26	3	P16636,Q03191,Q9WTT6
3716	1,2,5,7,18,19,22,24	3,4,6,8,20,21,23,25,26	7	P00762,P01835,P07150,P16636,P83121,Q6P6R2,Q9WTT6
3717	1,2,5,7,18,19,22,25	3,4,6,8,20,21,23,24,26	3	P00762,P01835,P97840
3718	1,2,5,7,18,19,22,26	3,4,6,8,20,21,23,24,25	6	P16636,Q03191,Q63751,Q63942,Q6P6R2,Q9WTT6
3719	1,2,5,7,18,19,23,24	3,4,6,8,20,21,22,25,26	4	P16636,Q03191,Q63532,Q9WTT6

3720	1,2,5,7,18,19,23,25	3,4,6,8,20,21,22,24,26	3	P02803,Q03191,Q99MH3
3721	1,2,5,7,18,19,23,26	3,4,6,8,20,21,22,24,25	5	P16636,Q03191,Q63661,Q63942,Q9QUL6
3722	1,2,5,7,18,19,24,25	3,4,6,8,20,21,22,23,26	4	P00762,P01835,P16636,Q03191
3723	1,2,5,7,18,19,24,26	3,4,6,8,20,21,22,23,25	4	P16636,Q03191,Q63942,Q9WTT6
3724	1,2,5,7,18,19,25,26	3,4,6,8,20,21,22,23,24	3	P16636,P82995,Q03191
3725	1,2,5,7,18,20,21,22	3,4,6,8,19,23,24,25,26	2	P25031,Q9QUL6
3726	1,2,5,7,18,20,21,23	3,4,6,8,19,22,24,25,26	5	P14668,P25031,P52590,Q63467,Q9QUL6
3727	1,2,5,7,18,20,21,24	3,4,6,8,19,22,23,25,26	1	Q9QUL6
3728	1,2,5,7,18,20,21,25	3,4,6,8,19,22,23,24,26	4	P02625,P25031,P31430,P52590
3729	1,2,5,7,18,20,21,26	3,4,6,8,19,22,23,24,25	5	P16636,P25031,P82995,P83748,Q9QUL6
3730	1,2,5,7,18,20,22,23	3,4,6,8,19,21,24,25,26	2	Q9QUL6,Q9QZK9
3731	1,2,5,7,18,20,22,24	3,4,6,8,19,21,23,25,26	7	P00762,P07150,P14669,P27213,P83121,Q9QZK9,Q9WTT6
3732	1,2,5,7,18,20,22,25	3,4,6,8,19,21,23,24,26	1	P00762
3733	1,2,5,7,18,20,22,26	3,4,6,8,19,21,23,24,25	5	P00762,P16636,Q6P6R2,Q99041,Q9QUL6
3734	1,2,5,7,18,20,23,24	3,4,6,8,19,21,22,25,26	2	Q9QUL6,Q9QZK9

3735	1,2,5,7,18,20,23,25	3,4,6,8,19,21,22,24,26	0	
3736	1,2,5,7,18,20,23,26	3,4,6,8,19,21,22,24,25	5	P16636,Q63942,Q6IFU8,Q6P6Q2,Q9QUL6
3737	1,2,5,7,18,20,24,25	3,4,6,8,19,21,22,23,26	2	P00762,P10715
3738	1,2,5,7,18,20,24,26	3,4,6,8,19,21,22,23,25	3	P00762,P16636,Q9QUL6
3739	1,2,5,7,18,20,25,26	3,4,6,8,19,21,22,23,24	4	P00762,P02625,P16636,P82995
3740	1,2,5,7,18,21,22,23	3,4,6,8,19,20,24,25,26	3	P14668,P52590,Q9QUL6
3741	1,2,5,7,18,21,22,24	3,4,6,8,19,20,23,25,26	0	
3742	1,2,5,7,18,21,22,25	3,4,6,8,19,20,23,24,26	4	P25031,P31430,P52590,P97840
3743	1,2,5,7,18,21,22,26	3,4,6,8,19,20,23,24,25	5	P16636,P25031,P82995,P83748,Q9QUL6
3744	1,2,5,7,18,21,23,24	3,4,6,8,19,20,22,25,26	4	D3ZUC6,P52590,Q03191,Q9QUL6
3745	1,2,5,7,18,21,23,25	3,4,6,8,19,20,22,24,26	6	P02803,P14668,P25031,P31430,P52590,Q99MH3
3746	1,2,5,7,18,21,23,26	3,4,6,8,19,20,22,24,25	12	D3ZUC6,P16636,P25031,P82995,P83748,Q03191,Q62636,Q63661,Q63942,Q6IFU8,Q6P6Q2,Q9QUL6
3747	1,2,5,7,18,21,24,25	3,4,6,8,19,20,22,23,26	3	P25031,P31430,P52590
3748	1,2,5,7,18,21,24,26	3,4,6,8,19,20,22,23,25	8	D3ZUC6,P16636,P25031,P55091,P82995,P83748,Q71MB6,Q9QUL6
3749	1,2,5,7,18,21,25,26	3,4,6,8,19,20,22,23,24	7	P16636,P25031,P31430,P55091,P82995,P83748,Q9QUL6

3750	1,2,5,7,18,22,23,24	3,4,6,8,19,20,21,25,26	2	P16636,Q9WTT6
3751	1,2,5,7,18,22,23,25	3,4,6,8,19,20,21,24,26	1	Q99MH3
3752	1,2,5,7,18,22,23,26	3,4,6,8,19,20,21,24,25	3	P16636,Q63942,Q9QUL6
3753	1,2,5,7,18,22,24,25	3,4,6,8,19,20,21,23,26	2	P00762,P01835
3754	1,2,5,7,18,22,24,26	3,4,6,8,19,20,21,23,25	4	P00762,P16636,Q9QUL6,Q9WTT6
3755	1,2,5,7,18,22,25,26	3,4,6,8,19,20,21,23,24	4	P00762,P16636,P55091,P82995
3756	1,2,5,7,18,23,24,25	3,4,6,8,19,20,21,22,26	1	Q99MH3
3757	1,2,5,7,18,23,24,26	3,4,6,8,19,20,21,22,25	6	P01946,P16636,Q03191,Q63942,Q6P6Q2,Q9QUL6
3758	1,2,5,7,18,23,25,26	3,4,6,8,19,20,21,22,24	8	P01946,P16636,P55091,P82995,Q63942,Q6P6Q2,Q99MH3,Q9QUL6
3759	1,2,5,7,18,24,25,26	3,4,6,8,19,20,21,22,23	5	P00762,P16636,P55091,P82995,Q7TPB1
3760	1,2,5,7,19,20,21,22	3,4,6,8,18,23,24,25,26	5	P00714,P00774,P12785,P35467,Q63751
3761	1,2,5,7,19,20,21,23	3,4,6,8,18,22,24,25,26	0	
3762	1,2,5,7,19,20,21,24	3,4,6,8,18,22,23,25,26	2	P00714,P35467
3763	1,2,5,7,19,20,21,25	3,4,6,8,18,22,23,24,26	5	O70417,P02625,P35467,P47967,P97840
3764	1,2,5,7,19,20,21,26	3,4,6,8,18,22,23,24,25	1	Q63751

3765	1,2,5,7,19,20,22,23	3,4,6,8,18,21,24,25,26	2	P00774,Q9WTT6
3766	1,2,5,7,19,20,22,24	3,4,6,8,18,21,23,25,26	12	P00714,P00762,P00774,P01835,P07150,P12785,P14669,P27213,P35467,P83121,Q6P6R2,Q9WTT6
3767	1,2,5,7,19,20,22,25	3,4,6,8,18,21,23,24,26	4	P00762,P00774,P12785,P35467
3768	1,2,5,7,19,20,22,26	3,4,6,8,18,21,23,24,25	5	P00762,P07150,Q63751,Q6P6R2,Q9WTT6
3769	1,2,5,7,19,20,23,24	3,4,6,8,18,21,22,25,26	2	P00774,Q9WTT6
3770	1,2,5,7,19,20,23,25	3,4,6,8,18,21,22,24,26	0	
3771	1,2,5,7,19,20,23,26	3,4,6,8,18,21,22,24,25	0	
3772	1,2,5,7,19,20,24,25	3,4,6,8,18,21,22,23,26	3	O70417,P00762,P35467
3773	1,2,5,7,19,20,24,26	3,4,6,8,18,21,22,23,25	2	O70417,Q9WTT6
3774	1,2,5,7,19,20,25,26	3,4,6,8,18,21,22,23,24	2	O70417,P02625
3775	1,2,5,7,19,21,22,23	3,4,6,8,18,20,24,25,26	0	
3776	1,2,5,7,19,21,22,24	3,4,6,8,18,20,23,25,26	5	P00714,P12785,P35467,Q4FZU2,Q6IG02
3777	1,2,5,7,19,21,22,25	3,4,6,8,18,20,23,24,26	6	P12785,P17046,P35467,P47967,P97840,Q6IG02
3778	1,2,5,7,19,21,22,26	3,4,6,8,18,20,23,24,25	2	P83748,Q63751
3779	1,2,5,7,19,21,23,24	3,4,6,8,18,20,22,25,26	0	

3780	1,2,5,7,19,21,23,25	3,4,6,8,18,20,22,24,26	1	P97840
3781	1,2,5,7,19,21,23,26	3,4,6,8,18,20,22,24,25	1	Q03191
3782	1,2,5,7,19,21,24,25	3,4,6,8,18,20,22,23,26	4	O70417,P35467,P97840,Q6IG02
3783	1,2,5,7,19,21,24,26	3,4,6,8,18,20,22,23,25	0	
3784	1,2,5,7,19,21,25,26	3,4,6,8,18,20,22,23,24	2	P82995,P97840
3785	1,2,5,7,19,22,23,24	3,4,6,8,18,20,21,25,26	4	P00774,P01835,Q63532,Q9WTT6
3786	1,2,5,7,19,22,23,25	3,4,6,8,18,20,21,24,26	2	P01835,P17046
3787	1,2,5,7,19,22,23,26	3,4,6,8,18,20,21,24,25	0	
3788	1,2,5,7,19,22,24,25	3,4,6,8,18,20,21,23,26	7	P00762,P01835,P12785,P17046,P35467,Q4FZU2,Q6IG02
3789	1,2,5,7,19,22,24,26	3,4,6,8,18,20,21,23,25	3	P01835,Q6P6R2,Q9WTT6
3790	1,2,5,7,19,22,25,26	3,4,6,8,18,20,21,23,24	4	P00762,P01835,P08644,Q5XI22
3791	1,2,5,7,19,23,24,25	3,4,6,8,18,20,21,22,26	2	P01835,Q63532
3792	1,2,5,7,19,23,24,26	3,4,6,8,18,20,21,22,25	4	P01946,P16636,Q03191,Q63532
3793	1,2,5,7,19,23,25,26	3,4,6,8,18,20,21,22,24	1	P01946
3794	1,2,5,7,19,24,25,26	3,4,6,8,18,20,21,22,23	3	O70417,P01835,Q7TPB1

3795	1,2,5,7,20,21,22,23	3,4,6,8,18,19,24,25,26	0	
3796	1,2,5,7,20,21,22,24	3,4,6,8,18,19,23,25,26	4	P00714,P12785,P35467,Q5XFX0
3797	1,2,5,7,20,21,22,25	3,4,6,8,18,19,23,24,26	3	P02625,P12785,P35467
3798	1,2,5,7,20,21,22,26	3,4,6,8,18,19,23,24,25	0	
3799	1,2,5,7,20,21,23,24	3,4,6,8,18,19,22,25,26	0	
3800	1,2,5,7,20,21,23,25	3,4,6,8,18,19,22,24,26	0	
3801	1,2,5,7,20,21,23,26	3,4,6,8,18,19,22,24,25	1	Q9QUL6
3802	1,2,5,7,20,21,24,25	3,4,6,8,18,19,22,23,26	2	P02625,P35467
3803	1,2,5,7,20,21,24,26	3,4,6,8,18,19,22,23,25	1	P02625
3804	1,2,5,7,20,21,25,26	3,4,6,8,18,19,22,23,24	1	P02625
3805	1,2,5,7,20,22,23,24	3,4,6,8,18,19,21,25,26	1	P00774
3806	1,2,5,7,20,22,23,25	3,4,6,8,18,19,21,24,26	0	
3807	1,2,5,7,20,22,23,26	3,4,6,8,18,19,21,24,25	0	
3808	1,2,5,7,20,22,24,25	3,4,6,8,18,19,21,23,26	4	P00762,P02625,P12785,P35467
3809	1,2,5,7,20,22,24,26	3,4,6,8,18,19,21,23,25	2	P00762,P07150

3810	1,2,5,7,20,22,25,26	3,4,6,8,18,19,21,23,24	3	P00762,P02625,Q5XI22
3811	1,2,5,7,20,23,24,25	3,4,6,8,18,19,21,22,26	0	
3812	1,2,5,7,20,23,24,26	3,4,6,8,18,19,21,22,25	1	P01946
3813	1,2,5,7,20,23,25,26	3,4,6,8,18,19,21,22,24	1	P01946
3814	1,2,5,7,20,24,25,26	3,4,6,8,18,19,21,22,23	2	P00762,P02625
3815	1,2,5,7,21,22,23,24	3,4,6,8,18,19,20,25,26	2	P00714,P35280
3816	1,2,5,7,21,22,23,25	3,4,6,8,18,19,20,24,26	0	
3817	1,2,5,7,21,22,23,26	3,4,6,8,18,19,20,24,25	1	Q9QUL6
3818	1,2,5,7,21,22,24,25	3,4,6,8,18,19,20,23,26	3	P12785,P35467,Q6IG02
3819	1,2,5,7,21,22,24,26	3,4,6,8,18,19,20,23,25	2	P00714,P35467
3820	1,2,5,7,21,22,25,26	3,4,6,8,18,19,20,23,24	2	P35467,Q5XI22
3821	1,2,5,7,21,23,24,25	3,4,6,8,18,19,20,22,26	1	P35280
3822	1,2,5,7,21,23,24,26	3,4,6,8,18,19,20,22,25	2	P01946,Q9QUL6
3823	1,2,5,7,21,23,25,26	3,4,6,8,18,19,20,22,24	1	P01946
3824	1,2,5,7,21,24,25,26	3,4,6,8,18,19,20,22,23	3	P02625,P22006,Q8CJD3

3825	1,2,5,7,22,23,24,25	3,4,6,8,18,19,20,21,26	2	P01835,P35280
3826	1,2,5,7,22,23,24,26	3,4,6,8,18,19,20,21,25	2	P00731,P01946
3827	1,2,5,7,22,23,25,26	3,4,6,8,18,19,20,21,24	1	P01946
3828	1,2,5,7,22,24,25,26	3,4,6,8,18,19,20,21,23	3	P00762,P01835,Q5XI22
3829	1,2,5,7,23,24,25,26	3,4,6,8,18,19,20,21,22	2	P01946,Q7TPB1
3830	1,2,5,8,18,19,20,21	3,4,6,7,22,23,24,25,26	6	P00731,P02803,P35281,P62804,P83121,Q63751
3831	1,2,5,8,18,19,20,22	3,4,6,7,21,23,24,25,26	4	P22006,P83121,Q63751,Q9QZK9
3832	1,2,5,8,18,19,20,23	3,4,6,7,21,22,24,25,26	7	P02803,P09605;P25809,P22006,P83121,Q03191,Q63942,Q9QZK9
3833	1,2,5,8,18,19,20,24	3,4,6,7,21,22,23,25,26	3	P09605;P25809,P83121,Q9QZK9
3834	1,2,5,8,18,19,20,25	3,4,6,7,21,22,23,24,26	2	P02803,P62815
3835	1,2,5,8,18,19,20,26	3,4,6,7,21,22,23,24,25	5	P09605;P25809,P22006,P62804,Q63751,Q63942
3836	1,2,5,8,18,19,21,22	3,4,6,7,20,23,24,25,26	3	A2RUV9,P83121,Q63751
3837	1,2,5,8,18,19,21,23	3,4,6,7,20,22,24,25,26	5	P02803,P05369,Q03191,Q63942,Q99MH3
3838	1,2,5,8,18,19,21,24	3,4,6,7,20,22,23,25,26	3	P05539,P83121,Q03191
3839	1,2,5,8,18,19,21,25	3,4,6,7,20,22,23,24,26	2	P02803,P31430

3840	1,2,5,8,18,19,21,26	3,4,6,7,20,22,23,24,25	4	P02803,Q03191,Q63751,Q63942
3841	1,2,5,8,18,19,22,23	3,4,6,7,20,21,24,25,26	5	P09605;P25809,P22006,P83121,Q03191,Q63942
3842	1,2,5,8,18,19,22,24	3,4,6,7,20,21,23,25,26	3	P01835,P09605;P25809,P83121
3843	1,2,5,8,18,19,22,25	3,4,6,7,20,21,23,24,26	2	P01835,P83121
3844	1,2,5,8,18,19,22,26	3,4,6,7,20,21,23,24,25	5	P09605;P25809,P83121,Q03191,Q63751,Q63942
3845	1,2,5,8,18,19,23,24	3,4,6,7,20,21,22,25,26	4	P09605;P25809,P83121,Q03191,Q63942
3846	1,2,5,8,18,19,23,25	3,4,6,7,20,21,22,24,26	4	P02803,Q03191,Q63942,Q99MH3
3847	1,2,5,8,18,19,23,26	3,4,6,7,20,21,22,24,25	6	P02803,P09605;P25809,P35467,Q03191,Q63942,Q6P6Q2
3848	1,2,5,8,18,19,24,25	3,4,6,7,20,21,22,23,26	2	P01835,P83121
3849	1,2,5,8,18,19,24,26	3,4,6,7,20,21,22,23,25	4	P09605;P25809,P83121,Q03191,Q63942
3850	1,2,5,8,18,19,25,26	3,4,6,7,20,21,22,23,24	3	P02803,Q03191,Q63942
3851	1,2,5,8,18,20,21,22	3,4,6,7,19,23,24,25,26	2	P83121,Q5FVI6
3852	1,2,5,8,18,20,21,23	3,4,6,7,19,22,24,25,26	0	
3853	1,2,5,8,18,20,21,24	3,4,6,7,19,22,23,25,26	3	P83121,Q5FVI6,Q9QZK9
3854	1,2,5,8,18,20,21,25	3,4,6,7,19,22,23,24,26	2	P02625,P31430

3855	1,2,5,8,18,20,21,26	3,4,6,7,19,22,23,24,25	3	P62804,Q63751,Q63942
3856	1,2,5,8,18,20,22,23	3,4,6,7,19,21,24,25,26	4	P22006,P83121,Q5FVI6,Q9QZK9
3857	1,2,5,8,18,20,22,24	3,4,6,7,19,21,23,25,26	5	P27213,P83121,Q5FVI6,Q99041,Q9QZK9
3858	1,2,5,8,18,20,22,25	3,4,6,7,19,21,23,24,26	3	P00762,P83121,Q5FVI6
3859	1,2,5,8,18,20,22,26	3,4,6,7,19,21,23,24,25	5	P22006,P83121,Q63751,Q63942,Q99041
3860	1,2,5,8,18,20,23,24	3,4,6,7,19,21,22,25,26	4	P09605;P25809,P83121,Q5FVI6,Q9QZK9
3861	1,2,5,8,18,20,23,25	3,4,6,7,19,21,22,24,26	1	Q99MH3
3862	1,2,5,8,18,20,23,26	3,4,6,7,19,21,22,24,25	6	P09605;P25809,P47967,Q63942,Q6IFU8,Q6IG02,Q6P6Q2
3863	1,2,5,8,18,20,24,25	3,4,6,7,19,21,22,23,26	2	P02625,P83121
3864	1,2,5,8,18,20,24,26	3,4,6,7,19,21,22,23,25	6	P09605;P25809,P47967,P83121,Q63942,Q99041,Q9QZK9
3865	1,2,5,8,18,20,25,26	3,4,6,7,19,21,22,23,24	2	P02625,Q63942
3866	1,2,5,8,18,21,22,23	3,4,6,7,19,20,24,25,26	1	Q5FVI6
3867	1,2,5,8,18,21,22,24	3,4,6,7,19,20,23,25,26	2	P83121,Q5FVI6
3868	1,2,5,8,18,21,22,25	3,4,6,7,19,20,23,24,26	2	P31430,Q5FVI6
3869	1,2,5,8,18,21,22,26	3,4,6,7,19,20,23,24,25	1	Q63942

3870	1,2,5,8,18,21,23,24	3,4,6,7,19,20,22,25,26	2	P05539,Q5FVI6
3871	1,2,5,8,18,21,23,25	3,4,6,7,19,20,22,24,26	3	P02803,P31430,Q99MH3
3872	1,2,5,8,18,21,23,26	3,4,6,7,19,20,22,24,25	7	P05369,Q03191,Q63942,Q6IFU8,Q6IFV1,Q6P6Q2,Q9QUL6
3873	1,2,5,8,18,21,24,25	3,4,6,7,19,20,22,23,26	3	P05539,P31430,Q5FVI6
3874	1,2,5,8,18,21,24,26	3,4,6,7,19,20,22,23,25	1	Q63942
3875	1,2,5,8,18,21,25,26	3,4,6,7,19,20,22,23,24	3	P31430,P55091,Q63942
3876	1,2,5,8,18,22,23,24	3,4,6,7,19,20,21,25,26	3	P09605;P25809,P83121,Q5FVI6
3877	1,2,5,8,18,22,23,25	3,4,6,7,19,20,21,24,26	2	Q5FVI6,Q99MH3
3878	1,2,5,8,18,22,23,26	3,4,6,7,19,20,21,24,25	4	P09605;P25809,P47967,Q63942,Q6P6Q2
3879	1,2,5,8,18,22,24,25	3,4,6,7,19,20,21,23,26	3	P01835,P83121,Q5FVI6
3880	1,2,5,8,18,22,24,26	3,4,6,7,19,20,21,23,25	5	P09605;P25809,P47967,P83121,Q63942,Q99041
3881	1,2,5,8,18,22,25,26	3,4,6,7,19,20,21,23,24	1	Q63942
3882	1,2,5,8,18,23,24,25	3,4,6,7,19,20,21,22,26	2	Q5FVI6,Q99MH3
3883	1,2,5,8,18,23,24,26	3,4,6,7,19,20,21,22,25	5	P09605;P25809,P47967,Q03191,Q63942,Q6P6Q2
3884	1,2,5,8,18,23,25,26	3,4,6,7,19,20,21,22,24	5	P55091,Q63942,Q6IFU8,Q6P6Q2,Q99MH3

3885	1,2,5,8,18,24,25,26	3,4,6,7,19,20,21,22,23	3	P55091,Q63942,Q7TPB1
3886	1,2,5,8,19,20,21,22	3,4,6,7,18,23,24,25,26	6	A2RUV9,P00714,P00774,P12785,P83121,Q63751
3887	1,2,5,8,19,20,21,23	3,4,6,7,18,22,24,25,26	0	
3888	1,2,5,8,19,20,21,24	3,4,6,7,18,22,23,25,26	3	P00714,P12785,P83121
3889	1,2,5,8,19,20,21,25	3,4,6,7,18,22,23,24,26	3	P02625,P12785,Q8VD89
3890	1,2,5,8,19,20,21,26	3,4,6,7,18,22,23,24,25	2	P62804,Q63751
3891	1,2,5,8,19,20,22,23	3,4,6,7,18,21,24,25,26	3	P00774,P22006,P83121
3892	1,2,5,8,19,20,22,24	3,4,6,7,18,21,23,25,26	5	P00714,P00774,P01835,P12785,P83121
3893	1,2,5,8,19,20,22,25	3,4,6,7,18,21,23,24,26	3	P00774,P12785,P83121
3894	1,2,5,8,19,20,22,26	3,4,6,7,18,21,23,24,25	3	P12785,P83121,Q63751
3895	1,2,5,8,19,20,23,24	3,4,6,7,18,21,22,25,26	4	P00774,P09605;P25809,P83121,Q9QZK9
3896	1,2,5,8,19,20,23,25	3,4,6,7,18,21,22,24,26	0	
3897	1,2,5,8,19,20,23,26	3,4,6,7,18,21,22,24,25	1	P09605;P25809
3898	1,2,5,8,19,20,24,25	3,4,6,7,18,21,22,23,26	3	P02625,P12785,P83121
3899	1,2,5,8,19,20,24,26	3,4,6,7,18,21,22,23,25	2	P09605;P25809,P83121

3900	1,2,5,8,19,20,25,26	3,4,6,7,18,21,22,23,24	1	P02625
3901	1,2,5,8,19,21,22,23	3,4,6,7,18,20,24,25,26	0	
3902	1,2,5,8,19,21,22,24	3,4,6,7,18,20,23,25,26	5	A2RUV9,P00714,P12785,P83121,Q4FZU2
3903	1,2,5,8,19,21,22,25	3,4,6,7,18,20,23,24,26	2	A2RUV9,P12785
3904	1,2,5,8,19,21,22,26	3,4,6,7,18,20,23,24,25	3	A2RUV9,P12785,Q63751
3905	1,2,5,8,19,21,23,24	3,4,6,7,18,20,22,25,26	0	
3906	1,2,5,8,19,21,23,25	3,4,6,7,18,20,22,24,26	2	P02803,P06760
3907	1,2,5,8,19,21,23,26	3,4,6,7,18,20,22,24,25	1	Q03191
3908	1,2,5,8,19,21,24,25	3,4,6,7,18,20,22,23,26	2	A2RUV9,P12785
3909	1,2,5,8,19,21,24,26	3,4,6,7,18,20,22,23,25	0	
3910	1,2,5,8,19,21,25,26	3,4,6,7,18,20,22,23,24	0	
3911	1,2,5,8,19,22,23,24	3,4,6,7,18,20,21,25,26	4	P00774,P01835,P09605;P25809,P83121
3912	1,2,5,8,19,22,23,25	3,4,6,7,18,20,21,24,26	1	P01835
3913	1,2,5,8,19,22,23,26	3,4,6,7,18,20,21,24,25	1	P09605;P25809
3914	1,2,5,8,19,22,24,25	3,4,6,7,18,20,21,23,26	5	A2RUV9,P01835,P12785,P83121,Q4FZU2

3915	1,2,5,8,19,22,24,26	3,4,6,7,18,20,21,23,25	4	P01835,P09605;P25809,P12785,P83121
3916	1,2,5,8,19,22,25,26	3,4,6,7,18,20,21,23,24	2	P01835,P12785
3917	1,2,5,8,19,23,24,25	3,4,6,7,18,20,21,22,26	1	P01835
3918	1,2,5,8,19,23,24,26	3,4,6,7,18,20,21,22,25	2	P09605;P25809,Q03191
3919	1,2,5,8,19,23,25,26	3,4,6,7,18,20,21,22,24	0	
3920	1,2,5,8,19,24,25,26	3,4,6,7,18,20,21,22,23	2	P01835,Q7TPB1
3921	1,2,5,8,20,21,22,23	3,4,6,7,18,19,24,25,26	1	Q5FVI6
3922	1,2,5,8,20,21,22,24	3,4,6,7,18,19,23,25,26	6	P00714,P12785,P35467,P83121,Q5FVI6,Q5XFX0
3923	1,2,5,8,20,21,22,25	3,4,6,7,18,19,23,24,26	4	P02625,P12785,P35467,Q5FVI6
3924	1,2,5,8,20,21,22,26	3,4,6,7,18,19,23,24,25	3	P12785,P62804,Q63751
3925	1,2,5,8,20,21,23,24	3,4,6,7,18,19,22,25,26	1	Q5FVI6
3926	1,2,5,8,20,21,23,25	3,4,6,7,18,19,22,24,26	1	P06760
3927	1,2,5,8,20,21,23,26	3,4,6,7,18,19,22,24,25	2	P62804,Q6IFV1
3928	1,2,5,8,20,21,24,25	3,4,6,7,18,19,22,23,26	5	P02625,P12785,P35467,Q5FVI6,Q5XFX0
3929	1,2,5,8,20,21,24,26	3,4,6,7,18,19,22,23,25	2	P02625,P62804

3930	1,2,5,8,20,21,25,26	3,4,6,7,18,19,22,23,24	2	P02625,P62804
3931	1,2,5,8,20,22,23,24	3,4,6,7,18,19,21,25,26	5	P00774,P47967,P83121,Q5FVI6,Q9QZK9
3932	1,2,5,8,20,22,23,25	3,4,6,7,18,19,21,24,26	1	Q5FVI6
3933	1,2,5,8,20,22,23,26	3,4,6,7,18,19,21,24,25	1	P47967
3934	1,2,5,8,20,22,24,25	3,4,6,7,18,19,21,23,26	7	P00762,P02625,P12785,P35467,P83121,Q5FVI6,Q5XFX0
3935	1,2,5,8,20,22,24,26	3,4,6,7,18,19,21,23,25	4	P12785,P47967,P83121,Q99041
3936	1,2,5,8,20,22,25,26	3,4,6,7,18,19,21,23,24	2	P02625,P12785
3937	1,2,5,8,20,23,24,25	3,4,6,7,18,19,21,22,26	1	P08649
3938	1,2,5,8,20,23,24,26	3,4,6,7,18,19,21,22,25	4	D4A1J4,P09605;P25809,P47967,P97840
3939	1,2,5,8,20,23,25,26	3,4,6,7,18,19,21,22,24	0	
3940	1,2,5,8,20,24,25,26	3,4,6,7,18,19,21,22,23	1	P02625
3941	1,2,5,8,21,22,23,24	3,4,6,7,18,19,20,25,26	2	P35280,Q5FVI6
3942	1,2,5,8,21,22,23,25	3,4,6,7,18,19,20,24,26	2	P06760,Q5FVI6
3943	1,2,5,8,21,22,23,26	3,4,6,7,18,19,20,24,25	0	
3944	1,2,5,8,21,22,24,25	3,4,6,7,18,19,20,23,26	4	P12785,P35467,Q5FVI6,Q5XFX0

3945	1,2,5,8,21,22,24,26	3,4,6,7,18,19,20,23,25	2	P12785,Q5FVI6
3946	1,2,5,8,21,22,25,26	3,4,6,7,18,19,20,23,24	1	P12785
3947	1,2,5,8,21,23,24,25	3,4,6,7,18,19,20,22,26	3	P06760,P35280,Q5FVI6
3948	1,2,5,8,21,23,24,26	3,4,6,7,18,19,20,22,25	1	Q6IFV1
3949	1,2,5,8,21,23,25,26	3,4,6,7,18,19,20,22,24	0	
3950	1,2,5,8,21,24,25,26	3,4,6,7,18,19,20,22,23	1	P02625
3951	1,2,5,8,22,23,24,25	3,4,6,7,18,19,20,21,26	3	P01835,P35280,Q5FVI6
3952	1,2,5,8,22,23,24,26	3,4,6,7,18,19,20,21,25	3	P00731,P09605;P25809,P47967
3953	1,2,5,8,22,23,25,26	3,4,6,7,18,19,20,21,24	0	
3954	1,2,5,8,22,24,25,26	3,4,6,7,18,19,20,21,23	2	P01835,P12785
3955	1,2,5,8,23,24,25,26	3,4,6,7,18,19,20,21,22	1	Q7TPB1
3956	1,2,5,18,19,20,21,22	3,4,6,7,8,23,24,25,26	5	P00714,P20761,P83121,Q63751,Q99041
3957	1,2,5,18,19,20,21,23	3,4,6,7,8,22,24,25,26	2	P20761,Q03191
3958	1,2,5,18,19,20,21,24	3,4,6,7,8,22,23,25,26	5	P06911,P20761,P83121,Q99041,Q9QZK9
3959	1,2,5,18,19,20,21,25	3,4,6,7,8,22,23,24,26	1	P20761
3960	1,2,5,18,19,20,21,26	3,4,6,7,8,22,23,24,25	5	P06911,P12020,Q63751,Q99041,Q9QUL6
3961	1,2,5,18,19,20,22,23	3,4,6,7,8,21,24,25,26	5	P20761,P22006,Q03191,Q9QZK9,Q9WTT6

3962	1,2,5,18,19,20,22,24	3,4,6,7,8,21,23,25,26	11	P00714,P07150,P14669,P20761,P83121,Q63751,Q6P6R2,Q99041,Q9QZK9,Q9WTT6,Q9Z0V6
3963	1,2,5,18,19,20,22,25	3,4,6,7,8,21,23,24,26	1	P20761
3964	1,2,5,18,19,20,22,26	3,4,6,7,8,21,23,24,25	6	P07150,P22006,Q63751,Q6P6R2,Q99041,Q9WTT6
3965	1,2,5,18,19,20,23,24	3,4,6,7,8,21,22,25,26	5	P09605;P25809,P20761,Q03191,Q9QZK9,Q9WTT6
3966	1,2,5,18,19,20,23,25	3,4,6,7,8,21,22,24,26	3	P20761,Q03191,Q99MH3
3967	1,2,5,18,19,20,23,26	3,4,6,7,8,21,22,24,25	6	P09605;P25809,Q03191,Q63942,Q6IFU8,Q6P6Q2,Q9QUL6
3968	1,2,5,18,19,20,24,25	3,4,6,7,8,21,22,23,26	3	O70417,P20761,P22282
3969	1,2,5,18,19,20,24,26	3,4,6,7,8,21,22,23,25	6	P06911,P09605;P25809,Q63751,Q99041,Q9QZK9,Q9WTT6
3970	1,2,5,18,19,20,25,26	3,4,6,7,8,21,22,23,24	1	Q63751
3971	1,2,5,18,19,21,22,23	3,4,6,7,8,20,24,25,26	2	P20761,Q03191
3972	1,2,5,18,19,21,22,24	3,4,6,7,8,20,23,25,26	3	P00714,P83121,Q99041
3973	1,2,5,18,19,21,22,25	3,4,6,7,8,20,23,24,26	3	P20761,P97840,Q63617
3974	1,2,5,18,19,21,22,26	3,4,6,7,8,20,23,24,25	3	Q63617,Q63751,Q99041
3975	1,2,5,18,19,21,23,24	3,4,6,7,8,20,22,25,26	3	D3ZUC6,P05369,Q03191
3976	1,2,5,18,19,21,23,25	3,4,6,7,8,20,22,24,26	5	P02803,P05369,P20761,Q03191,Q99MH3
3977	1,2,5,18,19,21,23,26	3,4,6,7,8,20,22,24,25	9	D3ZUC6,P05369,Q03191,Q63617,Q63661,Q63942,Q6IFU8,Q6P6Q2,Q9QUL6
3978	1,2,5,18,19,21,24,25	3,4,6,7,8,20,22,23,26	2	P20761,Q63617
3979	1,2,5,18,19,21,24,26	3,4,6,7,8,20,22,23,25	6	D3ZUC6,P06911,P12020,Q03191,Q63617,Q99041
3980	1,2,5,18,19,21,25,26	3,4,6,7,8,20,22,23,24	2	P31430,Q63617
3981	1,2,5,18,19,22,23,24	3,4,6,7,8,20,21,25,26	6	P09605;P25809,P20761,P83121,Q03191,Q9QZK9,Q9WTT6
3982	1,2,5,18,19,22,23,25	3,4,6,7,8,20,21,24,26	3	P20761,Q03191,Q99MH3
3983	1,2,5,18,19,22,23,26	3,4,6,7,8,20,21,24,25	3	P09605;P25809,Q03191,Q63942
3984	1,2,5,18,19,22,24,25	3,4,6,7,8,20,21,23,26	3	P01835,P20761,P83121

3985	1,2,5,18,19,22,24,26	3,4,6,7,8,20,21,23,25	7	P09605;P25809,P83121,Q63751,Q63942,Q6P6R2,Q99041,Q9WTT6
3986	1,2,5,18,19,22,25,26	3,4,6,7,8,20,21,23,24	2	O55145,Q63617
3987	1,2,5,18,19,23,24,25	3,4,6,7,8,20,21,22,26	3	P20761,Q03191,Q99MH3
3988	1,2,5,18,19,23,24,26	3,4,6,7,8,20,21,22,25	4	P09605;P25809,Q03191,Q63942,Q6IFU7
3989	1,2,5,18,19,23,25,26	3,4,6,7,8,20,21,22,24	4	Q03191,Q63942,Q6P6Q2,Q99MH3
3990	1,2,5,18,19,24,25,26	3,4,6,7,8,20,21,22,23	2	Q63617,Q7TPB1
3991	1,2,5,18,20,21,22,23	3,4,6,7,8,19,24,25,26	3	Q5FVI6,Q9QUL6,Q9QZK9
3992	1,2,5,18,20,21,22,24	3,4,6,7,8,19,23,25,26	5	P00714,P83121,Q5FVI6,Q99041,Q9QZK9
3993	1,2,5,18,20,21,22,25	3,4,6,7,8,19,23,24,26	3	P20761,P31430,Q99041
3994	1,2,5,18,20,21,22,26	3,4,6,7,8,19,23,24,25	3	Q63751,Q99041,Q9QUL6
3995	1,2,5,18,20,21,23,24	3,4,6,7,8,19,22,25,26	2	Q9QUL6,Q9QZK9
3996	1,2,5,18,20,21,23,25	3,4,6,7,8,19,22,24,26	2	P20761,P31430
3997	1,2,5,18,20,21,23,26	3,4,6,7,8,19,22,24,25	5	Q6IFU8,Q6IFV1,Q6IMF3,Q6P6Q2,Q9QUL6
3998	1,2,5,18,20,21,24,25	3,4,6,7,8,19,22,23,26	3	P02625,P20761,P31430
3999	1,2,5,18,20,21,24,26	3,4,6,7,8,19,22,23,25	4	P06911,P12020,Q99041,Q9QUL6
4000	1,2,5,18,20,21,25,26	3,4,6,7,8,19,22,23,24	4	P02625,P31430,Q99041,Q9QUL6
4001	1,2,5,18,20,22,23,24	3,4,6,7,8,19,21,25,26	5	P20761,P83121,Q5FVI6,Q9QZK9,Q9WTT6
4002	1,2,5,18,20,22,23,25	3,4,6,7,8,19,21,24,26	1	P20761
4003	1,2,5,18,20,22,23,26	3,4,6,7,8,19,21,24,25	5	Q6IFU7,Q6IFU8,Q6P6Q2,Q9QUL6,Q9QZK9
4004	1,2,5,18,20,22,24,25	3,4,6,7,8,19,21,23,26	3	P20761,Q5FVI6,Q99041
4005	1,2,5,18,20,22,24,26	3,4,6,7,8,19,21,23,25	6	P07150,Q6P6R2,Q99041,Q9QUL6,Q9QZK9,Q9WTT6
4006	1,2,5,18,20,22,25,26	3,4,6,7,8,19,21,23,24	1	Q99041
4007	1,2,5,18,20,23,24,25	3,4,6,7,8,19,21,22,26	1	P20761

4008	1,2,5,18,20,23,24,26 3,4,6,7,8,19,21,22,25	10	D4A1J4,P09605;P25809,P47967,Q63942,Q6IFU7,Q6IFU8,Q6P6Q2,Q9JJ50,Q9QUL6,Q9QZK9
4009	1,2,5,18,20,23,25,26 3,4,6,7,8,19,21,22,24	3	Q6IFU8,Q6P6Q2,Q9QUL6
4010	1,2,5,18,20,24,25,26 3,4,6,7,8,19,21,22,23	4	P02625,P16086,Q7TPB1,Q99041
4011	1,2,5,18,21,22,23,24 3,4,6,7,8,19,20,25,26	3	P35280,Q5FVI6,Q9QUL6
4012	1,2,5,18,21,22,23,25 3,4,6,7,8,19,20,24,26	4	P20761,P31430,Q5FVI6,Q99MH3
4013	1,2,5,18,21,22,23,26 3,4,6,7,8,19,20,24,25	4	Q6IFU8,Q6IFV1,Q6P6Q2,Q9QUL6
4014	1,2,5,18,21,22,24,25 3,4,6,7,8,19,20,23,26	2	P31430,Q5FVI6
4015	1,2,5,18,21,22,24,26 3,4,6,7,8,19,20,23,25	3	Q63617,Q99041,Q9QUL6
4016	1,2,5,18,21,22,25,26 3,4,6,7,8,19,20,23,24	4	O55145,P31430,Q63617,Q99041
4017	1,2,5,18,21,23,24,25 3,4,6,7,8,19,20,22,26	2	P31430,Q99MH3
4018	1,2,5,18,21,23,24,26 3,4,6,7,8,19,20,22,25	7	D3ZUC6,Q63942,Q6IFU7,Q6IFU8,Q6IFV1,Q6P6Q2,Q9QUL6
4019	1,2,5,18,21,23,25,26 3,4,6,7,8,19,20,22,24	7	P31430,P55091,Q63617,Q6IFU8,Q6P6Q2,Q99MH3,Q9QUL6
4020	1,2,5,18,21,24,25,26 3,4,6,7,8,19,20,22,23	4	P31430,P55091,Q63617,Q99041
4021	1,2,5,18,22,23,24,25 3,4,6,7,8,19,20,21,26	3	P20761,P35280,Q5FVI6
4022	1,2,5,18,22,23,24,26 3,4,6,7,8,19,20,21,25	6	P00731,P47967,Q63942,Q6IFU7,Q6P6Q2,Q9QUL6
4023	1,2,5,18,22,23,25,26 3,4,6,7,8,19,20,21,24	1	Q6P6Q2
4024	1,2,5,18,22,24,25,26 3,4,6,7,8,19,20,21,23	3	P16086,Q7TPB1,Q99041
4025	1,2,5,18,23,24,25,26 3,4,6,7,8,19,20,21,22	5	P55091,Q63942,Q6IFU7,Q6P6Q2,Q7TPB1
4026	1,2,5,19,20,21,22,23 3,4,6,7,8,18,24,25,26	2	P00714,P00774
4027	1,2,5,19,20,21,22,24 3,4,6,7,8,18,23,25,26	6	O70417,P00714,P00774,P12785,P35467,P83121
4028	1,2,5,19,20,21,22,25 3,4,6,7,8,18,23,24,26	5	O70417,P12785,P20761,P35467,Q8VD89
4029	1,2,5,19,20,21,22,26 3,4,6,7,8,18,23,24,25	3	P00714,Q63751,Q99041
4030	1,2,5,19,20,21,23,24 3,4,6,7,8,18,22,25,26	1	P00714

4031	1,2,5,19,20,21,23,25	3,4,6,7,8,18,22,24,26	3	O70417,P20761,Q8VD89
4032	1,2,5,19,20,21,23,26	3,4,6,7,8,18,22,24,25	0	
4033	1,2,5,19,20,21,24,25	3,4,6,7,8,18,22,23,26	5	O70417,P02625,P20761,P35467,Q8VD89
4034	1,2,5,19,20,21,24,26	3,4,6,7,8,18,22,23,25	5	O70417,P00714,P06911,Q63751,Q99041
4035	1,2,5,19,20,21,25,26	3,4,6,7,8,18,22,23,24	2	O70417,P02625
4036	1,2,5,19,20,22,23,24	3,4,6,7,8,18,21,25,26	7	P00714,P00774,P20761,P31430,P83121,Q9QZK9,Q9WTT6
4037	1,2,5,19,20,22,23,25	3,4,6,7,8,18,21,24,26	1	P20761
4038	1,2,5,19,20,22,23,26	3,4,6,7,8,18,21,24,25	2	O54728,Q62997
4039	1,2,5,19,20,22,24,25	3,4,6,7,8,18,21,23,26	6	O70417,P00774,P12785,P14669,P20761,P35467
4040	1,2,5,19,20,22,24,26	3,4,6,7,8,18,21,23,25	9	O70417,P00714,P07150,P14669,Q5XI22,Q63751,Q6P6R2,Q99041,Q9WTT6
4041	1,2,5,19,20,22,25,26	3,4,6,7,8,18,21,23,24	2	O70417,Q5XI22
4042	1,2,5,19,20,23,24,25	3,4,6,7,8,18,21,22,26	3	O70417,P11598,P20761
4043	1,2,5,19,20,23,24,26	3,4,6,7,8,18,21,22,25	2	D4A1J4,P09605;P25809
4044	1,2,5,19,20,23,25,26	3,4,6,7,8,18,21,22,24	1	O70417
4045	1,2,5,19,20,24,25,26	3,4,6,7,8,18,21,22,23	3	O70417,P02625,Q5XI22
4046	1,2,5,19,21,22,23,24	3,4,6,7,8,18,20,25,26	2	P00714,P35280
4047	1,2,5,19,21,22,23,25	3,4,6,7,8,18,20,24,26	1	P20761
4048	1,2,5,19,21,22,23,26	3,4,6,7,8,18,20,24,25	0	
4049	1,2,5,19,21,22,24,25	3,4,6,7,8,18,20,23,26	6	A2RUV9,O70417,P12785,P35467,Q4FZU2,Q6IG02
4050	1,2,5,19,21,22,24,26	3,4,6,7,8,18,20,23,25	5	O70377,P00714,Q5XI22,Q63617,Q99041
4051	1,2,5,19,21,22,25,26	3,4,6,7,8,18,20,23,24	3	O70417,Q5XI22,Q63617
4052	1,2,5,19,21,23,24,25	3,4,6,7,8,18,20,22,26	1	O70417
4053	1,2,5,19,21,23,24,26	3,4,6,7,8,18,20,22,25	0	

4054	1,2,5,19,2<br>1,23,25,26	3,4,6,7,8,1<br>8,20,22,24	1	Q63617
4055	1,2,5,19,2<br>1,24,25,26	3,4,6,7,8,1<br>8,20,22,23	3	O70417,Q5XI22,Q63617
4056	1,2,5,19,2<br>2,23,24,25	3,4,6,7,8,1<br>8,20,21,26	4	P01835,P11598,P20761,P82471
4057	1,2,5,19,2<br>2,23,24,26	3,4,6,7,8,1<br>8,20,21,25	2	P00731,P82471
4058	1,2,5,19,2<br>2,23,25,26	3,4,6,7,8,1<br>8,20,21,24	0	
4059	1,2,5,19,2<br>2,24,25,26	3,4,6,7,8,1<br>8,20,21,23	3	O70417,P01835,Q5XI22
4060	1,2,5,19,2<br>3,24,25,26	3,4,6,7,8,1<br>8,20,21,22	2	O70417,Q7TPB1
4061	1,2,5,20,2<br>1,22,23,24	3,4,6,7,8,1<br>8,19,25,26	4	P00714,P35280,Q5FVI6,Q9QZK9
4062	1,2,5,20,2<br>1,22,23,25	3,4,6,7,8,1<br>8,19,24,26	1	P20761
4063	1,2,5,20,2<br>1,22,23,26	3,4,6,7,8,1<br>8,19,24,25	1	Q9QUL6
4064	1,2,5,20,2<br>1,22,24,25	3,4,6,7,8,1<br>8,19,23,26	5	P02625,P12785,P35467,Q5FVI6,Q5XFX0
4065	1,2,5,20,2<br>1,22,24,26	3,4,6,7,8,1<br>8,19,23,25	6	O70377,P00714,P35467,Q5BJY9,Q5XI22,Q99041
4066	1,2,5,20,2<br>1,22,25,26	3,4,6,7,8,1<br>8,19,23,24	3	P02625,Q5XI22,Q99041
4067	1,2,5,20,2<br>1,23,24,25	3,4,6,7,8,1<br>8,19,22,26	0	
4068	1,2,5,20,2<br>1,23,24,26	3,4,6,7,8,1<br>8,19,22,25	1	Q9QUL6
4069	1,2,5,20,2<br>1,23,25,26	3,4,6,7,8,1<br>8,19,22,24	0	
4070	1,2,5,20,2<br>1,24,25,26	3,4,6,7,8,1<br>8,19,22,23	6	O70417,P02625,P16086,Q5XI22,Q8CJD3,Q99041
4071	1,2,5,20,2<br>2,23,24,25	3,4,6,7,8,1<br>8,19,21,26	5	P0C0A9,P11598,P20761,P35280,Q5FVI6
4072	1,2,5,20,2<br>2,23,24,26	3,4,6,7,8,1<br>8,19,21,25	3	P00731,P47967,Q9QZK9
4073	1,2,5,20,2<br>2,23,25,26	3,4,6,7,8,1<br>8,19,21,24	0	
4074	1,2,5,20,2<br>2,24,25,26	3,4,6,7,8,1<br>8,19,21,23	6	P00731,P02625,P0C0A9,P16086,Q5XI22,Q99041
4075	1,2,5,20,2<br>3,24,25,26	3,4,6,7,8,1<br>8,19,21,22	2	D4A1J4,Q7TPB1
4076	1,2,5,21,2<br>2,23,24,25	3,4,6,7,8,1<br>8,19,20,26	2	P35280,Q5FVI6

4077	1,2,5,21,22,23,24,26	3,4,6,7,8,18,19,20,25	3	P00714,P00731,P35280
4078	1,2,5,21,22,23,25,26	3,4,6,7,8,18,19,20,24	0	
4079	1,2,5,21,22,24,25,26	3,4,6,7,8,18,19,20,23	4	P35467,Q5XI22,Q63617,Q99041
4080	1,2,5,21,23,24,25,26	3,4,6,7,8,18,19,20,22	2	P35280,Q7TPB1
4081	1,2,5,22,23,24,25,26	3,4,6,7,8,18,19,20,21	4	P00731,P35280,P82471,Q7TPB1
4082	1,2,6,7,8,18,19,20	3,4,5,21,22,23,24,25,26	7	P00731,P02631,P02803,P10715,P35280,Q63532,Q91ZS3
4083	1,2,6,7,8,18,19,21	3,4,5,20,22,23,24,25,26	16	O35547,P00731,P02631,P02782,P02803,P05964,P08723,P25031,P35281,P53792,P97840,Q63532,Q6AYL6,Q6AYR8,Q91ZS3,Q9JI85
4084	1,2,6,7,8,18,19,22	3,4,5,20,21,23,24,25,26	4	D4A1J4,P02631,Q63532,Q8CJD3
4085	1,2,6,7,8,18,19,23	3,4,5,20,21,22,24,25,26	12	P02631,P02803,P05964,P15399,P31044,P97580,Q03191,Q30KJ2,Q63532,Q63942,Q8CJD3,Q99MH3
4086	1,2,6,7,8,18,19,24	3,4,5,20,21,22,23,25,26	2	P02631,Q63532
4087	1,2,6,7,8,18,19,25	3,4,5,20,21,22,23,24,26	15	P00731,P02631,P02803,P10715,P12020,P19629,P31044,P35280,P47967,P68370,P97840,Q5GRG2,Q62635,Q63532,Q91ZS3
4088	1,2,6,7,8,18,19,26	3,4,5,20,21,22,23,24,25	6	P02631,P02803,P35280,P83748,Q63532,Q63942
4089	1,2,6,7,8,18,20,21	3,4,5,19,22,23,24,25,26	11	P00731,P02631,P02782,P08723,P13432,P25031,P62804,Q6AYL6,Q6AYR8,Q91ZS3,Q9JI85
4090	1,2,6,7,8,18,20,22	3,4,5,19,21,23,24,25,26	4	P02631,P10715,Q91ZS3,Q9JI85
4091	1,2,6,7,8,18,20,23	3,4,5,19,21,22,24,25,26	4	P02631,P10715,Q8CJD3,Q91ZS3
4092	1,2,6,7,8,18,20,24	3,4,5,19,21,22,23,25,26	4	P02631,P10715,Q6AYR8,Q91ZS3
4093	1,2,6,7,8,18,20,25	3,4,5,19,21,22,23,24,26	9	P02631,P10715,P12020,P19629,P31044,P35280,P68370,Q5GRG2,Q91ZS3

4094	1,2,6,7,8,18,20,26	3,4,5,19,21,22,23,24,25	7	P01041,P10715,P35280,P62804,Q63942,Q6AYR8,Q91ZS3
4095	1,2,6,7,8,18,21,22	3,4,5,19,20,23,24,25,26	9	D4A1J4,P02631,P02782,P08723,P11598,P13432,P25031,Q91ZS3,Q9JI85
4096	1,2,6,7,8,18,21,23	3,4,5,19,20,22,24,25,26	9	P02631,P02782,P02803,P08723,P25031,Q6AYL6,Q6AYR8,Q91ZS3,Q9JI85
4097	1,2,6,7,8,18,21,24	3,4,5,19,20,22,23,25,26	8	O54728,P02631,P02782,P13432,Q6AYL6,Q6AYR8,Q91ZS3,Q9JI85
4098	1,2,6,7,8,18,21,25	3,4,5,19,20,22,23,24,26	21	O54728,P00731,P02631,P02803,P08723,P10715,P12020,P19629,P25031,P31044,P31430,P47967,P68370,P97840,P98089,Q5GRG2,Q6AYL6,Q6P6R2,Q6P9T8,Q91ZS3,Q9WTT6
4099	1,2,6,7,8,18,21,26	3,4,5,19,20,22,23,24,25	10	P02782,P11598,P25031,P55091,P82995,P83748,Q00715,Q63942,Q6AYR8,Q91ZS3
4100	1,2,6,7,8,18,22,23	3,4,5,19,20,21,24,25,26	2	P02631,Q8CJD3
4101	1,2,6,7,8,18,22,24	3,4,5,19,20,21,23,25,26	1	P02631
4102	1,2,6,7,8,18,22,25	3,4,5,19,20,21,23,24,26	10	P02631,P06911,P10715,P12020,P19629,P31044,P68370,P97840,Q5GRG2,Q91ZS3
4103	1,2,6,7,8,18,22,26	3,4,5,19,20,21,23,24,25	2	P83748,Q63942
4104	1,2,6,7,8,18,23,24	3,4,5,19,20,21,22,25,26	1	P02631
4105	1,2,6,7,8,18,23,25	3,4,5,19,20,21,22,24,26	13	P02631,P02803,P06911,P10715,P12020,P19629,P31044,P68370,Q5GRG2,Q6P6R2,Q6P9T8,Q91ZS3,Q99MH3
4106	1,2,6,7,8,18,23,26	3,4,5,19,20,21,22,24,25	3	P02631,Q63942,Q6P6Q2
4107	1,2,6,7,8,18,24,25	3,4,5,19,20,21,22,23,26	9	O54728,P02631,P10715,P12020,P19629,P31044,P68370,Q5GRG2,Q91ZS3
4108	1,2,6,7,8,18,24,26	3,4,5,19,20,21,22,23,25	3	P10715,P55091,Q63942

4109	1,2,6,7,8,18,25,26	3,4,5,19,20,21,22,23,24	12	P10715,P12020,P19629,P31044,P35280,P55091,P68370,P82995,Q5GRG2,Q63942,Q6P9T8,Q91ZS3
4110	1,2,6,7,8,19,20,21	3,4,5,18,22,23,24,25,26	12	P00731,P02631,P02782,P12785,P35281,P53792,P62804,Q63532,Q6AYL6,Q6AYR8,Q91ZS3,Q9JI85
4111	1,2,6,7,8,19,20,22	3,4,5,18,21,23,24,25,26	4	P00774,P02631,P12785,Q63532
4112	1,2,6,7,8,19,20,23	3,4,5,18,21,22,24,25,26	6	P02631,P15399,P97580,Q30KJ2,Q63532,Q8CJD3
4113	1,2,6,7,8,19,20,24	3,4,5,18,21,22,23,25,26	3	P02631,P12785,Q63532
4114	1,2,6,7,8,19,20,25	3,4,5,18,21,22,23,24,26	13	P02631,P10715,P12020,P12785,P19629,P31044,P35280,P47967,P68370,P97840,Q5GRG2,Q63532,Q91ZS3
4115	1,2,6,7,8,19,20,26	3,4,5,18,21,22,23,24,25	3	P35280,P62804,Q63532
4116	1,2,6,7,8,19,21,22	3,4,5,18,20,23,24,25,26	12	D4A1J4,P02631,P02782,P08723,P12785,P13432,P19218,P35281,P97840,Q63532,Q6IG02,Q9JI85
4117	1,2,6,7,8,19,21,23	3,4,5,18,20,22,24,25,26	11	P02631,P02782,P02803,P15399,P16086,P35281,P97580,Q30KJ2,Q63532,Q6AYL6,Q8CJD3
4118	1,2,6,7,8,19,21,24	3,4,5,18,20,22,23,25,26	7	P02631,P02782,P12785,P13432,P35281,Q63532,Q6IG02
4119	1,2,6,7,8,19,21,25	3,4,5,18,20,22,23,24,26	18	P00731,P02631,P02803,P10715,P12020,P12785,P19629,P31044,P47967,P53792,P68370,P97840,Q5GRG2,Q63532,Q6IG02,Q6P6R2,Q91ZS3,Q9QZK9
4120	1,2,6,7,8,19,21,26	3,4,5,18,20,22,23,24,25	6	P02782,P35281,P53792,P83748,Q00715,Q63532
4121	1,2,6,7,8,19,22,23	3,4,5,18,20,21,24,25,26	7	P02631,P15399,P50280,P97580,Q30KJ2,Q63532,Q8CJD3
4122	1,2,6,7,8,19,22,24	3,4,5,18,20,21,23,25,26	6	P01835,P02631,P12785,Q4FZU2,Q63532,Q6IG02
4123	1,2,6,7,8,19,22,25	3,4,5,18,20,21,23,24,26	15	P01835,P02631,P06911,P10715,P12020,P12785,P19629,P31044,P47967,P68370,P97840,Q4FZU2,Q5GRG2,Q63532,Q6IG02

4124	1,2,6,7,8,19,22,26	3,4,5,18,20,21,23,24,25	2	P12785,Q63532
4125	1,2,6,7,8,19,23,24	3,4,5,18,20,21,22,25,26	5	P02631,P15399,P97580,Q30KJ2,Q63532
4126	1,2,6,7,8,19,23,25	3,4,5,18,20,21,22,24,26	18	P02631,P02803,P06911,P10715,P12020,P15399,P19629,P31044,P50280,P68370,P97580,P97840,Q30KJ2,Q5GRG2,Q63532,Q6P6R2,Q6P9T8,Q99041
4127	1,2,6,7,8,19,23,26	3,4,5,18,20,21,22,24,25	5	P02631,P15399,P97580,Q30KJ2,Q63532
4128	1,2,6,7,8,19,24,25	3,4,5,18,20,21,22,23,26	14	P01835,P02631,P06911,P10715,P12020,P12785,P19629,P31044,P68370,P97840,Q4FZU2,Q5GRG2,Q63532,Q6IG02
4129	1,2,6,7,8,19,24,26	3,4,5,18,20,21,22,23,25	2	P02631,Q63532
4130	1,2,6,7,8,19,25,26	3,4,5,18,20,21,22,23,24	11	P10715,P12020,P19629,P31044,P35280,P68370,P97840,Q5GRG2,Q63532,Q91ZS3,Q9QZK9
4131	1,2,6,7,8,20,21,22	3,4,5,18,19,23,24,25,26	6	P02782,P08723,P12785,P13432,Q91ZS3,Q9JI85
4132	1,2,6,7,8,20,21,23	3,4,5,18,19,22,24,25,26	5	P02631,P62804,Q6AYL6,Q6AYR8,Q91ZS3
4133	1,2,6,7,8,20,21,24	3,4,5,18,19,22,23,25,26	6	P02782,P12785,P13432,Q6AYL6,Q6AYR8,Q91ZS3
4134	1,2,6,7,8,20,21,25	3,4,5,18,19,22,23,24,26	12	P10715,P12020,P12785,P19629,P47727,P47967,P68370,Q5GRG2,Q6AYL6,Q6P6R2,Q91ZS3,Q9WTT6
4135	1,2,6,7,8,20,21,26	3,4,5,18,19,22,23,24,25	6	P01041,P62804,P83748,Q00715,Q6AYR8,Q91ZS3
4136	1,2,6,7,8,20,22,23	3,4,5,18,19,21,24,25,26	2	P02631,Q8CJD3
4137	1,2,6,7,8,20,22,24	3,4,5,18,19,21,23,25,26	1	P12785
4138	1,2,6,7,8,20,22,25	3,4,5,18,19,21,23,24,26	9	P06911,P10715,P12020,P12785,P19629,P31044,P68370,Q5GRG2,Q91ZS3

4139	1,2,6,7,8,20,22,26	3,4,5,18,19,21,23,24,25	1	P12785
4140	1,2,6,7,8,20,23,24	3,4,5,18,19,21,22,25,26	1	P02631
4141	1,2,6,7,8,20,23,25	3,4,5,18,19,21,22,24,26	11	P02631,P06911,P10715,P12020,P19629,P31044,P47727,P68370,Q5GRG2,Q6P6R2,Q91ZS3
4142	1,2,6,7,8,20,23,26	3,4,5,18,19,21,22,24,25	1	Q91ZS3
4143	1,2,6,7,8,20,24,25	3,4,5,18,19,21,22,23,26	7	P10715,P12020,P12785,P19629,P68370,Q5GRG2,Q91ZS3
4144	1,2,6,7,8,20,24,26	3,4,5,18,19,21,22,23,25	1	P10715
4145	1,2,6,7,8,20,25,26	3,4,5,18,19,21,22,23,24	7	P10715,P12020,P19629,P35280,P68370,Q5GRG2,Q91ZS3
4146	1,2,6,7,8,21,22,23	3,4,5,18,19,20,24,25,26	4	P02631,P02782,P08723,P13432
4147	1,2,6,7,8,21,22,24	3,4,5,18,19,20,23,25,26	5	P02782,P12785,P13432,P35467,Q6IG02
4148	1,2,6,7,8,21,22,25	3,4,5,18,19,20,23,24,26	16	D4A1J4,P06911,P08723,P10715,P12020,P12785,P13432,P19629,P35467,P47967,P68370,P97840,Q5GRG2,Q6IG02,Q91ZS3,Q9WTT6
4149	1,2,6,7,8,21,22,26	3,4,5,18,19,20,23,24,25	5	P02782,P12785,P13432,P83748,Q00715
4150	1,2,6,7,8,21,23,24	3,4,5,18,19,20,22,25,26	3	P02631,P13432,Q6AYL6
4151	1,2,6,7,8,21,23,25	3,4,5,18,19,20,22,24,26	17	P02631,P06911,P07150,P10715,P12020,P19629,P31044,P47727,P50280,P68370,P97840,Q5GRG2,Q6AYL6,Q6P6R2,Q6P9T8,Q91ZS3,Q9WTT6
4152	1,2,6,7,8,21,23,26	3,4,5,18,19,20,22,24,25	4	P83748,Q00715,Q6IFV1,Q91ZS3
4153	1,2,6,7,8,21,24,25	3,4,5,18,19,20,22,23,26	15	O54728,P10715,P12020,P12785,P13432,P19629,P22006,P35467,P68370,P97840,Q5GRG2,Q6IG02,Q6P6R2,Q91ZS3,Q9WTT6

4154	1,2,6,7,8,21,24,26	3,4,5,18,19,20,22,23,25	2	P13432,P83748
4155	1,2,6,7,8,21,25,26	3,4,5,18,19,20,22,23,24	12	P10715,P12020,P19629,P68370,P83748,Q00715,Q5GRG2,Q6P6R2,Q6P9T8,Q91ZS3,Q9QZK9,Q9WTT6
4156	1,2,6,7,8,22,23,24	3,4,5,18,19,20,21,25,26	1	P02631
4157	1,2,6,7,8,22,23,25	3,4,5,18,19,20,21,24,26	9	P02631,P06911,P10715,P12020,P19629,P31044,P50280,P68370,Q5GRG2
4158	1,2,6,7,8,22,23,26	3,4,5,18,19,20,21,24,25	0	
4159	1,2,6,7,8,22,24,25	3,4,5,18,19,20,21,23,26	11	P01835,P06911,P10715,P12020,P12785,P19629,P35467,P68370,Q4FZU2,Q5GRG2,Q6IG02
4160	1,2,6,7,8,22,24,26	3,4,5,18,19,20,21,23,25	1	P12785
4161	1,2,6,7,8,22,25,26	3,4,5,18,19,20,21,23,24	9	P06911,P10715,P12020,P12785,P19629,P31044,P68370,Q5GRG2,Q91ZS3
4162	1,2,6,7,8,23,24,25	3,4,5,18,19,20,21,22,26	10	P02631,P06911,P10715,P12020,P19629,P31044,P50280,P68370,Q5GRG2,Q6P6R2
4163	1,2,6,7,8,23,24,26	3,4,5,18,19,20,21,22,25	0	
4164	1,2,6,7,8,23,25,26	3,4,5,18,19,20,21,22,24	12	P01946,P06911,P10715,P12020,P19629,P31044,P68370,Q5GRG2,Q6P6R2,Q6P9T8,Q91ZS3,Q9WTT6
4165	1,2,6,7,8,24,25,26	3,4,5,18,19,20,21,22,23	6	P10715,P12020,P19629,P68370,Q5GRG2,Q91ZS3
4166	1,2,6,7,18,19,20,21	3,4,5,8,22,23,24,25,26	8	O54861,P00731,P25031,P97840,Q63532,Q6AYL6,Q91ZS3,Q9JI85
4167	1,2,6,7,18,19,20,22	3,4,5,8,21,23,24,25,26	2	Q63532,Q6AYS4
4168	1,2,6,7,18,19,20,23	3,4,5,8,21,22,24,25,26	7	P02631,P15399,P97580,Q30KJ2,Q63532,Q6AYS4,Q8CJD3

4169	1,2,6,7,18,19,20,24	3,4,5,8,21,22,23,25,26	3	P02631,Q63532,Q6AYS4
4170	1,2,6,7,18,19,20,25	3,4,5,8,21,22,23,24,26	6	P12020,P35280,P47967,P97840,Q63532,Q91ZS3
4171	1,2,6,7,18,19,20,26	3,4,5,8,21,22,23,24,25	5	P06760,P35280,Q63532,Q63751,Q6AYS4
4172	1,2,6,7,18,19,21,22	3,4,5,8,20,23,24,25,26	6	D4A1J4,P08723,P19218,P25031,P97840,Q63532
4173	1,2,6,7,18,19,21,23	3,4,5,8,20,22,24,25,26	14	D3ZUC6,P02631,P05964,P14668,P15399,P25031,P97580,P97840,Q03191,Q30KJ2,Q63532,Q6AYL6,Q8CJD3,Q99MH3
4174	1,2,6,7,18,19,21,24	3,4,5,8,20,22,23,25,26	6	D3ZUC6,P02631,P13432,P97840,Q63532,Q6AYL6
4175	1,2,6,7,18,19,21,25	3,4,5,8,20,22,23,24,26	13	O54728,P00731,P02803,P25031,P31430,P47967,P97840,P98089,Q62635,Q63532,Q6AYL6,Q8CIZ5,Q91ZS3
4176	1,2,6,7,18,19,21,26	3,4,5,8,20,22,23,24,25	7	D3ZUC6,P25031,P82995,P83748,P97840,Q63532,Q63661
4177	1,2,6,7,18,19,22,23	3,4,5,8,20,21,24,25,26	6	P02631,P15399,P97580,Q30KJ2,Q63532,Q8CJD3
4178	1,2,6,7,18,19,22,24	3,4,5,8,20,21,23,25,26	3	P02631,Q63532,Q6AYS4
4179	1,2,6,7,18,19,22,25	3,4,5,8,20,21,23,24,26	6	P06911,P12020,P47967,P97840,Q5GRG2,Q63532
4180	1,2,6,7,18,19,22,26	3,4,5,8,20,21,23,24,25	3	P06760,Q63532,Q63942
4181	1,2,6,7,18,19,23,24	3,4,5,8,20,21,22,25,26	7	P02631,P15399,P97580,Q03191,Q30KJ2,Q63532,Q6AYS4
4182	1,2,6,7,18,19,23,25	3,4,5,8,20,21,22,24,26	13	P02631,P02803,P12020,P15399,P19629,P97580,P97840,Q30KJ2,Q5GRG2,Q62635,Q63532,Q8CJD3,Q99MH3
4183	1,2,6,7,18,19,23,26	3,4,5,8,20,21,22,24,25	7	P02631,P15399,P97580,Q03191,Q30KJ2,Q63532,Q63942

4184	1,2,6,7,18,19,24,25	3,4,5,8,20,21,22,23,26	5	P02631,P12020,P19629,P97840,Q63532
4185	1,2,6,7,18,19,24,26	3,4,5,8,20,21,22,23,25	4	P06760,Q63532,Q63942,Q6AYS4
4186	1,2,6,7,18,19,25,26	3,4,5,8,20,21,22,23,24	7	P12020,P35280,P55091,P82995,P97840,Q63532,Q91ZS3
4187	1,2,6,7,18,20,21,22	3,4,5,8,19,23,24,25,26	4	P13432,P25031,Q91ZS3,Q9JI85
4188	1,2,6,7,18,20,21,23	3,4,5,8,19,22,24,25,26	5	P14668,P25031,Q6AYL6,Q91ZS3,Q9QUL6
4189	1,2,6,7,18,20,21,24	3,4,5,8,19,22,23,25,26	6	O54728,O54861,P13432,P25031,Q6AYL6,Q91ZS3
4190	1,2,6,7,18,20,21,25	3,4,5,8,19,22,23,24,26	8	O54728,P25031,P31430,P47967,P97840,P98089,Q6AYL6,Q91ZS3
4191	1,2,6,7,18,20,21,26	3,4,5,8,19,22,23,24,25	7	O54861,P01041,P25031,P83748,Q6AYR8,Q91ZS3,Q9QUL6
4192	1,2,6,7,18,20,22,23	3,4,5,8,19,21,24,25,26	1	Q8CJD3
4193	1,2,6,7,18,20,22,24	3,4,5,8,19,21,23,25,26	1	Q6AYS4
4194	1,2,6,7,18,20,22,25	3,4,5,8,19,21,23,24,26	4	P12020,P97840,Q5GRG2,Q91ZS3
4195	1,2,6,7,18,20,22,26	3,4,5,8,19,21,23,24,25	2	P06760,Q9QUL6
4196	1,2,6,7,18,20,23,24	3,4,5,8,19,21,22,25,26	1	Q6AYS4
4197	1,2,6,7,18,20,23,25	3,4,5,8,19,21,22,24,26	4	P12020,P19629,Q5GRG2,Q91ZS3
4198	1,2,6,7,18,20,23,26	3,4,5,8,19,21,22,24,25	5	Q63942,Q6IFU8,Q6P6Q2,Q91ZS3,Q9QUL6

4199	1,2,6,7,18,20,24,25	3,4,5,8,19,21,22,23,26	4	O54728,P10715,P19629,Q91ZS3
4200	1,2,6,7,18,20,24,26	3,4,5,8,19,21,22,23,25	3	P06760,Q6AYS4,Q9QUL6
4201	1,2,6,7,18,20,25,26	3,4,5,8,19,21,22,23,24	3	P35280,P55091,Q91ZS3
4202	1,2,6,7,18,21,22,23	3,4,5,8,19,20,24,25,26	4	P13432,P14668,P25031,Q9QUL6
4203	1,2,6,7,18,21,22,24	3,4,5,8,19,20,23,25,26	1	P13432
4204	1,2,6,7,18,21,22,25	3,4,5,8,19,20,23,24,26	10	P09605;P25809,P12020,P19629,P25031,P31430,P47967,P97840,P98089,Q5GRG2,Q91ZS3
4205	1,2,6,7,18,21,22,26	3,4,5,8,19,20,23,24,25	4	P13432,P25031,P83748,Q9QUL6
4206	1,2,6,7,18,21,23,24	3,4,5,8,19,20,22,25,26	4	D3ZUC6,P13432,Q6AYL6,Q9QUL6
4207	1,2,6,7,18,21,23,25	3,4,5,8,19,20,22,24,26	12	O54728,P12020,P14668,P19629,P25031,P31430,P97840,P98089,Q6AYL6,Q6P6R2,Q91ZS3,Q99MH3
4208	1,2,6,7,18,21,23,26	3,4,5,8,19,20,22,24,25	10	D3ZUC6,P25031,P83748,Q63661,Q63942,Q6IFU8,Q6IFV1,Q6P6Q2,Q91ZS3,Q9QUL6
4209	1,2,6,7,18,21,24,25	3,4,5,8,19,20,22,23,26	10	O54728,P13432,P19629,P25031,P31430,P97840,P98089,Q62997,Q6AYL6,Q91ZS3
4210	1,2,6,7,18,21,24,26	3,4,5,8,19,20,22,23,25	6	D3ZUC6,P13432,P25031,P55091,P83748,Q9QUL6
4211	1,2,6,7,18,21,25,26	3,4,5,8,19,20,22,23,24	9	P19629,P25031,P31430,P55091,P82995,P83748,P97840,P98089,Q91ZS3
4212	1,2,6,7,18,22,23,24	3,4,5,8,19,20,21,25,26	0	
4213	1,2,6,7,18,22,23,25	3,4,5,8,19,20,21,24,26	5	P06911,P12020,P19629,P97840,Q5GRG2

4214	1,2,6,7,18,22,23,26	3,4,5,8,19,20,21,24,25	2	Q63942,Q9QUL6
4215	1,2,6,7,18,22,24,25	3,4,5,8,19,20,21,23,26	4	P12020,P19629,P97840,Q5GRG2
4216	1,2,6,7,18,22,24,26	3,4,5,8,19,20,21,23,25	0	
4217	1,2,6,7,18,22,25,26	3,4,5,8,19,20,21,23,24	6	P12020,P19629,P55091,P97840,Q5GRG2,Q91ZS3
4218	1,2,6,7,18,23,24,25	3,4,5,8,19,20,21,22,26	3	P12020,P19629,Q5GRG2
4219	1,2,6,7,18,23,24,26	3,4,5,8,19,20,21,22,25	3	Q63942,Q6IFU7,Q9QUL6
4220	1,2,6,7,18,23,25,26	3,4,5,8,19,20,21,22,24	7	P01946,P12020,P19629,P55091,Q63942,Q6P6Q2,Q91ZS3
4221	1,2,6,7,18,24,25,26	3,4,5,8,19,20,21,22,23	4	P19629,P55091,Q7TPB1,Q91ZS3
4222	1,2,6,7,19,20,21,22	3,4,5,8,18,23,24,25,26	5	P00714,P12785,P35467,P97840,Q6AYS4
4223	1,2,6,7,19,20,21,23	3,4,5,8,18,22,24,25,26	4	P15399,P97580,Q30KJ2,Q63532
4224	1,2,6,7,19,20,21,24	3,4,5,8,18,22,23,25,26	6	O54861,P00714,P13432,P35467,Q63532,Q6AYS4
4225	1,2,6,7,19,20,21,25	3,4,5,8,18,22,23,24,26	5	P12020,P35467,P47967,P97840,Q91ZS3
4226	1,2,6,7,19,20,21,26	3,4,5,8,18,22,23,24,25	4	O54861,P83748,Q6AYS4,Q91ZS3
4227	1,2,6,7,19,20,22,23	3,4,5,8,18,21,24,25,26	8	P00774,P15399,P31430,P97580,Q30KJ2,Q63532,Q6AYS4,Q8CJD3
4228	1,2,6,7,19,20,22,24	3,4,5,8,18,21,23,25,26	6	P00714,P00774,P12785,P35467,Q63532,Q6AYS4

4229	1,2,6,7,19,20,22,25	3,4,5,8,18,21,23,24,26	9	P06911,P12020,P12785,P35467,P47967,P97840,Q5GRG2,Q63532,Q91ZS3
4230	1,2,6,7,19,20,22,26	3,4,5,8,18,21,23,24,25	3	Q63532,Q63751,Q6AYS4
4231	1,2,6,7,19,20,23,24	3,4,5,8,18,21,22,25,26	6	P02631,P15399,P97580,Q30KJ2,Q63532,Q6AYS4
4232	1,2,6,7,19,20,23,25	3,4,5,8,18,21,22,24,26	8	P12020,P15399,P97580,P97840,Q30KJ2,Q5GRG2,Q63532,Q91ZS3
4233	1,2,6,7,19,20,23,26	3,4,5,8,18,21,22,24,25	5	P15399,P97580,Q30KJ2,Q63532,Q6AYS4
4234	1,2,6,7,19,20,24,25	3,4,5,8,18,21,22,23,26	6	P12020,P35467,P97840,Q63532,Q6AYS4,Q91ZS3
4235	1,2,6,7,19,20,24,26	3,4,5,8,18,21,22,23,25	2	Q63532,Q6AYS4
4236	1,2,6,7,19,20,25,26	3,4,5,8,18,21,22,23,24	5	P12020,P35280,P97840,Q63532,Q91ZS3
4237	1,2,6,7,19,21,22,23	3,4,5,8,18,20,24,25,26	7	P14668,P15399,P97580,P97840,Q30KJ2,Q63532,Q8CJD3
4238	1,2,6,7,19,21,22,24	3,4,5,8,18,20,23,25,26	9	P00714,P12785,P13432,P35467,P97840,Q4FZU2,Q63532,Q6AYS4,Q6IG02
4239	1,2,6,7,19,21,22,25	3,4,5,8,18,20,23,24,26	12	P06911,P09605;P25809,P12020,P12785,P19629,P35467,P47967,P97840,Q5GRG2,Q63532,Q6IG02,Q91ZS3
4240	1,2,6,7,19,21,22,26	3,4,5,8,18,20,23,24,25	3	P83748,P97840,Q63532
4241	1,2,6,7,19,21,23,24	3,4,5,8,18,20,22,25,26	6	P02631,P15399,P97580,P97840,Q30KJ2,Q63532
4242	1,2,6,7,19,21,23,25	3,4,5,8,18,20,22,24,26	11	P12020,P15399,P19629,P47967,P97580,P97840,Q30KJ2,Q5GRG2,Q63532,Q6P6R2,Q91ZS3
4243	1,2,6,7,19,21,23,26	3,4,5,8,18,20,22,24,25	4	P15399,P97580,Q30KJ2,Q63532

4244	1,2,6,7,19,21,24,25	3,4,5,8,18,20,22,23,26	9	P12020,P19629,P22006,P35467,P47967,P97840,Q63532,Q6IG02,Q91ZS3
4245	1,2,6,7,19,21,24,26	3,4,5,8,18,20,22,23,25	2	Q63532,Q6AYS4
4246	1,2,6,7,19,21,25,26	3,4,5,8,18,20,22,23,24	7	P12020,P47967,P83748,P97840,Q63532,Q91ZS3,Q9QZK9
4247	1,2,6,7,19,22,23,24	3,4,5,8,18,20,21,25,26	7	P02631,P15399,P31430,P97580,Q30KJ2,Q63532,Q6AYS4
4248	1,2,6,7,19,22,23,25	3,4,5,8,18,20,21,24,26	10	P06911,P12020,P15399,P19629,P50280,P97580,P97840,Q30KJ2,Q5GRG2,Q63532
4249	1,2,6,7,19,22,23,26	3,4,5,8,18,20,21,24,25	5	P15399,P97580,Q30KJ2,Q63532,Q6AYS4
4250	1,2,6,7,19,22,24,25	3,4,5,8,18,20,21,23,26	13	P01835,P06911,P12020,P12785,P19629,P35467,P97840,Q4FZU2,Q4FZU6,Q5GRG2,Q63532,Q6IG02,Q6P6Q2
4251	1,2,6,7,19,22,24,26	3,4,5,8,18,20,21,23,25	2	Q63532,Q6AYS4
4252	1,2,6,7,19,22,25,26	3,4,5,8,18,20,21,23,24	6	P06911,P12020,P19629,P97840,Q5GRG2,Q63532
4253	1,2,6,7,19,23,24,25	3,4,5,8,18,20,21,22,26	10	P02631,P06911,P12020,P15399,P19629,P97580,P97840,Q30KJ2,Q5GRG2,Q63532
4254	1,2,6,7,19,23,24,26	3,4,5,8,18,20,21,22,25	6	P01946,P15399,P97580,Q30KJ2,Q63532,Q6AYS4
4255	1,2,6,7,19,23,25,26	3,4,5,8,18,20,21,22,24	9	P01946,P12020,P15399,P19629,P97580,P97840,Q30KJ2,Q5GRG2,Q63532
4256	1,2,6,7,19,24,25,26	3,4,5,8,18,20,21,22,23	6	P12020,P14480,P19629,P97840,Q63532,Q7TPB1
4257	1,2,6,7,20,21,22,23	3,4,5,8,18,19,24,25,26	1	P13432
4258	1,2,6,7,20,21,22,24	3,4,5,8,18,19,23,25,26	6	P00714,P12785,P13432,P35467,Q5XFX0,Q64194

4259	1,2,6,7,20,21,22,25	3,4,5,8,18,19,23,24,26	9	P09605;P25809,P12020,P12785,P19629,P35467,P47967,P97840,Q5GRG2,Q91ZS3
4260	1,2,6,7,20,21,22,26	3,4,5,8,18,19,23,24,25	3	P13432,P83748,Q91ZS3
4261	1,2,6,7,20,21,23,24	3,4,5,8,18,19,22,25,26	1	P13432
4262	1,2,6,7,20,21,23,25	3,4,5,8,18,19,22,24,26	6	P12020,P19629,P97840,Q5GRG2,Q6P6R2,Q91ZS3
4263	1,2,6,7,20,21,23,26	3,4,5,8,18,19,22,24,25	2	Q91ZS3,Q9QUL6
4264	1,2,6,7,20,21,24,25	3,4,5,8,18,19,22,23,26	8	O54728,P09605;P25809,P13432,P19629,P35467,P97840,Q64194,Q91ZS3
4265	1,2,6,7,20,21,24,26	3,4,5,8,18,19,22,23,25	4	O54861,P13432,Q6AYS4,Q91ZS3
4266	1,2,6,7,20,21,25,26	3,4,5,8,18,19,22,23,24	1	Q91ZS3
4267	1,2,6,7,20,22,23,24	3,4,5,8,18,19,21,25,26	1	Q6AYS4
4268	1,2,6,7,20,22,23,25	3,4,5,8,18,19,21,24,26	5	P06911,P12020,P19629,Q5GRG2,Q91ZS3
4269	1,2,6,7,20,22,23,26	3,4,5,8,18,19,21,24,25	1	Q6AYS4
4270	1,2,6,7,20,22,24,25	3,4,5,8,18,19,21,23,26	8	P06911,P12020,P12785,P19629,P35467,Q5GRG2,Q64194,Q91ZS3
4271	1,2,6,7,20,22,24,26	3,4,5,8,18,19,21,23,25	1	Q6AYS4
4272	1,2,6,7,20,22,25,26	3,4,5,8,18,19,21,23,24	4	P12020,P19629,Q5GRG2,Q91ZS3
4273	1,2,6,7,20,23,24,25	3,4,5,8,18,19,21,22,26	4	P12020,P19629,Q5GRG2,Q91ZS3

4274	1,2,6,7,20,23,24,26	3,4,5,8,18,19,21,22,25	1	Q6AYS4
4275	1,2,6,7,20,23,25,26	3,4,5,8,18,19,21,22,24	5	P01946,P12020,P19629,Q5GRG2,Q91ZS3
4276	1,2,6,7,20,24,25,26	3,4,5,8,18,19,21,22,23	2	P19629,Q91ZS3
4277	1,2,6,7,21,22,23,24	3,4,5,8,18,19,20,25,26	1	P13432
4278	1,2,6,7,21,22,23,25	3,4,5,8,18,19,20,24,26	9	P06911,P09605;P25809,P12020,P19629,P70709,P97840,Q5GRG2,Q6P6R2,Q91ZS3
4279	1,2,6,7,21,22,23,26	3,4,5,8,18,19,20,24,25	1	P83748
4280	1,2,6,7,21,22,24,25	3,4,5,8,18,19,20,23,26	13	P06911,P09605;P25809,P12020,P12785,P13432,P19629,P22006,P35467,P97840,Q5GRG2,Q64194,Q6IG02,Q91ZS3
4281	1,2,6,7,21,22,24,26	3,4,5,8,18,19,20,23,25	4	P00714,P13432,P35467,P83748
4282	1,2,6,7,21,22,25,26	3,4,5,8,18,19,20,23,24	9	P09605;P25809,P12020,P19629,P35467,P83748,P97840,Q5GRG2,Q91ZS3,Q9QZK9
4283	1,2,6,7,21,23,24,25	3,4,5,8,18,19,20,22,26	7	P12020,P19629,P22006,P97840,Q5GRG2,Q6P6R2,Q91ZS3
4284	1,2,6,7,21,23,24,26	3,4,5,8,18,19,20,22,25	1	Q6IFV1
4285	1,2,6,7,21,23,25,26	3,4,5,8,18,19,20,22,24	9	P01946,P12020,P19629,P22006,P83121,P97840,Q5GRG2,Q6P6R2,Q91ZS3
4286	1,2,6,7,21,24,25,26	3,4,5,8,18,19,20,22,23	4	P19629,P22006,P97840,Q91ZS3
4287	1,2,6,7,22,23,24,25	3,4,5,8,18,19,20,21,26	5	P06911,P12020,P19629,Q4FZU6,Q5GRG2
4288	1,2,6,7,22,23,24,26	3,4,5,8,18,19,20,21,25	2	P00731,Q6AYS4

4289	1,2,6,7,22,23,25,26	3,4,5,8,18,19,20,21,24	5	P01946,P06911,P12020,P19629,Q5GRG2
4290	1,2,6,7,22,24,25,26	3,4,5,8,18,19,20,21,23	4	P06911,P12020,P19629,Q5GRG2
4291	1,2,6,7,23,24,25,26	3,4,5,8,18,19,20,21,22	5	P01946,P12020,P19629,Q5GRG2,Q7TPB1
4292	1,2,6,8,18,19,20,21	3,4,5,7,22,23,24,25,26	9	O35547,P00731,P02782,P08723,P35281,P62804,Q63751,Q6AYR8,Q8K4G9
4293	1,2,6,8,18,19,20,22	3,4,5,7,21,23,24,25,26	4	P22006,Q63751,Q8CJD3,Q8K4G9
4294	1,2,6,8,18,19,20,23	3,4,5,7,21,22,24,25,26	4	P15399,P22006,Q8CJD3,Q9Z1F2
4295	1,2,6,8,18,19,20,24	3,4,5,7,21,22,23,25,26	0	
4296	1,2,6,8,18,19,20,25	3,4,5,7,21,22,23,24,26	4	P35280,Q62635,Q8K4G9,Q91ZS3
4297	1,2,6,8,18,19,20,26	3,4,5,7,21,22,23,24,25	6	P35280,P62804,Q63355,Q63751,Q63942,Q8K4G9
4298	1,2,6,8,18,19,21,22	3,4,5,7,20,23,24,25,26	5	O35547,P02782,P08723,P35281,Q8CJD3
4299	1,2,6,8,18,19,21,23	3,4,5,7,20,22,24,25,26	11	O35547,P02782,P02803,P15399,P35281,Q03191,Q62635,Q63942,Q8CJD3,Q99MH3,Q9Z1F2
4300	1,2,6,8,18,19,21,24	3,4,5,7,20,22,23,25,26	5	O35547,P02782,P05539,P13432,P35281
4301	1,2,6,8,18,19,21,25	3,4,5,7,20,22,23,24,26	12	O35547,O54728,P00731,P02803,P31430,P35281,P47967,P97840,P98089,Q62635,Q8CIZ5,Q91ZS3
4302	1,2,6,8,18,19,21,26	3,4,5,7,20,22,23,24,25	6	P02782,P35281,Q00715,Q63751,Q63942,Q8K4G9
4303	1,2,6,8,18,19,22,23	3,4,5,7,20,21,24,25,26	4	P15399,Q63942,Q8CJD3,Q9Z1F2

4304	1,2,6,8,18,19,22,24	3,4,5,7,20,21,23,25,26	1	P83121
4305	1,2,6,8,18,19,22,25	3,4,5,7,20,21,23,24,26	4	P06911,P97840,Q4FZU6,Q62635
4306	1,2,6,8,18,19,22,26	3,4,5,7,20,21,23,24,25	3	Q63751,Q63942,Q8K4G9
4307	1,2,6,8,18,19,23,24	3,4,5,7,20,21,22,25,26	4	P05539,P15399,Q03191,Q63942
4308	1,2,6,8,18,19,23,25	3,4,5,7,20,21,22,24,26	7	P02803,P15399,P50280,Q62635,Q8CJD3,Q99MH3,Q9Z1F2
4309	1,2,6,8,18,19,23,26	3,4,5,7,20,21,22,24,25	6	P15399,P35467,Q03191,Q63942,Q6IFV1,Q6P6Q2
4310	1,2,6,8,18,19,24,25	3,4,5,7,20,21,22,23,26	3	P05539,Q4FZU6,Q62635
4311	1,2,6,8,18,19,24,26	3,4,5,7,20,21,22,23,25	1	Q63942
4312	1,2,6,8,18,19,25,26	3,4,5,7,20,21,22,23,24	4	P35280,Q62635,Q63942,Q8K4G9
4313	1,2,6,8,18,20,21,22	3,4,5,7,19,23,24,25,26	5	O35547,P02782,P08723,P13432,P62804
4314	1,2,6,8,18,20,21,23	3,4,5,7,19,22,24,25,26	5	O35547,P02782,P62804,Q6AYR8,Q8CJD3
4315	1,2,6,8,18,20,21,24	3,4,5,7,19,22,23,25,26	6	O54728,P02782,P05539,P13432,P62804,Q6AYR8
4316	1,2,6,8,18,20,21,25	3,4,5,7,19,22,23,24,26	8	O54728,P31430,P47727,P62804,P98089,Q8K4G9,Q91ZS3,Q9WTT6
4317	1,2,6,8,18,20,21,26	3,4,5,7,19,22,23,24,25	7	P02782,P62804,Q00715,Q63942,Q6AYR8,Q8K4G9,Q91ZS3
4318	1,2,6,8,18,20,22,23	3,4,5,7,19,21,24,25,26	2	P22006,Q8CJD3

4319	1,2,6,8,18,20,22,24	3,4,5,7,19,21,23,25,26	0	
4320	1,2,6,8,18,20,22,25	3,4,5,7,19,21,23,24,26	1	Q91ZS3
4321	1,2,6,8,18,20,22,26	3,4,5,7,19,21,23,24,25	2	Q63942,Q8K4G9
4322	1,2,6,8,18,20,23,24	3,4,5,7,19,21,22,25,26	0	
4323	1,2,6,8,18,20,23,25	3,4,5,7,19,21,22,24,26	1	Q91ZS3
4324	1,2,6,8,18,20,23,26	3,4,5,7,19,21,22,24,25	4	Q63942,Q6IFU8,Q6IFV1,Q6P6Q2
4325	1,2,6,8,18,20,24,25	3,4,5,7,19,21,22,23,26	2	O54728,Q91ZS3
4326	1,2,6,8,18,20,24,26	3,4,5,7,19,21,22,23,25	1	Q63942
4327	1,2,6,8,18,20,25,26	3,4,5,7,19,21,22,23,24	4	P35280,Q63942,Q8K4G9,Q91ZS3
4328	1,2,6,8,18,21,22,23	3,4,5,7,19,20,24,25,26	5	O35547,P02782,P08723,P13432,Q8CJD3
4329	1,2,6,8,18,21,22,24	3,4,5,7,19,20,23,25,26	3	P02782,P05539,P13432
4330	1,2,6,8,18,21,22,25	3,4,5,7,19,20,23,24,26	7	O35547,P02782,P08723,P31430,P97840,P98089,Q91ZS3
4331	1,2,6,8,18,21,22,26	3,4,5,7,19,20,23,24,25	4	P02782,P13432,Q00715,Q63942
4332	1,2,6,8,18,21,23,24	3,4,5,7,19,20,22,25,26	4	O54728,P02782,P05539,P13432
4333	1,2,6,8,18,21,23,25	3,4,5,7,19,20,22,24,26	10	O54728,P02803,P07150,P14669,P31430,P98089,Q6P6R2,Q91ZS3,Q99MH3,Q9WTT6

4334	1,2,6,8,18,21,23,26	3,4,5,7,19,20,22,24,25	7	P02782,P14669,Q00715,Q63942,Q6IFU8,Q6IFV1,Q6P6Q2
4335	1,2,6,8,18,21,24,25	3,4,5,7,19,20,22,23,26	9	O54728,P05539,P13432,P31430,P98089,Q62997,Q6P6R2,Q91ZS3,Q9WTT6
4336	1,2,6,8,18,21,24,26	3,4,5,7,19,20,22,23,25	3	P02782,P13432,Q63942
4337	1,2,6,8,18,21,25,26	3,4,5,7,19,20,22,23,24	7	P31430,P55091,P98089,Q00715,Q63942,Q91ZS3,Q9WTT6
4338	1,2,6,8,18,22,23,24	3,4,5,7,19,20,21,25,26	0	
4339	1,2,6,8,18,22,23,25	3,4,5,7,19,20,21,24,26	3	P06911,Q5GRG2,Q8CJD3
4340	1,2,6,8,18,22,23,26	3,4,5,7,19,20,21,24,25	4	Q63942,Q6IFU7,Q6IFV1,Q6P6Q2
4341	1,2,6,8,18,22,24,25	3,4,5,7,19,20,21,23,26	1	Q4FZU6
4342	1,2,6,8,18,22,24,26	3,4,5,7,19,20,21,23,25	1	Q63942
4343	1,2,6,8,18,22,25,26	3,4,5,7,19,20,21,23,24	1	Q63942
4344	1,2,6,8,18,23,24,25	3,4,5,7,19,20,21,22,26	3	O54728,P05539,Q6P6R2
4345	1,2,6,8,18,23,24,26	3,4,5,7,19,20,21,22,25	4	Q63942,Q6IFU7,Q6IFV1,Q6P6Q2
4346	1,2,6,8,18,23,25,26	3,4,5,7,19,20,21,22,24	4	P55091,Q63942,Q6IFU8,Q6P6Q2
4347	1,2,6,8,18,24,25,26	3,4,5,7,19,20,21,22,23	3	P55091,Q63942,Q7TPB1
4348	1,2,6,8,19,20,21,22	3,4,5,7,18,23,24,25,26	6	P00714,P02782,P12785,P35281,P62804,Q8K4G9

4349	1,2,6,8,19,20,21,23	3,4,5,7,18,22,24,25,26	6	P02782,P15399,P35281,P62804,Q8CJD3,Q8K4G9
4350	1,2,6,8,19,20,21,24	3,4,5,7,18,22,23,25,26	7	P00714,P02782,P12785,P13432,P35281,P62804,Q8K4G9
4351	1,2,6,8,19,20,21,25	3,4,5,7,18,22,23,24,26	8	P12785,P35281,P47967,P62804,P97840,Q8K4G9,Q8VD89,Q91ZS3
4352	1,2,6,8,19,20,21,26	3,4,5,7,18,22,23,24,25	6	P02782,P35281,P62804,Q00715,Q63751,Q8K4G9
4353	1,2,6,8,19,20,22,23	3,4,5,7,18,21,24,25,26	4	P00774,P15399,P22006,Q8CJD3
4354	1,2,6,8,19,20,22,24	3,4,5,7,18,21,23,25,26	3	P00714,P00774,P12785
4355	1,2,6,8,19,20,22,25	3,4,5,7,18,21,23,24,26	4	P06911,P12785,Q5GRG2,Q8K4G9
4356	1,2,6,8,19,20,22,26	3,4,5,7,18,21,23,24,25	3	P12785,Q63751,Q8K4G9
4357	1,2,6,8,19,20,23,24	3,4,5,7,18,21,22,25,26	1	P15399
4358	1,2,6,8,19,20,23,25	3,4,5,7,18,21,22,24,26	3	P15399,Q8CJD3,Q8K4G9
4359	1,2,6,8,19,20,23,26	3,4,5,7,18,21,22,24,25	4	P15399,P62804,Q66H69,Q8K4G9
4360	1,2,6,8,19,20,24,25	3,4,5,7,18,21,22,23,26	2	P12785,Q8K4G9
4361	1,2,6,8,19,20,24,26	3,4,5,7,18,21,22,23,25	1	Q8K4G9
4362	1,2,6,8,19,20,25,26	3,4,5,7,18,21,22,23,24	2	P35280,Q8K4G9
4363	1,2,6,8,19,21,22,23	3,4,5,7,18,20,24,25,26	5	O35547,P02782,P15399,P35281,Q8CJD3

4364	1,2,6,8,19,21,22,24	3,4,5,7,18,20,23,25,26	6	A2RUV9,P00714,P02782,P12785,P13432,Q6IG02
4365	1,2,6,8,19,21,22,25	3,4,5,7,18,20,23,24,26	9	A2RUV9,P02782,P06911,P12785,P47967,P97840,Q4FZU6,Q5GRG2,Q6IG02
4366	1,2,6,8,19,21,22,26	3,4,5,7,18,20,23,24,25	5	A2RUV9,P02782,P12785,Q00715,Q8K4G9
4367	1,2,6,8,19,21,23,24	3,4,5,7,18,20,22,25,26	4	P02782,P05539,P15399,P35281
4368	1,2,6,8,19,21,23,25	3,4,5,7,18,20,22,24,26	7	P02803,P15399,P35281,P50280,P97840,Q6P6R2,Q9WTT6
4369	1,2,6,8,19,21,23,26	3,4,5,7,18,20,22,24,25	5	P02782,P15399,P35281,Q00715,Q6IFV1
4370	1,2,6,8,19,21,24,25	3,4,5,7,18,20,22,23,26	5	P05539,P12785,P97840,Q4FZU6,Q6IG02
4371	1,2,6,8,19,21,24,26	3,4,5,7,18,20,22,23,25	1	P02782
4372	1,2,6,8,19,21,25,26	3,4,5,7,18,20,22,23,24	4	P97840,Q00715,Q8K4G9,Q9WTT6
4373	1,2,6,8,19,22,23,24	3,4,5,7,18,20,21,25,26	1	P15399
4374	1,2,6,8,19,22,23,25	3,4,5,7,18,20,21,24,26	7	P06911,P12020,P15399,P50280,Q4FZU6,Q5GRG2,Q8CJD3
4375	1,2,6,8,19,22,23,26	3,4,5,7,18,20,21,24,25	0	
4376	1,2,6,8,19,22,24,25	3,4,5,7,18,20,21,23,26	7	P01835,P06911,P12785,Q4FZU2,Q4FZU6,Q5GRG2,Q6IG02
4377	1,2,6,8,19,22,24,26	3,4,5,7,18,20,21,23,25	1	P12785
4378	1,2,6,8,19,22,25,26	3,4,5,7,18,20,21,23,24	3	P06911,P12785,Q8K4G9

4379	1,2,6,8,19,23,24,25	3,4,5,7,18,20,21,22,26	4	P15399,P50280,Q4FZU6,Q6P6R2
4380	1,2,6,8,19,23,24,26	3,4,5,7,18,20,21,22,25	1	P15399
4381	1,2,6,8,19,23,25,26	3,4,5,7,18,20,21,22,24	1	P15399
4382	1,2,6,8,19,24,25,26	3,4,5,7,18,20,21,22,23	0	
4383	1,2,6,8,20,21,22,23	3,4,5,7,18,19,24,25,26	3	P02782,P13432,Q8CJD3
4384	1,2,6,8,20,21,22,24	3,4,5,7,18,19,23,25,26	6	P00714,P02782,P12785,P13432,P35467,Q5XFX0
4385	1,2,6,8,20,21,22,25	3,4,5,7,18,19,23,24,26	6	P12785,P35467,P47727,Q8K4G9,Q91ZS3,Q9WTT6
4386	1,2,6,8,20,21,22,26	3,4,5,7,18,19,23,24,25	7	P02782,P12785,P13432,P62804,Q00715,Q8K4G9,Q99068
4387	1,2,6,8,20,21,23,24	3,4,5,7,18,19,22,25,26	2	P02782,P13432
4388	1,2,6,8,20,21,23,25	3,4,5,7,18,19,22,24,26	4	P47727,Q6P6R2,Q91ZS3,Q9WTT6
4389	1,2,6,8,20,21,23,26	3,4,5,7,18,19,22,24,25	3	P62804,Q00715,Q6IFV1
4390	1,2,6,8,20,21,24,25	3,4,5,7,18,19,22,23,26	8	O54728,P12785,P13432,P35467,Q5XFX0,Q6P6R2,Q91ZS3,Q9WTT6
4391	1,2,6,8,20,21,24,26	3,4,5,7,18,19,22,23,25	5	P02782,P13432,P62804,Q00715,Q6AYR8
4392	1,2,6,8,20,21,25,26	3,4,5,7,18,19,22,23,24	5	P62804,Q00715,Q8K4G9,Q91ZS3,Q9WTT6
4393	1,2,6,8,20,22,23,24	3,4,5,7,18,19,21,25,26	1	P82995

4394	1,2,6,8,20,22,23,25	3,4,5,7,18,19,21,24,26	2	P06911,Q5GRG2
4395	1,2,6,8,20,22,23,26	3,4,5,7,18,19,21,24,25	0	
4396	1,2,6,8,20,22,24,25	3,4,5,7,18,19,21,23,26	3	P12785,P35467,Q5XFX0
4397	1,2,6,8,20,22,24,26	3,4,5,7,18,19,21,23,25	1	P12785
4398	1,2,6,8,20,22,25,26	3,4,5,7,18,19,21,23,24	2	P12785,Q8K4G9
4399	1,2,6,8,20,23,24,25	3,4,5,7,18,19,21,22,26	1	Q6P6R2
4400	1,2,6,8,20,23,24,26	3,4,5,7,18,19,21,22,25	1	Q6IFV1
4401	1,2,6,8,20,23,25,26	3,4,5,7,18,19,21,22,24	2	Q6P6R2,Q9WTT6
4402	1,2,6,8,20,24,25,26	3,4,5,7,18,19,21,22,23	0	
4403	1,2,6,8,21,22,23,24	3,4,5,7,18,19,20,25,26	2	P02782,P13432
4404	1,2,6,8,21,22,23,25	3,4,5,7,18,19,20,24,26	6	P06911,P50280,P70709,Q5GRG2,Q6P6R2,Q9WTT6
4405	1,2,6,8,21,22,23,26	3,4,5,7,18,19,20,24,25	2	P02782,Q6IFV1
4406	1,2,6,8,21,22,24,25	3,4,5,7,18,19,20,23,26	6	P12785,P13432,P35467,Q4FZU6,Q5XFX0,Q6IG02
4407	1,2,6,8,21,22,24,26	3,4,5,7,18,19,20,23,25	3	P02782,P12785,P13432
4408	1,2,6,8,21,22,25,26	3,4,5,7,18,19,20,23,24	2	P12785,Q9WTT6

4409	1,2,6,8,21,23,24,25	3,4,5,7,18,19,20,22,26	5	O54728,P19629,Q5U2V4,Q6P6R2,Q9WTT6
4410	1,2,6,8,21,23,24,26	3,4,5,7,18,19,20,22,25	2	P02782,Q6IFV1
4411	1,2,6,8,21,23,25,26	3,4,5,7,18,19,20,22,24	6	P07150,P14669,Q6IFV1,Q6P6R2,Q91ZS3,Q9WTT6
4412	1,2,6,8,21,24,25,26	3,4,5,7,18,19,20,22,23	2	Q6P6R2,Q9WTT6
4413	1,2,6,8,22,23,24,25	3,4,5,7,18,19,20,21,26	5	P06911,P19629,P50280,Q4FZU6,Q5GRG2
4414	1,2,6,8,22,23,24,26	3,4,5,7,18,19,20,21,25	1	P00731
4415	1,2,6,8,22,23,25,26	3,4,5,7,18,19,20,21,24	2	P06911,Q5GRG2
4416	1,2,6,8,22,24,25,26	3,4,5,7,18,19,20,21,23	1	P12785
4417	1,2,6,8,23,24,25,26	3,4,5,7,18,19,20,21,22	2	Q6P6R2,Q7TPB1
4418	1,2,6,18,19,20,21,22	3,4,5,7,8,23,24,25,26	2	O35547,Q63751
4419	1,2,6,18,19,20,21,23	3,4,5,7,8,22,24,25,26	2	P15399,Q8CJD3
4420	1,2,6,18,19,20,21,24	3,4,5,7,8,22,23,25,26	2	P13432,Q6AYS4
4421	1,2,6,18,19,20,21,25	3,4,5,7,8,22,23,24,26	4	P47967,P97840,P98089,Q91ZS3
4422	1,2,6,18,19,20,21,26	3,4,5,7,8,22,23,24,25	4	P62804,Q63751,Q6AYS4,Q8K4G9
4423	1,2,6,18,19,20,22,23	3,4,5,7,8,21,24,25,26	4	P15399,P22006,Q6AYS4,Q8CJD3
4424	1,2,6,18,19,20,22,24	3,4,5,7,8,21,23,25,26	1	Q6AYS4
4425	1,2,6,18,19,20,22,25	3,4,5,7,8,21,23,24,26	1	P97840
4426	1,2,6,18,19,20,22,26	3,4,5,7,8,21,23,24,25	3	Q63751,Q6AYS4,Q99041

4427	1,2,6,18,19,20,23,24	3,4,5,7,8,21,22,25,26	3	P15399,Q6AYS4,Q6IFU7
4428	1,2,6,18,19,20,23,25	3,4,5,7,8,21,22,24,26	2	P15399,Q8CJD3
4429	1,2,6,18,19,20,23,26	3,4,5,7,8,21,22,24,25	6	P15399,Q63942,Q6AYS4,Q6IFU7,Q6IFU8,Q6P6Q2
4430	1,2,6,18,19,20,24,25	3,4,5,7,8,21,22,23,26	1	Q6AYS4
4431	1,2,6,18,19,20,24,26	3,4,5,7,8,21,22,23,25	2	Q6AYS4,Q99041
4432	1,2,6,18,19,20,25,26	3,4,5,7,8,21,22,23,24	2	P35280,Q8K4G9
4433	1,2,6,18,19,21,22,23	3,4,5,7,8,20,24,25,26	3	O35547,P15399,Q8CJD3
4434	1,2,6,18,19,21,22,24	3,4,5,7,8,20,23,25,26	2	P00714,P13432
4435	1,2,6,18,19,21,22,25	3,4,5,7,8,20,23,24,26	4	P47967,P97840,P98089,Q4FZU6
4436	1,2,6,18,19,21,22,26	3,4,5,7,8,20,23,24,25	1	Q63751
4437	1,2,6,18,19,21,23,24	3,4,5,7,8,20,22,25,26	3	D3ZUC6,P05539,P15399
4438	1,2,6,18,19,21,23,25	3,4,5,7,8,20,22,24,26	6	P15399,P97840,P98089,Q62635,Q6P6R2,Q99MH3
4439	1,2,6,18,19,21,23,26	3,4,5,7,8,20,22,24,25	10	D3ZUC6,P15399,Q03191,Q63661,Q63942,Q6IFU7,Q6IFU8,Q6IFV1,Q6P6Q2,Q9QUL6
4440	1,2,6,18,19,21,24,25	3,4,5,7,8,20,22,23,26	5	O54728,P05539,P97840,P98089,Q4FZU6
4441	1,2,6,18,19,21,24,26	3,4,5,7,8,20,22,23,25	2	D3ZUC6,Q6AYS4
4442	1,2,6,18,19,21,25,26	3,4,5,7,8,20,22,23,24	4	P31430,P97840,P98089,Q62635
4443	1,2,6,18,19,22,23,24	3,4,5,7,8,20,21,25,26	3	P15399,Q6AYS4,Q6IFU7
4444	1,2,6,18,19,22,23,25	3,4,5,7,8,20,21,24,26	5	P15399,P97840,Q4FZU6,Q8CJD3,Q99MH3
4445	1,2,6,18,19,22,23,26	3,4,5,7,8,20,21,24,25	3	Q63942,Q6AYS4,Q6IFU7
4446	1,2,6,18,19,22,24,25	3,4,5,7,8,20,21,23,26	2	P97840,Q4FZU6
4447	1,2,6,18,19,22,24,26	3,4,5,7,8,20,21,23,25	3	Q5M819,Q6AYS4,Q99041
4448	1,2,6,18,19,22,25,26	3,4,5,7,8,20,21,23,24	1	P97840
4449	1,2,6,18,19,23,24,25	3,4,5,7,8,20,21,22,26	3	P15399,Q4FZU6,Q99MH3

4450	1,2,6,18,19,23,24,26	3,4,5,7,8,20,21,22,25	5	P15399,Q03191,Q63942,Q6AYS4,Q6IFU7
4451	1,2,6,18,19,23,25,26	3,4,5,7,8,20,21,22,24	5	P15399,Q63942,Q6IFU8,Q6P6Q2,Q99MH3
4452	1,2,6,18,19,24,25,26	3,4,5,7,8,20,21,22,23	1	Q7TPB1
4453	1,2,6,18,20,21,22,23	3,4,5,7,8,19,24,25,26	2	Q8CJD3,Q9QUL6
4454	1,2,6,18,20,21,22,24	3,4,5,7,8,19,23,25,26	3	P00714,P13432,Q99041
4455	1,2,6,18,20,21,22,25	3,4,5,7,8,19,23,24,26	3	P97840,P98089,Q91ZS3
4456	1,2,6,18,20,21,22,26	3,4,5,7,8,19,23,24,25	3	Q63751,Q99041,Q9QUL6
4457	1,2,6,18,20,21,23,24	3,4,5,7,8,19,22,25,26	3	P13432,Q6IFU7,Q9QUL6
4458	1,2,6,18,20,21,23,25	3,4,5,7,8,19,22,24,26	4	O54728,P98089,Q6P6R2,Q91ZS3
4459	1,2,6,18,20,21,23,26	3,4,5,7,8,19,22,24,25	6	Q6IFU7,Q6IFU8,Q6IFV1,Q6IMF3,Q6P6Q2,Q9QUL6
4460	1,2,6,18,20,21,24,25	3,4,5,7,8,19,22,23,26	3	O54728,P98089,Q91ZS3
4461	1,2,6,18,20,21,24,26	3,4,5,7,8,19,22,23,25	4	P13432,Q6AYS4,Q99041,Q9QUL6
4462	1,2,6,18,20,21,25,26	3,4,5,7,8,19,22,23,24	3	P31430,P98089,Q91ZS3
4463	1,2,6,18,20,22,23,24	3,4,5,7,8,19,21,25,26	2	Q6AYS4,Q6IFU7
4464	1,2,6,18,20,22,23,25	3,4,5,7,8,19,21,24,26	1	Q8CJD3
4465	1,2,6,18,20,22,23,26	3,4,5,7,8,19,21,24,25	5	Q6AYS4,Q6IFU7,Q6IFU8,Q6P6Q2,Q9QUL6
4466	1,2,6,18,20,22,24,25	3,4,5,7,8,19,21,23,26	0	
4467	1,2,6,18,20,22,24,26	3,4,5,7,8,19,21,23,25	2	Q6AYS4,Q99041
4468	1,2,6,18,20,22,25,26	3,4,5,7,8,19,21,23,24	1	Q99041
4469	1,2,6,18,20,23,24,25	3,4,5,7,8,19,21,22,26	0	
4470	1,2,6,18,20,23,24,26	3,4,5,7,8,19,21,22,25	7	Q63942,Q6AYS4,Q6IFU7,Q6IFU8,Q6IFV1,Q6P6Q2,Q9QUL6
4471	1,2,6,18,20,23,25,26	3,4,5,7,8,19,21,22,24	3	Q6IFU7,Q6IFU8,Q6P6Q2
4472	1,2,6,18,20,24,25,26	3,4,5,7,8,19,21,22,23	2	Q7TPB1,Q99041

4473	1,2,6,18,2<br>1,22,23,24	3,4,5,7,8,1<br>9,20,25,26	2	P13432,Q6IFU7
4474	1,2,6,18,2<br>1,22,23,25	3,4,5,7,8,1<br>9,20,24,26	3	P97840,P98089,Q6P6R2
4475	1,2,6,18,2<br>1,22,23,26	3,4,5,7,8,1<br>9,20,24,25	5	Q6IFU7,Q6IFU8,Q6IFV1,Q6P6Q2,Q9QUL6
4476	1,2,6,18,2<br>1,22,24,25	3,4,5,7,8,1<br>9,20,23,26	5	O54728,P13432,P97840,P98089,Q4FZU6
4477	1,2,6,18,2<br>1,22,24,26	3,4,5,7,8,1<br>9,20,23,25	3	P13432,Q99041,Q9QUL6
4478	1,2,6,18,2<br>1,22,25,26	3,4,5,7,8,1<br>9,20,23,24	3	P31430,P97840,P98089
4479	1,2,6,18,2<br>1,23,24,25	3,4,5,7,8,1<br>9,20,22,26	3	O54728,P98089,Q6P6R2
4480	1,2,6,18,2<br>1,23,24,26	3,4,5,7,8,1<br>9,20,22,25	7	D3ZUC6,Q63942,Q6IFU7,Q6IFU8,Q6IFV1,Q6P6Q2,Q9QUL6
4481	1,2,6,18,2<br>1,23,25,26	3,4,5,7,8,1<br>9,20,22,24	9	P00774,P31430,P55091,P98089,Q6IFU8,Q6IFV1,Q6P6Q2,Q6P6R2,Q91ZS3
4482	1,2,6,18,2<br>1,24,25,26	3,4,5,7,8,1<br>9,20,22,23	3	P31430,P55091,P98089
4483	1,2,6,18,2<br>2,23,24,25	3,4,5,7,8,1<br>9,20,21,26	2	Q4FZU6,Q6IFU7
4484	1,2,6,18,2<br>2,23,24,26	3,4,5,7,8,1<br>9,20,21,25	5	P00731,Q63942,Q6IFU7,Q6IFV1,Q9QUL6
4485	1,2,6,18,2<br>2,23,25,26	3,4,5,7,8,1<br>9,20,21,24	2	Q6IFU7,Q6P6Q2
4486	1,2,6,18,2<br>2,24,25,26	3,4,5,7,8,1<br>9,20,21,23	3	Q4FZU6,Q7TPB1,Q99041
4487	1,2,6,18,2<br>3,24,25,26	3,4,5,7,8,1<br>9,20,21,22	6	P55091,Q63942,Q6IFU7,Q6IFU8,Q6P6Q2,Q7TPB1
4488	1,2,6,19,2<br>0,21,22,23	3,4,5,7,8,1<br>8,24,25,26	4	P00714,P15399,Q6AYS4,Q8CJD3
4489	1,2,6,19,2<br>0,21,22,24	3,4,5,7,8,1<br>8,23,25,26	6	P00714,P00774,P12785,P13432,P35467,Q6AYS4
4490	1,2,6,19,2<br>0,21,22,25	3,4,5,7,8,1<br>8,23,24,26	5	P12785,P35467,P47967,P97840,Q8VD89
4491	1,2,6,19,2<br>0,21,22,26	3,4,5,7,8,1<br>8,23,24,25	3	P00714,Q63751,Q6AYS4
4492	1,2,6,19,2<br>0,21,23,24	3,4,5,7,8,1<br>8,22,25,26	3	P00714,P15399,Q6AYS4
4493	1,2,6,19,2<br>0,21,23,25	3,4,5,7,8,1<br>8,22,24,26	4	P15399,P97840,Q6P6R2,Q8VD89
4494	1,2,6,19,2<br>0,21,23,26	3,4,5,7,8,1<br>8,22,24,25	3	P15399,Q6AYS4,Q6IFV1
4495	1,2,6,19,2<br>0,21,24,25	3,4,5,7,8,1<br>8,22,23,26	5	P35467,P97840,Q64194,Q6AYS4,Q8VD89

4496	1,2,6,19,20,21,24,26	3,4,5,7,8,18,22,23,25	2	P00714,Q6AYS4
4497	1,2,6,19,20,21,25,26	3,4,5,7,8,18,22,23,24	3	P97840,Q8K4G9,Q91ZS3
4498	1,2,6,19,20,22,23,24	3,4,5,7,8,18,21,25,26	6	P00714,P00774,P15399,P31430,P82995,Q6AYS4
4499	1,2,6,19,20,22,23,25	3,4,5,7,8,18,21,24,26	4	P06911,P15399,Q6AYS4,Q8CJD3
4500	1,2,6,19,20,22,23,26	3,4,5,7,8,18,21,24,25	1	Q6AYS4
4501	1,2,6,19,20,22,24,25	3,4,5,7,8,18,21,23,26	5	P12785,P35467,Q4FZU6,Q6AYS4,Q9ESW0
4502	1,2,6,19,20,22,24,26	3,4,5,7,8,18,21,23,25	4	P00714,P20646,Q6AYS4,Q99041
4503	1,2,6,19,20,22,25,26	3,4,5,7,8,18,21,23,24	1	Q6AYS4
4504	1,2,6,19,20,23,24,25	3,4,5,7,8,18,21,22,26	2	P15399,Q6AYS4
4505	1,2,6,19,20,23,24,26	3,4,5,7,8,18,21,22,25	2	P15399,Q6AYS4
4506	1,2,6,19,20,23,25,26	3,4,5,7,8,18,21,22,24	2	P15399,Q6AYS4
4507	1,2,6,19,20,24,25,26	3,4,5,7,8,18,21,22,23	1	Q6AYS4
4508	1,2,6,19,21,22,23,24	3,4,5,7,8,18,20,25,26	3	P00714,P15399,Q6AYS4
4509	1,2,6,19,21,22,23,25	3,4,5,7,8,18,20,24,26	4	P06911,P15399,P97840,Q4FZU6
4510	1,2,6,19,21,22,23,26	3,4,5,7,8,18,20,24,25	1	P15399
4511	1,2,6,19,21,22,24,25	3,4,5,7,8,18,20,23,26	5	P12785,P35467,P97840,Q4FZU6,Q6IG02
4512	1,2,6,19,21,22,24,26	3,4,5,7,8,18,20,23,25	4	O70377,P00714,P07171,Q6AYS4
4513	1,2,6,19,21,22,25,26	3,4,5,7,8,18,20,23,24	1	P97840
4514	1,2,6,19,21,23,24,25	3,4,5,7,8,18,20,22,26	4	P15399,P97840,Q4FZU6,Q6P6R2
4515	1,2,6,19,21,23,24,26	3,4,5,7,8,18,20,22,25	3	P15399,Q6AYS4,Q6IFV1
4516	1,2,6,19,21,23,25,26	3,4,5,7,8,18,20,22,24	3	P15399,P97840,Q6P6R2
4517	1,2,6,19,21,24,25,26	3,4,5,7,8,18,20,22,23	2	P14480,P97840
4518	1,2,6,19,22,23,24,25	3,4,5,7,8,18,20,21,26	6	P06911,P15399,P82471,Q4FZU6,Q6AYS4,Q9ESW0

4519	1,2,6,19,22,23,24,26	3,4,5,7,8,18,20,21,25	5	P00731,P20646,P82471,Q6AYS4,Q6IFU7
4520	1,2,6,19,22,23,25,26	3,4,5,7,8,18,20,21,24	3	P06911,P15399,Q4FZU6
4521	1,2,6,19,22,24,25,26	3,4,5,7,8,18,20,21,23	4	P14480,Q4FZU6,Q6AYS4,Q9ESW0
4522	1,2,6,19,23,24,25,26	3,4,5,7,8,18,20,21,22	5	P14480,P15399,Q4FZU6,Q6AYS4,Q7TPB1
4523	1,2,6,20,21,22,23,24	3,4,5,7,8,18,19,25,26	3	P00714,P13432,Q6AYS4
4524	1,2,6,20,21,22,23,25	3,4,5,7,8,18,19,24,26	2	P70709,Q6P6R2
4525	1,2,6,20,21,22,23,26	3,4,5,7,8,18,19,24,25	1	Q6IFV1
4526	1,2,6,20,21,22,24,25	3,4,5,7,8,18,19,23,26	6	P12785,P13432,P35467,P55016,Q5XFX0,Q64194
4527	1,2,6,20,21,22,24,26	3,4,5,7,8,18,19,23,25	7	O70377,P00714,P13432,P35467,P55016,Q6AYS4,Q99041
4528	1,2,6,20,21,22,25,26	3,4,5,7,8,18,19,23,24	1	Q91ZS3
4529	1,2,6,20,21,23,24,25	3,4,5,7,8,18,19,22,26	2	P70709,Q6P6R2
4530	1,2,6,20,21,23,24,26	3,4,5,7,8,18,19,22,25	2	Q6AYS4,Q6IFV1
4531	1,2,6,20,21,23,25,26	3,4,5,7,8,18,19,22,24	2	Q6P6R2,Q91ZS3
4532	1,2,6,20,21,24,25,26	3,4,5,7,8,18,19,22,23	2	P55016,Q91ZS3
4533	1,2,6,20,22,23,24,25	3,4,5,7,8,18,19,21,26	1	Q6AYS4
4534	1,2,6,20,22,23,24,26	3,4,5,7,8,18,19,21,25	3	P00731,Q6AYS4,Q6IFU7
4535	1,2,6,20,22,23,25,26	3,4,5,7,8,18,19,21,24	0	
4536	1,2,6,20,22,24,25,26	3,4,5,7,8,18,19,21,23	3	P00731,Q6AYS4,Q99041
4537	1,2,6,20,23,24,25,26	3,4,5,7,8,18,19,21,22	2	Q6AYS4,Q7TPB1
4538	1,2,6,21,22,23,24,25	3,4,5,7,8,18,19,20,26	3	P70709,Q4FZU6,Q6P6R2
4539	1,2,6,21,22,23,24,26	3,4,5,7,8,18,19,20,25	4	P00731,P13432,Q6IFU7,Q6IFV1
4540	1,2,6,21,22,23,25,26	3,4,5,7,8,18,19,20,24	1	Q6P6R2
4541	1,2,6,21,22,24,25,26	3,4,5,7,8,18,19,20,23	1	P35467

4542	1,2,6,21,23,24,25,26	3,4,5,7,8,18,19,20,22	3	Q6IFV1,Q6P6R2,Q7TPB1
4543	1,2,6,22,23,24,25,26	3,4,5,7,8,18,19,20,21	5	P00731,P82471,Q4FZU6,Q6IFU7,Q7TPB1
4544	1,2,7,8,18,19,20,21	3,4,5,6,22,23,24,25,26	7	P00731,P02803,P35281,P62804,Q63751,Q6AYL6,Q6AYR8
4545	1,2,7,8,18,19,20,22	3,4,5,6,21,23,24,25,26	2	P01835,Q63751
4546	1,2,7,8,18,19,20,23	3,4,5,6,21,22,24,25,26	2	Q03191,Q8CJD3
4547	1,2,7,8,18,19,20,24	3,4,5,6,21,22,23,25,26	2	P01835,P83121
4548	1,2,7,8,18,19,20,25	3,4,5,6,21,22,23,24,26	5	P01835,P02803,P10715,P35280,P47967
4549	1,2,7,8,18,19,20,26	3,4,5,6,21,22,23,24,25	5	P01835,P06760,P35280,P82995,Q63751
4550	1,2,7,8,18,19,21,22	3,4,5,6,20,23,24,25,26	3	D4A1J4,P01835,P97840
4551	1,2,7,8,18,19,21,23	3,4,5,6,20,22,24,25,26	7	D3ZUC6,P02803,P05964,P14668,Q03191,Q8CJD3,Q99MH3
4552	1,2,7,8,18,19,21,24	3,4,5,6,20,22,23,25,26	3	D3ZUC6,P01835,P05539
4553	1,2,7,8,18,19,21,25	3,4,5,6,20,22,23,24,26	8	P00731,P02803,P10715,P19629,P31430,P47967,P97840,P98089
4554	1,2,7,8,18,19,21,26	3,4,5,6,20,22,23,24,25	4	D3ZUC6,P02803,P82995,Q63751
4555	1,2,7,8,18,19,22,23	3,4,5,6,20,21,24,25,26	4	P01835,Q03191,Q8CJD3,Q99MH3
4556	1,2,7,8,18,19,22,24	3,4,5,6,20,21,23,25,26	3	P01835,P83121,Q4FZU2
4557	1,2,7,8,18,19,22,25	3,4,5,6,20,21,23,24,26	6	P01835,P10715,P19629,P47967,P97840,Q4FZU2

4558	1,2,7,8,18,19,22,26	3,4,5,6,20,21,23,24,25	4	P01835,P06760,P82995,Q63751
4559	1,2,7,8,18,19,23,24	3,4,5,6,20,21,22,25,26	4	P01835,Q03191,Q63532,Q99MH3
4560	1,2,7,8,18,19,23,25	3,4,5,6,20,21,22,24,26	7	P01835,P02803,P10715,P19629,P31044,Q03191,Q99MH3
4561	1,2,7,8,18,19,23,26	3,4,5,6,20,21,22,24,25	6	P01835,P02803,P82995,Q03191,Q63942,Q99MH3
4562	1,2,7,8,18,19,24,25	3,4,5,6,20,21,22,23,26	4	P01835,P10715,P19629,Q4FZU2
4563	1,2,7,8,18,19,24,26	3,4,5,6,20,21,22,23,25	3	P01835,P82995,Q63942
4564	1,2,7,8,18,19,25,26	3,4,5,6,20,21,22,23,24	7	P01835,P02803,P10715,P19629,P35280,P55091,P82995
4565	1,2,7,8,18,20,21,22	3,4,5,6,19,23,24,25,26	0	
4566	1,2,7,8,18,20,21,23	3,4,5,6,19,22,24,25,26	3	P14668,Q6AYL6,Q6AYR8
4567	1,2,7,8,18,20,21,24	3,4,5,6,19,22,23,25,26	2	Q6AYL6,Q6AYR8
4568	1,2,7,8,18,20,21,25	3,4,5,6,19,22,23,24,26	6	P10715,P19629,P31430,P98089,Q6AYL6,Q91ZS3
4569	1,2,7,8,18,20,21,26	3,4,5,6,19,22,23,24,25	5	P01041,P62804,P82995,Q00715,Q6AYR8
4570	1,2,7,8,18,20,22,23	3,4,5,6,19,21,24,25,26	1	Q8CJD3
4571	1,2,7,8,18,20,22,24	3,4,5,6,19,21,23,25,26	3	P01835,P10715,P83121
4572	1,2,7,8,18,20,22,25	3,4,5,6,19,21,23,24,26	3	P01835,P10715,P19629

4573	1,2,7,8,18,20,22,26	3,4,5,6,19,21,23,24,25	2	P01835,Q63751
4574	1,2,7,8,18,20,23,24	3,4,5,6,19,21,22,25,26	1	P10715
4575	1,2,7,8,18,20,23,25	3,4,5,6,19,21,22,24,26	3	P10715,P19629,Q99MH3
4576	1,2,7,8,18,20,23,26	3,4,5,6,19,21,22,24,25	0	
4577	1,2,7,8,18,20,24,25	3,4,5,6,19,21,22,23,26	3	P01835,P10715,P19629
4578	1,2,7,8,18,20,24,26	3,4,5,6,19,21,22,23,25	2	P01835,P10715
4579	1,2,7,8,18,20,25,26	3,4,5,6,19,21,22,23,24	5	P10715,P19629,P35280,P55091,P82995
4580	1,2,7,8,18,21,22,23	3,4,5,6,19,20,24,25,26	1	P14668
4581	1,2,7,8,18,21,22,24	3,4,5,6,19,20,23,25,26	1	P01835
4582	1,2,7,8,18,21,22,25	3,4,5,6,19,20,23,24,26	5	P10715,P19629,P31430,P97840,P98089
4583	1,2,7,8,18,21,22,26	3,4,5,6,19,20,23,24,25	2	P82995,Q80WL1
4584	1,2,7,8,18,21,23,24	3,4,5,6,19,20,22,25,26	2	D3ZUC6,P05539
4585	1,2,7,8,18,21,23,25	3,4,5,6,19,20,22,24,26	6	P02803,P10715,P19629,P31430,P98089,Q99MH3
4586	1,2,7,8,18,21,23,26	3,4,5,6,19,20,22,24,25	4	D3ZUC6,P55091,P82995,Q6IFV1
4587	1,2,7,8,18,21,24,25	3,4,5,6,19,20,22,23,26	7	O54728,P05539,P10715,P19629,P31430,P55091,P98089

4588	1,2,7,8,18,21,24,26	3,4,5,6,19,20,22,23,25	3	D3ZUC6,P55091,P82995
4589	1,2,7,8,18,21,25,26	3,4,5,6,19,20,22,23,24	7	P08721,P10715,P19629,P31430,P55091,P82995,P98089
4590	1,2,7,8,18,22,23,24	3,4,5,6,19,20,21,25,26	1	P01835
4591	1,2,7,8,18,22,23,25	3,4,5,6,19,20,21,24,26	5	P01835,P10715,P19629,Q5GRG2,Q99MH3
4592	1,2,7,8,18,22,23,26	3,4,5,6,19,20,21,24,25	1	P01835
4593	1,2,7,8,18,22,24,25	3,4,5,6,19,20,21,23,26	4	P01835,P10715,P19629,Q4FZU2
4594	1,2,7,8,18,22,24,26	3,4,5,6,19,20,21,23,25	1	P01835
4595	1,2,7,8,18,22,25,26	3,4,5,6,19,20,21,23,24	5	P01835,P10715,P19629,P55091,P82995
4596	1,2,7,8,18,23,24,25	3,4,5,6,19,20,21,22,26	4	P01835,P10715,P19629,Q99MH3
4597	1,2,7,8,18,23,24,26	3,4,5,6,19,20,21,22,25	4	P01835,P01946,P55091,Q63942
4598	1,2,7,8,18,23,25,26	3,4,5,6,19,20,21,22,24	8	P01835,P01946,P10715,P19629,P24594,P55091,P82995,Q99MH3
4599	1,2,7,8,18,24,25,26	3,4,5,6,19,20,21,22,23	6	P01835,P10715,P19629,P55091,P82995,Q7TPB1
4600	1,2,7,8,19,20,21,22	3,4,5,6,18,23,24,25,26	5	P00774,P12785,Q4FZU2,Q63751,Q6IFW6
4601	1,2,7,8,19,20,21,23	3,4,5,6,18,22,24,25,26	0	
4602	1,2,7,8,19,20,21,24	3,4,5,6,18,22,23,25,26	3	P12785,Q4FZU2,Q6IFW6

4603	1,2,7,8,19,20,21,25	3,4,5,6,18,22,23,24,26	7	P10715,P12785,P19629,P47967,P97840,Q4FZU2,Q6IFW6
4604	1,2,7,8,19,20,21,26	3,4,5,6,18,22,23,24,25	3	P62804,Q00715,Q63751
4605	1,2,7,8,19,20,22,23	3,4,5,6,18,21,24,25,26	4	P00774,P01835,Q08849,Q8CJD3
4606	1,2,7,8,19,20,22,24	3,4,5,6,18,21,23,25,26	10	P00774,P01835,P12785,P20646,P83121,Q4FZU2,Q6IFU8,Q6IFW6,Q6IMF3,Q6P6Q2
4607	1,2,7,8,19,20,22,25	3,4,5,6,18,21,23,24,26	10	P00774,P01835,P10715,P12785,P19629,Q4FZU2,Q5GRG2,Q6IFW6,Q6IMF3,Q6P6Q2
4608	1,2,7,8,19,20,22,26	3,4,5,6,18,21,23,24,25	3	P01835,P12785,Q63751
4609	1,2,7,8,19,20,23,24	3,4,5,6,18,21,22,25,26	2	P00774,P01835
4610	1,2,7,8,19,20,23,25	3,4,5,6,18,21,22,24,26	4	P01835,P10715,P19629,Q08849
4611	1,2,7,8,19,20,23,26	3,4,5,6,18,21,22,24,25	2	P01835,Q08849
4612	1,2,7,8,19,20,24,25	3,4,5,6,18,21,22,23,26	8	P01835,P10715,P12785,P19629,Q4FZU2,Q6IFW6,Q6IMF3,Q6P6Q2
4613	1,2,7,8,19,20,24,26	3,4,5,6,18,21,22,23,25	1	P01835
4614	1,2,7,8,19,20,25,26	3,4,5,6,18,21,22,23,24	3	P01835,P10715,P35280
4615	1,2,7,8,19,21,22,23	3,4,5,6,18,20,24,25,26	1	P01835
4616	1,2,7,8,19,21,22,24	3,4,5,6,18,20,23,25,26	8	P01835,P12785,Q4FZU2,Q6IFU8,Q6IFW6,Q6IG02,Q6IMF3,Q6P6Q2
4617	1,2,7,8,19,21,22,25	3,4,5,6,18,20,23,24,26	11	P01835,P12785,P19629,P47967,P97840,Q4FZU2,Q5GRG2,Q6IFW6,Q6IG02,Q6IMF3,Q6P6Q2

4618	1,2,7,8,19,21,22,26	3,4,5,6,18,20,23,24,25	3	P01835,P12785,Q63751
4619	1,2,7,8,19,21,23,24	3,4,5,6,18,20,22,25,26	2	D3ZUC6,P01835
4620	1,2,7,8,19,21,23,25	3,4,5,6,18,20,22,24,26	4	P02803,P19629,P47967,P97840
4621	1,2,7,8,19,21,23,26	3,4,5,6,18,20,22,24,25	1	D3ZUC6
4622	1,2,7,8,19,21,24,25	3,4,5,6,18,20,22,23,26	11	P01835,P10715,P12785,P19629,P22006,P47967,P97840,Q4FZU2,Q6IFW6,Q6IG02,Q6IMF3
4623	1,2,7,8,19,21,24,26	3,4,5,6,18,20,22,23,25	1	P01835
4624	1,2,7,8,19,21,25,26	3,4,5,6,18,20,22,23,24	3	P19629,P82995,P97840
4625	1,2,7,8,19,22,23,24	3,4,5,6,18,20,21,25,26	5	P00774,P01835,P20646,Q4FZU2,Q63532
4626	1,2,7,8,19,22,23,25	3,4,5,6,18,20,21,24,26	5	P01835,P06911,P19629,Q4FZU2,Q5GRG2
4627	1,2,7,8,19,22,23,26	3,4,5,6,18,20,21,24,25	1	P01835
4628	1,2,7,8,19,22,24,25	3,4,5,6,18,20,21,23,26	11	P01835,P10715,P12785,P19629,Q4FZU2,Q5GRG2,Q6IFU8,Q6IFW6,Q6IG02,Q6IMF3,Q6P6Q2
4629	1,2,7,8,19,22,24,26	3,4,5,6,18,20,21,23,25	3	P01835,P12785,P20646
4630	1,2,7,8,19,22,25,26	3,4,5,6,18,20,21,23,24	4	P01835,P12785,P19629,Q5GRG2
4631	1,2,7,8,19,23,24,25	3,4,5,6,18,20,21,22,26	4	P01835,P10715,P19629,Q4FZU2
4632	1,2,7,8,19,23,24,26	3,4,5,6,18,20,21,22,25	2	P01835,P01946

4633	1,2,7,8,19,23,25,26	3,4,5,6,18,20,21,22,24	3	P01835,P01946,P19629
4634	1,2,7,8,19,24,25,26	3,4,5,6,18,20,21,22,23	4	P01835,P10715,P19629,Q7TPB1
4635	1,2,7,8,20,21,22,23	3,4,5,6,18,19,24,25,26	0	
4636	1,2,7,8,20,21,22,24	3,4,5,6,18,19,23,25,26	6	P00714,P12785,P35467,Q4FZU2,Q5XFX0,Q6IFW6
4637	1,2,7,8,20,21,22,25	3,4,5,6,18,19,23,24,26	6	P10715,P12785,P19629,P35467,Q4FZU2,Q6IFW6
4638	1,2,7,8,20,21,22,26	3,4,5,6,18,19,23,24,25	1	P12785
4639	1,2,7,8,20,21,23,24	3,4,5,6,18,19,22,25,26	0	
4640	1,2,7,8,20,21,23,25	3,4,5,6,18,19,22,24,26	2	P10715,P19629
4641	1,2,7,8,20,21,23,26	3,4,5,6,18,19,22,24,25	0	
4642	1,2,7,8,20,21,24,25	3,4,5,6,18,19,22,23,26	8	P02625,P10715,P12785,P19629,P35467,Q4FZU2,Q5XFX0,Q6IFW6
4643	1,2,7,8,20,21,24,26	3,4,5,6,18,19,22,23,25	1	P02625
4644	1,2,7,8,20,21,25,26	3,4,5,6,18,19,22,23,24	5	P02625,P10715,P19629,P82995,Q91ZS3
4645	1,2,7,8,20,22,23,24	3,4,5,6,18,19,21,25,26	2	P00774,P01835
4646	1,2,7,8,20,22,23,25	3,4,5,6,18,19,21,24,26	6	P01835,P06911,P10715,P19629,Q08849,Q5GRG2
4647	1,2,7,8,20,22,23,26	3,4,5,6,18,19,21,24,25	2	P01835,Q08849

4648	1,2,7,8,20,<br>22,24,25	3,4,5,6,18,<br>19,21,23,2<br>6	9	P01835,P10715,P12785,P19629,P35467,Q4FZU2,Q5GRG2,Q6IFW6,Q6IMF3
4649	1,2,7,8,20,<br>22,24,26	3,4,5,6,18,<br>19,21,23,2<br>5	2	P01835,P12785
4650	1,2,7,8,20,<br>22,25,26	3,4,5,6,18,<br>19,21,23,2<br>4	4	P01835,P10715,P12785,P19629
4651	1,2,7,8,20,<br>23,24,25	3,4,5,6,18,<br>19,21,22,2<br>6	3	P01835,P10715,P19629
4652	1,2,7,8,20,<br>23,24,26	3,4,5,6,18,<br>19,21,22,2<br>5	2	P01835,P01946
4653	1,2,7,8,20,<br>23,25,26	3,4,5,6,18,<br>19,21,22,2<br>4	4	P01946,P10715,P19629,Q08849
4654	1,2,7,8,20,<br>24,25,26	3,4,5,6,18,<br>19,21,22,2<br>3	4	P01835,P02625,P10715,P19629
4655	1,2,7,8,21,<br>22,23,24	3,4,5,6,18,<br>19,20,25,2<br>6	1	P01835
4656	1,2,7,8,21,<br>22,23,25	3,4,5,6,18,<br>19,20,24,2<br>6	3	P06911,P19629,Q5GRG2
4657	1,2,7,8,21,<br>22,23,26	3,4,5,6,18,<br>19,20,24,2<br>5	0	
4658	1,2,7,8,21,<br>22,24,25	3,4,5,6,18,<br>19,20,23,2<br>6	10	P01835,P10715,P12785,P19629,P22006,P35467,Q4FZU2,Q6IFW6,Q6IG02,Q6IMF3
4659	1,2,7,8,21,<br>22,24,26	3,4,5,6,18,<br>19,20,23,2<br>5	2	P01835,P12785
4660	1,2,7,8,21,<br>22,25,26	3,4,5,6,18,<br>19,20,23,2<br>4	3	P12785,P19629,P82995
4661	1,2,7,8,21,<br>23,24,25	3,4,5,6,18,<br>19,20,22,2<br>6	3	P10715,P19629,P22006
4662	1,2,7,8,21,<br>23,24,26	3,4,5,6,18,<br>19,20,22,2<br>5	1	P01946

4663	1,2,7,8,21,23,25,26	3,4,5,6,18,19,20,22,24	4	P01946,P19629,P22006,P82995
4664	1,2,7,8,21,24,25,26	3,4,5,6,18,19,20,22,23	6	P02625,P10715,P19629,P22006,P55091,P82995
4665	1,2,7,8,22,23,24,25	3,4,5,6,18,19,20,21,26	6	P01835,P06911,P10715,P19629,Q4FZU2,Q5GRG2
4666	1,2,7,8,22,23,24,26	3,4,5,6,18,19,20,21,25	2	P00731,P01835
4667	1,2,7,8,22,23,25,26	3,4,5,6,18,19,20,21,24	6	P01835,P01946,P06911,P10715,P19629,Q5GRG2
4668	1,2,7,8,22,24,25,26	3,4,5,6,18,19,20,21,23	4	P01835,P10715,P12785,P19629
4669	1,2,7,8,23,24,25,26	3,4,5,6,18,19,20,21,22	5	P01835,P01946,P10715,P19629,Q7TPB1
4670	1,2,7,18,19,20,21,22	3,4,5,6,8,23,24,25,26	4	P06760,P97840,Q63751,Q9Z0V6
4671	1,2,7,18,19,20,21,23	3,4,5,6,8,22,24,25,26	4	D3ZUC6,P14668,Q03191,Q9Z0V6
4672	1,2,7,18,19,20,21,24	3,4,5,6,8,22,23,25,26	2	D3ZUC6,Q9Z0V6
4673	1,2,7,18,19,20,21,25	3,4,5,6,8,22,23,24,26	5	P02793;Q7TP54,P20760,P31430,P47967,P97840
4674	1,2,7,18,19,20,21,26	3,4,5,6,8,22,23,24,25	5	D3ZUC6,P06760,P82995,Q63751,Q9Z0V6
4675	1,2,7,18,19,20,22,23	3,4,5,6,8,21,24,25,26	4	P06760,Q8CJD3,Q9WTT6,Q9Z0V6
4676	1,2,7,18,19,20,22,24	3,4,5,6,8,21,23,25,26	8	P01835,P06760,P07150,P14669,P20646,Q6AYS4,Q9WTT6,Q9Z0V6
4677	1,2,7,18,19,20,22,25	3,4,5,6,8,21,23,24,26	5	P01835,P20760,P47967,P97840,Q9Z0V6
4678	1,2,7,18,19,20,22,26	3,4,5,6,8,21,23,24,25	7	P01835,P06760,P07150,P20646,Q63751,Q9WTT6,Q9Z0V6
4679	1,2,7,18,19,20,23,24	3,4,5,6,8,21,22,25,26	5	P06760,Q03191,Q6AYS4,Q9WTT6,Q9Z0V6
4680	1,2,7,18,19,20,23,25	3,4,5,6,8,21,22,24,26	2	P20760,Q99MH3
4681	1,2,7,18,19,20,23,26	3,4,5,6,8,21,22,24,25	3	P06760,Q03191,Q9Z0V6

4682	1,2,7,18,19,20,24,25	3,4,5,6,8,21,22,23,26	2	P01835,Q9Z0V6
4683	1,2,7,18,19,20,24,26	3,4,5,6,8,21,22,23,25	6	P01835,P06760,Q63751,Q6AYS4,Q9WTT6,Q9Z0V6
4684	1,2,7,18,19,20,25,26	3,4,5,6,8,21,22,23,24	2	P35280,P82995
4685	1,2,7,18,19,21,22,23	3,4,5,6,8,20,24,25,26	5	D3ZUC6,P14668,P97840,Q03191,Q8CJD3
4686	1,2,7,18,19,21,22,24	3,4,5,6,8,20,23,25,26	6	D3ZUC6,P01835,P97840,Q4FZU2,Q9WTT6,Q9Z0V6
4687	1,2,7,18,19,21,22,25	3,4,5,6,8,20,23,24,26	3	P20760,P47967,P97840
4688	1,2,7,18,19,21,22,26	3,4,5,6,8,20,23,24,25	7	D3ZUC6,P06760,P36374,P82995,P97840,Q63751,Q9Z0V6
4689	1,2,7,18,19,21,23,24	3,4,5,6,8,20,22,25,26	4	D3ZUC6,P14668,Q03191,Q99MH3
4690	1,2,7,18,19,21,23,25	3,4,5,6,8,20,22,24,26	8	D3ZUC6,P02803,P14668,P20760,P47967,P97840,Q03191,Q99MH3
4691	1,2,7,18,19,21,23,26	3,4,5,6,8,20,22,24,25	6	D3ZUC6,P14668,P82995,Q03191,Q63661,Q99MH3
4692	1,2,7,18,19,21,24,25	3,4,5,6,8,20,22,23,26	4	D3ZUC6,P31430,P47967,P97840
4693	1,2,7,18,19,21,24,26	3,4,5,6,8,20,22,23,25	3	D3ZUC6,P06760,P82995
4694	1,2,7,18,19,21,25,26	3,4,5,6,8,20,22,23,24	6	D3ZUC6,P31430,P47967,P55091,P82995,P97840
4695	1,2,7,18,19,22,23,24	3,4,5,6,8,20,21,25,26	7	P01835,P06760,P20646,Q03191,Q63532,Q9WTT6,Q9Z0V6
4696	1,2,7,18,19,22,23,25	3,4,5,6,8,20,21,24,26	4	P01835,P20760,P97840,Q99MH3
4697	1,2,7,18,19,22,23,26	3,4,5,6,8,20,21,24,25	5	P01835,P06760,P20646,Q03191,Q9WTT6
4698	1,2,7,18,19,22,24,25	3,4,5,6,8,20,21,23,26	3	P01835,P97840,Q4FZU2
4699	1,2,7,18,19,22,24,26	3,4,5,6,8,20,21,23,25	5	P01835,P06760,P20646,Q9WTT6,Q9Z0V6
4700	1,2,7,18,19,22,25,26	3,4,5,6,8,20,21,23,24	3	P01835,P82995,P97840
4701	1,2,7,18,19,23,24,25	3,4,5,6,8,20,21,22,26	4	P01835,Q03191,Q63532,Q99MH3
4702	1,2,7,18,19,23,24,26	3,4,5,6,8,20,21,22,25	6	P01835,P01946,P06760,Q03191,Q63532,Q9WTT6
4703	1,2,7,18,19,23,25,26	3,4,5,6,8,20,21,22,24	6	P01835,P01946,P55091,P82995,Q03191,Q99MH3
4704	1,2,7,18,19,24,25,26	3,4,5,6,8,20,21,22,23	4	P01835,P55091,P82995,Q7TPB1

4705	1,2,7,18,20,21,22,23	3,4,5,6,8,19,24,25,26	1	P14668
4706	1,2,7,18,20,21,22,24	3,4,5,6,8,19,23,25,26	1	Q9Z0V6
4707	1,2,7,18,20,21,22,25	3,4,5,6,8,19,23,24,26	3	P20760,P31430,P97840
4708	1,2,7,18,20,21,22,26	3,4,5,6,8,19,23,24,25	5	P06760,P82995,Q63751,Q99041,Q9Z0V6
4709	1,2,7,18,20,21,23,24	3,4,5,6,8,19,22,25,26	1	D3ZUC6
4710	1,2,7,18,20,21,23,25	3,4,5,6,8,19,22,24,26	4	P14668,P20760,P31430,Q99MH3
4711	1,2,7,18,20,21,23,26	3,4,5,6,8,19,22,24,25	3	D3ZUC6,P14668,Q9QUL6
4712	1,2,7,18,20,21,24,25	3,4,5,6,8,19,22,23,26	2	O54728,P31430
4713	1,2,7,18,20,21,24,26	3,4,5,6,8,19,22,23,25	2	D3ZUC6,P06760
4714	1,2,7,18,20,21,25,26	3,4,5,6,8,19,22,23,24	4	P31430,P55091,P82995,Q91ZS3
4715	1,2,7,18,20,22,23,24	3,4,5,6,8,19,21,25,26	4	P06760,Q9QZK9,Q9WTT6,Q9Z0V6
4716	1,2,7,18,20,22,23,25	3,4,5,6,8,19,21,24,26	0	
4717	1,2,7,18,20,22,23,26	3,4,5,6,8,19,21,24,25	1	P06760
4718	1,2,7,18,20,22,24,25	3,4,5,6,8,19,21,23,26	1	P01835
4719	1,2,7,18,20,22,24,26	3,4,5,6,8,19,21,23,25	5	P01835,P06760,Q99041,Q9WTT6,Q9Z0V6
4720	1,2,7,18,20,22,25,26	3,4,5,6,8,19,21,23,24	1	P82995
4721	1,2,7,18,20,23,24,25	3,4,5,6,8,19,21,22,26	0	
4722	1,2,7,18,20,23,24,26	3,4,5,6,8,19,21,22,25	2	P01946,P06760
4723	1,2,7,18,20,23,25,26	3,4,5,6,8,19,21,22,24	1	P01946
4724	1,2,7,18,20,24,25,26	3,4,5,6,8,19,21,22,23	2	P55091,Q7TPB1
4725	1,2,7,18,21,22,23,24	3,4,5,6,8,19,20,25,26	1	D3ZUC6
4726	1,2,7,18,21,22,23,25	3,4,5,6,8,19,20,24,26	5	P14668,P20760,P31430,P97840,Q99MH3
4727	1,2,7,18,21,22,23,26	3,4,5,6,8,19,20,24,25	4	D3ZUC6,P14668,P82995,Q9QUL6

4728	1,2,7,18,21,22,24,25	3,4,5,6,8,19,20,23,26	2	P31430,P97840
4729	1,2,7,18,21,22,24,26	3,4,5,6,8,19,20,23,25	3	D3ZUC6,P06760,P82995
4730	1,2,7,18,21,22,25,26	3,4,5,6,8,19,20,23,24	4	P31430,P55091,P82995,P97840
4731	1,2,7,18,21,23,24,25	3,4,5,6,8,19,20,22,26	4	D3ZUC6,P22006,P31430,Q99MH3
4732	1,2,7,18,21,23,24,26	3,4,5,6,8,19,20,22,25	5	D3ZUC6,P01946,P55091,P82995,Q9QUL6
4733	1,2,7,18,21,23,25,26	3,4,5,6,8,19,20,22,24	7	D3ZUC6,P01946,P22006,P31430,P55091,P82995,Q99MH3
4734	1,2,7,18,21,24,25,26	3,4,5,6,8,19,20,22,23	7	D3ZUC6,P22006,P31430,P55091,P82995,P98089,Q7TPB1
4735	1,2,7,18,22,23,24,25	3,4,5,6,8,19,20,21,26	1	P01835
4736	1,2,7,18,22,23,24,26	3,4,5,6,8,19,20,21,25	4	P00731,P01835,P06760,P20646
4737	1,2,7,18,22,23,25,26	3,4,5,6,8,19,20,21,24	4	P01835,P01946,P55091,P82995
4738	1,2,7,18,22,24,25,26	3,4,5,6,8,19,20,21,23	4	P01835,P55091,P82995,Q7TPB1
4739	1,2,7,18,23,24,25,26	3,4,5,6,8,19,20,21,22	4	P01835,P01946,P55091,Q7TPB1
4740	1,2,7,19,20,21,22,23	3,4,5,6,8,18,24,25,26	2	P00774,P14668
4741	1,2,7,19,20,21,22,24	3,4,5,6,8,18,23,25,26	11	P00714,P00774,P12785,P35467,Q4FZU2,Q6AYS4,Q6IFW6,Q6IG02,Q6IMF3,Q6P6Q2,Q9Z0V6
4742	1,2,7,19,20,21,22,25	3,4,5,6,8,18,23,24,26	9	P02793;Q7TP54,P12785,P35467,P47967,P97840,Q4FZU2,Q6IFW6,Q6IG02,Q6P6Q2
4743	1,2,7,19,20,21,22,26	3,4,5,6,8,18,23,24,25	2	Q63751,Q9Z0V6
4744	1,2,7,19,20,21,23,24	3,4,5,6,8,18,22,25,26	0	
4745	1,2,7,19,20,21,23,25	3,4,5,6,8,18,22,24,26	3	P14668,P47967,P97840
4746	1,2,7,19,20,21,23,26	3,4,5,6,8,18,22,24,25	0	
4747	1,2,7,19,20,21,24,25	3,4,5,6,8,18,22,23,26	8	P02793;Q7TP54,P35467,P47967,P97840,Q4FZU2,Q6IFW6,Q6IG02,Q6P6Q2
4748	1,2,7,19,20,21,24,26	3,4,5,6,8,18,22,23,25	1	Q6AYS4
4749	1,2,7,19,20,21,25,26	3,4,5,6,8,18,22,23,24	2	P47967,P97840
4750	1,2,7,19,20,22,23,24	3,4,5,6,8,18,21,25,26	7	P00774,P01835,P20646,P31430,Q6AYS4,Q9WTT6,Q9Z0V6

4751	1,2,7,19,20,22,23,25	3,4,5,6,8,18,21,24,26	1	P01835
4752	1,2,7,19,20,22,23,26	3,4,5,6,8,18,21,24,25	6	P01835,P06760,P20646,Q6AYS4,Q9WTT6,Q9Z0V6
4753	1,2,7,19,20,22,24,25	3,4,5,6,8,18,21,23,26	10	P00774,P01835,P12785,P35467,Q4FZU2,Q6IFU8,Q6IFW6,Q6IG02,Q6IMF3,Q6P6Q2
4754	1,2,7,19,20,22,24,26	3,4,5,6,8,18,21,23,25	9	P01835,P06760,P07150,P14669,P20646,Q63751,Q6AYS4,Q9WTT6,Q9Z0V6
4755	1,2,7,19,20,22,25,26	3,4,5,6,8,18,21,23,24	1	P01835
4756	1,2,7,19,20,23,24,25	3,4,5,6,8,18,21,22,26	1	P01835
4757	1,2,7,19,20,23,24,26	3,4,5,6,8,18,21,22,25	5	P01835,P01946,P06760,P20646,Q6AYS4
4758	1,2,7,19,20,23,25,26	3,4,5,6,8,18,21,22,24	1	P01946
4759	1,2,7,19,20,24,25,26	3,4,5,6,8,18,21,22,23	1	P01835
4760	1,2,7,19,21,22,23,24	3,4,5,6,8,18,20,25,26	3	D3ZUC6,P01835,Q4FZU2
4761	1,2,7,19,21,22,23,25	3,4,5,6,8,18,20,24,26	3	P14668,P47967,P97840
4762	1,2,7,19,21,22,23,26	3,4,5,6,8,18,20,24,25	1	D3ZUC6
4763	1,2,7,19,21,22,24,25	3,4,5,6,8,18,20,23,26	12	P01835,P12785,P22006,P35467,P47967,P97840,Q4FZU2,Q6IFU8,Q6IFW6,Q6IG02,Q6IMF3,Q6P6Q2
4764	1,2,7,19,21,22,24,26	3,4,5,6,8,18,20,23,25	2	D3ZUC6,P01835
4765	1,2,7,19,21,22,25,26	3,4,5,6,8,18,20,23,24	2	P47967,P97840
4766	1,2,7,19,21,23,24,25	3,4,5,6,8,18,20,22,26	2	P22006,P97840
4767	1,2,7,19,21,23,24,26	3,4,5,6,8,18,20,22,25	2	D3ZUC6,P01946
4768	1,2,7,19,21,23,25,26	3,4,5,6,8,18,20,22,24	3	P01946,P22006,P97840
4769	1,2,7,19,21,24,25,26	3,4,5,6,8,18,20,22,23	2	P22006,P97840
4770	1,2,7,19,22,23,24,25	3,4,5,6,8,18,20,21,26	3	P01835,P82471,Q4FZU2
4771	1,2,7,19,22,23,24,26	3,4,5,6,8,18,20,21,25	8	P00731,P01835,P01946,P06760,P20646,P82471,Q6AYS4,Q9WTT6
4772	1,2,7,19,22,23,25,26	3,4,5,6,8,18,20,21,24	2	P01835,P01946
4773	1,2,7,19,22,24,25,26	3,4,5,6,8,18,20,21,23	2	P01835,Q7TPB1

4774	1,2,7,19,23,24,25,26	3,4,5,6,8,18,20,21,22	4	P01835,P01946,P14480,Q7TPB1
4775	1,2,7,20,21,22,23,24	3,4,5,6,8,18,19,25,26	1	P00714
4776	1,2,7,20,21,22,23,25	3,4,5,6,8,18,19,24,26	0	
4777	1,2,7,20,21,22,23,26	3,4,5,6,8,18,19,24,25	0	
4778	1,2,7,20,21,22,24,25	3,4,5,6,8,18,19,23,26	8	P12785,P35467,Q4FZU2,Q5XFX0,Q64194,Q6IFW6,Q6IG02,Q6P6Q2
4779	1,2,7,20,21,22,24,26	3,4,5,6,8,18,19,23,25	2	P00714,P35467
4780	1,2,7,20,21,22,25,26	3,4,5,6,8,18,19,23,24	1	P35467
4781	1,2,7,20,21,23,24,25	3,4,5,6,8,18,19,22,26	0	
4782	1,2,7,20,21,23,24,26	3,4,5,6,8,18,19,22,25	1	P01946
4783	1,2,7,20,21,23,25,26	3,4,5,6,8,18,19,22,24	1	P01946
4784	1,2,7,20,21,24,25,26	3,4,5,6,8,18,19,22,23	3	P02625,P22006,P35467
4785	1,2,7,20,22,23,24,25	3,4,5,6,8,18,19,21,26	1	P01835
4786	1,2,7,20,22,23,24,26	3,4,5,6,8,18,19,21,25	4	P00731,P01835,P20646,Q6AYS4
4787	1,2,7,20,22,23,25,26	3,4,5,6,8,18,19,21,24	1	P01946
4788	1,2,7,20,22,24,25,26	3,4,5,6,8,18,19,21,23	3	P00731,P01835,P35467
4789	1,2,7,20,23,24,25,26	3,4,5,6,8,18,19,21,22	2	P01946,Q7TPB1
4790	1,2,7,21,22,23,24,25	3,4,5,6,8,18,19,20,26	1	P22006
4791	1,2,7,21,22,23,24,26	3,4,5,6,8,18,19,20,25	3	D3ZUC6,P00731,P01946
4792	1,2,7,21,22,23,25,26	3,4,5,6,8,18,19,20,24	2	P01946,P22006
4793	1,2,7,21,22,24,25,26	3,4,5,6,8,18,19,20,23	2	P22006,P35467
4794	1,2,7,21,23,24,25,26	3,4,5,6,8,18,19,20,22	3	P01946,P22006,Q7TPB1
4795	1,2,7,22,23,24,25,26	3,4,5,6,8,18,19,20,21	5	P00731,P01835,P01946,P82471,Q7TPB1
4796	1,2,8,18,19,20,21,22	3,4,5,6,7,23,24,25,26	3	P20760,Q63751,Q8K4G9

4797	1,2,8,18,19,20,21,23	3,4,5,6,7,22,24,25,26	5	P20760,P35281,Q03191,Q8CJD3,Q8K4G9
4798	1,2,8,18,19,20,21,24	3,4,5,6,7,22,23,25,26	4	P05539,P83121,Q63751,Q8K4G9
4799	1,2,8,18,19,20,21,25	3,4,5,6,7,22,23,24,26	4	P20760,P31430,P47967,Q8K4G9
4800	1,2,8,18,19,20,21,26	3,4,5,6,7,22,23,24,25	4	P62804,Q00715,Q63751,Q8K4G9
4801	1,2,8,18,19,20,22,23	3,4,5,6,7,21,24,25,26	5	P20646,P22006,Q8CJD3,Q8K4G9,Q9QZK9
4802	1,2,8,18,19,20,22,24	3,4,5,6,7,21,23,25,26	6	P01835,P20646,P83121,Q63751,Q8K4G9,Q9QZK9
4803	1,2,8,18,19,20,22,25	3,4,5,6,7,21,23,24,26	4	P01835,P20760,P20761,Q8K4G9
4804	1,2,8,18,19,20,22,26	3,4,5,6,7,21,23,24,25	4	P01835,P20646,Q63751,Q8K4G9
4805	1,2,8,18,19,20,23,24	3,4,5,6,7,21,22,25,26	4	P05539,P09605;P25809,Q03191,Q9QZK9
4806	1,2,8,18,19,20,23,25	3,4,5,6,7,21,22,24,26	5	P20760,P20761,Q8CJD3,Q8K4G9,Q99MH3
4807	1,2,8,18,19,20,23,26	3,4,5,6,7,21,22,24,25	4	P09605;P25809,Q03191,Q63942,Q8K4G9
4808	1,2,8,18,19,20,24,25	3,4,5,6,7,21,22,23,26	4	P01835,P05539,P20760,Q8K4G9
4809	1,2,8,18,19,20,24,26	3,4,5,6,7,21,22,23,25	4	P01835,P09605;P25809,Q63751,Q8K4G9
4810	1,2,8,18,19,20,25,26	3,4,5,6,7,21,22,23,24	3	P35280,Q63751,Q8K4G9
4811	1,2,8,18,19,21,22,23	3,4,5,6,7,20,24,25,26	4	P20760,Q03191,Q8CJD3,Q99MH3
4812	1,2,8,18,19,21,22,24	3,4,5,6,7,20,23,25,26	4	P01835,P05539,P83121,Q4FZU2
4813	1,2,8,18,19,21,22,25	3,4,5,6,7,20,23,24,26	5	P20760,P31430,P47967,P97840,Q8K4G9
4814	1,2,8,18,19,21,22,26	3,4,5,6,7,20,23,24,25	2	Q63751,Q8K4G9
4815	1,2,8,18,19,21,23,24	3,4,5,6,7,20,22,25,26	4	D3ZUC6,P05539,Q03191,Q99MH3
4816	1,2,8,18,19,21,23,25	3,4,5,6,7,20,22,24,26	6	P02803,P05539,P20760,P31430,Q03191,Q99MH3
4817	1,2,8,18,19,21,23,26	3,4,5,6,7,20,22,24,25	5	D3ZUC6,Q03191,Q63942,Q6IFV1,Q99MH3
4818	1,2,8,18,19,21,24,25	3,4,5,6,7,20,22,23,26	4	P05539,P20760,P31430,P98089
4819	1,2,8,18,19,21,24,26	3,4,5,6,7,20,22,23,25	2	D3ZUC6,Q63751

4820	1,2,8,18,19,21,25,26	3,4,5,6,7,20,22,23,24	4	P31430,P55091,P98089,Q8K4G9
4821	1,2,8,18,19,22,23,24	3,4,5,6,7,20,21,25,26	5	P01835,P05539,P20646,P83121,Q03191
4822	1,2,8,18,19,22,23,25	3,4,5,6,7,20,21,24,26	4	P01835,P20760,Q8CJD3,Q99MH3
4823	1,2,8,18,19,22,23,26	3,4,5,6,7,20,21,24,25	4	P01835,P20646,Q03191,Q63942
4824	1,2,8,18,19,22,24,25	3,4,5,6,7,20,21,23,26	2	P01835,Q4FZU2
4825	1,2,8,18,19,22,24,26	3,4,5,6,7,20,21,23,25	3	P01835,P20646,Q63751
4826	1,2,8,18,19,22,25,26	3,4,5,6,7,20,21,23,24	2	P01835,Q8K4G9
4827	1,2,8,18,19,23,24,25	3,4,5,6,7,20,21,22,26	5	P01835,P05539,P20760,Q03191,Q99MH3
4828	1,2,8,18,19,23,24,26	3,4,5,6,7,20,21,22,25	5	P01835,P09605;P25809,P20646,Q03191,Q63942
4829	1,2,8,18,19,23,25,26	3,4,5,6,7,20,21,22,24	4	P01835,Q03191,Q63942,Q99MH3
4830	1,2,8,18,19,24,25,26	3,4,5,6,7,20,21,22,23	3	P01835,P55091,Q7TPB1
4831	1,2,8,18,20,21,22,23	3,4,5,6,7,19,24,25,26	3	P20760,Q5FVI6,Q8CJD3
4832	1,2,8,18,20,21,22,24	3,4,5,6,7,19,23,25,26	4	P05539,P83121,Q5FVI6,Q99041
4833	1,2,8,18,20,21,22,25	3,4,5,6,7,19,23,24,26	4	P20760,P31430,Q5FVI6,Q8K4G9
4834	1,2,8,18,20,21,22,26	3,4,5,6,7,19,23,24,25	4	P62804,Q63751,Q8K4G9,Q99041
4835	1,2,8,18,20,21,23,24	3,4,5,6,7,19,22,25,26	3	P05539,Q5FVI6,Q9QZK9
4836	1,2,8,18,20,21,23,25	3,4,5,6,7,19,22,24,26	3	P20760,P31430,Q99MH3
4837	1,2,8,18,20,21,23,26	3,4,5,6,7,19,22,24,25	4	P62804,Q6IFV1,Q6P6Q2,Q8K4G9
4838	1,2,8,18,20,21,24,25	3,4,5,6,7,19,22,23,26	4	O54728,P05539,P20760,P31430
4839	1,2,8,18,20,21,24,26	3,4,5,6,7,19,22,23,25	3	P62804,Q8K4G9,Q99041
4840	1,2,8,18,20,21,25,26	3,4,5,6,7,19,22,23,24	4	P31430,P55091,P62804,Q8K4G9
4841	1,2,8,18,20,22,23,24	3,4,5,6,7,19,21,25,26	2	Q5FVI6,Q9QZK9
4842	1,2,8,18,20,22,23,25	3,4,5,6,7,19,21,24,26	1	P20760

4843	1,2,8,18,20,22,23,26	3,4,5,6,7,19,21,24,25	0	
4844	1,2,8,18,20,22,24,25	3,4,5,6,7,19,21,23,26	2	P01835,Q5FVI6
4845	1,2,8,18,20,22,24,26	3,4,5,6,7,19,21,23,25	3	P01835,P20646,Q99041
4846	1,2,8,18,20,22,25,26	3,4,5,6,7,19,21,23,24	1	Q8K4G9
4847	1,2,8,18,20,23,24,25	3,4,5,6,7,19,21,22,26	0	
4848	1,2,8,18,20,23,24,26	3,4,5,6,7,19,21,22,25	1	P09605;P25809
4849	1,2,8,18,20,23,25,26	3,4,5,6,7,19,21,22,24	2	Q6P6Q2,Q8K4G9
4850	1,2,8,18,20,24,25,26	3,4,5,6,7,19,21,22,23	3	P55091,Q7TPB1,Q8K4G9
4851	1,2,8,18,21,22,23,24	3,4,5,6,7,19,20,25,26	2	P05539,Q5FVI6
4852	1,2,8,18,21,22,23,25	3,4,5,6,7,19,20,24,26	4	P20760,P31430,Q5FVI6,Q99MH3
4853	1,2,8,18,21,22,23,26	3,4,5,6,7,19,20,24,25	1	Q6IFV1
4854	1,2,8,18,21,22,24,25	3,4,5,6,7,19,20,23,26	4	P05539,P20760,P31430,Q5FVI6
4855	1,2,8,18,21,22,24,26	3,4,5,6,7,19,20,23,25	1	Q99041
4856	1,2,8,18,21,22,25,26	3,4,5,6,7,19,20,23,24	2	P31430,P55091
4857	1,2,8,18,21,23,24,25	3,4,5,6,7,19,20,22,26	7	O54728,P05539,P20760,P31430,P98089,Q5FVI6,Q99MH3
4858	1,2,8,18,21,23,24,26	3,4,5,6,7,19,20,22,25	2	D3ZUC6,Q6IFV1
4859	1,2,8,18,21,23,25,26	3,4,5,6,7,19,20,22,24	7	P14669,P31430,P55091,P98089,Q6IFV1,Q6P6Q2,Q99MH3
4860	1,2,8,18,21,24,25,26	3,4,5,6,7,19,20,22,23	4	P31430,P55091,P98089,Q7TPB1
4861	1,2,8,18,22,23,24,25	3,4,5,6,7,19,20,21,26	2	P01835,Q5FVI6
4862	1,2,8,18,22,23,24,26	3,4,5,6,7,19,20,21,25	3	P00731,P01835,P20646
4863	1,2,8,18,22,23,25,26	3,4,5,6,7,19,20,21,24	1	P01835
4864	1,2,8,18,22,24,25,26	3,4,5,6,7,19,20,21,23	3	P01835,P55091,Q7TPB1
4865	1,2,8,18,23,24,25,26	3,4,5,6,7,19,20,21,22	3	P01835,P55091,Q7TPB1

4866	1,2,8,19,2 3,4,5,6,7,1<br>0,21,22,23 8,24,25,26	4	P00774,P61206;P84079;P84082,Q8CJD3,Q8K4G9
4867	1,2,8,19,2 3,4,5,6,7,1<br>0,21,22,24 8,23,25,26	8	P00714,P00774,P12785,P61206;P84079;P84082,P83121,Q4FZU2,Q6IFW6,Q8K4G9
4868	1,2,8,19,2 3,4,5,6,7,1<br>0,21,22,25 8,23,24,26	6	P12785,P20760,Q4FZU2,Q6IFW6,Q8K4G9,Q8VD89
4869	1,2,8,19,2 3,4,5,6,7,1<br>0,21,22,26 8,23,24,25	3	P62804,Q63751,Q8K4G9
4870	1,2,8,19,2 3,4,5,6,7,1<br>0,21,23,24 8,22,25,26	2	P05539,P61206;P84079;P84082
4871	1,2,8,19,2 3,4,5,6,7,1<br>0,21,23,25 8,22,24,26	3	P20760,Q8K4G9,Q8VD89
4872	1,2,8,19,2 3,4,5,6,7,1<br>0,21,23,26 8,22,24,25	2	P62804,Q8K4G9
4873	1,2,8,19,2 3,4,5,6,7,1<br>0,21,24,25 8,22,23,26	6	P05539,P12785,Q4FZU2,Q6IFW6,Q8K4G9,Q8VD89
4874	1,2,8,19,2 3,4,5,6,7,1<br>0,21,24,26 8,22,23,25	3	P62804,Q63751,Q8K4G9
4875	1,2,8,19,2 3,4,5,6,7,1<br>0,21,25,26 8,22,23,24	2	P62804,Q8K4G9
4876	1,2,8,19,2 3,4,5,6,7,1<br>0,22,23,24 8,21,25,26	5	P00774,P01835,P20646,P25031,P61206;P84079;P84082
4877	1,2,8,19,2 3,4,5,6,7,1<br>0,22,23,25 8,21,24,26	6	P01835,P20760,P25031,Q08849,Q8CJD3,Q8K4G9
4878	1,2,8,19,2 3,4,5,6,7,1<br>0,22,23,26 8,21,24,25	5	P01835,P20646,P25031,Q08849,Q8K4G9
4879	1,2,8,19,2 3,4,5,6,7,1<br>0,22,24,25 8,21,23,26	9	P00774,P01835,P12785,P25031,Q4FZU2,Q6IFW6,Q6IMF3,Q6P6Q2,Q8K4G9
4880	1,2,8,19,2 3,4,5,6,7,1<br>0,22,24,26 8,21,23,25	6	P01835,P12785,P20646,P25031,Q63751,Q8K4G9
4881	1,2,8,19,2 3,4,5,6,7,1<br>0,22,25,26 8,21,23,24	4	P01835,P12785,Q63751,Q8K4G9
4882	1,2,8,19,2 3,4,5,6,7,1<br>0,23,24,25 8,21,22,26	3	P01835,P25031,P83748
4883	1,2,8,19,2 3,4,5,6,7,1<br>0,23,24,26 8,21,22,25	4	P01835,P09605;P25809,P20646,P25031
4884	1,2,8,19,2 3,4,5,6,7,1<br>0,23,25,26 8,21,22,24	2	Q08849,Q8K4G9
4885	1,2,8,19,2 3,4,5,6,7,1<br>0,24,25,26 8,21,22,23	2	P01835,Q8K4G9
4886	1,2,8,19,2 3,4,5,6,7,1<br>1,22,23,24 8,20,25,26	2	P01835,P61206;P84079;P84082
4887	1,2,8,19,2 3,4,5,6,7,1<br>1,22,23,25 8,20,24,26	1	P20760
4888	1,2,8,19,2 3,4,5,6,7,1<br>1,22,23,26 8,20,24,25	0	

4889	1,2,8,19,2<br>1,22,24,25	3,4,5,6,7,1<br>8,20,23,26	6	P01835,P12785,Q4FZU2,Q6IFW6,Q6IG02,Q6IMF3
4890	1,2,8,19,2<br>1,22,24,26	3,4,5,6,7,1<br>8,20,23,25	2	P01835,P12785
4891	1,2,8,19,2<br>1,22,25,26	3,4,5,6,7,1<br>8,20,23,24	1	Q8K4G9
4892	1,2,8,19,2<br>1,23,24,25	3,4,5,6,7,1<br>8,20,22,26	1	P05539
4893	1,2,8,19,2<br>1,23,24,26	3,4,5,6,7,1<br>8,20,22,25	1	D3ZUC6
4894	1,2,8,19,2<br>1,23,25,26	3,4,5,6,7,1<br>8,20,22,24	1	Q8K4G9
4895	1,2,8,19,2<br>1,24,25,26	3,4,5,6,7,1<br>8,20,22,23	1	Q8K4G9
4896	1,2,8,19,2<br>2,23,24,25	3,4,5,6,7,1<br>8,20,21,26	4	P01835,P25031,P61206;P84079;P84082,Q4FZU2
4897	1,2,8,19,2<br>2,23,24,26	3,4,5,6,7,1<br>8,20,21,25	5	P00731,P01835,P20646,P25031,P61206;P84079;P84082
4898	1,2,8,19,2<br>2,23,25,26	3,4,5,6,7,1<br>8,20,21,24	1	P01835
4899	1,2,8,19,2<br>2,24,25,26	3,4,5,6,7,1<br>8,20,21,23	3	P01835,P12785,P25031
4900	1,2,8,19,2<br>3,24,25,26	3,4,5,6,7,1<br>8,20,21,22	3	P01835,P25031,Q7TPB1
4901	1,2,8,20,2<br>1,22,23,24	3,4,5,6,7,1<br>8,19,25,26	2	P61206;P84079;P84082,Q5FVI6
4902	1,2,8,20,2<br>1,22,23,25	3,4,5,6,7,1<br>8,19,24,26	2	P20760,Q5FVI6
4903	1,2,8,20,2<br>1,22,23,26	3,4,5,6,7,1<br>8,19,24,25	1	Q8K4G9
4904	1,2,8,20,2<br>1,22,24,25	3,4,5,6,7,1<br>8,19,23,26	6	P12785,P35467,Q4FZU2,Q5FVI6,Q5XFX0,Q6IFW6
4905	1,2,8,20,2<br>1,22,24,26	3,4,5,6,7,1<br>8,19,23,25	6	O70377,P00714,P12785,Q5XFX0,Q8K4G9,Q99041
4906	1,2,8,20,2<br>1,22,25,26	3,4,5,6,7,1<br>8,19,23,24	3	P02625,P12785,Q8K4G9
4907	1,2,8,20,2<br>1,23,24,25	3,4,5,6,7,1<br>8,19,22,26	0	
4908	1,2,8,20,2<br>1,23,24,26	3,4,5,6,7,1<br>8,19,22,25	1	Q6IFV1
4909	1,2,8,20,2<br>1,23,25,26	3,4,5,6,7,1<br>8,19,22,24	1	Q8K4G9
4910	1,2,8,20,2<br>1,24,25,26	3,4,5,6,7,1<br>8,19,22,23	2	P02625,Q8K4G9
4911	1,2,8,20,2<br>2,23,24,25	3,4,5,6,7,1<br>8,19,21,26	4	P01835,P25031,P83748,Q5FVI6

4912	1,2,8,20,22,23,24,26	3,4,5,6,7,18,19,21,25	5	P00731,P01835,P20646,P25031,P47967
4913	1,2,8,20,22,23,25,26	3,4,5,6,7,18,19,21,24	1	Q08849
4914	1,2,8,20,22,24,25,26	3,4,5,6,7,18,19,21,23	3	P00731,P01835,P12785
4915	1,2,8,20,23,24,25,26	3,4,5,6,7,18,19,21,22	2	P19218,Q7TPB1
4916	1,2,8,21,22,23,24,25	3,4,5,6,7,18,19,20,26	1	Q5FVI6
4917	1,2,8,21,22,23,24,26	3,4,5,6,7,18,19,20,25	2	P00731,Q6IFV1
4918	1,2,8,21,22,23,25,26	3,4,5,6,7,18,19,20,24	0	
4919	1,2,8,21,22,24,25,26	3,4,5,6,7,18,19,20,23	1	P12785
4920	1,2,8,21,23,24,25,26	3,4,5,6,7,18,19,20,22	1	Q7TPB1
4921	1,2,8,22,23,24,25,26	3,4,5,6,7,18,19,20,21	4	P00731,P01835,P25031,Q7TPB1
4922	1,2,18,19,20,21,22,23	3,4,5,6,7,8,24,25,26	4	P20760,P20761,Q8CJD3,Q9Z0V6
4923	1,2,18,19,20,21,22,24	3,4,5,6,7,8,23,25,26	7	P00714,P20760,P20761,Q63751,Q99041,Q9WTT6,Q9Z0V6
4924	1,2,18,19,20,21,22,25	3,4,5,6,7,8,23,24,26	5	P20760,P20761,P47967,P97840,Q63751
4925	1,2,18,19,20,21,22,26	3,4,5,6,7,8,23,24,25	7	O70377,P08937,P36374,Q63751,Q8K4G9,Q99041,Q9Z0V6
4926	1,2,18,19,20,21,23,24	3,4,5,6,7,8,22,25,26	6	D3ZUC6,P05539,P20760,P20761,Q9QZK9,Q9Z0V6
4927	1,2,18,19,20,21,23,25	3,4,5,6,7,8,22,24,26	3	P20760,P20761,Q99MH3
4928	1,2,18,19,20,21,23,26	3,4,5,6,7,8,22,24,25	1	D3ZUC6
4929	1,2,18,19,20,21,24,25	3,4,5,6,7,8,22,23,26	3	P05539,P20760,P20761

4930	1,2,18,19,20,21,24,26	3,4,5,6,7,8,22,23,25	7	D3ZUC6,P06911,P12020,Q63751,Q6AYS4,Q99041,Q9Z0V6
4931	1,2,18,19,20,21,25,26	3,4,5,6,7,8,22,23,24	3	P31430,Q63751,Q8K4G9
4932	1,2,18,19,20,22,23,24	3,4,5,6,7,8,21,25,26	6	P20646,P20761,Q6AYS4,Q9QZK9,Q9WTT6,Q9Z0V6
4933	1,2,18,19,20,22,23,25	3,4,5,6,7,8,21,24,26	3	P20760,P20761,Q8CJD3
4934	1,2,18,19,20,22,23,26	3,4,5,6,7,8,21,24,25	5	P06760,P20646,Q6AYS4,Q9WTT6,Q9Z0V6
4935	1,2,18,19,20,22,24,25	3,4,5,6,7,8,21,23,26	4	P01835,P20760,P20761,Q9Z0V6
4936	1,2,18,19,20,22,24,26	3,4,5,6,7,8,21,23,25	11	O70377,P01835,P06760,P07150,P20646,Q63751,Q6AYS4,Q6P6R2,Q99041,Q9WTT6,Q9Z0V6
4937	1,2,18,19,20,22,25,26	3,4,5,6,7,8,21,23,24	3	Q63751,Q8K4G9,Q99041
4938	1,2,18,19,20,23,24,25	3,4,5,6,7,8,21,22,26	3	P20760,P20761,P83748
4939	1,2,18,19,20,23,24,26	3,4,5,6,7,8,21,22,25	6	P06760,P20646,Q6AYS4,Q9QZK9,Q9WTT6,Q9Z0V6
4940	1,2,18,19,20,23,25,26	3,4,5,6,7,8,21,22,24	0	
4941	1,2,18,19,20,24,25,26	3,4,5,6,7,8,21,22,23	2	Q7TPB1,Q99041
4942	1,2,18,19,21,22,23,24	3,4,5,6,7,8,20,25,26	3	D3ZUC6,P20760,P20761
4943	1,2,18,19,21,22,23,25	3,4,5,6,7,8,20,24,26	4	P20760,P20761,P97840,Q99MH3
4944	1,2,18,19,21,22,23,26	3,4,5,6,7,8,20,24,25	1	D3ZUC6

4945	1,2,18,19,21,22,24,25	3,4,5,6,7,8,20,23,26	4	P20760,P20761,P97840,Q4FZU2
4946	1,2,18,19,21,22,24,26	3,4,5,6,7,8,20,23,25	6	D3ZUC6,O70377,Q63617,Q63751,Q99041,Q9Z0V6
4947	1,2,18,19,21,22,25,26	3,4,5,6,7,8,20,23,24	4	P31430,P97840,Q63617,Q63751
4948	1,2,18,19,21,23,24,25	3,4,5,6,7,8,20,22,26	5	D3ZUC6,P05539,P20760,P20761,Q99MH3
4949	1,2,18,19,21,23,24,26	3,4,5,6,7,8,20,22,25	2	D3ZUC6,Q03191
4950	1,2,18,19,21,23,25,26	3,4,5,6,7,8,20,22,24	4	D3ZUC6,P31430,Q63661,Q99MH3
4951	1,2,18,19,21,24,25,26	3,4,5,6,7,8,20,22,23	5	D3ZUC6,P31430,P55091,Q63617,Q7TPB1
4952	1,2,18,19,22,23,24,25	3,4,5,6,7,8,20,21,26	5	P01835,P20760,P20761,P82471,Q99MH3
4953	1,2,18,19,22,23,24,26	3,4,5,6,7,8,20,21,25	7	P00731,P01835,P06760,P20646,Q6AYS4,Q6IFU7,Q9WTT6
4954	1,2,18,19,22,23,25,26	3,4,5,6,7,8,20,21,24	2	P01835,Q99MH3
4955	1,2,18,19,22,24,25,26	3,4,5,6,7,8,20,21,23	3	P01835,Q7TPB1,Q99041
4956	1,2,18,19,23,24,25,26	3,4,5,6,7,8,20,21,22	4	P01835,Q03191,Q7TPB1,Q99MH3
4957	1,2,18,20,21,22,23,24	3,4,5,6,7,8,19,25,26	4	P20761,P55016,Q5FVI6,Q9QZK9
4958	1,2,18,20,21,22,23,25	3,4,5,6,7,8,19,24,26	2	P20760,P20761
4959	1,2,18,20,21,22,23,26	3,4,5,6,7,8,19,24,25	3	P55016,Q6IFV1,Q9QUL6

4960	1,2,18,20,21,22,24,25	3,4,5,6,7,8,19,23,26	5	P20760,P20761,P55016,Q5FVI6,Q99041
4961	1,2,18,20,21,22,24,26	3,4,5,6,7,8,19,23,25	5	O70377,P55016,Q63751,Q99041,Q9Z0V6
4962	1,2,18,20,21,22,25,26	3,4,5,6,7,8,19,23,24	3	P31430,P55016,Q99041
4963	1,2,18,20,21,23,24,25	3,4,5,6,7,8,19,22,26	2	P20760,P20761
4964	1,2,18,20,21,23,24,26	3,4,5,6,7,8,19,22,25	4	D3ZUC6,P55016,Q6IFV1,Q9QUL6
4965	1,2,18,20,21,23,25,26	3,4,5,6,7,8,19,22,24	2	P31430,Q6P6Q2
4966	1,2,18,20,21,24,25,26	3,4,5,6,7,8,19,22,23	4	P31430,P55016,P55091,Q99041
4967	1,2,18,20,22,23,24,25	3,4,5,6,7,8,19,21,26	4	P20760,P20761,P83748,Q5FVI6
4968	1,2,18,20,22,23,24,26	3,4,5,6,7,8,19,21,25	4	P00731,P20646,Q6IFU7,Q9QZK9
4969	1,2,18,20,22,23,25,26	3,4,5,6,7,8,19,21,24	0	
4970	1,2,18,20,22,24,25,26	3,4,5,6,7,8,19,21,23	3	P00731,Q7TPB1,Q99041
4971	1,2,18,20,23,24,25,26	3,4,5,6,7,8,19,21,22	2	P19218,Q7TPB1
4972	1,2,18,21,22,23,24,25	3,4,5,6,7,8,19,20,26	3	P20760,P20761,Q5FVI6
4973	1,2,18,21,22,23,24,26	3,4,5,6,7,8,19,20,25	4	D3ZUC6,P00731,Q6IFU7,Q6IFV1
4974	1,2,18,21,22,23,25,26	3,4,5,6,7,8,19,20,24	1	P31430

4975	1,2,18,21,22,24,25,26	3,4,5,6,7,8,19,20,23	3	P31430,P55091,Q99041
4976	1,2,18,21,23,24,25,26	3,4,5,6,7,8,19,20,22	4	D3ZUC6,P31430,P55091,Q7TPB1
4977	1,2,18,22,23,24,25,26	3,4,5,6,7,8,19,20,21	5	P00731,P01835,P82471,Q6IFU7,Q7TPB1
4978	1,2,19,20,21,22,23,24	3,4,5,6,7,8,18,25,26	5	P00714,P00774,P20761,P61206;P84079;P84082,Q6AYS4
4979	1,2,19,20,21,22,23,25	3,4,5,6,7,8,18,24,26	3	P20760,P20761,Q8VD89
4980	1,2,19,20,21,22,23,26	3,4,5,6,7,8,18,24,25	0	
4981	1,2,19,20,21,22,24,25	3,4,5,6,7,8,18,23,26	10	P12785,P20760,P20761,P35467,P55016,Q4FZU2,Q6IFW6,Q6IG02,Q6P6Q2,Q8VD89
4982	1,2,19,20,21,22,24,26	3,4,5,6,7,8,18,23,25	7	O70377,P00714,P55016,Q63751,Q6AYS4,Q99041,Q9Z0V6
4983	1,2,19,20,21,22,25,26	3,4,5,6,7,8,18,23,24	3	P55016,Q63751,Q8K4G9
4984	1,2,19,20,21,23,24,25	3,4,5,6,7,8,18,22,26	3	P20760,P20761,Q8VD89
4985	1,2,19,20,21,23,24,26	3,4,5,6,7,8,18,22,25	2	P55016,Q6AYS4
4986	1,2,19,20,21,23,25,26	3,4,5,6,7,8,18,22,24	0	
4987	1,2,19,20,21,24,25,26	3,4,5,6,7,8,18,22,23	1	P55016
4988	1,2,19,20,22,23,24,25	3,4,5,6,7,8,18,21,26	8	P00774,P01835,P20646,P20760,P20761,P25031,P83748,Q6AYS4
4989	1,2,19,20,22,23,24,26	3,4,5,6,7,8,18,21,25	8	P00731,P01835,P20646,P25031,P61206;P84079;P84082,Q6AYS4,Q9WTT6,Q9Z0V6

4990	1,2,19,20,22,23,25,26	3,4,5,6,7,8,18,21,24	0	
4991	1,2,19,20,22,24,25,26	3,4,5,6,7,8,18,21,23	4	P00731,P01835,P20646,Q6AYS4
4992	1,2,19,20,23,24,25,26	3,4,5,6,7,8,18,21,22	2	Q6AYS4,Q7TPB1
4993	1,2,19,21,22,23,24,25	3,4,5,6,7,8,18,20,26	3	P20760,P20761,P82471
4994	1,2,19,21,22,23,24,26	3,4,5,6,7,8,18,20,25	4	D3ZUC6,O70377,P00731,P61206;P84079;P84082
4995	1,2,19,21,22,23,25,26	3,4,5,6,7,8,18,20,24	0	
4996	1,2,19,21,22,24,25,26	3,4,5,6,7,8,18,20,23	1	P07171
4997	1,2,19,21,23,24,25,26	3,4,5,6,7,8,18,20,22	1	Q7TPB1
4998	1,2,19,22,23,24,25,26	3,4,5,6,7,8,18,20,21	6	P00731,P01835,P20646,P25031,P82471,Q7TPB1
4999	1,2,20,21,22,23,24,25	3,4,5,6,7,8,18,19,26	4	P20760,P20761,P55016,Q5FVI6
5000	1,2,20,21,22,23,24,26	3,4,5,6,7,8,18,19,25	4	O70377,P00714,P00731,P55016
5001	1,2,20,21,22,23,25,26	3,4,5,6,7,8,18,19,24	1	P55016
5002	1,2,20,21,22,24,25,26	3,4,5,6,7,8,18,19,23	5	P00731,P02625,P35467,P55016,Q99041
5003	1,2,20,21,23,24,25,26	3,4,5,6,7,8,18,19,22	2	P55016,Q7TPB1
5004	1,2,20,22,23,24,25,26	3,4,5,6,7,8,18,19,21	2	P00731,Q7TPB1

5005	1,2,21,22,23,24,25,26	3,4,5,6,7,8,18,19,20	2	P00731,Q7TPB1
5006	1,3,4,5,6,7,8,18	2,19,20,21,22,23,24,25,26	11	O55006,P00731,P01681,P02803,P18418,P52590,P61206;P84079;P84082,Q63617,Q6IRE4,Q8VD89,Q9ESW0
5007	1,3,4,5,6,7,8,19	2,18,20,21,22,23,24,25,26	9	O55006,P00731,P01681,P16086,P18418,P52590,Q30KJ2,Q5FVI6,Q63617
5008	1,3,4,5,6,7,8,20	2,18,19,21,22,23,24,25,26	8	O55006,P00731,P01681,P18418,P52590,Q63617,Q6IRE4,Q9ESW0
5009	1,3,4,5,6,7,8,21	2,18,19,20,22,23,24,25,26	12	O55006,P00731,P01681,P18418,P20646,P42854,P52590,P61206;P84079;P84082,Q63617,Q6IRE4,Q7TPB1,Q9ESW0
5010	1,3,4,5,6,7,8,22	2,18,19,20,21,23,24,25,26	6	O55006,P01681,P18418,P52590,Q63617,Q9QW07
5011	1,3,4,5,6,7,8,23	2,18,19,20,21,22,24,25,26	10	P01681,P18418,P28648,P52590,P97580,Q63617,Q63751,Q6IRE4,Q99041,Q9ESW0
5012	1,3,4,5,6,7,8,24	2,18,19,20,21,22,23,25,26	6	O55006,P01681,P18418,P52590,Q63617,Q6IRE4
5013	1,3,4,5,6,7,8,25	2,18,19,20,21,22,23,24,26	15	O55006,O70377,P01681,P06911,P10715,P12020,P18418,P19629,P52590,P61206;P84079;P84082,Q4AEF8,Q5GRG2,Q63617,Q6IRE4,Q9Z0V6
5014	1,3,4,5,6,7,8,26	2,18,19,20,21,22,23,24,25	17	O55006,P01681,P04041,P05539,P18418,P20760,P20761,P52590,P61206;P84079;P84082,P83748,Q4FZU2,Q5FVI6,Q63617,Q6AY33,Q6IRE4,Q8VD89,Q9ESW0
5015	1,3,4,5,6,7,18,19	2,8,20,21,22,23,24,25,26	7	O55006,P00731,P01681,P18418,P61206;P84079;P84082,Q5FVI6,Q9ESW0
5016	1,3,4,5,6,7,18,20	2,8,19,21,22,23,24,25,26	8	O55006,P00731,P01681,P18418,P61206;P84079;P84082,Q4FZU2,Q6IRE4,Q9ESW0
5017	1,3,4,5,6,7,18,21	2,8,19,20,22,23,24,25,26	11	P00731,P01681,P01835,P18418,P20646,P25031,P42854,P52590,P61206;P84079;P84082,Q6IRE4,Q9ESW0
5018	1,3,4,5,6,7,18,22	2,8,19,20,21,23,24,25,26	7	P01681,P18418,P61206;P84079;P84082,Q8K4G9,Q8VD89,Q9ESW0,Q9QW07
5019	1,3,4,5,6,7,18,23	2,8,19,20,21,22,24,25,26	14	P01681,P12785,P18418,P28648,P52590,P55053,P61206;P84079;P84082,Q4FZU2,Q6IFW6,Q6IG02,Q6IRE4,Q8K4G9,Q8VD89,Q9ESW0

5020	1,3,4,5,6,7,18,24	2,8,19,20,21,22,23,25,26	9	O55006,P01681,P18418,P52590,P61206;P84079;P84082,Q6IRE4,Q8K4G9,Q8VD89,Q9ESW0
5021	1,3,4,5,6,7,18,25	2,8,19,20,21,22,23,24,26	8	P00731,P01681,P18418,P25031,P52590,P61206;P84079;P84082,Q6IRE4,Q9ESW0
5022	1,3,4,5,6,7,18,26	2,8,19,20,21,22,23,24,25	15	P01681,P05539,P18418,P20760,P20761,P25031,P61206;P84079;P84082,P83748,Q4FZU2,Q5FVI6,Q6AY33,Q6IFW6,Q6IRE4,Q8VD89,Q9ESW0
5023	1,3,4,5,6,7,19,20	2,8,18,21,22,23,24,25,26	6	P00731,P01681,P18418,P55091,Q5FVI6,Q6IRE4
5024	1,3,4,5,6,7,19,21	2,8,18,20,22,23,24,25,26	8	P00731,P01681,P16086,P18418,P52590,Q30KJ2,Q5FVI6,Q6IRE4
5025	1,3,4,5,6,7,19,22	2,8,18,20,21,23,24,25,26	5	P01681,P18418,P55091,Q5FVI6,Q9QW07
5026	1,3,4,5,6,7,19,23	2,8,18,20,21,22,24,25,26	8	P01681,P15399,P16086,P18418,P97580,Q30KJ2,Q5FVI6,Q99041
5027	1,3,4,5,6,7,19,24	2,8,18,20,21,22,23,25,26	5	O55006,P01681,P18418,Q30KJ2,Q5FVI6
5028	1,3,4,5,6,7,19,25	2,8,18,20,21,22,23,24,26	8	P00731,P01681,P12020,P17046,P18418,P61206;P84079;P84082,Q30KJ2,Q5FVI6
5029	1,3,4,5,6,7,19,26	2,8,18,20,21,22,23,24,25	10	O54728,P01681,P05539,P18418,P20760,P20761,P83748,Q30KJ2,Q4FZU2,Q5FVI6
5030	1,3,4,5,6,7,20,21	2,8,18,19,22,23,24,25,26	10	P00731,P01681,P01835,P18418,P42854,P52590,P61206;P84079;P84082,Q6IRE4,Q7TPB1,Q9ESW0
5031	1,3,4,5,6,7,20,22	2,8,18,19,21,23,24,25,26	7	P01681,P18418,P55091,Q6IRE4,Q7TPB1,Q99MH3,Q9QW07
5032	1,3,4,5,6,7,20,23	2,8,18,19,21,22,24,25,26	3	P01681,Q4FZU2,Q6IRE4
5033	1,3,4,5,6,7,20,24	2,8,18,19,21,22,23,25,26	4	P01681,P18418,Q6IRE4,Q99MH3
5034	1,3,4,5,6,7,20,25	2,8,18,19,21,22,23,24,26	5	P01681,P18418,P55314,P61206;P84079;P84082,Q6IRE4

5035	1,3,4,5,6,7,20,26	2,8,18,19,21,22,23,24,25	11	O54728,P01681,P05539,P20760,P20761,P61206;P84079;P84082,P83748,Q4FZU2,Q5FVI6,Q6IRE4,Q99MH3
5036	1,3,4,5,6,7,21,22	2,8,18,19,20,23,24,25,26	8	P01681,P13432,P18418,P19218,P52590,Q6IRE4,Q7TPB1,Q9QW07
5037	1,3,4,5,6,7,21,23	2,8,18,19,20,22,24,25,26	9	P01681,P01835,P34901,P52590,Q4FZU2,Q63751,Q6IRE4,Q8K4G9,Q9ESW0
5038	1,3,4,5,6,7,21,24	2,8,18,19,20,22,23,25,26	6	P01681,P13432,P18418,P52590,Q6IRE4,Q8K4G9
5039	1,3,4,5,6,7,21,25	2,8,18,19,20,22,23,24,26	8	P00731,P01681,P01835,P18418,P20646,P52590,P61206;P84079;P84082,Q6IRE4
5040	1,3,4,5,6,7,21,26	2,8,18,19,20,22,23,24,25	12	P01681,P01835,P05539,P20760,P20761,P52590,P61206;P84079;P84082,P83748,Q4FZU2,Q5FVI6,Q6IRE4,Q9ESW0
5041	1,3,4,5,6,7,22,23	2,8,18,19,20,21,24,25,26	4	P01681,P34901,Q8K4G9,Q9QW07
5042	1,3,4,5,6,7,22,24	2,8,18,19,20,21,23,25,26	6	P01681,P08010,P18418,Q8K4G9,Q99MH3,Q9QW07
5043	1,3,4,5,6,7,22,25	2,8,18,19,20,21,23,24,26	8	P01681,P06911,P12020,P17046,P18418,P61206;P84079;P84082,Q5GRG2,Q9QW07
5044	1,3,4,5,6,7,22,26	2,8,18,19,20,21,23,24,25	9	O54728,P01681,P05539,P20760,P20761,P83748,Q8VD89,Q99MH3,Q9QW07
5045	1,3,4,5,6,7,23,24	2,8,18,19,20,21,22,25,26	6	P01681,P18418,P34901,Q63751,Q6IRE4,Q8K4G9
5046	1,3,4,5,6,7,23,25	2,8,18,19,20,21,22,24,26	10	P01681,P06911,P12020,P55314,P61206;P84079;P84082,Q5GRG2,Q63751,Q6IRE4,Q8K4G9,Q99041
5047	1,3,4,5,6,7,23,26	2,8,18,19,20,21,22,24,25	18	O54728,P01681,P01946,P02091,P05539,P20760,P20761,P83748,Q4FZU2,Q5FVI6,Q6IFU8,Q6IFW6,Q6IG02,Q6IMF3,Q6IRE4,Q6P6Q2,Q8K4G9,Q8VD89
5048	1,3,4,5,6,7,24,25	2,8,18,19,20,21,22,23,26	5	P01681,P18418,P61206;P84079;P84082,Q6IRE4,Q8K4G9
5049	1,3,4,5,6,7,24,26	2,8,18,19,20,21,22,23,25	14	P01681,P02091,P05539,P18418,P20760,P20761,P83748,Q4FZU2,Q5FVI6,Q6IRE4,Q8CJD3,Q8K4G9,Q8VD89,Q99MH3

5050	1,3,4,5,6,7,25,26	2,8,18,19,20,21,22,23,24	12	P01681,P02091,P05539,P20760,P20761,P55314,P61206;P84079;P84082,P83748,Q4FZU2,Q5FVI6,Q6AY33,Q6IRE4
5051	1,3,4,5,6,8,18,19	2,7,20,21,22,23,24,25,26	5	O55006,P00731,P01681,P02803,P22006
5052	1,3,4,5,6,8,18,20	2,7,19,21,22,23,24,25,26	7	O55006,P00731,P01681,P22006,Q4FZU2,Q6IRE4,Q9ESW0
5053	1,3,4,5,6,8,18,21	2,7,19,20,22,23,24,25,26	8	O35547,P00731,P01681,P01835,P0C0A9,P25031,Q4FZU2,Q9ESW0
5054	1,3,4,5,6,8,18,22	2,7,19,20,21,23,24,25,26	3	P01681,P22006,Q9QW07
5055	1,3,4,5,6,8,18,23	2,7,19,20,21,22,24,25,26	8	P01681,P12785,P35467,Q4FZU2,Q5XI22,Q6IFW6,Q6IG02,Q9ESW0
5056	1,3,4,5,6,8,18,24	2,7,19,20,21,22,23,25,26	2	O55006,P01681
5057	1,3,4,5,6,8,18,25	2,7,19,20,21,22,23,24,26	2	P00731,P01681
5058	1,3,4,5,6,8,18,26	2,7,19,20,21,22,23,24,25	11	P01681,P04041,P20760,P20761,Q4FZU2,Q63942,Q6AY33,Q6IFW6,Q6IG02,Q8VD89,Q9ESW0
5059	1,3,4,5,6,8,19,20	2,7,18,21,22,23,24,25,26	6	P00731,P01681,P22006,P35281,P55091,P82995
5060	1,3,4,5,6,8,19,21	2,7,18,20,22,23,24,25,26	5	O35547,P00731,P01681,P0C0A9,P35281
5061	1,3,4,5,6,8,19,22	2,7,18,20,21,23,24,25,26	4	P01681,P22006,P55091,Q9QW07
5062	1,3,4,5,6,8,19,23	2,7,18,20,21,22,24,25,26	6	P01681,P15399,P55091,P82995,P97580,Q30KJ2
5063	1,3,4,5,6,8,19,24	2,7,18,20,21,22,23,25,26	3	O55006,P01681,P82995
5064	1,3,4,5,6,8,19,25	2,7,18,20,21,22,23,24,26	1	P01681

5065	1,3,4,5,6,8,19,26	2,7,18,20,21,22,23,24,25	5	O54728,P01681,P20760,P20761,Q4FZU2
5066	1,3,4,5,6,8,20,21	2,7,18,19,22,23,24,25,26	5	P00731,P01681,P01835,Q6IRE4,Q7TPB1
5067	1,3,4,5,6,8,20,22	2,7,18,19,21,23,24,25,26	8	D3ZUC6,P01681,P22006,P55091,P82995,Q7TPB1,Q99MH3,Q9QW07
5068	1,3,4,5,6,8,20,23	2,7,18,19,21,22,24,25,26	8	P01681,P22006,P55091,P82995,P97840,Q4FZU2,Q6IG02,Q6IRE4
5069	1,3,4,5,6,8,20,24	2,7,18,19,21,22,23,25,26	5	P01681,P82995,P97840,Q6IRE4,Q99MH3
5070	1,3,4,5,6,8,20,25	2,7,18,19,21,22,23,24,26	3	D3ZUC6,P01681,Q6IRE4
5071	1,3,4,5,6,8,20,26	2,7,18,19,21,22,23,24,25	8	O54728,P01681,P20760,P20761,P97840,Q4FZU2,Q6IRE4,Q99MH3
5072	1,3,4,5,6,8,21,22	2,7,18,19,20,23,24,25,26	4	P01681,P13432,Q7TPB1,Q9QW07
5073	1,3,4,5,6,8,21,23	2,7,18,19,20,22,24,25,26	4	P01681,P01835,Q4FZU2,Q9Z0V6
5074	1,3,4,5,6,8,21,24	2,7,18,19,20,22,23,25,26	2	P01681,P13432
5075	1,3,4,5,6,8,21,25	2,7,18,19,20,22,23,24,26	7	P00731,P01681,P01835,P06760,P20646,Q9WTT6,Q9Z0V6
5076	1,3,4,5,6,8,21,26	2,7,18,19,20,22,23,24,25	6	P01681,P01835,P20760,P20761,Q4FZU2,Q5M872
5077	1,3,4,5,6,8,22,23	2,7,18,19,20,21,24,25,26	4	P01681,P55091,P82995,Q9QW07
5078	1,3,4,5,6,8,22,24	2,7,18,19,20,21,23,25,26	4	P01681,P82995,Q99MH3,Q9QW07
5079	1,3,4,5,6,8,22,25	2,7,18,19,20,21,23,24,26	4	D3ZUC6,P01681,P06911,Q9QW07

5080	1,3,4,5,6,8,22,26	2,7,18,19,20,21,23,24,25	7	O54728,P01681,P20760,P20761,Q4FZU2,Q99MH3,Q9QW07
5081	1,3,4,5,6,8,23,24	2,7,18,19,20,21,22,25,26	5	P01681,P34901,P82995,P97840,Q63751
5082	1,3,4,5,6,8,23,25	2,7,18,19,20,21,22,24,26	7	P01681,P06760,P06911,P55314,Q63751,Q9WTT6,Q9Z0V6
5083	1,3,4,5,6,8,23,26	2,7,18,19,20,21,22,24,25	15	O54728,P01681,P02793;Q7TP54,P20760,P20761,P47967,P97840,Q4FZU2,Q5I0D1,Q6IFU8,Q6IFW6,Q6IG02,Q6IMF3,Q6P6Q2,Q9Z0V6
5084	1,3,4,5,6,8,24,25	2,7,18,19,20,21,22,23,26	2	P01681,Q9Z0V6
5085	1,3,4,5,6,8,24,26	2,7,18,19,20,21,22,23,25	7	P01681,P20760,P20761,P47967,P97840,Q4FZU2,Q99MH3
5086	1,3,4,5,6,8,25,26	2,7,18,19,20,21,22,23,24	6	P01681,P20761,Q4FZU2,Q6AY33,Q9WTT6,Q9Z0V6
5087	1,3,4,5,6,18,19,20	2,7,8,21,22,23,24,25,26	4	P00731,P01681,P22006,Q4FZU2
5088	1,3,4,5,6,18,19,21	2,7,8,20,22,23,24,25,26	5	O35547,P00731,P01681,P01835,P25031
5089	1,3,4,5,6,18,19,22	2,7,8,20,21,23,24,25,26	3	P01681,P22006,Q9QW07
5090	1,3,4,5,6,18,19,23	2,7,8,20,21,22,24,25,26	8	P01681,P02625,P12785,P15399,P22006,P97580,Q4FZU2,Q6IFW6
5091	1,3,4,5,6,18,19,24	2,7,8,20,21,22,23,25,26	1	P01681
5092	1,3,4,5,6,18,19,25	2,7,8,20,21,22,23,24,26	2	P00731,P01681
5093	1,3,4,5,6,18,19,26	2,7,8,20,21,22,23,24,25	4	P01681,Q4FZU2,Q63942,Q6IFW6
5094	1,3,4,5,6,18,20,21	2,7,8,19,22,23,24,25,26	7	P00731,P01681,P01835,P25031,Q4FZU2,Q6IRE4,Q9ESW0

5095	1,3,4,5,6,18,20,22	2,7,8,19,21,23,24,25,26	4	P01681,P22006,Q4FZU2,Q9QW07
5096	1,3,4,5,6,18,20,23	2,7,8,19,21,22,24,25,26	9	P01681,P01835,P12785,P22006,Q4FZU2,Q6IFW6,Q6IG02,Q6IMF3,Q6IRE4
5097	1,3,4,5,6,18,20,24	2,7,8,19,21,22,23,25,26	3	P01681,Q4FZU2,Q6IRE4
5098	1,3,4,5,6,18,20,25	2,7,8,19,21,22,23,24,26	4	P01681,P01835,Q4FZU2,Q6IRE4
5099	1,3,4,5,6,18,20,26	2,7,8,19,21,22,23,24,25	7	P01681,P01835,Q4FZU2,Q6IFW6,Q6IG02,Q6IRE4,Q9ESW0
5100	1,3,4,5,6,18,21,22	2,7,8,19,20,23,24,25,26	5	O35547,P01681,P01835,P25031,Q9QW07
5101	1,3,4,5,6,18,21,23	2,7,8,19,20,22,24,25,26	10	P01681,P01835,P12785,P25031,P34901,Q4FZU2,Q6IFW6,Q6IG02,Q6IMF3,Q9ESW0
5102	1,3,4,5,6,18,21,24	2,7,8,19,20,22,23,25,26	3	P01681,P01835,P13432
5103	1,3,4,5,6,18,21,25	2,7,8,19,20,22,23,24,26	7	P00731,P01681,P01835,P20646,P25031,P31430,Q4FZU2
5104	1,3,4,5,6,18,21,26	2,7,8,19,20,22,23,24,25	6	P01681,P01835,P25031,Q4FZU2,Q6IFW6,Q9ESW0
5105	1,3,4,5,6,18,22,23	2,7,8,19,20,21,24,25,26	7	P01681,P02625,P12785,P34901,Q4FZU2,Q6IFW6,Q9QW07
5106	1,3,4,5,6,18,22,24	2,7,8,19,20,21,23,25,26	2	P01681,Q9QW07
5107	1,3,4,5,6,18,22,25	2,7,8,19,20,21,23,24,26	2	P01681,Q9QW07
5108	1,3,4,5,6,18,22,26	2,7,8,19,20,21,23,24,25	7	O54728,P01681,P05539,Q4FZU2,Q6IFW6,Q8VD89,Q9QW07
5109	1,3,4,5,6,18,23,24	2,7,8,19,20,21,22,25,26	7	P01681,P12785,P34901,Q4FZU2,Q63751,Q6IFU7,Q6IFW6

5110	1,3,4,5,6,18,23,25	2,7,8,19,20,21,22,24,26	7	P01681,P12785,P55314,Q4FZU2,Q63751,Q6IFW6,Q6IG02
5111	1,3,4,5,6,18,23,26	2,7,8,19,20,21,22,24,25	14	P01681,P01835,P12785,P35467,Q4FZU2,Q5I0D1,Q63942,Q6IFU7,Q6IFU8,Q6IFW6,Q6IG02,Q6IMF3,Q6P6Q2,Q8VD89
5112	1,3,4,5,6,18,24,25	2,7,8,19,20,21,22,23,26	1	P01681
5113	1,3,4,5,6,18,24,26	2,7,8,19,20,21,22,23,25	5	P01681,P20760,Q4FZU2,Q6IFW6,Q8VD89
5114	1,3,4,5,6,18,25,26	2,7,8,19,20,21,22,23,24	4	P01681,Q4FZU2,Q6AY33,Q6IFW6
5115	1,3,4,5,6,19,20,21	2,7,8,18,22,23,24,25,26	6	P00731,P01681,P01835,P55091,Q6IRE4,Q7TPB1
5116	1,3,4,5,6,19,20,22	2,7,8,18,21,23,24,25,26	8	O54728,P01681,P22006,P55091,P82995,Q62997,Q99MH3,Q9QW07
5117	1,3,4,5,6,19,20,23	2,7,8,18,21,22,24,25,26	7	P01681,P15399,P22006,P55091,P82995,P97580,Q4FZU2
5118	1,3,4,5,6,19,20,24	2,7,8,18,21,22,23,25,26	5	P01681,P55091,P82995,Q80WL1,Q99MH3
5119	1,3,4,5,6,19,20,25	2,7,8,18,21,22,23,24,26	1	P01681
5120	1,3,4,5,6,19,20,26	2,7,8,18,21,22,23,24,25	5	O54728,P01681,P05539,Q4FZU2,Q99MH3
5121	1,3,4,5,6,19,21,22	2,7,8,18,20,23,24,25,26	4	P01681,P55091,Q7TPB1,Q9QW07
5122	1,3,4,5,6,19,21,23	2,7,8,18,20,22,24,25,26	5	P01681,P01835,P15399,P55091,P97580
5123	1,3,4,5,6,19,21,24	2,7,8,18,20,22,23,25,26	1	P01681
5124	1,3,4,5,6,19,21,25	2,7,8,18,20,22,23,24,26	3	P00731,P01681,P01835

5125	1,3,4,5,6,19,21,26	2,7,8,18,20,22,23,24,25	3	P01681,P01835,Q4FZU2
5126	1,3,4,5,6,19,22,23	2,7,8,18,20,21,24,25,26	8	O54728,P01681,P02625,P15399,P55091,P82995,P97580,Q9QW07
5127	1,3,4,5,6,19,22,24	2,7,8,18,20,21,23,25,26	5	P01681,P55091,P82995,Q99MH3,Q9QW07
5128	1,3,4,5,6,19,22,25	2,7,8,18,20,21,23,24,26	3	P01681,P17046,Q9QW07
5129	1,3,4,5,6,19,22,26	2,7,8,18,20,21,23,24,25	5	O54728,P01681,P05539,Q99MH3,Q9QW07
5130	1,3,4,5,6,19,23,24	2,7,8,18,20,21,22,25,26	6	P01681,P15399,P34901,P55091,P82995,P97580
5131	1,3,4,5,6,19,23,25	2,7,8,18,20,21,22,24,26	5	P01681,P15399,P55314,P97580,Q6BEA2
5132	1,3,4,5,6,19,23,26	2,7,8,18,20,21,22,24,25	12	O54728,P01681,P05539,P15399,P97580,Q30KJ2,Q4FZU2,Q6IFU8,Q6IFW6,Q6IG02,Q6IMF3,Q6P6Q2
5133	1,3,4,5,6,19,24,25	2,7,8,18,20,21,22,23,26	1	P01681
5134	1,3,4,5,6,19,24,26	2,7,8,18,20,21,22,23,25	3	O54728,P01681,P20760
5135	1,3,4,5,6,19,25,26	2,7,8,18,20,21,22,23,24	3	P01681,P05539,Q4FZU2
5136	1,3,4,5,6,20,21,22	2,7,8,18,19,23,24,25,26	8	P01681,P01835,P55091,P82995,Q6IRE4,Q7TPB1,Q99MH3,Q9QW07
5137	1,3,4,5,6,20,21,23	2,7,8,18,19,22,24,25,26	6	P01681,P01835,P55091,P82995,Q4FZU2,Q6IRE4
5138	1,3,4,5,6,20,21,24	2,7,8,18,19,22,23,25,26	6	P01681,P01835,P13432,P82995,Q6IRE4,Q99MH3
5139	1,3,4,5,6,20,21,25	2,7,8,18,19,22,23,24,26	4	P01681,P01835,P20646,Q6IRE4

5140	1,3,4,5,6,20,21,26	2,7,8,18,19,22,23,24,25	5	P01681,P01835,Q4FZU2,Q6IRE4,Q99MH3
5141	1,3,4,5,6,20,22,23	2,7,8,18,19,21,24,25,26	6	P01681,P22006,P55091,P82995,Q4FZU2,Q9QW07
5142	1,3,4,5,6,20,22,24	2,7,8,18,19,21,23,25,26	5	P01681,P55091,P82995,Q99MH3,Q9QW07
5143	1,3,4,5,6,20,22,25	2,7,8,18,19,21,23,24,26	6	D3ZUC6,P01681,P82995,Q03191,Q99MH3,Q9QW07
5144	1,3,4,5,6,20,22,26	2,7,8,18,19,21,23,24,25	6	O54728,P01681,P05539,Q4FZU2,Q99MH3,Q9QW07
5145	1,3,4,5,6,20,23,24	2,7,8,18,19,21,22,25,26	8	P01681,P34901,P55091,P82995,P97840,Q4FZU2,Q6IRE4,Q80WL1
5146	1,3,4,5,6,20,23,25	2,7,8,18,19,21,22,24,26	7	P01681,P01835,P55314,P82995,Q4FZU2,Q63751,Q6IRE4
5147	1,3,4,5,6,20,23,26	2,7,8,18,19,21,22,24,25	13	O54728,P01681,P01835,P05539,P47967,P97840,Q4FZU2,Q6IFU8,Q6IFW6,Q6IG02,Q6IMF3,Q6IRE4,Q6P6Q2
5148	1,3,4,5,6,20,24,25	2,7,8,18,19,21,22,23,26	4	P01681,P82995,Q6IRE4,Q80WL1
5149	1,3,4,5,6,20,24,26	2,7,8,18,19,21,22,23,25	8	P01681,P20760,P47967,P97840,Q4FZU2,Q6IRE4,Q8CJD3,Q99MH3
5150	1,3,4,5,6,20,25,26	2,7,8,18,19,21,22,23,24	5	P01681,P01835,P05539,Q4FZU2,Q6IRE4
5151	1,3,4,5,6,21,22,23	2,7,8,18,19,20,24,25,26	6	P01681,P01835,P34901,P55091,P82995,Q9QW07
5152	1,3,4,5,6,21,22,24	2,7,8,18,19,20,23,25,26	5	P01681,P13432,P82995,Q99MH3,Q9QW07
5153	1,3,4,5,6,21,22,25	2,7,8,18,19,20,23,24,26	3	P01681,P01835,Q9QW07
5154	1,3,4,5,6,21,22,26	2,7,8,18,19,20,23,24,25	7	O54728,P01681,P01835,P05539,Q4FZU2,Q99MH3,Q9QW07

5155	1,3,4,5,6,21,23,24	2,7,8,18,19,20,22,25,26	5	P01681,P01835,P34901,P82995,Q63751
5156	1,3,4,5,6,21,23,25	2,7,8,18,19,20,22,24,26	9	P01681,P01835,P06760,P20646,P34901,P55314,Q4FZU2,Q63751,Q6P777
5157	1,3,4,5,6,21,23,26	2,7,8,18,19,20,22,24,25	9	P01681,P01835,P34901,Q4FZU2,Q6IFU8,Q6IFW6,Q6IG02,Q6IMF3,Q6P6Q2
5158	1,3,4,5,6,21,24,25	2,7,8,18,19,20,22,23,26	2	P01681,P01835
5159	1,3,4,5,6,21,24,26	2,7,8,18,19,20,22,23,25	6	P01681,P01835,P20760,Q4FZU2,Q8CJD3,Q99MH3
5160	1,3,4,5,6,21,25,26	2,7,8,18,19,20,22,23,24	4	P01681,P01835,P20646,Q4FZU2
5161	1,3,4,5,6,22,23,24	2,7,8,18,19,20,21,25,26	6	P01681,P34901,P55091,P82995,Q63751,Q9QW07
5162	1,3,4,5,6,22,23,25	2,7,8,18,19,20,21,24,26	7	P01681,P06911,P55314,P82995,Q63751,Q6P767,Q9QW07
5163	1,3,4,5,6,22,23,26	2,7,8,18,19,20,21,24,25	12	O54728,P01681,P05539,P34901,P47967,Q4FZU2,Q6IFU8,Q6IFW6,Q6IG02,Q6IMF3,Q6P6Q2,Q9QW07
5164	1,3,4,5,6,22,24,25	2,7,8,18,19,20,21,23,26	4	P01681,P62804,P82995,Q9QW07
5165	1,3,4,5,6,22,24,26	2,7,8,18,19,20,21,23,25	6	O54728,P01681,P20760,P47967,Q99MH3,Q9QW07
5166	1,3,4,5,6,22,25,26	2,7,8,18,19,20,21,23,24	5	O54728,P01681,P05539,Q4FZU2,Q9QW07
5167	1,3,4,5,6,23,24,25	2,7,8,18,19,20,21,22,26	5	P01681,P34901,P55314,P82995,Q63751
5168	1,3,4,5,6,23,24,26	2,7,8,18,19,20,21,22,25	14	O54728,P01681,P02091,P20760,P27590,P34901,P47967,P97840,Q4FZU2,Q6IFU8,Q6IFW6,Q6IG02,Q6IMF3,Q6P6Q2
5169	1,3,4,5,6,23,25,26	2,7,8,18,19,20,21,22,24	12	P01681,P02091,P05539,P55314,Q4FZU2,Q5I0D1,Q63751,Q6IFU8,Q6IFW6,Q6IG02,Q6IMF3,Q6P6Q2

5170	1,3,4,5,6,24,25,26	2,7,8,18,19,20,21,22,23	3	P01681,Q4FZU2,Q8CJD3
5171	1,3,4,5,7,8,18,19	2,6,20,21,22,23,24,25,26	5	P00731,P02803,P18418,P52590,Q9ESW0
5172	1,3,4,5,7,8,18,20	2,6,19,21,22,23,24,25,26	9	P00731,P01681,P10715,P18418,P52590,P97608,Q4FZU6,Q6IRE4,Q9ESW0
5173	1,3,4,5,7,8,18,21	2,6,19,20,22,23,24,25,26	10	P00731,P02803,P18418,P25031,P42854,P52590,Q4FZU6,Q6AYS4,Q6IRE4,Q9ESW0
5174	1,3,4,5,7,8,18,22	2,6,19,20,21,23,24,25,26	5	P18418,P52590,Q8VD89,Q9ESW0,Q9QW07
5175	1,3,4,5,7,8,18,23	2,6,19,20,21,22,24,25,26	12	P02803,P12785,P18418,P28648,P35467,P52590,Q4FZU6,Q5XI22,Q64194,Q6IG02,Q8VD89,Q9ESW0
5176	1,3,4,5,7,8,18,24	2,6,19,20,21,22,23,25,26	4	P18418,P52590,Q8VD89,Q9ESW0
5177	1,3,4,5,7,8,18,25	2,6,19,20,21,22,23,24,26	9	P00714,P02803,P08721,P10715,P18418,P52590,P61206;P84079;P84082,Q6AYS4,Q9ESW0
5178	1,3,4,5,7,8,18,26	2,6,19,20,21,22,23,24,25	11	P04041,P18418,P52590,Q4FZU2,Q4FZU6,Q6AY33,Q6IG02,Q8VD89,Q9ESW0,Q9QX79,Q9R1T1
5179	1,3,4,5,7,8,19,20	2,6,18,21,22,23,24,25,26	5	P01681,P18418,Q4FZU6,Q6IRE4,Q9ESW0
5180	1,3,4,5,7,8,19,21	2,6,18,20,22,23,24,25,26	6	P00731,P01681,P18418,P35281,P52590,Q9ESW0
5181	1,3,4,5,7,8,19,22	2,6,18,20,21,23,24,25,26	4	O54728,P18418,Q6P6R2,Q9QW07
5182	1,3,4,5,7,8,19,23	2,6,18,20,21,22,24,25,26	2	P18418,Q99041
5183	1,3,4,5,7,8,19,24	2,6,18,20,21,22,23,25,26	1	P18418
5184	1,3,4,5,7,8,19,25	2,6,18,20,21,22,23,24,26	3	P10715,P18418,Q9QUL6

5185	1,3,4,5,7,8,19,26	2,6,18,20,21,22,23,24,25	6	O54728,P01681,P18418,Q4FZU6,Q5FVI6,Q9ESW0
5186	1,3,4,5,7,8,20,21	2,6,18,19,22,23,24,25,26	8	P01681,P18418,P42854,P52590,Q4FZU6,Q6IRE4,Q7TPB1,Q9ESW0
5187	1,3,4,5,7,8,20,22	2,6,18,19,21,23,24,25,26	8	O54728,P01681,P18418,Q4FZU6,Q63617,Q6IRE4,Q9ESW0,Q9QW07
5188	1,3,4,5,7,8,20,23	2,6,18,19,21,22,24,25,26	6	P01681,P97840,Q4FZU6,Q63617,Q6IRE4,Q9ESW0
5189	1,3,4,5,7,8,20,24	2,6,18,19,21,22,23,25,26	5	P18418,P97840,Q4FZU6,Q63617,Q6IRE4
5190	1,3,4,5,7,8,20,25	2,6,18,19,21,22,23,24,26	4	P10715,P18418,Q6IRE4,Q9ESW0
5191	1,3,4,5,7,8,20,26	2,6,18,19,21,22,23,24,25	7	O54728,P01681,P05539,P97840,Q4FZU6,Q6IRE4,Q9ESW0
5192	1,3,4,5,7,8,21,22	2,6,18,19,20,23,24,25,26	5	P18418,P52590,Q7TPB1,Q9ESW0,Q9QW07
5193	1,3,4,5,7,8,21,23	2,6,18,19,20,22,24,25,26	6	P18418,P52590,Q4FZU6,Q6IRE4,Q99041,Q9ESW0
5194	1,3,4,5,7,8,21,24	2,6,18,19,20,22,23,25,26	4	P18418,P52590,Q6IRE4,Q9ESW0
5195	1,3,4,5,7,8,21,25	2,6,18,19,20,22,23,24,26	8	P08721,P10715,P18418,P20646,P52590,Q6AYS4,Q6IRE4,Q9ESW0
5196	1,3,4,5,7,8,21,26	2,6,18,19,20,22,23,24,25	4	P01681,P52590,Q4FZU6,Q9ESW0
5197	1,3,4,5,7,8,22,23	2,6,18,19,20,21,24,25,26	3	O54728,P18418,Q9QW07
5198	1,3,4,5,7,8,22,24	2,6,18,19,20,21,23,25,26	3	P08010,P18418,Q9QW07
5199	1,3,4,5,7,8,22,25	2,6,18,19,20,21,23,24,26	3	P10715,P18418,Q9QW07

5200	1,3,4,5,7,8,22,26	2,6,18,19,20,21,23,24,25	7	O54728,P05539,P18418,Q4FZU6,Q8VD89,Q9ESW0,Q9QW07
5201	1,3,4,5,7,8,23,24	2,6,18,19,20,21,22,25,26	4	P18418,P34901,P97840,Q63751
5202	1,3,4,5,7,8,23,25	2,6,18,19,20,21,22,24,26	8	P10715,P18418,P19629,P52590,P55314,Q63751,Q6AYS4,Q99041
5203	1,3,4,5,7,8,23,26	2,6,18,19,20,21,22,24,25	13	O54728,P01681,P01946,P02091,P05539,P97840,Q4FZU2,Q4FZU6,Q5I0D1,Q6IG02,Q8VD89,Q9ESW0,Q9QX79
5204	1,3,4,5,7,8,24,25	2,6,18,19,20,21,22,23,26	4	P10715,P18418,P19629,P52590
5205	1,3,4,5,7,8,24,26	2,6,18,19,20,21,22,23,25	7	O54728,P02091,P18418,P97840,Q4FZU6,Q8CJD3,Q8VD89
5206	1,3,4,5,7,8,25,26	2,6,18,19,20,21,22,23,24	4	P02091,P05539,P10715,Q9ESW0
5207	1,3,4,5,7,18,19,20	2,6,8,21,22,23,24,25,26	7	P00731,P07150,Q4FZU6,Q6IRE4,Q6P6R2,Q9ESW0,Q9WTT6
5208	1,3,4,5,7,18,19,21	2,6,8,20,22,23,24,25,26	3	P00731,P25031,Q9ESW0
5209	1,3,4,5,7,18,19,22	2,6,8,20,21,23,24,25,26	5	P07150,Q6P6R2,Q9ESW0,Q9QW07,Q9WTT6
5210	1,3,4,5,7,18,19,23	2,6,8,20,21,22,24,25,26	4	P02680,P12785,Q03191,Q9ESW0
5211	1,3,4,5,7,18,19,24	2,6,8,20,21,22,23,25,26	2	Q6P6R2,Q9WTT6
5212	1,3,4,5,7,18,19,25	2,6,8,20,21,22,23,24,26	2	P00731,Q9ESW0
5213	1,3,4,5,7,18,19,26	2,6,8,20,21,22,23,24,25	5	O54728,Q4FZU6,Q5FVI6,Q6P6R2,Q9ESW0
5214	1,3,4,5,7,18,20,21	2,6,8,19,22,23,24,25,26	7	P00731,P01681,P25031,P42854,Q4FZU6,Q6IRE4,Q9ESW0

5215	1,3,4,5,7,18,20,22	2,6,8,19,21,23,24,25,26	7	P07150,Q4FZU6,Q6IRE4,Q6P6R2,Q9ESW0,Q9QW07,Q9WTT6
5216	1,3,4,5,7,18,20,23	2,6,8,19,21,22,24,25,26	7	A2RUV9,P12785,Q4FZU2,Q4FZU6,Q6IG02,Q6IRE4,Q9ESW0
5217	1,3,4,5,7,18,20,24	2,6,8,19,21,22,23,25,26	5	Q4FZU6,Q6IRE4,Q6P6R2,Q9ESW0,Q9WTT6
5218	1,3,4,5,7,18,20,25	2,6,8,19,21,22,23,24,26	3	P61206;P84079;P84082,Q6IRE4,Q9ESW0
5219	1,3,4,5,7,18,20,26	2,6,8,19,21,22,23,24,25	9	O54728,P01681,P05539,Q4FZU2,Q4FZU6,Q6IG02,Q6IRE4,Q6P6R2,Q9ESW0
5220	1,3,4,5,7,18,21,22	2,6,8,19,20,23,24,25,26	3	P25031,Q9ESW0,Q9QW07
5221	1,3,4,5,7,18,21,23	2,6,8,19,20,22,24,25,26	7	P12785,P25031,P34901,Q4FZU6,Q62636,Q8K4G9,Q9ESW0
5222	1,3,4,5,7,18,21,24	2,6,8,19,20,22,23,25,26	4	P25031,Q6IRE4,Q8K4G9,Q9ESW0
5223	1,3,4,5,7,18,21,25	2,6,8,19,20,22,23,24,26	6	P00731,P08721,P25031,P31430,P61206;P84079;P84082,Q9ESW0
5224	1,3,4,5,7,18,21,26	2,6,8,19,20,22,23,24,25	6	P01681,P25031,P61206;P84079;P84082,Q4FZU2,Q4FZU6,Q9ESW0
5225	1,3,4,5,7,18,22,23	2,6,8,19,20,21,24,25,26	7	P12785,P34901,P62804,Q8K4G9,Q8VD89,Q9ESW0,Q9QW07
5226	1,3,4,5,7,18,22,24	2,6,8,19,20,21,23,25,26	7	P62804,Q00715,Q6P6R2,Q8K4G9,Q8VD89,Q9QW07,Q9WTT6
5227	1,3,4,5,7,18,22,25	2,6,8,19,20,21,23,24,26	2	P62804,Q9QW07
5228	1,3,4,5,7,18,22,26	2,6,8,19,20,21,23,24,25	7	O54728,P05539,Q4FZU6,Q6P6R2,Q8VD89,Q9ESW0,Q9QW07
5229	1,3,4,5,7,18,23,24	2,6,8,19,20,21,22,25,26	7	P12785,P34901,P62804,Q00715,Q8K4G9,Q8VD89,Q9ESW0

5230	1,3,4,5,7,18,23,25	2,6,8,19,20,21,22,24,26	7	P00714,P12785,P55314,P62804,Q63751,Q8K4G9,Q9ESW0
5231	1,3,4,5,7,18,23,26	2,6,8,19,20,21,22,24,25	16	O54728,P01946,P02091,P05539,P12785,P35467,Q4FZU2,Q4FZU6,Q64194,Q6IFU8,Q6IFW6,Q6IG02,Q6IMF3,Q6P6Q2,Q8VD89,Q9ESW0
5232	1,3,4,5,7,18,24,25	2,6,8,19,20,21,22,23,26	4	P08721,P62804,Q00715,Q8K4G9
5233	1,3,4,5,7,18,24,26	2,6,8,19,20,21,22,23,25	5	P02091,Q6P6R2,Q8CJD3,Q8VD89,Q9ESW0
5234	1,3,4,5,7,18,25,26	2,6,8,19,20,21,22,23,24	6	P02091,P05539,P08721,P61206;P84079;P84082,Q4FZU2,Q9ESW0
5235	1,3,4,5,7,19,20,21	2,6,8,18,22,23,24,25,26	6	P00731,P01681,P42854,Q4FZU6,Q6IRE4,Q9ESW0
5236	1,3,4,5,7,19,20,22	2,6,8,18,21,23,24,25,26	10	O54728,P00774,P07150,P14669,P55091,P98089,Q62997,Q6P6R2,Q9QW07,Q9WTT6
5237	1,3,4,5,7,19,20,23	2,6,8,18,21,22,24,25,26	5	O54728,P55091,P98089,Q4FZU6,Q6IRE4
5238	1,3,4,5,7,19,20,24	2,6,8,18,21,22,23,25,26	5	P07150,P14669,Q6IRE4,Q6P6R2,Q9WTT6
5239	1,3,4,5,7,19,20,25	2,6,8,18,21,22,23,24,26	1	Q6IRE4
5240	1,3,4,5,7,19,20,26	2,6,8,18,21,22,23,24,25	6	O54728,P01681,P05539,Q4FZU6,Q5FVI6,Q6P6R2
5241	1,3,4,5,7,19,21,22	2,6,8,18,20,23,24,25,26	1	Q9QW07
5242	1,3,4,5,7,19,21,23	2,6,8,18,20,22,24,25,26	0	
5243	1,3,4,5,7,19,21,24	2,6,8,18,20,22,23,25,26	0	
5244	1,3,4,5,7,19,21,25	2,6,8,18,20,22,23,24,26	1	P17046

5245	1,3,4,5,7,19,21,26	2,6,8,18,20,22,23,24,25	6	O54728,P01681,P05539,Q4FZU6,Q5FVI6,Q9ESW0
5246	1,3,4,5,7,19,22,23	2,6,8,18,20,21,24,25,26	6	O54728,P17046,P55091,P62804,Q9QW07,Q9WTT6
5247	1,3,4,5,7,19,22,24	2,6,8,18,20,21,23,25,26	7	O54728,P07150,P14669,P62804,Q6P6R2,Q9QW07,Q9WTT6
5248	1,3,4,5,7,19,22,25	2,6,8,18,20,21,23,24,26	3	P17046,P62804,Q9QW07
5249	1,3,4,5,7,19,22,26	2,6,8,18,20,21,23,24,25	5	O54728,P05539,Q6P6R2,Q9QW07,Q9WTT6
5250	1,3,4,5,7,19,23,24	2,6,8,18,20,21,22,25,26	4	P34901,P62804,Q00715,Q8K4G9
5251	1,3,4,5,7,19,23,25	2,6,8,18,20,21,22,24,26	4	P17046,P55314,P62804,Q99041
5252	1,3,4,5,7,19,23,26	2,6,8,18,20,21,22,24,25	6	O54728,P01946,P02091,P05539,Q4FZU2,Q5FVI6
5253	1,3,4,5,7,19,24,25	2,6,8,18,20,21,22,23,26	2	P17046,P62804
5254	1,3,4,5,7,19,24,26	2,6,8,18,20,21,22,23,25	4	O54728,P02091,Q6P6R2,Q8CJD3
5255	1,3,4,5,7,19,25,26	2,6,8,18,20,21,22,23,24	4	O54728,P02091,P05539,Q5FVI6
5256	1,3,4,5,7,20,21,22	2,6,8,18,19,23,24,25,26	7	P01681,Q4FZU6,Q6IRE4,Q7TPB1,Q99MH3,Q9ESW0,Q9QW07
5257	1,3,4,5,7,20,21,23	2,6,8,18,19,22,24,25,26	4	P01681,Q4FZU6,Q6IRE4,Q9ESW0
5258	1,3,4,5,7,20,21,24	2,6,8,18,19,22,23,25,26	4	P01681,Q4FZU6,Q6IRE4,Q9ESW0
5259	1,3,4,5,7,20,21,25	2,6,8,18,19,22,23,24,26	3	P01681,Q6IRE4,Q9ESW0

5260	1,3,4,5,7,20,21,26	2,6,8,18,19,22,23,24,25	6	O54728,P01681,P05539,Q4FZU6,Q6IRE4,Q9ESW0
5261	1,3,4,5,7,20,22,23	2,6,8,18,19,21,24,25,26	7	O54728,P55091,P98089,Q4FZU6,Q62997,Q6IRE4,Q9QW07
5262	1,3,4,5,7,20,22,24	2,6,8,18,19,21,23,25,26	9	P07150,P08010,P14669,Q62635,Q6IRE4,Q6P6R2,Q99MH3,Q9QW07,Q9WTT6
5263	1,3,4,5,7,20,22,25	2,6,8,18,19,21,23,24,26	2	Q6IRE4,Q9QW07
5264	1,3,4,5,7,20,22,26	2,6,8,18,19,21,23,24,25	8	O54728,P01681,P05539,Q4FZU6,Q62635,Q6P6R2,Q99MH3,Q9QW07
5265	1,3,4,5,7,20,23,24	2,6,8,18,19,21,22,25,26	5	P34901,P97840,Q4FZU6,Q6IRE4,Q8K4G9
5266	1,3,4,5,7,20,23,25	2,6,8,18,19,21,22,24,26	2	P55314,Q6IRE4
5267	1,3,4,5,7,20,23,26	2,6,8,18,19,21,22,24,25	13	O54728,P01681,P01946,P02091,P05539,P97840,Q4FZU2,Q4FZU6,Q6IFW6,Q6IG02,Q6IRE4,Q8CIZ5,Q9ESW0
5268	1,3,4,5,7,20,24,25	2,6,8,18,19,21,22,23,26	1	Q6IRE4
5269	1,3,4,5,7,20,24,26	2,6,8,18,19,21,22,23,25	11	O54728,P01681,P02091,P05539,P08010,P97840,Q4FZU6,Q62635,Q6IRE4,Q8CJD3,Q99MH3
5270	1,3,4,5,7,20,25,26	2,6,8,18,19,21,22,23,24	5	O54728,P01681,P02091,P05539,Q6IRE4
5271	1,3,4,5,7,21,22,23	2,6,8,18,19,20,24,25,26	3	P34901,Q8K4G9,Q9QW07
5272	1,3,4,5,7,21,22,24	2,6,8,18,19,20,23,25,26	2	Q8K4G9,Q9QW07
5273	1,3,4,5,7,21,22,25	2,6,8,18,19,20,23,24,26	2	P17046,Q9QW07
5274	1,3,4,5,7,21,22,26	2,6,8,18,19,20,23,24,25	6	O54728,P01681,P05539,Q4FZU6,Q9ESW0,Q9QW07

5275	1,3,4,5,7,21,23,24	2,6,8,18,19,20,22,25,26	4	P34901,Q63751,Q6IRE4,Q8K4G9
5276	1,3,4,5,7,21,23,25	2,6,8,18,19,20,22,24,26	4	P55314,Q63751,Q6IRE4,Q8K4G9
5277	1,3,4,5,7,21,23,26	2,6,8,18,19,20,22,24,25	9	O54728,P01681,P01946,P02091,P05539,P34901,Q4FZU2,Q4FZU6,Q9ESW0
5278	1,3,4,5,7,21,24,25	2,6,8,18,19,20,22,23,26	3	P22006,Q6IRE4,Q8K4G9
5279	1,3,4,5,7,21,24,26	2,6,8,18,19,20,22,23,25	4	P02091,Q4FZU6,Q8CJD3,Q9ESW0
5280	1,3,4,5,7,21,25,26	2,6,8,18,19,20,22,23,24	4	P01681,P02091,P05539,Q9ESW0
5281	1,3,4,5,7,22,23,24	2,6,8,18,19,20,21,25,26	6	O54728,P34901,P62804,Q00715,Q8K4G9,Q9QW07
5282	1,3,4,5,7,22,23,25	2,6,8,18,19,20,21,24,26	6	P17046,P55314,P62804,Q63751,Q8K4G9,Q9QW07
5283	1,3,4,5,7,22,23,26	2,6,8,18,19,20,21,24,25	8	O54728,P01946,P02091,P05539,Q4FZU2,Q8CIZ5,Q8K4G9,Q9QW07
5284	1,3,4,5,7,22,24,25	2,6,8,18,19,20,21,23,26	5	P17046,P62804,Q00715,Q8K4G9,Q9QW07
5285	1,3,4,5,7,22,24,26	2,6,8,18,19,20,21,23,25	11	O54728,P02091,P05539,P08010,Q62635,Q6P6R2,Q8CJD3,Q8K4G9,Q8VD89,Q99MH3,Q9QW07
5286	1,3,4,5,7,22,25,26	2,6,8,18,19,20,21,23,24	4	O54728,P02091,P05539,Q9QW07
5287	1,3,4,5,7,23,24,25	2,6,8,18,19,20,21,22,26	6	P34901,P55314,P62804,Q00715,Q63751,Q8K4G9
5288	1,3,4,5,7,23,24,26	2,6,8,18,19,20,21,22,25	9	O54728,P01946,P02091,P34901,P47967,P97840,Q4FZU2,Q8K4G9,Q8VD89
5289	1,3,4,5,7,23,25,26	2,6,8,18,19,20,21,22,24	6	O54728,P01946,P02091,P05539,P55314,Q4FZU2

5290	1,3,4,5,7,24,25,26	2,6,8,18,19,20,21,22,23	3	P01946,P02091,Q8CJD3
5291	1,3,4,5,8,18,19,20	2,6,7,21,22,23,24,25,26	5	P00731,P01681,P22006,P83121,Q9ESW0
5292	1,3,4,5,8,18,19,21	2,6,7,20,22,23,24,25,26	6	P00731,P01681,P02803,P10758,P35281,Q9ESW0
5293	1,3,4,5,8,18,19,22	2,6,7,20,21,23,24,25,26	4	P22006,P83121,Q6P6R2,Q9QW07
5294	1,3,4,5,8,18,19,23	2,6,7,20,21,22,24,25,26	8	P02803,P09605;P25809,P12785,P35467,Q03191,Q64194,Q6IG02,Q9ESW0
5295	1,3,4,5,8,18,19,24	2,6,7,20,21,22,23,25,26	2	P09605;P25809,P83121
5296	1,3,4,5,8,18,19,25	2,6,7,20,21,22,23,24,26	1	P02803
5297	1,3,4,5,8,18,19,26	2,6,7,20,21,22,23,24,25	6	O54728,P01681,P09605;P25809,Q4FZU2,Q63942,Q9ESW0
5298	1,3,4,5,8,18,20,21	2,6,7,19,22,23,24,25,26	7	P00731,P01681,P10758,P14480,Q4FZU6,Q6IRE4,Q9ESW0
5299	1,3,4,5,8,18,20,22	2,6,7,19,21,23,24,25,26	7	P01681,P22006,P83121,P97840,Q6P6R2,Q9ESW0,Q9QW07
5300	1,3,4,5,8,18,20,23	2,6,7,19,21,22,24,25,26	10	P01681,P12785,P22006,P35467,P97840,Q4FZU2,Q4FZU6,Q6IFW6,Q6IG02,Q9ESW0
5301	1,3,4,5,8,18,20,24	2,6,7,19,21,22,23,25,26	4	P83121,P97840,Q9ESW0,Q9QZK9
5302	1,3,4,5,8,18,20,25	2,6,7,19,21,22,23,24,26	1	Q9ESW0
5303	1,3,4,5,8,18,20,26	2,6,7,19,21,22,23,24,25	8	O54728,P01681,P97840,Q4FZU2,Q4FZU6,Q6IFW6,Q6IG02,Q9ESW0
5304	1,3,4,5,8,18,21,22	2,6,7,19,20,23,24,25,26	3	P10758,Q9ESW0,Q9QW07

5305	1,3,4,5,8,18,21,23	2,6,7,19,20,22,24,25,26	6	P01681,P10758,P12785,Q4FZU2,Q6IG02,Q9ESW0
5306	1,3,4,5,8,18,21,24	2,6,7,19,20,22,23,25,26	2	P10758,Q9ESW0
5307	1,3,4,5,8,18,21,25	2,6,7,19,20,22,23,24,26	5	P00731,P08721,P31430,Q6AYS4,Q9ESW0
5308	1,3,4,5,8,18,21,26	2,6,7,19,20,22,23,24,25	6	P01681,P10758,Q4FZU2,Q4FZU6,Q6IG02,Q9ESW0
5309	1,3,4,5,8,18,22,23	2,6,7,19,20,21,24,25,26	4	P97840,Q64194,Q6IG02,Q9QW07
5310	1,3,4,5,8,18,22,24	2,6,7,19,20,21,23,25,26	4	P83121,P97840,Q6P6R2,Q9QW07
5311	1,3,4,5,8,18,22,25	2,6,7,19,20,21,23,24,26	2	Q6AYS4,Q9QW07
5312	1,3,4,5,8,18,22,26	2,6,7,19,20,21,23,24,25	5	O54728,P97840,Q6P6R2,Q9ESW0,Q9QW07
5313	1,3,4,5,8,18,23,24	2,6,7,19,20,21,22,25,26	6	P09605;P25809,P12785,P34901,P47967,P97840,Q6IG02
5314	1,3,4,5,8,18,23,25	2,6,7,19,20,21,22,24,26	4	P00714,P12785,Q6AYS4,Q6IG02
5315	1,3,4,5,8,18,23,26	2,6,7,19,20,21,22,24,25	19	O54728,P01681,P09605;P25809,P12785,P35467,P47967,P97840,Q4FZU2,Q4FZU6,Q5I0D1,Q63942,Q64194,Q6AY61,Q6IFU8,Q6IFW6,Q6IG02,Q6IMF3,Q6P6Q2,Q9ESW0
5316	1,3,4,5,8,18,24,25	2,6,7,19,20,21,22,23,26	1	P08721
5317	1,3,4,5,8,18,24,26	2,6,7,19,20,21,22,23,25	6	P09605;P25809,P47967,P97840,Q4FZU2,Q8VD89,Q9ESW0
5318	1,3,4,5,8,18,25,26	2,6,7,19,20,21,22,23,24	6	P00714,P08721,Q4FZU2,Q6AY33,Q6IG02,Q9ESW0
5319	1,3,4,5,8,19,20,21	2,6,7,18,22,23,24,25,26	3	P01681,P35281,P55091

5320	1,3,4,5,8,19,20,22	2,6,7,18,21,23,24,25,26	11	O54728,P00774,P01681,P22006,P55091,P83121,P98089,Q62894,Q62997,Q6P6R2,Q9QW07
5321	1,3,4,5,8,19,20,23	2,6,7,18,21,22,24,25,26	4	P01681,P22006,P55091,P97840
5322	1,3,4,5,8,19,20,24	2,6,7,18,21,22,23,25,26	3	P55091,P83121,P97840
5323	1,3,4,5,8,19,20,25	2,6,7,18,21,22,23,24,26	2	P01681,Q9QUL6
5324	1,3,4,5,8,19,20,26	2,6,7,18,21,22,23,24,25	5	O54728,P01681,P97840,Q4FZU6,Q62894
5325	1,3,4,5,8,19,21,22	2,6,7,18,20,23,24,25,26	2	P01681,Q9QW07
5326	1,3,4,5,8,19,21,23	2,6,7,18,20,22,24,25,26	2	P01681,P35281
5327	1,3,4,5,8,19,21,24	2,6,7,18,20,22,23,25,26	0	
5328	1,3,4,5,8,19,21,25	2,6,7,18,20,22,23,24,26	1	Q9QUL6
5329	1,3,4,5,8,19,21,26	2,6,7,18,20,22,23,24,25	3	O54728,P01681,P35281
5330	1,3,4,5,8,19,22,23	2,6,7,18,20,21,24,25,26	3	O54728,P55091,Q9QW07
5331	1,3,4,5,8,19,22,24	2,6,7,18,20,21,23,25,26	4	O54728,P83121,Q6P6R2,Q9QW07
5332	1,3,4,5,8,19,22,25	2,6,7,18,20,21,23,24,26	2	Q9QUL6,Q9QW07
5333	1,3,4,5,8,19,22,26	2,6,7,18,20,21,23,24,25	4	O54728,P01681,Q6P6R2,Q9QW07
5334	1,3,4,5,8,19,23,24	2,6,7,18,20,21,22,25,26	1	P09605;P25809

5335	1,3,4,5,8,19,23,25	2,6,7,18,20,21,22,24,26	2	Q99041,Q9QUL6
5336	1,3,4,5,8,19,23,26	2,6,7,18,20,21,22,24,25	7	O54728,P01681,P09605;P25809,P35467,P97840,Q4FZU2,Q6IG02
5337	1,3,4,5,8,19,24,25	2,6,7,18,20,21,22,23,26	1	Q9QUL6
5338	1,3,4,5,8,19,24,26	2,6,7,18,20,21,22,23,25	3	O54728,P09605;P25809,P97840
5339	1,3,4,5,8,19,25,26	2,6,7,18,20,21,22,23,24	2	O54728,P01681
5340	1,3,4,5,8,20,21,22	2,6,7,18,19,23,24,25,26	4	P01681,P55091,Q7TPB1,Q9QW07
5341	1,3,4,5,8,20,21,23	2,6,7,18,19,22,24,25,26	5	P01681,P97840,Q4FZU6,Q6IRE4,Q9ESW0
5342	1,3,4,5,8,20,21,24	2,6,7,18,19,22,23,25,26	4	P01681,P97840,Q5XFX0,Q6IRE4
5343	1,3,4,5,8,20,21,25	2,6,7,18,19,22,23,24,26	2	P01681,Q6IRE4
5344	1,3,4,5,8,20,21,26	2,6,7,18,19,22,23,24,25	6	O54728,P01681,P97840,Q4FZU2,Q4FZU6,Q9ESW0
5345	1,3,4,5,8,20,22,23	2,6,7,18,19,21,24,25,26	7	O54728,P01681,P22006,P55091,P82995,P97840,Q9QW07
5346	1,3,4,5,8,20,22,24	2,6,7,18,19,21,23,25,26	6	P55091,P82995,P83121,P97840,Q99MH3,Q9QW07
5347	1,3,4,5,8,20,22,25	2,6,7,18,19,21,23,24,26	2	D3ZUC6,Q9QW07
5348	1,3,4,5,8,20,22,26	2,6,7,18,19,21,23,24,25	8	O54728,P01681,P05539,P97840,Q4FZU6,Q6P6R2,Q99MH3,Q9QW07
5349	1,3,4,5,8,20,23,24	2,6,7,18,19,21,22,25,26	3	P47967,P82995,P97840

5350	1,3,4,5,8,20,23,25	2,6,7,18,19,21,22,24,26	1	P01681
5351	1,3,4,5,8,20,23,26	2,6,7,18,19,21,22,24,25	11	O54728,P01681,P47967,P97840,Q4FZU2,Q4FZU6,Q6IFW6,Q6IG02,Q6IMF3,Q6P6Q2,Q8CIZ5
5352	1,3,4,5,8,20,24,25	2,6,7,18,19,21,22,23,26	0	
5353	1,3,4,5,8,20,24,26	2,6,7,18,19,21,22,23,25	7	O54728,P01681,P47967,P97840,Q4FZU6,Q8CJD3,Q99MH3
5354	1,3,4,5,8,20,25,26	2,6,7,18,19,21,22,23,24	3	O54728,P01681,P97840
5355	1,3,4,5,8,21,22,23	2,6,7,18,19,20,24,25,26	2	P01681,Q9QW07
5356	1,3,4,5,8,21,22,24	2,6,7,18,19,20,23,25,26	2	Q5XFX0,Q9QW07
5357	1,3,4,5,8,21,22,25	2,6,7,18,19,20,23,24,26	2	Q6AYS4,Q9QW07
5358	1,3,4,5,8,21,22,26	2,6,7,18,19,20,23,24,25	3	O54728,P01681,Q9QW07
5359	1,3,4,5,8,21,23,24	2,6,7,18,19,20,22,25,26	2	P34901,P97840
5360	1,3,4,5,8,21,23,25	2,6,7,18,19,20,22,24,26	3	P06760,Q63751,Q6AYS4
5361	1,3,4,5,8,21,23,26	2,6,7,18,19,20,22,24,25	10	O54728,P01681,P97840,Q4FZU2,Q4FZU6,Q6IFW6,Q6IG02,Q6IMF3,Q6P6Q2,Q9ESW0
5362	1,3,4,5,8,21,24,25	2,6,7,18,19,20,22,23,26	0	
5363	1,3,4,5,8,21,24,26	2,6,7,18,19,20,22,23,25	3	P01681,P97840,Q8CJD3
5364	1,3,4,5,8,21,25,26	2,6,7,18,19,20,22,23,24	2	P01681,Q6AYS4

5365	1,3,4,5,8,22,23,24	2,6,7,18,19,20,21,25,26	5	P34901,P47967,P82995,P97840,Q9QW07
5366	1,3,4,5,8,22,23,25	2,6,7,18,19,20,21,24,26	1	Q9QW07
5367	1,3,4,5,8,22,23,26	2,6,7,18,19,20,21,24,25	8	O54728,P01681,P47967,P97840,Q4FZU2,Q6IG02,Q8CIZ5,Q9QW07
5368	1,3,4,5,8,22,24,25	2,6,7,18,19,20,21,23,26	2	Q9QUL6,Q9QW07
5369	1,3,4,5,8,22,24,26	2,6,7,18,19,20,21,23,25	6	O54728,P47967,P97840,Q6P6R2,Q99MH3,Q9QW07
5370	1,3,4,5,8,22,25,26	2,6,7,18,19,20,21,23,24	2	O54728,Q9QW07
5371	1,3,4,5,8,23,24,25	2,6,7,18,19,20,21,22,26	1	Q63751
5372	1,3,4,5,8,23,24,26	2,6,7,18,19,20,21,22,25	9	O54728,P02091,P09605;P25809,P34901,P47967,P97840,Q4FZU2,Q5I0D1,Q6IG02
5373	1,3,4,5,8,23,25,26	2,6,7,18,19,20,21,22,24	7	O54728,P02091,Q4FZU2,Q5I0D1,Q6IFW6,Q6IG02,Q6P6Q2
5374	1,3,4,5,8,24,25,26	2,6,7,18,19,20,21,22,23	2	Q62902,Q8CJD3
5375	1,3,4,5,18,19,20,21	2,6,7,8,22,23,24,25,26	4	P00731,P01681,P10758,Q9ESW0
5376	1,3,4,5,18,19,20,22	2,6,7,8,21,23,24,25,26	8	O54728,P07150,P14669,P22006,Q62997,Q6P6R2,Q9QW07,Q9WTT6
5377	1,3,4,5,18,19,20,23	2,6,7,8,21,22,24,25,26	4	P12785,P22006,Q4FZU2,Q6IG02
5378	1,3,4,5,18,19,20,24	2,6,7,8,21,22,23,25,26	5	P07150,P83121,Q6P6R2,Q9QZK9,Q9WTT6
5379	1,3,4,5,18,19,20,25	2,6,7,8,21,22,23,24,26	0	

5380	1,3,4,5,18,19,20,26	2,6,7,8,21,22,23,24,25	6	O54728,P01681,Q4FZU2,Q4FZU6,Q6IG02,Q6P6R2
5381	1,3,4,5,18,19,21,22	2,6,7,8,20,23,24,25,26	2	P10758,Q9QW07
5382	1,3,4,5,18,19,21,23	2,6,7,8,20,22,24,25,26	3	P10758,P12785,Q9ESW0
5383	1,3,4,5,18,19,21,24	2,6,7,8,20,22,23,25,26	1	P10758
5384	1,3,4,5,18,19,21,25	2,6,7,8,20,22,23,24,26	2	P00731,P31430
5385	1,3,4,5,18,19,21,26	2,6,7,8,20,22,23,24,25	5	P01681,P10758,P12020,Q4FZU2,Q9ESW0
5386	1,3,4,5,18,19,22,23	2,6,7,8,20,21,24,25,26	4	O54728,P12785,Q9QW07,Q9WTT6
5387	1,3,4,5,18,19,22,24	2,6,7,8,20,21,23,25,26	6	P07150,P62804,P83121,Q6P6R2,Q9QW07,Q9WTT6
5388	1,3,4,5,18,19,22,25	2,6,7,8,20,21,23,24,26	1	Q9QW07
5389	1,3,4,5,18,19,22,26	2,6,7,8,20,21,23,24,25	4	O54728,Q6P6R2,Q9QW07,Q9WTT6
5390	1,3,4,5,18,19,23,24	2,6,7,8,20,21,22,25,26	5	P09605;P25809,P12785,P34901,Q03191,Q9WTT6
5391	1,3,4,5,18,19,23,25	2,6,7,8,20,21,22,24,26	2	P12785,Q6BEA2
5392	1,3,4,5,18,19,23,26	2,6,7,8,20,21,22,24,25	12	O54728,P09605;P25809,P12785,P35467,Q03191,Q4FZU2,Q64194,Q6IFU8,Q6IFW6,Q6IG02,Q6IMF3,Q6P6Q2
5393	1,3,4,5,18,19,24,25	2,6,7,8,20,21,22,23,26	0	
5394	1,3,4,5,18,19,24,26	2,6,7,8,20,21,22,23,25	4	O54728,P09605;P25809,Q6P6R2,Q9WTT6

5395	1,3,4,5,18,19,25,26	2,6,7,8,20,21,22,23,24	1	Q4FZU2
5396	1,3,4,5,18,20,21,22	2,6,7,8,19,23,24,25,26	4	P01681,P10758,Q9ESW0,Q9QW07
5397	1,3,4,5,18,20,21,23	2,6,7,8,19,22,24,25,26	8	P01681,P01835,P10758,P12785,Q4FZU2,Q4FZU6,Q6IG02,Q9ESW0
5398	1,3,4,5,18,20,21,24	2,6,7,8,19,22,23,25,26	4	P01681,P12020,Q6IRE4,Q9ESW0
5399	1,3,4,5,18,20,21,25	2,6,7,8,19,22,23,24,26	3	P01681,P31430,Q9ESW0
5400	1,3,4,5,18,20,21,26	2,6,7,8,19,22,23,24,25	8	P01681,P01835,P06911,P12020,Q4FZU2,Q4FZU6,Q6IG02,Q9ESW0
5401	1,3,4,5,18,20,22,23	2,6,7,8,19,21,24,25,26	7	P12785,P22006,P97840,Q4FZU2,Q6IG02,Q9QW07,Q9QZK9
5402	1,3,4,5,18,20,22,24	2,6,7,8,19,21,23,25,26	8	P07150,P14669,P83121,P97840,Q6P6R2,Q9QW07,Q9QZK9,Q9WTT6
5403	1,3,4,5,18,20,22,25	2,6,7,8,19,21,23,24,26	1	Q9QW07
5404	1,3,4,5,18,20,22,26	2,6,7,8,19,21,23,24,25	10	O54728,P01681,P05539,P97840,Q4FZU2,Q4FZU6,Q6IG02,Q6P6R2,Q99041,Q9QW07
5405	1,3,4,5,18,20,23,24	2,6,7,8,19,21,22,25,26	7	P12785,P34901,P47967,P97840,Q4FZU2,Q6IG02,Q9QZK9
5406	1,3,4,5,18,20,23,25	2,6,7,8,19,21,22,24,26	3	P12785,Q4FZU2,Q6IG02
5407	1,3,4,5,18,20,23,26	2,6,7,8,19,21,22,24,25	14	O54728,P01681,P12785,P35467,P47967,P97840,Q4FZU2,Q4FZU6,Q6IFU8,Q6IFW6,Q6IG02,Q6IMF3,Q6P6Q2,Q9ESW0
5408	1,3,4,5,18,20,24,25	2,6,7,8,19,21,22,23,26	0	
5409	1,3,4,5,18,20,24,26	2,6,7,8,19,21,22,23,25	10	P01681,P06911,P12020,P47967,P97840,Q4FZU2,Q4FZU6,Q6IG02,Q6P6R2,Q8CJD3

5410	1,3,4,5,18,20,25,26	2,6,7,8,19,21,22,23,24	3	P01681,Q4FZU2,Q6IG02
5411	1,3,4,5,18,21,22,23	2,6,7,8,19,20,24,25,26	4	P10758,P12785,P34901,Q9QW07
5412	1,3,4,5,18,21,22,24	2,6,7,8,19,20,23,25,26	1	Q9QW07
5413	1,3,4,5,18,21,22,25	2,6,7,8,19,20,23,24,26	2	P31430,Q9QW07
5414	1,3,4,5,18,21,22,26	2,6,7,8,19,20,23,24,25	5	O54728,P01681,Q4FZU2,Q9ESW0,Q9QW07
5415	1,3,4,5,18,21,23,24	2,6,7,8,19,20,22,25,26	2	P12785,P34901
5416	1,3,4,5,18,21,23,25	2,6,7,8,19,20,22,24,26	4	P12785,P31430,P34901,Q4FZU2
5417	1,3,4,5,18,21,23,26	2,6,7,8,19,20,22,24,25	11	P01681,P12785,P34901,Q4FZU2,Q62636,Q6IFU8,Q6IFW6,Q6IG02,Q6IMF3,Q6P6Q2,Q9ESW0
5418	1,3,4,5,18,21,24,25	2,6,7,8,19,20,22,23,26	2	P08721,P31430
5419	1,3,4,5,18,21,24,26	2,6,7,8,19,20,22,23,25	6	P01681,P06911,P12020,Q4FZU2,Q8CJD3,Q9ESW0
5420	1,3,4,5,18,21,25,26	2,6,7,8,19,20,22,23,24	5	P01681,P08721,P31430,Q4FZU2,Q9ESW0
5421	1,3,4,5,18,22,23,24	2,6,7,8,19,20,21,25,26	6	P12785,P34901,P62804,P97840,Q00715,Q9QW07
5422	1,3,4,5,18,22,23,25	2,6,7,8,19,20,21,24,26	3	P12785,P62804,Q9QW07
5423	1,3,4,5,18,22,23,26	2,6,7,8,19,20,21,24,25	12	O54728,P12785,P34901,P47967,P97840,Q4FZU2,Q6IFU8,Q6IFW6,Q6IG02,Q6IMF3,Q6P6Q2,Q9QW07
5424	1,3,4,5,18,22,24,25	2,6,7,8,19,20,21,23,26	3	P62804,Q00715,Q9QW07

5425	1,3,4,5,18,22,24,26	2,6,7,8,19,20,21,23,25	5	O54728,P47967,P97840,Q6P6R2,Q9QW07
5426	1,3,4,5,18,22,25,26	2,6,7,8,19,20,21,23,24	2	O54728,Q9QW07
5427	1,3,4,5,18,23,24,25	2,6,7,8,19,20,21,22,26	5	P12785,P34901,P62804,Q00715,Q63751
5428	1,3,4,5,18,23,24,26	2,6,7,8,19,20,21,22,25	14	O54728,P02091,P09605;P25809,P12785,P34901,P35467,P47967,P97840,Q4FZU2,Q6IFU8,Q6IFW6,Q6IG02,Q6IMF3,Q6P6Q2
5429	1,3,4,5,18,23,25,26	2,6,7,8,19,20,21,22,24	11	P00714,P02091,P12785,P35467,Q4FZU2,Q5I0D1,Q6IFU8,Q6IFW6,Q6IG02,Q6IMF3,Q6P6Q2
5430	1,3,4,5,18,24,25,26	2,6,7,8,19,20,21,22,23	3	Q4FZU2,Q62902,Q8CJD3
5431	1,3,4,5,19,20,21,22	2,6,7,8,18,23,24,25,26	5	O54728,P01681,P55091,Q7TPB1,Q9QW07
5432	1,3,4,5,19,20,21,23	2,6,7,8,18,22,24,25,26	2	P01681,P55091
5433	1,3,4,5,19,20,21,24	2,6,7,8,18,22,23,25,26	2	P01681,P55091
5434	1,3,4,5,19,20,21,25	2,6,7,8,18,22,23,24,26	1	P01681
5435	1,3,4,5,19,20,21,26	2,6,7,8,18,22,23,24,25	3	O54728,P01681,Q4FZU6
5436	1,3,4,5,19,20,22,23	2,6,7,8,18,21,24,25,26	9	O54728,P00774,P22006,P55091,P82995,P98089,Q62997,Q9QW07,Q9WTT6
5437	1,3,4,5,19,20,22,24	2,6,7,8,18,21,23,25,26	12	O54728,P00774,P07150,P14669,P55091,P82995,P83121,P98089,Q6P6R2,Q99MH3,Q9QW07,Q9WTT6
5438	1,3,4,5,19,20,22,25	2,6,7,8,18,21,23,24,26	3	O54728,P55091,Q9QW07
5439	1,3,4,5,19,20,22,26	2,6,7,8,18,21,23,24,25	10	O54728,P01681,P05539,P07150,P98089,Q62997,Q6P6R2,Q99MH3,Q9QW07,Q9WTT6

5440	1,3,4,5,19,20,23,24	2,6,7,8,18,21,22,25,26	4	P34901,P55091,P82995,P98089
5441	1,3,4,5,19,20,23,25	2,6,7,8,18,21,22,24,26	0	
5442	1,3,4,5,19,20,23,26	2,6,7,8,18,21,22,24,25	9	O54728,P01681,P05539,P97840,P98089,Q4FZU2,Q4FZU6,Q6IFW6,Q6IG02
5443	1,3,4,5,19,20,24,25	2,6,7,8,18,21,22,23,26	0	
5444	1,3,4,5,19,20,24,26	2,6,7,8,18,21,22,23,25	6	O54728,P01681,P97840,P98089,Q6P6R2,Q99MH3
5445	1,3,4,5,19,20,25,26	2,6,7,8,18,21,22,23,24	3	O54728,P01681,P05539
5446	1,3,4,5,19,21,22,23	2,6,7,8,18,20,24,25,26	4	O54728,P34901,P55091,Q9QW07
5447	1,3,4,5,19,21,22,24	2,6,7,8,18,20,23,25,26	1	Q9QW07
5448	1,3,4,5,19,21,22,25	2,6,7,8,18,20,23,24,26	2	P17046,Q9QW07
5449	1,3,4,5,19,21,22,26	2,6,7,8,18,20,23,24,25	3	O54728,P01681,Q9QW07
5450	1,3,4,5,19,21,23,24	2,6,7,8,18,20,22,25,26	1	P34901
5451	1,3,4,5,19,21,23,25	2,6,7,8,18,20,22,24,26	0	
5452	1,3,4,5,19,21,23,26	2,6,7,8,18,20,22,24,25	3	O54728,P01681,Q4FZU2
5453	1,3,4,5,19,21,24,25	2,6,7,8,18,20,22,23,26	0	
5454	1,3,4,5,19,21,24,26	2,6,7,8,18,20,22,23,25	3	O54728,P01681,Q8CJD3

5455	1,3,4,5,19,21,25,26	2,6,7,8,18,20,22,23,24	1	P01681
5456	1,3,4,5,19,22,23,24	2,6,7,8,18,20,21,25,26	10	O54728,P34901,P55091,P62804,P82471,P82995,P98089,Q00715,Q9QW07,Q9WTT6
5457	1,3,4,5,19,22,23,25	2,6,7,8,18,20,21,24,26	5	O54728,P17046,P62804,Q6AYR8,Q9QW07
5458	1,3,4,5,19,22,23,26	2,6,7,8,18,20,21,24,25	4	O54728,P05539,P98089,Q9QW07
5459	1,3,4,5,19,22,24,25	2,6,7,8,18,20,21,23,26	5	P17046,P62804,Q00715,Q6AYR8,Q9QW07
5460	1,3,4,5,19,22,24,26	2,6,7,8,18,20,21,23,25	6	O54728,P98089,Q6P6R2,Q99MH3,Q9QW07,Q9WTT6
5461	1,3,4,5,19,22,25,26	2,6,7,8,18,20,21,23,24	4	O54728,P05539,Q6AYR8,Q9QW07
5462	1,3,4,5,19,23,24,25	2,6,7,8,18,20,21,22,26	3	P34901,P62804,Q00715
5463	1,3,4,5,19,23,24,26	2,6,7,8,18,20,21,22,25	5	O54728,P02091,P09605;P25809,P34901,P98089
5464	1,3,4,5,19,23,25,26	2,6,7,8,18,20,21,22,24	3	O54728,P02091,Q4FZU2
5465	1,3,4,5,19,24,25,26	2,6,7,8,18,20,21,22,23	3	O54728,Q62902,Q8CJD3
5466	1,3,4,5,20,21,22,23	2,6,7,8,18,19,24,25,26	4	P01681,P34901,P55091,Q9QW07
5467	1,3,4,5,20,21,22,24	2,6,7,8,18,19,23,25,26	6	P01681,P55091,Q5XFX0,Q62635,Q99MH3,Q9QW07
5468	1,3,4,5,20,21,22,25	2,6,7,8,18,19,23,24,26	2	P01681,Q9QW07
5469	1,3,4,5,20,21,22,26	2,6,7,8,18,19,23,24,25	6	O54728,P01681,P05539,Q4FZU6,Q99MH3,Q9QW07

5470	1,3,4,5,20,21,23,24	2,6,7,8,18,19,22,25,26	5	P01681,P34901,P82995,P97840,Q6IRE4
5471	1,3,4,5,20,21,23,25	2,6,7,8,18,19,22,24,26	2	P01681,Q6IRE4
5472	1,3,4,5,20,21,23,26	2,6,7,8,18,19,22,24,25	11	O54728,P01681,P01835,P97840,Q4FZU2,Q4FZU6,Q6IFU8,Q6IFW6,Q6IG02,Q6IMF3,Q6P6Q2
5473	1,3,4,5,20,21,24,25	2,6,7,8,18,19,22,23,26	1	Q6IRE4
5474	1,3,4,5,20,21,24,26	2,6,7,8,18,19,22,23,25	6	P01681,P12020,P97840,Q4FZU6,Q8CJD3,Q99MH3
5475	1,3,4,5,20,21,25,26	2,6,7,8,18,19,22,23,24	2	P01681,Q4FZU2
5476	1,3,4,5,20,22,23,24	2,6,7,8,18,19,21,25,26	11	O54728,P34901,P47967,P55091,P82995,P97840,P98089,Q62635,Q8CIZ5,Q9QW07,Q9QZK9
5477	1,3,4,5,20,22,23,25	2,6,7,8,18,19,21,24,26	2	P82995,Q9QW07
5478	1,3,4,5,20,22,23,26	2,6,7,8,18,19,21,24,25	12	O54728,P01681,P05539,P47967,P97840,P98089,Q4FZU2,Q62997,Q6IFW6,Q6IG02,Q8CIZ5,Q9QW07
5479	1,3,4,5,20,22,24,25	2,6,7,8,18,19,21,23,26	2	P82995,Q9QW07
5480	1,3,4,5,20,22,24,26	2,6,7,8,18,19,21,23,25	11	O54728,P01681,P47967,P97840,P98089,Q62635,Q6P6R2,Q8CIZ5,Q8CJD3,Q99MH3,Q9QW07
5481	1,3,4,5,20,22,25,26	2,6,7,8,18,19,21,23,24	4	O54728,P01681,P05539,Q9QW07
5482	1,3,4,5,20,23,24,25	2,6,7,8,18,19,21,22,26	3	P34901,P82995,Q63751
5483	1,3,4,5,20,23,24,26	2,6,7,8,18,19,21,22,25	12	D4A1J4,O54728,P01681,P02091,P34901,P47967,P97840,P98089,Q4FZU2,Q6IFW6,Q6IG02,Q8CIZ5
5484	1,3,4,5,20,23,25,26	2,6,7,8,18,19,21,22,24	8	O54728,P01681,P02091,P05539,Q4FZU2,Q6IFW6,Q6IG02,Q6IMF3

5485	1,3,4,5,20,24,25,26	2,6,7,8,18,19,21,22,23	1	Q8CJD3
5486	1,3,4,5,21,22,23,24	2,6,7,8,18,19,20,25,26	3	P34901,P35280,Q9QW07
5487	1,3,4,5,21,22,23,25	2,6,7,8,18,19,20,24,26	3	P34901,P35280,Q9QW07
5488	1,3,4,5,21,22,23,26	2,6,7,8,18,19,20,24,25	5	O54728,P01681,P34901,Q4FZU2,Q9QW07
5489	1,3,4,5,21,22,24,25	2,6,7,8,18,19,20,23,26	2	P35280,Q9QW07
5490	1,3,4,5,21,22,24,26	2,6,7,8,18,19,20,23,25	5	O54728,P01681,Q8CJD3,Q99MH3,Q9QW07
5491	1,3,4,5,21,22,25,26	2,6,7,8,18,19,20,23,24	3	O54728,P01681,Q9QW07
5492	1,3,4,5,21,23,24,25	2,6,7,8,18,19,20,22,26	3	P34901,P35280,Q63751
5493	1,3,4,5,21,23,24,26	2,6,7,8,18,19,20,22,25	7	O54728,P01681,P02091,P34901,P47967,P97840,Q4FZU2
5494	1,3,4,5,21,23,25,26	2,6,7,8,18,19,20,22,24	3	P01681,Q4FZU2,Q6IMF3
5495	1,3,4,5,21,24,25,26	2,6,7,8,18,19,20,22,23	1	Q8CJD3
5496	1,3,4,5,22,23,24,25	2,6,7,8,18,19,20,21,26	8	P34901,P35280,P62804,P82471,Q00715,Q63751,Q6AYR8,Q9QW07
5497	1,3,4,5,22,23,24,26	2,6,7,8,18,19,20,21,25	11	O54728,P00731,P02091,P34901,P47967,P62804,P82471,P97840,P98089,Q8CIZ5,Q9QW07
5498	1,3,4,5,22,23,25,26	2,6,7,8,18,19,20,21,24	6	O54728,P02091,P05539,P62804,Q4FZU2,Q9QW07
5499	1,3,4,5,22,24,25,26	2,6,7,8,18,19,20,21,23	4	O54728,P62804,Q8CJD3,Q9QW07

5500	1,3,4,5,23,24,25,26	2,6,7,8,18,19,20,21,22	11	O54728,P01946,P02091,P34901,P60905,P62804,Q4FZU2,Q62902,Q63751,Q7TPB1,Q8CJD3
5501	1,3,4,6,7,8,18,19	2,5,20,21,22,23,24,25,26	10	O55006,P00731,P02803,P16086,P18418,P35280,P35281,Q30KJ2,Q5FVI6,Q63617
5502	1,3,4,6,7,8,18,20	2,5,19,21,22,23,24,25,26	8	O55006,O70417,P00731,P01681,P10715,P18418,P35280,Q63617
5503	1,3,4,6,7,8,18,21	2,5,19,20,22,23,24,25,26	18	D4A1J4,O70417,P00731,P01681,P08723,P0C0A9,P11598,P13432,P16086,P18418,P22282,P22283,P25031,P42854,Q63617,Q6AYL6,Q8CJ52,Q9JI85
5504	1,3,4,6,7,8,18,22	2,5,19,20,21,23,24,25,26	5	D4A1J4,O70417,P18418,Q63617,Q8VD89
5505	1,3,4,6,7,8,18,23	2,5,19,20,21,22,24,25,26	9	O70417,P12785,P16086,P18418,P35467,Q5XI22,Q63617,Q8VD89,Q99041
5506	1,3,4,6,7,8,18,24	2,5,19,20,21,22,23,25,26	5	O55006,O70417,P18418,Q63617,Q8VD89
5507	1,3,4,6,7,8,18,25	2,5,19,20,21,22,23,24,26	12	P00714,P00731,P06911,P08721,P10715,P12020,P18418,P19629,P31044,P35280,Q5GRG2,Q63617
5508	1,3,4,6,7,8,18,26	2,5,19,20,21,22,23,24,25	12	O70417,P01681,P02770,P11598,P18418,P20761,P35280,Q4FZU2,Q5FVI6,Q63617,Q6AY33,Q8VD89
5509	1,3,4,6,7,8,19,20	2,5,18,21,22,23,24,25,26	9	P00731,P01681,P16086,P18418,P35280,P35281,Q30KJ2,Q5FVI6,Q63617
5510	1,3,4,6,7,8,19,21	2,5,18,20,22,23,24,25,26	12	D4A1J4,P00731,P01681,P08723,P0C0A9,P16086,P18418,P22282,P35281,Q30KJ2,Q5FVI6,Q63617
5511	1,3,4,6,7,8,19,22	2,5,18,20,21,23,24,25,26	7	D4A1J4,P16086,P18418,P22282,Q30KJ2,Q63617,Q9QUL6
5512	1,3,4,6,7,8,19,23	2,5,18,20,21,22,24,25,26	8	P15399,P16086,P18418,P97580,Q30KJ2,Q5FVI6,Q63617,Q99041
5513	1,3,4,6,7,8,19,24	2,5,18,20,21,22,23,25,26	7	O55006,P16086,P18418,Q30KJ2,Q5FVI6,Q63617,Q9QUL6
5514	1,3,4,6,7,8,19,25	2,5,18,20,21,22,23,24,26	15	P00731,P06911,P10715,P12020,P16086,P18418,P19629,P31044,P35280,Q30KJ2,Q5FVI6,Q5GRG2,Q63617,Q9QUL6,Q9QZK9

5515	1,3,4,6,7,8,19,26	2,5,18,20,21,22,23,24,25	9	P01681,P02770,P16086,P18418,P20761,P35280,Q30KJ2,Q5FVI6,Q63617
5516	1,3,4,6,7,8,20,21	2,5,18,19,22,23,24,25,26	10	P00731,P01681,P08723,P13432,P16086,P18418,P42854,Q63617,Q7TPB1,Q9JI85
5517	1,3,4,6,7,8,20,22	2,5,18,19,21,23,24,25,26	5	P01681,P18418,Q03191,Q63617,Q99MH3
5518	1,3,4,6,7,8,20,23	2,5,18,19,21,22,24,25,26	5	P01681,P16086,P18418,Q63617,Q99041
5519	1,3,4,6,7,8,20,24	2,5,18,19,21,22,23,25,26	3	P18418,Q63617,Q99MH3
5520	1,3,4,6,7,8,20,25	2,5,18,19,21,22,23,24,26	9	P01681,P06911,P10715,P12020,P18418,P19629,P35280,Q5GRG2,Q63617
5521	1,3,4,6,7,8,20,26	2,5,18,19,21,22,23,24,25	8	P01681,P02770,P18418,P20761,P35280,Q5FVI6,Q63617,Q99MH3
5522	1,3,4,6,7,8,21,22	2,5,18,19,20,23,24,25,26	12	D4A1J4,O70417,P01681,P02782,P08723,P13432,P16086,P18418,P22282,Q63617,Q7TPB1,Q8CJ52
5523	1,3,4,6,7,8,21,23	2,5,18,19,20,22,24,25,26	8	O70417,P01681,P08723,P16086,P18418,P22282,Q63617,Q99041
5524	1,3,4,6,7,8,21,24	2,5,18,19,20,22,23,25,26	3	P13432,P18418,Q63617
5525	1,3,4,6,7,8,21,25	2,5,18,19,20,22,23,24,26	11	P00731,P06911,P08723,P10715,P12020,P18418,P19629,Q5GRG2,Q63617,Q9QZK9,Q9WTT6
5526	1,3,4,6,7,8,21,26	2,5,18,19,20,22,23,24,25	9	O35783,P01681,P02770,P18418,P20761,P22282,Q5FVI6,Q63617,Q9EQV9
5527	1,3,4,6,7,8,22,23	2,5,18,19,20,21,24,25,26	6	O70417,P16086,P18418,P22282,Q63617,Q99041
5528	1,3,4,6,7,8,22,24	2,5,18,19,20,21,23,25,26	3	P18418,Q63617,Q99MH3
5529	1,3,4,6,7,8,22,25	2,5,18,19,20,21,23,24,26	8	P06911,P10715,P12020,P18418,P19629,Q5GRG2,Q63617,Q9QUL6

5530	1,3,4,6,7,8,22,26	2,5,18,19,20,21,23,24,25	9	P01681,P02770,P05539,P18418,P20761,P22282,Q63617,Q8VD89,Q99MH3
5531	1,3,4,6,7,8,23,24	2,5,18,19,20,21,22,25,26	5	P16086,P18418,Q63617,Q63751,Q99041
5532	1,3,4,6,7,8,23,25	2,5,18,19,20,21,22,24,26	11	P06911,P10715,P12020,P16086,P18418,P19629,P31044,Q5GRG2,Q63617,Q63751,Q99041
5533	1,3,4,6,7,8,23,26	2,5,18,19,20,21,22,24,25	12	O70417,P01681,P01946,P02091,P02770,P18418,P20761,P22282,Q4FZU2,Q5FVI6,Q63617,Q8VD89
5534	1,3,4,6,7,8,24,25	2,5,18,19,20,21,22,23,26	8	P06911,P10715,P12020,P18418,P19629,Q5GRG2,Q63617,Q9QUL6
5535	1,3,4,6,7,8,24,26	2,5,18,19,20,21,22,23,25	7	P02091,P02770,P18418,P20761,Q63617,Q8VD89,Q99MH3
5536	1,3,4,6,7,8,25,26	2,5,18,19,20,21,22,23,24	13	P02091,P06911,P10715,P12020,P18418,P19629,P20761,P35280,Q5FVI6,Q5GRG2,Q63617,Q6AY33,Q9QZK9
5537	1,3,4,6,7,18,19,20	2,5,8,21,22,23,24,25,26	4	P00731,P16086,P35280,Q5FVI6
5538	1,3,4,6,7,18,19,21	2,5,8,20,22,23,24,25,26	7	P00731,P08723,P16086,P18418,P25031,P42854,Q5FVI6
5539	1,3,4,6,7,18,19,22	2,5,8,20,21,23,24,25,26	4	P02625,P16086,P18418,Q5FVI6
5540	1,3,4,6,7,18,19,23	2,5,8,20,21,22,24,25,26	9	P02625,P12785,P15399,P16086,P97580,Q30KJ2,Q5BJY9,Q5FVI6,Q5XI22
5541	1,3,4,6,7,18,19,24	2,5,8,20,21,22,23,25,26	2	P18418,Q5FVI6
5542	1,3,4,6,7,18,19,25	2,5,8,20,21,22,23,24,26	7	P00731,P12020,P16086,P18418,P35280,P47967,Q5FVI6
5543	1,3,4,6,7,18,19,26	2,5,8,20,21,22,23,24,25	4	P06760,P35280,Q30KJ2,Q5FVI6
5544	1,3,4,6,7,18,20,21	2,5,8,19,22,23,24,25,26	7	P00731,P01681,P01835,P13432,P25031,P42854,Q9JI85

5545	1,3,4,6,7,18,20,22	2,5,8,19,21,23,24,25,26	0	
5546	1,3,4,6,7,18,20,23	2,5,8,19,21,22,24,25,26	6	A2RUV9,P12785,P16086,Q4FZU2,Q5XI22,Q6IG02
5547	1,3,4,6,7,18,20,24	2,5,8,19,21,22,23,25,26	0	
5548	1,3,4,6,7,18,20,25	2,5,8,19,21,22,23,24,26	2	P35280,Q5FVI6
5549	1,3,4,6,7,18,20,26	2,5,8,19,21,22,23,24,25	5	P01681,P06760,P35280,Q4FZU2,Q5FVI6
5550	1,3,4,6,7,18,21,22	2,5,8,19,20,23,24,25,26	4	D4A1J4,P08723,P13432,P25031
5551	1,3,4,6,7,18,21,23	2,5,8,19,20,22,24,25,26	5	P12785,P16086,P25031,Q4FZU2,Q5XI22
5552	1,3,4,6,7,18,21,24	2,5,8,19,20,22,23,25,26	3	P13432,P18418,P25031
5553	1,3,4,6,7,18,21,25	2,5,8,19,20,22,23,24,26	4	P00731,P25031,P31430,P47967
5554	1,3,4,6,7,18,21,26	2,5,8,19,20,22,23,24,25	6	O35783,P01681,P20761,P25031,Q4FZU2,Q5FVI6
5555	1,3,4,6,7,18,22,23	2,5,8,19,20,21,24,25,26	4	P02625,P12785,P16086,Q8VD89
5556	1,3,4,6,7,18,22,24	2,5,8,19,20,21,23,25,26	2	P18418,Q8VD89
5557	1,3,4,6,7,18,22,25	2,5,8,19,20,21,23,24,26	3	P06911,P12020,Q5GRG2
5558	1,3,4,6,7,18,22,26	2,5,8,19,20,21,23,24,25	5	P05539,P06760,P20761,Q5FVI6,Q8VD89
5559	1,3,4,6,7,18,23,24	2,5,8,19,20,21,22,25,26	3	P12785,Q63751,Q8VD89

5560	1,3,4,6,7,18,23,25	2,5,8,19,20,21,22,24,26	5	P00714,P06911,P12020,P12785,Q63751
5561	1,3,4,6,7,18,23,26	2,5,8,19,20,21,22,24,25	15	A2RUV9,P01946,P02091,P12785,P20761,P35467,P55053,Q4FZU2,Q5FVI6,Q6IFU8,Q6IFW6,Q6IG02,Q6IMF3,Q6P6Q2,Q8VD89
5562	1,3,4,6,7,18,24,25	2,5,8,19,20,21,22,23,26	1	P18418
5563	1,3,4,6,7,18,24,26	2,5,8,19,20,21,22,23,25	5	P02091,P06760,P20761,Q5FVI6,Q8VD89
5564	1,3,4,6,7,18,25,26	2,5,8,19,20,21,22,23,24	6	P00714,P02091,P35280,P61206;P84079;P84082,Q4FZU2,Q5FVI6
5565	1,3,4,6,7,19,20,21	2,5,8,18,22,23,24,25,26	5	P00731,P01681,P16086,P42854,Q5FVI6
5566	1,3,4,6,7,19,20,22	2,5,8,18,21,23,24,25,26	4	P16086,P55091,Q5FVI6,Q99MH3
5567	1,3,4,6,7,19,20,23	2,5,8,18,21,22,24,25,26	6	P15399,P16086,P55091,P97580,Q30KJ2,Q5FVI6
5568	1,3,4,6,7,19,20,24	2,5,8,18,21,22,23,25,26	1	Q5FVI6
5569	1,3,4,6,7,19,20,25	2,5,8,18,21,22,23,24,26	3	P12020,P35280,Q5FVI6
5570	1,3,4,6,7,19,20,26	2,5,8,18,21,22,23,24,25	5	P01681,P05539,P35280,Q30KJ2,Q5FVI6
5571	1,3,4,6,7,19,21,22	2,5,8,18,20,23,24,25,26	4	D4A1J4,P08723,P16086,Q5FVI6
5572	1,3,4,6,7,19,21,23	2,5,8,18,20,22,24,25,26	6	P15399,P16086,P97580,Q30KJ2,Q5FVI6,Q99041
5573	1,3,4,6,7,19,21,24	2,5,8,18,20,22,23,25,26	3	P13432,P16086,Q5FVI6
5574	1,3,4,6,7,19,21,25	2,5,8,18,20,22,23,24,26	8	P00731,P12020,P15399,P16086,P47967,P97840,Q5FVI6,Q9QZK9

5575	1,3,4,6,7,19,21,26	2,5,8,18,20,22,23,24,25	5	P01681,P16086,P20761,Q30KJ2,Q5FVI6
5576	1,3,4,6,7,19,22,23	2,5,8,18,20,21,24,25,26	7	P02625,P15399,P16086,P55091,P97580,Q30KJ2,Q5FVI6
5577	1,3,4,6,7,19,22,24	2,5,8,18,20,21,23,25,26	0	
5578	1,3,4,6,7,19,22,25	2,5,8,18,20,21,23,24,26	6	P06911,P12020,P16086,P47967,Q5FVI6,Q5GRG2
5579	1,3,4,6,7,19,22,26	2,5,8,18,20,21,23,24,25	5	O54728,P05539,P20761,Q30KJ2,Q5FVI6
5580	1,3,4,6,7,19,23,24	2,5,8,18,20,21,22,25,26	5	P15399,P16086,P97580,Q30KJ2,Q5FVI6
5581	1,3,4,6,7,19,23,25	2,5,8,18,20,21,22,24,26	9	P06911,P12020,P15399,P16086,P97580,Q30KJ2,Q5FVI6,Q5GRG2,Q99041
5582	1,3,4,6,7,19,23,26	2,5,8,18,20,21,22,24,25	11	O54728,P01946,P02091,P05539,P15399,P16086,P20761,P97580,Q30KJ2,Q4FZU2,Q5FVI6
5583	1,3,4,6,7,19,24,25	2,5,8,18,20,21,22,23,26	2	P12020,Q5FVI6
5584	1,3,4,6,7,19,24,26	2,5,8,18,20,21,22,23,25	4	P02091,P20761,Q30KJ2,Q5FVI6
5585	1,3,4,6,7,19,25,26	2,5,8,18,20,21,22,23,24	7	P02091,P05539,P12020,P35280,Q30KJ2,Q5FVI6,Q9QZK9
5586	1,3,4,6,7,20,21,22	2,5,8,18,19,23,24,25,26	6	P01681,P13432,P42854,Q03191,Q7TPB1,Q99MH3
5587	1,3,4,6,7,20,21,23	2,5,8,18,19,22,24,25,26	3	P01681,P01835,P16086
5588	1,3,4,6,7,20,21,24	2,5,8,18,19,22,23,25,26	3	P01681,P13432,Q99MH3
5589	1,3,4,6,7,20,21,25	2,5,8,18,19,22,23,24,26	3	P01681,P09605;P25809,Q03191

5590	1,3,4,6,7,20,21,26	2,5,8,18,19,22,23,24,25	9	O35783,P01681,P01835,P01836,P05539,P20761,Q4FZU2,Q5FVI6,Q99MH3
5591	1,3,4,6,7,20,22,23	2,5,8,18,19,21,24,25,26	2	P16086,P55091
5592	1,3,4,6,7,20,22,24	2,5,8,18,19,21,23,25,26	2	Q03191,Q99MH3
5593	1,3,4,6,7,20,22,25	2,5,8,18,19,21,23,24,26	4	P06911,P12020,Q03191,Q5GRG2
5594	1,3,4,6,7,20,22,26	2,5,8,18,19,21,23,24,25	7	O54728,P01681,P05539,P20761,Q03191,Q5FVI6,Q99MH3
5595	1,3,4,6,7,20,23,24	2,5,8,18,19,21,22,25,26	1	Q63751
5596	1,3,4,6,7,20,23,25	2,5,8,18,19,21,22,24,26	5	P06911,P12020,Q5FVI6,Q5GRG2,Q63751
5597	1,3,4,6,7,20,23,26	2,5,8,18,19,21,22,24,25	10	A2RUV9,P01681,P01946,P02091,P05539,P20761,Q4FZU2,Q5FVI6,Q6IFW6,Q6IG02
5598	1,3,4,6,7,20,24,25	2,5,8,18,19,21,22,23,26	2	P12020,Q03191
5599	1,3,4,6,7,20,24,26	2,5,8,18,19,21,22,23,25	5	P01681,P02091,P20761,Q5FVI6,Q99MH3
5600	1,3,4,6,7,20,25,26	2,5,8,18,19,21,22,23,24	6	P01681,P02091,P05539,P35280,Q03191,Q5FVI6
5601	1,3,4,6,7,21,22,23	2,5,8,18,19,20,24,25,26	1	P16086
5602	1,3,4,6,7,21,22,24	2,5,8,18,19,20,23,25,26	2	P13432,Q99MH3
5603	1,3,4,6,7,21,22,25	2,5,8,18,19,20,23,24,26	6	P06911,P09605;P25809,P12020,Q03191,Q5GRG2,Q9QZK9
5604	1,3,4,6,7,21,22,26	2,5,8,18,19,20,23,24,25	5	P01681,P05539,P13432,P20761,Q99MH3

5605	1,3,4,6,7,21,23,24	2,5,8,18,19,20,22,25,26	2	P13432,Q63751
5606	1,3,4,6,7,21,23,25	2,5,8,18,19,20,22,24,26	9	P06911,P12020,P15399,P16086,P83121,Q5GRG2,Q63751,Q6P6R2,Q99041
5607	1,3,4,6,7,21,23,26	2,5,8,18,19,20,22,24,25	7	P01681,P01946,P02091,P20761,P83121,Q4FZU2,Q5FVI6
5608	1,3,4,6,7,21,24,25	2,5,8,18,19,20,22,23,26	3	P09605;P25809,P13432,P22006
5609	1,3,4,6,7,21,24,26	2,5,8,18,19,20,22,23,25	6	P01681,P02091,P13432,P20761,Q5FVI6,Q99MH3
5610	1,3,4,6,7,21,25,26	2,5,8,18,19,20,22,23,24	9	O35783,P01681,P02091,P09605;P25809,P20761,P22006,P83121,Q5FVI6,Q9QZK9
5611	1,3,4,6,7,22,23,24	2,5,8,18,19,20,21,25,26	2	P62804,Q63751
5612	1,3,4,6,7,22,23,25	2,5,8,18,19,20,21,24,26	5	P06911,P12020,P62804,Q5GRG2,Q63751
5613	1,3,4,6,7,22,23,26	2,5,8,18,19,20,21,24,25	8	O54728,P01946,P02091,P05539,P20761,Q4FZU2,Q5FVI6,Q8VD89
5614	1,3,4,6,7,22,24,25	2,5,8,18,19,20,21,23,26	5	P06911,P12020,P62804,Q03191,Q5GRG2
5615	1,3,4,6,7,22,24,26	2,5,8,18,19,20,21,23,25	4	P02091,P20761,Q8VD89,Q99MH3
5616	1,3,4,6,7,22,25,26	2,5,8,18,19,20,21,23,24	9	P02091,P05539,P06911,P12020,P20761,Q03191,Q5FVI6,Q5GRG2,Q9QZK9
5617	1,3,4,6,7,23,24,25	2,5,8,18,19,20,21,22,26	6	P06911,P12020,P19629,P62804,Q5GRG2,Q63751
5618	1,3,4,6,7,23,24,26	2,5,8,18,19,20,21,22,25	7	P01946,P02091,P20761,Q4FZU2,Q5FVI6,Q63751,Q8VD89
5619	1,3,4,6,7,23,25,26	2,5,8,18,19,20,21,22,24	12	P01946,P02091,P05539,P06911,P12020,P20761,P55314,P83121,Q4FZU2,Q5FVI6,Q5GRG2,Q63751

5620	1,3,4,6,7,24,25,26	2,5,8,18,19,20,21,22,23	3	P02091,P20761,Q5FVI6
5621	1,3,4,6,8,18,19,20	2,5,7,21,22,23,24,25,26	5	P00731,P01681,P22006,P35280,P35281
5622	1,3,4,6,8,18,19,21	2,5,7,20,22,23,24,25,26	8	O35547,P00731,P01681,P02782,P08723,P0C0A9,P10758,P35281
5623	1,3,4,6,8,18,19,22	2,5,7,20,21,23,24,25,26	1	P02625
5624	1,3,4,6,8,18,19,23	2,5,7,20,21,22,24,25,26	9	P02625,P12785,P15399,P35281,P35467,P97580,Q30KJ2,Q5BJY9,Q5XI22
5625	1,3,4,6,8,18,19,24	2,5,7,20,21,22,23,25,26	0	
5626	1,3,4,6,8,18,19,25	2,5,7,20,21,22,23,24,26	2	P00731,P35280
5627	1,3,4,6,8,18,19,26	2,5,7,20,21,22,23,24,25	5	P01681,P02770,P35280,Q4FZU2,Q63942
5628	1,3,4,6,8,18,20,21	2,5,7,19,22,23,24,25,26	7	O35547,P00731,P01681,P01835,P08723,P0C0A9,P10758
5629	1,3,4,6,8,18,20,22	2,5,7,19,21,23,24,25,26	3	O70417,P01681,P22006
5630	1,3,4,6,8,18,20,23	2,5,7,19,21,22,24,25,26	8	O70417,P01681,P12785,P22006,P35467,Q4FZU2,Q5XI22,Q6IG02
5631	1,3,4,6,8,18,20,24	2,5,7,19,21,22,23,25,26	0	
5632	1,3,4,6,8,18,20,25	2,5,7,19,21,22,23,24,26	2	P01681,P35280
5633	1,3,4,6,8,18,20,26	2,5,7,19,21,22,23,24,25	4	P01681,P35280,Q4FZU2,Q6IG02
5634	1,3,4,6,8,18,21,22	2,5,7,19,20,23,24,25,26	8	O35547,O70417,P01681,P02782,P08723,P0C0A9,P10758,P13432

5635	1,3,4,6,8,18,21,23	2,5,7,19,20,22,24,25,26	10	O35547,O70417,P01681,P08644,P08723,P0C0A9,P10758,P12785,Q4FZU2,Q5XI22
5636	1,3,4,6,8,18,21,24	2,5,7,19,20,22,23,25,26	2	P10758,P13432
5637	1,3,4,6,8,18,21,25	2,5,7,19,20,22,23,24,26	4	P00731,P08723,P31430,Q9WTT6
5638	1,3,4,6,8,18,21,26	2,5,7,19,20,22,23,24,25	8	O70417,P01681,P02770,P0C0A9,P10758,P20761,Q4FZU2,Q9EQV9
5639	1,3,4,6,8,18,22,23	2,5,7,19,20,21,24,25,26	3	O70417,P02625,Q5XI22
5640	1,3,4,6,8,18,22,24	2,5,7,19,20,21,23,25,26	0	
5641	1,3,4,6,8,18,22,25	2,5,7,19,20,21,23,24,26	1	P06911
5642	1,3,4,6,8,18,22,26	2,5,7,19,20,21,23,24,25	3	O70417,P01681,Q4FZU2
5643	1,3,4,6,8,18,23,24	2,5,7,19,20,21,22,25,26	3	O70417,P12785,Q5XI22
5644	1,3,4,6,8,18,23,25	2,5,7,19,20,21,22,24,26	4	P00714,P12785,Q5XI22,Q63751
5645	1,3,4,6,8,18,23,26	2,5,7,19,20,21,22,24,25	13	O70417,P01681,P12785,P35467,Q4FZU2,Q5XI22,Q63942,Q6AY61,Q6IFU8,Q6IFW6,Q6IG02,Q6IMF3,Q6P6Q2
5646	1,3,4,6,8,18,24,25	2,5,7,19,20,21,22,23,26	0	
5647	1,3,4,6,8,18,24,26	2,5,7,19,20,21,22,23,25	4	P02770,P20761,Q4FZU2,Q8VD89
5648	1,3,4,6,8,18,25,26	2,5,7,19,20,21,22,23,24	4	P00714,P35280,Q4FZU2,Q6AY33
5649	1,3,4,6,8,19,20,21	2,5,7,18,22,23,24,25,26	5	P00731,P01681,P08723,P35281,Q7TPB1

5650	1,3,4,6,8,19,20,22	2,5,7,18,21,23,24,25,26	4	P01681,P22006,P55091,P82995
5651	1,3,4,6,8,19,20,23	2,5,7,18,21,22,24,25,26	7	P01681,P15399,P35281,P55091,P82995,P97580,Q30KJ2
5652	1,3,4,6,8,19,20,24	2,5,7,18,21,22,23,25,26	2	P01681,P82995
5653	1,3,4,6,8,19,20,25	2,5,7,18,21,22,23,24,26	3	P01681,P35280,Q9QUL6
5654	1,3,4,6,8,19,20,26	2,5,7,18,21,22,23,24,25	2	P01681,P35280
5655	1,3,4,6,8,19,21,22	2,5,7,18,20,23,24,25,26	6	P01681,P02782,P08723,P0C0A9,P35281,Q7TPB1
5656	1,3,4,6,8,19,21,23	2,5,7,18,20,22,24,25,26	6	P01681,P0C0A9,P15399,P35281,P97580,Q30KJ2
5657	1,3,4,6,8,19,21,24	2,5,7,18,20,22,23,25,26	3	P02782,P13432,P35281
5658	1,3,4,6,8,19,21,25	2,5,7,18,20,22,23,24,26	5	P00731,P01681,P35281,Q9QUL6,Q9WTT6
5659	1,3,4,6,8,19,21,26	2,5,7,18,20,22,23,24,25	4	P01681,P0C0A9,P20761,P35281
5660	1,3,4,6,8,19,22,23	2,5,7,18,20,21,24,25,26	6	P02625,P15399,P55091,P82995,P97580,Q30KJ2
5661	1,3,4,6,8,19,22,24	2,5,7,18,20,21,23,25,26	2	P82995,Q9QUL6
5662	1,3,4,6,8,19,22,25	2,5,7,18,20,21,23,24,26	2	P06911,Q9QUL6
5663	1,3,4,6,8,19,22,26	2,5,7,18,20,21,23,24,25	2	O54728,P01681
5664	1,3,4,6,8,19,23,24	2,5,7,18,20,21,22,25,26	4	P15399,P82995,P97580,Q30KJ2

5665	1,3,4,6,8,19,23,25	2,5,7,18,20,21,22,24,26	6	P06911,P15399,P97580,Q30KJ2,Q99041,Q9QUL6
5666	1,3,4,6,8,19,23,26	2,5,7,18,20,21,22,24,25	6	P01681,P15399,P35467,P97580,Q30KJ2,Q4FZU2
5667	1,3,4,6,8,19,24,25	2,5,7,18,20,21,22,23,26	1	Q9QUL6
5668	1,3,4,6,8,19,24,26	2,5,7,18,20,21,22,23,25	2	P01681,P20761
5669	1,3,4,6,8,19,25,26	2,5,7,18,20,21,22,23,24	3	P01681,P35280,Q9QUL6
5670	1,3,4,6,8,20,21,22	2,5,7,18,19,23,24,25,26	8	P01681,P02782,P08723,P13432,P16636,Q03191,Q7TPB1,Q99MH3
5671	1,3,4,6,8,20,21,23	2,5,7,18,19,22,24,25,26	3	P01681,P01835,P16636
5672	1,3,4,6,8,20,21,24	2,5,7,18,19,22,23,25,26	4	P01681,P13432,Q5XFX0,Q99MH3
5673	1,3,4,6,8,20,21,25	2,5,7,18,19,22,23,24,26	4	P01681,P16636,Q03191,Q9WTT6
5674	1,3,4,6,8,20,21,26	2,5,7,18,19,22,23,24,25	5	P01681,P01835,P20761,Q4FZU2,Q99MH3
5675	1,3,4,6,8,20,22,23	2,5,7,18,19,21,24,25,26	3	P01681,P55091,P82995
5676	1,3,4,6,8,20,22,24	2,5,7,18,19,21,23,25,26	3	P82995,Q03191,Q99MH3
5677	1,3,4,6,8,20,22,25	2,5,7,18,19,21,23,24,26	5	P01681,P06911,P16636,Q03191,Q9QUL6
5678	1,3,4,6,8,20,22,26	2,5,7,18,19,21,23,24,25	5	O54728,P01681,P20761,Q03191,Q99MH3
5679	1,3,4,6,8,20,23,24	2,5,7,18,19,21,22,25,26	3	P01681,P82995,P97840

5680	1,3,4,6,8,20,23,25	2,5,7,18,19,21,22,24,26	4	P01681,P06911,P16636,Q63751
5681	1,3,4,6,8,20,23,26	2,5,7,18,19,21,22,24,25	8	P01681,P20761,P97840,Q4FZU2,Q6IFW6,Q6IG02,Q6IMF3,Q6P6Q2
5682	1,3,4,6,8,20,24,25	2,5,7,18,19,21,22,23,26	2	Q03191,Q9QUL6
5683	1,3,4,6,8,20,24,26	2,5,7,18,19,21,22,23,25	5	B0BNN3,P01681,P20761,P97840,Q99MH3
5684	1,3,4,6,8,20,25,26	2,5,7,18,19,21,22,23,24	3	P01681,P35280,Q03191
5685	1,3,4,6,8,21,22,23	2,5,7,18,19,20,24,25,26	4	O70417,P01681,P02782,P08723
5686	1,3,4,6,8,21,22,24	2,5,7,18,19,20,23,25,26	4	P02782,P13432,Q5XFX0,Q99MH3
5687	1,3,4,6,8,21,22,25	2,5,7,18,19,20,23,24,26	5	P06911,P08723,P16636,Q03191,Q9WTT6
5688	1,3,4,6,8,21,22,26	2,5,7,18,19,20,23,24,25	5	P01681,P02782,P20761,Q5QE79,Q99MH3
5689	1,3,4,6,8,21,23,24	2,5,7,18,19,20,22,25,26	2	P13432,Q63751
5690	1,3,4,6,8,21,23,25	2,5,7,18,19,20,22,24,26	7	P01681,P06911,P07150,P16636,Q63751,Q6P6R2,Q9WTT6
5691	1,3,4,6,8,21,23,26	2,5,7,18,19,20,22,24,25	6	P01681,P20761,Q4FZU2,Q5QE79,Q6IMF3,Q6P6Q2
5692	1,3,4,6,8,21,24,25	2,5,7,18,19,20,22,23,26	2	P13432,Q9WTT6
5693	1,3,4,6,8,21,24,26	2,5,7,18,19,20,22,23,25	5	P01681,P13432,P20761,Q99MH3,Q9EQV9
5694	1,3,4,6,8,21,25,26	2,5,7,18,19,20,22,23,24	5	P01681,P20761,Q5QE79,Q9QZK9,Q9WTT6

5695	1,3,4,6,8,22,23,24	2,5,7,18,19,20,21,25,26	2	P82995,Q63751
5696	1,3,4,6,8,22,23,25	2,5,7,18,19,20,21,24,26	3	P06911,Q5GRG2,Q63751
5697	1,3,4,6,8,22,23,26	2,5,7,18,19,20,21,24,25	4	O54728,P01681,P20761,Q4FZU2
5698	1,3,4,6,8,22,24,25	2,5,7,18,19,20,21,23,26	3	P06911,Q03191,Q9QUL6
5699	1,3,4,6,8,22,24,26	2,5,7,18,19,20,21,23,25	2	P20761,Q99MH3
5700	1,3,4,6,8,22,25,26	2,5,7,18,19,20,21,23,24	2	P06911,Q03191
5701	1,3,4,6,8,23,24,25	2,5,7,18,19,20,21,22,26	2	P06911,Q63751
5702	1,3,4,6,8,23,24,26	2,5,7,18,19,20,21,22,25	4	P20761,P27590,P97840,Q4FZU2
5703	1,3,4,6,8,23,25,26	2,5,7,18,19,20,21,22,24	4	P01681,Q4FZU2,Q63751,Q9WTT6
5704	1,3,4,6,8,24,25,26	2,5,7,18,19,20,21,22,23	1	P20761
5705	1,3,4,6,18,19,20,21	2,5,7,8,22,23,24,25,26	5	P00731,P01681,P01835,P10758,P35281
5706	1,3,4,6,18,19,20,22	2,5,7,8,21,23,24,25,26	1	P22006
5707	1,3,4,6,18,19,20,23	2,5,7,8,21,22,24,25,26	5	P02625,P12785,P15399,P22006,Q4FZU2
5708	1,3,4,6,18,19,20,24	2,5,7,8,21,22,23,25,26	0	
5709	1,3,4,6,18,19,20,25	2,5,7,8,21,22,23,24,26	1	P35280

5710	1,3,4,6,18,19,20,26	2,5,7,8,21,22,23,24,25	4	P01681,P35280,Q4FZU2,Q63355
5711	1,3,4,6,18,19,21,22	2,5,7,8,20,23,24,25,26	4	O35547,P02625,P08723,P10758
5712	1,3,4,6,18,19,21,23	2,5,7,8,20,22,24,25,26	4	P02625,P10758,P12785,P15399
5713	1,3,4,6,18,19,21,24	2,5,7,8,20,22,23,25,26	1	P10758
5714	1,3,4,6,18,19,21,25	2,5,7,8,20,22,23,24,26	2	P00731,P47967
5715	1,3,4,6,18,19,21,26	2,5,7,8,20,22,23,24,25	3	P01681,P10758,Q4FZU2
5716	1,3,4,6,18,19,22,23	2,5,7,8,20,21,24,25,26	3	P02625,P12785,P15399
5717	1,3,4,6,18,19,22,24	2,5,7,8,20,21,23,25,26	1	P02625
5718	1,3,4,6,18,19,22,25	2,5,7,8,20,21,23,24,26	0	
5719	1,3,4,6,18,19,22,26	2,5,7,8,20,21,23,24,25	1	P02625
5720	1,3,4,6,18,19,23,24	2,5,7,8,20,21,22,25,26	3	P02625,P12785,P15399
5721	1,3,4,6,18,19,23,25	2,5,7,8,20,21,22,24,26	3	P12785,P15399,P97580
5722	1,3,4,6,18,19,23,26	2,5,7,8,20,21,22,24,25	11	P02625,P12785,P15399,P35467,Q30KJ2,Q4FZU2,Q6IFU8,Q6IFW6,Q6IG02,Q6IMF3,Q6P6Q2
5723	1,3,4,6,18,19,24,25	2,5,7,8,20,21,22,23,26	0	
5724	1,3,4,6,18,19,24,26	2,5,7,8,20,21,22,23,25	0	

5725	1,3,4,6,18,19,25,26	2,5,7,8,20,21,22,23,24	2	P35280,Q4FZU2
5726	1,3,4,6,18,20,21,22	2,5,7,8,19,23,24,25,26	4	P01681,P01835,P10758,P13432
5727	1,3,4,6,18,20,21,23	2,5,7,8,19,22,24,25,26	6	P01681,P01835,P10758,P12785,Q4FZU2,Q6IG02
5728	1,3,4,6,18,20,21,24	2,5,7,8,19,22,23,25,26	3	P01681,P01835,P13432
5729	1,3,4,6,18,20,21,25	2,5,7,8,19,22,23,24,26	3	P01681,P01835,P31430
5730	1,3,4,6,18,20,21,26	2,5,7,8,19,22,23,24,25	3	P01681,P01835,Q4FZU2
5731	1,3,4,6,18,20,22,23	2,5,7,8,19,21,24,25,26	3	P02625,P12785,Q4FZU2
5732	1,3,4,6,18,20,22,24	2,5,7,8,19,21,23,25,26	0	
5733	1,3,4,6,18,20,22,25	2,5,7,8,19,21,23,24,26	0	
5734	1,3,4,6,18,20,22,26	2,5,7,8,19,21,23,24,25	2	P01681,Q4FZU2
5735	1,3,4,6,18,20,23,24	2,5,7,8,19,21,22,25,26	3	P12785,P82995,Q4FZU2
5736	1,3,4,6,18,20,23,25	2,5,7,8,19,21,22,24,26	3	P12785,Q4FZU2,Q6IG02
5737	1,3,4,6,18,20,23,26	2,5,7,8,19,21,22,24,25	9	P01681,P12785,P35467,Q4FZU2,Q6IFU8,Q6IFW6,Q6IG02,Q6IMF3,Q6P6Q2
5738	1,3,4,6,18,20,24,25	2,5,7,8,19,21,22,23,26	0	
5739	1,3,4,6,18,20,24,26	2,5,7,8,19,21,22,23,25	2	P01681,Q4FZU2

5740	1,3,4,6,18,20,25,26	2,5,7,8,19,21,22,23,24	3	P01681,P35280,Q4FZU2
5741	1,3,4,6,18,21,22,23	2,5,7,8,19,20,24,25,26	3	P02625,P10758,P12785
5742	1,3,4,6,18,21,22,24	2,5,7,8,19,20,23,25,26	1	P13432
5743	1,3,4,6,18,21,22,25	2,5,7,8,19,20,23,24,26	1	P31430
5744	1,3,4,6,18,21,22,26	2,5,7,8,19,20,23,24,25	2	P01681,Q4FZU2
5745	1,3,4,6,18,21,23,24	2,5,7,8,19,20,22,25,26	2	P12785,P13432
5746	1,3,4,6,18,21,23,25	2,5,7,8,19,20,22,24,26	4	P01835,P12785,P31430,Q4FZU2
5747	1,3,4,6,18,21,23,26	2,5,7,8,19,20,22,24,25	9	P01681,P01835,P12785,Q4FZU2,Q6IFU8,Q6IFW6,Q6IG02,Q6IMF3,Q6P6Q2
5748	1,3,4,6,18,21,24,25	2,5,7,8,19,20,22,23,26	1	P31430
5749	1,3,4,6,18,21,24,26	2,5,7,8,19,20,22,23,25	3	P01681,P13432,Q4FZU2
5750	1,3,4,6,18,21,25,26	2,5,7,8,19,20,22,23,24	5	P00774,P01681,P01835,P31430,Q4FZU2
5751	1,3,4,6,18,22,23,24	2,5,7,8,19,20,21,25,26	5	P02625,P12785,P16391,P62804,Q6IFU7
5752	1,3,4,6,18,22,23,25	2,5,7,8,19,20,21,24,26	2	P06911,P12785
5753	1,3,4,6,18,22,23,26	2,5,7,8,19,20,21,24,25	9	P02625,P12785,Q4FZU2,Q6IFU7,Q6IFU8,Q6IFW6,Q6IG02,Q6IMF3,Q6P6Q2
5754	1,3,4,6,18,22,24,25	2,5,7,8,19,20,21,23,26	1	P62804

5755	1,3,4,6,18,22,24,26	2,5,7,8,19,20,21,23,25	1	Q8VD89
5756	1,3,4,6,18,22,25,26	2,5,7,8,19,20,21,23,24	1	Q4FZU2
5757	1,3,4,6,18,23,24,25	2,5,7,8,19,20,21,22,26	3	P12785,P62804,Q63751
5758	1,3,4,6,18,23,24,26	2,5,7,8,19,20,21,22,25	11	P12785,P27590,P35467,Q4FZU2,Q6IFU7,Q6IFU8,Q6IFW6,Q6IG02,Q6IMF3,Q6P6Q2,Q8VD89
5759	1,3,4,6,18,23,25,26	2,5,7,8,19,20,21,22,24	10	P00714,P00774,P12785,P35467,Q4FZU2,Q6IFU8,Q6IFW6,Q6IG02,Q6IMF3,Q6P6Q2
5760	1,3,4,6,18,24,25,26	2,5,7,8,19,20,21,22,23	1	Q4FZU2
5761	1,3,4,6,19,20,21,22	2,5,7,8,18,23,24,25,26	5	P01681,P55091,P82995,Q7TPB1,Q99MH3
5762	1,3,4,6,19,20,21,23	2,5,7,8,18,22,24,25,26	5	P01681,P01835,P15399,P55091,P82995
5763	1,3,4,6,19,20,21,24	2,5,7,8,18,22,23,25,26	2	P01681,P82995
5764	1,3,4,6,19,20,21,25	2,5,7,8,18,22,23,24,26	3	P01681,P16636,P47967
5765	1,3,4,6,19,20,21,26	2,5,7,8,18,22,23,24,25	2	P01681,P01835
5766	1,3,4,6,19,20,22,23	2,5,7,8,18,21,24,25,26	5	P02625,P15399,P31430,P55091,P82995
5767	1,3,4,6,19,20,22,24	2,5,7,8,18,21,23,25,26	3	P55091,P82995,Q99MH3
5768	1,3,4,6,19,20,22,25	2,5,7,8,18,21,23,24,26	2	P55091,P82995
5769	1,3,4,6,19,20,22,26	2,5,7,8,18,21,23,24,25	4	O54728,P01681,P05539,Q99MH3

5770	1,3,4,6,19,20,23,24	2,5,7,8,18,21,22,25,26	3	P15399,P55091,P82995
5771	1,3,4,6,19,20,23,25	2,5,7,8,18,21,22,24,26	3	P15399,P82995,P97580
5772	1,3,4,6,19,20,23,26	2,5,7,8,18,21,22,24,25	6	O54728,P01681,P15399,Q30KJ2,Q4FZU2,Q6IG02
5773	1,3,4,6,19,20,24,25	2,5,7,8,18,21,22,23,26	1	P82995
5774	1,3,4,6,19,20,24,26	2,5,7,8,18,21,22,23,25	2	P01681,Q99MH3
5775	1,3,4,6,19,20,25,26	2,5,7,8,18,21,22,23,24	2	P01681,P35280
5776	1,3,4,6,19,21,22,23	2,5,7,8,18,20,24,25,26	4	P02625,P15399,P55091,P82995
5777	1,3,4,6,19,21,22,24	2,5,7,8,18,20,23,25,26	1	P13432
5778	1,3,4,6,19,21,22,25	2,5,7,8,18,20,23,24,26	1	P47967
5779	1,3,4,6,19,21,22,26	2,5,7,8,18,20,23,24,25	1	P01681
5780	1,3,4,6,19,21,23,24	2,5,7,8,18,20,22,25,26	3	P15399,P82995,P97580
5781	1,3,4,6,19,21,23,25	2,5,7,8,18,20,22,24,26	2	P15399,P97580
5782	1,3,4,6,19,21,23,26	2,5,7,8,18,20,22,24,25	5	P00762,P01681,P15399,Q30KJ2,Q4FZU2
5783	1,3,4,6,19,21,24,25	2,5,7,8,18,20,22,23,26	0	
5784	1,3,4,6,19,21,24,26	2,5,7,8,18,20,22,23,25	1	P01681

5785	1,3,4,6,19,21,25,26	2,5,7,8,18,20,22,23,24	2	P01681,Q9QZK9
5786	1,3,4,6,19,22,23,24	2,5,7,8,18,20,21,25,26	7	P02625,P15399,P31430,P55091,P62804,P82471,P82995
5787	1,3,4,6,19,22,23,25	2,5,7,8,18,20,21,24,26	4	P06911,P15399,P82995,P97580
5788	1,3,4,6,19,22,23,26	2,5,7,8,18,20,21,24,25	4	O54728,P02625,P15399,Q30KJ2
5789	1,3,4,6,19,22,24,25	2,5,7,8,18,20,21,23,26	4	P06911,P62804,P82995,Q9QUL6
5790	1,3,4,6,19,22,24,26	2,5,7,8,18,20,21,23,25	2	O54728,Q99MH3
5791	1,3,4,6,19,22,25,26	2,5,7,8,18,20,21,23,24	0	
5792	1,3,4,6,19,23,24,25	2,5,7,8,18,20,21,22,26	5	P15399,P62804,P82995,P97580,Q63751
5793	1,3,4,6,19,23,24,26	2,5,7,8,18,20,21,22,25	4	P02091,P15399,Q30KJ2,Q4FZU2
5794	1,3,4,6,19,23,25,26	2,5,7,8,18,20,21,22,24	5	P02091,P15399,P97580,Q30KJ2,Q4FZU2
5795	1,3,4,6,19,24,25,26	2,5,7,8,18,20,21,22,23	0	
5796	1,3,4,6,20,21,22,23	2,5,7,8,18,19,24,25,26	5	P01681,P01835,P16636,P55091,P82995
5797	1,3,4,6,20,21,22,24	2,5,7,8,18,19,23,25,26	7	P01681,P13432,P16636,P82995,Q03191,Q5XFX0,Q99MH3
5798	1,3,4,6,20,21,22,25	2,5,7,8,18,19,23,24,26	4	P01681,P09605;P25809,P16636,Q03191
5799	1,3,4,6,20,21,22,26	2,5,7,8,18,19,23,24,25	4	P01681,P01835,Q03191,Q99MH3

5800	1,3,4,6,20,21,23,24	2,5,7,8,18,19,22,25,26	4	P01681,P01835,P13432,P82995
5801	1,3,4,6,20,21,23,25	2,5,7,8,18,19,22,24,26	4	P01681,P01835,P16636,Q6P6R2
5802	1,3,4,6,20,21,23,26	2,5,7,8,18,19,22,24,25	7	P01681,P01835,Q4FZU2,Q6IFU8,Q6IFW6,Q6IG02,Q6IMF3
5803	1,3,4,6,20,21,24,25	2,5,7,8,18,19,22,23,26	3	P01681,P16636,Q03191
5804	1,3,4,6,20,21,24,26	2,5,7,8,18,19,22,23,25	6	B0BNN3,P01681,P01835,P13432,Q03191,Q99MH3
5805	1,3,4,6,20,21,25,26	2,5,7,8,18,19,22,23,24	4	P01681,P01835,Q03191,Q4FZU2
5806	1,3,4,6,20,22,23,24	2,5,7,8,18,19,21,25,26	2	P55091,P82995
5807	1,3,4,6,20,22,23,25	2,5,7,8,18,19,21,24,26	3	P06911,P16636,P82995
5808	1,3,4,6,20,22,23,26	2,5,7,8,18,19,21,24,25	5	O54728,P01681,P05539,Q4FZU2,Q6IG02
5809	1,3,4,6,20,22,24,25	2,5,7,8,18,19,21,23,26	3	P82995,Q03191,Q99MH3
5810	1,3,4,6,20,22,24,26	2,5,7,8,18,19,21,23,25	4	P01681,P02803,Q03191,Q99MH3
5811	1,3,4,6,20,22,25,26	2,5,7,8,18,19,21,23,24	4	P01681,P05539,Q03191,Q99MH3
5812	1,3,4,6,20,23,24,25	2,5,7,8,18,19,21,22,26	2	P82995,Q63751
5813	1,3,4,6,20,23,24,26	2,5,7,8,18,19,21,22,25	6	P01681,P97840,Q4FZU2,Q6IFW6,Q6IG02,Q6IMF3
5814	1,3,4,6,20,23,25,26	2,5,7,8,18,19,21,22,24	5	P01681,Q4FZU2,Q6IFW6,Q6IG02,Q6IMF3

5815	1,3,4,6,20,24,25,26	2,5,7,8,18,19,21,22,23	3	P01681,Q03191,Q99MH3
5816	1,3,4,6,21,22,23,24	2,5,7,8,18,19,20,25,26	2	P13432,P82995
5817	1,3,4,6,21,22,23,25	2,5,7,8,18,19,20,24,26	3	P06911,P16636,Q63751
5818	1,3,4,6,21,22,23,26	2,5,7,8,18,19,20,24,25	3	P01681,Q4FZU2,Q6IMF3
5819	1,3,4,6,21,22,24,25	2,5,7,8,18,19,20,23,26	2	P13432,Q03191
5820	1,3,4,6,21,22,24,26	2,5,7,8,18,19,20,23,25	4	P01681,P13432,Q03191,Q99MH3
5821	1,3,4,6,21,22,25,26	2,5,7,8,18,19,20,23,24	2	P01681,Q03191
5822	1,3,4,6,21,23,24,25	2,5,7,8,18,19,20,22,26	2	Q63751,Q6P6R2
5823	1,3,4,6,21,23,24,26	2,5,7,8,18,19,20,22,25	3	P01681,Q4FZU2,Q6IMF3
5824	1,3,4,6,21,23,25,26	2,5,7,8,18,19,20,22,24	7	P01681,P01835,P27213,P83121,Q4FZU2,Q6IFU8,Q6IMF3
5825	1,3,4,6,21,24,25,26	2,5,7,8,18,19,20,22,23	1	P01681
5826	1,3,4,6,22,23,24,25	2,5,7,8,18,19,20,21,26	5	P06911,P62804,P82471,P82995,Q63751
5827	1,3,4,6,22,23,24,26	2,5,7,8,18,19,20,21,25	3	O54728,P00731,P82471
5828	1,3,4,6,22,23,25,26	2,5,7,8,18,19,20,21,24	3	P05539,P06911,Q4FZU2
5829	1,3,4,6,22,24,25,26	2,5,7,8,18,19,20,21,23	2	Q03191,Q99MH3

5830	1,3,4,6,23,24,25,26	2,5,7,8,18,19,20,21,22	5	P02091,P27590,Q4FZU2,Q63751,Q7TPB1
5831	1,3,4,7,8,18,19,20	2,5,6,21,22,23,24,25,26	3	P00731,P18418,P35280
5832	1,3,4,7,8,18,19,21	2,5,6,20,22,23,24,25,26	6	P00731,P02803,P10758,P18418,P35281,P42854
5833	1,3,4,7,8,18,19,22	2,5,6,20,21,23,24,25,26	1	P18418
5834	1,3,4,7,8,18,19,23	2,5,6,20,21,22,24,25,26	6	P02803,P12785,P35467,Q5BJY9,Q5XI22,Q99041
5835	1,3,4,7,8,18,19,24	2,5,6,20,21,22,23,25,26	1	P18418
5836	1,3,4,7,8,18,19,25	2,5,6,20,21,22,23,24,26	7	P00714,P00731,P02803,P10715,P18418,P35280,Q9QUL6
5837	1,3,4,7,8,18,19,26	2,5,6,20,21,22,23,24,25	4	P02770,P06760,P35280,Q5FVI6
5838	1,3,4,7,8,18,20,21	2,5,6,19,22,23,24,25,26	5	P00731,P10758,P42854,Q4FZU6,Q9ESW0
5839	1,3,4,7,8,18,20,22	2,5,6,19,21,23,24,25,26	1	Q63617
5840	1,3,4,7,8,18,20,23	2,5,6,19,21,22,24,25,26	7	A2RUV9,P12785,P35467,Q4FZU6,Q5XI22,Q63617,Q6IG02
5841	1,3,4,7,8,18,20,24	2,5,6,19,21,22,23,25,26	3	P10715,P18418,Q63617
5842	1,3,4,7,8,18,20,25	2,5,6,19,21,22,23,24,26	4	P00714,P10715,P35280,Q63617
5843	1,3,4,7,8,18,20,26	2,5,6,19,21,22,23,24,25	3	P02770,P35280,Q4FZU6
5844	1,3,4,7,8,18,21,22	2,5,6,19,20,23,24,25,26	4	D4A1J4,P10758,P18418,Q80WL1

5845	1,3,4,7,8,18,21,23	2,5,6,19,20,22,24,25,26	4	P10758,P12785,Q5XI22,Q9ESW0
5846	1,3,4,7,8,18,21,24	2,5,6,19,20,22,23,25,26	2	P10758,P18418
5847	1,3,4,7,8,18,21,25	2,5,6,19,20,22,23,24,26	5	P00731,P08721,P10715,P18418,P31430
5848	1,3,4,7,8,18,21,26	2,5,6,19,20,22,23,24,25	7	P02770,P10758,P82995,Q4FZU6,Q9EQV9,Q9ESW0,Q9JID2
5849	1,3,4,7,8,18,22,23	2,5,6,19,20,21,24,25,26	3	Q5XI22,Q63617,Q8VD89
5850	1,3,4,7,8,18,22,24	2,5,6,19,20,21,23,25,26	2	P18418,Q8VD89
5851	1,3,4,7,8,18,22,25	2,5,6,19,20,21,23,24,26	3	P08721,P10715,P18418
5852	1,3,4,7,8,18,22,26	2,5,6,19,20,21,23,24,25	2	P02770,Q8VD89
5853	1,3,4,7,8,18,23,24	2,5,6,19,20,21,22,25,26	4	P12785,Q5XI22,Q63617,Q8VD89
5854	1,3,4,7,8,18,23,25	2,5,6,19,20,21,22,24,26	6	P00714,P10715,P12785,P19629,Q5XI22,Q99041
5855	1,3,4,7,8,18,23,26	2,5,6,19,20,21,22,24,25	14	A2RUV9,P01946,P02091,P02770,P12785,P35467,Q4FZU2,Q4FZU6,Q5XI22,Q6AY61,Q6IFW6,Q6IG02,Q6P6Q2,Q8VD89
5856	1,3,4,7,8,18,24,25	2,5,6,19,20,21,22,23,26	3	P08721,P10715,P18418
5857	1,3,4,7,8,18,24,26	2,5,6,19,20,21,22,23,25	3	P02091,P02770,Q8VD89
5858	1,3,4,7,8,18,25,26	2,5,6,19,20,21,22,23,24	7	B4F795,P00714,P02091,P08721,P10715,P35280,P82995
5859	1,3,4,7,8,19,20,21	2,5,6,18,22,23,24,25,26	3	P35281,P42854,Q6IFU7

5860	1,3,4,7,8,19,20,22	2,5,6,18,21,23,24,25,26	4	P00774,P55091,Q63617,Q9QUL6
5861	1,3,4,7,8,19,20,23	2,5,6,18,21,22,24,25,26	2	Q63617,Q99041
5862	1,3,4,7,8,19,20,24	2,5,6,18,21,22,23,25,26	2	Q63617,Q9QUL6
5863	1,3,4,7,8,19,20,25	2,5,6,18,21,22,23,24,26	5	P10715,P35280,Q63617,Q6IFU7,Q9QUL6
5864	1,3,4,7,8,19,20,26	2,5,6,18,21,22,23,24,25	4	O54728,P35280,Q4FZU6,Q5FVI6
5865	1,3,4,7,8,19,21,22	2,5,6,18,20,23,24,25,26	3	D4A1J4,P35281,Q9QUL6
5866	1,3,4,7,8,19,21,23	2,5,6,18,20,22,24,25,26	2	P35281,Q99041
5867	1,3,4,7,8,19,21,24	2,5,6,18,20,22,23,25,26	2	P35281,Q9QUL6
5868	1,3,4,7,8,19,21,25	2,5,6,18,20,22,23,24,26	3	P47967,Q6IFU7,Q9QUL6
5869	1,3,4,7,8,19,21,26	2,5,6,18,20,22,23,24,25	3	P02770,P35281,Q5FVI6
5870	1,3,4,7,8,19,22,23	2,5,6,18,20,21,24,25,26	2	Q99041,Q9QUL6
5871	1,3,4,7,8,19,22,24	2,5,6,18,20,21,23,25,26	1	Q9QUL6
5872	1,3,4,7,8,19,22,25	2,5,6,18,20,21,23,24,26	3	P06911,Q5GRG2,Q9QUL6
5873	1,3,4,7,8,19,22,26	2,5,6,18,20,21,23,24,25	1	O54728
5874	1,3,4,7,8,19,23,24	2,5,6,18,20,21,22,25,26	2	Q99041,Q9QUL6

5875	1,3,4,7,8,19,23,25	2,5,6,18,20,21,22,24,26	2	Q99041,Q9QUL6
5876	1,3,4,7,8,19,23,26	2,5,6,18,20,21,22,24,25	6	O54728,P01946,P02091,P35467,Q5FVI6,Q99041
5877	1,3,4,7,8,19,24,25	2,5,6,18,20,21,22,23,26	2	P10715,Q9QUL6
5878	1,3,4,7,8,19,24,26	2,5,6,18,20,21,22,23,25	2	P02091,P02770
5879	1,3,4,7,8,19,25,26	2,5,6,18,20,21,22,23,24	4	P02091,P35280,Q5FVI6,Q9QUL6
5880	1,3,4,7,8,20,21,22	2,5,6,18,19,23,24,25,26	3	P42854,Q63617,Q7TPB1
5881	1,3,4,7,8,20,21,23	2,5,6,18,19,22,24,25,26	2	Q4FZU6,Q63617
5882	1,3,4,7,8,20,21,24	2,5,6,18,19,22,23,25,26	1	Q63617
5883	1,3,4,7,8,20,21,25	2,5,6,18,19,22,23,24,26	3	P10715,Q6IFU7,Q9QUL6
5884	1,3,4,7,8,20,21,26	2,5,6,18,19,22,23,24,25	4	P01041,P01836,P42854,Q4FZU6
5885	1,3,4,7,8,20,22,23	2,5,6,18,19,21,24,25,26	1	Q63617
5886	1,3,4,7,8,20,22,24	2,5,6,18,19,21,23,25,26	2	Q63617,Q99MH3
5887	1,3,4,7,8,20,22,25	2,5,6,18,19,21,23,24,26	4	P10715,Q03191,Q63617,Q9QUL6
5888	1,3,4,7,8,20,22,26	2,5,6,18,19,21,23,24,25	5	O54728,P05539,Q4FZU6,Q63617,Q99MH3
5889	1,3,4,7,8,20,23,24	2,5,6,18,19,21,22,25,26	2	P97840,Q63617

5890	1,3,4,7,8,20,23,25	2,5,6,18,19,21,22,24,26	4	P10715,Q63617,Q99041,Q9QUL6
5891	1,3,4,7,8,20,23,26	2,5,6,18,19,21,22,24,25	8	A2RUV9,O54728,P01946,P02091,P97840,Q4FZU6,Q63617,Q6IG02
5892	1,3,4,7,8,20,24,25	2,5,6,18,19,21,22,23,26	3	P10715,Q63617,Q9QUL6
5893	1,3,4,7,8,20,24,26	2,5,6,18,19,21,22,23,25	5	P02091,P97840,Q4FZU6,Q63617,Q99MH3
5894	1,3,4,7,8,20,25,26	2,5,6,18,19,21,22,23,24	3	P02091,P10715,P35280
5895	1,3,4,7,8,21,22,23	2,5,6,18,19,20,24,25,26	0	
5896	1,3,4,7,8,21,22,24	2,5,6,18,19,20,23,25,26	0	
5897	1,3,4,7,8,21,22,25	2,5,6,18,19,20,23,24,26	2	P19629,Q9QUL6
5898	1,3,4,7,8,21,22,26	2,5,6,18,19,20,23,24,25	1	Q80WL1
5899	1,3,4,7,8,21,23,24	2,5,6,18,19,20,22,25,26	0	
5900	1,3,4,7,8,21,23,25	2,5,6,18,19,20,22,24,26	3	P19629,Q63751,Q99041
5901	1,3,4,7,8,21,23,26	2,5,6,18,19,20,22,24,25	3	P01946,P02091,Q4FZU6
5902	1,3,4,7,8,21,24,25	2,5,6,18,19,20,22,23,26	5	P08721,P10715,P19629,P22006,Q9QUL6
5903	1,3,4,7,8,21,24,26	2,5,6,18,19,20,22,23,25	3	P02091,P02770,Q9EQV9
5904	1,3,4,7,8,21,25,26	2,5,6,18,19,20,22,23,24	4	P02091,P08721,P19223,P22006

5905	1,3,4,7,8,22,23,24	2,5,6,18,19,20,21,25,26	1	Q63617
5906	1,3,4,7,8,22,23,25	2,5,6,18,19,20,21,24,26	7	P06911,P19629,Q5GRG2,Q63617,Q63751,Q99041,Q9QUL6
5907	1,3,4,7,8,22,23,26	2,5,6,18,19,20,21,24,25	3	O54728,P01946,P02091
5908	1,3,4,7,8,22,24,25	2,5,6,18,19,20,21,23,26	4	P06911,P10715,P19629,Q9QUL6
5909	1,3,4,7,8,22,24,26	2,5,6,18,19,20,21,23,25	4	O54728,P02091,P02770,Q99MH3
5910	1,3,4,7,8,22,25,26	2,5,6,18,19,20,21,23,24	3	P02091,P10715,Q9QUL6
5911	1,3,4,7,8,23,24,25	2,5,6,18,19,20,21,22,26	6	P10715,P19629,Q63617,Q63751,Q99041,Q9QUL6
5912	1,3,4,7,8,23,24,26	2,5,6,18,19,20,21,22,25	4	P01946,P02091,P97840,Q8VD89
5913	1,3,4,7,8,23,25,26	2,5,6,18,19,20,21,22,24	6	P00714,P01946,P02091,P10715,P19629,Q99041
5914	1,3,4,7,8,24,25,26	2,5,6,18,19,20,21,22,23	6	P01946,P02091,P08721,P10715,P19629,Q9QUL6
5915	1,3,4,7,18,19,20,21	2,5,6,8,22,23,24,25,26	4	P00731,P06760,P10758,P42854
5916	1,3,4,7,18,19,20,22	2,5,6,8,21,23,24,25,26	5	P06760,P07150,Q5U2V4,Q6P6R2,Q9WTT6
5917	1,3,4,7,18,19,20,23	2,5,6,8,21,22,24,25,26	3	P06760,P12785,Q9WTT6
5918	1,3,4,7,18,19,20,24	2,5,6,8,21,22,23,25,26	3	P06760,P07150,Q9WTT6
5919	1,3,4,7,18,19,20,25	2,5,6,8,21,22,23,24,26	1	P35280

5920	1,3,4,7,18,19,20,26	2,5,6,8,21,22,23,24,25	6	P06760,P35280,Q4FZU6,Q5FVI6,Q6P6R2,Q9WTT6
5921	1,3,4,7,18,19,21,22	2,5,6,8,20,23,24,25,26	2	P06760,P10758
5922	1,3,4,7,18,19,21,23	2,5,6,8,20,22,24,25,26	2	P10758,P12785
5923	1,3,4,7,18,19,21,24	2,5,6,8,20,22,23,25,26	1	P10758
5924	1,3,4,7,18,19,21,25	2,5,6,8,20,22,23,24,26	3	P00731,P31430,P47967
5925	1,3,4,7,18,19,21,26	2,5,6,8,20,22,23,24,25	3	P06760,P10758,Q5FVI6
5926	1,3,4,7,18,19,22,23	2,5,6,8,20,21,24,25,26	5	P02625,P06760,P12785,P62804,Q9WTT6
5927	1,3,4,7,18,19,22,24	2,5,6,8,20,21,23,25,26	4	P06760,P62804,Q6P6R2,Q9WTT6
5928	1,3,4,7,18,19,22,25	2,5,6,8,20,21,23,24,26	1	P62804
5929	1,3,4,7,18,19,22,26	2,5,6,8,20,21,23,24,25	5	O54728,P06760,Q5FVI6,Q6P6R2,Q9WTT6
5930	1,3,4,7,18,19,23,24	2,5,6,8,20,21,22,25,26	4	P06760,P12785,P62804,Q9WTT6
5931	1,3,4,7,18,19,23,25	2,5,6,8,20,21,22,24,26	3	P00714,P12785,P62804
5932	1,3,4,7,18,19,23,26	2,5,6,8,20,21,22,24,25	9	O54728,P01946,P02091,P06760,P12785,P35467,Q5FVI6,Q6IG02,Q6P6Q2
5933	1,3,4,7,18,19,24,25	2,5,6,8,20,21,22,23,26	1	P62804
5934	1,3,4,7,18,19,24,26	2,5,6,8,20,21,22,23,25	4	P02091,P06760,Q5FVI6,Q9WTT6

5935	1,3,4,7,18,19,25,26	2,5,6,8,20,21,22,23,24	4	P00714,P02091,P35280,Q5FVI6
5936	1,3,4,7,18,20,21,22	2,5,6,8,19,23,24,25,26	2	P10758,P42854
5937	1,3,4,7,18,20,21,23	2,5,6,8,19,22,24,25,26	5	A2RUV9,P10758,P12785,P42854,Q4FZU6
5938	1,3,4,7,18,20,21,24	2,5,6,8,19,22,23,25,26	0	
5939	1,3,4,7,18,20,21,25	2,5,6,8,19,22,23,24,26	2	P31430,P42854
5940	1,3,4,7,18,20,21,26	2,5,6,8,19,22,23,24,25	5	P01041,P01836,P06760,P42854,Q4FZU6
5941	1,3,4,7,18,20,22,23	2,5,6,8,19,21,24,25,26	4	A2RUV9,P06760,P12785,Q9WTT6
5942	1,3,4,7,18,20,22,24	2,5,6,8,19,21,23,25,26	4	P06760,P07150,Q6P6R2,Q9WTT6
5943	1,3,4,7,18,20,22,25	2,5,6,8,19,21,23,24,26	0	
5944	1,3,4,7,18,20,22,26	2,5,6,8,19,21,23,24,25	6	O54728,P05539,P06760,Q4FZU6,Q6P6R2,Q9WTT6
5945	1,3,4,7,18,20,23,24	2,5,6,8,19,21,22,25,26	3	A2RUV9,P06760,P12785
5946	1,3,4,7,18,20,23,25	2,5,6,8,19,21,22,24,26	2	A2RUV9,P12785
5947	1,3,4,7,18,20,23,26	2,5,6,8,19,21,22,24,25	13	A2RUV9,P01946,P02091,P06760,P12785,P35467,Q4FZU2,Q4FZU6,Q6IFU8,Q6IFW6,Q6IG02,Q6IMF3,Q6P6Q2
5948	1,3,4,7,18,20,24,25	2,5,6,8,19,21,22,23,26	0	
5949	1,3,4,7,18,20,24,26	2,5,6,8,19,21,22,23,25	3	P02091,P06760,Q4FZU6

5950	1,3,4,7,18,20,25,26	2,5,6,8,19,21,22,23,24	2	P02091,P35280
5951	1,3,4,7,18,21,22,23	2,5,6,8,19,20,24,25,26	2	P10758,P12785
5952	1,3,4,7,18,21,22,24	2,5,6,8,19,20,23,25,26	0	
5953	1,3,4,7,18,21,22,25	2,5,6,8,19,20,23,24,26	2	P08721,P31430
5954	1,3,4,7,18,21,22,26	2,5,6,8,19,20,23,24,25	2	P06760,Q9JID2
5955	1,3,4,7,18,21,23,24	2,5,6,8,19,20,22,25,26	1	P12785
5956	1,3,4,7,18,21,23,25	2,5,6,8,19,20,22,24,26	2	P12785,P31430
5957	1,3,4,7,18,21,23,26	2,5,6,8,19,20,22,24,25	10	A2RUV9,P01946,P02091,P12785,Q4FZU2,Q62636,Q6IFU8,Q6IG02,Q6IMF3,Q6P6Q2
5958	1,3,4,7,18,21,24,25	2,5,6,8,19,20,22,23,26	3	P08721,P22006,P31430
5959	1,3,4,7,18,21,24,26	2,5,6,8,19,20,22,23,25	3	P02091,P06760,Q9JID2
5960	1,3,4,7,18,21,25,26	2,5,6,8,19,20,22,23,24	6	O35783,P02091,P08721,P22006,P31430,P82995
5961	1,3,4,7,18,22,23,24	2,5,6,8,19,20,21,25,26	5	P06760,P12785,P62804,Q8VD89,Q9WTT6
5962	1,3,4,7,18,22,23,25	2,5,6,8,19,20,21,24,26	2	P12785,P62804
5963	1,3,4,7,18,22,23,26	2,5,6,8,19,20,21,24,25	9	A2RUV9,O54728,P01946,P02091,P06760,P12785,Q6IG02,Q6P6Q2,Q8VD89
5964	1,3,4,7,18,22,24,25	2,5,6,8,19,20,21,23,26	1	P62804

5965	1,3,4,7,18,22,24,26	2,5,6,8,19,20,21,23,25	5	P02091,P06760,Q6P6R2,Q8VD89,Q9WTT6
5966	1,3,4,7,18,22,25,26	2,5,6,8,19,20,21,23,24	1	P02091
5967	1,3,4,7,18,23,24,25	2,5,6,8,19,20,21,22,26	4	A2RUV9,P12785,P62804,Q63751
5968	1,3,4,7,18,23,24,26	2,5,6,8,19,20,21,22,25	8	A2RUV9,P01946,P02091,P06760,P12785,Q6IG02,Q6P6Q2,Q8VD89
5969	1,3,4,7,18,23,25,26	2,5,6,8,19,20,21,22,24	8	A2RUV9,P00714,P01946,P02091,P12785,Q4FZU2,Q6IG02,Q6P6Q2
5970	1,3,4,7,18,24,25,26	2,5,6,8,19,20,21,22,23	4	B4F795,P01946,P02091,P08721
5971	1,3,4,7,19,20,21,22	2,5,6,8,18,23,24,25,26	2	P55091,Q7TPB1
5972	1,3,4,7,19,20,21,23	2,5,6,8,18,22,24,25,26	0	
5973	1,3,4,7,19,20,21,24	2,5,6,8,18,22,23,25,26	0	
5974	1,3,4,7,19,20,21,25	2,5,6,8,18,22,23,24,26	1	P47967
5975	1,3,4,7,19,20,21,26	2,5,6,8,18,22,23,24,25	2	Q4FZU6,Q5FVI6
5976	1,3,4,7,19,20,22,23	2,5,6,8,18,21,24,25,26	4	O54728,P00774,P55091,Q9WTT6
5977	1,3,4,7,19,20,22,24	2,5,6,8,18,21,23,25,26	8	P00774,P06760,P07150,P14669,P55091,Q6P6R2,Q99MH3,Q9WTT6
5978	1,3,4,7,19,20,22,25	2,5,6,8,18,21,23,24,26	1	Q9QUL6
5979	1,3,4,7,19,20,22,26	2,5,6,8,18,21,23,24,25	7	O54728,P05539,P06760,Q5FVI6,Q6P6R2,Q99MH3,Q9WTT6

5980	1,3,4,7,19,20,23,24	2,5,6,8,18,21,22,25,26	1	Q9WTT6
5981	1,3,4,7,19,20,23,25	2,5,6,8,18,21,22,24,26	0	
5982	1,3,4,7,19,20,23,26	2,5,6,8,18,21,22,24,25	6	O54728,P01946,P02091,P05539,P06760,Q5FVI6
5983	1,3,4,7,19,20,24,25	2,5,6,8,18,21,22,23,26	1	Q9QUL6
5984	1,3,4,7,19,20,24,26	2,5,6,8,18,21,22,23,25	5	O54728,P02091,P06760,Q5FVI6,Q9WTT6
5985	1,3,4,7,19,20,25,26	2,5,6,8,18,21,22,23,24	4	P02091,P05539,P35280,Q5FVI6
5986	1,3,4,7,19,21,22,23	2,5,6,8,18,20,24,25,26	0	
5987	1,3,4,7,19,21,22,24	2,5,6,8,18,20,23,25,26	0	
5988	1,3,4,7,19,21,22,25	2,5,6,8,18,20,23,24,26	1	P47967
5989	1,3,4,7,19,21,22,26	2,5,6,8,18,20,23,24,25	1	O54728
5990	1,3,4,7,19,21,23,24	2,5,6,8,18,20,22,25,26	0	
5991	1,3,4,7,19,21,23,25	2,5,6,8,18,20,22,24,26	3	P15399,P47967,Q99041
5992	1,3,4,7,19,21,23,26	2,5,6,8,18,20,22,24,25	4	O54728,P01946,P02091,Q5FVI6
5993	1,3,4,7,19,21,24,25	2,5,6,8,18,20,22,23,26	2	P22006,P47967
5994	1,3,4,7,19,21,24,26	2,5,6,8,18,20,22,23,25	2	P02091,Q5FVI6

5995	1,3,4,7,19,21,25,26	2,5,6,8,18,20,22,23,24	3	P02091,P22006,Q5FVI6
5996	1,3,4,7,19,22,23,24	2,5,6,8,18,20,21,25,26	2	P62804,Q9WTT6
5997	1,3,4,7,19,22,23,25	2,5,6,8,18,20,21,24,26	2	P06911,P62804
5998	1,3,4,7,19,22,23,26	2,5,6,8,18,20,21,24,25	6	O54728,P01946,P02091,P05539,P06760,Q5FVI6
5999	1,3,4,7,19,22,24,25	2,5,6,8,18,20,21,23,26	2	P62804,Q9QUL6
6000	1,3,4,7,19,22,24,26	2,5,6,8,18,20,21,23,25	5	O54728,P02091,P06760,Q6P6R2,Q9WTT6
6001	1,3,4,7,19,22,25,26	2,5,6,8,18,20,21,23,24	4	O54728,P02091,P05539,Q5FVI6
6002	1,3,4,7,19,23,24,25	2,5,6,8,18,20,21,22,26	1	P62804
6003	1,3,4,7,19,23,24,26	2,5,6,8,18,20,21,22,25	5	O54728,P01946,P02091,P06760,Q5FVI6
6004	1,3,4,7,19,23,25,26	2,5,6,8,18,20,21,22,24	5	O54728,P01946,P02091,P05539,Q5FVI6
6005	1,3,4,7,19,24,25,26	2,5,6,8,18,20,21,22,23	3	P01946,P02091,Q5FVI6
6006	1,3,4,7,20,21,22,23	2,5,6,8,18,19,24,25,26	0	
6007	1,3,4,7,20,21,22,24	2,5,6,8,18,19,23,25,26	4	P01836,Q03191,Q63942,Q99MH3
6008	1,3,4,7,20,21,22,25	2,5,6,8,18,19,23,24,26	2	Q03191,Q63942
6009	1,3,4,7,20,21,22,26	2,5,6,8,18,19,23,24,25	5	O54728,P01836,P05539,Q4FZU6,Q99MH3

6010	1,3,4,7,20,21,23,24	2,5,6,8,18,19,22,25,26	0	
6011	1,3,4,7,20,21,23,25	2,5,6,8,18,19,22,24,26	0	
6012	1,3,4,7,20,21,23,26	2,5,6,8,18,19,22,24,25	6	A2RUV9,P01836,P01946,P02091,Q4FZU2,Q4FZU6
6013	1,3,4,7,20,21,24,25	2,5,6,8,18,19,22,23,26	2	P22006,Q63942
6014	1,3,4,7,20,21,24,26	2,5,6,8,18,19,22,23,25	4	P01836,P02091,Q4FZU6,Q99MH3
6015	1,3,4,7,20,21,25,26	2,5,6,8,18,19,22,23,24	2	P01836,P02091
6016	1,3,4,7,20,22,23,24	2,5,6,8,18,19,21,25,26	1	Q9WTT6
6017	1,3,4,7,20,22,23,25	2,5,6,8,18,19,21,24,26	0	
6018	1,3,4,7,20,22,23,26	2,5,6,8,18,19,21,24,25	6	A2RUV9,O54728,P01946,P02091,P05539,P06760
6019	1,3,4,7,20,22,24,25	2,5,6,8,18,19,21,23,26	2	Q03191,Q63942
6020	1,3,4,7,20,22,24,26	2,5,6,8,18,19,21,23,25	10	O54728,P01836,P02091,P02803,P05539,P06760,Q62635,Q6P6R2,Q99MH3,Q9WTT6
6021	1,3,4,7,20,22,25,26	2,5,6,8,18,19,21,23,24	4	O54728,P02091,P05539,Q03191
6022	1,3,4,7,20,23,24,25	2,5,6,8,18,19,21,22,26	2	A2RUV9,Q63751
6023	1,3,4,7,20,23,24,26	2,5,6,8,18,19,21,22,25	5	A2RUV9,O54728,P01946,P02091,P97840
6024	1,3,4,7,20,23,25,26	2,5,6,8,18,19,21,22,24	4	A2RUV9,P01946,P02091,P05539

6025	1,3,4,7,20,24,25,26	2,5,6,8,18,19,21,22,23	2	P01946,P02091
6026	1,3,4,7,21,22,23,24	2,5,6,8,18,19,20,25,26	1	P62804
6027	1,3,4,7,21,22,23,25	2,5,6,8,18,19,20,24,26	1	P62804
6028	1,3,4,7,21,22,23,26	2,5,6,8,18,19,20,24,25	4	O54728,P01946,P02091,P05539
6029	1,3,4,7,21,22,24,25	2,5,6,8,18,19,20,23,26	3	P22006,P62804,Q63942
6030	1,3,4,7,21,22,24,26	2,5,6,8,18,19,20,23,25	3	P01836,P02091,Q99MH3
6031	1,3,4,7,21,22,25,26	2,5,6,8,18,19,20,23,24	3	P02091,P05539,P22006
6032	1,3,4,7,21,23,24,25	2,5,6,8,18,19,20,22,26	3	P22006,P62804,Q63751
6033	1,3,4,7,21,23,24,26	2,5,6,8,18,19,20,22,25	3	P01946,P02091,P22006
6034	1,3,4,7,21,23,25,26	2,5,6,8,18,19,20,22,24	5	P01946,P02091,P22006,P27213,P83121
6035	1,3,4,7,21,24,25,26	2,5,6,8,18,19,20,22,23	3	P01946,P02091,P22006
6036	1,3,4,7,22,23,24,25	2,5,6,8,18,19,20,21,26	4	P62804,P82471,Q00715,Q63751
6037	1,3,4,7,22,23,24,26	2,5,6,8,18,19,20,21,25	5	O54728,P00731,P01946,P02091,P62804
6038	1,3,4,7,22,23,25,26	2,5,6,8,18,19,20,21,24	5	O54728,P01946,P02091,P05539,P62804
6039	1,3,4,7,22,24,25,26	2,5,6,8,18,19,20,21,23	3	P01946,P02091,P62804

6040	1,3,4,7,23,24,25,26	2,5,6,8,18,19,20,21,22	6	A2RUV9,P01946,P02091,P62804,Q63751,Q7TPB1
6041	1,3,4,8,18,19,20,21	2,5,6,7,22,23,24,25,26	3	P00731,P10758,P35281
6042	1,3,4,8,18,19,20,22	2,5,6,7,21,23,24,25,26	2	P22006,Q6P6R2
6043	1,3,4,8,18,19,20,23	2,5,6,7,21,22,24,25,26	5	P12785,P35467,P83748,Q5XI22,Q6IG02
6044	1,3,4,8,18,19,20,24	2,5,6,7,21,22,23,25,26	1	P83748
6045	1,3,4,8,18,19,20,25	2,5,6,7,21,22,23,24,26	3	P35280,P83748,Q9QUL6
6046	1,3,4,8,18,19,20,26	2,5,6,7,21,22,23,24,25	2	P35280,Q64268
6047	1,3,4,8,18,19,21,22	2,5,6,7,20,23,24,25,26	2	P10758,P35281
6048	1,3,4,8,18,19,21,23	2,5,6,7,20,22,24,25,26	5	P10758,P12785,P35281,P35467,Q5XI22
6049	1,3,4,8,18,19,21,24	2,5,6,7,20,22,23,25,26	2	P10758,P35281
6050	1,3,4,8,18,19,21,25	2,5,6,7,20,22,23,24,26	4	P00731,P10758,P31430,P35281
6051	1,3,4,8,18,19,21,26	2,5,6,7,20,22,23,24,25	3	P10758,P35281,Q64268
6052	1,3,4,8,18,19,22,23	2,5,6,7,20,21,24,25,26	4	P02625,P12785,P35467,Q5XI22
6053	1,3,4,8,18,19,22,24	2,5,6,7,20,21,23,25,26	1	P83121
6054	1,3,4,8,18,19,22,25	2,5,6,7,20,21,23,24,26	1	Q9QUL6

6055	1,3,4,8,18,19,22,26	2,5,6,7,20,21,23,24,25	2	O54728,Q6P6R2
6056	1,3,4,8,18,19,23,24	2,5,6,7,20,21,22,25,26	5	P09605;P25809,P12785,P35467,P83748,Q5XI22
6057	1,3,4,8,18,19,23,25	2,5,6,7,20,21,22,24,26	6	P00714,P12785,P35467,P83748,Q5XI22,Q9QUL6
6058	1,3,4,8,18,19,23,26	2,5,6,7,20,21,22,24,25	9	P09605;P25809,P12785,P35467,Q4FZU2,Q64194,Q6AY61,Q6IFW6,Q6IG02,Q6P6Q2
6059	1,3,4,8,18,19,24,25	2,5,6,7,20,21,22,23,26	2	P83748,Q9QUL6
6060	1,3,4,8,18,19,24,26	2,5,6,7,20,21,22,23,25	0	
6061	1,3,4,8,18,19,25,26	2,5,6,7,20,21,22,23,24	2	P00714,P35280
6062	1,3,4,8,18,20,21,22	2,5,6,7,19,23,24,25,26	1	P10758
6063	1,3,4,8,18,20,21,23	2,5,6,7,19,22,24,25,26	4	P10758,P12785,Q5XI22,Q6IG02
6064	1,3,4,8,18,20,21,24	2,5,6,7,19,22,23,25,26	1	P10758
6065	1,3,4,8,18,20,21,25	2,5,6,7,19,22,23,24,26	2	P10758,P31430
6066	1,3,4,8,18,20,21,26	2,5,6,7,19,22,23,24,25	4	P10758,Q4FZU2,Q4FZU6,Q6IG02
6067	1,3,4,8,18,20,22,23	2,5,6,7,19,21,24,25,26	4	P83748,P97840,Q5XI22,Q6IG02
6068	1,3,4,8,18,20,22,24	2,5,6,7,19,21,23,25,26	3	P83121,P83748,P97840
6069	1,3,4,8,18,20,22,25	2,5,6,7,19,21,23,24,26	0	

6070	1,3,4,8,18,20,22,26	2,5,6,7,19,21,23,24,25	2	O54728,P97840
6071	1,3,4,8,18,20,23,24	2,5,6,7,19,21,22,25,26	6	P12785,P83748,P97840,Q5XI22,Q6IG02,Q9QZK9
6072	1,3,4,8,18,20,23,25	2,5,6,7,19,21,22,24,26	4	P00714,P12785,P83748,Q6IG02
6073	1,3,4,8,18,20,23,26	2,5,6,7,19,21,22,24,25	13	A2RUV9,P12785,P19218,P35467,P97840,Q4FZU2,Q4FZU6,Q6AY61,Q6IFU8,Q6IFW6,Q6IG02,Q6IMF3,Q6P6Q2
6074	1,3,4,8,18,20,24,25	2,5,6,7,19,21,22,23,26	1	P83748
6075	1,3,4,8,18,20,24,26	2,5,6,7,19,21,22,23,25	1	P97840
6076	1,3,4,8,18,20,25,26	2,5,6,7,19,21,22,23,24	3	P00714,P35280,Q6IG02
6077	1,3,4,8,18,21,22,23	2,5,6,7,19,20,24,25,26	2	P10758,Q5XI22
6078	1,3,4,8,18,21,22,24	2,5,6,7,19,20,23,25,26	1	P10758
6079	1,3,4,8,18,21,22,25	2,5,6,7,19,20,23,24,26	3	P08721,P10758,P31430
6080	1,3,4,8,18,21,22,26	2,5,6,7,19,20,23,24,25	2	P10758,Q9JID2
6081	1,3,4,8,18,21,23,24	2,5,6,7,19,20,22,25,26	3	P10758,P12785,Q5XI22
6082	1,3,4,8,18,21,23,25	2,5,6,7,19,20,22,24,26	3	P10758,P12785,P31430
6083	1,3,4,8,18,21,23,26	2,5,6,7,19,20,22,24,25	10	P10758,P12785,P35467,Q4FZU2,Q6AY61,Q6IFU8,Q6IFW6,Q6IG02,Q6IMF3,Q6P6Q2
6084	1,3,4,8,18,21,24,25	2,5,6,7,19,20,22,23,26	2	P08721,P31430

6085	1,3,4,8,18,21,24,26	2,5,6,7,19,20,22,23,25	3	P10758,Q9EQV9,Q9JID2
6086	1,3,4,8,18,21,25,26	2,5,6,7,19,20,22,23,24	2	P08721,P31430
6087	1,3,4,8,18,22,23,24	2,5,6,7,19,20,21,25,26	2	P83748,P97840
6088	1,3,4,8,18,22,23,25	2,5,6,7,19,20,21,24,26	1	P83748
6089	1,3,4,8,18,22,23,26	2,5,6,7,19,20,21,24,25	9	O54728,P12785,P35467,P97840,Q4FZU2,Q6AY61,Q6IFW6,Q6IG02,Q6P6Q2
6090	1,3,4,8,18,22,24,25	2,5,6,7,19,20,21,23,26	2	P83748,Q9QUL6
6091	1,3,4,8,18,22,24,26	2,5,6,7,19,20,21,23,25	1	P97840
6092	1,3,4,8,18,22,25,26	2,5,6,7,19,20,21,23,24	0	
6093	1,3,4,8,18,23,24,25	2,5,6,7,19,20,21,22,26	3	P12785,P83748,Q63751
6094	1,3,4,8,18,23,24,26	2,5,6,7,19,20,21,22,25	8	P12785,P35467,P47967,P97840,Q4FZU2,Q6IFW6,Q6IG02,Q6P6Q2
6095	1,3,4,8,18,23,25,26	2,5,6,7,19,20,21,22,24	9	P00714,P12785,P35467,Q4FZU2,Q6AY61,Q6IFU8,Q6IFW6,Q6IG02,Q6P6Q2
6096	1,3,4,8,18,24,25,26	2,5,6,7,19,20,21,22,23	2	B4F795,P08721
6097	1,3,4,8,19,20,21,22	2,5,6,7,18,23,24,25,26	5	P10758,P35281,P55091,Q7TPB1,Q9QUL6
6098	1,3,4,8,19,20,21,23	2,5,6,7,18,22,24,25,26	3	P10758,P35281,P55091
6099	1,3,4,8,19,20,21,24	2,5,6,7,18,22,23,25,26	2	P35281,Q9QUL6

6100	1,3,4,8,19,20,21,25	2,5,6,7,18,22,23,24,26	3	P16636,P35281,Q9QUL6
6101	1,3,4,8,19,20,21,26	2,5,6,7,18,22,23,24,25	1	P35281
6102	1,3,4,8,19,20,22,23	2,5,6,7,18,21,24,25,26	7	O54728,P00774,P25031,P55091,P82995,P83748,Q9QUL6
6103	1,3,4,8,19,20,22,24	2,5,6,7,18,21,23,25,26	7	P00774,P25031,P55091,P82995,P83121,P83748,Q9QUL6
6104	1,3,4,8,19,20,22,25	2,5,6,7,18,21,23,24,26	2	P83748,Q9QUL6
6105	1,3,4,8,19,20,22,26	2,5,6,7,18,21,23,24,25	2	O54728,Q6P6R2
6106	1,3,4,8,19,20,23,24	2,5,6,7,18,21,22,25,26	5	P25031,P55091,P82995,P83748,Q9QUL6
6107	1,3,4,8,19,20,23,25	2,5,6,7,18,21,22,24,26	2	P83748,Q9QUL6
6108	1,3,4,8,19,20,23,26	2,5,6,7,18,21,22,24,25	4	O54728,P35467,P97840,Q6IG02
6109	1,3,4,8,19,20,24,25	2,5,6,7,18,21,22,23,26	3	P25031,P83748,Q9QUL6
6110	1,3,4,8,19,20,24,26	2,5,6,7,18,21,22,23,25	1	P97840
6111	1,3,4,8,19,20,25,26	2,5,6,7,18,21,22,23,24	2	P35280,Q9QUL6
6112	1,3,4,8,19,21,22,23	2,5,6,7,18,20,24,25,26	2	P10758,P35281
6113	1,3,4,8,19,21,22,24	2,5,6,7,18,20,23,25,26	1	Q9QUL6
6114	1,3,4,8,19,21,22,25	2,5,6,7,18,20,23,24,26	1	Q9QUL6

6115	1,3,4,8,19,21,22,26	2,5,6,7,18,20,23,24,25	1	O54728
6116	1,3,4,8,19,21,23,24	2,5,6,7,18,20,22,25,26	1	P35281
6117	1,3,4,8,19,21,23,25	2,5,6,7,18,20,22,24,26	3	P35281,Q99041,Q9QUL6
6118	1,3,4,8,19,21,23,26	2,5,6,7,18,20,22,24,25	1	P35281
6119	1,3,4,8,19,21,24,25	2,5,6,7,18,20,22,23,26	1	Q9QUL6
6120	1,3,4,8,19,21,24,26	2,5,6,7,18,20,22,23,25	1	Q9EQV9
6121	1,3,4,8,19,21,25,26	2,5,6,7,18,20,22,23,24	1	Q9QUL6
6122	1,3,4,8,19,22,23,24	2,5,6,7,18,20,21,25,26	3	P25031,P83748,Q9QUL6
6123	1,3,4,8,19,22,23,25	2,5,6,7,18,20,21,24,26	4	P06911,P25031,P83748,Q9QUL6
6124	1,3,4,8,19,22,23,26	2,5,6,7,18,20,21,24,25	1	O54728
6125	1,3,4,8,19,22,24,25	2,5,6,7,18,20,21,23,26	3	P25031,P83748,Q9QUL6
6126	1,3,4,8,19,22,24,26	2,5,6,7,18,20,21,23,25	3	O54728,P25031,Q9QUL6
6127	1,3,4,8,19,22,25,26	2,5,6,7,18,20,21,23,24	2	O54728,Q9QUL6
6128	1,3,4,8,19,23,24,25	2,5,6,7,18,20,21,22,26	3	P25031,P83748,Q9QUL6
6129	1,3,4,8,19,23,24,26	2,5,6,7,18,20,21,22,25	2	O54728,P02091

6130	1,3,4,8,19,23,25,26	2,5,6,7,18,20,21,22,24	1	Q9QUL6
6131	1,3,4,8,19,24,25,26	2,5,6,7,18,20,21,22,23	1	Q9QUL6
6132	1,3,4,8,20,21,22,23	2,5,6,7,18,19,24,25,26	1	P16636
6133	1,3,4,8,20,21,22,24	2,5,6,7,18,19,23,25,26	3	Q03191,Q5XFX0,Q99MH3
6134	1,3,4,8,20,21,22,25	2,5,6,7,18,19,23,24,26	3	P16636,Q03191,Q9QUL6
6135	1,3,4,8,20,21,22,26	2,5,6,7,18,19,23,24,25	2	O54728,Q99MH3
6136	1,3,4,8,20,21,23,24	2,5,6,7,18,19,22,25,26	2	P83748,P97840
6137	1,3,4,8,20,21,23,25	2,5,6,7,18,19,22,24,26	2	P16636,P83748
6138	1,3,4,8,20,21,23,26	2,5,6,7,18,19,22,24,25	4	P97840,Q4FZU2,Q4FZU6,Q6IG02
6139	1,3,4,8,20,21,24,25	2,5,6,7,18,19,22,23,26	4	P16636,Q03191,Q5XFX0,Q9QUL6
6140	1,3,4,8,20,21,24,26	2,5,6,7,18,19,22,23,25	4	B0BNN3,P97840,Q99MH3,Q9EQV9
6141	1,3,4,8,20,21,25,26	2,5,6,7,18,19,22,23,24	0	
6142	1,3,4,8,20,22,23,24	2,5,6,7,18,19,21,25,26	4	P25031,P82995,P83748,P97840
6143	1,3,4,8,20,22,23,25	2,5,6,7,18,19,21,24,26	3	P16636,P83748,Q9QUL6
6144	1,3,4,8,20,22,23,26	2,5,6,7,18,19,21,24,25	3	O54728,P97840,Q6IG02

6145	1,3,4,8,20,22,24,25	2,5,6,7,18,19,21,23,26	5	P25031,P83748,Q03191,Q5XFX0,Q9QUL6
6146	1,3,4,8,20,22,24,26	2,5,6,7,18,19,21,23,25	4	O54728,P97840,Q64573,Q99MH3
6147	1,3,4,8,20,22,25,26	2,5,6,7,18,19,21,23,24	2	O54728,Q03191
6148	1,3,4,8,20,23,24,25	2,5,6,7,18,19,21,22,26	3	P25031,P83748,Q9QUL6
6149	1,3,4,8,20,23,24,26	2,5,6,7,18,19,21,22,25	4	P19218,P47967,P97840,Q6IG02
6150	1,3,4,8,20,23,25,26	2,5,6,7,18,19,21,22,24	2	P19218,Q6IG02
6151	1,3,4,8,20,24,25,26	2,5,6,7,18,19,21,22,23	0	
6152	1,3,4,8,21,22,23,24	2,5,6,7,18,19,20,25,26	0	
6153	1,3,4,8,21,22,23,25	2,5,6,7,18,19,20,24,26	2	P16636,Q9QUL6
6154	1,3,4,8,21,22,23,26	2,5,6,7,18,19,20,24,25	1	O54728
6155	1,3,4,8,21,22,24,25	2,5,6,7,18,19,20,23,26	2	Q5XFX0,Q9QUL6
6156	1,3,4,8,21,22,24,26	2,5,6,7,18,19,20,23,25	1	Q99MH3
6157	1,3,4,8,21,22,25,26	2,5,6,7,18,19,20,23,24	0	
6158	1,3,4,8,21,23,24,25	2,5,6,7,18,19,20,22,26	3	P83748,Q63751,Q9QUL6
6159	1,3,4,8,21,23,24,26	2,5,6,7,18,19,20,22,25	1	P97840

6160	1,3,4,8,21,23,25,26	2,5,6,7,18,19,20,22,24	1	P27213
6161	1,3,4,8,21,24,25,26	2,5,6,7,18,19,20,22,23	0	
6162	1,3,4,8,22,23,24,25	2,5,6,7,18,19,20,21,26	5	P25031,P62804,P83748,Q63751,Q9QUL6
6163	1,3,4,8,22,23,24,26	2,5,6,7,18,19,20,21,25	5	O54728,P00731,P25031,P47967,P97840
6164	1,3,4,8,22,23,25,26	2,5,6,7,18,19,20,21,24	1	O54728
6165	1,3,4,8,22,24,25,26	2,5,6,7,18,19,20,21,23	1	Q9QUL6
6166	1,3,4,8,23,24,25,26	2,5,6,7,18,19,20,21,22	2	P02091,Q7TPB1
6167	1,3,4,18,19,20,21,22	2,5,6,7,8,23,24,25,26	2	P10758,P36374
6168	1,3,4,18,19,20,21,23	2,5,6,7,8,22,24,25,26	2	P10758,P12785
6169	1,3,4,18,19,20,21,24	2,5,6,7,8,22,23,25,26	1	P10758
6170	1,3,4,18,19,20,21,25	2,5,6,7,8,22,23,24,26	2	P10758,P31430
6171	1,3,4,18,19,20,21,26	2,5,6,7,8,22,23,24,25	3	P10758,P12020,P36374
6172	1,3,4,18,19,20,22,23	2,5,6,7,8,21,24,25,26	3	P12785,P83748,Q9WTT6
6173	1,3,4,18,19,20,22,24	2,5,6,7,8,21,23,25,26	6	P06760,P07150,P14669,P83748,Q6P6R2,Q9WTT6
6174	1,3,4,18,19,20,22,25	2,5,6,7,8,21,23,24,26	1	P83748
6175	1,3,4,18,19,20,22,26	2,5,6,7,8,21,23,24,25	4	O54728,P06760,Q6P6R2,Q9WTT6
6176	1,3,4,18,19,20,23,24	2,5,6,7,8,21,22,25,26	4	P12785,P83748,Q9QZK9,Q9WTT6
6177	1,3,4,18,19,20,23,25	2,5,6,7,8,21,22,24,26	2	P12785,P83748
6178	1,3,4,18,19,20,23,26	2,5,6,7,8,21,22,24,25	9	O54728,P06760,P12785,P35467,Q4FZU2,Q6IFU8,Q6IFW6,Q6IG02,Q6P6Q2

6179	1,3,4,18,19,20,24,25	2,5,6,7,8,21,22,23,26	1	P83748
6180	1,3,4,18,19,20,24,26	2,5,6,7,8,21,22,23,25	3	P06760,Q6P6R2,Q9WTT6
6181	1,3,4,18,19,20,25,26	2,5,6,7,8,21,22,23,24	1	P35280
6182	1,3,4,18,19,21,22,23	2,5,6,7,8,20,24,25,26	2	P10758,P12785
6183	1,3,4,18,19,21,22,24	2,5,6,7,8,20,23,25,26	1	P10758
6184	1,3,4,18,19,21,22,25	2,5,6,7,8,20,23,24,26	1	P10758
6185	1,3,4,18,19,21,22,26	2,5,6,7,8,20,23,24,25	2	P10758,P36374
6186	1,3,4,18,19,21,23,24	2,5,6,7,8,20,22,25,26	2	P10758,P12785
6187	1,3,4,18,19,21,23,25	2,5,6,7,8,20,22,24,26	2	P10758,P12785
6188	1,3,4,18,19,21,23,26	2,5,6,7,8,20,22,24,25	8	P10758,P12785,P35467,Q4FZU2,Q6IFU8,Q6IG02,Q6IMF3,Q6P6Q2
6189	1,3,4,18,19,21,24,25	2,5,6,7,8,20,22,23,26	1	P31430
6190	1,3,4,18,19,21,24,26	2,5,6,7,8,20,22,23,25	2	P10758,P12020
6191	1,3,4,18,19,21,25,26	2,5,6,7,8,20,22,23,24	1	P31430
6192	1,3,4,18,19,22,23,24	2,5,6,7,8,20,21,25,26	5	P02625,P12785,P62804,P83748,Q9WTT6
6193	1,3,4,18,19,22,23,25	2,5,6,7,8,20,21,24,26	3	P12785,P62804,P83748
6194	1,3,4,18,19,22,23,26	2,5,6,7,8,20,21,24,25	8	O54728,P02625,P06760,P12785,P35467,Q6IG02,Q6P6Q2,Q9WTT6
6195	1,3,4,18,19,22,24,25	2,5,6,7,8,20,21,23,26	2	P62804,P83748
6196	1,3,4,18,19,22,24,26	2,5,6,7,8,20,21,23,25	4	O54728,P06760,Q6P6R2,Q9WTT6
6197	1,3,4,18,19,22,25,26	2,5,6,7,8,20,21,23,24	0	
6198	1,3,4,18,19,23,24,25	2,5,6,7,8,20,21,22,26	3	P12785,P62804,P83748
6199	1,3,4,18,19,23,24,26	2,5,6,7,8,20,21,22,25	6	P02091,P06760,P12785,P35467,Q6IG02,Q6P6Q2
6200	1,3,4,18,19,23,25,26	2,5,6,7,8,20,21,22,24	6	P00714,P12785,P35467,Q4FZU2,Q6IG02,Q6P6Q2
6201	1,3,4,18,19,24,25,26	2,5,6,7,8,20,21,22,23	0	

6202	1,3,4,18,20,21,22,23	2,5,6,7,8,19,24,25,26	2	P10758,P12785
6203	1,3,4,18,20,21,22,24	2,5,6,7,8,19,23,25,26	1	P10758
6204	1,3,4,18,20,21,22,25	2,5,6,7,8,19,23,24,26	1	P31430
6205	1,3,4,18,20,21,22,26	2,5,6,7,8,19,23,24,25	2	P10758,P36374
6206	1,3,4,18,20,21,23,24	2,5,6,7,8,19,22,25,26	3	P10758,P12785,P83748
6207	1,3,4,18,20,21,23,25	2,5,6,7,8,19,22,24,26	2	P12785,P31430
6208	1,3,4,18,20,21,23,26	2,5,6,7,8,19,22,24,25	10	A2RUV9,P10758,P12785,Q4FZU2,Q4FZU6,Q6IFU8,Q6IFW6,Q6IG02,Q6IMF3,Q6P6Q2
6209	1,3,4,18,20,21,24,25	2,5,6,7,8,19,22,23,26	1	P31430
6210	1,3,4,18,20,21,24,26	2,5,6,7,8,19,22,23,25	2	P06911,P12020
6211	1,3,4,18,20,21,25,26	2,5,6,7,8,19,22,23,24	2	P31430,Q4FZU2
6212	1,3,4,18,20,22,23,24	2,5,6,7,8,19,21,25,26	5	P12785,P83748,P97840,Q9QZK9,Q9WTT6
6213	1,3,4,18,20,22,23,25	2,5,6,7,8,19,21,24,26	2	P12785,P83748
6214	1,3,4,18,20,22,23,26	2,5,6,7,8,19,21,24,25	11	A2RUV9,O54728,P06760,P12785,P97840,Q4FZU2,Q6IFU8,Q6IFW6,Q6IG02,Q6IMF3,Q6P6Q2
6215	1,3,4,18,20,22,24,25	2,5,6,7,8,19,21,23,26	1	P83748
6216	1,3,4,18,20,22,24,26	2,5,6,7,8,19,21,23,25	5	P06760,P97840,Q6P6R2,Q99041,Q9WTT6
6217	1,3,4,18,20,22,25,26	2,5,6,7,8,19,21,23,24	0	
6218	1,3,4,18,20,23,24,25	2,5,6,7,8,19,21,22,26	3	A2RUV9,P12785,P83748
6219	1,3,4,18,20,23,24,26	2,5,6,7,8,19,21,22,25	11	A2RUV9,P12785,P19218,P47967,P97840,Q4FZU2,Q6IFU8,Q6IFW6,Q6IG02,Q6IMF3,Q6P6Q2
6220	1,3,4,18,20,23,25,26	2,5,6,7,8,19,21,22,24	10	A2RUV9,P12785,P19218,P35467,Q4FZU2,Q6IFU8,Q6IFW6,Q6IG02,Q6IMF3,Q6P6Q2
6221	1,3,4,18,20,24,25,26	2,5,6,7,8,19,21,22,23	0	
6222	1,3,4,18,21,22,23,24	2,5,6,7,8,19,20,25,26	2	P10758,P12785
6223	1,3,4,18,21,22,23,25	2,5,6,7,8,19,20,24,26	2	P12785,P31430
6224	1,3,4,18,21,22,23,26	2,5,6,7,8,19,20,24,25	7	P10758,P12785,Q4FZU2,Q6IFU8,Q6IG02,Q6IMF3,Q6P6Q2

6225	1,3,4,18,21,22,24,25	2,5,6,7,8,19,20,23,26	1	P31430
6226	1,3,4,18,21,22,24,26	2,5,6,7,8,19,20,23,25	1	Q9JID2
6227	1,3,4,18,21,22,25,26	2,5,6,7,8,19,20,23,24	1	P31430
6228	1,3,4,18,21,23,24,25	2,5,6,7,8,19,20,22,26	3	P12785,P31430,P83748
6229	1,3,4,18,21,23,24,26	2,5,6,7,8,19,20,22,25	7	P12785,Q4FZU2,Q6IFU8,Q6IG02,Q6IMF3,Q6P6Q2,Q9JID2
6230	1,3,4,18,21,23,25,26	2,5,6,7,8,19,20,22,24	10	P00774,P12785,P27213,P31430,Q4FZU2,Q6IFU8,Q6IFW6,Q6IG02,Q6IMF3,Q6P6Q2
6231	1,3,4,18,21,24,25,26	2,5,6,7,8,19,20,22,23	2	P08721,P31430
6232	1,3,4,18,22,23,24,25	2,5,6,7,8,19,20,21,26	5	P12785,P62804,P82471,P83748,Q00715
6233	1,3,4,18,22,23,24,26	2,5,6,7,8,19,20,21,25	8	O54728,P00731,P12785,P47967,P62804,P97840,Q6IG02,Q6P6Q2
6234	1,3,4,18,22,23,25,26	2,5,6,7,8,19,20,21,24	5	P12785,P62804,Q4FZU2,Q6IG02,Q6P6Q2
6235	1,3,4,18,22,24,25,26	2,5,6,7,8,19,20,21,23	1	P62804
6236	1,3,4,18,23,24,25,26	2,5,6,7,8,19,20,21,22	9	A2RUV9,P02091,P12785,P62804,Q4FZU2,Q6IFU8,Q6IG02,Q6P6Q2,Q7TPB1
6237	1,3,4,19,20,21,22,23	2,5,6,7,8,18,24,25,26	1	P55091
6238	1,3,4,19,20,21,22,24	2,5,6,7,8,18,23,25,26	3	P00774,P55091,Q99MH3
6239	1,3,4,19,20,21,22,25	2,5,6,7,8,18,23,24,26	2	P16636,Q63942
6240	1,3,4,19,20,21,22,26	2,5,6,7,8,18,23,24,25	2	O54728,Q99MH3
6241	1,3,4,19,20,21,23,24	2,5,6,7,8,18,22,25,26	2	P55091,P83748
6242	1,3,4,19,20,21,23,25	2,5,6,7,8,18,22,24,26	2	P16636,P83748
6243	1,3,4,19,20,21,23,26	2,5,6,7,8,18,22,24,25	1	O54728
6244	1,3,4,19,20,21,24,25	2,5,6,7,8,18,22,23,26	1	P83748
6245	1,3,4,19,20,21,24,26	2,5,6,7,8,18,22,23,25	0	
6246	1,3,4,19,20,21,25,26	2,5,6,7,8,18,22,23,24	0	
6247	1,3,4,19,20,22,23,24	2,5,6,7,8,18,21,25,26	8	P00774,P25031,P31430,P55091,P82995,P83748,P98089,Q9WTT6

6248	1,3,4,19,20,22,23,25	2,5,6,7,8,18,21,24,26	2	P55091,P83748
6249	1,3,4,19,20,22,23,26	2,5,6,7,8,18,21,24,25	3	O54728,P98089,Q62997
6250	1,3,4,19,20,22,24,25	2,5,6,7,8,18,21,23,26	3	P25031,P83748,Q9QUL6
6251	1,3,4,19,20,22,24,26	2,5,6,7,8,18,21,23,25	9	O54728,P06760,P07150,P14669,P20646,P98089,Q6P6R2,Q99MH3,Q9WTT6
6252	1,3,4,19,20,22,25,26	2,5,6,7,8,18,21,23,24	1	O54728
6253	1,3,4,19,20,23,24,25	2,5,6,7,8,18,21,22,26	3	P25031,P82995,P83748
6254	1,3,4,19,20,23,24,26	2,5,6,7,8,18,21,22,25	3	O54728,P02091,P98089
6255	1,3,4,19,20,23,25,26	2,5,6,7,8,18,21,22,24	2	O54728,P02091
6256	1,3,4,19,20,24,25,26	2,5,6,7,8,18,21,22,23	0	
6257	1,3,4,19,21,22,23,24	2,5,6,7,8,18,20,25,26	1	P83748
6258	1,3,4,19,21,22,23,25	2,5,6,7,8,18,20,24,26	0	
6259	1,3,4,19,21,22,23,26	2,5,6,7,8,18,20,24,25	1	O54728
6260	1,3,4,19,21,22,24,25	2,5,6,7,8,18,20,23,26	1	Q9QUL6
6261	1,3,4,19,21,22,24,26	2,5,6,7,8,18,20,23,25	1	O54728
6262	1,3,4,19,21,22,25,26	2,5,6,7,8,18,20,23,24	0	
6263	1,3,4,19,21,23,24,25	2,5,6,7,8,18,20,22,26	1	P83748
6264	1,3,4,19,21,23,24,26	2,5,6,7,8,18,20,22,25	0	
6265	1,3,4,19,21,23,25,26	2,5,6,7,8,18,20,22,24	1	P27213
6266	1,3,4,19,21,24,25,26	2,5,6,7,8,18,20,22,23	0	
6267	1,3,4,19,22,23,24,25	2,5,6,7,8,18,20,21,26	6	P25031,P62804,P82471,P83748,Q00715,Q9QUL6
6268	1,3,4,19,22,23,24,26	2,5,6,7,8,18,20,21,25	9	O54728,P00731,P02091,P20646,P25031,P62804,P82471,P98089,Q9WTT6
6269	1,3,4,19,22,23,25,26	2,5,6,7,8,18,20,21,24	4	O54728,P02091,P62804,P82471
6270	1,3,4,19,22,24,25,26	2,5,6,7,8,18,20,21,23	2	O54728,P62804

6271	1,3,4,19,2 3,24,25,26	2,5,6,7,8,1 8,20,21,22	5	P01946,P02091,P62804,P82471,Q7TPB1
6272	1,3,4,20,2 1,22,23,24	2,5,6,7,8,1 8,19,25,26	3	P16636,P82995,P83748
6273	1,3,4,20,2 1,22,23,25	2,5,6,7,8,1 8,19,24,26	3	P16636,P83748,Q63942
6274	1,3,4,20,2 1,22,23,26	2,5,6,7,8,1 8,19,24,25	1	O54728
6275	1,3,4,20,2 1,22,24,25	2,5,6,7,8,1 8,19,23,26	5	P16636,P83748,Q03191,Q5XFX0,Q63942
6276	1,3,4,20,2 1,22,24,26	2,5,6,7,8,1 8,19,23,25	3	P02803,Q03191,Q99MH3
6277	1,3,4,20,2 1,22,25,26	2,5,6,7,8,1 8,19,23,24	1	Q03191
6278	1,3,4,20,2 1,23,24,25	2,5,6,7,8,1 8,19,22,26	2	P16636,P83748
6279	1,3,4,20,2 1,23,24,26	2,5,6,7,8,1 8,19,22,25	1	P97840
6280	1,3,4,20,2 1,23,25,26	2,5,6,7,8,1 8,19,22,24	1	Q4FZU2
6281	1,3,4,20,2 1,24,25,26	2,5,6,7,8,1 8,19,22,23	1	Q03191
6282	1,3,4,20,2 2,23,24,25	2,5,6,7,8,1 8,19,21,26	3	P25031,P82995,P83748
6283	1,3,4,20,2 2,23,24,26	2,5,6,7,8,1 8,19,21,25	9	O54728,P00731,P02803,P47967,P53792,P97840,P98089,Q62635,Q8CIZ5
6284	1,3,4,20,2 2,23,25,26	2,5,6,7,8,1 8,19,21,24	2	O54728,P05539
6285	1,3,4,20,2 2,24,25,26	2,5,6,7,8,1 8,19,21,23	4	P00731,P02803,Q03191,Q99MH3
6286	1,3,4,20,2 3,24,25,26	2,5,6,7,8,1 8,19,21,22	3	P02091,P19218,Q7TPB1
6287	1,3,4,21,2 2,23,24,25	2,5,6,7,8,1 8,19,20,26	3	P62804,P83748,Q63751
6288	1,3,4,21,2 2,23,24,26	2,5,6,7,8,1 8,19,20,25	2	O54728,P00731
6289	1,3,4,21,2 2,23,25,26	2,5,6,7,8,1 8,19,20,24	0	
6290	1,3,4,21,2 2,24,25,26	2,5,6,7,8,1 8,19,20,23	1	Q03191
6291	1,3,4,21,2 3,24,25,26	2,5,6,7,8,1 8,19,20,22	4	P02091,P22006,P27213,Q7TPB1
6292	1,3,4,22,2 3,24,25,26	2,5,6,7,8,1 8,19,20,21	6	O54728,P00731,P02091,P62804,P82471,Q7TPB1

6293	1,3,5,6,7,8,18,19	2,4,20,21,22,23,24,25,26	5	O55006,P00731,P51907,P53792,Q6IRE4
6294	1,3,5,6,7,8,18,20	2,4,19,21,22,23,24,25,26	6	O55006,P00731,P10715,P27213,Q6IRE4,Q9ET32
6295	1,3,5,6,7,8,18,21	2,4,19,20,22,23,24,25,26	6	P00731,P19218,P25031,P53792,Q6IRE4,Q9ET32
6296	1,3,5,6,7,8,18,22	2,4,19,20,21,23,24,25,26	4	P27213,Q6IRE4,Q9ET32,Q9QW07
6297	1,3,5,6,7,8,18,23	2,4,19,20,21,22,24,25,26	3	P51907,Q6IRE4,Q9ET32
6298	1,3,5,6,7,8,18,24	2,4,19,20,21,22,23,25,26	4	O55006,P10715,Q6IRE4,Q9ET32
6299	1,3,5,6,7,8,18,25	2,4,19,20,21,22,23,24,26	6	P00731,P01015,P10715,P19629,Q6IRE4,Q9ET32
6300	1,3,5,6,7,8,18,26	2,4,19,20,21,22,23,24,25	6	P04041,P83748,Q63942,Q6IRE4,Q8VD89,Q9ET32
6301	1,3,5,6,7,8,19,20	2,4,18,21,22,23,24,25,26	5	O55006,P53792,P55091,P98089,Q6IRE4
6302	1,3,5,6,7,8,19,21	2,4,18,20,22,23,24,25,26	6	P00731,P16086,P19218,P35281,P53792,Q6IRE4
6303	1,3,5,6,7,8,19,22	2,4,18,20,21,23,24,25,26	5	P17046,P19218,P55091,Q6IRE4,Q9QW07
6304	1,3,5,6,7,8,19,23	2,4,18,20,21,22,24,25,26	8	O70594,P15399,P16086,P51907,P97580,Q30KJ2,Q6IRE4,Q99041
6305	1,3,5,6,7,8,19,24	2,4,18,20,21,22,23,25,26	3	O55006,P53792,Q6IRE4
6306	1,3,5,6,7,8,19,25	2,4,18,20,21,22,23,24,26	6	P10715,P12020,P17046,P19629,P53792,Q6IRE4
6307	1,3,5,6,7,8,19,26	2,4,18,20,21,22,23,24,25	3	O54728,P53792,Q6IRE4

6308	1,3,5,6,7,8,20,21	2,4,18,19,22,23,24,25,26	5	P01681,P53792,Q6IRE4,Q99MH3,Q9ET32
6309	1,3,5,6,7,8,20,22	2,4,18,19,21,23,24,25,26	8	D3ZUC6,P27213,P55091,P98089,Q6IRE4,Q99MH3,Q9ET32,Q9QW07
6310	1,3,5,6,7,8,20,23	2,4,18,19,21,22,24,25,26	3	P98089,Q6IRE4,Q9ET32
6311	1,3,5,6,7,8,20,24	2,4,18,19,21,22,23,25,26	5	P10715,P27213,Q6IRE4,Q99MH3,Q9ET32
6312	1,3,5,6,7,8,20,25	2,4,18,19,21,22,23,24,26	6	D3ZUC6,P10715,P12020,P19629,Q6IRE4,Q9ET32
6313	1,3,5,6,7,8,20,26	2,4,18,19,21,22,23,24,25	4	P01681,Q6IRE4,Q99MH3,Q9ET32
6314	1,3,5,6,7,8,21,22	2,4,18,19,20,23,24,25,26	5	P13432,P19218,Q6IRE4,Q9ET32,Q9QW07
6315	1,3,5,6,7,8,21,23	2,4,18,19,20,22,24,25,26	3	P51907,Q6IRE4,Q9ET32
6316	1,3,5,6,7,8,21,24	2,4,18,19,20,22,23,25,26	4	P13432,P53792,Q6IRE4,Q9ET32
6317	1,3,5,6,7,8,21,25	2,4,18,19,20,22,23,24,26	8	P10715,P19218,P19629,P53792,Q6IRE4,Q9ET32,Q9QZK9,Q9WTT6
6318	1,3,5,6,7,8,21,26	2,4,18,19,20,22,23,24,25	6	F1LM93,P01681,P53792,P83748,Q6IRE4,Q9ET32
6319	1,3,5,6,7,8,22,23	2,4,18,19,20,21,24,25,26	4	P51907,Q6IRE4,Q9ET32,Q9QW07
6320	1,3,5,6,7,8,22,24	2,4,18,19,20,21,23,25,26	4	P27213,Q6IRE4,Q99MH3,Q9QW07
6321	1,3,5,6,7,8,22,25	2,4,18,19,20,21,23,24,26	10	D3ZUC6,P06911,P10715,P12020,P17046,P19629,Q5GRG2,Q6IRE4,Q9ET32,Q9QW07
6322	1,3,5,6,7,8,22,26	2,4,18,19,20,21,23,24,25	4	O54728,Q6IRE4,Q99MH3,Q9QW07

6323	1,3,5,6,7,8,23,24	2,4,18,19,20,21,22,25,26	5	P08937,P51907,Q63751,Q6IRE4,Q9ET32
6324	1,3,5,6,7,8,23,25	2,4,18,19,20,21,22,24,26	13	O70377,P01015,P06911,P10715,P12020,P19629,P51907,P55314,Q5GRG2,Q63751,Q6IRE4,Q99041,Q9ET32
6325	1,3,5,6,7,8,23,26	2,4,18,19,20,21,22,24,25	7	P01946,P02091,P51907,Q5I0D1,Q6IG02,Q6IRE4,Q9ET32
6326	1,3,5,6,7,8,24,25	2,4,18,19,20,21,22,23,26	4	P10715,P19629,Q6IRE4,Q9ET32
6327	1,3,5,6,7,8,24,26	2,4,18,19,20,21,22,23,25	4	P02091,Q6IRE4,Q8CJD3,Q99MH3
6328	1,3,5,6,7,8,25,26	2,4,18,19,20,21,22,23,24	5	P02091,P10715,P19629,Q6IRE4,Q9QZK9
6329	1,3,5,6,7,18,19,20	2,4,8,21,22,23,24,25,26	5	P00731,P53792,Q5U2V4,Q6IRE4,Q80WL1
6330	1,3,5,6,7,18,19,21	2,4,8,20,22,23,24,25,26	5	P00731,P19218,P25031,P53792,Q6IRE4
6331	1,3,5,6,7,18,19,22	2,4,8,20,21,23,24,25,26	5	P17046,P19218,Q5U2V4,Q6IRE4,Q9QW07
6332	1,3,5,6,7,18,19,23	2,4,8,20,21,22,24,25,26	6	P15399,P16086,P97580,Q03191,Q30KJ2,Q6IRE4
6333	1,3,5,6,7,18,19,24	2,4,8,20,21,22,23,25,26	3	P53792,Q6IRE4,Q80WL1
6334	1,3,5,6,7,18,19,25	2,4,8,20,21,22,23,24,26	5	P00731,P01015,P17046,P53792,Q6IRE4
6335	1,3,5,6,7,18,19,26	2,4,8,20,21,22,23,24,25	3	P53792,Q5U2V4,Q6IRE4
6336	1,3,5,6,7,18,20,21	2,4,8,19,22,23,24,25,26	4	P00731,P01835,P25031,Q6IRE4
6337	1,3,5,6,7,18,20,22	2,4,8,19,21,23,24,25,26	4	P27213,Q5U2V4,Q6IRE4,Q9QW07

6338	1,3,5,6,7,18,20,23	2,4,8,19,21,22,24,25,26	3	Q6IG02,Q6IRE4,Q80WL1
6339	1,3,5,6,7,18,20,24	2,4,8,19,21,22,23,25,26	3	P27213,Q6IRE4,Q80WL1
6340	1,3,5,6,7,18,20,25	2,4,8,19,21,22,23,24,26	2	Q6IRE4,Q80WL1
6341	1,3,5,6,7,18,20,26	2,4,8,19,21,22,23,24,25	4	Q4FZU2,Q6IG02,Q6IRE4,Q80WL1
6342	1,3,5,6,7,18,21,22	2,4,8,19,20,23,24,25,26	5	P13432,P19218,P25031,Q6IRE4,Q9QW07
6343	1,3,5,6,7,18,21,23	2,4,8,19,20,22,24,25,26	2	P25031,Q6IRE4
6344	1,3,5,6,7,18,21,24	2,4,8,19,20,22,23,25,26	4	P13432,P25031,Q62997,Q6IRE4
6345	1,3,5,6,7,18,21,25	2,4,8,19,20,22,23,24,26	4	P00731,P25031,P53792,Q6IRE4
6346	1,3,5,6,7,18,21,26	2,4,8,19,20,22,23,24,25	4	P25031,P83748,Q4FZU2,Q6IRE4
6347	1,3,5,6,7,18,22,23	2,4,8,19,20,21,24,25,26	3	P01015,Q6IRE4,Q9QW07
6348	1,3,5,6,7,18,22,24	2,4,8,19,20,21,23,25,26	3	P27213,Q6IRE4,Q9QW07
6349	1,3,5,6,7,18,22,25	2,4,8,19,20,21,23,24,26	4	P01015,P17046,Q6IRE4,Q9QW07
6350	1,3,5,6,7,18,22,26	2,4,8,19,20,21,23,24,25	5	O54728,Q5U2V4,Q6IRE4,Q8VD89,Q9QW07
6351	1,3,5,6,7,18,23,24	2,4,8,19,20,21,22,25,26	2	Q6IRE4,Q80WL1
6352	1,3,5,6,7,18,23,25	2,4,8,19,20,21,22,24,26	5	P01015,P17046,P55314,Q63751,Q6IRE4

6353	1,3,5,6,7,18,23,26	2,4,8,19,20,21,22,24,25	12	P01946,P02091,P35467,Q4FZU2,Q63942,Q6IFU8,Q6IFW6,Q6IG02,Q6IMF3,Q6IRE4,Q6P6Q2,Q8VD89
6354	1,3,5,6,7,18,24,25	2,4,8,19,20,21,22,23,26	3	P17046,Q6IRE4,Q80WL1
6355	1,3,5,6,7,18,24,26	2,4,8,19,20,21,22,23,25	5	P02091,Q6IRE4,Q80WL1,Q8CJD3,Q8VD89
6356	1,3,5,6,7,18,25,26	2,4,8,19,20,21,22,23,24	3	P02091,P61206;P84079;P84082,Q6IRE4
6357	1,3,5,6,7,19,20,21	2,4,8,18,22,23,24,25,26	6	P00714,P00731,P01681,P53792,P55091,Q6IRE4
6358	1,3,5,6,7,19,20,22	2,4,8,18,21,23,24,25,26	13	P00714,P00774,P07150,P14669,P17046,P27213,P31430,P55091,P98089,Q5U2V4,Q6IRE4,Q99MH3,Q9QW07
6359	1,3,5,6,7,19,20,23	2,4,8,18,21,22,24,25,26	9	P15399,P16086,P31430,P55091,P97580,P98089,Q30KJ2,Q6IRE4,Q80WL1
6360	1,3,5,6,7,19,20,24	2,4,8,18,21,22,23,25,26	8	P00714,P31430,P53792,P55091,P98089,Q6IRE4,Q80WL1,Q99MH3
6361	1,3,5,6,7,19,20,25	2,4,8,18,21,22,23,24,26	4	P17046,P53792,Q6IRE4,Q80WL1
6362	1,3,5,6,7,19,20,26	2,4,8,18,21,22,23,24,25	8	O54728,P01681,P53792,P98089,Q5U2V4,Q6IRE4,Q80WL1,Q99MH3
6363	1,3,5,6,7,19,21,22	2,4,8,18,20,23,24,25,26	7	P00714,P17046,P19218,P55091,Q6IRE4,Q6TMA8,Q9QW07
6364	1,3,5,6,7,19,21,23	2,4,8,18,20,22,24,25,26	6	P15399,P16086,P17046,P97580,Q30KJ2,Q6IRE4
6365	1,3,5,6,7,19,21,24	2,4,8,18,20,22,23,25,26	5	P00714,P19218,P53792,Q6IRE4,Q6TMA8
6366	1,3,5,6,7,19,21,25	2,4,8,18,20,22,23,24,26	7	P00731,P17046,P19218,P53792,P97840,Q6IRE4,Q9QZK9
6367	1,3,5,6,7,19,21,26	2,4,8,18,20,22,23,24,25	3	P53792,P83748,Q6IRE4

6368	1,3,5,6,7,19,22,23	2,4,8,18,20,21,24,25,26	10	P15399,P16086,P17046,P31430,P55091,P97580,P98089,Q30KJ2,Q6IRE4,Q9QW07
6369	1,3,5,6,7,19,22,24	2,4,8,18,20,21,23,25,26	9	P00714,P17046,P31430,P55091,P98089,Q6IRE4,Q6TMA8,Q99MH3,Q9QW07
6370	1,3,5,6,7,19,22,25	2,4,8,18,20,21,23,24,26	6	P06911,P12020,P17046,Q6IRE4,Q6TMA8,Q9QW07
6371	1,3,5,6,7,19,22,26	2,4,8,18,20,21,23,24,25	7	O54728,P17046,P98089,Q5U2V4,Q6TMA8,Q99MH3,Q9QW07
6372	1,3,5,6,7,19,23,24	2,4,8,18,20,21,22,25,26	9	P15399,P17046,P19223,P31430,P97580,P98089,Q30KJ2,Q6IRE4,Q80WL1
6373	1,3,5,6,7,19,23,25	2,4,8,18,20,21,22,24,26	9	P01015,P12020,P15399,P17046,P55314,P97580,Q30KJ2,Q6IRE4,Q80WL1
6374	1,3,5,6,7,19,23,26	2,4,8,18,20,21,22,24,25	8	O54728,P01946,P02091,P15399,P97580,P98089,Q30KJ2,Q6IRE4
6375	1,3,5,6,7,19,24,25	2,4,8,18,20,21,22,23,26	5	P17046,P53792,Q6IRE4,Q6TMA8,Q80WL1
6376	1,3,5,6,7,19,24,26	2,4,8,18,20,21,22,23,25	6	P02091,P98089,Q6IRE4,Q6TMA8,Q80WL1,Q99MH3
6377	1,3,5,6,7,19,25,26	2,4,8,18,20,21,22,23,24	5	P02091,P17046,P53792,Q6IRE4,Q9QZK9
6378	1,3,5,6,7,20,21,22	2,4,8,18,19,23,24,25,26	8	P00714,P13432,P27213,P55091,Q6IRE4,Q7TPB1,Q99MH3,Q9QW07
6379	1,3,5,6,7,20,21,23	2,4,8,18,19,22,24,25,26	2	P01835,Q6IRE4
6380	1,3,5,6,7,20,21,24	2,4,8,18,19,22,23,25,26	4	P00714,P13432,Q6IRE4,Q99MH3
6381	1,3,5,6,7,20,21,25	2,4,8,18,19,22,23,24,26	1	Q6IRE4
6382	1,3,5,6,7,20,21,26	2,4,8,18,19,22,23,24,25	3	P01681,Q6IRE4,Q99MH3

6383	1,3,5,6,7,20,22,23	2,4,8,18,19,21,24,25,26	6	P27213,P31430,P55091,P98089,Q6IRE4,Q9QW07
6384	1,3,5,6,7,20,22,24	2,4,8,18,19,21,23,25,26	10	P00714,P14669,P27213,P31430,P55091,P98089,Q6IRE4,Q80WL1,Q99MH3,Q9QW07
6385	1,3,5,6,7,20,22,25	2,4,8,18,19,21,23,24,26	5	D3ZUC6,P17046,Q6IRE4,Q99MH3,Q9QW07
6386	1,3,5,6,7,20,22,26	2,4,8,18,19,21,23,24,25	6	O54728,P98089,Q5U2V4,Q6IRE4,Q99MH3,Q9QW07
6387	1,3,5,6,7,20,23,24	2,4,8,18,19,21,22,25,26	4	P31430,P98089,Q6IRE4,Q80WL1
6388	1,3,5,6,7,20,23,25	2,4,8,18,19,21,22,24,26	5	P17046,P55314,Q63751,Q6IRE4,Q80WL1
6389	1,3,5,6,7,20,23,26	2,4,8,18,19,21,22,24,25	9	O54728,P01681,P01946,P02091,P98089,Q4FZU2,Q6IG02,Q6IRE4,Q80WL1
6390	1,3,5,6,7,20,24,25	2,4,8,18,19,21,22,23,26	4	P17046,Q6IRE4,Q80WL1,Q99MH3
6391	1,3,5,6,7,20,24,26	2,4,8,18,19,21,22,23,25	6	P02091,P98089,Q6IRE4,Q80WL1,Q8CJD3,Q99MH3
6392	1,3,5,6,7,20,25,26	2,4,8,18,19,21,22,23,24	5	D3ZUC6,P02091,Q6IRE4,Q80WL1,Q99MH3
6393	1,3,5,6,7,21,22,23	2,4,8,18,19,20,24,25,26	3	P17046,Q6IRE4,Q9QW07
6394	1,3,5,6,7,21,22,24	2,4,8,18,19,20,23,25,26	7	P00714,P13432,P19218,Q6IRE4,Q6TMA8,Q99MH3,Q9QW07
6395	1,3,5,6,7,21,22,25	2,4,8,18,19,20,23,24,26	4	P17046,P19218,Q6IRE4,Q9QW07
6396	1,3,5,6,7,21,22,26	2,4,8,18,19,20,23,24,25	5	O54728,P83748,Q6IRE4,Q99MH3,Q9QW07
6397	1,3,5,6,7,21,23,24	2,4,8,18,19,20,22,25,26	2	Q63751,Q6IRE4

6398	1,3,5,6,7,21,23,25	2,4,8,18,19,20,22,24,26	5	P17046,P19629,P55314,Q63751,Q6IRE4
6399	1,3,5,6,7,21,23,26	2,4,8,18,19,20,22,24,25	4	P01946,P02091,Q4FZU2,Q6IRE4
6400	1,3,5,6,7,21,24,25	2,4,8,18,19,20,22,23,26	4	P17046,P19629,Q62997,Q6IRE4
6401	1,3,5,6,7,21,24,26	2,4,8,18,19,20,22,23,25	5	P02091,P13432,Q6IRE4,Q8CJD3,Q99MH3
6402	1,3,5,6,7,21,25,26	2,4,8,18,19,20,22,23,24	4	P02091,P83748,Q6IRE4,Q9QZK9
6403	1,3,5,6,7,22,23,24	2,4,8,18,19,20,21,25,26	8	P17046,P19223,P31430,P62804,P98089,Q63751,Q6IRE4,Q9QW07
6404	1,3,5,6,7,22,23,25	2,4,8,18,19,20,21,24,26	10	P01015,P06911,P12020,P17046,P19629,P55314,Q5GRG2,Q63751,Q6IRE4,Q9QW07
6405	1,3,5,6,7,22,23,26	2,4,8,18,19,20,21,24,25	6	O54728,P01946,P02091,P98089,Q6IRE4,Q9QW07
6406	1,3,5,6,7,22,24,25	2,4,8,18,19,20,21,23,26	6	P17046,P19629,P62804,Q6IRE4,Q6TMA8,Q9QW07
6407	1,3,5,6,7,22,24,26	2,4,8,18,19,20,21,23,25	8	O54728,P02091,P98089,Q6IRE4,Q6TMA8,Q8CJD3,Q99MH3,Q9QW07
6408	1,3,5,6,7,22,25,26	2,4,8,18,19,20,21,23,24	6	O54728,P02091,P17046,Q6IRE4,Q6TMA8,Q9QW07
6409	1,3,5,6,7,23,24,25	2,4,8,18,19,20,21,22,26	8	P08937,P17046,P19629,P55314,P62804,Q63751,Q6IRE4,Q80WL1
6410	1,3,5,6,7,23,24,26	2,4,8,18,19,20,21,22,25	5	P01946,P02091,P98089,Q6IRE4,Q80WL1
6411	1,3,5,6,7,23,25,26	2,4,8,18,19,20,21,22,24	6	P01946,P02091,P17046,P55314,Q63751,Q6IRE4
6412	1,3,5,6,7,24,25,26	2,4,8,18,19,20,21,22,23	7	P01946,P02091,P17046,Q6IRE4,Q6TMA8,Q80WL1,Q8CJD3

6413	1,3,5,6,8,18,19,20	2,4,7,21,22,23,24,25,26	4	P00731,P22006,P83121,Q6IRE4
6414	1,3,5,6,8,18,19,21	2,4,7,20,22,23,24,25,26	5	O35547,P00731,P35281,P53792,Q6IRE4
6415	1,3,5,6,8,18,19,22	2,4,7,20,21,23,24,25,26	3	P22006,P83121,Q9QW07
6416	1,3,5,6,8,18,19,23	2,4,7,20,21,22,24,25,26	5	O70594,P22006,P35467,P51907,Q9Z1F2
6417	1,3,5,6,8,18,19,24	2,4,7,20,21,22,23,25,26	2	P83121,Q920P0
6418	1,3,5,6,8,18,19,25	2,4,7,20,21,22,23,24,26	1	P00731
6419	1,3,5,6,8,18,19,26	2,4,7,20,21,22,23,24,25	1	Q63942
6420	1,3,5,6,8,18,20,21	2,4,7,19,22,23,24,25,26	6	O35547,P00731,P01681,P01835,P14480,Q6IRE4
6421	1,3,5,6,8,18,20,22	2,4,7,19,21,23,24,25,26	6	P14480,P22006,P27213,P83121,Q6IRE4,Q9QW07
6422	1,3,5,6,8,18,20,23	2,4,7,19,21,22,24,25,26	4	P22006,Q4FZU2,Q6IG02,Q6IRE4
6423	1,3,5,6,8,18,20,24	2,4,7,19,21,22,23,25,26	3	P83121,Q6IRE4,Q80WL1
6424	1,3,5,6,8,18,20,25	2,4,7,19,21,22,23,24,26	2	D3ZUC6,Q6IRE4
6425	1,3,5,6,8,18,20,26	2,4,7,19,21,22,23,24,25	6	P01681,P22006,Q4FZU2,Q63942,Q6IG02,Q6IRE4
6426	1,3,5,6,8,18,21,22	2,4,7,19,20,23,24,25,26	6	O35547,P13432,P14480,P19218,Q6IRE4,Q9QW07
6427	1,3,5,6,8,18,21,23	2,4,7,19,20,22,24,25,26	4	O35547,O70594,P51907,Q6IRE4

6428	1,3,5,6,8,18,21,24	2,4,7,19,20,22,23,25,26	3	P13432,Q62997,Q6IRE4
6429	1,3,5,6,8,18,21,25	2,4,7,19,20,22,23,24,26	4	P00731,P31430,Q6IRE4,Q9WTT6
6430	1,3,5,6,8,18,21,26	2,4,7,19,20,22,23,24,25	4	P01681,Q4FZU2,Q63942,Q6IRE4
6431	1,3,5,6,8,18,22,23	2,4,7,19,20,21,24,25,26	3	P22006,P51907,Q9QW07
6432	1,3,5,6,8,18,22,24	2,4,7,19,20,21,23,25,26	2	P83121,Q9QW07
6433	1,3,5,6,8,18,22,25	2,4,7,19,20,21,23,24,26	2	P01015,Q9QW07
6434	1,3,5,6,8,18,22,26	2,4,7,19,20,21,23,24,25	2	Q63942,Q9QW07
6435	1,3,5,6,8,18,23,24	2,4,7,19,20,21,22,25,26	3	P47967,P51907,Q6IRE4
6436	1,3,5,6,8,18,23,25	2,4,7,19,20,21,22,24,26	4	P01015,P51907,P55314,Q6IRE4
6437	1,3,5,6,8,18,23,26	2,4,7,19,20,21,22,24,25	12	P35467,P47967,P51907,Q4FZU2,Q5I0D1,Q63942,Q6IFU8,Q6IFV1,Q6IFW6,Q6IG02,Q6IMF3,Q6P6Q2
6438	1,3,5,6,8,18,24,25	2,4,7,19,20,21,22,23,26	1	Q6IRE4
6439	1,3,5,6,8,18,24,26	2,4,7,19,20,21,22,23,25	3	P47967,Q63942,Q6IRE4
6440	1,3,5,6,8,18,25,26	2,4,7,19,20,21,22,23,24	1	Q63942
6441	1,3,5,6,8,19,20,21	2,4,7,18,22,23,24,25,26	5	P01681,P35281,P53792,P55091,Q6IRE4
6442	1,3,5,6,8,19,20,22	2,4,7,18,21,23,24,25,26	12	D3ZUC6,P00774,P22006,P27213,P31430,P55091,P82995,P83121,P98089,Q6IRE4,Q99MH3,Q9QW07

6443	1,3,5,6,8,19,20,23	2,4,7,18,21,22,24,25,26	6	P22006,P31430,P55091,P82995,P98089,Q6IRE4
6444	1,3,5,6,8,19,20,24	2,4,7,18,21,22,23,25,26	8	P31430,P55091,P82995,P83121,P98089,Q6IRE4,Q80WL1,Q99MH3
6445	1,3,5,6,8,19,20,25	2,4,7,18,21,22,23,24,26	2	D3ZUC6,Q6IRE4
6446	1,3,5,6,8,19,20,26	2,4,7,18,21,22,23,24,25	5	O54728,P01681,P98089,Q6IRE4,Q99MH3
6447	1,3,5,6,8,19,21,22	2,4,7,18,20,23,24,25,26	6	O35547,P00714,P19218,P35281,P55091,Q9QW07
6448	1,3,5,6,8,19,21,23	2,4,7,18,20,22,24,25,26	5	O70594,P35281,P51907,P55091,Q6IRE4
6449	1,3,5,6,8,19,21,24	2,4,7,18,20,22,23,25,26	4	P00714,P35281,P53792,Q6IRE4
6450	1,3,5,6,8,19,21,25	2,4,7,18,20,22,23,24,26	4	P06760,P35281,P53792,Q6IRE4
6451	1,3,5,6,8,19,21,26	2,4,7,18,20,22,23,24,25	3	P01681,P35281,P53792
6452	1,3,5,6,8,19,22,23	2,4,7,18,20,21,24,25,26	7	P22006,P31430,P51907,P55091,P82995,P98089,Q9QW07
6453	1,3,5,6,8,19,22,24	2,4,7,18,20,21,23,25,26	8	P00714,P31430,P55091,P82995,P83121,P98089,Q99MH3,Q9QW07
6454	1,3,5,6,8,19,22,25	2,4,7,18,20,21,23,24,26	3	D3ZUC6,P17046,Q9QW07
6455	1,3,5,6,8,19,22,26	2,4,7,18,20,21,23,24,25	4	O54728,P98089,Q99MH3,Q9QW07
6456	1,3,5,6,8,19,23,24	2,4,7,18,20,21,22,25,26	5	P31430,P51907,P55091,P82995,P98089
6457	1,3,5,6,8,19,23,25	2,4,7,18,20,21,22,24,26	4	O70594,P01015,P51907,P55314

6458	1,3,5,6,8,19,23,26	2,4,7,18,20,21,22,24,25	5	O54728,P35467,P51907,P98089,Q6IG02
6459	1,3,5,6,8,19,24,25	2,4,7,18,20,21,22,23,26	0	
6460	1,3,5,6,8,19,24,26	2,4,7,18,20,21,22,23,25	2	P98089,Q99MH3
6461	1,3,5,6,8,19,25,26	2,4,7,18,20,21,22,23,24	0	
6462	1,3,5,6,8,20,21,22	2,4,7,18,19,23,24,25,26	11	P00714,P01681,P13432,P14480,P55091,P82995,Q5XFX0,Q6IRE4,Q7TPB1,Q99MH3,Q9QW07
6463	1,3,5,6,8,20,21,23	2,4,7,18,19,22,24,25,26	5	P01681,P01835,P55091,P82995,Q6IRE4
6464	1,3,5,6,8,20,21,24	2,4,7,18,19,22,23,25,26	7	P00714,P01681,P13432,P82995,Q5XFX0,Q6IRE4,Q99MH3
6465	1,3,5,6,8,20,21,25	2,4,7,18,19,22,23,24,26	4	P01681,P06760,Q6IRE4,Q9WTT6
6466	1,3,5,6,8,20,21,26	2,4,7,18,19,22,23,24,25	3	P01681,Q6IRE4,Q99MH3
6467	1,3,5,6,8,20,22,23	2,4,7,18,19,21,24,25,26	8	D3ZUC6,P22006,P31430,P55091,P82995,P98089,Q6IRE4,Q9QW07
6468	1,3,5,6,8,20,22,24	2,4,7,18,19,21,23,25,26	11	D3ZUC6,P00714,P27213,P55091,P82995,P83121,P98089,Q5XFX0,Q6IRE4,Q99MH3,Q9QW07
6469	1,3,5,6,8,20,22,25	2,4,7,18,19,21,23,24,26	5	D3ZUC6,P82995,Q6IRE4,Q99MH3,Q9QW07
6470	1,3,5,6,8,20,22,26	2,4,7,18,19,21,23,24,25	7	D3ZUC6,O54728,P01681,P98089,Q6IRE4,Q99MH3,Q9QW07
6471	1,3,5,6,8,20,23,24	2,4,7,18,19,21,22,25,26	7	P47967,P55091,P82995,P97840,P98089,Q6IRE4,Q80WL1
6472	1,3,5,6,8,20,23,25	2,4,7,18,19,21,22,24,26	5	D3ZUC6,P06760,P55314,P82995,Q6IRE4

6473	1,3,5,6,8,20,23,26	2,4,7,18,19,21,22,24,25	11	O54728,P01681,P47967,P97840,P98089,Q4FZU2,Q6IFW6,Q6IG02,Q6IMF3,Q6IRE4,Q6P6Q2
6474	1,3,5,6,8,20,24,25	2,4,7,18,19,21,22,23,26	4	D3ZUC6,P82995,Q6IRE4,Q80WL1
6475	1,3,5,6,8,20,24,26	2,4,7,18,19,21,22,23,25	8	P01681,P47967,P97840,P98089,Q6IRE4,Q80WL1,Q8CJD3,Q99MH3
6476	1,3,5,6,8,20,25,26	2,4,7,18,19,21,22,23,24	3	D3ZUC6,P01681,Q6IRE4
6477	1,3,5,6,8,21,22,23	2,4,7,18,19,20,24,25,26	5	P51907,P55091,P82995,Q6IRE4,Q9QW07
6478	1,3,5,6,8,21,22,24	2,4,7,18,19,20,23,25,26	7	P00714,P13432,P82995,Q5XFX0,Q6IRE4,Q99MH3,Q9QW07
6479	1,3,5,6,8,21,22,25	2,4,7,18,19,20,23,24,26	4	P06760,Q6IRE4,Q9QW07,Q9WTT6
6480	1,3,5,6,8,21,22,26	2,4,7,18,19,20,23,24,25	3	P01681,Q99MH3,Q9QW07
6481	1,3,5,6,8,21,23,24	2,4,7,18,19,20,22,25,26	3	P51907,P82995,Q6IRE4
6482	1,3,5,6,8,21,23,25	2,4,7,18,19,20,22,24,26	8	O70594,P06760,P51907,P55314,Q63751,Q6IRE4,Q6P6R2,Q9WTT6
6483	1,3,5,6,8,21,23,26	2,4,7,18,19,20,22,24,25	8	P01681,P51907,Q4FZU2,Q6IFV1,Q6IG02,Q6IMF3,Q6IRE4,Q6P6Q2
6484	1,3,5,6,8,21,24,25	2,4,7,18,19,20,22,23,26	5	P06760,Q5XFX0,Q62997,Q6IRE4,Q9WTT6
6485	1,3,5,6,8,21,24,26	2,4,7,18,19,20,22,23,25	4	P01681,Q6IRE4,Q8CJD3,Q99MH3
6486	1,3,5,6,8,21,25,26	2,4,7,18,19,20,22,23,24	5	P01681,P06760,Q6IRE4,Q9QZK9,Q9WTT6
6487	1,3,5,6,8,22,23,24	2,4,7,18,19,20,21,25,26	7	P31430,P47967,P51907,P55091,P82995,P98089,Q9QW07

6488	1,3,5,6,8,22,23,25	2,4,7,18,19,20,21,24,26	8	D3ZUC6,P01015,P06760,P06911,P51907,P55314,P82995,Q9QW07
6489	1,3,5,6,8,22,23,26	2,4,7,18,19,20,21,24,25	5	O54728,P47967,P51907,P98089,Q9QW07
6490	1,3,5,6,8,22,24,25	2,4,7,18,19,20,21,23,26	3	D3ZUC6,P82995,Q9QW07
6491	1,3,5,6,8,22,24,26	2,4,7,18,19,20,21,23,25	5	O54728,P47967,P98089,Q99MH3,Q9QW07
6492	1,3,5,6,8,22,25,26	2,4,7,18,19,20,21,23,24	2	D3ZUC6,Q9QW07
6493	1,3,5,6,8,23,24,25	2,4,7,18,19,20,21,22,26	7	P06760,P36374,P51907,P55314,P82995,Q63751,Q6IRE4
6494	1,3,5,6,8,23,24,26	2,4,7,18,19,20,21,22,25	8	P02091,P47967,P51907,P97840,P98089,Q5I0D1,Q6IG02,Q6IRE4
6495	1,3,5,6,8,23,25,26	2,4,7,18,19,20,21,22,24	8	P02091,P51907,P55314,Q4FZU2,Q5I0D1,Q6IG02,Q6P6Q2,Q9WTT6
6496	1,3,5,6,8,24,25,26	2,4,7,18,19,20,21,22,23	2	Q6IRE4,Q8CJD3
6497	1,3,5,6,18,19,20,21	2,4,7,8,22,23,24,25,26	5	P00731,P01681,P01835,Q02253,Q6IRE4
6498	1,3,5,6,18,19,20,22	2,4,7,8,21,23,24,25,26	9	P07150,P22006,P27213,P55091,Q02253,Q5U2V4,Q6IRE4,Q80WL1,Q9QW07
6499	1,3,5,6,18,19,20,23	2,4,7,8,21,22,24,25,26	5	P22006,P82995,Q6IG02,Q6IRE4,Q80WL1
6500	1,3,5,6,18,19,20,24	2,4,7,8,21,22,23,25,26	3	P82995,Q6IRE4,Q80WL1
6501	1,3,5,6,18,19,20,25	2,4,7,8,21,22,23,24,26	3	Q02253,Q6IRE4,Q80WL1
6502	1,3,5,6,18,19,20,26	2,4,7,8,21,22,23,24,25	6	P01681,P22006,Q4FZU2,Q6IG02,Q6IRE4,Q80WL1

6503	1,3,5,6,18,19,21,22	2,4,7,8,20,23,24,25,26	3	O35547,P19218,Q9QW07
6504	1,3,5,6,18,19,21,23	2,4,7,8,20,22,24,25,26	2	O35547,Q6IRE4
6505	1,3,5,6,18,19,21,24	2,4,7,8,20,22,23,25,26	1	Q6IRE4
6506	1,3,5,6,18,19,21,25	2,4,7,8,20,22,23,24,26	2	P00731,P53792
6507	1,3,5,6,18,19,21,26	2,4,7,8,20,22,23,24,25	2	P01681,Q4FZU2
6508	1,3,5,6,18,19,22,23	2,4,7,8,20,21,24,25,26	3	P19223,P22006,Q9QW07
6509	1,3,5,6,18,19,22,24	2,4,7,8,20,21,23,25,26	3	P83121,Q80WL1,Q9QW07
6510	1,3,5,6,18,19,22,25	2,4,7,8,20,21,23,24,26	3	P01015,P17046,Q9QW07
6511	1,3,5,6,18,19,22,26	2,4,7,8,20,21,23,24,25	2	O54728,Q9QW07
6512	1,3,5,6,18,19,23,24	2,4,7,8,20,21,22,25,26	3	P19223,P30836,Q80WL1
6513	1,3,5,6,18,19,23,25	2,4,7,8,20,21,22,24,26	4	P01015,P17046,P55314,Q80WL1
6514	1,3,5,6,18,19,23,26	2,4,7,8,20,21,22,24,25	10	P35467,Q07523,Q4FZU2,Q63942,Q6IFU8,Q6IFW6,Q6IG02,Q6IMF3,Q6P6Q2,Q80WL1
6515	1,3,5,6,18,19,24,25	2,4,7,8,20,21,22,23,26	2	P30836,Q80WL1
6516	1,3,5,6,18,19,24,26	2,4,7,8,20,21,22,23,25	1	Q80WL1
6517	1,3,5,6,18,19,25,26	2,4,7,8,20,21,22,23,24	1	Q80WL1

6518	1,3,5,6,18,20,21,22	2,4,7,8,19,23,24,25,26	5	P00714,P01835,P14480,Q6IRE4,Q9QW07
6519	1,3,5,6,18,20,21,23	2,4,7,8,19,22,24,25,26	5	P01681,P01835,Q4FZU2,Q6IG02,Q6IRE4
6520	1,3,5,6,18,20,21,24	2,4,7,8,19,22,23,25,26	5	P01835,P13432,P20766,Q6IRE4,Q80WL1
6521	1,3,5,6,18,20,21,25	2,4,7,8,19,22,23,24,26	2	P01835,Q6IRE4
6522	1,3,5,6,18,20,21,26	2,4,7,8,19,22,23,24,25	6	P01681,P01835,Q4FZU2,Q6IG02,Q6IRE4,Q99041
6523	1,3,5,6,18,20,22,23	2,4,7,8,19,21,24,25,26	6	P22006,P82995,Q6IG02,Q6IRE4,Q80WL1,Q9QW07
6524	1,3,5,6,18,20,22,24	2,4,7,8,19,21,23,25,26	8	P00714,P27213,P82995,P83121,Q6IRE4,Q80WL1,Q99041,Q9QW07
6525	1,3,5,6,18,20,22,25	2,4,7,8,19,21,23,24,26	4	D3ZUC6,Q6IRE4,Q80WL1,Q9QW07
6526	1,3,5,6,18,20,22,26	2,4,7,8,19,21,23,24,25	6	O54728,P22006,Q4FZU2,Q6IRE4,Q99041,Q9QW07
6527	1,3,5,6,18,20,23,24	2,4,7,8,19,21,22,25,26	4	P82995,Q6IG02,Q6IRE4,Q80WL1
6528	1,3,5,6,18,20,23,25	2,4,7,8,19,21,22,24,26	5	P55314,Q4FZU2,Q6IG02,Q6IRE4,Q80WL1
6529	1,3,5,6,18,20,23,26	2,4,7,8,19,21,22,24,25	11	P01681,P35467,P47967,Q4FZU2,Q6IFU8,Q6IFW6,Q6IG02,Q6IMF3,Q6IRE4,Q6P6Q2,Q80WL1
6530	1,3,5,6,18,20,24,25	2,4,7,8,19,21,22,23,26	2	Q6IRE4,Q80WL1
6531	1,3,5,6,18,20,24,26	2,4,7,8,19,21,22,23,25	6	P47967,Q4FZU2,Q6IG02,Q6IRE4,Q80WL1,Q99041
6532	1,3,5,6,18,20,25,26	2,4,7,8,19,21,22,23,24	4	Q4FZU2,Q6IG02,Q6IRE4,Q80WL1

6533	1,3,5,6,18,21,22,23	2,4,7,8,19,20,24,25,26	1	Q9QW07
6534	1,3,5,6,18,21,22,24	2,4,7,8,19,20,23,25,26	4	P00714,P13432,Q6IRE4,Q9QW07
6535	1,3,5,6,18,21,22,25	2,4,7,8,19,20,23,24,26	1	Q9QW07
6536	1,3,5,6,18,21,22,26	2,4,7,8,19,20,23,24,25	3	Q4FZU2,Q99041,Q9QW07
6537	1,3,5,6,18,21,23,24	2,4,7,8,19,20,22,25,26	1	Q6IRE4
6538	1,3,5,6,18,21,23,25	2,4,7,8,19,20,22,24,26	2	P55314,Q6IRE4
6539	1,3,5,6,18,21,23,26	2,4,7,8,19,20,22,24,25	9	P01835,Q4FZU2,Q6IFU8,Q6IFV1,Q6IFW6,Q6IG02,Q6IMF3,Q6IRE4,Q6P6Q2
6540	1,3,5,6,18,21,24,25	2,4,7,8,19,20,22,23,26	2	Q62997,Q6IRE4
6541	1,3,5,6,18,21,24,26	2,4,7,8,19,20,22,23,25	4	Q4FZU2,Q6IRE4,Q8CJD3,Q99041
6542	1,3,5,6,18,21,25,26	2,4,7,8,19,20,22,23,24	3	P31430,Q4FZU2,Q6IRE4
6543	1,3,5,6,18,22,23,24	2,4,7,8,19,20,21,25,26	5	P19223,P82995,Q6IFU7,Q80WL1,Q9QW07
6544	1,3,5,6,18,22,23,25	2,4,7,8,19,20,21,24,26	5	P01015,P02761,P17046,P55314,Q9QW07
6545	1,3,5,6,18,22,23,26	2,4,7,8,19,20,21,24,25	10	O54728,P47967,Q4FZU2,Q6IFU7,Q6IFU8,Q6IFW6,Q6IG02,Q6IMF3,Q6P6Q2,Q9QW07
6546	1,3,5,6,18,22,24,25	2,4,7,8,19,20,21,23,26	2	Q80WL1,Q9QW07
6547	1,3,5,6,18,22,24,26	2,4,7,8,19,20,21,23,25	3	P47967,Q99041,Q9QW07

6548	1,3,5,6,18,22,25,26	2,4,7,8,19,20,21,23,24	1	Q9QW07
6549	1,3,5,6,18,23,24,25	2,4,7,8,19,20,21,22,26	4	P55314,Q63751,Q6IRE4,Q80WL1
6550	1,3,5,6,18,23,24,26	2,4,7,8,19,20,21,22,25	10	P02091,P47967,Q4FZU2,Q6IFU7,Q6IFU8,Q6IFW6,Q6IG02,Q6IMF3,Q6P6Q2,Q80WL1
6551	1,3,5,6,18,23,25,26	2,4,7,8,19,20,21,22,24	10	P02091,P55314,Q4FZU2,Q5I0D1,Q6IFU8,Q6IFW6,Q6IG02,Q6IMF3,Q6P6Q2,Q80WL1
6552	1,3,5,6,18,24,25,26	2,4,7,8,19,20,21,22,23	2	Q80WL1,Q8CJD3
6553	1,3,5,6,19,20,21,22	2,4,7,8,18,23,24,25,26	7	P00714,P01681,P55091,P82995,Q6IRE4,Q99MH3,Q9QW07
6554	1,3,5,6,19,20,21,23	2,4,7,8,18,22,24,25,26	7	P00714,P01681,P01835,P55091,P82995,Q6IRE4,Q80WL1
6555	1,3,5,6,19,20,21,24	2,4,7,8,18,22,23,25,26	7	P00714,P20766,P55091,P82995,Q6IRE4,Q80WL1,Q99MH3
6556	1,3,5,6,19,20,21,25	2,4,7,8,18,22,23,24,26	3	P53792,Q6IRE4,Q80WL1
6557	1,3,5,6,19,20,21,26	2,4,7,8,18,22,23,24,25	4	P00714,P01681,Q6IRE4,Q99MH3
6558	1,3,5,6,19,20,22,23	2,4,7,8,18,21,24,25,26	12	O54728,P00714,P00774,P19223,P22006,P31430,P55091,P82995,P98089,Q6IRE4,Q80WL1,Q9QW07
6559	1,3,5,6,19,20,22,24	2,4,7,8,18,21,23,25,26	14	P00714,P00774,P07150,P14669,P24594,P27213,P31430,P55091,P82995,P98089,Q64057,Q6IRE4,Q80WL1,Q99MH3
6560	1,3,5,6,19,20,22,25	2,4,7,8,18,21,23,24,26	7	D3ZUC6,P17046,P55091,P82995,Q6IRE4,Q80WL1,Q9QW07
6561	1,3,5,6,19,20,22,26	2,4,7,8,18,21,23,24,25	9	O54728,P00714,P01681,P31430,P55091,P98089,Q80WL1,Q99MH3,Q9QW07
6562	1,3,5,6,19,20,23,24	2,4,7,8,18,21,22,25,26	8	P00714,P19223,P31430,P55091,P82995,P98089,Q6IRE4,Q80WL1

6563	1,3,5,6,19,20,23,25	2,4,7,8,18,21,22,24,26	6	P17046,P55091,P55314,P82995,Q6IRE4,Q80WL1
6564	1,3,5,6,19,20,23,26	2,4,7,8,18,21,22,24,25	8	O54728,P01681,P31430,P98089,Q4FZU2,Q6IG02,Q6IRE4,Q80WL1
6565	1,3,5,6,19,20,24,25	2,4,7,8,18,21,22,23,26	3	P82995,Q6IRE4,Q80WL1
6566	1,3,5,6,19,20,24,26	2,4,7,8,18,21,22,23,25	6	P00714,P31430,P98089,Q6IRE4,Q80WL1,Q99MH3
6567	1,3,5,6,19,20,25,26	2,4,7,8,18,21,22,23,24	4	D3ZUC6,P01681,Q6IRE4,Q80WL1
6568	1,3,5,6,19,21,22,23	2,4,7,8,18,20,24,25,26	4	P00714,P55091,P82995,Q9QW07
6569	1,3,5,6,19,21,22,24	2,4,7,8,18,20,23,25,26	7	P00714,P13432,P19218,P55091,P82995,Q99MH3,Q9QW07
6570	1,3,5,6,19,21,22,25	2,4,7,8,18,20,23,24,26	3	P17046,P19218,Q9QW07
6571	1,3,5,6,19,21,22,26	2,4,7,8,18,20,23,24,25	5	O54728,P00714,P01681,Q99MH3,Q9QW07
6572	1,3,5,6,19,21,23,24	2,4,7,8,18,20,22,25,26	5	P00714,P55091,P82995,Q6IRE4,Q80WL1
6573	1,3,5,6,19,21,23,25	2,4,7,8,18,20,22,24,26	2	P17046,Q6IRE4
6574	1,3,5,6,19,21,23,26	2,4,7,8,18,20,22,24,25	2	P01681,Q4FZU2
6575	1,3,5,6,19,21,24,25	2,4,7,8,18,20,22,23,26	3	P17046,Q6IRE4,Q80WL1
6576	1,3,5,6,19,21,24,26	2,4,7,8,18,20,22,23,25	3	P00714,Q8CJD3,Q99MH3
6577	1,3,5,6,19,21,25,26	2,4,7,8,18,20,22,23,24	1	P53792

6578	1,3,5,6,19,22,23,24	2,4,7,8,18,20,21,25,26	8	P00714,P17046,P19223,P31430,P55091,P82995,P98089,Q80WL1
6579	1,3,5,6,19,22,23,25	2,4,7,8,18,20,21,24,26	7	P01015,P02761,P17046,P55091,P55314,P82995,Q9QW07
6580	1,3,5,6,19,22,23,26	2,4,7,8,18,20,21,24,25	4	O54728,P31430,P98089,Q9QW07
6581	1,3,5,6,19,22,24,25	2,4,7,8,18,20,21,23,26	4	P17046,P82995,Q6TMA8,Q80WL1
6582	1,3,5,6,19,22,24,26	2,4,7,8,18,20,21,23,25	7	O54728,P00714,P31430,P98089,Q6TMA8,Q80WL1,Q99MH3
6583	1,3,5,6,19,22,25,26	2,4,7,8,18,20,21,23,24	3	O54728,P17046,Q9QW07
6584	1,3,5,6,19,23,24,25	2,4,7,8,18,20,21,22,26	3	P17046,P82995,Q80WL1
6585	1,3,5,6,19,23,24,26	2,4,7,8,18,20,21,22,25	5	O54728,P02091,P31430,P98089,Q80WL1
6586	1,3,5,6,19,23,25,26	2,4,7,8,18,20,21,22,24	4	P02091,P17046,P55314,Q80WL1
6587	1,3,5,6,19,24,25,26	2,4,7,8,18,20,21,22,23	2	Q80WL1,Q8CJD3
6588	1,3,5,6,20,21,22,23	2,4,7,8,18,19,24,25,26	7	P00714,P01681,P01835,P55091,P82995,Q6IRE4,Q9QW07
6589	1,3,5,6,20,21,22,24	2,4,7,8,18,19,23,25,26	8	P00714,P13432,P55091,P82995,Q5XFX0,Q6IRE4,Q99MH3,Q9QW07
6590	1,3,5,6,20,21,22,25	2,4,7,8,18,19,23,24,26	4	Q03191,Q6IRE4,Q99MH3,Q9QW07
6591	1,3,5,6,20,21,22,26	2,4,7,8,18,19,23,24,25	6	O54728,P00714,P01681,Q6IRE4,Q99MH3,Q9QW07
6592	1,3,5,6,20,21,23,24	2,4,7,8,18,19,22,25,26	7	P00714,P01835,P20766,P55091,P82995,Q6IRE4,Q80WL1

6593	1,3,5,6,20,21,23,25	2,4,7,8,18,19,22,24,26	5	P01835,P55314,P82995,Q6IRE4,Q80WL1
6594	1,3,5,6,20,21,23,26	2,4,7,8,18,19,22,24,25	9	P01681,P01835,Q4FZU2,Q6IFU8,Q6IFV1,Q6IG02,Q6IMF3,Q6IRE4,Q6P6Q2
6595	1,3,5,6,20,21,24,25	2,4,7,8,18,19,22,23,26	5	P82995,Q5XFX0,Q6IRE4,Q80WL1,Q99MH3
6596	1,3,5,6,20,21,24,26	2,4,7,8,18,19,22,23,25	6	P00714,P01681,Q6IRE4,Q80WL1,Q8CJD3,Q99MH3
6597	1,3,5,6,20,21,25,26	2,4,7,8,18,19,22,23,24	4	P01681,P01835,Q6IRE4,Q99MH3
6598	1,3,5,6,20,22,23,24	2,4,7,8,18,19,21,25,26	10	P00714,P19223,P31430,P47967,P55091,P82995,P98089,Q6IRE4,Q80WL1,Q9QW07
6599	1,3,5,6,20,22,23,25	2,4,7,8,18,19,21,24,26	8	D3ZUC6,P17046,P55091,P55314,P82995,Q6IRE4,Q80WL1,Q9QW07
6600	1,3,5,6,20,22,23,26	2,4,7,8,18,19,21,24,25	10	O54728,P01681,P31430,P47967,P98089,Q4FZU2,Q6IG02,Q6IRE4,Q80WL1,Q9QW07
6601	1,3,5,6,20,22,24,25	2,4,7,8,18,19,21,23,26	8	D3ZUC6,P82995,Q03191,Q5XFX0,Q64057,Q6IRE4,Q80WL1,Q99MH3
6602	1,3,5,6,20,22,24,26	2,4,7,8,18,19,21,23,25	11	O54728,P00714,P02803,P47967,P98089,Q64057,Q6IRE4,Q80WL1,Q99041,Q99MH3,Q9QW07
6603	1,3,5,6,20,22,25,26	2,4,7,8,18,19,21,23,24	6	D3ZUC6,O54728,Q6IRE4,Q80WL1,Q99MH3,Q9QW07
6604	1,3,5,6,20,23,24,25	2,4,7,8,18,19,21,22,26	5	P55314,P82995,Q63751,Q6IRE4,Q80WL1
6605	1,3,5,6,20,23,24,26	2,4,7,8,18,19,21,22,25	9	P02091,P47967,P97840,P98089,Q4FZU2,Q6IG02,Q6IMF3,Q6IRE4,Q80WL1
6606	1,3,5,6,20,23,25,26	2,4,7,8,18,19,21,22,24	10	D3ZUC6,P02091,P55314,Q4FZU2,Q6IFU8,Q6IFW6,Q6IG02,Q6IMF3,Q6IRE4,Q80WL1
6607	1,3,5,6,20,24,25,26	2,4,7,8,18,19,21,22,23	5	D3ZUC6,Q6IRE4,Q80WL1,Q8CJD3,Q99MH3

6608	1,3,5,6,21,22,23,24	2,4,7,8,18,19,20,25,26	7	P00714,P13432,P35280,P55091,P82995,Q6IRE4,Q9QW07
6609	1,3,5,6,21,22,23,25	2,4,7,8,18,19,20,24,26	5	P17046,P35280,P55314,Q6IRE4,Q9QW07
6610	1,3,5,6,21,22,23,26	2,4,7,8,18,19,20,24,25	4	O54728,Q4FZU2,Q6IMF3,Q9QW07
6611	1,3,5,6,21,22,24,25	2,4,7,8,18,19,20,23,26	6	P13432,P17046,P35280,Q5XFX0,Q6IRE4,Q9QW07
6612	1,3,5,6,21,22,24,26	2,4,7,8,18,19,20,23,25	5	P00714,P13432,Q8CJD3,Q99MH3,Q9QW07
6613	1,3,5,6,21,22,25,26	2,4,7,8,18,19,20,23,24	1	Q9QW07
6614	1,3,5,6,21,23,24,25	2,4,7,8,18,19,20,22,26	4	P35280,Q63751,Q6IRE4,Q80WL1
6615	1,3,5,6,21,23,24,26	2,4,7,8,18,19,20,22,25	5	P02091,Q4FZU2,Q6IFV1,Q6IMF3,Q6IRE4
6616	1,3,5,6,21,23,25,26	2,4,7,8,18,19,20,22,24	6	P02091,P55314,Q4FZU2,Q6IFU8,Q6IMF3,Q6IRE4
6617	1,3,5,6,21,24,25,26	2,4,7,8,18,19,20,22,23	2	Q6IRE4,Q8CJD3
6618	1,3,5,6,22,23,24,25	2,4,7,8,18,19,20,21,26	7	P17046,P35280,P55314,P62804,P82995,Q63751,Q80WL1
6619	1,3,5,6,22,23,24,26	2,4,7,8,18,19,20,21,25	7	O54728,P00731,P02091,P31430,P47967,P98089,Q80WL1
6620	1,3,5,6,22,23,25,26	2,4,7,8,18,19,20,21,24	5	O54728,P02091,P02761,P55314,Q9QW07
6621	1,3,5,6,22,24,25,26	2,4,7,8,18,19,20,21,23	4	Q64057,Q80WL1,Q8CJD3,Q99MH3
6622	1,3,5,6,23,24,25,26	2,4,7,8,18,19,20,21,22	6	P01946,P02091,P55314,Q63751,Q7TPB1,Q80WL1

6623	1,3,5,7,8,18,19,20	2,4,6,21,22,23,24,25,26	4	P25236,P83121,Q6IRE4,Q6P6R2
6624	1,3,5,7,8,18,19,21	2,4,6,20,22,23,24,25,26	3	P00731,P53792,Q6IRE4
6625	1,3,5,7,8,18,19,22	2,4,6,20,21,23,24,25,26	4	P25236,P83121,Q6P6R2,Q9QW07
6626	1,3,5,7,8,18,19,23	2,4,6,20,21,22,24,25,26	4	P09605;P25809,P25236,P35467,Q03191
6627	1,3,5,7,8,18,19,24	2,4,6,20,21,22,23,25,26	4	P09605;P25809,P25236,P83121,Q6IRE4
6628	1,3,5,7,8,18,19,25	2,4,6,20,21,22,23,24,26	5	P01015,P10715,P25236,P32755,P62815
6629	1,3,5,7,8,18,19,26	2,4,6,20,21,22,23,24,25	4	O54728,P09605;P25809,P70709,Q6P6R2
6630	1,3,5,7,8,18,20,21	2,4,6,19,22,23,24,25,26	4	P14480,Q4FZU6,Q6IRE4,Q9ET32
6631	1,3,5,7,8,18,20,22	2,4,6,19,21,23,24,25,26	7	P14480,P25236,P27213,P83121,Q6IRE4,Q6P6R2,Q9QW07
6632	1,3,5,7,8,18,20,23	2,4,6,19,21,22,24,25,26	6	P14480,P25236,Q4FZU6,Q6IG02,Q6IRE4,Q9ET32
6633	1,3,5,7,8,18,20,24	2,4,6,19,21,22,23,25,26	5	P10715,P25236,P27213,P83121,Q6IRE4
6634	1,3,5,7,8,18,20,25	2,4,6,19,21,22,23,24,26	3	P10715,P25236,Q6IRE4
6635	1,3,5,7,8,18,20,26	2,4,6,19,21,22,23,24,25	5	P10715,P50280,Q4FZU6,Q6IG02,Q6IRE4
6636	1,3,5,7,8,18,21,22	2,4,6,19,20,23,24,25,26	3	P14480,Q6IRE4,Q9QW07
6637	1,3,5,7,8,18,21,23	2,4,6,19,20,22,24,25,26	1	Q6IRE4

6638	1,3,5,7,8,18,21,24	2,4,6,19,20,22,23,25,26	1	Q6IRE4
6639	1,3,5,7,8,18,21,25	2,4,6,19,20,22,23,24,26	4	P08721,P10715,P31430,Q6IRE4
6640	1,3,5,7,8,18,21,26	2,4,6,19,20,22,23,24,25	2	Q4FZU6,Q6IRE4
6641	1,3,5,7,8,18,22,23	2,4,6,19,20,21,24,25,26	3	P01015,P25236,Q9QW07
6642	1,3,5,7,8,18,22,24	2,4,6,19,20,21,23,25,26	6	P25236,P27213,P83121,Q6IRE4,Q6P6R2,Q9QW07
6643	1,3,5,7,8,18,22,25	2,4,6,19,20,21,23,24,26	5	P01015,P10715,P19629,P25236,Q9QW07
6644	1,3,5,7,8,18,22,26	2,4,6,19,20,21,23,24,25	5	O54728,P25236,Q6P6R2,Q8VD89,Q9QW07
6645	1,3,5,7,8,18,23,24	2,4,6,19,20,21,22,25,26	3	P09605;P25809,P25236,Q6IRE4
6646	1,3,5,7,8,18,23,25	2,4,6,19,20,21,22,24,26	5	P01015,P10715,P19629,P25236,Q6IRE4
6647	1,3,5,7,8,18,23,26	2,4,6,19,20,21,22,24,25	11	O54728,P01946,P02091,P09605;P25809,P35467,Q4FZU6,Q6IFW6,Q6IG02,Q6IRE4,Q6P6Q2,Q8VD89
6648	1,3,5,7,8,18,24,25	2,4,6,19,20,21,22,23,26	4	P10715,P19629,P25236,Q6IRE4
6649	1,3,5,7,8,18,24,26	2,4,6,19,20,21,22,23,25	6	P02091,P09605;P25809,P25236,Q6IRE4,Q8CJD3,Q8VD89
6650	1,3,5,7,8,18,25,26	2,4,6,19,20,21,22,23,24	4	P02091,P10715,P32755,Q6IRE4
6651	1,3,5,7,8,19,20,21	2,4,6,18,22,23,24,25,26	3	P53792,Q4FZU6,Q6IRE4
6652	1,3,5,7,8,19,20,22	2,4,6,18,21,23,24,25,26	10	O54728,P00774,P25236,P27213,P55091,P83121,P98089,Q6IRE4,Q6P6R2,Q9QW07

6653	1,3,5,7,8,19,20,23	2,4,6,18,21,22,24,25,26	4	P00774,P55091,P98089,Q6IRE4
6654	1,3,5,7,8,19,20,24	2,4,6,18,21,22,23,25,26	5	P00774,P25236,P83121,P98089,Q6IRE4
6655	1,3,5,7,8,19,20,25	2,4,6,18,21,22,23,24,26	5	P10715,P17046,P25236,Q6IFU7,Q6IRE4
6656	1,3,5,7,8,19,20,26	2,4,6,18,21,22,23,24,25	4	O54728,P98089,Q4FZU6,Q6IRE4
6657	1,3,5,7,8,19,21,22	2,4,6,18,20,23,24,25,26	2	P19218,Q9QW07
6658	1,3,5,7,8,19,21,23	2,4,6,18,20,22,24,25,26	2	O70594,Q6IRE4
6659	1,3,5,7,8,19,21,24	2,4,6,18,20,22,23,25,26	2	P53792,Q6IRE4
6660	1,3,5,7,8,19,21,25	2,4,6,18,20,22,23,24,26	4	P17046,P53792,Q6IFU7,Q6IRE4
6661	1,3,5,7,8,19,21,26	2,4,6,18,20,22,23,24,25	3	O54728,P53792,Q6IRE4
6662	1,3,5,7,8,19,22,23	2,4,6,18,20,21,24,25,26	7	O54728,P00774,P17046,P25236,P55091,P98089,Q9QW07
6663	1,3,5,7,8,19,22,24	2,4,6,18,20,21,23,25,26	7	P00774,P25236,P83121,P98089,Q6P6R2,Q6TMA8,Q9QW07
6664	1,3,5,7,8,19,22,25	2,4,6,18,20,21,23,24,26	4	P10715,P17046,P25236,Q9QW07
6665	1,3,5,7,8,19,22,26	2,4,6,18,20,21,23,24,25	4	O54728,P98089,Q6P6R2,Q9QW07
6666	1,3,5,7,8,19,23,24	2,4,6,18,20,21,22,25,26	4	P09605;P25809,P25236,P98089,Q6IRE4
6667	1,3,5,7,8,19,23,25	2,4,6,18,20,21,22,24,26	5	O70594,P01015,P17046,P19629,Q99041

6668	1,3,5,7,8,19,23,26	2,4,6,18,20,21,22,24,25	5	O54728,P01946,P02091,P09605;P25809,P98089
6669	1,3,5,7,8,19,24,25	2,4,6,18,20,21,22,23,26	5	P10715,P17046,P19629,P25236,Q6IRE4
6670	1,3,5,7,8,19,24,26	2,4,6,18,20,21,22,23,25	5	O54728,P02091,P25236,P98089,Q8CJD3
6671	1,3,5,7,8,19,25,26	2,4,6,18,20,21,22,23,24	4	O54728,P02091,P10715,P32755
6672	1,3,5,7,8,20,21,22	2,4,6,18,19,23,24,25,26	4	P14480,Q6IRE4,Q99MH3,Q9QW07
6673	1,3,5,7,8,20,21,23	2,4,6,18,19,22,24,25,26	3	P14480,Q4FZU6,Q6IRE4
6674	1,3,5,7,8,20,21,24	2,4,6,18,19,22,23,25,26	3	Q5XFX0,Q6IRE4,Q99MH3
6675	1,3,5,7,8,20,21,25	2,4,6,18,19,22,23,24,26	3	P10715,Q6IFU7,Q6IRE4
6676	1,3,5,7,8,20,21,26	2,4,6,18,19,22,23,24,25	3	Q4FZU6,Q6IRE4,Q99MH3
6677	1,3,5,7,8,20,22,23	2,4,6,18,19,21,24,25,26	9	O54728,P00774,P14480,P25236,P55091,P98089,Q6IRE4,Q8CIZ5,Q9QW07
6678	1,3,5,7,8,20,22,24	2,4,6,18,19,21,23,25,26	10	P00774,P25236,P27213,P83121,P98089,Q5XFX0,Q6IRE4,Q6P6R2,Q99MH3,Q9QW07
6679	1,3,5,7,8,20,22,25	2,4,6,18,19,21,23,24,26	5	D3ZUC6,P10715,P25236,Q6IRE4,Q9QW07
6680	1,3,5,7,8,20,22,26	2,4,6,18,19,21,23,24,25	8	O54728,P98089,Q4FZU6,Q6IRE4,Q6P6R2,Q8CIZ5,Q99MH3,Q9QW07
6681	1,3,5,7,8,20,23,24	2,4,6,18,19,21,22,25,26	5	P25236,P97840,P98089,Q6IRE4,Q8CIZ5
6682	1,3,5,7,8,20,23,25	2,4,6,18,19,21,22,24,26	3	P10715,P19629,Q6IRE4

6683	1,3,5,7,8,20,23,26	2,4,6,18,19,21,22,24,25	9	O54728,P01946,P02091,P97840,P98089,Q4FZU6,Q6IG02,Q6IRE4,Q8CIZ5
6684	1,3,5,7,8,20,24,25	2,4,6,18,19,21,22,23,26	4	P10715,P19629,P25236,Q6IRE4
6685	1,3,5,7,8,20,24,26	2,4,6,18,19,21,22,23,25	10	O54728,P02091,P25236,P97840,P98089,Q4FZU6,Q6IRE4,Q8CIZ5,Q8CJD3,Q99MH3
6686	1,3,5,7,8,20,25,26	2,4,6,18,19,21,22,23,24	3	P02091,P10715,Q6IRE4
6687	1,3,5,7,8,21,22,23	2,4,6,18,19,20,24,25,26	2	Q6IRE4,Q9QW07
6688	1,3,5,7,8,21,22,24	2,4,6,18,19,20,23,25,26	3	Q5XFX0,Q6IRE4,Q9QW07
6689	1,3,5,7,8,21,22,25	2,4,6,18,19,20,23,24,26	5	P10715,P17046,P19629,Q6IRE4,Q9QW07
6690	1,3,5,7,8,21,22,26	2,4,6,18,19,20,23,24,25	3	O54728,Q6IRE4,Q9QW07
6691	1,3,5,7,8,21,23,24	2,4,6,18,19,20,22,25,26	1	Q6IRE4
6692	1,3,5,7,8,21,23,25	2,4,6,18,19,20,22,24,26	2	P19629,Q6IRE4
6693	1,3,5,7,8,21,23,26	2,4,6,18,19,20,22,24,25	4	P01946,P02091,Q4FZU6,Q6IRE4
6694	1,3,5,7,8,21,24,25	2,4,6,18,19,20,22,23,26	4	P10715,P19629,Q62997,Q6IRE4
6695	1,3,5,7,8,21,24,26	2,4,6,18,19,20,22,23,25	3	P02091,Q6IRE4,Q8CJD3
6696	1,3,5,7,8,21,25,26	2,4,6,18,19,20,22,23,24	4	P02091,P10715,P19629,Q6IRE4
6697	1,3,5,7,8,22,23,24	2,4,6,18,19,20,21,25,26	5	P25236,P98089,Q6IRE4,Q8CIZ5,Q9QW07

6698	1,3,5,7,8,22,23,25	2,4,6,18,19,20,21,24,26	6	P01015,P10715,P17046,P19629,P25236,Q9QW07
6699	1,3,5,7,8,22,23,26	2,4,6,18,19,20,21,24,25	6	O54728,P01946,P02091,P98089,Q8CIZ5,Q9QW07
6700	1,3,5,7,8,22,24,25	2,4,6,18,19,20,21,23,26	6	P10715,P17046,P19629,P25236,Q6IRE4,Q9QW07
6701	1,3,5,7,8,22,24,26	2,4,6,18,19,20,21,23,25	9	O54728,P02091,P25236,P98089,Q6P6R2,Q8CIZ5,Q8CJD3,Q99MH3,Q9QW07
6702	1,3,5,7,8,22,25,26	2,4,6,18,19,20,21,23,24	5	O54728,P02091,P10715,P19629,Q9QW07
6703	1,3,5,7,8,23,24,25	2,4,6,18,19,20,21,22,26	5	P10715,P19629,P25236,Q63751,Q6IRE4
6704	1,3,5,7,8,23,24,26	2,4,6,18,19,20,21,22,25	8	O54728,P01946,P02091,P47967,P97840,P98089,Q6IRE4,Q8CIZ5
6705	1,3,5,7,8,23,25,26	2,4,6,18,19,20,21,22,24	6	O54728,P01946,P02091,P10715,P19629,Q6IRE4
6706	1,3,5,7,8,24,25,26	2,4,6,18,19,20,21,22,23	7	P01946,P02091,P10715,P19629,P25236,Q6IRE4,Q8CJD3
6707	1,3,5,7,18,19,20,21	2,4,6,8,22,23,24,25,26	2	P00731,Q6IRE4
6708	1,3,5,7,18,19,20,22	2,4,6,8,21,23,24,25,26	10	P07150,P14669,P25236,P27213,Q5U2V4,Q6IRE4,Q6P6R2,Q9QW07,Q9WTT6,Q9Z0V6
6709	1,3,5,7,18,19,20,23	2,4,6,8,21,22,24,25,26	4	P25236,Q6IRE4,Q80WL1,Q9WTT6
6710	1,3,5,7,18,19,20,24	2,4,6,8,21,22,23,25,26	10	P07150,P14669,P25236,P50280,P83121,Q6IRE4,Q6P6R2,Q80WL1,Q9WTT6,Q9Z0V6
6711	1,3,5,7,18,19,20,25	2,4,6,8,21,22,23,24,26	4	P17046,P25236,P62815,Q6IRE4
6712	1,3,5,7,18,19,20,26	2,4,6,8,21,22,23,24,25	7	O54728,P50280,Q4FZU6,Q5U2V4,Q6IRE4,Q6P6R2,Q9WTT6

6713	1,3,5,7,18,19,21,22	2,4,6,8,20,23,24,25,26	3	P17046,P19218,Q9QW07
6714	1,3,5,7,18,19,21,23	2,4,6,8,20,22,24,25,26	3	P14668,Q03191,Q6IRE4
6715	1,3,5,7,18,19,21,24	2,4,6,8,20,22,23,25,26	1	Q6IRE4
6716	1,3,5,7,18,19,21,25	2,4,6,8,20,22,23,24,26	4	P00731,P17046,P53792,Q6IRE4
6717	1,3,5,7,18,19,21,26	2,4,6,8,20,22,23,24,25	1	Q6IRE4
6718	1,3,5,7,18,19,22,23	2,4,6,8,20,21,24,25,26	5	P01015,P17046,P25236,Q9QW07,Q9WTT6
6719	1,3,5,7,18,19,22,24	2,4,6,8,20,21,23,25,26	11	P07150,P14669,P17046,P25236,P62804,P83121,Q00715,Q6P6R2,Q9QW07,Q9WTT6,Q9Z0V6
6720	1,3,5,7,18,19,22,25	2,4,6,8,20,21,23,24,26	5	P01015,P17046,P25236,P62804,Q9QW07
6721	1,3,5,7,18,19,22,26	2,4,6,8,20,21,23,24,25	6	O54728,P25236,Q5U2V4,Q6P6R2,Q9QW07,Q9WTT6
6722	1,3,5,7,18,19,23,24	2,4,6,8,20,21,22,25,26	5	P25236,Q00715,Q03191,Q80WL1,Q9WTT6
6723	1,3,5,7,18,19,23,25	2,4,6,8,20,21,22,24,26	3	P01015,P17046,P25236
6724	1,3,5,7,18,19,23,26	2,4,6,8,20,21,22,24,25	9	O54728,P01946,P02091,P09605;P25809,P35467,Q03191,Q07523,Q6IG02,Q6P6Q2
6725	1,3,5,7,18,19,24,25	2,4,6,8,20,21,22,23,26	5	P17046,P25236,P62804,Q00715,Q80WL1
6726	1,3,5,7,18,19,24,26	2,4,6,8,20,21,22,23,25	4	P02091,P25236,Q6P6R2,Q9WTT6
6727	1,3,5,7,18,19,25,26	2,4,6,8,20,21,22,23,24	3	P02091,P17046,P32755

6728	1,3,5,7,18,20,21,22	2,4,6,8,19,23,24,25,26	4	P14480,P50280,Q6IRE4,Q9QW07
6729	1,3,5,7,18,20,21,23	2,4,6,8,19,22,24,25,26	3	P14480,Q4FZU6,Q6IRE4
6730	1,3,5,7,18,20,21,24	2,4,6,8,19,22,23,25,26	2	P50280,Q6IRE4
6731	1,3,5,7,18,20,21,25	2,4,6,8,19,22,23,24,26	1	Q6IRE4
6732	1,3,5,7,18,20,21,26	2,4,6,8,19,22,23,24,25	4	P06911,P50280,Q4FZU6,Q6IRE4
6733	1,3,5,7,18,20,22,23	2,4,6,8,19,21,24,25,26	6	P14480,P25236,Q6IRE4,Q8CIZ5,Q9QW07,Q9WTT6
6734	1,3,5,7,18,20,22,24	2,4,6,8,19,21,23,25,26	11	P07150,P14669,P25236,P27213,P50280,P83121,Q6IRE4,Q6P6R2,Q9QW07,Q9WTT6,Q9Z0V6
6735	1,3,5,7,18,20,22,25	2,4,6,8,19,21,23,24,26	4	P17046,P25236,Q6IRE4,Q9QW07
6736	1,3,5,7,18,20,22,26	2,4,6,8,19,21,23,24,25	10	O54728,P07150,P50280,Q4FZU6,Q5U2V4,Q6IRE4,Q6P6R2,Q8CIZ5,Q9QW07,Q9WTT6
6737	1,3,5,7,18,20,23,24	2,4,6,8,19,21,22,25,26	3	P25236,Q6IRE4,Q80WL1
6738	1,3,5,7,18,20,23,25	2,4,6,8,19,21,22,24,26	2	Q6IRE4,Q80WL1
6739	1,3,5,7,18,20,23,26	2,4,6,8,19,21,22,24,25	13	A2RUV9,O54728,P01946,P02091,Q4FZU2,Q4FZU6,Q6IFU8,Q6IFW6,Q6IG02,Q6IMF3,Q6IRE4,Q6P6Q2,Q8CIZ5
6740	1,3,5,7,18,20,24,25	2,4,6,8,19,21,22,23,26	3	P25236,Q6IRE4,Q80WL1
6741	1,3,5,7,18,20,24,26	2,4,6,8,19,21,22,23,25	8	P02091,P25236,P50280,Q4FZU6,Q6IRE4,Q6P6R2,Q80WL1,Q8CJD3
6742	1,3,5,7,18,20,25,26	2,4,6,8,19,21,22,23,24	2	P02091,Q6IRE4

6743	1,3,5,7,18,21,22,23	2,4,6,8,19,20,24,25,26	2	Q6IRE4,Q9QW07
6744	1,3,5,7,18,21,22,24	2,4,6,8,19,20,23,25,26	2	Q6IRE4,Q9QW07
6745	1,3,5,7,18,21,22,25	2,4,6,8,19,20,23,24,26	3	P17046,Q6IRE4,Q9QW07
6746	1,3,5,7,18,21,22,26	2,4,6,8,19,20,23,24,25	4	O54728,P50280,Q6IRE4,Q9QW07
6747	1,3,5,7,18,21,23,24	2,4,6,8,19,20,22,25,26	1	Q6IRE4
6748	1,3,5,7,18,21,23,25	2,4,6,8,19,20,22,24,26	3	P01015,P17046,Q6IRE4
6749	1,3,5,7,18,21,23,26	2,4,6,8,19,20,22,24,25	9	P01946,P02091,Q4FZU2,Q4FZU6,Q6IFU8,Q6IG02,Q6IMF3,Q6IRE4,Q6P6Q2
6750	1,3,5,7,18,21,24,25	2,4,6,8,19,20,22,23,26	2	Q62997,Q6IRE4
6751	1,3,5,7,18,21,24,26	2,4,6,8,19,20,22,23,25	4	P02091,P50280,Q6IRE4,Q8CJD3
6752	1,3,5,7,18,21,25,26	2,4,6,8,19,20,22,23,24	3	P02091,P31430,Q6IRE4
6753	1,3,5,7,18,22,23,24	2,4,6,8,19,20,21,25,26	5	P25236,P62804,Q00715,Q9QW07,Q9WTT6
6754	1,3,5,7,18,22,23,25	2,4,6,8,19,20,21,24,26	6	P01015,P17046,P25236,P62804,Q00715,Q9QW07
6755	1,3,5,7,18,22,23,26	2,4,6,8,19,20,21,24,25	8	O54728,P01946,P02091,Q6IG02,Q6P6Q2,Q8CIZ5,Q8VD89,Q9QW07
6756	1,3,5,7,18,22,24,25	2,4,6,8,19,20,21,23,26	5	P17046,P25236,P62804,Q00715,Q9QW07
6757	1,3,5,7,18,22,24,26	2,4,6,8,19,20,21,23,25	9	O54728,P02091,P25236,P50280,Q6P6R2,Q8CJD3,Q8VD89,Q9QW07,Q9WTT6

6758	1,3,5,7,18,22,25,26	2,4,6,8,19,20,21,23,24	4	O54728,P02091,P17046,Q9QW07
6759	1,3,5,7,18,23,24,25	2,4,6,8,19,20,21,22,26	8	P01015,P17046,P25236,P62804,Q00715,Q63751,Q6IRE4,Q80WL1
6760	1,3,5,7,18,23,24,26	2,4,6,8,19,20,21,22,25	9	P01946,P02091,P47967,Q07523,Q6IG02,Q6IRE4,Q6P6Q2,Q8CIZ5,Q8VD89
6761	1,3,5,7,18,23,25,26	2,4,6,8,19,20,21,22,24	5	P01946,P02091,Q6IG02,Q6IRE4,Q6P6Q2
6762	1,3,5,7,18,24,25,26	2,4,6,8,19,20,21,22,23	5	P01946,P02091,P25236,Q6IRE4,Q8CJD3
6763	1,3,5,7,19,20,21,22	2,4,6,8,18,23,24,25,26	6	P00714,P00774,P17046,P55091,Q6IRE4,Q9QW07
6764	1,3,5,7,19,20,21,23	2,4,6,8,18,22,24,25,26	2	P55091,Q6IRE4
6765	1,3,5,7,19,20,21,24	2,4,6,8,18,22,23,25,26	3	P00714,P00774,Q6IRE4
6766	1,3,5,7,19,20,21,25	2,4,6,8,18,22,23,24,26	3	P17046,P53792,Q6IRE4
6767	1,3,5,7,19,20,21,26	2,4,6,8,18,22,23,24,25	4	O54728,P50280,Q4FZU6,Q6IRE4
6768	1,3,5,7,19,20,22,23	2,4,6,8,18,21,24,25,26	10	O54728,P00774,P17046,P25236,P31430,P55091,P98089,Q6IRE4,Q9QW07,Q9WTT6
6769	1,3,5,7,19,20,22,24	2,4,6,8,18,21,23,25,26	17	O54728,P00714,P00774,P07150,P14669,P17046,P25236,P27213,P31430,P55091,P83121,P98089,Q6IRE4,Q6P6R2,Q99MH3,Q9WTT6,Q9Z0V6
6770	1,3,5,7,19,20,22,25	2,4,6,8,18,21,23,24,26	6	P00774,P14669,P17046,P25236,Q6IRE4,Q9QW07
6771	1,3,5,7,19,20,22,26	2,4,6,8,18,21,23,24,25	10	O54728,P07150,P14669,P98089,Q5U2V4,Q6IRE4,Q6P6R2,Q99MH3,Q9QW07,Q9WTT6
6772	1,3,5,7,19,20,23,24	2,4,6,8,18,21,22,25,26	8	P00774,P25236,P31430,P55091,P98089,Q6IRE4,Q80WL1,Q9WTT6

6773	1,3,5,7,19,20,23,25	2,4,6,8,18,21,22,24,26	3	P17046,Q6IRE4,Q80WL1
6774	1,3,5,7,19,20,23,26	2,4,6,8,18,21,22,24,25	7	O54728,P01946,P02091,P98089,Q4FZU6,Q6IRE4,Q80WL1
6775	1,3,5,7,19,20,24,25	2,4,6,8,18,21,22,23,26	4	P17046,P25236,Q6IRE4,Q80WL1
6776	1,3,5,7,19,20,24,26	2,4,6,8,18,21,22,23,25	13	O54728,P02091,P07150,P14669,P25236,P50280,P98089,Q6IRE4,Q6P6R2,Q80WL1,Q8CJD3,Q99MH3,Q9WTT6
6777	1,3,5,7,19,20,25,26	2,4,6,8,18,21,22,23,24	5	O54728,P02091,P17046,Q6IRE4,Q80WL1
6778	1,3,5,7,19,21,22,23	2,4,6,8,18,20,24,25,26	3	P17046,P55091,Q9QW07
6779	1,3,5,7,19,21,22,24	2,4,6,8,18,20,23,25,26	6	P00714,P00774,P17046,P19218,Q6TMA8,Q9QW07
6780	1,3,5,7,19,21,22,25	2,4,6,8,18,20,23,24,26	4	P17046,P19218,Q6TMA8,Q9QW07
6781	1,3,5,7,19,21,22,26	2,4,6,8,18,20,23,24,25	4	O54728,P17046,Q6TMA8,Q9QW07
6782	1,3,5,7,19,21,23,24	2,4,6,8,18,20,22,25,26	2	P17046,Q6IRE4
6783	1,3,5,7,19,21,23,25	2,4,6,8,18,20,22,24,26	2	P17046,Q6IRE4
6784	1,3,5,7,19,21,23,26	2,4,6,8,18,20,22,24,25	4	O54728,P01946,P02091,Q6IRE4
6785	1,3,5,7,19,21,24,25	2,4,6,8,18,20,22,23,26	3	P17046,Q6IRE4,Q6TMA8
6786	1,3,5,7,19,21,24,26	2,4,6,8,18,20,22,23,25	4	P02091,Q6IRE4,Q6TMA8,Q8CJD3
6787	1,3,5,7,19,21,25,26	2,4,6,8,18,20,22,23,24	3	P02091,P17046,Q6IRE4

6788	1,3,5,7,19,22,23,24	2,4,6,8,18,20,21,25,26	11	O54728,P00774,P17046,P19223,P25236,P31430,P55091,P62804,P98089,Q00715,Q9WTT6
6789	1,3,5,7,19,22,23,25	2,4,6,8,18,20,21,24,26	5	P01015,P17046,P25236,P62804,Q6AYR8
6790	1,3,5,7,19,22,23,26	2,4,6,8,18,20,21,24,25	7	O54728,P01946,P02091,P17046,P98089,Q9QW07,Q9WTT6
6791	1,3,5,7,19,22,24,25	2,4,6,8,18,20,21,23,26	5	P17046,P25236,P62804,Q00715,Q6TMA8
6792	1,3,5,7,19,22,24,26	2,4,6,8,18,20,21,23,25	11	O54728,P02091,P14669,P17046,P25236,P98089,Q6P6R2,Q6TMA8,Q8CJD3,Q99MH3,Q9WTT6
6793	1,3,5,7,19,22,25,26	2,4,6,8,18,20,21,23,24	6	O54728,P02091,P08644,P17046,Q6TMA8,Q9QW07
6794	1,3,5,7,19,23,24,25	2,4,6,8,18,20,21,22,26	5	P17046,P25236,P62804,Q00715,Q80WL1
6795	1,3,5,7,19,23,24,26	2,4,6,8,18,20,21,22,25	5	O54728,P01946,P02091,P98089,Q80WL1
6796	1,3,5,7,19,23,25,26	2,4,6,8,18,20,21,22,24	4	O54728,P01946,P02091,P17046
6797	1,3,5,7,19,24,25,26	2,4,6,8,18,20,21,22,23	7	P01946,P02091,P17046,P25236,Q6TMA8,Q80WL1,Q8CJD3
6798	1,3,5,7,20,21,22,23	2,4,6,8,18,19,24,25,26	3	P55091,Q6IRE4,Q9QW07
6799	1,3,5,7,20,21,22,24	2,4,6,8,18,19,23,25,26	7	P00714,P00774,Q5XFX0,Q62635,Q6IRE4,Q99MH3,Q9QW07
6800	1,3,5,7,20,21,22,25	2,4,6,8,18,19,23,24,26	3	P17046,Q6IRE4,Q9QW07
6801	1,3,5,7,20,21,22,26	2,4,6,8,18,19,23,24,25	6	O54728,P50280,Q4FZU6,Q6IRE4,Q99MH3,Q9QW07
6802	1,3,5,7,20,21,23,24	2,4,6,8,18,19,22,25,26	1	Q6IRE4

6803	1,3,5,7,20,21,23,25	2,4,6,8,18,19,22,24,26	2	P17046,Q6IRE4
6804	1,3,5,7,20,21,23,26	2,4,6,8,18,19,22,24,25	5	O54728,P01946,P02091,Q4FZU6,Q6IRE4
6805	1,3,5,7,20,21,24,25	2,4,6,8,18,19,22,23,26	1	Q6IRE4
6806	1,3,5,7,20,21,24,26	2,4,6,8,18,19,22,23,25	6	P02091,P50280,Q4FZU6,Q6IRE4,Q8CJD3,Q99MH3
6807	1,3,5,7,20,21,25,26	2,4,6,8,18,19,22,23,24	2	P02091,Q6IRE4
6808	1,3,5,7,20,22,23,24	2,4,6,8,18,19,21,25,26	10	P00774,P25236,P31430,P55091,P98089,Q62635,Q6IRE4,Q80WL1,Q8CIZ5,Q9WTT6
6809	1,3,5,7,20,22,23,25	2,4,6,8,18,19,21,24,26	4	P17046,P25236,Q6IRE4,Q9QW07
6810	1,3,5,7,20,22,23,26	2,4,6,8,18,19,21,24,25	9	O54728,P01946,P02091,P98089,Q4FZU6,Q62635,Q6IRE4,Q8CIZ5,Q9QW07
6811	1,3,5,7,20,22,24,25	2,4,6,8,18,19,21,23,26	6	P0C0A9,P14669,P17046,P25236,Q6IRE4,Q99MH3
6812	1,3,5,7,20,22,24,26	2,4,6,8,18,19,21,23,25	17	O54728,P02091,P02803,P07150,P14669,P25236,P50280,P98089,Q62635,Q63598,Q6IRE4,Q6P6R2,Q8CIZ5,Q8CJD3,Q99MH3,Q9QW07,Q9WTT6
6813	1,3,5,7,20,22,25,26	2,4,6,8,18,19,21,23,24	6	O54728,P02091,P17046,Q6IRE4,Q99MH3,Q9QW07
6814	1,3,5,7,20,23,24,25	2,4,6,8,18,19,21,22,26	6	P0C0A9,P17046,P25236,Q63751,Q6IRE4,Q80WL1
6815	1,3,5,7,20,23,24,26	2,4,6,8,18,19,21,22,25	11	O54728,P01946,P02091,P47967,P97840,P98089,Q4FZU6,Q62635,Q6IRE4,Q80WL1,Q8CIZ5
6816	1,3,5,7,20,23,25,26	2,4,6,8,18,19,21,22,24	6	O54728,P01946,P02091,Q6IG02,Q6IRE4,Q80WL1
6817	1,3,5,7,20,24,25,26	2,4,6,8,18,19,21,22,23	7	P01946,P02091,P0C0A9,Q6IRE4,Q80WL1,Q8CJD3,Q99MH3

6818	1,3,5,7,21,22,23,24	2,4,6,8,18,19,20,25,26	3	P35280,Q6IRE4,Q9QW07
6819	1,3,5,7,21,22,23,25	2,4,6,8,18,19,20,24,26	4	P17046,P35280,Q6IRE4,Q9QW07
6820	1,3,5,7,21,22,23,26	2,4,6,8,18,19,20,24,25	5	O54728,P01946,P02091,Q6IRE4,Q9QW07
6821	1,3,5,7,21,22,24,25	2,4,6,8,18,19,20,23,26	6	P17046,P35280,Q63355,Q6IRE4,Q6TMA8,Q9QW07
6822	1,3,5,7,21,22,24,26	2,4,6,8,18,19,20,23,25	8	O54728,P00714,P02091,Q6IRE4,Q6TMA8,Q8CJD3,Q99MH3,Q9QW07
6823	1,3,5,7,21,22,25,26	2,4,6,8,18,19,20,23,24	5	O54728,P02091,P17046,Q6IRE4,Q9QW07
6824	1,3,5,7,21,23,24,25	2,4,6,8,18,19,20,22,26	5	P17046,P22006,P35280,Q63751,Q6IRE4
6825	1,3,5,7,21,23,24,26	2,4,6,8,18,19,20,22,25	4	P01946,P02091,Q6IRE4,Q8CJD3
6826	1,3,5,7,21,23,25,26	2,4,6,8,18,19,20,22,24	3	P01946,P02091,Q6IRE4
6827	1,3,5,7,21,24,25,26	2,4,6,8,18,19,20,22,23	5	P01946,P02091,P22006,Q6IRE4,Q8CJD3
6828	1,3,5,7,22,23,24,25	2,4,6,8,18,19,20,21,26	9	P01015,P0C0A9,P17046,P25236,P35280,P62804,Q00715,Q63751,Q6AYR9
6829	1,3,5,7,22,23,24,26	2,4,6,8,18,19,20,21,25	10	O54728,P00731,P01946,P02091,P47967,P62804,P98089,Q62635,Q6AYR9,Q8CIZ5
6830	1,3,5,7,22,23,25,26	2,4,6,8,18,19,20,21,24	6	O54728,P01946,P02091,P17046,P62804,Q9QW07
6831	1,3,5,7,22,24,25,26	2,4,6,8,18,19,20,21,23	12	O54728,P01946,P02091,P0C0A9,P17046,P24368,P25236,P62804,Q5XI22,Q6AYR9,Q6TMA8,Q8CJD3
6832	1,3,5,7,23,24,25,26	2,4,6,8,18,19,20,21,22	11	P01946,P02091,P0C0A9,P17046,P62804,Q63751,Q6AYR9,Q6IRE4,Q7TPB1,Q80WL1,Q8CJD3

6833	1,3,5,8,18,19,20,21	2,4,6,7,22,23,24,25,26	5	P00731,P14480,P35281,P83121,Q6IRE4
6834	1,3,5,8,18,19,20,22	2,4,6,7,21,23,24,25,26	7	P09605;P25809,P14480,P22006,P25236,P83121,Q6P6R2,Q9QW07
6835	1,3,5,8,18,19,20,23	2,4,6,7,21,22,24,25,26	5	P09605;P25809,P22006,P35467,P83121,Q6IG02
6836	1,3,5,8,18,19,20,24	2,4,6,7,21,22,23,25,26	5	P09605;P25809,P25236,P83121,Q6IRE4,Q6P6R2
6837	1,3,5,8,18,19,20,25	2,4,6,7,21,22,23,24,26	2	P25236,P62815
6838	1,3,5,8,18,19,20,26	2,4,6,7,21,22,23,24,25	4	O54728,P09605;P25809,Q6IG02,Q6P6R2
6839	1,3,5,8,18,19,21,22	2,4,6,7,20,23,24,25,26	2	P83121,Q9QW07
6840	1,3,5,8,18,19,21,23	2,4,6,7,20,22,24,25,26	3	O70594,P35467,Q03191
6841	1,3,5,8,18,19,21,24	2,4,6,7,20,22,23,25,26	2	P05539,P83121
6842	1,3,5,8,18,19,21,25	2,4,6,7,20,22,23,24,26	0	
6843	1,3,5,8,18,19,21,26	2,4,6,7,20,22,23,24,25	1	P12020
6844	1,3,5,8,18,19,22,23	2,4,6,7,20,21,24,25,26	6	P09605;P25809,P22006,P25236,P35467,P83121,Q9QW07
6845	1,3,5,8,18,19,22,24	2,4,6,7,20,21,23,25,26	5	P09605;P25809,P25236,P83121,Q6P6R2,Q9QW07
6846	1,3,5,8,18,19,22,25	2,4,6,7,20,21,23,24,26	4	P01015,P25236,P83121,Q9QW07
6847	1,3,5,8,18,19,22,26	2,4,6,7,20,21,23,24,25	5	O54728,P09605;P25809,P83121,Q6P6R2,Q9QW07

6848	1,3,5,8,18,19,23,24	2,4,6,7,20,21,22,25,26	5	P09605;P25809,P25236,P35467,P83121,Q03191
6849	1,3,5,8,18,19,23,25	2,4,6,7,20,21,22,24,26	3	O70594,P01015,P35467
6850	1,3,5,8,18,19,23,26	2,4,6,7,20,21,22,24,25	9	O54728,P09605;P25809,P35467,Q03191,Q07523,Q63942,Q64194,Q6IG02,Q6P6Q2
6851	1,3,5,8,18,19,24,25	2,4,6,7,20,21,22,23,26	2	P25236,P83121
6852	1,3,5,8,18,19,24,26	2,4,6,7,20,21,22,23,25	4	P09605;P25809,P25236,P83121,Q6P6R2
6853	1,3,5,8,18,19,25,26	2,4,6,7,20,21,22,23,24	1	P32755
6854	1,3,5,8,18,20,21,22	2,4,6,7,19,23,24,25,26	4	P14480,P83121,Q6IRE4,Q9QW07
6855	1,3,5,8,18,20,21,23	2,4,6,7,19,22,24,25,26	3	P14480,Q6IG02,Q6IRE4
6856	1,3,5,8,18,20,21,24	2,4,6,7,19,22,23,25,26	3	P12020,P83121,Q6IRE4
6857	1,3,5,8,18,20,21,25	2,4,6,7,19,22,23,24,26	3	P31430,Q63532,Q6IRE4
6858	1,3,5,8,18,20,21,26	2,4,6,7,19,22,23,24,25	7	P06911,P12020,P50280,Q4FZU6,Q63532,Q6IG02,Q6IRE4
6859	1,3,5,8,18,20,22,23	2,4,6,7,19,21,24,25,26	9	P09605;P25809,P14480,P22006,P83121,P97840,Q6IG02,Q8CIZ5,Q9QW07,Q9QZK9
6860	1,3,5,8,18,20,22,24	2,4,6,7,19,21,23,25,26	8	P25236,P27213,P83121,P97840,Q6IRE4,Q6P6R2,Q9QW07,Q9QZK9
6861	1,3,5,8,18,20,22,25	2,4,6,7,19,21,23,24,26	3	P25236,P83121,Q9QW07
6862	1,3,5,8,18,20,22,26	2,4,6,7,19,21,23,24,25	9	O54728,P47967,P50280,P83121,P97840,Q6IG02,Q6P6R2,Q99041,Q9QW07

6863	1,3,5,8,18,20,23,24	2,4,6,7,19,21,22,25,26	8	P09605;P25809,P25236,P47967,P83121,P97840,Q6IG02,Q6IRE4,Q9QZK9
6864	1,3,5,8,18,20,23,25	2,4,6,7,19,21,22,24,26	2	Q6IG02,Q6IRE4
6865	1,3,5,8,18,20,23,26	2,4,6,7,19,21,22,24,25	14	O54728,P09605;P25809,P35467,P47967,P97840,Q4FZU2,Q4FZU6,Q6IFU8,Q6IFW6,Q6IG02,Q6IMF3,Q6IRE4,Q6P6Q2,Q8CIZ5
6866	1,3,5,8,18,20,24,25	2,4,6,7,19,21,22,23,26	4	P08649,P25236,P83121,Q6IRE4
6867	1,3,5,8,18,20,24,26	2,4,6,7,19,21,22,23,25	11	P06911,P09605;P25809,P12020,P47967,P50280,P83121,P97840,Q6IG02,Q6IRE4,Q8CJD3,Q99041
6868	1,3,5,8,18,20,25,26	2,4,6,7,19,21,22,23,24	2	Q6IG02,Q6IRE4
6869	1,3,5,8,18,21,22,23	2,4,6,7,19,20,24,25,26	2	P14480,Q9QW07
6870	1,3,5,8,18,21,22,24	2,4,6,7,19,20,23,25,26	2	P83121,Q9QW07
6871	1,3,5,8,18,21,22,25	2,4,6,7,19,20,23,24,26	1	Q9QW07
6872	1,3,5,8,18,21,22,26	2,4,6,7,19,20,23,24,25	1	Q9QW07
6873	1,3,5,8,18,21,23,24	2,4,6,7,19,20,22,25,26	0	
6874	1,3,5,8,18,21,23,25	2,4,6,7,19,20,22,24,26	0	
6875	1,3,5,8,18,21,23,26	2,4,6,7,19,20,22,24,25	8	P35467,Q4FZU2,Q6IFU8,Q6IFV1,Q6IFW6,Q6IG02,Q6IMF3,Q6P6Q2
6876	1,3,5,8,18,21,24,25	2,4,6,7,19,20,22,23,26	2	P31430,Q62997
6877	1,3,5,8,18,21,24,26	2,4,6,7,19,20,22,23,25	3	P06911,P12020,Q8CJD3

6878	1,3,5,8,18,21,25,26	2,4,6,7,19,20,22,23,24	1	P31430
6879	1,3,5,8,18,22,23,24	2,4,6,7,19,20,21,25,26	6	P09605;P25809,P25236,P47967,P83121,P97840,Q9QW07
6880	1,3,5,8,18,22,23,25	2,4,6,7,19,20,21,24,26	3	P01015,P25236,Q9QW07
6881	1,3,5,8,18,22,23,26	2,4,6,7,19,20,21,24,25	9	O54728,P09605;P25809,P35467,P47967,P97840,Q6IG02,Q6P6Q2,Q8CIZ5,Q9QW07
6882	1,3,5,8,18,22,24,25	2,4,6,7,19,20,21,23,26	3	P25236,P83121,Q9QW07
6883	1,3,5,8,18,22,24,26	2,4,6,7,19,20,21,23,25	9	O54728,P09605;P25809,P25236,P47967,P83121,P97840,Q6P6R2,Q99041,Q9QW07
6884	1,3,5,8,18,22,25,26	2,4,6,7,19,20,21,23,24	1	Q9QW07
6885	1,3,5,8,18,23,24,25	2,4,6,7,19,20,21,22,26	1	P25236
6886	1,3,5,8,18,23,24,26	2,4,6,7,19,20,21,22,25	8	P02091,P09605;P25809,P35467,P47967,P97840,Q6IFW6,Q6IG02,Q6P6Q2
6887	1,3,5,8,18,23,25,26	2,4,6,7,19,20,21,22,24	7	P02091,P35467,Q5I0D1,Q6IFU8,Q6IFW6,Q6IG02,Q6P6Q2
6888	1,3,5,8,18,24,25,26	2,4,6,7,19,20,21,22,23	2	B4F795,Q8CJD3
6889	1,3,5,8,19,20,21,22	2,4,6,7,18,23,24,25,26	6	P00714,P00774,P14480,P55091,P83121,Q9QW07
6890	1,3,5,8,19,20,21,23	2,4,6,7,18,22,24,25,26	2	P55091,Q6IRE4
6891	1,3,5,8,19,20,21,24	2,4,6,7,18,22,23,25,26	5	P00714,P00774,P55091,P83121,Q6IRE4
6892	1,3,5,8,19,20,21,25	2,4,6,7,18,22,23,24,26	1	Q6IRE4

6893	1,3,5,8,19,20,21,26	2,4,6,7,18,22,23,24,25	2	O54728,Q6IRE4
6894	1,3,5,8,19,20,22,23	2,4,6,7,18,21,24,25,26	11	O54728,P00774,P09605;P25809,P22006,P25031,P31430,P55091,P82995,P83121,P98089,Q9QW07
6895	1,3,5,8,19,20,22,24	2,4,6,7,18,21,23,25,26	12	P00714,P00774,P25031,P25236,P31430,P55091,P82995,P83121,P98089,Q6P6R2,Q99MH3,Q9QW07
6896	1,3,5,8,19,20,22,25	2,4,6,7,18,21,23,24,26	6	D3ZUC6,P00774,P25236,P55091,P83121,Q9QW07
6897	1,3,5,8,19,20,22,26	2,4,6,7,18,21,23,24,25	6	O54728,P83121,P98089,Q6P6R2,Q99MH3,Q9QW07
6898	1,3,5,8,19,20,23,24	2,4,6,7,18,21,22,25,26	12	P00774,P09605;P25809,P25031,P25236,P31430,P55091,P82995,P83121,P83748,P98089,Q6IRE4,Q80WL1
6899	1,3,5,8,19,20,23,25	2,4,6,7,18,21,22,24,26	1	P83748
6900	1,3,5,8,19,20,23,26	2,4,6,7,18,21,22,24,25	6	O54728,P09605;P25809,P97840,P98089,Q08849,Q6IG02
6901	1,3,5,8,19,20,24,25	2,4,6,7,18,21,22,23,26	4	P25236,P83121,P83748,Q6IRE4
6902	1,3,5,8,19,20,24,26	2,4,6,7,18,21,22,23,25	7	O54728,P09605;P25809,P83121,P97840,P98089,Q6P6R2,Q99MH3
6903	1,3,5,8,19,20,25,26	2,4,6,7,18,21,22,23,24	1	O54728
6904	1,3,5,8,19,21,22,23	2,4,6,7,18,20,24,25,26	2	P55091,Q9QW07
6905	1,3,5,8,19,21,22,24	2,4,6,7,18,20,23,25,26	4	P00714,P00774,P83121,Q9QW07
6906	1,3,5,8,19,21,22,25	2,4,6,7,18,20,23,24,26	2	P17046,Q9QW07
6907	1,3,5,8,19,21,22,26	2,4,6,7,18,20,23,24,25	2	O54728,Q9QW07

6908	1,3,5,8,19,21,23,24	2,4,6,7,18,20,22,25,26	0	
6909	1,3,5,8,19,21,23,25	2,4,6,7,18,20,22,24,26	1	O70594
6910	1,3,5,8,19,21,23,26	2,4,6,7,18,20,22,24,25	1	O54728
6911	1,3,5,8,19,21,24,25	2,4,6,7,18,20,22,23,26	0	
6912	1,3,5,8,19,21,24,26	2,4,6,7,18,20,22,23,25	1	Q8CJD3
6913	1,3,5,8,19,21,25,26	2,4,6,7,18,20,22,23,24	0	
6914	1,3,5,8,19,22,23,24	2,4,6,7,18,20,21,25,26	11	O54728,P00774,P09605;P25809,P19223,P25031,P25236,P31430,P55091,P82995,P83121,P98089
6915	1,3,5,8,19,22,23,25	2,4,6,7,18,20,21,24,26	3	P01015,P17046,Q9QW07
6916	1,3,5,8,19,22,23,26	2,4,6,7,18,20,21,24,25	4	O54728,P09605;P25809,P98089,Q9QW07
6917	1,3,5,8,19,22,24,25	2,4,6,7,18,20,21,23,26	4	P17046,P25031,P25236,P83121
6918	1,3,5,8,19,22,24,26	2,4,6,7,18,20,21,23,25	8	O54728,P09605;P25809,P25236,P83121,P98089,Q6P6R2,Q99MH3,Q9QW07
6919	1,3,5,8,19,22,25,26	2,4,6,7,18,20,21,23,24	2	O54728,Q9QW07
6920	1,3,5,8,19,23,24,25	2,4,6,7,18,20,21,22,26	3	P25031,P25236,P83748
6921	1,3,5,8,19,23,24,26	2,4,6,7,18,20,21,22,25	5	O54728,P02091,P09605;P25809,P47967,P98089
6922	1,3,5,8,19,23,25,26	2,4,6,7,18,20,21,22,24	2	O54728,P02091

6923	1,3,5,8,19,24,25,26	2,4,6,7,18,20,21,22,23	1	Q8CJD3
6924	1,3,5,8,20,21,22,23	2,4,6,7,18,19,24,25,26	4	P14480,P55091,Q6IRE4,Q9QW07
6925	1,3,5,8,20,21,22,24	2,4,6,7,18,19,23,25,26	7	P00714,P00774,P83121,Q5XFX0,Q6IRE4,Q99MH3,Q9QW07
6926	1,3,5,8,20,21,22,25	2,4,6,7,18,19,23,24,26	4	Q5XFX0,Q63532,Q6IRE4,Q9QW07
6927	1,3,5,8,20,21,22,26	2,4,6,7,18,19,23,24,25	5	O54728,Q63532,Q6IRE4,Q99MH3,Q9QW07
6928	1,3,5,8,20,21,23,24	2,4,6,7,18,19,22,25,26	3	P82995,P97840,Q6IRE4
6929	1,3,5,8,20,21,23,25	2,4,6,7,18,19,22,24,26	2	P06760,Q6IRE4
6930	1,3,5,8,20,21,23,26	2,4,6,7,18,19,22,24,25	5	O54728,P97840,Q4FZU6,Q6IG02,Q6IRE4
6931	1,3,5,8,20,21,24,25	2,4,6,7,18,19,22,23,26	2	Q5XFX0,Q6IRE4
6932	1,3,5,8,20,21,24,26	2,4,6,7,18,19,22,23,25	6	P12020,P50280,P97840,Q6IRE4,Q8CJD3,Q99MH3
6933	1,3,5,8,20,21,25,26	2,4,6,7,18,19,22,23,24	2	Q63532,Q6IRE4
6934	1,3,5,8,20,22,23,24	2,4,6,7,18,19,21,25,26	13	P00774,P25031,P25236,P47967,P55091,P82995,P83121,P83748,P97840,P98089,Q6IRE4,Q8CIZ5,Q9QW07
6935	1,3,5,8,20,22,23,25	2,4,6,7,18,19,21,24,26	2	D3ZUC6,Q9QW07
6936	1,3,5,8,20,22,23,26	2,4,6,7,18,19,21,24,25	7	O54728,P47967,P97840,P98089,Q6IG02,Q8CIZ5,Q9QW07
6937	1,3,5,8,20,22,24,25	2,4,6,7,18,19,21,23,26	8	D3ZUC6,P25031,P25236,P83121,Q5XFX0,Q6IRE4,Q99MH3,Q9QW07

6938	1,3,5,8,20,22,24,26	2,4,6,7,18,19,21,23,25	11	O54728,P47967,P83121,P97840,P98089,Q62635,Q6P6R2,Q8CIZ5,Q8CJD3,Q99MH3,Q9QW07
6939	1,3,5,8,20,22,25,26	2,4,6,7,18,19,21,23,24	5	D3ZUC6,O54728,Q63532,Q99MH3,Q9QW07
6940	1,3,5,8,20,23,24,25	2,4,6,7,18,19,21,22,26	6	P08649,P25236,P82995,P83748,Q6IRE4,Q80WL1
6941	1,3,5,8,20,23,24,26	2,4,6,7,18,19,21,22,25	9	O54728,P02091,P09605;P25809,P47967,P97840,P98089,Q6IG02,Q6IRE4,Q8CIZ5
6942	1,3,5,8,20,23,25,26	2,4,6,7,18,19,21,22,24	4	O54728,P02091,Q6IG02,Q6IRE4
6943	1,3,5,8,20,24,25,26	2,4,6,7,18,19,21,22,23	2	Q6IRE4,Q8CJD3
6944	1,3,5,8,21,22,23,24	2,4,6,7,18,19,20,25,26	2	P35280,Q9QW07
6945	1,3,5,8,21,22,23,25	2,4,6,7,18,19,20,24,26	3	P06760,P35280,Q9QW07
6946	1,3,5,8,21,22,23,26	2,4,6,7,18,19,20,24,25	2	O54728,Q9QW07
6947	1,3,5,8,21,22,24,25	2,4,6,7,18,19,20,23,26	3	P35280,Q5XFX0,Q9QW07
6948	1,3,5,8,21,22,24,26	2,4,6,7,18,19,20,23,25	4	O54728,Q8CJD3,Q99MH3,Q9QW07
6949	1,3,5,8,21,22,25,26	2,4,6,7,18,19,20,23,24	2	Q63532,Q9QW07
6950	1,3,5,8,21,23,24,25	2,4,6,7,18,19,20,22,26	3	P06760,P35280,Q63751
6951	1,3,5,8,21,23,24,26	2,4,6,7,18,19,20,22,25	4	P02091,P47967,P97840,Q9QZQ5
6952	1,3,5,8,21,23,25,26	2,4,6,7,18,19,20,22,24	0	

6953	1,3,5,8,21,24,25,26	2,4,6,7,18,19,20,22,23	1	Q8CJD3
6954	1,3,5,8,22,23,24,25	2,4,6,7,18,19,20,21,26	4	P25031,P25236,P35280,P83748
6955	1,3,5,8,22,23,24,26	2,4,6,7,18,19,20,21,25	9	O54728,P00731,P02091,P09605;P25809,P47967,P97840,P98089,Q8CIZ5,Q9QW07
6956	1,3,5,8,22,23,25,26	2,4,6,7,18,19,20,21,24	3	O54728,P02091,Q9QW07
6957	1,3,5,8,22,24,25,26	2,4,6,7,18,19,20,21,23	4	O54728,P25236,Q8CJD3,Q9QW07
6958	1,3,5,8,23,24,25,26	2,4,6,7,18,19,20,21,22	5	P01946,P02091,Q5I0D1,Q7TPB1,Q8CJD3
6959	1,3,5,18,19,20,21,22	2,4,6,7,8,23,24,25,26	6	P12020,P50280,P83121,Q02253,Q6P6R2,Q9QW07
6960	1,3,5,18,19,20,21,23	2,4,6,7,8,22,24,25,26	2	P12020,Q6IRE4
6961	1,3,5,18,19,20,21,24	2,4,6,7,8,22,23,25,26	7	P06911,P12020,P20766,P50280,P83121,Q5GRG2,Q6IRE4
6962	1,3,5,18,19,20,21,25	2,4,6,7,8,22,23,24,26	4	P20760,P20761,Q02253,Q6IRE4
6963	1,3,5,18,19,20,21,26	2,4,6,7,8,22,23,24,25	5	P06911,P12020,P50280,Q5GRG2,Q6IRE4
6964	1,3,5,18,19,20,22,23	2,4,6,7,8,21,24,25,26	8	O54728,P20761,P22006,P55091,Q6P6R2,Q9QW07,Q9QZK9,Q9WTT6
6965	1,3,5,18,19,20,22,24	2,4,6,7,8,21,23,25,26	13	P07150,P12020,P14669,P25236,P27213,P50280,P83121,Q6P6R2,Q80WL1,Q99041,Q9QZK9,Q9WTT6,Q9Z0V6
6966	1,3,5,18,19,20,22,25	2,4,6,7,8,21,23,24,26	4	P17046,P20761,P20762,P25236
6967	1,3,5,18,19,20,22,26	2,4,6,7,8,21,23,24,25	10	O54728,P07150,P12020,P50280,Q5U2V4,Q6P6R2,Q99041,Q9QW07,Q9WTT6,Q9Z0V6
6968	1,3,5,18,19,20,23,24	2,4,6,7,8,21,22,25,26	8	P09605;P25809,P12020,P19223,P25236,P83748,Q80WL1,Q9QZK9,Q9WTT6
6969	1,3,5,18,19,20,23,25	2,4,6,7,8,21,22,24,26	2	P20761,Q80WL1
6970	1,3,5,18,19,20,23,26	2,4,6,7,8,21,22,24,25	10	O54728,P09605;P25809,P12020,P35467,Q4FZU2,Q6IFU8,Q6IFW6,Q6IG02,Q6P6Q2,Q80WL1
6971	1,3,5,18,19,20,24,25	2,4,6,7,8,21,22,23,26	5	P20761,P20762,P22282,P25236,Q80WL1
6972	1,3,5,18,19,20,24,26	2,4,6,7,8,21,22,23,25	10	O54728,P06911,P09605;P25809,P12020,P50280,Q5GRG2,Q6P6R2,Q80WL1,Q99041,Q9WTT6

6973	1,3,5,18,1<br>9,20,25,26	2,4,6,7,8,2<br>1,22,23,24	1	Q80WL1
6974	1,3,5,18,1<br>9,21,22,23	2,4,6,7,8,2<br>0,24,25,26	1	Q9QW07
6975	1,3,5,18,1<br>9,21,22,24	2,4,6,7,8,2<br>0,23,25,26	6	P00714,P12020,P20762,P83121,Q6P6R2,Q9QW07
6976	1,3,5,18,1<br>9,21,22,25	2,4,6,7,8,2<br>0,23,24,26	6	P17046,P20760,P20761,P20762,Q63617,Q9QW07
6977	1,3,5,18,1<br>9,21,22,26	2,4,6,7,8,2<br>0,23,24,25	5	O54728,P12020,Q63617,Q6P6R2,Q9QW07
6978	1,3,5,18,1<br>9,21,23,24	2,4,6,7,8,2<br>0,22,25,26	1	P12020
6979	1,3,5,18,1<br>9,21,23,25	2,4,6,7,8,2<br>0,22,24,26	2	P20760,P20761
6980	1,3,5,18,1<br>9,21,23,26	2,4,6,7,8,2<br>0,22,24,25	10	P12020,P35467,Q03191,Q07523,Q4FZU2,Q63617,Q6IFU8,Q6IG02,Q6IMF3,Q6P6Q2
6981	1,3,5,18,1<br>9,21,24,25	2,4,6,7,8,2<br>0,22,23,26	2	P20762,Q63617
6982	1,3,5,18,1<br>9,21,24,26	2,4,6,7,8,2<br>0,22,23,25	6	P06911,P12020,P50280,Q5GRG2,Q63617,Q8CJD3
6983	1,3,5,18,1<br>9,21,25,26	2,4,6,7,8,2<br>0,22,23,24	1	Q63617
6984	1,3,5,18,1<br>9,22,23,24	2,4,6,7,8,2<br>0,21,25,26	8	P09605;P25809,P19223,P25236,P62804,P83121,Q00715,Q6P6R2,Q9WTT6
6985	1,3,5,18,1<br>9,22,23,25	2,4,6,7,8,2<br>0,21,24,26	9	O55004,P01015,P02761,P17046,P20761,P20762,P25236,P62804,Q6AYR8
6986	1,3,5,18,1<br>9,22,23,26	2,4,6,7,8,2<br>0,21,24,25	7	O54728,P09605;P25809,P35467,Q6IG02,Q6P6Q2,Q6P6R2,Q9WTT6
6987	1,3,5,18,1<br>9,22,24,25	2,4,6,7,8,2<br>0,21,23,26	7	P17046,P20761,P20762,P25236,P62804,P83121,Q00715
6988	1,3,5,18,1<br>9,22,24,26	2,4,6,7,8,2<br>0,21,23,25	9	O54728,P09605;P25809,P12020,P25236,P50280,P83121,Q6P6R2,Q99041,Q9WTT6
6989	1,3,5,18,1<br>9,22,25,26	2,4,6,7,8,2<br>0,21,23,24	2	O54728,Q63617
6990	1,3,5,18,1<br>9,23,24,25	2,4,6,7,8,2<br>0,21,22,26	10	O55004,P01015,P20761,P20762,P25236,P30836,P62804,P83748,Q00715,Q80WL1
6991	1,3,5,18,1<br>9,23,24,26	2,4,6,7,8,2<br>0,21,22,25	10	O54728,P02091,P09605;P25809,P12020,P35467,Q03191,Q07523,Q6IG02,Q6P6Q2,Q80WL1
6992	1,3,5,18,1<br>9,23,25,26	2,4,6,7,8,2<br>0,21,22,24	6	P02091,P35467,Q07523,Q6IFU8,Q6IG02,Q6P6Q2
6993	1,3,5,18,1<br>9,24,25,26	2,4,6,7,8,2<br>0,21,22,23	5	P20762,Q63617,Q7TPB1,Q80WL1,Q8CJD3
6994	1,3,5,18,2<br>0,21,22,23	2,4,6,7,8,1<br>9,24,25,26	3	P14480,Q6IRE4,Q9QW07
6995	1,3,5,18,2<br>0,21,22,24	2,4,6,7,8,1<br>9,23,25,26	7	P00714,P12020,P50280,P83121,Q6IRE4,Q99041,Q9QW07

6996	1,3,5,18,20,21,22,25	2,4,6,7,8,19,23,24,26	3	Q63532,Q6IRE4,Q9QW07
6997	1,3,5,18,20,21,22,26	2,4,6,7,8,19,23,24,25	9	O54728,P02631,P12020,P50280,Q63532,Q6IRE4,Q6P6R2,Q99041,Q9QW07
6998	1,3,5,18,20,21,23,24	2,4,6,7,8,19,22,25,26	4	P12020,P20766,Q6IRE4,Q9QZK9
6999	1,3,5,18,20,21,23,25	2,4,6,7,8,19,22,24,26	1	Q6IRE4
7000	1,3,5,18,20,21,23,26	2,4,6,7,8,19,22,24,25	11	P06911,P12020,Q4FZU2,Q4FZU6,Q6IFU8,Q6IFW6,Q6IG02,Q6IMF3,Q6IRE4,Q6P6Q2,Q9QZQ5
7001	1,3,5,18,20,21,24,25	2,4,6,7,8,19,22,23,26	1	Q6IRE4
7002	1,3,5,18,20,21,24,26	2,4,6,7,8,19,22,23,25	7	P06911,P12020,P50280,Q5GRG2,Q6IRE4,Q8CJD3,Q99041
7003	1,3,5,18,20,21,25,26	2,4,6,7,8,19,22,23,24	5	P02631,P31430,P50280,Q63532,Q6IRE4
7004	1,3,5,18,20,22,23,24	2,4,6,7,8,19,21,25,26	10	P19223,P25236,P47967,P82995,P83121,P97840,Q80WL1,Q8CIZ5,Q9QZK9,Q9WTT6
7005	1,3,5,18,20,22,23,25	2,4,6,7,8,19,21,24,26	1	P20761
7006	1,3,5,18,20,22,23,26	2,4,6,7,8,19,21,24,25	12	O54728,P47967,P97840,Q4FZU2,Q6IFU8,Q6IFW6,Q6IG02,Q6IMF3,Q6P6Q2,Q6P6R2,Q8CIZ5,Q9QW07
7007	1,3,5,18,20,22,24,25	2,4,6,7,8,19,21,23,26	5	P0C0A9,P20762,P25236,Q80WL1,Q99041
7008	1,3,5,18,20,22,24,26	2,4,6,7,8,19,21,23,25	13	O54728,P02803,P07150,P12020,P25236,P47967,P50280,P97840,Q6P6R2,Q8CIZ5,Q8CJD3,Q99041,Q9WTT6
7009	1,3,5,18,20,22,25,26	2,4,6,7,8,19,21,23,24	4	O54728,P02631,Q99041,Q9QW07
7010	1,3,5,18,20,23,24,25	2,4,6,7,8,19,21,22,26	4	P25236,P83748,Q6IRE4,Q80WL1
7011	1,3,5,18,20,23,24,26	2,4,6,7,8,19,21,22,25	17	D4A1J4,P02091,P06911,P09605;P25809,P12020,P47967,P97840,Q4FZU2,Q6IFU8,Q6IFW6,Q6IG02,Q6IMF3,Q6IRE4,Q6P6Q2,Q80WL1,Q8CIZ5,Q9QZQ5
7012	1,3,5,18,20,23,25,26	2,4,6,7,8,19,21,22,24	9	P02091,Q4FZU2,Q6IFU8,Q6IFW6,Q6IG02,Q6IMF3,Q6IRE4,Q6P6Q2,Q80WL1
7013	1,3,5,18,20,24,25,26	2,4,6,7,8,19,21,22,23	6	P22282,P50280,Q6IRE4,Q80WL1,Q8CJD3,Q99041
7014	1,3,5,18,21,22,23,24	2,4,6,7,8,19,20,25,26	2	P35280,Q9QW07
7015	1,3,5,18,21,22,23,25	2,4,6,7,8,19,20,24,26	3	P01015,P35280,Q9QW07
7016	1,3,5,18,21,22,23,26	2,4,6,7,8,19,20,24,25	9	O54728,P12020,Q4FZU2,Q6IFU8,Q6IG02,Q6IMF3,Q6P6Q2,Q9QW07,Q9QZQ5
7017	1,3,5,18,21,22,24,25	2,4,6,7,8,19,20,23,26	3	P20762,P35280,Q9QW07
7018	1,3,5,18,21,22,24,26	2,4,6,7,8,19,20,23,25	6	P12020,P50280,Q63617,Q8CJD3,Q99041,Q9QW07

7019	1,3,5,18,2<br>1,22,25,26	2,4,6,7,8,1<br>9,20,23,24	4	P02631,Q63532,Q63617,Q9QW07
7020	1,3,5,18,2<br>1,23,24,25	2,4,6,7,8,1<br>9,20,22,26	1	P35280
7021	1,3,5,18,2<br>1,23,24,26	2,4,6,7,8,1<br>9,20,22,25	10	P02091,P06911,P12020,P47967,Q4FZU2,Q6IFU8,Q6IG02,Q6IMF3,Q6P6Q2,Q9QZQ5
7022	1,3,5,18,2<br>1,23,25,26	2,4,6,7,8,1<br>9,20,22,24	7	Q4FZU2,Q63617,Q6IFU8,Q6IG02,Q6IMF3,Q6P6Q2,Q9QZQ5
7023	1,3,5,18,2<br>1,24,25,26	2,4,6,7,8,1<br>9,20,22,23	3	P31430,Q63617,Q8CJD3
7024	1,3,5,18,2<br>2,23,24,25	2,4,6,7,8,1<br>9,20,21,26	7	O55004,P01015,P20762,P25236,P35280,P62804,Q00715
7025	1,3,5,18,2<br>2,23,24,26	2,4,6,7,8,1<br>9,20,21,25	10	O54728,P00731,P02091,P09605;P25809,P47967,P97840,Q6IG02,Q6P6Q2,Q8CIZ5,Q9QZQ5
7026	1,3,5,18,2<br>2,23,25,26	2,4,6,7,8,1<br>9,20,21,24	5	O54728,P02091,Q6IG02,Q6P6Q2,Q9QZQ5
7027	1,3,5,18,2<br>2,24,25,26	2,4,6,7,8,1<br>9,20,21,23	3	P25236,Q8CJD3,Q99041
7028	1,3,5,18,2<br>3,24,25,26	2,4,6,7,8,1<br>9,20,21,22	8	P01946,P02091,Q6IFU8,Q6IG02,Q6P6Q2,Q7TPB1,Q80WL1,Q9QZQ5
7029	1,3,5,19,2<br>0,21,22,23	2,4,6,7,8,1<br>8,24,25,26	7	O54728,P00714,P00774,P55091,P82995,P98089,Q9QW07
7030	1,3,5,19,2<br>0,21,22,24	2,4,6,7,8,1<br>8,23,25,26	9	P00714,P00774,P12020,P55091,P82995,P83121,P98089,Q99MH3,Q9QW07
7031	1,3,5,19,2<br>0,21,22,25	2,4,6,7,8,1<br>8,23,24,26	3	P17046,P55091,Q9QW07
7032	1,3,5,19,2<br>0,21,22,26	2,4,6,7,8,1<br>8,23,24,25	8	O54728,P00714,P12020,P50280,P98089,Q6P6R2,Q99MH3,Q9QW07
7033	1,3,5,19,2<br>0,21,23,24	2,4,6,7,8,1<br>8,22,25,26	7	P00714,P20766,P55091,P82995,P98089,Q6IRE4,Q80WL1
7034	1,3,5,19,2<br>0,21,23,25	2,4,6,7,8,1<br>8,22,24,26	1	Q6IRE4
7035	1,3,5,19,2<br>0,21,23,26	2,4,6,7,8,1<br>8,22,24,25	3	O54728,P98089,Q6IRE4
7036	1,3,5,19,2<br>0,21,24,25	2,4,6,7,8,1<br>8,22,23,26	3	P20762,Q6IRE4,Q80WL1
7037	1,3,5,19,2<br>0,21,24,26	2,4,6,7,8,1<br>8,22,23,25	9	P00714,P06911,P12020,P50280,P98089,Q5GRG2,Q6IRE4,Q8CJD3,Q99MH3
7038	1,3,5,19,2<br>0,21,25,26	2,4,6,7,8,1<br>8,22,23,24	1	Q6IRE4
7039	1,3,5,19,2<br>0,22,23,24	2,4,6,7,8,1<br>8,21,25,26	16	O54728,P00714,P00774,P11598,P14669,P19223,P25031,P25236,P31430,P55091,P82995,P83121,P83748,P98089,Q80WL1,Q9WTT6
7040	1,3,5,19,2<br>0,22,23,25	2,4,6,7,8,1<br>8,21,24,26	9	O54728,P00774,P11598,P17046,P20761,P55091,P82995,P83748,Q80WL1
7041	1,3,5,19,2<br>0,22,23,26	2,4,6,7,8,1<br>8,21,24,25	6	O54728,P31430,P55091,P98089,Q6P6R2,Q9WTT6

7042	1,3,5,19,2 0,22,24,25	2,4,6,7,8,1 8,21,23,26	13	P00774,P0C0A9,P11598,P14669,P17046,P20762,P25031,P25236,P55091,P82995,P83748,Q64057,Q80WL1
7043	1,3,5,19,2 0,22,24,26	2,4,6,7,8,1 8,21,23,25	17	O54728,P00714,P00774,P02803,P07150,P11598,P12020,P14669,P31430,P50280,P98089,Q64057,Q6P6R2,Q80WL1,Q99041,Q99MH3,Q9WTT6
7044	1,3,5,19,2 0,22,25,26	2,4,6,7,8,1 8,21,23,24	2	O54728,P17046
7045	1,3,5,19,2 0,23,24,25	2,4,6,7,8,1 8,21,22,26	8	P0C0A9,P11598,P17046,P25236,P82995,P83748,Q80WL1,Q9R0T3
7046	1,3,5,19,2 0,23,24,26	2,4,6,7,8,1 8,21,22,25	9	D4A1J4,O54728,P02091,P09605;P25809,P11598,P31430,P47967,P98089,Q80WL1
7047	1,3,5,19,2 0,23,25,26	2,4,6,7,8,1 8,21,22,24	3	O54728,P02091,Q80WL1
7048	1,3,5,19,2 0,24,25,26	2,4,6,7,8,1 8,21,22,23	5	O54728,P0C0A9,P11598,Q80WL1,Q8CJD3
7049	1,3,5,19,2 1,22,23,24	2,4,6,7,8,1 8,20,25,26	6	P00714,P35280,P55091,P82995,P98089,Q9QW07
7050	1,3,5,19,2 1,22,23,25	2,4,6,7,8,1 8,20,24,26	3	P17046,P35280,Q9QW07
7051	1,3,5,19,2 1,22,23,26	2,4,6,7,8,1 8,20,24,25	4	O54728,P98089,Q9QW07,Q9QZQ5
7052	1,3,5,19,2 1,22,24,25	2,4,6,7,8,1 8,20,23,26	4	P17046,P20762,P35280,Q9QW07
7053	1,3,5,19,2 1,22,24,26	2,4,6,7,8,1 8,20,23,25	10	O54728,P00714,P07171,P12020,P98089,Q63617,Q6P6R2,Q8CJD3,Q99MH3,Q9QW07
7054	1,3,5,19,2 1,22,25,26	2,4,6,7,8,1 8,20,23,24	4	O54728,P17046,Q63617,Q9QW07
7055	1,3,5,19,2 1,23,24,25	2,4,6,7,8,1 8,20,22,26	4	P11598,P17046,P20762,P35280
7056	1,3,5,19,2 1,23,24,26	2,4,6,7,8,1 8,20,22,25	3	O54728,P02091,Q9QZQ5
7057	1,3,5,19,2 1,23,25,26	2,4,6,7,8,1 8,20,22,24	3	P02091,Q63617,Q9QZQ5
7058	1,3,5,19,2 1,24,25,26	2,4,6,7,8,1 8,20,22,23	2	Q63617,Q8CJD3
7059	1,3,5,19,2 2,23,24,25	2,4,6,7,8,1 8,20,21,26	15	O55004,P01041,P11598,P17046,P20762,P25031,P25236,P35280,P62804,P82471,P82995,P83748,Q00715,Q6AYR8,Q80WL1
7060	1,3,5,19,2 2,23,24,26	2,4,6,7,8,1 8,20,21,25	13	O54728,P00731,P02091,P09605;P25809,P11598,P20646,P31430,P47967,P82471,P98089,Q6P6R2,Q9QZQ5,Q9WTT6
7061	1,3,5,19,2 2,23,25,26	2,4,6,7,8,1 8,20,21,24	5	O54728,P02091,P17046,P82471,Q6AYR8
7062	1,3,5,19,2 2,24,25,26	2,4,6,7,8,1 8,20,21,23	11	O54728,P11598,P17046,P20762,P24368,P25236,Q5XI22,Q6AYR8,Q6AYR9,Q6TMA8,Q8CJD3
7063	1,3,5,19,2 3,24,25,26	2,4,6,7,8,1 8,20,21,22	8	O54728,P01946,P02091,P11598,P42854,Q7TPB1,Q80WL1,Q9QZQ5
7064	1,3,5,20,2 1,22,23,24	2,4,6,7,8,1 8,19,25,26	8	P00714,P35280,P55091,P82995,P98089,Q62635,Q6IRE4,Q9QW07

7065	1,3,5,20,21,22,23,25	2,4,6,7,8,18,19,24,26	3	P35280,Q6IRE4,Q9QW07
7066	1,3,5,20,21,22,23,26	2,4,6,7,8,18,19,24,25	6	O54728,P02631,P98089,Q6IRE4,Q9QW07,Q9QZQ5
7067	1,3,5,20,21,22,24,25	2,4,6,7,8,18,19,23,26	6	P02631,P35280,Q5XFX0,Q6IRE4,Q99MH3,Q9QW07
7068	1,3,5,20,21,22,24,26	2,4,6,7,8,18,19,23,25	14	O54728,P00714,P02631,P02803,P12020,P50280,P98089,Q5BJY9,Q62635,Q6IRE4,Q8CJD3,Q99041,Q99MH3,Q9QW07
7069	1,3,5,20,21,22,25,26	2,4,6,7,8,18,19,23,24	7	O54728,P02631,Q5BJY9,Q63532,Q6IRE4,Q99MH3,Q9QW07
7070	1,3,5,20,21,23,24,25	2,4,6,7,8,18,19,22,26	4	P11598,P35280,Q6IRE4,Q80WL1
7071	1,3,5,20,21,23,24,26	2,4,6,7,8,18,19,22,25	8	P02091,P12020,P47967,P97840,P98089,Q6IRE4,Q8CJD3,Q9QZQ5
7072	1,3,5,20,21,23,25,26	2,4,6,7,8,18,19,22,24	6	P02091,P02631,Q63532,Q6IG02,Q6IRE4,Q9QZQ5
7073	1,3,5,20,21,24,25,26	2,4,6,7,8,18,19,22,23	5	P02631,P50280,Q6IRE4,Q8CJD3,Q99MH3
7074	1,3,5,20,22,23,24,25	2,4,6,7,8,18,19,21,26	9	P0C0A9,P11598,P25031,P25236,P35280,P82995,P83748,Q00715,Q80WL1
7075	1,3,5,20,22,23,24,26	2,4,6,7,8,18,19,21,25	14	O54728,P00731,P02091,P02803,P0C0A9,P11598,P47967,P97840,P98089,Q62635,Q64057,Q80WL1,Q8CIZ5,Q9QZQ5
7076	1,3,5,20,22,23,25,26	2,4,6,7,8,18,19,21,24	5	O54728,P02091,P02631,P0C0A9,Q9QZQ5
7077	1,3,5,20,22,24,25,26	2,4,6,7,8,18,19,21,23	13	O54728,P00731,P02631,P02803,P05964,P0C0A9,P11598,Q5XI22,Q64057,Q80WL1,Q8CJD3,Q99041,Q99MH3
7078	1,3,5,20,23,24,25,26	2,4,6,7,8,18,19,21,22	11	D4A1J4,P01946,P02091,P07647,P0C0A9,P11598,Q6IRE4,Q7TPB1,Q80WL1,Q8CJD3,Q9QZQ5
7079	1,3,5,21,22,23,24,25	2,4,6,7,8,18,19,20,26	6	P11598,P17046,P35280,P62804,Q9QW07,Q9QZQ5
7080	1,3,5,21,22,23,24,26	2,4,6,7,8,18,19,20,25	8	O54728,P00731,P02091,P35280,P47967,P98089,Q9QW07,Q9QZQ5
7081	1,3,5,21,22,23,25,26	2,4,6,7,8,18,19,20,24	6	O54728,P02091,P02631,P35280,Q9QW07,Q9QZQ5
7082	1,3,5,21,22,24,25,26	2,4,6,7,8,18,19,20,23	8	P02631,P35280,Q498D9,Q5BJY9,Q5XI22,Q63617,Q8CJD3,Q9QW07
7083	1,3,5,21,23,24,25,26	2,4,6,7,8,18,19,20,22	6	P01946,P02091,P35280,Q7TPB1,Q8CJD3,Q9QZQ5
7084	1,3,5,22,23,24,25,26	2,4,6,7,8,18,19,20,21	15	O54728,P00731,P01946,P02091,P0C0A9,P11598,P35280,P42854,P62804,P82471,Q6AYR8,Q6AYR9,Q7TPB1,Q8CJD3,Q9QZQ5
7085	1,3,6,7,8,18,19,20	2,4,5,21,22,23,24,25,26	3	P00731,P16086,P35280
7086	1,3,6,7,8,18,19,21	2,4,5,20,22,23,24,25,26	6	D4A1J4,P00731,P08723,P16086,P35281,P53792

7087	1,3,6,7,8,18,19,22	2,4,5,20,21,23,24,25,26	2	D4A1J4,P16086
7088	1,3,6,7,8,18,19,23	2,4,5,20,21,22,24,25,26	7	P15399,P16086,P35467,P51907,P97580,Q30KJ2,Q5XI22
7089	1,3,6,7,8,18,19,24	2,4,5,20,21,22,23,25,26	0	
7090	1,3,6,7,8,18,19,25	2,4,5,20,21,22,23,24,26	7	P00731,P10715,P12020,P16086,P19629,P31044,P35280
7091	1,3,6,7,8,18,19,26	2,4,5,20,21,22,23,24,25	2	P02770,P35280
7092	1,3,6,7,8,18,20,21	2,4,5,19,22,23,24,25,26	4	P00731,P08723,P13432,Q6AYL6
7093	1,3,6,7,8,18,20,22	2,4,5,19,21,23,24,25,26	0	
7094	1,3,6,7,8,18,20,23	2,4,5,19,21,22,24,25,26	2	P16086,Q5XI22
7095	1,3,6,7,8,18,20,24	2,4,5,19,21,22,23,25,26	1	P10715
7096	1,3,6,7,8,18,20,25	2,4,5,19,21,22,23,24,26	3	P10715,P19629,P35280
7097	1,3,6,7,8,18,20,26	2,4,5,19,21,22,23,24,25	2	P10715,P35280
7098	1,3,6,7,8,18,21,22	2,4,5,19,20,23,24,25,26	4	D4A1J4,P08723,P13432,P19218
7099	1,3,6,7,8,18,21,23	2,4,5,19,20,22,24,25,26	5	P08723,P16086,P51907,Q5XI22,Q6AYL6
7100	1,3,6,7,8,18,21,24	2,4,5,19,20,22,23,25,26	2	P13432,Q62997
7101	1,3,6,7,8,18,21,25	2,4,5,19,20,22,23,24,26	6	P00731,P10715,P19629,P31430,Q62997,Q9WTT6

7102	1,3,6,7,8,18,21,26	2,4,5,19,20,22,23,24,25	2	P02770,Q62997
7103	1,3,6,7,8,18,22,23	2,4,5,19,20,21,24,25,26	2	P16086,P51907
7104	1,3,6,7,8,18,22,24	2,4,5,19,20,21,23,25,26	0	
7105	1,3,6,7,8,18,22,25	2,4,5,19,20,21,23,24,26	5	P06911,P10715,P12020,P19629,Q5GRG2
7106	1,3,6,7,8,18,22,26	2,4,5,19,20,21,23,24,25	1	Q8VD89
7107	1,3,6,7,8,18,23,24	2,4,5,19,20,21,22,25,26	1	P51907
7108	1,3,6,7,8,18,23,25	2,4,5,19,20,21,22,24,26	7	P06911,P10715,P12020,P19629,P51907,Q5GRG2,Q63751
7109	1,3,6,7,8,18,23,26	2,4,5,19,20,21,22,24,25	7	P01946,P02091,P35467,P51907,Q6IG02,Q6P6Q2,Q8VD89
7110	1,3,6,7,8,18,24,25	2,4,5,19,20,21,22,23,26	3	P10715,P19629,Q62997
7111	1,3,6,7,8,18,24,26	2,4,5,19,20,21,22,23,25	3	P02091,P02770,Q8VD89
7112	1,3,6,7,8,18,25,26	2,4,5,19,20,21,22,23,24	4	P02091,P10715,P19629,P35280
7113	1,3,6,7,8,19,20,21	2,4,5,18,22,23,24,25,26	4	P00731,P16086,P35281,P53792
7114	1,3,6,7,8,19,20,22	2,4,5,18,21,23,24,25,26	5	P00774,P16086,P31430,P55091,Q99MH3
7115	1,3,6,7,8,19,20,23	2,4,5,18,21,22,24,25,26	6	P15399,P16086,P31430,P55091,P97580,Q30KJ2
7116	1,3,6,7,8,19,20,24	2,4,5,18,21,22,23,25,26	1	Q99MH3

7117	1,3,6,7,8,19,20,25	2,4,5,18,21,22,23,24,26	4	P10715,P12020,P19629,P35280
7118	1,3,6,7,8,19,20,26	2,4,5,18,21,22,23,24,25	2	P35280,Q99MH3
7119	1,3,6,7,8,19,21,22	2,4,5,18,20,23,24,25,26	7	D4A1J4,P02782,P08723,P13432,P16086,P19218,P35281
7120	1,3,6,7,8,19,21,23	2,4,5,18,20,22,24,25,26	8	O70594,P15399,P16086,P35281,P51907,P97580,Q30KJ2,Q99041
7121	1,3,6,7,8,19,21,24	2,4,5,18,20,22,23,25,26	4	P13432,P16086,P35281,P53792
7122	1,3,6,7,8,19,21,25	2,4,5,18,20,22,23,24,26	8	P12020,P16086,P19629,P35281,P47967,P53792,P97840,Q9QZK9
7123	1,3,6,7,8,19,21,26	2,4,5,18,20,22,23,24,25	3	P16086,P35281,P53792
7124	1,3,6,7,8,19,22,23	2,4,5,18,20,21,24,25,26	7	P15399,P16086,P31430,P51907,P55091,P97580,Q30KJ2
7125	1,3,6,7,8,19,22,24	2,4,5,18,20,21,23,25,26	1	P31430
7126	1,3,6,7,8,19,22,25	2,4,5,18,20,21,23,24,26	6	P06911,P10715,P12020,P16086,P19629,Q5GRG2
7127	1,3,6,7,8,19,22,26	2,4,5,18,20,21,23,24,25	1	O54728
7128	1,3,6,7,8,19,23,24	2,4,5,18,20,21,22,25,26	6	P15399,P16086,P31430,P51907,P97580,Q30KJ2
7129	1,3,6,7,8,19,23,25	2,4,5,18,20,21,22,24,26	11	O70594,P06911,P12020,P15399,P16086,P19629,P51907,P97580,Q30KJ2,Q5GRG2,Q99041
7130	1,3,6,7,8,19,23,26	2,4,5,18,20,21,22,24,25	7	P01946,P02091,P15399,P16086,P51907,P97580,Q30KJ2
7131	1,3,6,7,8,19,24,25	2,4,5,18,20,21,22,23,26	3	P10715,P12020,P19629

7132	1,3,6,7,8,19,24,26	2,4,5,18,20,21,22,23,25	1	P02091
7133	1,3,6,7,8,19,25,26	2,4,5,18,20,21,22,23,24	6	P02091,P10715,P12020,P19629,P35280,Q9QZK9
7134	1,3,6,7,8,20,21,22	2,4,5,18,19,23,24,25,26	6	P08723,P13432,Q03191,Q5XFX0,Q7TPB1,Q99MH3
7135	1,3,6,7,8,20,21,23	2,4,5,18,19,22,24,25,26	1	P16086
7136	1,3,6,7,8,20,21,24	2,4,5,18,19,22,23,25,26	3	P13432,Q5XFX0,Q99MH3
7137	1,3,6,7,8,20,21,25	2,4,5,18,19,22,23,24,26	4	P10715,P19629,Q03191,Q9WTT6
7138	1,3,6,7,8,20,21,26	2,4,5,18,19,22,23,24,25	1	Q99MH3
7139	1,3,6,7,8,20,22,23	2,4,5,18,19,21,24,25,26	2	P16086,P55091
7140	1,3,6,7,8,20,22,24	2,4,5,18,19,21,23,25,26	3	Q03191,Q5XFX0,Q99MH3
7141	1,3,6,7,8,20,22,25	2,4,5,18,19,21,23,24,26	7	D3ZUC6,P06911,P10715,P12020,P19629,Q03191,Q5GRG2
7142	1,3,6,7,8,20,22,26	2,4,5,18,19,21,23,24,25	1	Q99MH3
7143	1,3,6,7,8,20,23,24	2,4,5,18,19,21,22,25,26	0	
7144	1,3,6,7,8,20,23,25	2,4,5,18,19,21,22,24,26	5	P06911,P10715,P12020,P19629,Q5GRG2
7145	1,3,6,7,8,20,23,26	2,4,5,18,19,21,22,24,25	3	P01946,P02091,Q6IG02
7146	1,3,6,7,8,20,24,25	2,4,5,18,19,21,22,23,26	3	P10715,P12020,P19629

7147	1,3,6,7,8,20,24,26	2,4,5,18,19,21,22,23,25	2	P02091,Q99MH3
7148	1,3,6,7,8,20,25,26	2,4,5,18,19,21,22,23,24	4	P02091,P10715,P19629,P35280
7149	1,3,6,7,8,21,22,23	2,4,5,18,19,20,24,25,26	3	P08723,P13432,P16086
7150	1,3,6,7,8,21,22,24	2,4,5,18,19,20,23,25,26	4	P13432,P14408,Q5XFX0,Q99MH3
7151	1,3,6,7,8,21,22,25	2,4,5,18,19,20,23,24,26	9	D4A1J4,P06911,P10715,P12020,P13432,P19218,P19629,Q5GRG2,Q9QZK9
7152	1,3,6,7,8,21,22,26	2,4,5,18,19,20,23,24,25	2	P13432,Q99MH3
7153	1,3,6,7,8,21,23,24	2,4,5,18,19,20,22,25,26	3	P13432,Q62997,Q63751
7154	1,3,6,7,8,21,23,25	2,4,5,18,19,20,22,24,26	12	P06911,P12020,P16086,P19629,Q5GRG2,Q62997,Q63751,Q6P6R2,Q6P9T8,Q99041,Q9QZK9,Q9WTT6
7155	1,3,6,7,8,21,23,26	2,4,5,18,19,20,22,24,25	2	P01946,P02091
7156	1,3,6,7,8,21,24,25	2,4,5,18,19,20,22,23,26	6	P10715,P13432,P19629,P22006,Q62997,Q9WTT6
7157	1,3,6,7,8,21,24,26	2,4,5,18,19,20,22,23,25	4	P02091,P13432,Q62997,Q99MH3
7158	1,3,6,7,8,21,25,26	2,4,5,18,19,20,22,23,24	7	P02091,P10715,P19629,Q62997,Q6P9T8,Q9QZK9,Q9WTT6
7159	1,3,6,7,8,22,23,24	2,4,5,18,19,20,21,25,26	1	P51907
7160	1,3,6,7,8,22,23,25	2,4,5,18,19,20,21,24,26	7	P06911,P10715,P12020,P19629,P51907,Q5GRG2,Q63751
7161	1,3,6,7,8,22,23,26	2,4,5,18,19,20,21,24,25	4	O54728,P01946,P02091,P51907

7162	1,3,6,7,8,22,24,25	2,4,5,18,19,20,21,23,26	5	P06911,P10715,P12020,P19629,Q5GRG2
7163	1,3,6,7,8,22,24,26	2,4,5,18,19,20,21,23,25	2	P02091,Q99MH3
7164	1,3,6,7,8,22,25,26	2,4,5,18,19,20,21,23,24	7	P02091,P06911,P10715,P12020,P19629,Q5GRG2,Q9QZK9
7165	1,3,6,7,8,23,24,25	2,4,5,18,19,20,21,22,26	7	P06911,P10715,P12020,P19629,P51907,Q5GRG2,Q63751
7166	1,3,6,7,8,23,24,26	2,4,5,18,19,20,21,22,25	3	P01946,P02091,P51907
7167	1,3,6,7,8,23,25,26	2,4,5,18,19,20,21,22,24	10	P01946,P02091,P06911,P10715,P12020,P19629,P51907,Q5GRG2,Q6P9T8,Q9QZK9
7168	1,3,6,7,8,24,25,26	2,4,5,18,19,20,21,22,23	5	P01946,P02091,P10715,P19629,Q9QZK9
7169	1,3,6,7,18,19,20,21	2,4,5,8,22,23,24,25,26	2	P00731,P16086
7170	1,3,6,7,18,19,20,22	2,4,5,8,21,23,24,25,26	4	P06760,P16086,Q5U2V4,Q6AYS4
7171	1,3,6,7,18,19,20,23	2,4,5,8,21,22,24,25,26	5	P15399,P16086,P97580,Q6AYS4,Q80WL1
7172	1,3,6,7,18,19,20,24	2,4,5,8,21,22,23,25,26	3	P06760,Q6AYS4,Q80WL1
7173	1,3,6,7,18,19,20,25	2,4,5,8,21,22,23,24,26	2	P35280,Q80WL1
7174	1,3,6,7,18,19,20,26	2,4,5,8,21,22,23,24,25	4	P06760,P35280,Q5U2V4,Q6AYS4
7175	1,3,6,7,18,19,21,22	2,4,5,8,20,23,24,25,26	4	D4A1J4,P16086,P19218,P97840
7176	1,3,6,7,18,19,21,23	2,4,5,8,20,22,24,25,26	4	P14668,P15399,P16086,P97580

7177	1,3,6,7,18,19,21,24	2,4,5,8,20,22,23,25,26	2	P13432,Q62997
7178	1,3,6,7,18,19,21,25	2,4,5,8,20,22,23,24,26	3	P00731,P47967,P97840
7179	1,3,6,7,18,19,21,26	2,4,5,8,20,22,23,24,25	0	
7180	1,3,6,7,18,19,22,23	2,4,5,8,20,21,24,25,26	3	P15399,P16086,P97580
7181	1,3,6,7,18,19,22,24	2,4,5,8,20,21,23,25,26	2	P06760,Q6AYS4
7182	1,3,6,7,18,19,22,25	2,4,5,8,20,21,23,24,26	2	P17046,P97840
7183	1,3,6,7,18,19,22,26	2,4,5,8,20,21,23,24,25	2	P06760,Q5U2V4
7184	1,3,6,7,18,19,23,24	2,4,5,8,20,21,22,25,26	5	P15399,P16086,P97580,Q6AYS4,Q80WL1
7185	1,3,6,7,18,19,23,25	2,4,5,8,20,21,22,24,26	4	P15399,P16086,P97580,Q30KJ2
7186	1,3,6,7,18,19,23,26	2,4,5,8,20,21,22,24,25	9	P01946,P02091,P15399,P16086,P35467,P97580,Q30KJ2,Q6IG02,Q6P6Q2
7187	1,3,6,7,18,19,24,25	2,4,5,8,20,21,22,23,26	1	Q80WL1
7188	1,3,6,7,18,19,24,26	2,4,5,8,20,21,22,23,25	3	P02091,P06760,Q6AYS4
7189	1,3,6,7,18,19,25,26	2,4,5,8,20,21,22,23,24	2	P02091,P35280
7190	1,3,6,7,18,20,21,22	2,4,5,8,19,23,24,25,26	1	P13432
7191	1,3,6,7,18,20,21,23	2,4,5,8,19,22,24,25,26	1	P16086

7192	1,3,6,7,18,20,21,24	2,4,5,8,19,22,23,25,26	1	P13432
7193	1,3,6,7,18,20,21,25	2,4,5,8,19,22,23,24,26	0	
7194	1,3,6,7,18,20,21,26	2,4,5,8,19,22,23,24,25	0	
7195	1,3,6,7,18,20,22,23	2,4,5,8,19,21,24,25,26	0	
7196	1,3,6,7,18,20,22,24	2,4,5,8,19,21,23,25,26	2	P27213,Q6AYS4
7197	1,3,6,7,18,20,22,25	2,4,5,8,19,21,23,24,26	0	
7198	1,3,6,7,18,20,22,26	2,4,5,8,19,21,23,24,25	2	P06760,Q5U2V4
7199	1,3,6,7,18,20,23,24	2,4,5,8,19,21,22,25,26	3	A2RUV9,Q6AYS4,Q80WL1
7200	1,3,6,7,18,20,23,25	2,4,5,8,19,21,22,24,26	2	A2RUV9,Q80WL1
7201	1,3,6,7,18,20,23,26	2,4,5,8,19,21,22,24,25	9	A2RUV9,P01946,P02091,Q4FZU2,Q6IFU8,Q6IFW6,Q6IG02,Q6IMF3,Q6P6Q2
7202	1,3,6,7,18,20,24,25	2,4,5,8,19,21,22,23,26	1	Q80WL1
7203	1,3,6,7,18,20,24,26	2,4,5,8,19,21,22,23,25	4	P02091,P06760,Q6AYS4,Q80WL1
7204	1,3,6,7,18,20,25,26	2,4,5,8,19,21,22,23,24	2	P02091,P35280
7205	1,3,6,7,18,21,22,23	2,4,5,8,19,20,24,25,26	1	P16086
7206	1,3,6,7,18,21,22,24	2,4,5,8,19,20,23,25,26	1	P13432

7207	1,3,6,7,18,21,22,25	2,4,5,8,19,20,23,24,26	1	P97840
7208	1,3,6,7,18,21,22,26	2,4,5,8,19,20,23,24,25	0	
7209	1,3,6,7,18,21,23,24	2,4,5,8,19,20,22,25,26	2	P13432,Q62997
7210	1,3,6,7,18,21,23,25	2,4,5,8,19,20,22,24,26	2	P19629,Q62997
7211	1,3,6,7,18,21,23,26	2,4,5,8,19,20,22,24,25	7	P01946,P02091,Q4FZU2,Q6IFU8,Q6IG02,Q6IMF3,Q6P6Q2
7212	1,3,6,7,18,21,24,25	2,4,5,8,19,20,22,23,26	4	P13432,P19629,P22006,Q62997
7213	1,3,6,7,18,21,24,26	2,4,5,8,19,20,22,23,25	3	P02091,P13432,Q62997
7214	1,3,6,7,18,21,25,26	2,4,5,8,19,20,22,23,24	5	P02091,P25031,P31430,Q62997,Q9QZK9
7215	1,3,6,7,18,22,23,24	2,4,5,8,19,20,21,25,26	1	P62804
7216	1,3,6,7,18,22,23,25	2,4,5,8,19,20,21,24,26	5	P01015,P06911,P12020,P19629,P62804
7217	1,3,6,7,18,22,23,26	2,4,5,8,19,20,21,24,25	4	P01946,P02091,Q6P6Q2,Q8VD89
7218	1,3,6,7,18,22,24,25	2,4,5,8,19,20,21,23,26	2	P19629,P62804
7219	1,3,6,7,18,22,24,26	2,4,5,8,19,20,21,23,25	3	P02091,P06760,Q8VD89
7220	1,3,6,7,18,22,25,26	2,4,5,8,19,20,21,23,24	1	P02091
7221	1,3,6,7,18,23,24,25	2,4,5,8,19,20,21,22,26	4	P19629,P62804,Q63751,Q80WL1

7222	1,3,6,7,18,23,24,26	2,4,5,8,19,20,21,22,25	6	A2RUV9,P01946,P02091,Q6IG02,Q6P6Q2,Q8VD89
7223	1,3,6,7,18,23,25,26	2,4,5,8,19,20,21,22,24	5	A2RUV9,P01946,P02091,Q6IG02,Q6P6Q2
7224	1,3,6,7,18,24,25,26	2,4,5,8,19,20,21,22,23	2	P01946,P02091
7225	1,3,6,7,19,20,21,22	2,4,5,8,18,23,24,25,26	5	P00714,P16086,P55091,Q6AYS4,Q99MH3
7226	1,3,6,7,19,20,21,23	2,4,5,8,18,22,24,25,26	5	P15399,P16086,P55091,P97580,Q6AYS4
7227	1,3,6,7,19,20,21,24	2,4,5,8,18,22,23,25,26	4	P00714,P13432,Q6AYS4,Q99MH3
7228	1,3,6,7,19,20,21,25	2,4,5,8,18,22,23,24,26	2	P47967,P97840
7229	1,3,6,7,19,20,21,26	2,4,5,8,18,22,23,24,25	2	Q6AYS4,Q99MH3
7230	1,3,6,7,19,20,22,23	2,4,5,8,18,21,24,25,26	8	P00774,P15399,P16086,P31430,P55091,P97580,P98089,Q6AYS4
7231	1,3,6,7,19,20,22,24	2,4,5,8,18,21,23,25,26	9	P00714,P00774,P14669,P31430,P55091,P98089,Q6AYS4,Q80WL1,Q99MH3
7232	1,3,6,7,19,20,22,25	2,4,5,8,18,21,23,24,26	2	P12020,P17046
7233	1,3,6,7,19,20,22,26	2,4,5,8,18,21,23,24,25	7	O54728,P06760,P31430,P98089,Q5U2V4,Q6AYS4,Q99MH3
7234	1,3,6,7,19,20,23,24	2,4,5,8,18,21,22,25,26	8	P15399,P16086,P31430,P55091,P97580,P98089,Q6AYS4,Q80WL1
7235	1,3,6,7,19,20,23,25	2,4,5,8,18,21,22,24,26	6	P12020,P15399,P16086,P97580,Q30KJ2,Q80WL1
7236	1,3,6,7,19,20,23,26	2,4,5,8,18,21,22,24,25	9	P01946,P02091,P15399,P31430,P97580,P98089,Q30KJ2,Q6AYS4,Q80WL1

7237	1,3,6,7,19,20,24,25	2,4,5,8,18,21,22,23,26	2	Q6AYS4,Q80WL1
7238	1,3,6,7,19,20,24,26	2,4,5,8,18,21,22,23,25	5	P02091,P98089,Q6AYS4,Q80WL1,Q99MH3
7239	1,3,6,7,19,20,25,26	2,4,5,8,18,21,22,23,24	4	P02091,P35280,Q6AYS4,Q80WL1
7240	1,3,6,7,19,21,22,23	2,4,5,8,18,20,24,25,26	4	P15399,P16086,P55091,P97580
7241	1,3,6,7,19,21,22,24	2,4,5,8,18,20,23,25,26	6	P00714,P13432,P19218,Q6AYS4,Q6TMA8,Q99MH3
7242	1,3,6,7,19,21,22,25	2,4,5,8,18,20,23,24,26	7	D4A1J4,P06911,P17046,P19218,P47967,P97840,Q9QZK9
7243	1,3,6,7,19,21,22,26	2,4,5,8,18,20,23,24,25	1	Q99MH3
7244	1,3,6,7,19,21,23,24	2,4,5,8,18,20,22,25,26	5	P15399,P16086,P97580,Q30KJ2,Q6AYS4
7245	1,3,6,7,19,21,23,25	2,4,5,8,18,20,22,24,26	9	P12020,P15399,P16086,P17046,P47967,P97580,P97840,Q30KJ2,Q9QZK9
7246	1,3,6,7,19,21,23,26	2,4,5,8,18,20,22,24,25	6	P01946,P02091,P15399,P16086,P97580,Q30KJ2
7247	1,3,6,7,19,21,24,25	2,4,5,8,18,20,22,23,26	4	P22006,P47967,P97840,Q62997
7248	1,3,6,7,19,21,24,26	2,4,5,8,18,20,22,23,25	2	P02091,Q6AYS4
7249	1,3,6,7,19,21,25,26	2,4,5,8,18,20,22,23,24	3	P02091,P97840,Q9QZK9
7250	1,3,6,7,19,22,23,24	2,4,5,8,18,20,21,25,26	9	P15399,P16086,P31430,P55091,P62804,P97580,P98089,Q30KJ2,Q6AYS4
7251	1,3,6,7,19,22,23,25	2,4,5,8,18,20,21,24,26	9	P06911,P12020,P15399,P16086,P17046,P62804,P97580,Q30KJ2,Q5GRG2

7252	1,3,6,7,19,22,23,26	2,4,5,8,18,20,21,24,25	10	O54728,P01946,P02091,P15399,P16086,P31430,P97580,P98089,Q30KJ2,Q6AYS4
7253	1,3,6,7,19,22,24,25	2,4,5,8,18,20,21,23,26	5	P06911,P12020,P17046,P62804,Q6TMA8
7254	1,3,6,7,19,22,24,26	2,4,5,8,18,20,21,23,25	6	P02091,P31430,P98089,Q6AYS4,Q6TMA8,Q99MH3
7255	1,3,6,7,19,22,25,26	2,4,5,8,18,20,21,23,24	3	P02091,P17046,Q9QZK9
7256	1,3,6,7,19,23,24,25	2,4,5,8,18,20,21,22,26	9	P12020,P15399,P17046,P19629,P62804,P97580,Q30KJ2,Q63751,Q80WL1
7257	1,3,6,7,19,23,24,26	2,4,5,8,18,20,21,22,25	9	P01946,P02091,P15399,P31430,P97580,P98089,Q30KJ2,Q6AYS4,Q80WL1
7258	1,3,6,7,19,23,25,26	2,4,5,8,18,20,21,22,24	7	P01946,P02091,P12020,P15399,P97580,Q30KJ2,Q9QZK9
7259	1,3,6,7,19,24,25,26	2,4,5,8,18,20,21,22,23	4	P01946,P02091,Q80WL1,Q9QZK9
7260	1,3,6,7,20,21,22,23	2,4,5,8,18,19,24,25,26	1	P55091
7261	1,3,6,7,20,21,22,24	2,4,5,8,18,19,23,25,26	7	P00714,P02803,P13432,Q03191,Q5XFX0,Q6AYS4,Q99MH3
7262	1,3,6,7,20,21,22,25	2,4,5,8,18,19,23,24,26	2	P09605;P25809,Q03191
7263	1,3,6,7,20,21,22,26	2,4,5,8,18,19,23,24,25	2	Q03191,Q99MH3
7264	1,3,6,7,20,21,23,24	2,4,5,8,18,19,22,25,26	1	P13432
7265	1,3,6,7,20,21,23,25	2,4,5,8,18,19,22,24,26	0	
7266	1,3,6,7,20,21,23,26	2,4,5,8,18,19,22,24,25	2	P01946,P02091

7267	1,3,6,7,20,21,24,25	2,4,5,8,18,19,22,23,26	2	P13432,Q03191
7268	1,3,6,7,20,21,24,26	2,4,5,8,18,19,22,23,25	4	P02091,P13432,Q6AYS4,Q99MH3
7269	1,3,6,7,20,21,25,26	2,4,5,8,18,19,22,23,24	2	P02091,Q03191
7270	1,3,6,7,20,22,23,24	2,4,5,8,18,19,21,25,26	6	P02803,P31430,P55091,P98089,Q6AYS4,Q80WL1
7271	1,3,6,7,20,22,23,25	2,4,5,8,18,19,21,24,26	3	P06911,P12020,Q5GRG2
7272	1,3,6,7,20,22,23,26	2,4,5,8,18,19,21,24,25	5	O54728,P01946,P02091,P98089,Q6AYS4
7273	1,3,6,7,20,22,24,25	2,4,5,8,18,19,21,23,26	3	P02803,Q03191,Q99MH3
7274	1,3,6,7,20,22,24,26	2,4,5,8,18,19,21,23,25	7	P02091,P02803,P98089,Q03191,Q63598,Q6AYS4,Q99MH3
7275	1,3,6,7,20,22,25,26	2,4,5,8,18,19,21,23,24	3	P02091,Q03191,Q99MH3
7276	1,3,6,7,20,23,24,25	2,4,5,8,18,19,21,22,26	3	P19629,Q63751,Q80WL1
7277	1,3,6,7,20,23,24,26	2,4,5,8,18,19,21,22,25	5	P01946,P02091,P98089,Q6AYS4,Q80WL1
7278	1,3,6,7,20,23,25,26	2,4,5,8,18,19,21,22,24	3	P01946,P02091,Q80WL1
7279	1,3,6,7,20,24,25,26	2,4,5,8,18,19,21,22,23	5	P01946,P02091,Q03191,Q80WL1,Q99MH3
7280	1,3,6,7,21,22,23,24	2,4,5,8,18,19,20,25,26	1	P13432
7281	1,3,6,7,21,22,23,25	2,4,5,8,18,19,20,24,26	5	P06911,P12020,P17046,P19629,Q5GRG2

7282	1,3,6,7,21,22,23,26	2,4,5,8,18,19,20,24,25	2	P01946,P02091
7283	1,3,6,7,21,22,24,25	2,4,5,8,18,19,20,23,26	5	P09605;P25809,P13432,P19629,P22006,Q03191
7284	1,3,6,7,21,22,24,26	2,4,5,8,18,19,20,23,25	3	P02091,P13432,Q99MH3
7285	1,3,6,7,21,22,25,26	2,4,5,8,18,19,20,23,24	4	P02091,P22006,Q03191,Q9QZK9
7286	1,3,6,7,21,23,24,25	2,4,5,8,18,19,20,22,26	5	P19629,P22006,Q62997,Q63751,Q6P6R2
7287	1,3,6,7,21,23,24,26	2,4,5,8,18,19,20,22,25	2	P01946,P02091
7288	1,3,6,7,21,23,25,26	2,4,5,8,18,19,20,22,24	6	P01946,P02091,P19629,P22006,P83121,Q9QZK9
7289	1,3,6,7,21,24,25,26	2,4,5,8,18,19,20,22,23	6	P01946,P02091,P19629,P22006,Q62997,Q9QZK9
7290	1,3,6,7,22,23,24,25	2,4,5,8,18,19,20,21,26	7	P06911,P12020,P17046,P19629,P62804,Q5GRG2,Q63751
7291	1,3,6,7,22,23,24,26	2,4,5,8,18,19,20,21,25	5	P00731,P01946,P02091,P98089,Q6AYS4
7292	1,3,6,7,22,23,25,26	2,4,5,8,18,19,20,21,24	5	P01946,P02091,P06911,P12020,P19629
7293	1,3,6,7,22,24,25,26	2,4,5,8,18,19,20,21,23	4	P02091,P19629,Q03191,Q99MH3
7294	1,3,6,7,23,24,25,26	2,4,5,8,18,19,20,21,22	6	P01946,P02091,P19629,Q63751,Q7TPB1,Q80WL1
7295	1,3,6,8,18,19,20,21	2,4,5,7,22,23,24,25,26	4	O35547,P00731,P35281,P36376
7296	1,3,6,8,18,19,20,22	2,4,5,7,21,23,24,25,26	1	P22006

7297	1,3,6,8,18,19,20,23	2,4,5,7,21,22,24,25,26	2	P22006,P35467
7298	1,3,6,8,18,19,20,24	2,4,5,7,21,22,23,25,26	0	
7299	1,3,6,8,18,19,20,25	2,4,5,7,21,22,23,24,26	1	P35280
7300	1,3,6,8,18,19,20,26	2,4,5,7,21,22,23,24,25	1	P35280
7301	1,3,6,8,18,19,21,22	2,4,5,7,20,23,24,25,26	5	O35547,P02782,P08723,P14408,P35281
7302	1,3,6,8,18,19,21,23	2,4,5,7,20,22,24,25,26	5	O35547,O70594,P35281,P35467,P51907
7303	1,3,6,8,18,19,21,24	2,4,5,7,20,22,23,25,26	6	P05539,P13432,P14408,P35281,P36376,Q62997
7304	1,3,6,8,18,19,21,25	2,4,5,7,20,22,23,24,26	3	O35547,P00731,P35281
7305	1,3,6,8,18,19,21,26	2,4,5,7,20,22,23,24,25	1	P35281
7306	1,3,6,8,18,19,22,23	2,4,5,7,20,21,24,25,26	2	P35467,P51907
7307	1,3,6,8,18,19,22,24	2,4,5,7,20,21,23,25,26	1	P83121
7308	1,3,6,8,18,19,22,25	2,4,5,7,20,21,23,24,26	0	
7309	1,3,6,8,18,19,22,26	2,4,5,7,20,21,23,24,25	0	
7310	1,3,6,8,18,19,23,24	2,4,5,7,20,21,22,25,26	2	P35467,P51907
7311	1,3,6,8,18,19,23,25	2,4,5,7,20,21,22,24,26	3	O70594,P35467,P51907

7312	1,3,6,8,18,19,23,26	2,4,5,7,20,21,22,24,25	5	P35467,P51907,Q63942,Q6IG02,Q6P6Q2
7313	1,3,6,8,18,19,24,25	2,4,5,7,20,21,22,23,26	0	
7314	1,3,6,8,18,19,24,26	2,4,5,7,20,21,22,23,25	0	
7315	1,3,6,8,18,19,25,26	2,4,5,7,20,21,22,23,24	1	P35280
7316	1,3,6,8,18,20,21,22	2,4,5,7,19,23,24,25,26	5	O35547,P02782,P08723,P13432,P14480
7317	1,3,6,8,18,20,21,23	2,4,5,7,19,22,24,25,26	1	O35547
7318	1,3,6,8,18,20,21,24	2,4,5,7,19,22,23,25,26	3	P05539,P13432,P36376
7319	1,3,6,8,18,20,21,25	2,4,5,7,19,22,23,24,26	1	Q9WTT6
7320	1,3,6,8,18,20,21,26	2,4,5,7,19,22,23,24,25	0	
7321	1,3,6,8,18,20,22,23	2,4,5,7,19,21,24,25,26	1	P22006
7322	1,3,6,8,18,20,22,24	2,4,5,7,19,21,23,25,26	0	
7323	1,3,6,8,18,20,22,25	2,4,5,7,19,21,23,24,26	0	
7324	1,3,6,8,18,20,22,26	2,4,5,7,19,21,23,24,25	0	
7325	1,3,6,8,18,20,23,24	2,4,5,7,19,21,22,25,26	1	P82995
7326	1,3,6,8,18,20,23,25	2,4,5,7,19,21,22,24,26	0	

7327	1,3,6,8,18,20,23,26	2,4,5,7,19,21,22,24,25	7	P35467,Q4FZU2,Q6IFU8,Q6IFW6,Q6IG02,Q6IMF3,Q6P6Q2
7328	1,3,6,8,18,20,24,25	2,4,5,7,19,21,22,23,26	0	
7329	1,3,6,8,18,20,24,26	2,4,5,7,19,21,22,23,25	0	
7330	1,3,6,8,18,20,25,26	2,4,5,7,19,21,22,23,24	1	P35280
7331	1,3,6,8,18,21,22,23	2,4,5,7,19,20,24,25,26	3	O35547,P02782,P08723
7332	1,3,6,8,18,21,22,24	2,4,5,7,19,20,23,25,26	3	P02782,P13432,P14408
7333	1,3,6,8,18,21,22,25	2,4,5,7,19,20,23,24,26	1	P14408
7334	1,3,6,8,18,21,22,26	2,4,5,7,19,20,23,24,25	2	P02782,P14408
7335	1,3,6,8,18,21,23,24	2,4,5,7,19,20,22,25,26	3	P05539,P13432,Q62997
7336	1,3,6,8,18,21,23,25	2,4,5,7,19,20,22,24,26	4	P07150,Q62997,Q6P6R2,Q9WTT6
7337	1,3,6,8,18,21,23,26	2,4,5,7,19,20,22,24,25	7	P35467,Q4FZU2,Q6IFU8,Q6IFV1,Q6IG02,Q6IMF3,Q6P6Q2
7338	1,3,6,8,18,21,24,25	2,4,5,7,19,20,22,23,26	4	P05539,P13432,P14408,Q62997
7339	1,3,6,8,18,21,24,26	2,4,5,7,19,20,22,23,25	3	P13432,P14408,Q62997
7340	1,3,6,8,18,21,25,26	2,4,5,7,19,20,22,23,24	3	P31430,Q62997,Q9WTT6
7341	1,3,6,8,18,22,23,24	2,4,5,7,19,20,21,25,26	1	P51907

7342	1,3,6,8,18,22,23,25	2,4,5,7,19,20,21,24,26	2	P06911,P51907
7343	1,3,6,8,18,22,23,26	2,4,5,7,19,20,21,24,25	4	P35467,P51907,Q6IG02,Q6P6Q2
7344	1,3,6,8,18,22,24,25	2,4,5,7,19,20,21,23,26	1	P14408
7345	1,3,6,8,18,22,24,26	2,4,5,7,19,20,21,23,25	0	
7346	1,3,6,8,18,22,25,26	2,4,5,7,19,20,21,23,24	0	
7347	1,3,6,8,18,23,24,25	2,4,5,7,19,20,21,22,26	2	P51907,Q63751
7348	1,3,6,8,18,23,24,26	2,4,5,7,19,20,21,22,25	7	P35467,P47967,P51907,Q6IFV1,Q6IG02,Q6IMF3,Q6P6Q2
7349	1,3,6,8,18,23,25,26	2,4,5,7,19,20,21,22,24	8	P35467,P51907,Q4FZU2,Q6IFU8,Q6IFW6,Q6IG02,Q6IMF3,Q6P6Q2
7350	1,3,6,8,18,24,25,26	2,4,5,7,19,20,21,22,23	1	P08494
7351	1,3,6,8,19,20,21,22	2,4,5,7,18,23,24,25,26	6	P00714,P02782,P35281,P55091,P82995,Q99MH3
7352	1,3,6,8,19,20,21,23	2,4,5,7,18,22,24,25,26	3	P35281,P55091,P82995
7353	1,3,6,8,19,20,21,24	2,4,5,7,18,22,23,25,26	5	P00714,P13432,P35281,P82995,Q99MH3
7354	1,3,6,8,19,20,21,25	2,4,5,7,18,22,23,24,26	2	P16636,P35281
7355	1,3,6,8,19,20,21,26	2,4,5,7,18,22,23,24,25	2	P35281,Q99MH3
7356	1,3,6,8,19,20,22,23	2,4,5,7,18,21,24,25,26	6	P00774,P22006,P31430,P55091,P82995,P98089

7357	1,3,6,8,19,20,22,24	2,4,5,7,18,21,23,25,26	9	P00714,P00774,P25031,P31430,P55091,P82995,P98089,Q62636,Q99MH3
7358	1,3,6,8,19,20,22,25	2,4,5,7,18,21,23,24,26	2	D3ZUC6,P82995
7359	1,3,6,8,19,20,22,26	2,4,5,7,18,21,23,24,25	3	O54728,P98089,Q99MH3
7360	1,3,6,8,19,20,23,24	2,4,5,7,18,21,22,25,26	6	P25031,P31430,P55091,P82995,P98089,Q80WL1
7361	1,3,6,8,19,20,23,25	2,4,5,7,18,21,22,24,26	1	P82995
7362	1,3,6,8,19,20,23,26	2,4,5,7,18,21,22,24,25	2	P98089,Q6IG02
7363	1,3,6,8,19,20,24,25	2,4,5,7,18,21,22,23,26	2	P82995,Q80WL1
7364	1,3,6,8,19,20,24,26	2,4,5,7,18,21,22,23,25	2	P98089,Q99MH3
7365	1,3,6,8,19,20,25,26	2,4,5,7,18,21,22,23,24	1	P35280
7366	1,3,6,8,19,21,22,23	2,4,5,7,18,20,24,25,26	4	P02782,P35281,P51907,P55091
7367	1,3,6,8,19,21,22,24	2,4,5,7,18,20,23,25,26	4	P00714,P02782,P13432,P14408
7368	1,3,6,8,19,21,22,25	2,4,5,7,18,20,23,24,26	0	
7369	1,3,6,8,19,21,22,26	2,4,5,7,18,20,23,24,25	2	P02782,P35281
7370	1,3,6,8,19,21,23,24	2,4,5,7,18,20,22,25,26	3	P35281,P51907,P82995
7371	1,3,6,8,19,21,23,25	2,4,5,7,18,20,22,24,26	6	O70594,P15399,P35281,P51907,Q6P6R2,Q9WTT6

7372	1,3,6,8,19,21,23,26	2,4,5,7,18,20,22,24,25	2	P35281,Q6IFV1
7373	1,3,6,8,19,21,24,25	2,4,5,7,18,20,22,23,26	1	Q62997
7374	1,3,6,8,19,21,24,26	2,4,5,7,18,20,22,23,25	0	
7375	1,3,6,8,19,21,25,26	2,4,5,7,18,20,22,23,24	2	Q9QZK9,Q9WTT6
7376	1,3,6,8,19,22,23,24	2,4,5,7,18,20,21,25,26	6	P25031,P31430,P51907,P55091,P82995,P98089
7377	1,3,6,8,19,22,23,25	2,4,5,7,18,20,21,24,26	3	P06911,P51907,Q71MB6
7378	1,3,6,8,19,22,23,26	2,4,5,7,18,20,21,24,25	3	O54728,P51907,P98089
7379	1,3,6,8,19,22,24,25	2,4,5,7,18,20,21,23,26	1	P06911
7380	1,3,6,8,19,22,24,26	2,4,5,7,18,20,21,23,25	2	P98089,Q99MH3
7381	1,3,6,8,19,22,25,26	2,4,5,7,18,20,21,23,24	0	
7382	1,3,6,8,19,23,24,25	2,4,5,7,18,20,21,22,26	2	P51907,P82995
7383	1,3,6,8,19,23,24,26	2,4,5,7,18,20,21,22,25	3	P02091,P51907,P98089
7384	1,3,6,8,19,23,25,26	2,4,5,7,18,20,21,22,24	2	P02091,P51907
7385	1,3,6,8,19,24,25,26	2,4,5,7,18,20,21,22,23	0	
7386	1,3,6,8,20,21,22,23	2,4,5,7,18,19,24,25,26	3	P16636,P55091,P82995

7387	1,3,6,8,20,21,22,24	2,4,5,7,18,19,23,25,26	7	P00714,P02782,P13432,P82995,Q03191,Q5XFX0,Q99MH3
7388	1,3,6,8,20,21,22,25	2,4,5,7,18,19,23,24,26	3	P16636,Q03191,Q5XFX0
7389	1,3,6,8,20,21,22,26	2,4,5,7,18,19,23,24,25	3	Q03191,Q99068,Q99MH3
7390	1,3,6,8,20,21,23,24	2,4,5,7,18,19,22,25,26	2	P13432,P82995
7391	1,3,6,8,20,21,23,25	2,4,5,7,18,19,22,24,26	4	P16636,P46720,Q6P6R2,Q9WTT6
7392	1,3,6,8,20,21,23,26	2,4,5,7,18,19,22,24,25	2	Q6IFV1,Q6IG02
7393	1,3,6,8,20,21,24,25	2,4,5,7,18,19,22,23,26	4	P13432,P16636,Q03191,Q5XFX0
7394	1,3,6,8,20,21,24,26	2,4,5,7,18,19,22,23,25	2	P13432,Q99MH3
7395	1,3,6,8,20,21,25,26	2,4,5,7,18,19,22,23,24	2	Q03191,Q9WTT6
7396	1,3,6,8,20,22,23,24	2,4,5,7,18,19,21,25,26	5	P25031,P31430,P55091,P82995,P98089
7397	1,3,6,8,20,22,23,25	2,4,5,7,18,19,21,24,26	4	D3ZUC6,P06911,P16636,P82995
7398	1,3,6,8,20,22,23,26	2,4,5,7,18,19,21,24,25	2	O54728,P98089
7399	1,3,6,8,20,22,24,25	2,4,5,7,18,19,21,23,26	5	D3ZUC6,P82995,Q03191,Q5XFX0,Q99MH3
7400	1,3,6,8,20,22,24,26	2,4,5,7,18,19,21,23,25	4	P02803,P98089,Q03191,Q99MH3
7401	1,3,6,8,20,22,25,26	2,4,5,7,18,19,21,23,24	3	D3ZUC6,Q03191,Q99MH3

7402	1,3,6,8,20,23,24,25	2,4,5,7,18,19,21,22,26	4	P82995,P83748,Q63751,Q80WL1
7403	1,3,6,8,20,23,24,26	2,4,5,7,18,19,21,22,25	4	P47967,P97840,P98089,Q6IG02
7404	1,3,6,8,20,23,25,26	2,4,5,7,18,19,21,22,24	1	Q6IG02
7405	1,3,6,8,20,24,25,26	2,4,5,7,18,19,21,22,23	2	Q03191,Q99MH3
7406	1,3,6,8,21,22,23,24	2,4,5,7,18,19,20,25,26	3	P13432,P14408,P82995
7407	1,3,6,8,21,22,23,25	2,4,5,7,18,19,20,24,26	3	P06911,P16636,Q9WTT6
7408	1,3,6,8,21,22,23,26	2,4,5,7,18,19,20,24,25	1	Q6IFV1
7409	1,3,6,8,21,22,24,25	2,4,5,7,18,19,20,23,26	4	P13432,P14408,Q03191,Q5XFX0
7410	1,3,6,8,21,22,24,26	2,4,5,7,18,19,20,23,25	3	P13432,P14408,Q99MH3
7411	1,3,6,8,21,22,25,26	2,4,5,7,18,19,20,23,24	3	Q03191,Q9QZK9,Q9WTT6
7412	1,3,6,8,21,23,24,25	2,4,5,7,18,19,20,22,26	4	Q62997,Q63751,Q6P6R2,Q9WTT6
7413	1,3,6,8,21,23,24,26	2,4,5,7,18,19,20,22,25	1	Q6IFV1
7414	1,3,6,8,21,23,25,26	2,4,5,7,18,19,20,22,24	5	P07150,P14669,Q6P6R2,Q9QZK9,Q9WTT6
7415	1,3,6,8,21,24,25,26	2,4,5,7,18,19,20,22,23	2	Q62997,Q9WTT6
7416	1,3,6,8,22,23,24,25	2,4,5,7,18,19,20,21,26	4	P06911,P51907,P82995,Q63751

7417	1,3,6,8,22,23,24,26	2,4,5,7,18,19,20,21,25	3	P00731,P47967,P98089
7418	1,3,6,8,22,23,25,26	2,4,5,7,18,19,20,21,24	1	P06911
7419	1,3,6,8,22,24,25,26	2,4,5,7,18,19,20,21,23	2	Q03191,Q99MH3
7420	1,3,6,8,23,24,25,26	2,4,5,7,18,19,20,21,22	4	P02091,P51907,Q63751,Q7TPB1
7421	1,3,6,18,19,20,21,22	2,4,5,7,8,23,24,25,26	2	Q02253,Q6AYS4
7422	1,3,6,18,19,20,21,23	2,4,5,7,8,22,24,25,26	0	
7423	1,3,6,18,19,20,21,24	2,4,5,7,8,22,23,25,26	2	P36376,Q6AYS4
7424	1,3,6,18,19,20,21,25	2,4,5,7,8,22,23,24,26	1	Q02253
7425	1,3,6,18,19,20,21,26	2,4,5,7,8,22,23,24,25	1	Q6AYS4
7426	1,3,6,18,19,20,22,23	2,4,5,7,8,21,24,25,26	4	P22006,P55091,P82995,Q6AYS4
[truncated: 2,815,236 more chars]
