# Supplementary material for: Dynamic Changes of Urine Proteome in Rat Models Inoculated with Two Different Hepatoma Cell Lines
Source: J Oncol. 2021 Jan 7;2021:8895330. doi: 10.1155/2021/8895330 (PMC7810548; doi:10.1155/2021/8895330)
Supplement: Supplementary Materials — Supplementary Figure 1. Functional analysis of differentially expressed proteins at days 5, 7, 14, and 28 in two models. (a) Cell component for the CBRH-7919 model. (b) Molecular function for the CBRH-7919 model. (c) Cell component for the RH-35 model. (d) Molecular function for the RH-35 model. Supplementary Table 1. All urinary proteins identified in the CBRH-7919 model. Supplementary Table 2. All urinary proteins identified in the RH-35 model. Supplementary Table 3. The details of 6435 random allocations in the CBRH-7919 model. Supplementary Table 4. The occurrence of the protein in 6435 random allocations in the CBRH-7919 model. Supplementary Table 5. The details of 12155 random allocations in the RH-35 model. Supplementary Table 6. The occurrence of the protein in 12155 random allocations in the RH-35 model. [file 8895330.f1.zip › 8895330.f1/Supplementary Table 6.pdf]

Title: Dynamic changes of urine proteome in rat models inoculated with two different hepatoma cell lines. Full author: Yameng Zhang, Yufei Gao, Jing Wei & Youhe Gao*.

Table S6 In RH-35 model, the occurance of the protein in 12155 random allocations.

Day 5		Day 7		Day 14		Day 28	
Protein ID	Occurance	Protein ID	Occurance	Protein ID	Occurance	Protein ID	Occurance
P10715	736	P34901	758	Q8CJD3	763	P97605	814
Q6AYS4	717	P00731	756	P02631	699	P34901	733
P02091	715	P19629	753	O54728	686	Q6AYS4	729
P02631	702	P13432	718	Q6IRE4	686	Q6IE51	711
P01835	702	P02631	710	Q4FZU6	677	P08010	691
Q6IRE4	695	Q6IRE4	705	P01835	668	P02631	690
P12020	691	P18418	695	P97840	667	P10715	687
P18418	687	P12785	694	P97605	663	Q01205	685
P19629	685	P01946	684	Q9WTT6	655	P51907	678
P06760	683	P10715	683	Q63942	648	P36374	675
P20766	677	P02091	665	Q9QZQ5	645	Q6AYL6	673
P20762	674	P00774	656	Q6IE51	642	Q6IRE4	673
P01836	669	P22006	645	P01681	638	Q63467	653
Q80WL1	668	Q9QW07	643	P52590	636	P52590	647
P08723	664	P01836	641	P01836	625	P60905	647
P83748	662	P97840	639	P25236	621	P97840	638
P13432	652	Q80WL1	638	Q7TP52	611	Q80WL1	637
Q5GRG2	641	Q9WTT6	637	P02625	611	P01681	630
P06911	641	P52590	636	P47967	606	P01836	625
P97840	627	O54728	635	P04182	605	P50115	625
P34901	626	P01681	631	P20766	595	P06760	624
Q8CJ52	626	P20766	627	P16086	594	P01835	622
P47727	620	P20760	626	P06911	594	O35547	617
Q9JHB9	619	P12020	618	Q99041	590	P50116	617
P52590	618	P01835	615	Q80WL1	580	Q7TPB1	616
P19223	618	Q99MH3	615	P14480	575	P19629	613
P20761	616	P15399	608	P06760	570	P13432	610
P01681	613	P83748	608	Q63617	567	P00714	606
P02782	612	Q4FZU6	606	P02803	566	P08753	602
O54728	603	Q6P6Q2	595	Q09030	561	O54728	598
Q99041	601	P83121	595	P55314	546	Q99041	594
P14668	601	P16086	579	G3V686	531	Q9QZQ5	593
Q62635	601	P00714	575	Q5HZV9	525	P83748	588
P22282	599	P20762	571	Q5GRG2	525	Q09030	585
Q9QZK9	597	P31430	562	P08723	515	P16086	580
P16086	596	P35467	562	Q62997	503	P01946	577
Q9Z0V6	592	P02803	559	P00714	502	Q62635	569
P02803	586	P98089	556	P83748	501	Q7TP52	563
P22283	581	P06760	555	Q6AYS4	501	P12020	557
P30120	580	P25031	548	P08753	493	Q63617	555
P31044	579	P06911	546	P10715	477	P20762	550
P16636	577	P20761	536	Q561R9	472	P85971	544
Q99MH3	573	P25236	536	P51907	460	B0BNN3	534
Q9JI85	570	Q63532	532	P12020	459	P83121	529
P35280	566	Q99041	530	Q9QW07	453	P20766	526
P36374	564	Q63617	517	P00507	452	P55314	521
P25236	561	P82995	514	P02793;Q7TI	444	G3V686	515
P50115	557	Q6AYS4	506	Q9JHB9	438	Q5GRG2	515
P14669	551	Q63942	497	P05539	431	P02091	512
D3ZHA0	546	P55091	496	Q9QUL6	427	P25236	509
P51907	545	Q4FZU2	495	P02781	424	P07171	507
Q6IG02	537	Q5GRG2	494	Q30KJ2	423	P11598	505
P07647	530	P05539	488	Q63532	416	Q9QUL6	502
Q5FVI6	527	P35280	474	P01946	413	P05539	492
P20760	526	Q6IG02	473	P83121	399	P98089	470
Q9QUL6	519	Q91ZS3	463	P02782	398	P00774	454
P08937	511	P47967	462	P07171	383	P27213	450
P0C0A9	510	Q30KJ2	457	P82471	376	Q63942	437
P06761	504	Q03191	438	P15399	374	Q8CIZ5	435
Q4G075	500	P97580	435	O35547	366	Q4FZU6	433
P55314	498	Q6P6R2	434	A2RUV9	362	Q6IFV1	429
P01946	496	Q7TPB1	389	P0C0A9	343	Q812E4	422
P09605;P258	494	Q5FVI6	378	Q6AYR9	342	P02625	421

Q9EQS0	490	P62804	376	P47727	340	Q9QW07	421
Q561R9	477	Q8CJD3	363	Q9Z0V6	331	P06911	417
Q63942	472	P35281	351	B0BNN3	320	P15399	416
P55091	461	Q63751	343	Q6AYQ8	314	Q561R9	410
B0BNN3	454	Q9QZK9	342	P14669	311	Q8CJD3	409
Q4FZU6	444	Q6IFU8	320	P13432	310	Q91ZS3	402
P27213	442	Q9Z0V6	318	P36374	298	Q99MH3	399
Q63617	439	Q7TP52	317	P19629	293	P19218	397
P02761	435	Q62997	317	Q6TMA8	279	P22006	396
Q9R0T3	435	Q9QUL6	317	Q4FZU2	278	P11883	385
A2RUV9	433	P42854	315	P24368	276	P36376	376
Q4FZU2	430	Q5XI22	306	P97580	265	P97580	372
P61206;P840	421	Q6IFU7	297	Q62894	258	Q30KJ2	364
Q6TMA8	415	D3ZUC6	296	Q63661	248	Q6AYR9	362
P15399	414	Q63661	282	Q9JI85	248	P08649	359
Q63751	409	P02782	280	Q99MH3	248	Q63532	343
Q6IFW6	406	P09605;P258(	277	P02770	241	Q6P6R2	341
Q30KJ2	405	P20646	270	P09605;P258	241	Q05695	335
Q63532	402	P17046	270	O55145	231	P16636	334
P00714	401	Q6IMF3	264	P60905	229	P47727	327
P07171	392	P61206;P840	258	P14408	229	P82471	324
P62804	391	P08723	256	Q8CJ52	225	Q63661	319
Q6P6Q2	385	P55314	247	F1LM93	221	P08290	317
Q9Z1F2	375	Q6IFV1	242	P08010	217	P00762	314
P06866	372	Q6IFW6	236	Q6P6R2	217	P53792	306
Q6IFU7	370	Q63475	235	Q08849	216	Q64268	306
Q10758	359	P07150	234	P53792	216	D3ZUK3	301
P11598	356	Q9ESW0	227	Q63514	215	Q9JJS8	295
Q62902	355	Q6IE51	225	P05369	212	P12785	285
P53792	353	A2RUV9	220	P02091	210	Q9Z1F2	283
O88267	336	P10758	215	P09006	210	P54921	273
Q6IMF3	335	P36374	213	Q6IFU7	209	P31044	269
P17046	321	P36376	209	P20762	199	Q5HZV9	268
P22006	315	P27213	208	P19218	198	P04905	255
P98089	303	P0C0A9	202	P42854	191	Q62975	255
P07150	301	Q08849	180	P97584	188	P82995	252
P83121	301	Q64057	175	Q63355	185	P17046	250
P23593	297	P02761	171	P62804	184	D3ZUC6	242
P19218	294	Q8VD89	165	Q9JJ22	178	Q9QX79	240
Q6AYR9	286	P16636	164	D3ZUC6	178	Q9Z0V6	231
P02780	284	P50280	162	Q62975	177	P13596	229
P05539	271	P02625	161	D3ZUK3	176	Q5M819	229
Q9QW07	270	O35547	160	P31044	167	P11762	228
Q5QE79	264	Q8K4G9	158	P30120	167	Q9JHB9	226
P68370	260	P53792	156	P08649	166	P0C0A9	223
P97580	258	P01041	154	Q10758	163	P62804	220
Q8VD89	248	Q9QZQ5	153	Q5FVI6	163	P35467	218
Q9JJS8	246	P19218	153	Q00715	160	P25031	212
Q6P6R2	246	P14669	146	Q63467	157	P06866	212
P47967	242	Q9EQV9	139	P04905	156	Q63357	209
P15999	238	Q5XFX0	139	O08815	154	Q6AYR8	207
P05371	235	P51907	135	P17046	152	P08723	206
Q9ESW0	235	Q00715	134	P09456	146	P02803	205
P04218	233	P11598	125	P18418	143	Q63475	197
P36970	219	O35783	121	Q06000	140	Q6IFU7	197
P08649	218	Q5QE79	121	Q6IG02	132	Q62753	186
Q64093	215	P05964	118	Q03191	131	Q5XI73	185
Q6IFV1	207	Q6AYL6	116	P36970	130	P35281	185
Q63493	206	P28648	115	Q9R0T3	129	P20759	180
Q6PCU2	203	Q9JI85	106	Q62902	124	Q66H69	178
P10536	198	P02770	105	P20760	123	P55091	176
P08721	196	P19223	105	P35281	119	O54858	162
P02625	189	Q6AYQ8	103	Q5M819	117	P02781	159
O55004	186	P00762	103	Q6P777	116	P35444	159
P02781	185	P82471	102	Q64057	109	P14480	154
P10758	181	P08010	99	P34901	107	P23593	153
Q6IFU8	179	P22282	94	P01041	107	P49744	152
Q5M8C6	175	P01015	93	Q9Z1F2	105	P02782	151

P20646	171	O55006	89	P35280	104	Q9QZK9	150
Q64361	170	P08721	88	P11598	101	Q4AEF8	149
Q9WTT6	169	Q8CIZ5	87	Q8CHN8	100	Q9QZ76	149
P35467	167	P14408	83	Q6P6Q2	97	P18418	146
P28648	166	P63036	80	Q6AY61	96	P97554	145
Q00715	163	P31044	78	P27791	96	Q62902	137
P01041	152	D4A1J4	76	Q99068	94	P14408	131
P50280	150	P14668	75	P35467	90	P80201	128
Q9QZA6	142	Q6TMA8	75	Q08464	89	P55016	126
P04905	141	Q07523	75	P06866	89	Q07116	125
P17475	140	P10959	73	P70709	88	F1LM93	122
P62815	139	P14480	73	P04218	84	P15999	120
P31430	133	Q6AY61	70	P10758	83	Q8K4G9	120
O35547	133	O89117	68	Q812E4	83	Q6TMA8	119
Q62997	129	Q64194	68	P16636	81	P00731	118
Q09030	126	P68370	68	P02780	80	O88267	117
P08753	126	Q8CJ52	68	P12785	77	Q68FS4	114
Q9WUW8	123	Q09030	66	P17559	74	P38444	113
F1LM93	121	O54800;Q5D	66	P36376	73	P16391	113
P06757	119	Q6AYR8	66	Q07116	71	P05964	110
Q91ZS3	116	P55016	60	P11883	70	P47967	106
P00762	116	Q498D9	57	P82995	69	Q9ESW0	104
Q63317	114	Q62902	55	P97615	68	O08815	102
P02793;Q7TF	111	Q63355	54	P22283	66	P80020	101
P14480	111	Q9QZA6	51	P20761	62	P05369	99
Q63355	110	P36970	47	P38444	62	P36970	98
P14408	107	Q6AY33	46	P49744	60	P70709	95
P11883	107	O70417	46	Q8K4G9	59	P10758	94
Q08849	103	Q99068	45	Q8VD89	58	P18427	92
D3ZUC6	102	P55159	45	P52303	57	P08644	91
Q498R7	101	Q62635	45	Q498R7	51	P22283	91
O55006	99	P47727	43	Q9ESW0	51	Q6P6T1	91
P82471	95	P70709	40	P35446	49	Q62740	90
Q63475	94	Q63317	38	P07647	46	Q5ZQU0	86
Q5XI22	94	Q9Z1F2	38	O88267	45	Q9JID2	85
Q566E6	90	Q6AYR9	38	Q6NX65	45	Q63514	84
Q8CJD3	90	P22283	38	O70417	45	Q8VD89	84
Q5I0D1	89	Q5BJY9	38	P13852	42	O35783	83
Q7TP52	88	Q9JID2	37	Q6AYL6	42	Q8CJ52	83
Q498D9	88	Q8N7M5	35	Q5QE79	41	Q64057	81
Q6AYL6	85	Q9ET32	35	Q6IFW6	39	O70417	73
P08689	83	P05371	33	P14668	39	Q99PP0	69
Q63661	82	Q9R0T3	33	P02761	38	P02793;Q7TP5	69
P97615	81	Q62636	31	P24388	38	P35434	67
G3V686	77	Q5U2V4	31	P22282	36	Q00715	66
O08557	77	P30120	30	Q920G2	35	Q03191	60
P14173	74	G3V686	29	P11762	34	Q5M871	59
Q63357	73	P80020	29	P06761	34	P07897	58
B4F795	68	Q6P9T8	29	P55016	34	Q9ET32	54
Q63598	68	O70594	29	P00762	29	P11030	53
Q9QX79	67	P14173	28	P04762	28	O54800;Q5DW	46
P09456	67	F1LM93	28	P05964	28	P50280	44
P23928	64	O70377	27	Q64335	28	O35568	43
P02770	62	P05369	27	Q63357	28	P80202	43
P97571	61	Q63598	24	Q9QZK9	27	P02780	42
P05964	60	P07171	24	P16296	27	P09456	42
P60905	56	P07647	21	P50280	27	Q99068	40
Q64194	55	Q5I0D1	20	P17475	26	A2RUV9	40
P29975	54	D3ZUK3	17	Q6IFV1	25	Q6PCU2	39
O54858	53	Q63467	16	Q6PCU2	24	P51886	38
Q62894	52	P36953	16	P85971	23	Q9WUW3	38
P24368	52	Q02253	16	P22985	23	P02680	37
Q7TPB1	52	Q9JJS8	15	P08644	20	P09605;P25809	35
Q8K4G9	51	D3ZHA0	14	P19132	20	P08721	34
P12368	51	P08753	14	P18427	20	Q5U2V4	33
O54715	50	P08644	14	Q6IE52	19	Q62894	32
P55018	49	P55053	13	Q01205	19	Q5QE79	32
P82995	48	P02793;Q7TP	12	O54861	19	Q64611	31

Q5BJY9	47	D4A5U3	11	Q9QZA6	19	Q80YN4	30
P12346	46	P15978	10	Q8N7M5	17	P22282	30
Q6AYQ8	44	O54861	10	Q91ZS3	17	Q6Q7Y5	29
P16391	41	Q561R9	10	P13596	16	P23785	29
Q6AYS7	40	Q9R1T1	9	Q5I0D1	16	P22734	28
Q5XFX0	40	P27590	9	P10719	16	P30120	27
P36376	40	P30836	9	O35568	15	P01015	27
P97574	39	Q01205	9	Q9QX79	15	Q9JI85	26
P35053	37	Q66H69	8	P20646	15	P11442	26
P08010	35	P30904	8	P10959	14	Q9R1T5	25
Q03191	35	P62815	8	Q5XI22	14	P08494	24
P35281	34	O55004	8	P61206;P84	14	P05371	23
P25031	33	Q5M819	8	Q71UF4	14	Q5I0D1	23
P01015	32	B4F795	8	Q63317	13	P42854	22
P18427	32	Q68FV8	7	P22006	13	Q6GMN2	21
P97605	32	Q71MB6	7	O55004	12	P24368	20
P63029	29	O35956	7	P05942	12	Q3T1K5	19
Q923M1	25	Q4V885	6	P02680	12	Q920P0	19
O08815	25	Q6P767	6	P0C0K7	11	Q8N7M5	18
P04041	24	P24594	6	O35783	11	Q99376	18
P46844	24	Q05175	6	Q5M8C6	11	P26772	18
Q05175	24	P06761	6	P07150	10	Q8CG08	17
Q5HZV9	23	P97605	6	Q5RLM2	10	Q6P777	17
P00731	23	P11883	6	Q9ET32	10	O89117	17
Q07936	22	P50398	5	Q63556	9	Q9QZA6	17
O35783	21	Q6P777	5	P26772	9	P35446	16
O54861	21	P08649	5	Q9JID2	9	Q6BEA2	16
Q08463	19	P51886	5	Q4V885	8	Q8CHN8	16
P12785	19	Q66HD0	5	Q80WY6	8	Q498S8	15
P46462	18	P08937	5	P46462	8	P15978	14
Q62636	17	P04041	5	Q63751	8	Q6P9T8	14
Q9EQV9	17	P32755	5	Q4AEF8	8	P10960	13
Q62687	17	Q10758	5	Q64268	7	Q63598	13
P02764	16	P38444	5	F1M3L7	7	Q64605	13
P05369	16	Q99PP0	4	Q6IMF3	7	Q64335	12
Q06496	15	Q9R044	4	Q62753	7	B5DFC9	11
P50116	14	Q9JJ50	4	Q3T1K5	6	Q63355	11
O70417	14	P24368	4	P01026	6	Q08849	11
Q9ET32	14	O88267	4	P05712	6	O88600	11
Q99068	14	O55145	4	P08721	5	Q63416	10
P11762	13	Q9WUF4	4	P36953	5	P28648	10
Q99PP0	13	P16391	4	P80020	5	O70594	10
P08932	12	P80201	4	P01015	5	P19223	10
P70545	12	Q9QX79	3	P25031	5	Q566E6	10
Q68FV8	12	Q62894	3	Q6IFU8	5	Q63317	9
Q63424	12	P60905	3	Q05175	5	P63322	9
P04073	12	Q8K1G0	3	Q64361	4	O08557	9
P40241	11	Q64573	3	P68370	4	P02651	9
Q5U2V4	11	Q4G075	3	P29288	3	P14668	9
P70709	10	Q63556	3	P27590	3	P07943	9
P42854	10	P0DP29;P0DF	3	Q66H69	3	P02761	9
Q80W57	10	B0BNN3	3	P80204	3	Q5XFX0	9
P01048	9	P97608	2	O70594	3	P60571	8
P05712	8	P19939	2	Q02253	3	P14669	8
D4A5U3	8	Q6IE52	2	P16391	3	P07861	8
Q812E4	8	P02780	2	P02696	3	Q4V885	7
Q8K1G0	7	P08494	2	Q63493	3	Q80WY6	7
P35434	7	P80204	2	Q9JLS4	3	Q8CFN2	7
Q68FS4	7	P06757	2	Q5ZQU0	2	O55145	7
Q6IE51	7	Q6BEA2	2	P63322	2	Q01177	7
Q9EPB1	7	Q64268	2	P15978	2	P21581	7
P48199	7	P17559	2	P55159	2	Q9WUD9	6
O70594	7	Q64361	1	Q9EQV9	2	Q9R044	6
Q920P0	7	P00507	1	Q9QXQ0	2	P40241	6
Q5M819	7	Q1WIM3	1	Q920P0	2	P0DMW0;P0DM	5
Q923S2	6	P70502	1	P23739	2	P63095	5
P80201	6	O88797	1	P04073	2	Q06000	5
O35077	6	Q4AEF8	1	Q6P9T8	2	Q5FVI6	5

P53790	5	Q5M872	1	P69897	2	B0LT89	5
Q64335	5	P02680	1	P02454	1	Q9WUC4	4
P80254	5	Q920P0	1	P08290	1	P04897	4
Q64611	5	P46720	1	P0DMW0;P0	1	P30836	4
P63036	5	Q9Z0J6	1	P55053	1	P15473	4
P29288	4	Q07936	1	P21743	1	P61206;P84079	4
Q6AYR8	4	Q63357	1	P48037	1	P68370	4
Q8R431	4	P04905	1	Q9EQV6	1	O54861	4
P16573	4	P04073	1	P20171	1	Q08464	3
P55016	4	Q63203	1	P28648	1	Q3MIE4	3
Q63618	4	P35053	1	Q63618	1	Q64093	3
Q6P767	3	A4KWA5;A4K	0	P04355	1	P30919	3
P63095	3	B0BNA5	0	O70377	1	Q62636	3
Q9QYU4	3	B0BND0	0	Q9WTW7	1	Q6IFW6	3
P24388	3	B0BNE5	0	Q62687	1	Q9WUF4	3
P14046	3	B0LT89	0	Q9QWJ9	1	P12368	3
Q6GMN2	3	B1H234	0	P80201	1	P38659	2
P02696	3	B2RYG6	0	A4KWA5;A4I	0	Q5I0D5	2
P19814	2	B5DEN9	0	B0BNA5	0	P19939	2
P97584	2	B5DFC9	0	B0BND0	0	Q91XN4	2
Q9R1T1	2	D3Z8L7	0	B0BNE5	0	P04276	2
P13265	2	D3ZTD8	0	B0LT89	0	P01041	2
Q6Q0N1	2	D3ZTE0	0	B1H234	0	P97574	2
P38918	2	D3ZTV3	0	B2RYG6	0	Q5BJP3	2
P46720	2	D3ZTX0	0	B4F795	0	P05942	2
P17559	2	D4A1R8	0	B5DEN9	0	P04355	2
P62898	2	E9PT87;P2068	0	B5DFC9	0	P70545	2
P09527	2	F1M3L7	0	D3Z8L7	0	P69897	2
P32755	2	G3V7W1	0	D3ZHA0	0	Q32KJ6	2
Q64319	2	H1UBN0;Q5B	0	D3ZTD8	0	P04937	2
O55096	2	O08557	0	D3ZTE0	0	Q9EQV6	1
Q80WY6	1	O08628	0	D3ZTV3	0	P97584	1
P08289	1	O08815	0	D3ZTX0	0	P21743	1
P10959	1	O09175	0	D4A1J4	0	P61589	1
P52303	1	O35077	0	D4A1R8	0	Q6RUV5	1
P55053	1	O35112	0	D4A5U3	0	P16573	1
Q8CFN2	1	O35142	0	E9PT87;P206	0	Q68FV8	1
Q9QZK8	1	O35217	0	G3V7W1	0	P15087	1
Q6P9T8	1	O35244	0	H1UBN0;Q5I	0	P46844	1
P30919	1	O35264	0	O08557	0	P32755	1
Q64057	1	O35331	0	O08628	0	D4A5U3	1
O89117	1	O35509;P624	0	O09175	0	Q9QXQ0	1
Q5RLM2	1	O35568	0	O35077	0	Q9WTT6	1
P23785	1	O35760	0	O35112	0	P08689	1
Q9JJ50	1	O35763	0	O35142	0	P18484	1
P07151	1	O35952	0	O35217	0	P97608	1
Q6RUV5	1	O54715	0	O35244	0	Q5XI22	1
P08644	1	O54858	0	O35264	0	Q63150	1
Q62975	1	O54975	0	O35331	0	Q63072	1
P04636	1	O55096	0	O35509;P62	0	P17559	1
Q6JE36	1	O70244	0	O35760	0	P68511	1
A4KWA5;A4K	0	O70352	0	O35763	0	A4KWA5;A4KW	0
B0BNA5	0	O70489	0	O35952	0	B0BNA5	0
B0BND0	0	O70513	0	O35956	0	B0BND0	0
B0BNE5	0	O70535	0	O54715	0	B0BNE5	0
B0LT89	0	O70540	0	O54800;Q5D	0	B1H234	0
B1H234	0	O88204	0	O54858	0	B2RYG6	0
B2RYG6	0	O88600	0	O54975	0	B4F795	0
B5DEN9	0	O88766	0	O55006	0	B5DEN9	0
B5DFC9	0	O88767	0	O55096	0	D3Z8L7	0
D3Z8L7	0	O88917	0	O70244	0	D3ZHA0	0
D3ZTD8	0	O88989	0	O70352	0	D3ZTD8	0
D3ZTE0	0	O89049	0	O70489	0	D3ZTE0	0
D3ZTV3	0	P00502	0	O70513	0	D3ZTV3	0
D3ZTX0	0	P00689	0	O70535	0	D3ZTX0	0
D3ZUK3	0	P00758	0	O70540	0	D4A1J4	0
D4A1J4	0	P00786	0	O88204	0	D4A1R8	0
D4A1R8	0	P00787	0	O88600	0	E9PT87;P20689	0

E9PT87;P206	0	P00884	0	O88766	0	F1M3L7	0
F1M3L7	0	P01026	0	O88767	0	G3V7W1	0
G3V7W1	0	P01048	0	O88797	0	H1UBN0;Q5BJS	0
H1UBN0;Q5E	0	P01830	0	O88917	0	O08628	0
O08628	0	P02454	0	O88989	0	O09175	0
O09175	0	P02650	0	O89049	0	O35077	0
O35112	0	P02651	0	O89117	0	O35112	0
O35142	0	P02696	0	P00502	0	O35142	0
O35217	0	P02764	0	P00689	0	O35217	0
O35244	0	P02767	0	P00731	0	O35244	0
O35264	0	P02781	0	P00758	0	O35264	0
O35331	0	P03994	0	P00774	0	O35331	0
O35509;P624	0	P04176	0	P00786	0	O35509;P62494	0
O35568	0	P04182	0	P00787	0	O35760	0
O35760	0	P04218	0	P00884	0	O35763	0
O35763	0	P04276	0	P01048	0	O35952	0
O35952	0	P04355	0	P01830	0	O35956	0
O35956	0	P04636	0	P02650	0	O54715	0
O54800;Q5D	0	P04639	0	P02651	0	O54975	0
O54975	0	P04642	0	P02764	0	O55004	0
O55145	0	P04762	0	P02767	0	O55006	0
O70244	0	P04764	0	P03994	0	O55096	0
O70352	0	P04785	0	P04041	0	O70244	0
O70377	0	P04797	0	P04176	0	O70352	0
O70489	0	P04897	0	P04276	0	O70377	0
O70513	0	P04903	0	P04636	0	O70489	0
O70535	0	P04904	0	P04639	0	O70513	0
O70540	0	P04906	0	P04642	0	O70535	0
O88204	0	P04916	0	P04764	0	O70540	0
O88600	0	P04937	0	P04785	0	O88204	0
O88766	0	P05065	0	P04797	0	O88766	0
O88767	0	P05197	0	P04897	0	O88767	0
O88797	0	P05544	0	P04903	0	O88797	0
O88917	0	P05545	0	P04904	0	O88917	0
O88989	0	P05712	0	P04906	0	O88989	0
O89049	0	P05942	0	P04916	0	O89049	0
P00502	0	P06399	0	P04937	0	P00502	0
P00507	0	P06685	0	P05065	0	P00507	0
P00689	0	P06866	0	P05197	0	P00689	0
P00758	0	P07151	0	P05371	0	P00758	0
P00774	0	P07154	0	P05544	0	P00786	0
P00786	0	P07314	0	P05545	0	P00787	0
P00787	0	P07340	0	P06399	0	P00884	0
P00884	0	P07379	0	P06685	0	P01026	0
P01026	0	P07483	0	P06757	0	P01048	0
P01830	0	P07522	0	P07151	0	P01830	0
P02454	0	P07632	0	P07154	0	P02454	0
P02650	0	P07861	0	P07314	0	P02650	0
P02651	0	P07897	0	P07340	0	P02696	0
P02680	0	P07943	0	P07379	0	P02764	0
P02767	0	P08289	0	P07483	0	P02767	0
P03994	0	P08290	0	P07522	0	P02770	0
P04176	0	P08460	0	P07632	0	P03994	0
P04182	0	P08592	0	P07861	0	P04041	0
P04276	0	P08650	0	P07897	0	P04073	0
P04355	0	P08689	0	P07943	0	P04176	0
P04639	0	P08932	0	P08289	0	P04182	0
P04642	0	P08934	0	P08460	0	P04218	0
P04762	0	P09006	0	P08494	0	P04636	0
P04764	0	P09034	0	P08592	0	P04639	0
P04785	0	P09456	0	P08650	0	P04642	0
P04797	0	P09527	0	P08689	0	P04762	0
P04897	0	P09606	0	P08932	0	P04764	0
P04903	0	P0C0K7	0	P08934	0	P04785	0
P04904	0	P0CG51;P629	0	P08937	0	P04797	0
P04906	0	P0DMW0;P0D	0	P09034	0	P04903	0
P04916	0	P10111	0	P09527	0	P04904	0
P04937	0	P10247	0	P09606	0	P04906	0

P05065	0	P10252	0	P0CG51;P629	0	P04916	0
P05197	0	P10536	0	P0DP29;P0D	0	P05065	0
P05544	0	P10719	0	P10111	0	P05197	0
P05545	0	P10760	0	P10247	0	P05544	0
P05942	0	P10960	0	P10252	0	P05545	0
P06399	0	P11030	0	P10536	0	P05712	0
P06685	0	P11232	0	P10760	0	P06399	0
P07154	0	P11348	0	P10960	0	P06685	0
P07314	0	P11442	0	P11030	0	P06757	0
P07340	0	P11762	0	P11232	0	P06761	0
P07379	0	P11980	0	P11348	0	P07150	0
P07483	0	P12346	0	P11442	0	P07151	0
P07522	0	P12368	0	P11980	0	P07154	0
P07632	0	P13221	0	P12346	0	P07314	0
P07861	0	P13265	0	P12368	0	P07340	0
P07897	0	P13596	0	P13221	0	P07379	0
P07943	0	P13635	0	P13265	0	P07483	0
P08290	0	P13852	0	P13635	0	P07522	0
P08460	0	P14046	0	P14046	0	P07632	0
P08494	0	P14562	0	P14173	0	P07647	0
P08592	0	P14630	0	P14562	0	P08289	0
P08650	0	P14740	0	P14630	0	P08460	0
P08934	0	P14841	0	P14740	0	P08592	0
P09006	0	P14925	0	P14841	0	P08650	0
P09034	0	P14942	0	P14925	0	P08932	0
P09606	0	P15083	0	P14942	0	P08934	0
P0C0K7	0	P15087	0	P15083	0	P08937	0
P0CG51;P629	0	P15473	0	P15087	0	P09006	0
P0DMW0;P0I	0	P15684	0	P15473	0	P09034	0
P0DP29;P0DI	0	P15800	0	P15684	0	P09527	0
P10111	0	P15943	0	P15800	0	P09606	0
P10247	0	P15999	0	P15943	0	P0C0K7	0
P10252	0	P16228	0	P15999	0	P0CG51;P62982	0
P10719	0	P16296	0	P16228	0	P0DP29;P0DP3	0
P10760	0	P16310	0	P16310	0	P10111	0
P10960	0	P16573	0	P16573	0	P10247	0
P11030	0	P16617	0	P16617	0	P10252	0
P11232	0	P17164	0	P17164	0	P10536	0
P11348	0	P17475	0	P18292	0	P10719	0
P11442	0	P18292	0	P18297	0	P10760	0
P11980	0	P18297	0	P18484	0	P10959	0
P13221	0	P18427	0	P18757	0	P11232	0
P13596	0	P18484	0	P19112	0	P11348	0
P13635	0	P18757	0	P19223	0	P11980	0
P13852	0	P19112	0	P19468	0	P12346	0
P14562	0	P19132	0	P19804	0	P13221	0
P14630	0	P19468	0	P19814	0	P13265	0
P14740	0	P19804	0	P19939	0	P13635	0
P14841	0	P19814	0	P20059	0	P13852	0
P14925	0	P20059	0	P20611	0	P14046	0
P14942	0	P20171	0	P20673	0	P14173	0
P15083	0	P20611	0	P20759	0	P14562	0
P15087	0	P20673	0	P20767	0	P14630	0
P15473	0	P20759	0	P20786	0	P14740	0
P15684	0	P20767	0	P20961	0	P14841	0
P15800	0	P20786	0	P21581	0	P14925	0
P15943	0	P20961	0	P21674	0	P14942	0
P15978	0	P21581	0	P21704	0	P15083	0
P16228	0	P21674	0	P22057	0	P15684	0
P16296	0	P21704	0	P22273	0	P15800	0
P16310	0	P21743	0	P22734	0	P15943	0
P16617	0	P22057	0	P23377	0	P16228	0
P17164	0	P22273	0	P23593	0	P16296	0
P18292	0	P22734	0	P23680	0	P16310	0
P18297	0	P22985	0	P23764	0	P16617	0
P18484	0	P23377	0	P23785	0	P17164	0
P18757	0	P23593	0	P23928	0	P17475	0
P19112	0	P23680	0	P24090	0	P18292	0

P19132	0	P23739	0	P24268	0	P18297	0
P19468	0	P23764	0	P24594	0	P18757	0
P19804	0	P23785	0	P25093	0	P19112	0
P19939	0	P23928	0	P25113	0	P19132	0
P20059	0	P24090	0	P25304	0	P19468	0
P20171	0	P24268	0	P26051	0	P19804	0
P20611	0	P24388	0	P26342	0	P19814	0
P20673	0	P25093	0	P26453	0	P20059	0
P20759	0	P25113	0	P26644	0	P20171	0
P20767	0	P25304	0	P27139	0	P20611	0
P20786	0	P26051	0	P27213	0	P20646	0
P20961	0	P26342	0	P27274	0	P20673	0
P21581	0	P26453	0	P27605	0	P20760	0
P21674	0	P26644	0	P27867	0	P20761	0
P21704	0	P26772	0	P28037	0	P20767	0
P21743	0	P27139	0	P28494	0	P20786	0
P22057	0	P27274	0	P28826	0	P20961	0
P22273	0	P27605	0	P29534	0	P21674	0
P22734	0	P27791	0	P29598	0	P21704	0
P22985	0	P27867	0	P29975	0	P22057	0
P23377	0	P28037	0	P30121	0	P22273	0
P23680	0	P28494	0	P30152	0	P22985	0
P23739	0	P28826	0	P30713	0	P23377	0
P23764	0	P29288	0	P30836	0	P23680	0
P24090	0	P29534	0	P30904	0	P23739	0
P24268	0	P29598	0	P30919	0	P23764	0
P24594	0	P29975	0	P31211	0	P23928	0
P25093	0	P30121	0	P31430	0	P24090	0
P25113	0	P30152	0	P31977	0	P24268	0
P25304	0	P30713	0	P32038	0	P24388	0
P26051	0	P30919	0	P32755	0	P24594	0
P26342	0	P31211	0	P34058	0	P25093	0
P26453	0	P31977	0	P34080	0	P25113	0
P26644	0	P32038	0	P35053	0	P25304	0
P26772	0	P34058	0	P35213	0	P26051	0
P27139	0	P34080	0	P35434	0	P26342	0
P27274	0	P35213	0	P35444	0	P26453	0
P27590	0	P35434	0	P35704	0	P26644	0
P27605	0	P35444	0	P35859	0	P27139	0
P27791	0	P35446	0	P35952	0	P27274	0
P27867	0	P35704	0	P36373	0	P27590	0
P28037	0	P35859	0	P38438	0	P27605	0
P28494	0	P35952	0	P38652	0	P27791	0
P28826	0	P36373	0	P38659	0	P27867	0
P29534	0	P38438	0	P38918	0	P28037	0
P29598	0	P38652	0	P39069	0	P28494	0
P30121	0	P38659	0	P40241	0	P28826	0
P30152	0	P38918	0	P41498	0	P29288	0
P30713	0	P39069	0	P41562	0	P29534	0
P30836	0	P40241	0	P42123	0	P29598	0
P30904	0	P41498	0	P43303	0	P29975	0
P31211	0	P41562	0	P43427	0	P30121	0
P31977	0	P42123	0	P45479	0	P30152	0
P32038	0	P43303	0	P45592	0	P30713	0
P34058	0	P43427	0	P46413	0	P30904	0
P34080	0	P45479	0	P46720	0	P31211	0
P35213	0	P45592	0	P46844	0	P31430	0
P35444	0	P46413	0	P46953	0	P31977	0
P35446	0	P46462	0	P47820	0	P32038	0
P35704	0	P46844	0	P47853	0	P34058	0
P35859	0	P46953	0	P48032	0	P34080	0
P35952	0	P47820	0	P48199	0	P35053	0
P36373	0	P47853	0	P48284	0	P35213	0
P36953	0	P48032	0	P48500	0	P35280	0
P38438	0	P48037	0	P48508	0	P35704	0
P38444	0	P48199	0	P49002	0	P35859	0
P38652	0	P48284	0	P49134	0	P35952	0
P38659	0	P48500	0	P50115	0	P36373	0

P39069	0	P48508	0	P50116	0	P36953	0
P41498	0	P49002	0	P50123	0	P38438	0
P41562	0	P49134	0	P50137	0	P38652	0
P42123	0	P49744	0	P50398	0	P38918	0
P43303	0	P50115	0	P50399	0	P39069	0
P43427	0	P50116	0	P50430	0	P41498	0
P45479	0	P50123	0	P50503	0	P41562	0
P45592	0	P50137	0	P50609	0	P42123	0
P46413	0	P50399	0	P51635	0	P43303	0
P46953	0	P50430	0	P51647	0	P43427	0
P47820	0	P50503	0	P51886	0	P45479	0
P47853	0	P50609	0	P52759	0	P45592	0
P48032	0	P51635	0	P52796	0	P46413	0
P48037	0	P51647	0	P52847	0	P46462	0
P48284	0	P52303	0	P53369	0	P46720	0
P48500	0	P52759	0	P53790	0	P46953	0
P48508	0	P52796	0	P53813	0	P47820	0
P49002	0	P52847	0	P54311	0	P47853	0
P49134	0	P53369	0	P54313	0	P48032	0
P49744	0	P53790	0	P54921	0	P48037	0
P50123	0	P53813	0	P55018	0	P48199	0
P50137	0	P54311	0	P55091	0	P48284	0
P50398	0	P54313	0	P55146	0	P48500	0
P50399	0	P54921	0	P55260	0	P48508	0
P50430	0	P55018	0	P55281	0	P49002	0
P50503	0	P55146	0	P57097	0	P49134	0
P50609	0	P55260	0	P57113	0	P50123	0
P51635	0	P55281	0	P58195	0	P50137	0
P51647	0	P57097	0	P60571	0	P50398	0
P51886	0	P57113	0	P60711;P632	0	P50399	0
P52759	0	P58195	0	P61107	0	P50430	0
P52796	0	P60571	0	P61459	0	P50503	0
P52847	0	P60711;P632	0	P61589	0	P50609	0
P53369	0	P61107	0	P61943	0	P51635	0
P53813	0	P61459	0	P61972	0	P51647	0
P54311	0	P61589	0	P61983	0	P52303	0
P54313	0	P61943	0	P62260	0	P52759	0
P54921	0	P61972	0	P62630	0	P52796	0
P55146	0	P61983	0	P62815	0	P52847	0
P55159	0	P62260	0	P62836	0	P53369	0
P55260	0	P62630	0	P62898	0	P53790	0
P55281	0	P62836	0	P62959	0	P53813	0
P57097	0	P62898	0	P62963	0	P54311	0
P57113	0	P62959	0	P63018	0	P54313	0
P58195	0	P62963	0	P63029	0	P55018	0
P60571	0	P63018	0	P63036	0	P55053	0
P60711;P632	0	P63029	0	P63095	0	P55146	0
P61107	0	P63095	0	P63102	0	P55159	0
P61459	0	P63102	0	P63331	0	P55260	0
P61589	0	P63322	0	P68035;P681	0	P55281	0
P61943	0	P63331	0	P68255	0	P57097	0
P61972	0	P68035;P681	0	P68511	0	P57113	0
P61983	0	P68255	0	P70470	0	P58195	0
P62260	0	P68511	0	P70490	0	P60711;P63259	0
P62630	0	P69897	0	P70502	0	P61107	0
P62836	0	P70470	0	P70545	0	P61459	0
P62959	0	P70490	0	P70619	0	P61943	0
P62963	0	P70545	0	P80067	0	P61972	0
P63018	0	P70619	0	P80202	0	P61983	0
P63102	0	P80067	0	P80254	0	P62260	0
P63322	0	P80202	0	P81827	0	P62630	0
P63331	0	P80254	0	P81828	0	P62815	0
P68035;P681	0	P81827	0	P82450	0	P62836	0
P68255	0	P81828	0	P84039	0	P62898	0
P68511	0	P82450	0	P85968	0	P62959	0
P69897	0	P84039	0	P85973	0	P62963	0
P70470	0	P85968	0	P97532	0	P63018	0
P70490	0	P85971	0	P97546	0	P63029	0

P70502	0	P85973	0	P97553	0	P63036	0
P70619	0	P97532	0	P97554	0	P63102	0
P80020	0	P97546	0	P97571	0	P63331	0
P80067	0	P97553	0	P97574	0	P68035;P68136	0
P80202	0	P97554	0	P97603	0	P68255	0
P80204	0	P97571	0	P97608	0	P70470	0
P81827	0	P97574	0	P97675	0	P70490	0
P81828	0	P97584	0	P97697	0	P70502	0
P82450	0	P97603	0	P97710	0	P70619	0
P84039	0	P97615	0	P97829	0	P80067	0
P85968	0	P97675	0	P98089	0	P80204	0
P85971	0	P97697	0	P98106	0	P80254	0
P85973	0	P97710	0	P98158	0	P81827	0
P97532	0	P97829	0	Q00238	0	P81828	0
P97546	0	P98106	0	Q00495	0	P82450	0
P97553	0	P98158	0	Q00657	0	P84039	0
P97554	0	Q00238	0	Q01177	0	P85968	0
P97603	0	Q00495	0	Q01460	0	P85973	0
P97608	0	Q00657	0	Q02765	0	P97532	0
P97675	0	Q01177	0	Q02974	0	P97546	0
P97697	0	Q01460	0	Q03336	0	P97553	0
P97710	0	Q02765	0	Q03626	0	P97571	0
P97829	0	Q02974	0	Q04589	0	P97603	0
P98106	0	Q03336	0	Q04807	0	P97615	0
P98158	0	Q03626	0	Q05030	0	P97675	0
Q00238	0	Q04589	0	Q05511	0	P97697	0
Q00495	0	Q04807	0	Q05695	0	P97710	0
Q00657	0	Q05030	0	Q05820	0	P97829	0
Q01177	0	Q05511	0	Q06496	0	P98106	0
Q01205	0	Q05695	0	Q06880	0	P98158	0
Q01460	0	Q05820	0	Q07523	0	Q00238	0
Q02253	0	Q06000	0	Q07936	0	Q00495	0
Q02765	0	Q06496	0	Q08406	0	Q00657	0
Q02974	0	Q06880	0	Q08415	0	Q01460	0
Q03336	0	Q07116	0	Q08420	0	Q02253	0
Q03626	0	Q08406	0	Q08463	0	Q02765	0
Q04589	0	Q08415	0	Q09326	0	Q02974	0
Q04807	0	Q08420	0	Q0PMD2	0	Q03336	0
Q05030	0	Q08463	0	Q1WIM1	0	Q03626	0
Q05511	0	Q08464	0	Q1WIM3	0	Q04589	0
Q05695	0	Q09326	0	Q32KJ6	0	Q04807	0
Q05820	0	Q0PMD2	0	Q32PY2	0	Q05030	0
Q06000	0	Q1WIM1	0	Q3KRC4	0	Q05175	0
Q06880	0	Q32KJ6	0	Q3MIE4	0	Q05511	0
Q07116	0	Q32PY2	0	Q3T1J1	0	Q05820	0
Q07523	0	Q3KRC4	0	Q3T1J9	0	Q06496	0
Q08406	0	Q3MIE4	0	Q3ZAV1	0	Q06880	0
Q08415	0	Q3T1J1	0	Q497B0	0	Q07523	0
Q08420	0	Q3T1J9	0	Q498D9	0	Q07936	0
Q08464	0	Q3T1K5	0	Q498S8	0	Q08406	0
Q09326	0	Q3ZAV1	0	Q499T2	0	Q08415	0
Q0PMD2	0	Q497B0	0	Q4FZV0	0	Q08420	0
Q1WIM1	0	Q498R7	0	Q4G075	0	Q08463	0
Q1WIM3	0	Q498S8	0	Q4KLZ6	0	Q09326	0
Q32KJ6	0	Q499T2	0	Q4KM73	0	Q0PMD2	0
Q32PY2	0	Q4FZV0	0	Q4QQV8	0	Q10758	0
Q3KRC4	0	Q4KLZ6	0	Q4QQW8	0	Q1WIM1	0
Q3MIE4	0	Q4KM73	0	Q4TU93	0	Q1WIM3	0
Q3T1J1	0	Q4QQV8	0	Q4V8I1	0	Q32PY2	0
Q3T1J9	0	Q4QQW8	0	Q4V8K5	0	Q3KRC4	0
Q3T1K5	0	Q4TU93	0	Q562C9	0	Q3T1J1	0
Q3ZAV1	0	Q4V8I1	0	Q566E6	0	Q3T1J9	0
Q497B0	0	Q4V8K5	0	Q568Z6	0	Q3ZAV1	0
Q498S8	0	Q562C9	0	Q5BJP3	0	Q497B0	0
Q499T2	0	Q566E6	0	Q5BJY9	0	Q498D9	0
Q4AEF8	0	Q568Z6	0	Q5BK81	0	Q498R7	0
Q4FZV0	0	Q5BJP3	0	Q5EGZ1	0	Q499T2	0
Q4KLZ6	0	Q5BK81	0	Q5FVF9	0	Q4FZU2	0

Q4KM73	0	Q5EGZ1	0	Q5FVH2	0	Q4FZV0	0
Q4QQV8	0	Q5FVF9	0	Q5FVR0	0	Q4G075	0
Q4QQW8	0	Q5FVH2	0	Q5FVR3	0	Q4KLZ6	0
Q4TU93	0	Q5FVR0	0	Q5HZW5	0	Q4KM73	0
Q4V885	0	Q5FVR3	0	Q5I0D5	0	Q4QQV8	0
Q4V8I1	0	Q5HZV9	0	Q5I0D7	0	Q4QQW8	0
Q4V8K5	0	Q5HZW5	0	Q5I0E9	0	Q4TU93	0
Q562C9	0	Q5I0D5	0	Q5M7T9	0	Q4V8I1	0
Q568Z6	0	Q5I0D7	0	Q5M871	0	Q4V8K5	0
Q5BJP3	0	Q5I0E9	0	Q5M872	0	Q562C9	0
Q5BK81	0	Q5M7T9	0	Q5M876	0	Q568Z6	0
Q5EGZ1	0	Q5M871	0	Q5RJL6	0	Q5BJY9	0
Q5FVF9	0	Q5M876	0	Q5RJP0	0	Q5BK81	0
Q5FVH2	0	Q5M8C6	0	Q5RKH6	0	Q5EGZ1	0
Q5FVR0	0	Q5RJL6	0	Q5RKI0	0	Q5FVF9	0
Q5FVR3	0	Q5RJP0	0	Q5RKI7	0	Q5FVH2	0
Q5HZW5	0	Q5RKH6	0	Q5U2Q3	0	Q5FVR0	0
Q5I0D5	0	Q5RKI0	0	Q5U2V4	0	Q5FVR3	0
Q5I0D7	0	Q5RKI7	0	Q5U300	0	Q5HZW5	0
Q5I0E9	0	Q5RLM2	0	Q5U367	0	Q5I0D7	0
Q5M7T9	0	Q5U2Q3	0	Q5XFX0	0	Q5I0E9	0
Q5M871	0	Q5U300	0	Q5XI20	0	Q5M7T9	0
Q5M872	0	Q5U367	0	Q5XI32	0	Q5M872	0
Q5M876	0	Q5XI20	0	Q5XI43	0	Q5M876	0
Q5RJL6	0	Q5XI32	0	Q5XI73	0	Q5M8C6	0
Q5RJP0	0	Q5XI43	0	Q5XI89	0	Q5RJL6	0
Q5RKH6	0	Q5XI73	0	Q5XIE8	0	Q5RJP0	0
Q5RKI0	0	Q5XI89	0	Q5Y4N8	0	Q5RKH6	0
Q5RKI7	0	Q5XIE8	0	Q62632	0	Q5RKI0	0
Q5U2Q3	0	Q5Y4N8	0	Q62635	0	Q5RKI7	0
Q5U300	0	Q5ZQU0	0	Q62636	0	Q5RLM2	0
Q5U367	0	Q62632	0	Q62638	0	Q5U2Q3	0
Q5XI20	0	Q62638	0	Q62740	0	Q5U300	0
Q5XI32	0	Q62687	0	Q62745	0	Q5U367	0
Q5XI43	0	Q62740	0	Q62786	0	Q5XI20	0
Q5XI73	0	Q62745	0	Q62795	0	Q5XI32	0
Q5XI89	0	Q62753	0	Q62812	0	Q5XI43	0
Q5XIE8	0	Q62786	0	Q62867	0	Q5XI89	0
Q5Y4N8	0	Q62795	0	Q62930	0	Q5XIE8	0
Q5ZQU0	0	Q62812	0	Q62946	0	Q5Y4N8	0
Q62632	0	Q62867	0	Q63041	0	Q62632	0
Q62638	0	Q62930	0	Q63072	0	Q62638	0
Q62740	0	Q62946	0	Q63083	0	Q62687	0
Q62745	0	Q62975	0	Q63150	0	Q62745	0
Q62753	0	Q63041	0	Q63203	0	Q62786	0
Q62786	0	Q63072	0	Q63257	0	Q62795	0
Q62795	0	Q63083	0	Q63270	0	Q62812	0
Q62812	0	Q63150	0	Q63416	0	Q62867	0
Q62867	0	Q63257	0	Q63424	0	Q62930	0
Q62930	0	Q63270	0	Q63474	0	Q62946	0
Q62946	0	Q63416	0	Q63475	0	Q62997	0
Q63041	0	Q63424	0	Q63515	0	Q63041	0
Q63072	0	Q63474	0	Q63530	0	Q63083	0
Q63083	0	Q63493	0	Q63598	0	Q63203	0
Q63150	0	Q63514	0	Q63610	0	Q63257	0
Q63203	0	Q63515	0	Q63621	0	Q63270	0
Q63257	0	Q63530	0	Q63678	0	Q63424	0
Q63270	0	Q63610	0	Q63691	0	Q63474	0
Q63416	0	Q63618	0	Q63716	0	Q63493	0
Q63467	0	Q63621	0	Q63772	0	Q63515	0
Q63474	0	Q63678	0	Q63797	0	Q63530	0
Q63514	0	Q63691	0	Q64093	0	Q63556	0
Q63515	0	Q63716	0	Q64119	0	Q63610	0
Q63530	0	Q63772	0	Q64194	0	Q63618	0
Q63556	0	Q63797	0	Q641X3	0	Q63621	0
Q63610	0	Q64093	0	Q641Z6	0	Q63678	0
Q63621	0	Q64119	0	Q641Z7	0	Q63691	0
Q63678	0	Q641X3	0	Q641Z8	0	Q63716	0

Q63691	0	Q641Z6	0	Q64230	0	Q63751	0
Q63716	0	Q641Z7	0	Q64240	0	Q63772	0
Q63772	0	Q641Z8	0	Q642A7	0	Q63797	0
Q63797	0	Q64230	0	Q64319	0	Q64119	0
Q64119	0	Q64240	0	Q64537	0	Q64194	0
Q641X3	0	Q642A7	0	Q64573	0	Q641X3	0
Q641Z6	0	Q64319	0	Q64602	0	Q641Z6	0
Q641Z7	0	Q64335	0	Q64604	0	Q641Z7	0
Q641Z8	0	Q64537	0	Q64605	0	Q641Z8	0
Q64230	0	Q64602	0	Q64611	0	Q64230	0
Q64240	0	Q64604	0	Q64640	0	Q64240	0
Q64268	0	Q64605	0	Q66H12	0	Q642A7	0
Q642A7	0	Q64611	0	Q66H94	0	Q64319	0
Q64537	0	Q64640	0	Q66HD0	0	Q64361	0
Q64573	0	Q66H12	0	Q66HG4	0	Q64537	0
Q64602	0	Q66H94	0	Q675A5	0	Q64573	0
Q64604	0	Q66HG4	0	Q68FP1	0	Q64602	0
Q64605	0	Q675A5	0	Q68FQ2	0	Q64604	0
Q64640	0	Q68FP1	0	Q68FS4	0	Q64640	0
Q66H12	0	Q68FQ2	0	Q68FT5	0	Q66H12	0
Q66H69	0	Q68FS4	0	Q68FV8	0	Q66H94	0
Q66H94	0	Q68FT5	0	Q6AXR4	0	Q66HD0	0
Q66HD0	0	Q6AXR4	0	Q6AXS4	0	Q66HG4	0
Q66HG4	0	Q6AXS4	0	Q6AY33	0	Q675A5	0
Q675A5	0	Q6AY41	0	Q6AY41	0	Q68FP1	0
Q68FP1	0	Q6AYD4	0	Q6AYD4	0	Q68FQ2	0
Q68FQ2	0	Q6AYE5	0	Q6AYE5	0	Q68FT5	0
Q68FT5	0	Q6AYP5	0	Q6AYP5	0	Q6AXR4	0
Q6AXR4	0	Q6AYR6	0	Q6AYR6	0	Q6AXS4	0
Q6AXS4	0	Q6AYS7	0	Q6AYR8	0	Q6AY33	0
Q6AY33	0	Q6AYT0	0	Q6AYS7	0	Q6AY41	0
Q6AY41	0	Q6B345	0	Q6AYT0	0	Q6AY61	0
Q6AY61	0	Q6DGG1	0	Q6B345	0	Q6AYD4	0
Q6AYD4	0	Q6GMN2	0	Q6BEA2	0	Q6AYE5	0
Q6AYE5	0	Q6IE64	0	Q6DGG1	0	Q6AYP5	0
Q6AYP5	0	Q6IFW2	0	Q6GMN2	0	Q6AYQ8	0
Q6AYR6	0	Q6IRK9	0	Q6IE64	0	Q6AYR6	0
Q6AYT0	0	Q6IUU3	0	Q6IFW2	0	Q6AYS7	0
Q6B345	0	Q6JE36	0	Q6IRK9	0	Q6AYT0	0
Q6BEA2	0	Q6MG61	0	Q6IUU3	0	Q6B345	0
Q6DGG1	0	Q6MG71	0	Q6JE36	0	Q6DGG1	0
Q6IE52	0	Q6NX65	0	Q6MG61	0	Q6IE52	0
Q6IE64	0	Q6NYB7	0	Q6MG71	0	Q6IE64	0
Q6IFW2	0	Q6P6S9	0	Q6NYB7	0	Q6IFU8	0
Q6IRK9	0	Q6P6T1	0	Q6P6S9	0	Q6IFW2	0
Q6IUU3	0	Q6P6V0	0	Q6P6T1	0	Q6IG02	0
Q6MG61	0	Q6P734	0	Q6P6V0	0	Q6IMF3	0
Q6MG71	0	Q6P7A9	0	Q6P734	0	Q6IRK9	0
Q6NX65	0	Q6P7Q4	0	Q6P767	0	Q6IUU3	0
Q6NYB7	0	Q6P7S1	0	Q6P7A9	0	Q6JE36	0
Q6P6S9	0	Q6PCU2	0	Q6P7Q4	0	Q6MG61	0
Q6P6T1	0	Q6PEC4	0	Q6P7S1	0	Q6MG71	0
Q6P6V0	0	Q6Q0N1	0	Q6PEC4	0	Q6NX65	0
Q6P734	0	Q6Q7Y5	0	Q6Q0N1	0	Q6NYB7	0
Q6P777	0	Q6RUV5	0	Q6Q7Y5	0	Q6P6Q2	0
Q6P7A9	0	Q6RY07	0	Q6RUV5	0	Q6P6S9	0
Q6P7Q4	0	Q6TUD4	0	Q6RY07	0	Q6P6V0	0
Q6P7S1	0	Q6VBQ5	0	Q6TUD4	0	Q6P734	0
Q6PEC4	0	Q6X936	0	Q6VBQ5	0	Q6P767	0
Q6Q7Y5	0	Q711G3	0	Q6X936	0	Q6P7A9	0
Q6RY07	0	Q71UF4	0	Q711G3	0	Q6P7Q4	0
Q6TUD4	0	Q76HN1	0	Q71MB6	0	Q6P7S1	0
Q6VBQ5	0	Q793F9	0	Q76HN1	0	Q6PEC4	0
Q6X936	0	Q794F9	0	Q793F9	0	Q6Q0N1	0
Q711G3	0	Q7M0E3	0	Q794F9	0	Q6RY07	0
Q71MB6	0	Q7TPB4	0	Q7M0E3	0	Q6TUD4	0
Q71UF4	0	Q7TQ94	0	Q7TPB1	0	Q6VBQ5	0
Q76HN1	0	Q80W57	0	Q7TPB4	0	Q6X936	0

Q793F9	0	Q80WD0	0	Q7TQ94	0	Q711G3	0
Q794F9	0	Q80WD1	0	Q80W57	0	Q71MB6	0
Q7M0E3	0	Q80WF4	0	Q80WD0	0	Q71UF4	0
Q7TPB4	0	Q80WY6	0	Q80WD1	0	Q76HN1	0
Q7TQ94	0	Q80YN4	0	Q80WF4	0	Q793F9	0
Q80WD0	0	Q810F4	0	Q80YN4	0	Q794F9	0
Q80WD1	0	Q811A3	0	Q810F4	0	Q7M0E3	0
Q80WF4	0	Q811M5	0	Q811A3	0	Q7TPB4	0
Q80YN4	0	Q811X6	0	Q811M5	0	Q7TQ94	0
Q810F4	0	Q812E4	0	Q811X6	0	Q80W57	0
Q811A3	0	Q812E9	0	Q812E9	0	Q80WD0	0
Q811M5	0	Q8CFN2	0	Q8CFN2	0	Q80WD1	0
Q811X6	0	Q8CG08	0	Q8CG08	0	Q80WF4	0
Q812E9	0	Q8CG45	0	Q8CG45	0	Q810F4	0
Q8CG08	0	Q8CGS4	0	Q8CGS4	0	Q811A3	0
Q8CG45	0	Q8CHN3	0	Q8CHN3	0	Q811M5	0
Q8CGS4	0	Q8CHN8	0	Q8CIZ5	0	Q811X6	0
Q8CHN3	0	Q8JZQ0	0	Q8JZQ0	0	Q812E9	0
Q8CHN8	0	Q8K3V3	0	Q8K1G0	0	Q8CG45	0
Q8CIZ5	0	Q8R431	0	Q8K3V3	0	Q8CGS4	0
Q8JZQ0	0	Q8R491	0	Q8R431	0	Q8CHN3	0
Q8K3V3	0	Q8R4E1	0	Q8R491	0	Q8JZQ0	0
Q8N7M5	0	Q8R5M3	0	Q8R4E1	0	Q8K1G0	0
Q8R491	0	Q8R5M5	0	Q8R5M3	0	Q8K3V3	0
Q8R4E1	0	Q8VIF7	0	Q8R5M5	0	Q8R431	0
Q8R5M3	0	Q91XN4	0	Q8VIF7	0	Q8R491	0
Q8R5M5	0	Q91XT9	0	Q91XN4	0	Q8R4E1	0
Q8VIF7	0	Q920A6	0	Q91XT9	0	Q8R5M3	0
Q91XN4	0	Q920G2	0	Q920A6	0	Q8R5M5	0
Q91XT9	0	Q923M1	0	Q923M1	0	Q8VIF7	0
Q920A6	0	Q923S2	0	Q923S2	0	Q91XT9	0
Q920G2	0	Q923V8	0	Q923V8	0	Q920A6	0
Q923V8	0	Q924C3	0	Q924C3	0	Q920G2	0
Q924C3	0	Q99376	0	Q99376	0	Q923M1	0
Q99376	0	Q99J86	0	Q99J86	0	Q923S2	0
Q99J86	0	Q99M75	0	Q99M75	0	Q923V8	0
Q99M75	0	Q99MA2	0	Q99MA2	0	Q924C3	0
Q99MA2	0	Q99MF4	0	Q99MF4	0	Q99J86	0
Q99MF4	0	Q99PS8	0	Q99PP0	0	Q99M75	0
Q99PS8	0	Q99PW3	0	Q99PS8	0	Q99MA2	0
Q99PW3	0	Q99PW7	0	Q99PW3	0	Q99MF4	0
Q99PW7	0	Q9EPB1	0	Q99PW7	0	Q99PS8	0
Q9EPF2	0	Q9EPF2	0	Q9EPB1	0	Q99PW3	0
Q9EQV6	0	Q9EQS0	0	Q9EPF2	0	Q99PW7	0
Q9EQX9	0	Q9EQV6	0	Q9EQS0	0	Q9EPB1	0
Q9ERA7	0	Q9EQX9	0	Q9EQX9	0	Q9EPF2	0
Q9ES87	0	Q9ERA7	0	Q9ERA7	0	Q9EQS0	0
Q9ESG3	0	Q9ES87	0	Q9ES87	0	Q9EQV9	0
Q9ESS6	0	Q9ESG3	0	Q9ESG3	0	Q9EQX9	0
Q9JHW1	0	Q9ESS6	0	Q9ESS6	0	Q9ERA7	0
Q9JHY1	0	Q9JHB9	0	Q9JHW1	0	Q9ES87	0
Q9JI92	0	Q9JHW1	0	Q9JHY1	0	Q9ESG3	0
Q9JID2	0	Q9JHY1	0	Q9JI92	0	Q9ESS6	0
Q9JIK1	0	Q9JI92	0	Q9JIK1	0	Q9JHW1	0
Q9JJ19	0	Q9JIK1	0	Q9JJ19	0	Q9JHY1	0
Q9JJ22	0	Q9JJ19	0	Q9JJ40	0	Q9JI92	0
Q9JJ40	0	Q9JJ22	0	Q9JJ50	0	Q9JIK1	0
Q9JJ73	0	Q9JJ40	0	Q9JJ73	0	Q9JJ19	0
Q9JLJ3	0	Q9JJ73	0	Q9JJS8	0	Q9JJ22	0
Q9JLS4	0	Q9JLJ3	0	Q9JLJ3	0	Q9JJ40	0
Q9QW30	0	Q9JLS4	0	Q9QW30	0	Q9JJ50	0
Q9QWJ9	0	Q9QW30	0	Q9QY17	0	Q9JJ73	0
Q9QXQ0	0	Q9QWJ9	0	Q9QYU4	0	Q9JLJ3	0
Q9QY17	0	Q9QXQ0	0	Q9QZ76	0	Q9JLS4	0
Q9QZ76	0	Q9QY17	0	Q9QZA2	0	Q9QW30	0
Q9QZA2	0	Q9QYU4	0	Q9QZH0	0	Q9QWJ9	0
Q9QZH0	0	Q9QZ76	0	Q9QZK8	0	Q9QY17	0
Q9QZQ5	0	Q9QZA2	0	Q9R044	0	Q9QYU4	0

Q9R044	0	Q9QZH0	0	Q9R063	0	Q9QZA2	0
Q9R063	0	Q9QZK8	0	Q9R066	0	Q9QZH0	0
Q9R066	0	Q9R063	0	Q9R0D6	0	Q9QZK8	0
Q9R0D6	0	Q9R066	0	Q9R0J8	0	Q9R063	0
Q9R0J8	0	Q9R0D6	0	Q9R0T4	0	Q9R066	0
Q9R0T4	0	Q9R0J8	0	Q9R141	0	Q9R0D6	0
Q9R141	0	Q9R0T4	0	Q9R1T1	0	Q9R0J8	0
Q9R1T3	0	Q9R141	0	Q9R1T3	0	Q9R0T3	0
Q9R1T5	0	Q9R1T3	0	Q9R1T5	0	Q9R0T4	0
Q9WTQ2	0	Q9R1T5	0	Q9WTQ2	0	Q9R141	0
Q9WTW7	0	Q9WTQ2	0	Q9WUC4	0	Q9R1T1	0
Q9WUC4	0	Q9WTW7	0	Q9WUD9	0	Q9R1T3	0
Q9WUD9	0	Q9WUC4	0	Q9WUF4	0	Q9WTQ2	0
Q9WUF4	0	Q9WUD9	0	Q9WUK5	0	Q9WTW7	0
Q9WUK5	0	Q9WUK5	0	Q9WUW3	0	Q9WUK5	0
Q9WUW3	0	Q9WUW3	0	Q9WUW8	0	Q9WUW8	0
Q9WUW9	0	Q9WUW8	0	Q9WUW9	0	Q9WUW9	0
Q9WVH8	0	Q9WUW9	0	Q9WVH8	0	Q9WVH8	0
Q9Z0J6	0	Q9WVH8	0	Q9Z0J6	0	Q9Z0J6	0
Q9Z0T0	0	Q9Z0T0	0	Q9Z0T0	0	Q9Z0T0	0
Q9Z0W7	0	Q9Z0W7	0	Q9Z0W7	0	Q9Z0W7	0
Q9Z1Y3	0	Q9Z1Y3	0	Q9Z1Y3	0	Q9Z1Y3	0
Q9Z2Y9	0	Q9Z2Y9	0	Q9Z2Y9	0	Q9Z2Y9	0
Q9Z339	0	Q9Z339	0	Q9Z339	0	Q9Z339	0
