# Supplementary figures and images for: Dynamic Changes of Urine Proteome in Rat Models Inoculated with Two Different Hepatoma Cell Lines
Source: J Oncol. 2021 Jan 7;2021:8895330. doi: 10.1155/2021/8895330 (PMC7810548; doi:10.1155/2021/8895330)

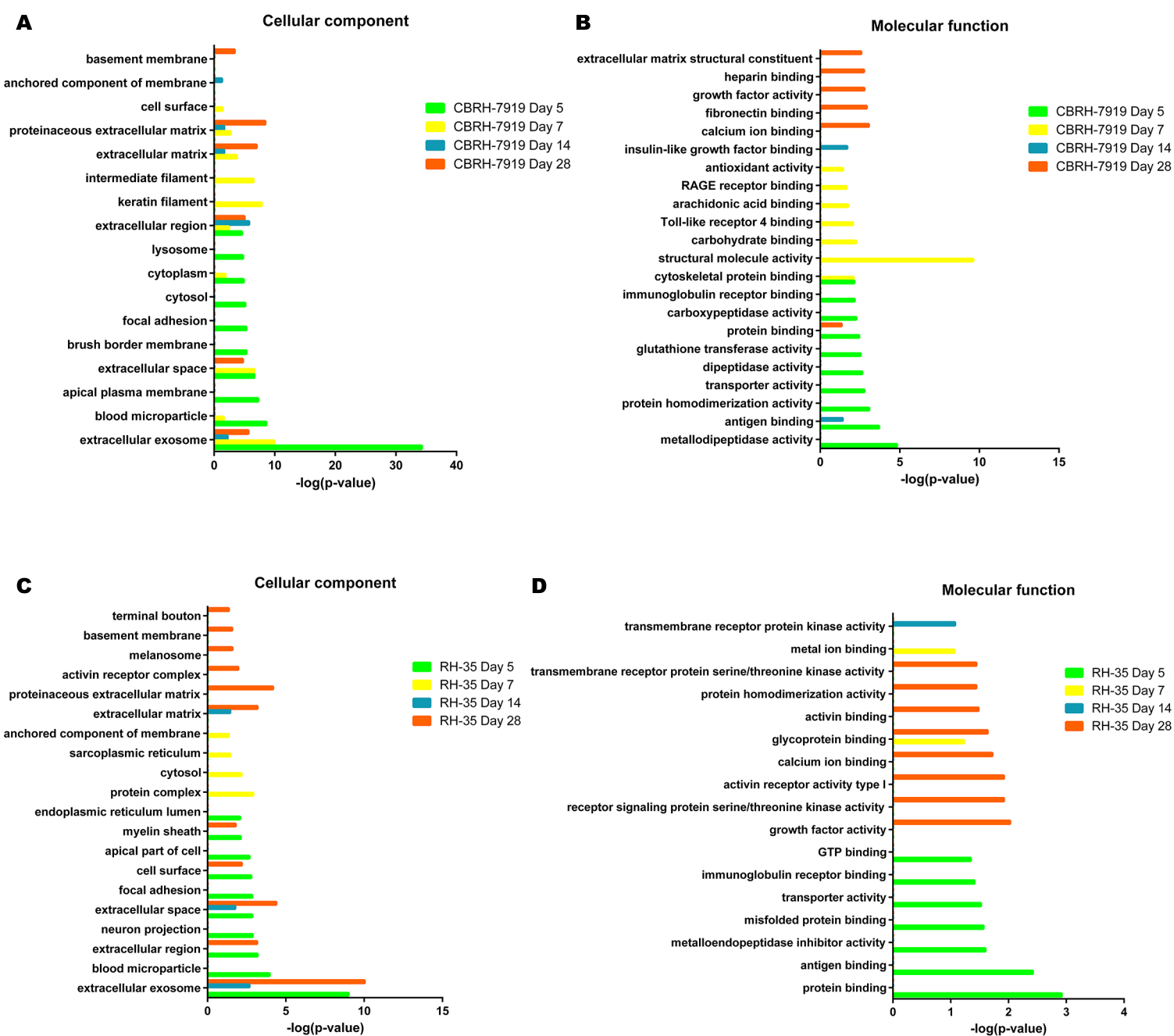

Supplement: Supplementary Materials — Supplementary Figure 1. Functional analysis of differentially expressed proteins at days 5, 7, 14, and 28 in two models. (a) Cell component for the CBRH-7919 model. (b) Molecular function for the CBRH-7919 model. (c) Cell component for the RH-35 model. (d) Molecular function for the RH-35 model. Supplementary Table 1. All urinary proteins identified in the CBRH-7919 model. Supplementary Table 2. All urinary proteins identified in the RH-35 model. Supplementary Table 3. The details of 6435 random allocations in the CBRH-7919 model. Supplementary Table 4. The occurrence of the protein in 6435 random allocations in the CBRH-7919 model. Supplementary Table 5. The details of 12155 random allocations in the RH-35 model. Supplementary Table 6. The occurrence of the protein in 12155 random allocations in the RH-35 model. [file 8895330.f1.zip › 8895330.f1/Supplementary Figure 1 (1).pdf]
